# Supplementary material for: Mitochondrial dysfunction rapidly modulates the abundance and thermal stability of cellular proteins
Source: Life Sci Alliance. 2023 Mar 20;6(6):e202201805. doi: 10.26508/lsa.202201805 (PMC10027898; doi:10.26508/lsa.202201805)
Supplement: Supplementary file 2 [file LSA-2022-01805_Supplemental_Data_1.pdf]

AAC1 − average.top3: 7.02106936170213 − max.qupm: 9

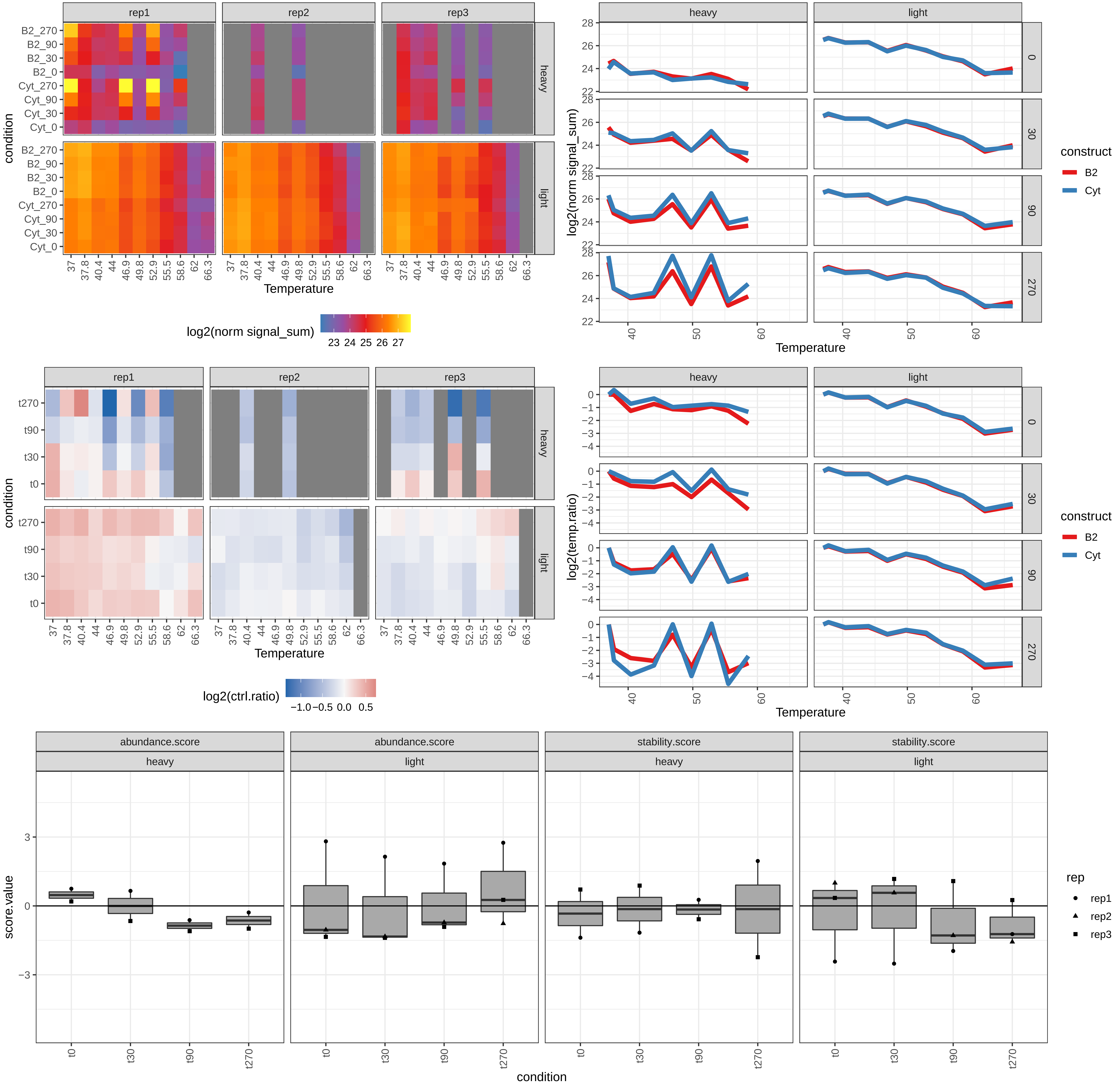

P04710 − ADP,ATP carrier protein 1 OS=Saccharomyces cerevisiae (strain ATCC 204508 / S288c) GN=AAC1 PE=1 SV=1

AAC3 − average.top3: 7.36031037037037 − max.qupm: 3

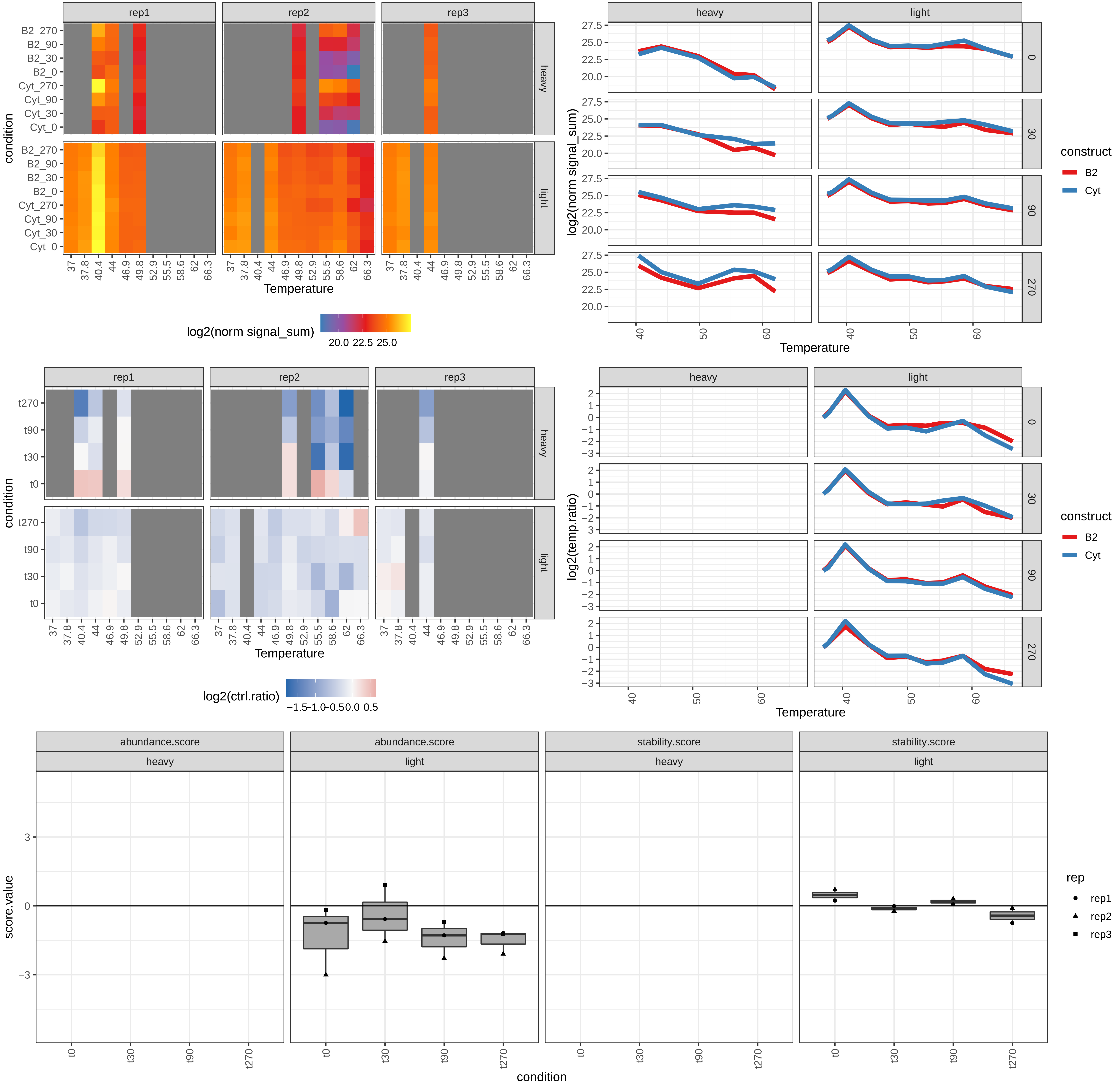

P18238 − ADP,ATP carrier protein 3 OS=Saccharomyces cerevisiae (strain ATCC 204508 / S288c) GN=AAC3 PE=1 SV=1

AAD16 − average.top3: 6.37756047619048 − max.qupm: 5

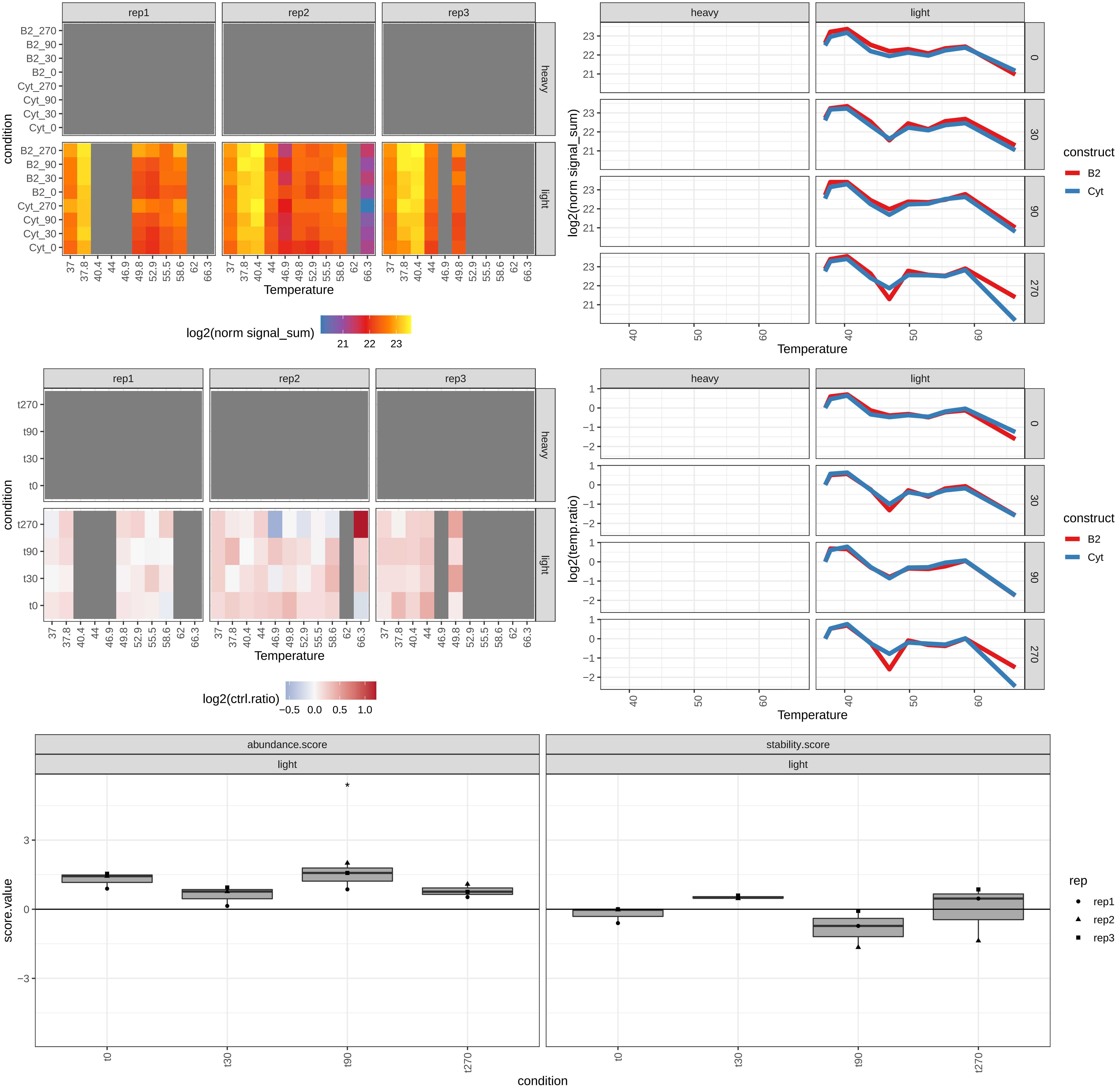

Q02895 − Putative aryl−alcohol dehydrogenase AAD16 OS=Saccharomyces cerevisiae (strain ATCC 204508 / S288c) GN=AAD16 PE=1 SV=1

AAP1 − average.top3: 7.33845392156863 − max.qupm: 27

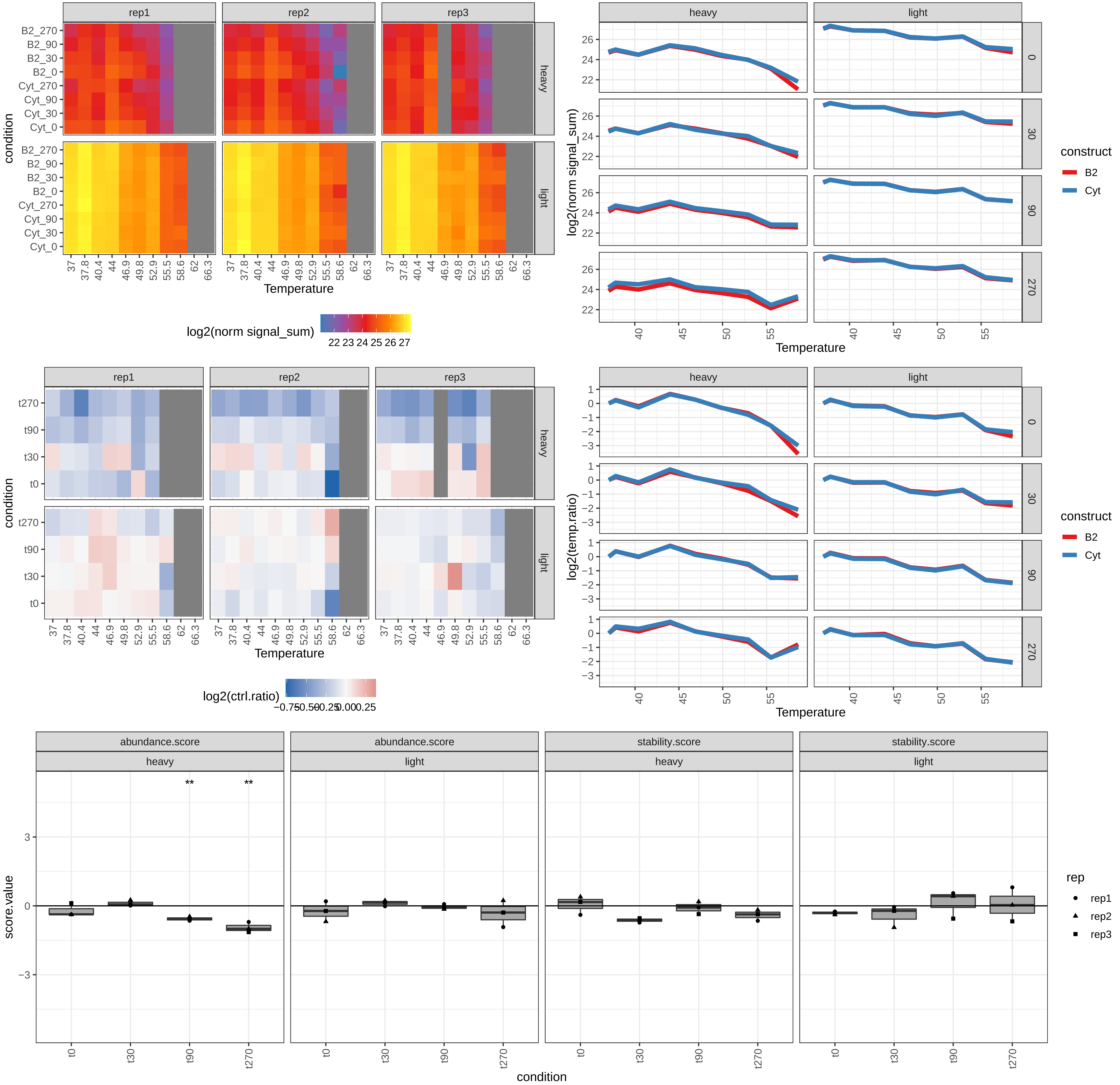

P37898 − Alanine/arginine aminopeptidase OS=Saccharomyces cerevisiae (strain ATCC 204508 / S288c) GN=AAP1 PE=1 SV=2

AAR2 − average.top3: 6.17357375 − max.qupm: 4

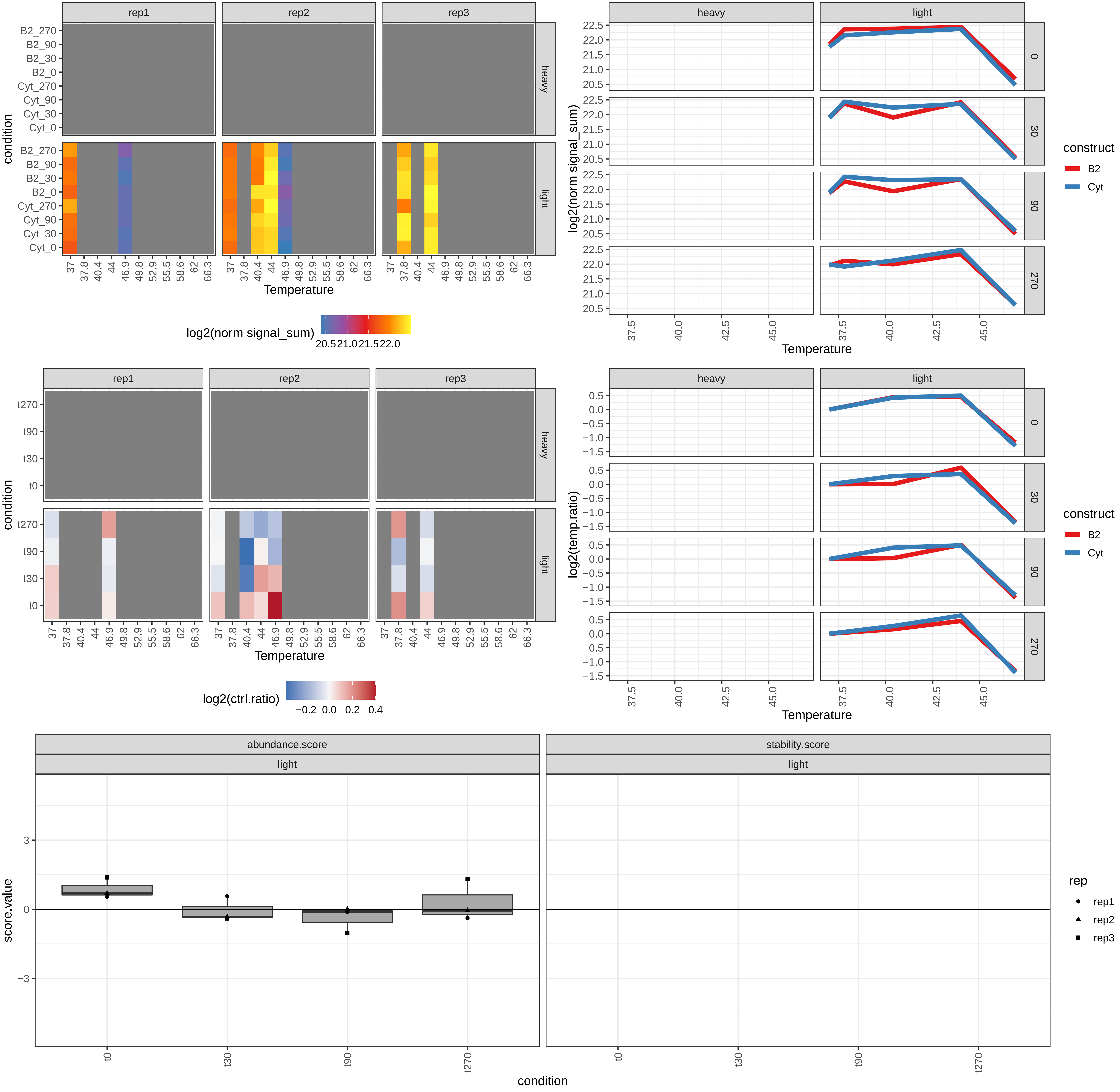

P32357 − A1 cistron−splicing factor AAR2 OS=Saccharomyces cerevisiae (strain ATCC 204508 / S288c) GN=AAR2 PE=1 SV=1

AAT1 − average.top3: 6.53717210526316 − max.qupm: 8

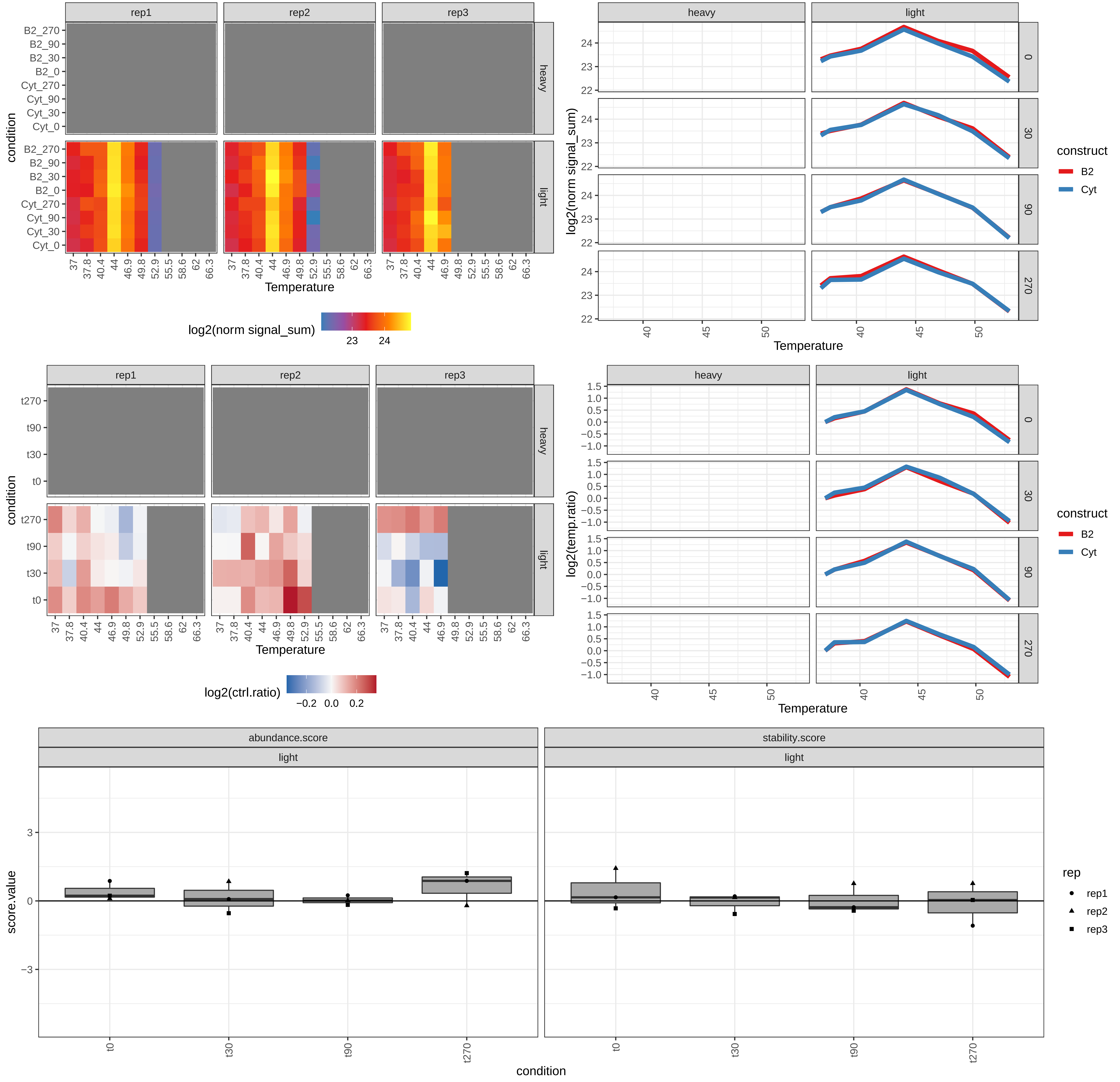

Q01802 − Aspartate aminotransferase, mitochondrial OS=Saccharomyces cerevisiae (strain ATCC 204508 / S288c) GN=AAT1 PE=1 SV=2

AAT2 − average.top3: 7.76223328125 − max.qupm: 14

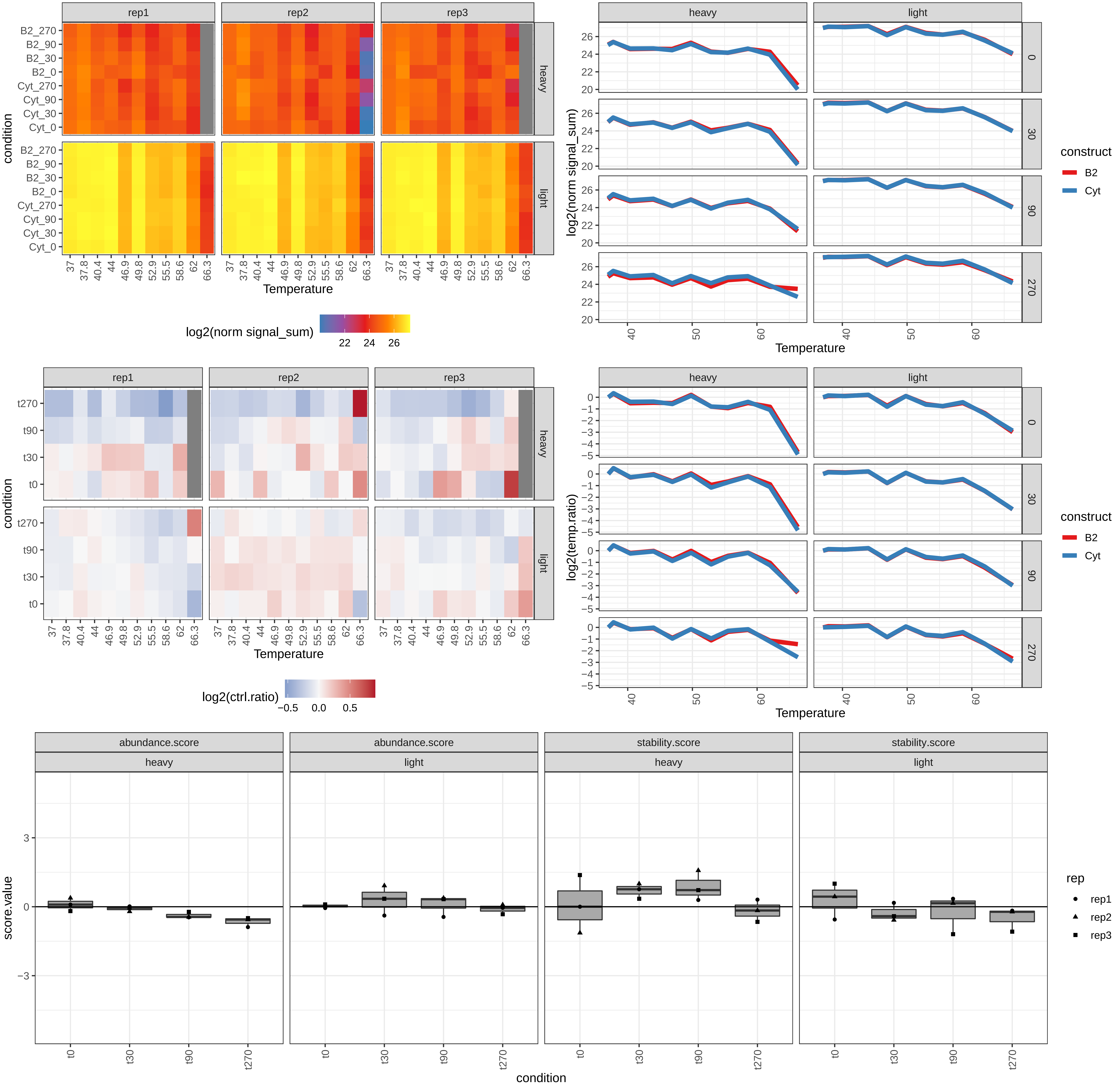

P23542 − Aspartate aminotransferase, cytoplasmic OS=Saccharomyces cerevisiae (strain ATCC 204508 / S288c) GN=AAT2 PE=1 SV=3

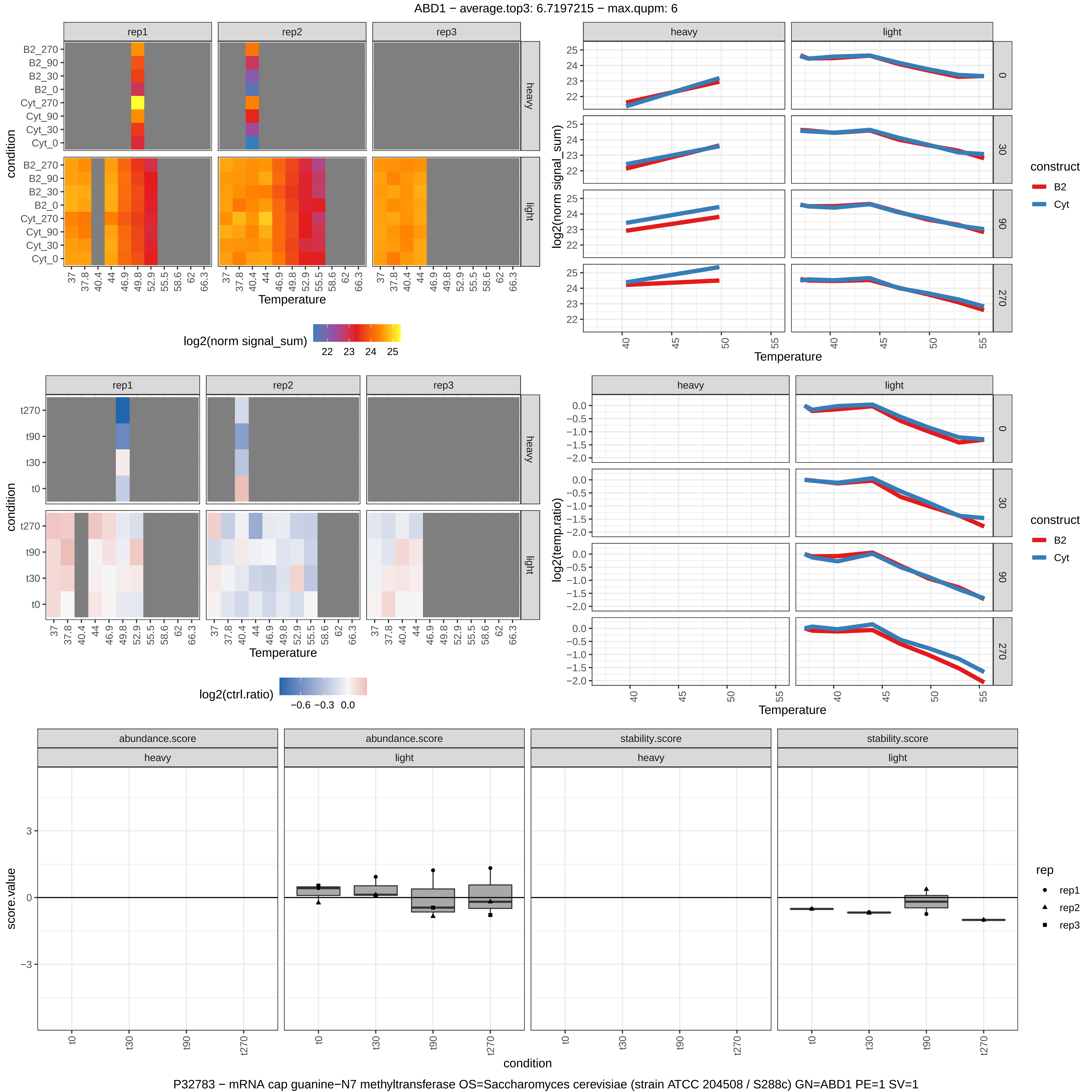
ABD1 − average.top3: 6.7197215 − max.qupm: 6
rep1
rep2
rep3
heavy
light
condition
B2_270
B2_90
B2_30
B2_0
Cyt_270
Cyt_90
Cyt_30
Cyt_0
Temperature
37
37.8
40.4
44
46.9
49.8
52.9
55.5
58.6
62
66.3
log2(norm signal_sum)
22
23
24
25
0
30
90
270
construct
B2
Cyt
40
45
50
55
t270
t90
t30
t0
log2(ctrl.ratio)
−0.6
−0.3
0.0
log2(temp.ratio)
−0.5
−1.0
−1.5
−2.0
abundance.score
stability.score
score.value
3
−3
rep
P32783 − mRNA cap guanine−N7 methyltransferase OS=Saccharomyces cerevisiae (strain ATCC 204508 / S288c) GN=ABD1 PE=1 SV=1

ABF1 − average.top3: 6.99063476190476 − max.qupm: 6

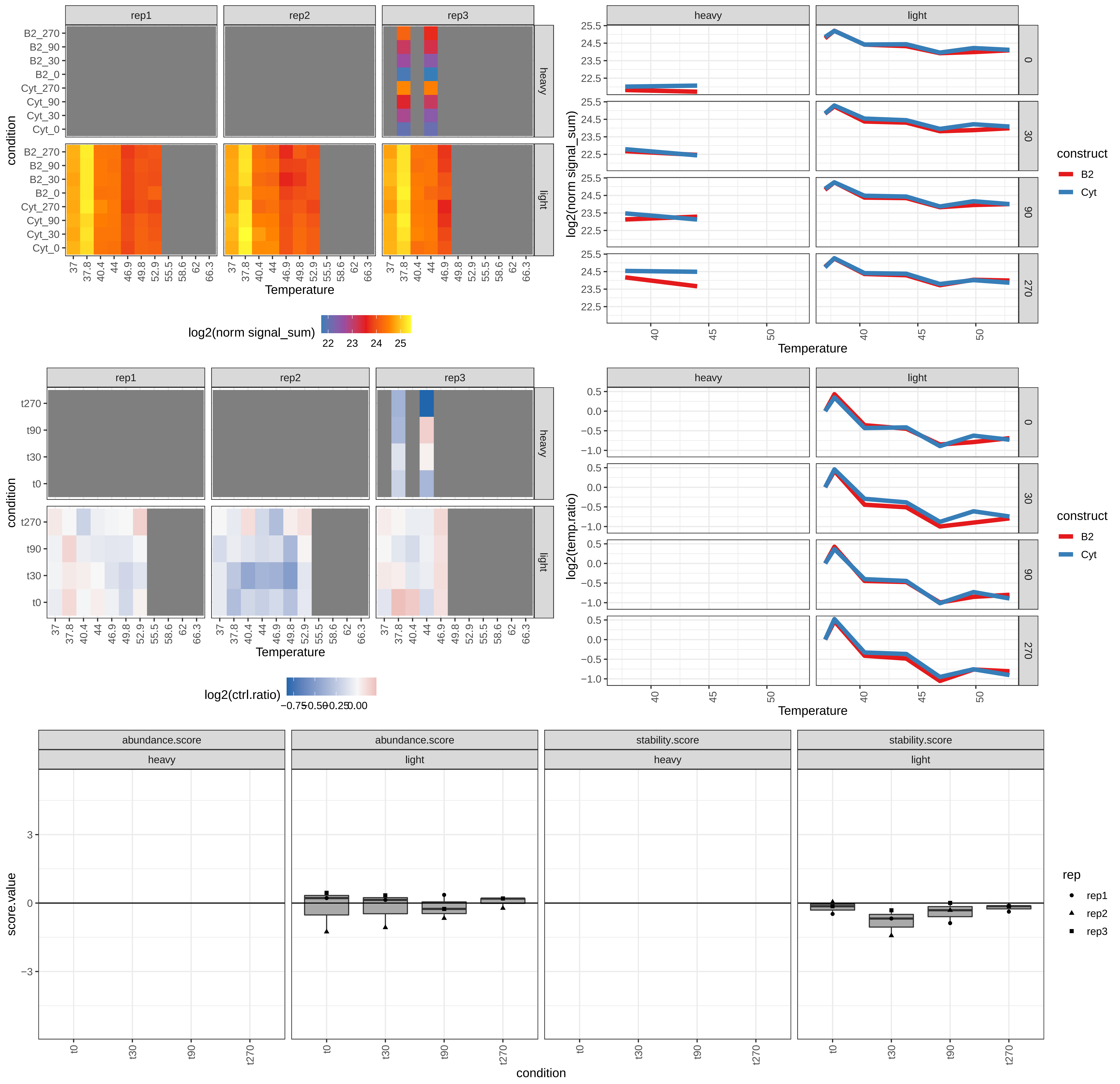

P14164 − ARS−binding factor 1 OS=Saccharomyces cerevisiae (strain ATCC 204508 / S288c) GN=ABF1 PE=1 SV=4

ABF2 − average.top3: 7.24907882352941 − max.qupm: 11

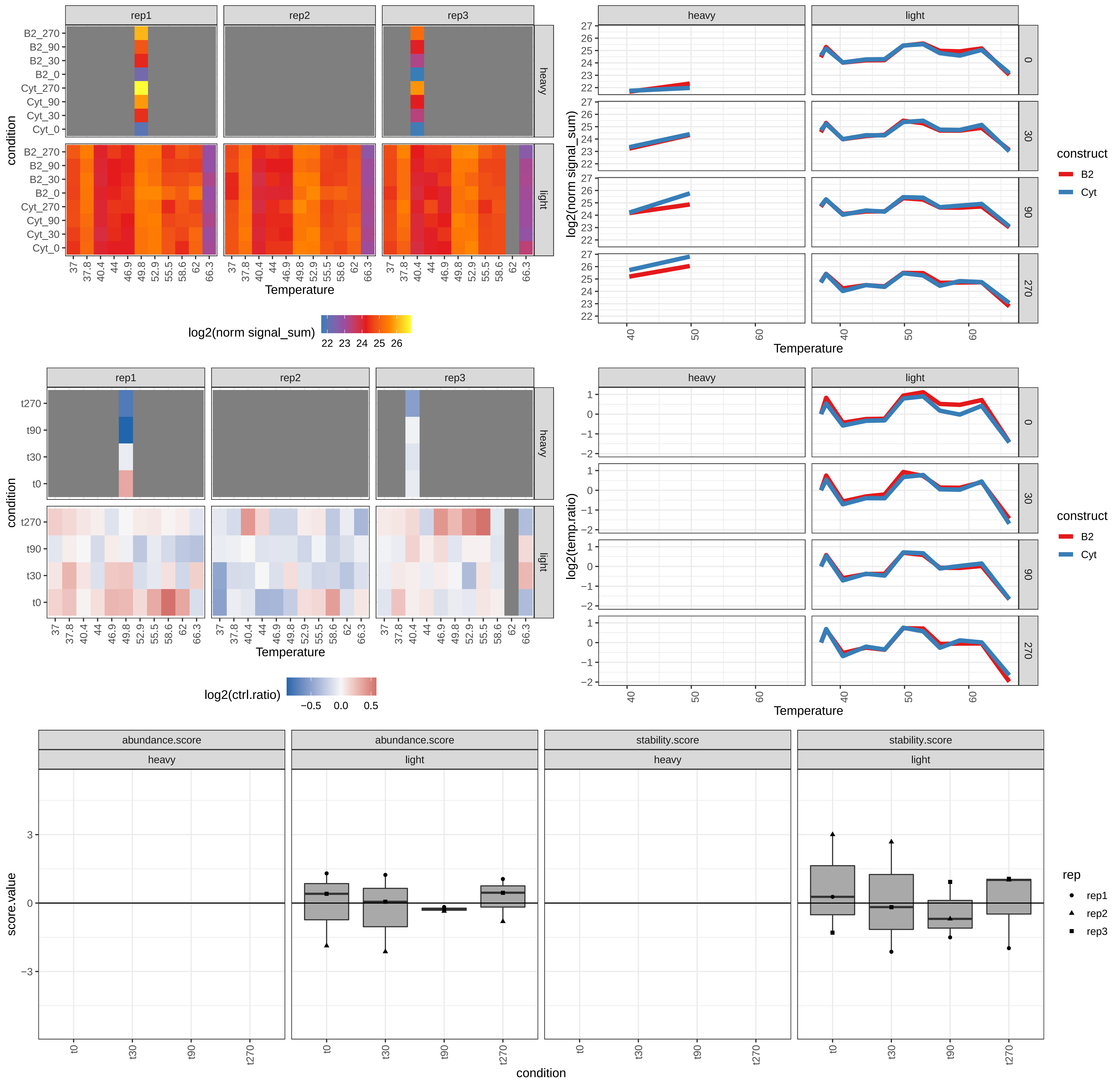

Q02486 − ARS−binding factor 2, mitochondrial OS=Saccharomyces cerevisiae (strain ATCC 204508 / S288c) GN=ABF2 PE=1 SV=1

ABP1 − average.top3: 7.56896666666667 − max.qupm: 11

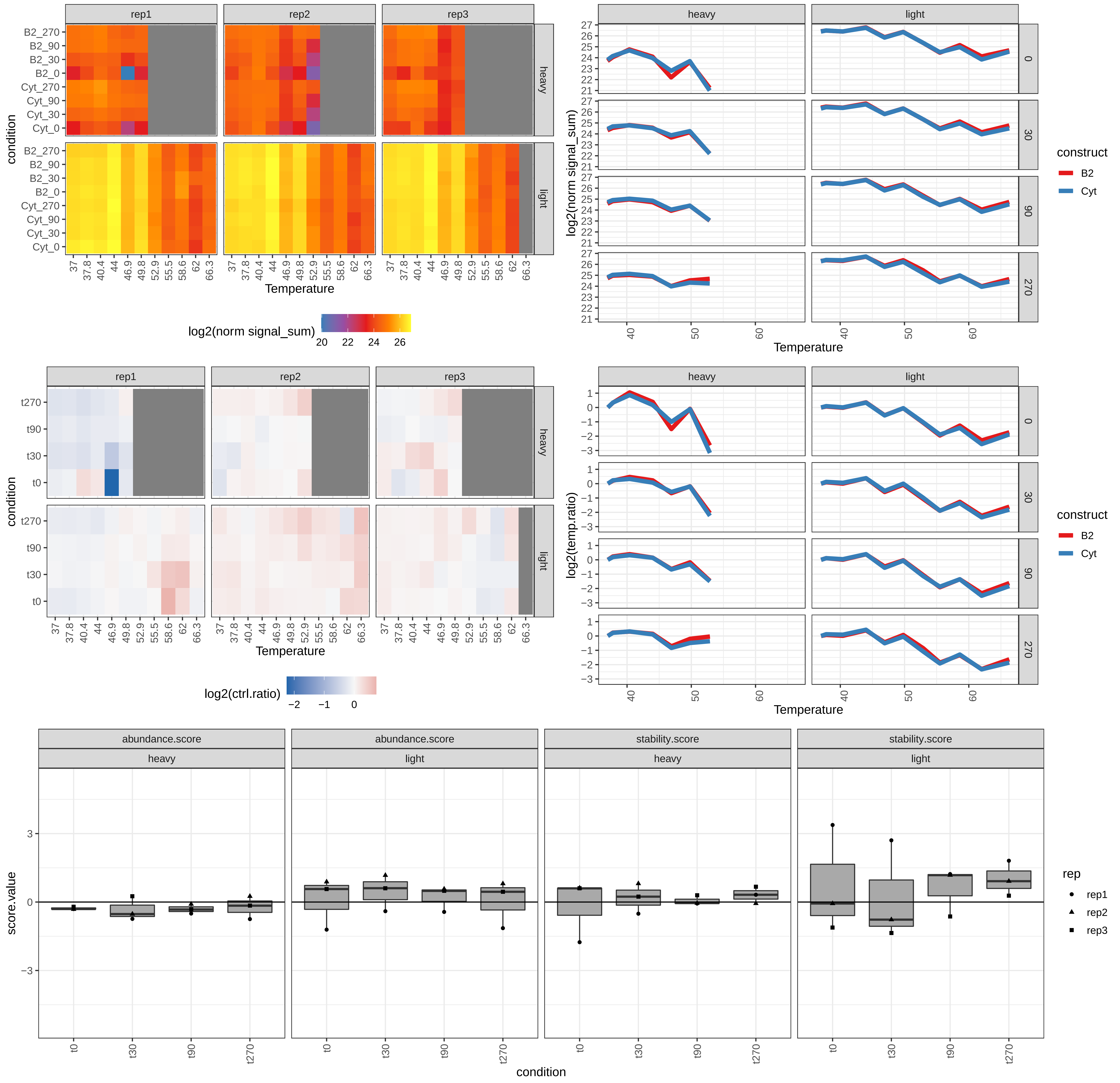

P15891 − Actin−binding protein OS=Saccharomyces cerevisiae (strain ATCC 204508 / S288c) GN=ABP1 PE=1 SV=4

ABP140 − average.top3: 6.96124606060606 − max.qupm: 9

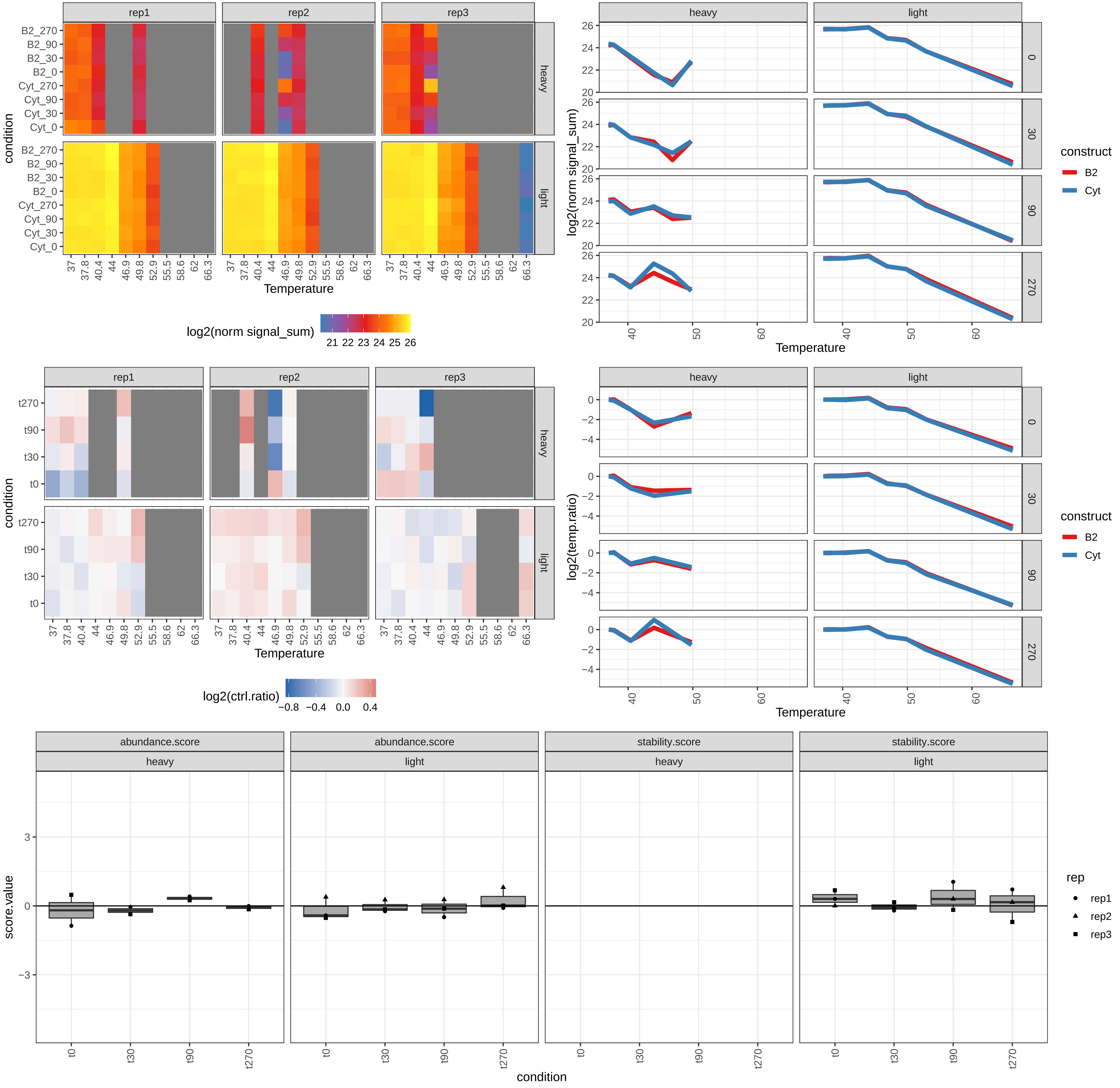

Q08641 − tRNA(Thr) (cytosine(32)−N(3))−methyltransferase OS=Saccharomyces cerevisiae (strain ATCC 204508 / S288c) GN=ABP140 PE=1 SV=3

ABZ1 − average.top3: 6.491655 − max.qupm: 6

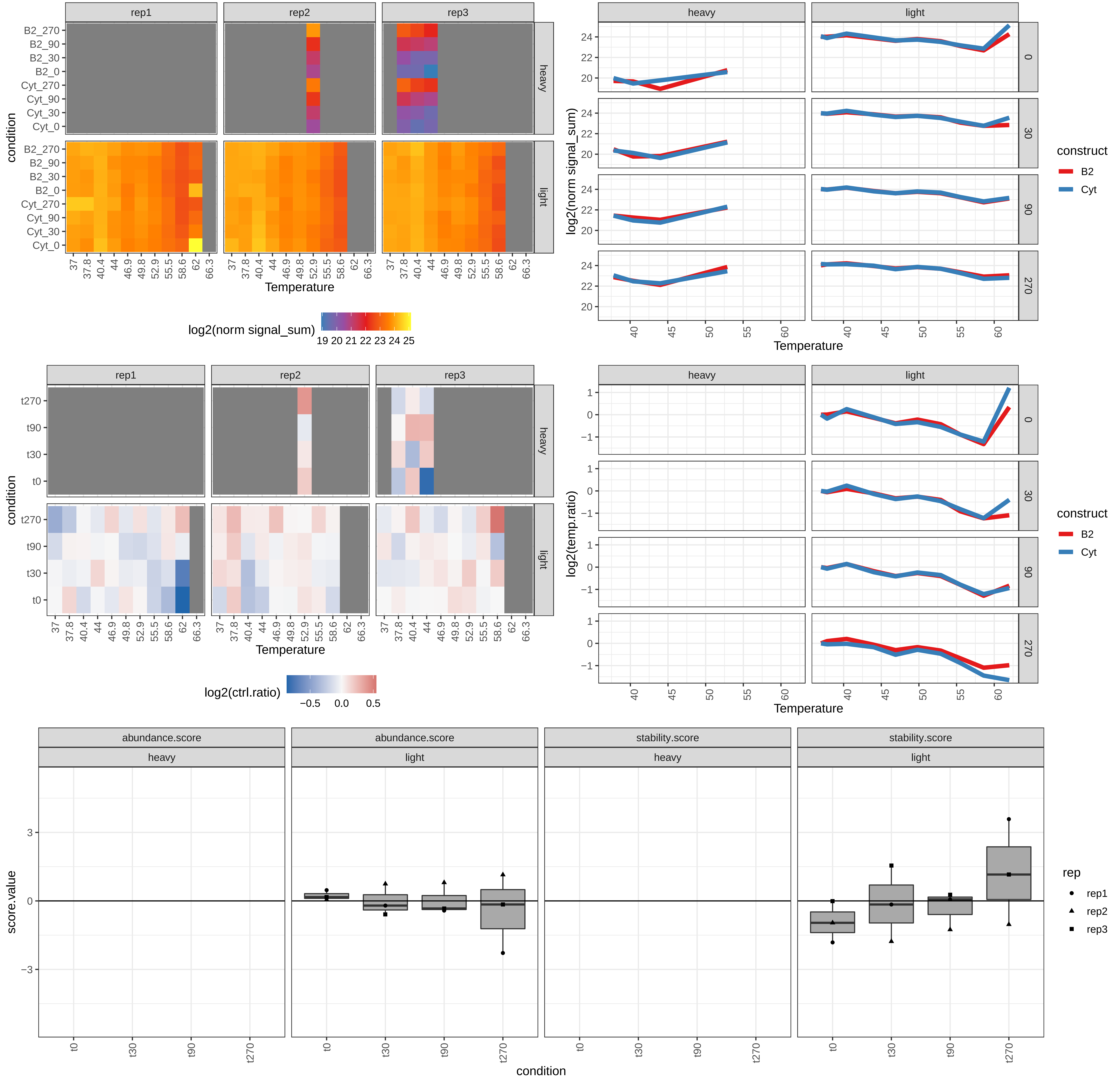

P37254 − Aminodeoxychorismate synthase OS=Saccharomyces cerevisiae (strain ATCC 204508 / S288c) GN=ABZ1 PE=1 SV=4

ABZ2 − average.top3: 6.87592285714286 − max.qupm: 12

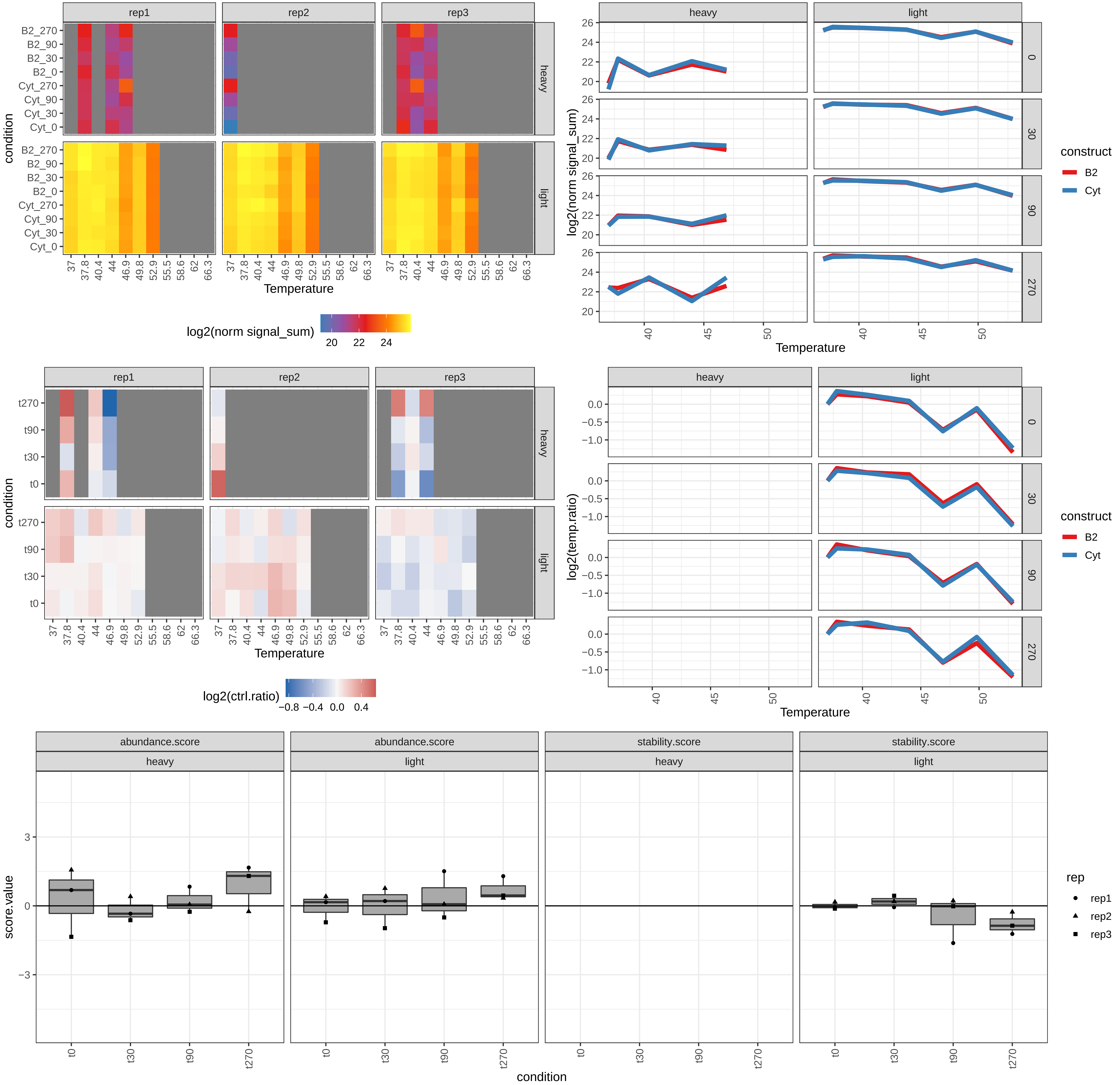

Q03266 − Aminodeoxychorismate lyase OS=Saccharomyces cerevisiae (strain ATCC 204508 / S288c) GN=ABZ2 PE=1 SV=1

ACB1 − average.top3: 7.18229758064516 − max.qupm: 5

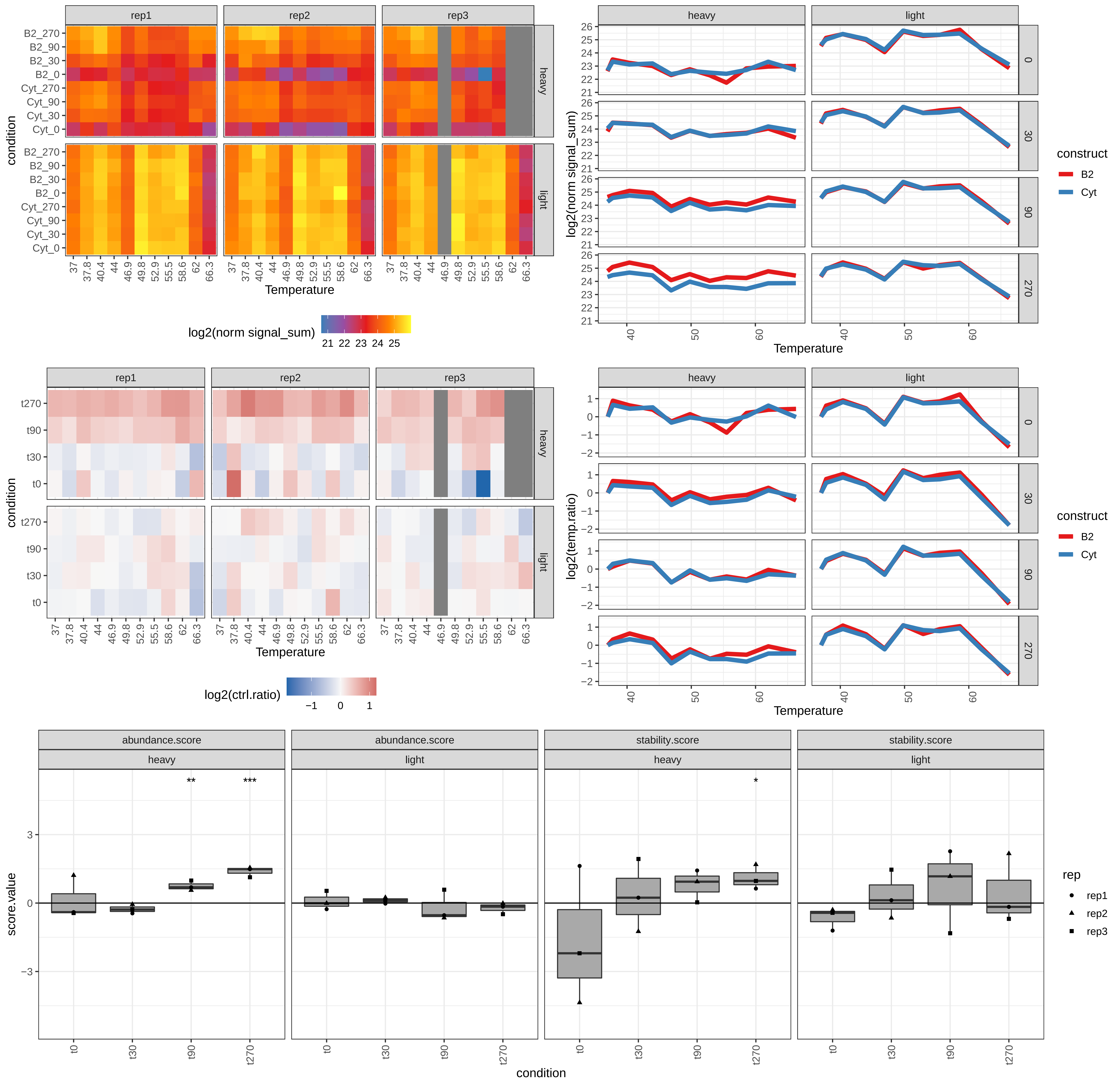

P31787 − Acyl−CoA−binding protein OS=Saccharomyces cerevisiae (strain ATCC 204508 / S288c) GN=ACB1 PE=1 SV=3

ACC1 − average.top3: 7.73302357142857 − max.qupm: 87

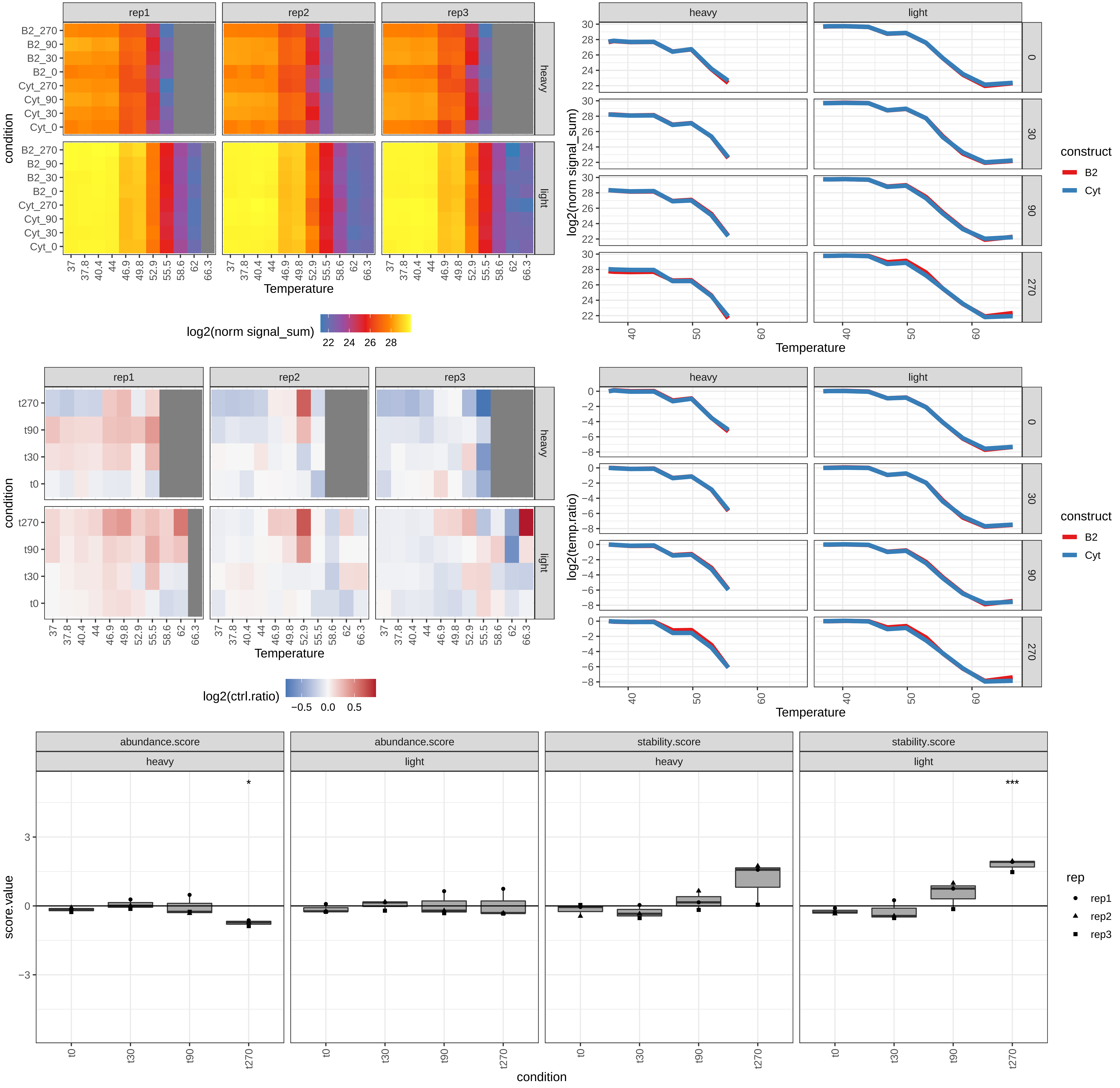

Q00955 − Acetyl−CoA carboxylase OS=Saccharomyces cerevisiae (strain ATCC 204508 / S288c) GN=ACC1 PE=1 SV=2

ACF2 − average.top3: 6.87303473684211 − max.qupm: 13

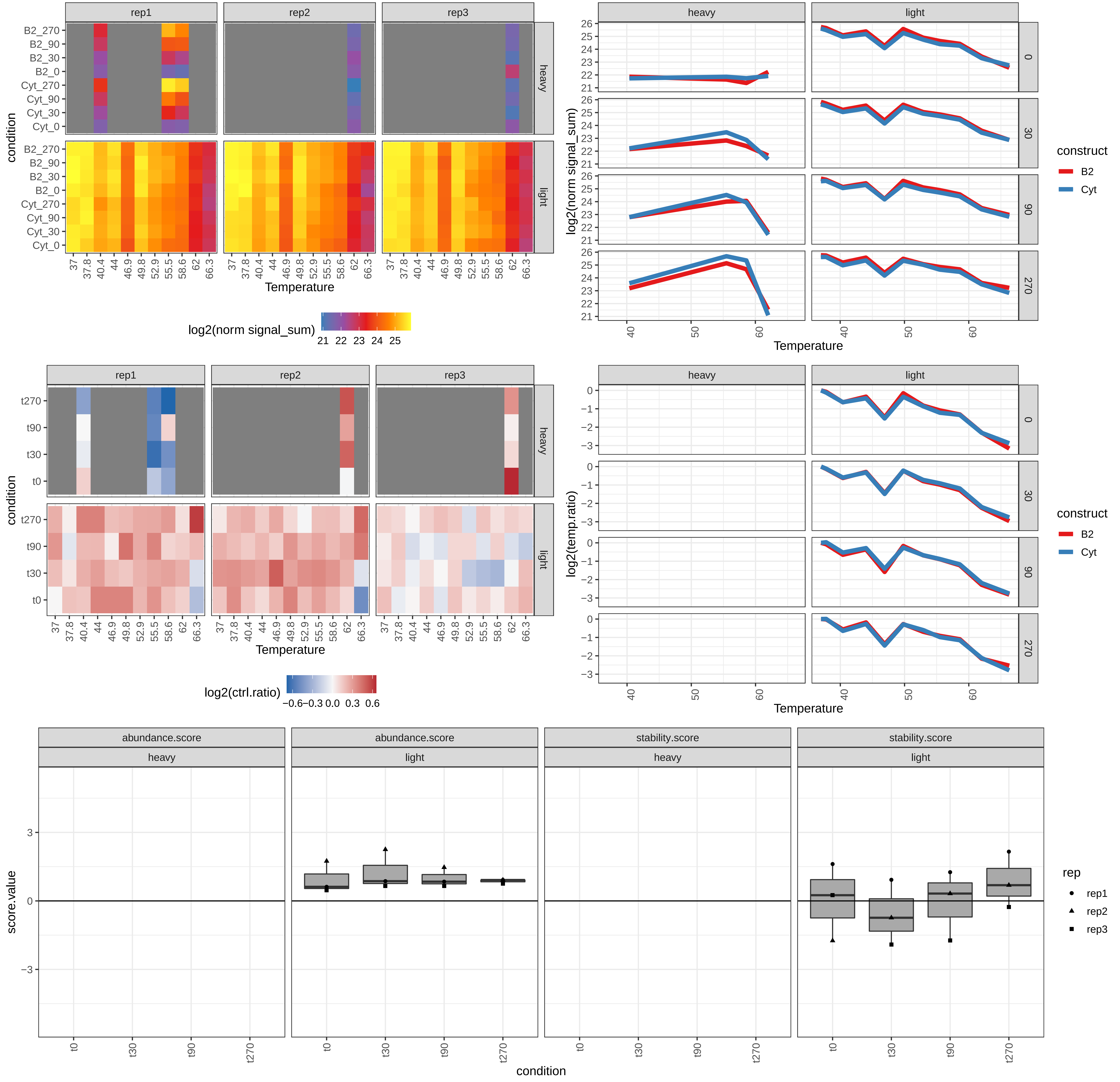

Q12168 − Endo−1,3(4)−beta−glucanase 2 OS=Saccharomyces cerevisiae (strain ATCC 204508 / S288c) GN=ACF2 PE=1 SV=1

ACH1 − average.top3: 8.00738196969697 − max.qupm: 26

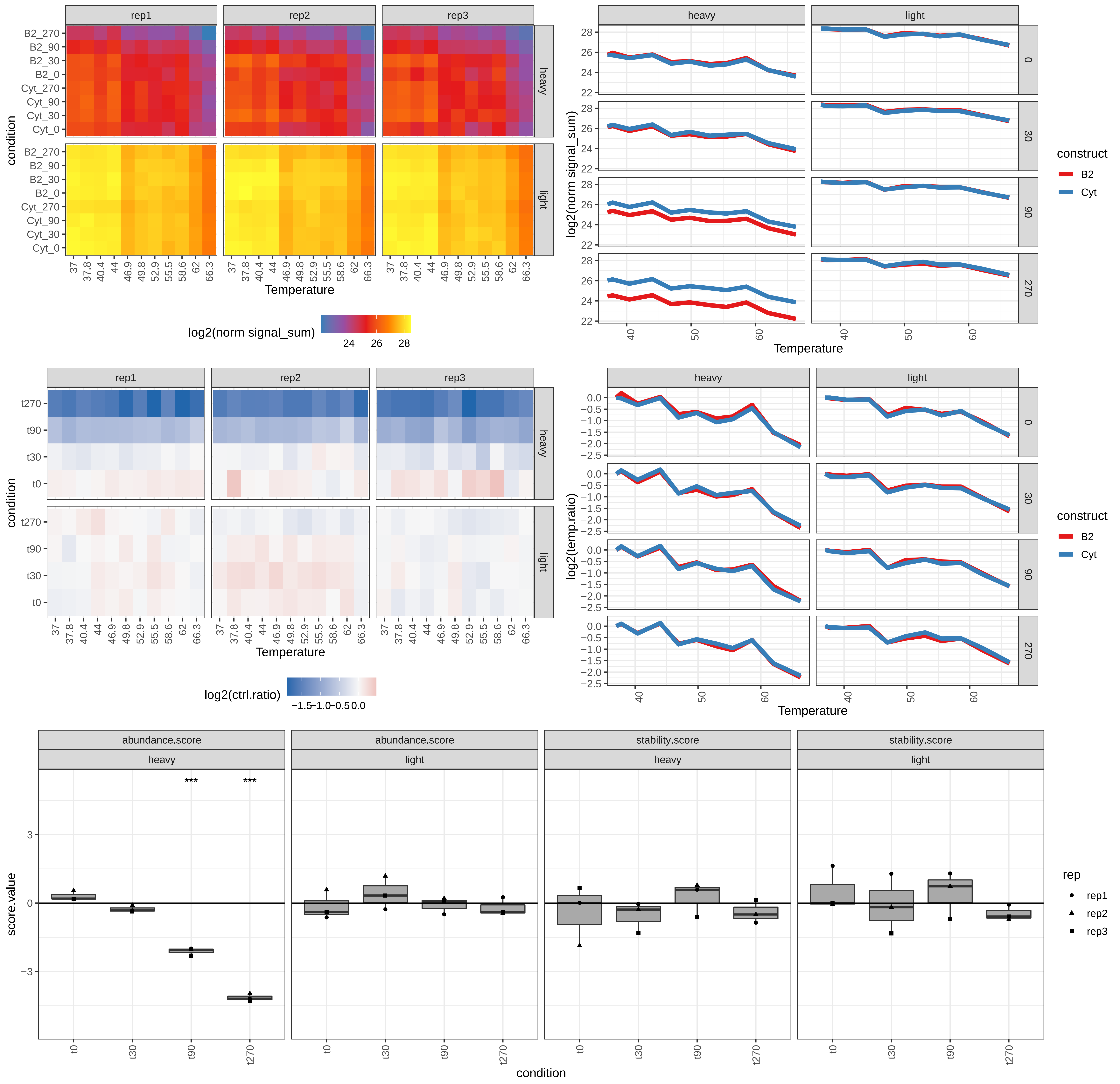

P32316 − Acetyl−CoA hydrolase OS=Saccharomyces cerevisiae (strain ATCC 204508 / S288c) GN=ACH1 PE=1 SV=2

ACO1 − average.top3: 8.26797274193548 − max.qupm: 45

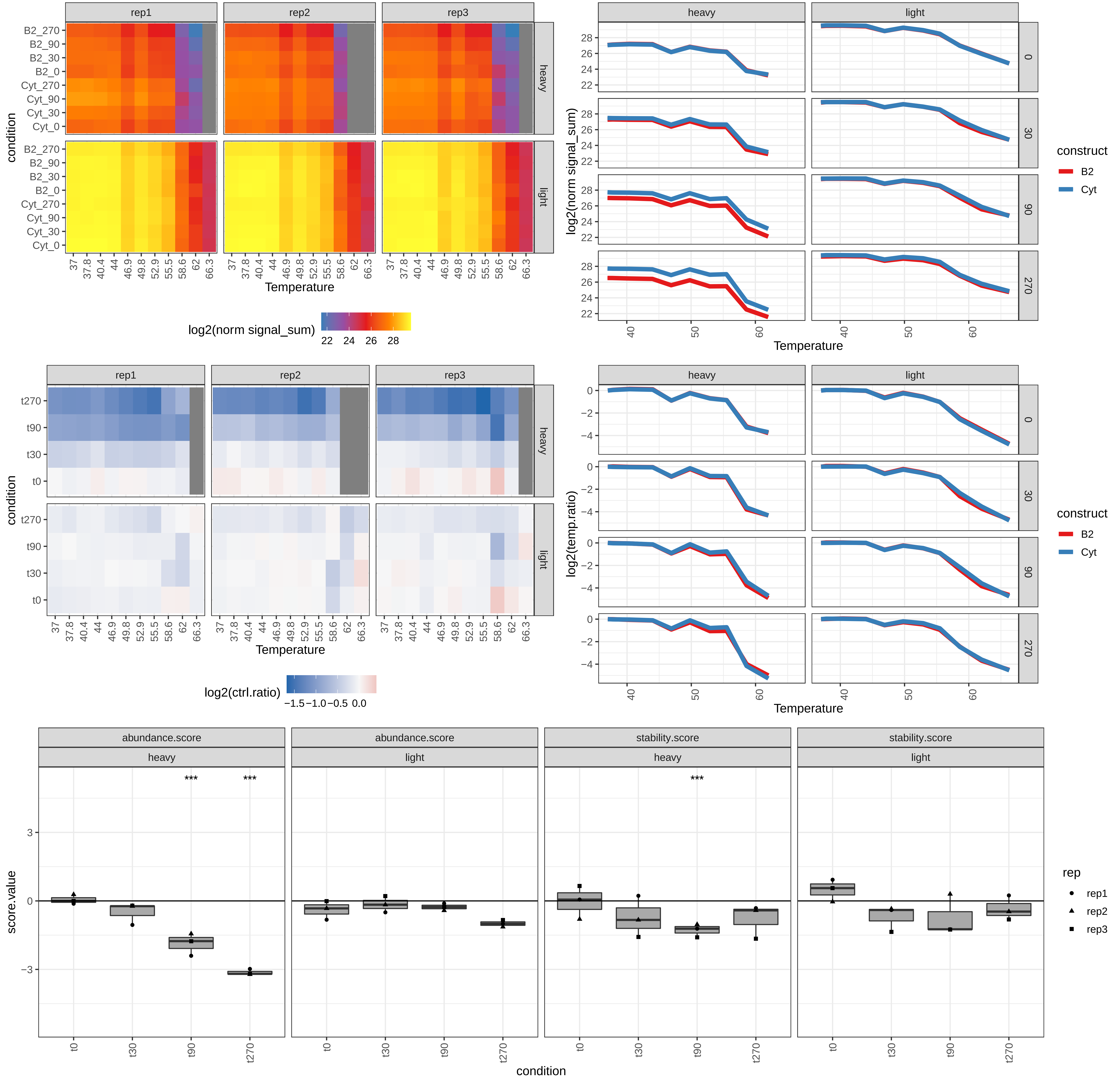

P19414 − Aconitate hydratase, mitochondrial OS=Saccharomyces cerevisiae (strain ATCC 204508 / S288c) GN=ACO1 PE=1 SV=2

ACO2 − average.top3: 6.8909575 − max.qupm: 9

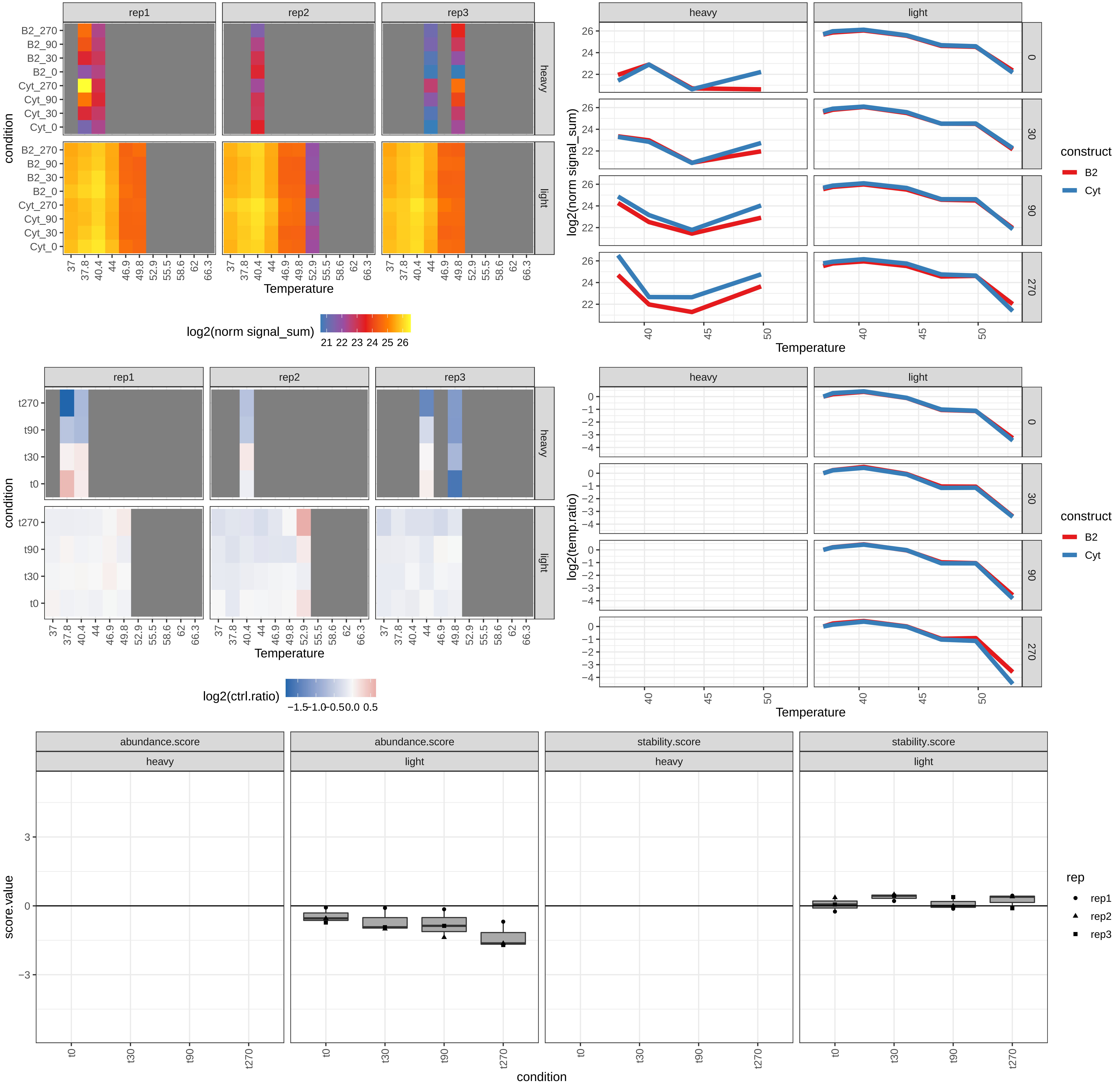

P39533 − Homocitrate dehydratase, mitochondrial OS=Saccharomyces cerevisiae (strain ATCC 204508 / S288c) GN=ACO2 PE=1 SV=1

ACS1 − average.top3: 8.03596017857143 − max.qupm: 24

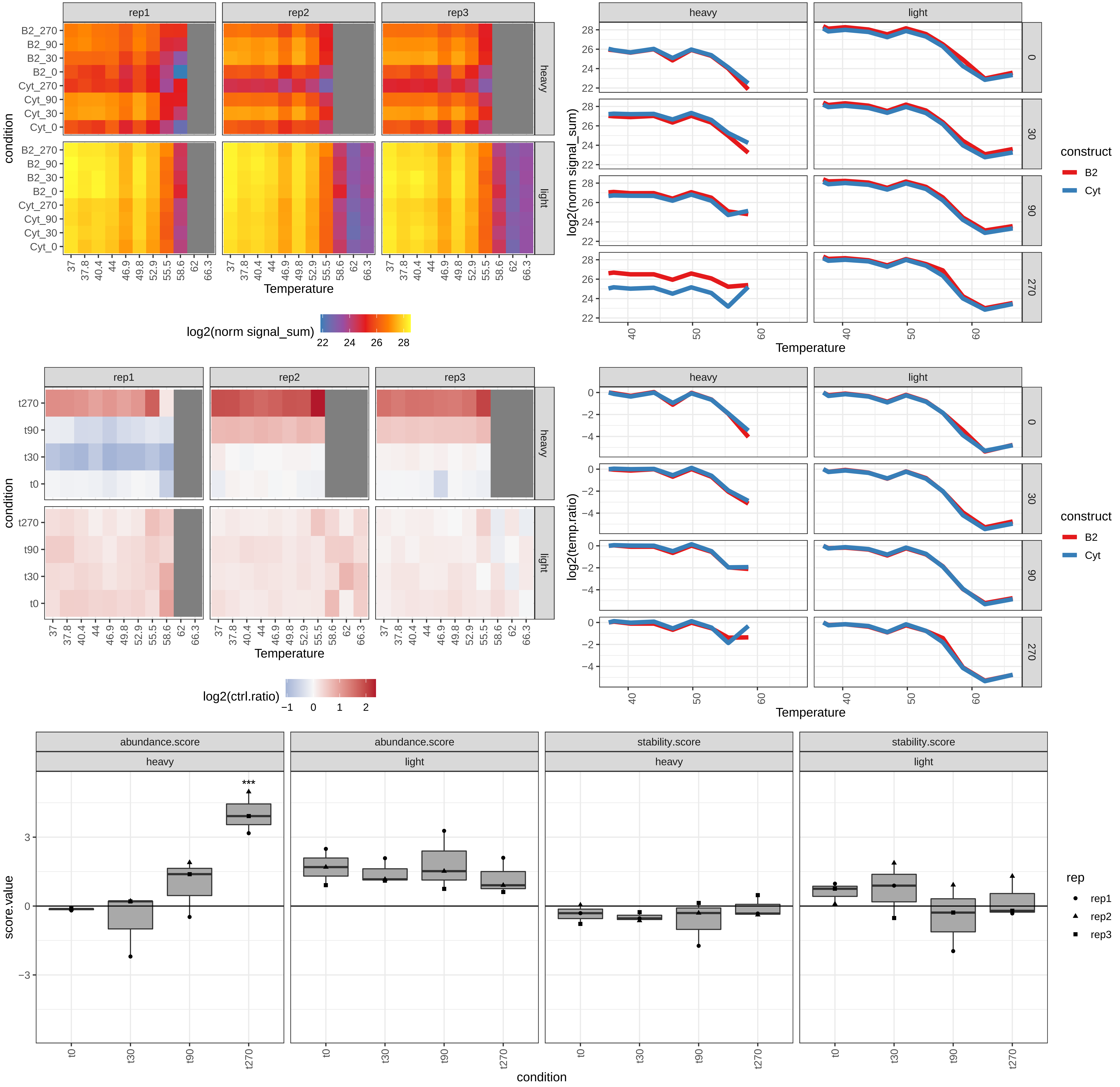

Q01574 − Acetyl−coenzyme A synthetase 1 OS=Saccharomyces cerevisiae (strain ATCC 204508 / S288c) GN=ACS1 PE=1 SV=2

ACS2 − average.top3: 7.68399755102041 − max.qupm: 29

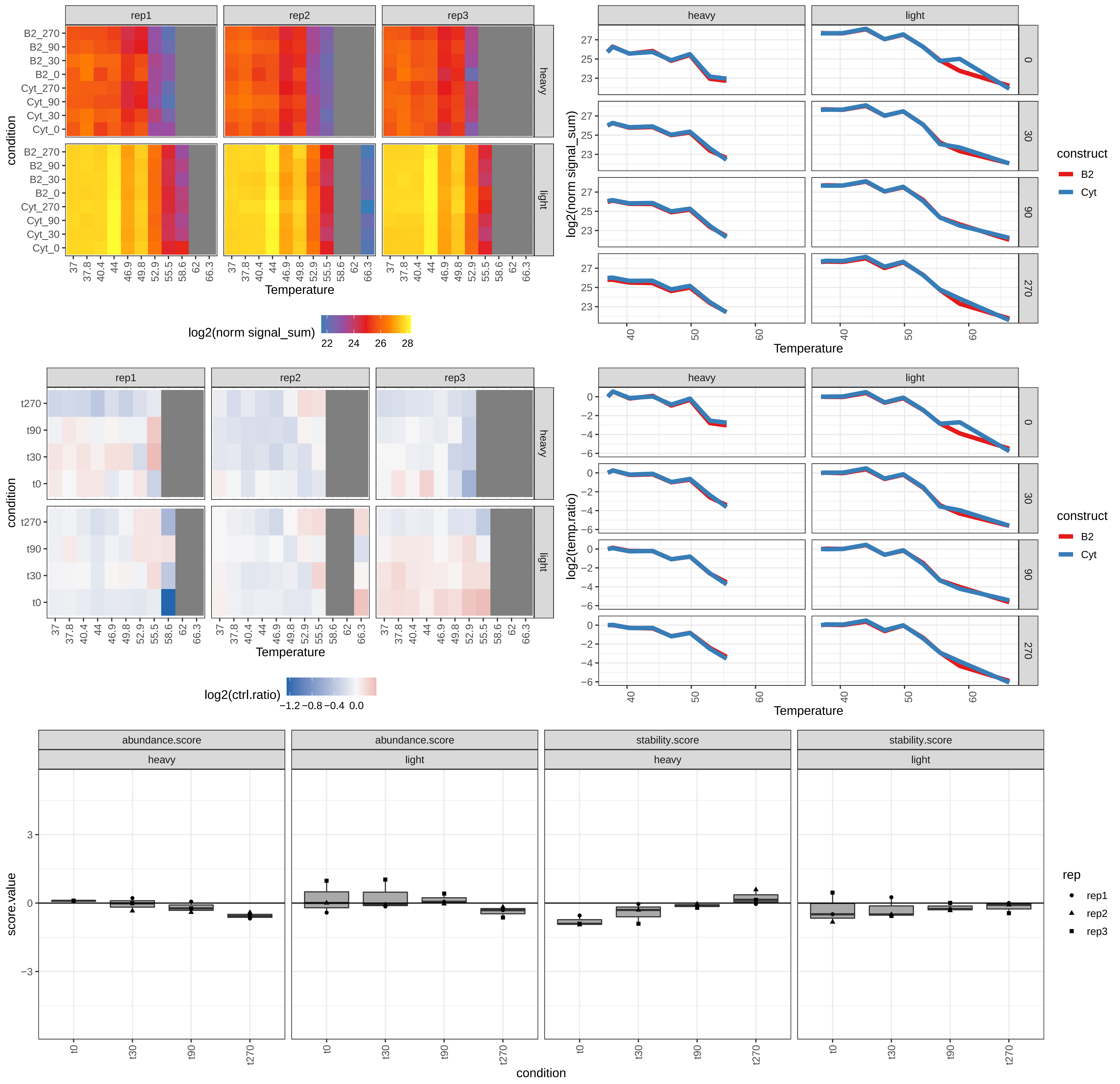

P52910 − Acetyl−coenzyme A synthetase 2 OS=Saccharomyces cerevisiae (strain ATCC 204508 / S288c) GN=ACS2 PE=1 SV=1

ACT1 − average.top3: 7.9069865625 − max.qupm: 13

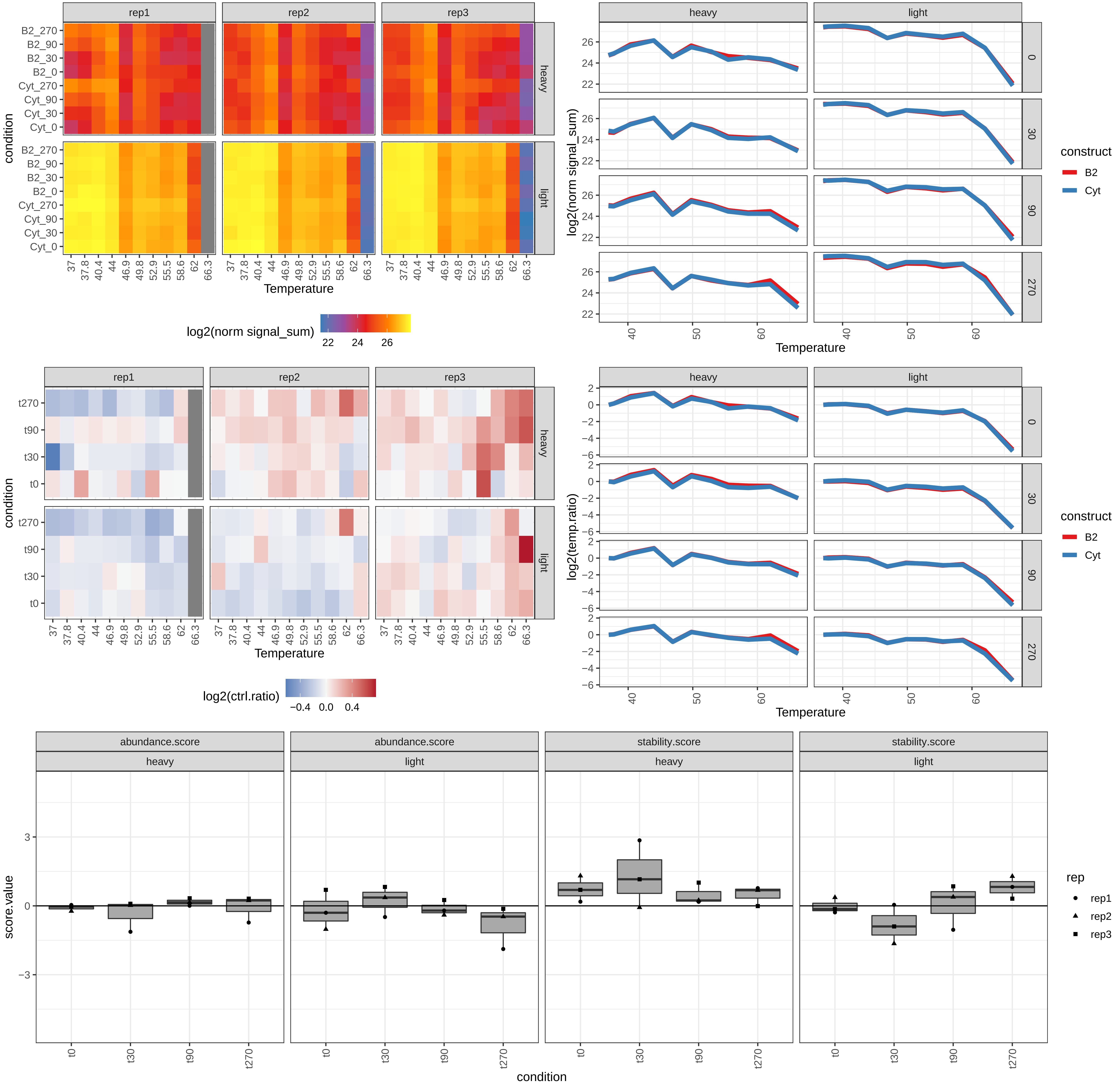

P60010 − Actin OS=Saccharomyces cerevisiae (strain ATCC 204508 / S288c) GN=ACT1 PE=1 SV=1

ADD66 − average.top3: 6.70212166666667 − max.qupm: 4

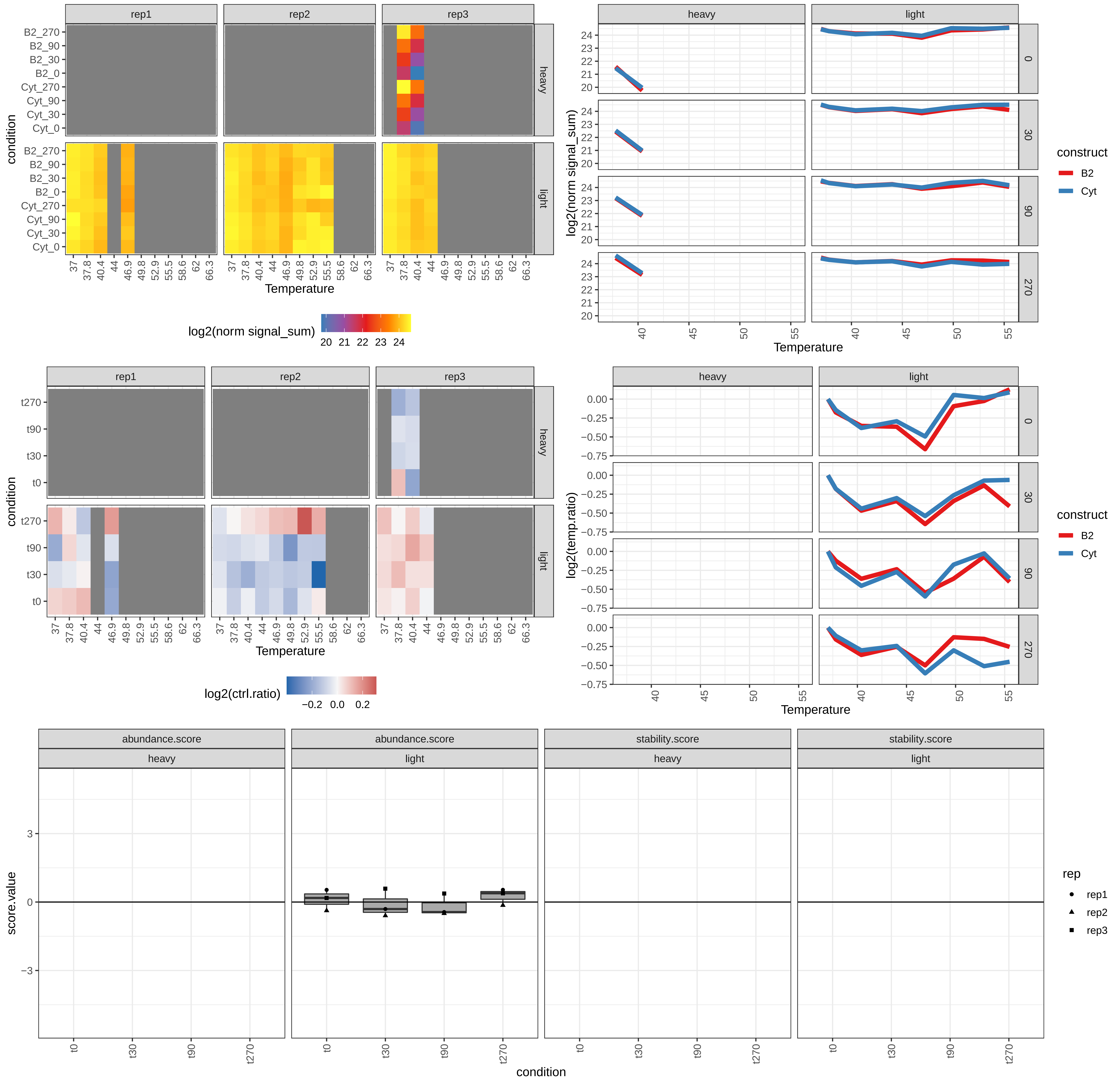

P36040 − Proteasome assembly chaperone 2 OS=Saccharomyces cerevisiae (strain ATCC 204508 / S288c) GN=ADD66 PE=1 SV=1

ADE1 − average.top3: 7.44216772727273 − max.qupm: 17

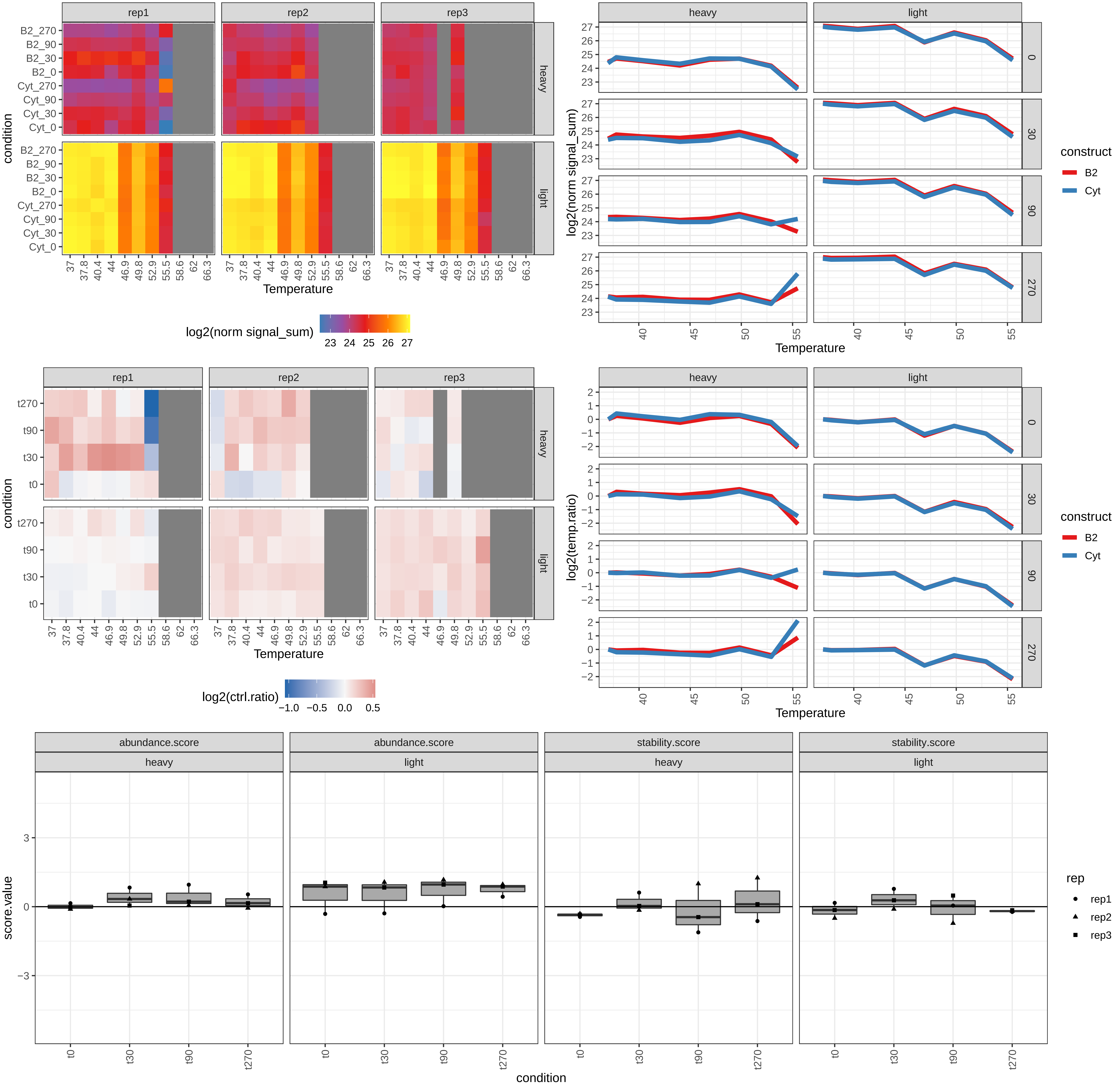

P27616 − Phosphoribosylaminoimidazole−succinocarboxamide synthase OS=Saccharomyces cerevisiae (strain ATCC 204508 / S288c) GN=ADE1 PE=1 SV=2

ADE12 − average.top3: 7.31965466666667 − max.qupm: 18

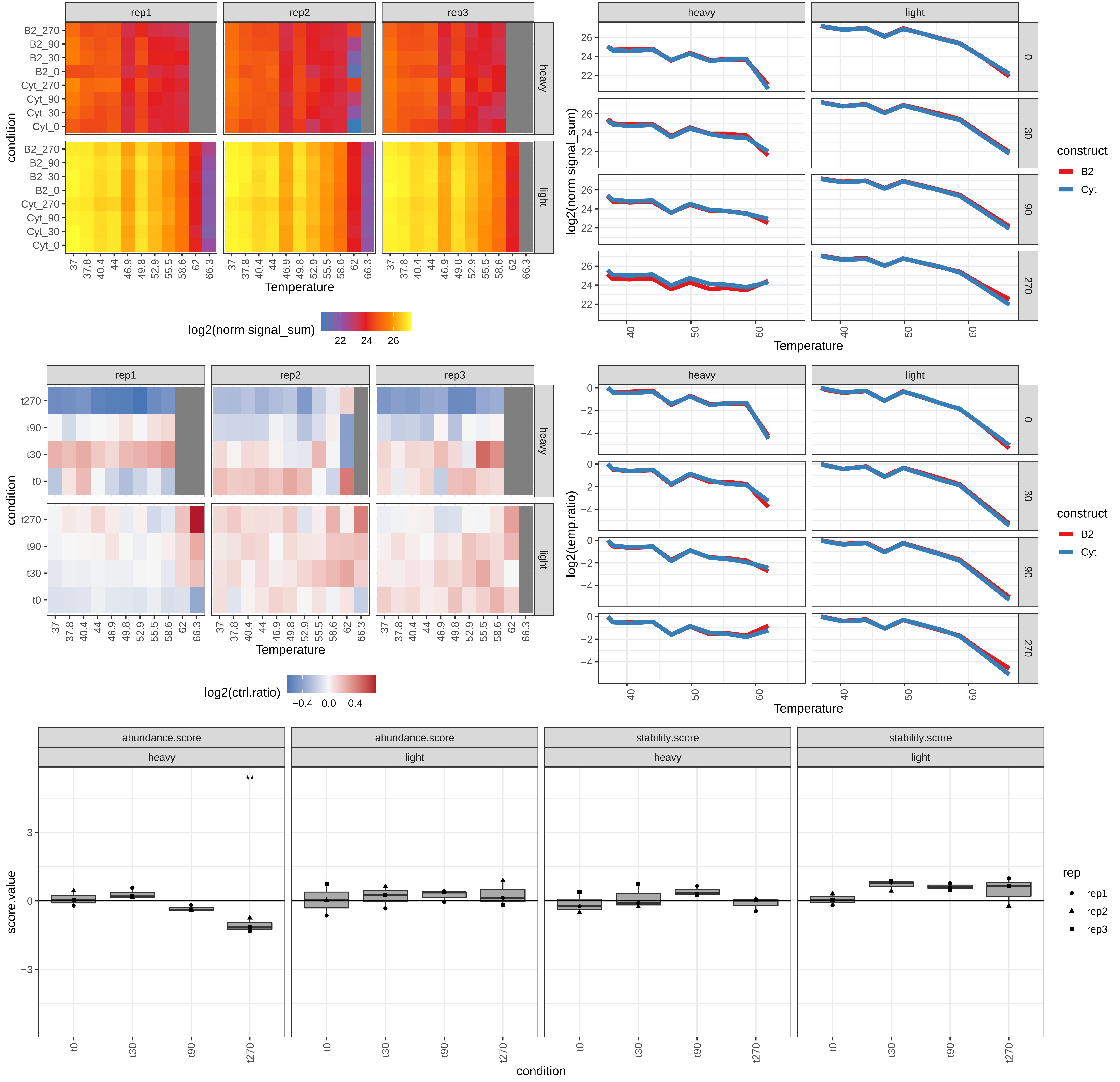

P80210 − Adenylosuccinate synthetase OS=Saccharomyces cerevisiae (strain ATCC 204508 / S288c) GN=ADE12 PE=1 SV=3

ADE13 − average.top3: 7.55580696969697 − max.qupm: 18

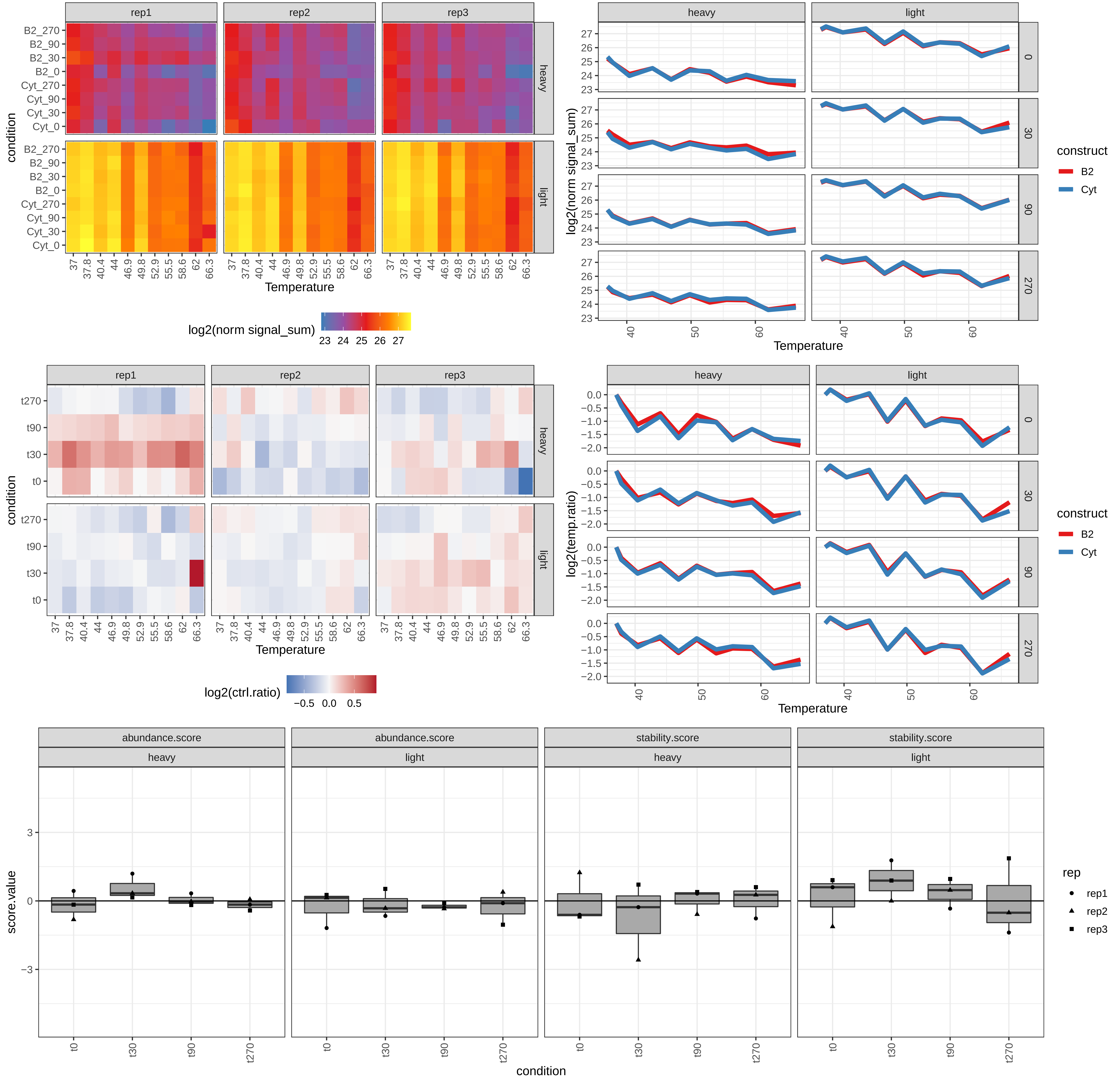

Q05911 − Adenylosuccinate lyase OS=Saccharomyces cerevisiae (strain ATCC 204508 / S288c) GN=ADE13 PE=1 SV=1

ADE16 − average.top3: 7.23666416666667 − max.qupm: 17

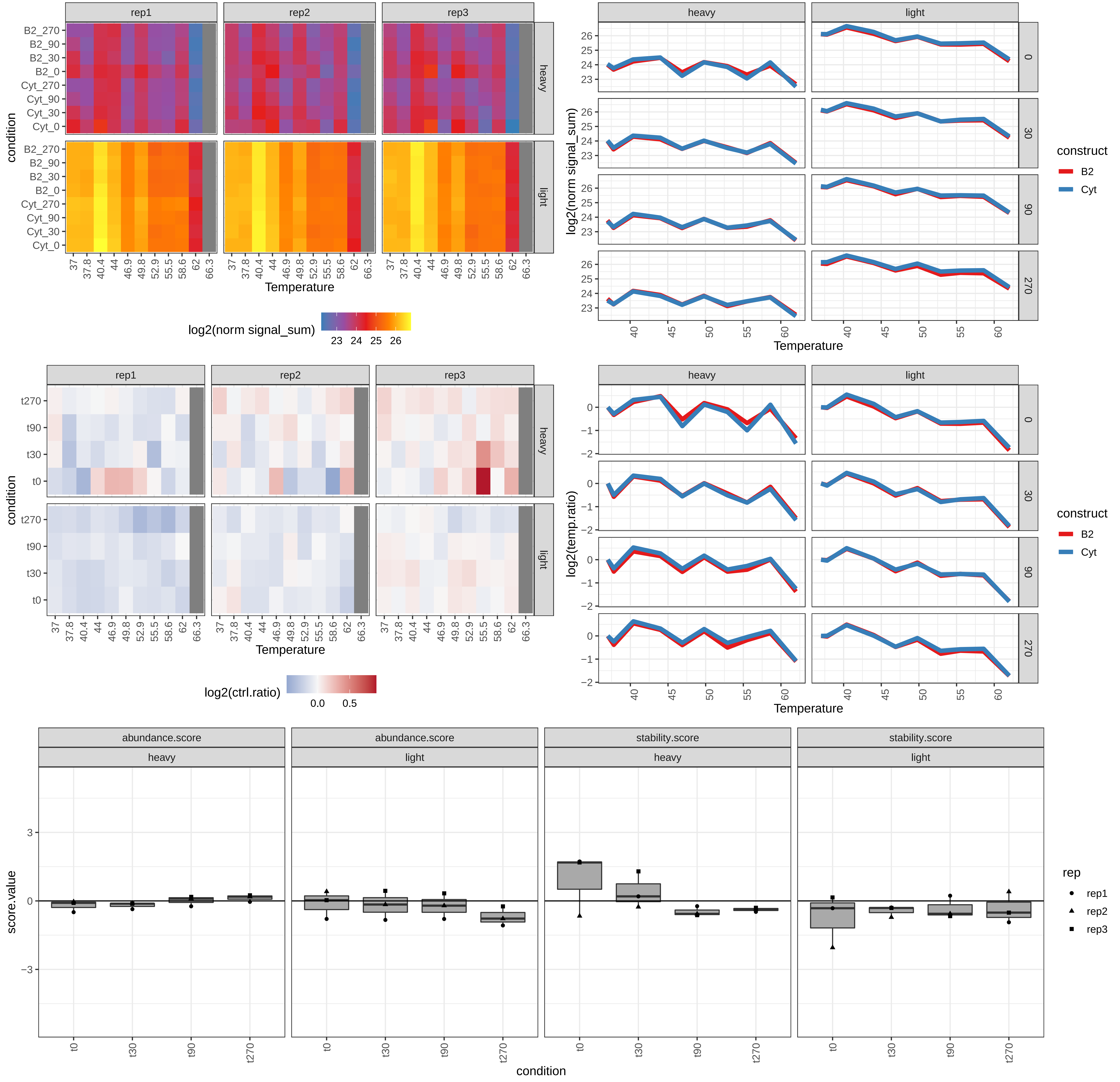

P54113 − Bifunctional purine biosynthesis protein ADE16 OS=Saccharomyces cerevisiae (strain ATCC 204508 / S288c) GN=ADE16 PE=1 SV=1

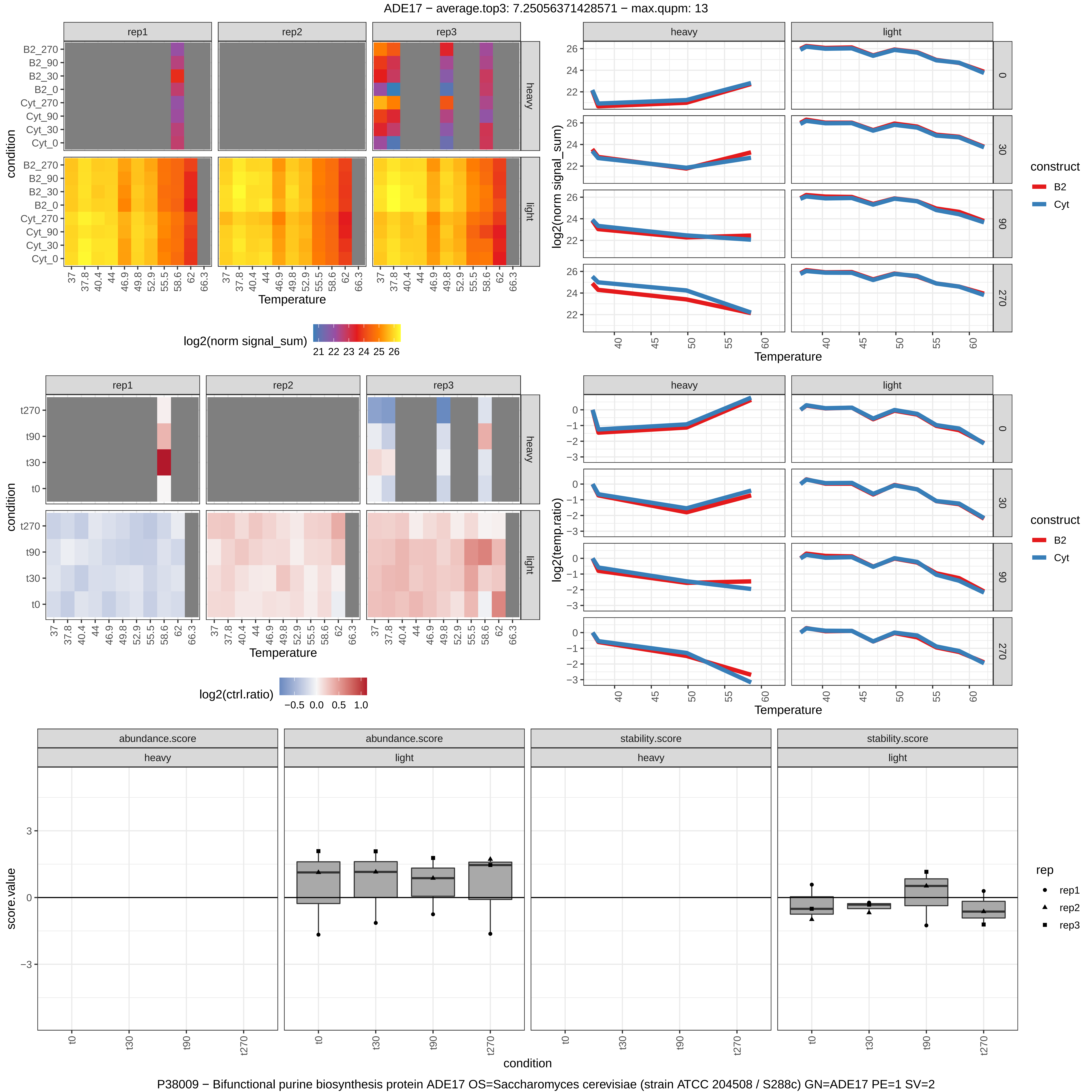

ADE17 − average.top3: 7.25056371428571 − max.qupm: 13
rep1
rep2
rep3
heavy
light
condition
Temperature
log2(norm signal_sum)
construct
B2
Cyt
log2(ctrl.ratio)
log2(temp.ratio)
abundance.score
stability.score
score.value
rep
P38009 − Bifunctional purine biosynthesis protein ADE17 OS=Saccharomyces cerevisiae (strain ATCC 204508 / S288c) GN=ADE17 PE=1 SV=2

ADE3 − average.top3: 7.33845933333333 − max.qupm: 26

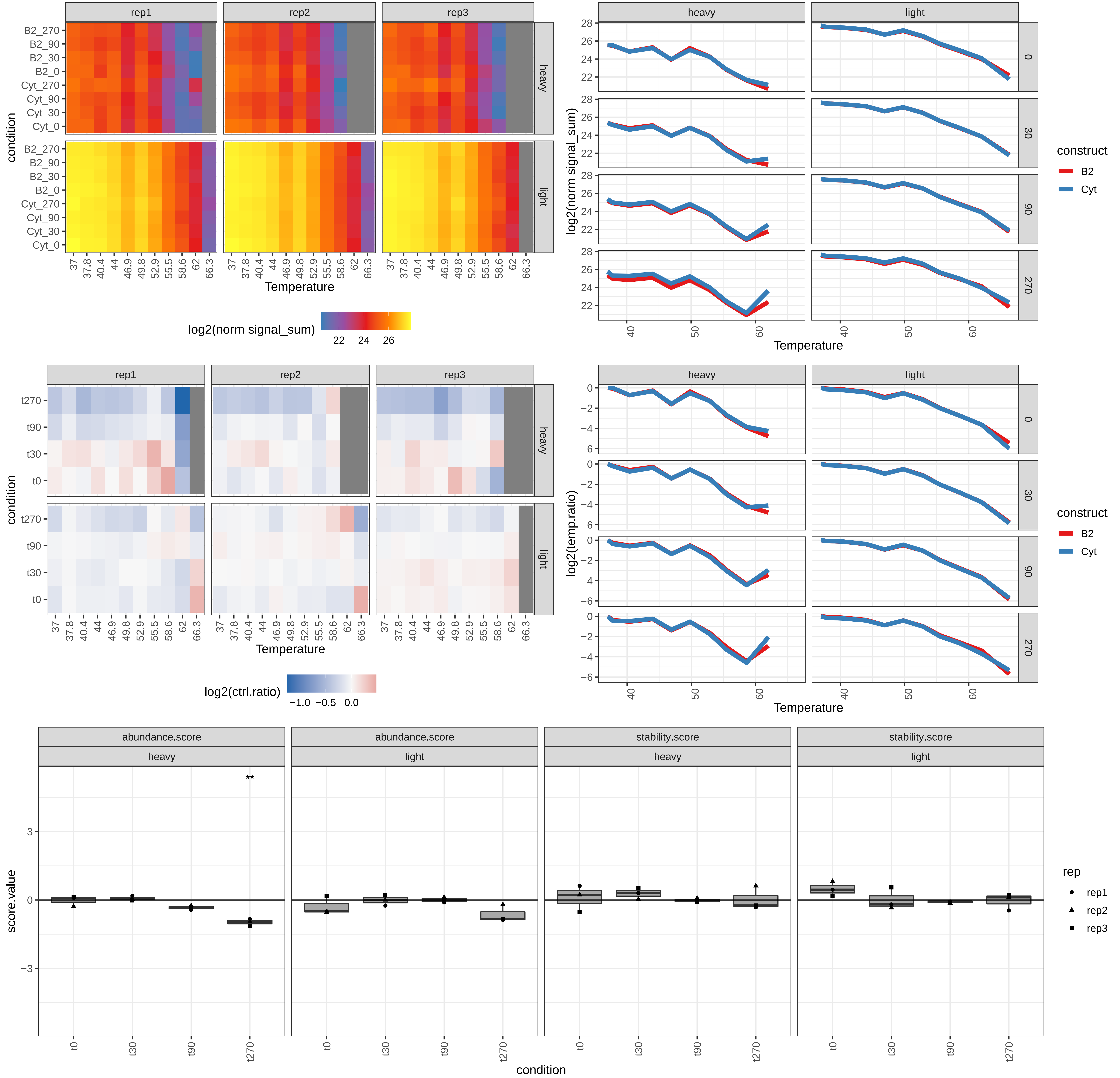

P07245 − C−1−tetrahydrofolate synthase, cytoplasmic OS=Saccharomyces cerevisiae (strain ATCC 204508 / S288c) GN=ADE3 PE=1 SV=1

ADE4 − average.top3: 6.9109212195122 − max.qupm: 17

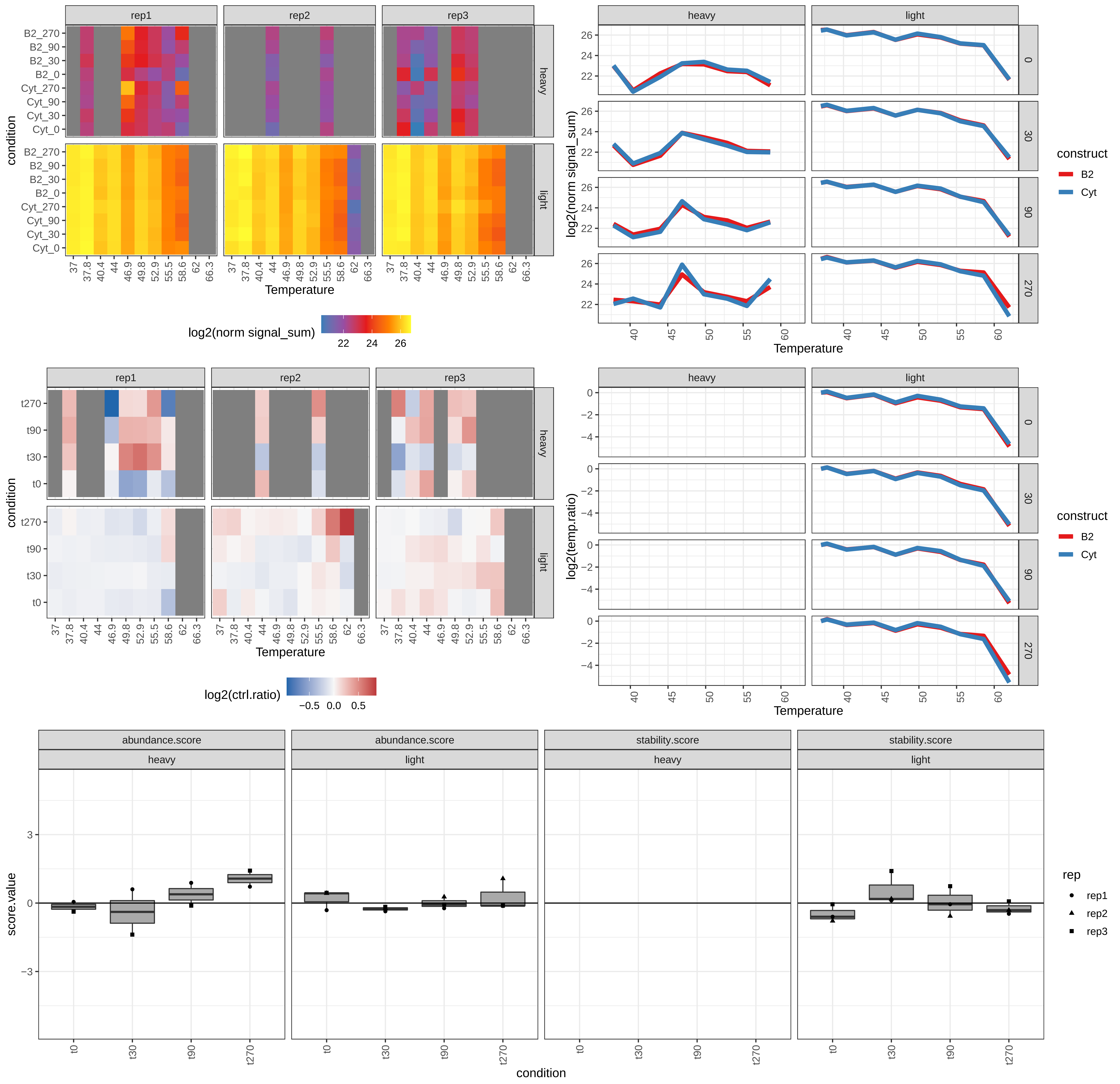

P04046 − Amidophosphoribosyltransferase OS=Saccharomyces cerevisiae (strain ATCC 204508 / S288c) GN=ADE4 PE=1 SV=2

ADE5 − average.top3: 7.54967096153846 − max.qupm: 26

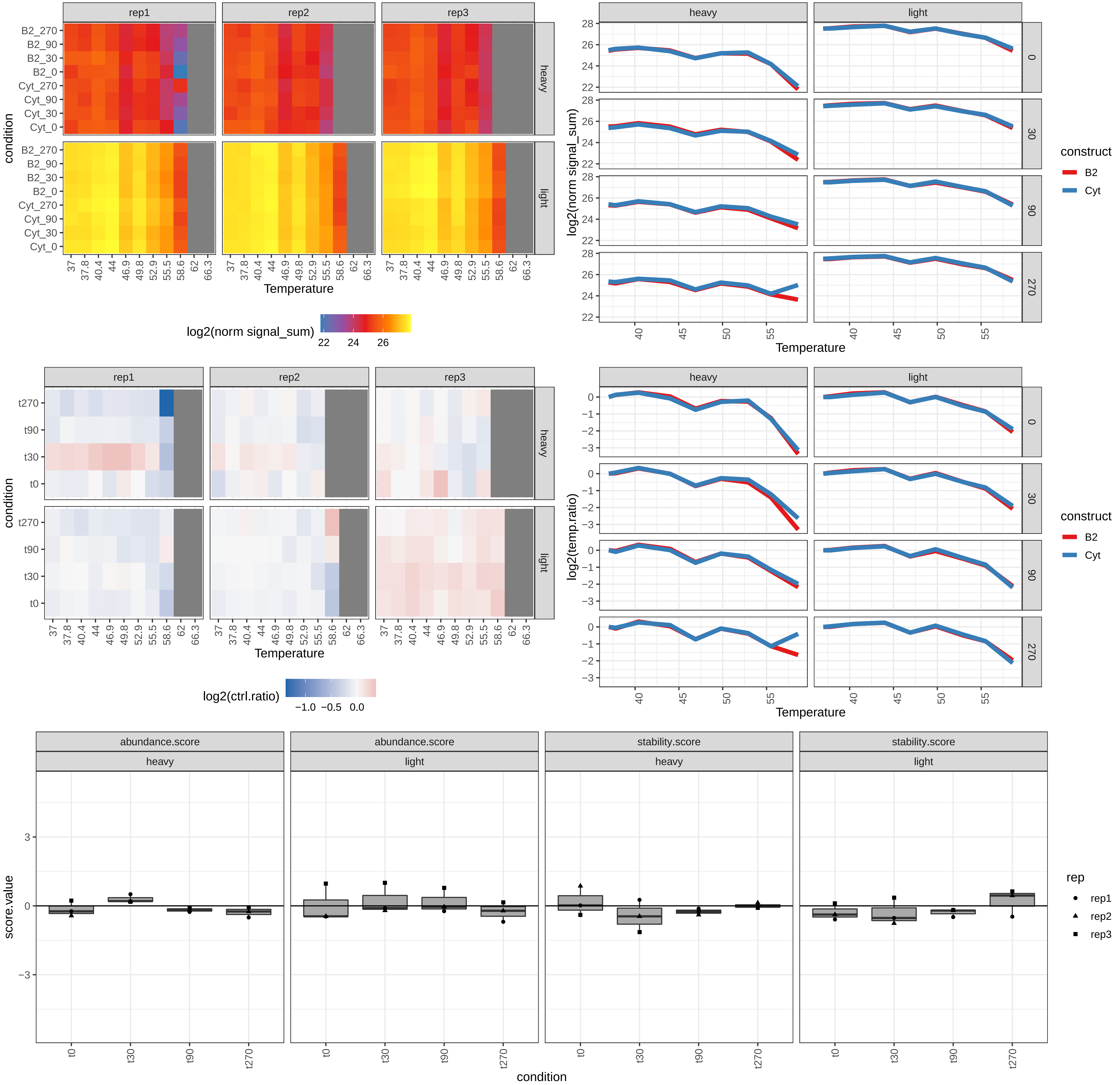

P07244 − Bifunctional purine biosynthetic protein ADE5,7 OS=Saccharomyces cerevisiae (strain ATCC 204508 / S288c) GN=ADE5,7 PE=1 SV=1

ADE6 − average.top3: 7.40557230769231 − max.qupm: 22

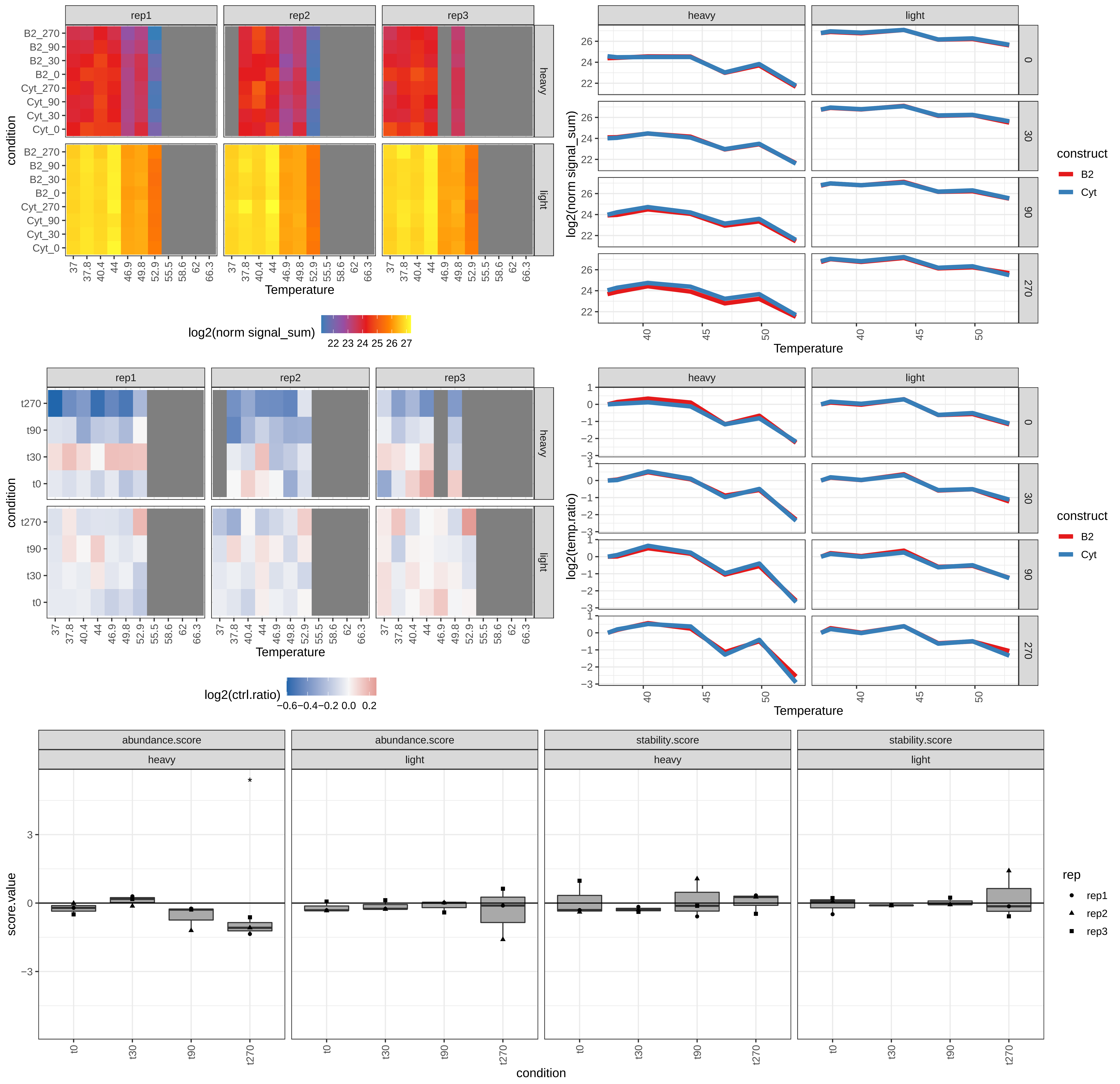

P38972 − Phosphoribosylformylglycinamidine synthase OS=Saccharomyces cerevisiae (strain ATCC 204508 / S288c) GN=ADE6 PE=1 SV=2

ADE8 − average.top3: 7.0619925 − max.qupm: 4

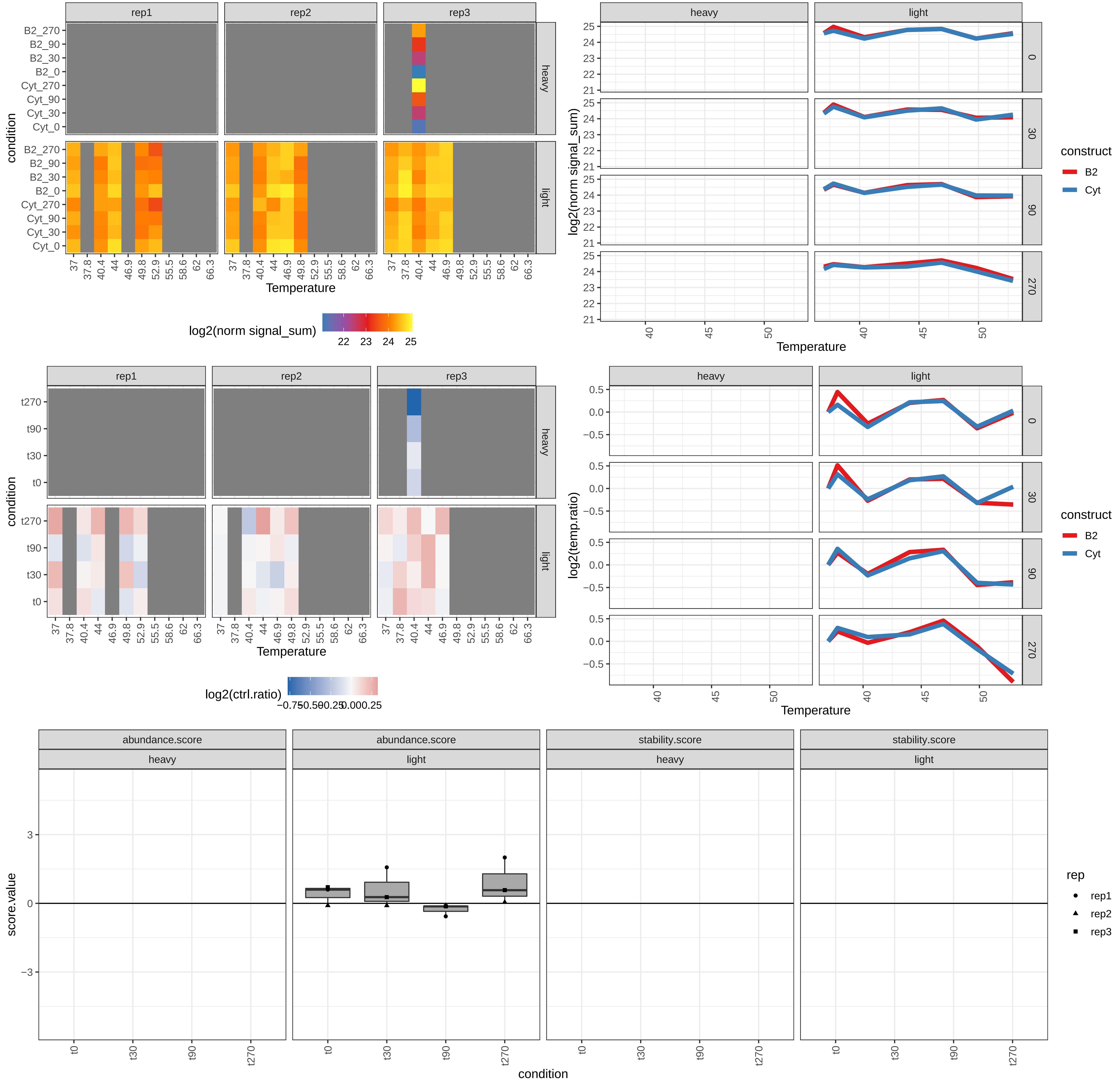

P04161 − Phosphoribosylglycinamide formyltransferase OS=Saccharomyces cerevisiae (strain ATCC 204508 / S288c) GN=ADE8 PE=1 SV=1

ADH1 − average.top3: 8.66905136363636 − max.qupm: 11

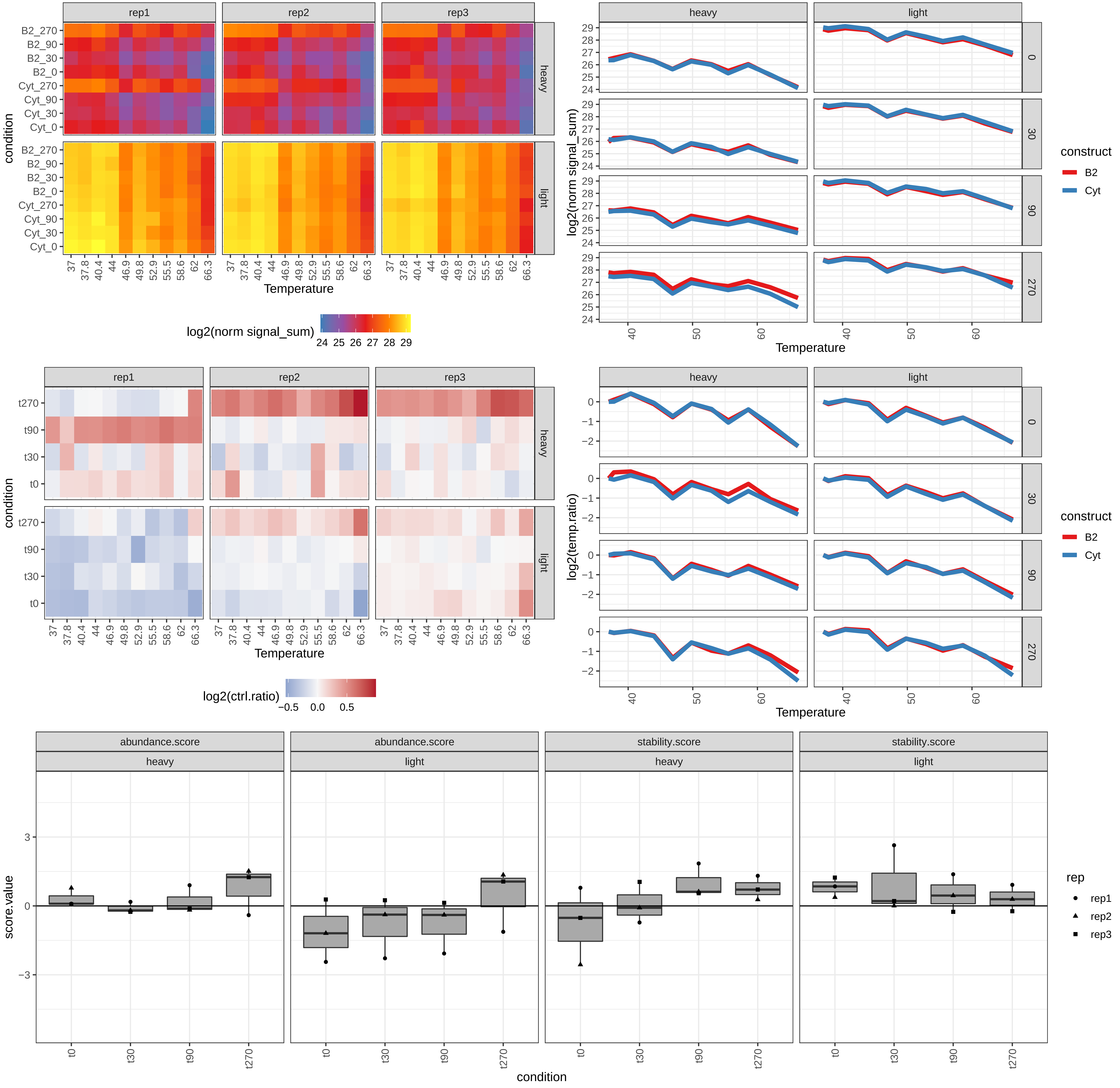

P00330 − Alcohol dehydrogenase 1 OS=Saccharomyces cerevisiae (strain ATCC 204508 / S288c) GN=ADH1 PE=1 SV=5

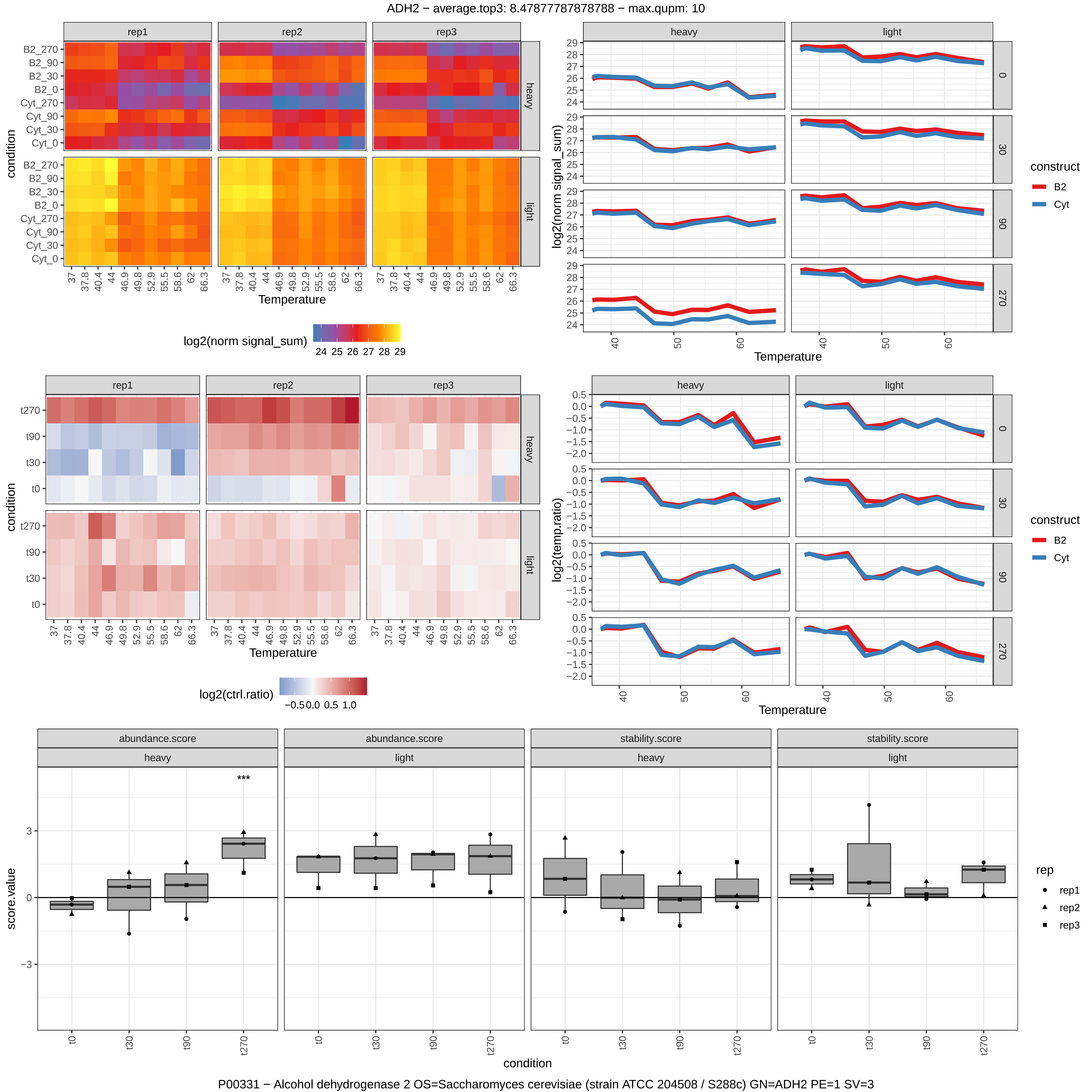
ADH2 − average.top3: 8.47877787878788 − max.qupm: 10
rep1
rep2
rep3
heavy
light
B2_270
B2_90
B2_30
B2_0
Cyt_270
Cyt_90
Cyt_30
Cyt_0
condition
Temperature
log2(norm signal_sum)
24 25 26 27 28 29
construct
B2
Cyt
0
30
90
270
t270
t90
t30
t0
log2(ctrl.ratio)
−0.5 0.0 0.5 1.0
log2(temp.ratio)
abundance.score
stability.score
***
score.value
rep
rep1
rep2
rep3
P00331 − Alcohol dehydrogenase 2 OS=Saccharomyces cerevisiae (strain ATCC 204508 / S288c) GN=ADH2 PE=1 SV=3

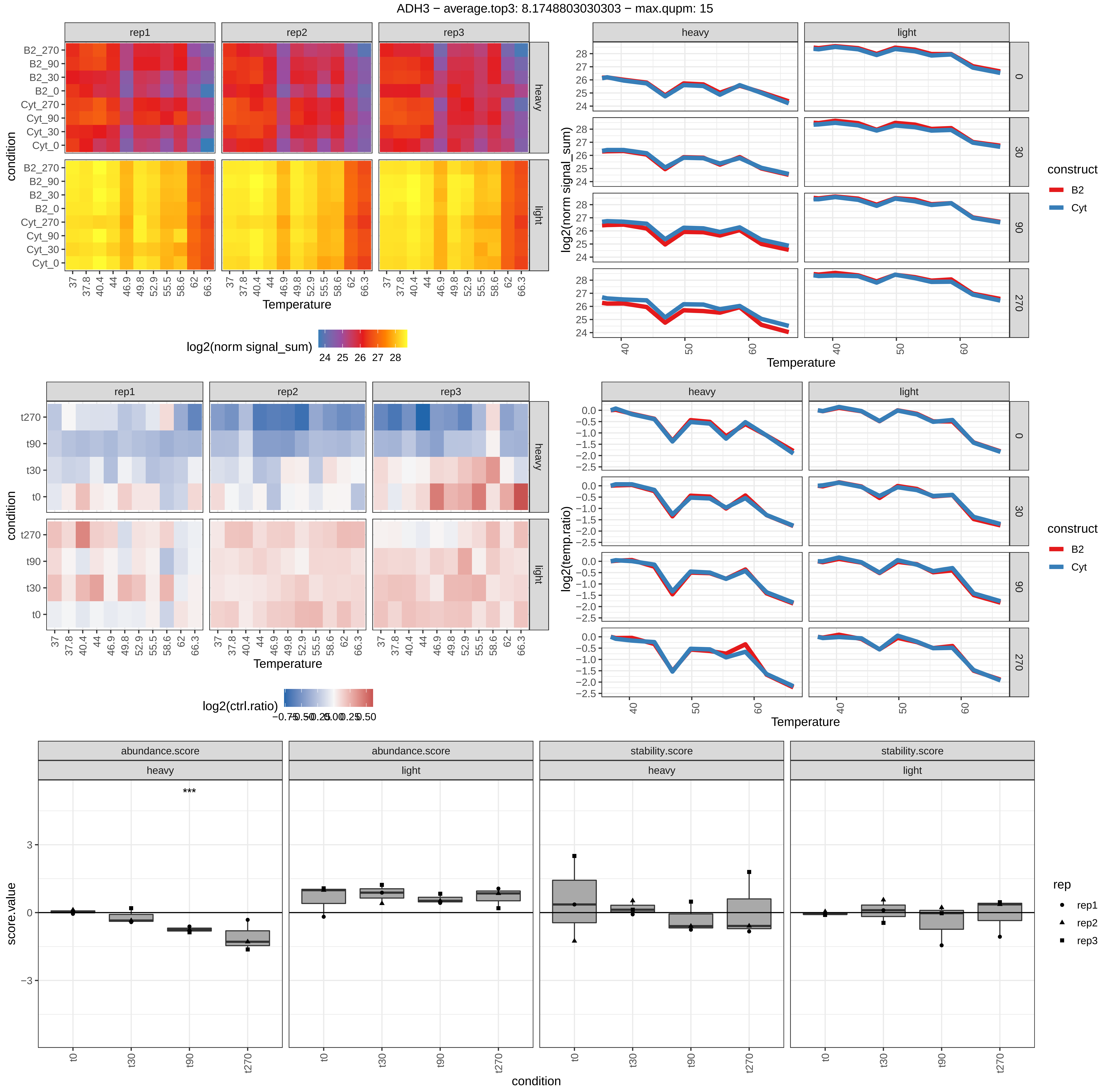

P07246 − Alcohol dehydrogenase 3, mitochondrial OS=Saccharomyces cerevisiae (strain ATCC 204508 / S288c) GN=ADH3 PE=1 SV=2

ADH5 − average.top3: 7.11946666666667 − max.qupm: 5

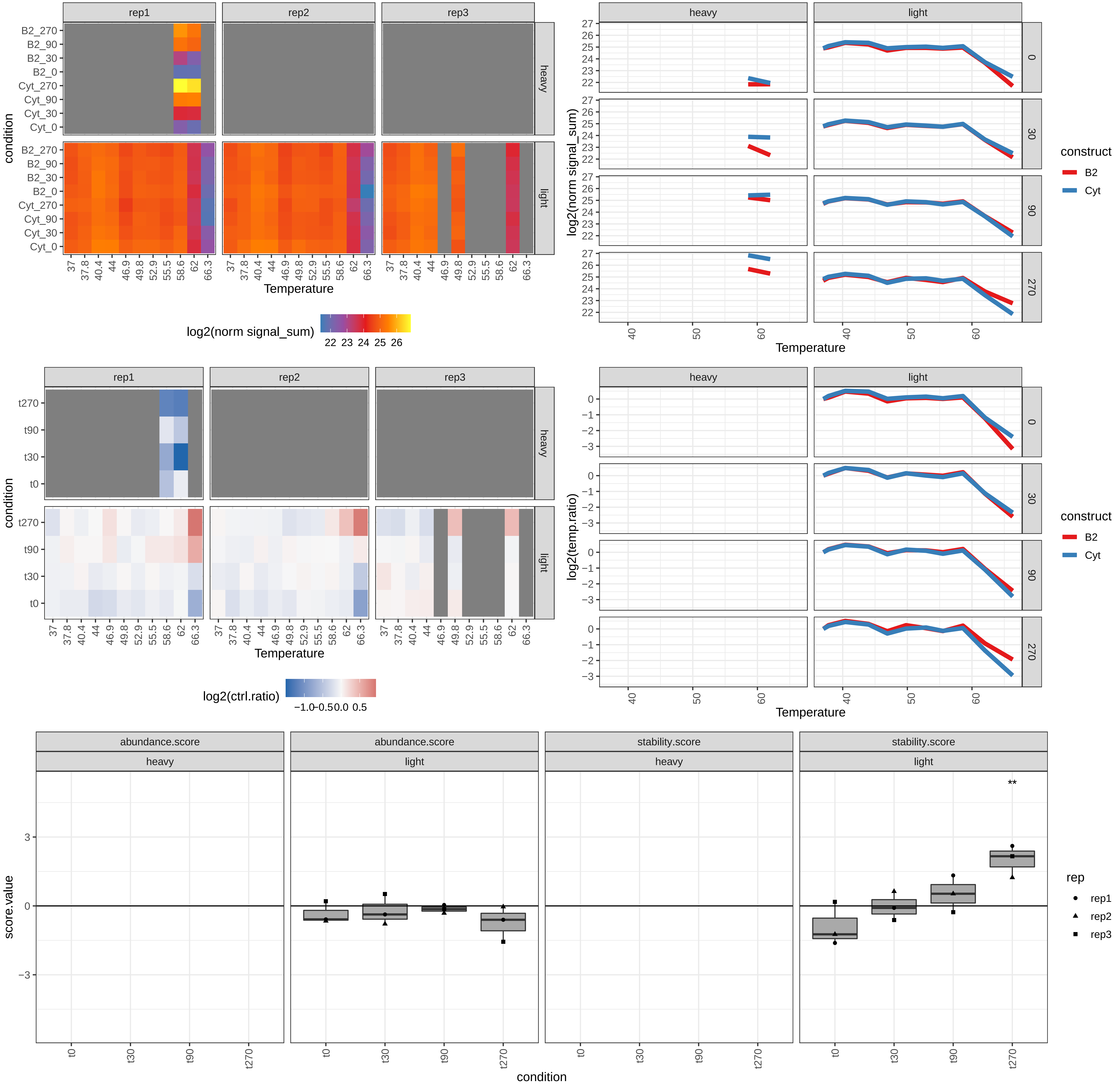

P38113 − Alcohol dehydrogenase 5 OS=Saccharomyces cerevisiae (strain ATCC 204508 / S288c) GN=ADH5 PE=1 SV=1

ADH6 − average.top3: 7.41478310344828 − max.qupm: 13

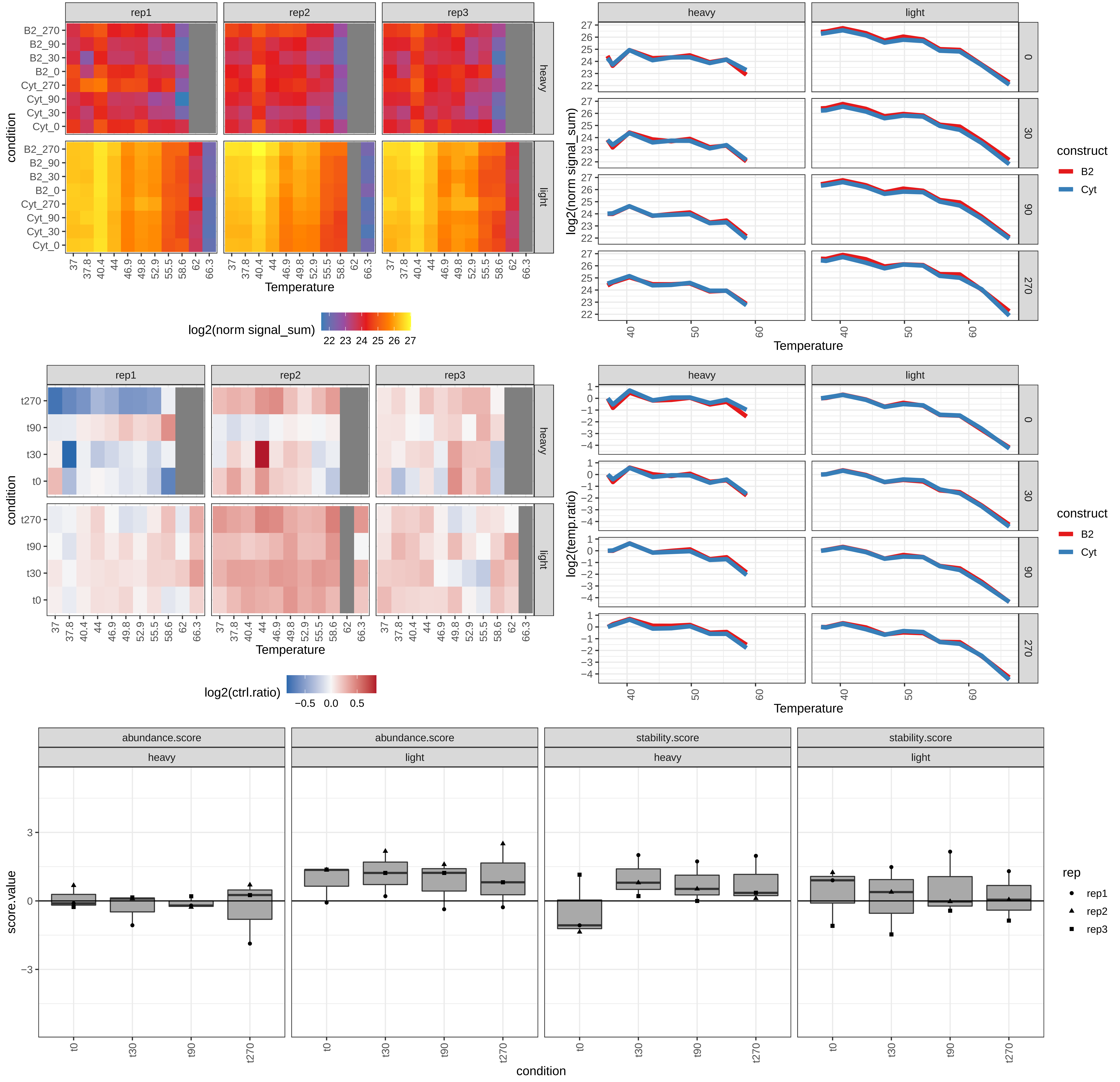

Q04894 − NADP−dependent alcohol dehydrogenase 6 OS=Saccharomyces cerevisiae (strain ATCC 204508 / S288c) GN=ADH6 PE=1 SV=1

ADI1 − average.top3: 7.04864181818182 − max.qupm: 9

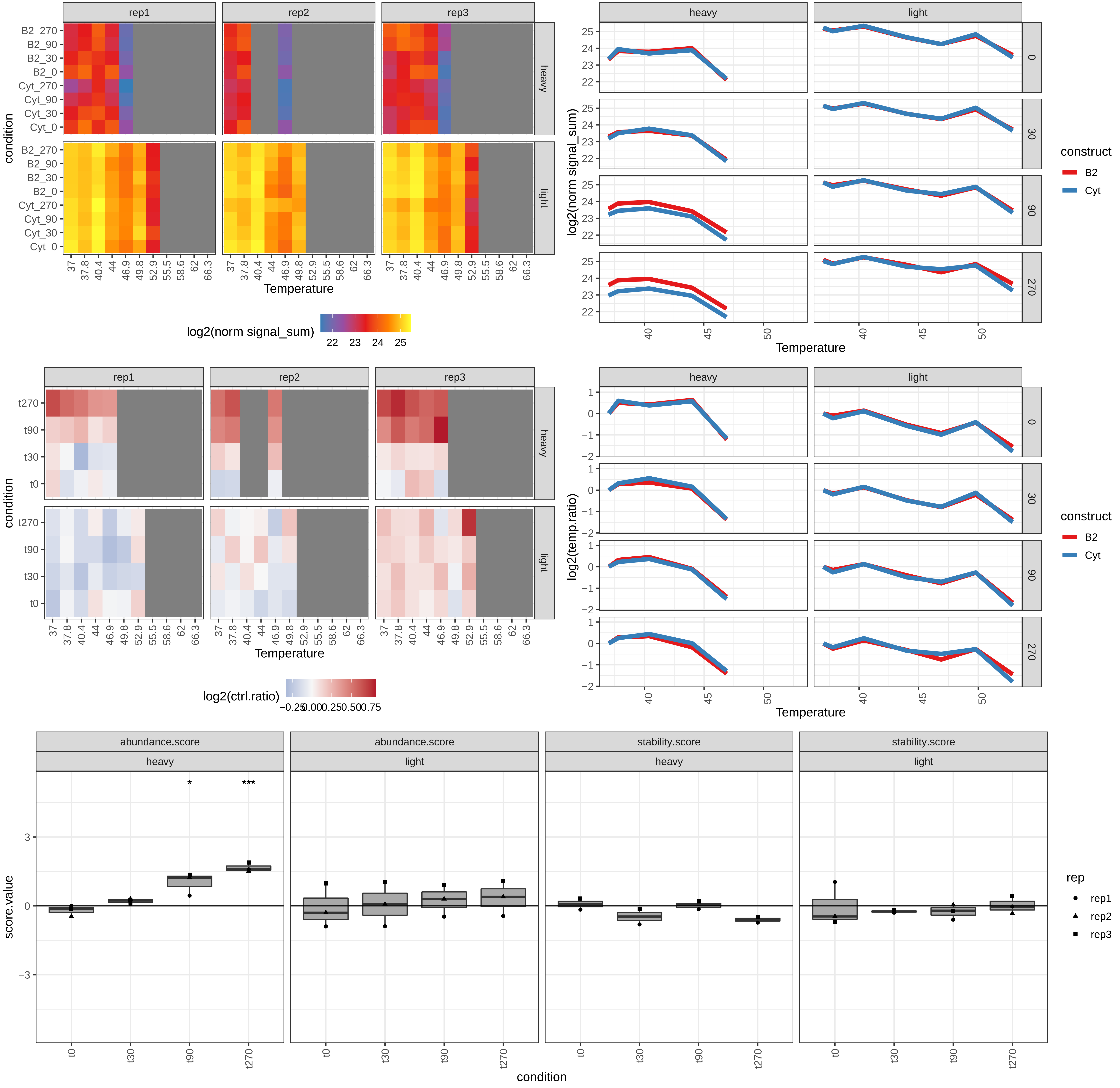

Q03677 − 1,2−dihydroxy−3−keto−5−methylthiopentene dioxygenase OS=Saccharomyces cerevisiae (strain ATCC 204508 / S288c) GN=ADI1 PE=1 SV=1

ADK1 − average.top3: 7.66525714285714 − max.qupm: 16

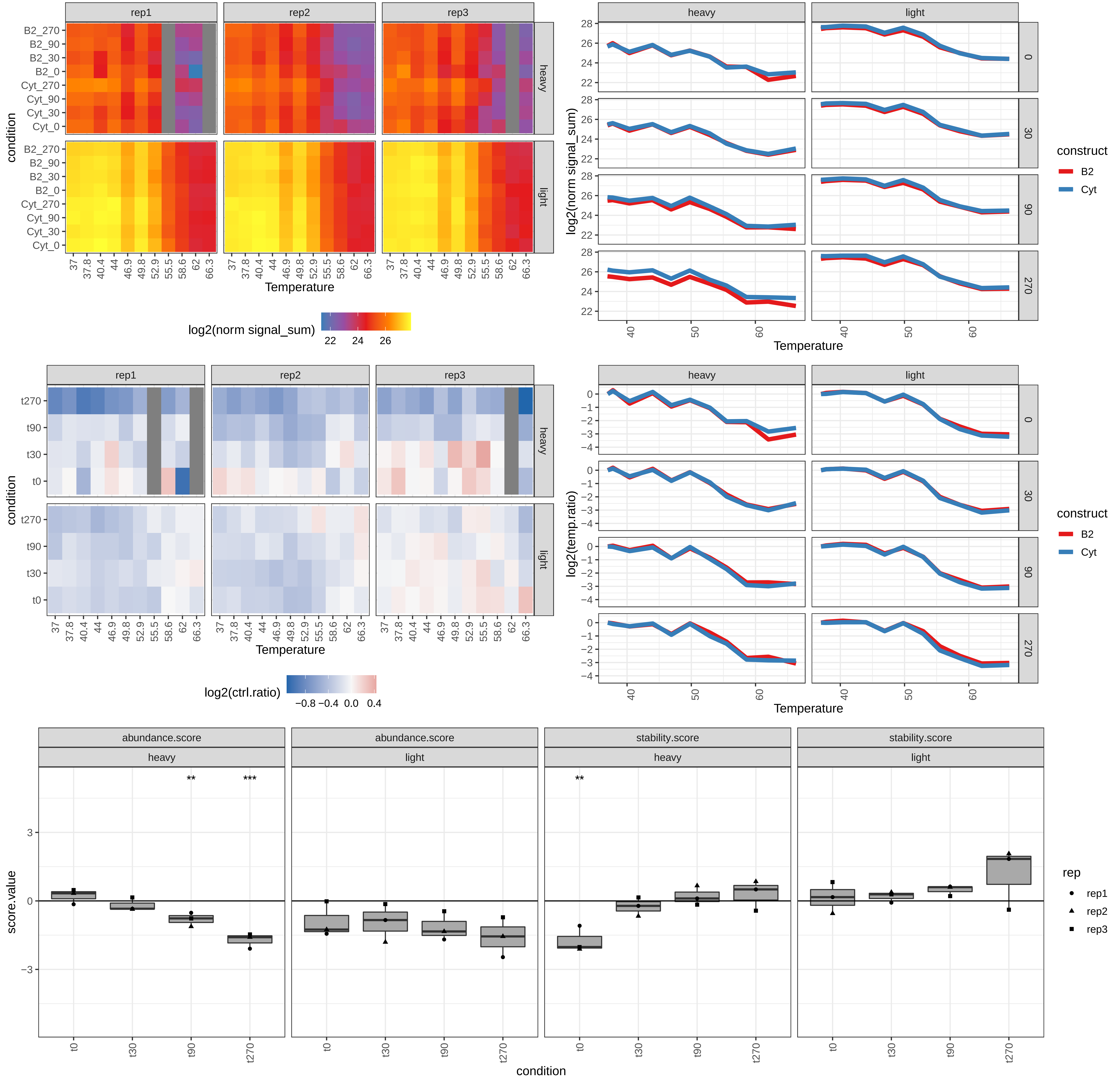

P07170 − Adenylate kinase OS=Saccharomyces cerevisiae (strain ATCC 204508 / S288c) GN=ADK1 PE=1 SV=2

ADO1 − average.top3: 7.54275333333333 − max.qupm: 12

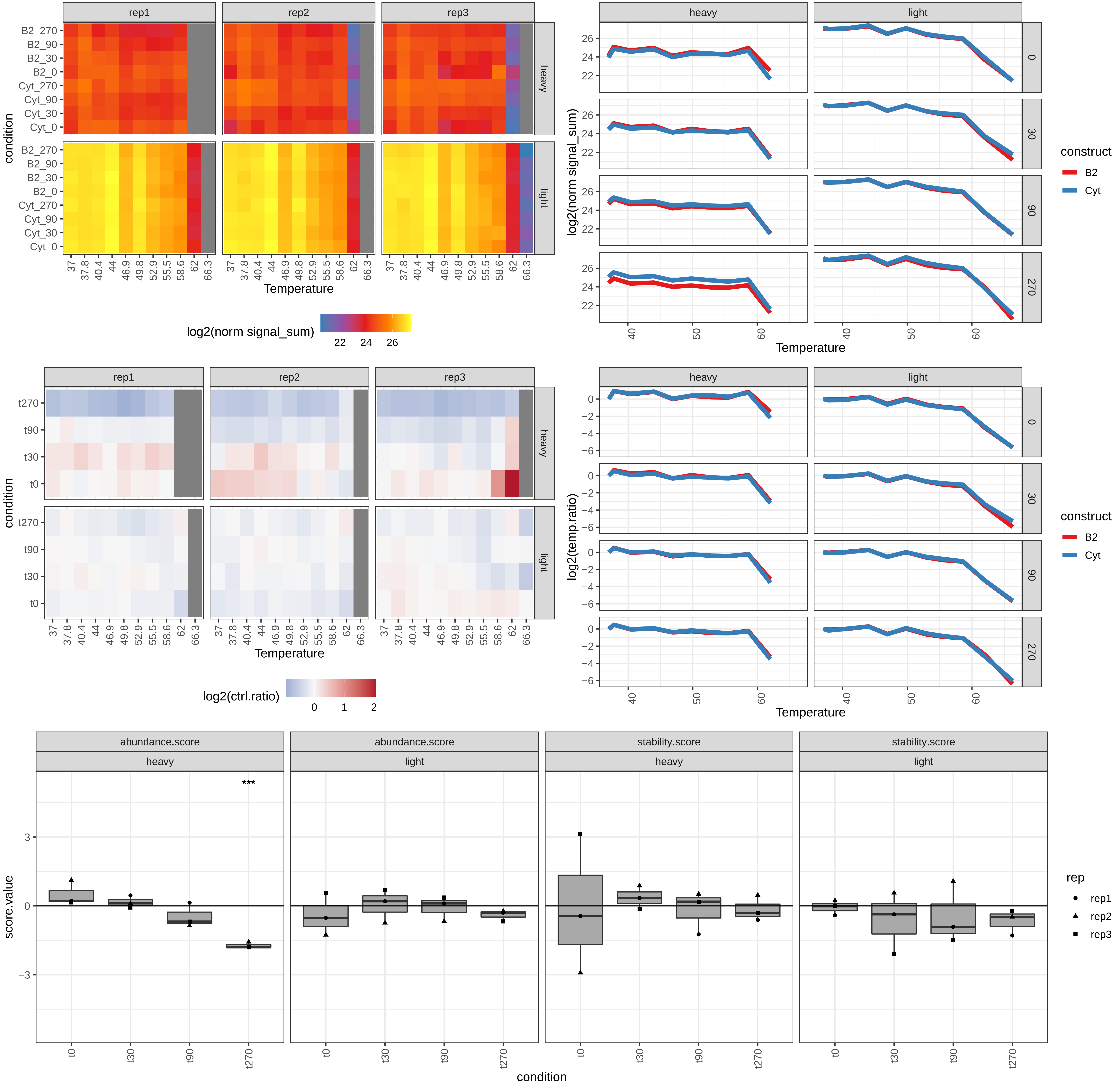

P47143 − Adenosine kinase OS=Saccharomyces cerevisiae (strain ATCC 204508 / S288c) GN=ADO1 PE=1 SV=1

ADP1 − average.top3: 7.01689361111111 − max.qupm: 11

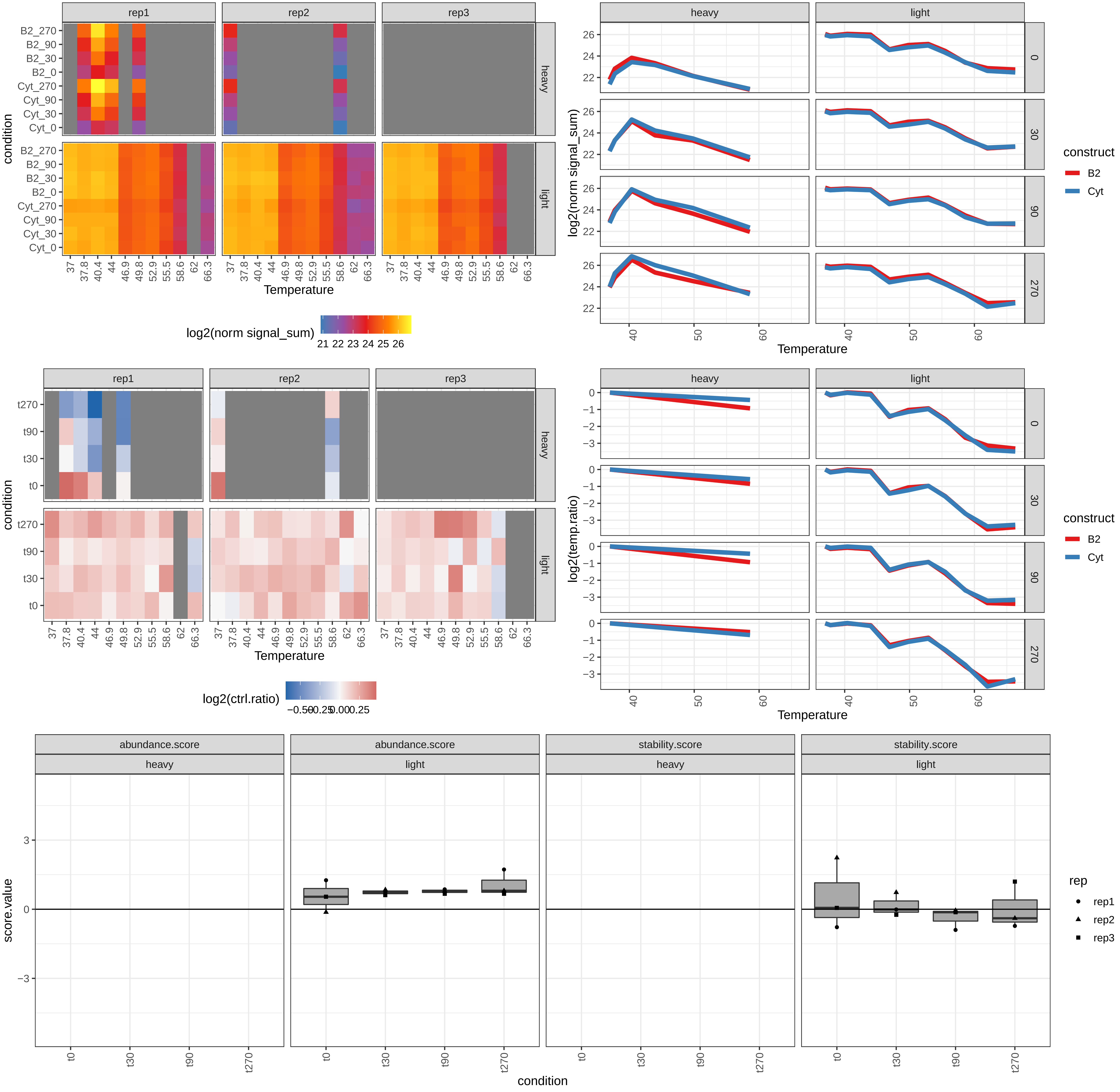

P25371 − Probable ATP−dependent permease OS=Saccharomyces cerevisiae (strain ATCC 204508 / S288c) GN=ADP1 PE=1 SV=2

ADR1 − average.top3: 6.39747444444444 − max.qupm: 7

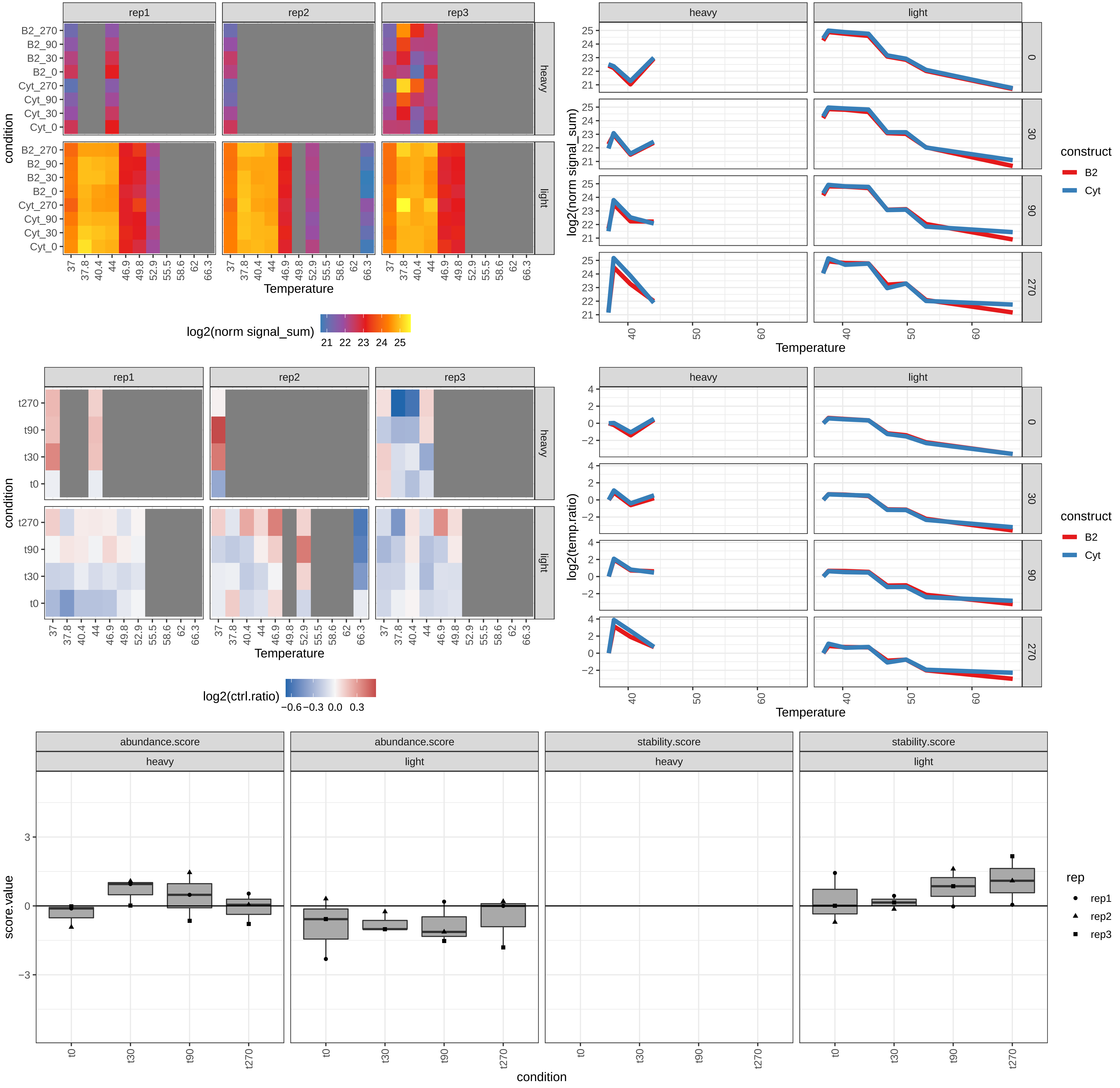

P07248 − Regulatory protein ADR1 OS=Saccharomyces cerevisiae (strain ATCC 204508 / S288c) GN=ADR1 PE=1 SV=2

ADY2 − average.top3: 7.46895170731707 − max.qupm: 4

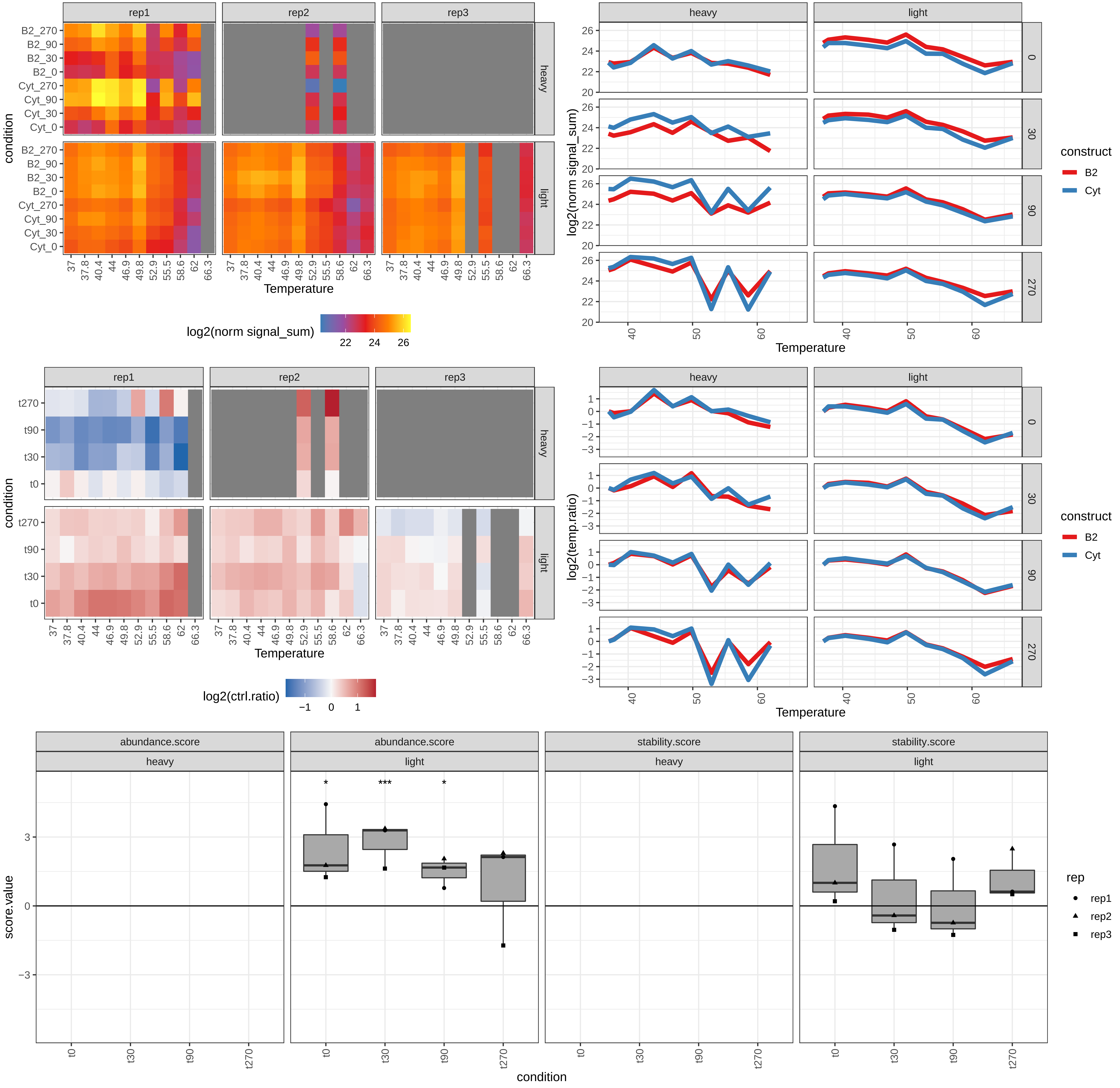

P25613 − Accumulation of dyads protein 2 OS=Saccharomyces cerevisiae (strain ATCC 204508 / S288c) GN=ADY2 PE=1 SV=1

AEP1 − average.top3: 6.401494375 − max.qupm: 5

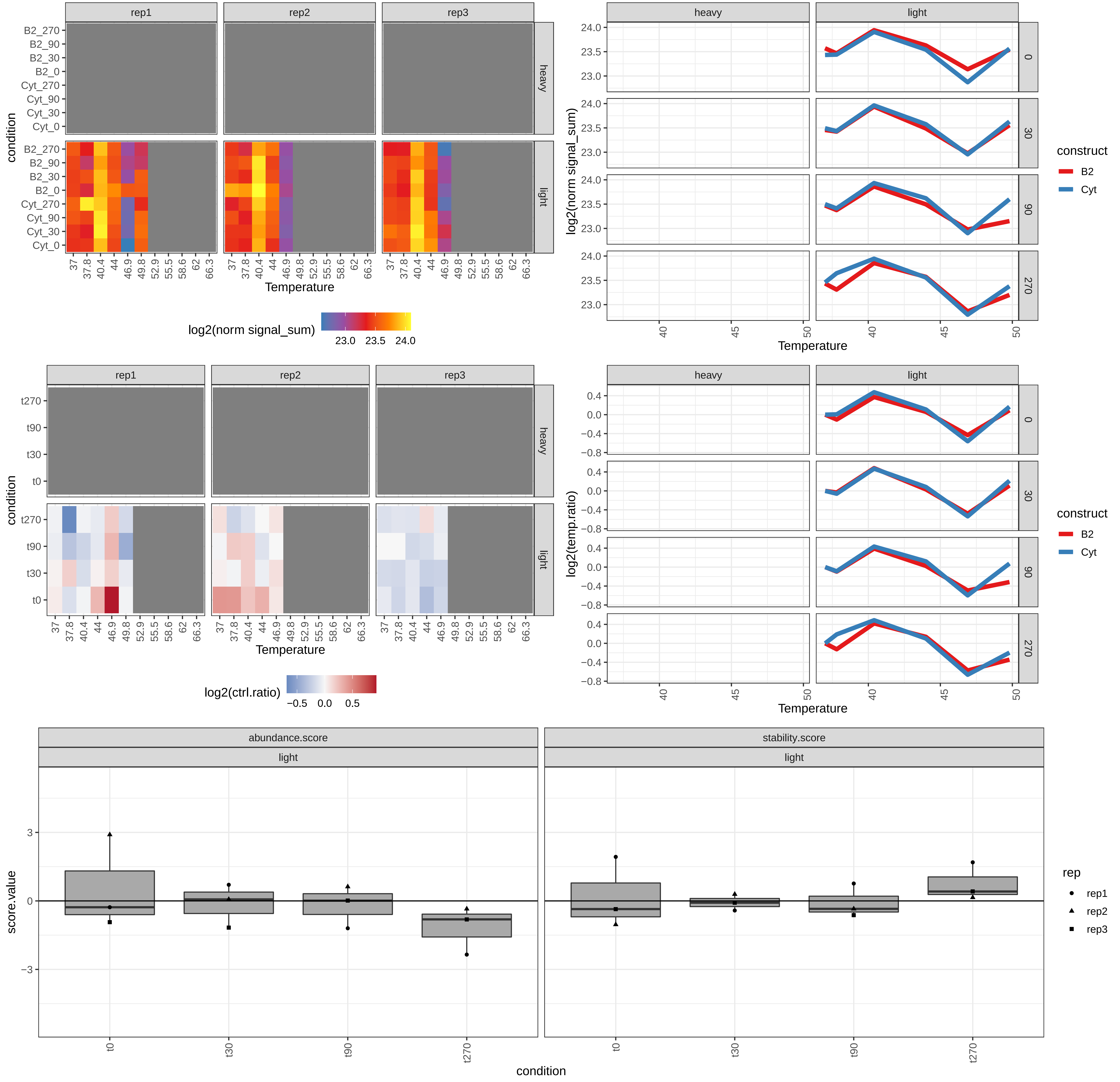

P32493 − ATPase expression protein 1, mitochondrial OS=Saccharomyces cerevisiae (strain ATCC 204508 / S288c) GN=AEP1 PE=1 SV=2

AEP2 − average.top3: 6.57467823529412 − max.qupm: 8

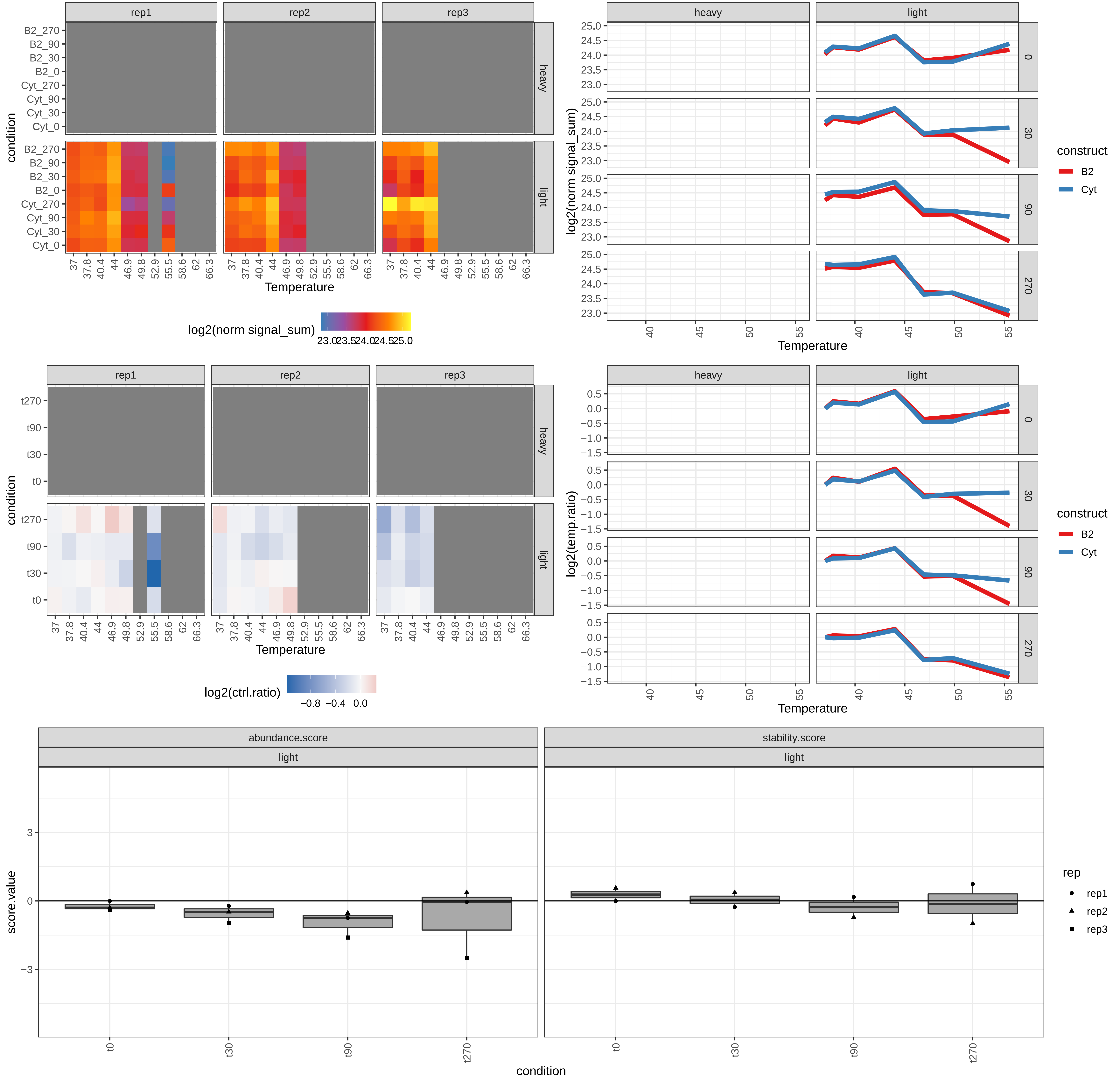

P22136 − ATPase expression protein 2, mitochondrial OS=Saccharomyces cerevisiae (strain ATCC 204508 / S288c) GN=AEP2 PE=1 SV=3

AFG1 − average.top3: 6.80708823529412 − max.qupm: 8

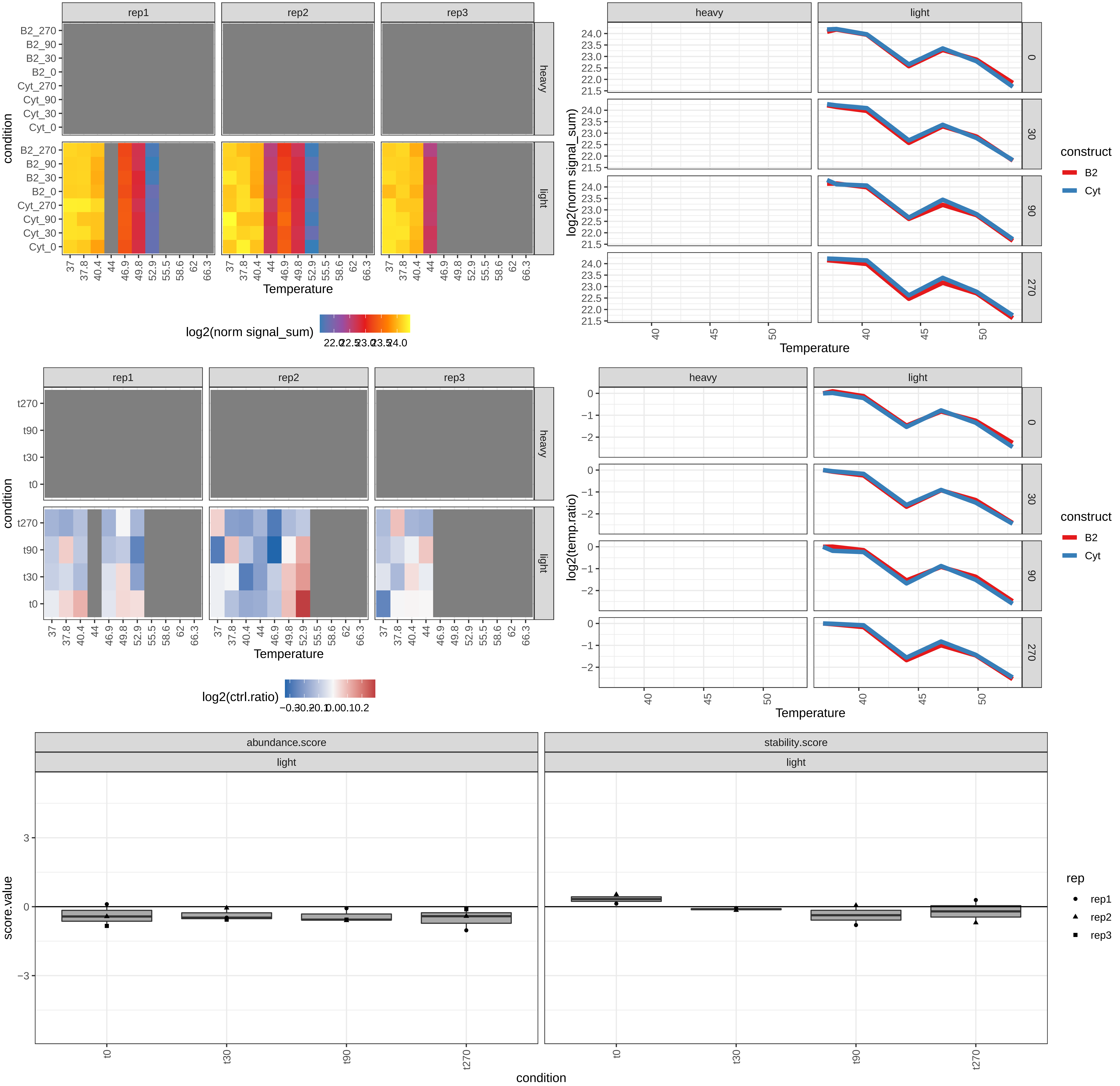

P32317 − Protein AFG1 OS=Saccharomyces cerevisiae (strain ATCC 204508 / S288c) GN=AFG1 PE=1 SV=1

AFG2 − average.top3: 6.8647415 − max.qupm: 10

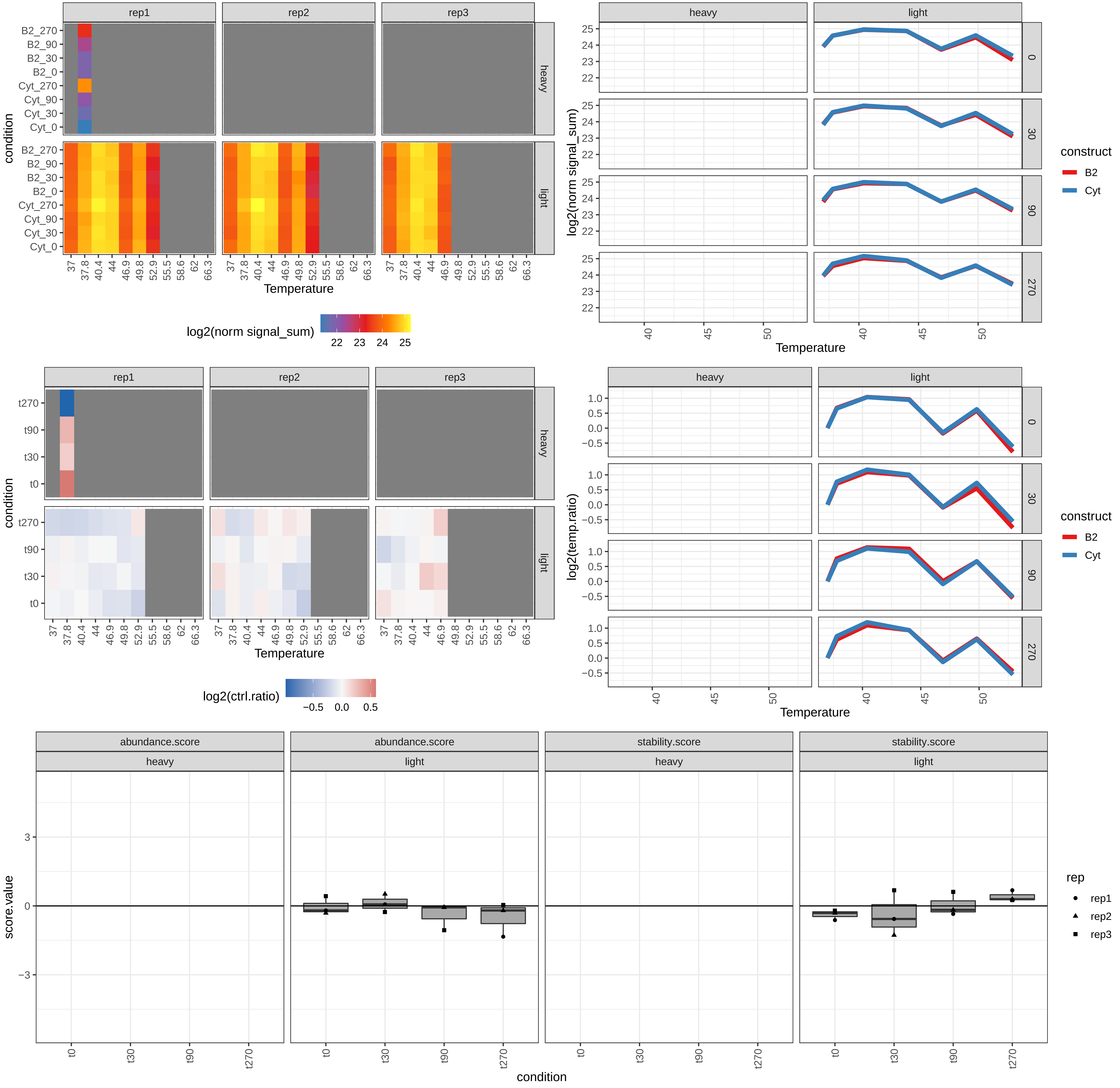

P32794 − ATPase family gene 2 protein OS=Saccharomyces cerevisiae (strain ATCC 204508 / S288c) GN=AFG2 PE=1 SV=1

AFG3 − average.top3: 6.97625566666667 − max.qupm: 12

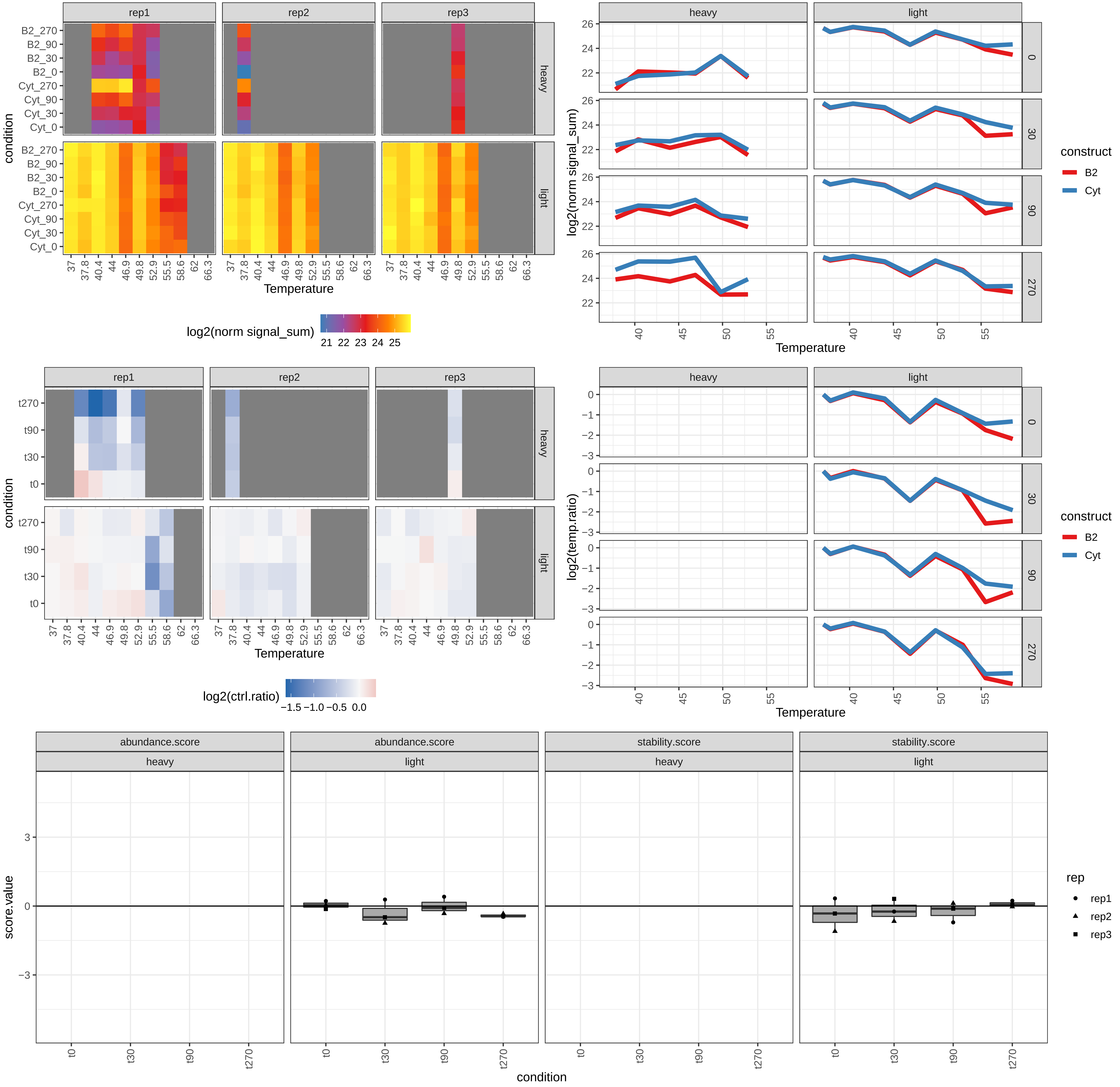

P39925 − Mitochondrial respiratory chain complexes assembly protein AFG3 OS=Saccharomyces cerevisiae (strain ATCC 204508 / S288c) GN=AFG3 PE=1 SV=1

AGC1 − average.top3: 6.44571125 − max.qupm: 7

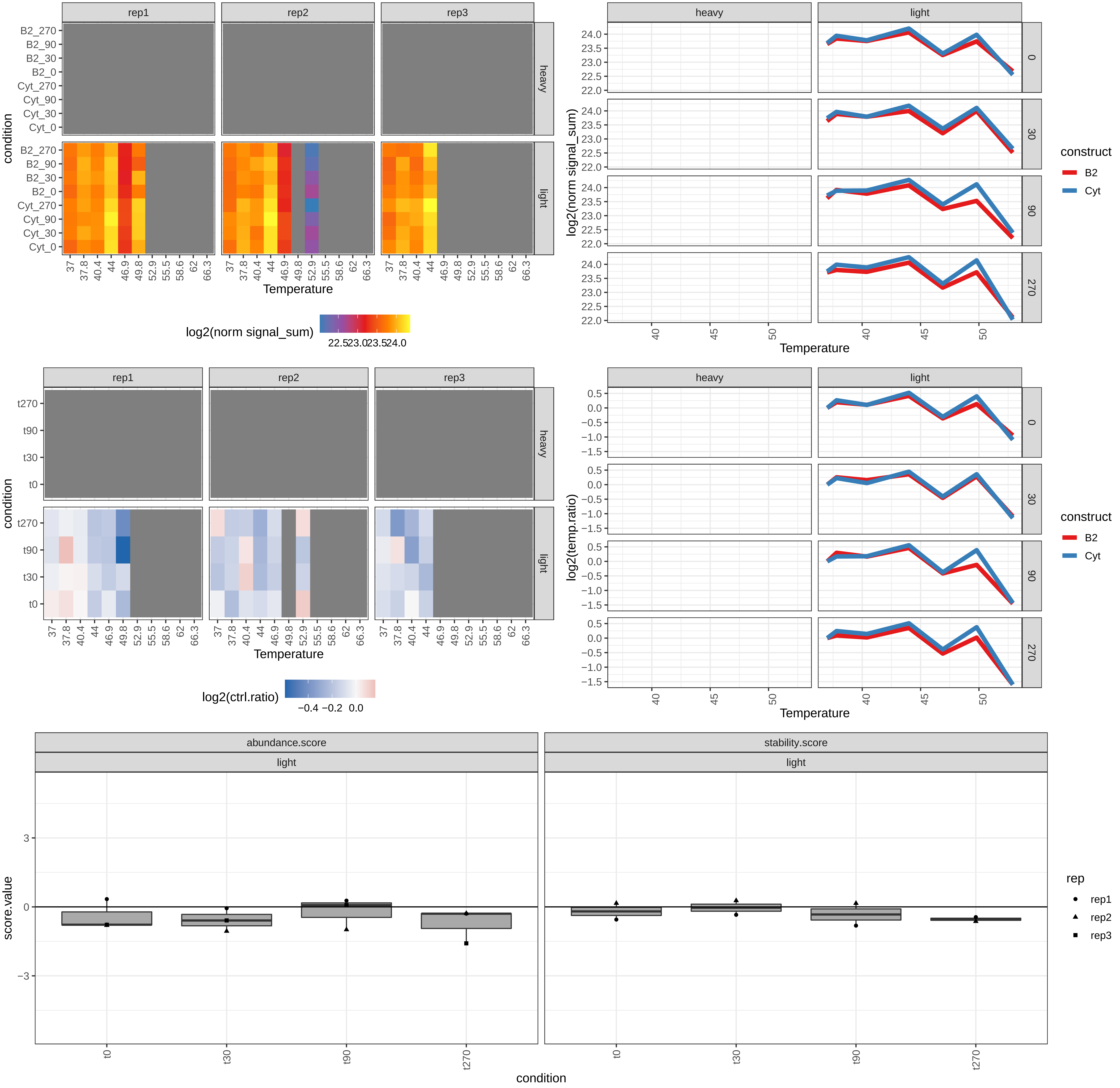

Q12482 − Mitochondrial aspartate−glutamate transporter AGC1 OS=Saccharomyces cerevisiae (strain ATCC 204508 / S288c) GN=AGC1 PE=3 SV=1

AGE2 − average.top3: 6.65083333333333 − max.qupm: 4

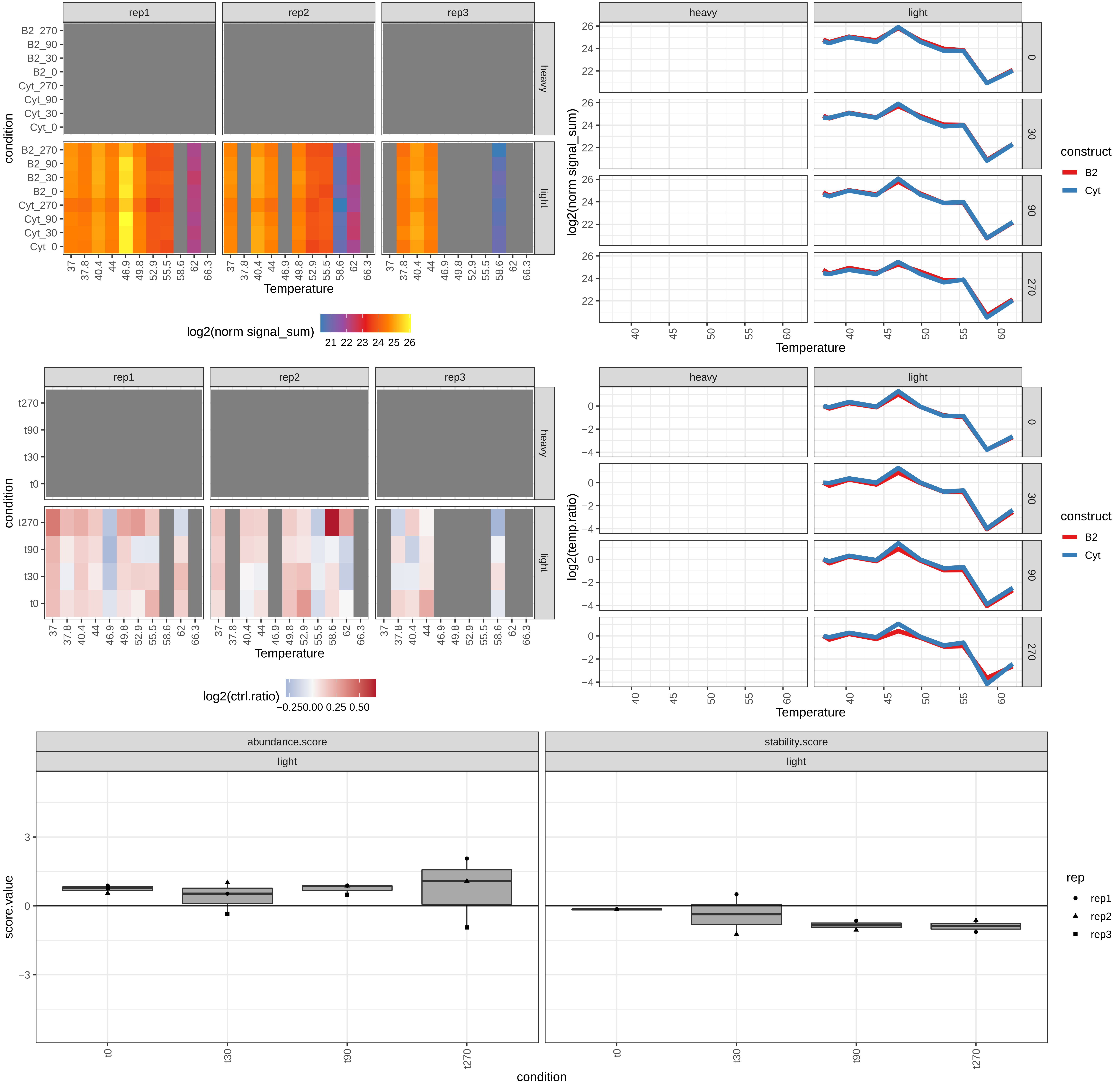

P40529 − ADP−ribosylation factor GTPase−activating protein effector protein 2 OS=Saccharomyces cerevisiae (strain ATCC 204508 / S288c) GN=AGE2 PE=1 SV=1

AGP1 − average.top3: 6.48043941176471 − max.qupm: 7

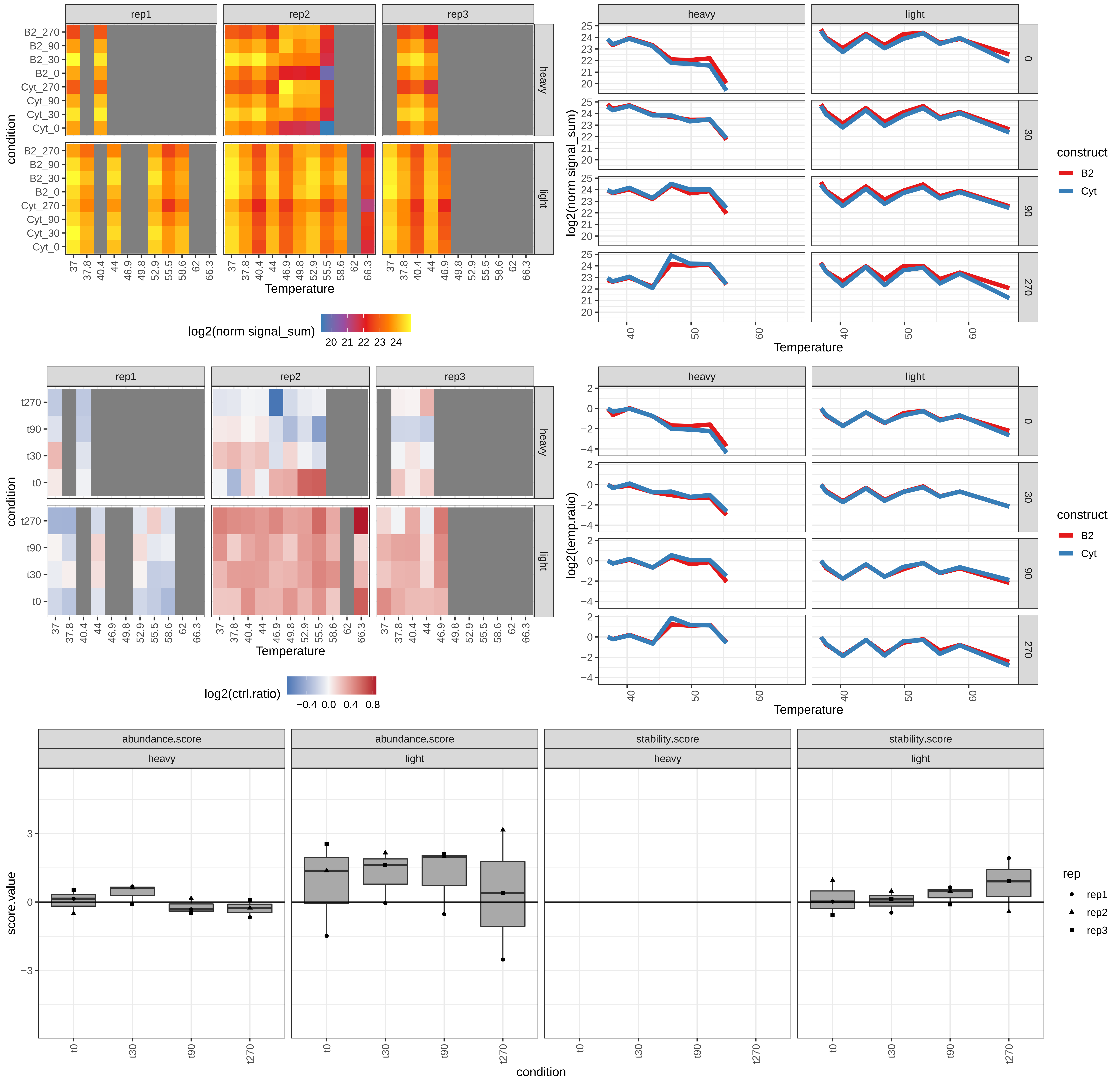

P25376 − General amino acid permease AGP1 OS=Saccharomyces cerevisiae (strain ATCC 204508 / S288c) GN=AGP1 PE=1 SV=3

AGP2 − average.top3: 7.11546666666667 − max.qupm: 6

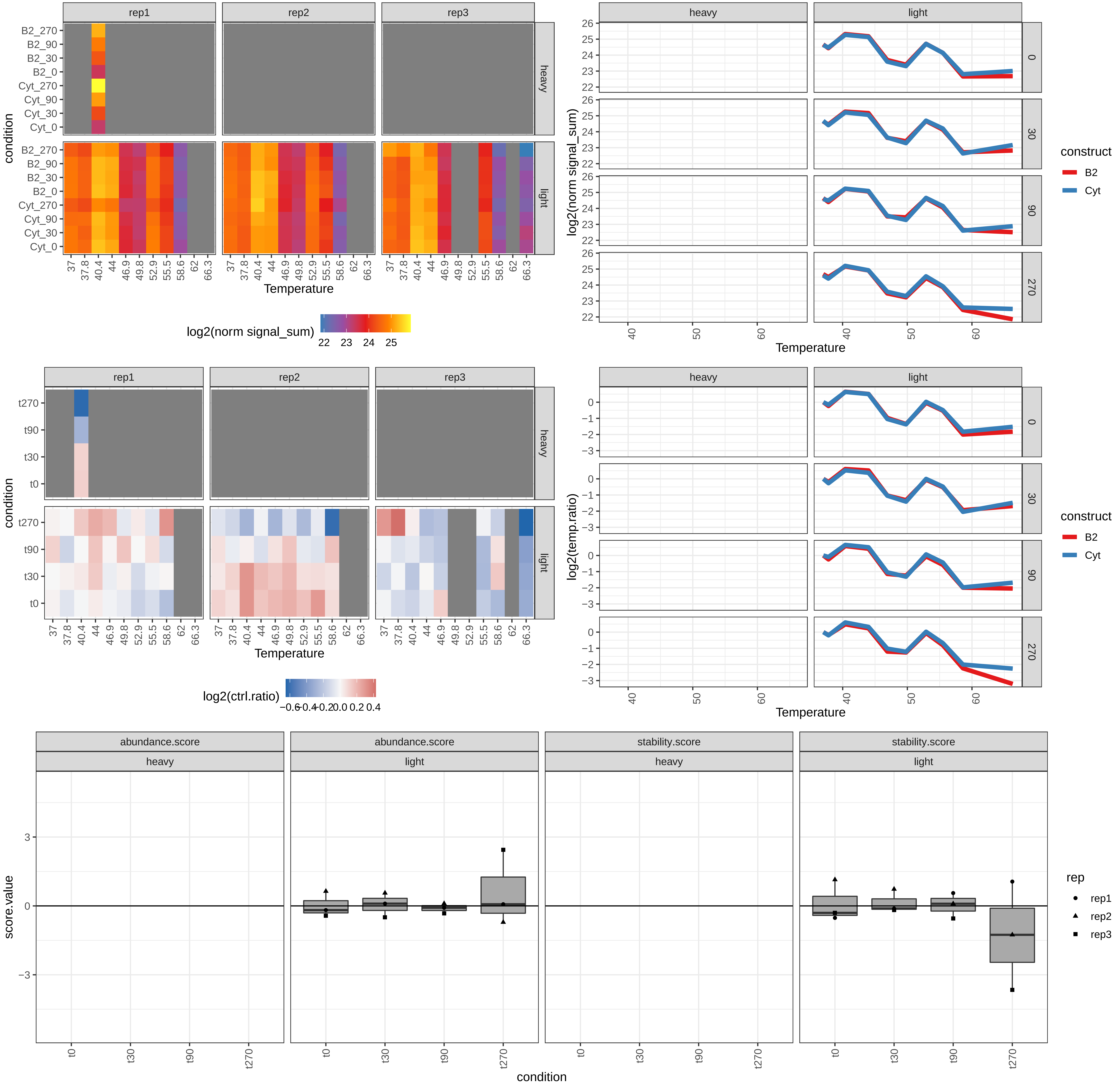

P38090 − General amino acid permease AGP2 OS=Saccharomyces cerevisiae (strain ATCC 204508 / S288c) GN=AGP2 PE=1 SV=1

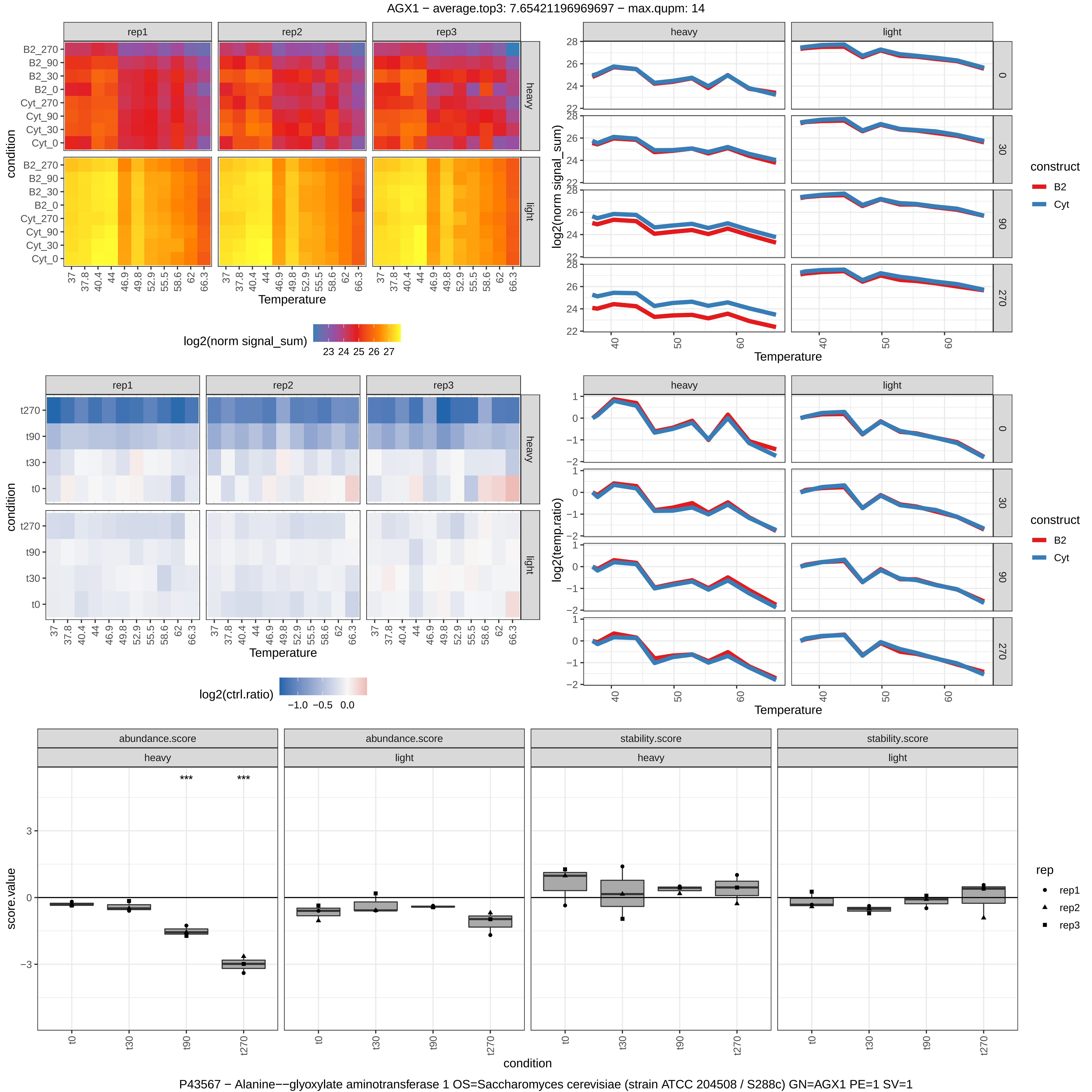
AGX1 − average.top3: 7.65421196969697 − max.qupm: 14
rep1
rep2
rep3
heavy
light
condition
Temperature
log2(norm signal_sum)
log2(ctrl.ratio)
log2(temp.ratio)
construct
B2
Cyt
abundance.score
stability.score
score.value
rep
rep1
rep2
rep3
***
P43567 − Alanine−−glyoxylate aminotransferase 1 OS=Saccharomyces cerevisiae (strain ATCC 204508 / S288c) GN=AGX1 PE=1 SV=1

AHA1 − average.top3: 7.41922622641509 − max.qupm: 11

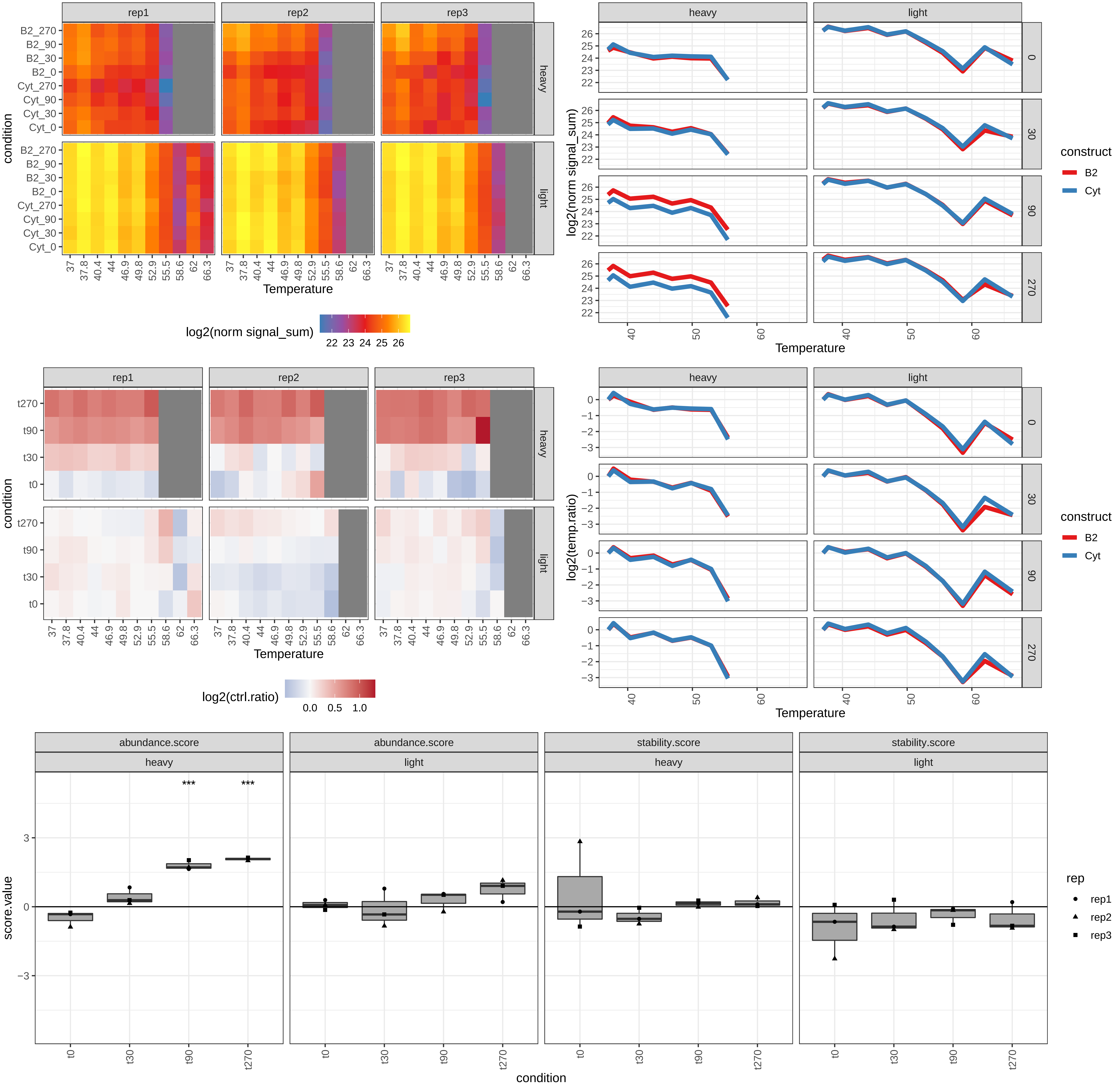

Q12449 − Hsp90 co−chaperone AHA1 OS=Saccharomyces cerevisiae (strain ATCC 204508 / S288c) GN=AHA1 PE=1 SV=1

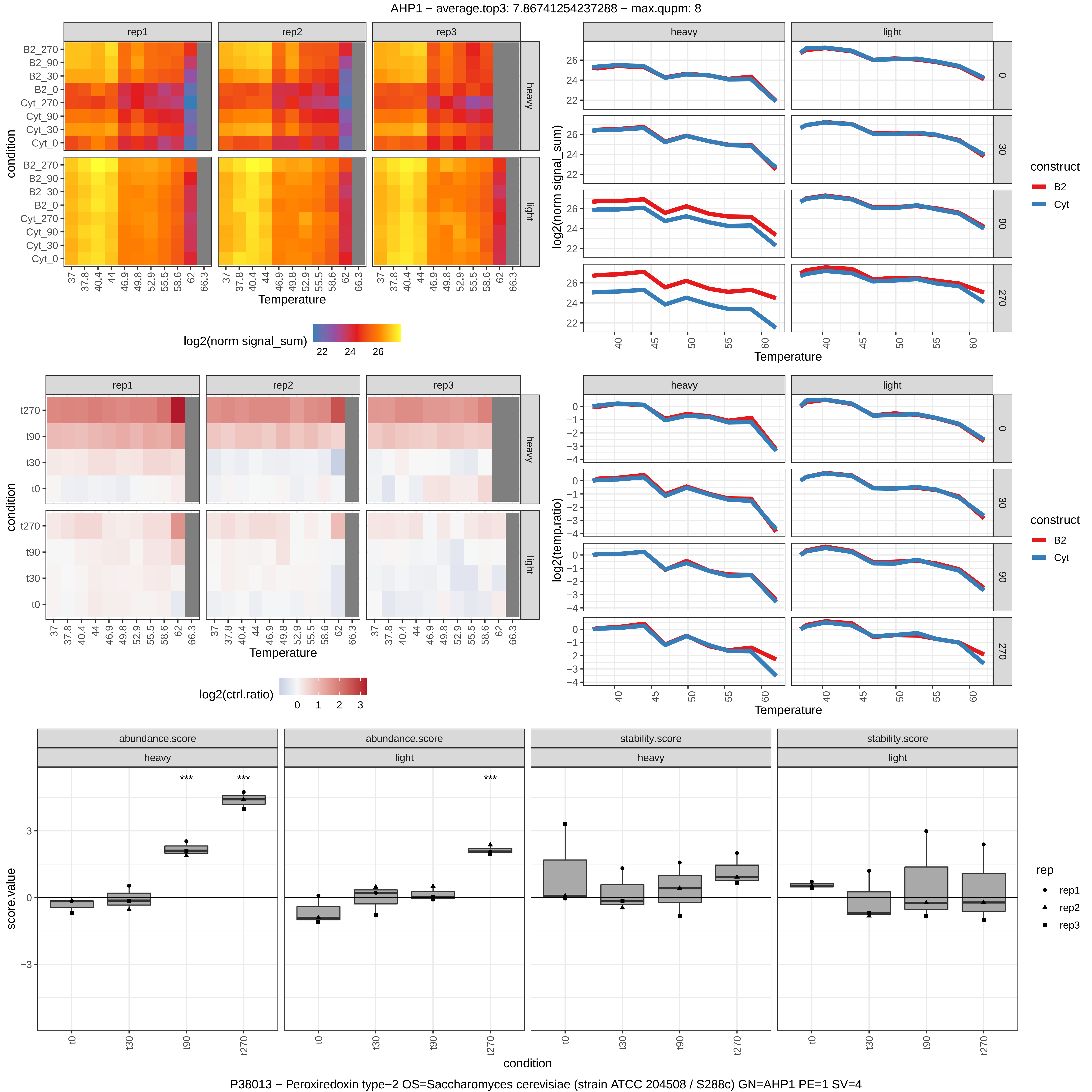

AHP1 − average.top3: 7.86741254237288 − max.qupm: 8
rep1
rep2
rep3
heavy
light
condition
Temperature
log2(norm signal_sum)
log2(temp.ratio)
log2(ctrl.ratio)
construct
B2
Cyt
abundance.score
stability.score
score.value
rep
rep1
rep2
rep3
***
P38013 − Peroxiredoxin type−2 OS=Saccharomyces cerevisiae (strain ATCC 204508 / S288c) GN=AHP1 PE=1 SV=4

AI1 − average.top3: 6.16208375 − max.qupm: 5

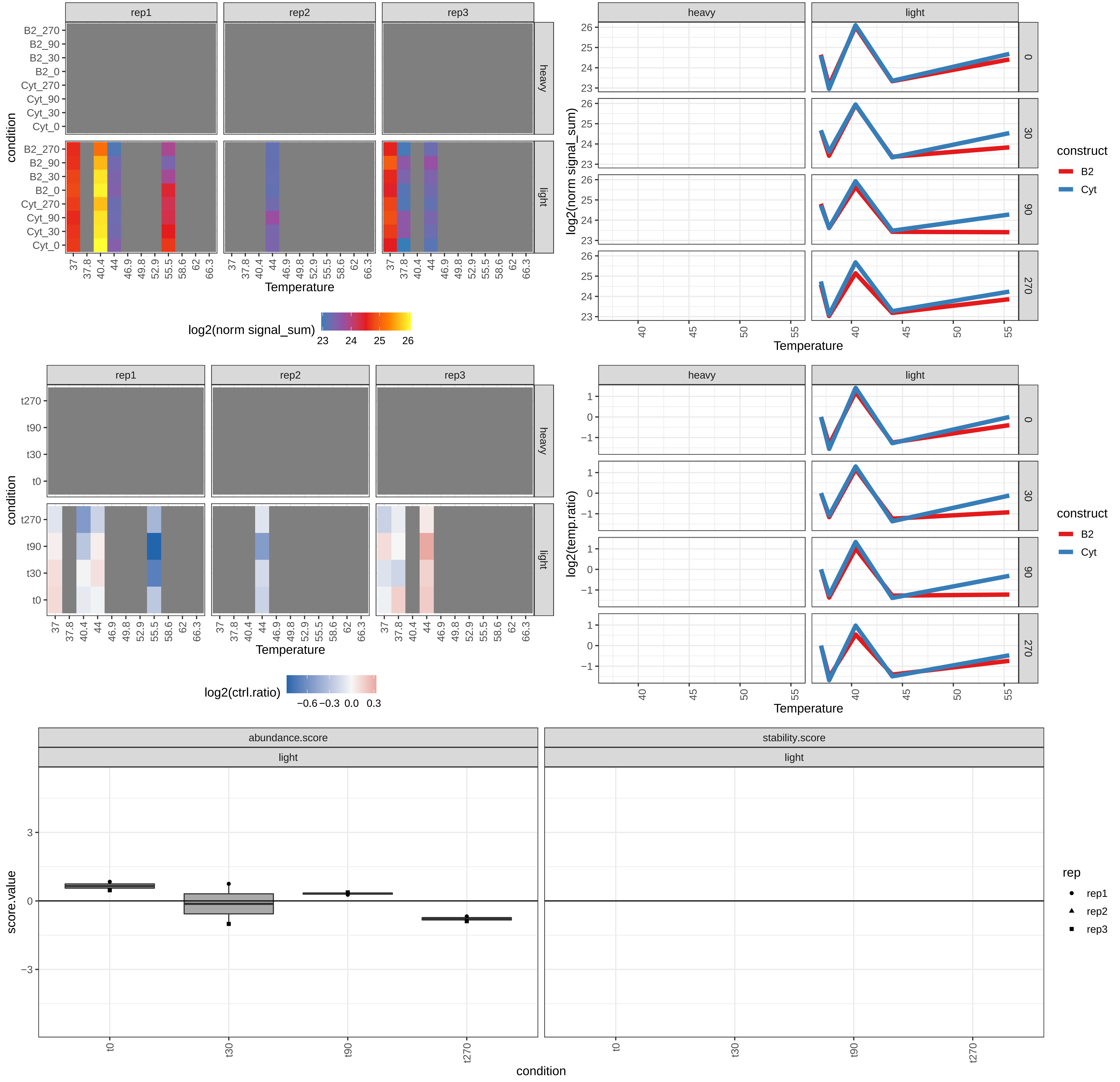

P03875 − Putative COX1/OXI3 intron 1 protein OS=Saccharomyces cerevisiae (strain ATCC 204508 / S288c) GN=AI1 PE=3 SV=2

AIF1 − average.top3: 6.651140625 − max.qupm: 6

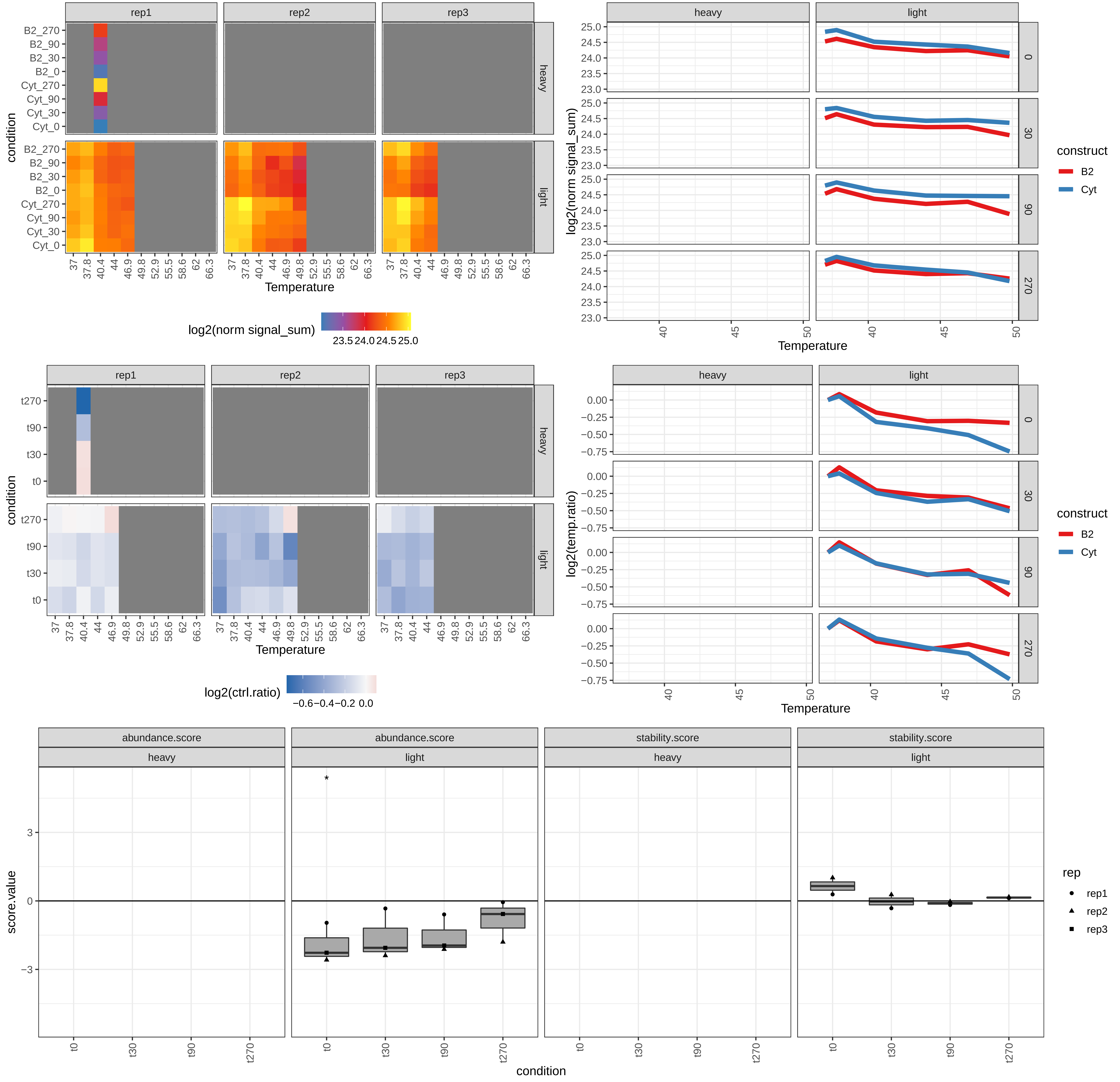

P52923 − Apoptosis−inducing factor 1 OS=Saccharomyces cerevisiae (strain ATCC 204508 / S288c) GN=AIF1 PE=1 SV=1

AIM10 − average.top3: 6.8083975 − max.qupm: 6

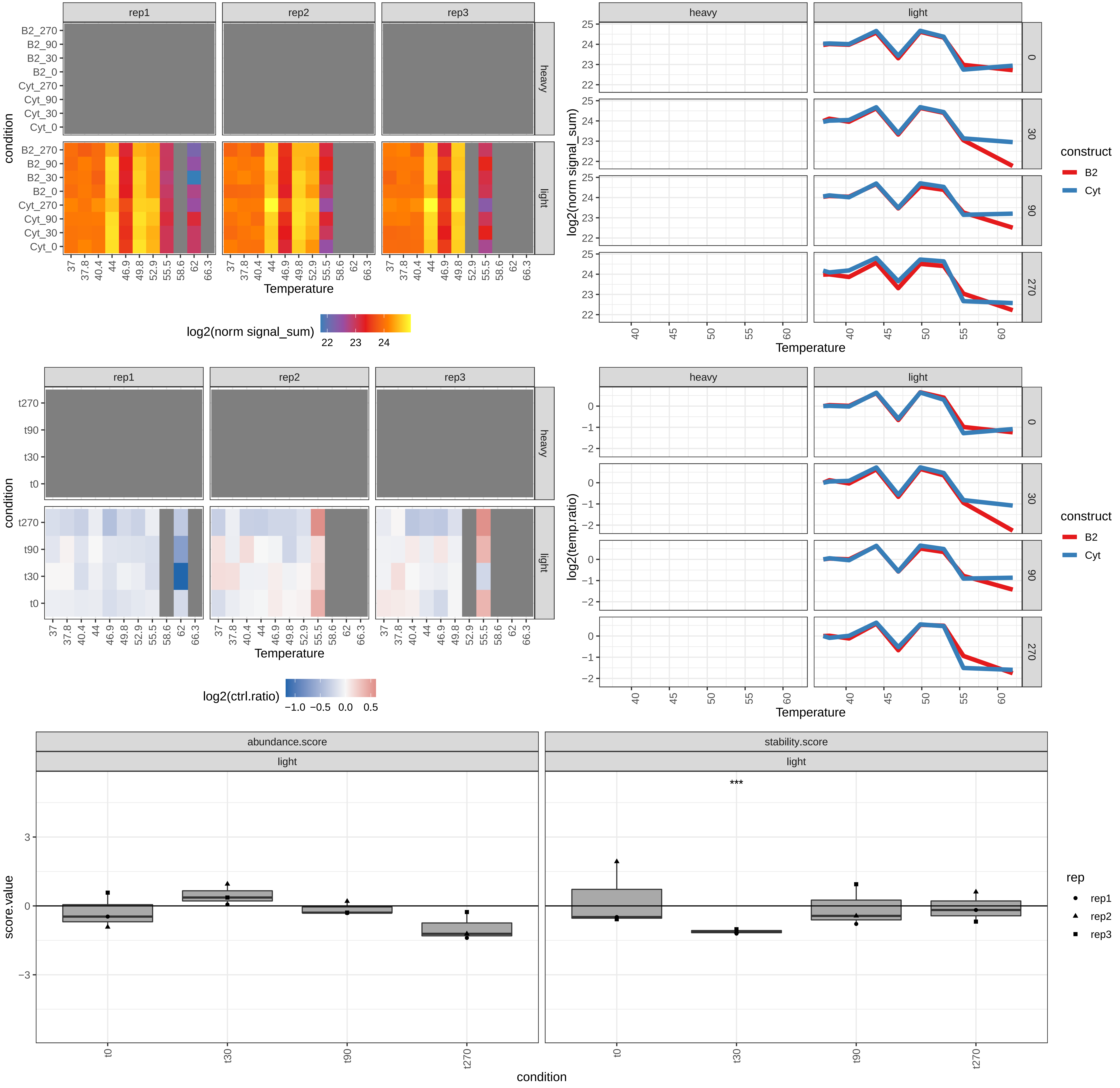

P39965 − Probable proline−−tRNA ligase, mitochondrial OS=Saccharomyces cerevisiae (strain ATCC 204508 / S288c) GN=AIM10 PE=1 SV=1

AIM14 − average.top3: 6.83517823529412 − max.qupm: 3

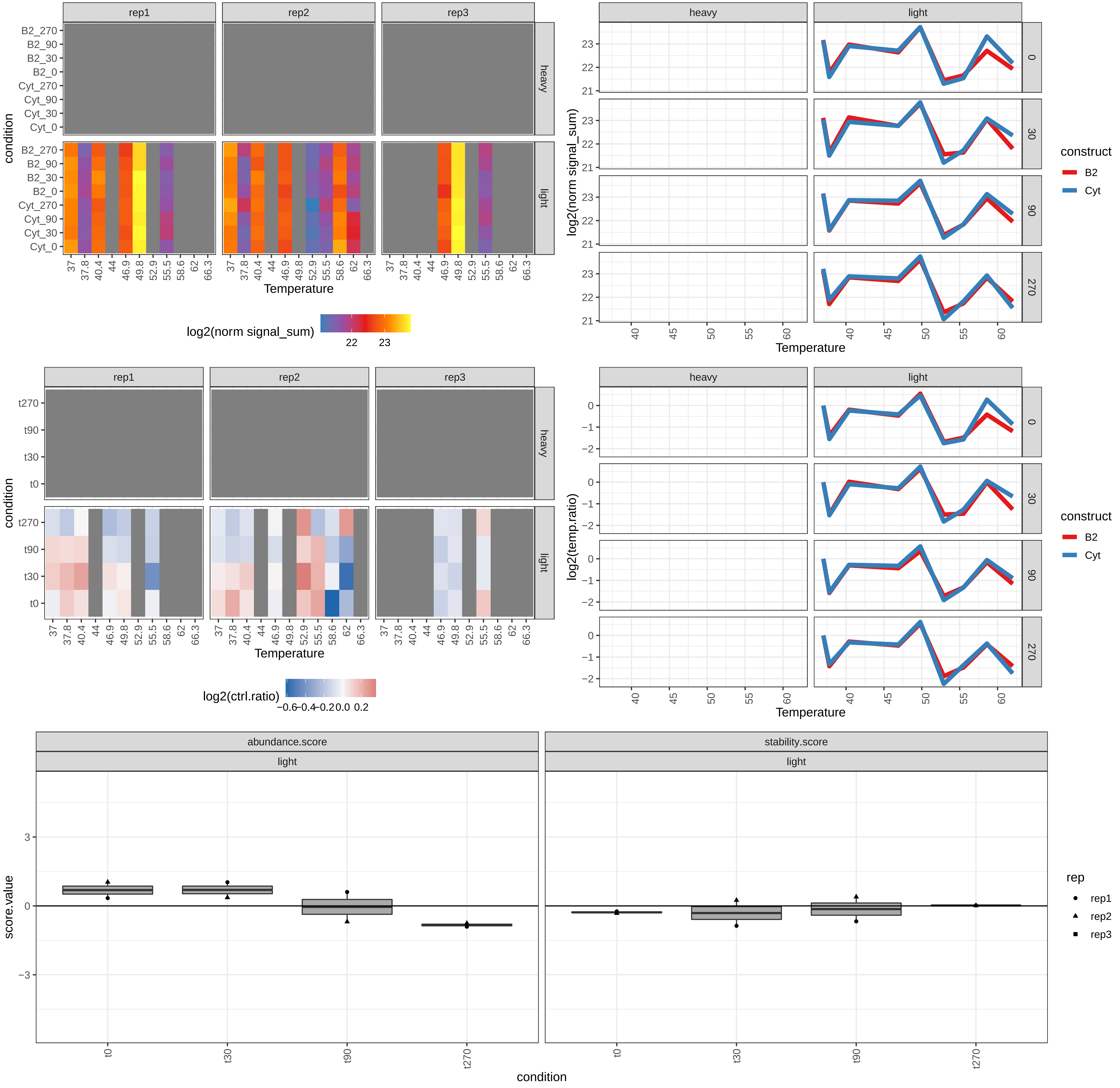

P53109 − Probable metalloreductase AIM14 OS=Saccharomyces cerevisiae (strain ATCC 204508 / S288c) GN=AIM14 PE=1 SV=1

AIM17 − average.top3: 6.96709181818182 − max.qupm: 8

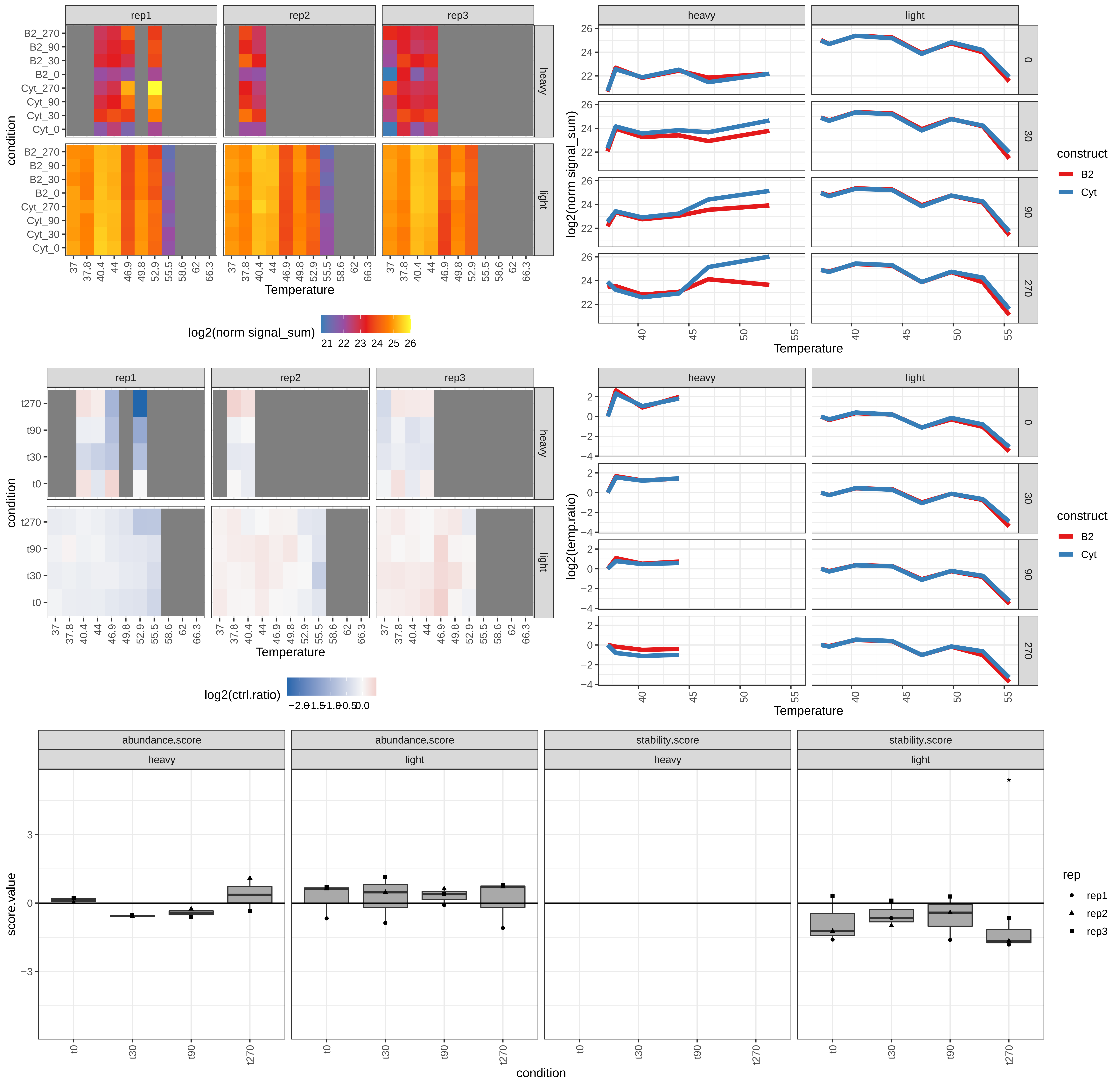

P23180 − Probable oxidoreductase AIM17 OS=Saccharomyces cerevisiae (strain ATCC 204508 / S288c) GN=AIM17 PE=1 SV=2

AIM18 − average.top3: 7.1563609375 − max.qupm: 7

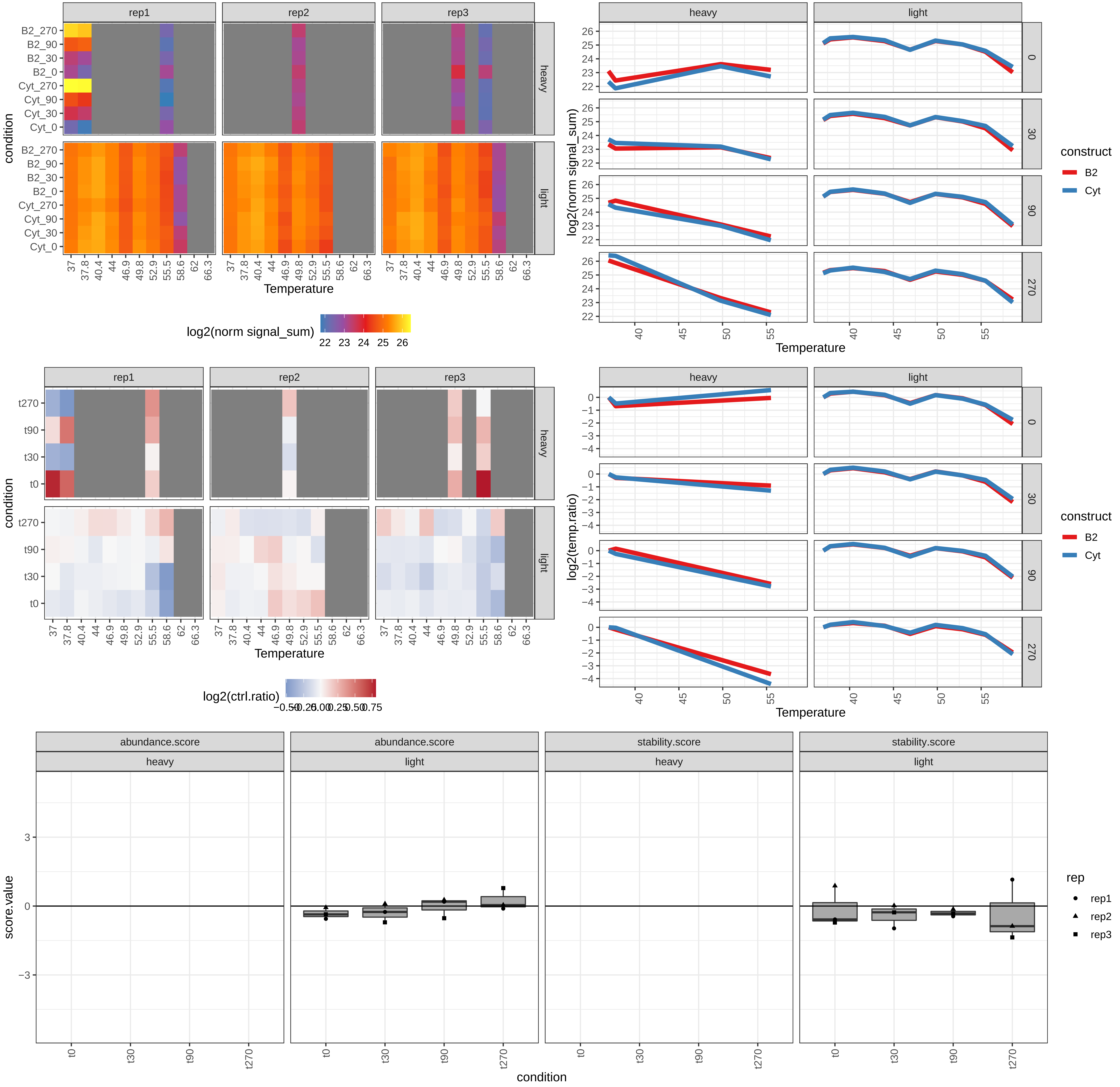

P38884 − Altered inheritance of mitochondria protein 18, mitochondrial OS=Saccharomyces cerevisiae (strain ATCC 204508 / S288c) GN=AIM18 PE=1 SV=1

AIM2 − average.top3: 7.11073803571429 − max.qupm: 8

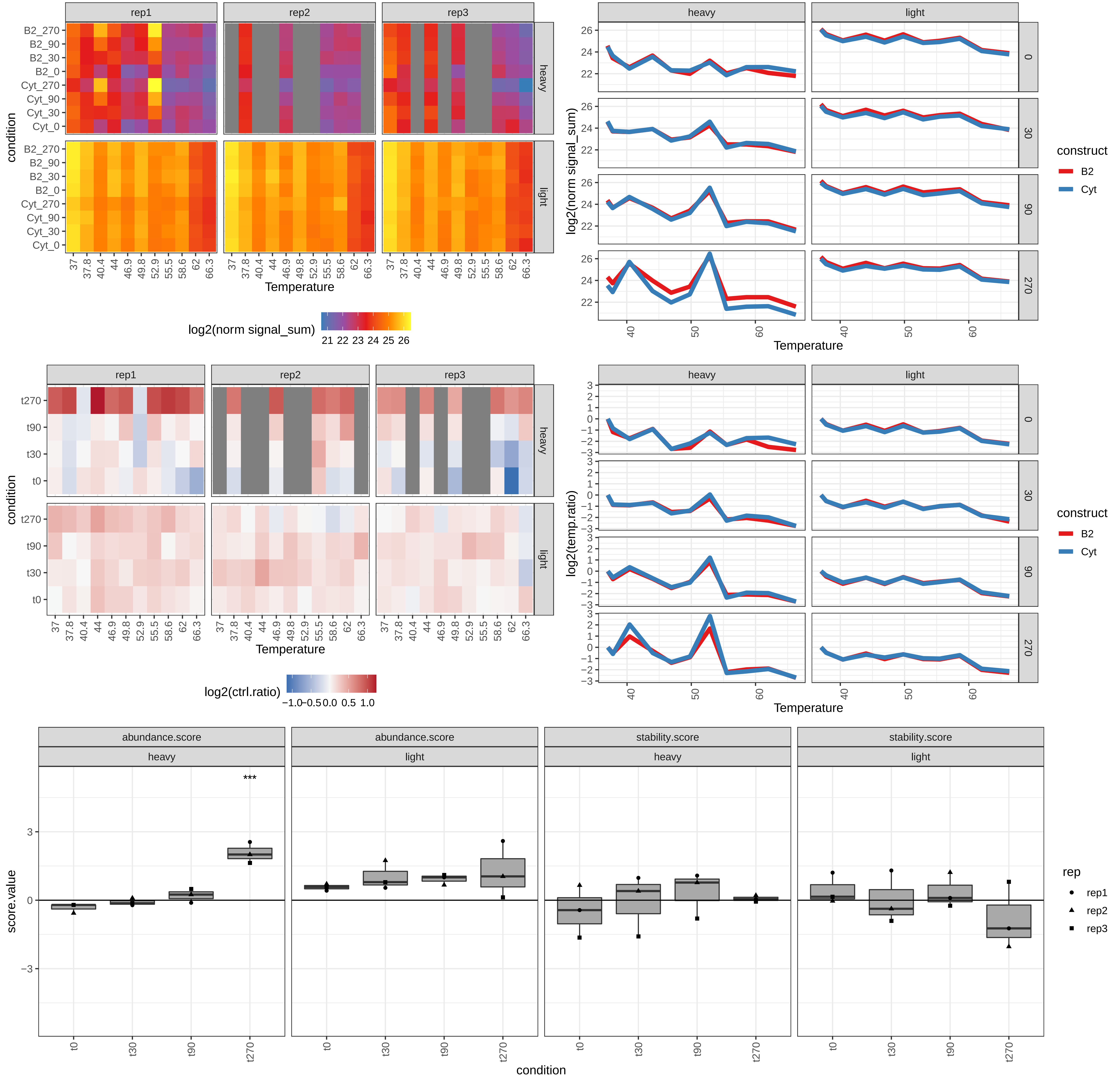

P39721 − Protein AIM2 OS=Saccharomyces cerevisiae (strain ATCC 204508 / S288c) GN=AIM2 PE=1 SV=1

AIM22 − average.top3: 6.55917047619048 − max.qupm: 5

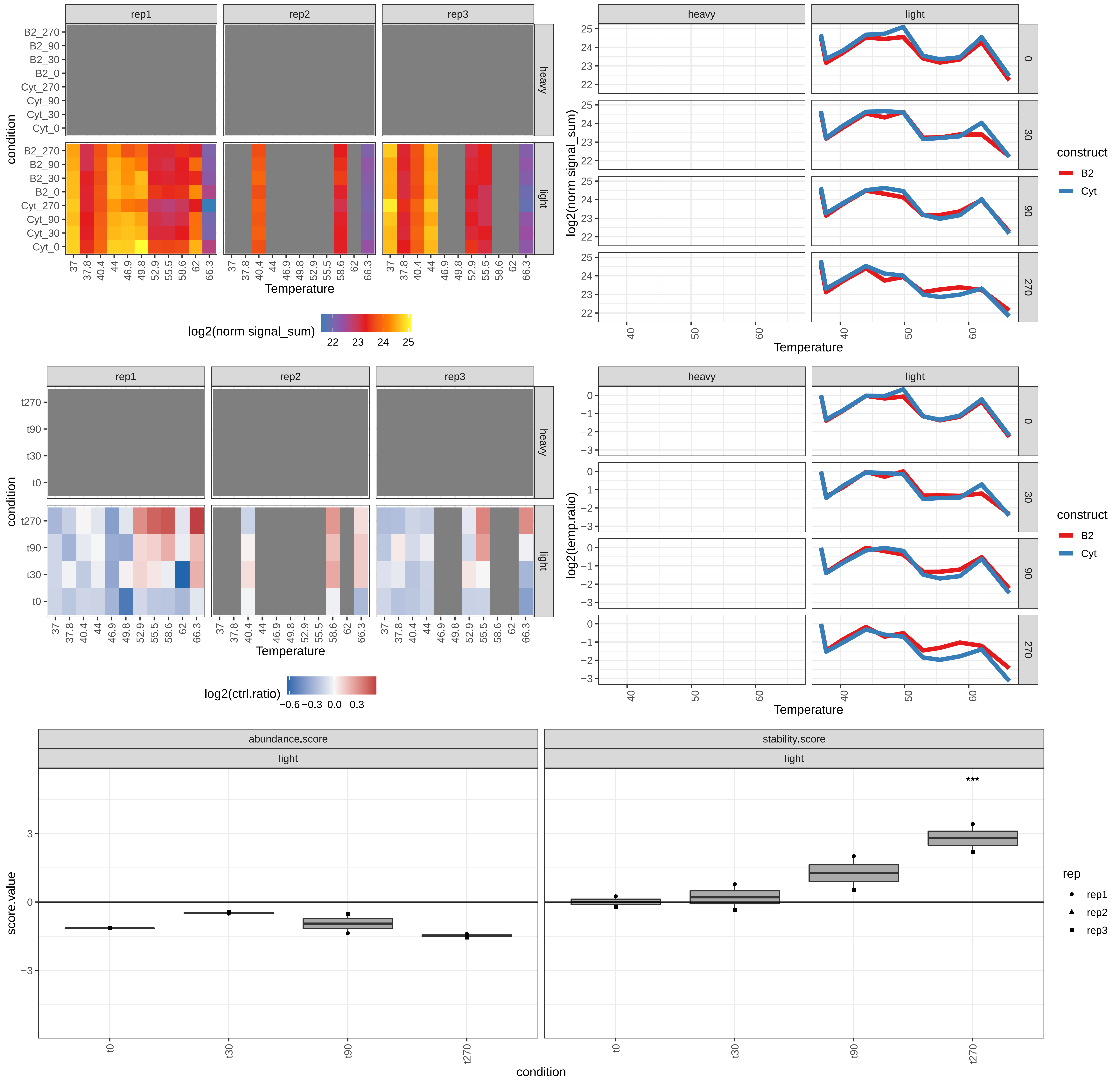

P47051 − Putative lipoate−protein ligase A OS=Saccharomyces cerevisiae (strain ATCC 204508 / S288c) GN=AIM22 PE=3 SV=2

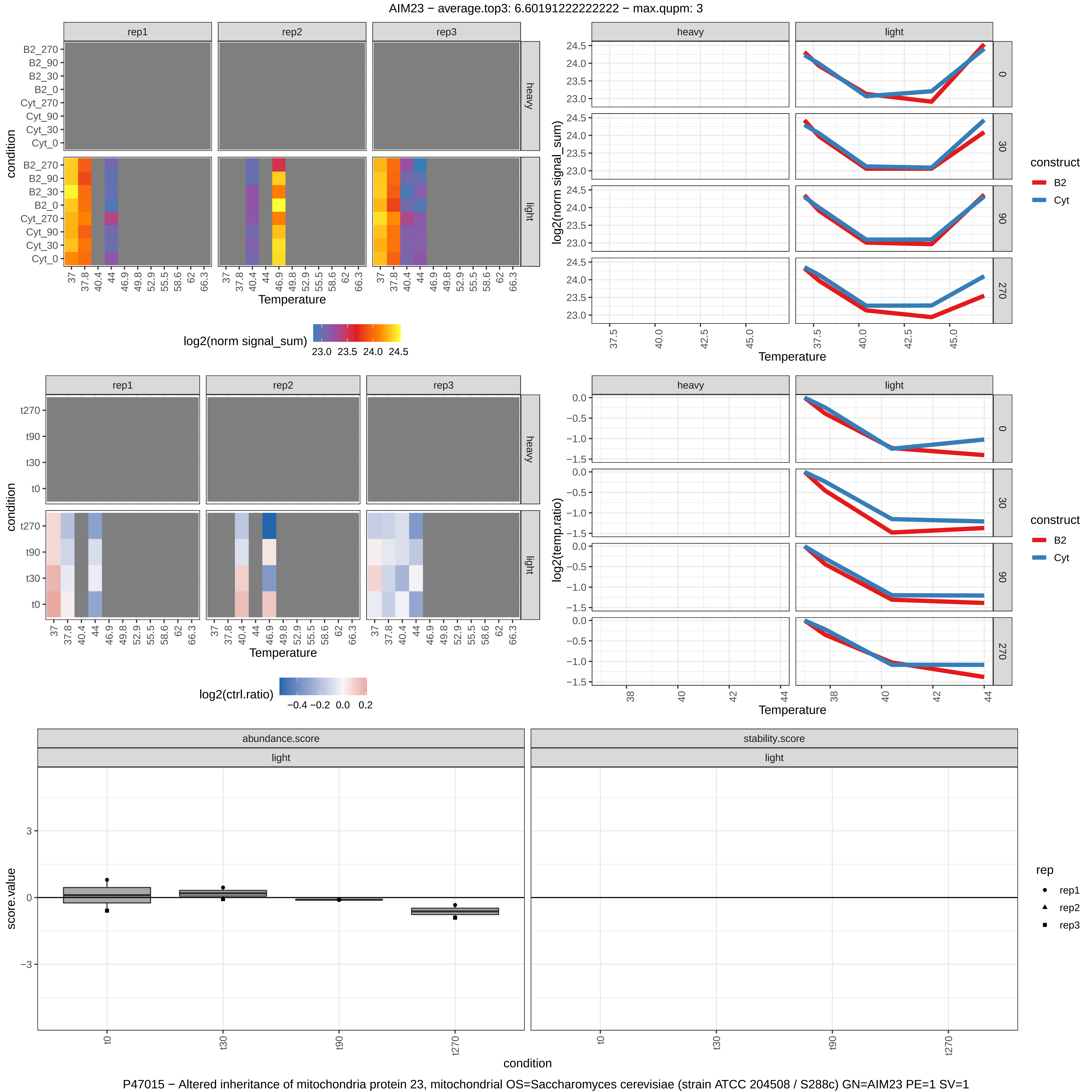

AIM23 − average.top3: 6.60191222222222 − max.qupm: 3
rep1
rep2
rep3
heavy
light
condition
Temperature
log2(norm signal_sum)
log2(temp.ratio)
log2(ctrl.ratio)
construct
B2
Cyt
abundance.score
stability.score
score.value
rep
rep1
rep2
rep3
P47015 − Altered inheritance of mitochondria protein 23, mitochondrial OS=Saccharomyces cerevisiae (strain ATCC 204508 / S288c) GN=AIM23 PE=1 SV=1

AIM24 − average.top3: 7.06288548387097 − max.qupm: 10

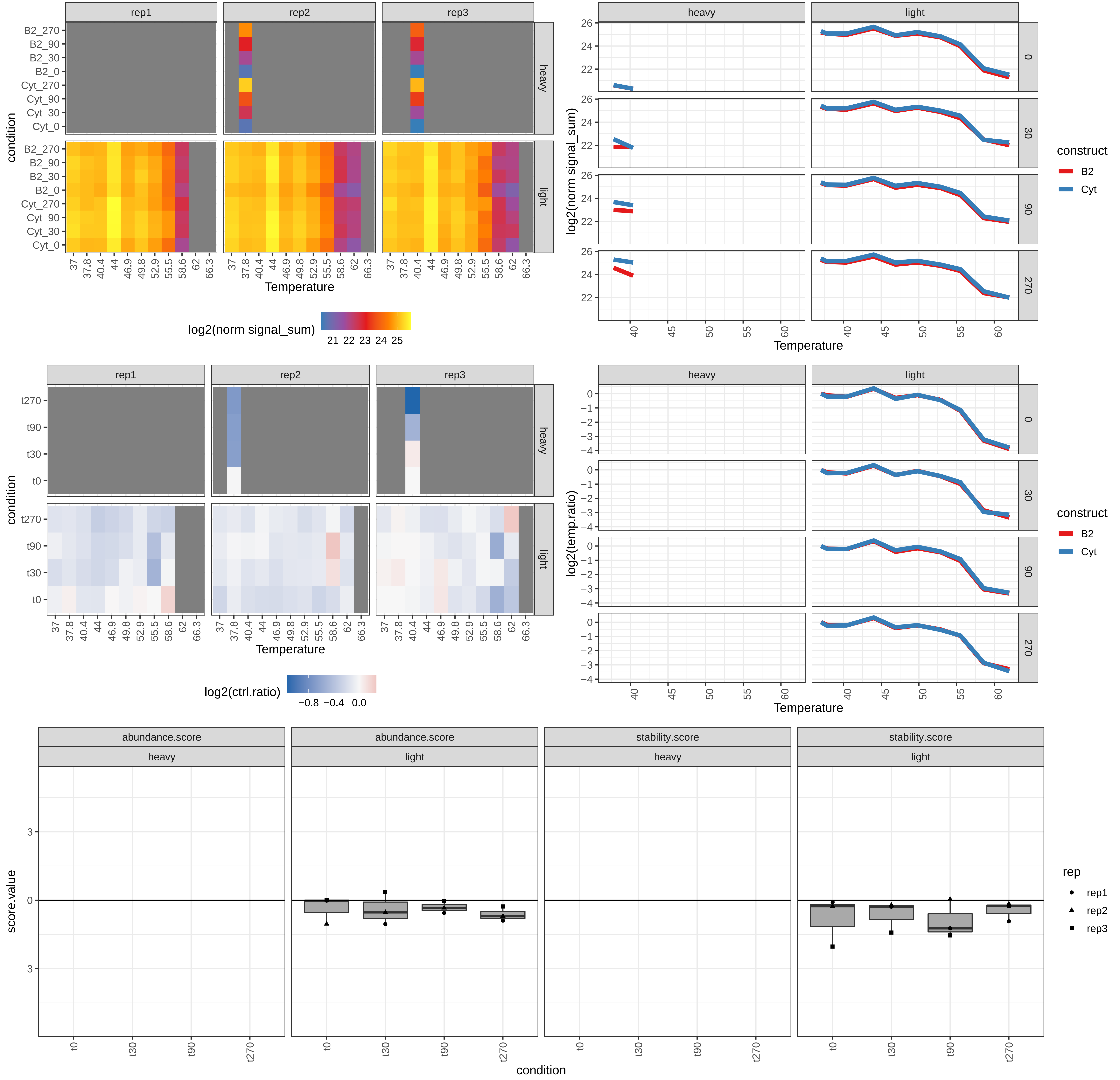

P47127 − Altered inheritance of mitochondria protein 24, mitochondrial OS=Saccharomyces cerevisiae (strain ATCC 204508 / S288c) GN=AIM24 PE=1 SV=1

AIM25 − average.top3: 6.77409 − max.qupm: 6

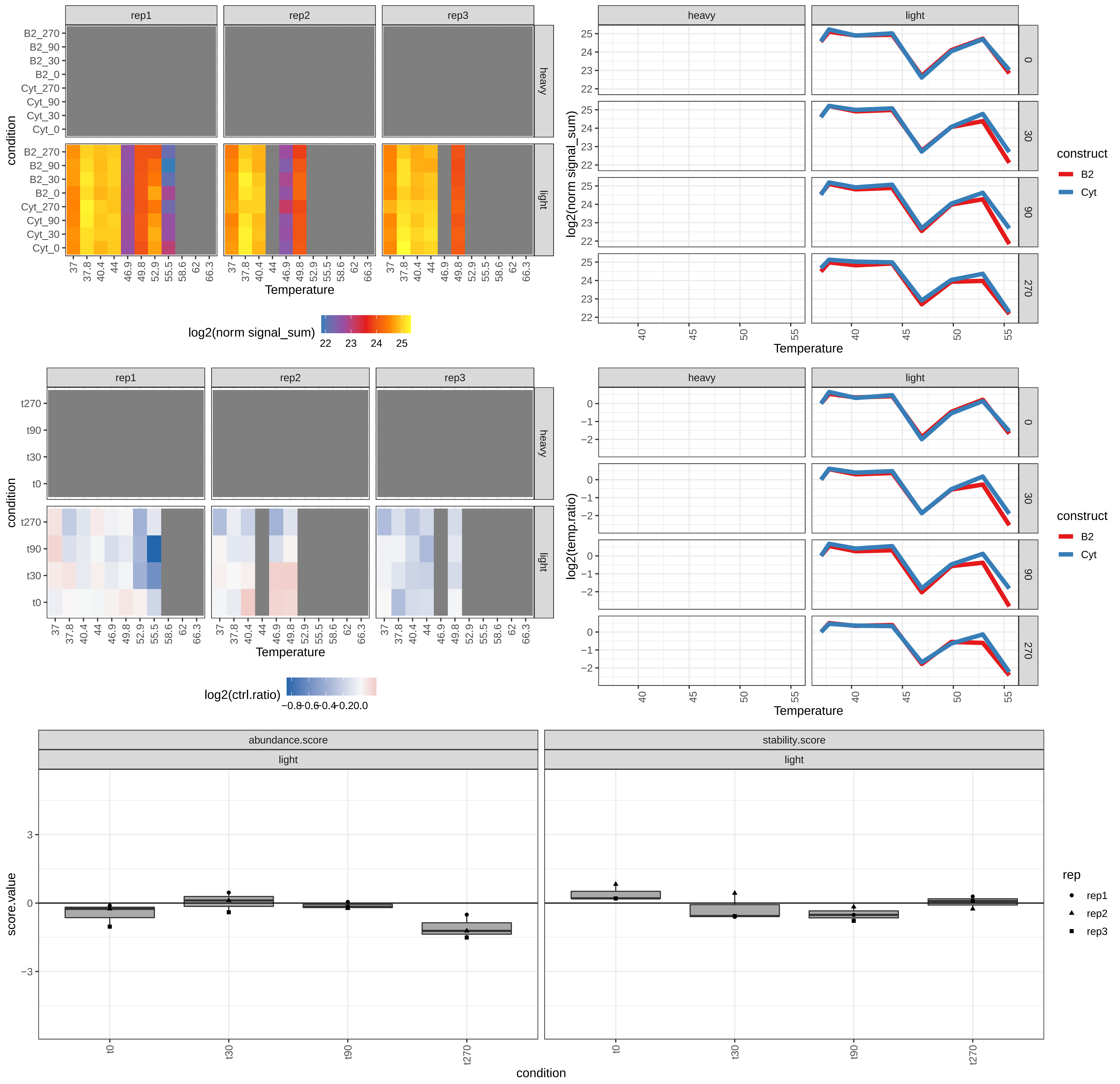

P47140 − Altered inheritance rate of mitochondria protein 25 OS=Saccharomyces cerevisiae (strain ATCC 204508 / S288c) GN=AIM25 PE=3 SV=1

AIM29 − average.top3: 7.36899583333333 − max.qupm: 6

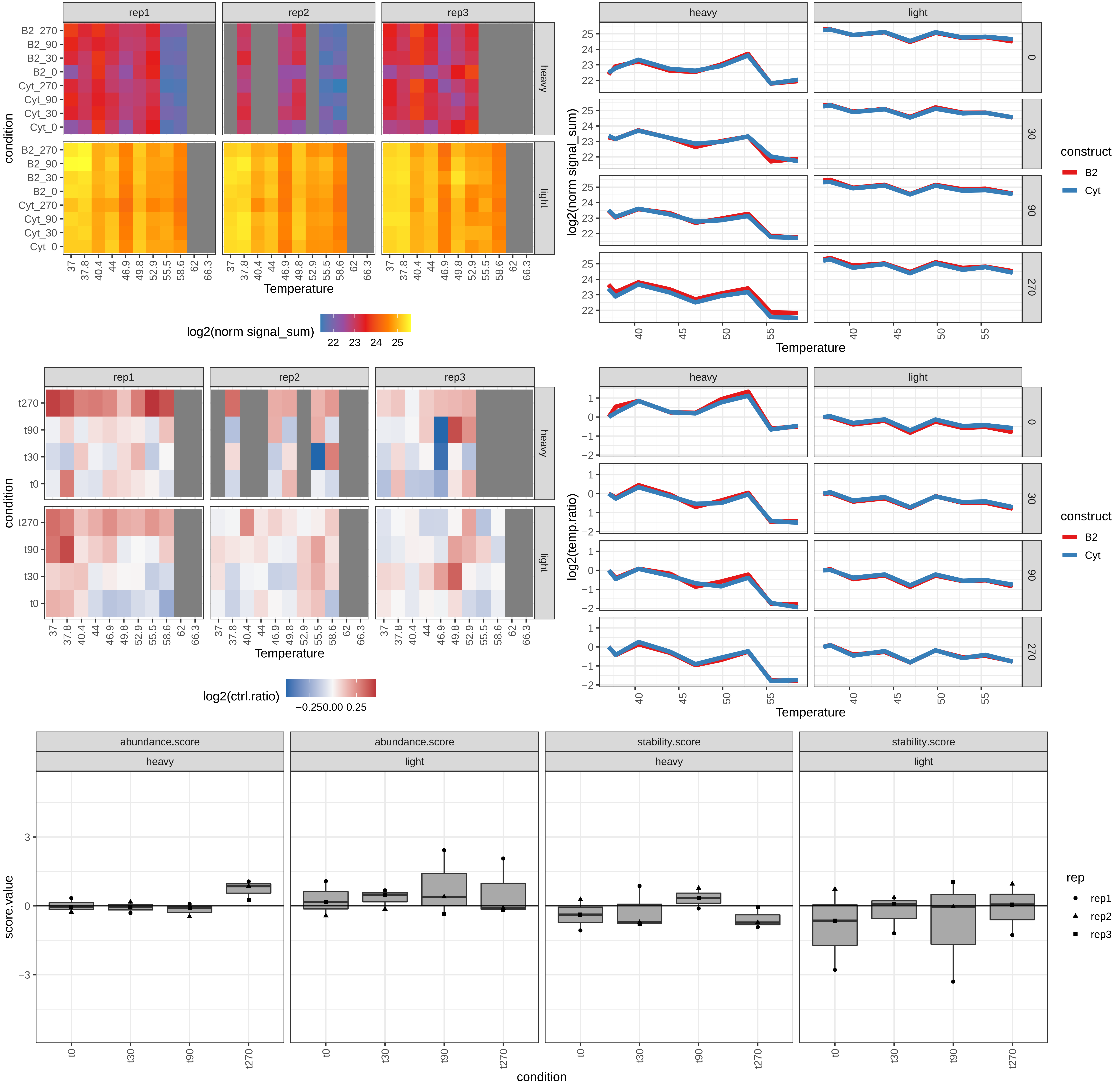

P36154 − Altered inheritance rate of mitochondria protein 29 OS=Saccharomyces cerevisiae (strain ATCC 204508 / S288c) GN=AIM29 PE=1 SV=1

AIM32 − average.top3: 6.78214909090909 − max.qupm: 7

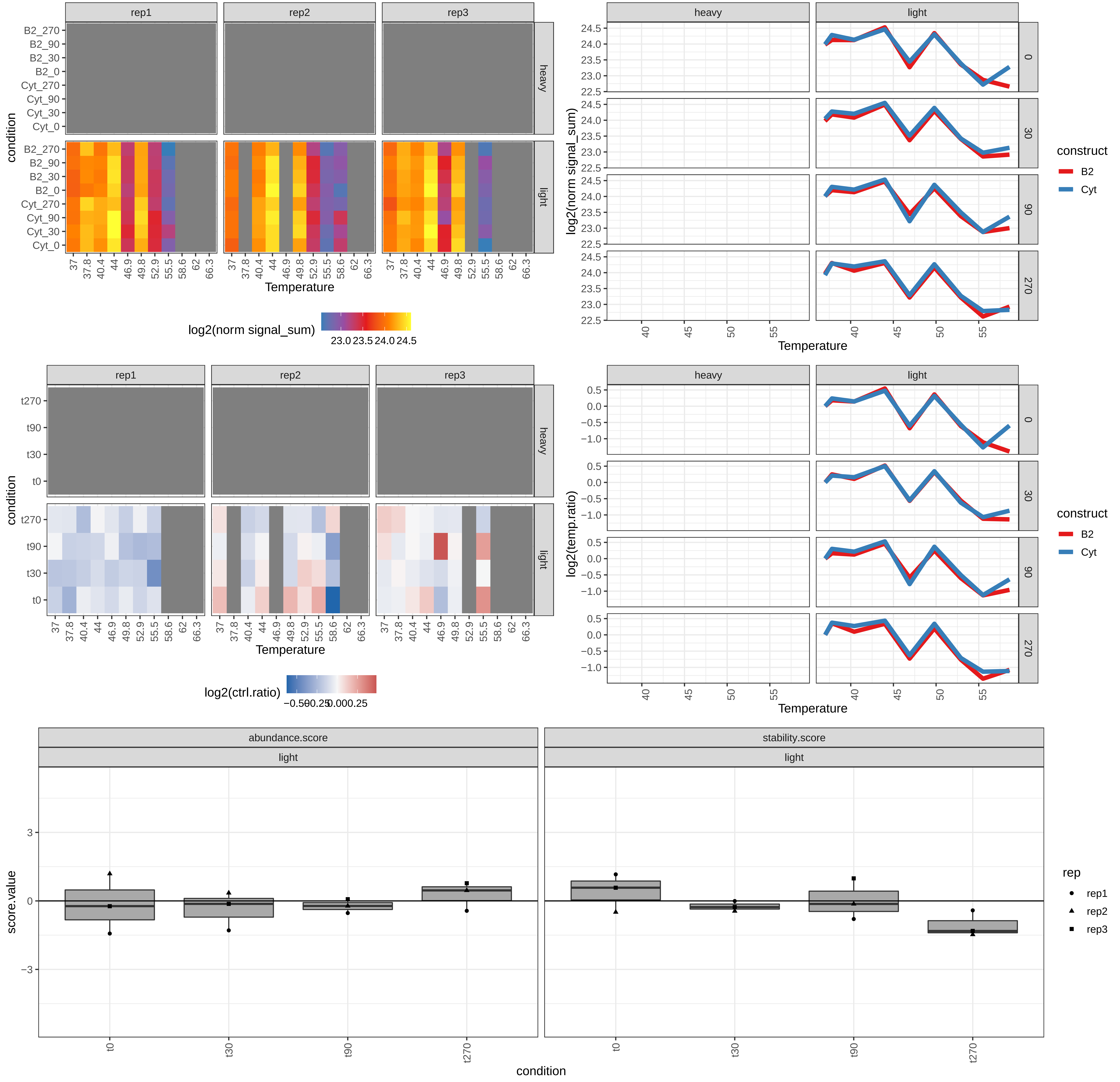

Q04689 − Altered inheritance of mitochondria protein 32 OS=Saccharomyces cerevisiae (strain ATCC 204508 / S288c) GN=AIM32 PE=3 SV=1

AIM36 − average.top3: 6.56257166666667 − max.qupm: 5

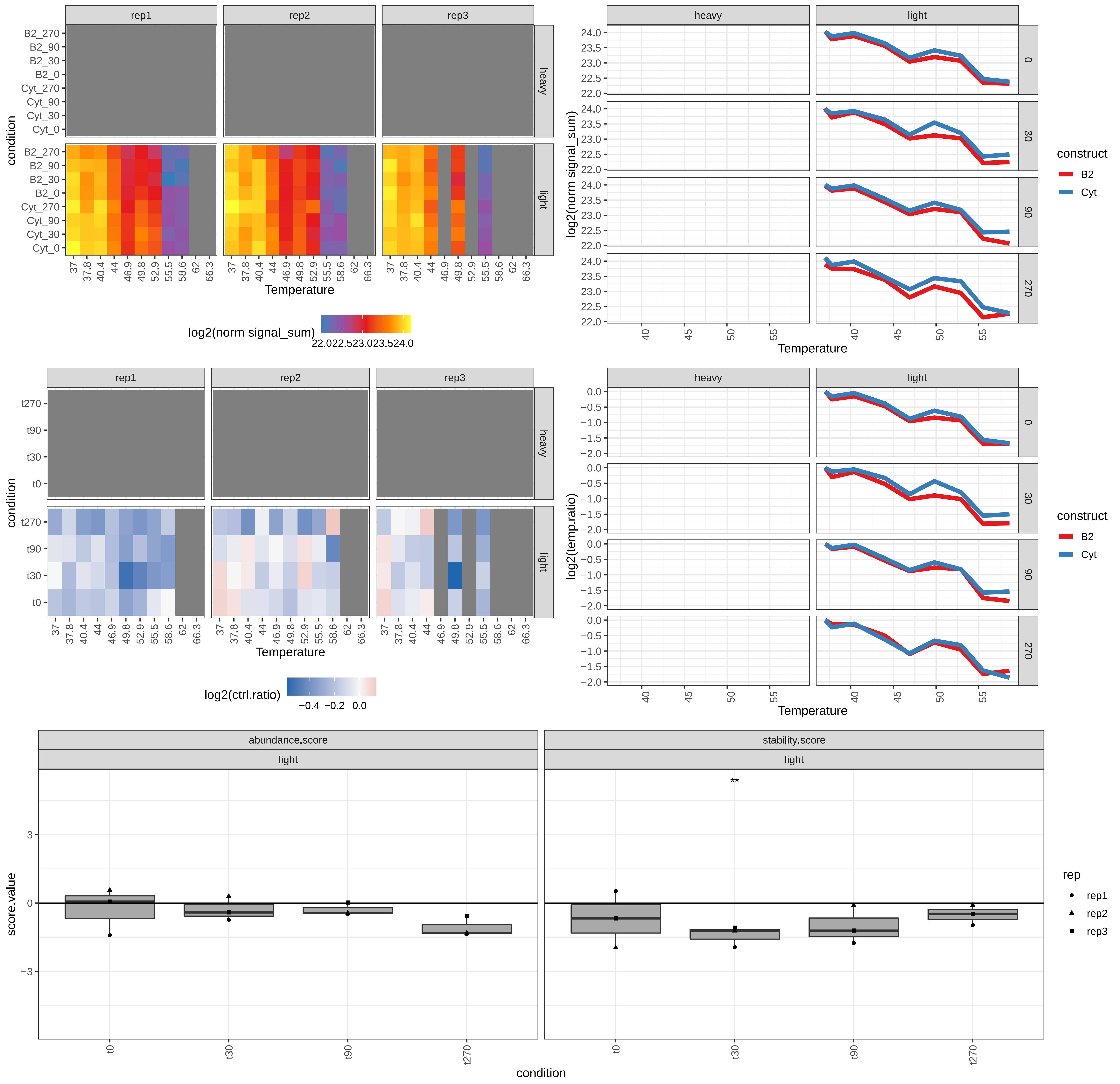

Q03798 − Altered inheritance of mitochondria protein 36, mitochondrial OS=Saccharomyces cerevisiae (strain ATCC 204508 / S288c) GN=AIM36 PE=1 SV=1

AIM39 − average.top3: 6.896028 − max.qupm: 6

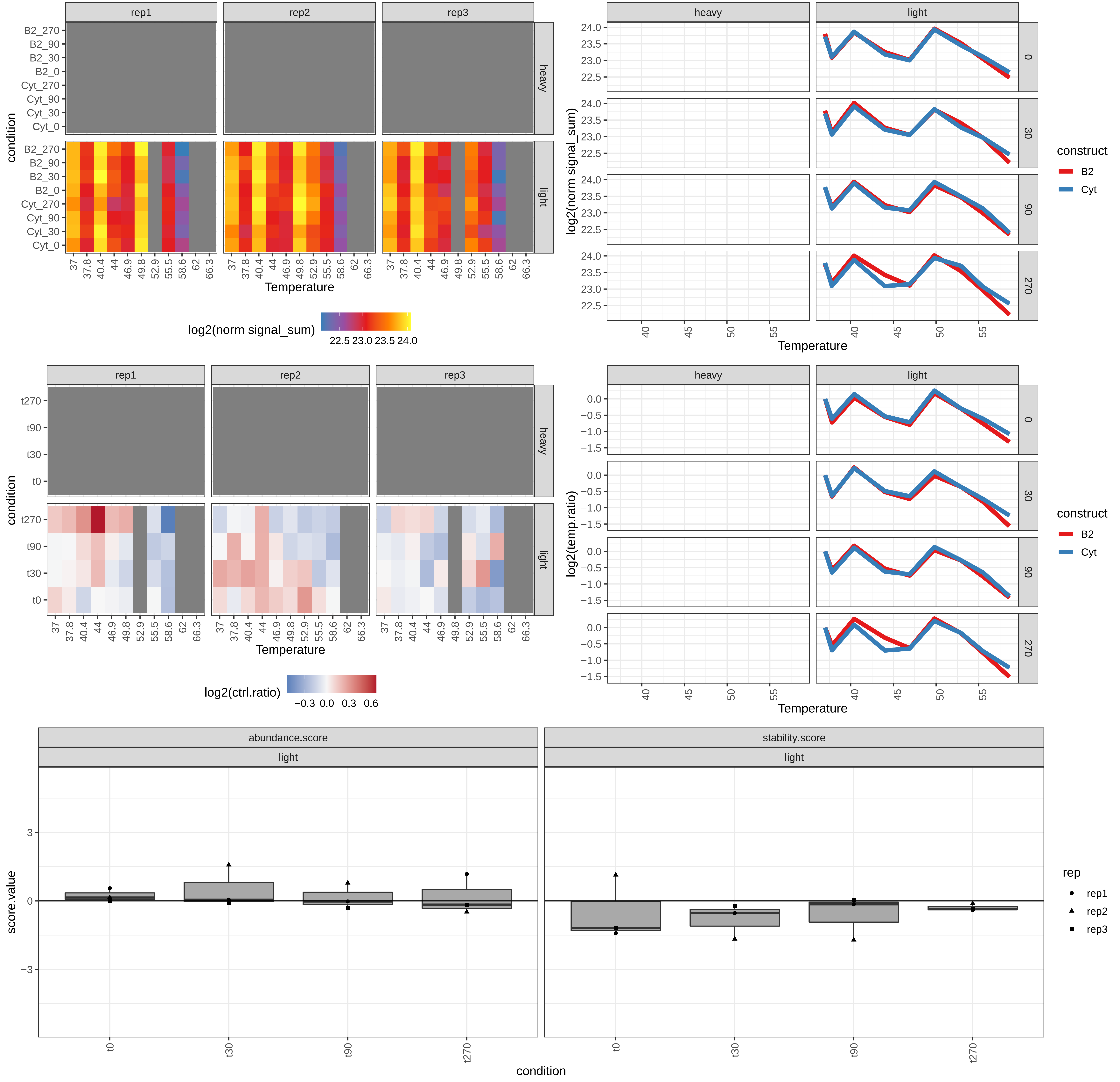

Q08223 − Altered inheritance of mitochondria protein 39, mitochondrial OS=Saccharomyces cerevisiae (strain ATCC 204508 / S288c) GN=AIM39 PE=3 SV=1

AIM41 − average.top3: 6.96095603448276 − max.qupm: 11

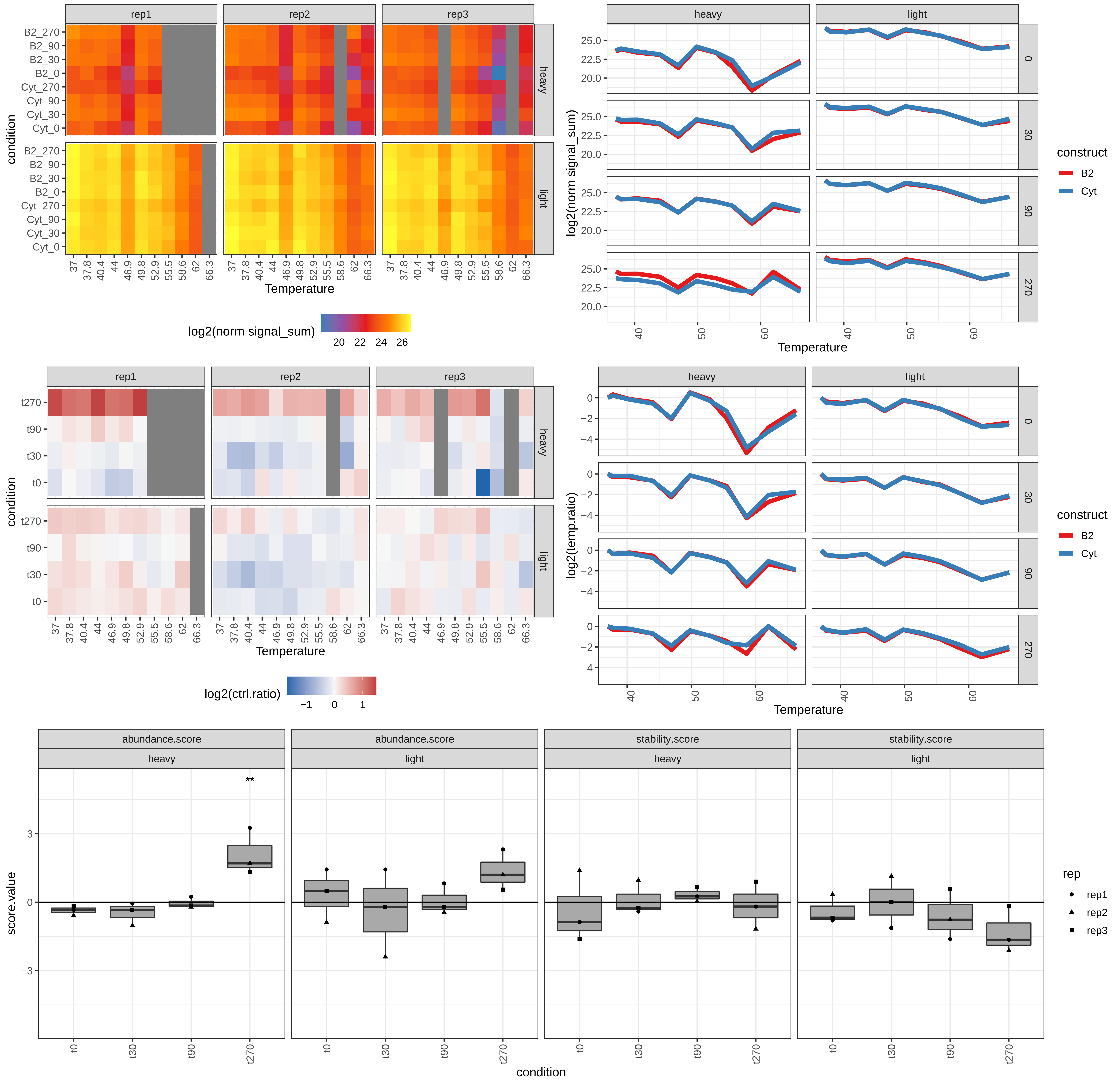

Q12032 − Altered inheritance of mitochondria protein 41, mitochondrial OS=Saccharomyces cerevisiae (strain ATCC 204508 / S288c) GN=AIM41 PE=1 SV=1

AIM45 − average.top3: 6.83325424242424 − max.qupm: 8

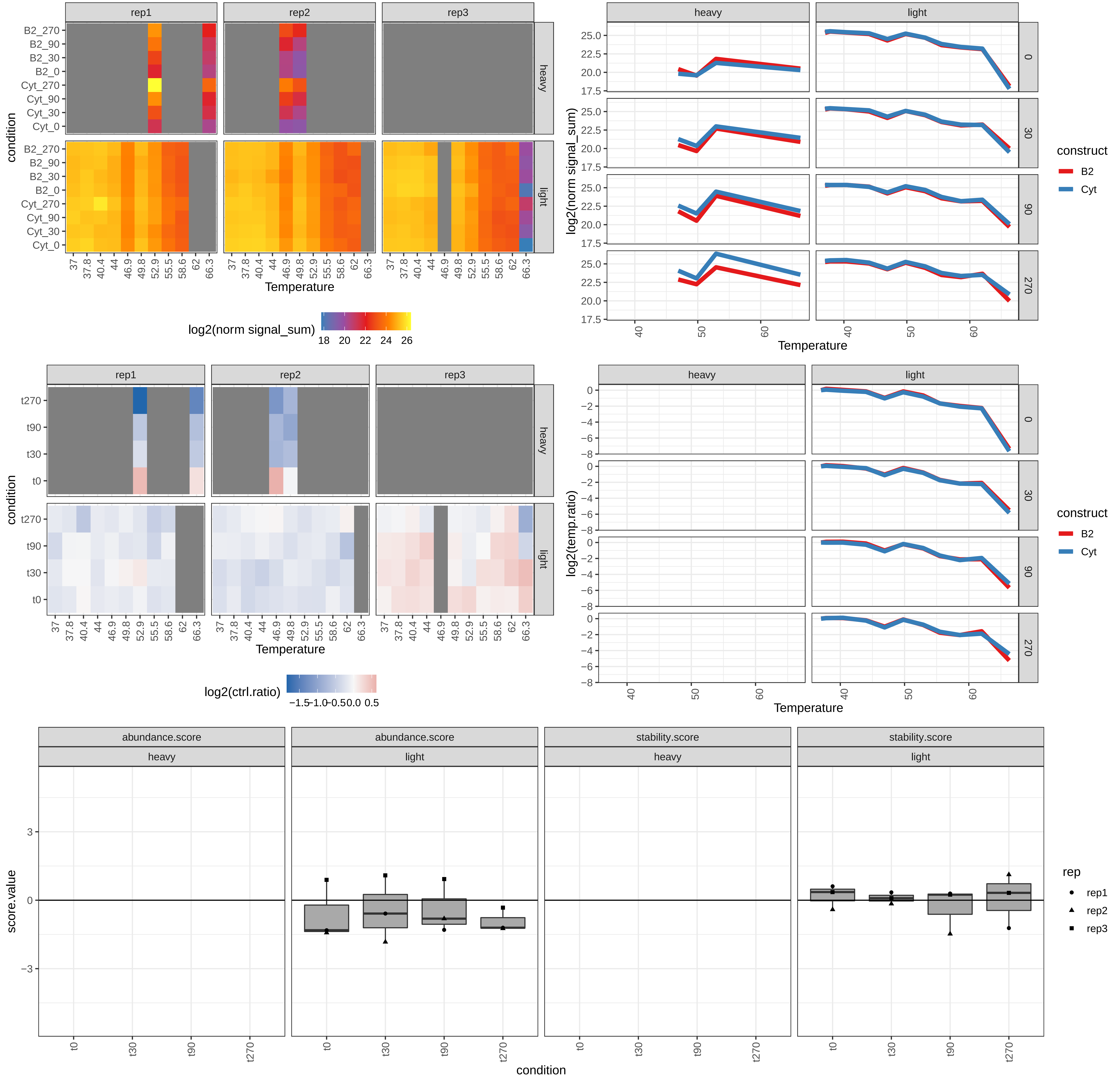

Q12480 − Probable electron transfer flavoprotein subunit alpha, mitochondrial OS=Saccharomyces cerevisiae (strain ATCC 204508 / S288c) GN=AIM45 PE=1 SV=1

AIM46 − average.top3: 6.8119 − max.qupm: 7

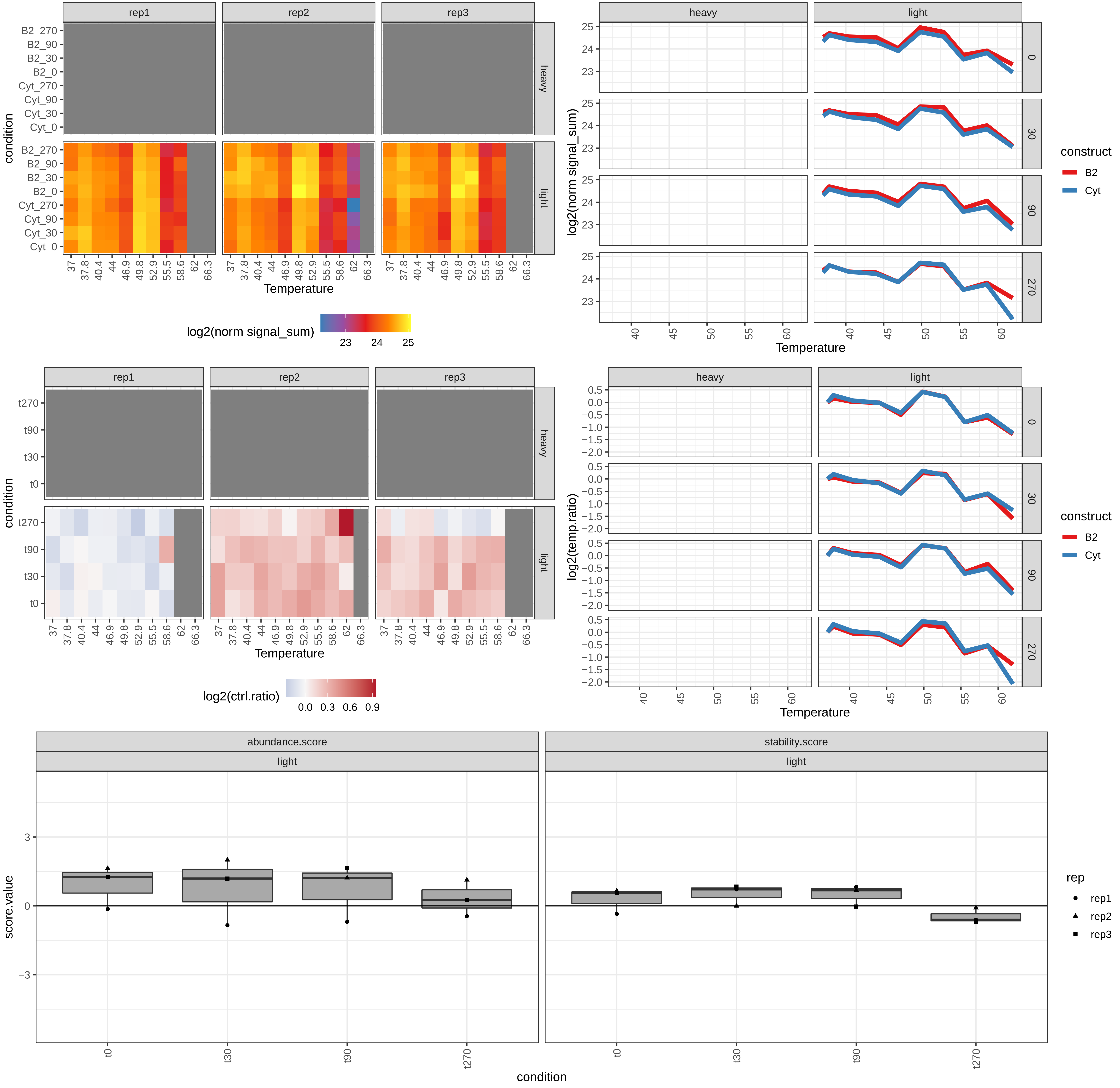

P38885 − Altered inheritance of mitochondria protein 46, mitochondrial OS=Saccharomyces cerevisiae (strain ATCC 204508 / S288c) GN=AIM46 PE=1 SV=1

AIM6 − average.top3: 7.02058280701754 − max.qupm: 8

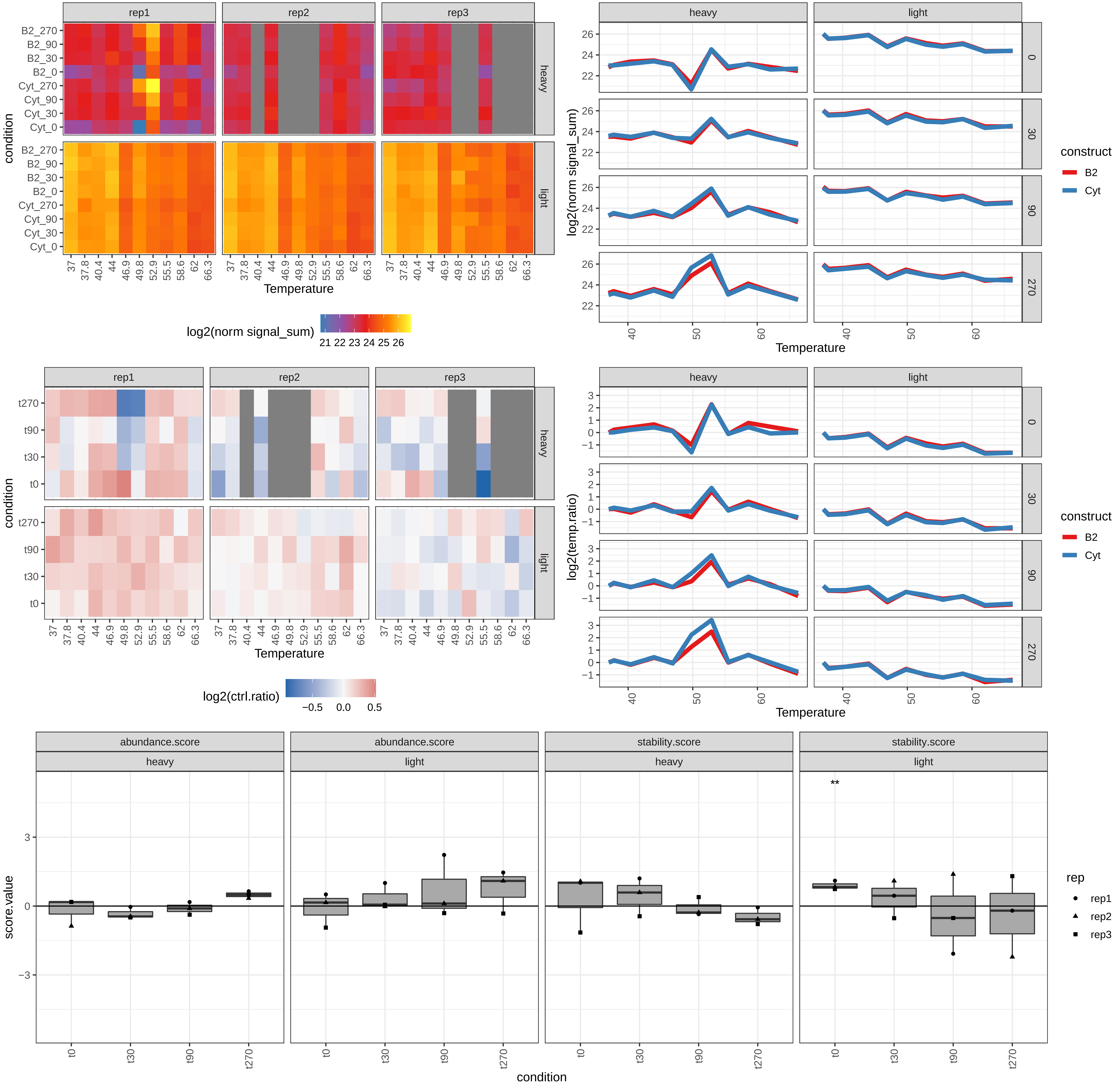

Q07716 − Altered inheritance of mitochondria protein 6 OS=Saccharomyces cerevisiae (strain ATCC 204508 / S288c) GN=AIM6 PE=3 SV=2

AIM9 − average.top3: 7.05131666666667 − max.qupm: 18

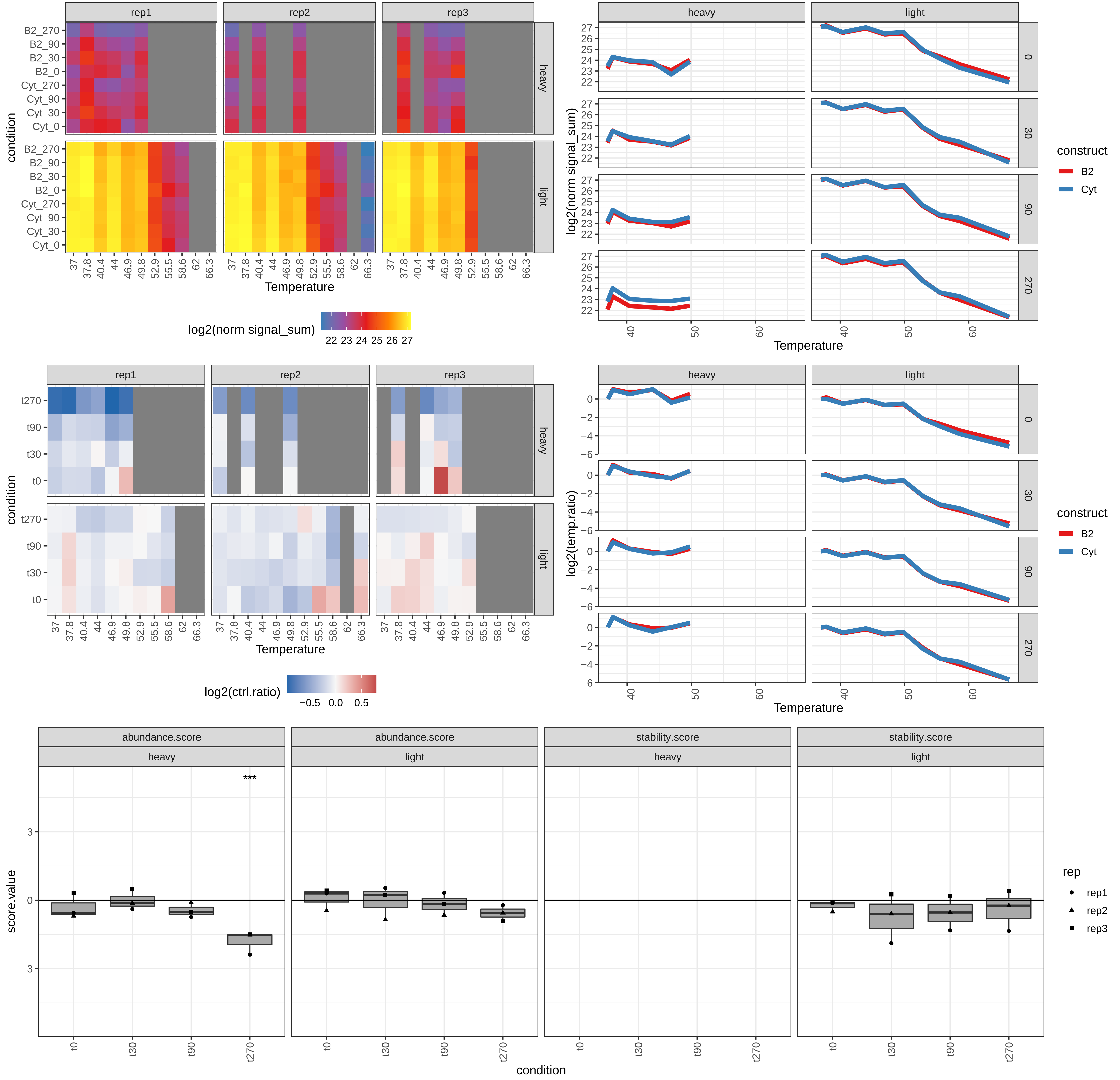

P40053 − Altered inheritance of mitochondria protein 9, mitochondrial OS=Saccharomyces cerevisiae (strain ATCC 204508 / S288c) GN=AIM9 PE=1 SV=1

AIP1 − average.top3: 7.41492178571429 − max.qupm: 16

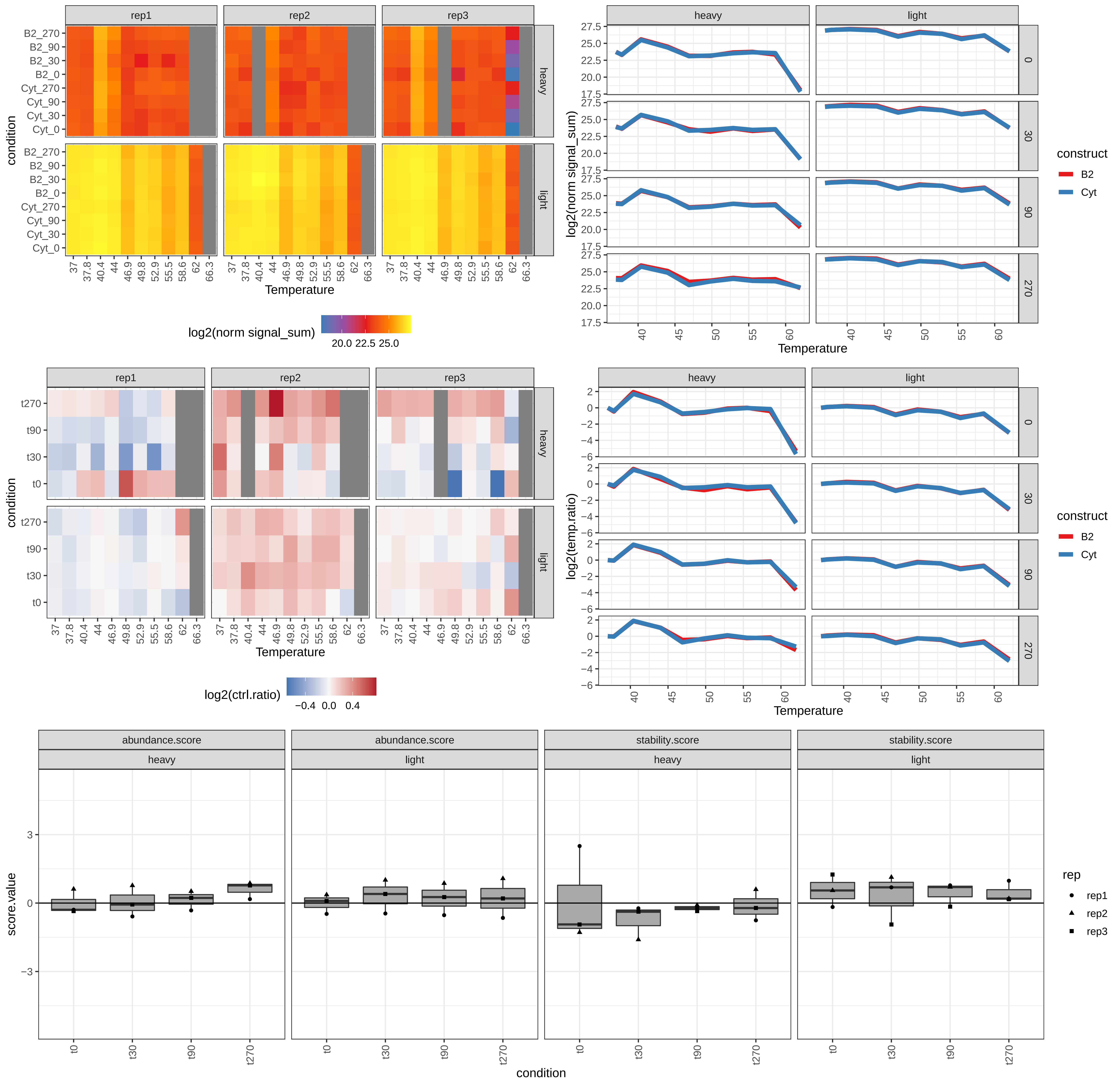

P46680 − Actin−interacting protein 1 OS=Saccharomyces cerevisiae (strain ATCC 204508 / S288c) GN=AIP1 PE=1 SV=1

AKL1 − average.top3: 6.95687692307692 − max.qupm: 9

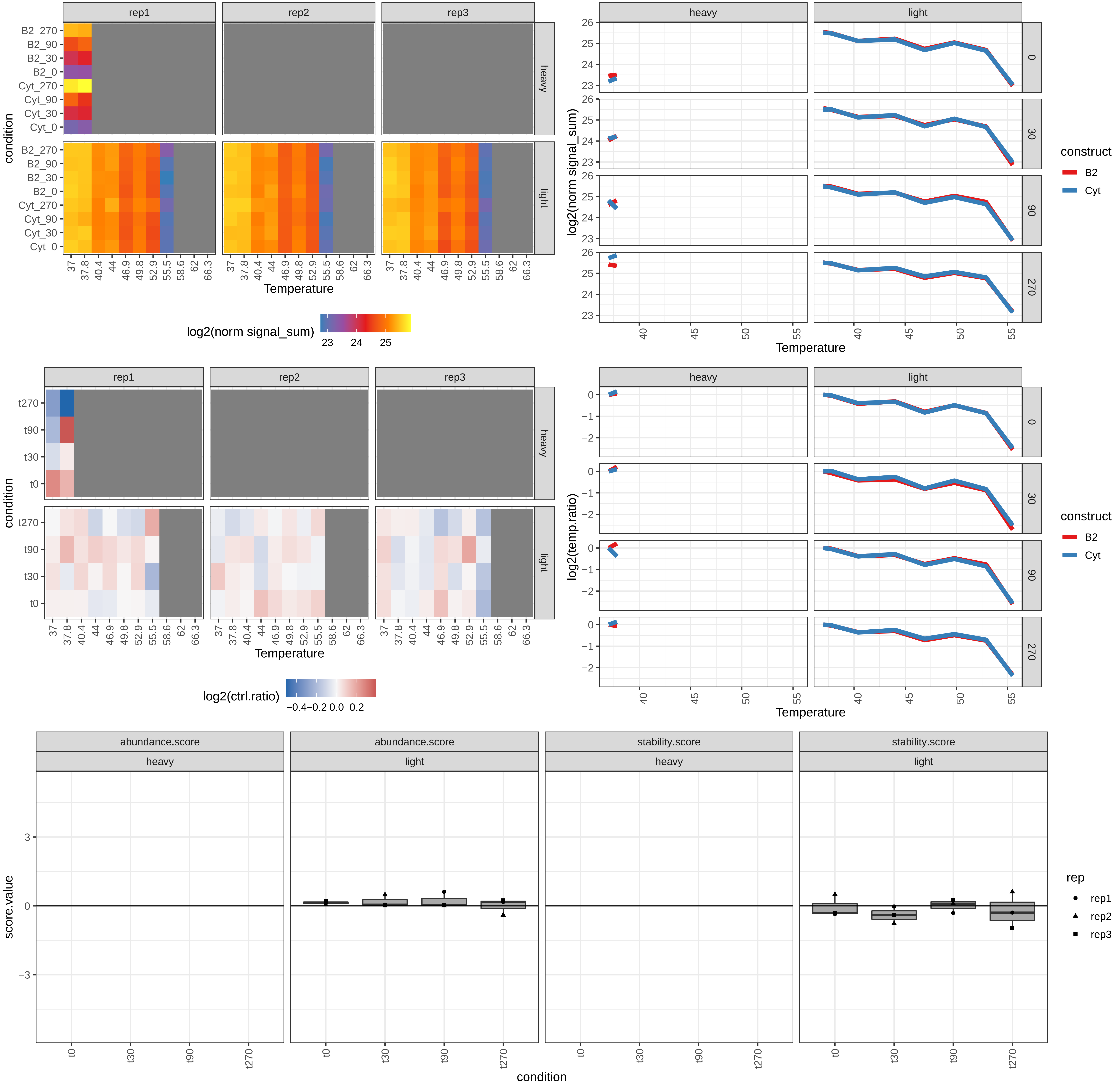

P38080 − Serine/threonine−protein kinase AKL1 OS=Saccharomyces cerevisiae (strain ATCC 204508 / S288c) GN=AKL1 PE=1 SV=1

AKR1 − average.top3: 6.88653958333333 − max.qupm: 7

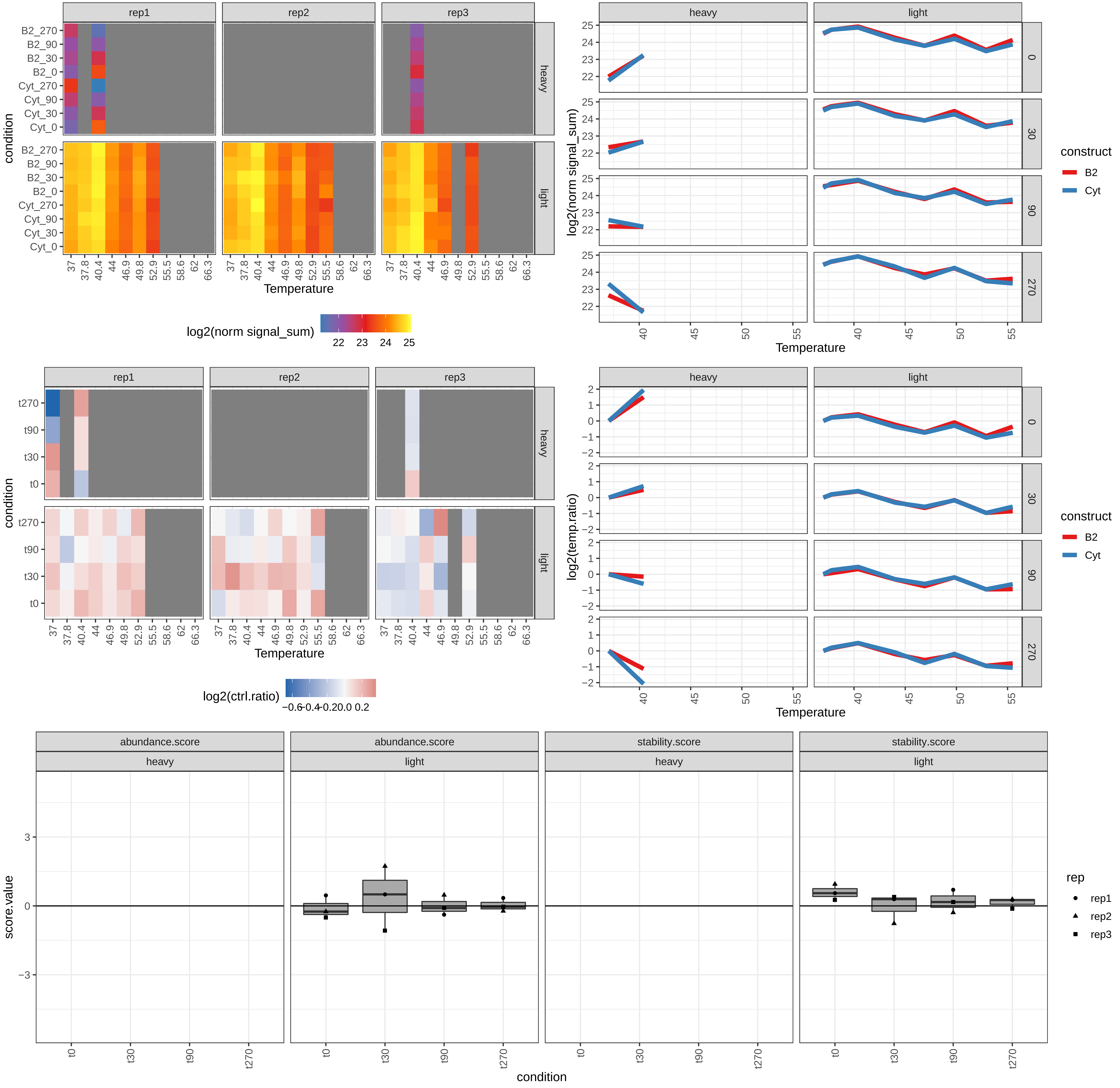

P39010 − Palmitoyltransferase AKR1 OS=Saccharomyces cerevisiae (strain ATCC 204508 / S288c) GN=AKR1 PE=1 SV=1

ALA1 − average.top3: 7.47279346153846 − max.qupm: 48

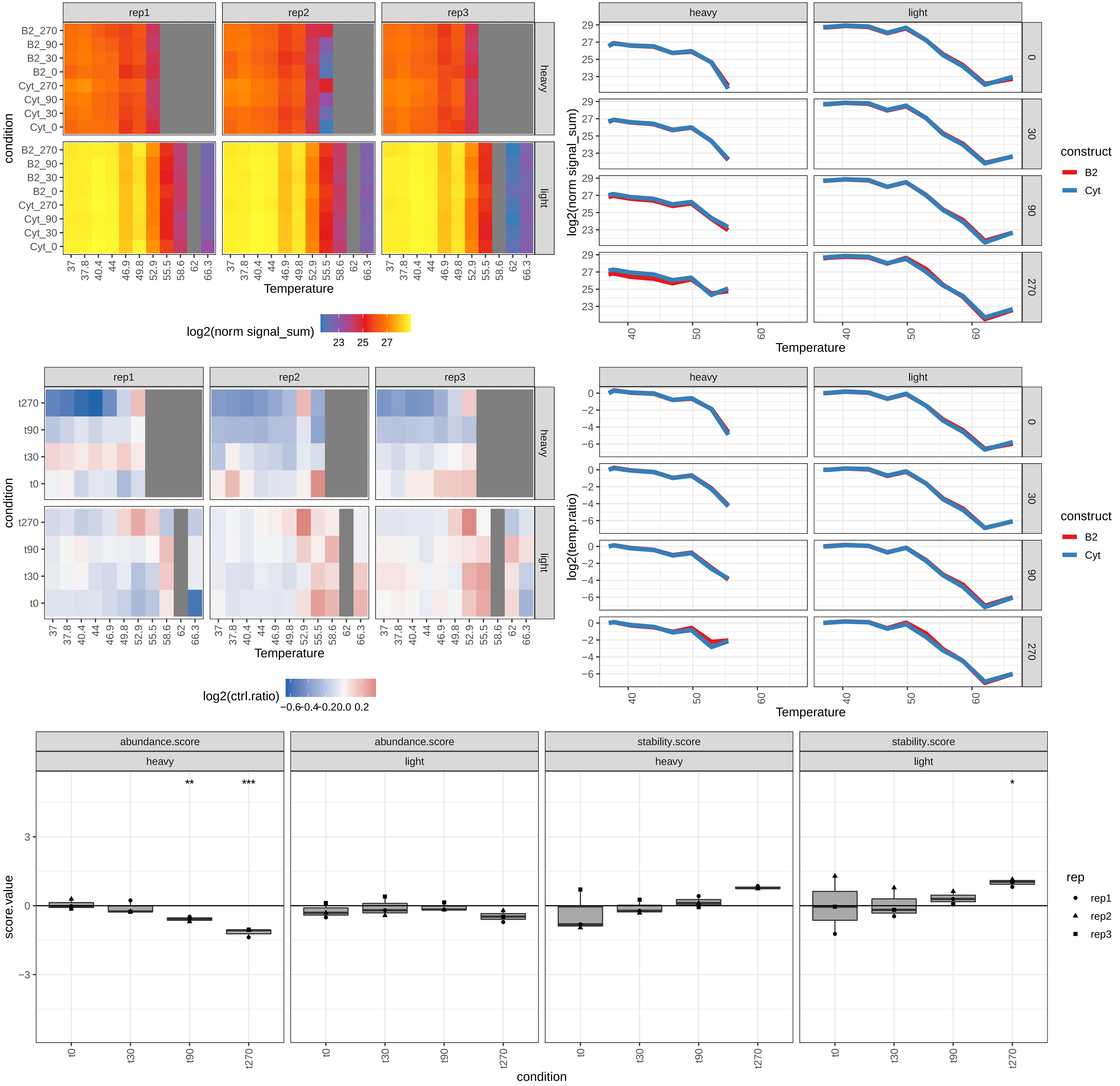

ligase, mitochondrial OS=Saccharomyces cerevisiae (strain ATCC 204508 / S288c) GN=ALA1 PE=1 SV=3|Isoform Cytoplasmic of Alanine−−tRNA ligase, mitochondrial OS=Saccharomyces cerevisiae (str

ALD2 − average.top3: 6.88584595744681 − max.qupm: 10

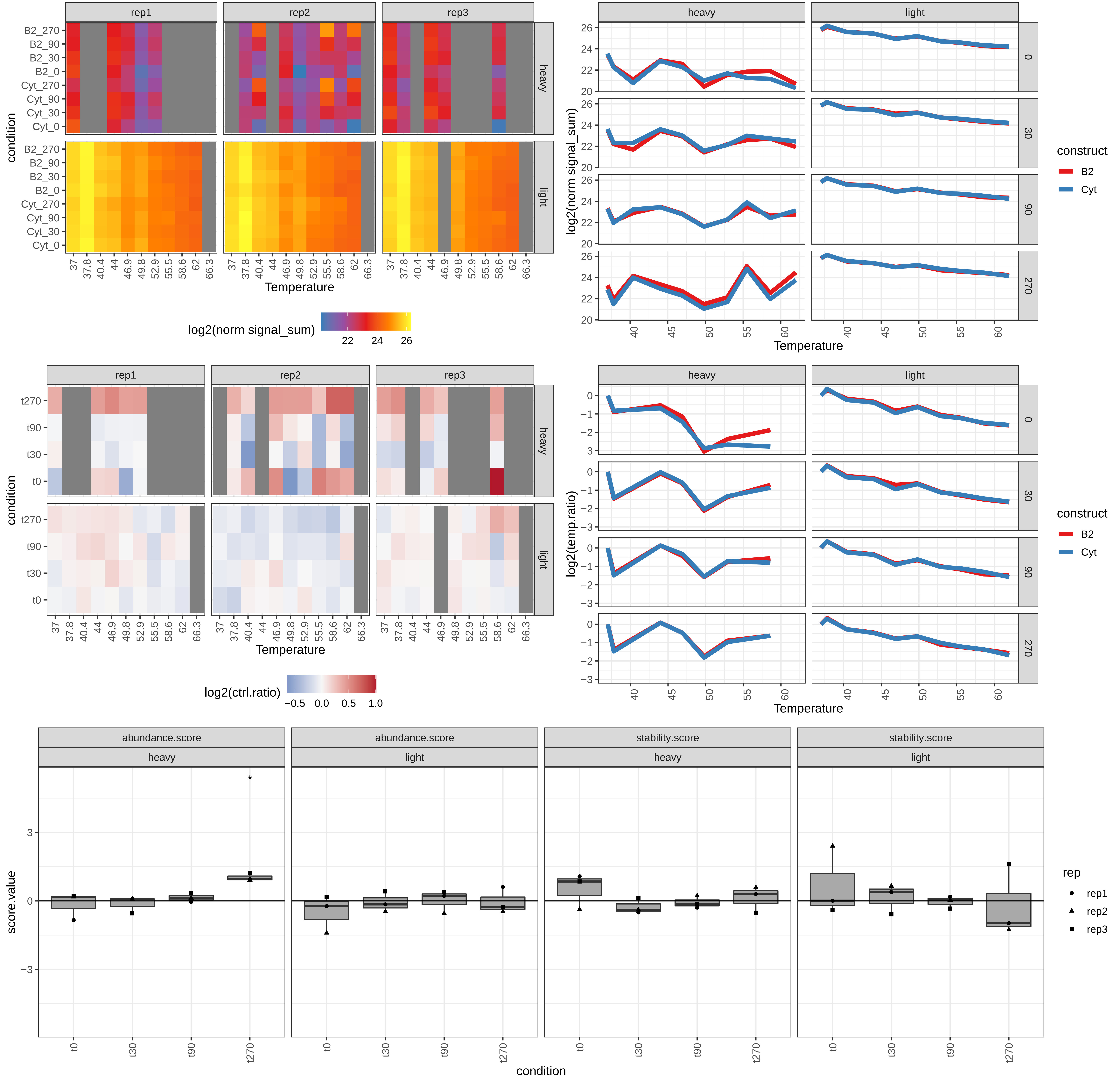

P47771 − Aldehyde dehydrogenase [NAD(P)+] 1 OS=Saccharomyces cerevisiae (strain ATCC 204508 / S288c) GN=ALD2 PE=1 SV=2

ALD3 − average.top3: 6.34964636363636 − max.qupm: 4

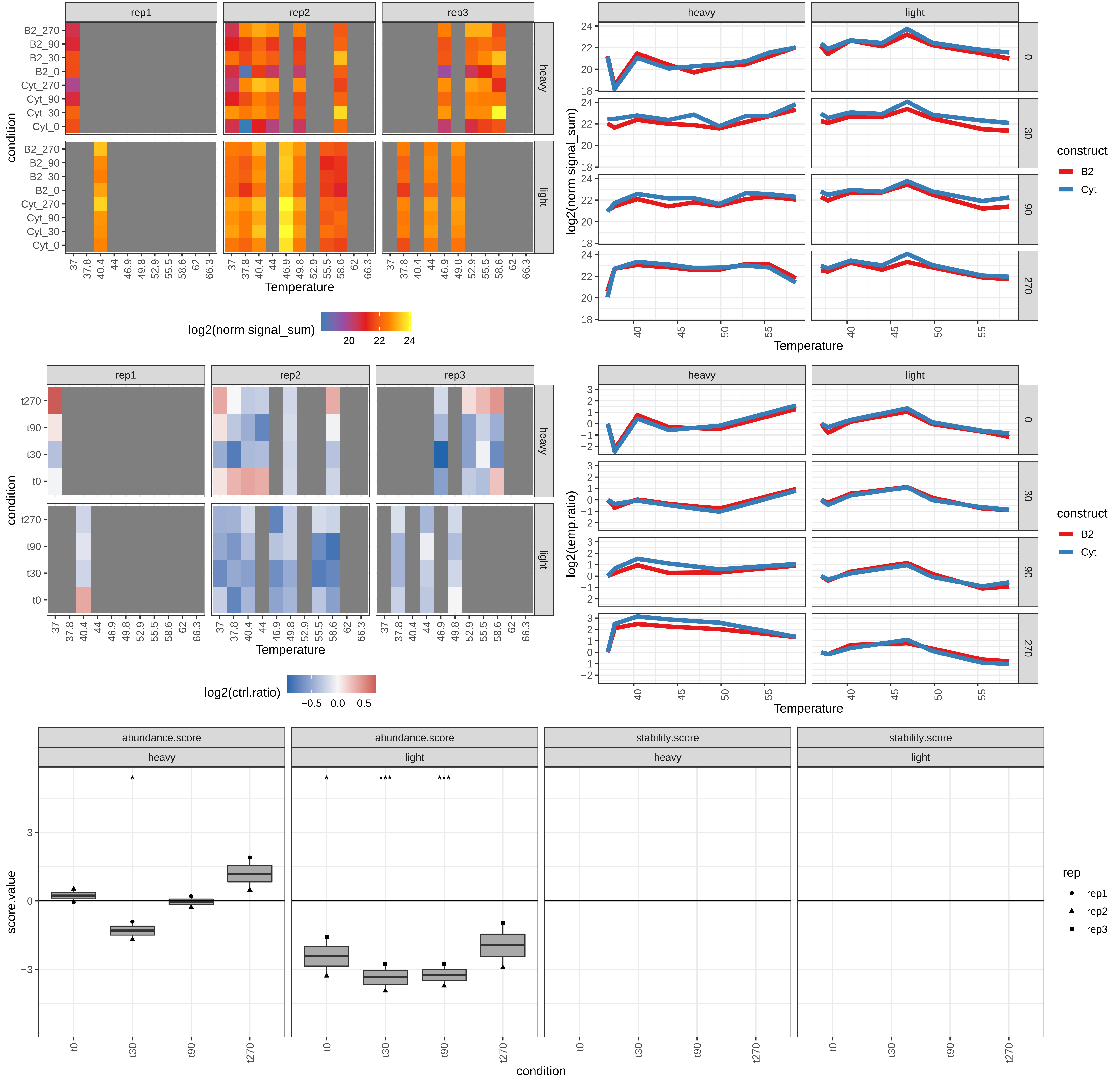

P54114 − Aldehyde dehydrogenase [NAD(P)+] 2 OS=Saccharomyces cerevisiae (strain ATCC 204508 / S288c) GN=ALD3 PE=1 SV=1

ALD4 − average.top3: 8.445740454545 − max.qupm: 26

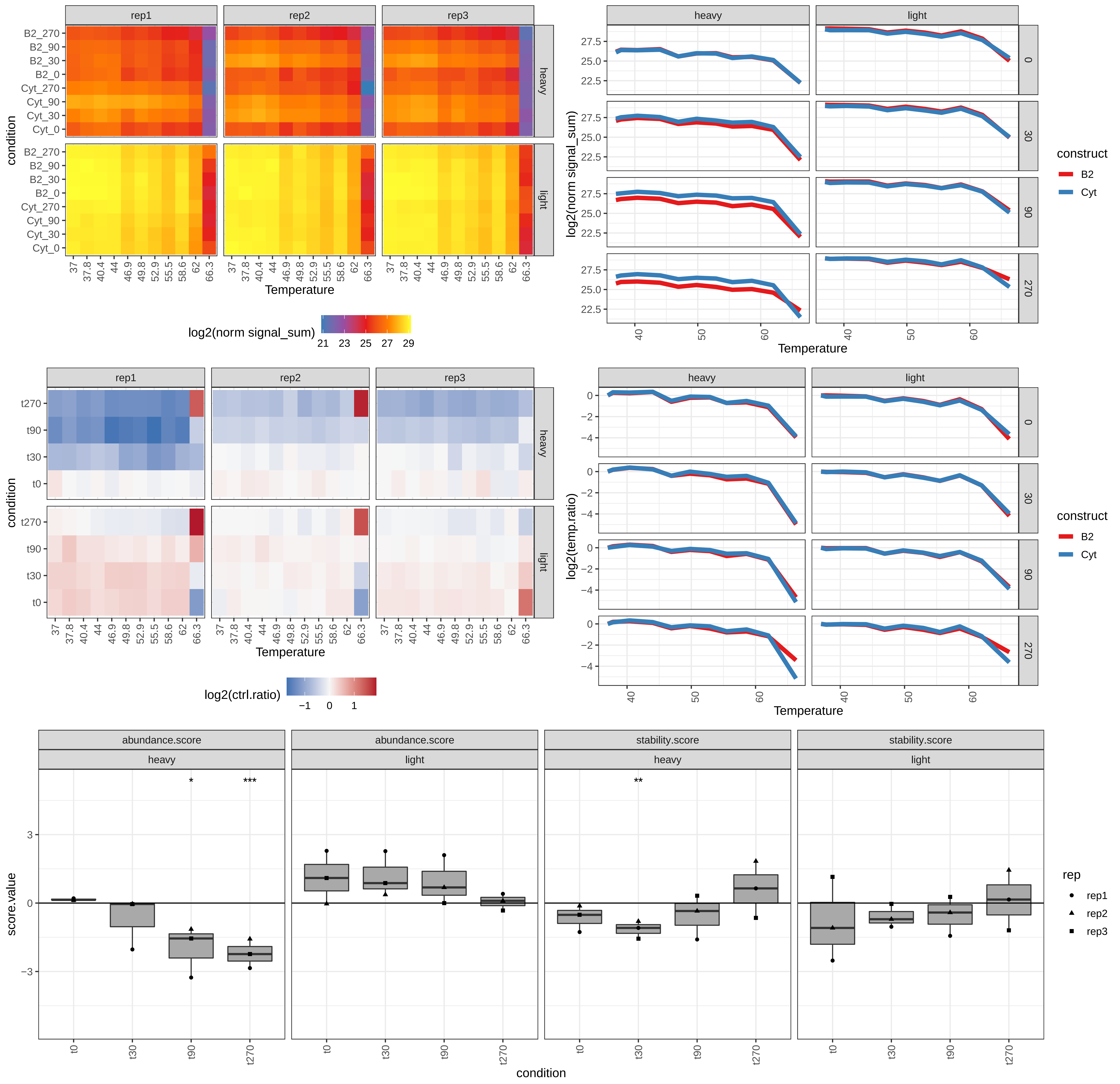

P46367 − Potassium−activated aldehyde dehydrogenase, mitochondrial OS=Saccharomyces cerevisiae (strain ATCC 204508 / S288c) GN=ALD4 PE=1 SV=2

ALD5 − average.top3: 7.15192593220339 − max.qupm: 15

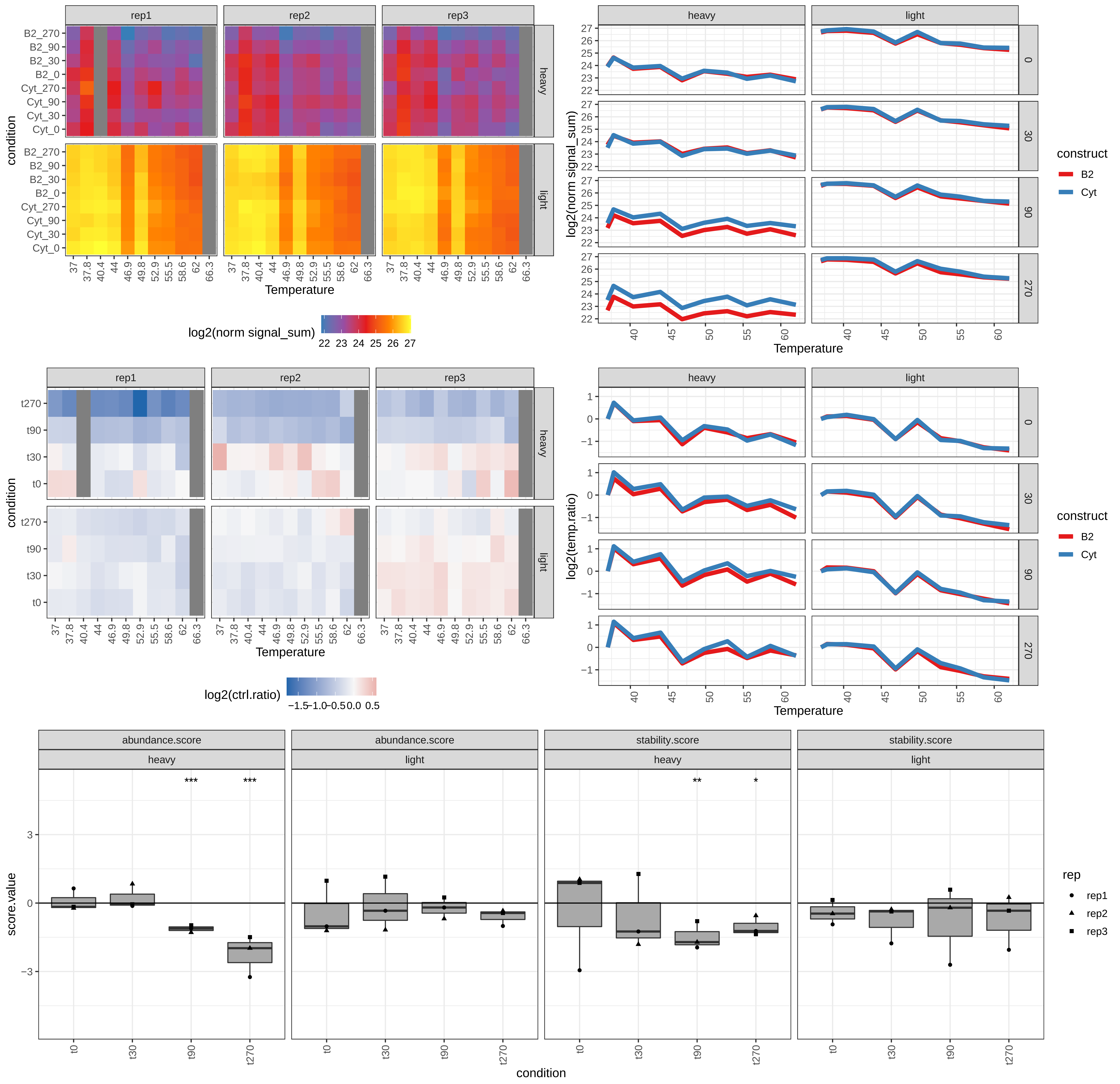

P40047 − Aldehyde dehydrogenase 5, mitochondrial OS=Saccharomyces cerevisiae (strain ATCC 204508 / S288c) GN=ALD5 PE=1 SV=4

ALD6 − average.top3: 8.12980365079365 − max.qupm: 26

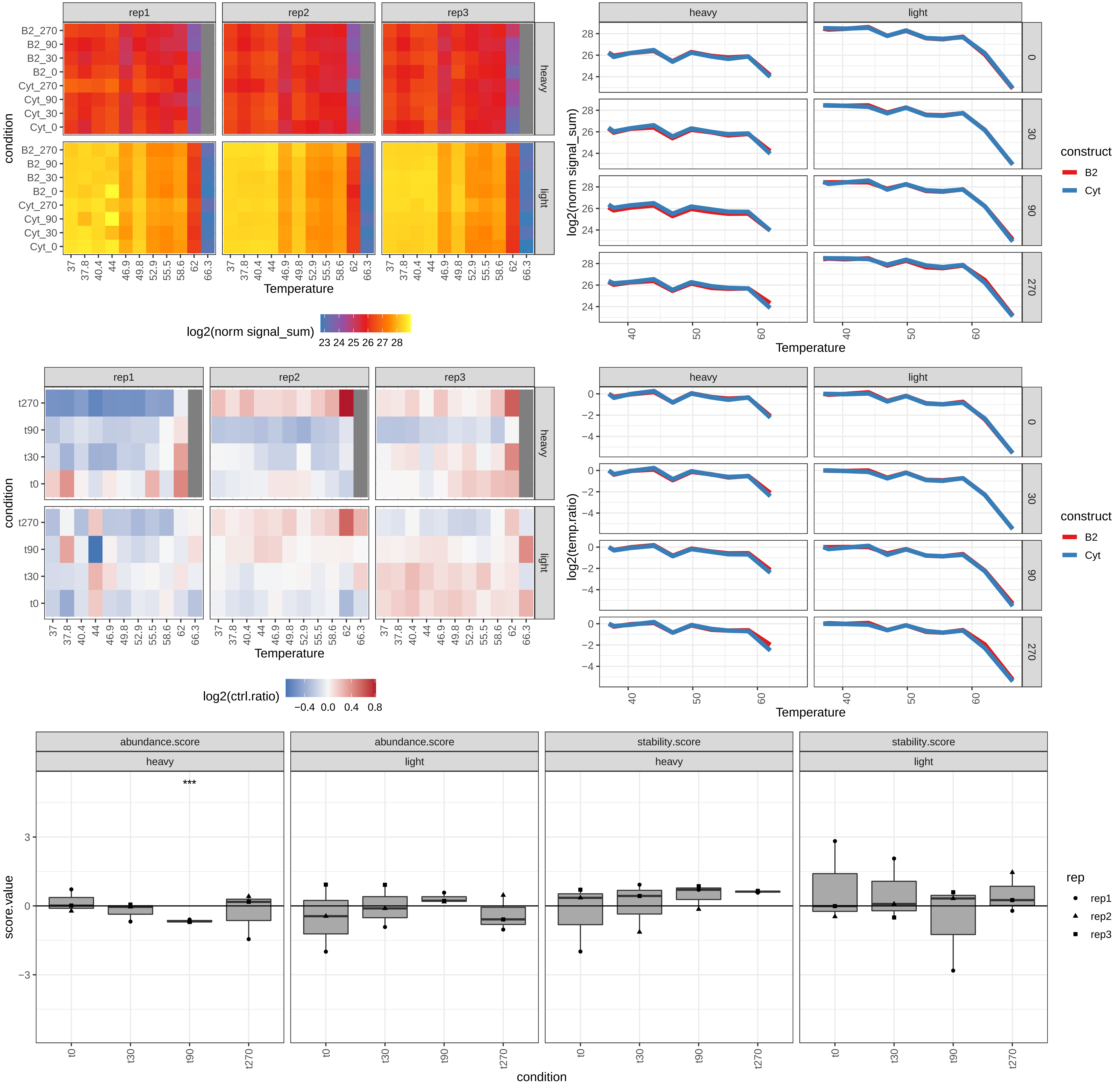

P54115 − Magnesium−activated aldehyde dehydrogenase, cytosolic OS=Saccharomyces cerevisiae (strain ATCC 204508 / S288c) GN=ALD6 PE=1 SV=4

ALE1 − average.top3: 6.8682430952381 − max.qupm: 10

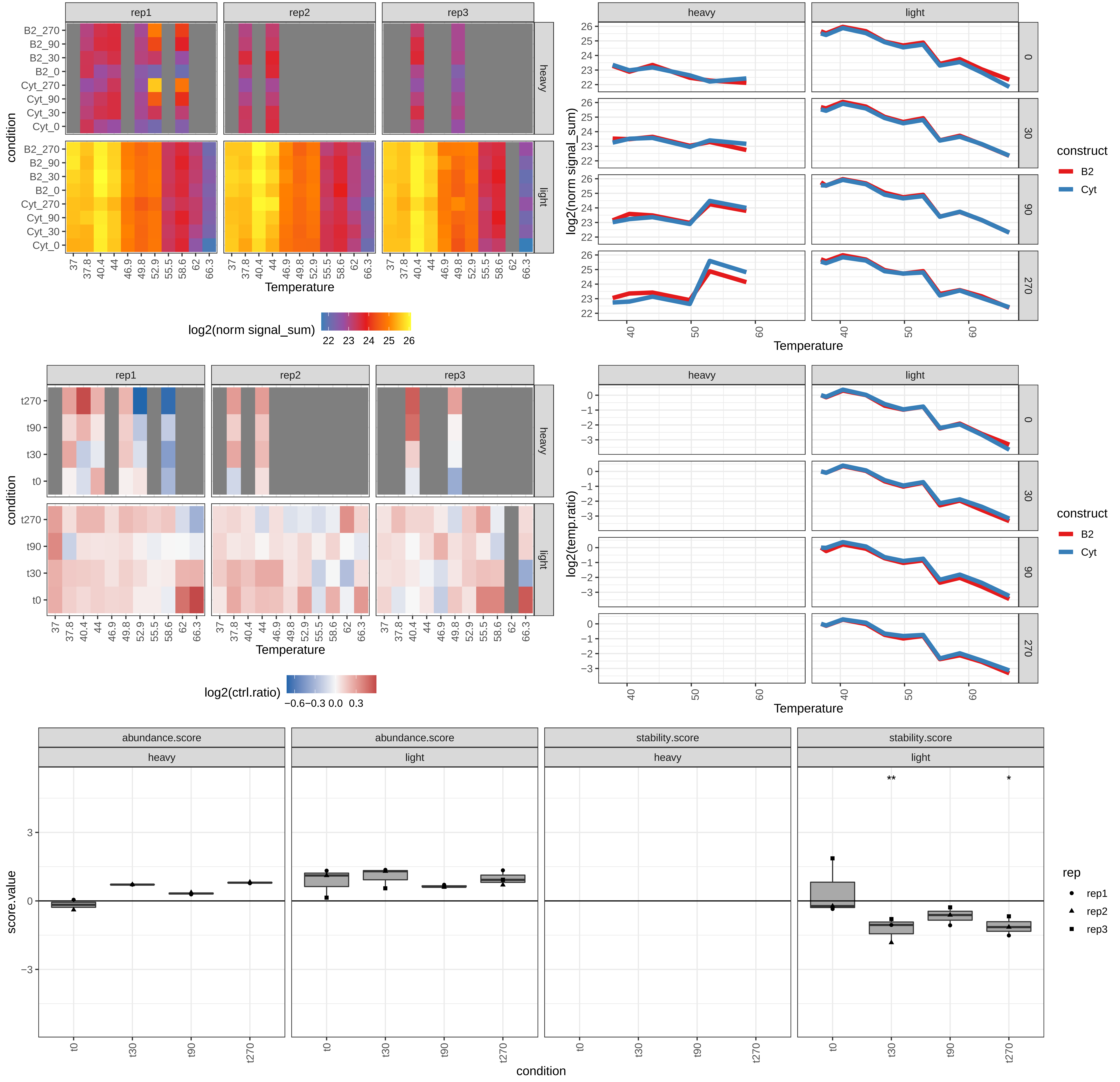

Q08548 − Lysophospholipid acyltransferase OS=Saccharomyces cerevisiae (strain ATCC 204508 / S288c) GN=ALE1 PE=1 SV=1

ALG1 − average.top3: 7.14918555555556 − max.qupm: 7

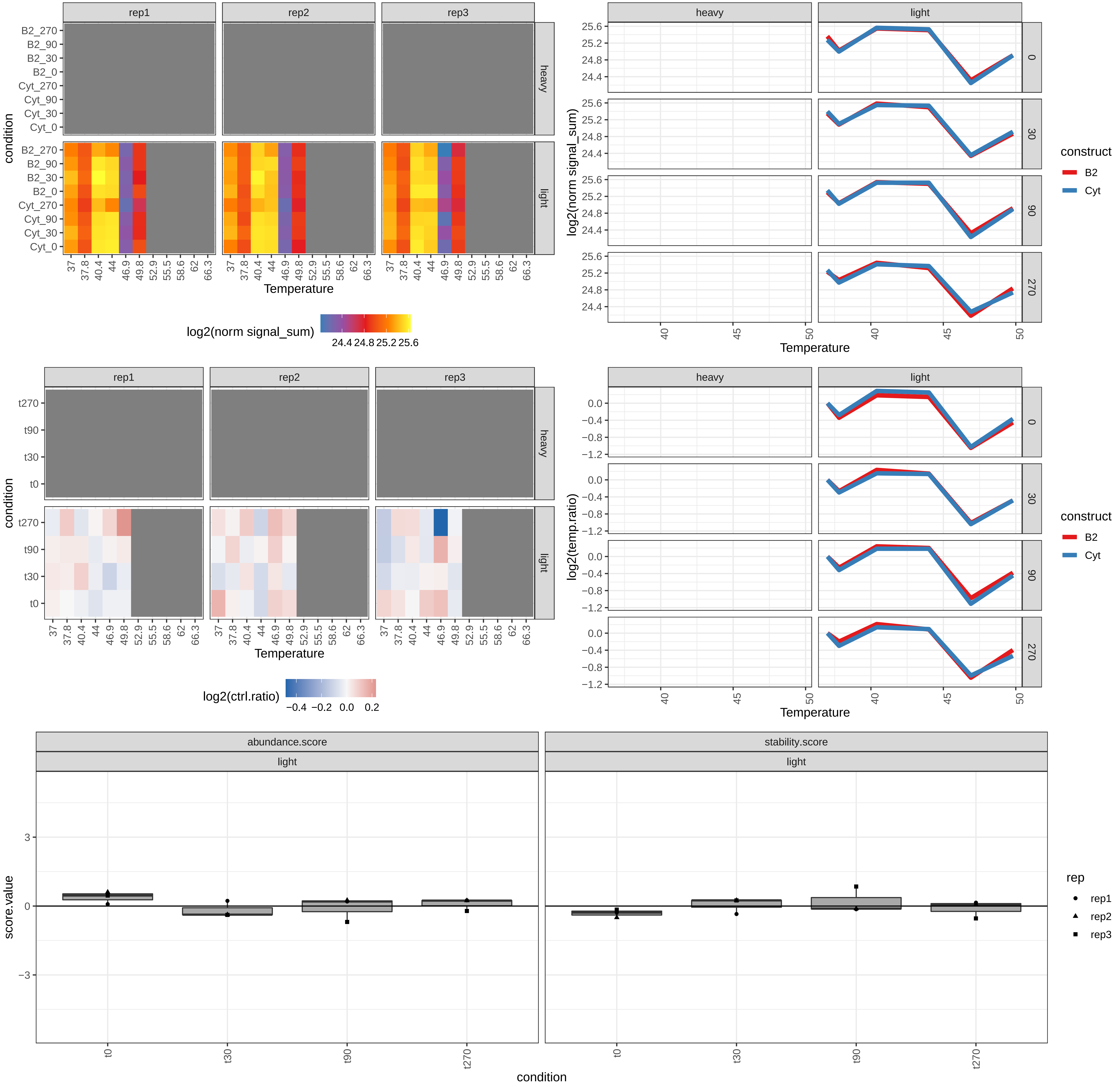

P16661 − Chitobiosyldiphosphodolichol beta−mannosyltransferase OS=Saccharomyces cerevisiae (strain ATCC 204508 / S288c) GN=ALG1 PE=1 SV=1

ALG11 − average.top3: 6.90612730769231 − max.qupm: 13

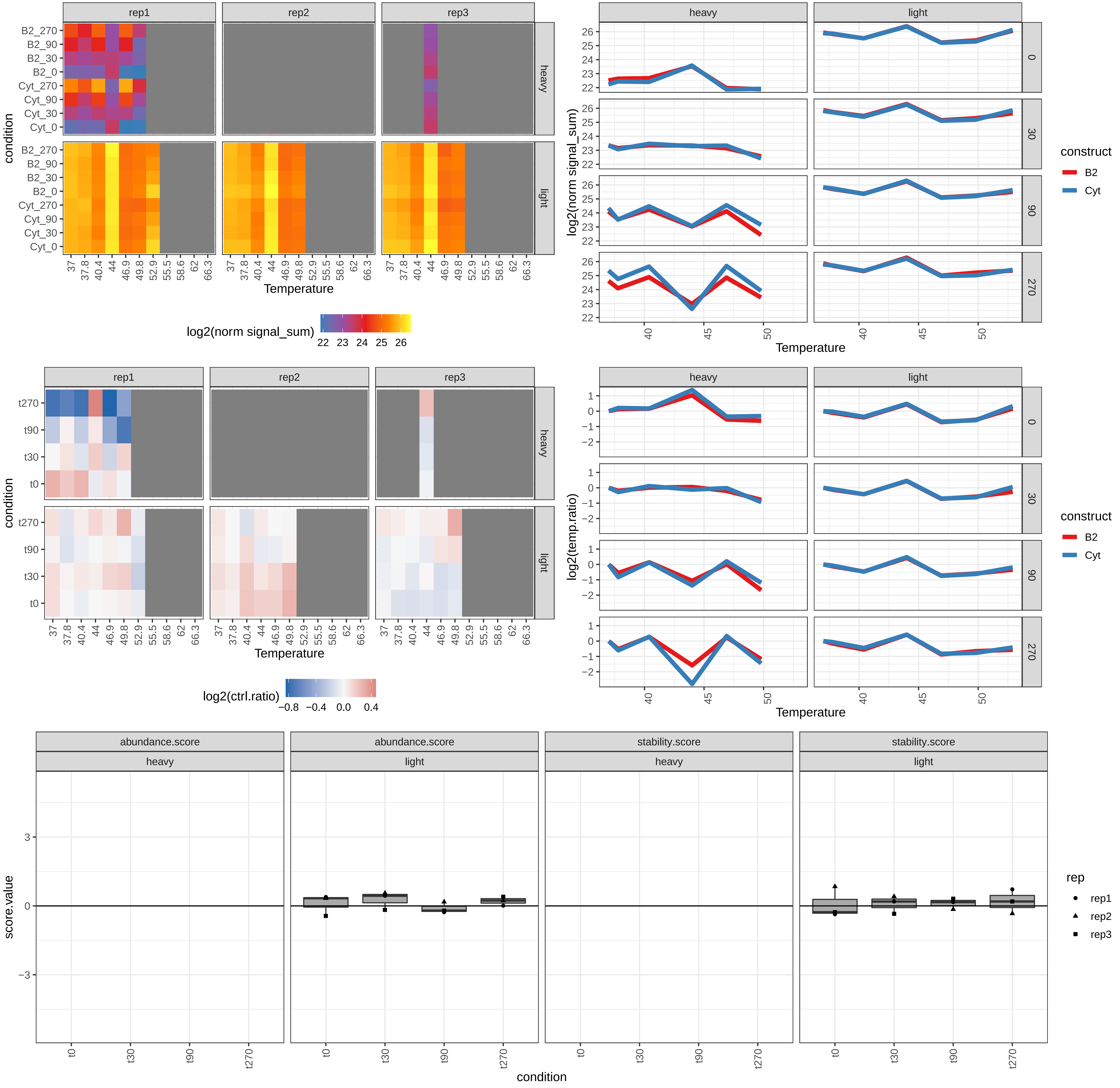

P53954 − GDP−Man:Man(3)GlcNAc(2)−PP−Dol alpha−1,2−mannosyltransferase OS=Saccharomyces cerevisiae (strain ATCC 204508 / S288c) GN=ALG11 PE=1 SV=1

ALG12 − average.top3: 6.55959277777778 − max.qupm: 5

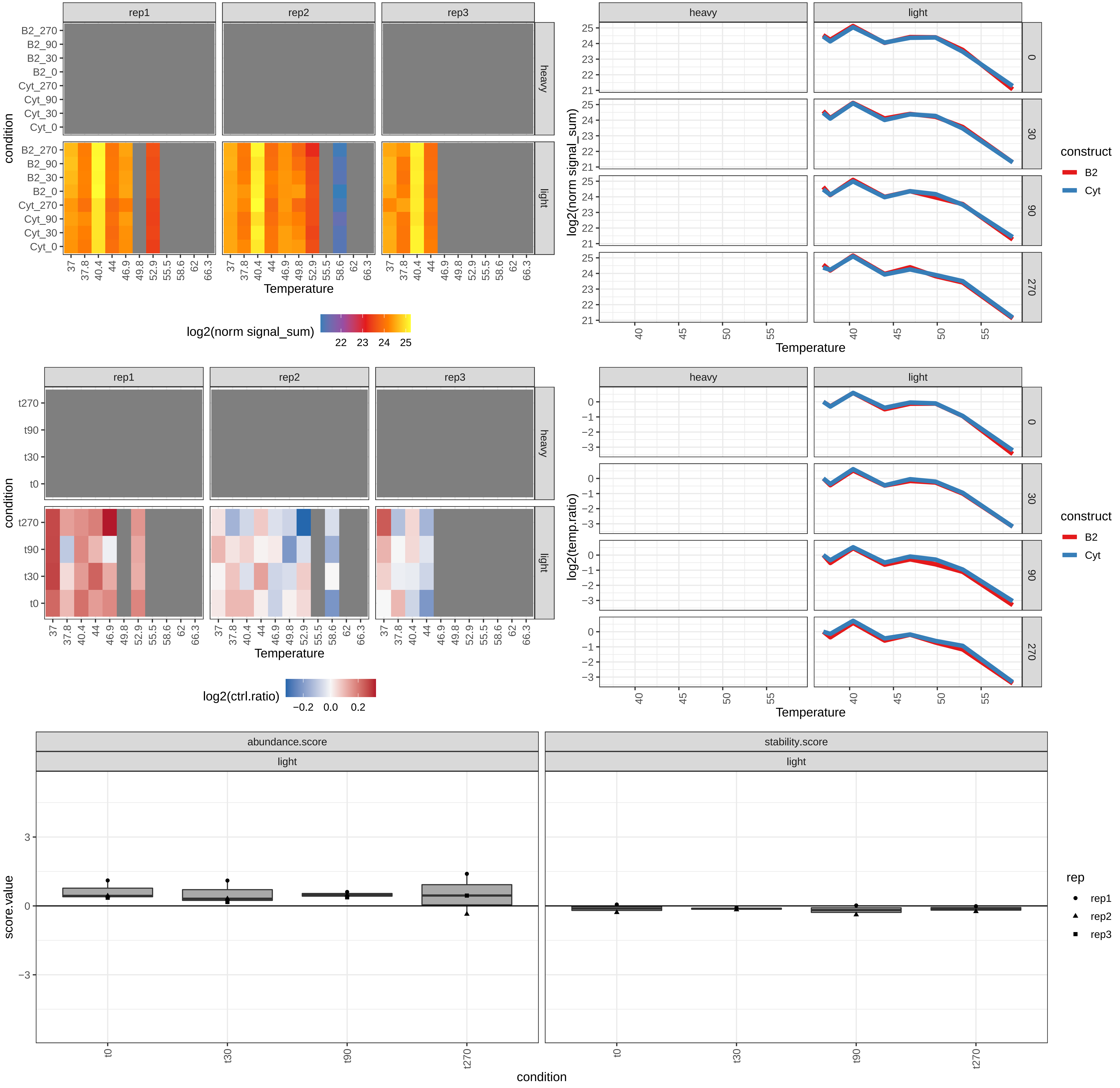

P53730 − Dol−P−Man:Man(7)GlcNAc(2)−PP−Dol alpha−1,6−mannosyltransferase OS=Saccharomyces cerevisiae (strain ATCC 204508 / S288c) GN=ALG12 PE=1 SV=1

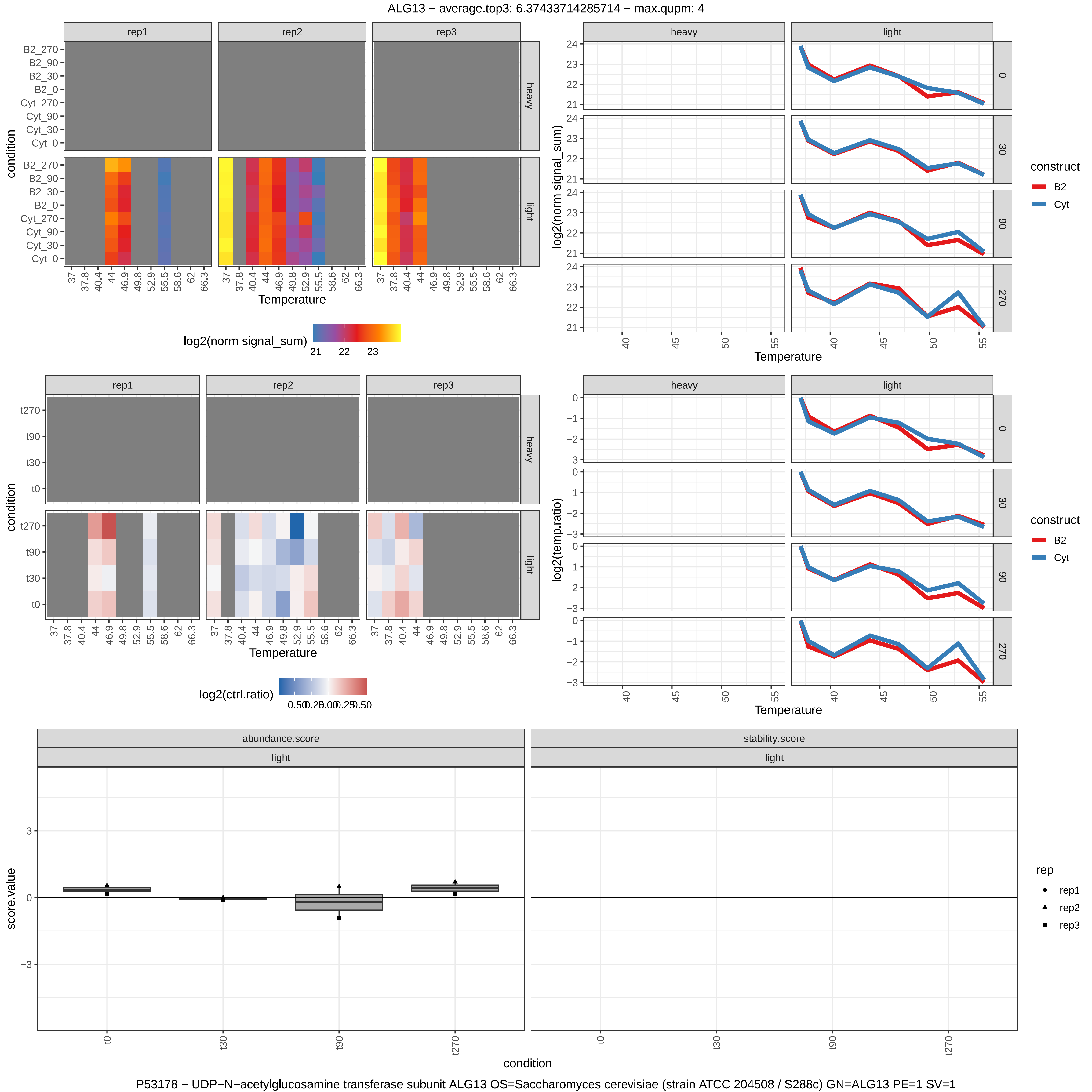
ALG13 − average.top3: 6.37433714285714 − max.qupm: 4
rep1
rep2
rep3
heavy
light
condition
Temperature
log2(norm signal_sum)
construct
B2
Cyt
log2(temp.ratio)
log2(ctrl.ratio)
abundance.score
stability.score
score.value
rep
rep1
rep2
rep3
P53178 − UDP−N−acetylglucosamine transferase subunit ALG13 OS=Saccharomyces cerevisiae (strain ATCC 204508 / S288c) GN=ALG13 PE=1 SV=1

ALG14 − average.top3: 6.83163545454545 − max.qupm: 3

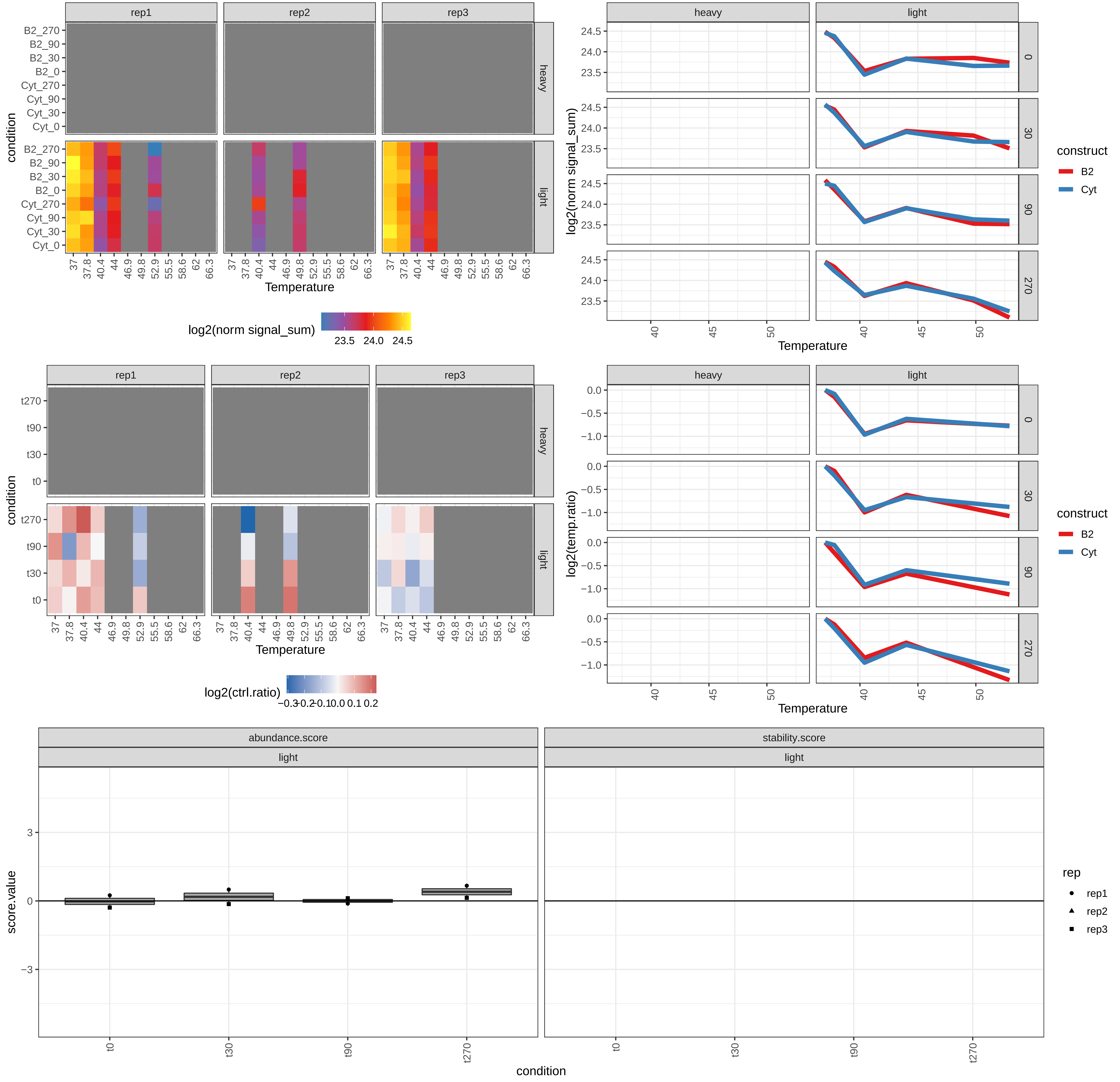

P38242 − UDP−N−acetylglucosamine transferase subunit ALG14 OS=Saccharomyces cerevisiae (strain ATCC 204508 / S288c) GN=ALG14 PE=1 SV=1

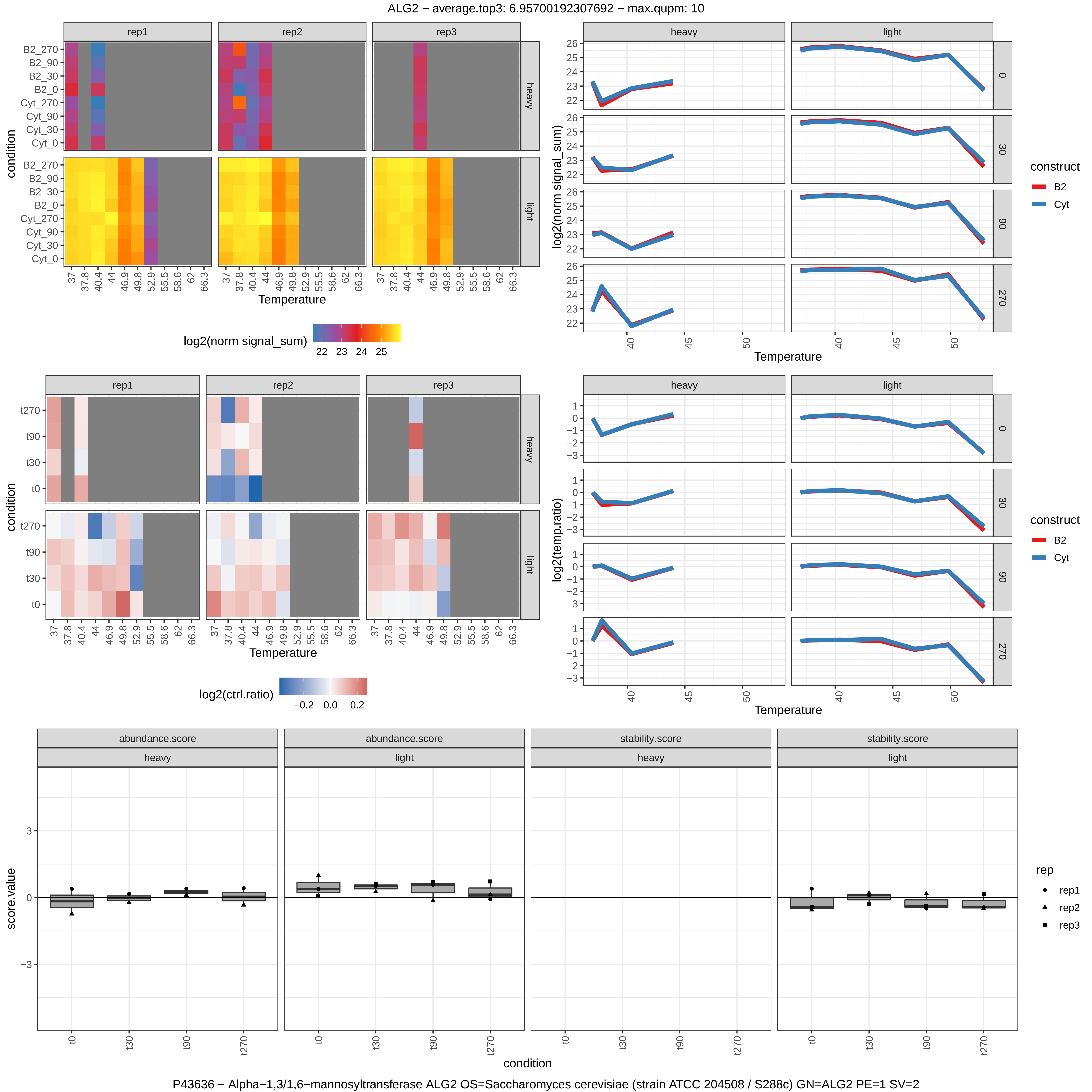
ALG2 − average.top3: 6.95700192307692 − max.qupm: 10
rep1
rep2
rep3
heavy
light
condition
Temperature
log2(norm signal_sum)
log2(ctrl.ratio)
log2(temp.ratio)
construct
B2
Cyt
abundance.score
stability.score
score.value
rep
rep1
rep2
rep3
P43636 − Alpha−1,3/1,6−mannosyltransferase ALG2 OS=Saccharomyces cerevisiae (strain ATCC 204508 / S288c) GN=ALG2 PE=1 SV=2

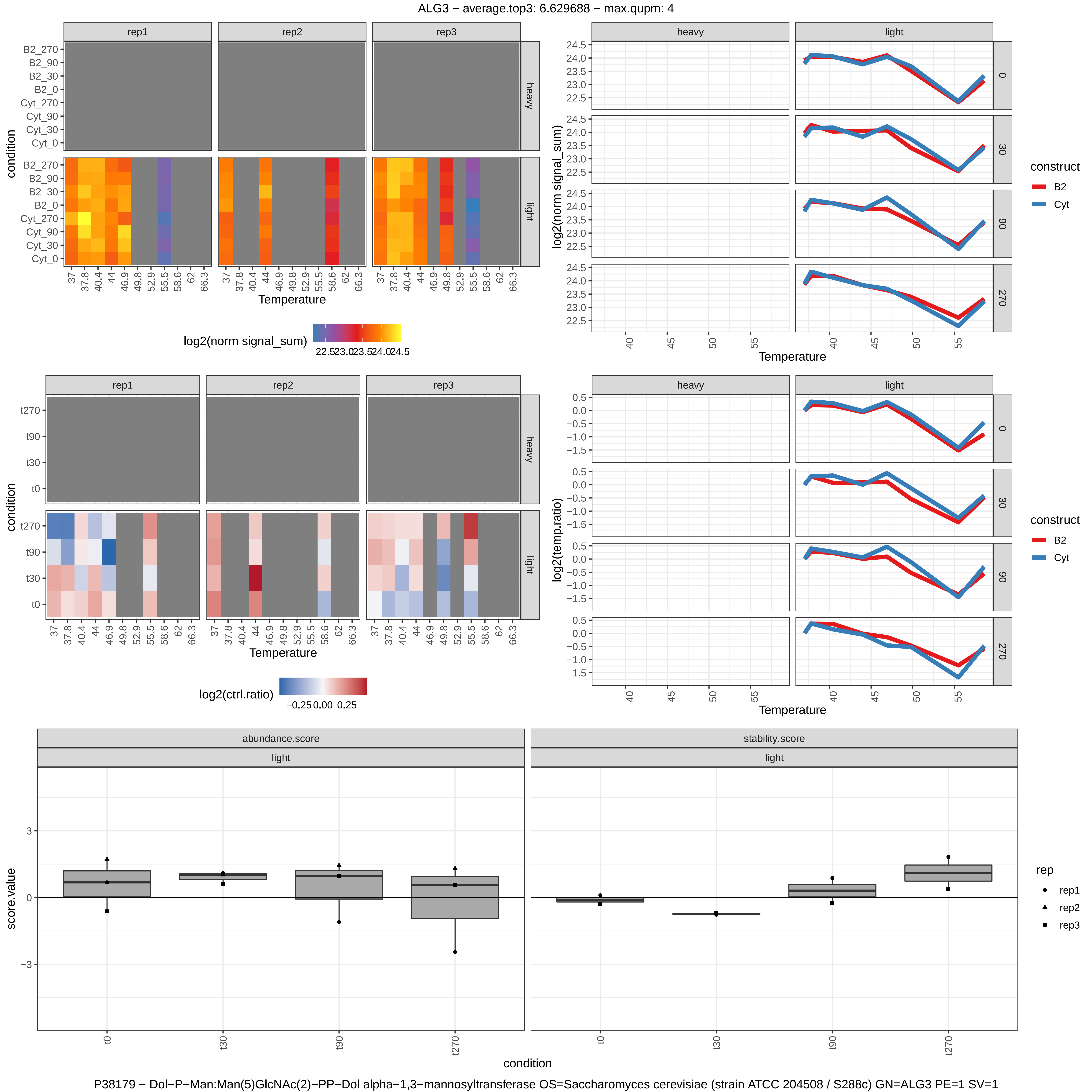
ALG3 − average.top3: 6.629688 − max.qupm: 4
rep1
rep2
rep3
heavy
light
condition
Temperature
log2(norm signal_sum)
log2(temp.ratio)
log2(ctrl.ratio)
construct
B2
Cyt
abundance.score
stability.score
score.value
rep
rep1
rep2
rep3
P38179 − Dol−P−Man:Man(5)GlcNAc(2)−PP−Dol alpha−1,3−mannosyltransferase OS=Saccharomyces cerevisiae (strain ATCC 204508 / S288c) GN=ALG3 PE=1 SV=1

ALG5 − average.top3: 7.725270625 − max.qupm: 4

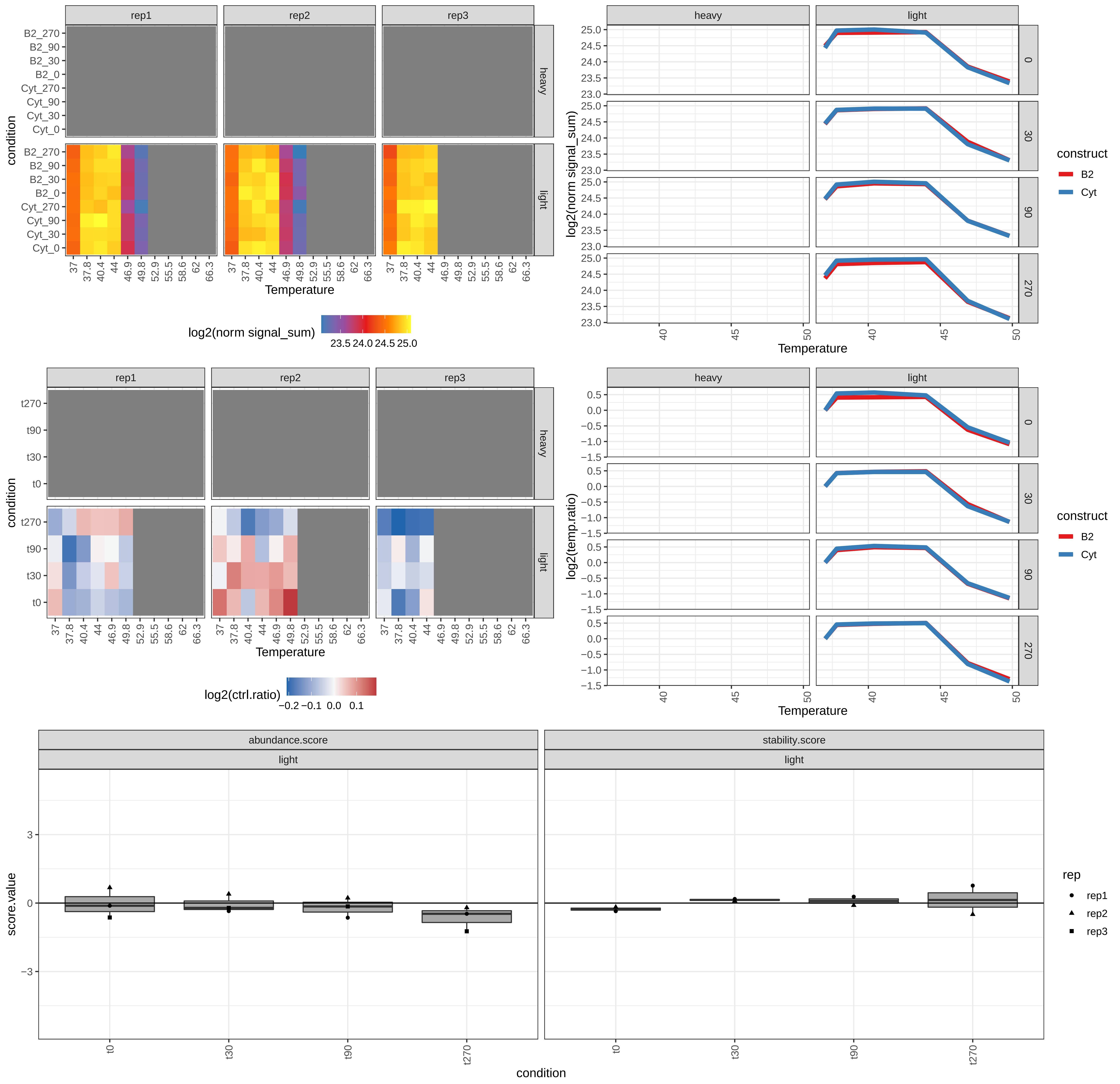

P40350 − Dolichyl−phosphate beta−glucosyltransferase OS=Saccharomyces cerevisiae (strain ATCC 204508 / S288c) GN=ALG5 PE=1 SV=1

ALG6 − average.top3: 6.95733333333333 − max.qupm: 3

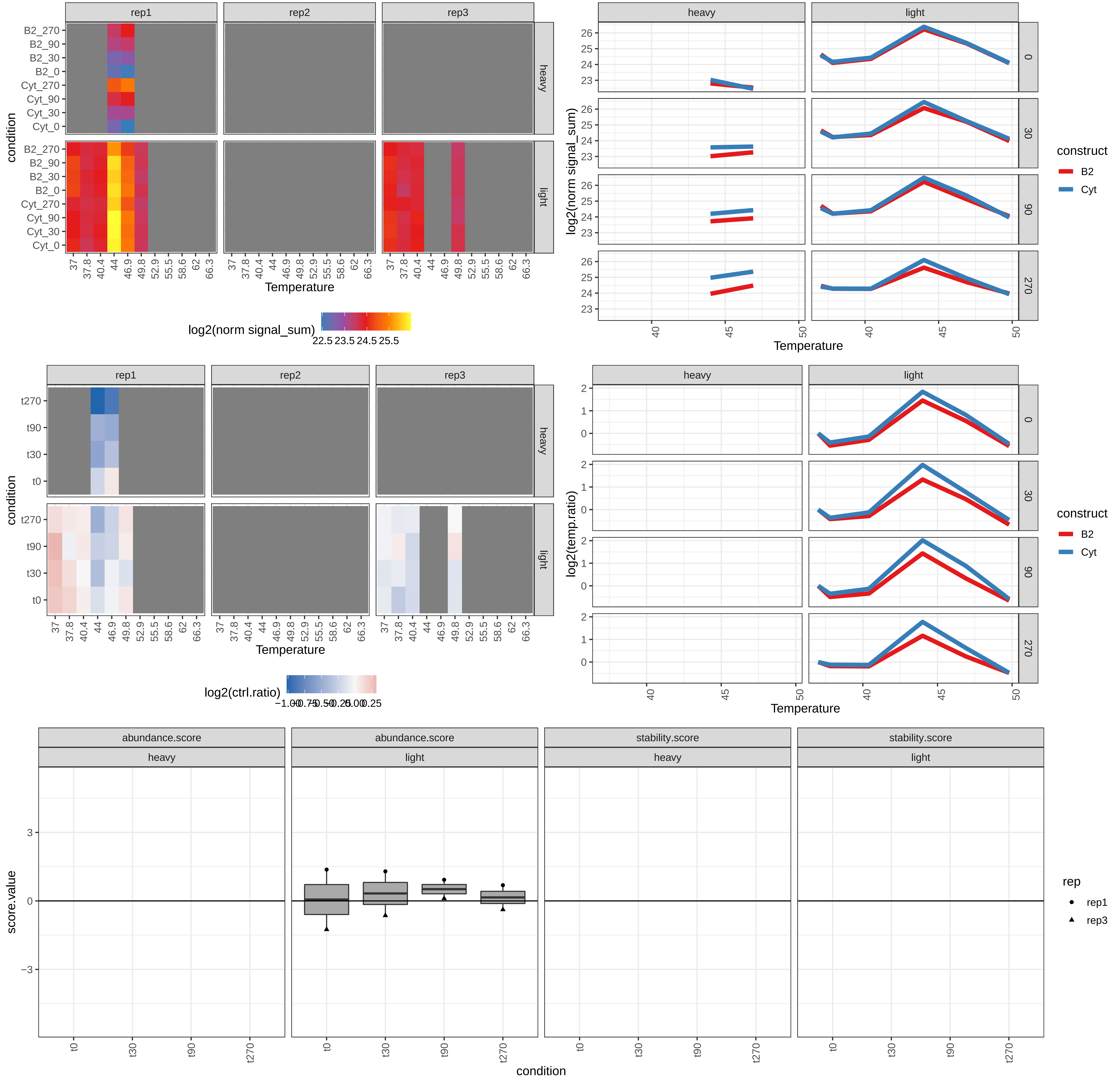

Q12001 − Dolichyl pyrophosphate Man9GlcNAc2 alpha−1,3−glucosyltransferase OS=Saccharomyces cerevisiae (strain ATCC 204508 / S288c) GN=ALG6 PE=1 SV=1

ALG8 − average.top3: 6.76692176470588 − max.qupm: 3

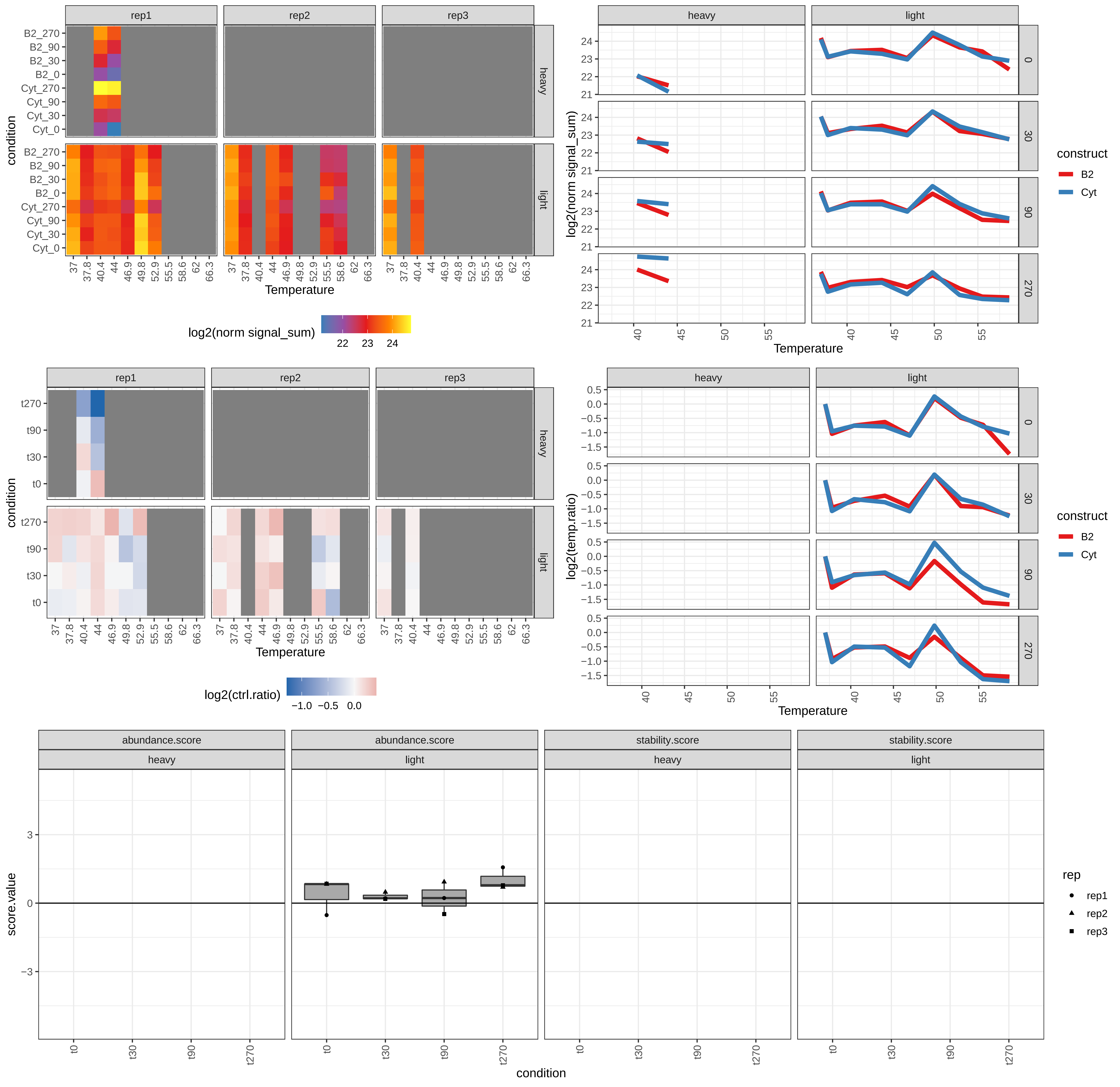

P40351 − Dolichyl pyrophosphate Glc1Man9GlcNAc2 alpha−1,3−glucosyltransferase OS=Saccharomyces cerevisiae (strain ATCC 204508 / S288c) GN=ALG8 PE=1 SV=1

ALG9 − average.top3: 6.70790551724138 − max.qupm: 6

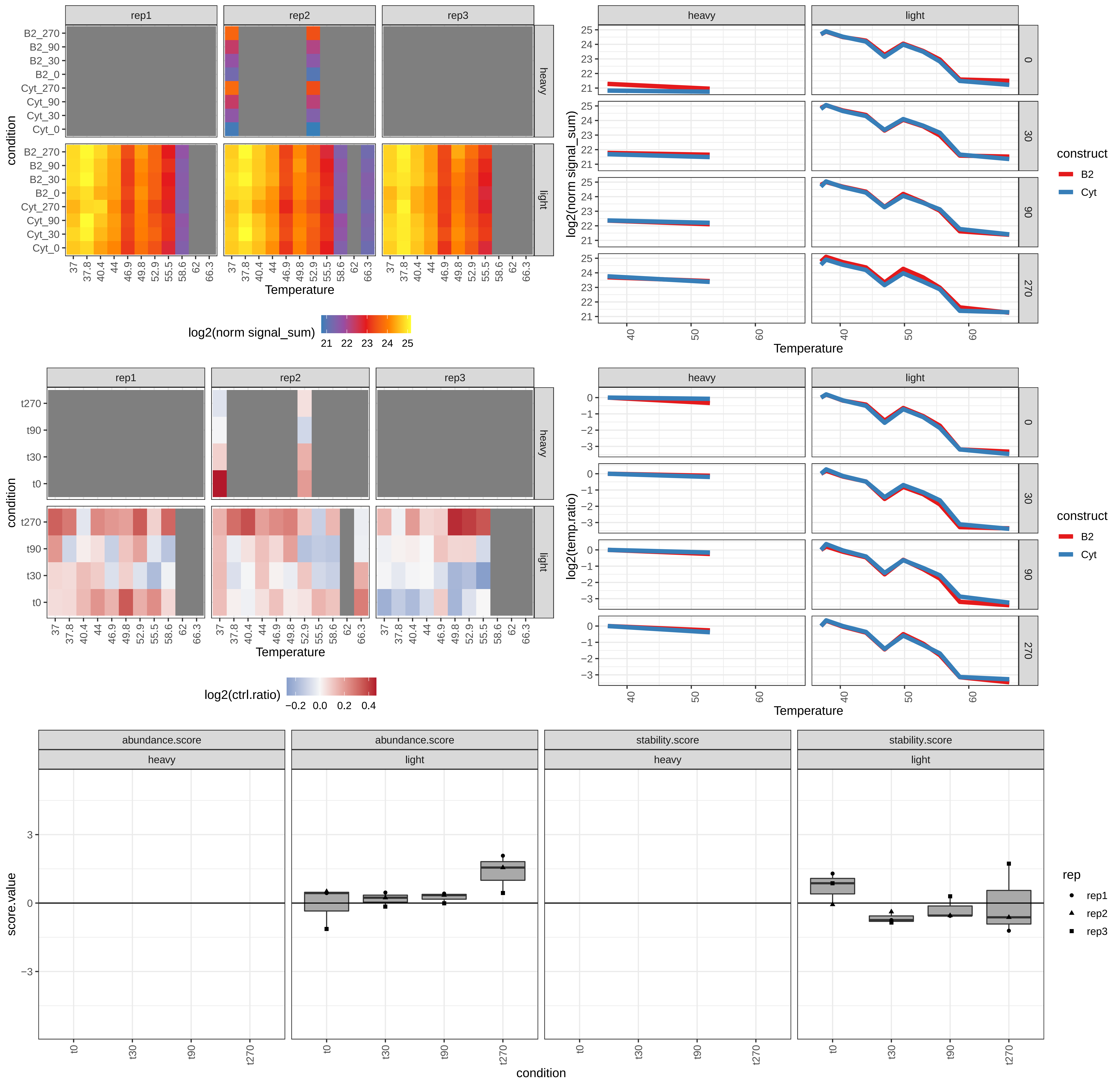

P53868 − Alpha−1,2−mannosyltransferase ALG9 OS=Saccharomyces cerevisiae (strain ATCC 204508 / S288c) GN=ALG9 PE=1 SV=1

ALO1 − average.top3: 7.09679629032258 − max.qupm: 14

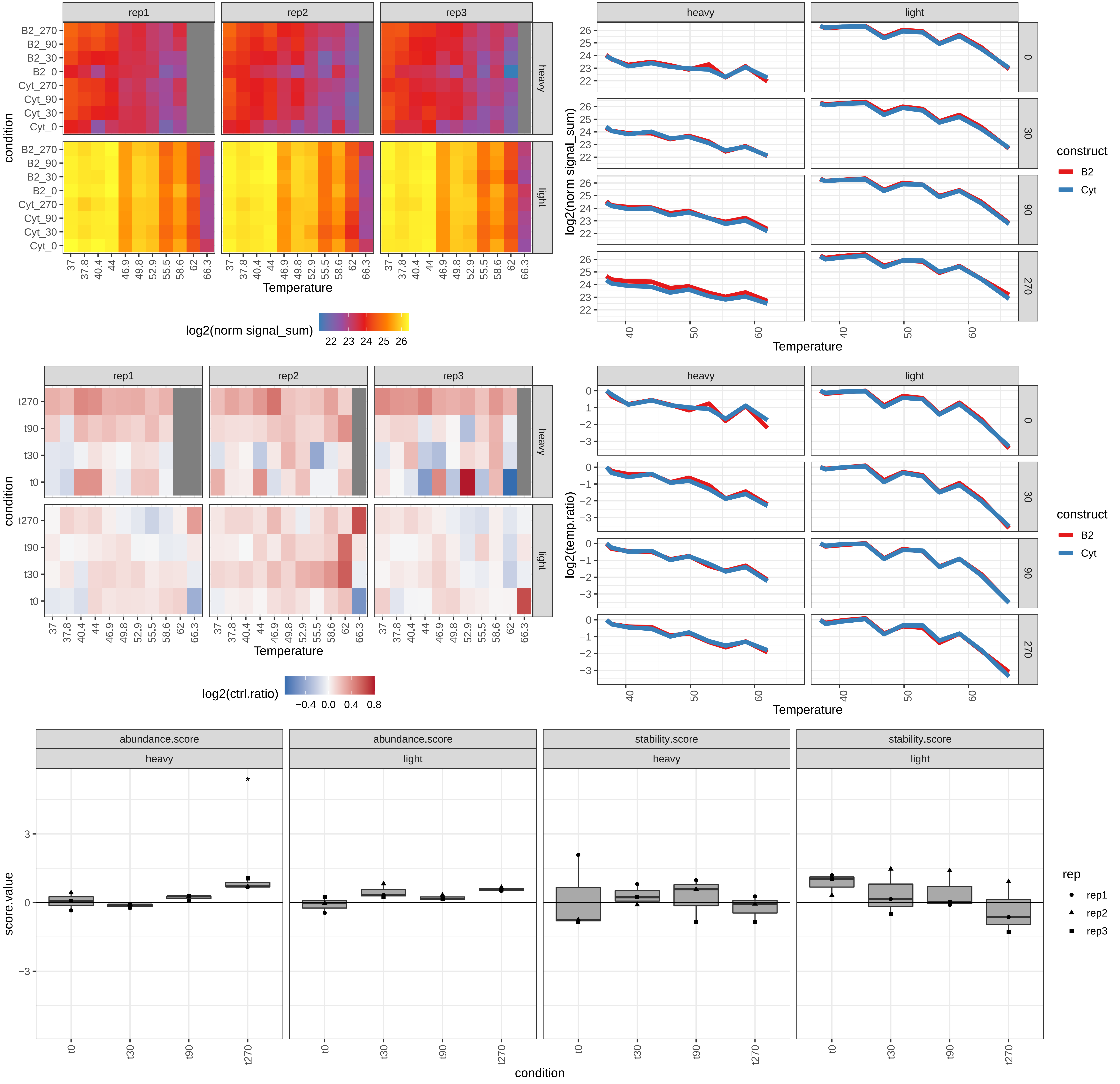

P54783 − D−arabinono−1,4−lactone oxidase OS=Saccharomyces cerevisiae (strain ATCC 204508 / S288c) GN=ALO1 PE=1 SV=1

ALP1 − average.top3: 6.51608954545455 − max.qupm: 5

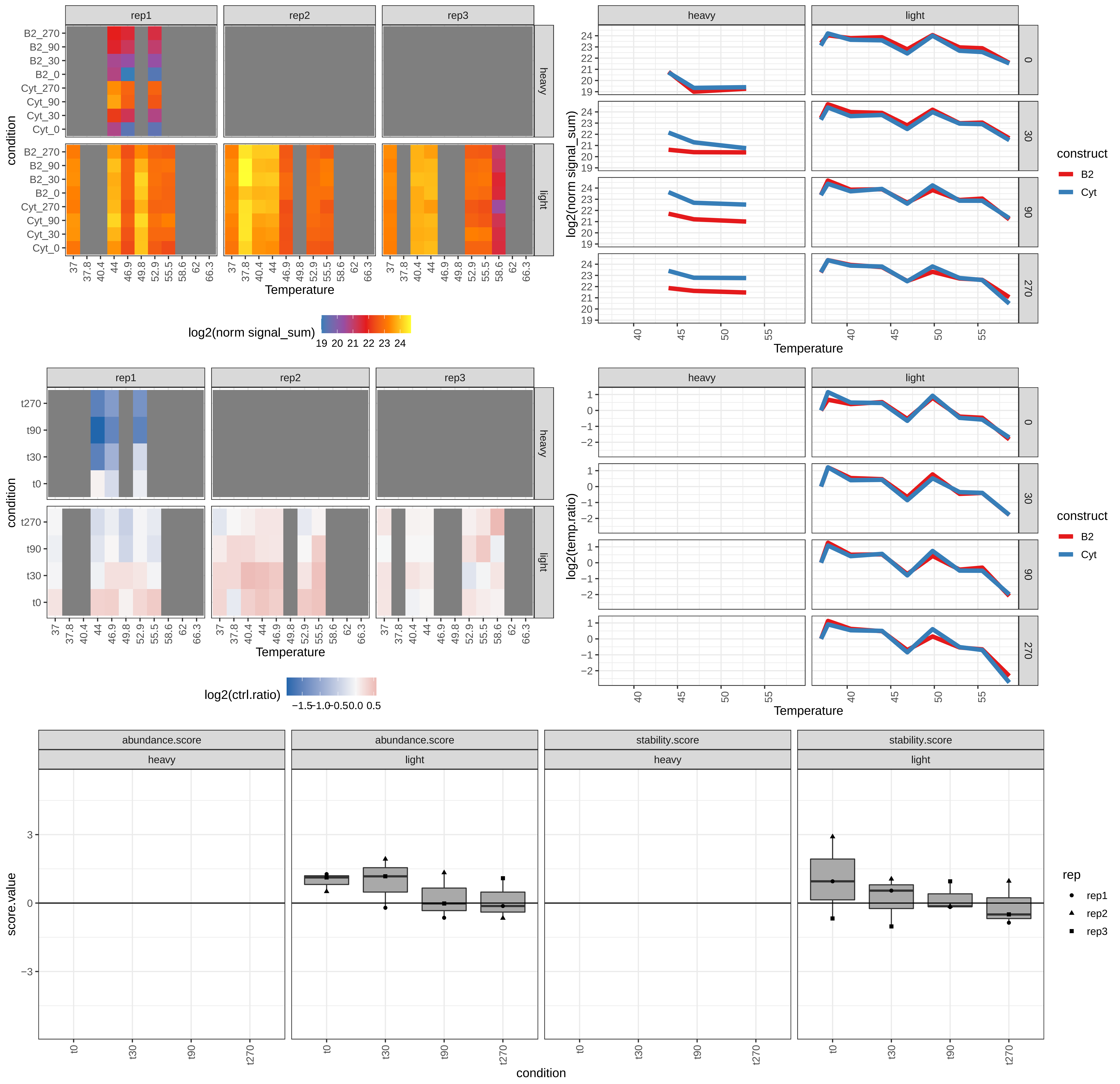

P38971 − Basic amino−acid permease OS=Saccharomyces cerevisiae (strain ATCC 204508 / S288c) GN=ALP1 PE=1 SV=2

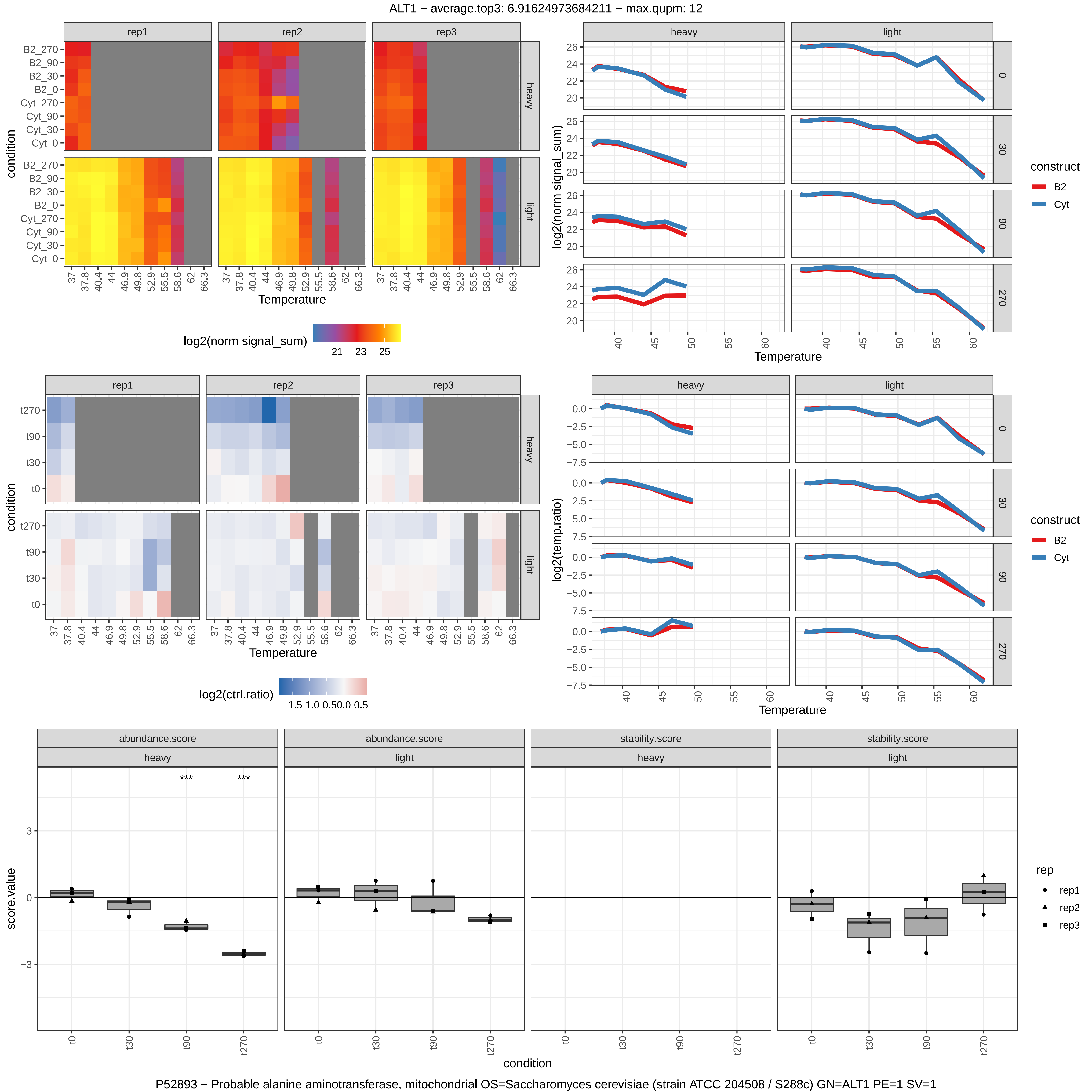
ALT1 − average.top3: 6.91624973684211 − max.qupm: 12
rep1
rep2
rep3
heavy
light
condition
Temperature
log2(norm signal_sum)
log2(ctrl.ratio)
log2(temp.ratio)
construct
B2
Cyt
abundance.score
stability.score
score.value
rep
condition
P52893 − Probable alanine aminotransferase, mitochondrial OS=Saccharomyces cerevisiae (strain ATCC 204508 / S288c) GN=ALT1 PE=1 SV=1

ALY2 − average.top3: 6.8780875 − max.qupm: 8

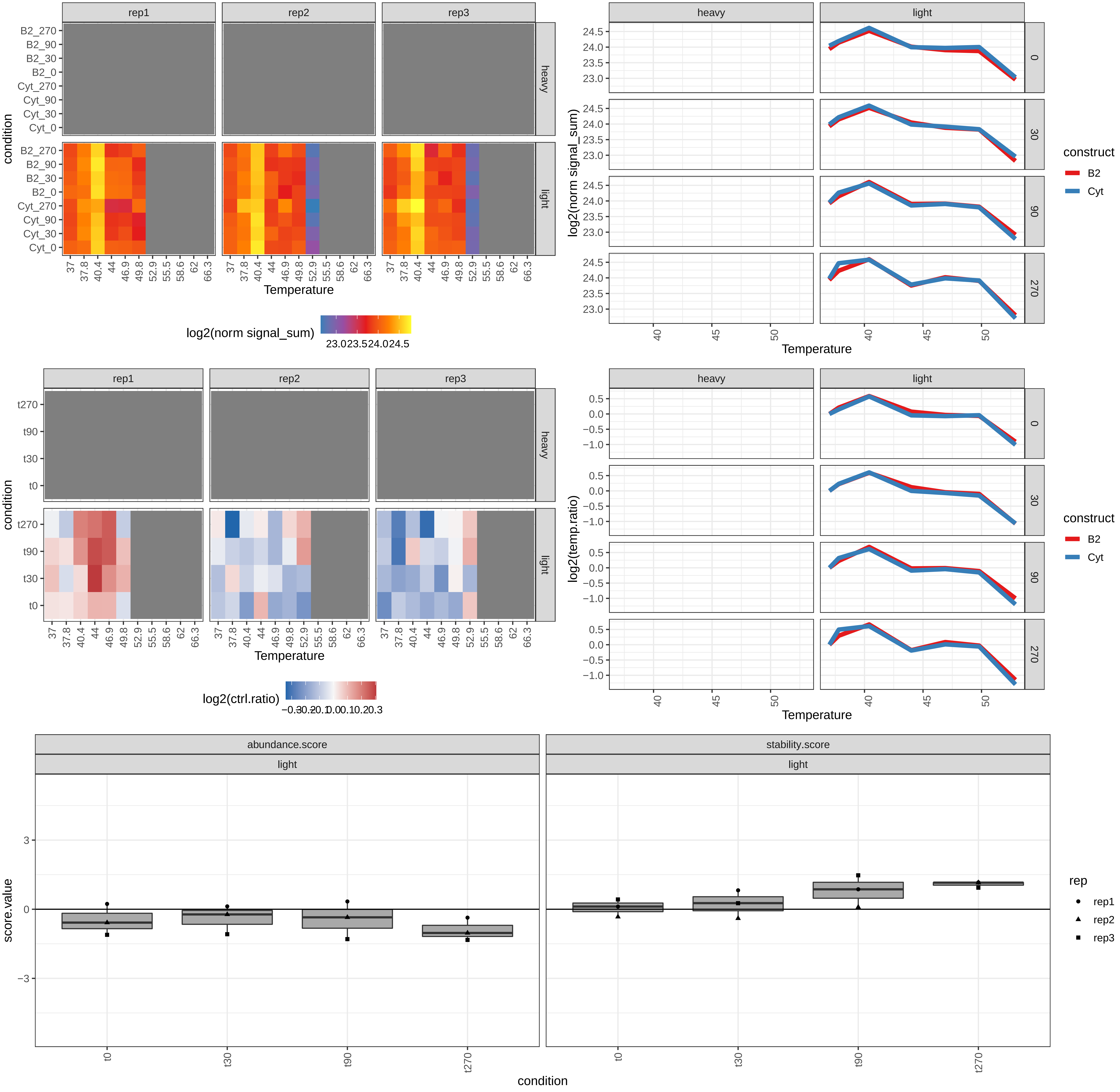

P47029 − Arrestin−related trafficking adapter 3 OS=Saccharomyces cerevisiae (strain ATCC 204508 / S288c) GN=ALY2 PE=1 SV=2

AMD1 − average.top3: 6.92116763157895 − max.qupm: 16

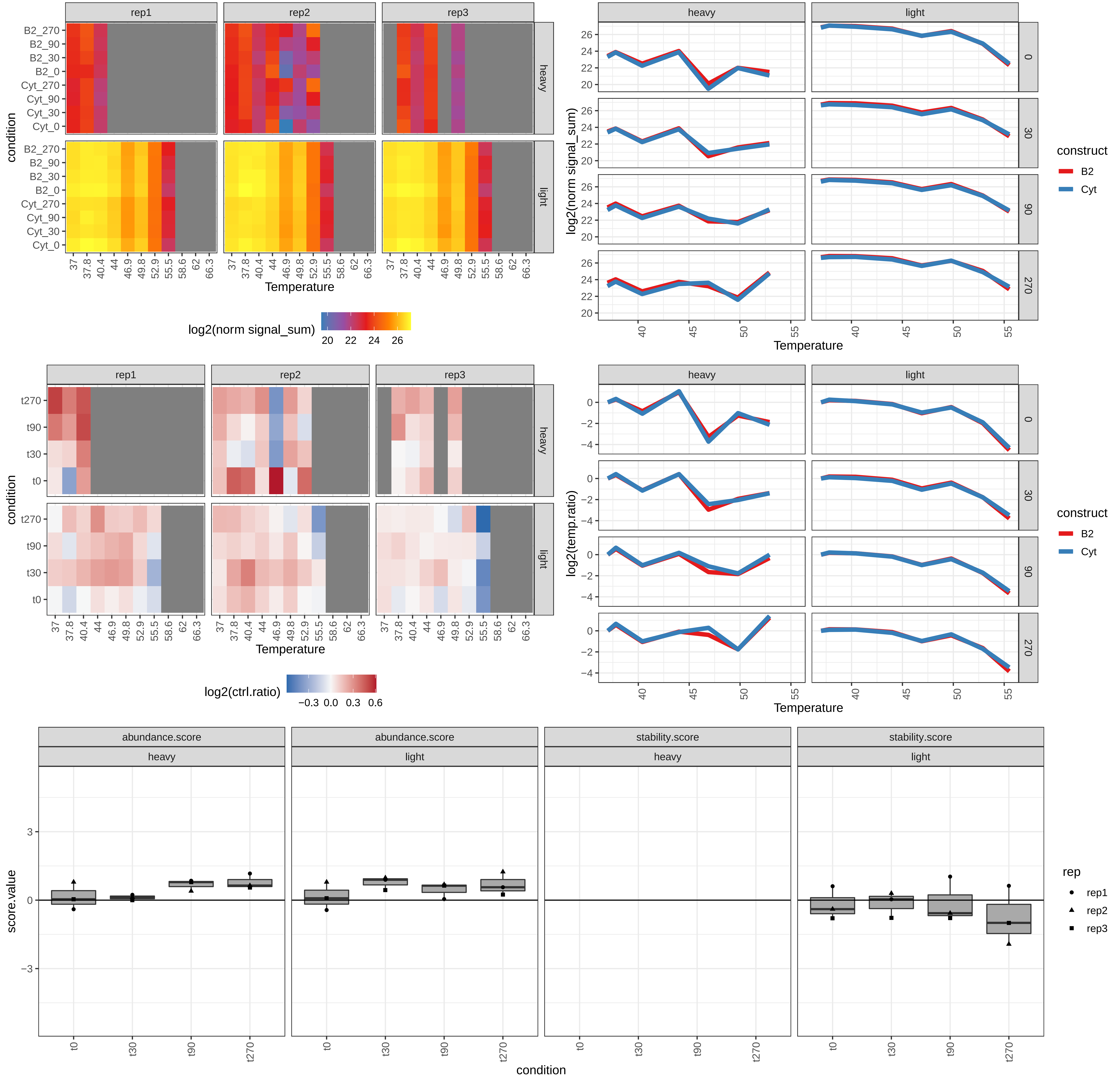

P15274 − AMP deaminase OS=Saccharomyces cerevisiae (strain ATCC 204508 / S288c) GN=AMD1 PE=1 SV=2

AMS1 − average.top3: 7.44173754098361 − max.qupm: 49

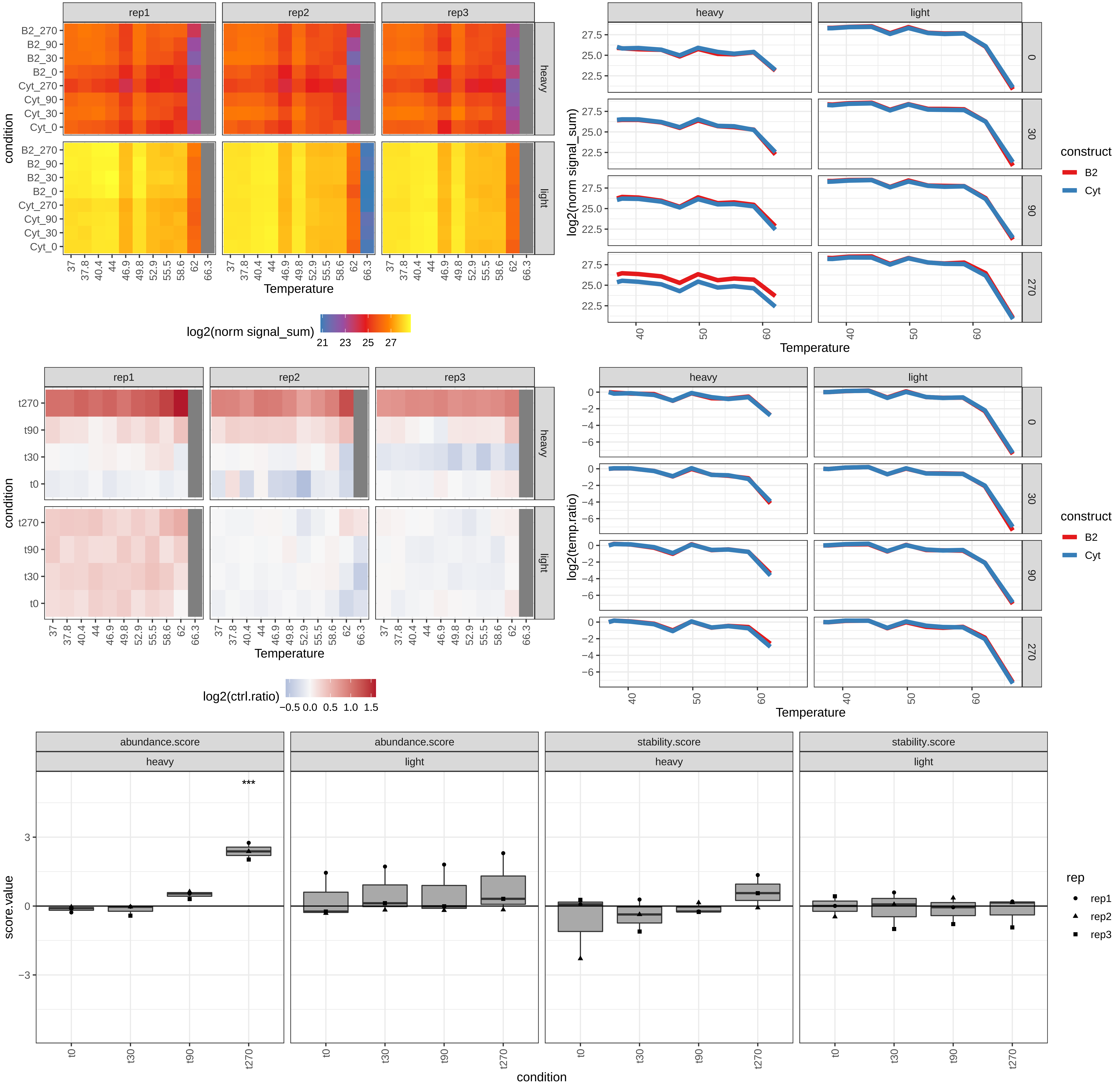

P22855 − Alpha−mannosidase OS=Saccharomyces cerevisiae (strain ATCC 204508 / S288c) GN=AMS1 PE=1 SV=2

ANP1 − average.top3: 6.82103178571429 − max.qupm: 9

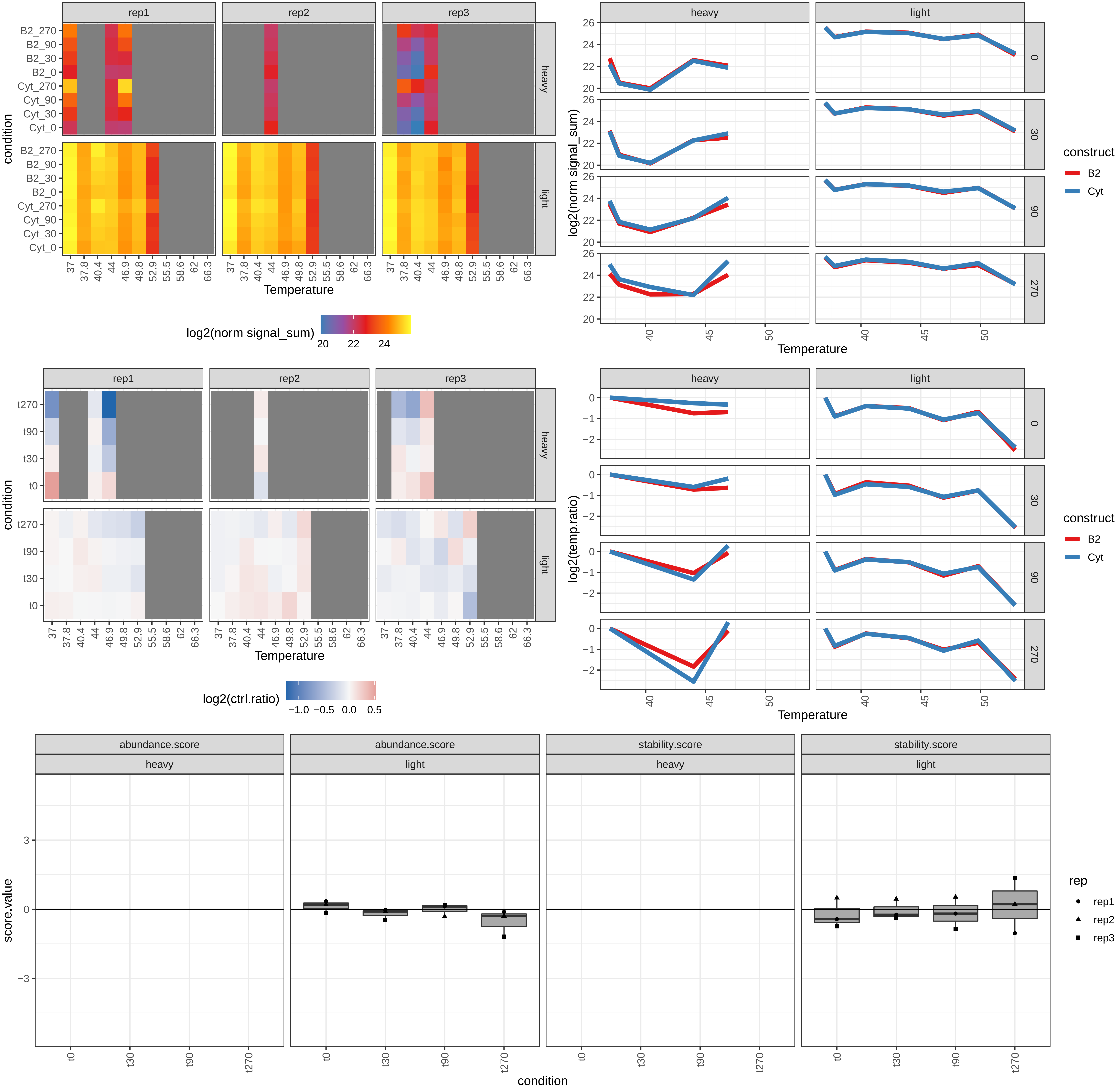

P32629 − Mannan polymerase II complex ANP1 subunit OS=Saccharomyces cerevisiae (strain ATCC 204508 / S288c) GN=ANP1 PE=1 SV=3

ANR2 − average.top3: 6.61164125 − max.qupm: 6

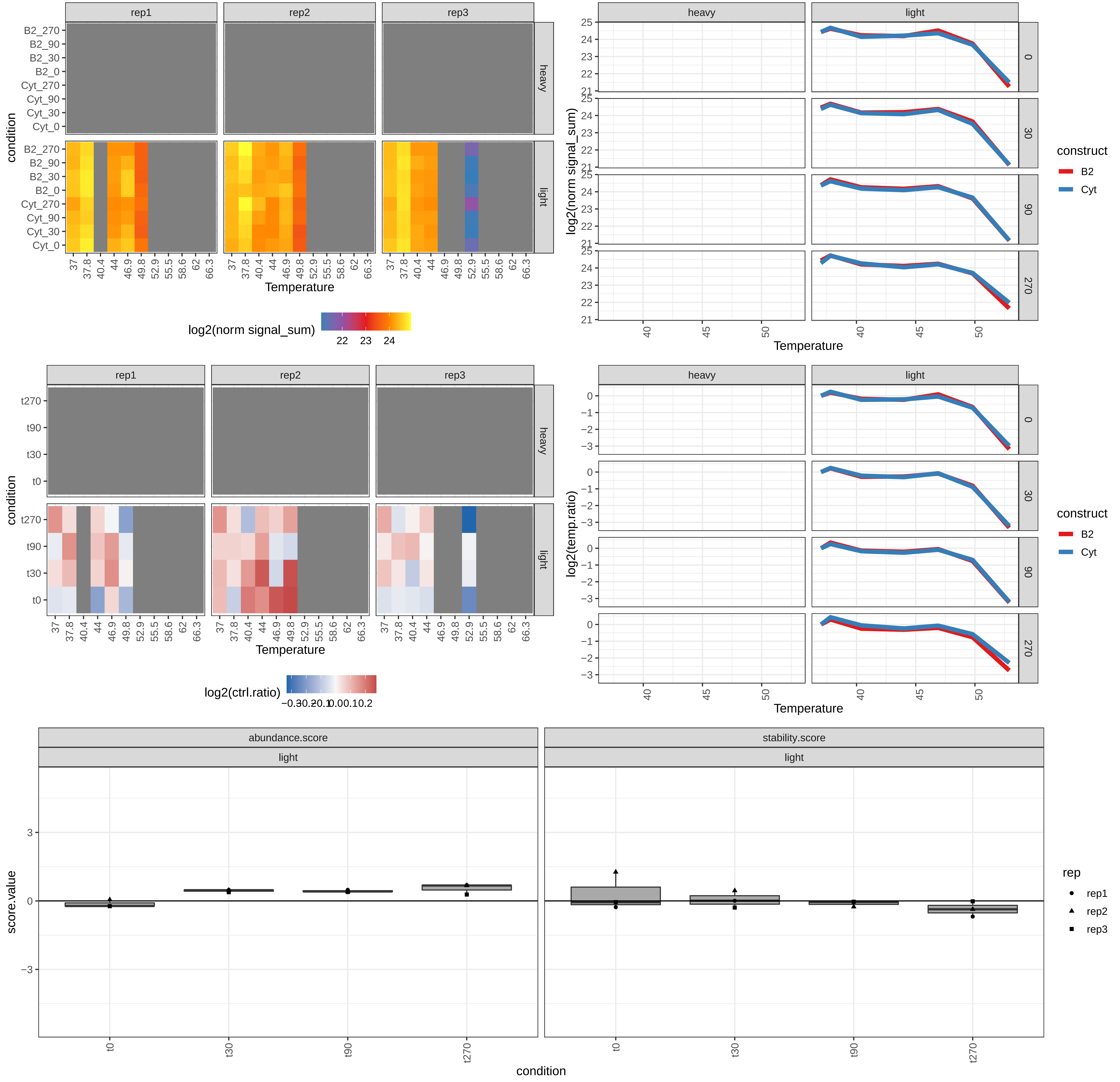

P36090 − Uncharacterized protein ANR2 OS=Saccharomyces cerevisiae (strain ATCC 204508 / S288c) GN=ANR2 PE=1 SV=1

AOS1 − average.top3: 6.72588 − max.qupm: 4

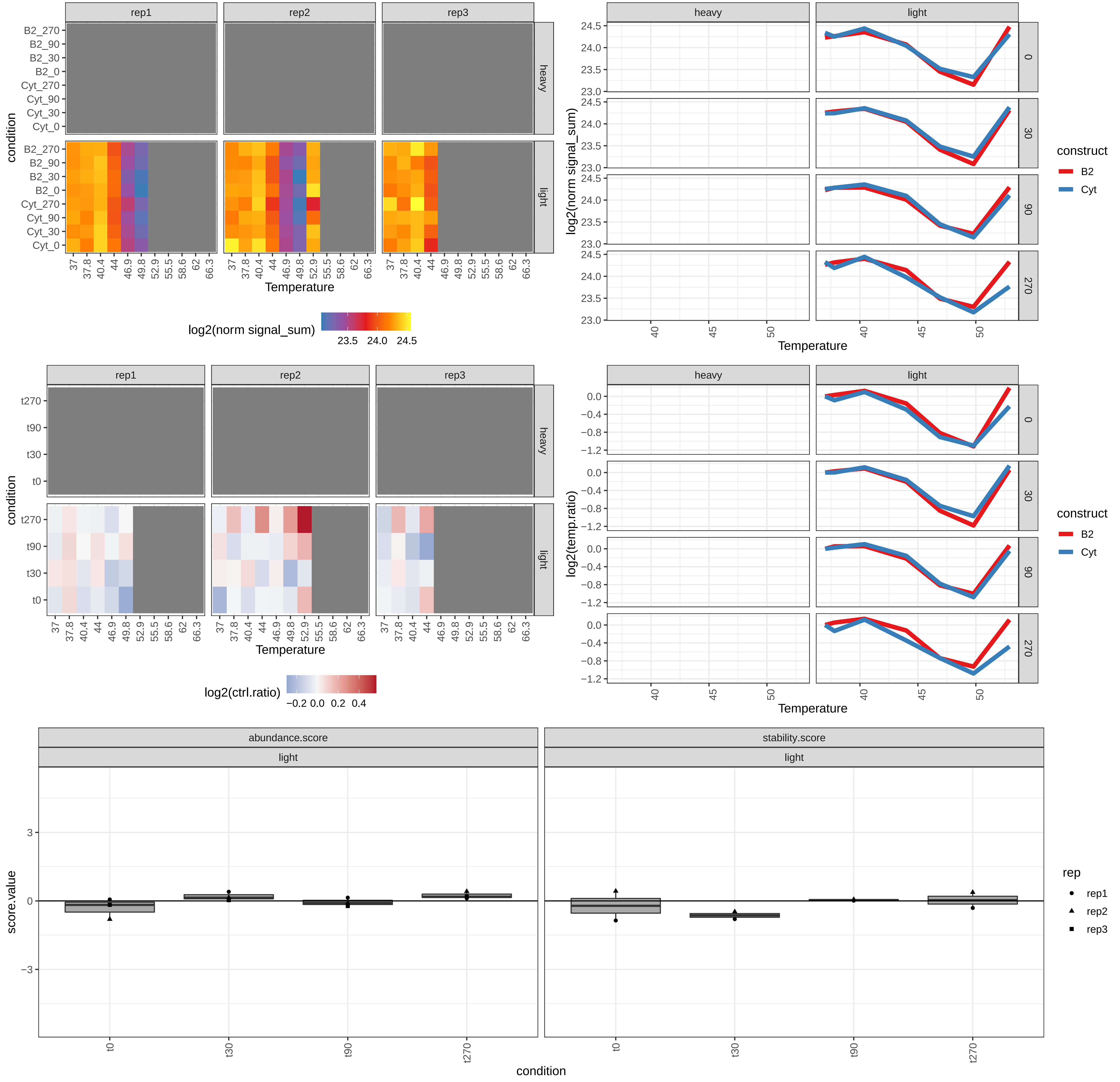

Q06624 − DNA damage tolerance protein RHC31 OS=Saccharomyces cerevisiae (strain ATCC 204508 / S288c) GN=AOS1 PE=1 SV=1

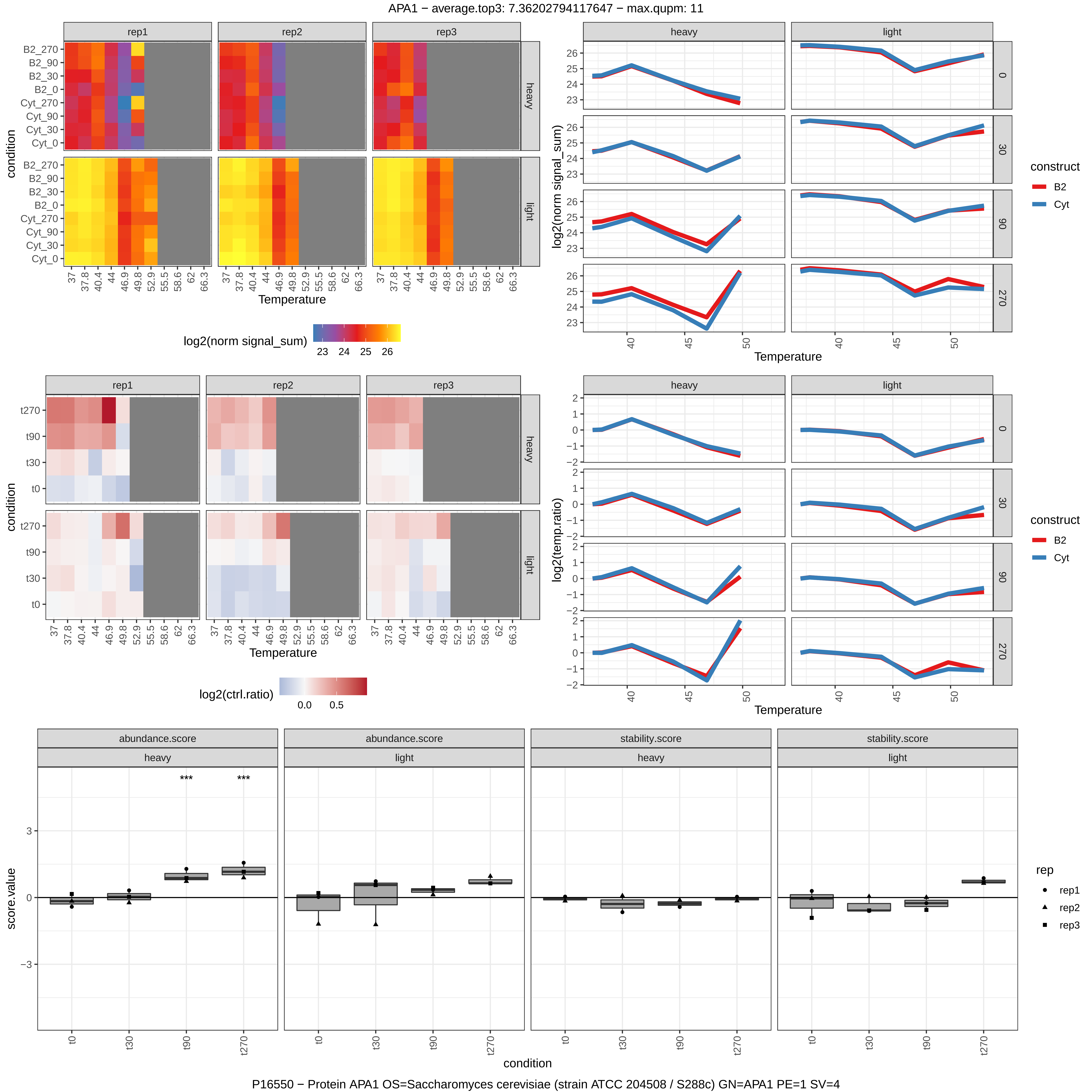

APA1 − average.top3: 7.36202794117647 − max.qupm: 11
rep1
rep2
rep3
heavy
light
condition
Temperature
log2(norm signal_sum)
construct
B2
Cyt
log2(temp.ratio)
log2(ctrl.ratio)
abundance.score
stability.score
score.value
rep
***
P16550 − Protein APA1 OS=Saccharomyces cerevisiae (strain ATCC 204508 / S288c) GN=APA1 PE=1 SV=4

APA2 − average.top3: 6.86365692307692 − max.qupm: 8

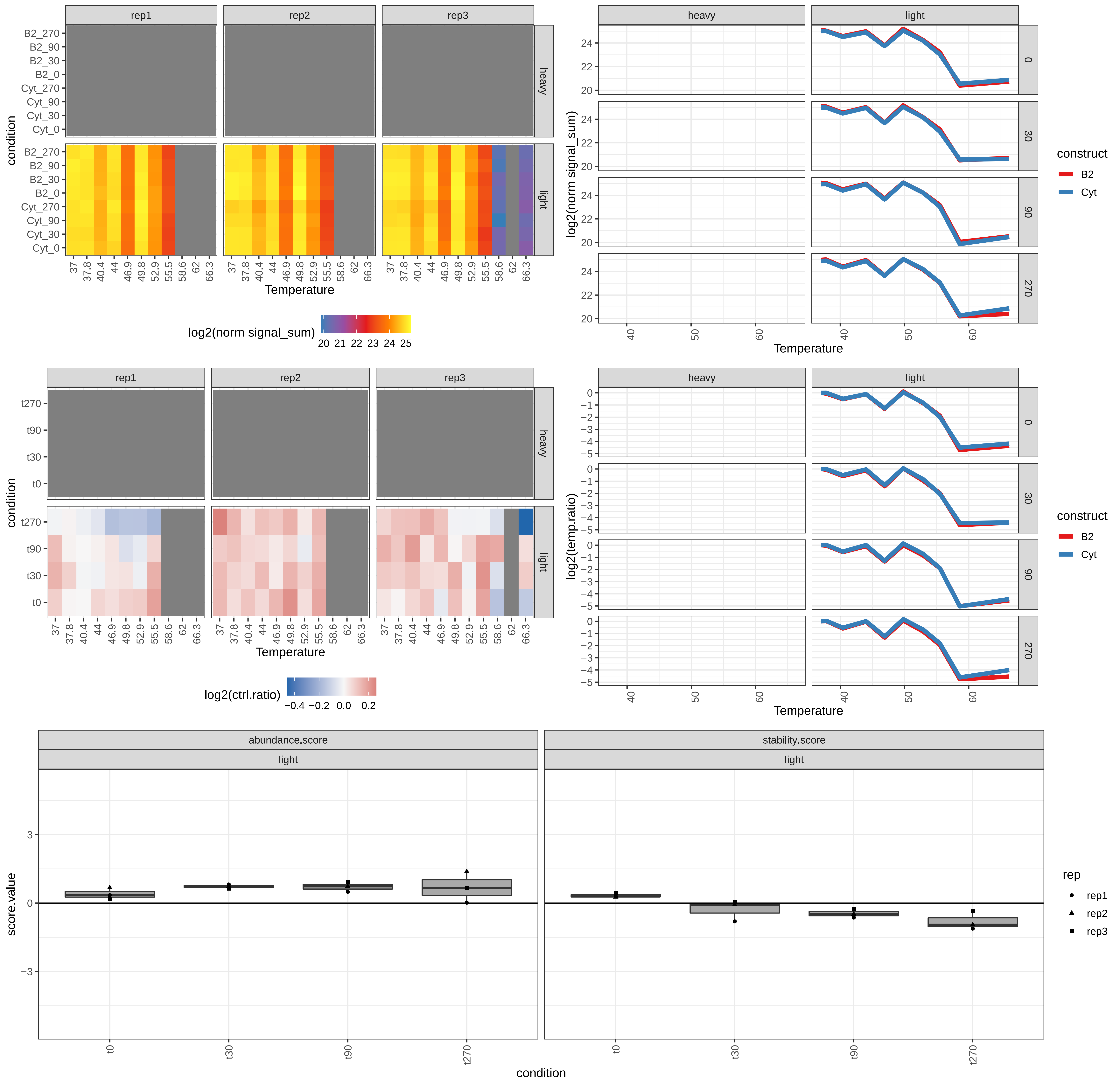

P22108 − Diadenosine 5',5'''−P1,P4−tetraphosphate phosphorylase 2 OS=Saccharomyces cerevisiae (strain ATCC 204508 / S288c) GN=APA2 PE=1 SV=1

APD1 − average.top3: 7.03786483870968 − max.qupm: 9

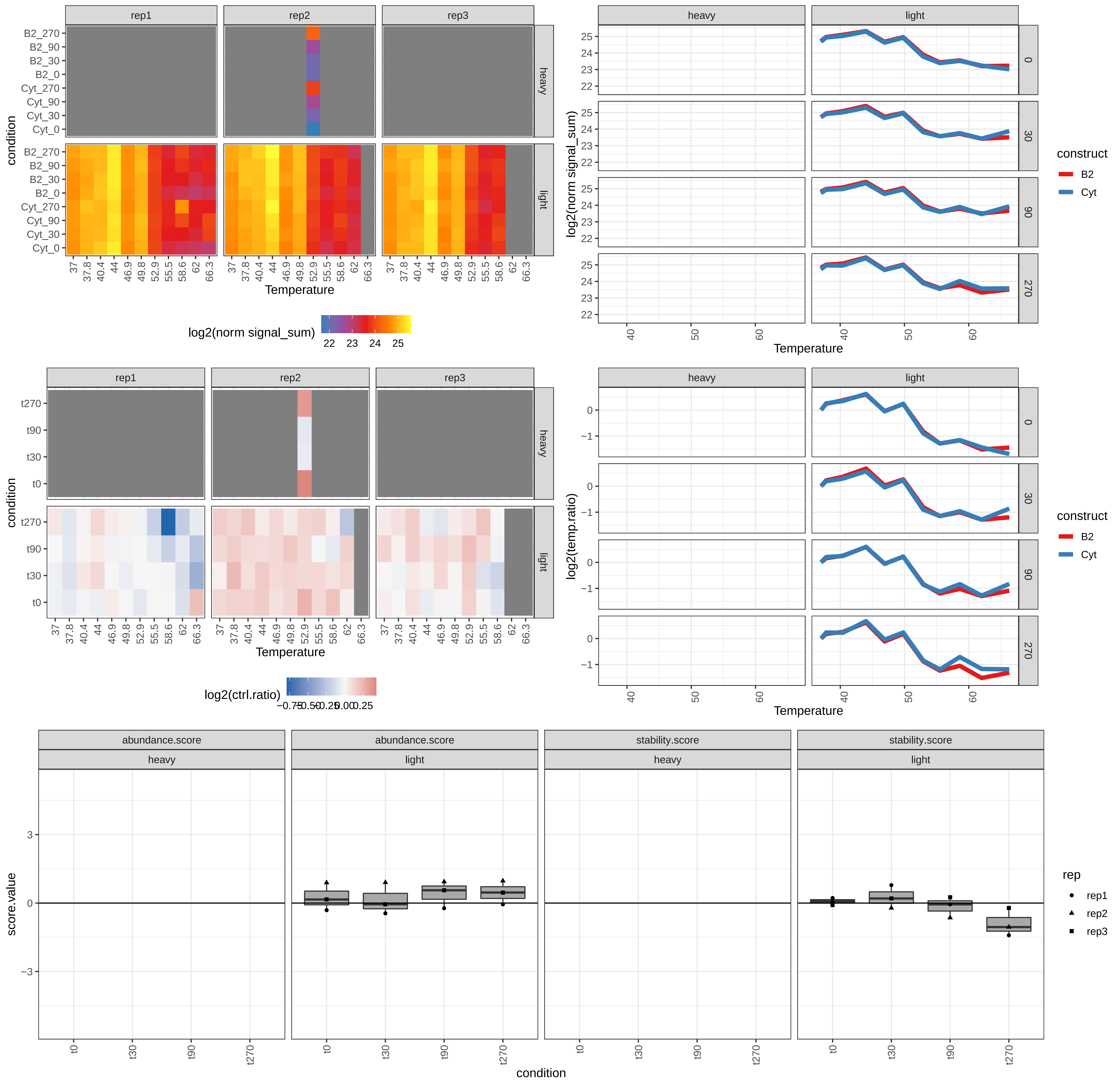

P38281 − Actin patches distal protein 1 OS=Saccharomyces cerevisiae (strain ATCC 204508 / S288c) GN=APD1 PE=1 SV=1

APE1 − average.top3: 7.80317590909091 − max.qupm: 17

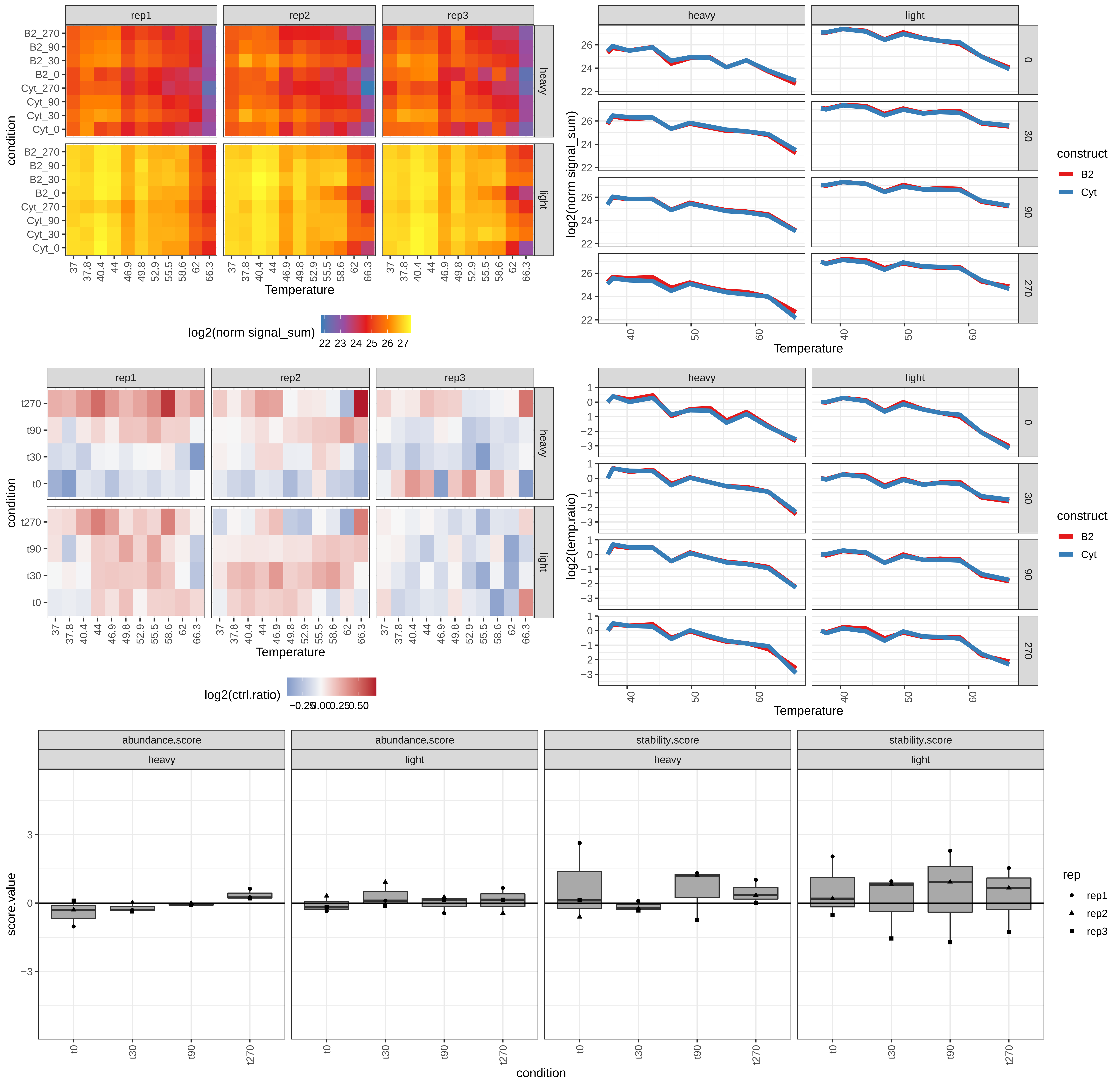

P14904 − Vacuolar aminopeptidase 1 OS=Saccharomyces cerevisiae (strain ATCC 204508 / S288c) GN=APE1 PE=1 SV=2

APE2 − average.top3: 7.57460646153846 − max.qupm: 34

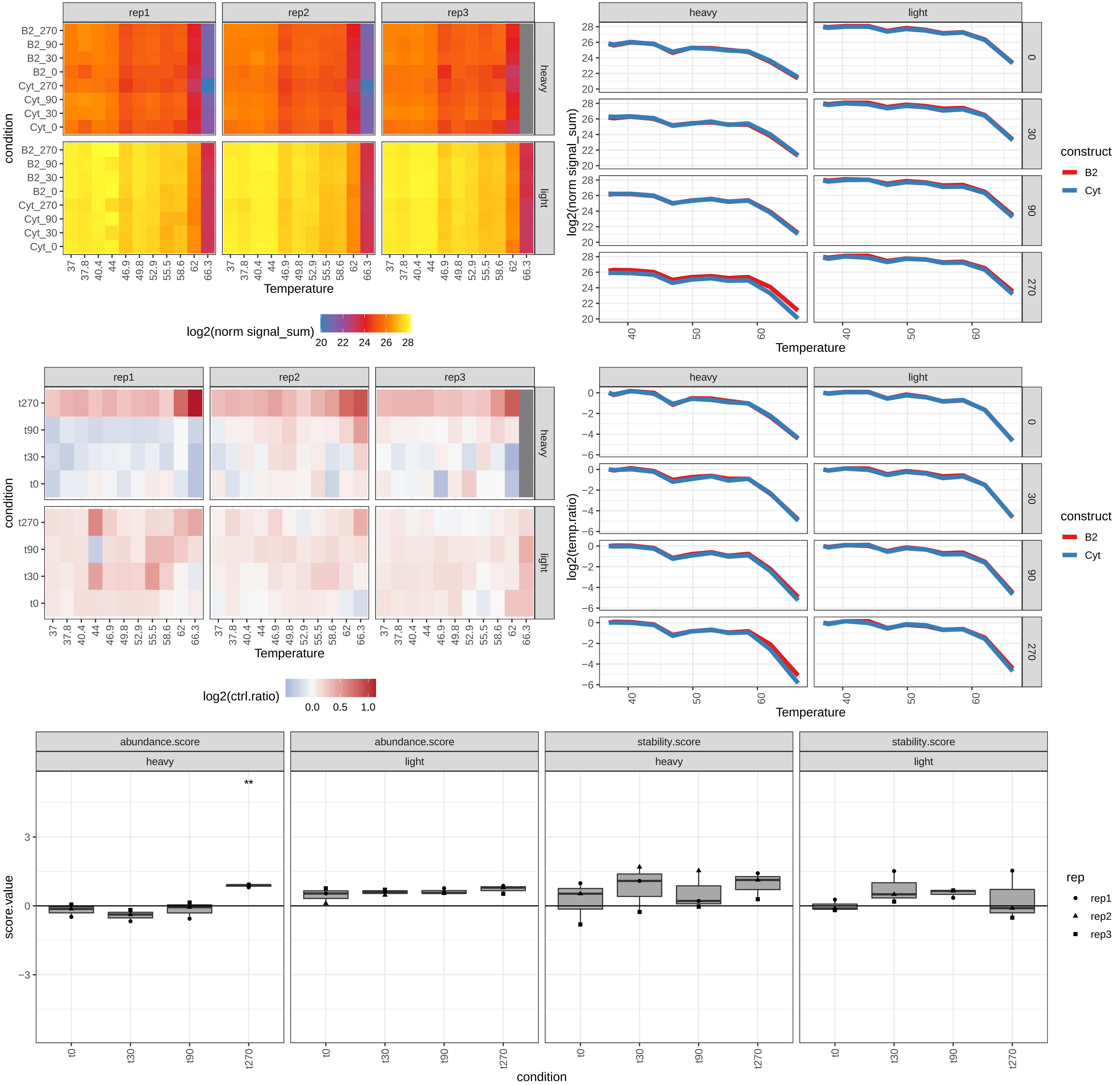

P32454 − Aminopeptidase 2, mitochondrial OS=Saccharomyces cerevisiae (strain ATCC 204508 / S288c) GN=APE2 PE=1 SV=4

APE3 − average.top3: 7.68435573770492 − max.qupm: 16

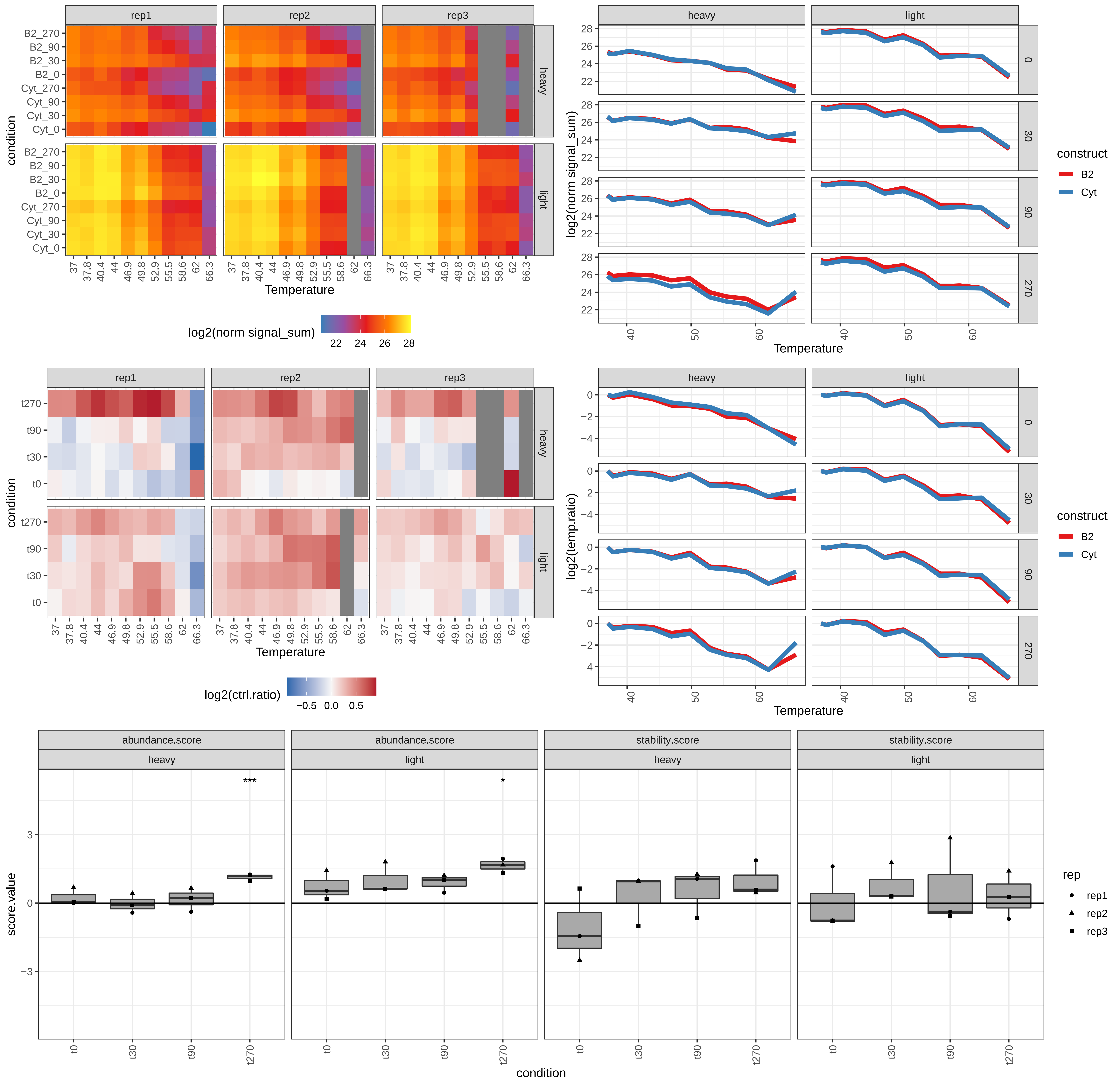

P37302 − Aminopeptidase Y OS=Saccharomyces cerevisiae (strain ATCC 204508 / S288c) GN=APE3 PE=1 SV=1

APE4 − average.top3: 7.24645181818182 − max.qupm: 17

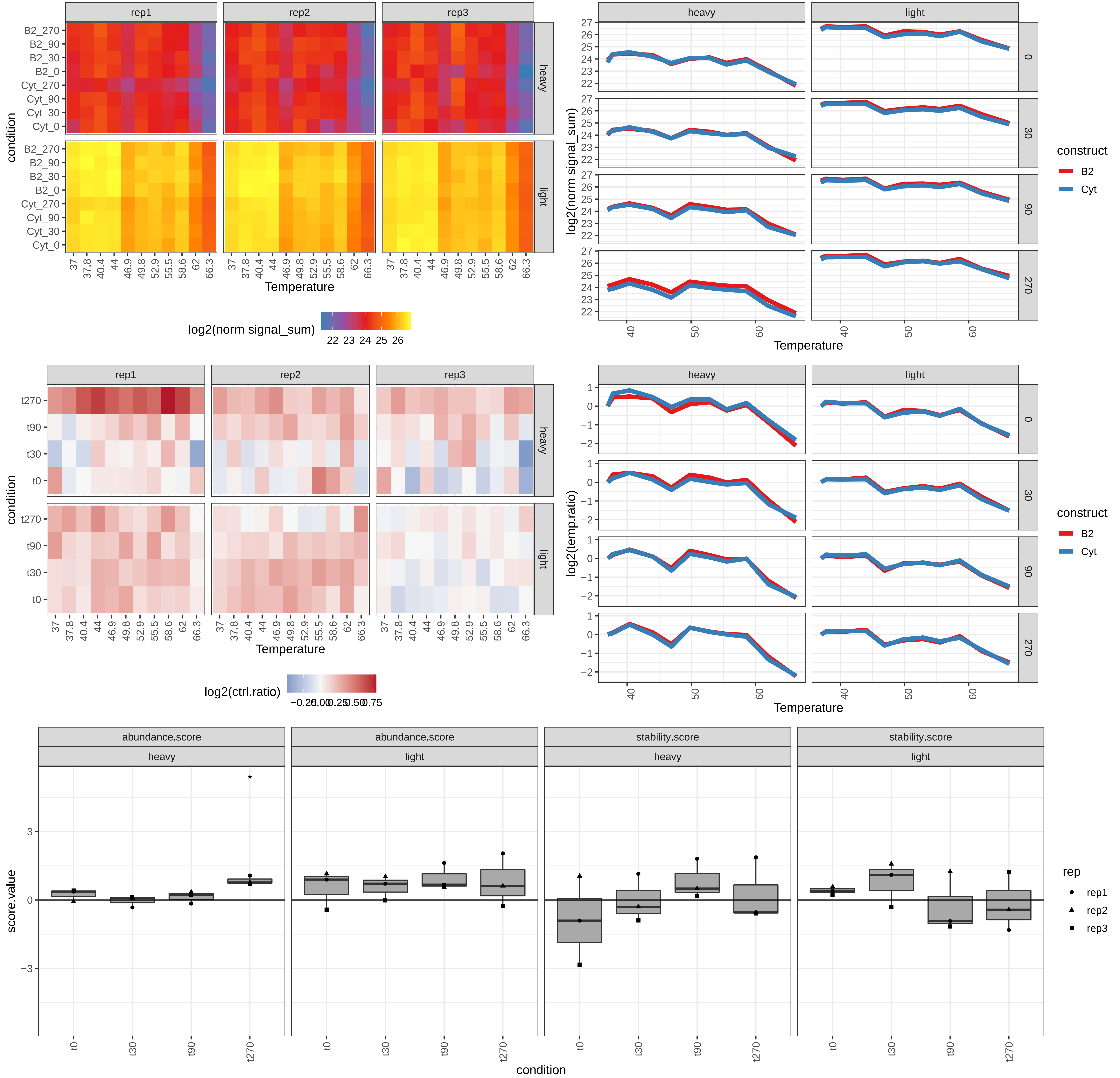

P38821 − Aspartyl aminopeptidase 4 OS=Saccharomyces cerevisiae (strain ATCC 204508 / S288c) GN=APE4 PE=1 SV=1

APL1 − average.top3: 6.64635111111111 − max.qupm: 8

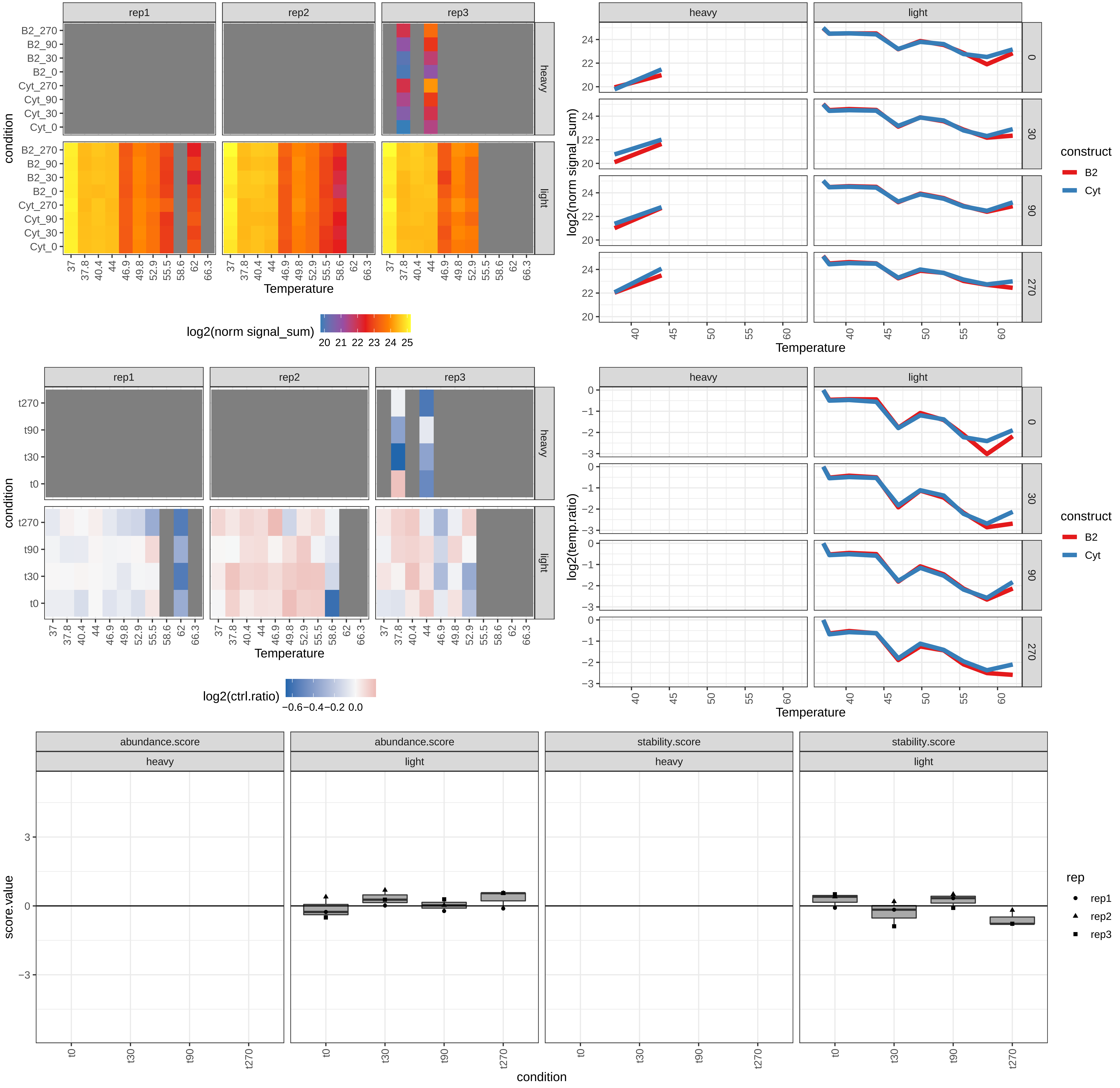

P27351 − AP−2 complex subunit beta OS=Saccharomyces cerevisiae (strain ATCC 204508 / S288c) GN=APL1 PE=1 SV=2

APL2 − average.top3: 6.80455697674419 − max.qupm: 12

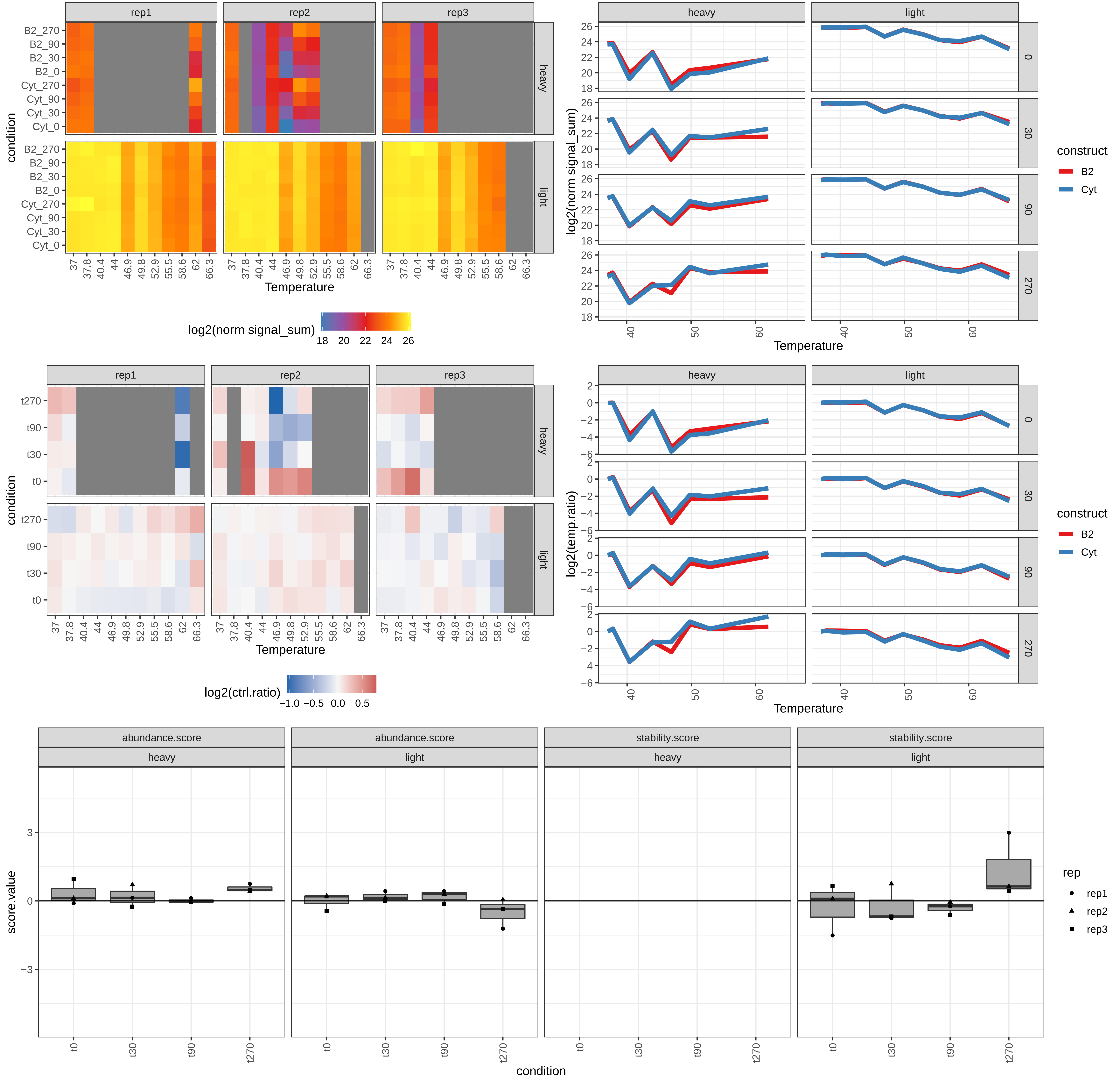

P36000 − AP−1 complex subunit beta−1 OS=Saccharomyces cerevisiae (strain ATCC 204508 / S288c) GN=APL2 PE=1 SV=1

APL3 − average.top3: 6.91448970588235 − max.qupm: 27

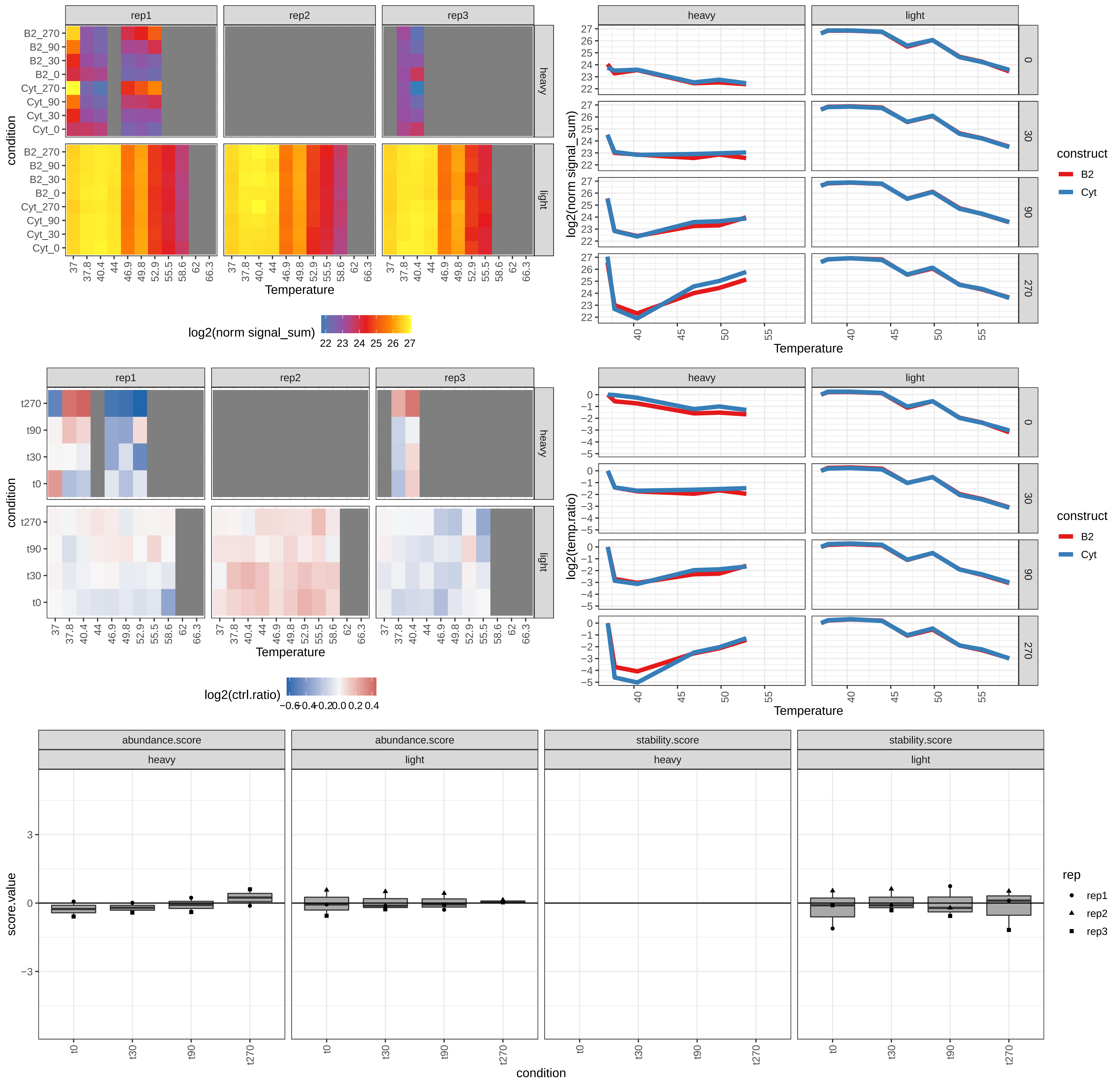

P38065 − AP−2 complex subunit alpha OS=Saccharomyces cerevisiae (strain ATCC 204508 / S288c) GN=APL3 PE=1 SV=1

APL4 − average.top3: 6.81469450980392 − max.qupm: 14

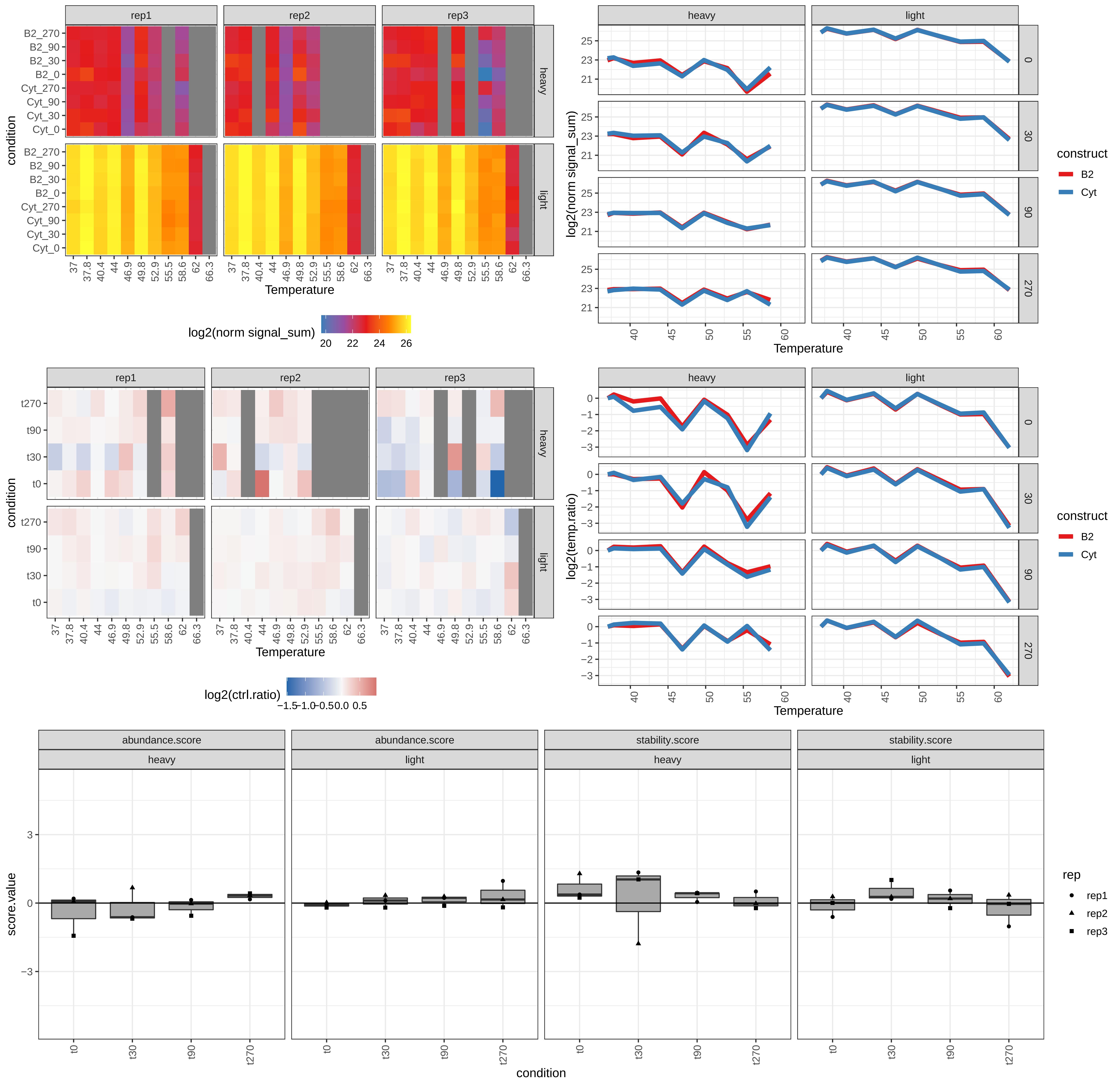

Q12028 − AP−1 complex subunit gamma−1 OS=Saccharomyces cerevisiae (strain ATCC 204508 / S288c) GN=APL4 PE=1 SV=1

APL5 − average.top3: 6.64543857142857 − max.qupm: 10

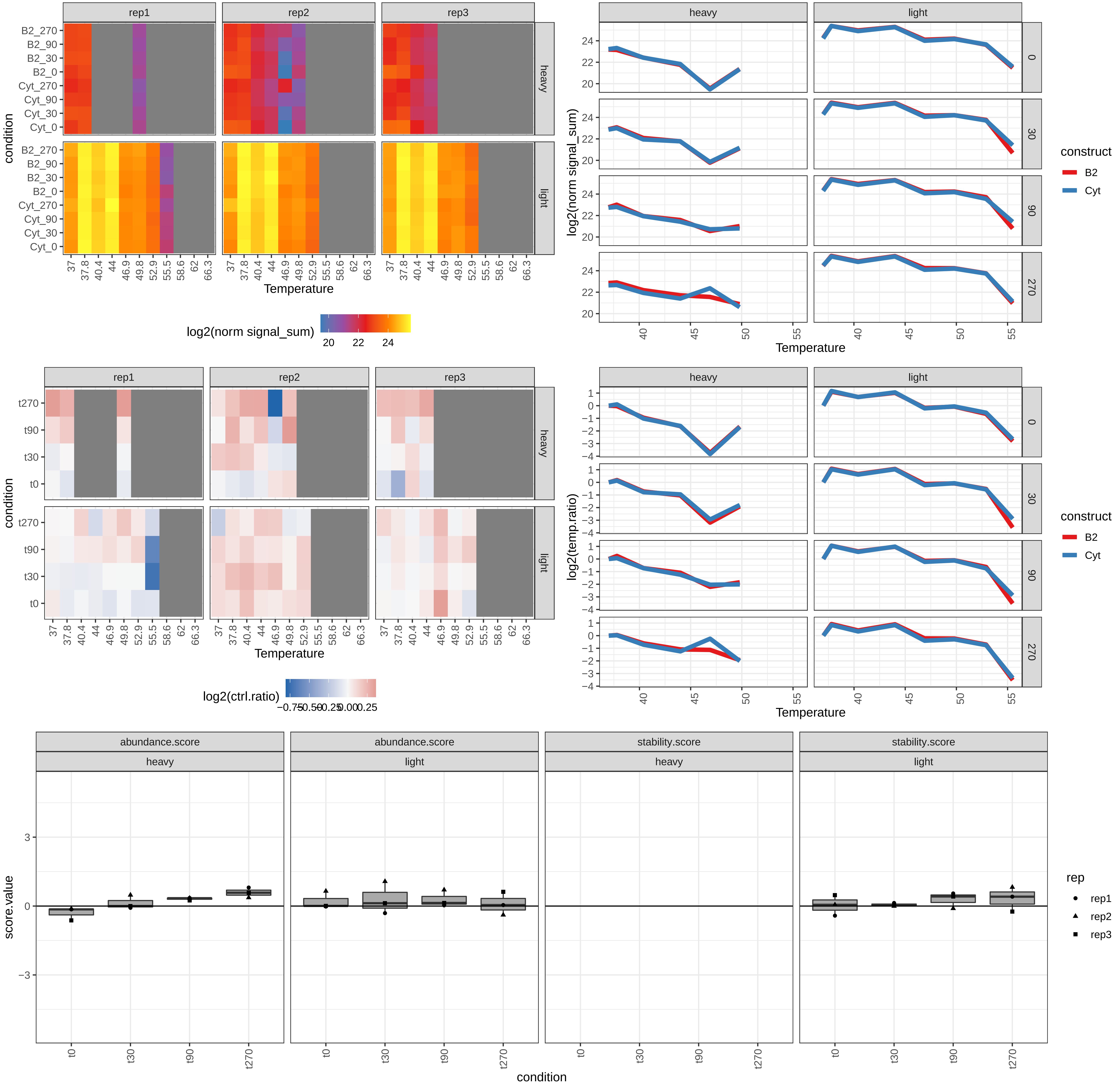

Q08951 − AP−3 complex subunit delta OS=Saccharomyces cerevisiae (strain ATCC 204508 / S288c) GN=APL5 PE=1 SV=1

APL6 − average.top3: 6.94024615384615 − max.qupm: 10

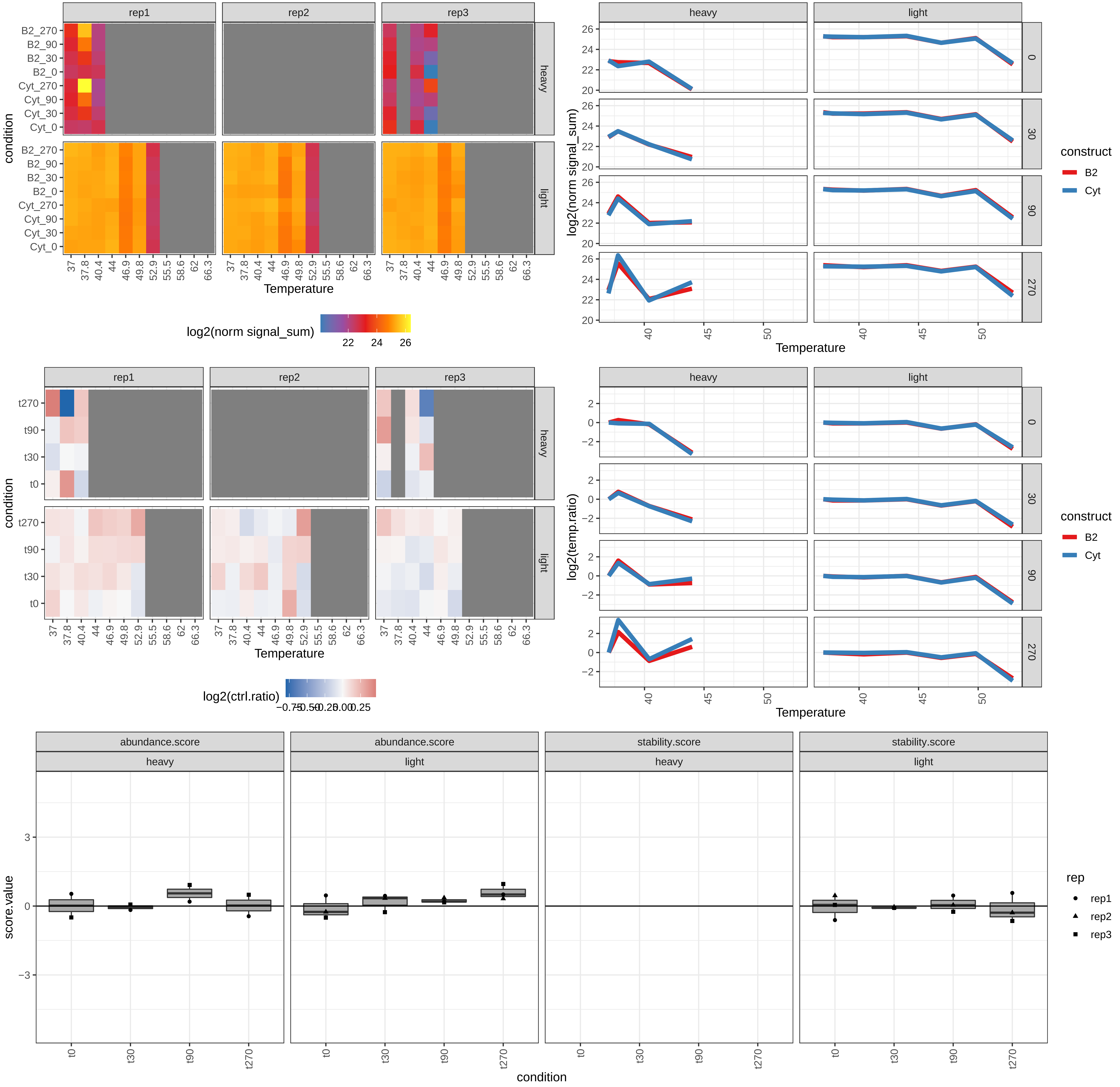

P46682 − AP−3 complex subunit beta OS=Saccharomyces cerevisiae (strain ATCC 204508 / S288c) GN=APL6 PE=1 SV=2

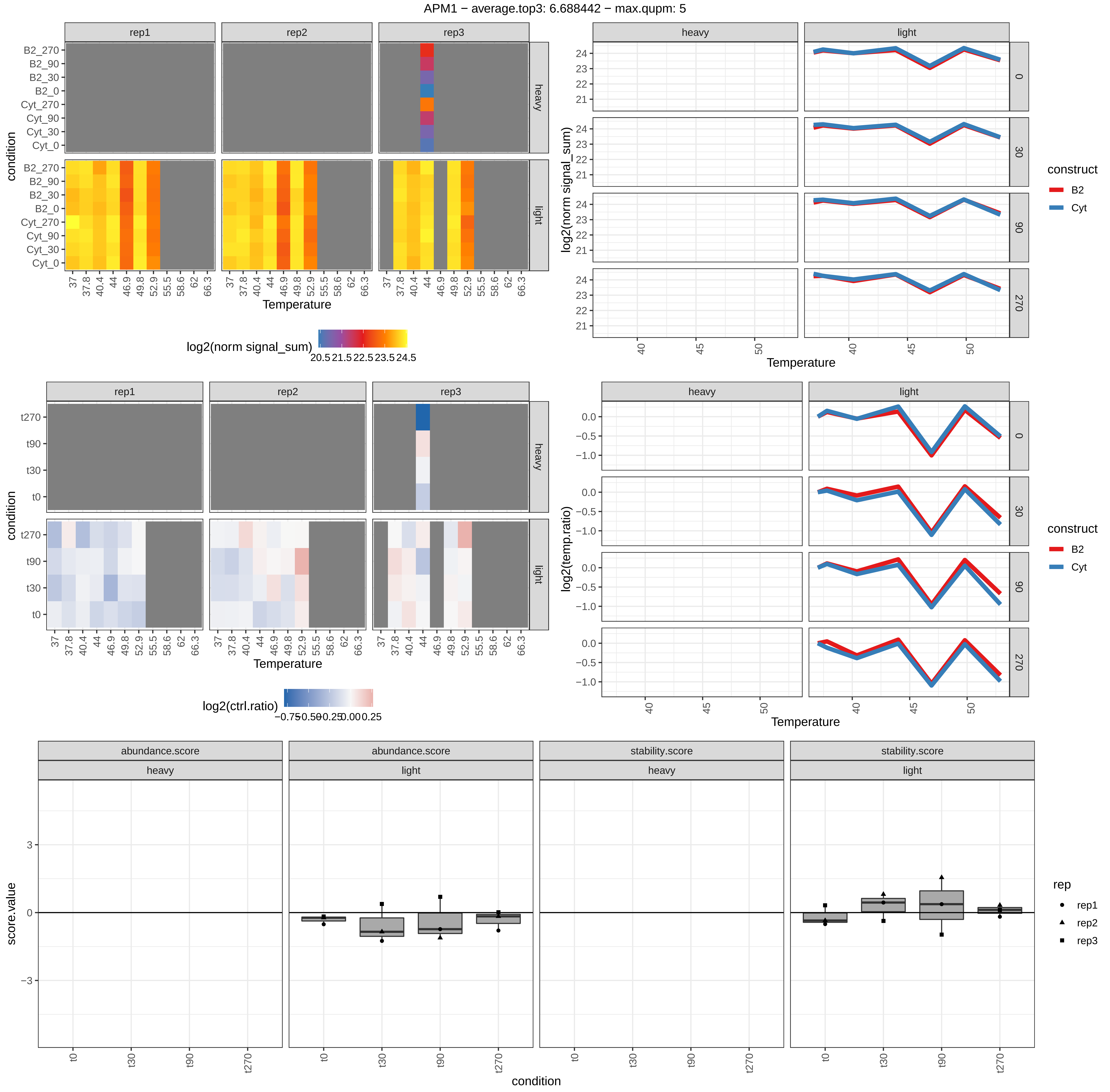

Q00776 − AP−1 complex subunit mu−1−I OS=Saccharomyces cerevisiae (strain ATCC 204508 / S288c) GN=APM1 PE=1 SV=2

APM3 − average.top3: 6.95299631578947 − max.qupm: 9

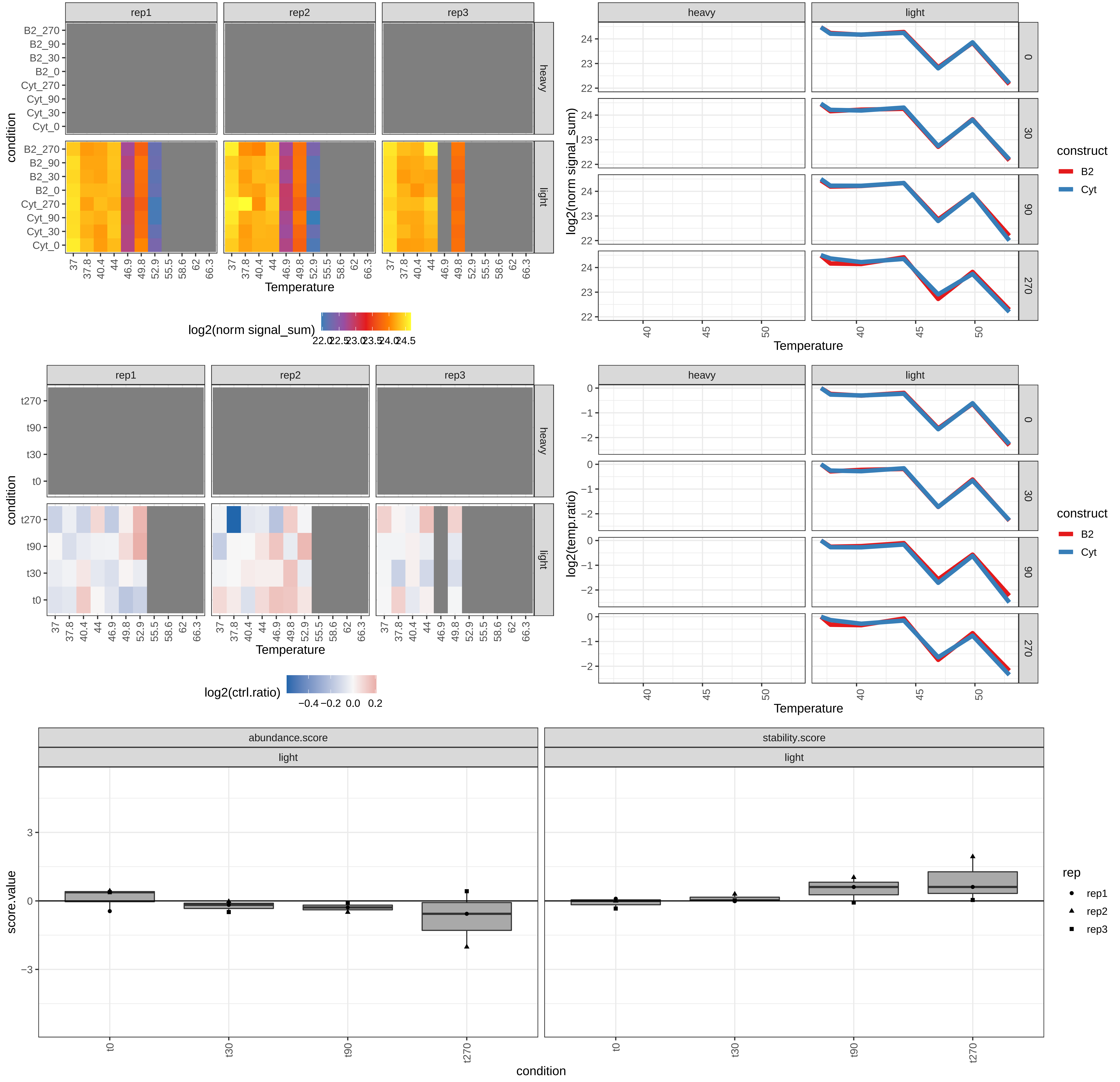

P38153 − AP−3 complex subunit mu OS=Saccharomyces cerevisiae (strain ATCC 204508 / S288c) GN=APM3 PE=1 SV=2

APM4 − average.top3: 6.95541454545455 − max.qupm: 7

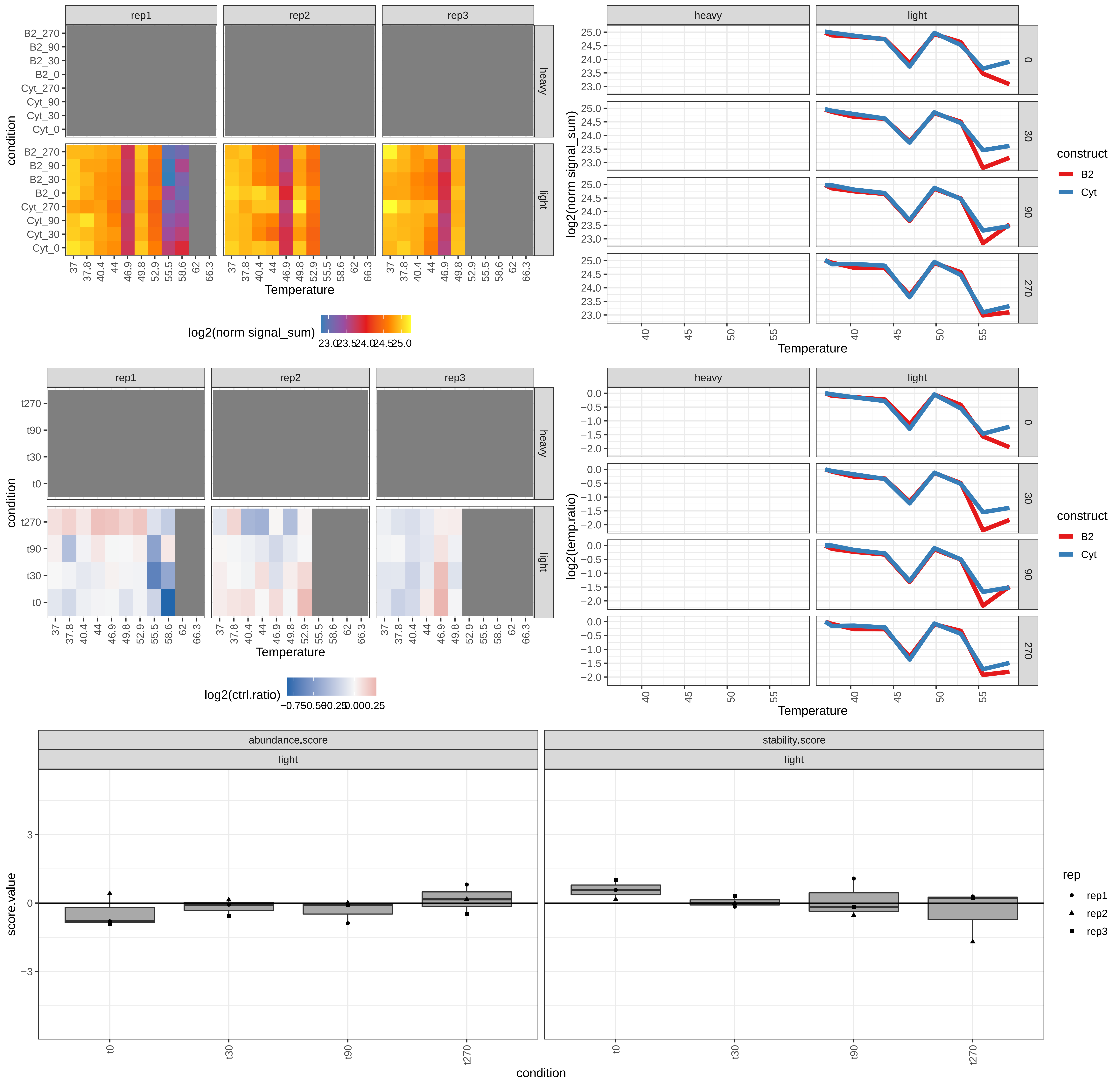

Q99186 − AP−2 complex subunit mu OS=Saccharomyces cerevisiae (strain ATCC 204508 / S288c) GN=APM4 PE=1 SV=1

APN1 − average.top3: 6.79803052631579 − max.qupm: 10

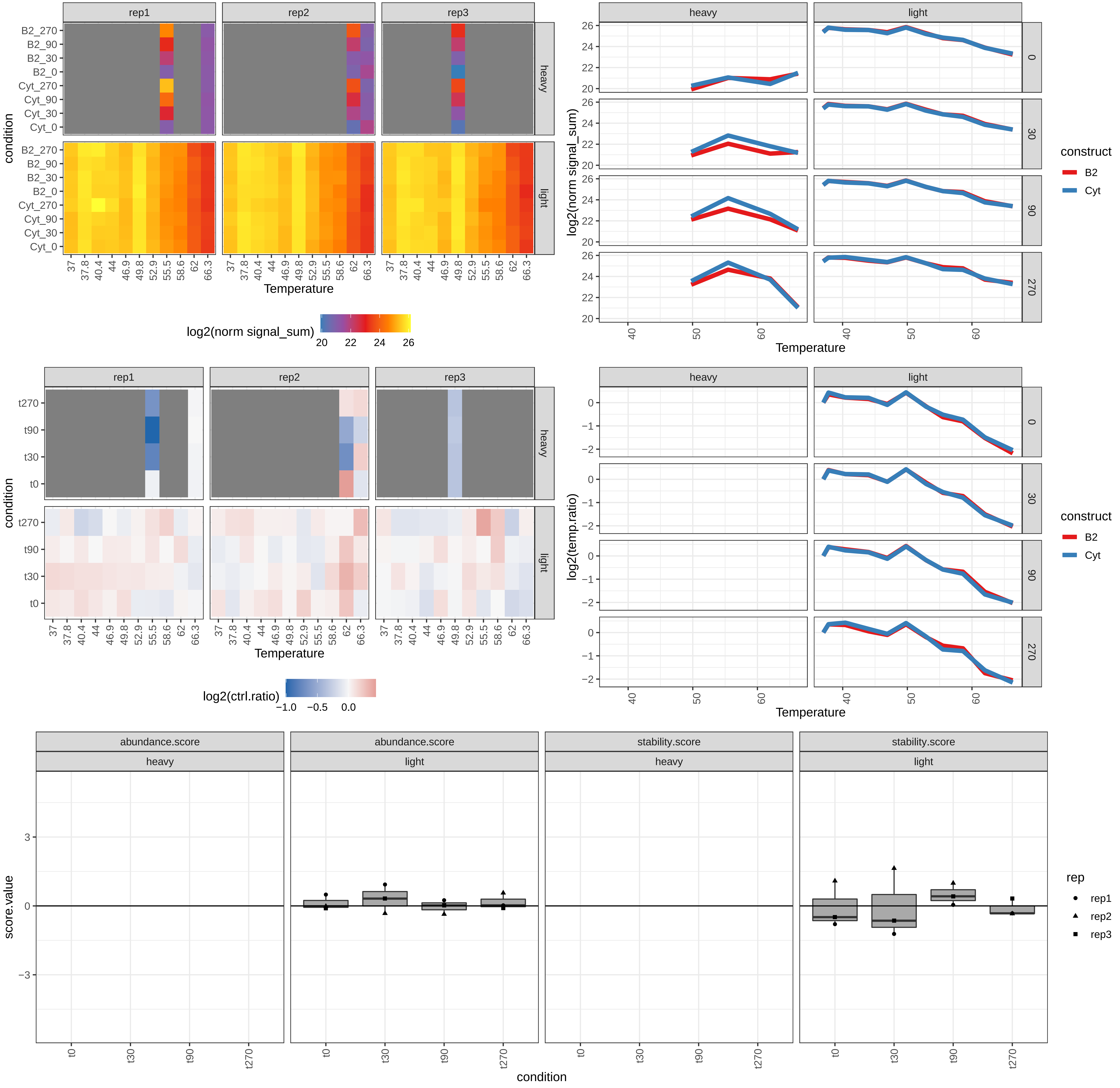

P22936 − DNA−(apurinic or apyrimidinic site) lyase 1 OS=Saccharomyces cerevisiae (strain ATCC 204508 / S288c) GN=APN1 PE=1 SV=4

APS2 − average.top3: 6.90778 − max.qupm: 5

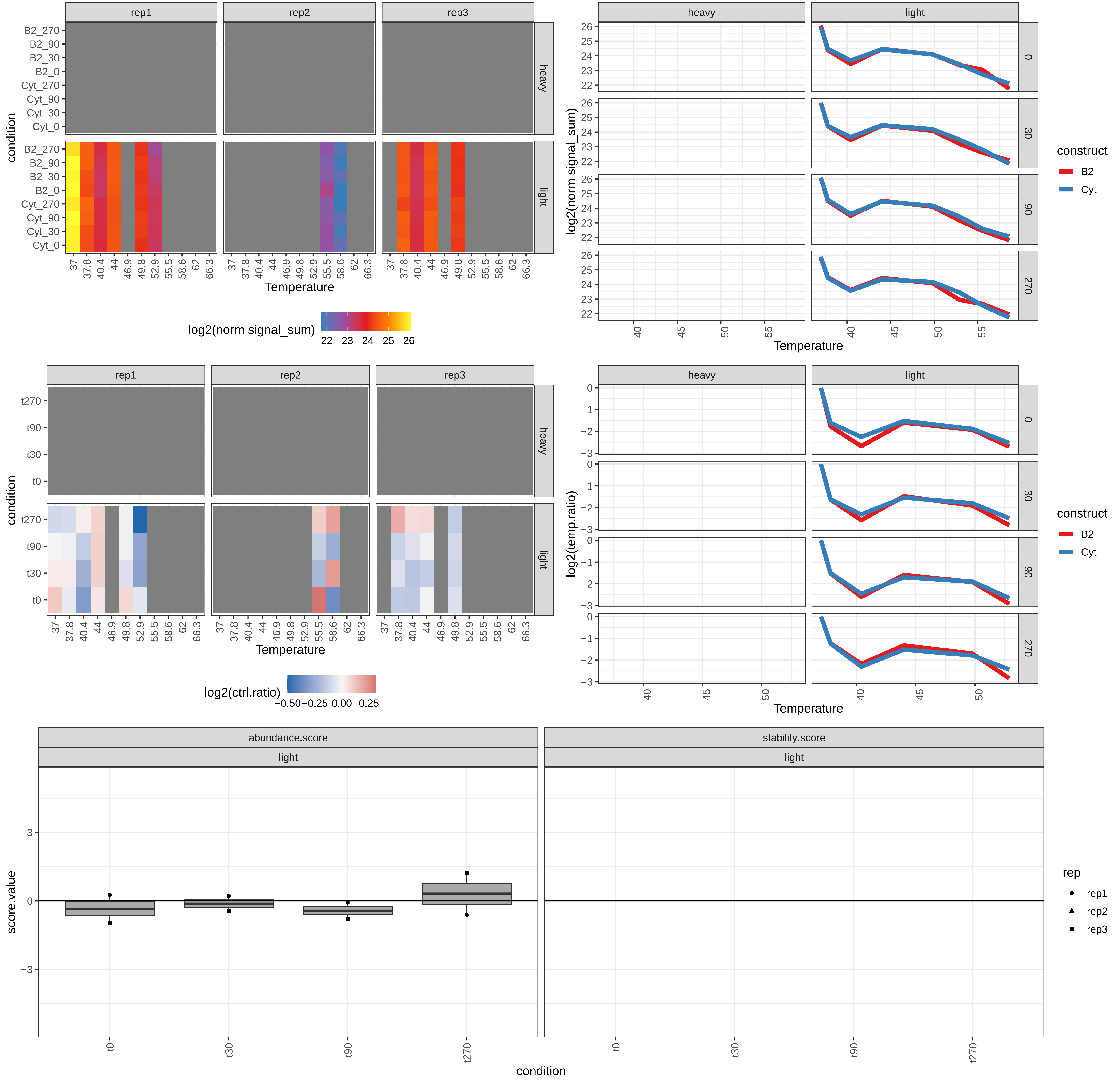

Q00381 − AP−2 complex subunit sigma OS=Saccharomyces cerevisiae (strain ATCC 204508 / S288c) GN=APS2 PE=1 SV=1

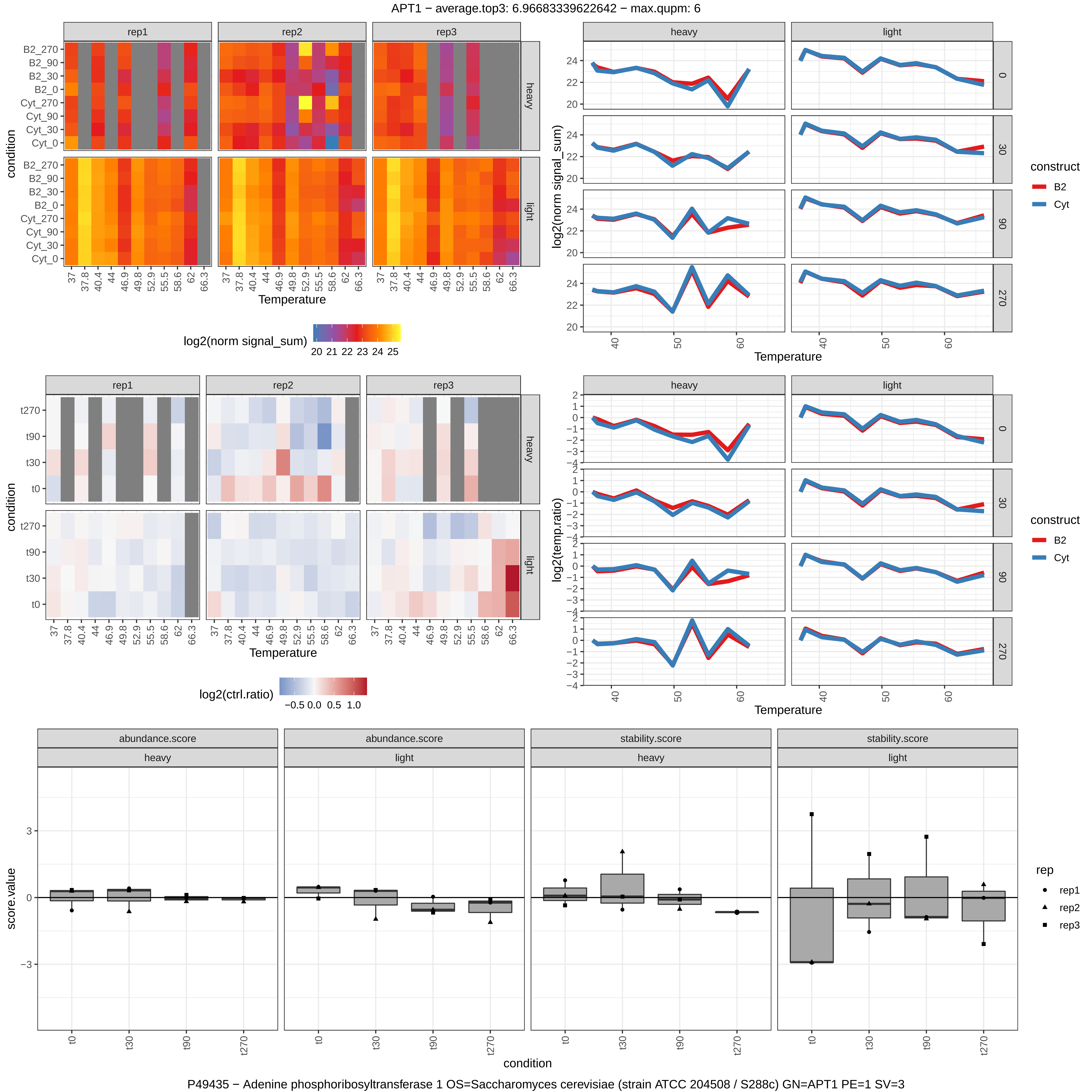
APT1 − average.top3: 6.96683339622642 − max.qupm: 6
rep1
rep2
rep3
heavy
light
condition
Temperature
log2(norm signal_sum)
log2(norm signal_sum)
construct
B2
Cyt
log2(ctrl.ratio)
log2(temp.ratio)
abundance.score
stability.score
score.value
rep
rep1
rep2
rep3
P49435 − Adenine phosphoribosyltransferase 1 OS=Saccharomyces cerevisiae (strain ATCC 204508 / S288c) GN=APT1 PE=1 SV=3

ARA1 − average.top3: 7.76122696969697 − max.qupm: 17

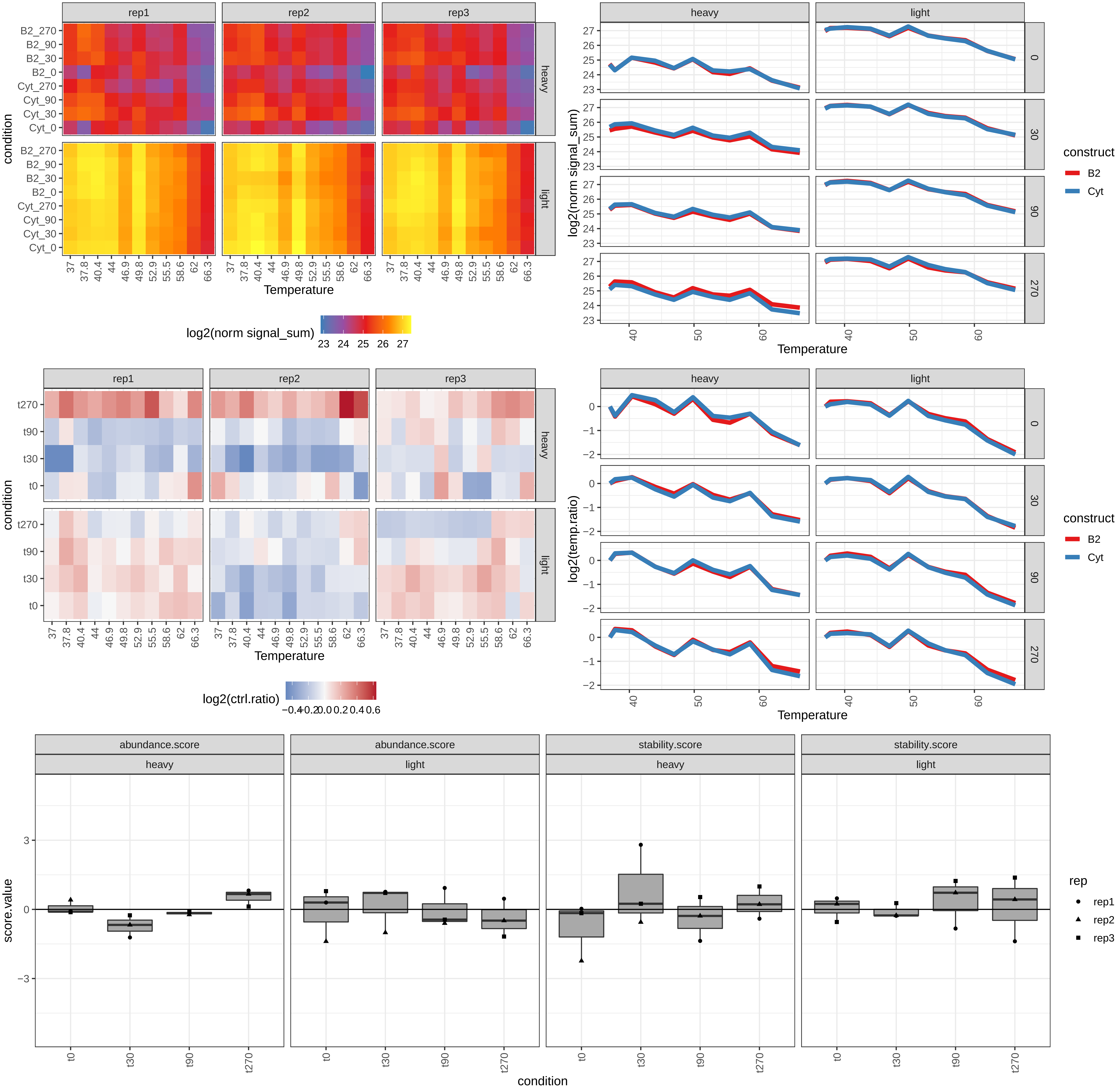

P38115 − D−arabinose dehydrogenase [NAD(P)+] heavy chain OS=Saccharomyces cerevisiae (strain ATCC 204508 / S288c) GN=ARA1 PE=1 SV=1

ARA2 − average.top3: 6.83447 − max.qupm: 6

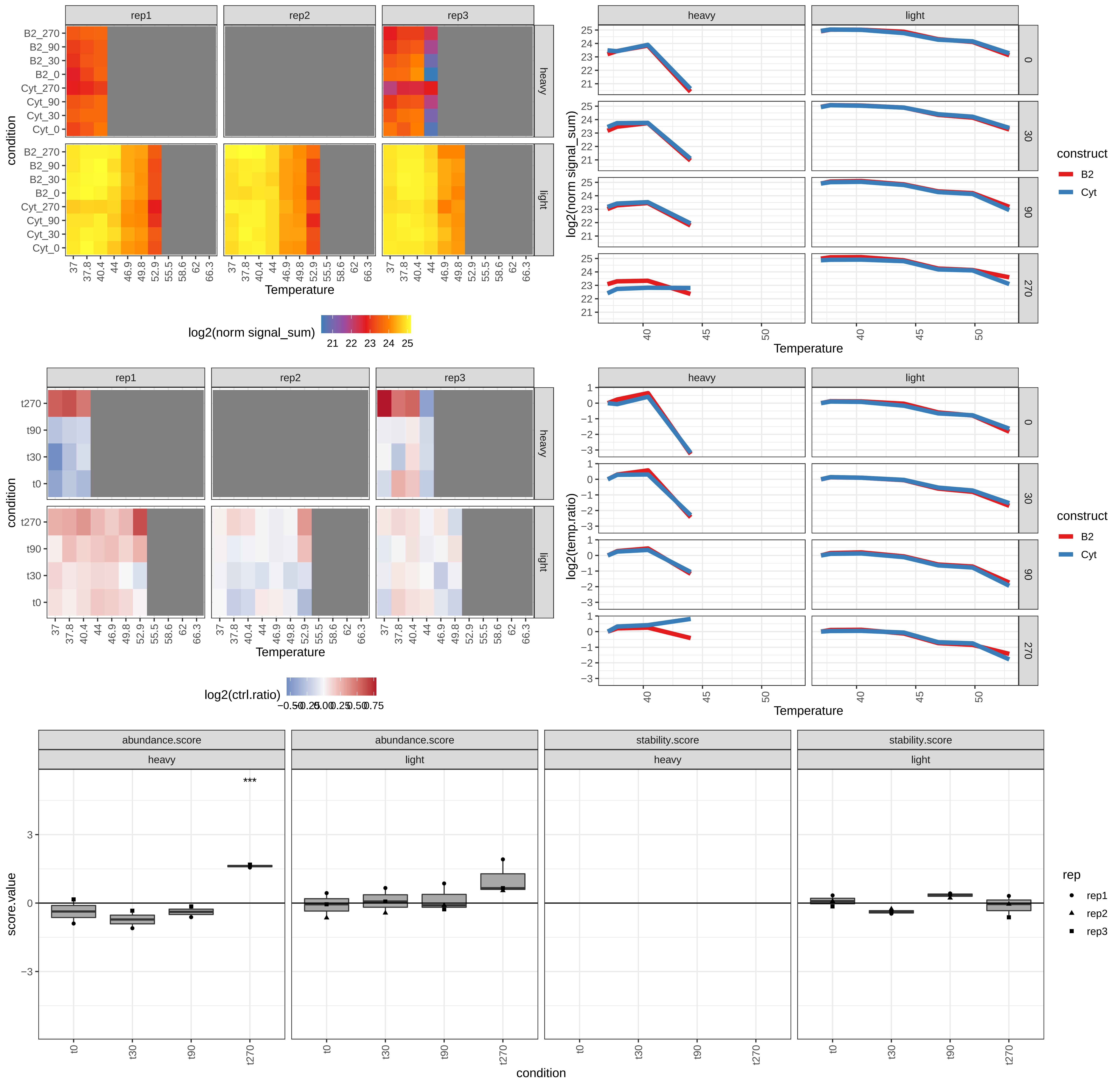

Q04212 − D−arabinose 1−dehydrogenase OS=Saccharomyces cerevisiae (strain ATCC 204508 / S288c) GN=ARA2 PE=1 SV=1

ARB1 − average.top3: 6.86321052631579 − max.qupm: 11

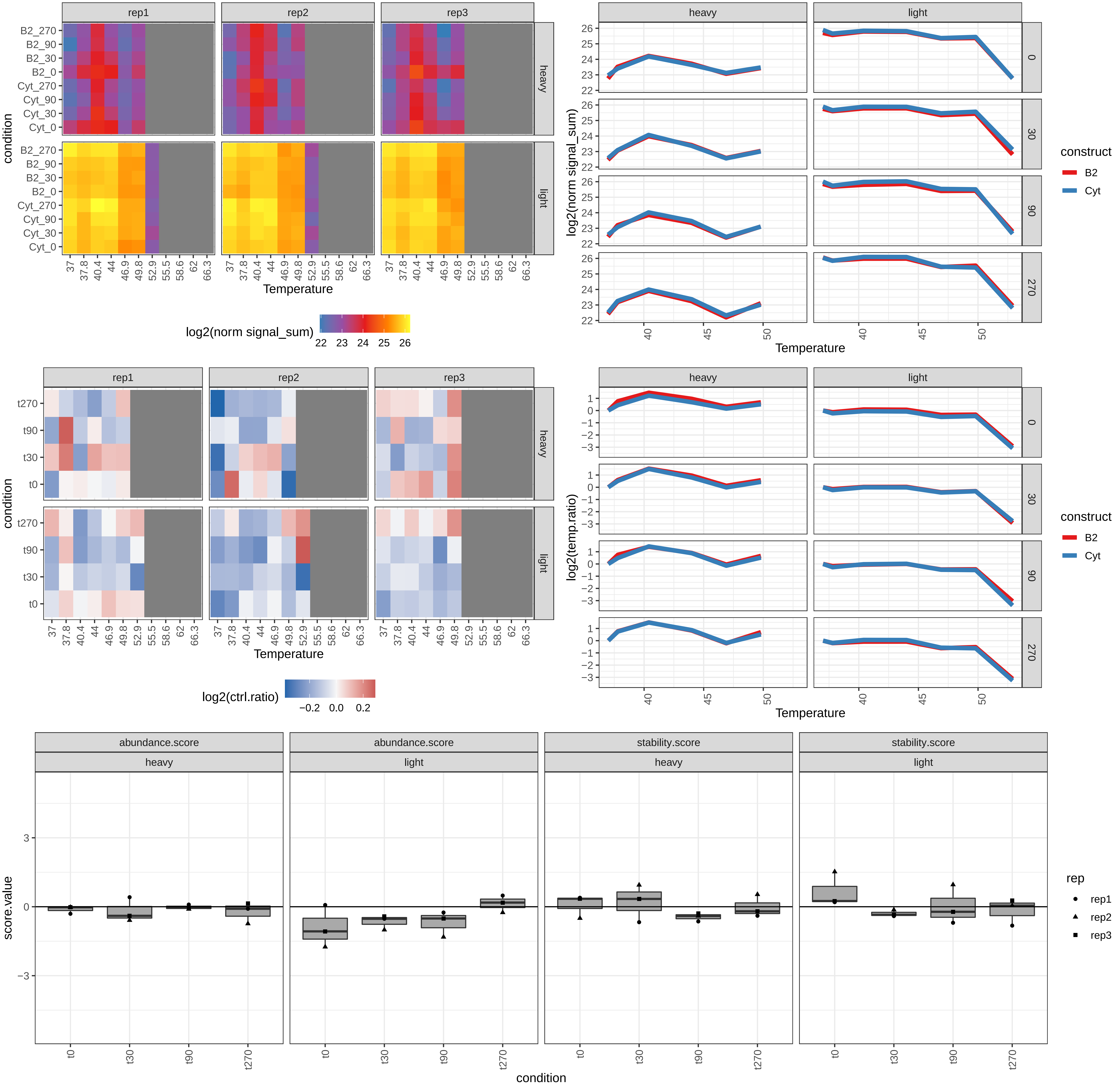

P40024 − ABC transporter ATP−binding protein ARB1 OS=Saccharomyces cerevisiae (strain ATCC 204508 / S288c) GN=ARB1 PE=1 SV=1

ARC1 − average.top3: 7.15871226415094 − max.qupm: 15

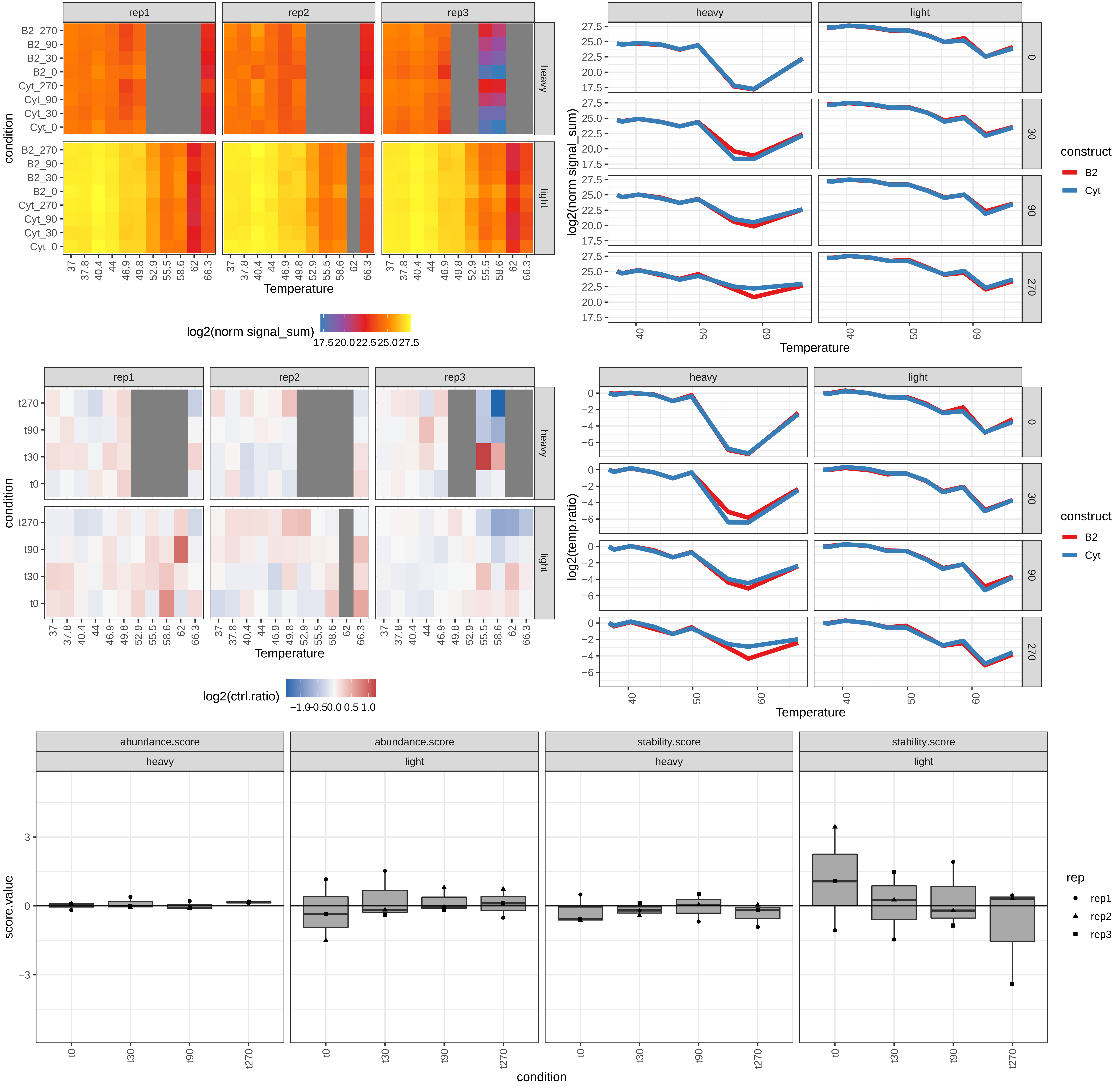

P46672 − tRNA−aminoacylation cofactor ARC1 OS=Saccharomyces cerevisiae (strain ATCC 204508 / S288c) GN=ARC1 PE=1 SV=2

ARC15 − average.top3: 6.89345625 − max.qupm: 6

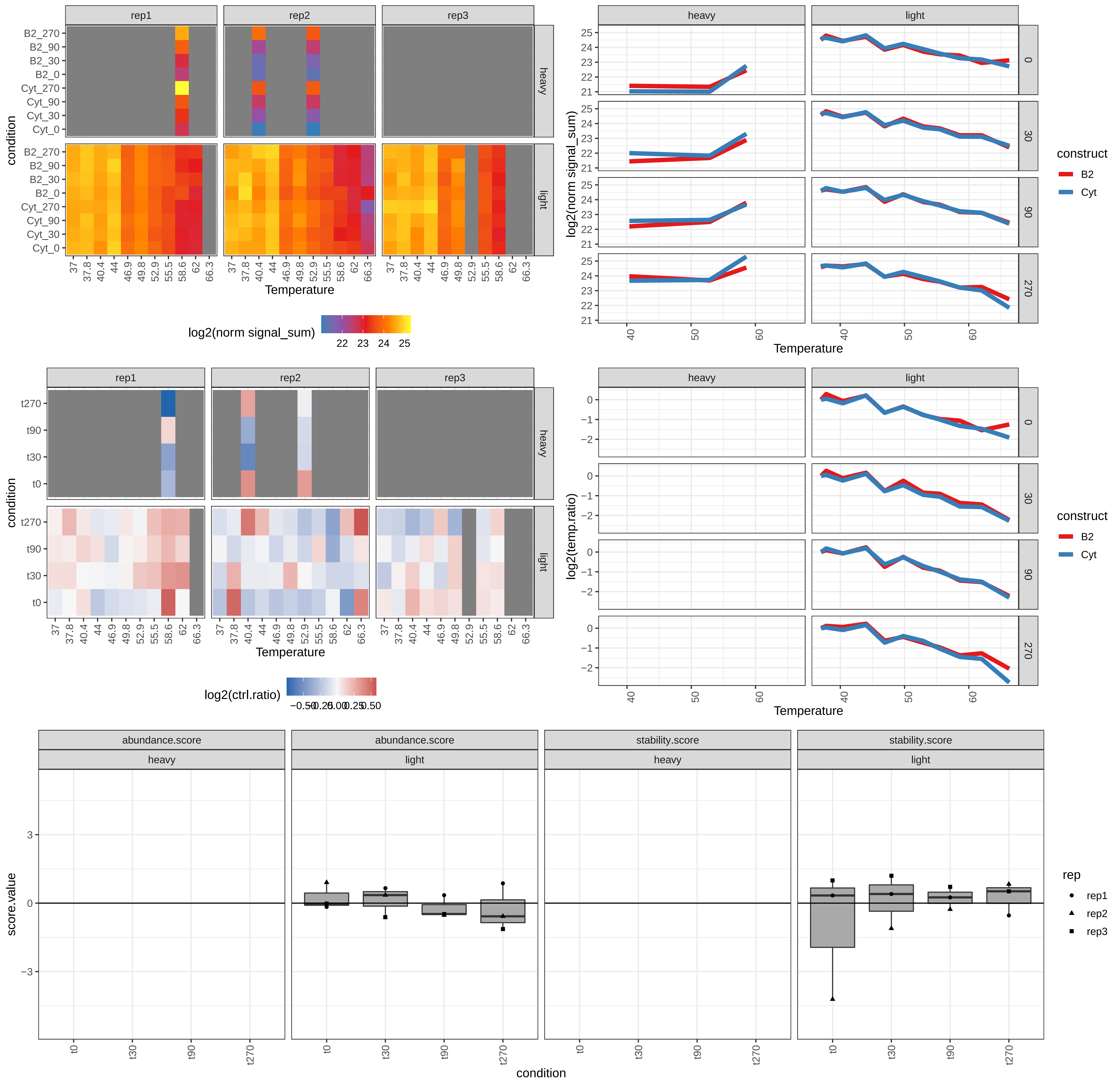

P40518 − Actin−related protein 2/3 complex subunit 5 OS=Saccharomyces cerevisiae (strain ATCC 204508 / S288c) GN=ARC15 PE=1 SV=1

ARC18 − average.top3: 7.3548196969697 − max.qupm: 5

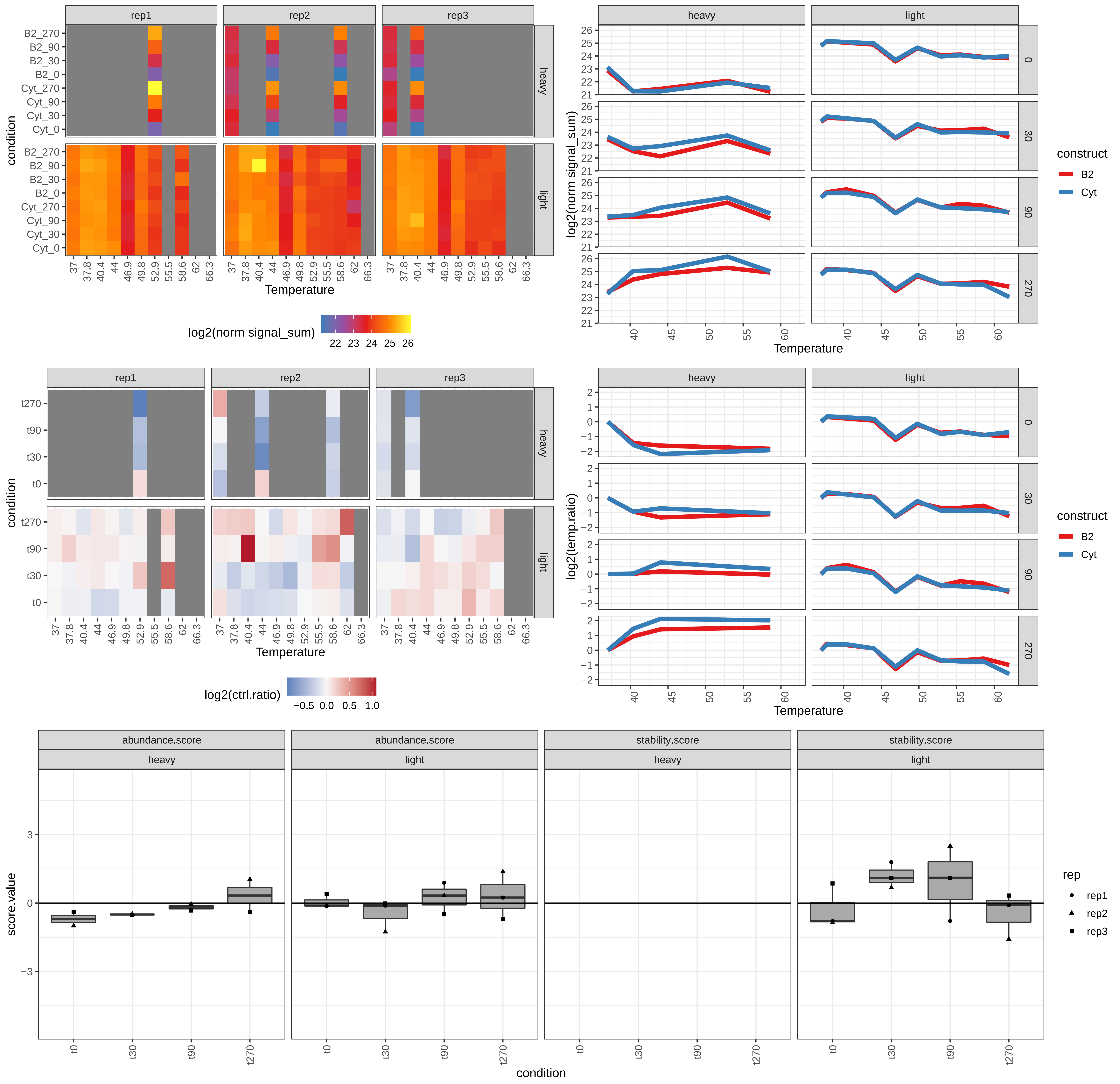

Q05933 − Actin−related protein 2/3 complex subunit 3 OS=Saccharomyces cerevisiae (strain ATCC 204508 / S288c) GN=ARC18 PE=1 SV=1

ARC19 − average.top3: 7.30189041666667 − max.qupm: 5

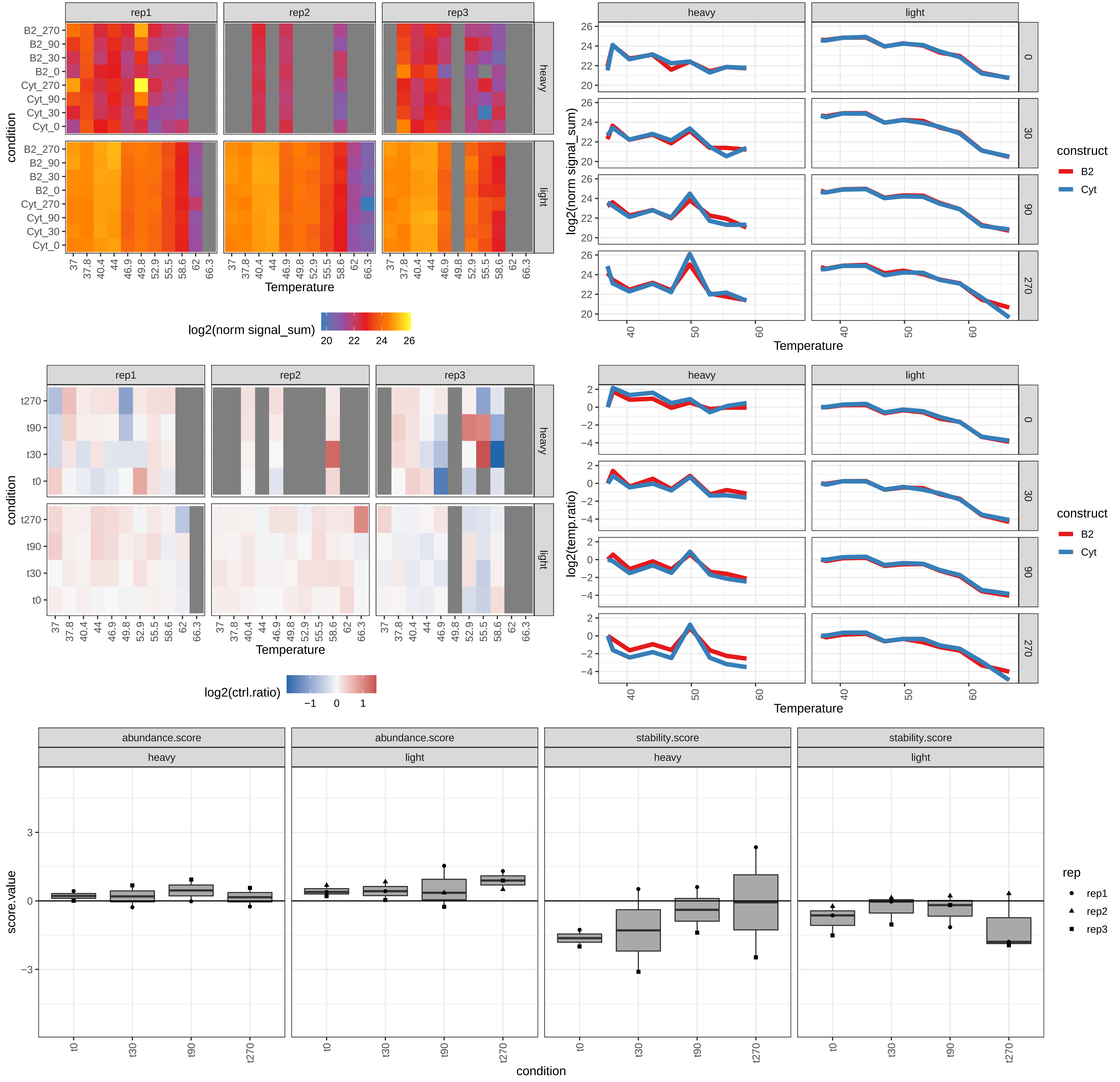

P33204 − Actin−related protein 2/3 complex subunit 4 OS=Saccharomyces cerevisiae (strain ATCC 204508 / S288c) GN=ARC19 PE=1 SV=2

ARC35 − average.top3: 7.21631037037037 − max.qupm: 12

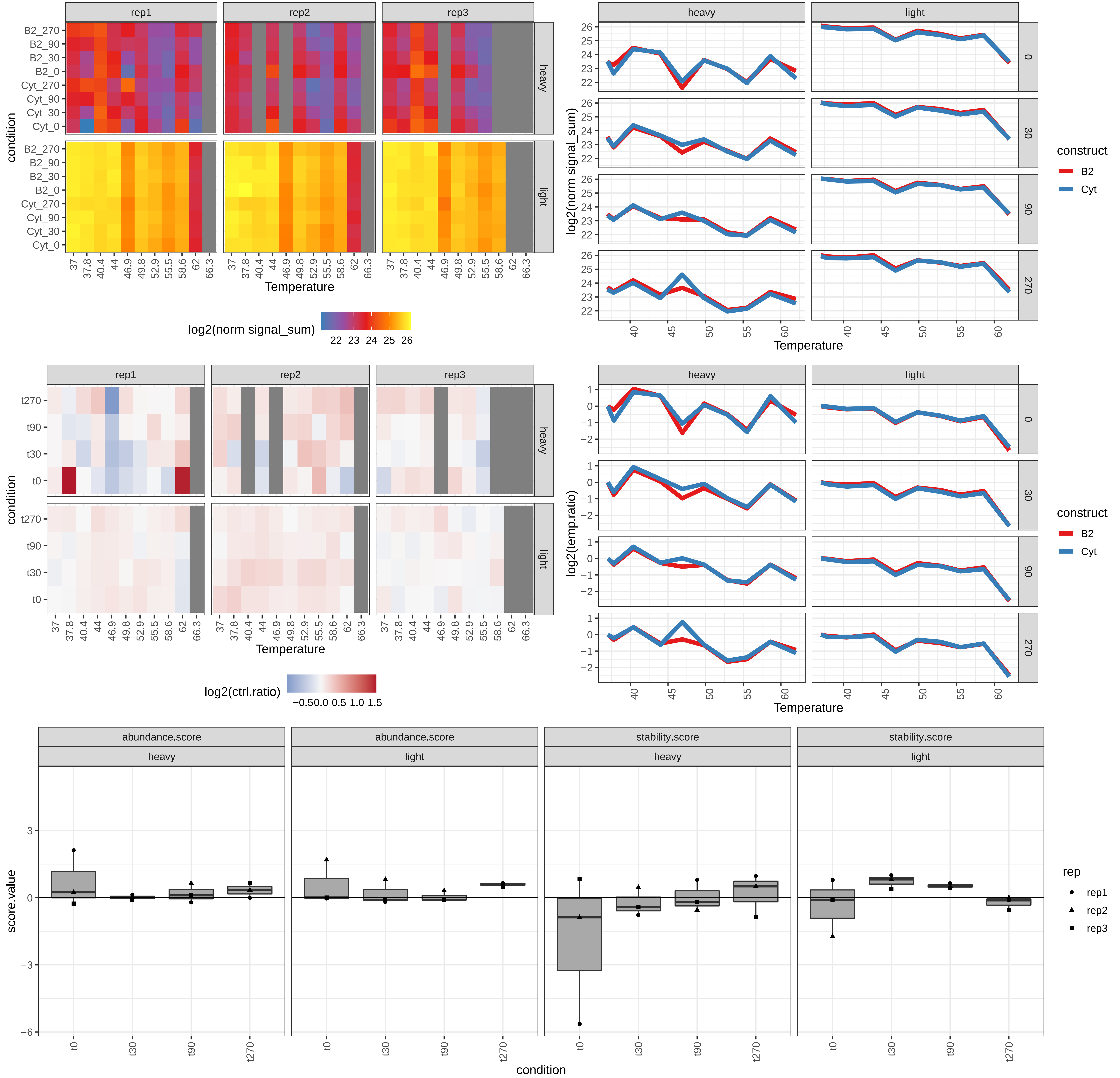

P53731 − Actin−related protein 2/3 complex subunit 2 OS=Saccharomyces cerevisiae (strain ATCC 204508 / S288c) GN=ARC35 PE=1 SV=1

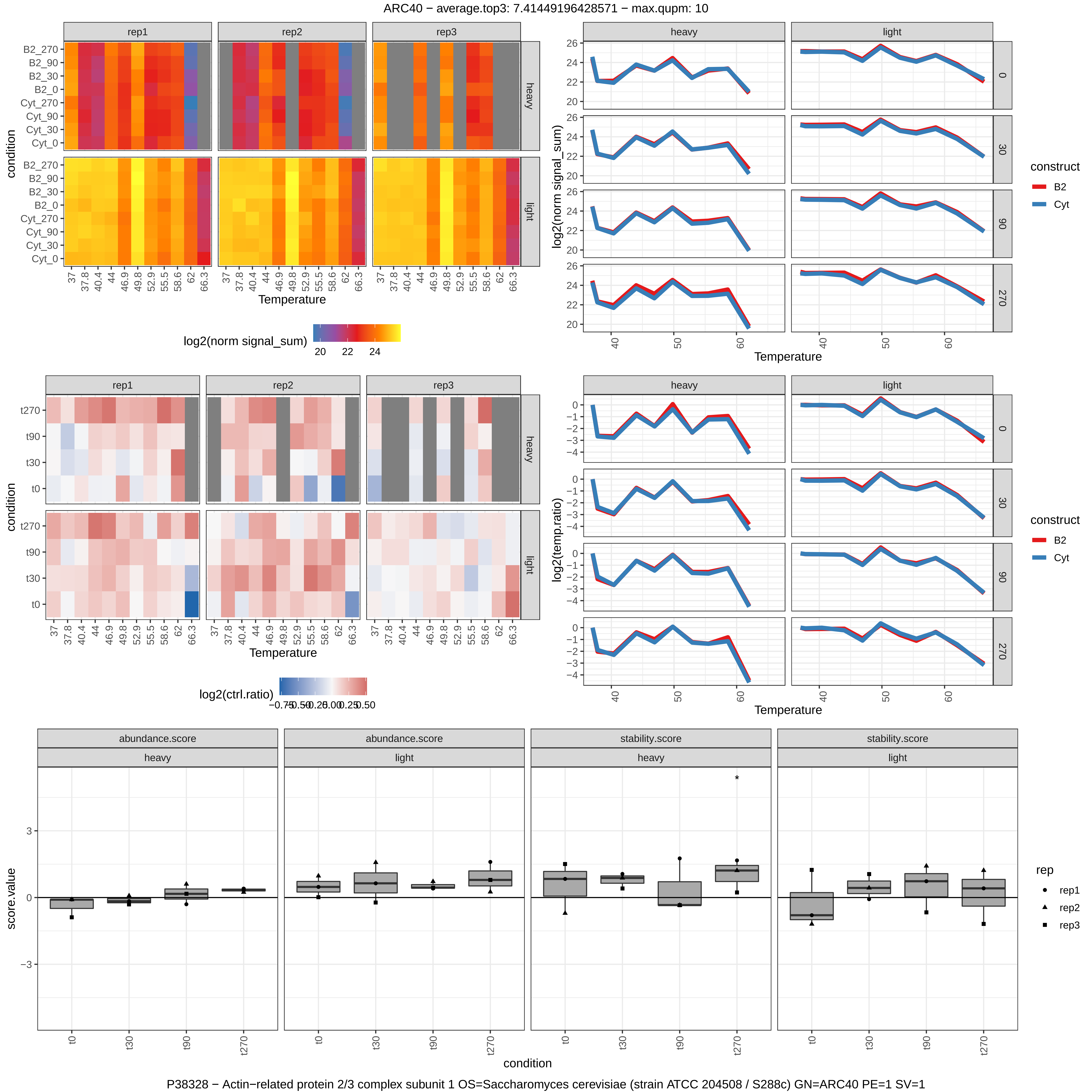
ARC40 − average.top3: 7.41449196428571 − max.qupm: 10
rep1
rep2
rep3
heavy
light
condition
Temperature
log2(norm signal_sum)
log2(ctrl.ratio)
log2(temp.ratio)
construct
B2
Cyt
abundance.score
stability.score
score.value
rep
rep1
rep2
rep3
P38328 − Actin−related protein 2/3 complex subunit 1 OS=Saccharomyces cerevisiae (strain ATCC 204508 / S288c) GN=ARC40 PE=1 SV=1

ARD1 − average.top3: 6.63024666666667 − max.qupm: 4

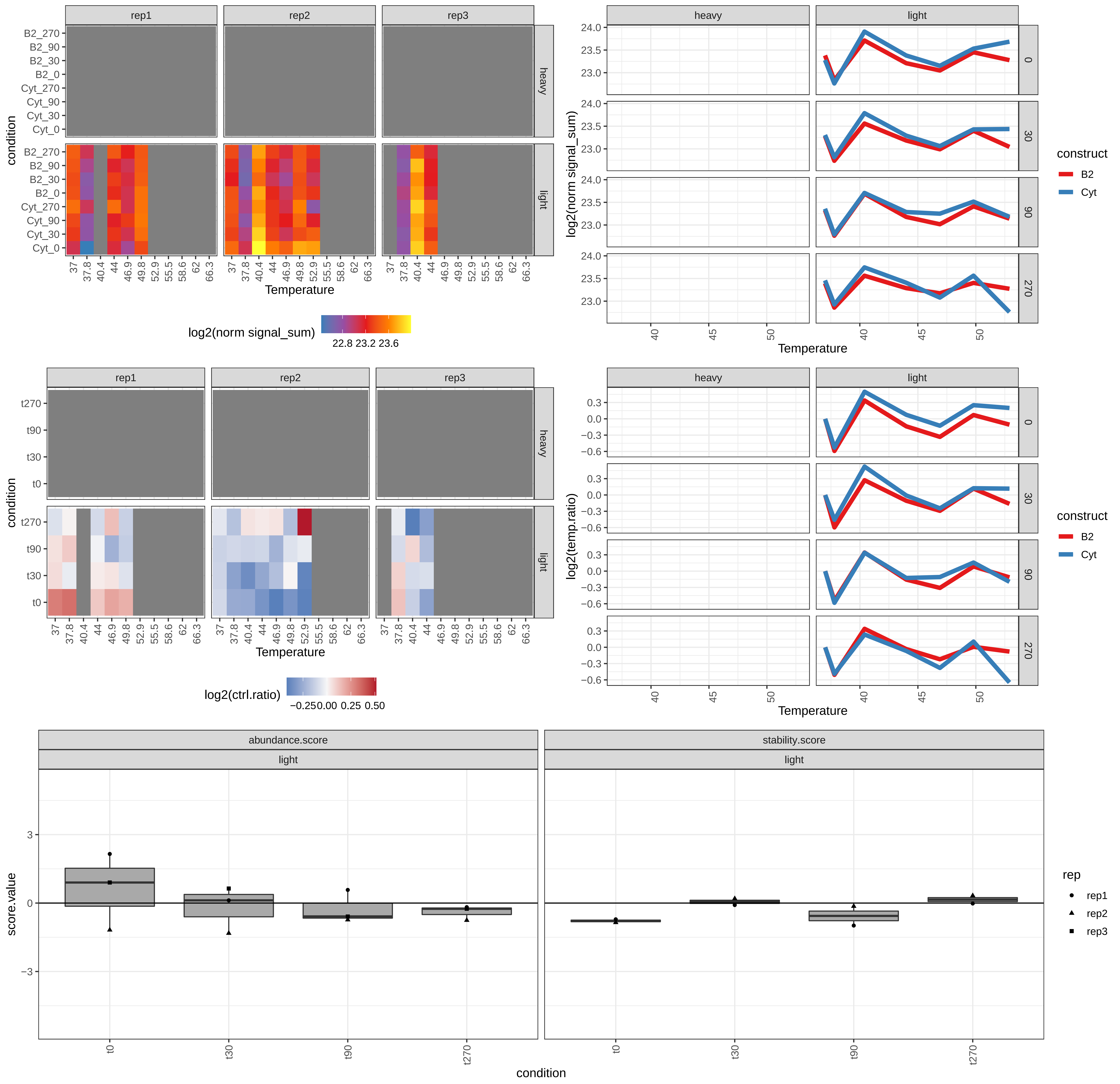

P07347 − N−terminal acetyltransferase A complex catalytic subunit ARD1 OS=Saccharomyces cerevisiae (strain ATCC 204508 / S288c) GN=ARD1 PE=1 SV=2

ARE2 − average.top3: 6.95620444444444 − max.qupm: 5

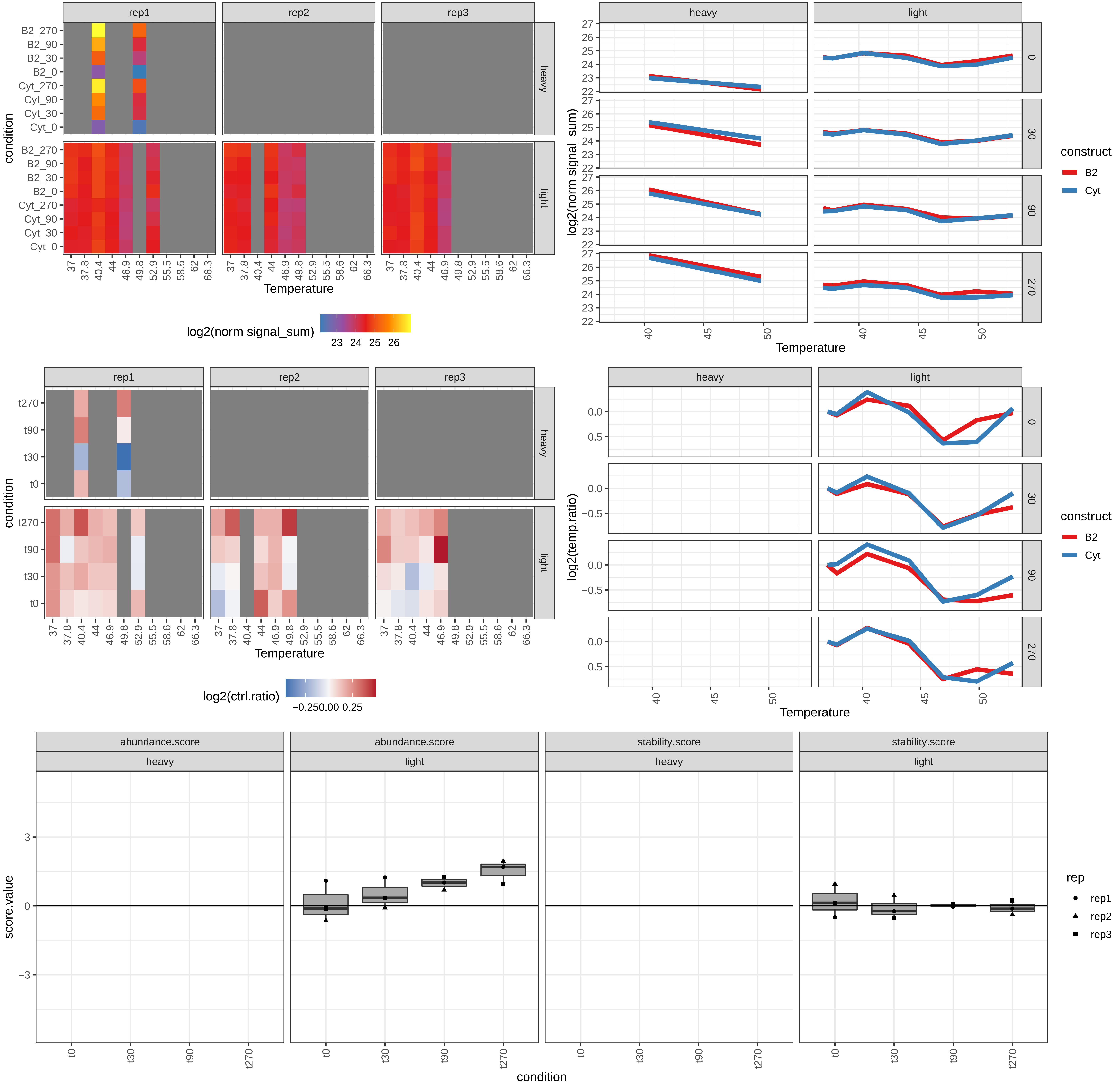

P53629 − Sterol O−acyltransferase 2 OS=Saccharomyces cerevisiae (strain ATCC 204508 / S288c) GN=ARE2 PE=1 SV=1

ARG1 − average.top3: 8.39585515151515 − max.qupm: 22

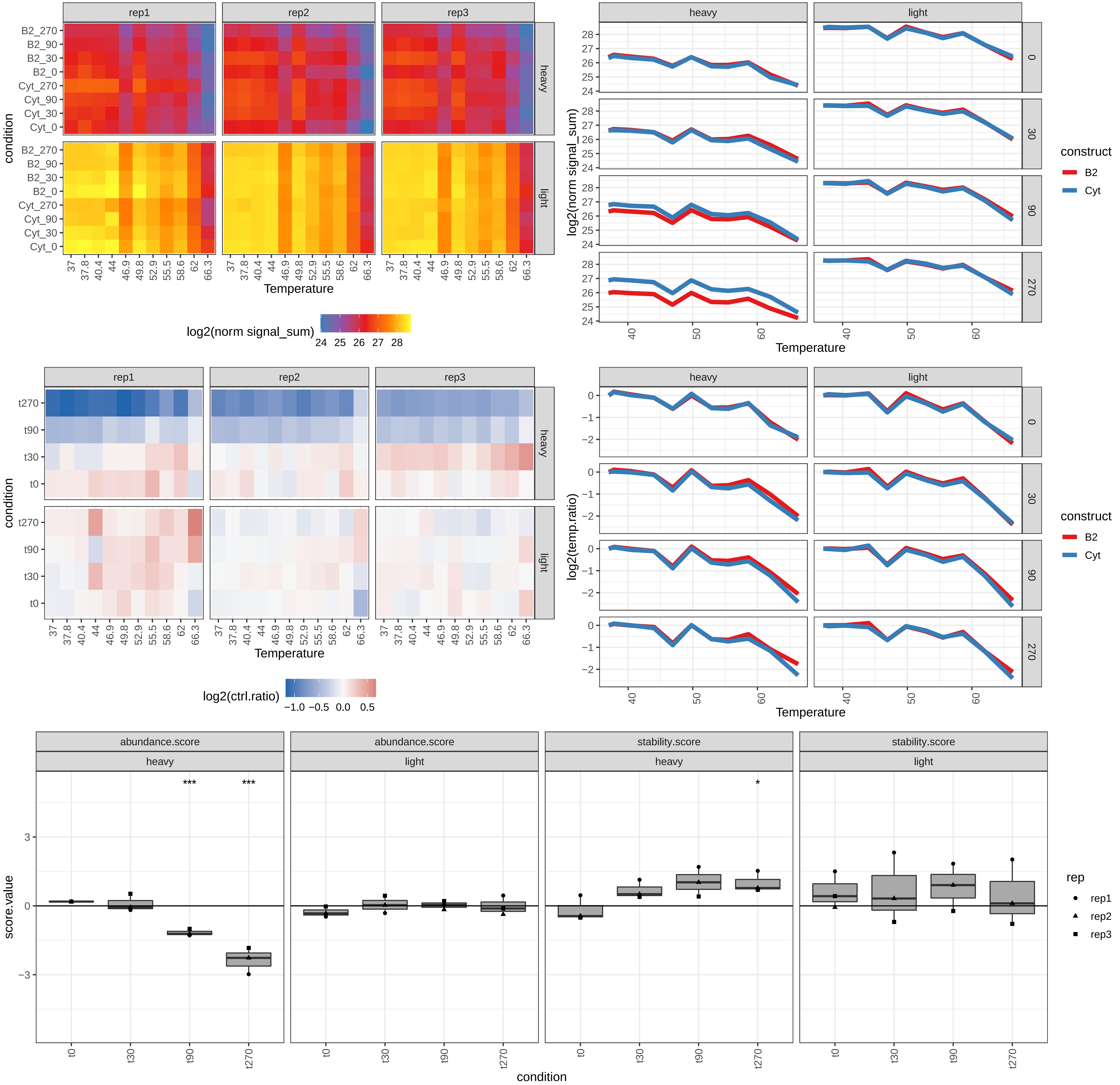

P22768 − Argininosuccinate synthase OS=Saccharomyces cerevisiae (strain ATCC 204508 / S288c) GN=ARG1 PE=1 SV=3

ARG2 − average.top3: 6.760780625 − max.qupm: 4

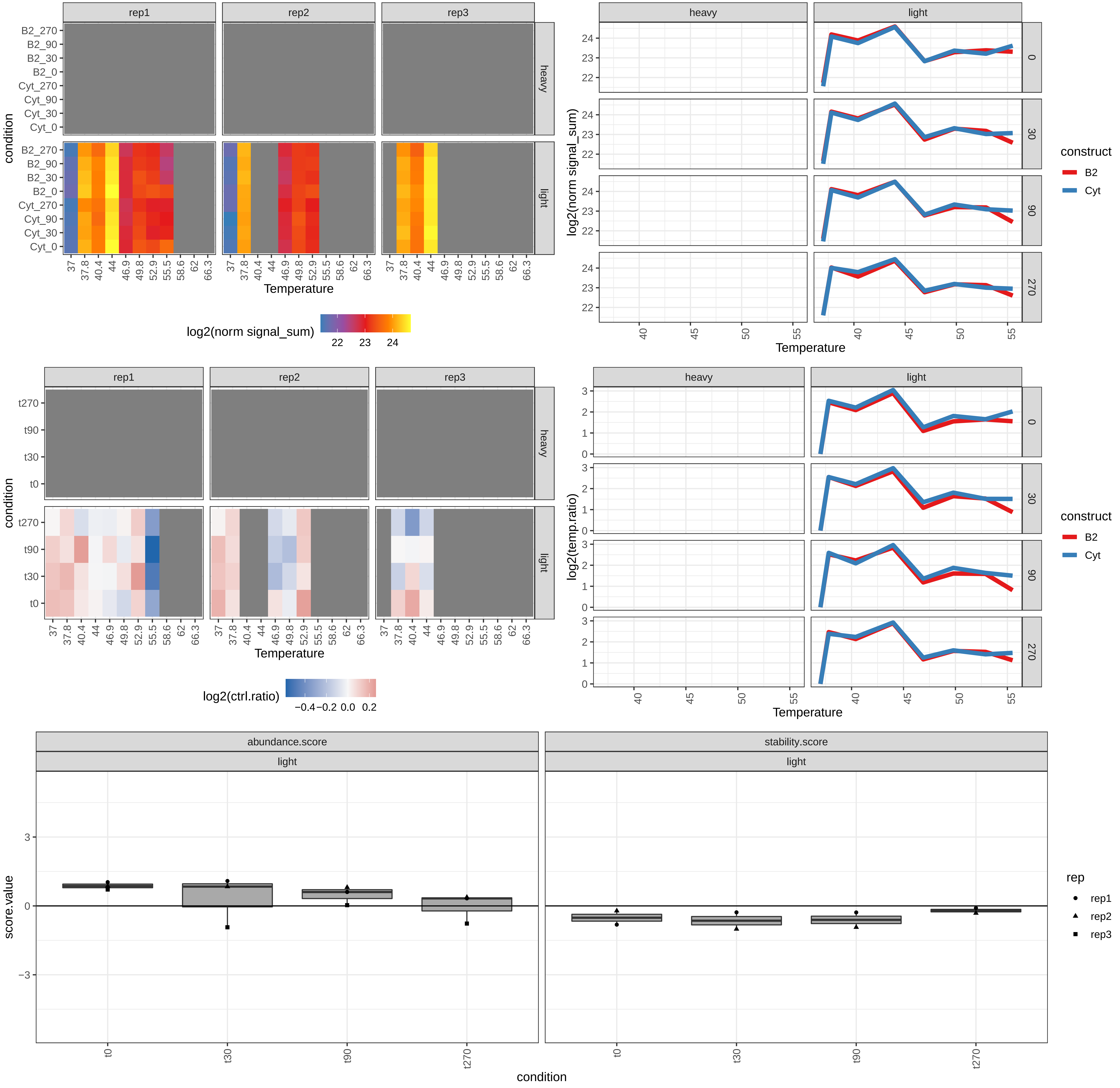

P40360 − Amino−acid acetyltransferase, mitochondrial OS=Saccharomyces cerevisiae (strain ATCC 204508 / S288c) GN=ARG2 PE=1 SV=1

ARG3 − average.top3: 7.40064060606061 − max.qupm: 11

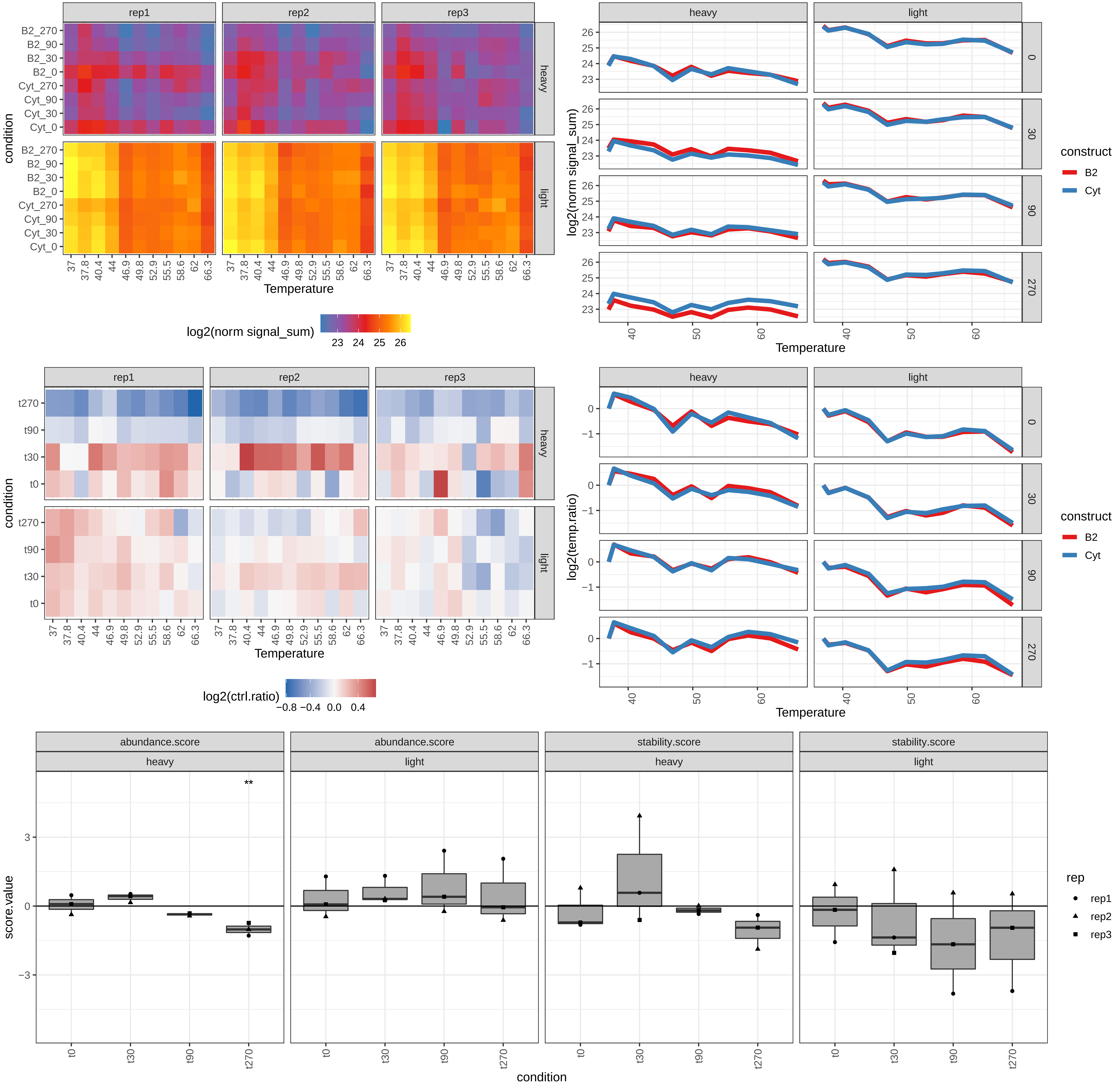

P05150 − Ornithine carbamoyltransferase OS=Saccharomyces cerevisiae (strain ATCC 204508 / S288c) GN=ARG3 PE=1 SV=1

ARG5 − average.top3: 7.51348648148148 − max.qupm: 25

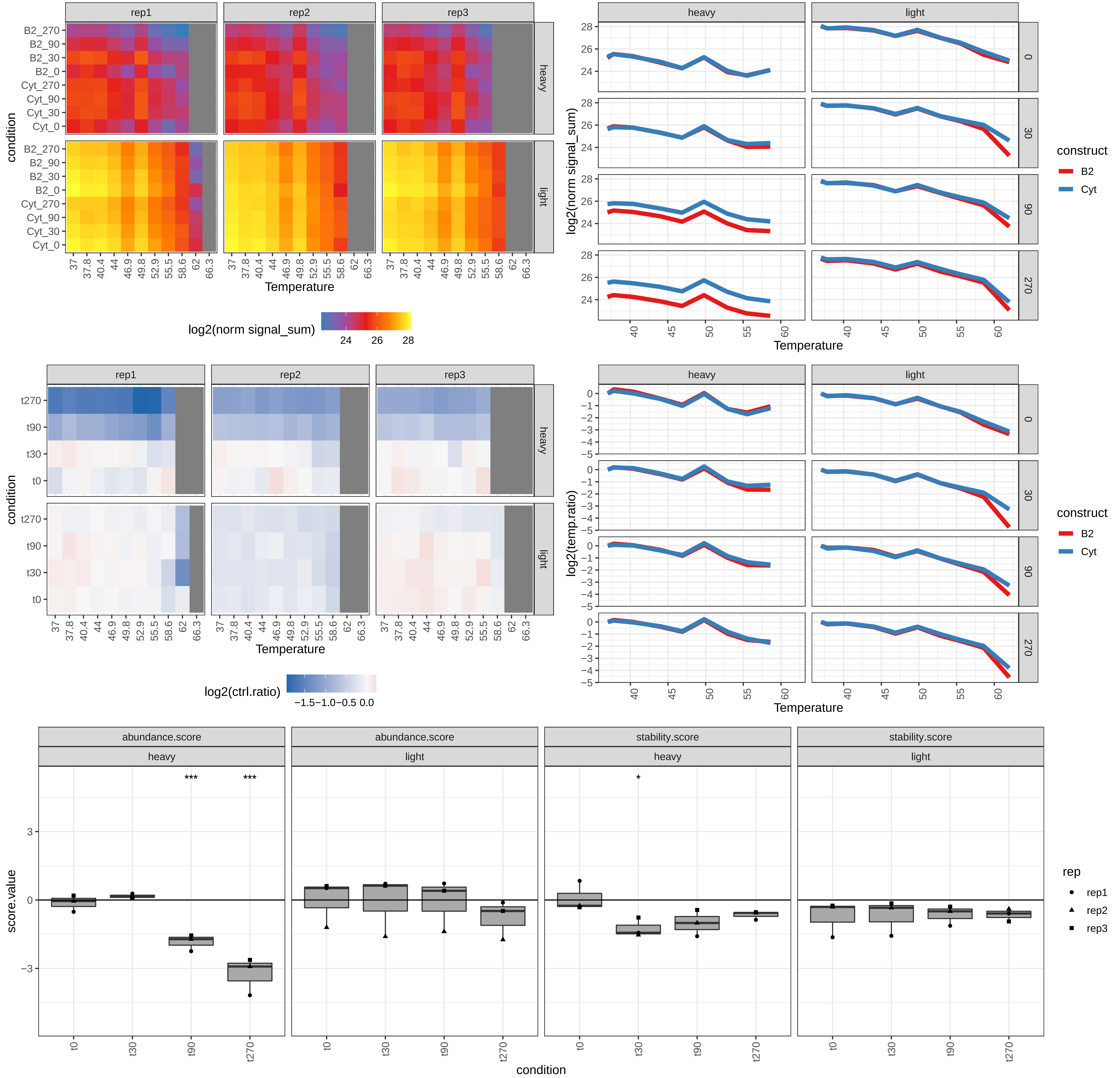

Q01217 − Protein ARG5,6, mitochondrial OS=Saccharomyces cerevisiae (strain ATCC 204508 / S288c) GN=ARG5,6 PE=1 SV=1

ARG7 − average.top3: 7.30870148148148 − max.qupm: 19

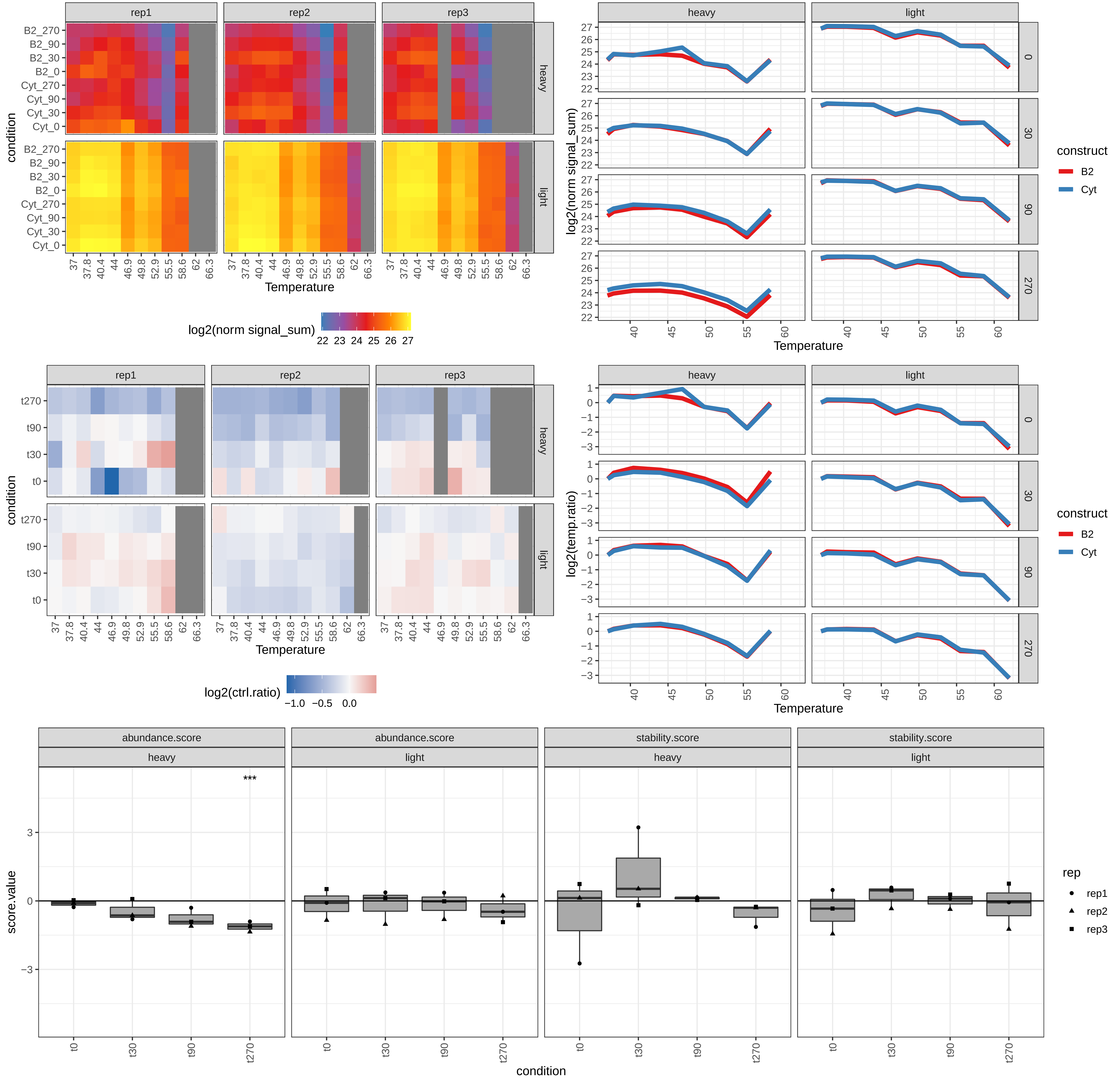

Q04728 − Arginine biosynthesis bifunctional protein ArgJ, mitochondrial OS=Saccharomyces cerevisiae (strain ATCC 204508 / S288c) GN=ARG7 PE=1 SV=1

ARG8 − average.top3: 7.28050442622951 − max.qupm: 11

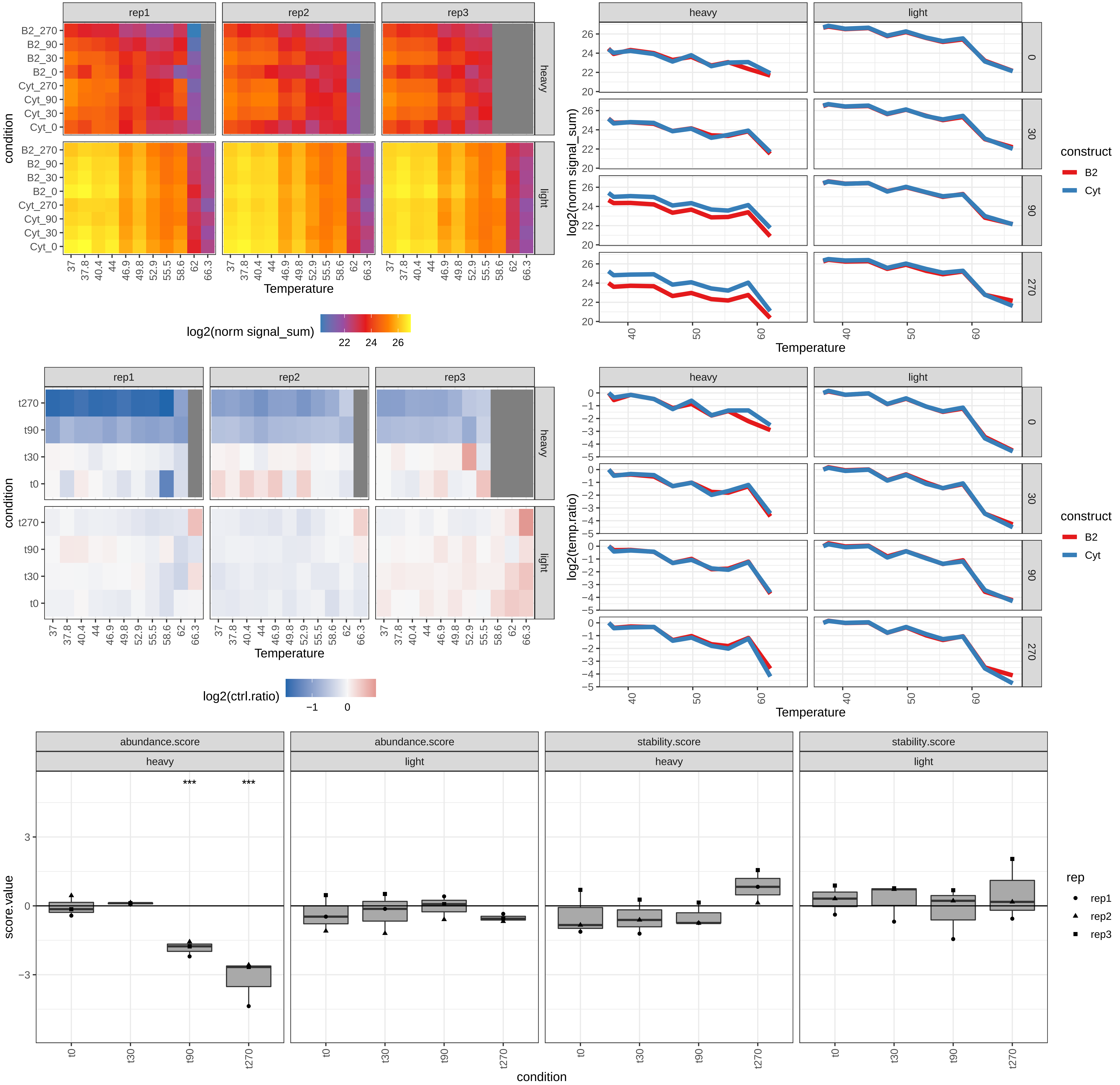

P18544 − Acetylornithine aminotransferase, mitochondrial OS=Saccharomyces cerevisiae (strain ATCC 204508 / S288c) GN=ARG8 PE=1 SV=1

ARH1 − average.top3: 6.75718166666667 − max.qupm: 8

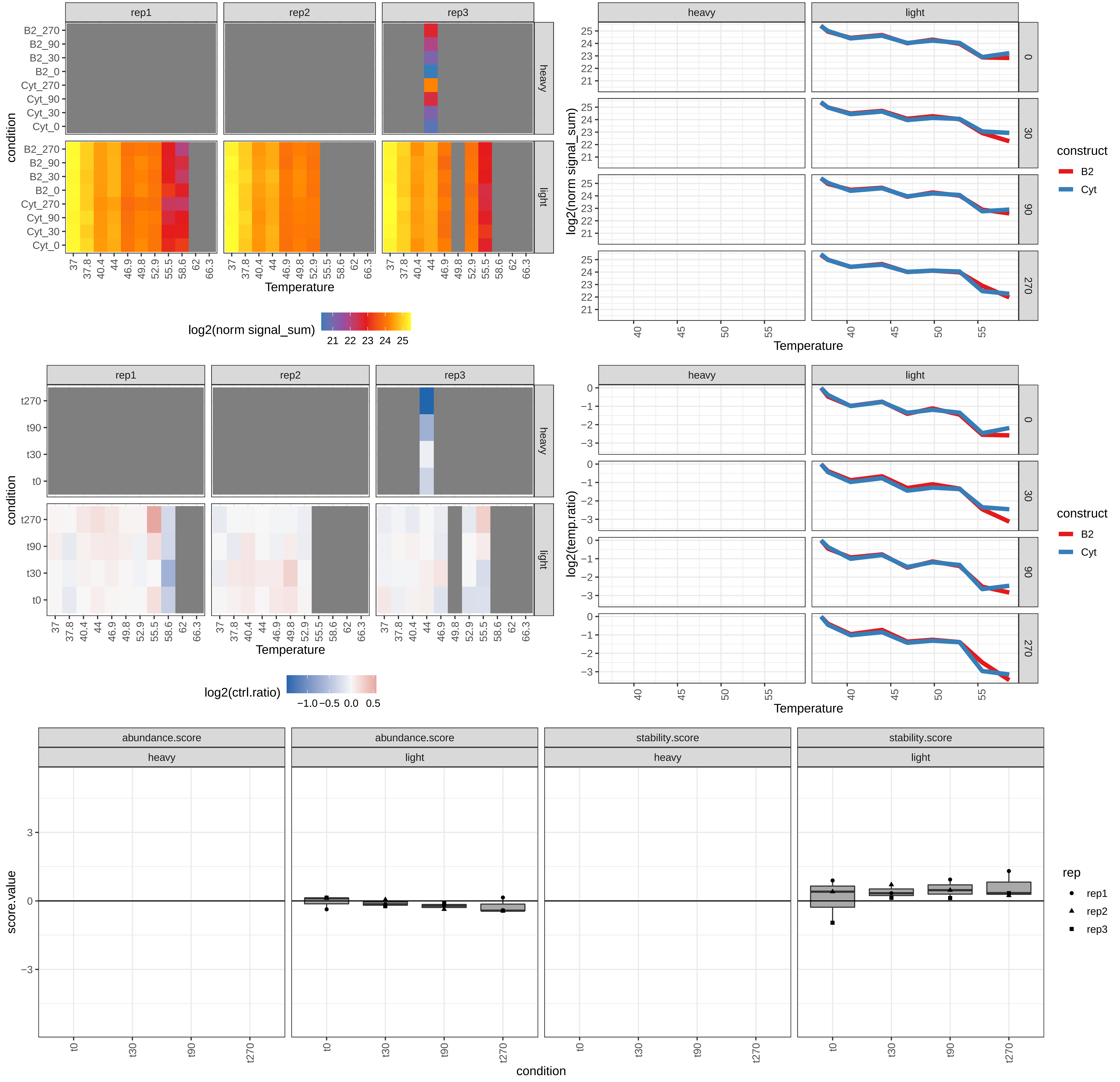

P48360 − Probable NADPH:adrenodoxin oxidoreductase, mitochondrial OS=Saccharomyces cerevisiae (strain ATCC 204508 / S288c) GN=ARH1 PE=1 SV=1

ARO1 − average.top3: 7.70777909090909 − max.qupm: 51

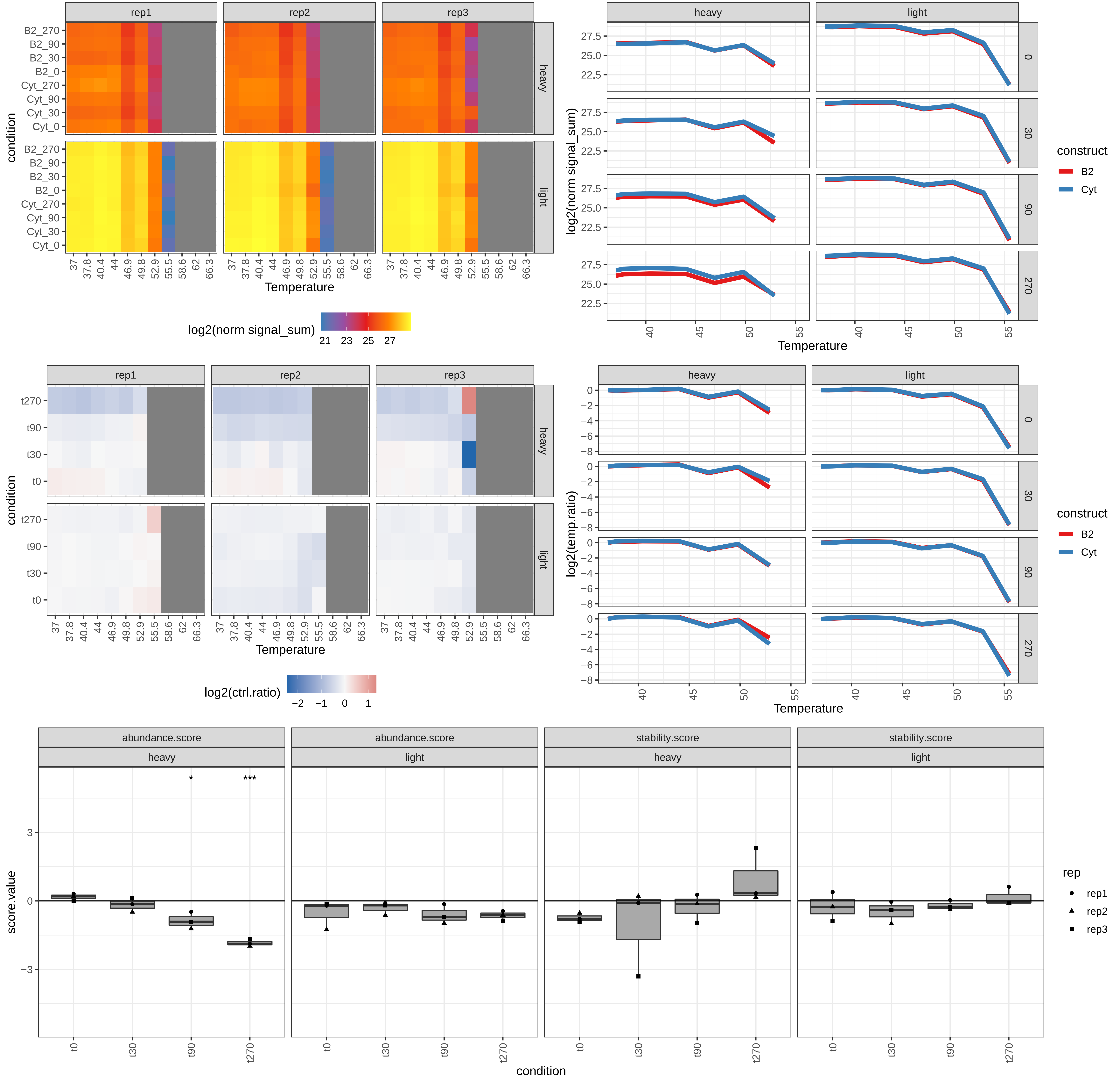

P08566 − Pentafunctional AROM polypeptide OS=Saccharomyces cerevisiae (strain ATCC 204508 / S288c) GN=ARO1 PE=1 SV=1

ARO10 − average.top3: 7.290296 − max.qupm: 16

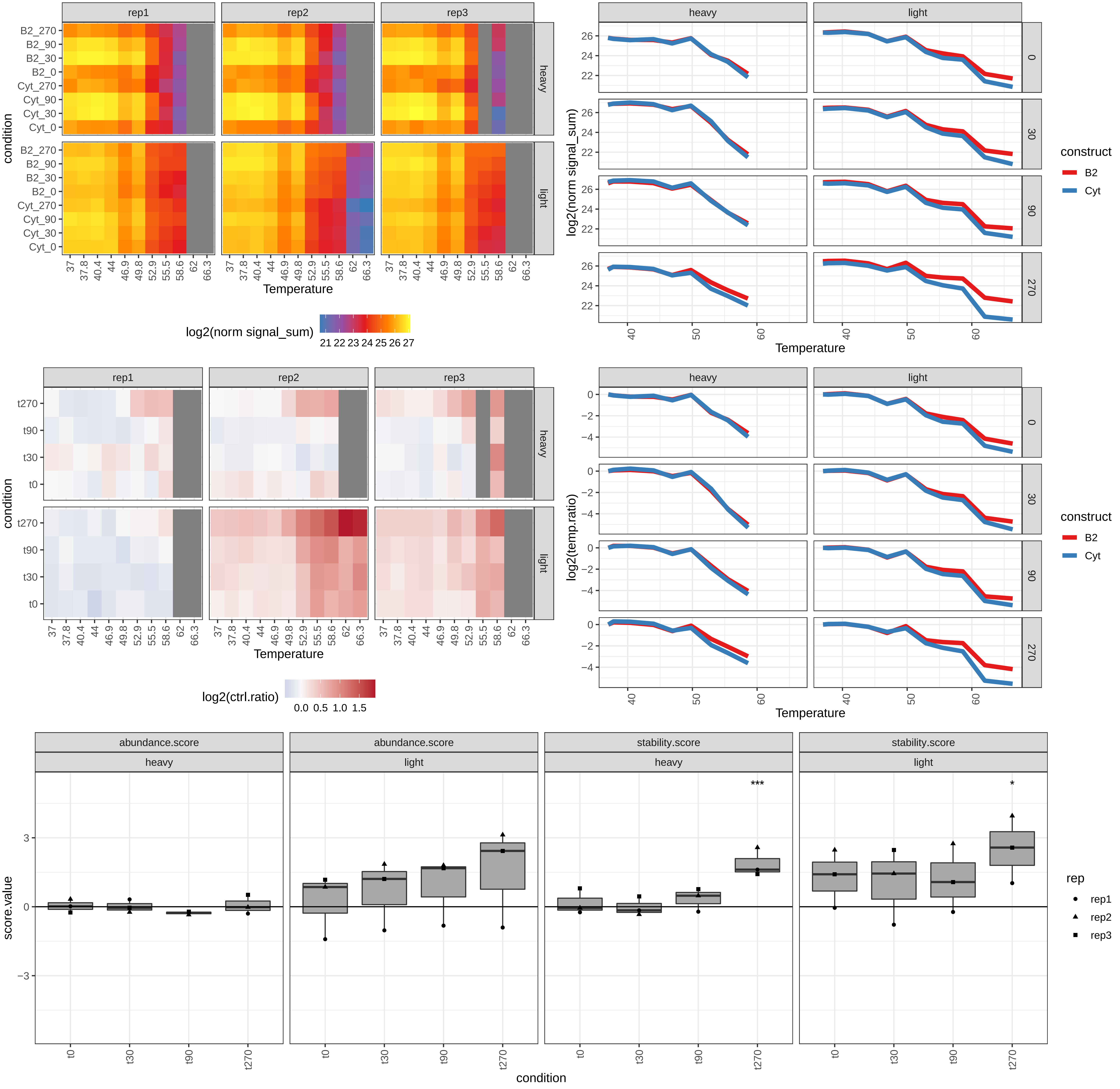

Q06408 − Transaminated amino acid decarboxylase OS=Saccharomyces cerevisiae (strain ATCC 204508 / S288c) GN=ARO10 PE=1 SV=1

ARO2 − average.top3: 7.27736830769231 − max.qupm: 16

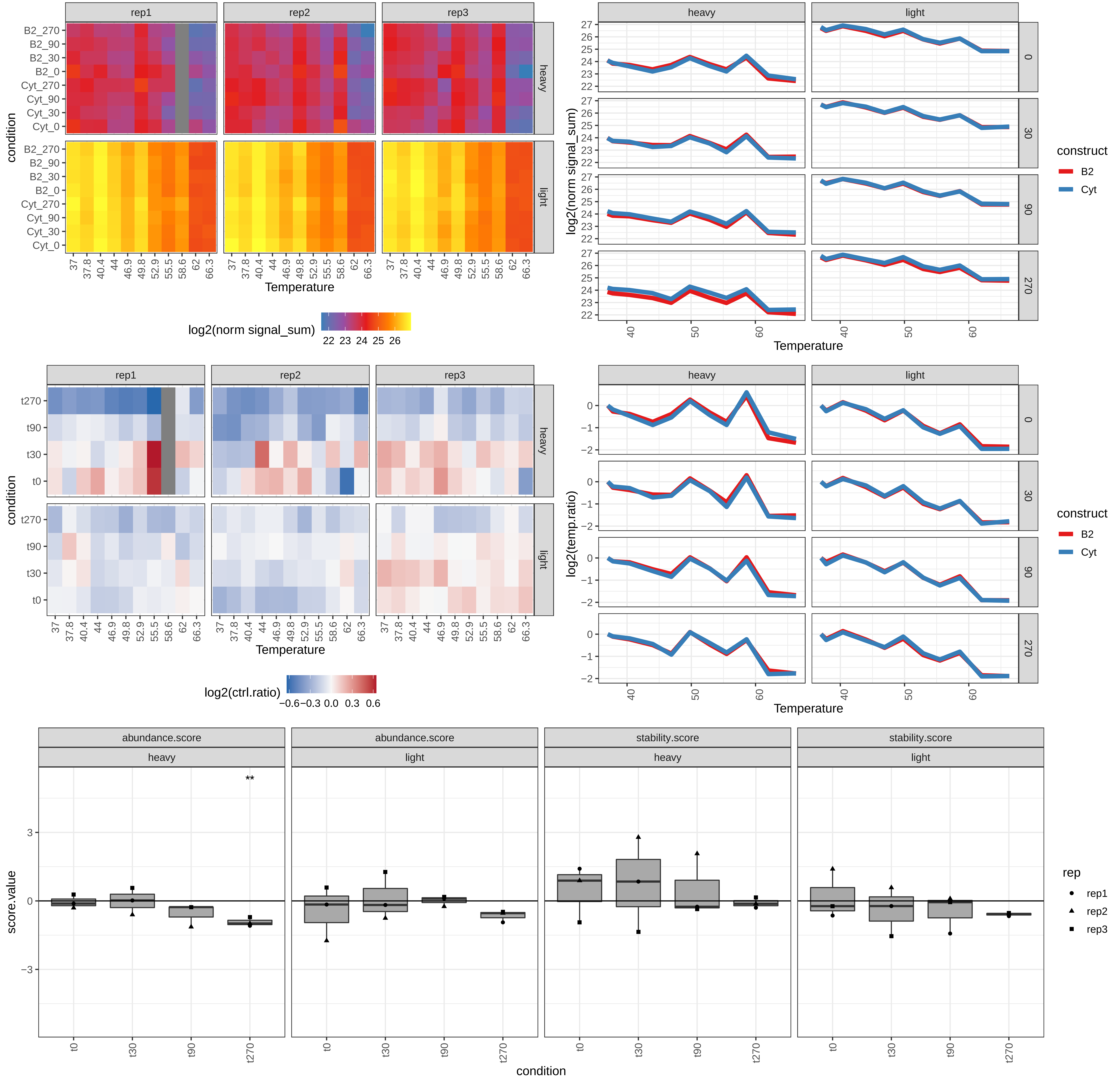

P28777 − Chorismate synthase OS=Saccharomyces cerevisiae (strain ATCC 204508 / S288c) GN=ARO2 PE=1 SV=1

ARO3 − average.top3: 7.66589865384615 − max.qupm: 16

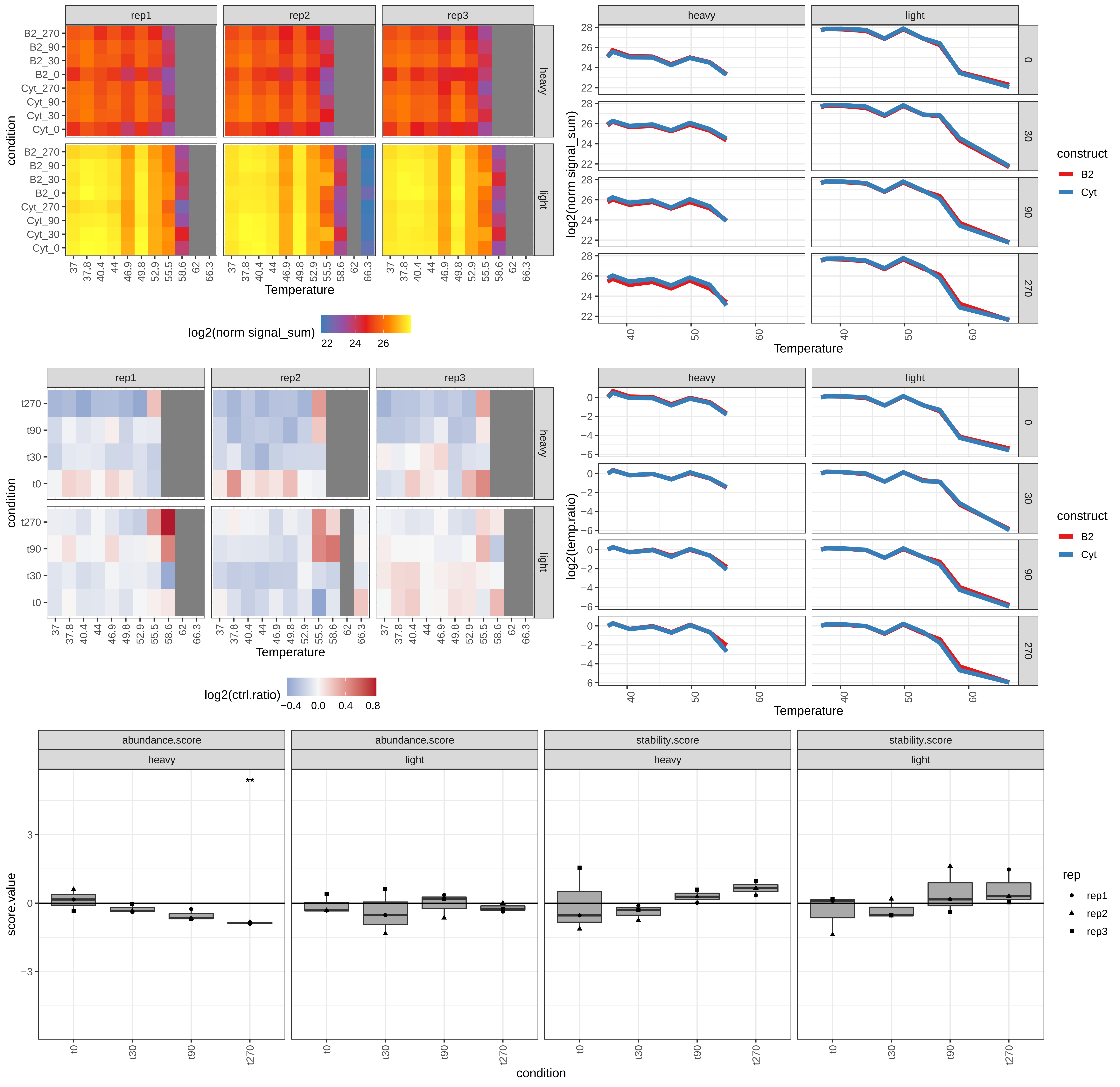

P14843 − Phospho−2−dehydro−3−deoxyheptonate aldolase, phenylalanine−inhibited OS=Saccharomyces cerevisiae (strain ATCC 204508 / S288c) GN=ARO3 PE=1 SV=1

ARO4 − average.top3: 7.91122666666667 − max.qupm: 17

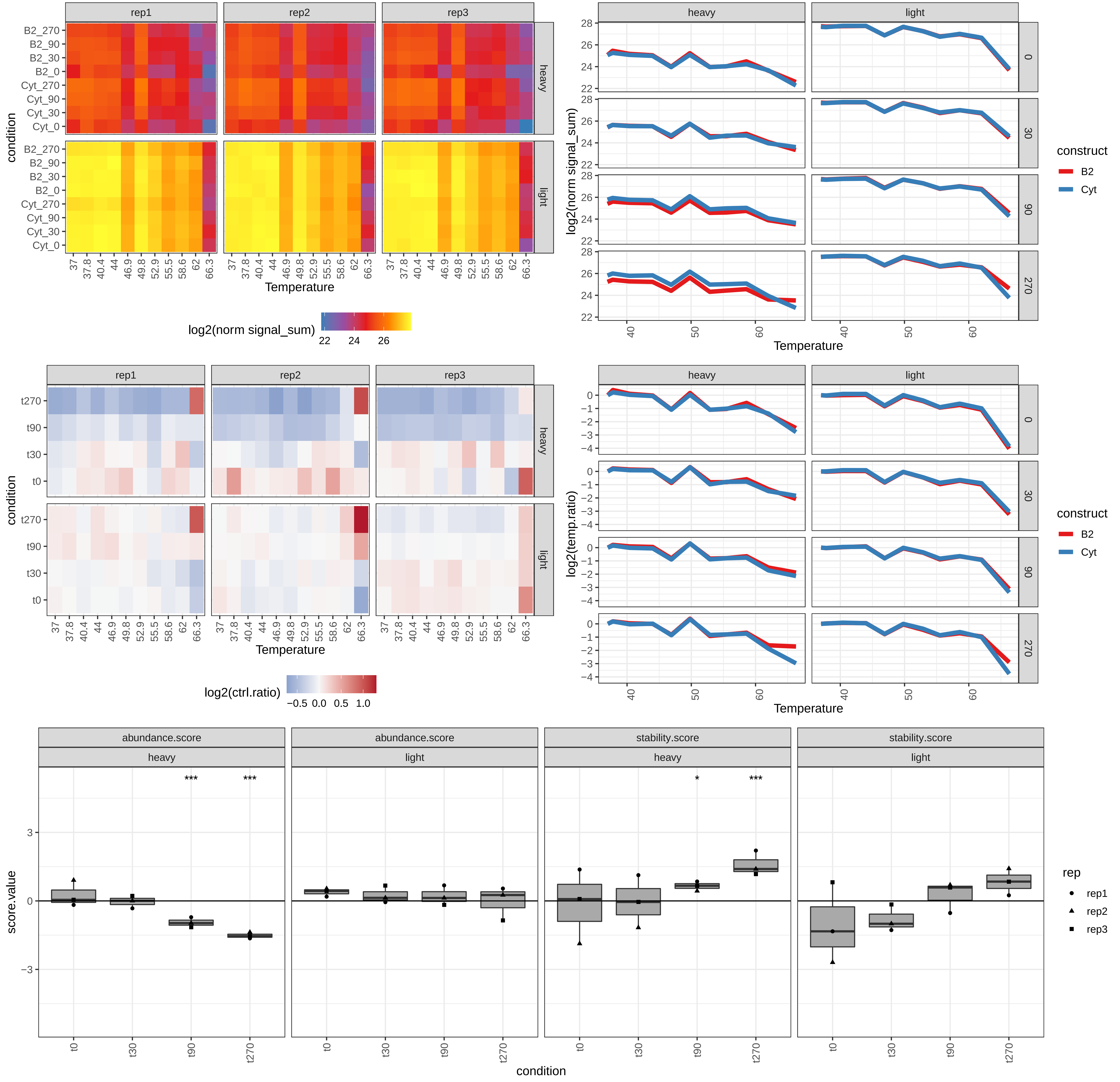

P32449 − Phospho−2−dehydro−3−deoxyheptonate aldolase, tyrosine−inhibited OS=Saccharomyces cerevisiae (strain ATCC 204508 / S288c) GN=ARO4 PE=1 SV=2

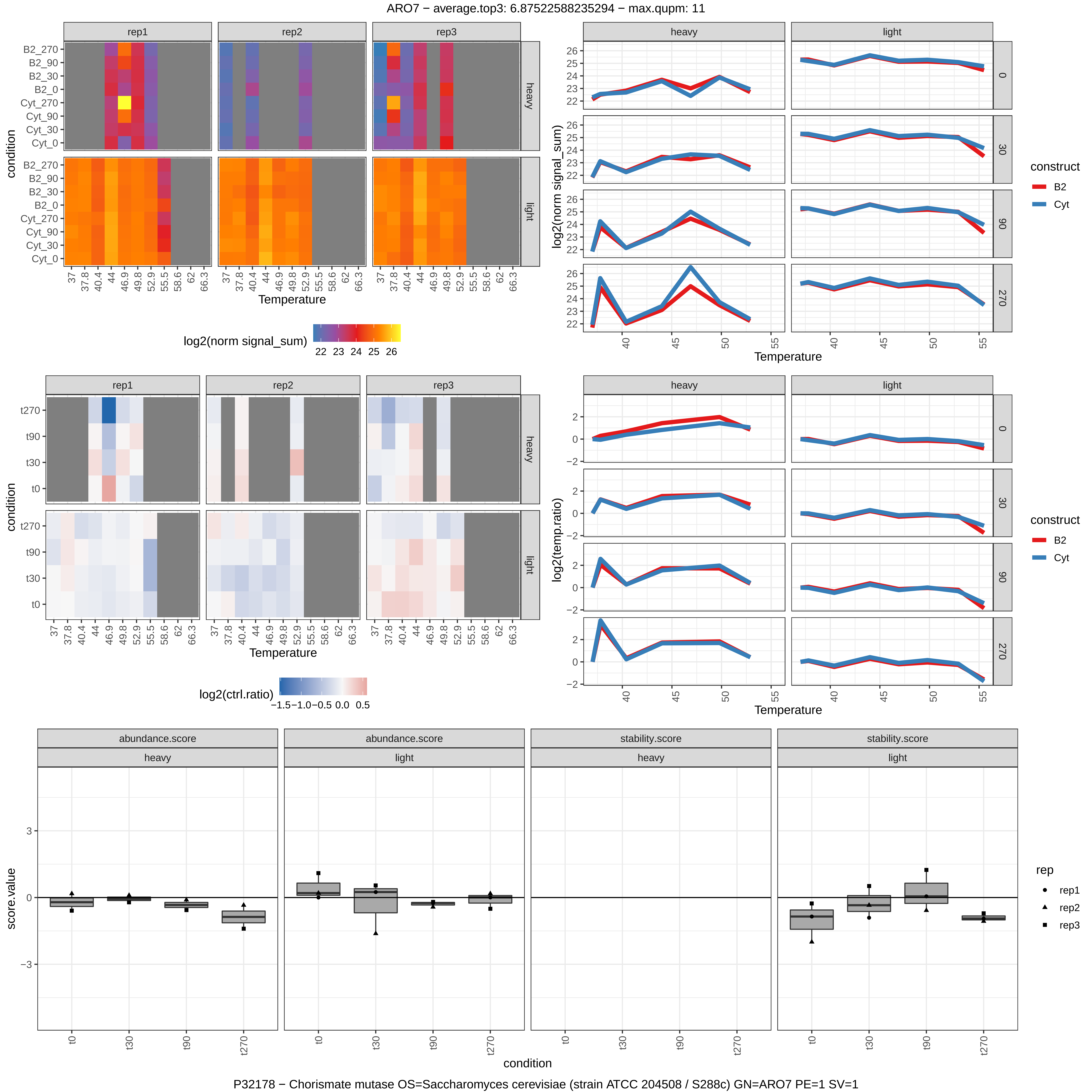
ARO7 − average.top3: 6.87522588235294 − max.qupm: 11
rep1
rep2
rep3
heavy
light
condition
Temperature
log2(norm signal_sum)
log2(ctrl.ratio)
log2(temp.ratio)
construct
B2
Cyt
abundance.score
stability.score
score.value
rep
rep1
rep2
rep3
P32178 − Chorismate mutase OS=Saccharomyces cerevisiae (strain ATCC 204508 / S288c) GN=ARO7 PE=1 SV=1

ARO8 − average.top3: 7.91644878787879 − max.qupm: 19

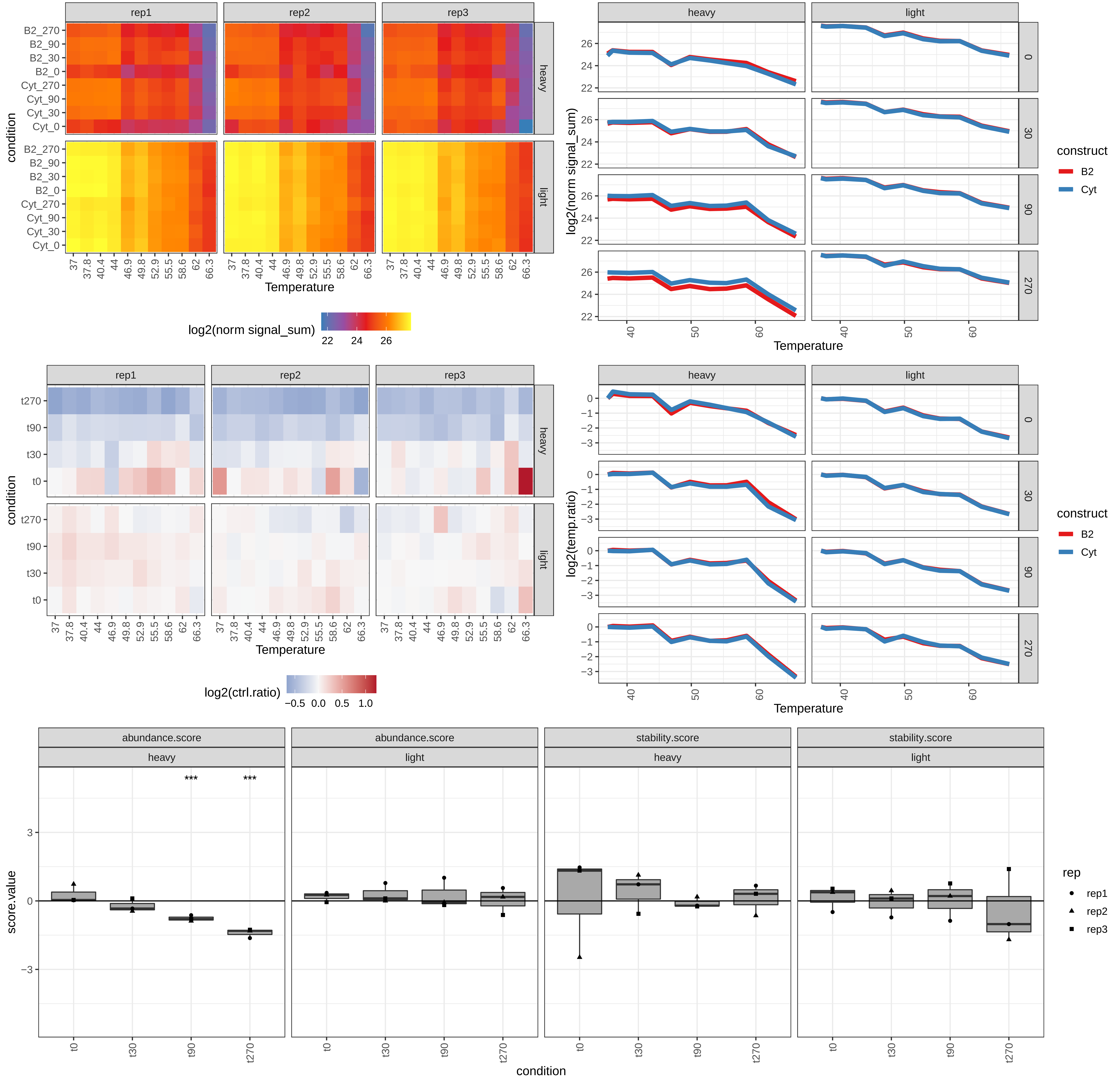

P53090 − Aromatic/aminoadipate aminotransferase 1 OS=Saccharomyces cerevisiae (strain ATCC 204508 / S288c) GN=ARO8 PE=1 SV=1

ARO9 − average.top3: 7.92594615384615 − max.qupm: 25

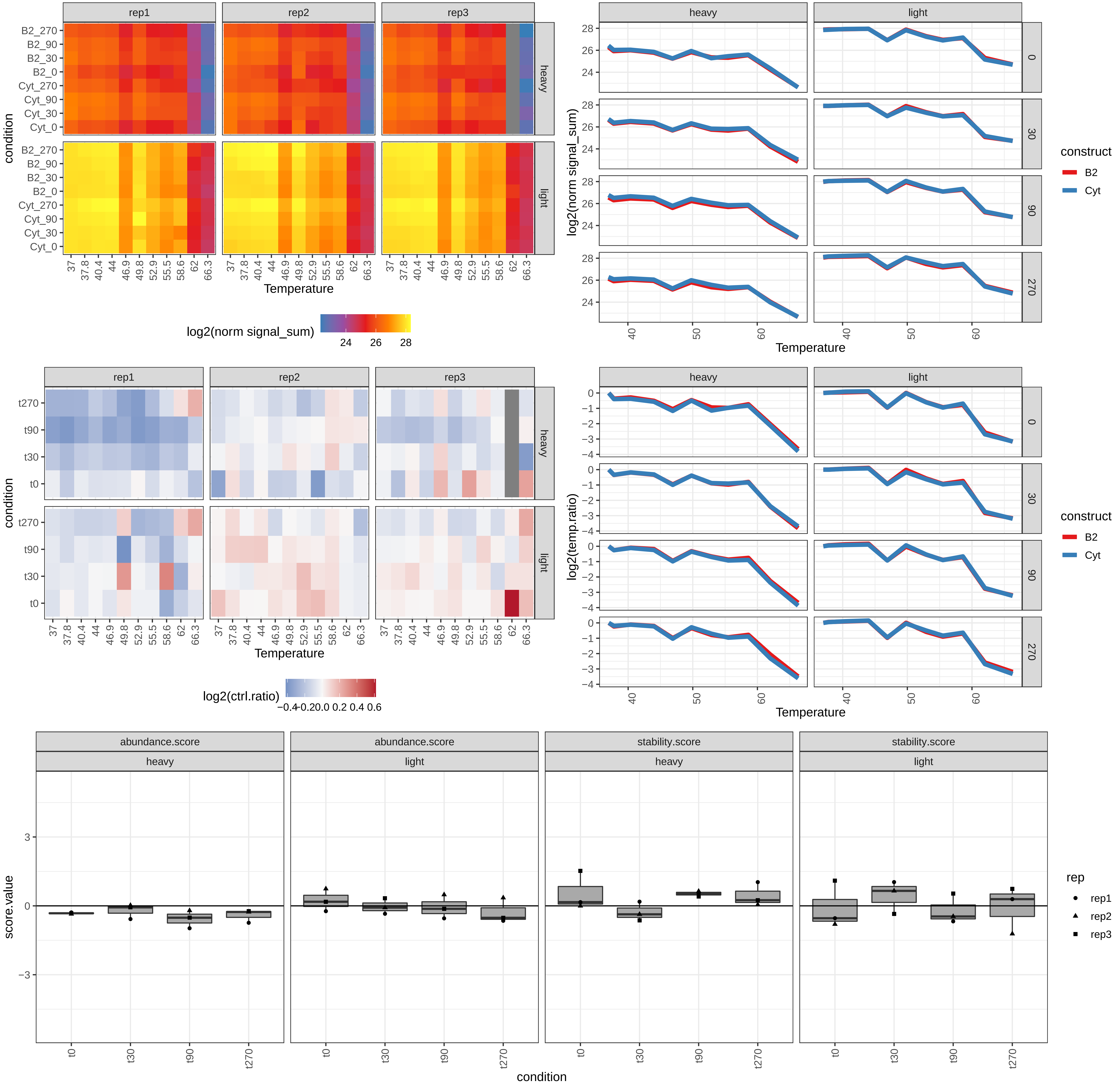

P38840 − Aromatic amino acid aminotransferase 2 OS=Saccharomyces cerevisiae (strain ATCC 204508 / S288c) GN=ARO9 PE=1 SV=3

ARP2 − average.top3: 7.30650915254237 − max.qupm: 14

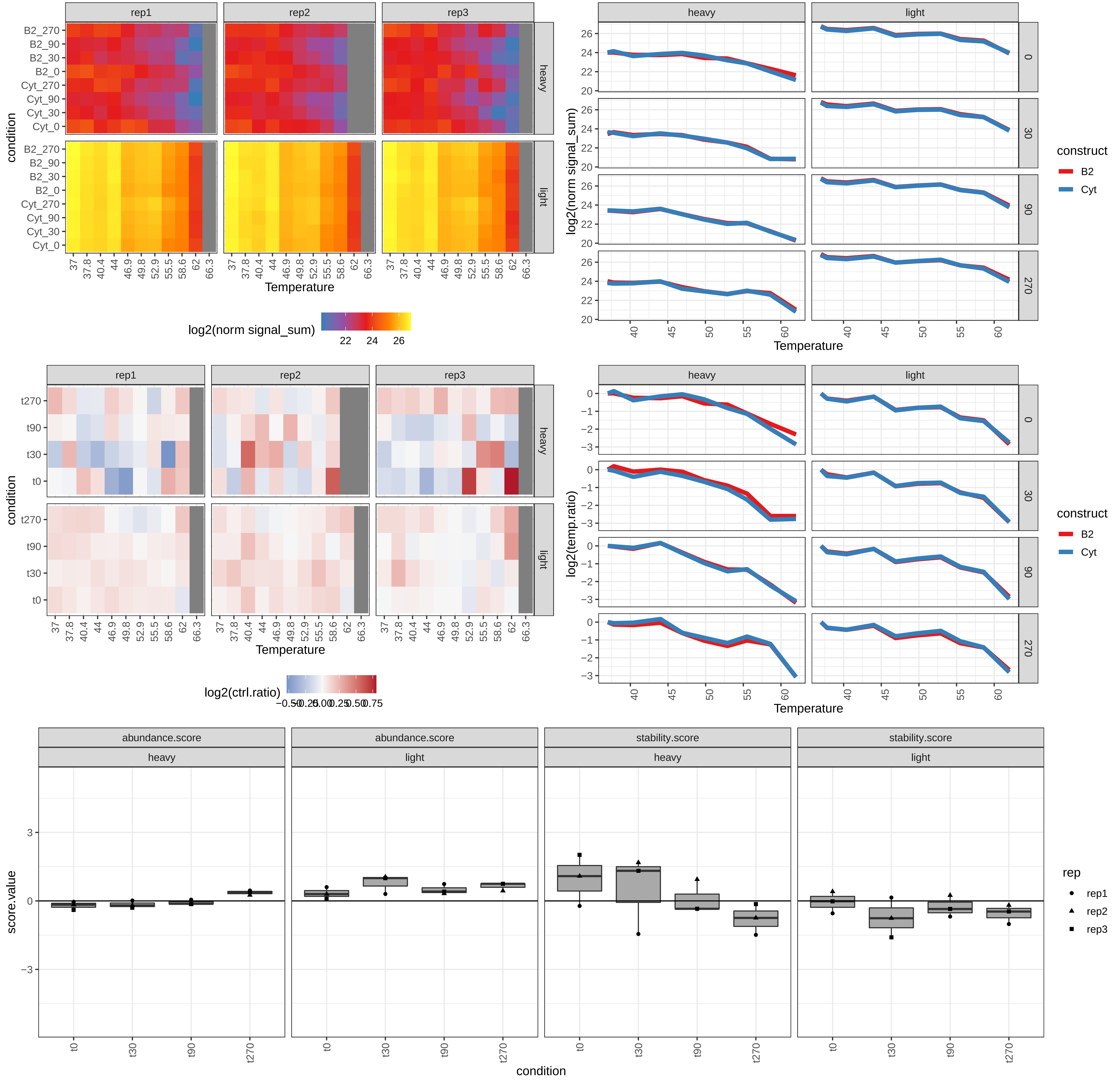

P32381 − Actin−related protein 2 OS=Saccharomyces cerevisiae (strain ATCC 204508 / S288c) GN=ARP2 PE=1 SV=1

ARP3 − average.top3: 7.41742033333333 − max.qupm: 14

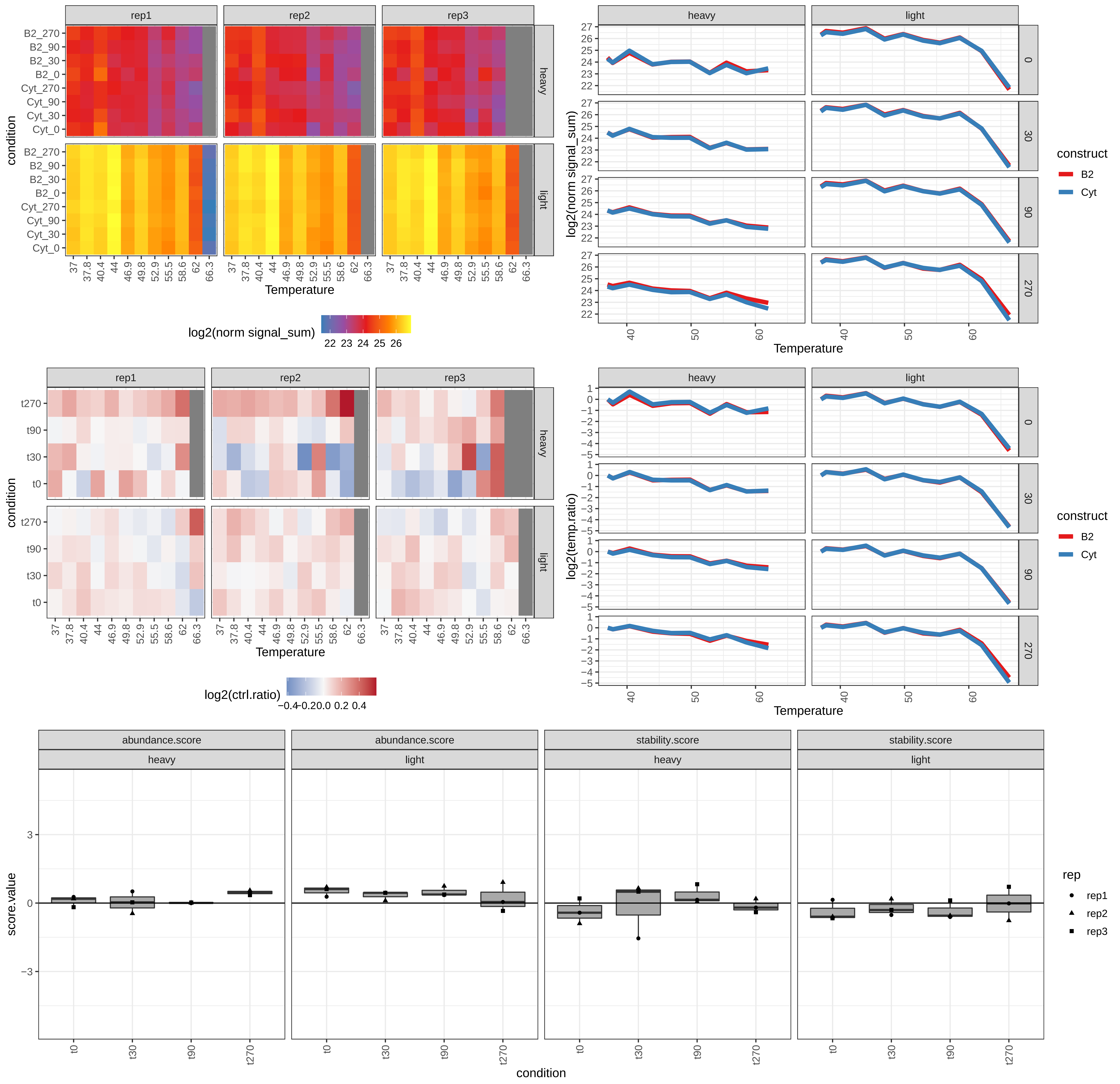

P47117 − Actin−related protein 3 OS=Saccharomyces cerevisiae (strain ATCC 204508 / S288c) GN=ARP3 PE=1 SV=1

ARP4 − average.top3: 7.07915913043478 − max.qupm: 10

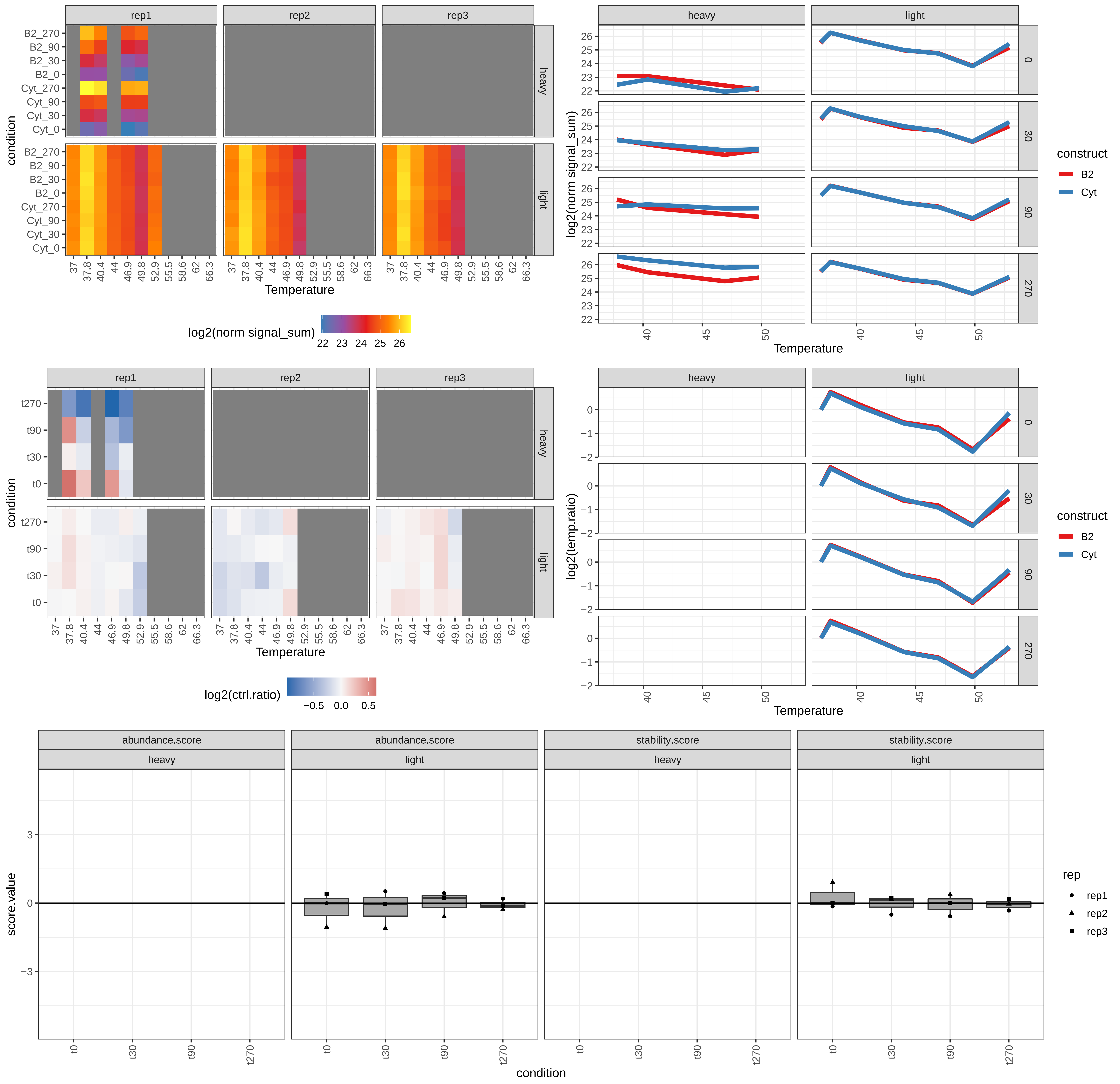

P80428 − Actin−related protein 4 OS=Saccharomyces cerevisiae (strain ATCC 204508 / S288c) GN=ARP4 PE=1 SV=1

ARP5 − average.top3: 6.3987332 − max.qupm: 8

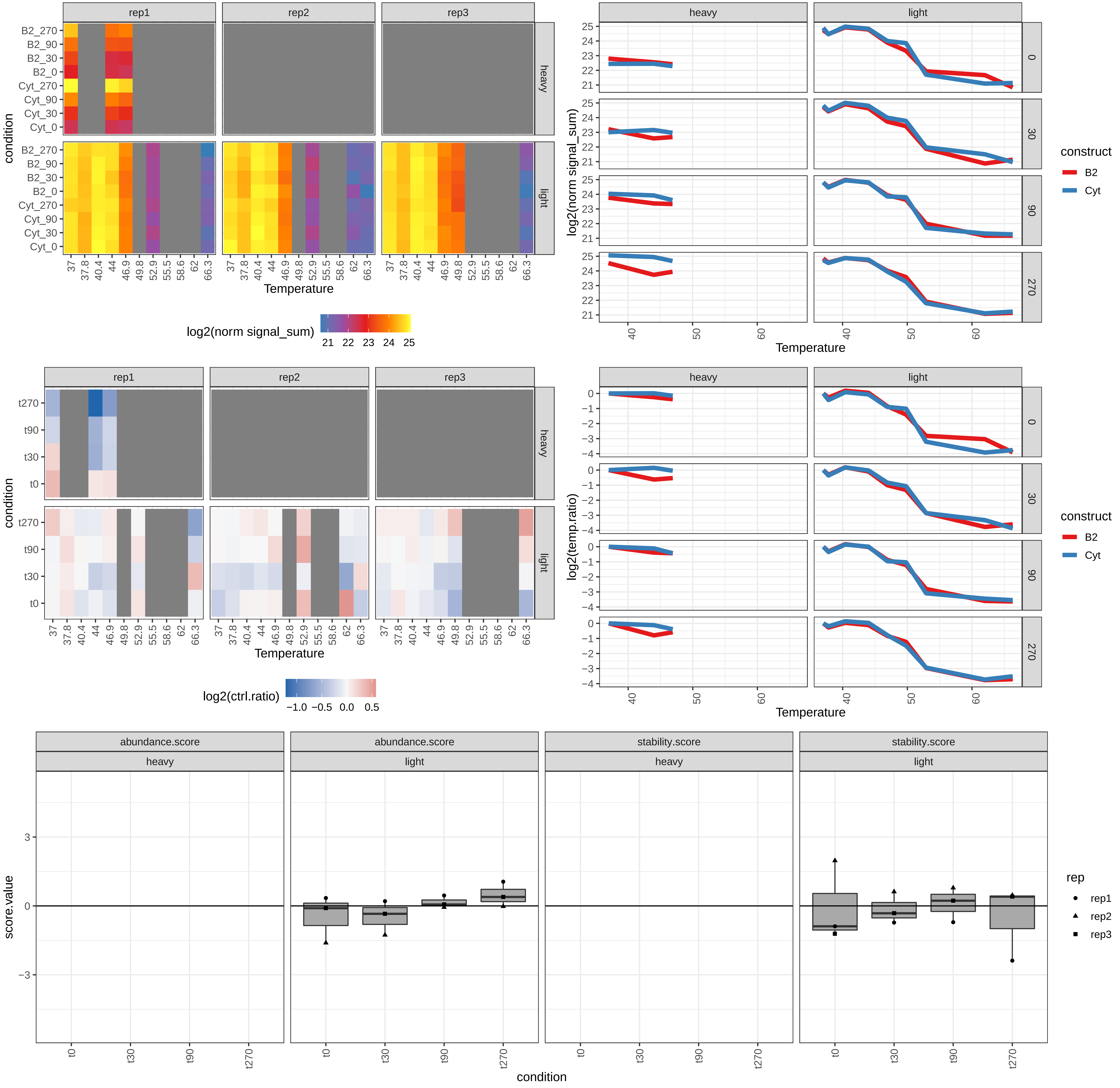

P53946 − Actin−related protein 5 OS=Saccharomyces cerevisiae (strain ATCC 204508 / S288c) GN=ARP5 PE=1 SV=1

ARP6 − average.top3: 6.71268625 − max.qupm: 4

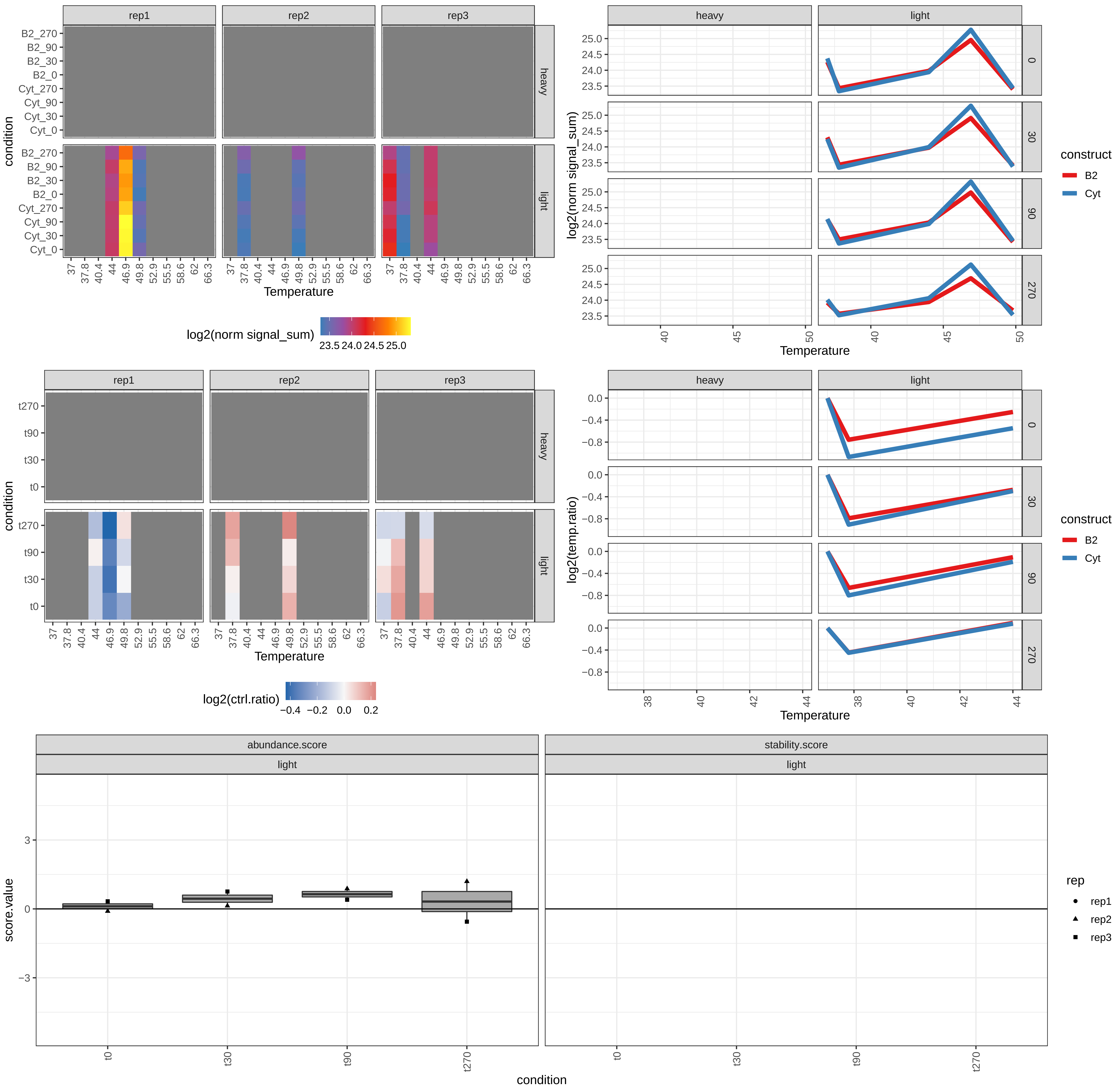

Q12509 − Actin−like protein ARP6 OS=Saccharomyces cerevisiae (strain ATCC 204508 / S288c) GN=ARP6 PE=1 SV=1

ARP7 − average.top3: 6.69337529411765 − max.qupm: 6

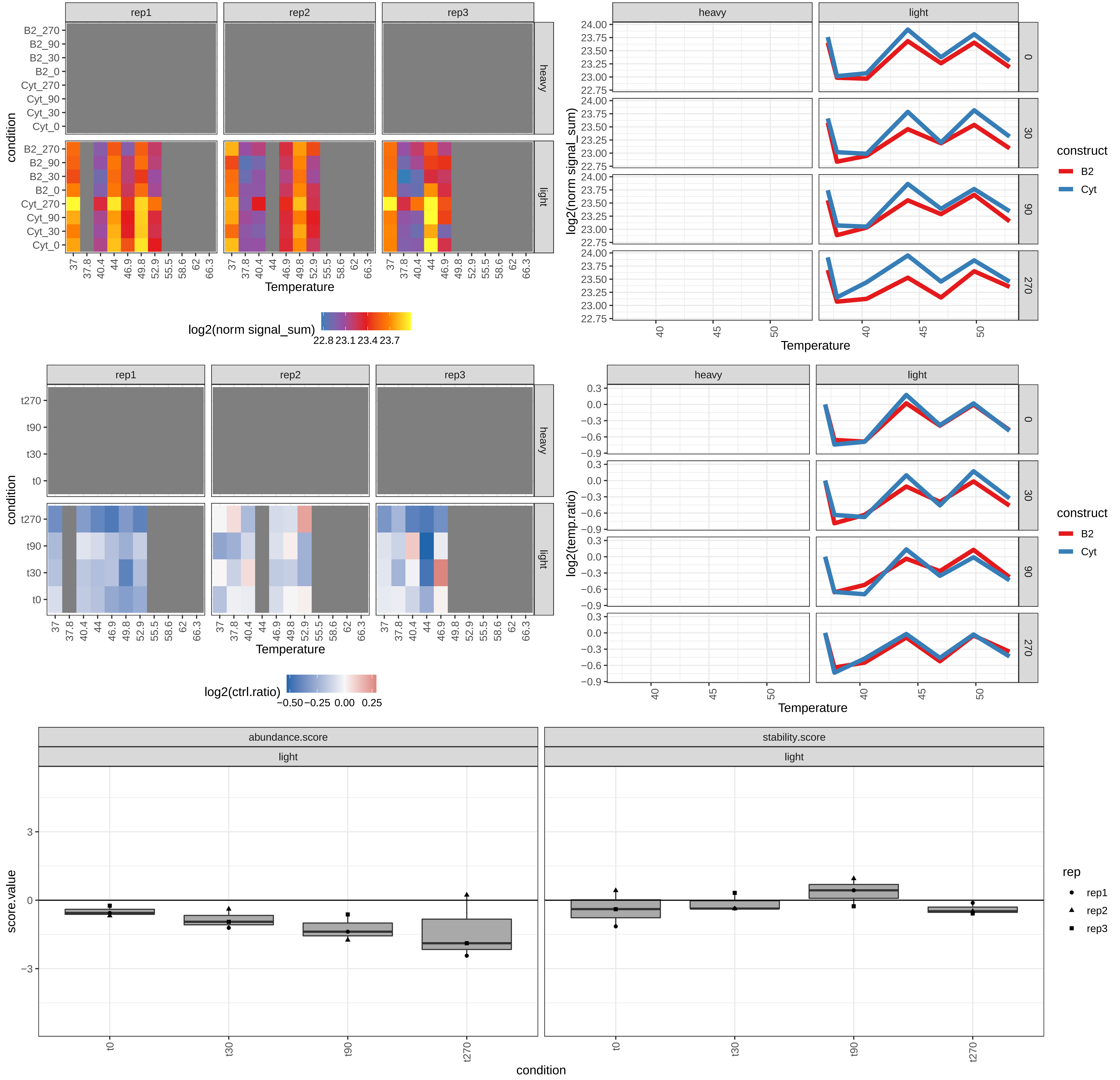

Q12406 − Actin−related protein 7 OS=Saccharomyces cerevisiae (strain ATCC 204508 / S288c) GN=ARP7 PE=1 SV=1

ARP8 − average.top3: 6.790403125 − max.qupm: 9

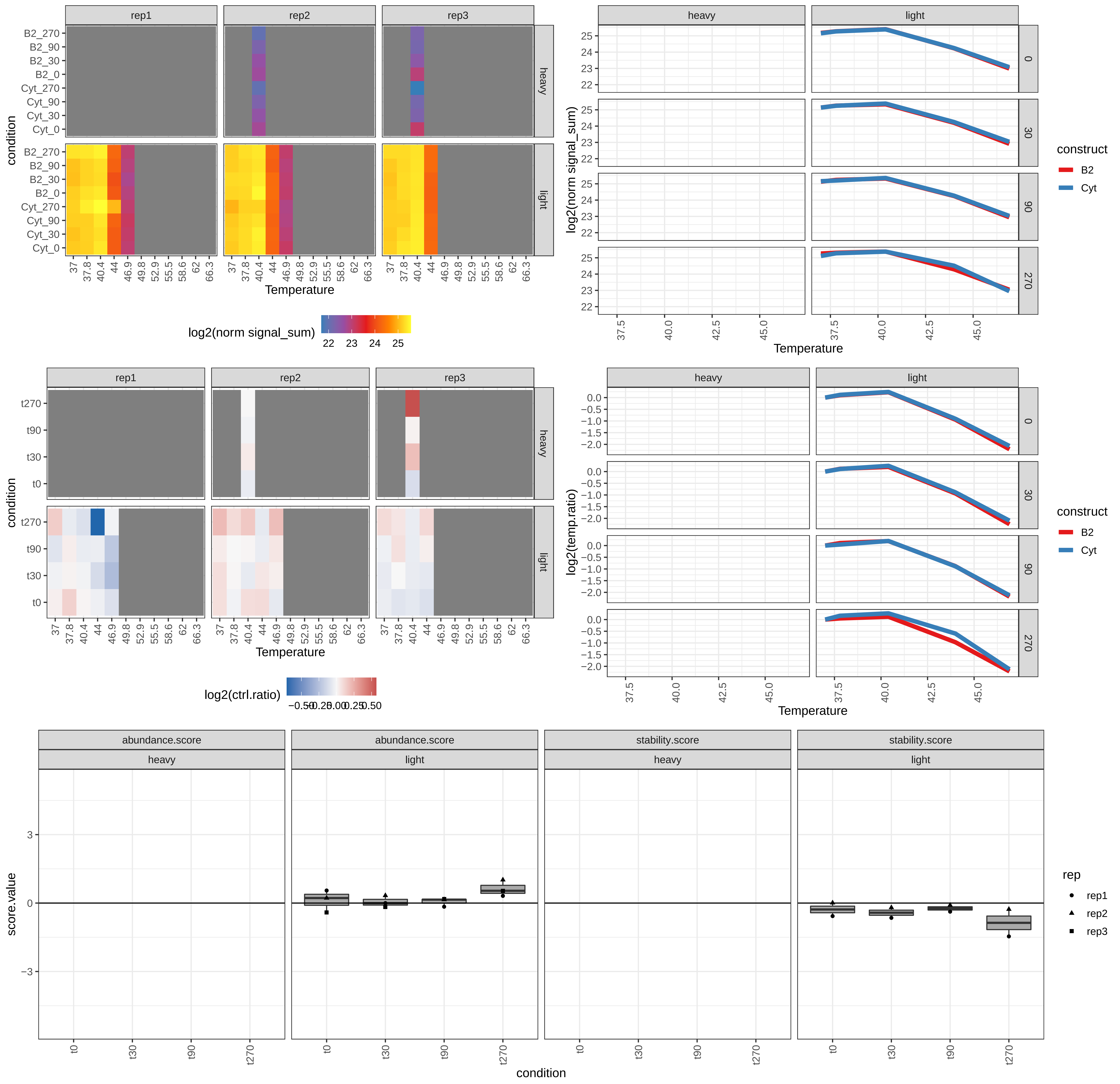

Q12386 − Actin−like protein ARP8 OS=Saccharomyces cerevisiae (strain ATCC 204508 / S288c) GN=ARP8 PE=1 SV=1

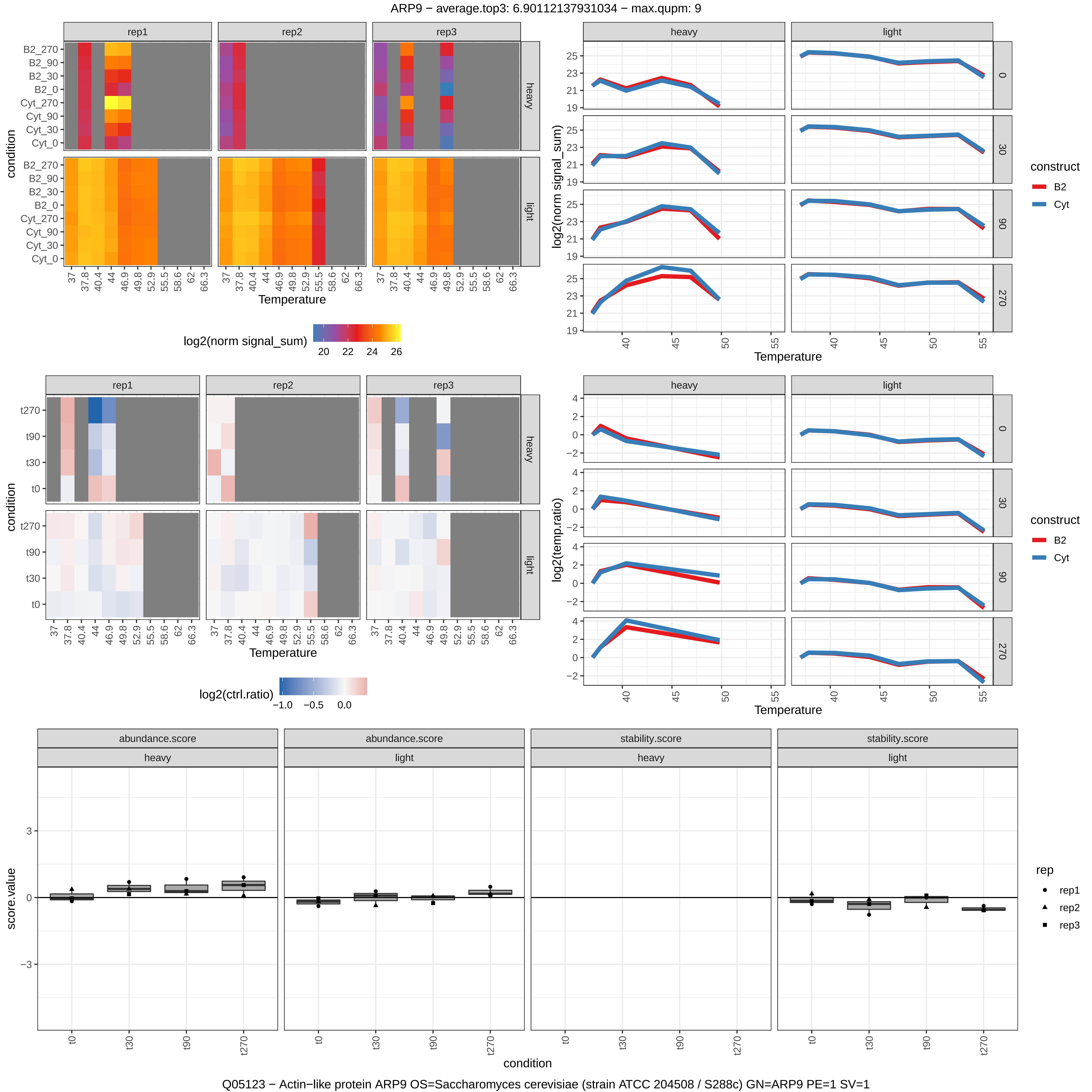

ARP9 − average.top3: 6.90112137931034 − max.qupm: 9
rep1
rep2
rep3
heavy
light
condition
Temperature
log2(norm signal_sum)
construct
B2
Cyt
log2(ctrl.ratio)
log2(temp.ratio)
abundance.score
stability.score
score.value
rep
rep1
rep2
rep3
Q05123 − Actin−like protein ARP9 OS=Saccharomyces cerevisiae (strain ATCC 204508 / S288c) GN=ARP9 PE=1 SV=1

ARX1 − average.top3: 7.11193939393939 − max.qupm: 6

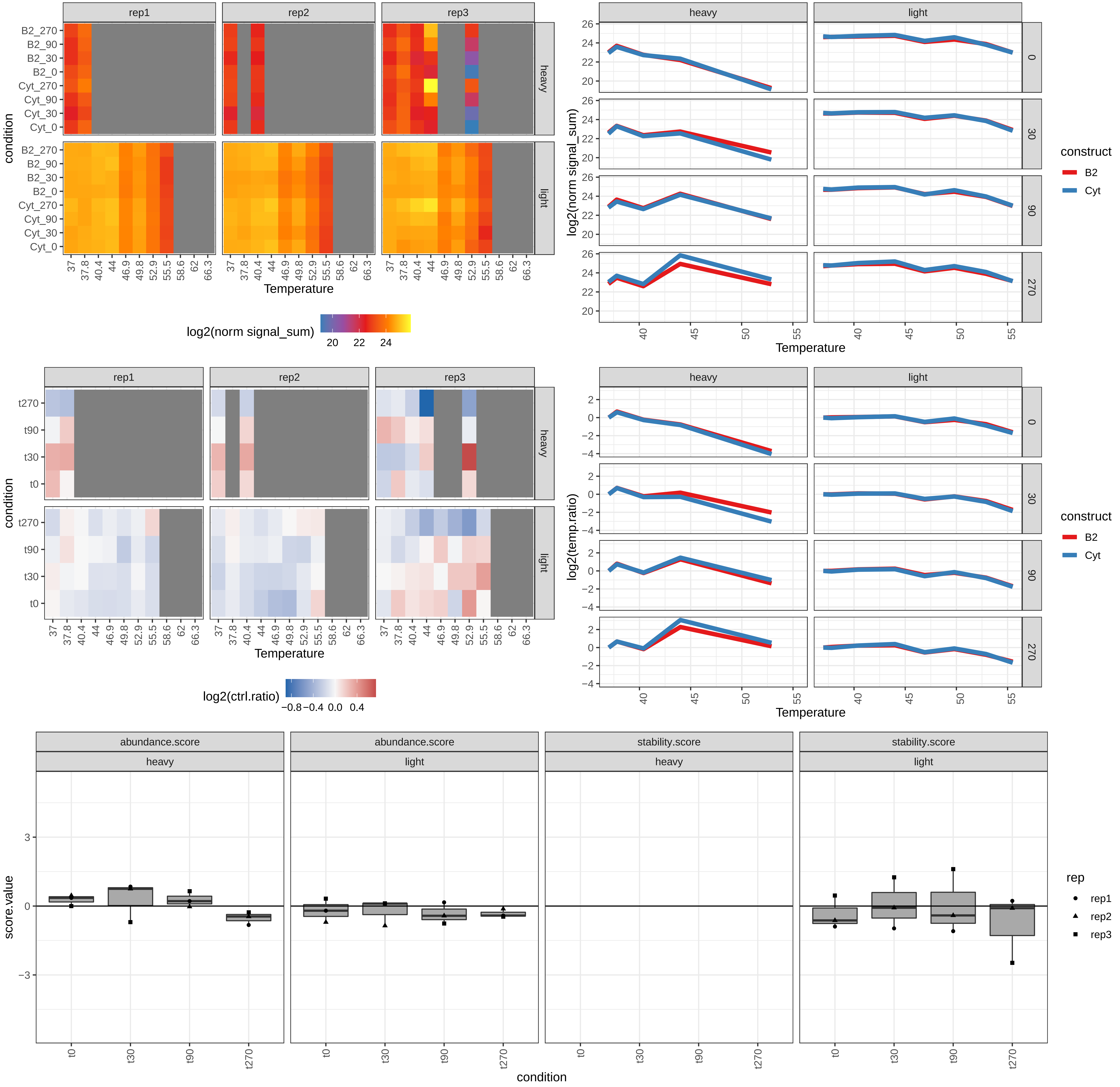

Q03862 − Probable metalloprotease ARX1 OS=Saccharomyces cerevisiae (strain ATCC 204508 / S288c) GN=ARX1 PE=1 SV=1

ASC1 − average.top3: 8.0289846031746 − max.qupm: 12

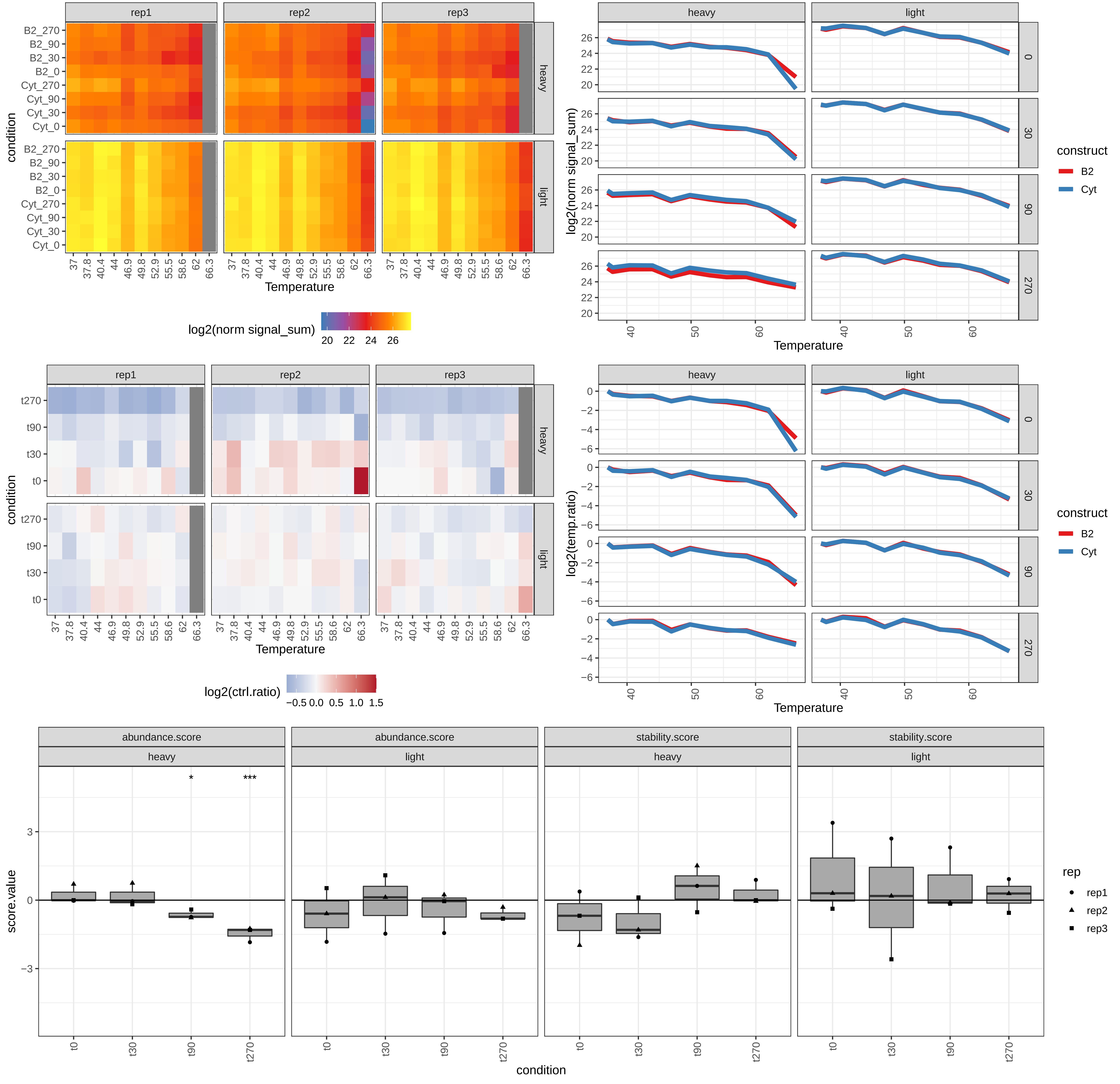

P38011 − Guanine nucleotide−binding protein subunit beta−like protein OS=Saccharomyces cerevisiae (strain ATCC 204508 / S288c) GN=ASC1 PE=1 SV=4

ASF1 − average.top3: 7.1143728 − max.qupm: 7

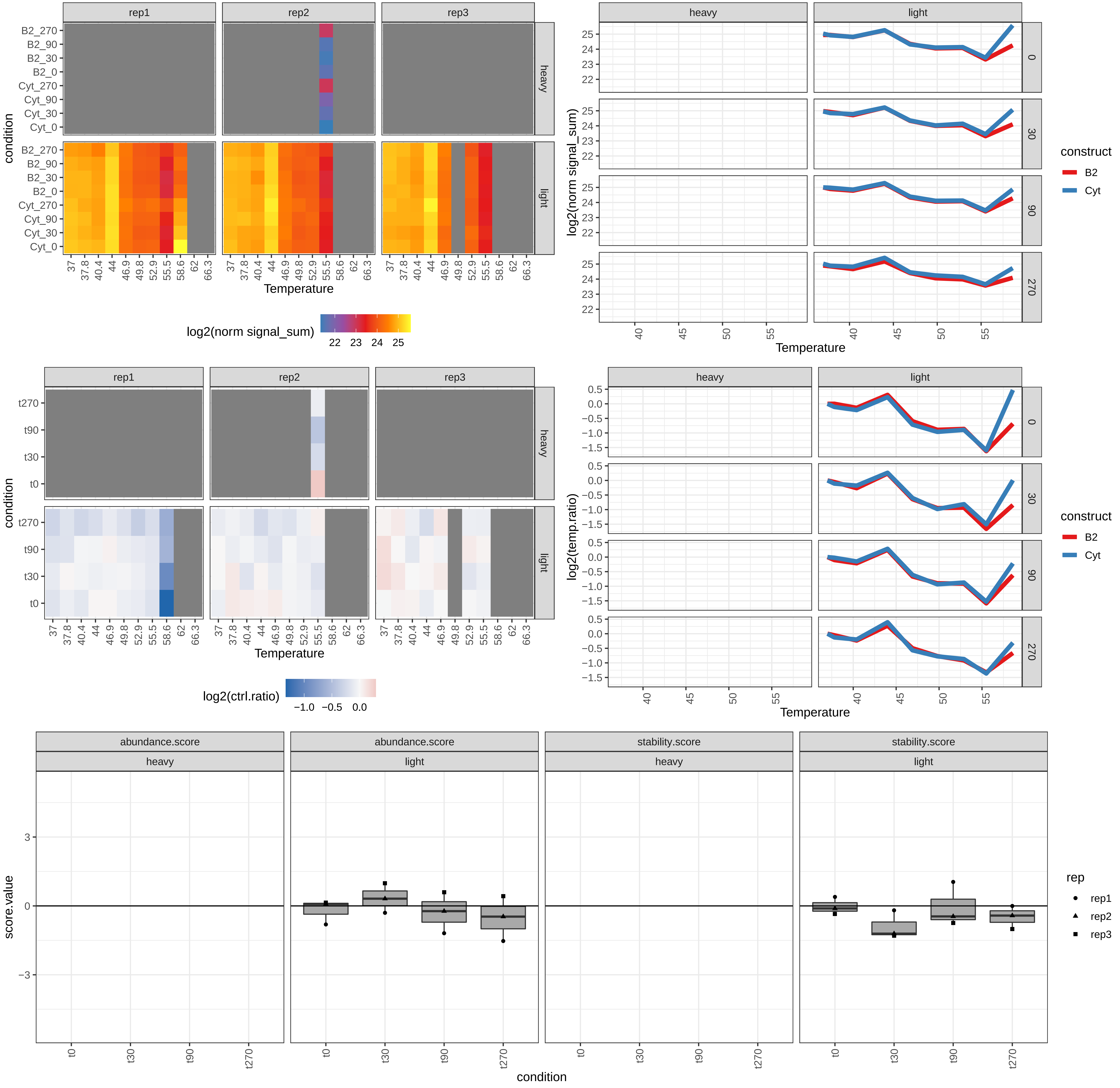

P32447 − Histone chaperone ASF1 OS=Saccharomyces cerevisiae (strain ATCC 204508 / S288c) GN=ASF1 PE=1 SV=1

ASN1 − average.top3: 7.49033454545455 − max.qupm: 20

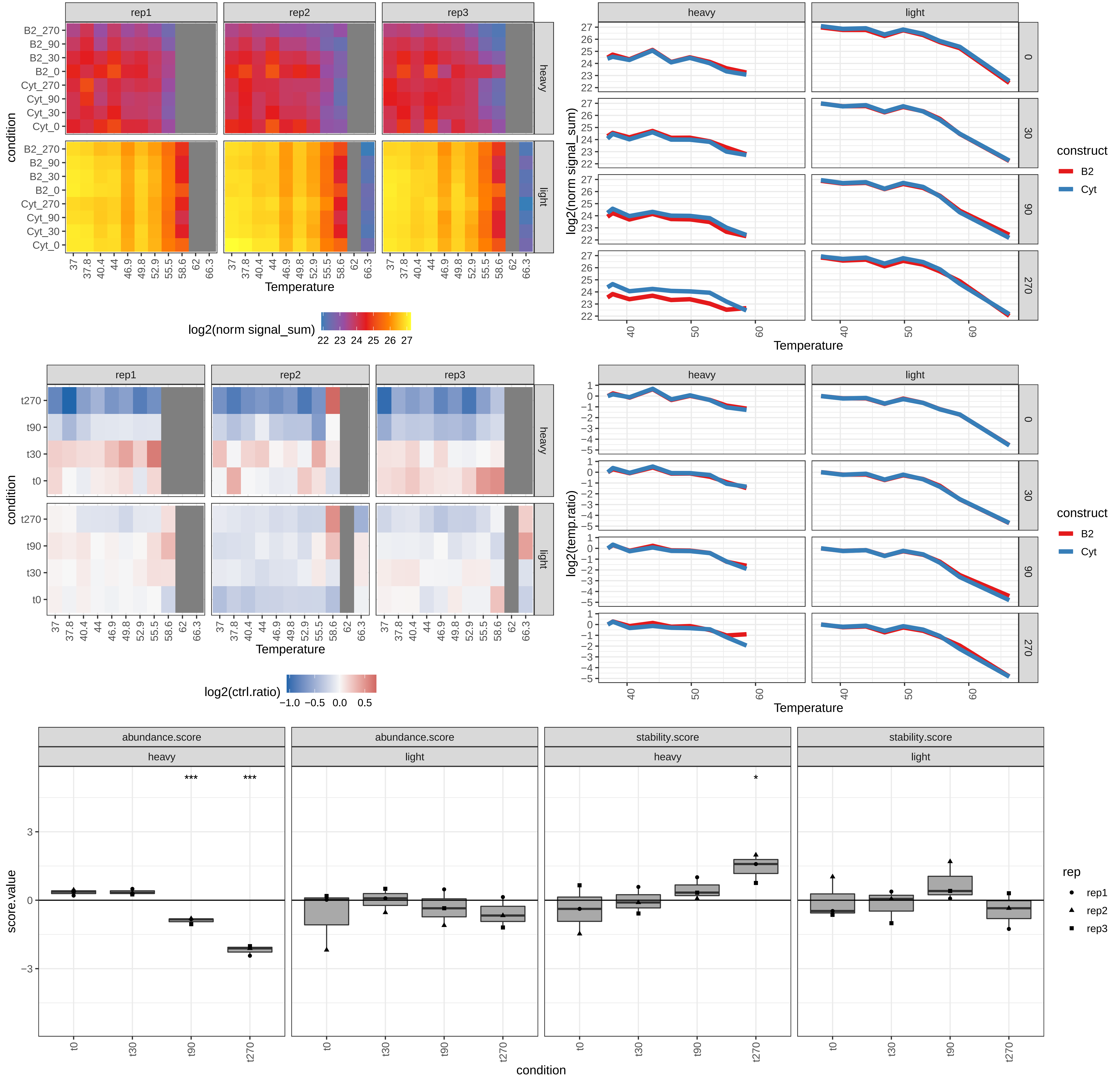

P49089 − Asparagine synthetase [glutamine−hydrolyzing] 1 OS=Saccharomyces cerevisiae (strain ATCC 204508 / S288c) GN=ASN1 PE=1 SV=2

ASN2 − average.top3: 7.15443816326531 − max.qupm: 12

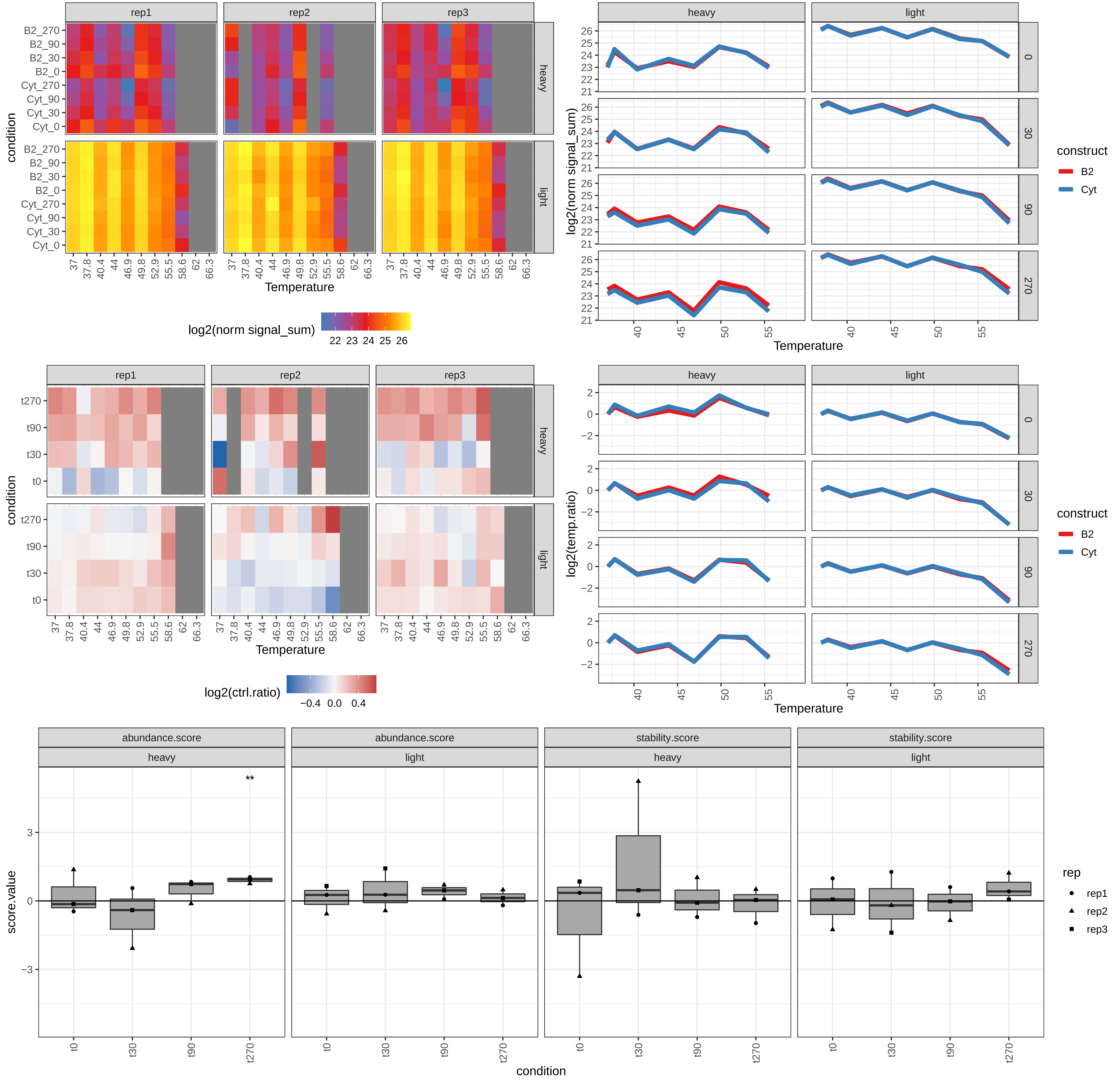

P49090 − Asparagine synthetase [glutamine−hydrolyzing] 2 OS=Saccharomyces cerevisiae (strain ATCC 204508 / S288c) GN=ASN2 PE=1 SV=2

ASP1 − average.top3: 6.63516125 − max.qupm: 5

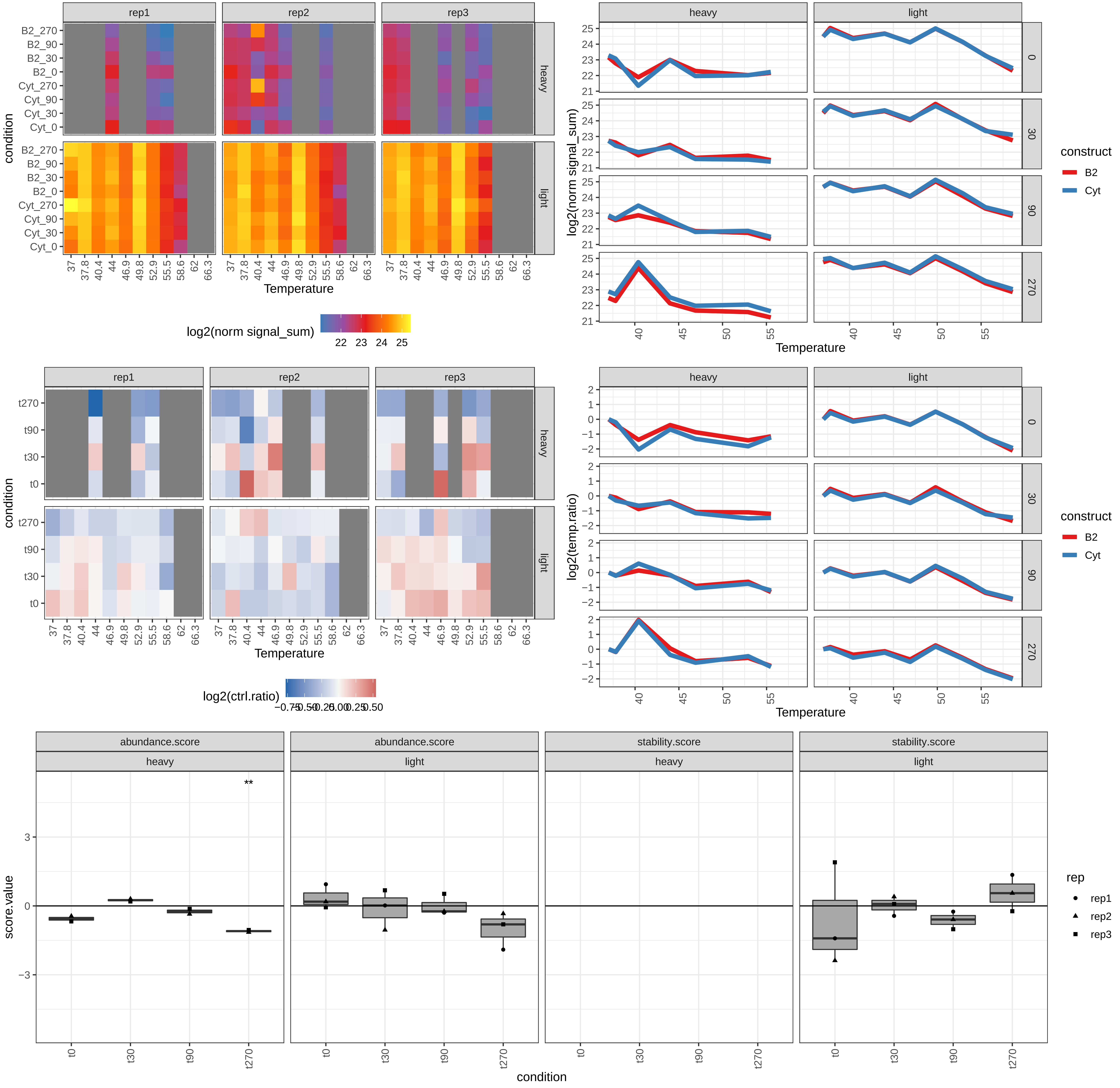

P38986 − L−asparaginase 1 OS=Saccharomyces cerevisiae (strain ATCC 204508 / S288c) GN=ASP1 PE=1 SV=1

AST2 − average.top3: 6.42520352941176 − max.qupm: 6

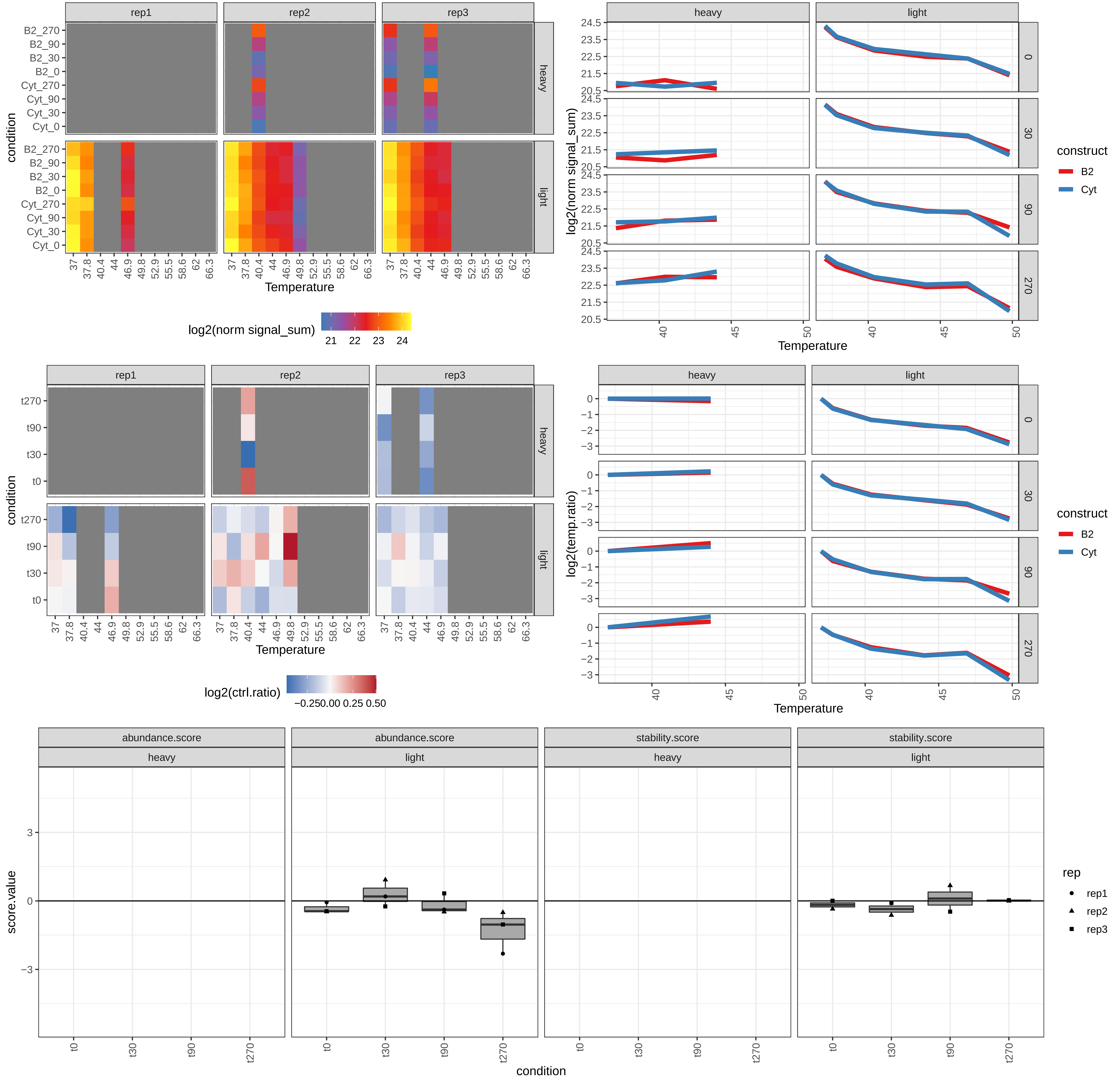

P39945 − Protein AST2 OS=Saccharomyces cerevisiae (strain ATCC 204508 / S288c) GN=AST2 PE=1 SV=1

ATE1 − average.top3: 6.403999 − max.qupm: 3

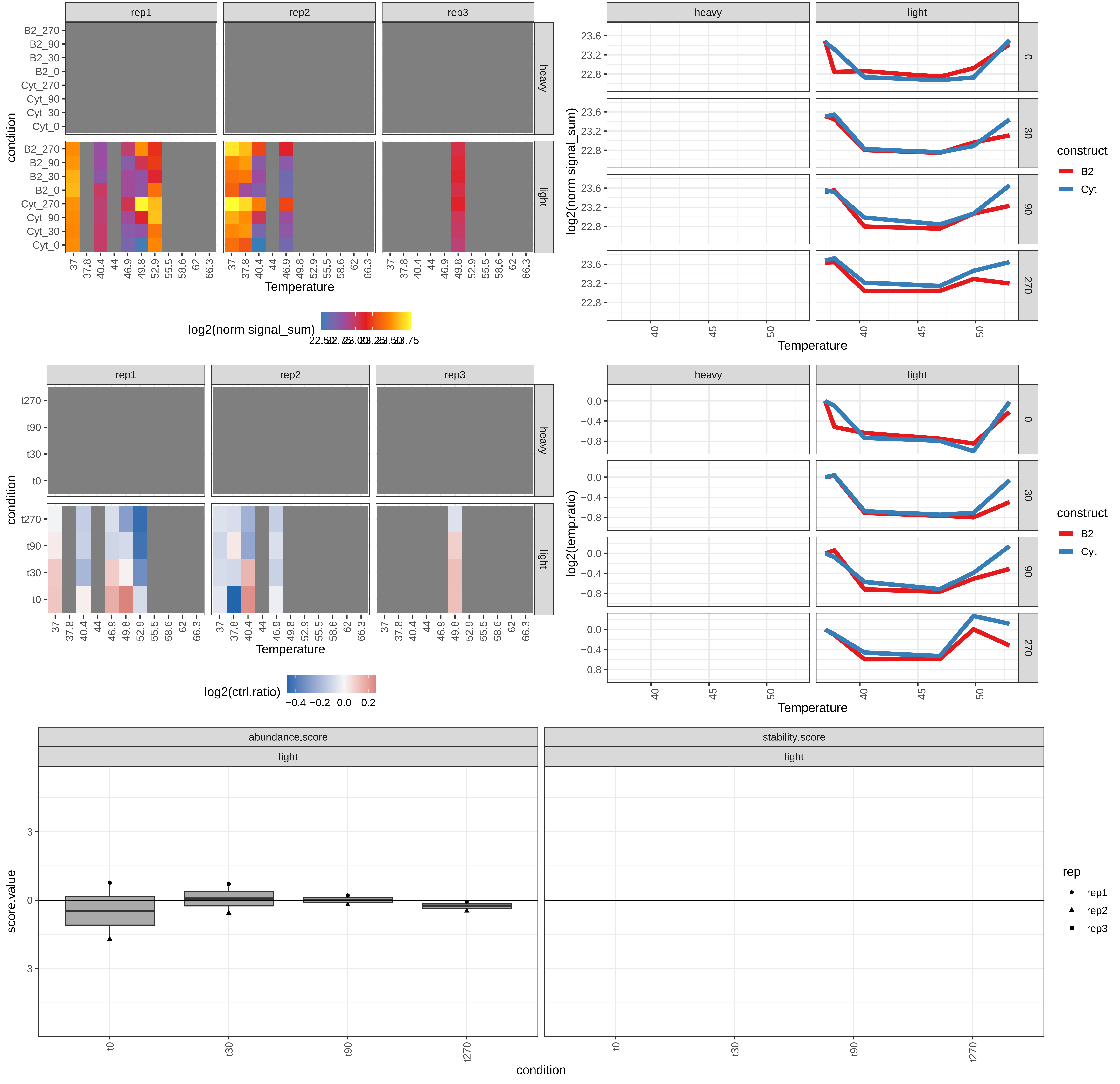

P16639 − Arginyl−tRNA−−protein transferase 1 OS=Saccharomyces cerevisiae (strain ATCC 204508 / S288c) GN=ATE1 PE=1 SV=2

ATG11 − average.top3: 6.30262266666667 − max.qupm: 5

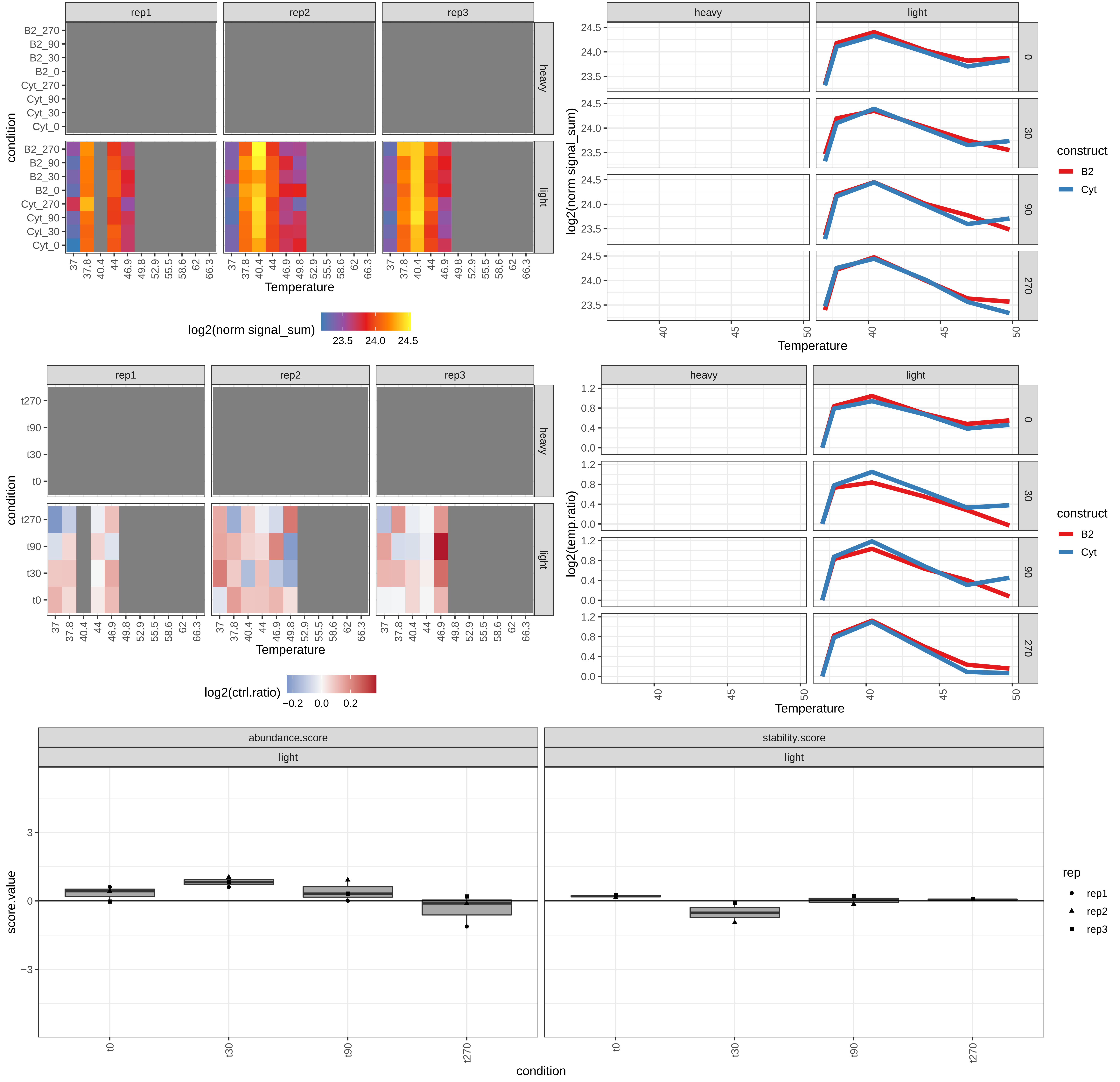

Q12527 − Autophagy−related protein 11 OS=Saccharomyces cerevisiae (strain ATCC 204508 / S288c) GN=ATG11 PE=1 SV=1

ATG17 − average.top3: 6.43375888888889 − max.qupm: 4

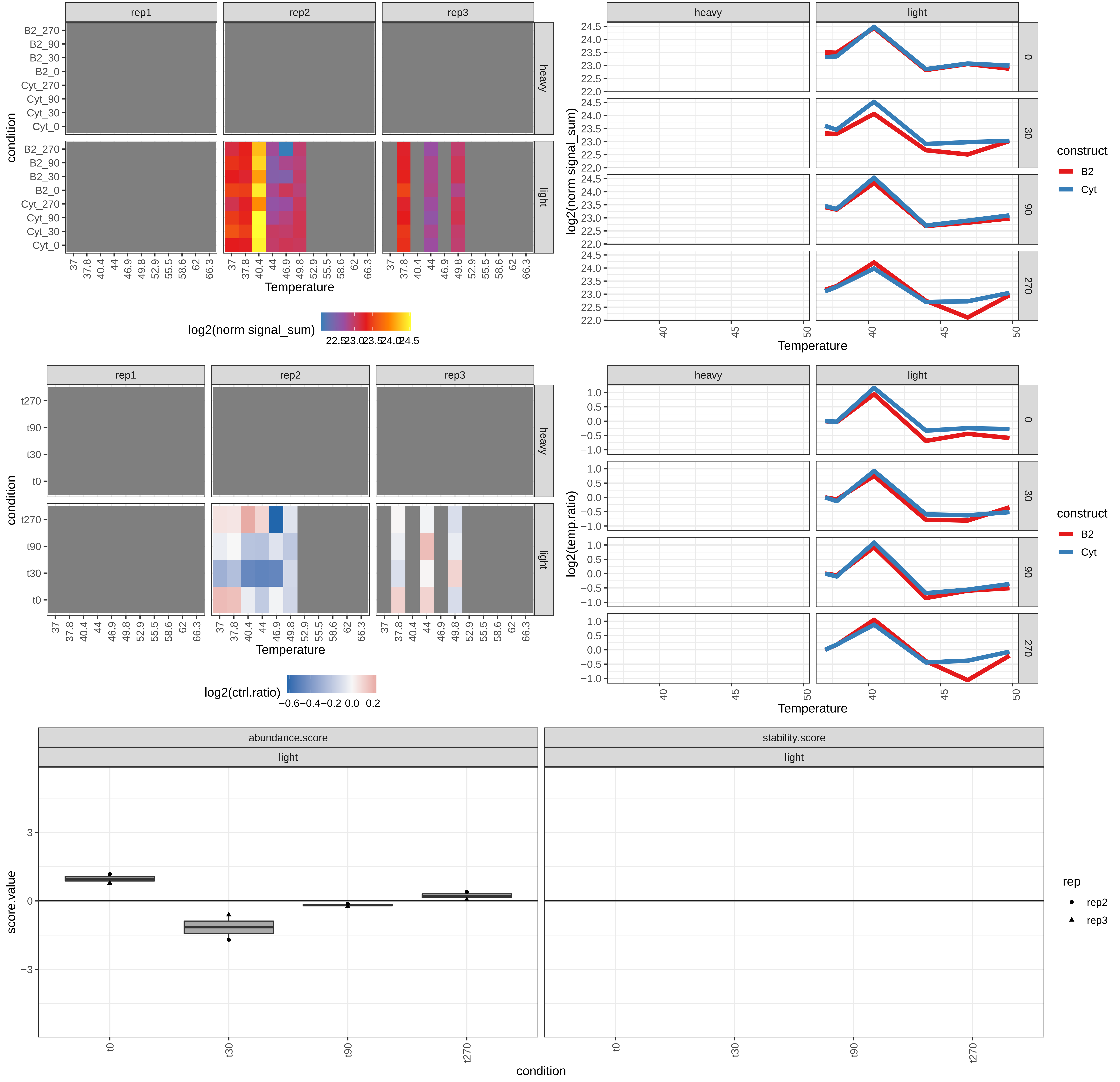

Q06410 − Autophagy−related protein 17 OS=Saccharomyces cerevisiae (strain ATCC 204508 / S288c) GN=ATG17 PE=1 SV=1

ATG18 − average.top3: 6.72802863636364 − max.qupm: 5

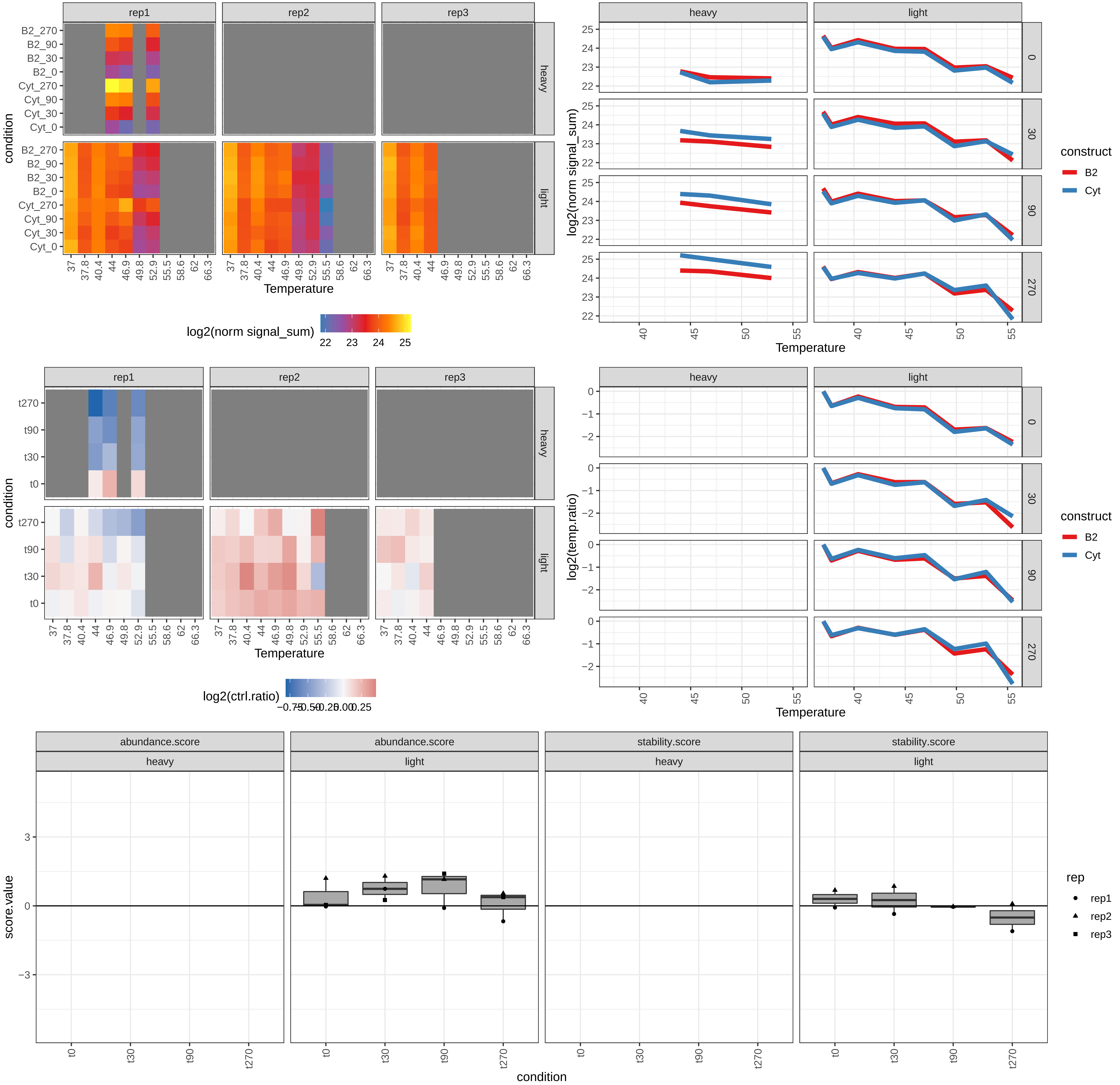

P43601 − Autophagy−related protein 18 OS=Saccharomyces cerevisiae (strain ATCC 204508 / S288c) GN=ATG18 PE=1 SV=1

ATG21 − average.top3: 6.481752 − max.qupm: 5

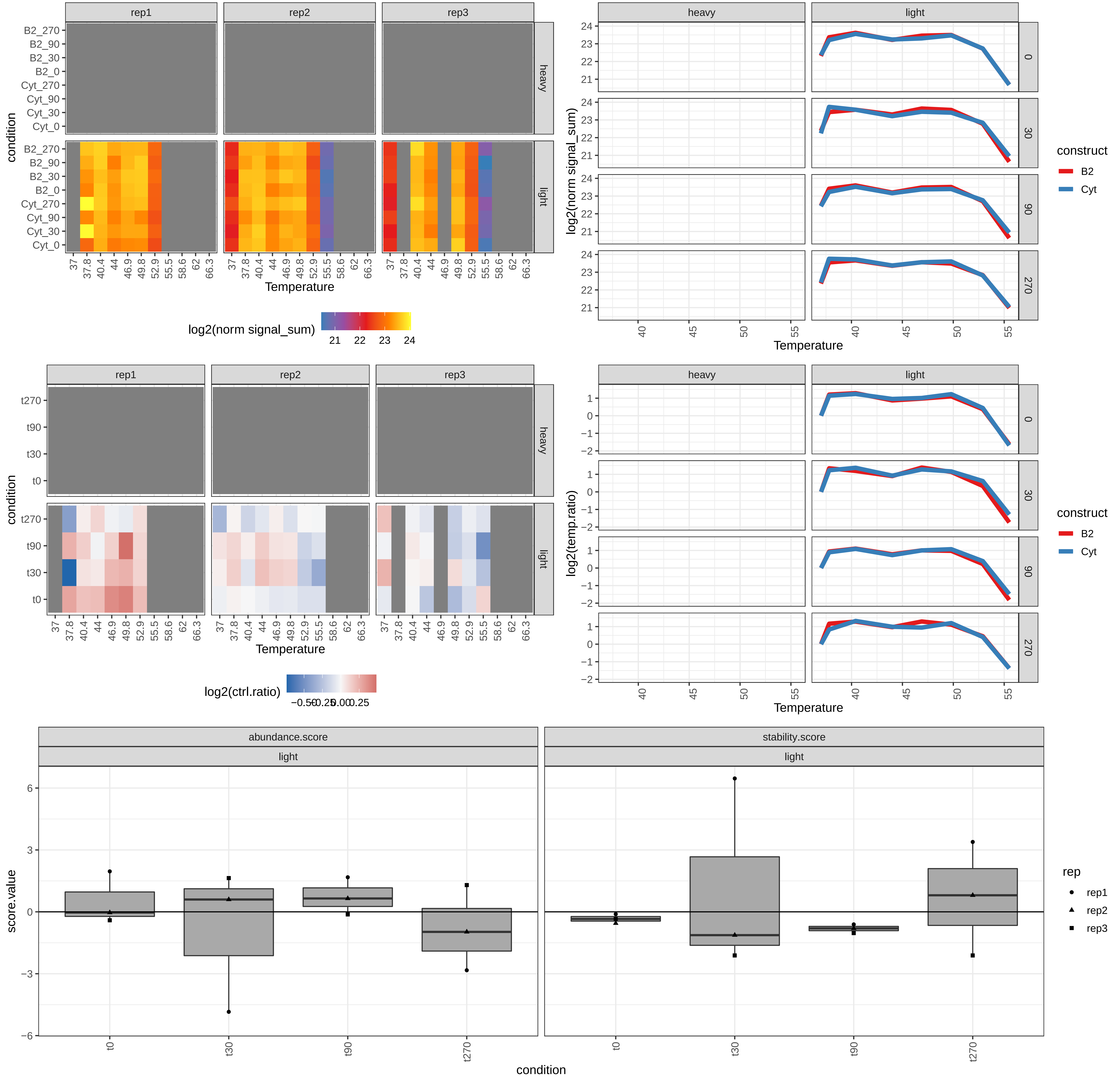

Q02887 − Autophagy−related protein 21 OS=Saccharomyces cerevisiae (strain ATCC 204508 / S288c) GN=ATG21 PE=1 SV=1

ATG27 − average.top3: 7.13716934782609 − max.qupm: 10

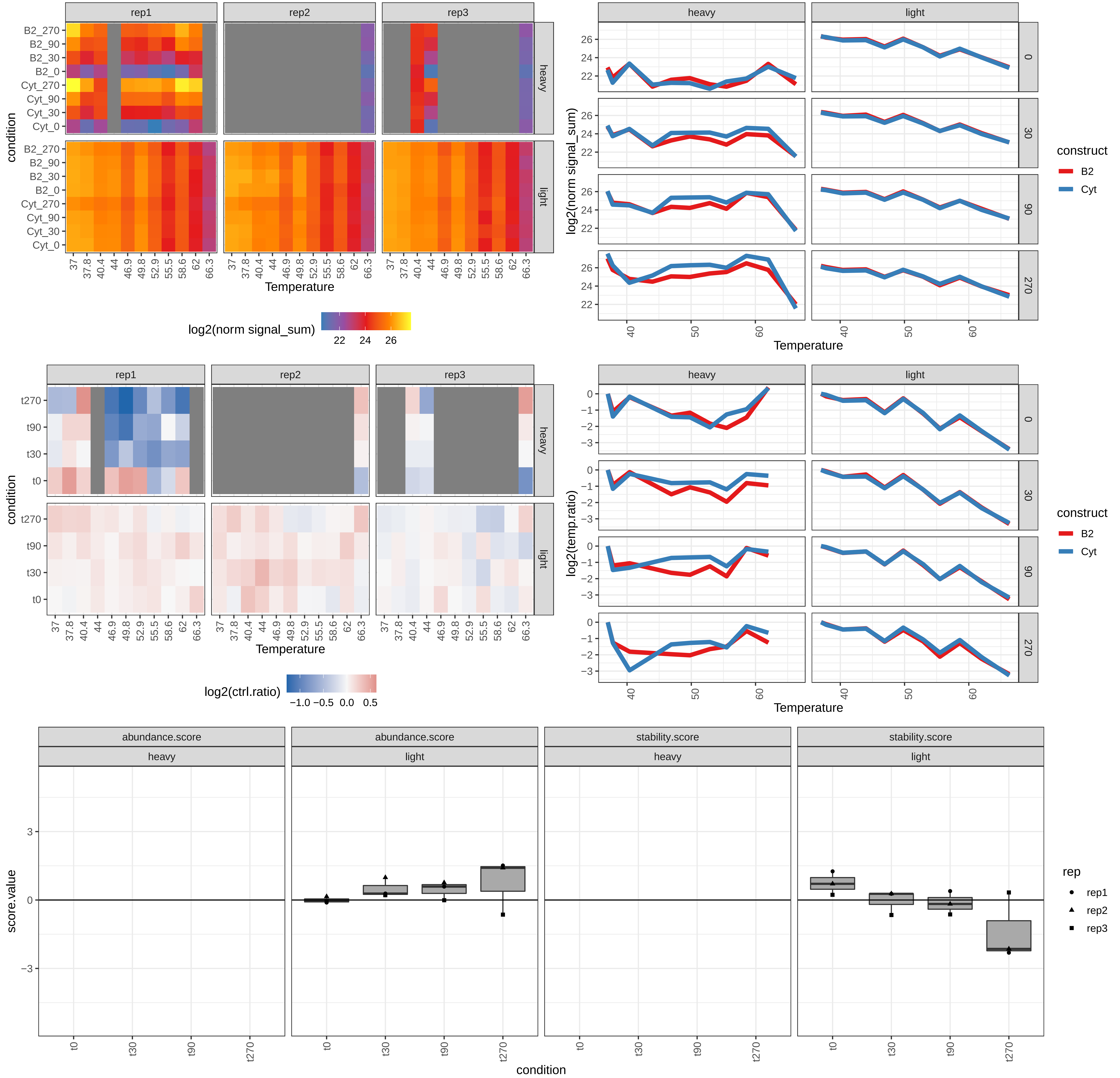

P46989 − Autophagy−related protein 27 OS=Saccharomyces cerevisiae (strain ATCC 204508 / S288c) GN=ATG27 PE=1 SV=2

ATG40 − average.top3: 7.06573523809524 − max.qupm: 4

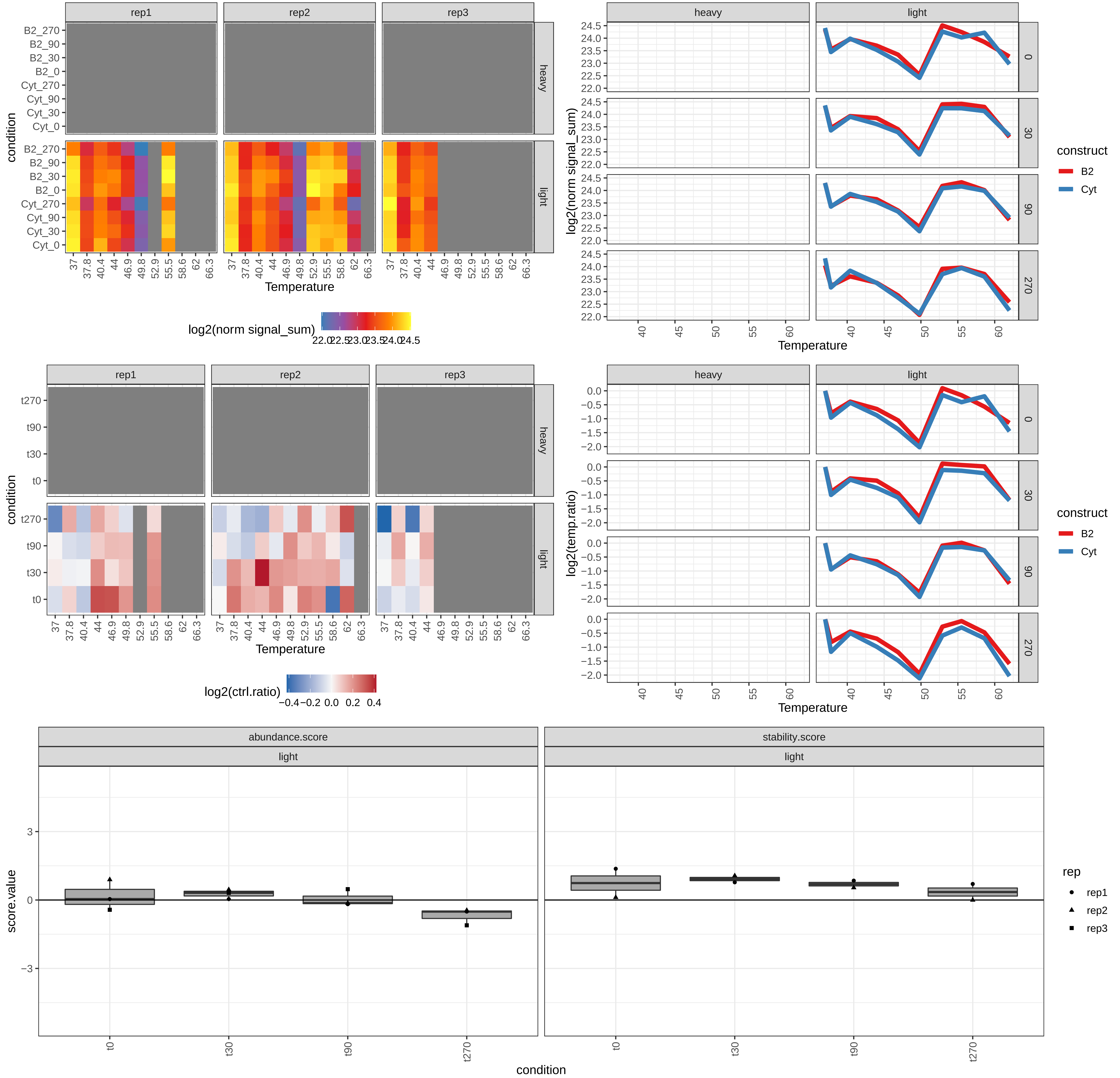

Q99325 − Autophagy−related protein 40 OS=Saccharomyces cerevisiae (strain ATCC 204508 / S288c) GN=ATG40 PE=1 SV=1

ATG7 − average.top3: 6.7024305 − max.qupm: 8

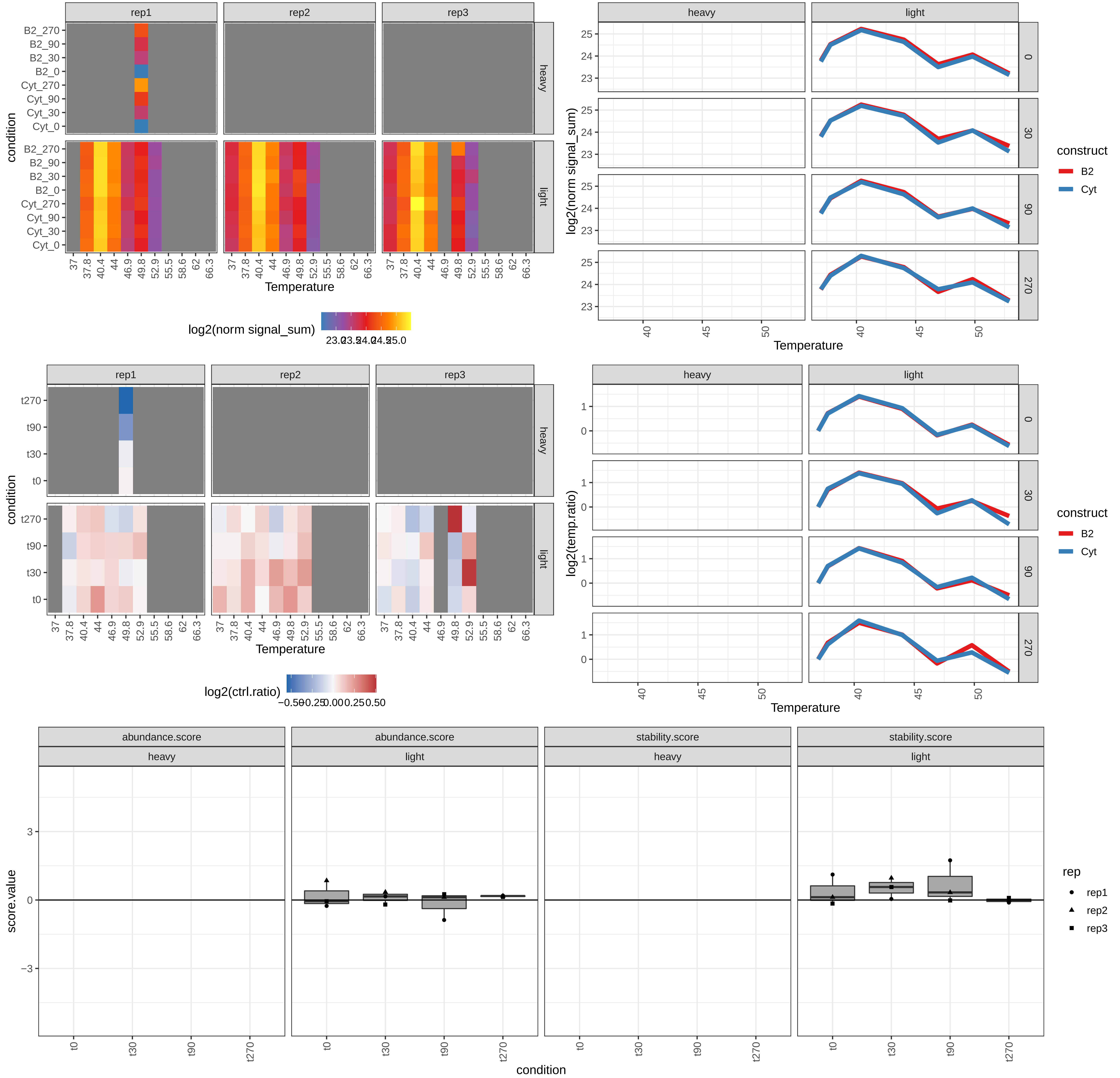

P38862 − Ubiquitin−like modifier−activating enzyme ATG7 OS=Saccharomyces cerevisiae (strain ATCC 204508 / S288c) GN=ATG7 PE=1 SV=1

ATG8 − average.top3: 6.77515533333333 − max.qupm: 3

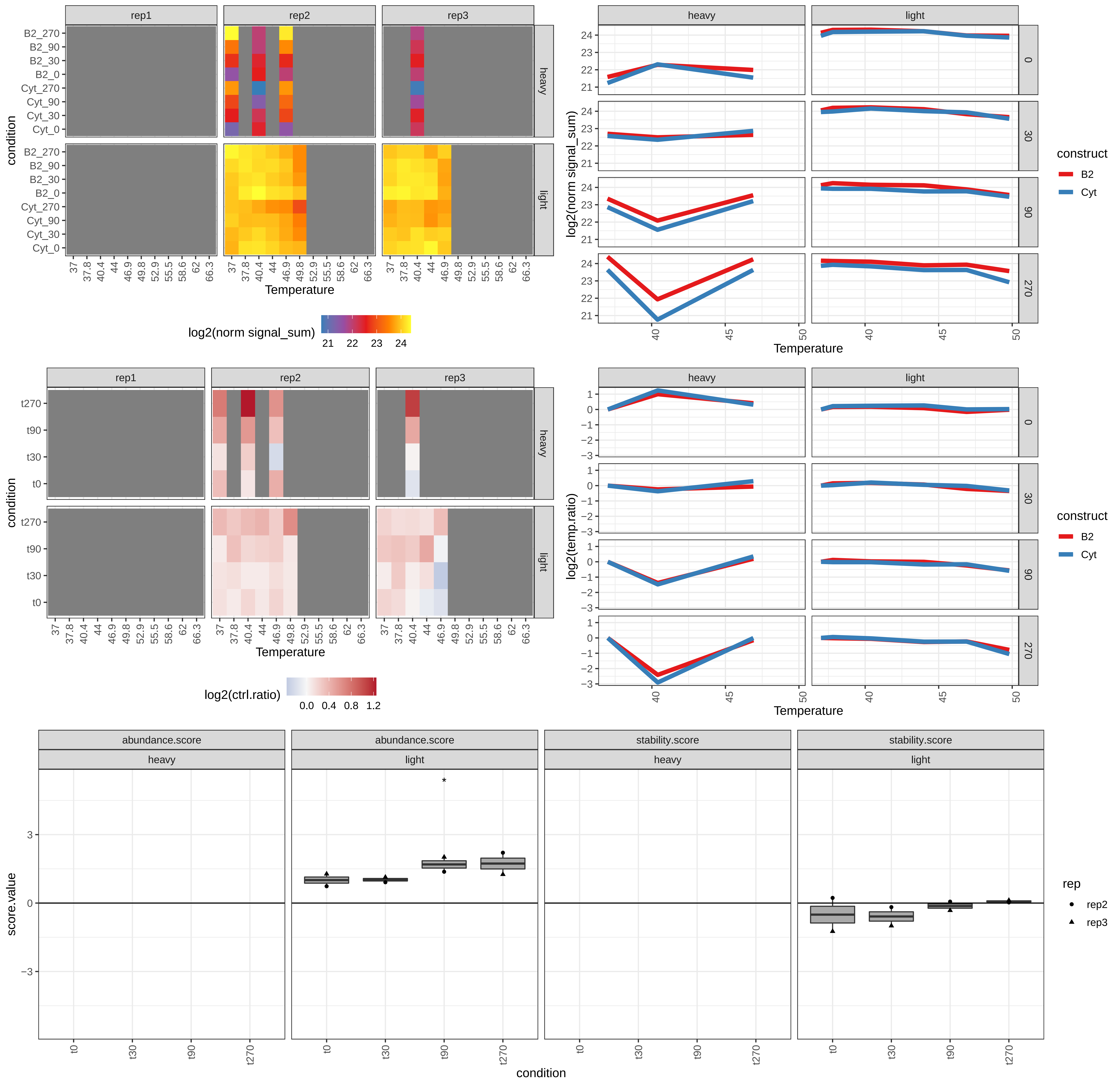

P38182 − Autophagy−related protein 8 OS=Saccharomyces cerevisiae (strain ATCC 204508 / S288c) GN=ATG8 PE=1 SV=1

ATG9 − average.top3: 6.28841083333333 − max.qupm: 5

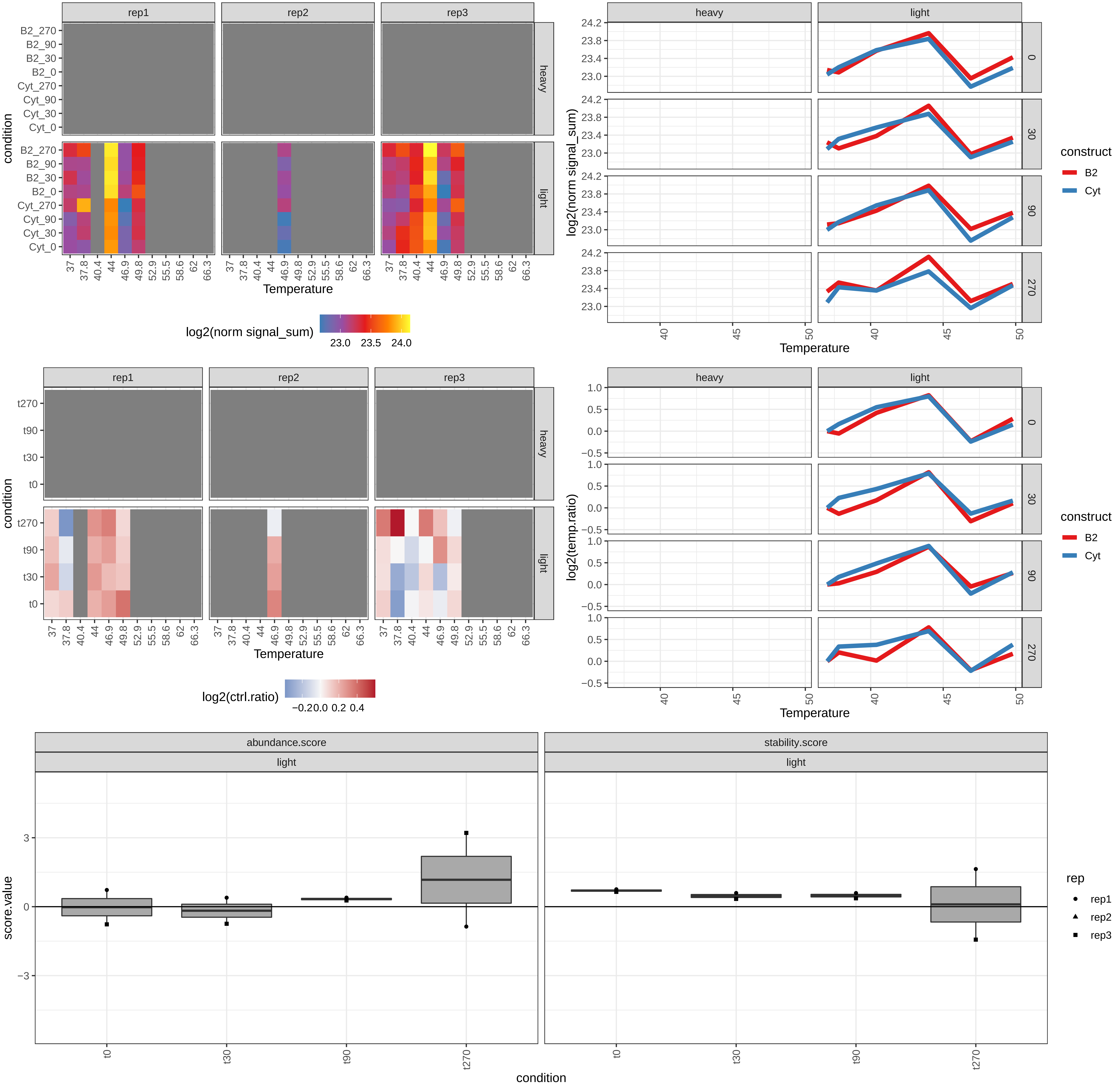

Q12142 − Autophagy−related protein 9 OS=Saccharomyces cerevisiae (strain ATCC 204508 / S288c) GN=ATG9 PE=1 SV=1

ATH1 − average.top3: 6.4255688 − max.qupm: 7

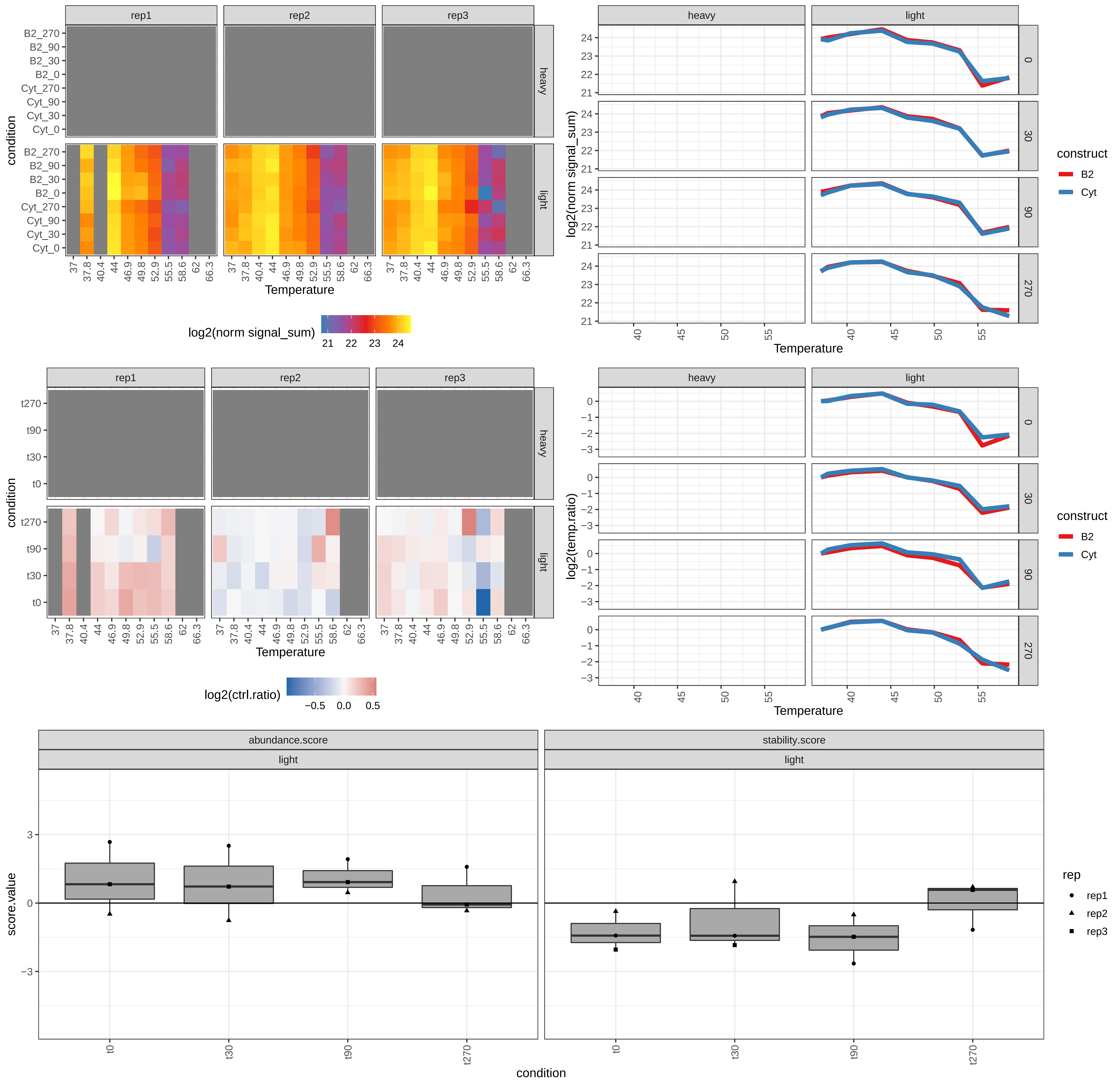

P48016 − Vacuolar acid trehalase OS=Saccharomyces cerevisiae (strain ATCC 204508 / S288c) GN=ATH1 PE=1 SV=1

ATM1 − average.top3: 7.32176769230769 − max.qupm: 14

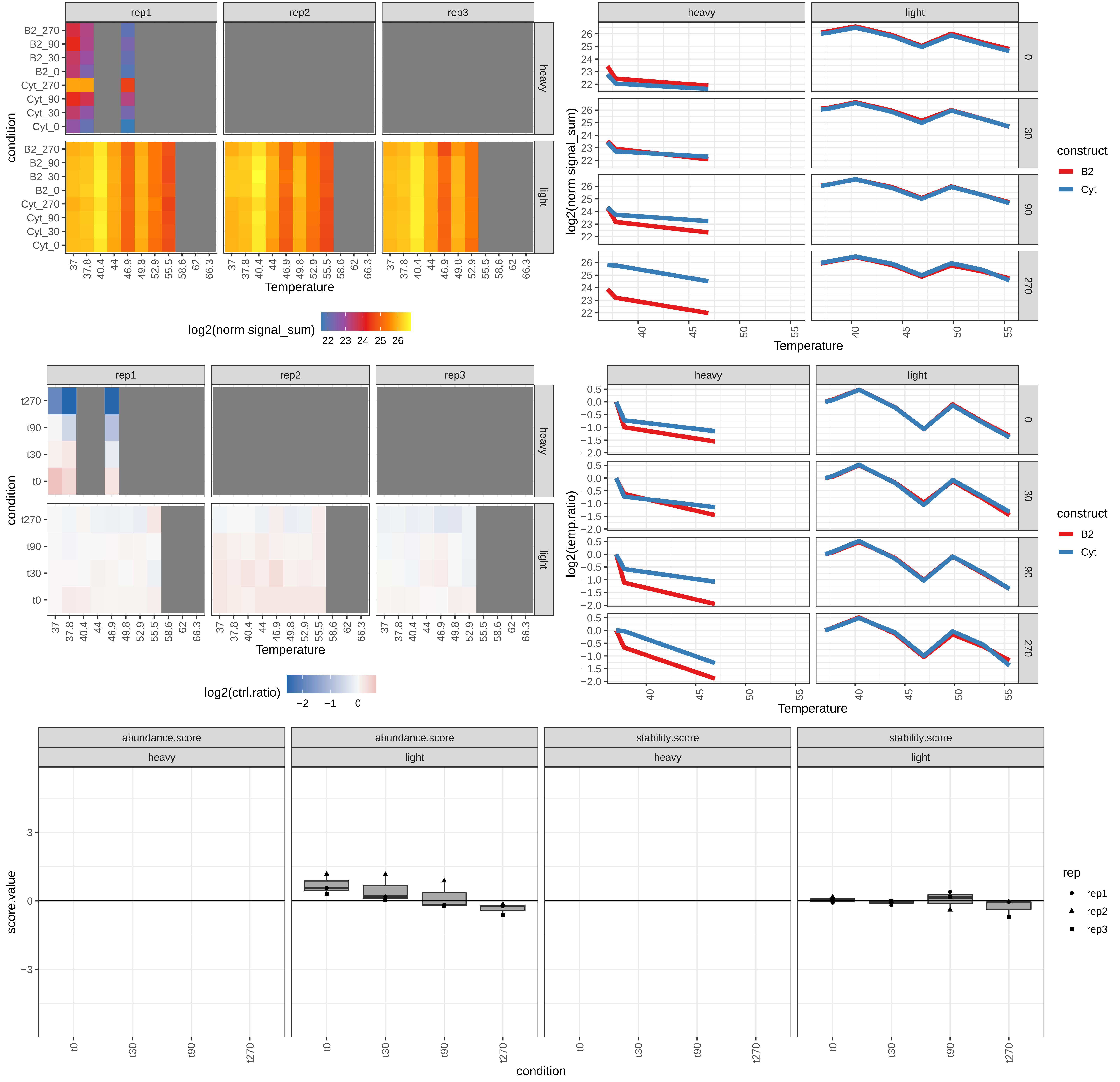

P40416 − Iron−sulfur clusters transporter ATM1, mitochondrial OS=Saccharomyces cerevisiae (strain ATCC 204508 / S288c) GN=ATM1 PE=1 SV=2

ATO2 − average.top3: 7.00113625 − max.qupm: 4

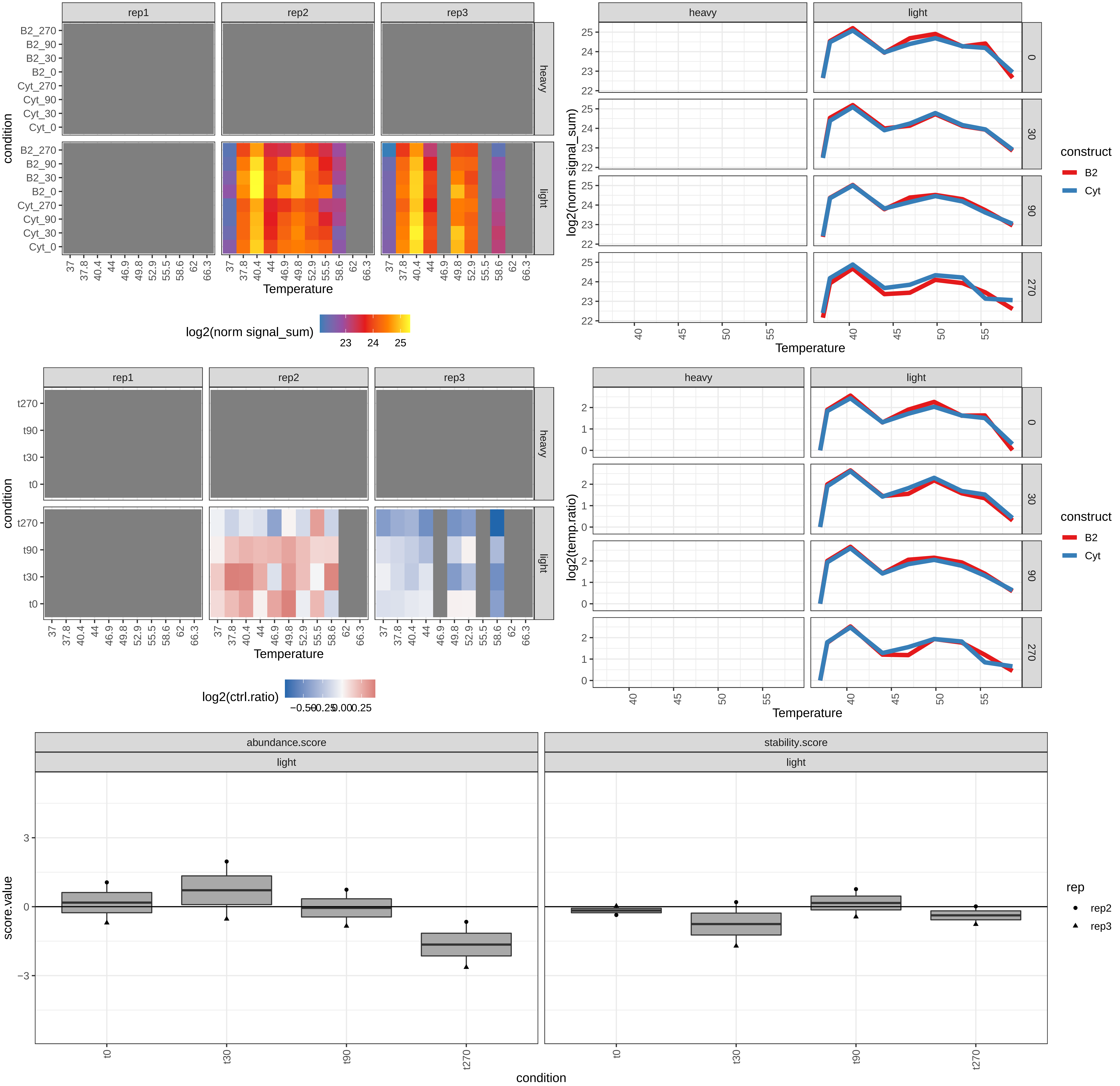

P32907 − Ammonia transport outward protein 2 OS=Saccharomyces cerevisiae (strain ATCC 204508 / S288c) GN=ATO2 PE=1 SV=1

ATO3 − average.top3: 7.57537568965517 − max.qupm: 5

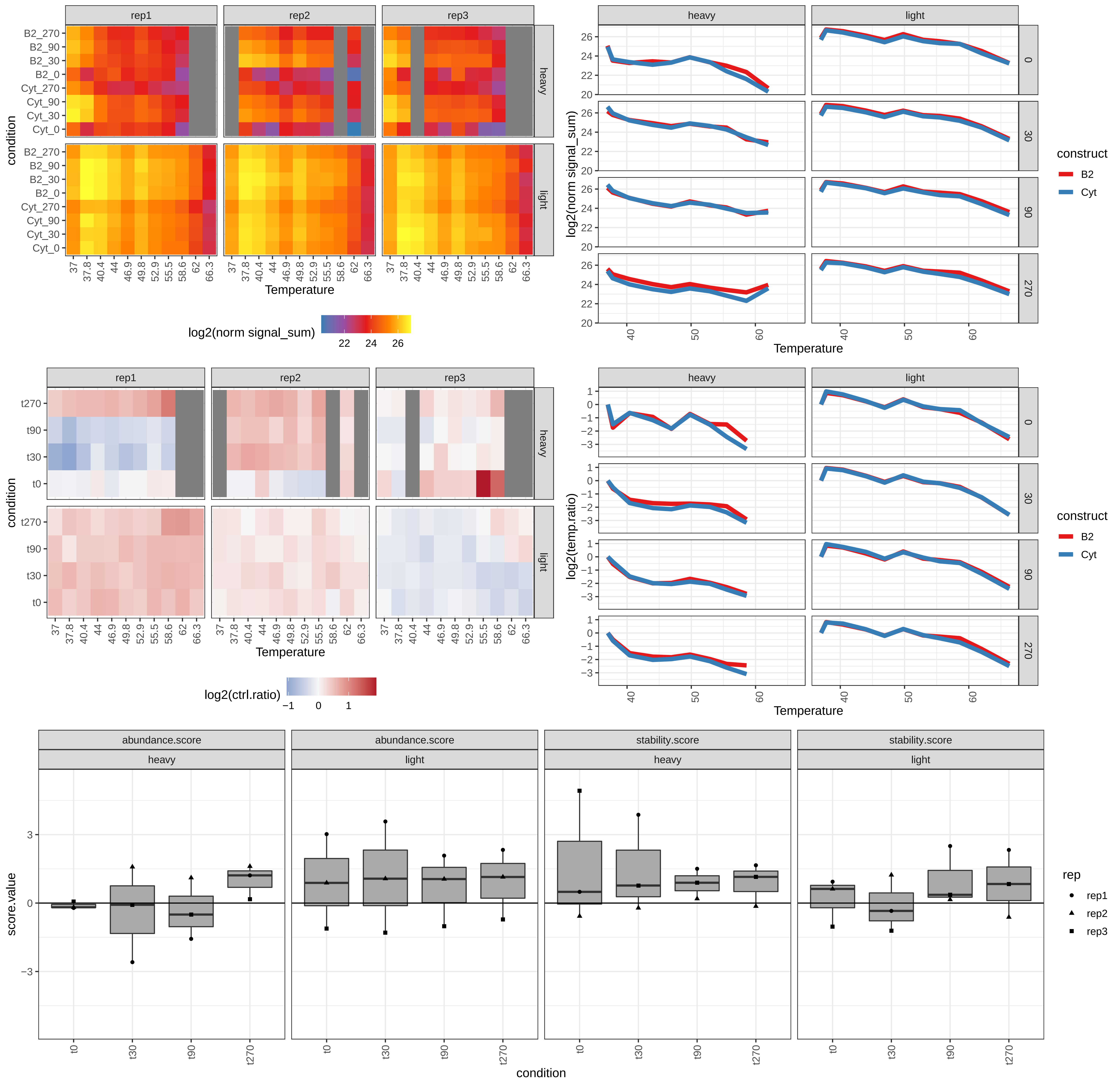

Q12359 − Ammonia transport outward protein 3 OS=Saccharomyces cerevisiae (strain ATCC 204508 / S288c) GN=ATO3 PE=1 SV=1

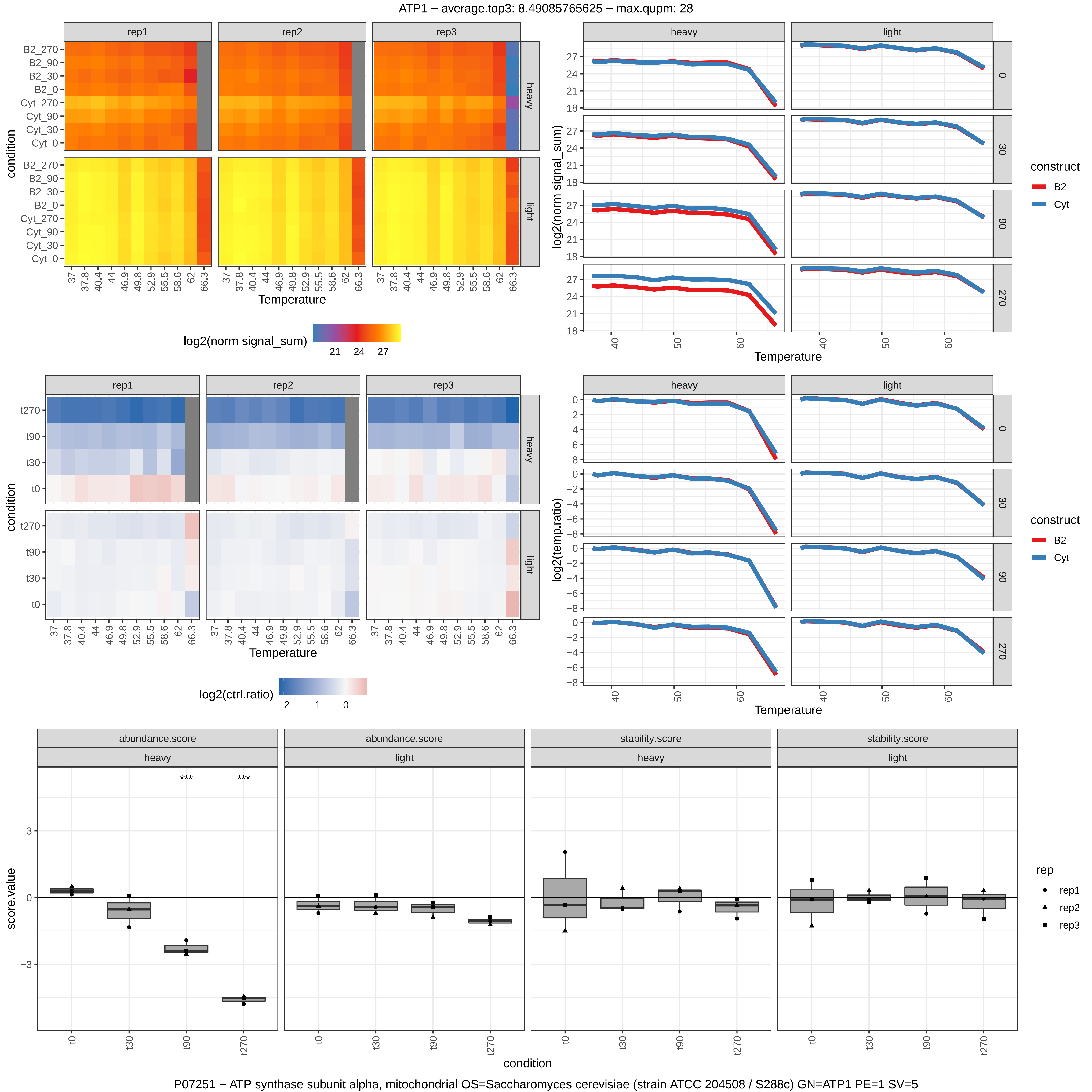
ATP1 − average.top3: 8.49085765625 − max.qupm: 28
rep1
rep2
rep3
heavy
light
B2_270
B2_90
B2_30
B2_0
Cyt_270
Cyt_90
Cyt_30
Cyt_0
condition
Temperature
37
37.8
40.4
44
46.9
49.8
52.9
55.5
58.6
62
66.3
log2(norm signal_sum)
21
24
27
log2(norm signal_sum)
construct
B2
Cyt
0
30
90
270
40
50
60
t270
t90
t30
t0
log2(ctrl.ratio)
−2
−1
0
log2(temp.ratio)
abundance.score
stability.score
score.value
***
rep
rep1
rep2
rep3
P07251 − ATP synthase subunit alpha, mitochondrial OS=Saccharomyces cerevisiae (strain ATCC 204508 / S288c) GN=ATP1 PE=1 SV=5

ATP10 − average.top3: 6.703532 − max.qupm: 8

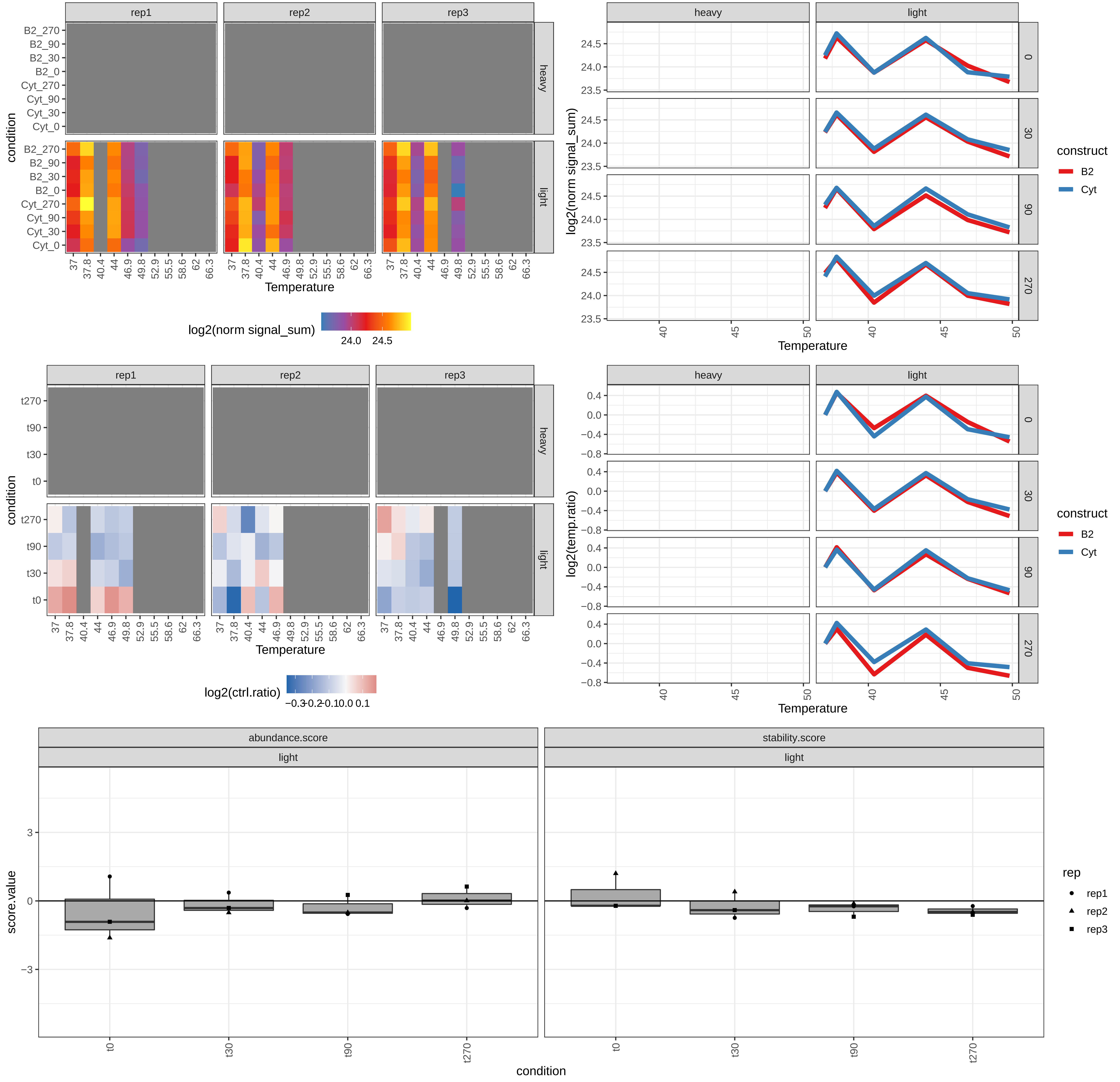

P18496 − Mitochondrial ATPase complex subunit ATP10 OS=Saccharomyces cerevisiae (strain ATCC 204508 / S288c) GN=ATP10 PE=1 SV=2

ATP11 − average.top3: 6.71835435897436 − max.qupm: 10

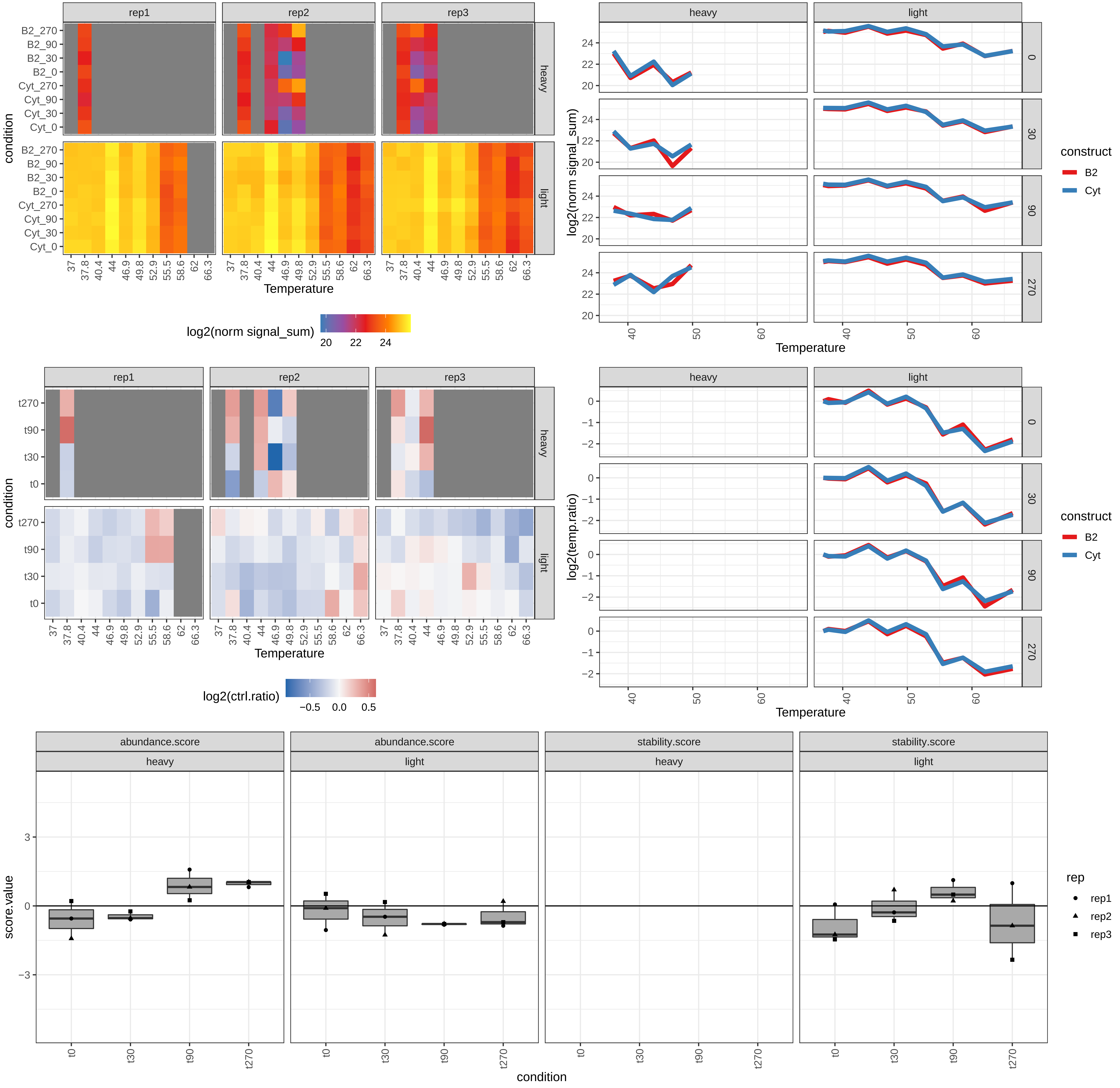

P32453 − Protein ATP11, mitochondrial OS=Saccharomyces cerevisiae (strain ATCC 204508 / S288c) GN=ATP11 PE=1 SV=1

ATP12 − average.top3: 6.71647075 − max.qupm: 9

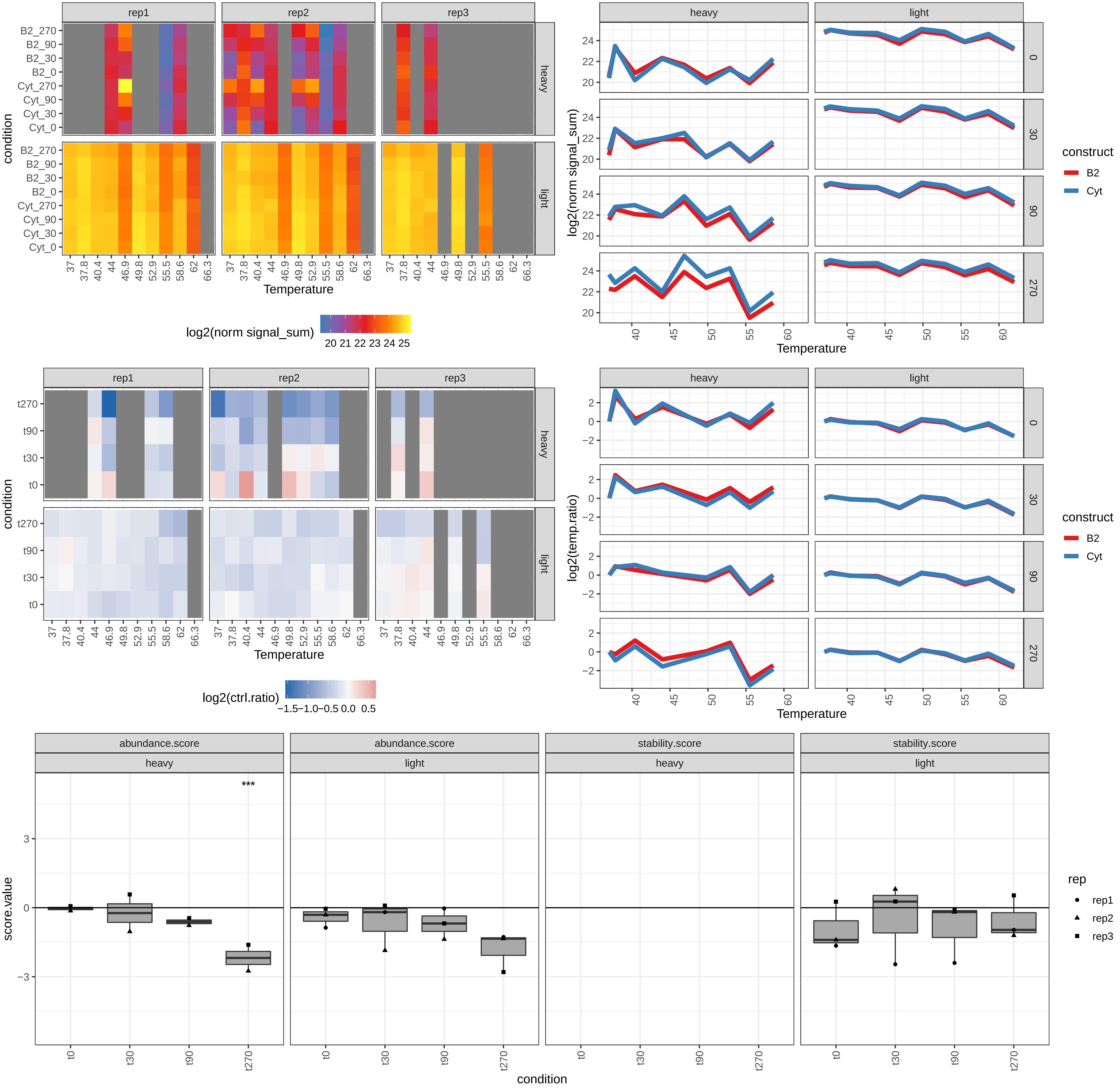

P22135 − Protein ATP12, mitochondrial OS=Saccharomyces cerevisiae (strain ATCC 204508 / S288c) GN=ATP12 PE=1 SV=2

ATP15 − average.top3: 6.891144375 − max.qupm: 3

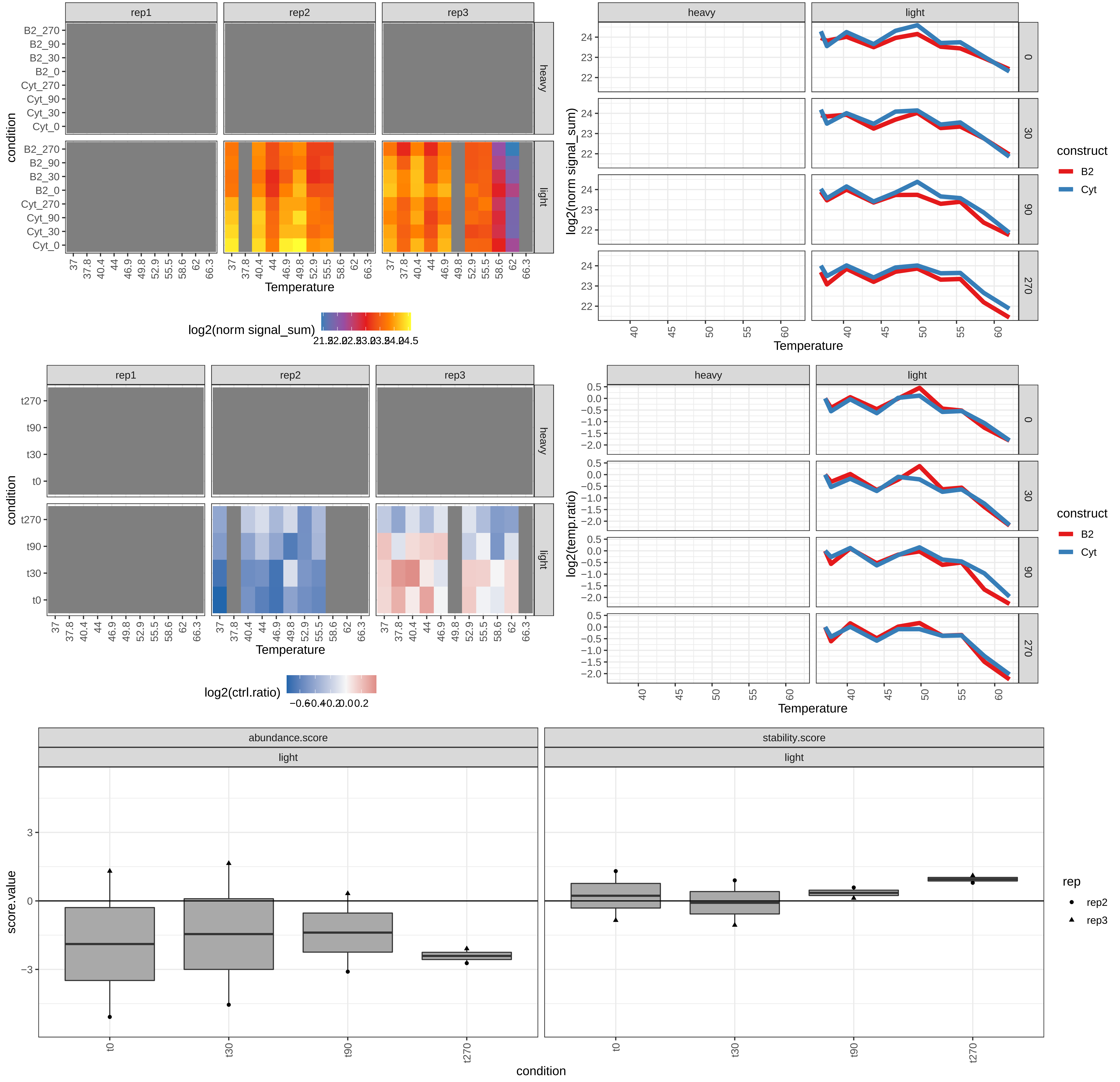

P21306 − ATP synthase subunit epsilon, mitochondrial OS=Saccharomyces cerevisiae (strain ATCC 204508 / S288c) GN=ATP15 PE=1 SV=2

ATP17 − average.top3: 7.49627357142857 − max.qupm: 4

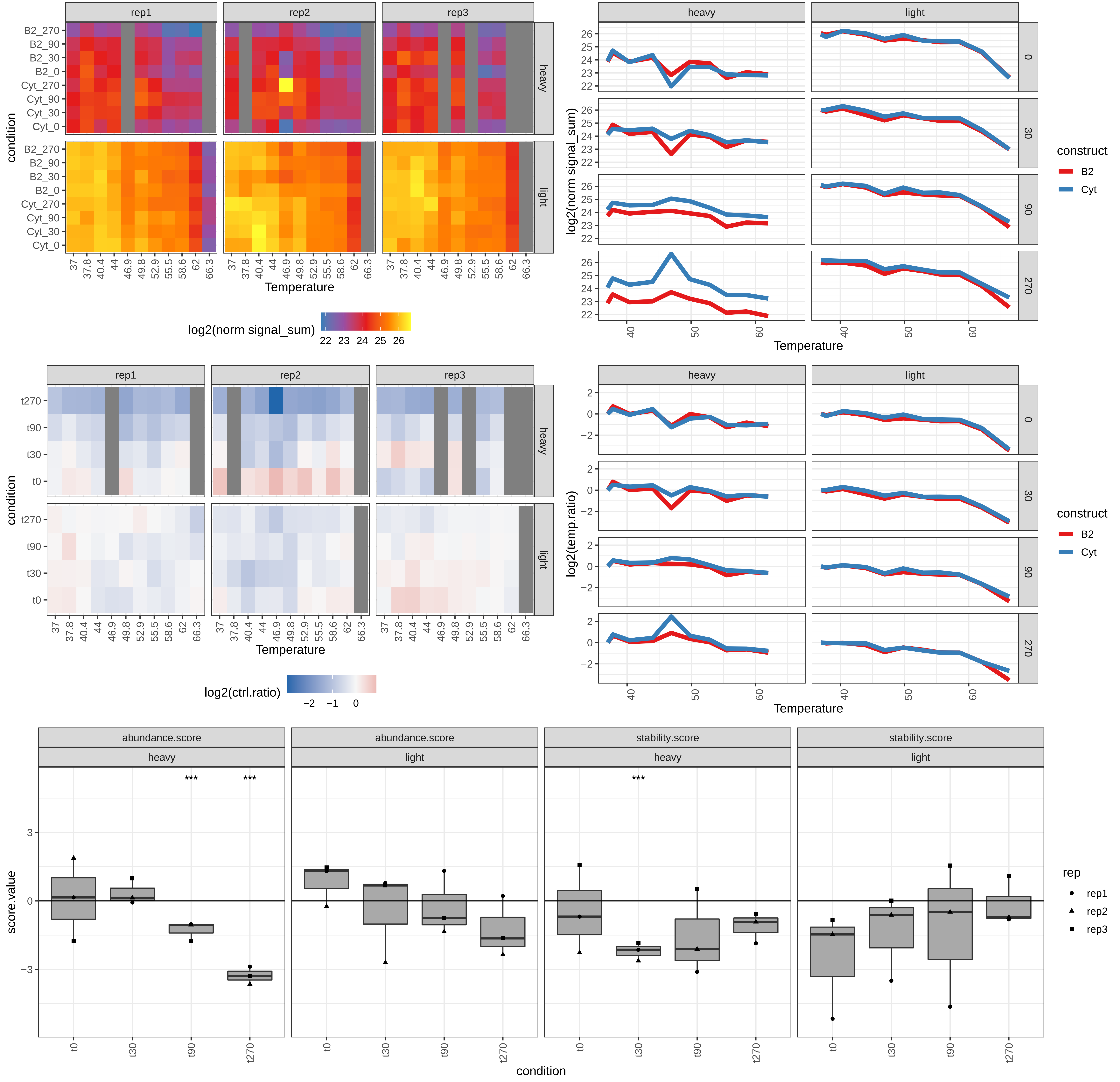

Q06405 − ATP synthase subunit f, mitochondrial OS=Saccharomyces cerevisiae (strain ATCC 204508 / S288c) GN=ATP17 PE=1 SV=1

ATP18 − average.top3: 7.49389975 − max.qupm: 4

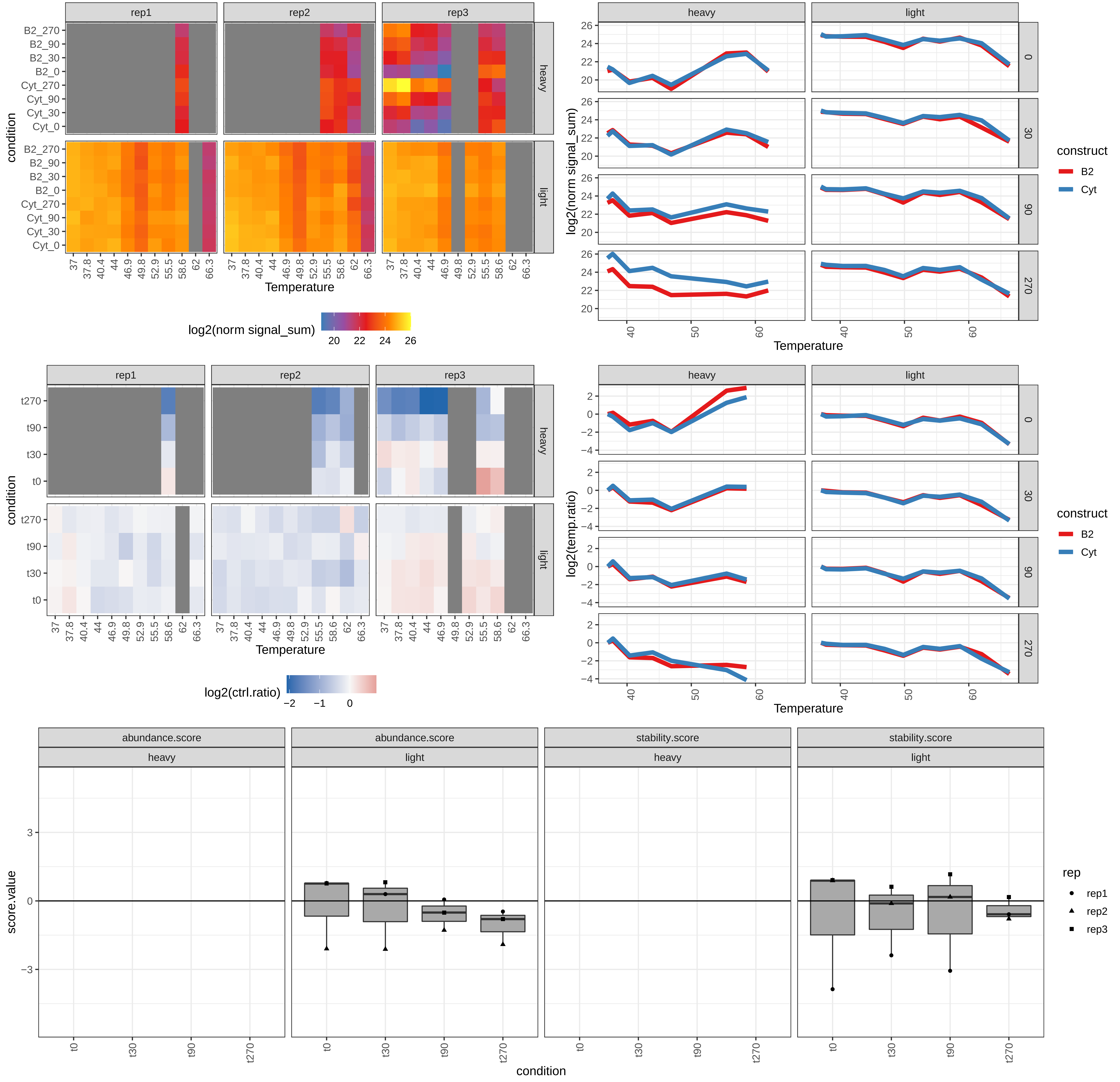

P81450 − ATP synthase subunit J, mitochondrial OS=Saccharomyces cerevisiae (strain ATCC 204508 / S288c) GN=ATP18 PE=1 SV=1

ATP2 − average.top3: 8.18797030769231 − max.qupm: 22

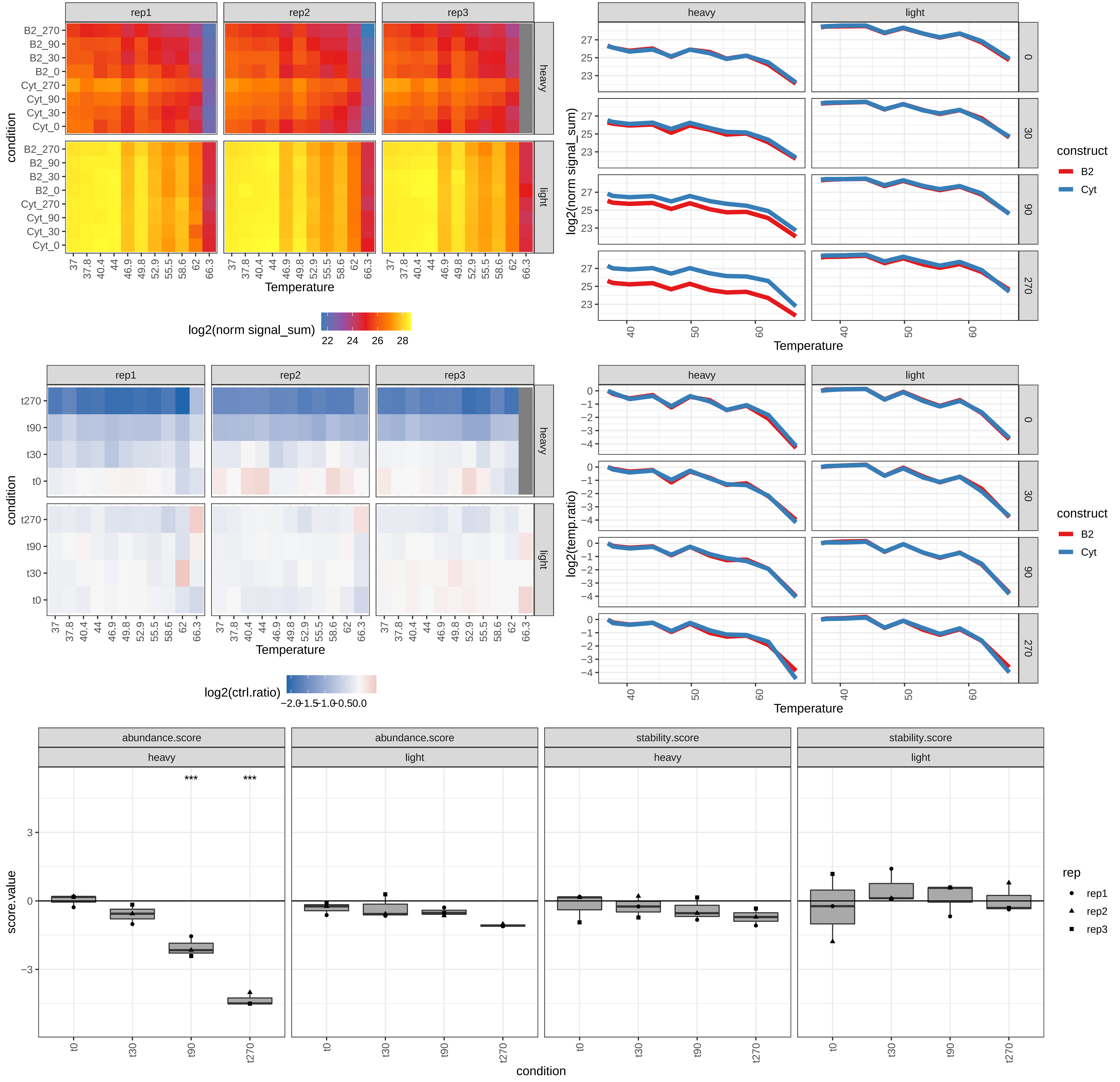

P00830 − ATP synthase subunit beta, mitochondrial OS=Saccharomyces cerevisiae (strain ATCC 204508 / S288c) GN=ATP2 PE=1 SV=2

ATP20 − average.top3: 6.90660914285714 − max.qupm: 5

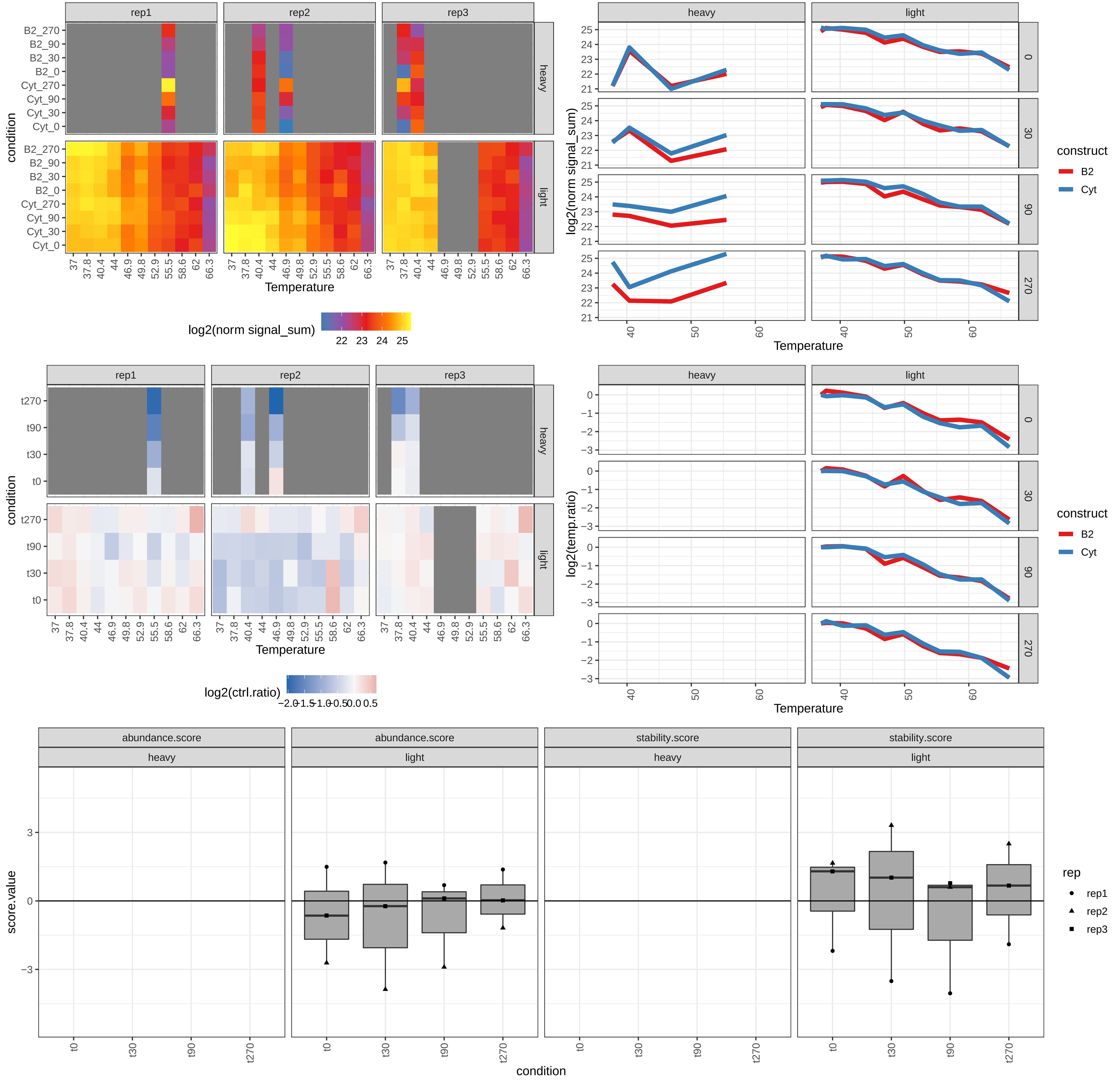

Q12233 − ATP synthase subunit g, mitochondrial OS=Saccharomyces cerevisiae (strain ATCC 204508 / S288c) GN=ATP20 PE=1 SV=1

ATP23 − average.top3: 6.46610538461538 − max.qupm: 5

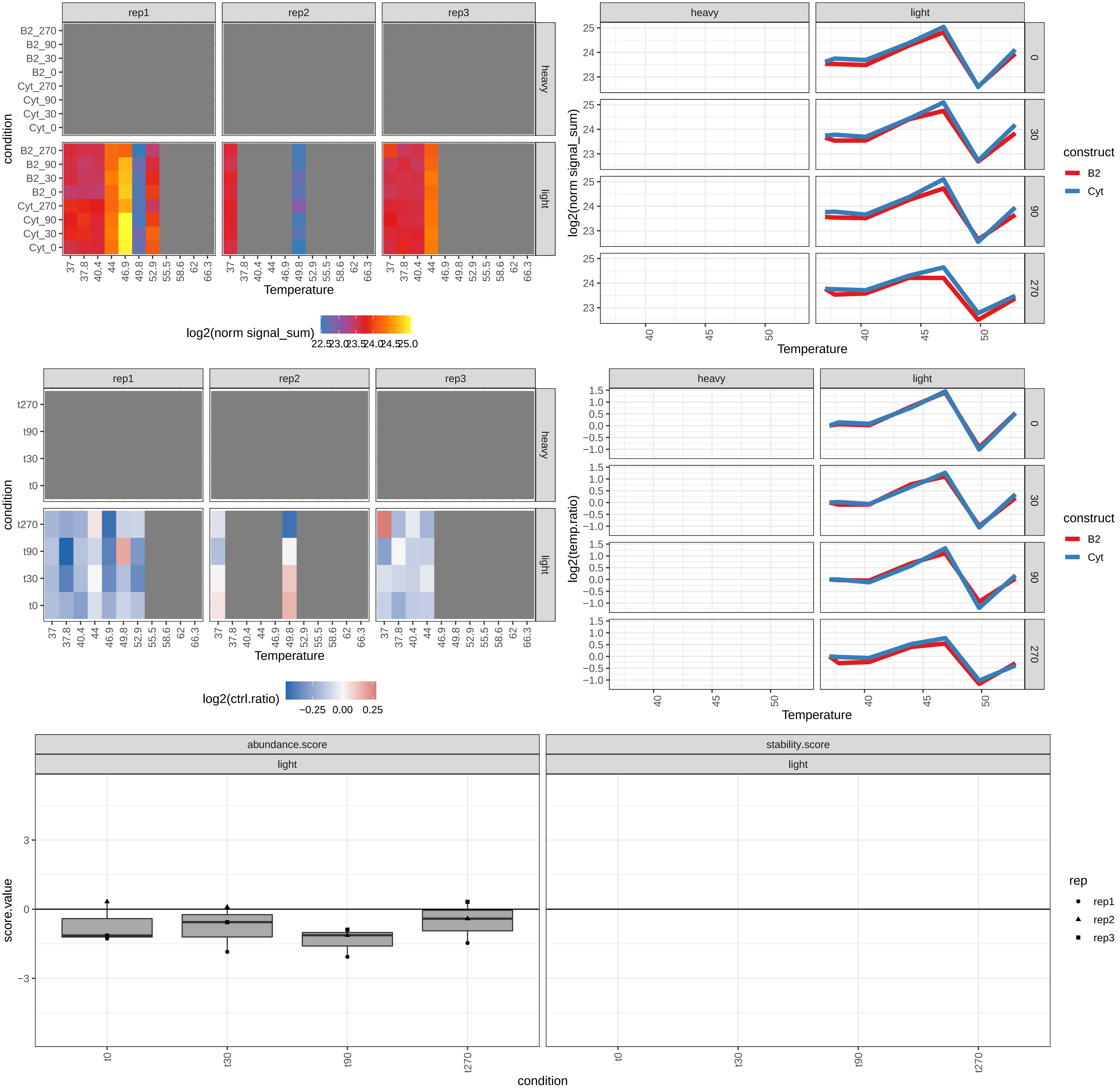

P53722 − Mitochondrial inner membrane protease ATP23 OS=Saccharomyces cerevisiae (strain ATCC 204508 / S288c) GN=ATP23 PE=1 SV=2

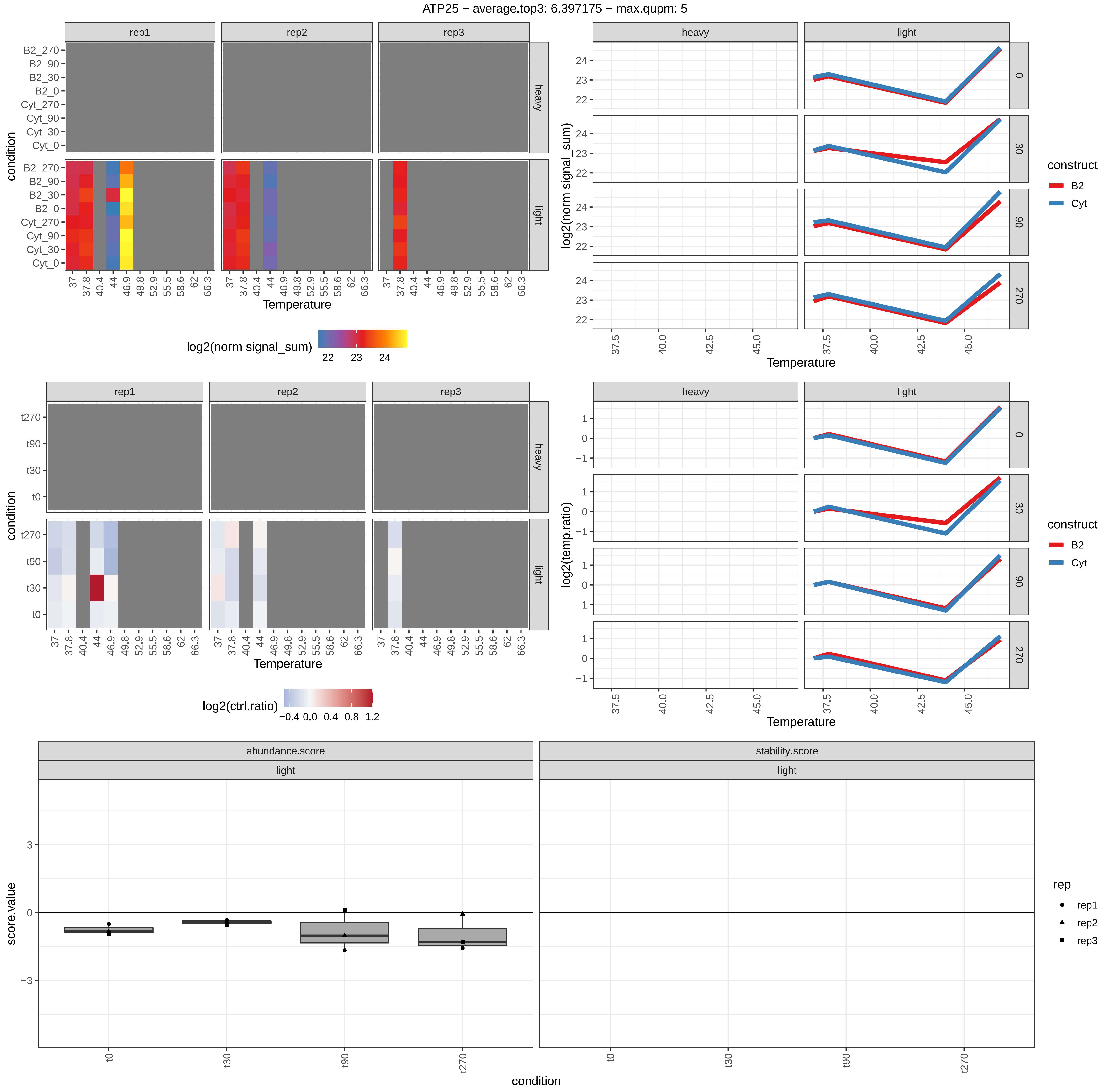

Q03153 − ATPase synthesis protein 25, mitochondrial OS=Saccharomyces cerevisiae (strain ATCC 204508 / S288c) GN=ATP25 PE=1 SV=1

ATP3 − average.top3: 7.54036193548387 − max.qupm: 17

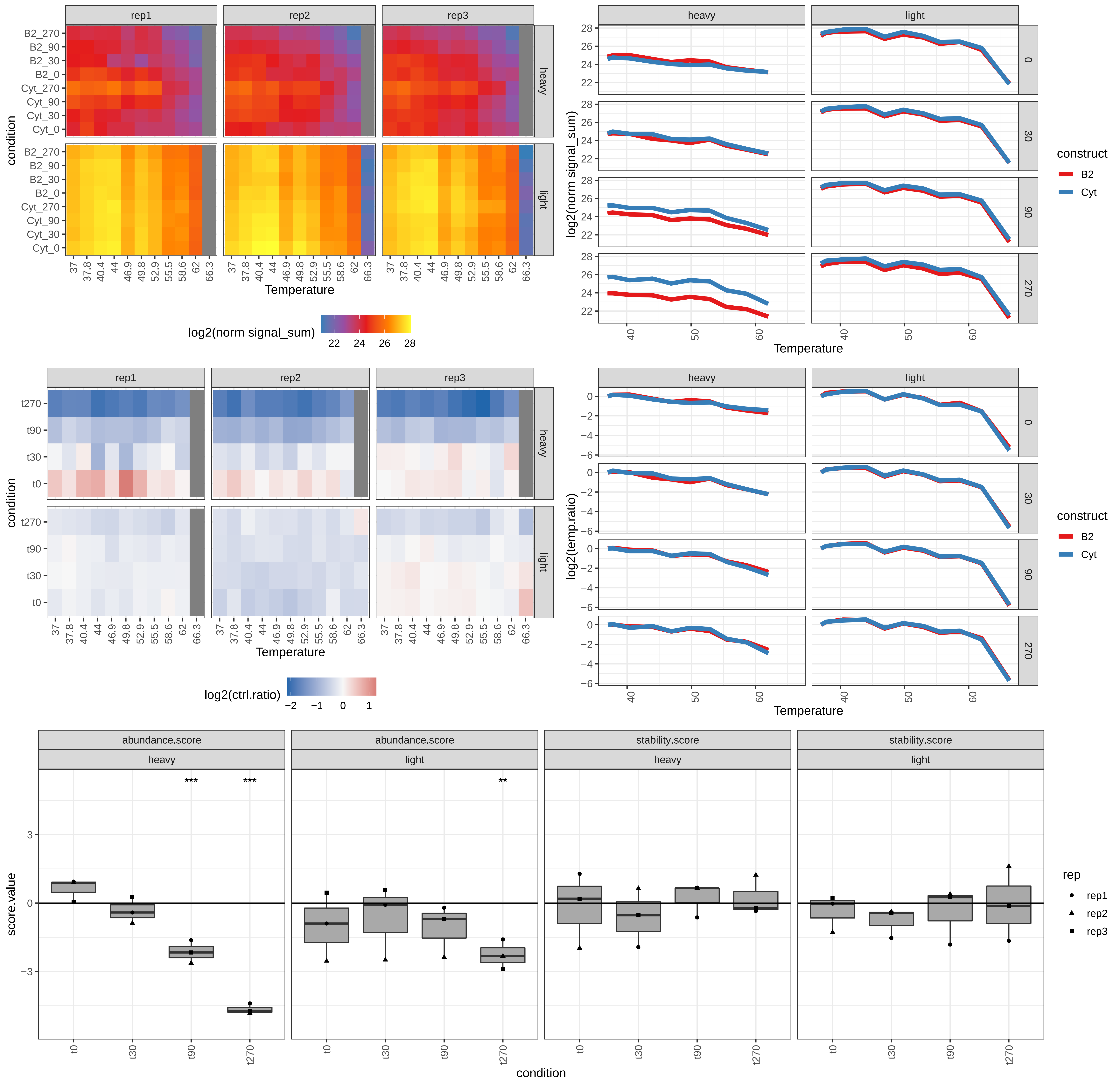

P38077 − ATP synthase subunit gamma, mitochondrial OS=Saccharomyces cerevisiae (strain ATCC 204508 / S288c) GN=ATP3 PE=1 SV=1

ATP4 − average.top3: 7.89402049180328 − max.qupm: 15

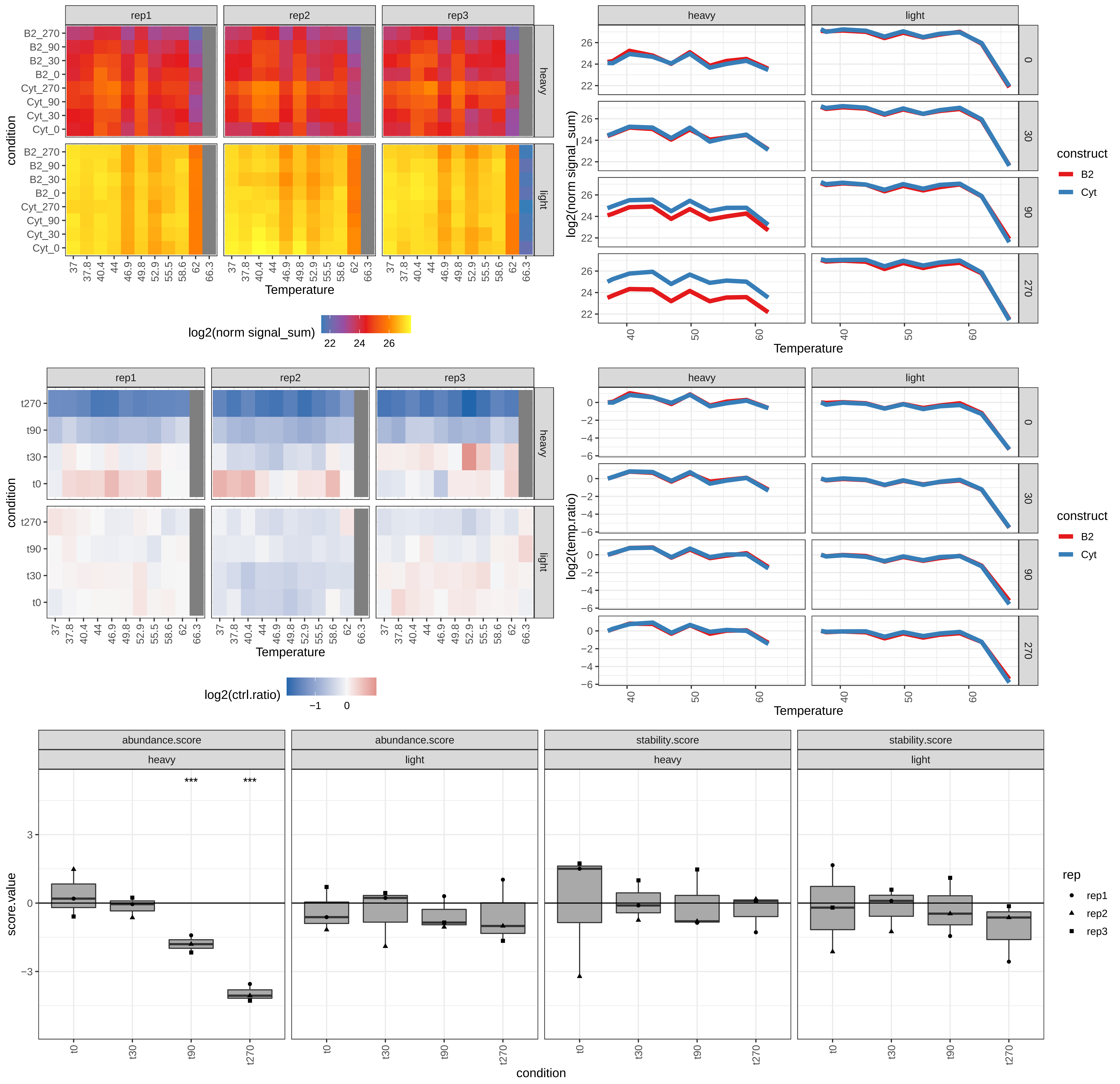

P05626 − ATP synthase subunit 4, mitochondrial OS=Saccharomyces cerevisiae (strain ATCC 204508 / S288c) GN=ATP4 PE=1 SV=2

ATP5 − average.top3: 7.75754557377049 − max.qupm: 14

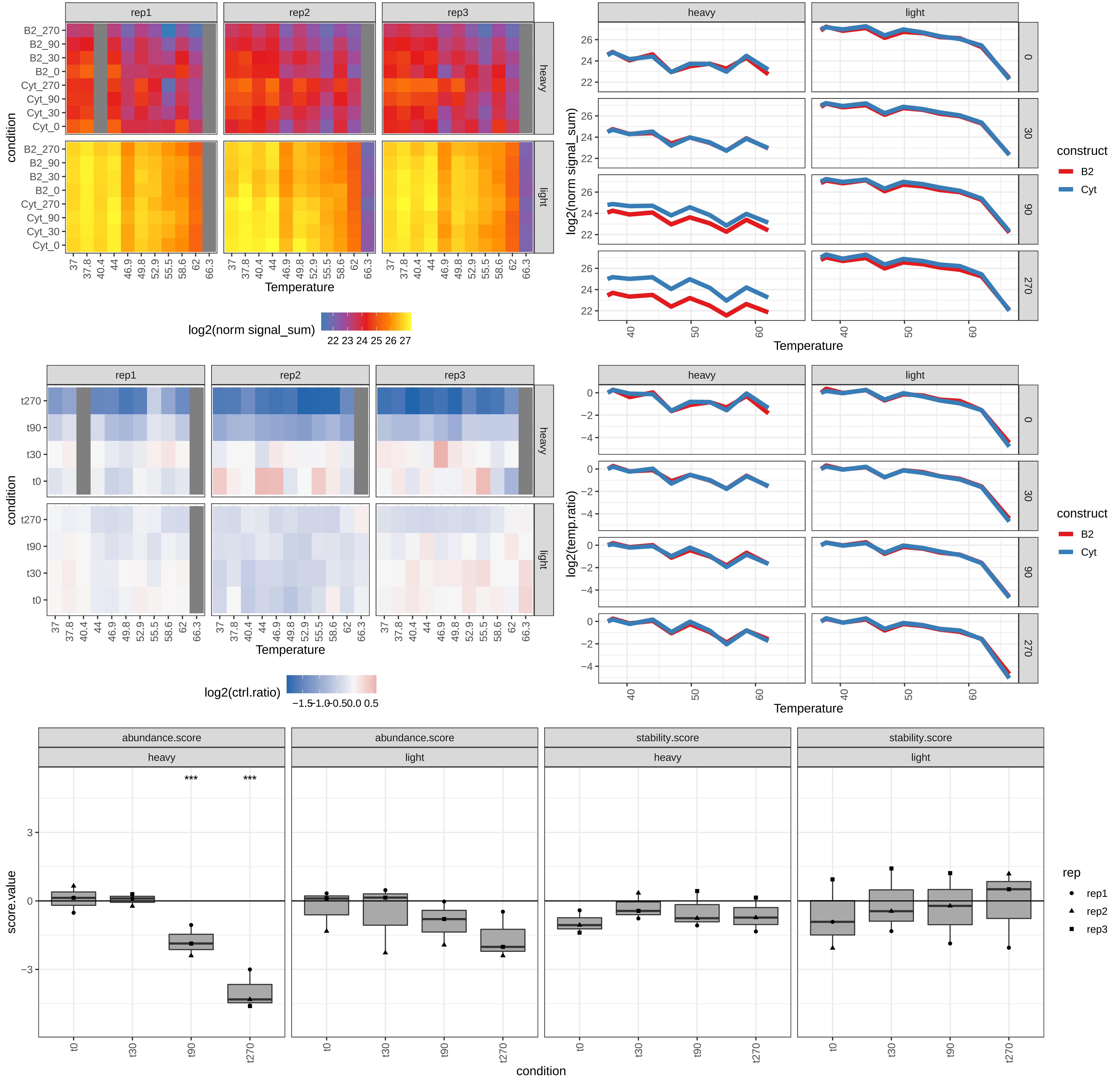

P09457 − ATP synthase subunit 5, mitochondrial OS=Saccharomyces cerevisiae (strain ATCC 204508 / S288c) GN=ATP5 PE=1 SV=1

ATP7 − average.top3: 7.3618455 − max.qupm: 14

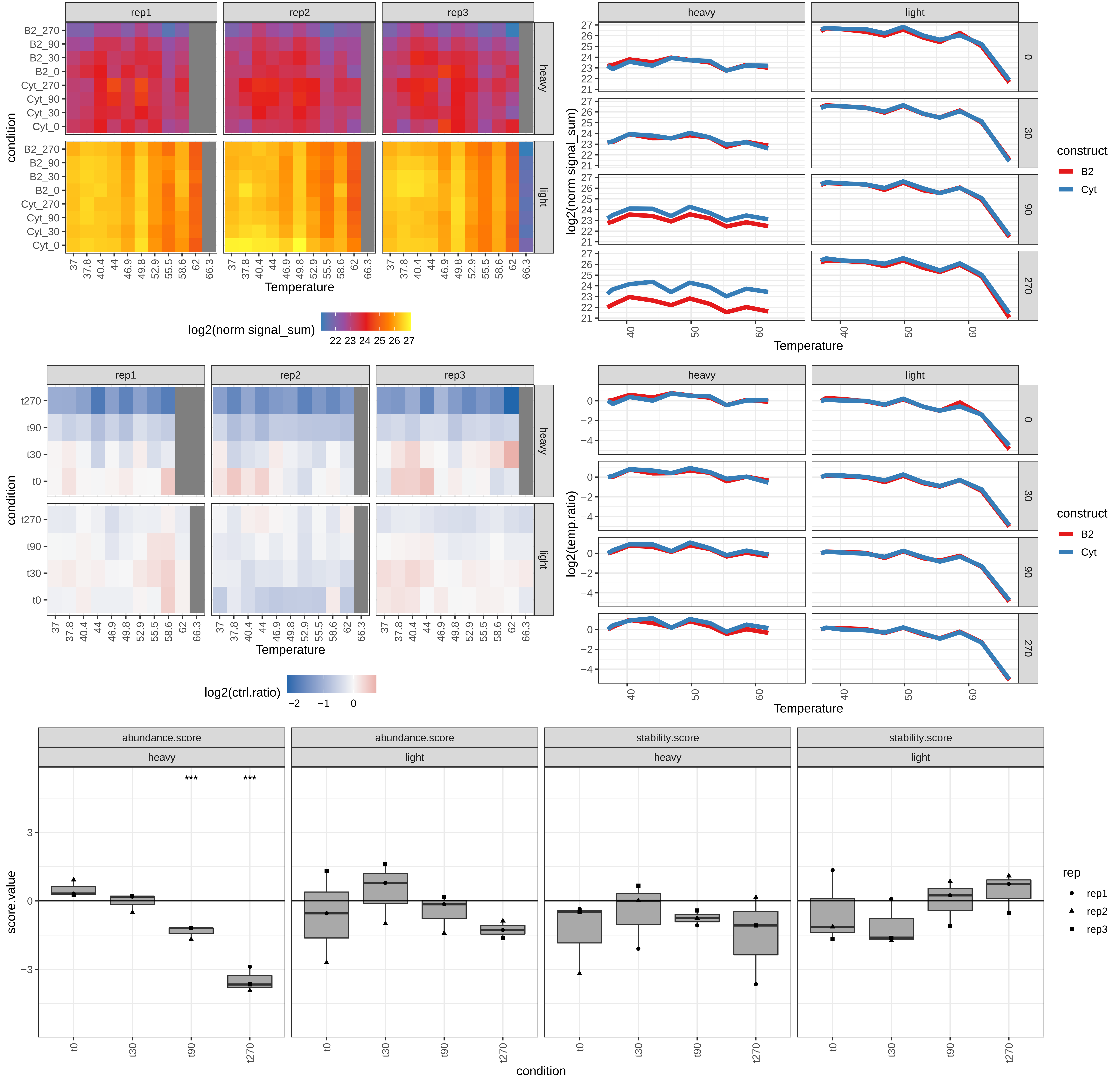

P30902 − ATP synthase subunit d, mitochondrial OS=Saccharomyces cerevisiae (strain ATCC 204508 / S288c) GN=ATP7 PE=1 SV=2

ATS1 − average.top3: 6.58552466666667 − max.qupm: 7

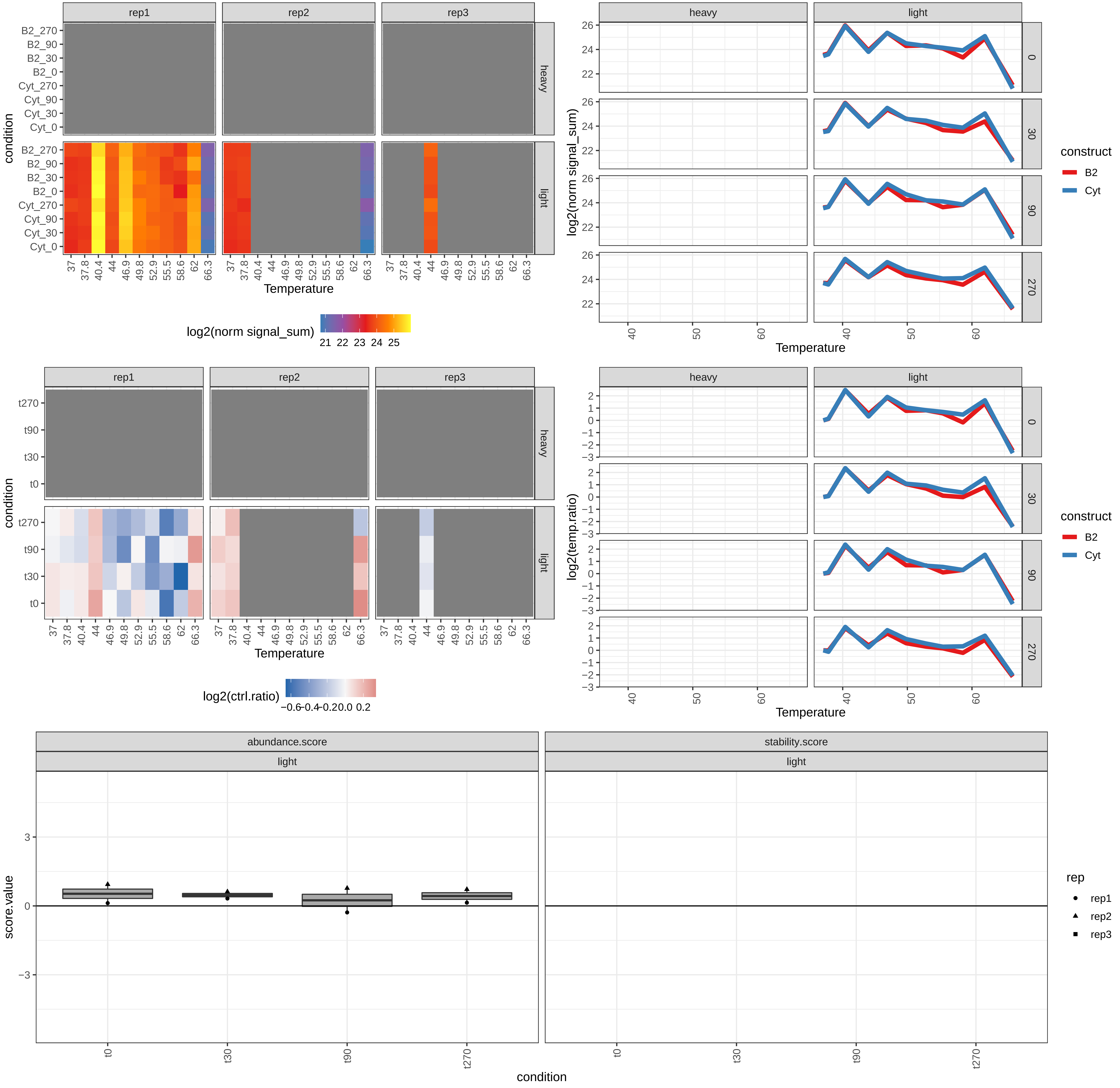

P31386 − Protein ATS1 OS=Saccharomyces cerevisiae (strain ATCC 204508 / S288c) GN=ATS1 PE=1 SV=2

AVL9 − average.top3: 6.86520105263158 − max.qupm: 9

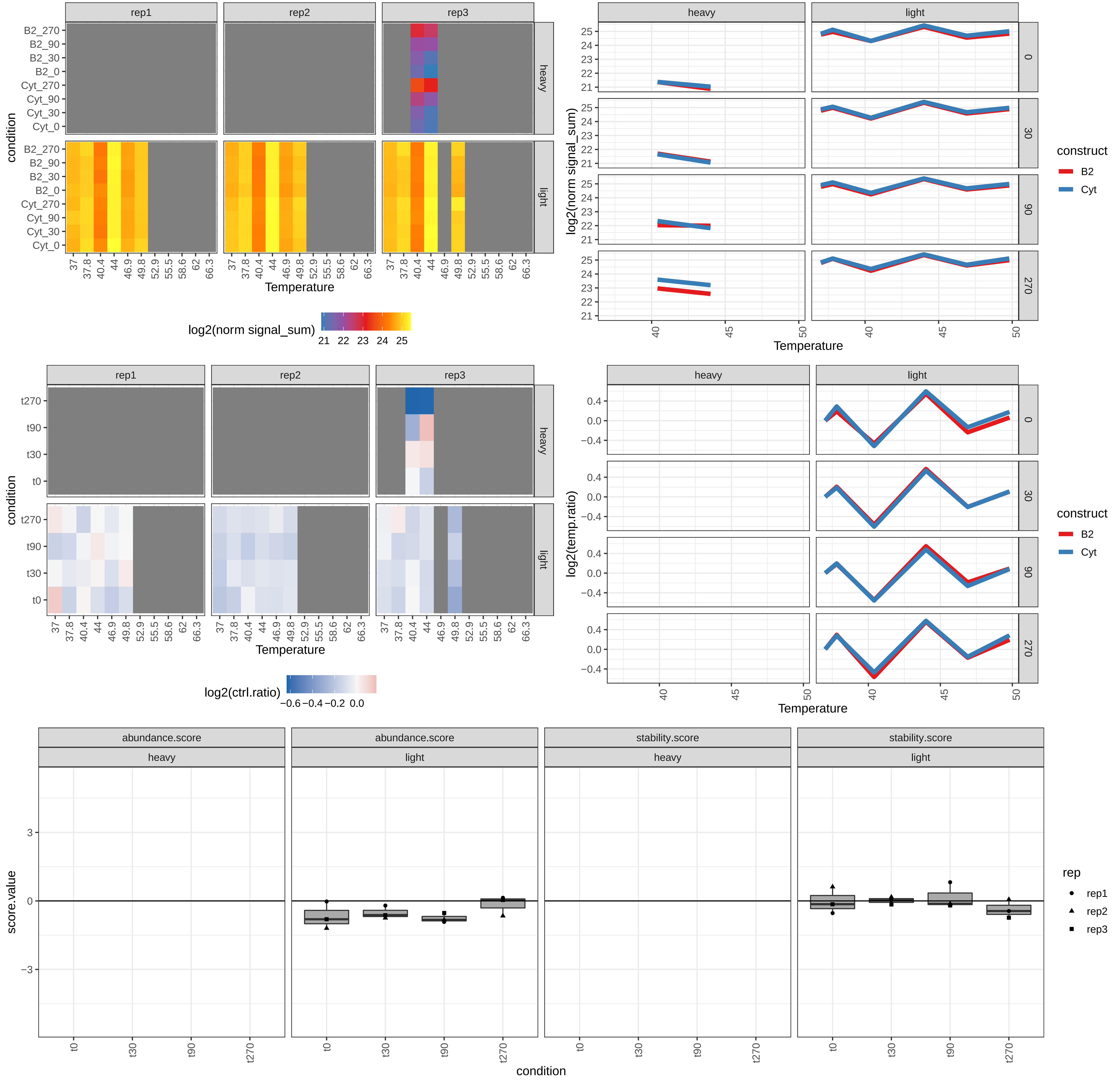

Q12500 − Late secretory pathway protein AVL9 OS=Saccharomyces cerevisiae (strain ATCC 204508 / S288c) GN=AVL9 PE=1 SV=1

AVT3 − average.top3: 6.5055265 − max.qupm: 5

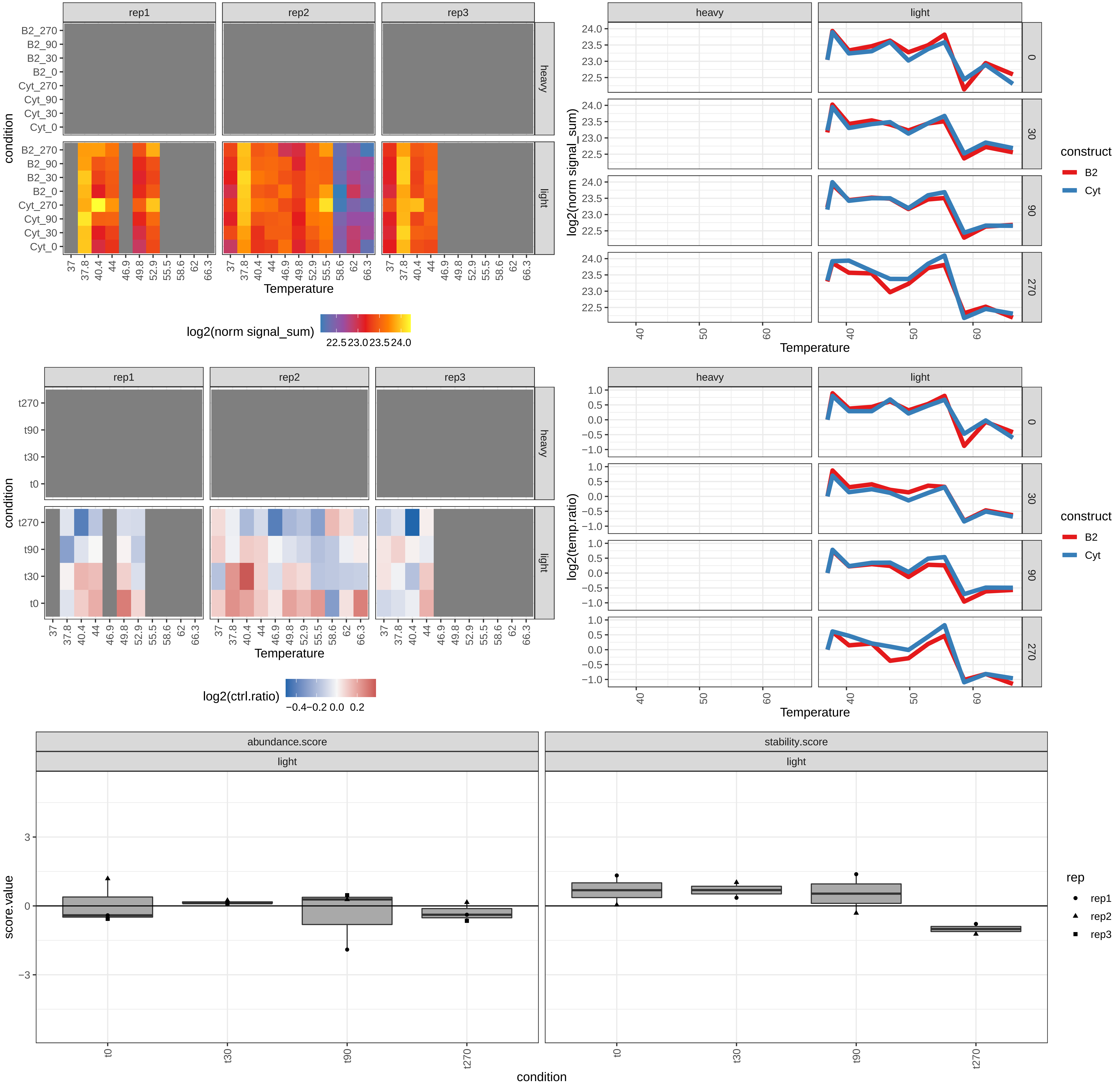

P36062 − Vacuolar amino acid transporter 3 OS=Saccharomyces cerevisiae (strain ATCC 204508 / S288c) GN=AVT3 PE=1 SV=1

AYR1 − average.top3: 7.5631309375 − max.qupm: 8

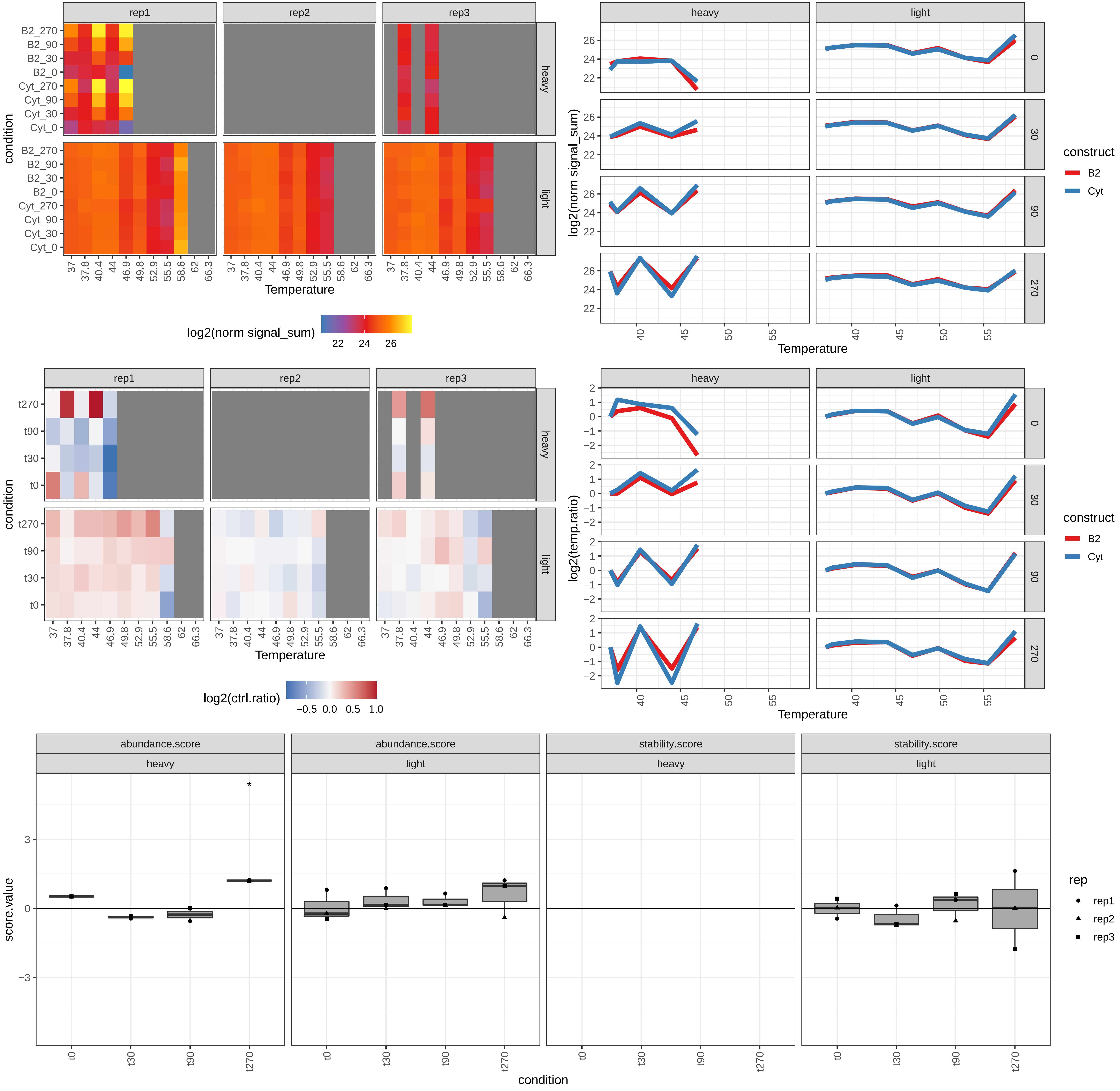

P40471 − NADPH−dependent 1−acyldihydroxyacetone phosphate reductase OS=Saccharomyces cerevisiae (strain ATCC 204508 / S288c) GN=AYR1 PE=1 SV=1

BAP2 − average.top3: 6.8858204 − max.qupm: 6

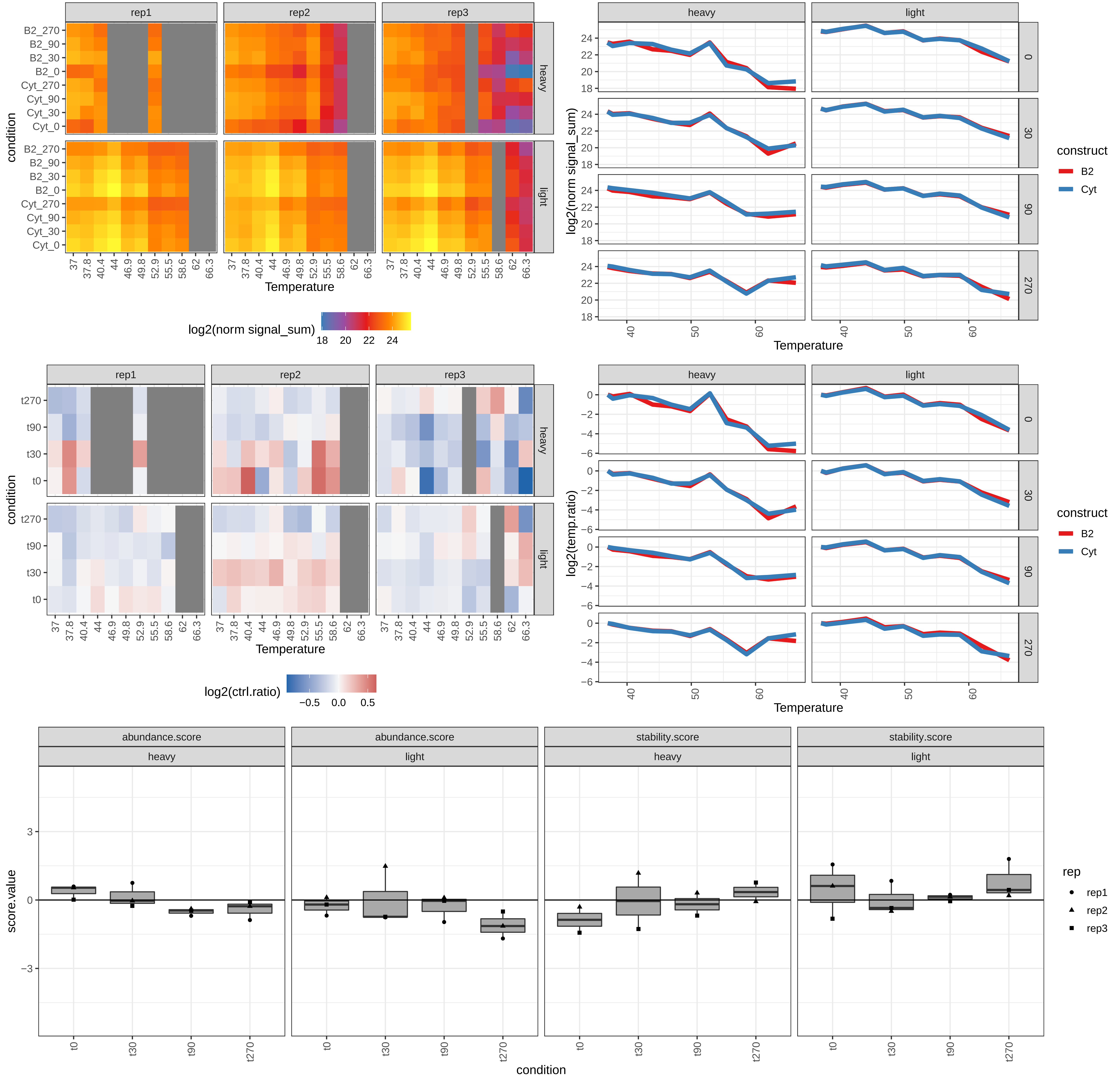

P38084 − Leu/Val/Ile amino−acid permease OS=Saccharomyces cerevisiae (strain ATCC 204508 / S288c) GN=BAP2 PE=1 SV=2

BAR1 − average.top3: 6.309379 − max.qupm: 3

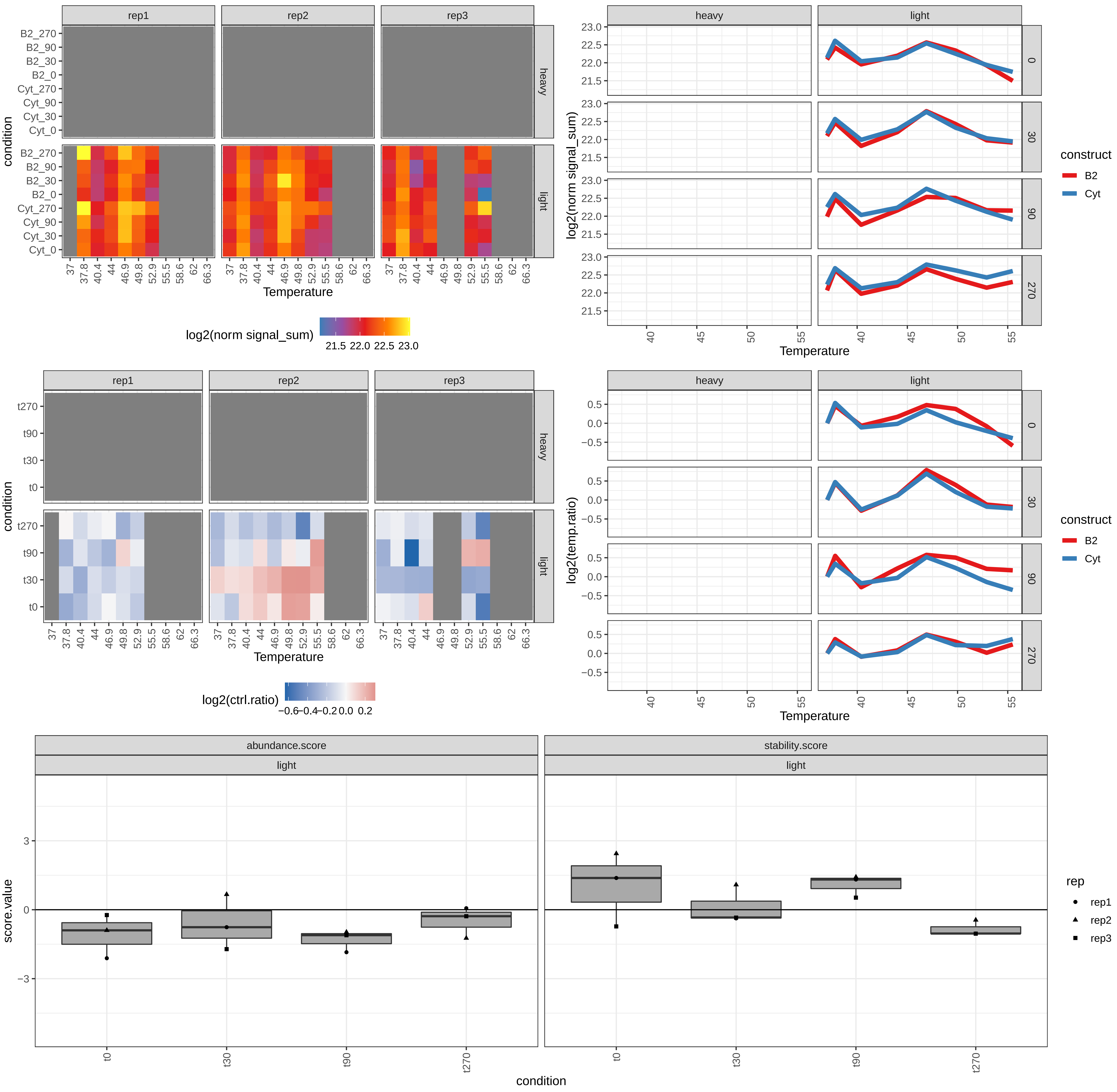

P12630 − Barrierpepsin OS=Saccharomyces cerevisiae (strain ATCC 204508 / S288c) GN=BAR1 PE=1 SV=1

BAT1 − average.top3: 7.51443583333333 − max.qupm: 13

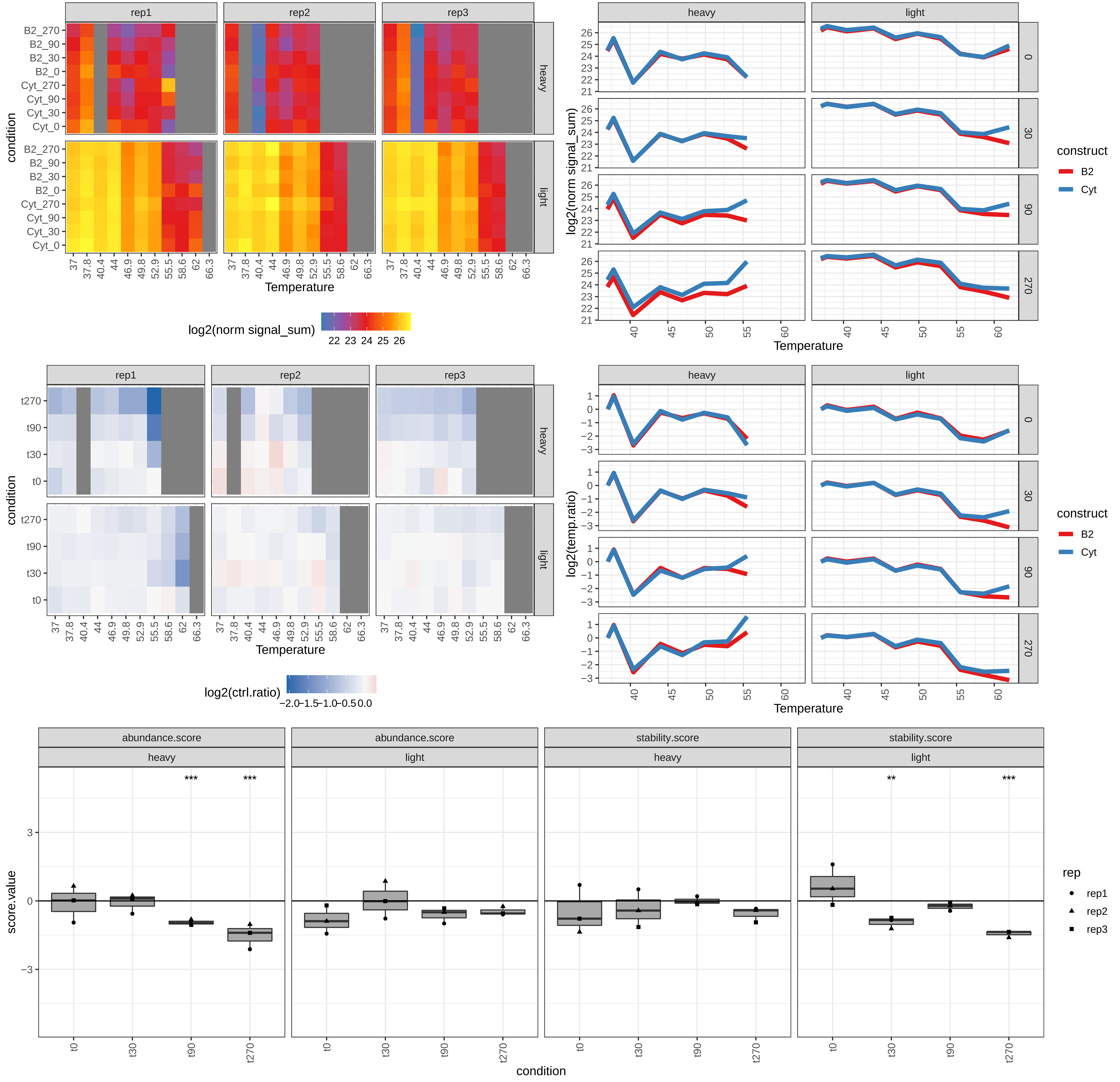

P38891 − Branched−chain−amino−acid aminotransferase, mitochondrial OS=Saccharomyces cerevisiae (strain ATCC 204508 / S288c) GN=BAT1 PE=1 SV=1

BAT2 − average.top3: 7.68490261904762 − max.qupm: 14

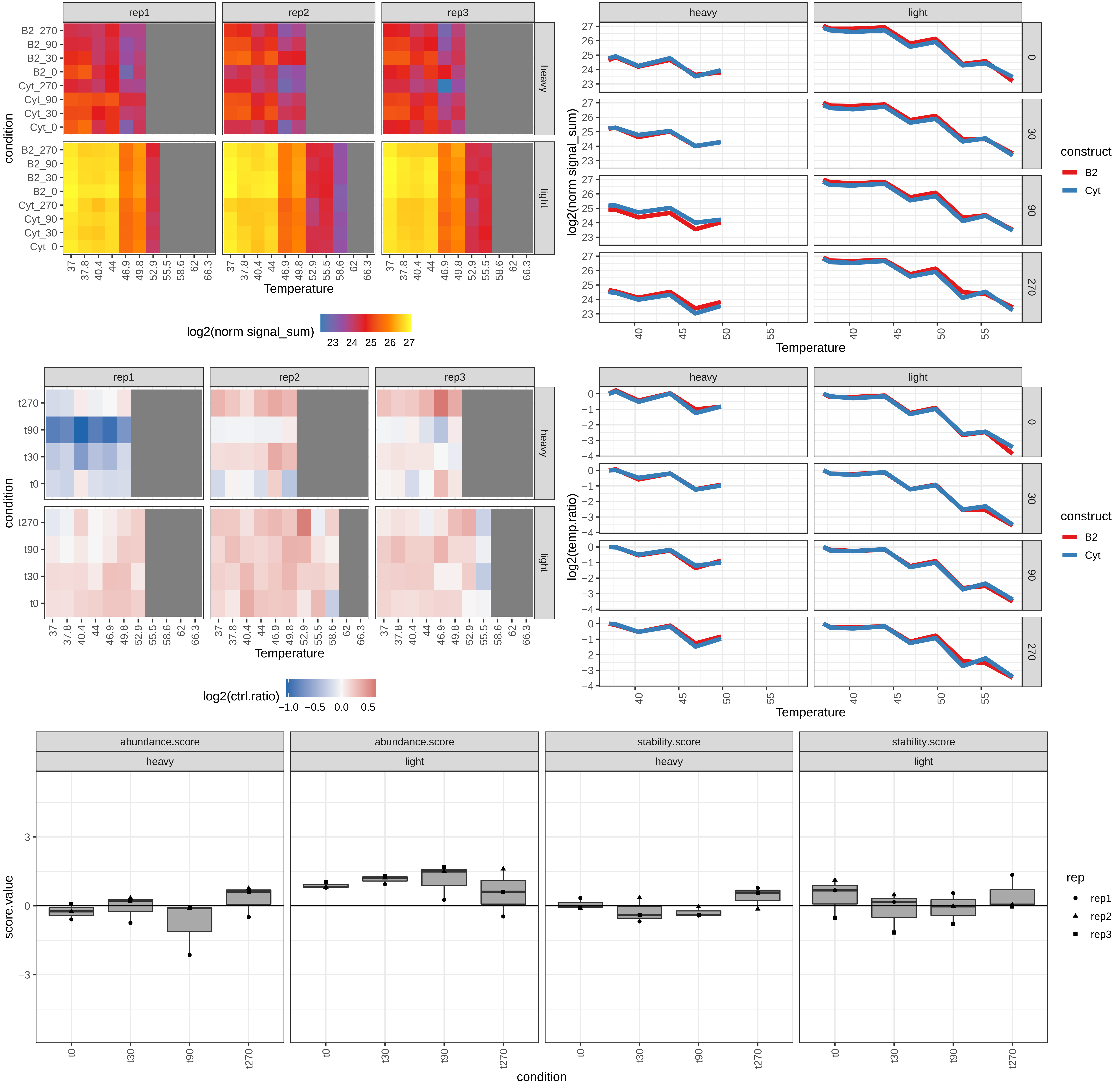

P47176 − Branched−chain−amino−acid aminotransferase, cytosolic OS=Saccharomyces cerevisiae (strain ATCC 204508 / S288c) GN=BAT2 PE=1 SV=1

BBC1 − average.top3: 7.21376586206897 − max.qupm: 17

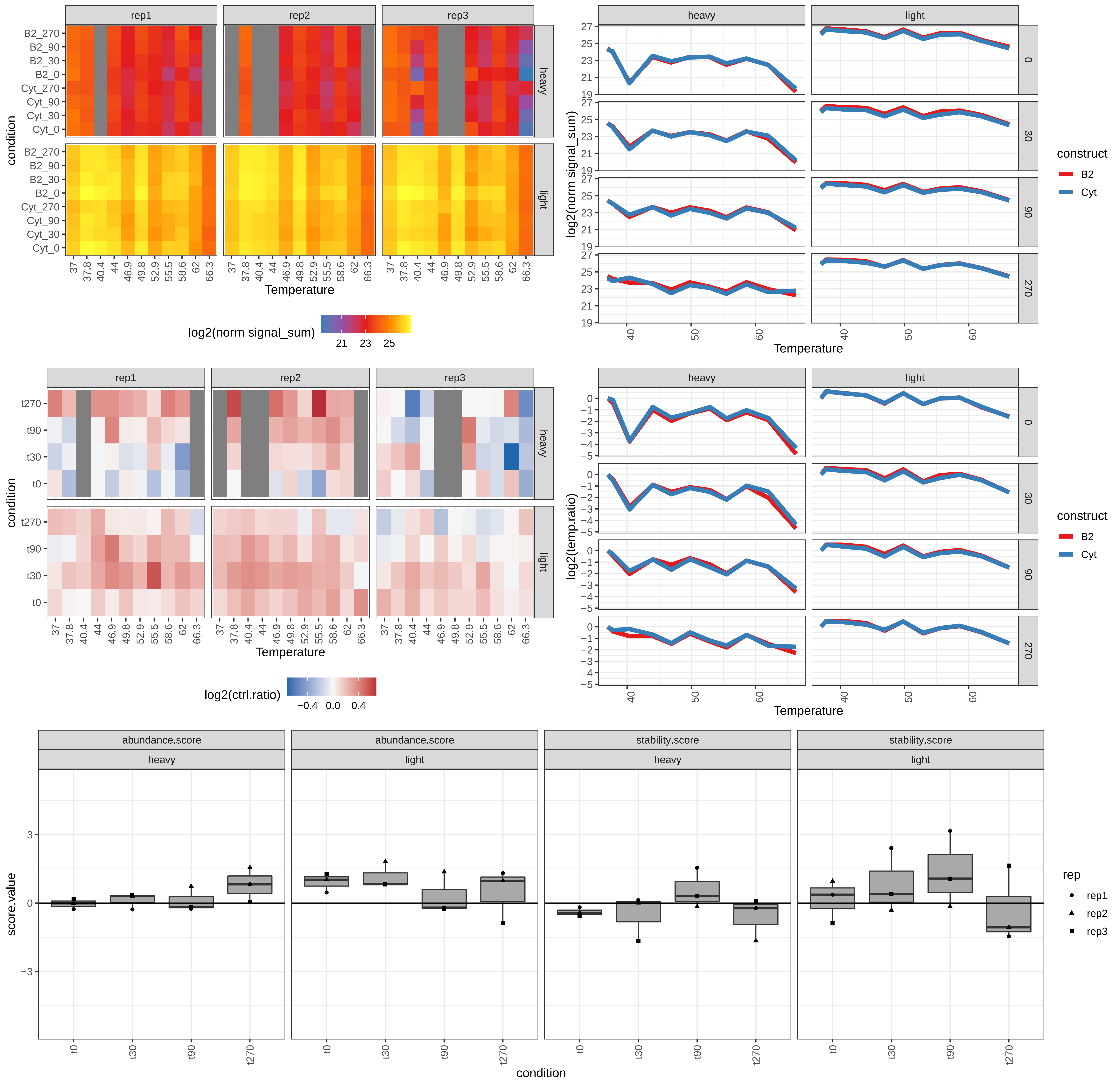

P47068 − Myosin tail region−interacting protein MTI1 OS=Saccharomyces cerevisiae (strain ATCC 204508 / S288c) GN=BBC1 PE=1 SV=2

BCH1 − average.top3: 7.106907 − max.qupm: 6

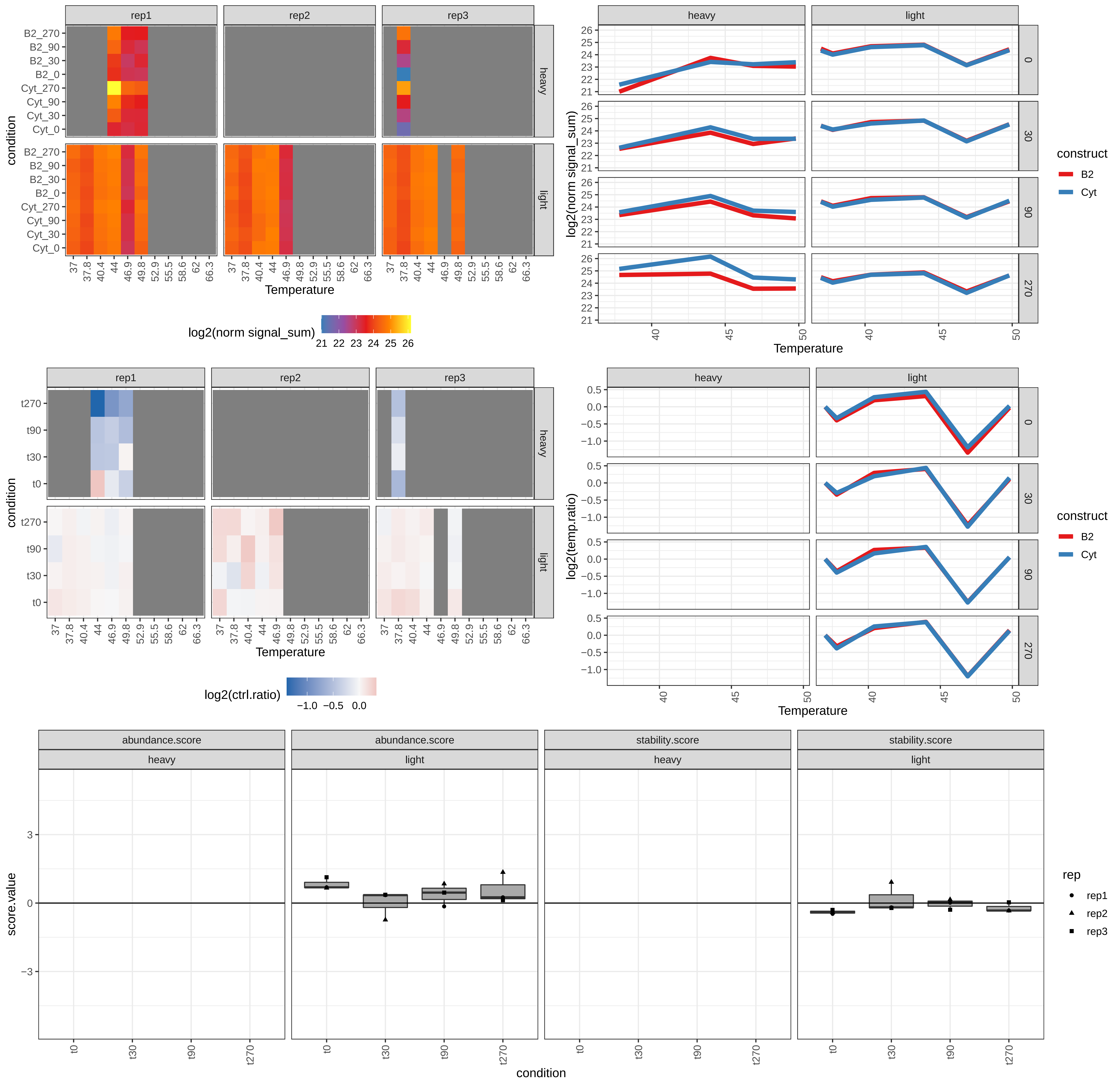

Q05029 − Protein BCH1 OS=Saccharomyces cerevisiae (strain ATCC 204508 / S288c) GN=BCH1 PE=1 SV=1

BCH2 − average.top3: 6.54804588235294 − max.qupm: 7

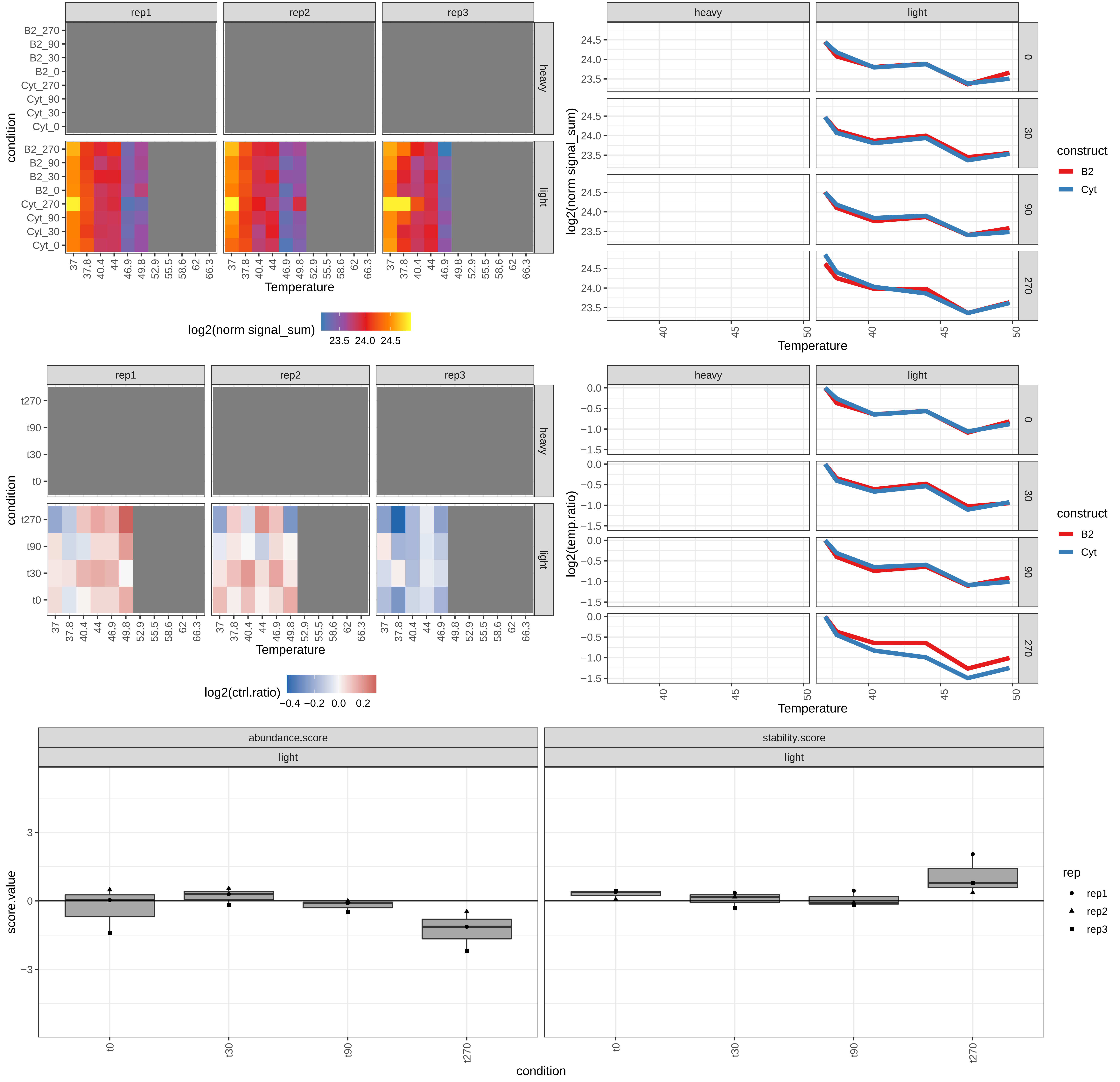

P36122 − Protein BCH2 OS=Saccharomyces cerevisiae (strain ATCC 204508 / S288c) GN=BCH2 PE=1 SV=1

BCP1 − average.top3: 6.61744357142857 − max.qupm: 6

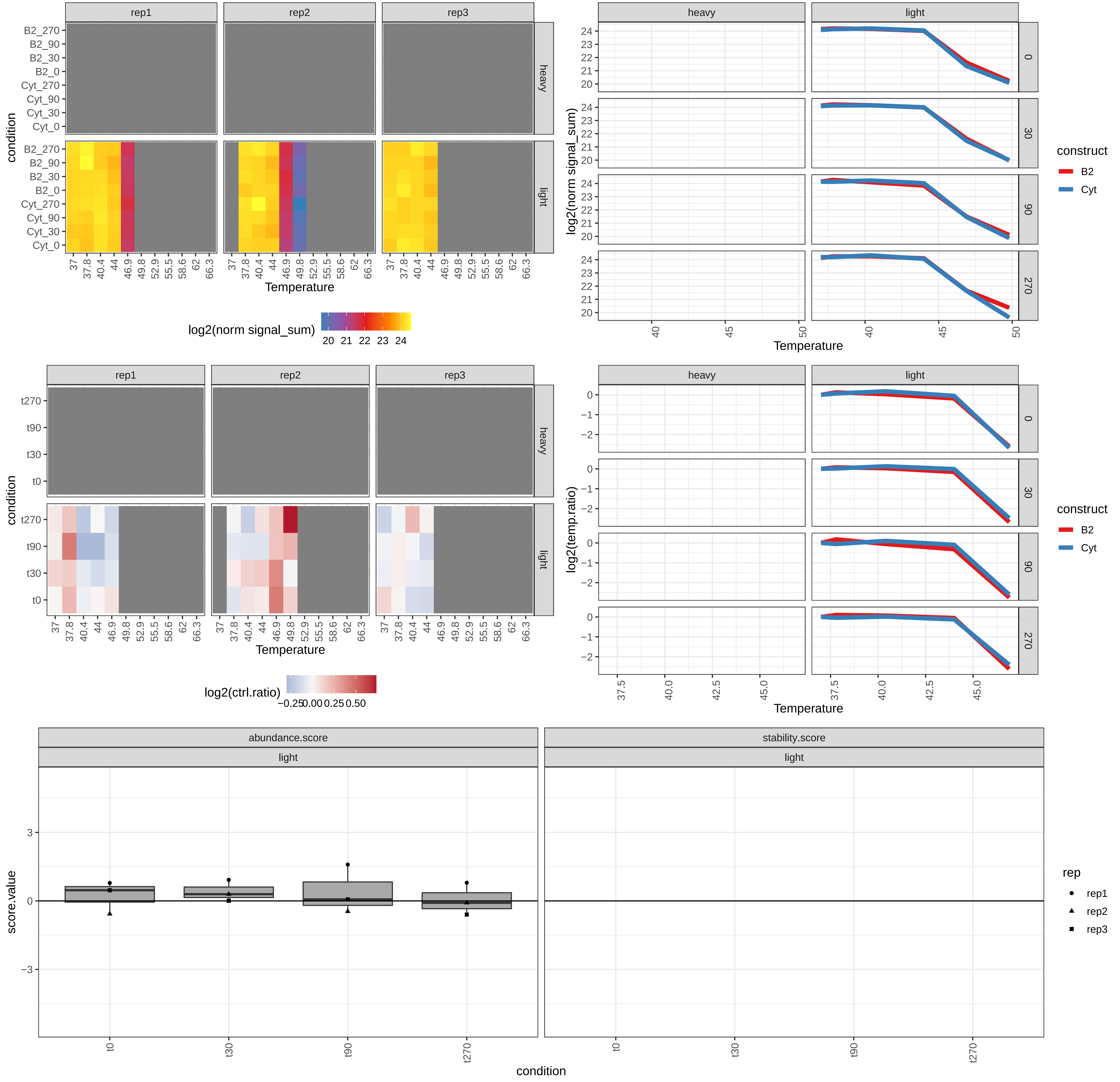

Q06338 − Protein BCP1 OS=Saccharomyces cerevisiae (strain ATCC 204508 / S288c) GN=BCP1 PE=1 SV=1

BCS1 − average.top3: 6.47479230769231 − max.qupm: 5

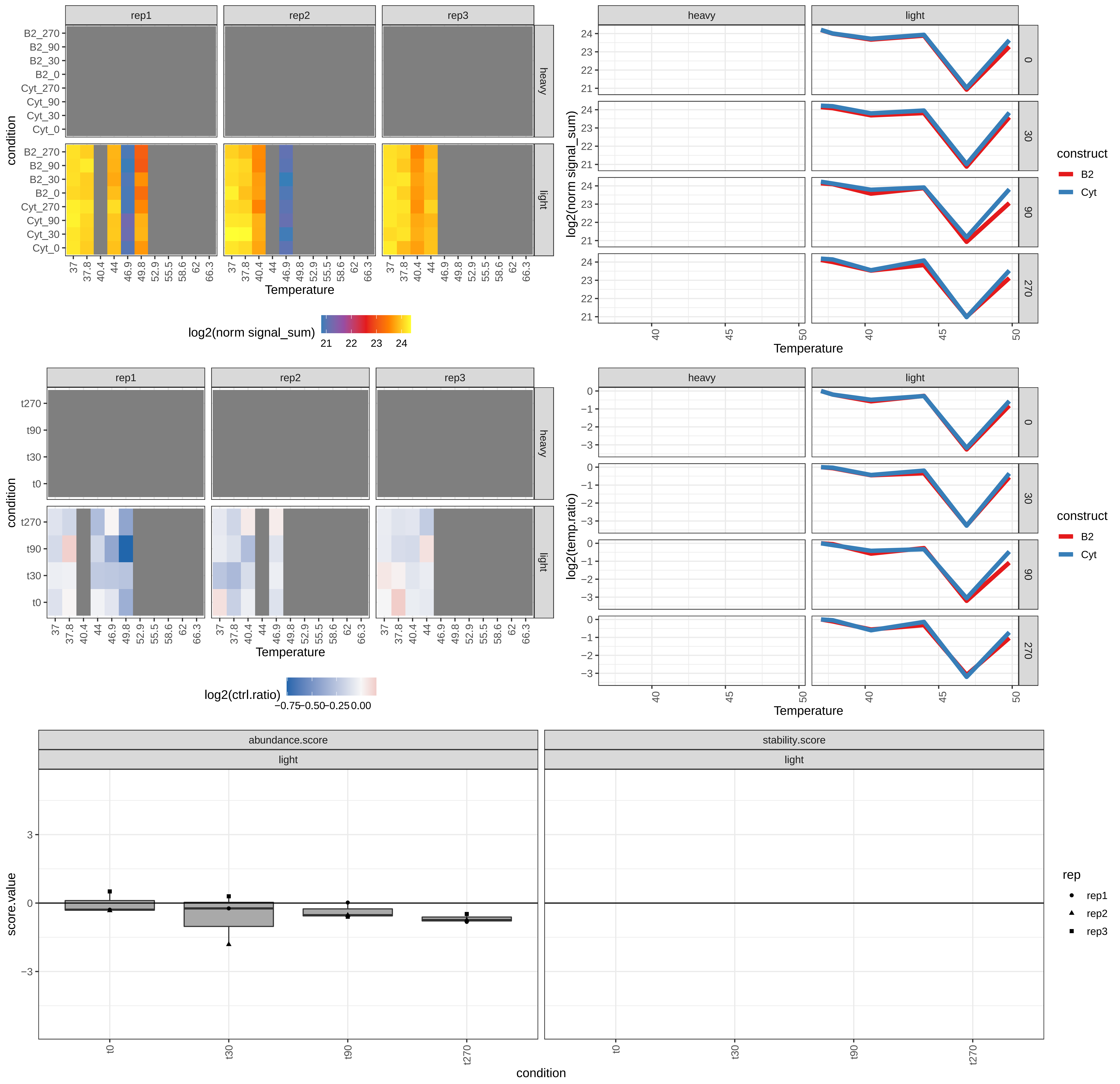

P32839 − Mitochondrial chaperone BCS1 OS=Saccharomyces cerevisiae (strain ATCC 204508 / S288c) GN=BCS1 PE=1 SV=2

BCY1 − average.top3: 7.24621166666667 − max.qupm: 13

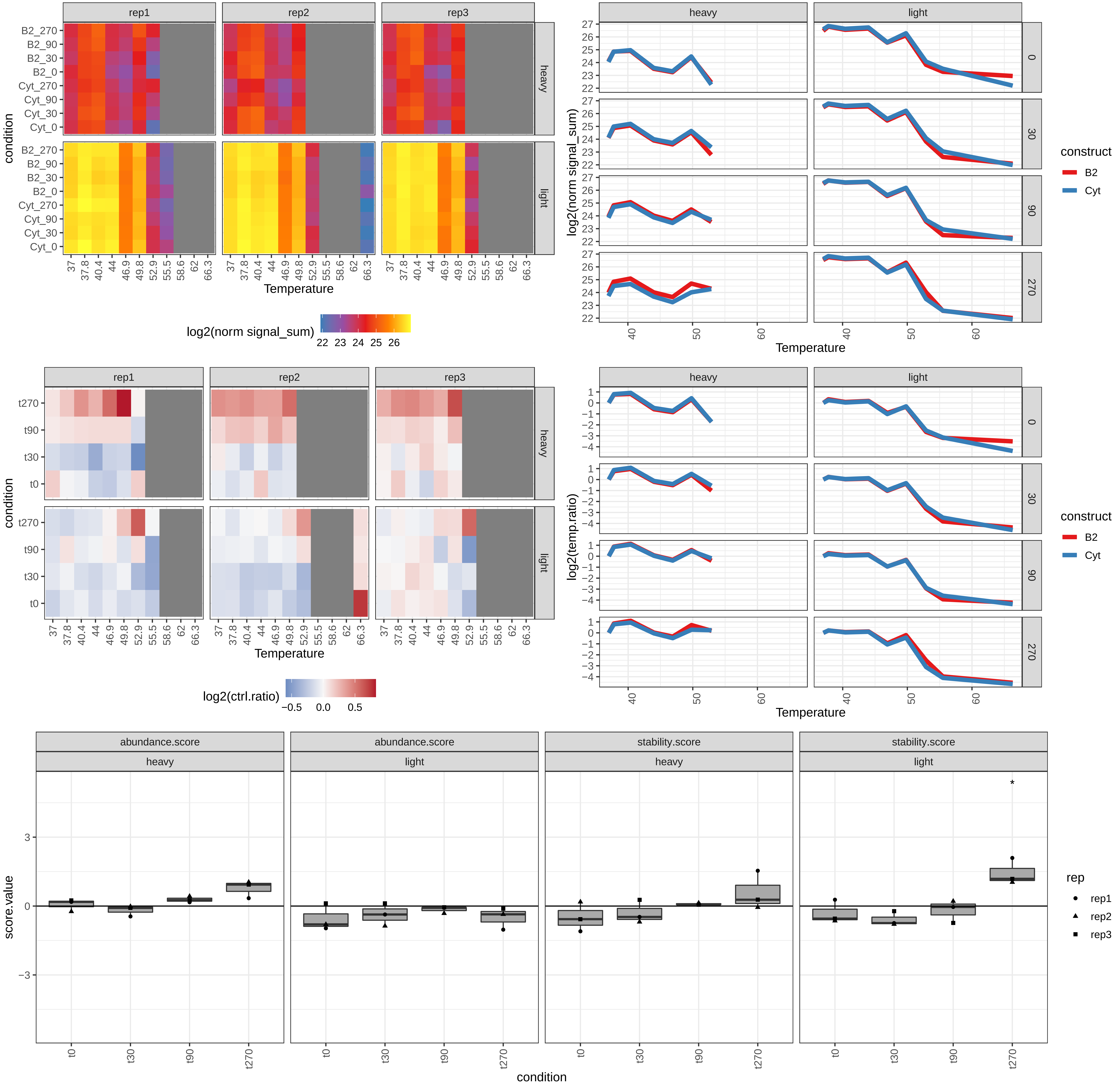

P07278 − cAMP−dependent protein kinase regulatory subunit OS=Saccharomyces cerevisiae (strain ATCC 204508 / S288c) GN=BCY1 PE=1 SV=4

BDF1 − average.top3: 6.97761944444444 − max.qupm: 7

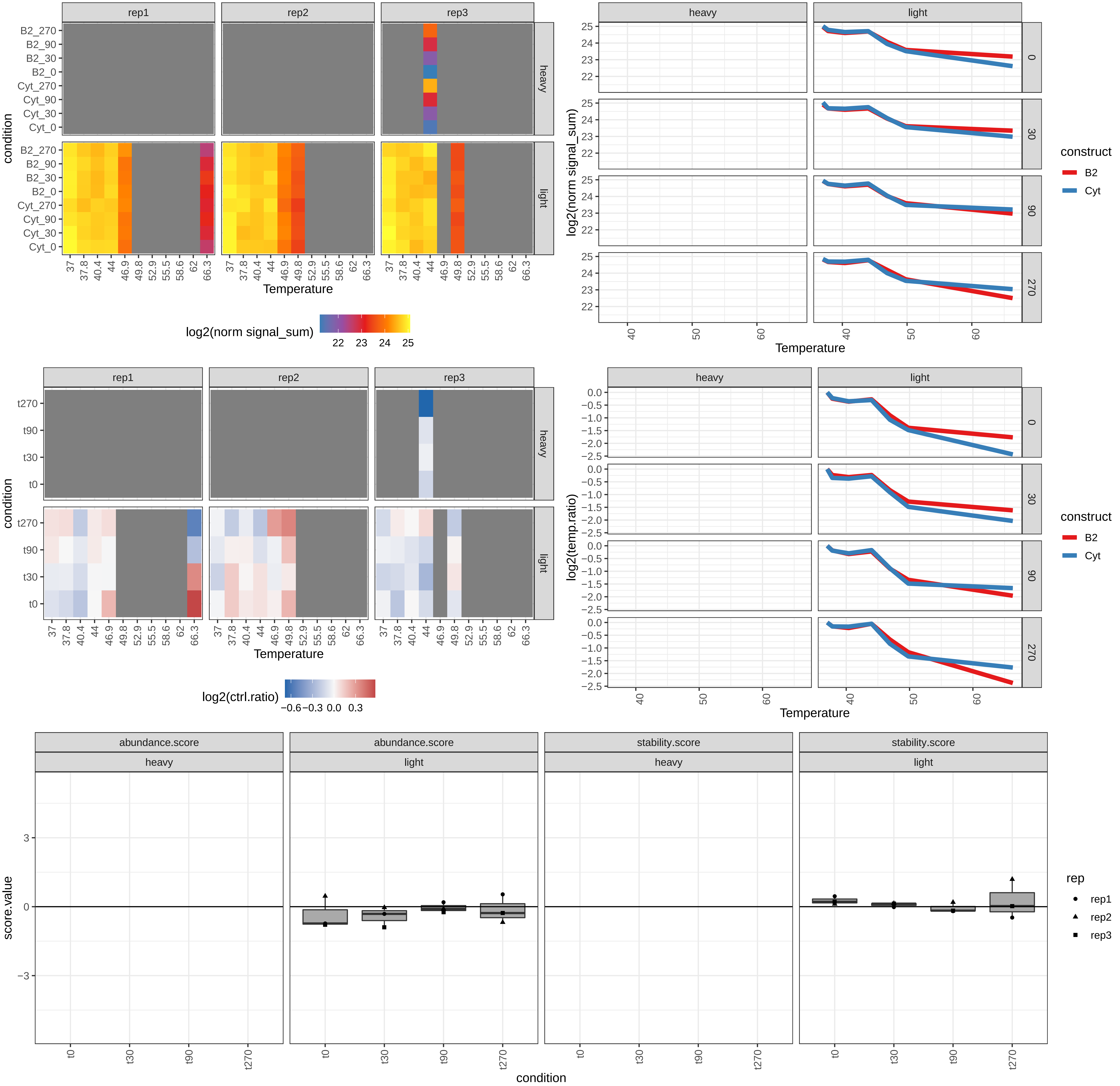

P35817 − Bromodomain−containing factor 1 OS=Saccharomyces cerevisiae (strain ATCC 204508 / S288c) GN=BDF1 PE=1 SV=3

BDH1 − average.top3: 7.27838711111111 − max.qupm: 11

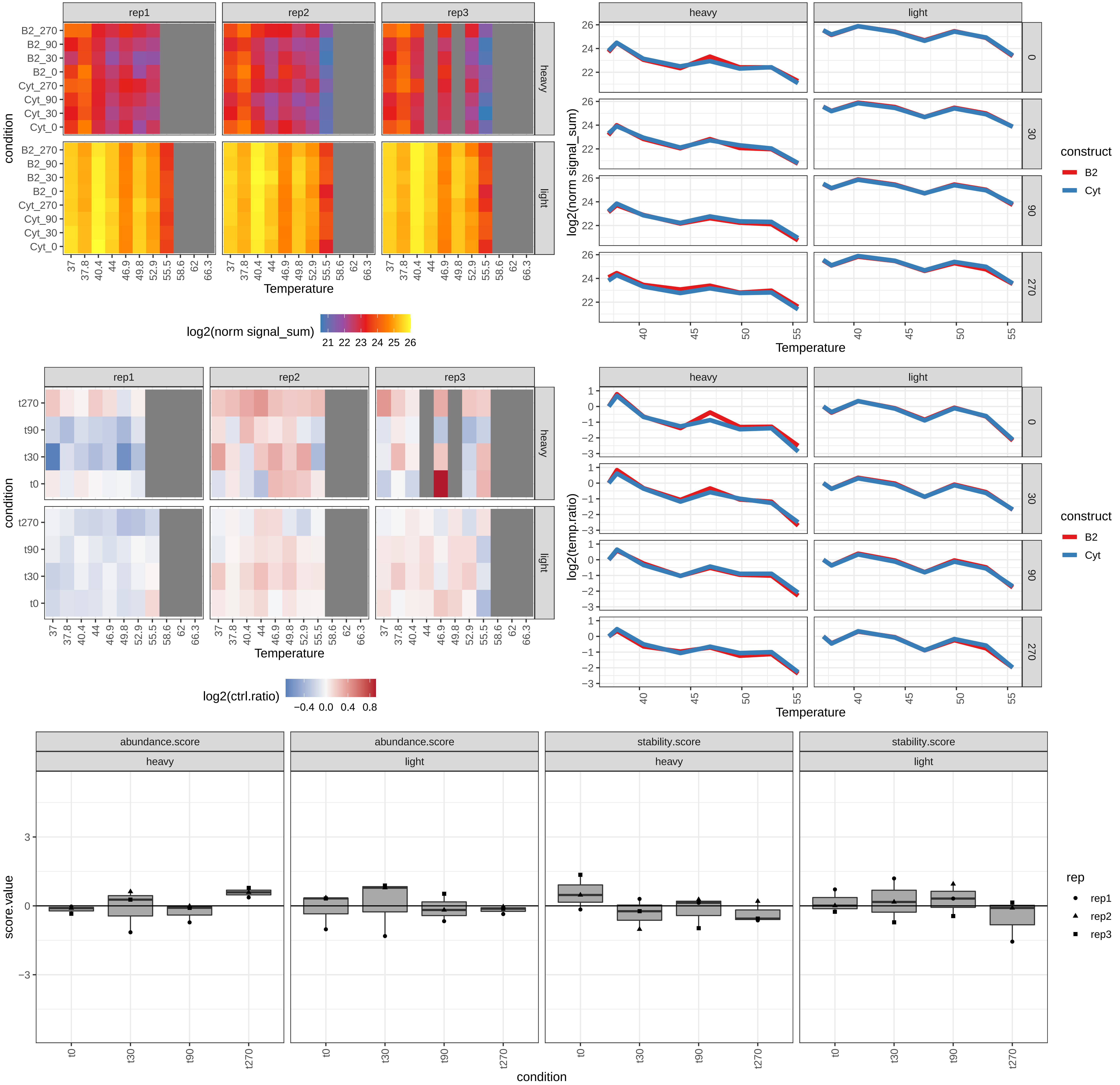

P39714 − (R,R)−butanediol dehydrogenase OS=Saccharomyces cerevisiae (strain ATCC 204508 / S288c) GN=BDH1 PE=1 SV=2

BDH2 − average.top3: 6.3847805 − max.qupm: 4

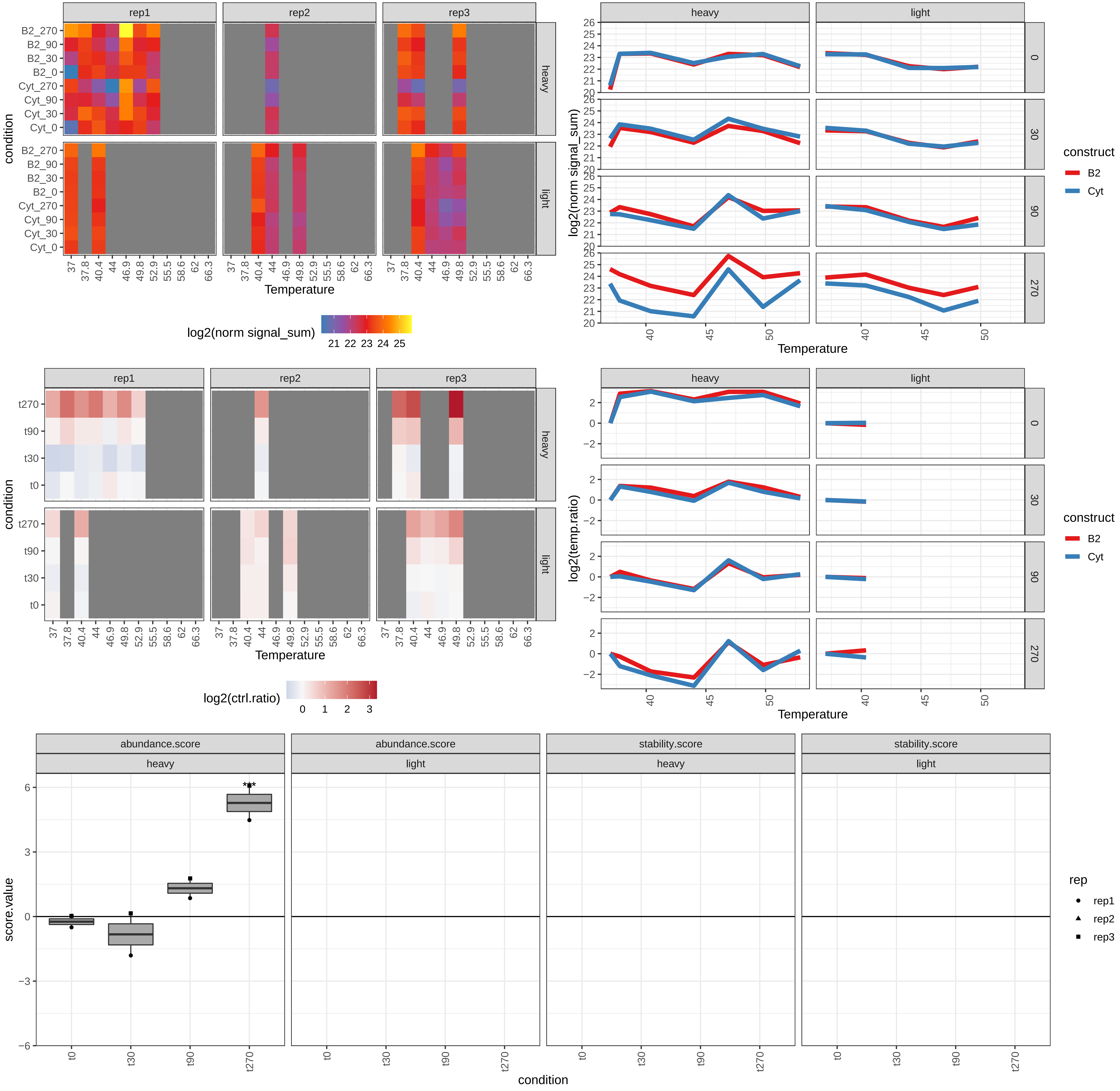

P39713 − Probable diacetyl reductase [(R)−acetoin forming] 2 OS=Saccharomyces cerevisiae (strain ATCC 204508 / S288c) GN=BDH2 PE=1 SV=1

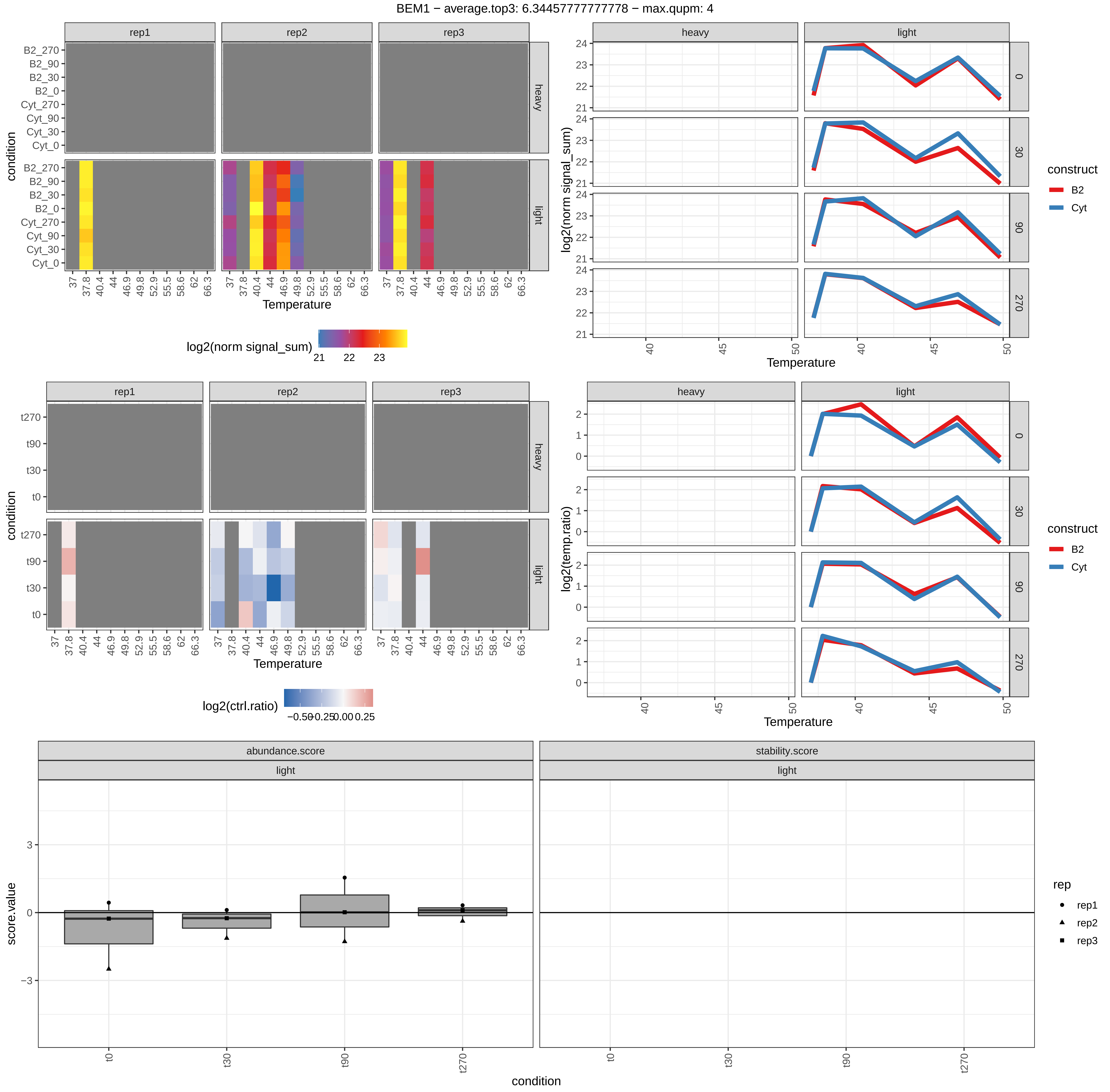

P29366 − Bud emergence protein 1 OS=Saccharomyces cerevisiae (strain ATCC 204508 / S288c) GN=BEM1 PE=1 SV=1

BEM2 − average.top3: 6.73132322580645 − max.qupm: 20

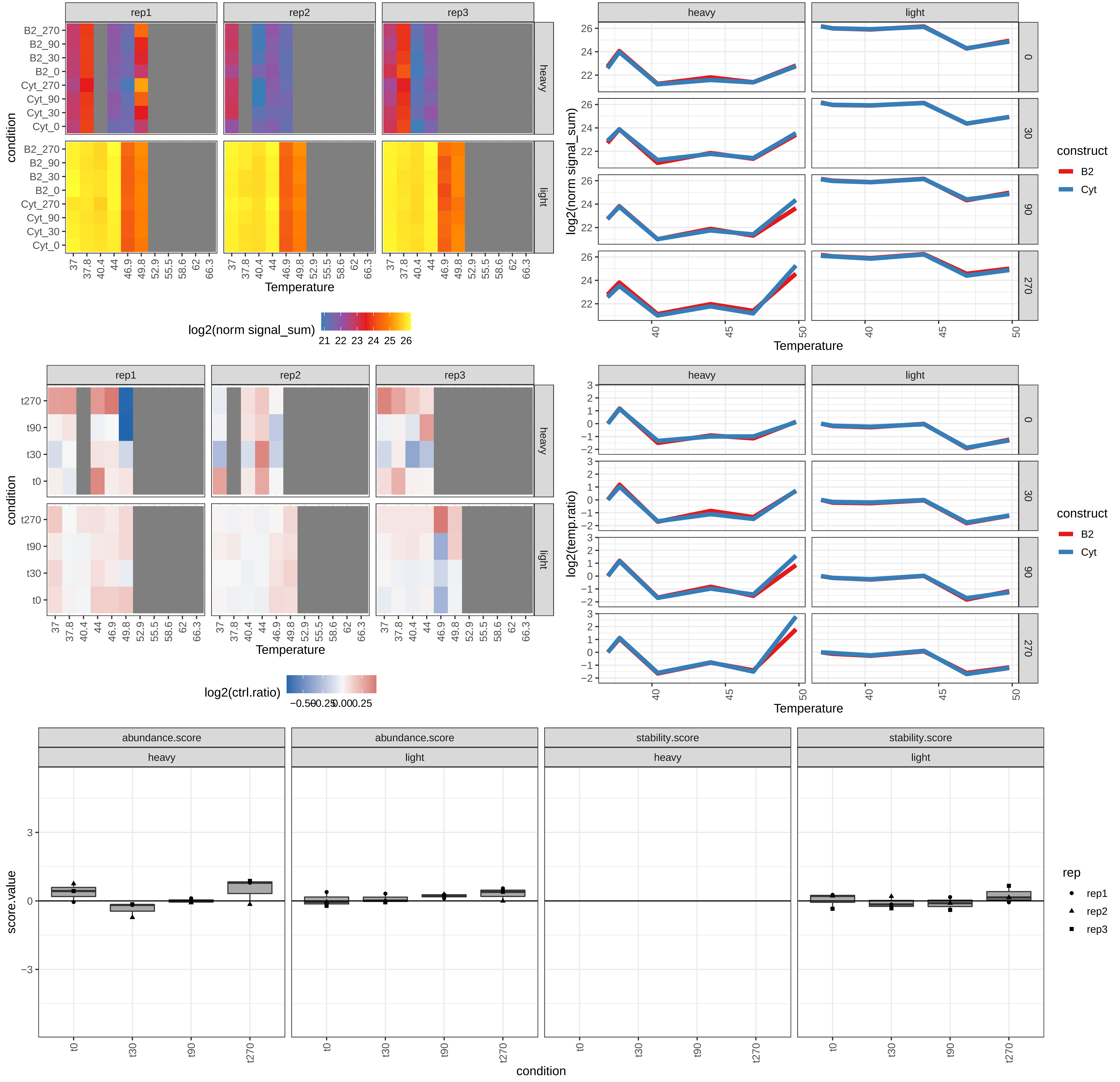

P39960 − GTPase−activating protein BEM2/IPL2 OS=Saccharomyces cerevisiae (strain ATCC 204508 / S288c) GN=BEM2 PE=1 SV=1

BEM3 − average.top3: 6.16470818181818 − max.qupm: 3

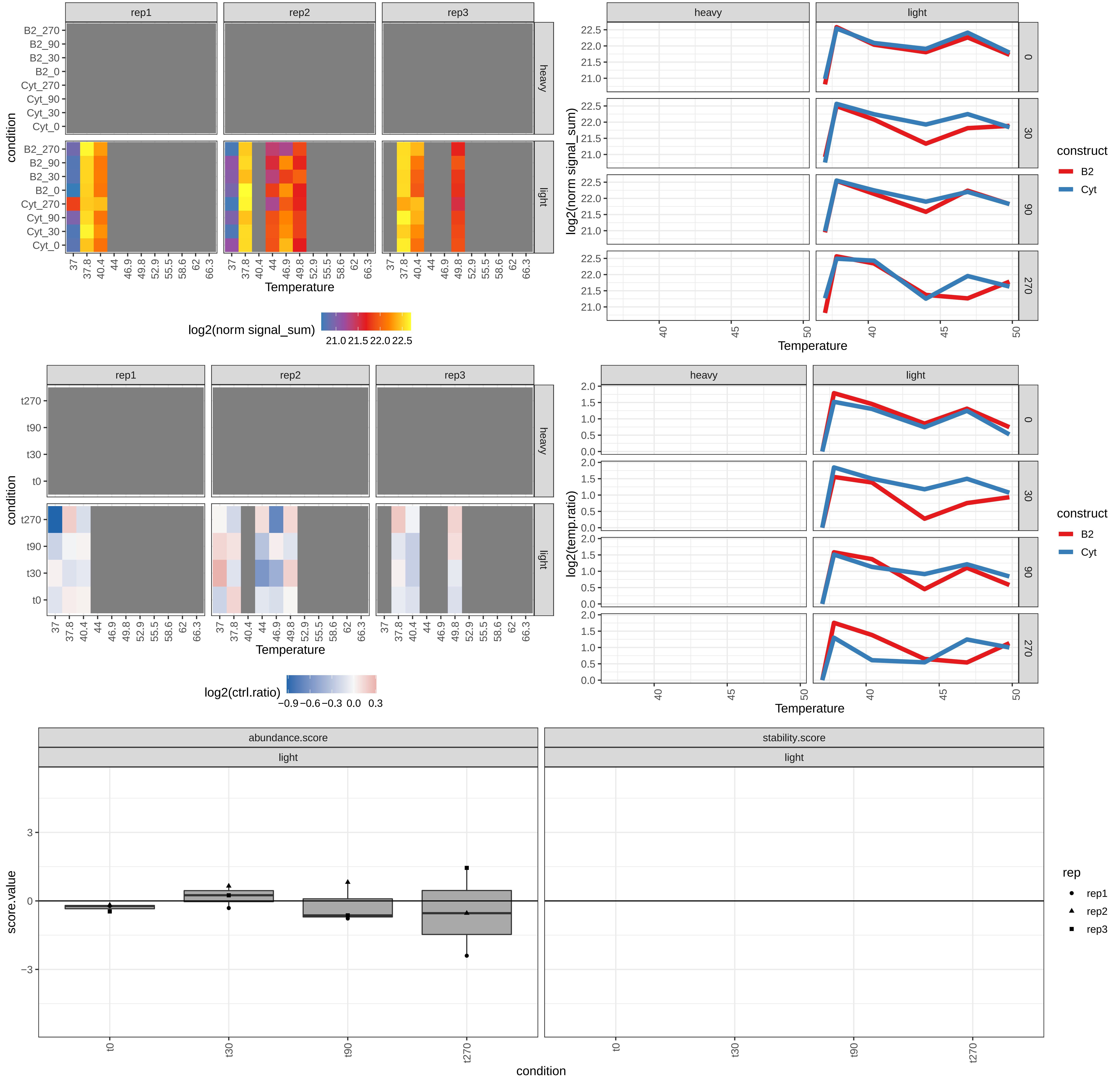

P32873 − GTPase−activating protein BEM3 OS=Saccharomyces cerevisiae (strain ATCC 204508 / S288c) GN=BEM3 PE=1 SV=3

BEM4 − average.top3: 6.59645 − max.qupm: 6

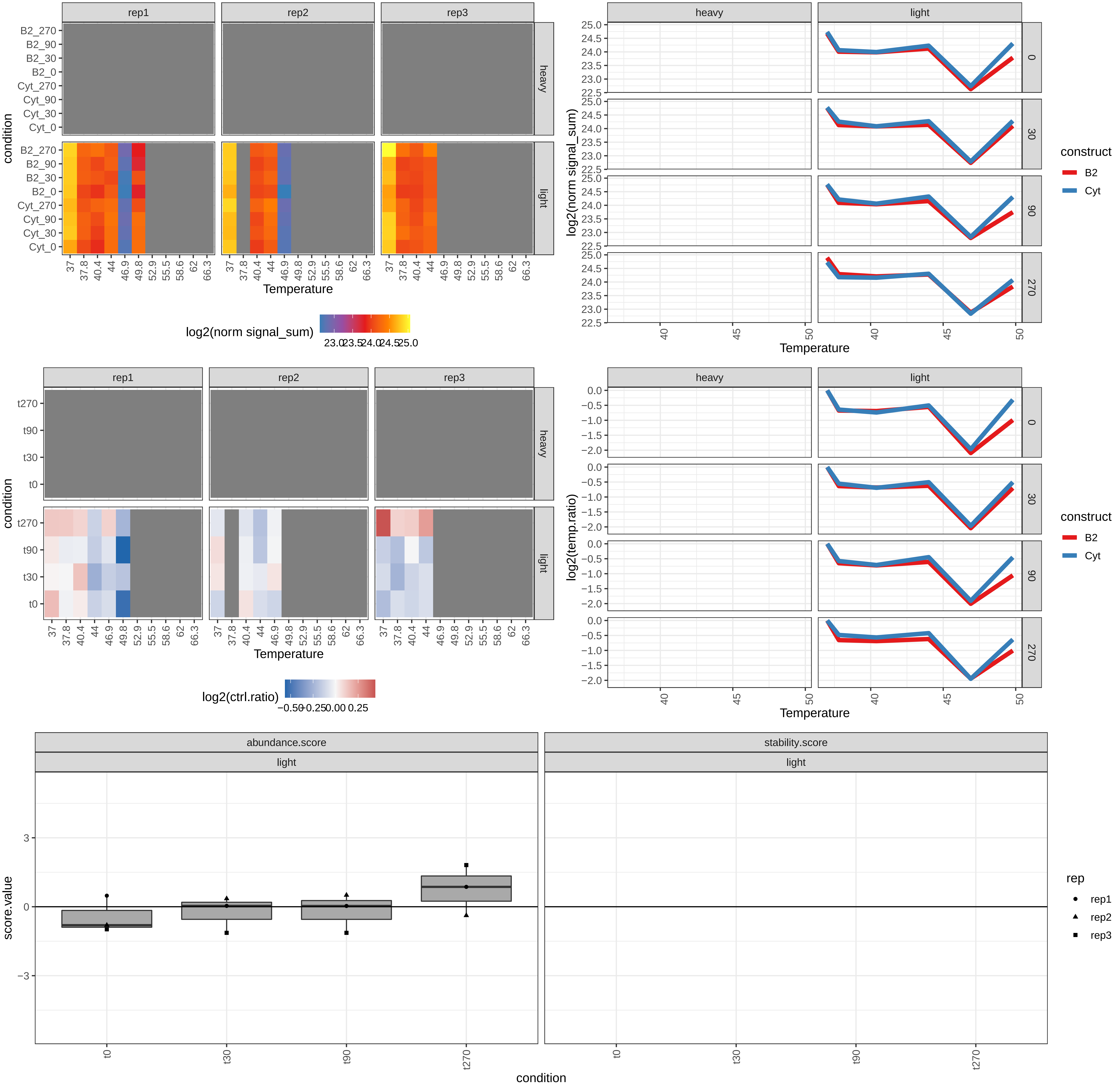

P39011 − Bud emergence protein 4 OS=Saccharomyces cerevisiae (strain ATCC 204508 / S288c) GN=BEM4 PE=1 SV=1

BER1 − average.top3: 6.30881444444444 − max.qupm: 5

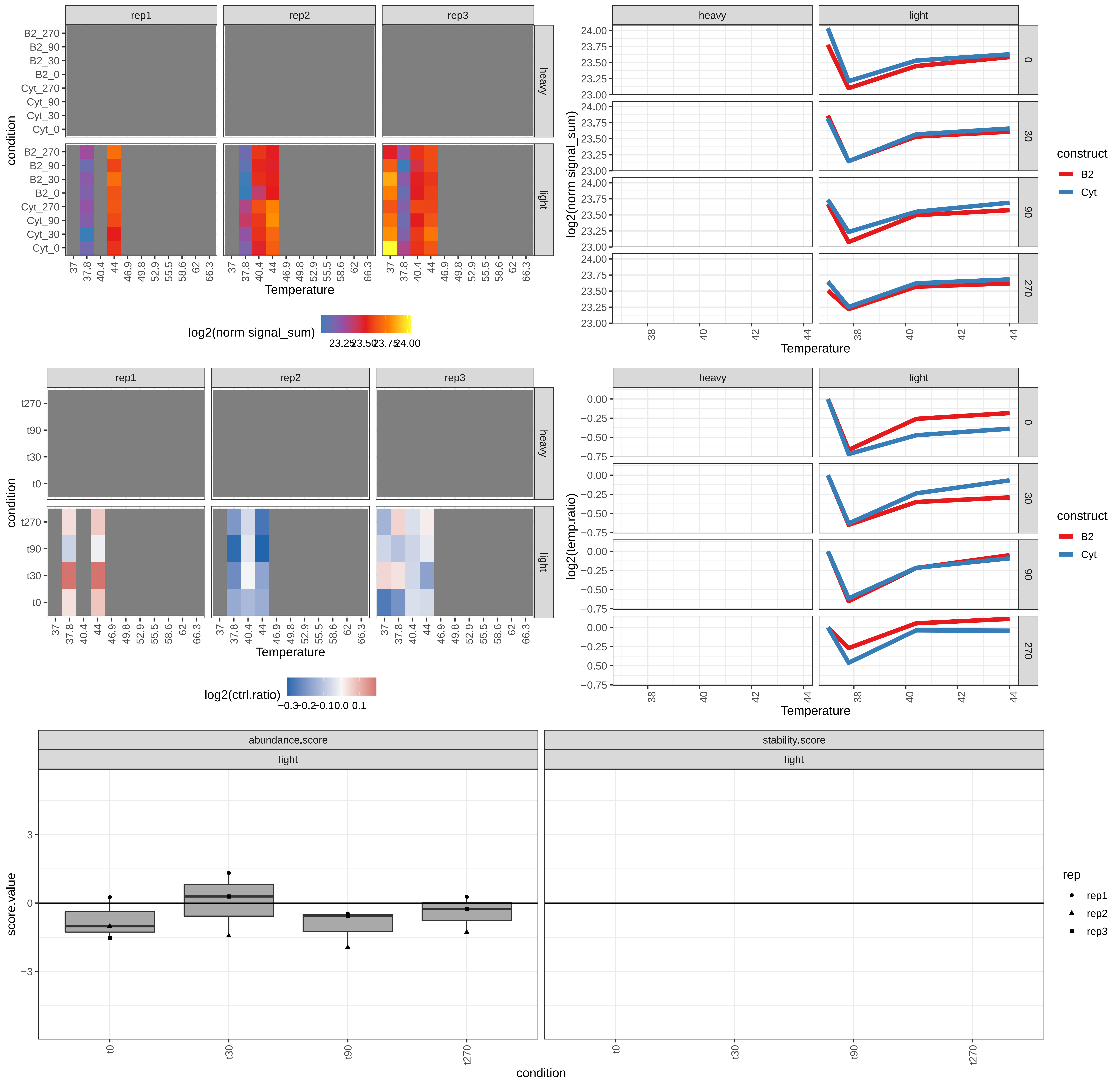

Q06688 − SRR1−like protein BER1 OS=Saccharomyces cerevisiae (strain ATCC 204508 / S288c) GN=BER1 PE=1 SV=1

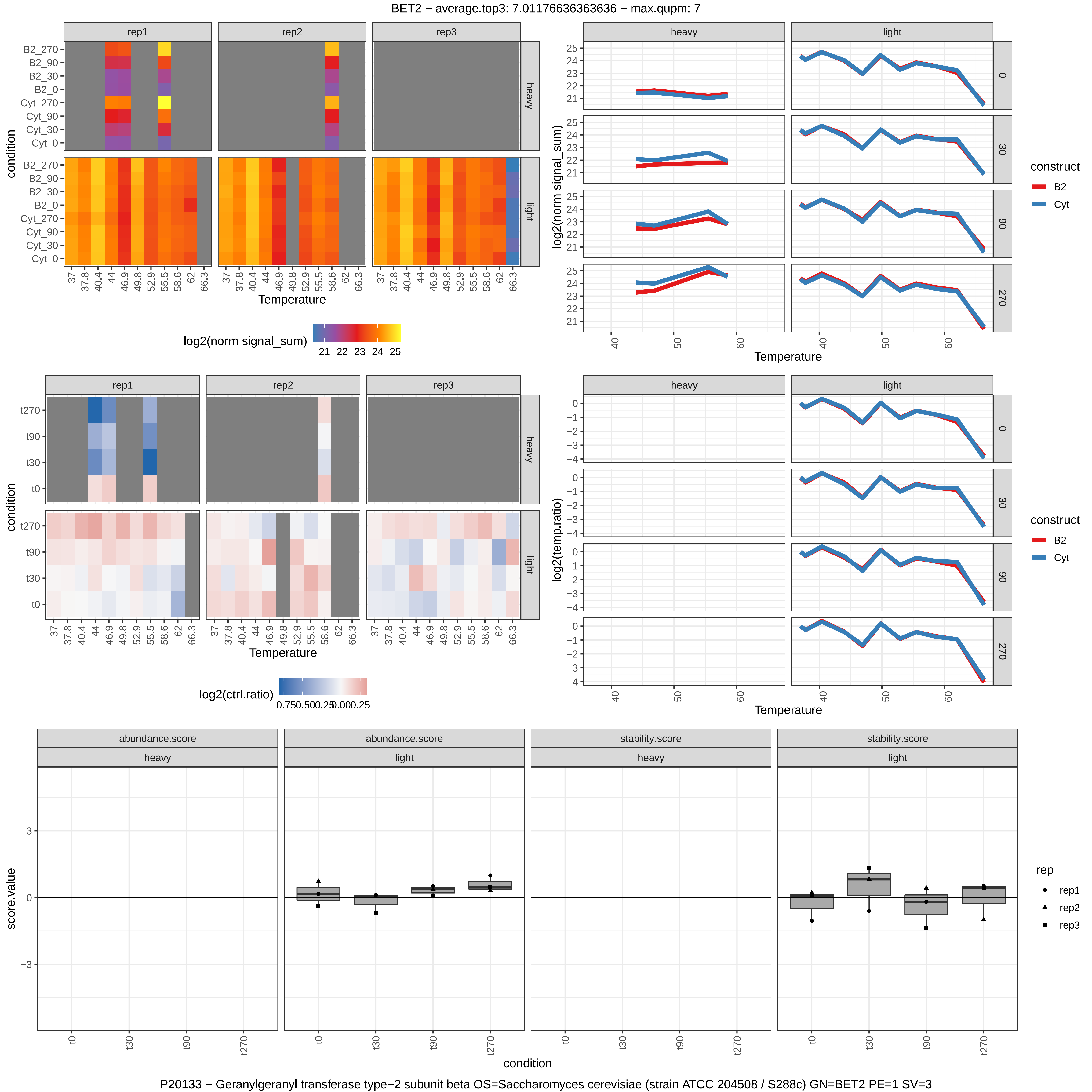

BET2 − average.top3: 7.01176636363636 − max.qupm: 7
rep1
rep2
rep3
heavy
light
condition
Temperature
log2(norm signal_sum)
construct
B2
Cyt
log2(ctrl.ratio)
log2(temp.ratio)
abundance.score
stability.score
score.value
rep
rep1
rep2
rep3
P20133 − Geranylgeranyl transferase type−2 subunit beta OS=Saccharomyces cerevisiae (strain ATCC 204508 / S288c) GN=BET2 PE=1 SV=3

BET3 − average.top3: 6.96339666666667 − max.qupm: 5

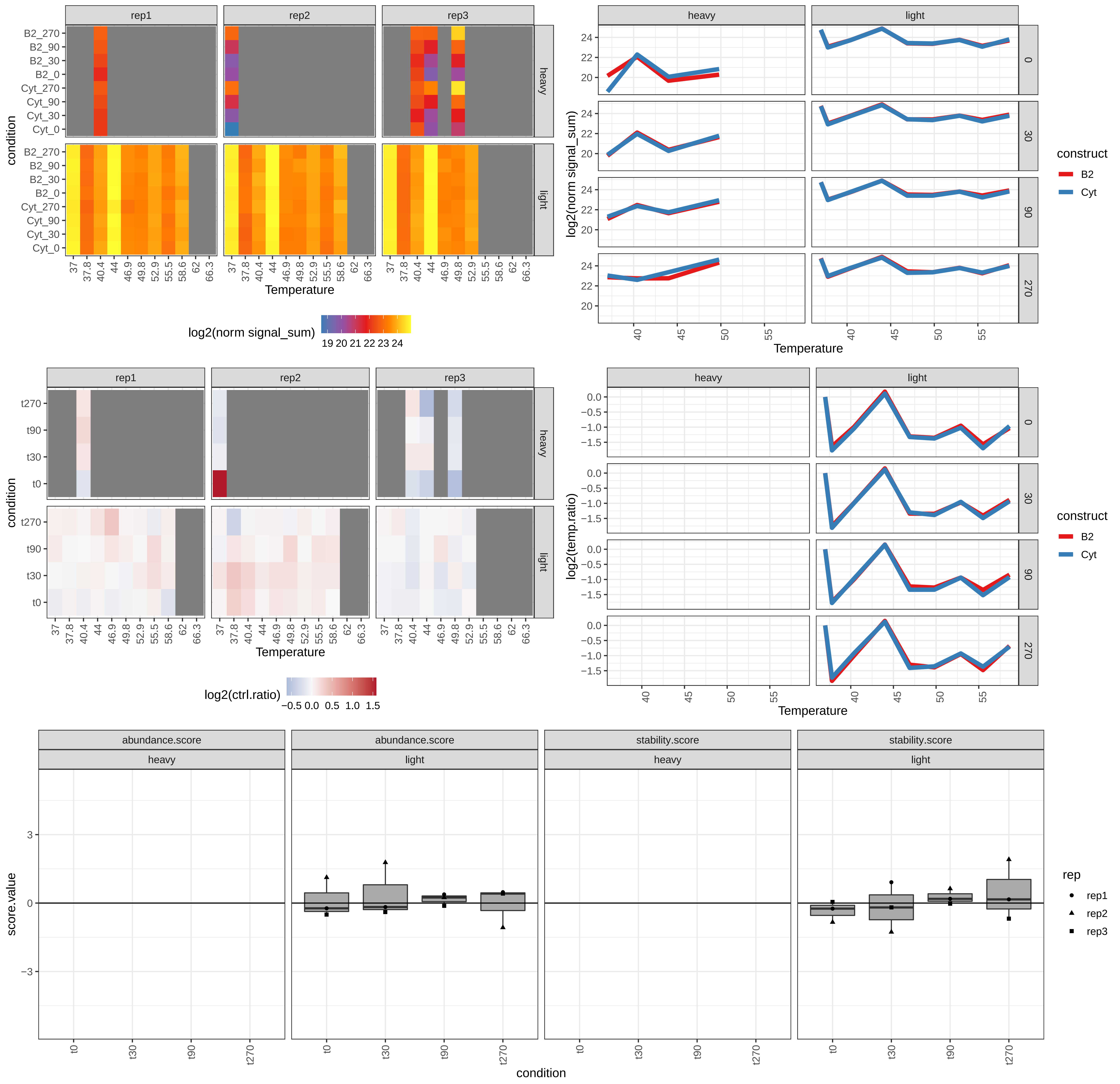

P36149 − Trafficking protein particle complex subunit BET3 OS=Saccharomyces cerevisiae (strain ATCC 204508 / S288c) GN=BET3 PE=1 SV=1

BET4 − average.top3: 6.84927763157895 − max.qupm: 9

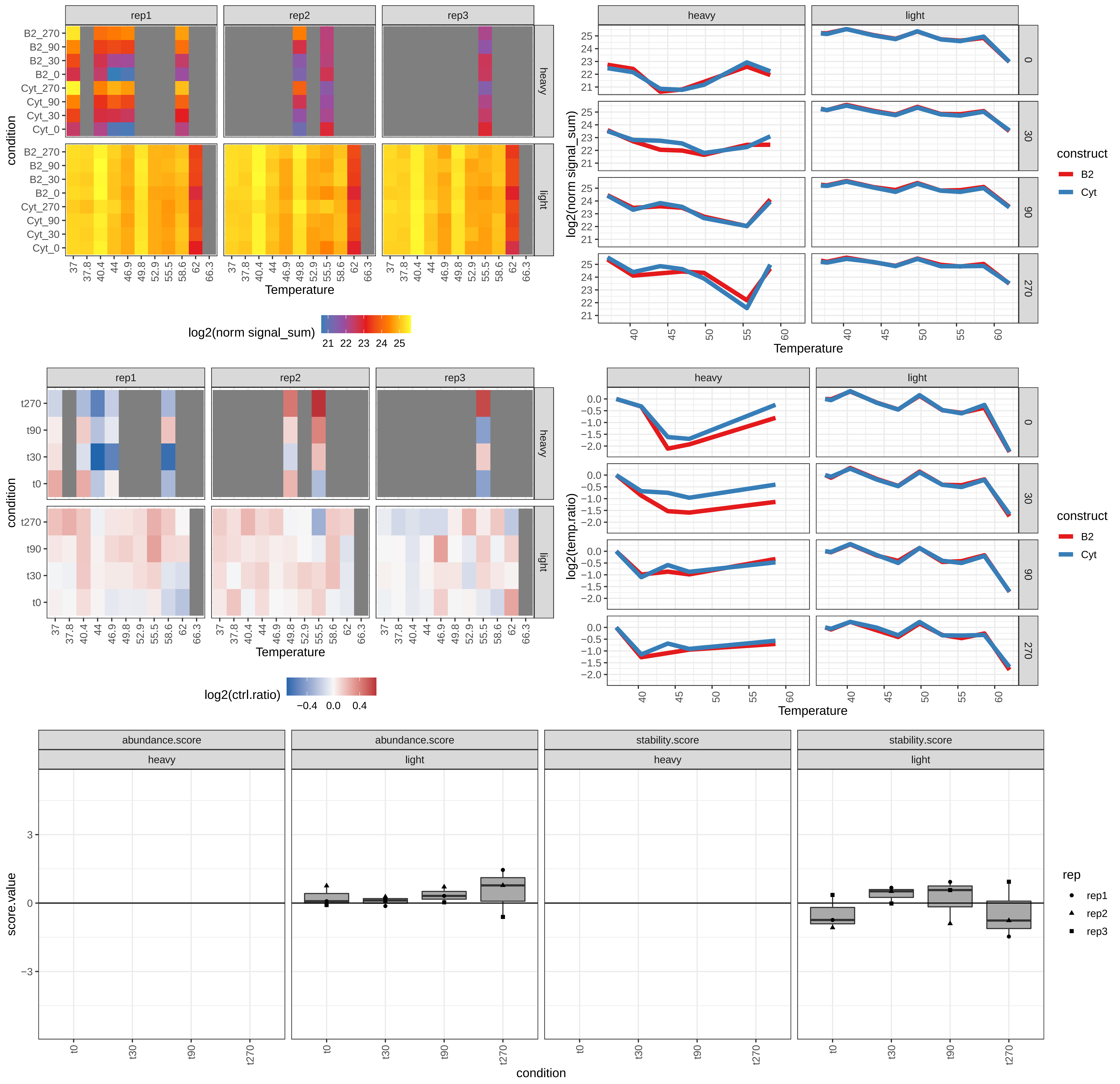

Q00618 − Geranylgeranyl transferase type−2 subunit alpha OS=Saccharomyces cerevisiae (strain ATCC 204508 / S288c) GN=BET4 PE=1 SV=3

BFR1 − average.top3: 7.27796765625 − max.qupm: 24

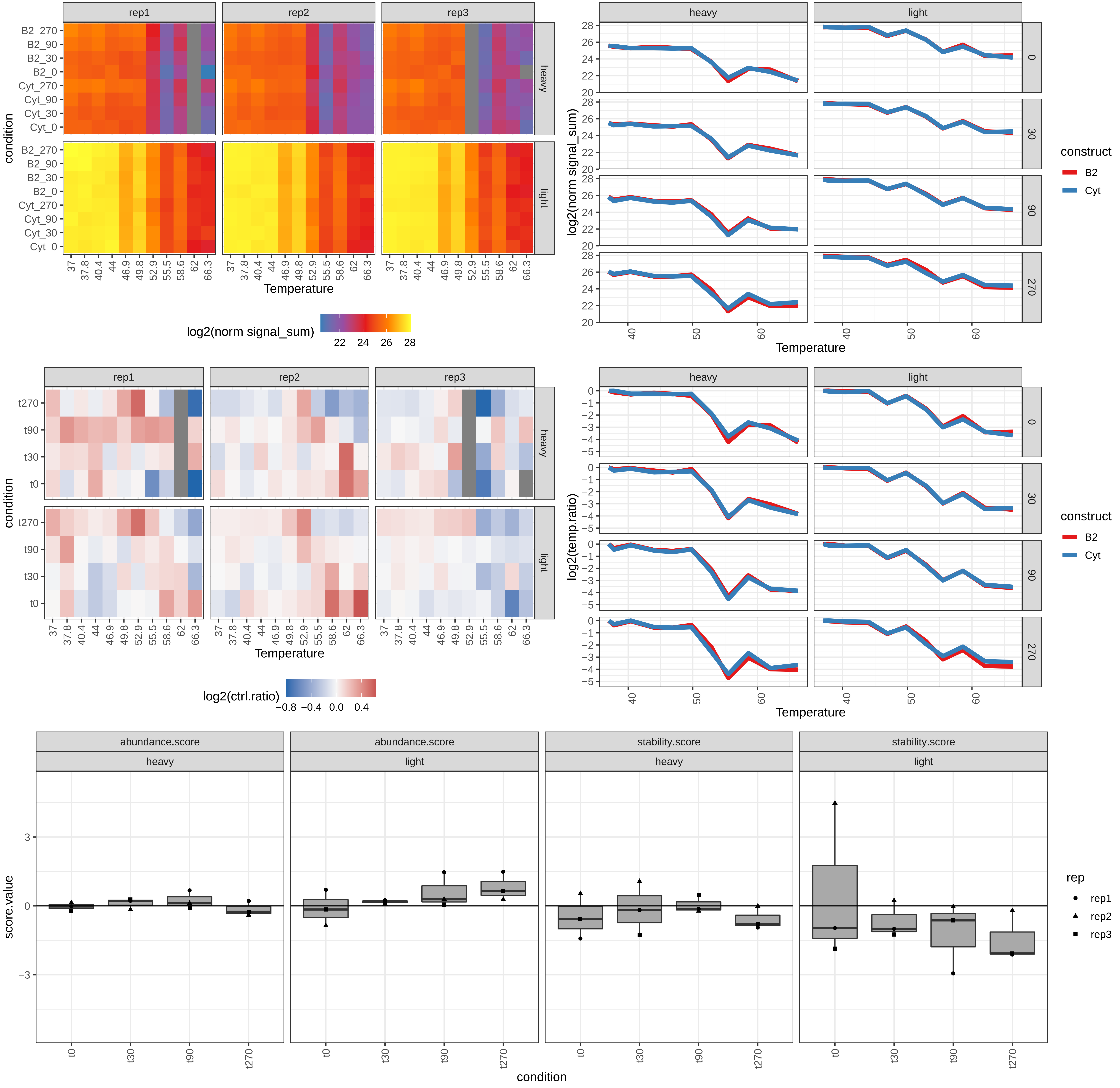

P38934 − Nuclear segregation protein BFR1 OS=Saccharomyces cerevisiae (strain ATCC 204508 / S288c) GN=BFR1 PE=1 SV=1

BGL2 − average.top3: 8.37644484848485 − max.qupm: 11

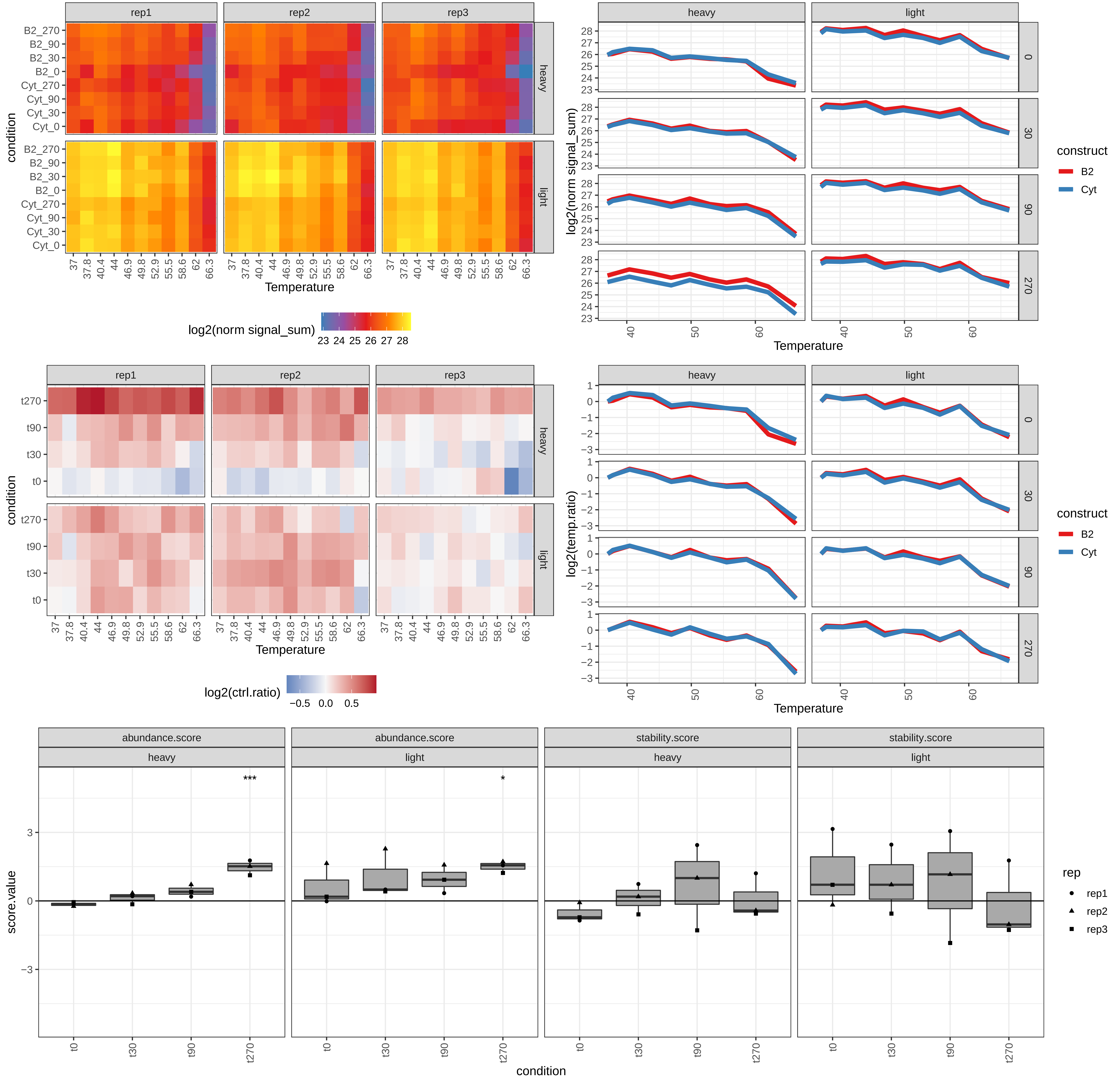

P15703 − Glucan 1,3−beta−glucosidase OS=Saccharomyces cerevisiae (strain ATCC 204508 / S288c) GN=BGL2 PE=1 SV=1

BIG1 − average.top3: 6.3105288 − max.qupm: 4

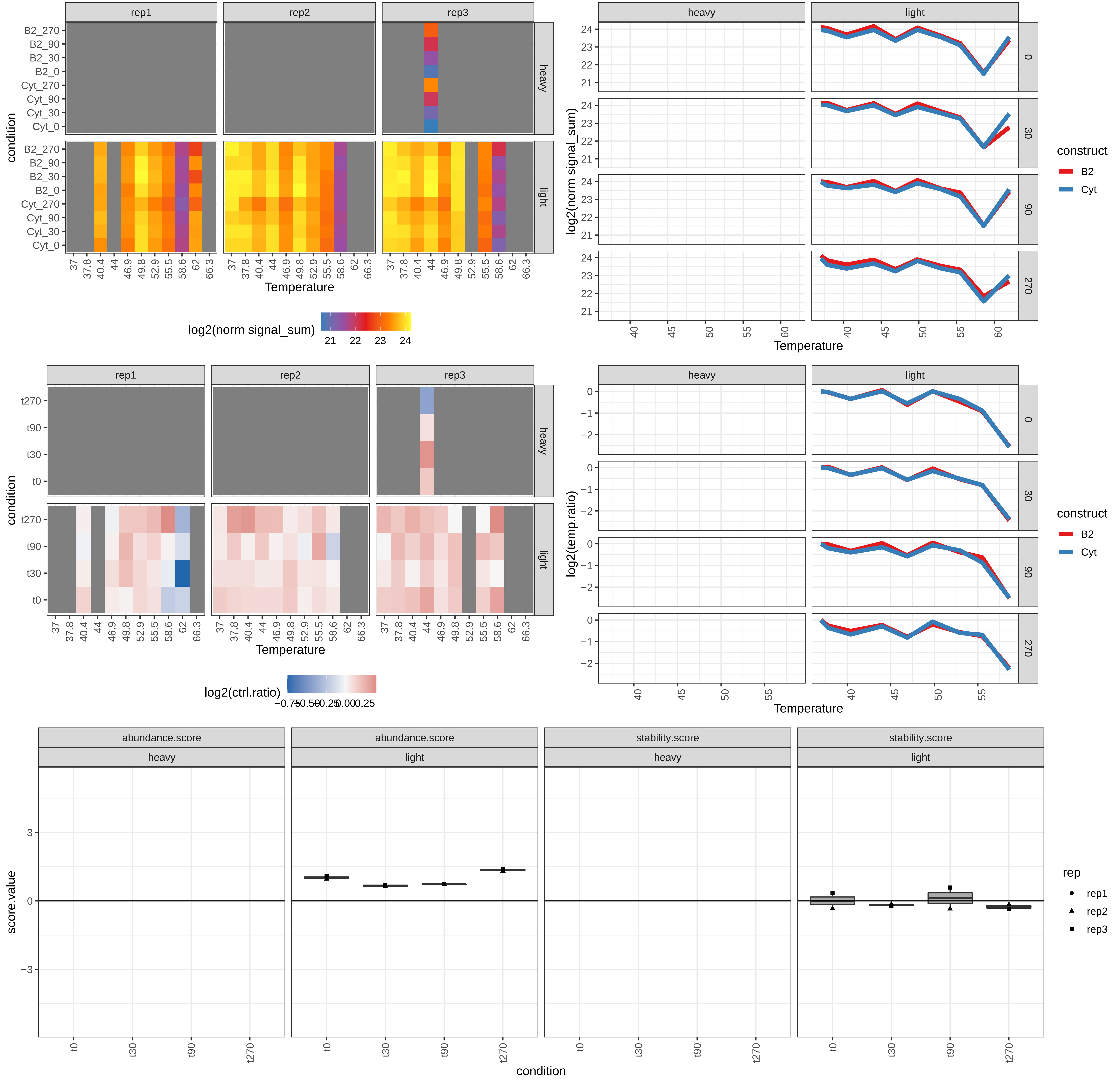

P38813 − Protein BIG1 OS=Saccharomyces cerevisiae (strain ATCC 204508 / S288c) GN=BIG1 PE=1 SV=1

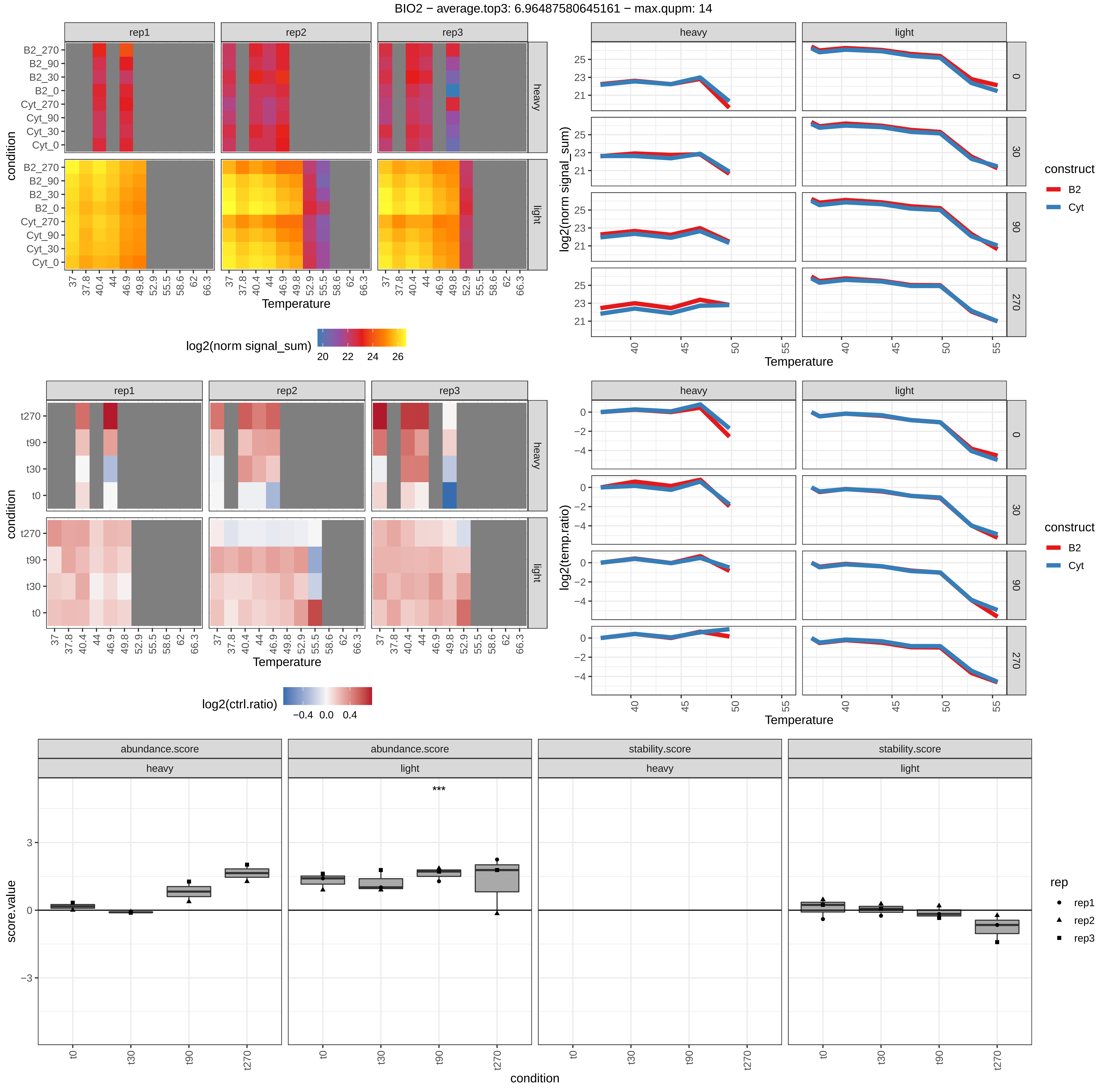

P32451 − Biotin synthase, mitochondrial OS=Saccharomyces cerevisiae (strain ATCC 204508 / S288c) GN=BIO2 PE=1 SV=2

BIO3 − average.top3: 6.86905 − max.qupm: 10

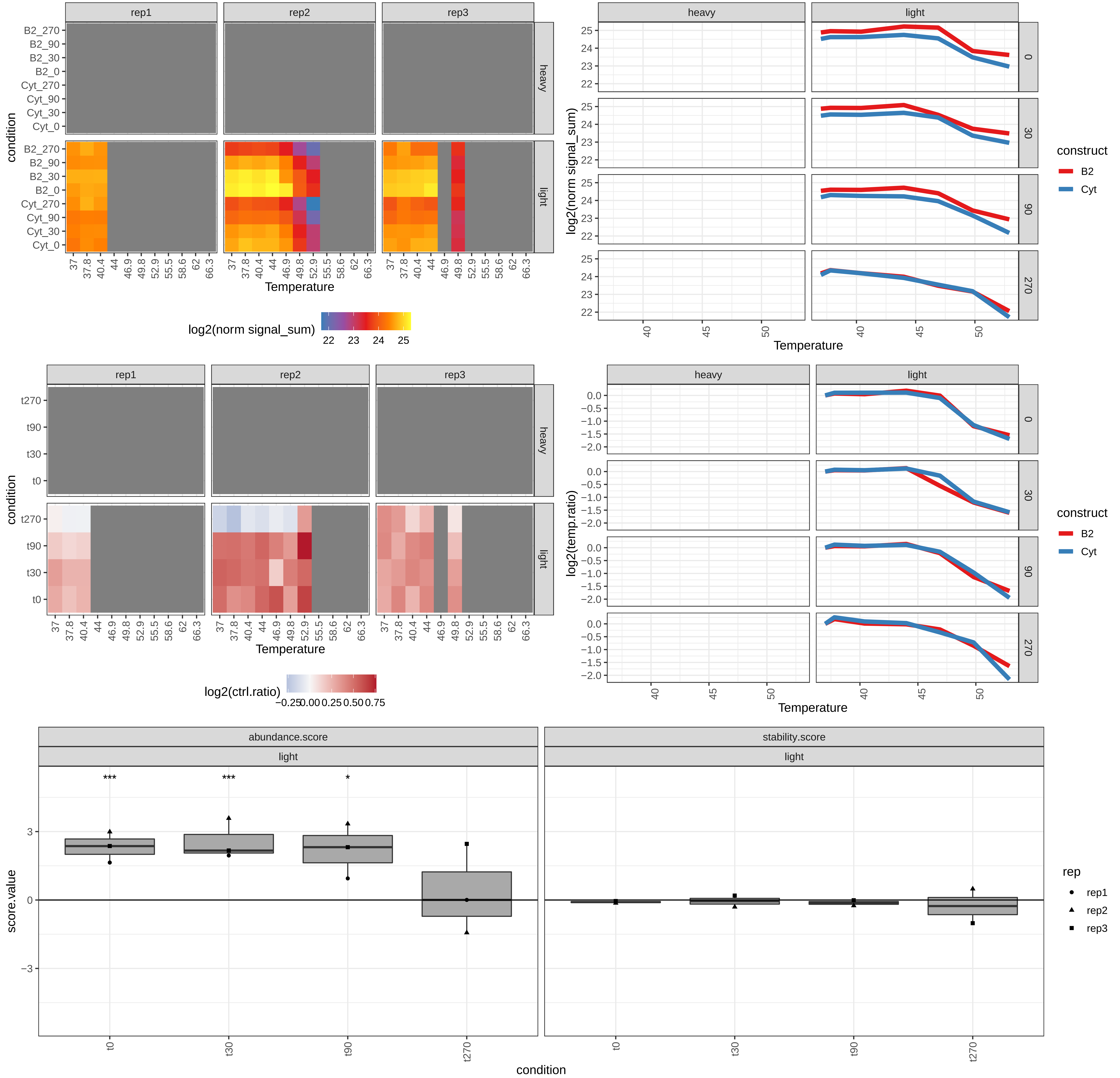

P50277 − Adenosylmethionine−8−amino−7−oxononanoate aminotransferase OS=Saccharomyces cerevisiae (strain ATCC 204508 / S288c) GN=BIO3 PE=3 SV=1

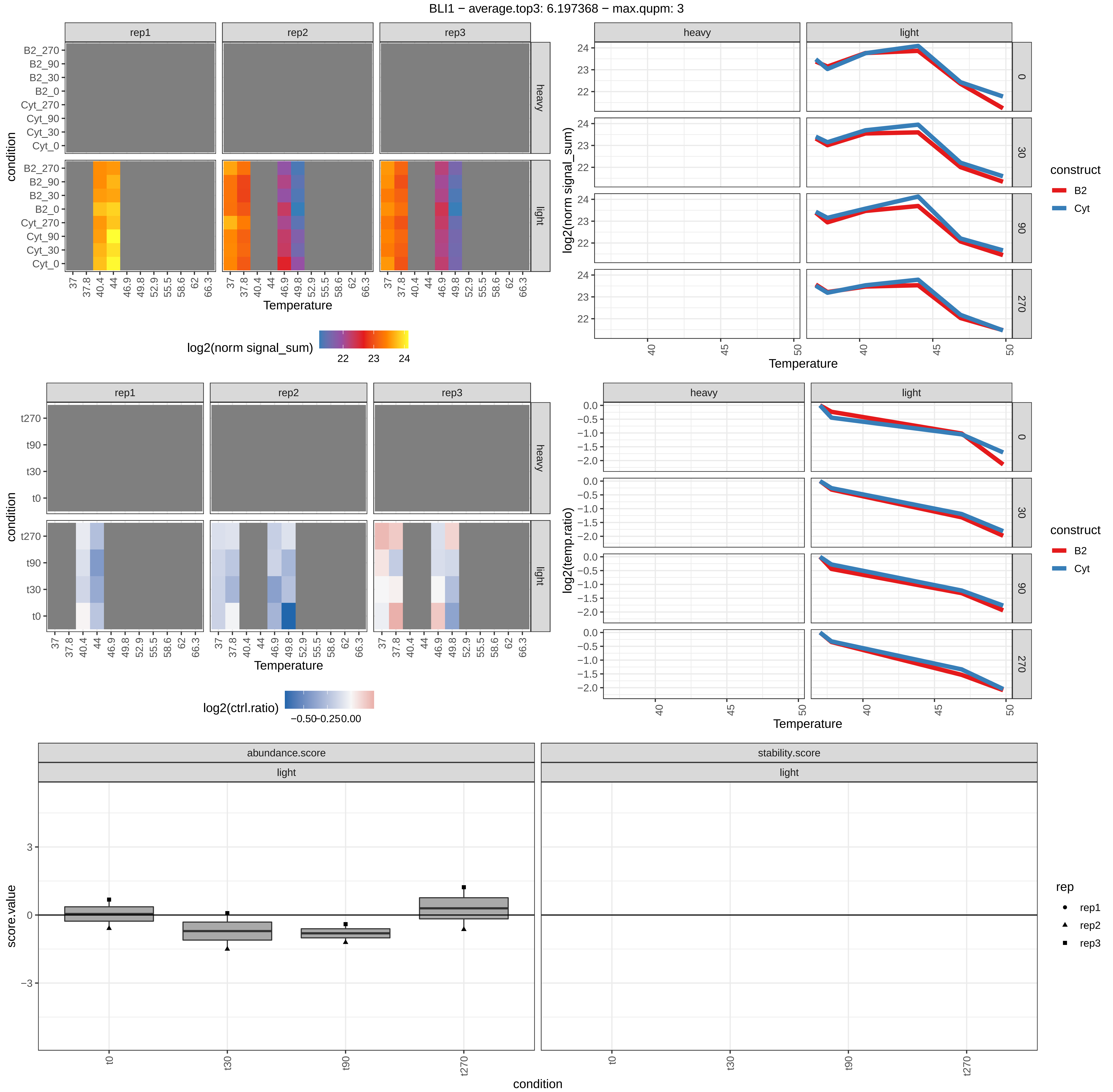

P35727 − Biogenesis of lysosome−related organelles complex 1 subunit BLI1 OS=Saccharomyces cerevisiae (strain ATCC 204508 / S288c) GN=BLI1 PE=1 SV=1

BLM10 − average.top3: 6.56959228571429 − max.qupm: 25

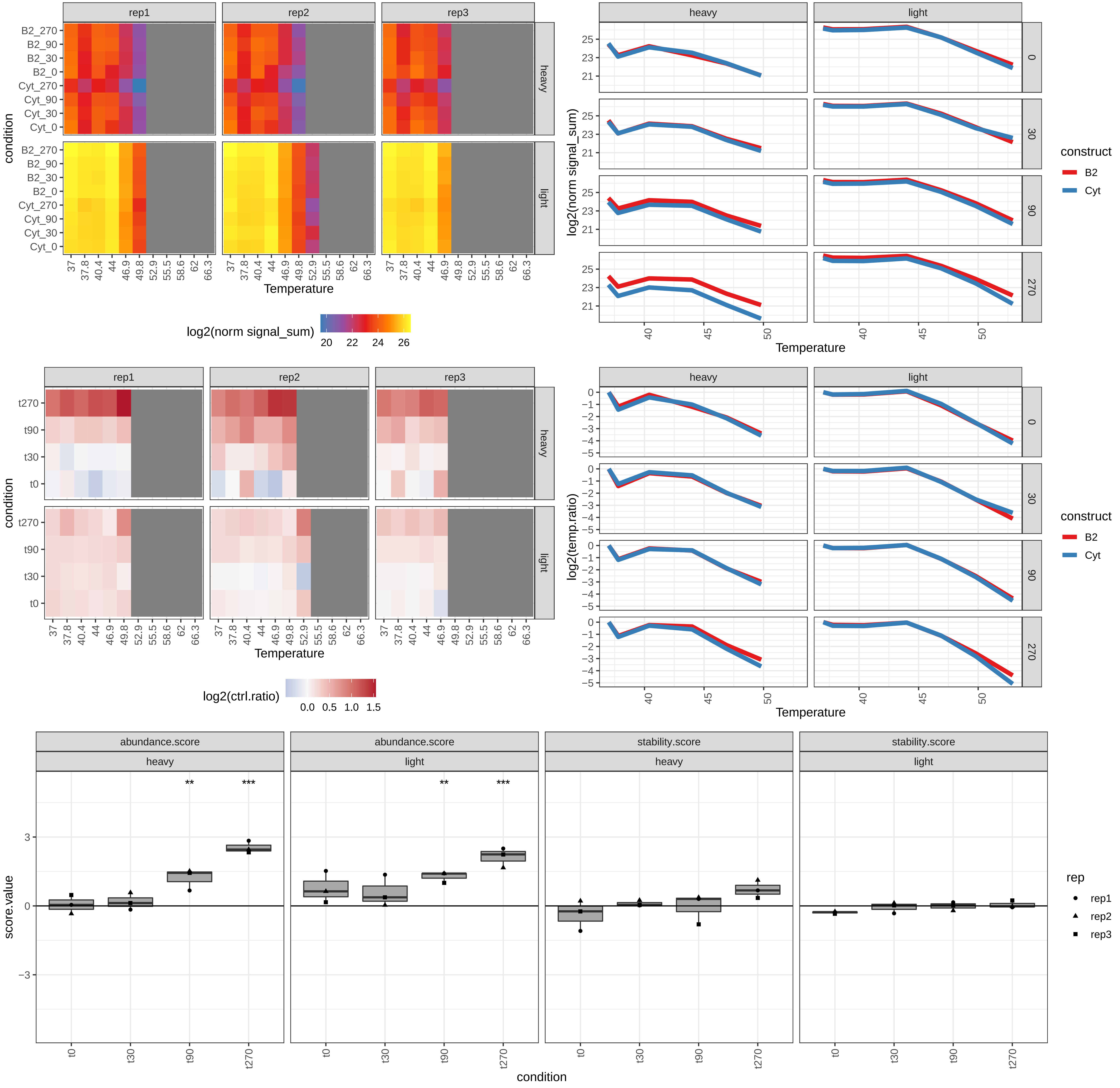

P43583 − Proteasome activator BLM10 OS=Saccharomyces cerevisiae (strain ATCC 204508 / S288c) GN=BLM10 PE=1 SV=2

BMH1 − average.top3: 7.11330387096774 − max.qupm: 8

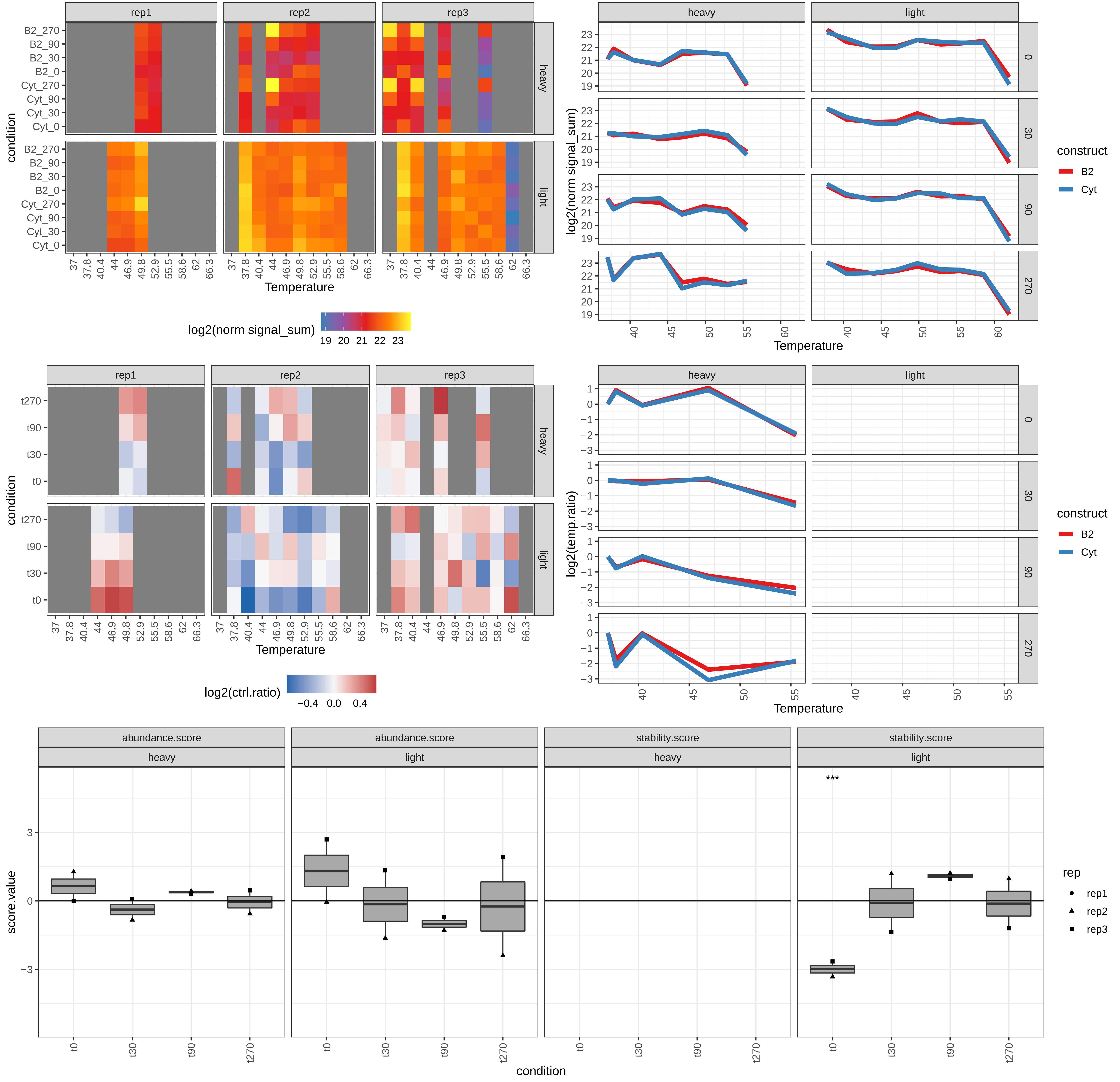

P29311 − Protein BMH1 OS=Saccharomyces cerevisiae (strain ATCC 204508 / S288c) GN=BMH1 PE=1 SV=4

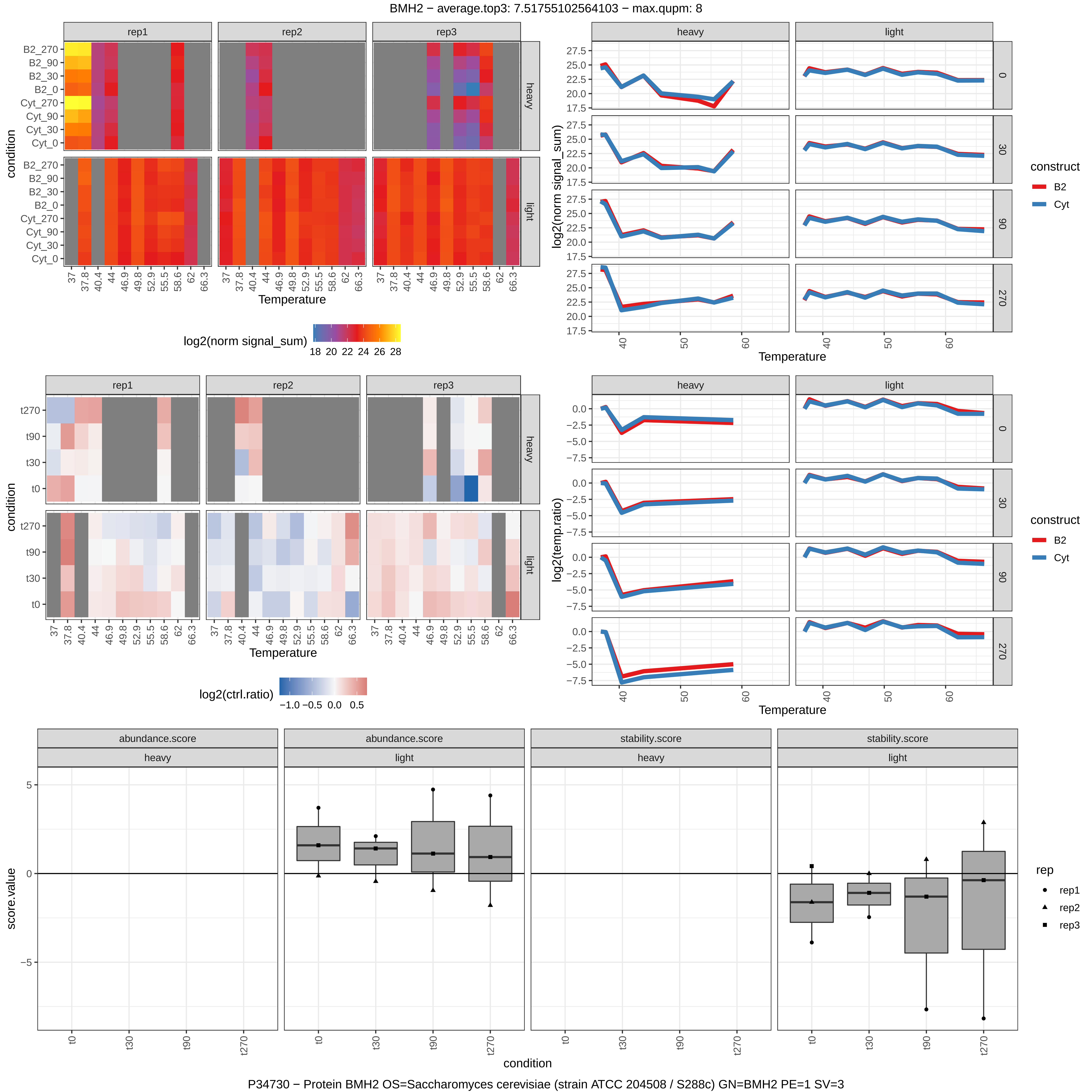
BMH2 − average.top3: 7.51755102564103 − max.qupm: 8
rep1
rep2
rep3
heavy
light
condition
Temperature
log2(norm signal_sum)
log2(ctrl.ratio)
log2(temp.ratio)
construct
B2
Cyt
abundance.score
stability.score
score.value
rep
rep1
rep2
rep3
P34730 − Protein BMH2 OS=Saccharomyces cerevisiae (strain ATCC 204508 / S288c) GN=BMH2 PE=1 SV=3

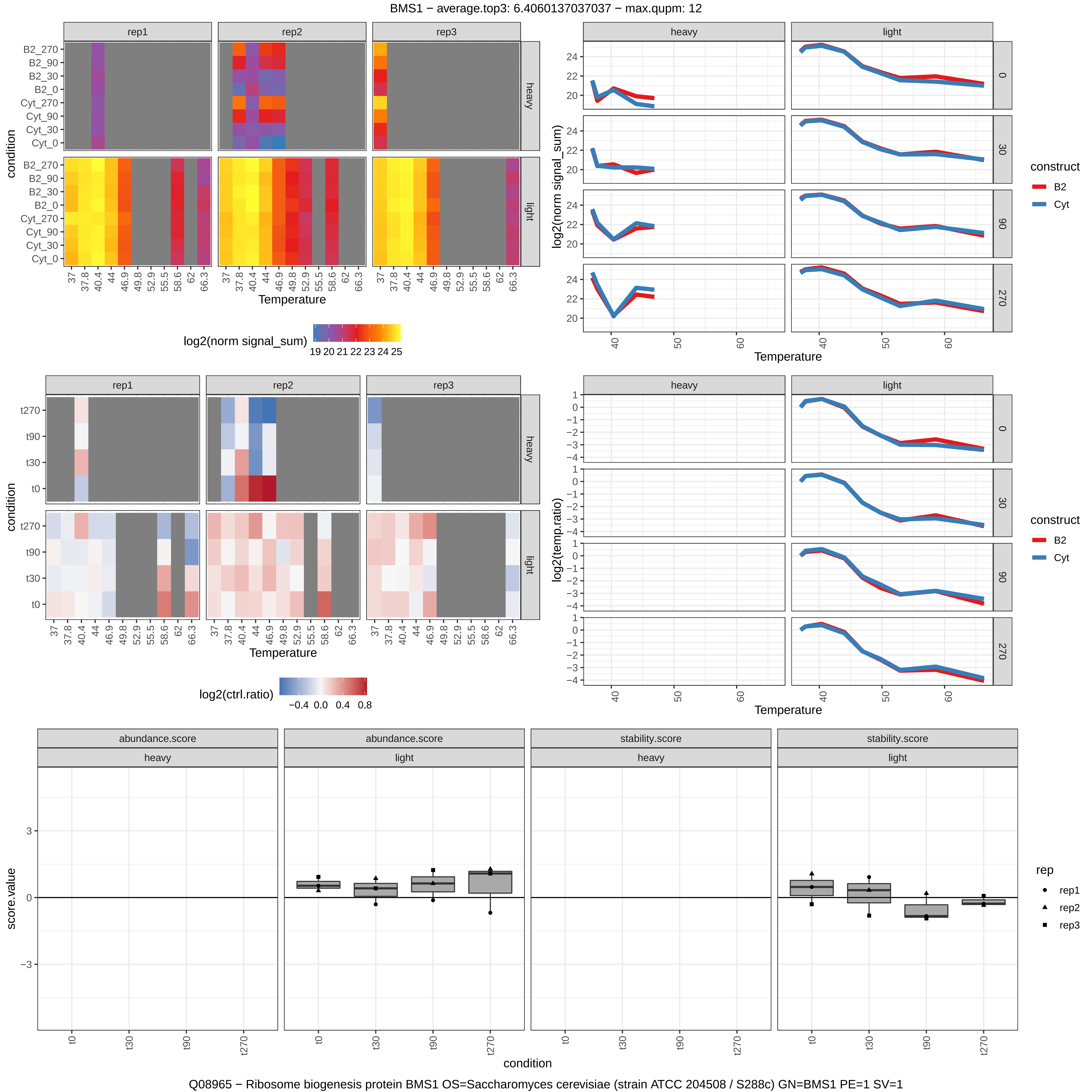
BMS1 − average.top3: 6.4060137037037 − max.qupm: 12
rep1
rep2
rep3
heavy
light
condition
Temperature
log2(norm signal_sum)
construct
B2
Cyt
log2(ctrl.ratio)
log2(temp.ratio)
abundance.score
stability.score
score.value
rep
rep1
rep2
rep3
Q08965 − Ribosome biogenesis protein BMS1 OS=Saccharomyces cerevisiae (strain ATCC 204508 / S288c) GN=BMS1 PE=1 SV=1

BMT5 − average.top3: 6.55840583333333 − max.qupm: 5

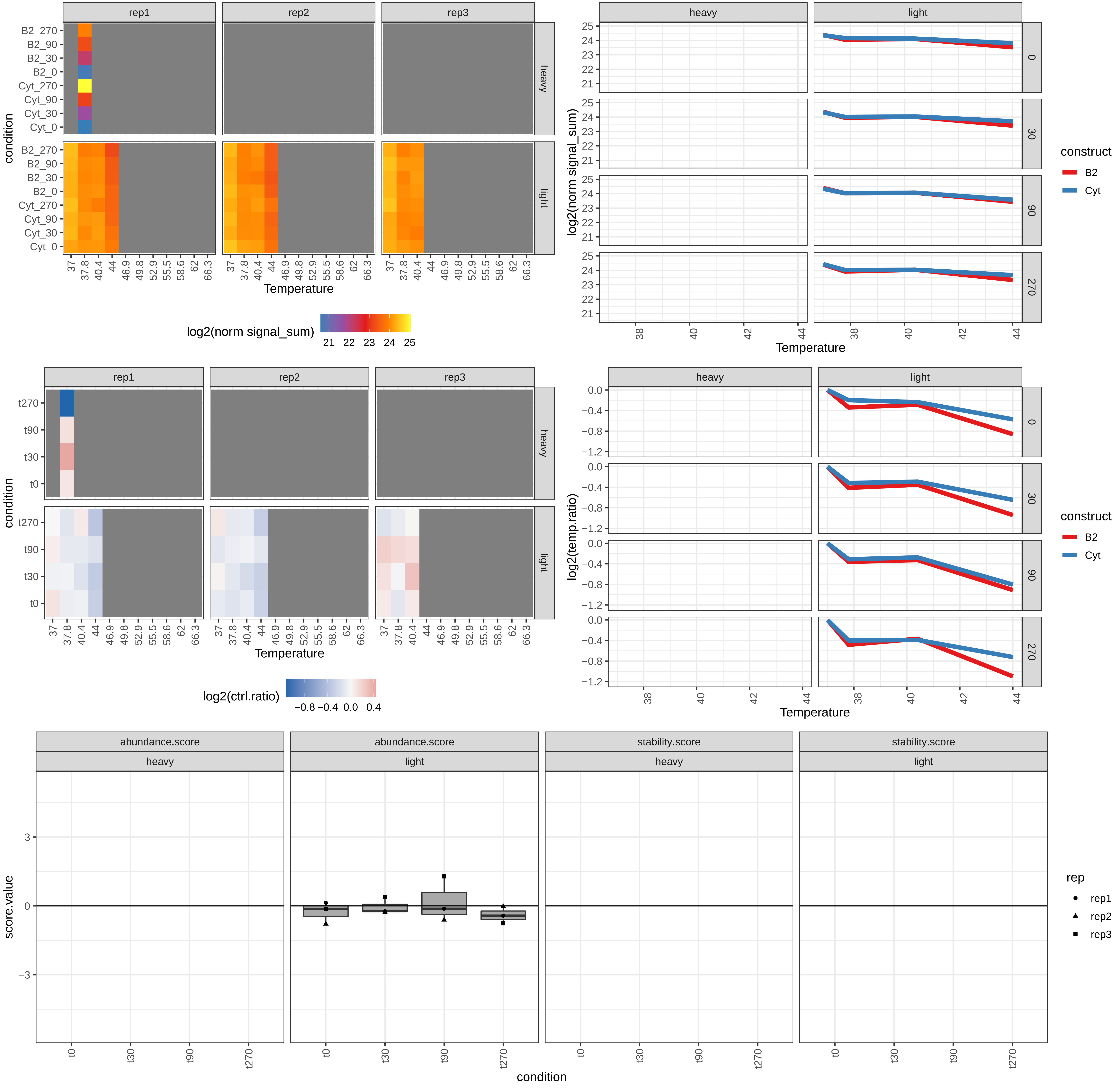

P40493 − 25S rRNA (uridine(2634)−N(3))−methyltransferase OS=Saccharomyces cerevisiae (strain ATCC 204508 / S288c) GN=BMT5 PE=1 SV=1

BNA1 − average.top3: 7.15499738461538 − max.qupm: 10

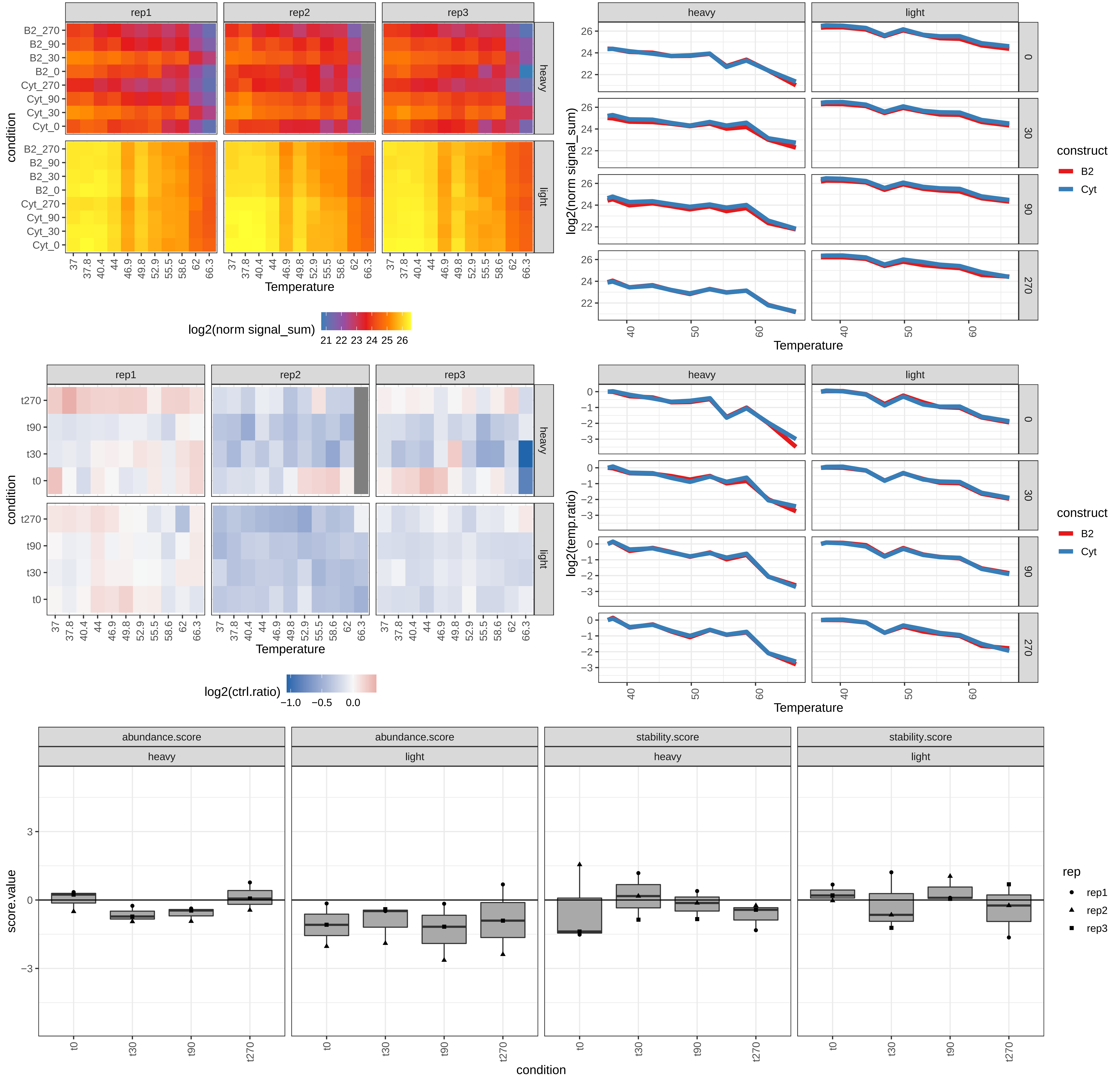

P47096 − 3−hydroxyanthranilate 3,4−dioxygenase OS=Saccharomyces cerevisiae (strain ATCC 204508 / S288c) GN=BNA1 PE=1 SV=1

BNA3 − average.top3: 6.87519588235294 − max.qupm: 8

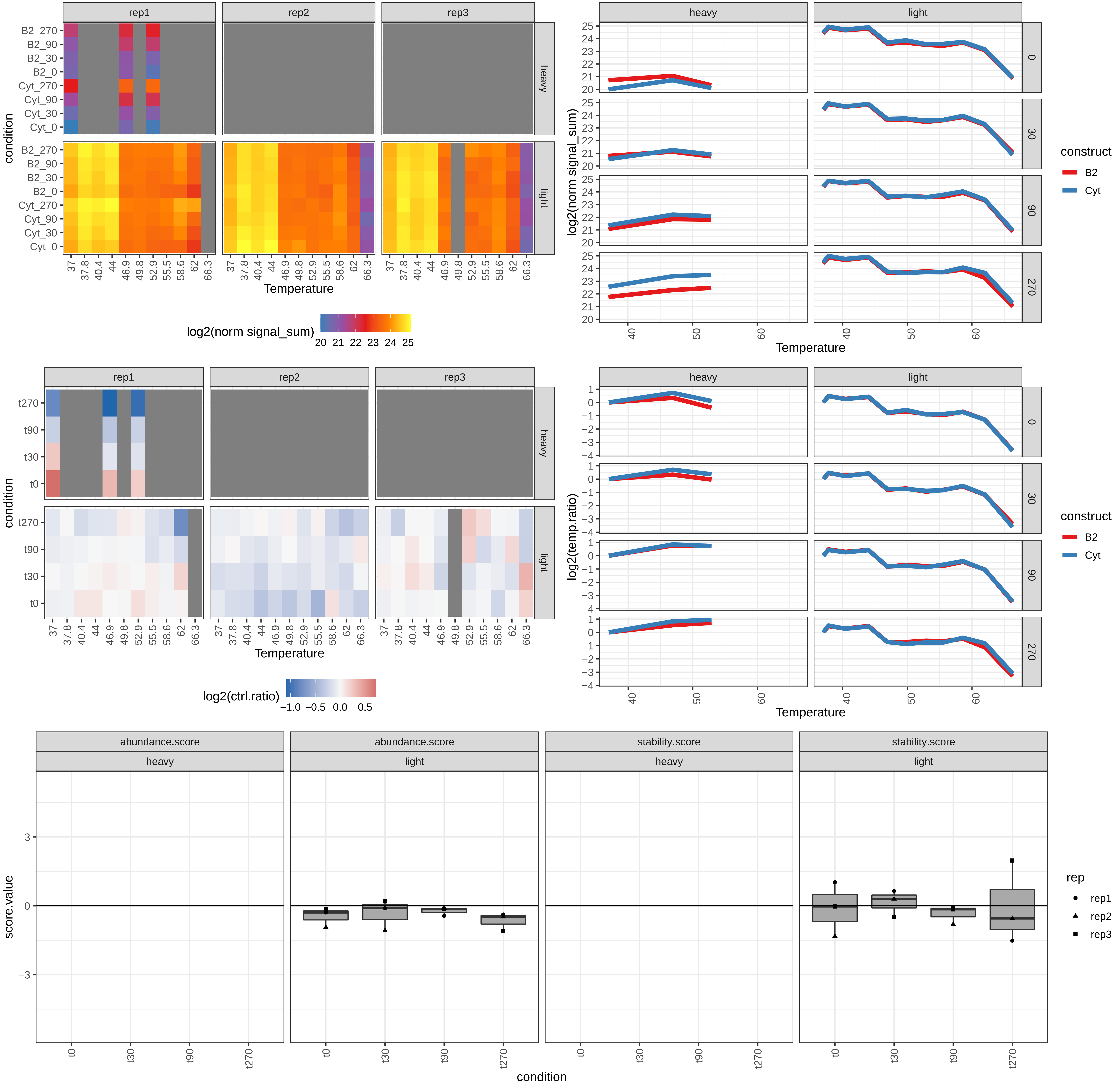

P47039 − Probable kynurenine−−oxoglutarate transaminase BNA3 OS=Saccharomyces cerevisiae (strain ATCC 204508 / S288c) GN=BNA3 PE=1 SV=1

BNA4 − average.top3: 6.91679 − max.qupm: 8

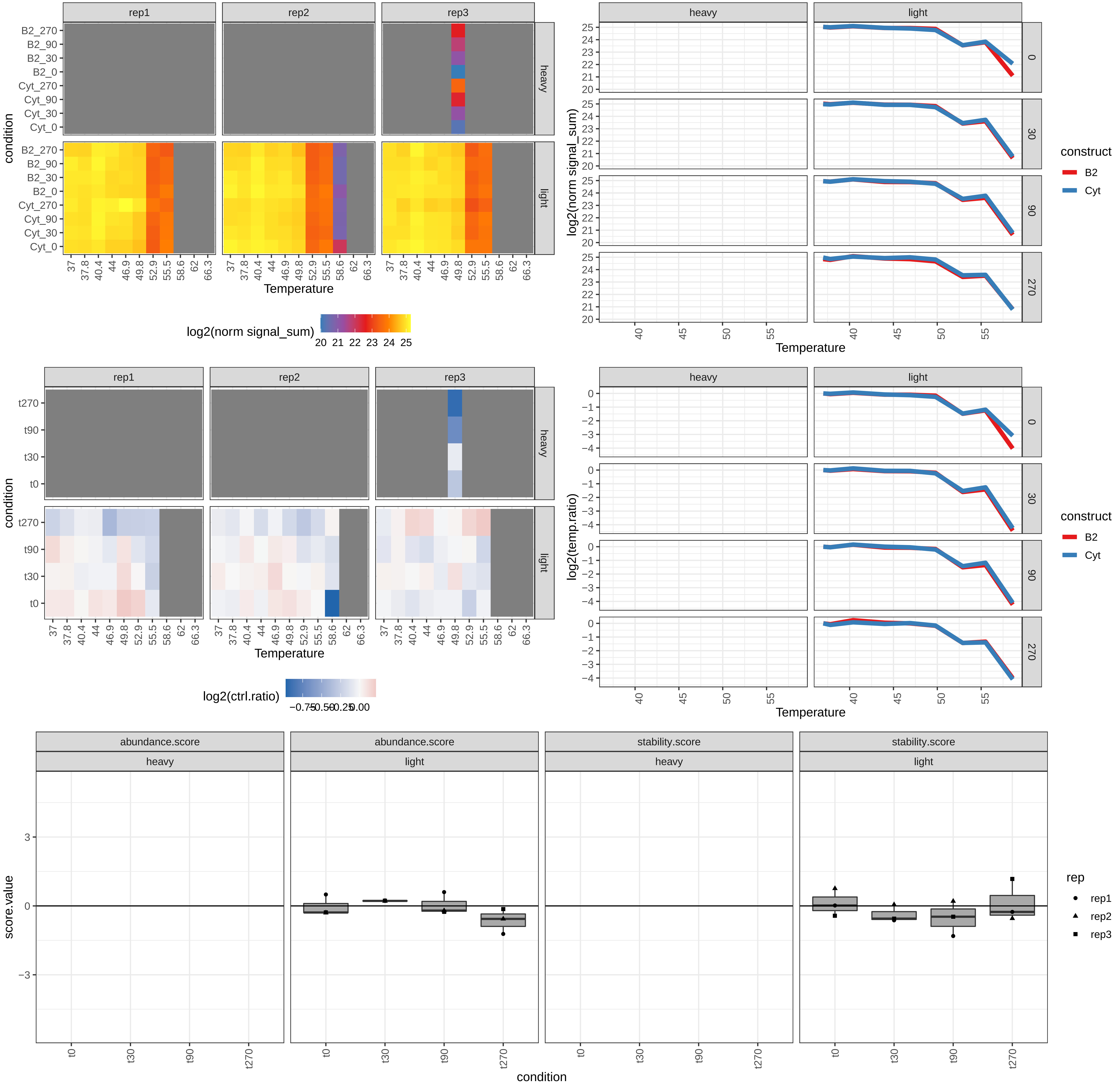

P38169 − Kynurenine 3−monooxygenase OS=Saccharomyces cerevisiae (strain ATCC 204508 / S288c) GN=BNA4 PE=1 SV=1

BNA5 − average.top3: 7.07298818181818 − max.qupm: 6

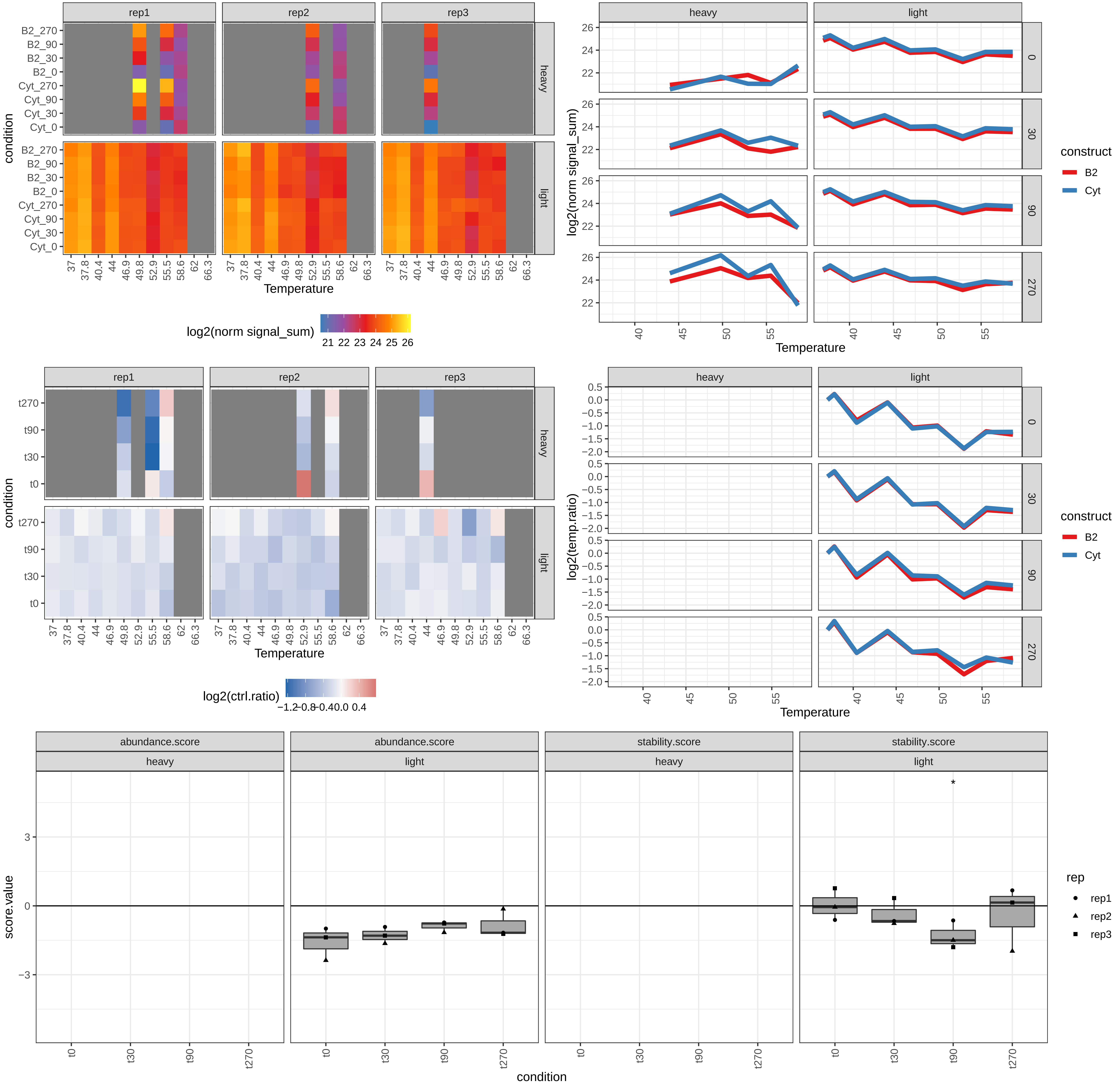

Q05979 − Kynureninase OS=Saccharomyces cerevisiae (strain ATCC 204508 / S288c) GN=BNA5 PE=1 SV=1

BNA6 − average.top3: 7.60516129032258 − max.qupm: 11

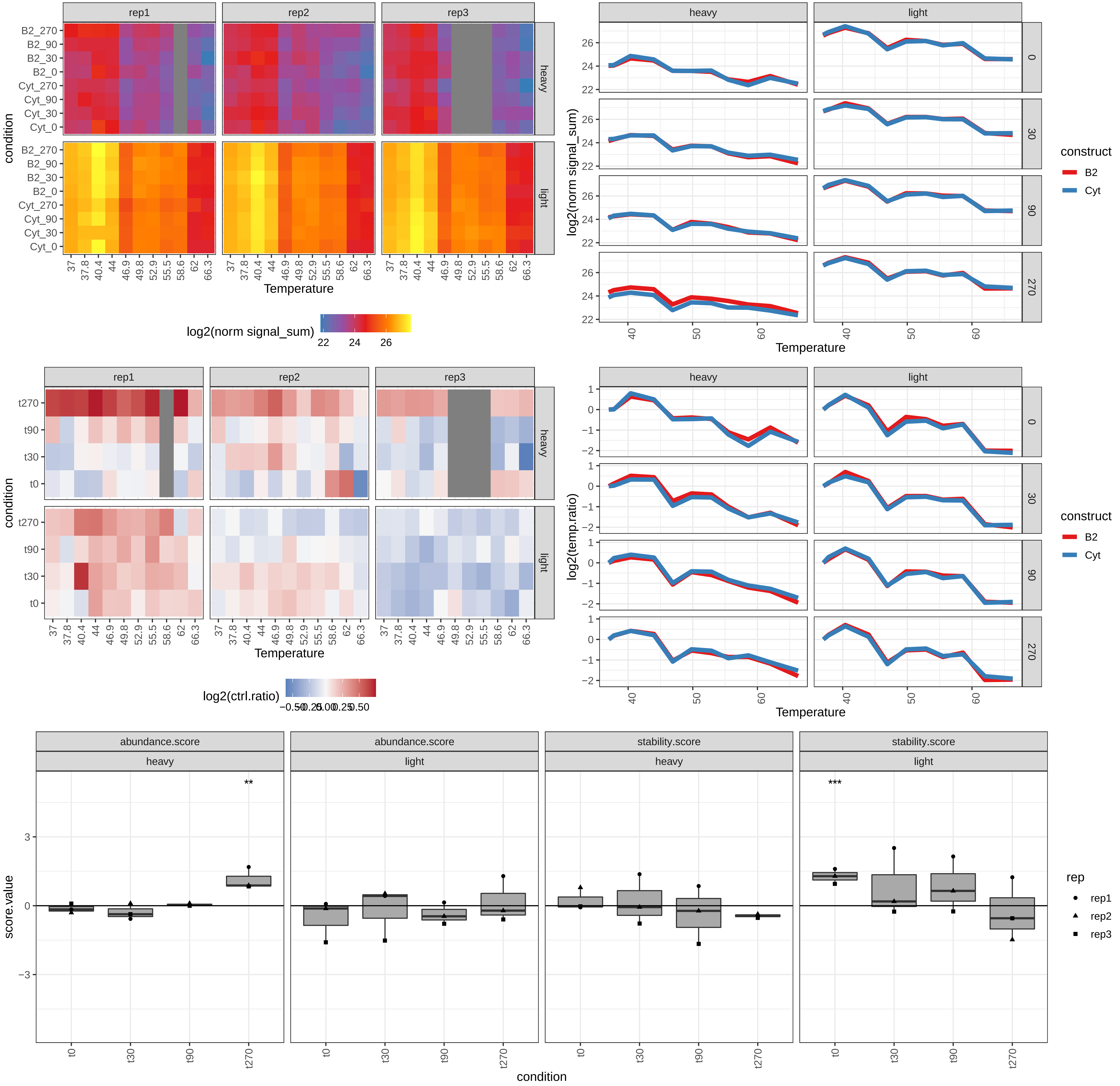

P43619 − Nicotinate−nucleotide pyrophosphorylase [carboxylating] OS=Saccharomyces cerevisiae (strain ATCC 204508 / S288c) GN=BNA6 PE=1 SV=1

BNA7 − average.top3: 6.32367346153846 − max.qupm: 4

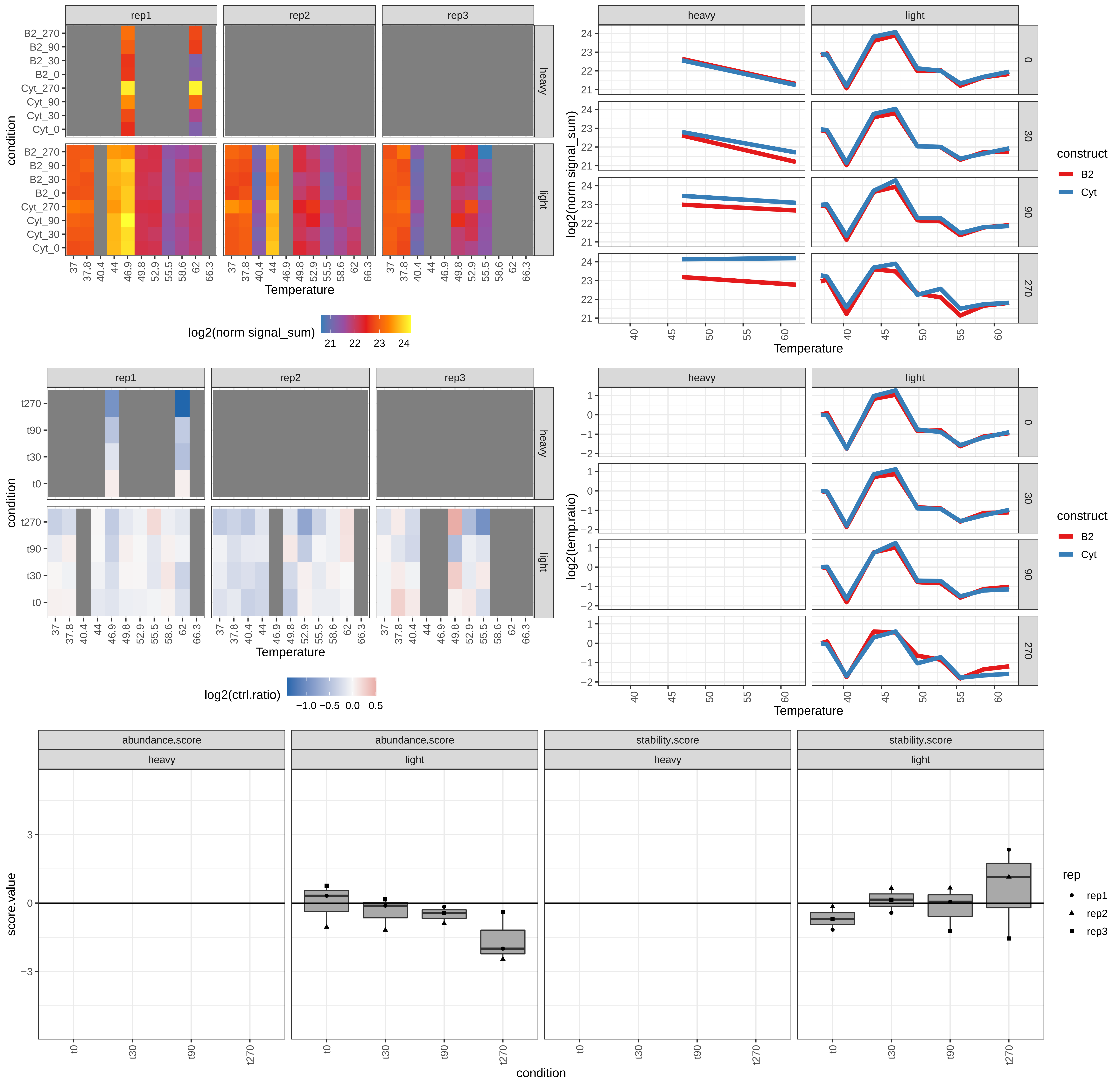

Q04066 − Kynurenine formamidase OS=Saccharomyces cerevisiae (strain ATCC 204508 / S288c) GN=BNA7 PE=1 SV=1

BNI1 − average.top3: 6.23408285714286 − max.qupm: 8

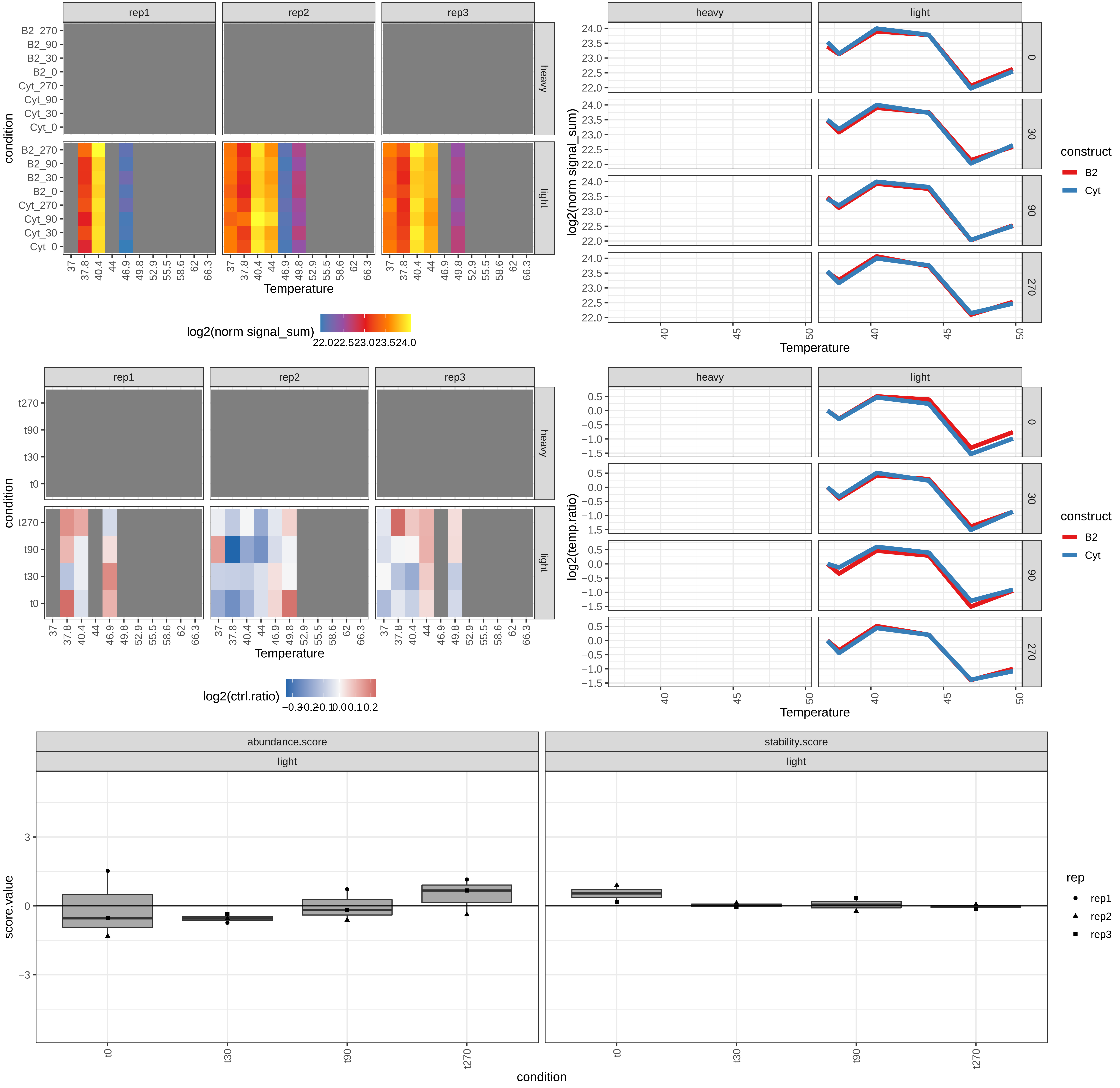

P41832 − Protein BNI1 OS=Saccharomyces cerevisiae (strain ATCC 204508 / S288c) GN=BNI1 PE=1 SV=3

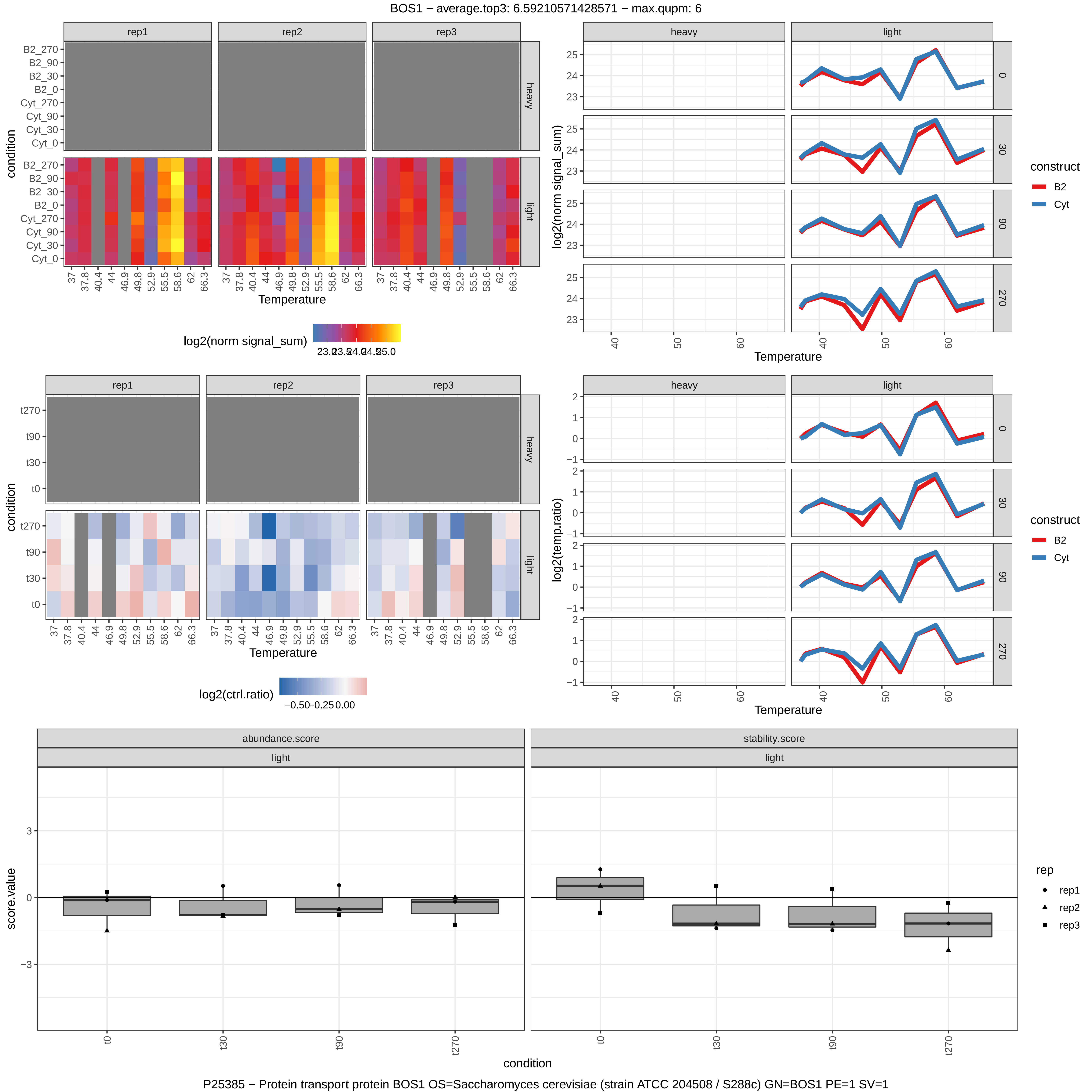
BOS1 − average.top3: 6.59210571428571 − max.qupm: 6
rep1
rep2
rep3
heavy
light
condition
Temperature
log2(norm signal_sum)
construct
B2
Cyt
log2(temp.ratio)
log2(ctrl.ratio)
abundance.score
stability.score
score.value
rep
rep1
rep2
rep3
P25385 − Protein transport protein BOS1 OS=Saccharomyces cerevisiae (strain ATCC 204508 / S288c) GN=BOS1 PE=1 SV=1

BPH1 − average.top3: 6.63036333333333 − max.qupm: 8

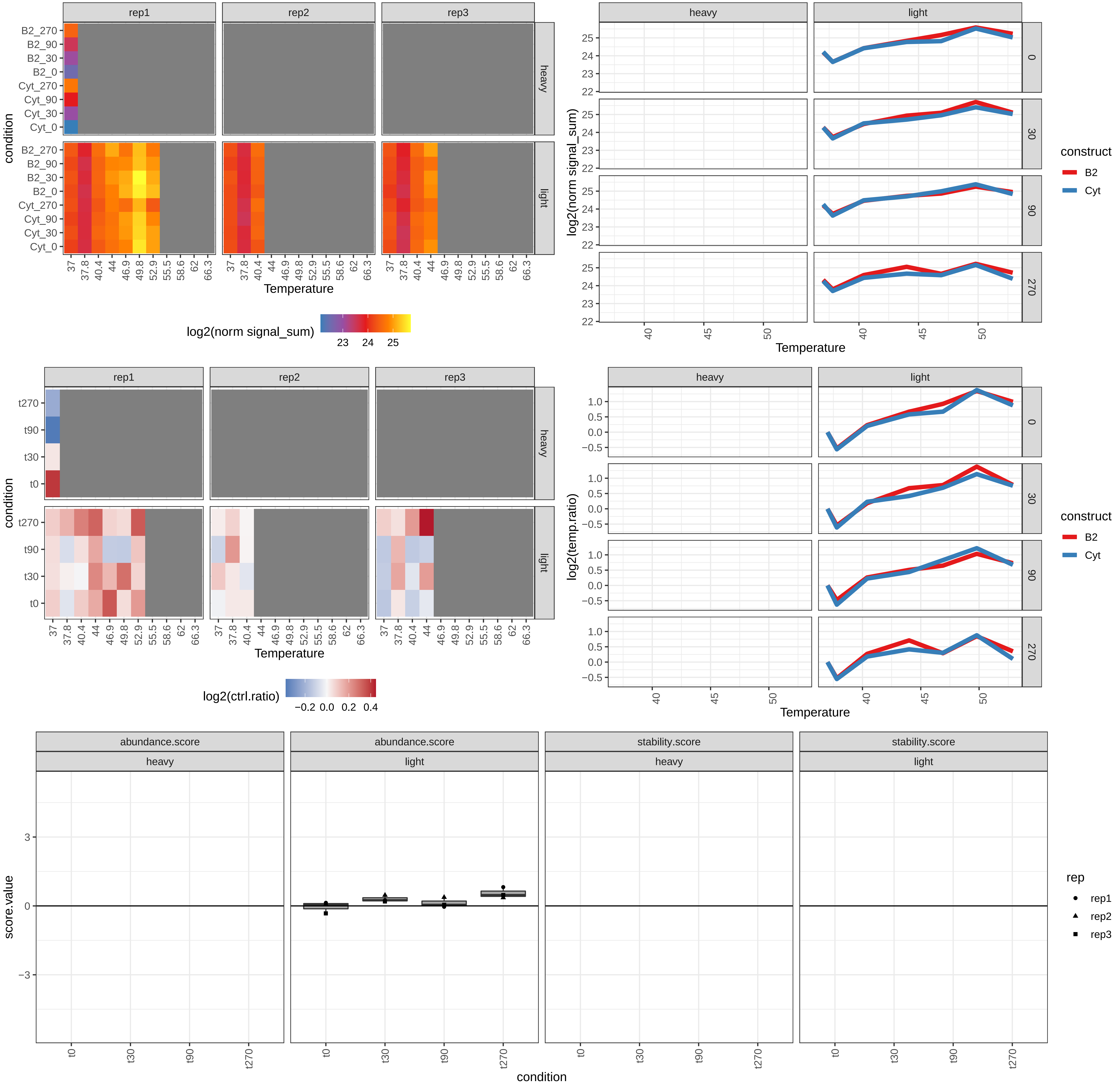

P25356 − Beige protein homolog 1 OS=Saccharomyces cerevisiae (strain ATCC 204508 / S288c) GN=BPH1 PE=1 SV=2

BPL1 − average.top3: 6.73735526315789 − max.qupm: 7

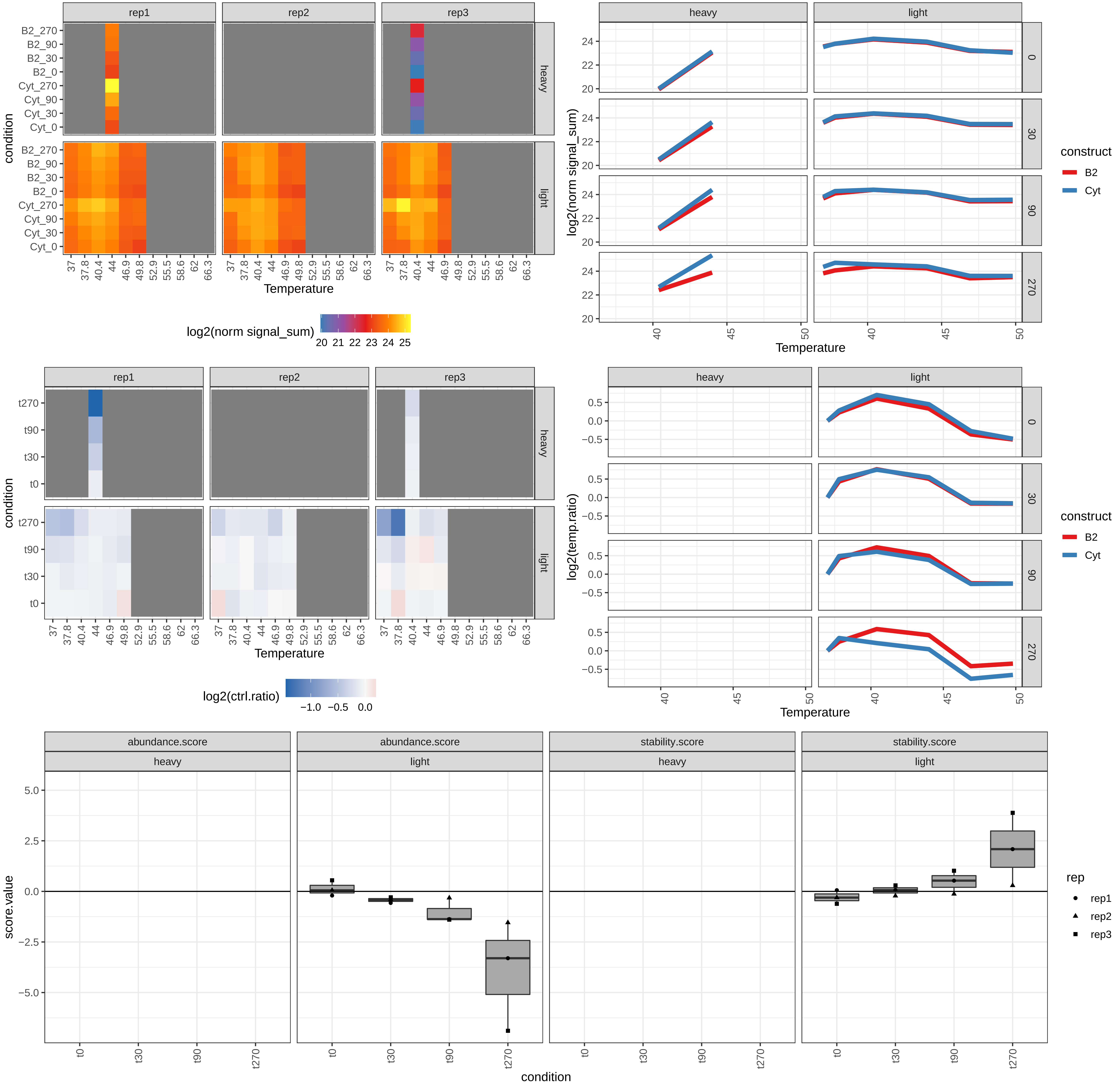

P48445 − Biotin−−protein ligase OS=Saccharomyces cerevisiae (strain ATCC 204508 / S288c) GN=BPL1 PE=1 SV=1

BPT1 − average.top3: 6.52442214285714 − max.qupm: 10

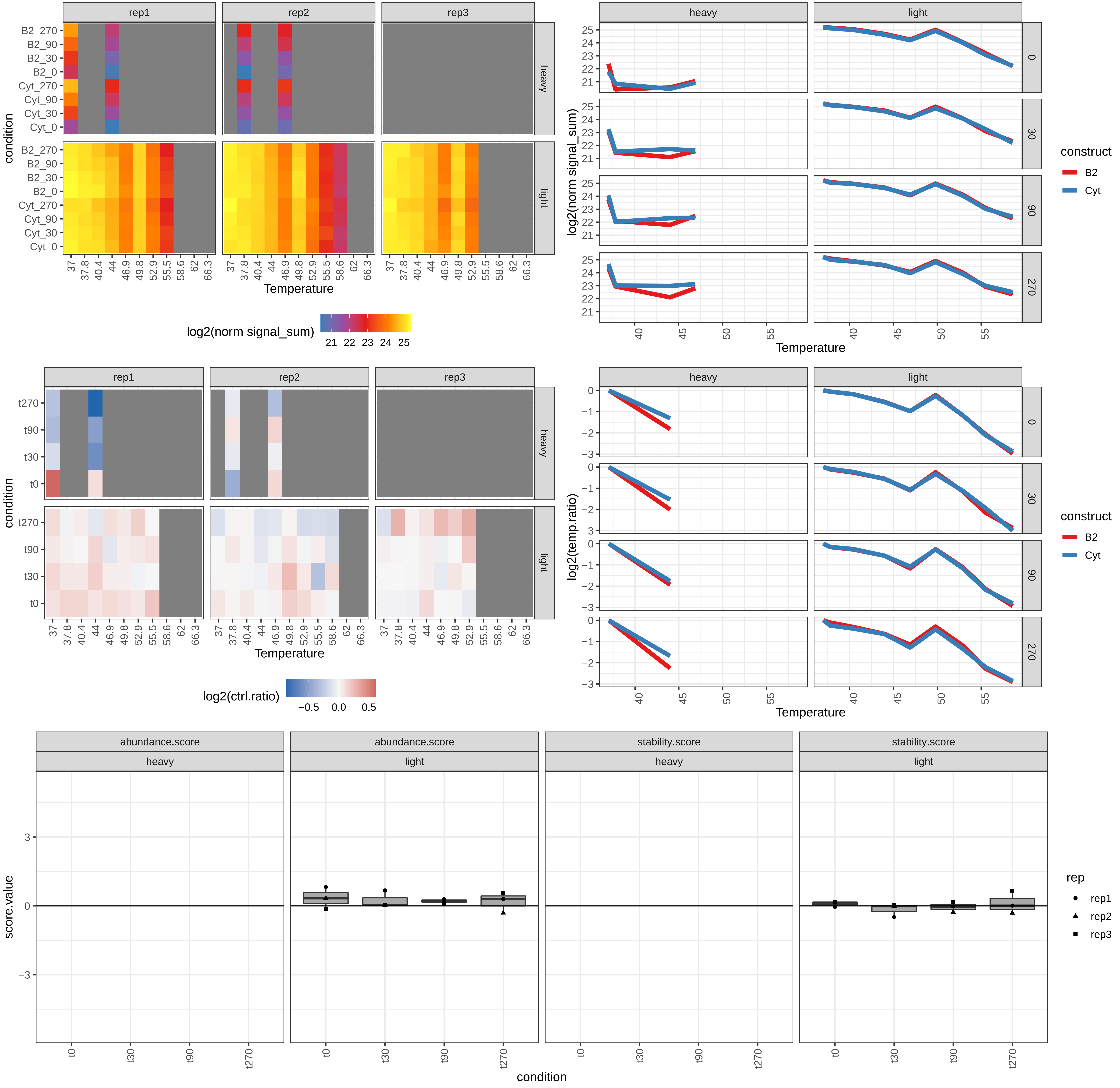

P14772 − Bile pigment transporter 1 OS=Saccharomyces cerevisiae (strain ATCC 204508 / S288c) GN=BPT1 PE=1 SV=2

BRE1 − average.top3: 6.68740925925926 − max.qupm: 10

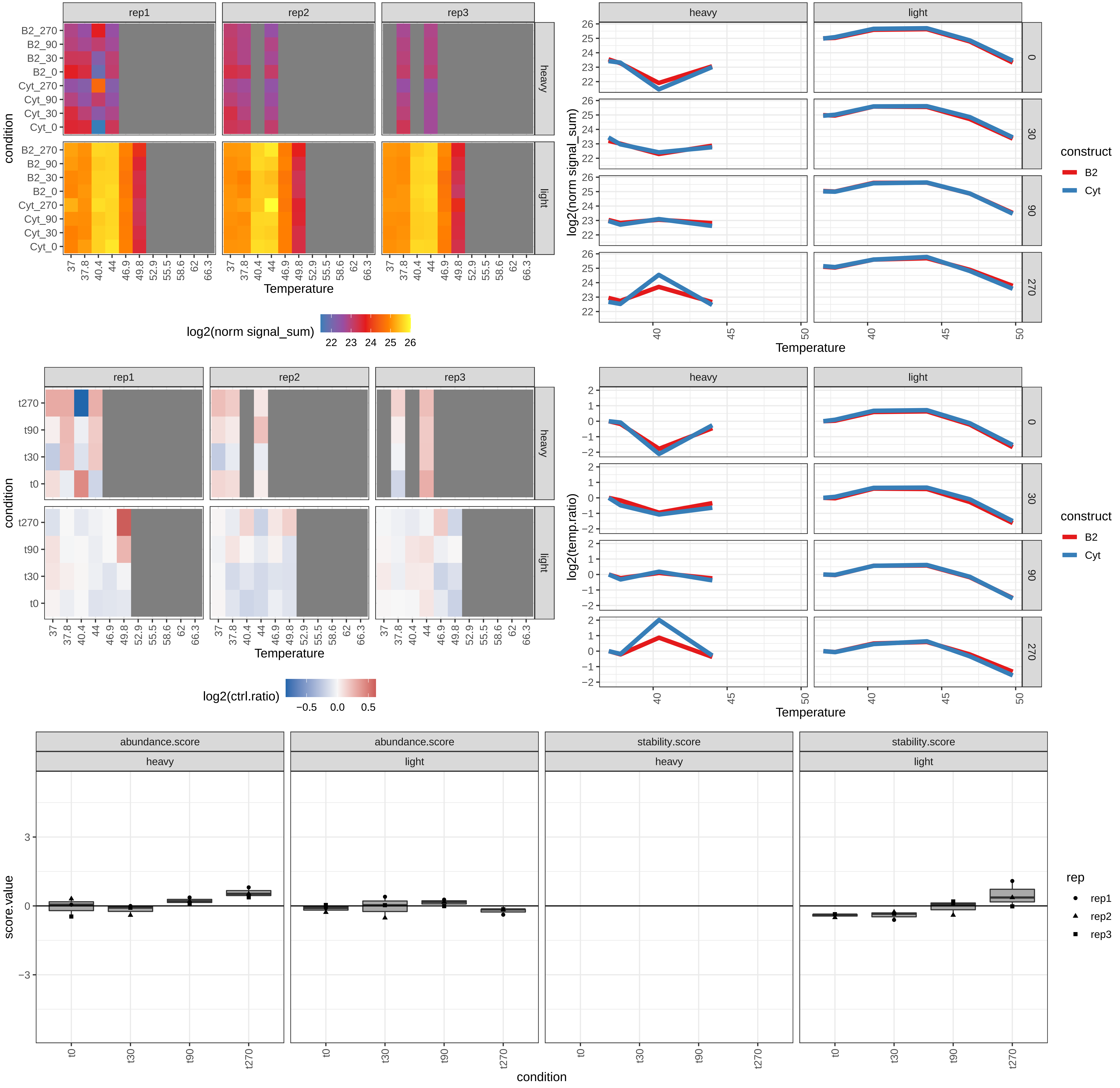

Q07457 − E3 ubiquitin−protein ligase BRE1 OS=Saccharomyces cerevisiae (strain ATCC 204508 / S288c) GN=BRE1 PE=1 SV=1

BRE2 − average.top3: 6.57408842105263 − max.qupm: 8

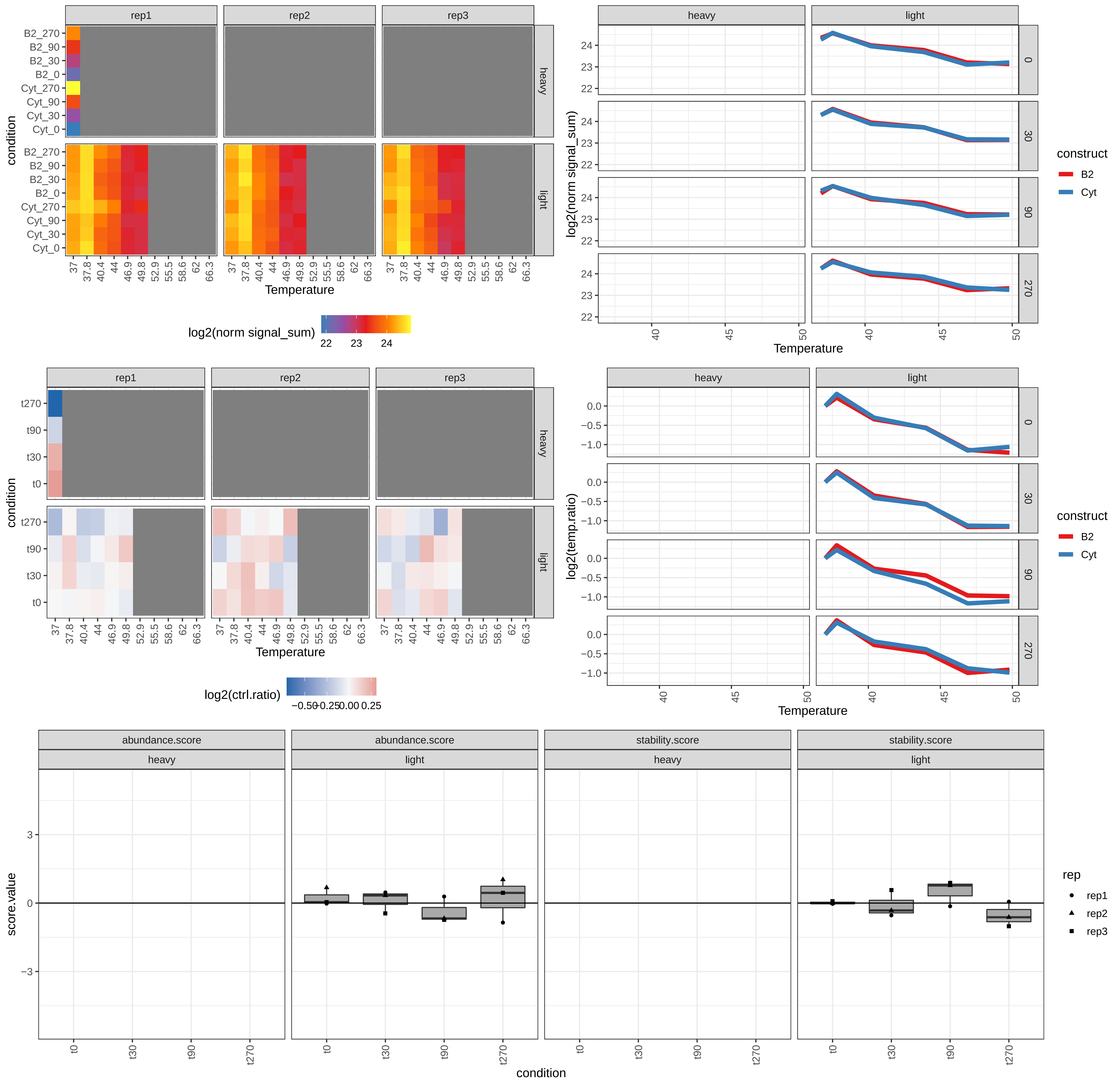

P43132 − COMPASS component BRE2 OS=Saccharomyces cerevisiae (strain ATCC 204508 / S288c) GN=BRE2 PE=1 SV=2

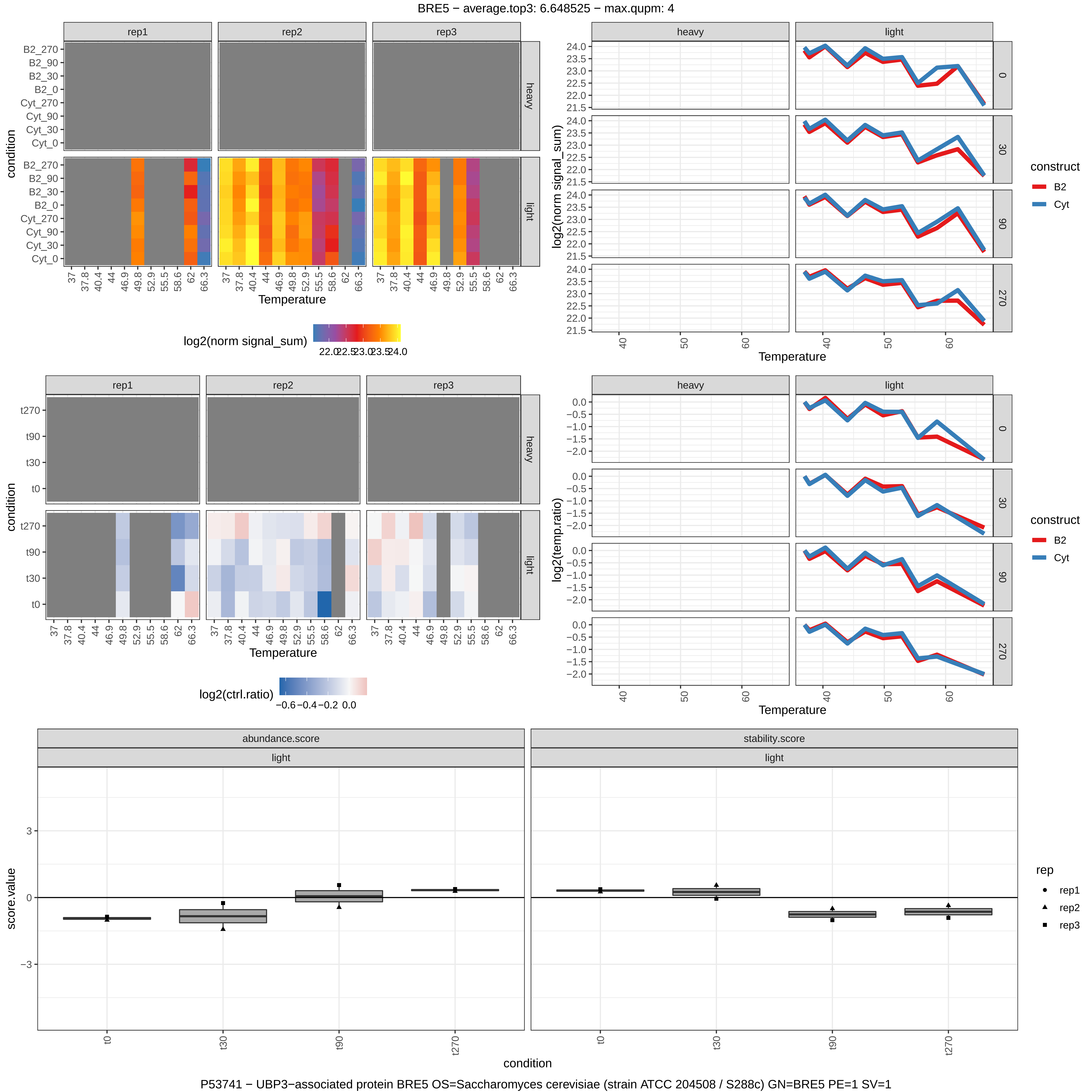

BRE5 − average.top3: 6.648525 − max.qupm: 4
rep1
rep2
rep3
heavy
light
condition
Temperature
log2(norm signal_sum)
log2(ctrl.ratio)
log2(temp.ratio)
construct
B2
Cyt
abundance.score
stability.score
score.value
rep
rep1
rep2
rep3
P53741 − UBP3−associated protein BRE5 OS=Saccharomyces cerevisiae (strain ATCC 204508 / S288c) GN=BRE5 PE=1 SV=1

BRO1 − average.top3: 7.01315727272727 − max.qupm: 15

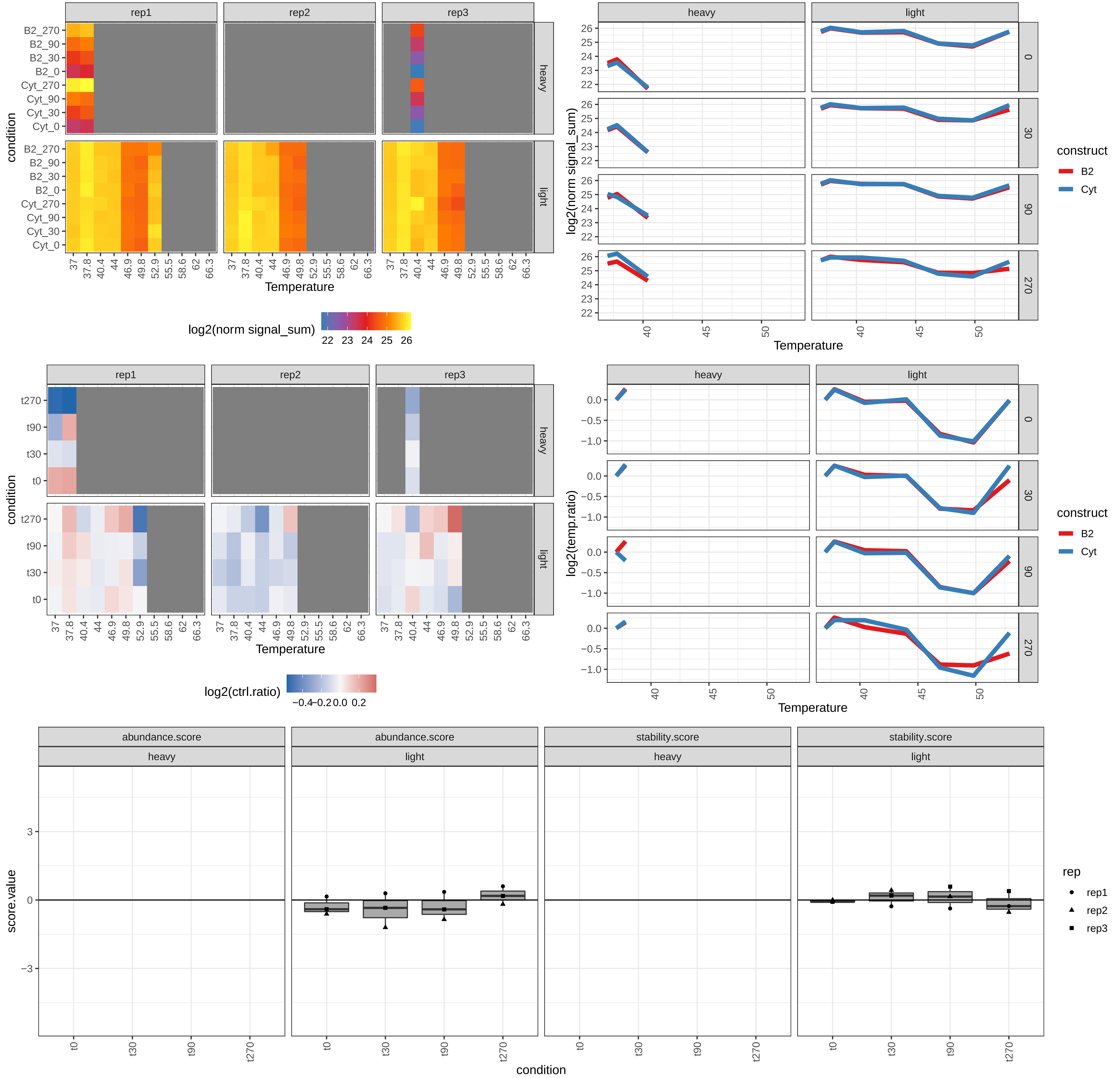

P48582 − Vacuolar−sorting protein BRO1 OS=Saccharomyces cerevisiae (strain ATCC 204508 / S288c) GN=BRO1 PE=1 SV=2

BRR2 − average.top3: 6.4278096969697 − max.qupm: 16

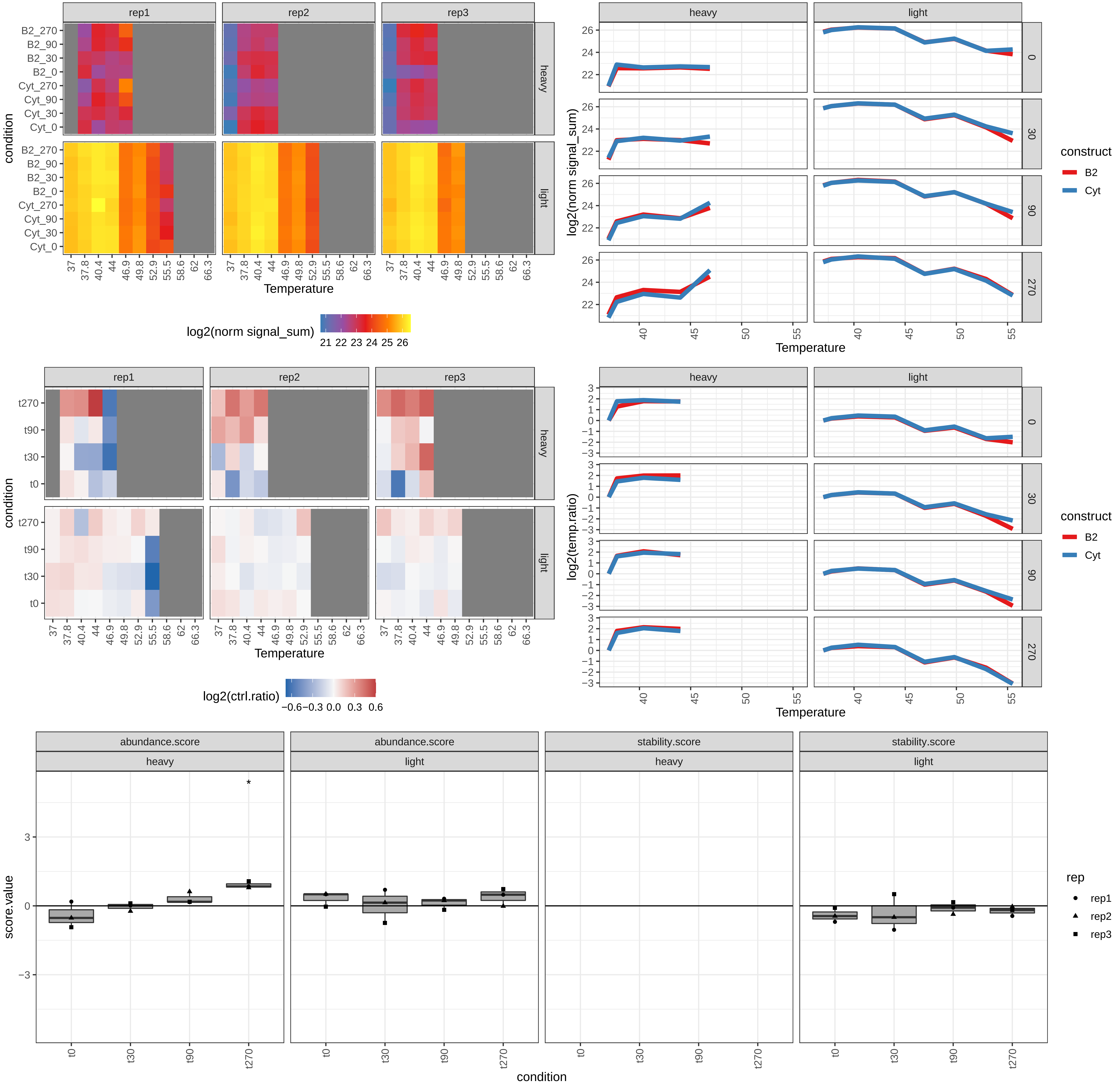

P32639 − Pre−mRNA−splicing helicase BRR2 OS=Saccharomyces cerevisiae (strain ATCC 204508 / S288c) GN=BRR2 PE=1 SV=2

BRX1 − average.top3: 7.44330476190476 − max.qupm: 8

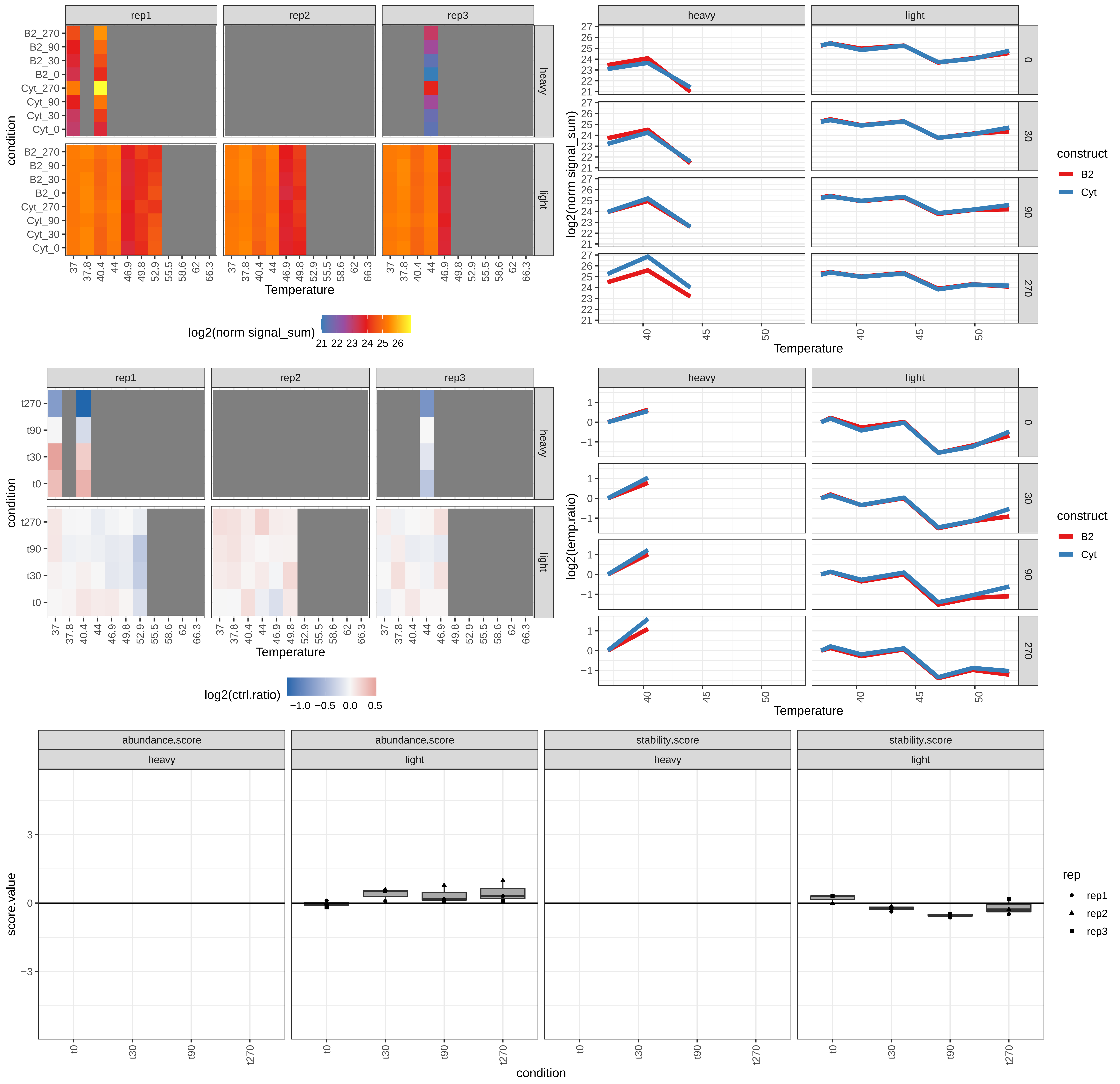

Q08235 − Ribosome biogenesis protein BRX1 OS=Saccharomyces cerevisiae (strain ATCC 204508 / S288c) GN=BRX1 PE=1 SV=2

BUB3 − average.top3: 6.64777071428571 − max.qupm: 6

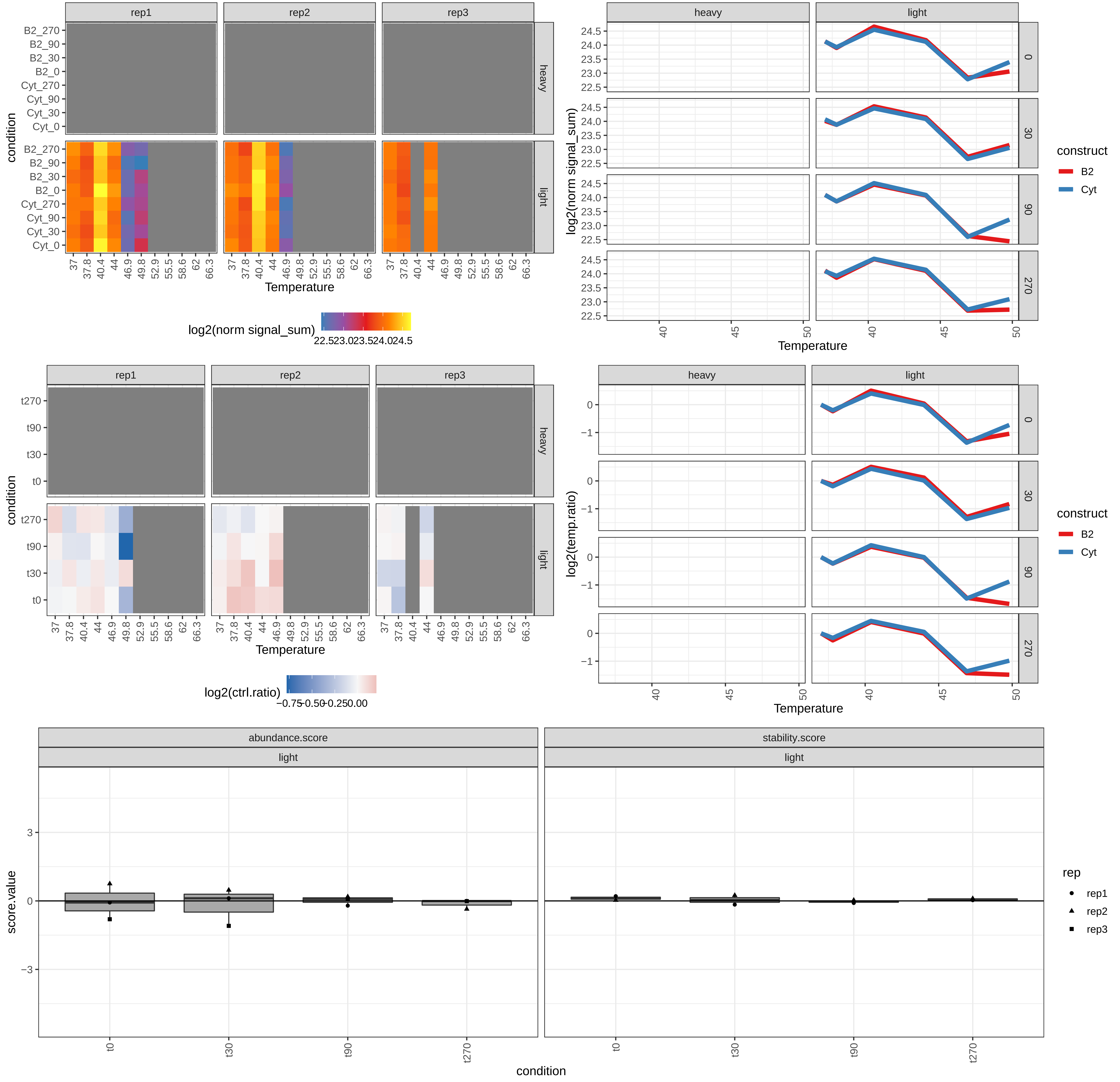

P26449 − Cell cycle arrest protein BUB3 OS=Saccharomyces cerevisiae (strain ATCC 204508 / S288c) GN=BUB3 PE=1 SV=1

BUD16 − average.top3: 6.425603 − max.qupm: 2

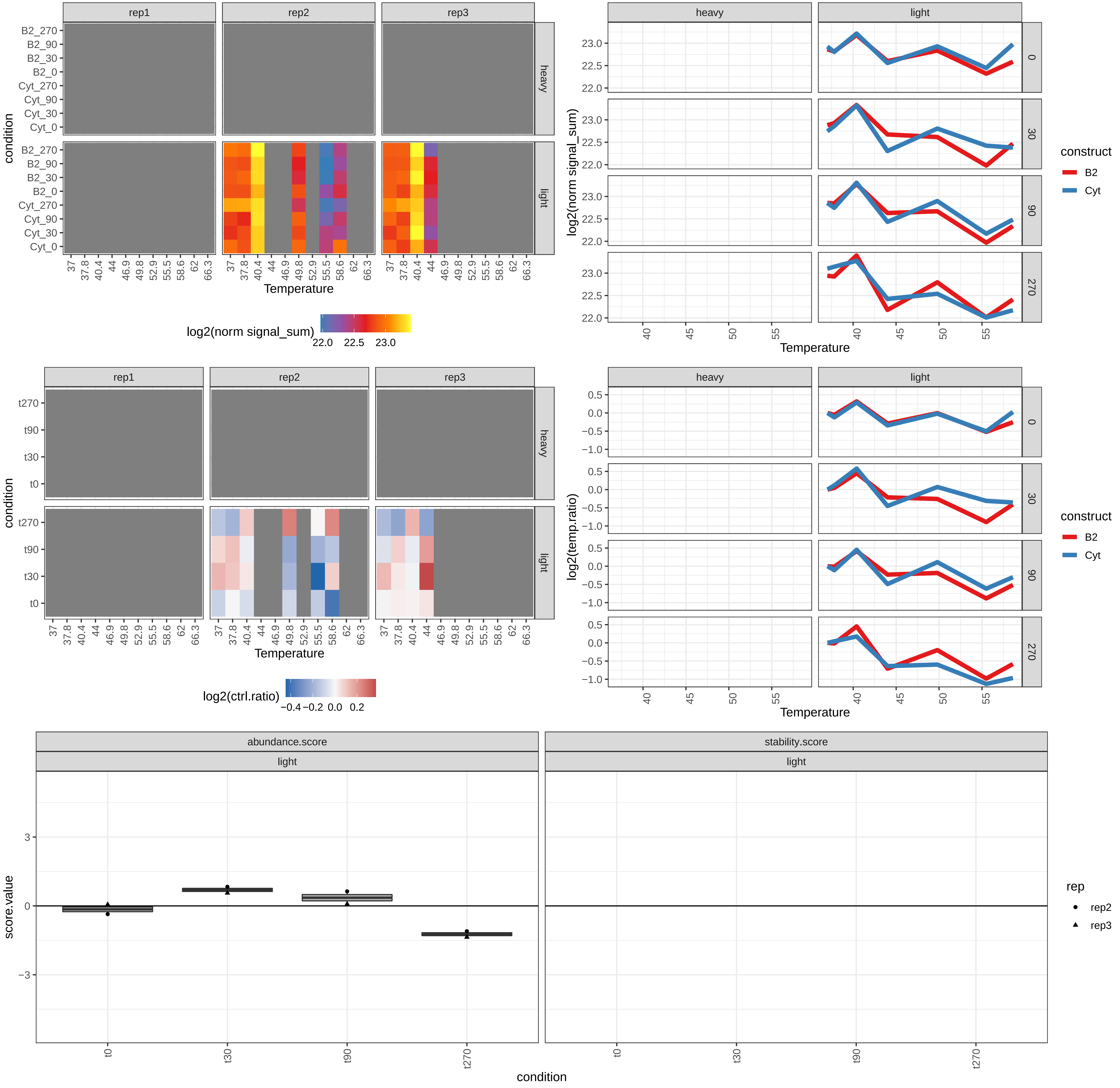

P39988 − Putative pyridoxal kinase BUD16 OS=Saccharomyces cerevisiae (strain ATCC 204508 / S288c) GN=BUD16 PE=1 SV=1

BUD17 − average.top3: 6.53991473684211 − max.qupm: 5

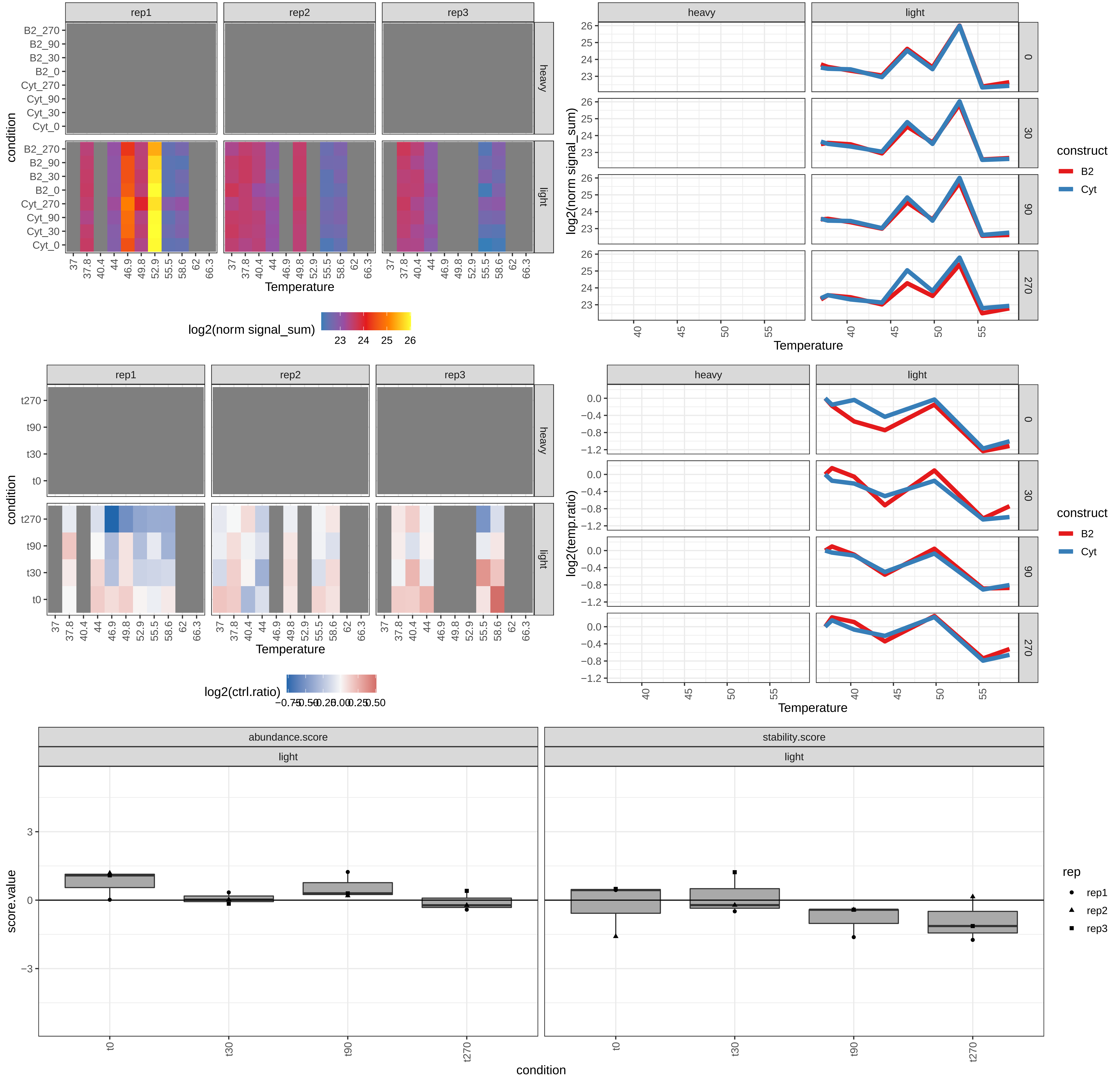

P53727 − Putative pyridoxal kinase BUD17 OS=Saccharomyces cerevisiae (strain ATCC 204508 / S288c) GN=BUD17 PE=1 SV=1

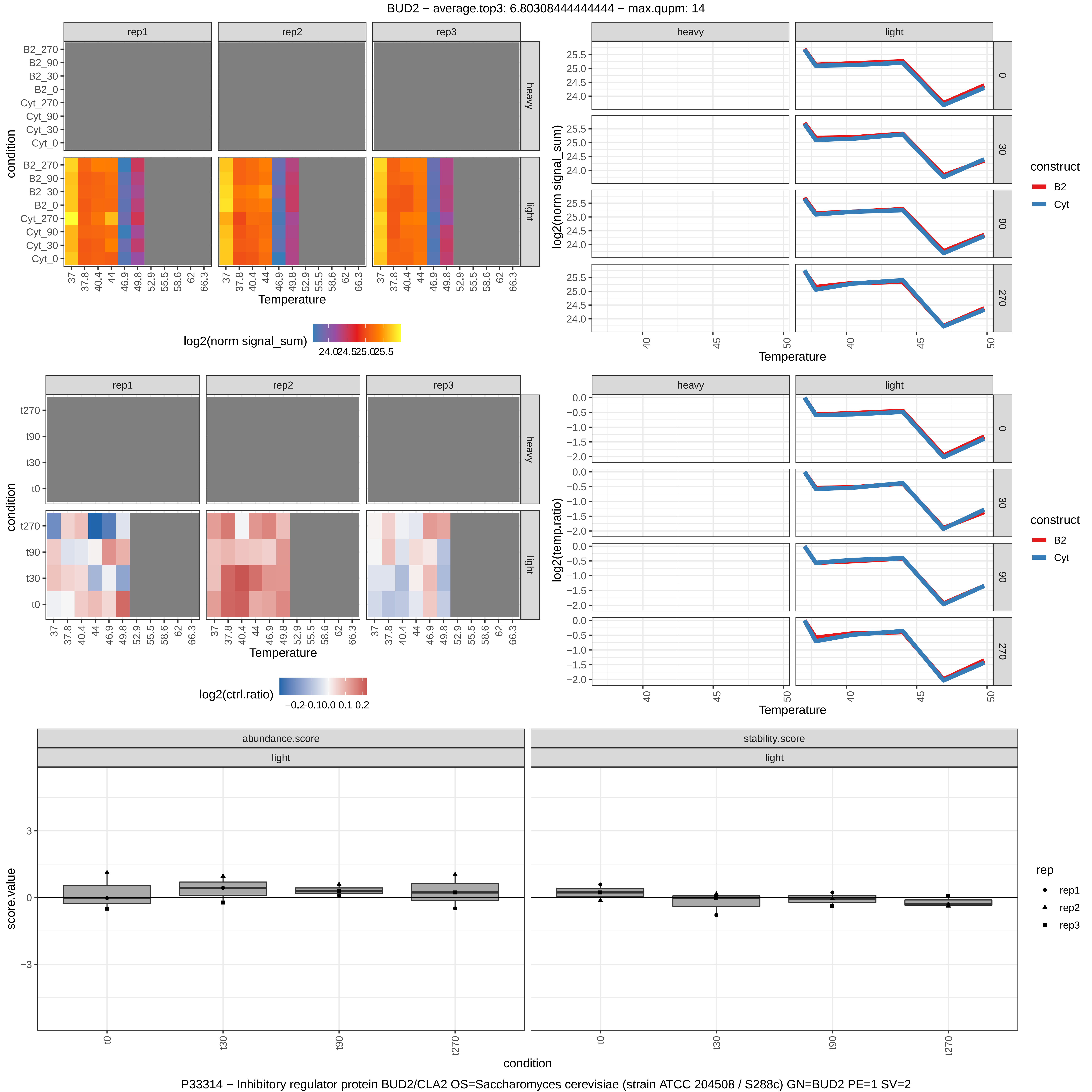

BUD2 − average.top3: 6.80308444444444 − max.qupm: 14
rep1
rep2
rep3
heavy
light
condition
Temperature
log2(norm signal_sum)
log2(ctrl.ratio)
log2(temp.ratio)
construct
B2
Cyt
abundance.score
stability.score
score.value
rep
rep1
rep2
rep3
P33314 − Inhibitory regulator protein BUD2/CLA2 OS=Saccharomyces cerevisiae (strain ATCC 204508 / S288c) GN=BUD2 PE=1 SV=2

BUD22 − average.top3: 6.671754375 − max.qupm: 5

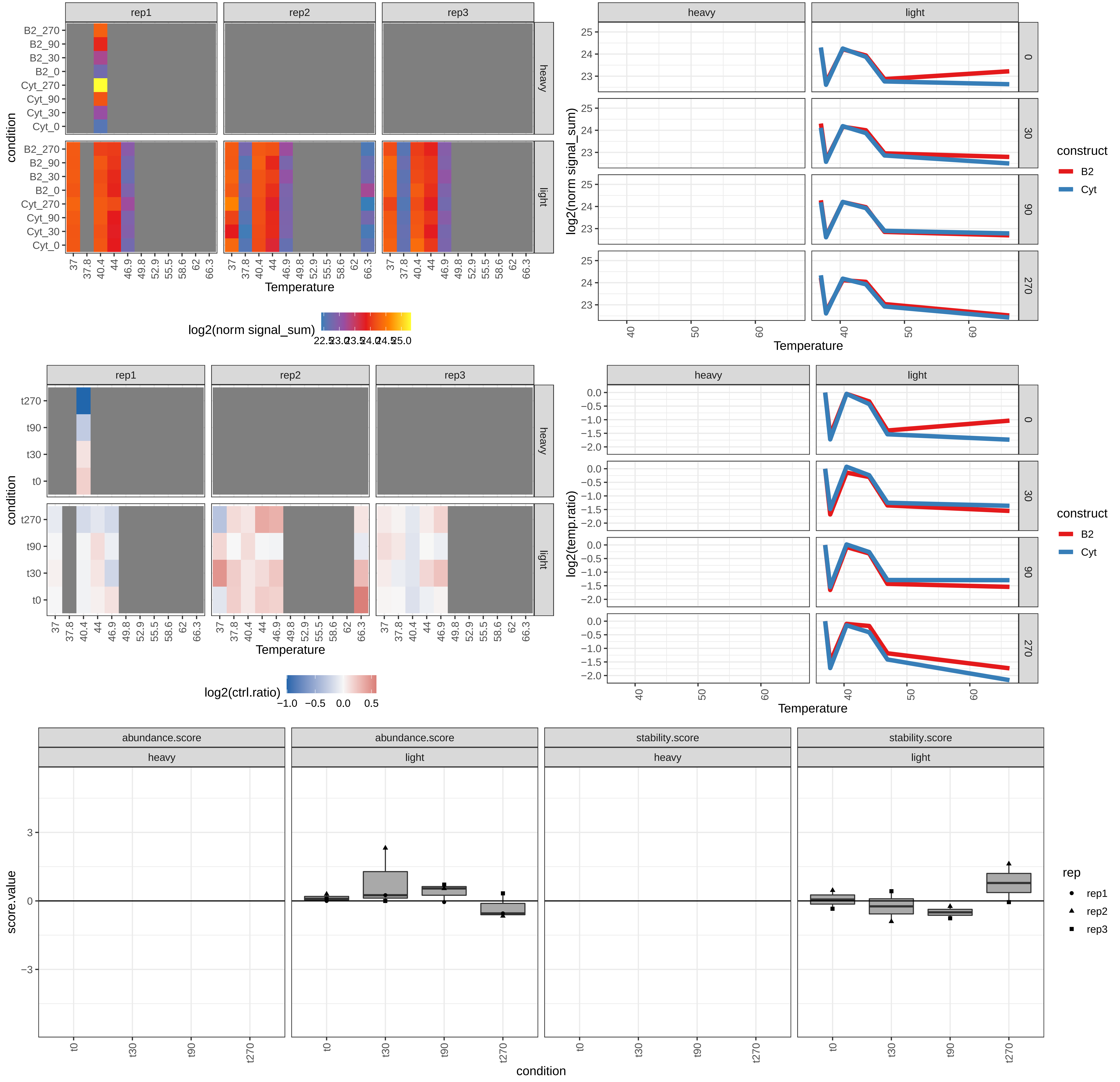

Q04347 − Bud site selection protein 22 OS=Saccharomyces cerevisiae (strain ATCC 204508 / S288c) GN=BUD22 PE=1 SV=1

BUD3 − average.top3: 6.50921277777778 − max.qupm: 6

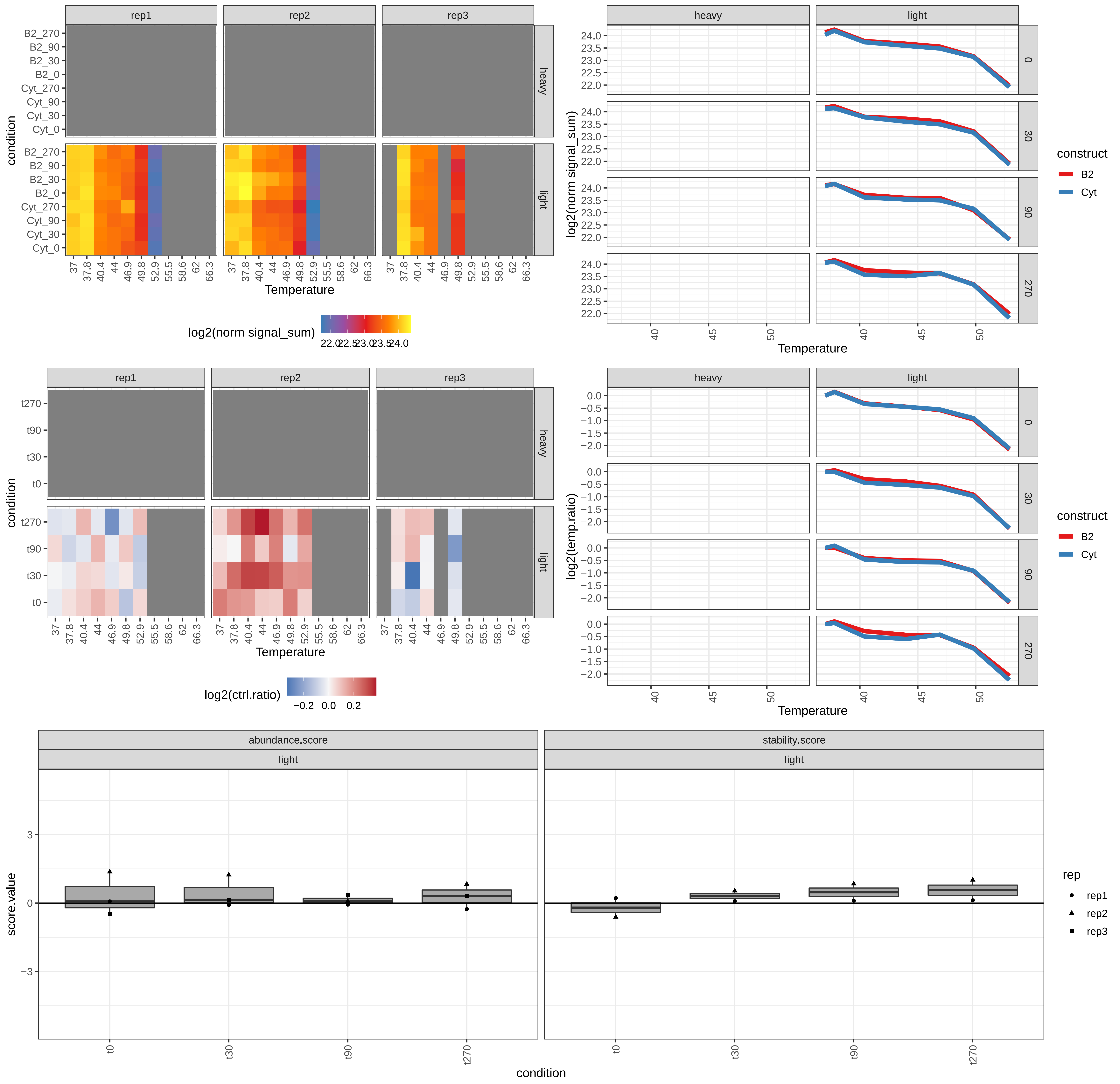

P25558 − Bud site selection protein 3 OS=Saccharomyces cerevisiae (strain ATCC 204508 / S288c) GN=BUD3 PE=1 SV=2

BUD31 − average.top3: 6.86959384615385 − max.qupm: 2

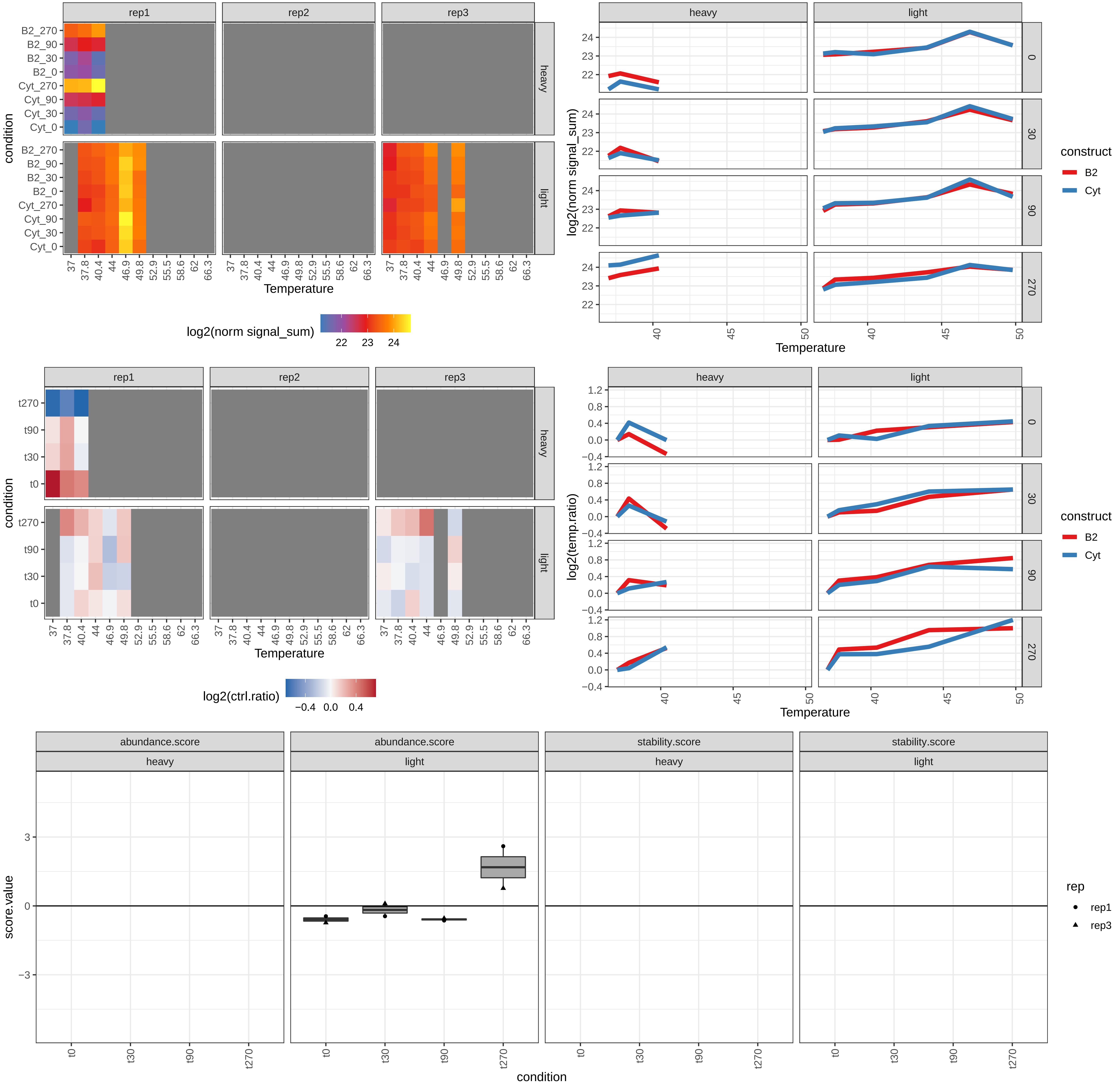

P25337 − Pre−mRNA−splicing factor BUD31 OS=Saccharomyces cerevisiae (strain ATCC 204508 / S288c) GN=BUD31 PE=1 SV=1

BUD4 − average.top3: 6.04808833333333 − max.qupm: 3

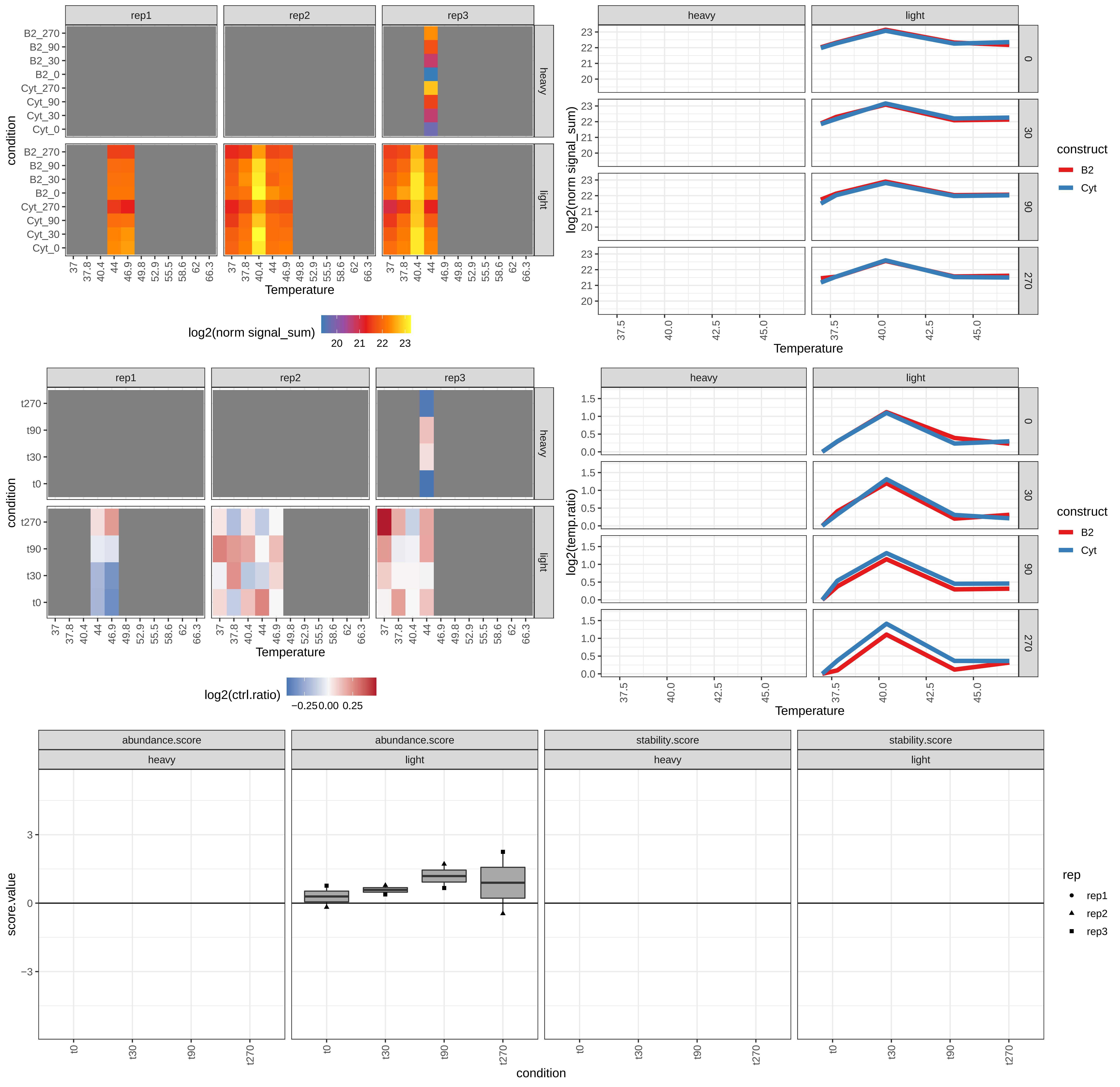

P47136 − Bud site selection protein 4 OS=Saccharomyces cerevisiae (strain ATCC 204508 / S288c) GN=BUD4 PE=1 SV=2

BUD6 − average.top3: 6.41261555555556 − max.qupm: 8

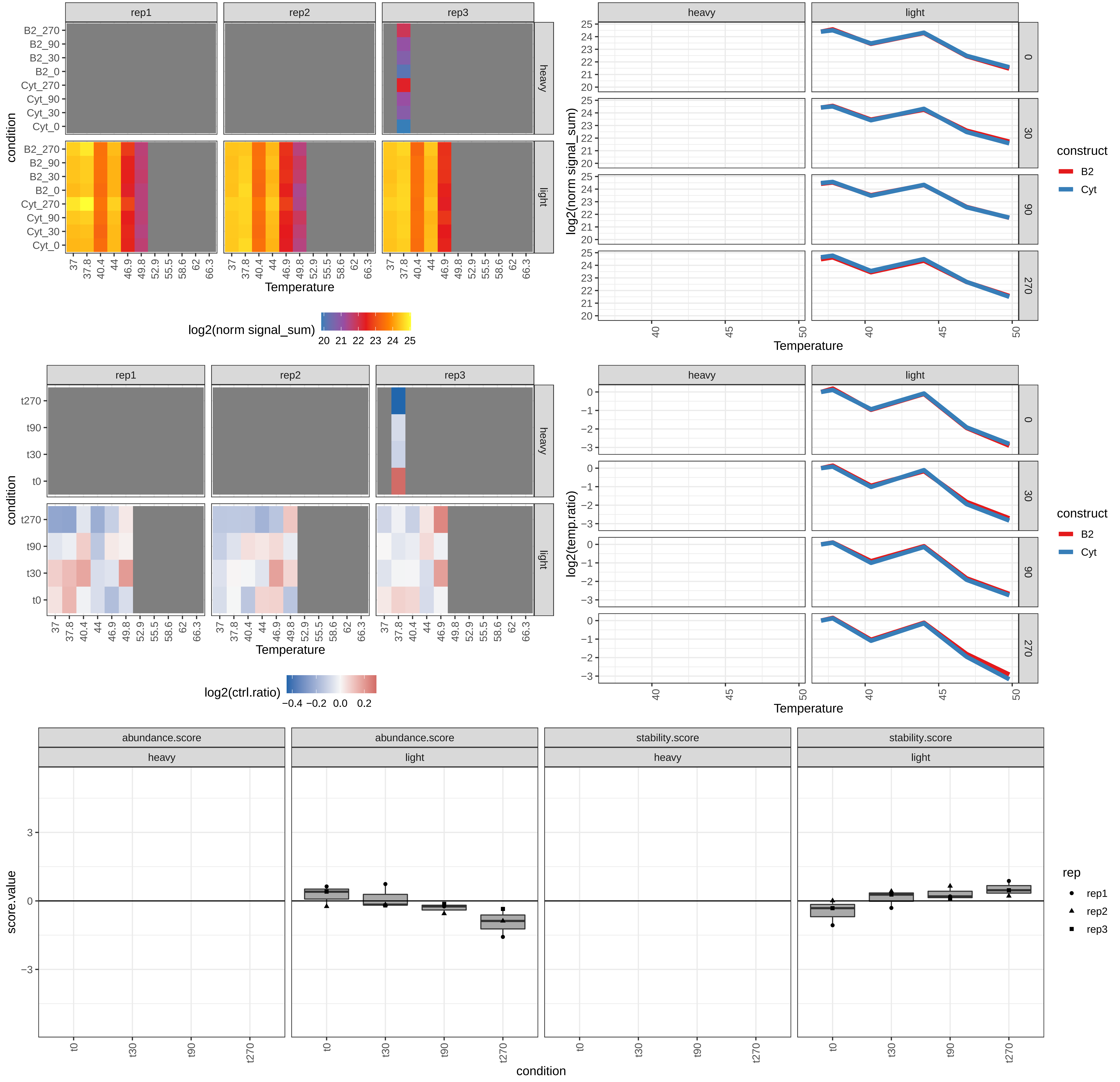

P41697 − Bud site selection protein 6 OS=Saccharomyces cerevisiae (strain ATCC 204508 / S288c) GN=BUD6 PE=1 SV=1

BUG1 − average.top3: 6.93311 − max.qupm: 4

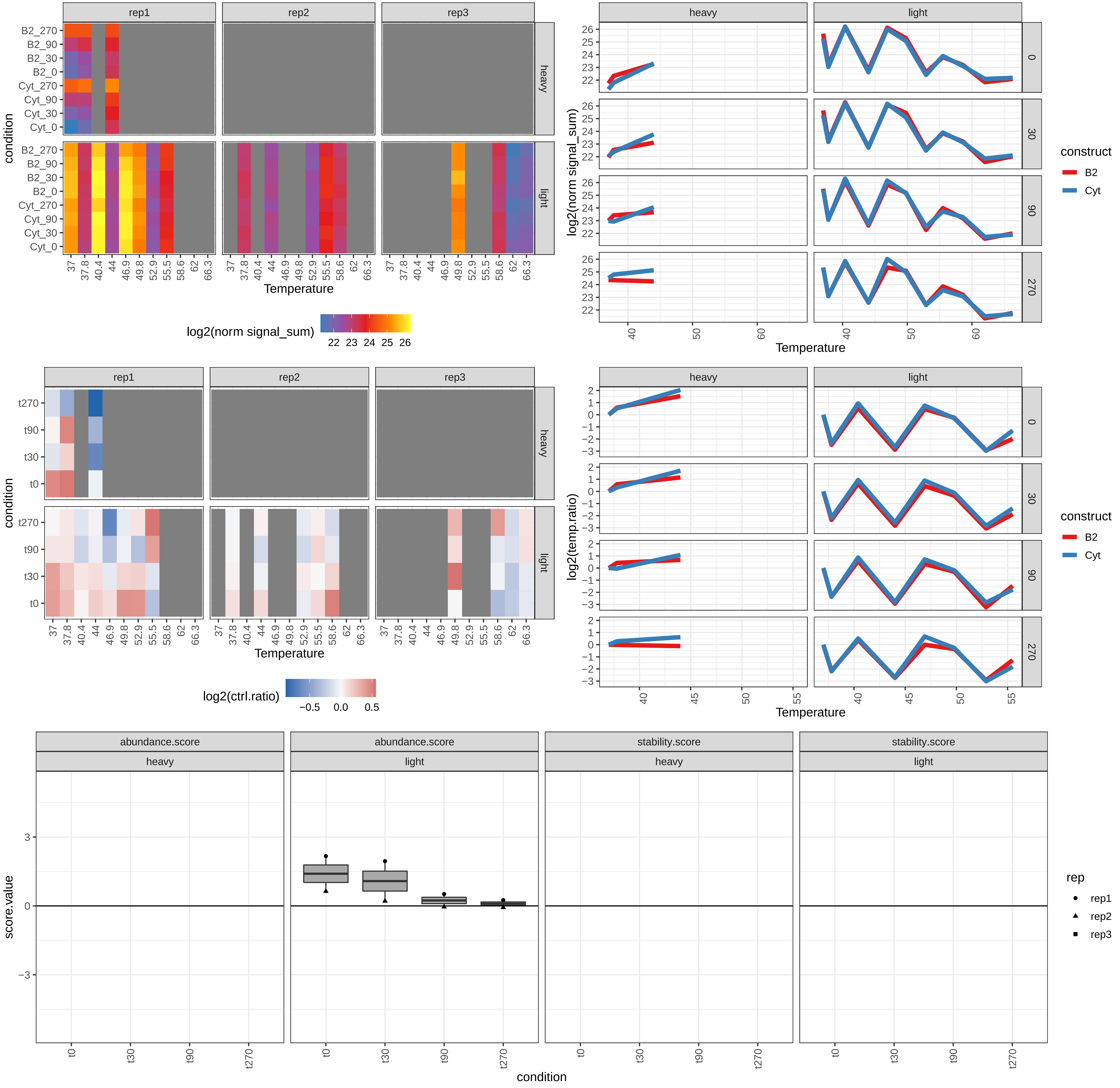

Q12191 − Binder of USO1 and GRH1 protein 1 OS=Saccharomyces cerevisiae (strain ATCC 204508 / S288c) GN=BUG1 PE=1 SV=1

BUL1 − average.top3: 6.44556111111111 − max.qupm: 5

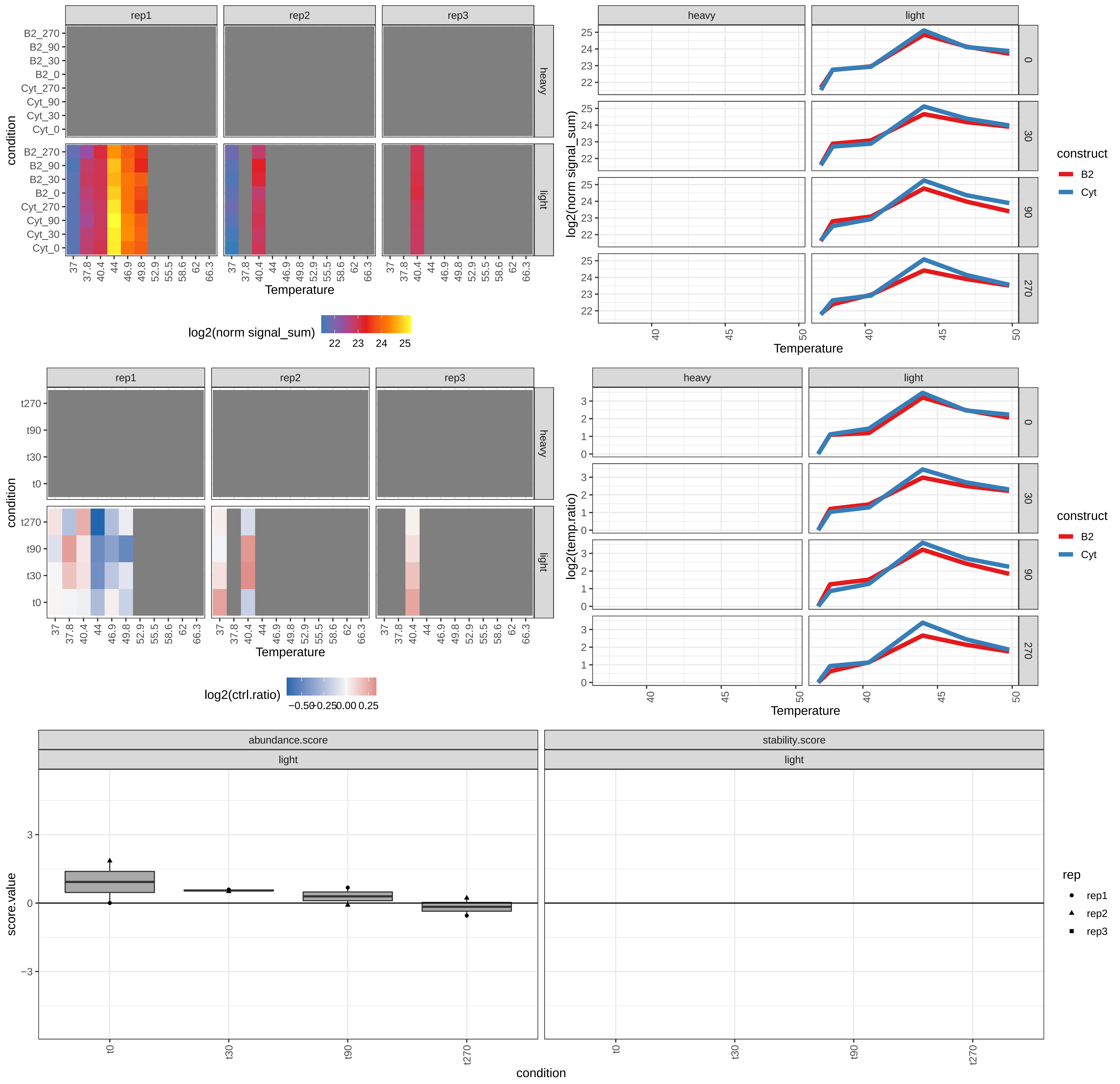

P48524 − Ubiquitin ligase−binding protein BUL1 OS=Saccharomyces cerevisiae (strain ATCC 204508 / S288c) GN=BUL1 PE=1 SV=1

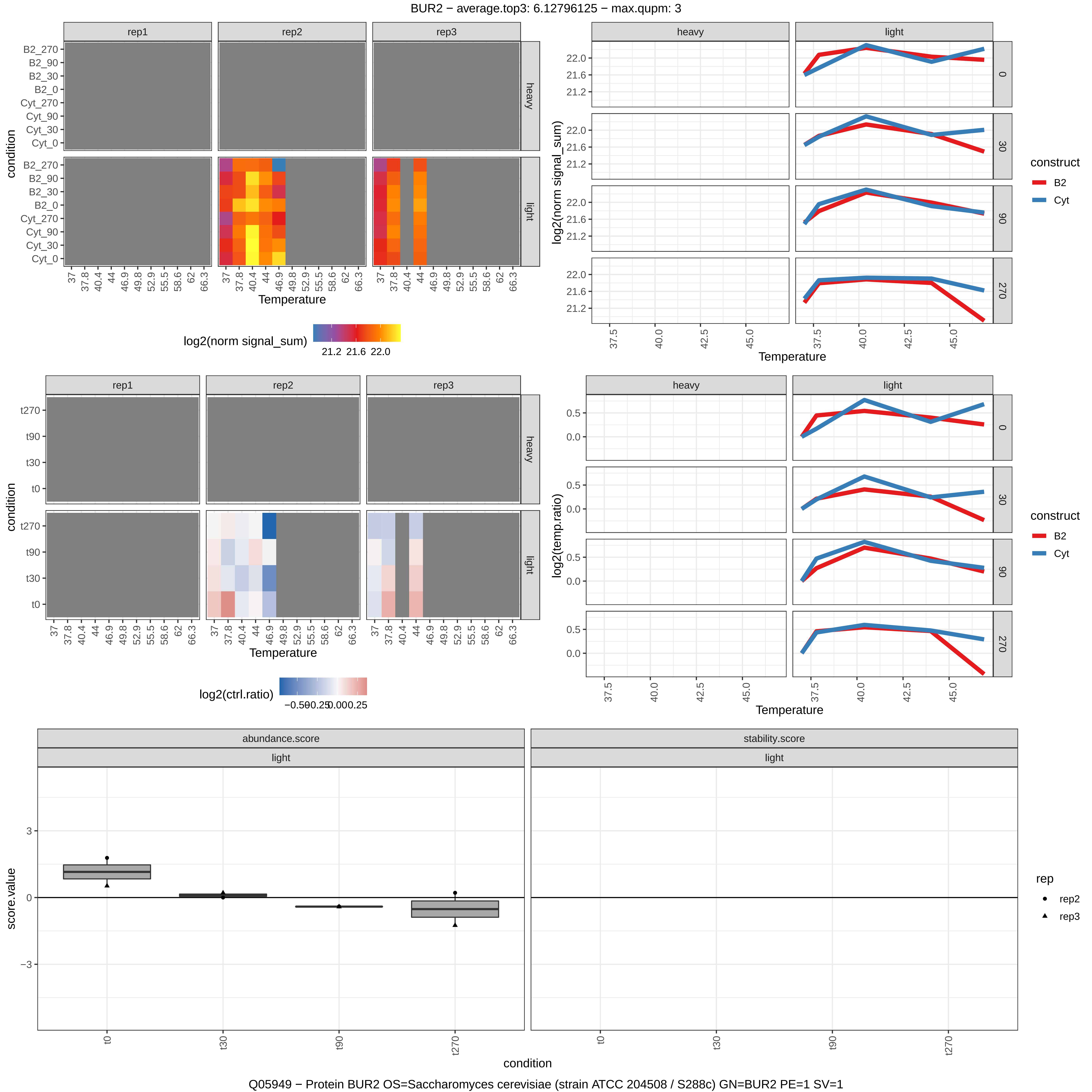
BUR2 − average.top3: 6.12796125 − max.qupm: 3
rep1
rep2
rep3
heavy
light
condition
Temperature
log2(norm signal_sum)
construct
B2
Cyt
log2(ctrl.ratio)
log2(temp.ratio)
abundance.score
stability.score
score.value
rep
rep2
rep3
Q05949 − Protein BUR2 OS=Saccharomyces cerevisiae (strain ATCC 204508 / S288c) GN=BUR2 PE=1 SV=1

BXI1 − average.top3: 6.98839055555556 − max.qupm: 3

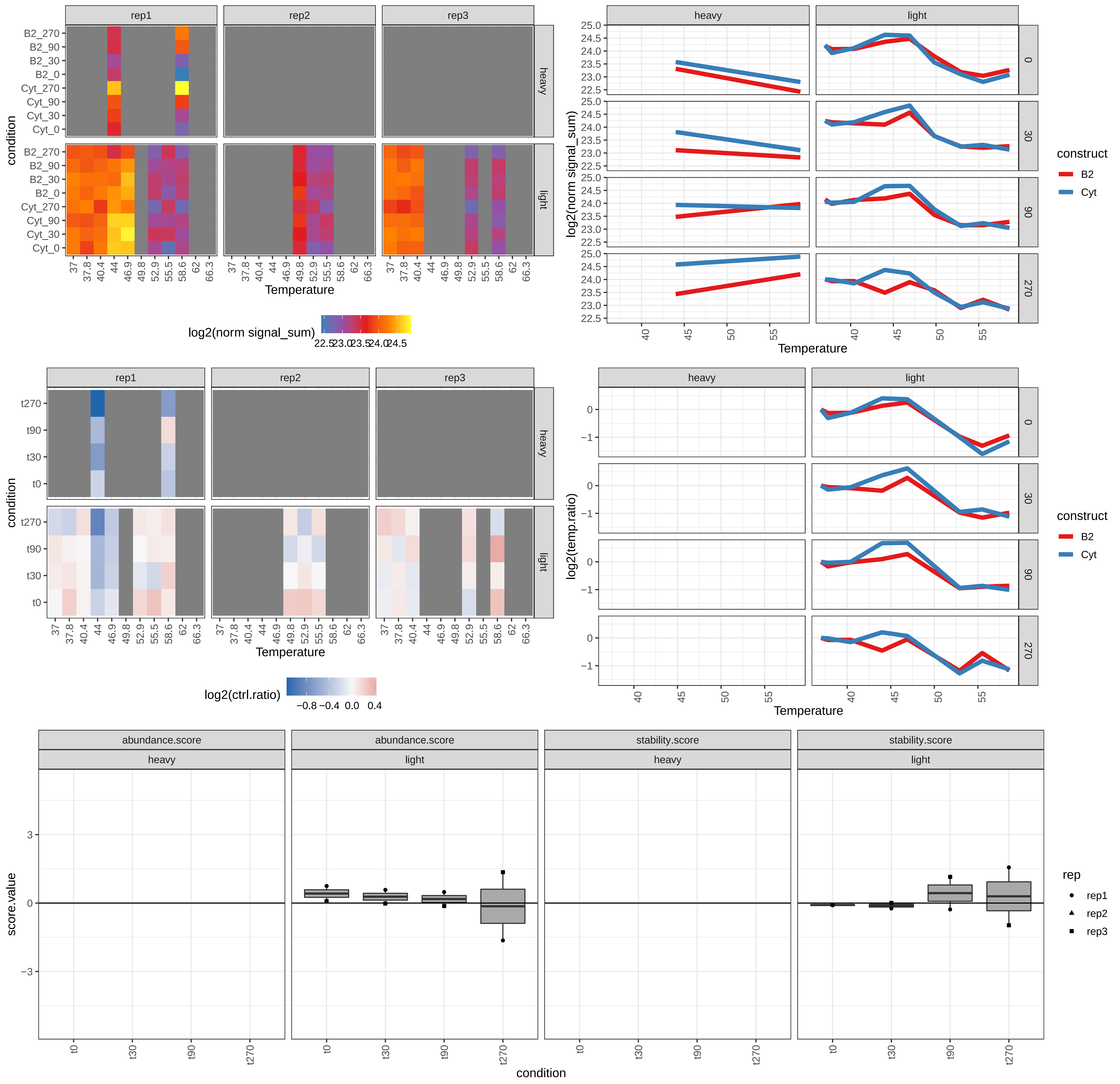

P48558 − Bax inhibitor 1 OS=Saccharomyces cerevisiae (strain ATCC 204508 / S288c) GN=BXI1 PE=1 SV=1

BYE1 − average.top3: 6.55031416666667 − max.qupm: 6

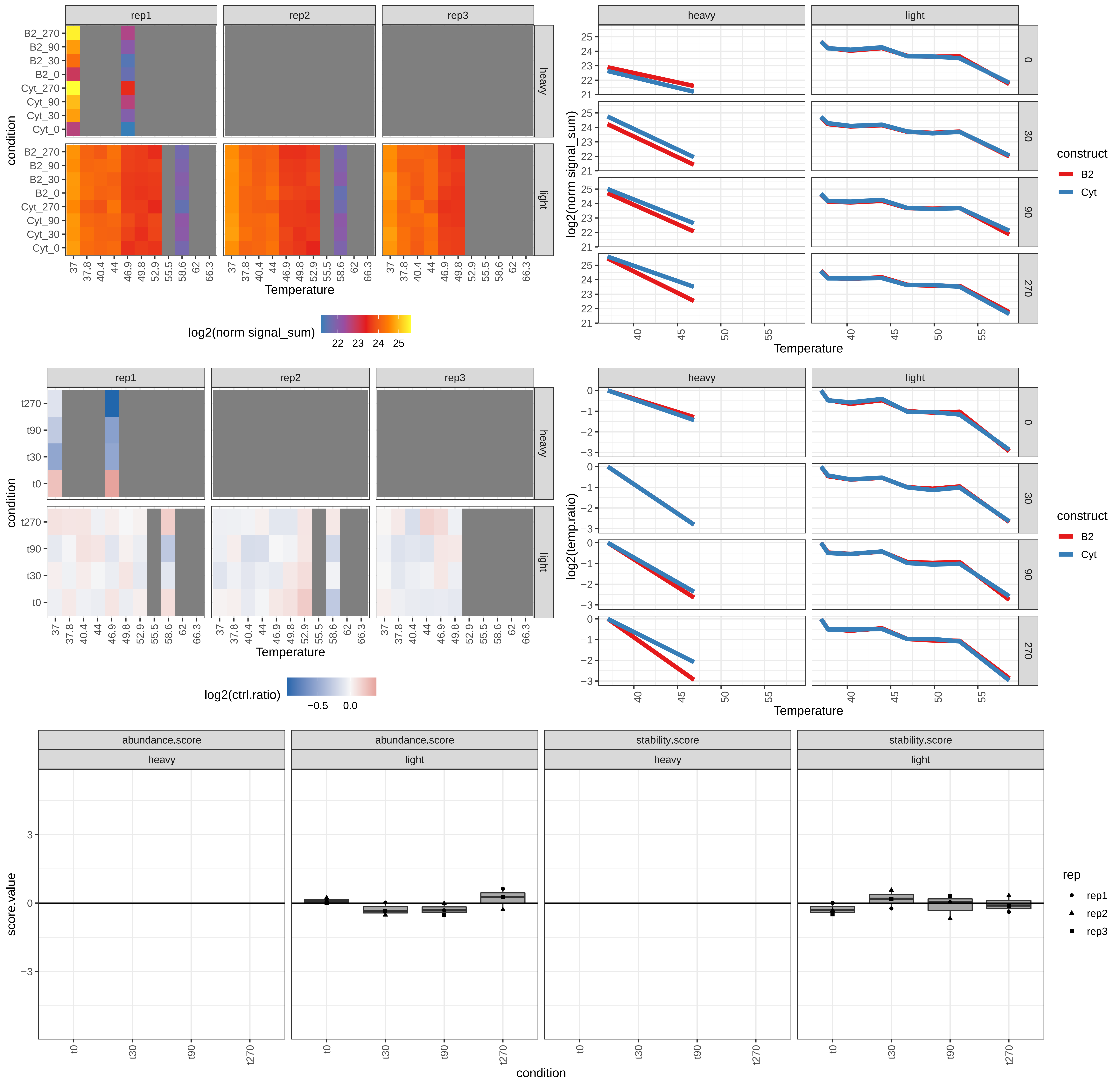

P36106 − Transcription factor BYE1 OS=Saccharomyces cerevisiae (strain ATCC 204508 / S288c) GN=BYE1 PE=1 SV=1

BZZ1 − average.top3: 6.64028947368421 − max.qupm: 10

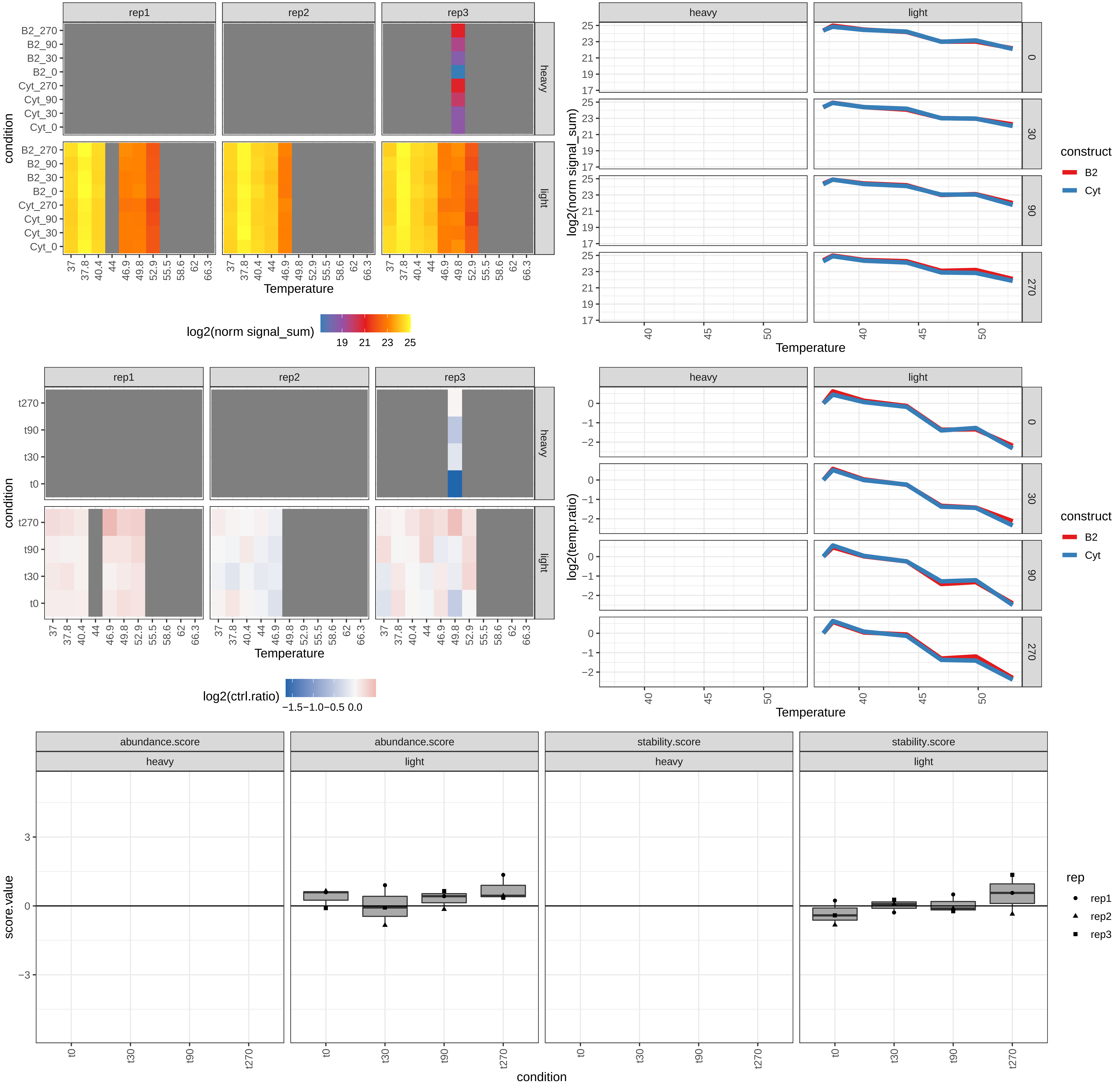

P38822 − Protein BZZ1 OS=Saccharomyces cerevisiae (strain ATCC 204508 / S288c) GN=BZZ1 PE=1 SV=1

CAB1 − average.top3: 6.71023111111111 − max.qupm: 5

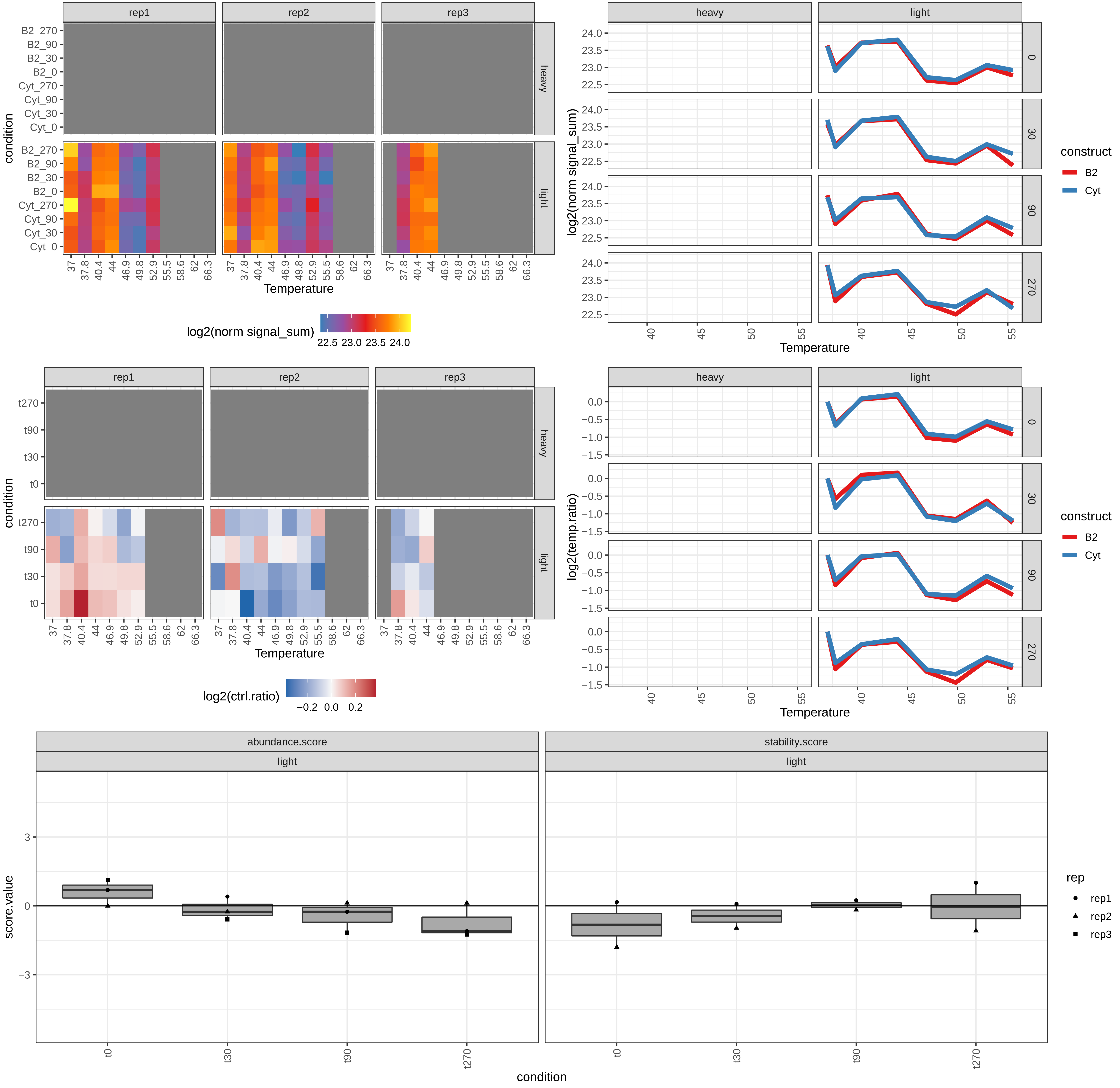

Q04430 − Pantothenate kinase CAB1 OS=Saccharomyces cerevisiae (strain ATCC 204508 / S288c) GN=CAB1 PE=1 SV=1

CAB2 − average.top3: 7.10706688888889 − max.qupm: 9

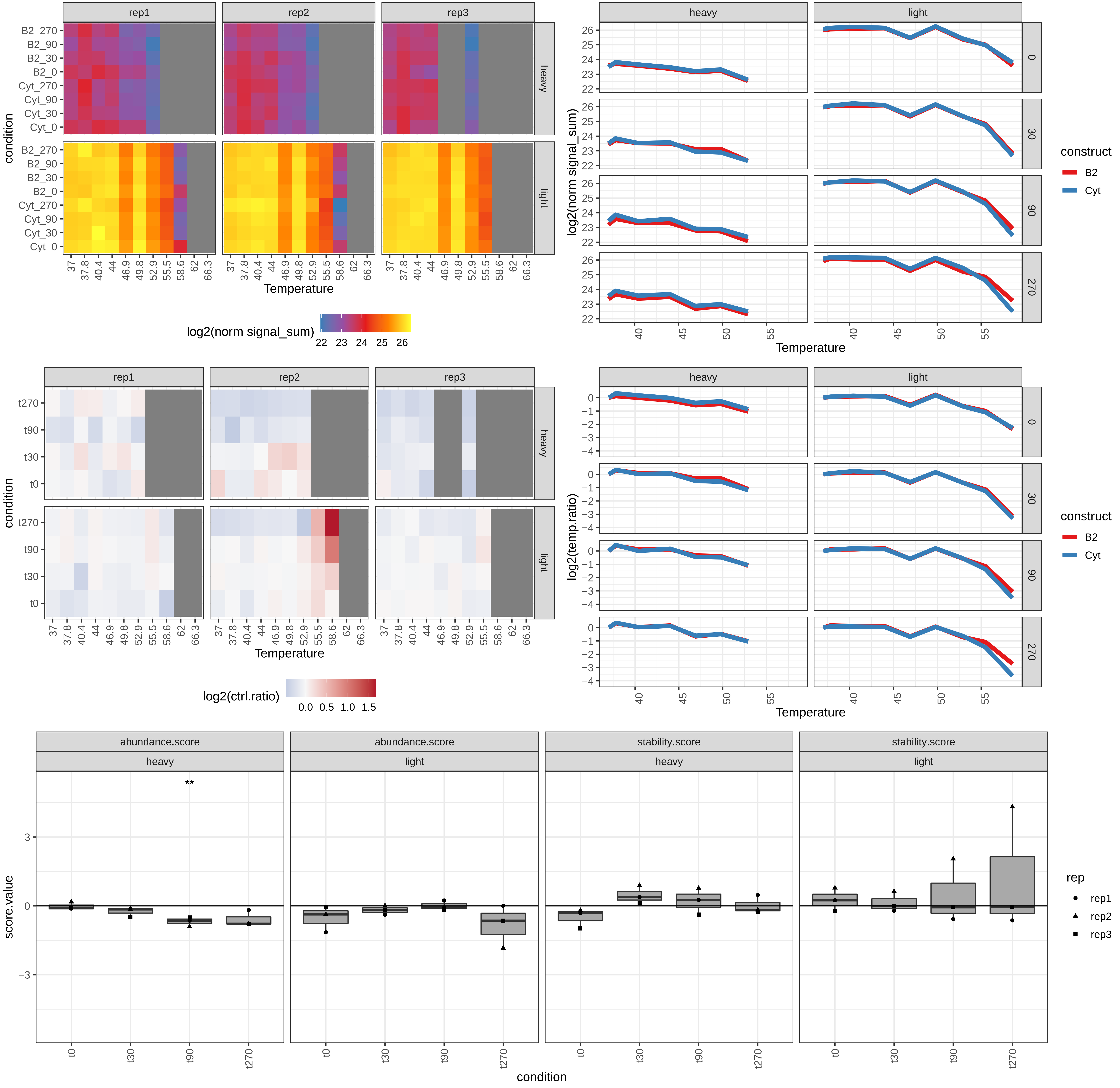

P40506 − Phosphopantothenate−−cysteine ligase CAB2 OS=Saccharomyces cerevisiae (strain ATCC 204508 / S288c) GN=CAB2 PE=1 SV=2

CAB3 − average.top3: 6.61859058823529 − max.qupm: 4

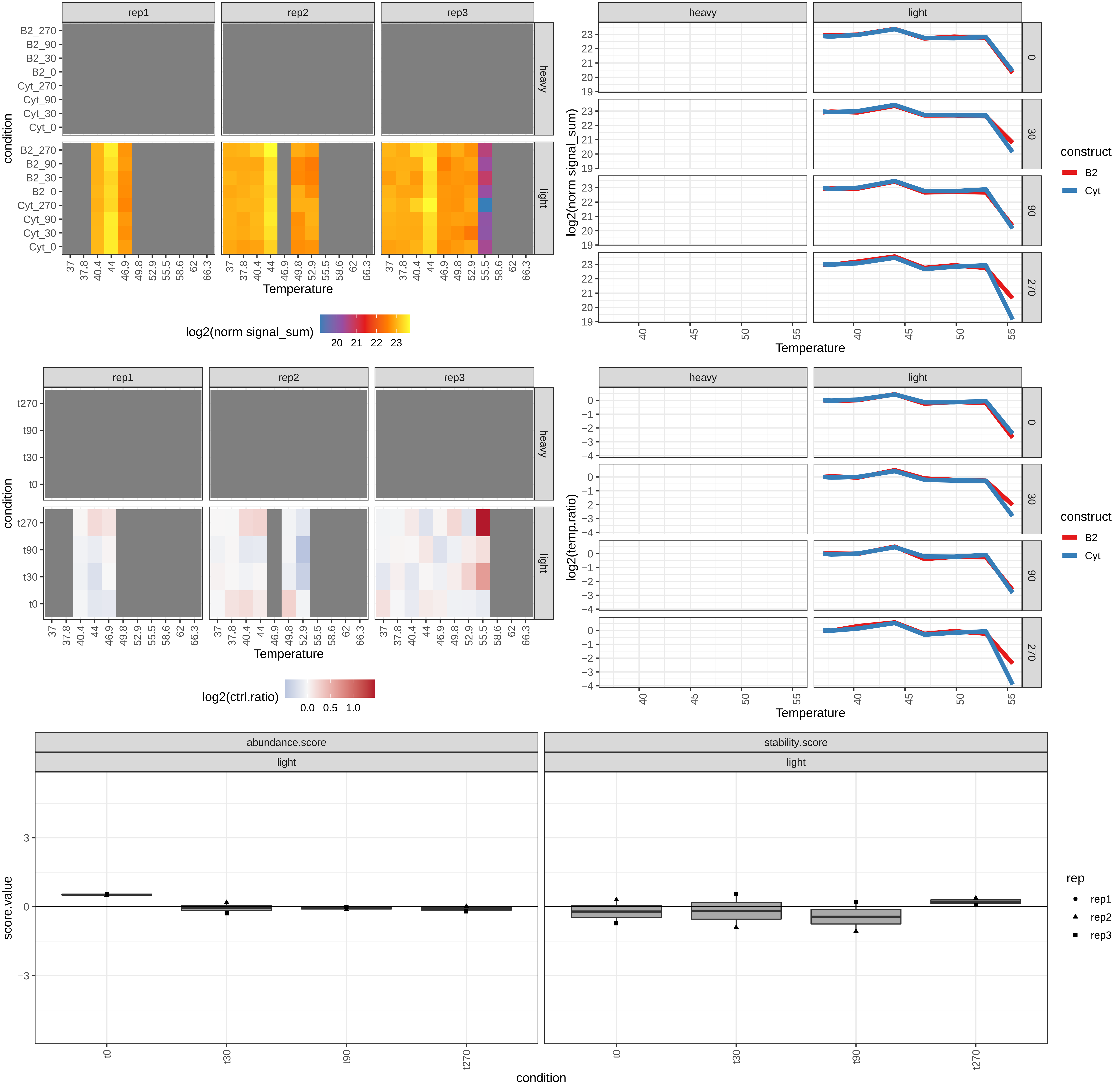

P36076 − Coenzyme A biosynthesis protein 3 OS=Saccharomyces cerevisiae (strain ATCC 204508 / S288c) GN=CAB3 PE=1 SV=1

CAB4 − average.top3: 6.79816651162791 − max.qupm: 7

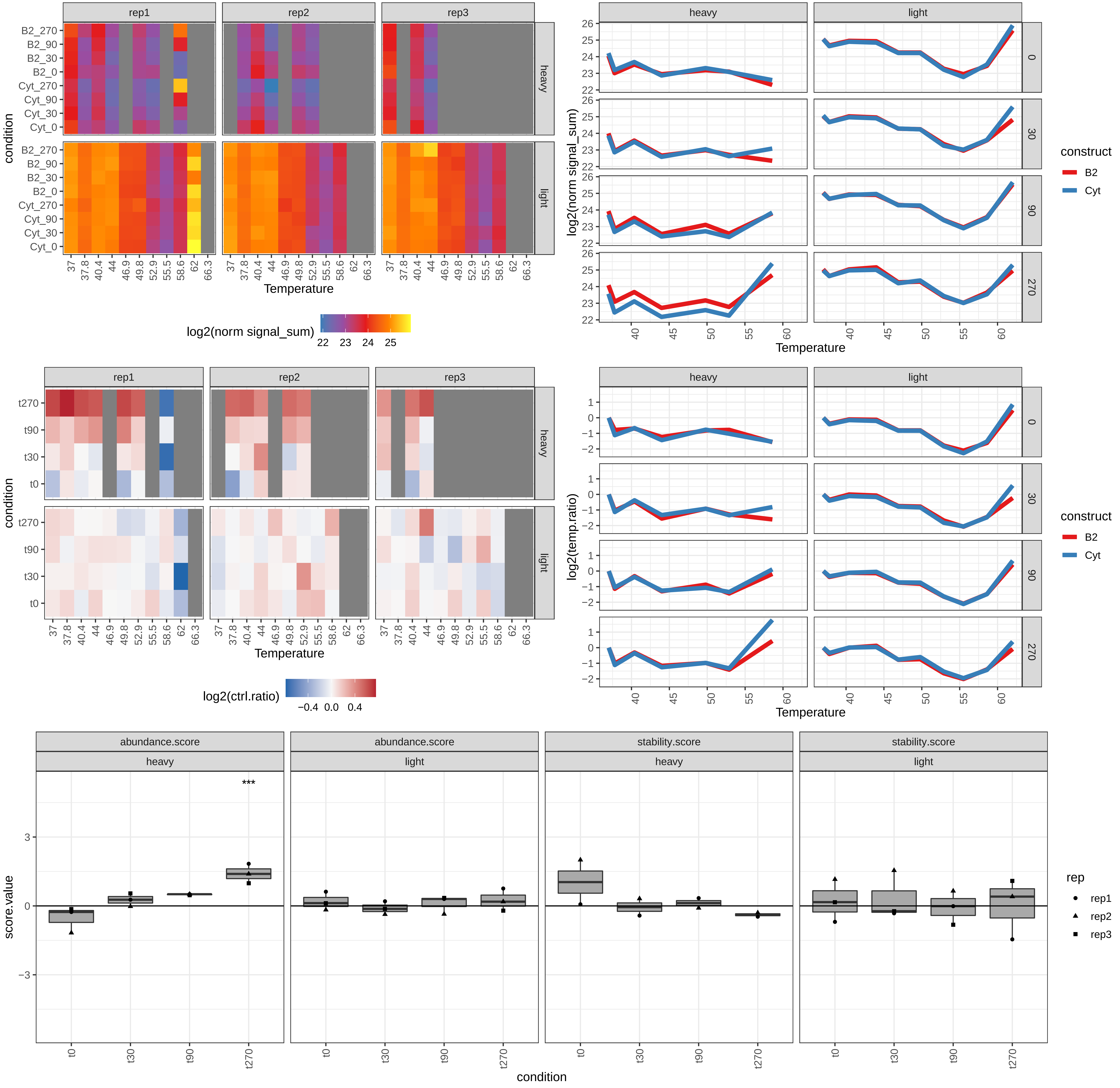

P53332 − Phosphopantetheine adenylyltransferase OS=Saccharomyces cerevisiae (strain ATCC 204508 / S288c) GN=CAB4 PE=1 SV=1

CAB5 − average.top3: 6.73098909090909 − max.qupm: 6

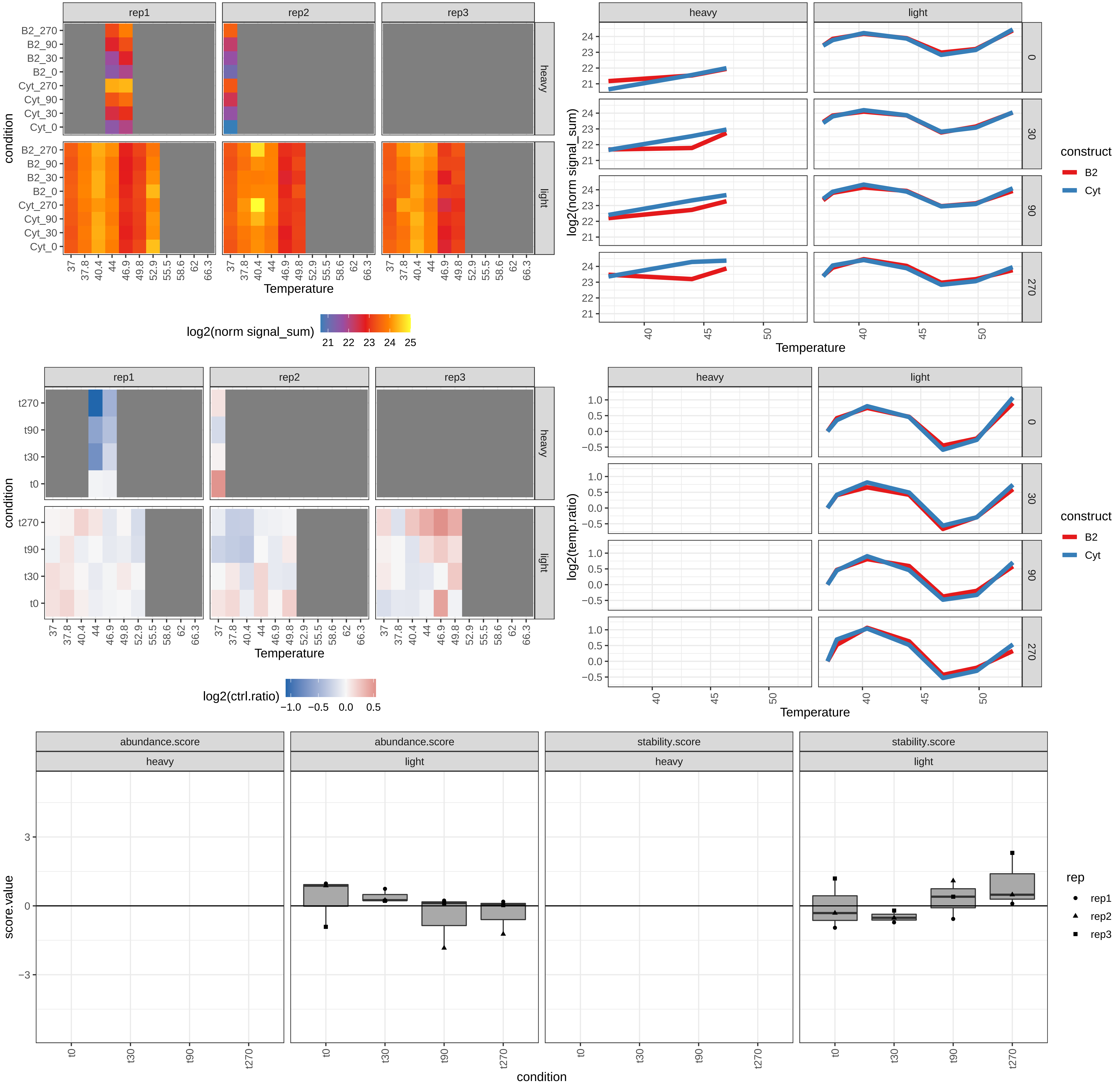

Q03941 − Dephospho−CoA kinase CAB5 OS=Saccharomyces cerevisiae (strain ATCC 204508 / S288c) GN=CAB5 PE=1 SV=1

CAC2 − average.top3: 6.55355421052632 − max.qupm: 4

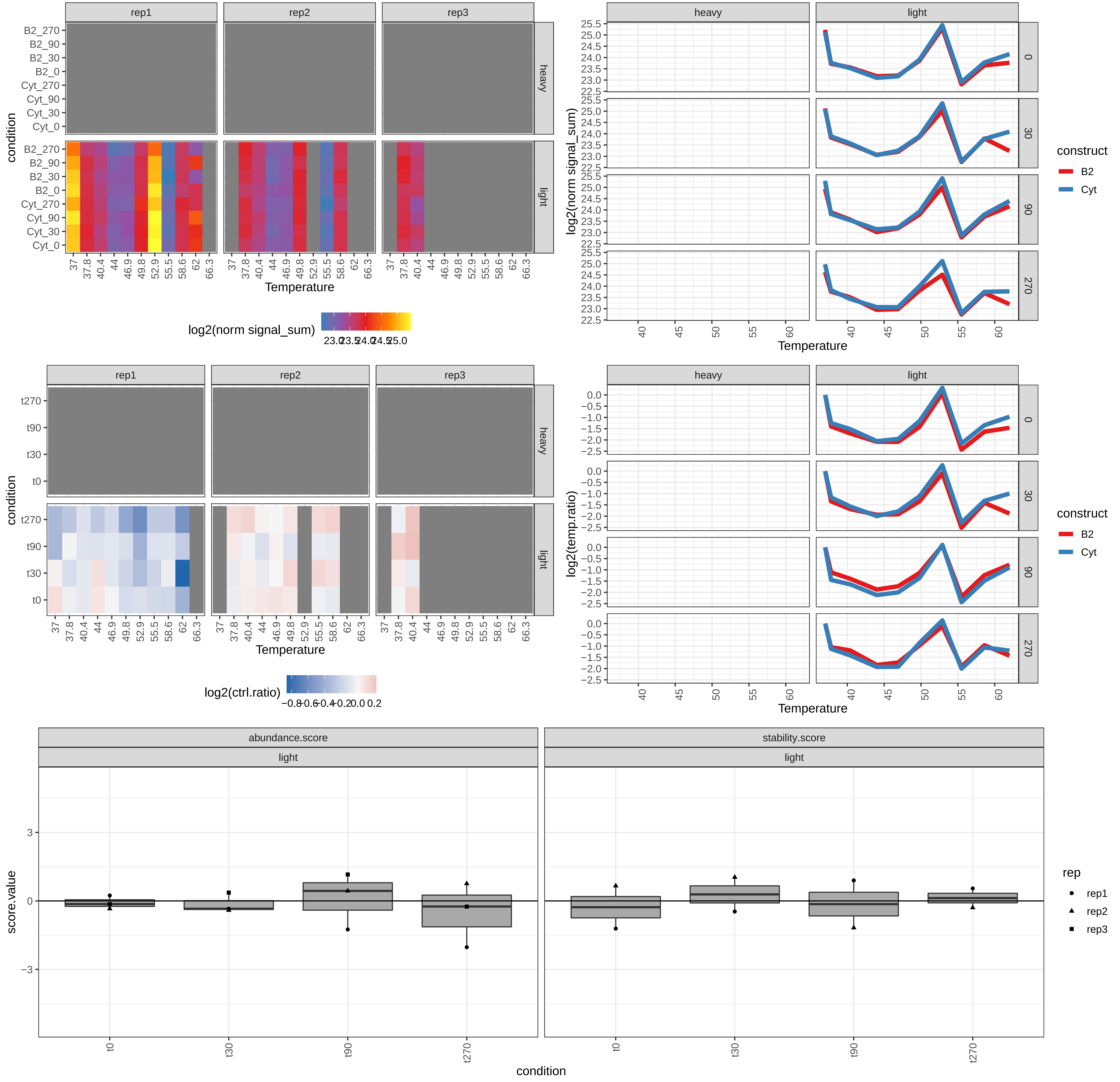

Q04199 − Chromatin assembly factor 1 subunit p60 OS=Saccharomyces cerevisiae (strain ATCC 204508 / S288c) GN=CAC2 PE=1 SV=1

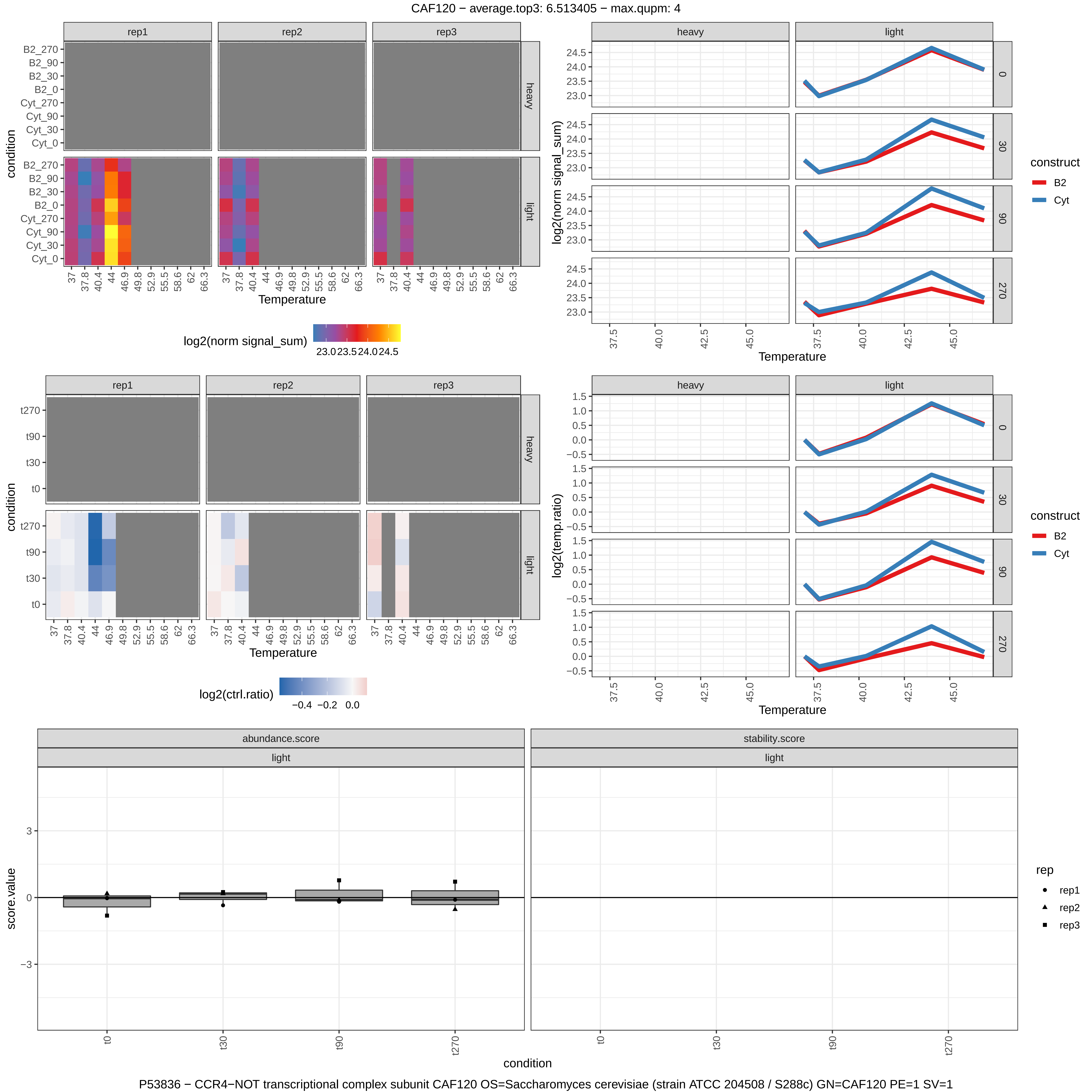
CAF120 − average.top3: 6.513405 − max.qupm: 4
rep1
rep2
rep3
heavy
light
condition
Temperature
log2(norm signal_sum)
log2(norm signal_sum)
construct
B2
Cyt
log2(ctrl.ratio)
log2(temp.ratio)
abundance.score
stability.score
score.value
rep
rep1
rep2
rep3
P53836 − CCR4−NOT transcriptional complex subunit CAF120 OS=Saccharomyces cerevisiae (strain ATCC 204508 / S288c) GN=CAF120 PE=1 SV=1

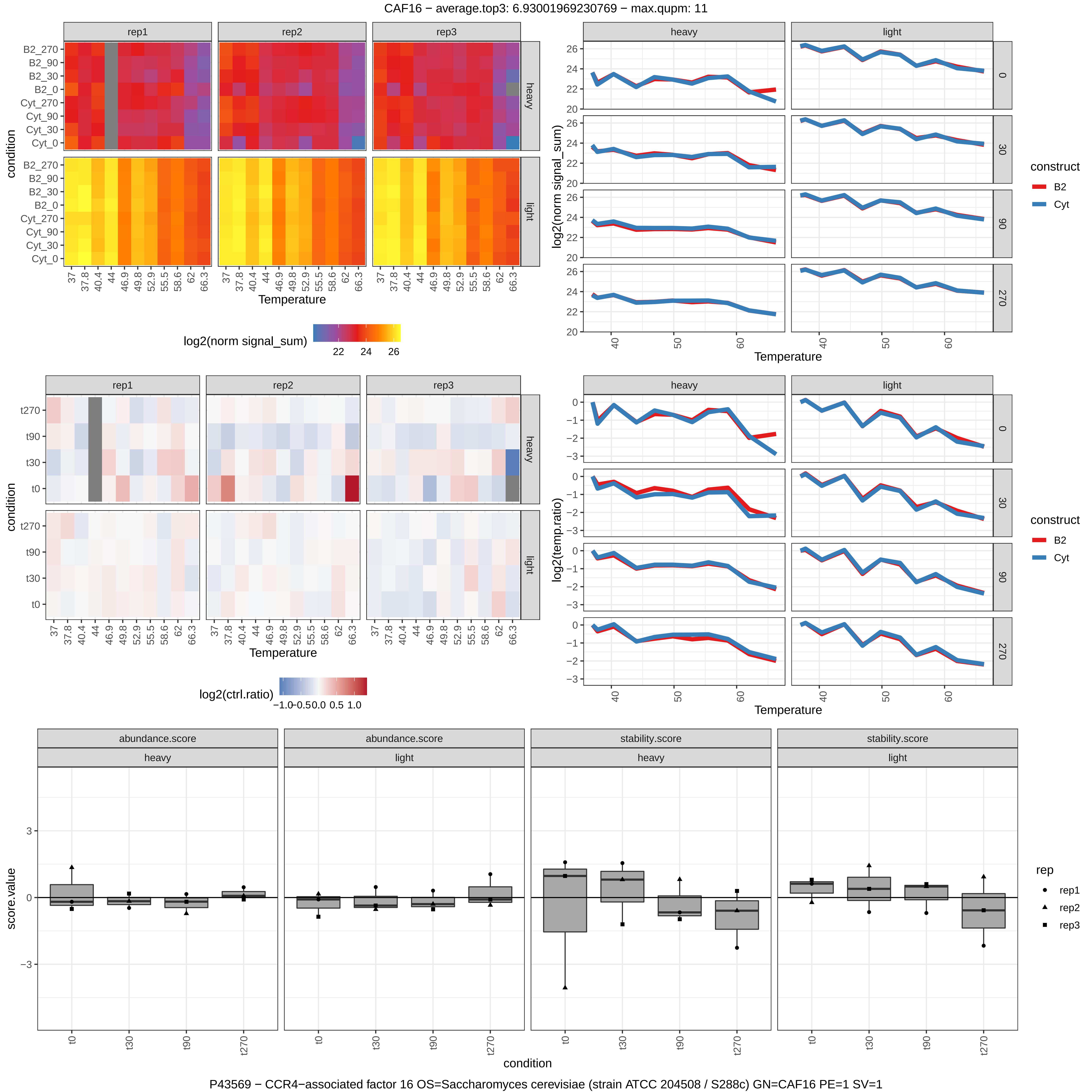

CAF16 − average.top3: 6.93001969230769 − max.qupm: 11
rep1
rep2
rep3
heavy
light
condition
Temperature
log2(norm signal_sum)
construct
B2
Cyt
log2(ctrl.ratio)
log2(temp.ratio)
abundance.score
stability.score
score.value
rep
rep1
rep2
rep3
P43569 − CCR4−associated factor 16 OS=Saccharomyces cerevisiae (strain ATCC 204508 / S288c) GN=CAF16 PE=1 SV=1

CAF40 − average.top3: 6.76290523809524 − max.qupm: 6

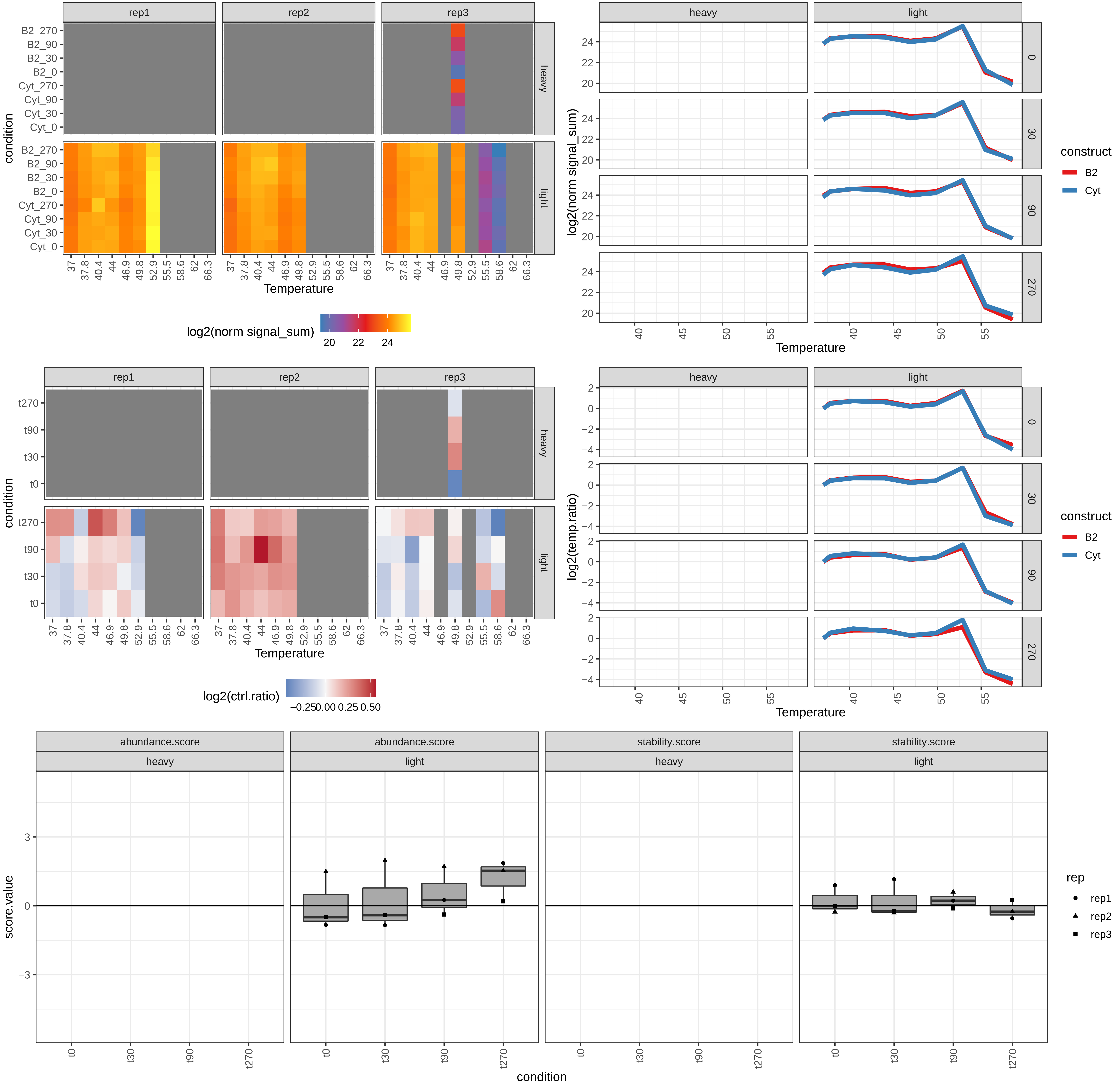

P53829 − Protein CAF40 OS=Saccharomyces cerevisiae (strain ATCC 204508 / S288c) GN=CAF40 PE=1 SV=1

CAJ1 − average.top3: 7.23584142857143 − max.qupm: 10

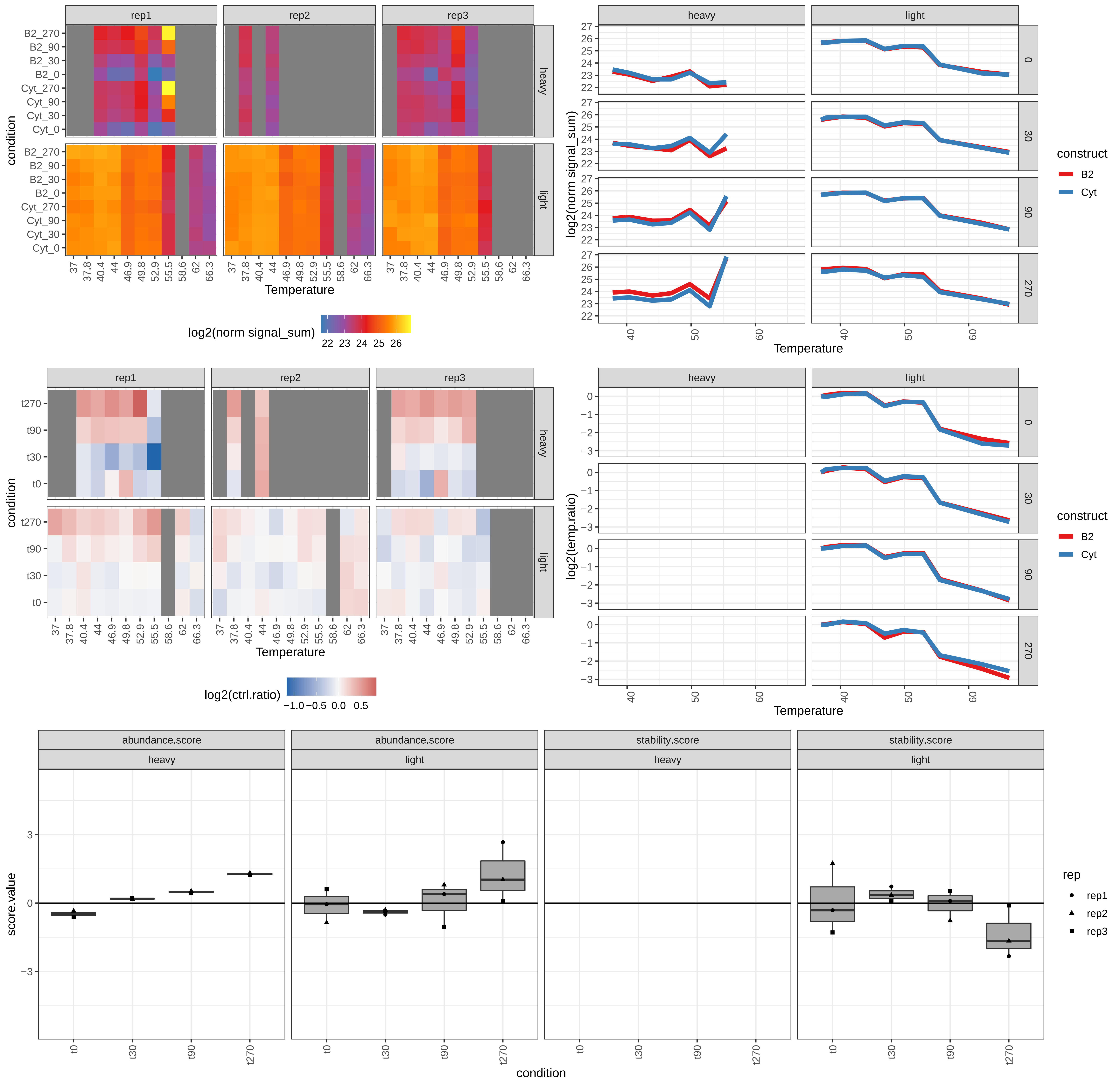

P39101 − Protein CAJ1 OS=Saccharomyces cerevisiae (strain ATCC 204508 / S288c) GN=CAJ1 PE=1 SV=1

CAK1 − average.top3: 6.28415769230769 − max.qupm: 4

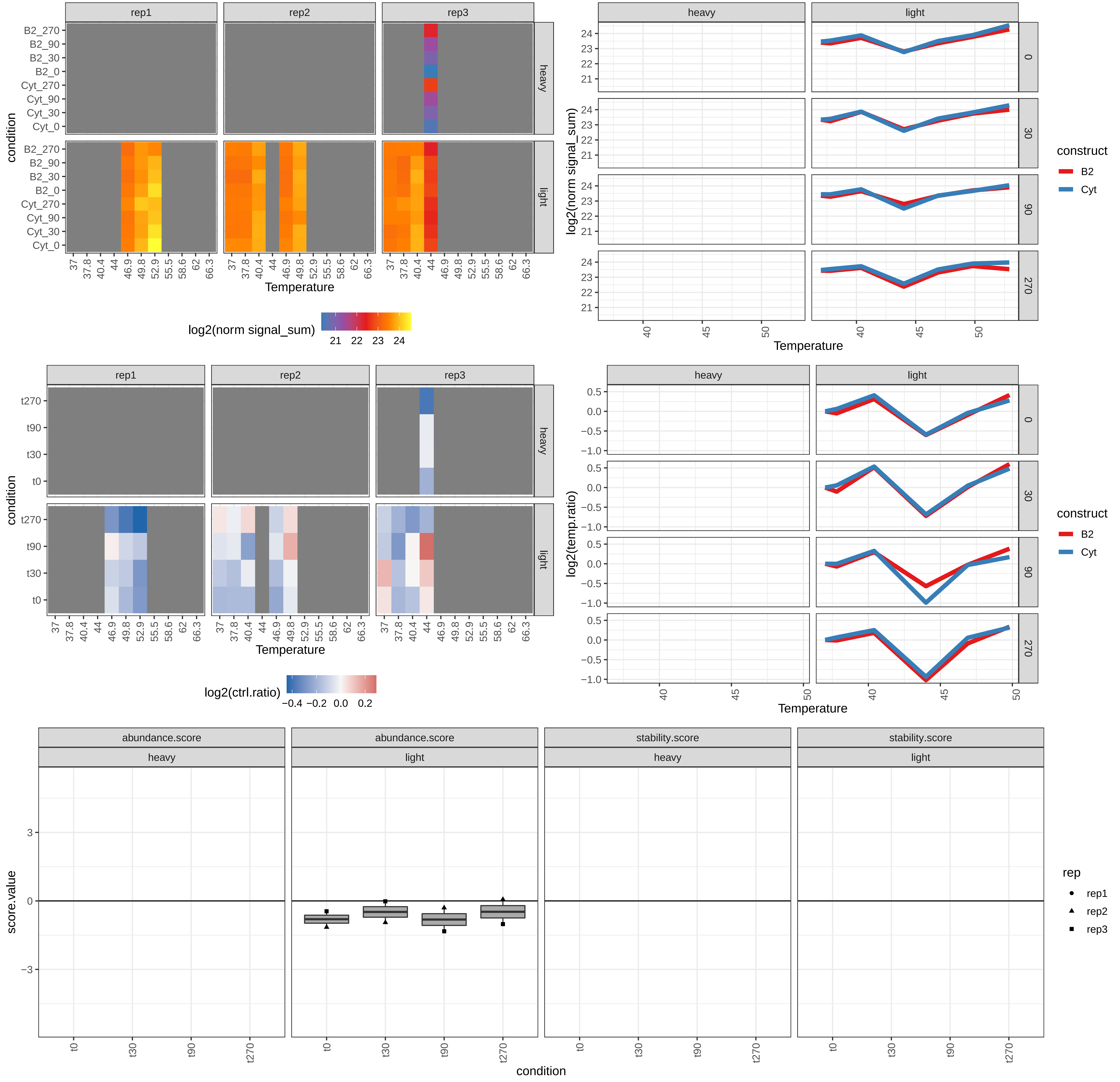

P43568 − Serine/threonine−protein kinase CAK1 OS=Saccharomyces cerevisiae (strain ATCC 204508 / S288c) GN=CAK1 PE=1 SV=1

CAM1 − average.top3: 7.47872578947368 − max.qupm: 12

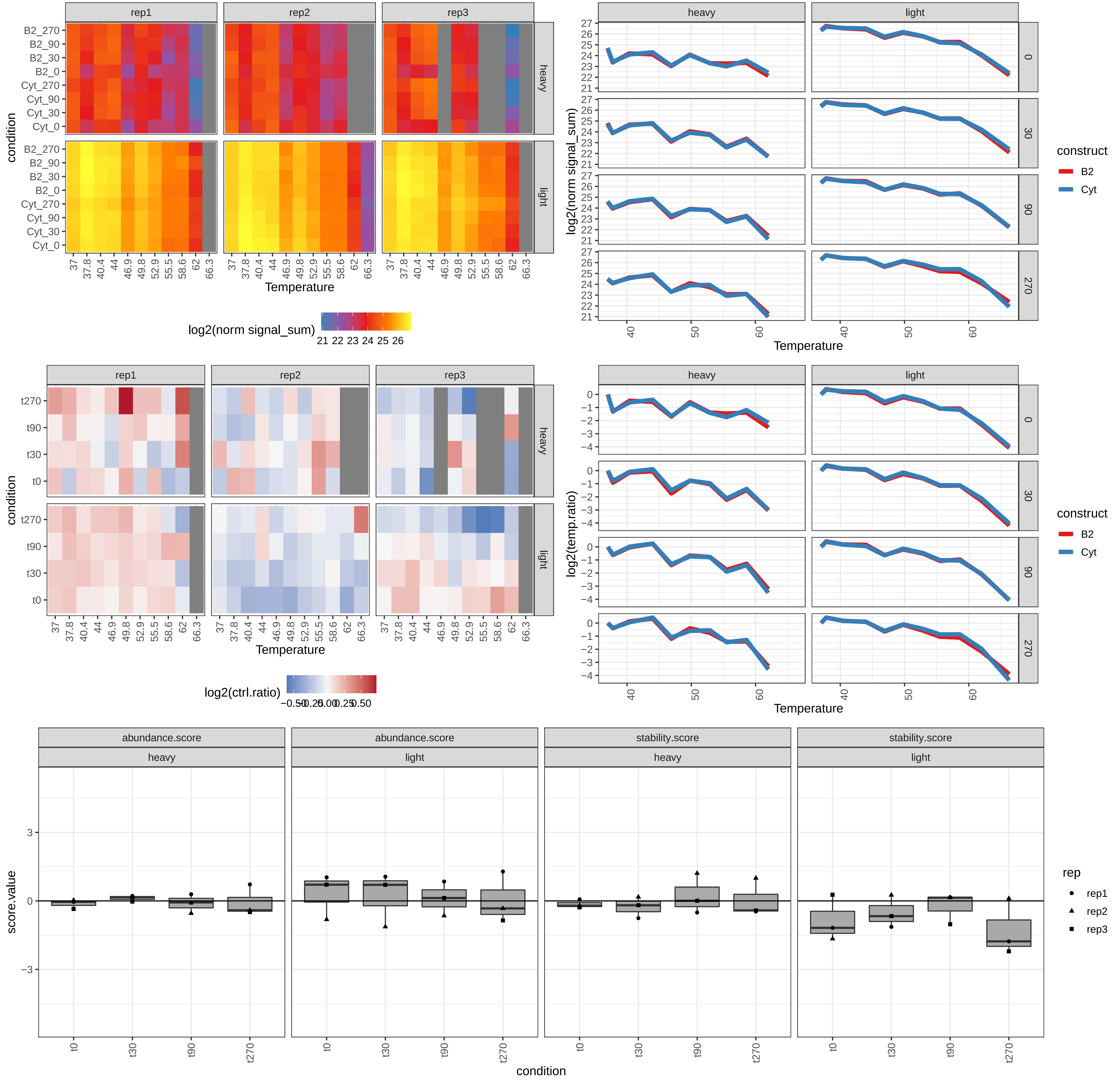

P29547 − Elongation factor 1−gamma 1 OS=Saccharomyces cerevisiae (strain ATCC 204508 / S288c) GN=CAM1 PE=1 SV=2

CAP1 − average.top3: 7.04331859649123 − max.qupm: 8

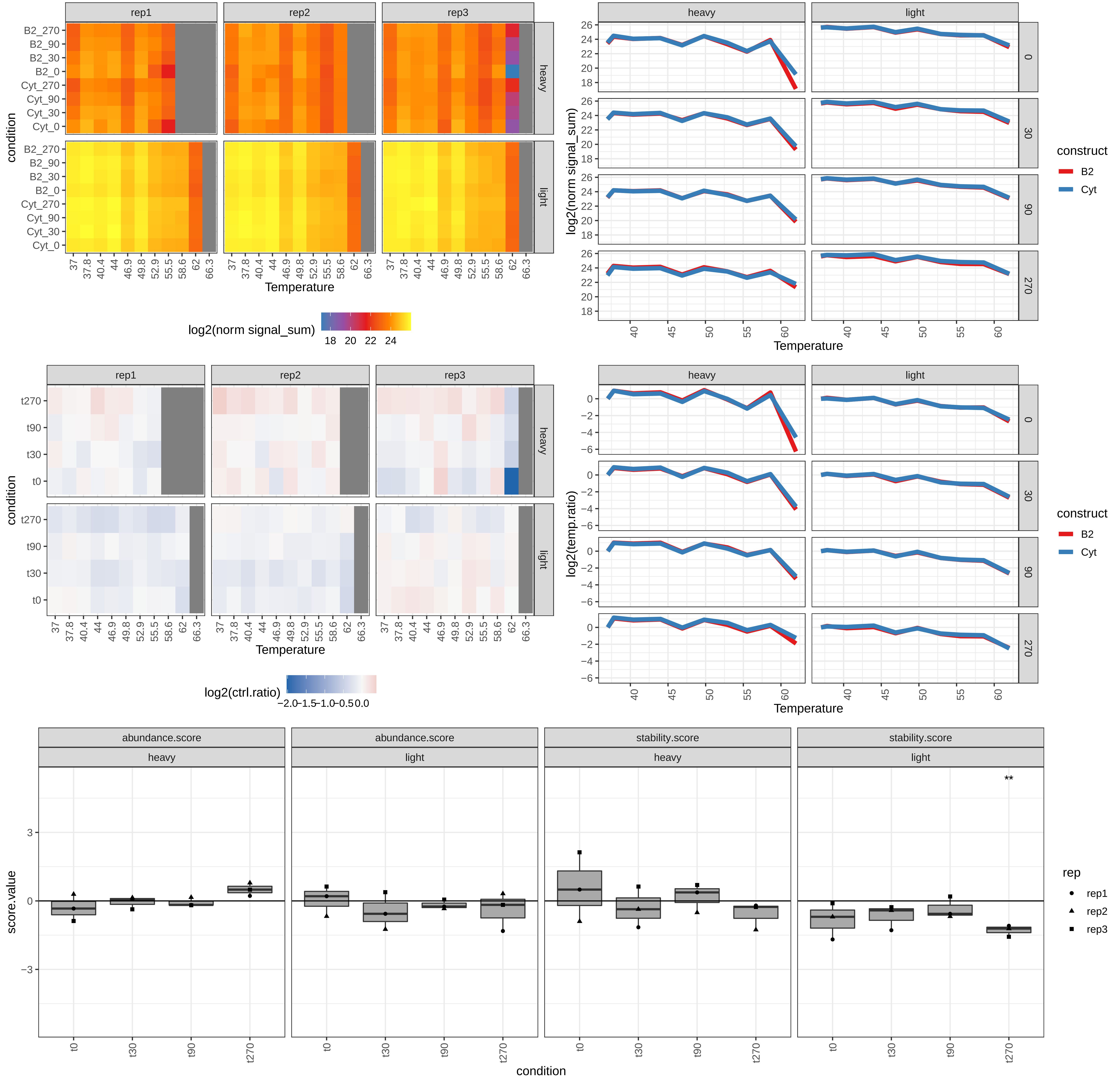

P28495 − F−actin−capping protein subunit alpha OS=Saccharomyces cerevisiae (strain ATCC 204508 / S288c) GN=CAP1 PE=1 SV=1

CAP2 − average.top3: 6.94363387755102 − max.qupm: 6

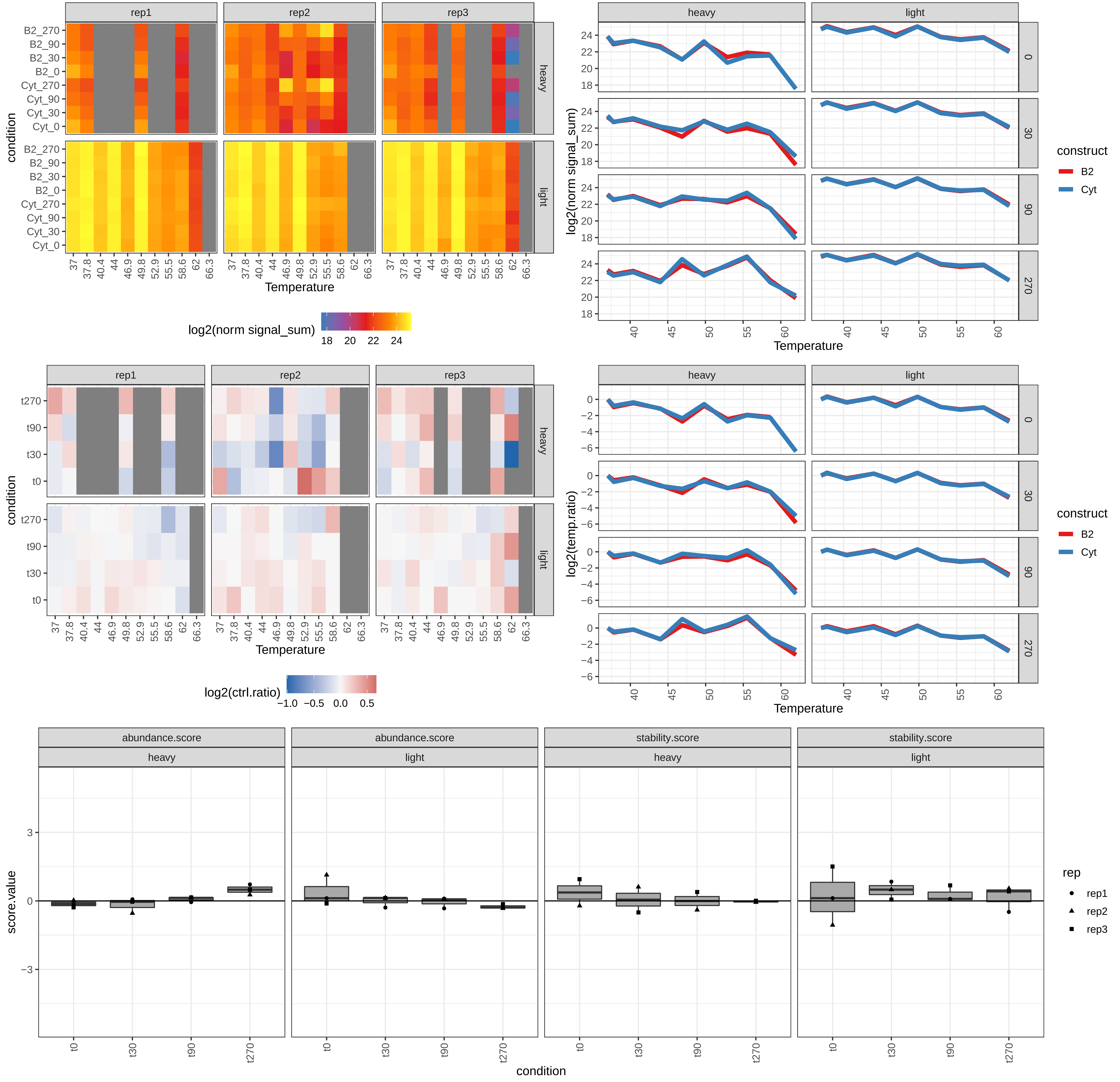

P13517 − F−actin−capping protein subunit beta OS=Saccharomyces cerevisiae (strain ATCC 204508 / S288c) GN=CAP2 PE=1 SV=3

CAR1 − average.top3: 7.60596640625 − max.qupm: 12

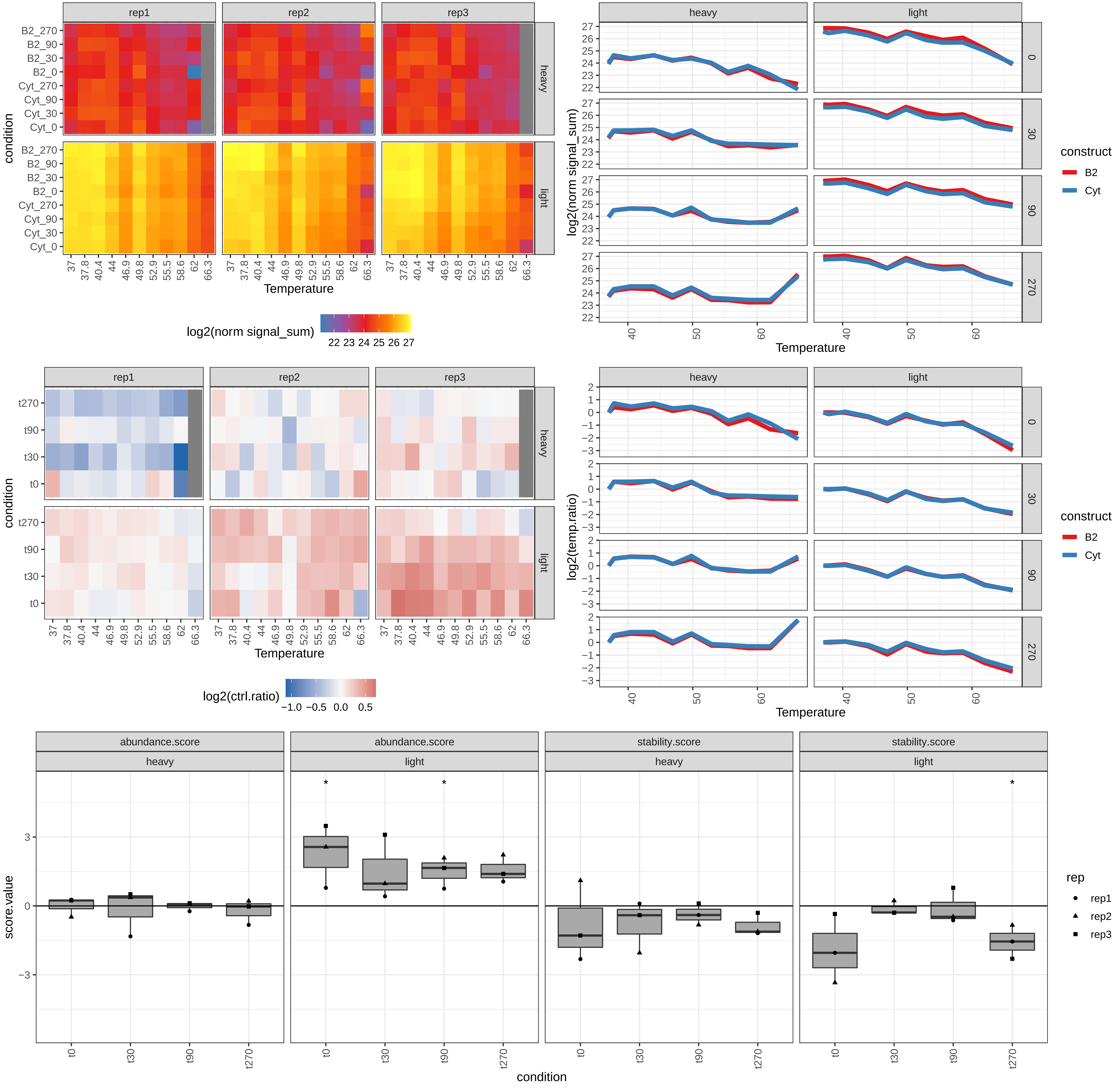

P00812 − Arginase OS=Saccharomyces cerevisiae (strain ATCC 204508 / S288c) GN=CAR1 PE=1 SV=1

CAR2 − average.top3: 7.60845694915254 − max.qupm: 13

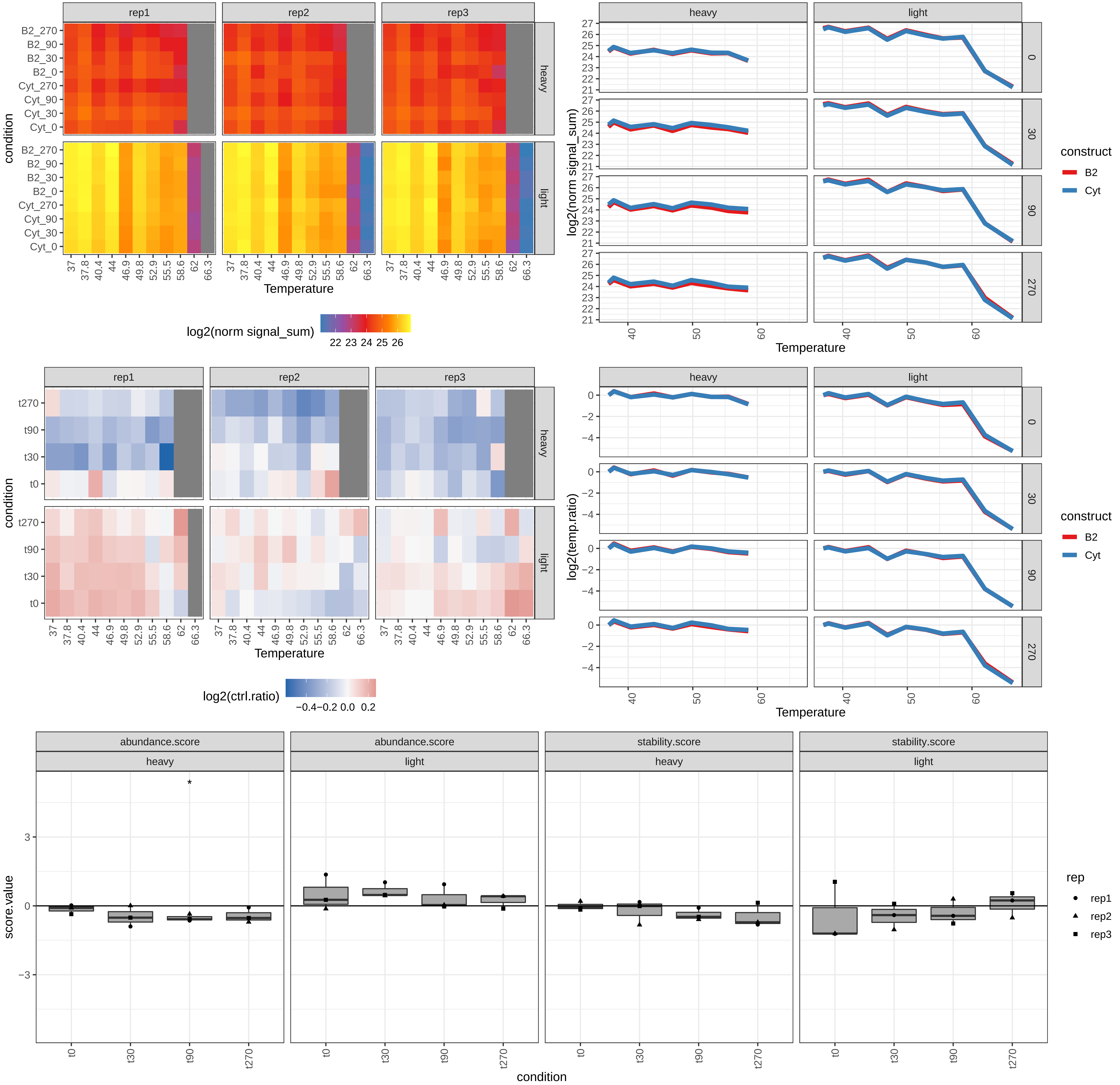

P07991 − Ornithine aminotransferase OS=Saccharomyces cerevisiae (strain ATCC 204508 / S288c) GN=CAR2 PE=1 SV=2

CAT2 − average.top3: 7.39602224489796 − max.qupm: 26

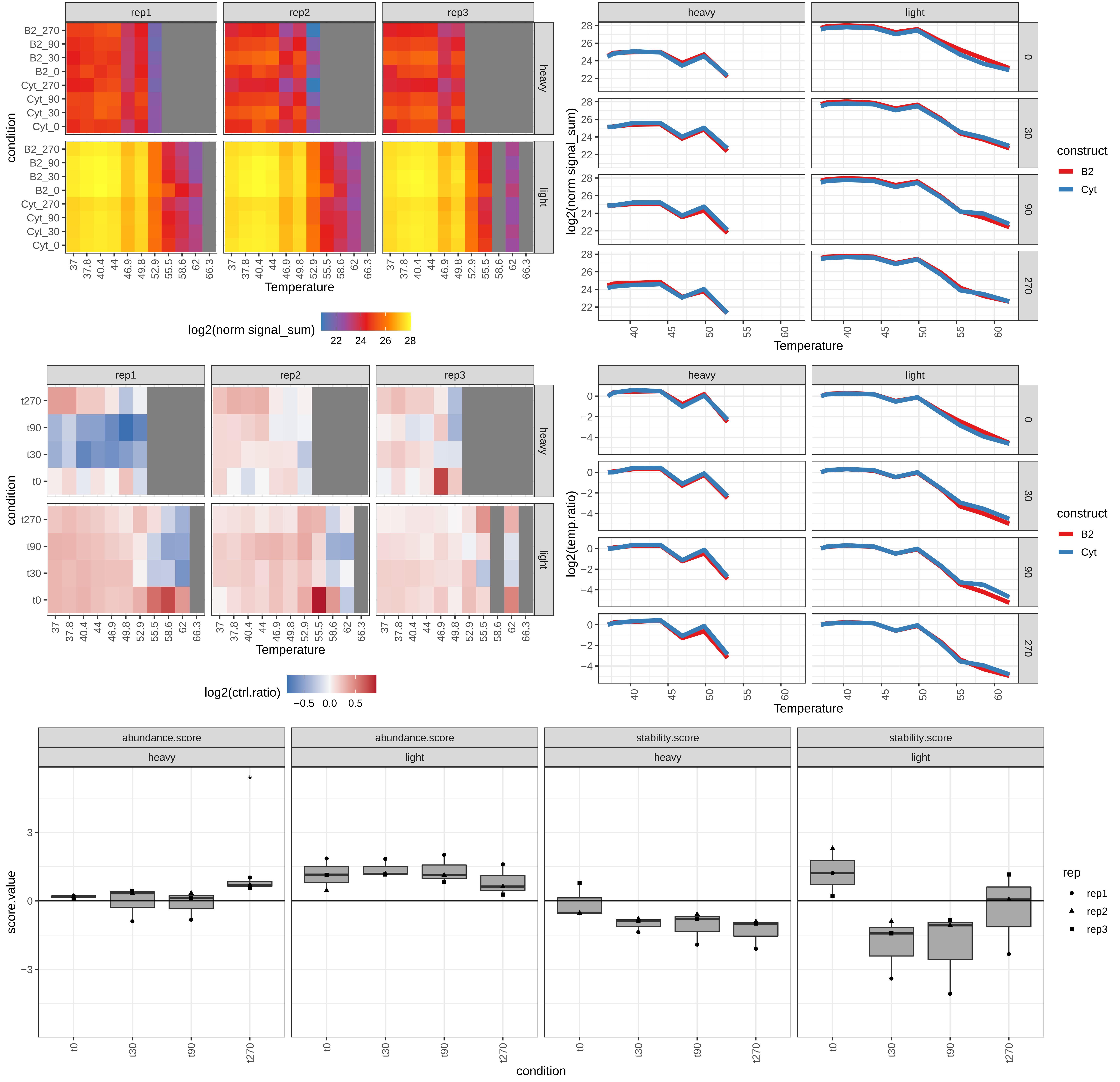

ferase, mitochondrial OS=Saccharomyces cerevisiae (strain ATCC 204508 / S288c) GN=CAT2 PE=1 SV=2|Isoform Peroxisomal of Carnitine O−acetyltransferase, mitochondrial OS=Saccharomyces cerevis

CAT8 − average.top3: 6.9247775 − max.qupm: 6

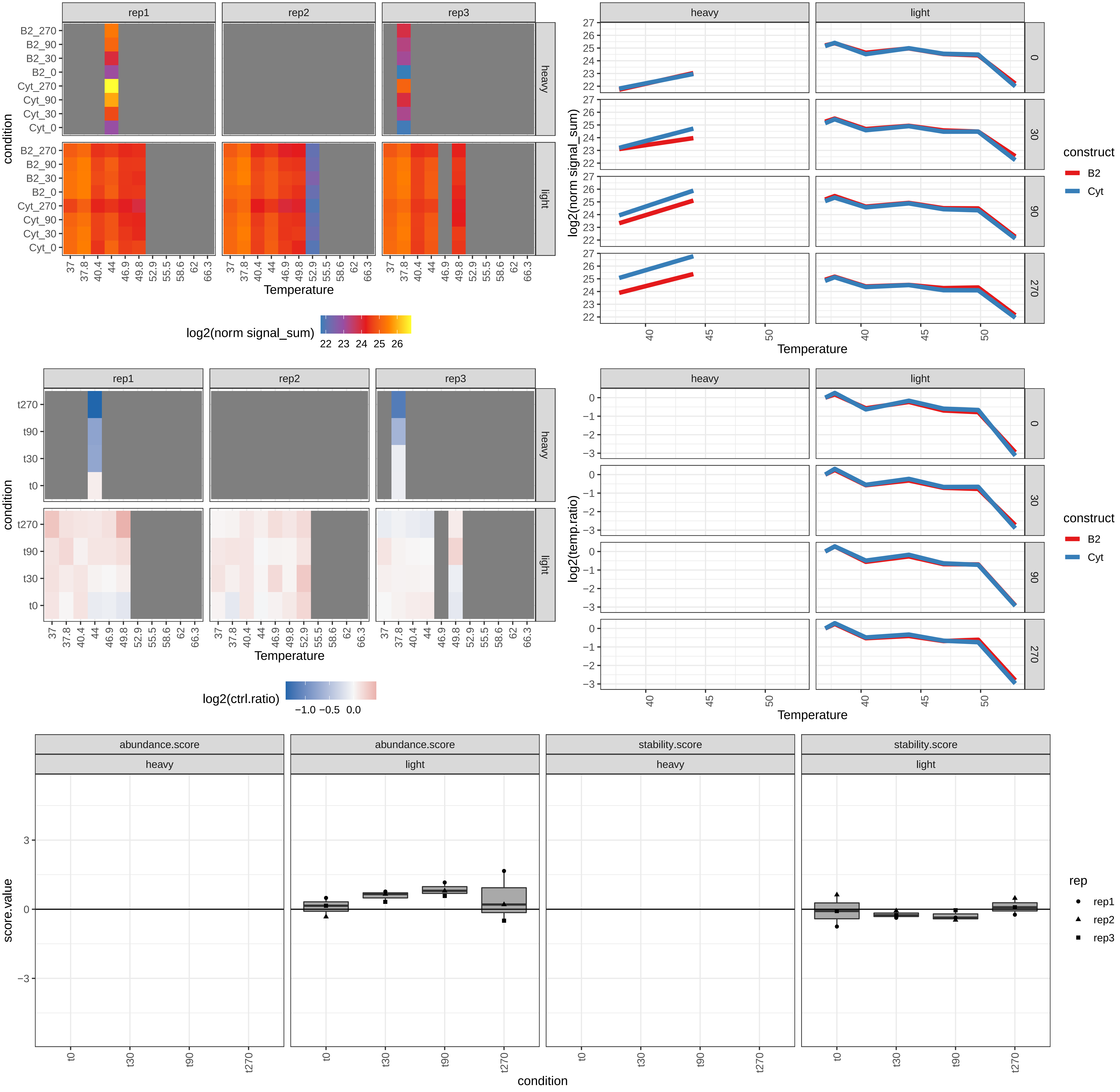

P39113 − Regulatory protein CAT8 OS=Saccharomyces cerevisiae (strain ATCC 204508 / S288c) GN=CAT8 PE=1 SV=2

CBC2 − average.top3: 7.20100294117647 − max.qupm: 6

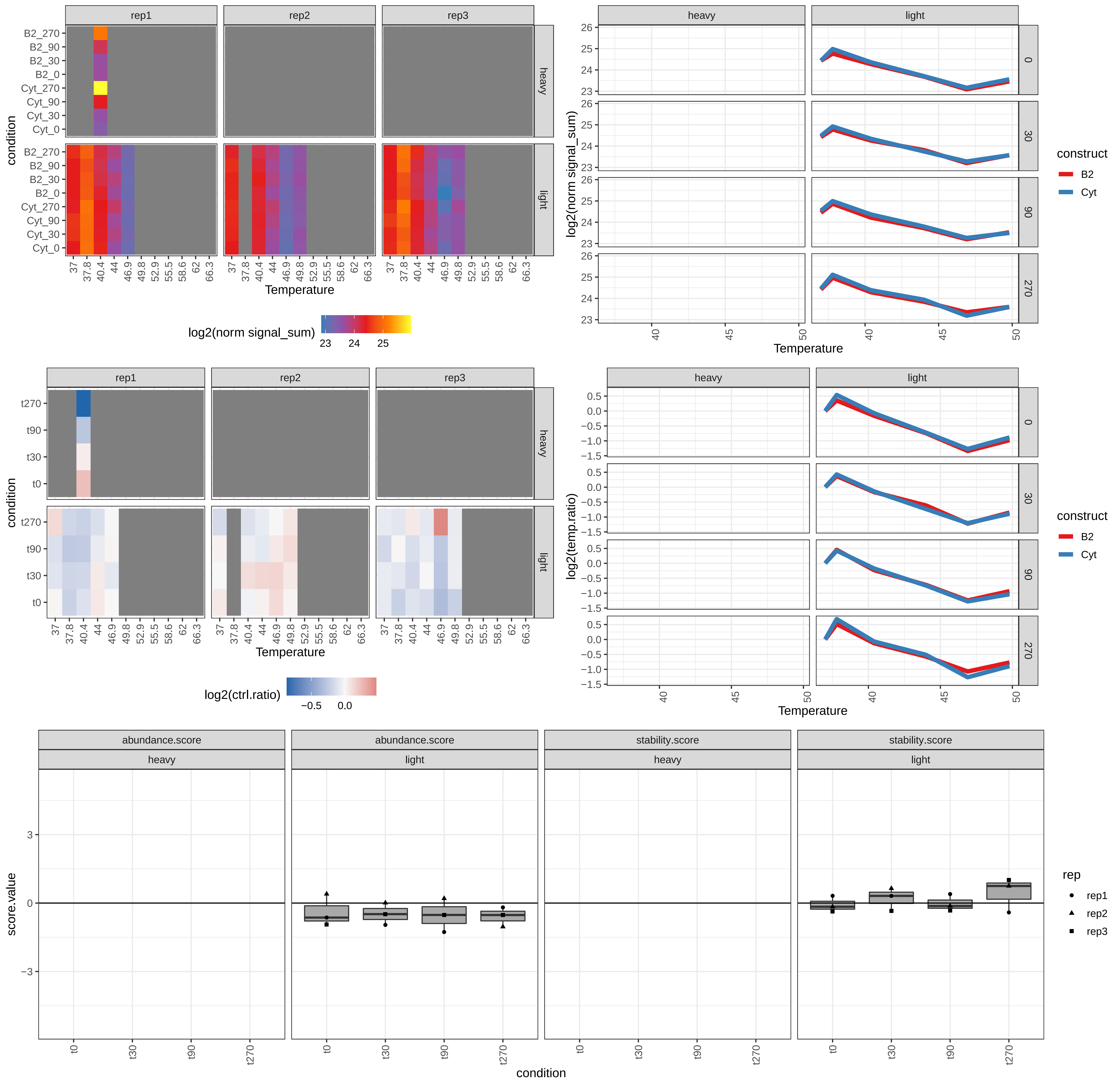

Q08920 − Nuclear cap−binding protein subunit 2 OS=Saccharomyces cerevisiae (strain ATCC 204508 / S288c) GN=CBC2 PE=1 SV=1

CBF5 − average.top3: 7.2674912962963 − max.qupm: 15

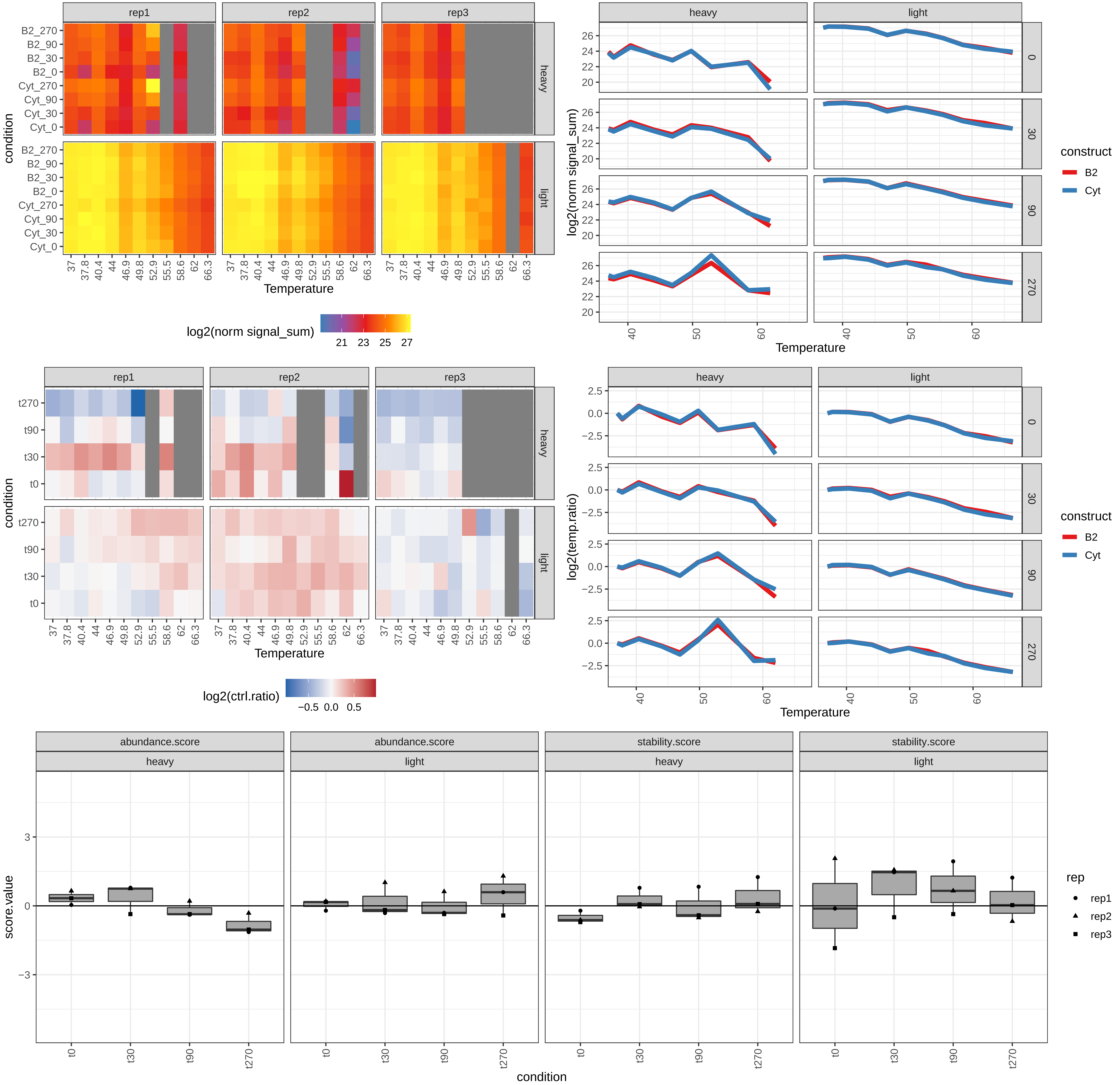

P33322 − H/ACA ribonucleoprotein complex subunit 4 OS=Saccharomyces cerevisiae (strain ATCC 204508 / S288c) GN=CBF5 PE=1 SV=1

CBK1 − average.top3: 6.9245955 − max.qupm: 9

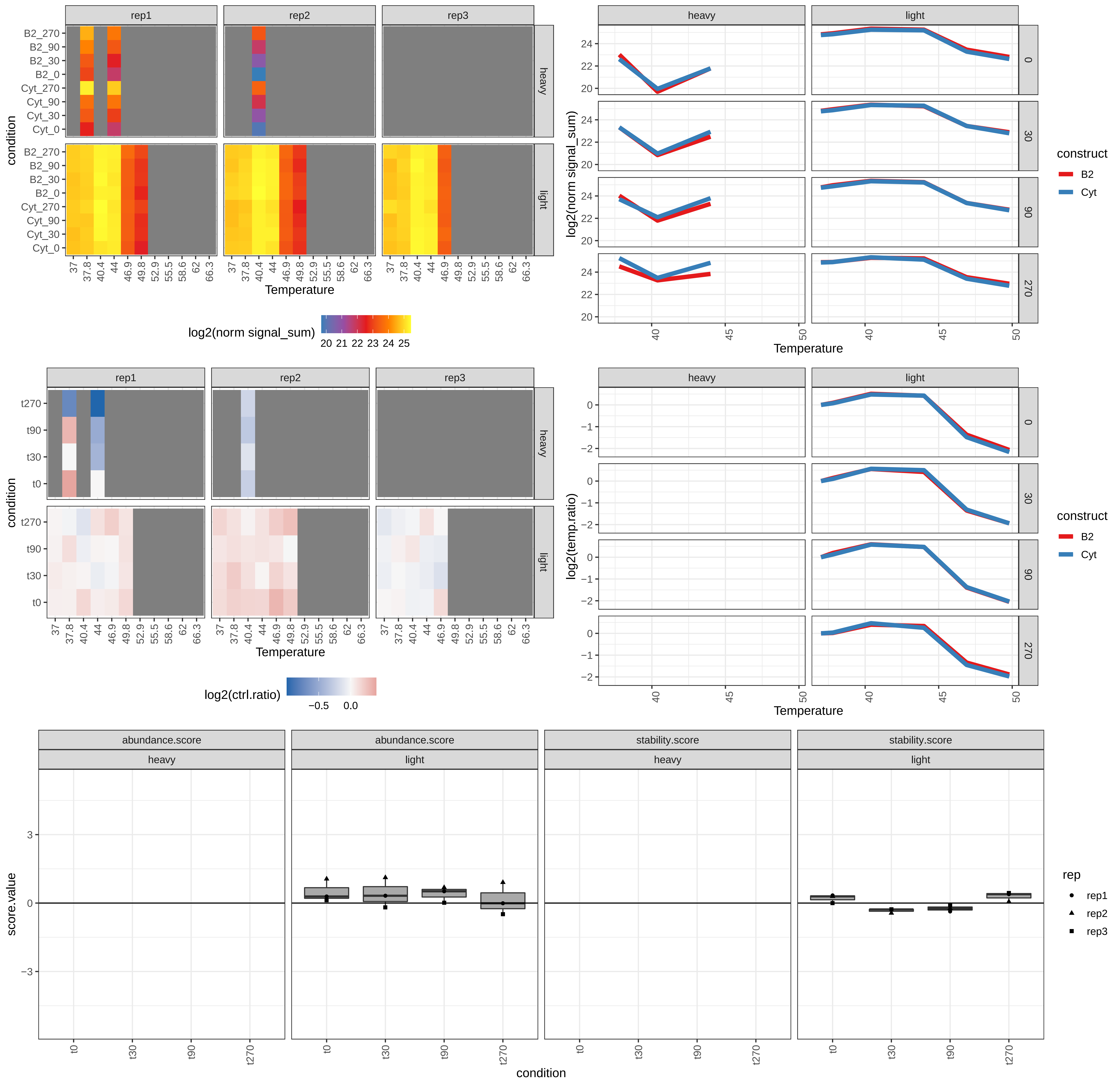

P53894 − Serine/threonine−protein kinase CBK1 OS=Saccharomyces cerevisiae (strain ATCC 204508 / S288c) GN=CBK1 PE=1 SV=1

CBP2 − average.top3: 6.795070625 − max.qupm: 7

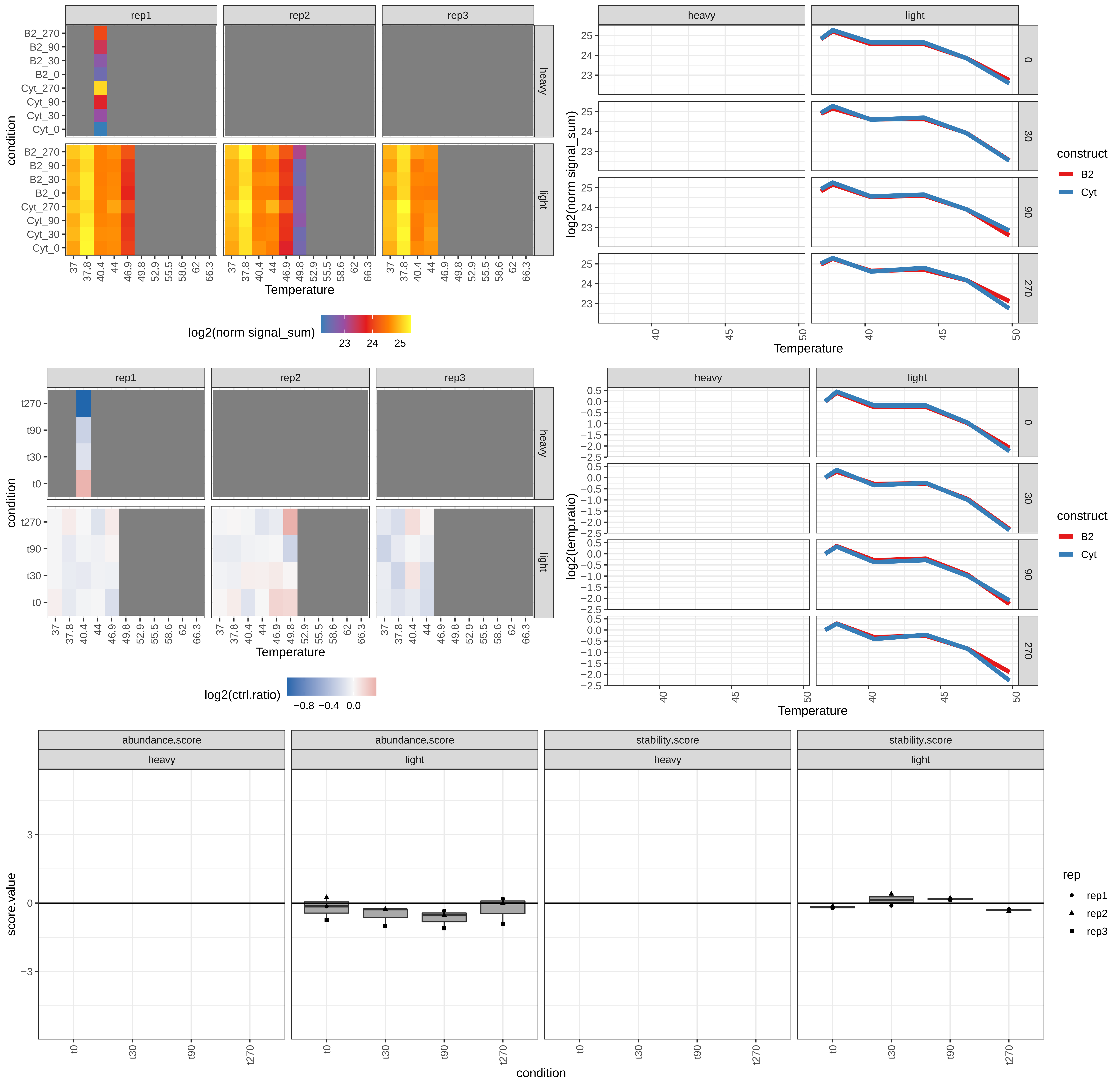

P03874 − Cytochrome B pre−mRNA−processing protein 2 OS=Saccharomyces cerevisiae (strain ATCC 204508 / S288c) GN=CBP2 PE=1 SV=2

CBP3 − average.top3: 7.09500909090909 − max.qupm: 7

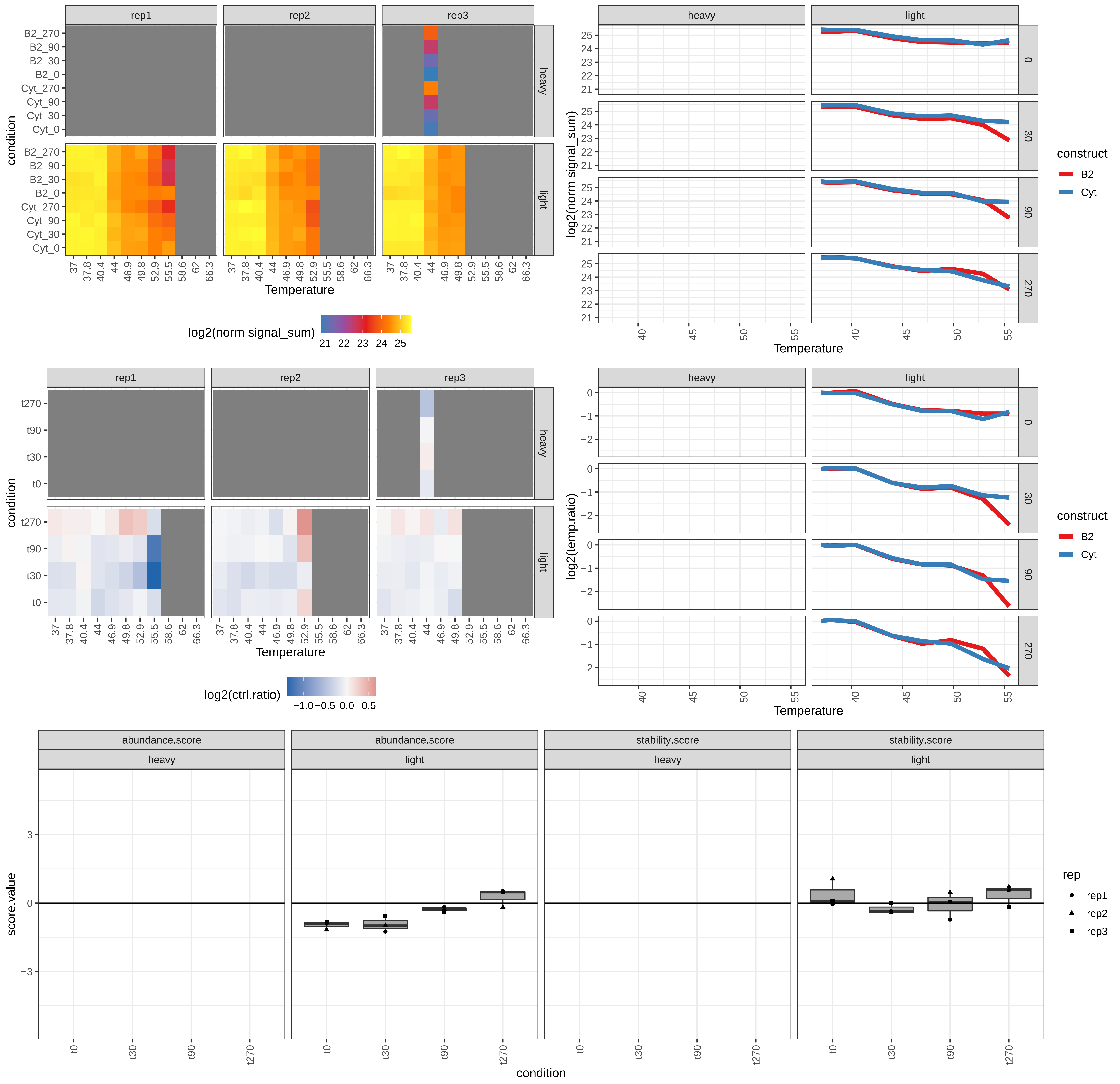

P21560 − Protein CBP3, mitochondrial OS=Saccharomyces cerevisiae (strain ATCC 204508 / S288c) GN=CBP3 PE=1 SV=1

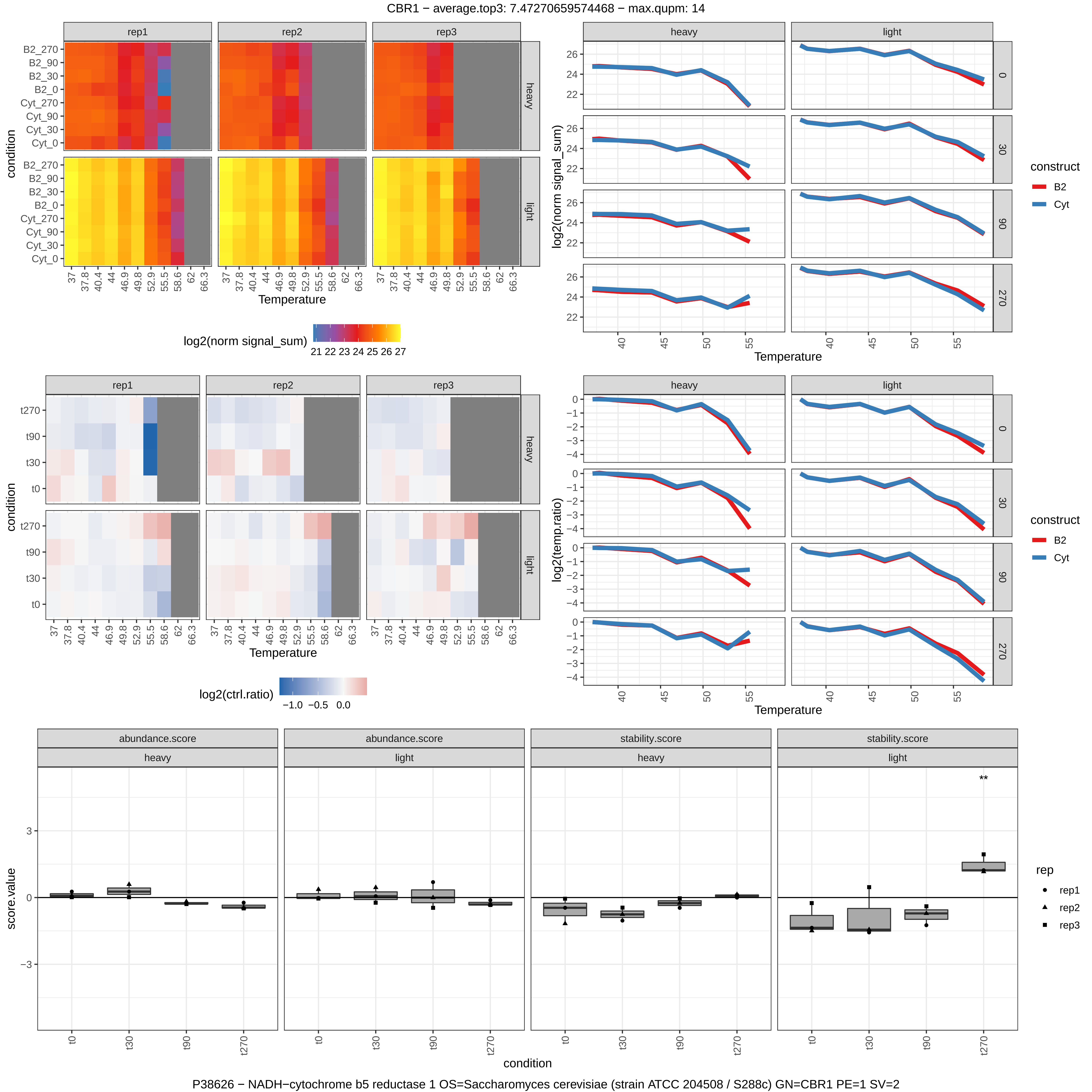
CBR1 − average.top3: 7.47270659574468 − max.qupm: 14
rep1
rep2
rep3
heavy
light
condition
B2_270
B2_90
B2_30
B2_0
Cyt_270
Cyt_90
Cyt_30
Cyt_0
Temperature
log2(norm signal_sum)
21 22 23 24 25 26 27
construct
B2
Cyt
log2(ctrl.ratio)
−1.0 −0.5 0.0
log2(temp.ratio)
abundance.score
stability.score
score.value
condition
rep
rep1
rep2
rep3
**
P38626 − NADH−cytochrome b5 reductase 1 OS=Saccharomyces cerevisiae (strain ATCC 204508 / S288c) GN=CBR1 PE=1 SV=2

CCA1 − average.top3: 6.47134333333333 − max.qupm: 4

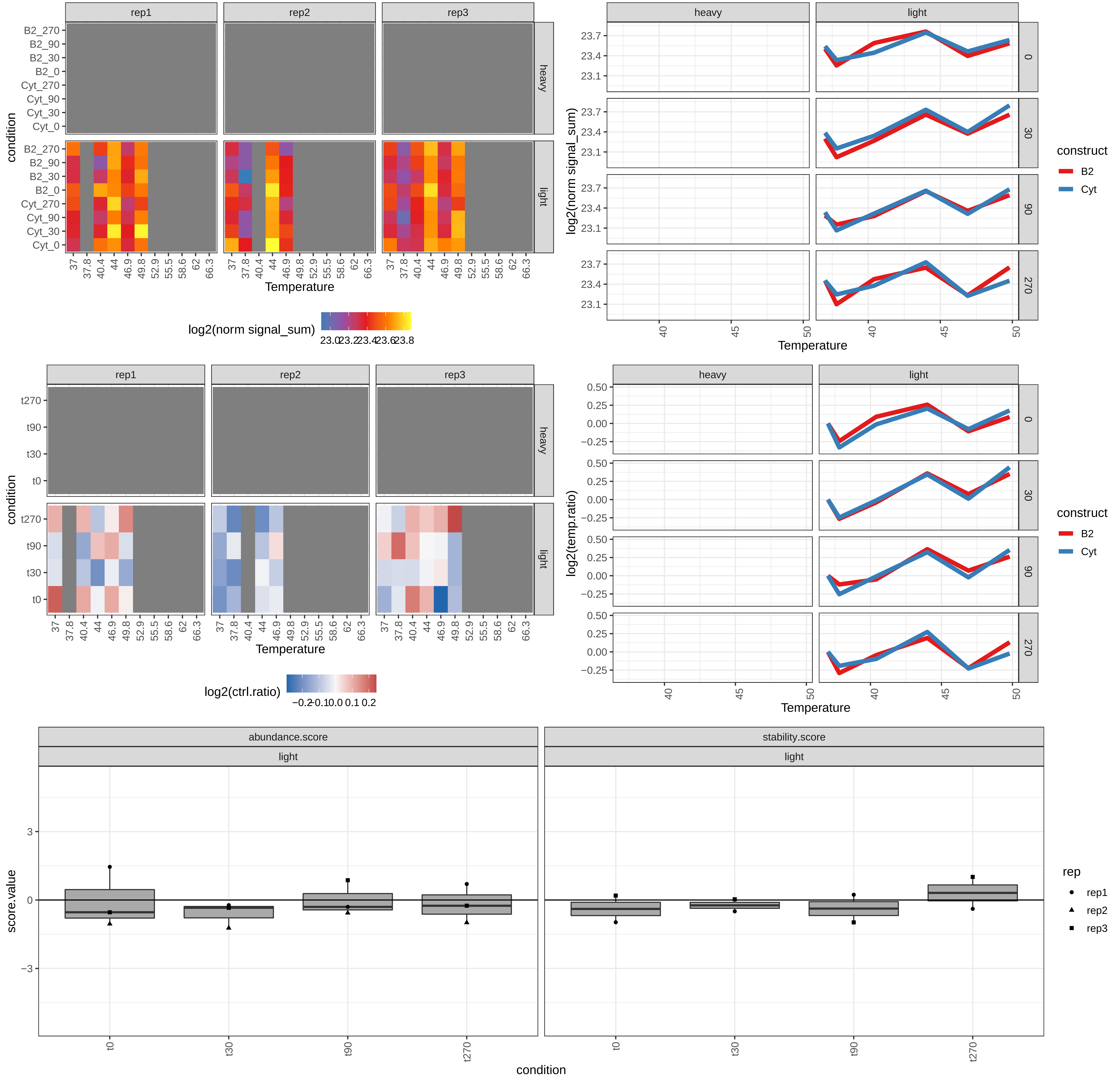

88c) GN=CCA1 PE=1 SV=1|Isoform Cytoplasmic+nuclear 1 of CCA tRNA nucleotidyltransferase, mitochondrial OS=Saccharomyces cerevisiae (strain ATCC 204508 / S288c) GN=CCA1|Isoform Cytoplasr

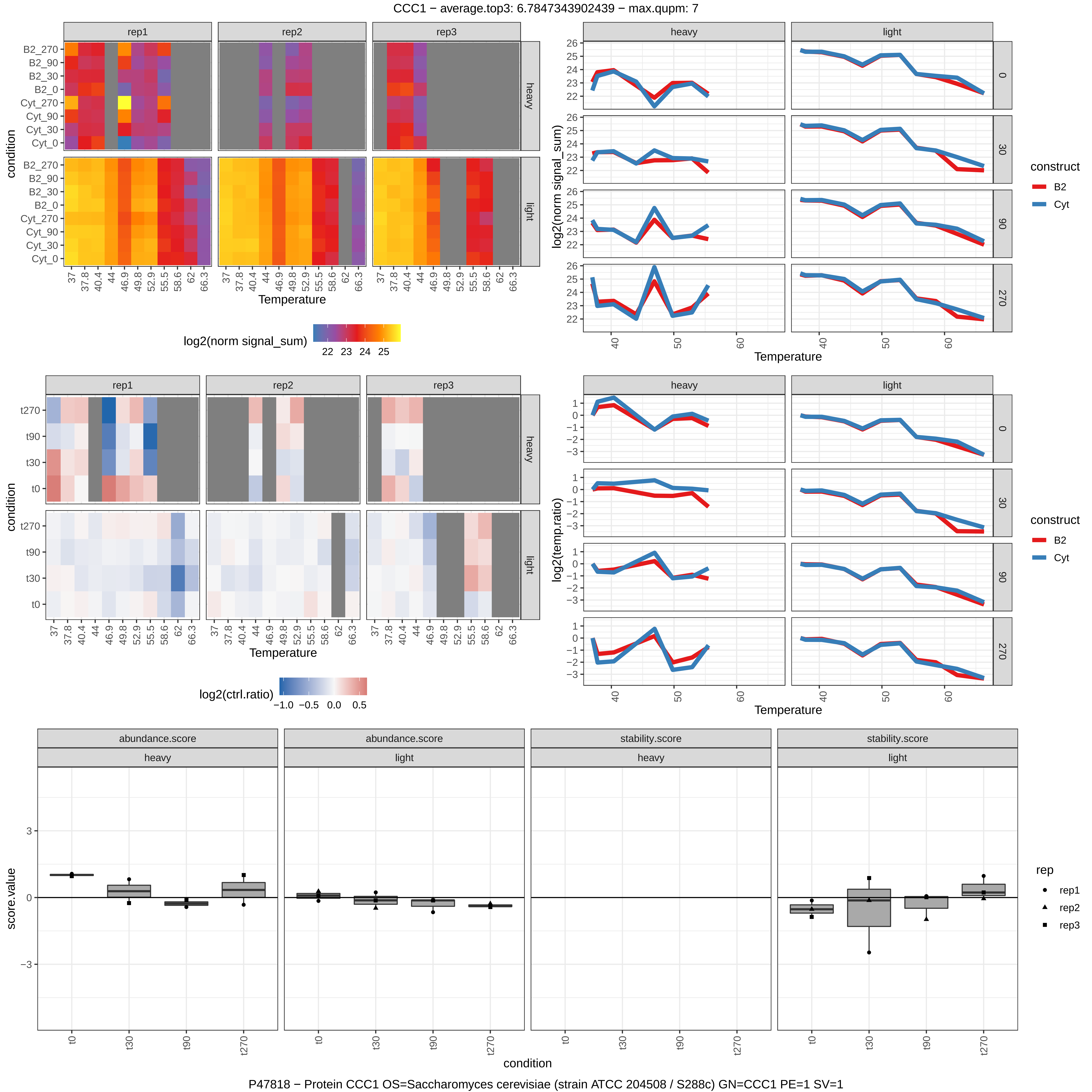

CCC1 − average.top3: 6.7847343902439 − max.qupm: 7
rep1
rep2
rep3
heavy
light
condition
Temperature
log2(norm signal_sum)
log2(ctrl.ratio)
log2(temp.ratio)
construct
B2
Cyt
abundance.score
stability.score
score.value
rep
rep1
rep2
rep3
P47818 − Protein CCC1 OS=Saccharomyces cerevisiae (strain ATCC 204508 / S288c) GN=CCC1 PE=1 SV=1

CCM1 − average.top3: 6.47512666666667 − max.qupm: 4

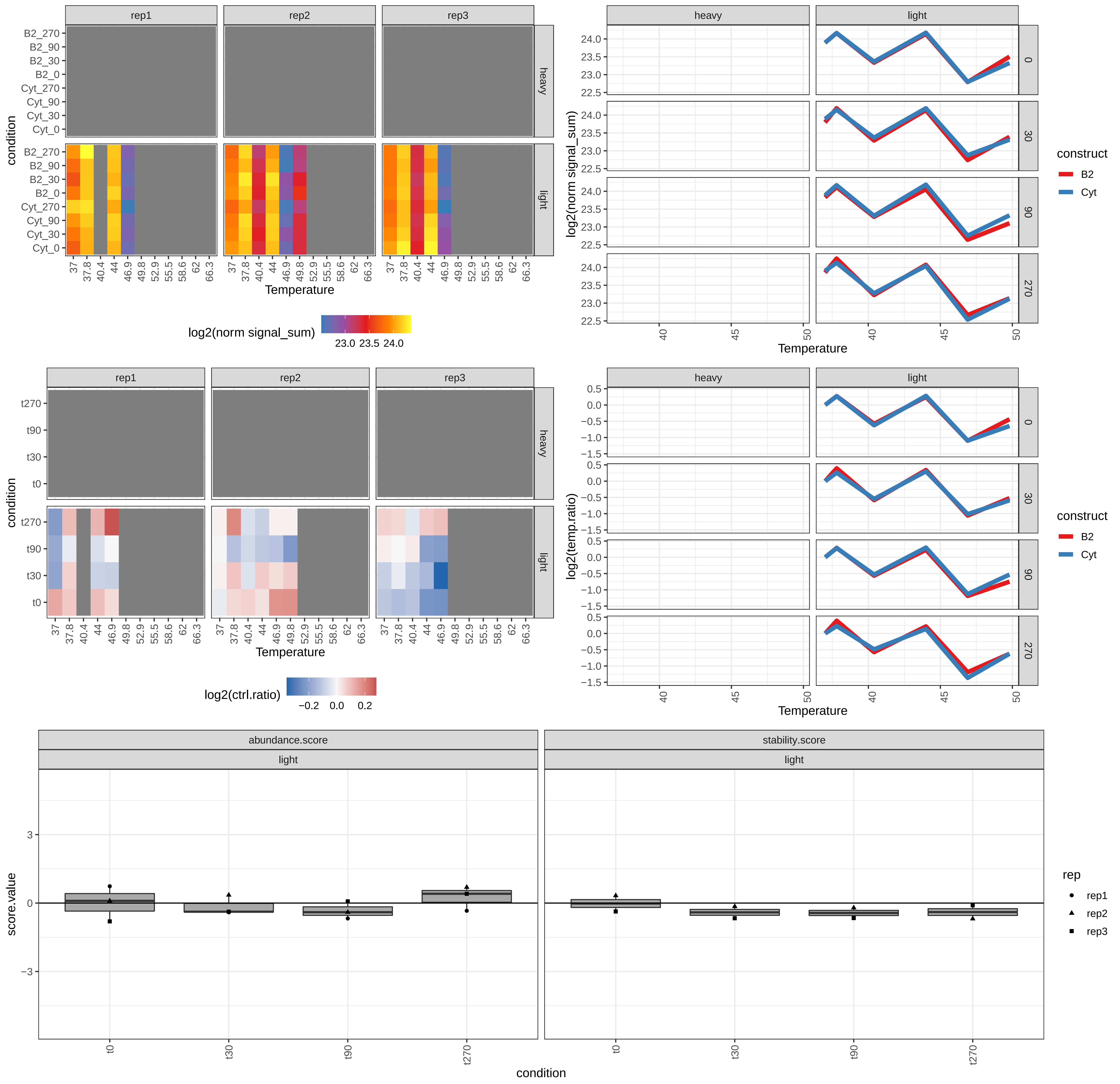

P48237 − Mitochondrial group I intron splicing factor CCM1 OS=Saccharomyces cerevisiae (strain ATCC 204508 / S288c) GN=CCM1 PE=1 SV=1

CCP1 − average.top3: 7.4758675862069 − max.qupm: 20

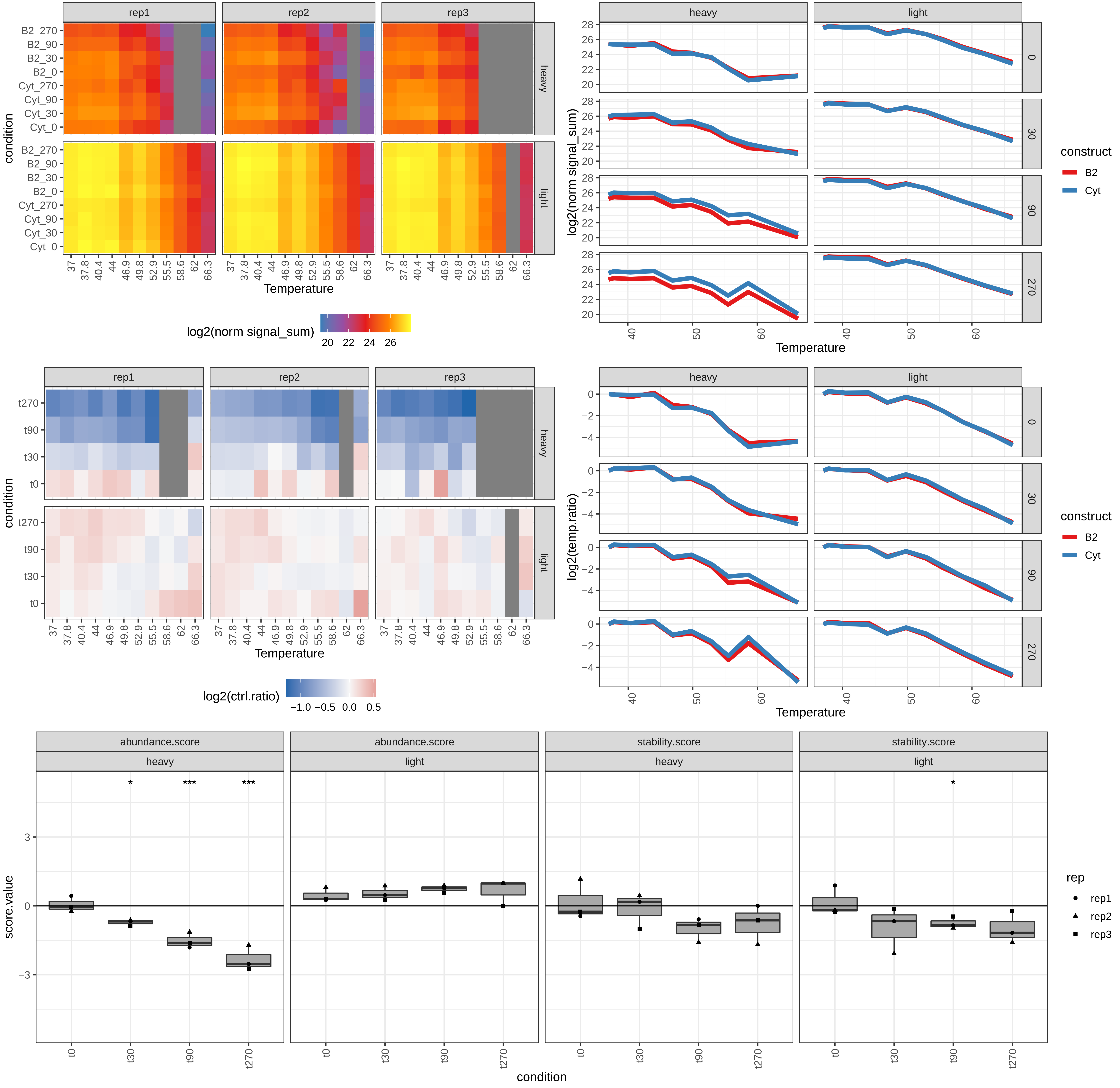

P00431 − Cytochrome c peroxidase, mitochondrial OS=Saccharomyces cerevisiae (strain ATCC 204508 / S288c) GN=CCP1 PE=1 SV=2

CCR4 − average.top3: 6.9659603125 − max.qupm: 10

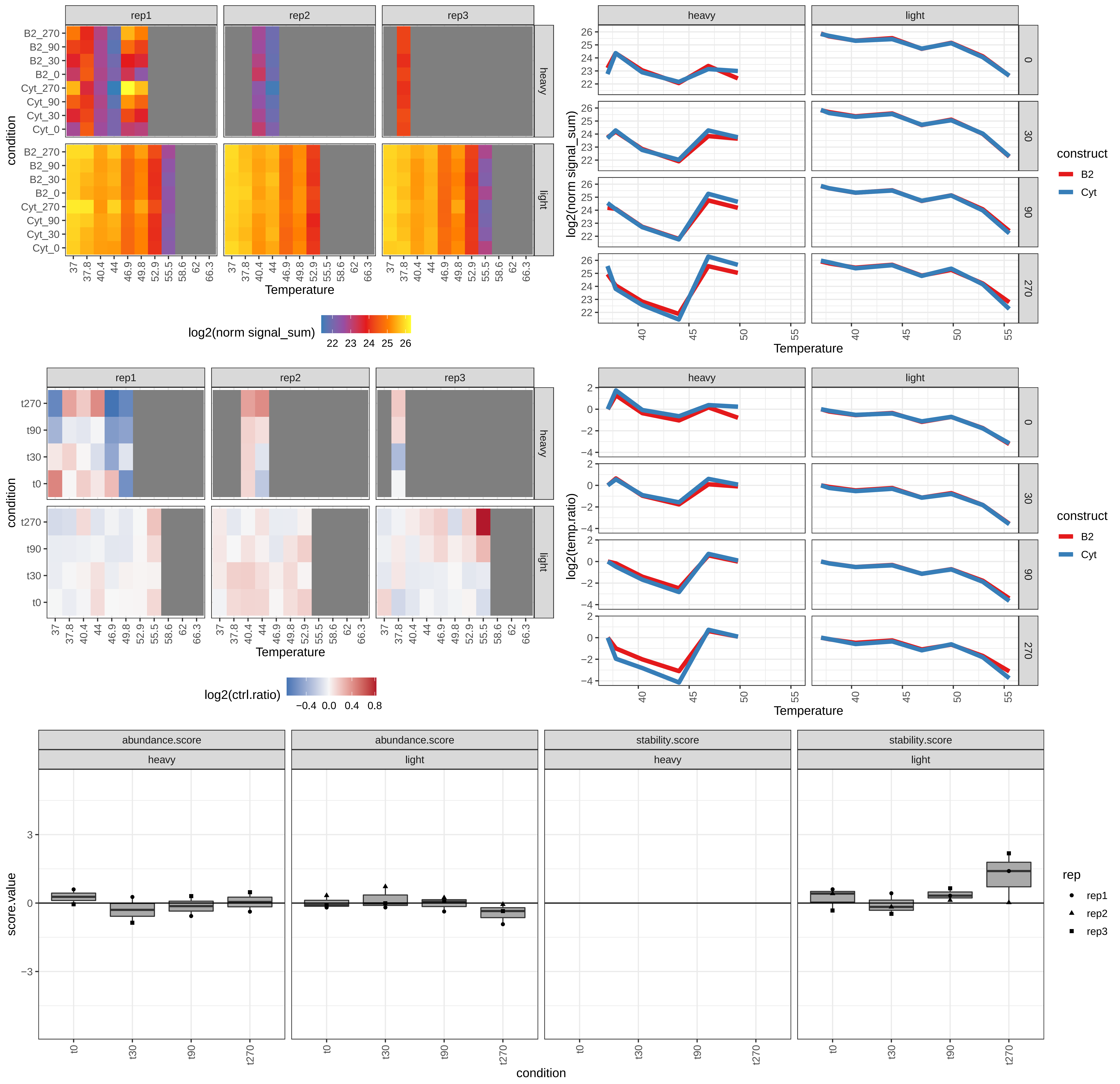

P31384 − Glucose−repressible alcohol dehydrogenase transcriptional effector OS=Saccharomyces cerevisiae (strain ATCC 204508 / S288c) GN=CCR4 PE=1 SV=2

CCS1 − average.top3: 6.82075111111111 − max.qupm: 7

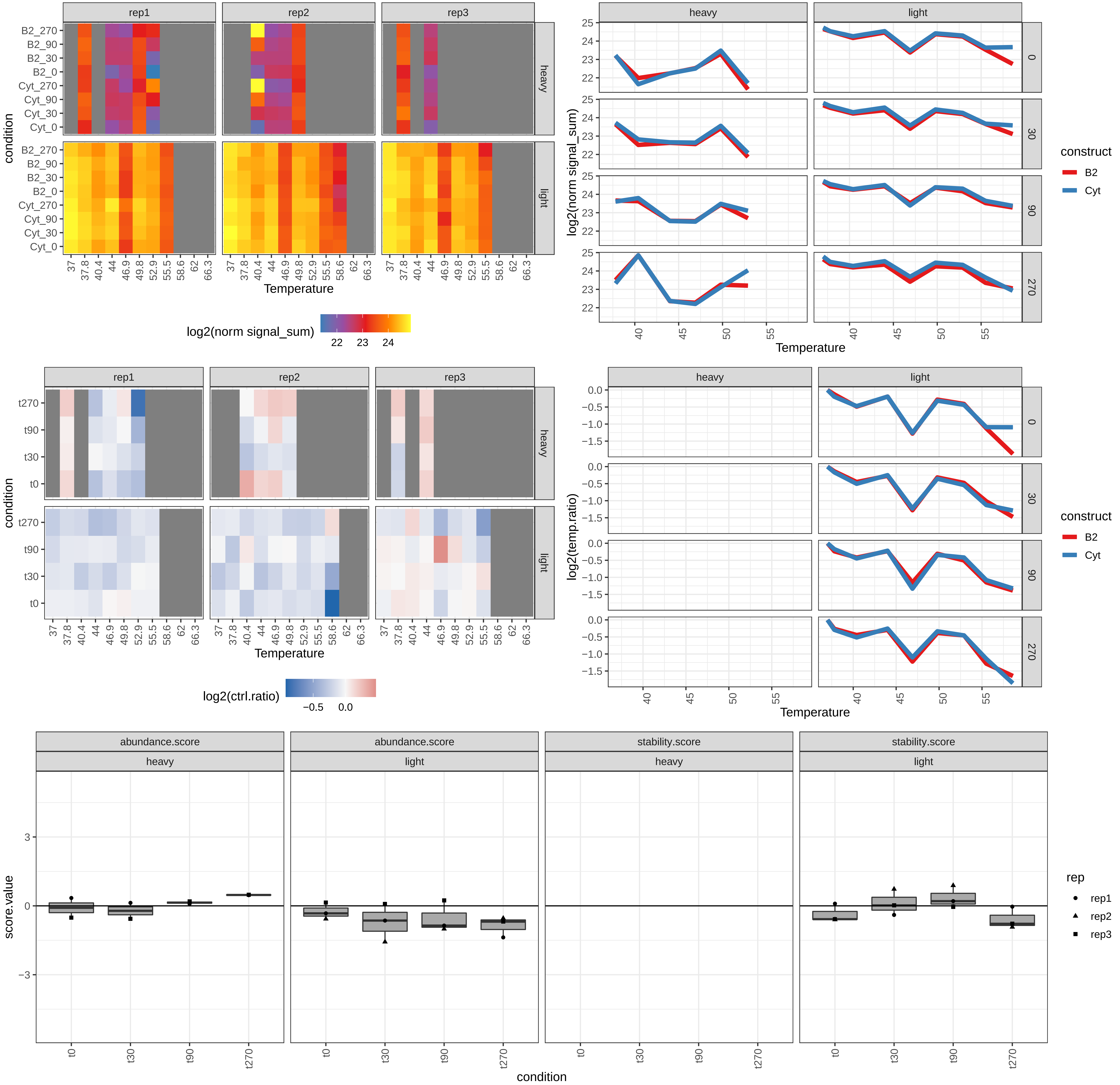

P40202 − Superoxide dismutase 1 copper chaperone OS=Saccharomyces cerevisiae (strain ATCC 204508 / S288c) GN=CCS1 PE=1 SV=1

CCT2 − average.top3: 7.26553068181818 − max.qupm: 13

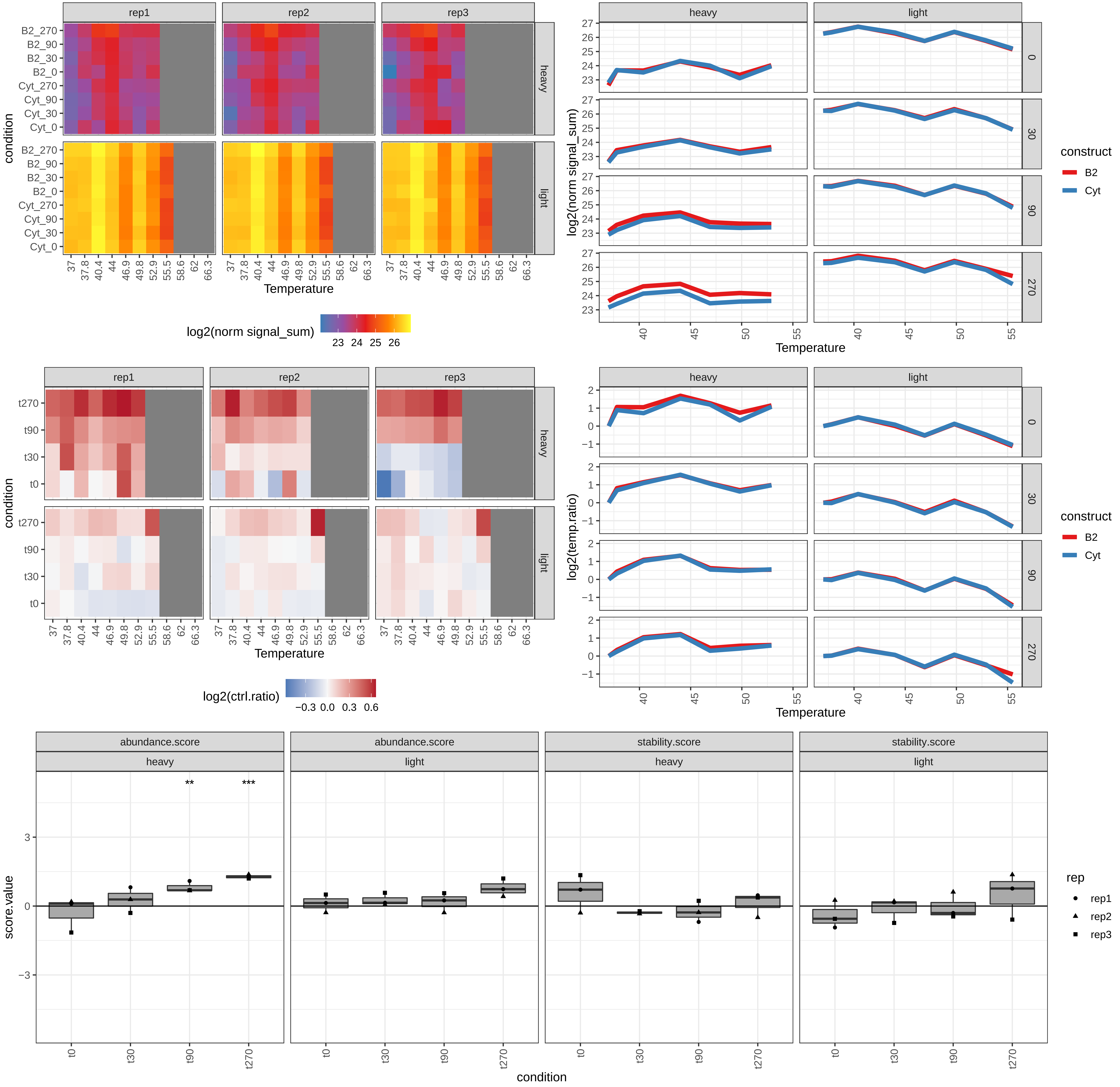

P39076 − T−complex protein 1 subunit beta OS=Saccharomyces cerevisiae (strain ATCC 204508 / S288c) GN=CCT2 PE=1 SV=1

CCT3 − average.top3: 7.26291897959184 − max.qupm: 23

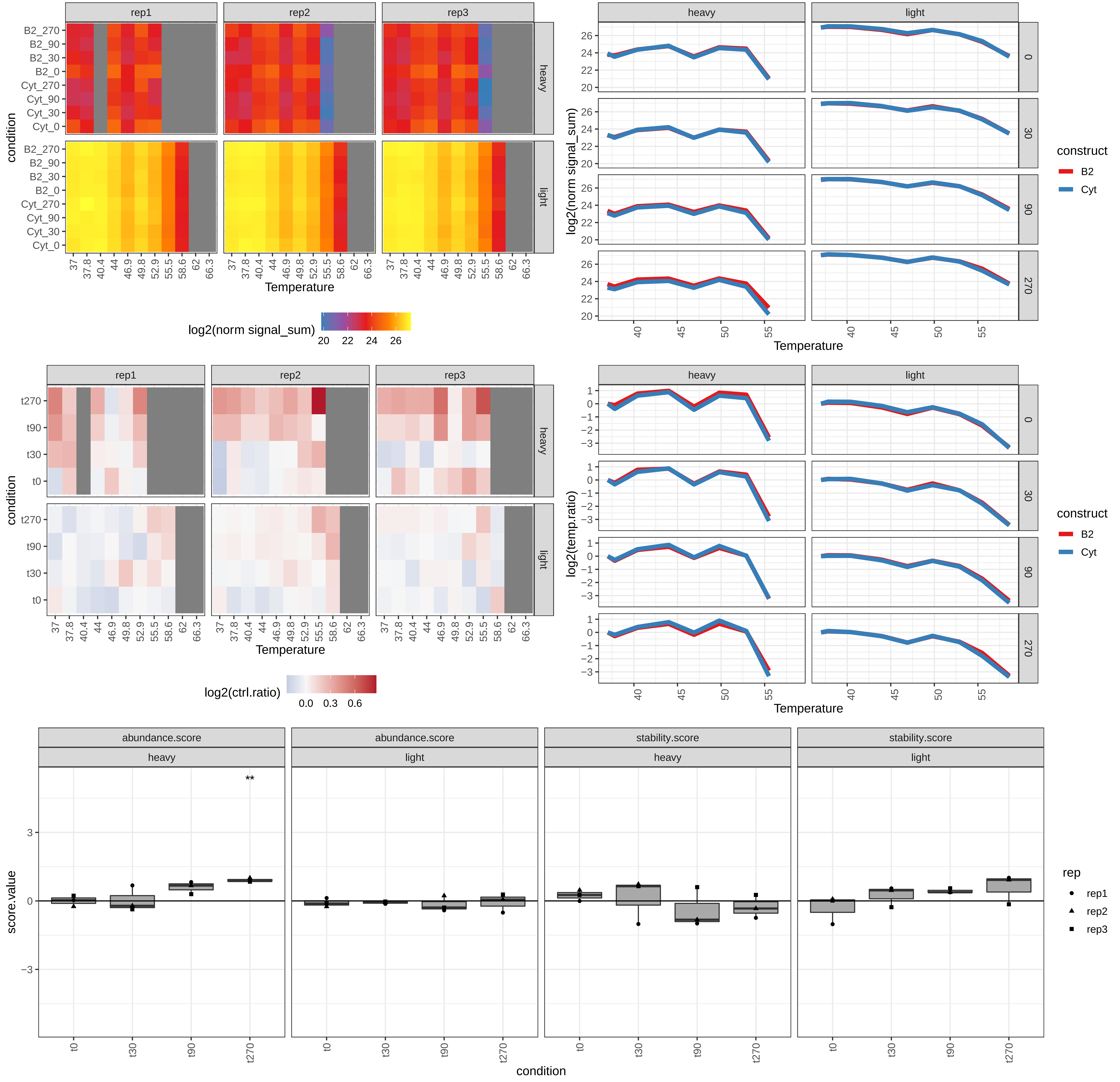

P39077 − T−complex protein 1 subunit gamma OS=Saccharomyces cerevisiae (strain ATCC 204508 / S288c) GN=CCT3 PE=1 SV=2

CCT4 − average.top3: 7.04719565217391 − max.qupm: 20

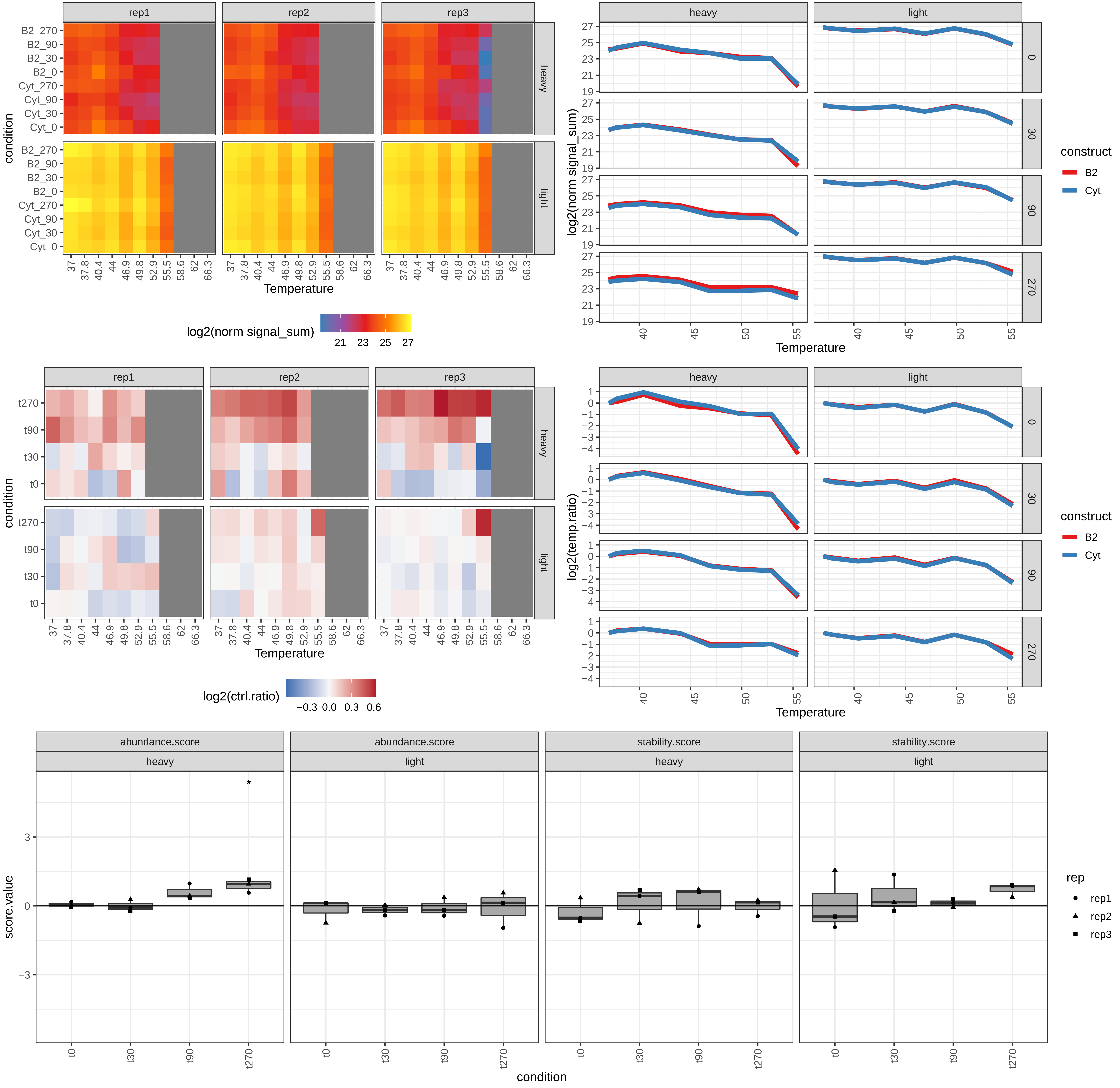

P39078 − T−complex protein 1 subunit delta OS=Saccharomyces cerevisiae (strain ATCC 204508 / S288c) GN=CCT4 PE=1 SV=2

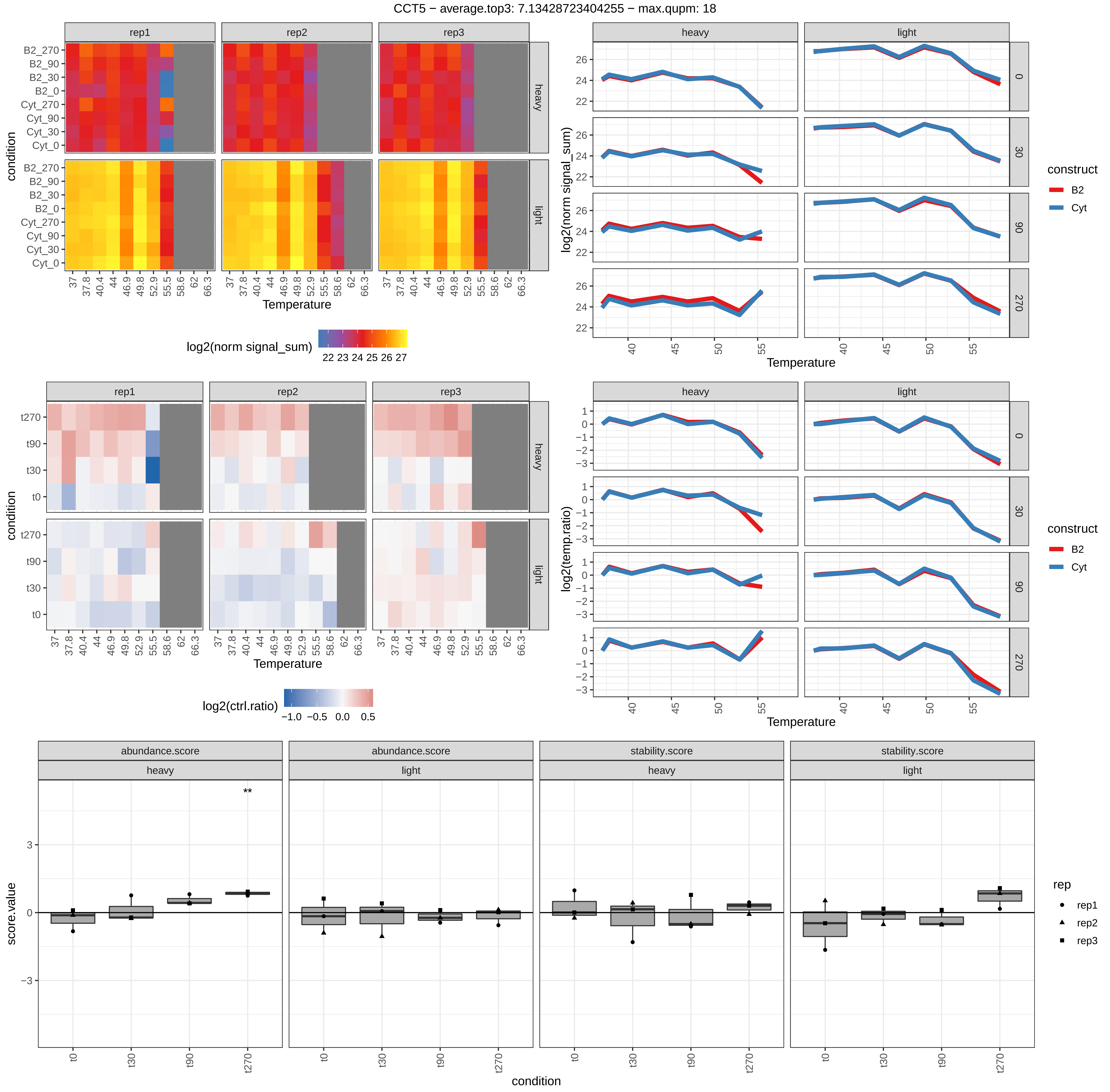

P40413 − T−complex protein 1 subunit epsilon OS=Saccharomyces cerevisiae (strain ATCC 204508 / S288c) GN=CCT5 PE=1 SV=3

CCT6 − average.top3: 7.26919477272727 − max.qupm: 17

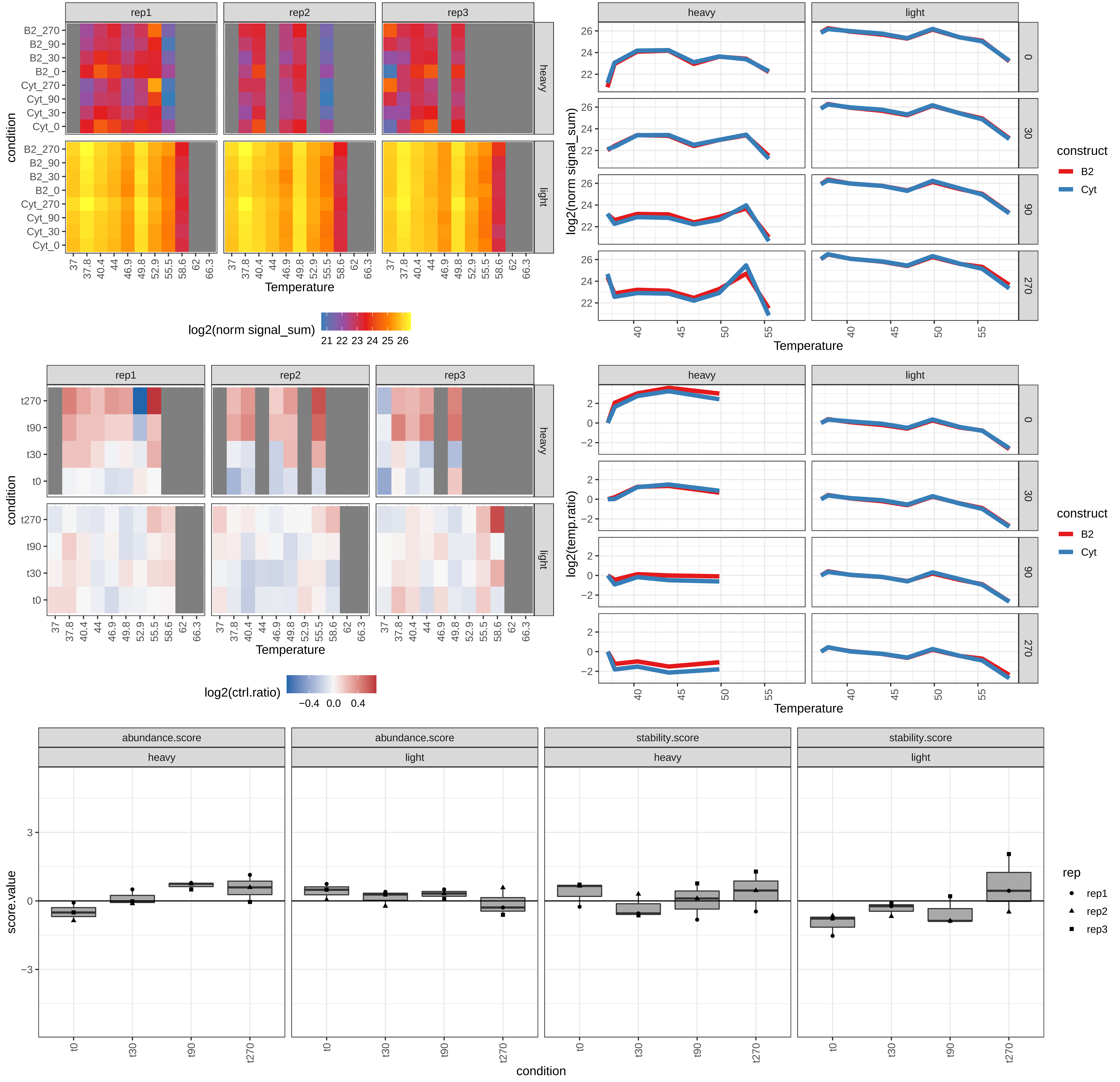

P39079 − T−complex protein 1 subunit zeta OS=Saccharomyces cerevisiae (strain ATCC 204508 / S288c) GN=CCT6 PE=1 SV=1

CCT7 − average.top3: 7.07963155555556 − max.qupm: 21

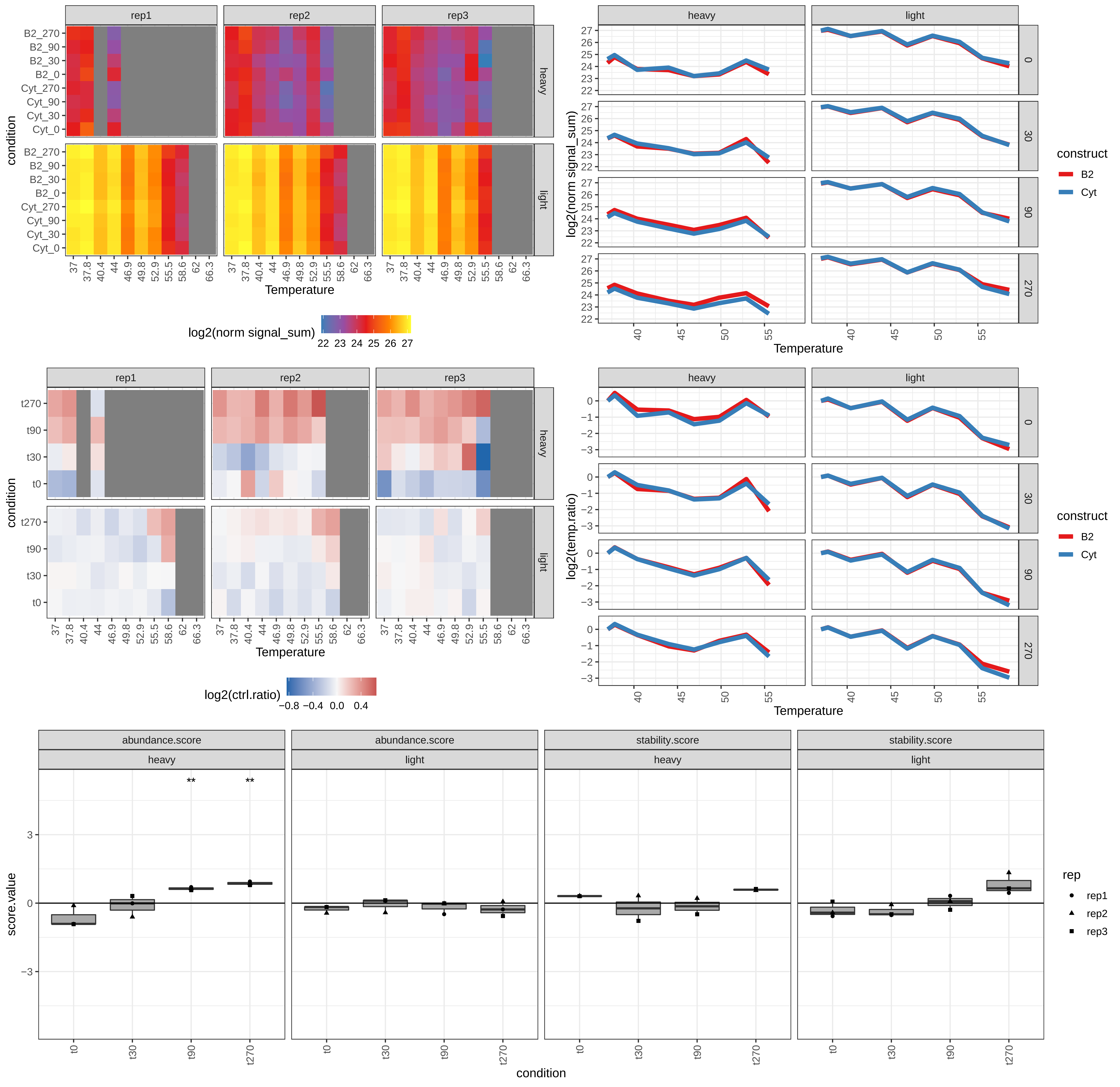

P42943 − T−complex protein 1 subunit eta OS=Saccharomyces cerevisiae (strain ATCC 204508 / S288c) GN=CCT7 PE=1 SV=1

CCT8 − average.top3: 7.02569075 − max.qupm: 15

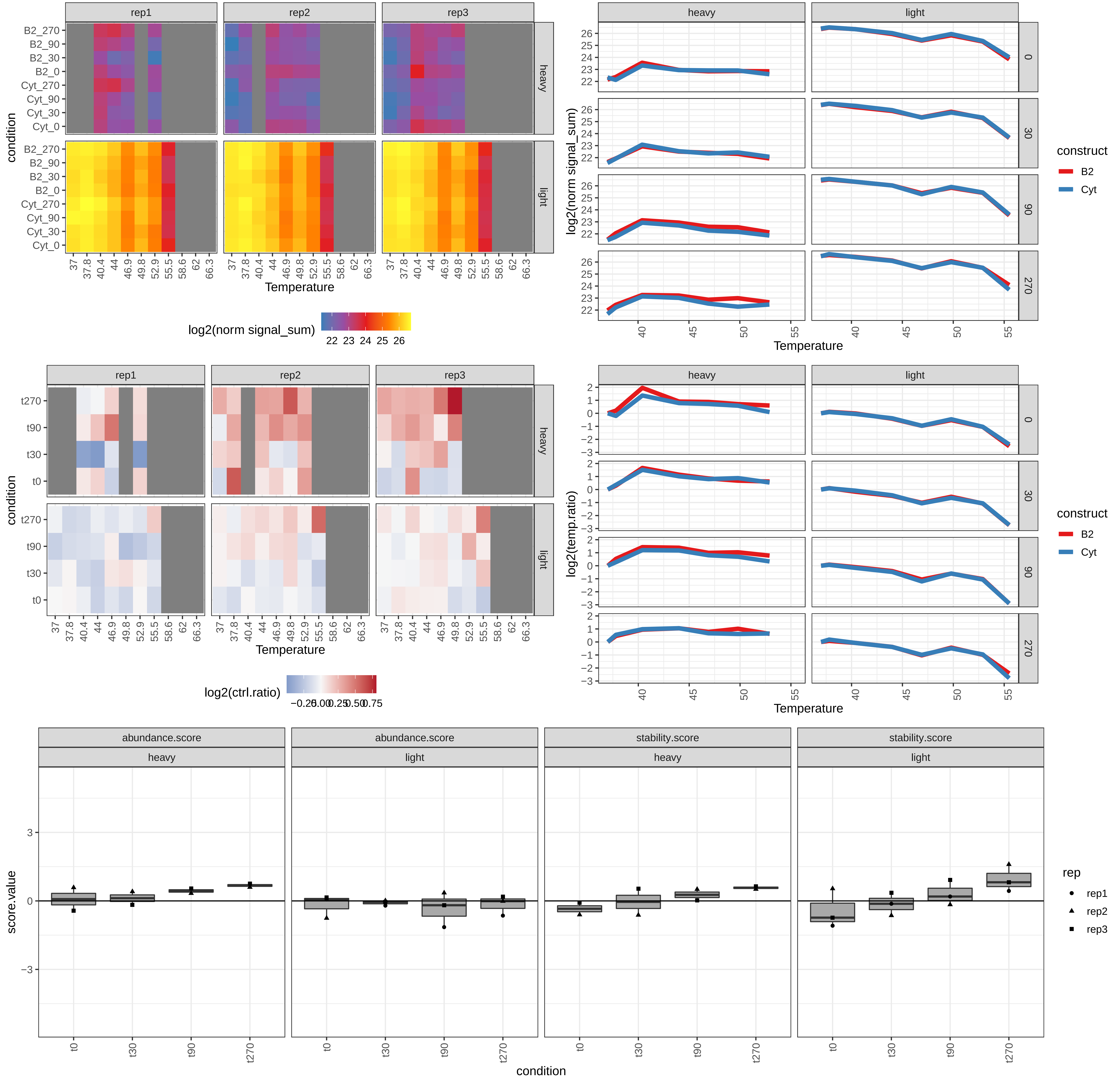

P47079 − T−complex protein 1 subunit theta OS=Saccharomyces cerevisiae (strain ATCC 204508 / S288c) GN=CCT8 PE=1 SV=1

CCZ1 − average.top3: 6.117258 − max.qupm: 4

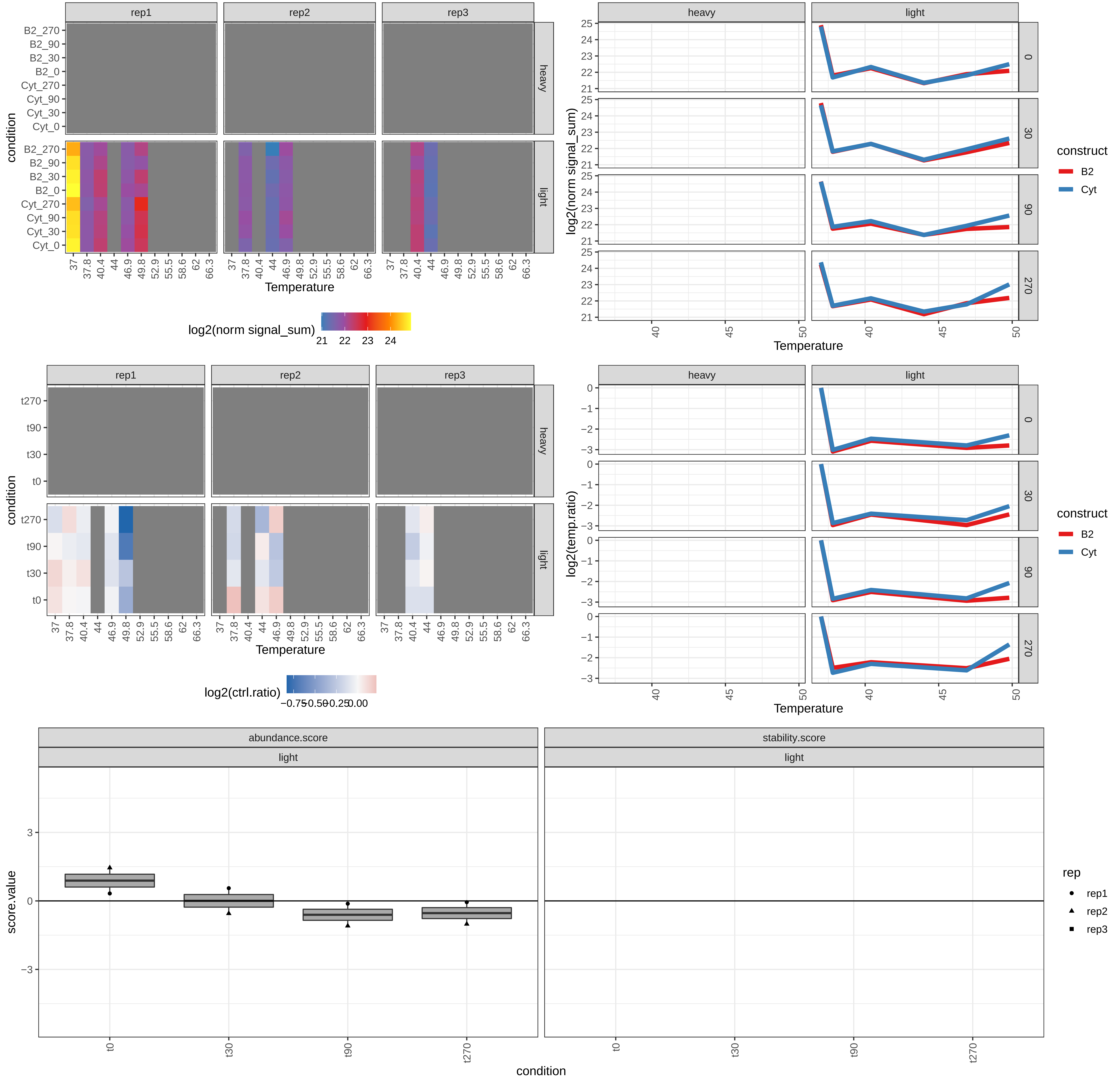

P38273 − Vacuolar fusion protein CCZ1 OS=Saccharomyces cerevisiae (strain ATCC 204508 / S288c) GN=CCZ1 PE=1 SV=1

CDC1 − average.top3: 6.65667125 − max.qupm: 4

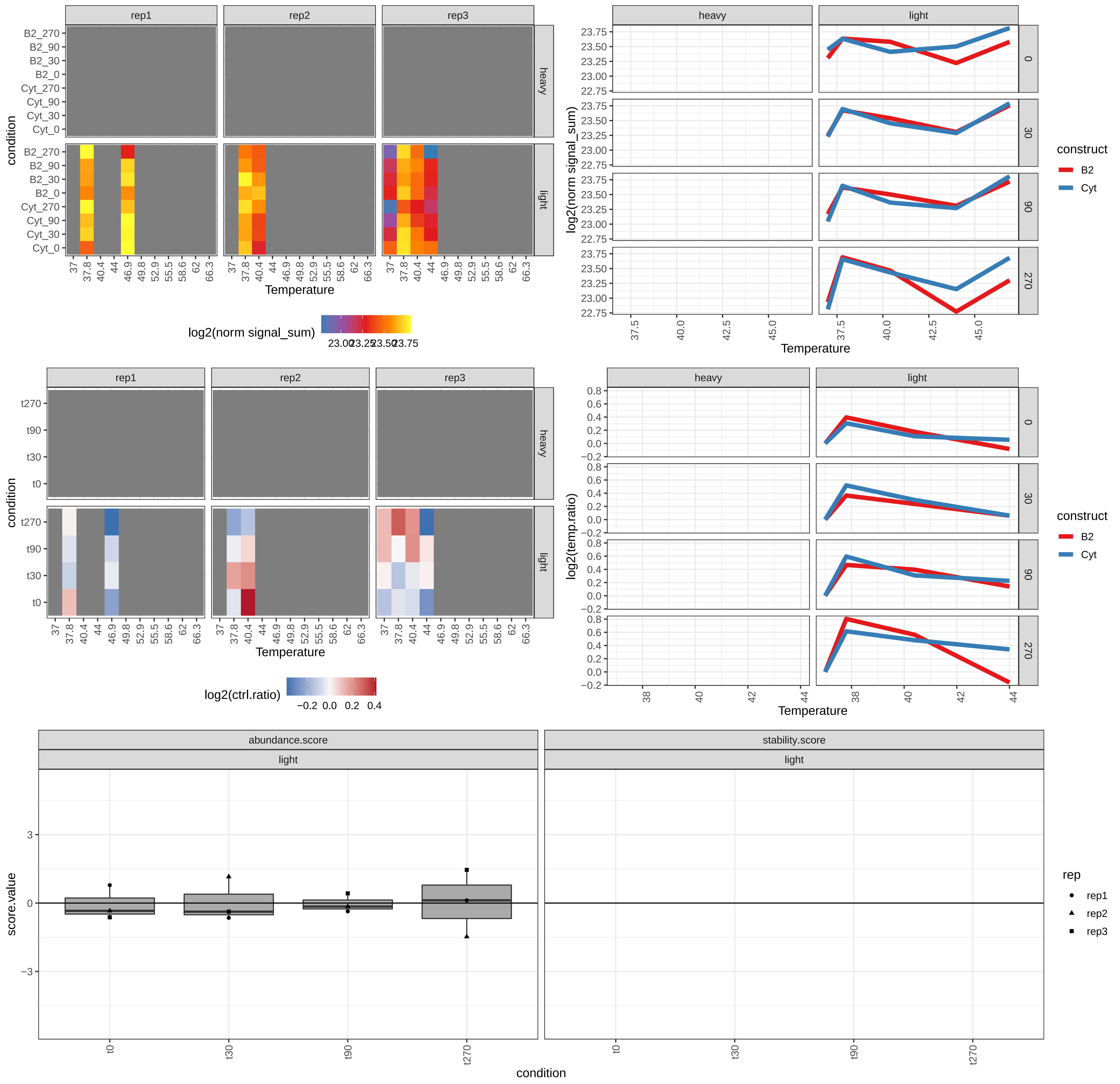

P40986 − Cell division control protein 1 OS=Saccharomyces cerevisiae (strain ATCC 204508 / S288c) GN=CDC1 PE=1 SV=2

CDC10 − average.top3: 7.46208902439024 − max.qupm: 10

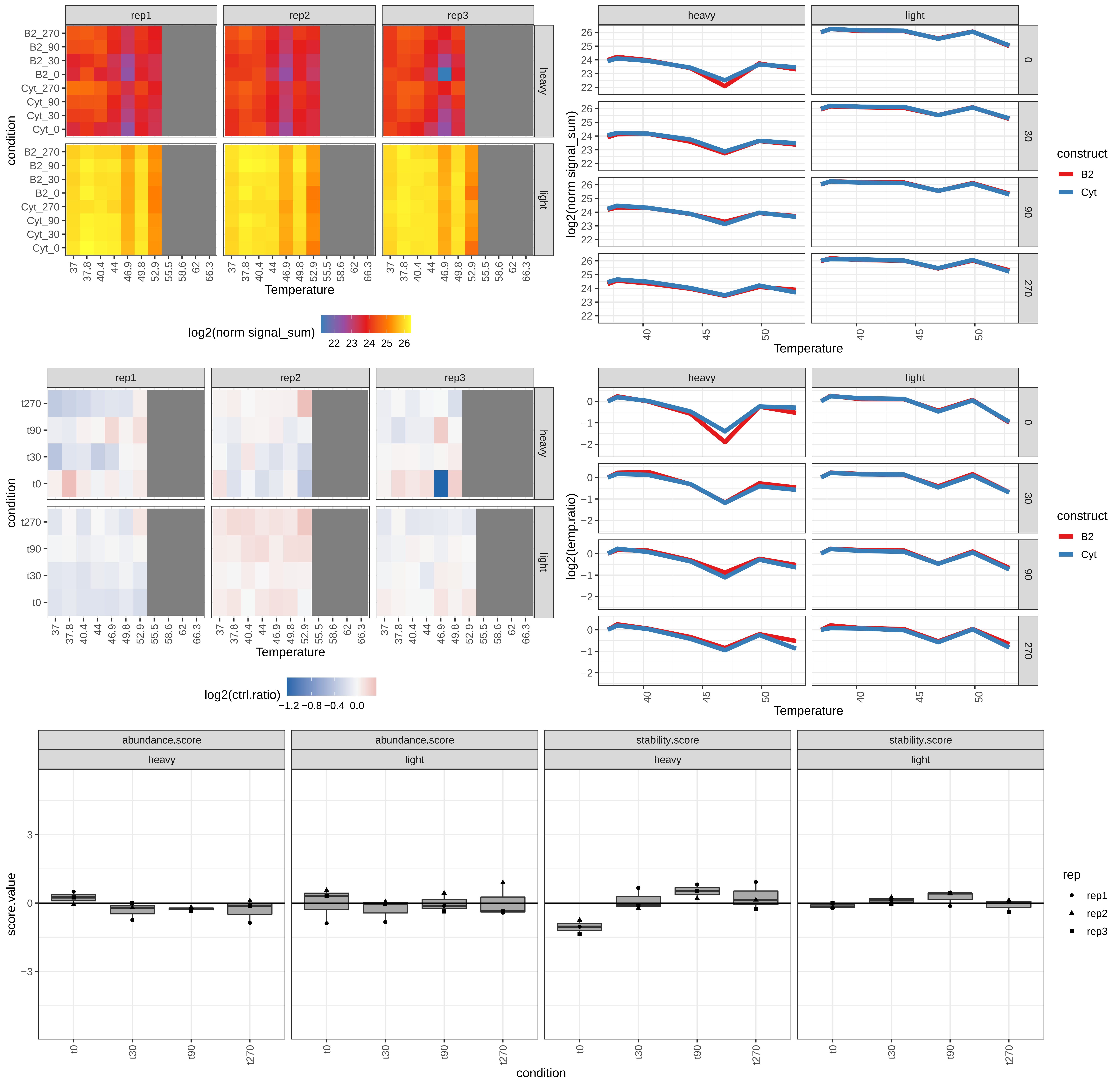

P25342 − Cell division control protein 10 OS=Saccharomyces cerevisiae (strain ATCC 204508 / S288c) GN=CDC10 PE=1 SV=1

CDC11 − average.top3: 7.06469423076923 − max.qupm: 8

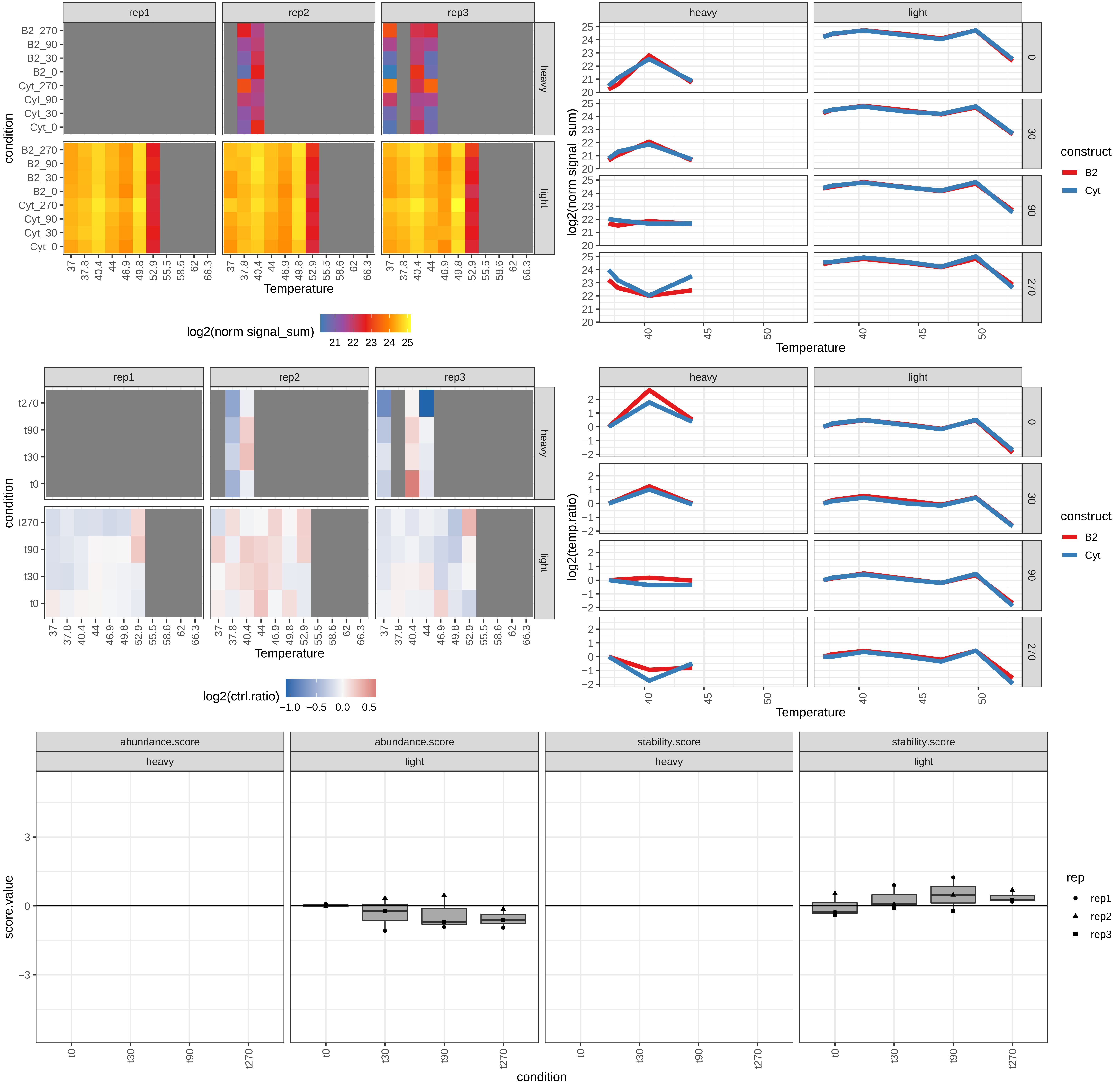

P32458 − Cell division control protein 11 OS=Saccharomyces cerevisiae (strain ATCC 204508 / S288c) GN=CDC11 PE=1 SV=1

CDC12 − average.top3: 7.0886258974359 − max.qupm: 14

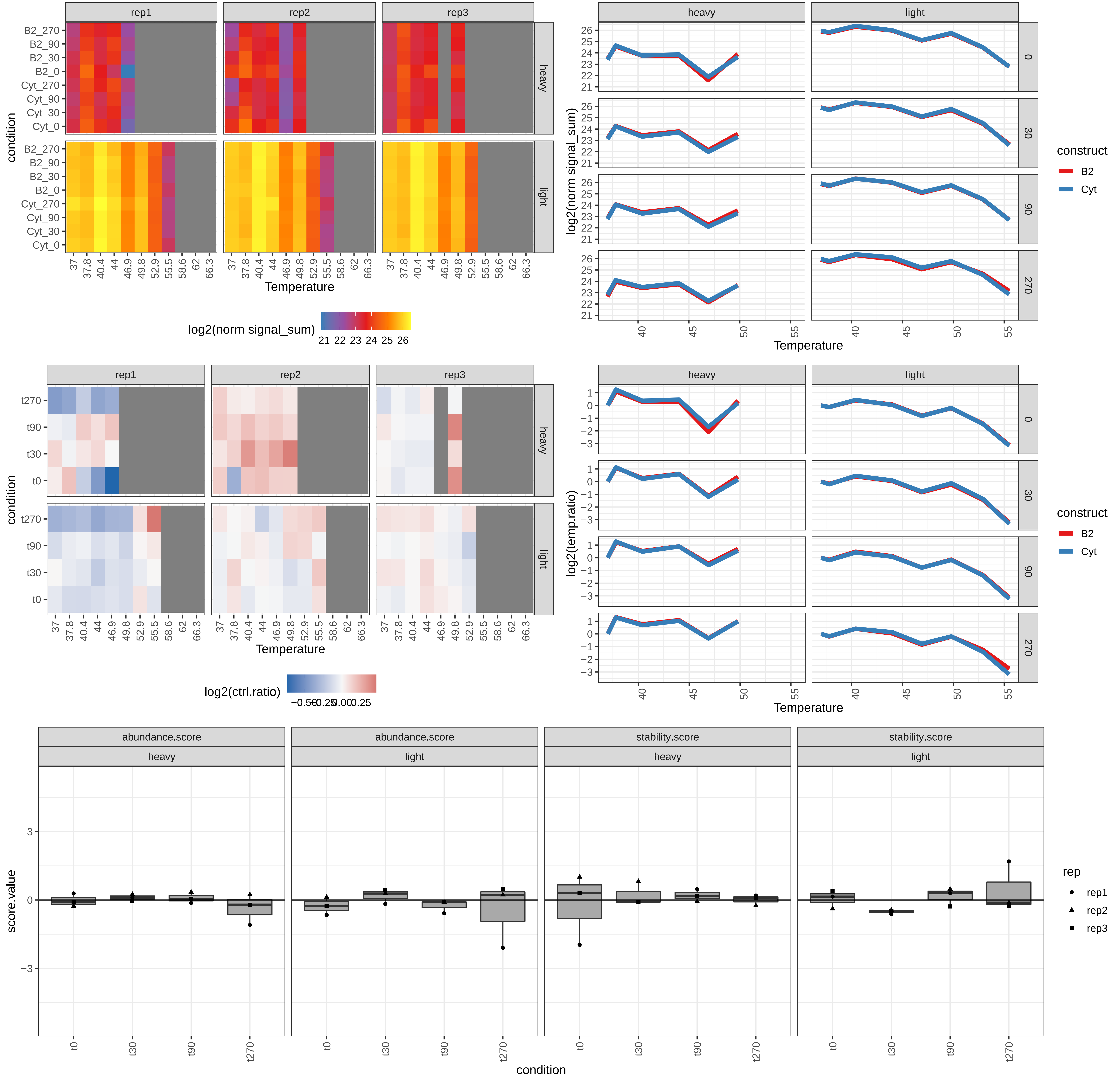

P32468 − Cell division control protein 12 OS=Saccharomyces cerevisiae (strain ATCC 204508 / S288c) GN=CDC12 PE=1 SV=1

CDC13 − average.top3: 7.105083 − max.qupm: 3

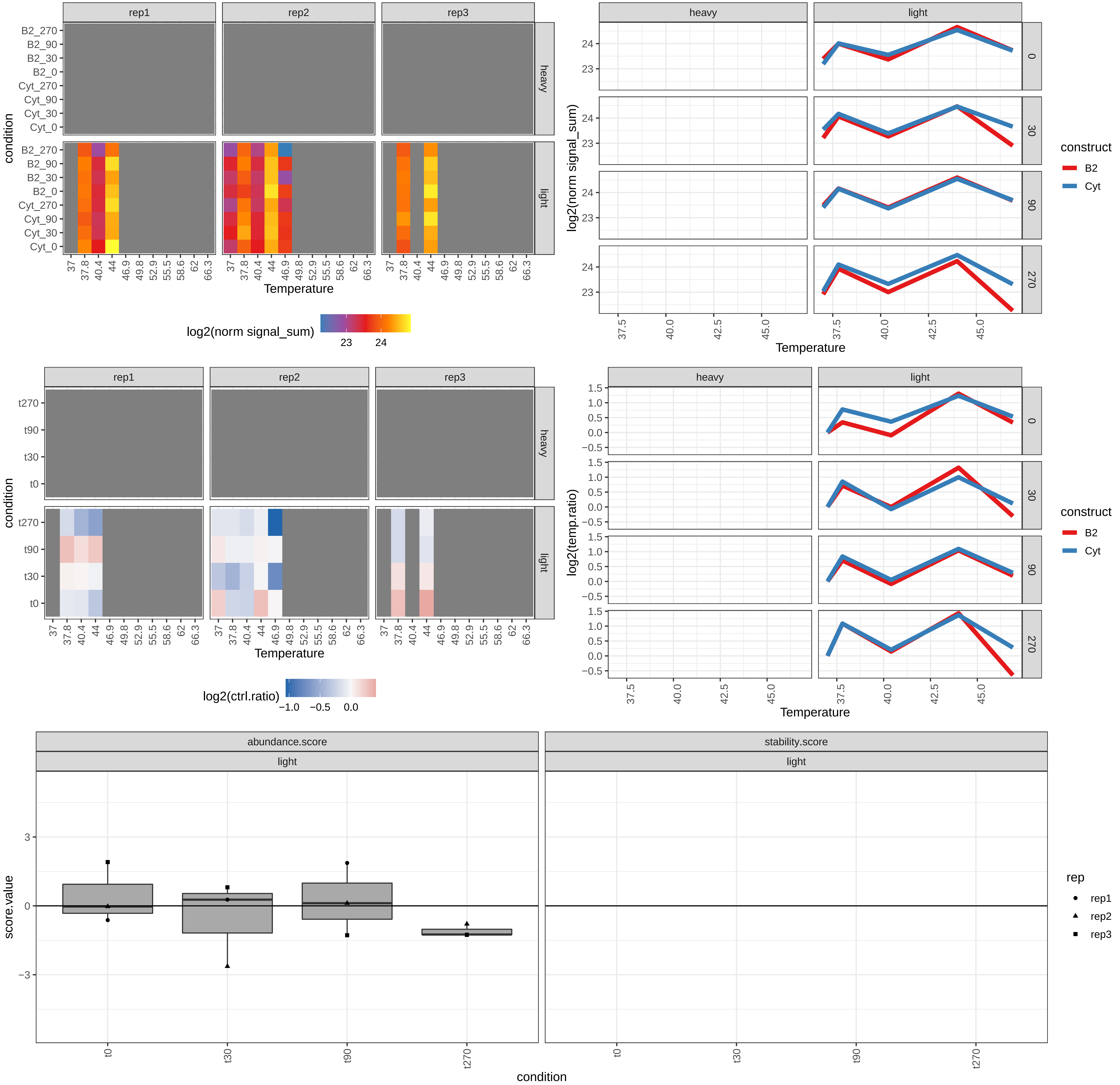

P32797 − Cell division control protein 13 OS=Saccharomyces cerevisiae (strain ATCC 204508 / S288c) GN=CDC13 PE=1 SV=2

CDC19 − average.top3: 8.70242031746032 − max.qupm: 36

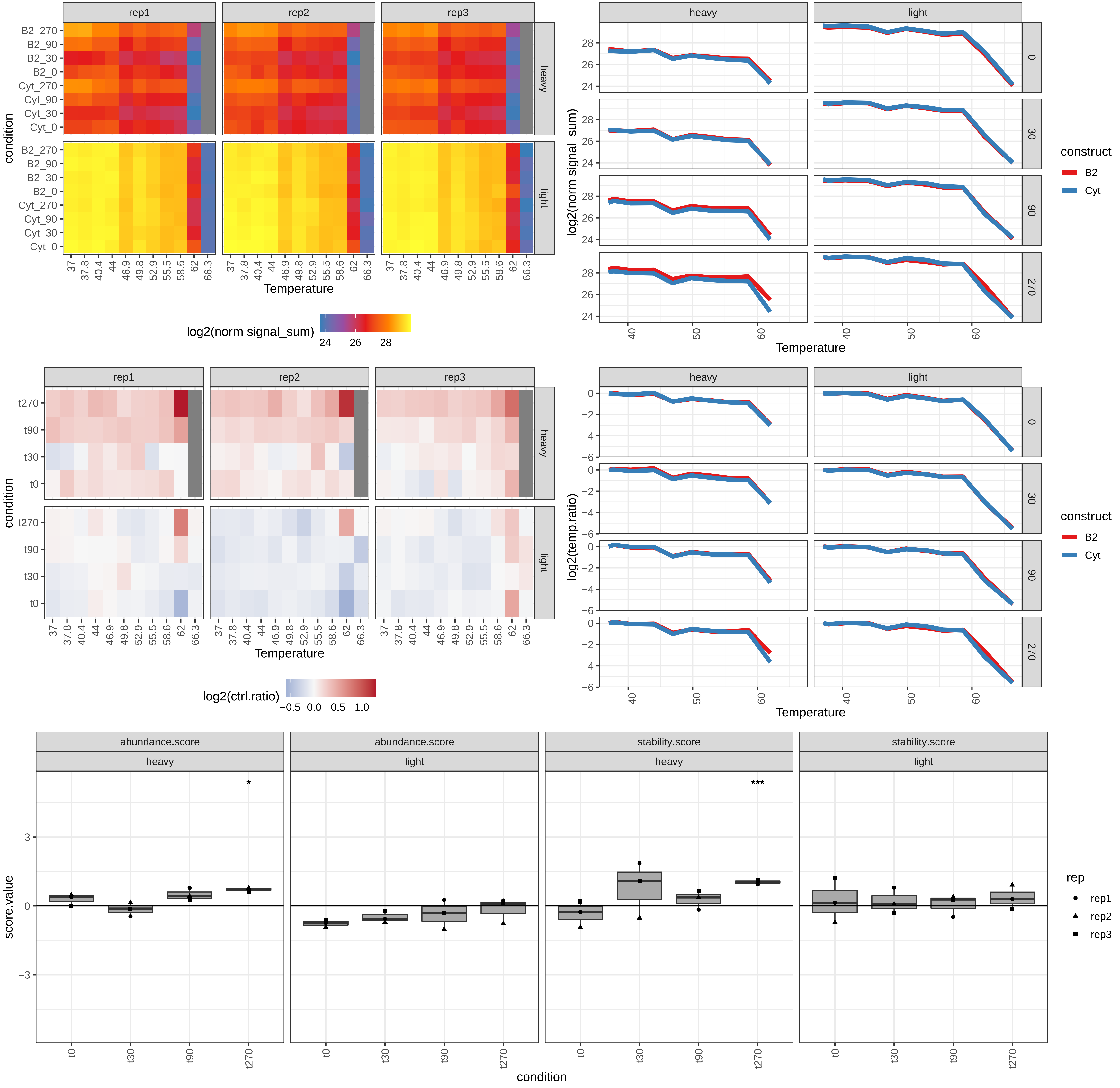

P00549 − Pyruvate kinase 1 OS=Saccharomyces cerevisiae (strain ATCC 204508 / S288c) GN=CDC19 PE=1 SV=2

CDC21 − average.top3: 6.78782586206897 − max.qupm: 9

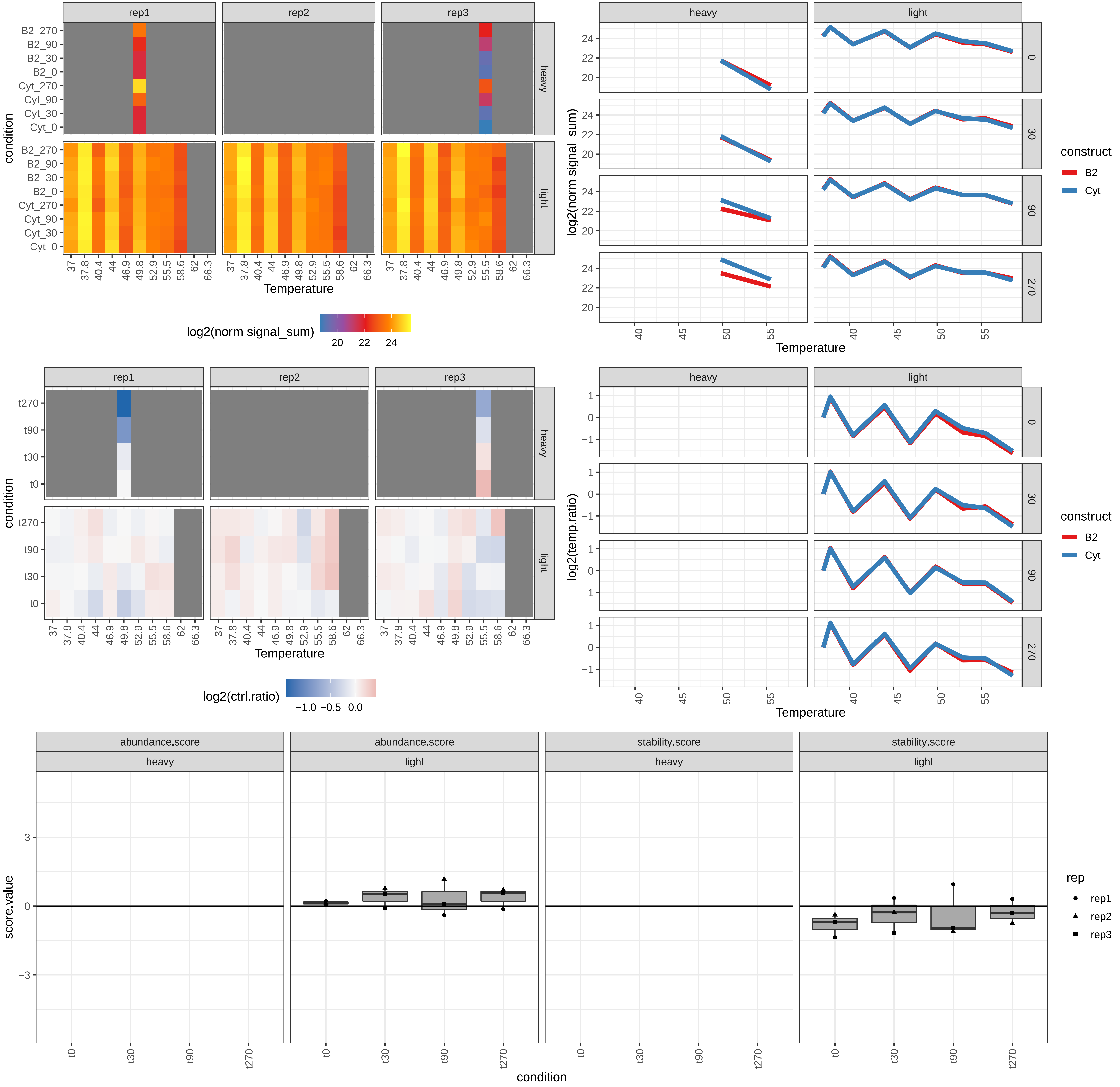

P06785 − Thymidylate synthase OS=Saccharomyces cerevisiae (strain ATCC 204508 / S288c) GN=CDC21 PE=1 SV=1

CDC24 − average.top3: 6.68396764705882 − max.qupm: 6

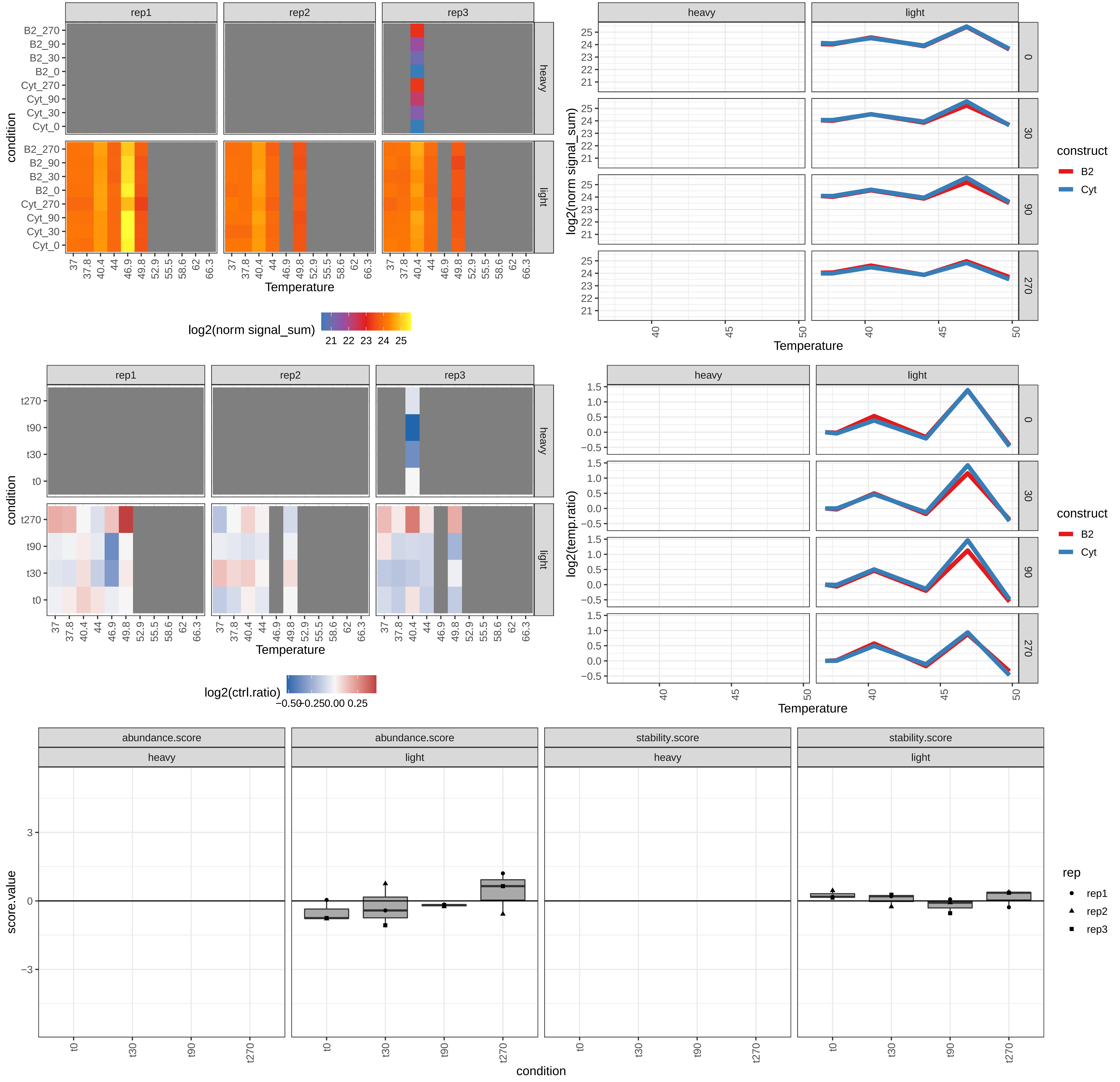

P11433 − Cell division control protein 24 OS=Saccharomyces cerevisiae (strain ATCC 204508 / S288c) GN=CDC24 PE=1 SV=2

CDC25 − average.top3: 7.35674181818182 − max.qupm: 8

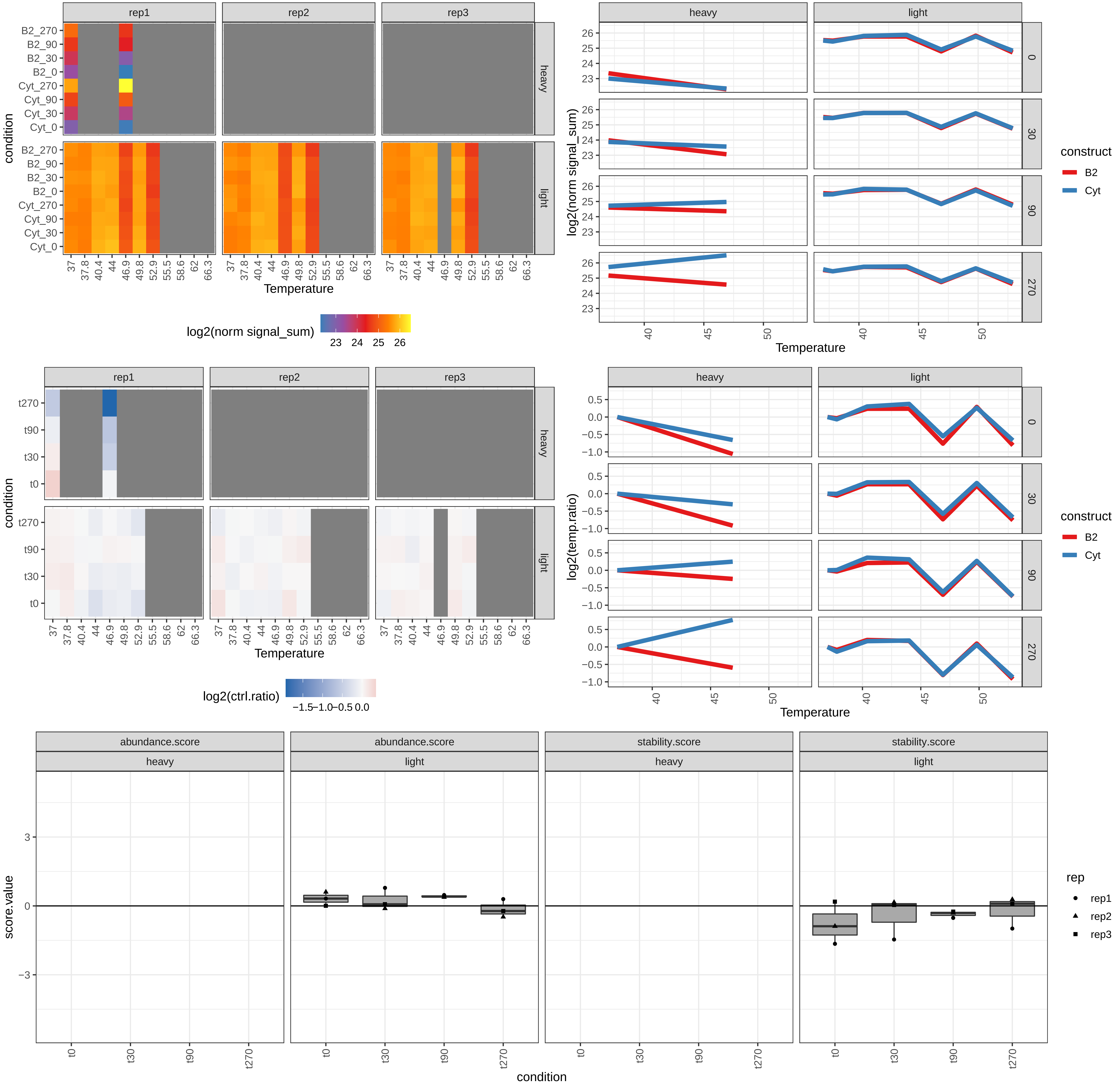

P04821 − Cell division control protein 25 OS=Saccharomyces cerevisiae (strain ATCC 204508 / S288c) GN=CDC25 PE=1 SV=1

CDC28 − average.top3: 7.1101604 − max.qupm: 10

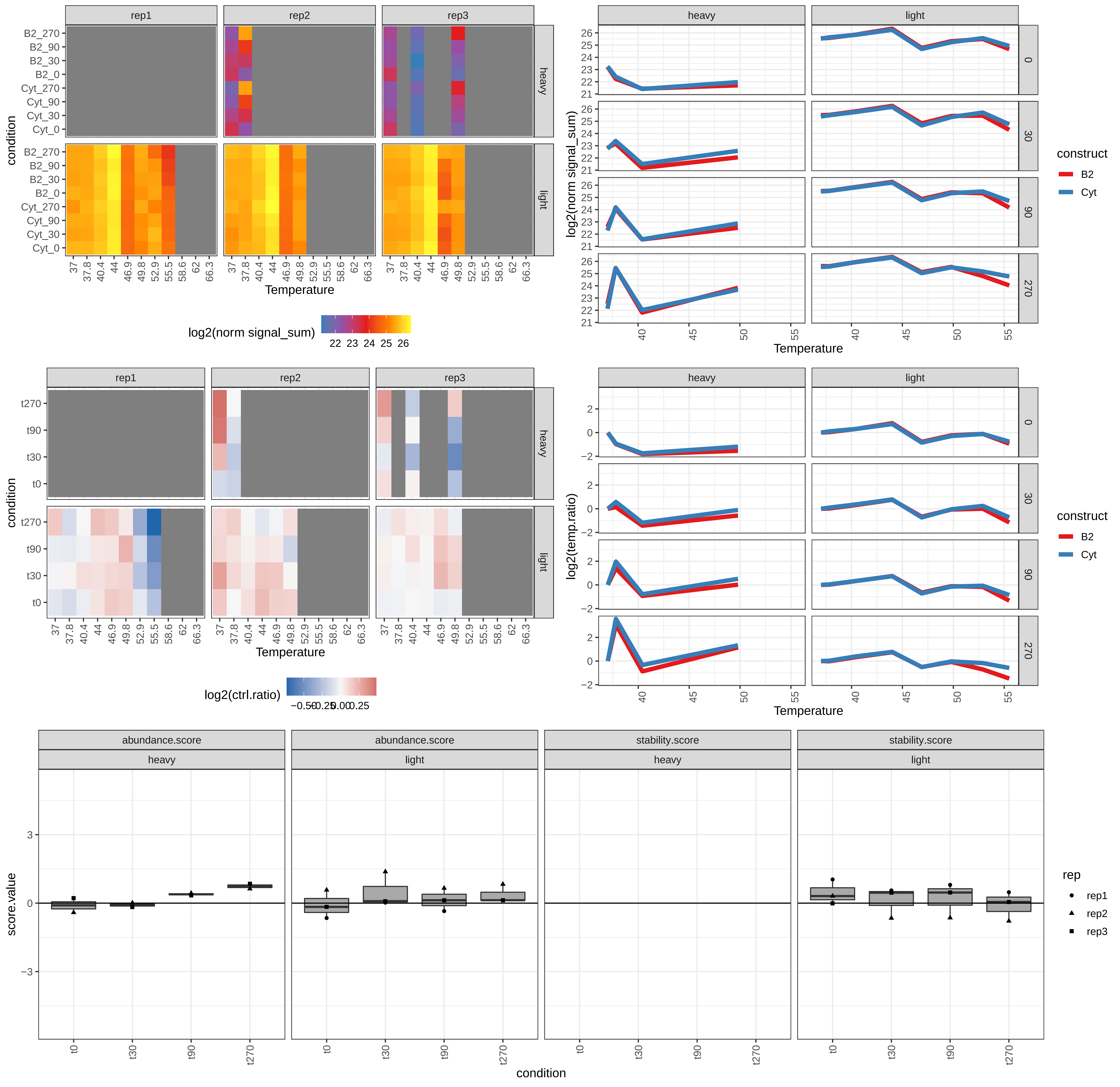

P00546 − Cyclin−dependent kinase 1 OS=Saccharomyces cerevisiae (strain ATCC 204508 / S288c) GN=CDC28 PE=1 SV=1

CDC3 − average.top3: 7.24132926829268 − max.qupm: 13

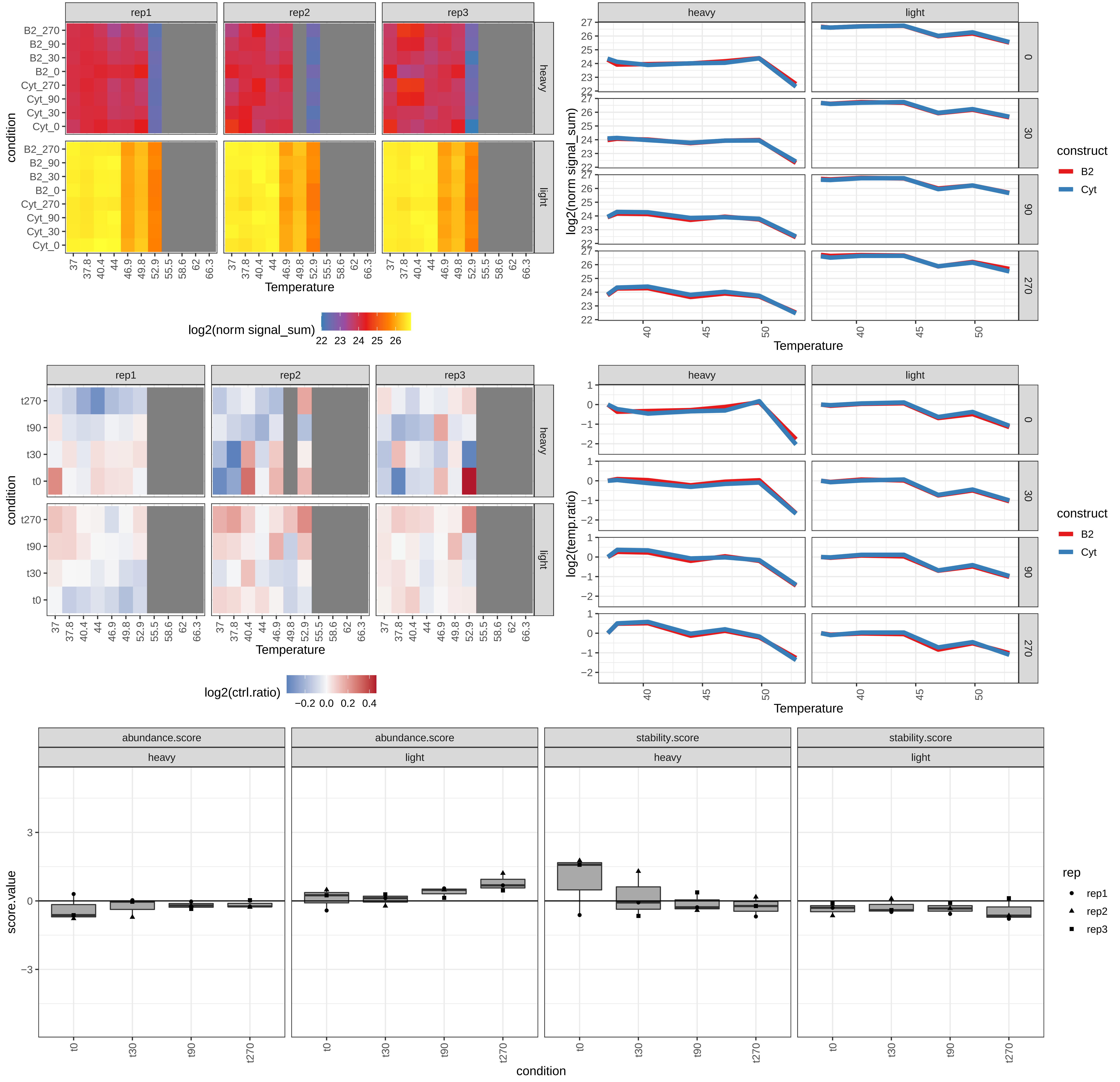

P32457 − Cell division control protein 3 OS=Saccharomyces cerevisiae (strain ATCC 204508 / S288c) GN=CDC3 PE=1 SV=3

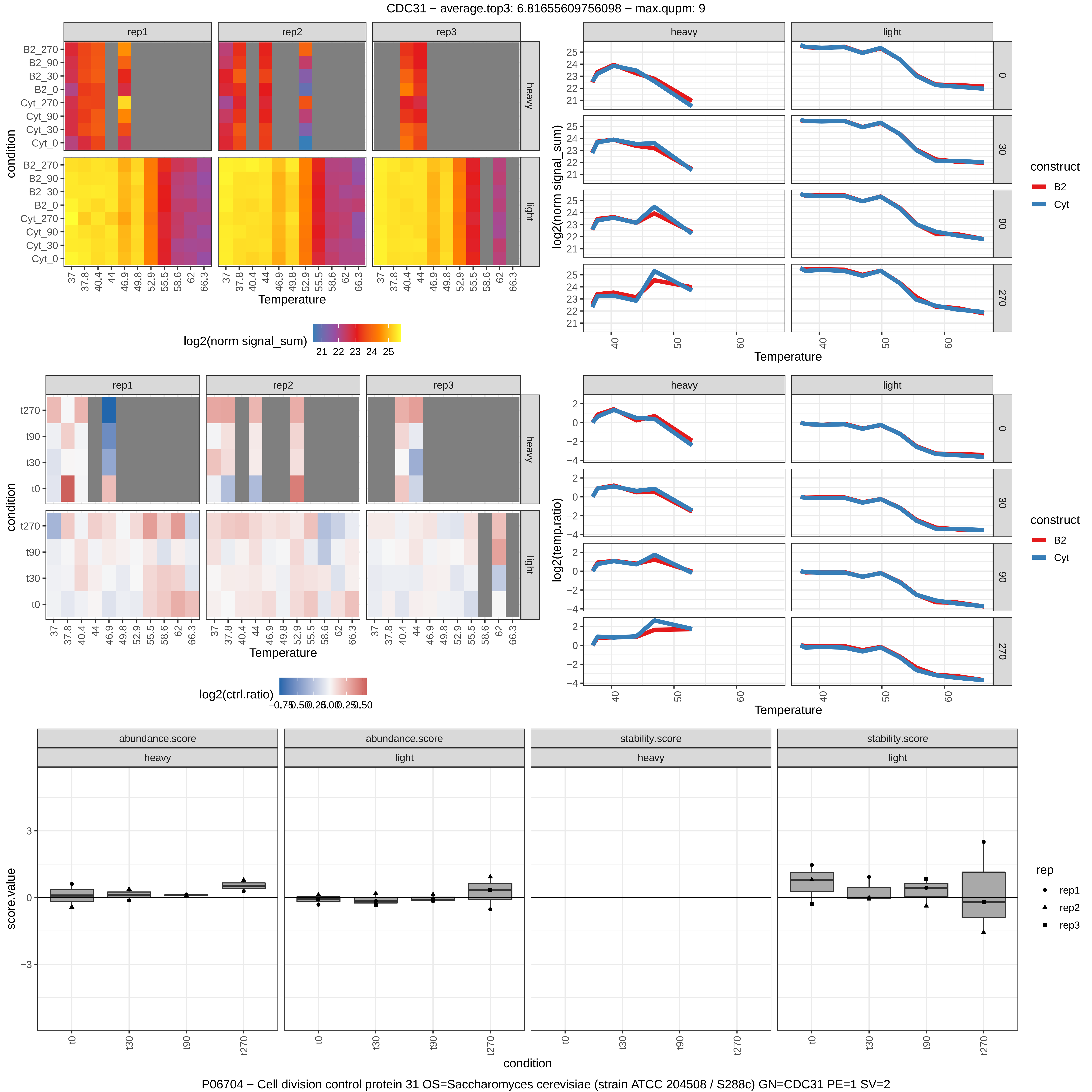

CDC31 − average.top3: 6.81655609756098 − max.qupm: 9
rep1
rep2
rep3
heavy
light
B2_270
B2_90
B2_30
B2_0
Cyt_270
Cyt_90
Cyt_30
Cyt_0
condition
Temperature
log2(norm signal_sum)
log2(norm signal_sum)
construct
B2
Cyt
t270
t90
t30
t0
log2(ctrl.ratio)
log2(temp.ratio)
abundance.score
stability.score
score.value
condition
rep
rep1
rep2
rep3
P06704 − Cell division control protein 31 OS=Saccharomyces cerevisiae (strain ATCC 204508 / S288c) GN=CDC31 PE=1 SV=2

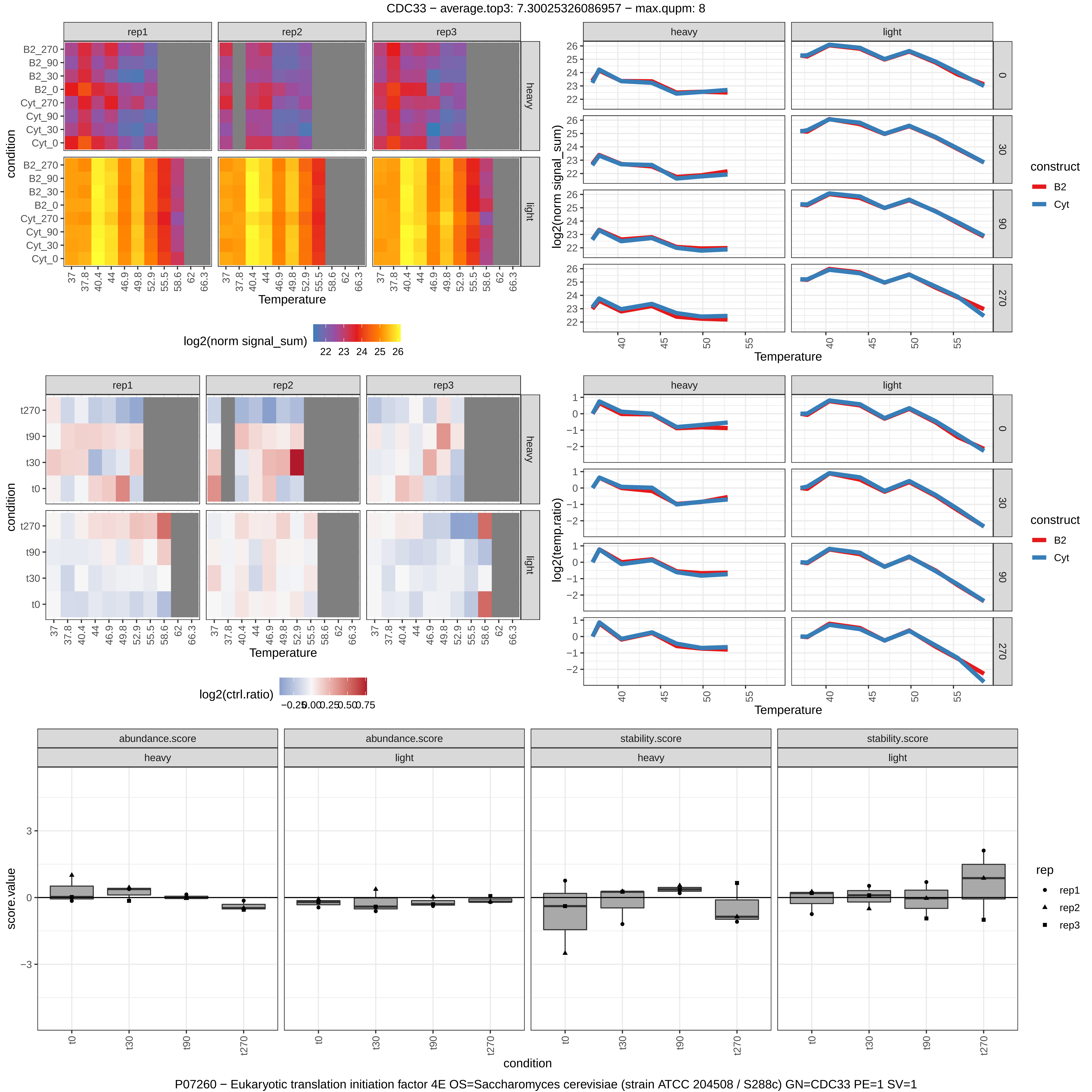
CDC33 − average.top3: 7.30025326086957 − max.qupm: 8
rep1
rep2
rep3
heavy
light
condition
Temperature
log2(norm signal_sum)
log2(temp.ratio)
log2(ctrl.ratio)
construct
B2
Cyt
abundance.score
stability.score
score.value
rep
P07260 − Eukaryotic translation initiation factor 4E OS=Saccharomyces cerevisiae (strain ATCC 204508 / S288c) GN=CDC33 PE=1 SV=1

CDC37 − average.top3: 6.80793509433962 − max.qupm: 11

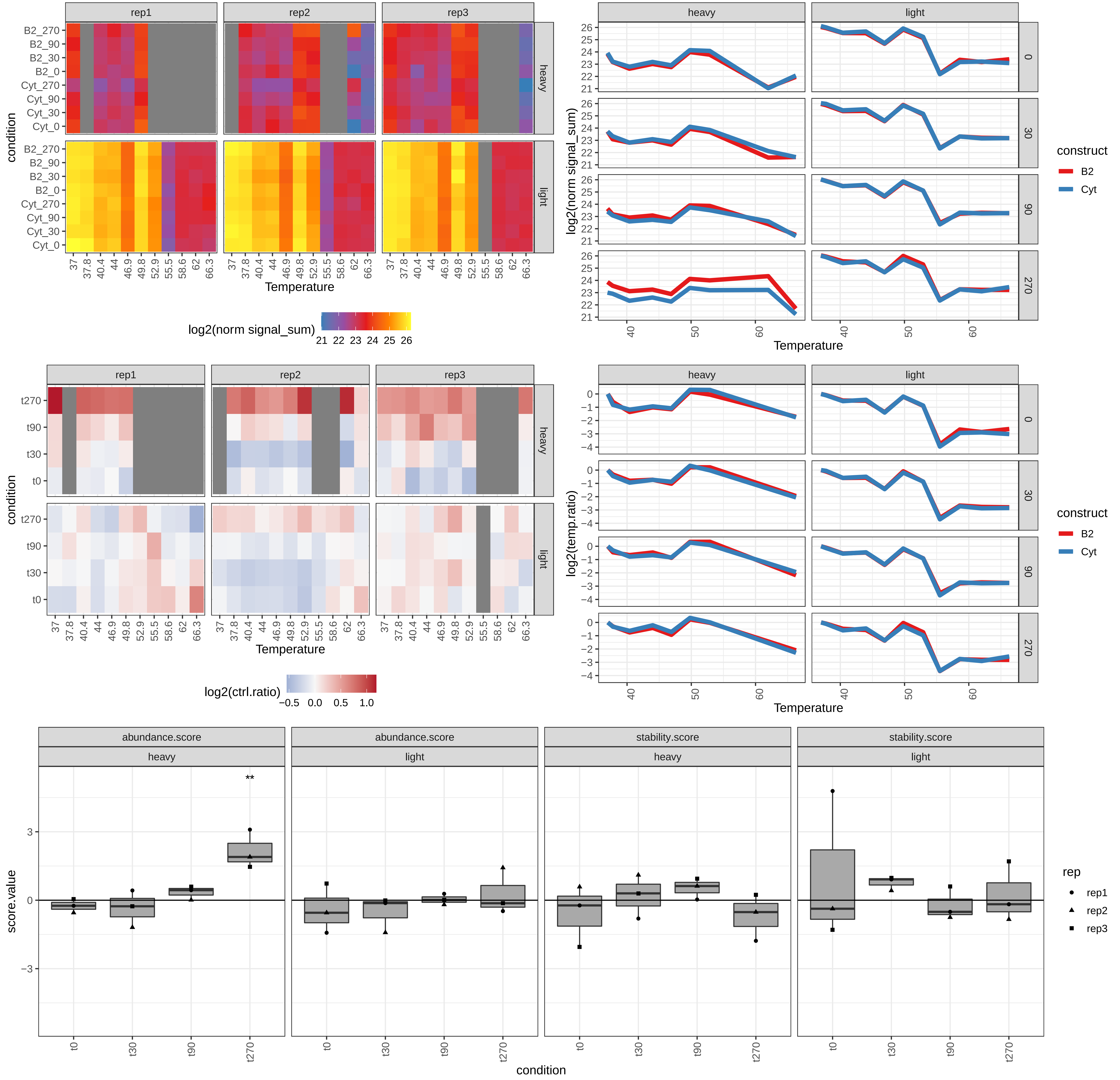

P06101 − Hsp90 co−chaperone Cdc37 OS=Saccharomyces cerevisiae (strain ATCC 204508 / S288c) GN=CDC37 PE=1 SV=2

CDC39 − average.top3: 6.75198454545455 − max.qupm: 24

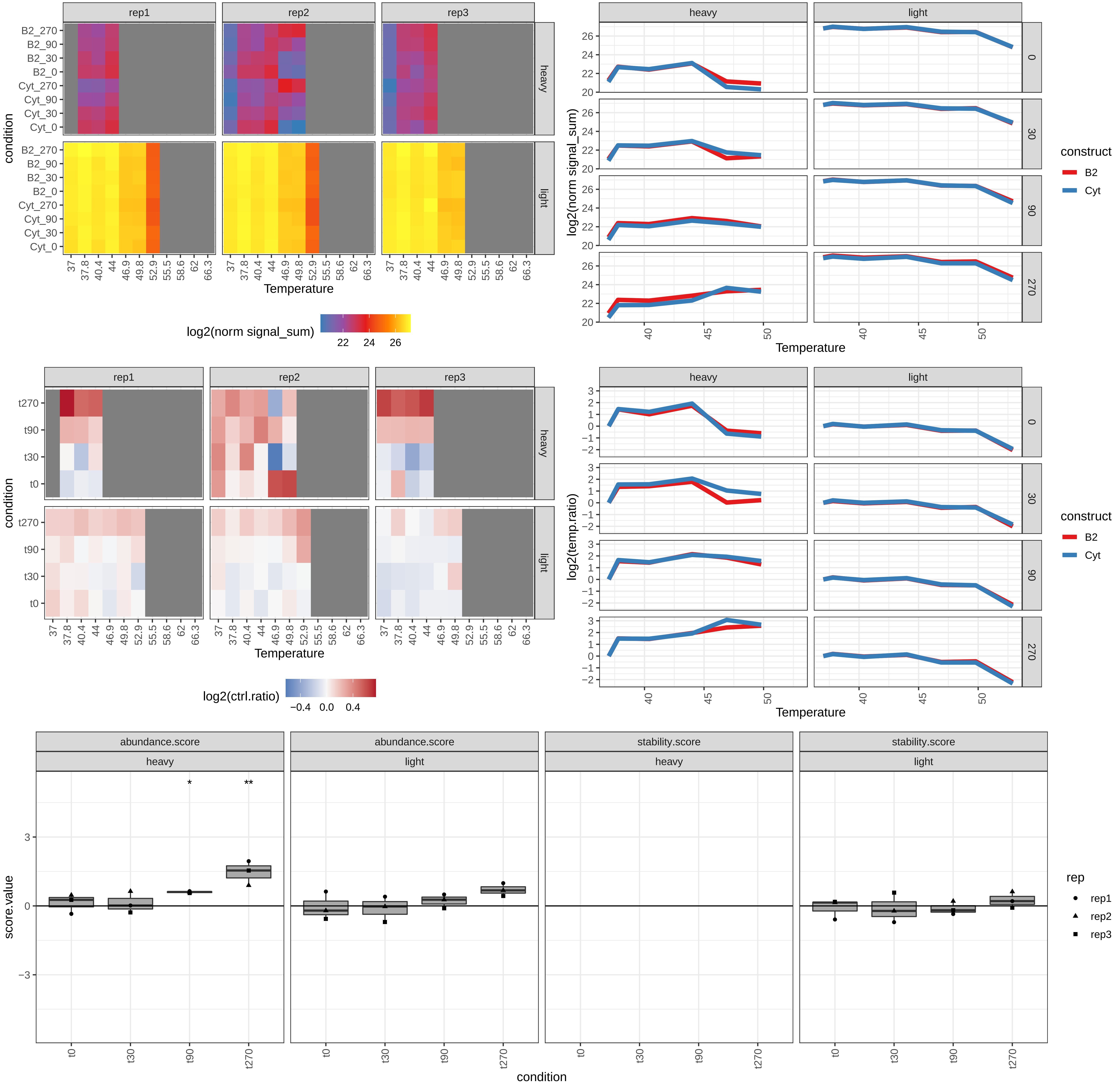

P25655 − General negative regulator of transcription subunit 1 OS=Saccharomyces cerevisiae (strain ATCC 204508 / S288c) GN=CDC39 PE=1 SV=3

CDC40 − average.top3: 6.45390214285714 − max.qupm: 3

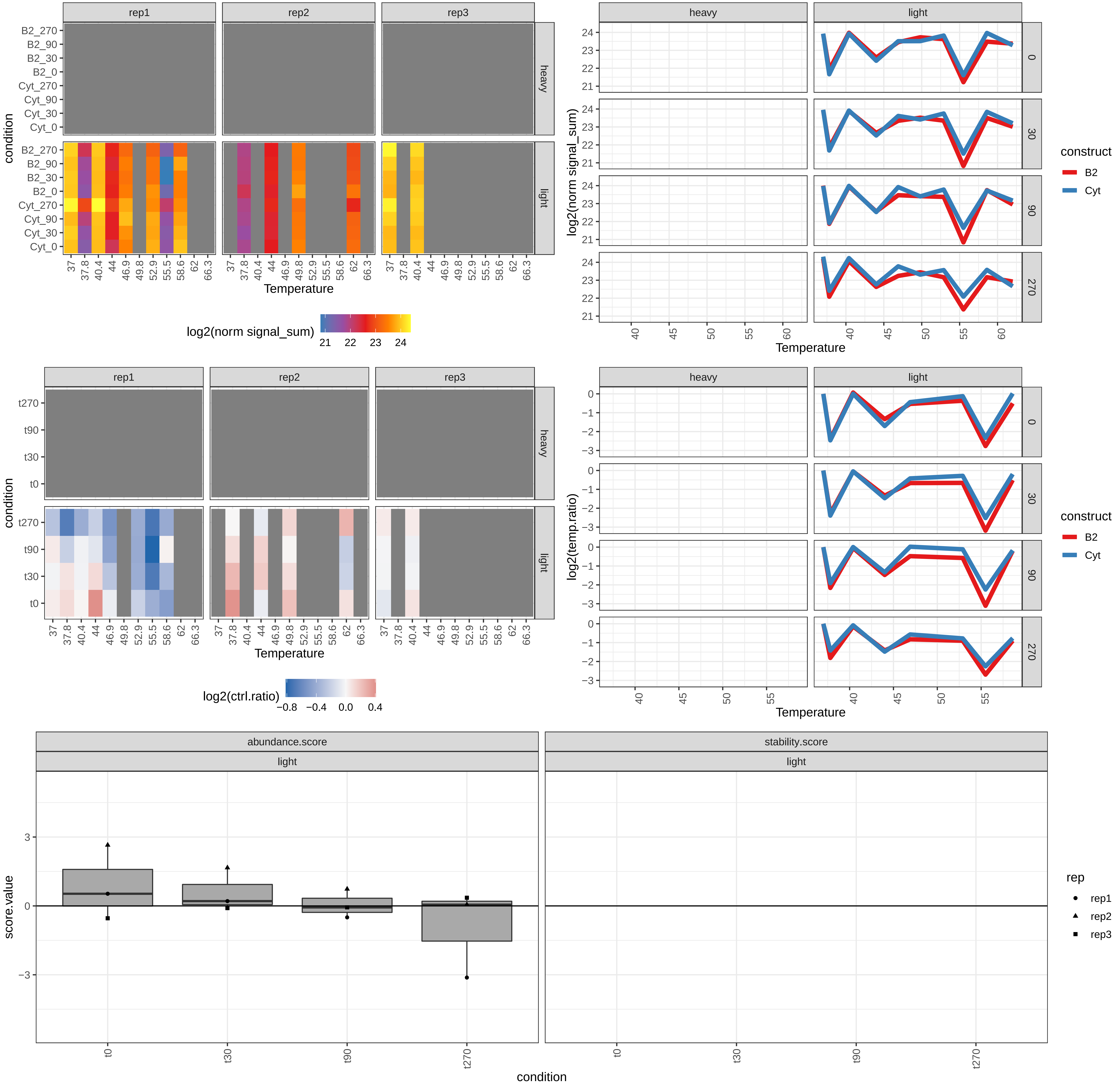

P40968 − Pre−mRNA−processing factor 17 OS=Saccharomyces cerevisiae (strain ATCC 204508 / S288c) GN=CDC40 PE=1 SV=2

CDC42 − average.top3: 7.313224 − max.qupm: 4

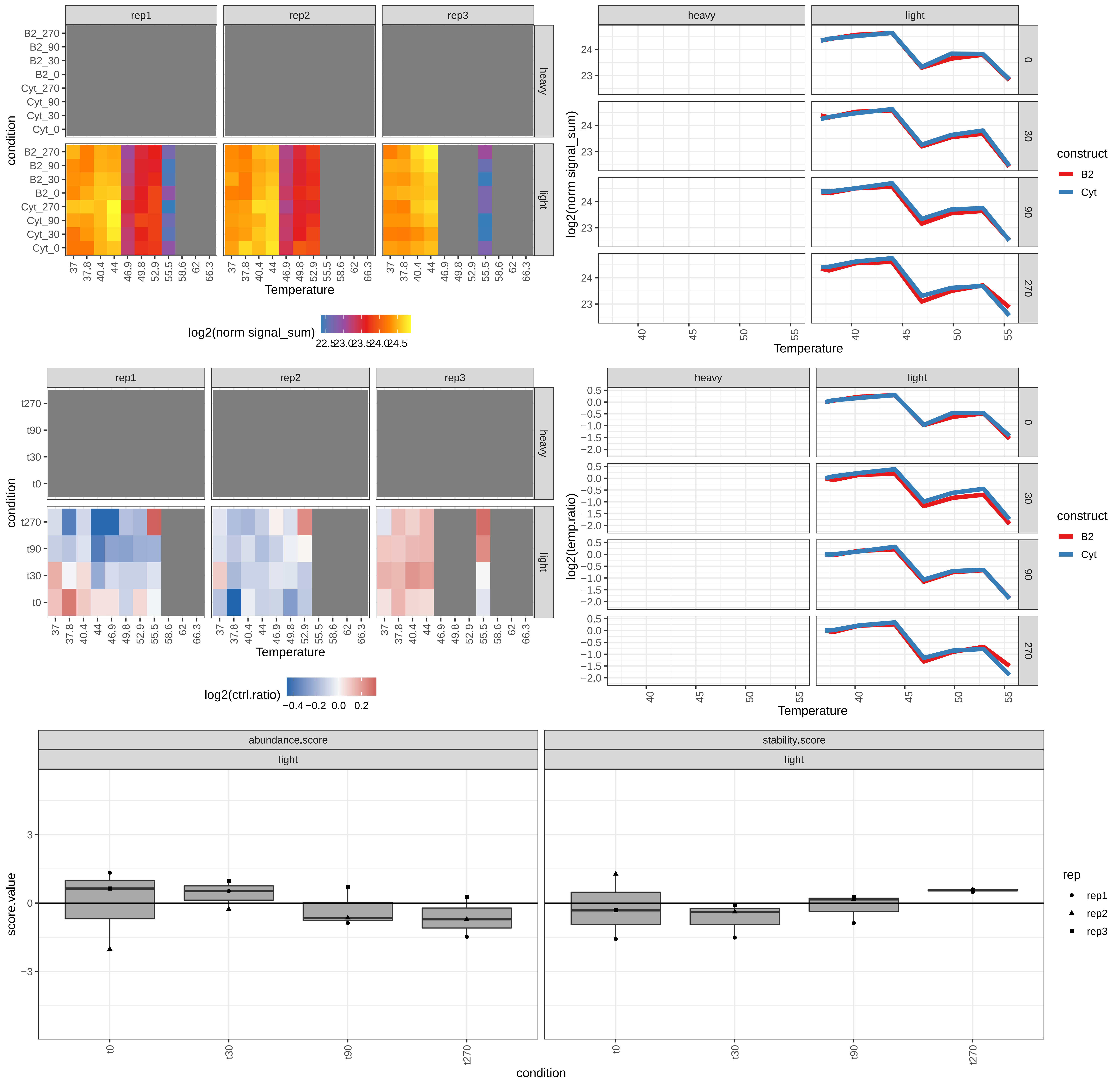

P19073 − Cell division control protein 42 OS=Saccharomyces cerevisiae (strain ATCC 204508 / S288c) GN=CDC42 PE=1 SV=2

CDC48 − average.top3: 7.751295 − max.qupm: 33

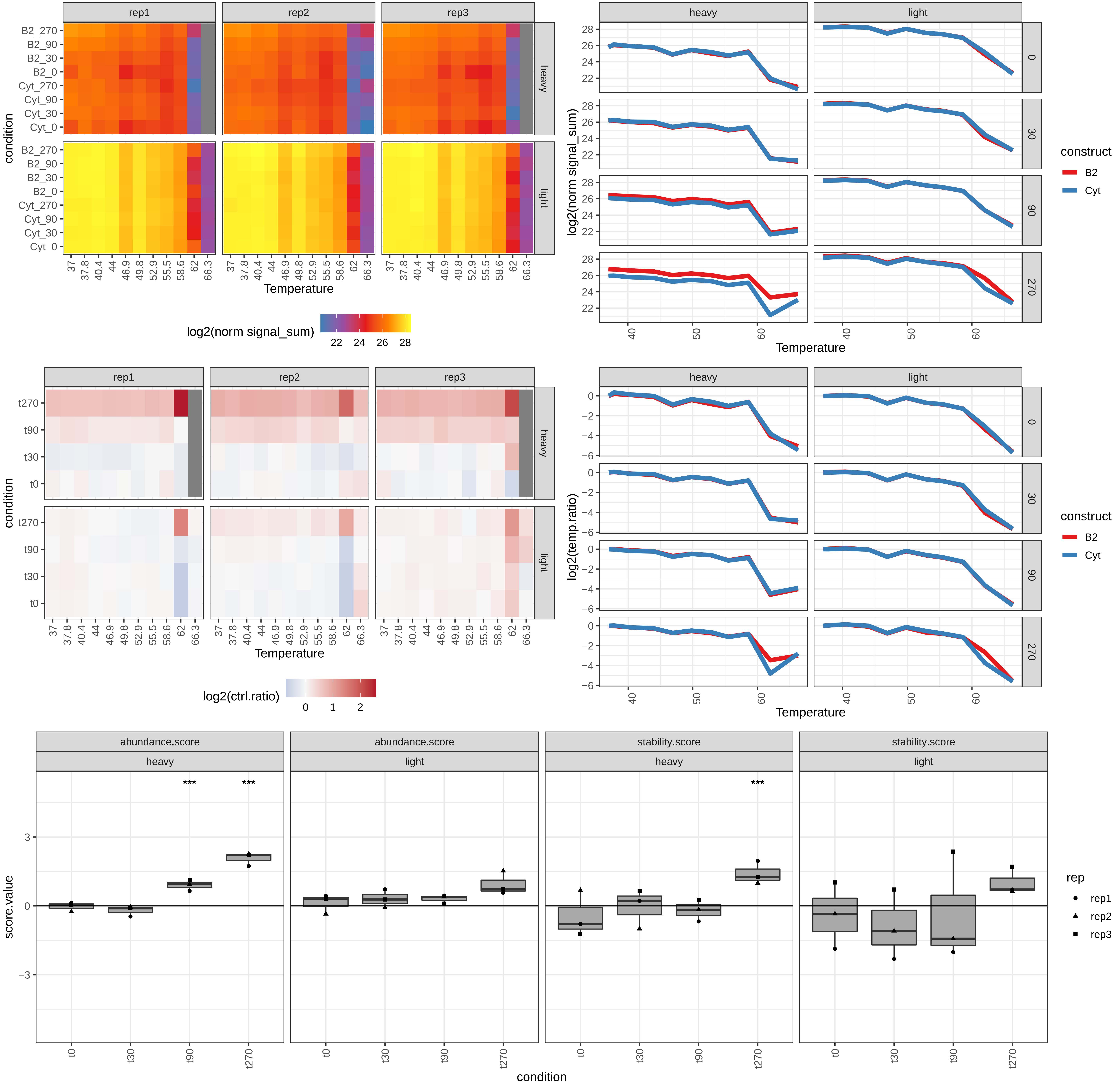

P25694 − Cell division control protein 48 OS=Saccharomyces cerevisiae (strain ATCC 204508 / S288c) GN=CDC48 PE=1 SV=3

CDC50 − average.top3: 6.66717411764706 − max.qupm: 4

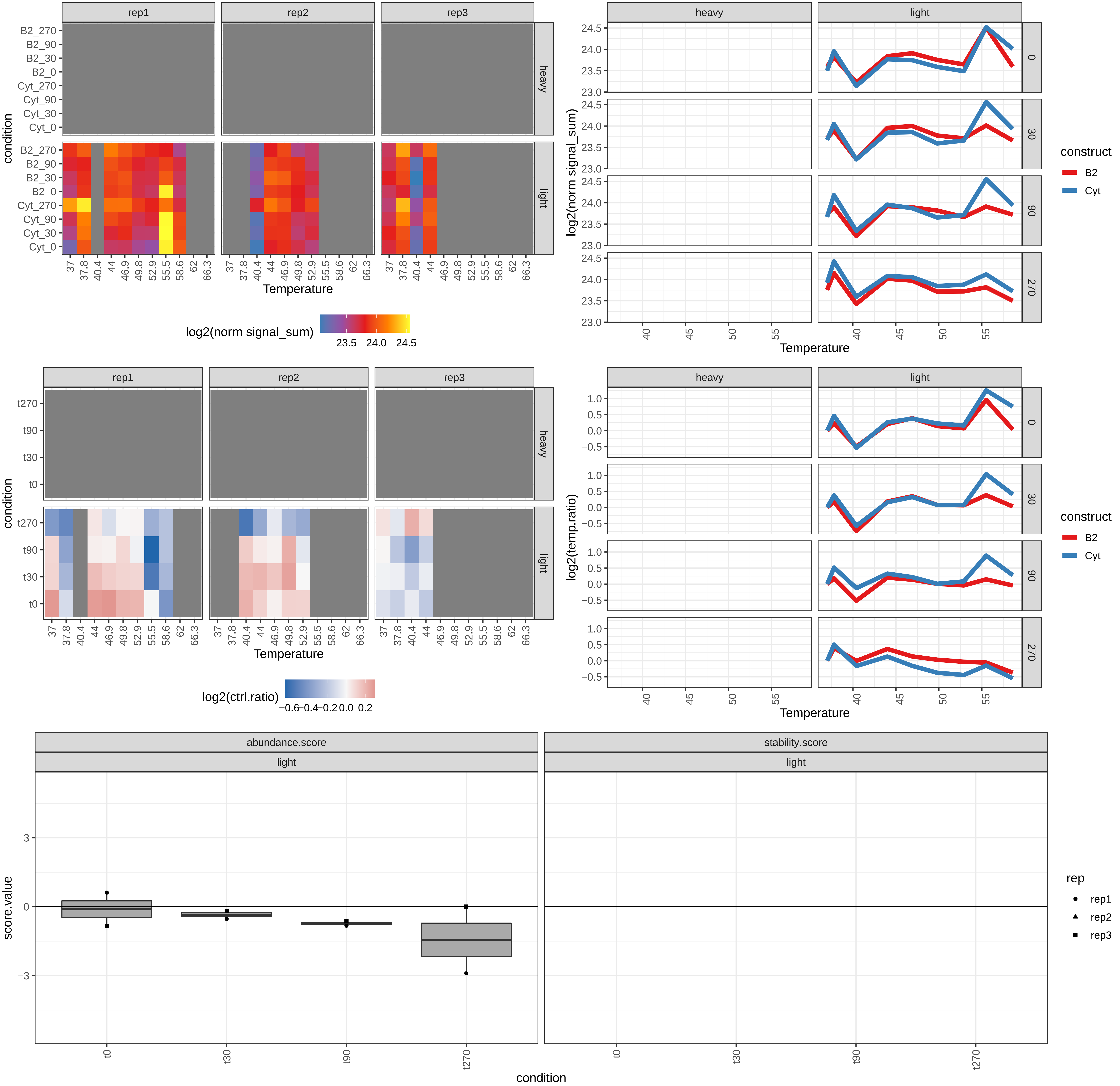

P25656 − Cell division control protein 50 OS=Saccharomyces cerevisiae (strain ATCC 204508 / S288c) GN=CDC50 PE=1 SV=1

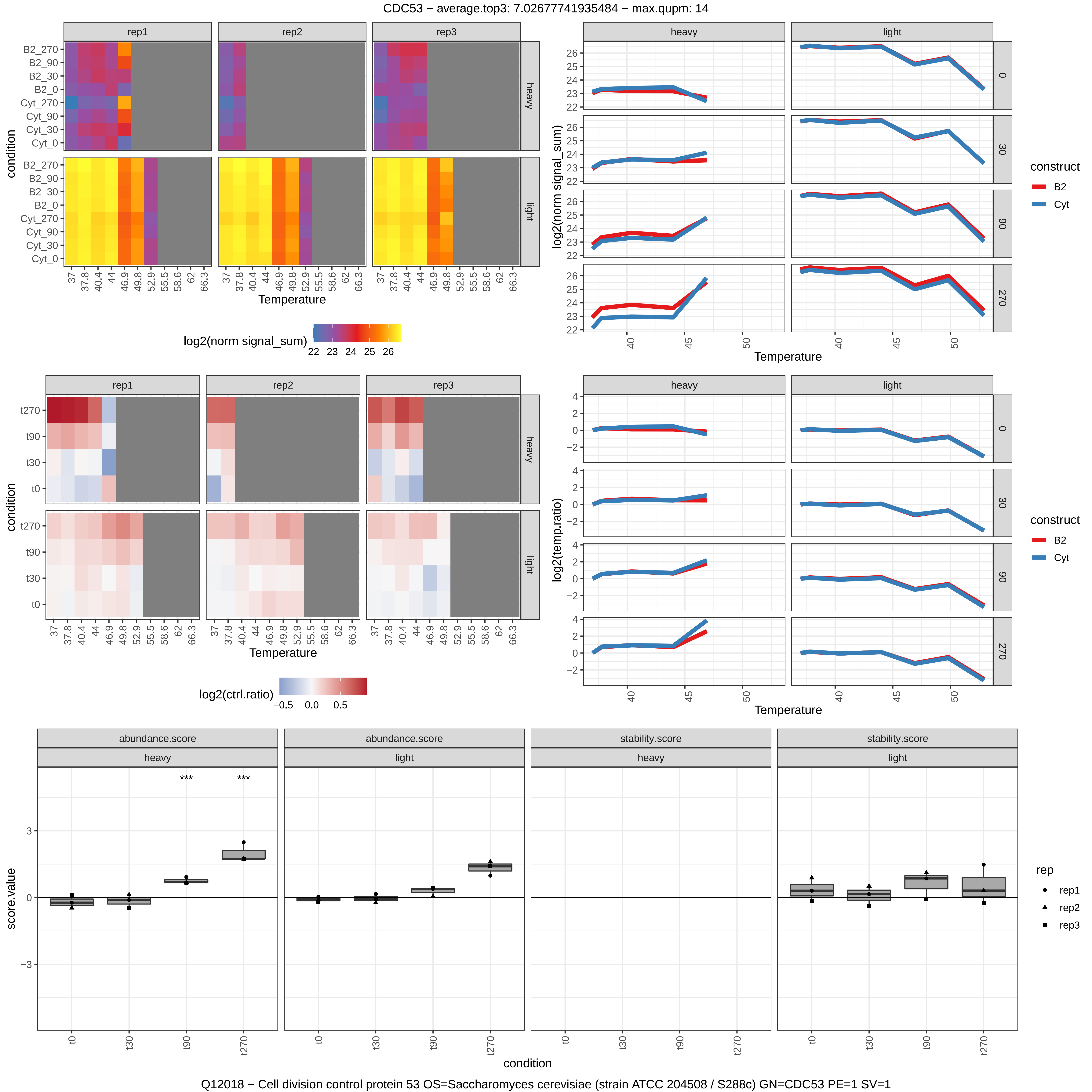

CDC53 − average.top3: 7.02677741935484 − max.qupm: 14
rep1
rep2
rep3
heavy
light
condition
Temperature
log2(norm signal_sum)
log2(ctrl.ratio)
log2(temp.ratio)
construct
B2
Cyt
abundance.score
stability.score
score.value
rep
rep1
rep2
rep3
***
***
Q12018 − Cell division control protein 53 OS=Saccharomyces cerevisiae (strain ATCC 204508 / S288c) GN=CDC53 PE=1 SV=1

CDC55 − average.top3: 6.99344971428571 − max.qupm: 11

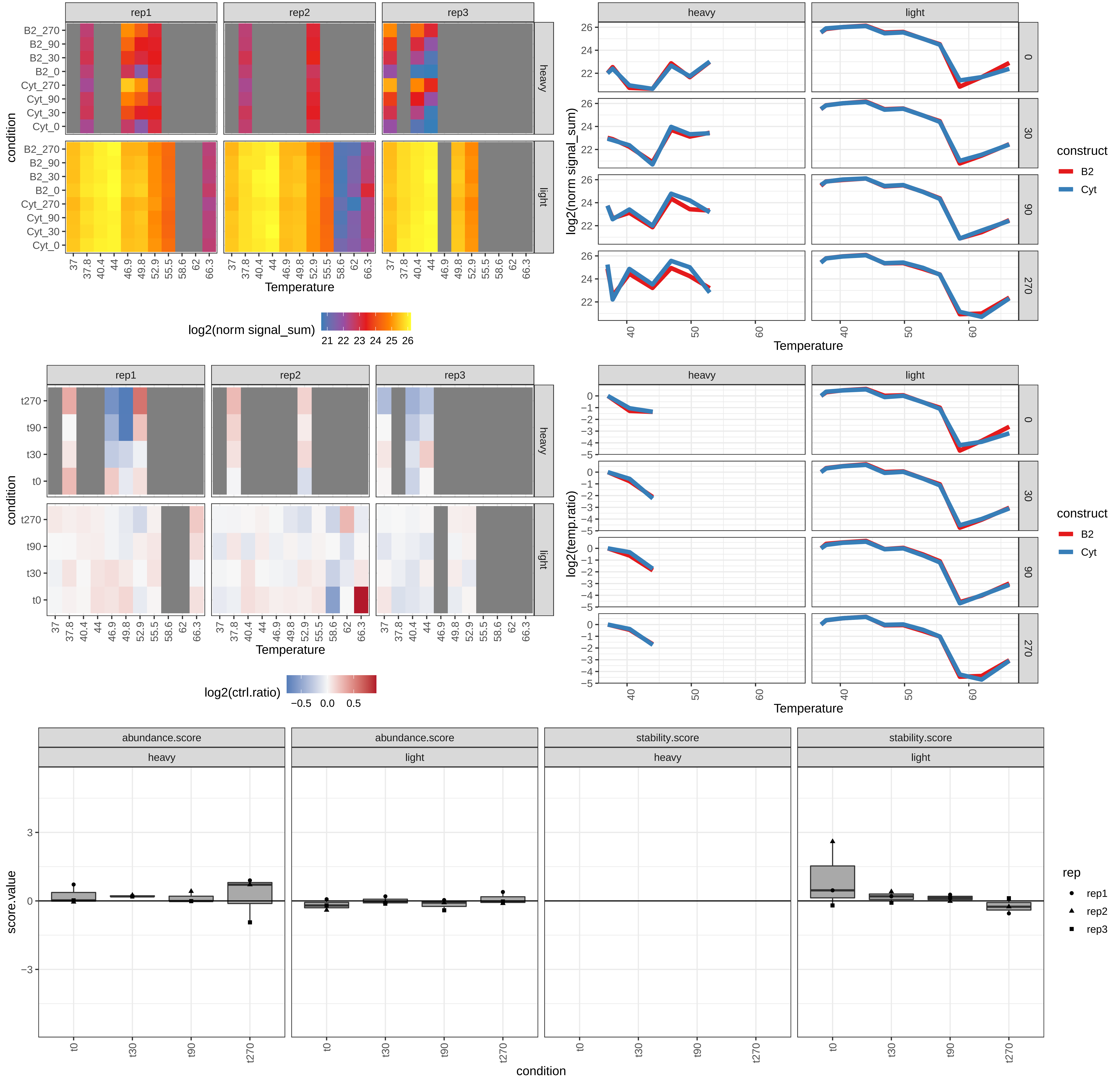

Q00362 − Protein phosphatase PP2A regulatory subunit B OS=Saccharomyces cerevisiae (strain ATCC 204508 / S288c) GN=CDC55 PE=1 SV=2

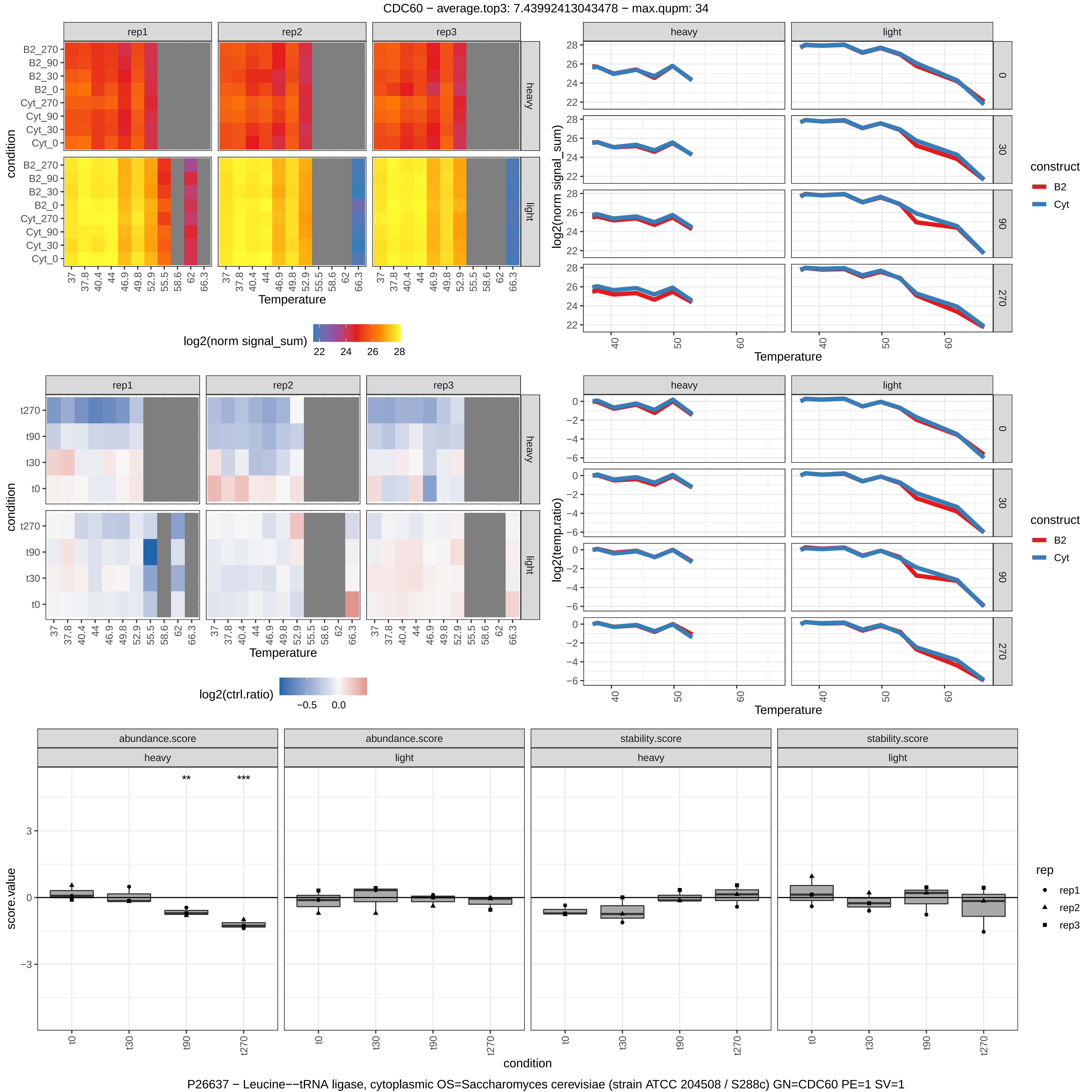

CDC60 − average.top3: 7.43992413043478 − max.qupm: 34
rep1
rep2
rep3
heavy
light
condition
Temperature
log2(norm signal_sum)
log2(norm signal_sum)
construct
B2
Cyt
log2(ctrl.ratio)
log2(temp.ratio)
abundance.score
stability.score
score.value
rep
rep1
rep2
rep3
P26637 − Leucine−−tRNA ligase, cytoplasmic OS=Saccharomyces cerevisiae (strain ATCC 204508 / S288c) GN=CDC60 PE=1 SV=1

CDC73 − average.top3: 7.25308368421053 − max.qupm: 7

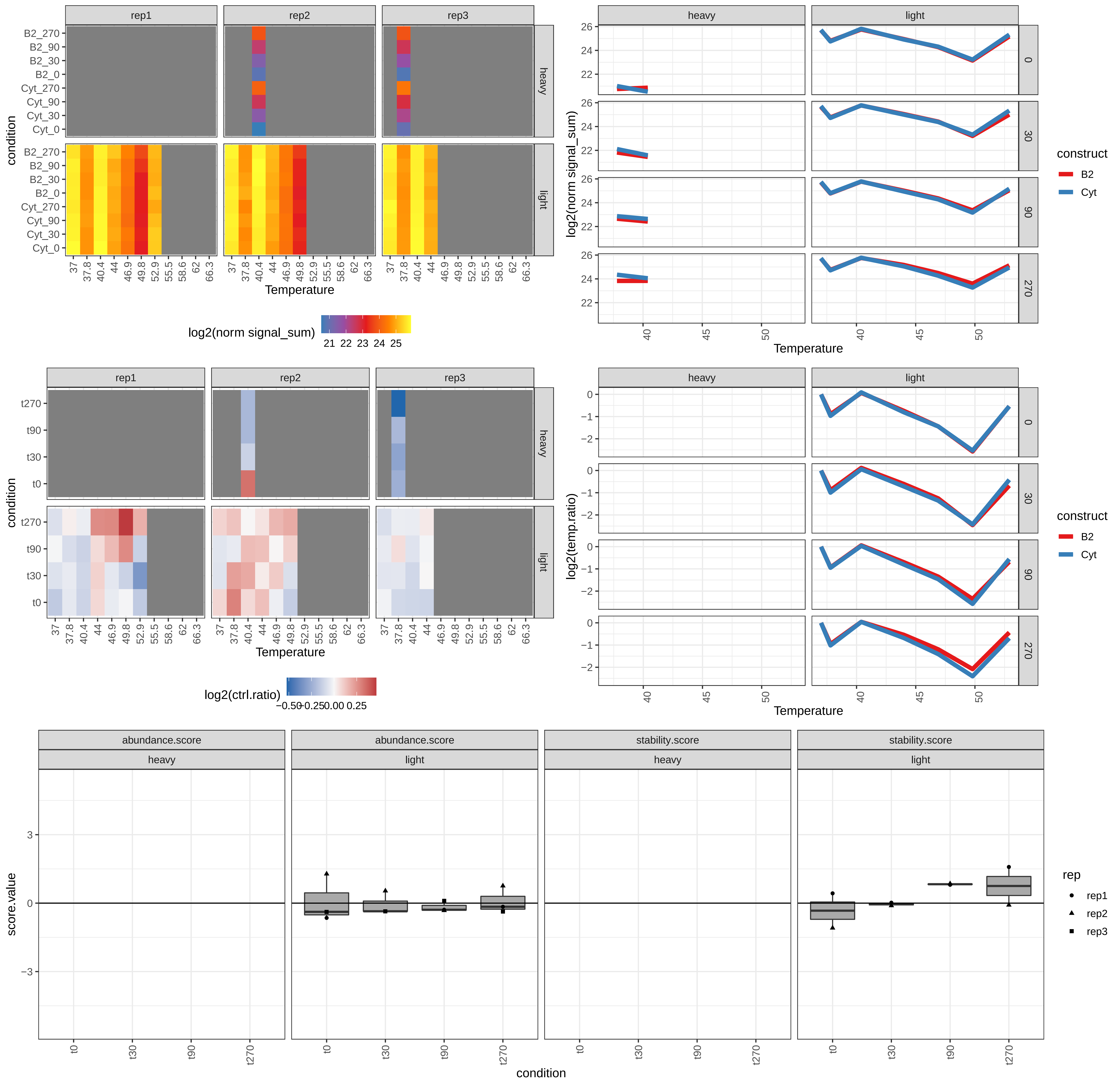

Q06697 − Cell division control protein 73 OS=Saccharomyces cerevisiae (strain ATCC 204508 / S288c) GN=CDC73 PE=1 SV=1

CDC9 − average.top3: 6.57581277777778 − max.qupm: 5

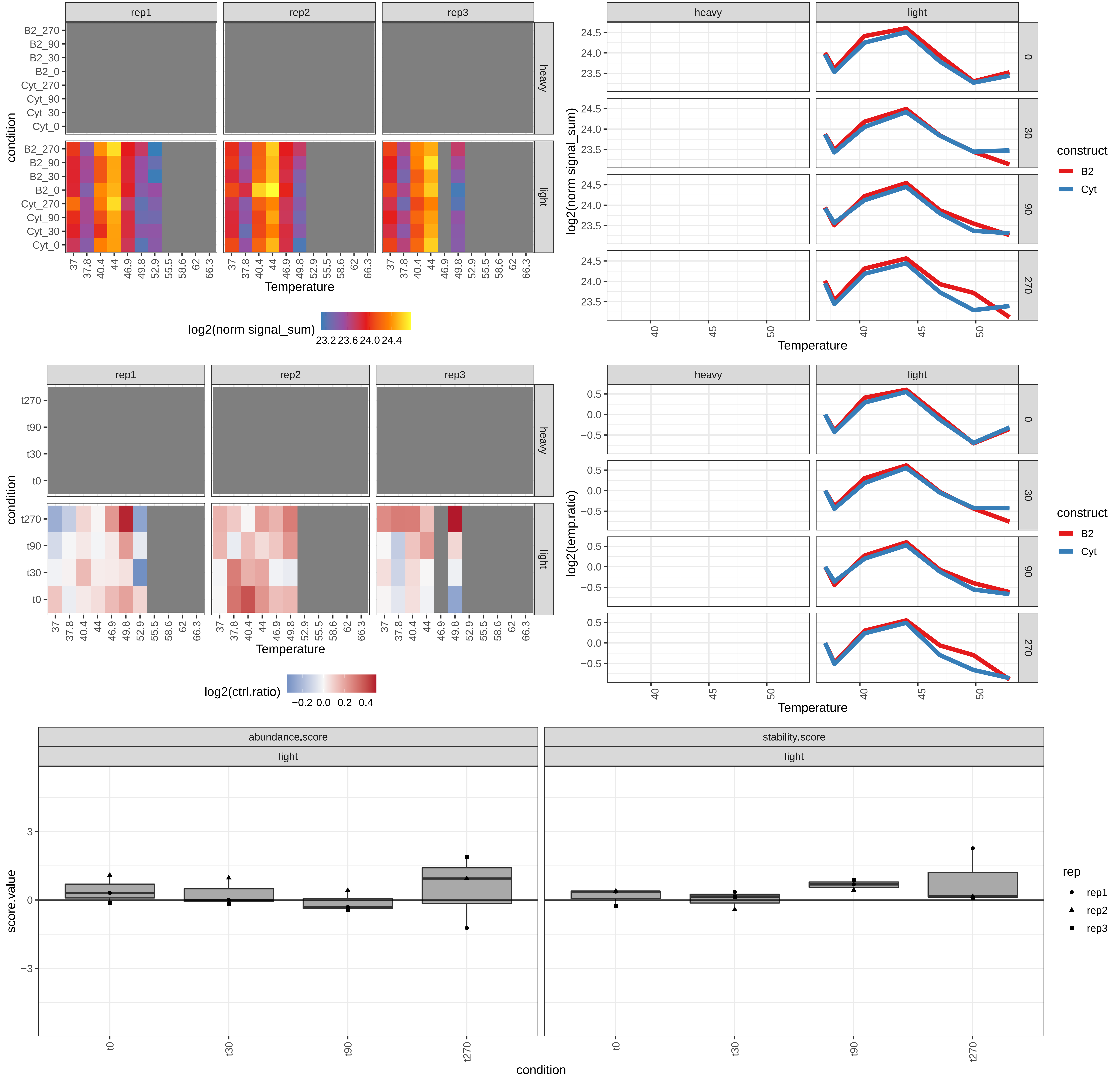

819−2 − DNA ligase 1 OS=Saccharomyces cerevisiae (strain ATCC 204508 / S288c) GN=CDC9 PE=1 SV=2|Isoform Nuclear of DNA ligase 1 OS=Saccharomyces cerevisiae (strain ATCC 204508 / S288c

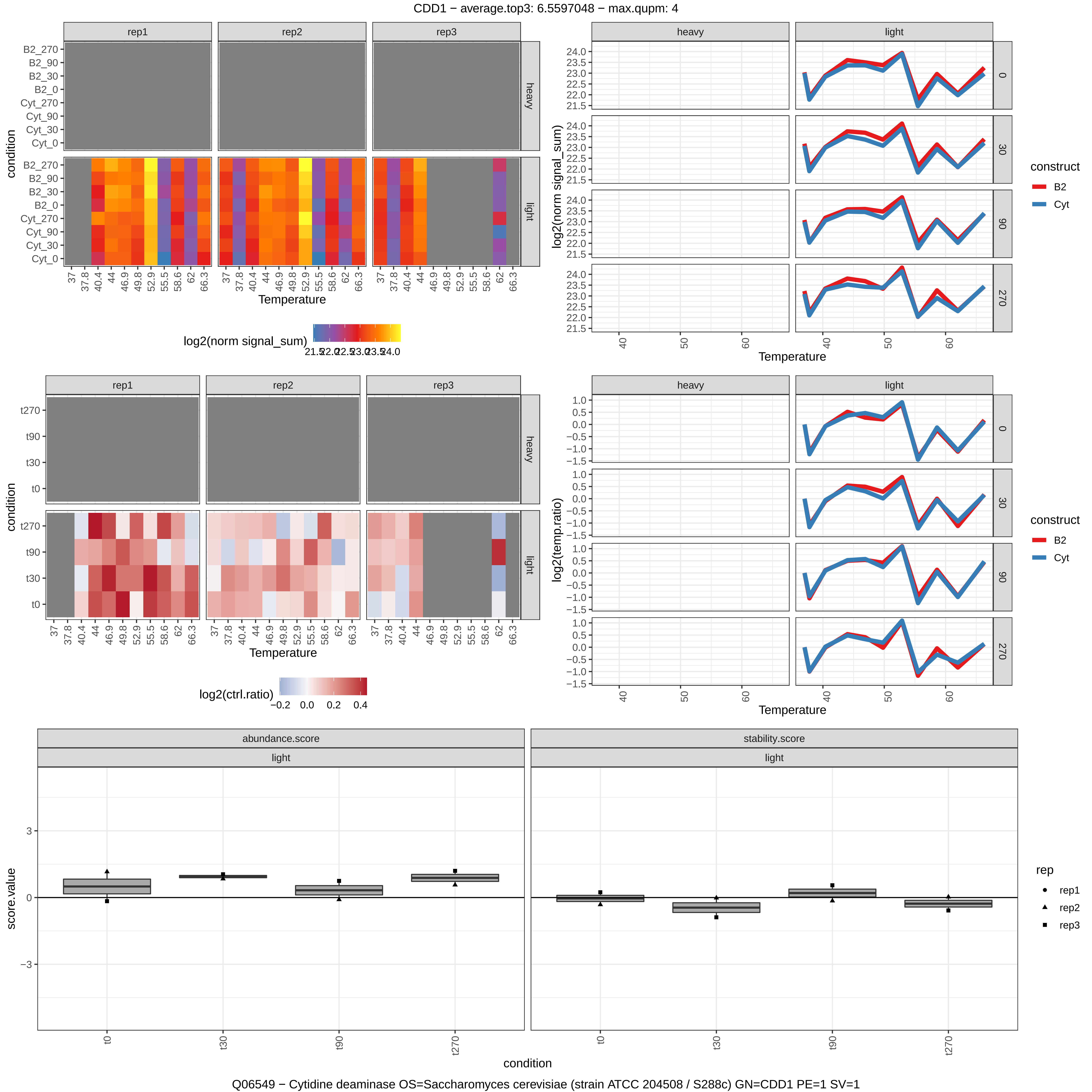
CDD1 − average.top3: 6.5597048 − max.qupm: 4
rep1
rep2
rep3
heavy
light
condition
Temperature
log2(norm signal_sum)
construct
B2
Cyt
log2(temp.ratio)
log2(ctrl.ratio)
abundance.score
stability.score
score.value
rep
rep1
rep2
rep3
Q06549 − Cytidine deaminase OS=Saccharomyces cerevisiae (strain ATCC 204508 / S288c) GN=CDD1 PE=1 SV=1

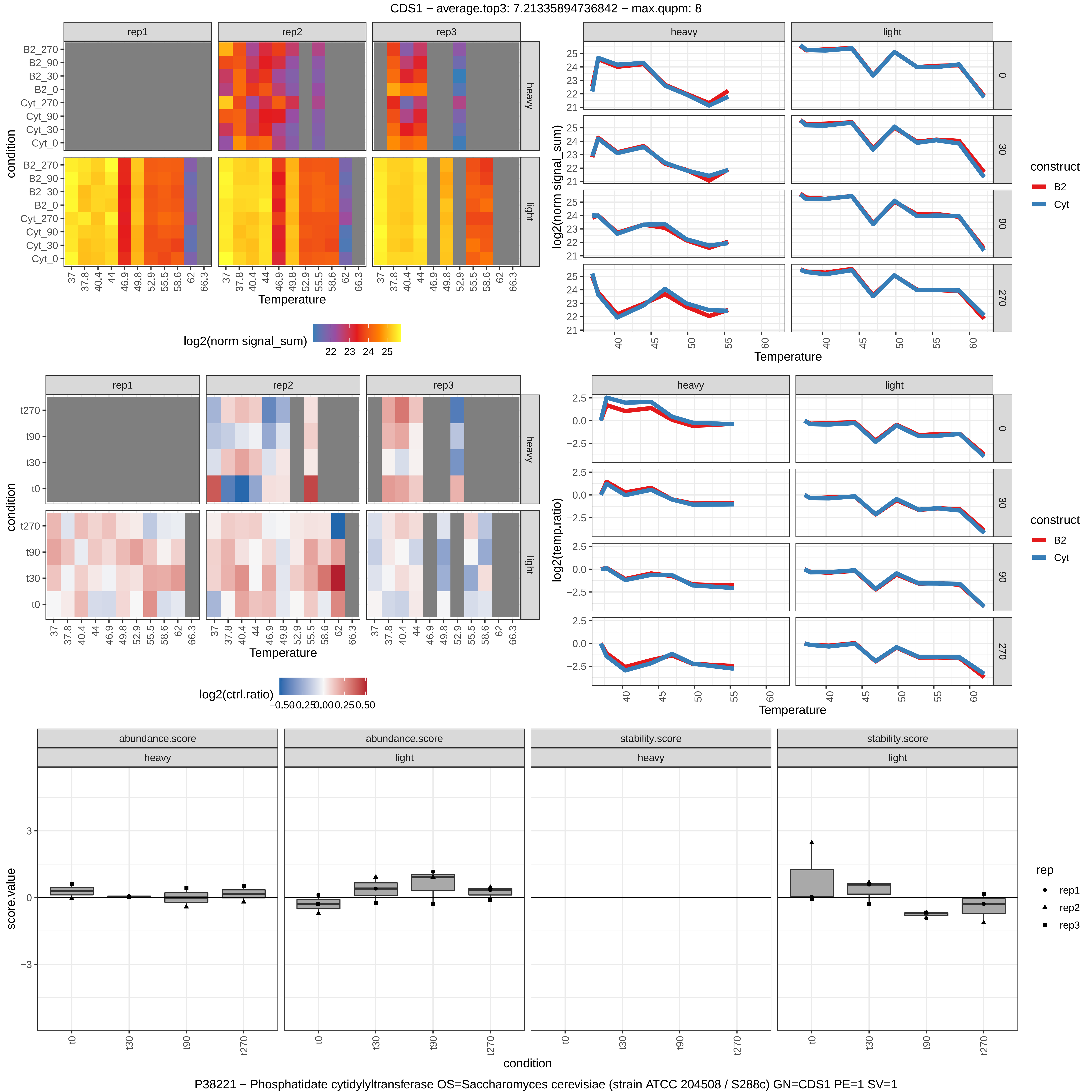
CDS1 − average.top3: 7.21335894736842 − max.qupm: 8
rep1
rep2
rep3
heavy
light
condition
Temperature
log2(norm signal_sum)
construct
B2
Cyt
log2(ctrl.ratio)
log2(temp.ratio)
abundance.score
stability.score
score.value
rep
rep1
rep2
rep3
P38221 − Phosphatidate cytidylyltransferase OS=Saccharomyces cerevisiae (strain ATCC 204508 / S288c) GN=CDS1 PE=1 SV=1

CEG1 − average.top3: 6.915656 − max.qupm: 10

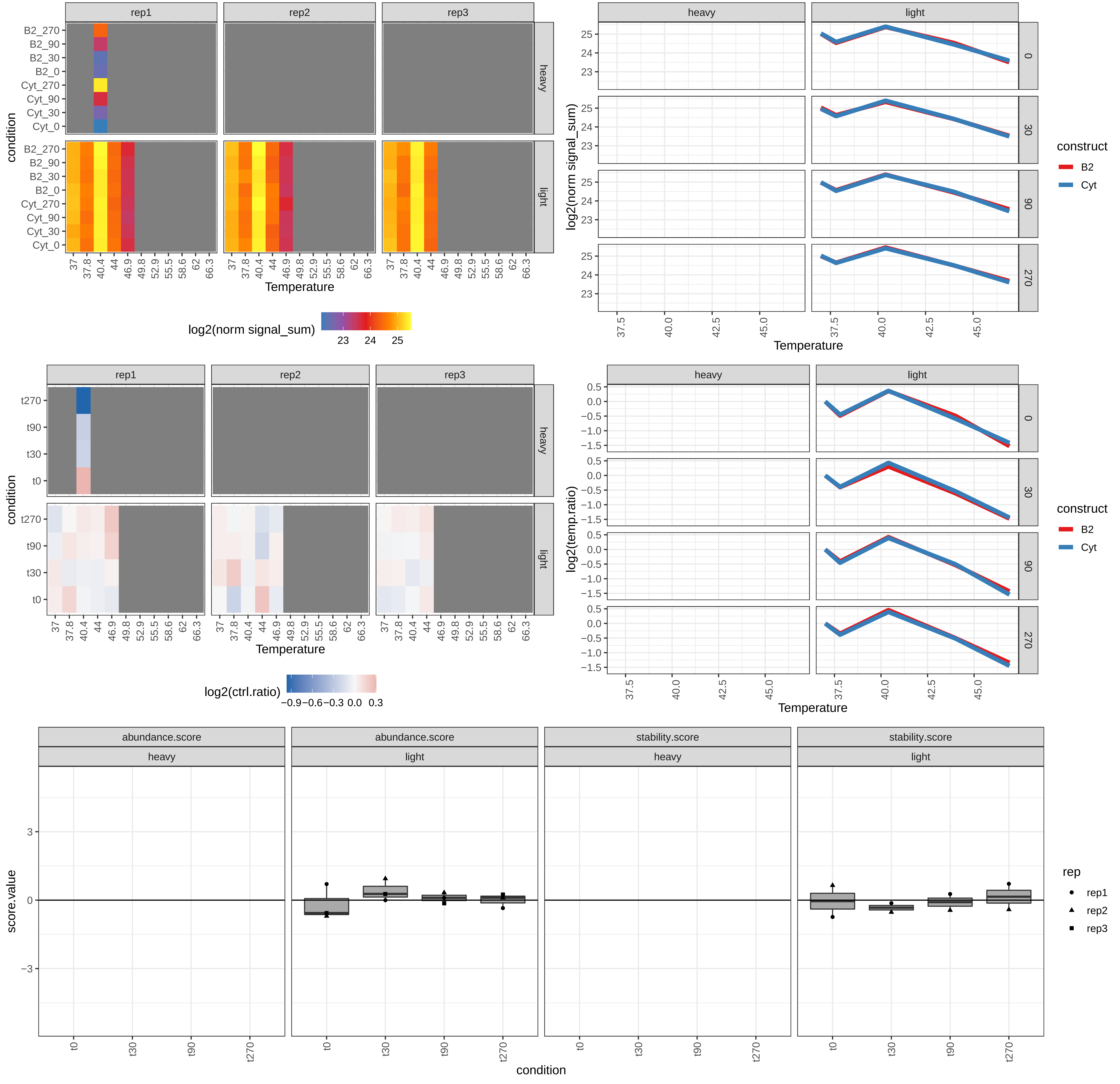

Q01159 − mRNA−capping enzyme subunit alpha OS=Saccharomyces cerevisiae (strain ATCC 204508 / S288c) GN=CEG1 PE=1 SV=2

CEM1 − average.top3: 6.60896461538462 − max.qupm: 2

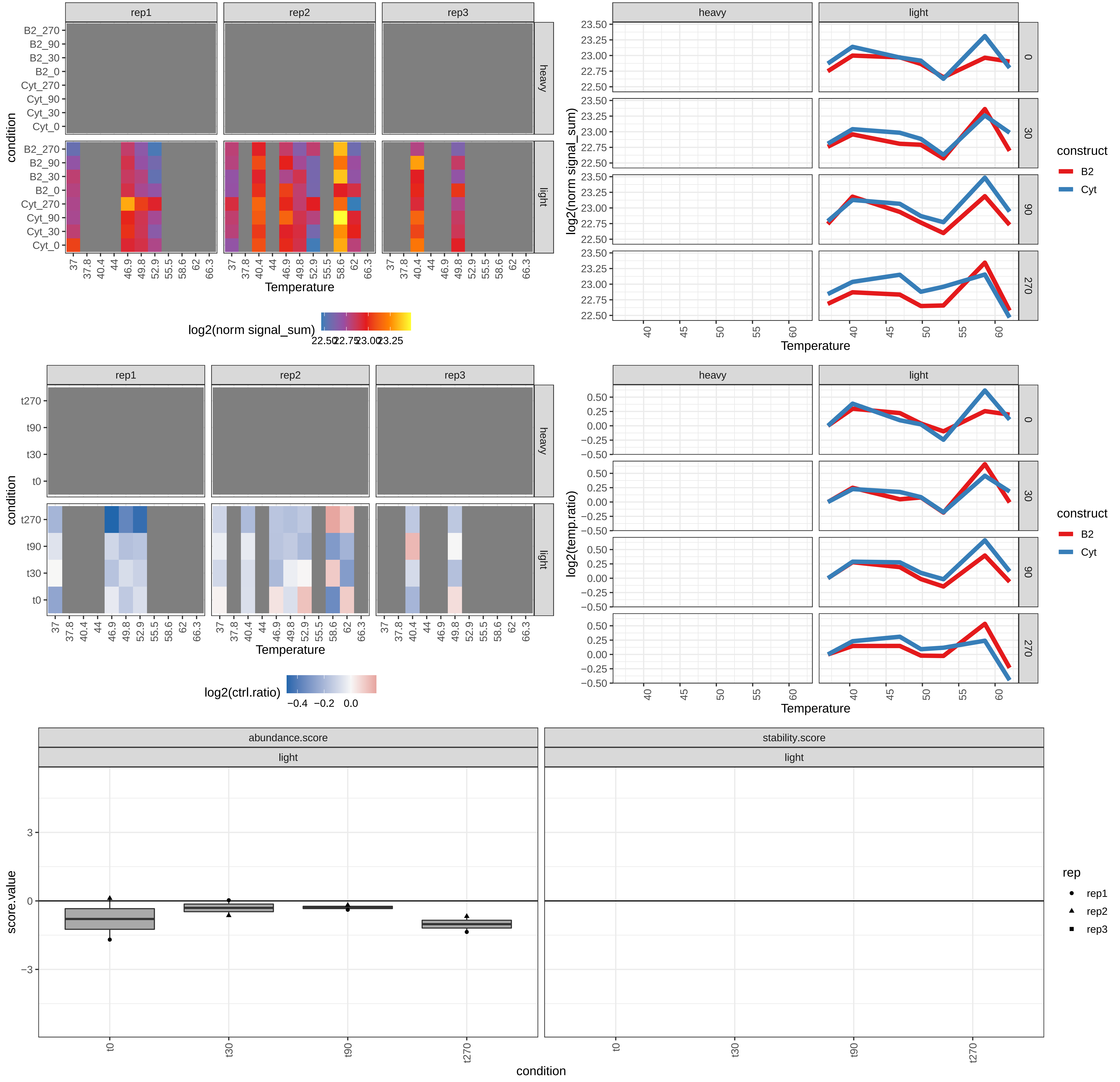

P39525 − 3−oxoacyl−[acyl−carrier−protein] synthase homolog OS=Saccharomyces cerevisiae (strain ATCC 204508 / S288c) GN=CEM1 PE=1 SV=1

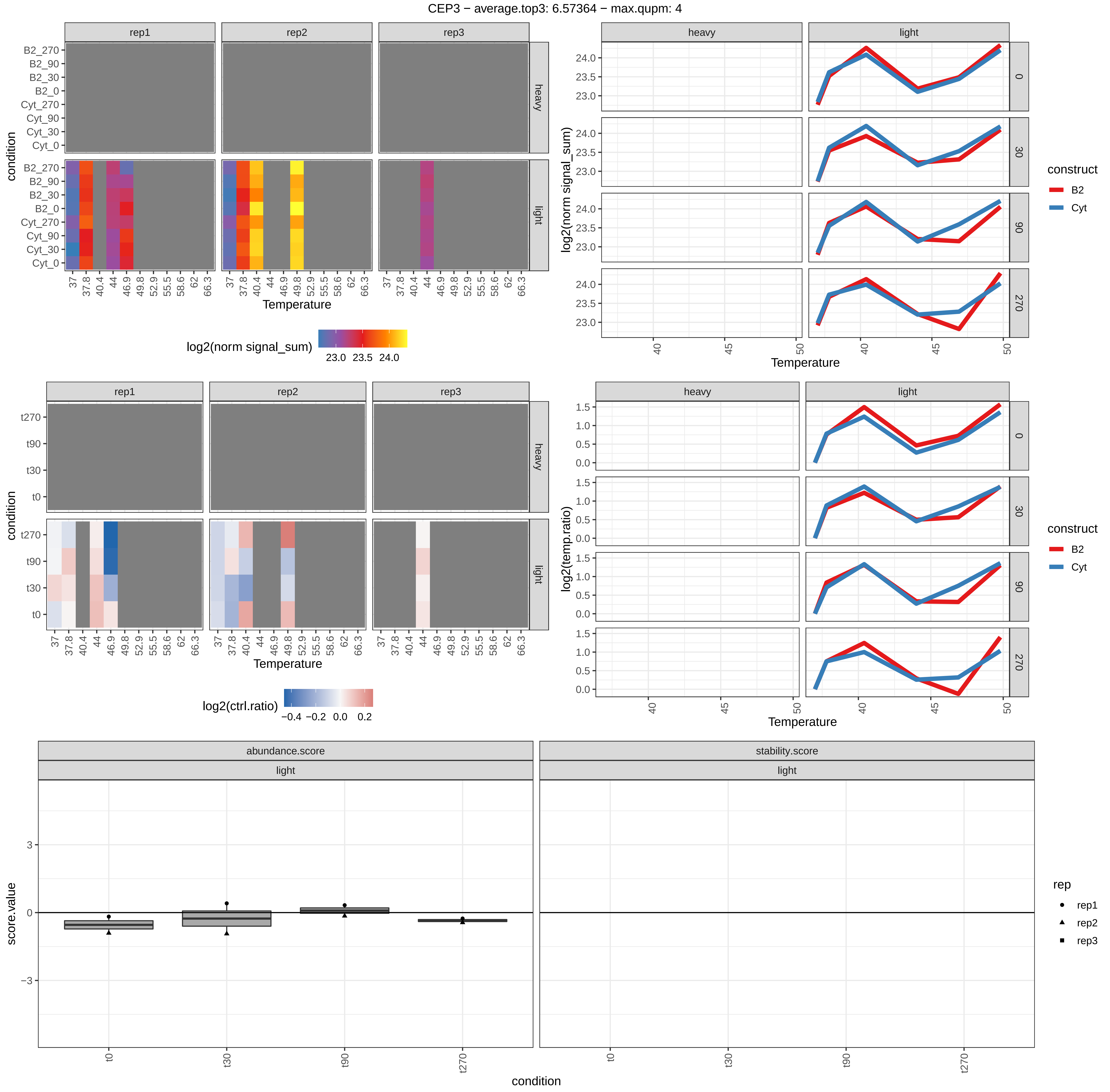

P40969 − Centromere DNA−binding protein complex CBF3 subunit B OS=Saccharomyces cerevisiae (strain ATCC 204508 / S288c) GN=CEP3 PE=1 SV=1

CET1 − average.top3: 7.1671164 − max.qupm: 10

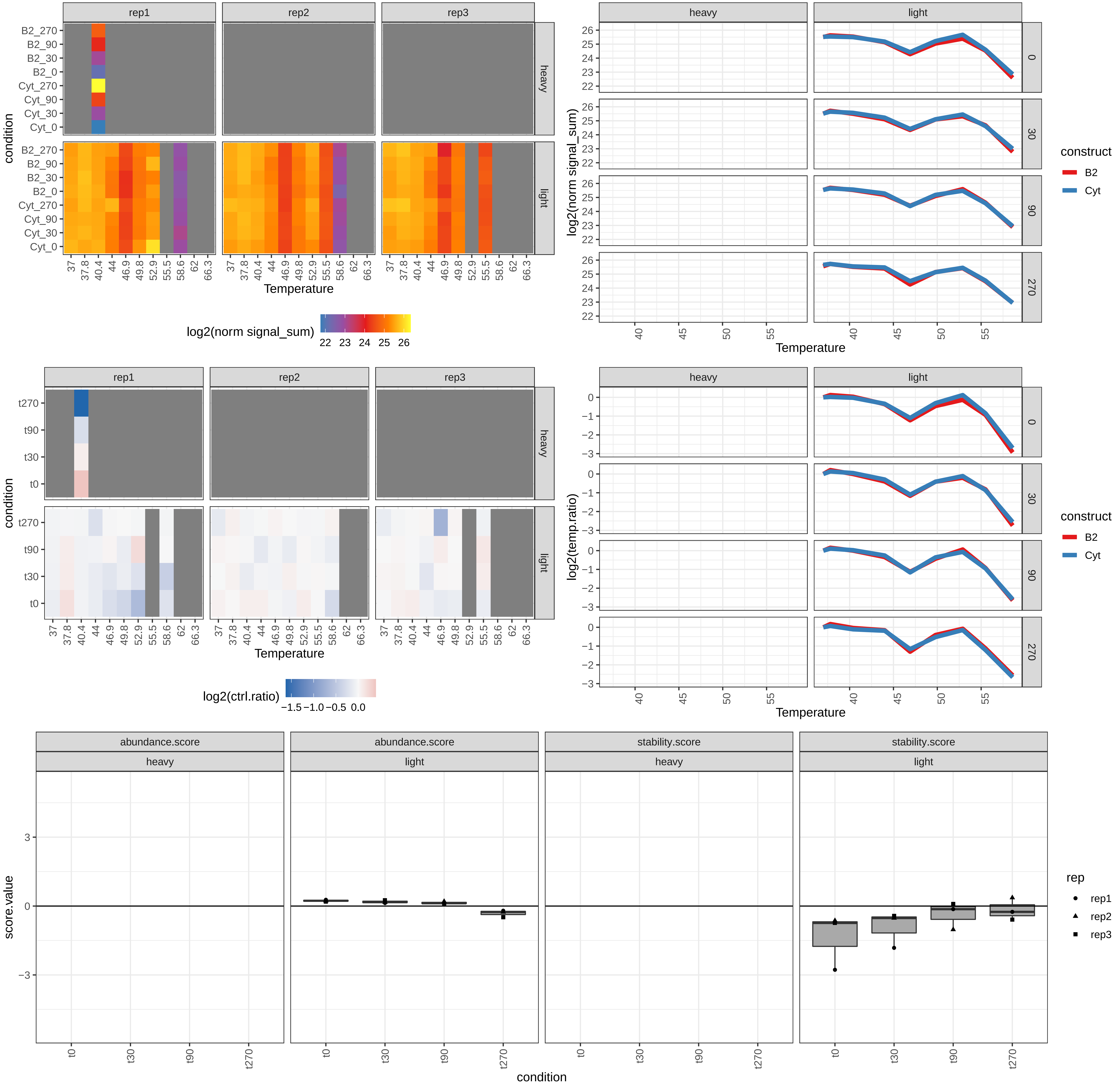

O13297 − mRNA−capping enzyme subunit beta OS=Saccharomyces cerevisiae (strain ATCC 204508 / S288c) GN=CET1 PE=1 SV=2

CEX1 − average.top3: 7.06555708333333 − max.qupm: 9

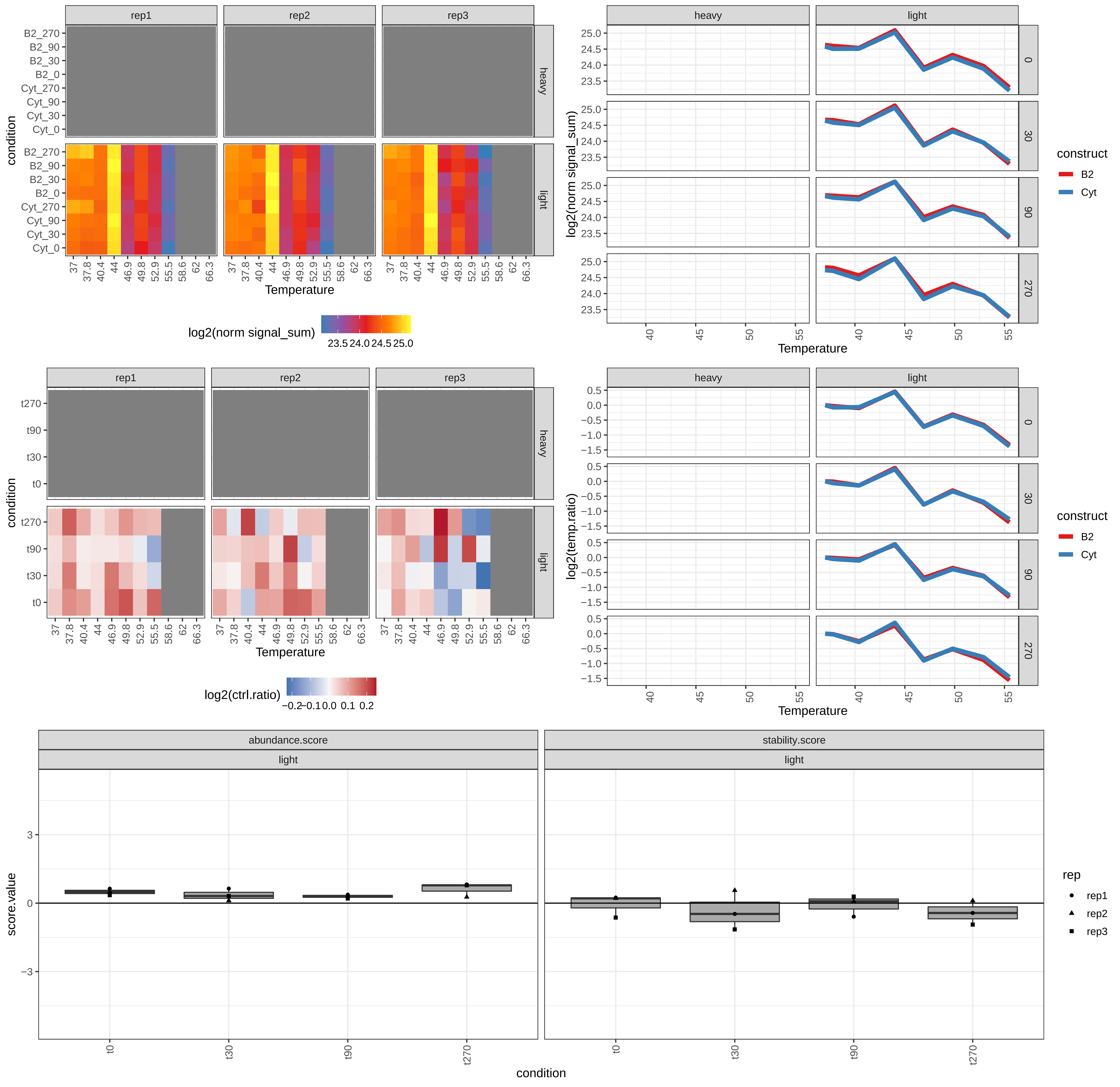

Q12453 − Cytoplasmic export protein 1 OS=Saccharomyces cerevisiae (strain ATCC 204508 / S288c) GN=CEX1 PE=1 SV=1

CFT1 − average.top3: 6.96402722222222 − max.qupm: 9

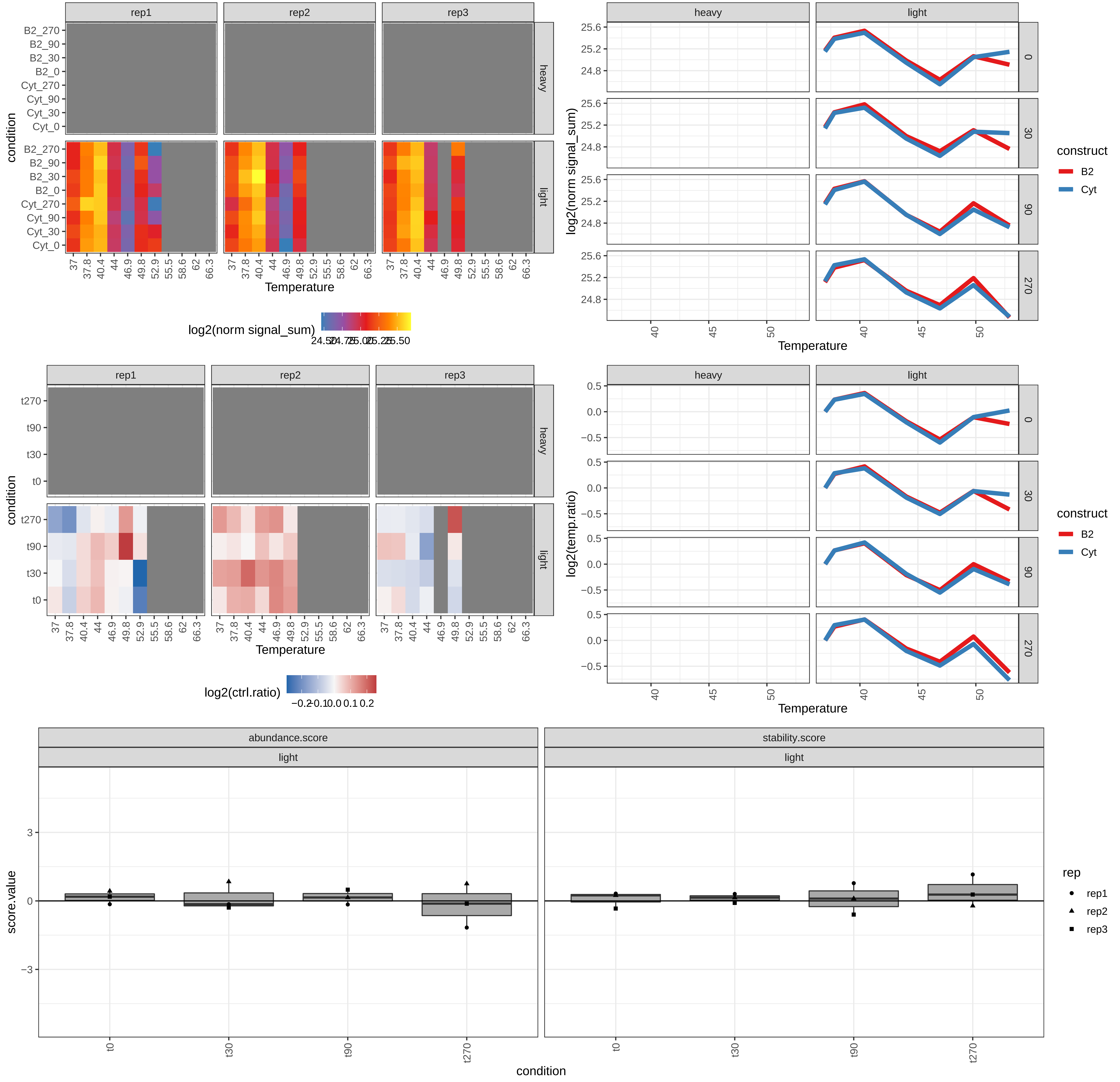

Q06632 − Protein CFT1 OS=Saccharomyces cerevisiae (strain ATCC 204508 / S288c) GN=CFT1 PE=1 SV=1

CFT2 − average.top3: 6.55693869565217 − max.qupm: 7

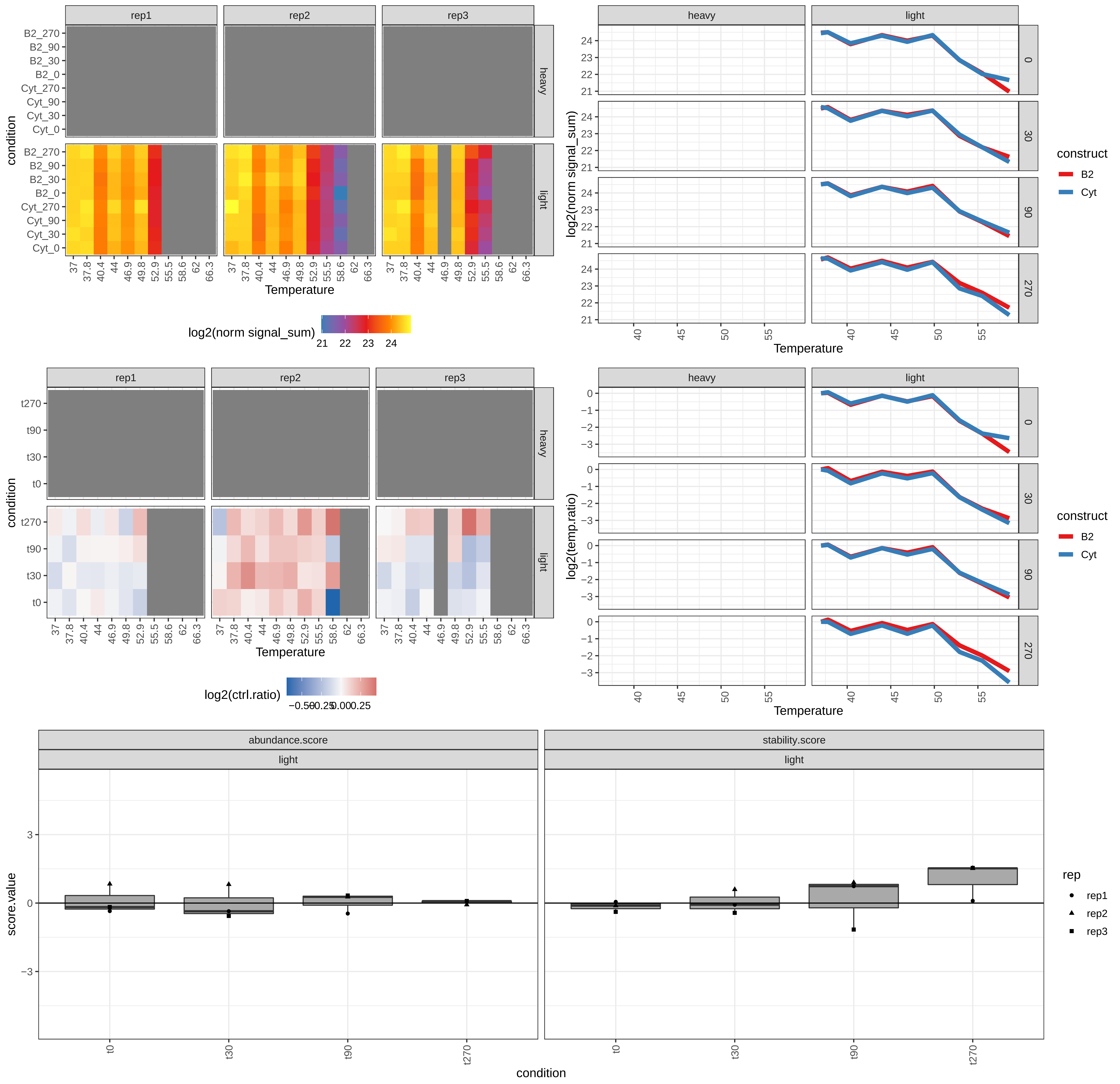

Q12102 − Cleavage factor two protein 2 OS=Saccharomyces cerevisiae (strain ATCC 204508 / S288c) GN=CFT2 PE=1 SV=1

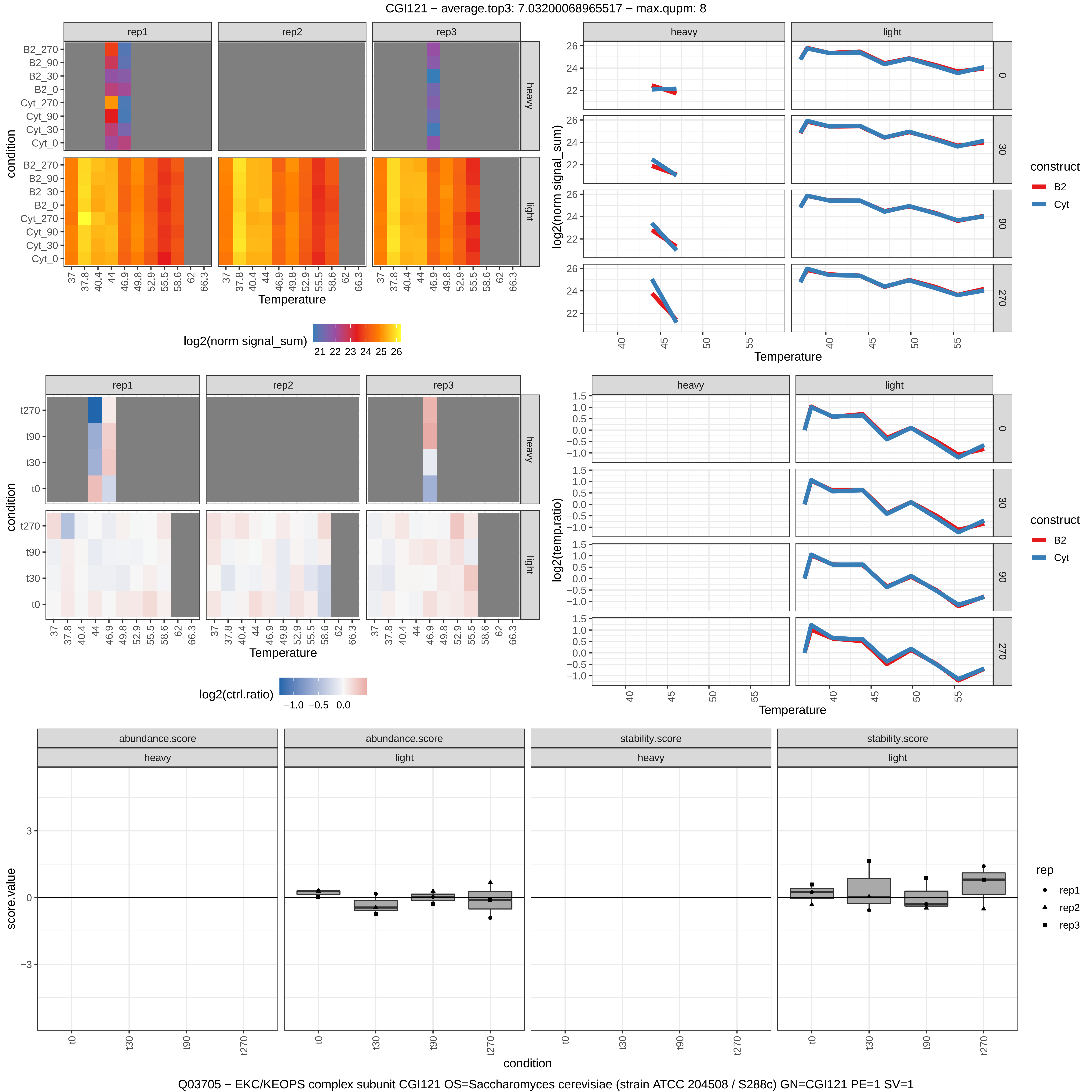

CGI121 − average.top3: 7.03200068965517 − max.qupm: 8
rep1
rep2
rep3
heavy
light
condition
Temperature
log2(norm signal_sum)
log2(norm signal_sum)
construct
B2
Cyt
log2(ctrl.ratio)
log2(temp.ratio)
abundance.score
stability.score
score.value
rep
rep1
rep2
rep3
Q03705 − EKC/KEOPS complex subunit CGI121 OS=Saccharomyces cerevisiae (strain ATCC 204508 / S288c) GN=CGI121 PE=1 SV=1

CHA1 − average.top3: 6.86032538461538 − max.qupm: 5

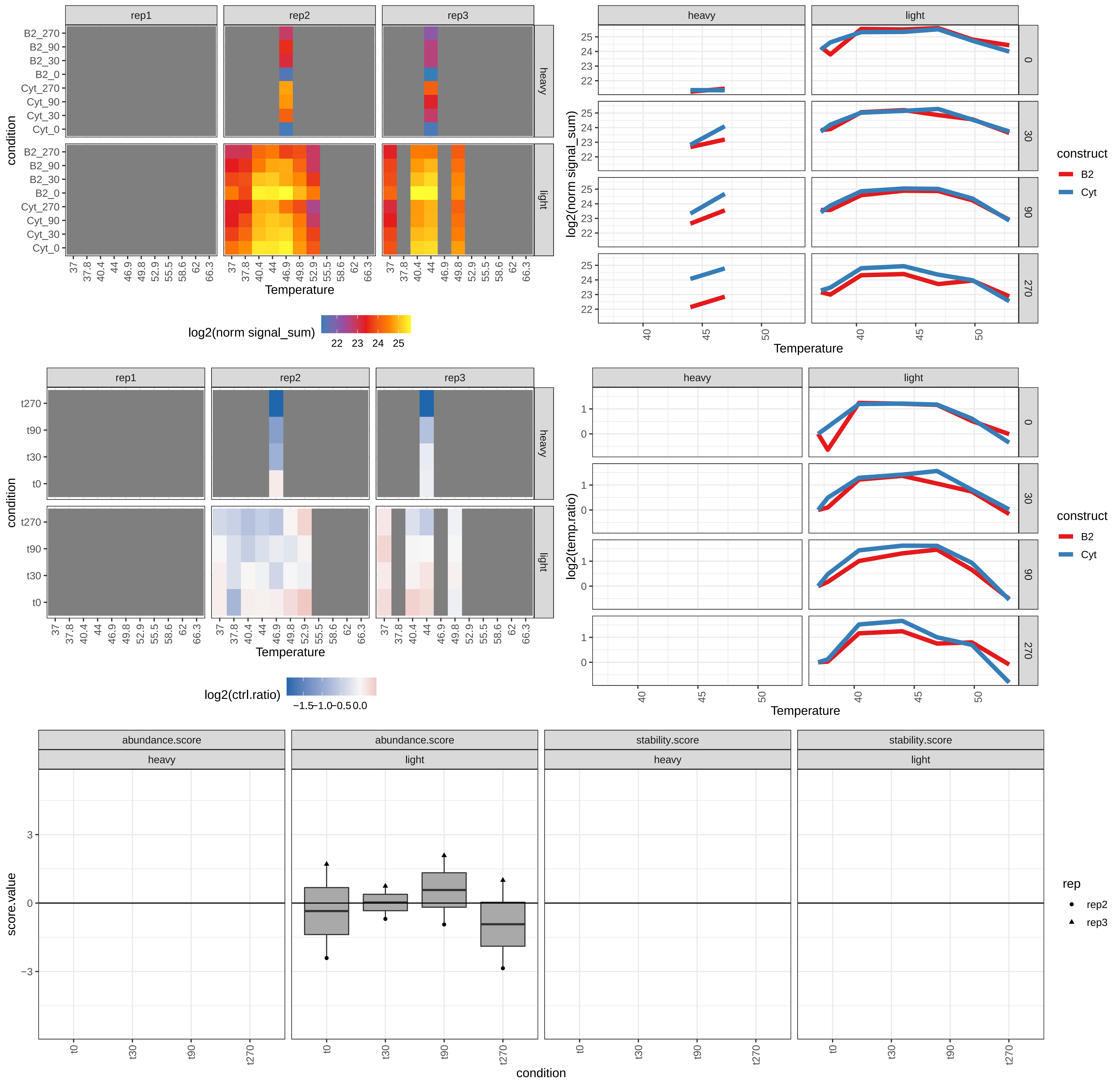

P25379 − Catabolic L−serine/threonine dehydratase OS=Saccharomyces cerevisiae (strain ATCC 204508 / S288c) GN=CHA1 PE=1 SV=2

CHC1 − average.top3: 7.42777916666667 − max.qupm: 48

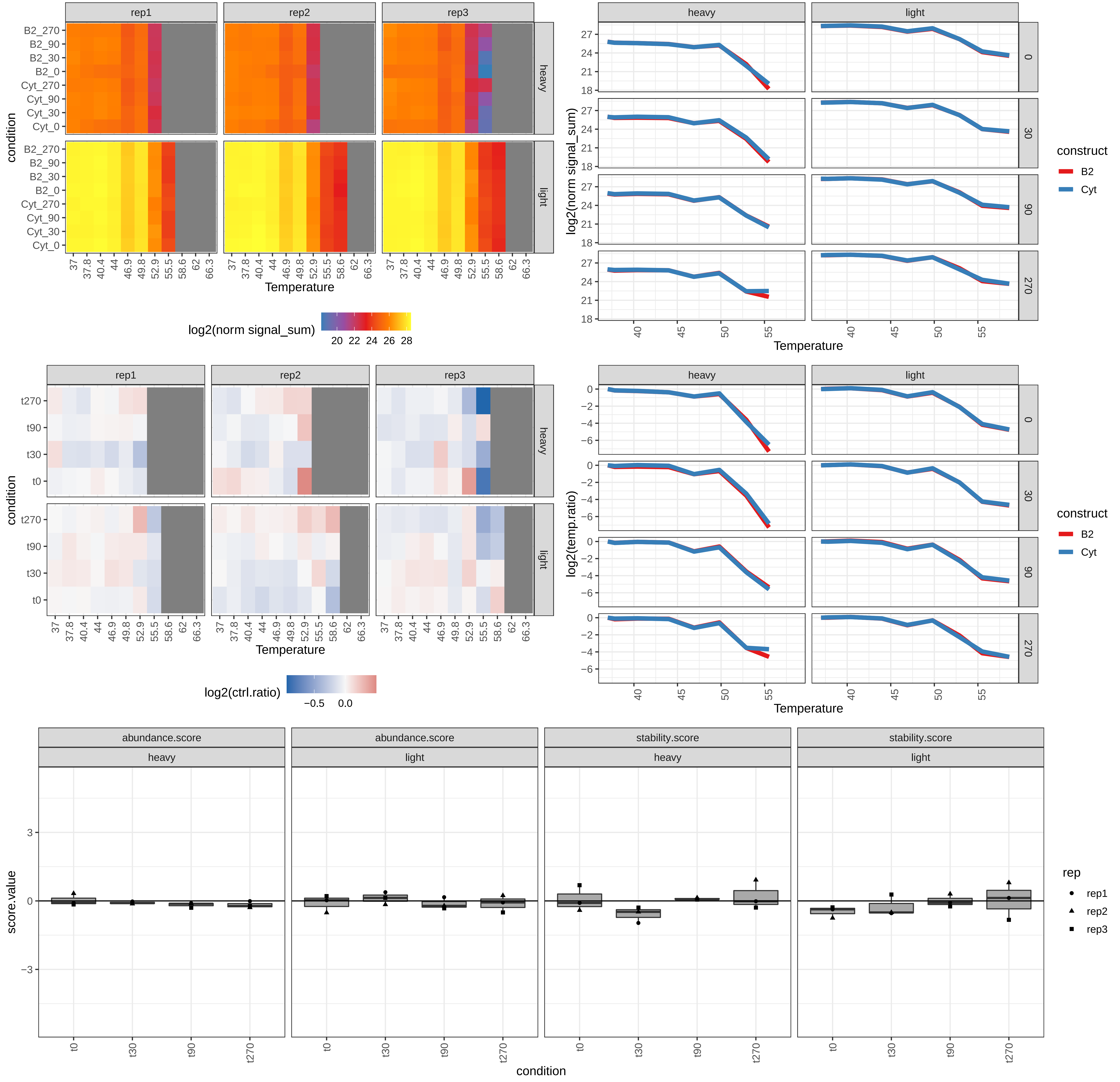

P22137 − Clathrin heavy chain OS=Saccharomyces cerevisiae (strain ATCC 204508 / S288c) GN=CHC1 PE=1 SV=1

CHD1 − average.top3: 6.72061461538462 − max.qupm: 13

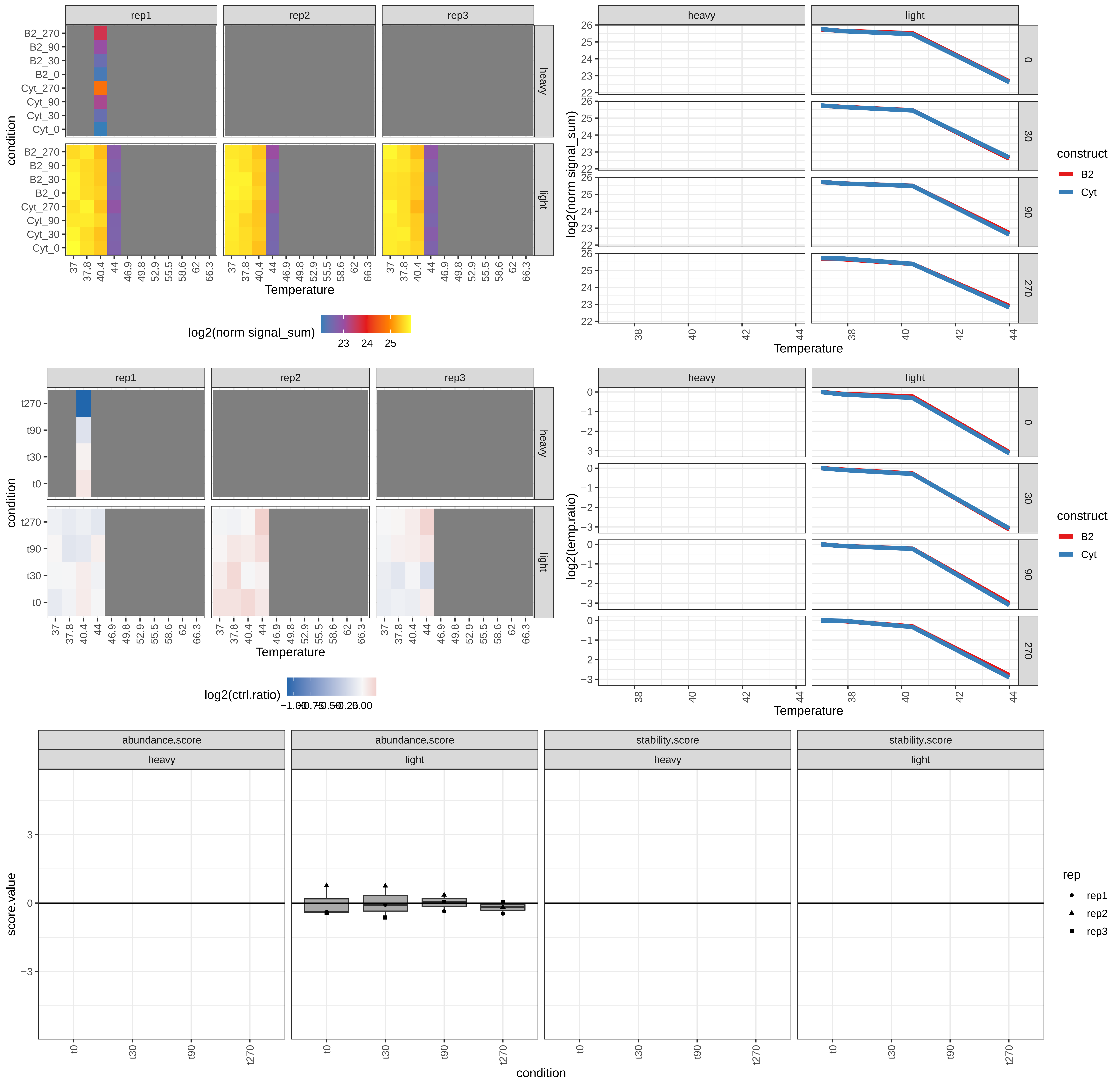

P32657 − Chromo domain−containing protein 1 OS=Saccharomyces cerevisiae (strain ATCC 204508 / S288c) GN=CHD1 PE=1 SV=1

CHO1 − average.top3: 6.80160095238095 − max.qupm: 4

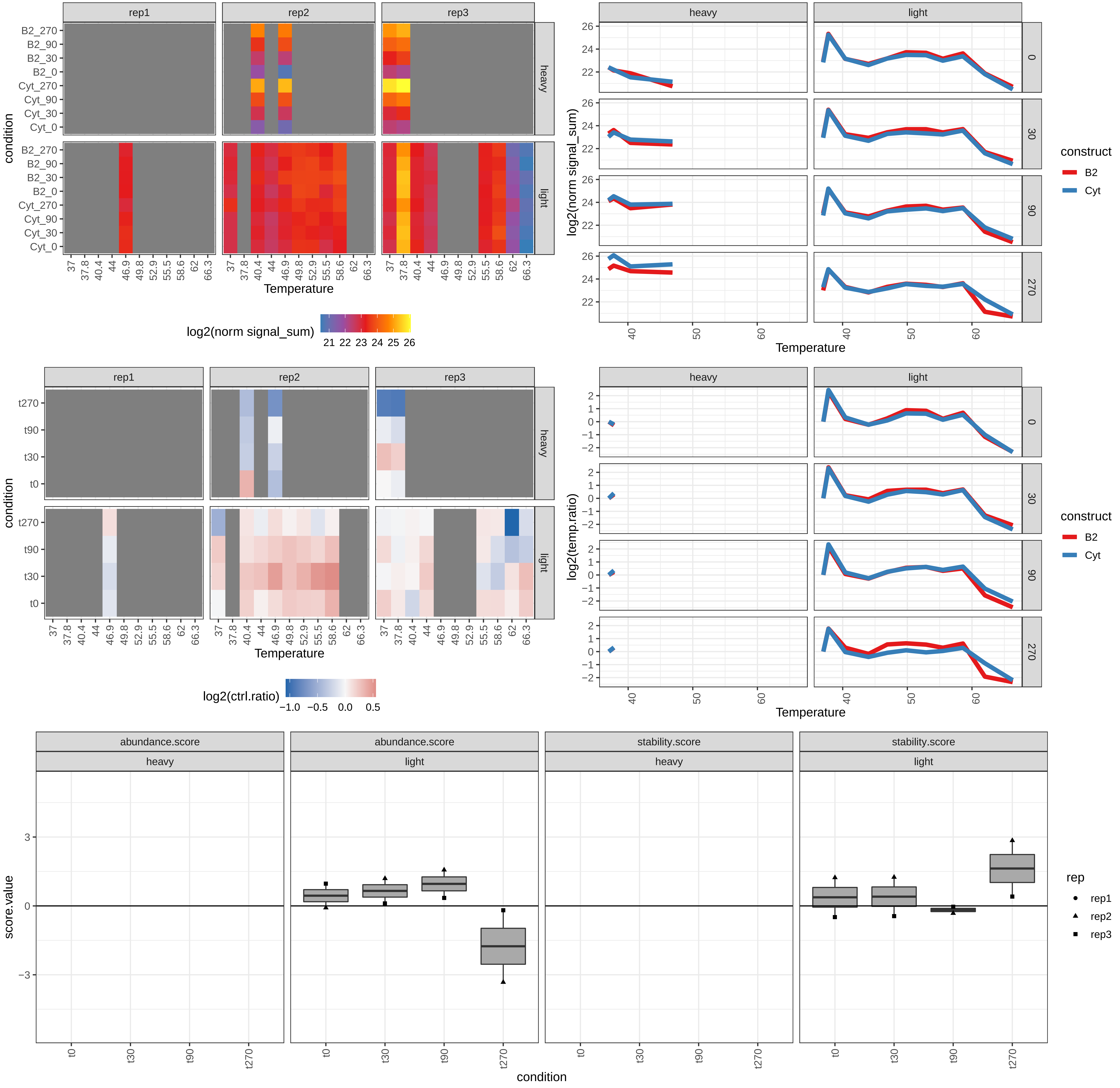

P08456 − CDP−diacylglycerol−−serine O−phosphatidyltransferase OS=Saccharomyces cerevisiae (strain ATCC 204508 / S288c) GN=CHO1 PE=1 SV=3

CHO2 − average.top3: 7.02438911111111 − max.qupm: 22

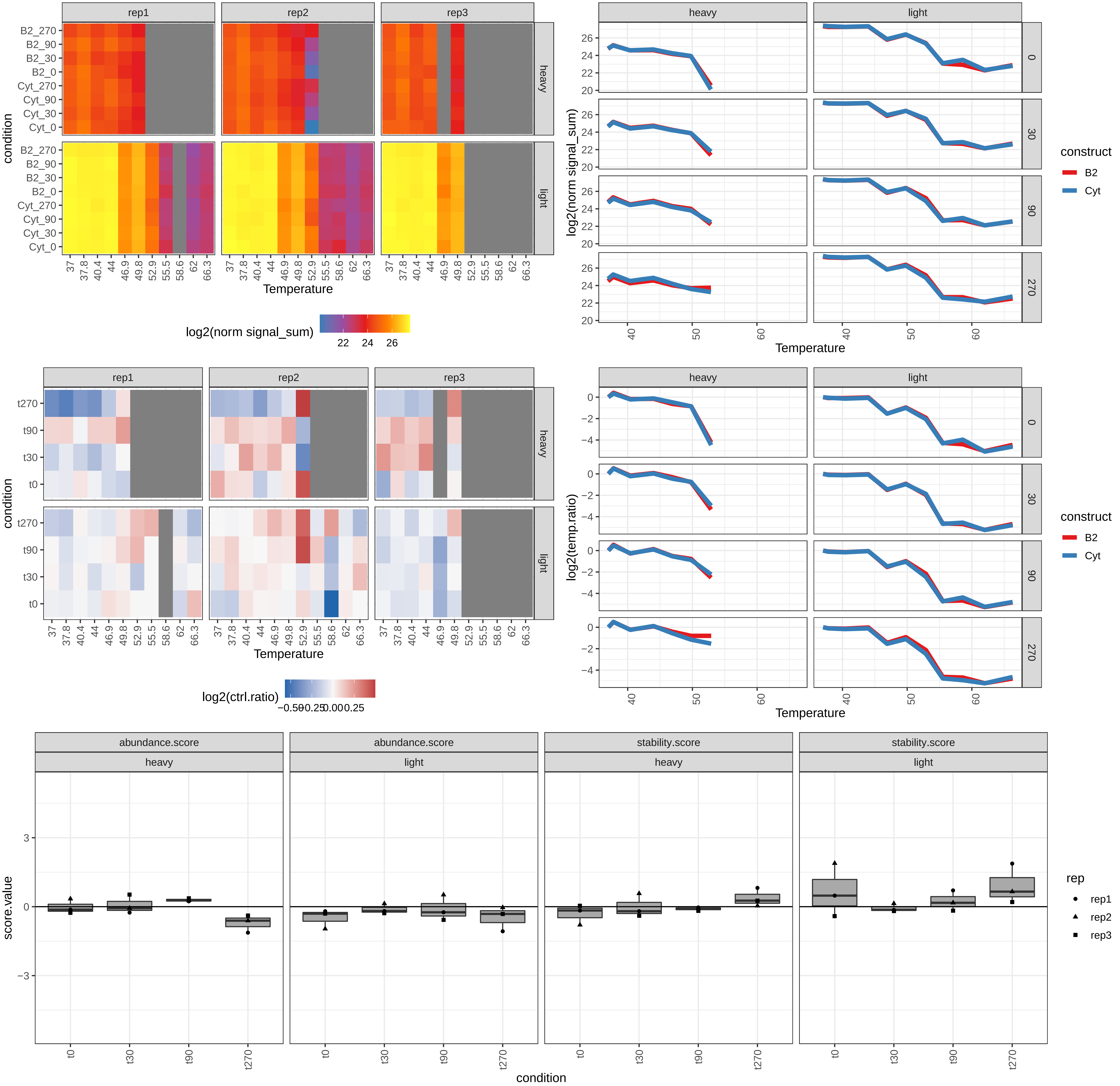

P05374 − Phosphatidylethanolamine N−methyltransferase OS=Saccharomyces cerevisiae (strain ATCC 204508 / S288c) GN=CHO2 PE=1 SV=1

CHS1 − average.top3: 6.5893268 − max.qupm: 4

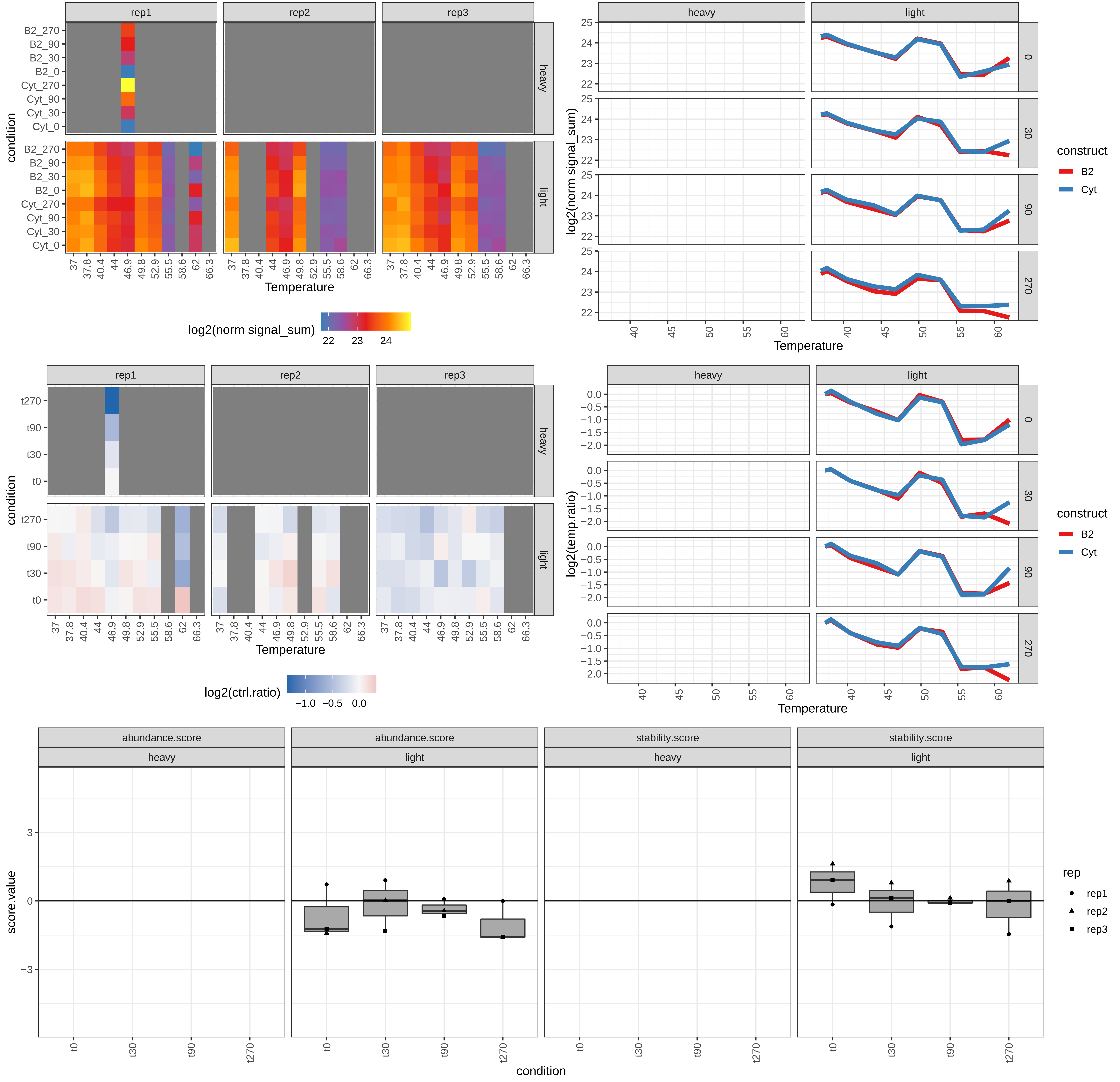

P08004 − Chitin synthase 1 OS=Saccharomyces cerevisiae (strain ATCC 204508 / S288c) GN=CHS1 PE=1 SV=2

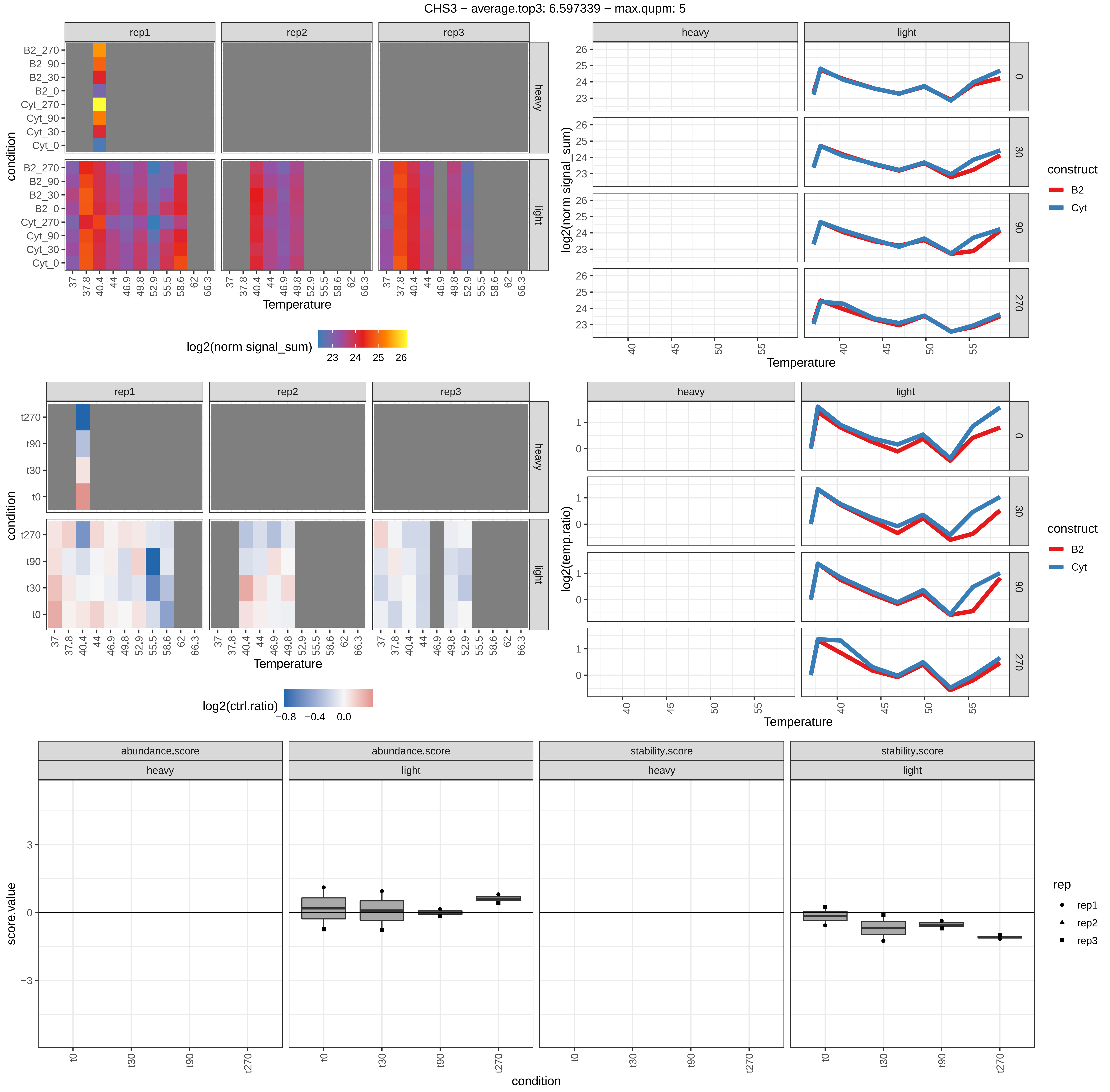

P29465 − Chitin synthase 3 OS=Saccharomyces cerevisiae (strain ATCC 204508 / S288c) GN=CHS3 PE=1 SV=3

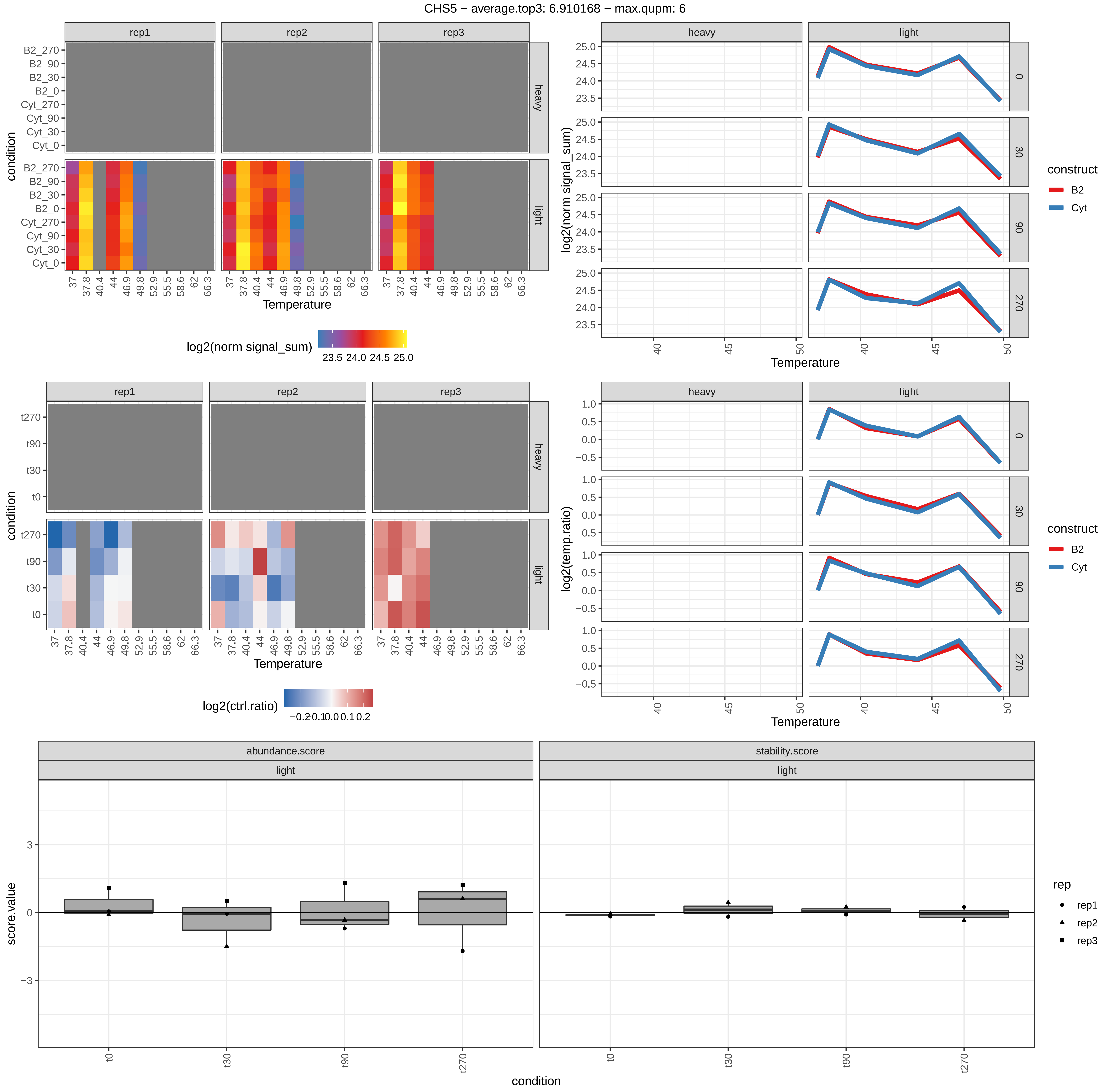

Q12114 − Chitin biosynthesis protein CHS5 OS=Saccharomyces cerevisiae (strain ATCC 204508 / S288c) GN=CHS5 PE=1 SV=1

CHS6 − average.top3: 6.487317 − max.qupm: 6

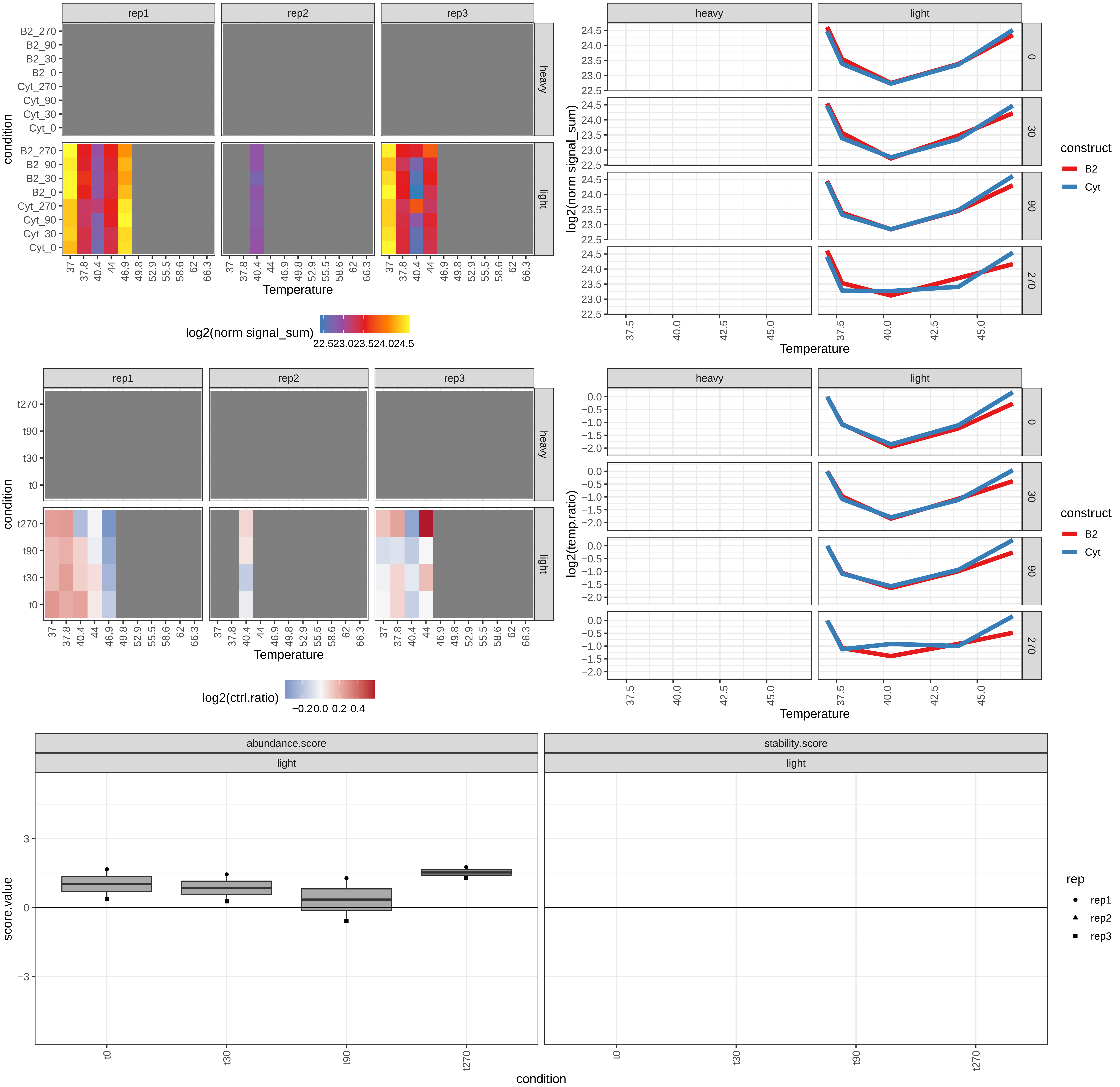

P40955 − Chitin biosynthesis protein CHS6 OS=Saccharomyces cerevisiae (strain ATCC 204508 / S288c) GN=CHS6 PE=1 SV=1

CIA1 − average.top3: 6.76655666666667 − max.qupm: 4

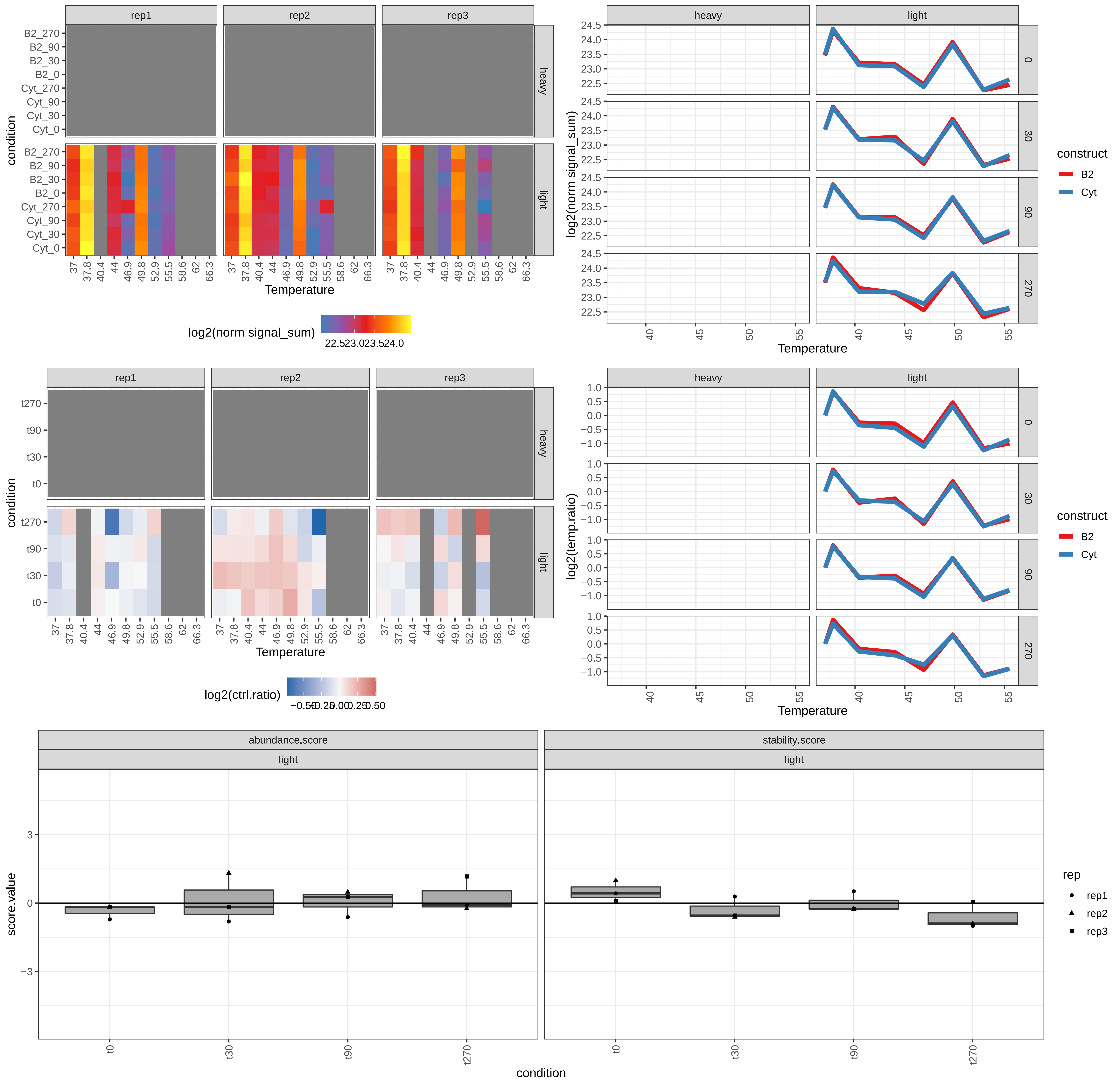

Q05583 − Cytosolic iron−sulfur protein assembly protein 1 OS=Saccharomyces cerevisiae (strain ATCC 204508 / S288c) GN=CIA1 PE=1 SV=1

CIC1 − average.top3: 6.816168 − max.qupm: 10

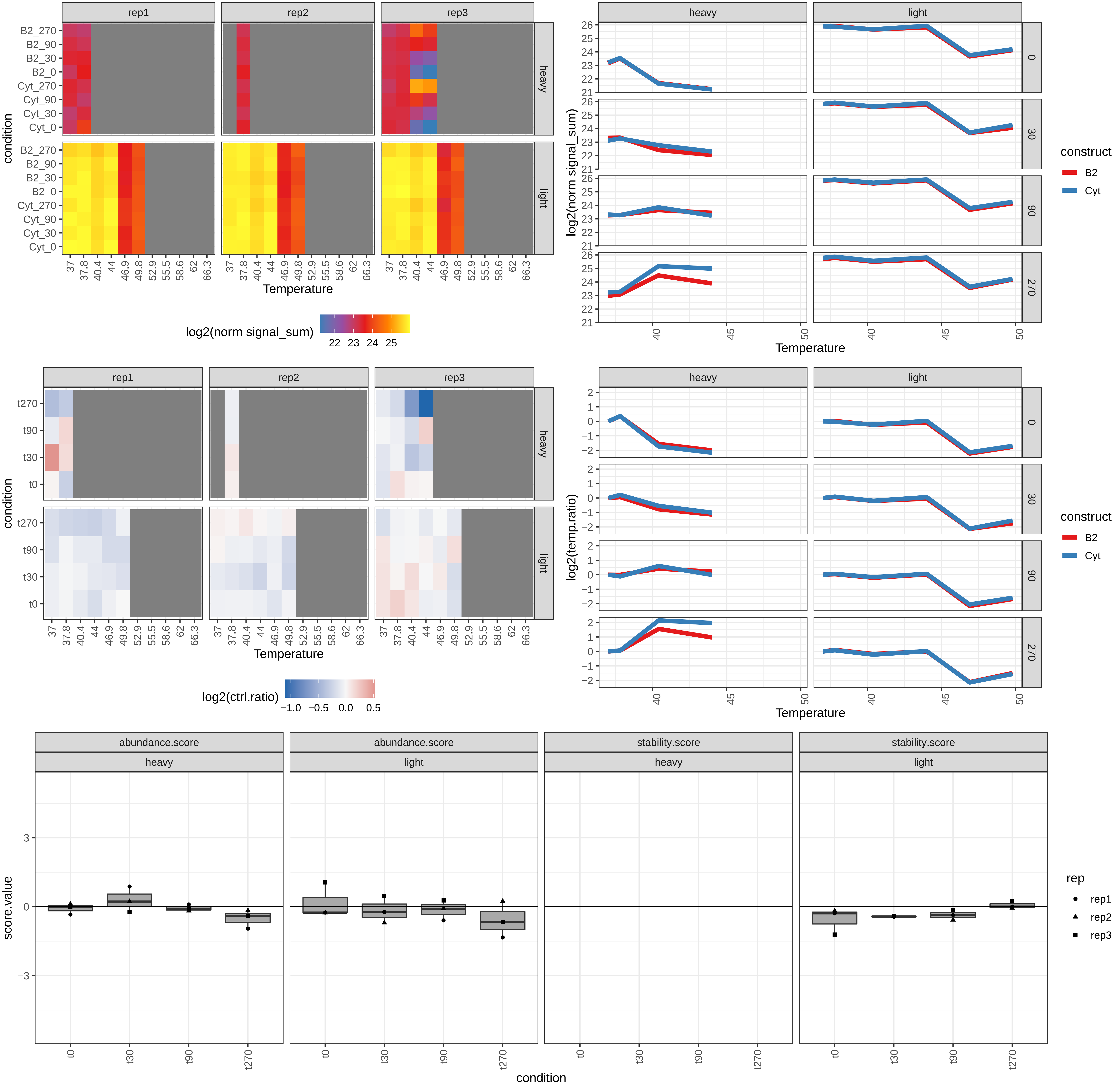

P38779 − Proteasome−interacting protein CIC1 OS=Saccharomyces cerevisiae (strain ATCC 204508 / S288c) GN=CIC1 PE=1 SV=1

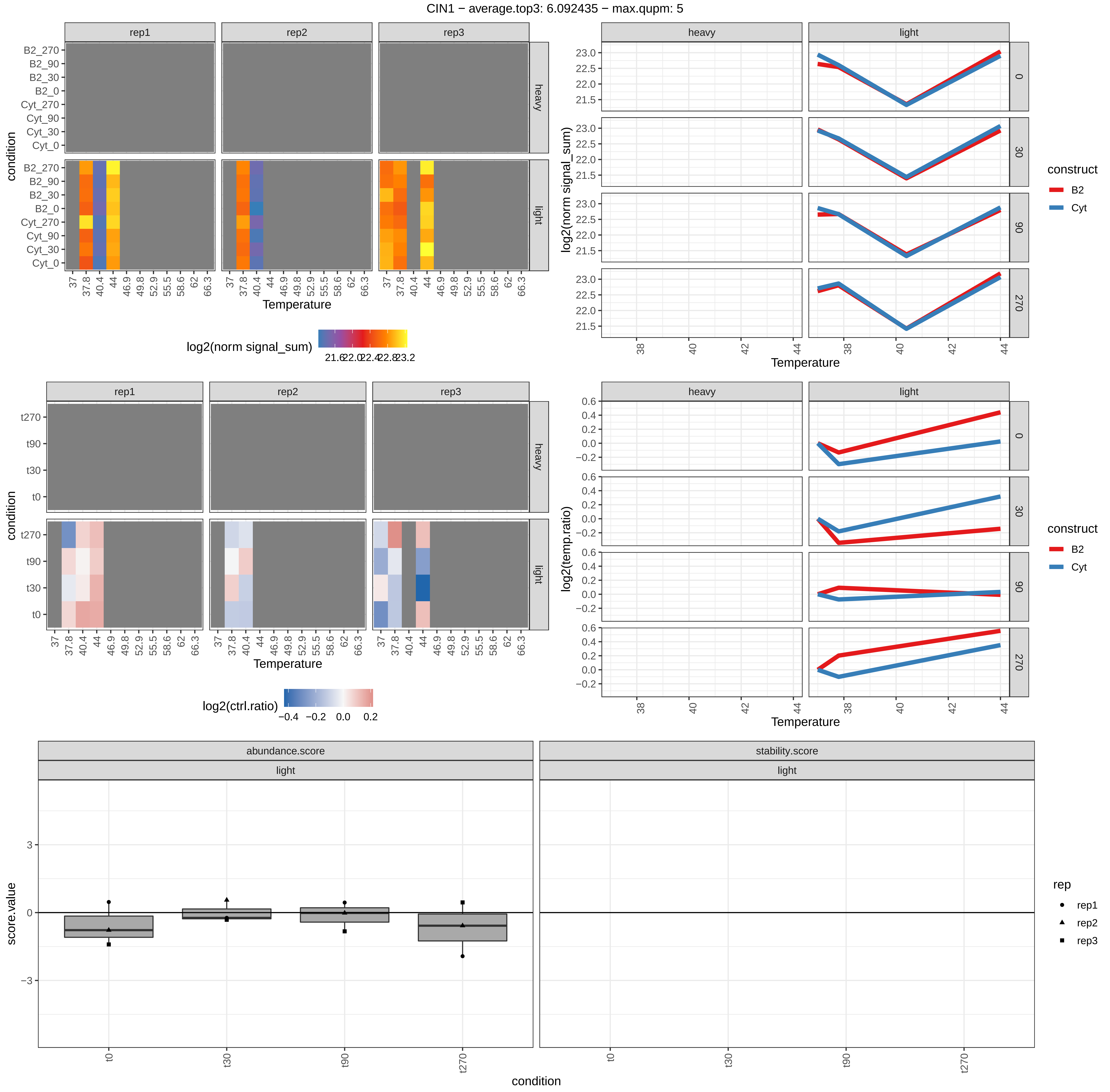

P40987 − Chromosome instability protein 1 OS=Saccharomyces cerevisiae (strain ATCC 204508 / S288c) GN=CIN1 PE=1 SV=1

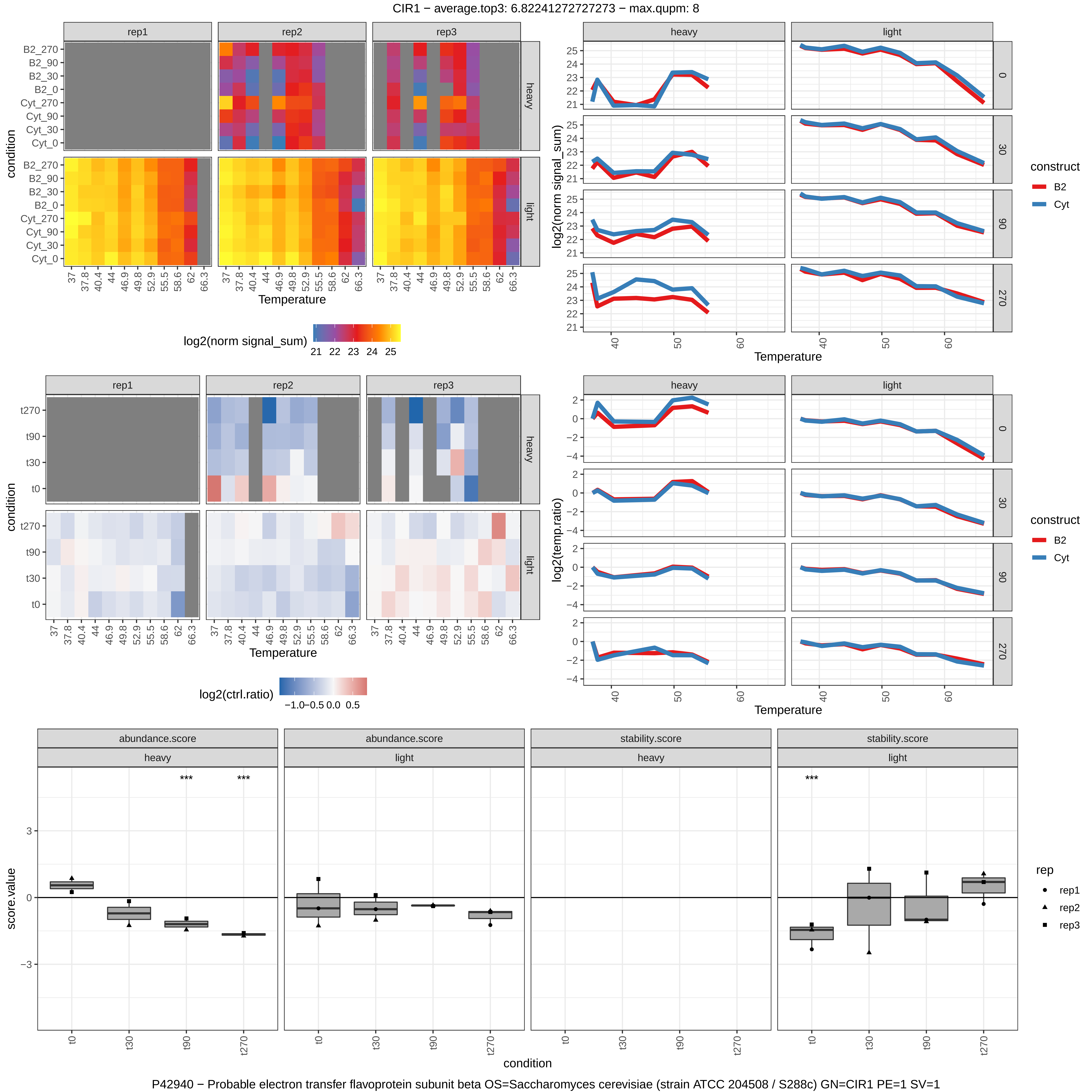
CIR1 − average.top3: 6.82241272727273 − max.qupm: 8
rep1
rep2
rep3
heavy
light
condition
Temperature
log2(norm signal_sum)
construct
B2
Cyt
log2(ctrl.ratio)
log2(temp.ratio)
abundance.score
stability.score
score.value
rep
rep1
rep2
rep3
***
P42940 − Probable electron transfer flavoprotein subunit beta OS=Saccharomyces cerevisiae (strain ATCC 204508 / S288c) GN=CIR1 PE=1 SV=1

CIR2 − average.top3: 7.14002206349206 − max.qupm: 22

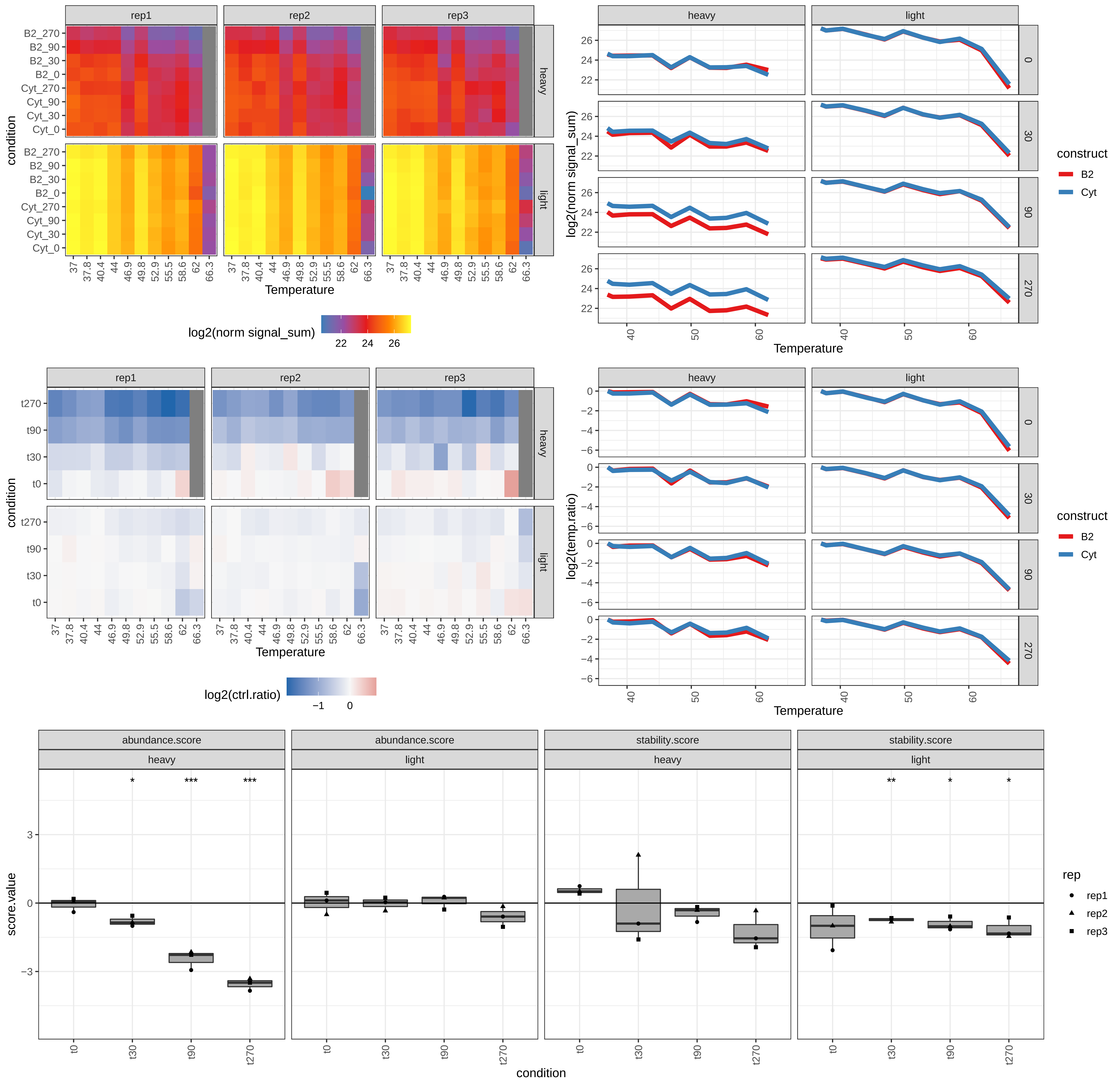

Q08822 − Probable electron transfer flavoprotein−ubiquinone oxidoreductase, mitochondrial OS=Saccharomyces cerevisiae (strain ATCC 204508 / S288c) GN=CIR2 PE=1 SV=1

CIS3 − average.top3: 7.78450696428571 − max.qupm: 2

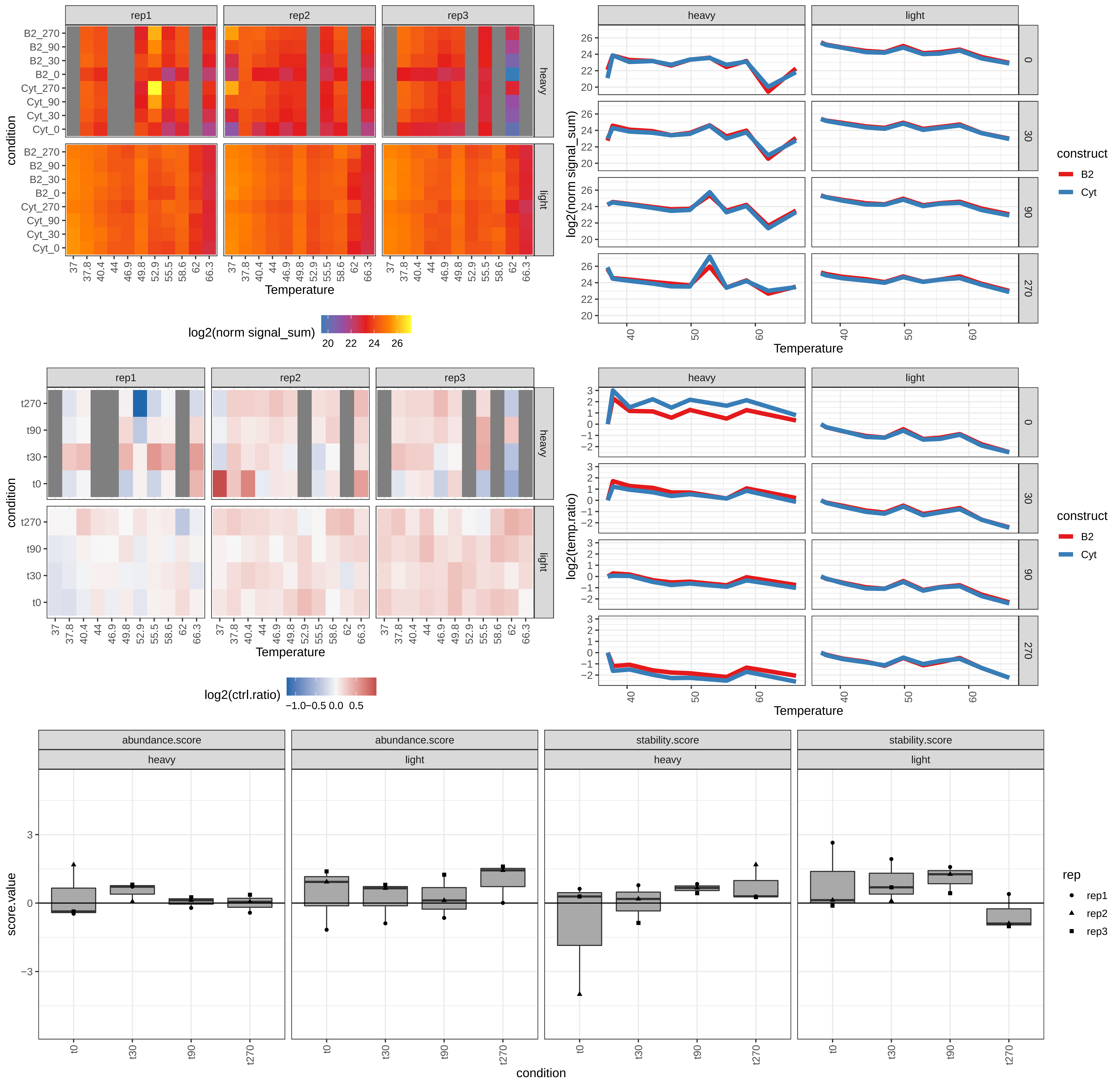

P47001 − Cell wall mannoprotein CIS3 OS=Saccharomyces cerevisiae (strain ATCC 204508 / S288c) GN=CIS3 PE=1 SV=1

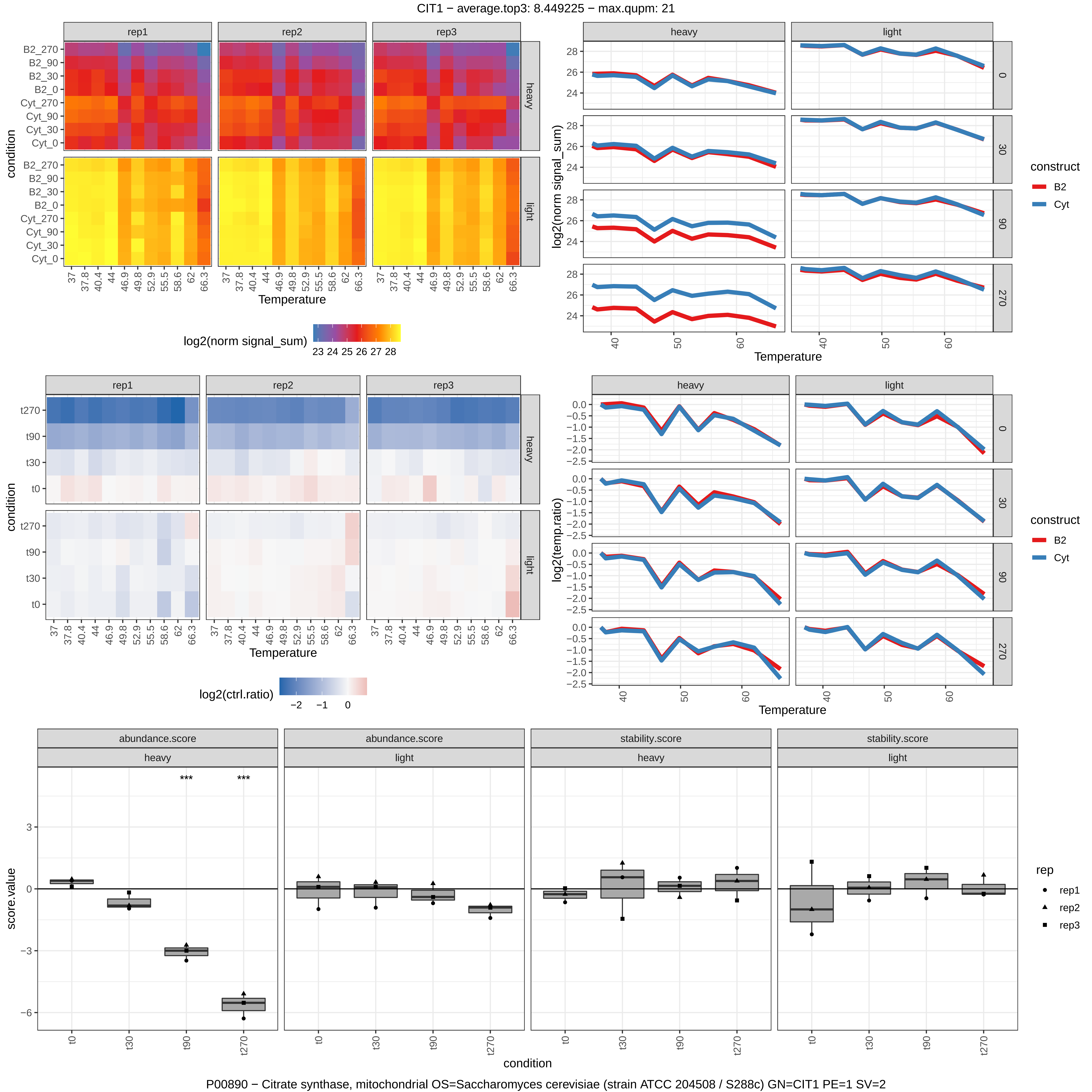
CIT1 − average.top3: 8.449225 − max.qupm: 21
rep1
rep2
rep3
heavy
light
condition
Temperature
log2(norm signal_sum)
construct
B2
Cyt
log2(ctrl.ratio)
log2(temp.ratio)
abundance.score
stability.score
score.value
rep
rep1
rep2
rep3
***
***
P00890 − Citrate synthase, mitochondrial OS=Saccharomyces cerevisiae (strain ATCC 204508 / S288c) GN=CIT1 PE=1 SV=2

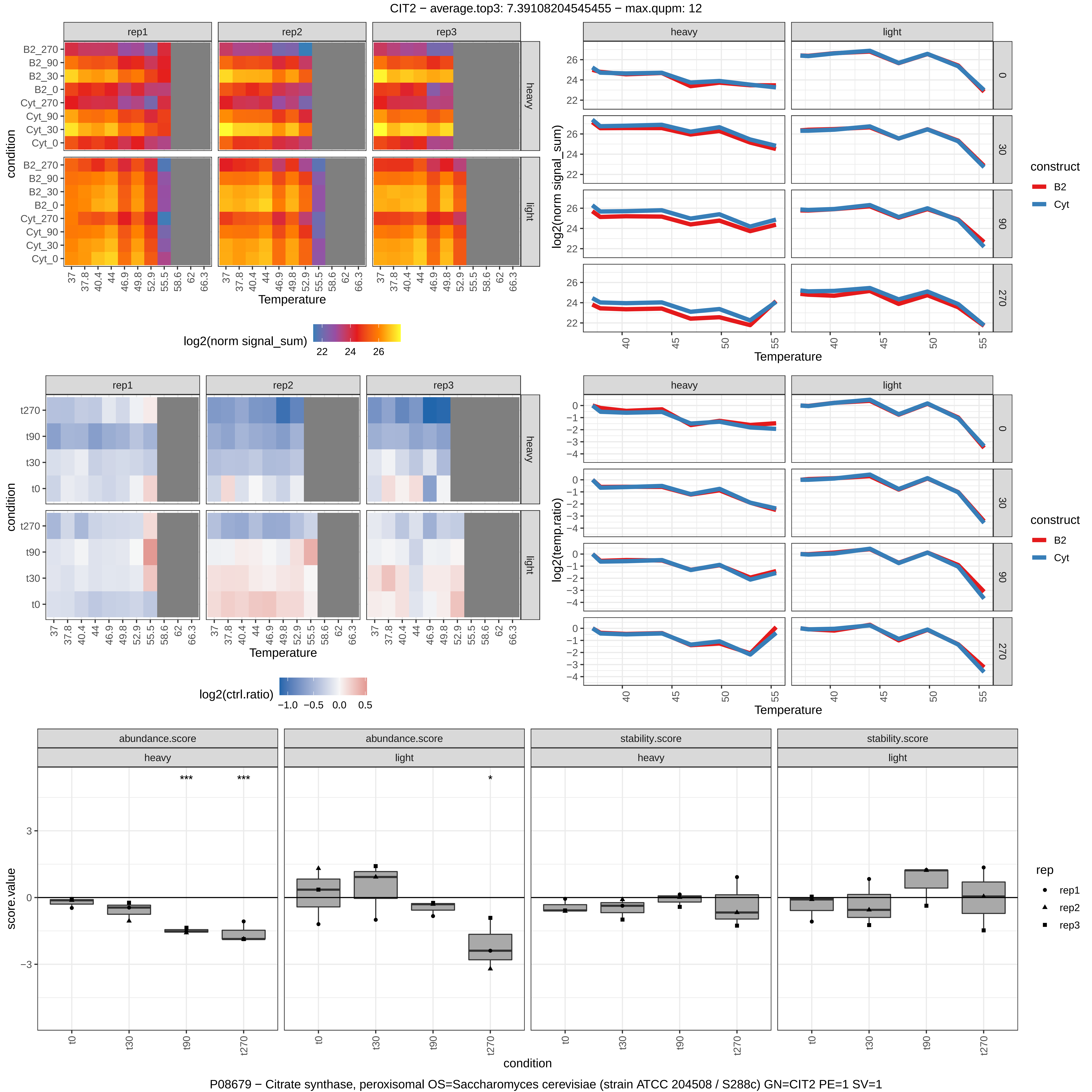

CIT2 − average.top3: 7.39108204545455 − max.qupm: 12
rep1
rep2
rep3
heavy
light
condition
B2_270
B2_90
B2_30
B2_0
Cyt_270
Cyt_90
Cyt_30
Cyt_0
Temperature
log2(norm signal_sum)
22 24 26
log2(norm signal_sum)
construct
B2
Cyt
0
30
90
270
t270
t90
t30
t0
log2(ctrl.ratio)
−1.0 −0.5 0.0 0.5
log2(temp.ratio)
abundance.score
stability.score
score.value
***
*
rep
rep1
rep2
rep3
condition
P08679 − Citrate synthase, peroxisomal OS=Saccharomyces cerevisiae (strain ATCC 204508 / S288c) GN=CIT2 PE=1 SV=1

CIT3 − average.top3: 7.53457314814815 − max.qupm: 17

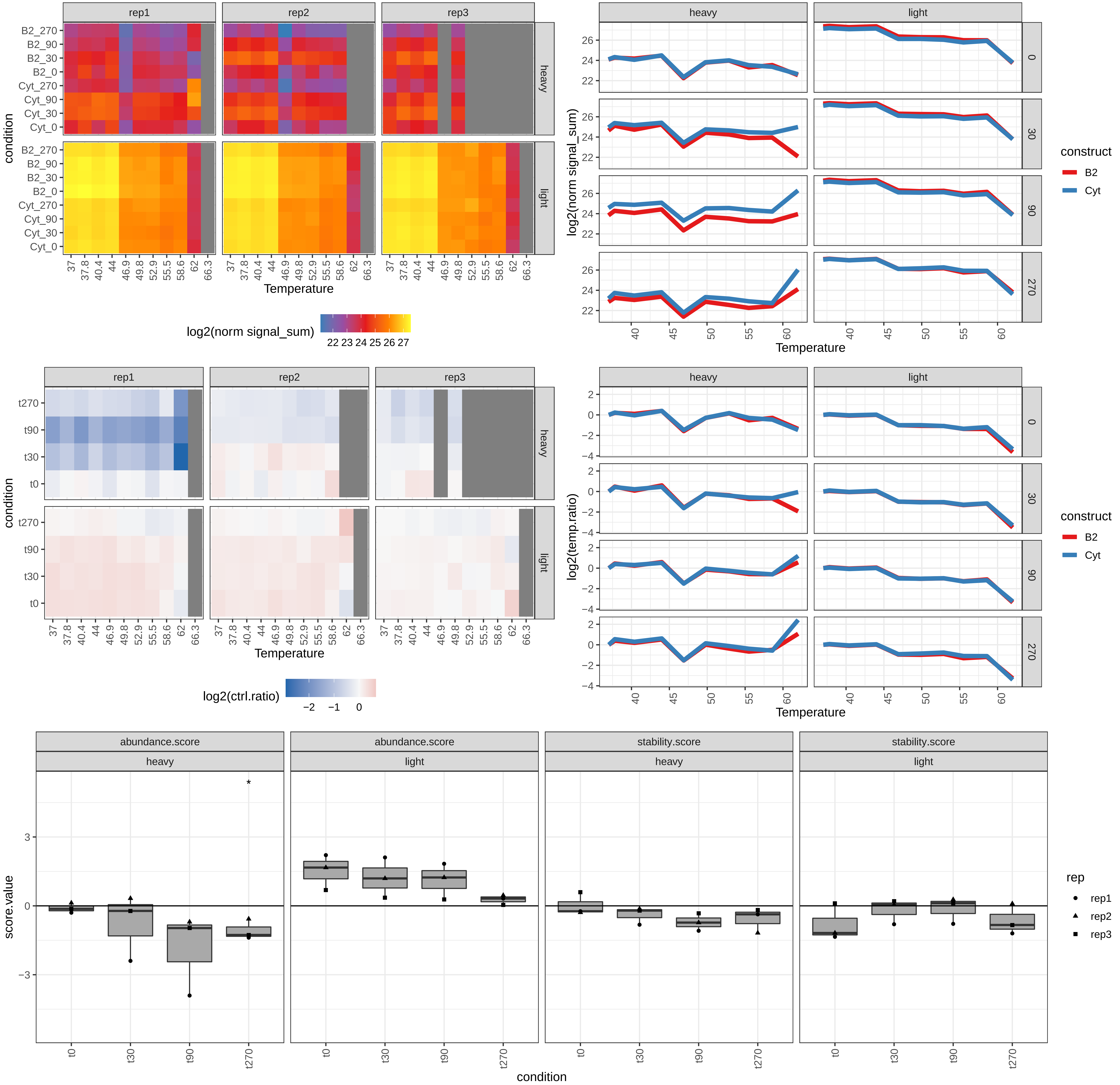

P43635 − Citrate synthase 3, mitochondrial OS=Saccharomyces cerevisiae (strain ATCC 204508 / S288c) GN=CIT3 PE=1 SV=1

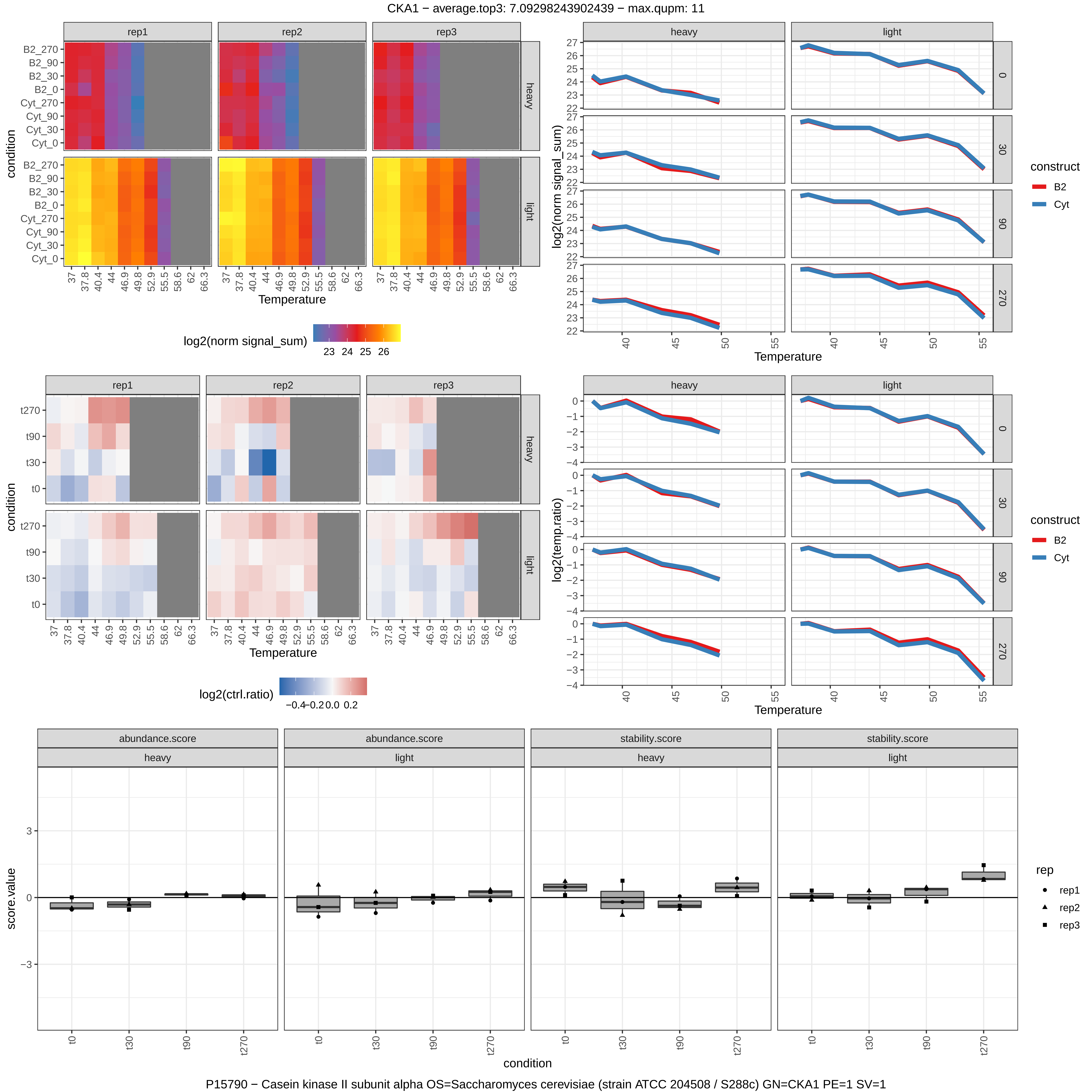

CKA1 − average.top3: 7.09298243902439 − max.qupm: 11
rep1
rep2
rep3
heavy
light
condition
Temperature
log2(norm signal_sum)
log2(norm signal_sum)
construct
B2
Cyt
log2(ctrl.ratio)
log2(temp.ratio)
abundance.score
stability.score
score.value
rep
rep1
rep2
rep3
P15790 − Casein kinase II subunit alpha OS=Saccharomyces cerevisiae (strain ATCC 204508 / S288c) GN=CKA1 PE=1 SV=1

CKA2 − average.top3: 7.20623484848485 − max.qupm: 9

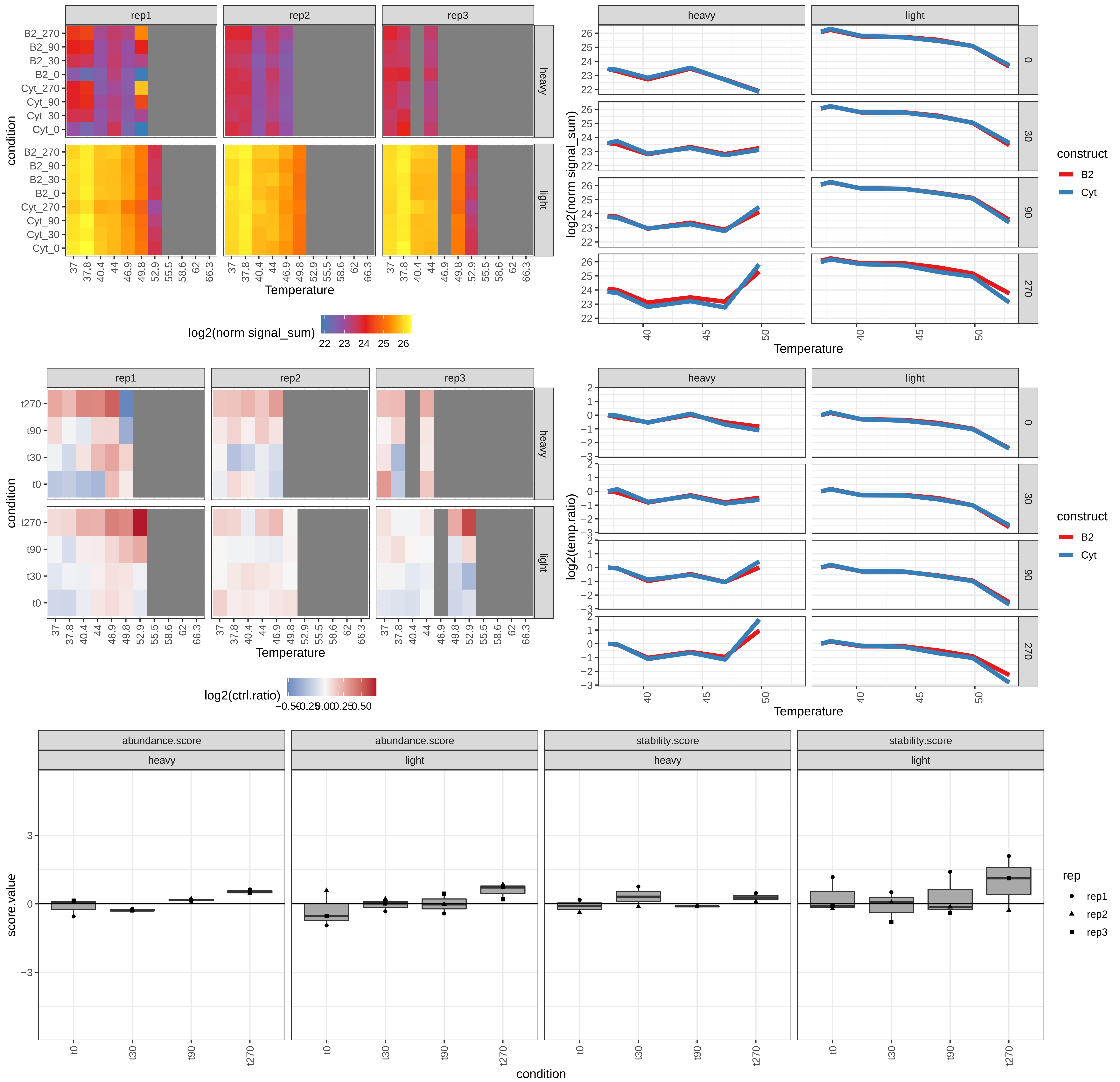

P19454 − Casein kinase II subunit alpha' OS=Saccharomyces cerevisiae (strain ATCC 204508 / S288c) GN=CKA2 PE=1 SV=2

CKB1 − average.top3: 7.39975368421053 − max.qupm: 6

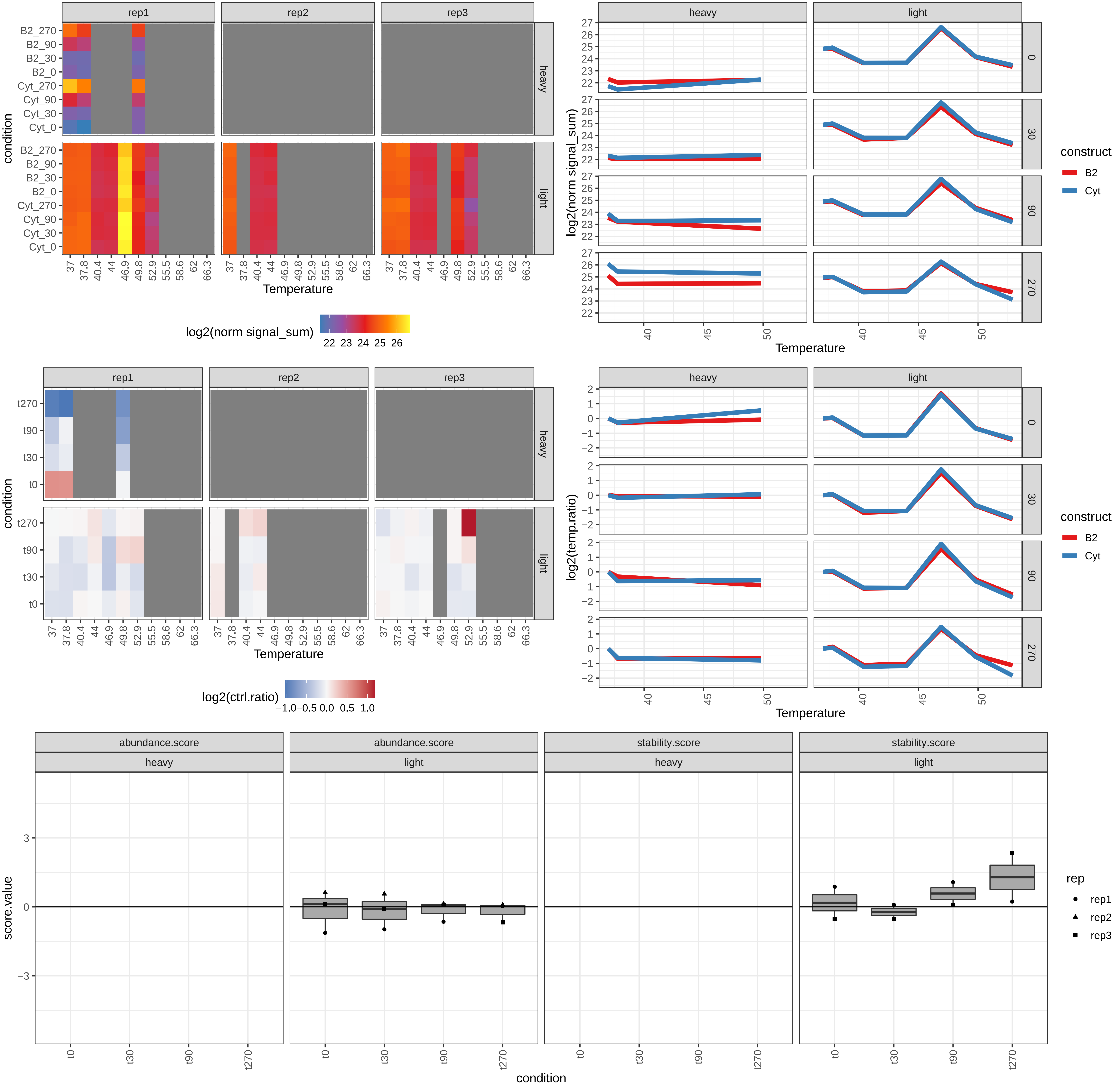

P43639 − Casein kinase II subunit beta OS=Saccharomyces cerevisiae (strain ATCC 204508 / S288c) GN=CKB1 PE=1 SV=1

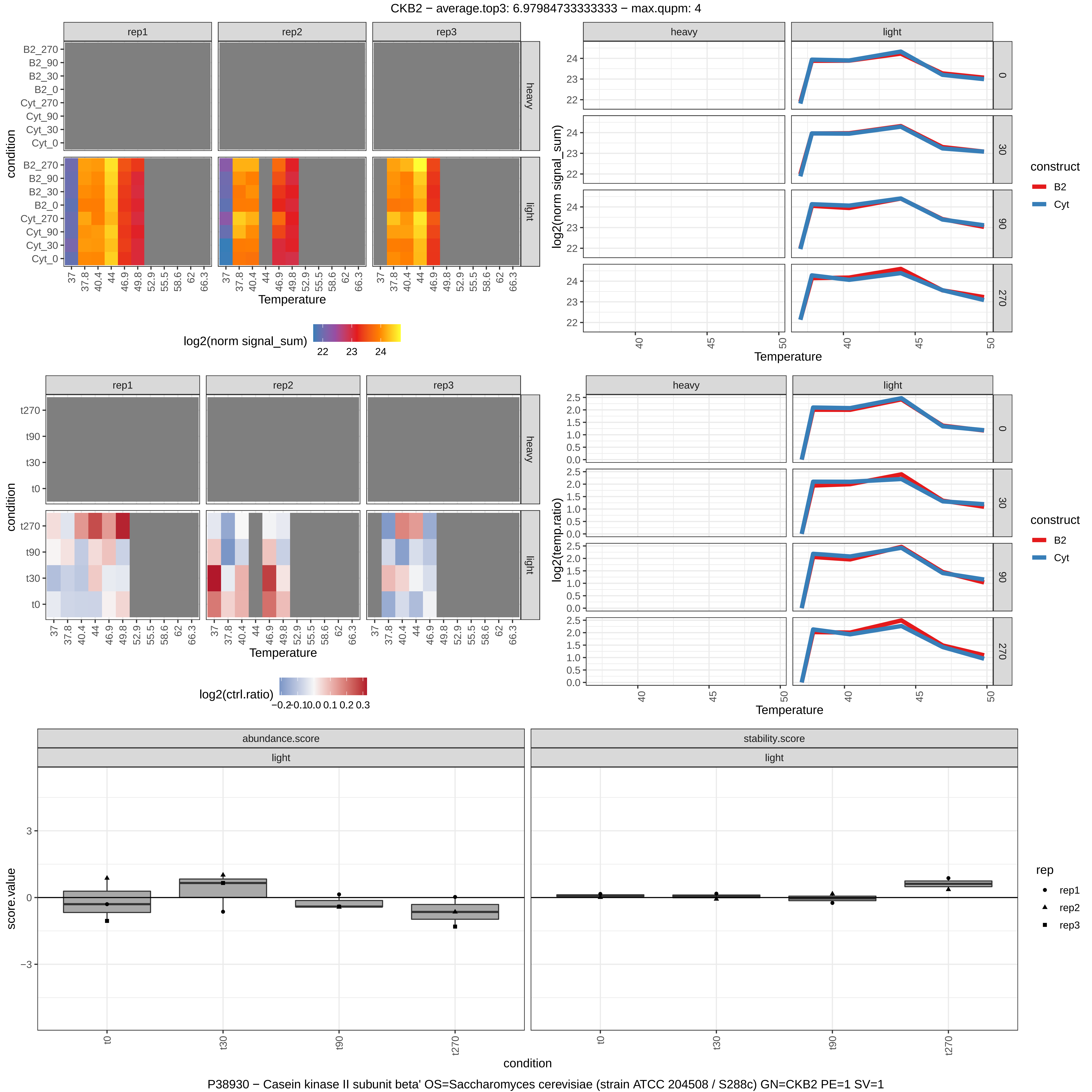
CKB2 − average.top3: 6.97984733333333 − max.qupm: 4
rep1
rep2
rep3
heavy
light
condition
Temperature
log2(norm signal_sum)
construct
B2
Cyt
log2(ctrl.ratio)
log2(temp.ratio)
abundance.score
stability.score
score.value
rep
rep1
rep2
rep3
P38930 − Casein kinase II subunit beta' OS=Saccharomyces cerevisiae (strain ATCC 204508 / S288c) GN=CKB2 PE=1 SV=1

CKI1 − average.top3: 6.57835526315789 − max.qupm: 8

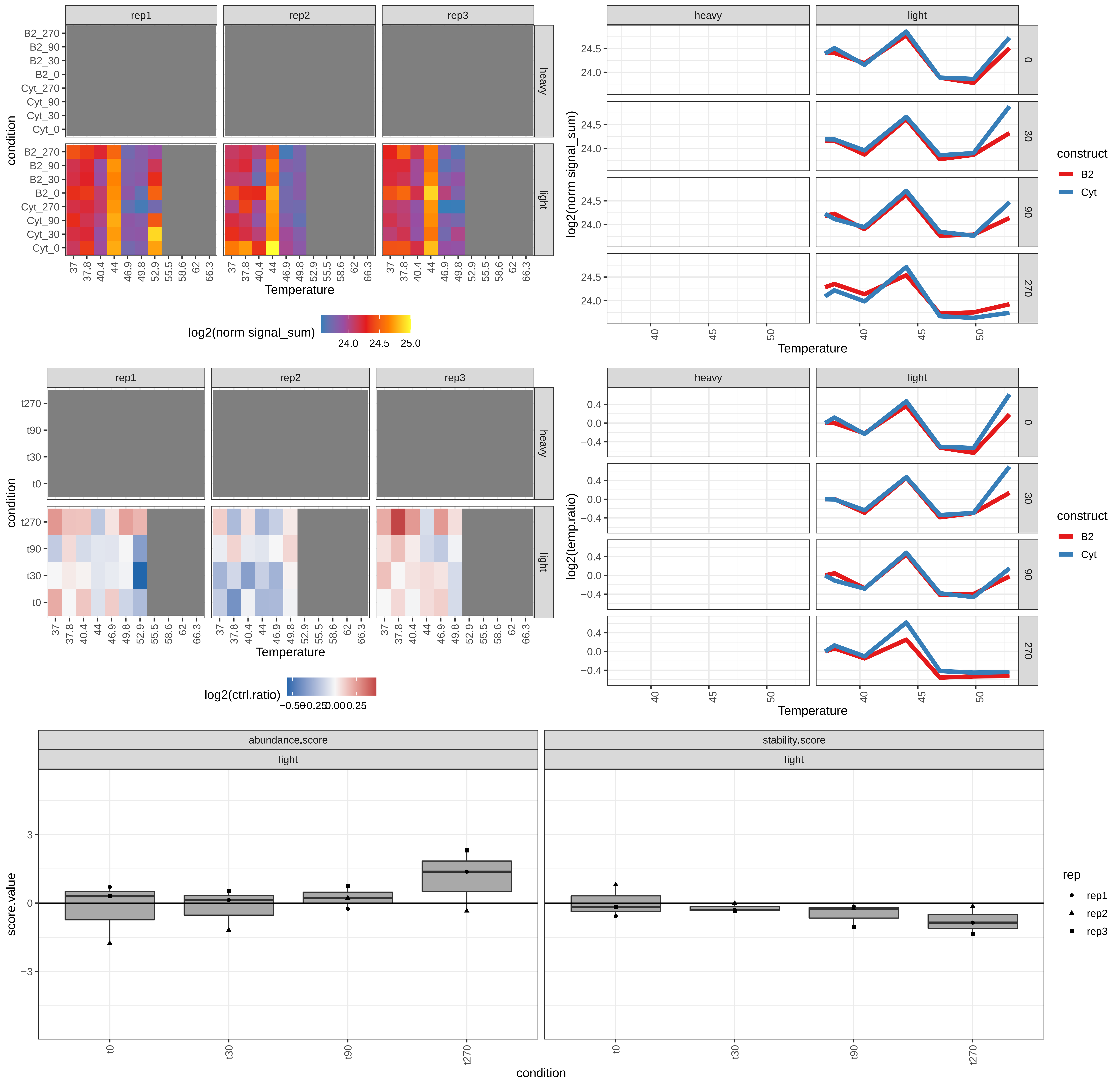

P20485 − Choline kinase OS=Saccharomyces cerevisiae (strain ATCC 204508 / S288c) GN=CKI1 PE=1 SV=1

CKS1 − average.top3: 6.843793 − max.qupm: 6

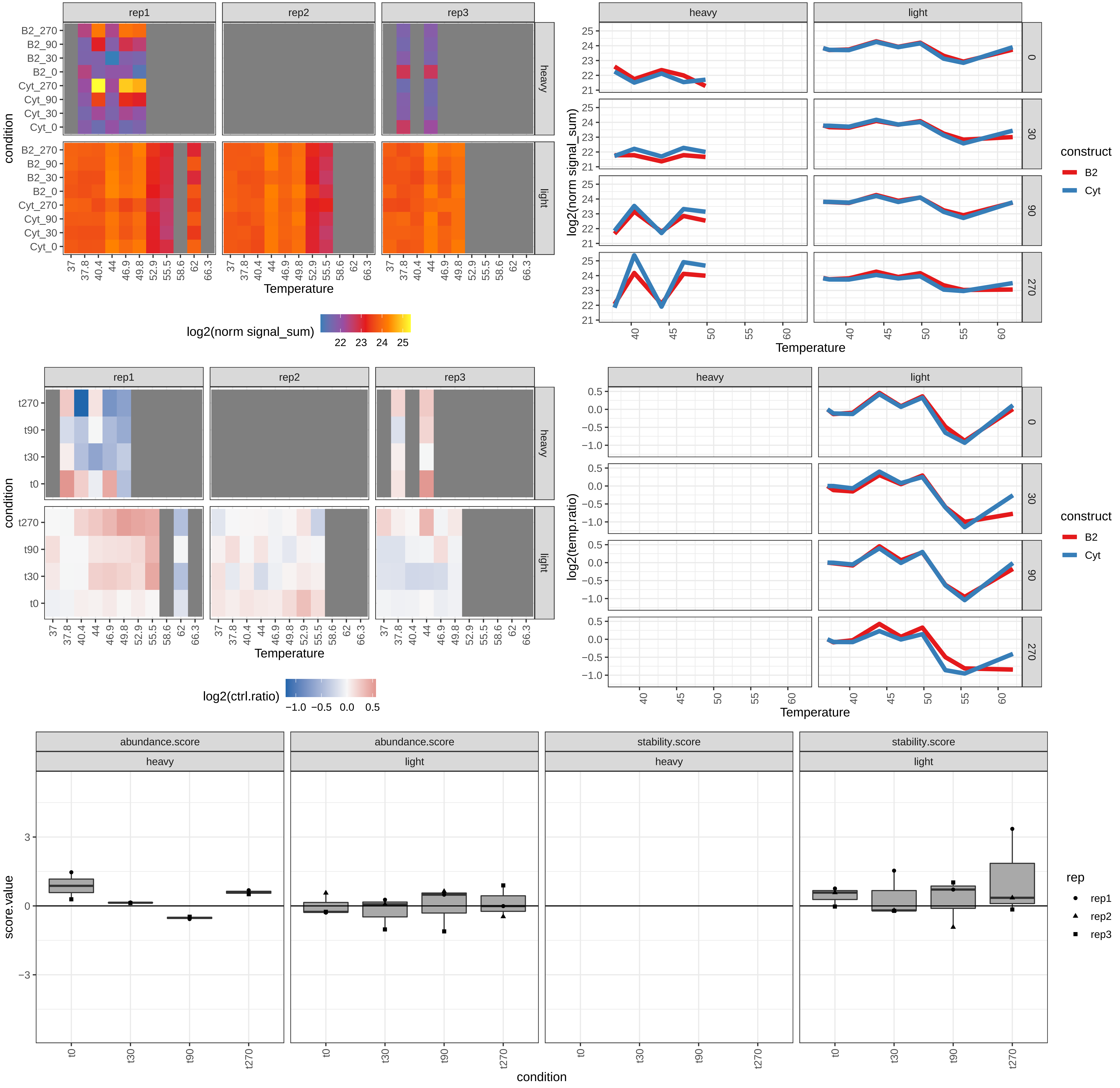

P20486 − Cyclin−dependent kinases regulatory subunit OS=Saccharomyces cerevisiae (strain ATCC 204508 / S288c) GN=CKS1 PE=1 SV=1

CLC1 − average.top3: 7.41775861538462 − max.qupm: 11

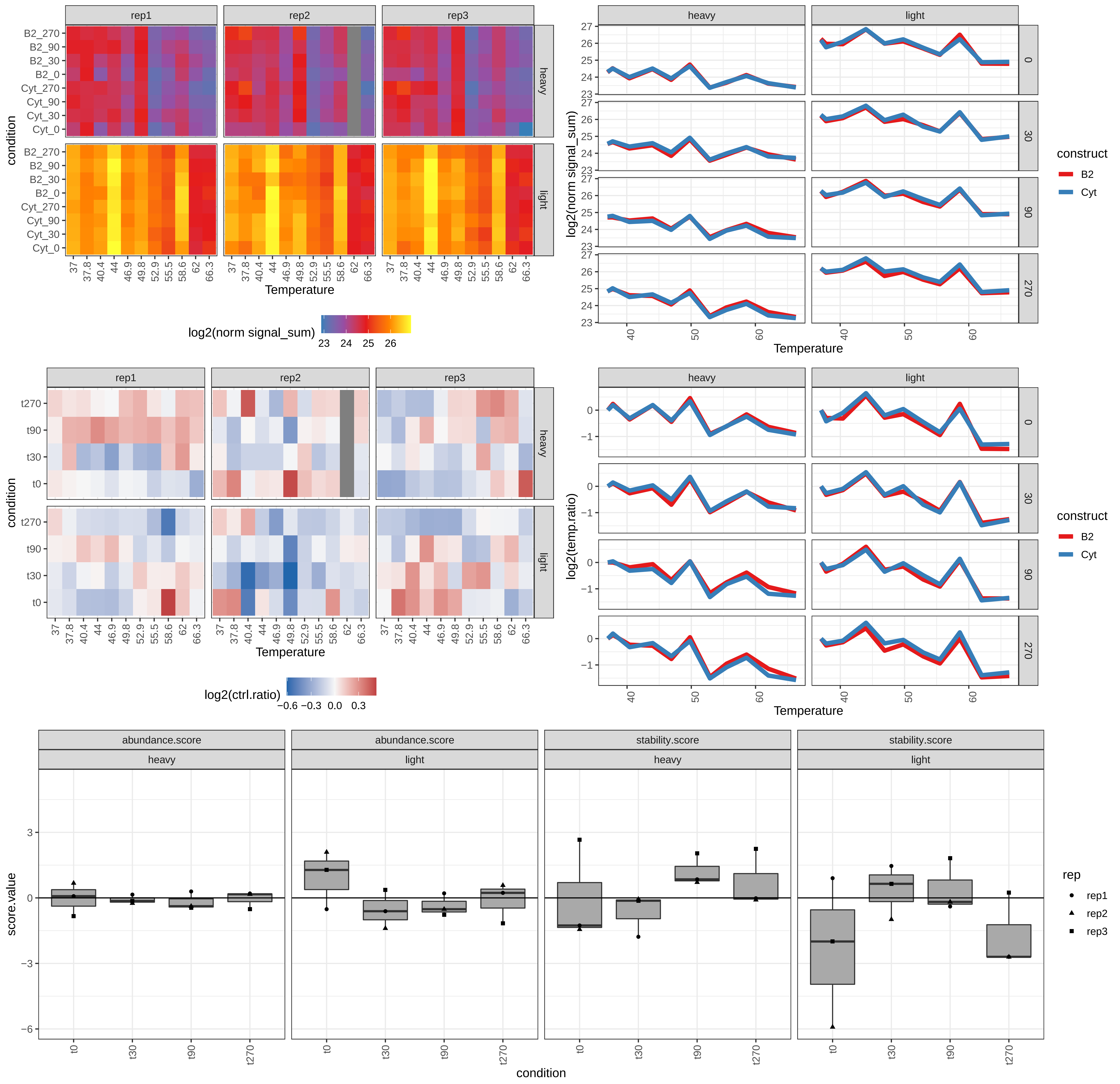

P17891 − Clathrin light chain OS=Saccharomyces cerevisiae (strain ATCC 204508 / S288c) GN=CLC1 PE=1 SV=1

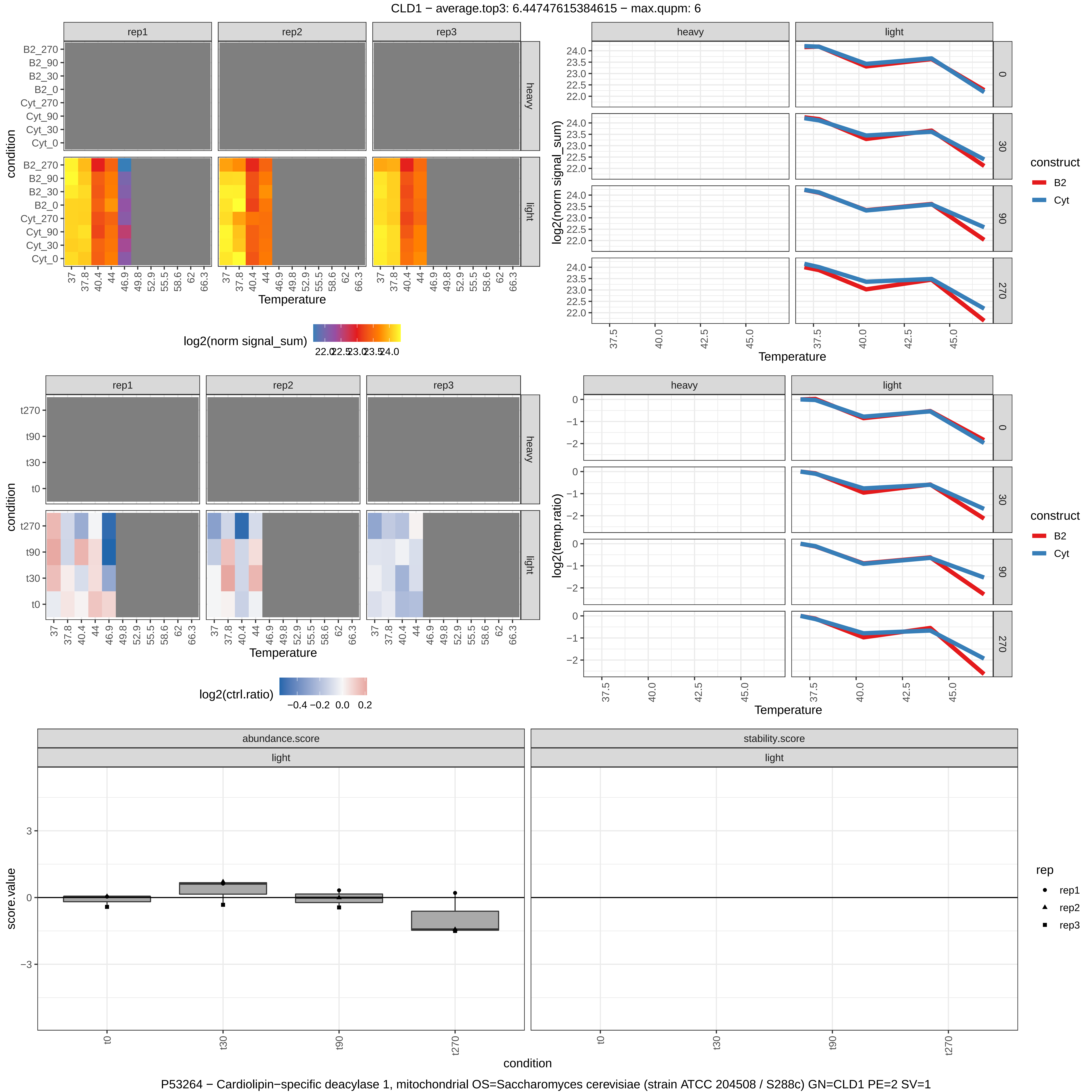

CLD1 − average.top3: 6.44747615384615 − max.qupm: 6
rep1
rep2
rep3
heavy
light
condition
B2_270
B2_90
B2_30
B2_0
Cyt_270
Cyt_90
Cyt_30
Cyt_0
Temperature
37
37.8
40.4
44
46.9
49.8
52.9
55.5
58.6
62
66.3
log2(norm signal_sum)
22.0
22.5
23.0
23.5
24.0
log2(norm signal_sum)
construct
B2
Cyt
0
30
90
270
37.5
40.0
42.5
45.0
t270
t90
t30
t0
log2(ctrl.ratio)
−0.4
−0.2
0.0
0.2
log2(temp.ratio)
0
−1
−2
abundance.score
stability.score
score.value
3
0
−3
condition
rep
rep1
rep2
rep3
P53264 − Cardiolipin−specific deacylase 1, mitochondrial OS=Saccharomyces cerevisiae (strain ATCC 204508 / S288c) GN=CLD1 PE=2 SV=1

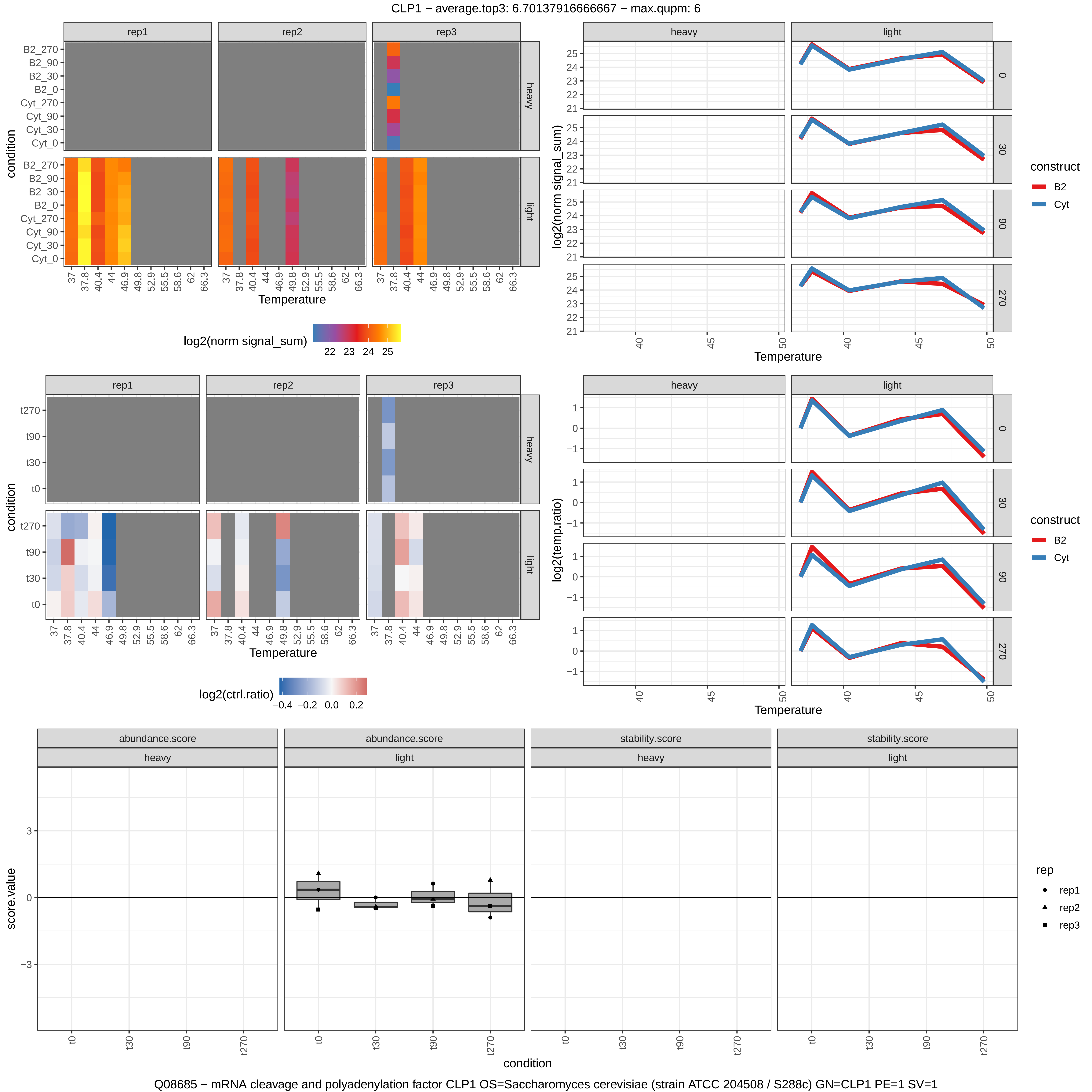
CLP1 − average.top3: 6.70137916666667 − max.qupm: 6
rep1
rep2
rep3
heavy
light
B2_270
B2_90
B2_30
B2_0
Cyt_270
Cyt_90
Cyt_30
Cyt_0
condition
37 37.8 40.4 44 46.9 49.8 52.9 55.5 58.6 62 66.3
Temperature
log2(norm signal_sum)
22 23 24 25
log2(norm signal_sum)
construct
B2
Cyt
0
30
90
270
40 45 50
t270
t90
t30
t0
log2(ctrl.ratio)
−0.4 −0.2 0.0 0.2
log2(temp.ratio)
abundance.score
stability.score
score.value
3
0
−3
condition
rep
rep1
rep2
rep3
Q08685 − mRNA cleavage and polyadenylation factor CLP1 OS=Saccharomyces cerevisiae (strain ATCC 204508 / S288c) GN=CLP1 PE=1 SV=1

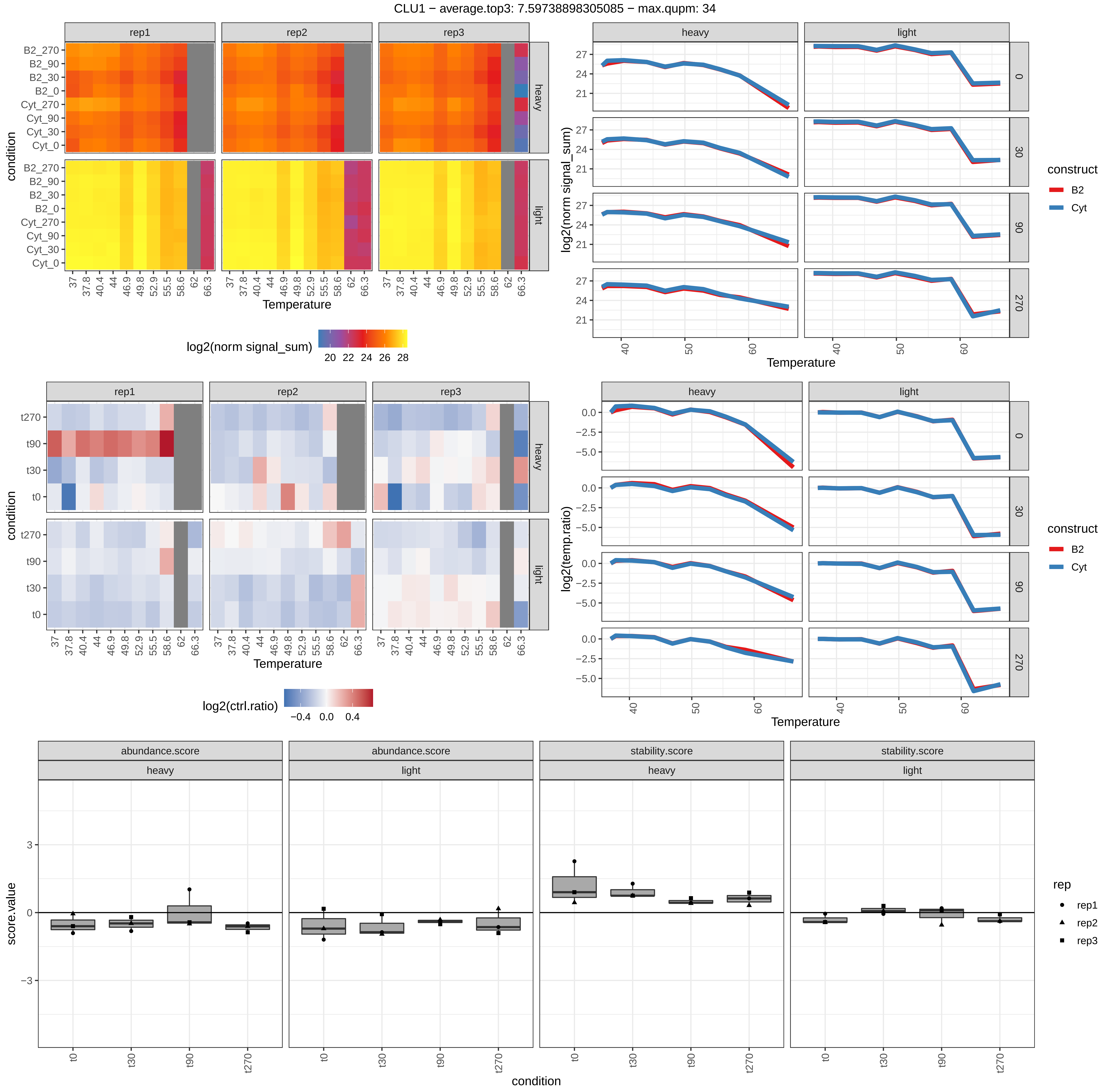

Q03690 − Clustered mitochondria protein 1 OS=Saccharomyces cerevisiae (strain ATCC 204508 / S288c) GN=CLU1 PE=1 SV=1

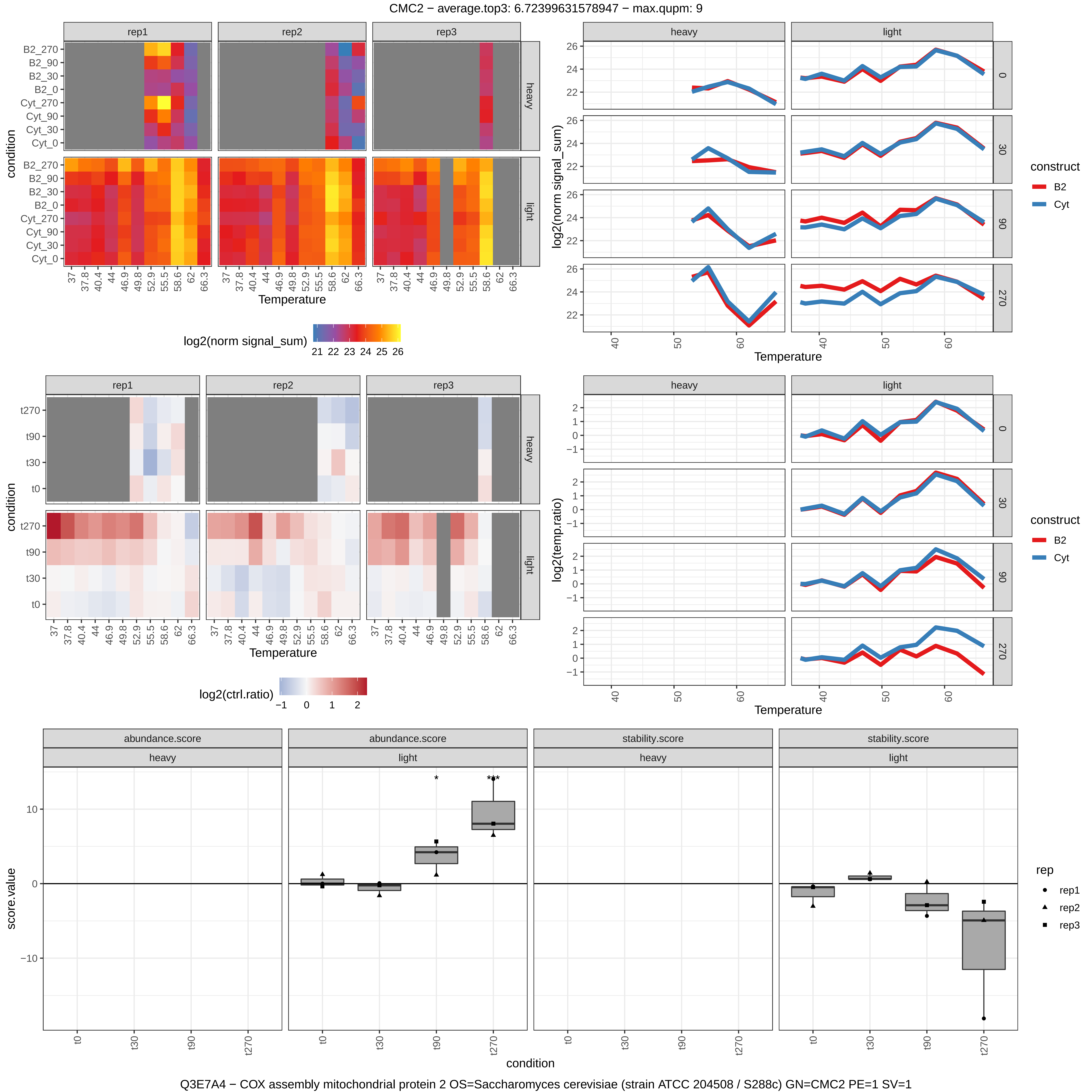
CMC2 − average.top3: 6.72399631578947 − max.qupm: 9
rep1
rep2
rep3
heavy
light
condition
Temperature
log2(norm signal_sum)
log2(ctrl.ratio)
log2(temp.ratio)
construct
B2
Cyt
abundance.score
stability.score
score.value
rep
rep1
rep2
rep3
Q3E7A4 − COX assembly mitochondrial protein 2 OS=Saccharomyces cerevisiae (strain ATCC 204508 / S288c) GN=CMC2 PE=1 SV=1

CMD1 − average.top3: 6.65196018518519 − max.qupm: 6

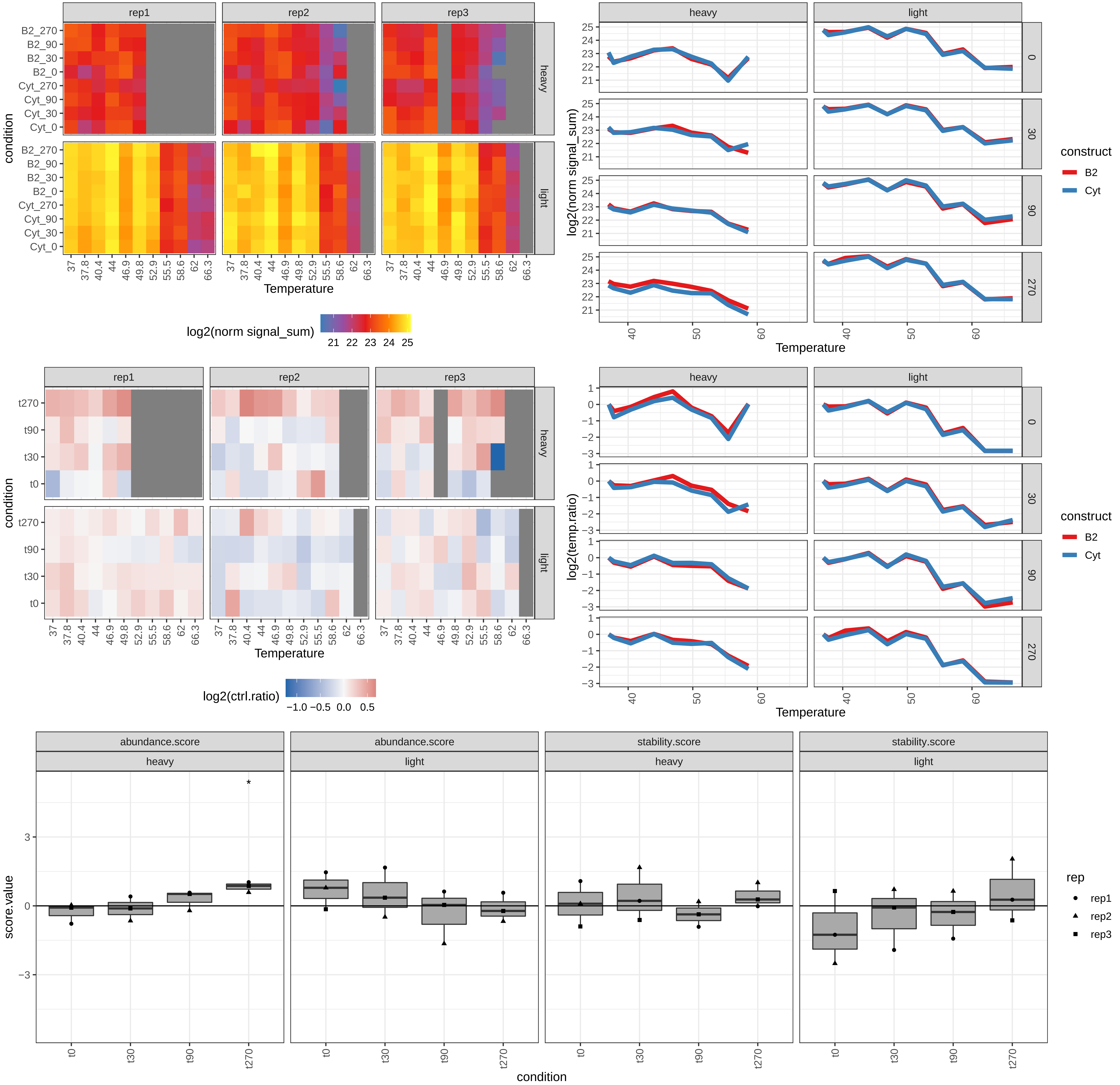

P06787 − Calmodulin OS=Saccharomyces cerevisiae (strain ATCC 204508 / S288c) GN=CMD1 PE=1 SV=1

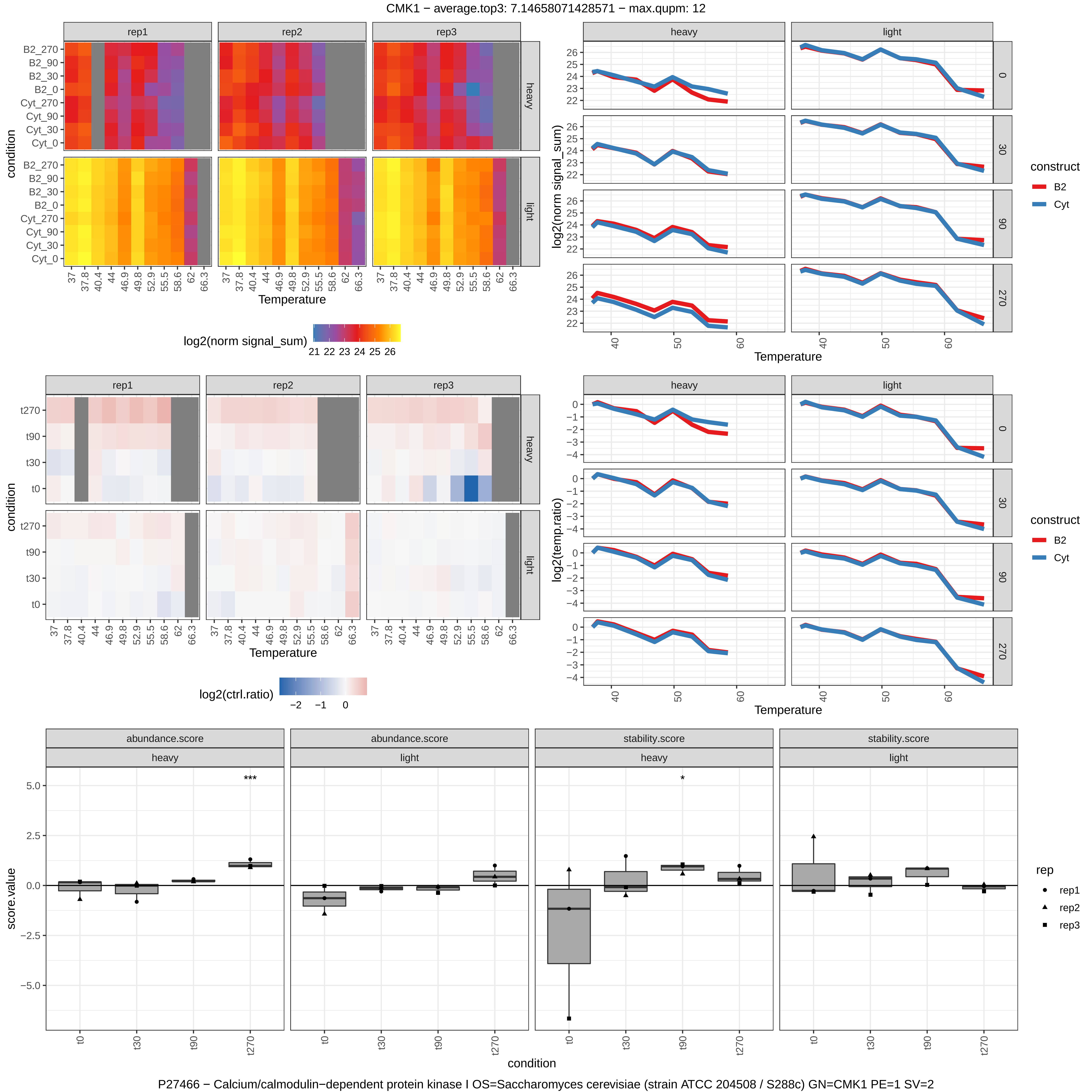

CMK1 − average.top3: 7.14658071428571 − max.qupm: 12
rep1
rep2
rep3
heavy
light
condition
Temperature
log2(norm signal_sum)
21 22 23 24 25 26
log2(norm signal_sum)
construct
B2
Cyt
log2(ctrl.ratio)
−2 −1 0
log2(temp.ratio)
abundance.score
stability.score
score.value
rep
***
*
P27466 − Calcium/calmodulin−dependent protein kinase I OS=Saccharomyces cerevisiae (strain ATCC 204508 / S288c) GN=CMK1 PE=1 SV=2

CMK2 − average.top3: 6.8830459375 − max.qupm: 8

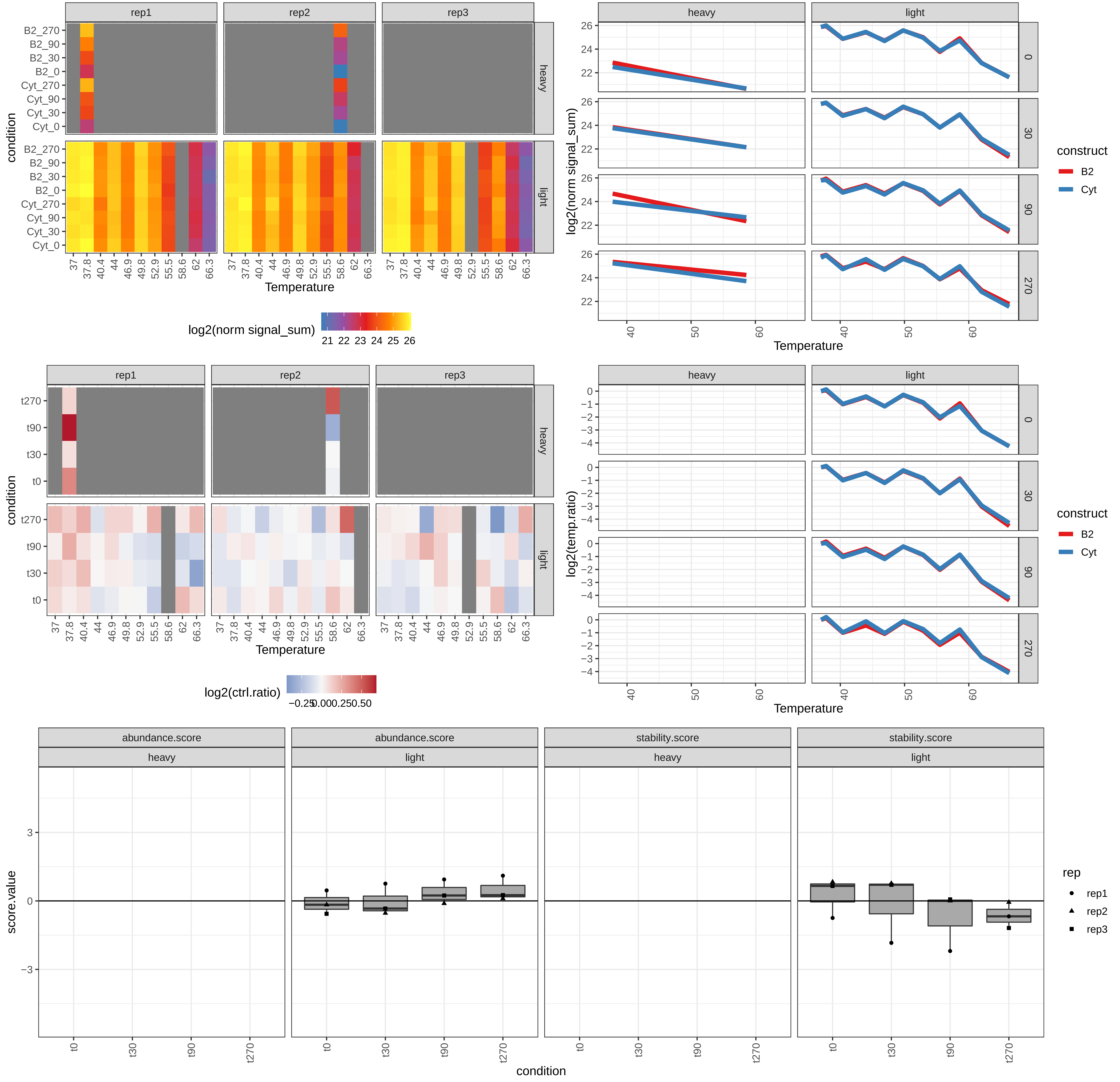

P22517 − Calcium/calmodulin−dependent protein kinase II OS=Saccharomyces cerevisiae (strain ATCC 204508 / S288c) GN=CMK2 PE=1 SV=2

CMP2 − average.top3: 7.0327896969697 − max.qupm: 8

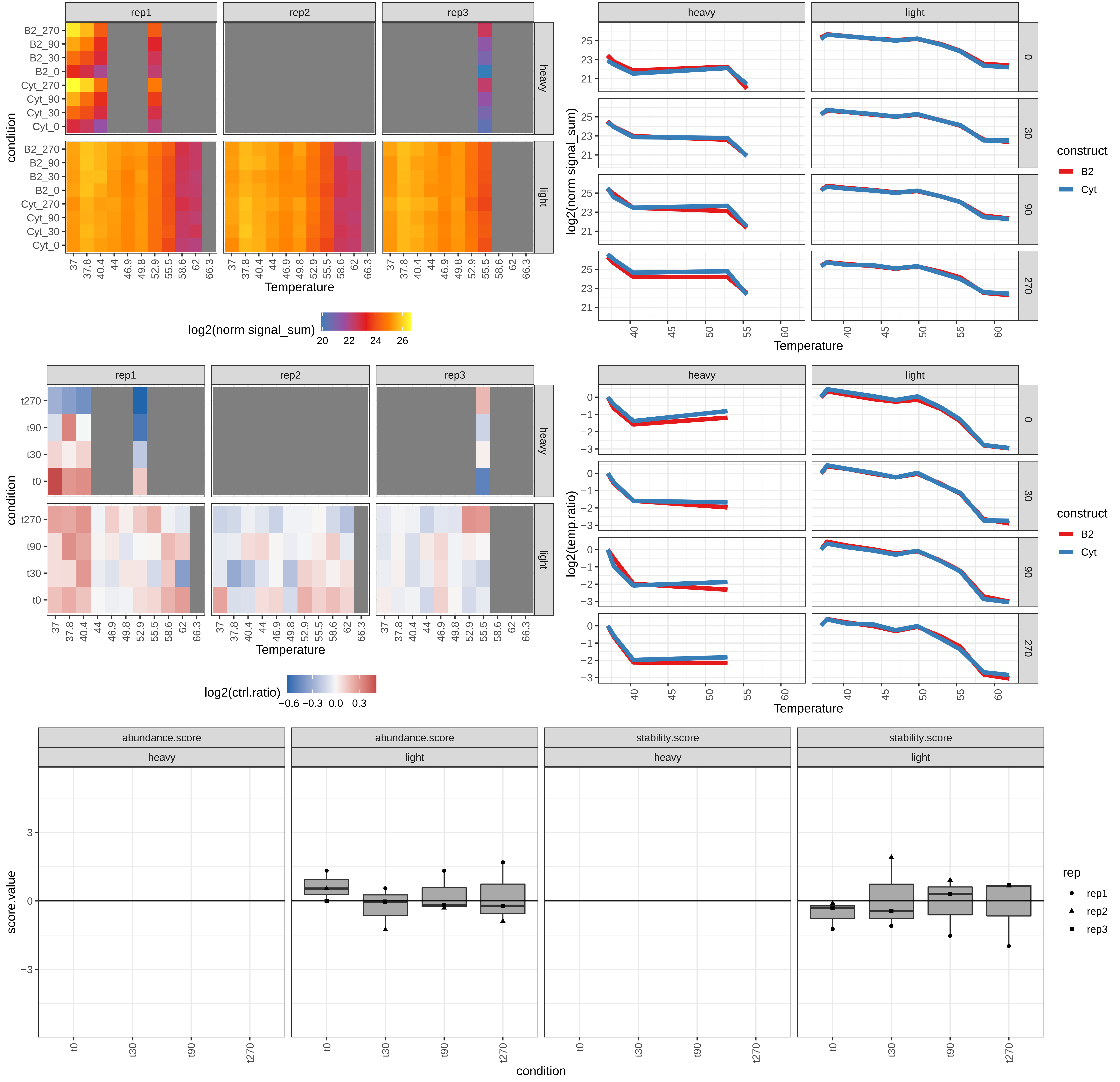

P14747 − Serine/threonine−protein phosphatase 2B catalytic subunit A2 OS=Saccharomyces cerevisiae (strain ATCC 204508 / S288c) GN=CMP2 PE=1 SV=2

CMR1 − average.top3: 6.397032 − max.qupm: 5

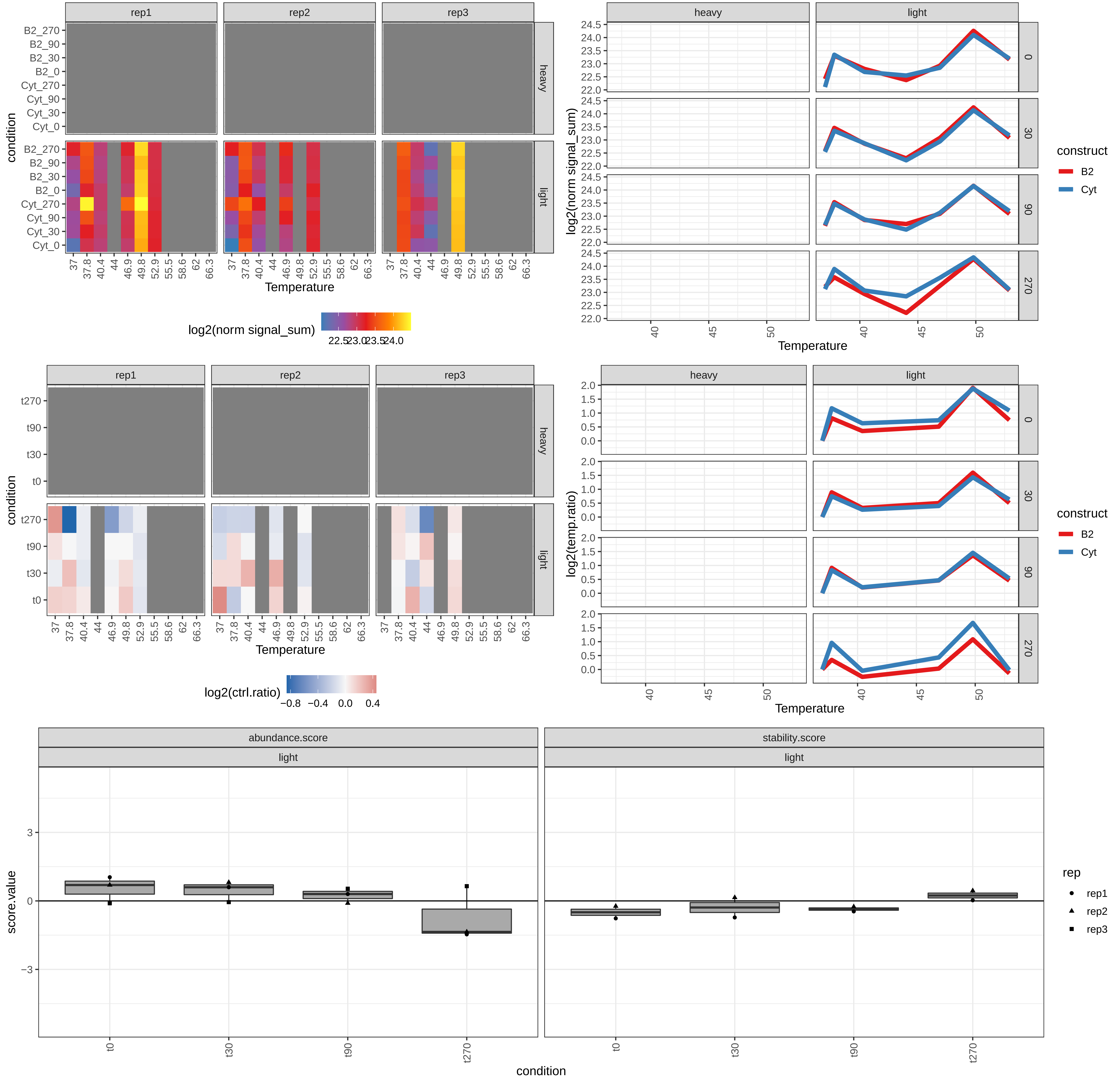

Q12510 − DNA damage−binding protein CMR1 OS=Saccharomyces cerevisiae (strain ATCC 204508 / S288c) GN=CMR1 PE=1 SV=1

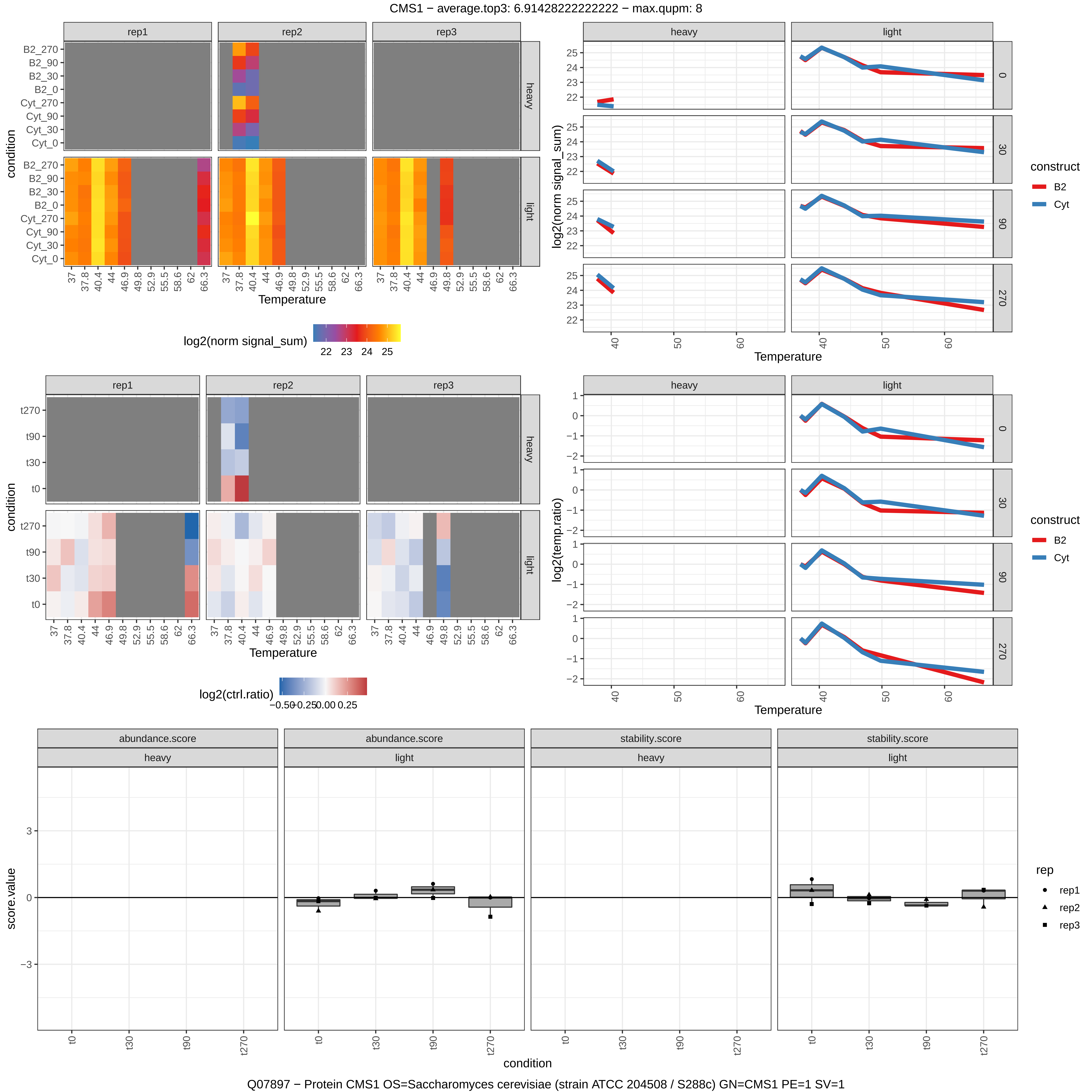
CMS1 − average.top3: 6.91428222222222 − max.qupm: 8
rep1
rep2
rep3
heavy
light
condition
B2_270
B2_90
B2_30
B2_0
Cyt_270
Cyt_90
Cyt_30
Cyt_0
Temperature
log2(norm signal_sum)
construct
B2
Cyt
t270
t90
t30
t0
log2(ctrl.ratio)
log2(temp.ratio)
abundance.score
stability.score
score.value
rep
condition
Q07897 − Protein CMS1 OS=Saccharomyces cerevisiae (strain ATCC 204508 / S288c) GN=CMS1 PE=1 SV=1

CNA1 − average.top3: 6.8445 − max.qupm: 10

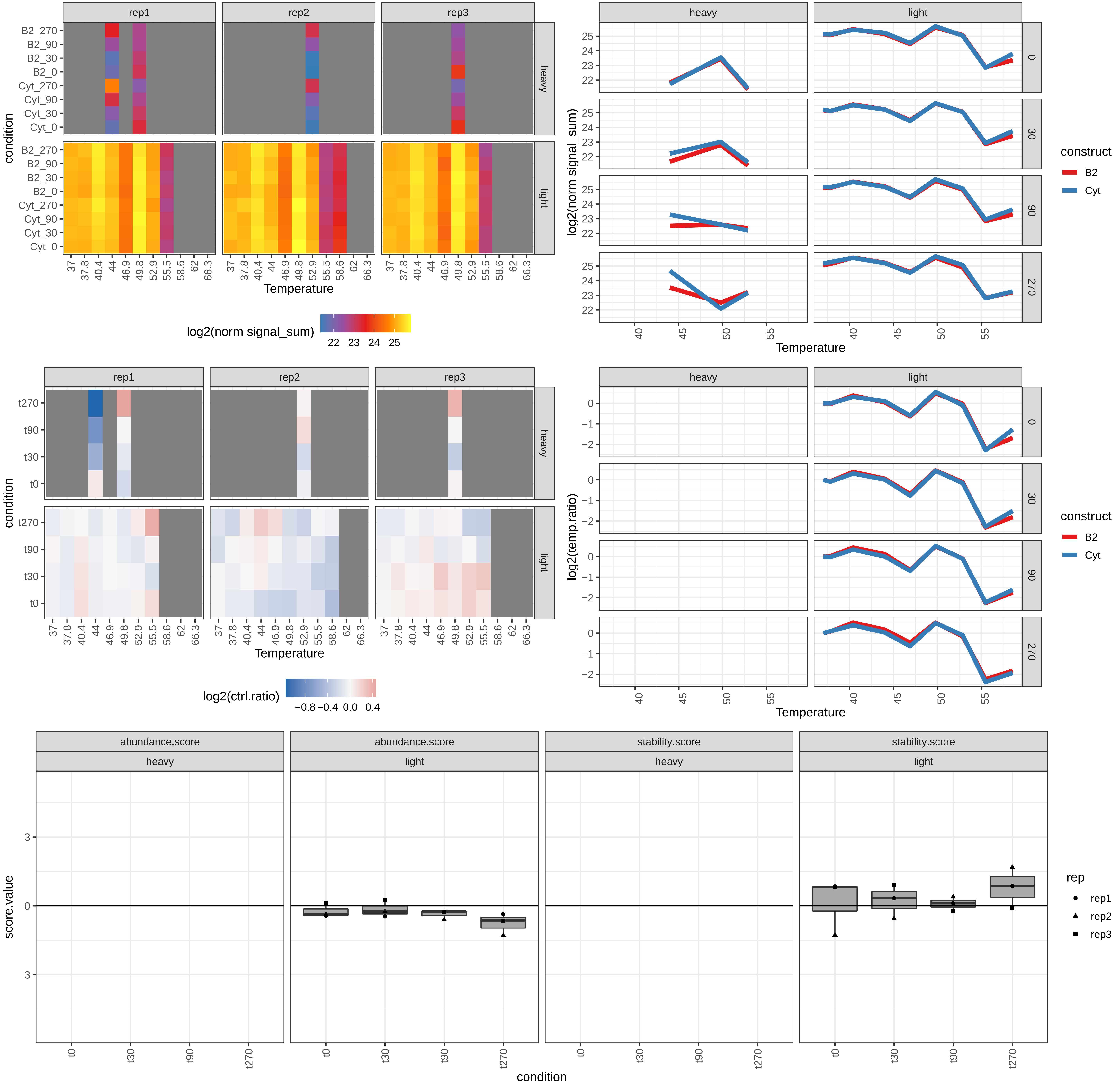

P23287 − Serine/threonine−protein phosphatase 2B catalytic subunit A1 OS=Saccharomyces cerevisiae (strain ATCC 204508 / S288c) GN=CNA1 PE=1 SV=2

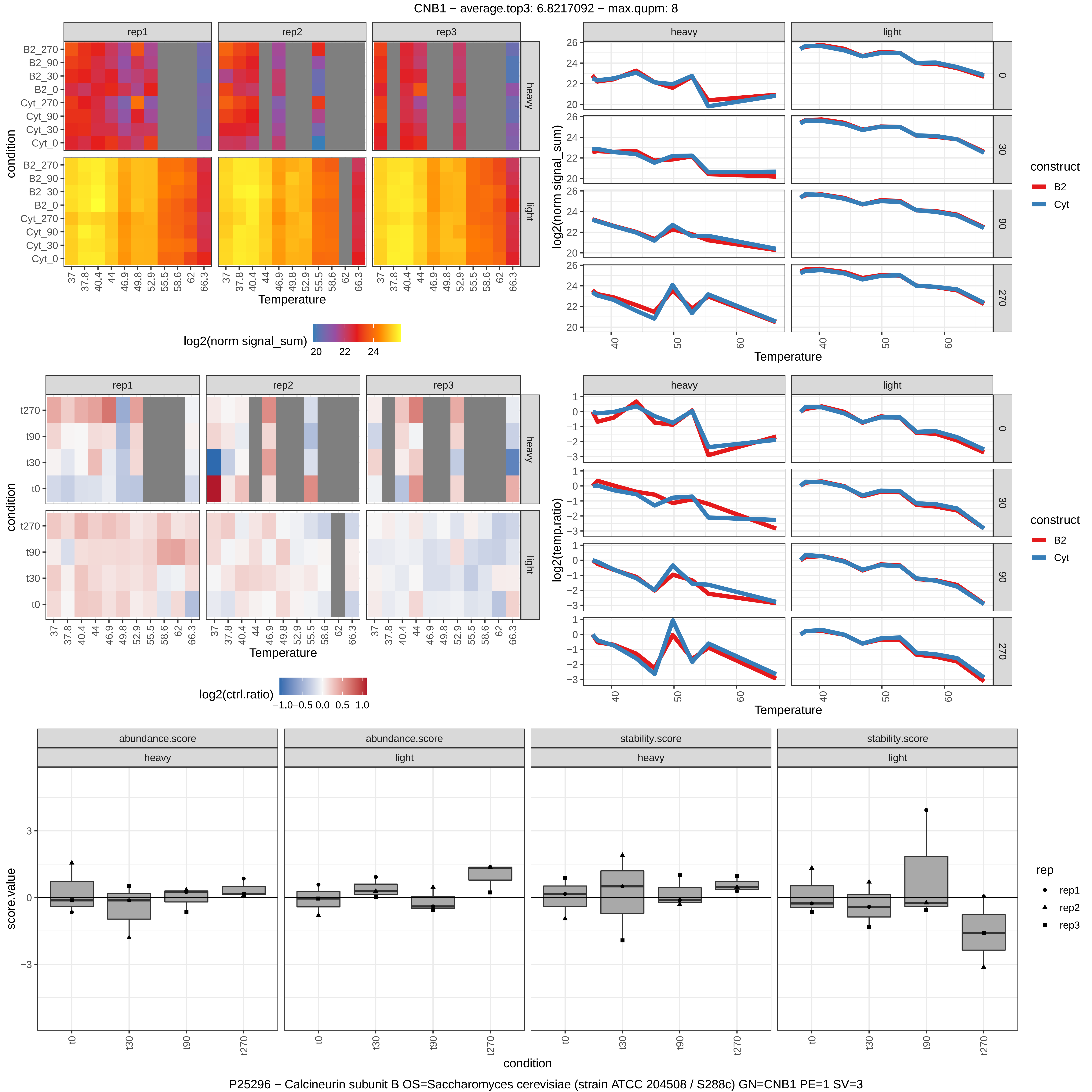

CNB1 − average.top3: 6.8217092 − max.qupm: 8
rep1
rep2
rep3
heavy
light
condition
Temperature
log2(norm signal_sum)
construct
B2
Cyt
log2(ctrl.ratio)
log2(temp.ratio)
abundance.score
stability.score
score.value
rep
rep1
rep2
rep3
P25296 − Calcineurin subunit B OS=Saccharomyces cerevisiae (strain ATCC 204508 / S288c) GN=CNB1 PE=1 SV=3

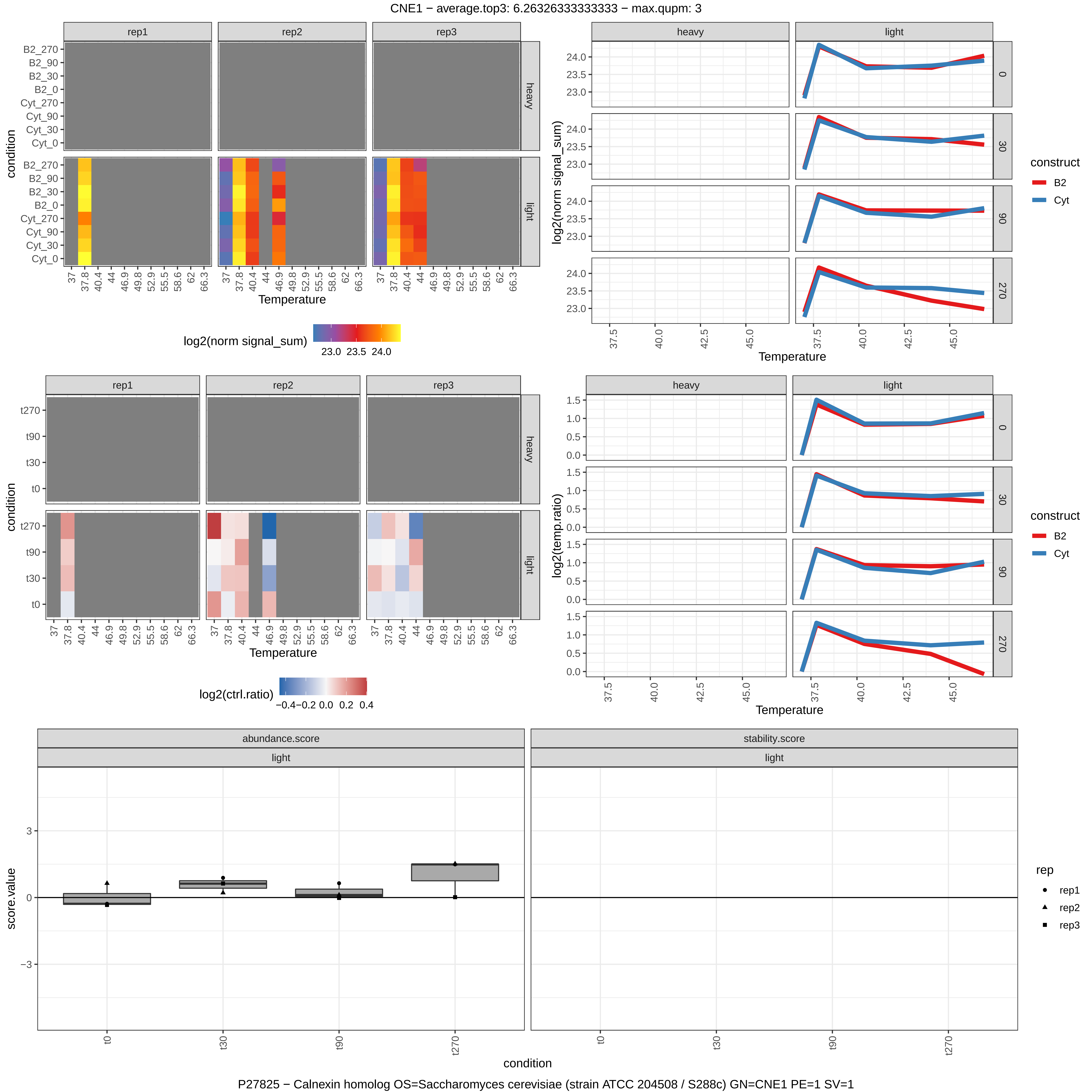
CNE1 − average.top3: 6.26326333333333 − max.qupm: 3
rep1
rep2
rep3
heavy
light
condition
Temperature
log2(norm signal_sum)
log2(ctrl.ratio)
log2(temp.ratio)
construct
B2
Cyt
abundance.score
stability.score
score.value
rep
rep1
rep2
rep3
P27825 − Calnexin homolog OS=Saccharomyces cerevisiae (strain ATCC 204508 / S288c) GN=CNE1 PE=1 SV=1

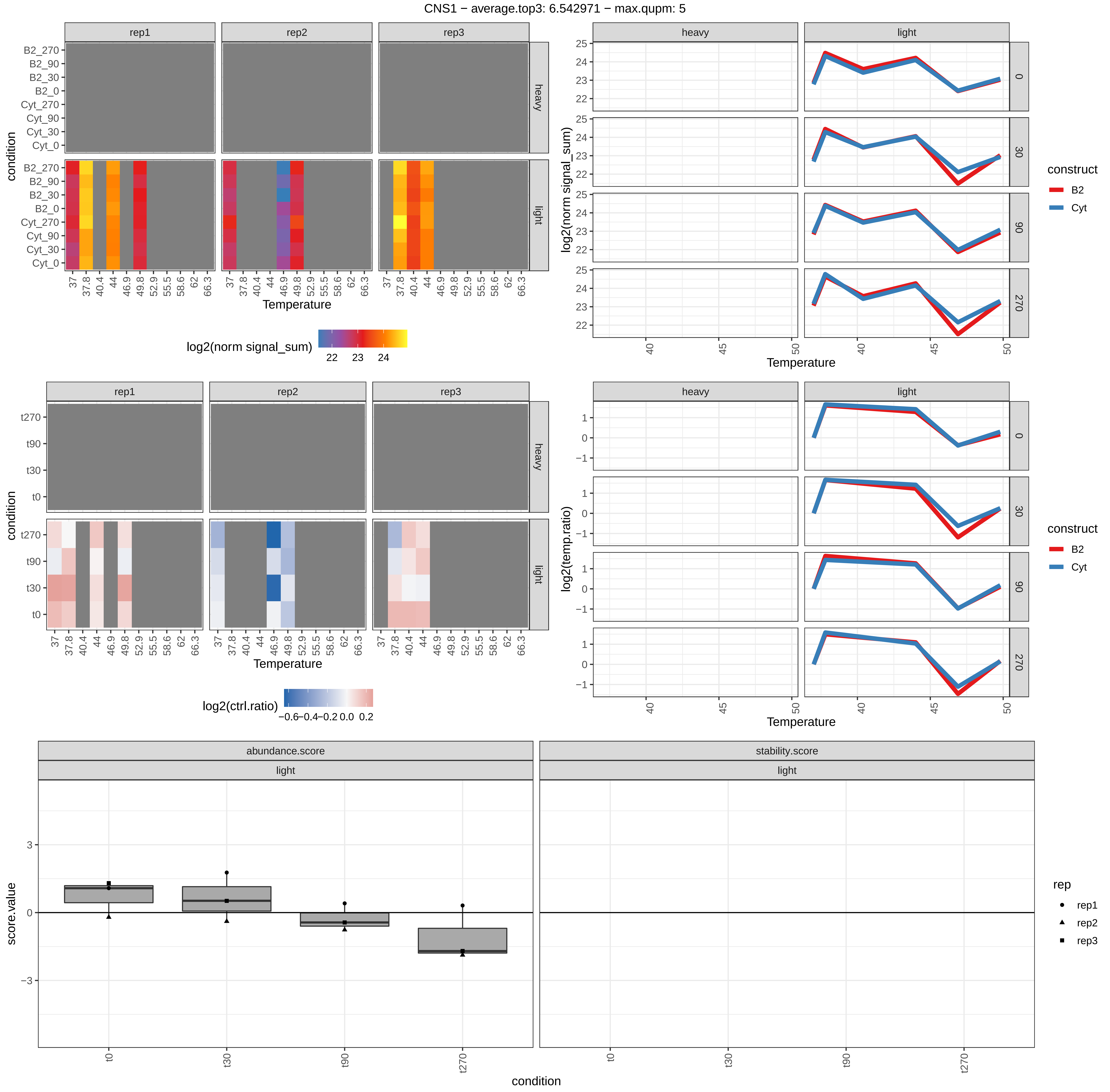

P33313 − Hsp70/Hsp90 co−chaperone CNS1 OS=Saccharomyces cerevisiae (strain ATCC 204508 / S288c) GN=CNS1 PE=1 SV=1

COA1 − average.top3: 7.47226428571429 − max.qupm: 3

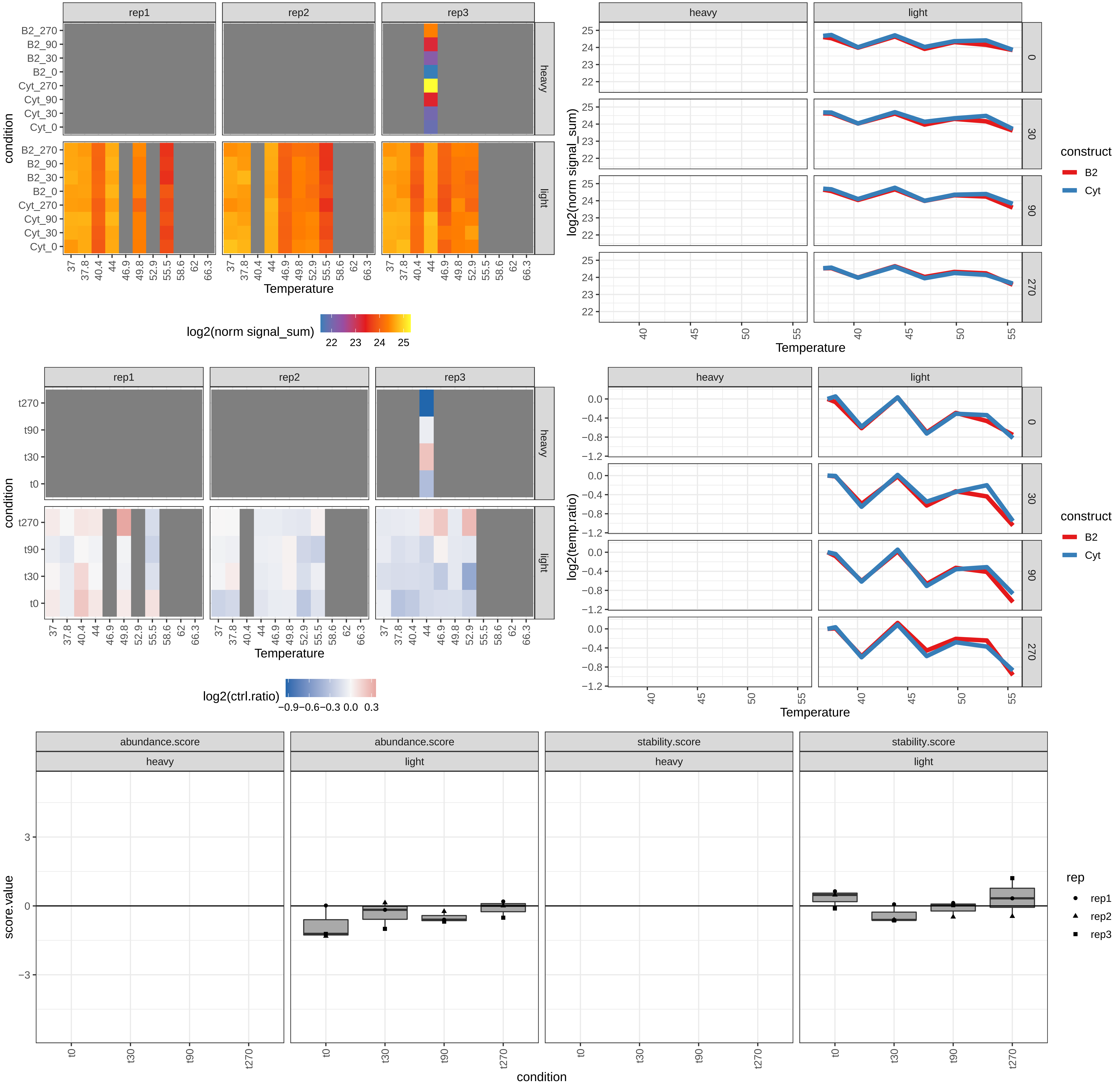

P40452 − Cytochrome c oxidase assembly factor 1 OS=Saccharomyces cerevisiae (strain ATCC 204508 / S288c) GN=COA1 PE=1 SV=1

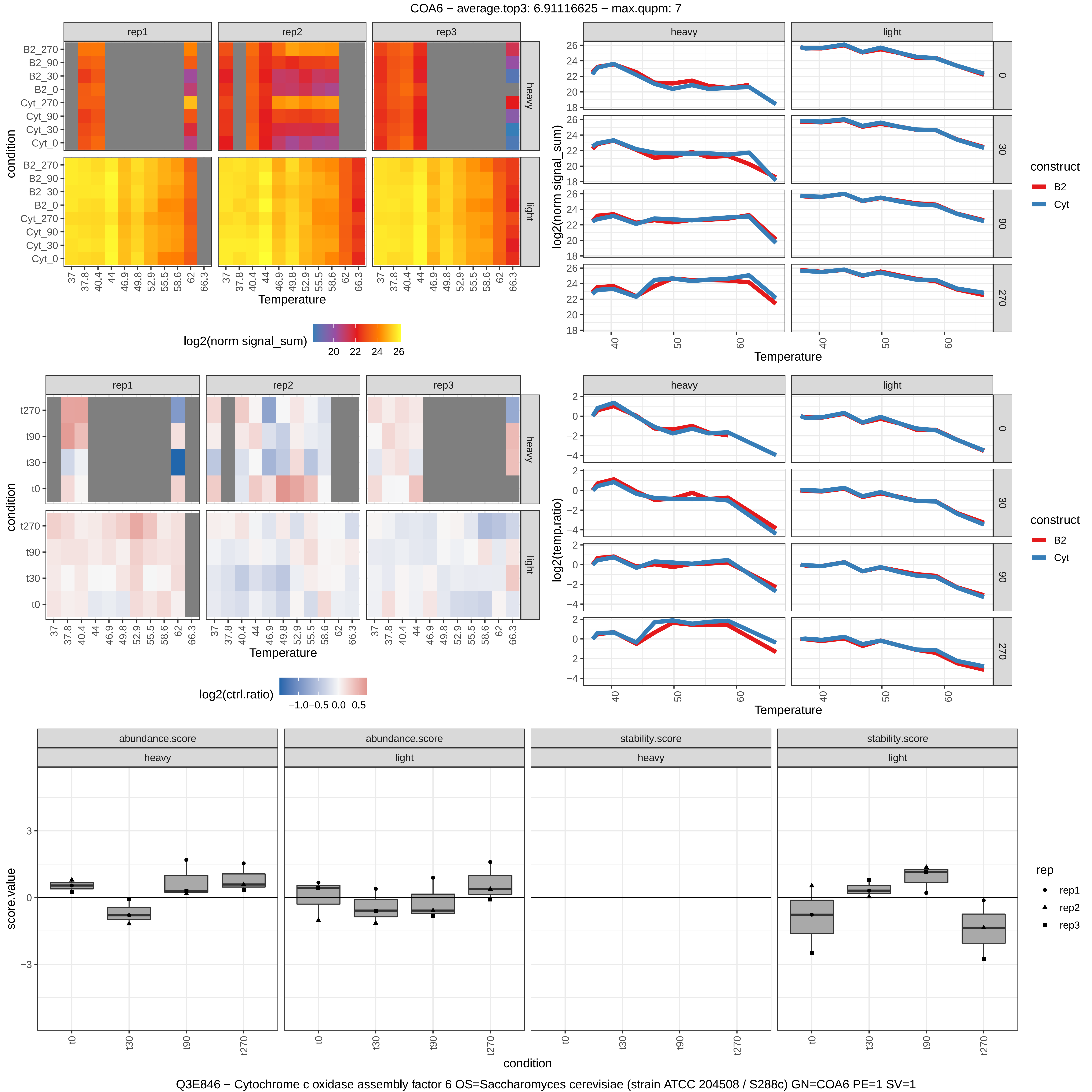

COA6 − average.top3: 6.91116625 − max.qupm: 7
rep1
rep2
rep3
heavy
light
condition
Temperature
log2(norm signal_sum)
log2(ctrl.ratio)
log2(temp.ratio)
construct
B2
Cyt
abundance.score
stability.score
score.value
rep
rep1
rep2
rep3
Q3E846 − Cytochrome c oxidase assembly factor 6 OS=Saccharomyces cerevisiae (strain ATCC 204508 / S288c) GN=COA6 PE=1 SV=1

COB|BI2|BI4|BI3 − average.top3: 7.85112571428571 − max.qupm: 2

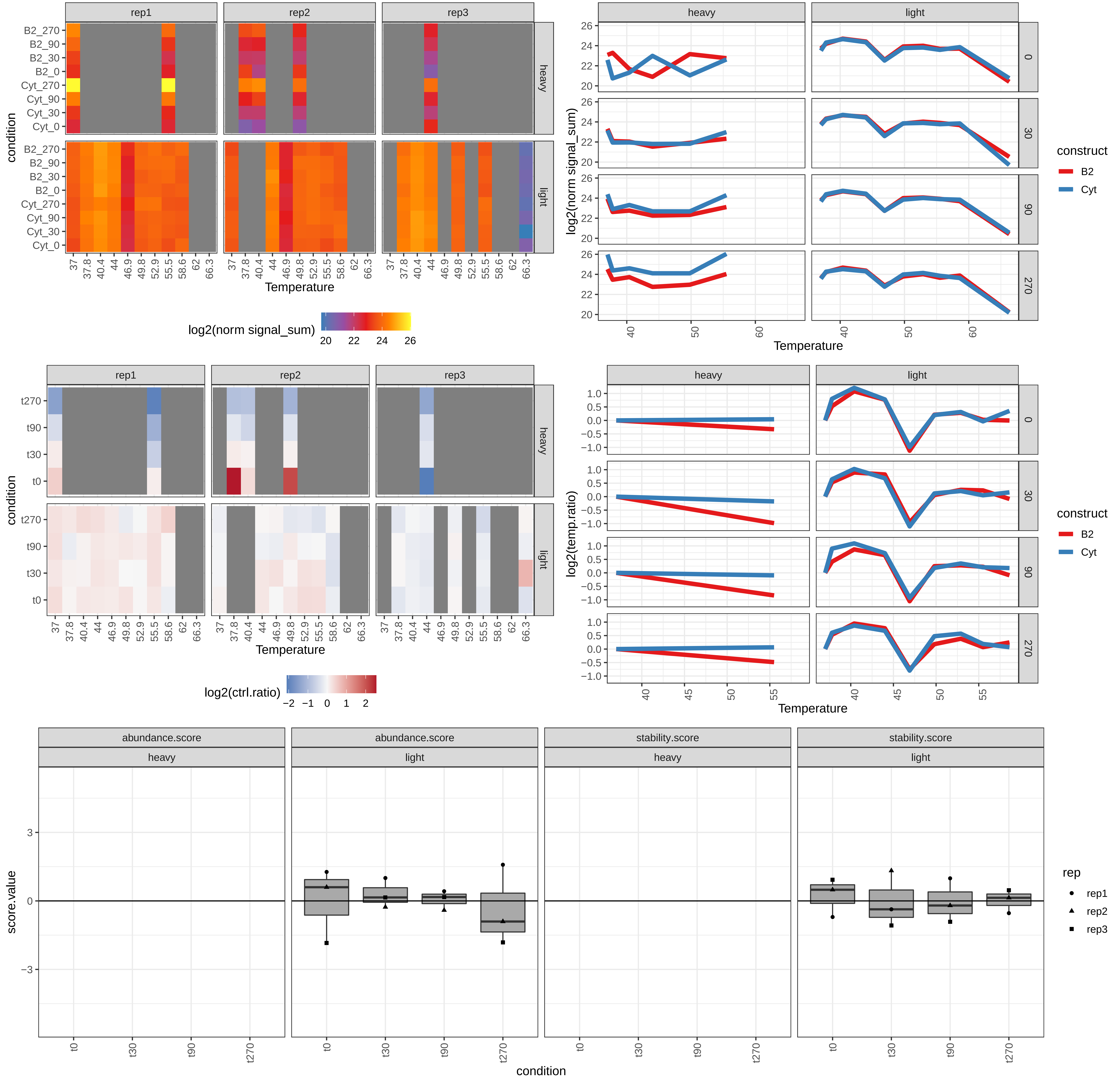

me b mRNA maturase bI2 OS=Saccharomyces cerevisiae (strain ATCC 204508 / S288c) GN=BI2 PE=1 SV=2|Intron−encoded RNA maturase bI4 OS=Saccharomyces cerevisiae (strain ATCC 204508 / S2

COF1 − average.top3: 7.26701214285714 − max.qupm: 6

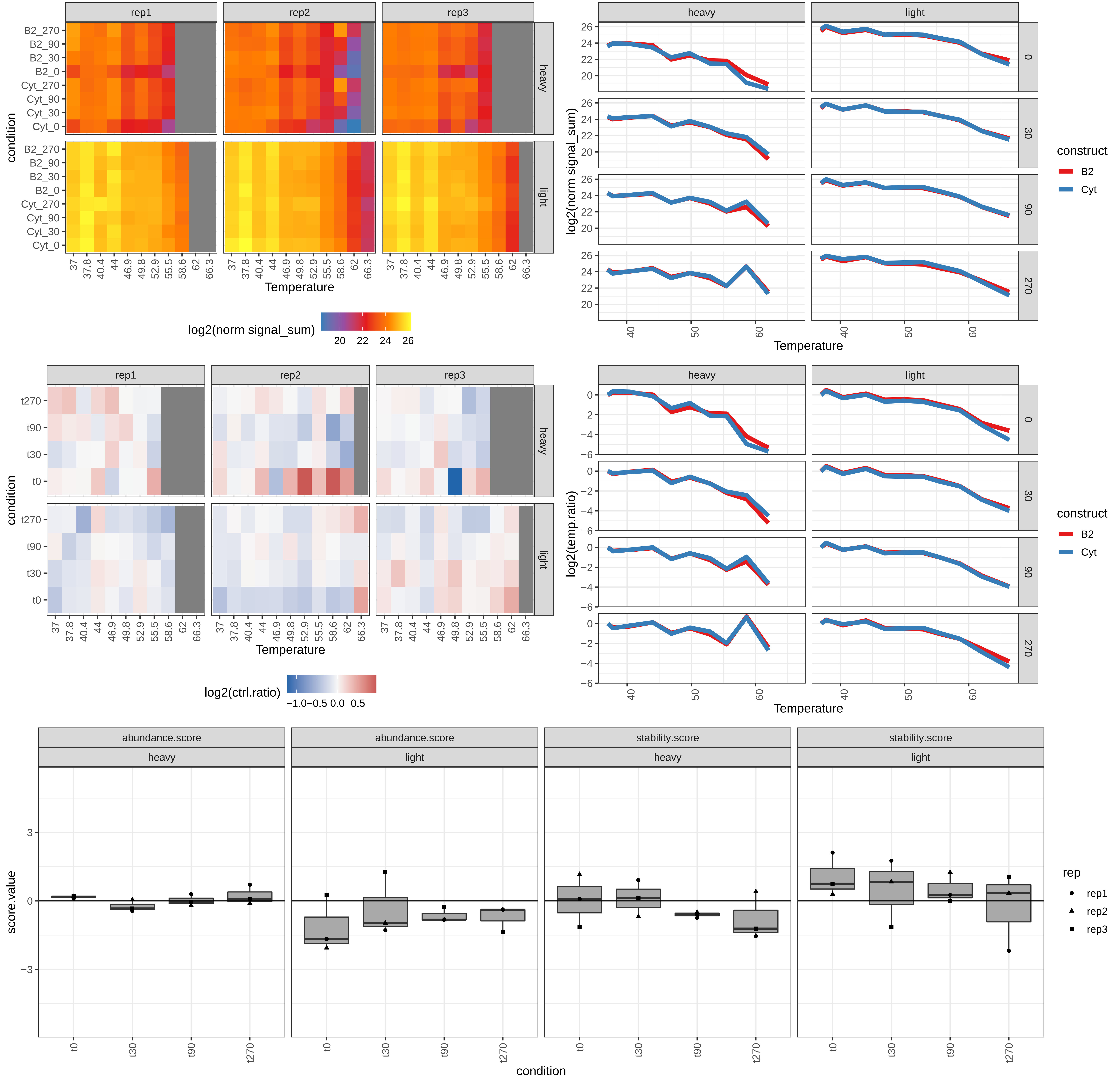

Q03048 − Cofilin OS=Saccharomyces cerevisiae (strain ATCC 204508 / S288c) GN=COF1 PE=1 SV=1

COG4 − average.top3: 6.28682642857143 − max.qupm: 6

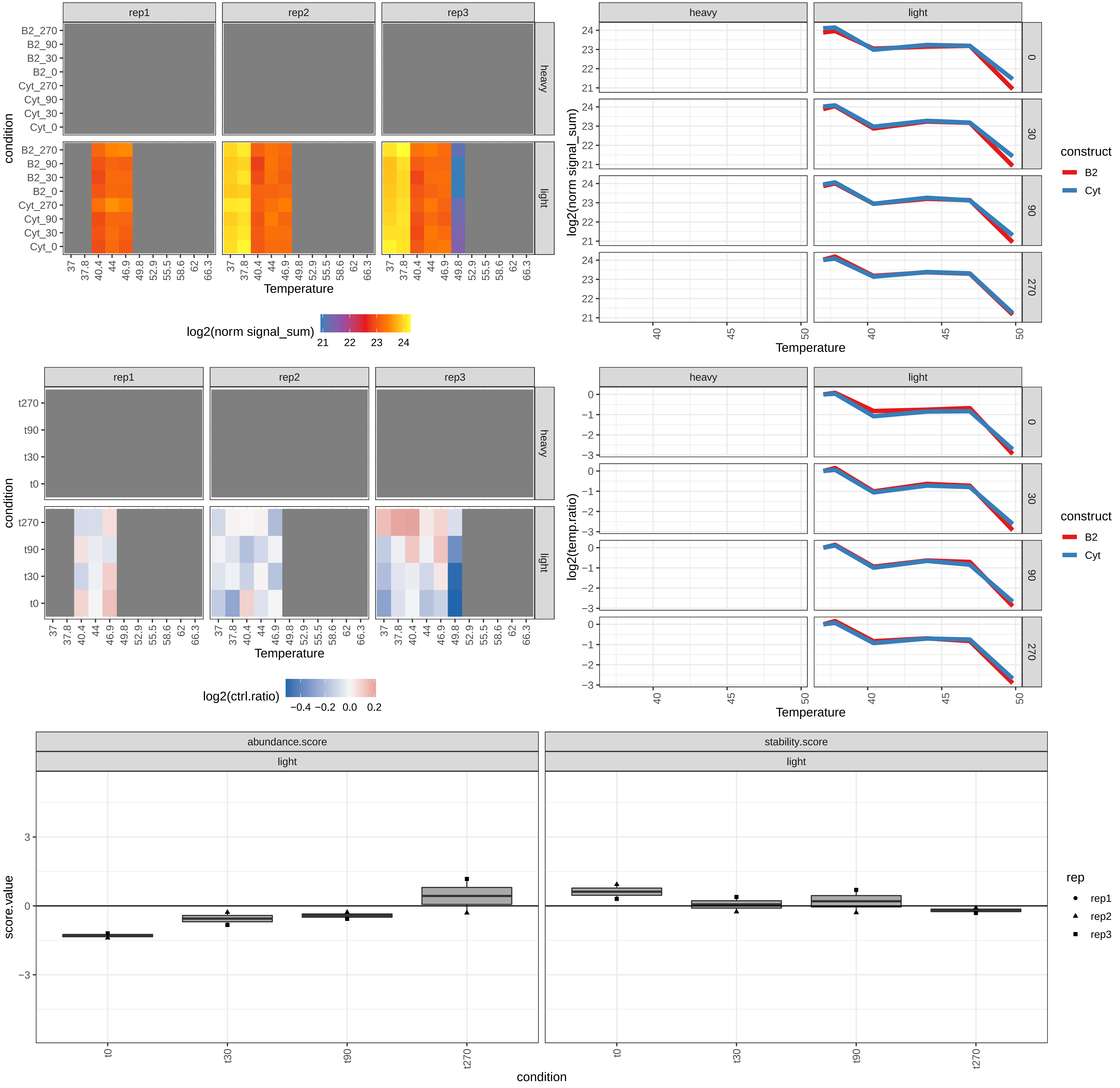

Q06096 − Conserved oligomeric Golgi complex subunit 4 OS=Saccharomyces cerevisiae (strain ATCC 204508 / S288c) GN=COG4 PE=1 SV=1

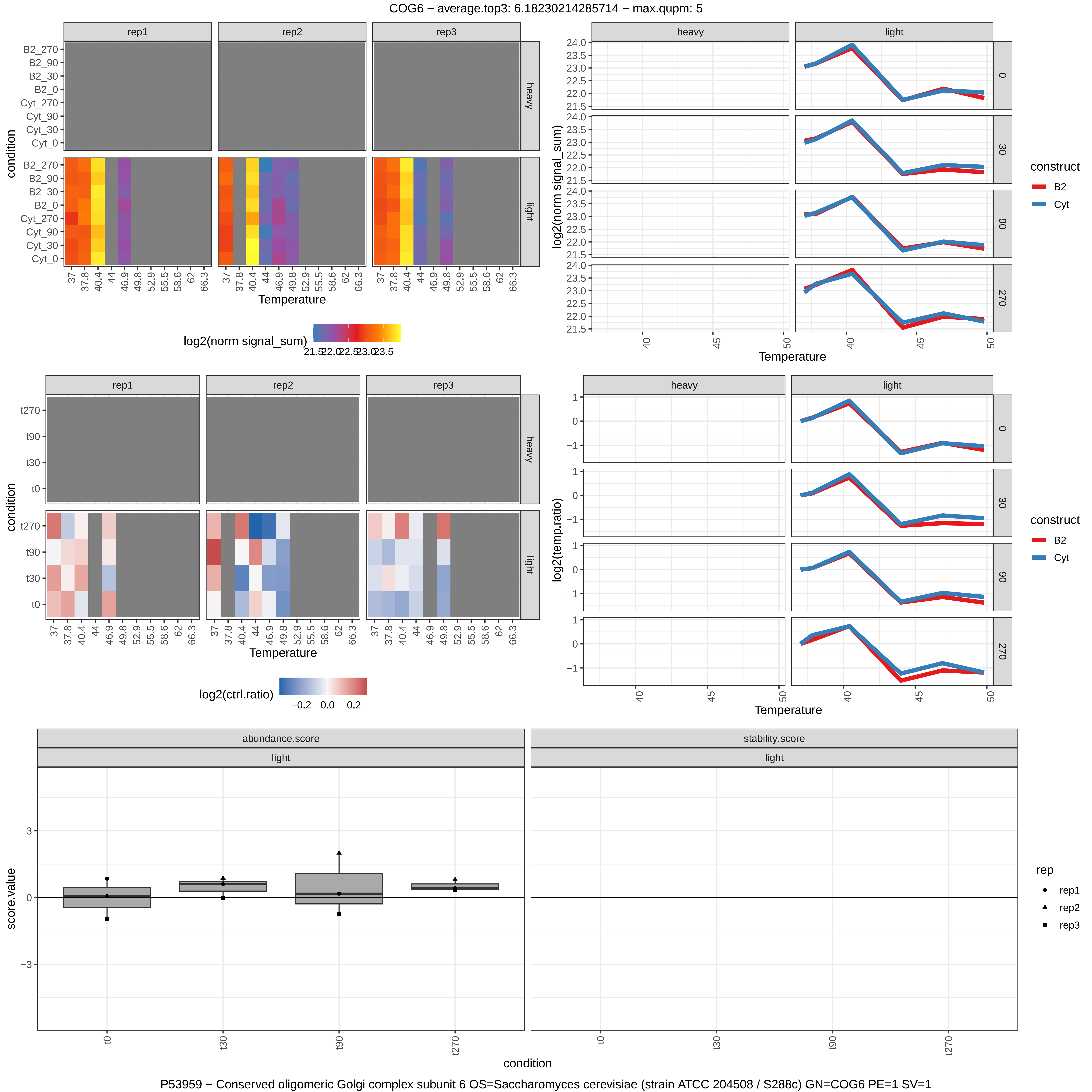

COG6 − average.top3: 6.18230214285714 − max.qupm: 5
rep1
rep2
rep3
heavy
light
condition
Temperature
log2(norm signal_sum)
construct
B2
Cyt
log2(ctrl.ratio)
log2(temp.ratio)
abundance.score
stability.score
score.value
rep
rep1
rep2
rep3
P53959 − Conserved oligomeric Golgi complex subunit 6 OS=Saccharomyces cerevisiae (strain ATCC 204508 / S288c) GN=COG6 PE=1 SV=1

COP1 − average.top3: 7.31586740740741 − max.qupm: 31

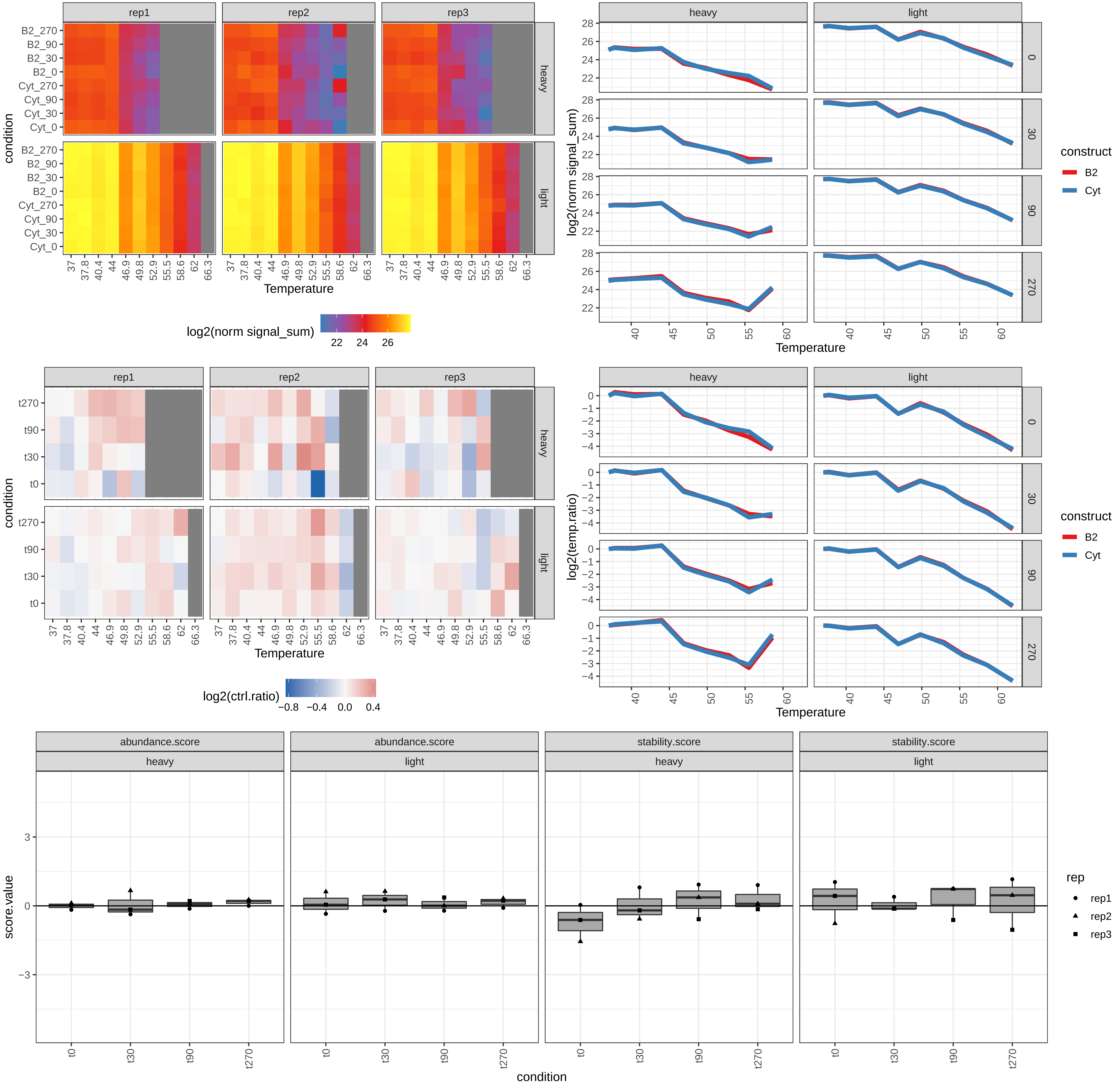

P53622 − Coatomer subunit alpha OS=Saccharomyces cerevisiae (strain ATCC 204508 / S288c) GN=COP1 PE=1 SV=2

COQ11 − average.top3: 6.85398285714286 − max.qupm: 6

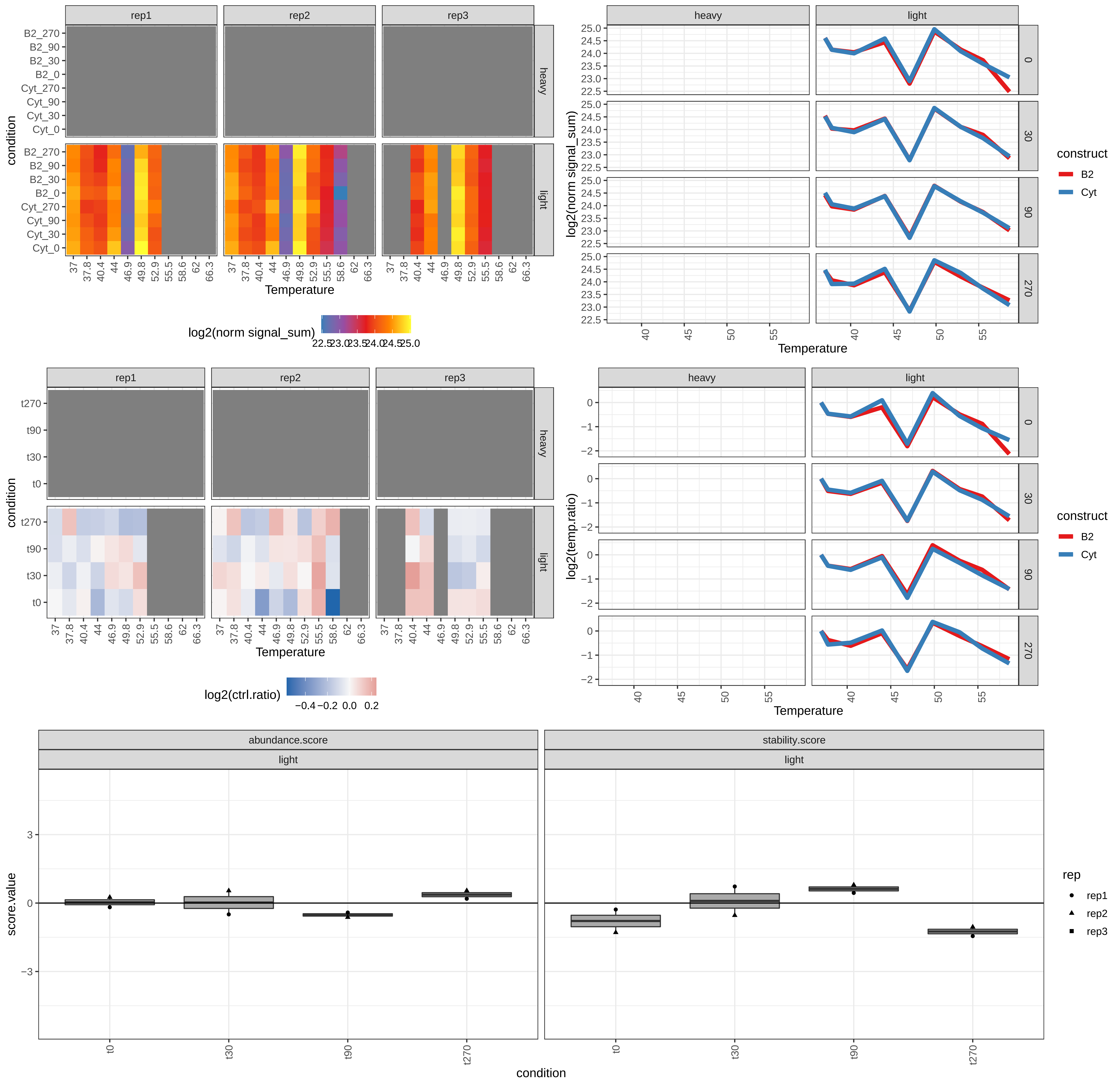

Q05892 − MIOREX complex component 2 OS=Saccharomyces cerevisiae (strain ATCC 204508 / S288c) GN=COQ11 PE=1 SV=1

COQ2 − average.top3: 6.64968176470588 − max.qupm: 4

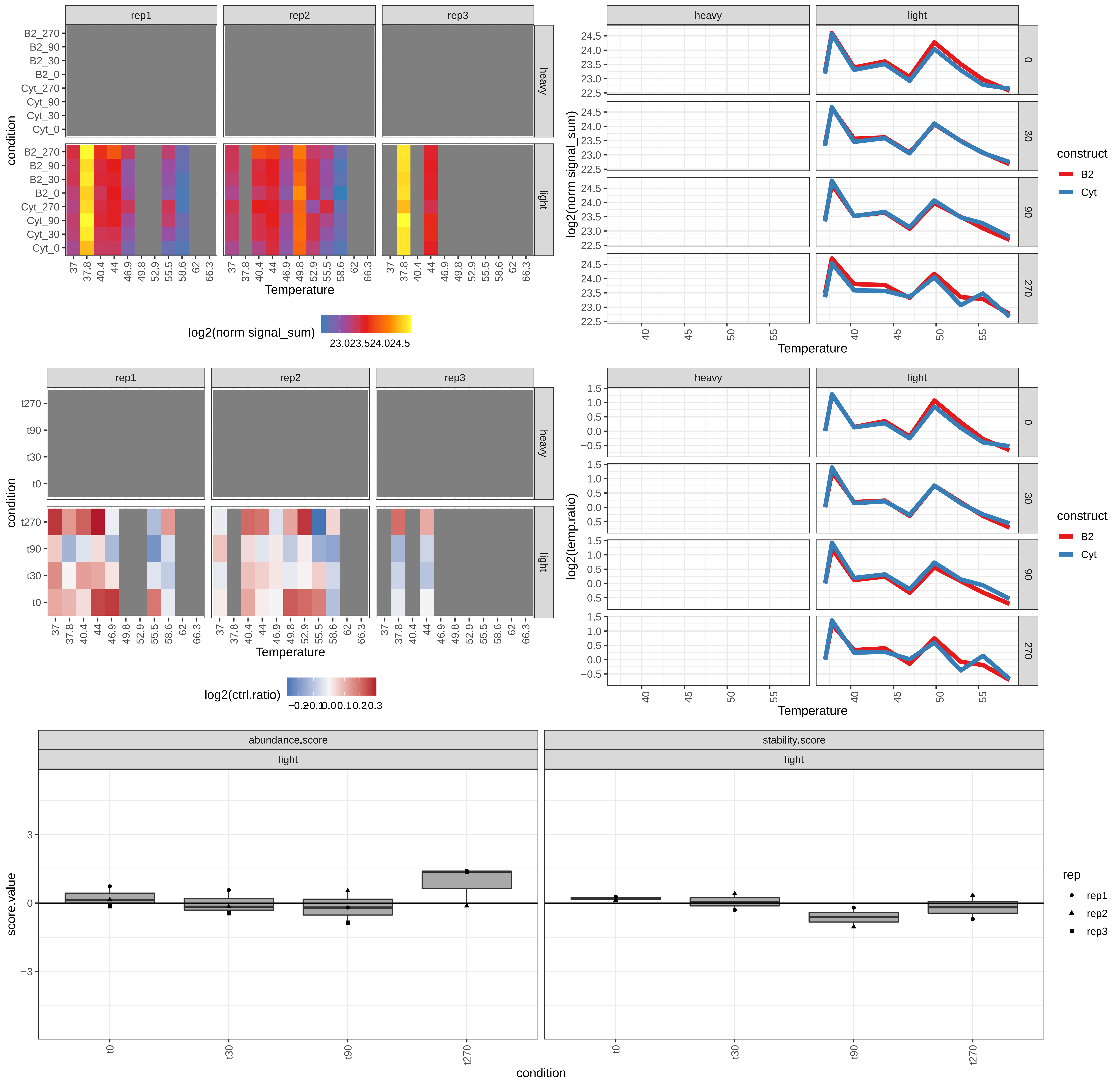

P32378 − 4−hydroxybenzoate polyprenyltransferase, mitochondrial OS=Saccharomyces cerevisiae (strain ATCC 204508 / S288c) GN=COQ2 PE=1 SV=1

COQ4 − average.top3: 6.50207692307692 − max.qupm: 3

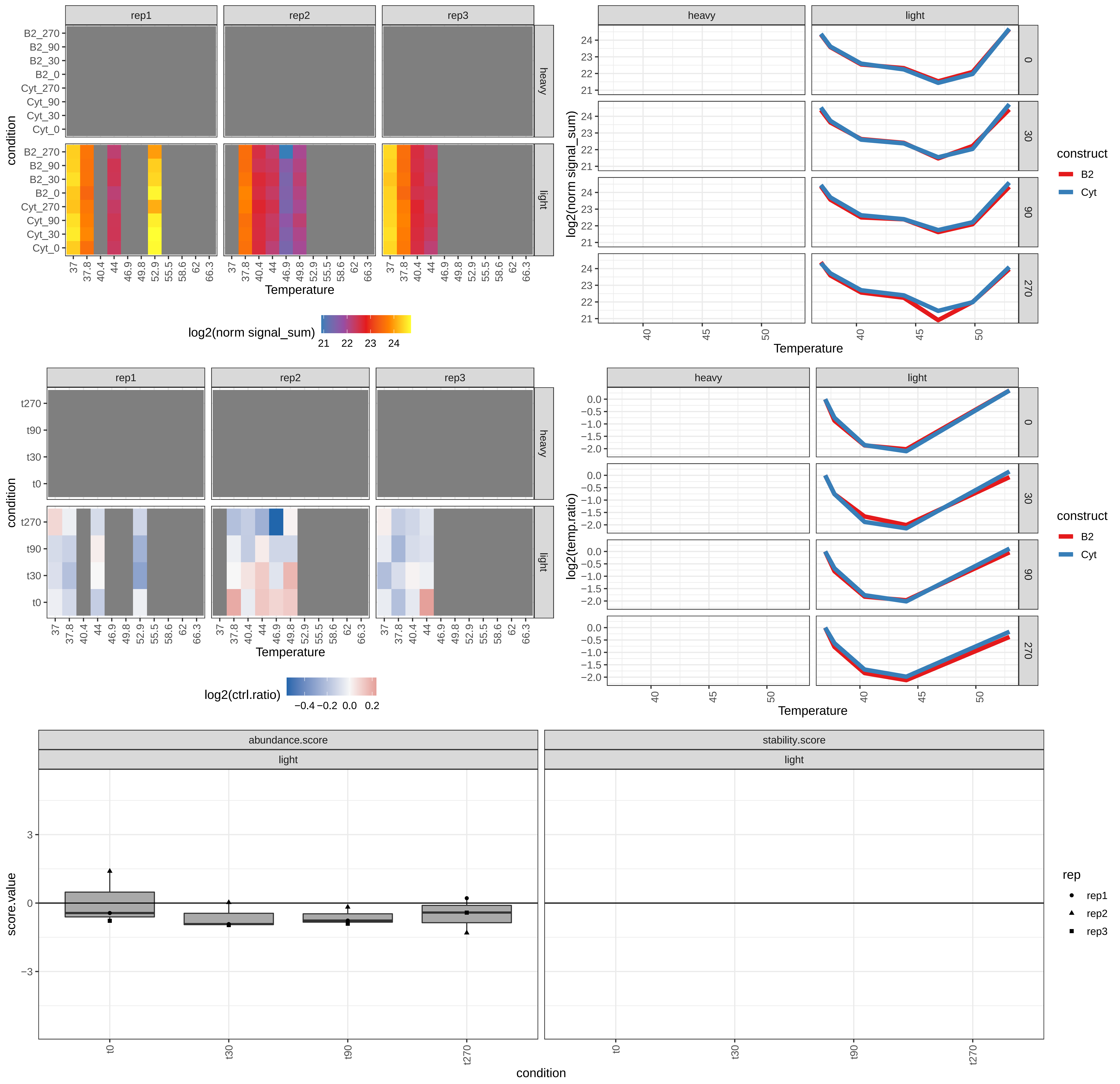

O13525 − Ubiquinone biosynthesis protein COQ4, mitochondrial OS=Saccharomyces cerevisiae (strain ATCC 204508 / S288c) GN=COQ4 PE=1 SV=2

COQ5 − average.top3: 7.32555666666667 − max.qupm: 7

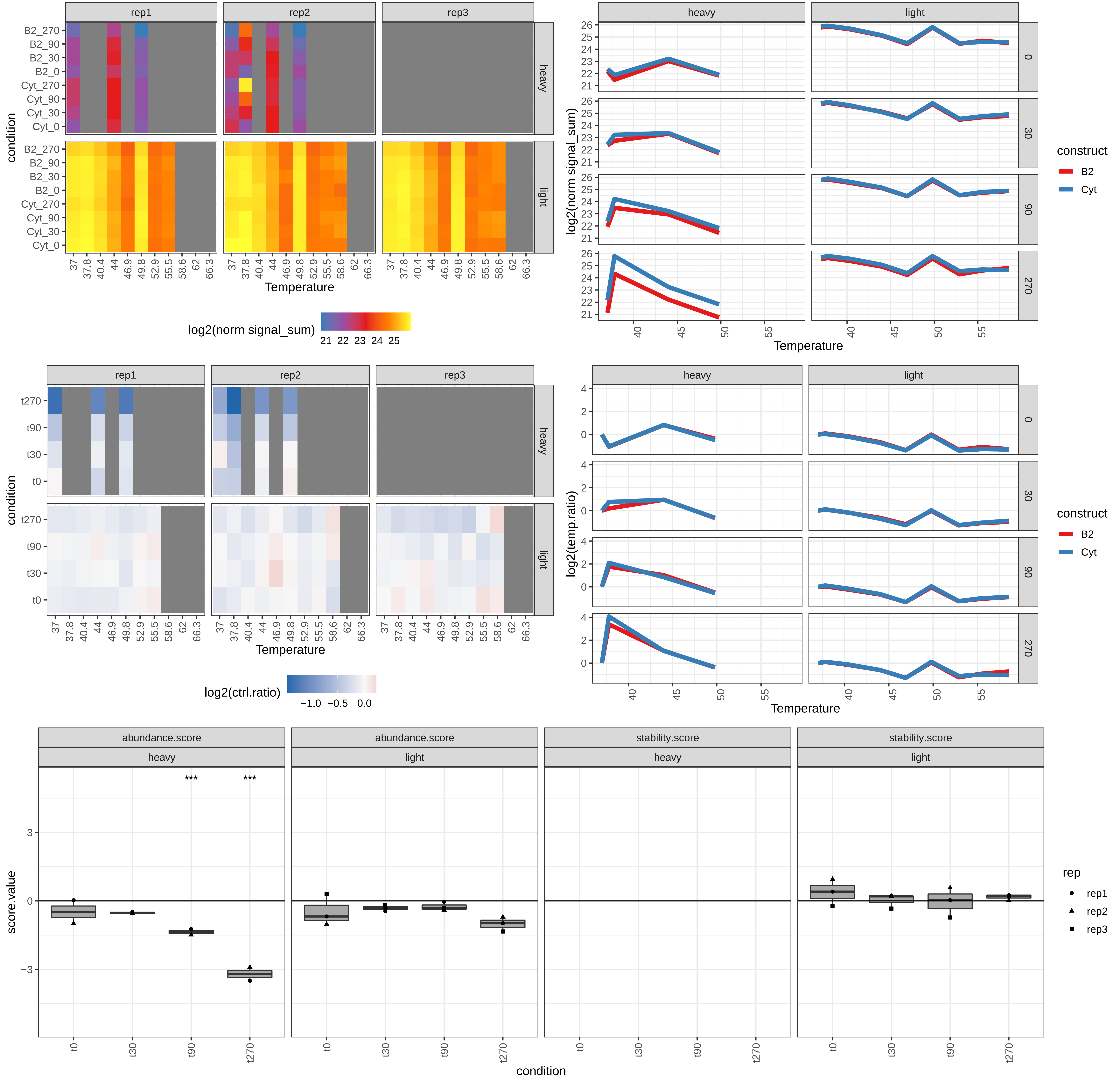

P49017 − 2−methoxy−6−polyprenyl−1,4−benzoquinol methylase, mitochondrial OS=Saccharomyces cerevisiae (strain ATCC 204508 / S288c) GN=COQ5 PE=1 SV=1

COQ6 − average.top3: 6.44369166666667 − max.qupm: 6

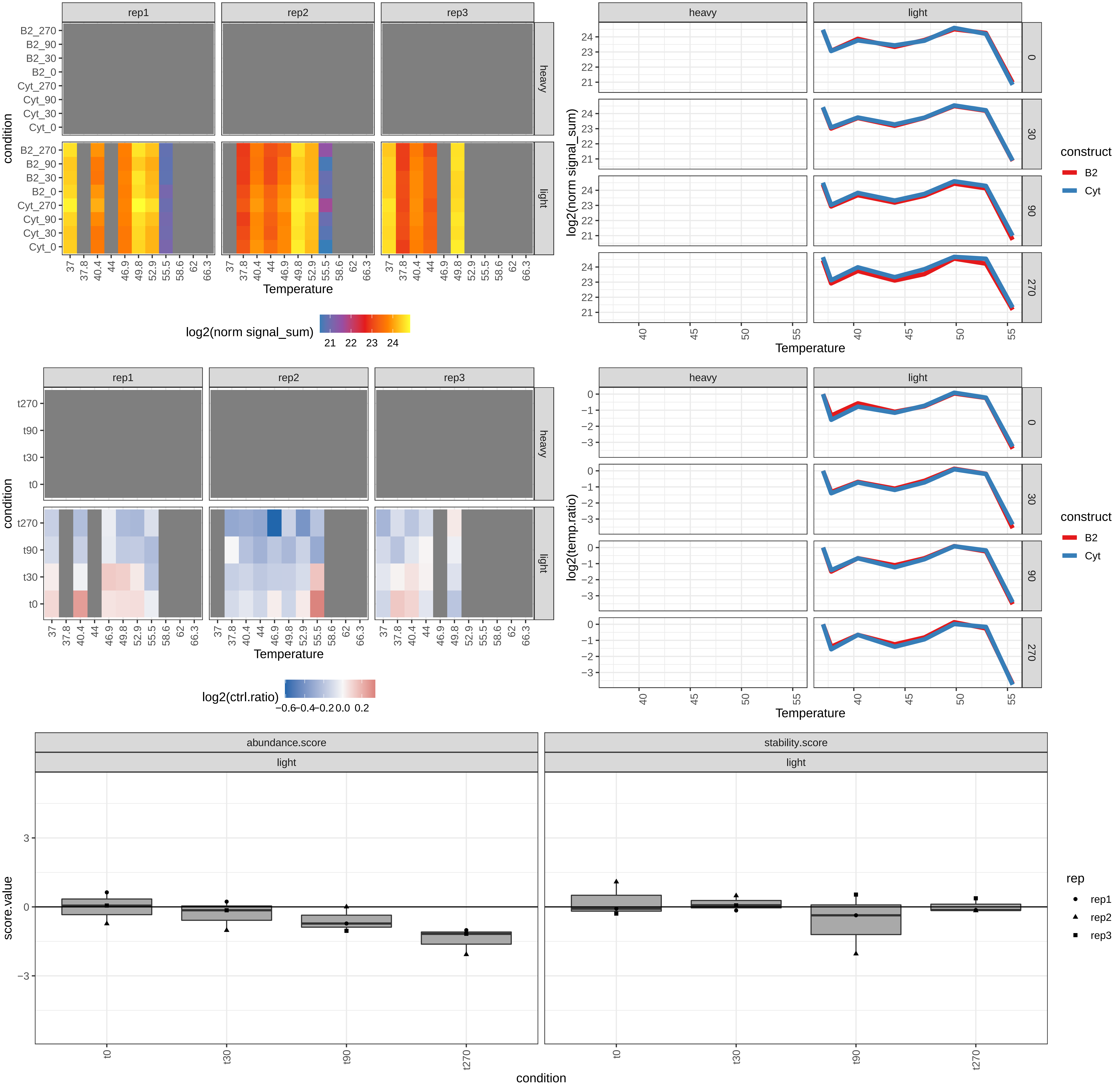

P53318 − Ubiquinone biosynthesis monooxygenase COQ6, mitochondrial OS=Saccharomyces cerevisiae (strain ATCC 204508 / S288c) GN=COQ6 PE=1 SV=1

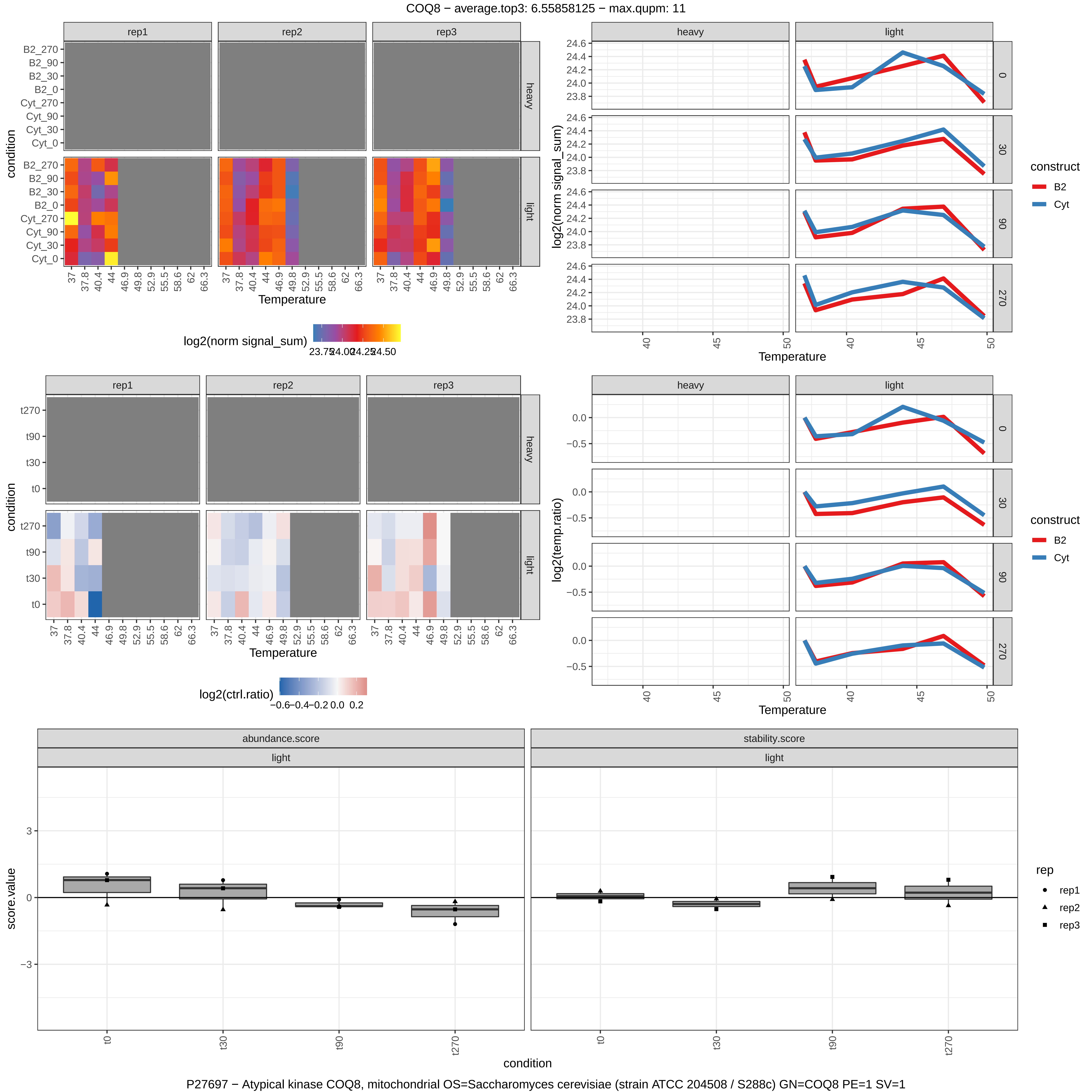
COQ8 − average.top3: 6.55858125 − max.qupm: 11
rep1
rep2
rep3
heavy
light
B2_270
B2_90
B2_30
B2_0
Cyt_270
Cyt_90
Cyt_30
Cyt_0
condition
37
37.8
40.4
44
46.9
49.8
52.9
55.5
58.6
62
66.3
Temperature
log2(norm signal_sum)
23.75
24.00
24.25
24.50
24.6
24.4
24.2
24.0
23.8
0
30
90
270
40
45
50
construct
B2
Cyt
t270
t90
t30
t0
log2(ctrl.ratio)
−0.6
−0.4
−0.2
0.0
0.2
log2(temp.ratio)
−0.5
abundance.score
stability.score
score.value
3
−3
condition
rep
rep1
rep2
rep3
P27697 − Atypical kinase COQ8, mitochondrial OS=Saccharomyces cerevisiae (strain ATCC 204508 / S288c) GN=COQ8 PE=1 SV=1

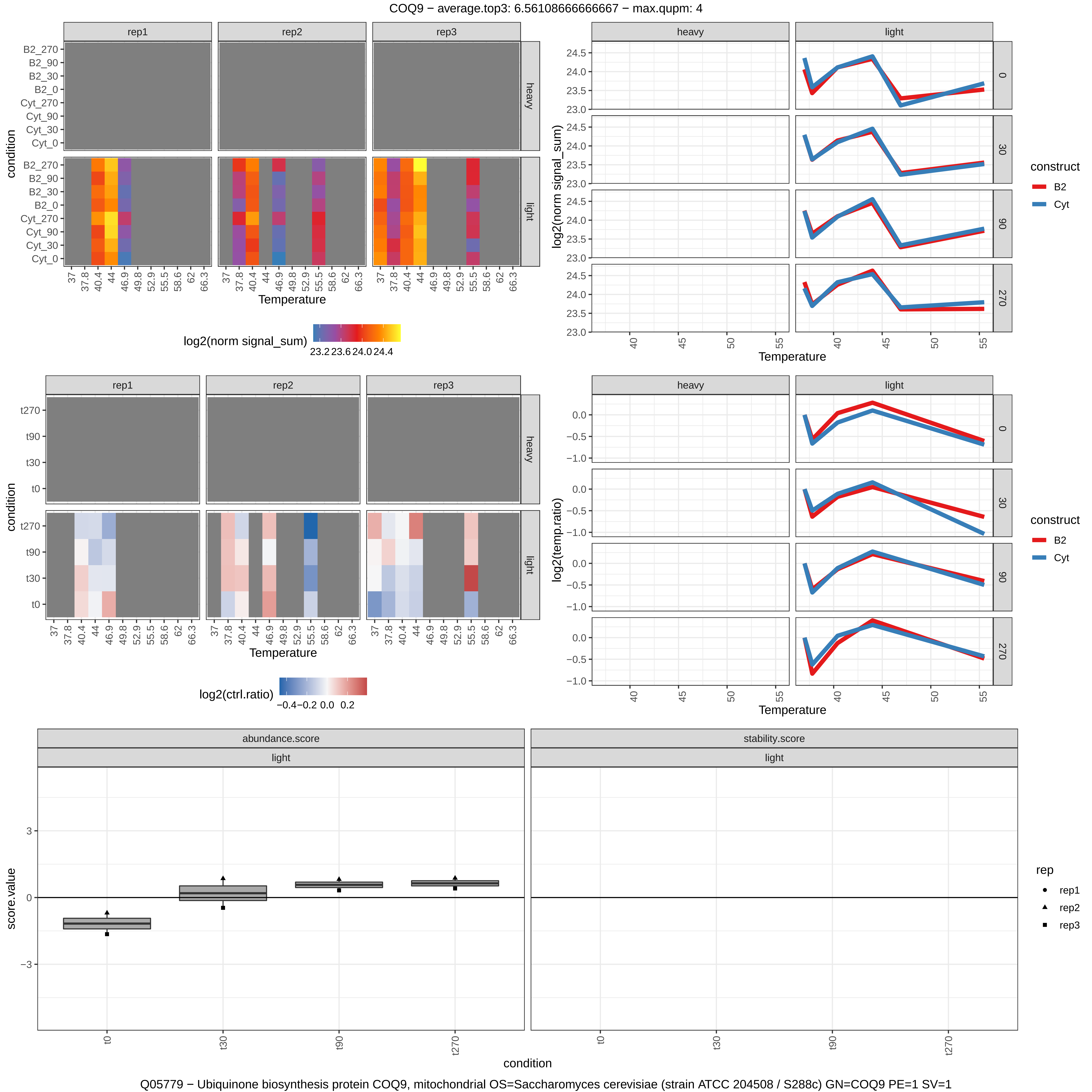
COQ9 − average.top3: 6.56108666666667 − max.qupm: 4
rep1
rep2
rep3
heavy
light
condition
Temperature
log2(norm signal_sum)
log2(temp.ratio)
log2(ctrl.ratio)
construct
B2
Cyt
abundance.score
stability.score
score.value
rep
Q05779 − Ubiquinone biosynthesis protein COQ9, mitochondrial OS=Saccharomyces cerevisiae (strain ATCC 204508 / S288c) GN=COQ9 PE=1 SV=1

COR1 − average.top3: 7.91011963636364 − max.qupm: 17

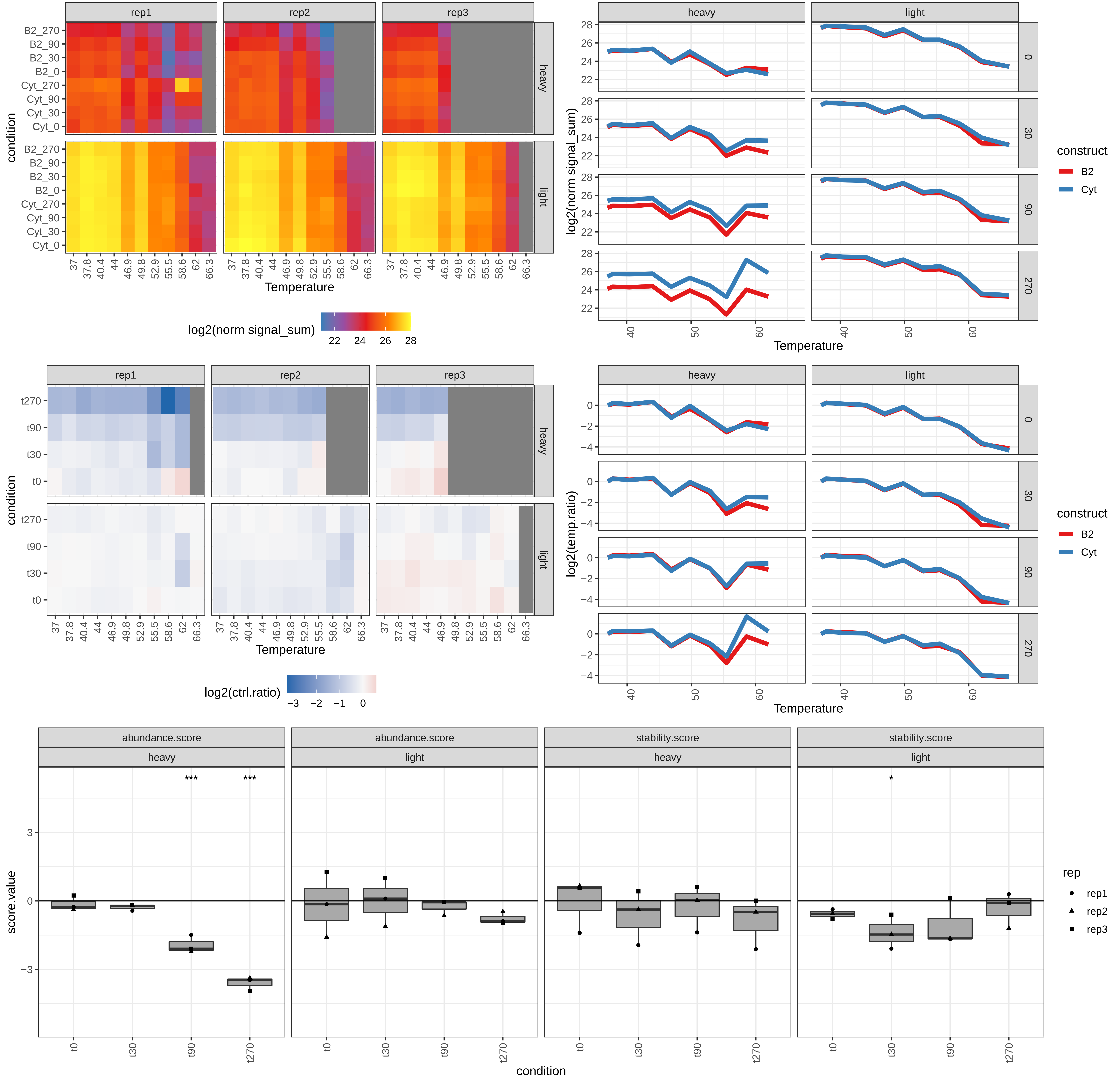

P07256 − Cytochrome b−c1 complex subunit 1, mitochondrial OS=Saccharomyces cerevisiae (strain ATCC 204508 / S288c) GN=COR1 PE=1 SV=1

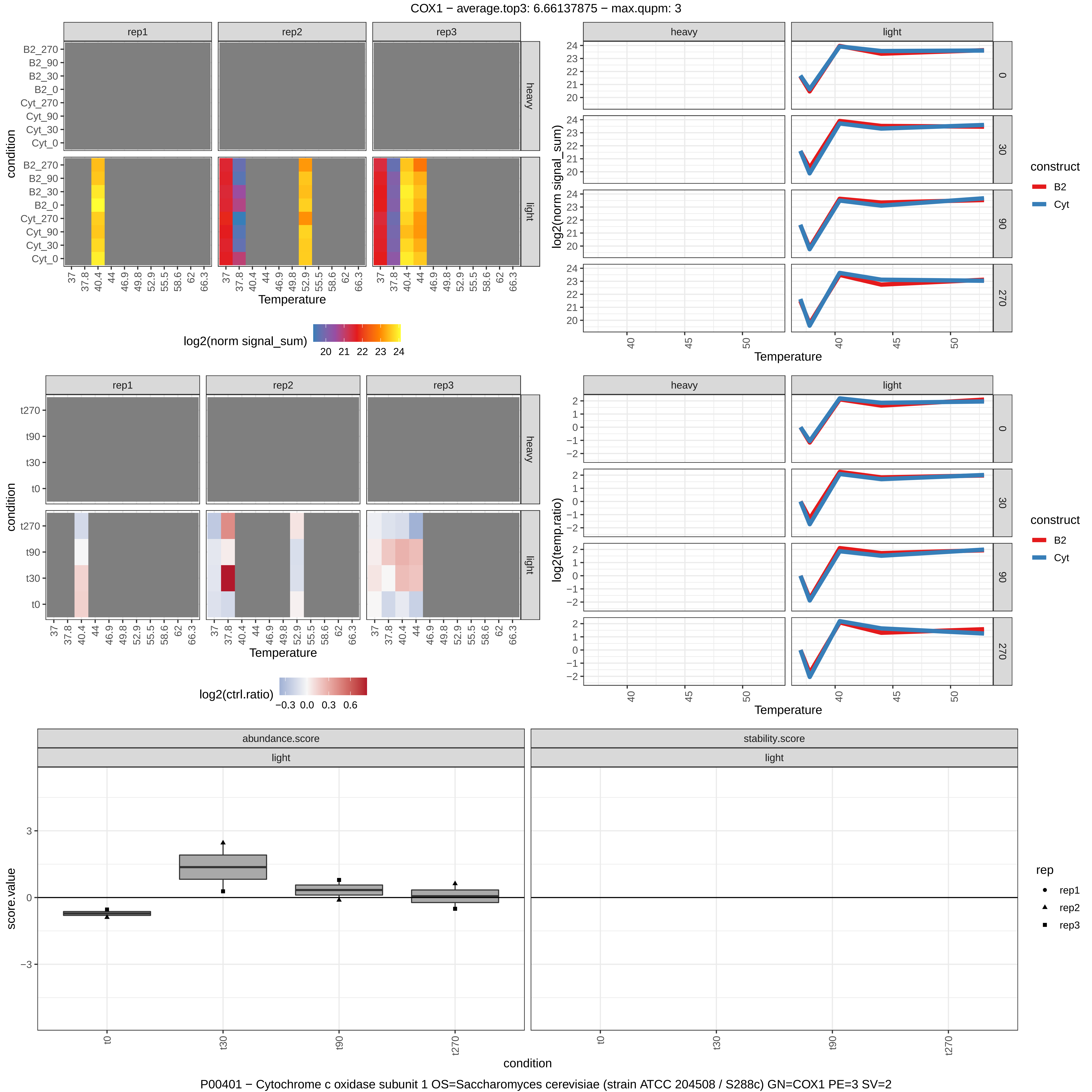

COX1 − average.top3: 6.66137875 − max.qupm: 3
rep1
rep2
rep3
heavy
light
condition
B2_270
B2_90
B2_30
B2_0
Cyt_270
Cyt_90
Cyt_30
Cyt_0
Temperature
log2(norm signal_sum)
20 21 22 23 24
log2(norm signal_sum)
construct
B2
Cyt
t270
t90
t30
t0
log2(ctrl.ratio)
−0.3 0.0 0.3 0.6
log2(temp.ratio)
abundance.score
stability.score
score.value
rep
rep1
rep2
rep3
condition
P00401 − Cytochrome c oxidase subunit 1 OS=Saccharomyces cerevisiae (strain ATCC 204508 / S288c) GN=COX1 PE=3 SV=2

COX12 − average.top3: 7.19828771929825 − max.qupm: 4

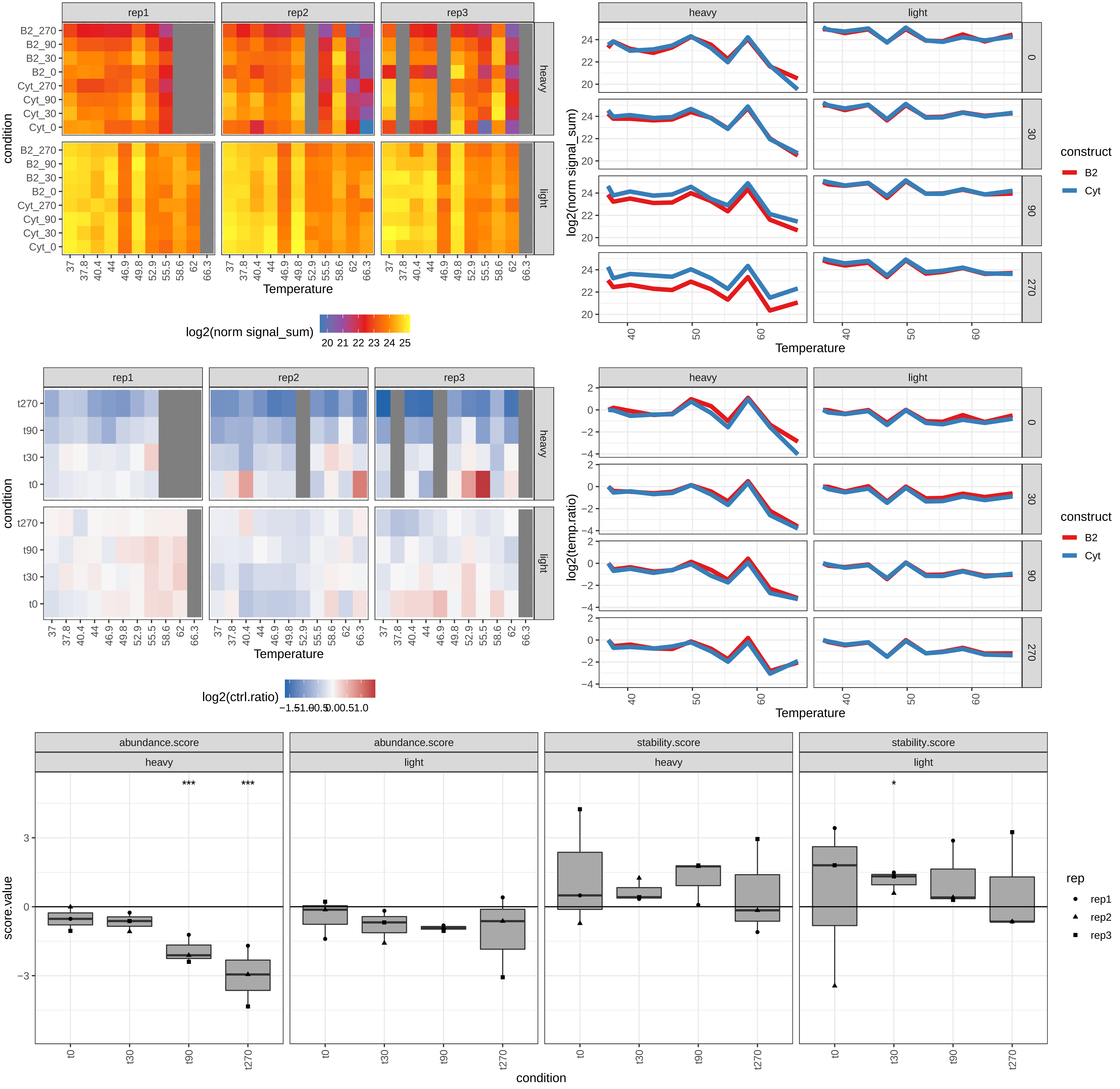

Q01519 − Cytochrome c oxidase subunit 6B OS=Saccharomyces cerevisiae (strain ATCC 204508 / S288c) GN=COX12 PE=1 SV=2

COX13 − average.top3: 7.43145365384615 − max.qupm: 7

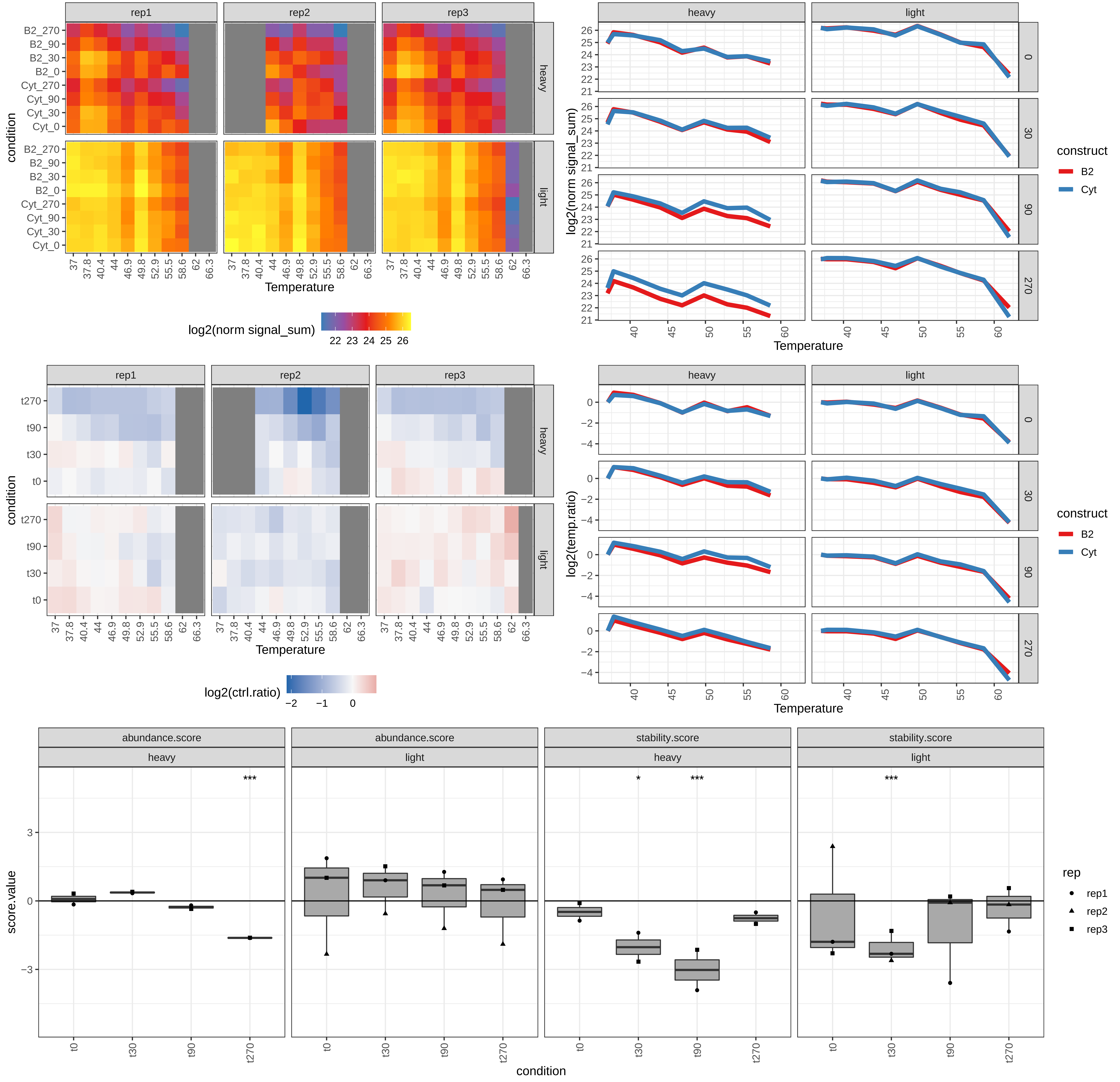

P32799 − Cytochrome c oxidase subunit 6A, mitochondrial OS=Saccharomyces cerevisiae (strain ATCC 204508 / S288c) GN=COX13 PE=1 SV=1

COX15 − average.top3: 7.03531742857143 − max.qupm: 8

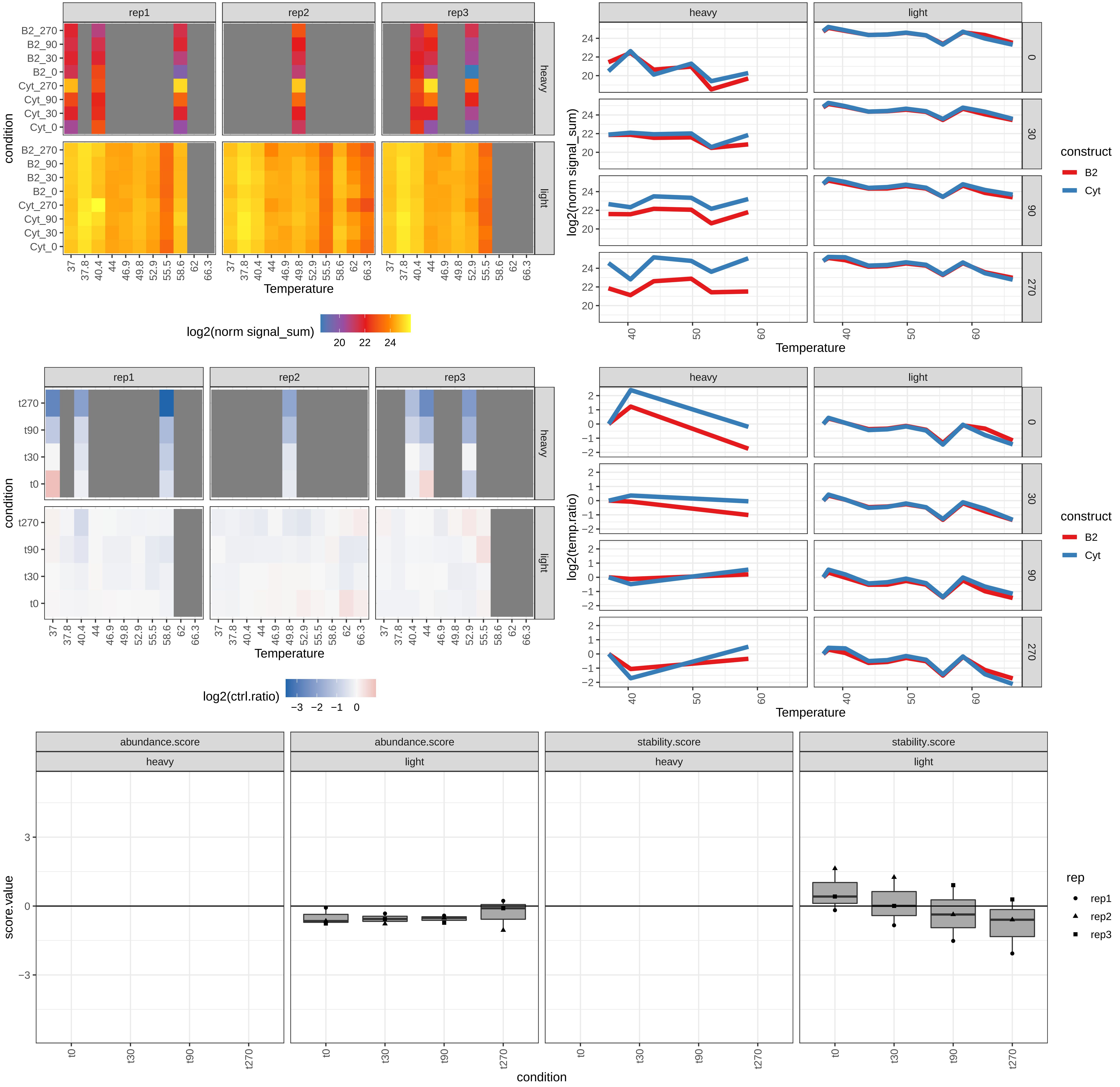

P40086 − Cytochrome c oxidase assembly protein COX15 OS=Saccharomyces cerevisiae (strain ATCC 204508 / S288c) GN=COX15 PE=3 SV=1

COX2 − average.top3: 7.283144 − max.qupm: 5

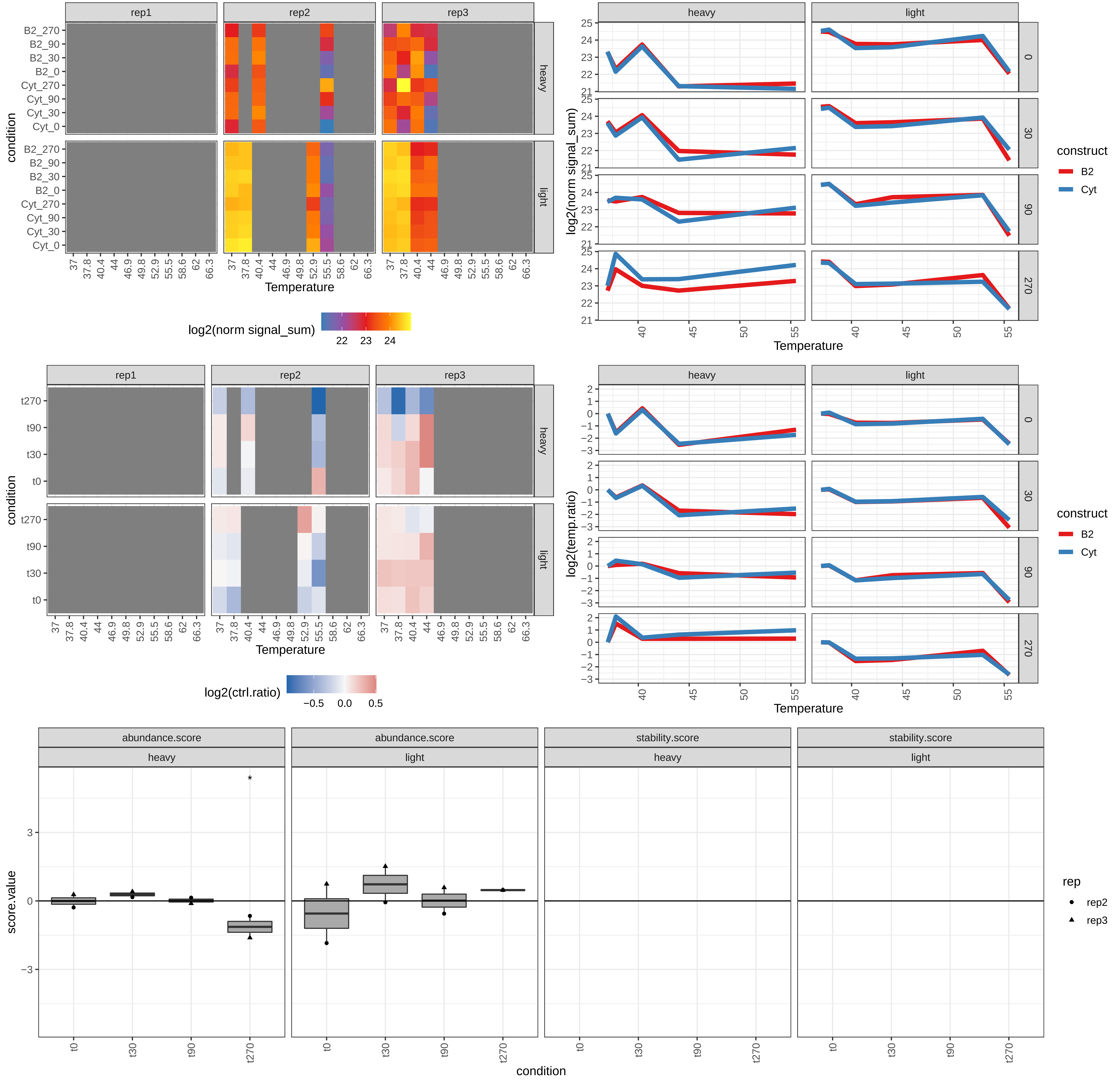

P00410 − Cytochrome c oxidase subunit 2 OS=Saccharomyces cerevisiae (strain ATCC 204508 / S288c) GN=COX2 PE=1 SV=1

COX4 − average.top3: 7.11868709677419 − max.qupm: 10

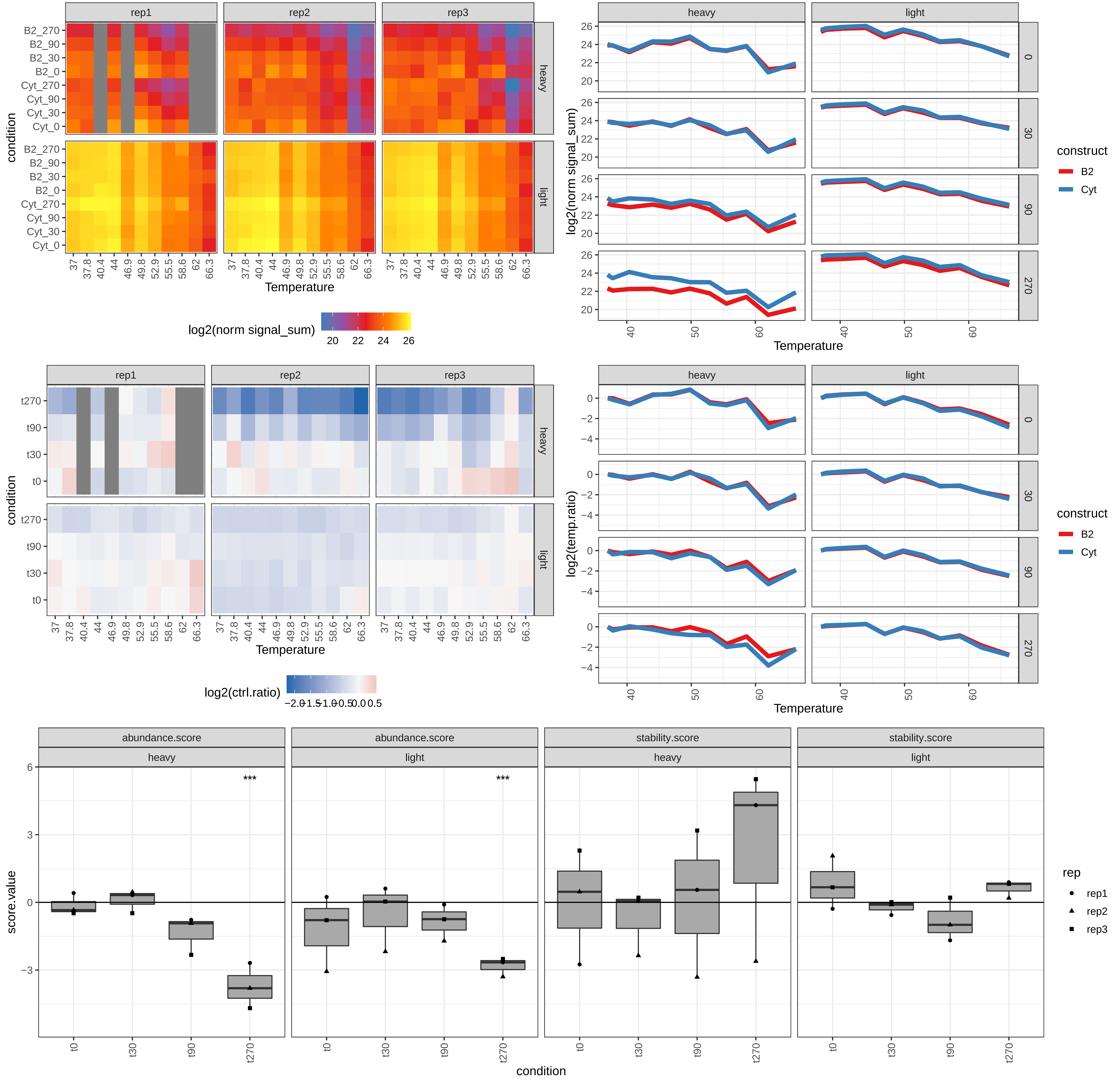

P04037 − Cytochrome c oxidase subunit 4, mitochondrial OS=Saccharomyces cerevisiae (strain ATCC 204508 / S288c) GN=COX4 PE=1 SV=1

COX5A − average.top3: 7.40806333333333 − max.qupm: 5

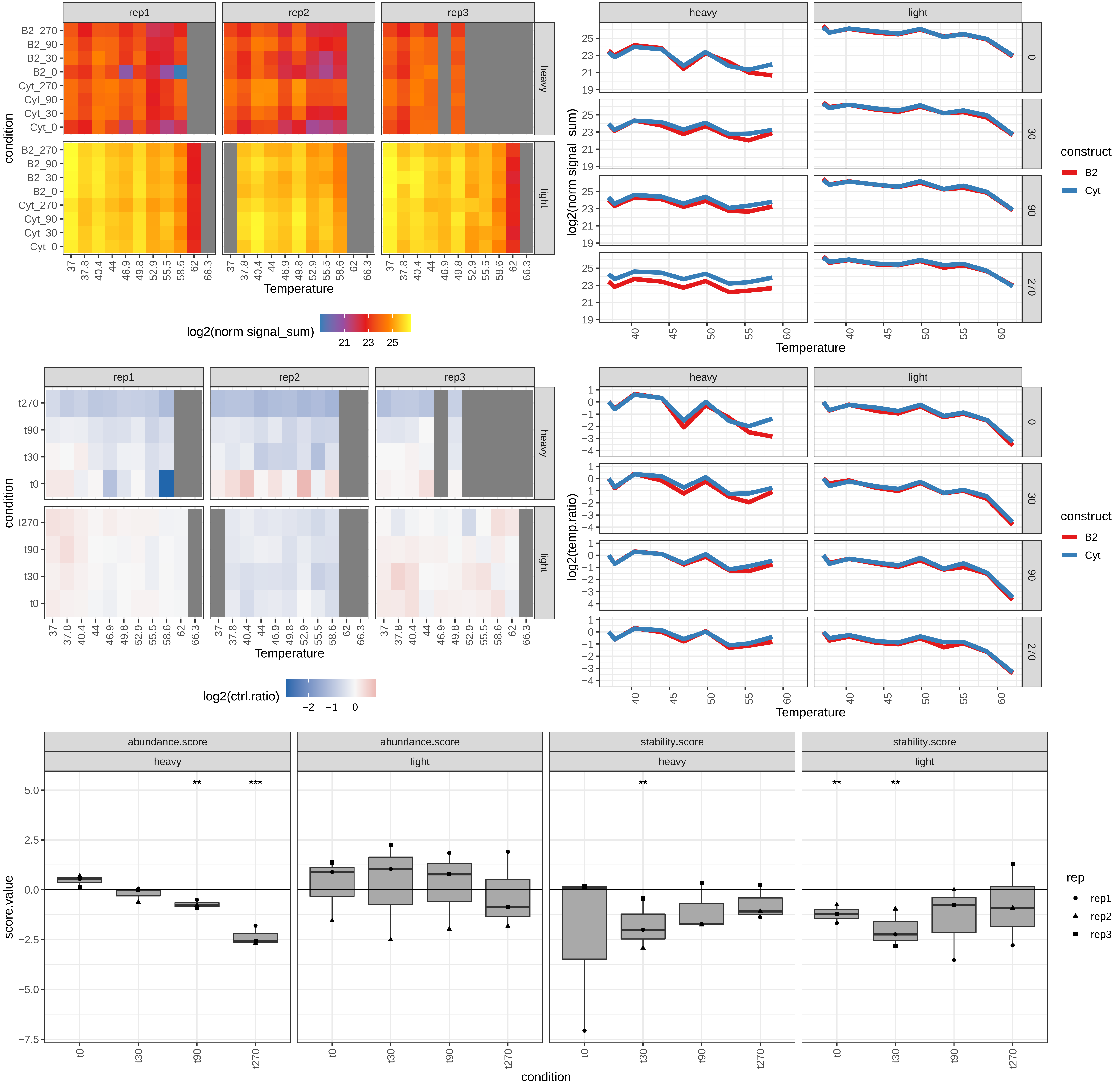

P00424 − Cytochrome c oxidase polypeptide 5A, mitochondrial OS=Saccharomyces cerevisiae (strain ATCC 204508 / S288c) GN=COX5A PE=1 SV=1

COX5B − average.top3: 6.70184333333333 − max.qupm: 5

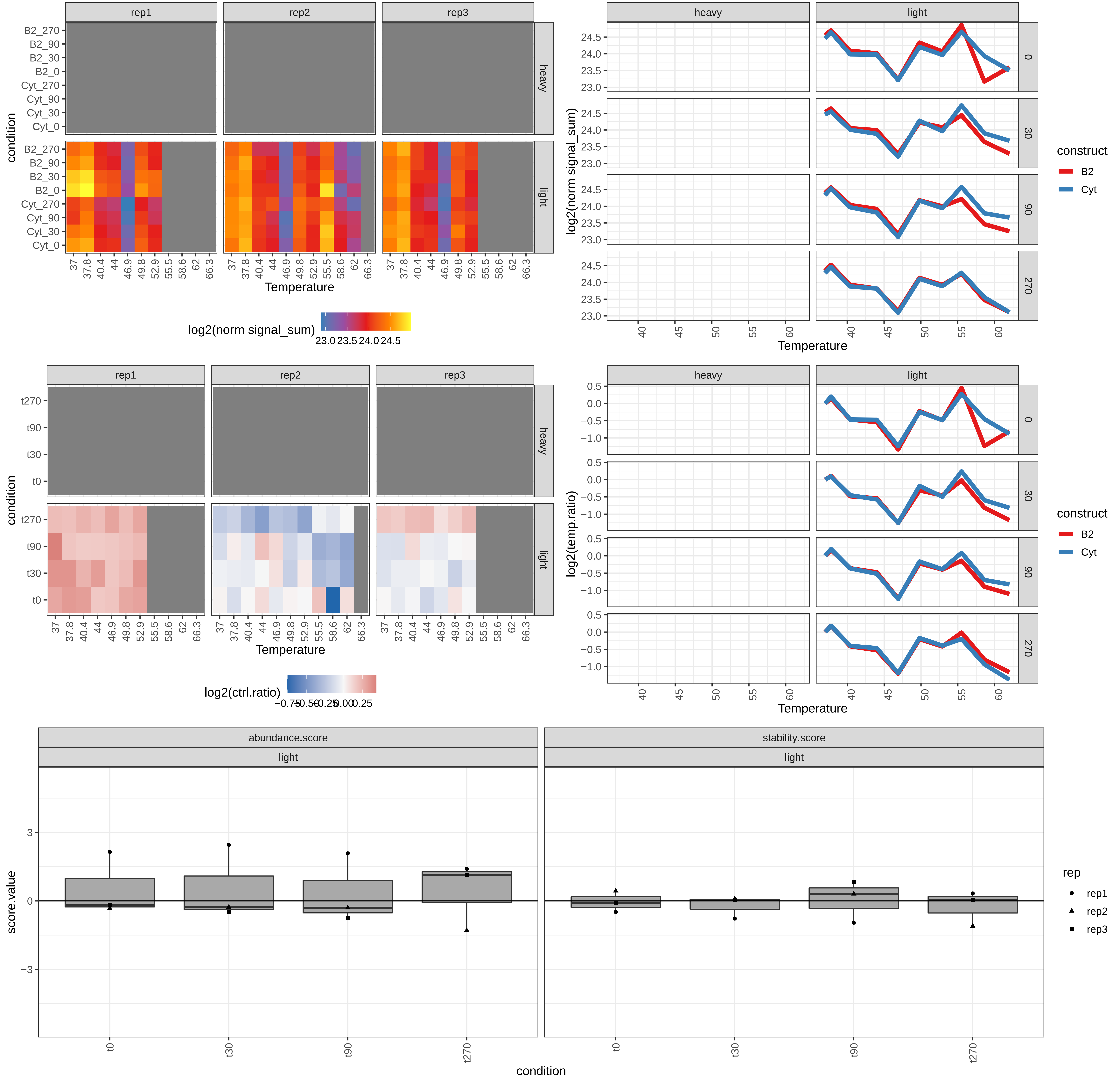

P00425 − Cytochrome c oxidase polypeptide 5B, mitochondrial OS=Saccharomyces cerevisiae (strain ATCC 204508 / S288c) GN=COX5B PE=1 SV=3

COX6 − average.top3: 7.40004283333333 − max.qupm: 11

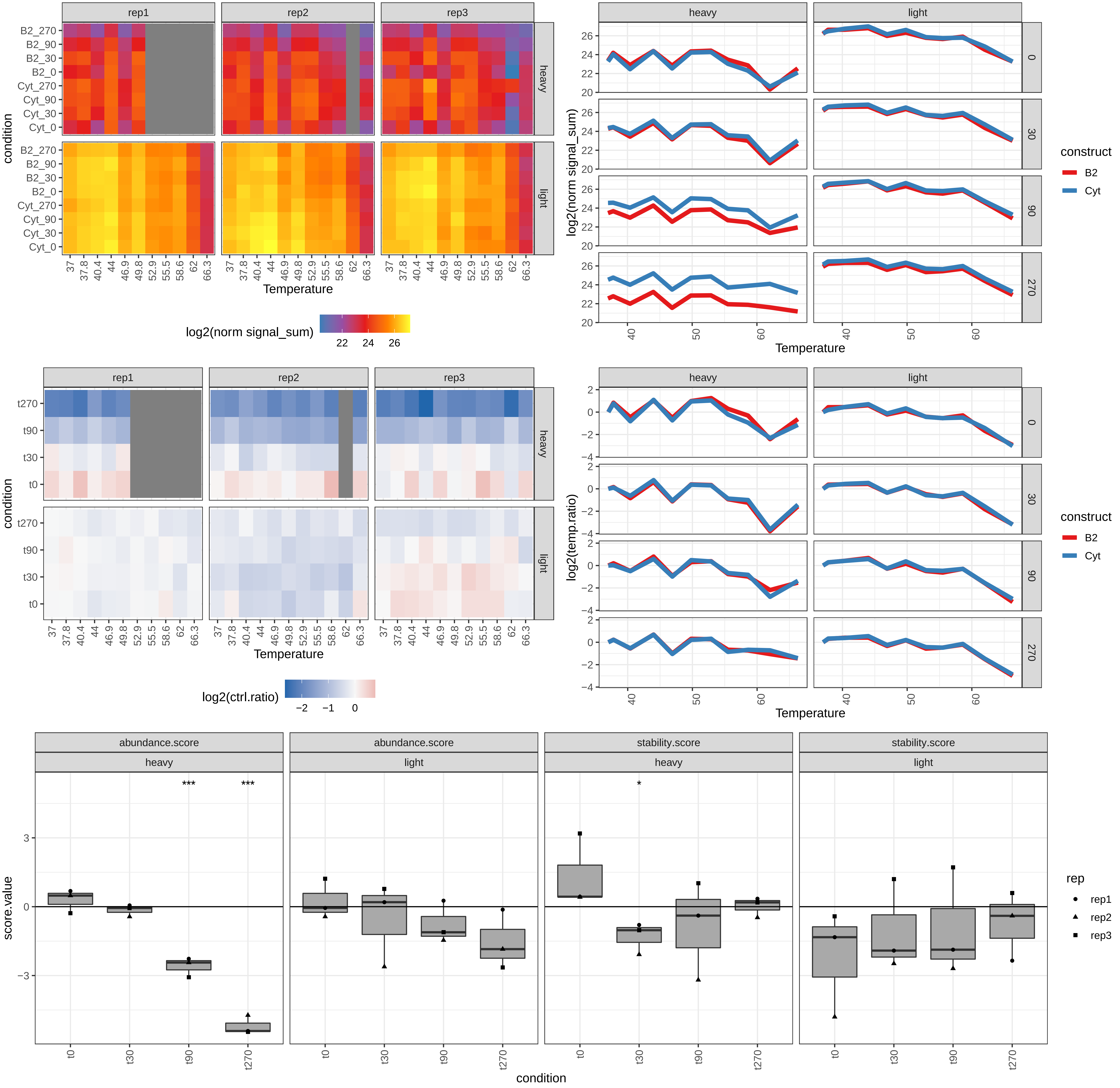

P00427 − Cytochrome c oxidase subunit 6, mitochondrial OS=Saccharomyces cerevisiae (strain ATCC 204508 / S288c) GN=COX6 PE=1 SV=1

COX9 − average.top3: 7.5459176 − max.qupm: 4

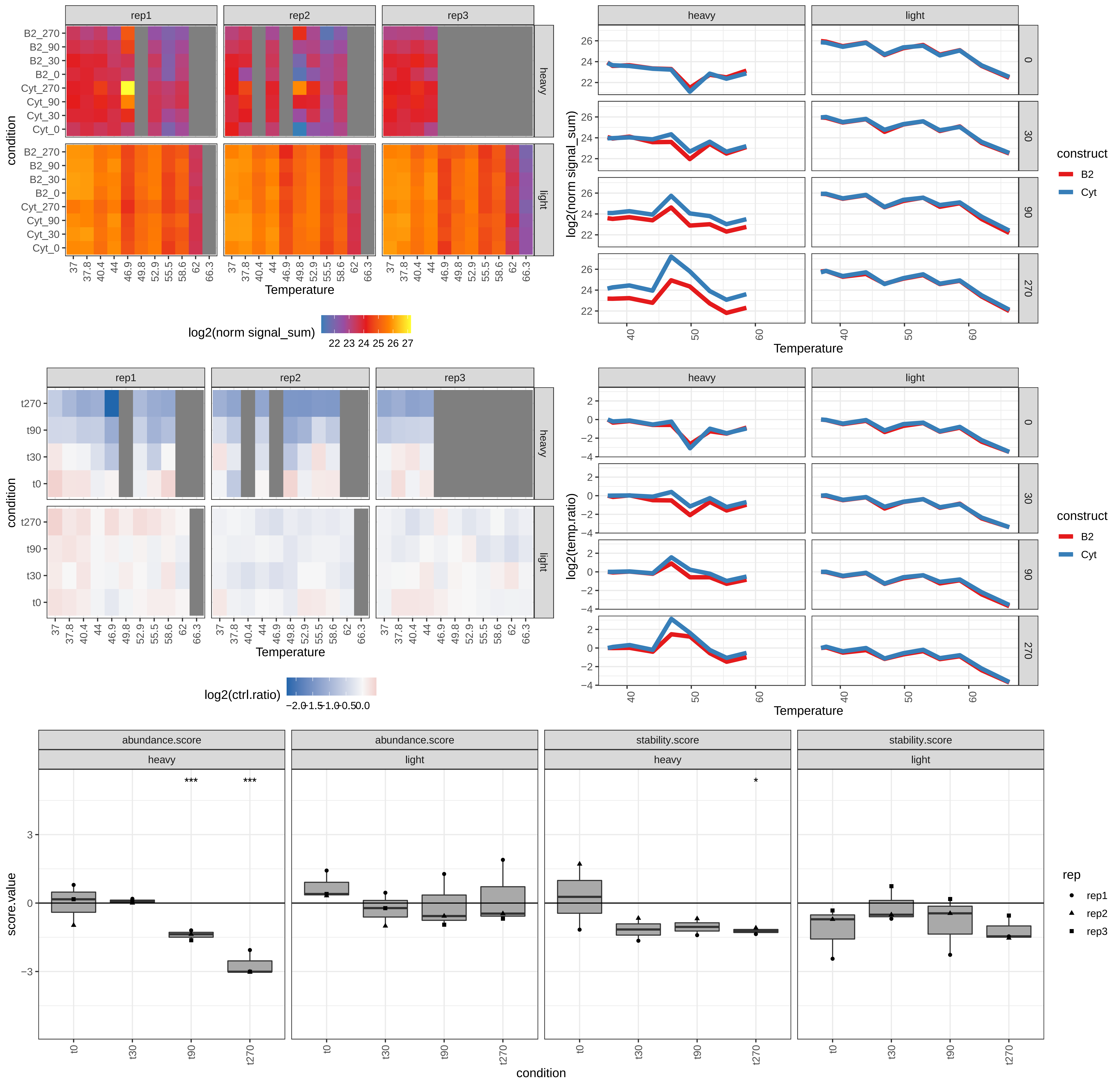

P07255 − Cytochrome c oxidase subunit 7A OS=Saccharomyces cerevisiae (strain ATCC 204508 / S288c) GN=COX9 PE=1 SV=2

COY1 − average.top3: 6.73982235294118 − max.qupm: 9

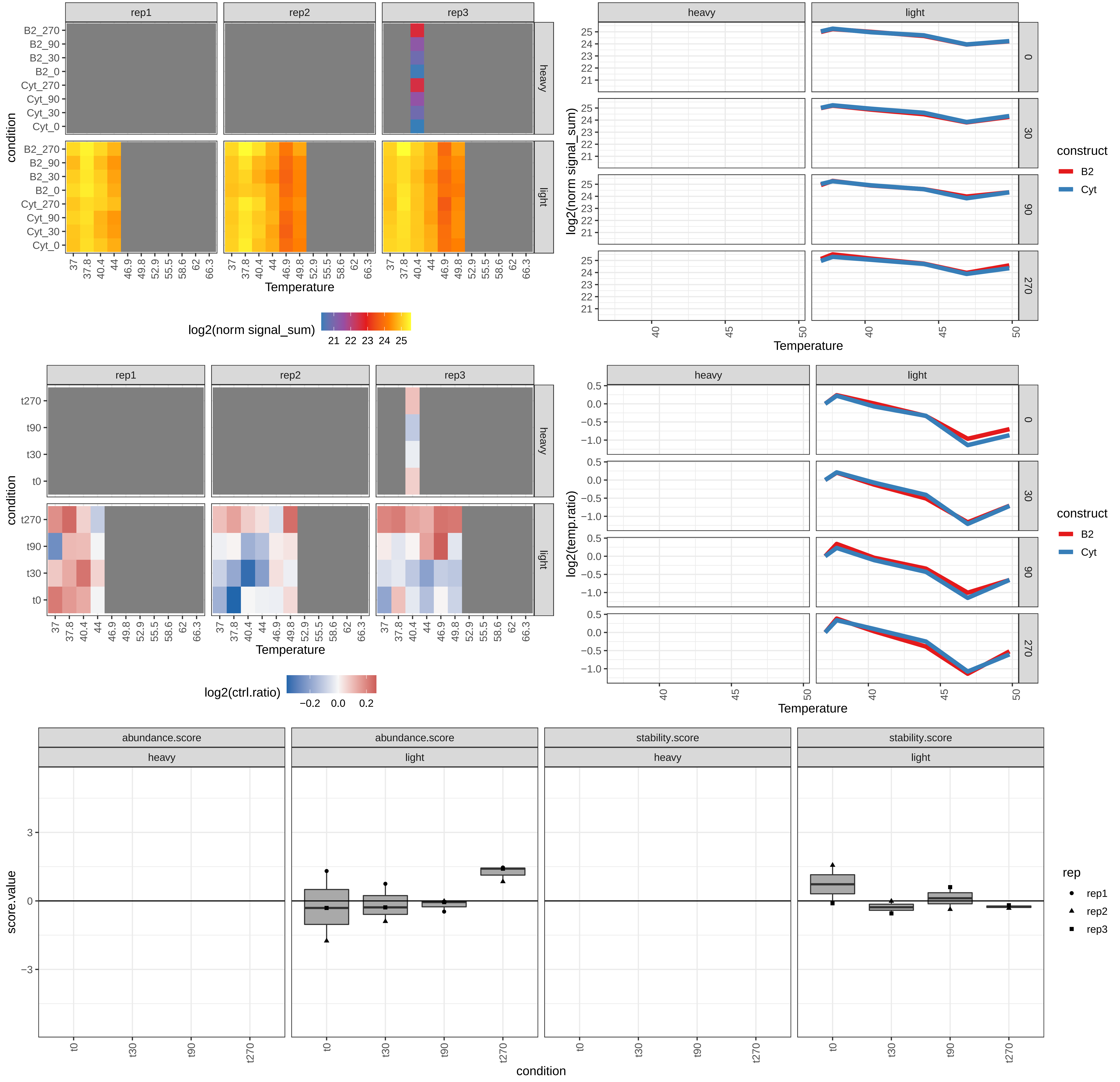

P34237 − Protein CASP OS=Saccharomyces cerevisiae (strain ATCC 204508 / S288c) GN=COY1 PE=1 SV=2

CPA1 − average.top3: 6.35036631578947 − max.qupm: 3

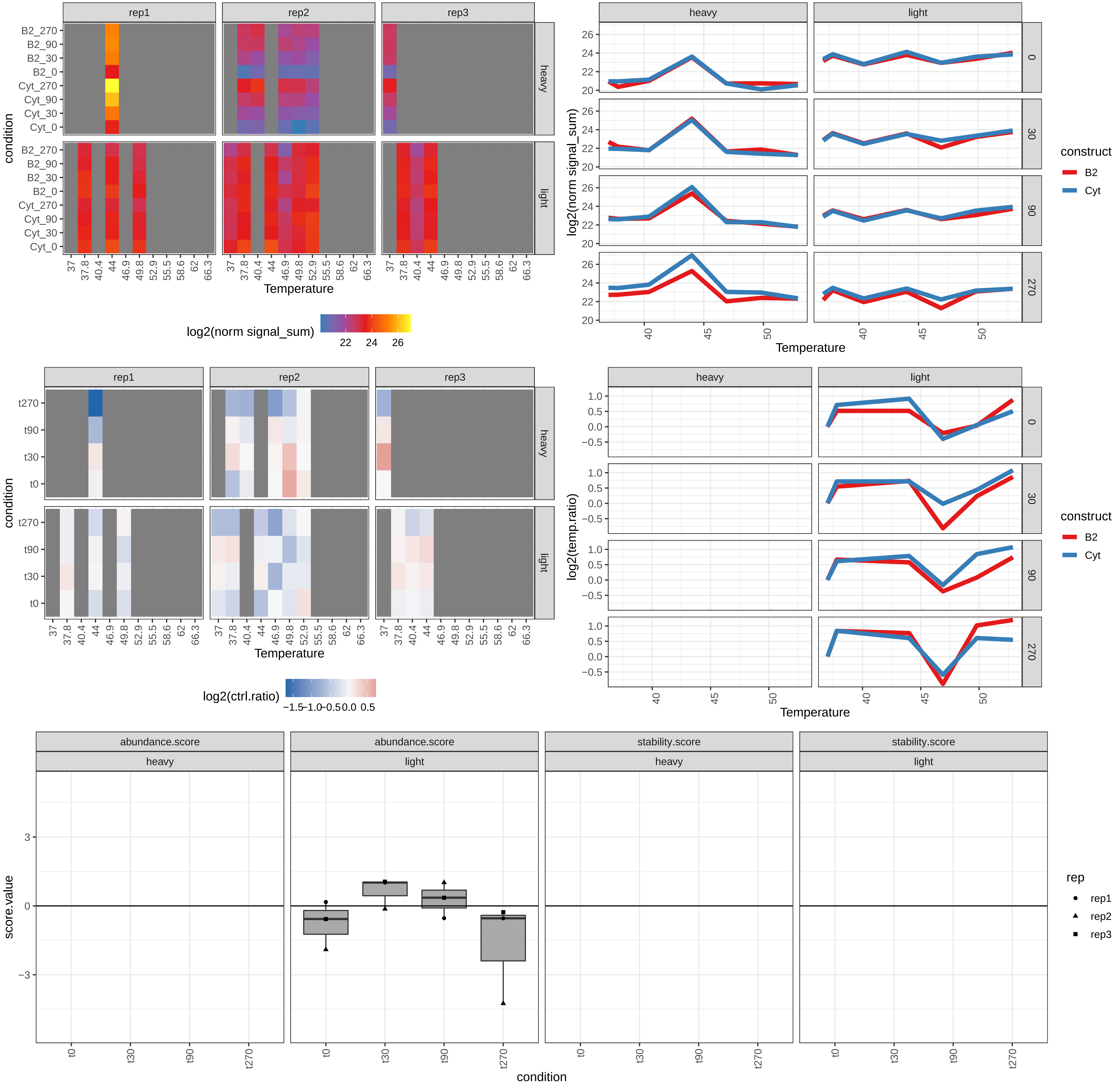

P07258 − Carbamoyl−phosphate synthase arginine−specific small chain OS=Saccharomyces cerevisiae (strain ATCC 204508 / S288c) GN=CPA1 PE=1 SV=1

CPA2 − average.top3: 7.7615049122807 − max.qupm: 37

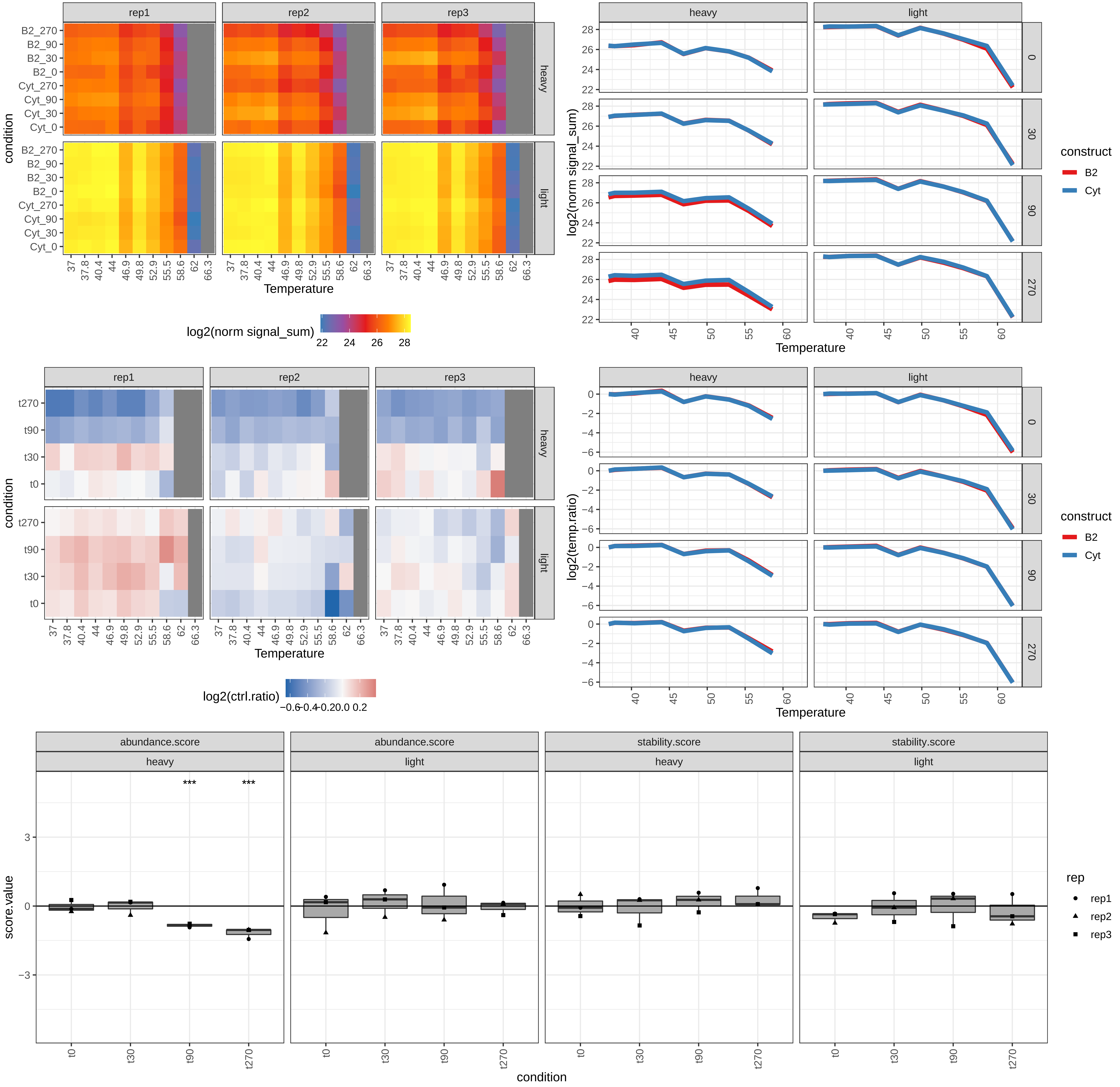

P03965 − Carbamoyl−phosphate synthase arginine−specific large chain OS=Saccharomyces cerevisiae (strain ATCC 204508 / S288c) GN=CPA2 PE=1 SV=1

CPD1 − average.top3: 6.41466666666667 − max.qupm: 3

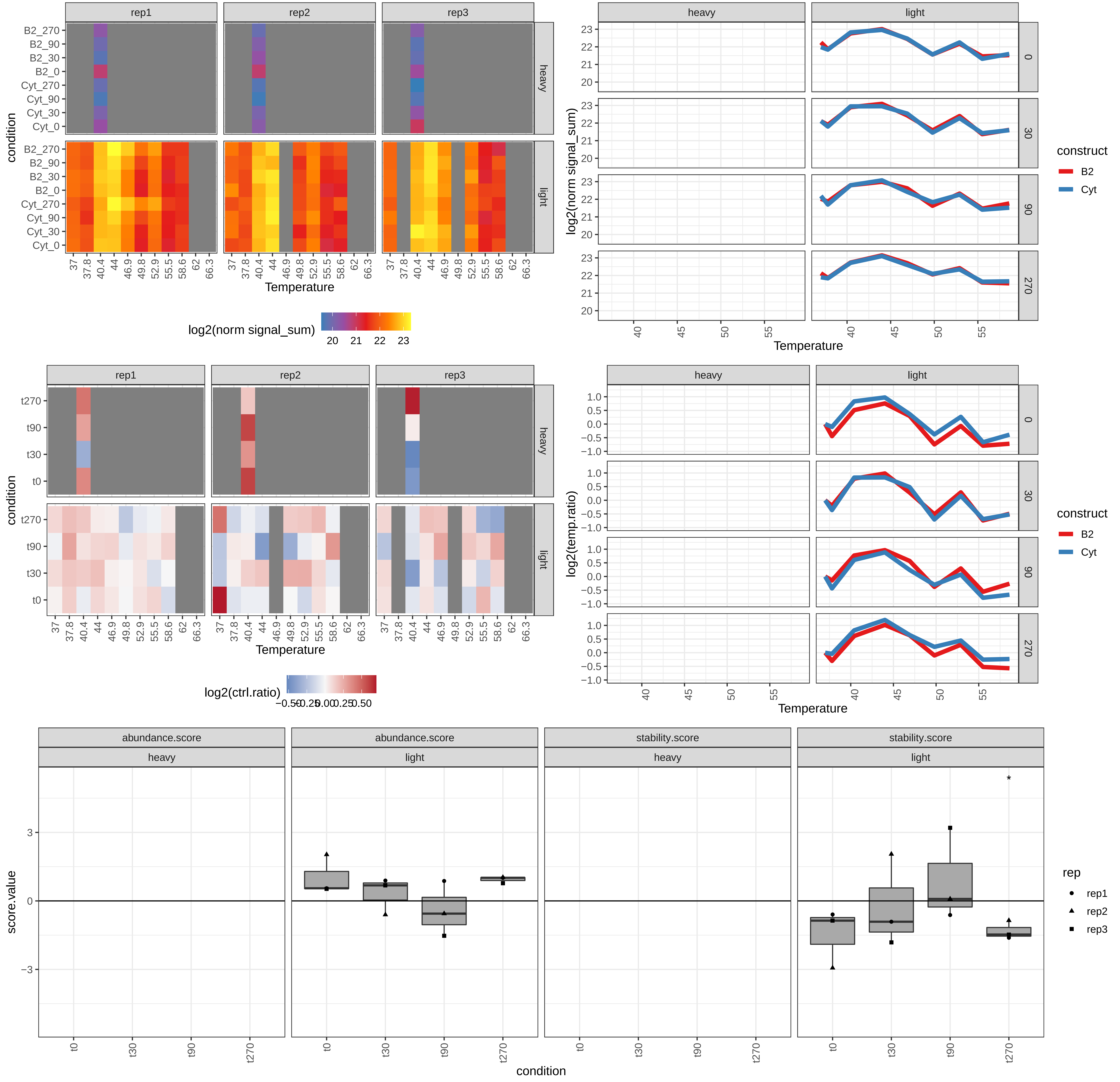

P53314 − 2',3'−cyclic−nucleotide 3'−phosphodiesterase OS=Saccharomyces cerevisiae (strain ATCC 204508 / S288c) GN=CPD1 PE=1 SV=1

CPR1 − average.top3: 7.7050546969697 − max.qupm: 9

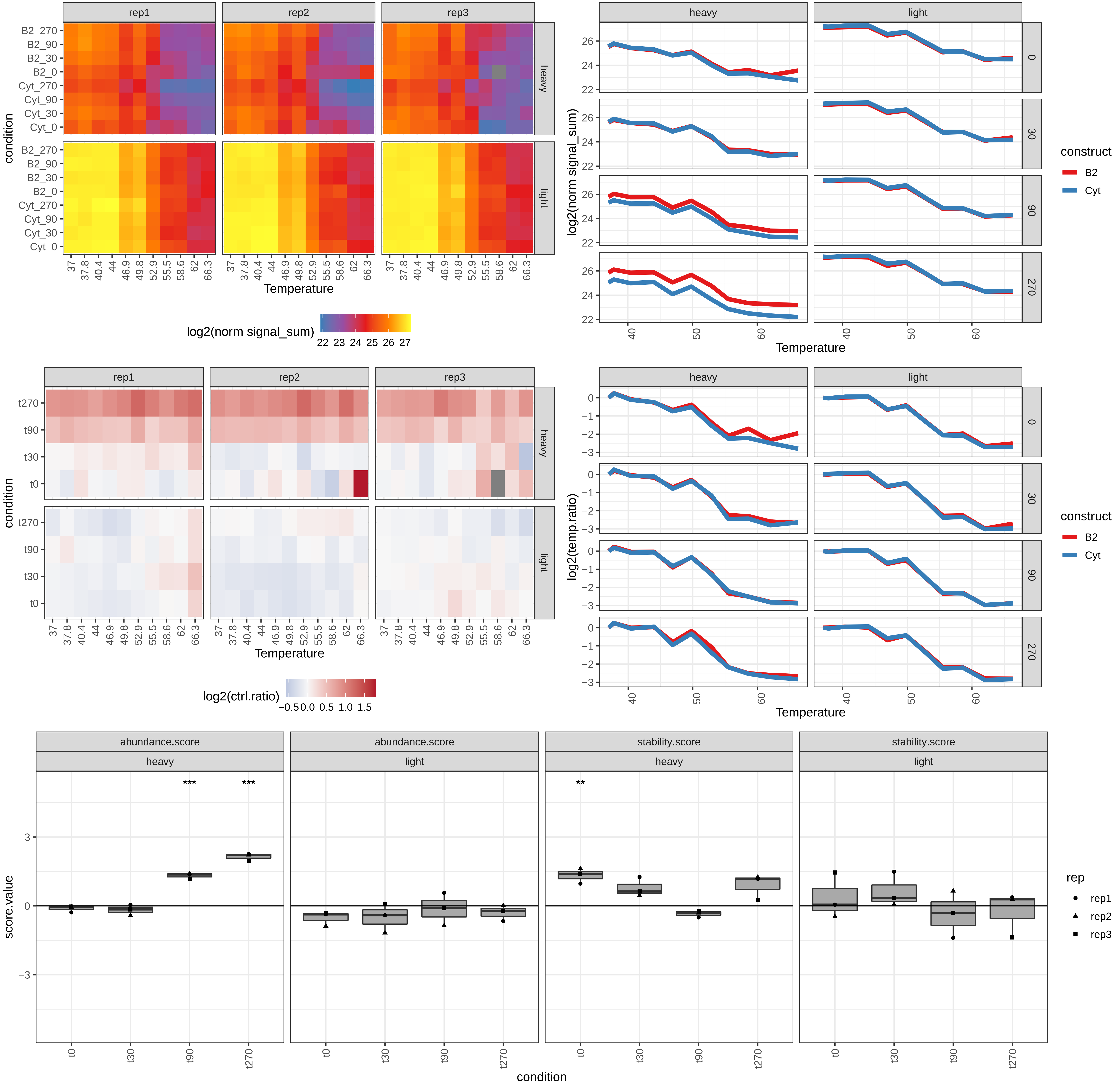

P14832 − Peptidyl−prolyl cis−trans isomerase OS=Saccharomyces cerevisiae (strain ATCC 204508 / S288c) GN=CPR1 PE=1 SV=3

CPR3 − average.top3: 7.48083557692308 − max.qupm: 9

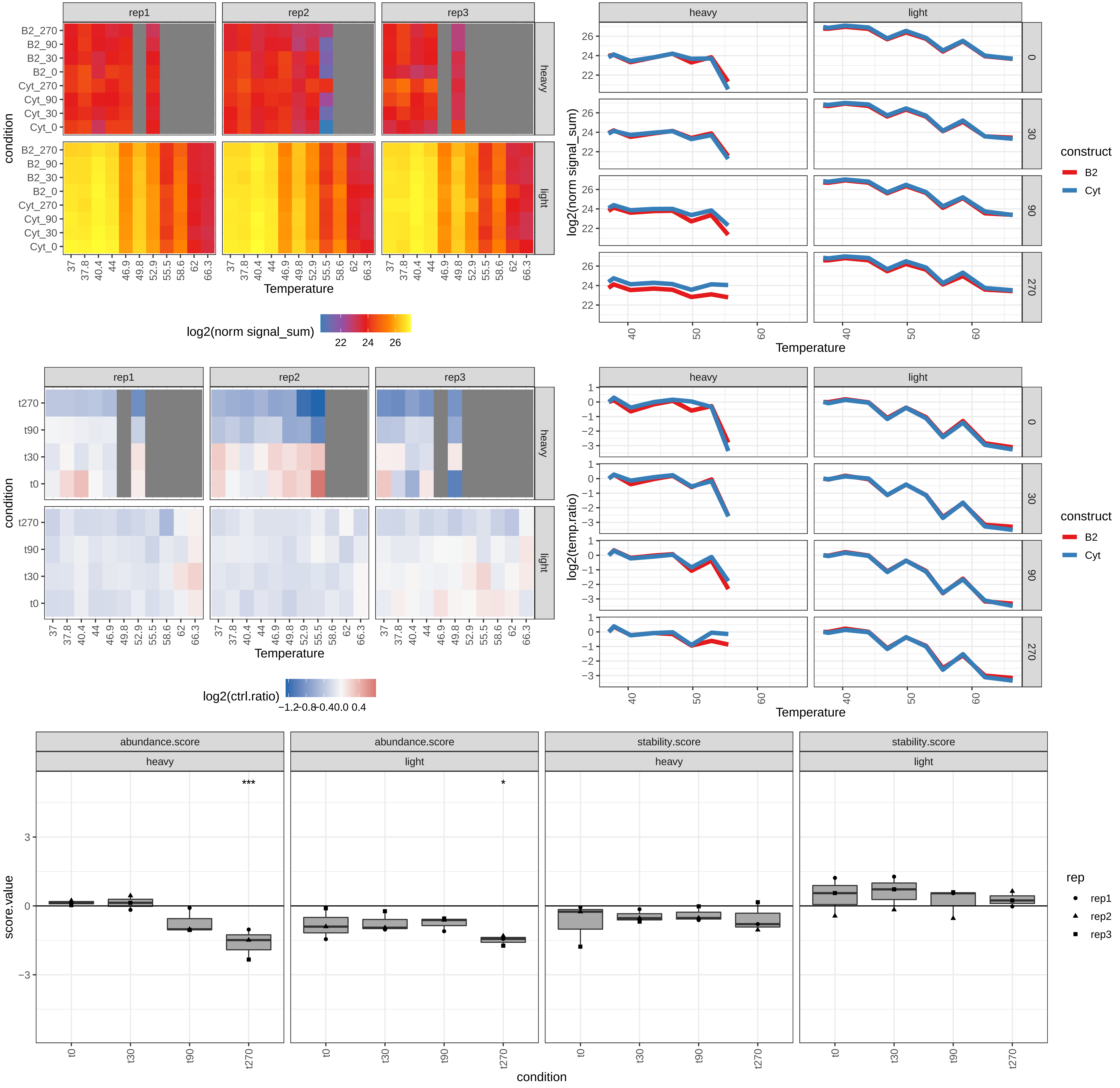

P25719 − Peptidyl−prolyl cis−trans isomerase C, mitochondrial OS=Saccharomyces cerevisiae (strain ATCC 204508 / S288c) GN=CPR3 PE=1 SV=1

CPR5 − average.top3: 7.31878788461538 − max.qupm: 6

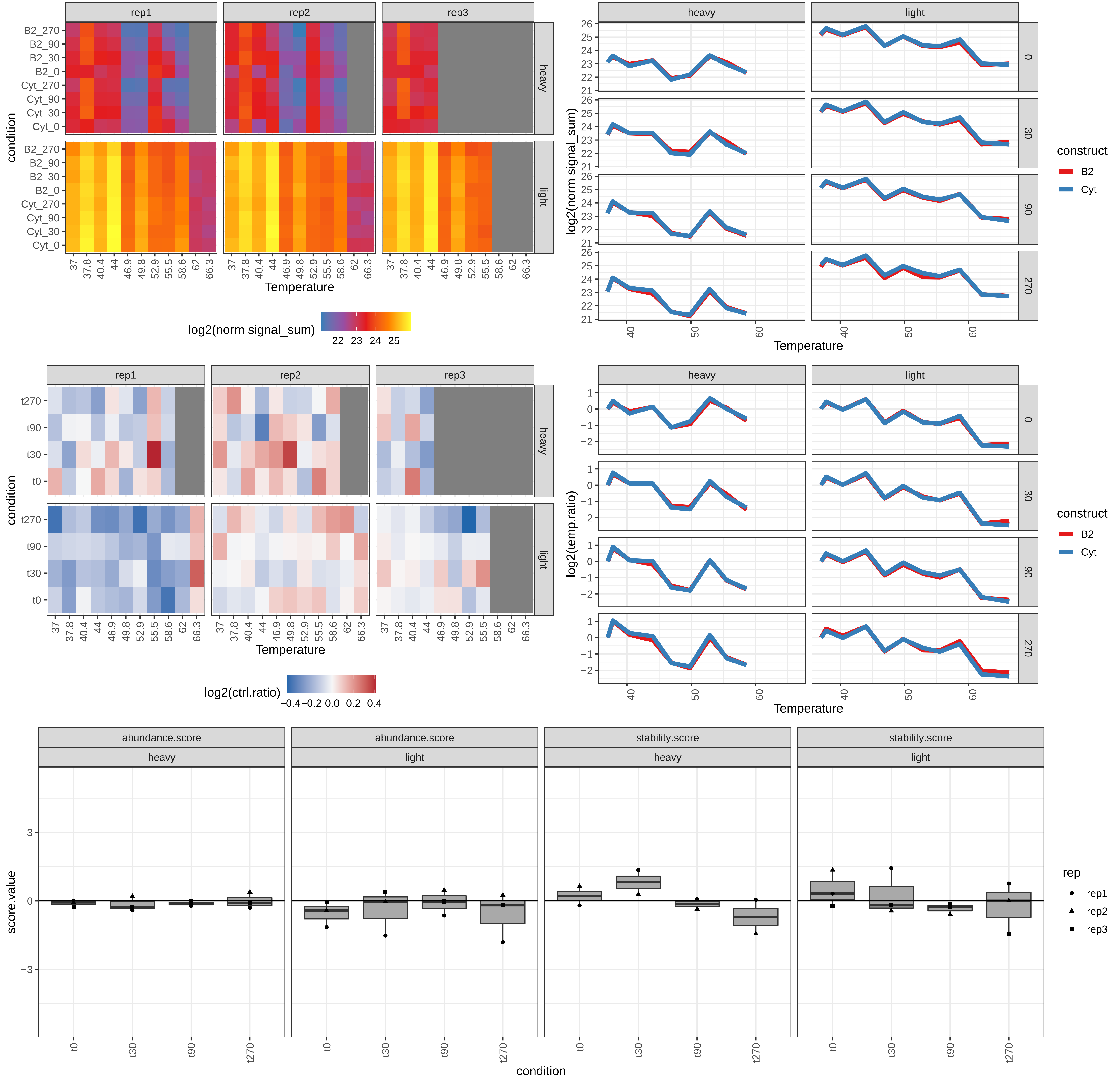

P35176 − Peptidyl−prolyl cis−trans isomerase D OS=Saccharomyces cerevisiae (strain ATCC 204508 / S288c) GN=CPR5 PE=2 SV=1

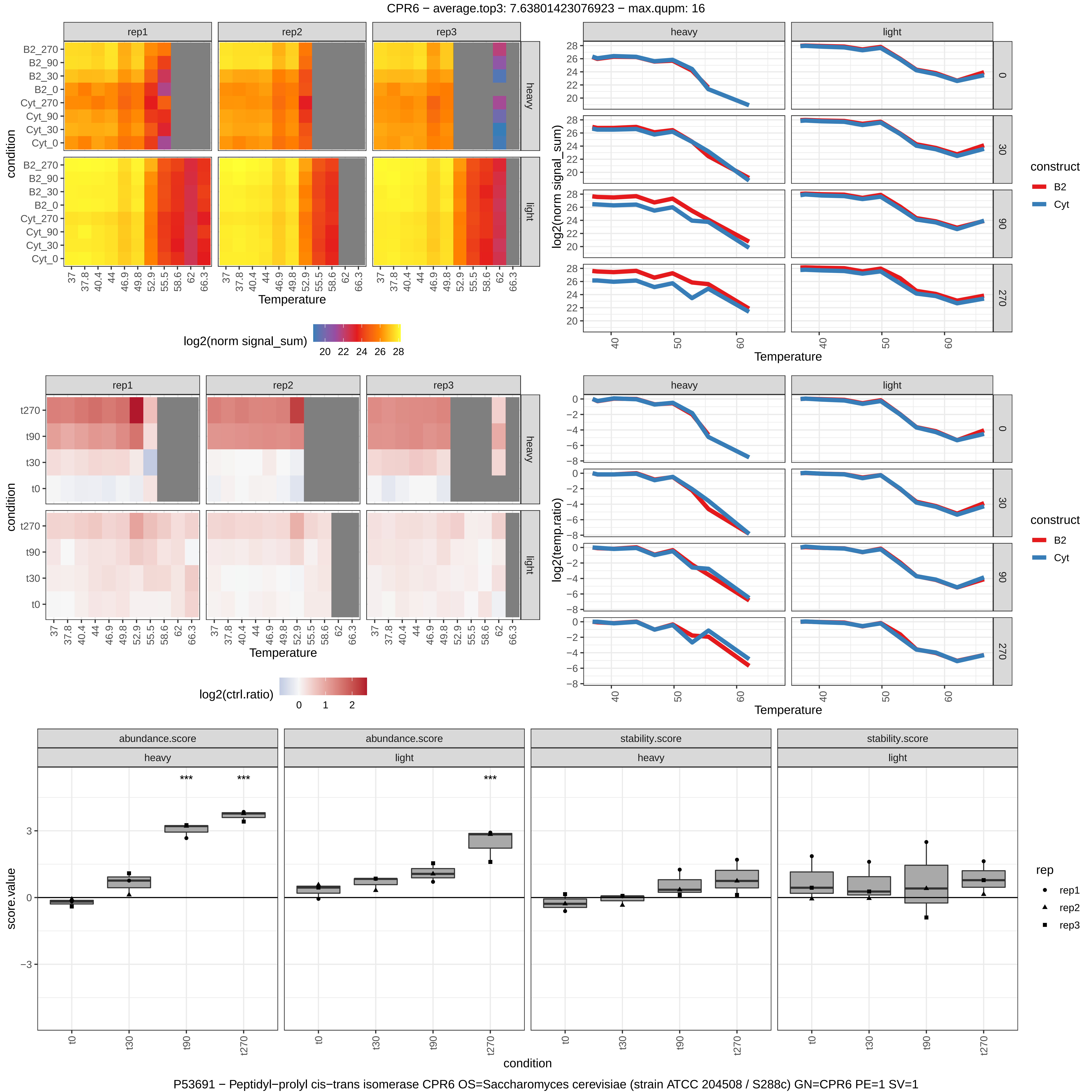
CPR6 − average.top3: 7.63801423076923 − max.qupm: 16
rep1
rep2
rep3
heavy
light
condition
Temperature
log2(norm signal_sum)
construct
B2
Cyt
log2(ctrl.ratio)
log2(temp.ratio)
abundance.score
stability.score
score.value
rep
rep1
rep2
rep3
P53691 − Peptidyl−prolyl cis−trans isomerase CPR6 OS=Saccharomyces cerevisiae (strain ATCC 204508 / S288c) GN=CPR6 PE=1 SV=1

CPR7 − average.top3: 7.17386631578947 − max.qupm: 7

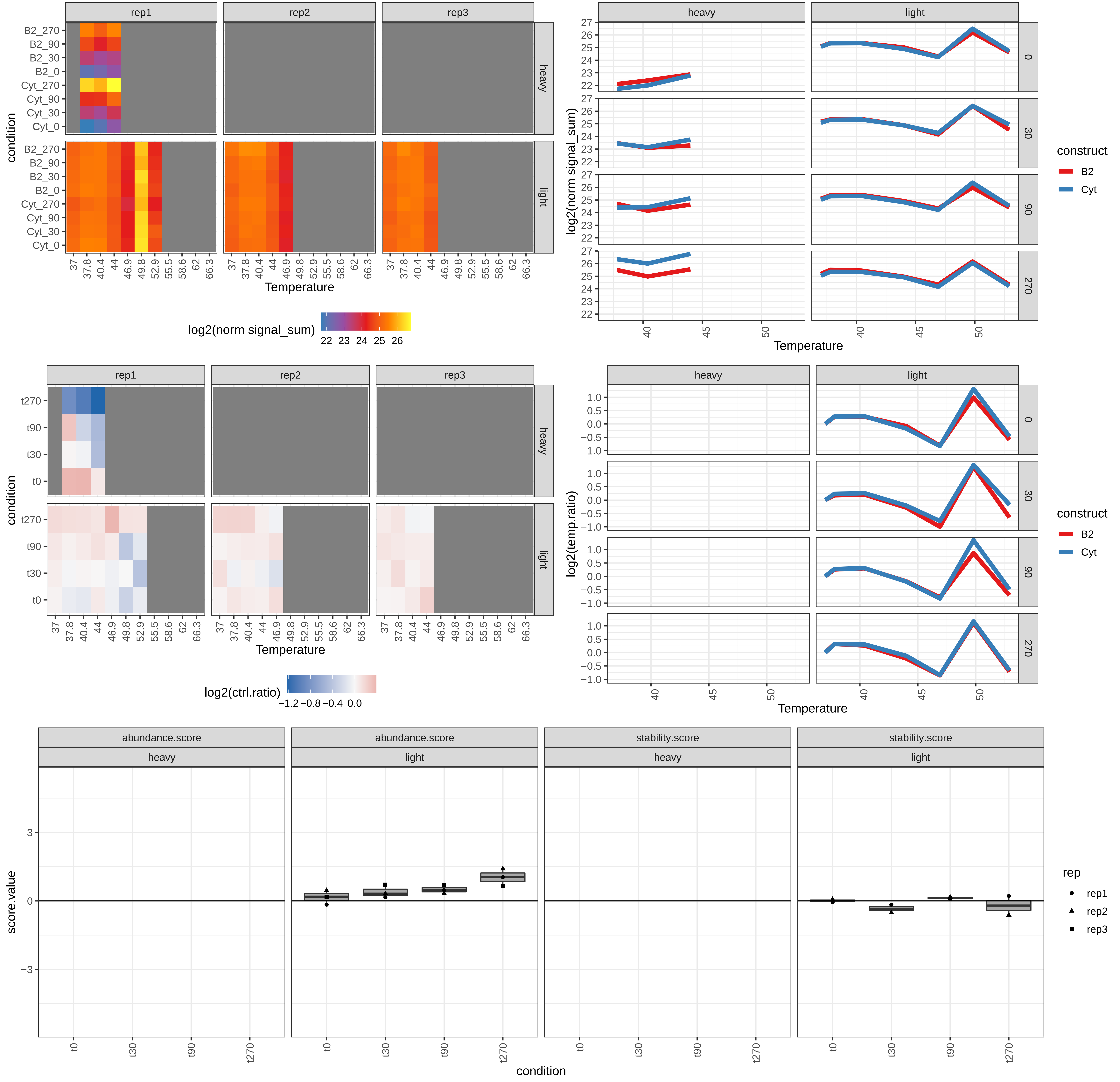

P47103 − Peptidyl−prolyl cis−trans isomerase CYP7 OS=Saccharomyces cerevisiae (strain ATCC 204508 / S288c) GN=CPR7 PE=1 SV=1

CPS1 − average.top3: 7.59139123076923 − max.qupm: 17

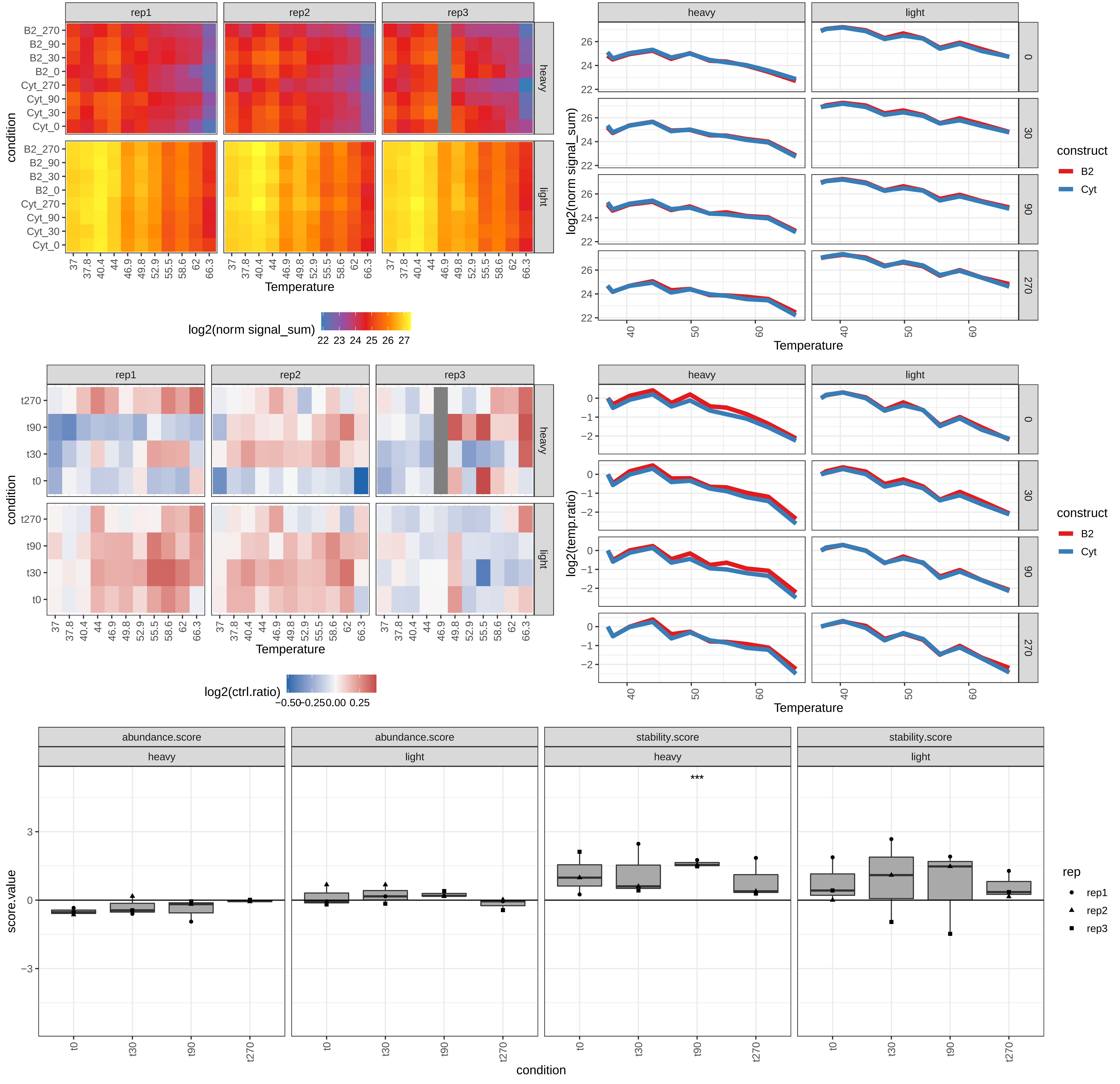

P27614 − Carboxypeptidase S OS=Saccharomyces cerevisiae (strain ATCC 204508 / S288c) GN=CPS1 PE=1 SV=2

CPT1 − average.top3: 7.00785961538462 − max.qupm: 3

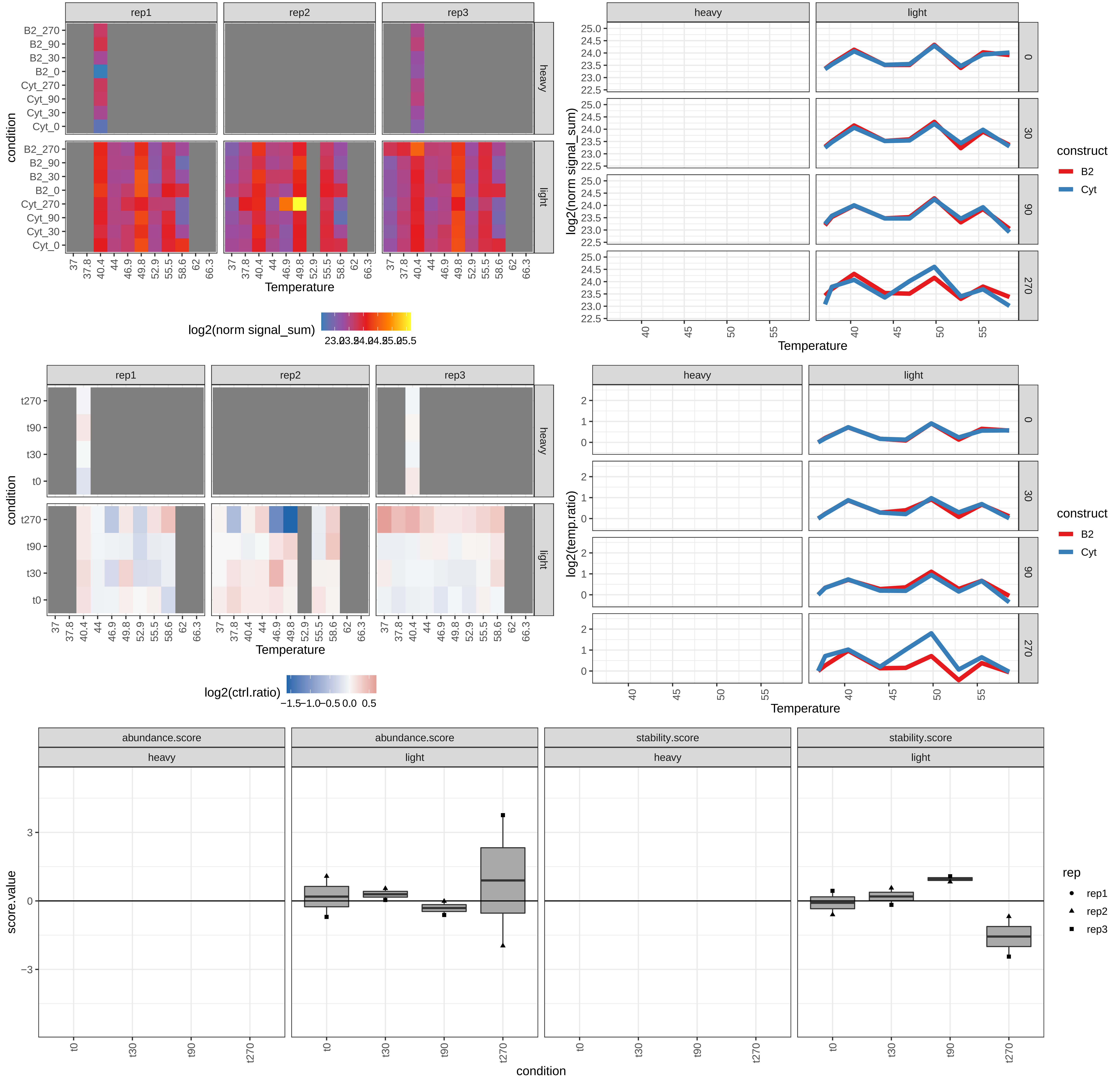

P17898 − Cholinephosphotransferase 1 OS=Saccharomyces cerevisiae (strain ATCC 204508 / S288c) GN=CPT1 PE=1 SV=3

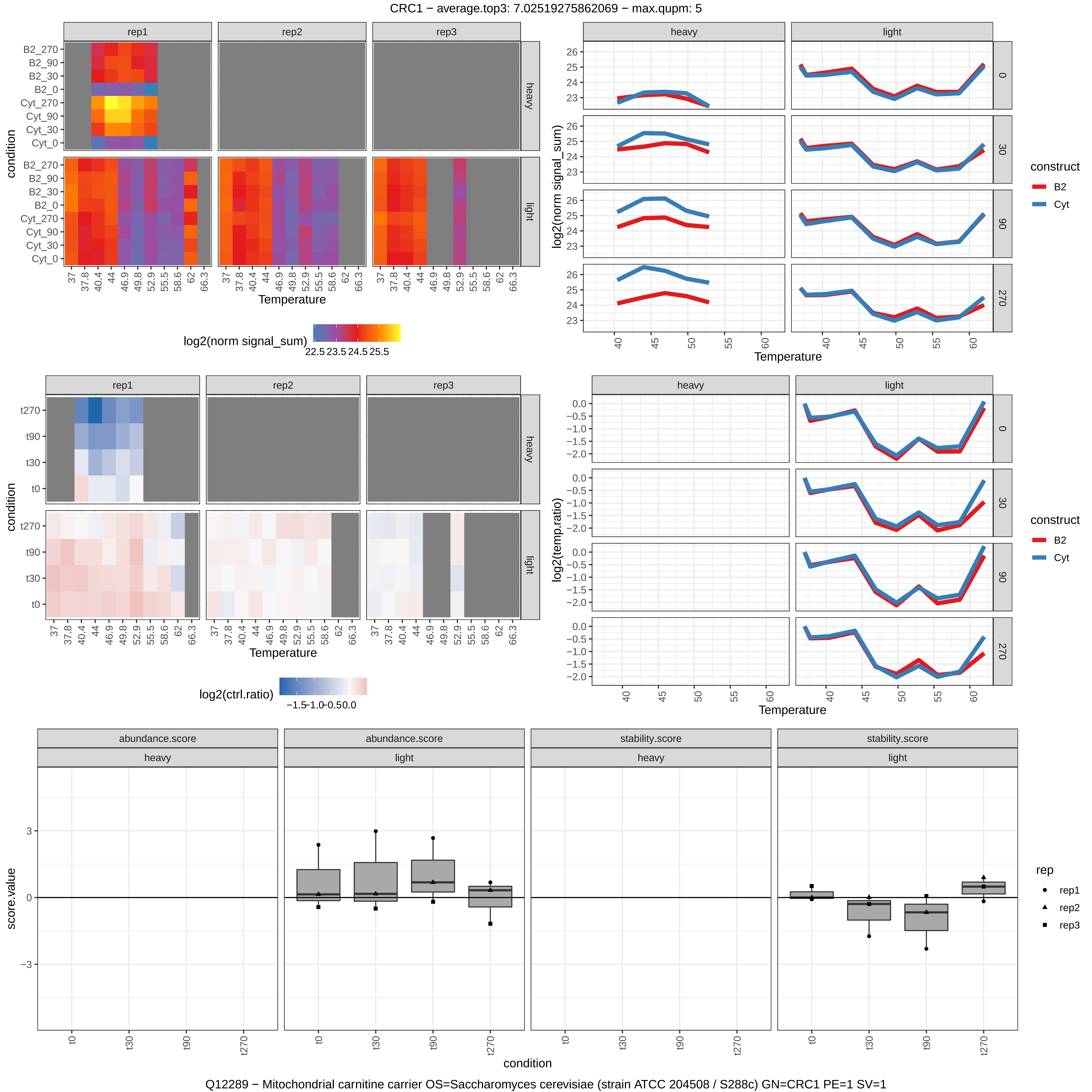
CRC1 − average.top3: 7.02519275862069 − max.qupm: 5
rep1
rep2
rep3
heavy
light
condition
Temperature
log2(norm signal_sum)
log2(ctrl.ratio)
log2(temp.ratio)
construct
B2
Cyt
abundance.score
stability.score
score.value
rep
Q12289 − Mitochondrial carnitine carrier OS=Saccharomyces cerevisiae (strain ATCC 204508 / S288c) GN=CRC1 PE=1 SV=1

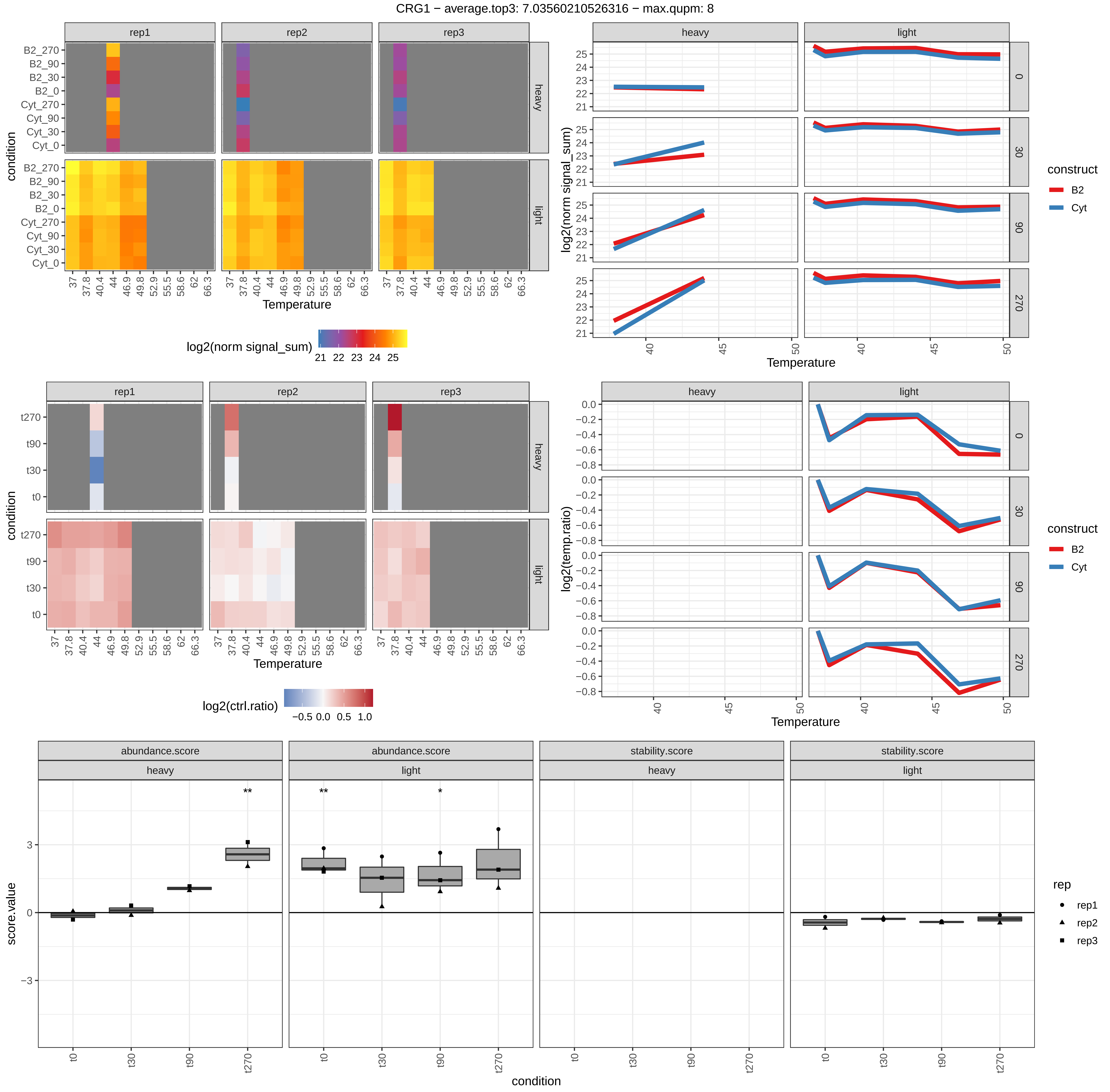

P38892 − Probable S−adenosylmethionine−dependent methyltransferase CRG1 OS=Saccharomyces cerevisiae (strain ATCC 204508 / S288c) GN=CRG1 PE=1 SV=1

CRH1 − average.top3: 7.50195796875 − max.qupm: 13

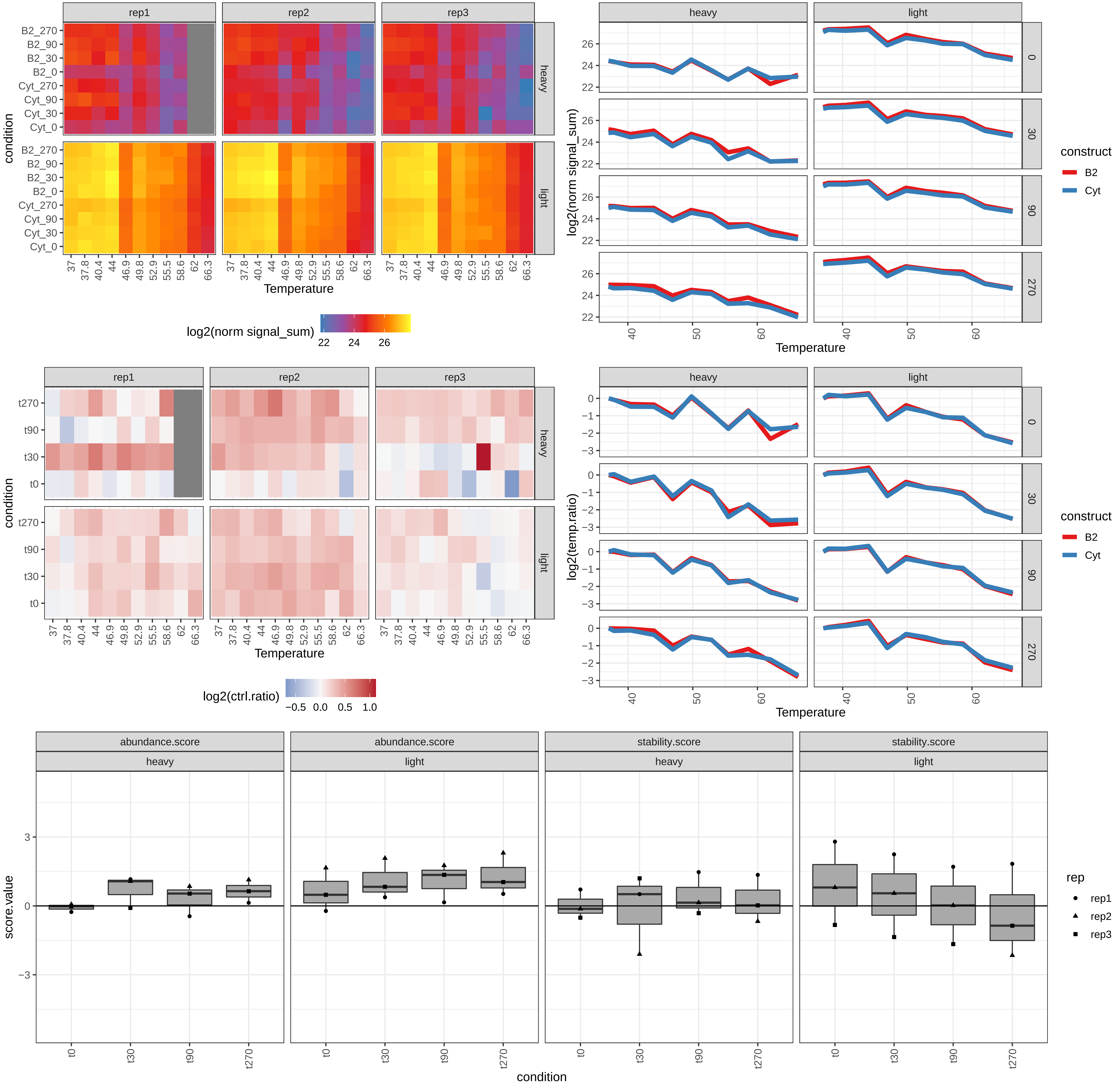

P53301 − Probable glycosidase CRH1 OS=Saccharomyces cerevisiae (strain ATCC 204508 / S288c) GN=CRH1 PE=1 SV=1

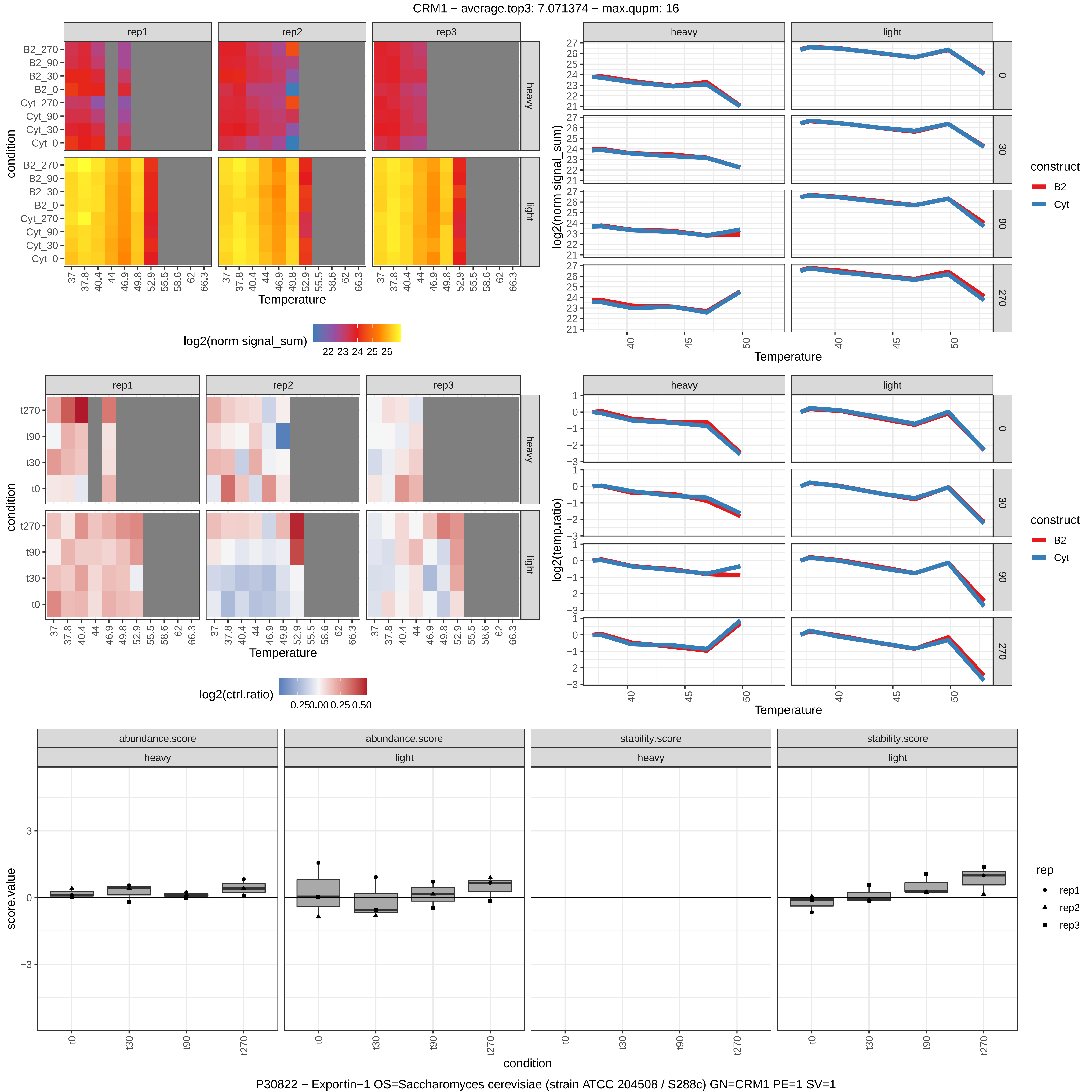

CRM1 − average.top3: 7.071374 − max.qupm: 16
rep1
rep2
rep3
heavy
light
condition
Temperature
log2(norm signal_sum)
construct
B2
Cyt
log2(ctrl.ratio)
log2(temp.ratio)
abundance.score
stability.score
score.value
rep
rep1
rep2
rep3
P30822 − Exportin−1 OS=Saccharomyces cerevisiae (strain ATCC 204508 / S288c) GN=CRM1 PE=1 SV=1

CRN1 − average.top3: 7.18967305555556 − max.qupm: 12

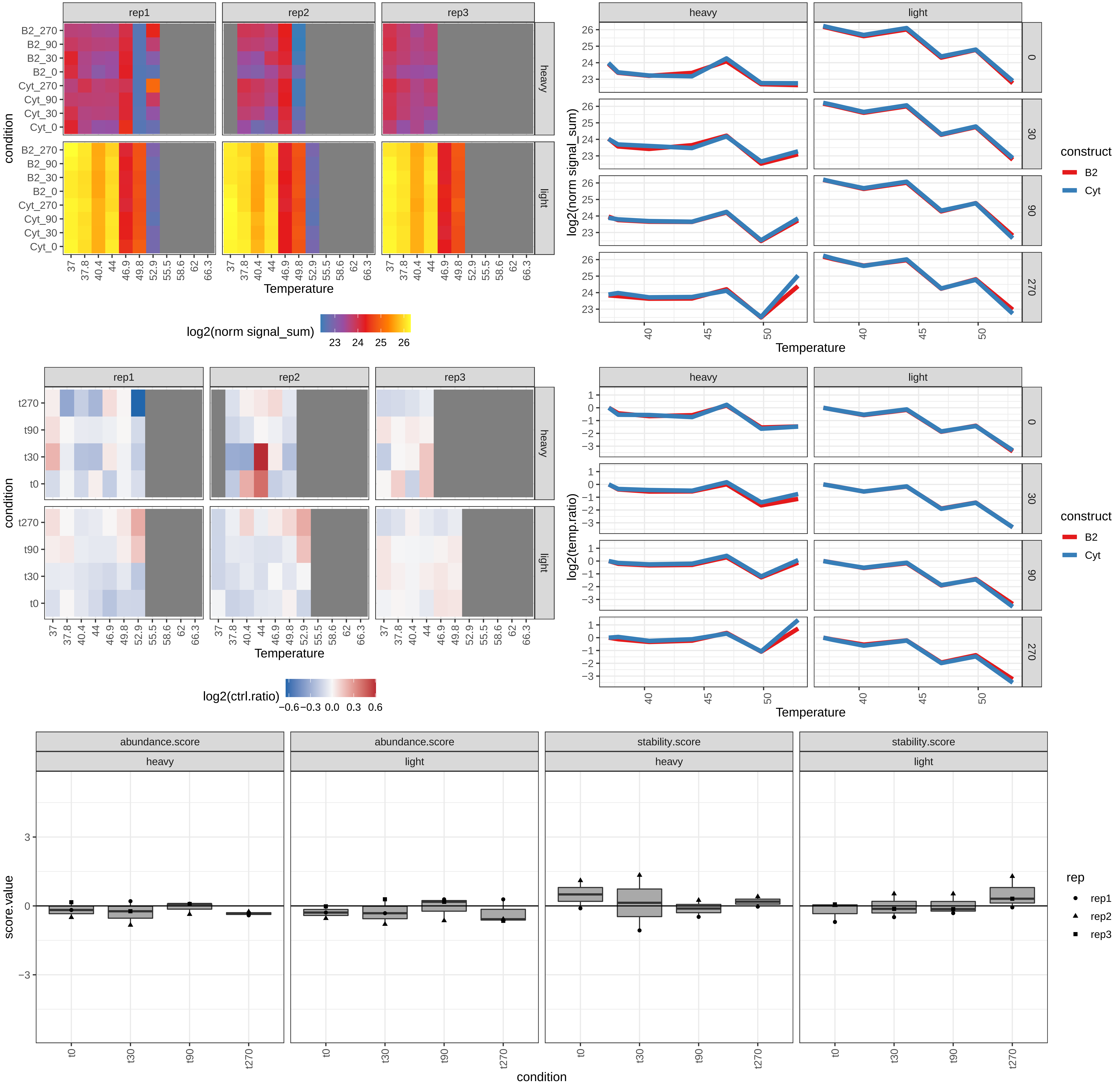

Q06440 − Coronin−like protein OS=Saccharomyces cerevisiae (strain ATCC 204508 / S288c) GN=CRN1 PE=1 SV=1

CRP1 − average.top3: 7.12792381818182 − max.qupm: 10

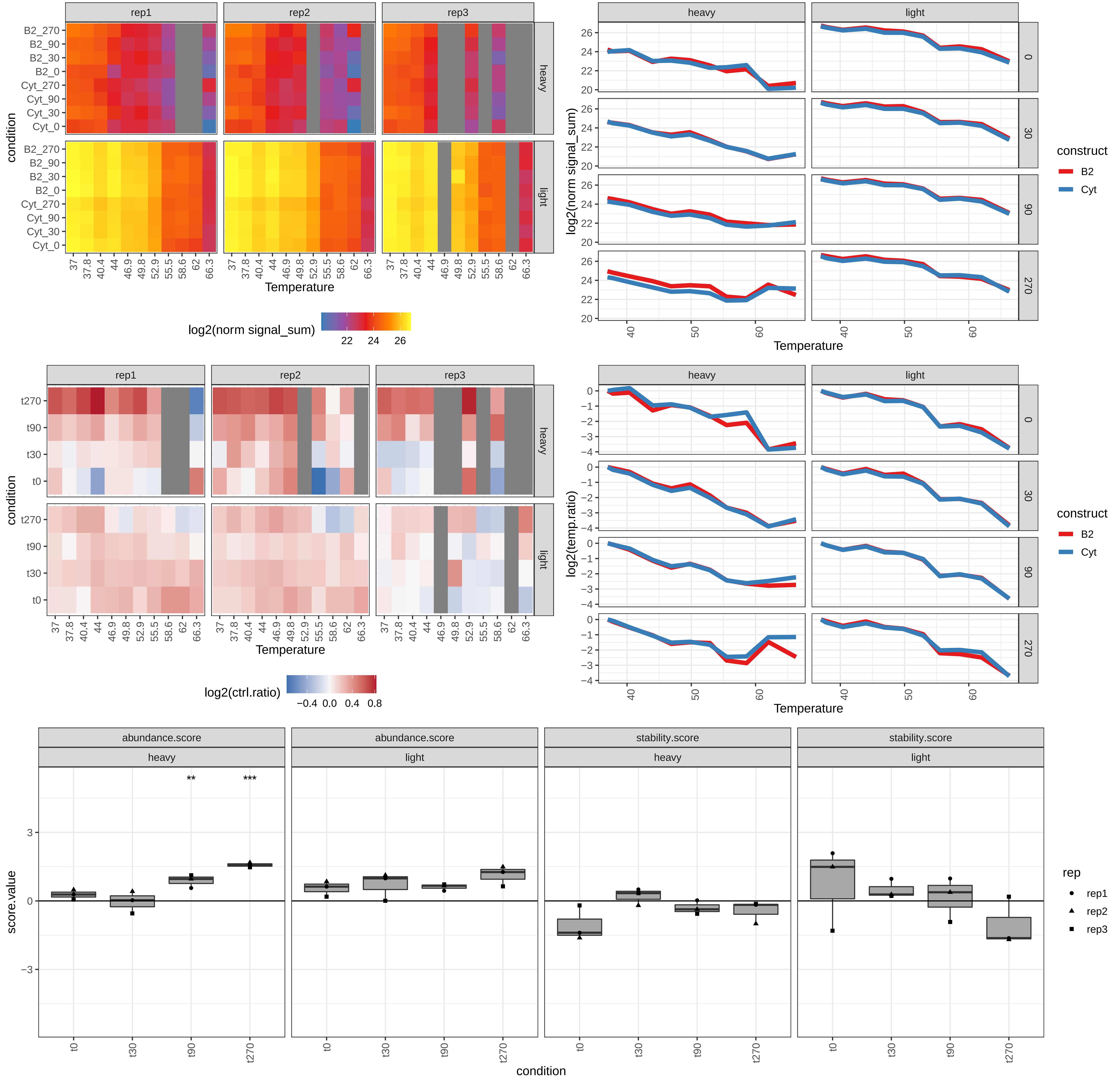

P38845 − Cruciform DNA−recognizing protein 1 OS=Saccharomyces cerevisiae (strain ATCC 204508 / S288c) GN=CRP1 PE=1 SV=1

CRZ1 − average.top3: 6.63643928571429 − max.qupm: 4

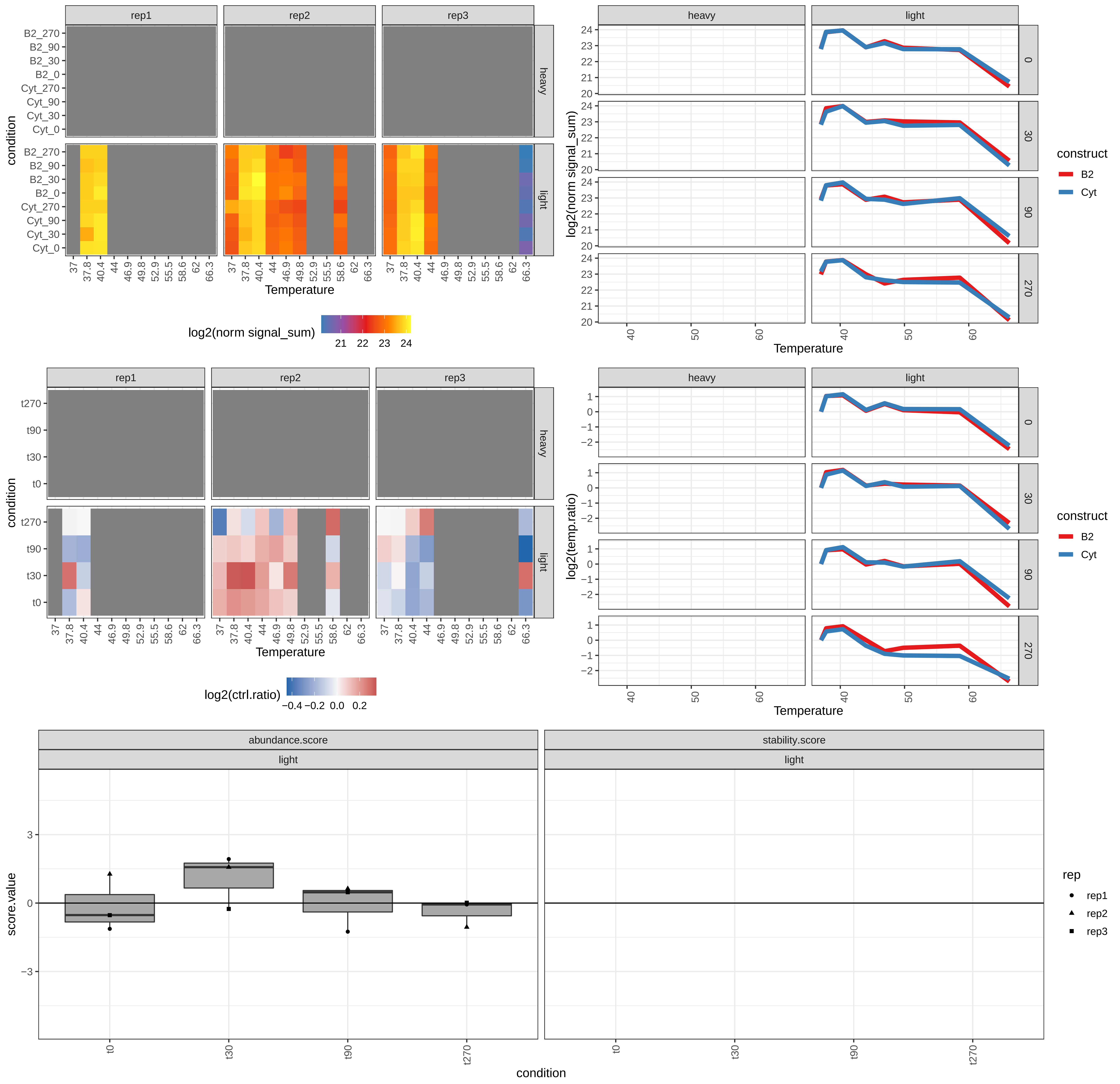

P53968 − Transcriptional regulator CRZ1 OS=Saccharomyces cerevisiae (strain ATCC 204508 / S288c) GN=CRZ1 PE=1 SV=1

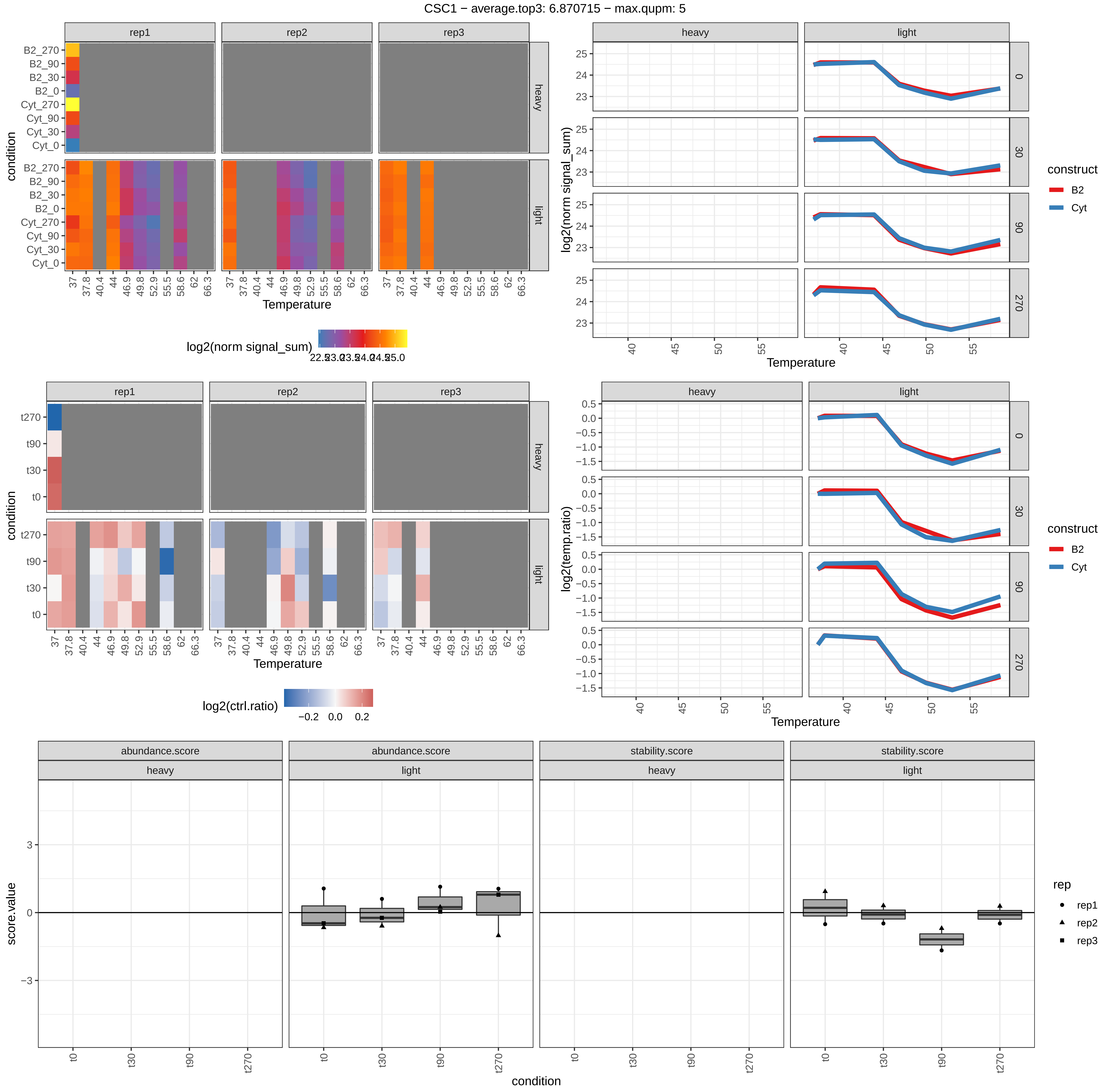

Q06538 − Calcium permeable stress−gated cation channel 1 OS=Saccharomyces cerevisiae (strain ATCC 204508 / S288c) GN=CSC1 PE=1 SV=1

CSE1 − average.top3: 6.78327967741935 − max.qupm: 11

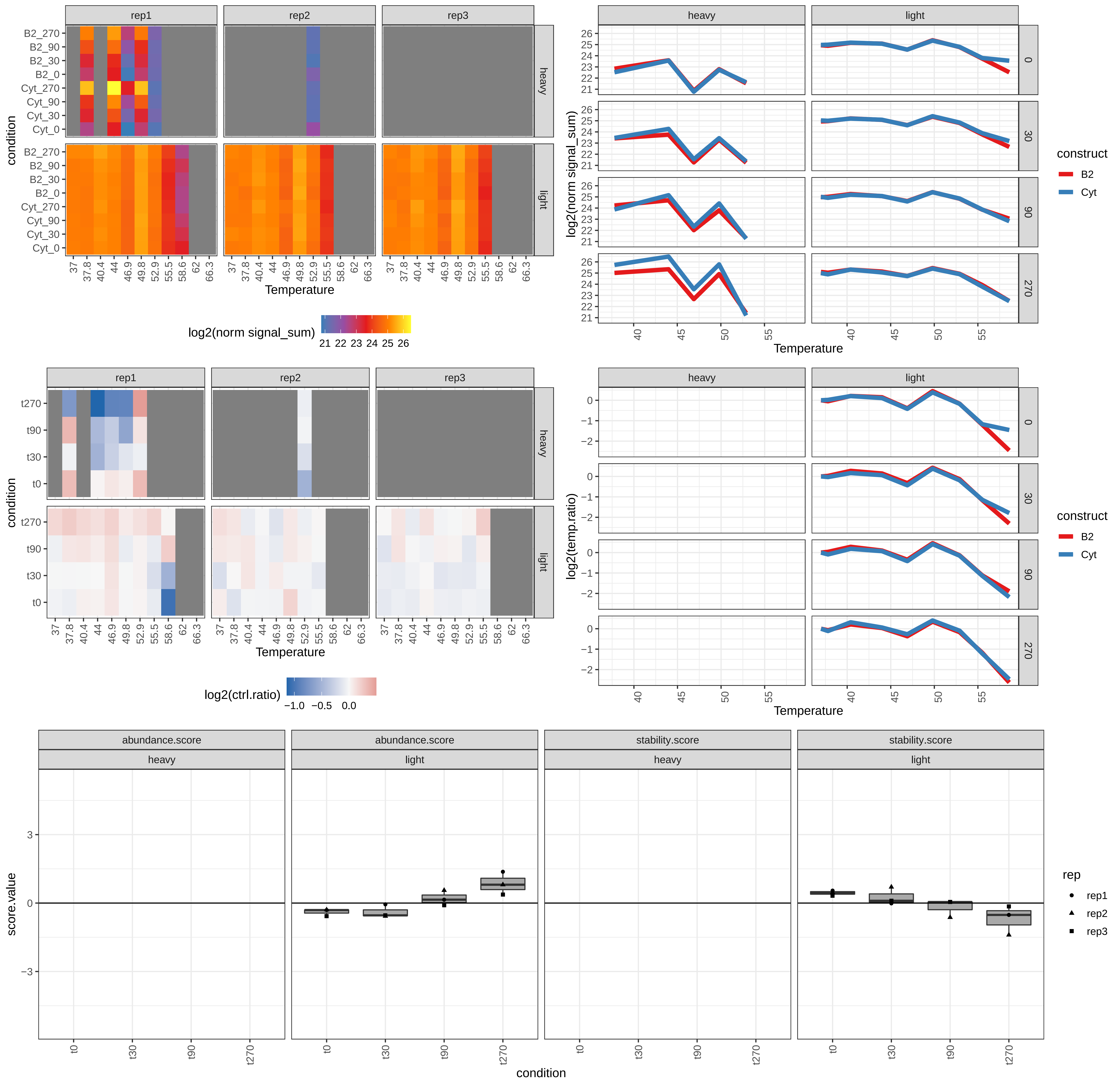

P33307 − Importin alpha re−exporter OS=Saccharomyces cerevisiae (strain ATCC 204508 / S288c) GN=CSE1 PE=1 SV=2

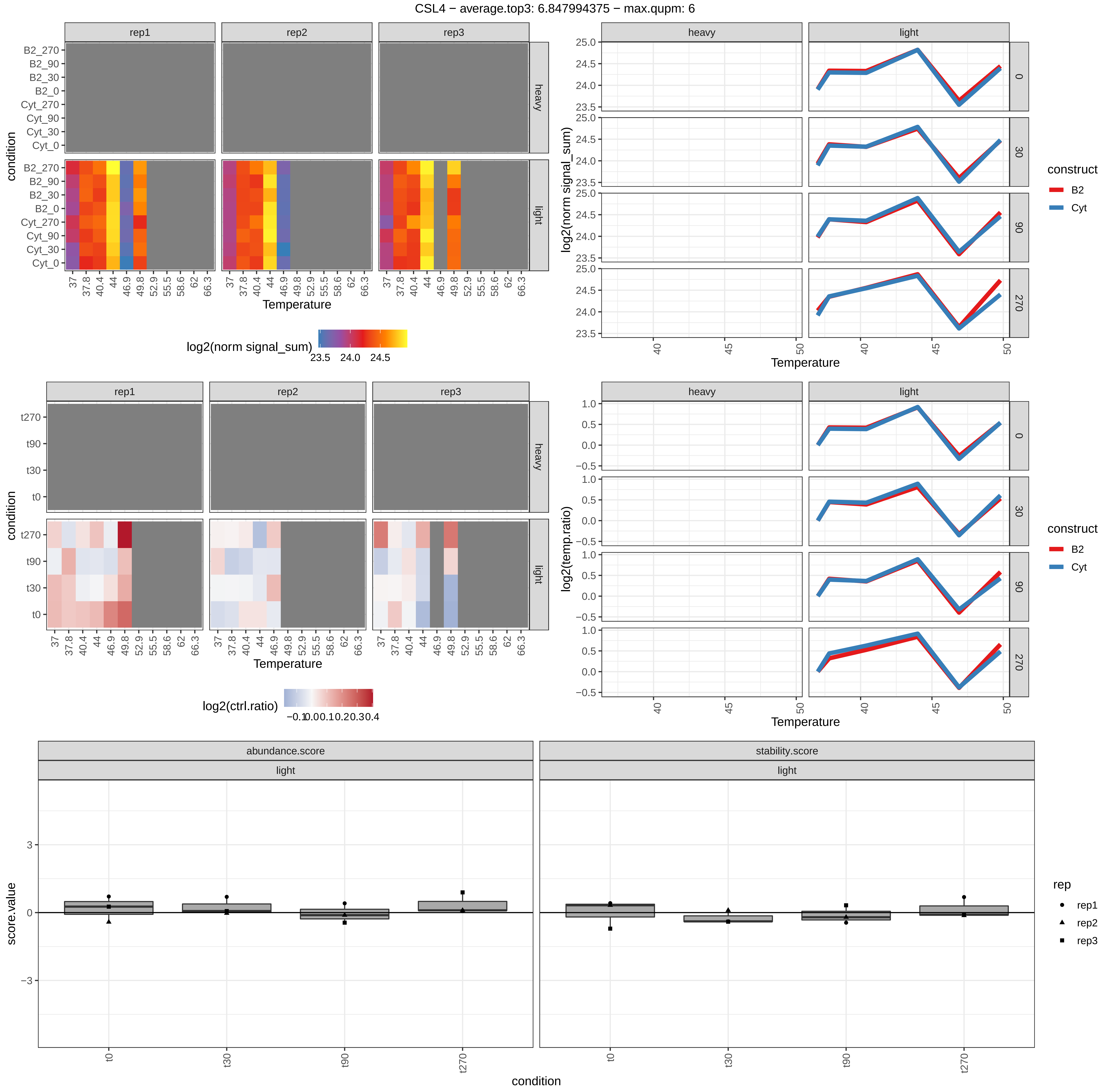

P53859 − Exosome complex component CSL4 OS=Saccharomyces cerevisiae (strain ATCC 204508 / S288c) GN=CSL4 PE=1 SV=1

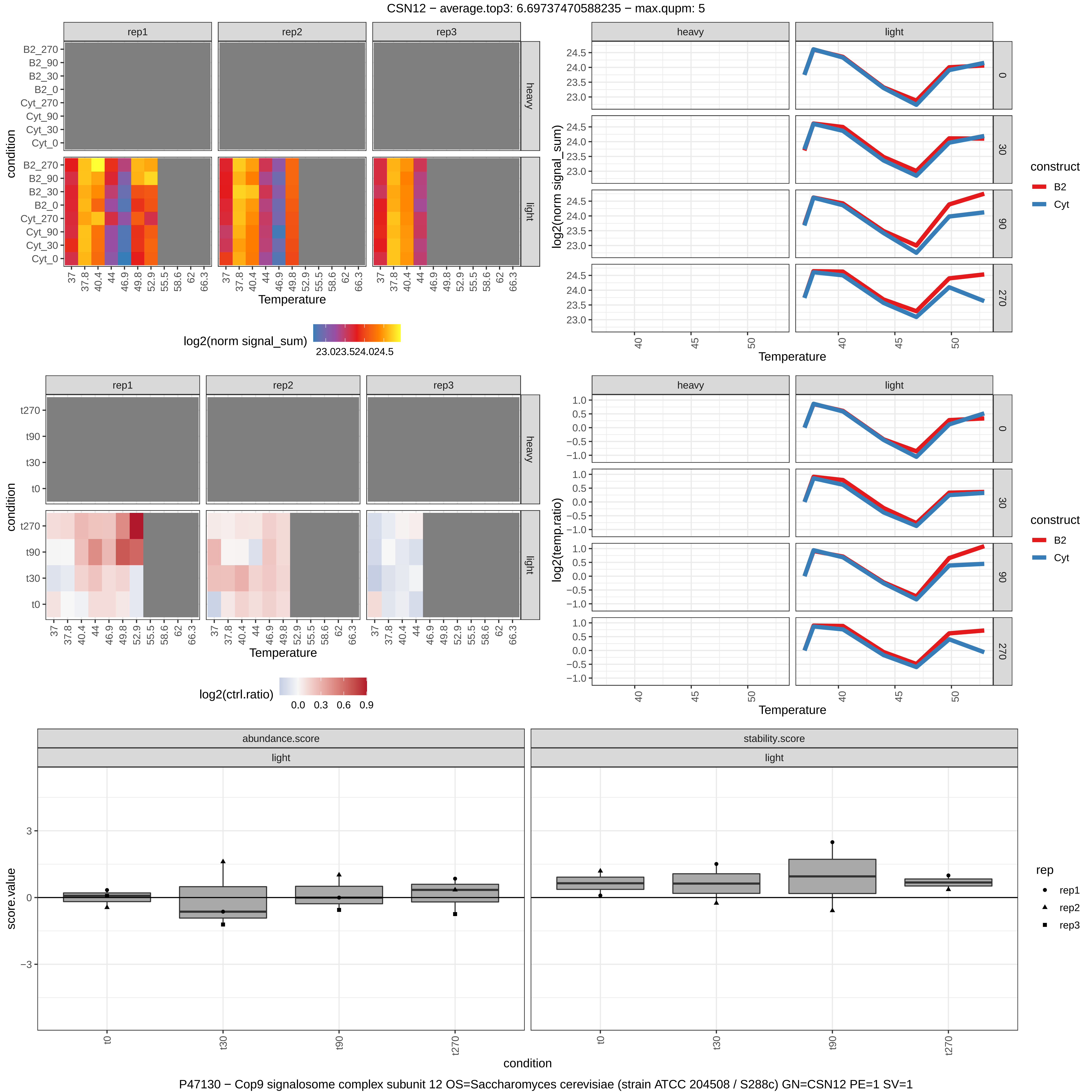
CSN12 − average.top3: 6.69737470588235 − max.qupm: 5
rep1
rep2
rep3
heavy
light
condition
Temperature
log2(norm signal_sum)
log2(ctrl.ratio)
log2(temp.ratio)
construct
B2
Cyt
abundance.score
stability.score
score.value
rep
rep1
rep2
rep3
P47130 − Cop9 signalosome complex subunit 12 OS=Saccharomyces cerevisiae (strain ATCC 204508 / S288c) GN=CSN12 PE=1 SV=1

CSR1 − average.top3: 7.087263125 − max.qupm: 10

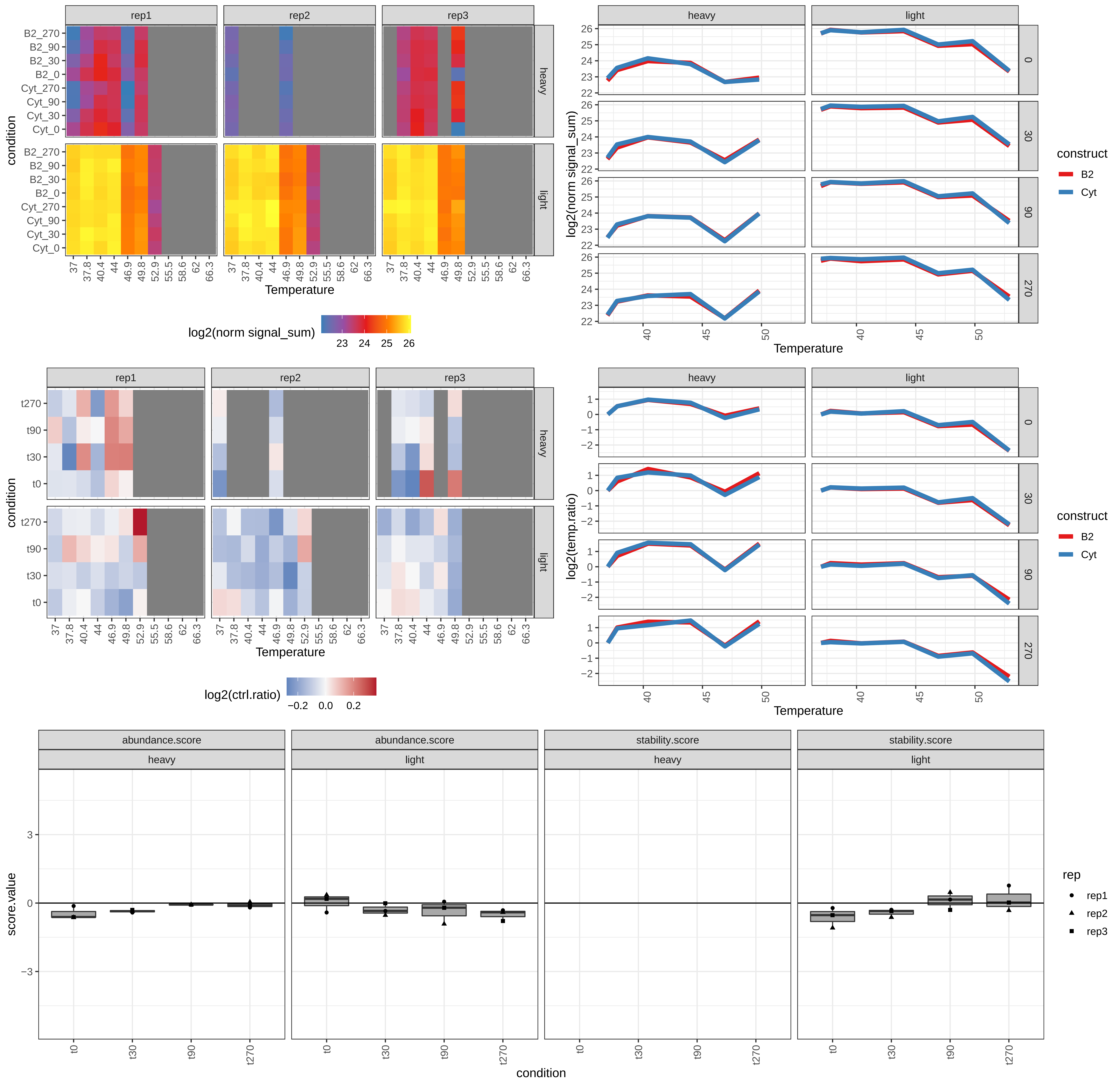

Q06705 − Phosphatidylinositol transfer protein CSR1 OS=Saccharomyces cerevisiae (strain ATCC 204508 / S288c) GN=CSR1 PE=1 SV=1

CTA1 − average.top3: 7.83311957446809 − max.qupm: 15

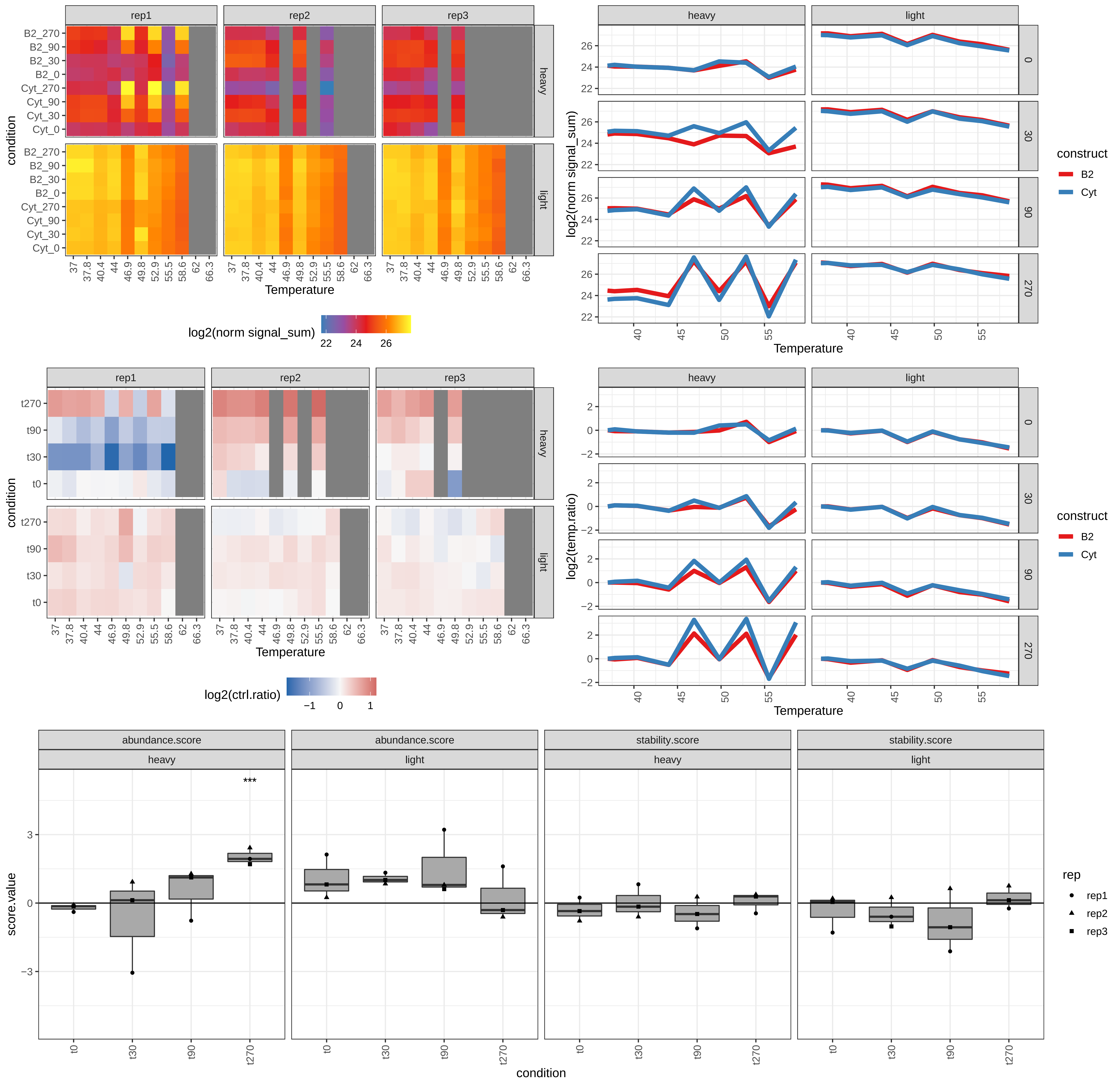

P15202 − Peroxisomal catalase A OS=Saccharomyces cerevisiae (strain ATCC 204508 / S288c) GN=CTA1 PE=1 SV=1

CTF4 − average.top3: 6.70923925 − max.qupm: 10

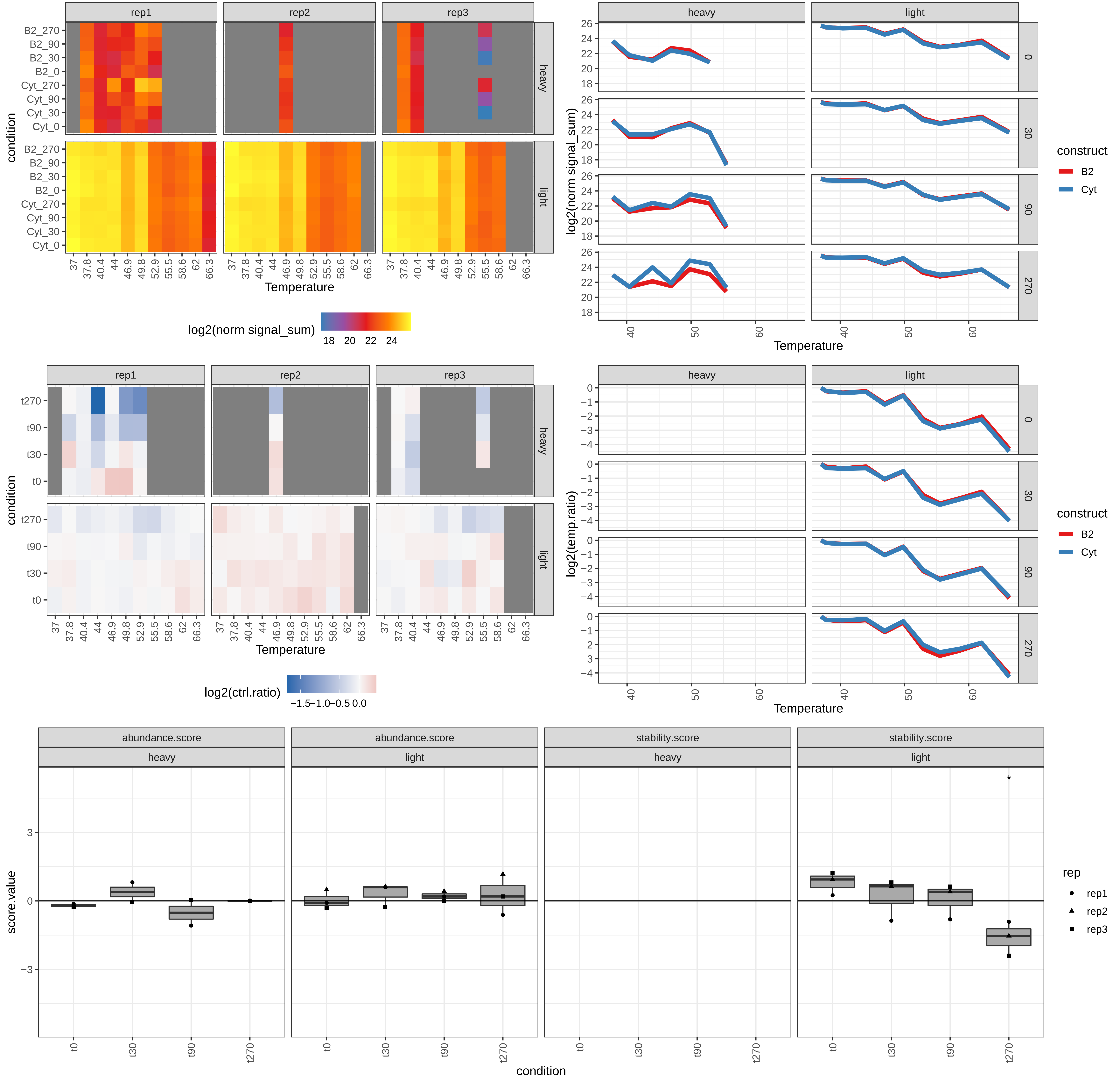

Q01454 − DNA polymerase alpha−binding protein OS=Saccharomyces cerevisiae (strain ATCC 204508 / S288c) GN=CTF4 PE=1 SV=2

CTK1 − average.top3: 6.62611384615385 − max.qupm: 6

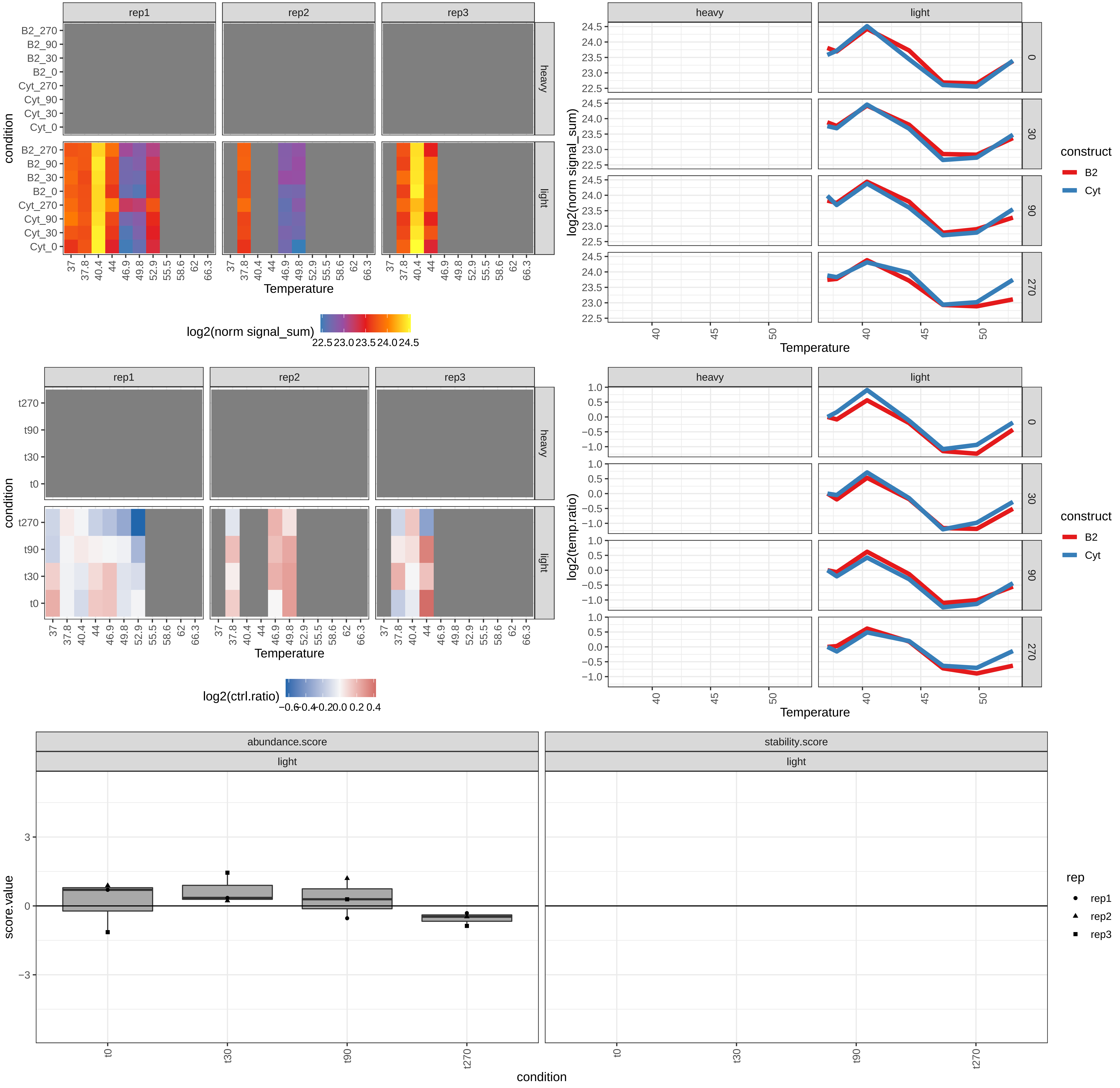

Q03957 − CTD kinase subunit alpha OS=Saccharomyces cerevisiae (strain ATCC 204508 / S288c) GN=CTK1 PE=1 SV=1

CTK2 − average.top3: 6.4700675 − max.qupm: 5

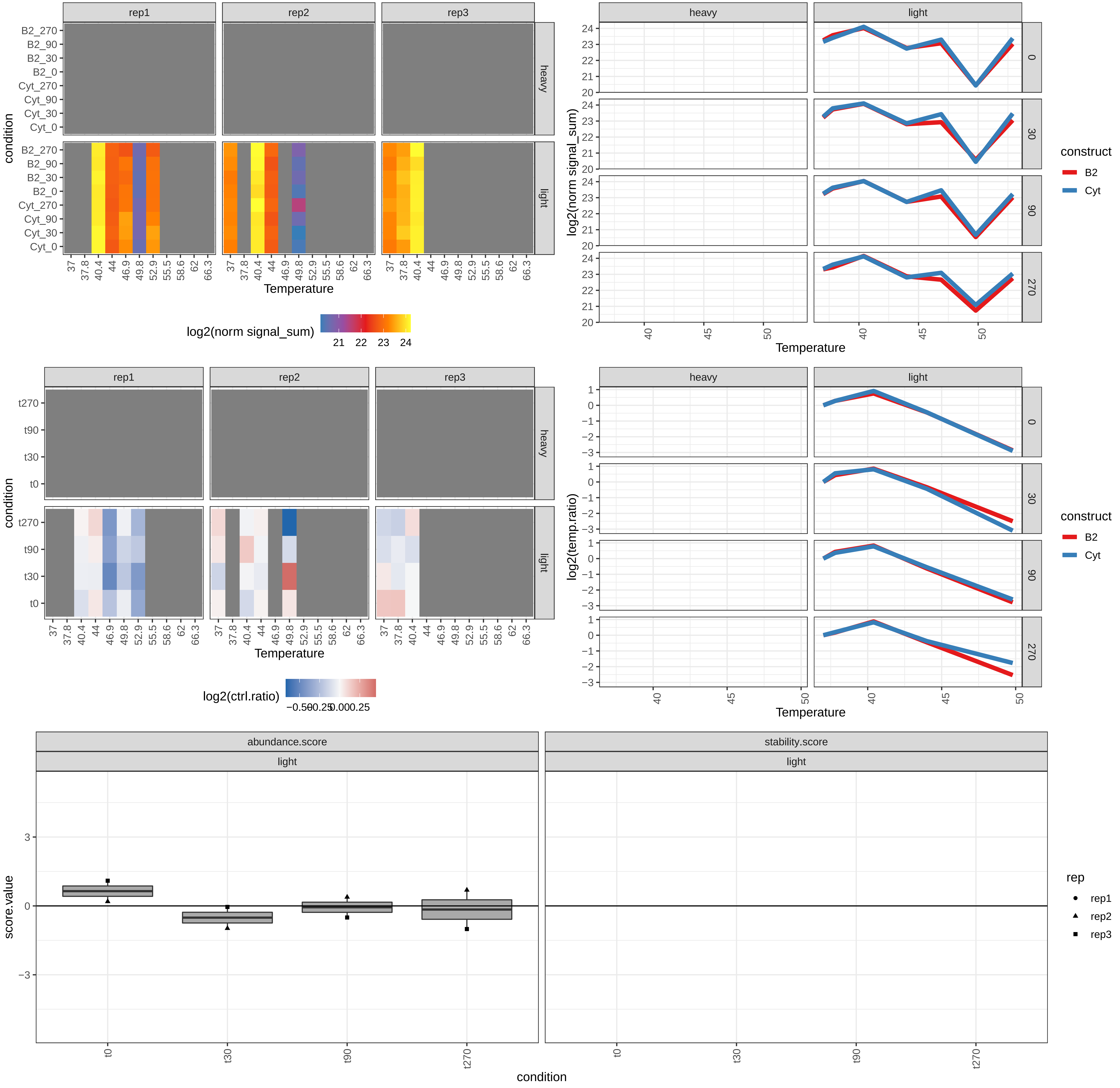

P46962 − CTD kinase subunit beta OS=Saccharomyces cerevisiae (strain ATCC 204508 / S288c) GN=CTK2 PE=1 SV=1

CTR1 − average.top3: 6.71578 − max.qupm: 3

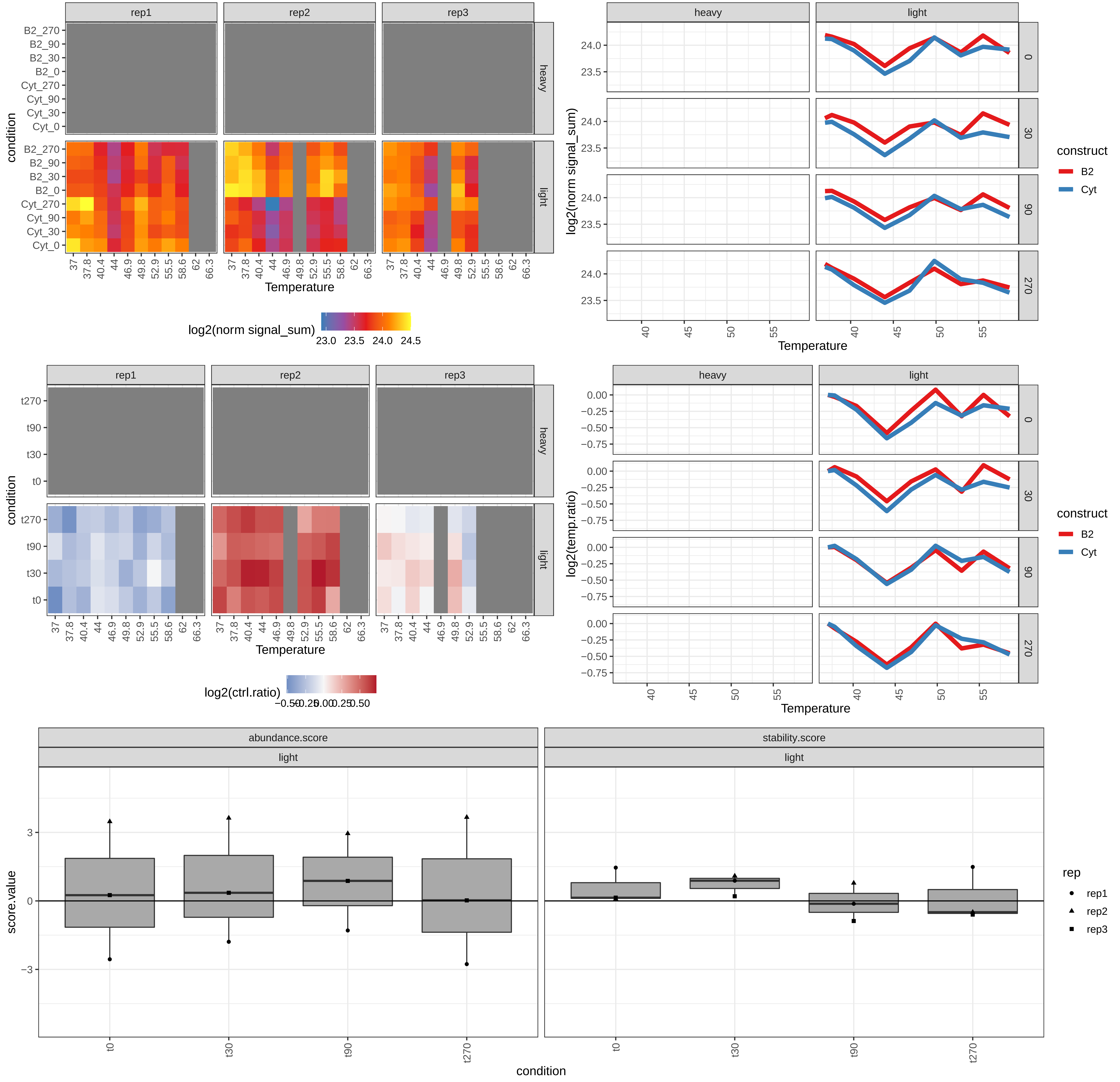

P49573 − Copper transport protein CTR1 OS=Saccharomyces cerevisiae (strain ATCC 204508 / S288c) GN=CTR1 PE=1 SV=1

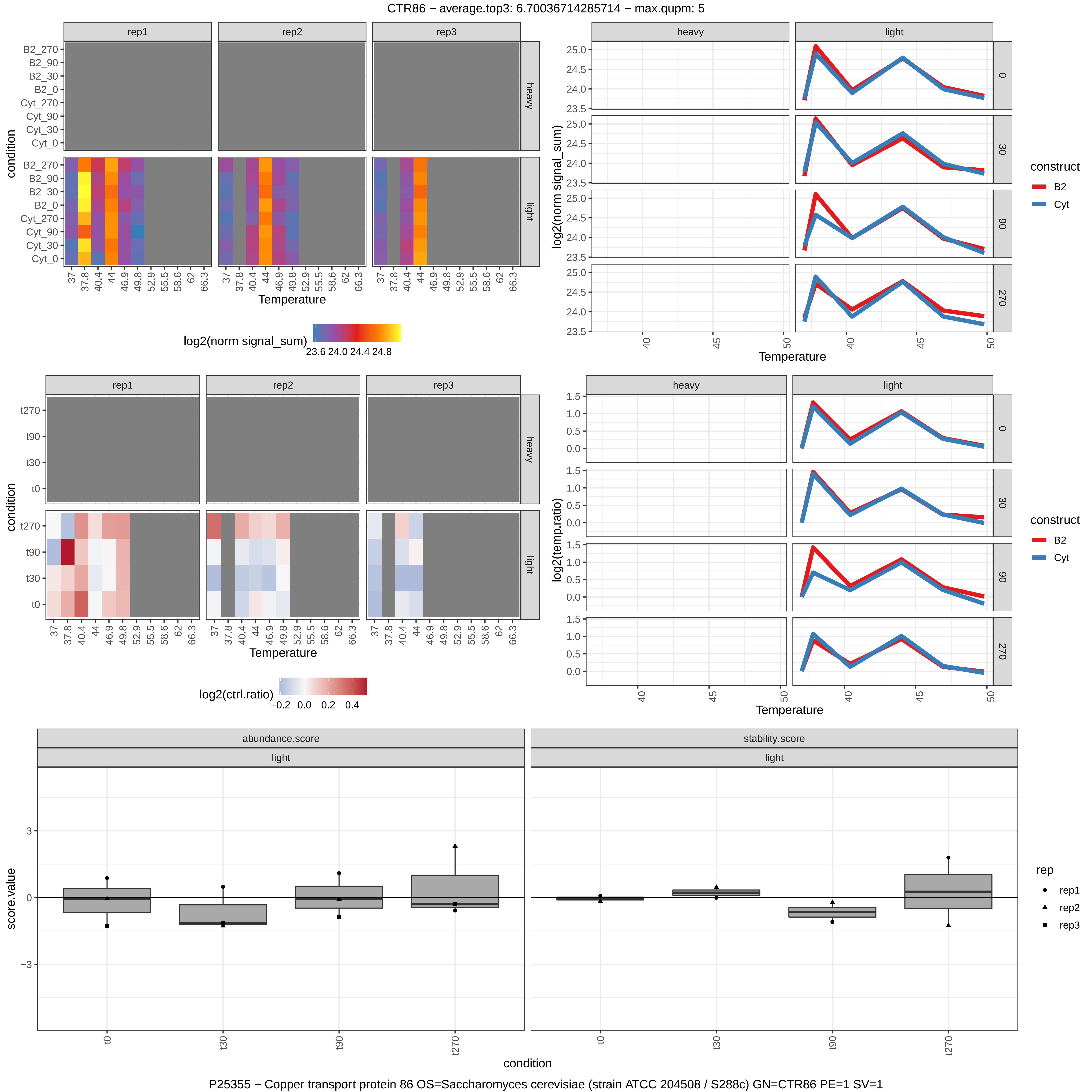
CTR86 − average.top3: 6.70036714285714 − max.qupm: 5
rep1
rep2
rep3
heavy
light
condition
Temperature
log2(norm signal_sum)
23.6 24.0 24.4 24.8
log2(norm signal_sum)
construct
B2
Cyt
log2(ctrl.ratio)
−0.2 0.0 0.2 0.4
log2(temp.ratio)
abundance.score
stability.score
score.value
rep
rep1
rep2
rep3
P25355 − Copper transport protein 86 OS=Saccharomyces cerevisiae (strain ATCC 204508 / S288c) GN=CTR86 PE=1 SV=1

CTR9 − average.top3: 6.87207355555556 − max.qupm: 27

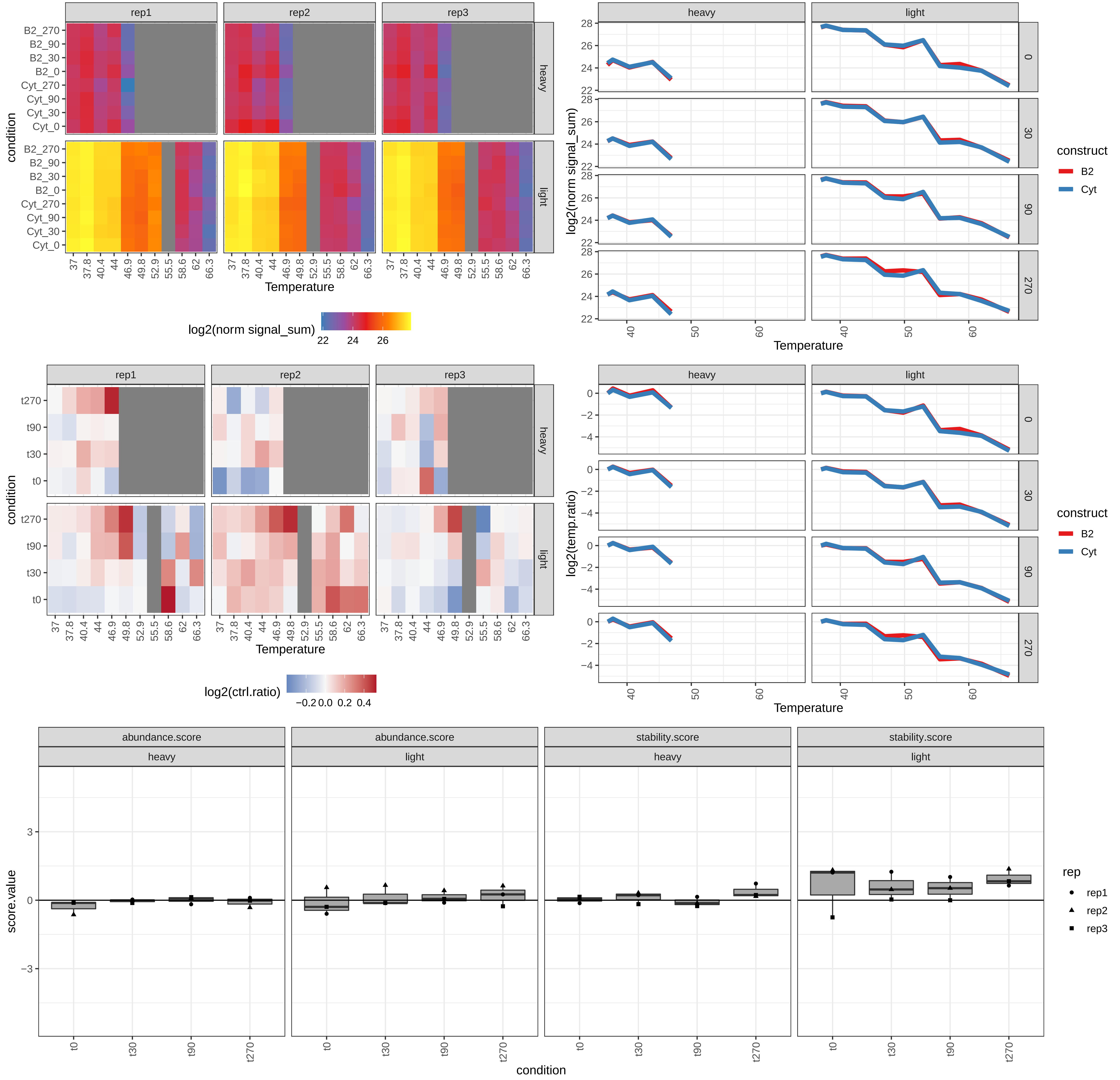

P89105 − RNA polymerase−associated protein CTR9 OS=Saccharomyces cerevisiae (strain ATCC 204508 / S288c) GN=CTR9 PE=1 SV=3

CTT1 − average.top3: 7.22379795918367 − max.qupm: 17

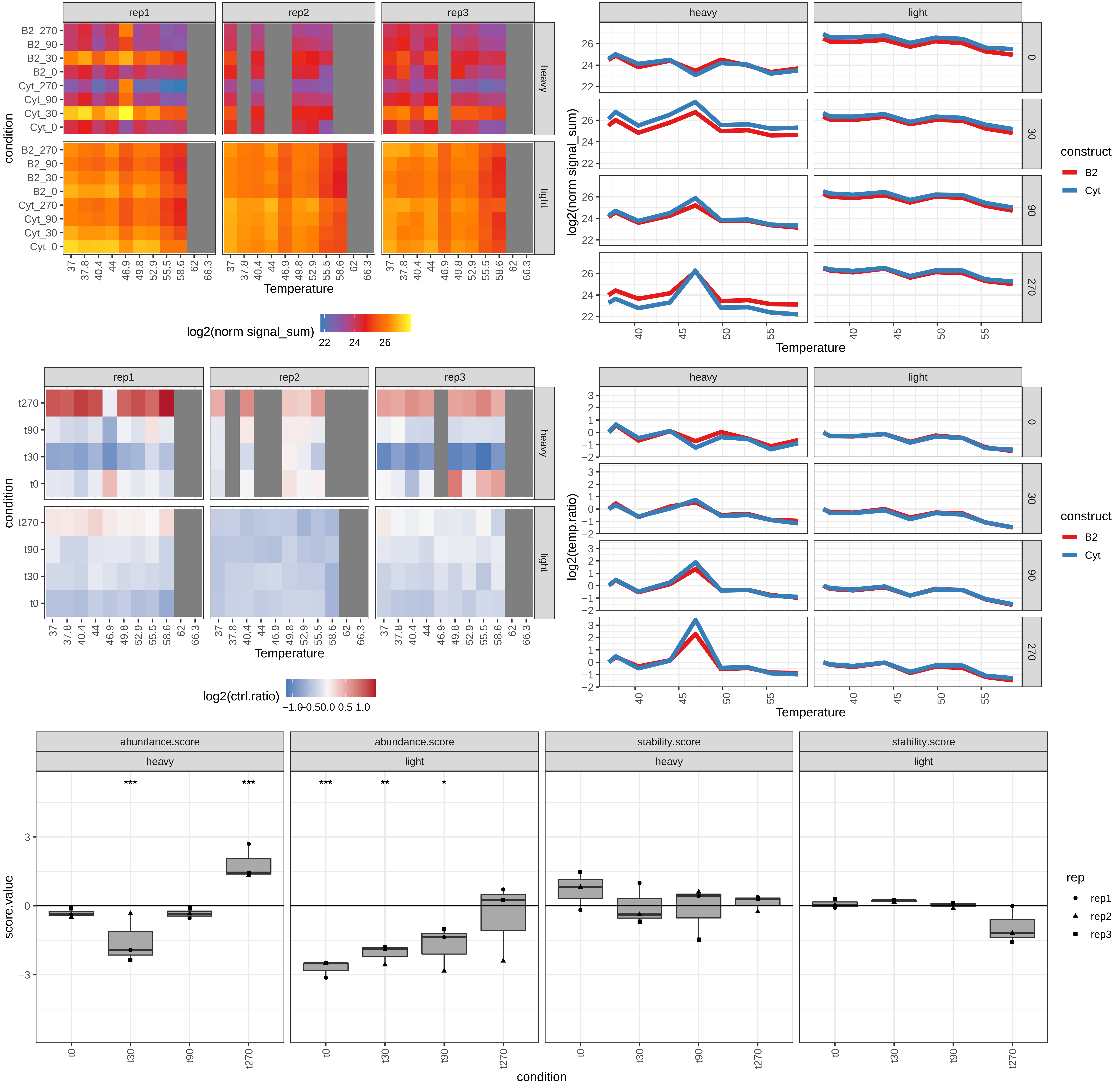

P06115 − Catalase T OS=Saccharomyces cerevisiae (strain ATCC 204508 / S288c) GN=CTT1 PE=1 SV=3

CUE3 − average.top3: 6.59697 − max.qupm: 3

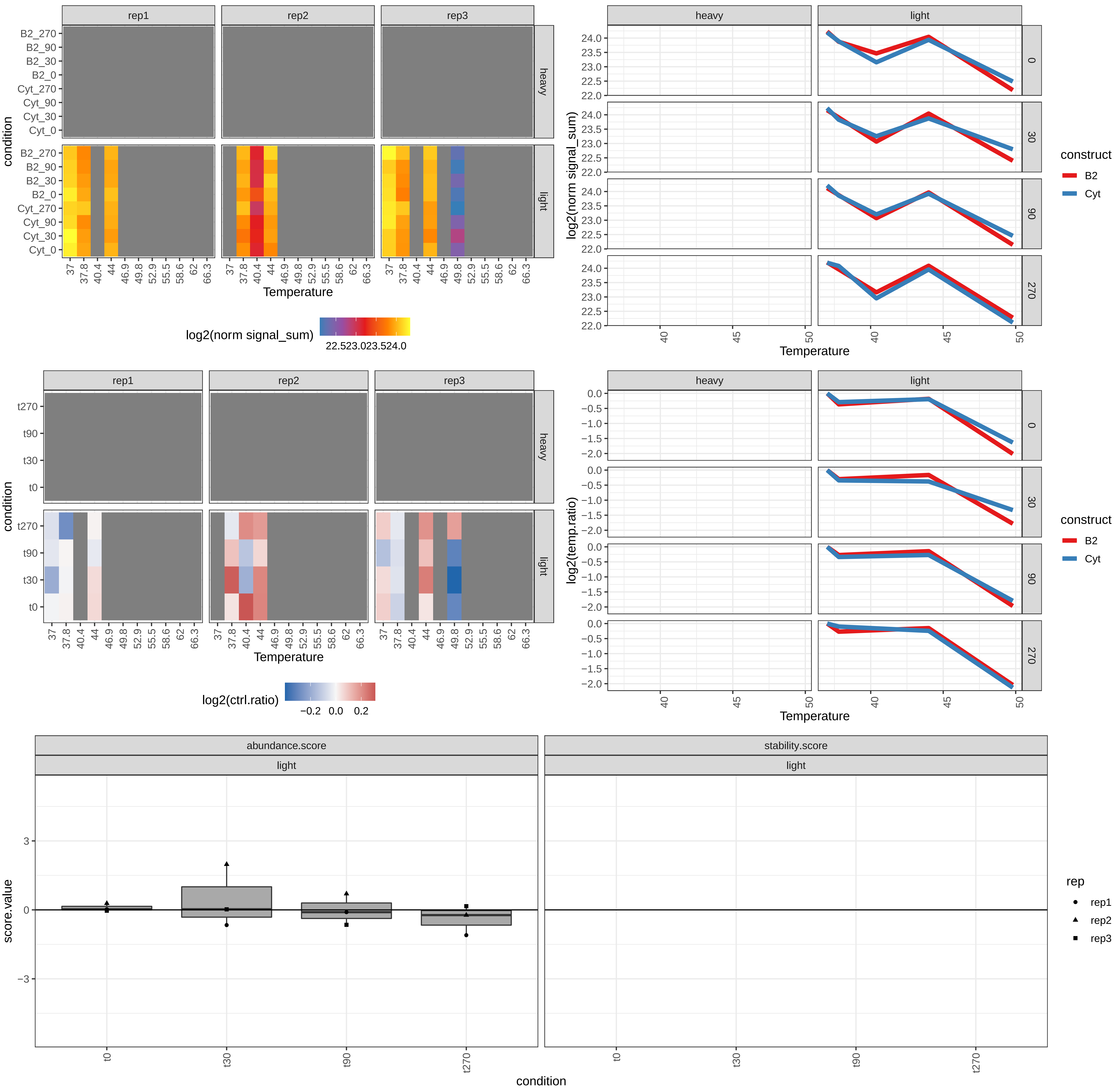

P53137 − CUE domain−containing protein 3 OS=Saccharomyces cerevisiae (strain ATCC 204508 / S288c) GN=CUE3 PE=1 SV=1

CUE5 − average.top3: 7.0336186 − max.qupm: 10

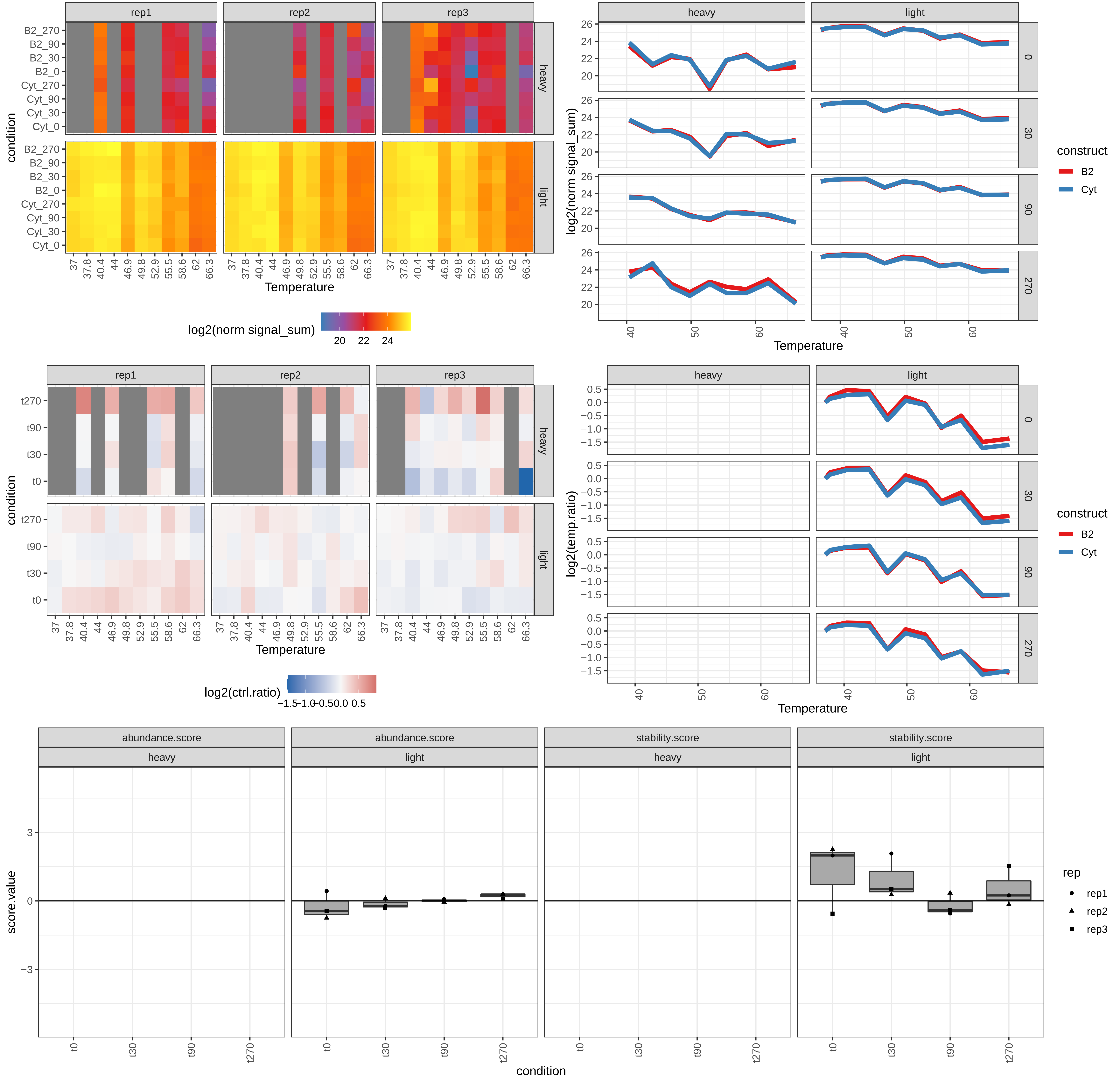

Q08412 − Ubiquitin−binding protein CUE5 OS=Saccharomyces cerevisiae (strain ATCC 204508 / S288c) GN=CUE5 PE=1 SV=1

CUP1 − average.top3: 6.93459681818182 − max.qupm: 4

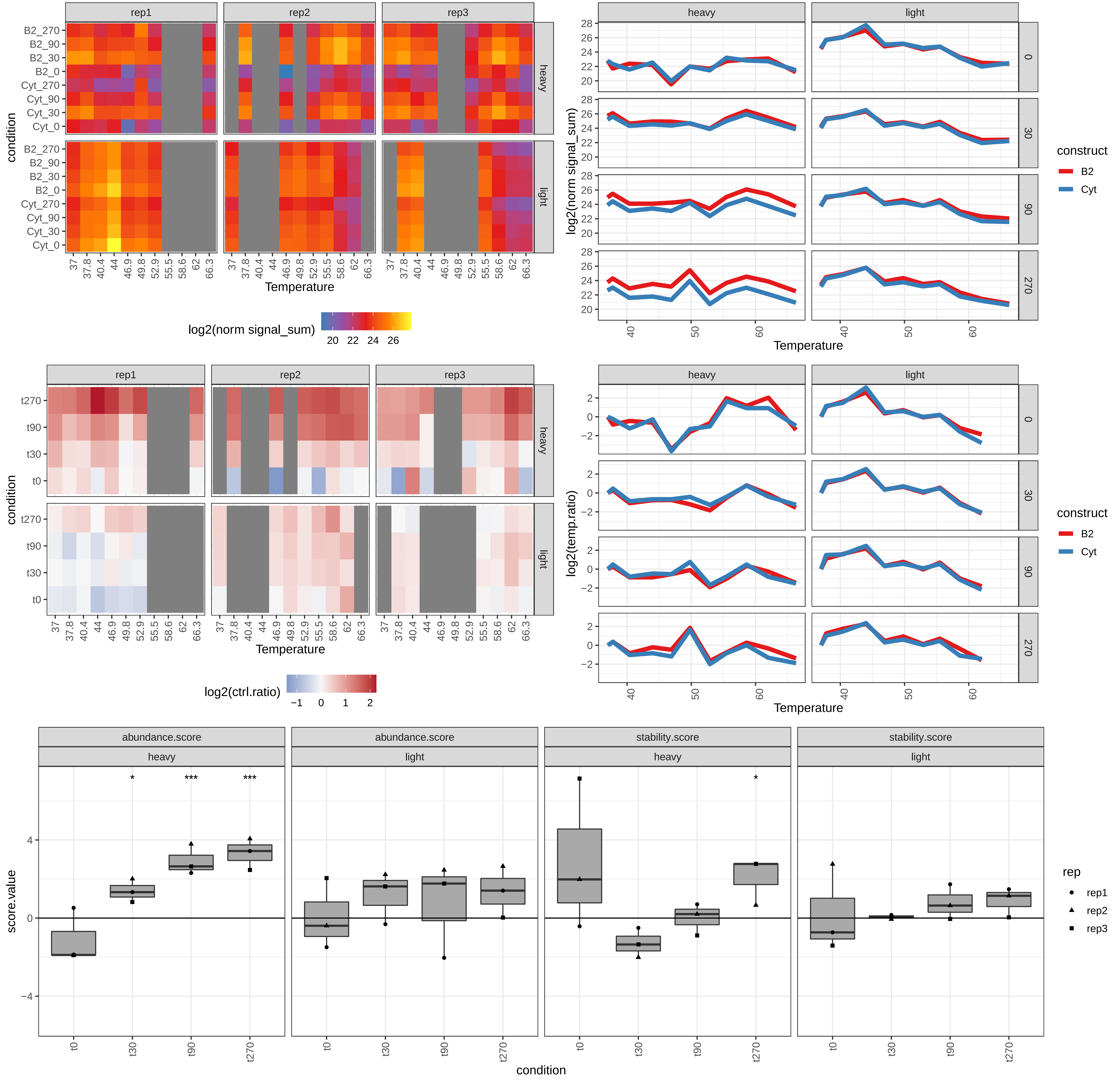

er metallothionein 1−1 OS=Saccharomyces cerevisiae (strain ATCC 204508 / S288c) GN=CUP1−1 PE=1 SV=1|Copper metallothionein 1−2 OS=Saccharomyces cerevisiae (strain ATCC 204508 / S288c) G

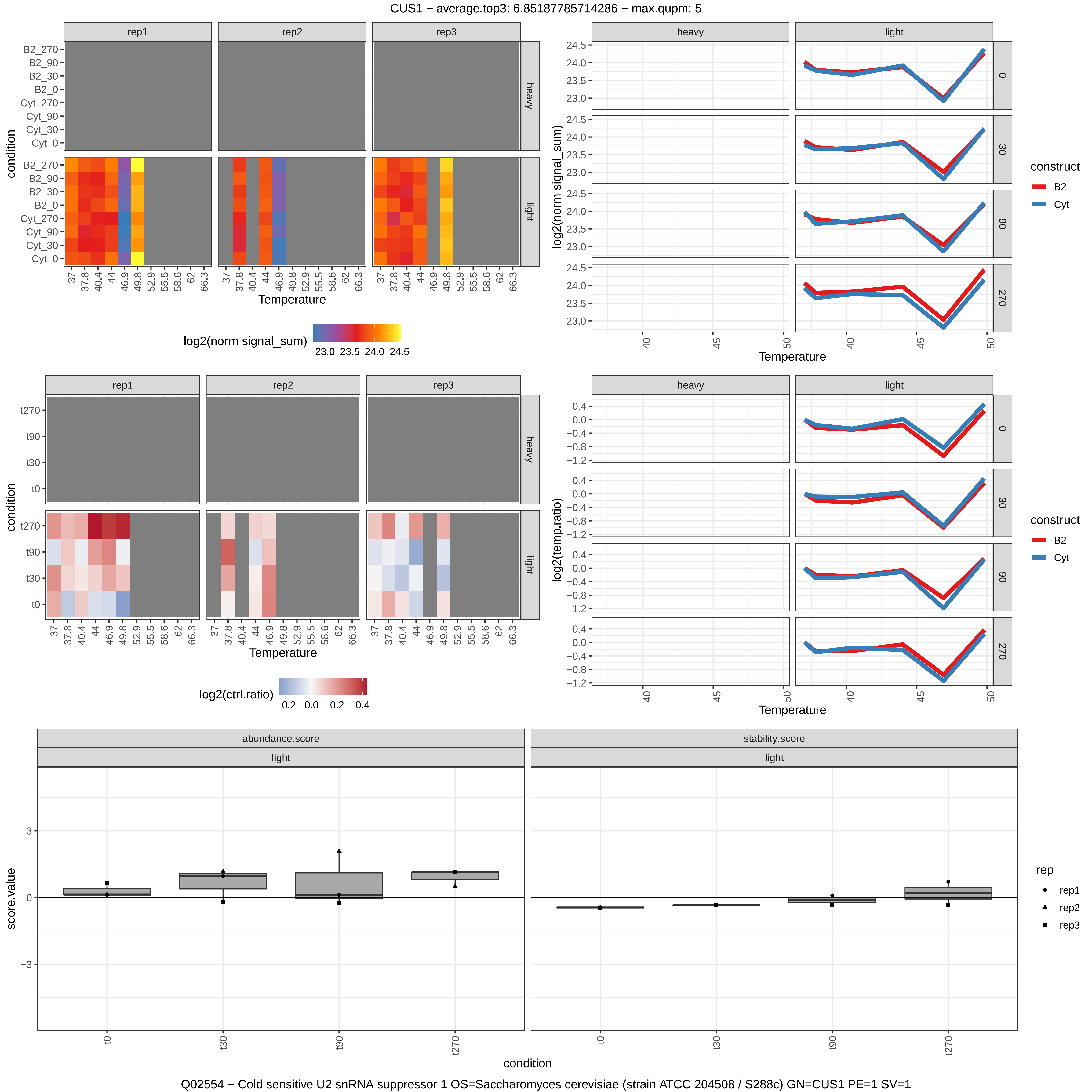

CUS1 − average.top3: 6.85187785714286 − max.qupm: 5
rep1
rep2
rep3
heavy
light
condition
Temperature
log2(norm signal_sum)
construct
B2
Cyt
log2(temp.ratio)
log2(ctrl.ratio)
abundance.score
stability.score
score.value
rep
rep1
rep2
rep3
Q02554 − Cold sensitive U2 snRNA suppressor 1 OS=Saccharomyces cerevisiae (strain ATCC 204508 / S288c) GN=CUS1 PE=1 SV=1

CUZ1 − average.top3: 6.43107272727273 − max.qupm: 3

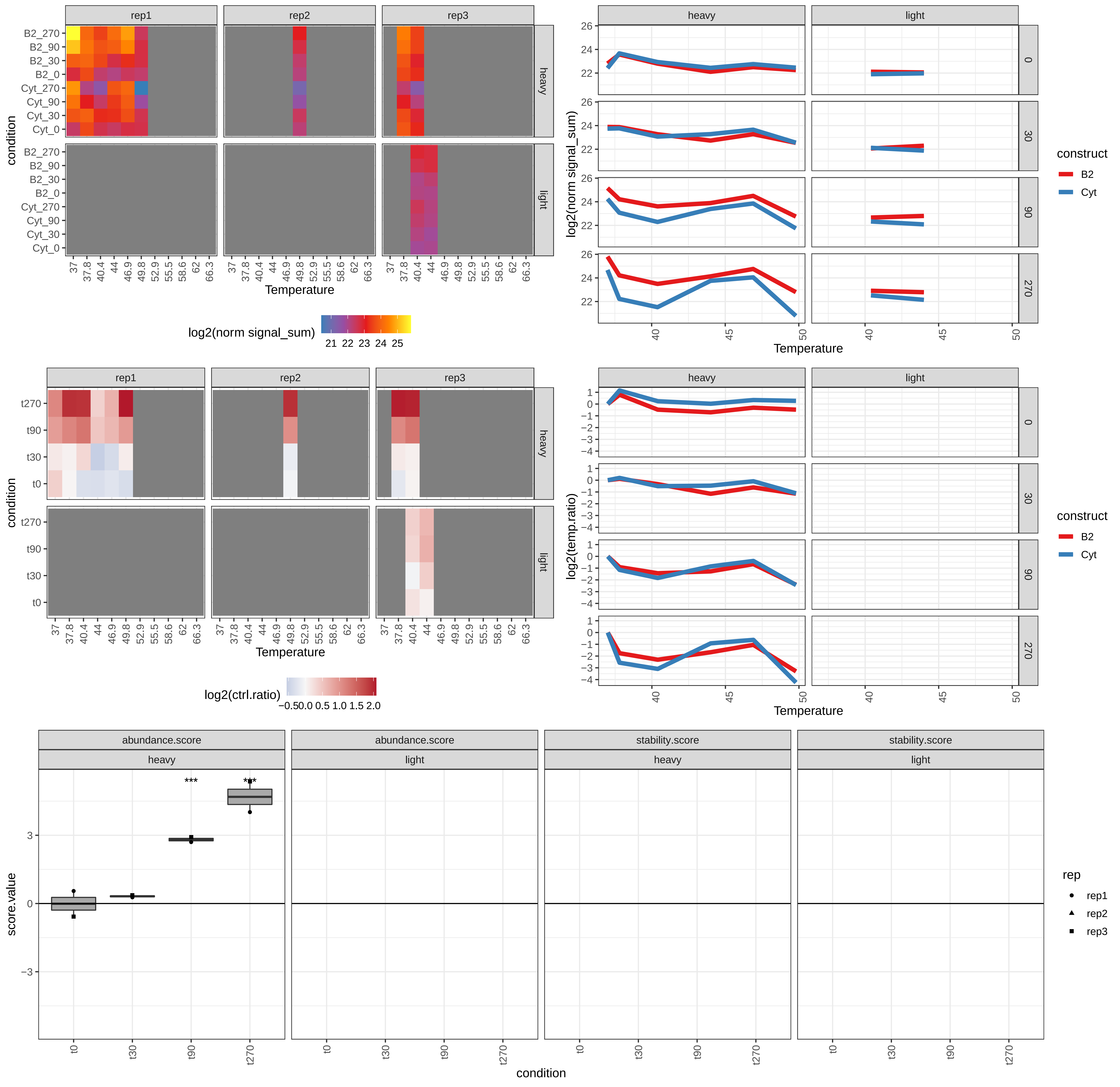

P53899 − CDC48−associated ubiquitin−like/zinc finger protein 1 OS=Saccharomyces cerevisiae (strain ATCC 204508 / S288c) GN=CUZ1 PE=1 SV=1

CWC22 − average.top3: 6.38806928571429 − max.qupm: 7

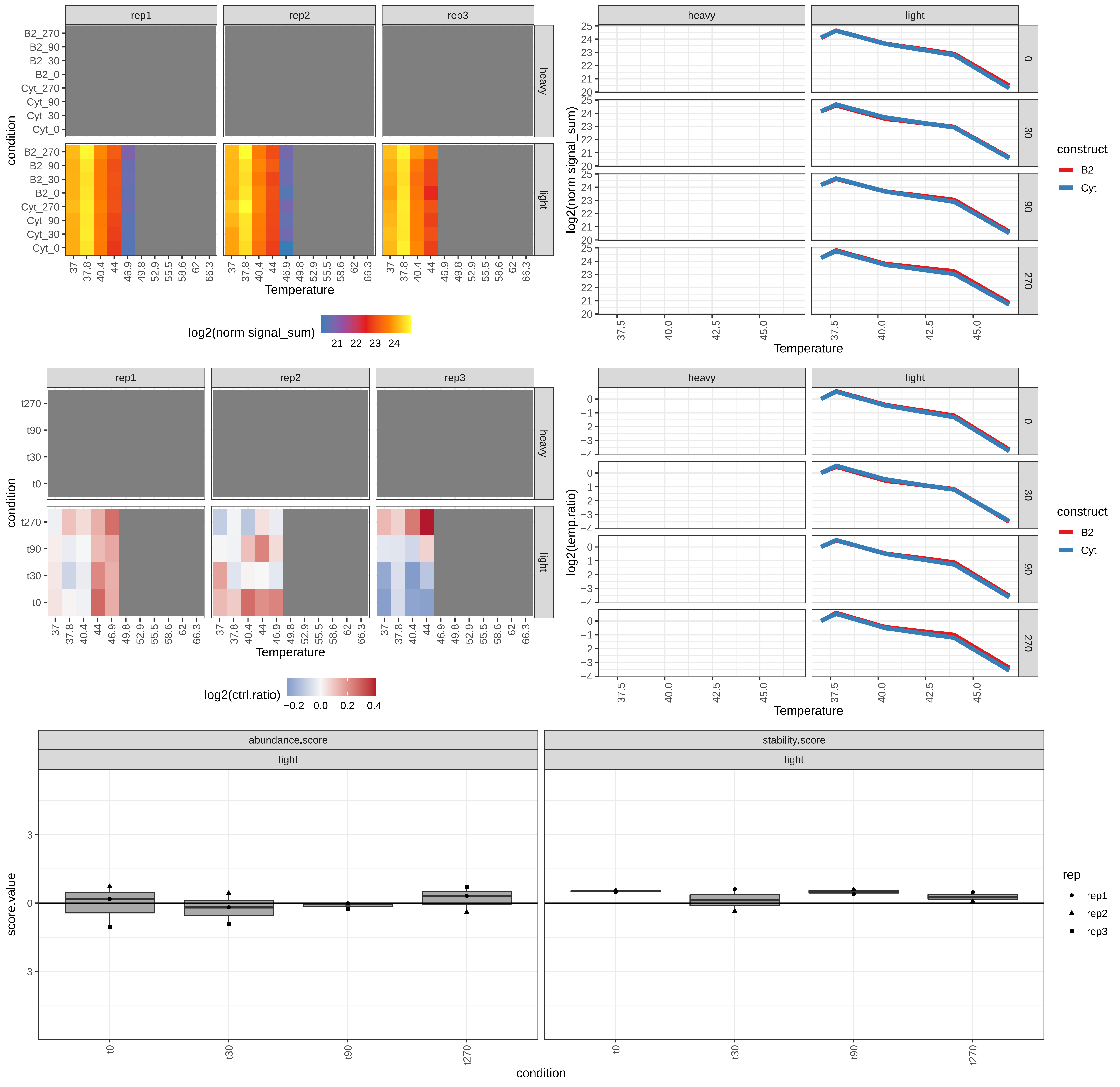

P53333 − Pre−mRNA−splicing factor CWC22 OS=Saccharomyces cerevisiae (strain ATCC 204508 / S288c) GN=CWC22 PE=1 SV=1

CWH41 − average.top3: 7.12485666666667 − max.qupm: 22

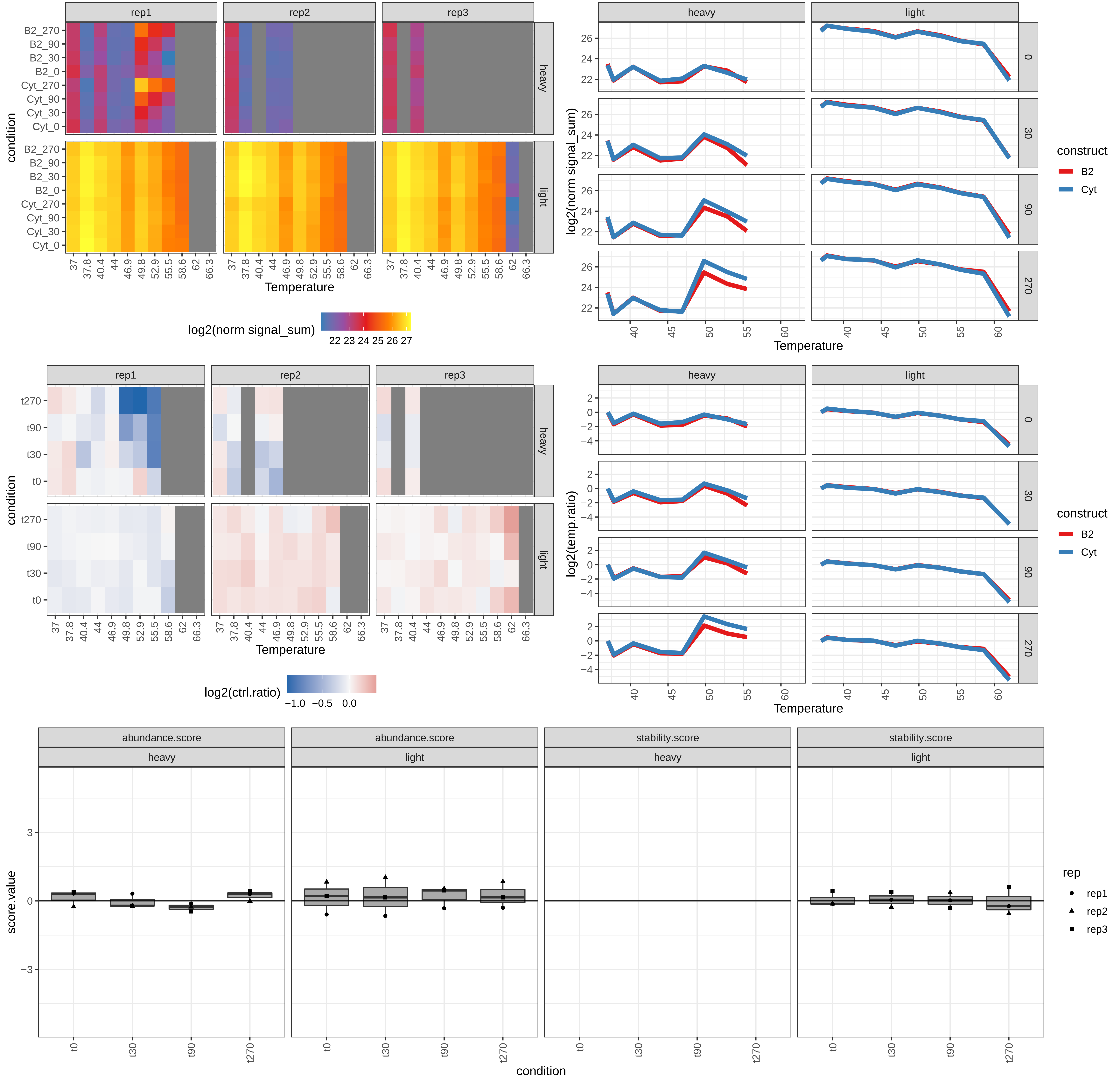

P53008 − Mannosyl−oligosaccharide glucosidase OS=Saccharomyces cerevisiae (strain ATCC 204508 / S288c) GN=CWH41 PE=1 SV=1

CWH43 − average.top3: 7.1575225 − max.qupm: 9

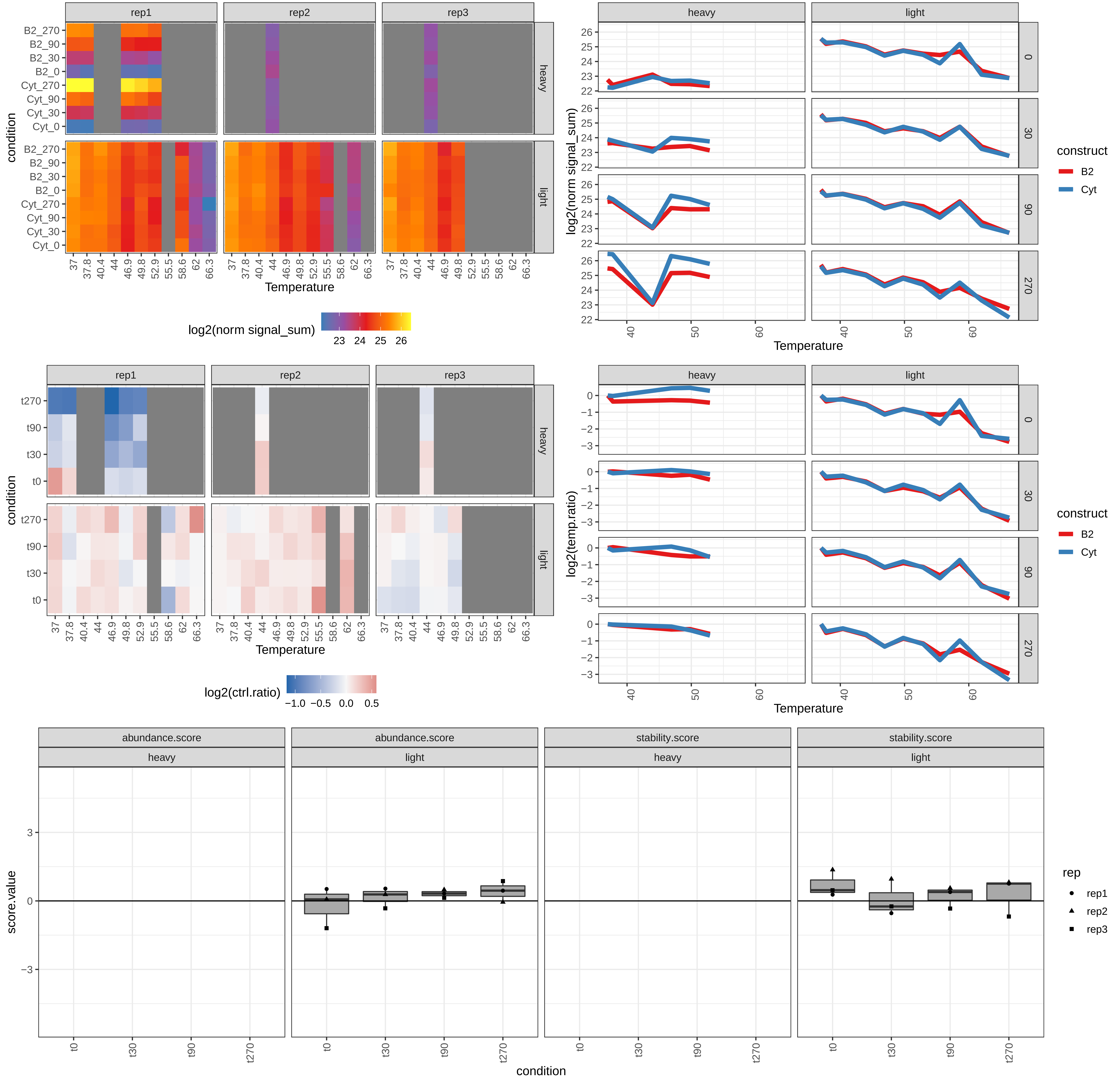

P25618 − Protein CWH43 OS=Saccharomyces cerevisiae (strain ATCC 204508 / S288c) GN=CWH43 PE=1 SV=2

CWP1 − average.top3: 7.69485742424242 − max.qupm: 10

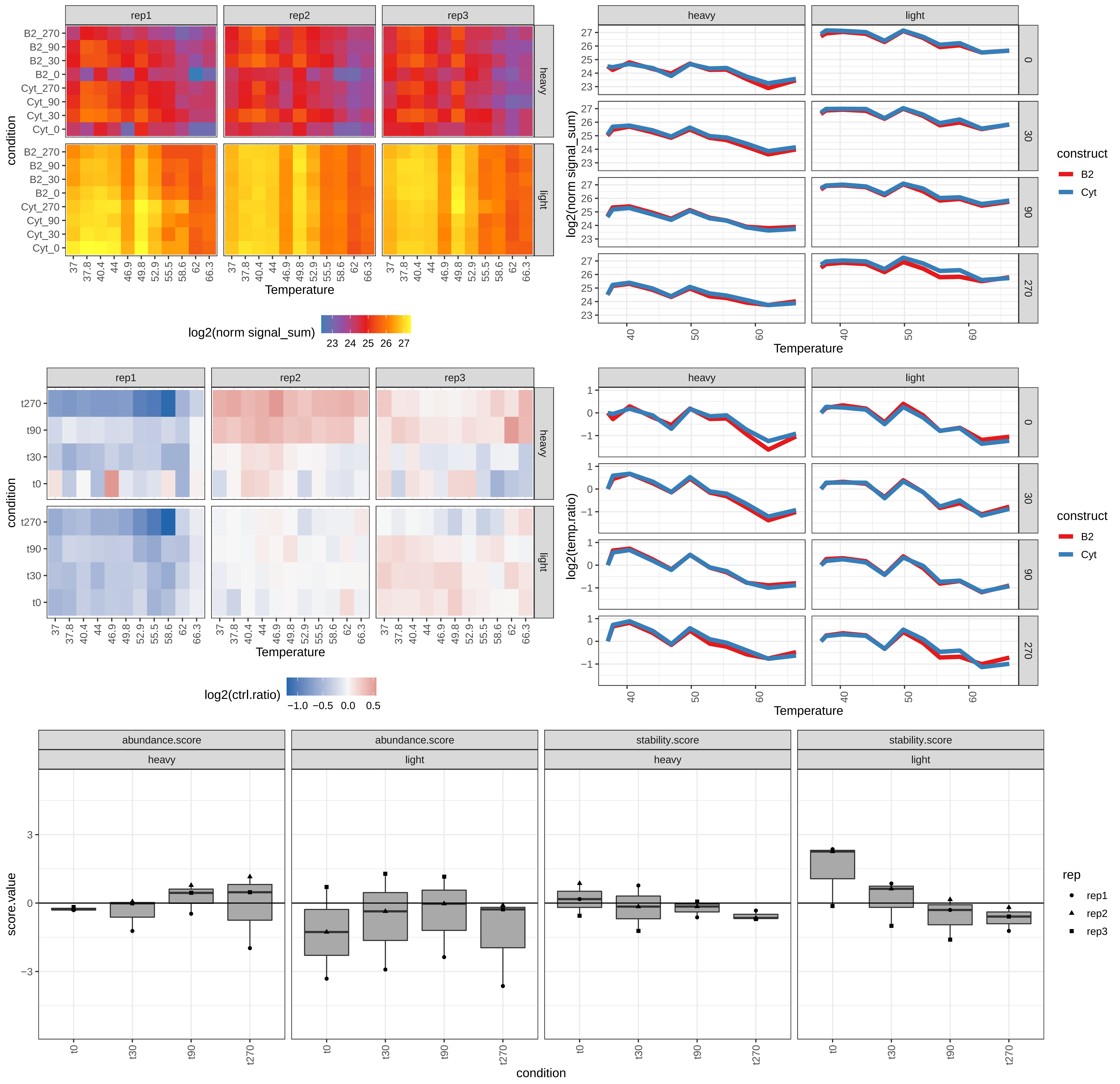

P28319 − Cell wall protein CWP1 OS=Saccharomyces cerevisiae (strain ATCC 204508 / S288c) GN=CWP1 PE=1 SV=2

CYB2 − average.top3: 8.06147921875 − max.qupm: 32

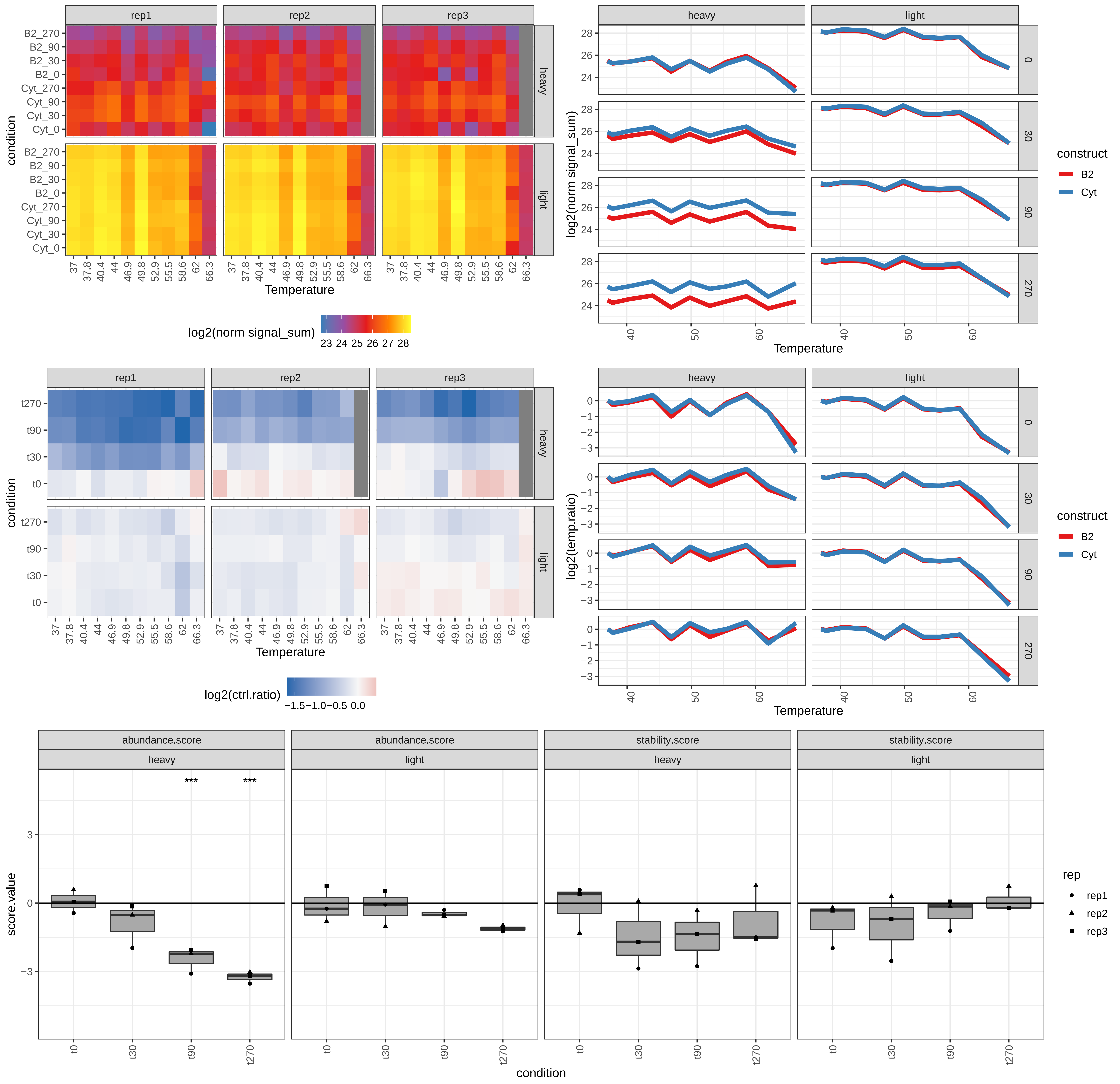

P00175 − Cytochrome b2, mitochondrial OS=Saccharomyces cerevisiae (strain ATCC 204508 / S288c) GN=CYB2 PE=1 SV=1

CYC1 − average.top3: 7.67951353846154 − max.qupm: 9

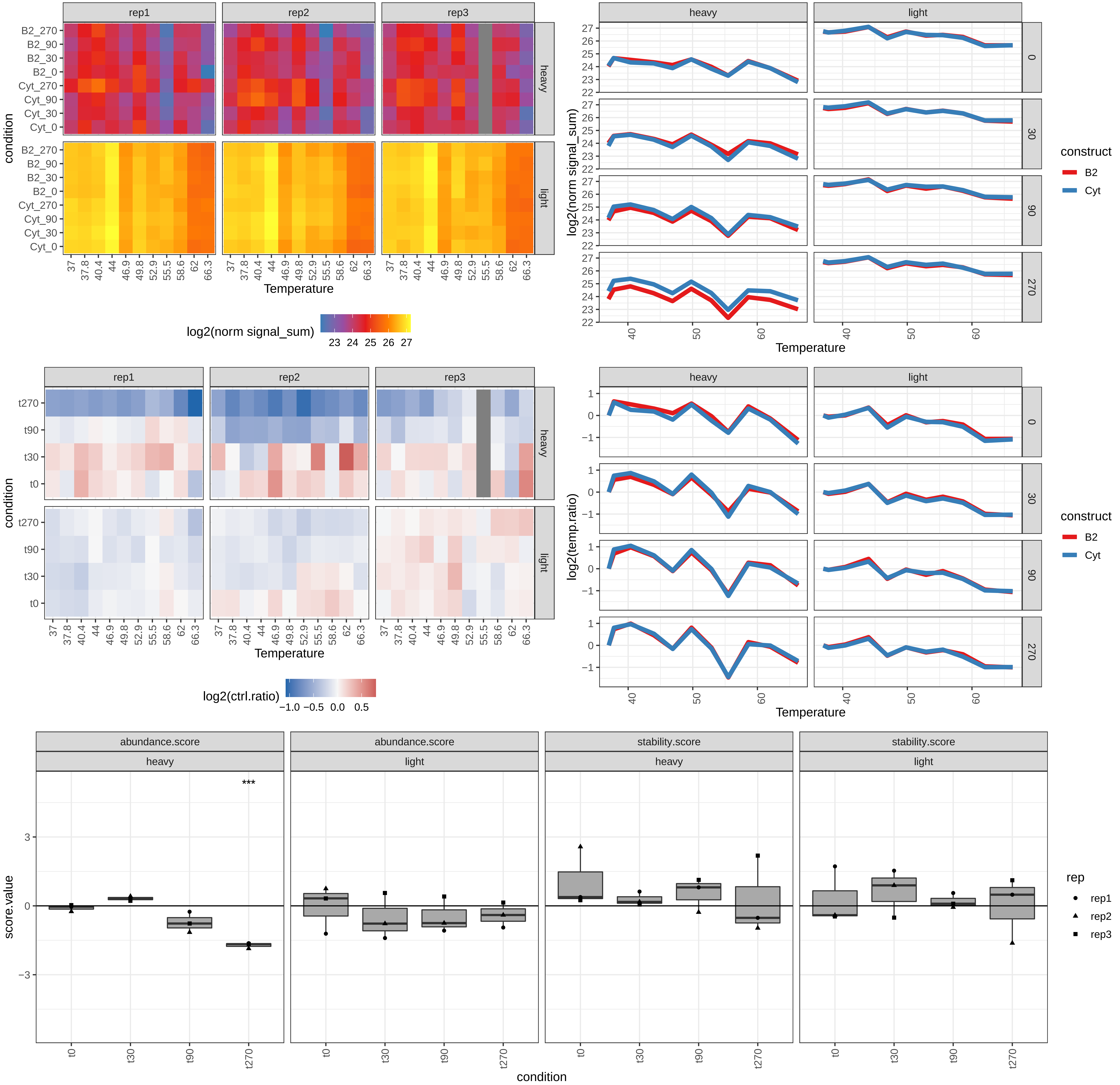

P00044 − Cytochrome c iso−1 OS=Saccharomyces cerevisiae (strain ATCC 204508 / S288c) GN=CYC1 PE=1 SV=2

CYC3 − average.top3: 7.16911307692308 − max.qupm: 4

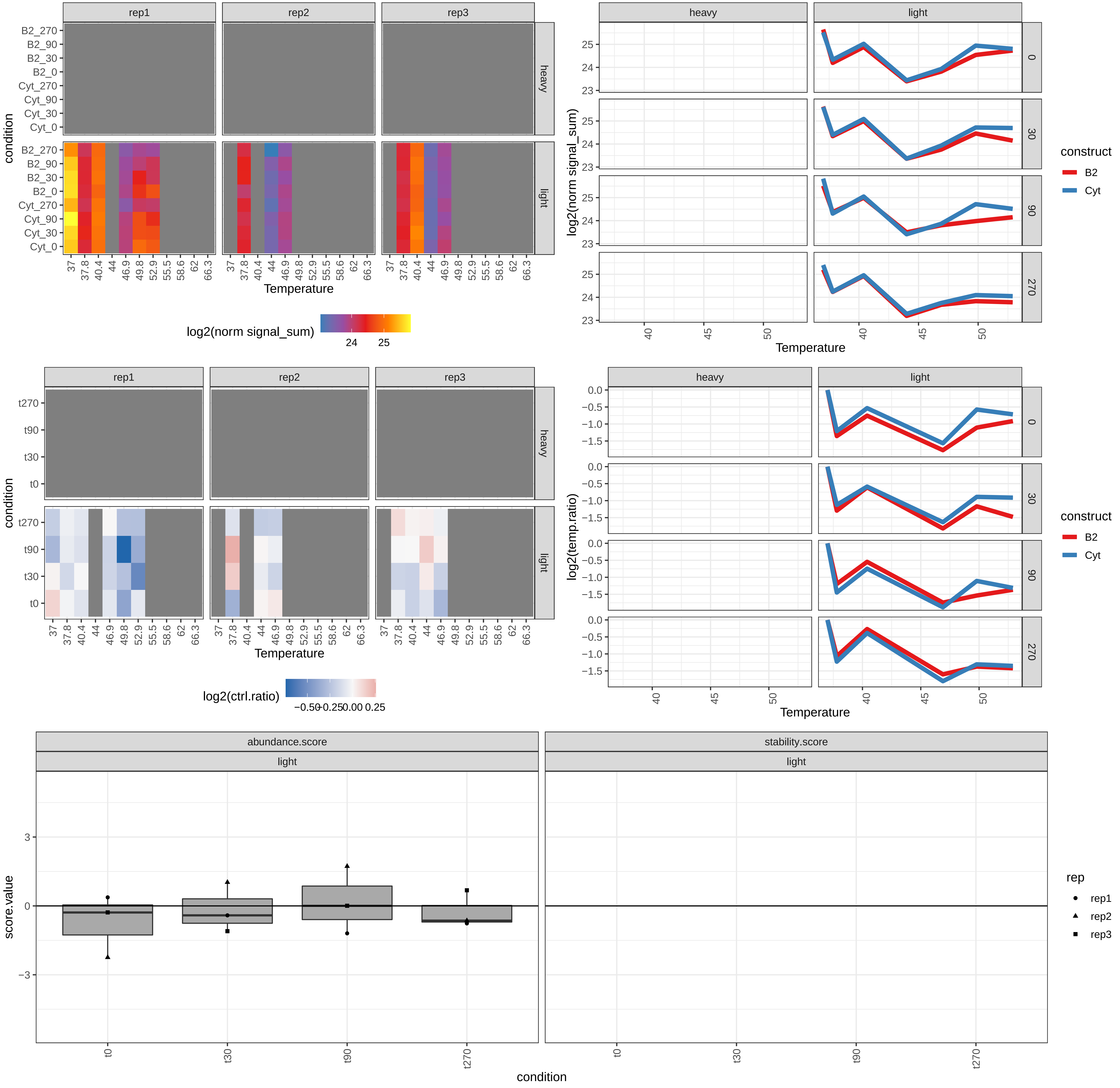

P06182 − Cytochrome c heme lyase OS=Saccharomyces cerevisiae (strain ATCC 204508 / S288c) GN=CYC3 PE=1 SV=1

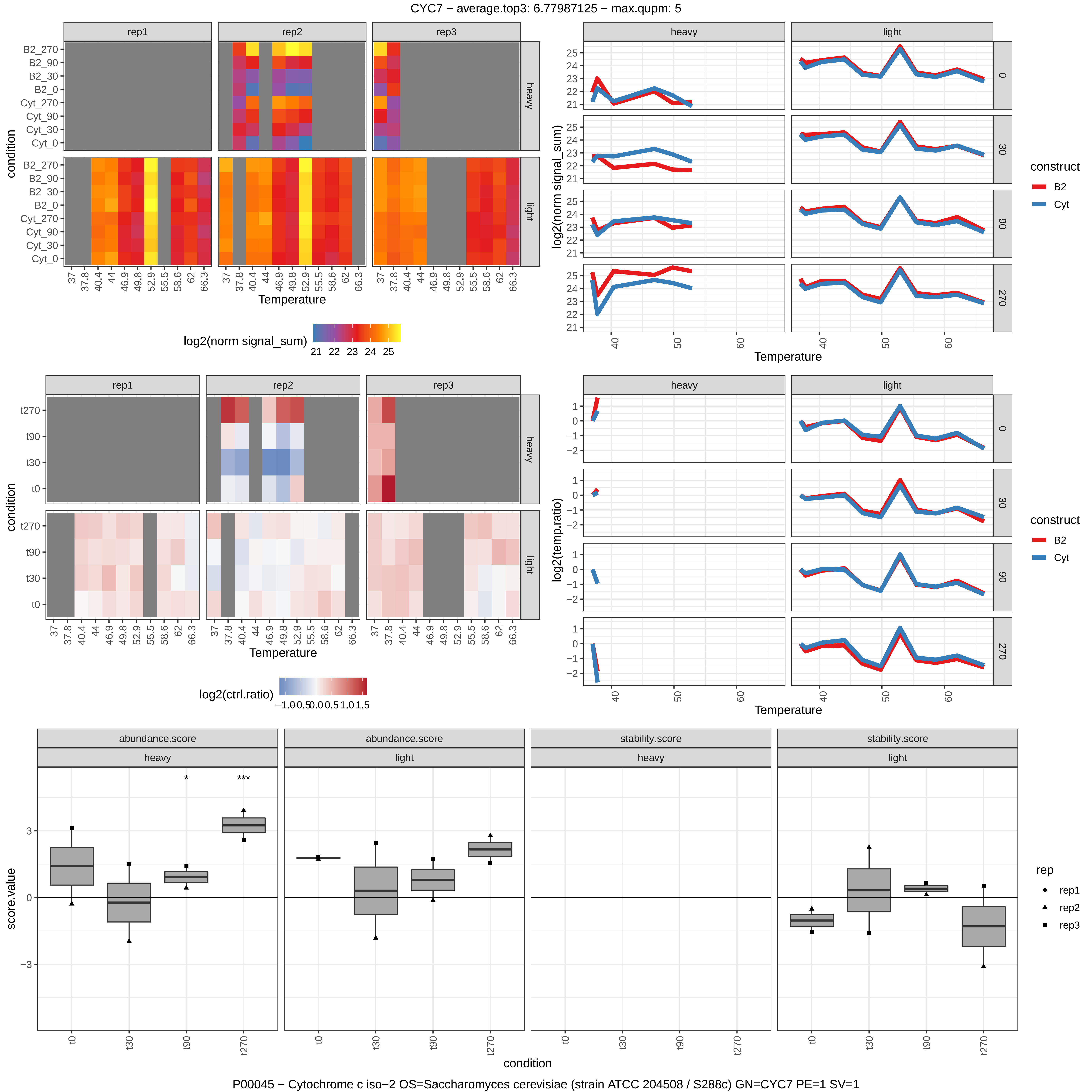

CYC7 − average.top3: 6.77987125 − max.qupm: 5
rep1
rep2
rep3
heavy
light
condition
Temperature
log2(norm signal_sum)
log2(temp.ratio)
log2(ctrl.ratio)
construct
B2
Cyt
abundance.score
stability.score
score.value
rep
rep1
rep2
rep3
P00045 − Cytochrome c iso−2 OS=Saccharomyces cerevisiae (strain ATCC 204508 / S288c) GN=CYC7 PE=1 SV=1

CYC8 − average.top3: 7.02492451612903 − max.qupm: 8

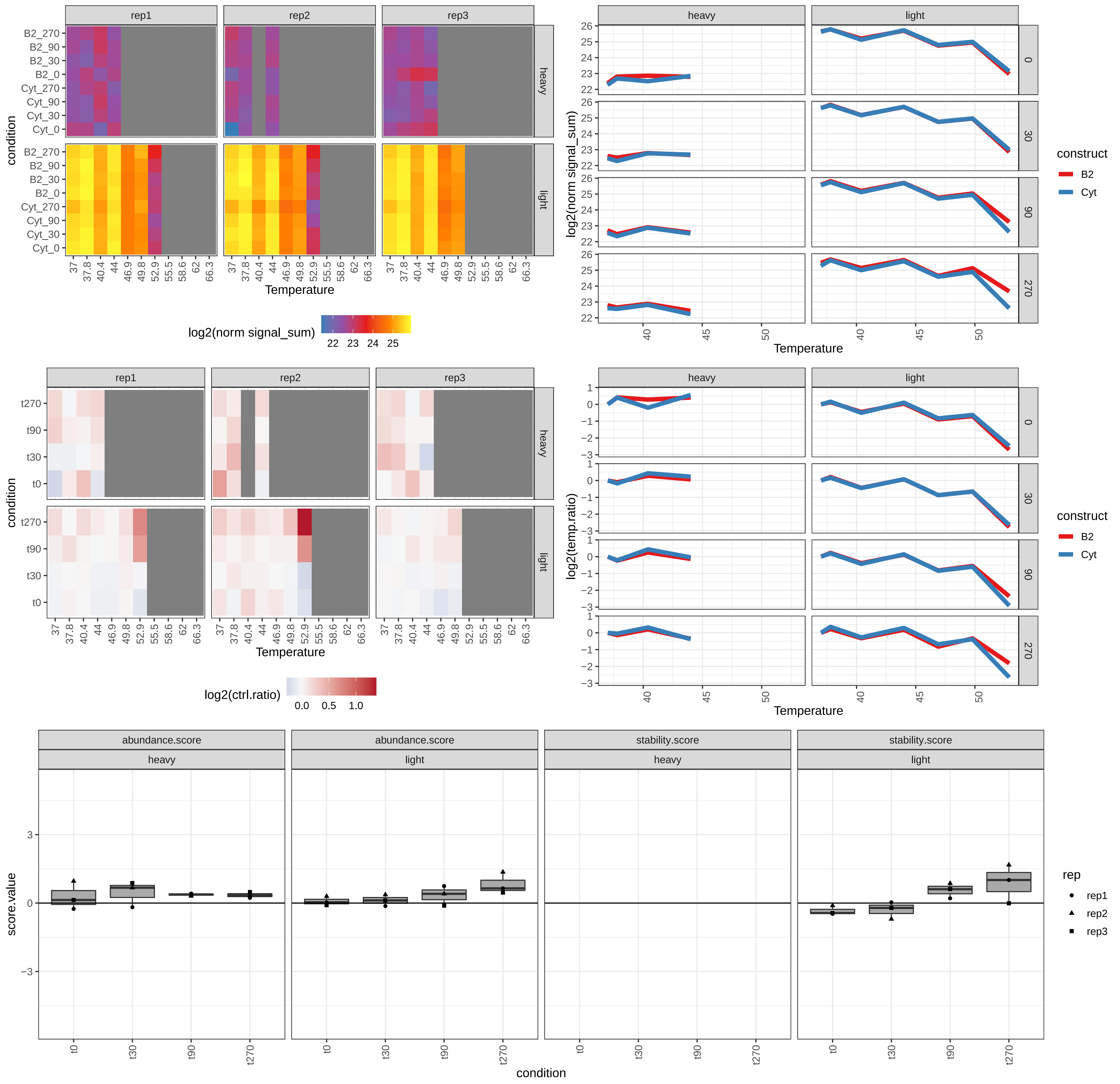

P14922 − General transcriptional corepressor CYC8 OS=Saccharomyces cerevisiae (strain ATCC 204508 / S288c) GN=CYC8 PE=1 SV=2

CYM1 − average.top3: 6.78199 − max.qupm: 24

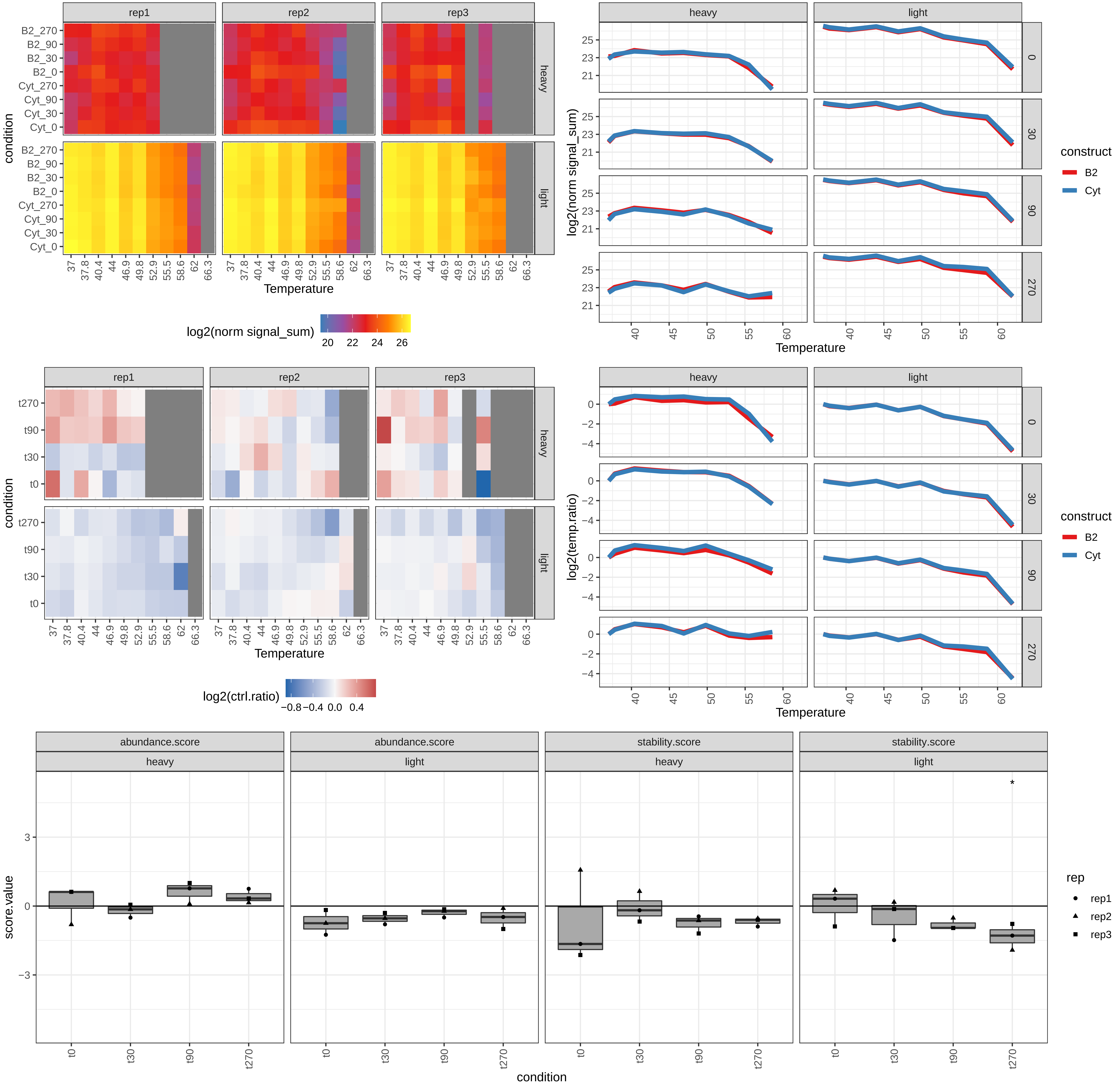

P32898 − Mitochondrial presequence protease OS=Saccharomyces cerevisiae (strain ATCC 204508 / S288c) GN=CYM1 PE=1 SV=2

CYR1 − average.top3: 6.81371060606061 − max.qupm: 17

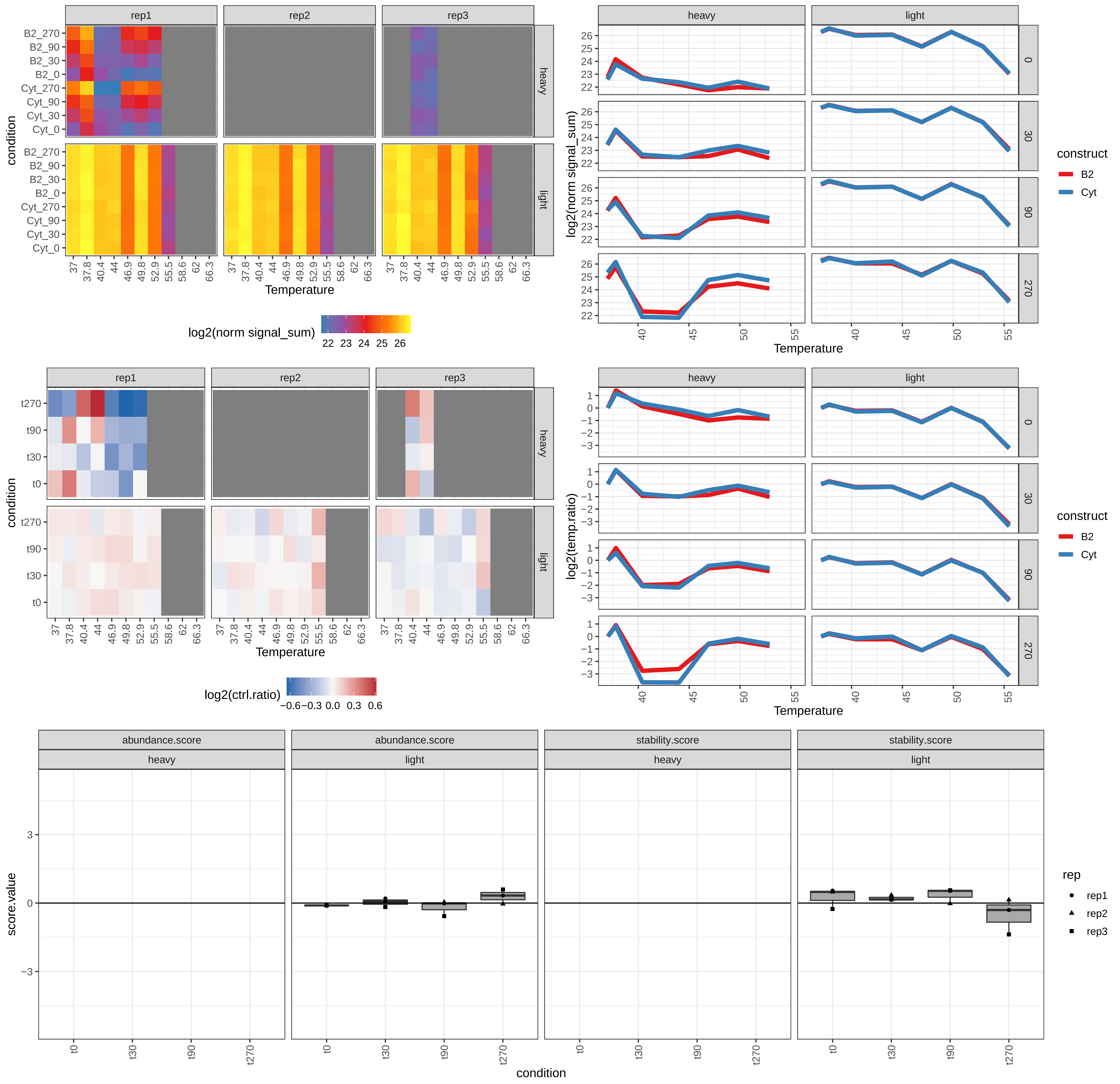

P08678 − Adenylate cyclase OS=Saccharomyces cerevisiae (strain ATCC 204508 / S288c) GN=CYR1 PE=1 SV=2

CYS3 − average.top3: 7.78431287878788 − max.qupm: 14

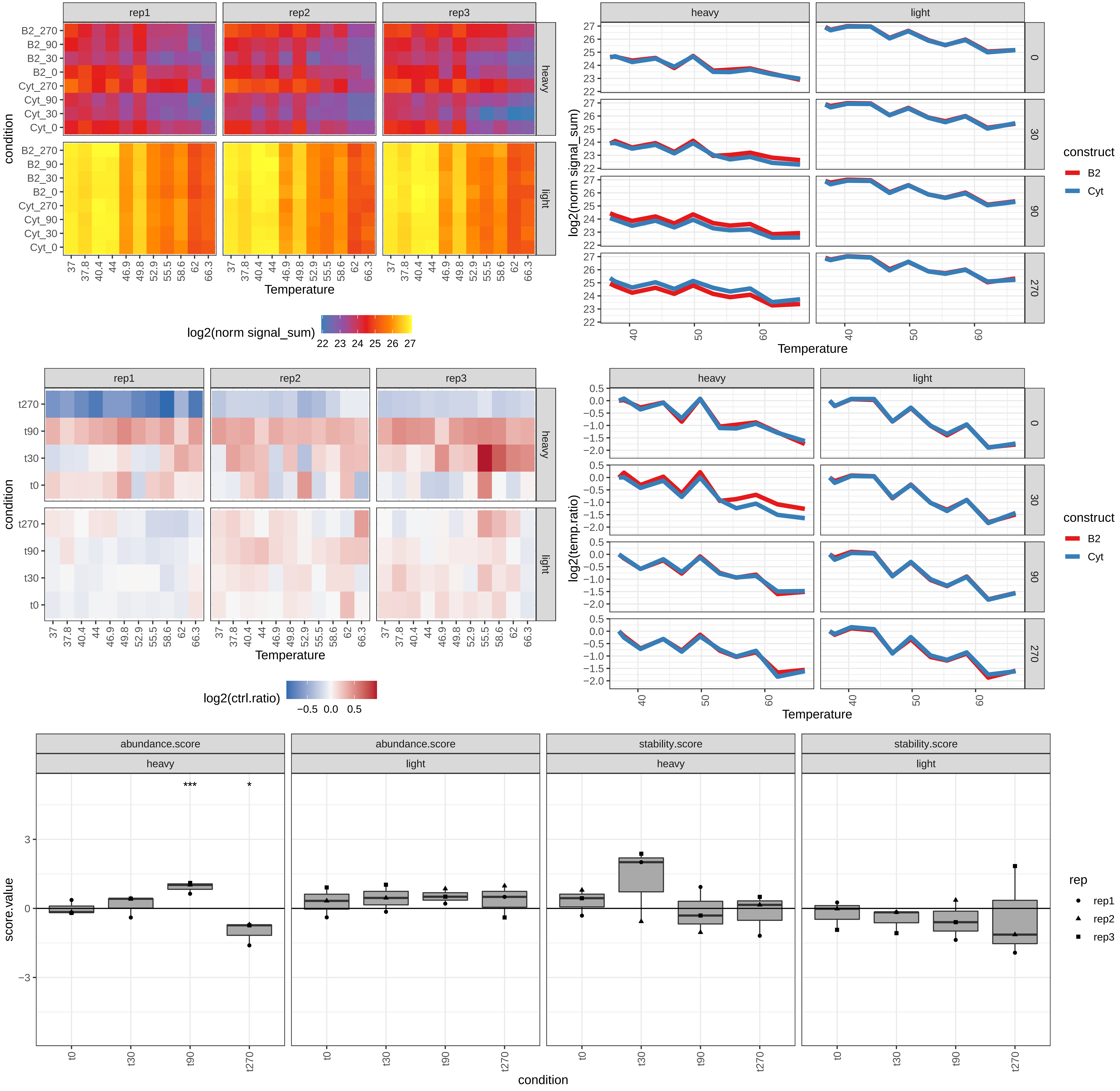

P31373 − Cystathionine gamma−lyase OS=Saccharomyces cerevisiae (strain ATCC 204508 / S288c) GN=CYS3 PE=1 SV=2

CYS4 − average.top3: 7.50721296296296 − max.qupm: 29

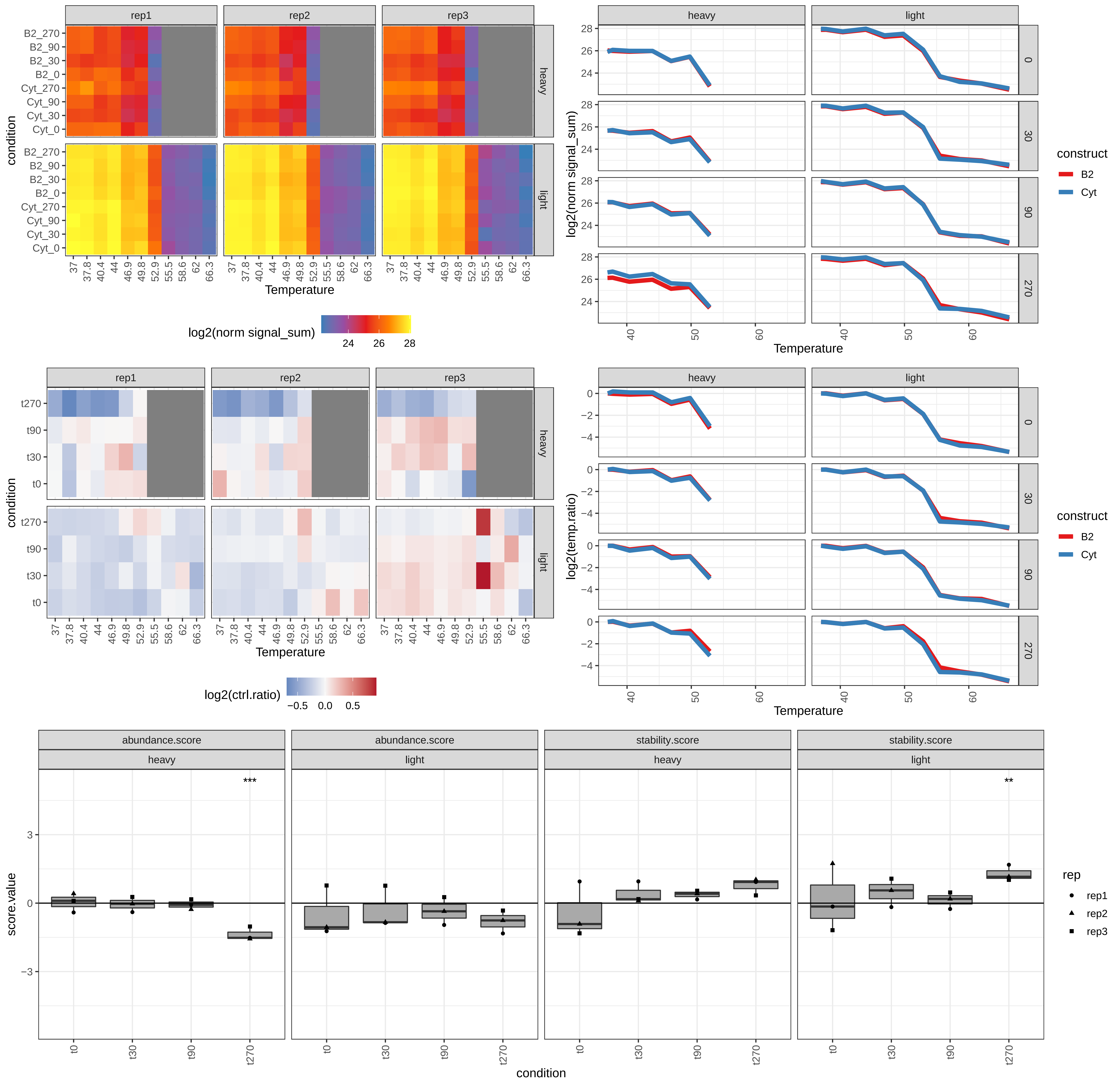

P32582 − Cystathionine beta−synthase OS=Saccharomyces cerevisiae (strain ATCC 204508 / S288c) GN=CYS4 PE=1 SV=1

CYT1 − average.top3: 6.87596971428571 − max.qupm: 6

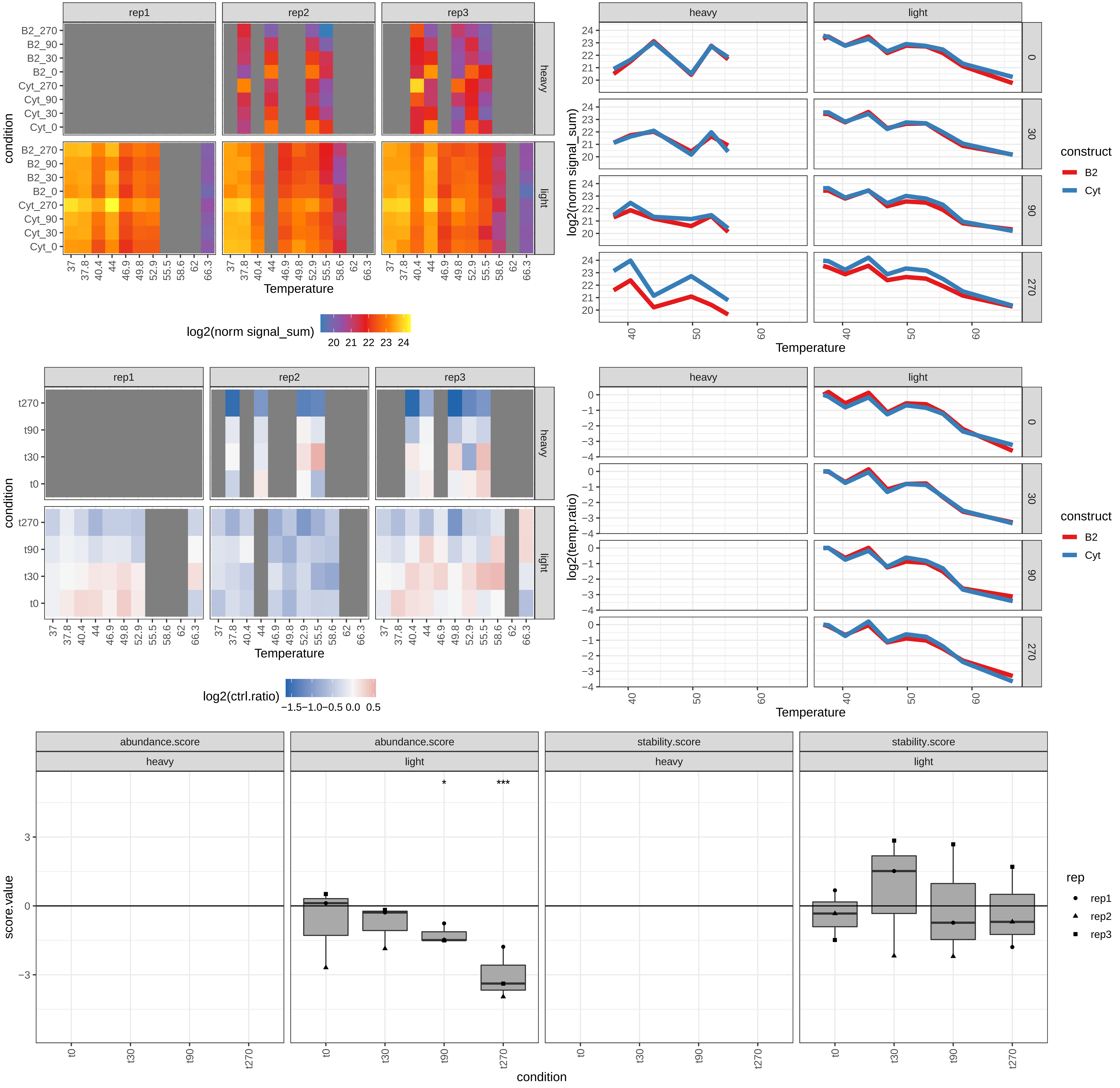

P07143 − Cytochrome c1, heme protein, mitochondrial OS=Saccharomyces cerevisiae (strain ATCC 204508 / S288c) GN=CYT1 PE=1 SV=1

CYT2 − average.top3: 6.9101712 − max.qupm: 6

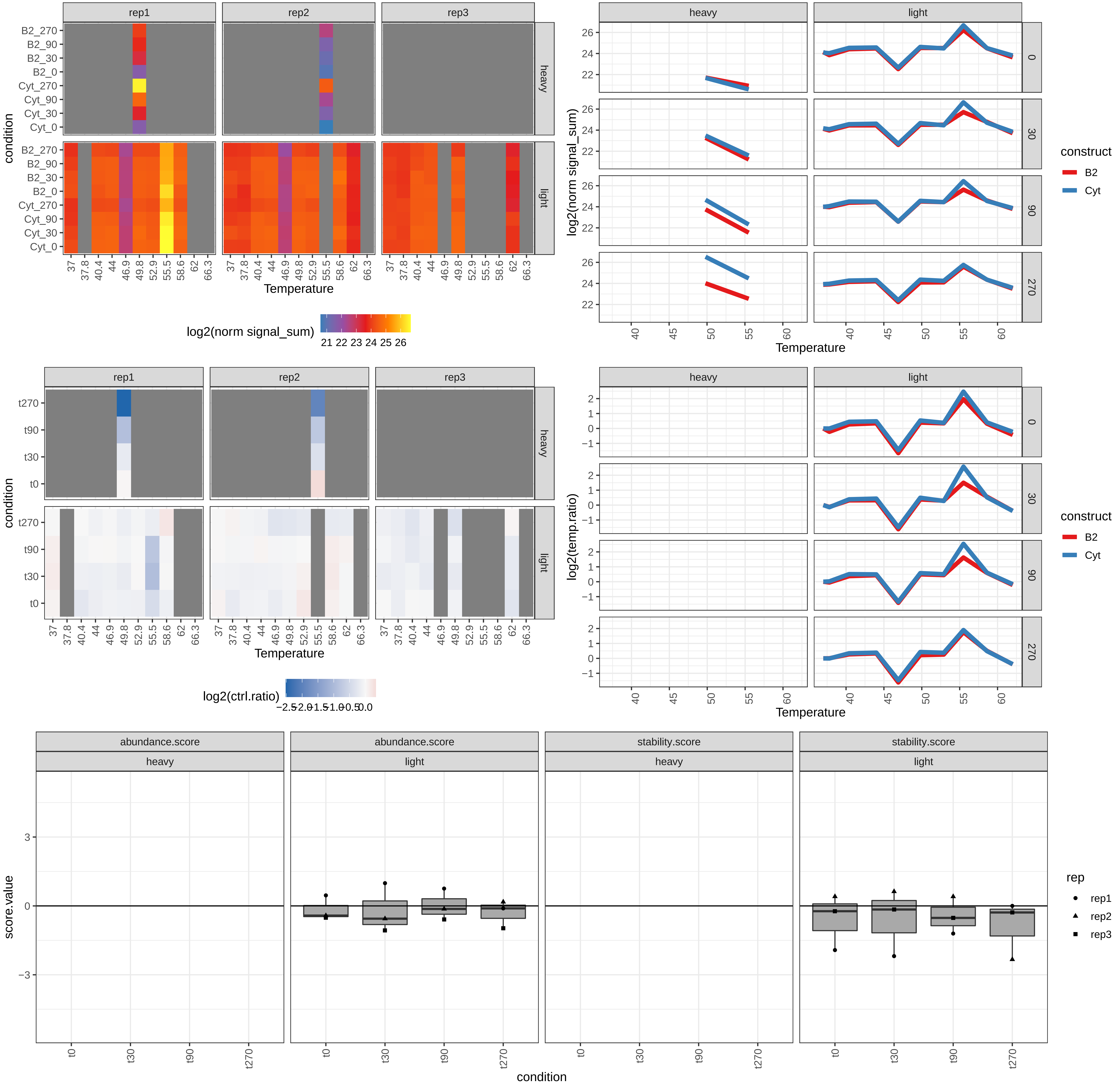

Q00873 − Cytochrome c1 heme lyase OS=Saccharomyces cerevisiae (strain ATCC 204508 / S288c) GN=CYT2 PE=3 SV=1

DAK1 − average.top3: 7.7975013559322 − max.qupm: 25

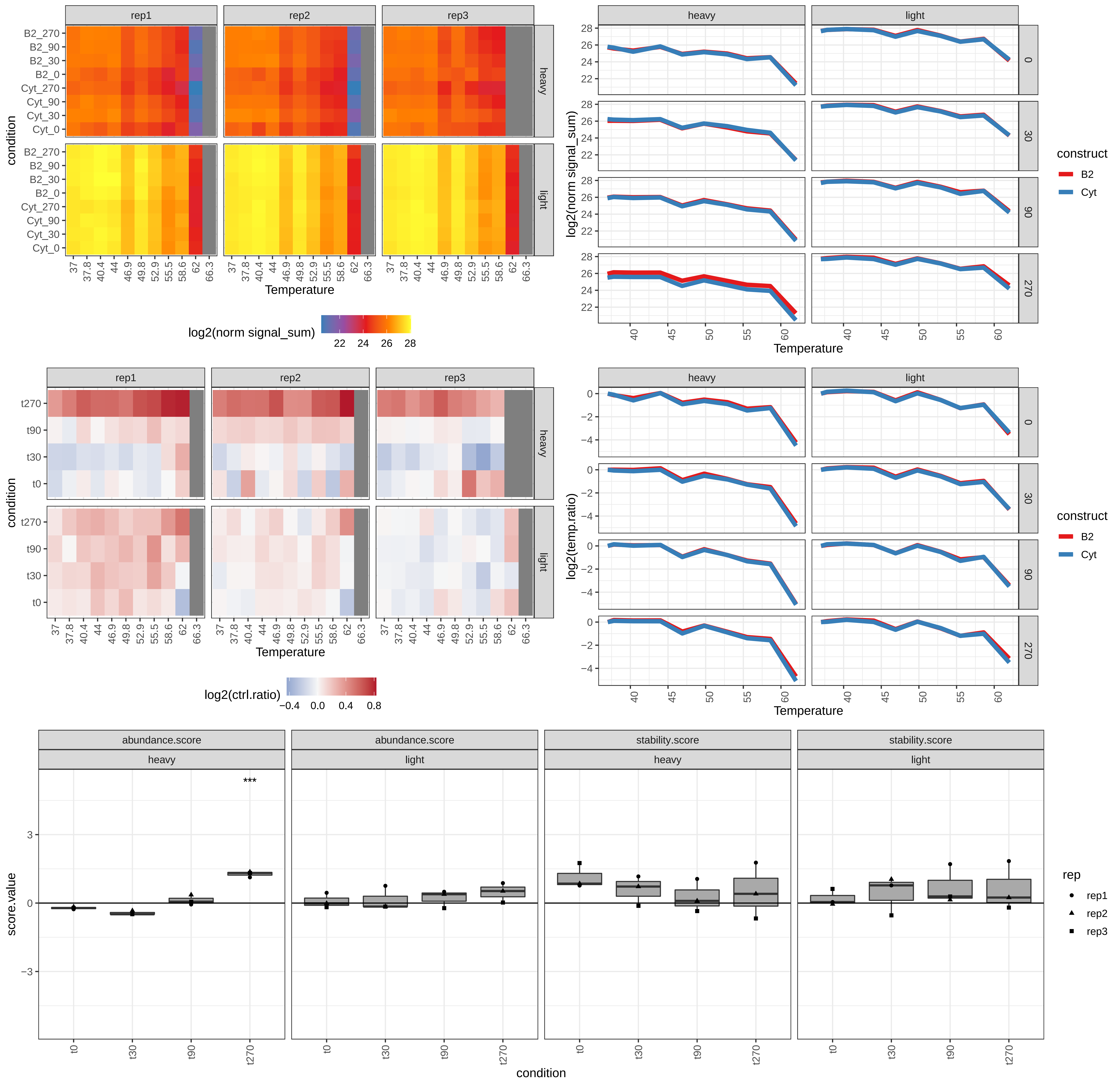

P54838 − Dihydroxyacetone kinase 1 OS=Saccharomyces cerevisiae (strain ATCC 204508 / S288c) GN=DAK1 PE=1 SV=1

DAP1 − average.top3: 6.98093076923077 − max.qupm: 4

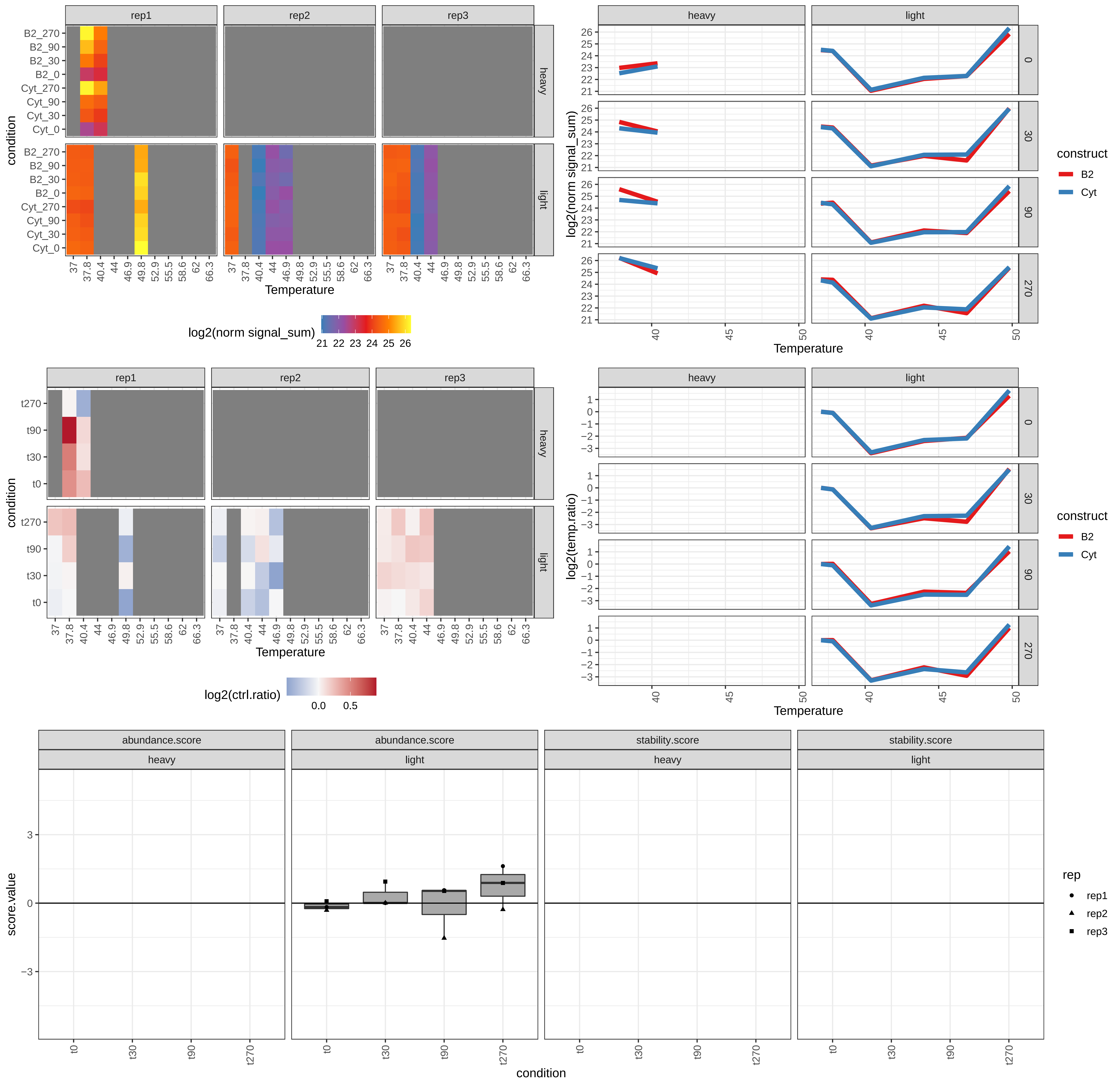

Q12091 − Damage response protein 1 OS=Saccharomyces cerevisiae (strain ATCC 204508 / S288c) GN=DAP1 PE=1 SV=1

DAP2 − average.top3: 6.9374 − max.qupm: 14

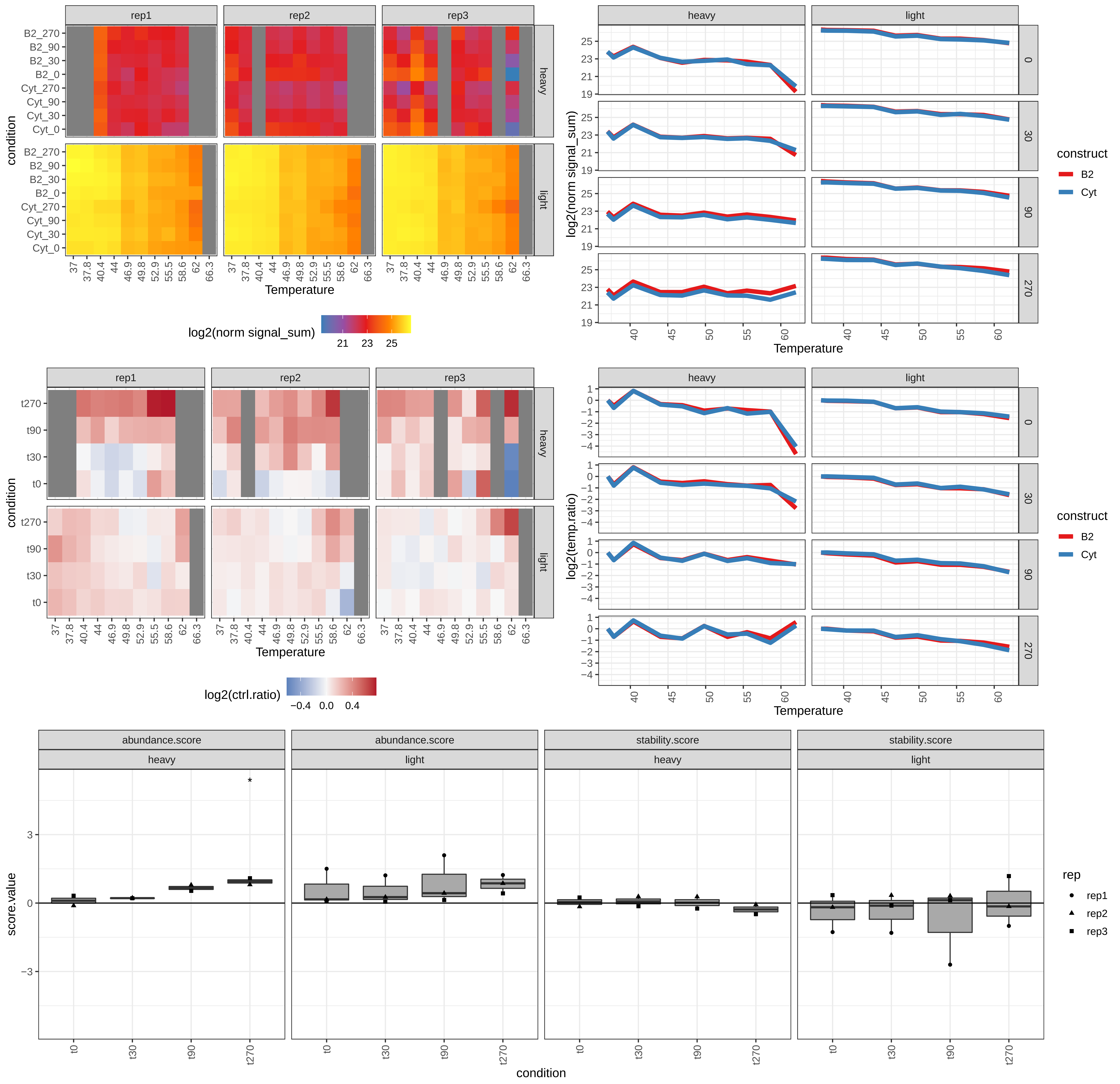

P18962 − Dipeptidyl aminopeptidase B OS=Saccharomyces cerevisiae (strain ATCC 204508 / S288c) GN=DAP2 PE=1 SV=2

DBP10 − average.top3: 6.6607395 − max.qupm: 8

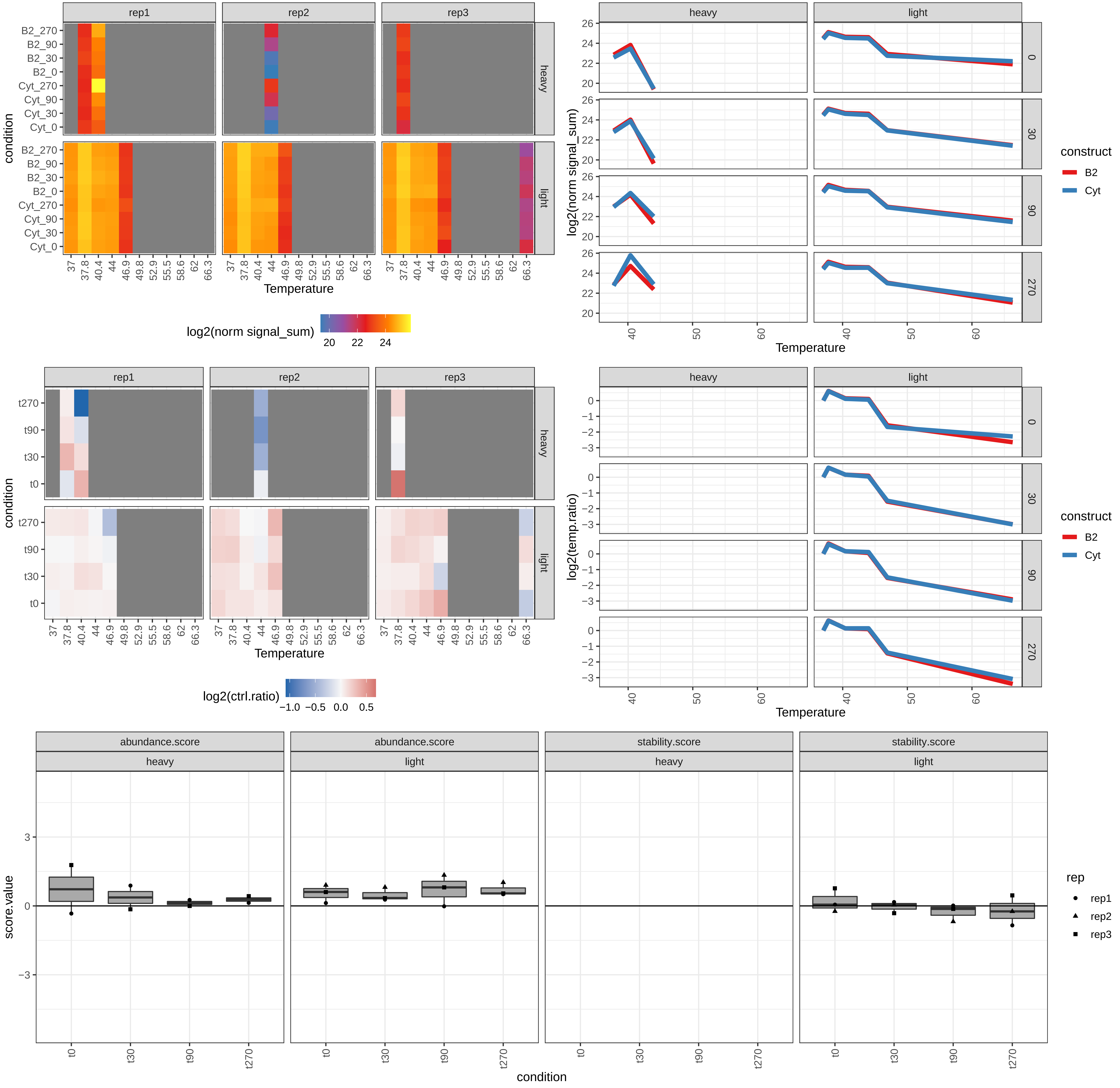

Q12389 − ATP−dependent RNA helicase DBP10 OS=Saccharomyces cerevisiae (strain ATCC 204508 / S288c) GN=DBP10 PE=1 SV=2

DBP2 − average.top3: 7.05255972972973 − max.qupm: 16

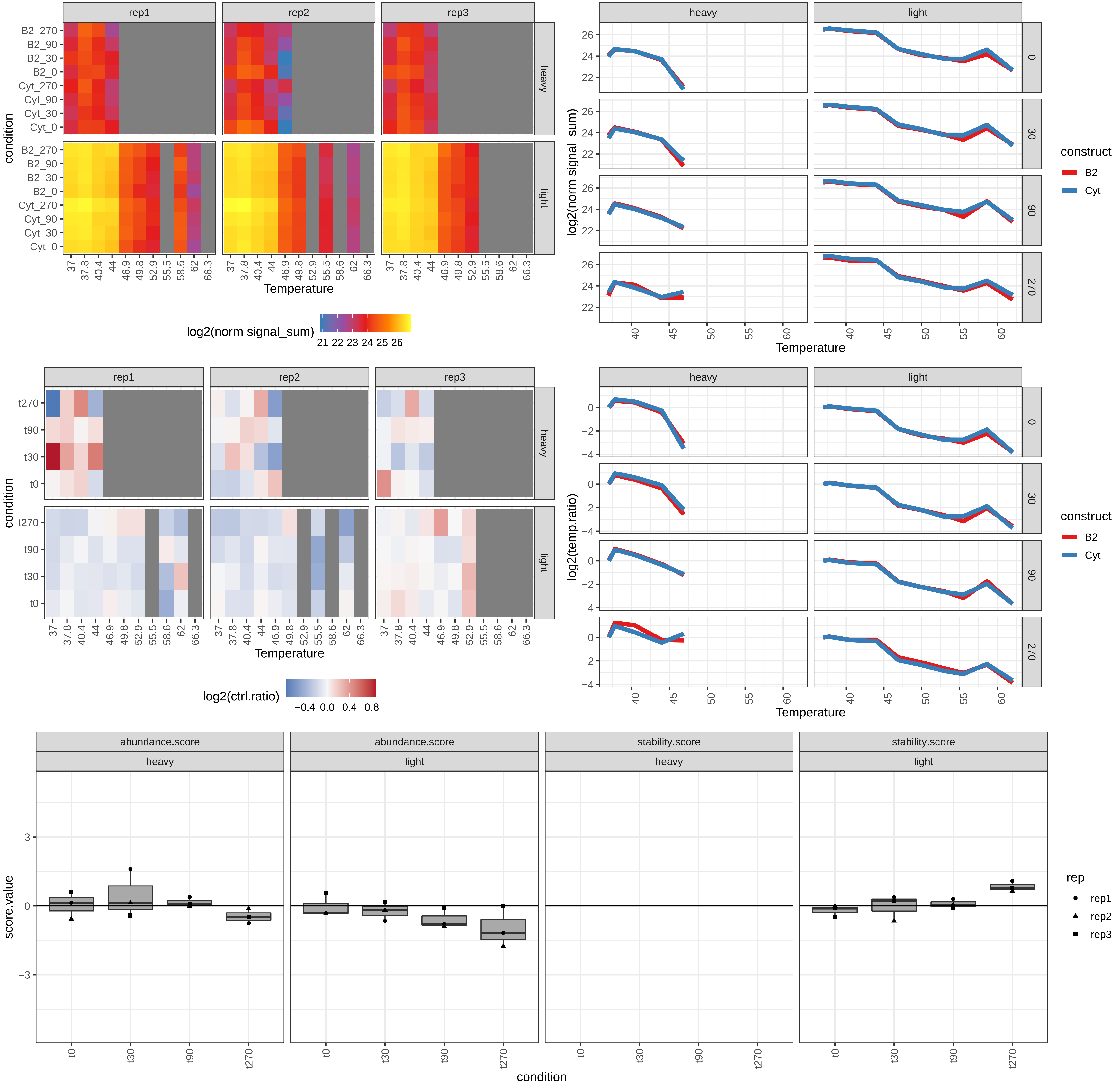

P24783 − ATP−dependent RNA helicase DBP2 OS=Saccharomyces cerevisiae (strain ATCC 204508 / S288c) GN=DBP2 PE=1 SV=1

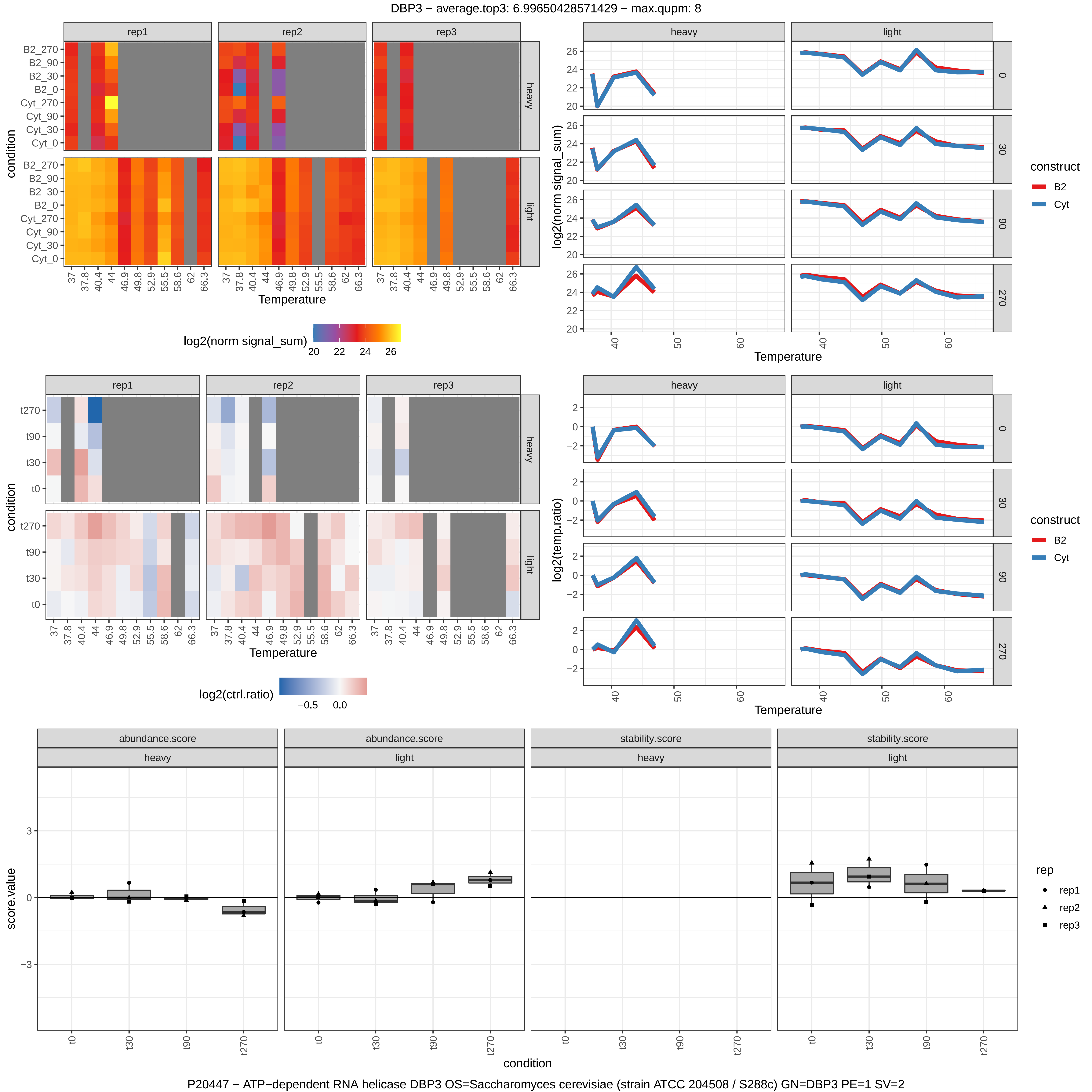

DBP3 − average.top3: 6.99650428571429 − max.qupm: 8
rep1
rep2
rep3
heavy
light
condition
Temperature
log2(norm signal_sum)
log2(ctrl.ratio)
log2(temp.ratio)
construct
B2
Cyt
abundance.score
stability.score
score.value
rep
rep1
rep2
rep3
P20447 − ATP−dependent RNA helicase DBP3 OS=Saccharomyces cerevisiae (strain ATCC 204508 / S288c) GN=DBP3 PE=1 SV=2

DBP5 − average.top3: 6.83480028571429 − max.qupm: 13

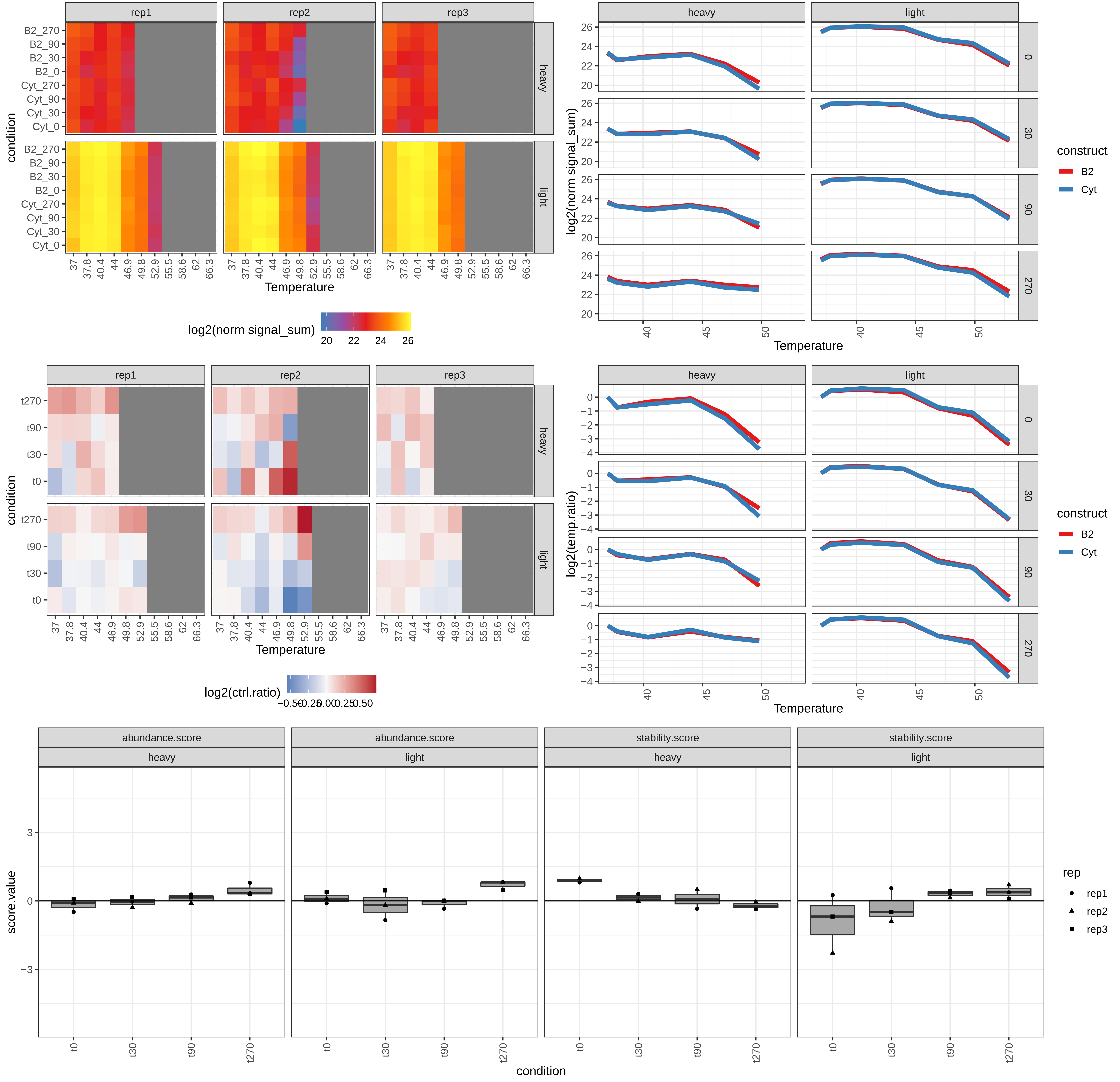

P20449 − ATP−dependent RNA helicase DBP5 OS=Saccharomyces cerevisiae (strain ATCC 204508 / S288c) GN=DBP5 PE=1 SV=2

DBP6 − average.top3: 6.64128615384615 − max.qupm: 5

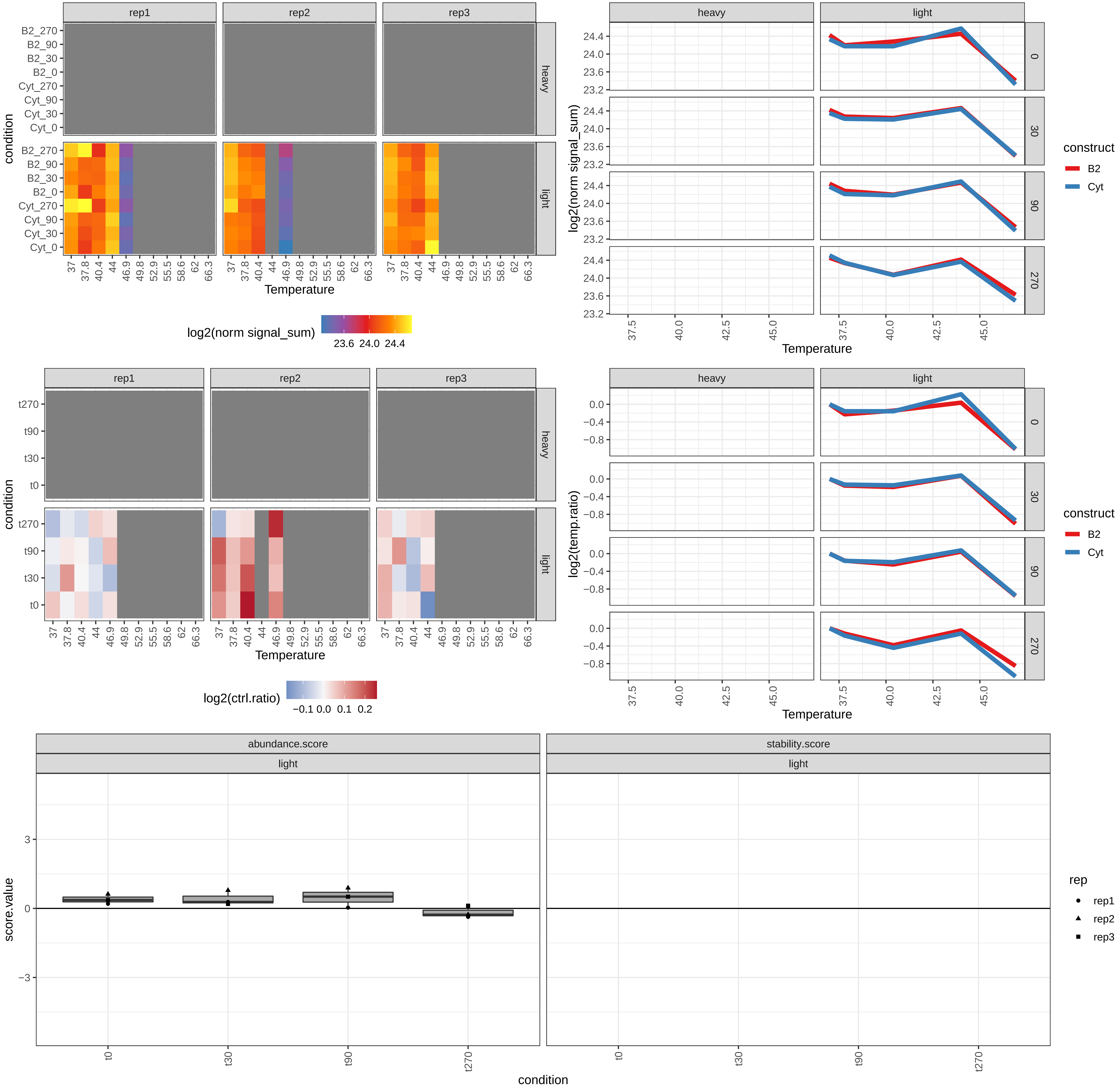

P53734 − ATP−dependent RNA helicase DBP6 OS=Saccharomyces cerevisiae (strain ATCC 204508 / S288c) GN=DBP6 PE=1 SV=1

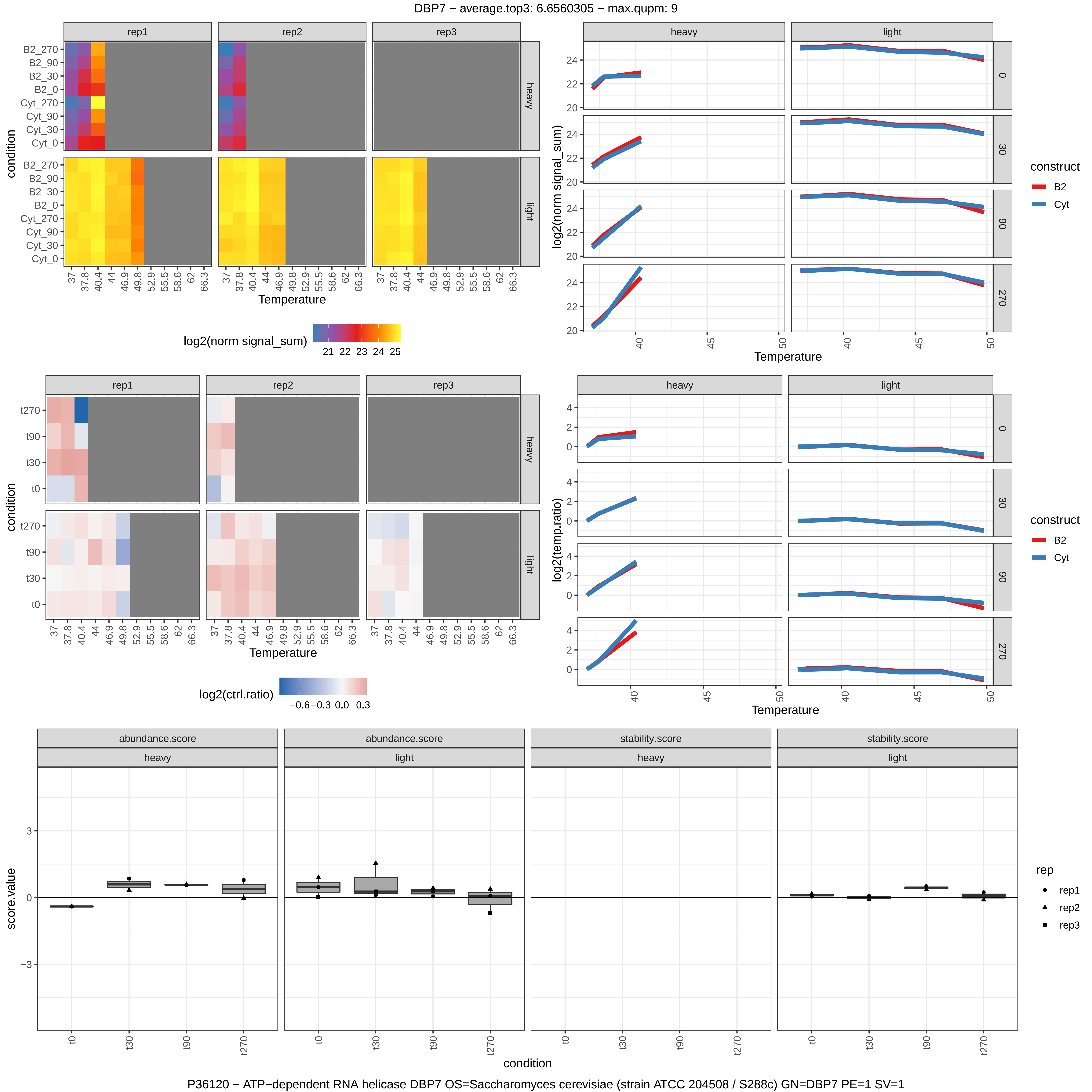
DBP7 − average.top3: 6.6560305 − max.qupm: 9
rep1
rep2
rep3
heavy
light
condition
B2_270
B2_90
B2_30
B2_0
Cyt_270
Cyt_90
Cyt_30
Cyt_0
Temperature
37
37.8
40.4
44
46.9
49.8
52.9
55.5
58.6
62
66.3
log2(norm signal_sum)
21 22 23 24 25
log2(norm signal_sum)
0
30
90
270
construct
B2
Cyt
t270
t90
t30
t0
log2(ctrl.ratio)
−0.6 −0.3 0.0 0.3
log2(temp.ratio)
abundance.score
stability.score
score.value
rep
rep1
rep2
rep3
P36120 − ATP−dependent RNA helicase DBP7 OS=Saccharomyces cerevisiae (strain ATCC 204508 / S288c) GN=DBP7 PE=1 SV=1

DBP9 − average.top3: 6.85342333333333 − max.qupm: 11

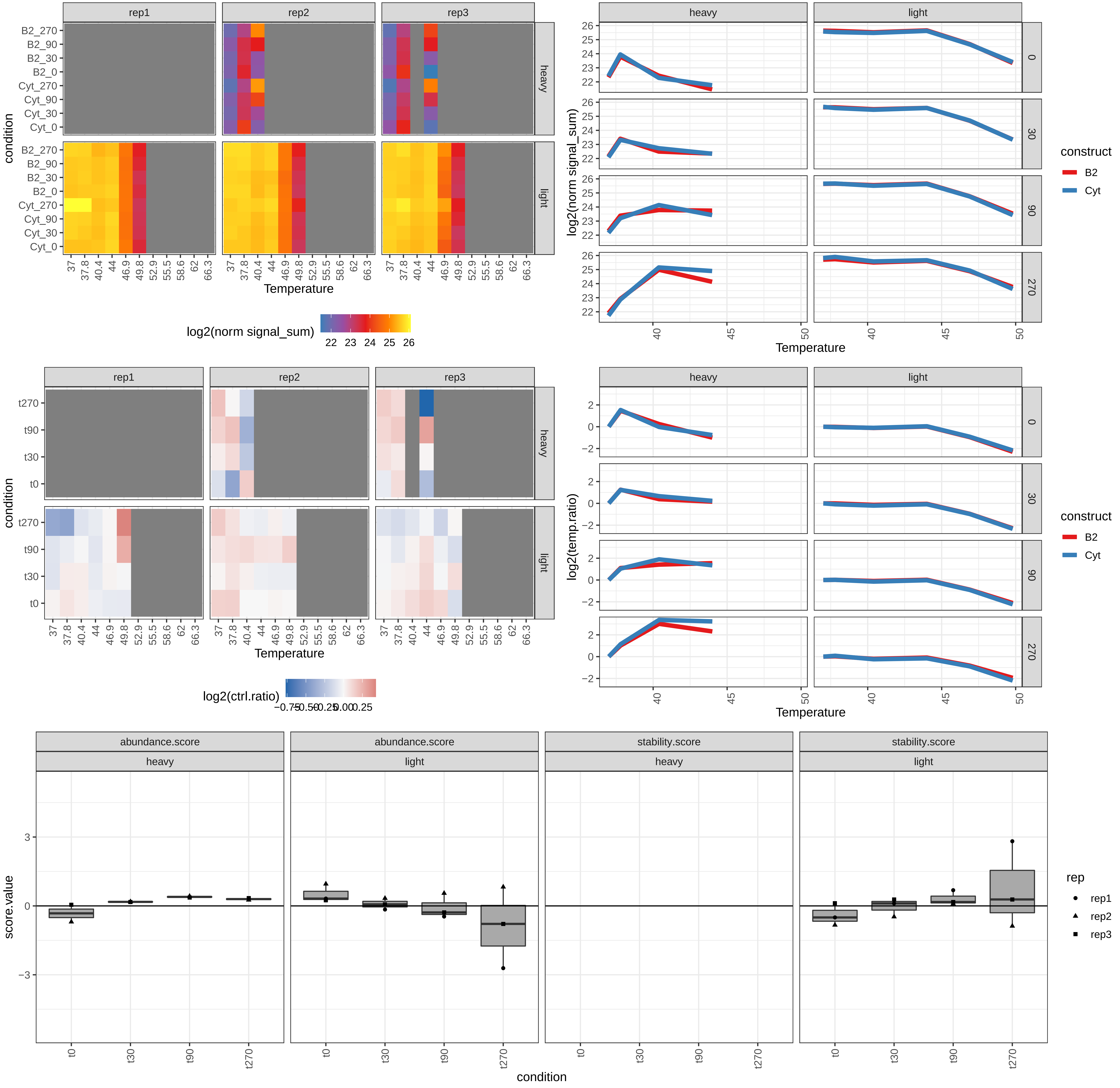

Q06218 − ATP−dependent RNA helicase DBP9 OS=Saccharomyces cerevisiae (strain ATCC 204508 / S288c) GN=DBP9 PE=1 SV=1

DCD1 − average.top3: 6.69781482758621 − max.qupm: 5

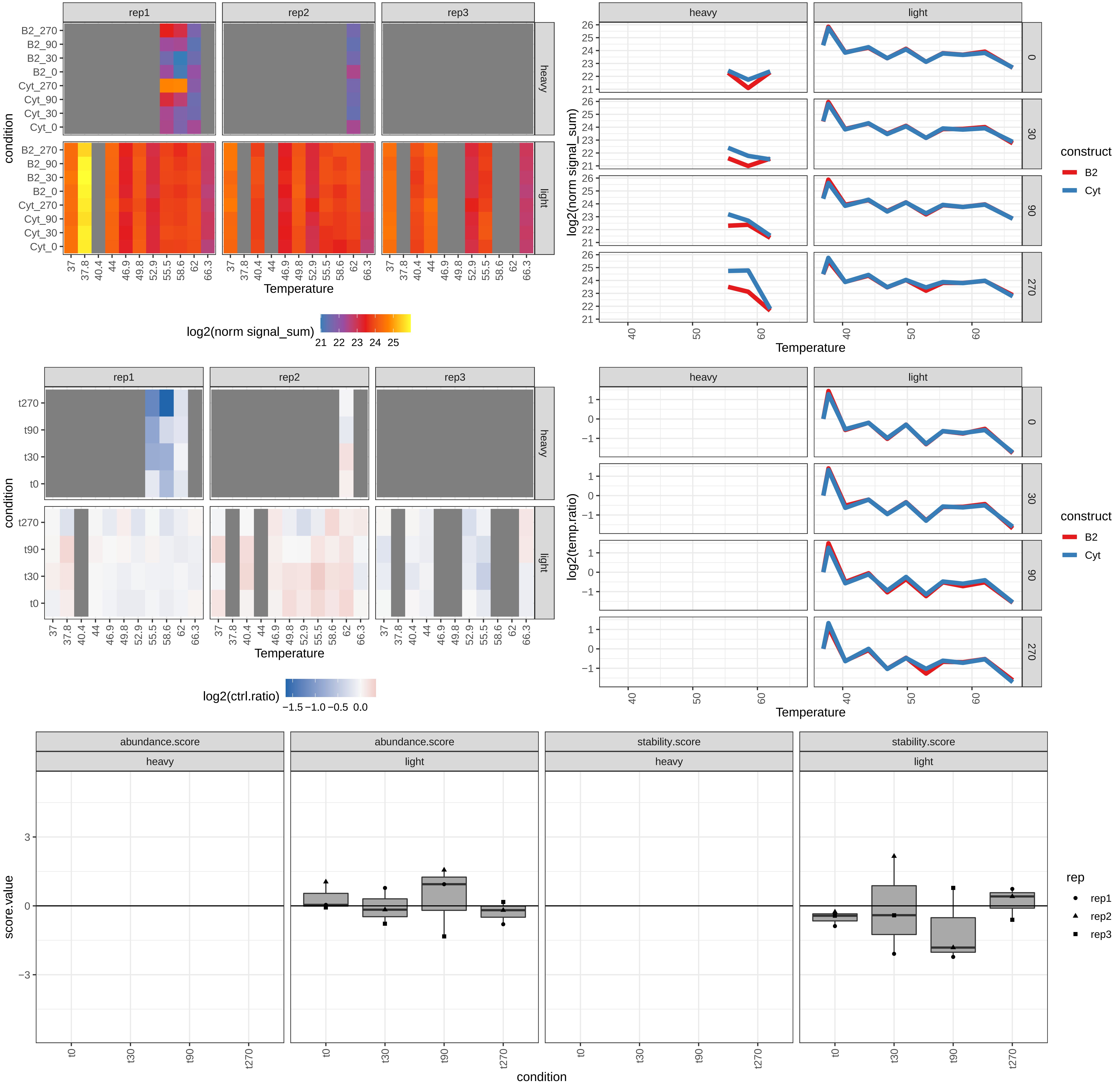

P06773 − Deoxycytidylate deaminase OS=Saccharomyces cerevisiae (strain ATCC 204508 / S288c) GN=DCD1 PE=1 SV=2

DCP1 − average.top3: 6.622595 − max.qupm: 3

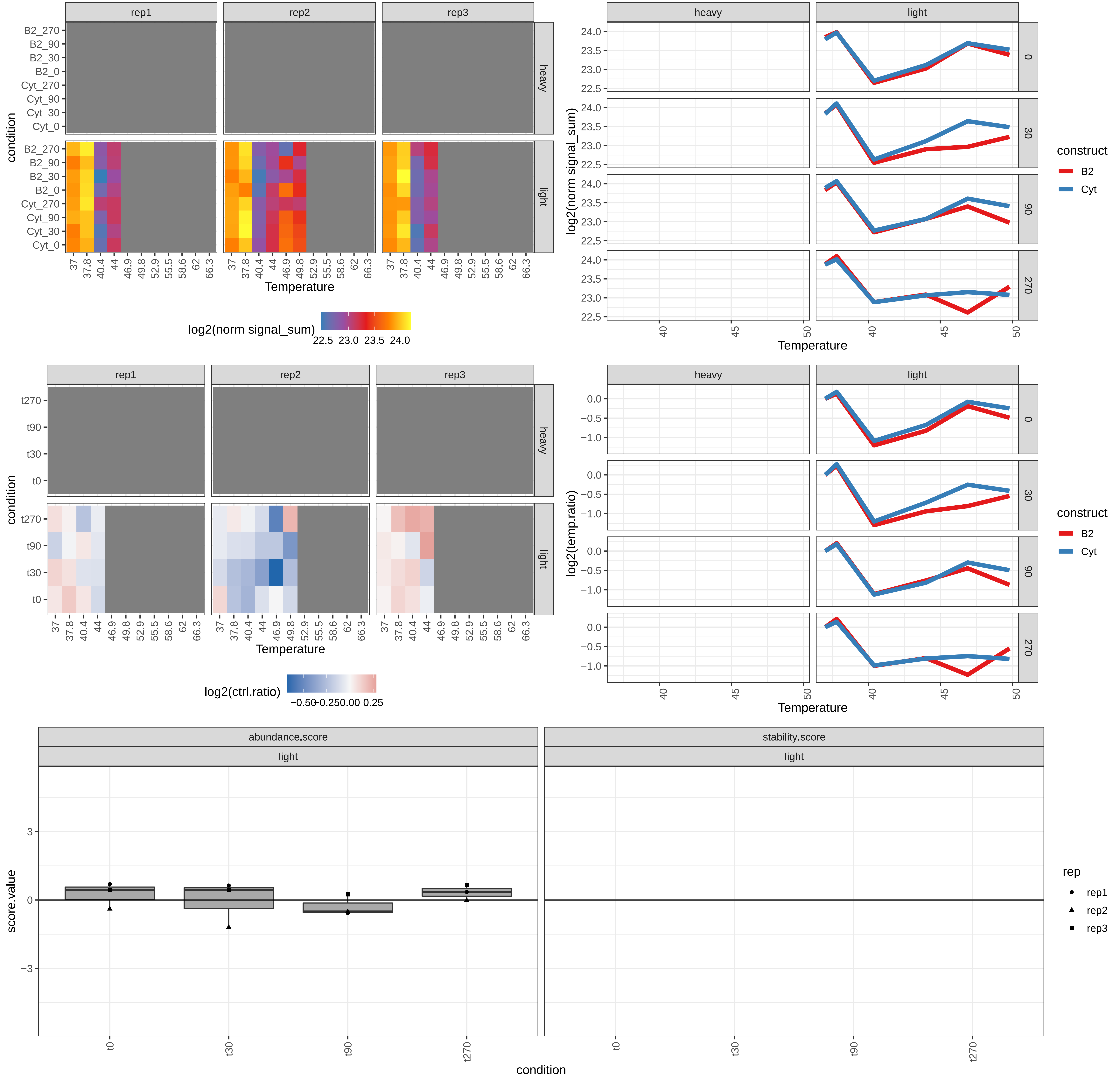

Q12517 − mRNA−decapping enzyme subunit 1 OS=Saccharomyces cerevisiae (strain ATCC 204508 / S288c) GN=DCP1 PE=1 SV=1

DCP2 − average.top3: 6.94950076923077 − max.qupm: 8

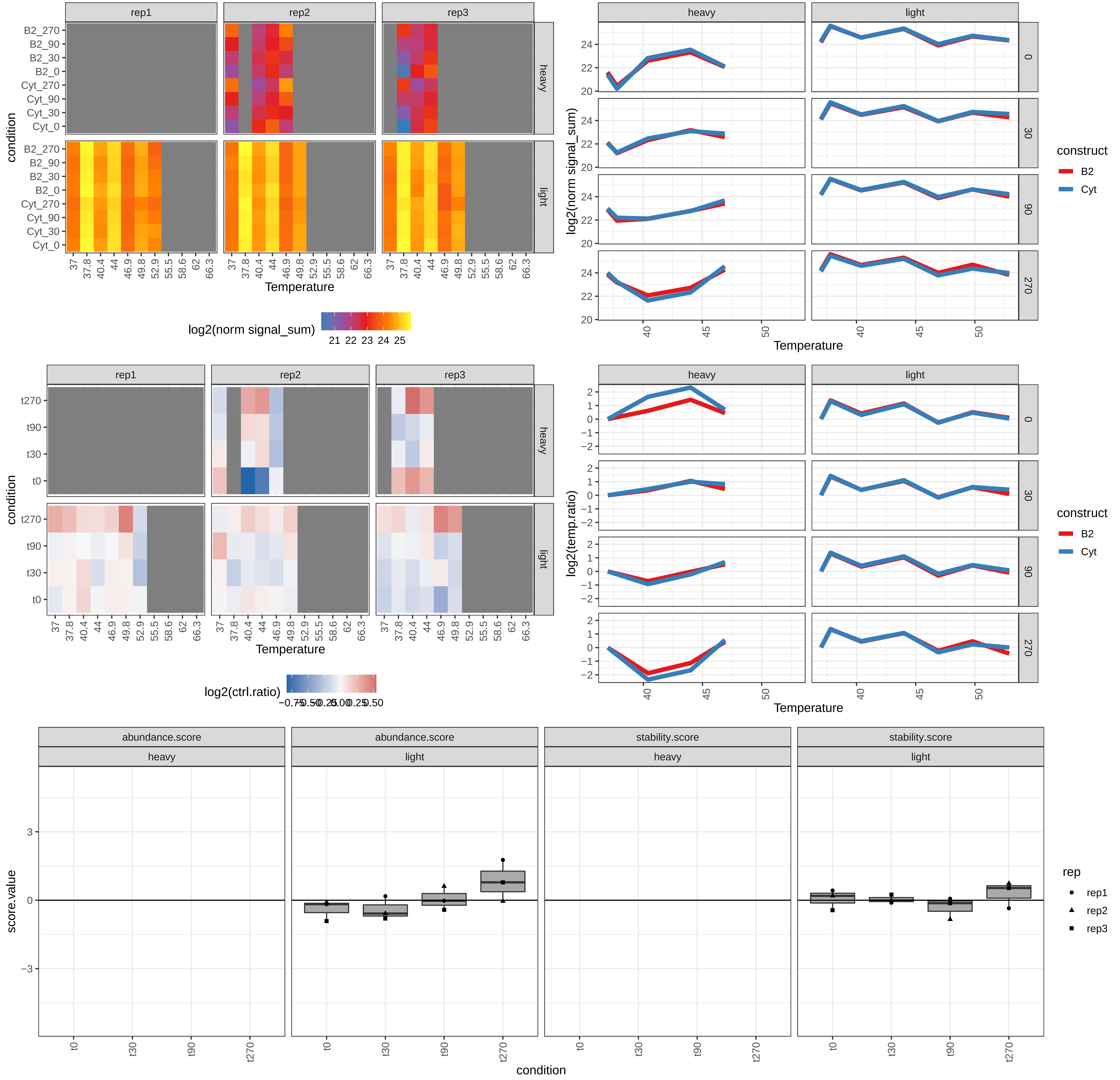

P53550 − m7GpppN−mRNA hydrolase OS=Saccharomyces cerevisiae (strain ATCC 204508 / S288c) GN=DCP2 PE=1 SV=1

DCS1 − average.top3: 7.49379193548387 − max.qupm: 17

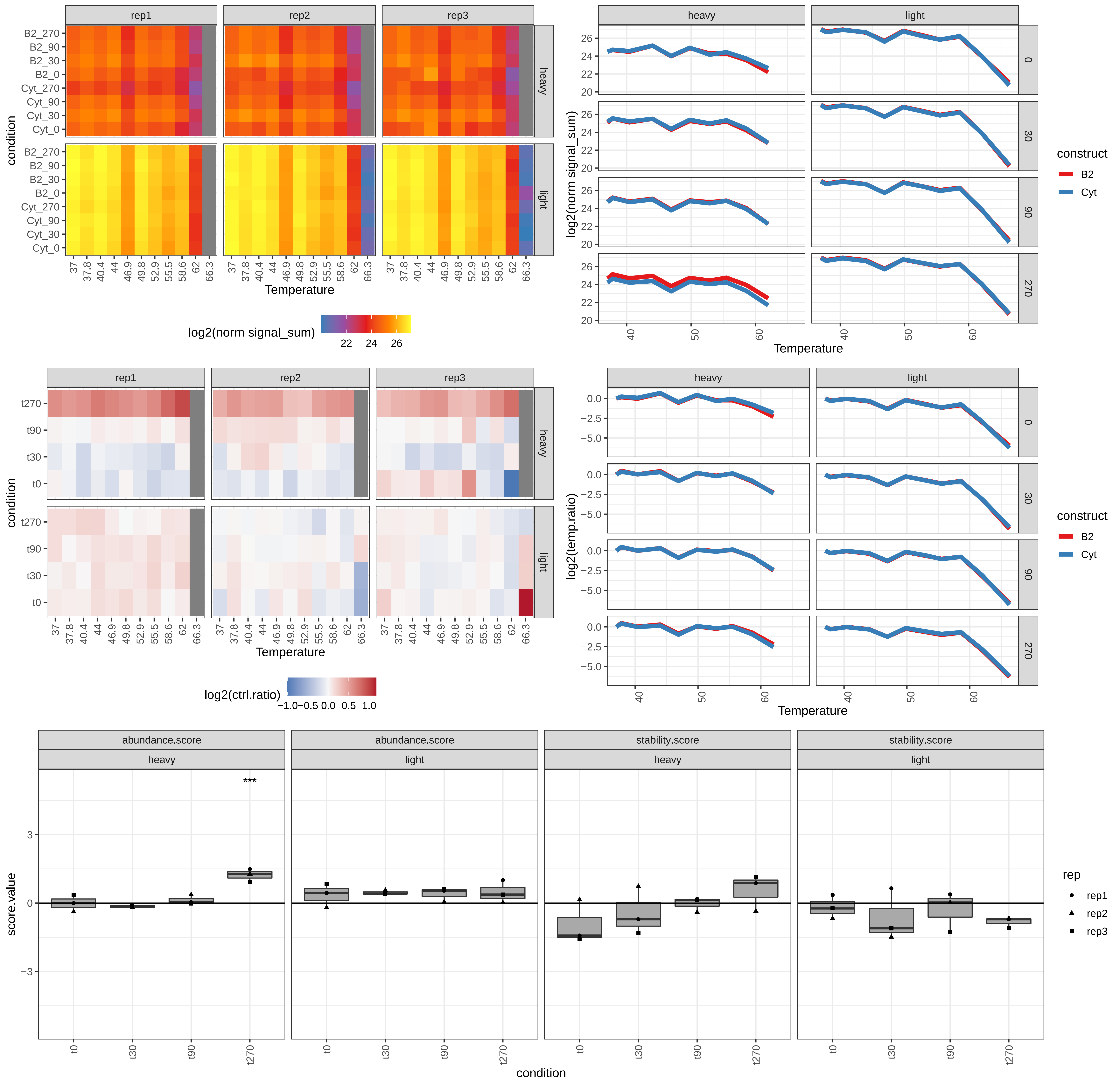

Q06151 − m7GpppX diphosphatase OS=Saccharomyces cerevisiae (strain ATCC 204508 / S288c) GN=DCS1 PE=1 SV=1

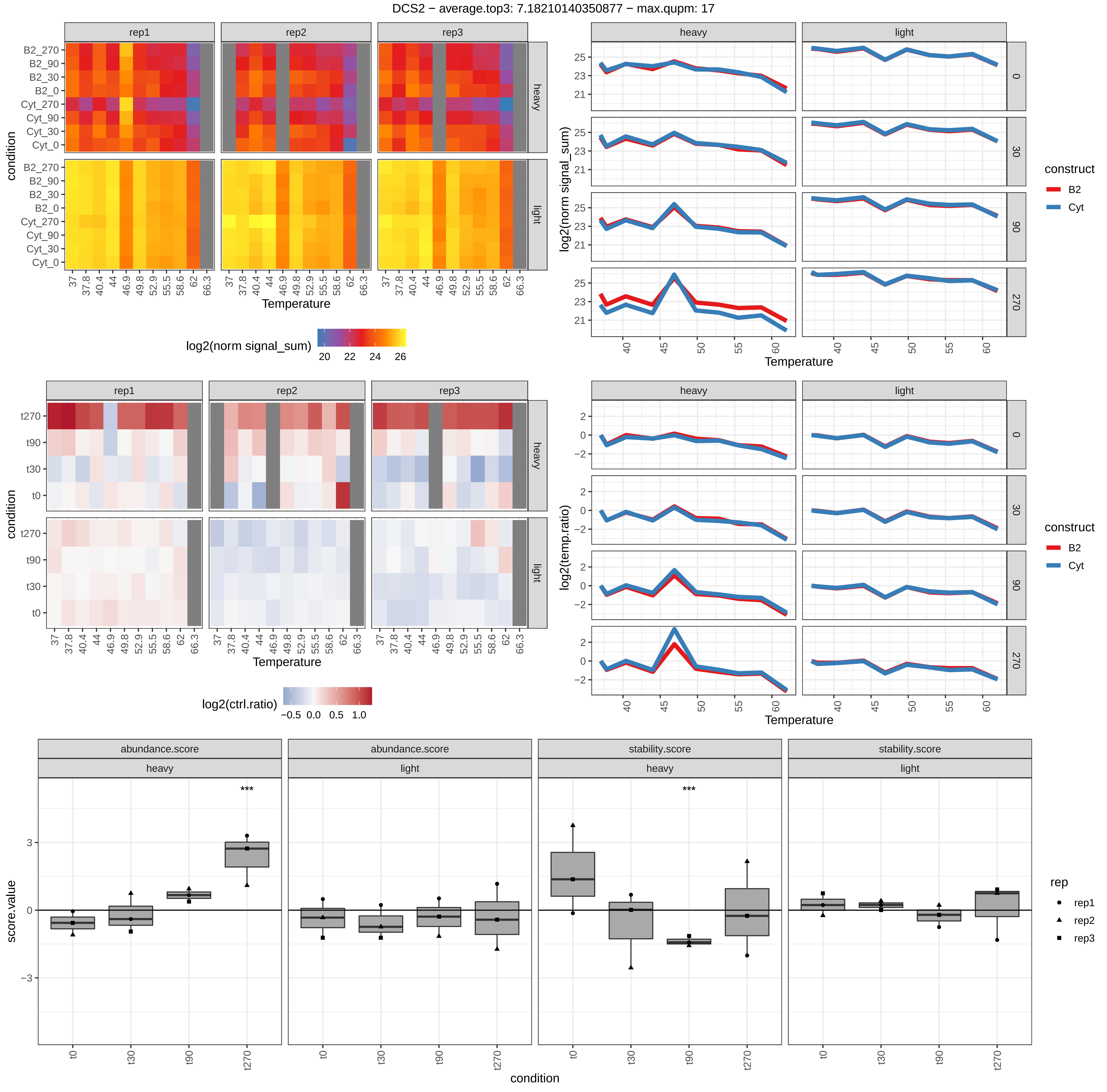

Q12123 − Inactive diphosphatase DCS2 OS=Saccharomyces cerevisiae (strain ATCC 204508 / S288c) GN=DCS2 PE=1 SV=3

DDI1 − average.top3: 6.61333944444444 − max.qupm: 5

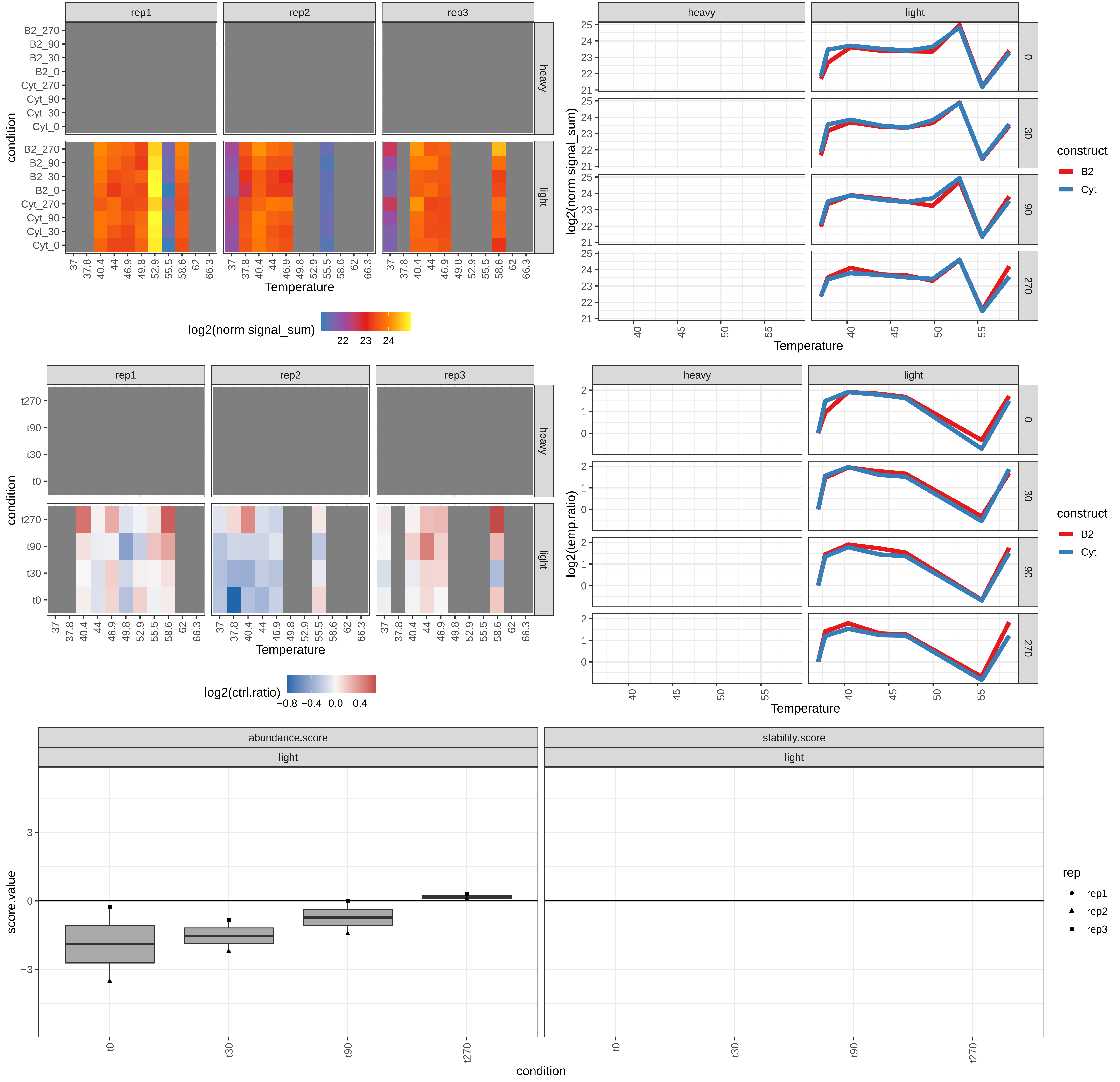

P40087 − DNA damage−inducible protein 1 OS=Saccharomyces cerevisiae (strain ATCC 204508 / S288c) GN=DDI1 PE=1 SV=1

DDP1 − average.top3: 7.25656517241379 − max.qupm: 9

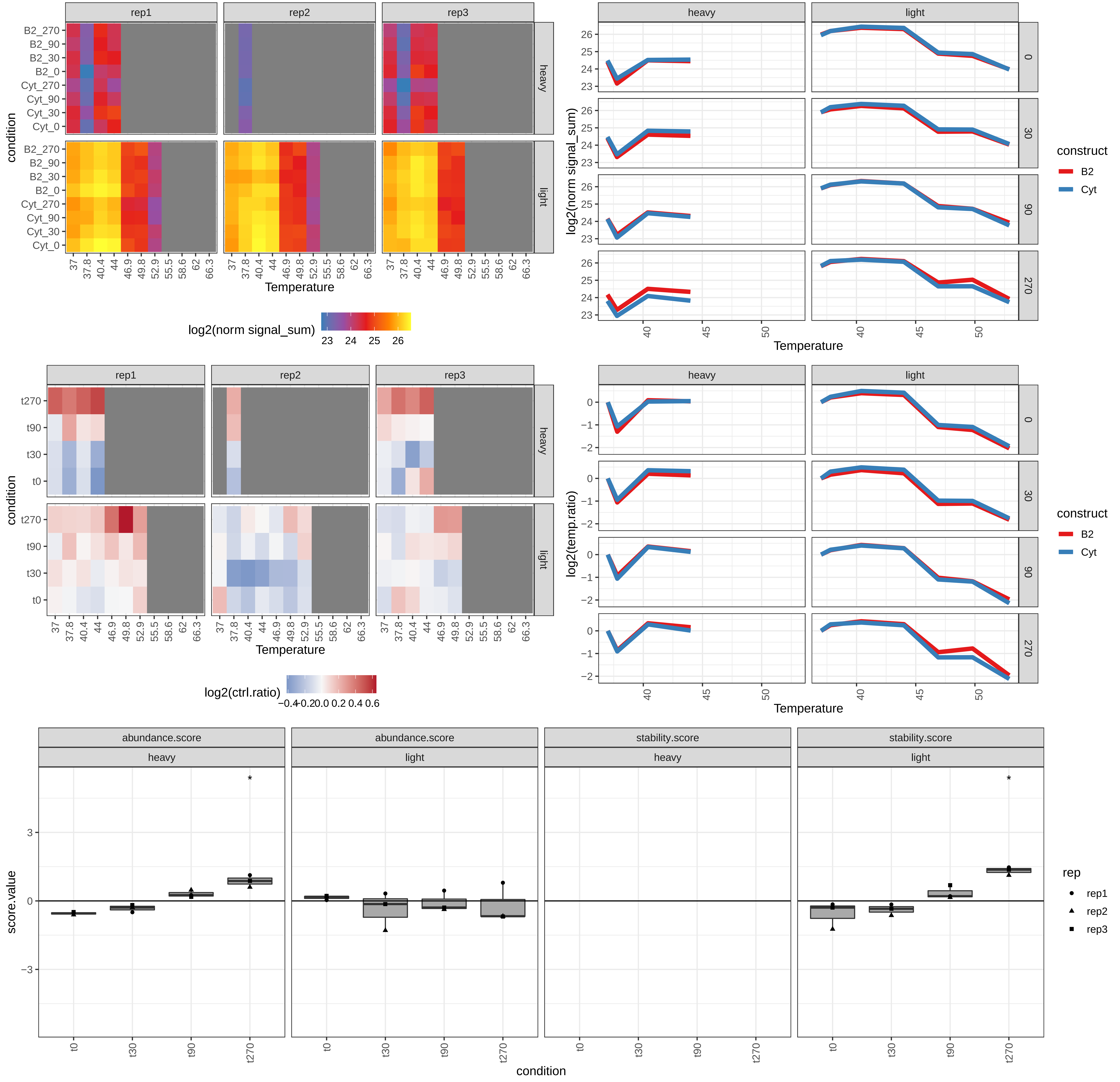

Q99321 − Diphosphoinositol polyphosphate phosphohydrolase DDP1 OS=Saccharomyces cerevisiae (strain ATCC 204508 / S288c) GN=DDP1 PE=1 SV=3

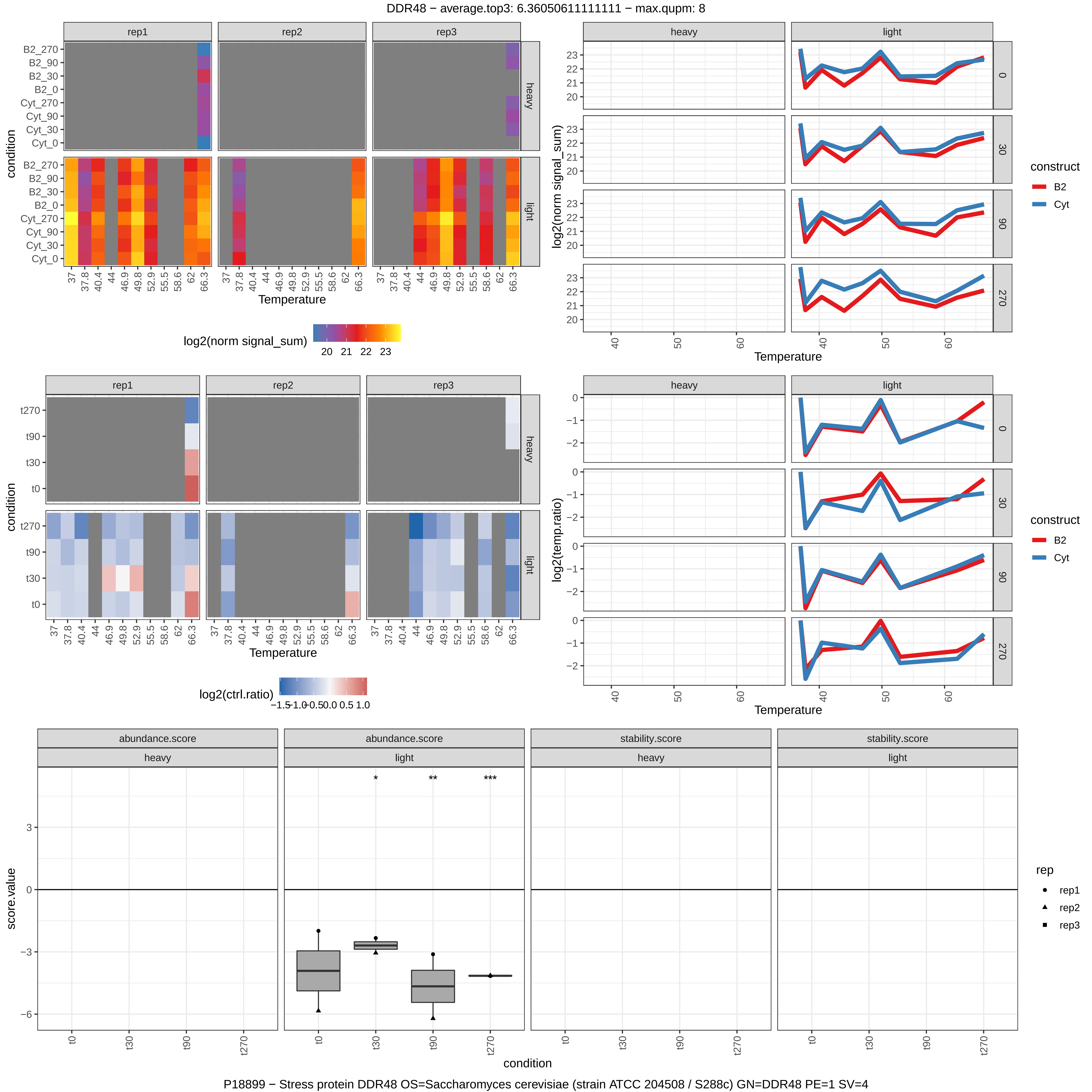
DDR48 − average.top3: 6.36050611111111 − max.qupm: 8
rep1
rep2
rep3
heavy
light
condition
Temperature
log2(norm signal_sum)
log2(ctrl.ratio)
log2(temp.ratio)
construct
B2
Cyt
abundance.score
stability.score
score.value
rep
rep1
rep2
rep3
condition
P18899 − Stress protein DDR48 OS=Saccharomyces cerevisiae (strain ATCC 204508 / S288c) GN=DDR48 PE=1 SV=4

DED1 − average.top3: 7.30990307692308 − max.qupm: 11

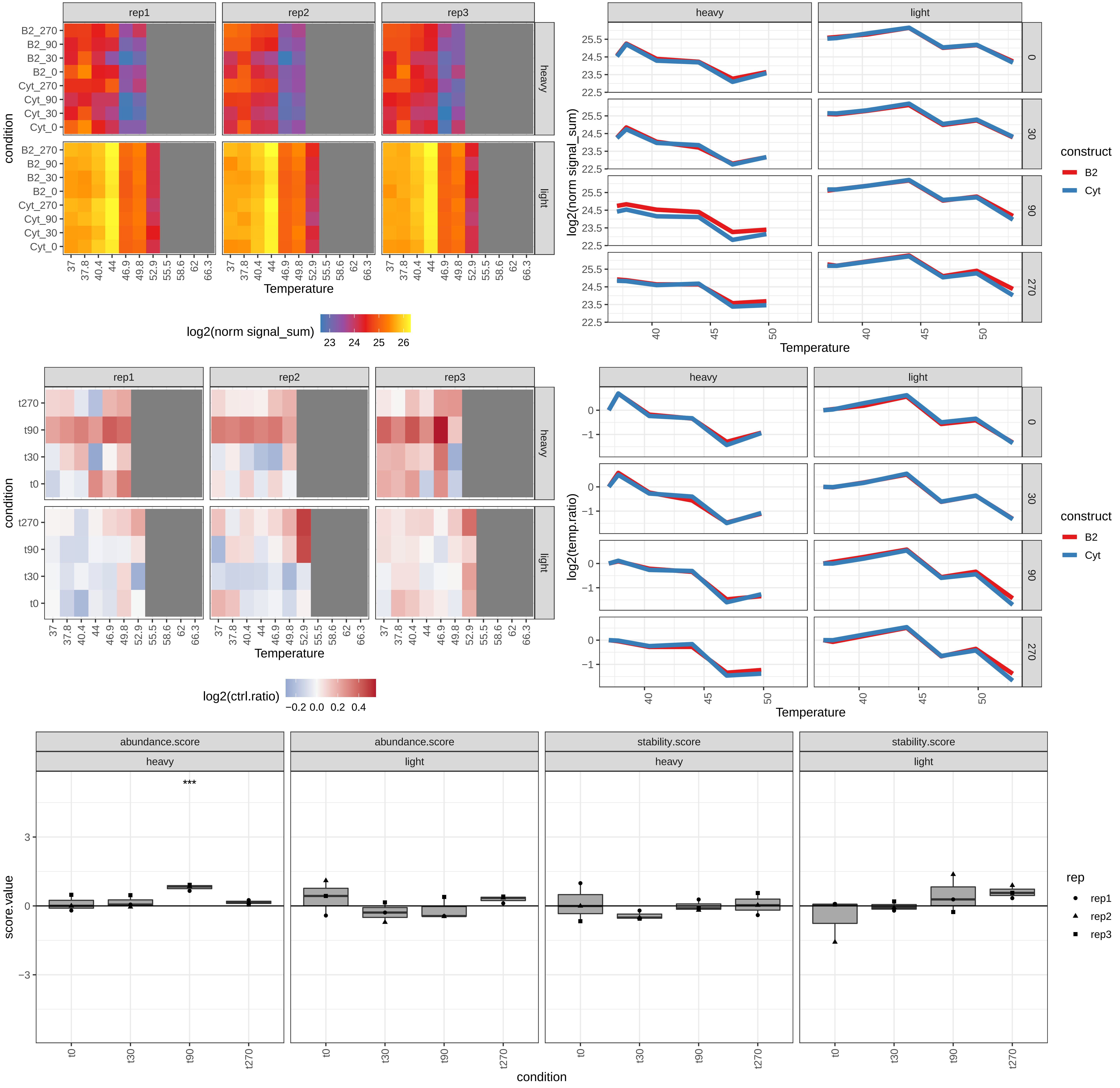

P06634 − ATP−dependent RNA helicase DED1 OS=Saccharomyces cerevisiae (strain ATCC 204508 / S288c) GN=DED1 PE=1 SV=2

DED81 − average.top3: 7.66017066666667 − max.qupm: 24

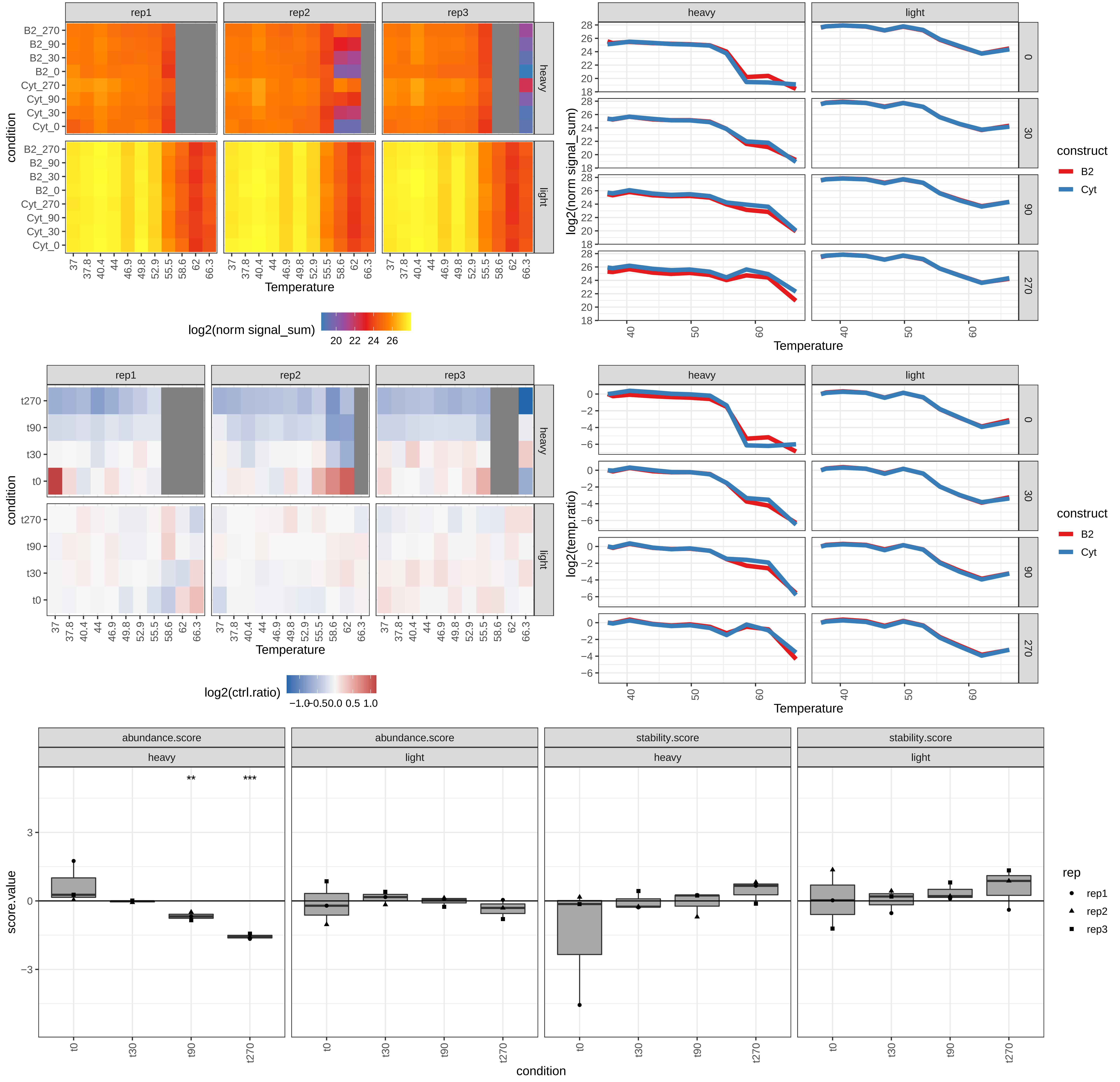

P38707 − Asparagine−−tRNA ligase, cytoplasmic OS=Saccharomyces cerevisiae (strain ATCC 204508 / S288c) GN=DED81 PE=1 SV=1

DEF1 − average.top3: 6.27289222222222 − max.qupm: 4

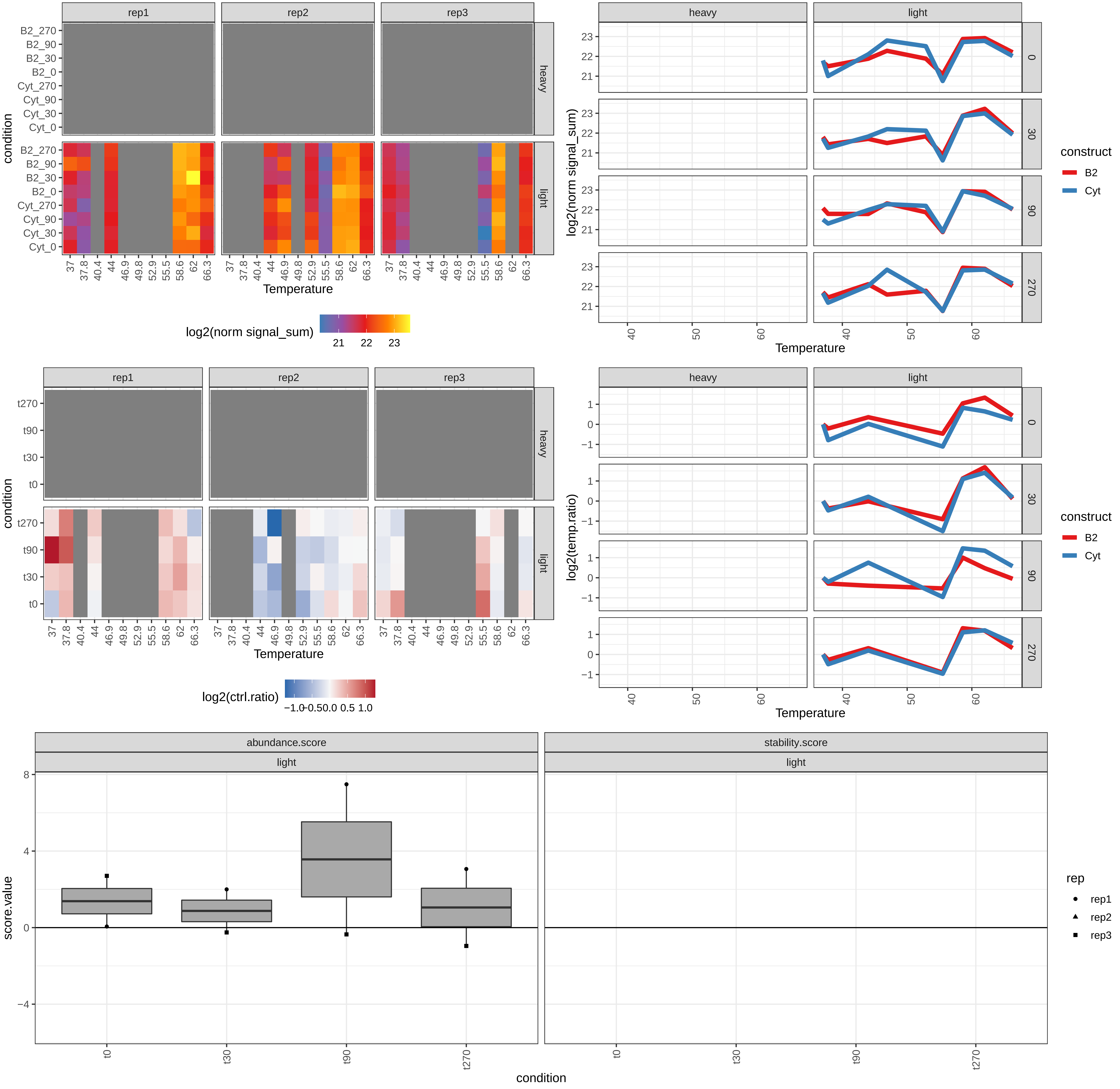

P35732 − RNA polymerase II degradation factor 1 OS=Saccharomyces cerevisiae (strain ATCC 204508 / S288c) GN=DEF1 PE=1 SV=1

DEG1 − average.top3: 6.92416294117647 − max.qupm: 5

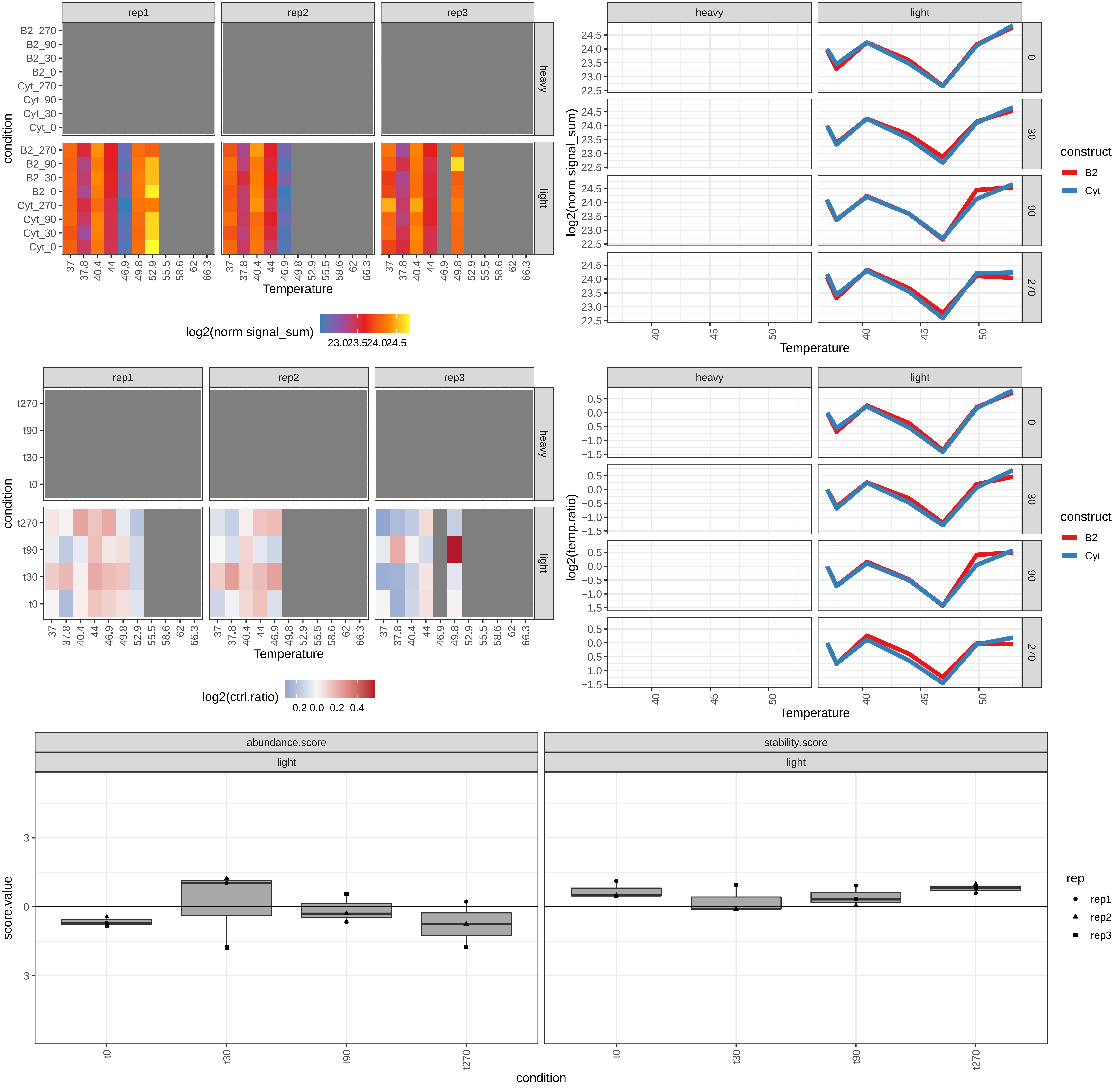

P31115 − tRNA pseudouridine(38/39) synthase OS=Saccharomyces cerevisiae (strain ATCC 204508 / S288c) GN=DEG1 PE=1 SV=1

DEP1 − average.top3: 5.96874466666667 − max.qupm: 3

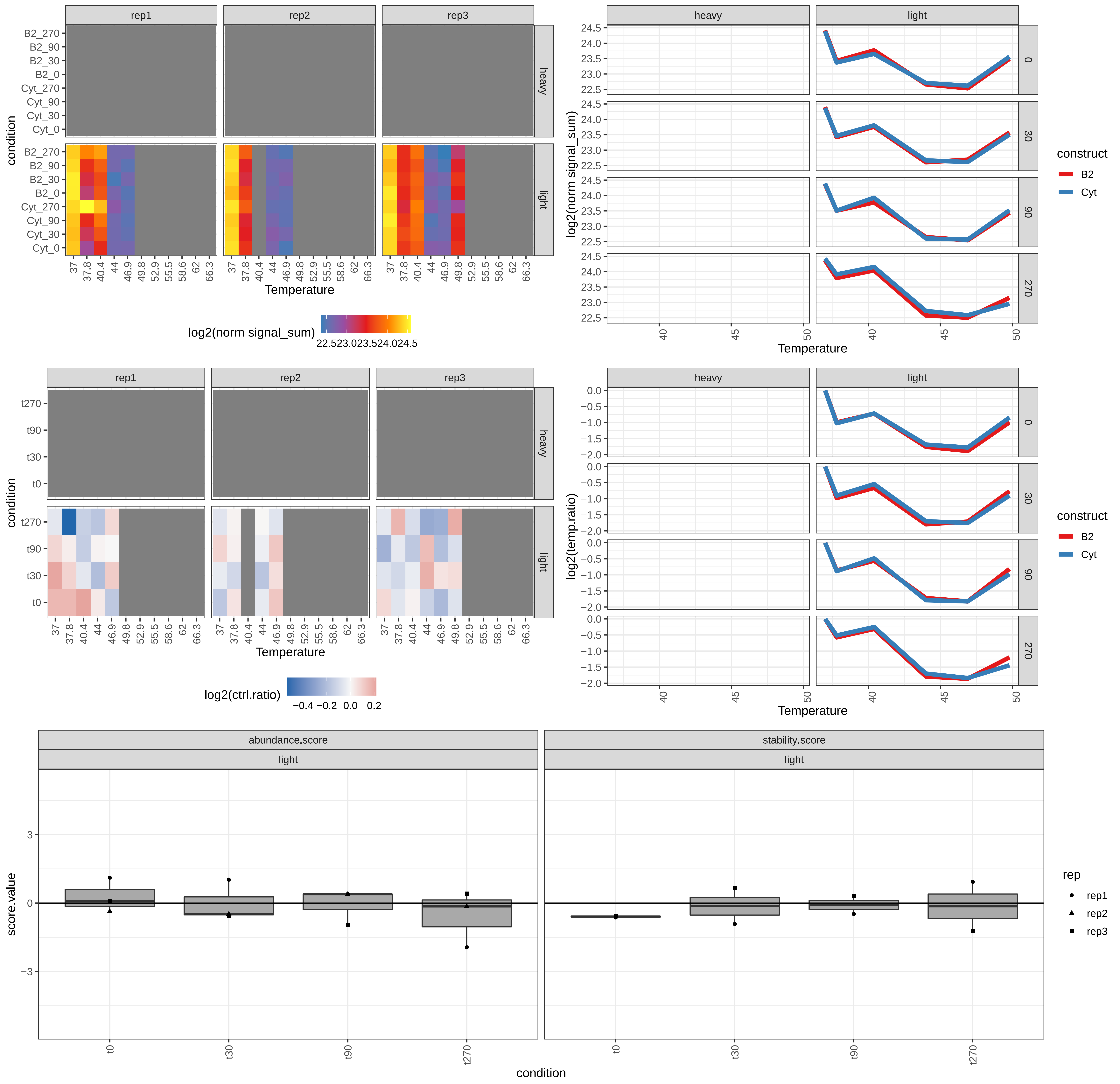

P31385 − Transcriptional regulatory protein DEP1 OS=Saccharomyces cerevisiae (strain ATCC 204508 / S288c) GN=DEP1 PE=1 SV=3

DET1 − average.top3: 6.85189333333333 − max.qupm: 13

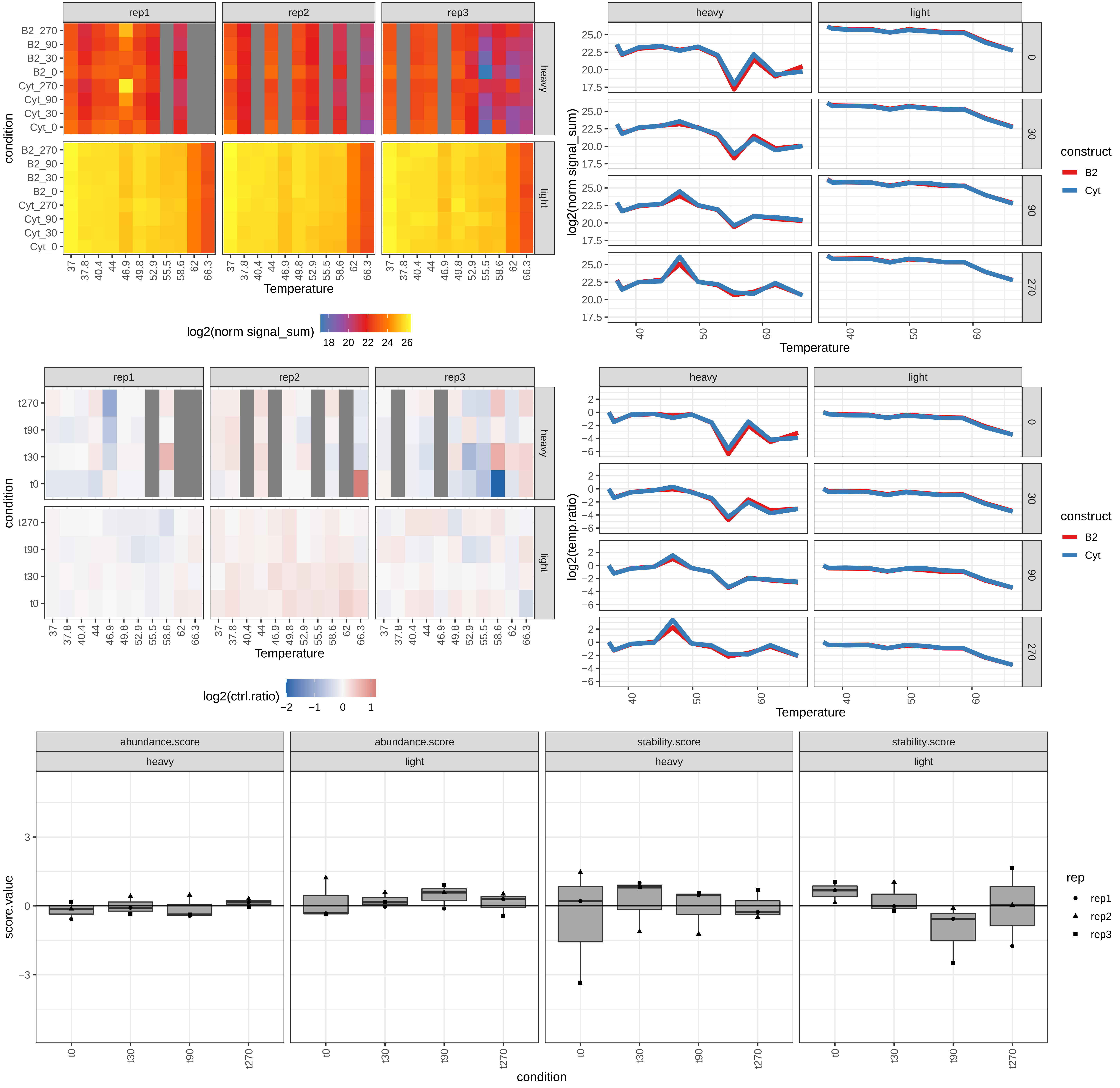

Q99288 − Broad−range acid phosphatase DET1 OS=Saccharomyces cerevisiae (strain ATCC 204508 / S288c) GN=DET1 PE=1 SV=1

DFG5 − average.top3: 6.79302476190476 − max.qupm: 2

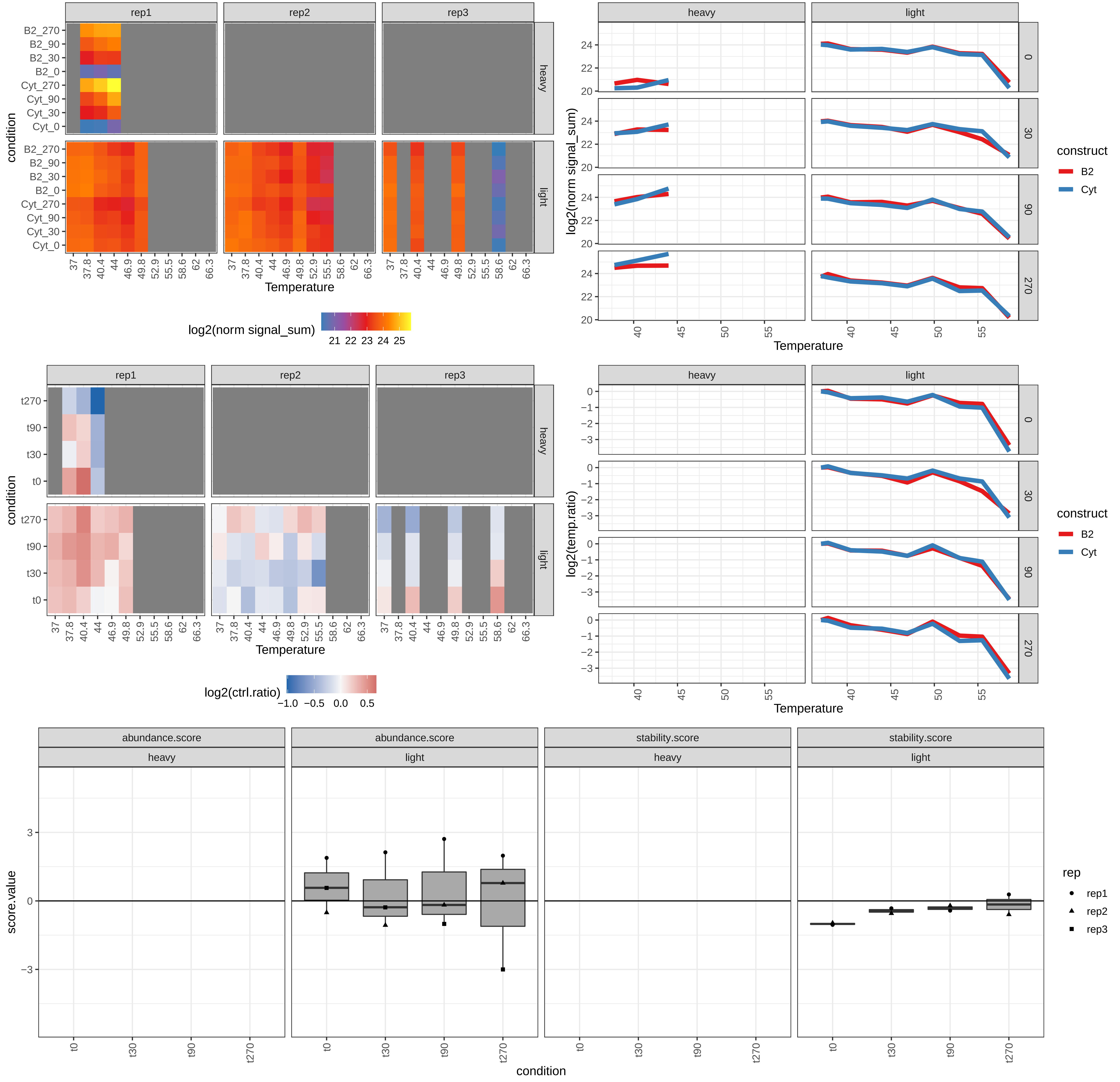

Q05031 − Mannan endo−1,6−alpha−mannosidase DFG5 OS=Saccharomyces cerevisiae (strain ATCC 204508 / S288c) GN=DFG5 PE=1 SV=1

DFM1 − average.top3: 6.914558 − max.qupm: 4

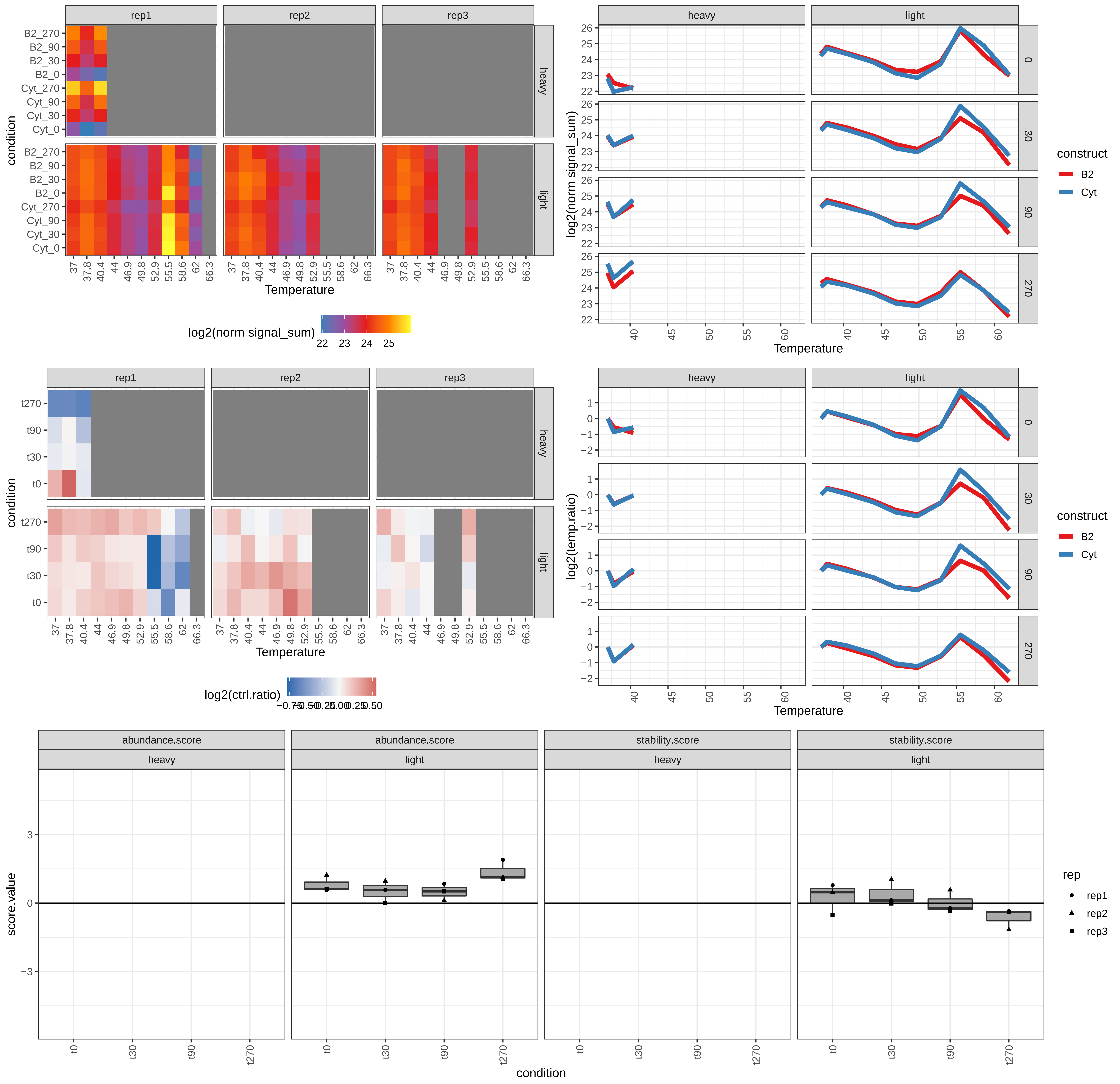

Q12743 − DER1−like family member protein 1 OS=Saccharomyces cerevisiae (strain ATCC 204508 / S288c) GN=DFM1 PE=1 SV=1

DFR1 − average.top3: 6.87264153846154 − max.qupm: 5

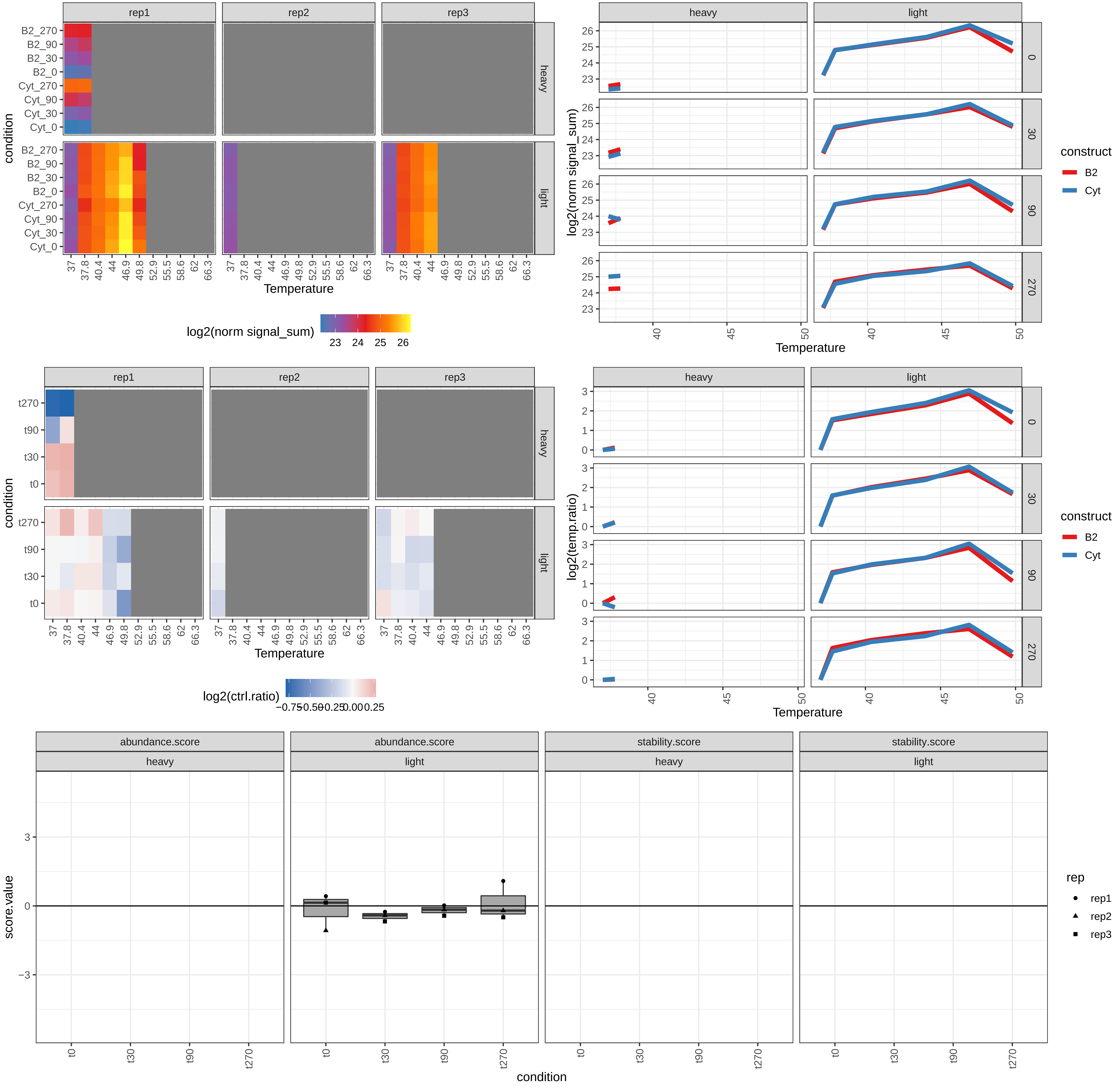

P07807 − Dihydrofolate reductase OS=Saccharomyces cerevisiae (strain ATCC 204508 / S288c) GN=DFR1 PE=1 SV=2

DGK1 − average.top3: 6.52665111111111 − max.qupm: 3

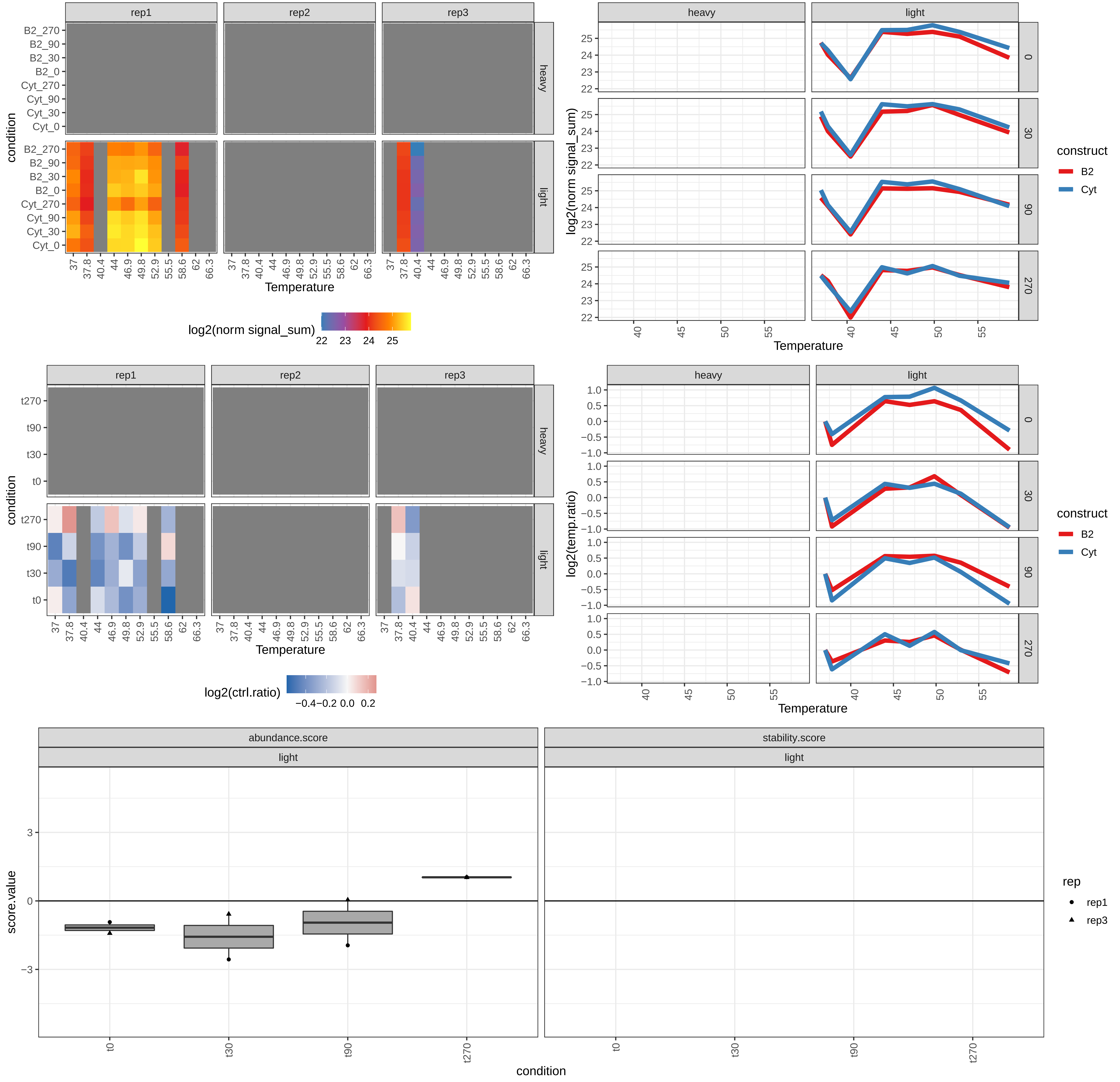

Q12382 − CTP−dependent diacylglycerol kinase 1 OS=Saccharomyces cerevisiae (strain ATCC 204508 / S288c) GN=DGK1 PE=1 SV=1

DHH1 − average.top3: 7.30982868421053 − max.qupm: 9

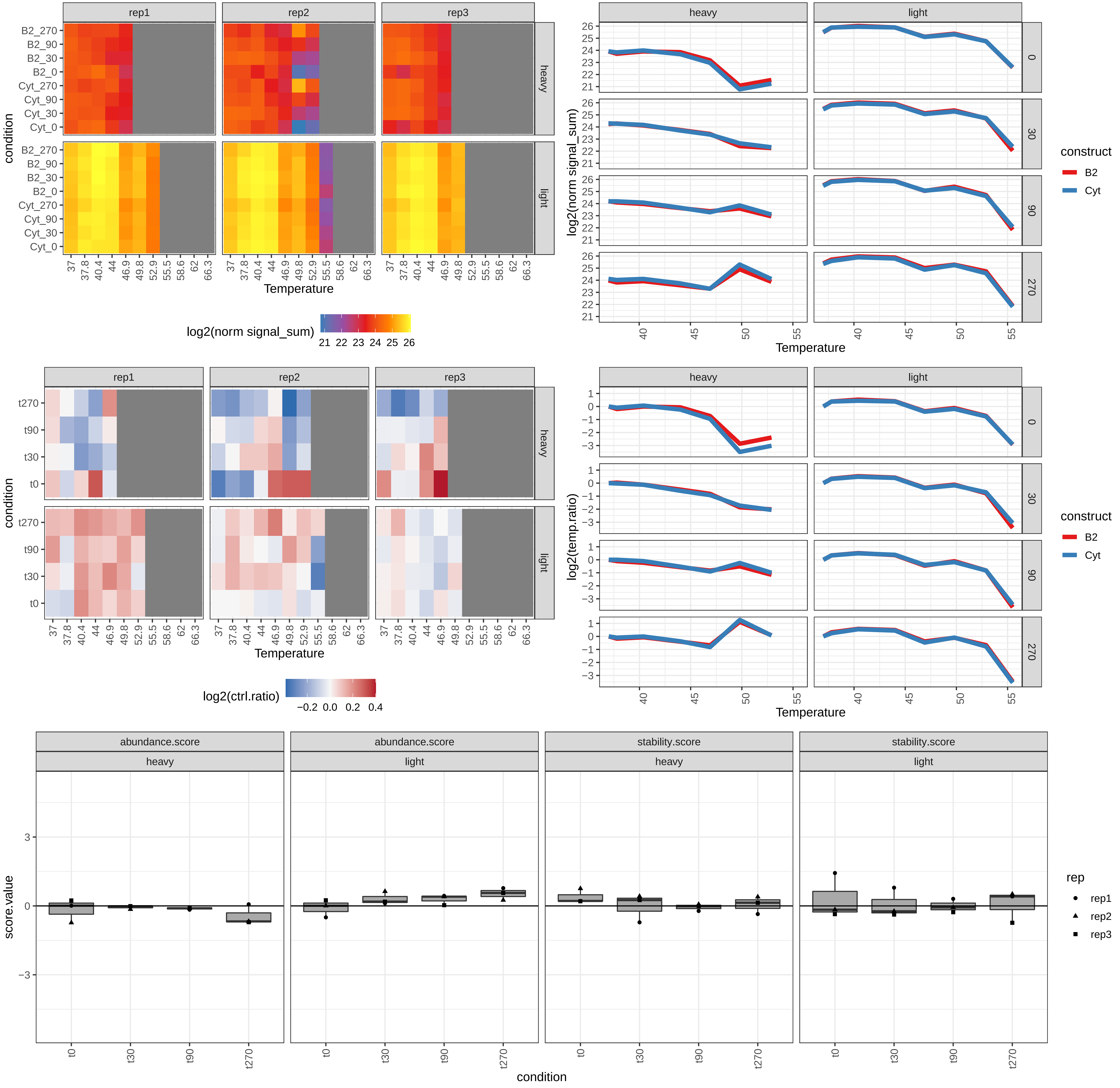

P39517 − ATP−dependent RNA helicase DHH1 OS=Saccharomyces cerevisiae (strain ATCC 204508 / S288c) GN=DHH1 PE=1 SV=1

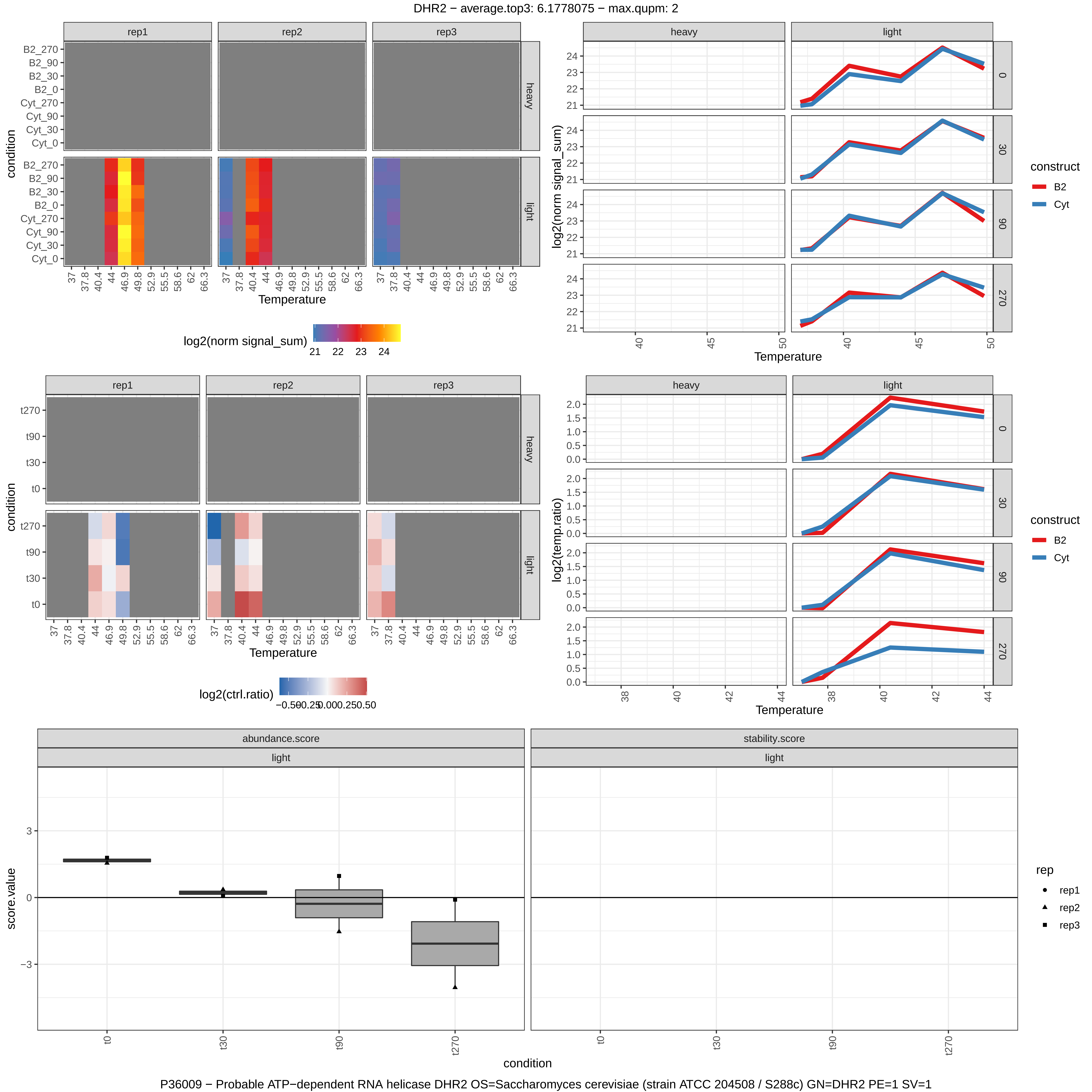
DHR2 − average.top3: 6.1778075 − max.qupm: 2
rep1
rep2
rep3
heavy
light
condition
Temperature
log2(norm signal_sum)
log2(ctrl.ratio)
log2(temp.ratio)
construct
B2
Cyt
abundance.score
stability.score
score.value
rep
rep1
rep2
rep3
P36009 − Probable ATP−dependent RNA helicase DHR2 OS=Saccharomyces cerevisiae (strain ATCC 204508 / S288c) GN=DHR2 PE=1 SV=1

DIA4 − average.top3: 6.31167555555556 − max.qupm: 3

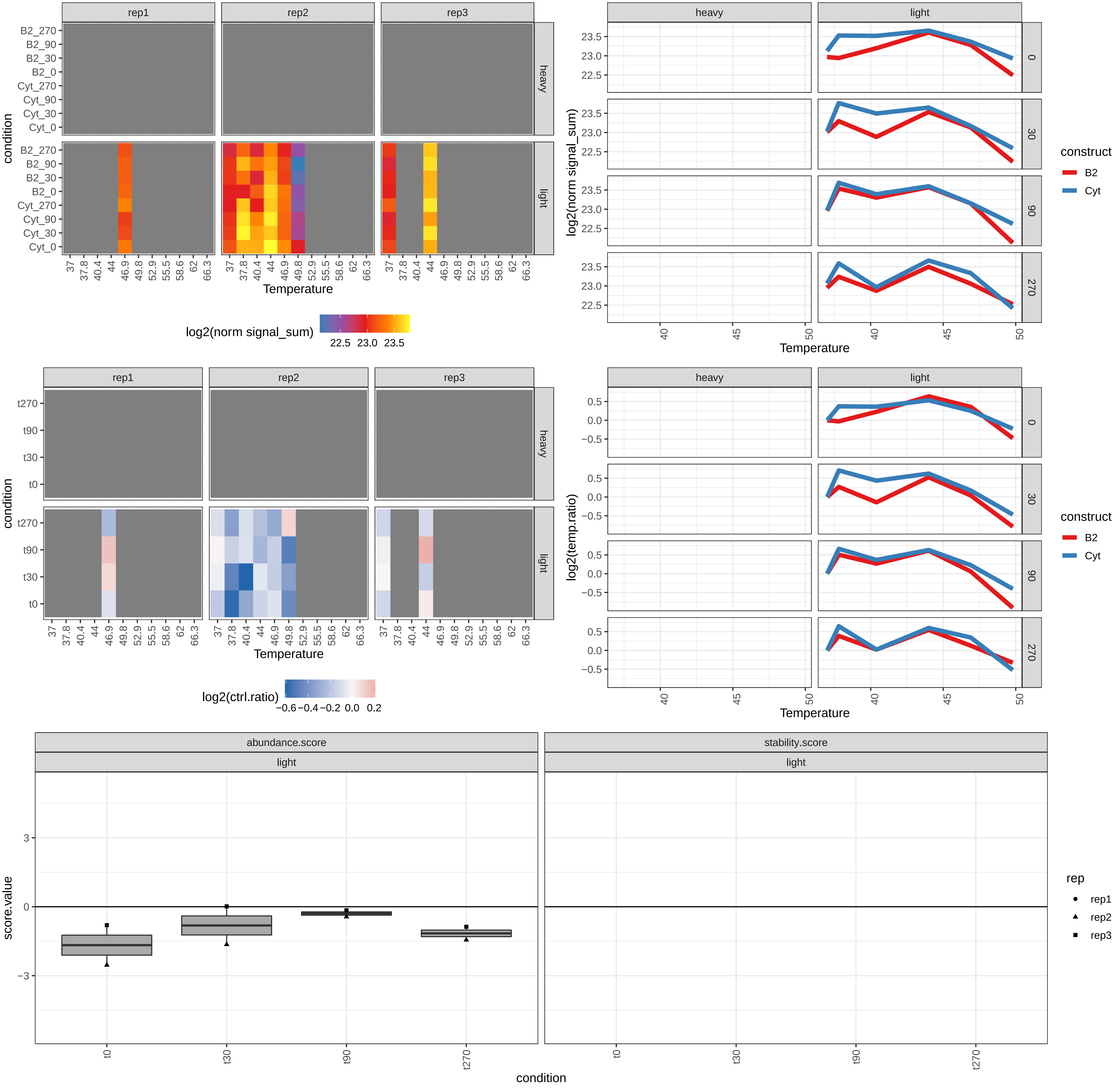

P38705 − Serine−−tRNA ligase, mitochondrial OS=Saccharomyces cerevisiae (strain ATCC 204508 / S288c) GN=DIA4 PE=1 SV=1

DIC1 − average.top3: 6.99821782608696 − max.qupm: 5

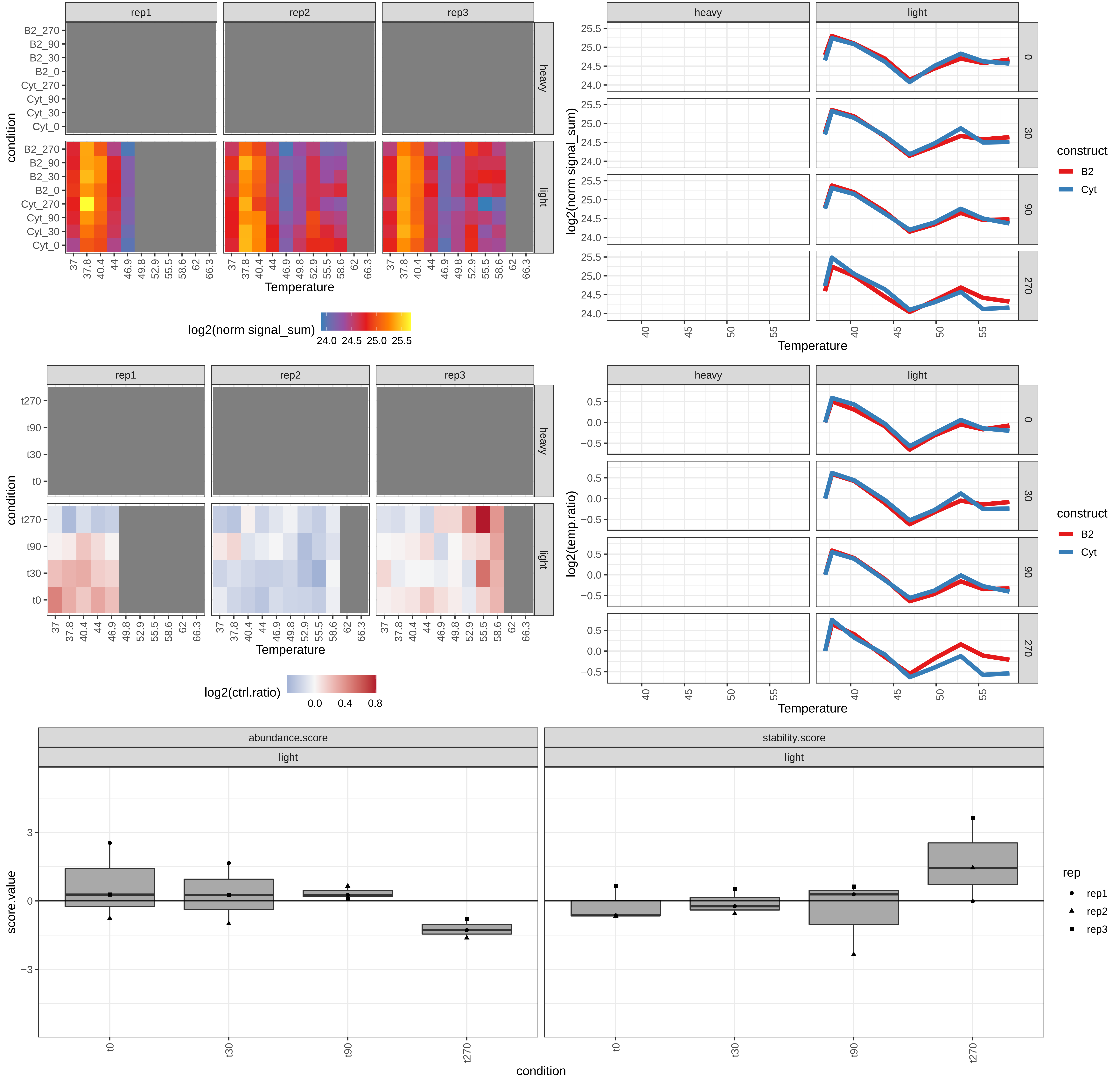

Q06143 − Mitochondrial dicarboxylate transporter OS=Saccharomyces cerevisiae (strain ATCC 204508 / S288c) GN=DIC1 PE=1 SV=1

DID4 − average.top3: 7.08581961538462 − max.qupm: 6

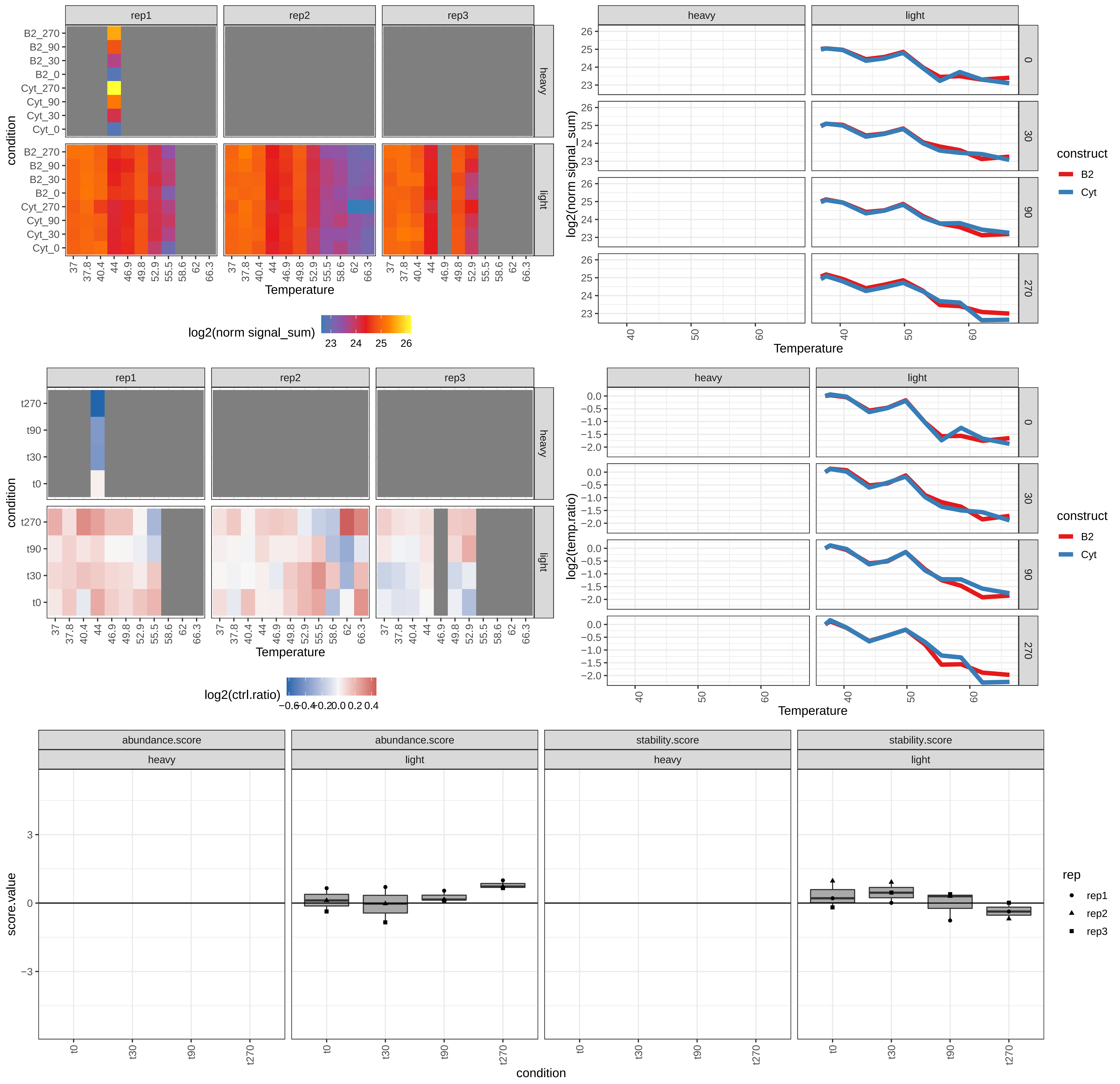

P36108 − DOA4−independent degradation protein 4 OS=Saccharomyces cerevisiae (strain ATCC 204508 / S288c) GN=DID4 PE=1 SV=2

DIM1 − average.top3: 6.974927 − max.qupm: 8

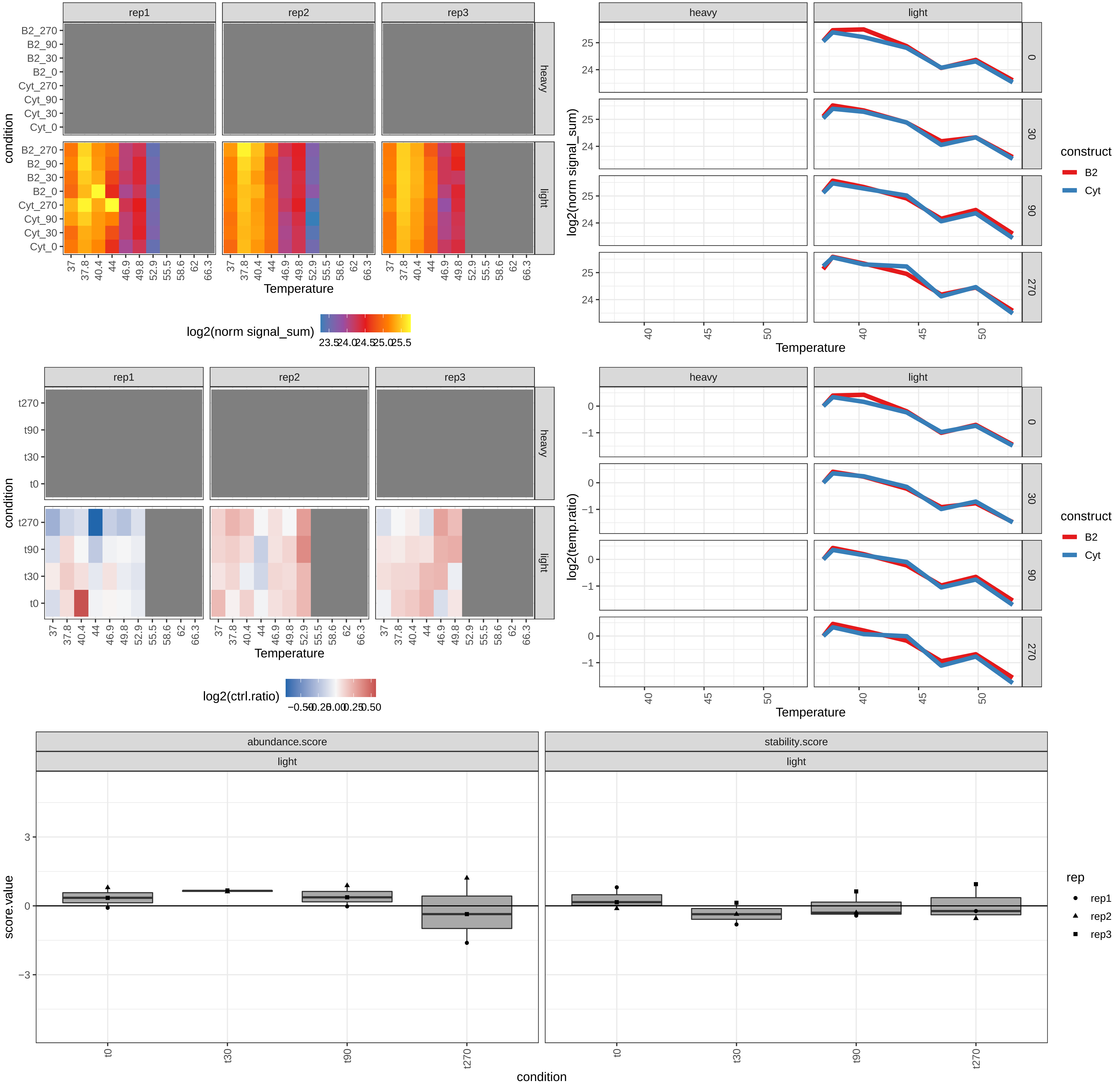

P41819 − Dimethyladenosine transferase OS=Saccharomyces cerevisiae (strain ATCC 204508 / S288c) GN=DIM1 PE=3 SV=1

DIP2 − average.top3: 6.94013405405405 − max.qupm: 15

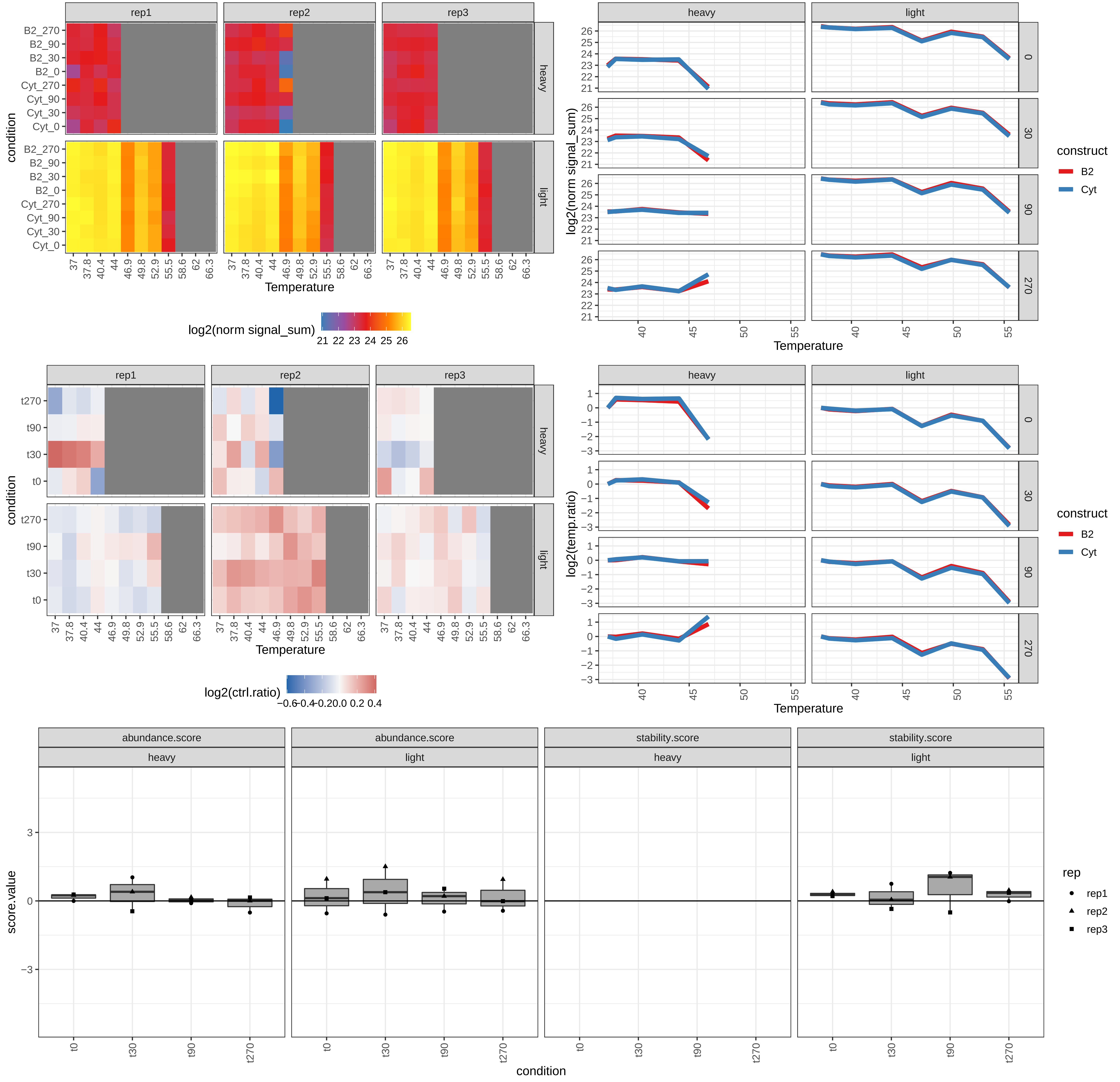

Q12220 − U3 small nucleolar RNA−associated protein 12 OS=Saccharomyces cerevisiae (strain ATCC 204508 / S288c) GN=DIP2 PE=1 SV=1

DIP5 − average.top3: 6.83848222222222 − max.qupm: 8

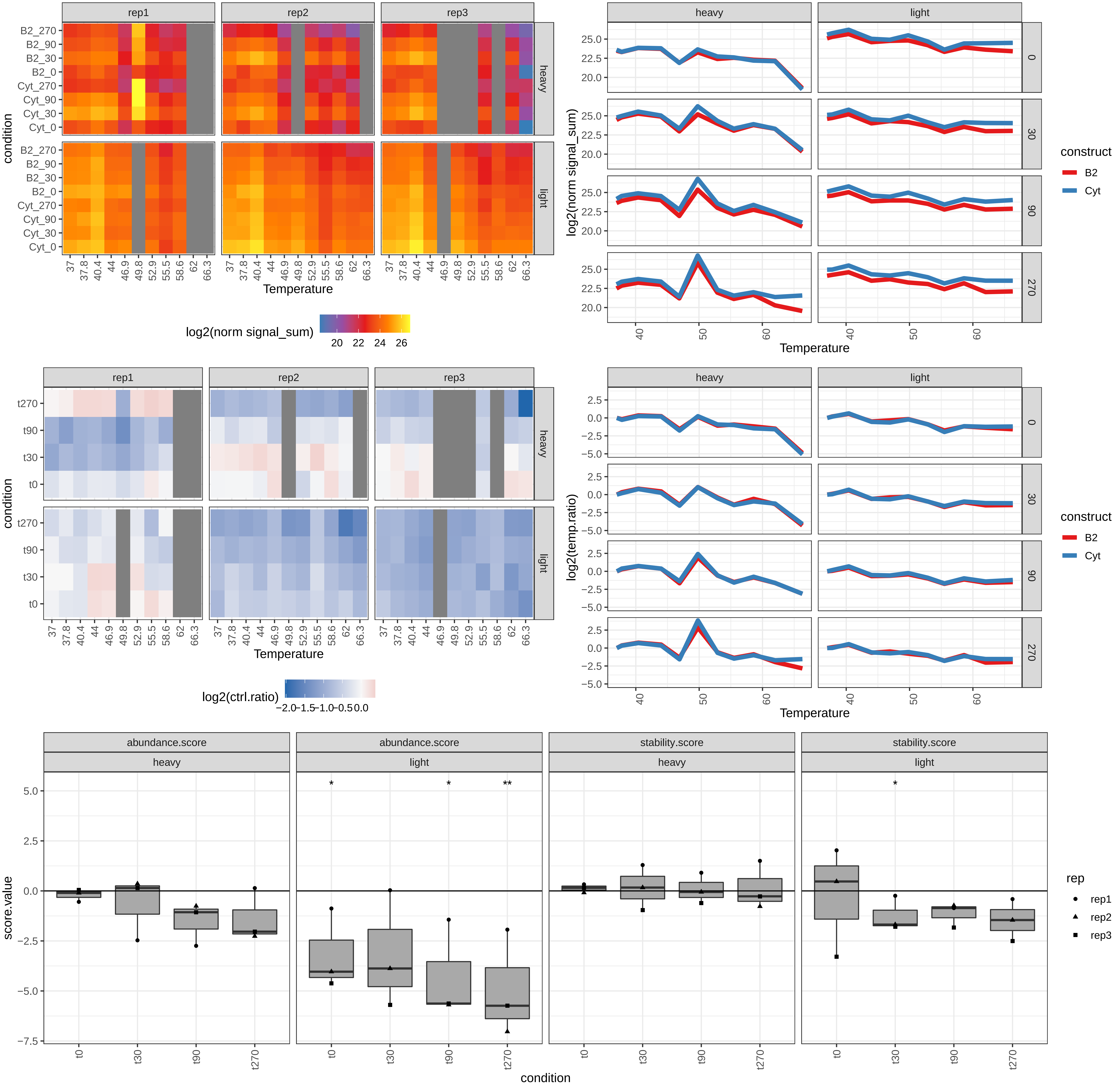

P53388 − Dicarboxylic amino acid permease OS=Saccharomyces cerevisiae (strain ATCC 204508 / S288c) GN=DIP5 PE=1 SV=1

DIS3 − average.top3: 6.97621526315789 − max.qupm: 21

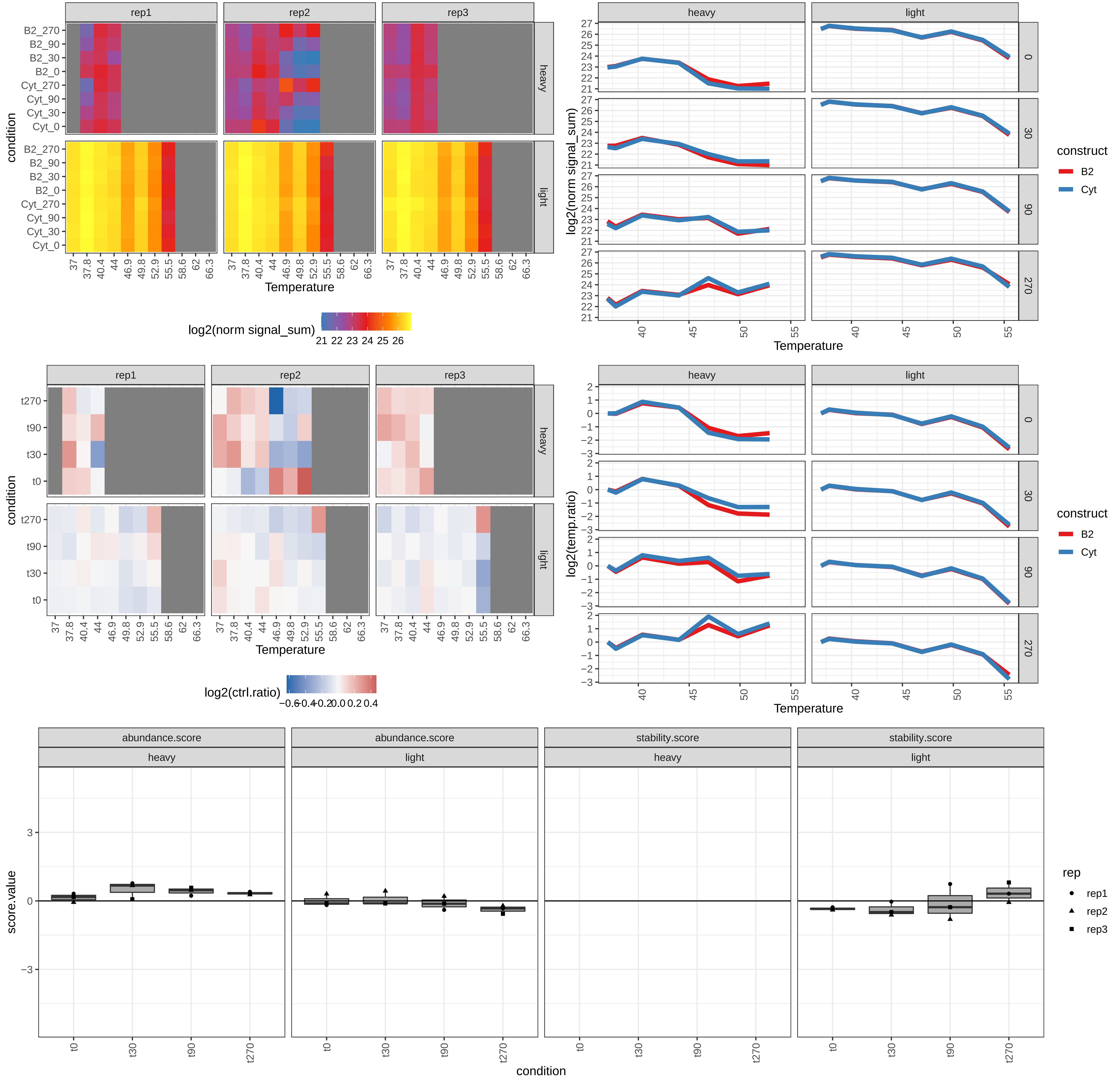

Q08162 − Exosome complex exonuclease DIS3 OS=Saccharomyces cerevisiae (strain ATCC 204508 / S288c) GN=DIS3 PE=1 SV=1

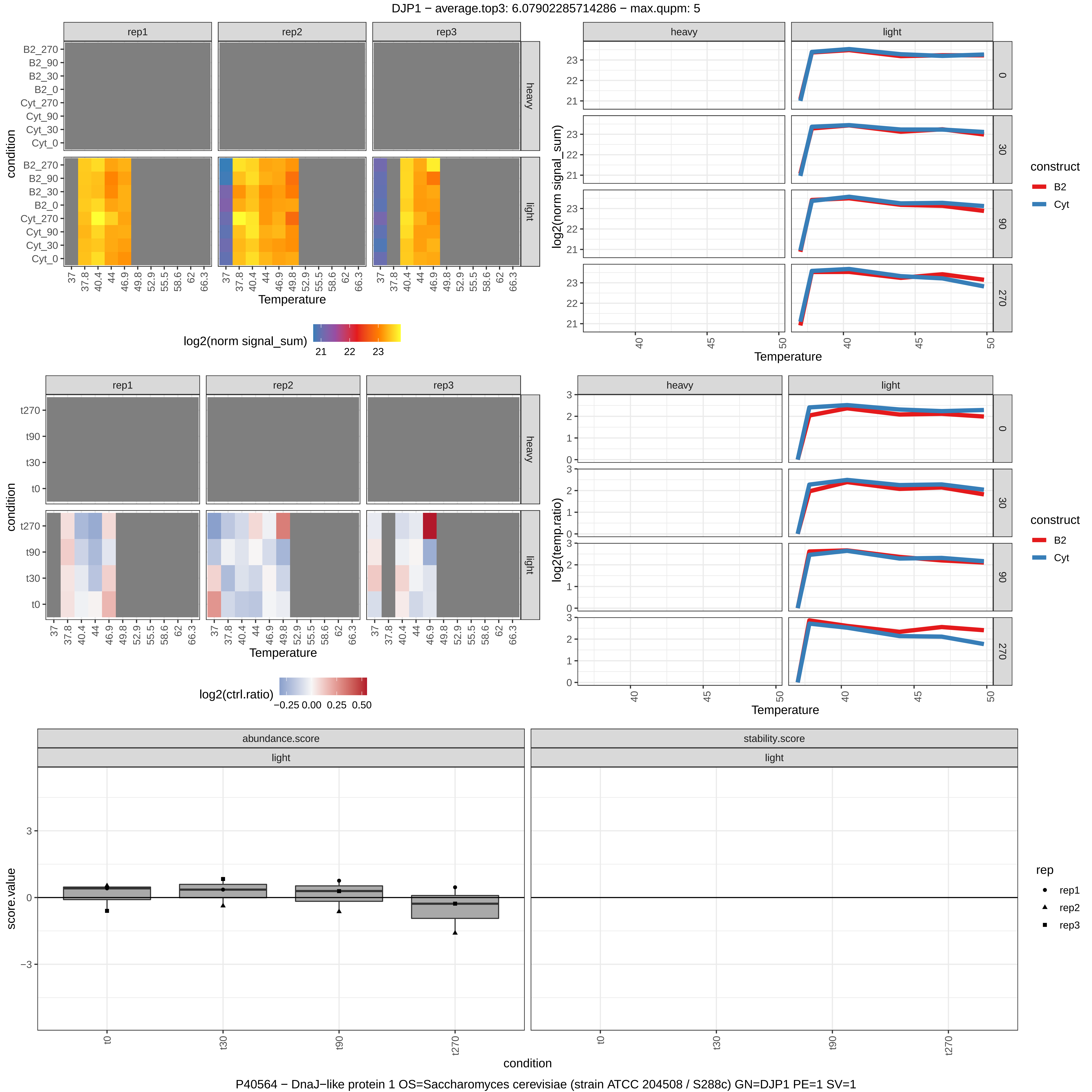
DJP1 − average.top3: 6.07902285714286 − max.qupm: 5
rep1
rep2
rep3
heavy
light
condition
Temperature
log2(norm signal_sum)
log2(ctrl.ratio)
log2(temp.ratio)
construct
B2
Cyt
abundance.score
stability.score
score.value
rep
P40564 − DnaJ−like protein 1 OS=Saccharomyces cerevisiae (strain ATCC 204508 / S288c) GN=DJP1 PE=1 SV=1

DLD1 − average.top3: 7.88669651515151 − max.qupm: 28

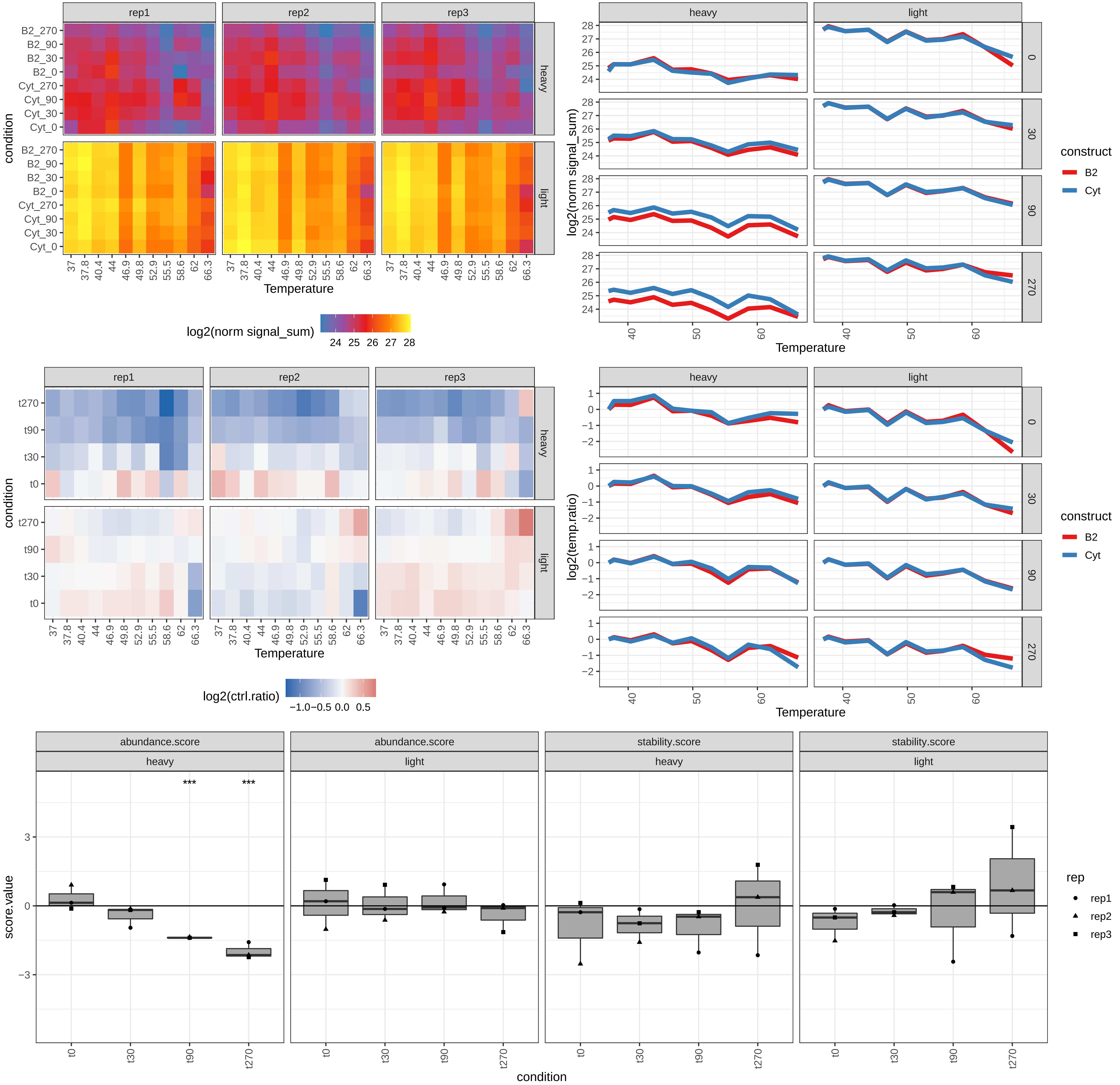

P32891 − D−lactate dehydrogenase [cytochrome] 1, mitochondrial OS=Saccharomyces cerevisiae (strain ATCC 204508 / S288c) GN=DLD1 PE=1 SV=2

DLD2 − average.top3: 6.8656862962963 − max.qupm: 13

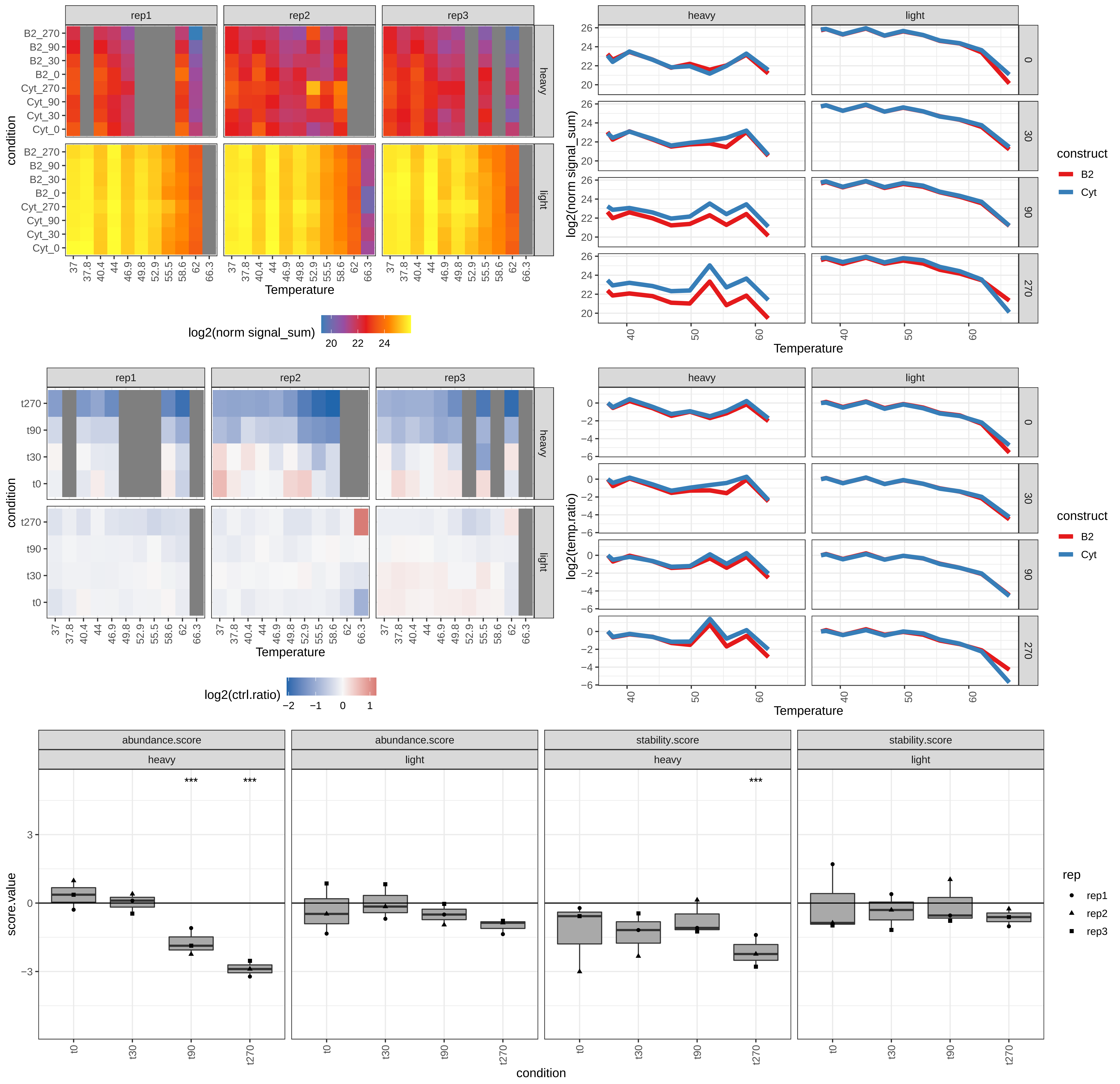

P46681 − D−lactate dehydrogenase [cytochrome] 2, mitochondrial OS=Saccharomyces cerevisiae (strain ATCC 204508 / S288c) GN=DLD2 PE=1 SV=1

DLD3 − average.top3: 6.74746140350877 − max.qupm: 10

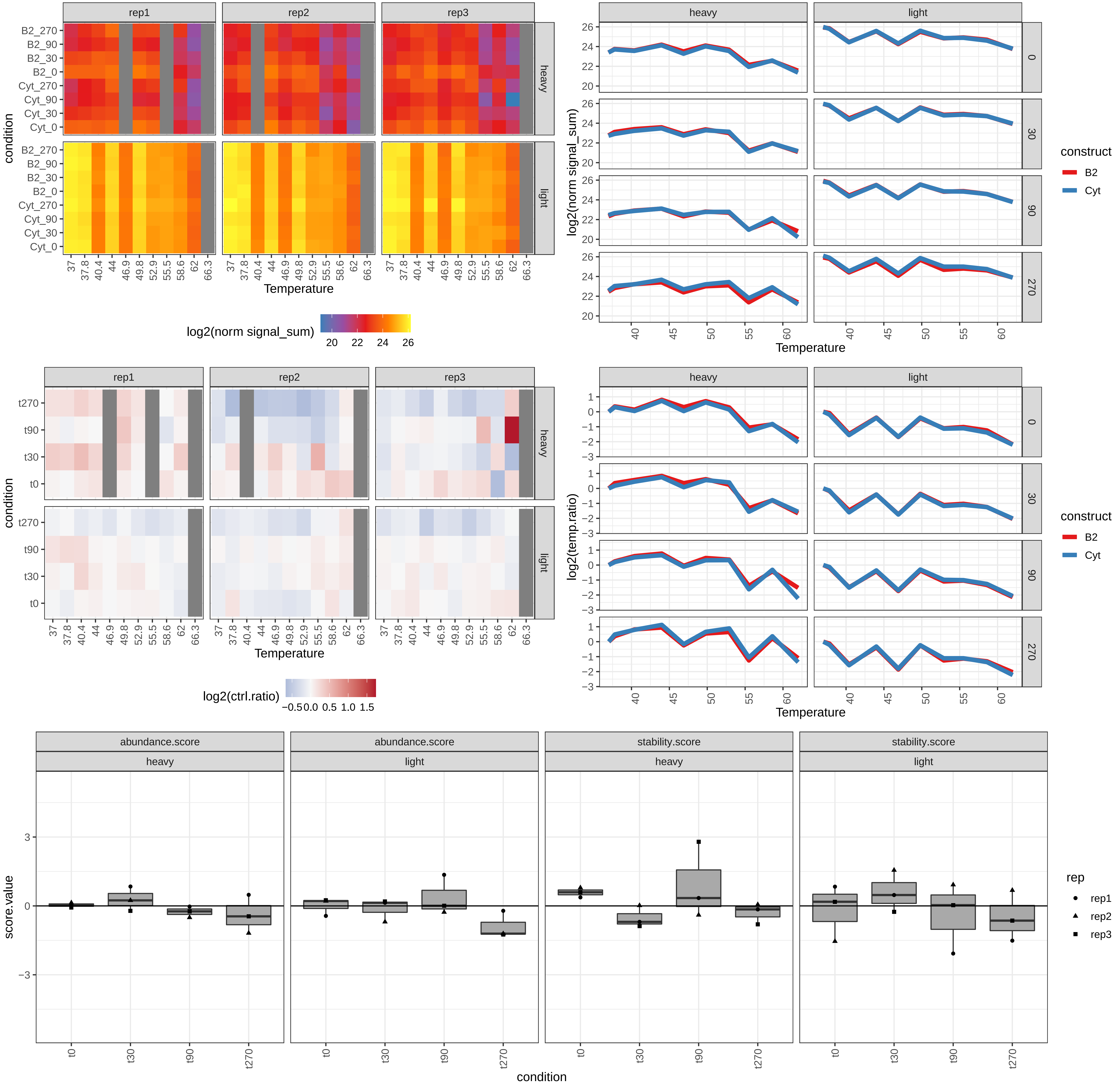

P39976 − D−lactate dehydrogenase [cytochrome] 3 OS=Saccharomyces cerevisiae (strain ATCC 204508 / S288c) GN=DLD3 PE=1 SV=1

DMA2 − average.top3: 6.47656642857143 − max.qupm: 4

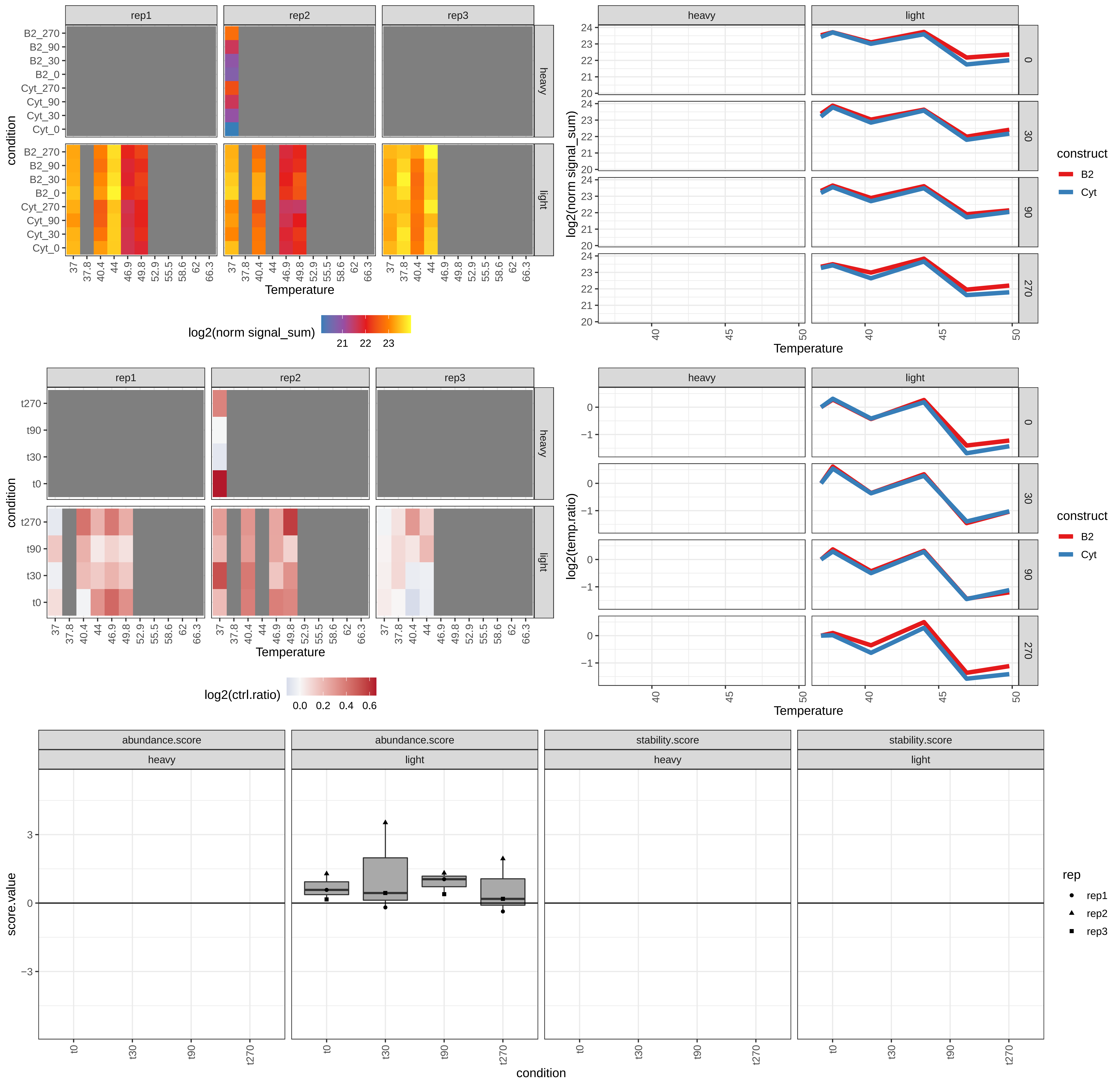

P53924 − E3 ubiquitin−protein ligase DMA2 OS=Saccharomyces cerevisiae (strain ATCC 204508 / S288c) GN=DMA2 PE=1 SV=1

DNF1 − average.top3: 6.79490366666667 − max.qupm: 11

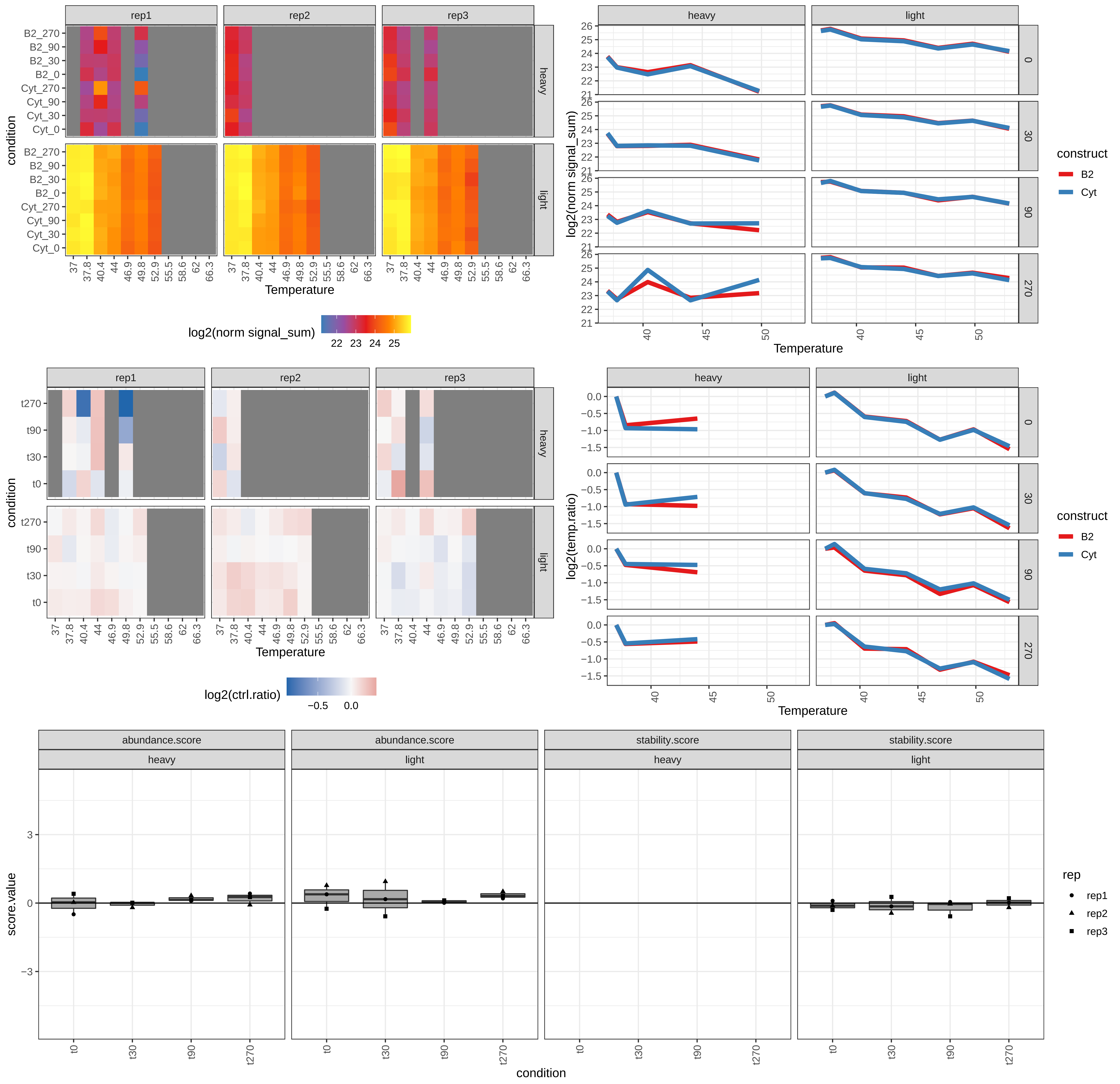

P32660 − Phospholipid−transporting ATPase DNF1 OS=Saccharomyces cerevisiae (strain ATCC 204508 / S288c) GN=DNF1 PE=1 SV=2

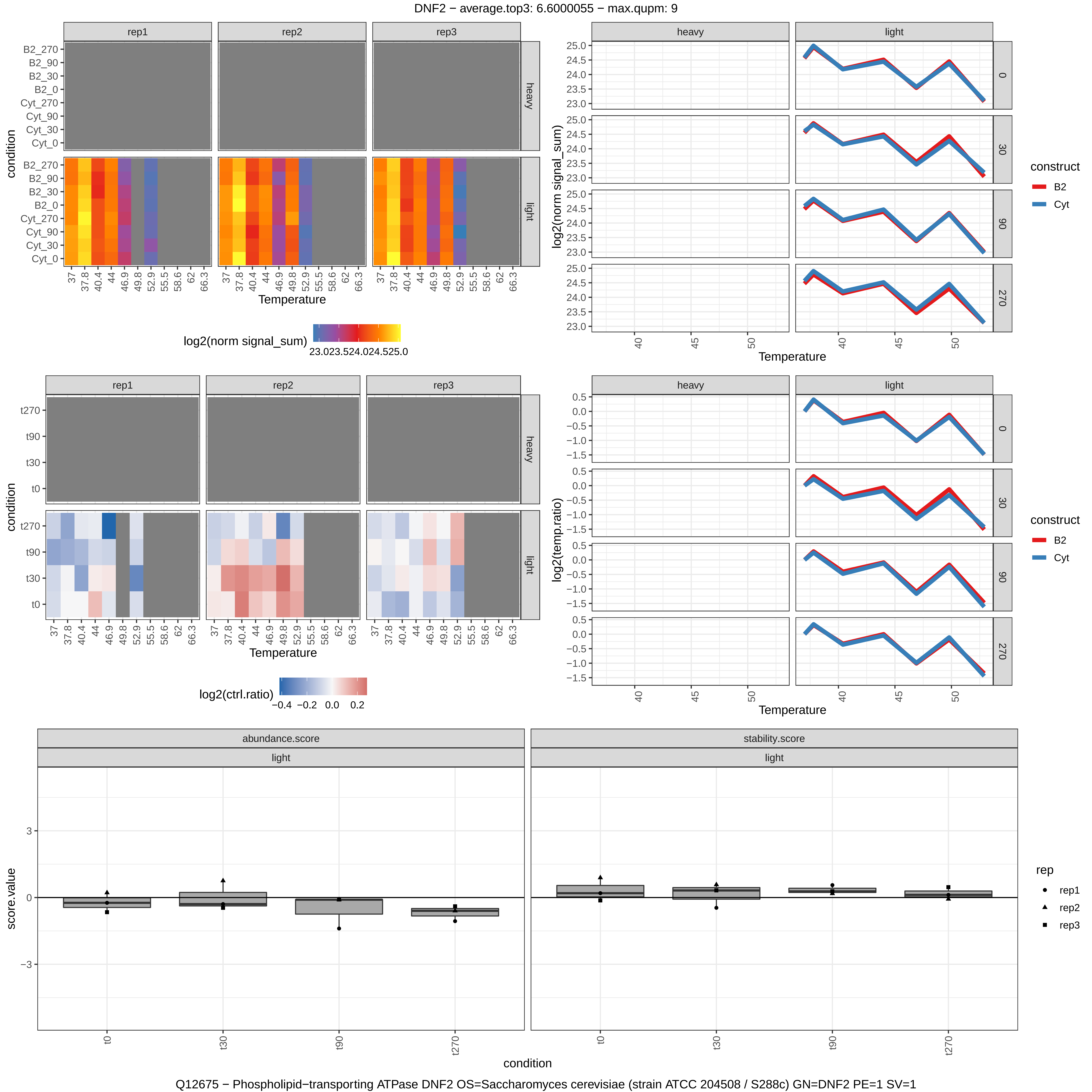

DNF2 − average.top3: 6.6000055 − max.qupm: 9
rep1
rep2
rep3
heavy
light
condition
Temperature
log2(norm signal_sum)
log2(ctrl.ratio)
log2(temp.ratio)
construct
B2
Cyt
abundance.score
stability.score
score.value
rep
rep1
rep2
rep3
Q12675 − Phospholipid−transporting ATPase DNF2 OS=Saccharomyces cerevisiae (strain ATCC 204508 / S288c) GN=DNF2 PE=1 SV=1

DNM1 − average.top3: 6.96971371428571 − max.qupm: 21

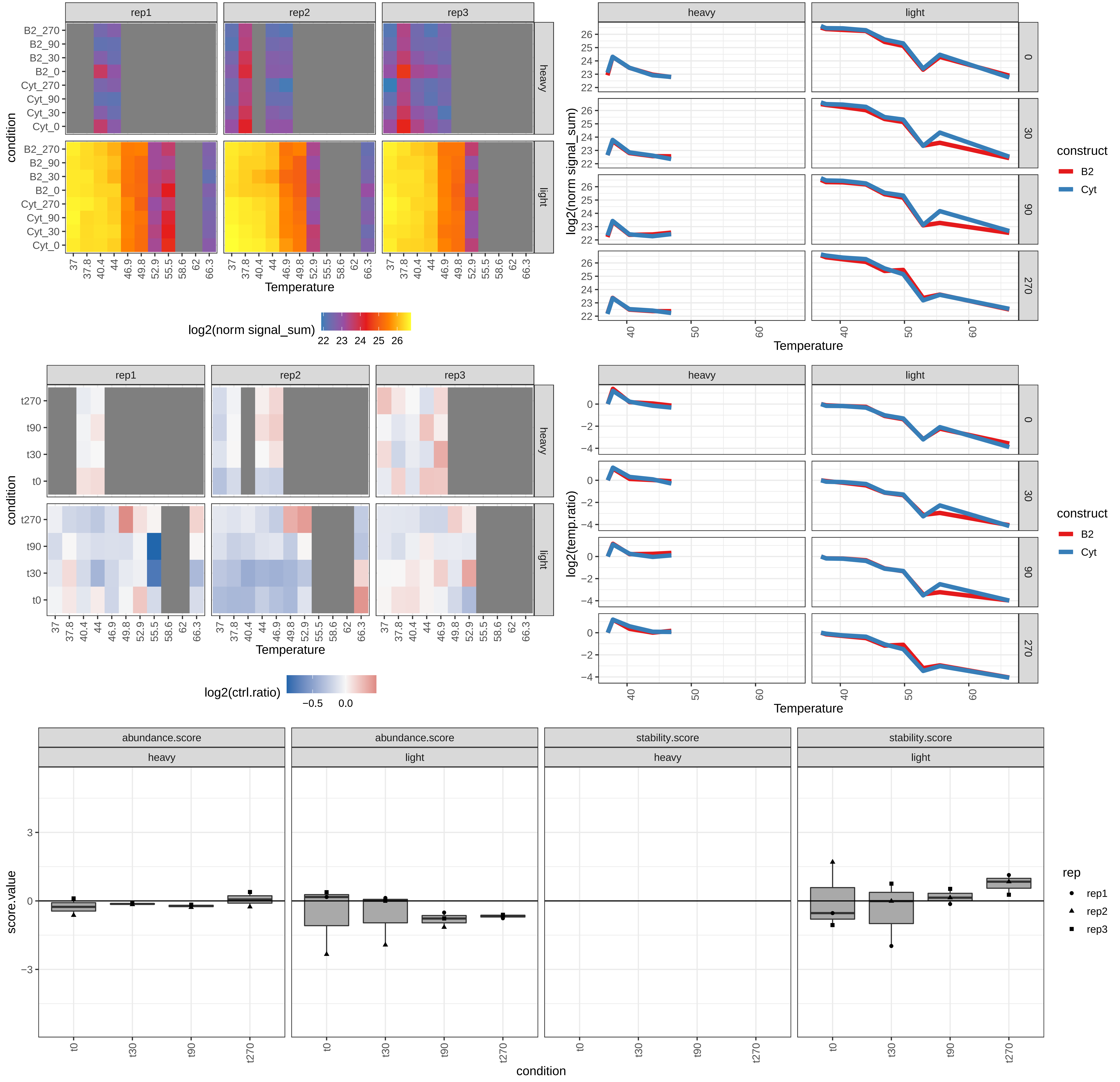

P54861 − Dynamin−related protein DNM1 OS=Saccharomyces cerevisiae (strain ATCC 204508 / S288c) GN=DNM1 PE=1 SV=1

DOA1 − average.top3: 7.0976426984127 − max.qupm: 20

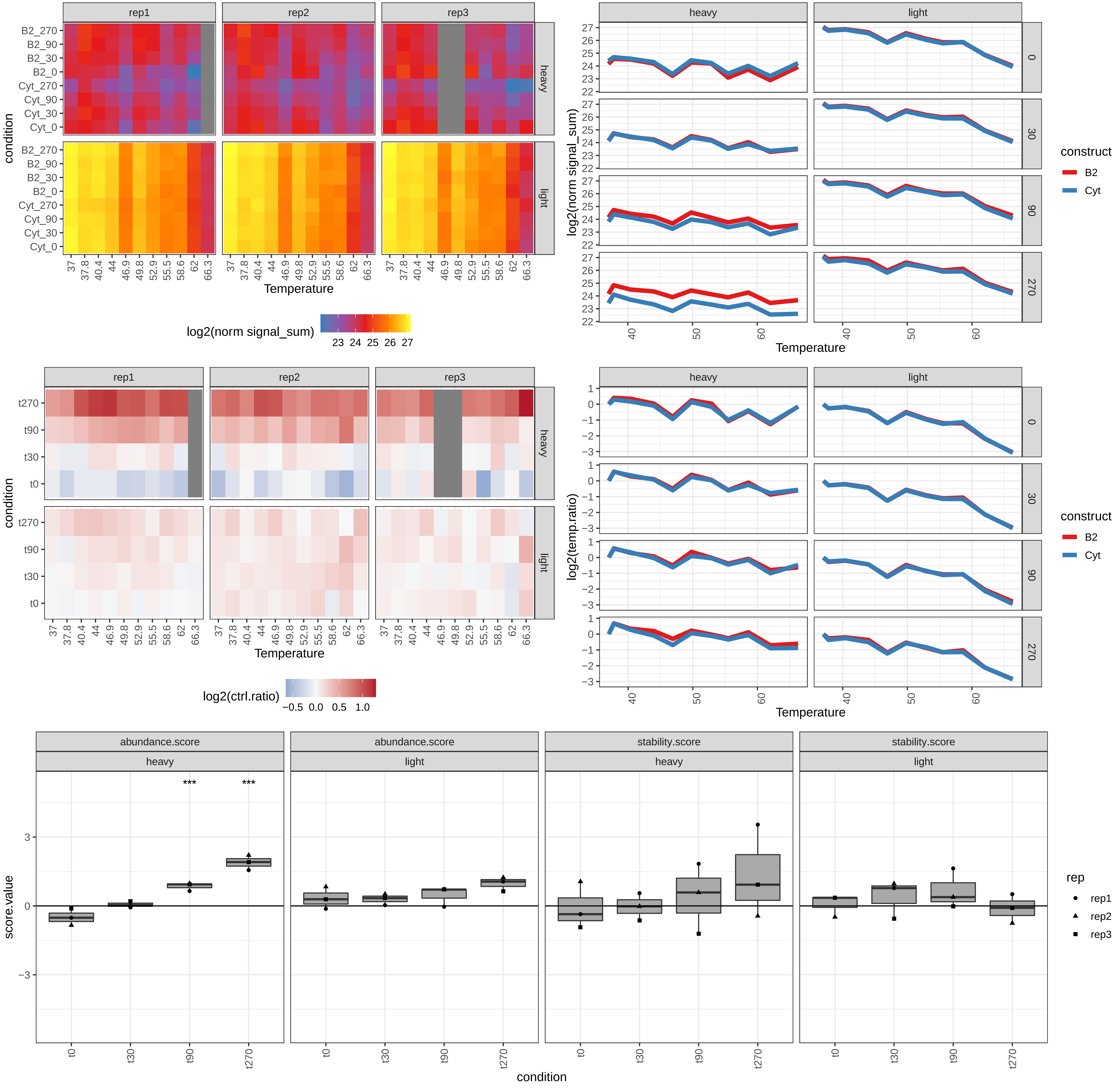

P36037 − Protein DOA1 OS=Saccharomyces cerevisiae (strain ATCC 204508 / S288c) GN=DOA1 PE=1 SV=1

DOA4 − average.top3: 6.25722625 − max.qupm: 4

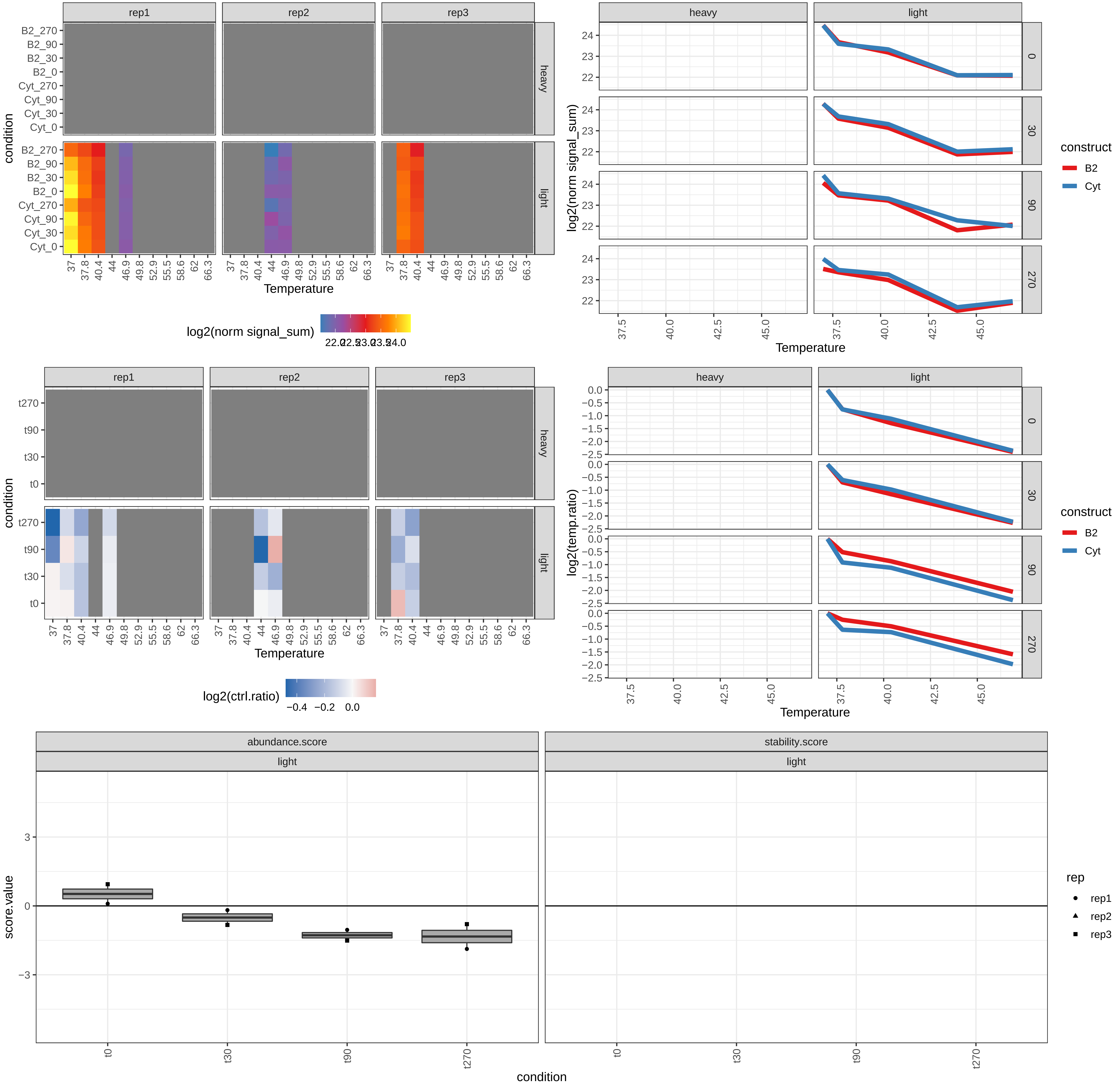

P32571 − Ubiquitin carboxyl−terminal hydrolase 4 OS=Saccharomyces cerevisiae (strain ATCC 204508 / S288c) GN=DOA4 PE=1 SV=2

DOG2 − average.top3: 7.16403666666667 − max.qupm: 5

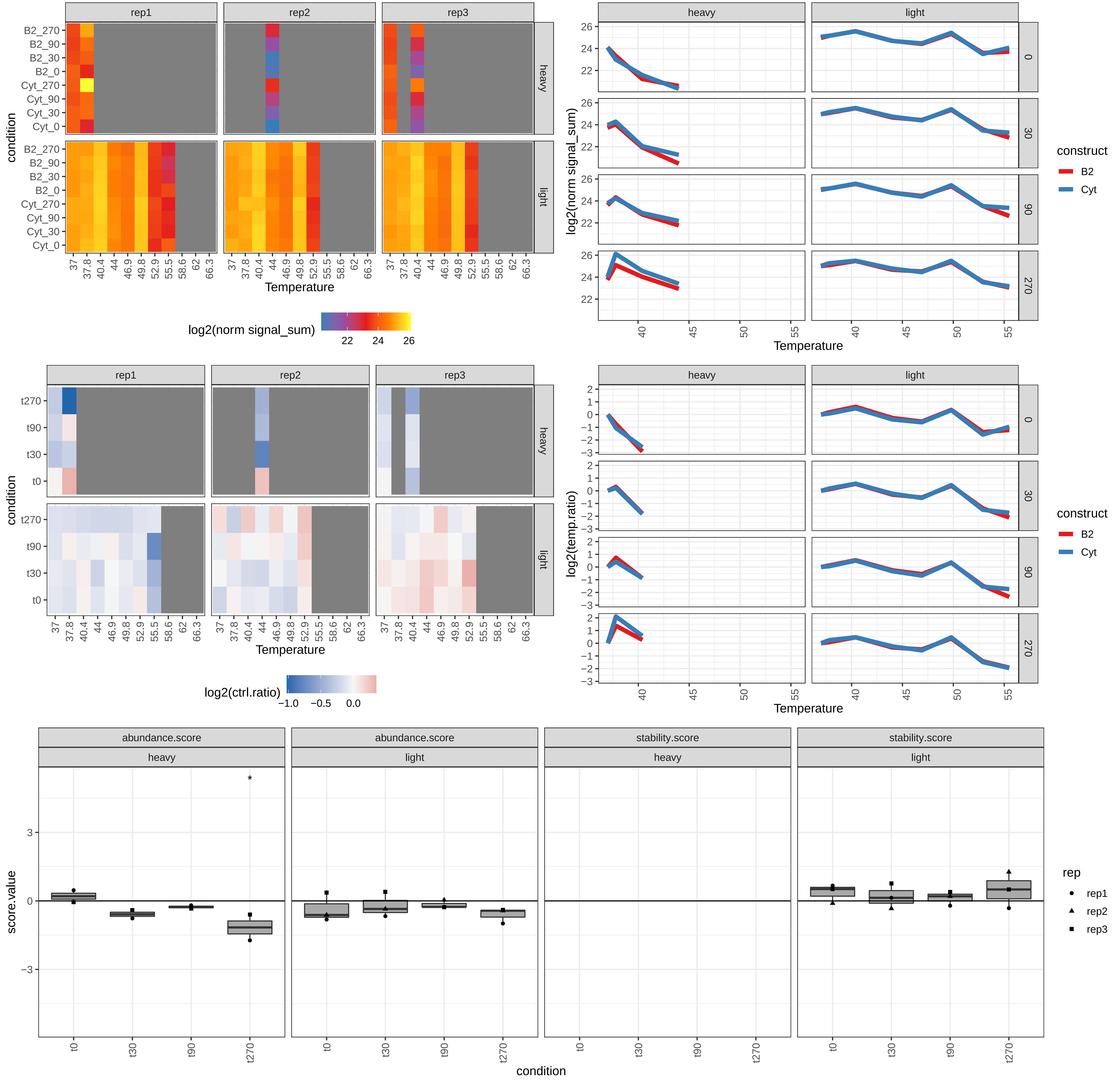

P38773 − 2−deoxyglucose−6−phosphate phosphatase 2 OS=Saccharomyces cerevisiae (strain ATCC 204508 / S288c) GN=DOG2 PE=1 SV=1

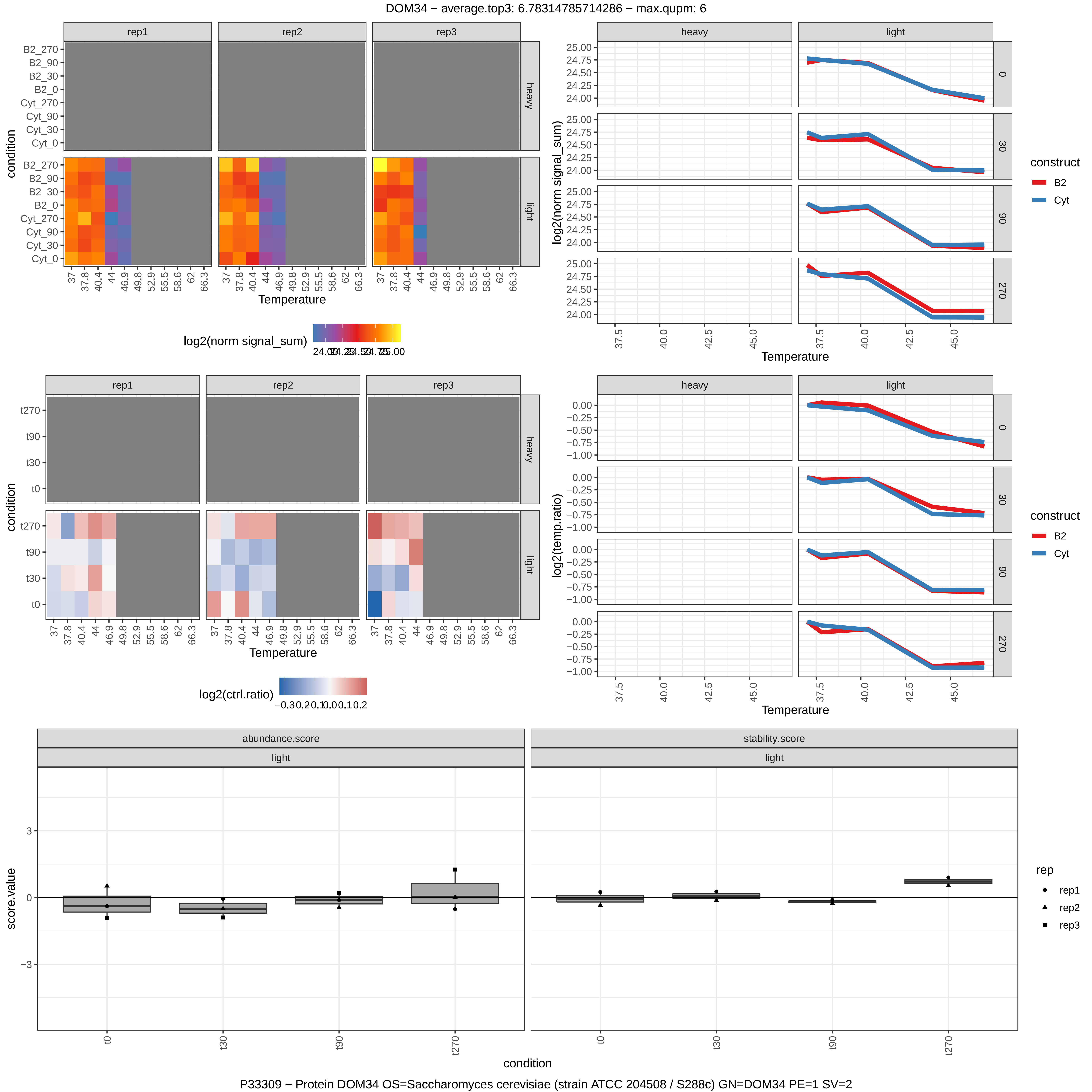

DOM34 − average.top3: 6.78314785714286 − max.qupm: 6
rep1
rep2
rep3
heavy
light
condition
Temperature
log2(norm signal_sum)
log2(ctrl.ratio)
log2(temp.ratio)
construct
B2
Cyt
abundance.score
stability.score
score.value
rep
rep1
rep2
rep3
P33309 − Protein DOM34 OS=Saccharomyces cerevisiae (strain ATCC 204508 / S288c) GN=DOM34 PE=1 SV=2

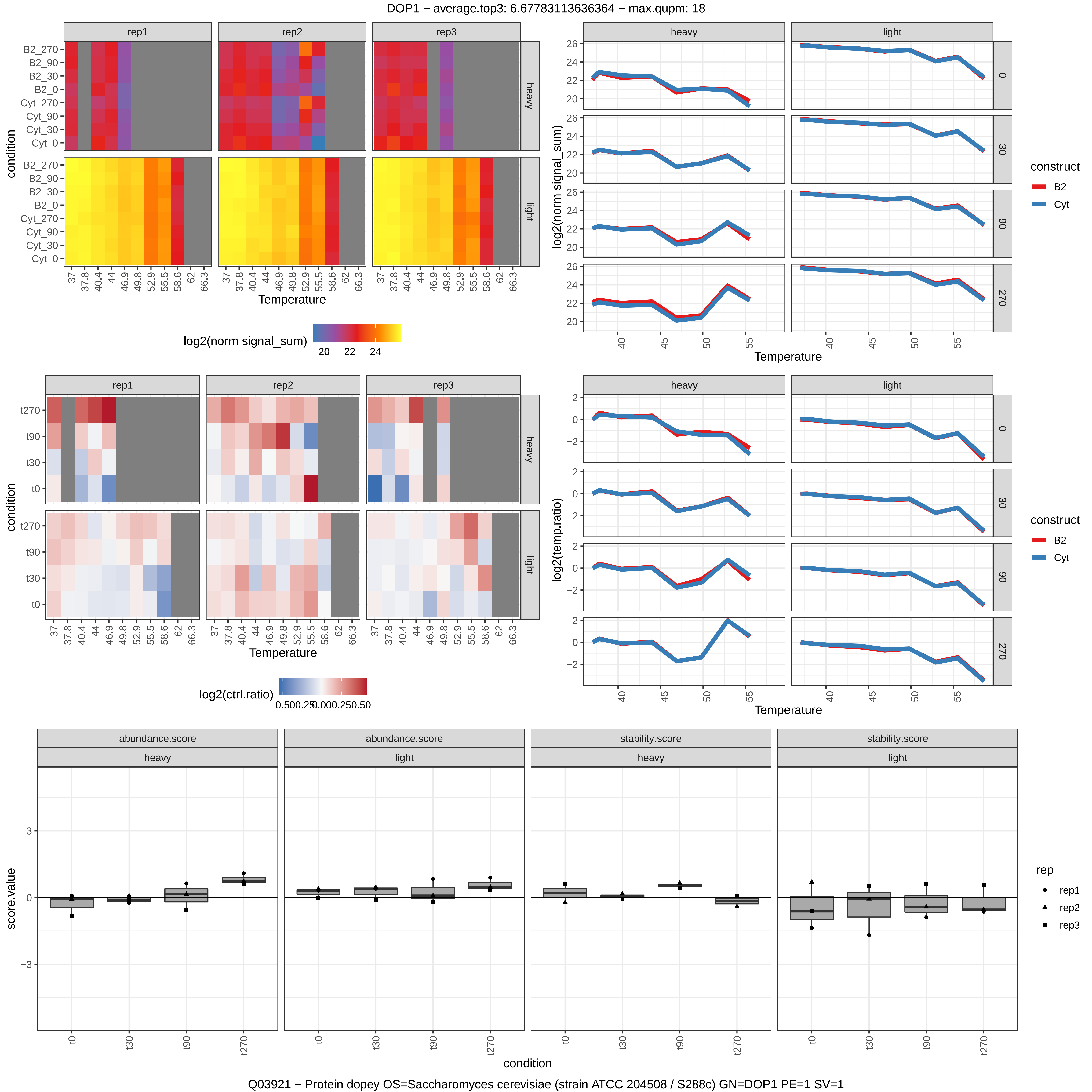

DOP1 − average.top3: 6.67783113636364 − max.qupm: 18
rep1
rep2
rep3
heavy
light
condition
B2_270
B2_90
B2_30
B2_0
Cyt_270
Cyt_90
Cyt_30
Cyt_0
Temperature
log2(norm signal_sum)
construct
B2
Cyt
t270
t90
t30
t0
log2(ctrl.ratio)
log2(temp.ratio)
abundance.score
stability.score
score.value
rep
rep1
rep2
rep3
Q03921 − Protein dopey OS=Saccharomyces cerevisiae (strain ATCC 204508 / S288c) GN=DOP1 PE=1 SV=1

DOS2 − average.top3: 6.55612666666667 − max.qupm: 4

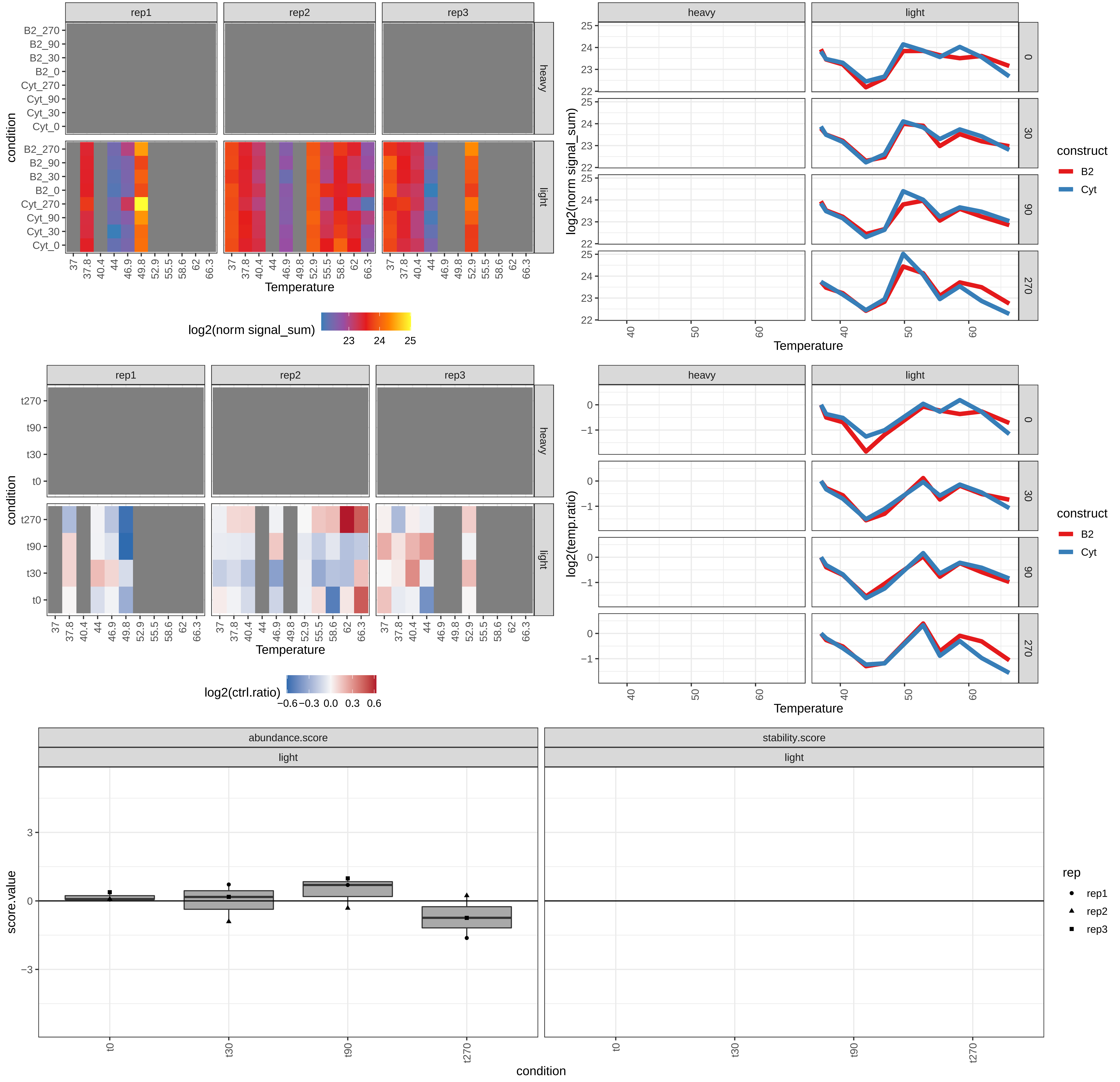

P54858 − Protein DOS2 OS=Saccharomyces cerevisiae (strain ATCC 204508 / S288c) GN=DOS2 PE=1 SV=1

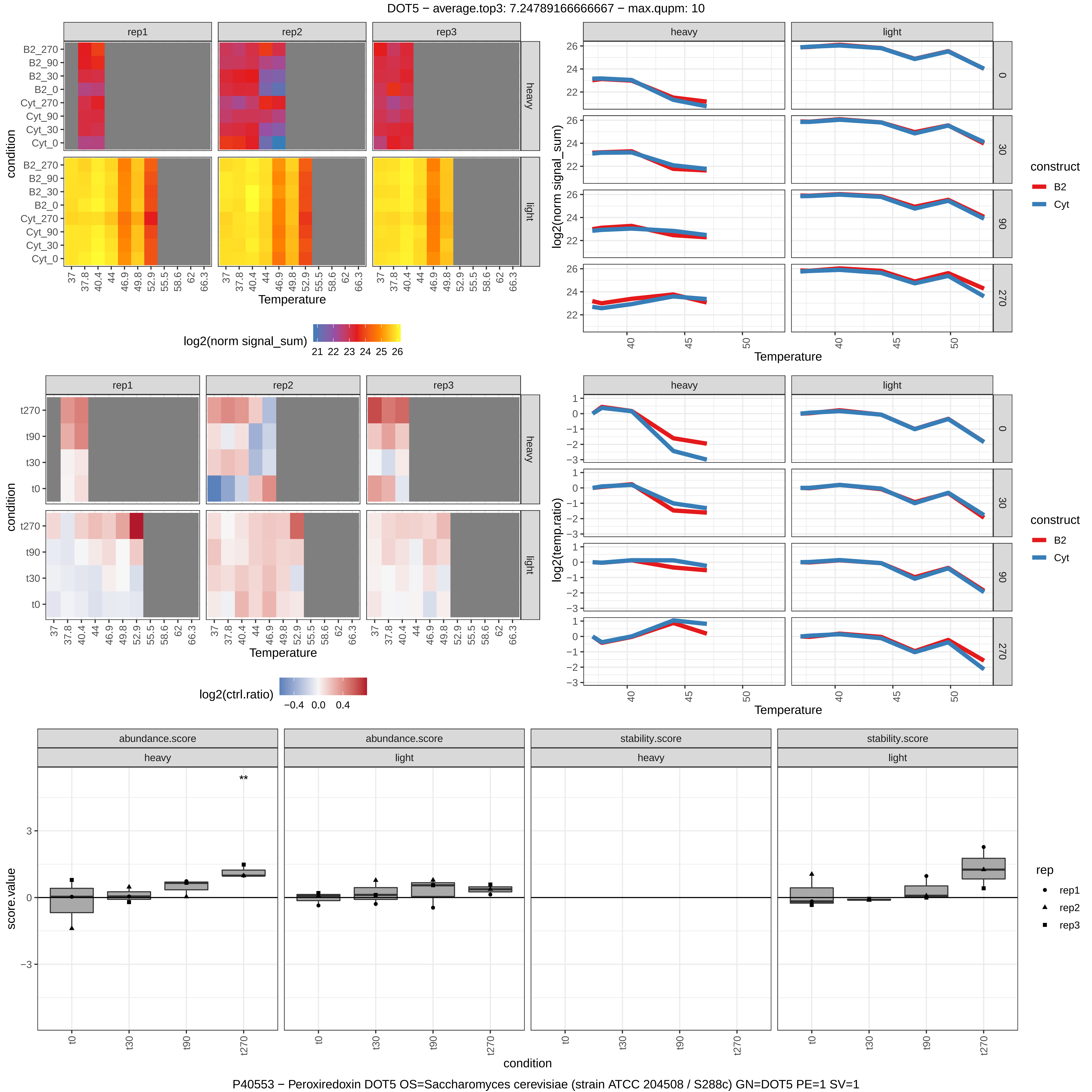
DOT5 − average.top3: 7.24789166666667 − max.qupm: 10
rep1
rep2
rep3
heavy
light
condition
Temperature
log2(norm signal_sum)
construct
B2
Cyt
log2(temp.ratio)
log2(ctrl.ratio)
abundance.score
stability.score
score.value
rep
rep1
rep2
rep3
**
P40553 − Peroxiredoxin DOT5 OS=Saccharomyces cerevisiae (strain ATCC 204508 / S288c) GN=DOT5 PE=1 SV=1

DPB4 − average.top3: 6.65471 − max.qupm: 6

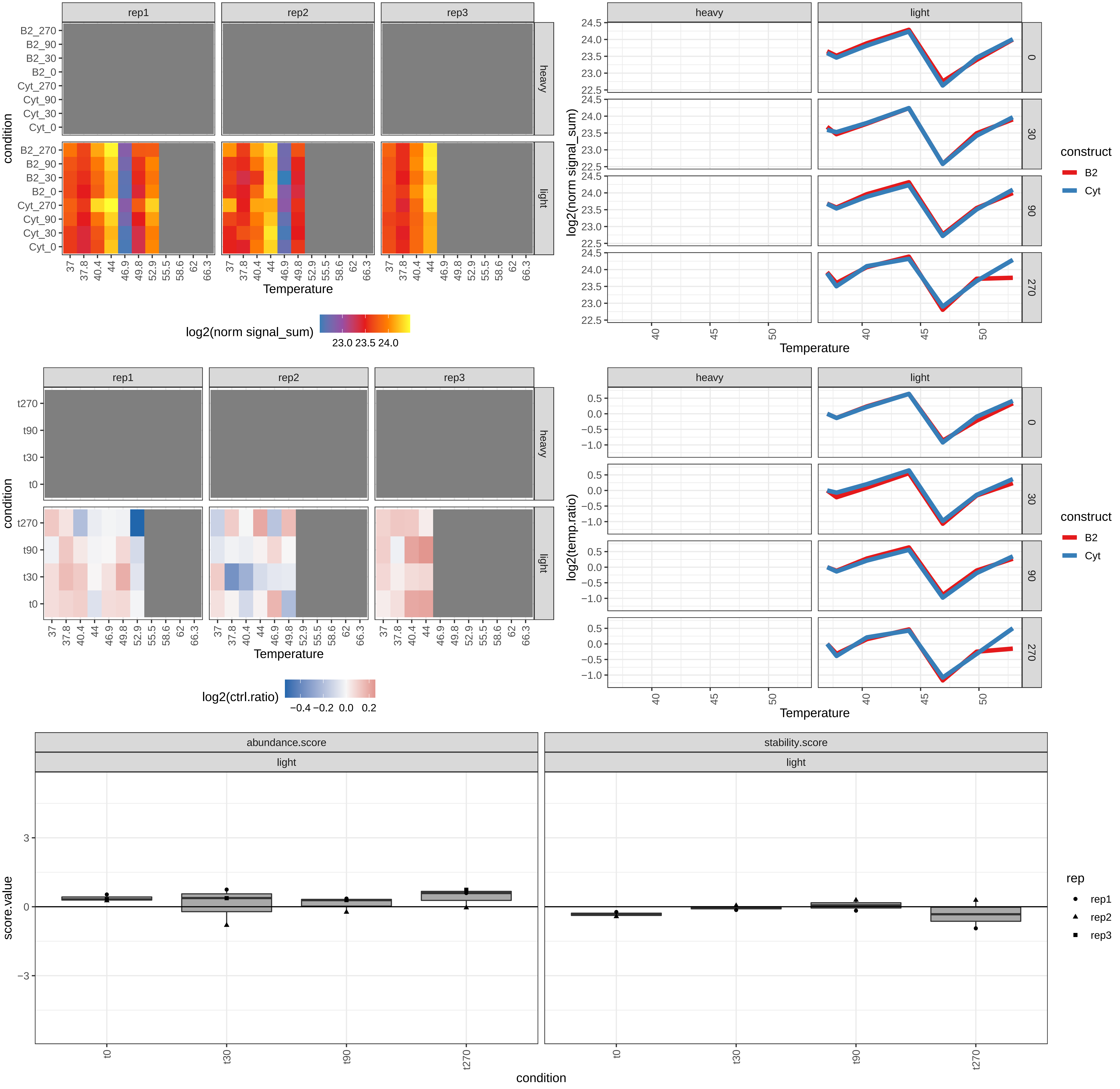

Q04603 − DNA polymerase epsilon subunit D OS=Saccharomyces cerevisiae (strain ATCC 204508 / S288c) GN=DPB4 PE=1 SV=1

DPH1 − average.top3: 6.8777775 − max.qupm: 6

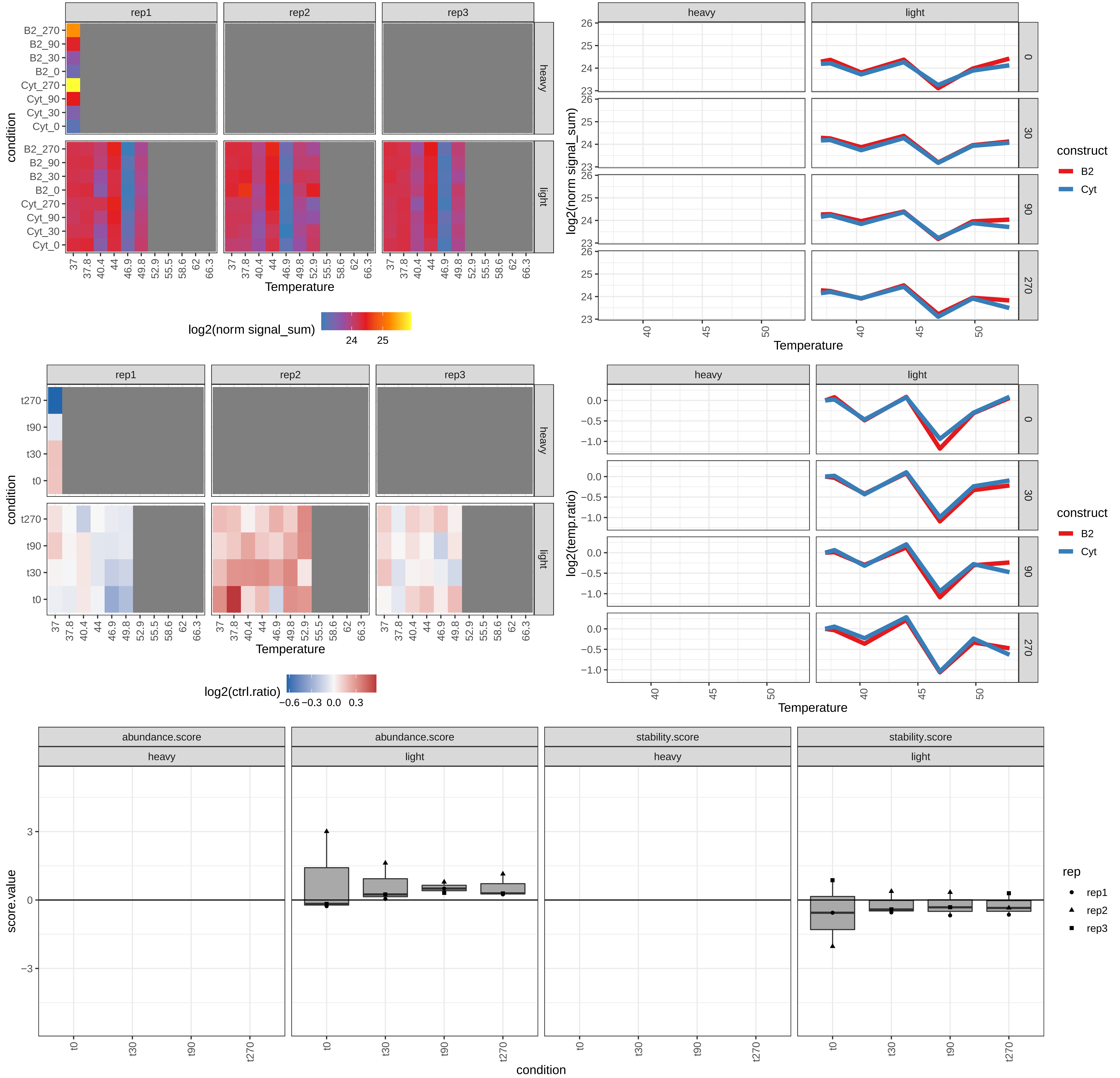

P40487 − Diphthamide biosynthesis protein 1 OS=Saccharomyces cerevisiae (strain ATCC 204508 / S288c) GN=DPH1 PE=1 SV=1

DPH2 − average.top3: 7.05243444444444 − max.qupm: 5

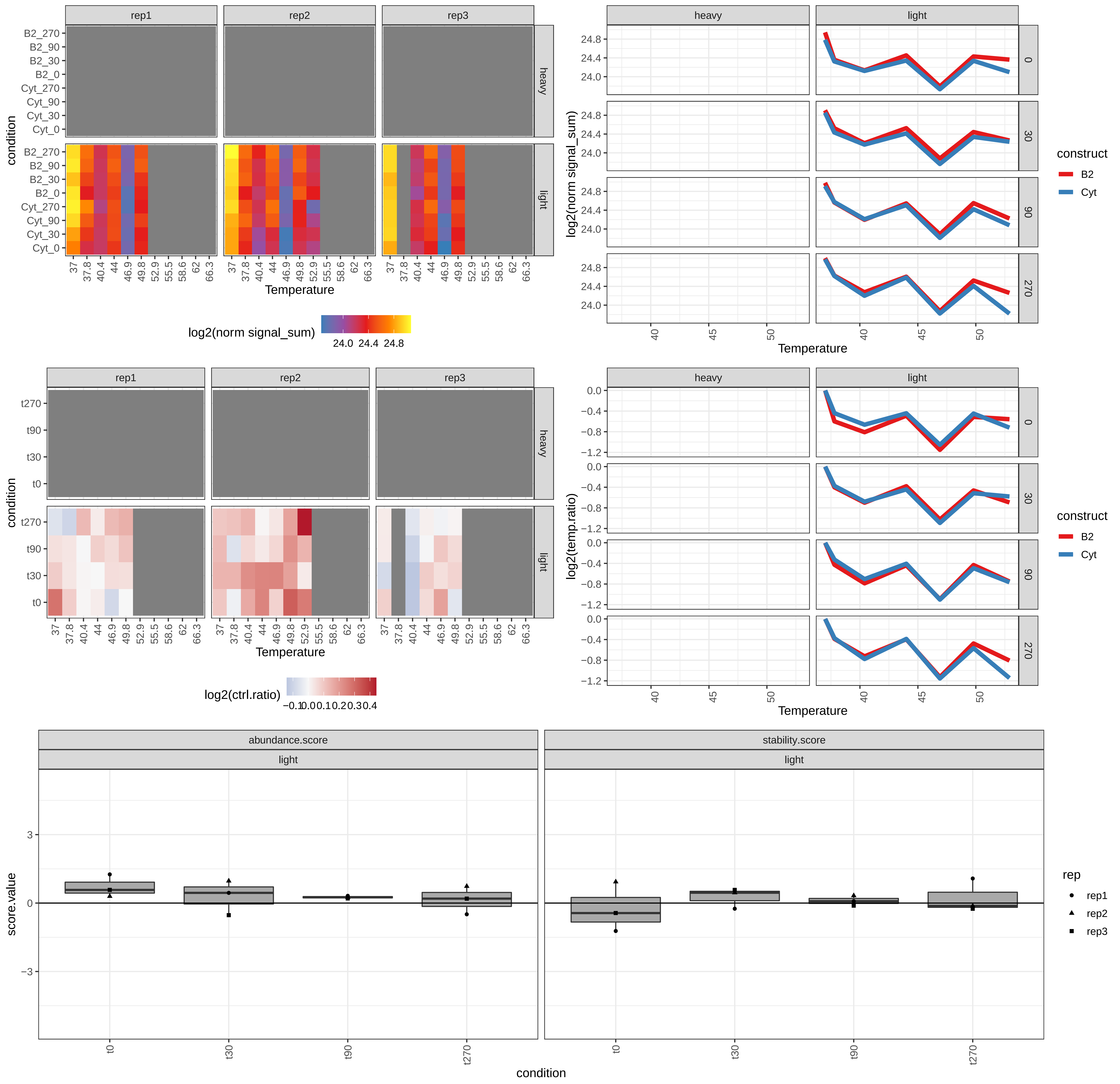

P32461 − Diphthamide biosynthesis protein 2 OS=Saccharomyces cerevisiae (strain ATCC 204508 / S288c) GN=DPH2 PE=1 SV=1

DPH5 − average.top3: 7.17045298245614 − max.qupm: 8

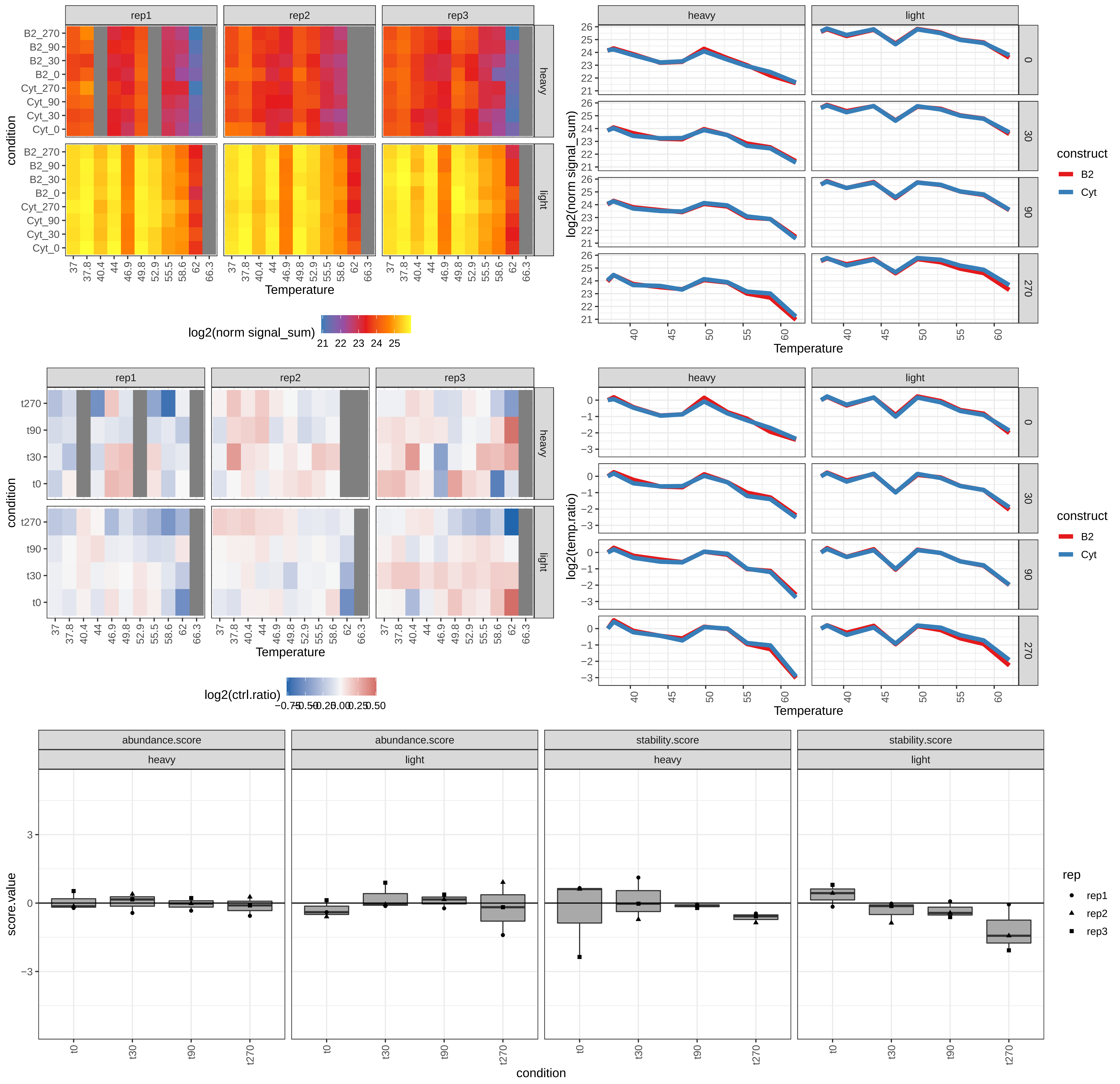

P32469 − Diphthine methyl ester synthase OS=Saccharomyces cerevisiae (strain ATCC 204508 / S288c) GN=DPH5 PE=1 SV=1

DPH6 − average.top3: 6.155225 − max.qupm: 5

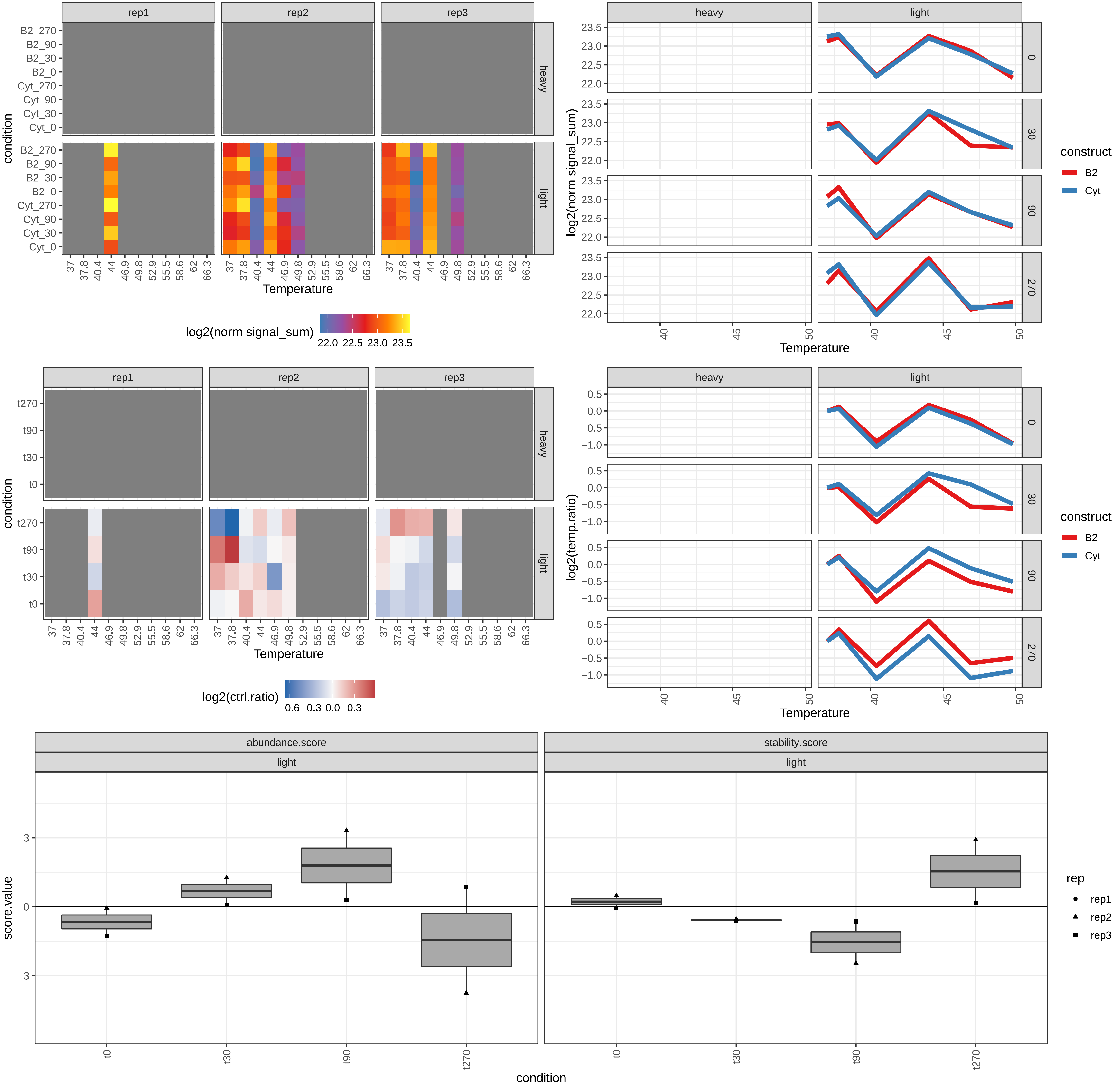

Q12429 − Diphthine−−ammonia ligase OS=Saccharomyces cerevisiae (strain ATCC 204508 / S288c) GN=DPH6 PE=1 SV=1

DPL1 − average.top3: 7.00267911111111 − max.qupm: 10

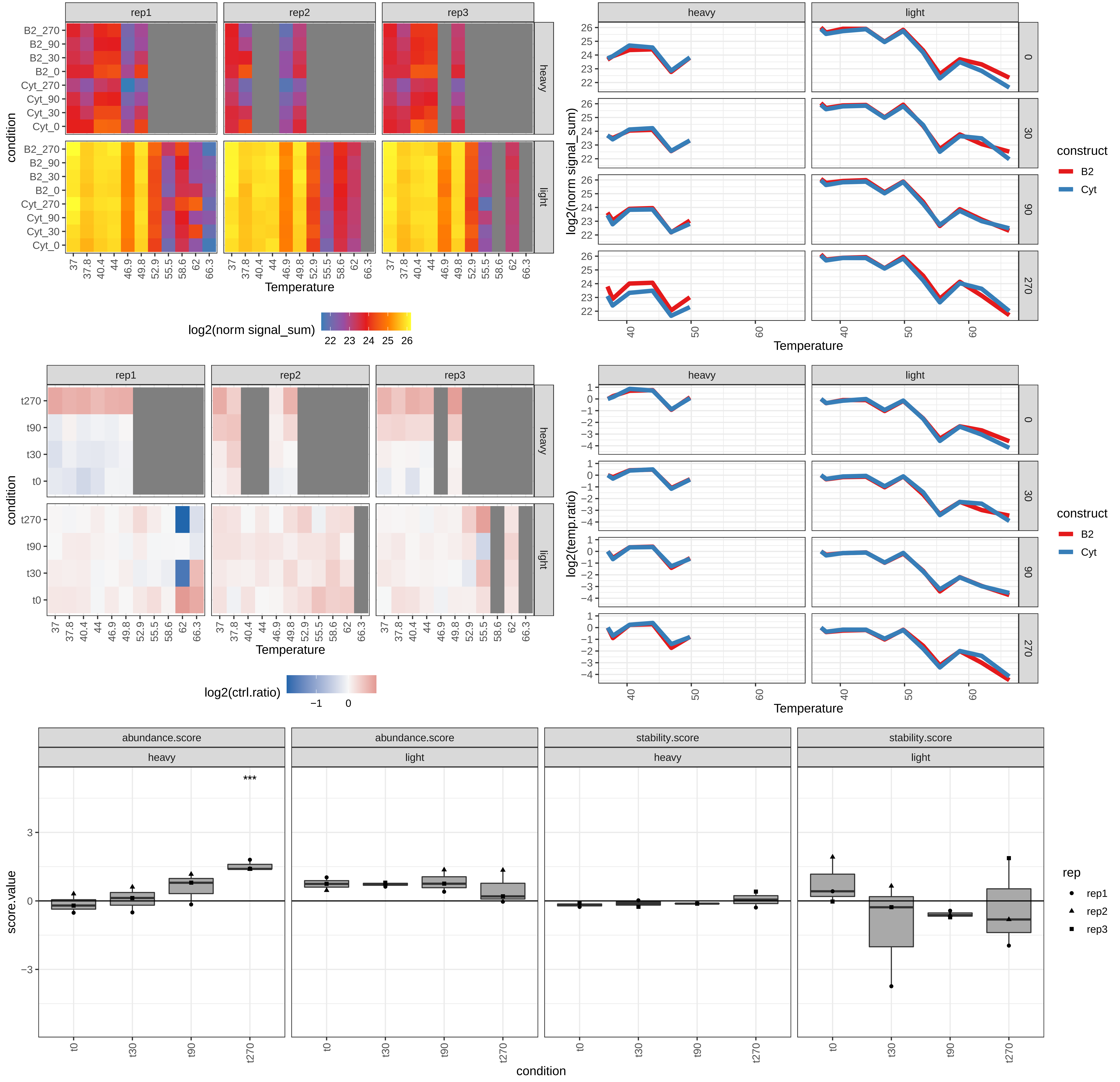

Q05567 − Sphingosine−1−phosphate lyase OS=Saccharomyces cerevisiae (strain ATCC 204508 / S288c) GN=DPL1 PE=1 SV=1

DPM1 − average.top3: 7.23865571428571 − max.qupm: 8

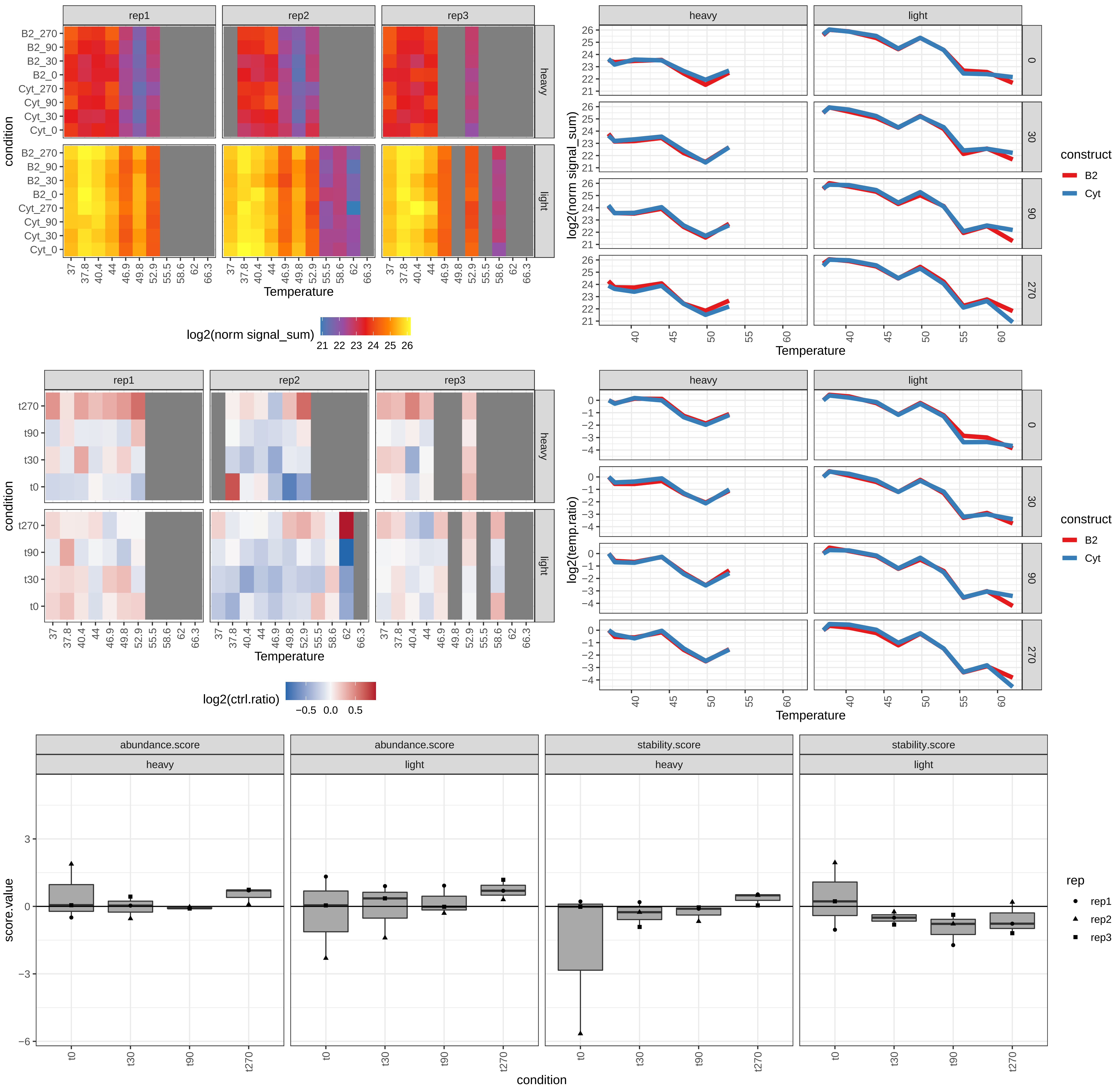

P14020 − Dolichol−phosphate mannosyltransferase OS=Saccharomyces cerevisiae (strain ATCC 204508 / S288c) GN=DPM1 PE=1 SV=3

DPP1 − average.top3: 6.94641176470588 − max.qupm: 5

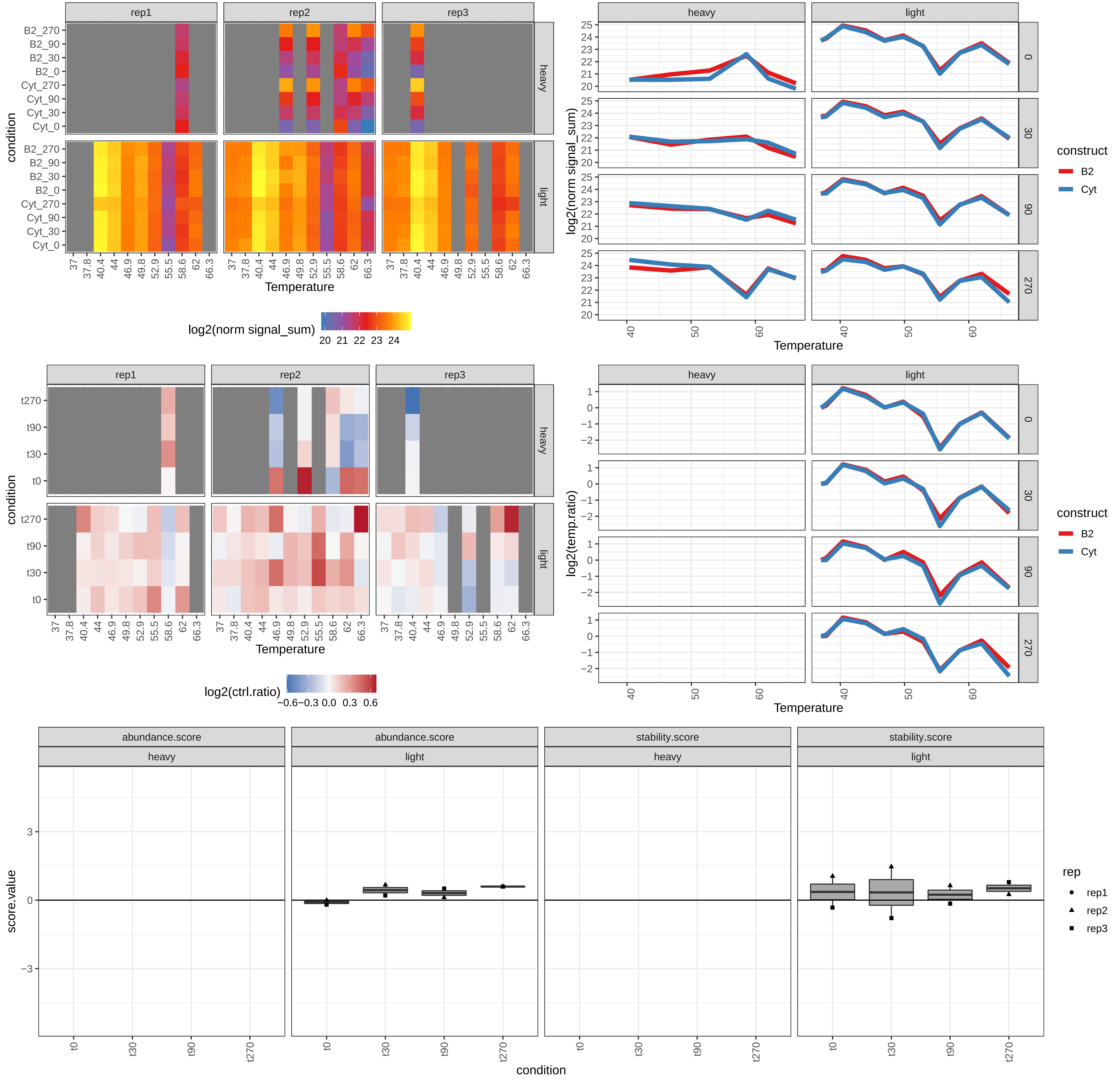

Q05521 − Diacylglycerol pyrophosphate phosphatase 1 OS=Saccharomyces cerevisiae (strain ATCC 204508 / S288c) GN=DPP1 PE=1 SV=1

DPS1 − average.top3: 8.08895676923077 − max.qupm: 35

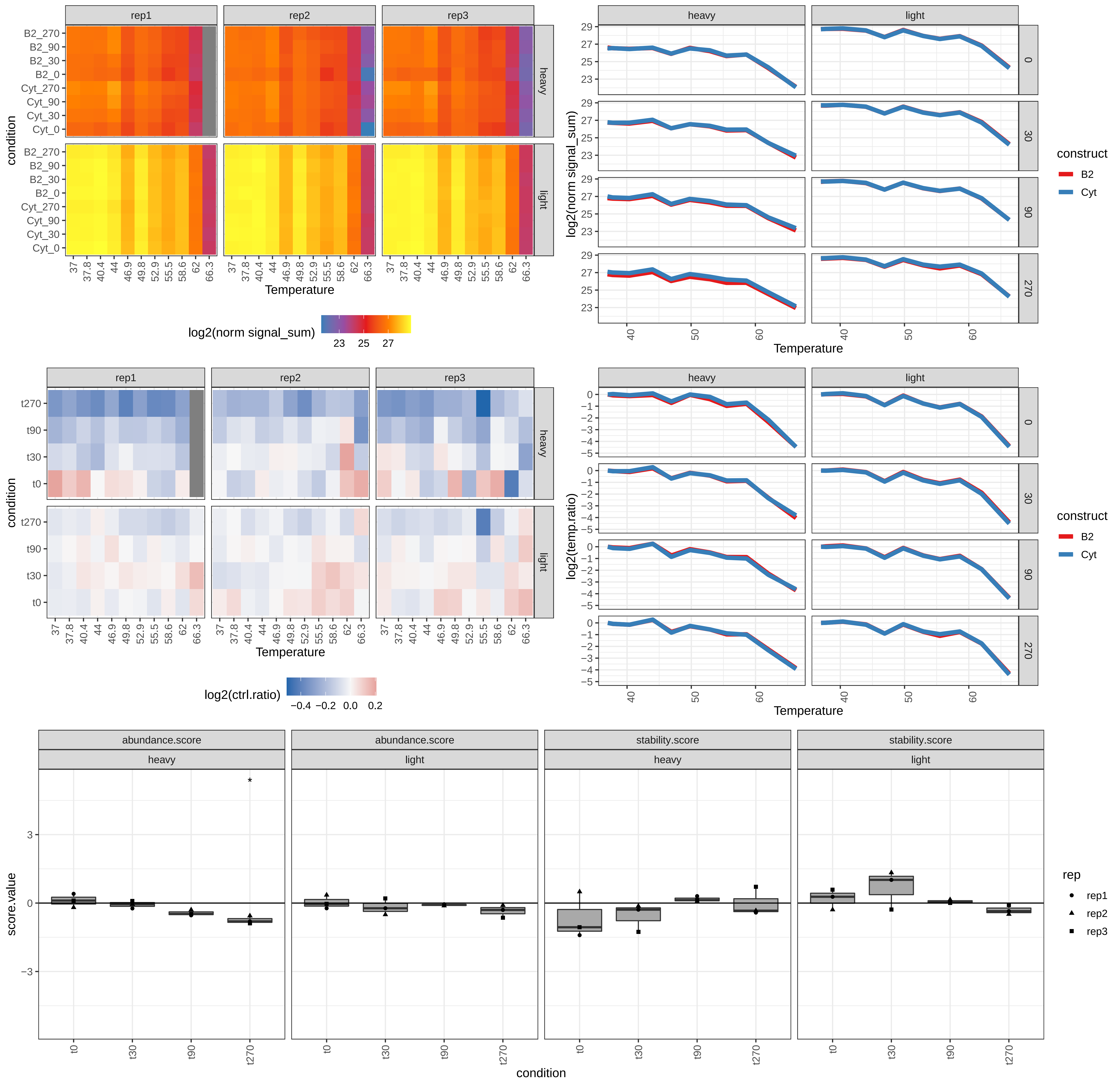

P04802 − Aspartate−−tRNA ligase, cytoplasmic OS=Saccharomyces cerevisiae (strain ATCC 204508 / S288c) GN=DPS1 PE=1 SV=3

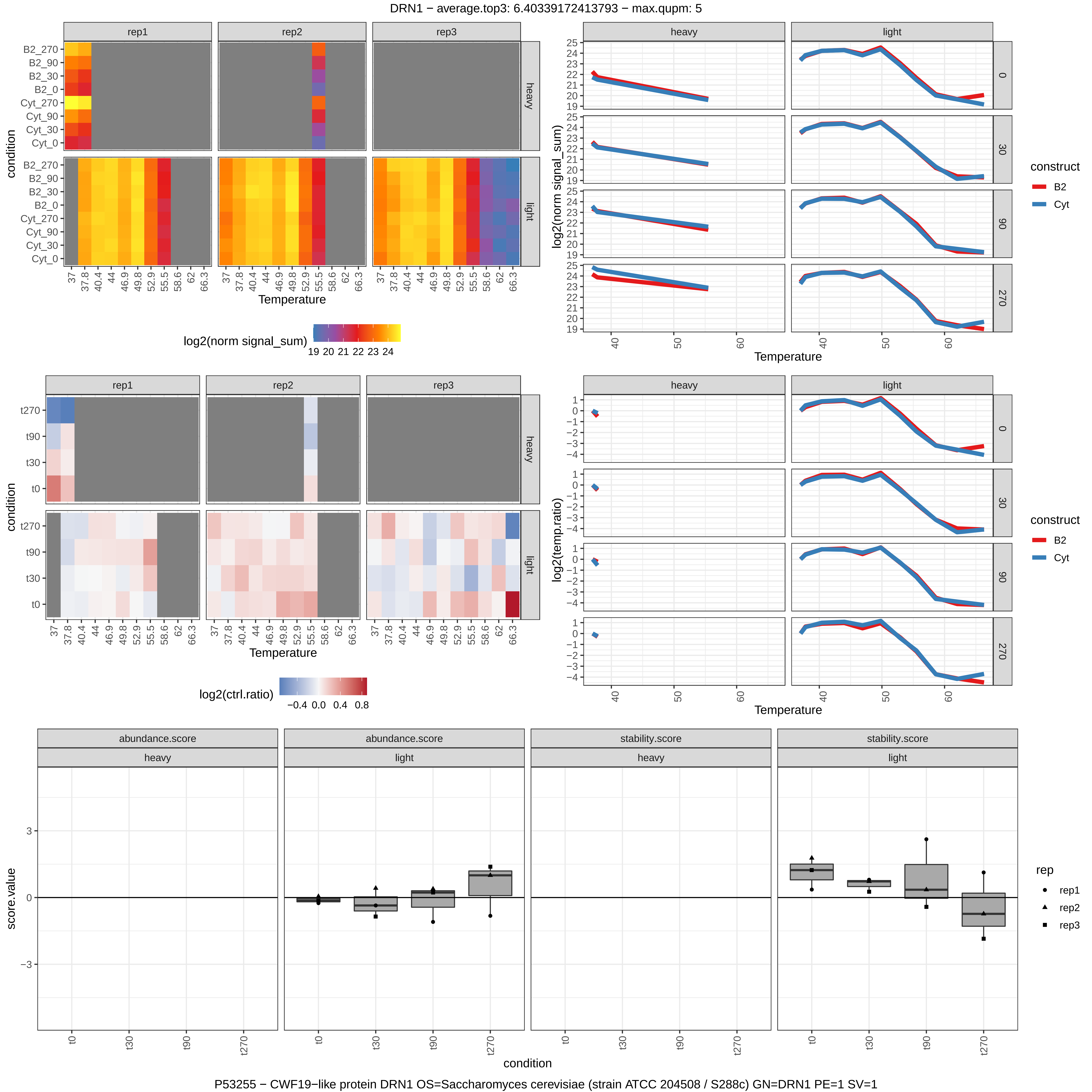
DRN1 − average.top3: 6.40339172413793 − max.qupm: 5
rep1
rep2
rep3
heavy
light
condition
Temperature
log2(norm signal_sum)
log2(ctrl.ratio)
log2(temp.ratio)
construct
B2
Cyt
abundance.score
stability.score
score.value
rep
rep1
rep2
rep3
P53255 − CWF19−like protein DRN1 OS=Saccharomyces cerevisiae (strain ATCC 204508 / S288c) GN=DRN1 PE=1 SV=1

DRS1 − average.top3: 6.9228075 − max.qupm: 8

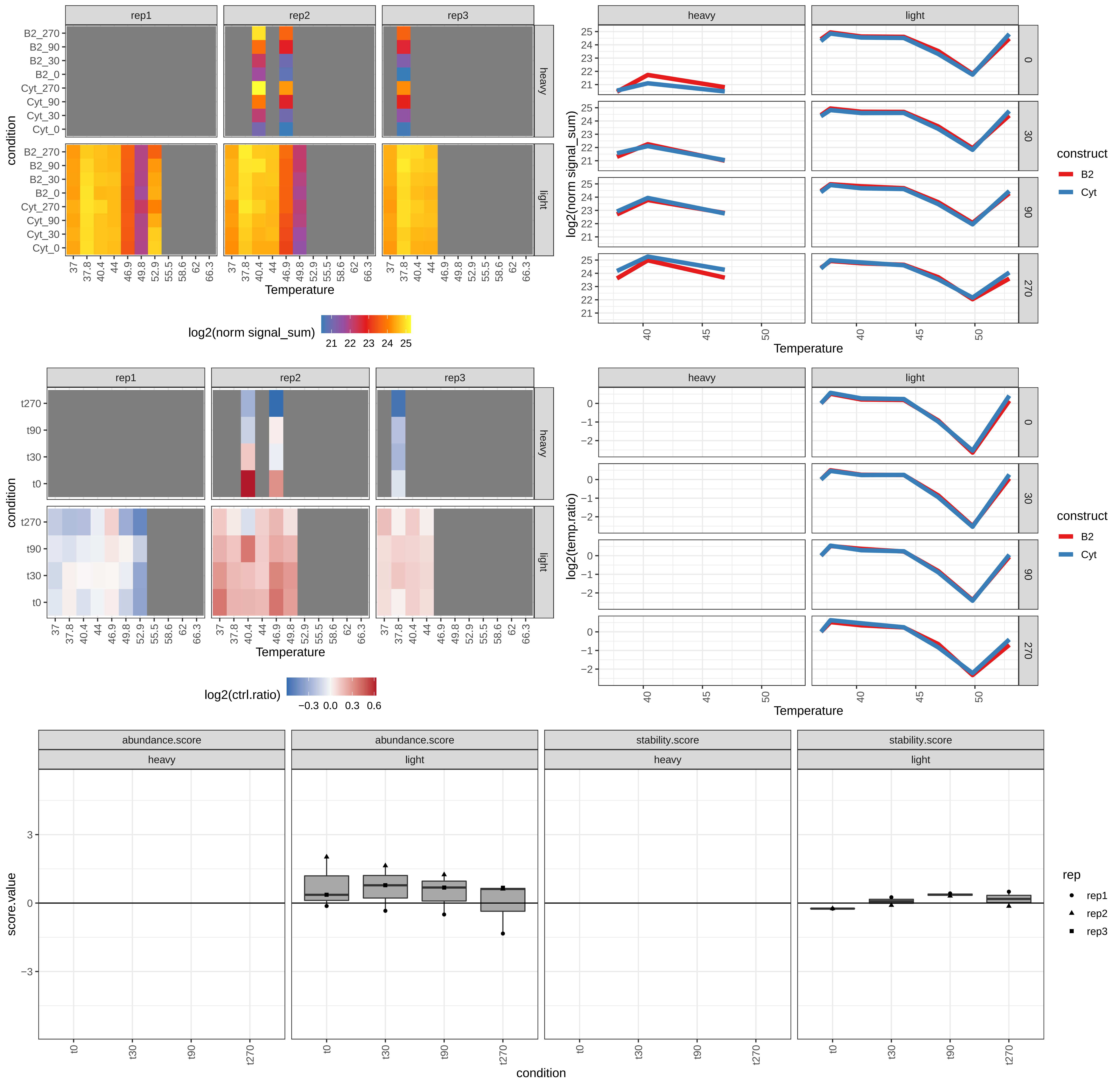

P32892 − ATP−dependent RNA helicase DRS1 OS=Saccharomyces cerevisiae (strain ATCC 204508 / S288c) GN=DRS1 PE=1 SV=2

DRS2 − average.top3: 6.53145652173913 − max.qupm: 8

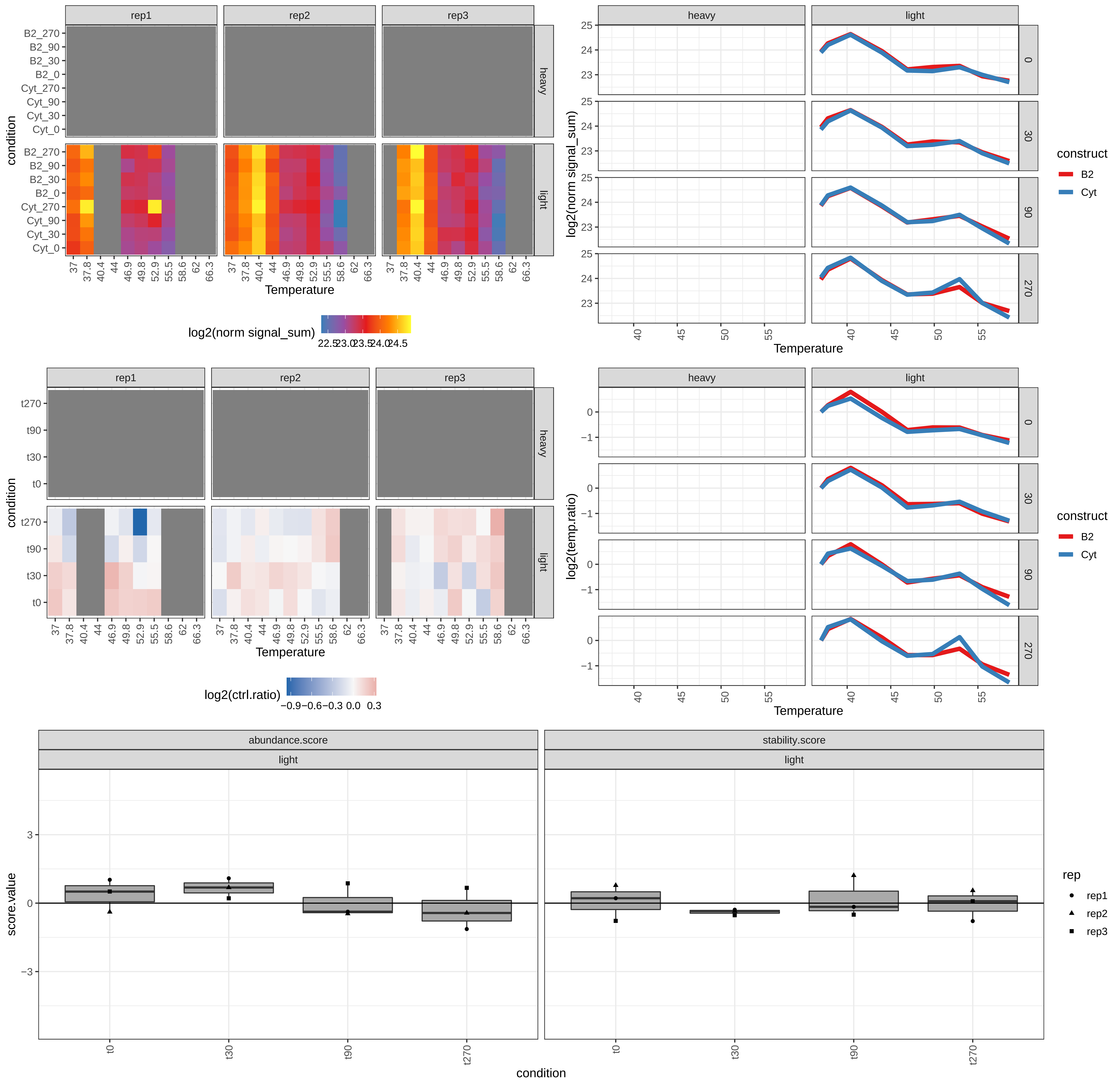

P39524 − Probable phospholipid−transporting ATPase DRS2 OS=Saccharomyces cerevisiae (strain ATCC 204508 / S288c) GN=DRS2 PE=1 SV=2

DSD1 − average.top3: 7.0182675 − max.qupm: 9

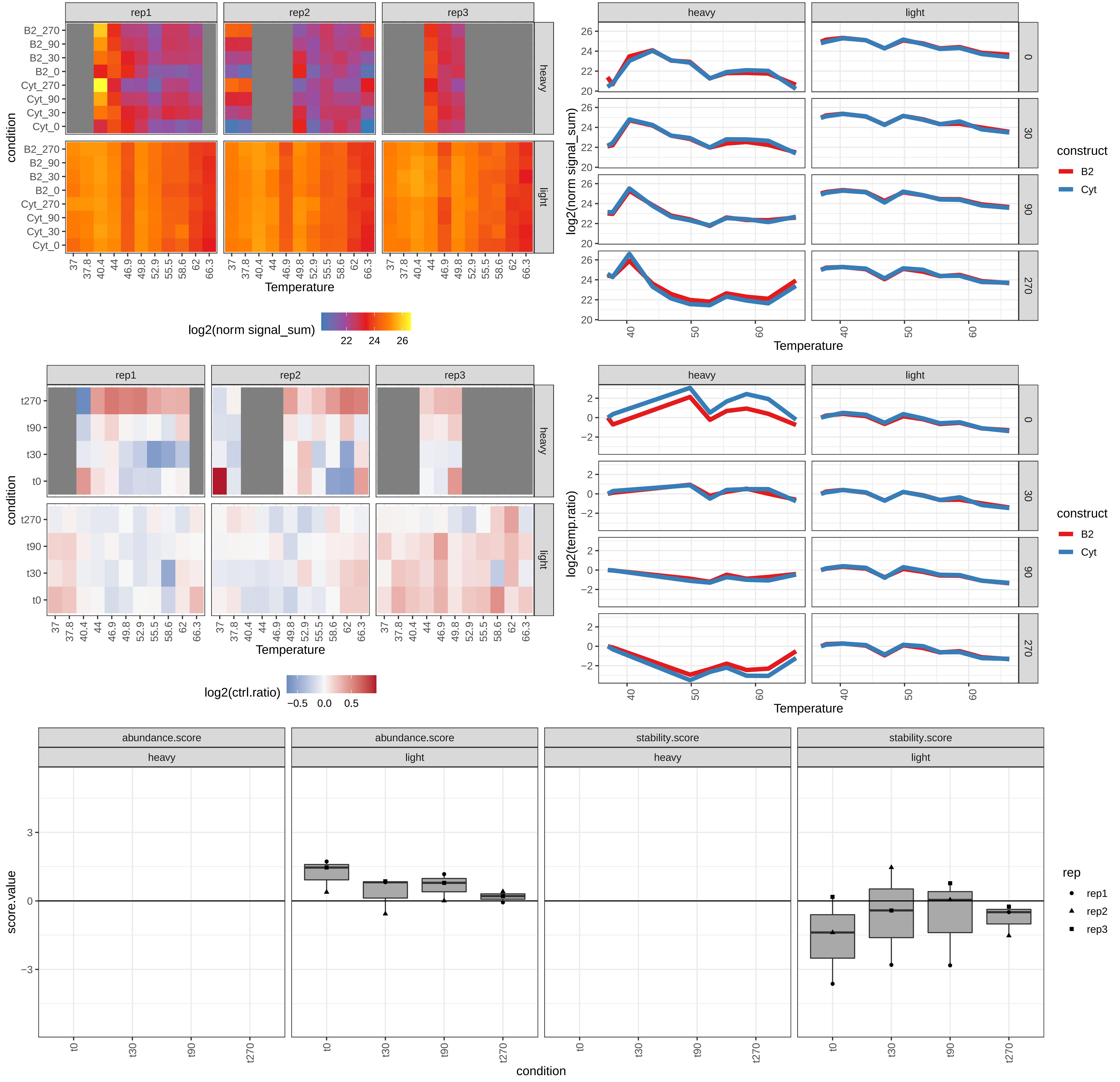

P53095 − D−serine dehydratase OS=Saccharomyces cerevisiae (strain ATCC 204508 / S288c) GN=DSD1 PE=1 SV=2

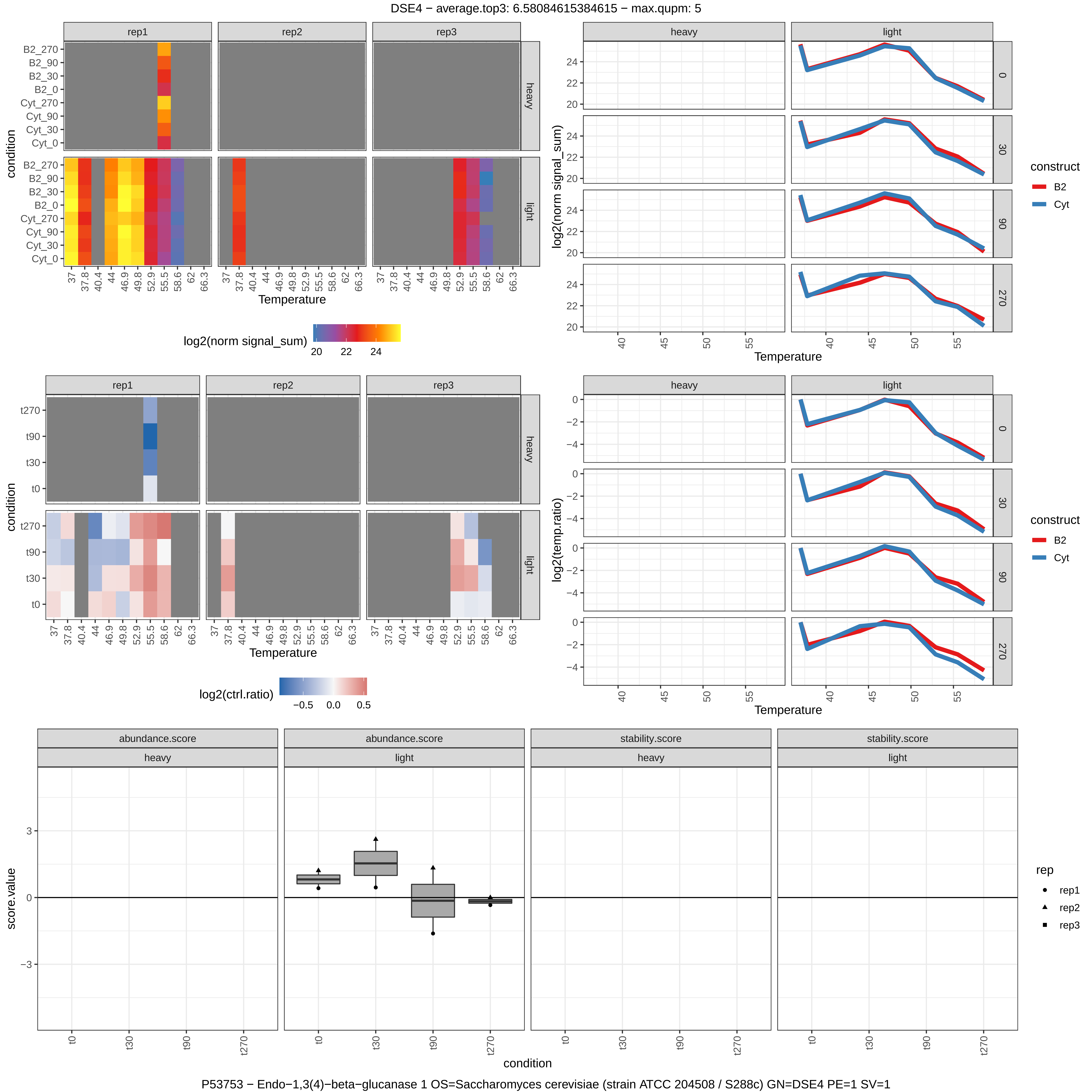

DSE4 − average.top3: 6.58084615384615 − max.qupm: 5
rep1
rep2
rep3
heavy
light
condition
Temperature
log2(norm signal_sum)
log2(ctrl.ratio)
log2(temp.ratio)
construct
B2
Cyt
abundance.score
stability.score
score.value
rep
rep1
rep2
rep3
P53753 − Endo−1,3(4)−beta−glucanase 1 OS=Saccharomyces cerevisiae (strain ATCC 204508 / S288c) GN=DSE4 PE=1 SV=1

DSF1|YNR073C − average.top3: 6.2735737037037 − max.qupm: 6

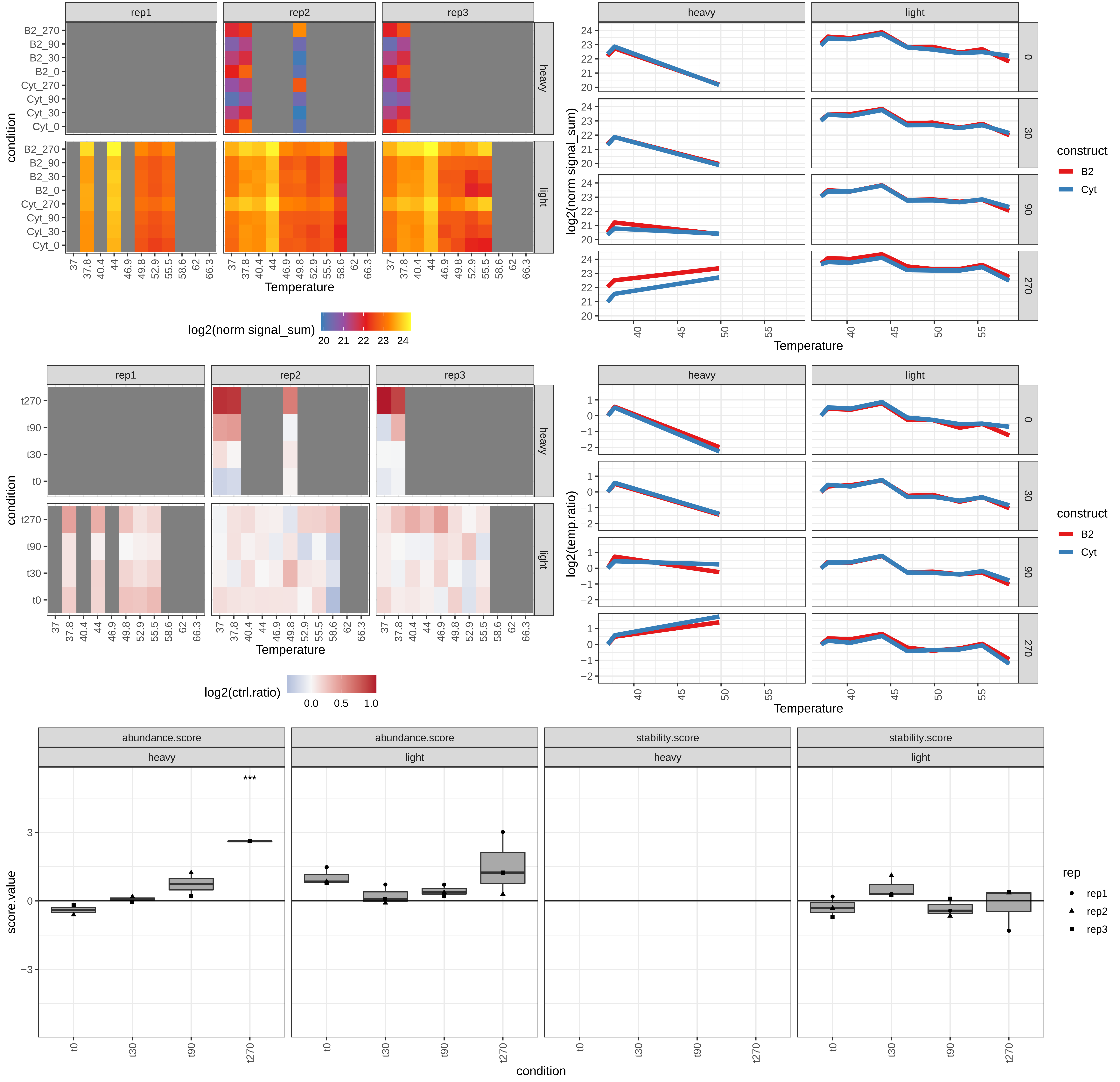

dehydrogenase DSF1 OS=Saccharomyces cerevisiae (strain ATCC 204508 / S288c) GN=DSF1 PE=1 SV=1|Mannitol dehydrogenase YNR073C OS=Saccharomyces cerevisiae (strain ATCC 204508 / S288

DSK2 − average.top3: 6.79306096774194 − max.qupm: 5

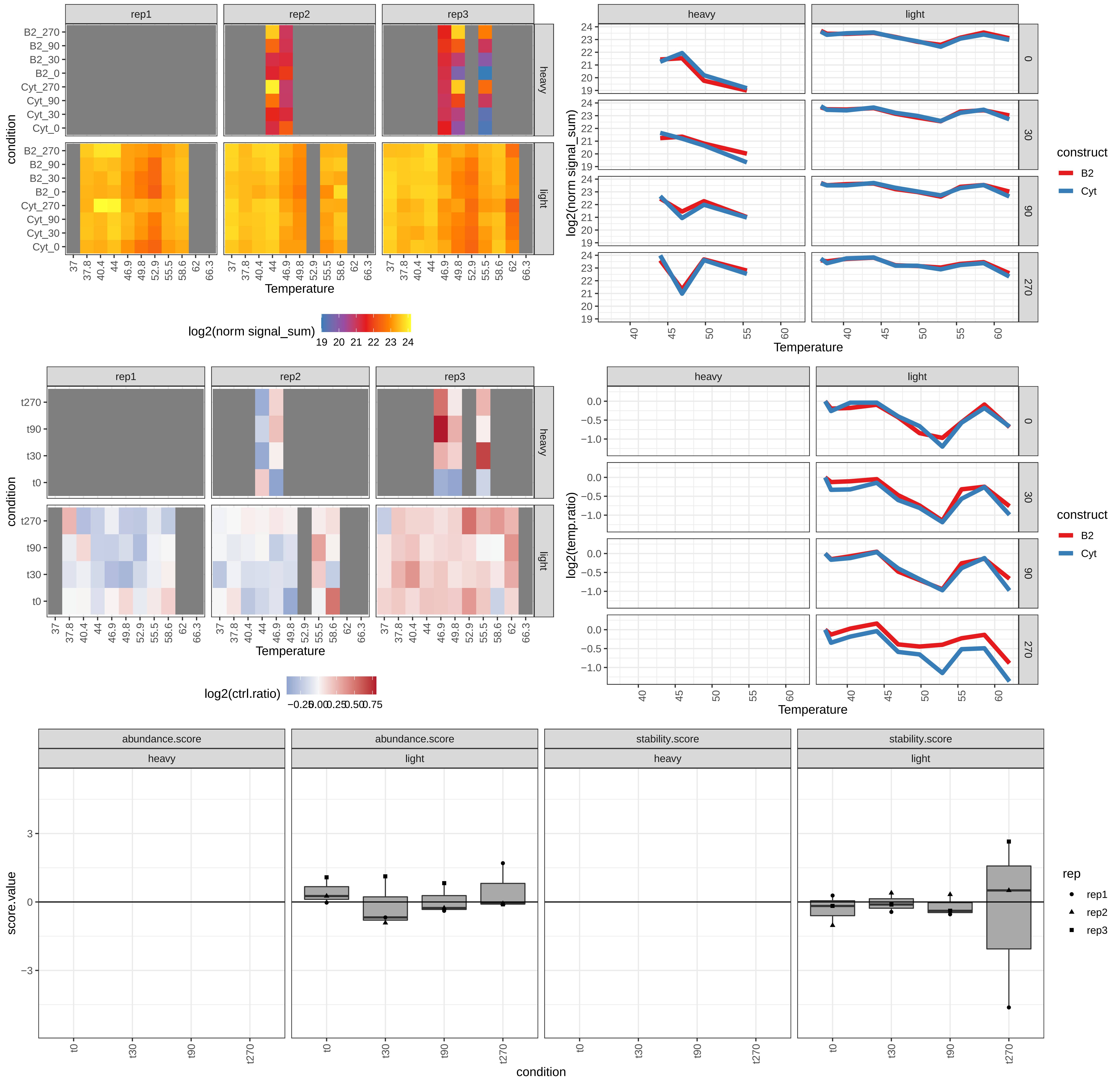

P48510 − Ubiquitin domain−containing protein DSK2 OS=Saccharomyces cerevisiae (strain ATCC 204508 / S288c) GN=DSK2 PE=1 SV=2

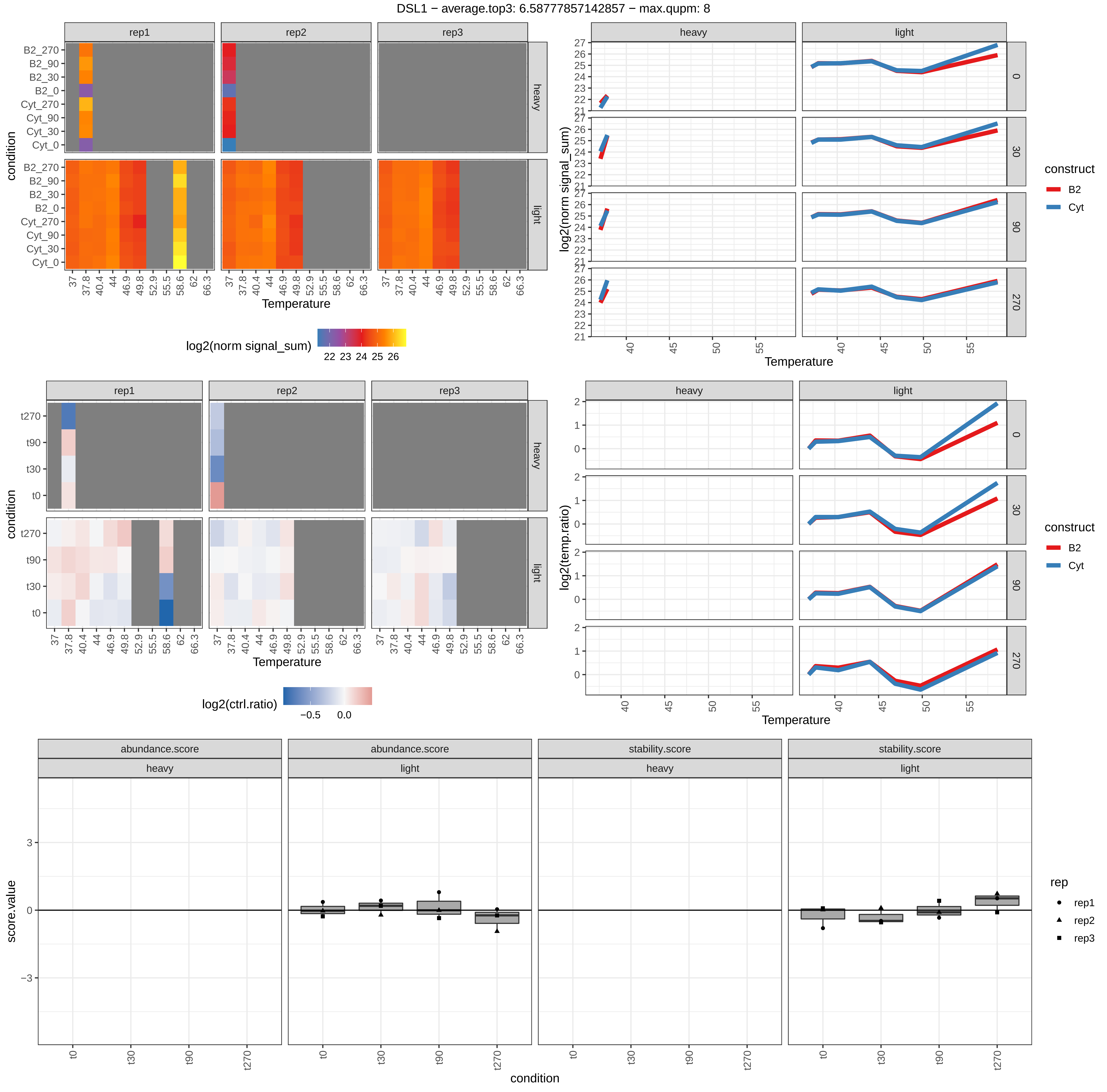

P53847 − Protein transport protein DSL1 OS=Saccharomyces cerevisiae (strain ATCC 204508 / S288c) GN=DSL1 PE=1 SV=1

DSS1 − average.top3: 6.352535 − max.qupm: 3

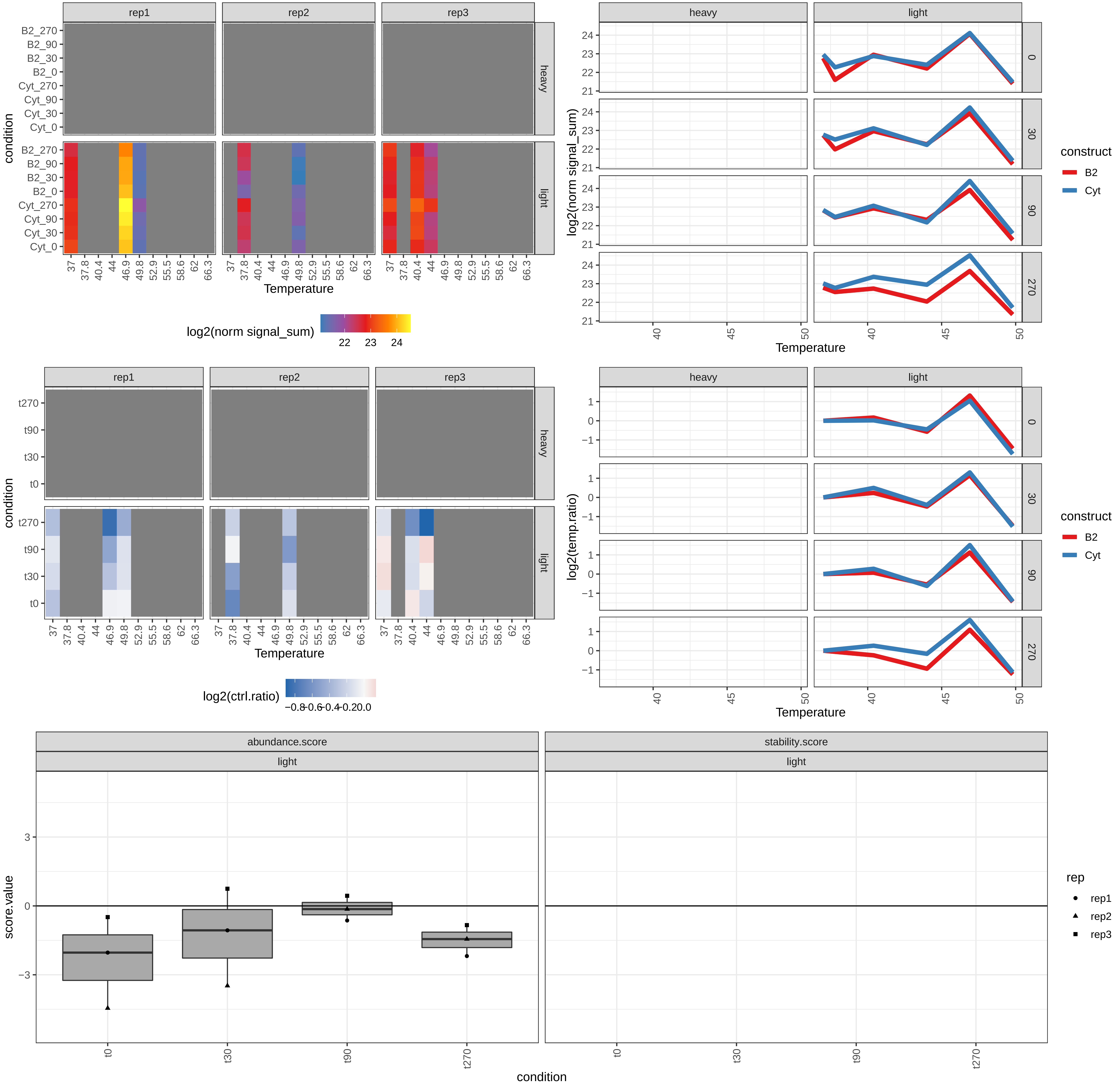

P39112 − Exoribonuclease II, mitochondrial OS=Saccharomyces cerevisiae (strain ATCC 204508 / S288c) GN=DSS1 PE=1 SV=2

DST1 − average.top3: 6.94553975 − max.qupm: 11

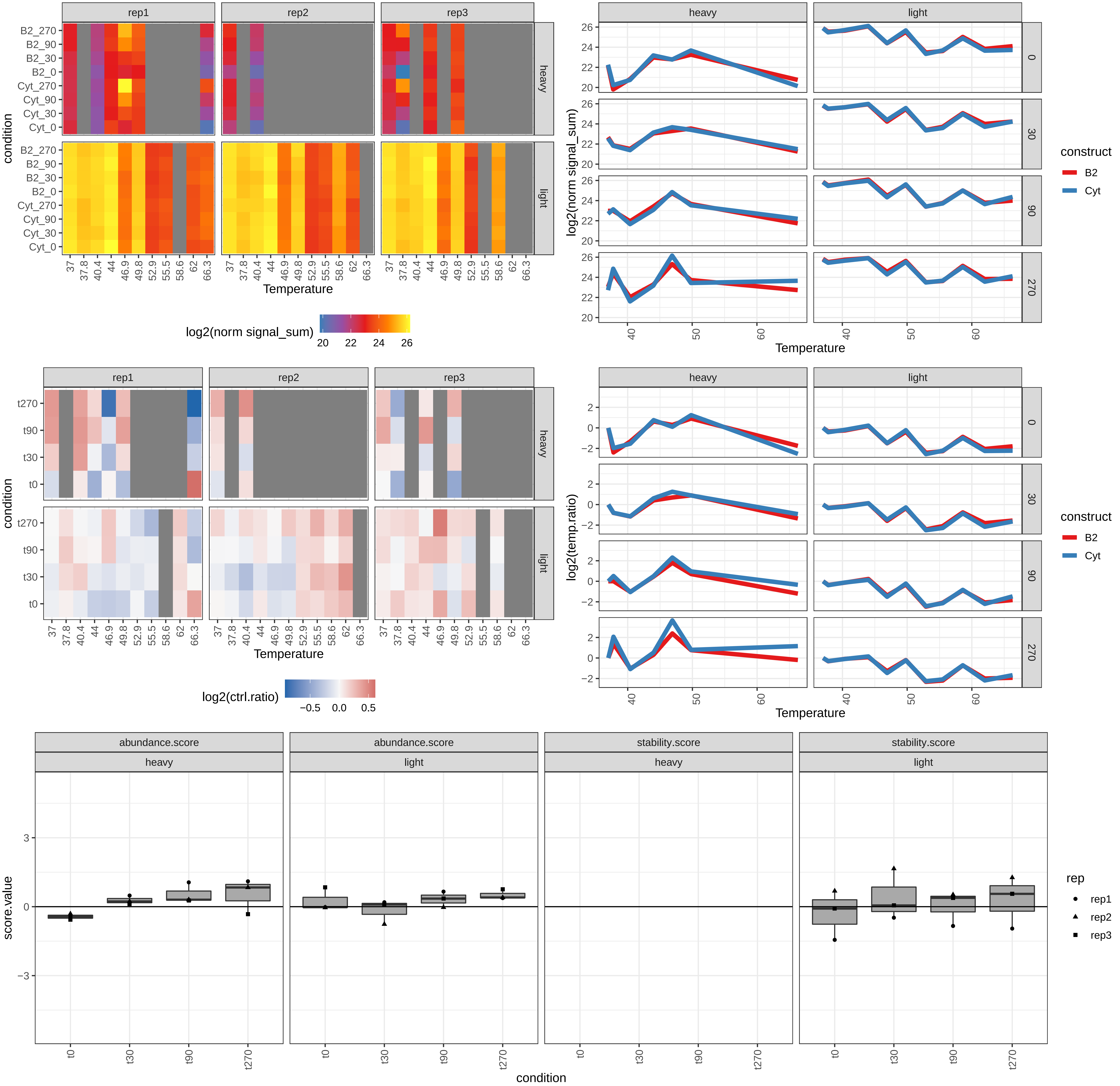

P07273 − Transcription elongation factor S−II OS=Saccharomyces cerevisiae (strain ATCC 204508 / S288c) GN=DST1 PE=1 SV=4

DTD1 − average.top3: 7.290066 − max.qupm: 5

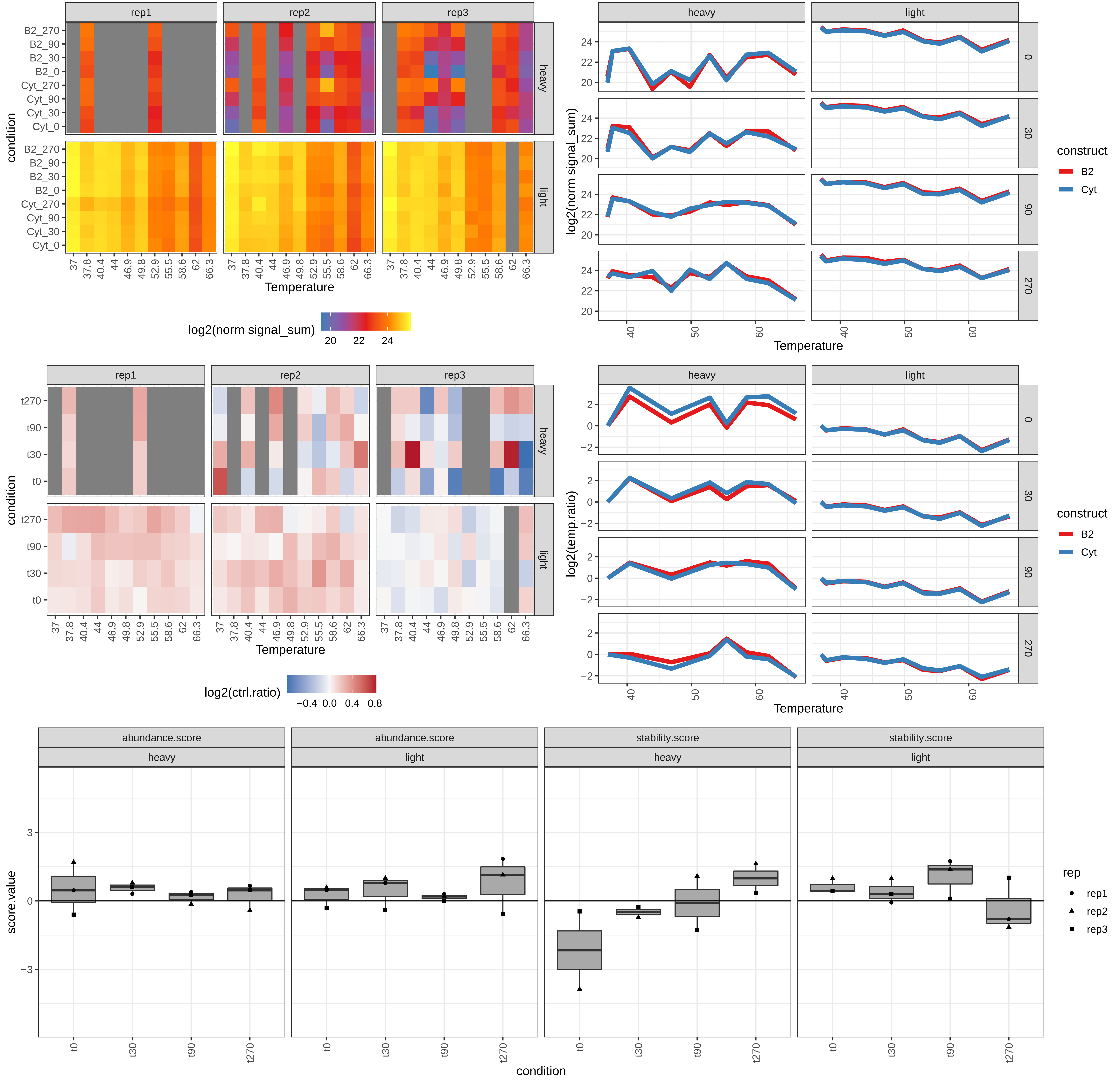

Q07648 − D−tyrosyl−tRNA(Tyr) deacylase OS=Saccharomyces cerevisiae (strain ATCC 204508 / S288c) GN=DTD1 PE=1 SV=1

DUG1 − average.top3: 7.80405166666667 − max.qupm: 21

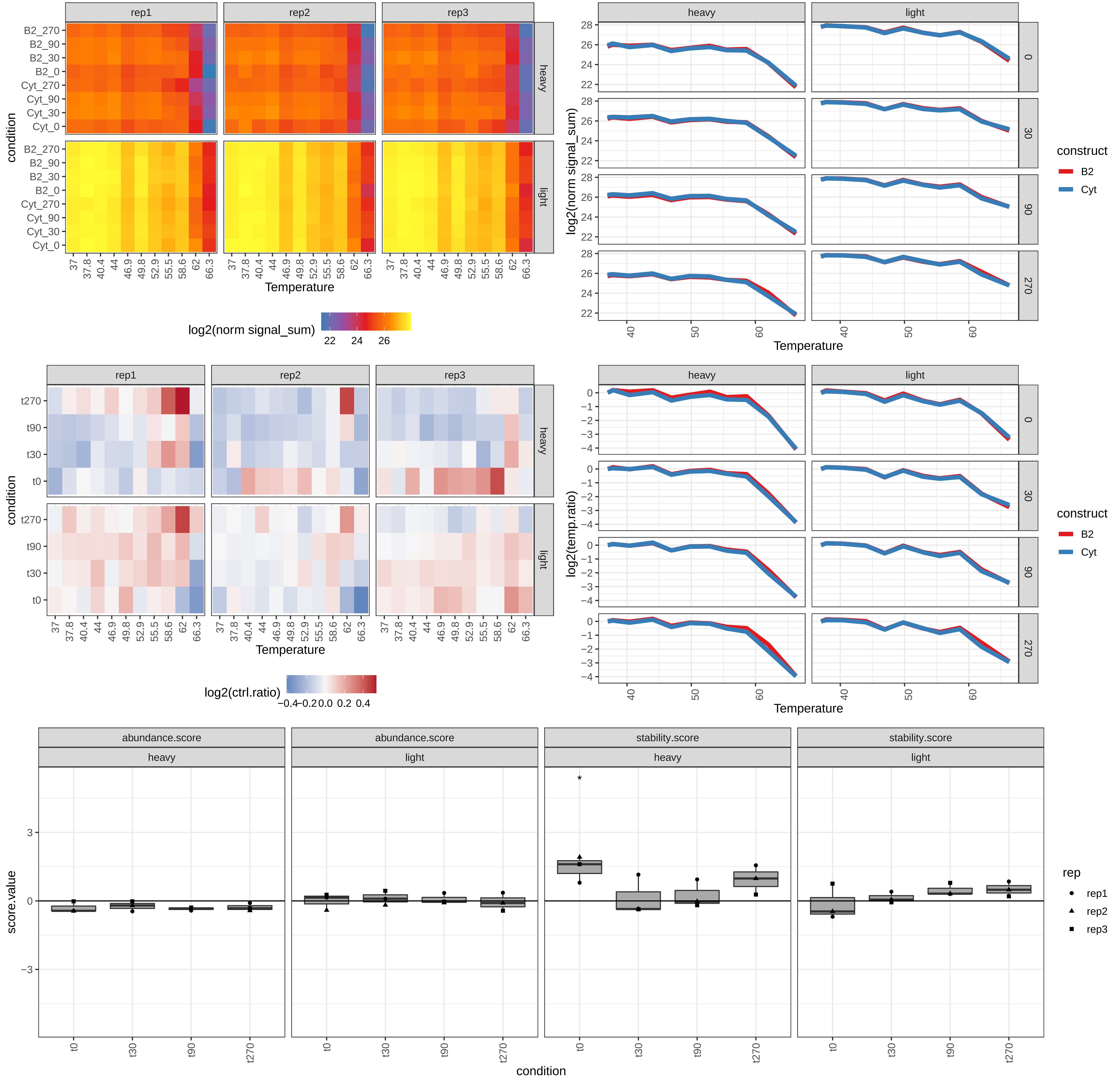

P43616 − Cys−Gly metallodipeptidase DUG1 OS=Saccharomyces cerevisiae (strain ATCC 204508 / S288c) GN=DUG1 PE=1 SV=1

DUG2 − average.top3: 6.86127314285714 − max.qupm: 10

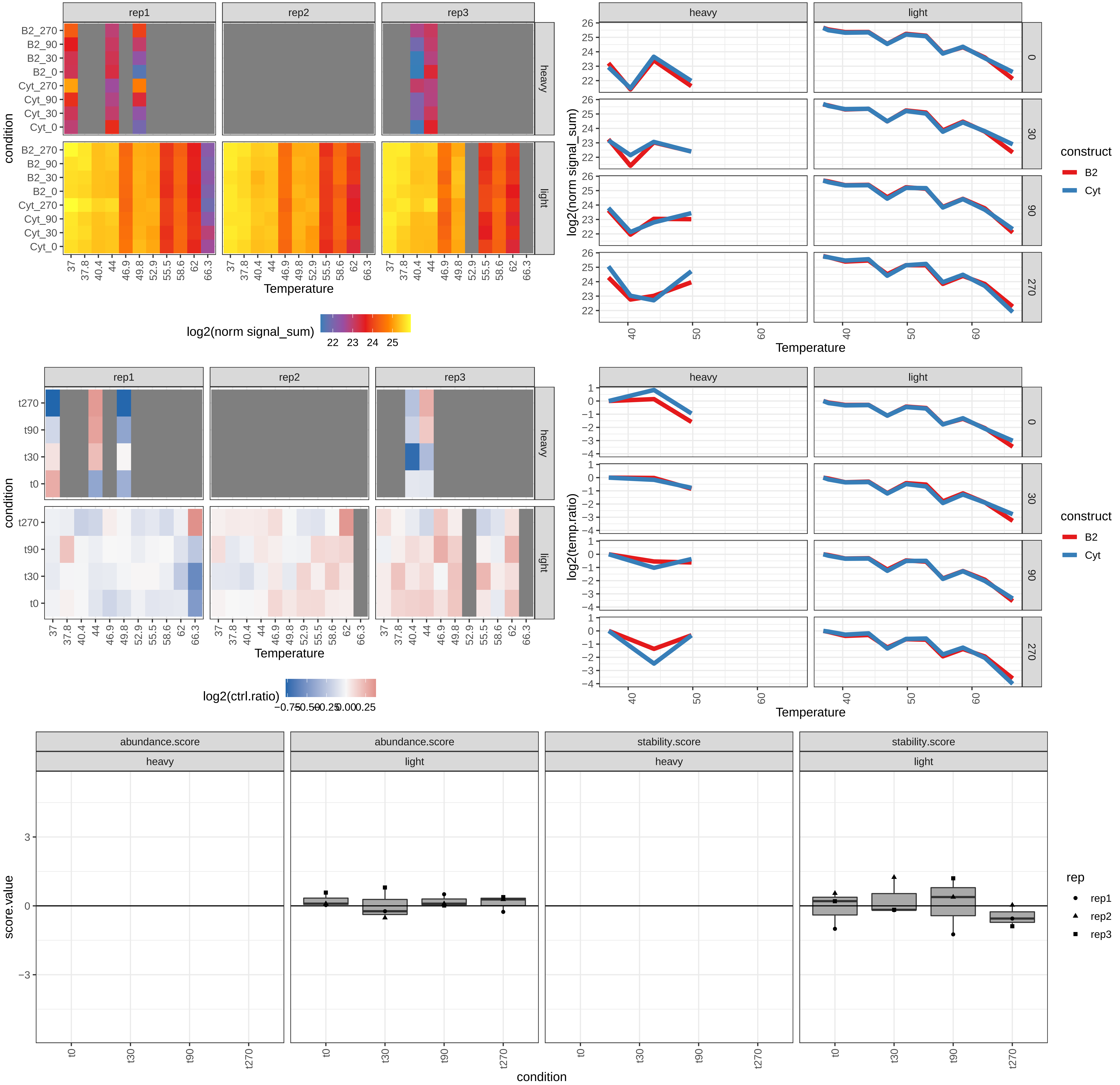

P38149 − Probable di− and tripeptidase DUG2 OS=Saccharomyces cerevisiae (strain ATCC 204508 / S288c) GN=DUG2 PE=1 SV=1

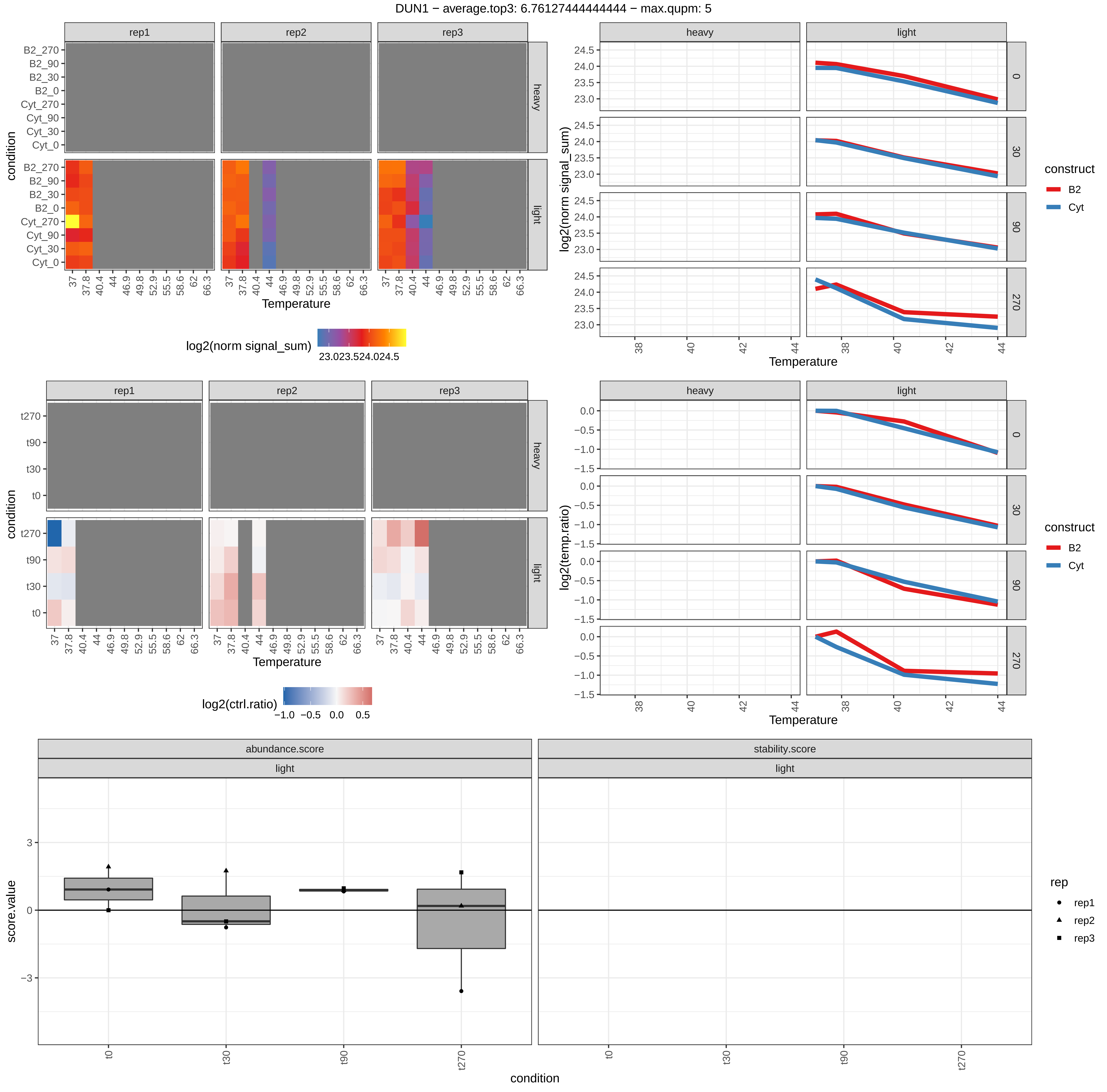

P39009 − DNA damage response protein kinase DUN1 OS=Saccharomyces cerevisiae (strain ATCC 204508 / S288c) GN=DUN1 PE=1 SV=1

DUR1 − average.top3: 6.688292 − max.qupm: 11

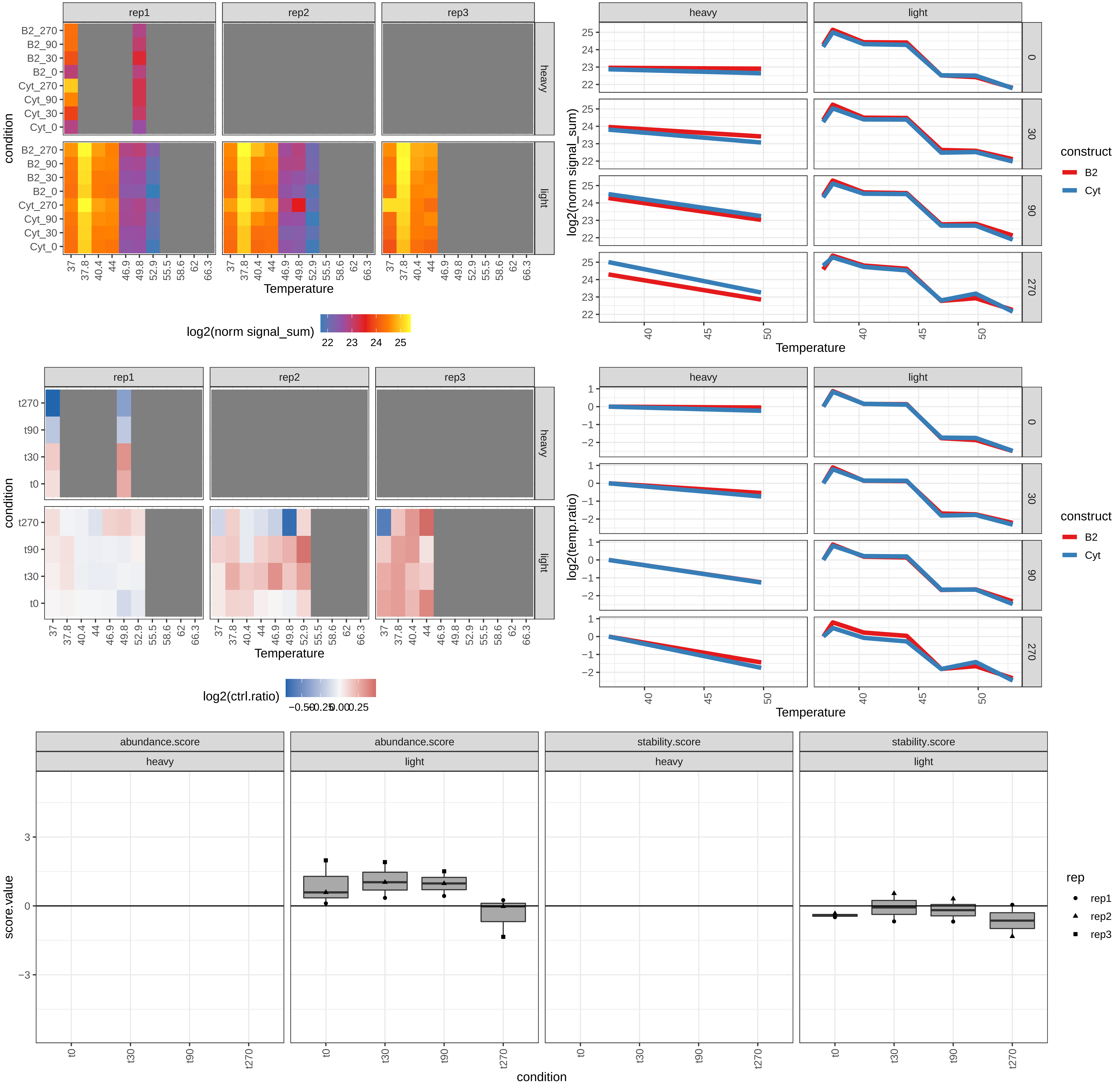

P32528 − Urea amidolyase OS=Saccharomyces cerevisiae (strain ATCC 204508 / S288c) GN=DUR1,2 PE=1 SV=2

DUS3 − average.top3: 6.79459 − max.qupm: 12

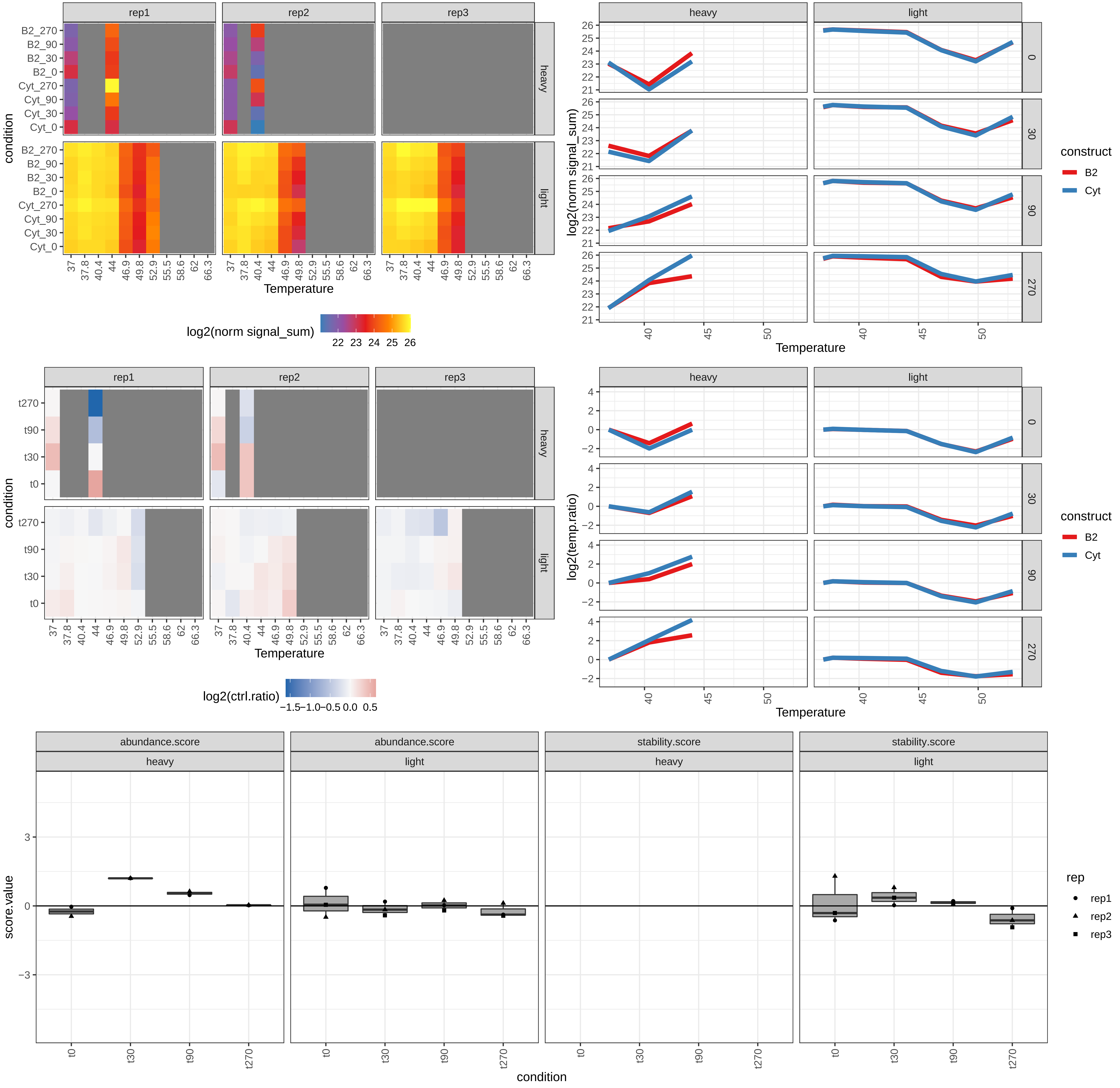

Q06053 − tRNA−dihydrouridine(47) synthase [NAD(P)(+)] OS=Saccharomyces cerevisiae (strain ATCC 204508 / S288c) GN=DUS3 PE=1 SV=2

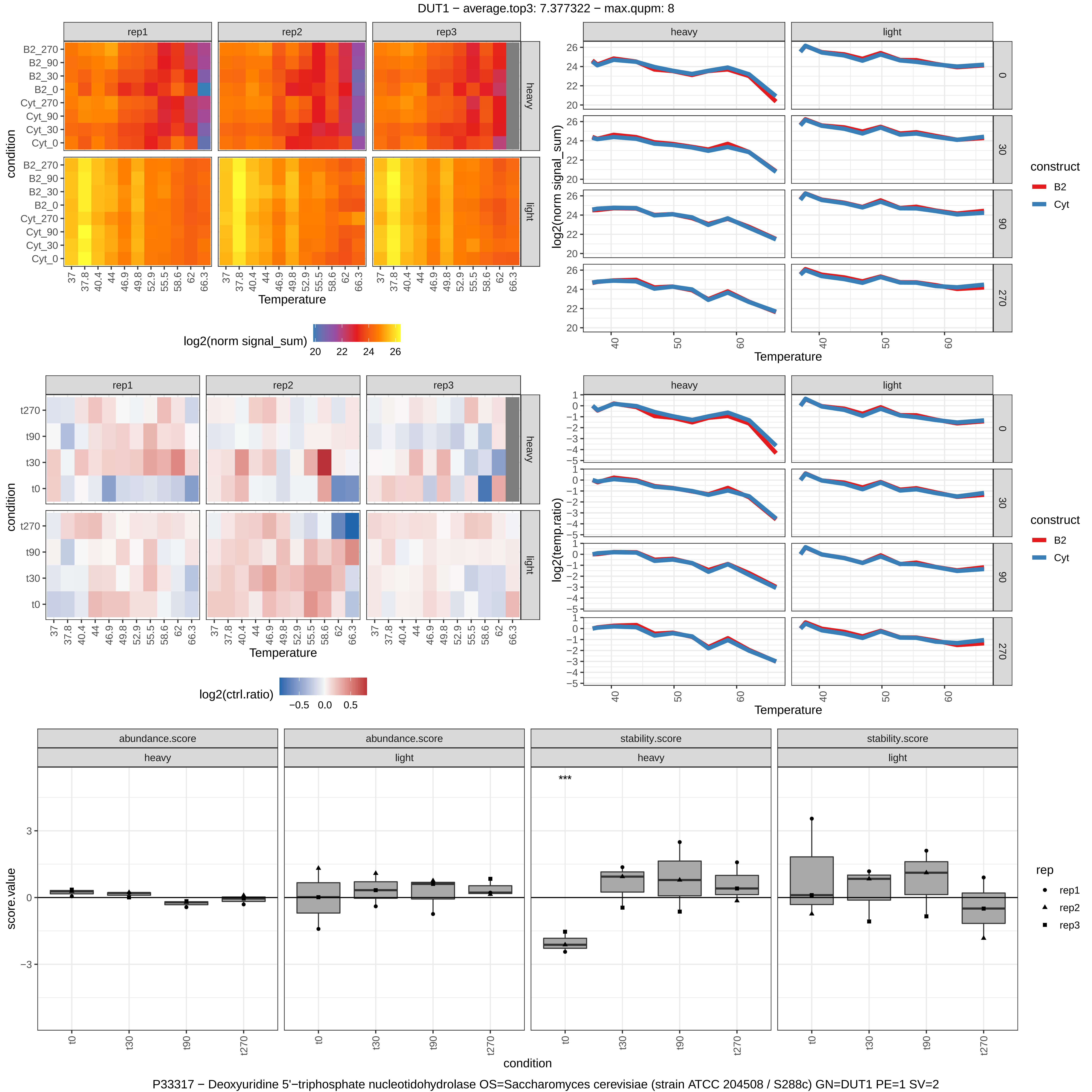

DUT1 − average.top3: 7.377322 − max.qupm: 8
rep1
rep2
rep3
heavy
light
condition
Temperature
log2(norm signal_sum)
log2(ctrl.ratio)
log2(temp.ratio)
construct
B2
Cyt
abundance.score
stability.score
score.value
rep
rep1
rep2
rep3
***
P33317 − Deoxyuridine 5'−triphosphate nucleotidohydrolase OS=Saccharomyces cerevisiae (strain ATCC 204508 / S288c) GN=DUT1 PE=1 SV=2

DYN1 − average.top3: 6.65864157894737 − max.qupm: 8

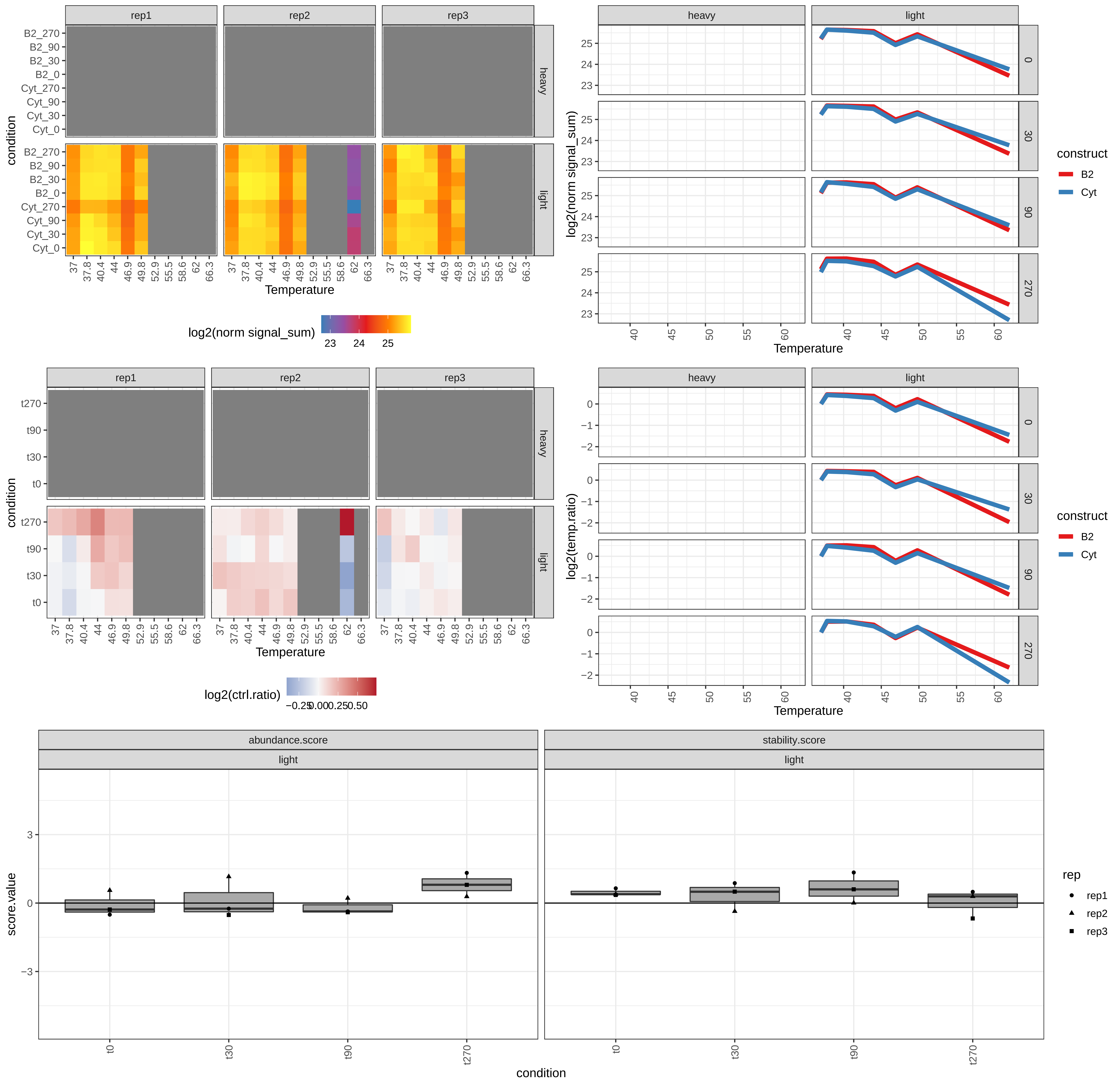

P36022 − Dynein heavy chain, cytoplasmic OS=Saccharomyces cerevisiae (strain ATCC 204508 / S288c) GN=DYN1 PE=1 SV=1

DYN2 − average.top3: 7.51415810810811 − max.qupm: 6

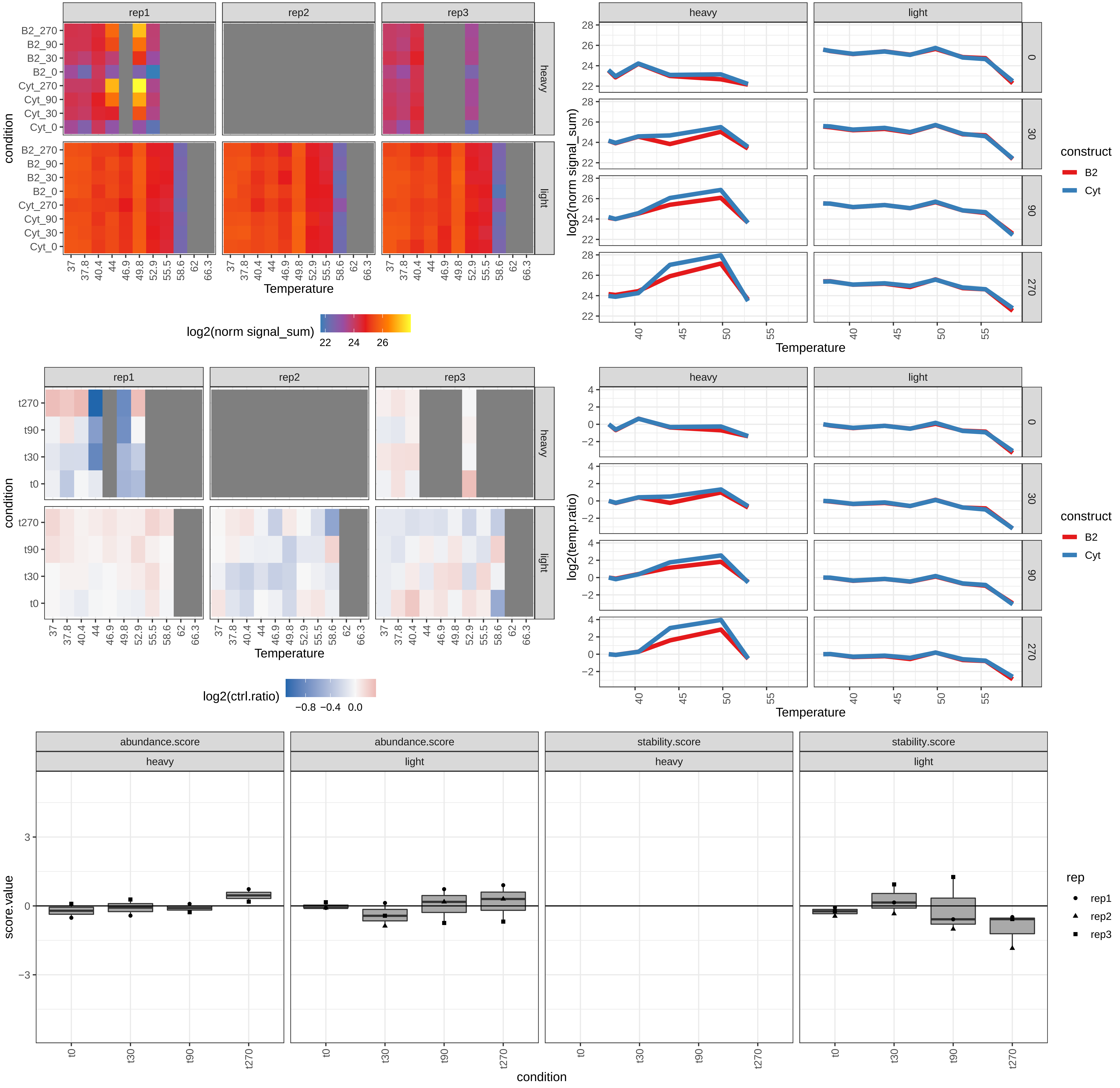

Q02647 − Dynein light chain 1, cytoplasmic OS=Saccharomyces cerevisiae (strain ATCC 204508 / S288c) GN=DYN2 PE=1 SV=1

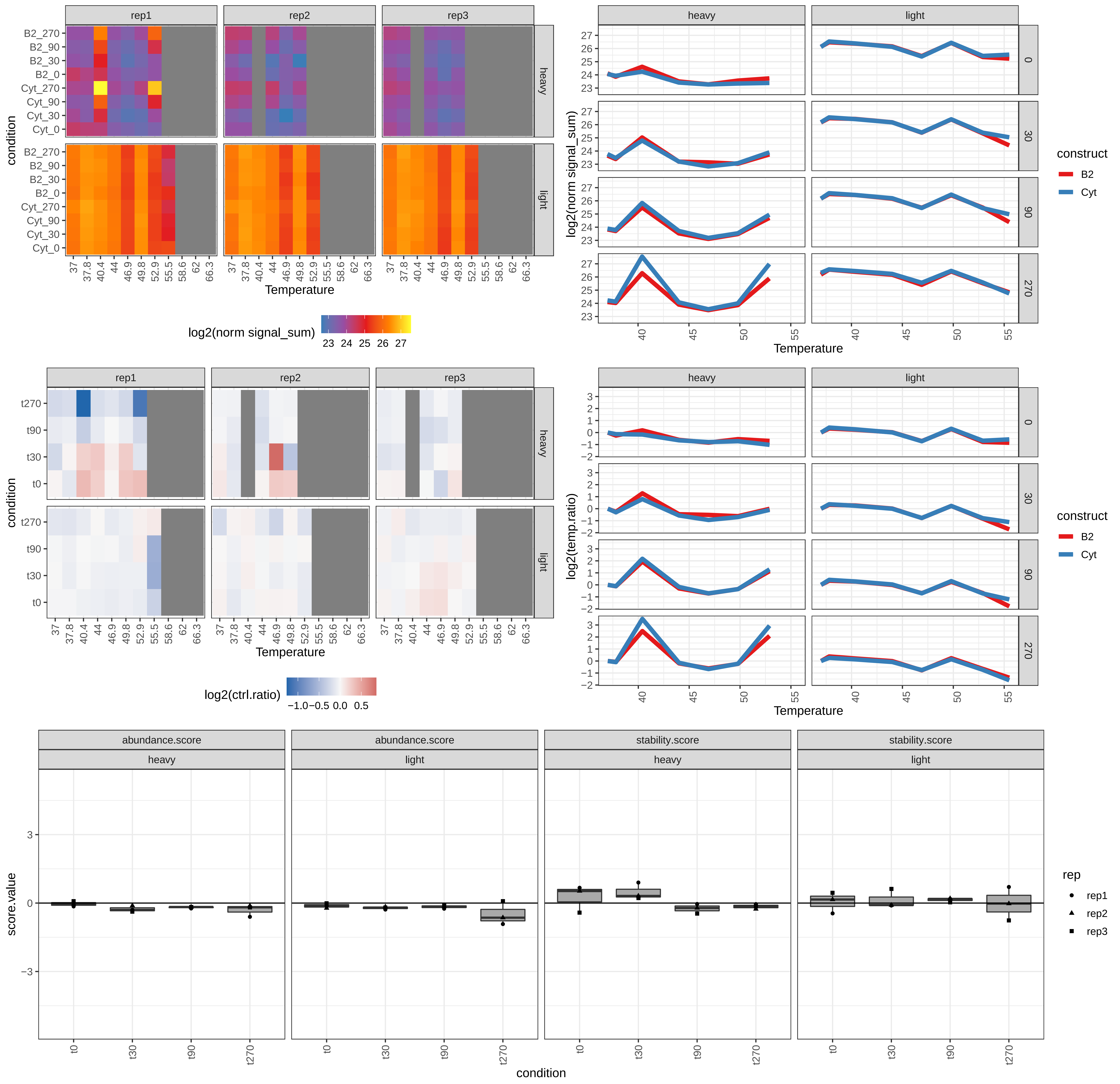

P38791 − Deoxyhypusine synthase OS=Saccharomyces cerevisiae (strain ATCC 204508 / S288c) GN=DYS1 PE=1 SV=1

EAF3 − average.top3: 6.45539 − max.qupm: 5

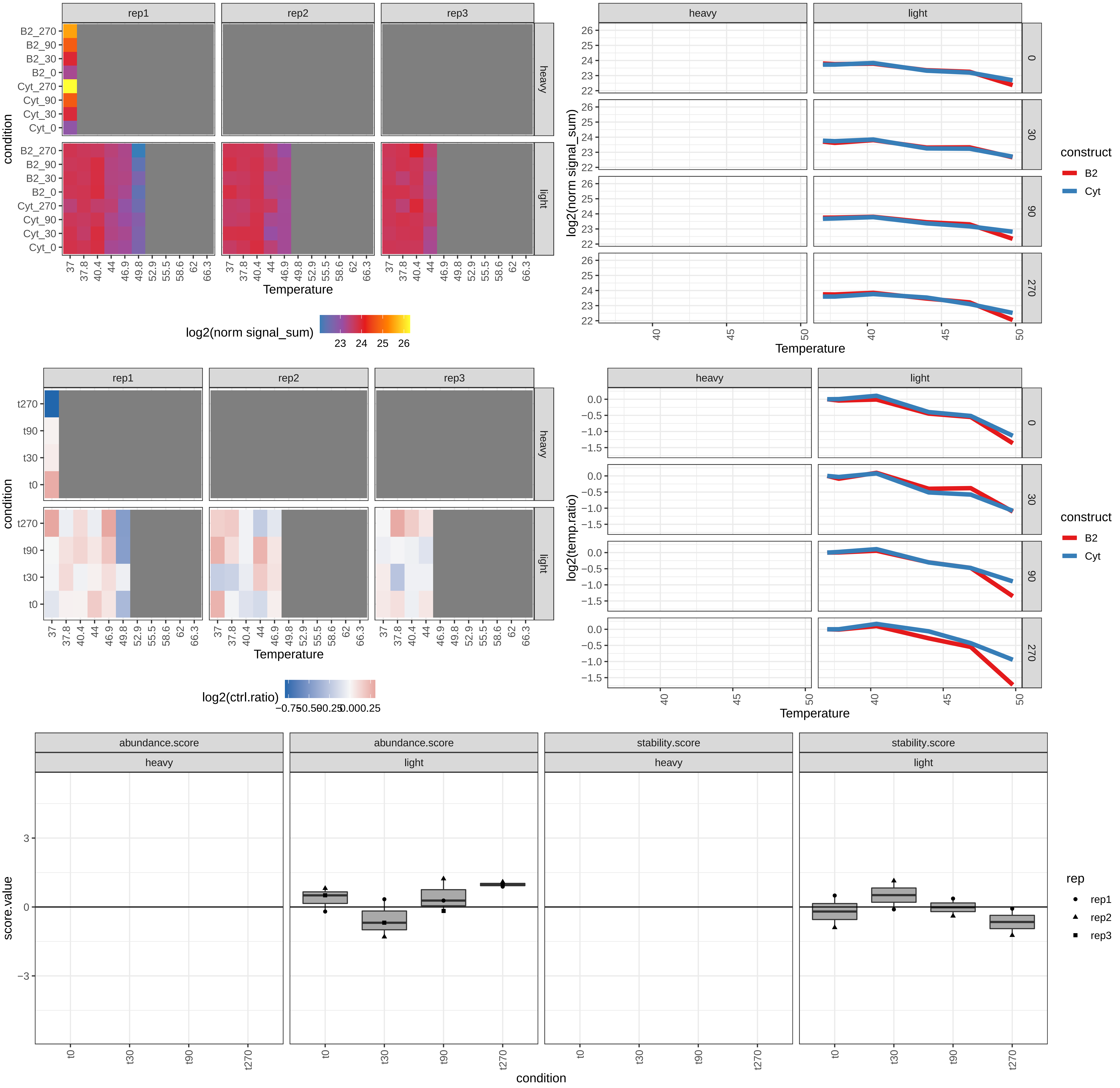

Q12432 − Chromatin modification−related protein EAF3 OS=Saccharomyces cerevisiae (strain ATCC 204508 / S288c) GN=EAF3 PE=1 SV=1

EAR1 − average.top3: 6.6698575 − max.qupm: 4

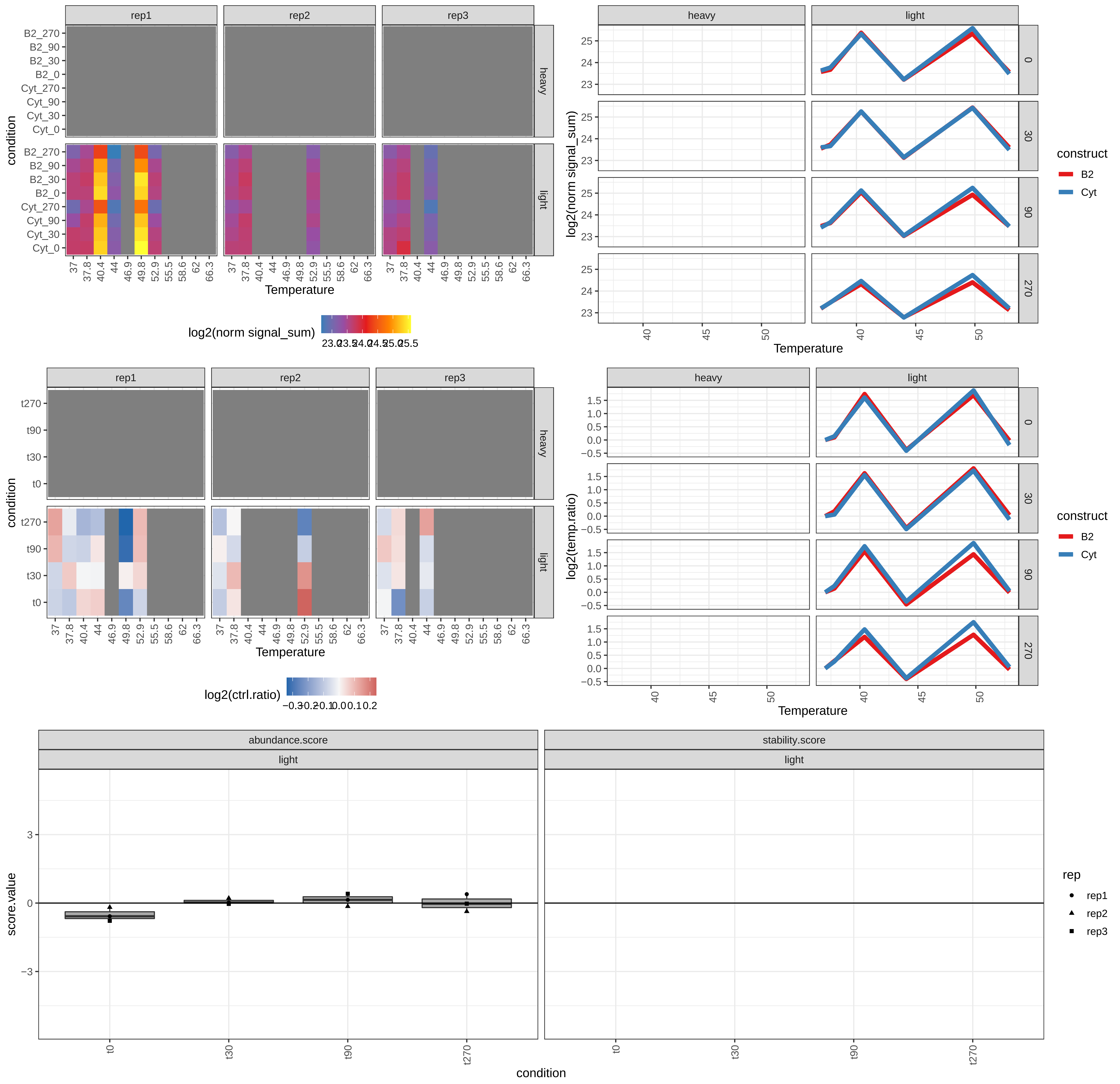

Q03212 − Protein EAR1 OS=Saccharomyces cerevisiae (strain ATCC 204508 / S288c) GN=EAR1 PE=1 SV=1

EBP2 − average.top3: 6.83107314285714 − max.qupm: 7

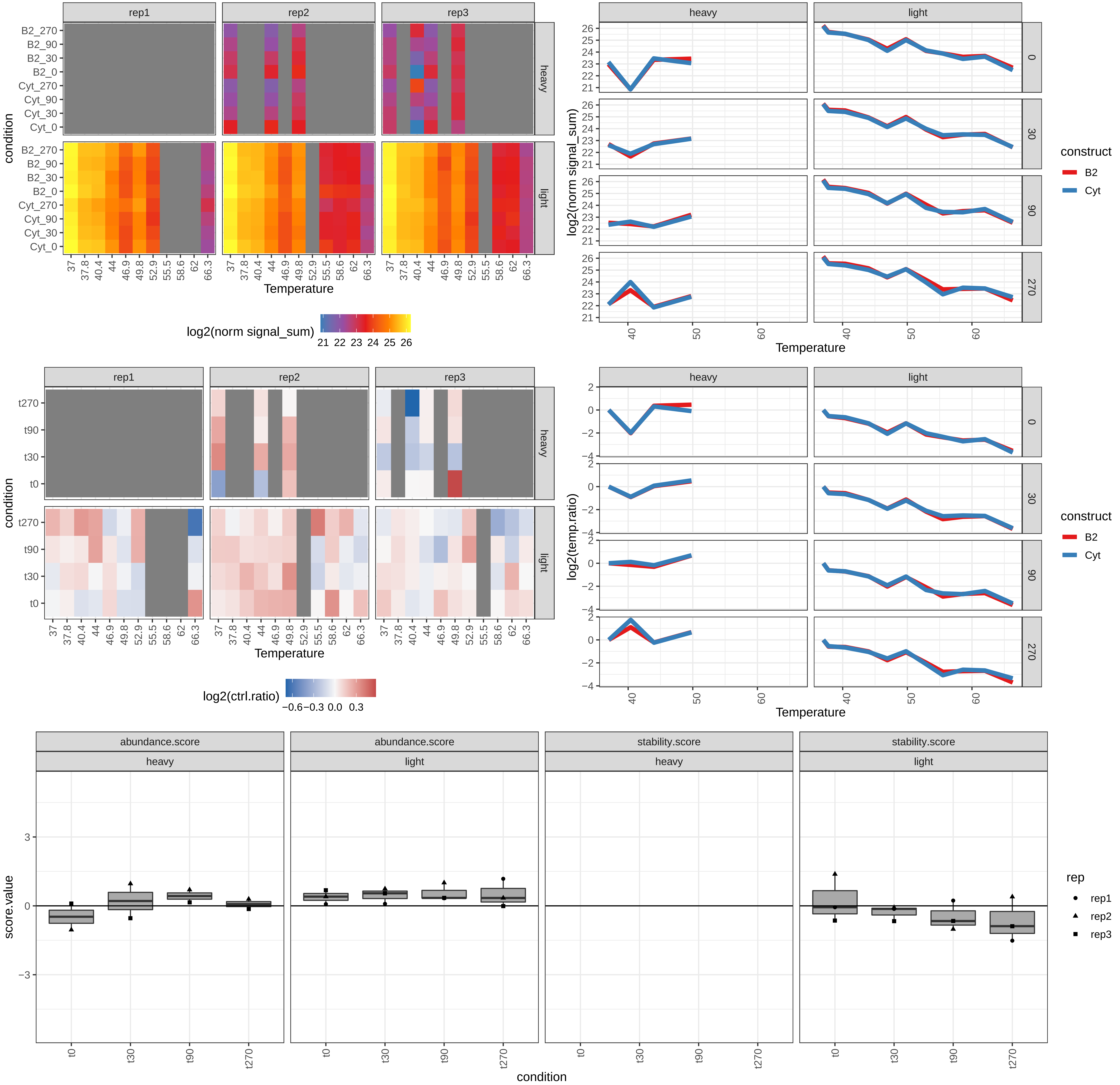

P36049 − rRNA−processing protein EBP2 OS=Saccharomyces cerevisiae (strain ATCC 204508 / S288c) GN=EBP2 PE=1 SV=1

EBS1 − average.top3: 6.57656944444444 − max.qupm: 5

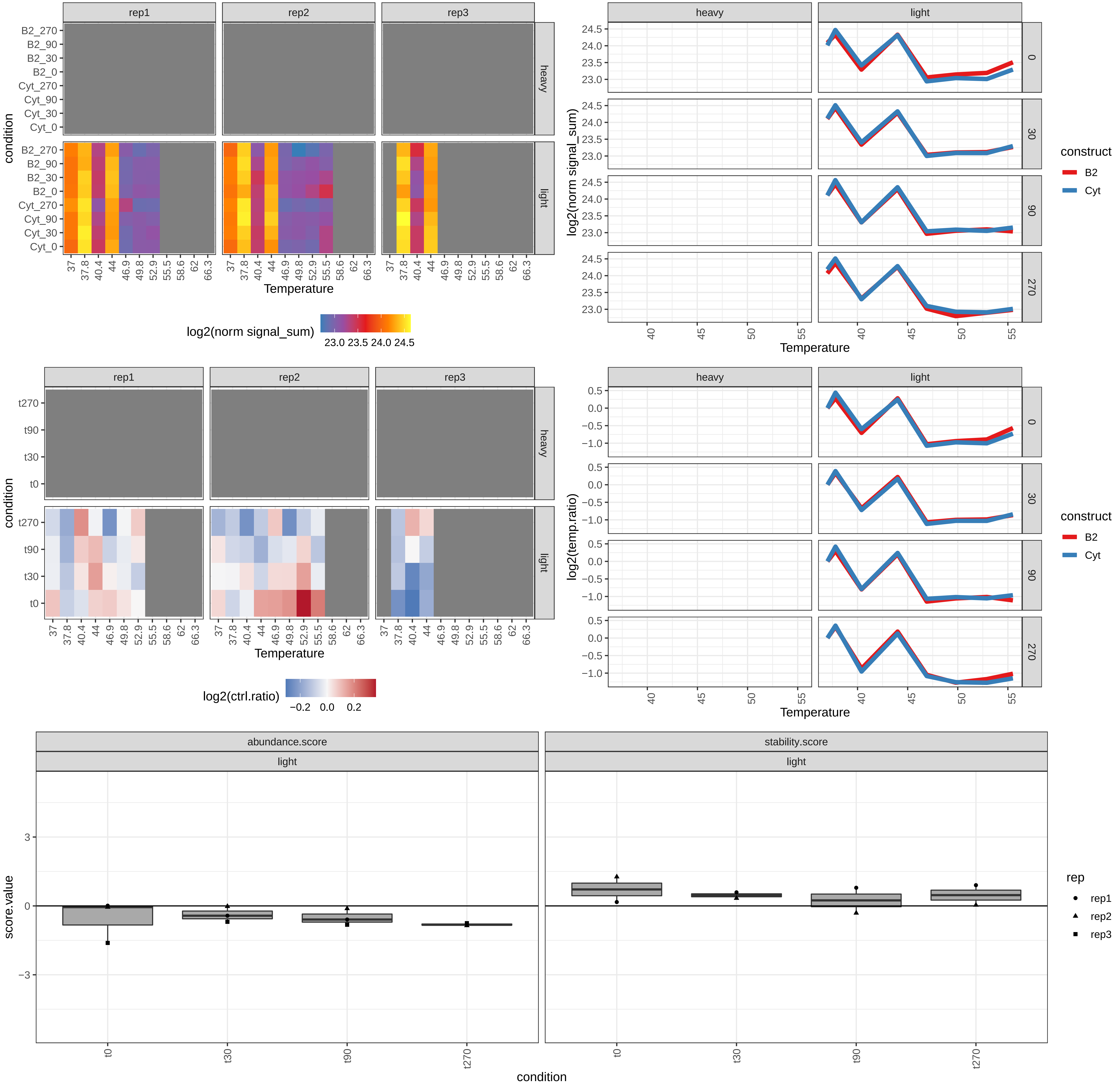

Q03466 − Protein EBS1 OS=Saccharomyces cerevisiae (strain ATCC 204508 / S288c) GN=EBS1 PE=3 SV=1

ECI1 − average.top3: 6.64611689655172 − max.qupm: 7

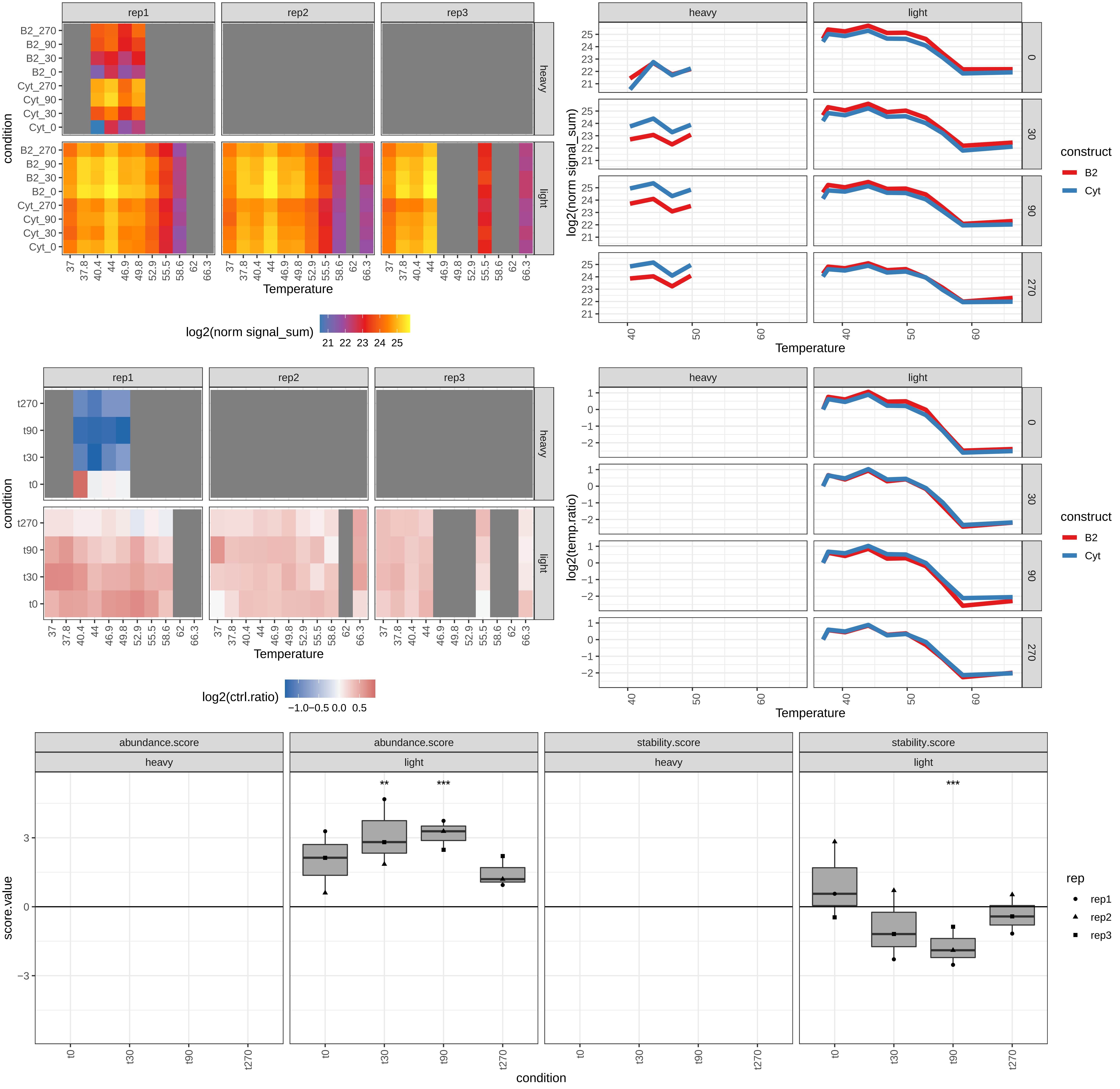

Q05871 − 3,2−trans−enoyl−CoA isomerase OS=Saccharomyces cerevisiae (strain ATCC 204508 / S288c) GN=ECI1 PE=1 SV=1

ECM14 − average.top3: 7.33911604166667 − max.qupm: 8

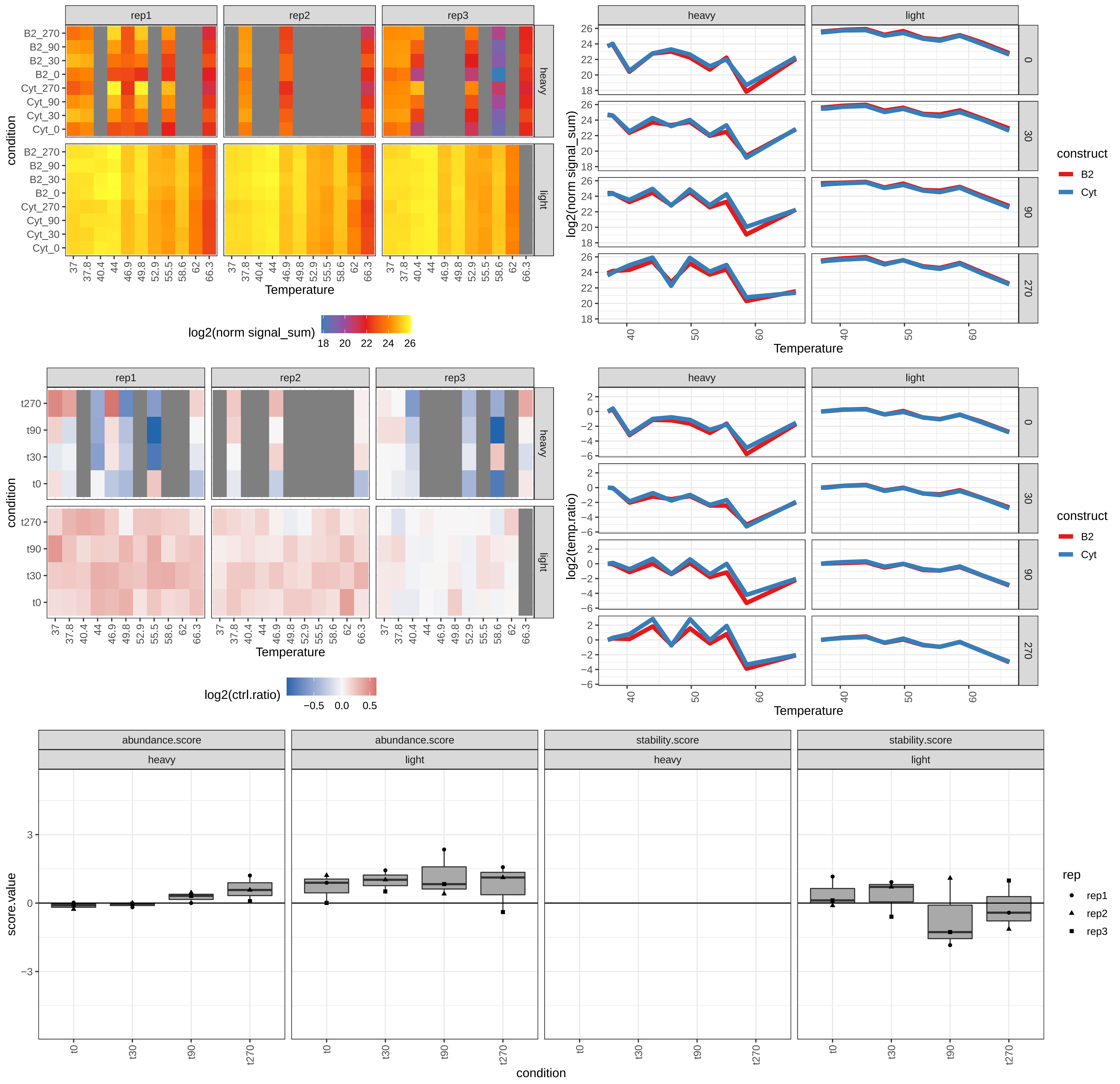

P38836 − Putative metallocarboxypeptidase ECM14 OS=Saccharomyces cerevisiae (strain ATCC 204508 / S288c) GN=ECM14 PE=1 SV=1

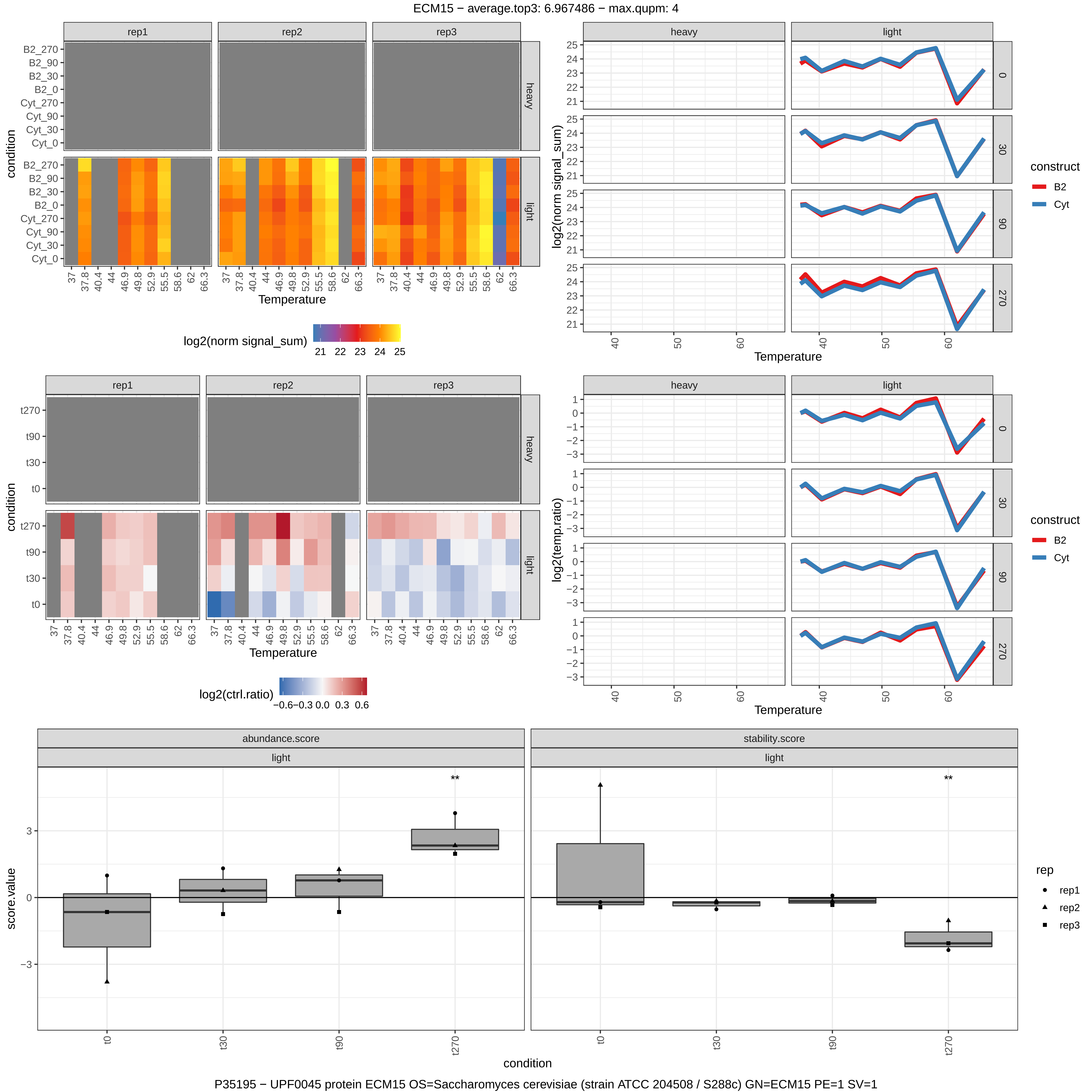

ECM15 − average.top3: 6.967486 − max.qupm: 4
rep1
rep2
rep3
heavy
light
condition
Temperature
log2(norm signal_sum)
log2(ctrl.ratio)
construct
B2
Cyt
log2(temp.ratio)
abundance.score
stability.score
score.value
rep
rep1
rep2
rep3
**
P35195 − UPF0045 protein ECM15 OS=Saccharomyces cerevisiae (strain ATCC 204508 / S288c) GN=ECM15 PE=1 SV=1

ECM16 − average.top3: 6.93346782608696 − max.qupm: 14

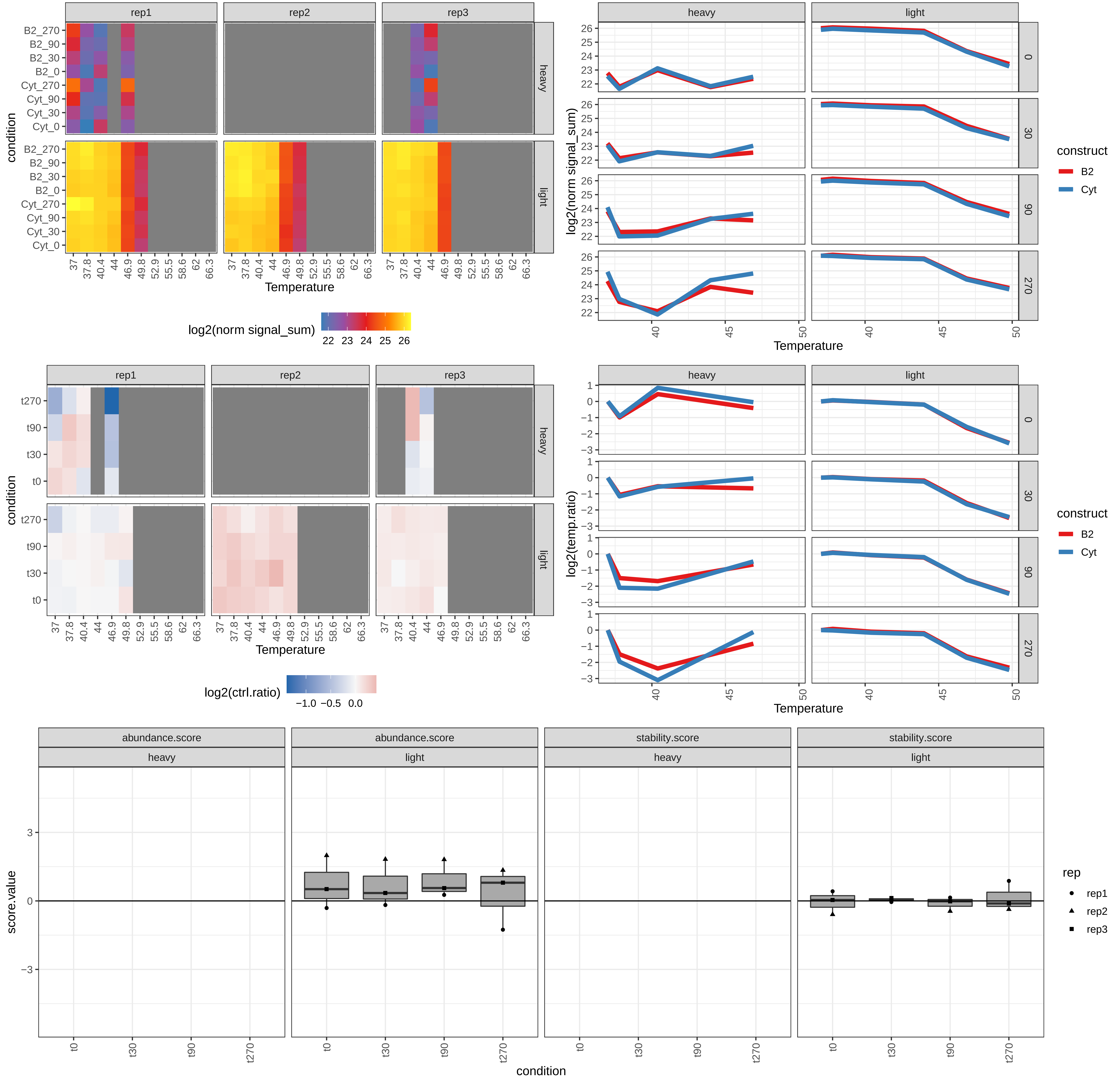

Q04217 − Probable ATP−dependent RNA helicase DHR1 OS=Saccharomyces cerevisiae (strain ATCC 204508 / S288c) GN=ECM16 PE=1 SV=1

ECM21 − average.top3: 6.42778166666667 − max.qupm: 6

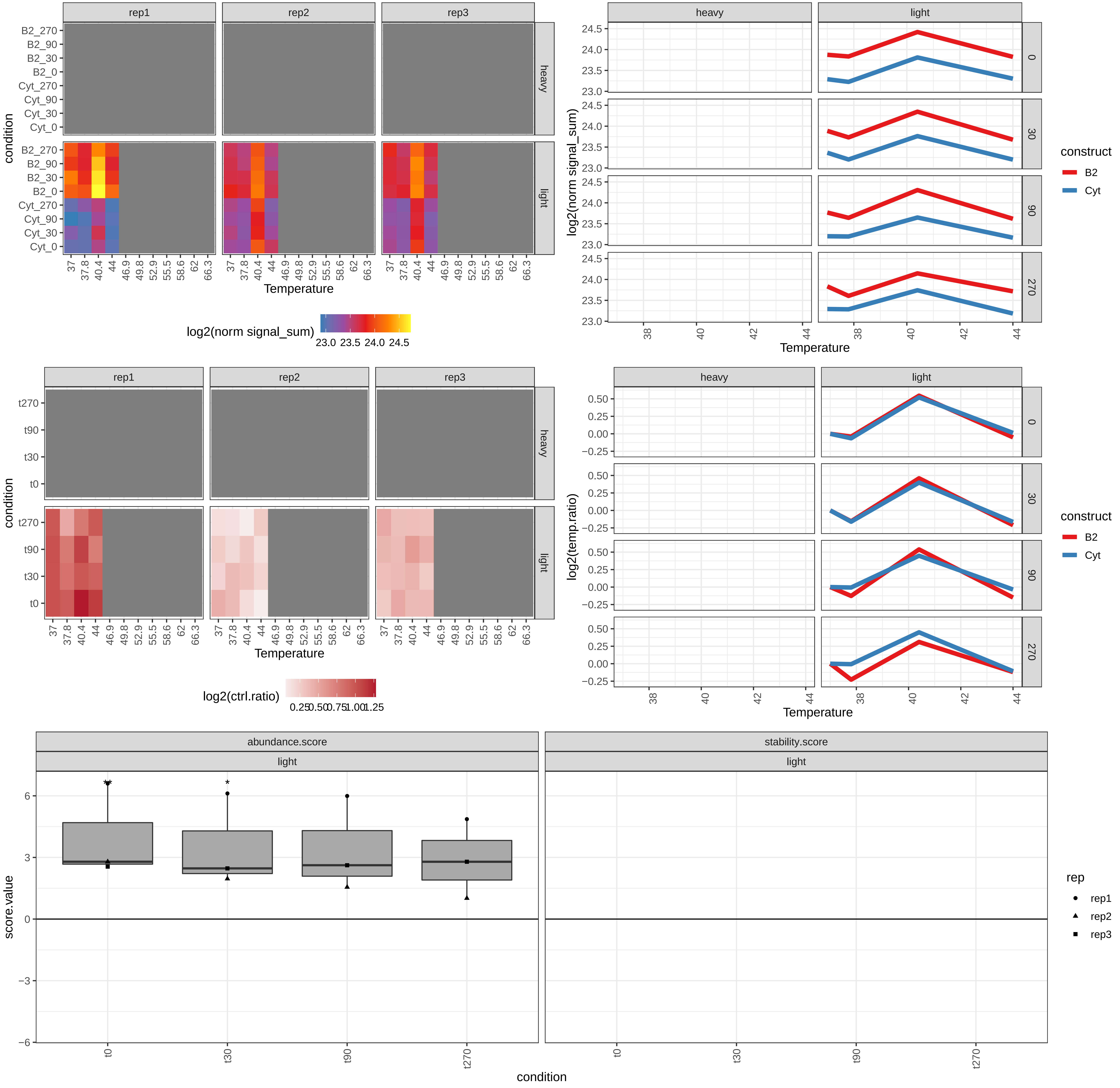

P38167 − Protein ECM21 OS=Saccharomyces cerevisiae (strain ATCC 204508 / S288c) GN=ECM21 PE=1 SV=2

ECM29 − average.top3: 6.62975885714286 − max.qupm: 16

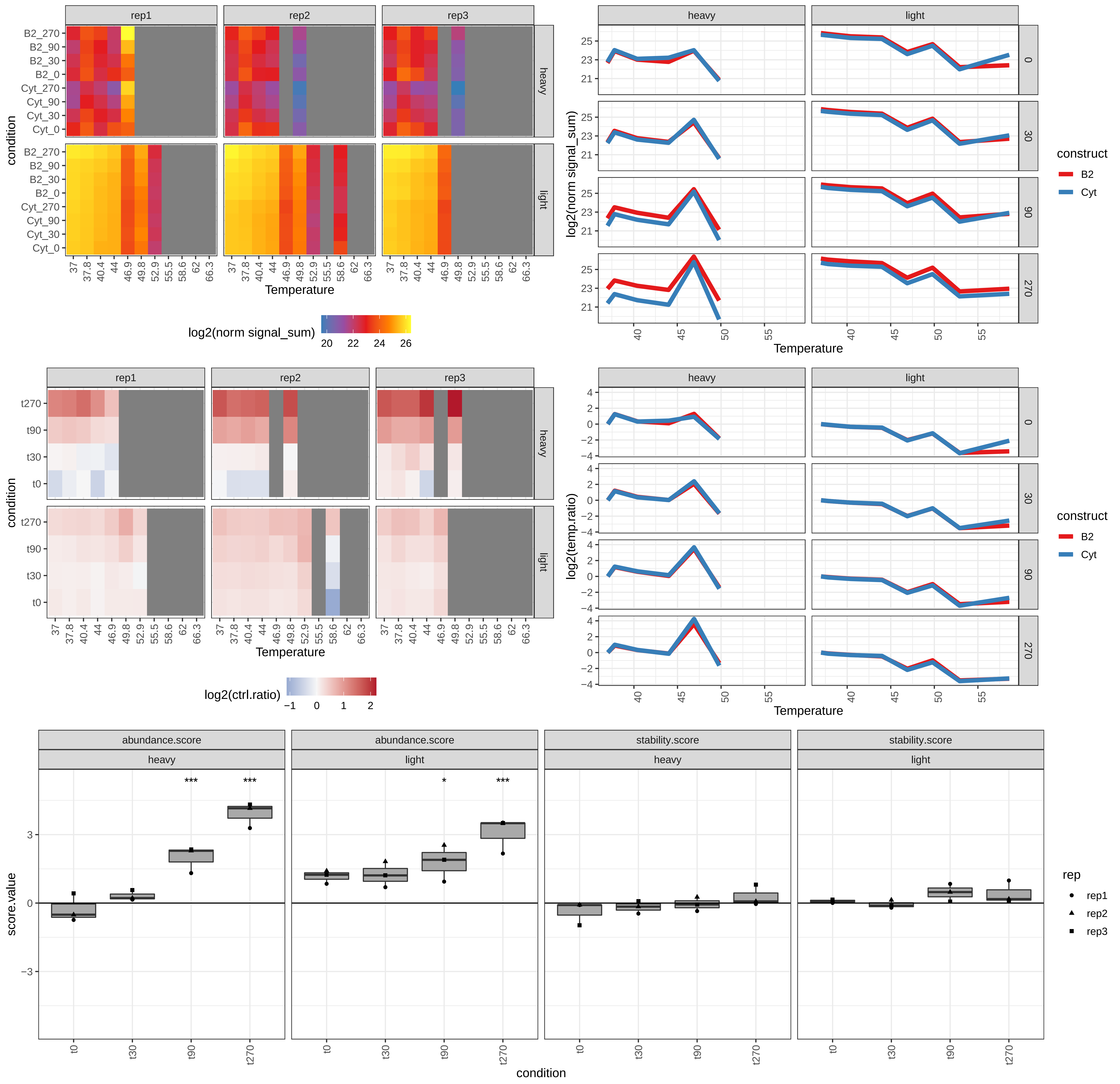

P38737 − Proteasome component ECM29 OS=Saccharomyces cerevisiae (strain ATCC 204508 / S288c) GN=ECM29 PE=1 SV=1

ECM31 − average.top3: 7.00187433333333 − max.qupm: 7

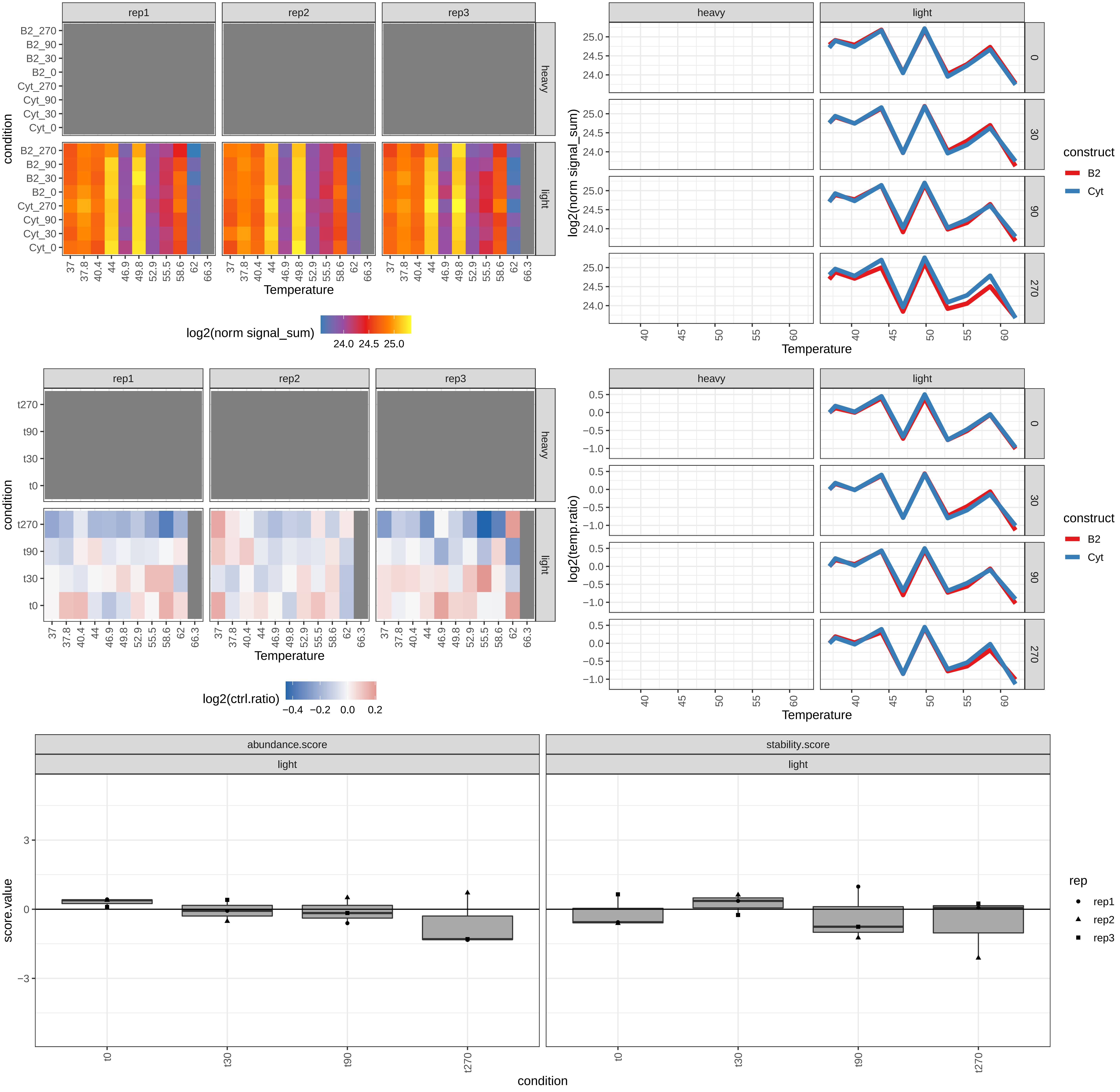

P38122 − 3−methyl−2−oxobutanoate hydroxymethyltransferase OS=Saccharomyces cerevisiae (strain ATCC 204508 / S288c) GN=ECM31 PE=1 SV=1

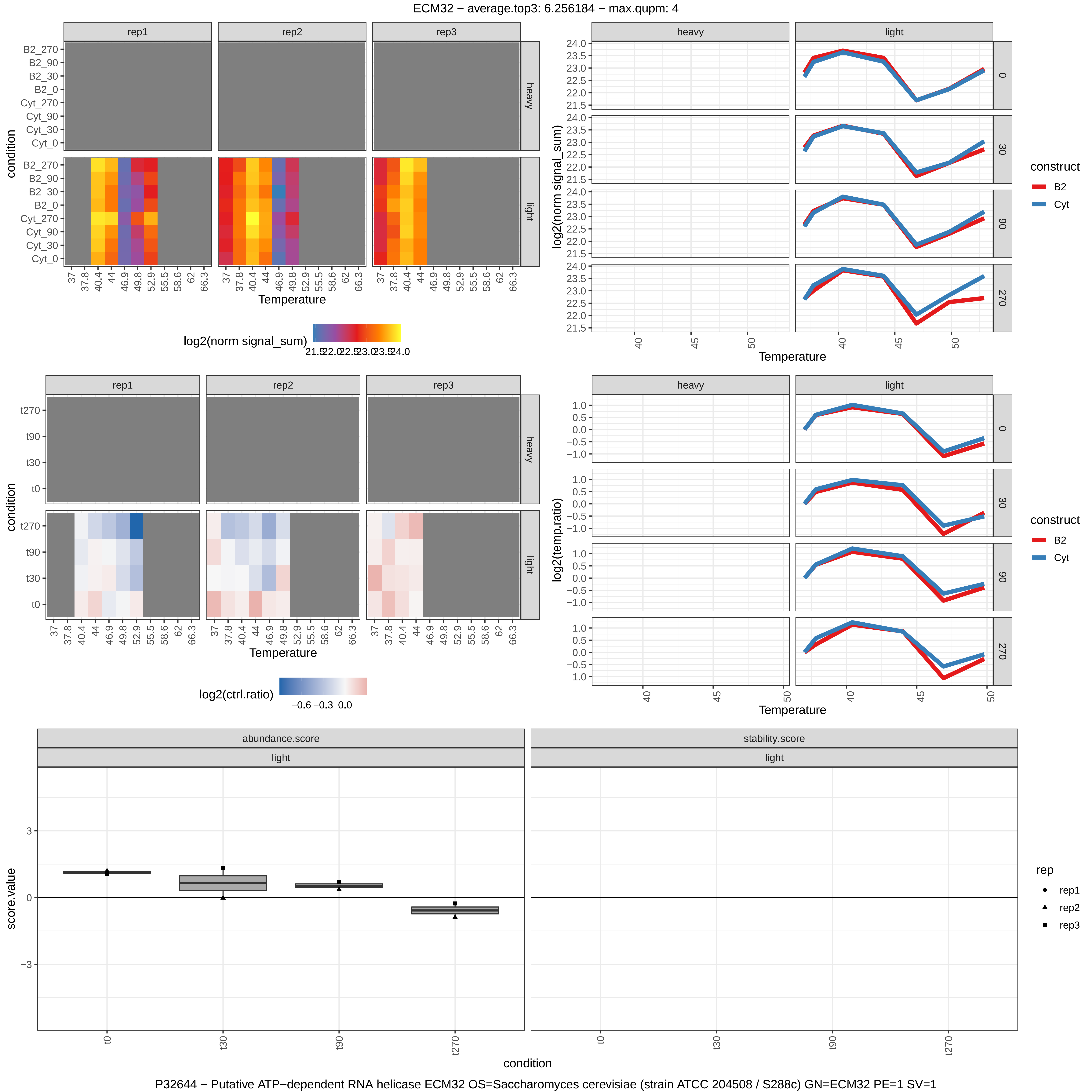

ECM32 − average.top3: 6.256184 − max.qupm: 4
rep1
rep2
rep3
heavy
light
condition
Temperature
log2(norm signal_sum)
log2(ctrl.ratio)
log2(temp.ratio)
construct
B2
Cyt
abundance.score
stability.score
score.value
rep
condition
P32644 − Putative ATP−dependent RNA helicase ECM32 OS=Saccharomyces cerevisiae (strain ATCC 204508 / S288c) GN=ECM32 PE=1 SV=1

ECM33 − average.top3: 7.47680724137931 − max.qupm: 6

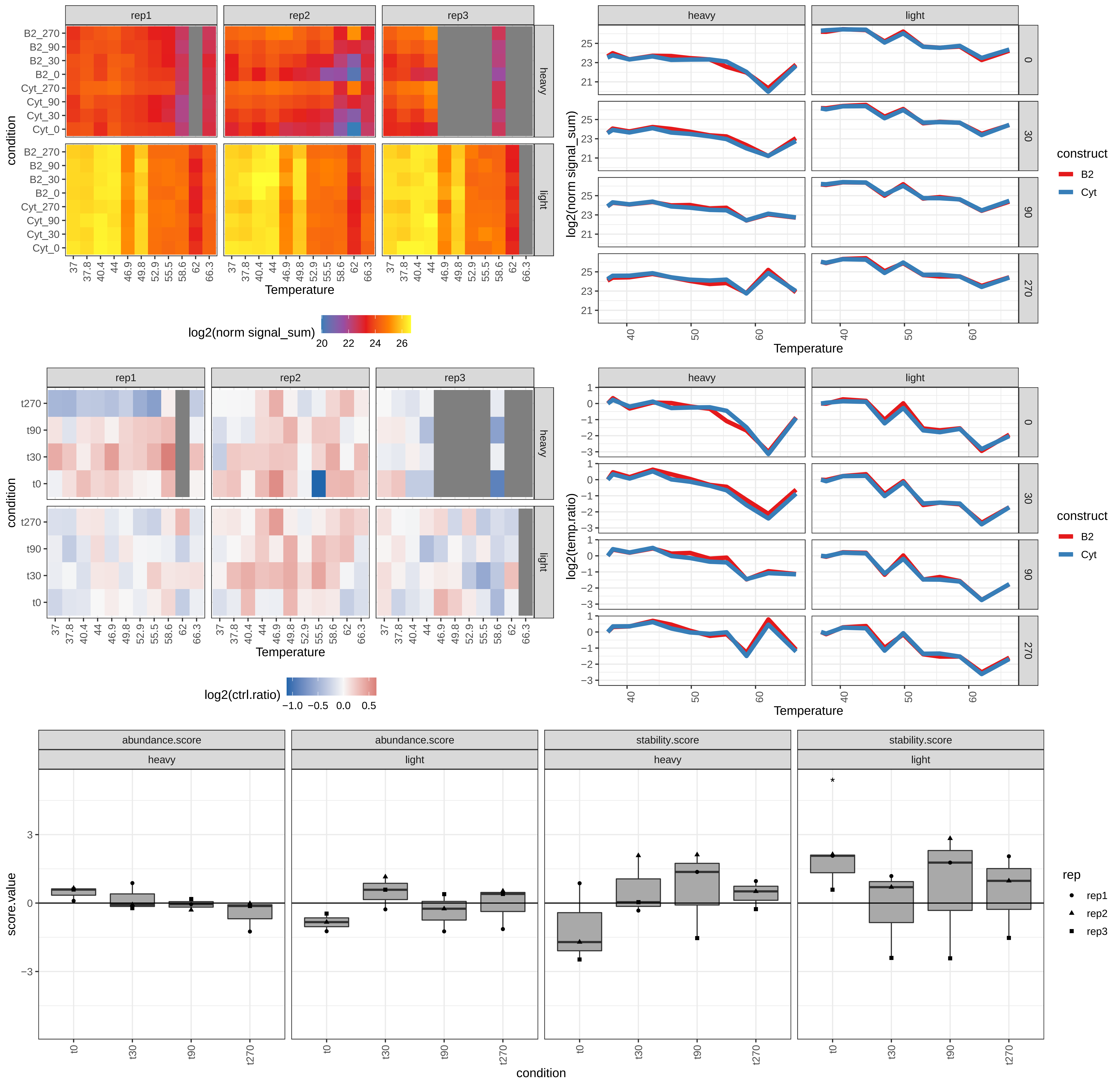

P38248 − Cell wall protein ECM33 OS=Saccharomyces cerevisiae (strain ATCC 204508 / S288c) GN=ECM33 PE=1 SV=3

ECM4 − average.top3: 7.1886404 − max.qupm: 14

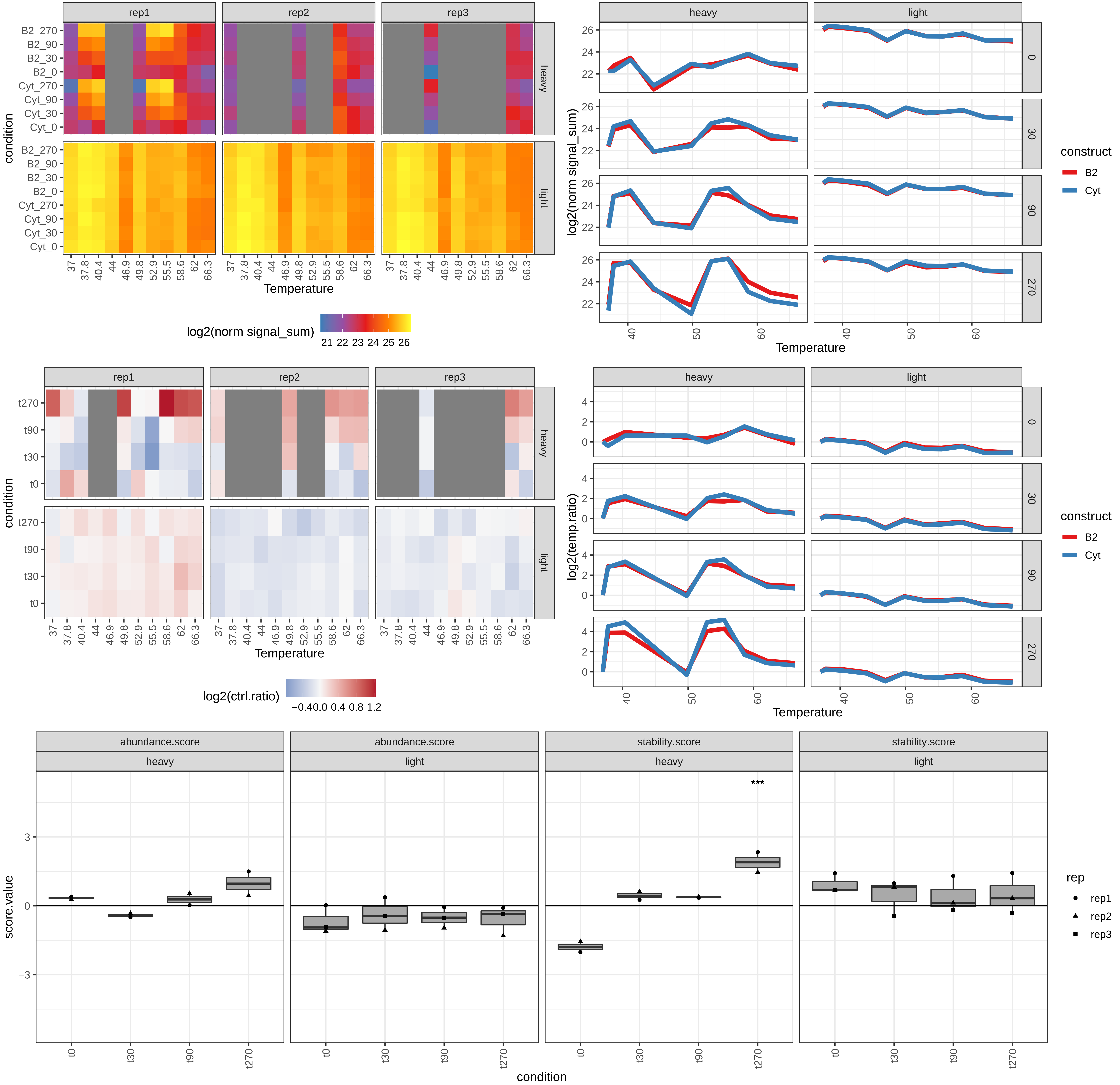

P36156 − Glutathione S−transferase omega−like 2 OS=Saccharomyces cerevisiae (strain ATCC 204508 / S288c) GN=ECM4 PE=1 SV=1

ECT1 − average.top3: 6.70028 − max.qupm: 3

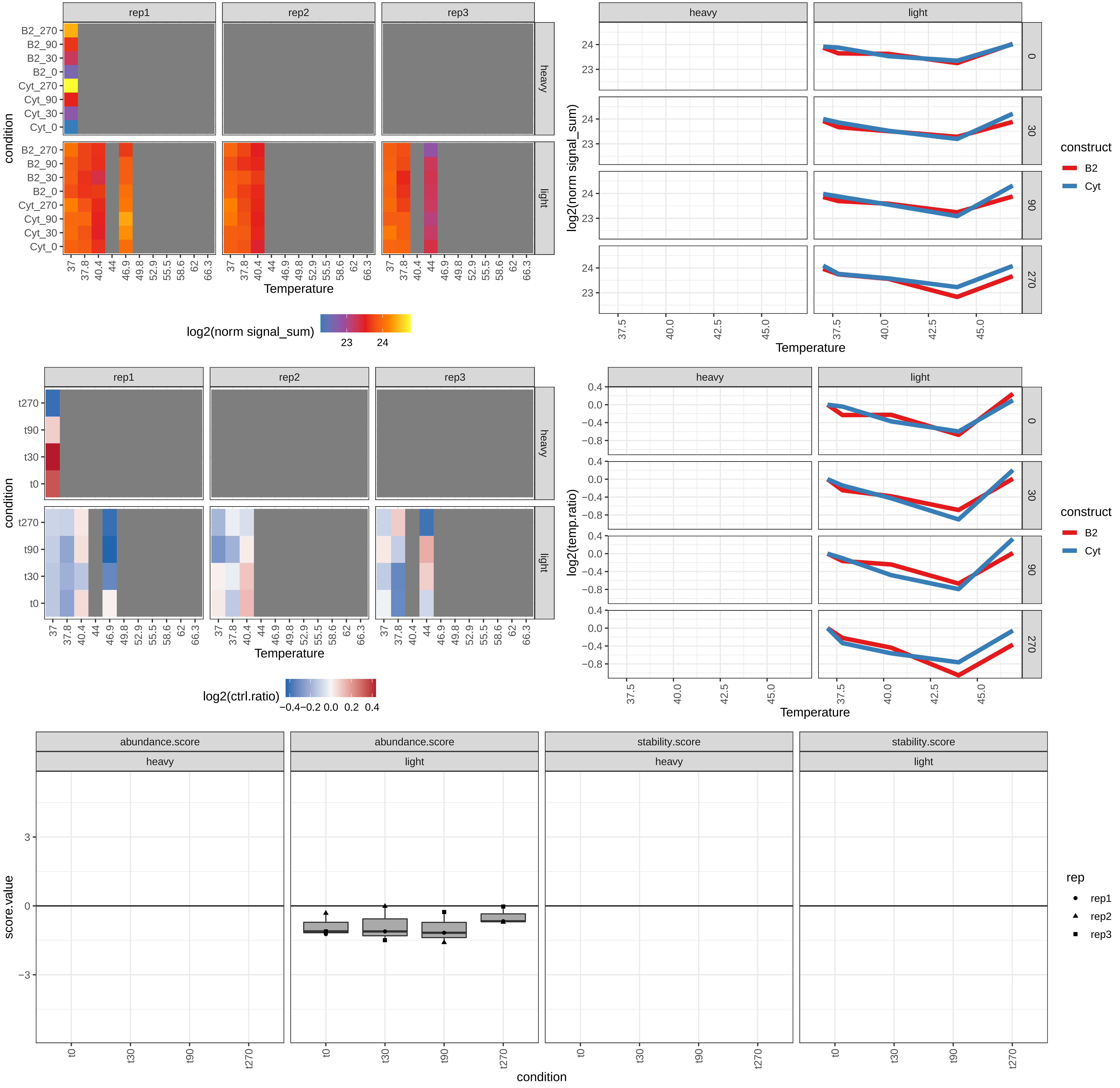

P33412 − Ethanolamine−phosphate cytidylyltransferase OS=Saccharomyces cerevisiae (strain ATCC 204508 / S288c) GN=ECT1 PE=1 SV=1

EDC3 − average.top3: 6.87348407407407 − max.qupm: 4

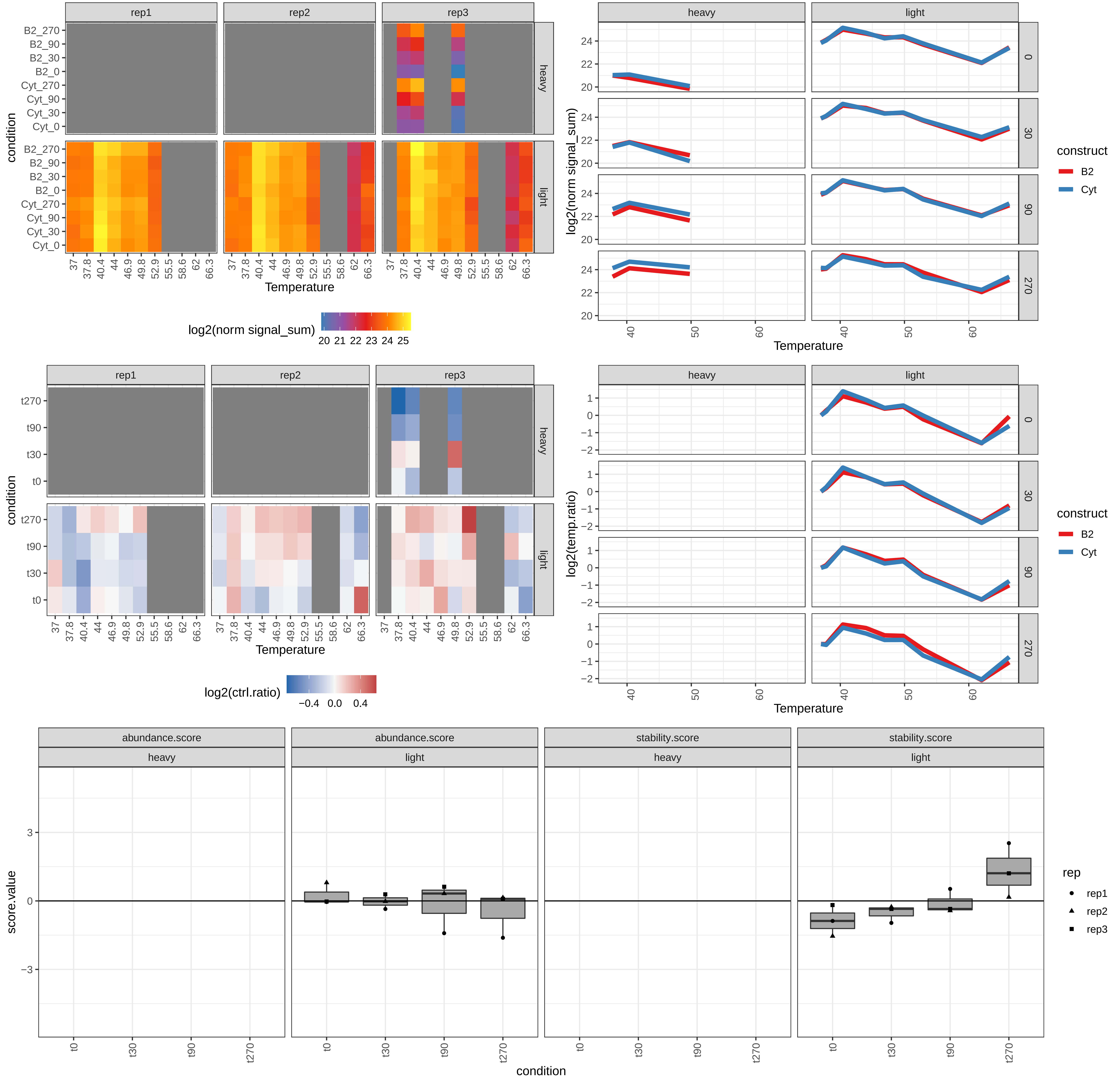

P39998 − Enhancer of mRNA−decapping protein 3 OS=Saccharomyces cerevisiae (strain ATCC 204508 / S288c) GN=EDC3 PE=1 SV=1

EDE1 − average.top3: 6.82598848484848 − max.qupm: 13

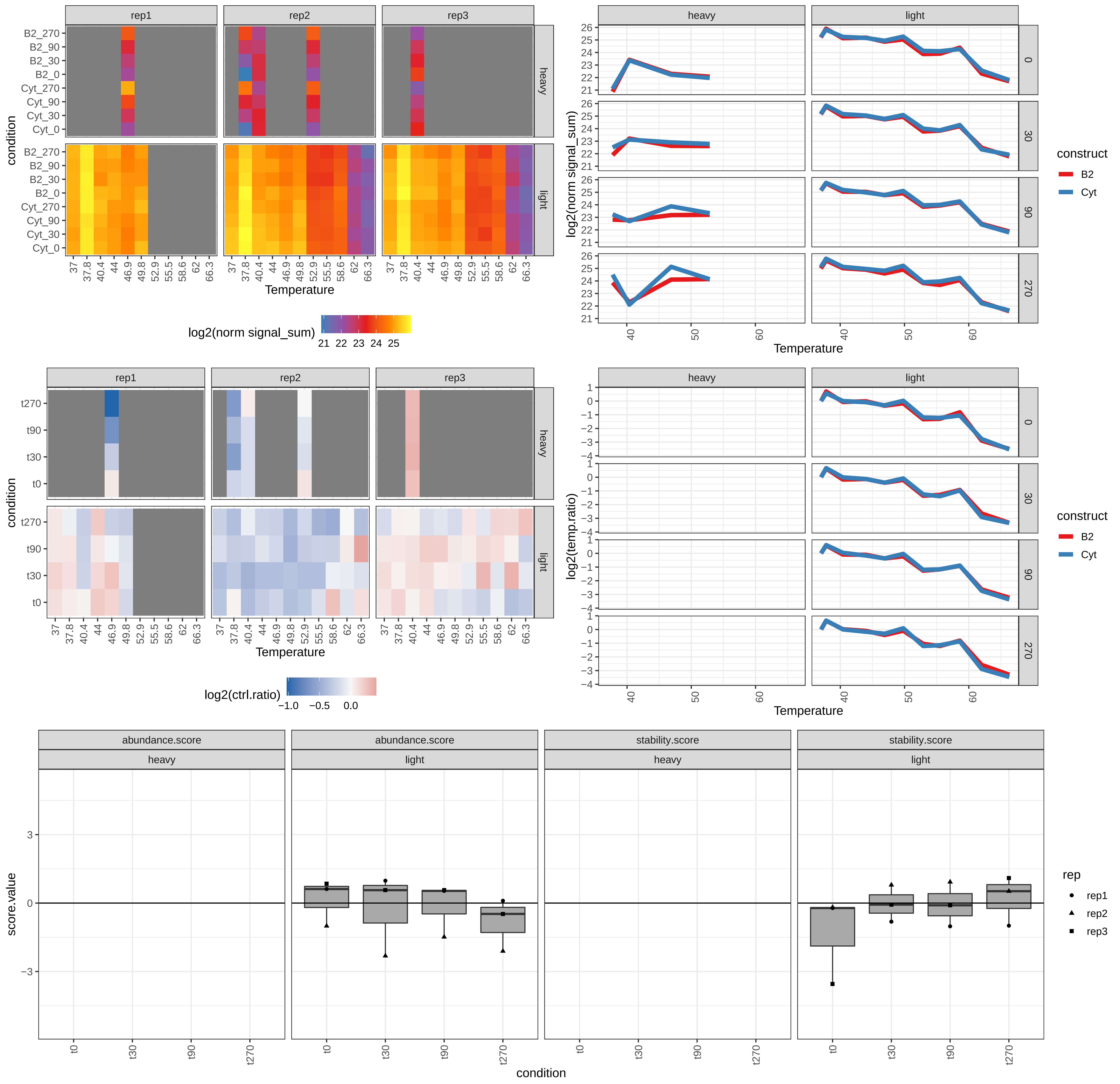

P34216 − EH domain−containing and endocytosis protein 1 OS=Saccharomyces cerevisiae (strain ATCC 204508 / S288c) GN=EDE1 PE=1 SV=2

EFB1 − average.top3: 7.19388321428571 − max.qupm: 5

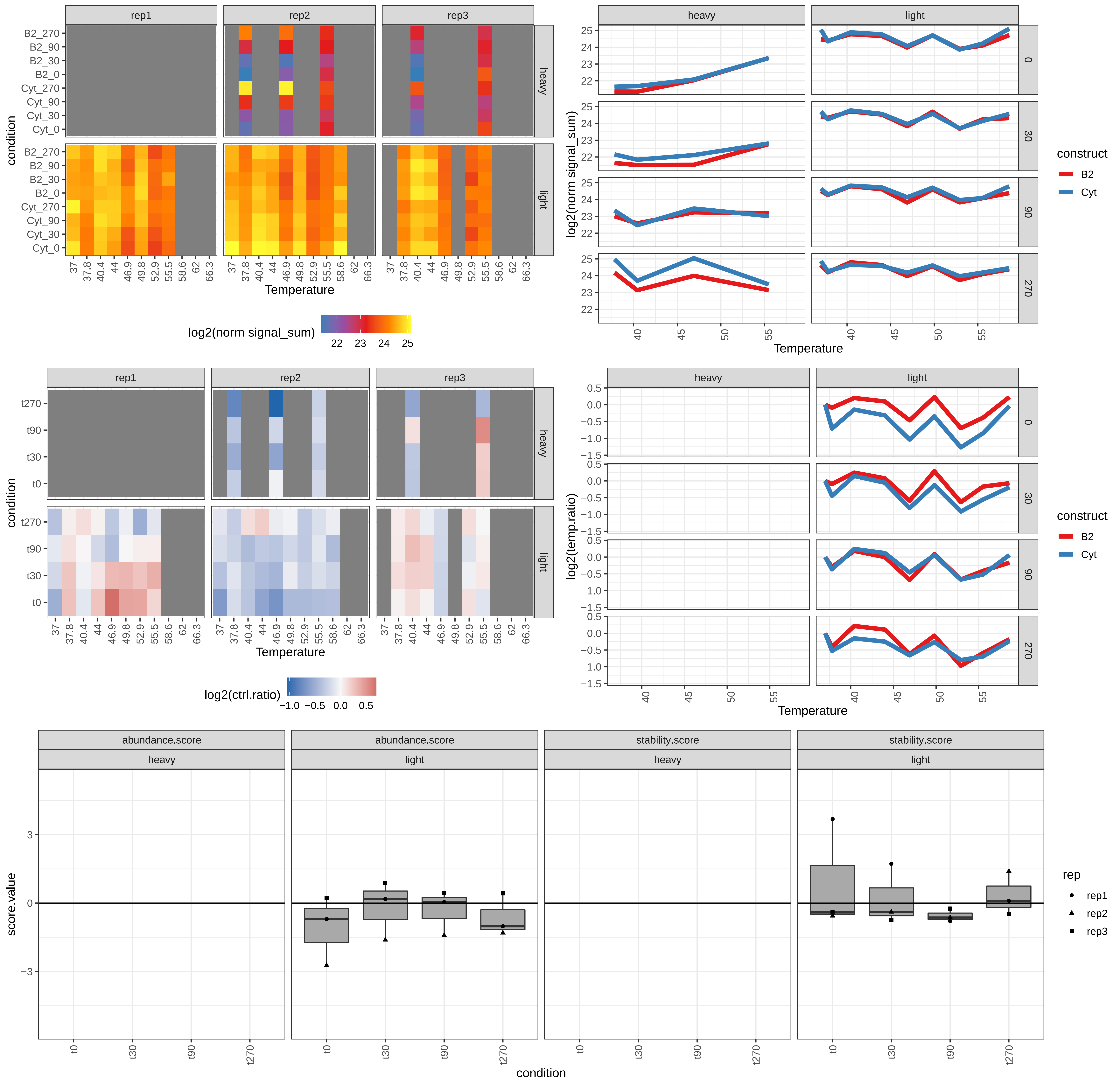

P32471 − Elongation factor 1−beta OS=Saccharomyces cerevisiae (strain ATCC 204508 / S288c) GN=EFB1 PE=1 SV=4

EFG1 − average.top3: 6.836125 − max.qupm: 5

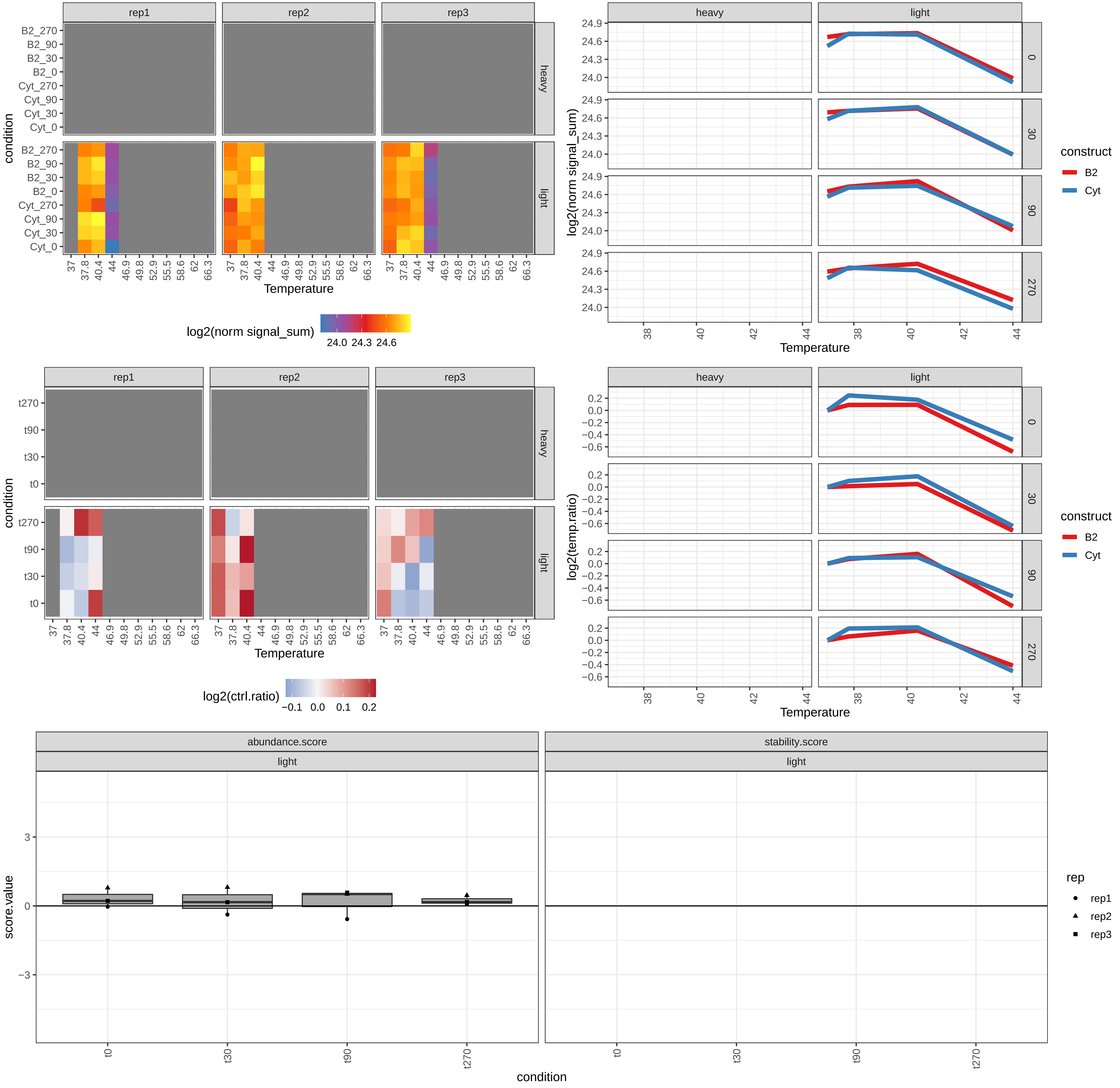

Q3E705 − rRNA−processing protein EFG1 OS=Saccharomyces cerevisiae (strain ATCC 204508 / S288c) GN=EFG1 PE=1 SV=2

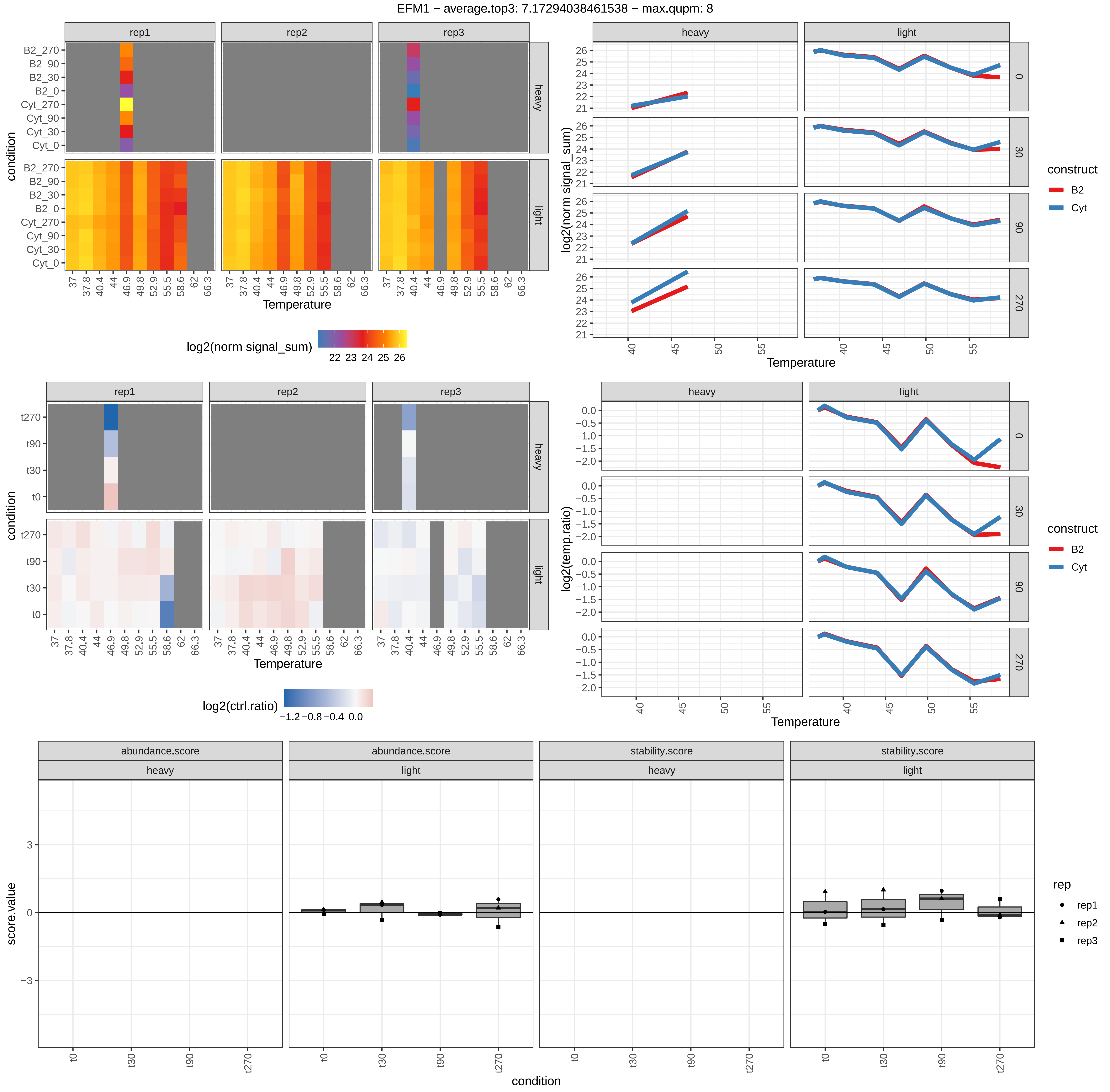

P38732 − Protein−lysine N−methyltransferase EFM1 OS=Saccharomyces cerevisiae (strain ATCC 204508 / S288c) GN=EFM1 PE=1 SV=1

EFM4 − average.top3: 6.85461413793103 − max.qupm: 6

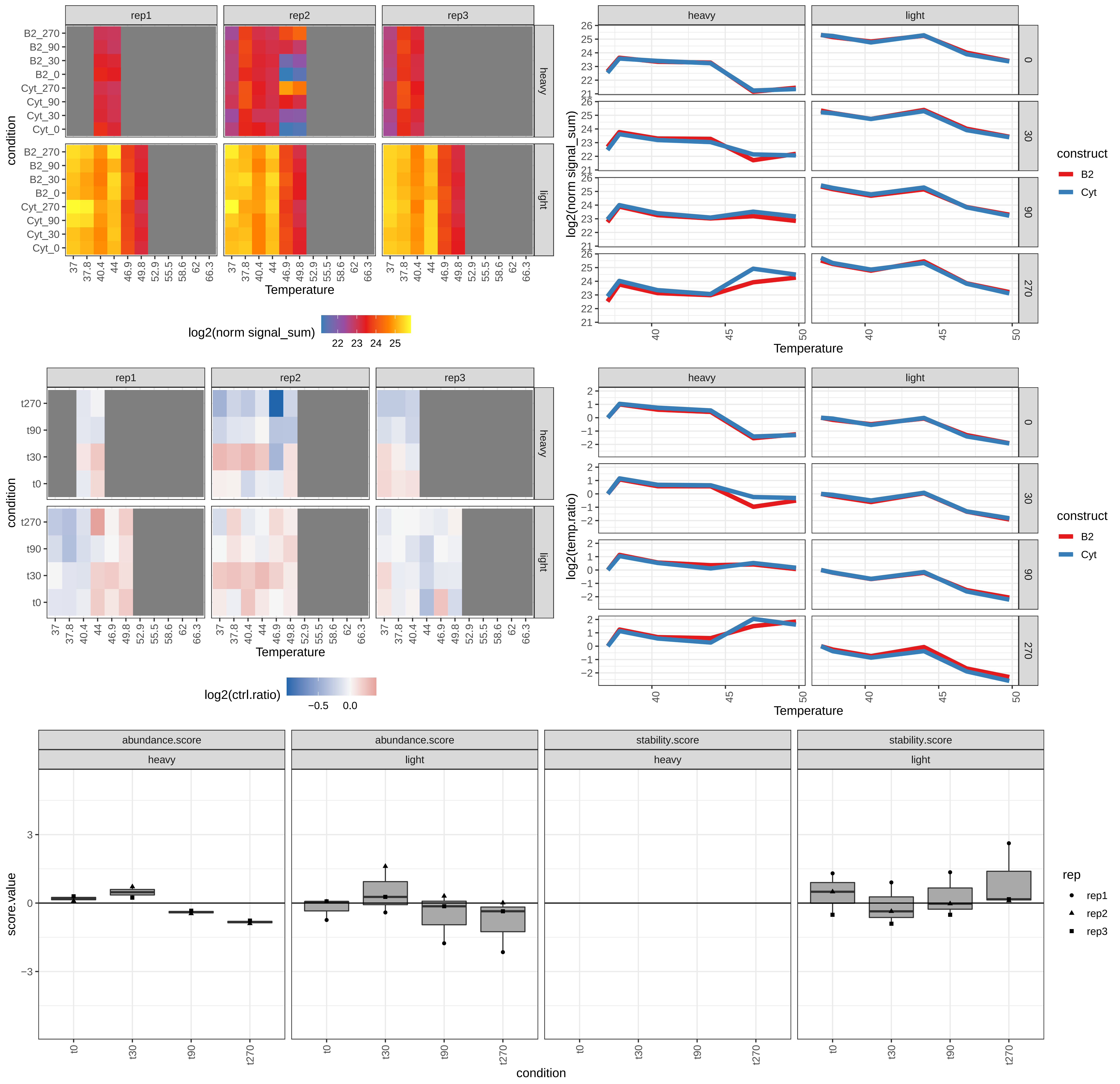

P40516 − Protein−lysine N−methyltransferase EFM4 OS=Saccharomyces cerevisiae (strain ATCC 204508 / S288c) GN=EFM4 PE=1 SV=1

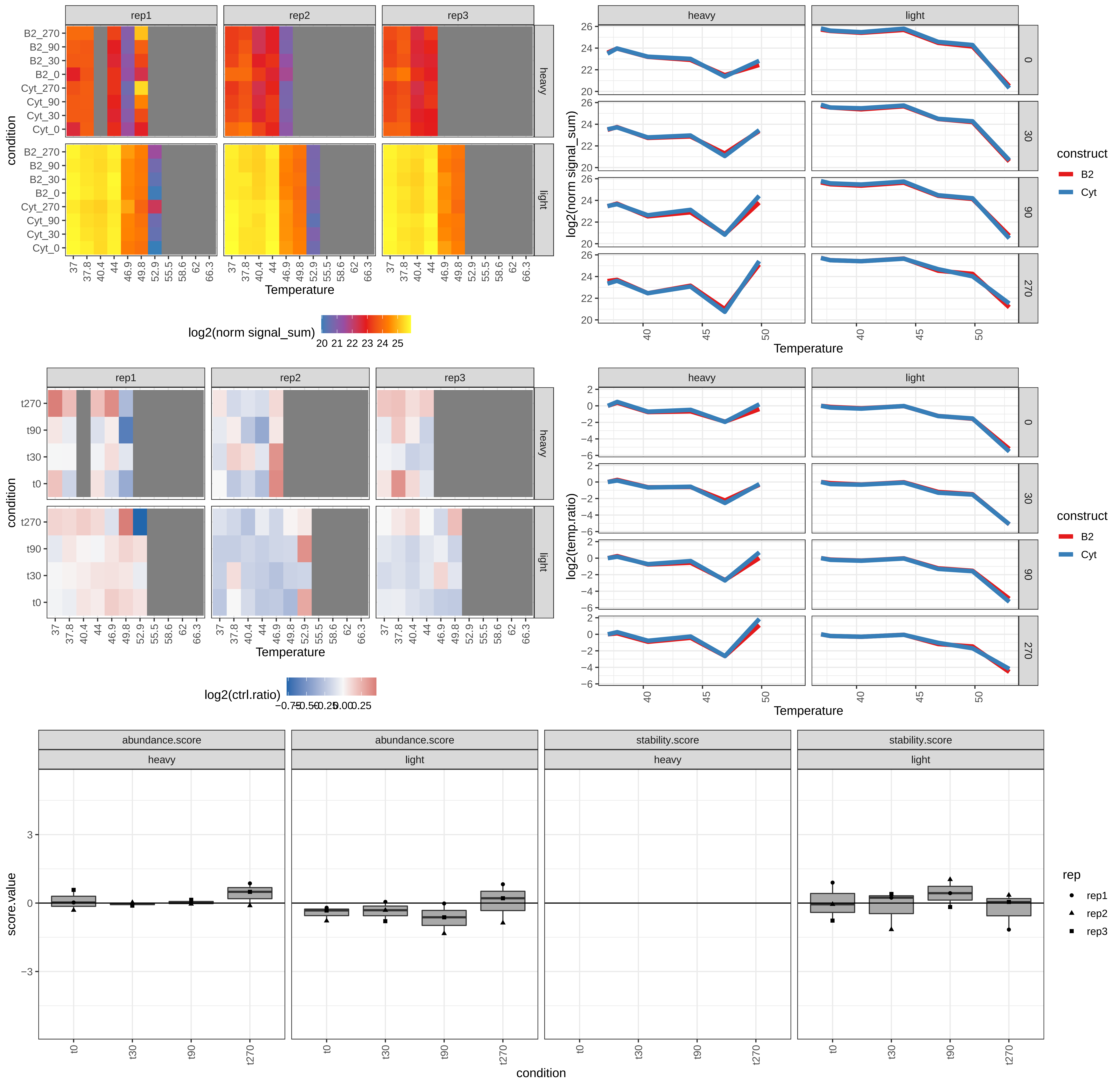

P53200 − Protein−lysine N−methyltransferase EFM5 OS=Saccharomyces cerevisiae (strain ATCC 204508 / S288c) GN=EFM5 PE=1 SV=2

EFR3 − average.top3: 6.58151133333333 − max.qupm: 5

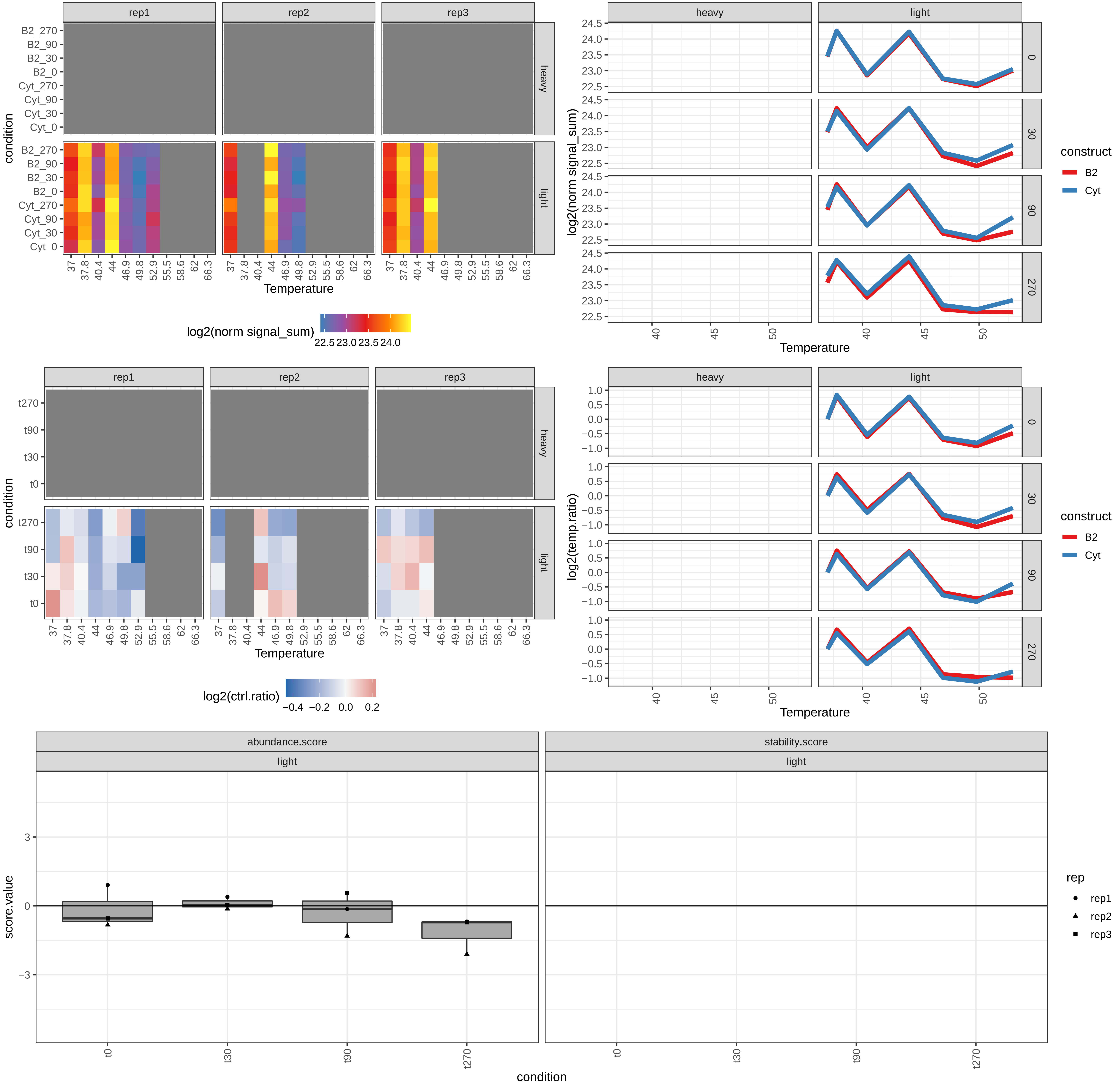

Q03653 − Protein EFR3 OS=Saccharomyces cerevisiae (strain ATCC 204508 / S288c) GN=EFR3 PE=1 SV=1

EFT1 − average.top3: 8.23859227272727 − max.qupm: 42

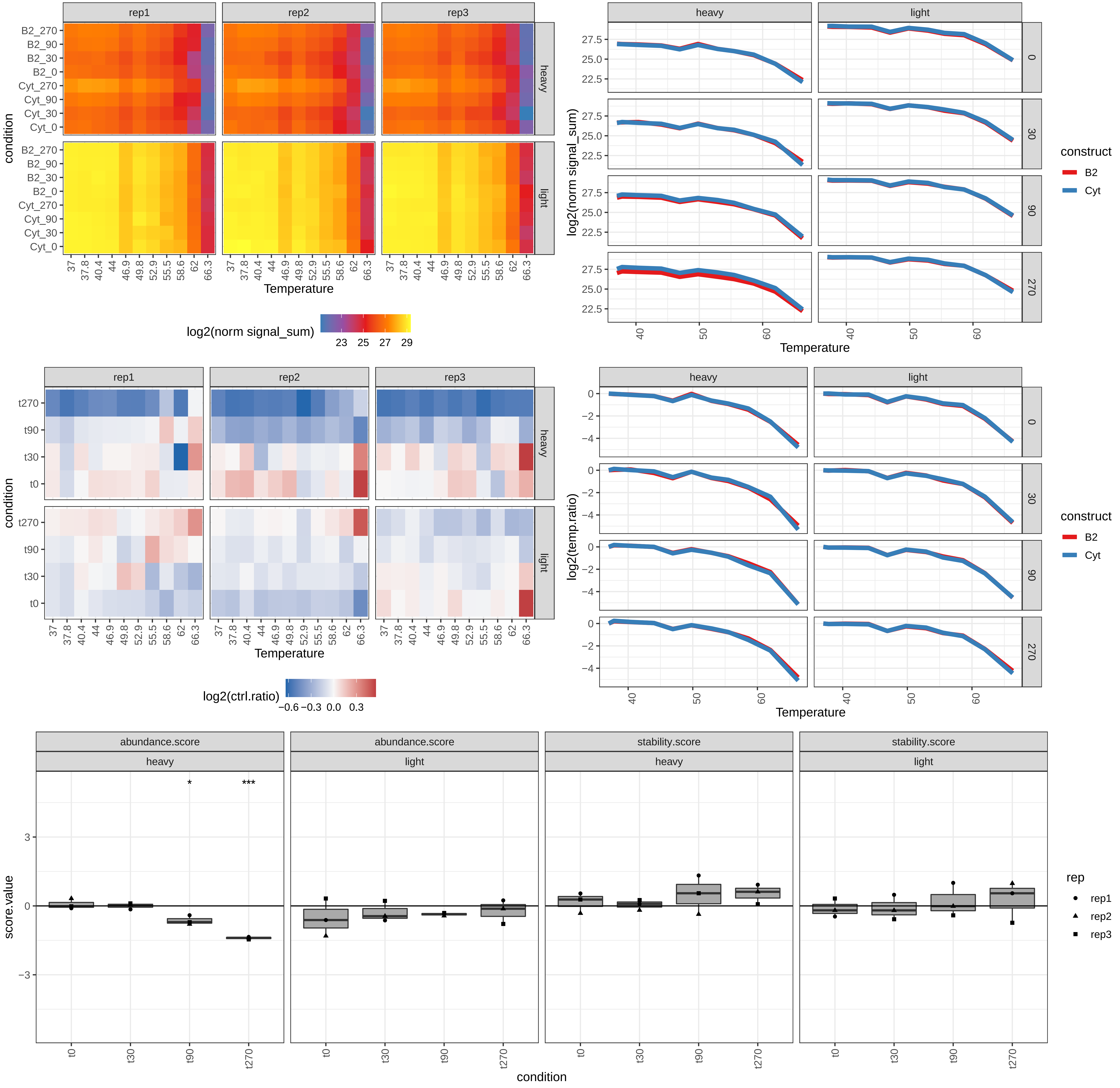

P32324 − Elongation factor 2 OS=Saccharomyces cerevisiae (strain ATCC 204508 / S288c) GN=EFT1 PE=1 SV=1

EGD1 − average.top3: 7.53874650793651 − max.qupm: 7

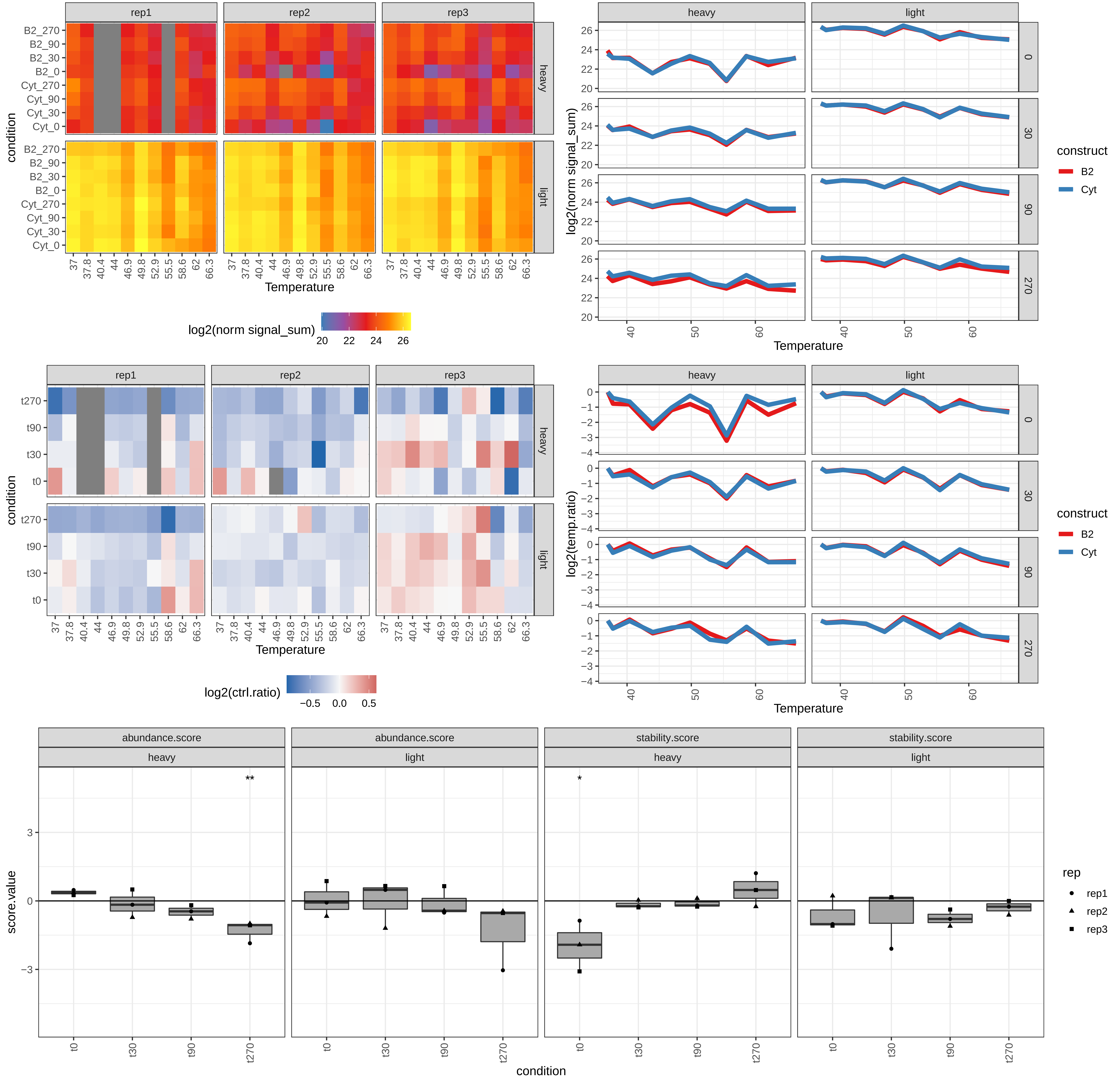

Q02642 − Nascent polypeptide−associated complex subunit beta−1 OS=Saccharomyces cerevisiae (strain ATCC 204508 / S288c) GN=EGD1 PE=1 SV=2

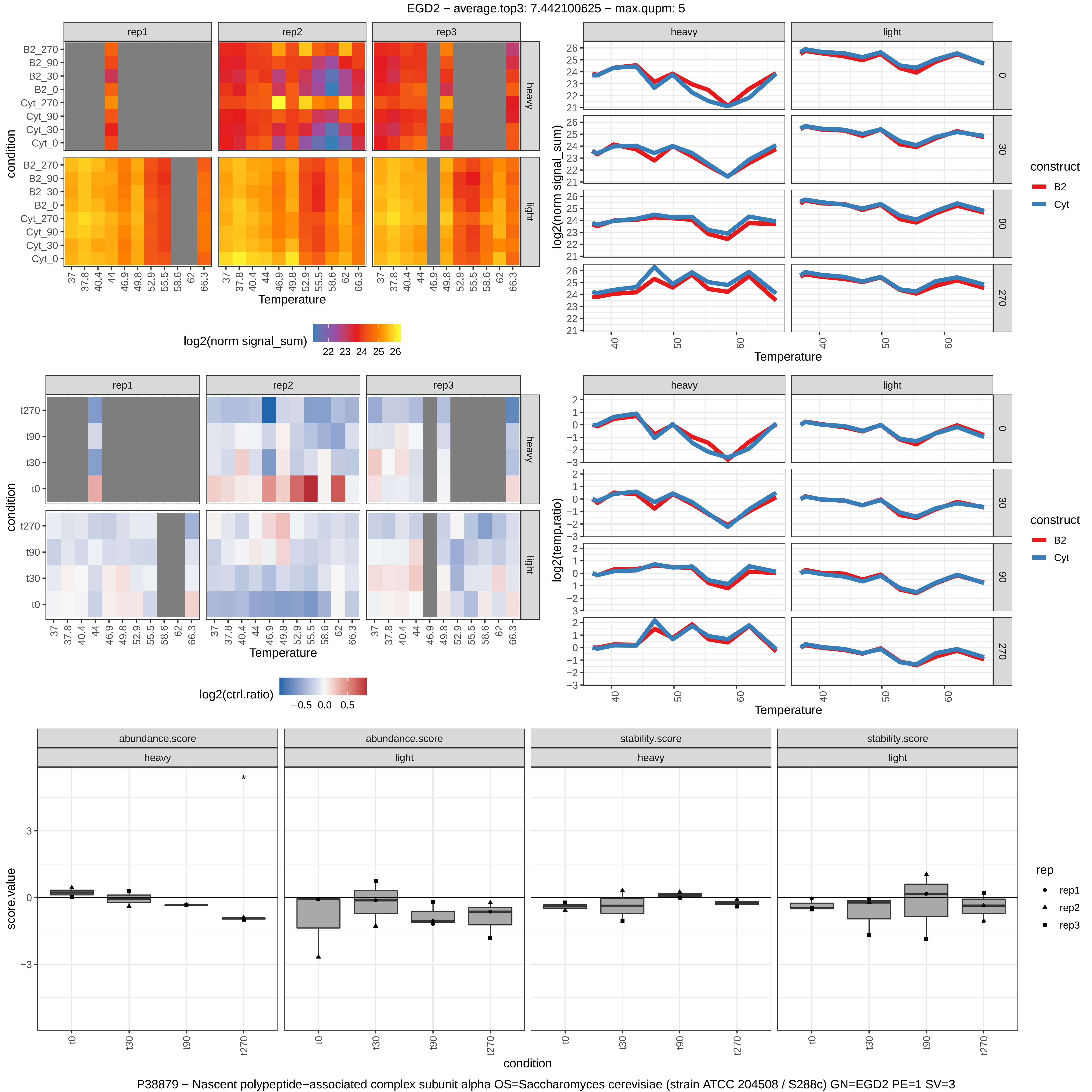
EGD2 − average.top3: 7.442100625 − max.qupm: 5
rep1
rep2
rep3
heavy
light
condition
Temperature
log2(norm signal_sum)
log2(ctrl.ratio)
log2(temp.ratio)
construct
B2
Cyt
abundance.score
stability.score
score.value
rep
rep1
rep2
rep3
P38879 − Nascent polypeptide−associated complex subunit alpha OS=Saccharomyces cerevisiae (strain ATCC 204508 / S288c) GN=EGD2 PE=1 SV=3

EHD3 − average.top3: 7.25619481481481 − max.qupm: 11

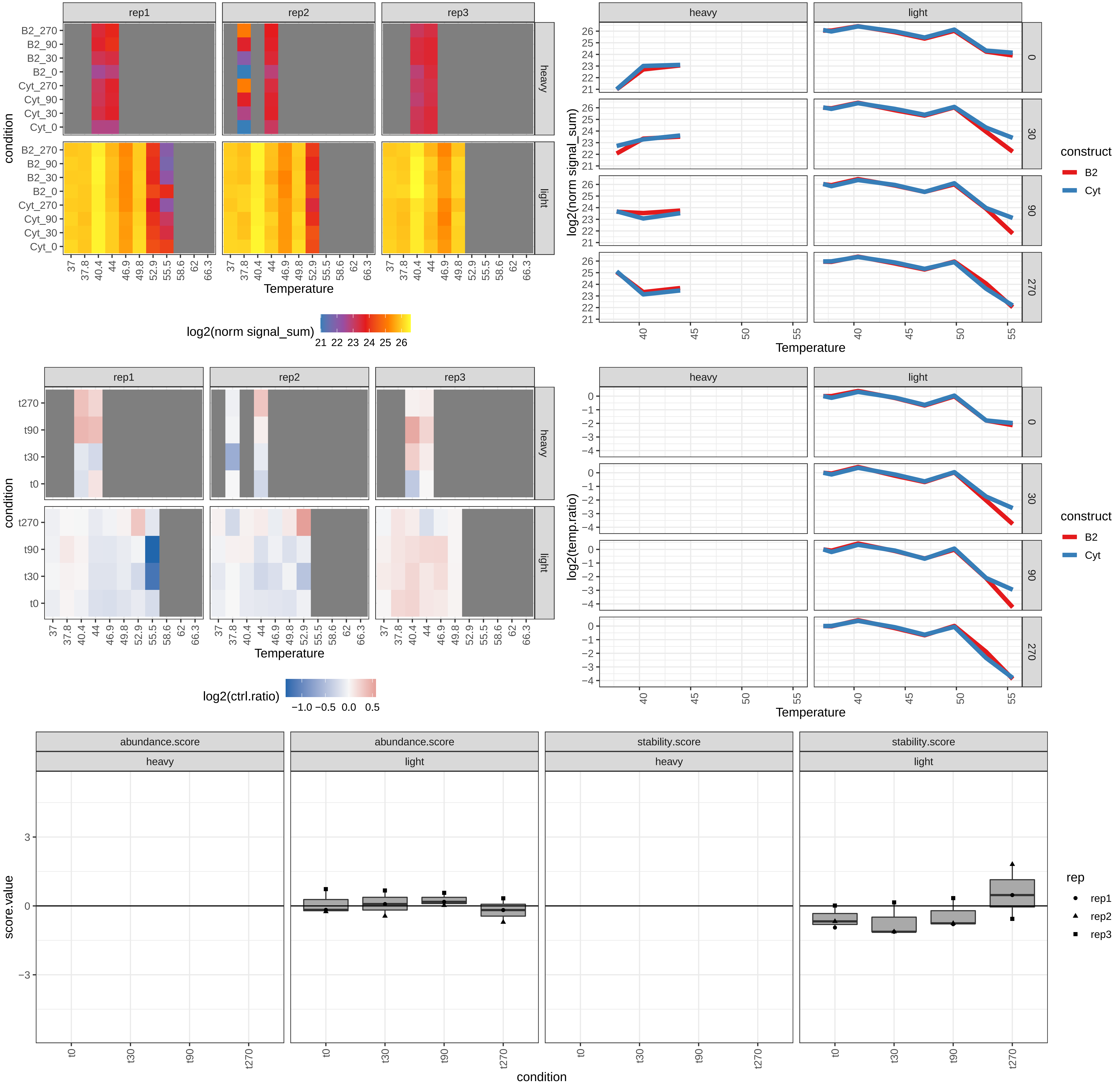

P28817 − 3−hydroxyisobutyryl−CoA hydrolase, mitochondrial OS=Saccharomyces cerevisiae (strain ATCC 204508 / S288c) GN=EHD3 PE=1 SV=2

EHT1 − average.top3: 7.28395186046512 − max.qupm: 15

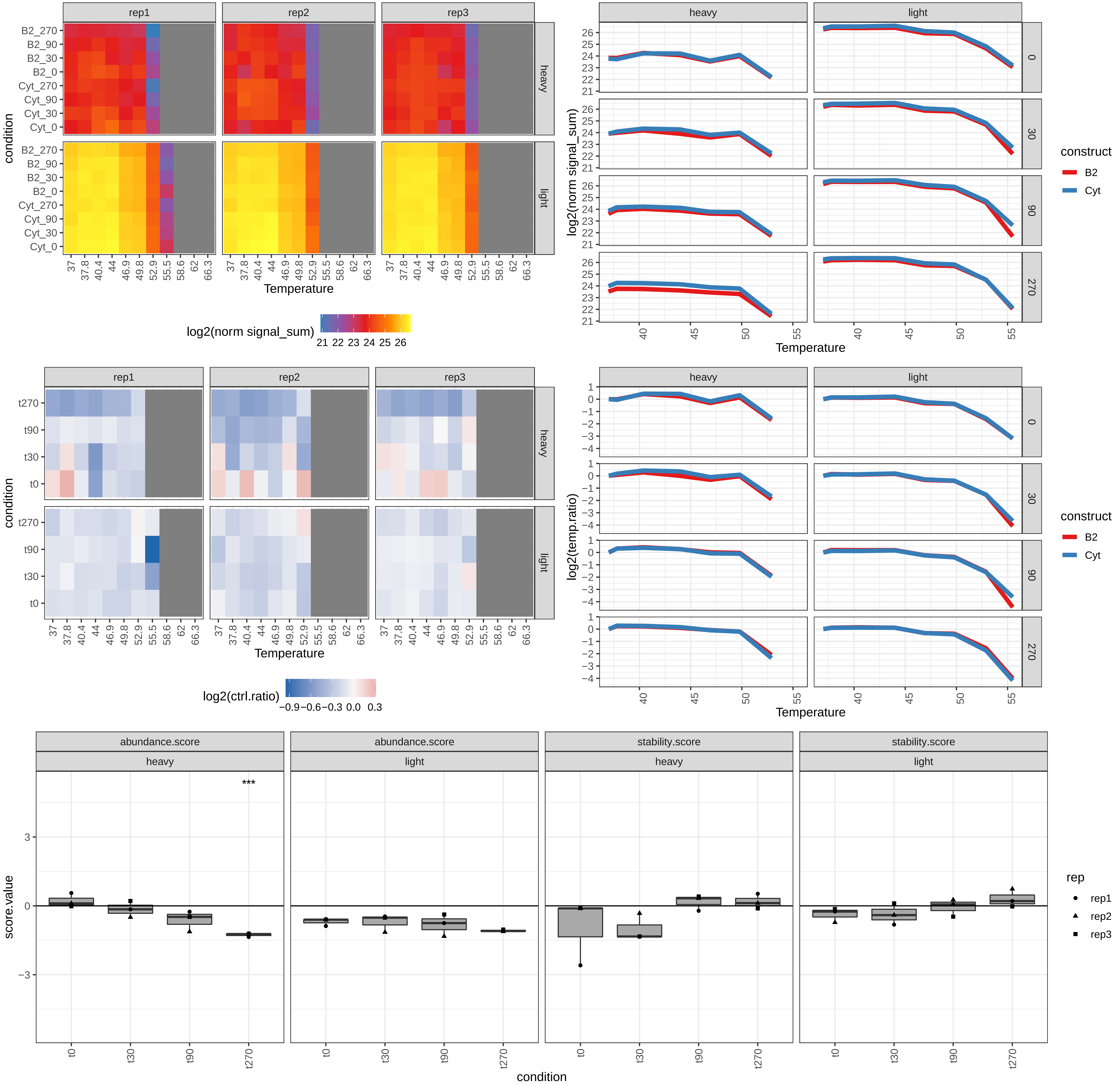

P38295 − Medium−chain fatty acid ethyl ester synthase/esterase 2 OS=Saccharomyces cerevisiae (strain ATCC 204508 / S288c) GN=EHT1 PE=1 SV=1

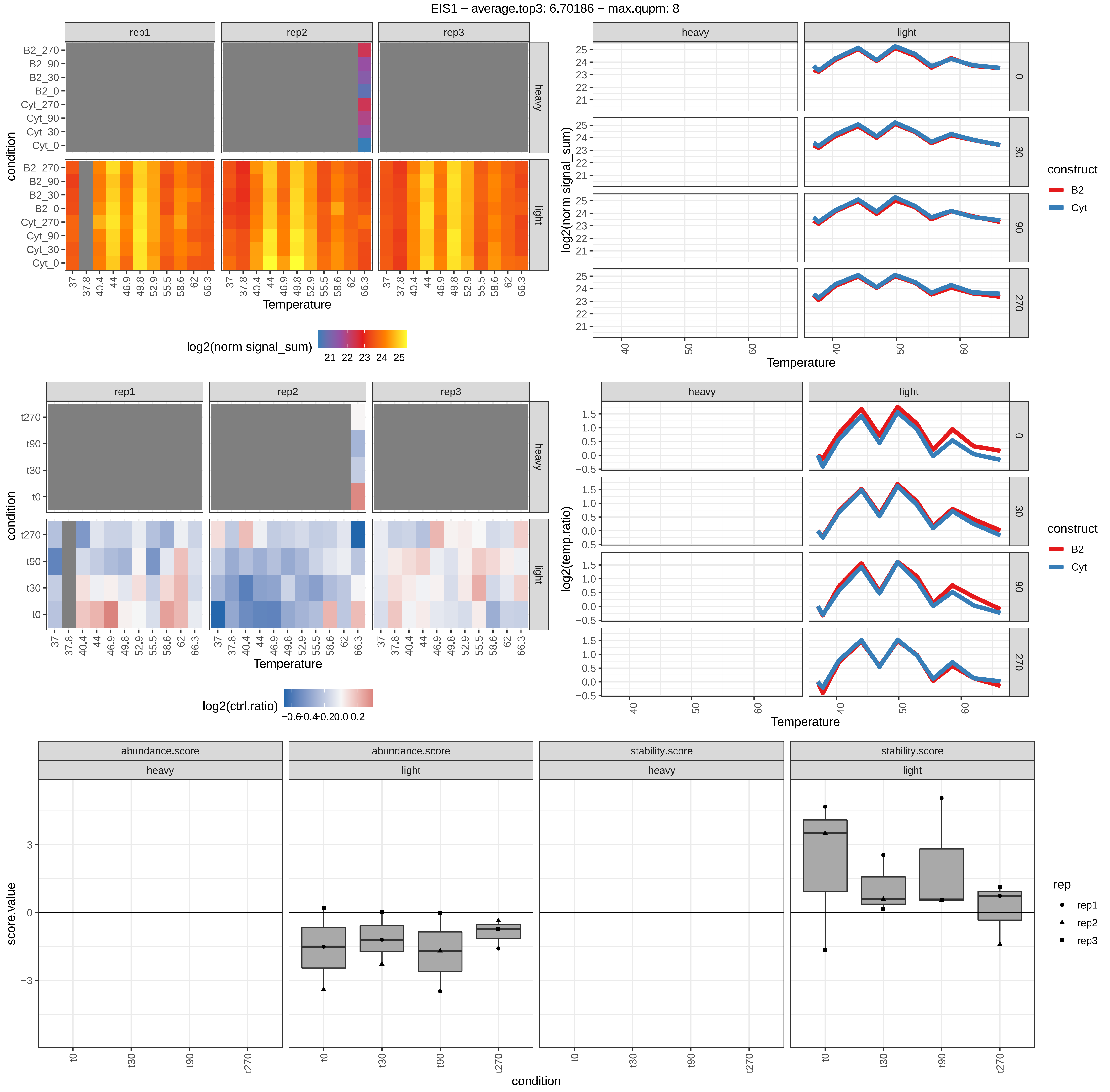

Q05050 − Eisosome protein 1 OS=Saccharomyces cerevisiae (strain ATCC 204508 / S288c) GN=EIS1 PE=1 SV=1

ELF1 − average.top3: 7.24054538461538 − max.qupm: 5

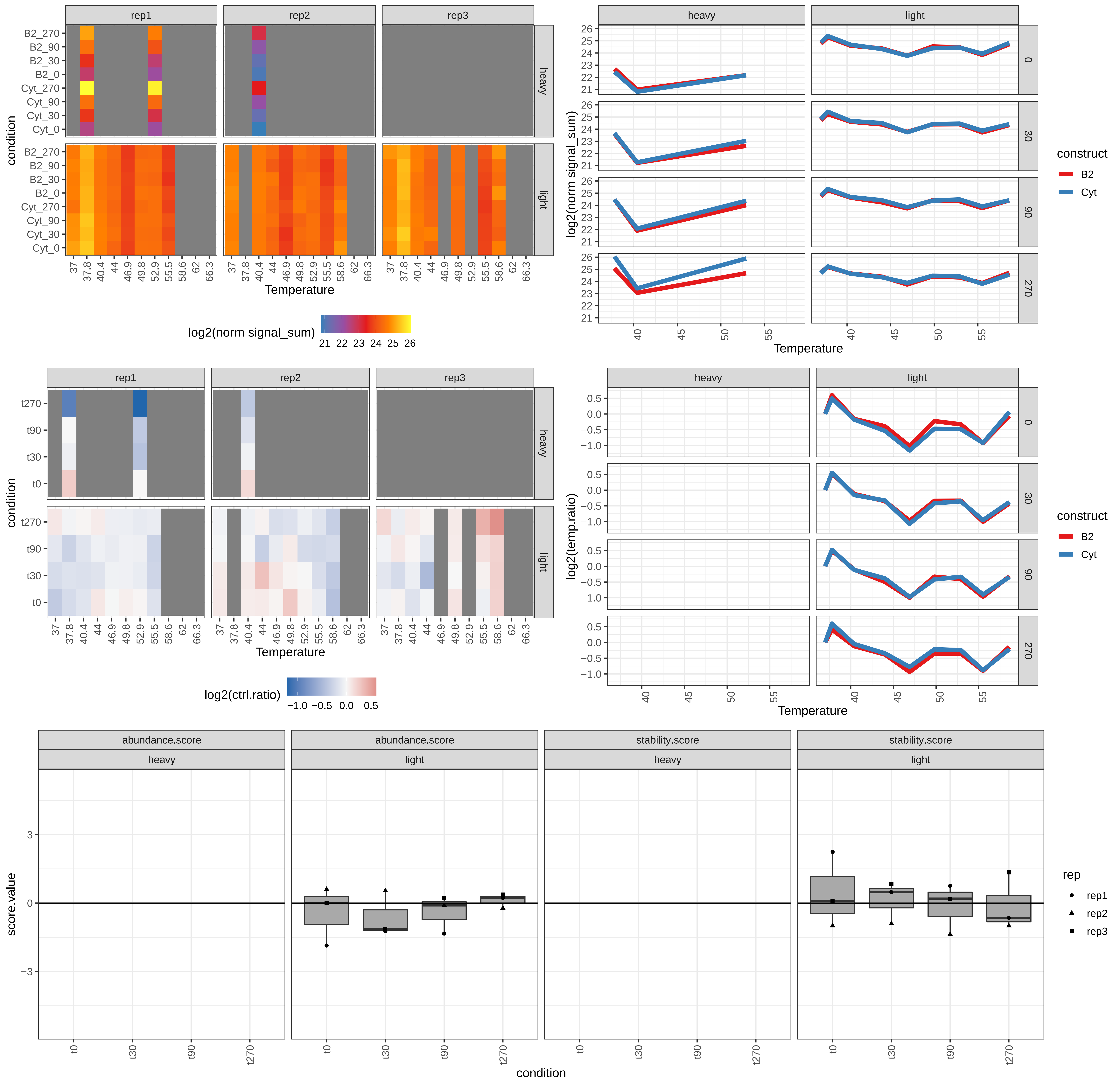

P36053 − Transcription elongation factor 1 OS=Saccharomyces cerevisiae (strain ATCC 204508 / S288c) GN=ELF1 PE=1 SV=1

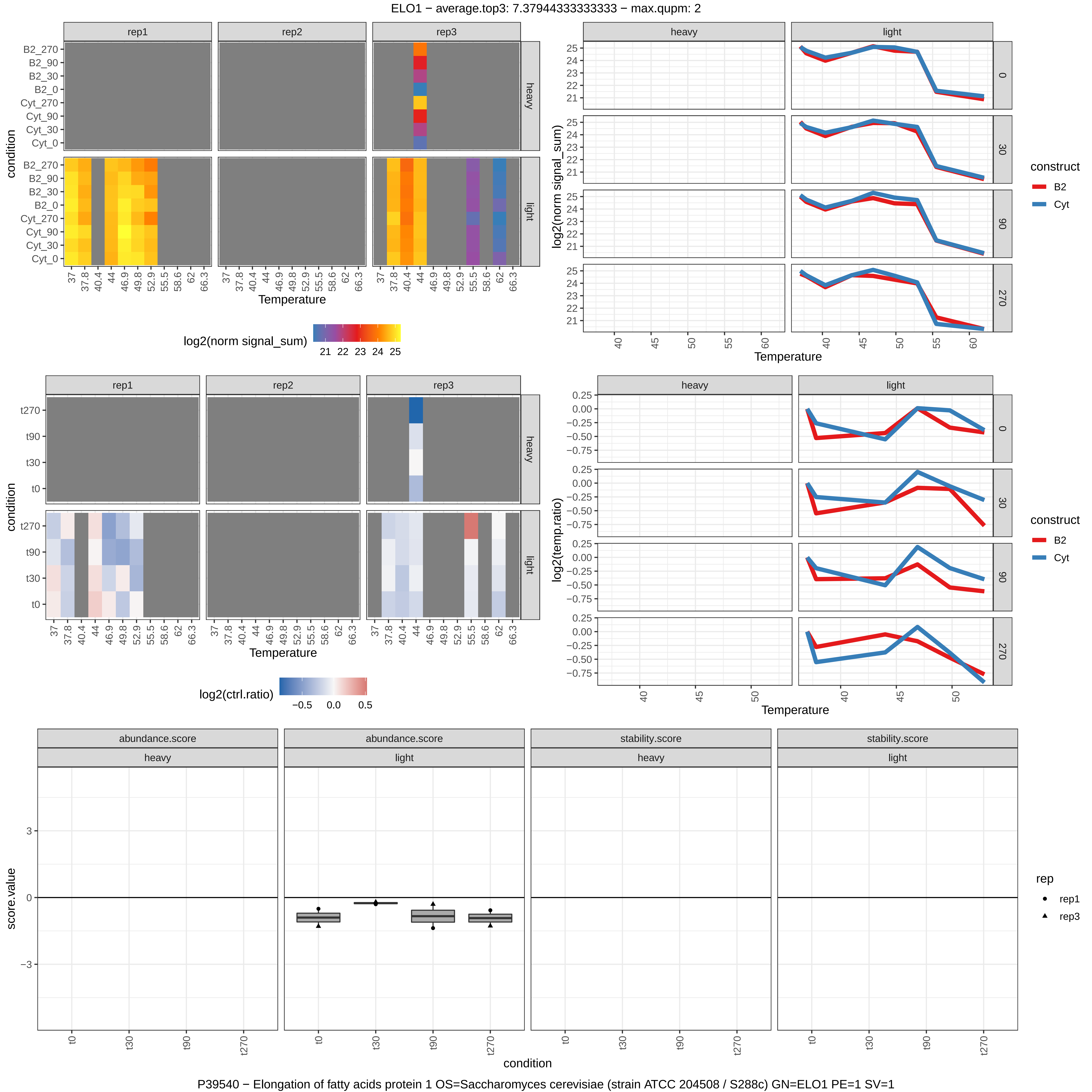
ELO1 − average.top3: 7.37944333333333 − max.qupm: 2
rep1
rep2
rep3
heavy
light
condition
Temperature
log2(norm signal_sum)
log2(norm signal_sum)
construct
B2
Cyt
log2(ctrl.ratio)
log2(temp.ratio)
abundance.score
stability.score
score.value
rep
rep1
rep3
condition
P39540 − Elongation of fatty acids protein 1 OS=Saccharomyces cerevisiae (strain ATCC 204508 / S288c) GN=ELO1 PE=1 SV=1

ELO3 − average.top3: 7.39644642857143 − max.qupm: 3

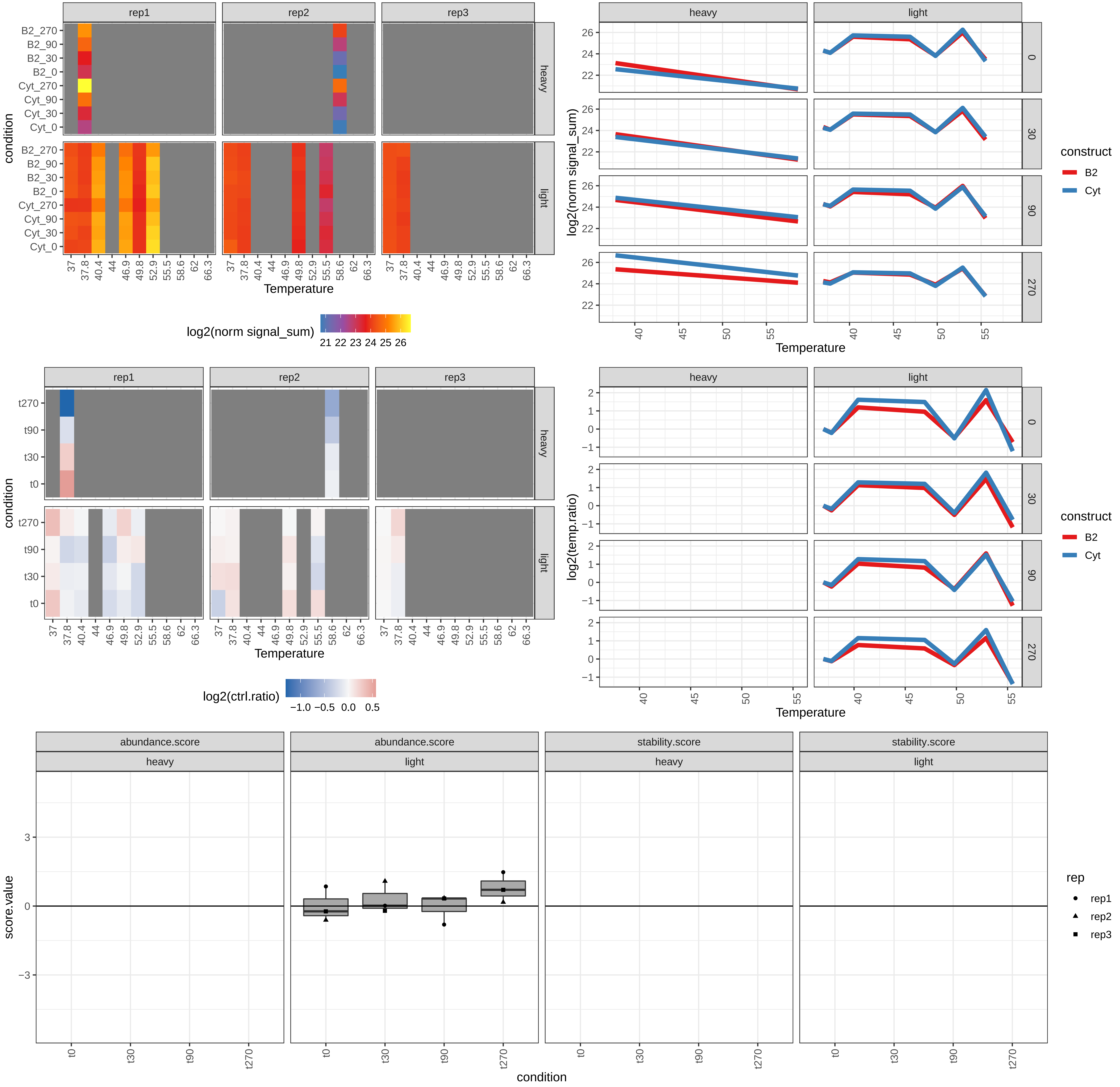

P40319 − Elongation of fatty acids protein 3 OS=Saccharomyces cerevisiae (strain ATCC 204508 / S288c) GN=ELO3 PE=1 SV=1

ELP2 − average.top3: 6.90494018518519 − max.qupm: 14

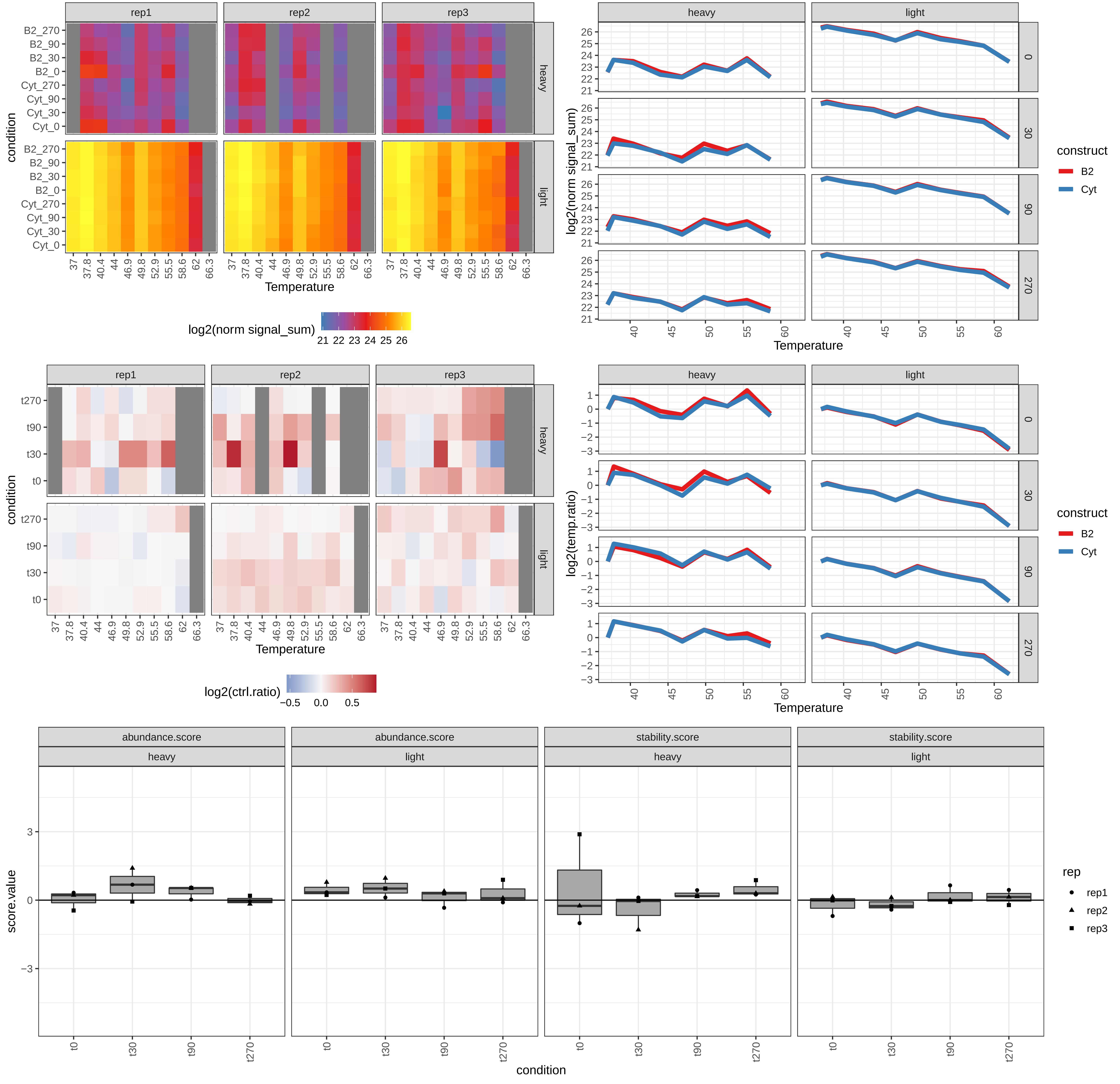

P42935 − Elongator complex protein 2 OS=Saccharomyces cerevisiae (strain ATCC 204508 / S288c) GN=ELP2 PE=1 SV=1

ELP3 − average.top3: 6.78132263157895 − max.qupm: 13

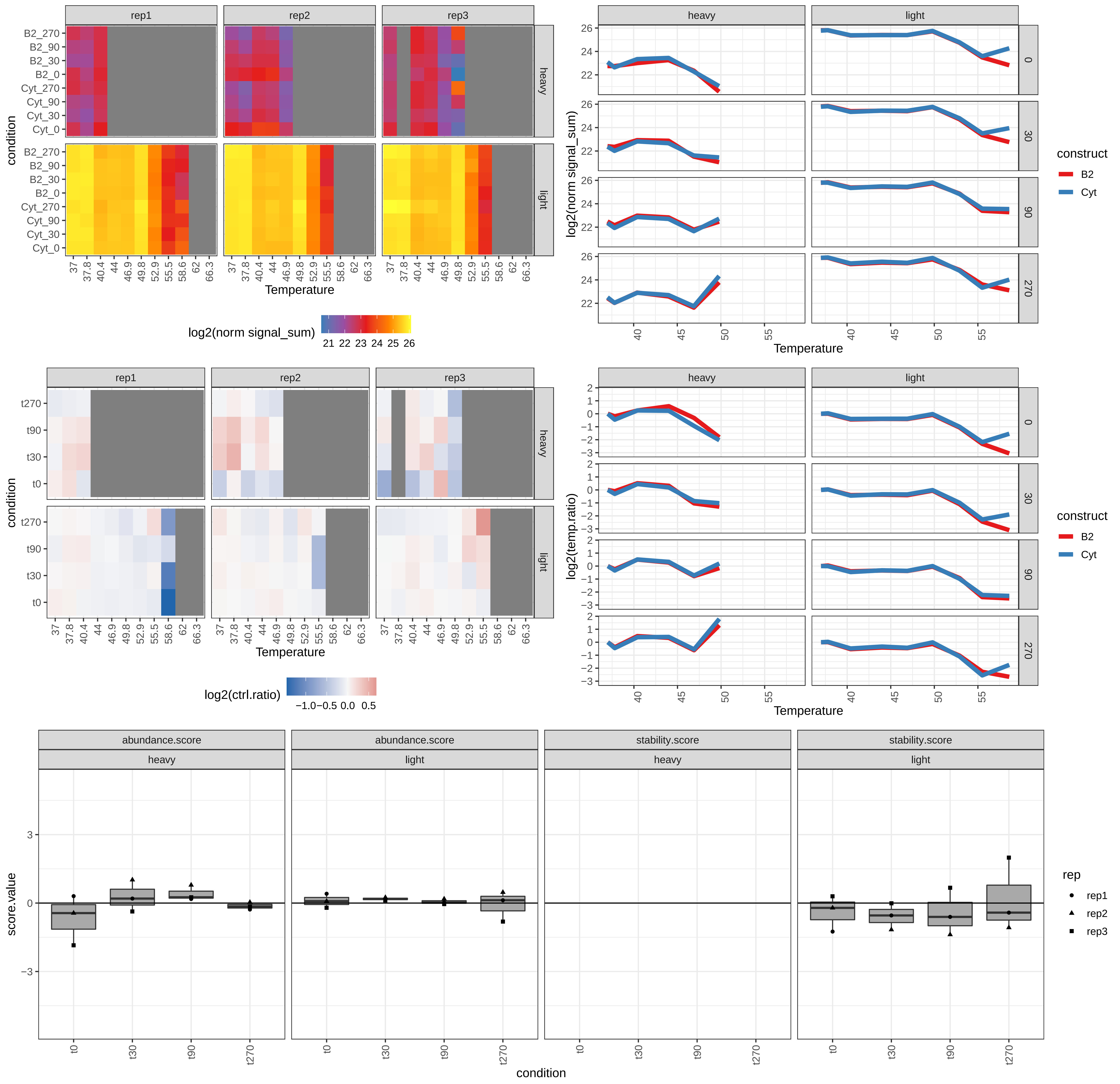

Q02908 − Elongator complex protein 3 OS=Saccharomyces cerevisiae (strain ATCC 204508 / S288c) GN=ELP3 PE=1 SV=1

ELP4 − average.top3: 7.04165421052632 − max.qupm: 5

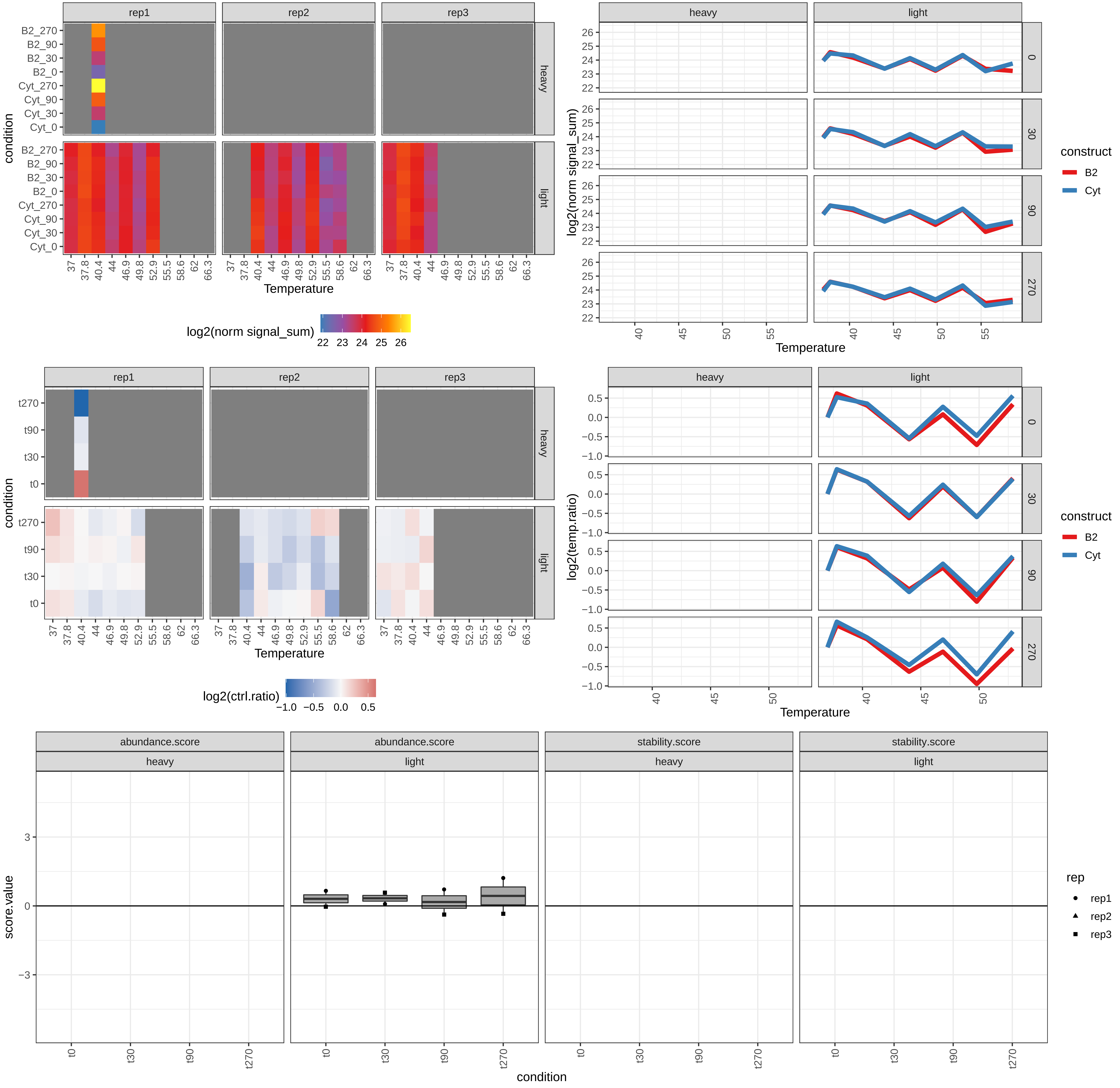

Q02884 − Elongator complex protein 4 OS=Saccharomyces cerevisiae (strain ATCC 204508 / S288c) GN=ELP4 PE=1 SV=1

EMC1 − average.top3: 7.027275 − max.qupm: 13

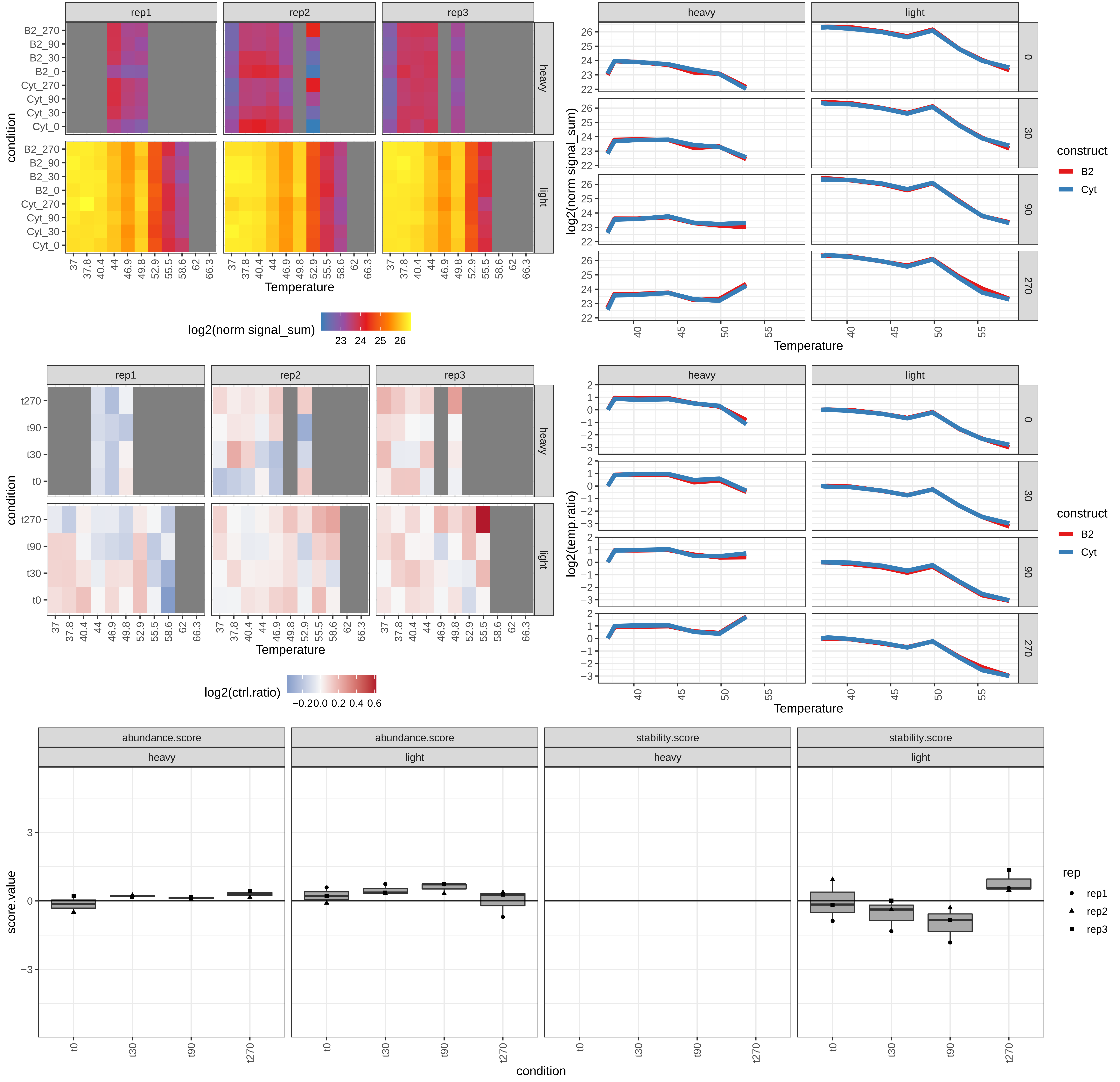

P25574 − ER membrane protein complex subunit 1 OS=Saccharomyces cerevisiae (strain ATCC 204508 / S288c) GN=EMC1 PE=1 SV=1

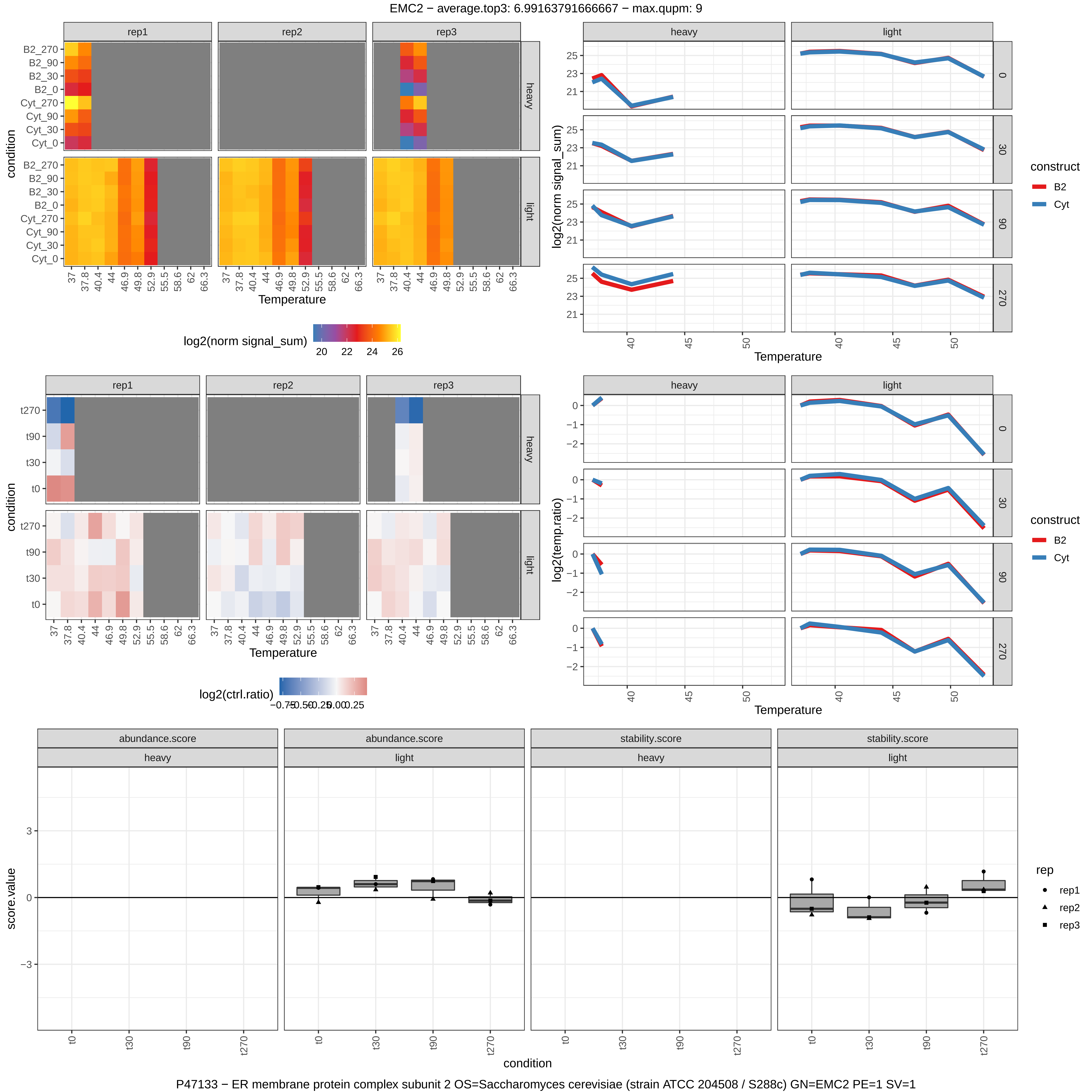

EMC2 − average.top3: 6.99163791666667 − max.qupm: 9
rep1
rep2
rep3
heavy
light
condition
Temperature
log2(norm signal_sum)
construct
B2
Cyt
log2(ctrl.ratio)
log2(temp.ratio)
abundance.score
stability.score
score.value
rep
rep1
rep2
rep3
P47133 − ER membrane protein complex subunit 2 OS=Saccharomyces cerevisiae (strain ATCC 204508 / S288c) GN=EMC2 PE=1 SV=1

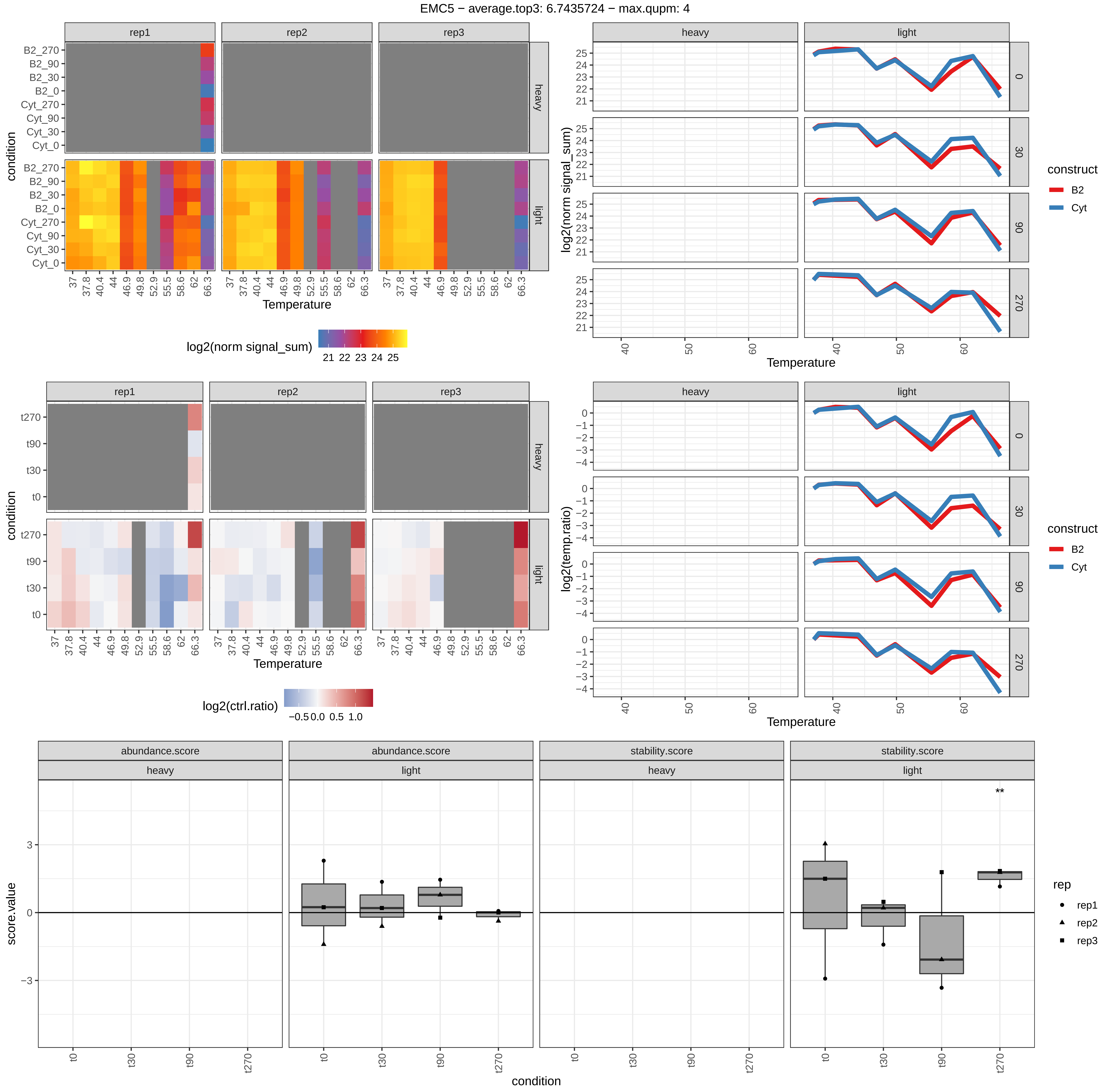

P40540 − ER membrane protein complex subunit 5 OS=Saccharomyces cerevisiae (strain ATCC 204508 / S288c) GN=EMC5 PE=1 SV=1

EMG1 − average.top3: 7.04723475 − max.qupm: 7

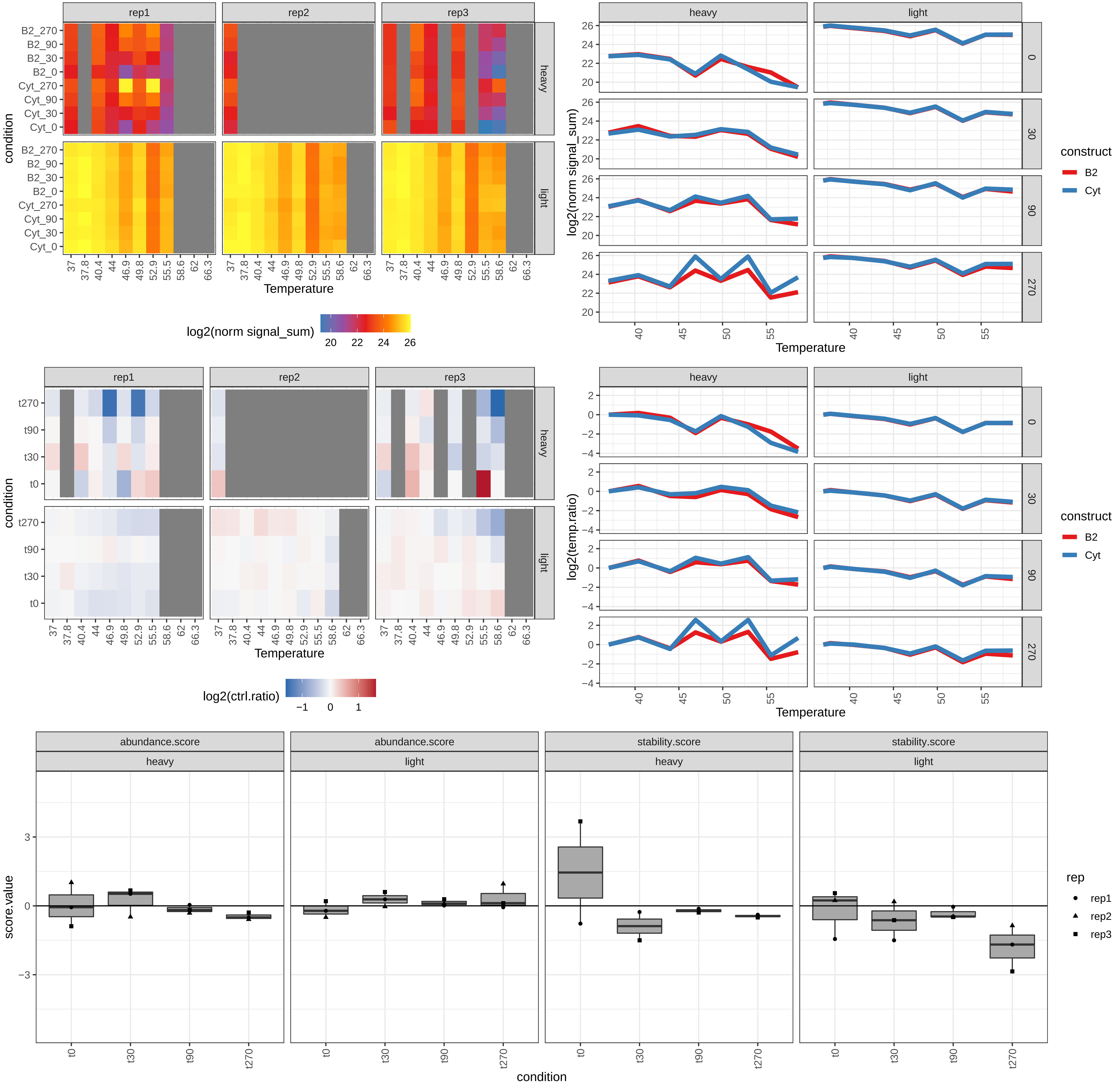

Q06287 − Ribosomal RNA small subunit methyltransferase NEP1 OS=Saccharomyces cerevisiae (strain ATCC 204508 / S288c) GN=EMG1 PE=1 SV=1

EMI2 − average.top3: 7.298228 − max.qupm: 16

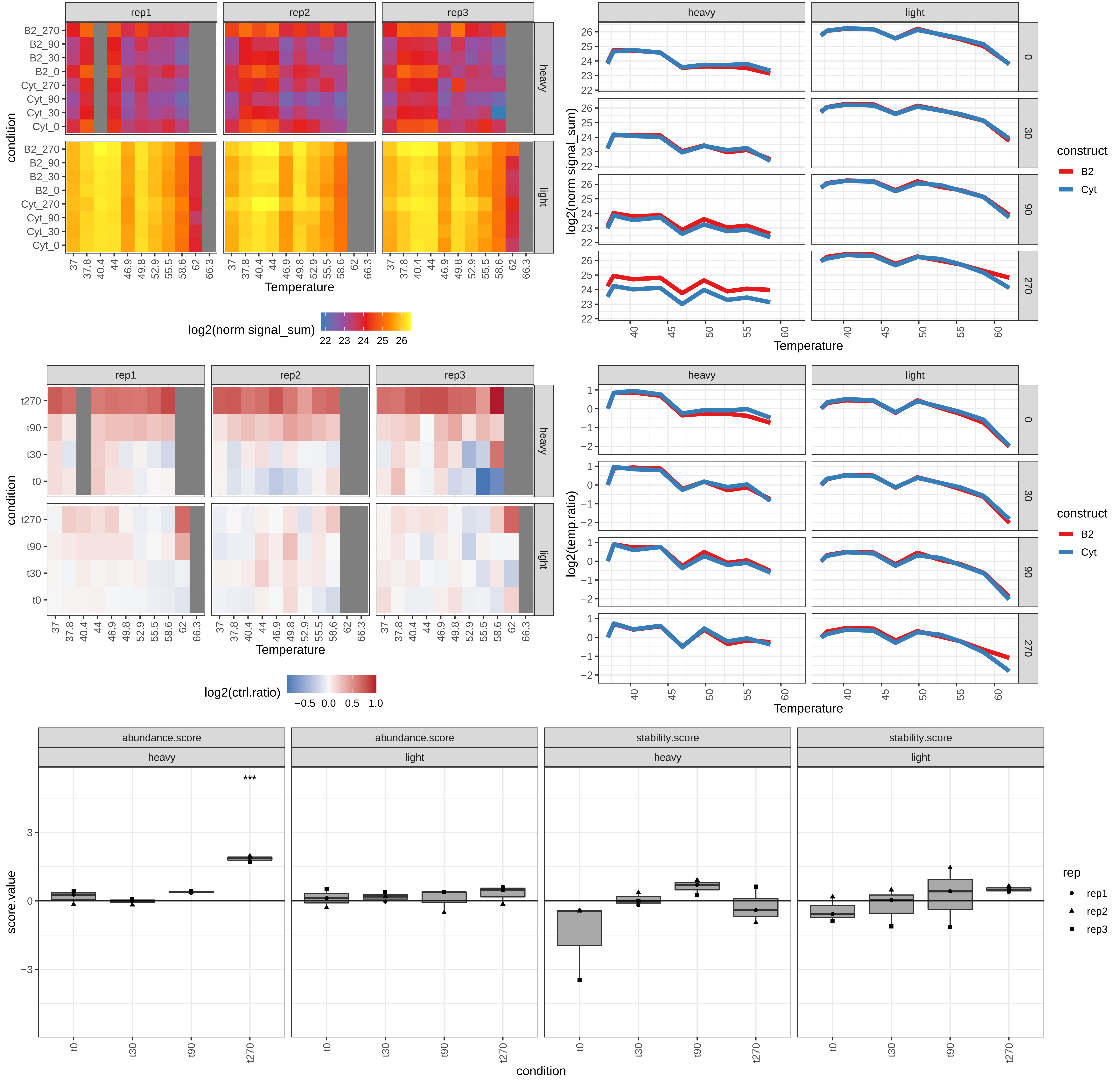

Q04409 − Putative glucokinase−2 OS=Saccharomyces cerevisiae (strain ATCC 204508 / S288c) GN=EMI2 PE=1 SV=1

EMP24 − average.top3: 7.10462543478261 − max.qupm: 7

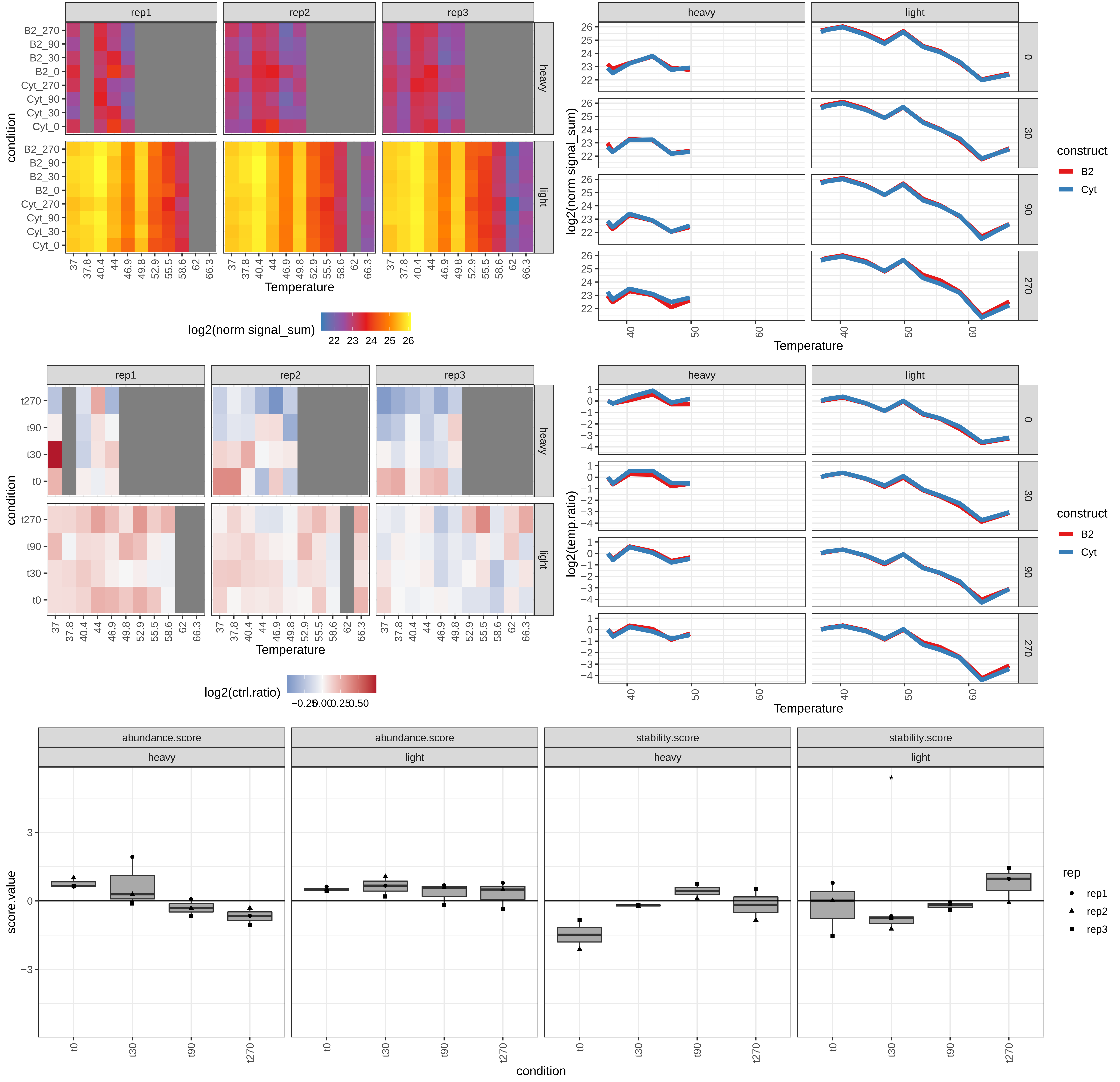

P32803 − Endosomal protein P24B OS=Saccharomyces cerevisiae (strain ATCC 204508 / S288c) GN=EMP24 PE=1 SV=1

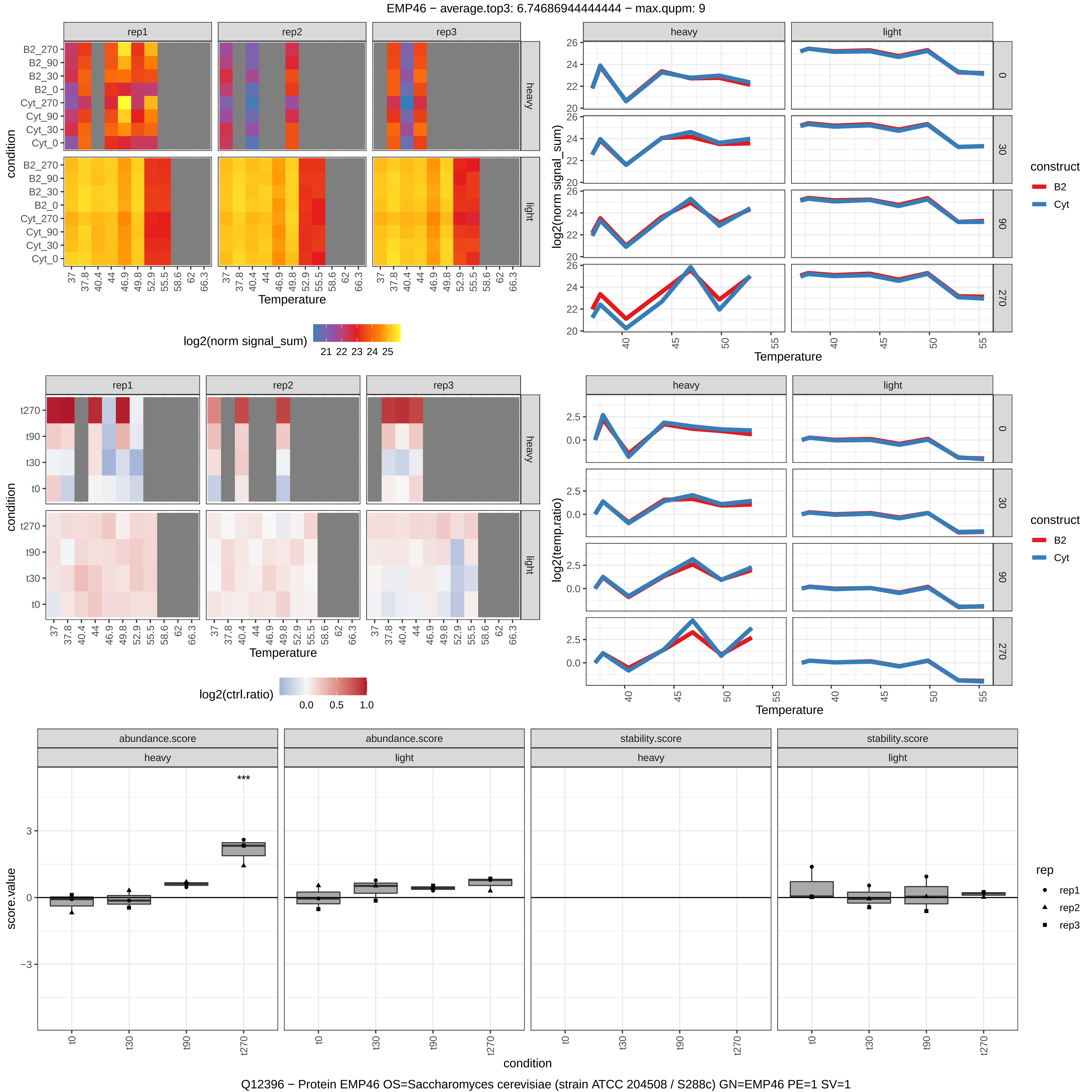
EMP46 − average.top3: 6.74686944444444 − max.qupm: 9
rep1
rep2
rep3
heavy
light
condition
Temperature
log2(norm signal_sum)
construct
B2
Cyt
log2(ctrl.ratio)
log2(temp.ratio)
abundance.score
stability.score
score.value
rep
rep1
rep2
rep3
***
Q12396 − Protein EMP46 OS=Saccharomyces cerevisiae (strain ATCC 204508 / S288c) GN=EMP46 PE=1 SV=1

EMP47 − average.top3: 7.131945 − max.qupm: 13

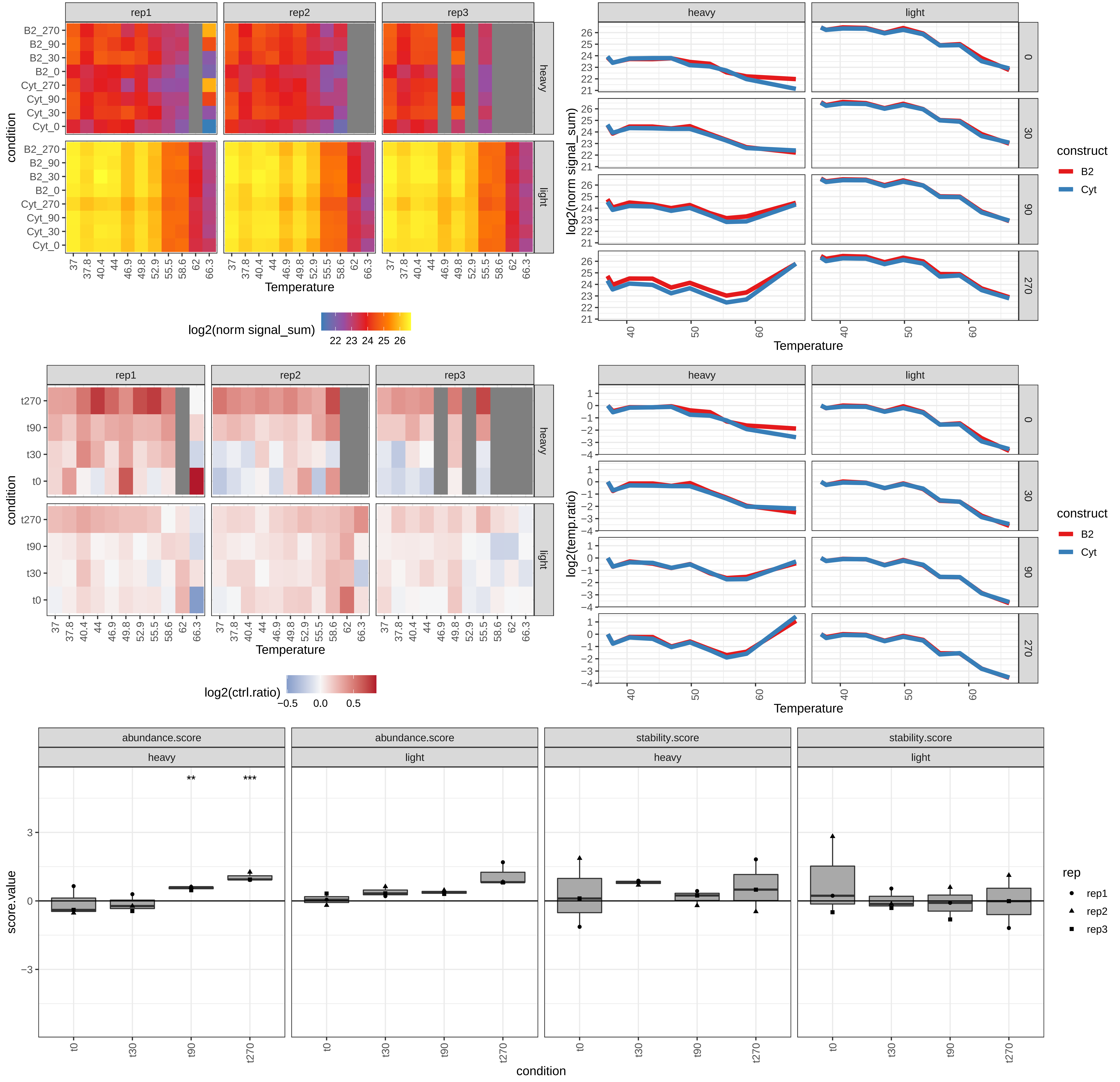

P43555 − Protein EMP47 OS=Saccharomyces cerevisiae (strain ATCC 204508 / S288c) GN=EMP47 PE=1 SV=1

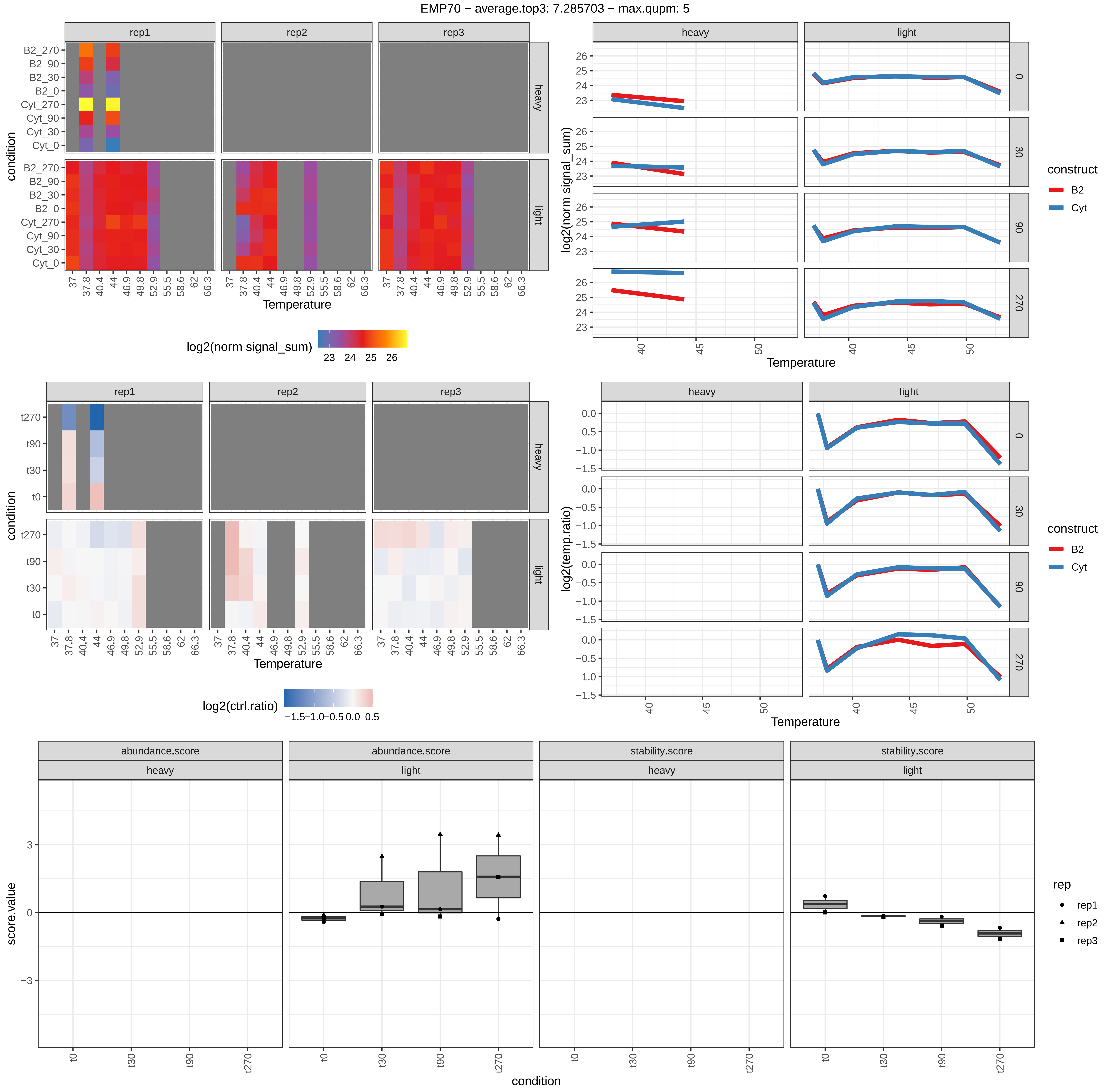

P32802 − Transmembrane 9 superfamily member 1 OS=Saccharomyces cerevisiae (strain ATCC 204508 / S288c) GN=EMP70 PE=1 SV=2

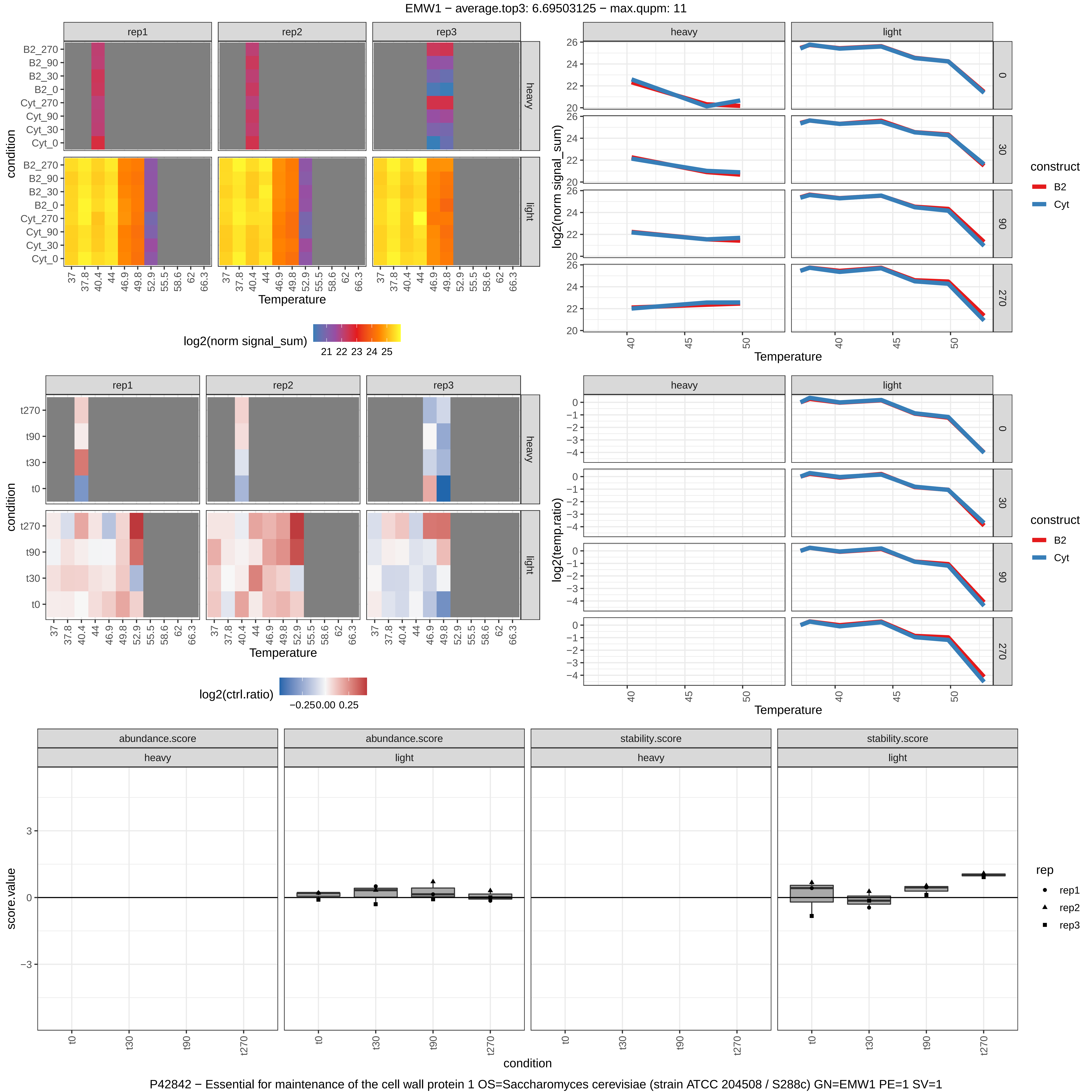
EMW1 − average.top3: 6.69503125 − max.qupm: 11
rep1
rep2
rep3
heavy
light
condition
Temperature
log2(norm signal_sum)
log2(temp.ratio)
log2(ctrl.ratio)
construct
B2
Cyt
abundance.score
stability.score
score.value
rep
rep1
rep2
rep3
P42842 − Essential for maintenance of the cell wall protein 1 OS=Saccharomyces cerevisiae (strain ATCC 204508 / S288c) GN=EMW1 PE=1 SV=1

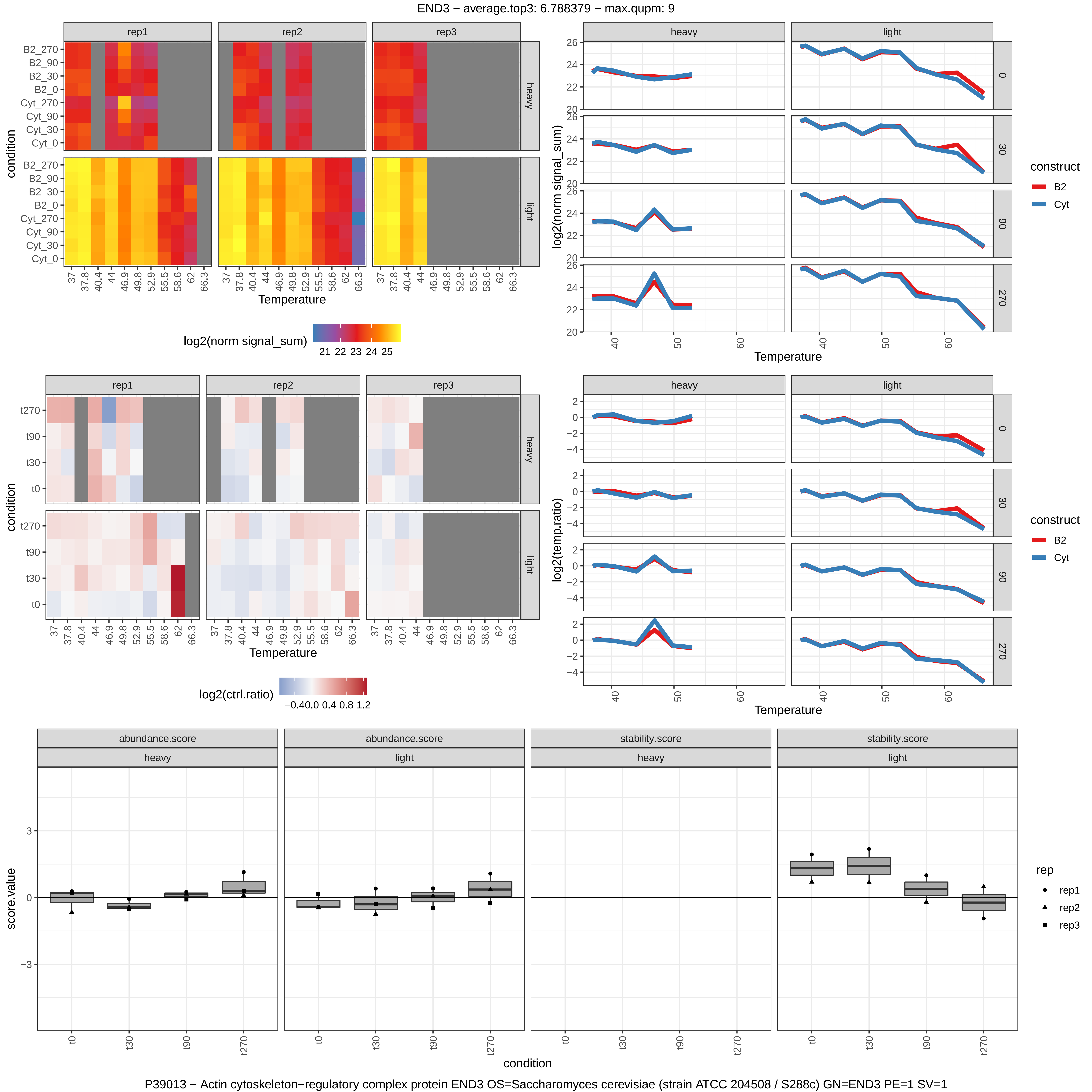
END3 − average.top3: 6.788379 − max.qupm: 9
rep1
rep2
rep3
heavy
light
condition
Temperature
log2(norm signal_sum)
log2(ctrl.ratio)
log2(temp.ratio)
construct
B2
Cyt
abundance.score
stability.score
score.value
rep
rep1
rep2
rep3
P39013 − Actin cytoskeleton−regulatory complex protein END3 OS=Saccharomyces cerevisiae (strain ATCC 204508 / S288c) GN=END3 PE=1 SV=1

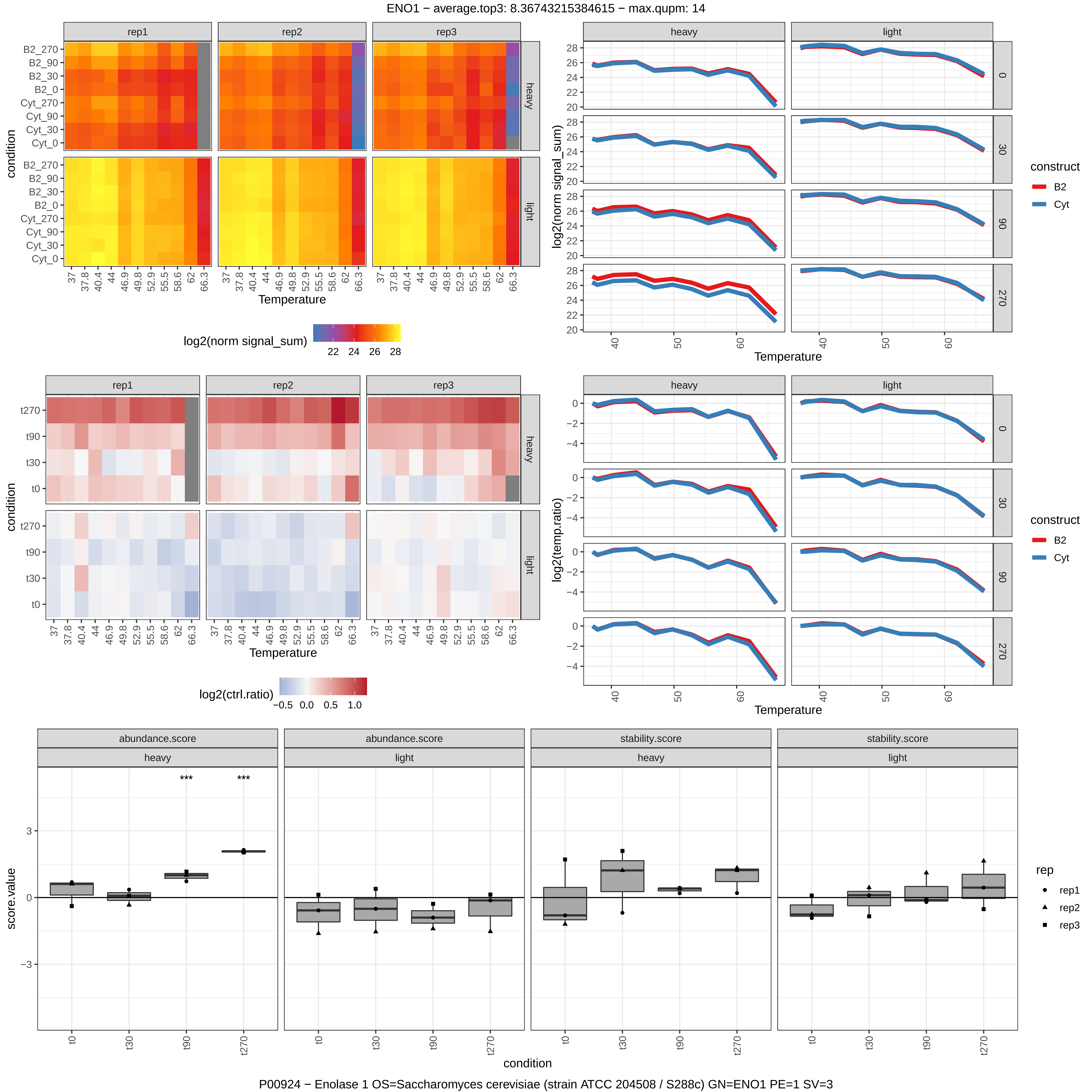

ENO1 − average.top3: 8.36743215384615 − max.qupm: 14
rep1
rep2
rep3
heavy
light
condition
Temperature
log2(norm signal_sum)
log2(ctrl.ratio)
construct
B2
Cyt
log2(temp.ratio)
abundance.score
stability.score
score.value
rep
***
P00924 − Enolase 1 OS=Saccharomyces cerevisiae (strain ATCC 204508 / S288c) GN=ENO1 PE=1 SV=3

ENO2 − average.top3: 8.2491390625 − max.qupm: 17

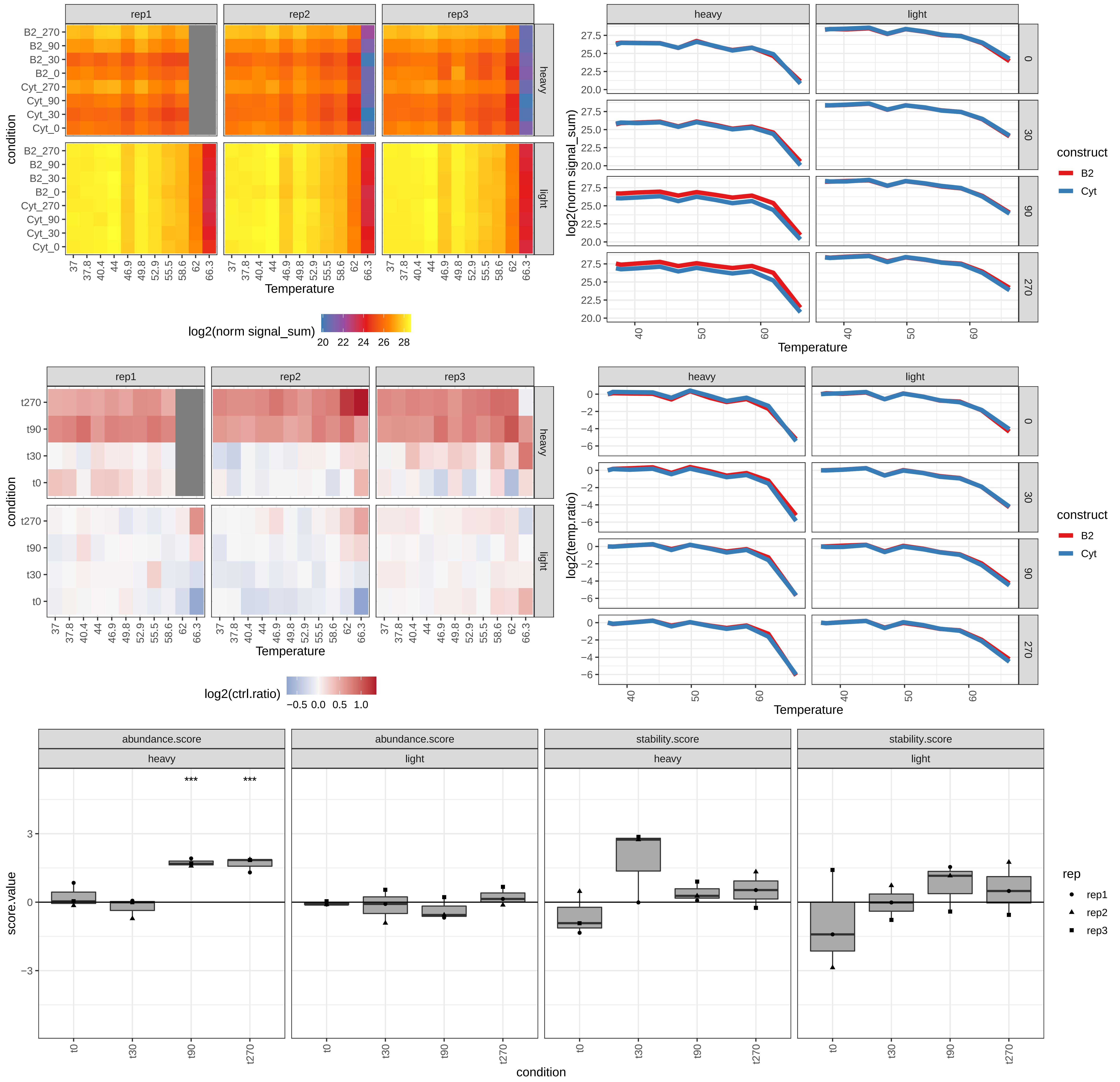

P00925 − Enolase 2 OS=Saccharomyces cerevisiae (strain ATCC 204508 / S288c) GN=ENO2 PE=1 SV=2

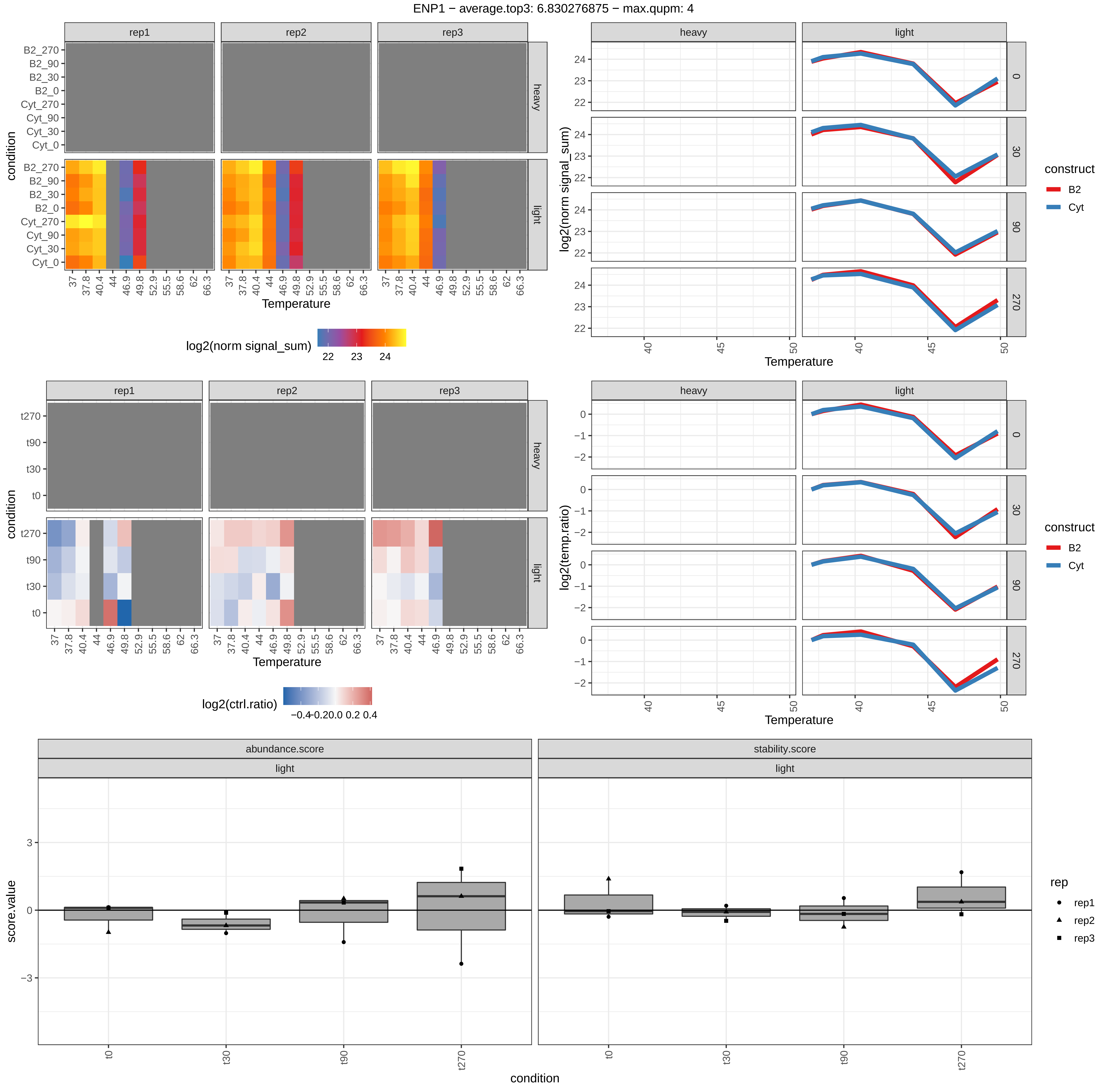

P38333 − Essential nuclear protein 1 OS=Saccharomyces cerevisiae (strain ATCC 204508 / S288c) GN=ENP1 PE=1 SV=1

ENP2 − average.top3: 6.5873265 − max.qupm: 5

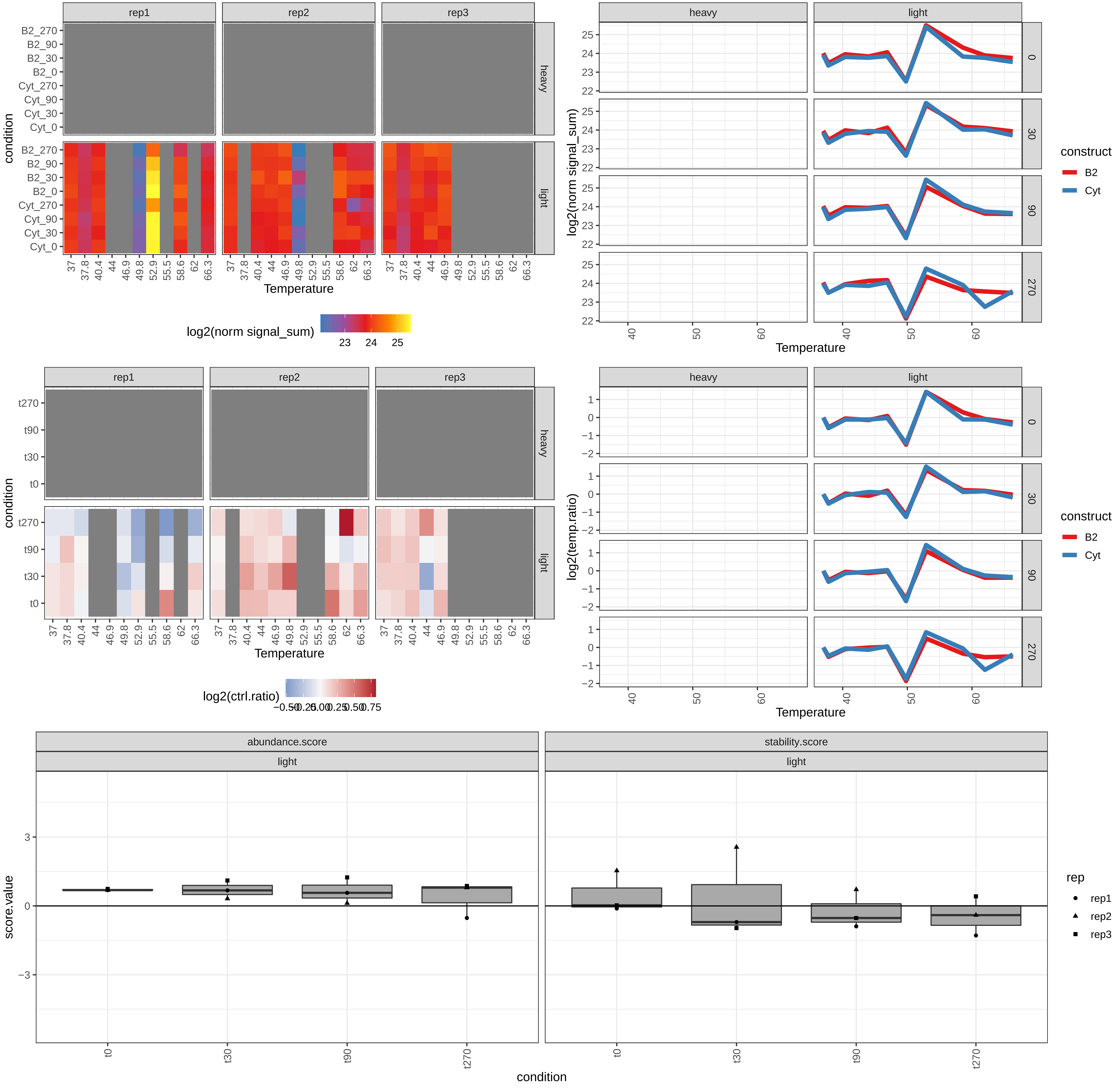

P48234 − Ribosome biogenesis protein ENP2 OS=Saccharomyces cerevisiae (strain ATCC 204508 / S288c) GN=ENP2 PE=1 SV=2

ENT1 − average.top3: 6.9179053125 − max.qupm: 7

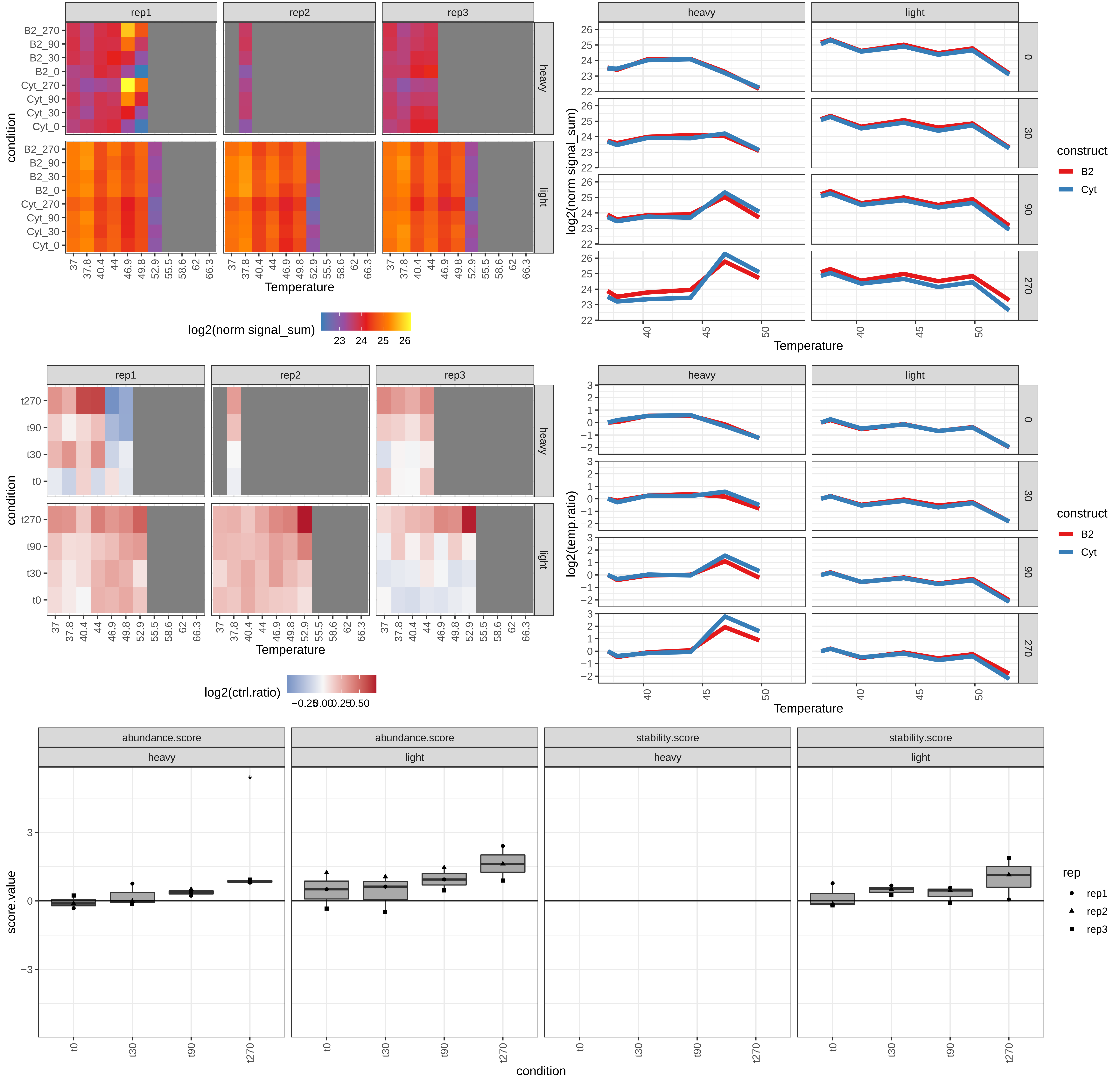

Q12518 − Epsin−1 OS=Saccharomyces cerevisiae (strain ATCC 204508 / S288c) GN=ENT1 PE=1 SV=1

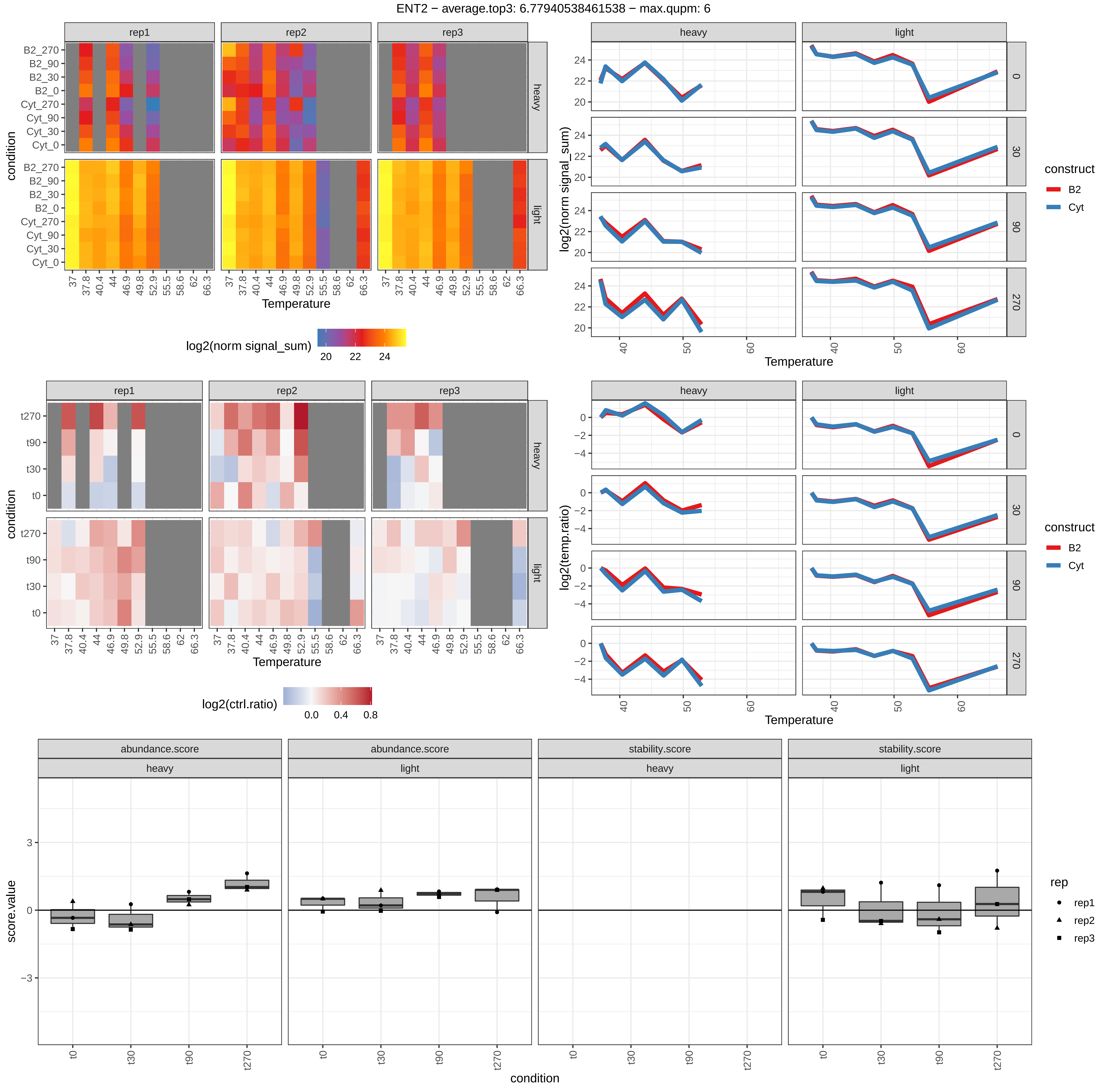

Q05785 − Epsin−2 OS=Saccharomyces cerevisiae (strain ATCC 204508 / S288c) GN=ENT2 PE=1 SV=1

ENT3 − average.top3: 7.15111 − max.qupm: 8

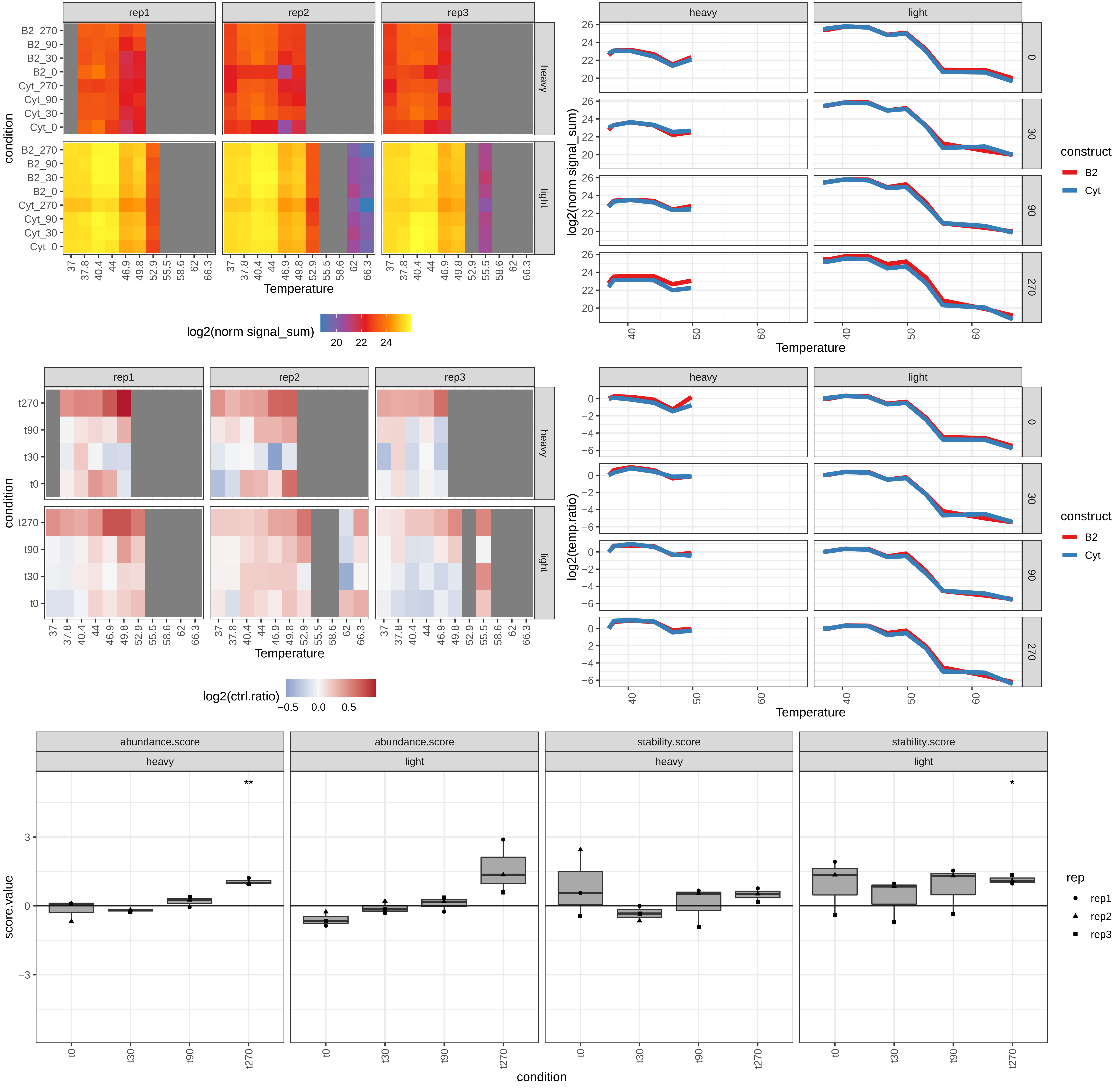

P47160 − Epsin−3 OS=Saccharomyces cerevisiae (strain ATCC 204508 / S288c) GN=ENT3 PE=1 SV=1

ENT5 − average.top3: 7.04844260869565 − max.qupm: 6

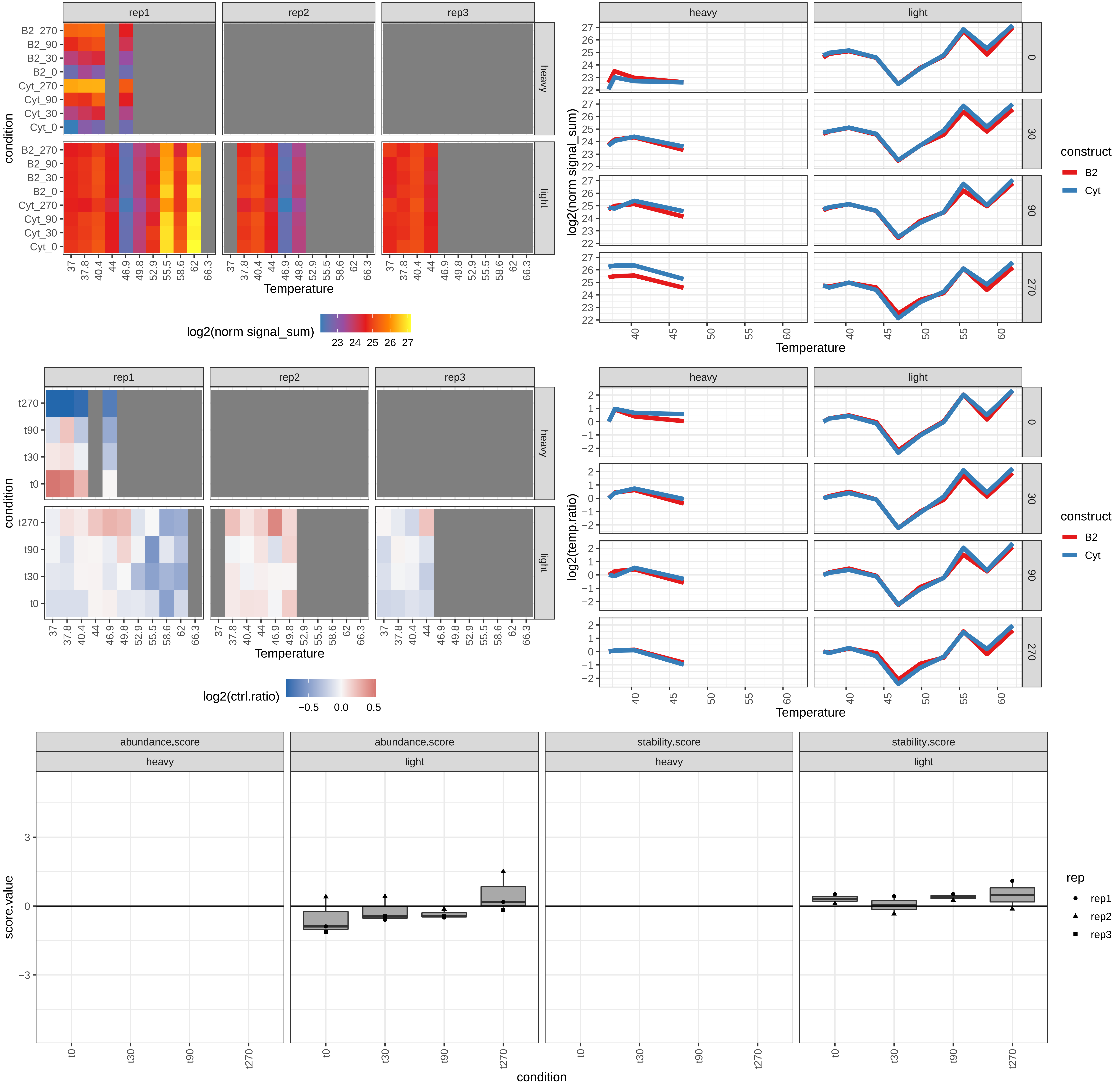

Q03769 − Epsin−5 OS=Saccharomyces cerevisiae (strain ATCC 204508 / S288c) GN=ENT5 PE=1 SV=1

ENV10 − average.top3: 6.94499 − max.qupm: 4

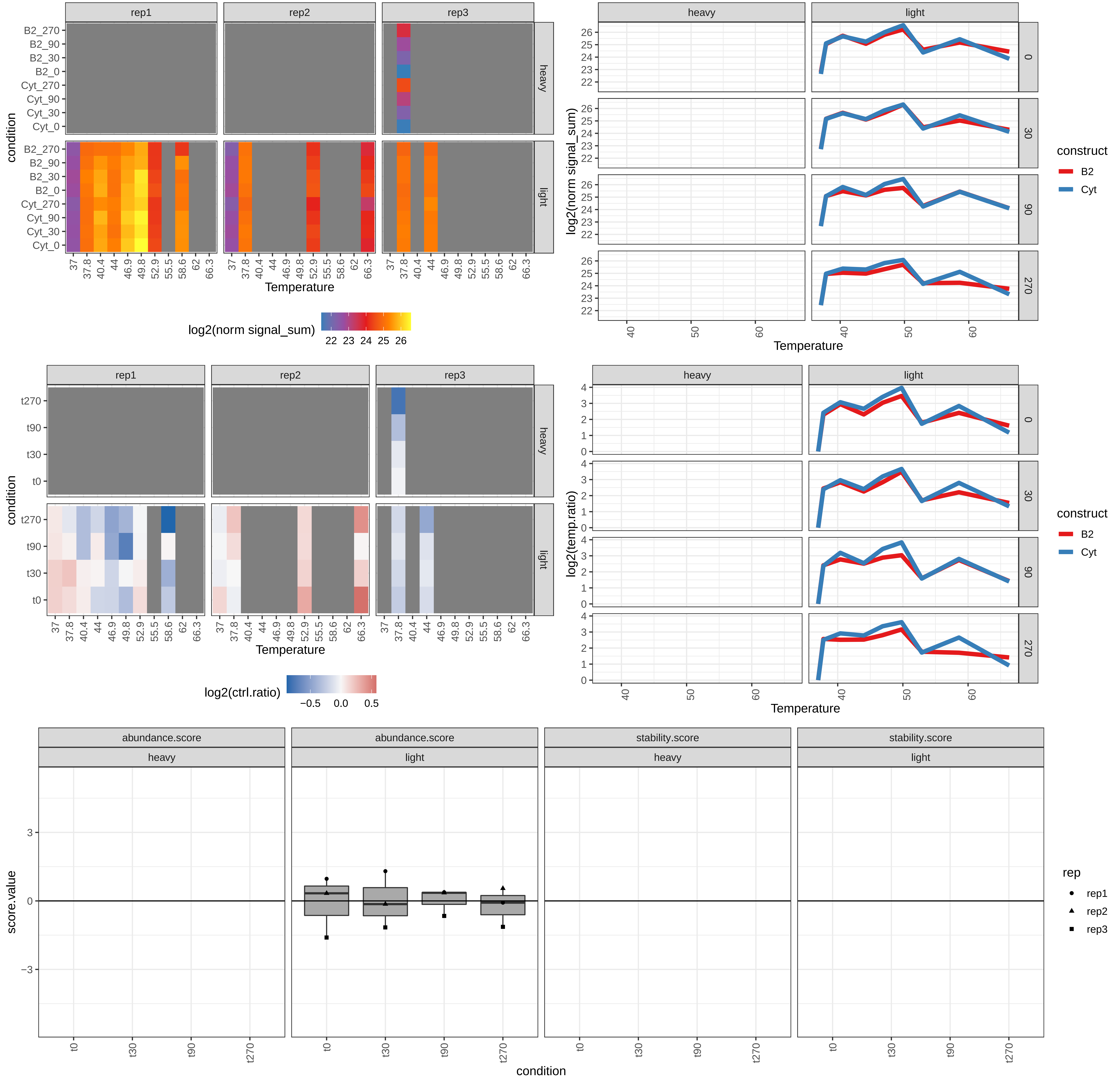

Q99382 − Late endosome and vacuole interface protein 10 OS=Saccharomyces cerevisiae (strain ATCC 204508 / S288c) GN=ENV10 PE=1 SV=1

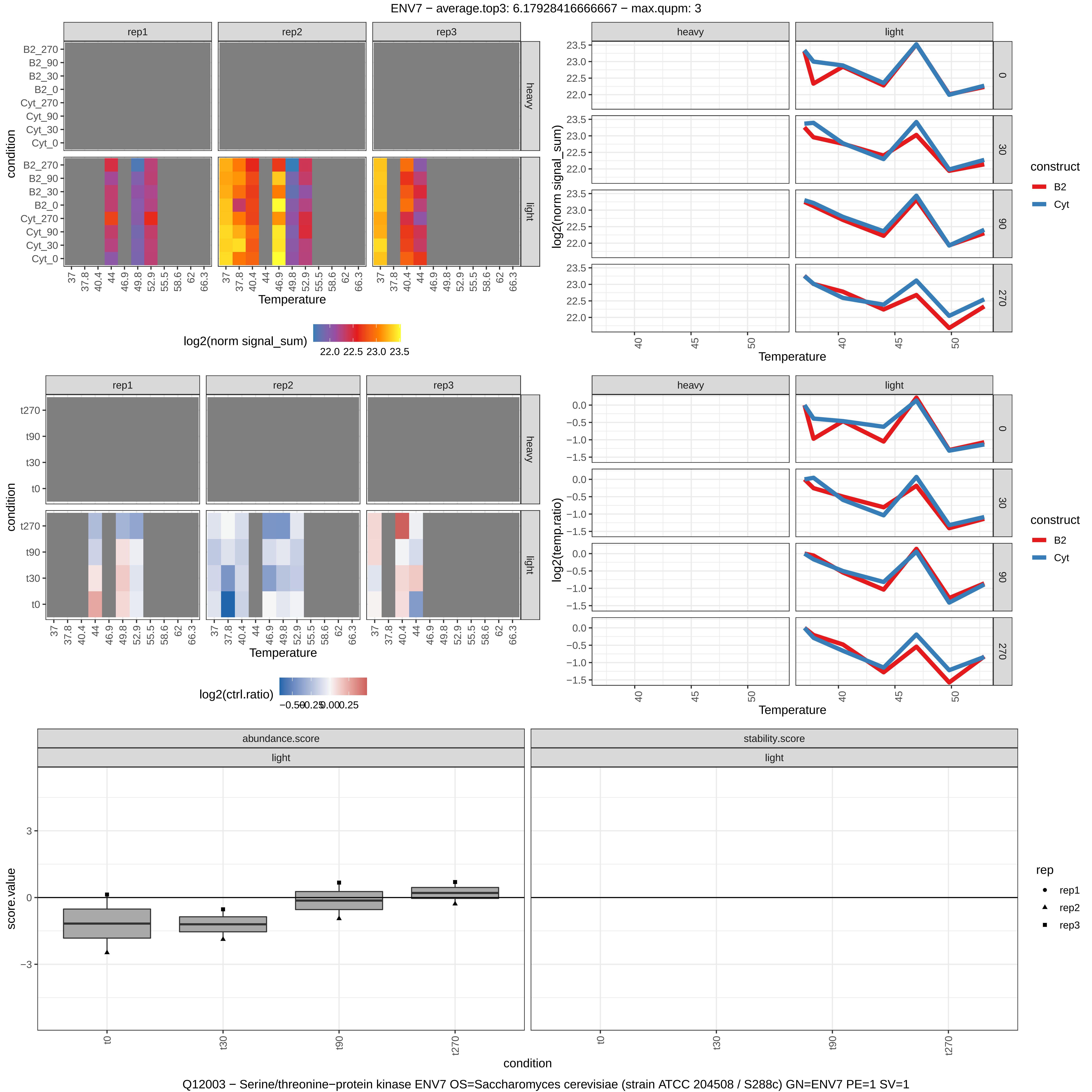

ENV7 − average.top3: 6.17928416666667 − max.qupm: 3
rep1
rep2
rep3
heavy
light
condition
Temperature
log2(norm signal_sum)
construct
B2
Cyt
log2(ctrl.ratio)
log2(temp.ratio)
abundance.score
stability.score
score.value
rep
rep1
rep2
rep3
Q12003 − Serine/threonine−protein kinase ENV7 OS=Saccharomyces cerevisiae (strain ATCC 204508 / S288c) GN=ENV7 PE=1 SV=1

EPS1 − average.top3: 6.82290193548387 − max.qupm: 12

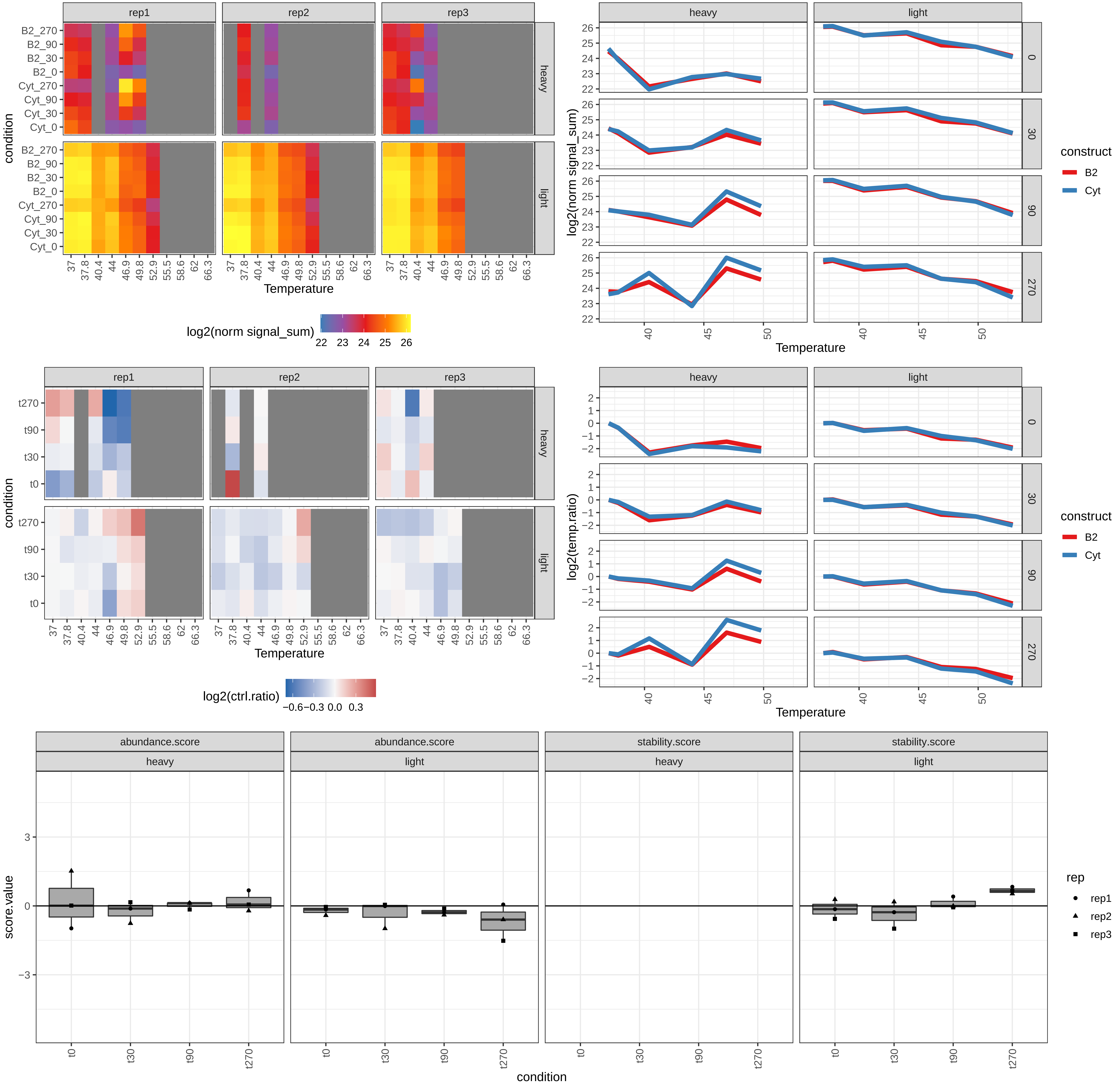

P40557 − ER−retained PMA1−suppressing protein 1 OS=Saccharomyces cerevisiae (strain ATCC 204508 / S288c) GN=EPS1 PE=1 SV=1

EPT1 − average.top3: 6.80289888888889 − max.qupm: 3

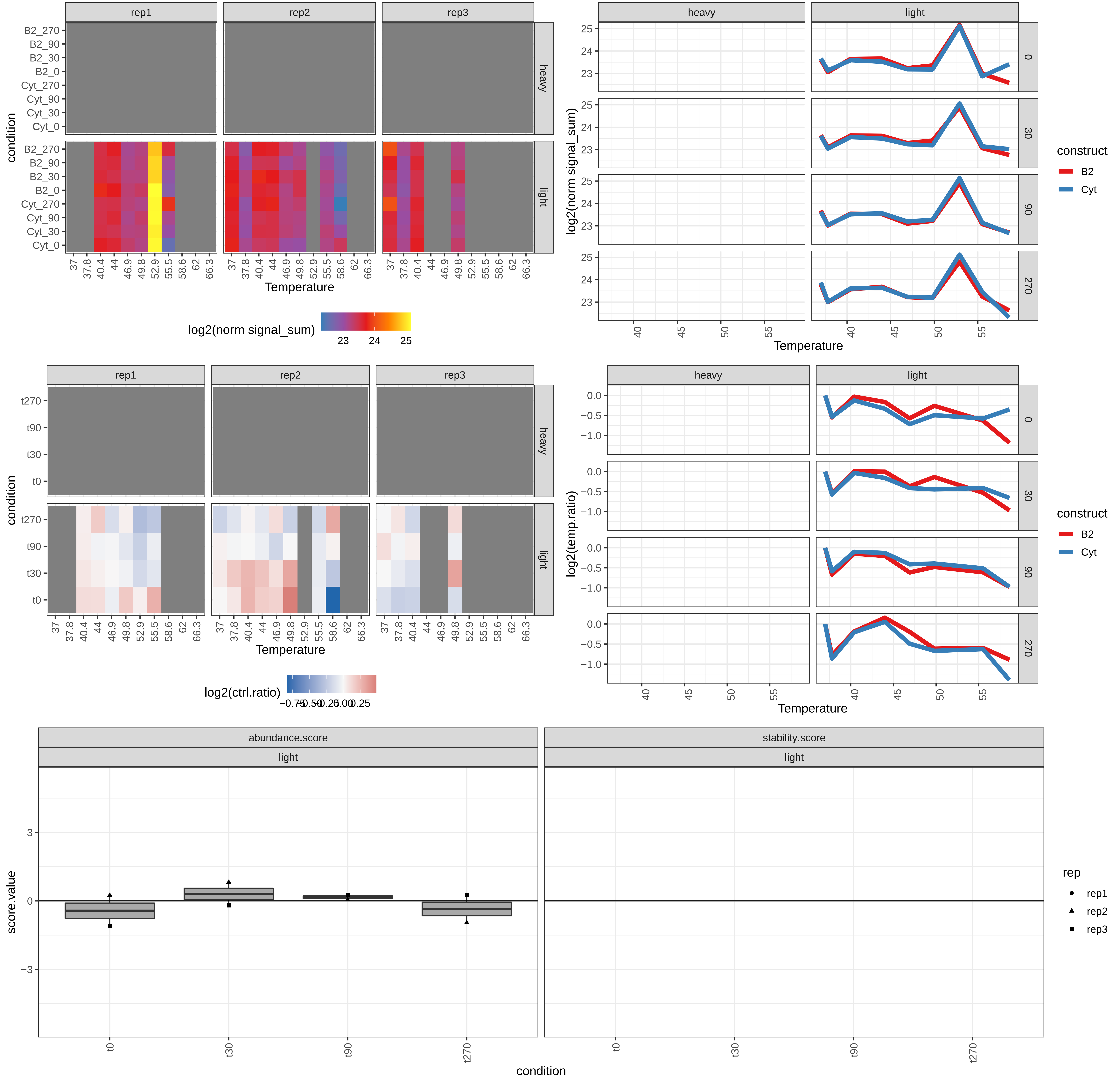

P22140 − Choline/ethanolaminephosphotransferase 1 OS=Saccharomyces cerevisiae (strain ATCC 204508 / S288c) GN=EPT1 PE=1 SV=2

ERB1 − average.top3: 6.91763894736842 − max.qupm: 9

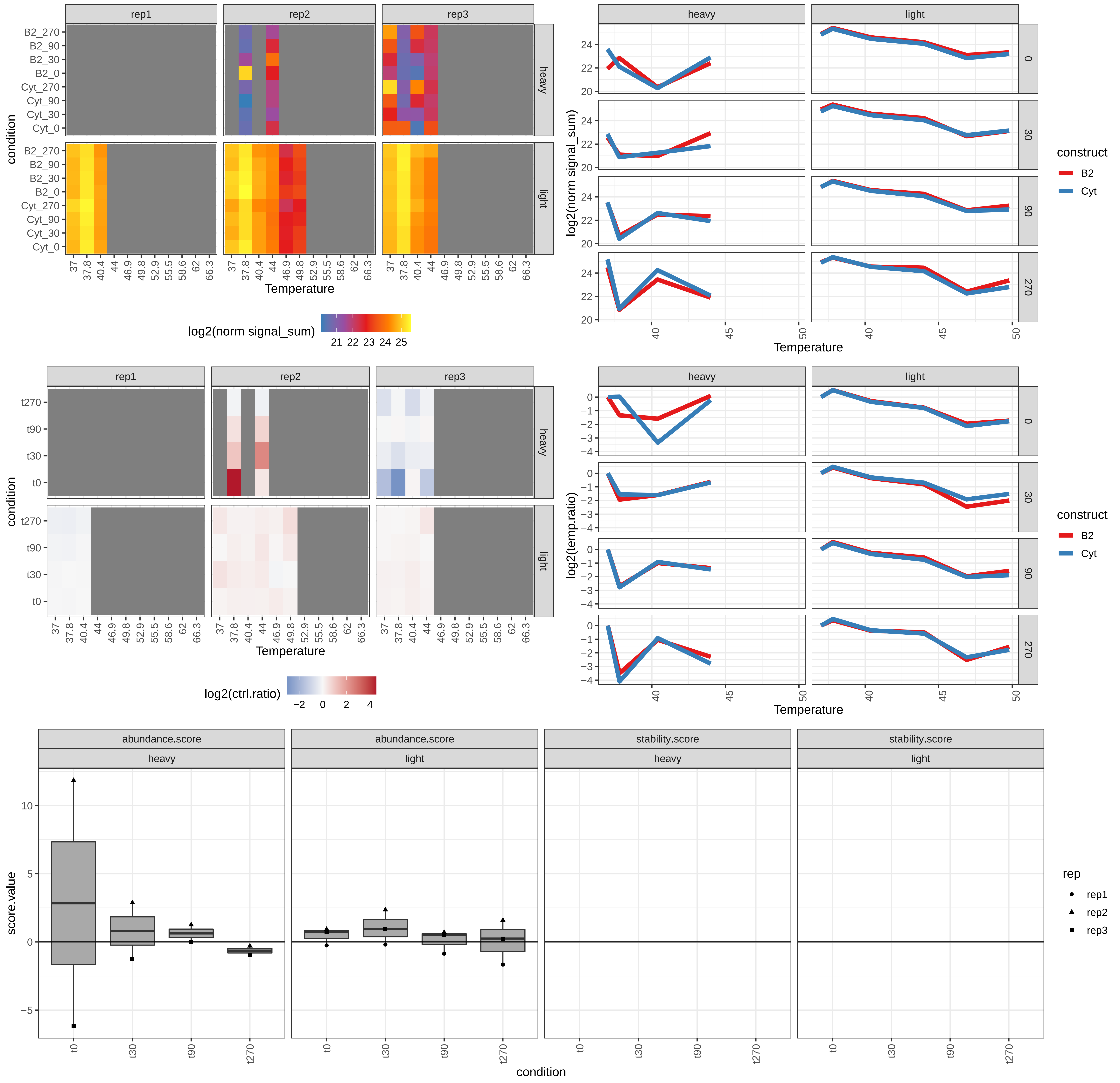

Q04660 − Ribosome biogenesis protein ERB1 OS=Saccharomyces cerevisiae (strain ATCC 204508 / S288c) GN=ERB1 PE=1 SV=1

ERD2 − average.top3: 7.33938076923077 − max.qupm: 2

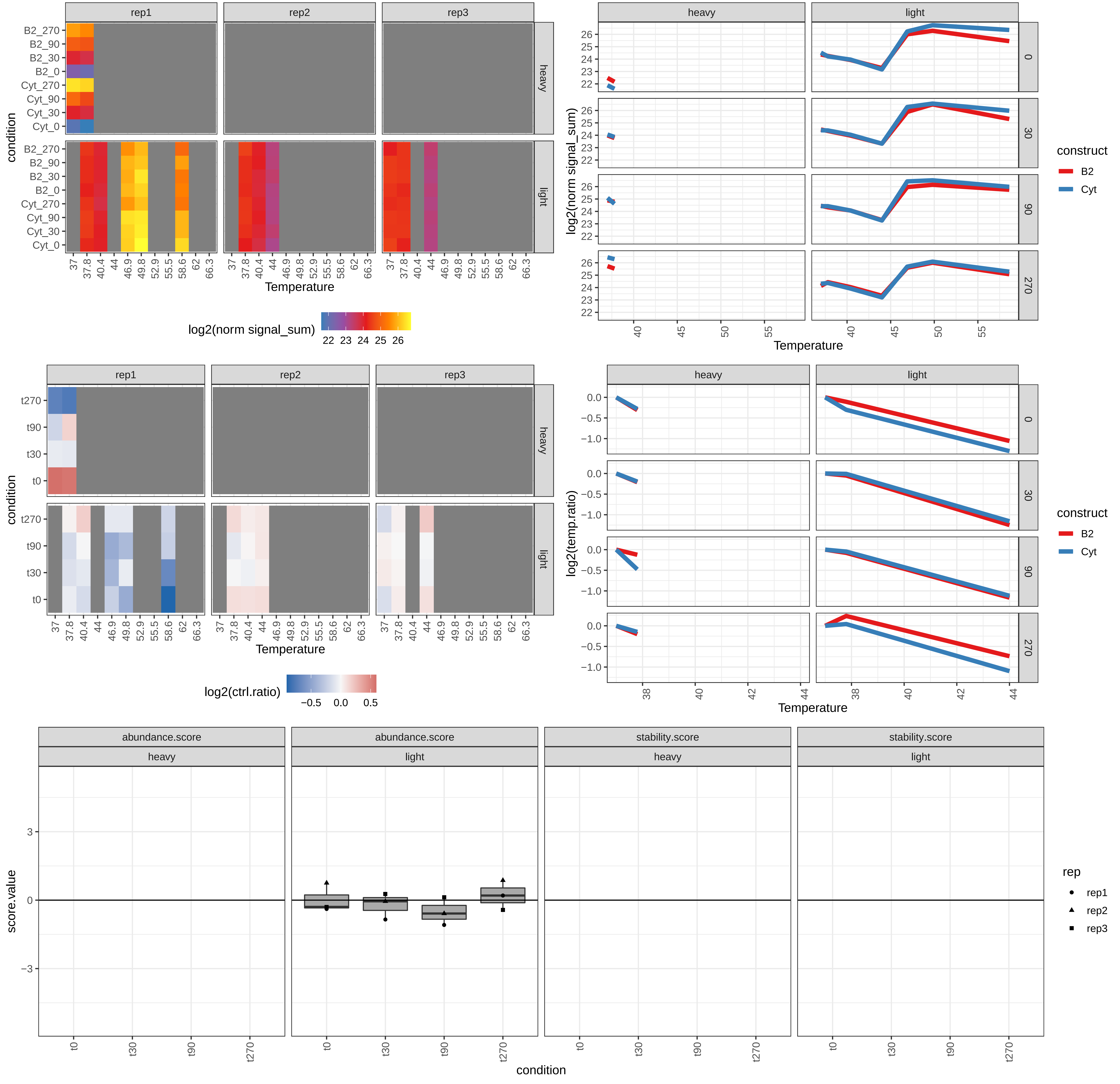

P18414 − ER lumen protein−retaining receptor OS=Saccharomyces cerevisiae (strain ATCC 204508 / S288c) GN=ERD2 PE=3 SV=1

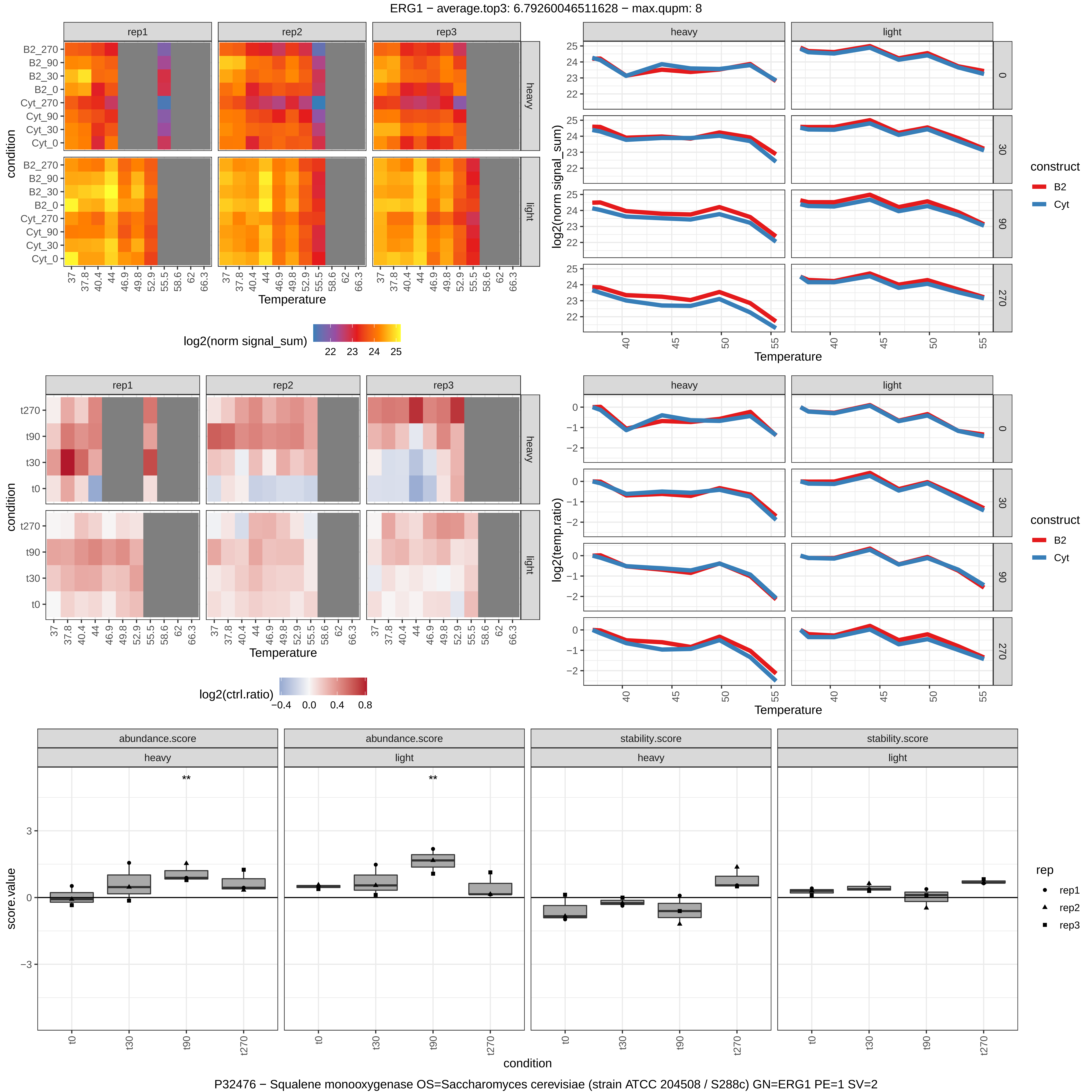
ERG1 − average.top3: 6.79260046511628 − max.qupm: 8
rep1
rep2
rep3
heavy
light
condition
Temperature
log2(norm signal_sum)
log2(norm signal_sum)
construct
B2
Cyt
log2(ctrl.ratio)
log2(temp.ratio)
abundance.score
stability.score
score.value
rep
rep1
rep2
rep3
P32476 − Squalene monooxygenase OS=Saccharomyces cerevisiae (strain ATCC 204508 / S288c) GN=ERG1 PE=1 SV=2

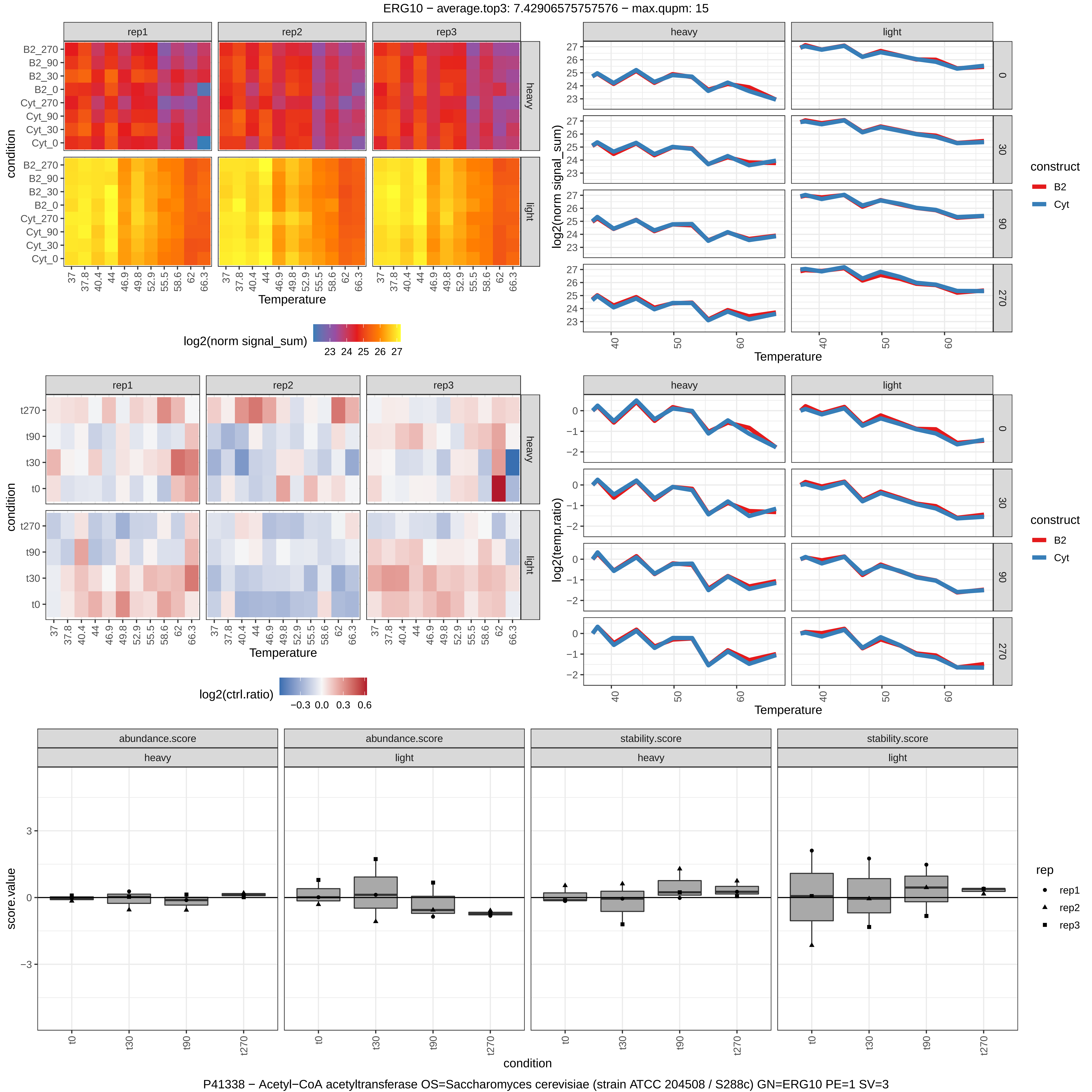
ERG10 − average.top3: 7.42906575757576 − max.qupm: 15
rep1
rep2
rep3
heavy
light
condition
Temperature
log2(norm signal_sum)
log2(norm signal_sum)
construct
B2
Cyt
log2(ctrl.ratio)
log2(temp.ratio)
abundance.score
stability.score
score.value
rep
rep1
rep2
rep3
P41338 − Acetyl−CoA acetyltransferase OS=Saccharomyces cerevisiae (strain ATCC 204508 / S288c) GN=ERG10 PE=1 SV=3

ERG11 − average.top3: 6.84014272727273 − max.qupm: 10

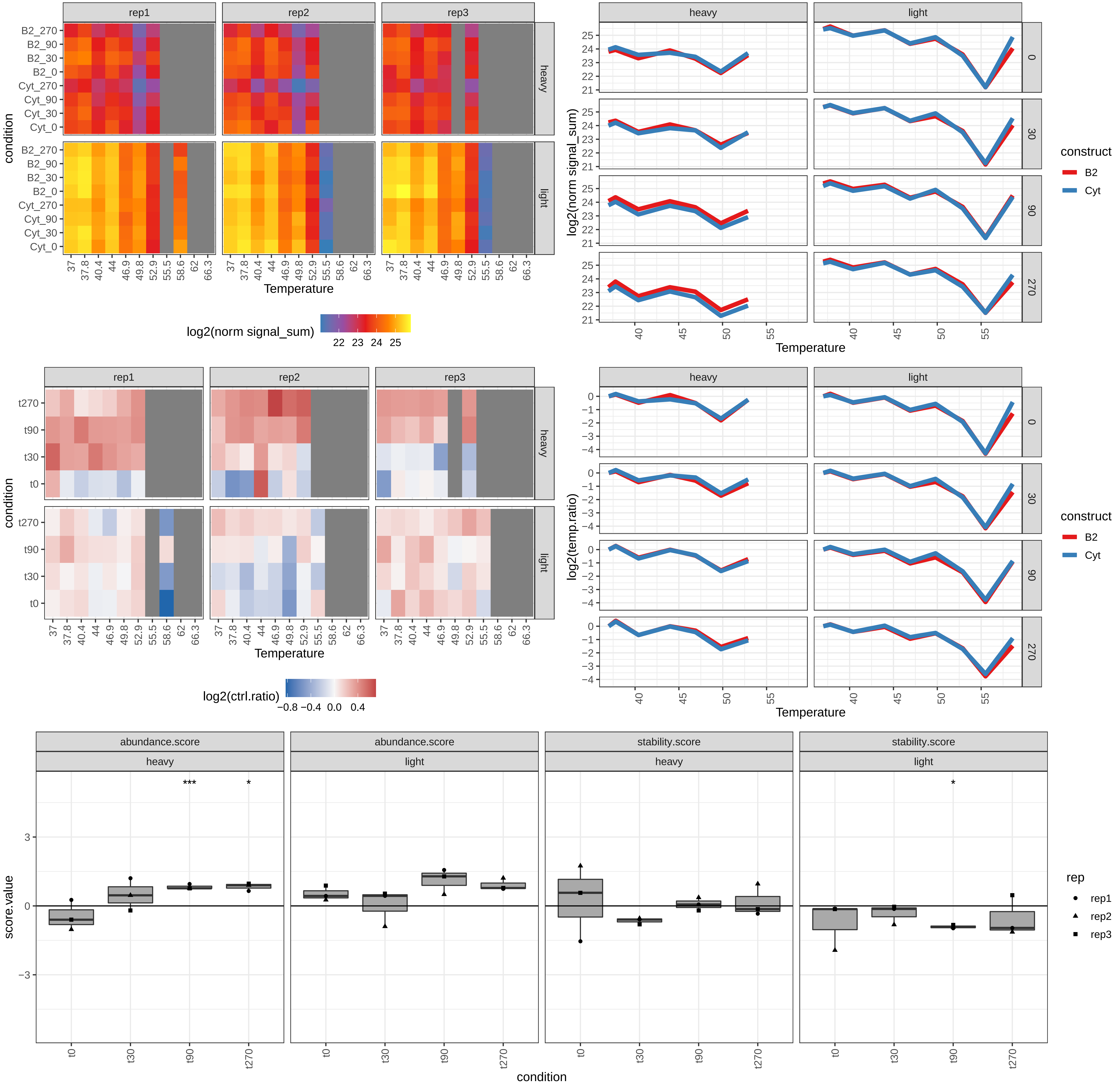

P10614 − Lanosterol 14−alpha demethylase OS=Saccharomyces cerevisiae (strain ATCC 204508 / S288c) GN=ERG11 PE=1 SV=1

ERG12 − average.top3: 7.0981784375 − max.qupm: 8

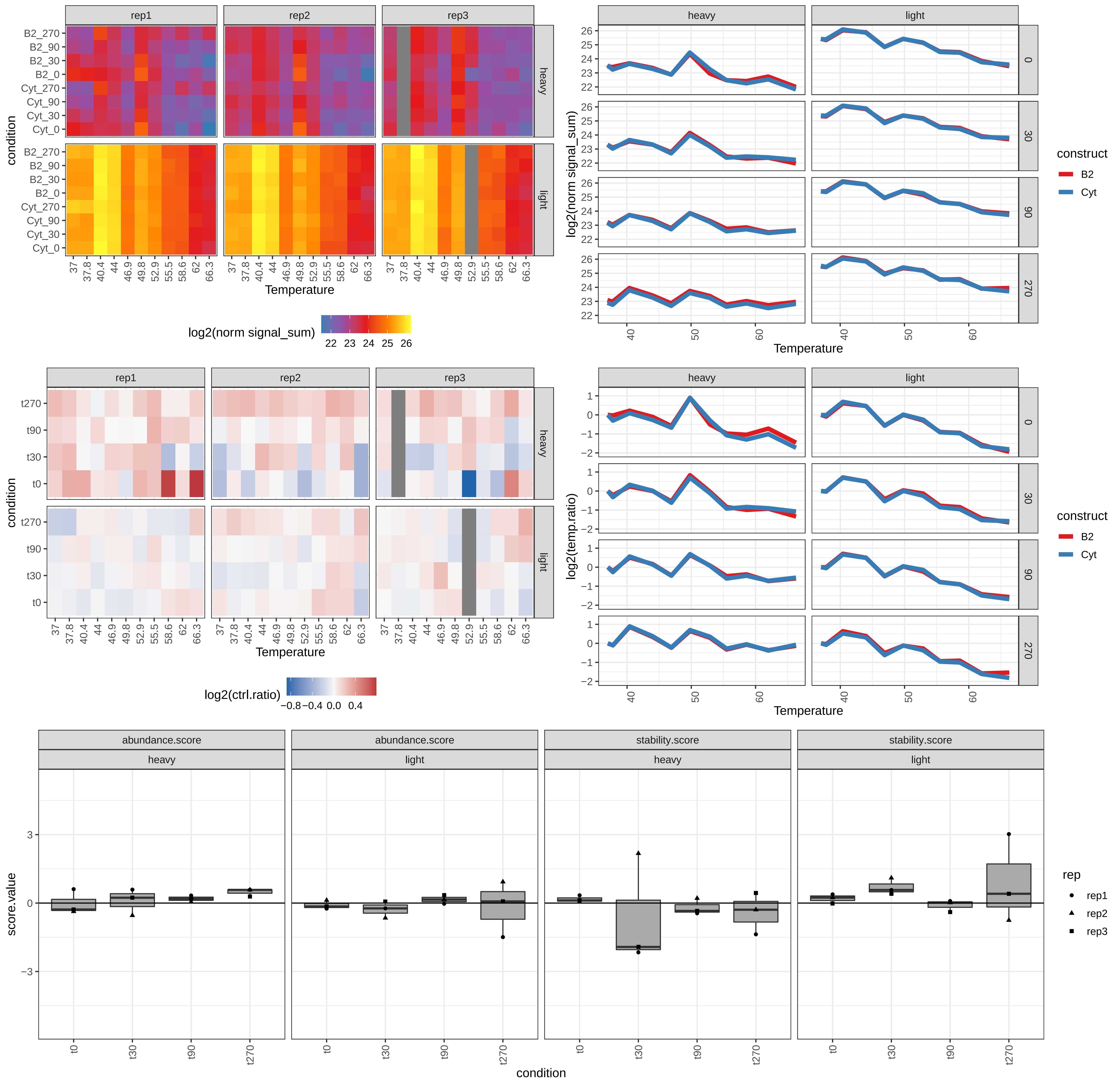

P07277 − Mevalonate kinase OS=Saccharomyces cerevisiae (strain ATCC 204508 / S288c) GN=ERG12 PE=1 SV=1

ERG13 − average.top3: 7.95811428571429 − max.qupm: 18

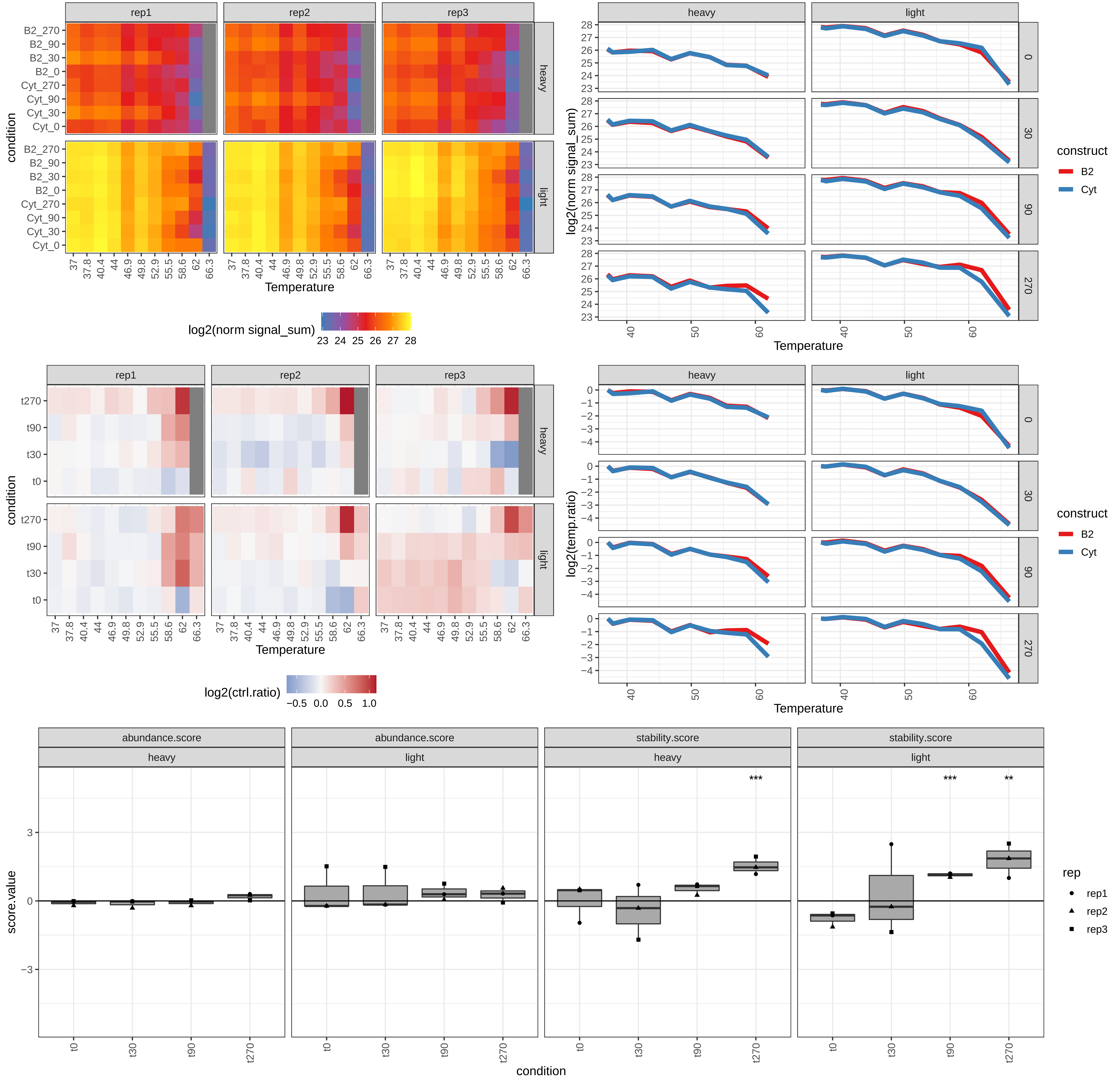

P54839 − Hydroxymethylglutaryl−CoA synthase OS=Saccharomyces cerevisiae (strain ATCC 204508 / S288c) GN=ERG13 PE=1 SV=1

ERG2 − average.top3: 7.12882431372549 − max.qupm: 4

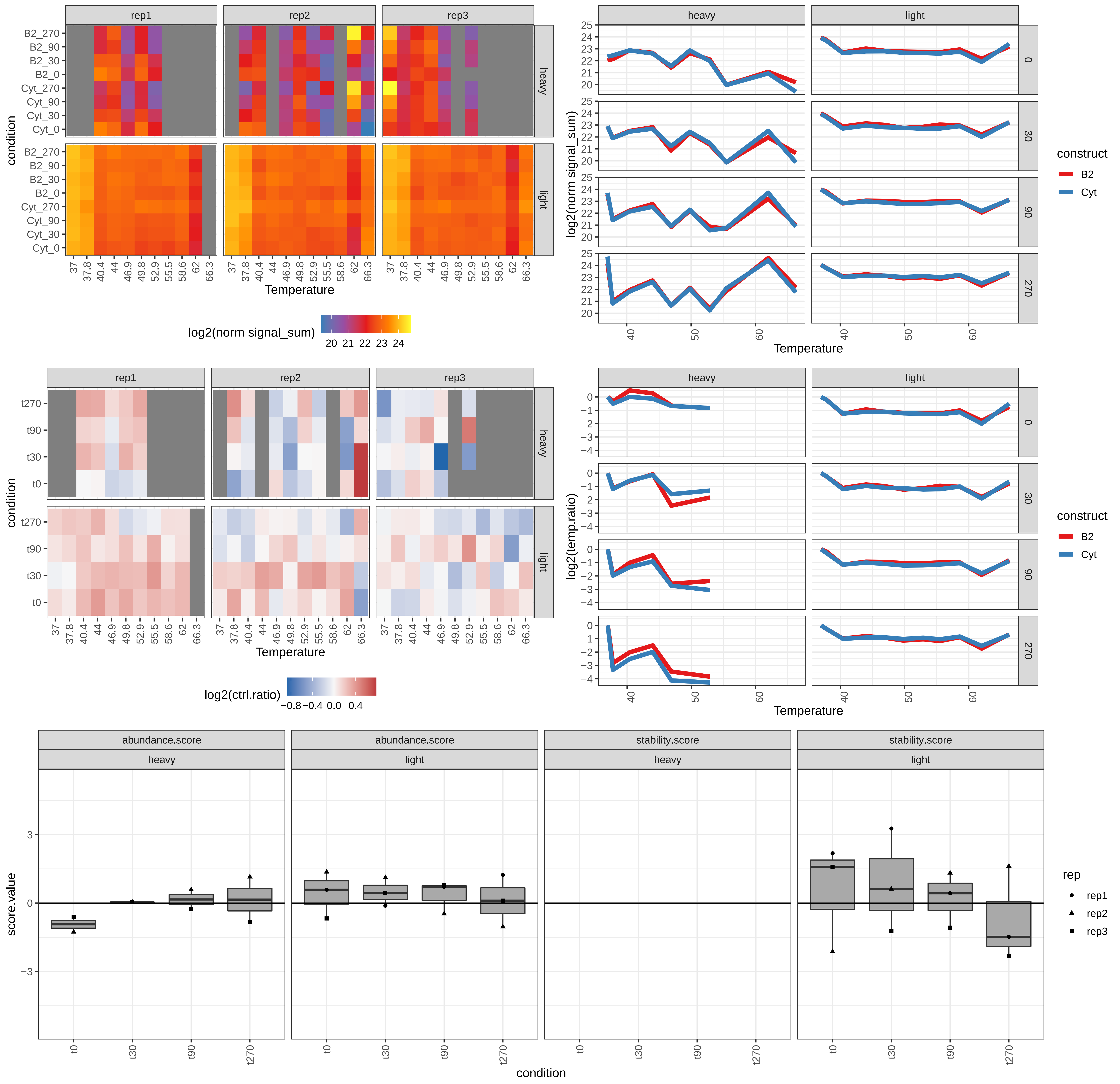

P32352 − C−8 sterol isomerase OS=Saccharomyces cerevisiae (strain ATCC 204508 / S288c) GN=ERG2 PE=1 SV=1

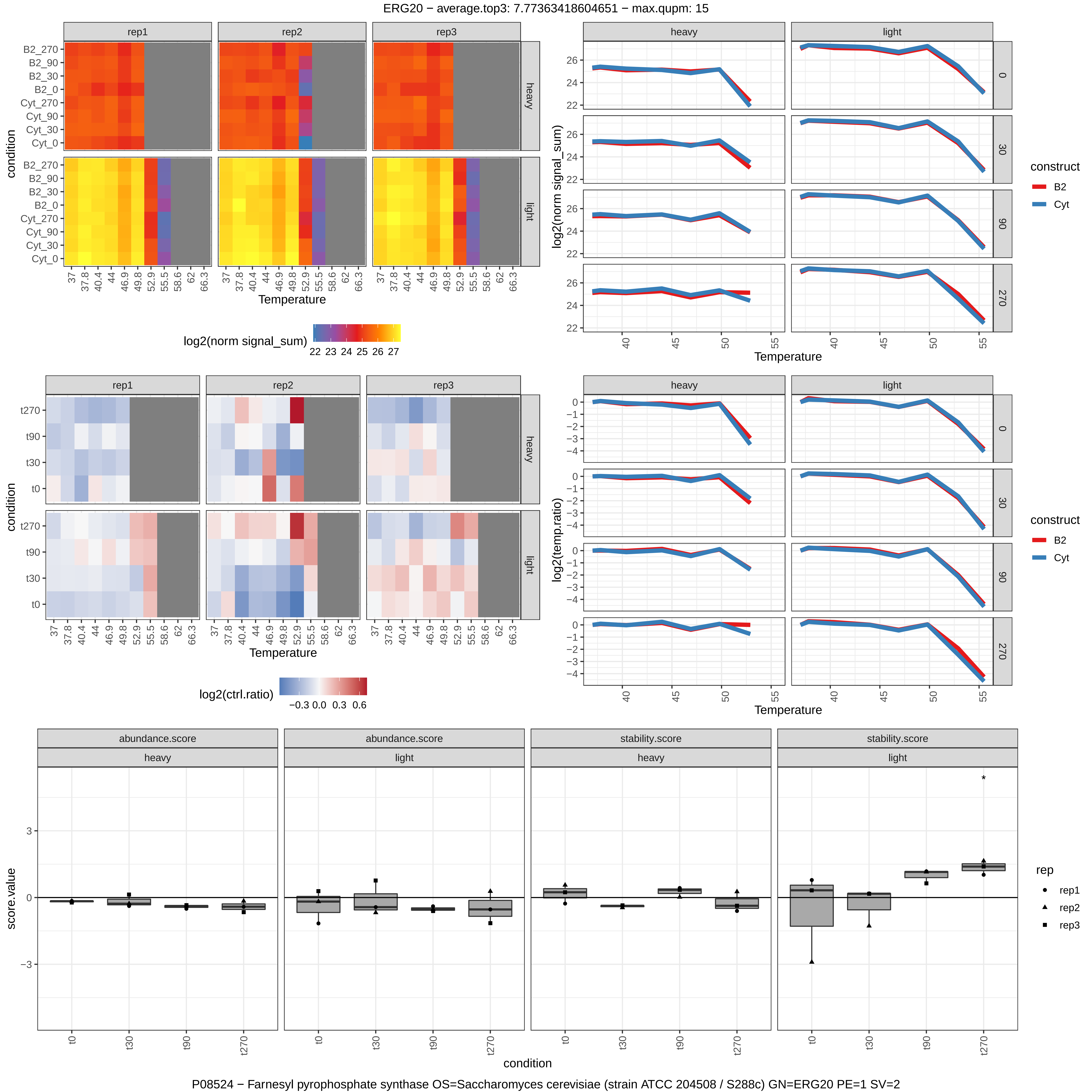
ERG20 − average.top3: 7.77363418604651 − max.qupm: 15
rep1
rep2
rep3
heavy
light
condition
Temperature
log2(norm signal_sum)
log2(temp.ratio)
log2(ctrl.ratio)
construct
B2
Cyt
abundance.score
stability.score
score.value
rep
rep1
rep2
rep3
P08524 − Farnesyl pyrophosphate synthase OS=Saccharomyces cerevisiae (strain ATCC 204508 / S288c) GN=ERG20 PE=1 SV=2

ERG24 − average.top3: 7.08421875 − max.qupm: 4

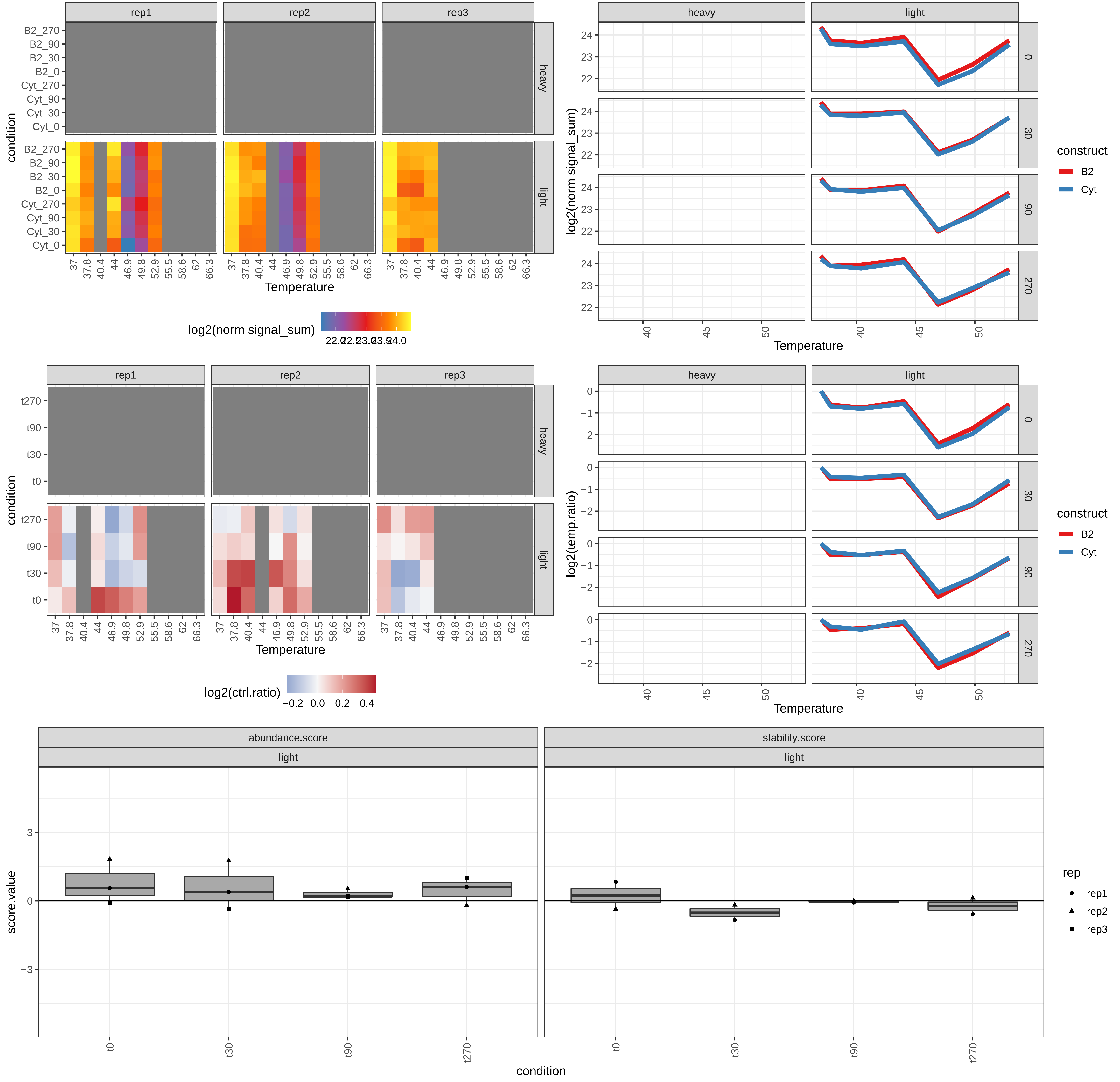

P32462 − Delta(14)−sterol reductase OS=Saccharomyces cerevisiae (strain ATCC 204508 / S288c) GN=ERG24 PE=1 SV=1

ERG25 − average.top3: 6.97522285714286 − max.qupm: 3

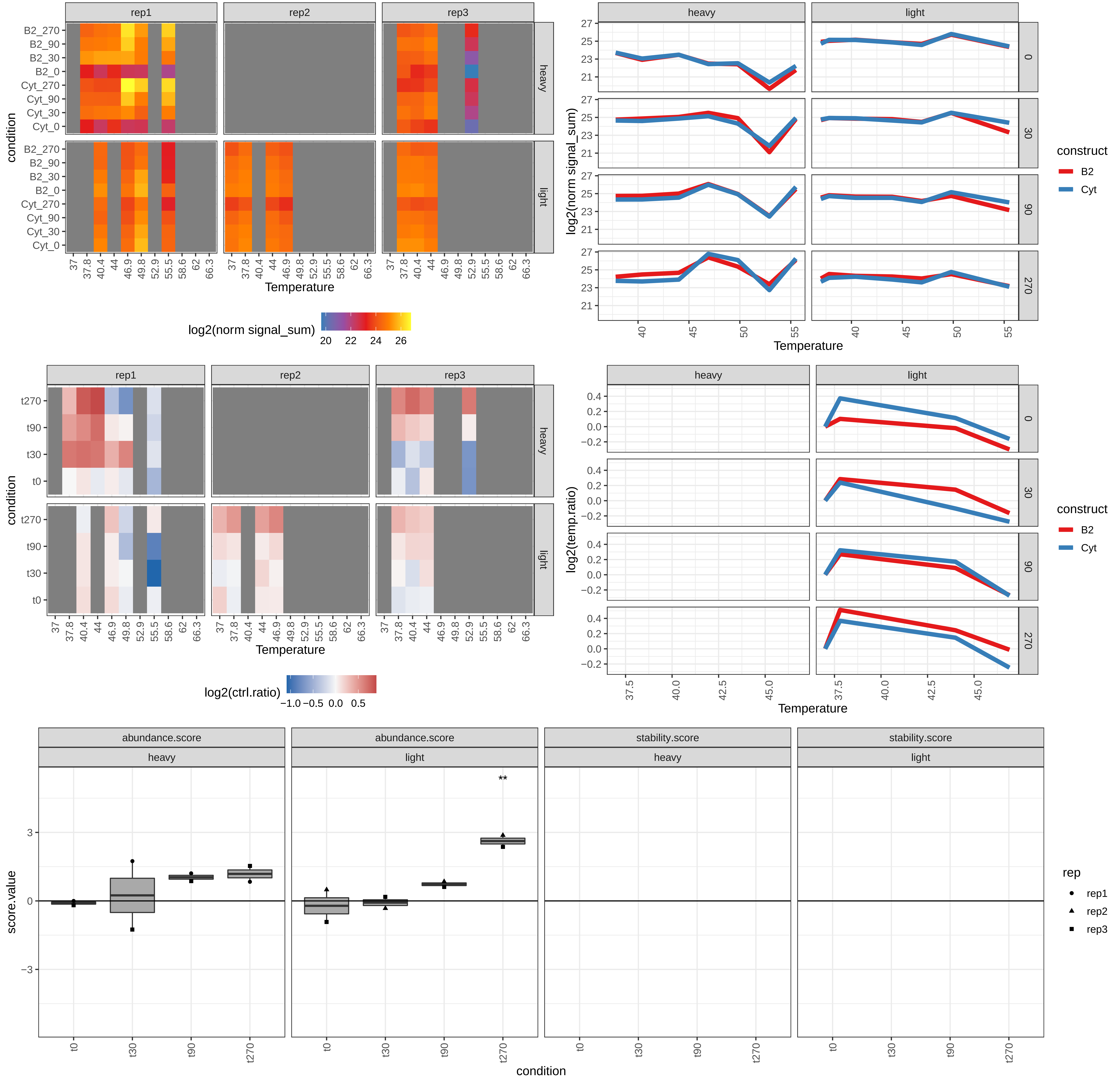

P53045 − Methylsterol monooxygenase OS=Saccharomyces cerevisiae (strain ATCC 204508 / S288c) GN=ERG25 PE=1 SV=1

ERG26 − average.top3: 7.07542277777778 − max.qupm: 11

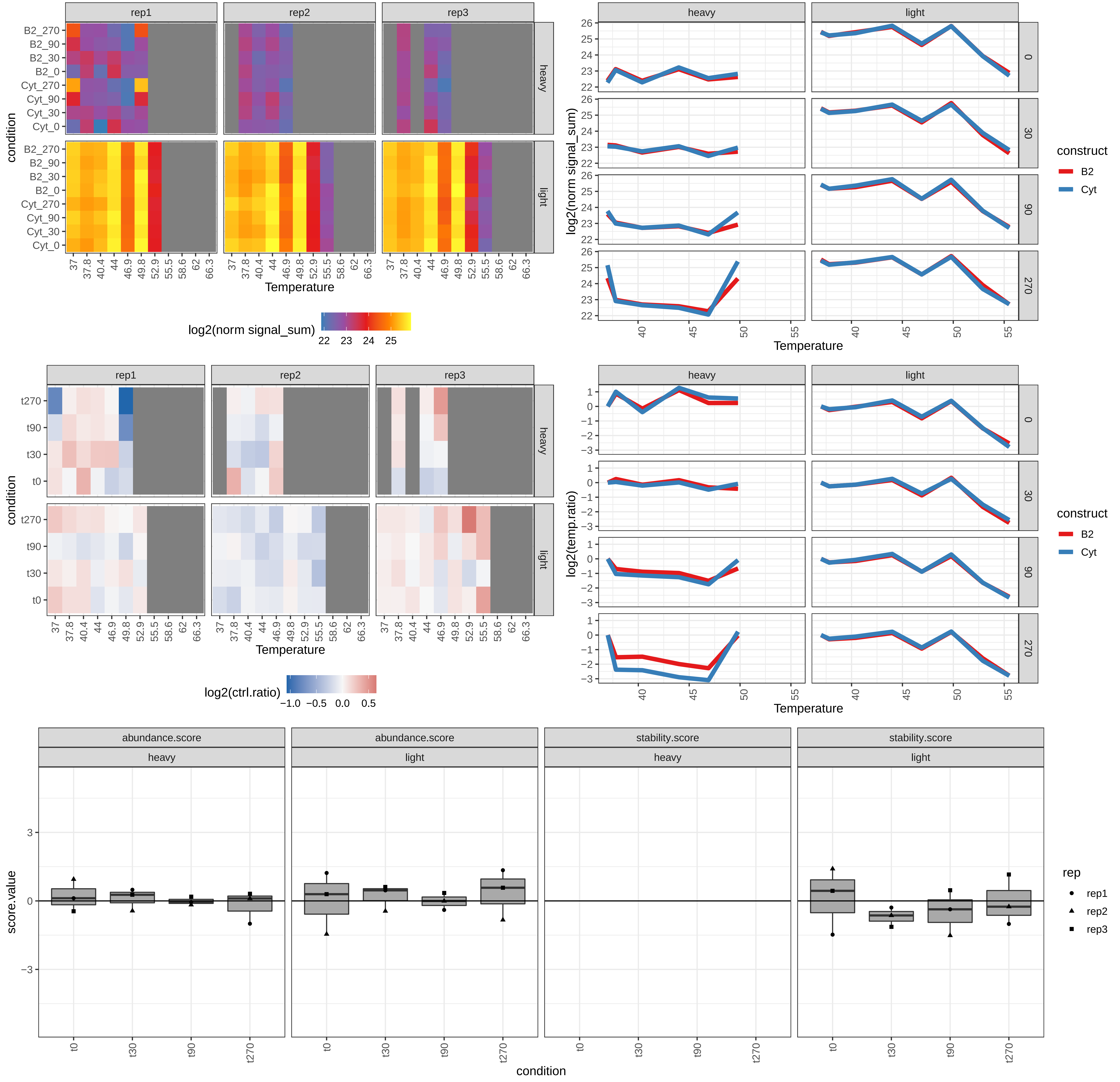

P53199 − Sterol−4−alpha−carboxylate 3−dehydrogenase, decarboxylating OS=Saccharomyces cerevisiae (strain ATCC 204508 / S288c) GN=ERG26 PE=1 SV=1

ERG27 − average.top3: 7.09116433333333 − max.qupm: 6

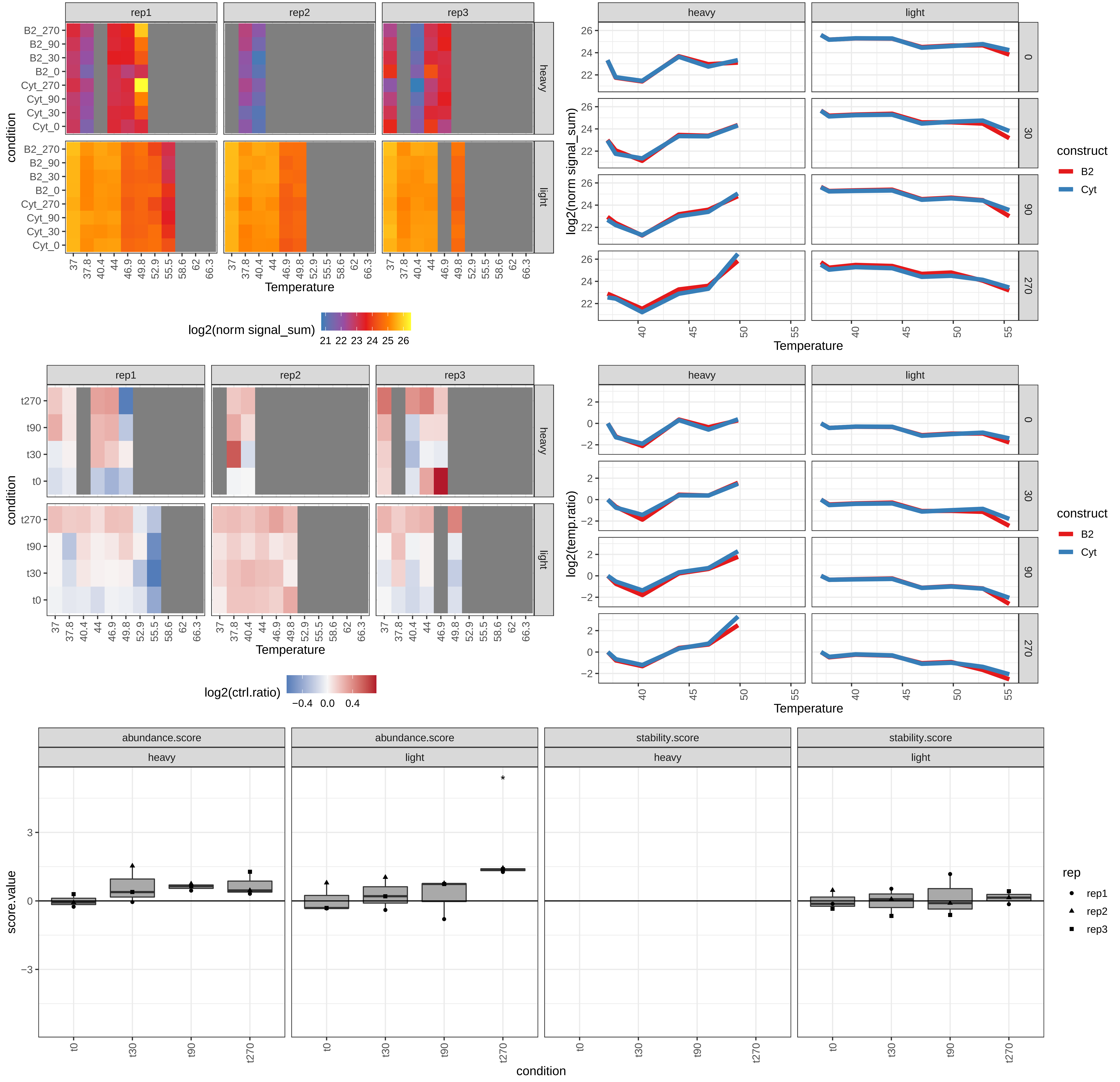

Q12452 − 3−keto−steroid reductase OS=Saccharomyces cerevisiae (strain ATCC 204508 / S288c) GN=ERG27 PE=1 SV=1

ERG3 − average.top3: 6.96874235294118 − max.qupm: 6

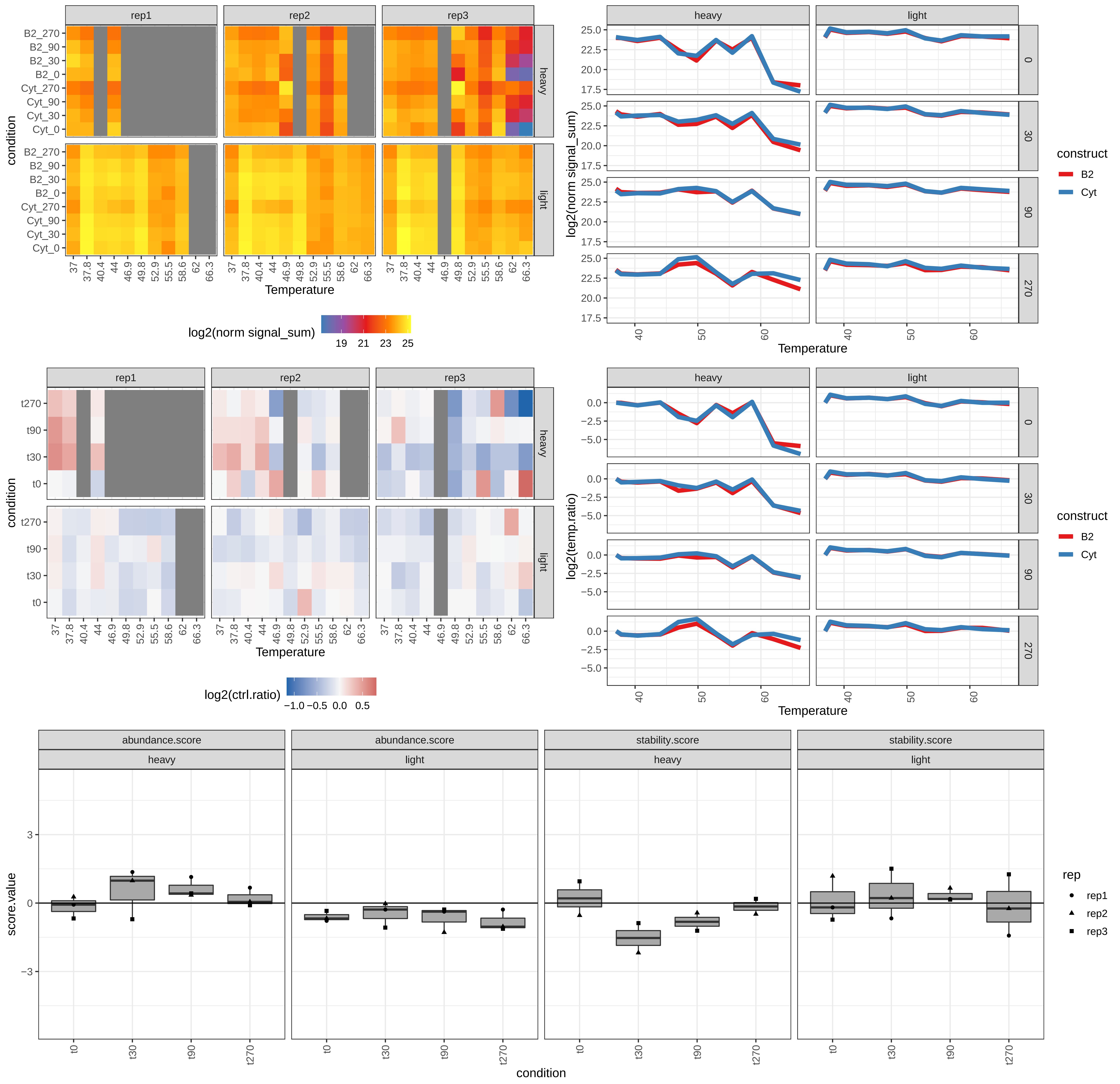

P32353 − Delta(7)−sterol 5(6)−desaturase OS=Saccharomyces cerevisiae (strain ATCC 204508 / S288c) GN=ERG3 PE=1 SV=1

ERG4 − average.top3: 6.99582576923077 − max.qupm: 4

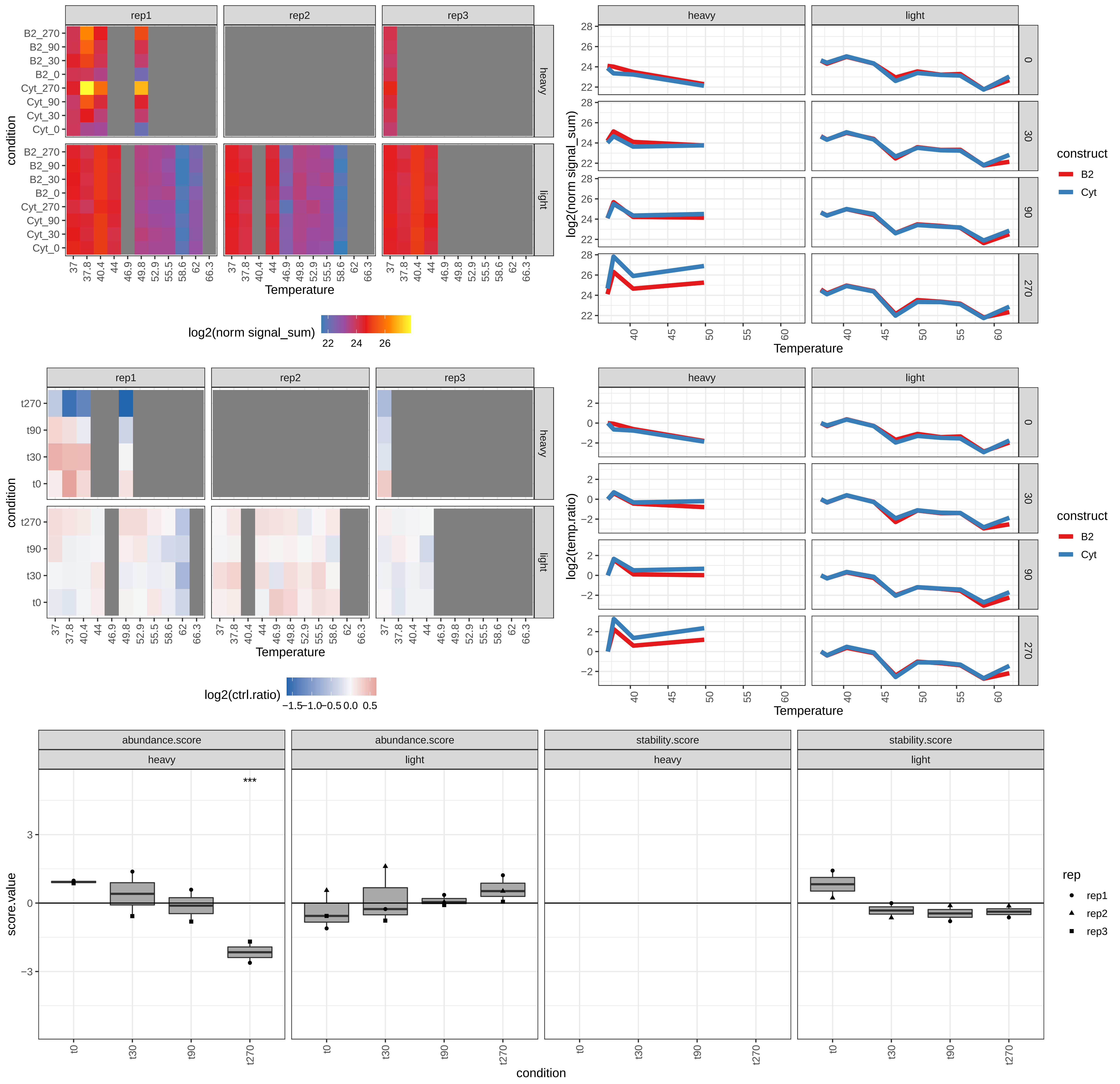

P25340 − Delta(24(24(1)))−sterol reductase OS=Saccharomyces cerevisiae (strain ATCC 204508 / S288c) GN=ERG4 PE=1 SV=2

ERG5 − average.top3: 6.79585260869565 − max.qupm: 9

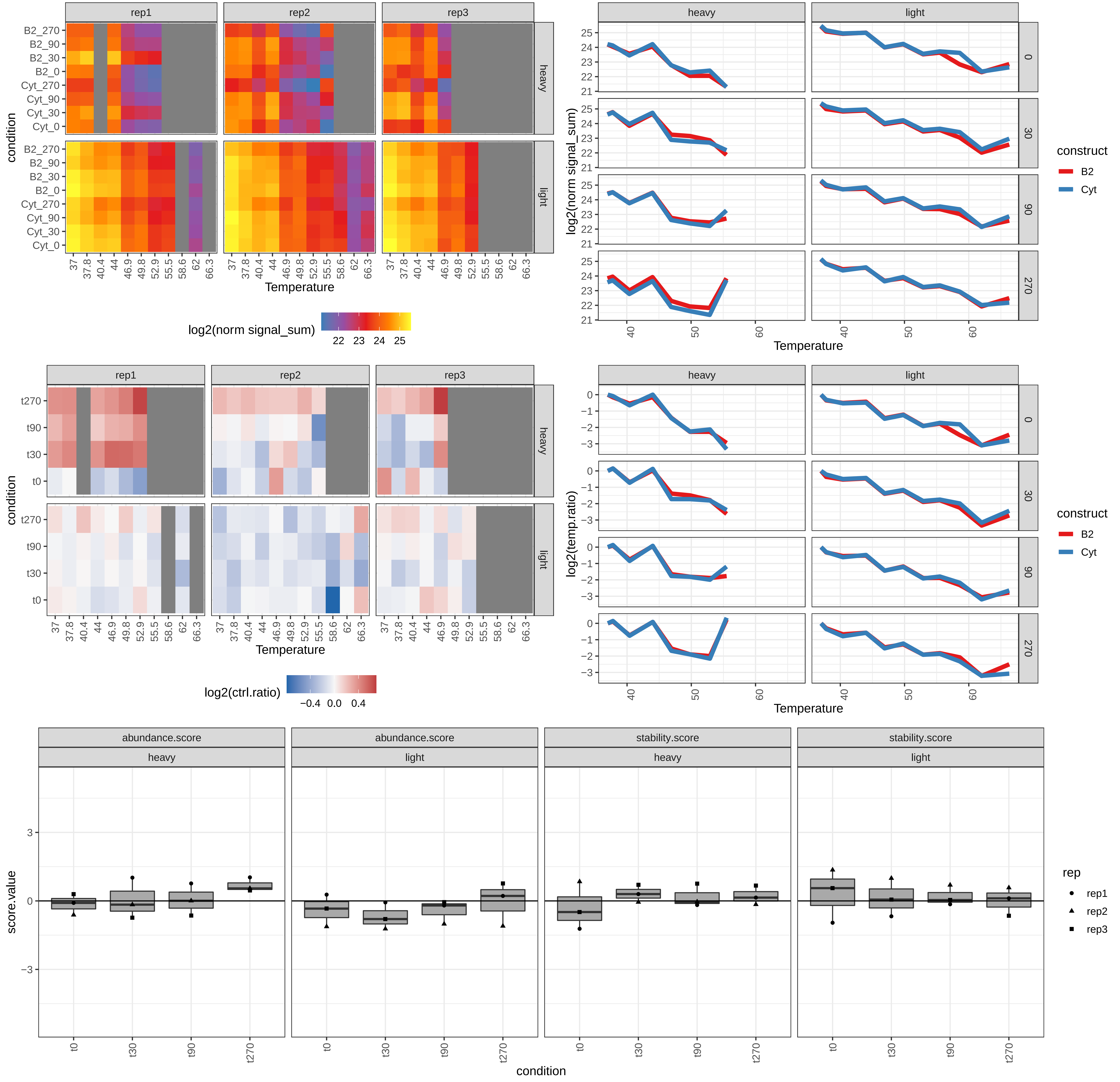

P54781 − Cytochrome P450 61 OS=Saccharomyces cerevisiae (strain ATCC 204508 / S288c) GN=ERG5 PE=1 SV=1

ERG6 − average.top3: 7.58109634615385 − max.qupm: 18

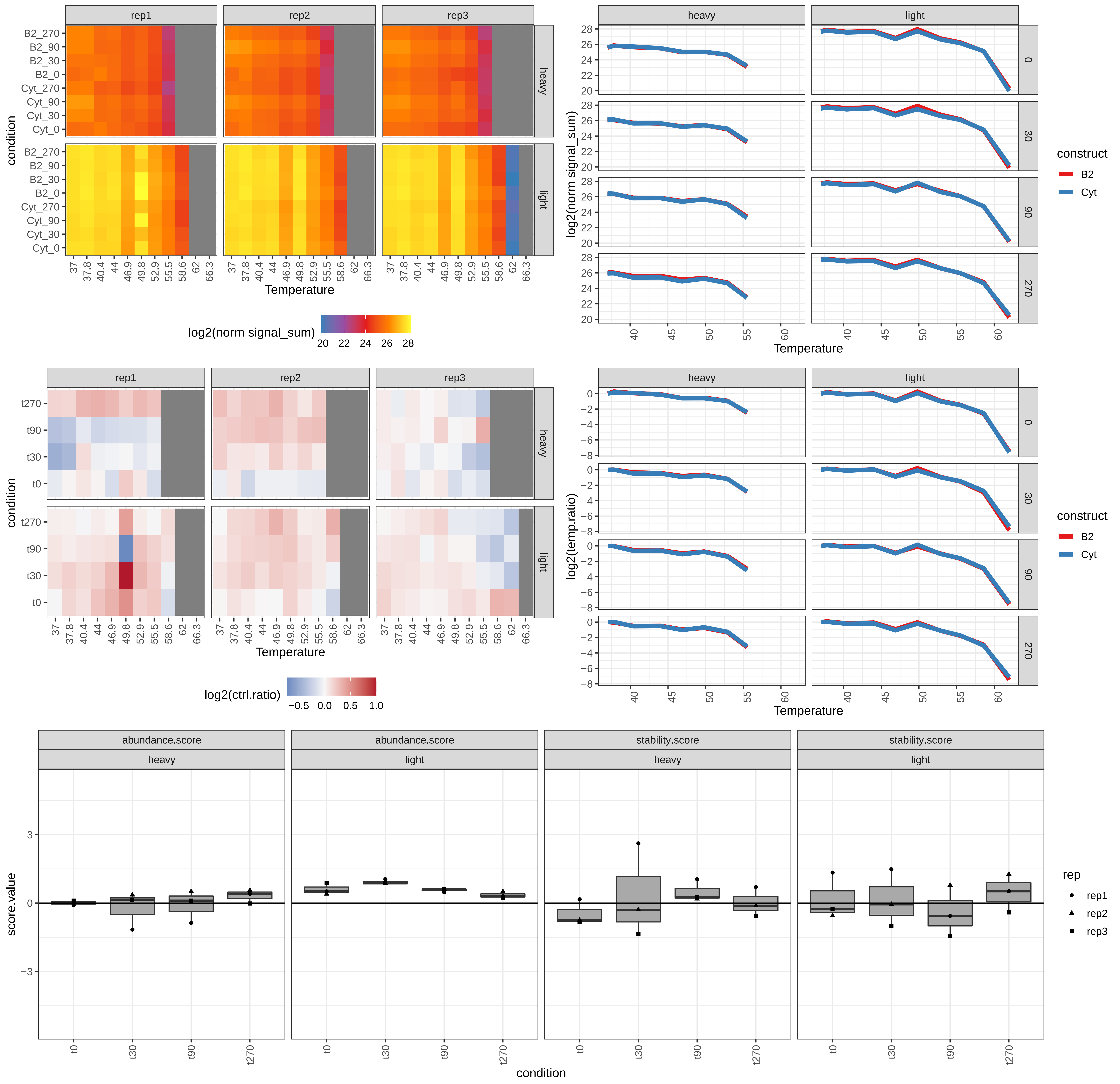

P25087 − Sterol 24−C−methyltransferase OS=Saccharomyces cerevisiae (strain ATCC 204508 / S288c) GN=ERG6 PE=1 SV=4

ERG7 − average.top3: 6.67011761904762 − max.qupm: 9

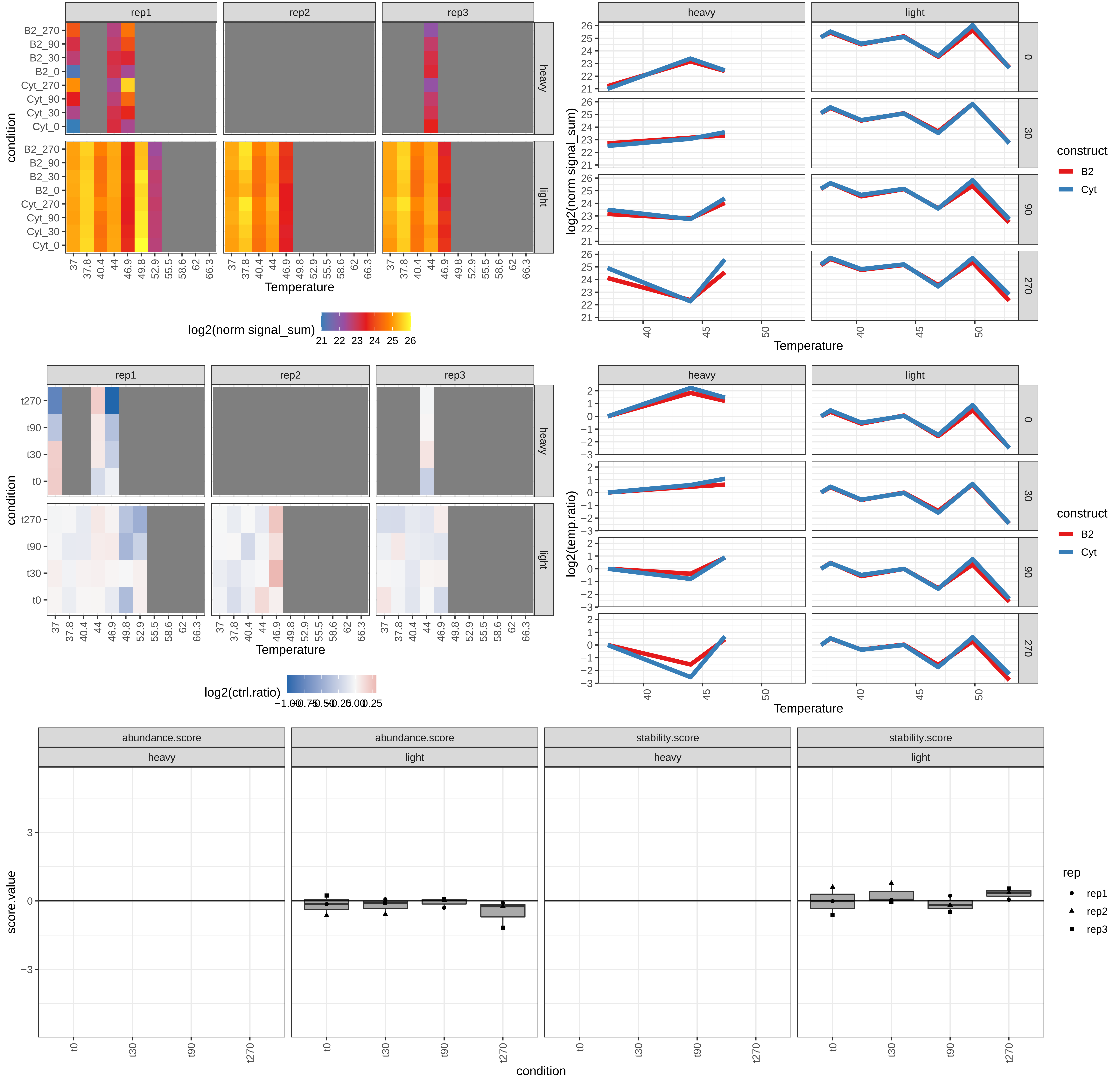

P38604 − Lanosterol synthase OS=Saccharomyces cerevisiae (strain ATCC 204508 / S288c) GN=ERG7 PE=1 SV=6

ERG8 − average.top3: 6.95046111111111 − max.qupm: 5

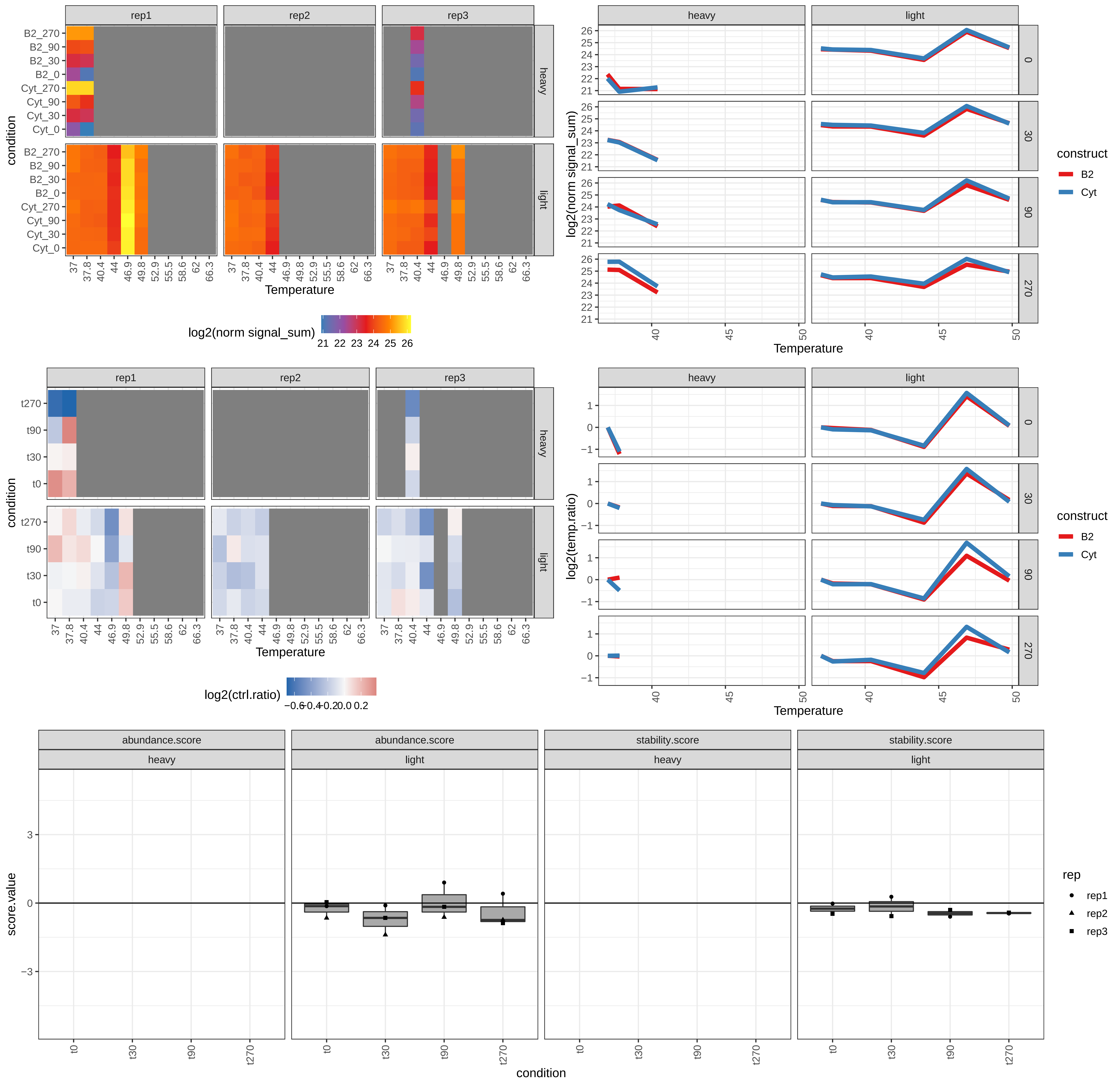

P24521 − Phosphomevalonate kinase OS=Saccharomyces cerevisiae (strain ATCC 204508 / S288c) GN=ERG8 PE=1 SV=2

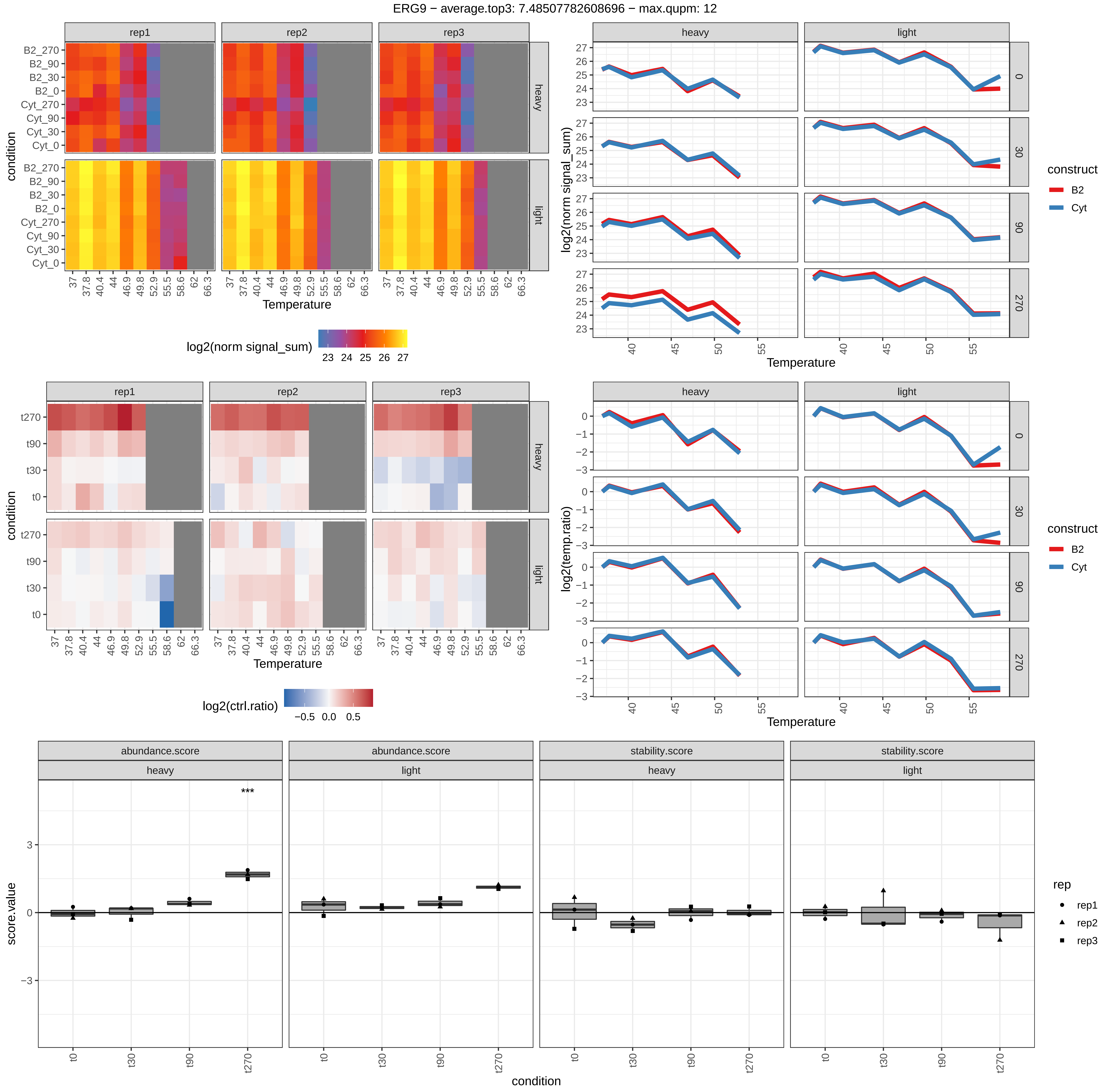

P29704 − Squalene synthase OS=Saccharomyces cerevisiae (strain ATCC 204508 / S288c) GN=ERG9 PE=1 SV=2

ERJ5 − average.top3: 6.82896368421053 − max.qupm: 3

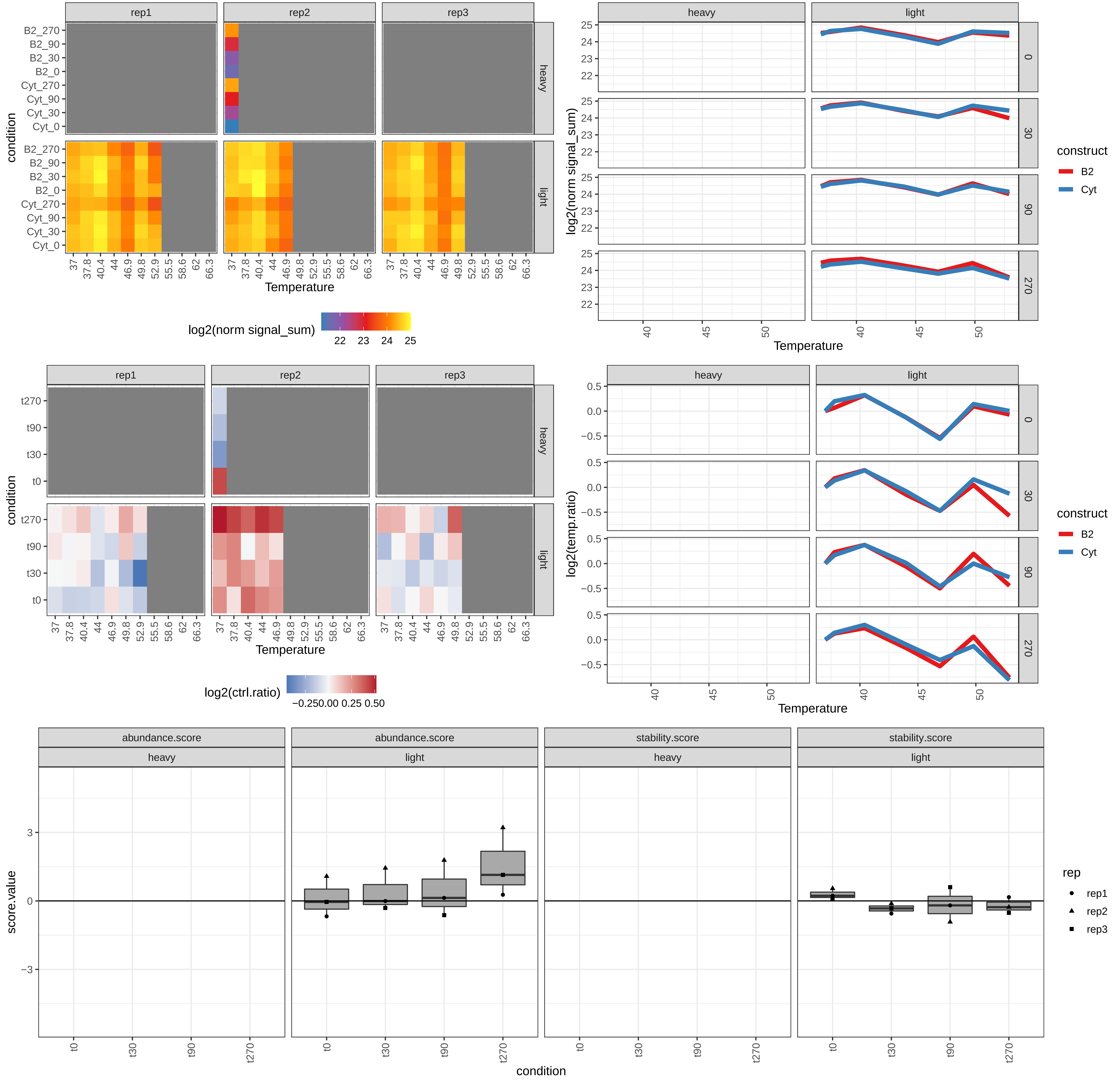

P43613 − ER−localized J domain−containing protein 5 OS=Saccharomyces cerevisiae (strain ATCC 204508 / S288c) GN=ERJ5 PE=1 SV=1

ERO1 − average.top3: 6.81395918367347 − max.qupm: 10

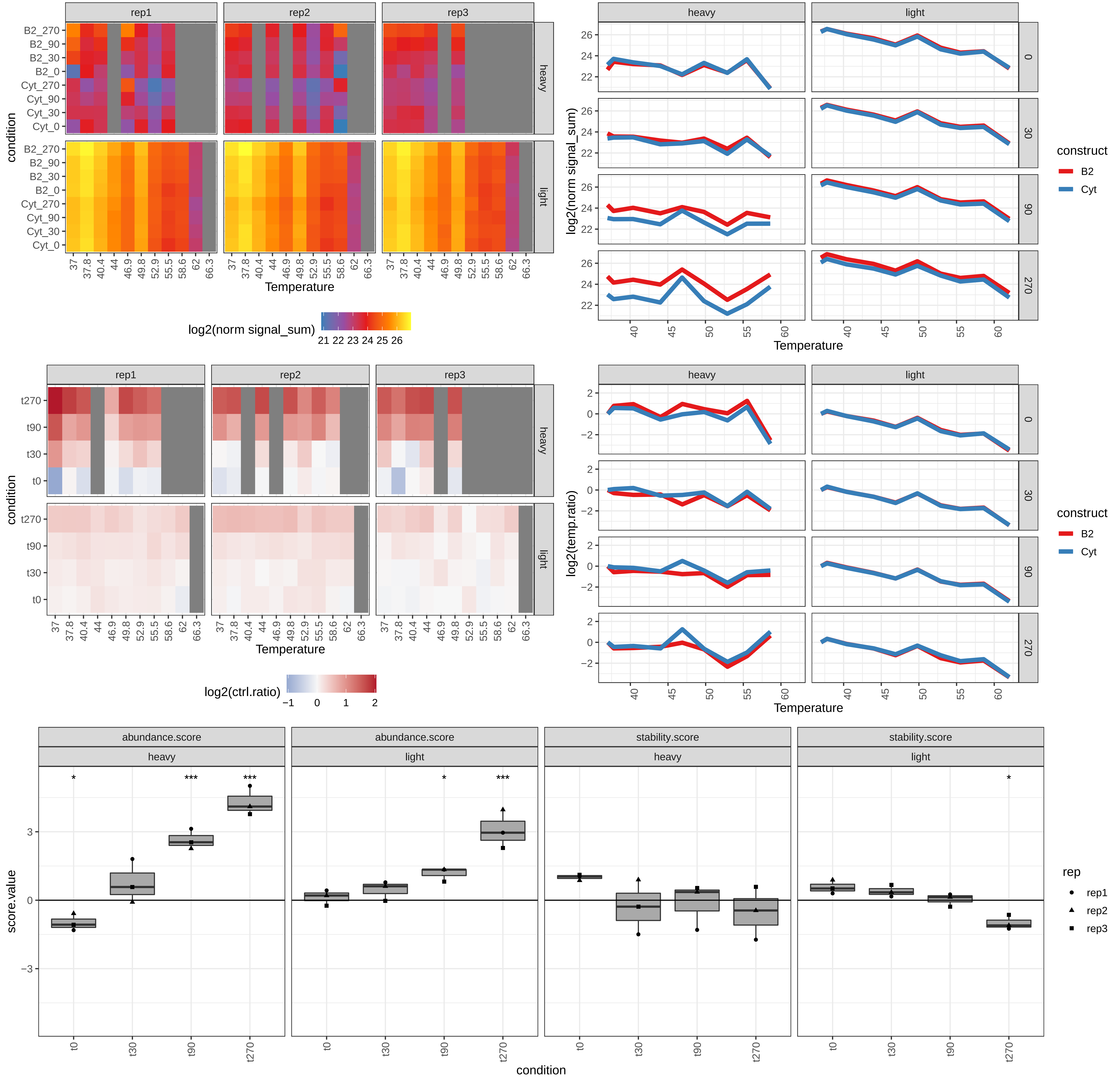

Q03103 − Endoplasmic oxidoreductin−1 OS=Saccharomyces cerevisiae (strain ATCC 204508 / S288c) GN=ERO1 PE=1 SV=1

ERP1 − average.top3: 7.25679351851852 − max.qupm: 7

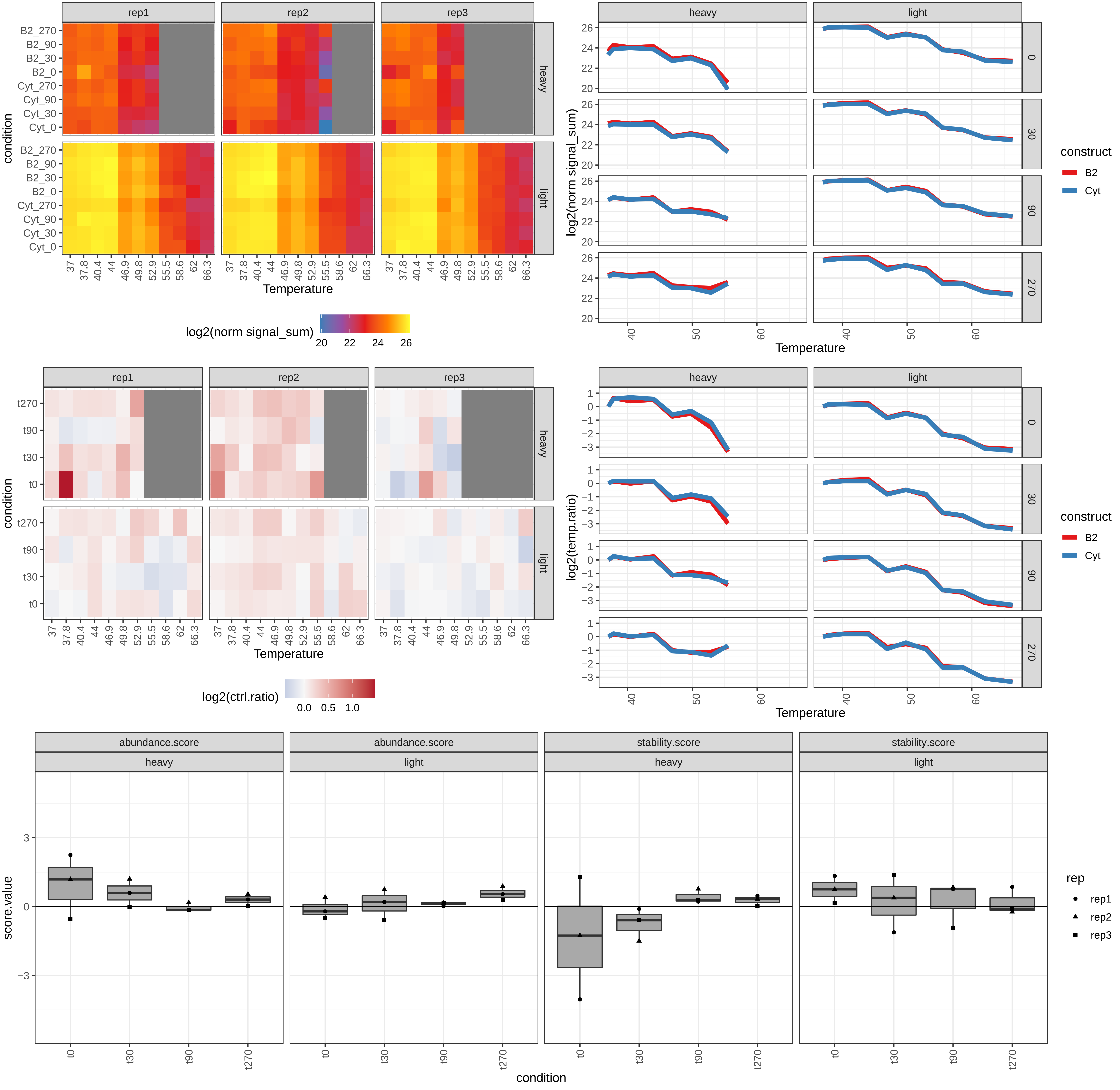

Q05359 − Protein ERP1 OS=Saccharomyces cerevisiae (strain ATCC 204508 / S288c) GN=ERP1 PE=1 SV=1

ERP3 − average.top3: 6.8584215 − max.qupm: 5

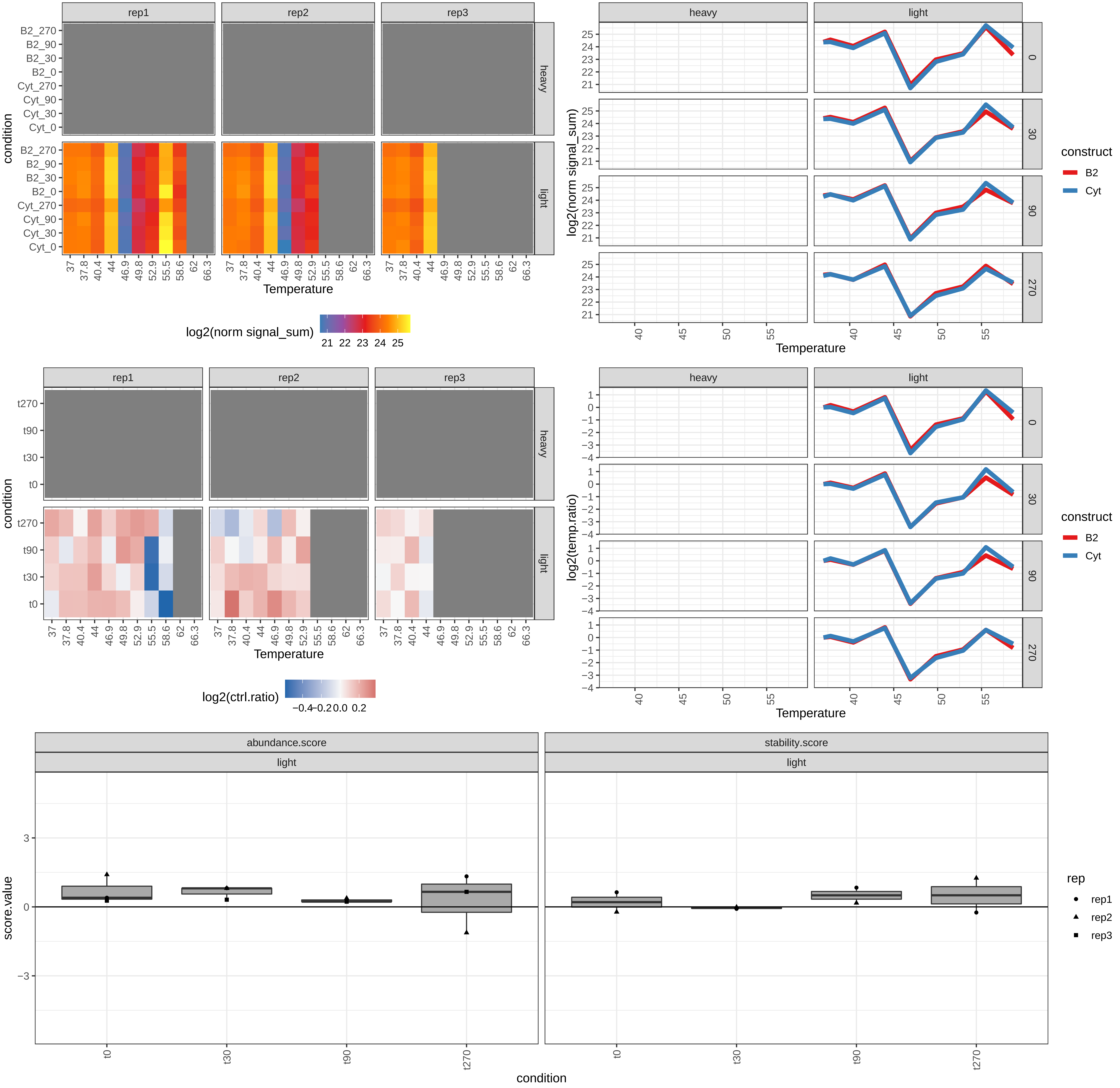

Q12403 − Protein ERP3 OS=Saccharomyces cerevisiae (strain ATCC 204508 / S288c) GN=ERP3 PE=1 SV=1

ERP4 − average.top3: 6.93765764705882 − max.qupm: 4

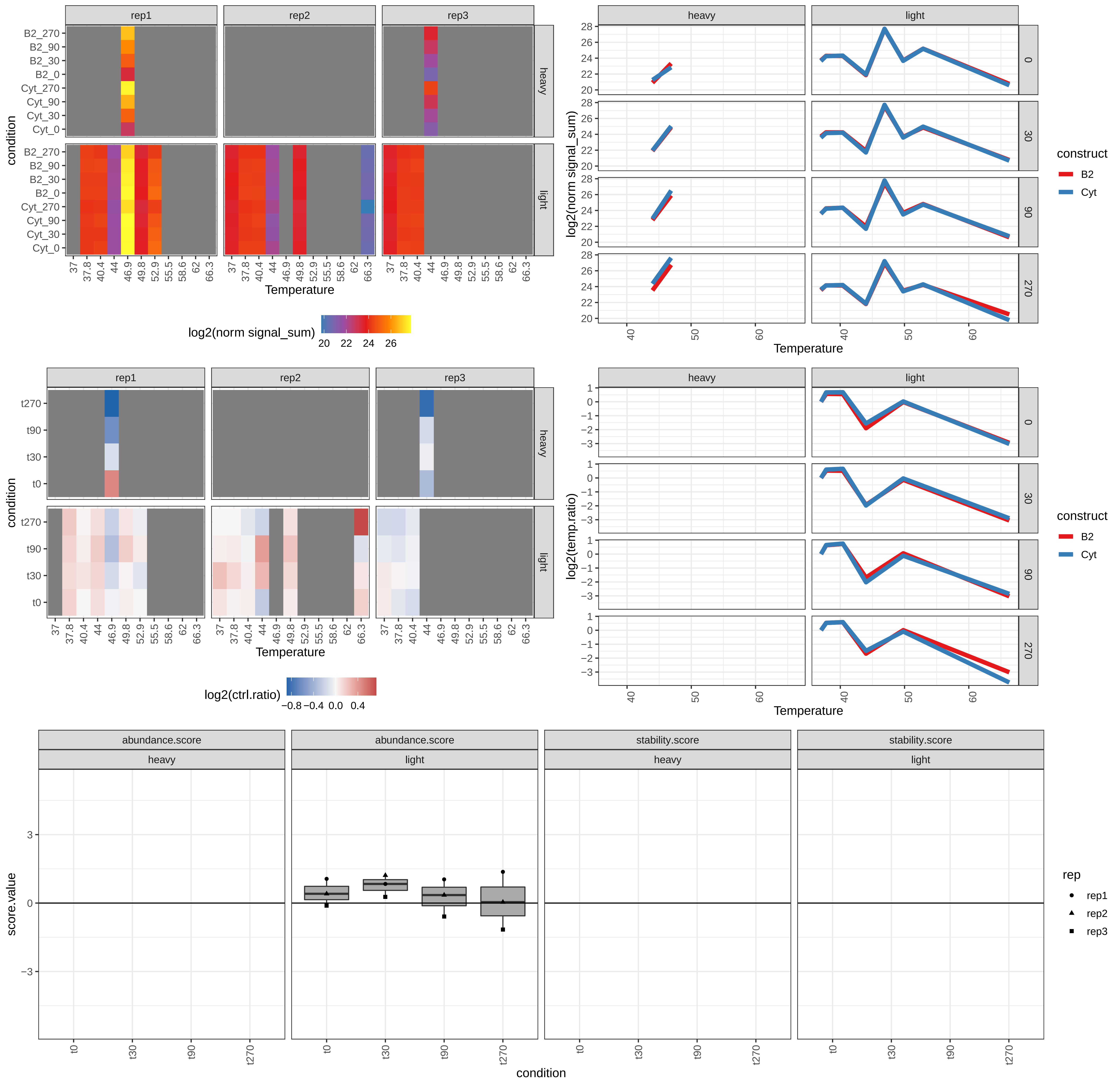

Q12450 − Protein ERP4 OS=Saccharomyces cerevisiae (strain ATCC 204508 / S288c) GN=ERP4 PE=1 SV=1

ERP5 − average.top3: 6.82088384615385 − max.qupm: 4

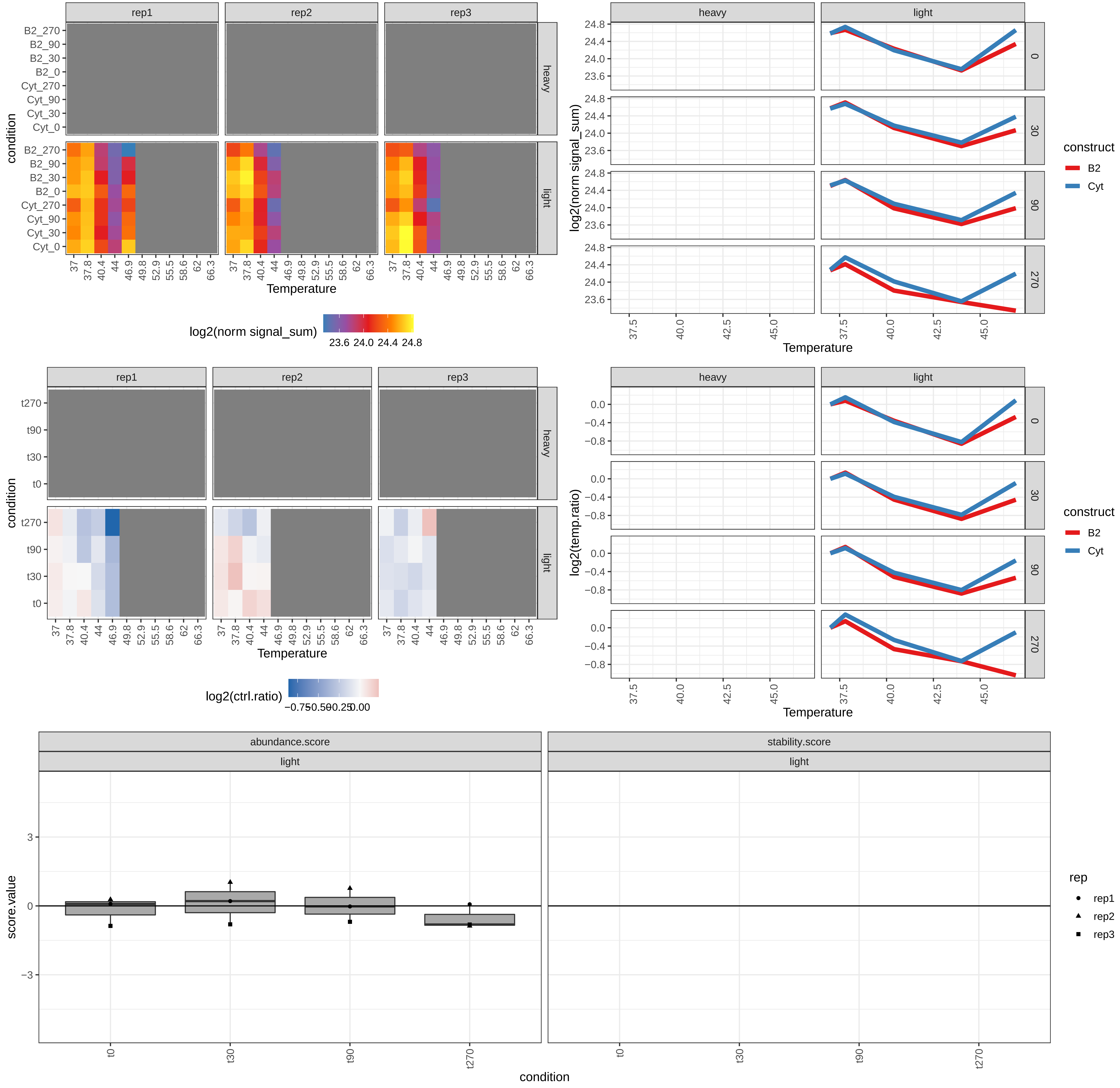

P38819 − Protein ERP5 OS=Saccharomyces cerevisiae (strain ATCC 204508 / S288c) GN=ERP5 PE=1 SV=1

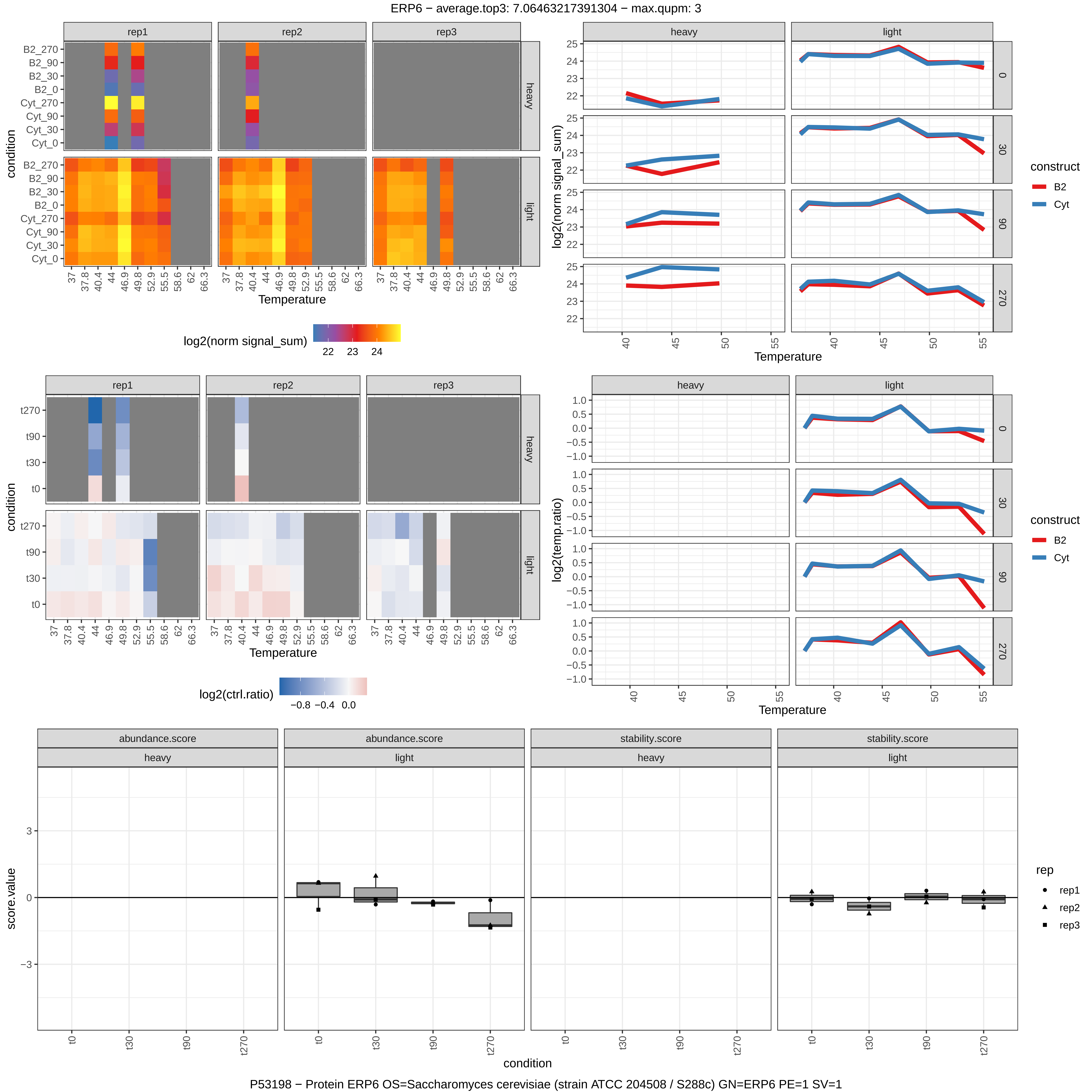

ERP6 − average.top3: 7.06463217391304 − max.qupm: 3
rep1
rep2
rep3
heavy
light
condition
B2_270
B2_90
B2_30
B2_0
Cyt_270
Cyt_90
Cyt_30
Cyt_0
Temperature
log2(norm signal_sum)
log2(ctrl.ratio)
log2(temp.ratio)
construct
B2
Cyt
t270
t90
t30
t0
abundance.score
stability.score
score.value
rep
rep1
rep2
rep3
P53198 − Protein ERP6 OS=Saccharomyces cerevisiae (strain ATCC 204508 / S288c) GN=ERP6 PE=1 SV=1

ERV1 − average.top3: 7.03244857142857 − max.qupm: 5

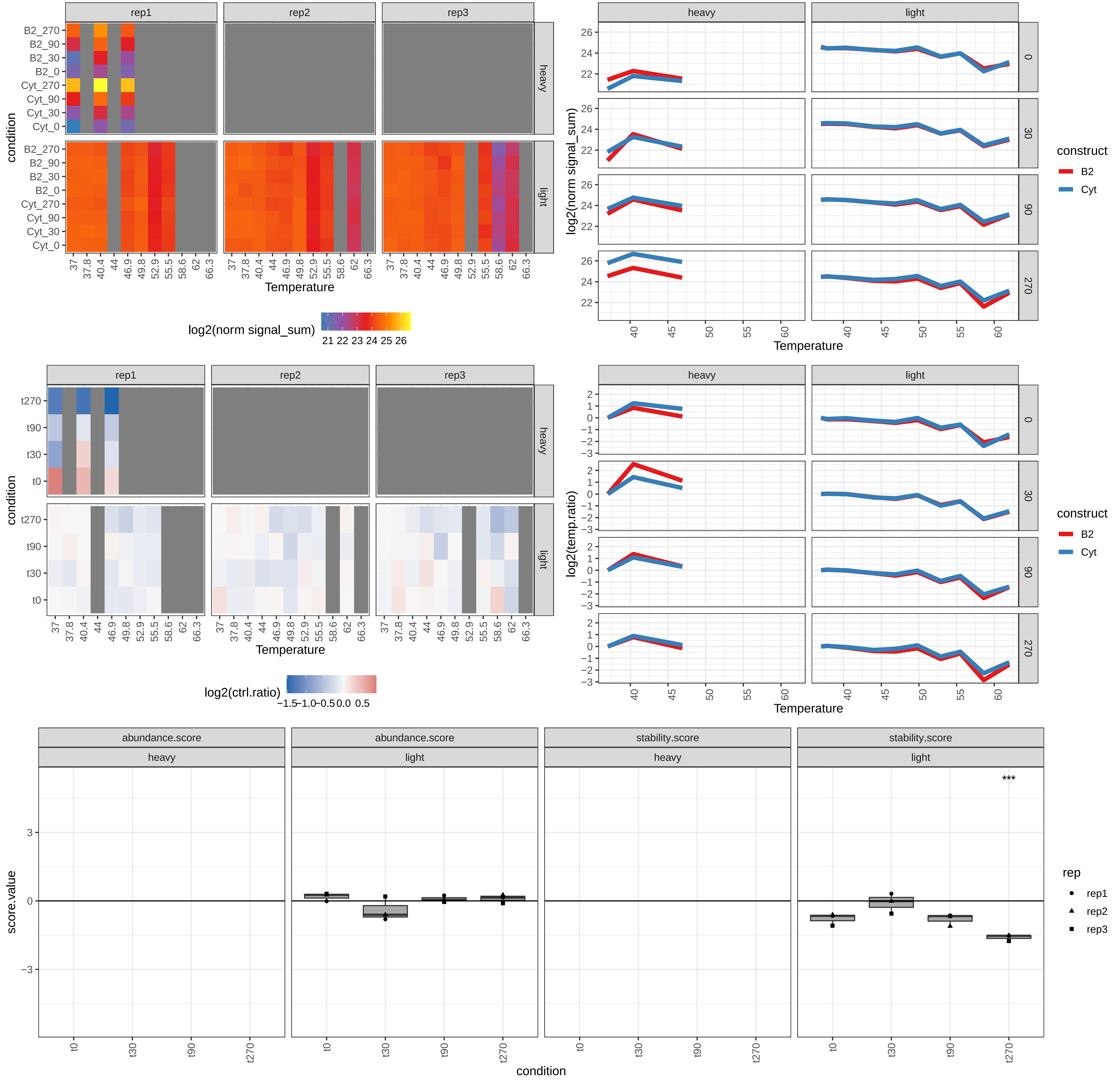

P27882 − Mitochondrial FAD−linked sulfhydryl oxidase ERV1 OS=Saccharomyces cerevisiae (strain ATCC 204508 / S288c) GN=ERV1 PE=1 SV=2

ERV25 − average.top3: 7.34470468085106 − max.qupm: 6

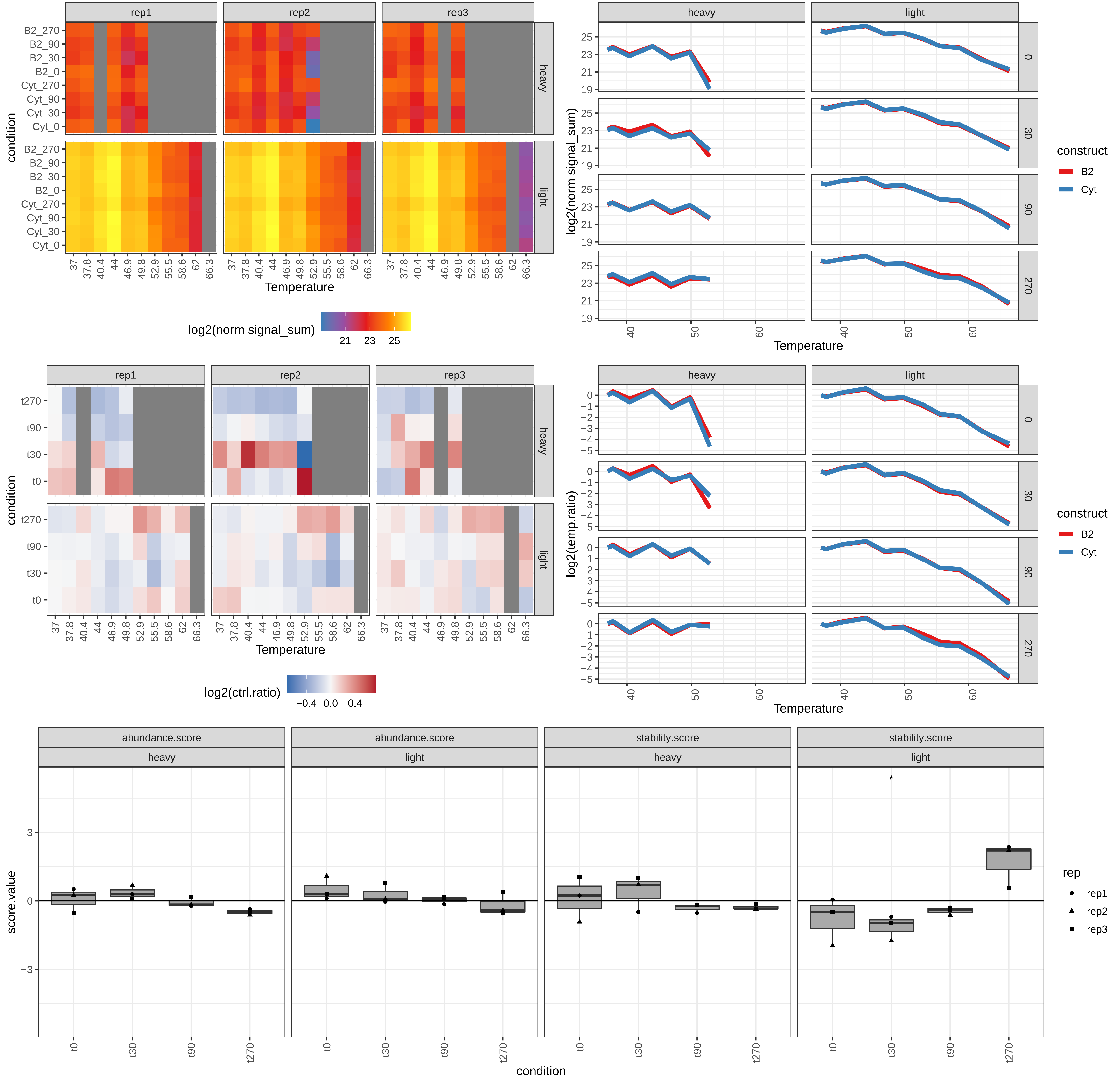

P54837 − Endoplasmic reticulum vesicle protein 25 OS=Saccharomyces cerevisiae (strain ATCC 204508 / S288c) GN=ERV25 PE=1 SV=1

ERV29 − average.top3: 7.06218396825397 − max.qupm: 4

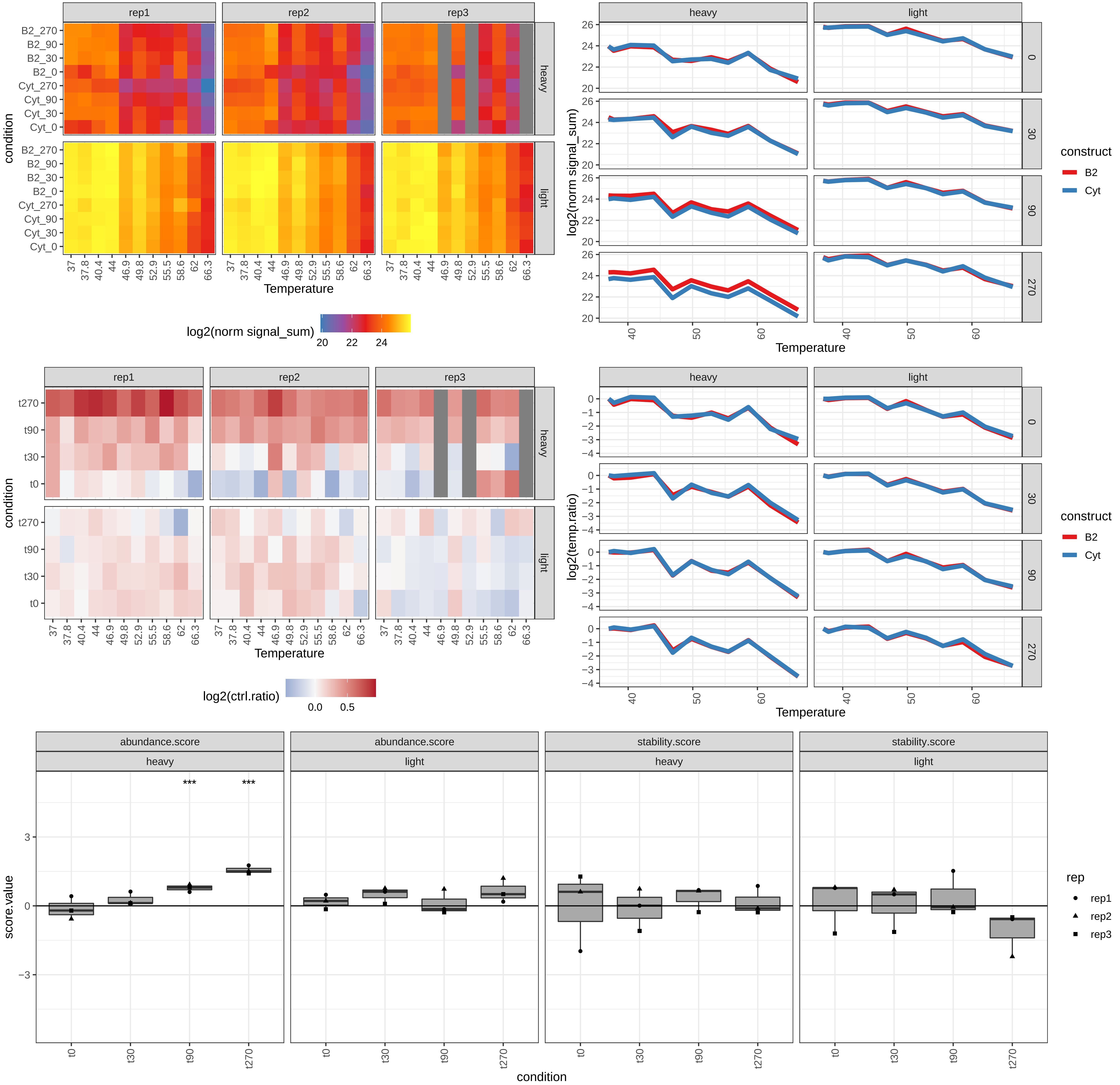

P53337 − ER−derived vesicles protein ERV29 OS=Saccharomyces cerevisiae (strain ATCC 204508 / S288c) GN=ERV29 PE=1 SV=1

ERV41 − average.top3: 6.68248772727273 − max.qupm: 6

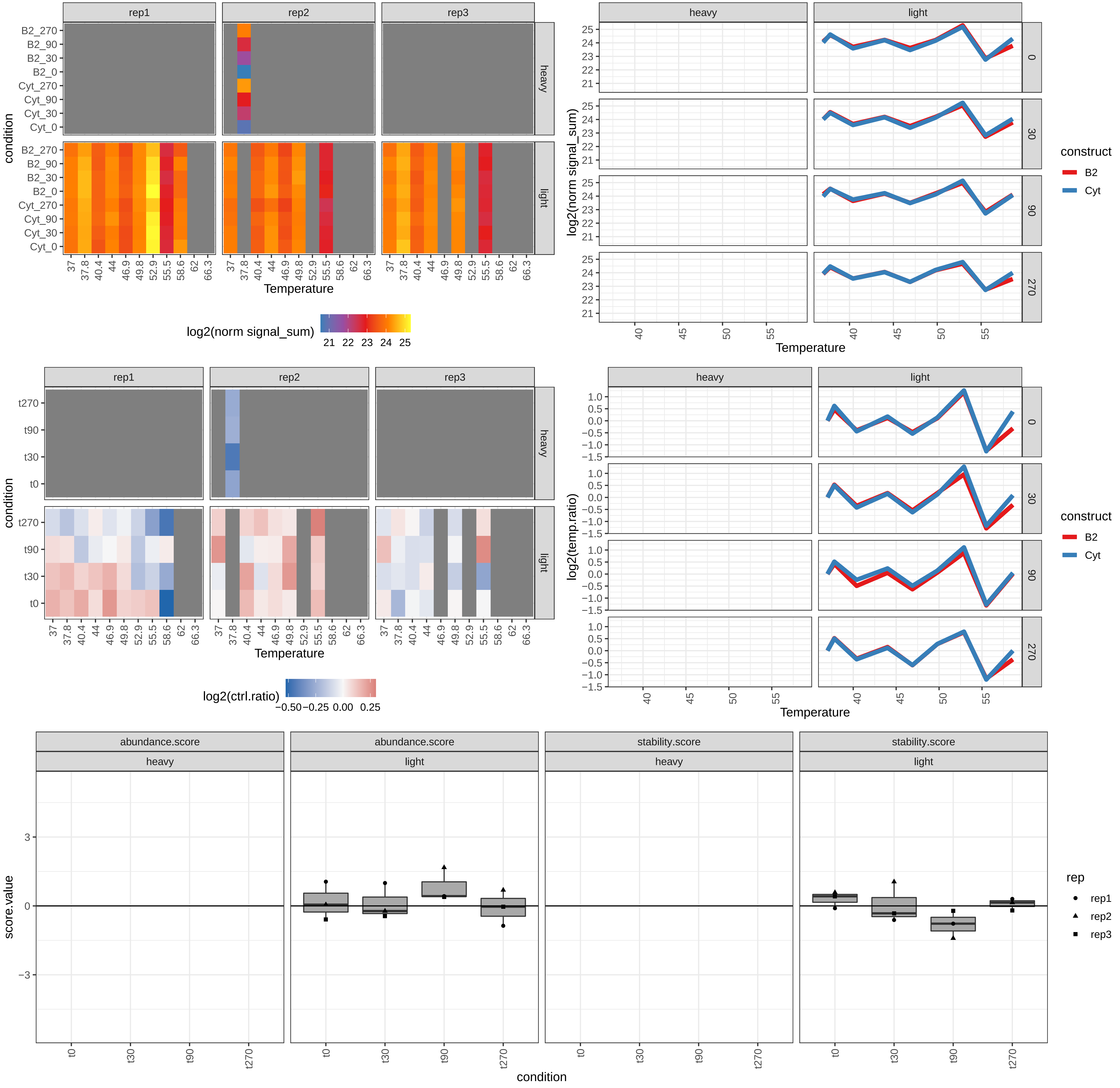

Q04651 − ER−derived vesicles protein ERV41 OS=Saccharomyces cerevisiae (strain ATCC 204508 / S288c) GN=ERV41 PE=1 SV=1

ERV46 − average.top3: 7.15973447368421 − max.qupm: 7

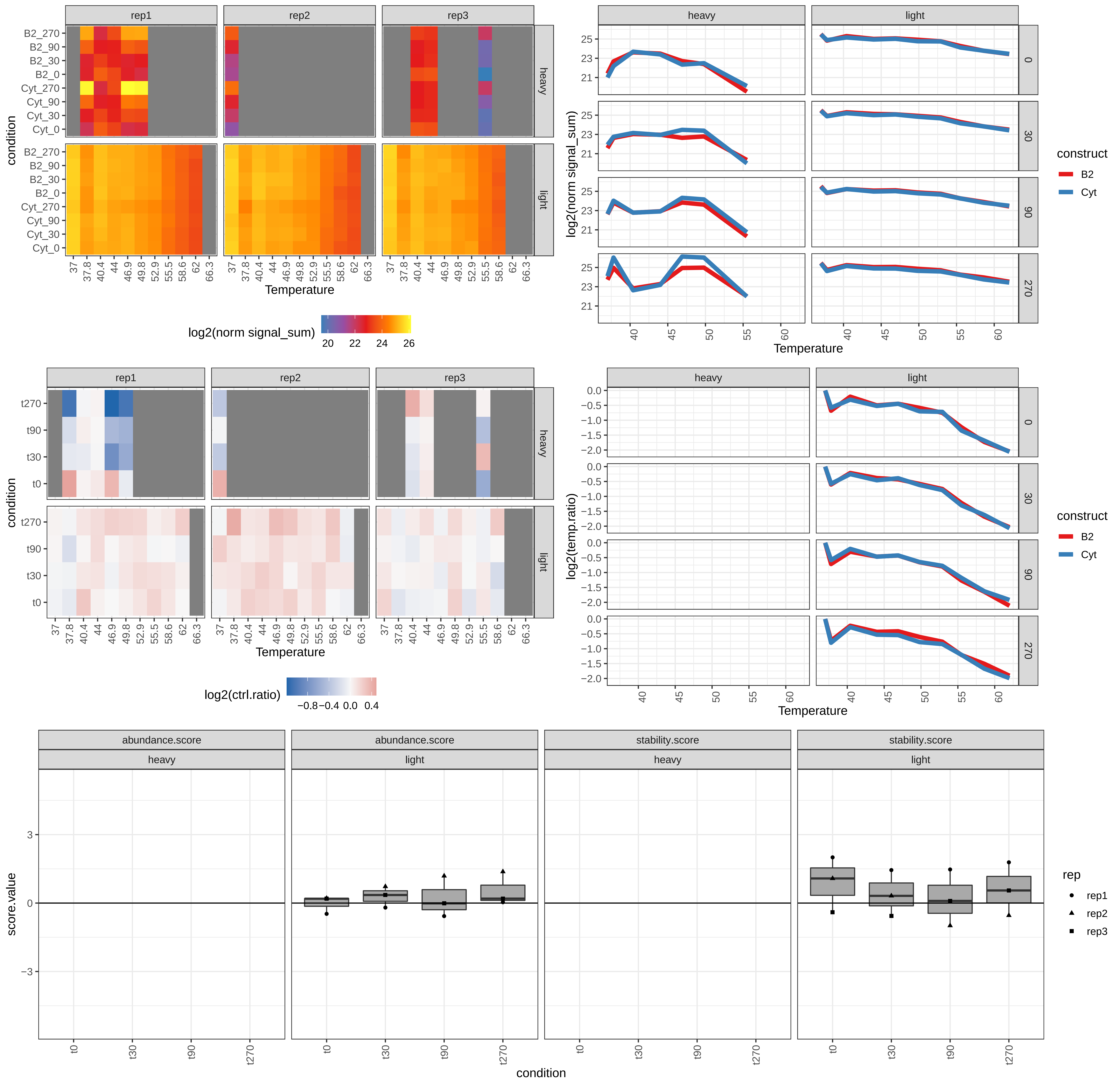

P39727 − ER−derived vesicles protein ERV46 OS=Saccharomyces cerevisiae (strain ATCC 204508 / S288c) GN=ERV46 PE=1 SV=2

ESBP6 − average.top3: 6.58235928571429 − max.qupm: 3

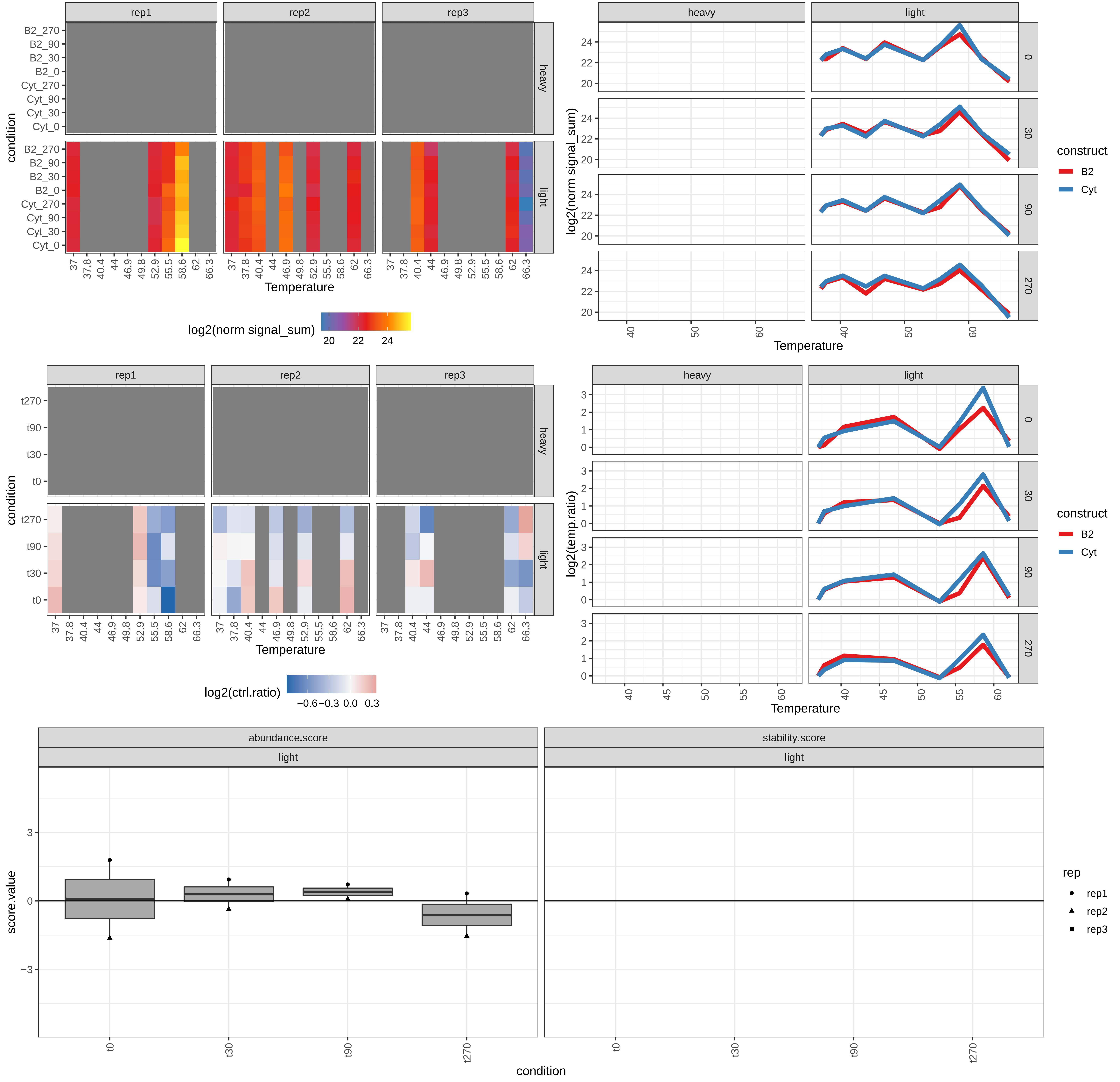

P53918 − Uncharacterized transporter ESBP6 OS=Saccharomyces cerevisiae (strain ATCC 204508 / S288c) GN=ESBP6 PE=1 SV=1

ESF1 − average.top3: 6.59994456521739 − max.qupm: 8

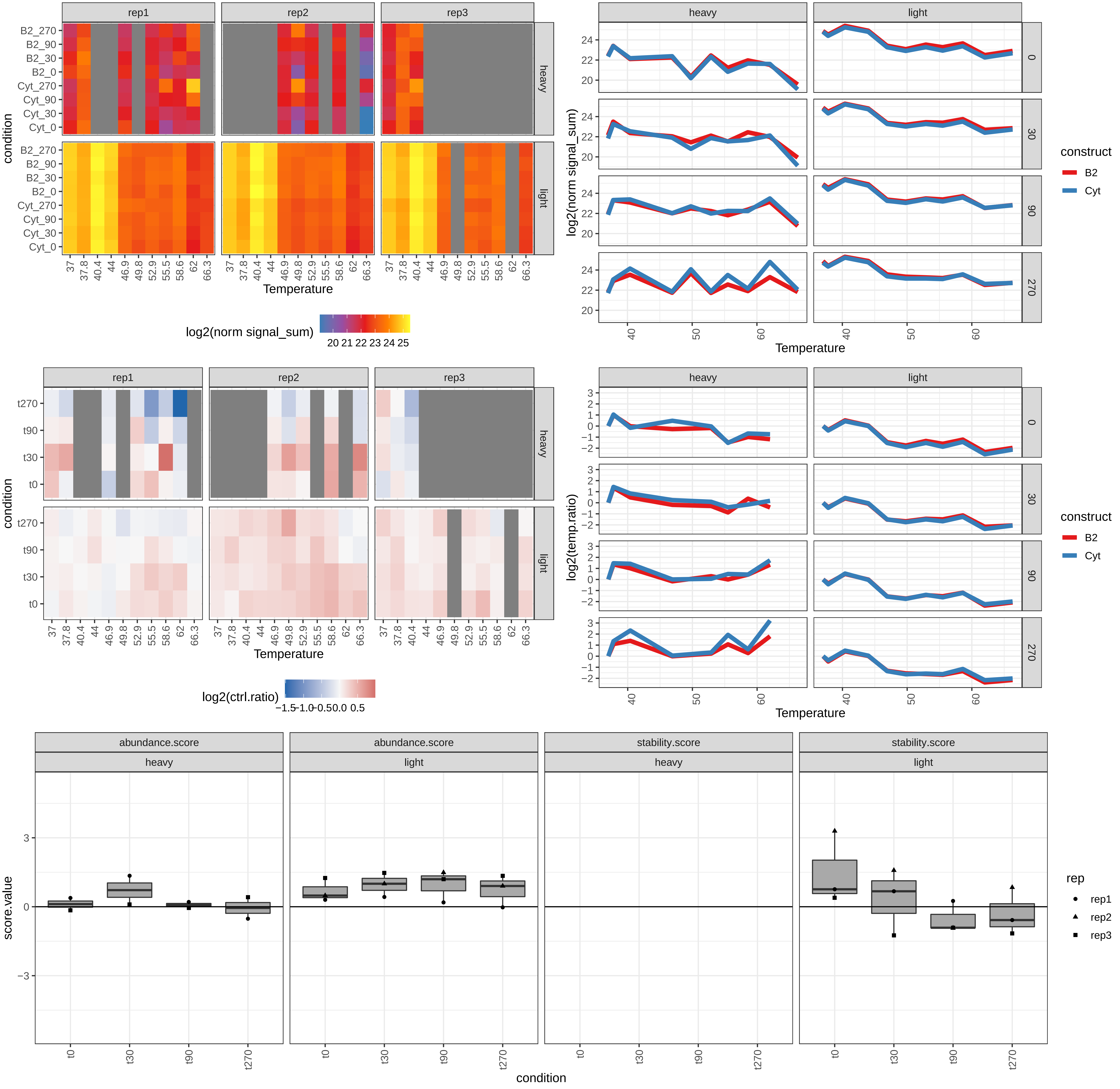

Q06344 − Pre−rRNA−processing protein ESF1 OS=Saccharomyces cerevisiae (strain ATCC 204508 / S288c) GN=ESF1 PE=1 SV=1

ESF2 − average.top3: 6.55867888888889 − max.qupm: 5

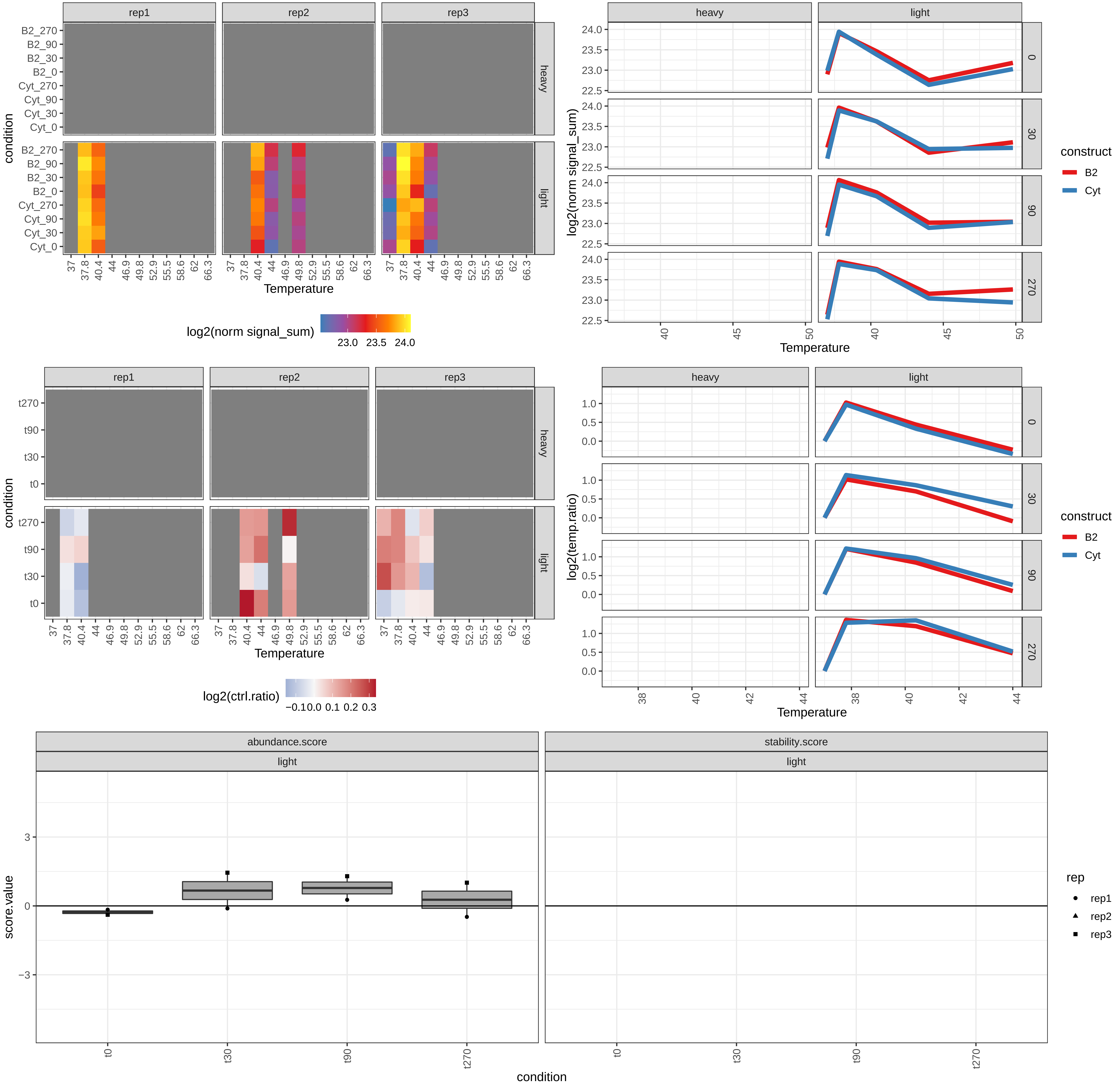

P53743 − Pre−rRNA−processing protein ESF2 OS=Saccharomyces cerevisiae (strain ATCC 204508 / S288c) GN=ESF2 PE=1 SV=1

ESL2 − average.top3: 6.91210866666667 − max.qupm: 6

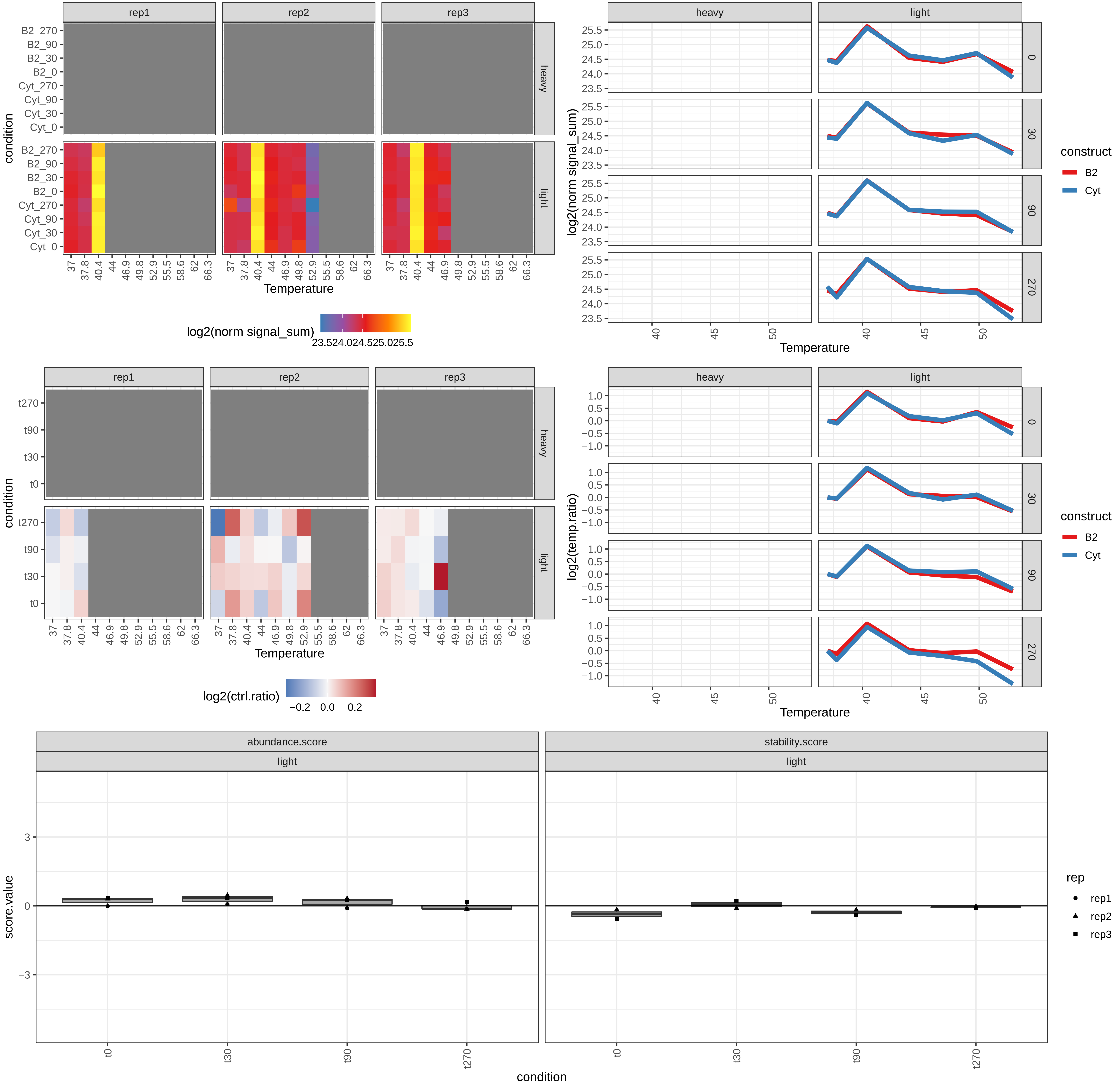

P36168 − EST/SMG−like protein 1 OS=Saccharomyces cerevisiae (strain ATCC 204508 / S288c) GN=ESL2 PE=1 SV=1

ESS1 − average.top3: 6.96449785714286 − max.qupm: 8

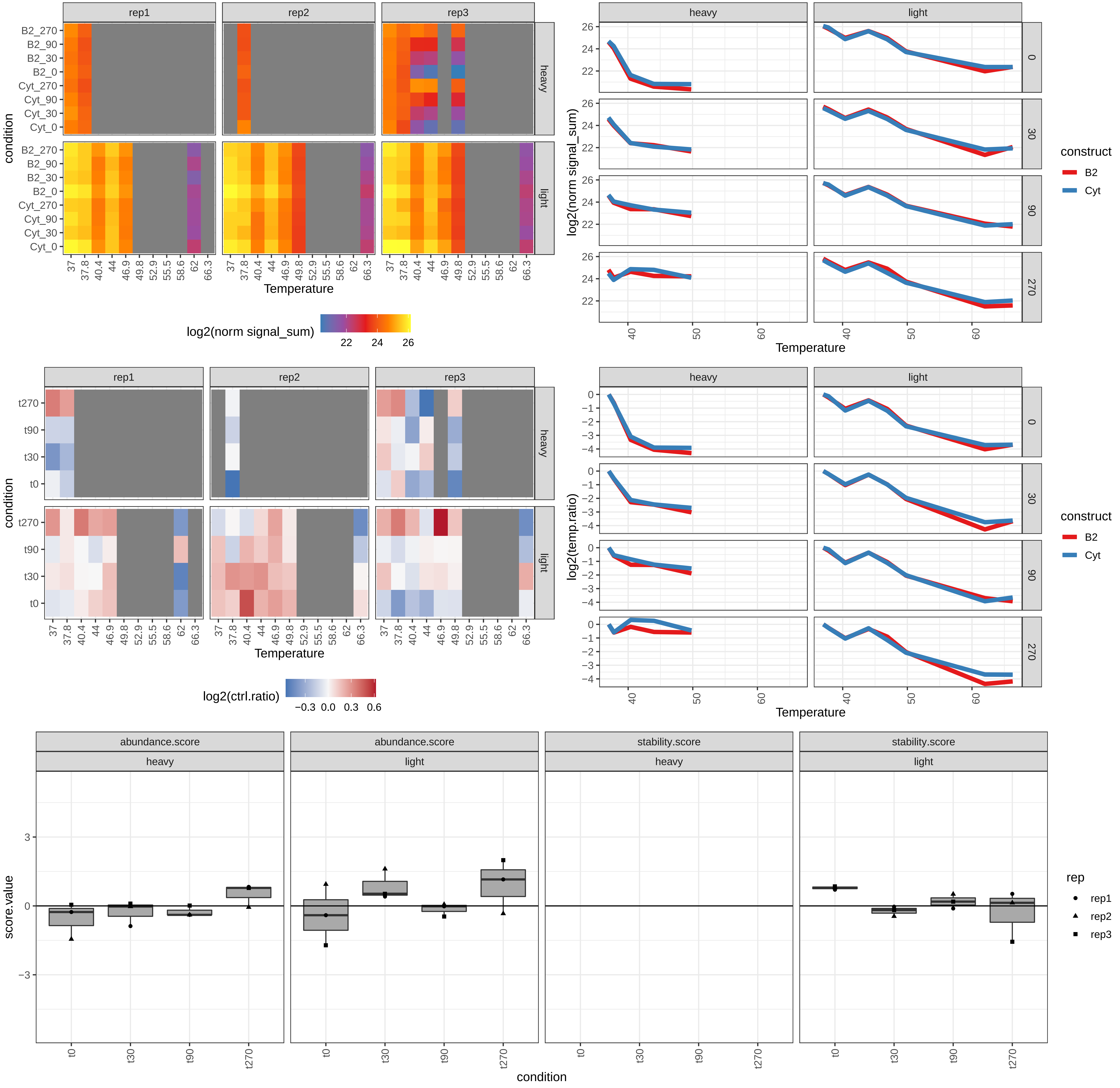

P22696 − Peptidyl−prolyl cis−trans isomerase ESS1 OS=Saccharomyces cerevisiae (strain ATCC 204508 / S288c) GN=ESS1 PE=1 SV=3

ETR1 − average.top3: 7.74765111111111 − max.qupm: 16

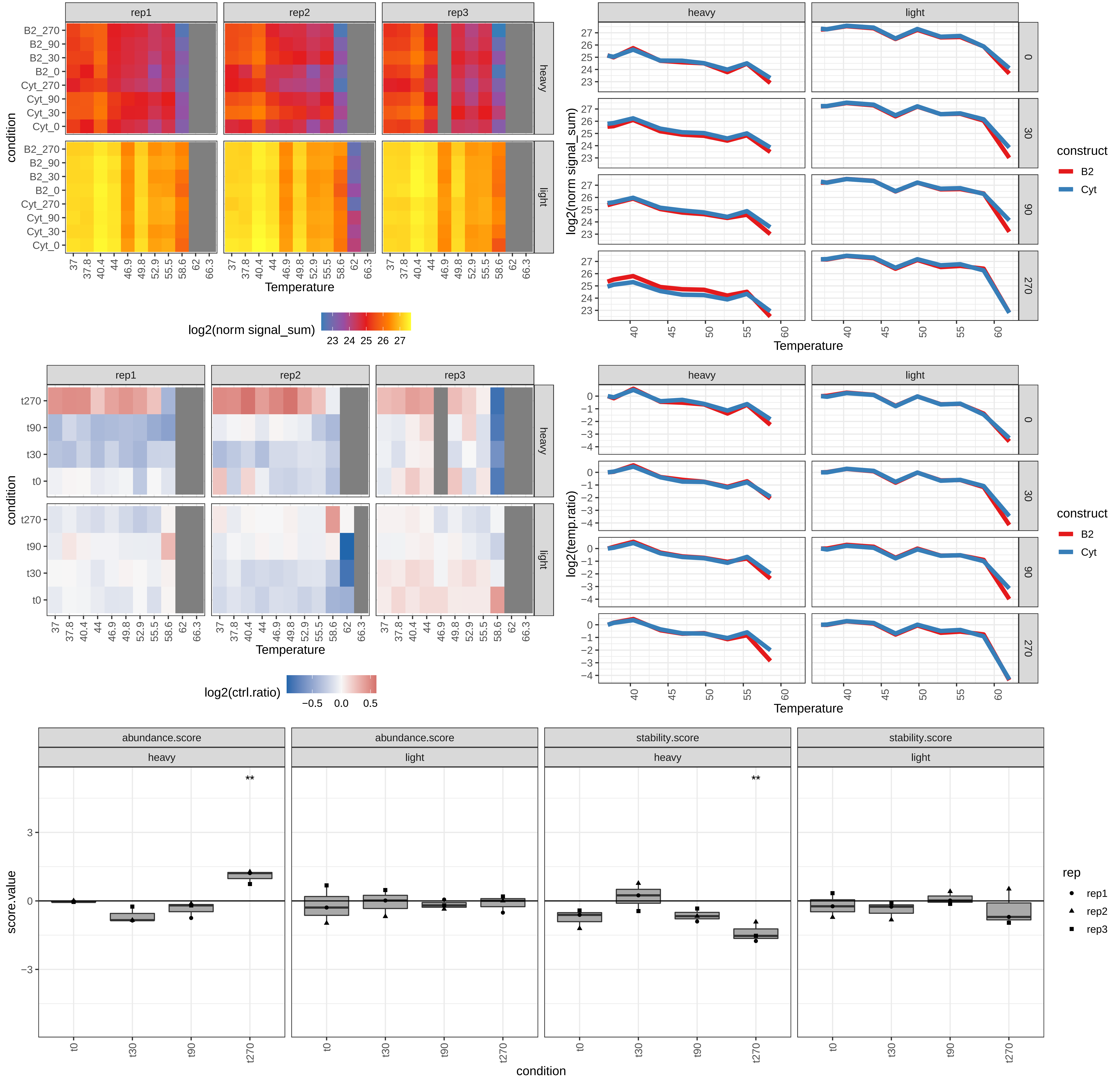

P38071 − Enoyl−[acyl−carrier protein] reductase [NADPH, B−specific], mitochondrial OS=Saccharomyces cerevisiae (strain ATCC 204508 / S288c) GN=ETR1 PE=1 SV=3

ETT1 − average.top3: 6.534316 − max.qupm: 6

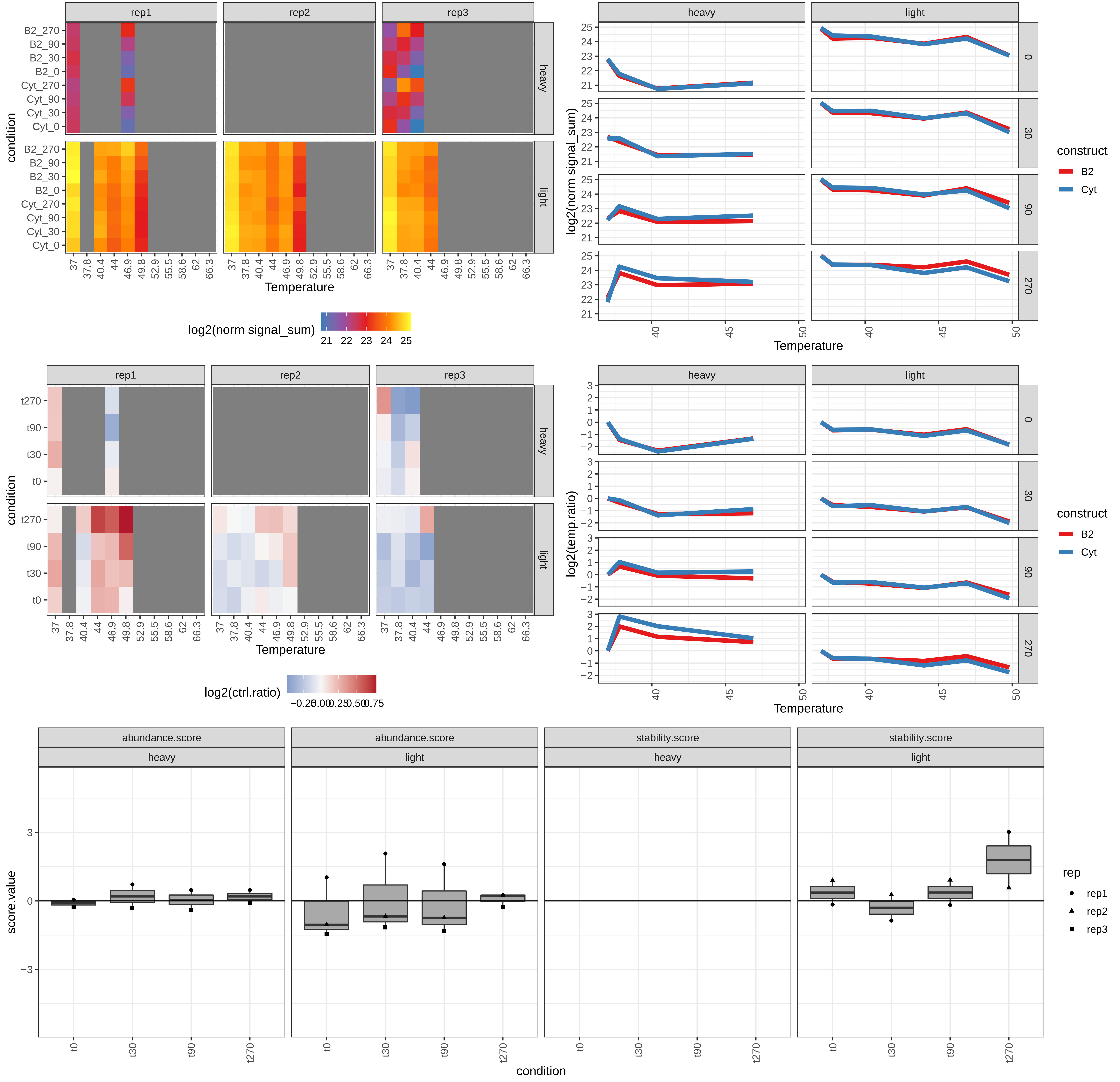

Q08421 − Enhancer of translation termination 1 OS=Saccharomyces cerevisiae (strain ATCC 204508 / S288c) GN=ETT1 PE=1 SV=1

EUG1 − average.top3: 6.55718 − max.qupm: 5

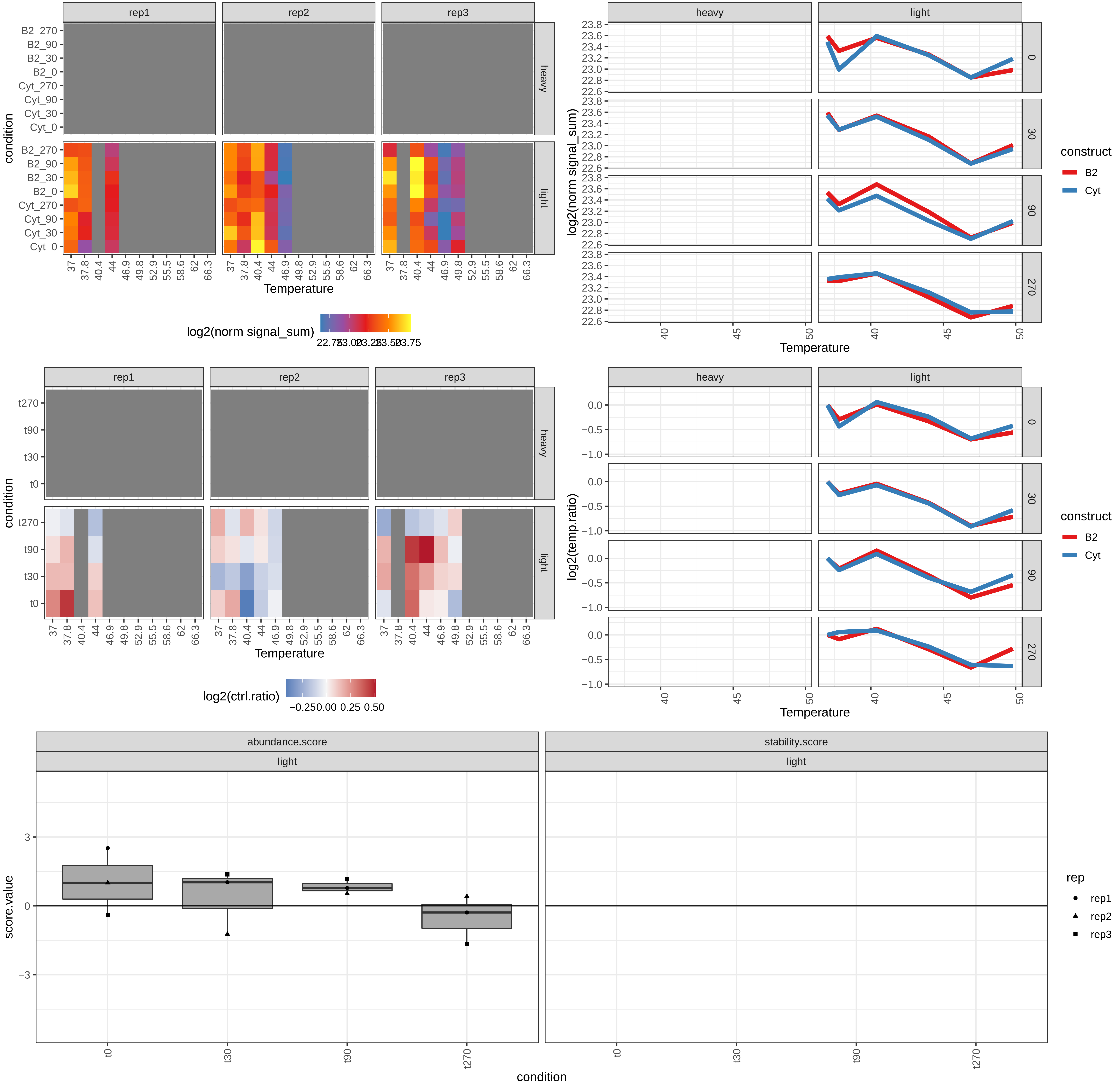

P32474 − Protein disulfide−isomerase EUG1 OS=Saccharomyces cerevisiae (strain ATCC 204508 / S288c) GN=EUG1 PE=1 SV=1

EXG1 − average.top3: 6.7313806 − max.qupm: 7

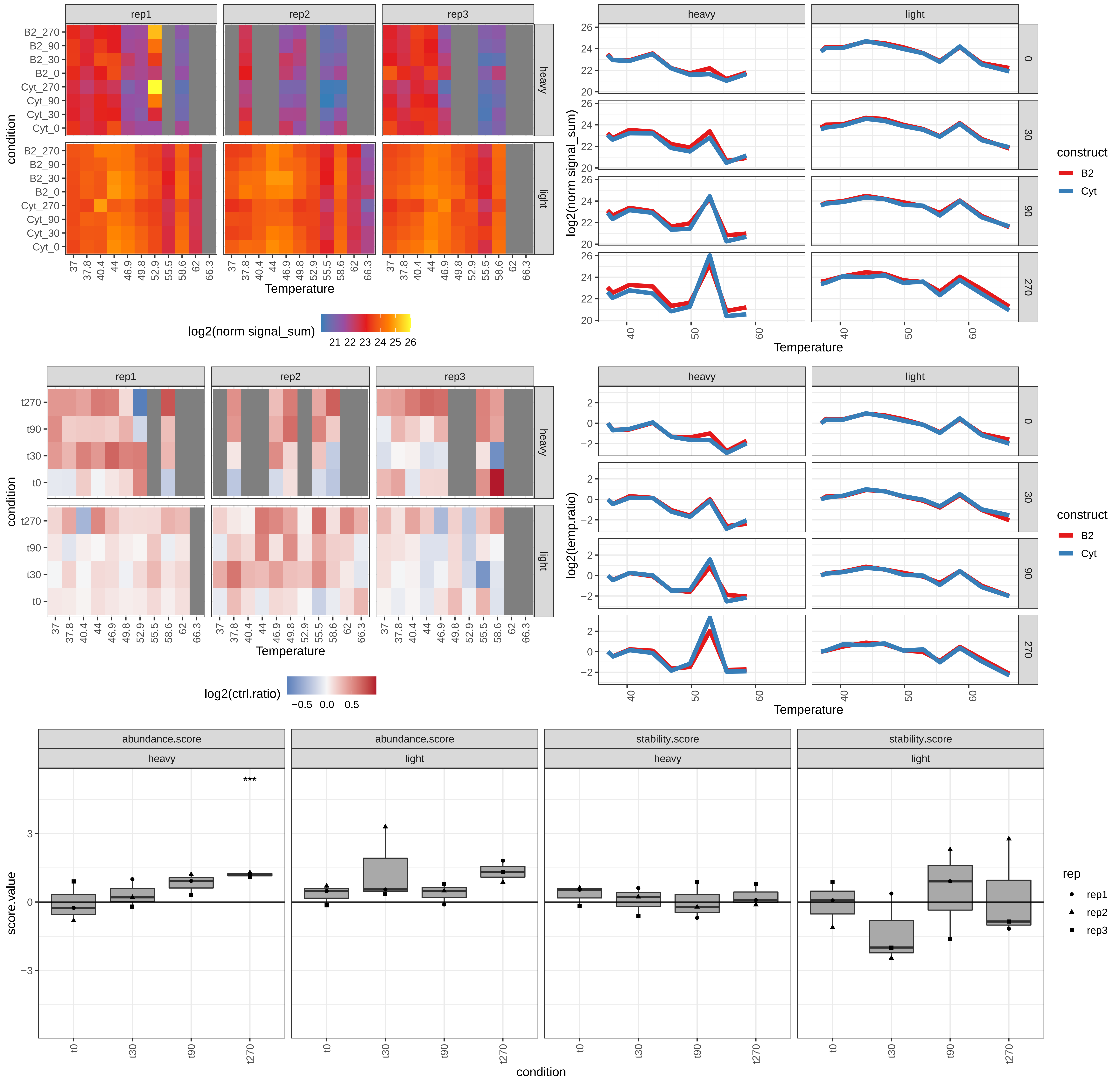

P23776 − Glucan 1,3−beta−glucosidase I/II OS=Saccharomyces cerevisiae (strain ATCC 204508 / S288c) GN=EXG1 PE=1 SV=1

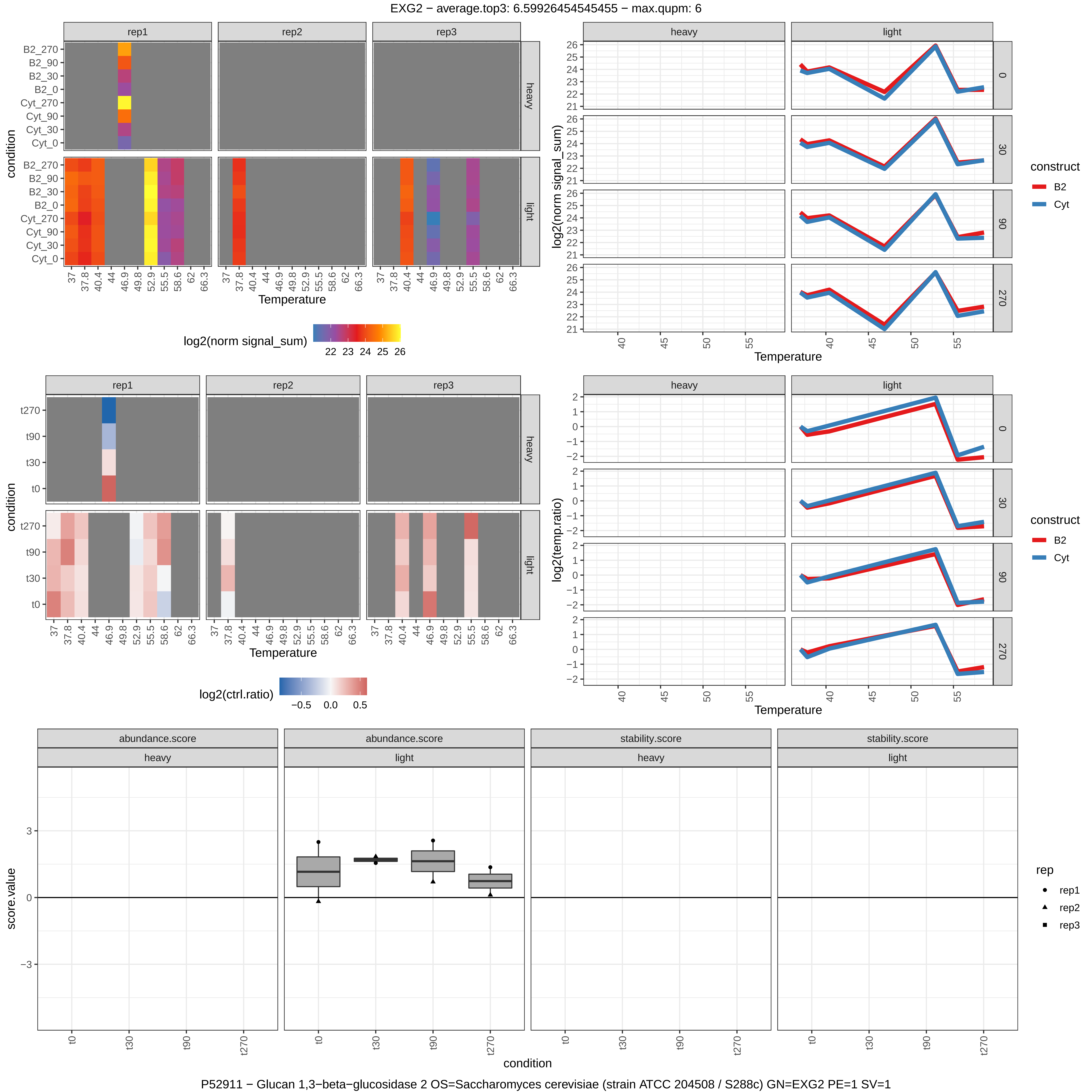
EXG2 − average.top3: 6.59926454545455 − max.qupm: 6
rep1
rep2
rep3
heavy
light
condition
Temperature
log2(norm signal_sum)
log2(ctrl.ratio)
log2(temp.ratio)
construct
B2
Cyt
abundance.score
stability.score
score.value
rep
rep1
rep2
rep3
P52911 − Glucan 1,3−beta−glucosidase 2 OS=Saccharomyces cerevisiae (strain ATCC 204508 / S288c) GN=EXG2 PE=1 SV=1

EXO70 − average.top3: 6.467198 − max.qupm: 6

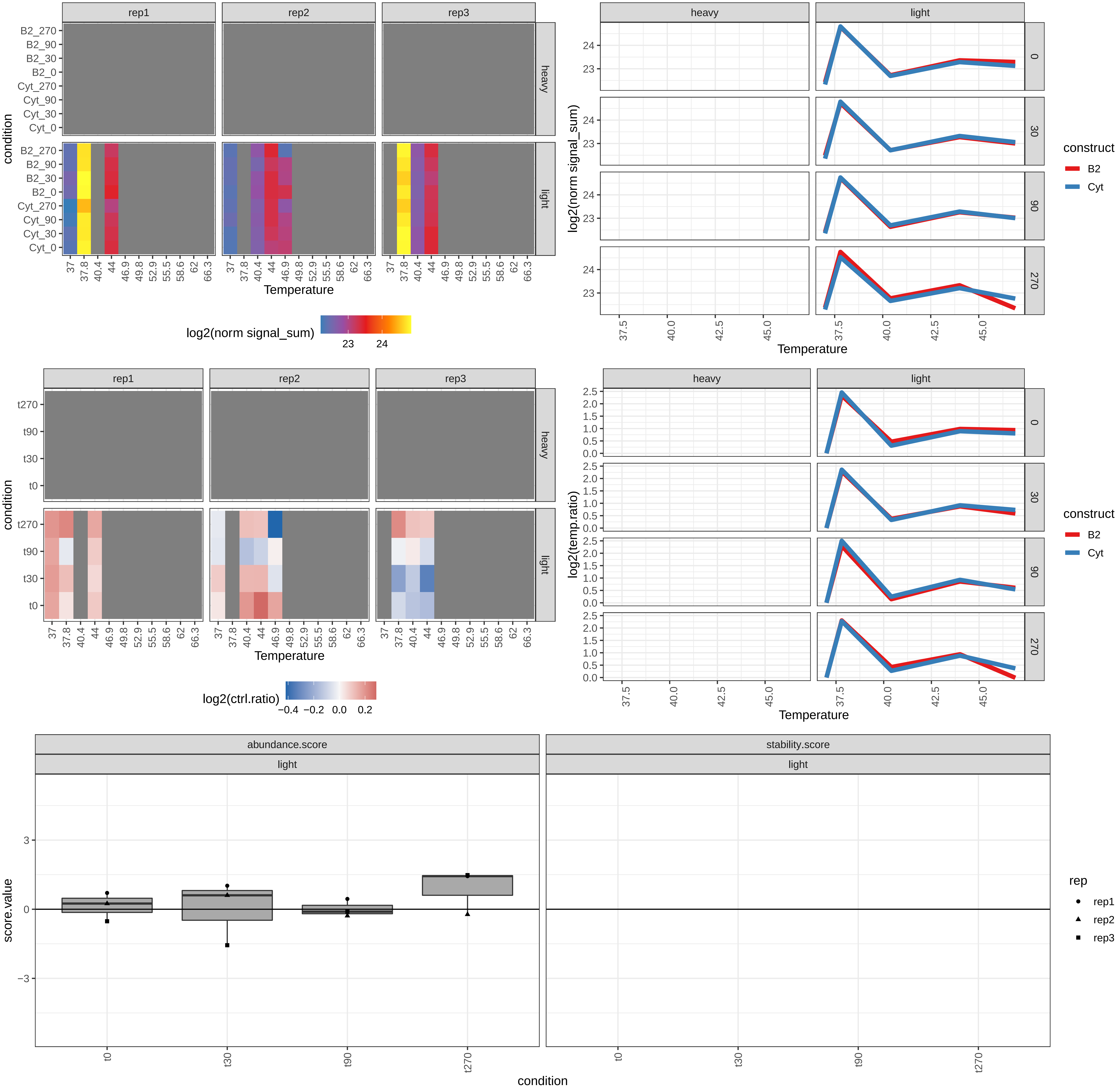

P19658 − Exocyst complex component EXO70 OS=Saccharomyces cerevisiae (strain ATCC 204508 / S288c) GN=EXO70 PE=1 SV=1

EXO84 − average.top3: 6.313634375 − max.qupm: 6

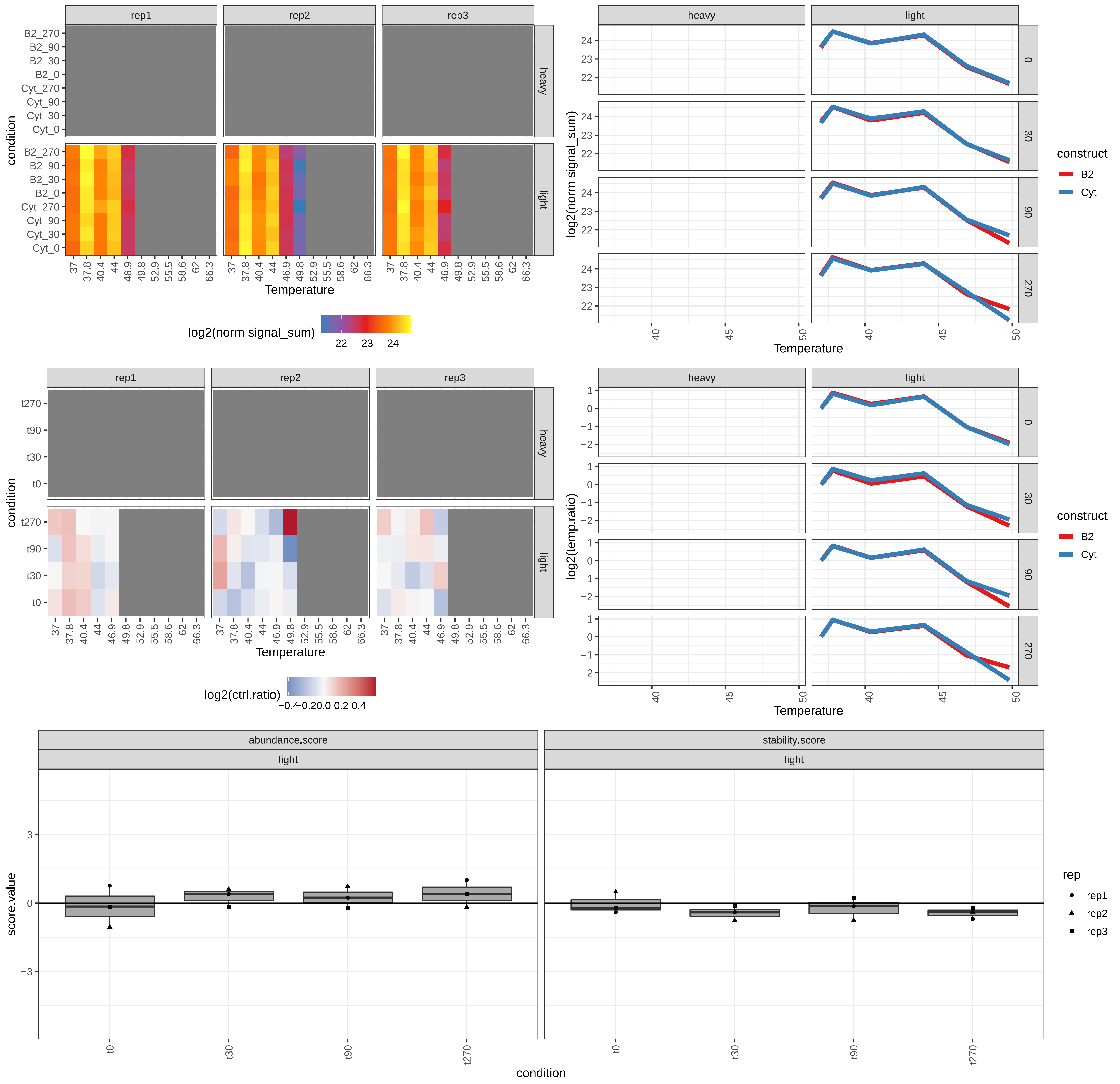

P38261 − Exocyst complex component EXO84 OS=Saccharomyces cerevisiae (strain ATCC 204508 / S288c) GN=EXO84 PE=1 SV=1

FAA1 − average.top3: 7.45229073170732 − max.qupm: 17

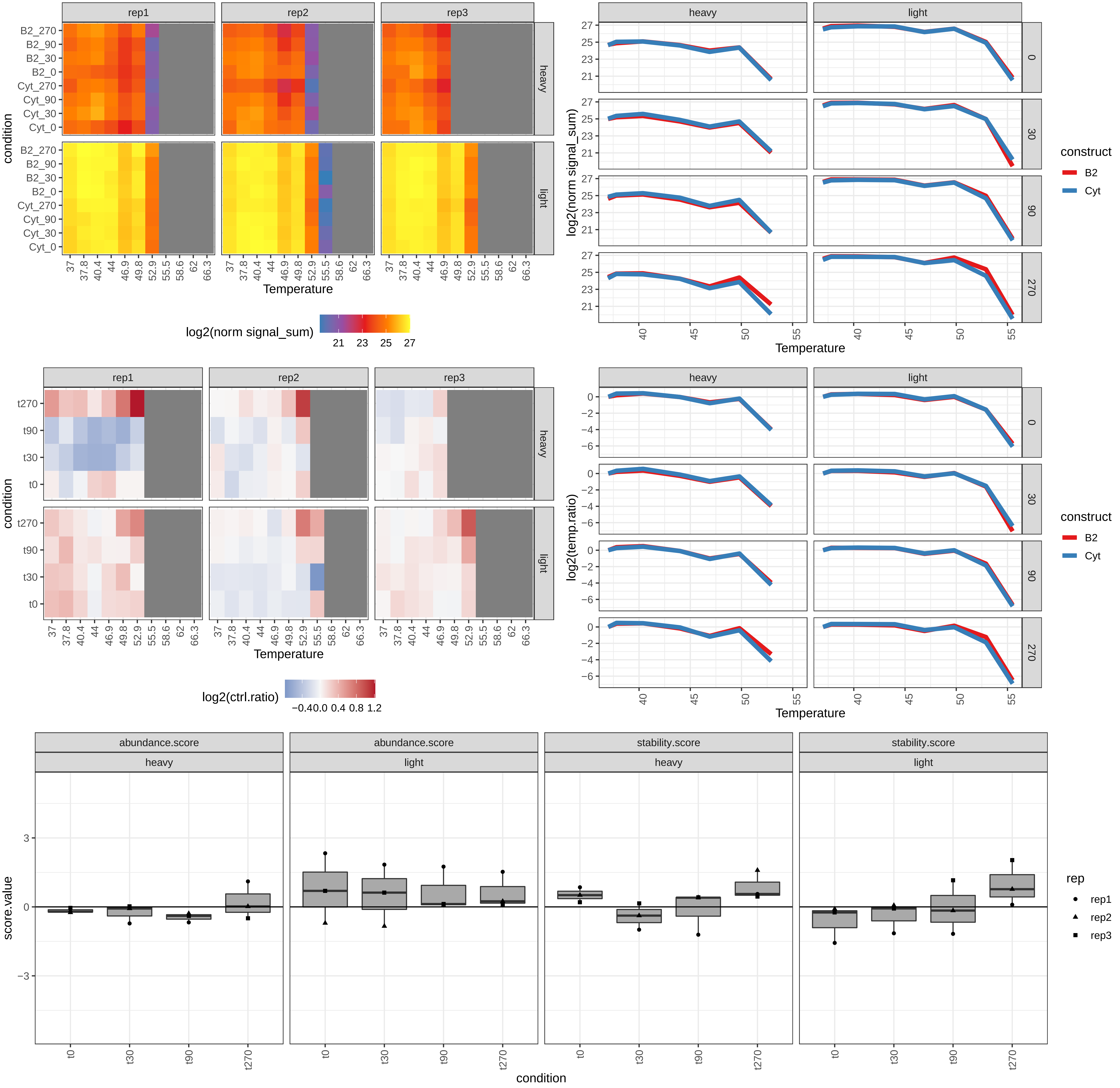

P30624 − Long−chain−fatty−acid−−CoA ligase 1 OS=Saccharomyces cerevisiae (strain ATCC 204508 / S288c) GN=FAA1 PE=1 SV=1

FAA2 − average.top3: 7.25673372093023 − max.qupm: 18

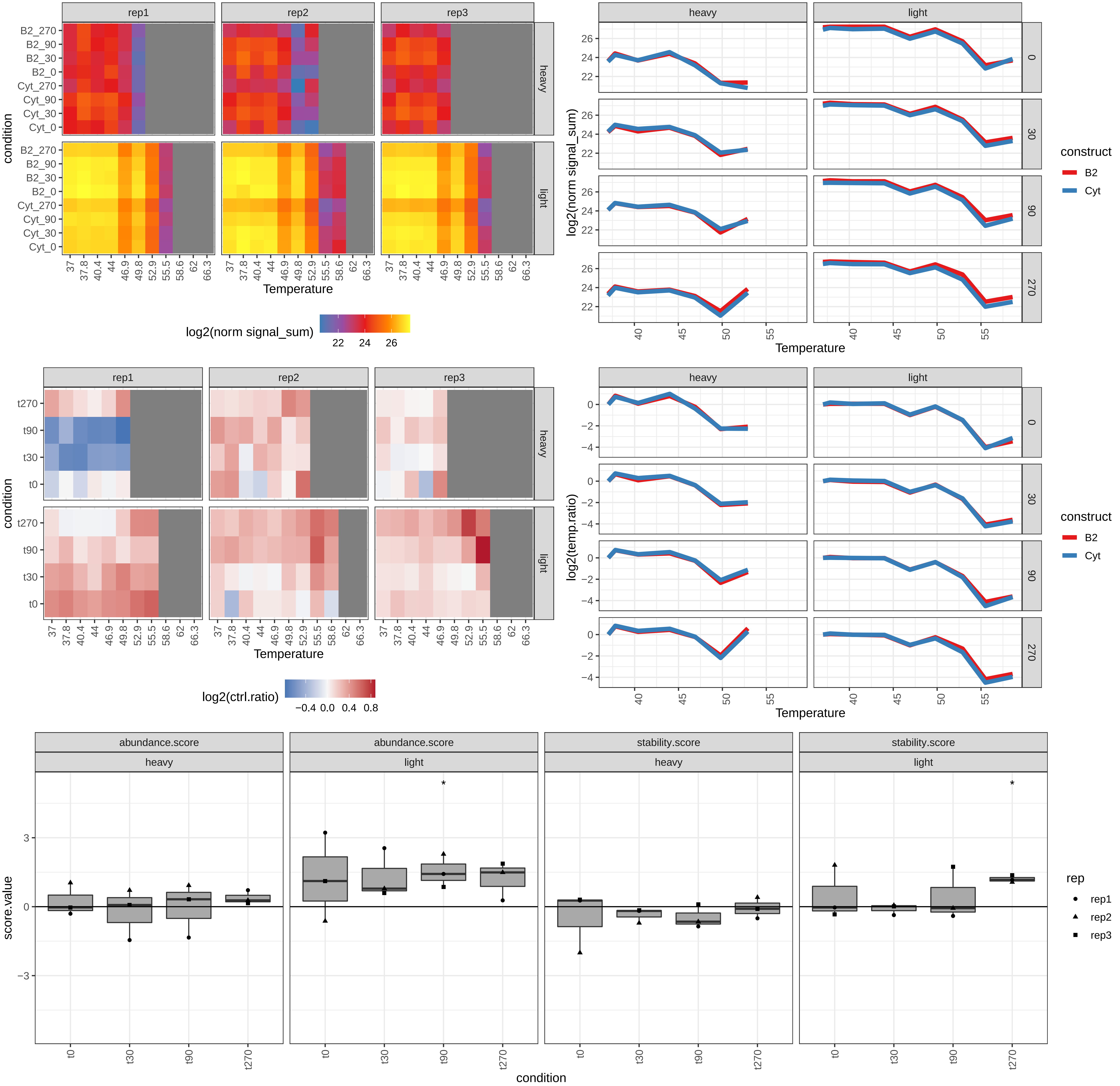

P39518 − Long−chain−fatty−acid−−CoA ligase 2 OS=Saccharomyces cerevisiae (strain ATCC 204508 / S288c) GN=FAA2 PE=1 SV=1

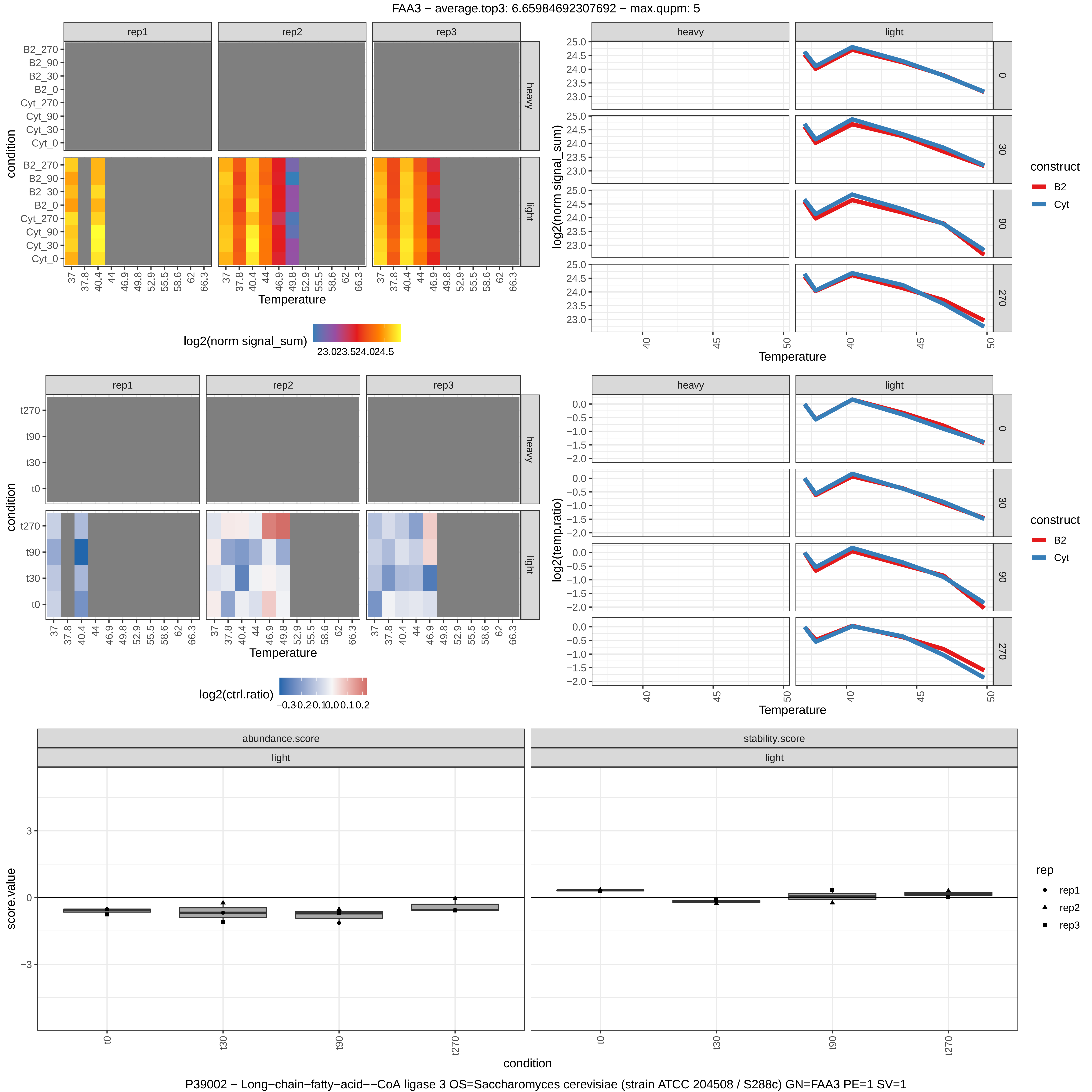

FAA3 − average.top3: 6.65984692307692 − max.qupm: 5
rep1
rep2
rep3
heavy
light
condition
B2_270
B2_90
B2_30
B2_0
Cyt_270
Cyt_90
Cyt_30
Cyt_0
37
37.8
40.4
44
46.9
49.8
52.9
55.5
58.6
62
66.3
Temperature
log2(norm signal_sum)
23.0
23.5
24.0
24.5
log2(norm signal_sum)
25.0
24.5
24.0
23.5
23.0
0
30
90
270
40
45
50
construct
B2
Cyt
t270
t90
t30
t0
log2(ctrl.ratio)
−0.3
−0.2
−0.1
0.0
0.1
0.2
log2(temp.ratio)
0.0
−0.5
−1.0
−1.5
−2.0
abundance.score
stability.score
score.value
3
0
−3
condition
rep
rep1
rep2
rep3
P39002 − Long−chain−fatty−acid−−CoA ligase 3 OS=Saccharomyces cerevisiae (strain ATCC 204508 / S288c) GN=FAA3 PE=1 SV=1

FAA4 − average.top3: 7.02069888888889 − max.qupm: 10

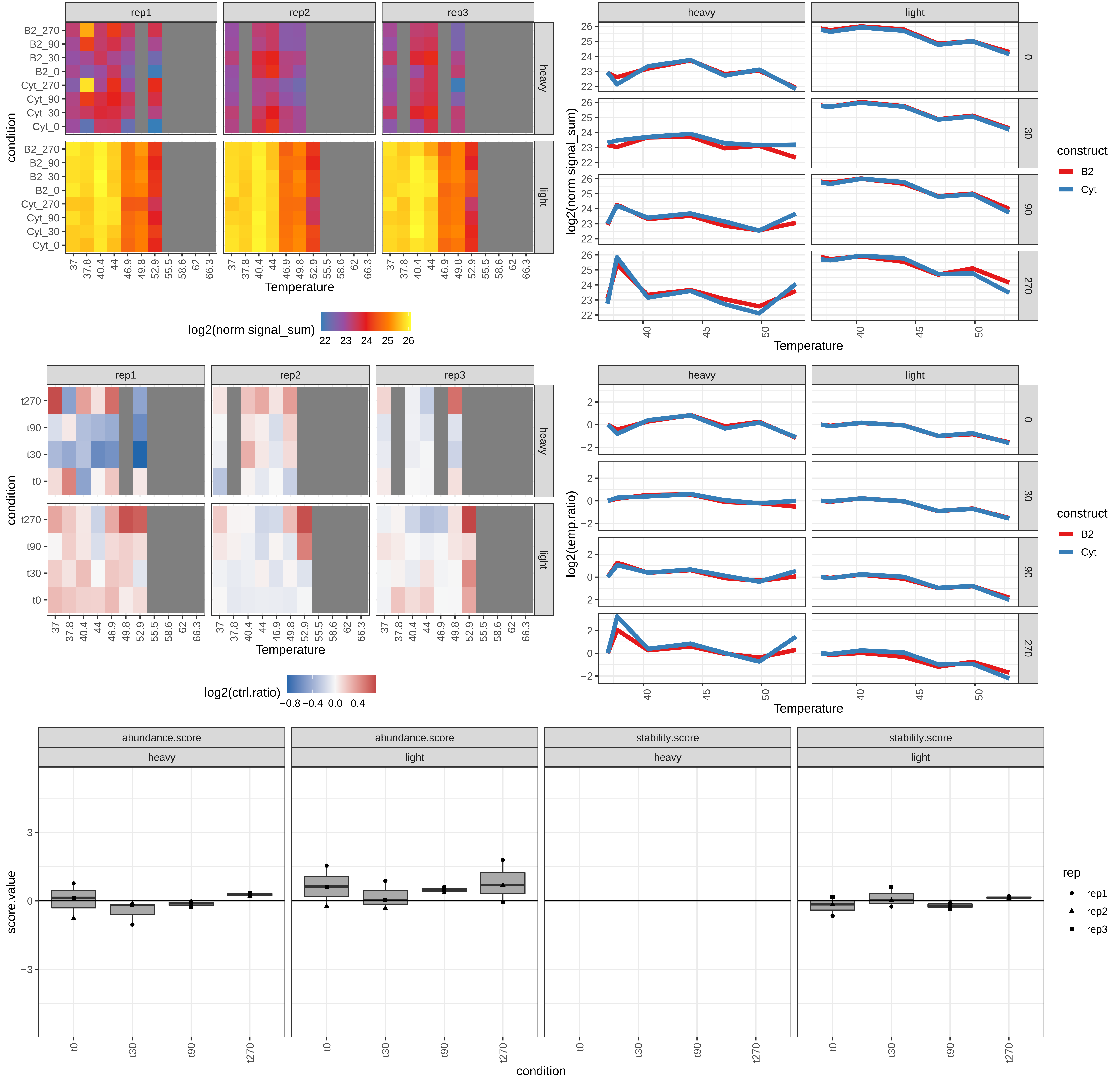

P47912 − Long−chain−fatty−acid−−CoA ligase 4 OS=Saccharomyces cerevisiae (strain ATCC 204508 / S288c) GN=FAA4 PE=1 SV=1

FAB1 − average.top3: 6.57166705882353 − max.qupm: 7

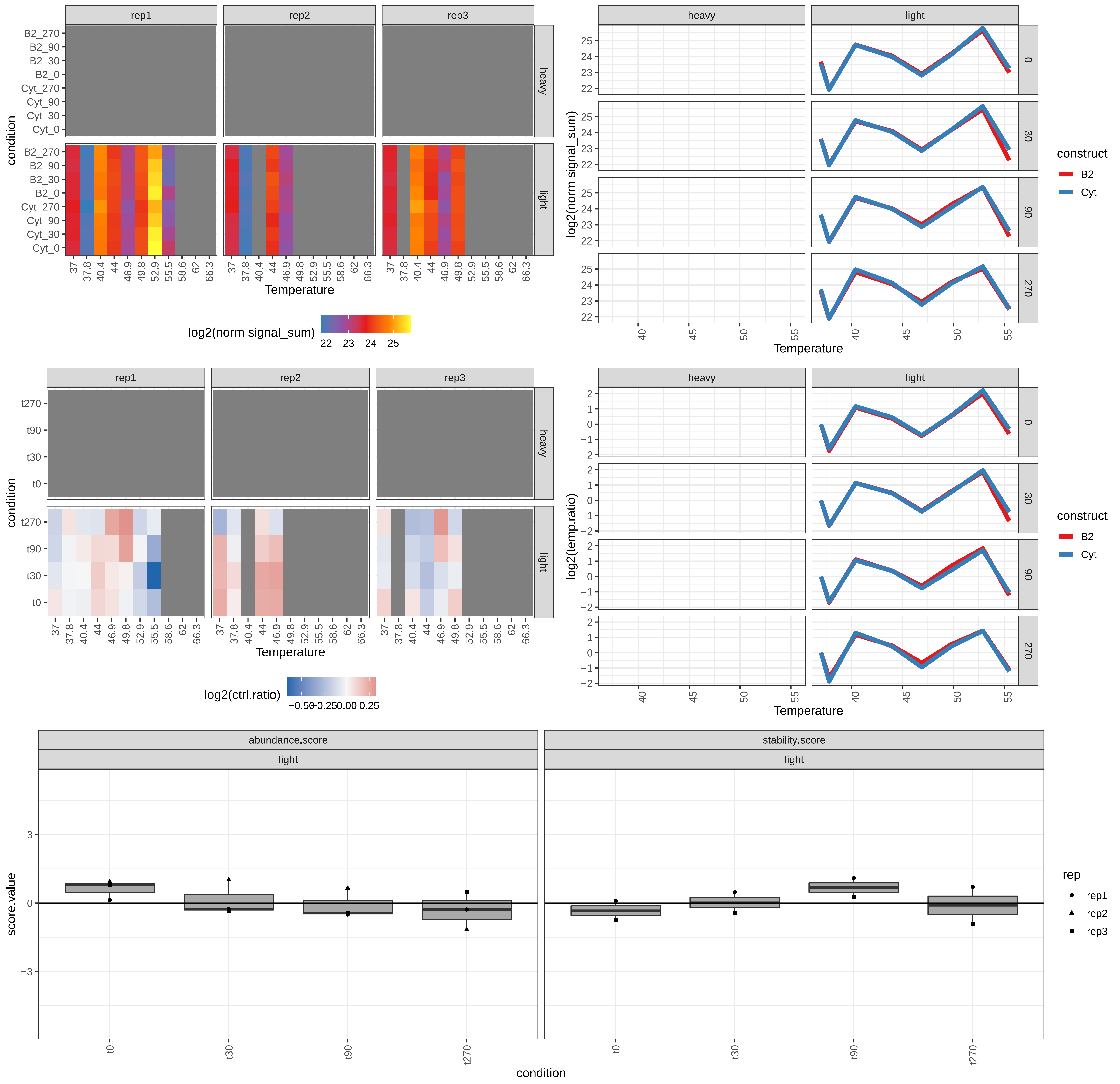

P34756 − 1−phosphatidylinositol 3−phosphate 5−kinase FAB1 OS=Saccharomyces cerevisiae (strain ATCC 204508 / S288c) GN=FAB1 PE=1 SV=3

FAD1 − average.top3: 6.66089526315789 − max.qupm: 7

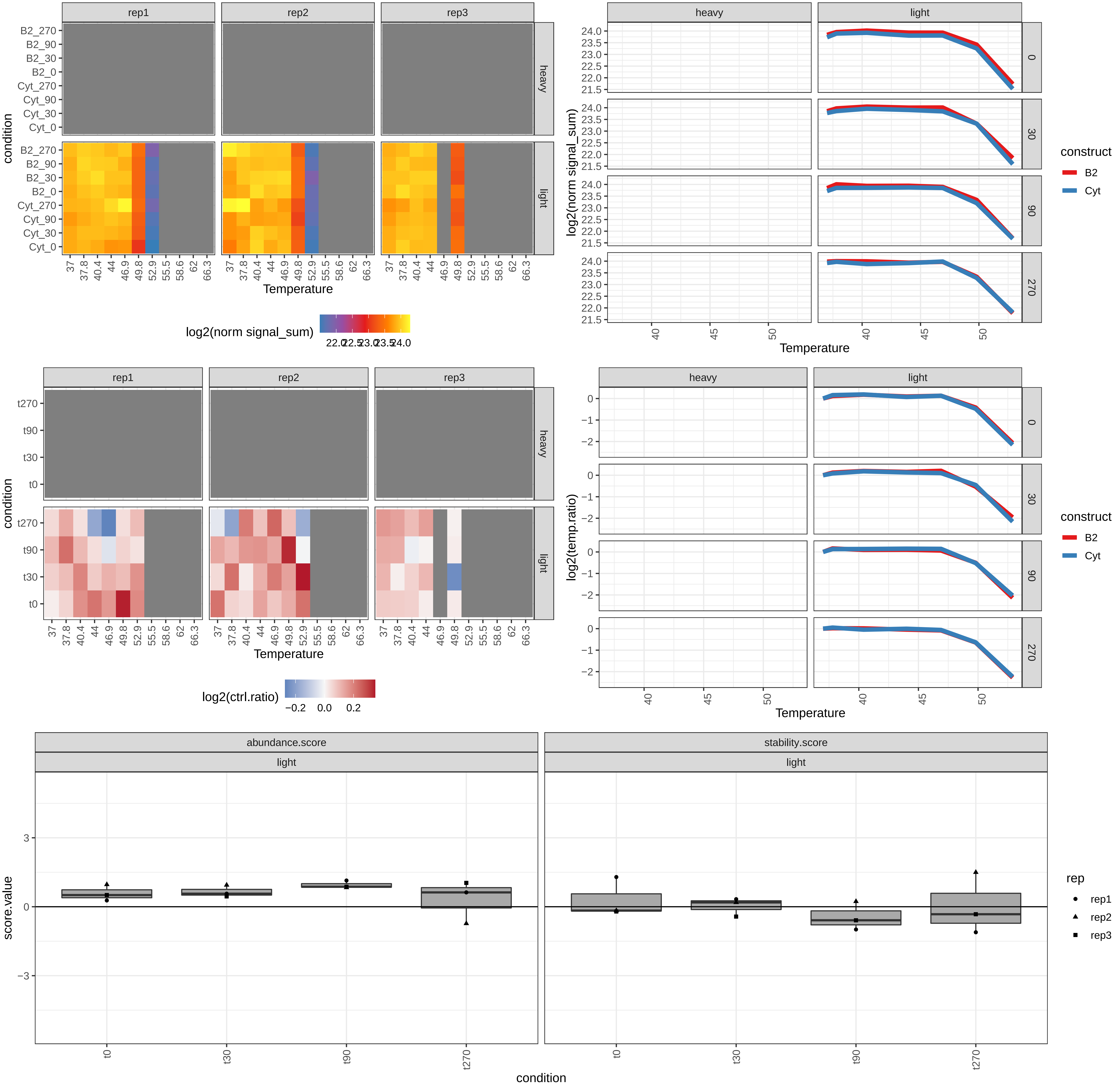

P38913 − FAD synthase OS=Saccharomyces cerevisiae (strain ATCC 204508 / S288c) GN=FAD1 PE=1 SV=1

FAP1 − average.top3: 6.654235 − max.qupm: 5

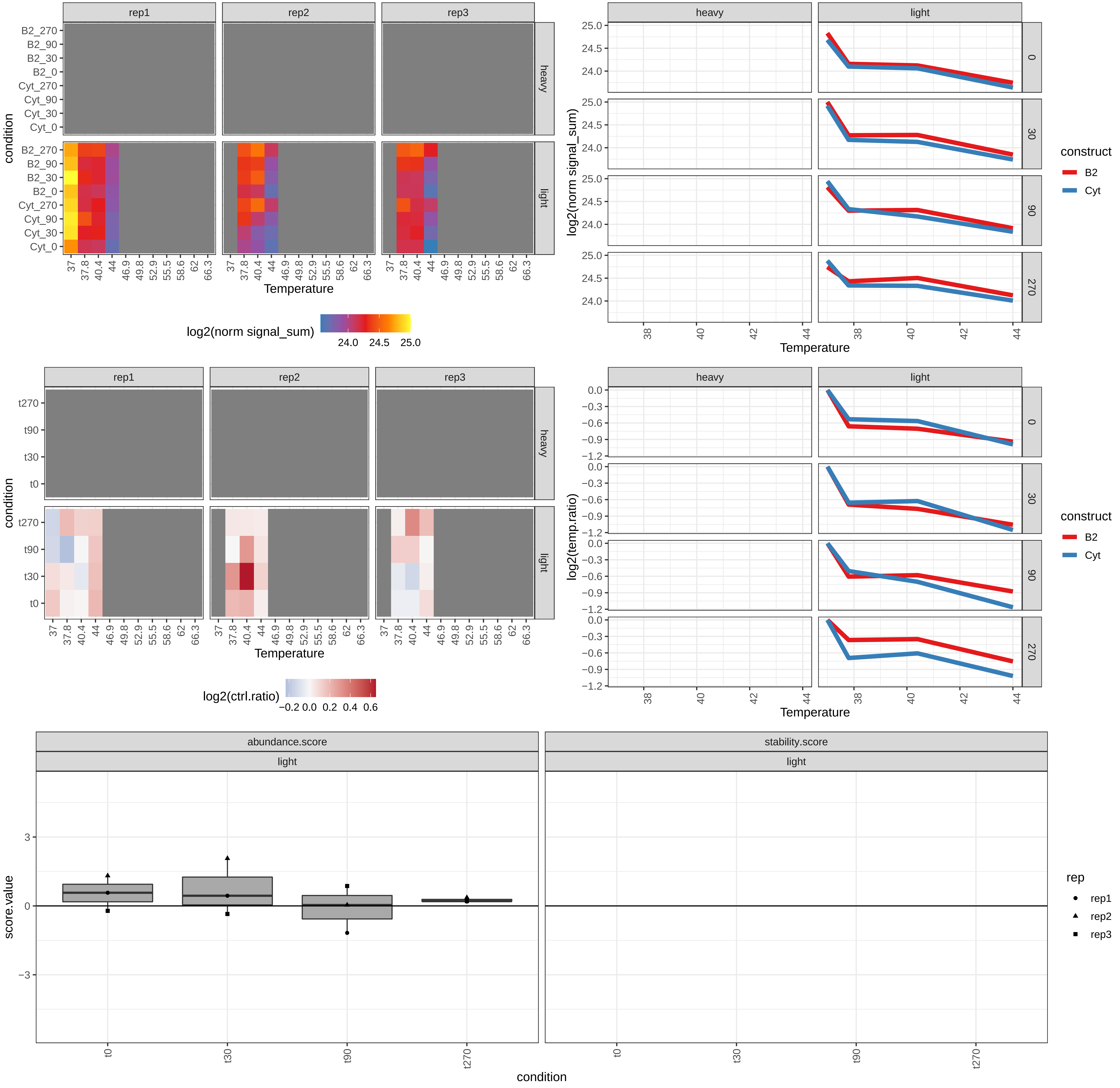

P53971 − FKBP12−associated protein 1 OS=Saccharomyces cerevisiae (strain ATCC 204508 / S288c) GN=FAP1 PE=1 SV=1

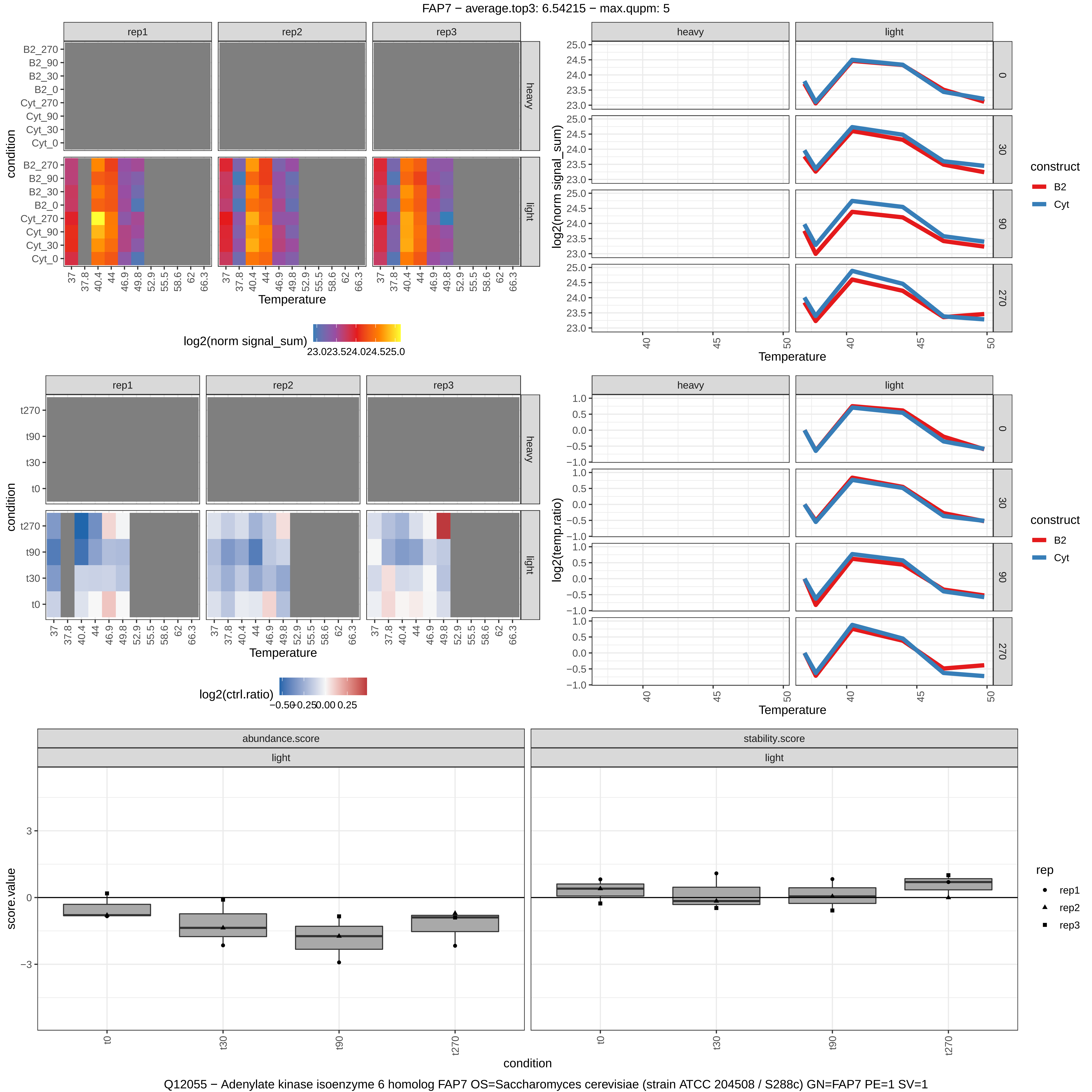
FAP7 − average.top3: 6.54215 − max.qupm: 5
rep1
rep2
rep3
heavy
light
condition
Temperature
log2(norm signal_sum)
log2(ctrl.ratio)
log2(temp.ratio)
construct
B2
Cyt
abundance.score
stability.score
score.value
rep
rep1
rep2
rep3
Q12055 − Adenylate kinase isoenzyme 6 homolog FAP7 OS=Saccharomyces cerevisiae (strain ATCC 204508 / S288c) GN=FAP7 PE=1 SV=1

FAR8 − average.top3: 6.833895 − max.qupm: 8

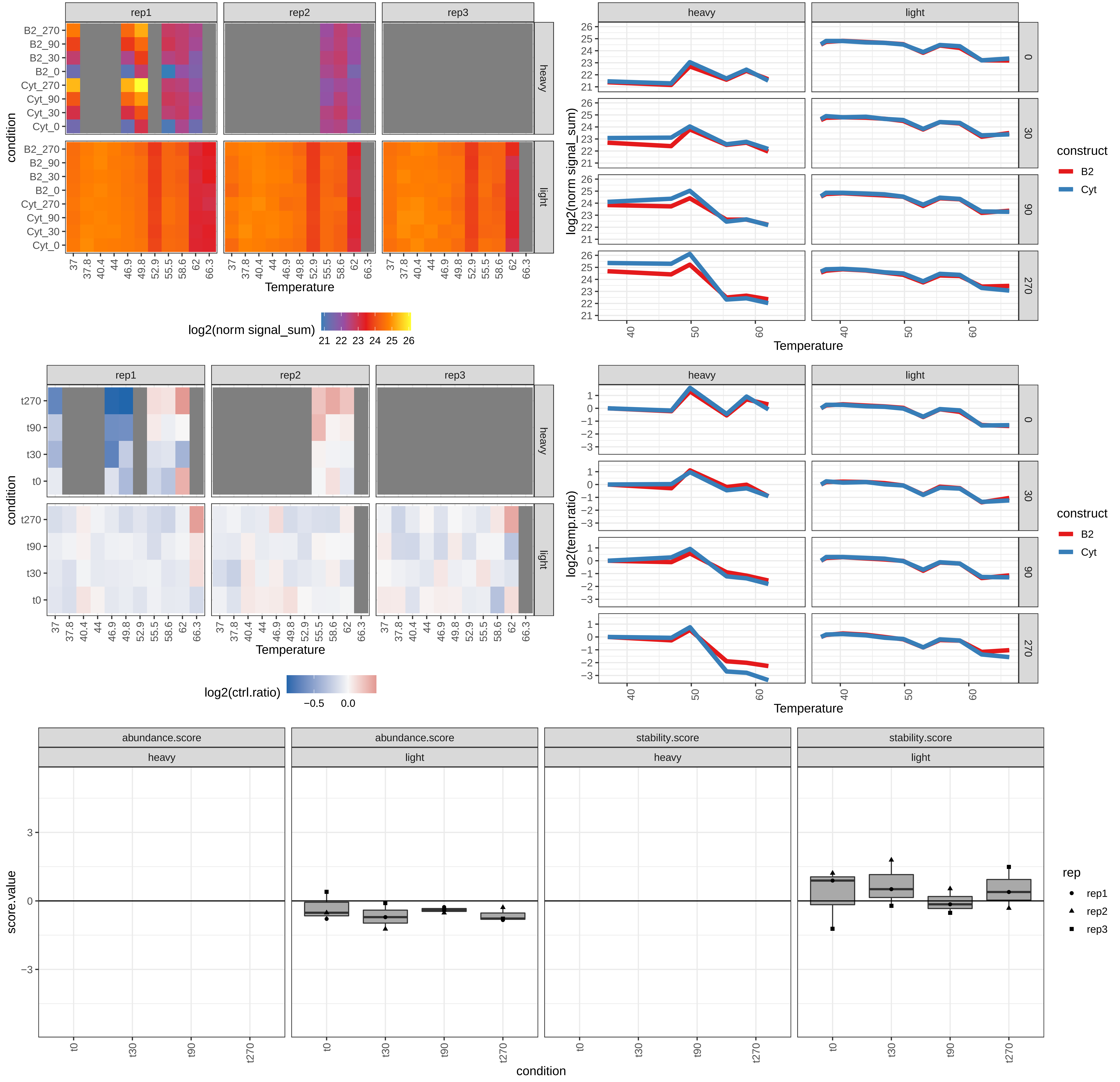

Q05040 − Factor arrest protein 8 OS=Saccharomyces cerevisiae (strain ATCC 204508 / S288c) GN=FAR8 PE=1 SV=1

FAS1 − average.top3: 8.12460827586207 − max.qupm: 74

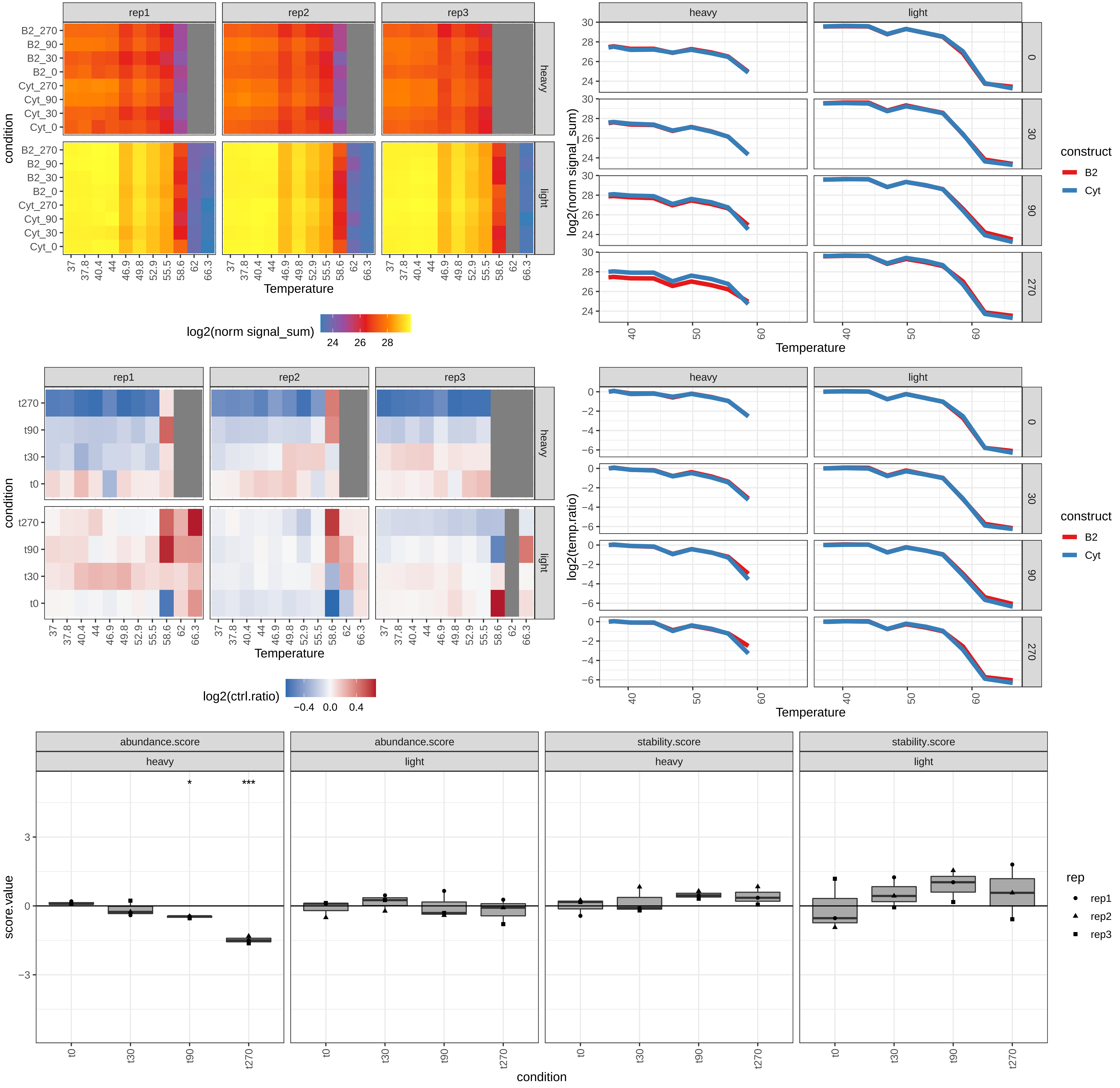

P07149 − Fatty acid synthase subunit beta OS=Saccharomyces cerevisiae (strain ATCC 204508 / S288c) GN=FAS1 PE=1 SV=2

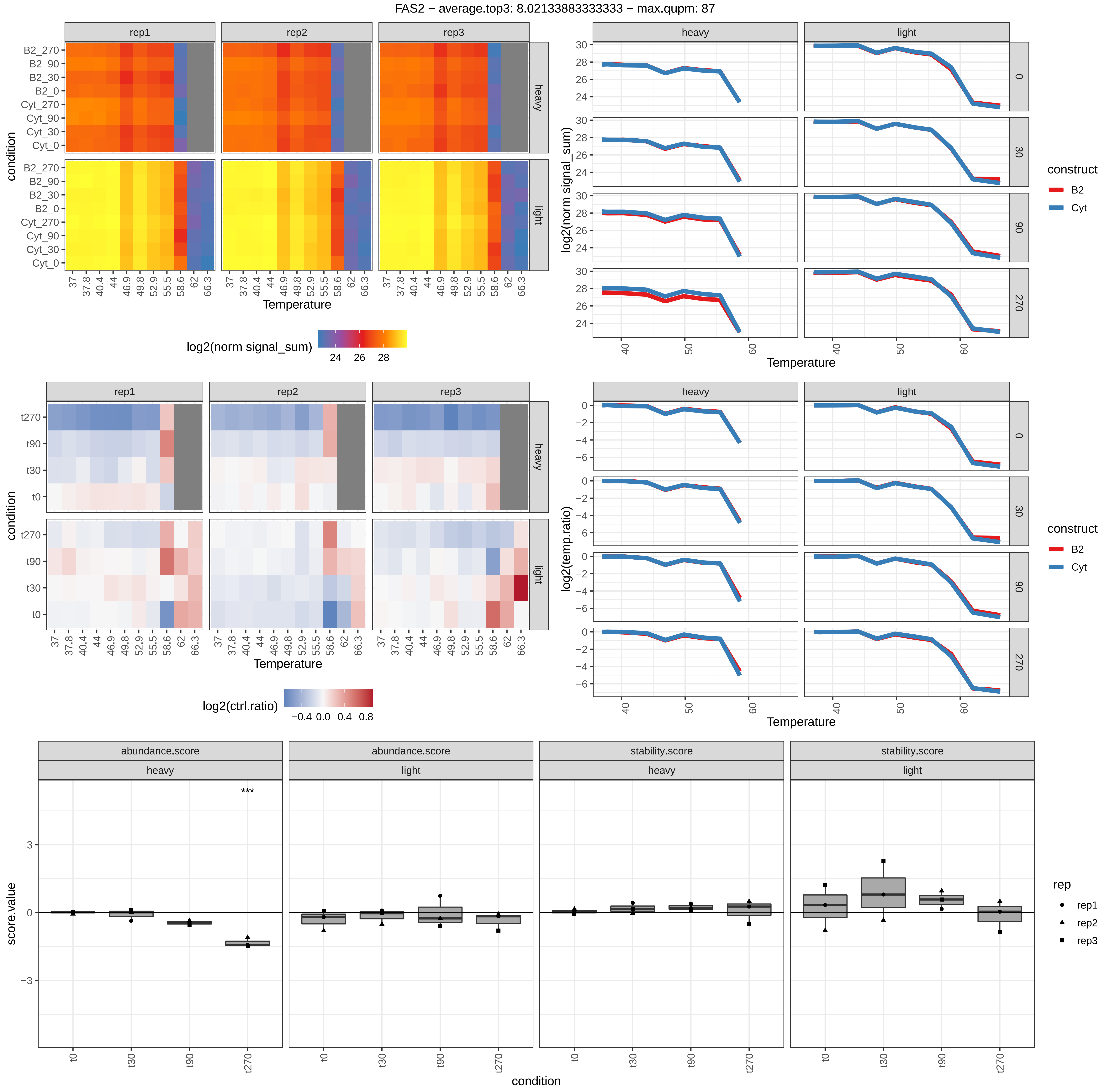

P19097 − Fatty acid synthase subunit alpha OS=Saccharomyces cerevisiae (strain ATCC 204508 / S288c) GN=FAS2 PE=1 SV=2

FAT1 − average.top3: 7.10311636363636 − max.qupm: 9

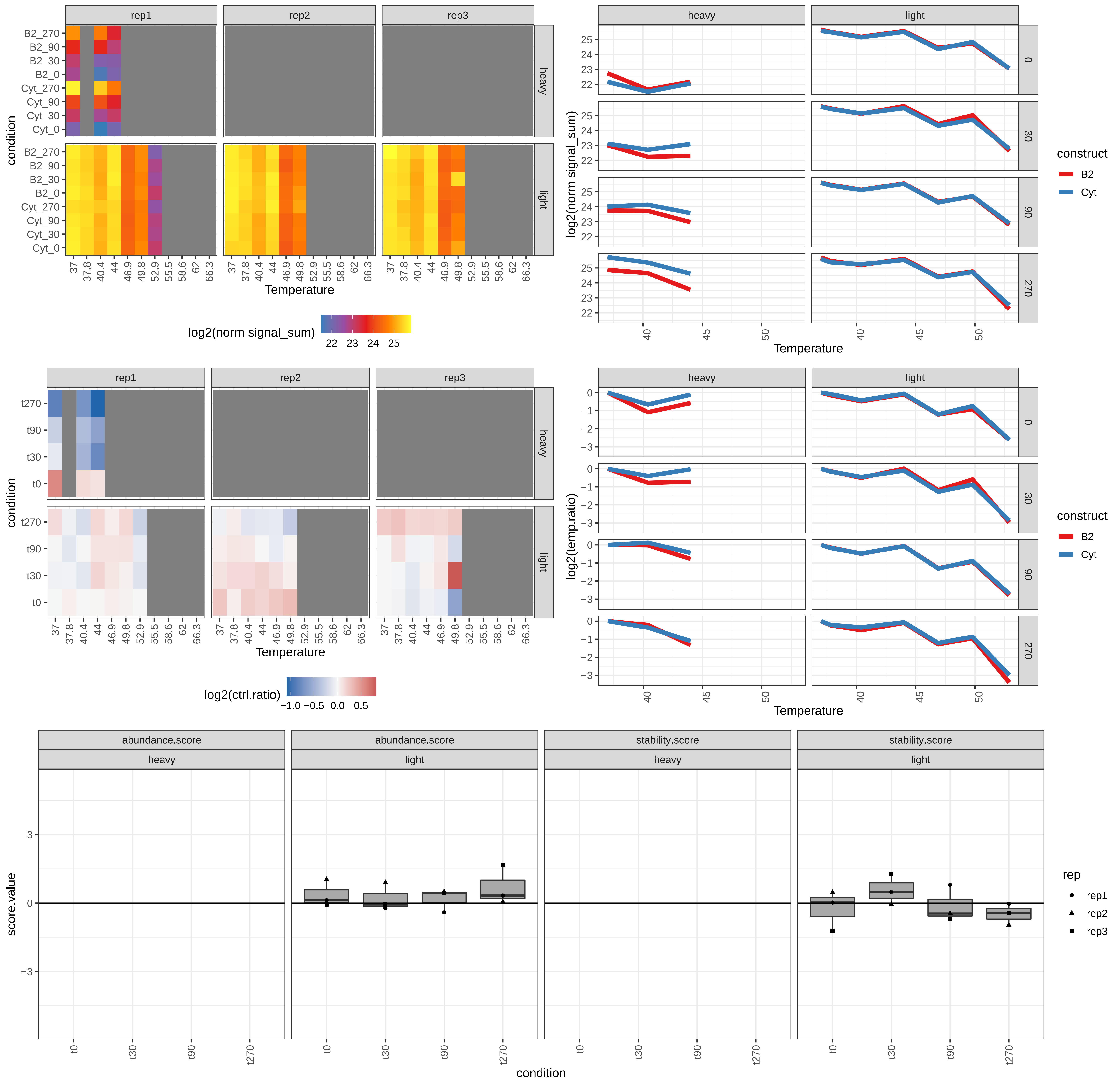

P38225 − Very long−chain fatty acid transport protein OS=Saccharomyces cerevisiae (strain ATCC 204508 / S288c) GN=FAT1 PE=1 SV=2

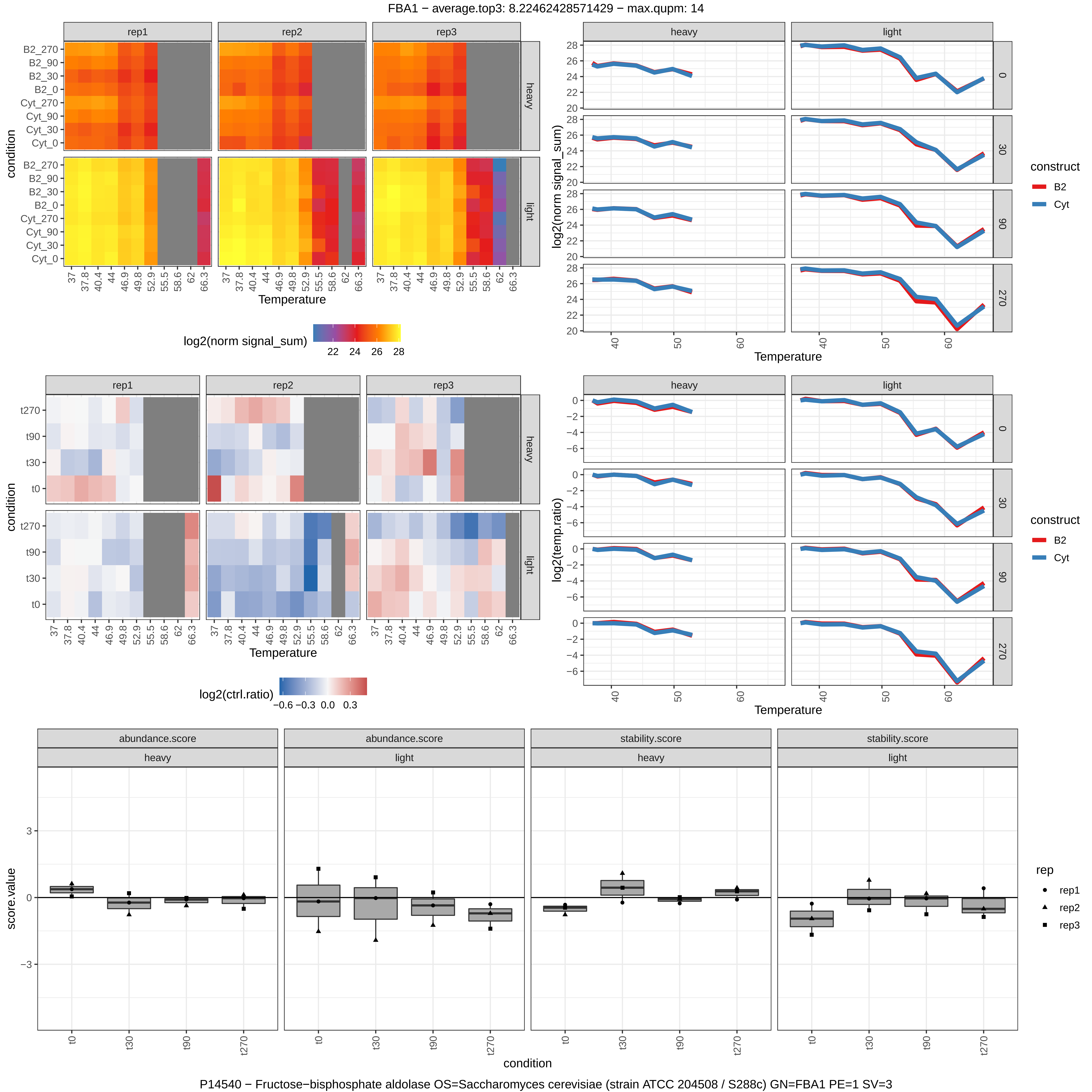
FBA1 − average.top3: 8.22462428571429 − max.qupm: 14
rep1
rep2
rep3
heavy
light
condition
Temperature
log2(norm signal_sum)
log2(ctrl.ratio)
log2(temp.ratio)
construct
B2
Cyt
abundance.score
stability.score
score.value
rep
rep1
rep2
rep3
P14540 − Fructose−bisphosphate aldolase OS=Saccharomyces cerevisiae (strain ATCC 204508 / S288c) GN=FBA1 PE=1 SV=3

FBP1 − average.top3: 7.75085793103448 − max.qupm: 15

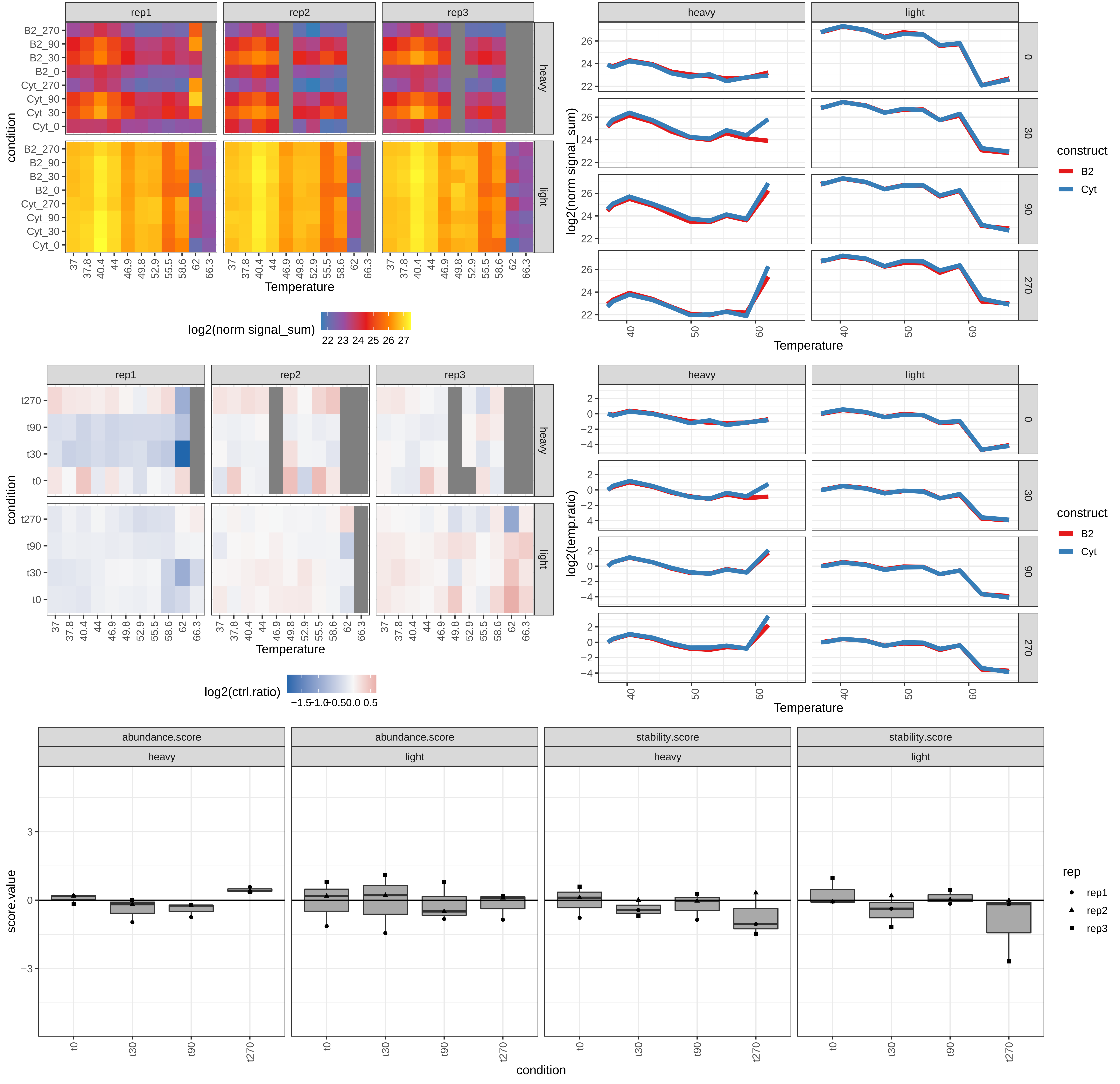

P09201 − Fructose−1,6−bisphosphatase OS=Saccharomyces cerevisiae (strain ATCC 204508 / S288c) GN=FBP1 PE=1 SV=2

FBP26 − average.top3: 7.1817972 − max.qupm: 10

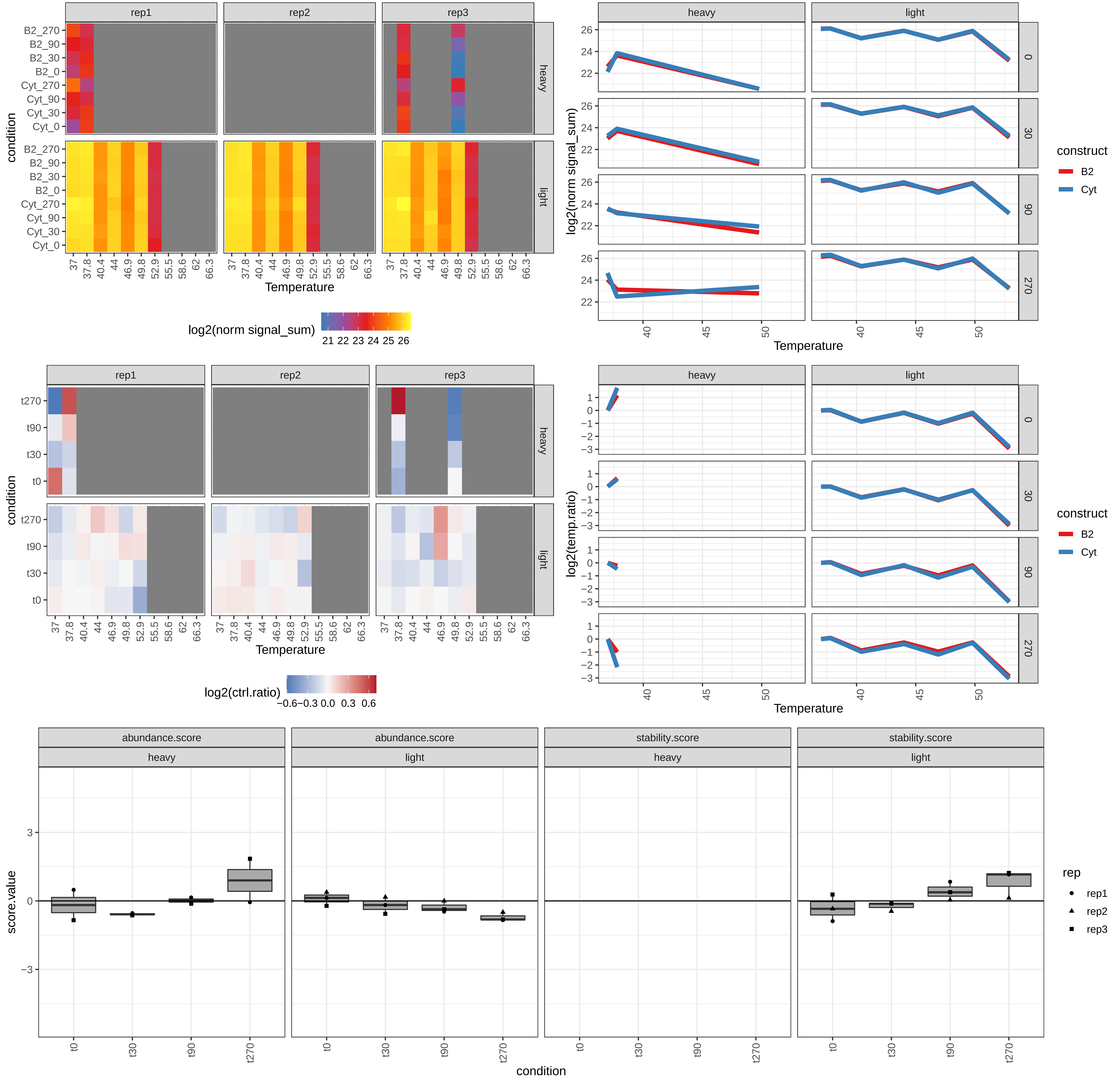

P32604 − Fructose−2,6−bisphosphatase OS=Saccharomyces cerevisiae (strain ATCC 204508 / S288c) GN=FBP26 PE=1 SV=2

FCF2 − average.top3: 6.224196 − max.qupm: 4

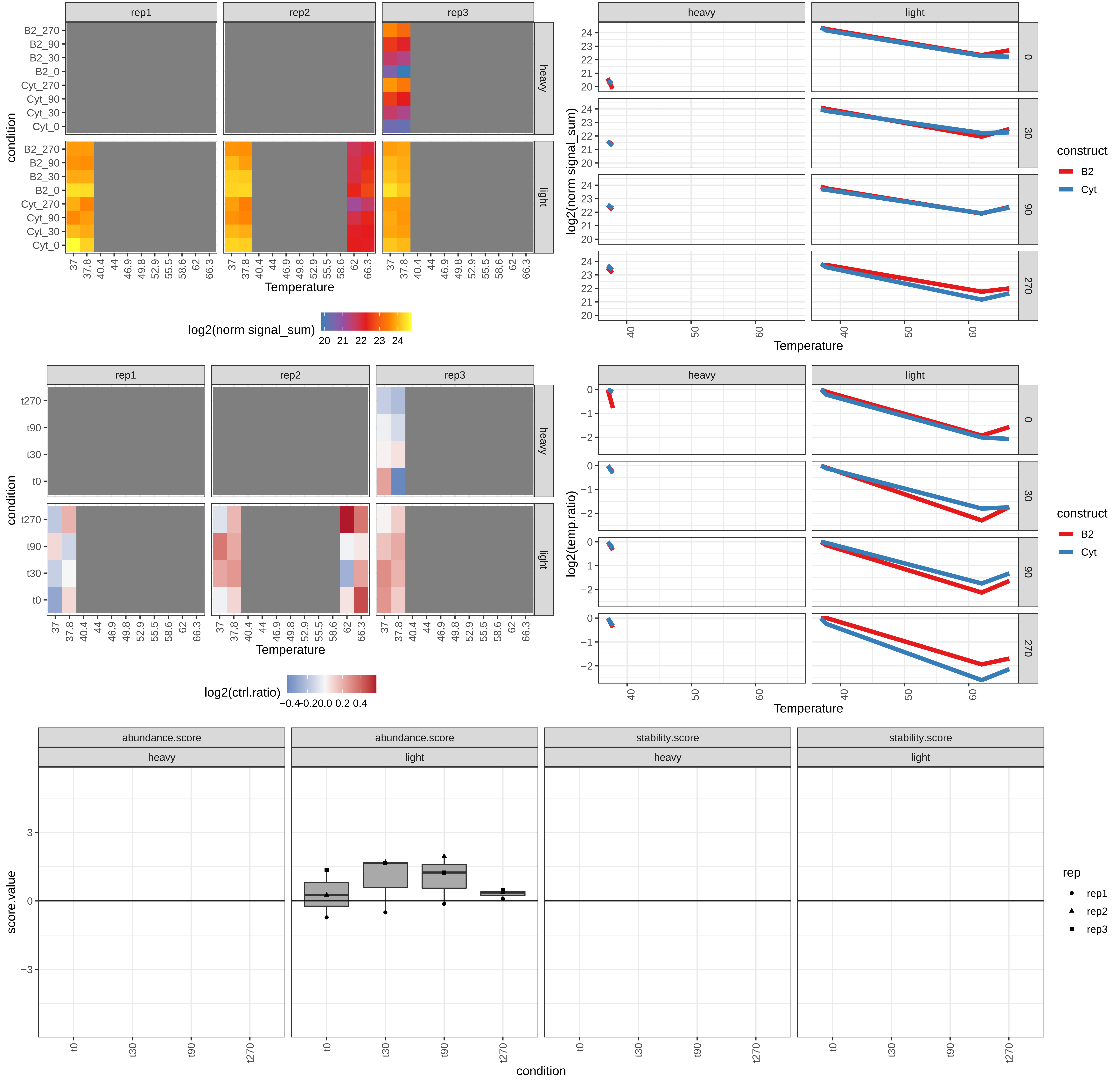

Q12035 − rRNA−processing protein FCF2 OS=Saccharomyces cerevisiae (strain ATCC 204508 / S288c) GN=FCF2 PE=1 SV=1

FCP1 − average.top3: 6.70255722222222 − max.qupm: 4

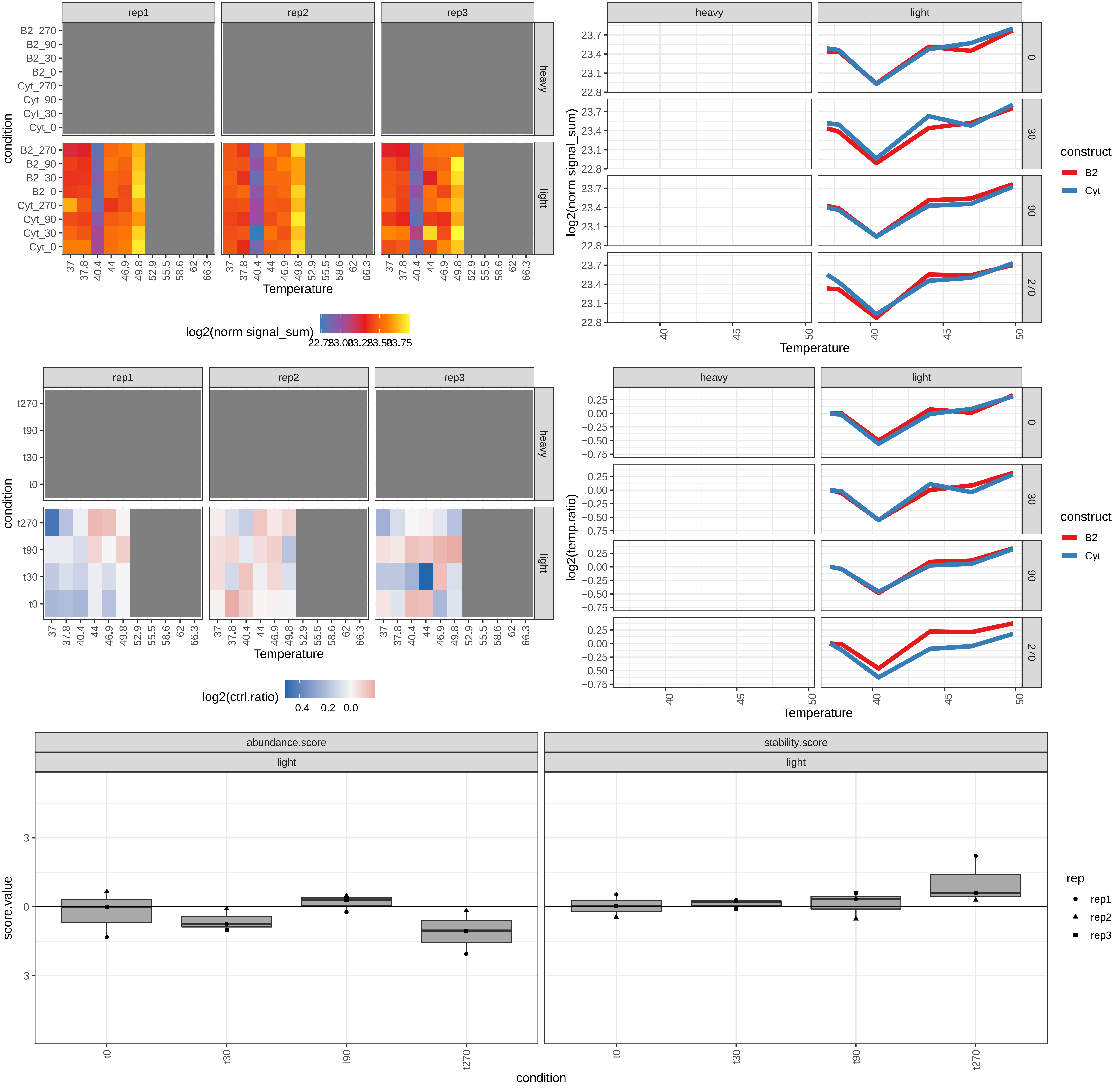

Q03254 − RNA polymerase II subunit A C−terminal domain phosphatase OS=Saccharomyces cerevisiae (strain ATCC 204508 / S288c) GN=FCP1 PE=1 SV=1

FCY1 − average.top3: 7.09825181818182 − max.qupm: 4

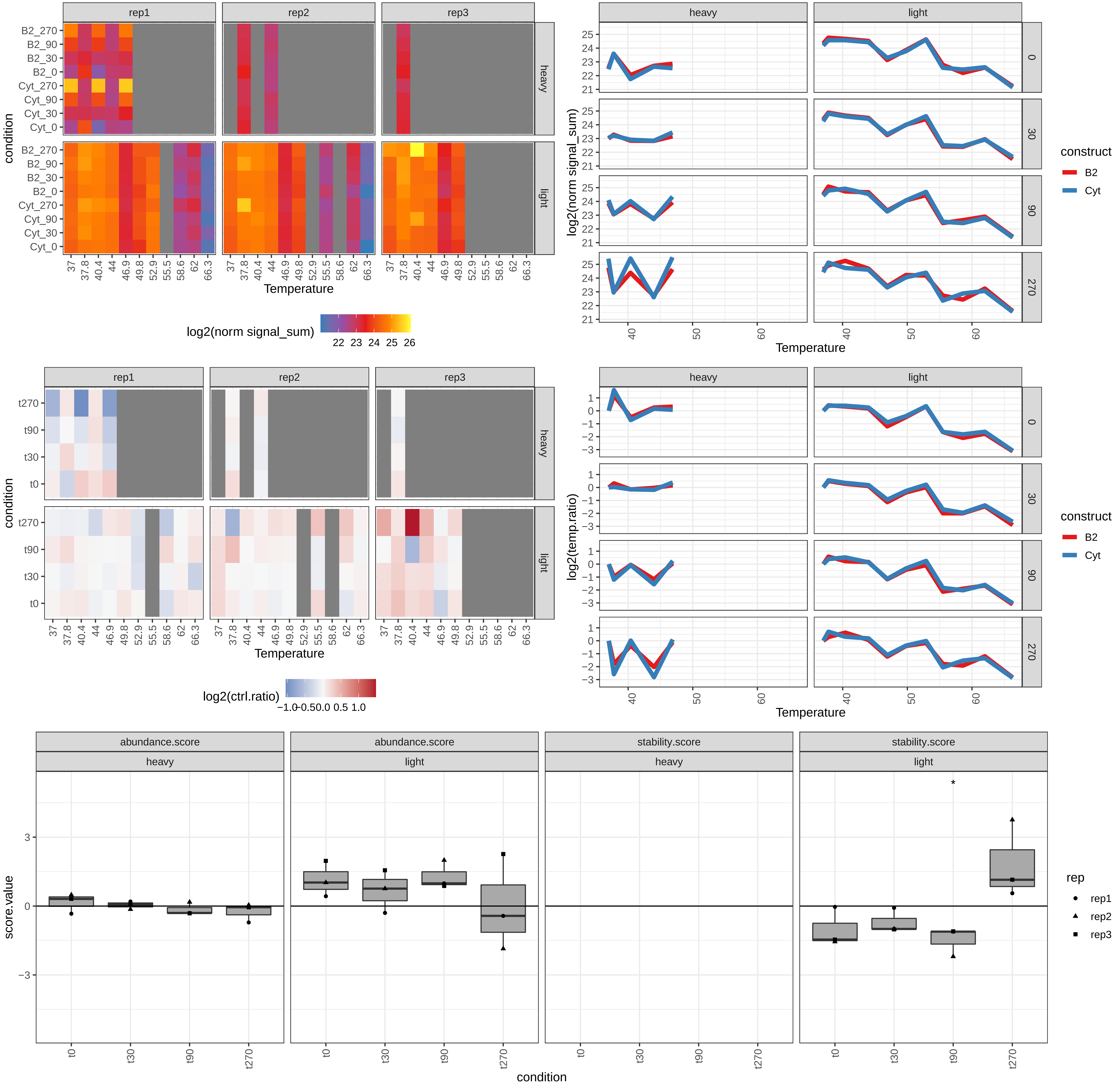

Q12178 − Cytosine deaminase OS=Saccharomyces cerevisiae (strain ATCC 204508 / S288c) GN=FCY1 PE=1 SV=1

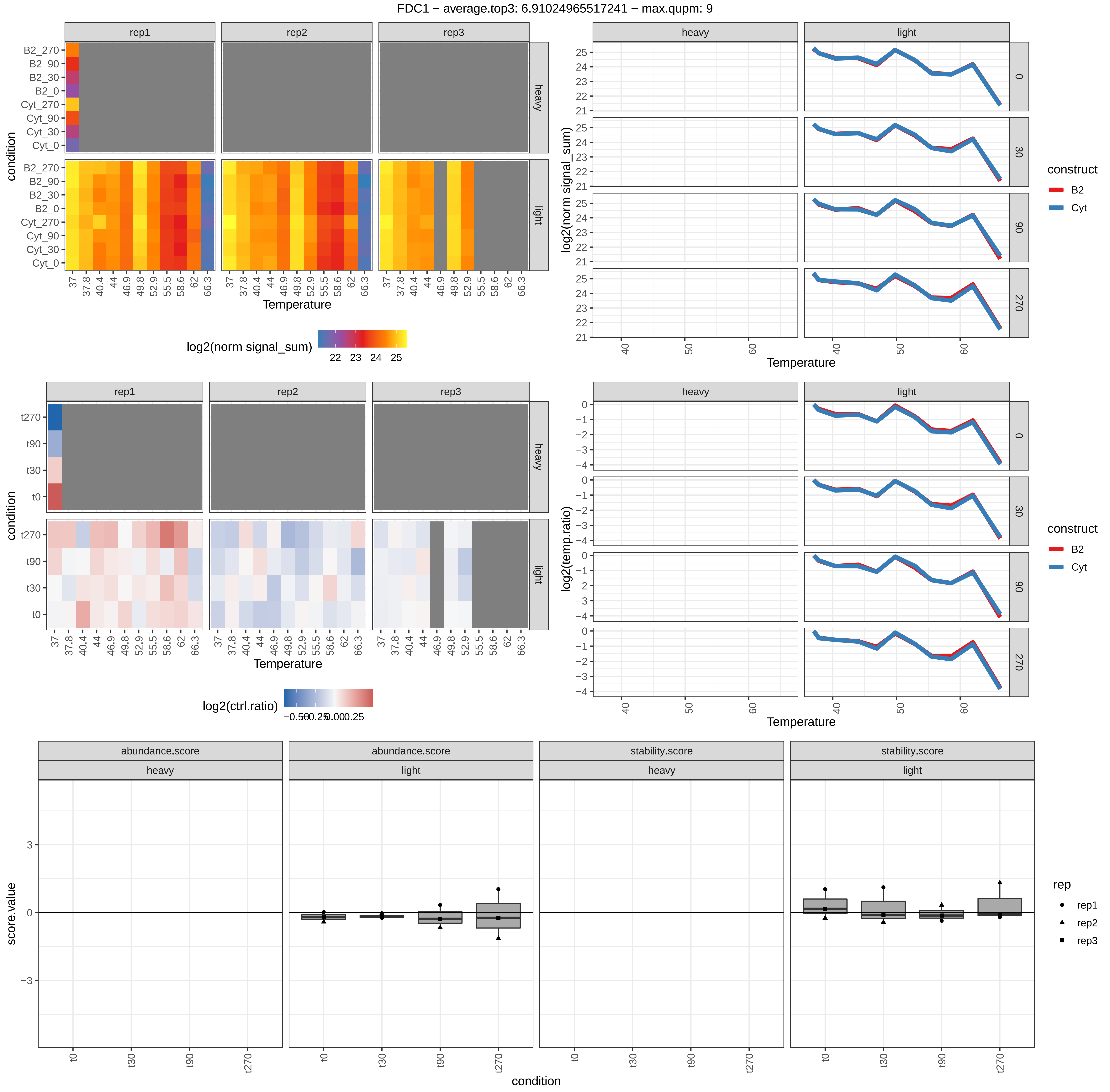

Q03034 − Ferulic acid decarboxylase 1 OS=Saccharomyces cerevisiae (strain ATCC 204508 / S288c) GN=FDC1 PE=1 SV=1

FDH1 − average.top3: 7.207186 − max.qupm: 14

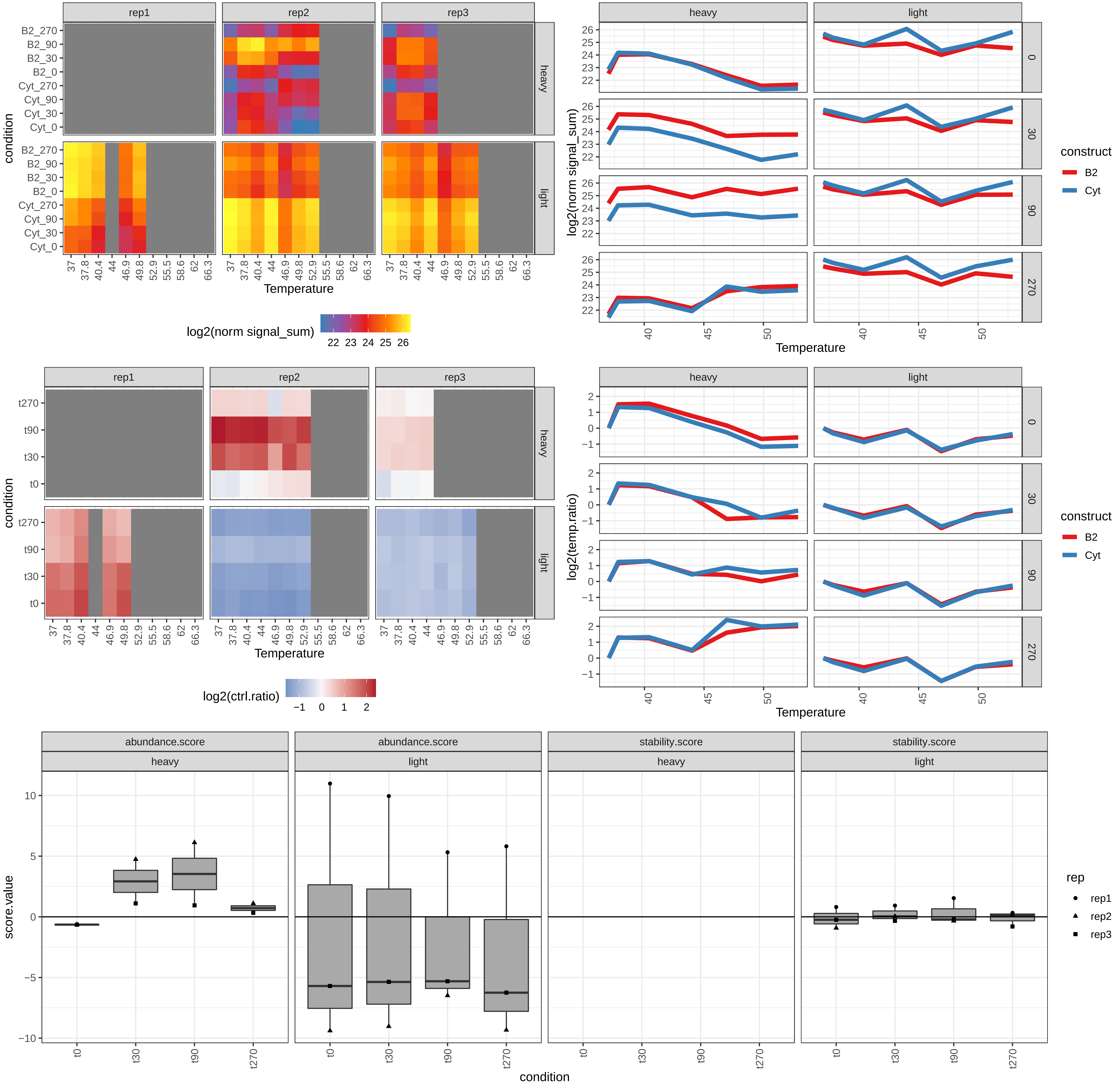

Q08911 − Formate dehydrogenase 1 OS=Saccharomyces cerevisiae (strain ATCC 204508 / S288c) GN=FDH1 PE=1 SV=1

FES1 − average.top3: 6.8198770212766 − max.qupm: 5

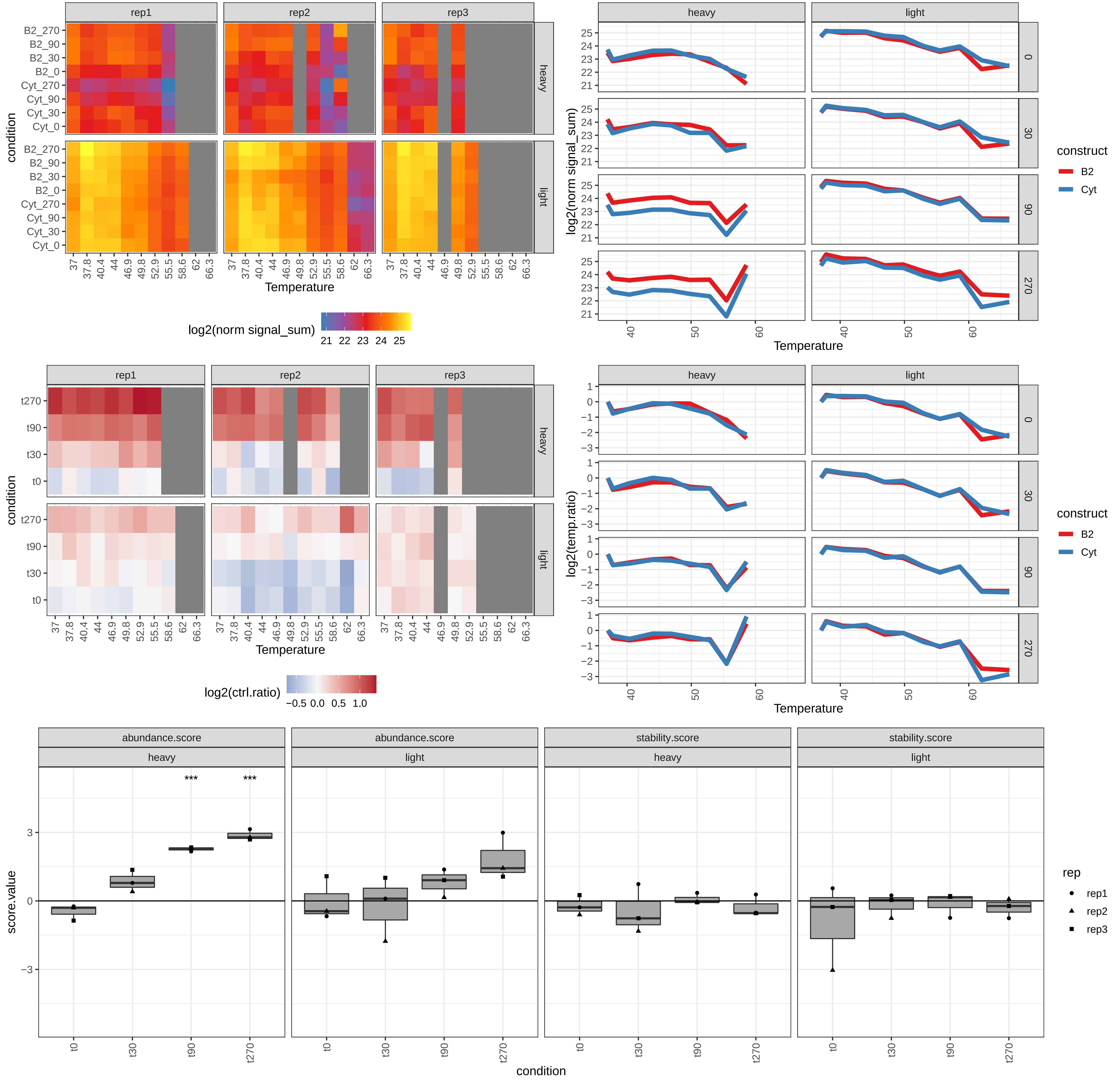

P38260 − Hsp70 nucleotide exchange factor FES1 OS=Saccharomyces cerevisiae (strain ATCC 204508 / S288c) GN=FES1 PE=1 SV=1

FET3 − average.top3: 7.02165085714286 − max.qupm: 6

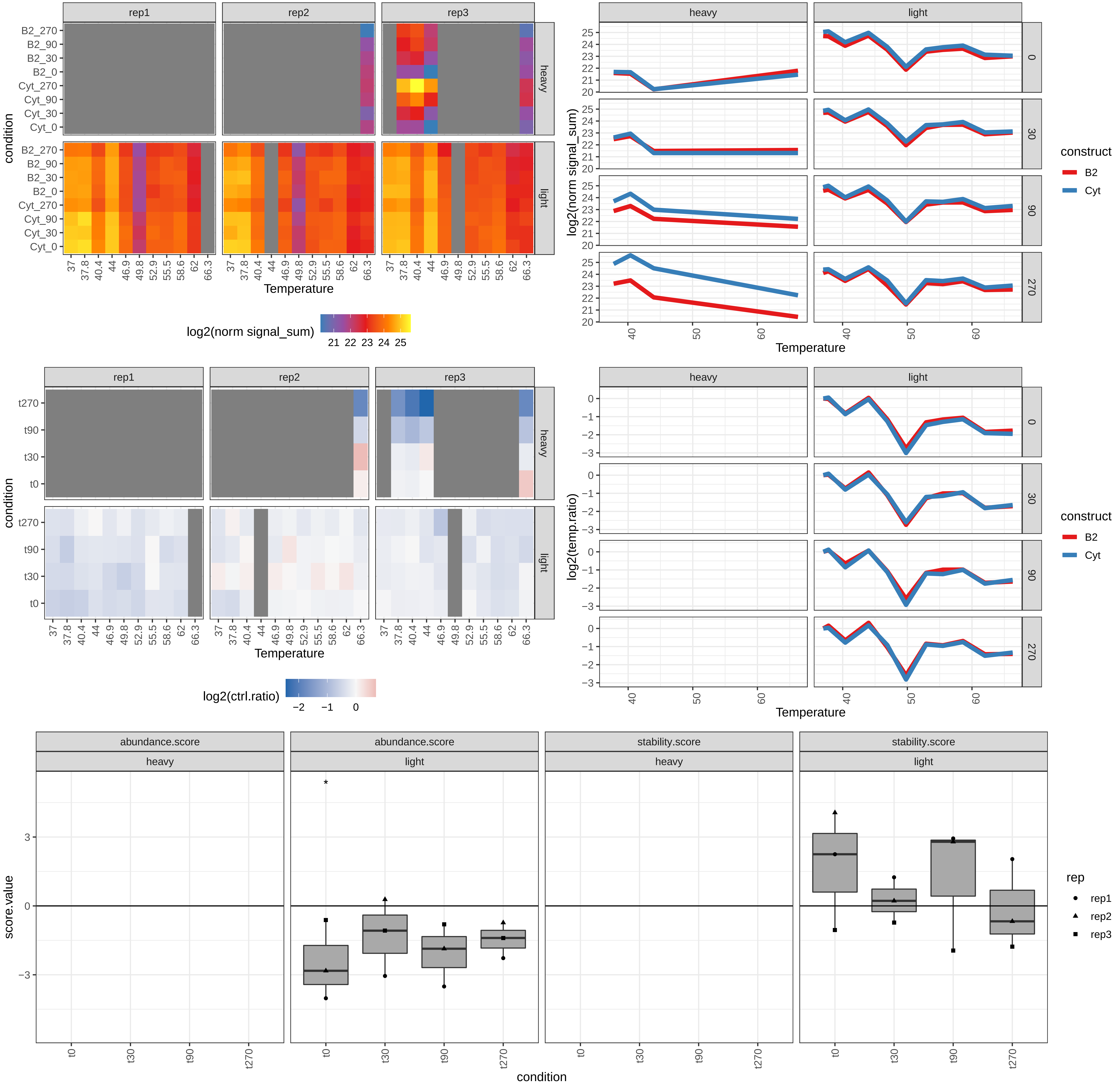

P38993 − Iron transport multicopper oxidase FET3 OS=Saccharomyces cerevisiae (strain ATCC 204508 / S288c) GN=FET3 PE=1 SV=2

FET5 − average.top3: 6.878227 − max.qupm: 13

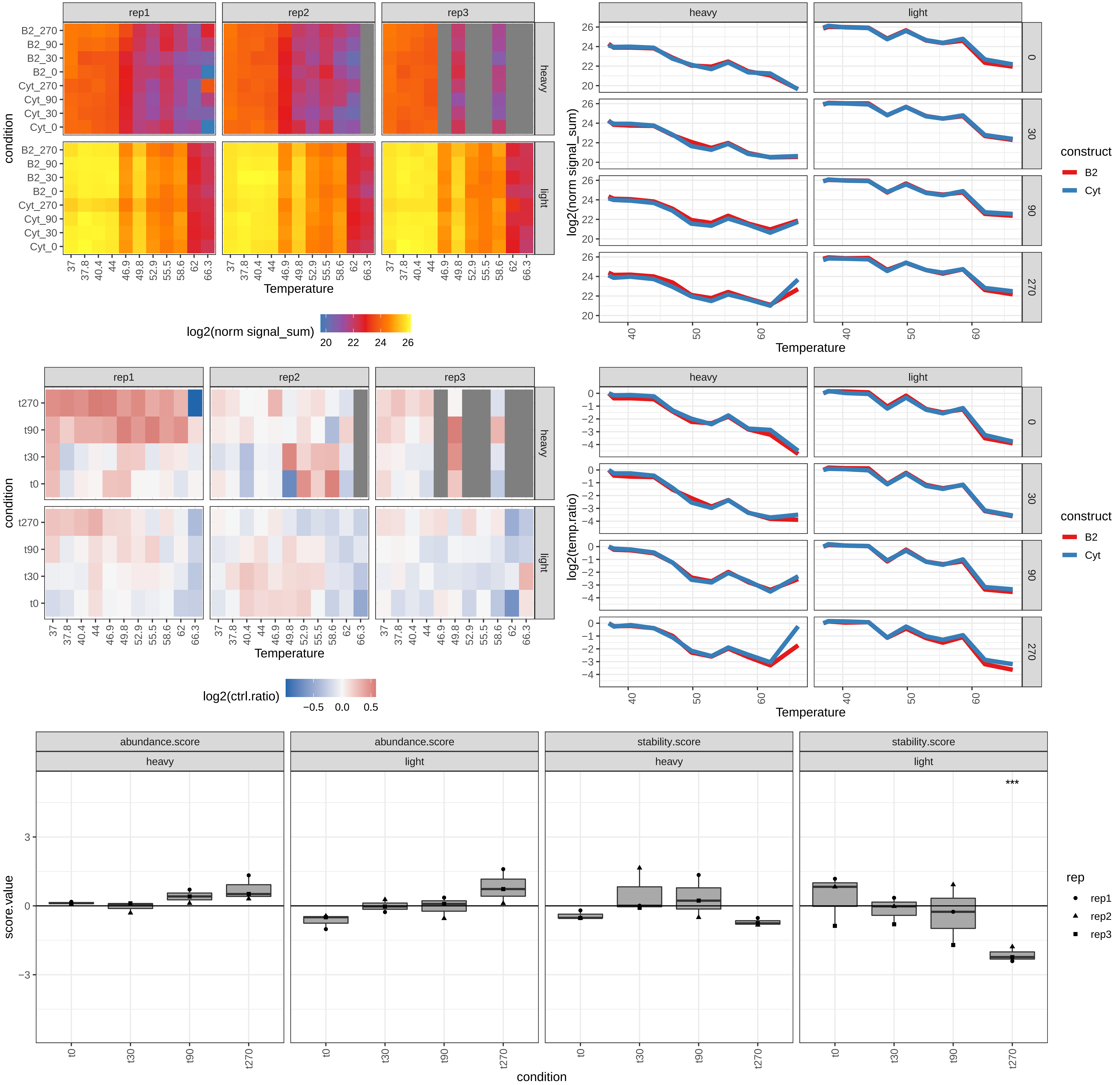

P43561 − Iron transport multicopper oxidase FET5 OS=Saccharomyces cerevisiae (strain ATCC 204508 / S288c) GN=FET5 PE=1 SV=1

FIS1 − average.top3: 7.12466826923077 − max.qupm: 8

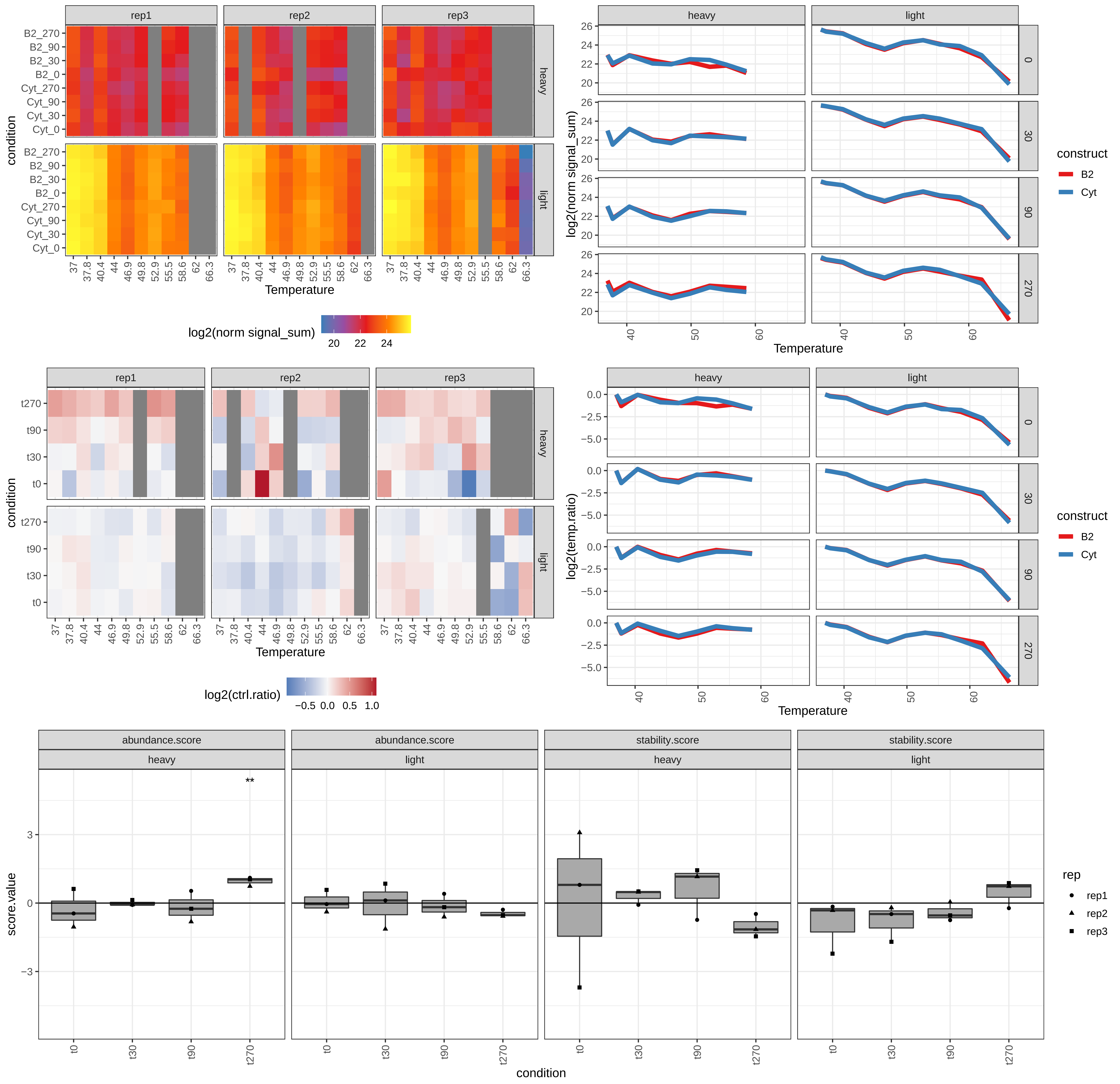

P40515 − Mitochondria fission 1 protein OS=Saccharomyces cerevisiae (strain ATCC 204508 / S288c) GN=FIS1 PE=1 SV=1

FKS1 − average.top3: 7.0558186 − max.qupm: 12

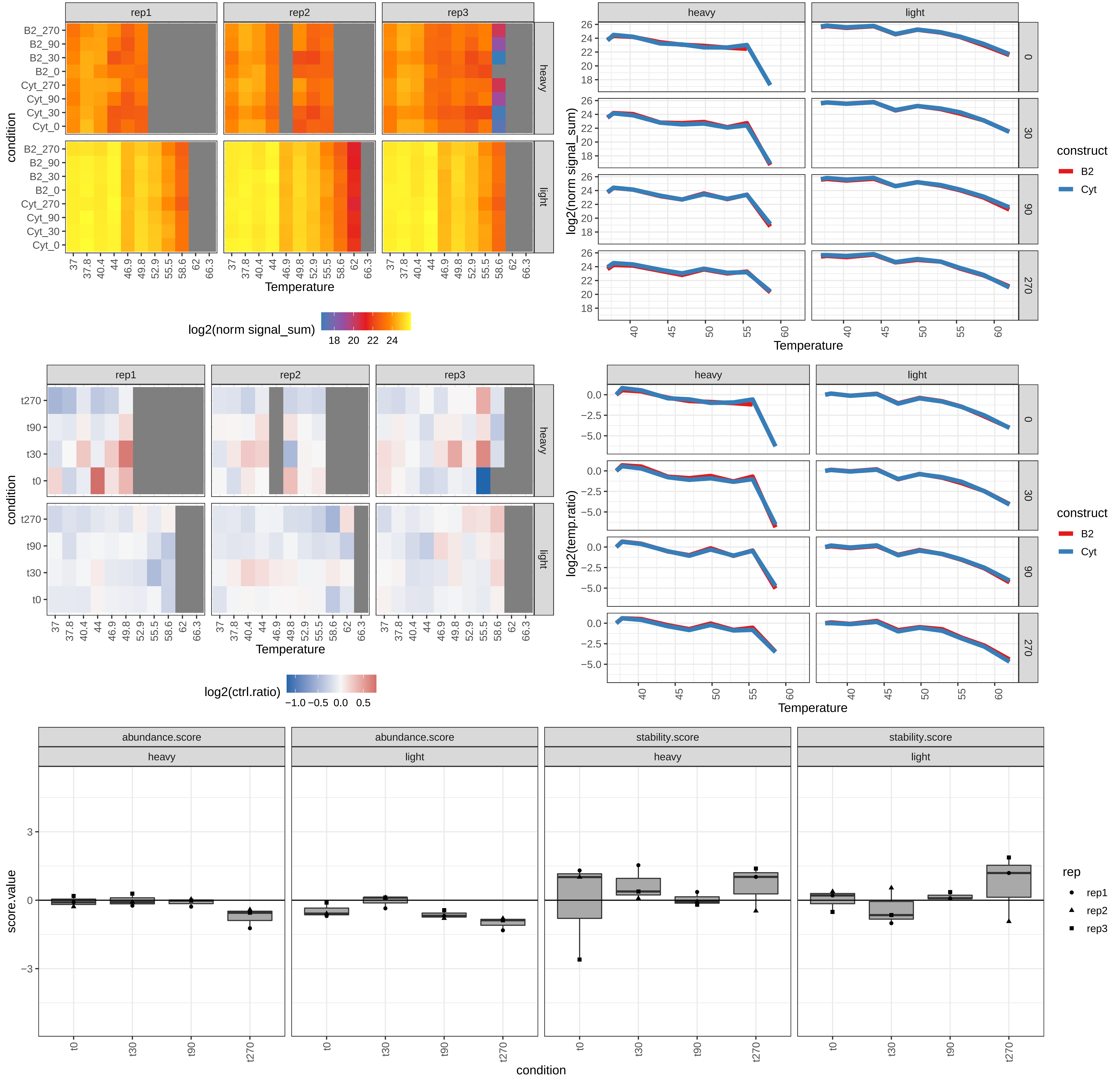

P38631 − 1,3−beta−glucan synthase component FKS1 OS=Saccharomyces cerevisiae (strain ATCC 204508 / S288c) GN=FKS1 PE=1 SV=2

FLC2 − average.top3: 6.56327692307692 − max.qupm: 3

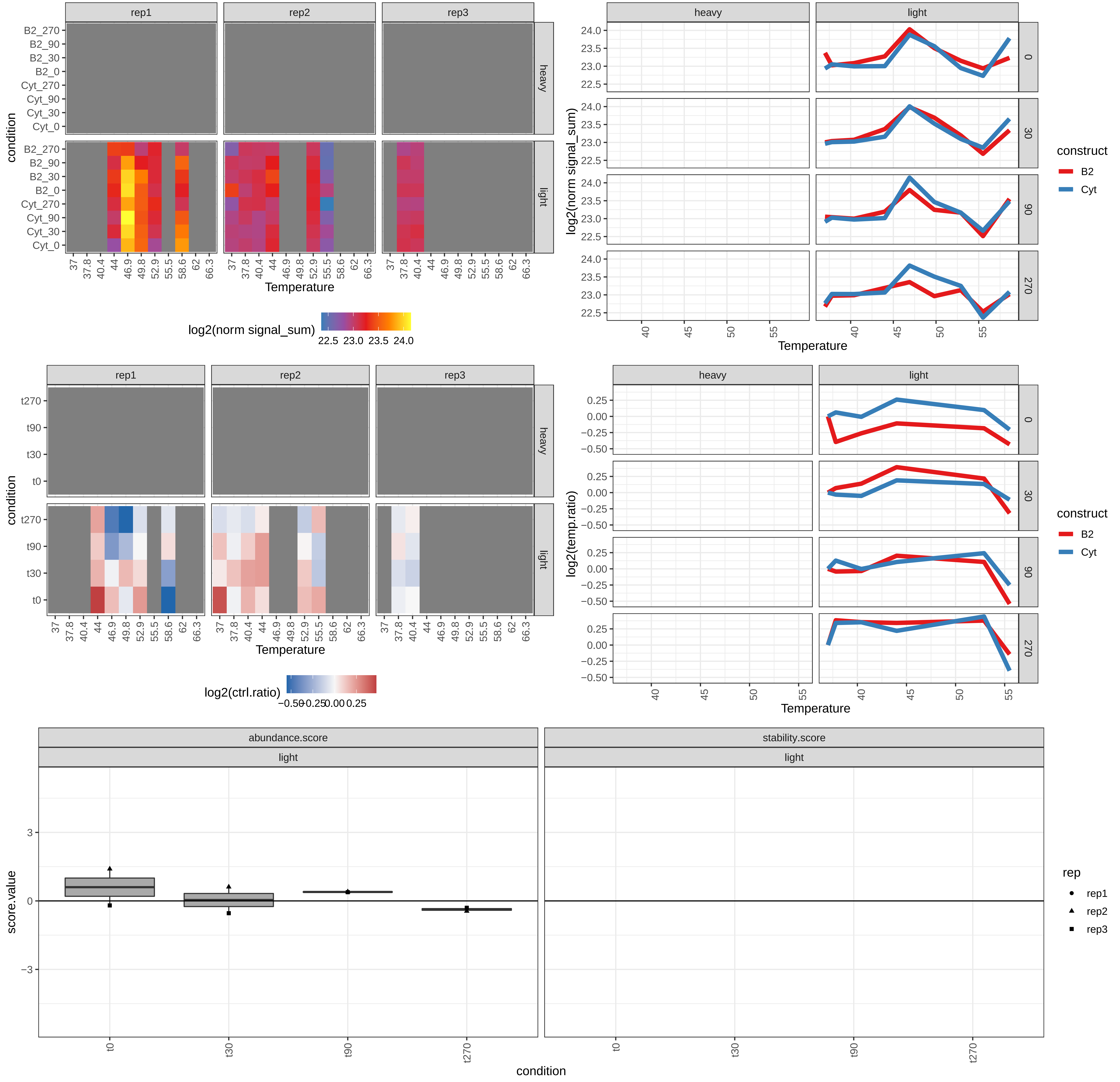

P39719 − Flavin carrier protein 2 OS=Saccharomyces cerevisiae (strain ATCC 204508 / S288c) GN=FLC2 PE=1 SV=2

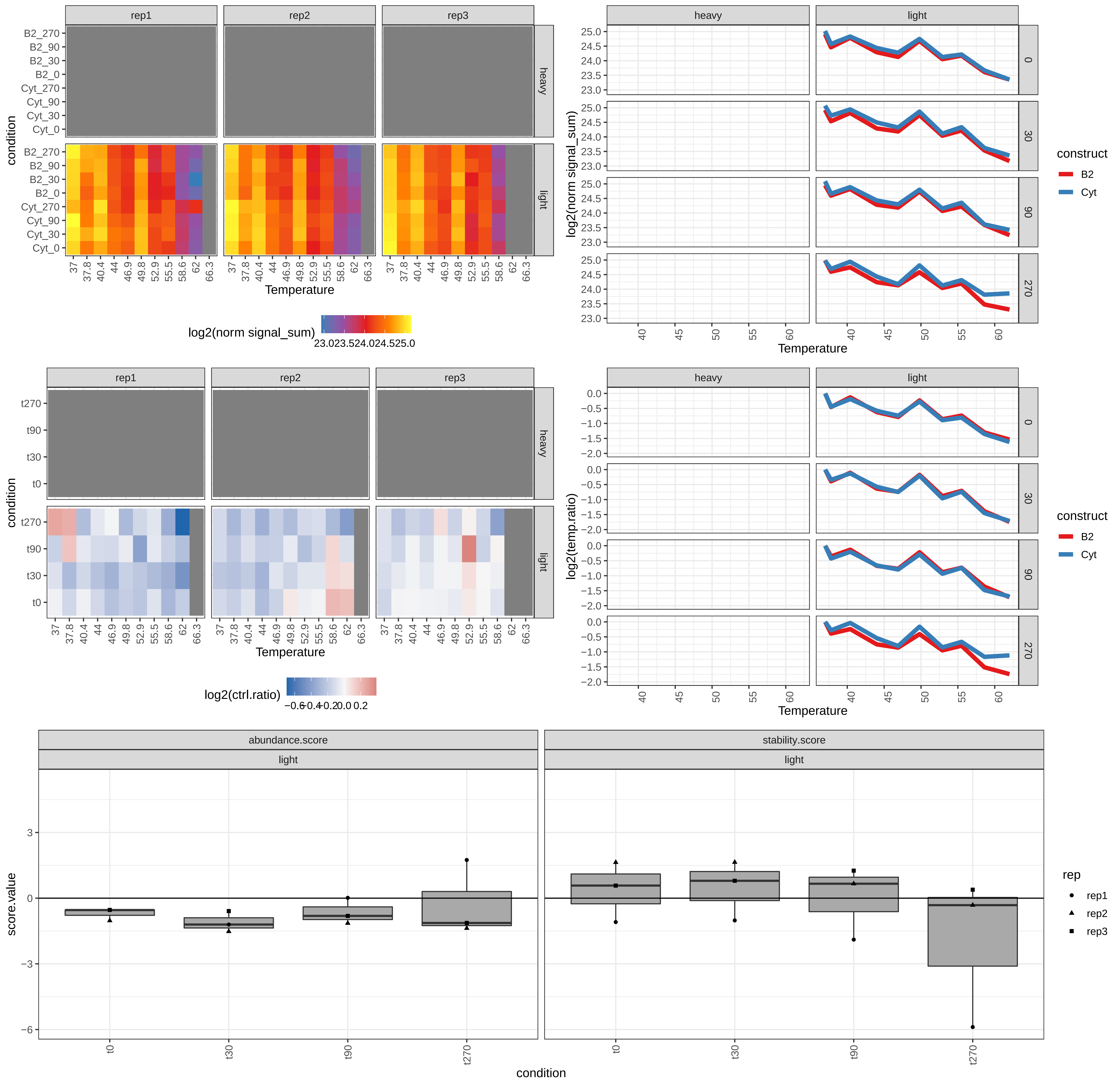

P40491 − ATP synthase assembly factor FMC1, mitochondrial OS=Saccharomyces cerevisiae (strain ATCC 204508 / S288c) GN=FMC1 PE=1 SV=1

FMN1 − average.top3: 6.47107882352941 − max.qupm: 4

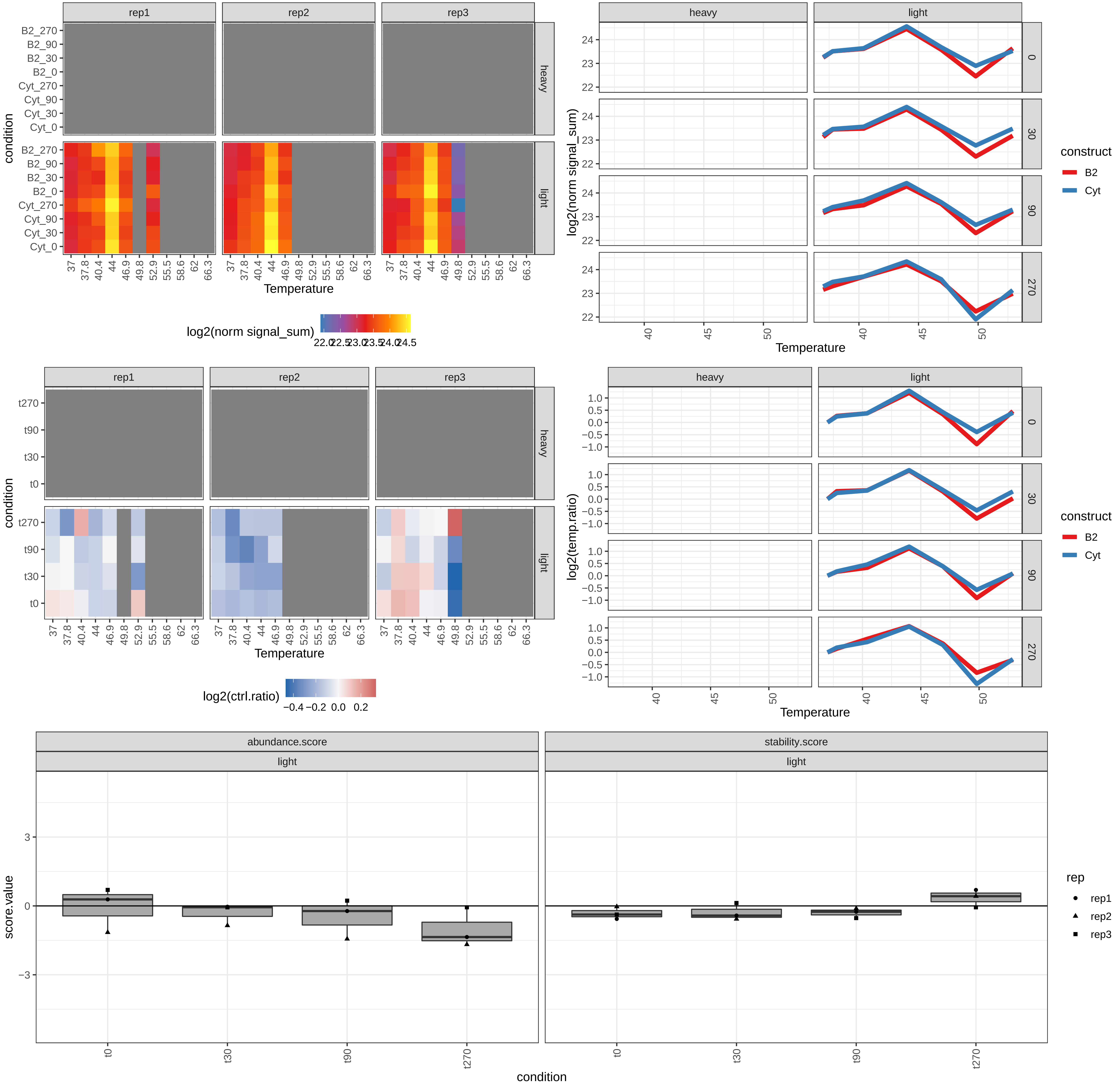

Q03778 − Riboflavin kinase OS=Saccharomyces cerevisiae (strain ATCC 204508 / S288c) GN=FMN1 PE=1 SV=1

FMP10 − average.top3: 7.08195785714286 − max.qupm: 4

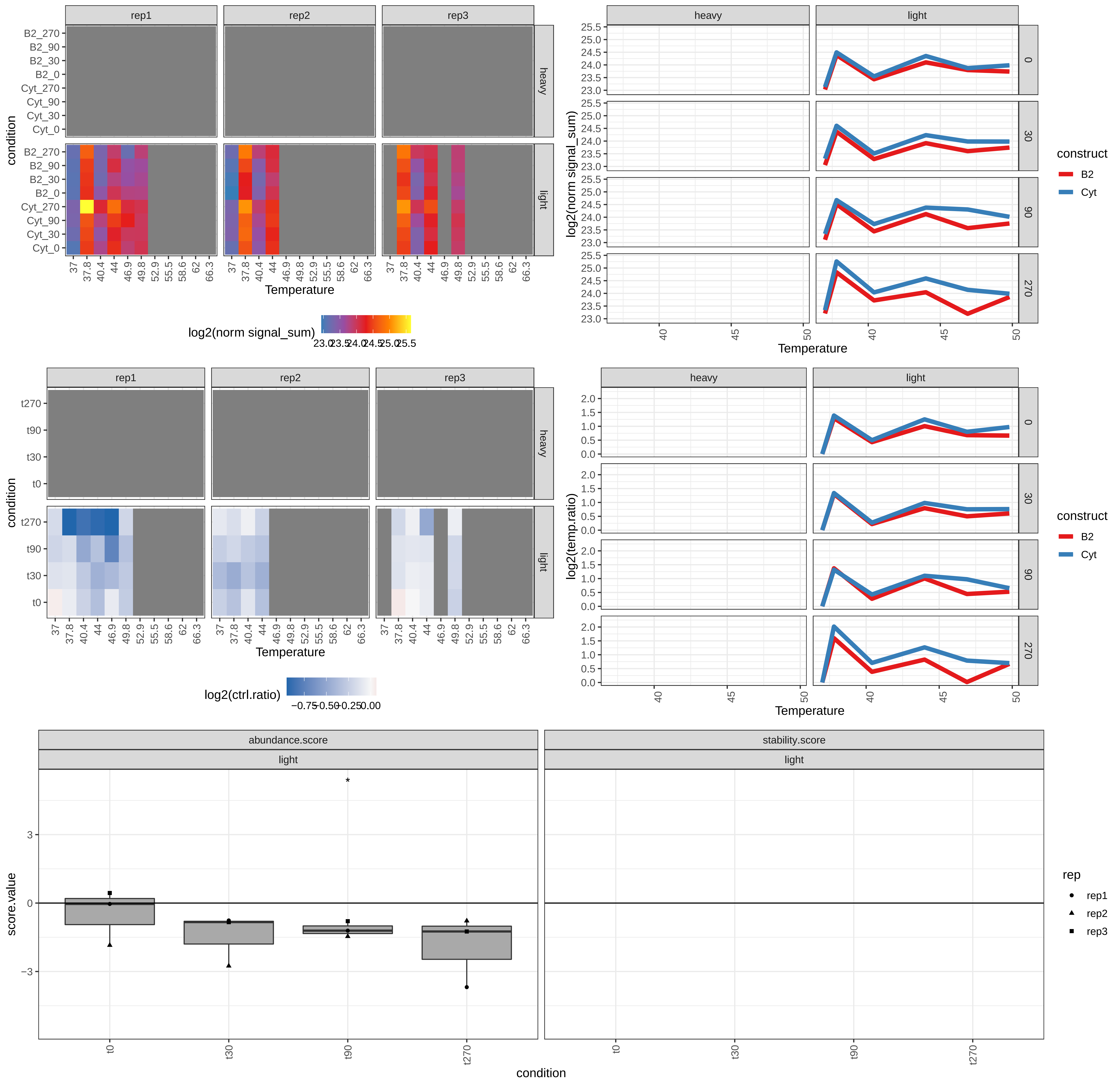

P40098 − Uncharacterized mitochondrial membrane protein FMP10 OS=Saccharomyces cerevisiae (strain ATCC 204508 / S288c) GN=FMP10 PE=1 SV=1

FMP16 − average.top3: 7.02661431818182 − max.qupm: 7

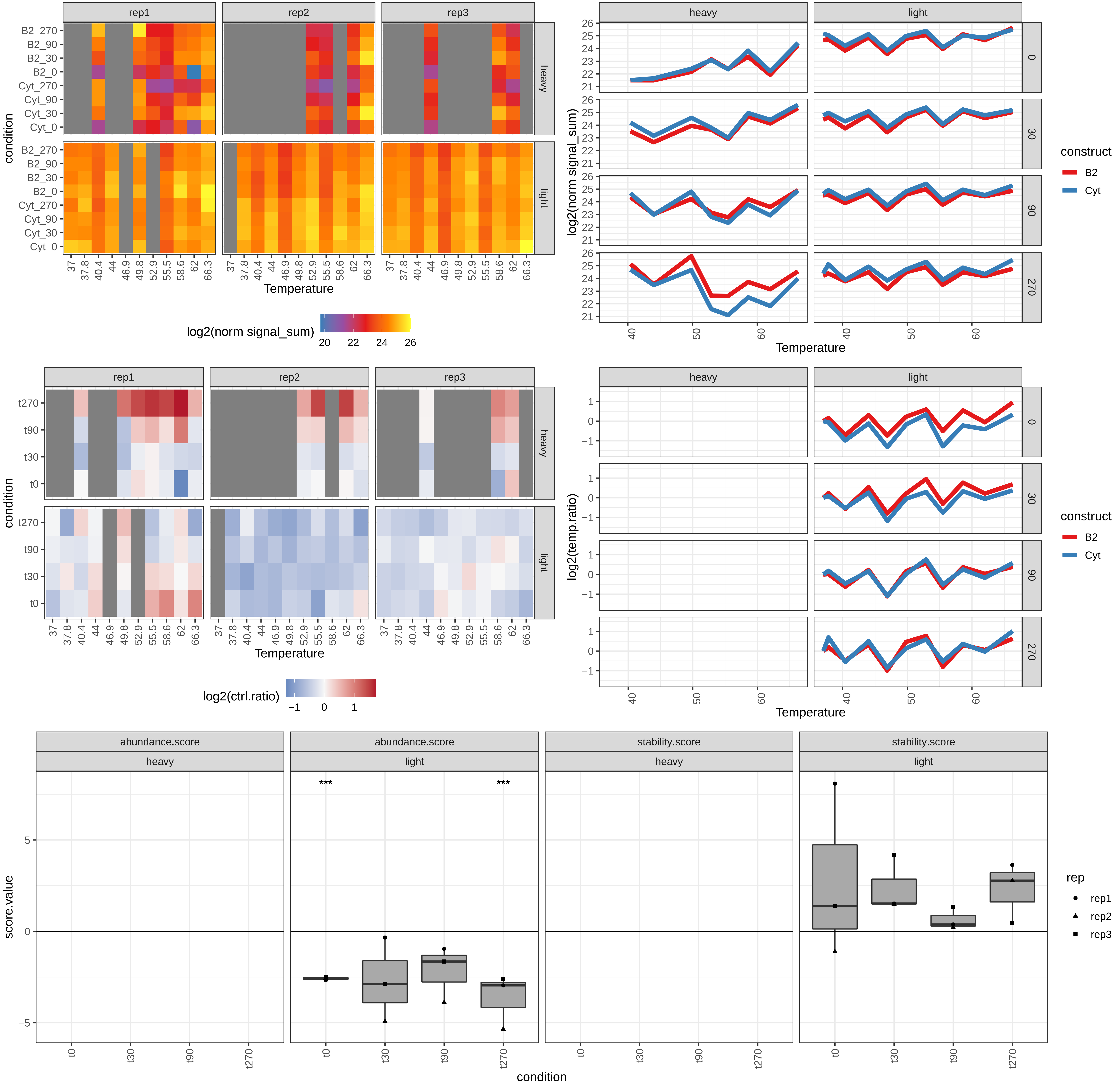

Q12497 − Protein FMP16, mitochondrial OS=Saccharomyces cerevisiae (strain ATCC 204508 / S288c) GN=FMP16 PE=1 SV=1

FMP25 − average.top3: 7.04964115384615 − max.qupm: 12

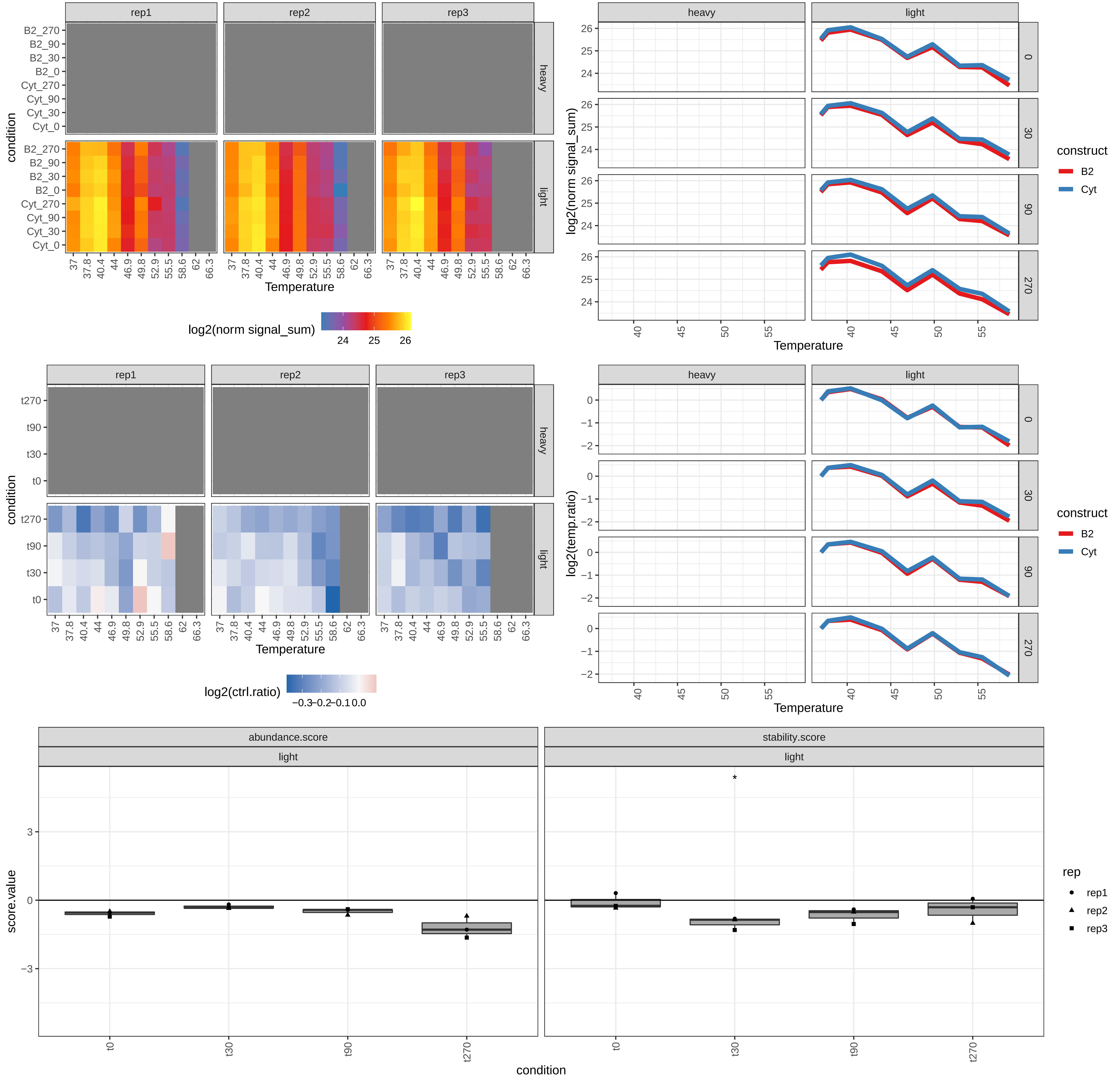

Q08023 − Protein FMP25, mitochondrial OS=Saccharomyces cerevisiae (strain ATCC 204508 / S288c) GN=FMP25 PE=4 SV=1

FMP27 − average.top3: 6.70082211538462 − max.qupm: 32

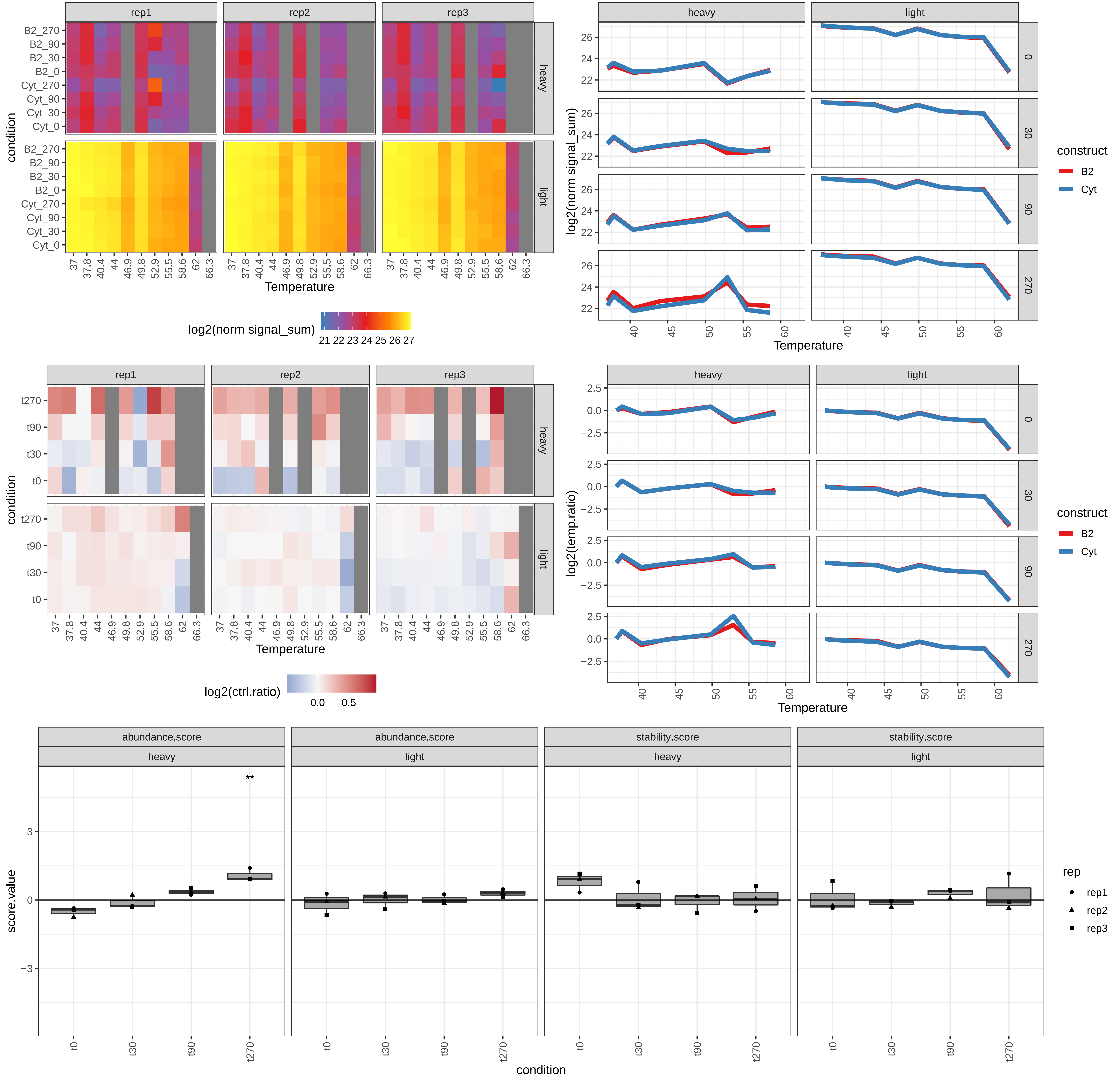

Q06179 − Protein FMP27, mitochondrial OS=Saccharomyces cerevisiae (strain ATCC 204508 / S288c) GN=FMP27 PE=1 SV=1

FMP30 − average.top3: 6.26922888888889 − max.qupm: 4

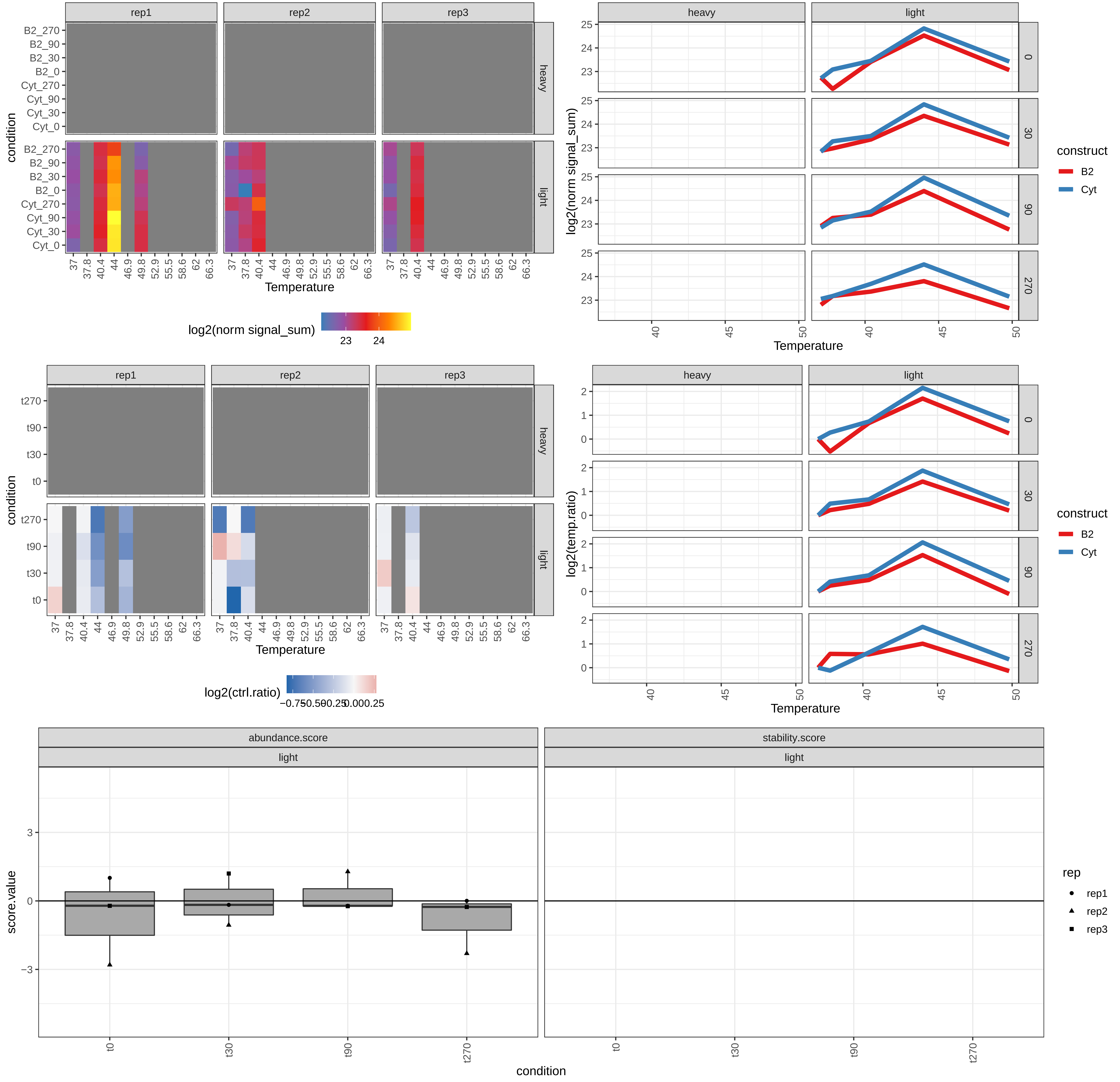

Q02883 − N−acyl−phosphatidylethanolamine−hydrolyzing phospholipase D, mitochondrial OS=Saccharomyces cerevisiae (strain ATCC 204508 / S288c) GN=FMP30 PE=1 SV=1

FMP32 − average.top3: 6.67886115384615 − max.qupm: 5

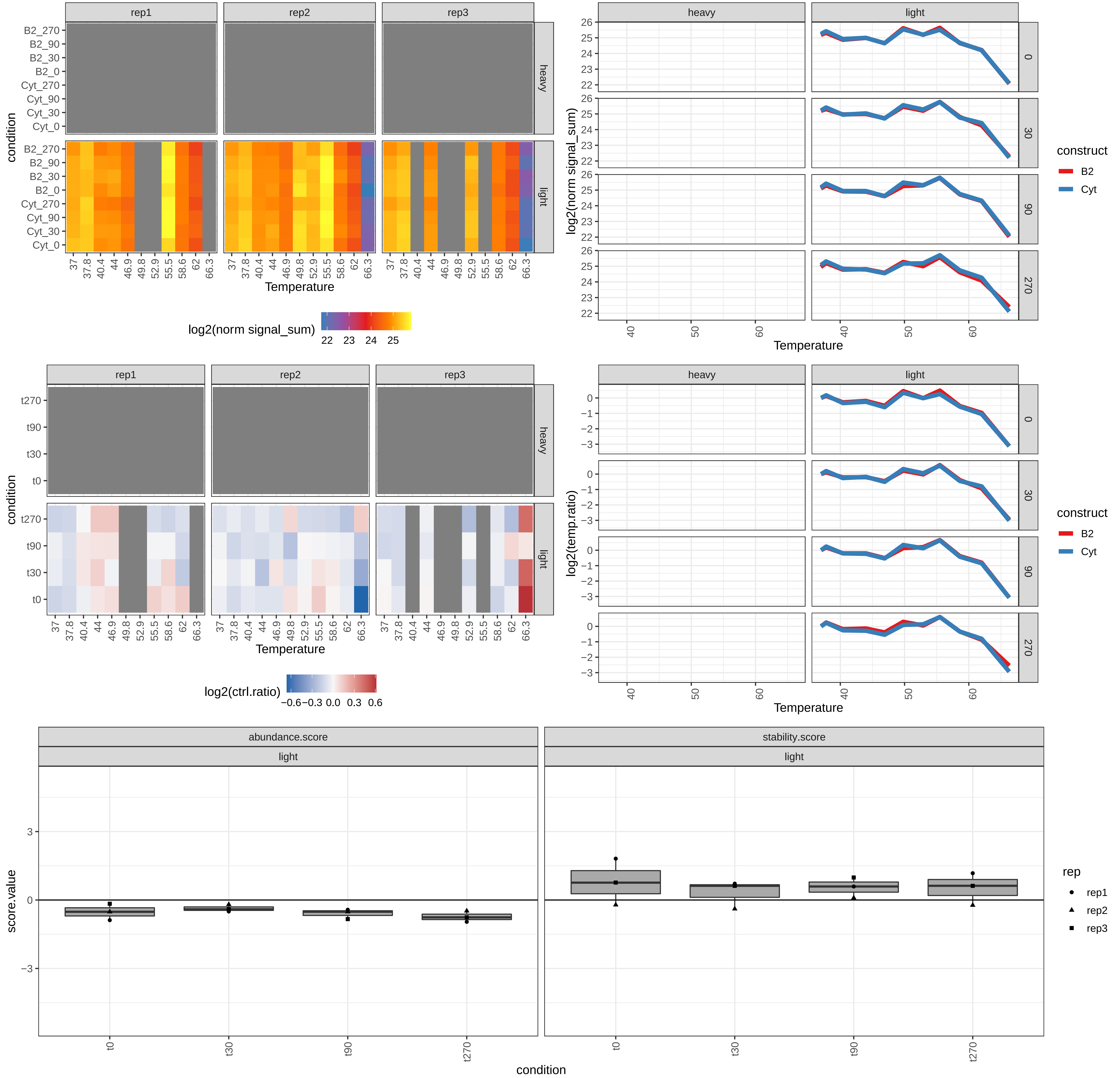

P43557 − Protein FMP32, mitochondrial OS=Saccharomyces cerevisiae (strain ATCC 204508 / S288c) GN=FMP32 PE=1 SV=1

FMP40 − average.top3: 6.87157928571429 − max.qupm: 11

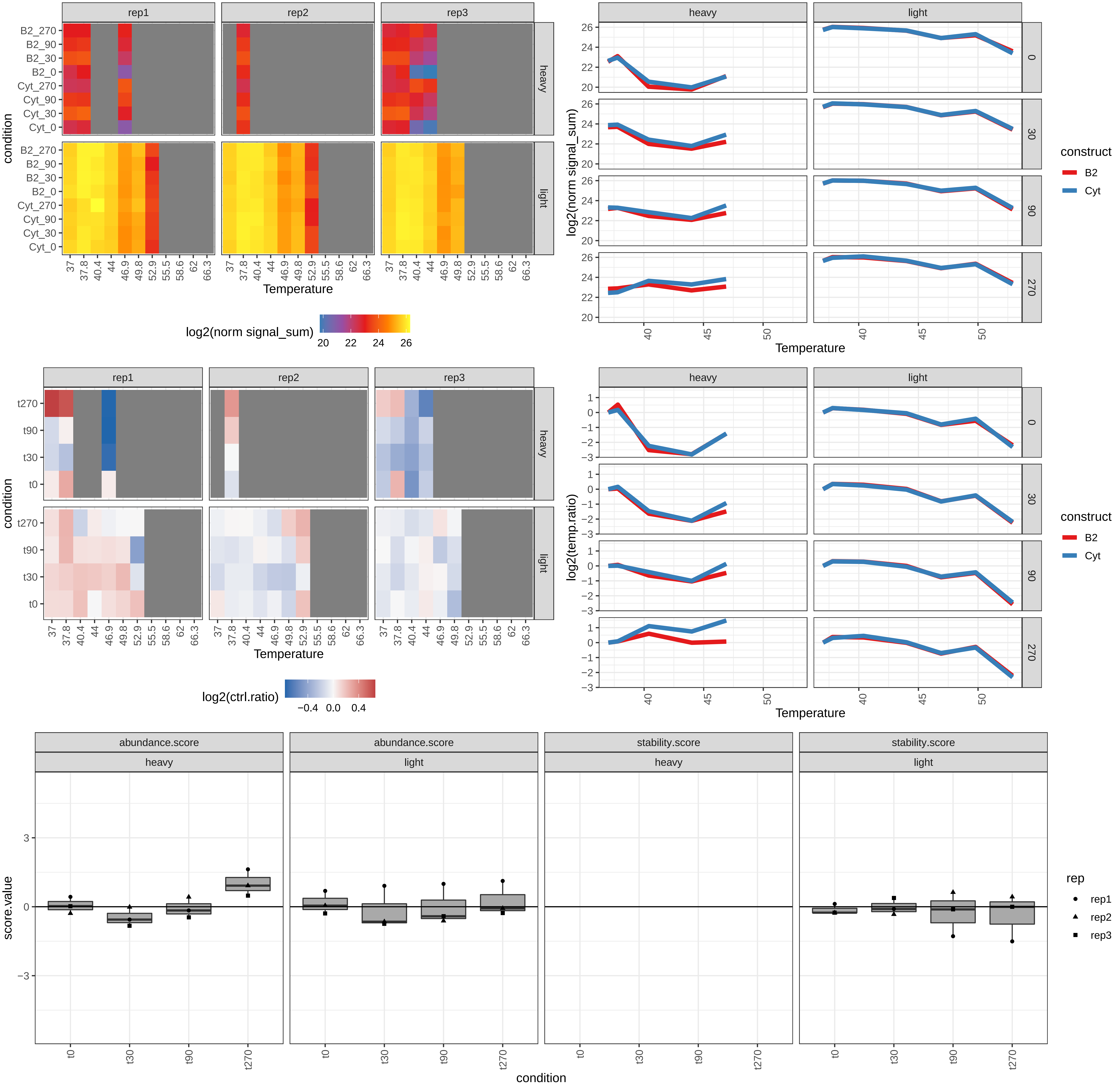

Q08968 − UPF0061 protein FMP40 OS=Saccharomyces cerevisiae (strain ATCC 204508 / S288c) GN=FMP40 PE=1 SV=1

FMP41 − average.top3: 7.40391485714286 − max.qupm: 7

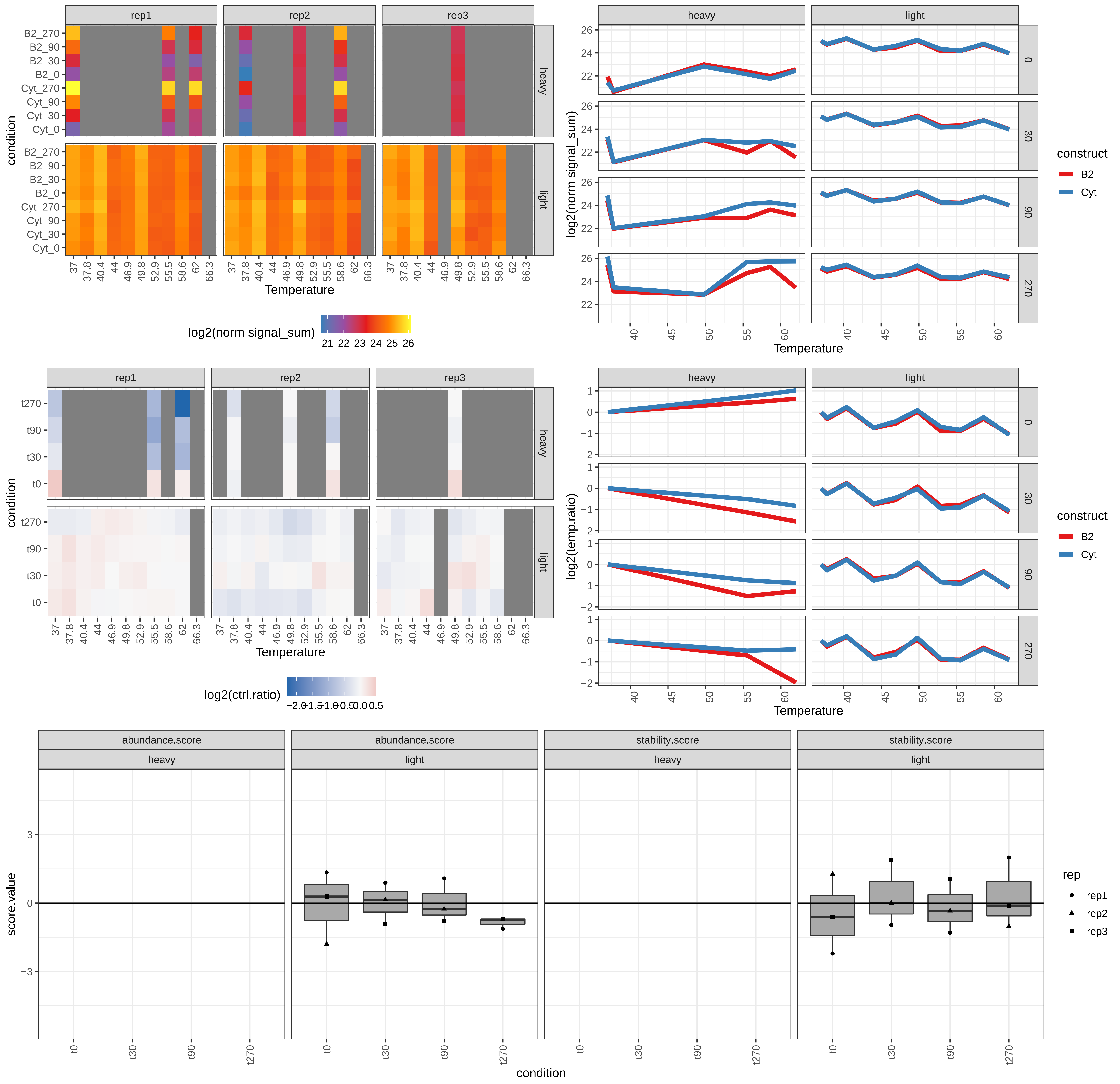

P53889 − Uncharacterized mitochondrial hydrolase FMP41 OS=Saccharomyces cerevisiae (strain ATCC 204508 / S288c) GN=FMP41 PE=1 SV=1

FMP42 − average.top3: 6.32442411764706 − max.qupm: 4

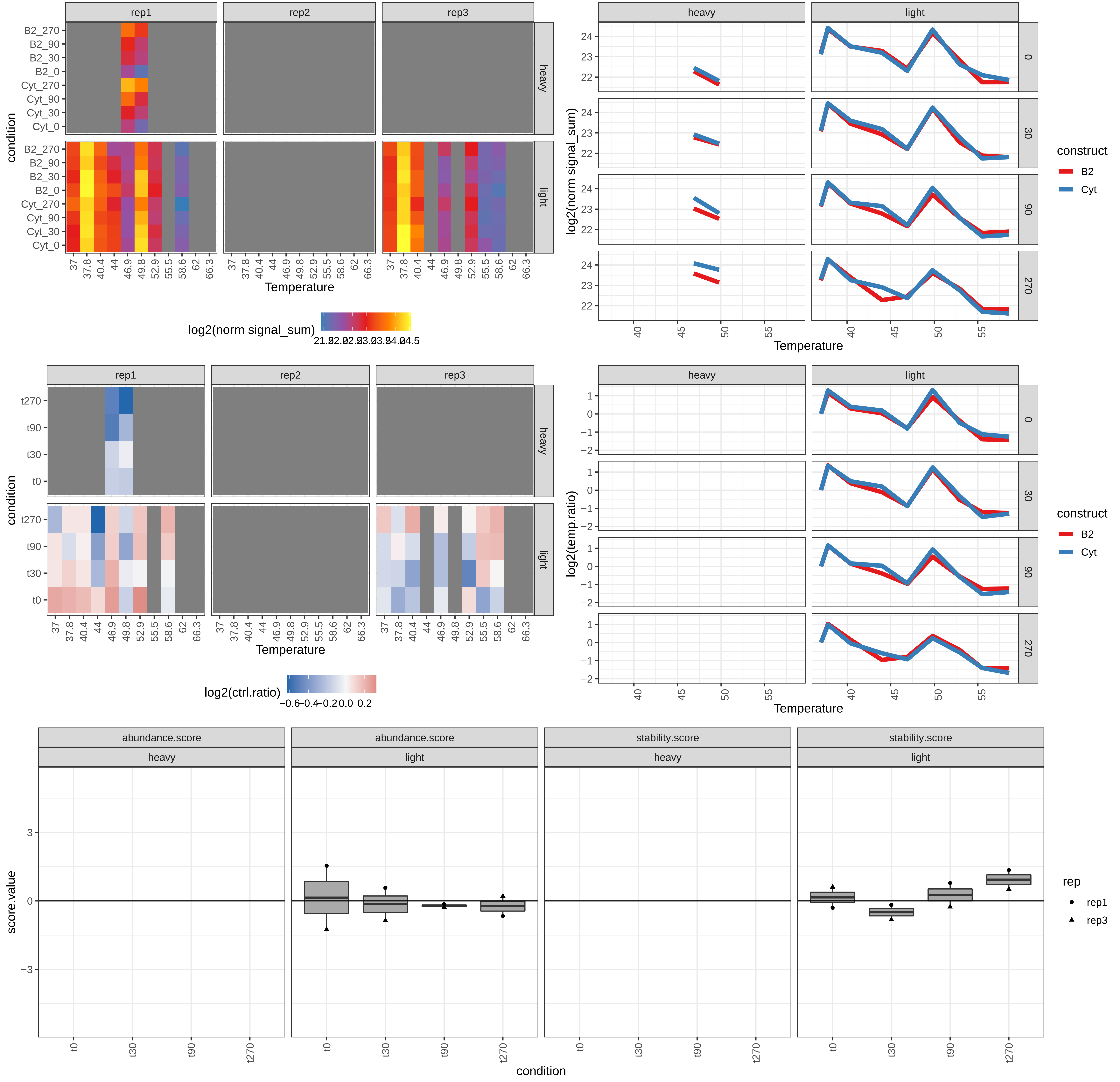

Q04991 − Protein FMP42 OS=Saccharomyces cerevisiae (strain ATCC 204508 / S288c) GN=FMP42 PE=1 SV=1

FMP46 − average.top3: 6.92807764705882 − max.qupm: 7

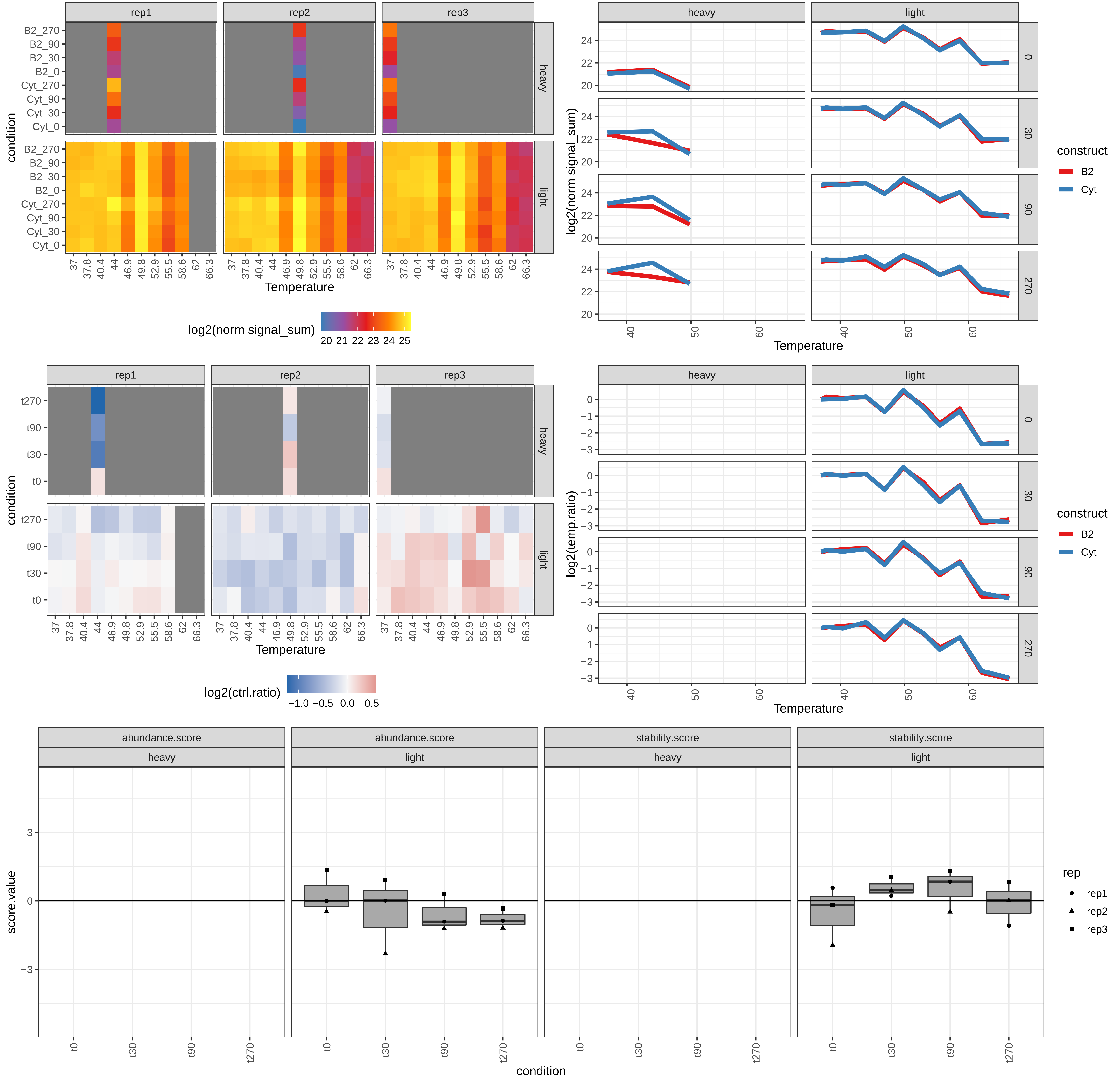

P36141 − Putative redox protein FMP46, mitochondrial OS=Saccharomyces cerevisiae (strain ATCC 204508 / S288c) GN=FMP46 PE=1 SV=1

FMP52 − average.top3: 7.44637453125 − max.qupm: 12

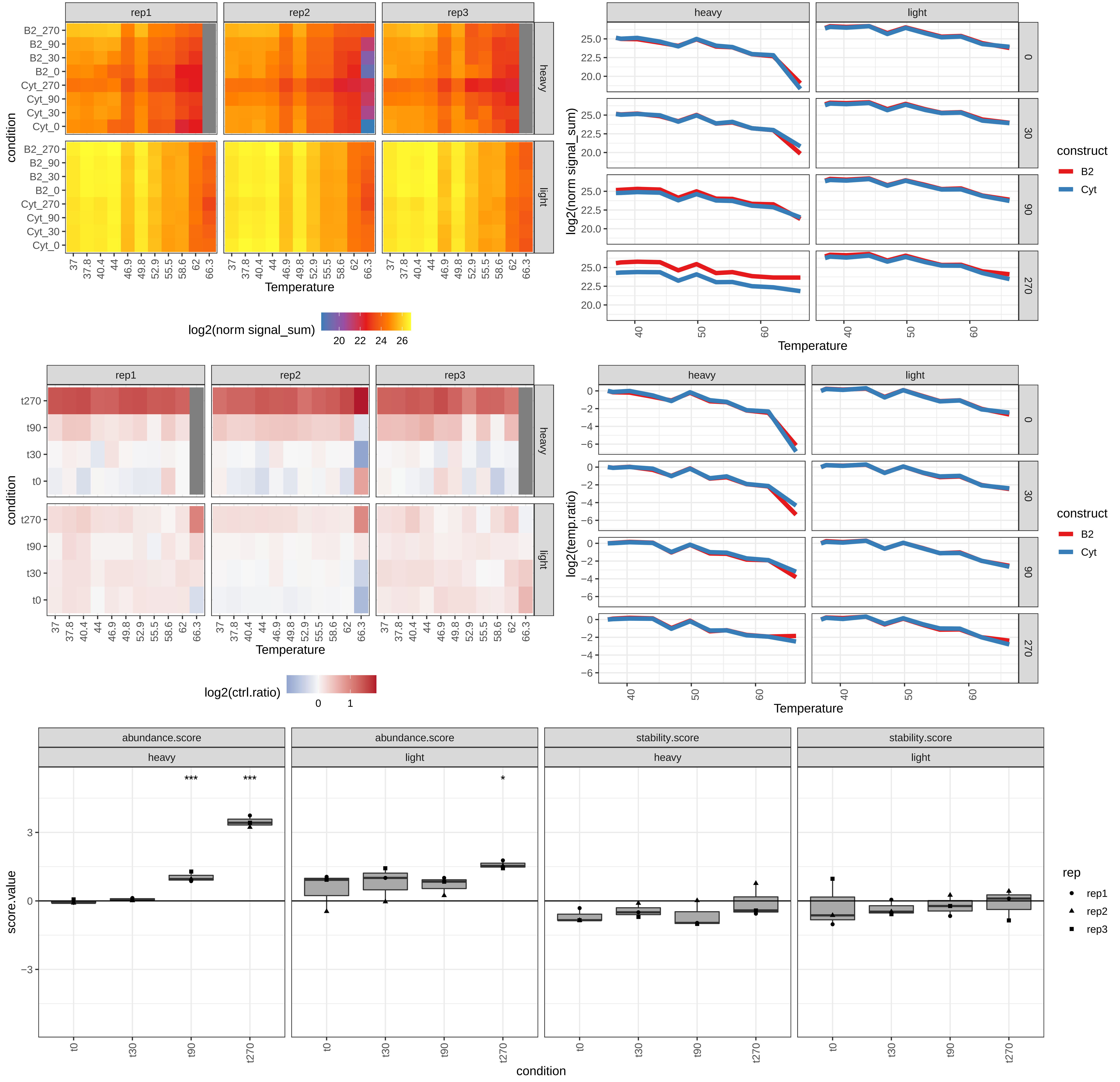

P40008 − Protein FMP52, mitochondrial OS=Saccharomyces cerevisiae (strain ATCC 204508 / S288c) GN=FMP52 PE=1 SV=1

FMT1 − average.top3: 6.73787714285714 − max.qupm: 6

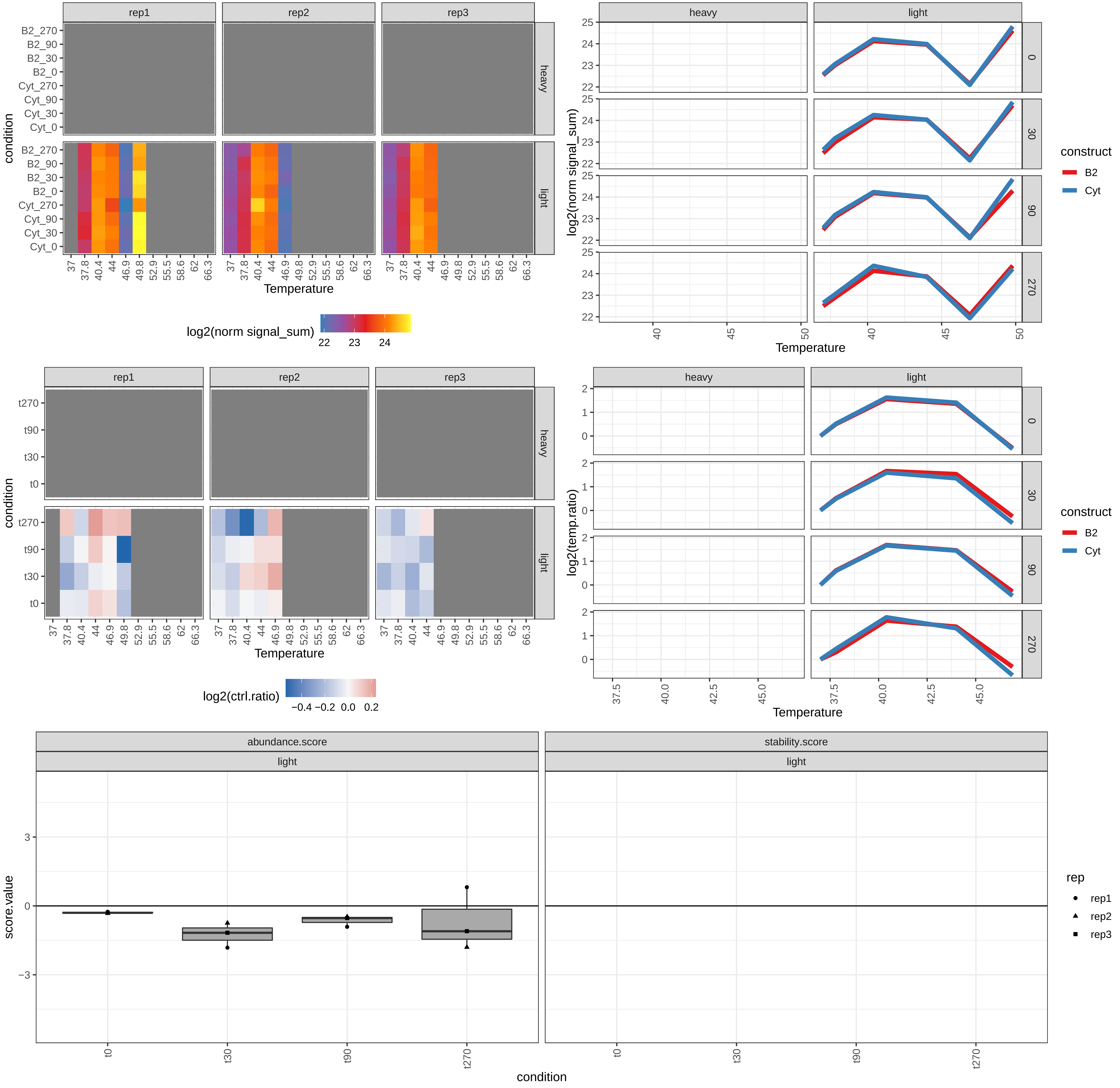

P32785 − Methionyl−tRNA formyltransferase, mitochondrial OS=Saccharomyces cerevisiae (strain ATCC 204508 / S288c) GN=FMT1 PE=1 SV=2

FOB1 − average.top3: 6.53032666666667 − max.qupm: 3

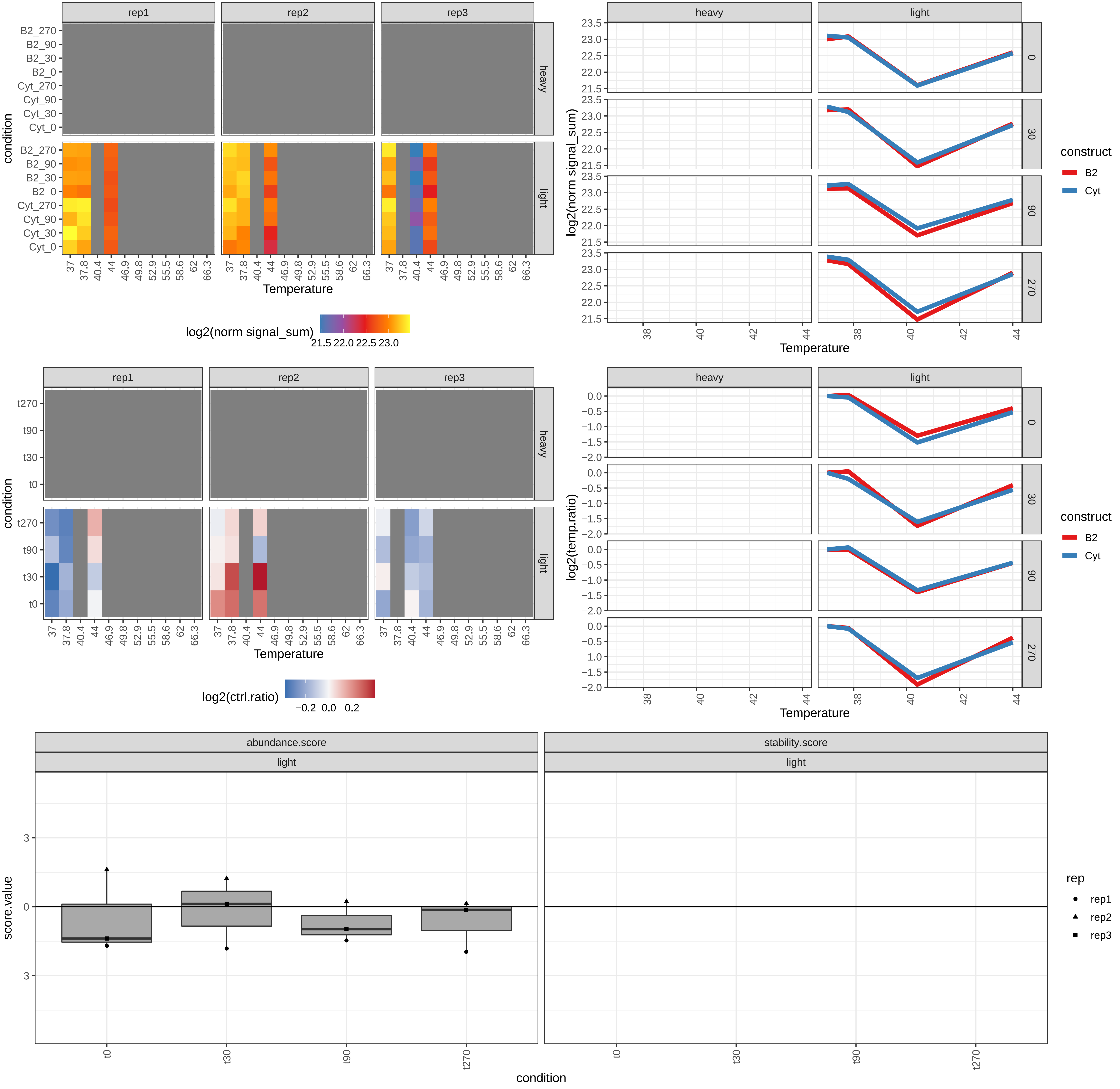

O13329 − DNA replication fork−blocking protein FOB1 OS=Saccharomyces cerevisiae (strain ATCC 204508 / S288c) GN=FOB1 PE=1 SV=2

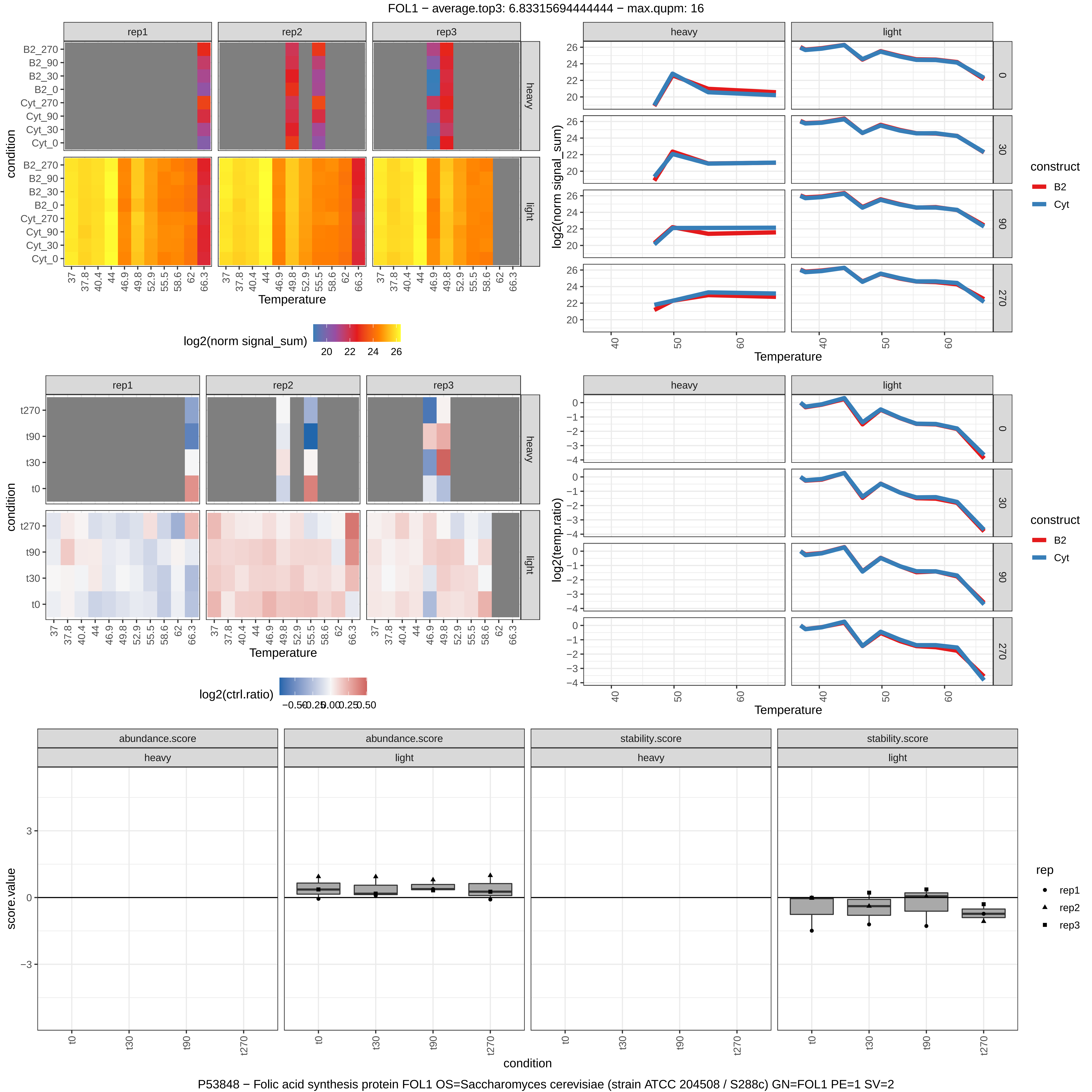
FOL1 − average.top3: 6.83315694444444 − max.qupm: 16
rep1
rep2
rep3
heavy
light
condition
Temperature
log2(norm signal_sum)
construct
B2
Cyt
log2(temp.ratio)
log2(ctrl.ratio)
abundance.score
stability.score
score.value
rep
rep1
rep2
rep3
P53848 − Folic acid synthesis protein FOL1 OS=Saccharomyces cerevisiae (strain ATCC 204508 / S288c) GN=FOL1 PE=1 SV=2

FOL2 − average.top3: 6.92296694444444 − max.qupm: 10

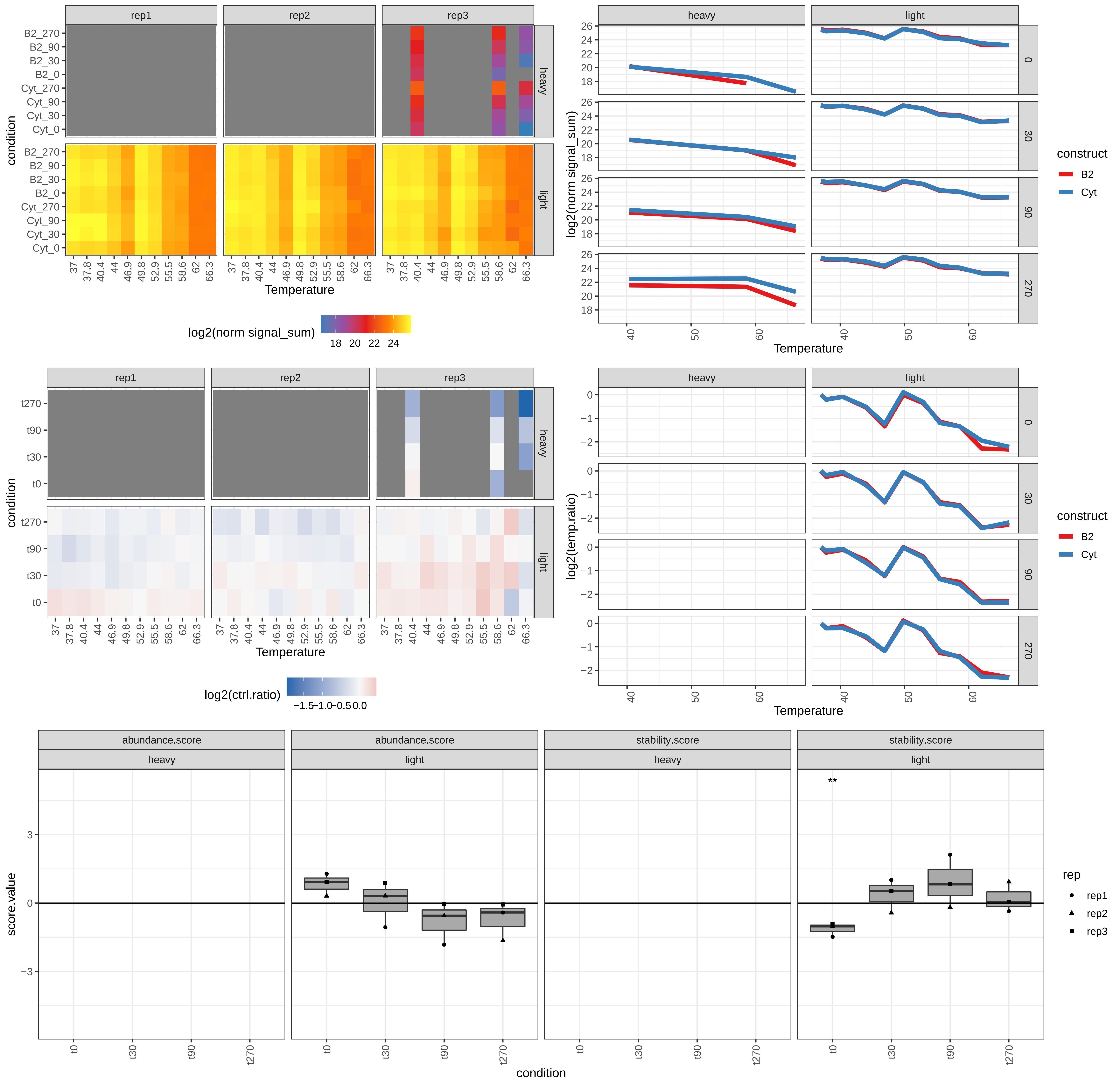

P51601 − GTP cyclohydrolase 1 OS=Saccharomyces cerevisiae (strain ATCC 204508 / S288c) GN=FOL2 PE=1 SV=1

FOX2 − average.top3: 7.71289413043478 − max.qupm: 51

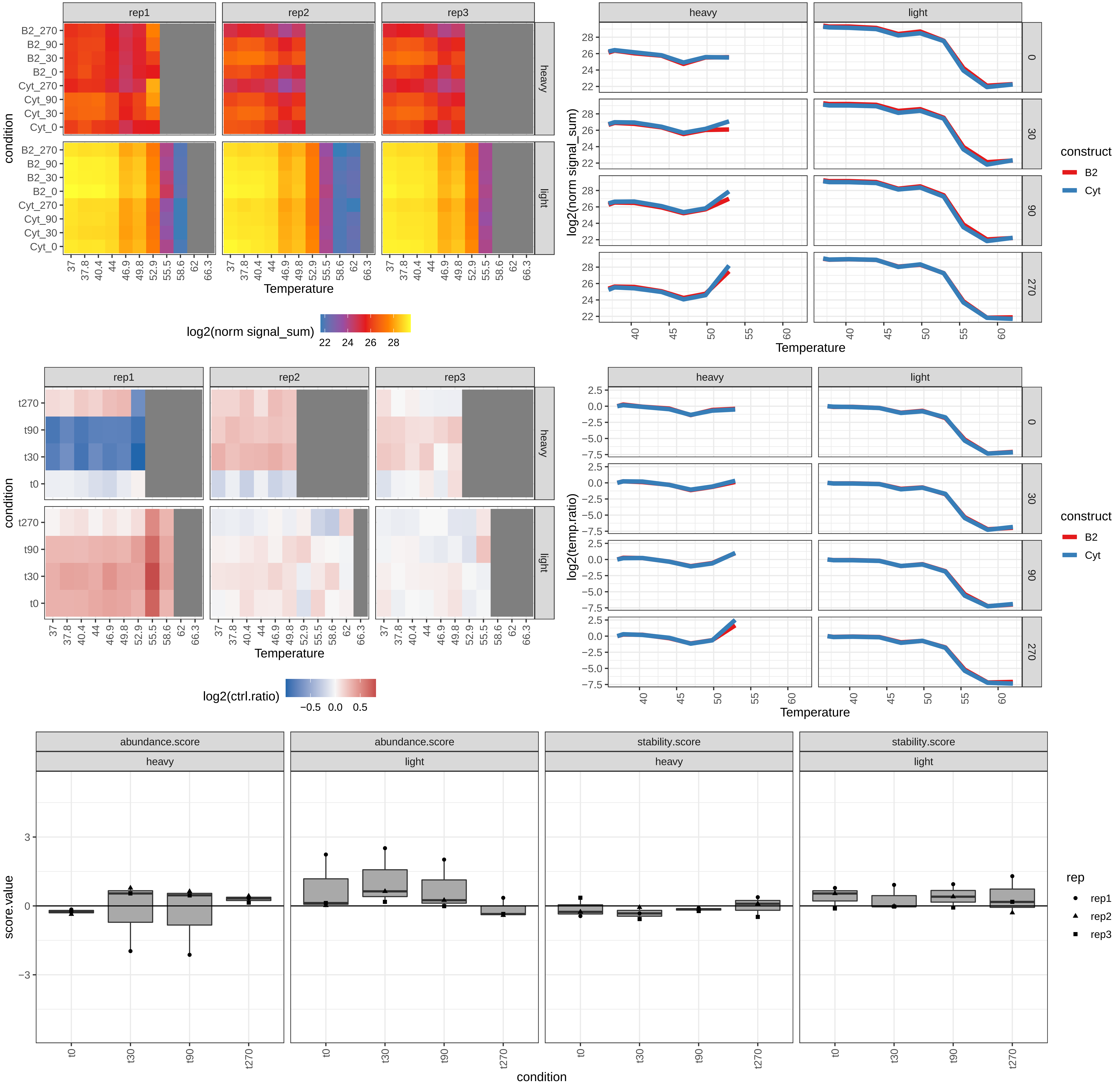

Q02207 − Peroxisomal hydratase−dehydrogenase−epimerase OS=Saccharomyces cerevisiae (strain ATCC 204508 / S288c) GN=FOX2 PE=2 SV=1

FPK1 − average.top3: 6.477745625 − max.qupm: 4

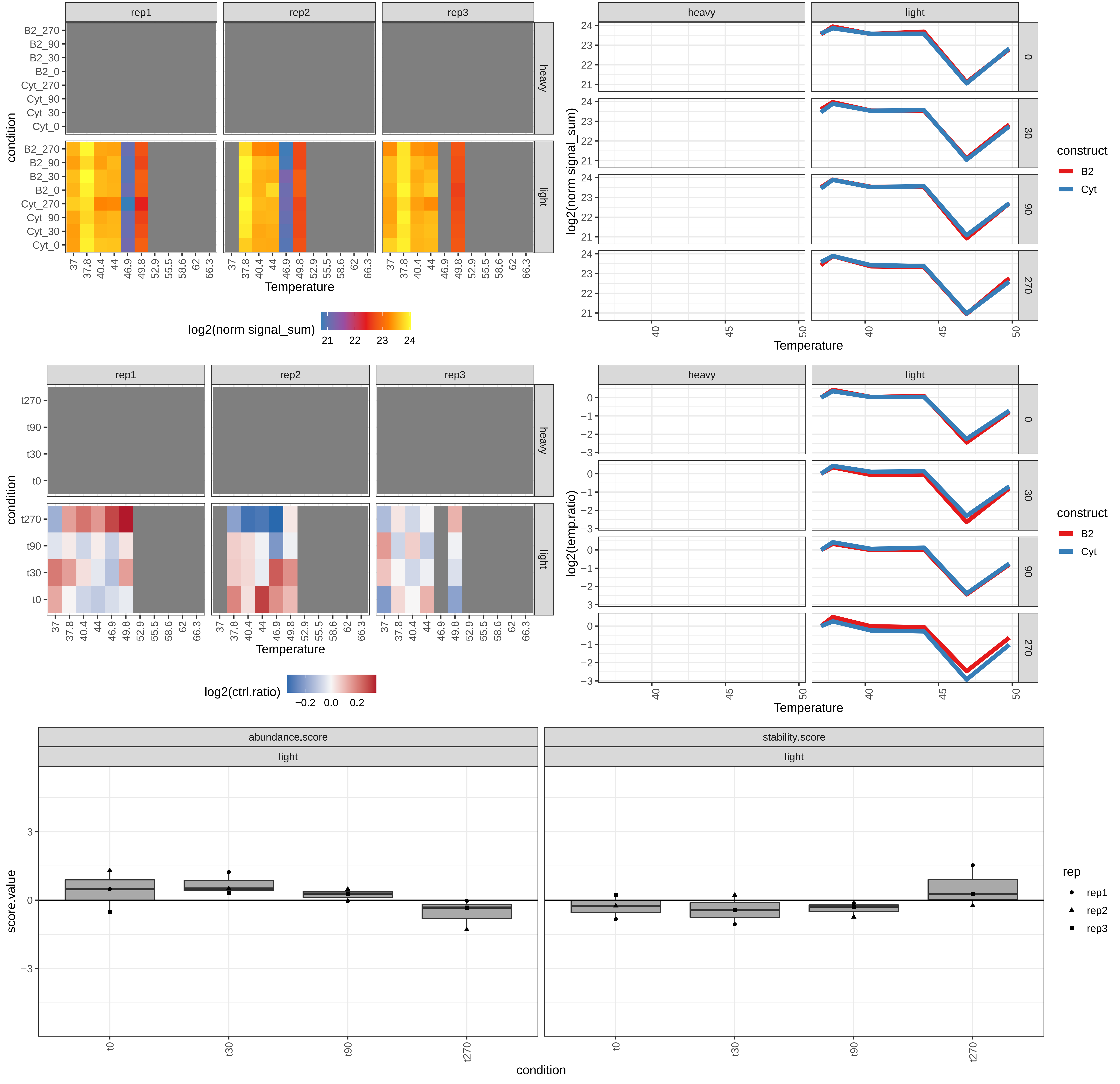

P53739 − Flippase kinase 1 OS=Saccharomyces cerevisiae (strain ATCC 204508 / S288c) GN=FPK1 PE=1 SV=1

FPR1 − average.top3: 7.57614466666667 − max.qupm: 8

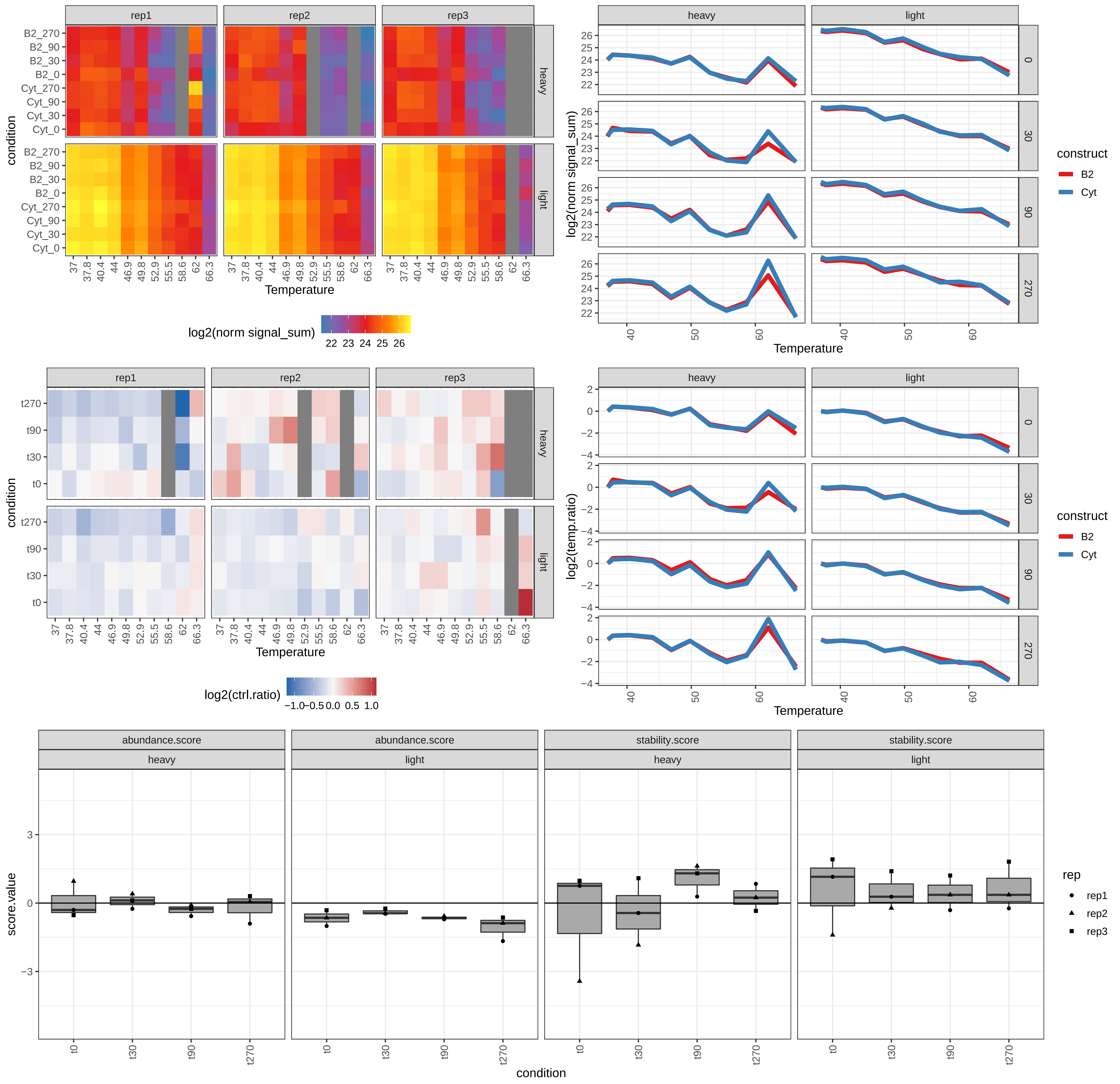

P20081 − FK506−binding protein 1 OS=Saccharomyces cerevisiae (strain ATCC 204508 / S288c) GN=FPR1 PE=1 SV=2

FPR2 − average.top3: 7.30920815789474 − max.qupm: 6

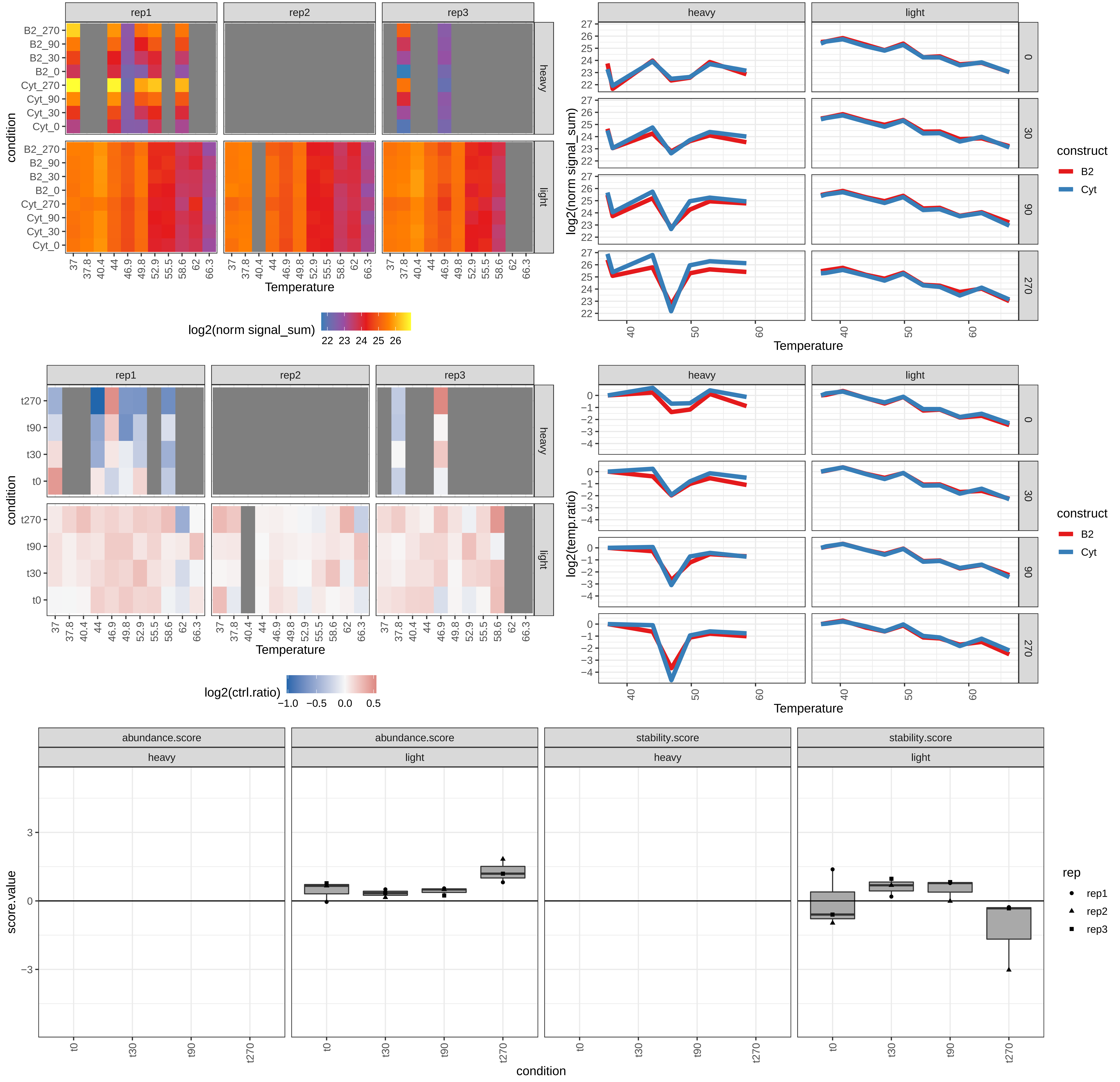

P32472 − Peptidyl−prolyl cis−trans isomerase FPR2 OS=Saccharomyces cerevisiae (strain ATCC 204508 / S288c) GN=FPR2 PE=1 SV=1

FPR3 − average.top3: 6.89626603773585 − max.qupm: 9

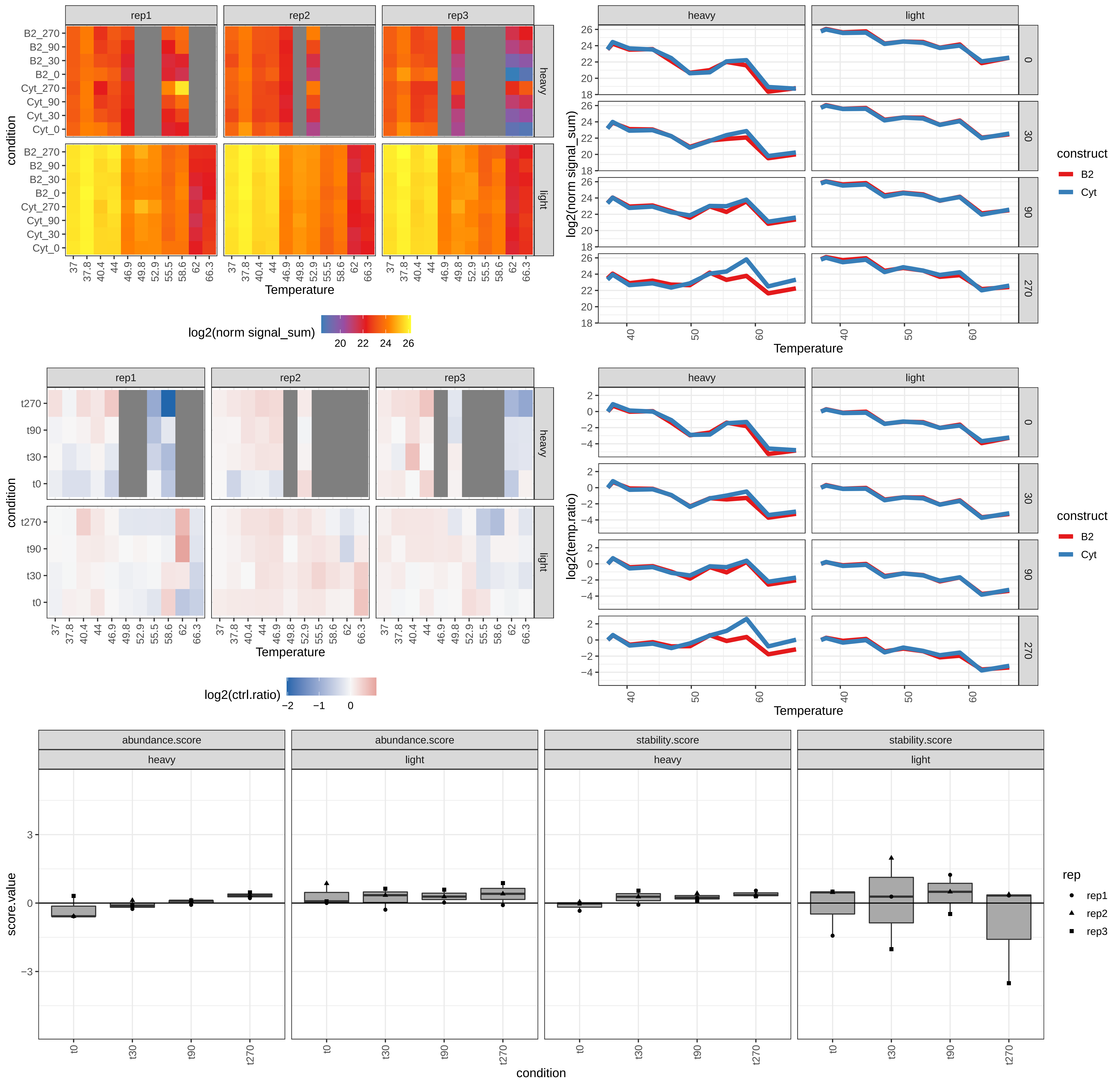

P38911 − FK506−binding nuclear protein OS=Saccharomyces cerevisiae (strain ATCC 204508 / S288c) GN=FPR3 PE=1 SV=2

FPR4 − average.top3: 7.18256083333333 − max.qupm: 3

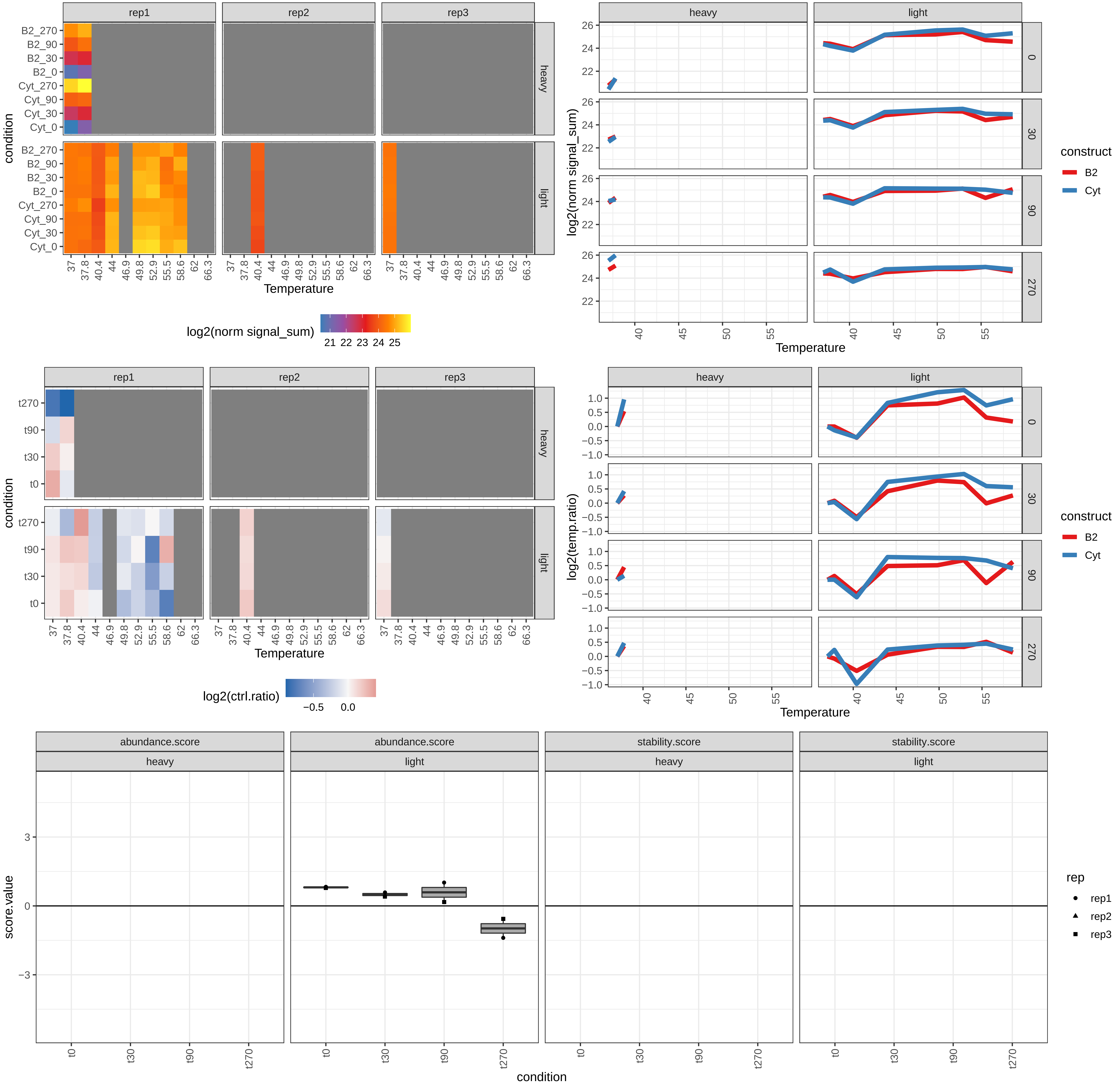

Q06205 − FK506−binding protein 4 OS=Saccharomyces cerevisiae (strain ATCC 204508 / S288c) GN=FPR4 PE=1 SV=1

FRA1 − average.top3: 7.28929517241379 − max.qupm: 17

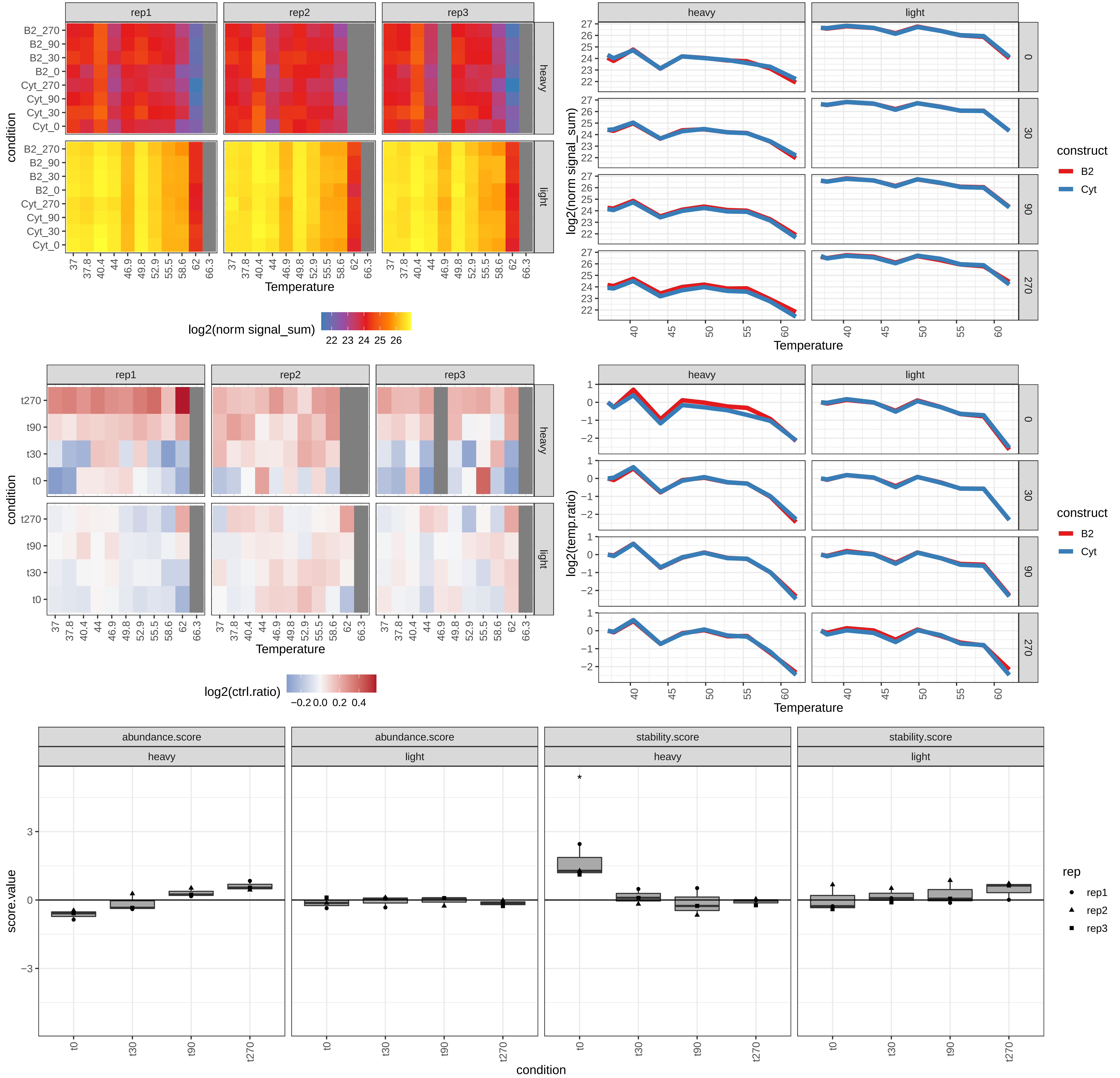

Q07825 − Putative Xaa−Pro aminopeptidase FRA1 OS=Saccharomyces cerevisiae (strain ATCC 204508 / S288c) GN=FRA1 PE=1 SV=1

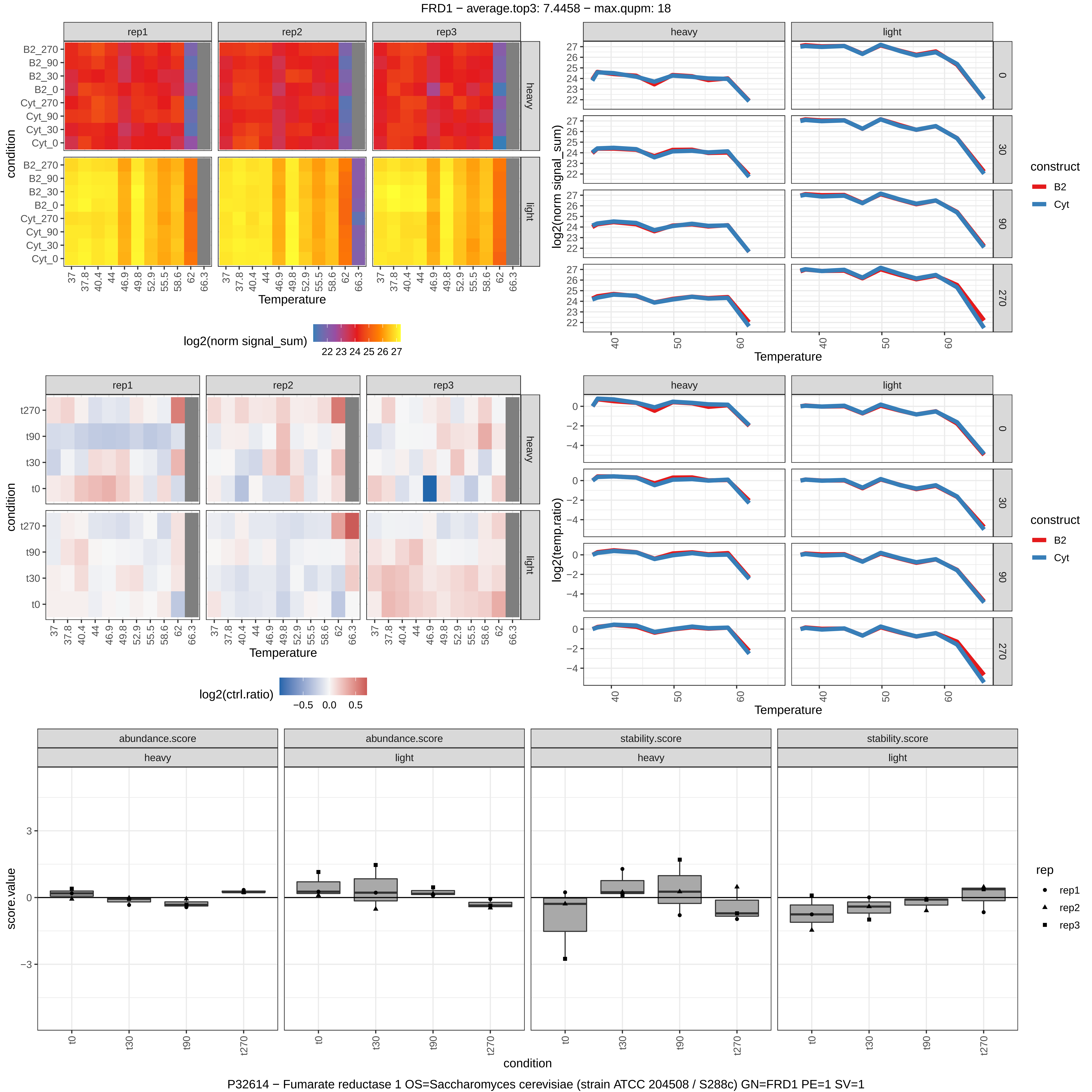
FRD1 − average.top3: 7.4458 − max.qupm: 18
rep1
rep2
rep3
heavy
light
condition
Temperature
log2(norm signal_sum)
22 23 24 25 26 27
log2(norm signal_sum)
construct
B2
Cyt
log2(ctrl.ratio)
−0.5 0.0 0.5
log2(temp.ratio)
abundance.score
stability.score
score.value
condition
rep
rep1
rep2
rep3
P32614 − Fumarate reductase 1 OS=Saccharomyces cerevisiae (strain ATCC 204508 / S288c) GN=FRD1 PE=1 SV=1

FRQ1 − average.top3: 6.92777722222222 − max.qupm: 5

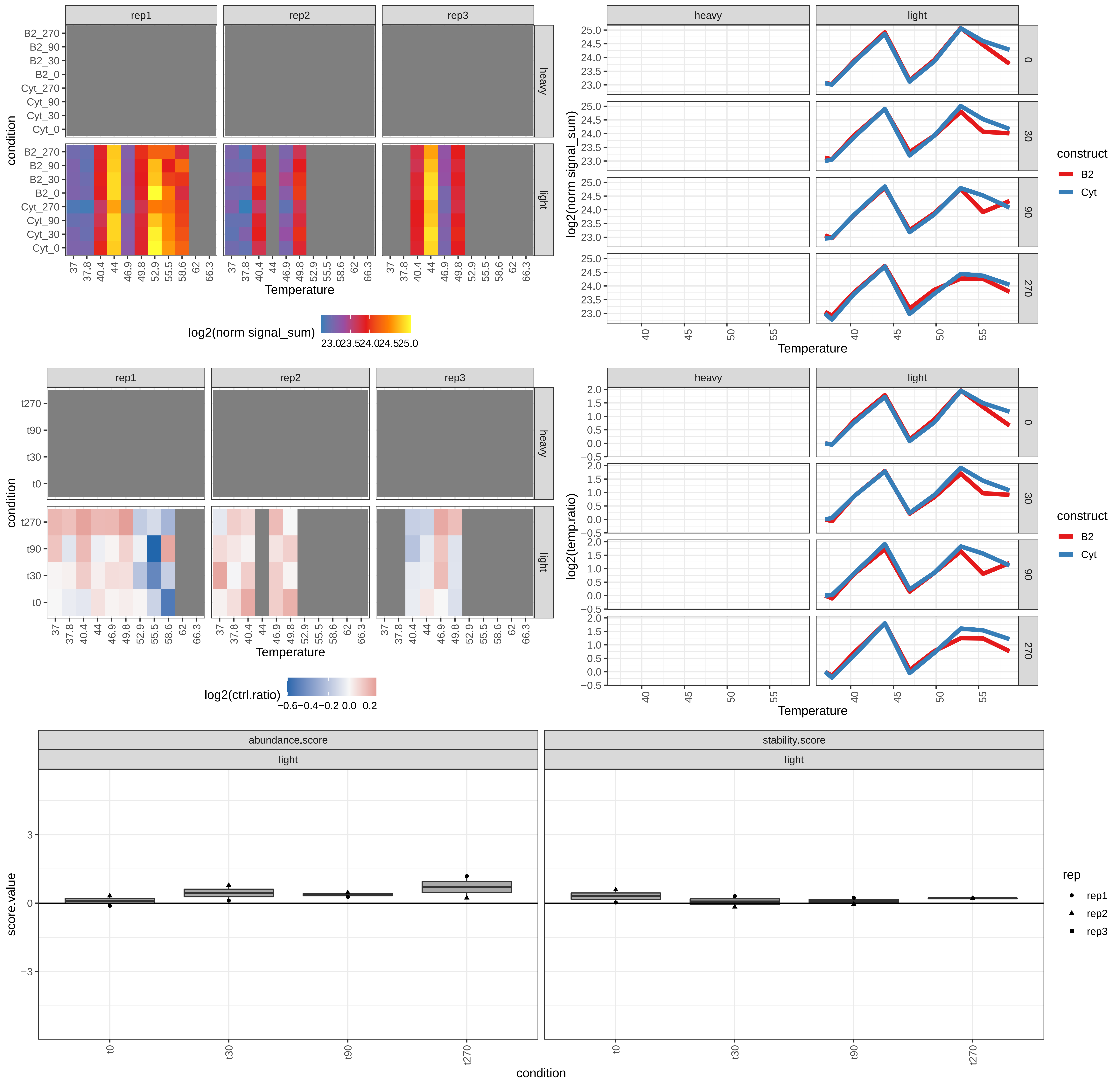

Q06389 − Calcium−binding protein NCS−1 OS=Saccharomyces cerevisiae (strain ATCC 204508 / S288c) GN=FRQ1 PE=1 SV=2

FRS1 − average.top3: 7.37318690909091 − max.qupm: 19

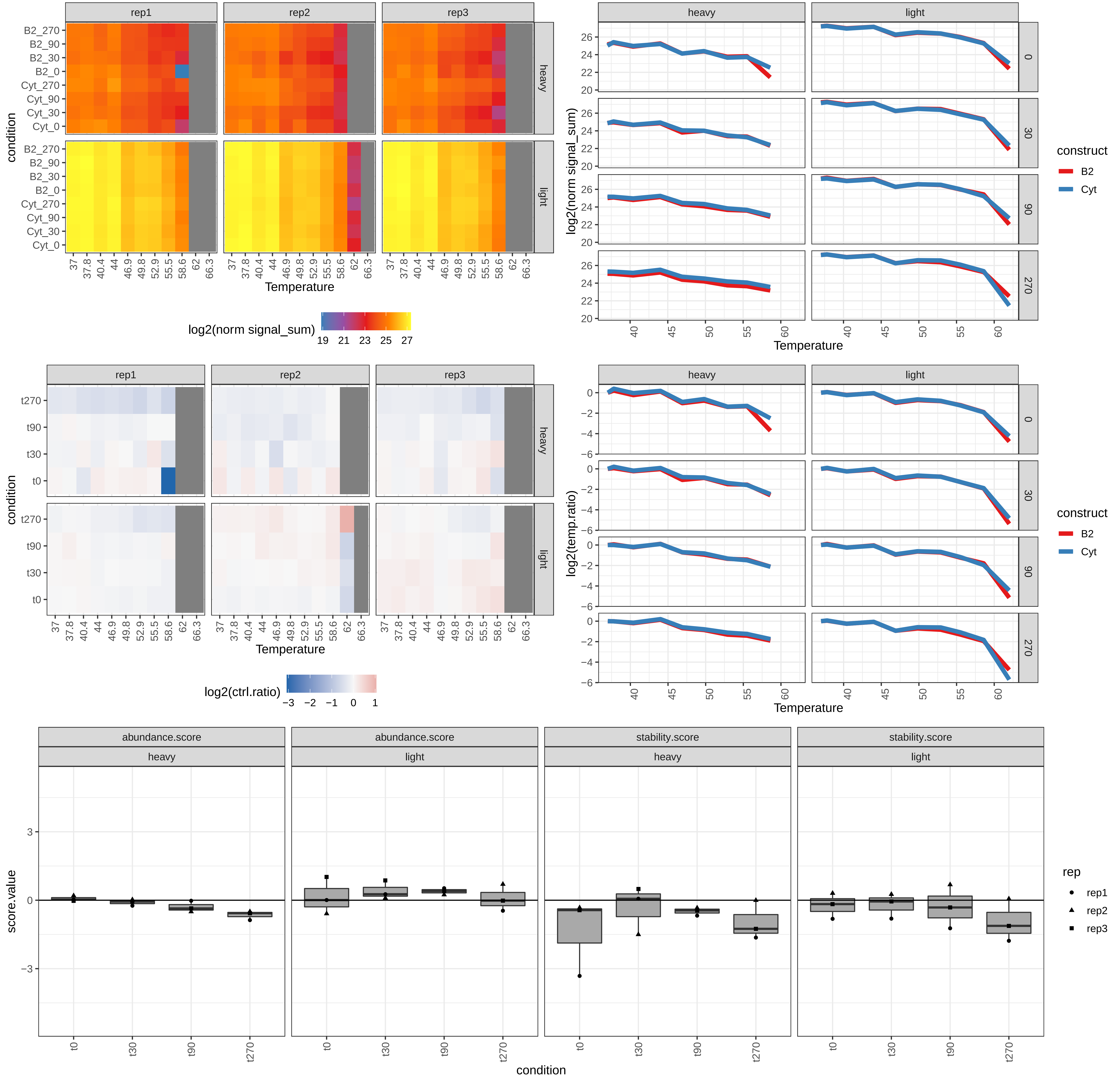

P15624 − Phenylalanine−−tRNA ligase beta subunit OS=Saccharomyces cerevisiae (strain ATCC 204508 / S288c) GN=FRS1 PE=1 SV=3

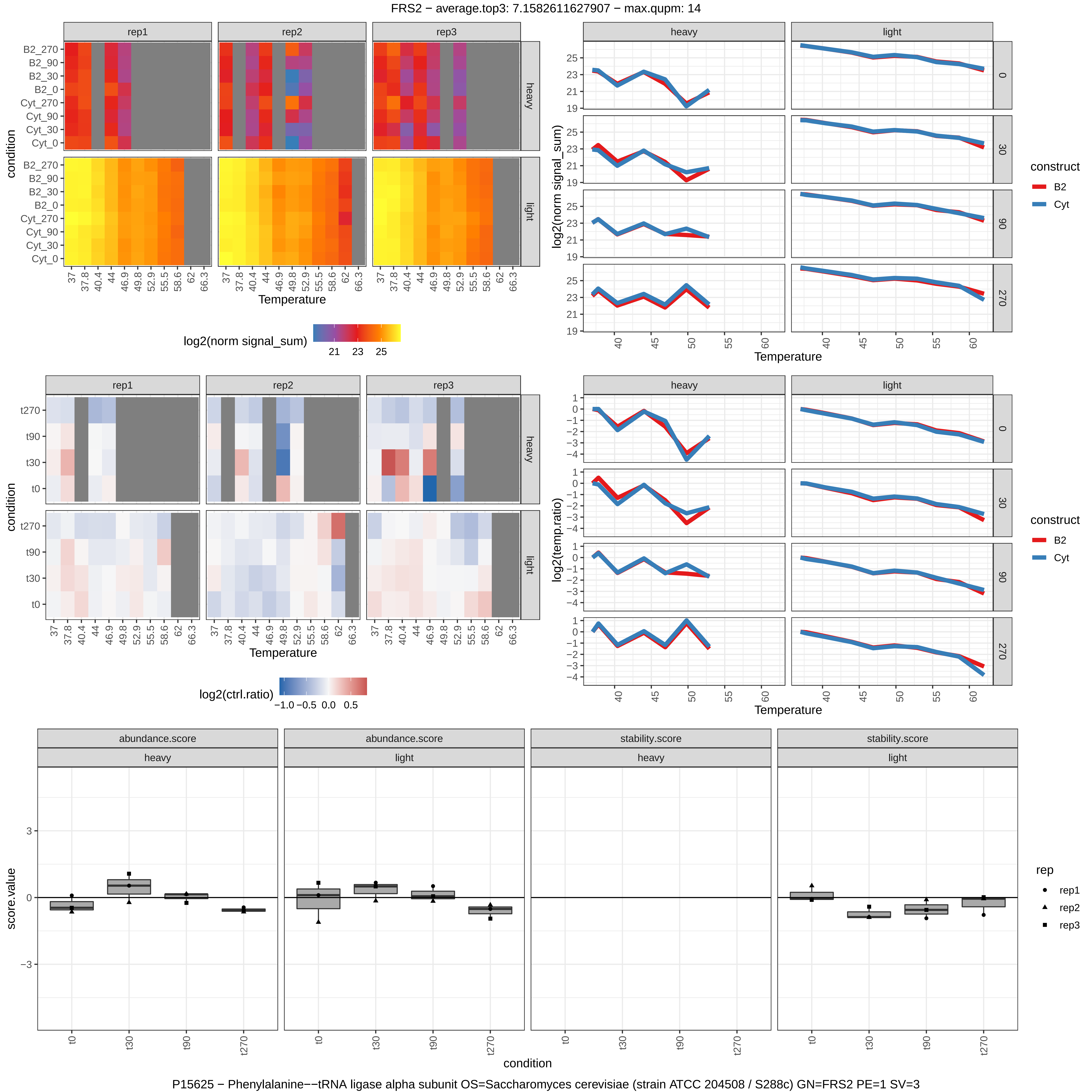
FRS2 − average.top3: 7.1582611627907 − max.qupm: 14
rep1
rep2
rep3
heavy
light
condition
Temperature
log2(norm signal_sum)
log2(temp.ratio)
log2(ctrl.ratio)
construct
B2
Cyt
abundance.score
stability.score
score.value
rep
rep1
rep2
rep3
P15625 − Phenylalanine−−tRNA ligase alpha subunit OS=Saccharomyces cerevisiae (strain ATCC 204508 / S288c) GN=FRS2 PE=1 SV=3

FSF1 − average.top3: 6.64214692307692 − max.qupm: 4

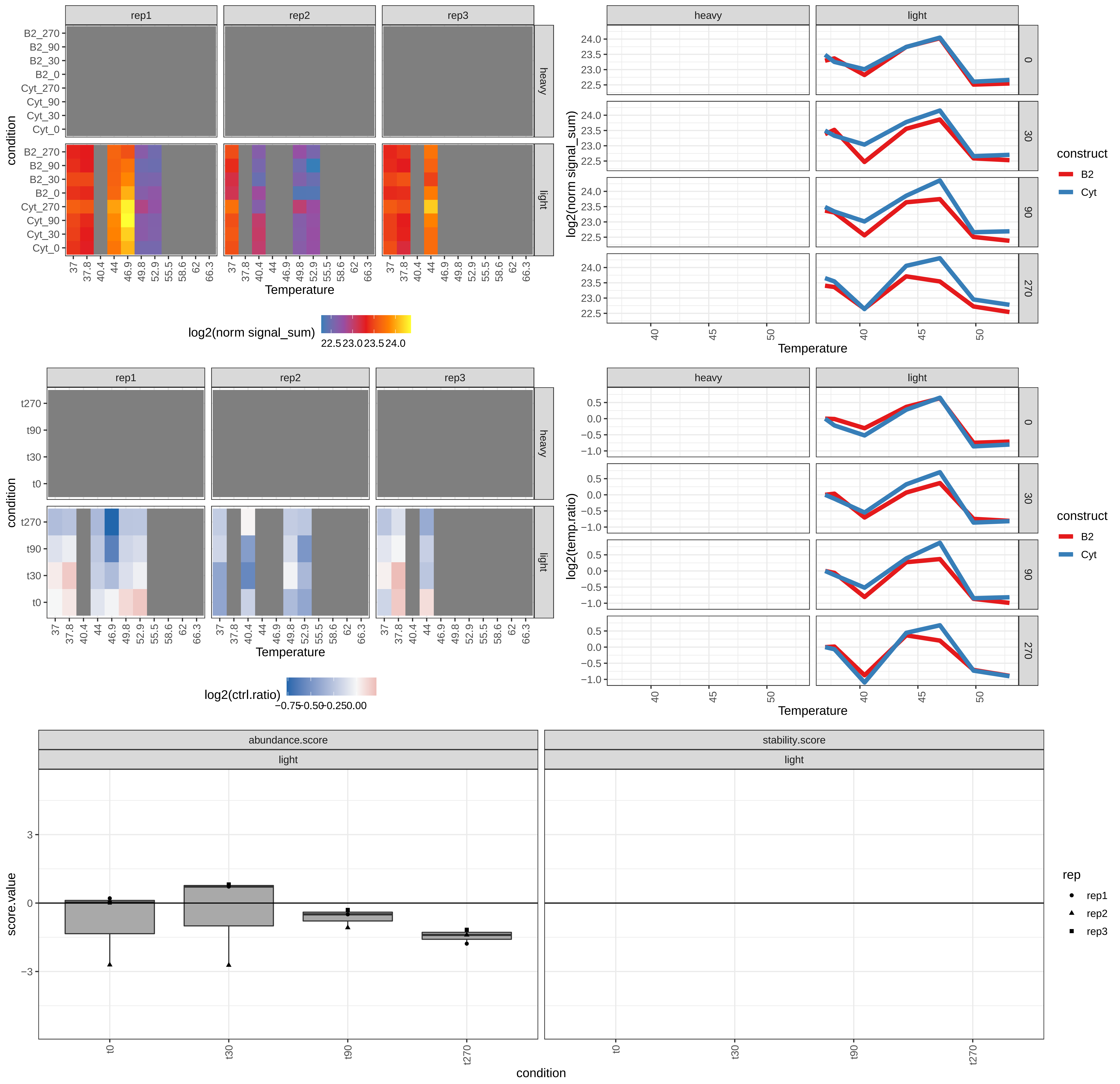

Q12029 − Probable mitochondrial transport protein FSF1 OS=Saccharomyces cerevisiae (strain ATCC 204508 / S288c) GN=FSF1 PE=1 SV=1

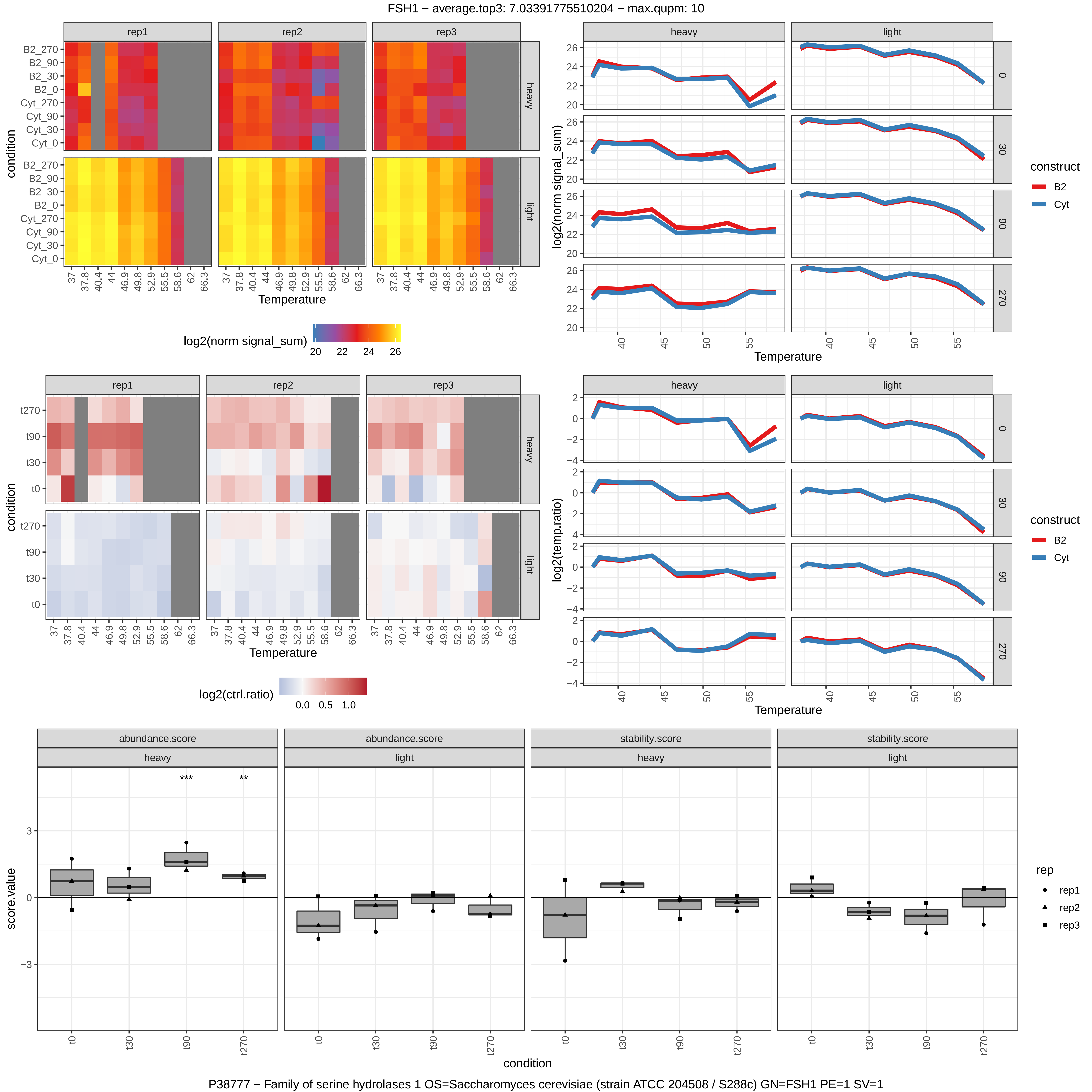

FSH1 − average.top3: 7.03391775510204 − max.qupm: 10
rep1
rep2
rep3
heavy
light
condition
B2_270
B2_90
B2_30
B2_0
Cyt_270
Cyt_90
Cyt_30
Cyt_0
Temperature
37 37.8 40.4 44 46.9 49.8 52.9 55.5 58.6 62 66.3
log2(norm signal_sum)
20 22 24 26
log2(norm signal_sum)
construct
B2
Cyt
0
30
90
270
t270
t90
t30
t0
log2(ctrl.ratio)
0.0 0.5 1.0
log2(temp.ratio)
abundance.score
stability.score
score.value
***
**
rep
rep1
rep2
rep3
condition
P38777 − Family of serine hydrolases 1 OS=Saccharomyces cerevisiae (strain ATCC 204508 / S288c) GN=FSH1 PE=1 SV=1

FSH3 − average.top3: 6.93521964285714 − max.qupm: 10

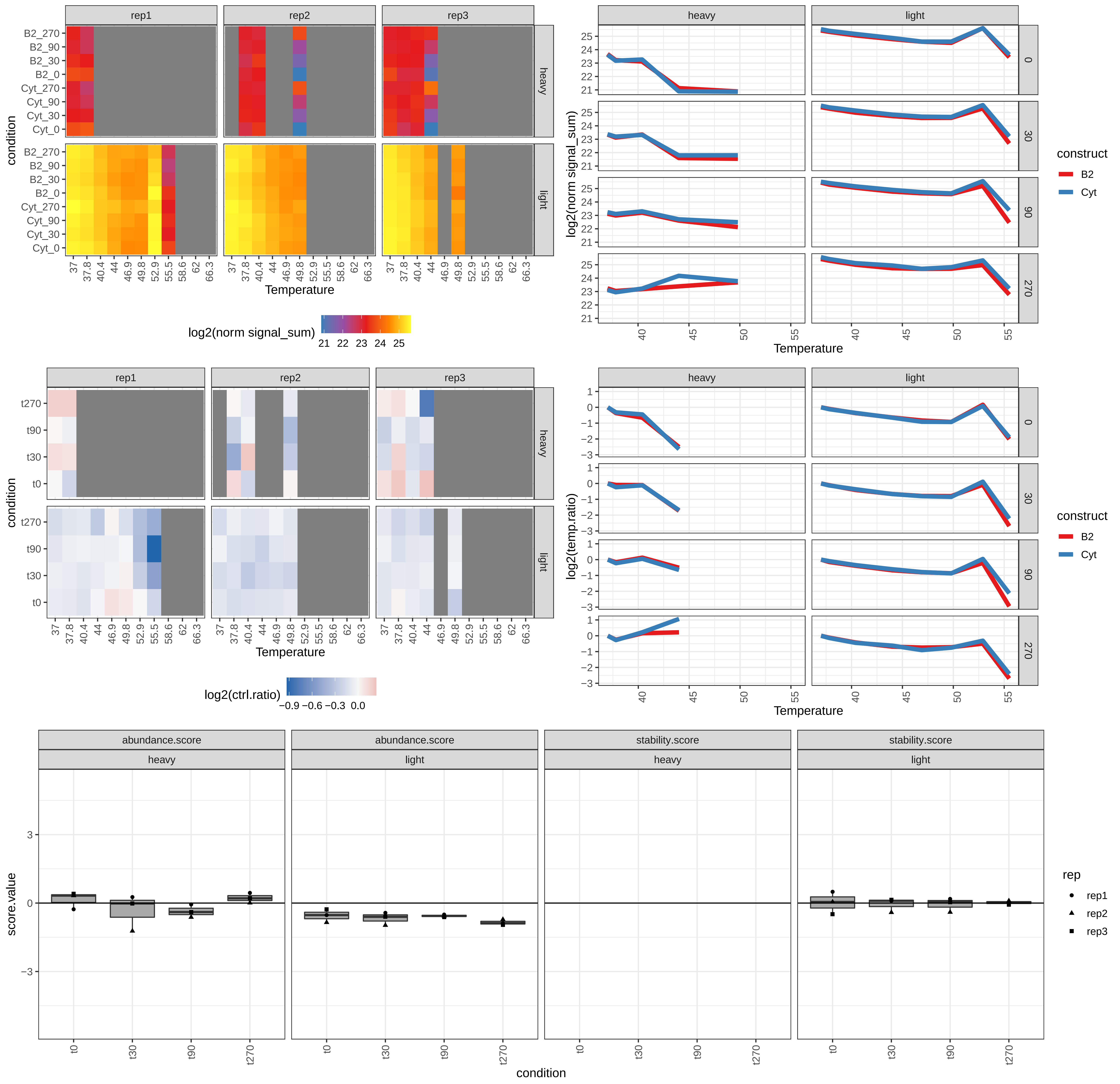

Q99369 − Family of serine hydrolases 3 OS=Saccharomyces cerevisiae (strain ATCC 204508 / S288c) GN=FSH3 PE=2 SV=1

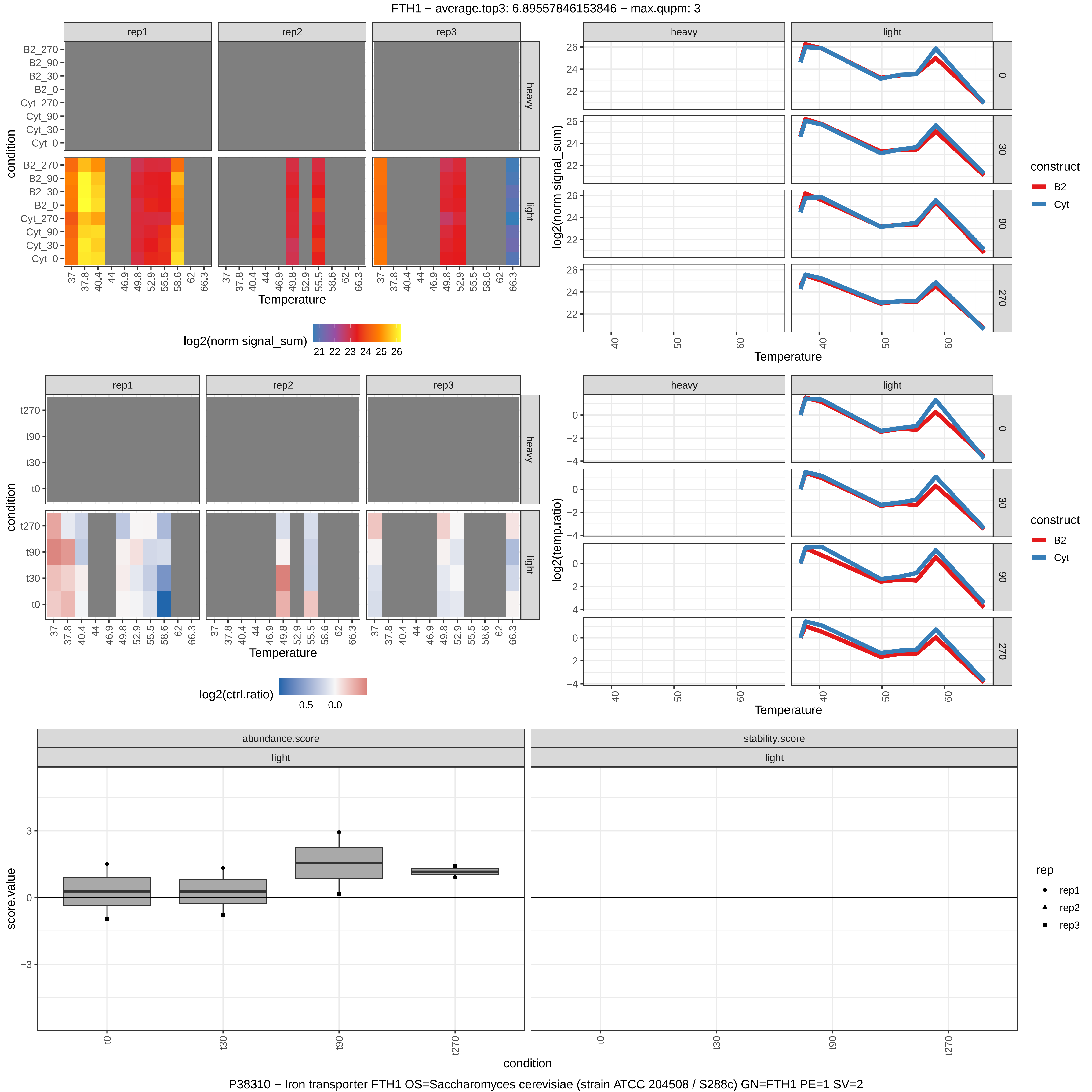

FTH1 − average.top3: 6.89557846153846 − max.qupm: 3
rep1
rep2
rep3
heavy
light
B2_270
B2_90
B2_30
B2_0
Cyt_270
Cyt_90
Cyt_30
Cyt_0
condition
37 37.8 40.4 44 46.9 49.8 52.9 55.5 58.6 62 66.3
Temperature
log2(norm signal_sum)
21 22 23 24 25 26
log2(norm signal_sum)
0
30
90
270
construct
B2
Cyt
t270
t90
t30
t0
log2(ctrl.ratio)
−0.5 0.0
log2(temp.ratio)
abundance.score
stability.score
score.value
rep
rep1
rep2
rep3
condition
P38310 − Iron transporter FTH1 OS=Saccharomyces cerevisiae (strain ATCC 204508 / S288c) GN=FTH1 PE=1 SV=2

FTR1 − average.top3: 7.02694032258065 − max.qupm: 6

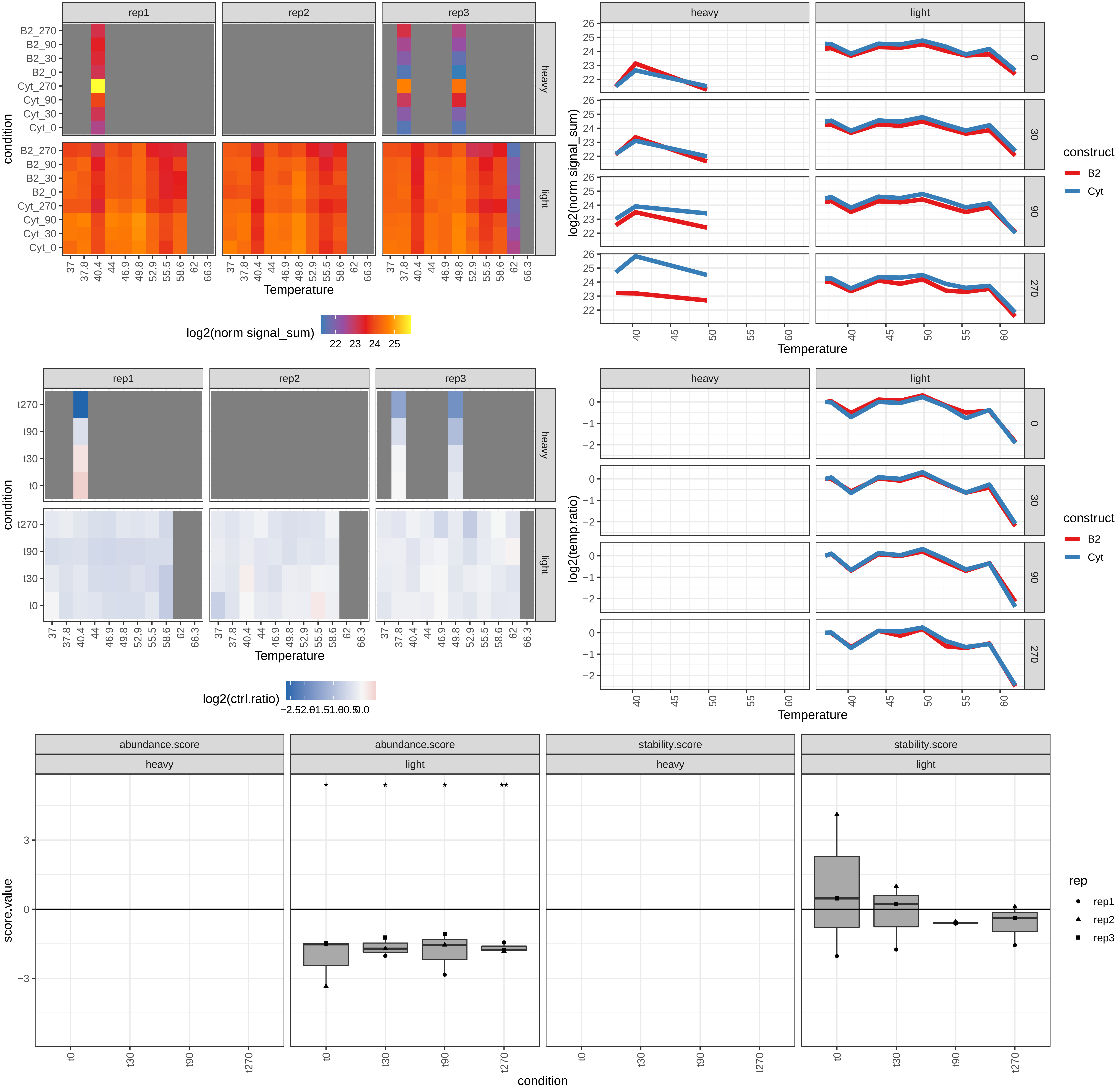

P40088 − Plasma membrane iron permease OS=Saccharomyces cerevisiae (strain ATCC 204508 / S288c) GN=FTR1 PE=1 SV=1

FUB1 − average.top3: 6.56566666666667 − max.qupm: 4

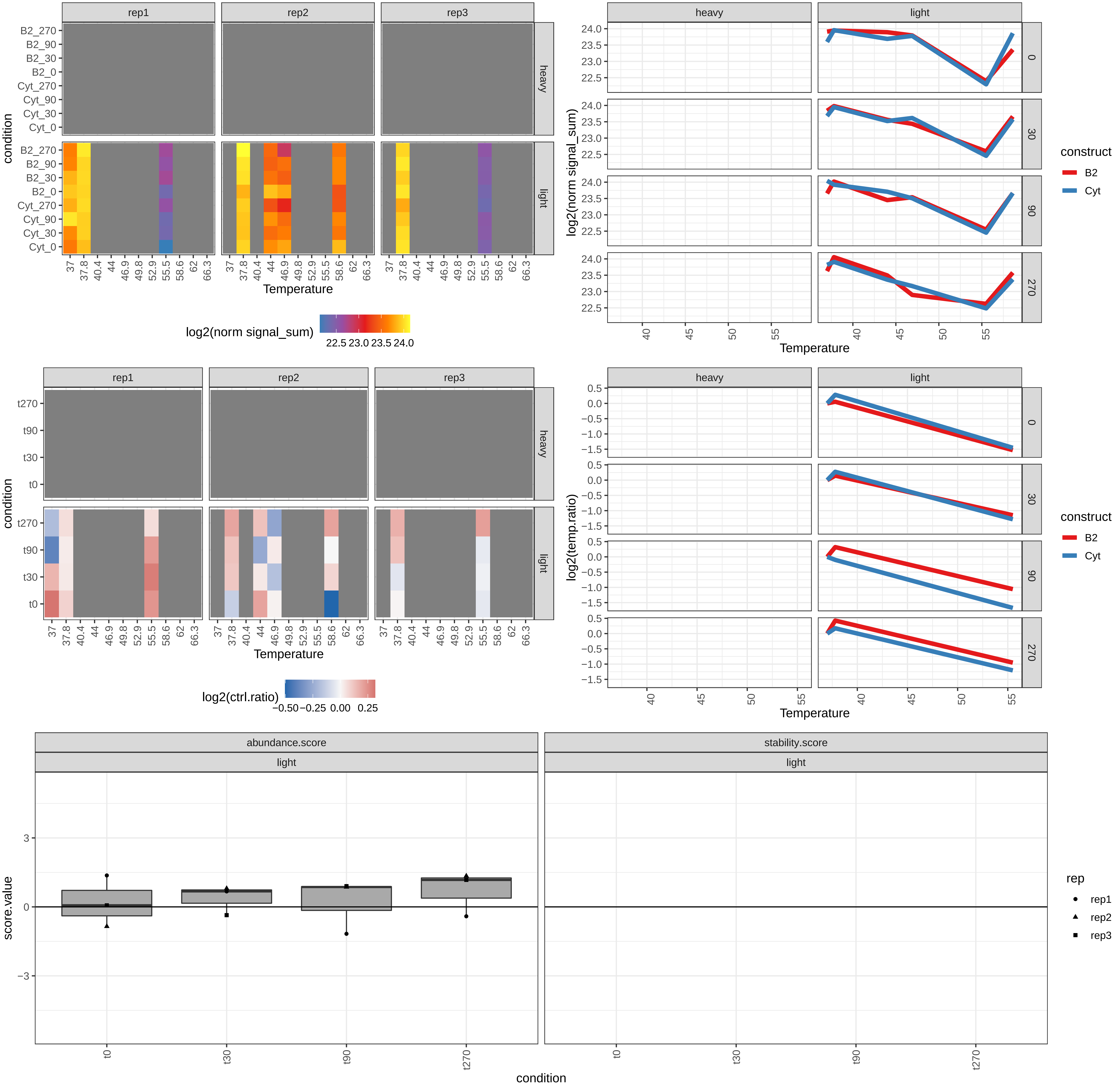

P25659 − Silencing boundary−establishment protein FUB1 OS=Saccharomyces cerevisiae (strain ATCC 204508 / S288c) GN=FUB1 PE=1 SV=1

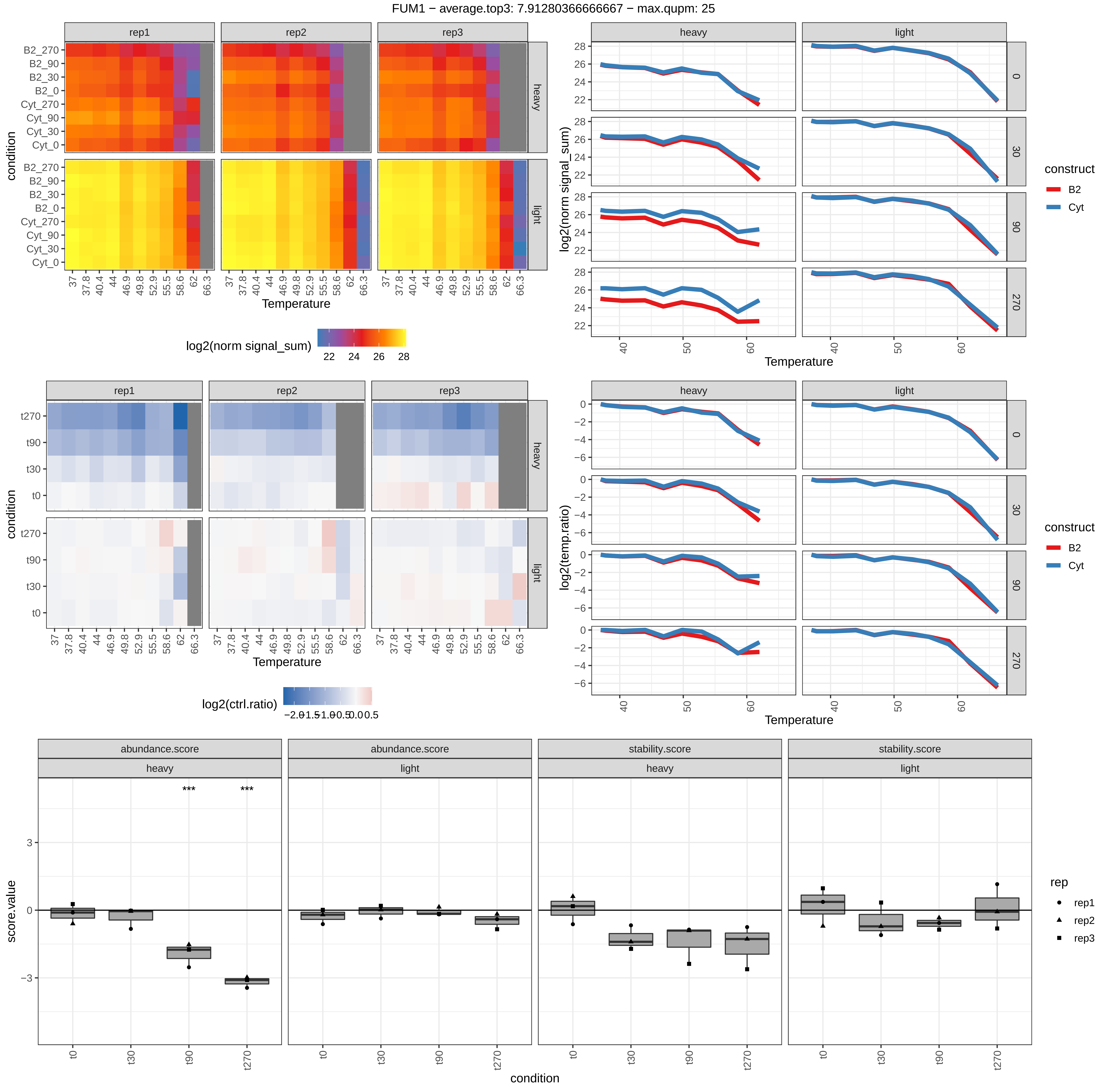

P08417 − Fumarate hydratase, mitochondrial OS=Saccharomyces cerevisiae (strain ATCC 204508 / S288c) GN=FUM1 PE=1 SV=2

FUN12 − average.top3: 7.355626 − max.qupm: 25

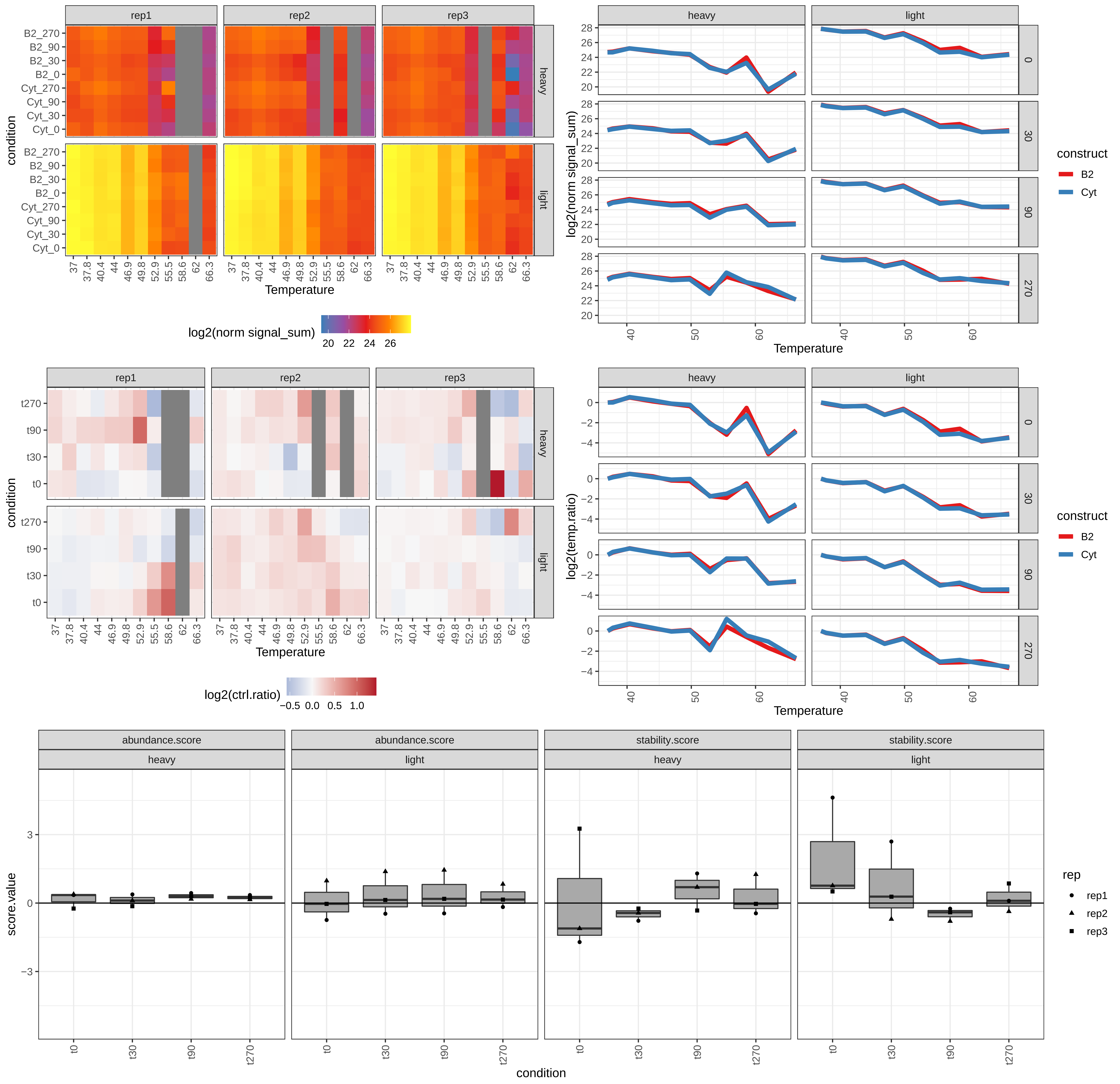

P39730 − Eukaryotic translation initiation factor 5B OS=Saccharomyces cerevisiae (strain ATCC 204508 / S288c) GN=FUN12 PE=1 SV=2

FUN30 − average.top3: 6.49970166666667 − max.qupm: 4

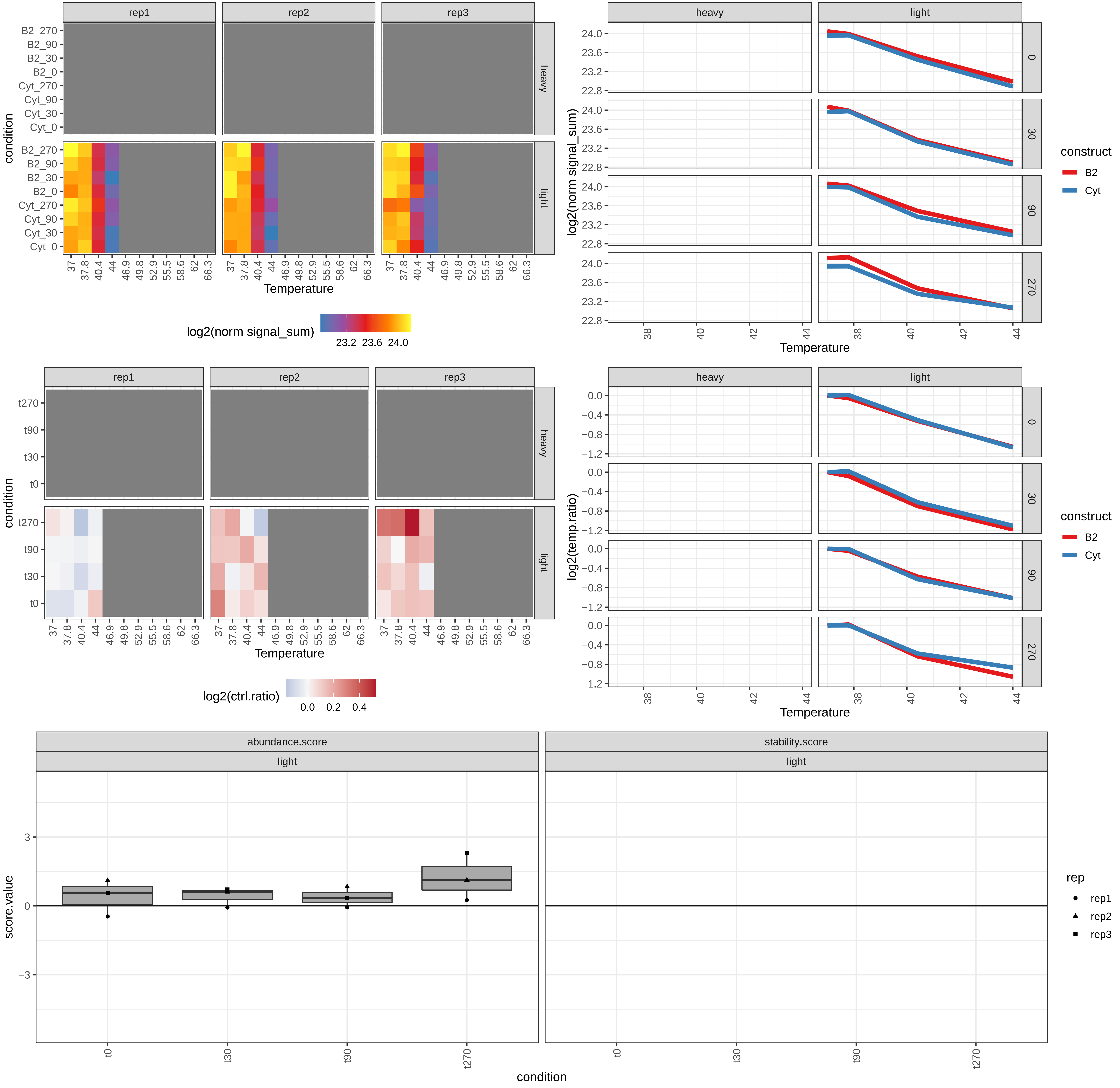

P31380 − ATP−dependent helicase FUN30 OS=Saccharomyces cerevisiae (strain ATCC 204508 / S288c) GN=FUN30 PE=1 SV=1

FUR1 − average.top3: 7.36252909090909 − max.qupm: 9

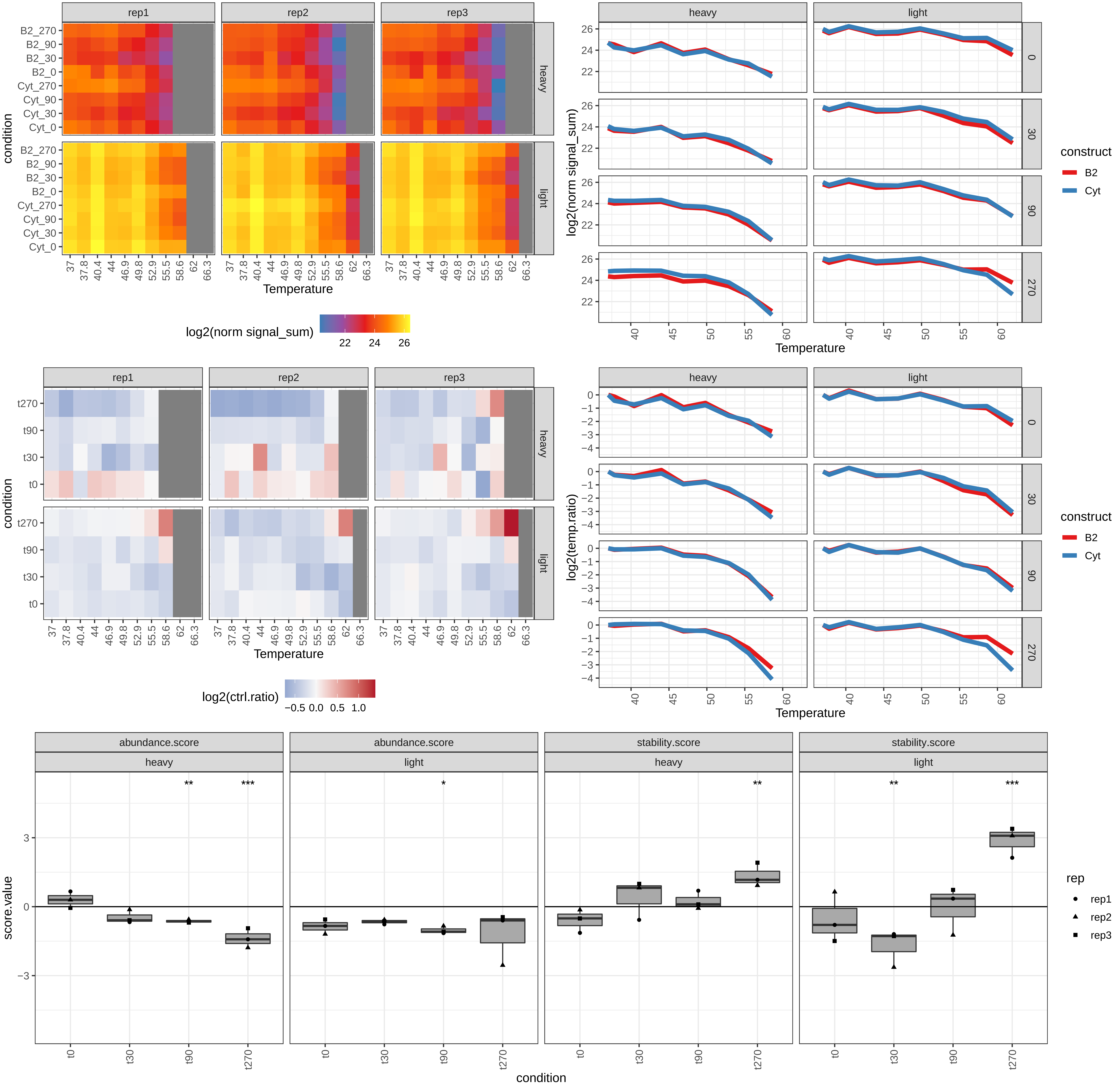

P18562 − Uracil phosphoribosyltransferase OS=Saccharomyces cerevisiae (strain ATCC 204508 / S288c) GN=FUR1 PE=1 SV=2

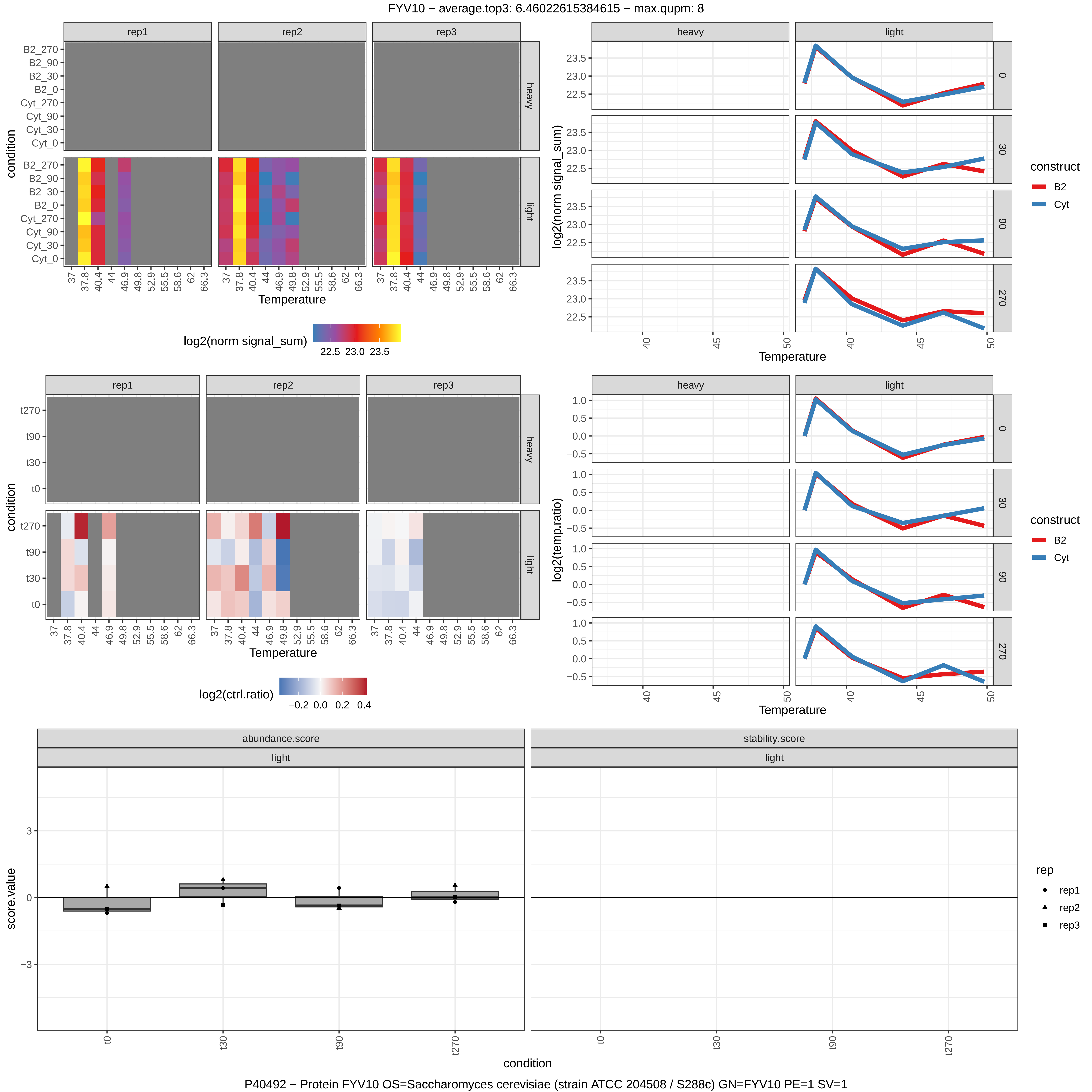

FYV10 − average.top3: 6.46022615384615 − max.qupm: 8
rep1
rep2
rep3
heavy
light
condition
Temperature
log2(norm signal_sum)
log2(ctrl.ratio)
log2(temp.ratio)
construct
B2
Cyt
abundance.score
stability.score
score.value
rep
rep1
rep2
rep3
P40492 − Protein FYV10 OS=Saccharomyces cerevisiae (strain ATCC 204508 / S288c) GN=FYV10 PE=1 SV=1

FZO1 − average.top3: 6.36341227272727 − max.qupm: 6

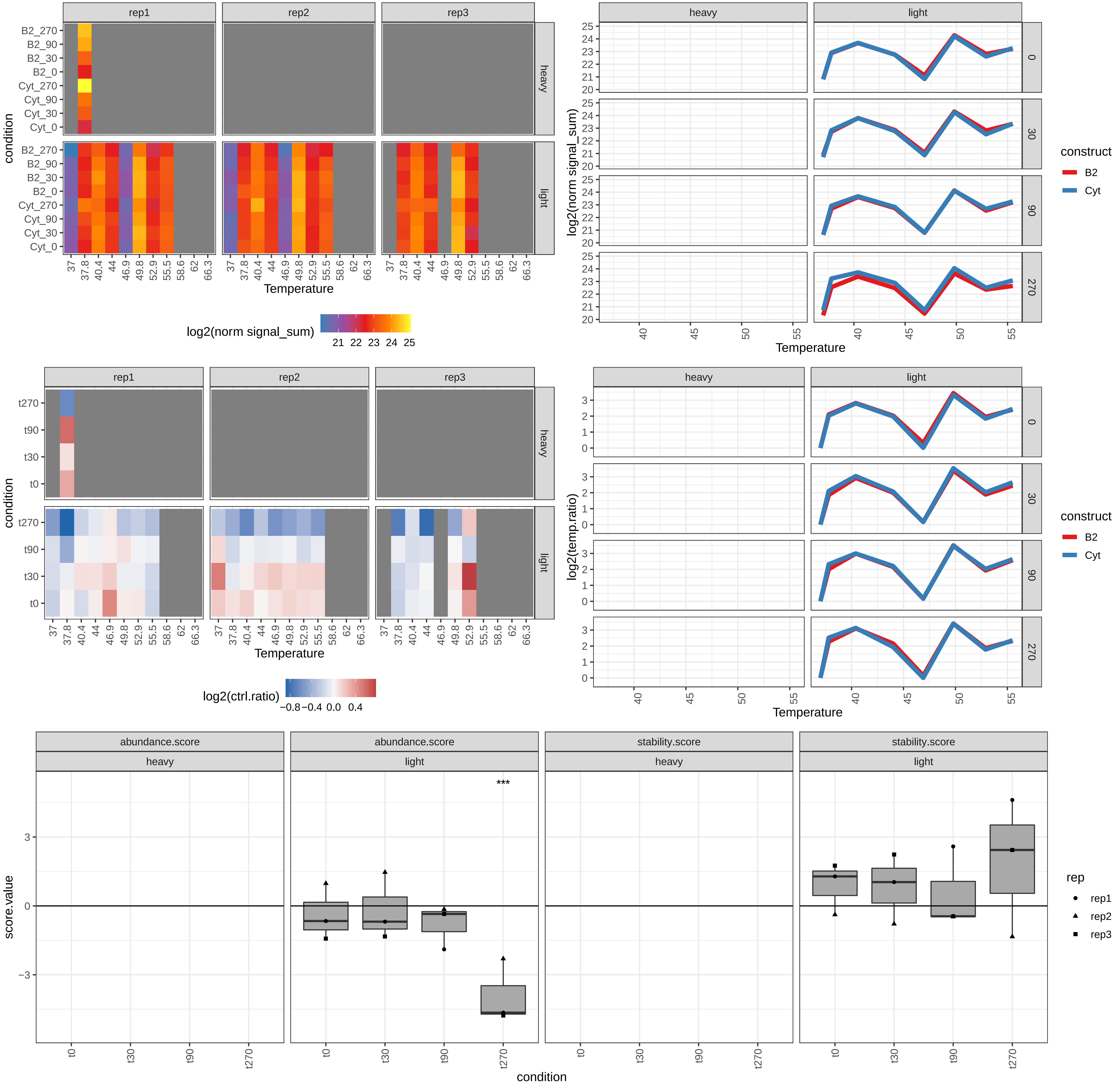

P38297 − Mitofusin FZO1 OS=Saccharomyces cerevisiae (strain ATCC 204508 / S288c) GN=FZO1 PE=1 SV=1

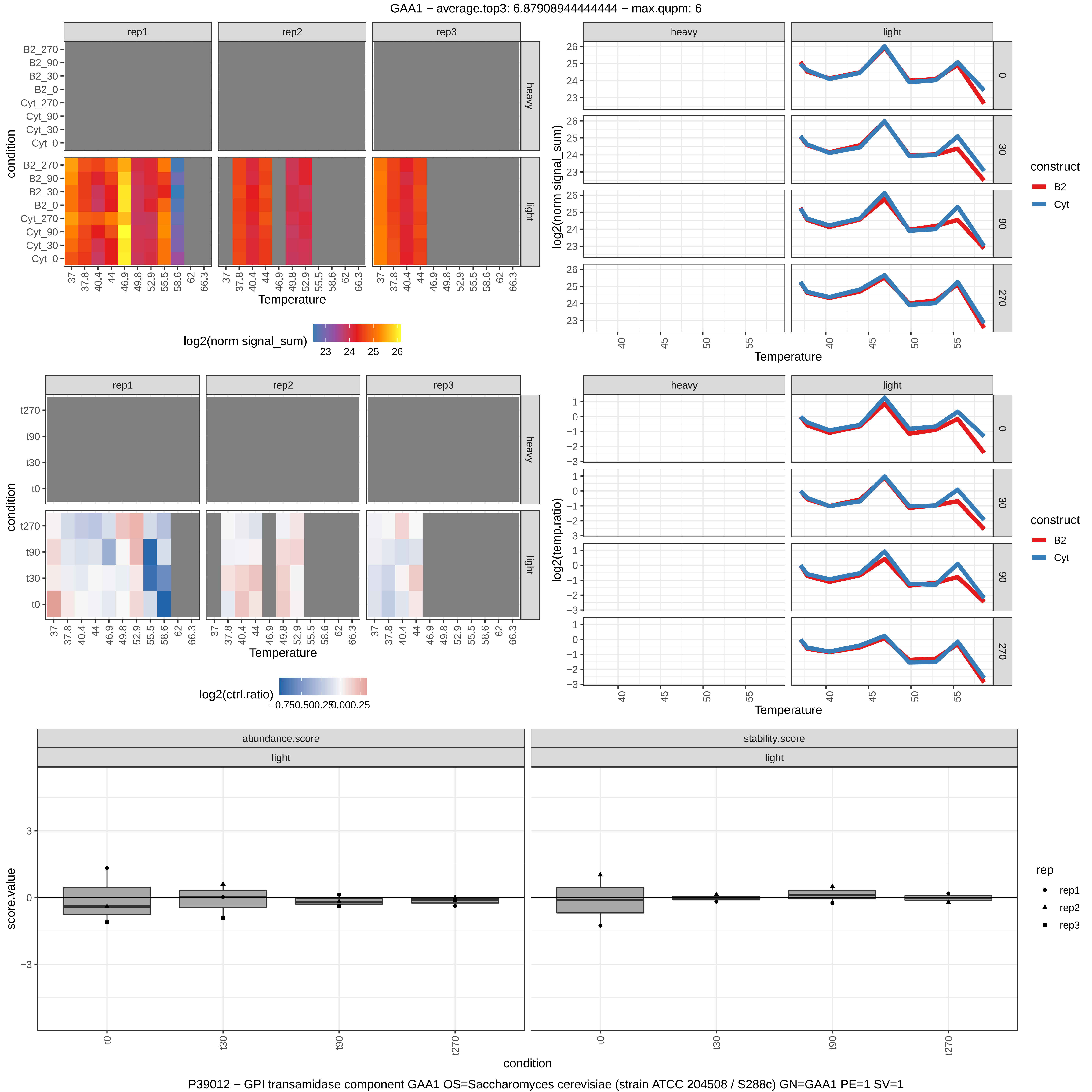
GAA1 − average.top3: 6.87908944444444 − max.qupm: 6
rep1
rep2
rep3
heavy
light
condition
Temperature
log2(norm signal_sum)
construct
B2
Cyt
log2(ctrl.ratio)
log2(temp.ratio)
abundance.score
stability.score
score.value
rep
rep1
rep2
rep3
P39012 − GPI transamidase component GAA1 OS=Saccharomyces cerevisiae (strain ATCC 204508 / S288c) GN=GAA1 PE=1 SV=1

GAB1 − average.top3: 6.82428 − max.qupm: 3

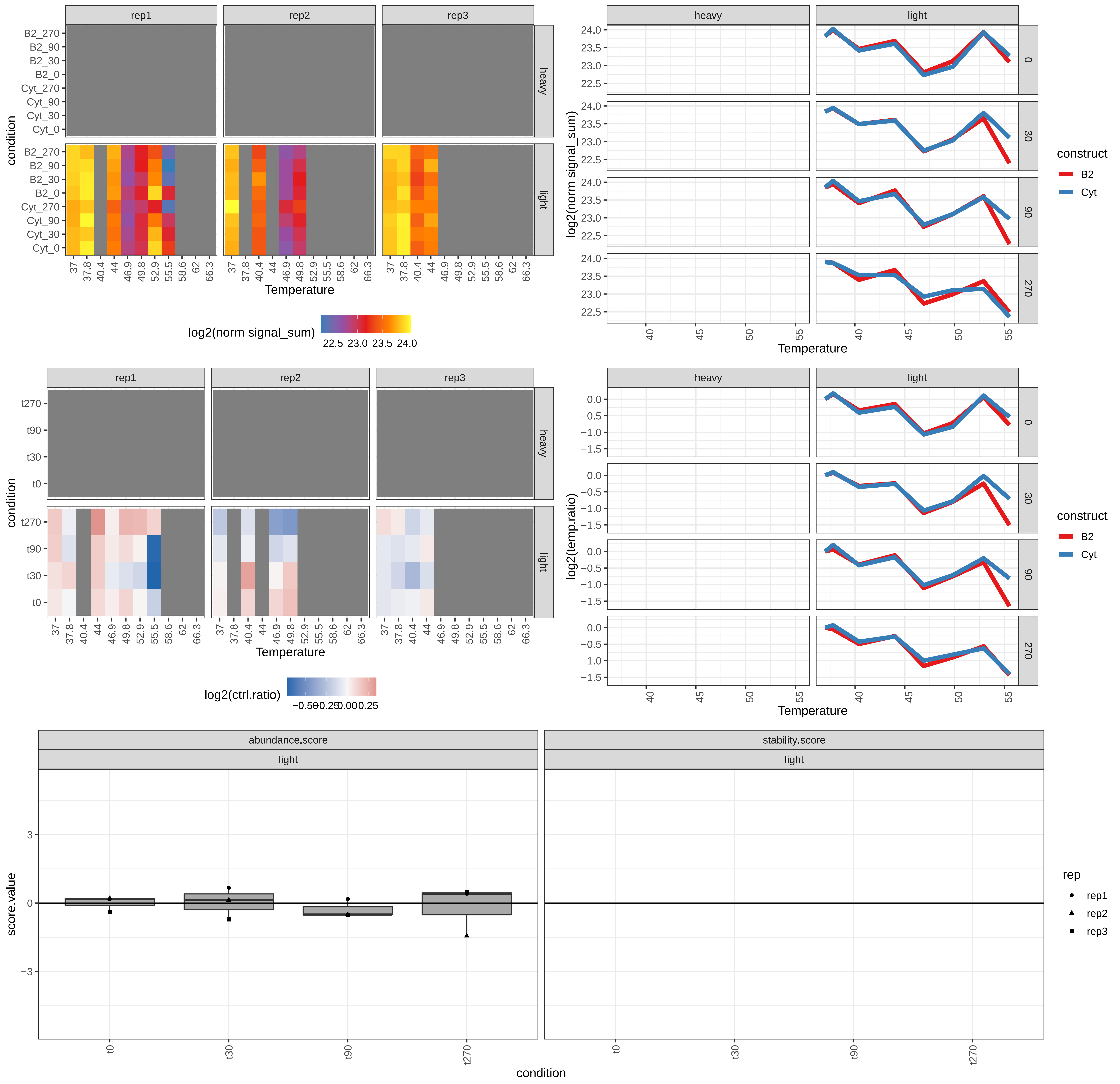

P41733 − GPI transamidase component GAB1 OS=Saccharomyces cerevisiae (strain ATCC 204508 / S288c) GN=GAB1 PE=1 SV=1

GAD1 − average.top3: 7.0534075 − max.qupm: 10

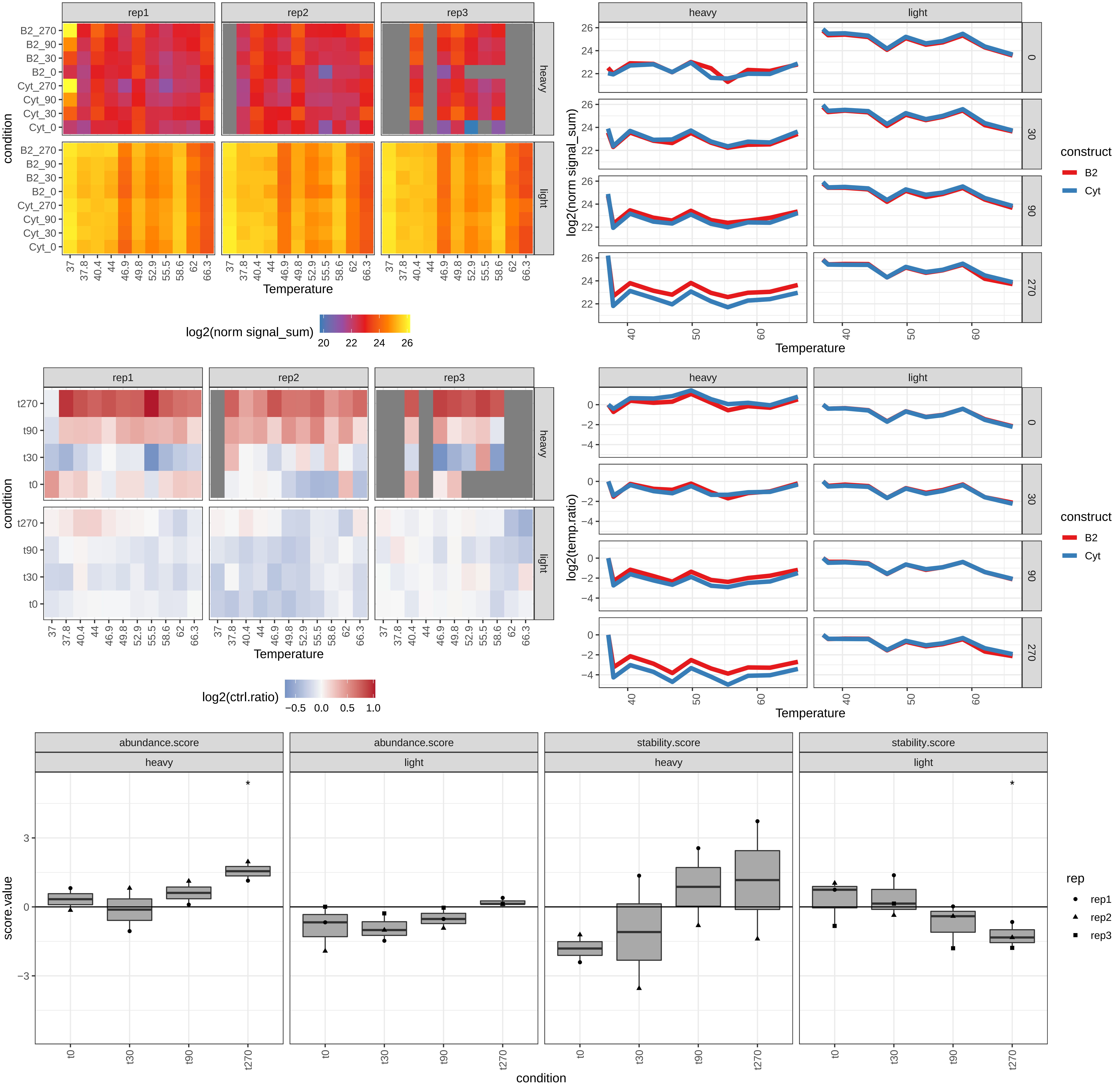

Q04792 − Glutamate decarboxylase OS=Saccharomyces cerevisiae (strain ATCC 204508 / S288c) GN=GAD1 PE=1 SV=1

GAL1 − average.top3: 7.47467685185185 − max.qupm: 18

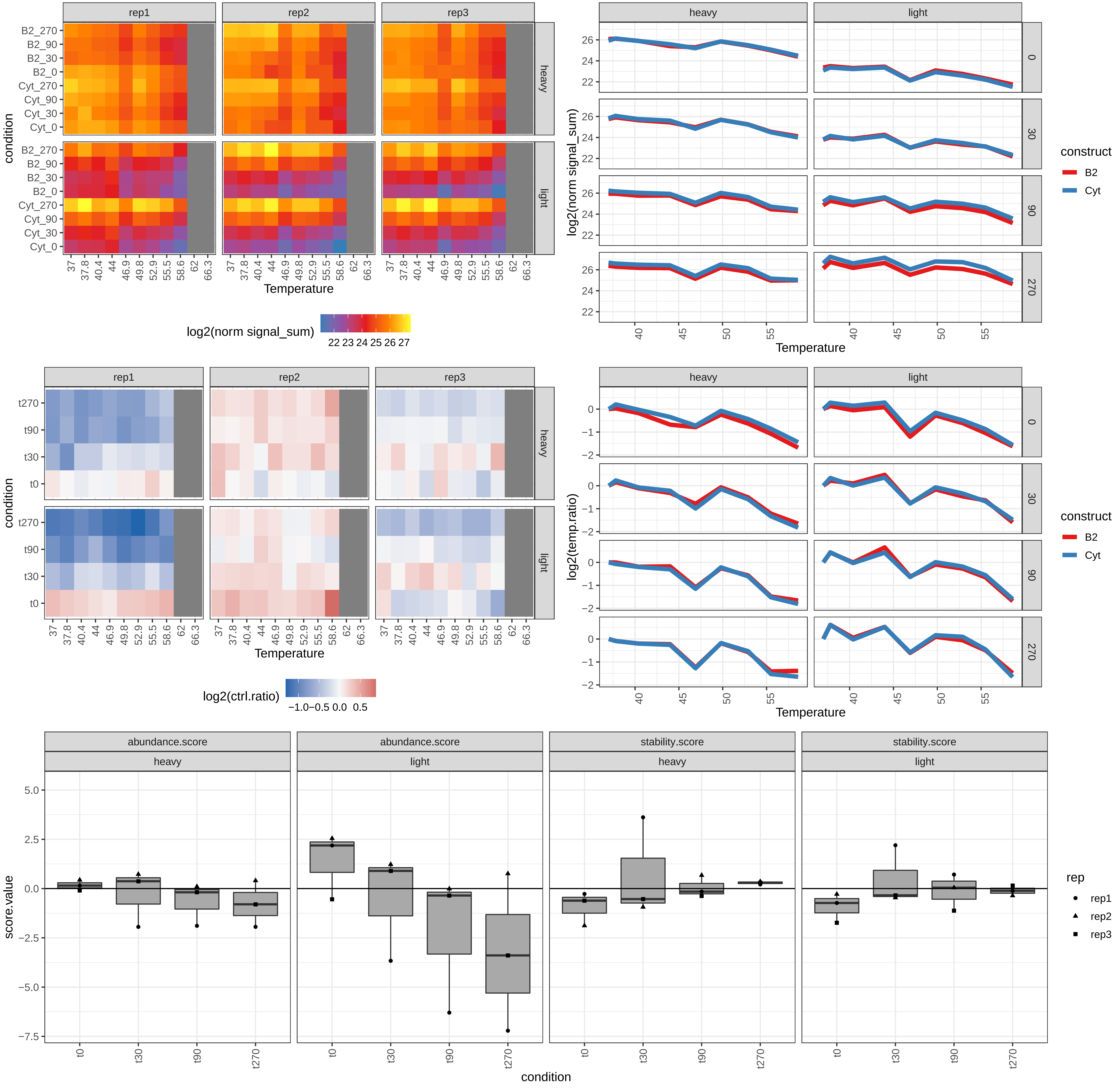

P04385 − Galactokinase OS=Saccharomyces cerevisiae (strain ATCC 204508 / S288c) GN=GAL1 PE=1 SV=4

GAL10 − average.top3: 7.69597125 − max.qupm: 24

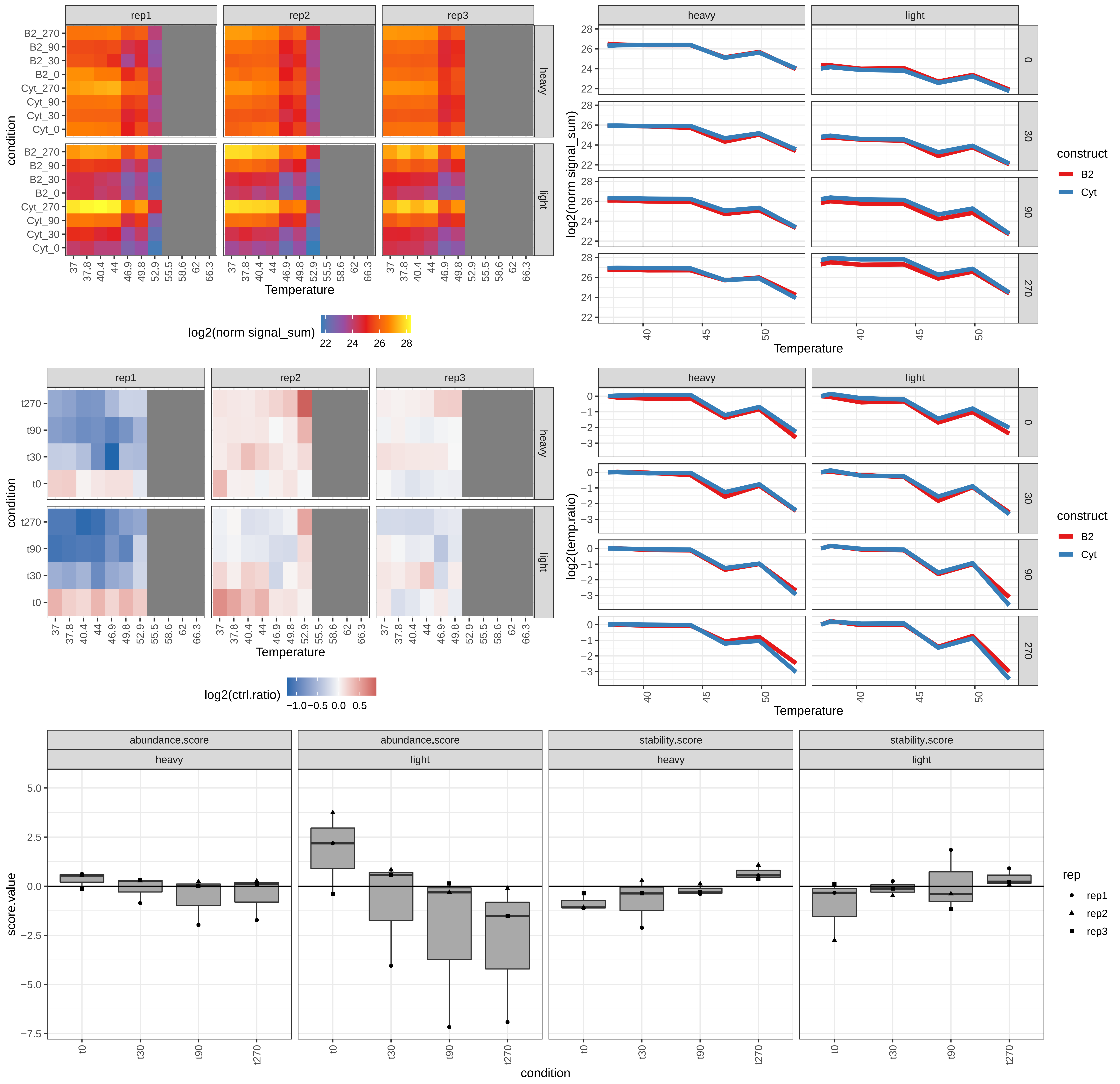

P04397 − Bifunctional protein GAL10 OS=Saccharomyces cerevisiae (strain ATCC 204508 / S288c) GN=GAL10 PE=1 SV=2

GAL2 − average.top3: 6.95700875 − max.qupm: 4

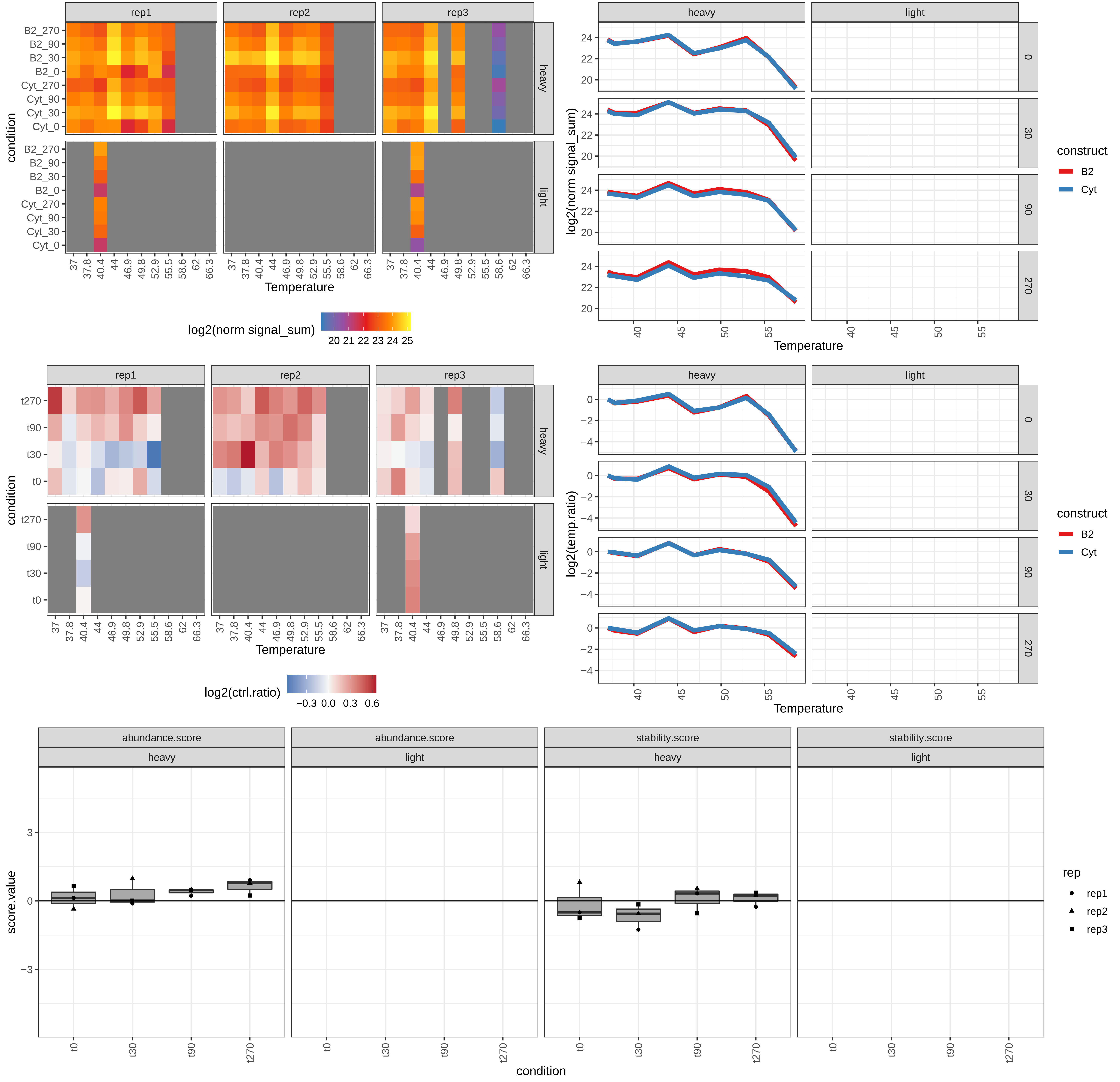

P13181 − Galactose transporter OS=Saccharomyces cerevisiae (strain ATCC 204508 / S288c) GN=GAL2 PE=1 SV=3

GAL3 − average.top3: 6.55158857142857 − max.qupm: 7

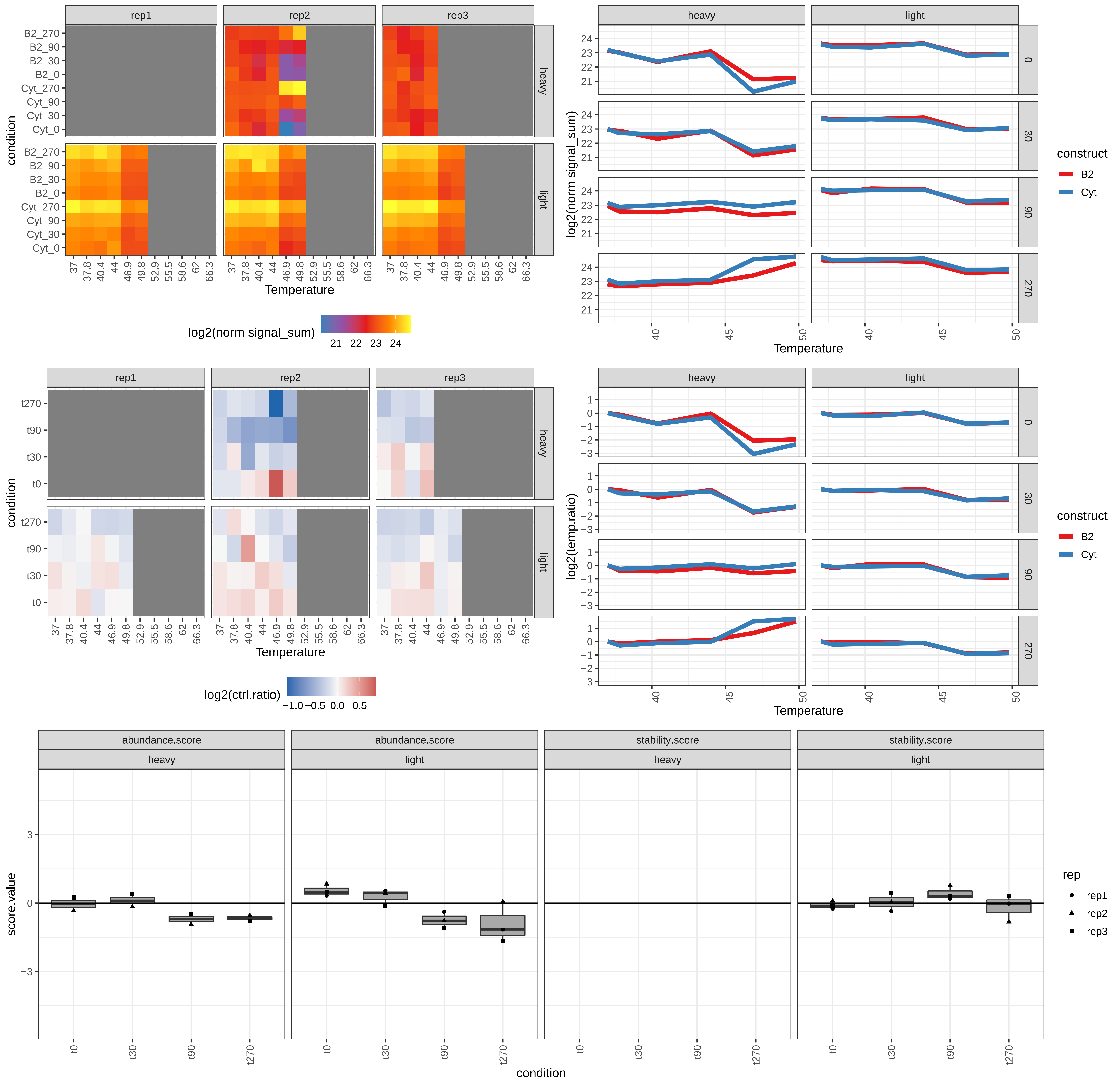

P13045 − Protein GAL3 OS=Saccharomyces cerevisiae (strain ATCC 204508 / S288c) GN=GAL3 PE=1 SV=2

GAL7 − average.top3: 7.56900196969697 − max.qupm: 10

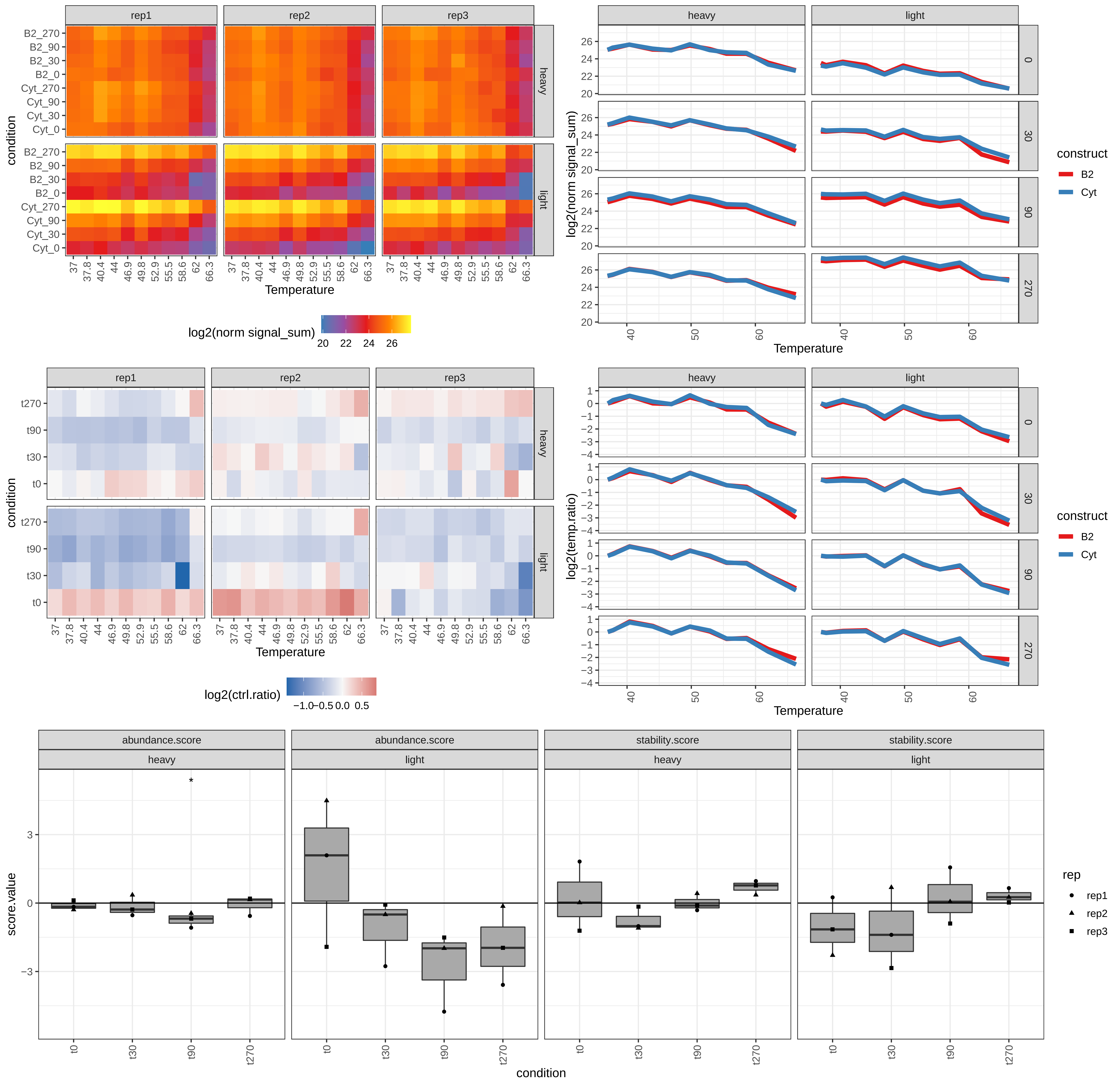

P08431 − Galactose−1−phosphate uridylyltransferase OS=Saccharomyces cerevisiae (strain ATCC 204508 / S288c) GN=GAL7 PE=1 SV=4

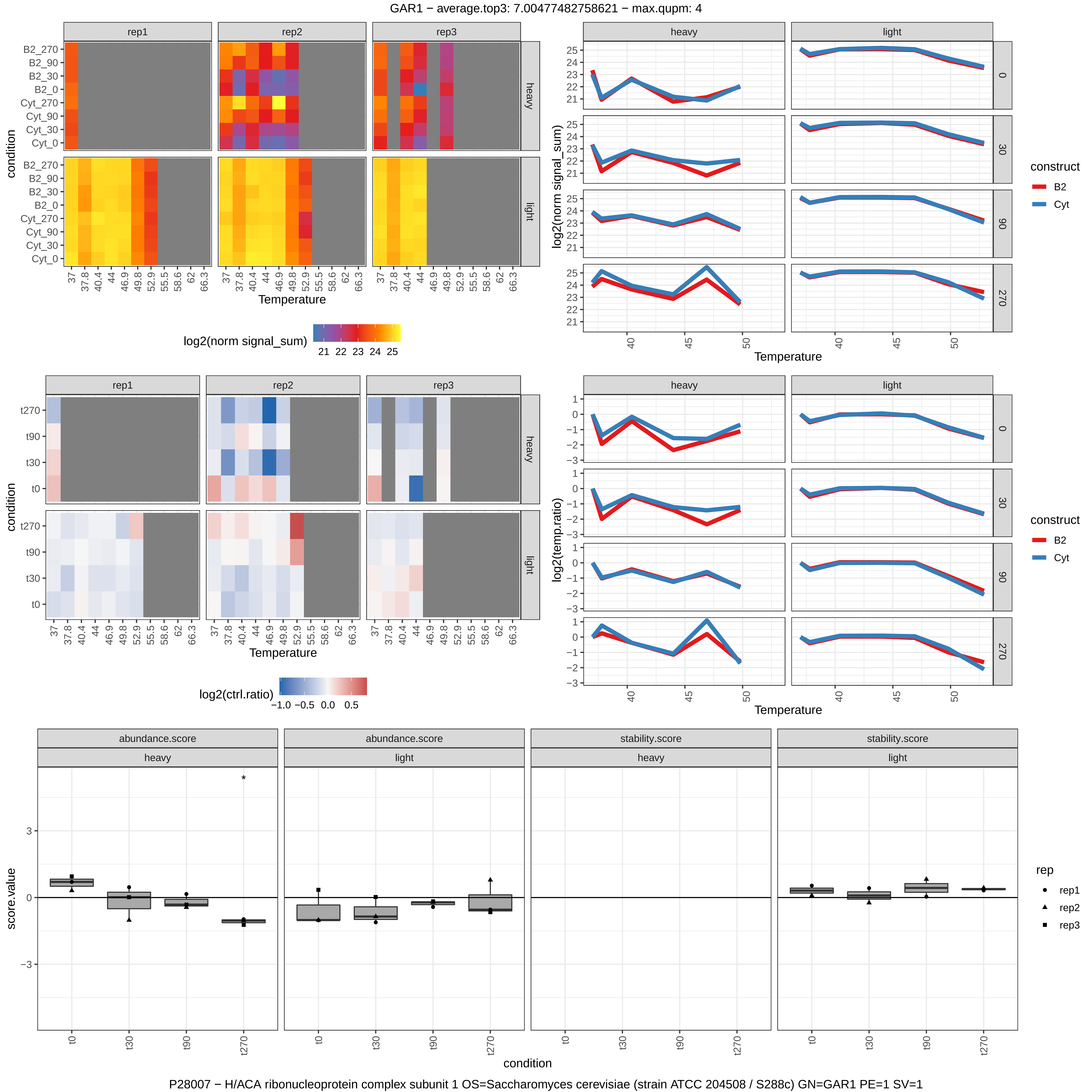
GAR1 − average.top3: 7.00477482758621 − max.qupm: 4
rep1
rep2
rep3
heavy
light
condition
Temperature
log2(norm signal_sum)
log2(ctrl.ratio)
log2(temp.ratio)
construct
B2
Cyt
abundance.score
stability.score
score.value
rep
rep1
rep2
rep3
P28007 − H/ACA ribonucleoprotein complex subunit 1 OS=Saccharomyces cerevisiae (strain ATCC 204508 / S288c) GN=GAR1 PE=1 SV=1

GAS1 − average.top3: 7.77057061538462 − max.qupm: 13

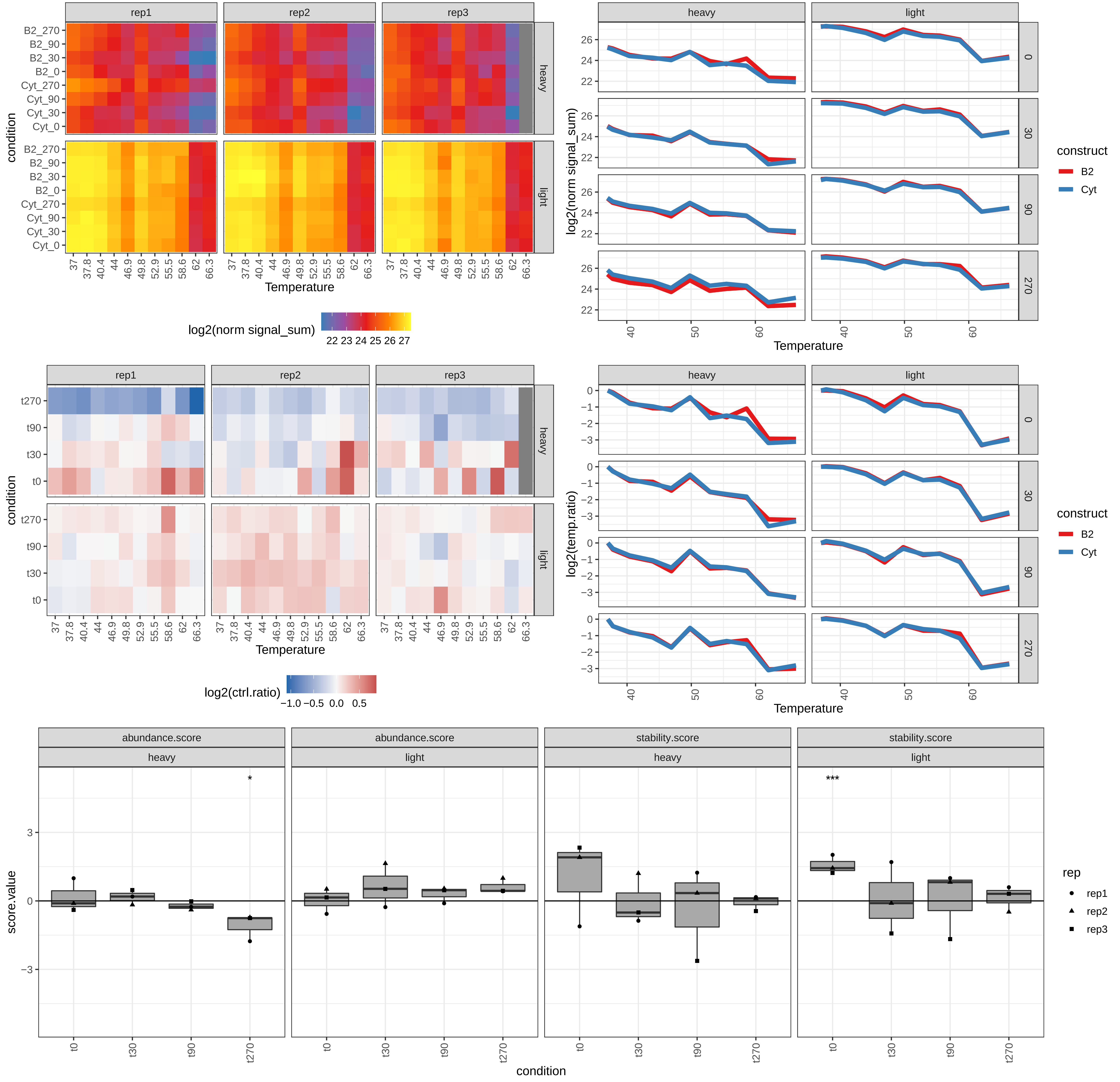

P22146 − 1,3−beta−glucanosyltransferase GAS1 OS=Saccharomyces cerevisiae (strain ATCC 204508 / S288c) GN=GAS1 PE=1 SV=2

GAS3 − average.top3: 7.14125031746032 − max.qupm: 14

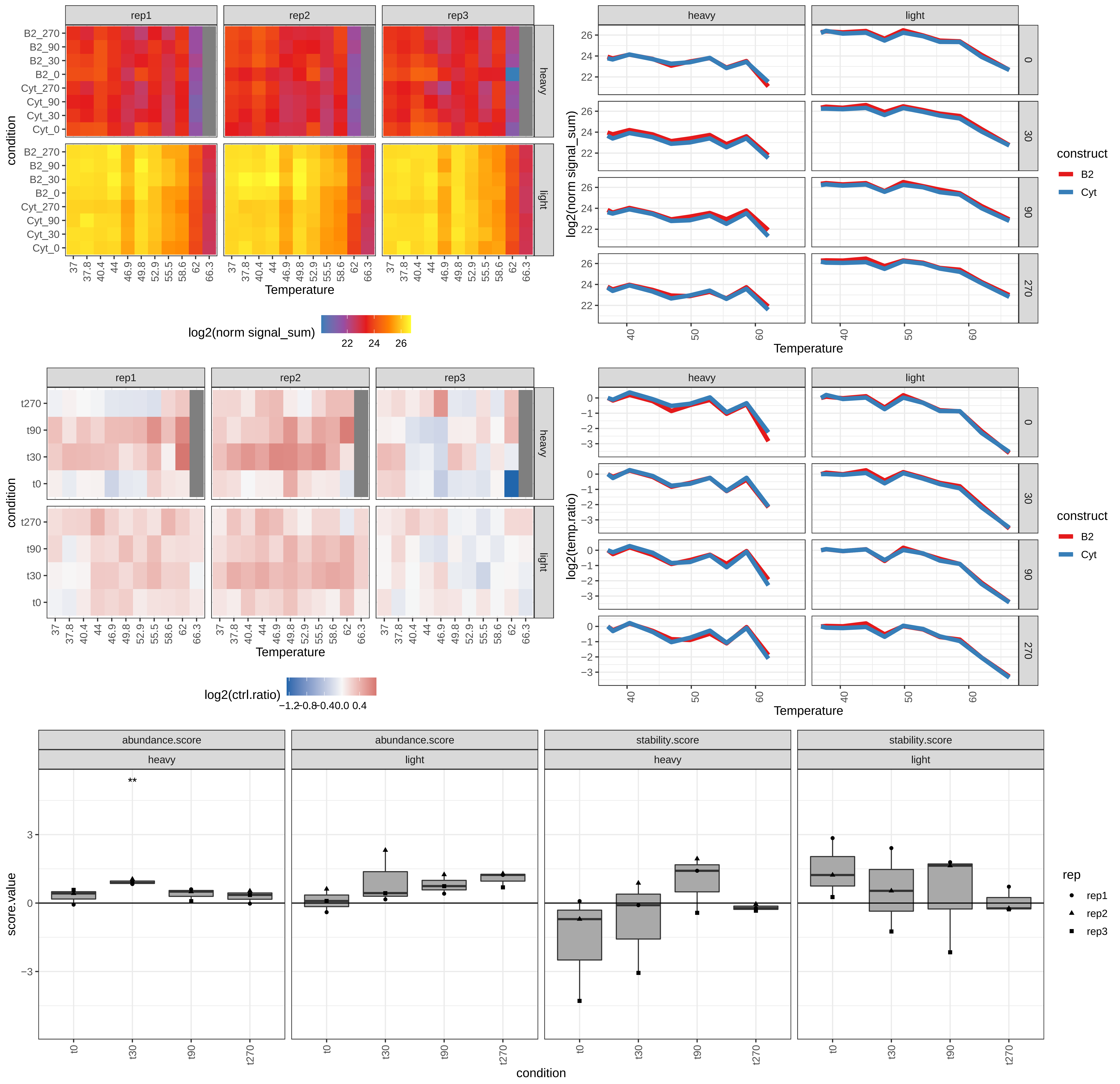

Q03655 − Probable 1,3−beta−glucanosyltransferase GAS3 OS=Saccharomyces cerevisiae (strain ATCC 204508 / S288c) GN=GAS3 PE=1 SV=1

GAS5 − average.top3: 7.64670016393443 − max.qupm: 8

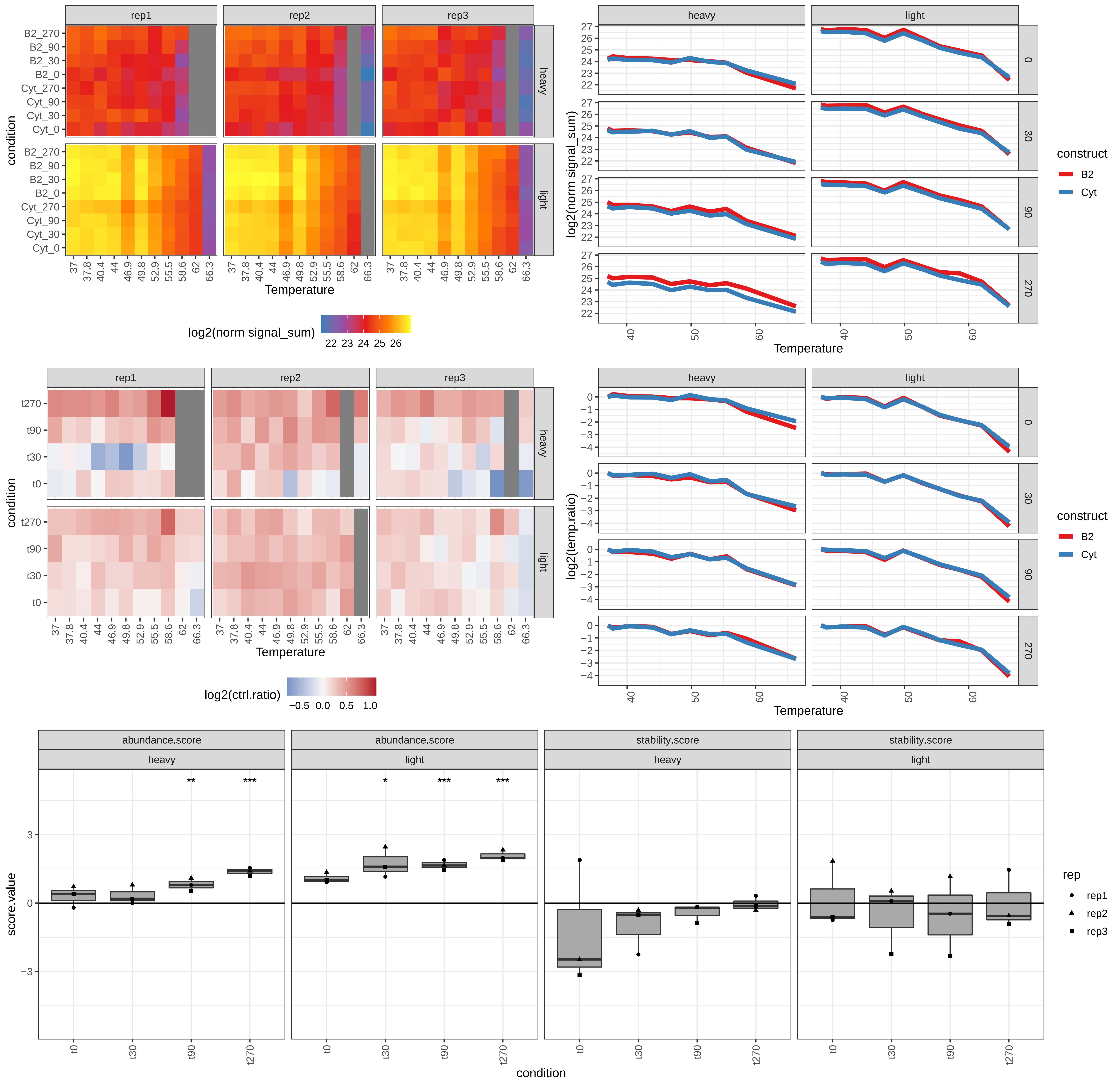

Q08193 − 1,3−beta−glucanosyltransferase GAS5 OS=Saccharomyces cerevisiae (strain ATCC 204508 / S288c) GN=GAS5 PE=1 SV=1

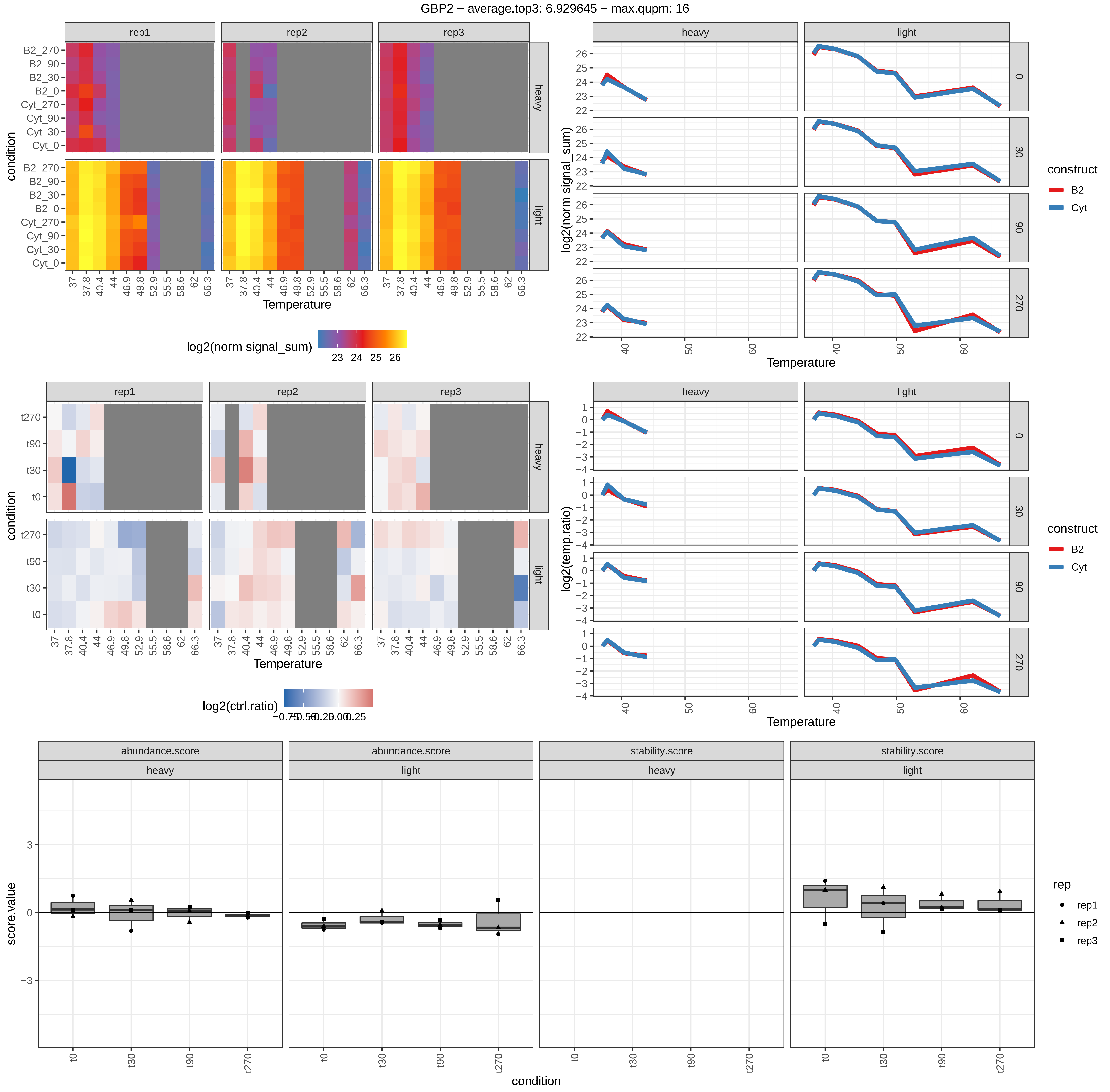

P25555 − Single−strand telomeric DNA−binding protein GBP2 OS=Saccharomyces cerevisiae (strain ATCC 204508 / S288c) GN=GBP2 PE=1 SV=1

GCD1 − average.top3: 6.64276 − max.qupm: 6

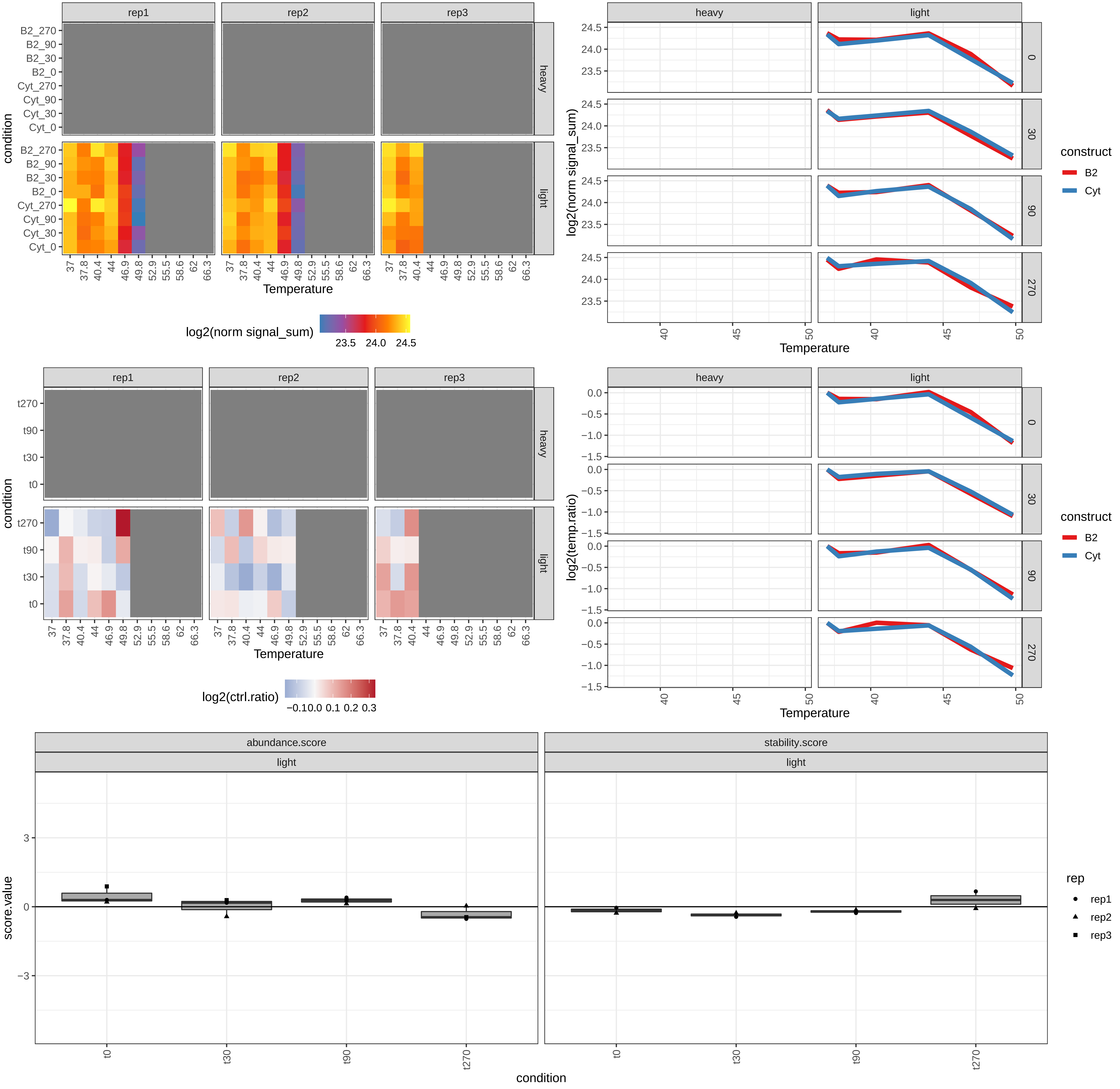

P09032 − Translation initiation factor eIF−2B subunit gamma OS=Saccharomyces cerevisiae (strain ATCC 204508 / S288c) GN=GCD1 PE=1 SV=3

GCD11 − average.top3: 7.46914525423729 − max.qupm: 19

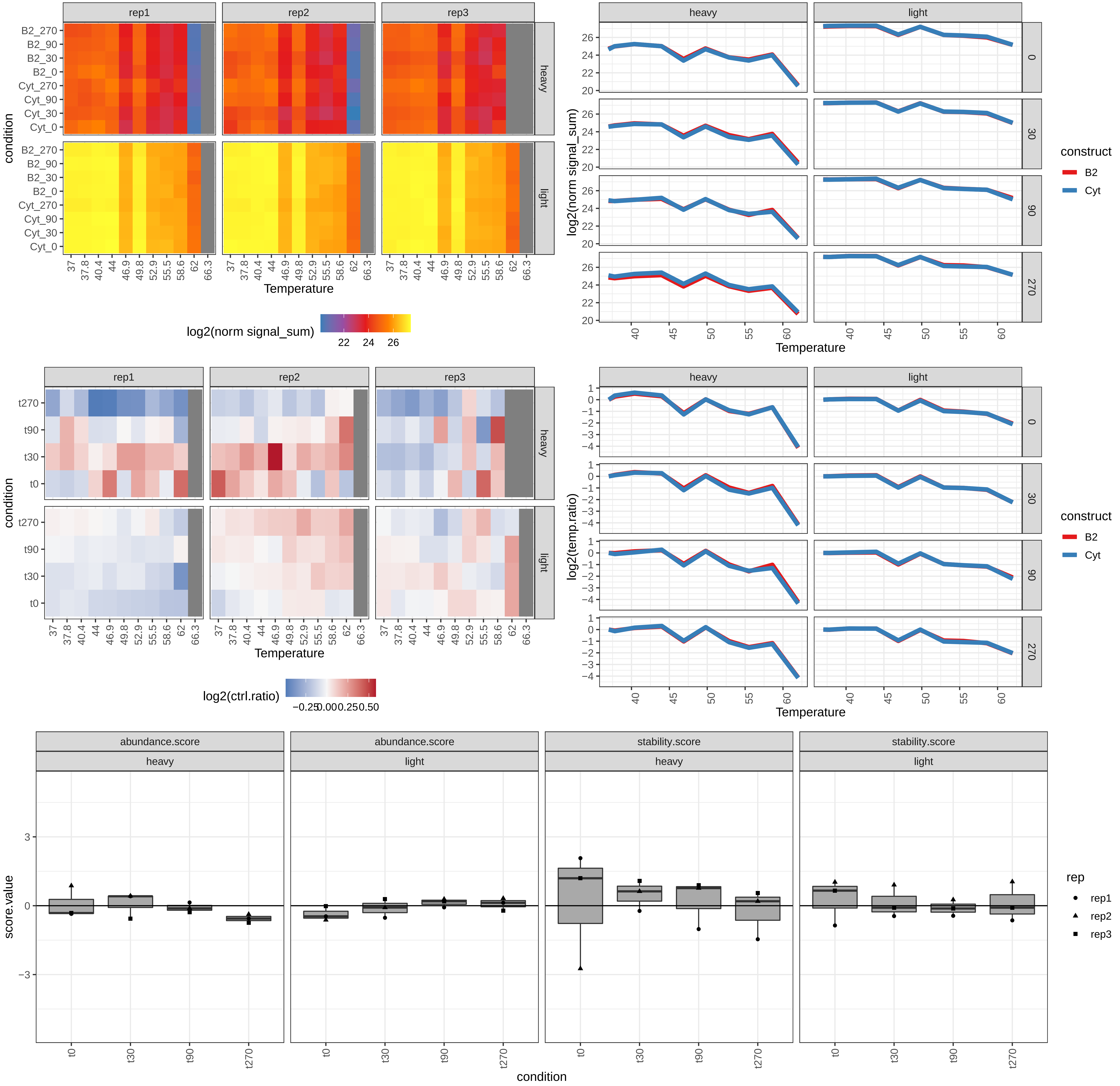

P32481 − Eukaryotic translation initiation factor 2 subunit gamma OS=Saccharomyces cerevisiae (strain ATCC 204508 / S288c) GN=GCD11 PE=1 SV=1

GCD14 − average.top3: 6.978973 − max.qupm: 8

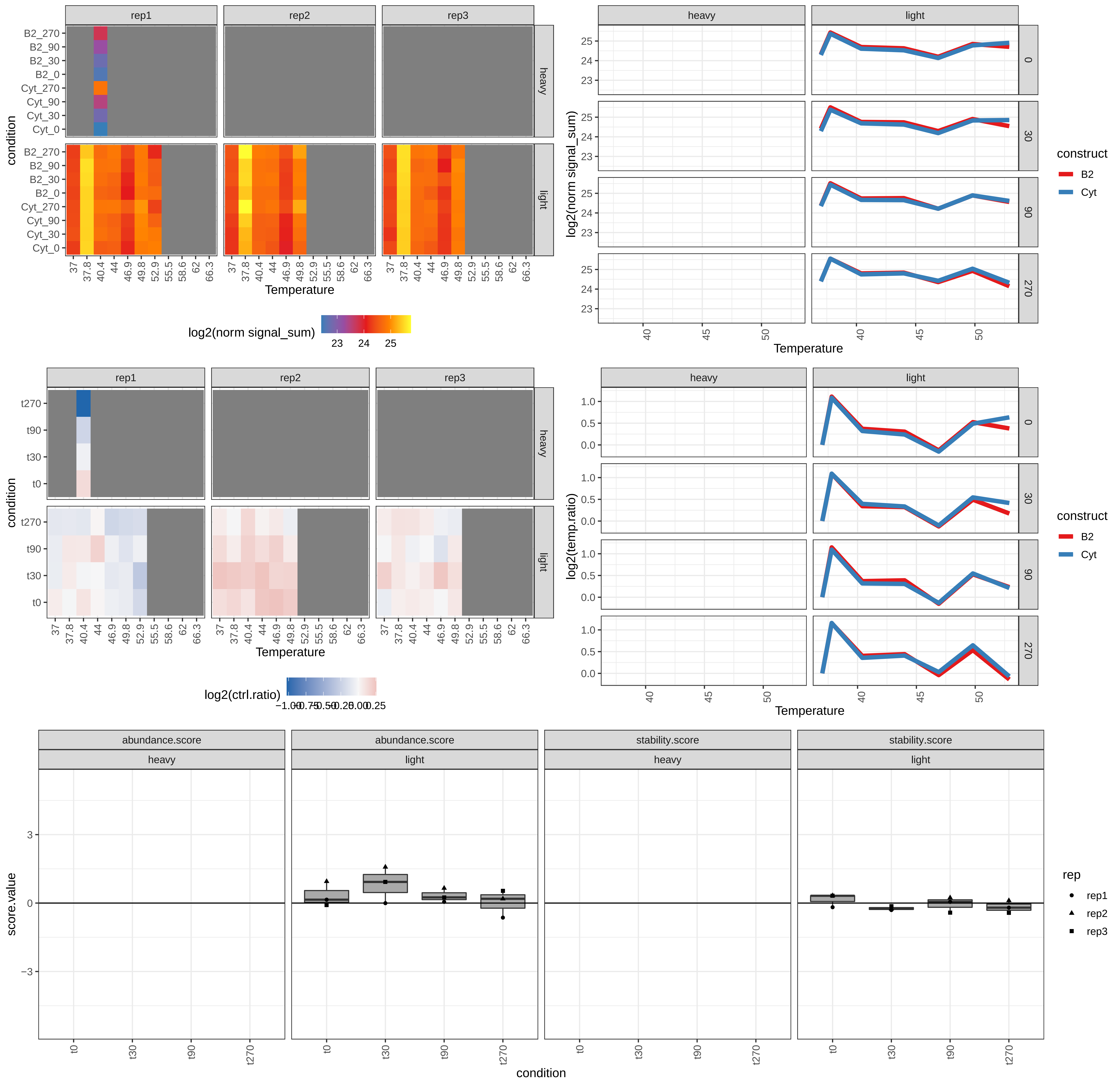

P46959 − tRNA (adenine(58)−N(1))−methyltransferase catalytic subunit TRM61 OS=Saccharomyces cerevisiae (strain ATCC 204508 / S288c) GN=GCD14 PE=1 SV=1

GCD2 − average.top3: 7.007156 − max.qupm: 6

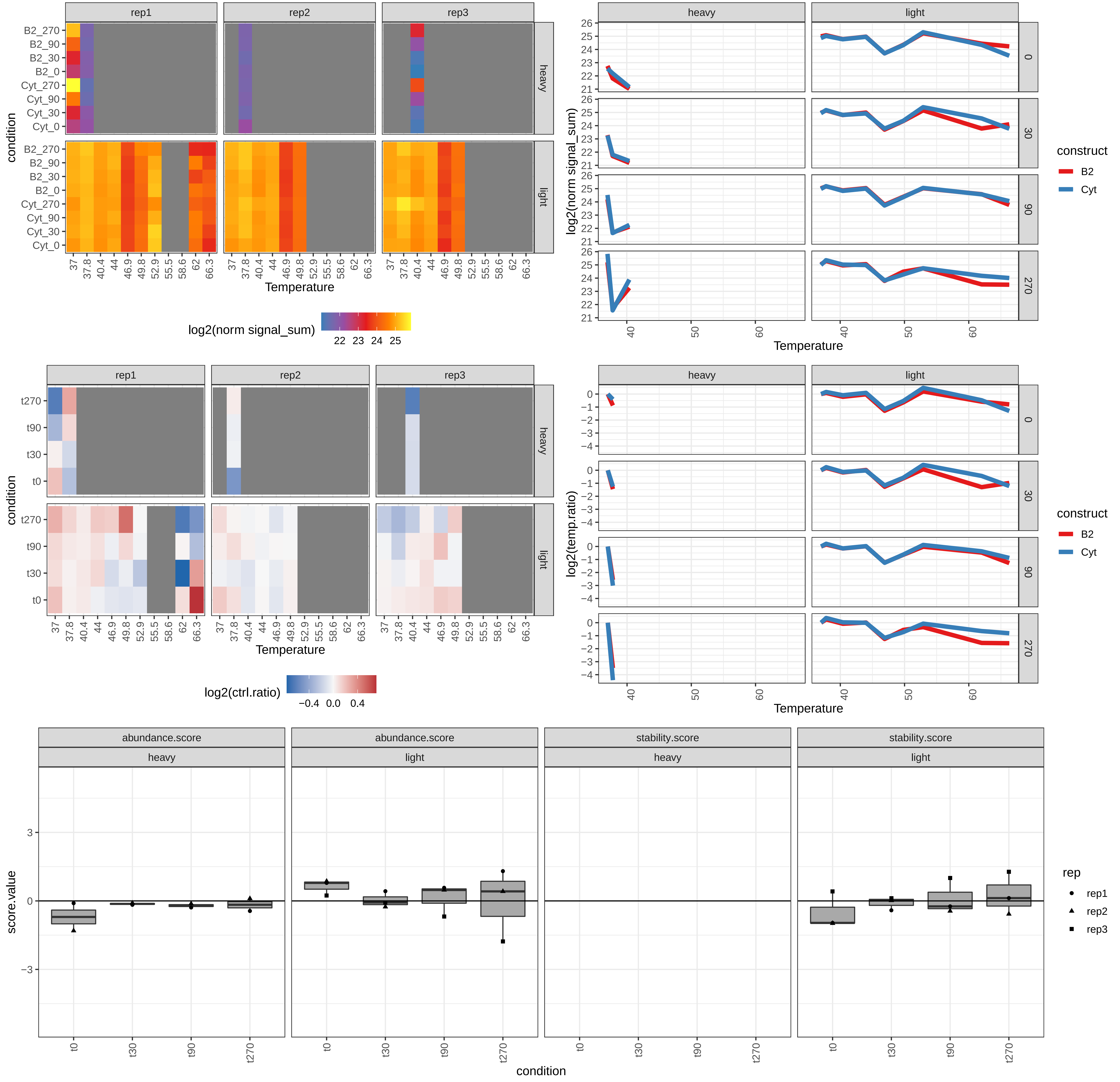

P12754 − Translation initiation factor eIF−2B subunit delta OS=Saccharomyces cerevisiae (strain ATCC 204508 / S288c) GN=GCD2 PE=1 SV=1

GCD6 − average.top3: 7.06821233333333 − max.qupm: 13

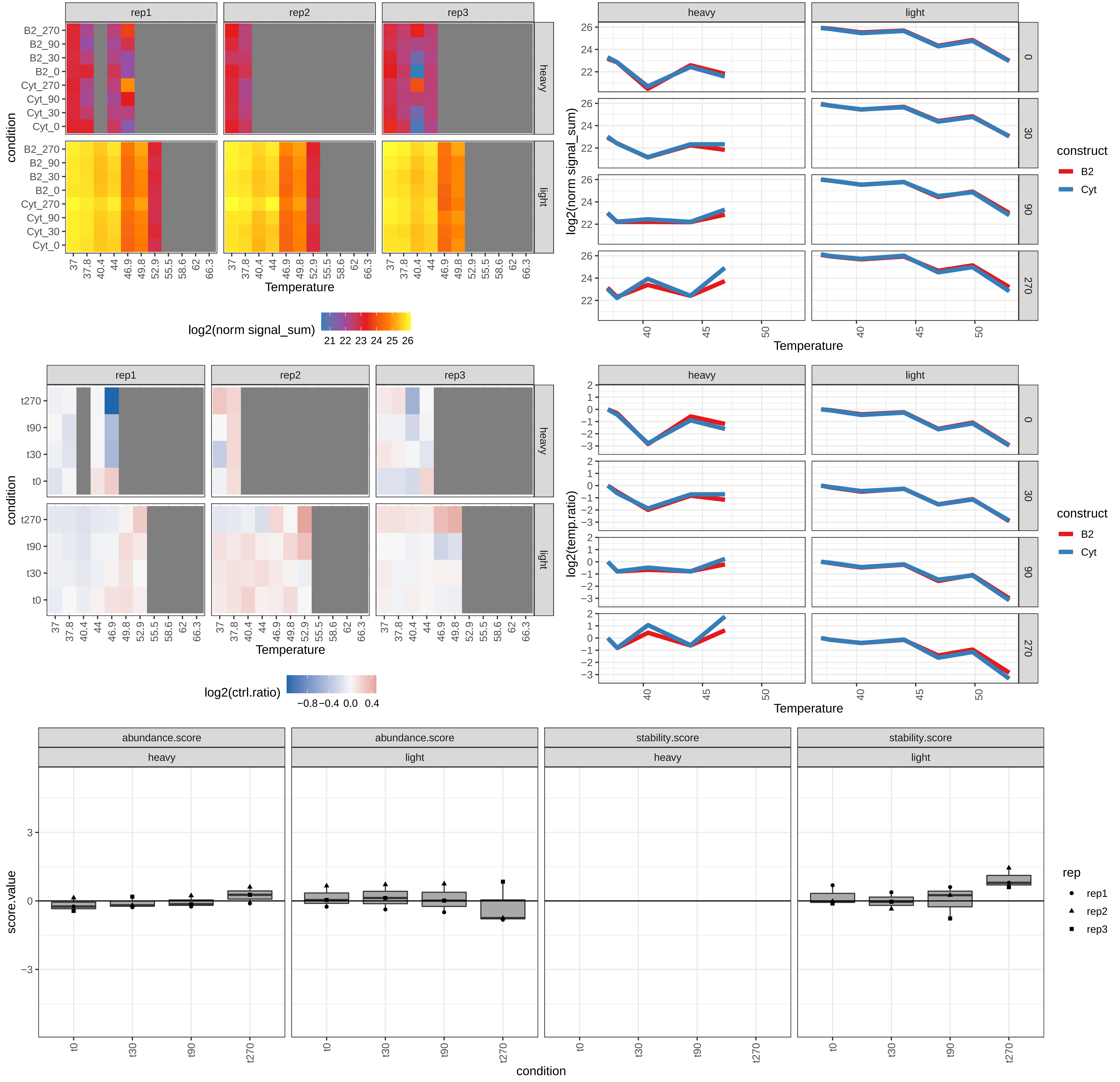

P32501 − Translation initiation factor eIF−2B subunit epsilon OS=Saccharomyces cerevisiae (strain ATCC 204508 / S288c) GN=GCD6 PE=1 SV=1

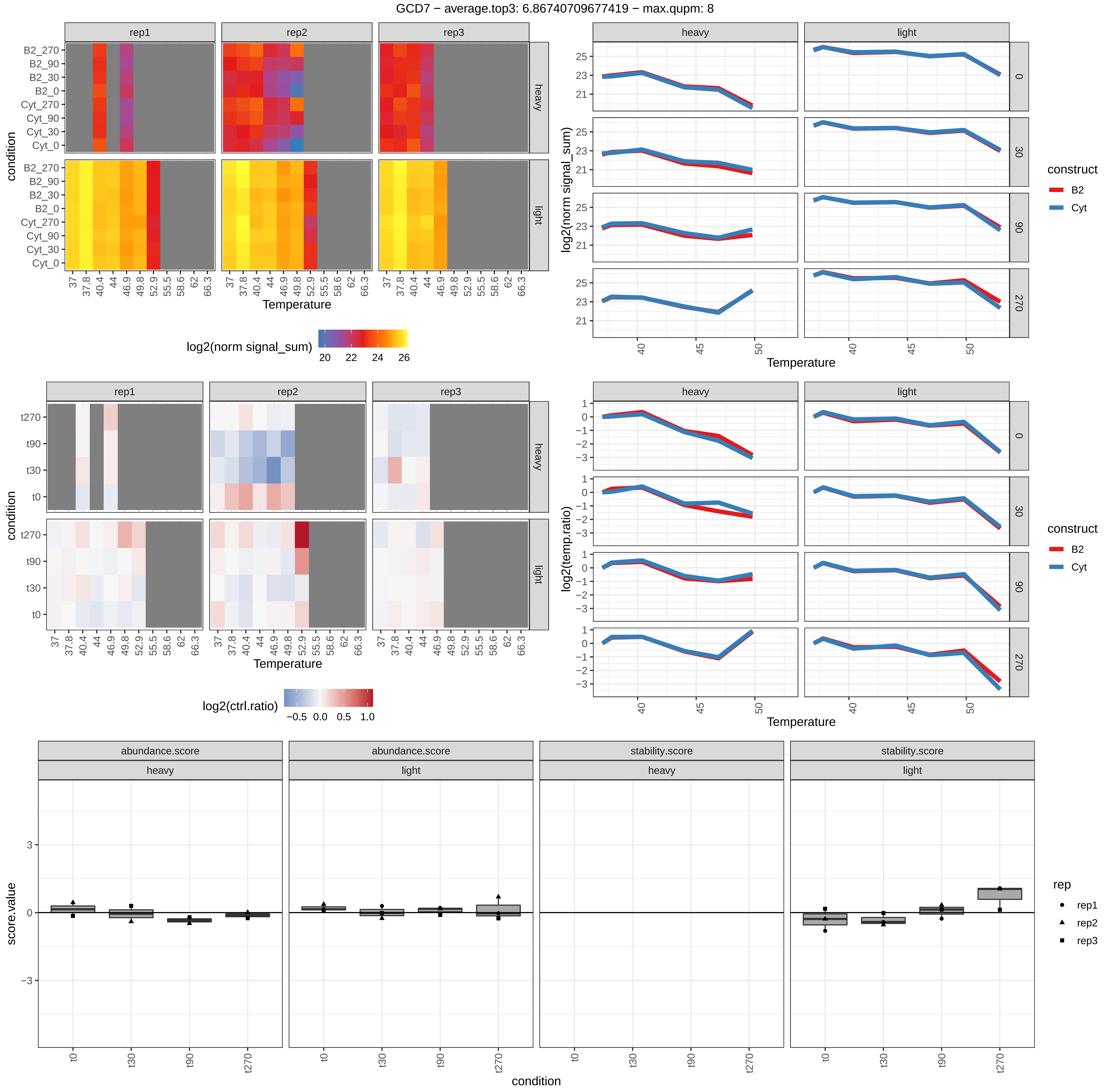

P32502 − Translation initiation factor eIF−2B subunit beta OS=Saccharomyces cerevisiae (strain ATCC 204508 / S288c) GN=GCD7 PE=1 SV=1

GCN1 − average.top3: 7.26595361702128 − max.qupm: 64

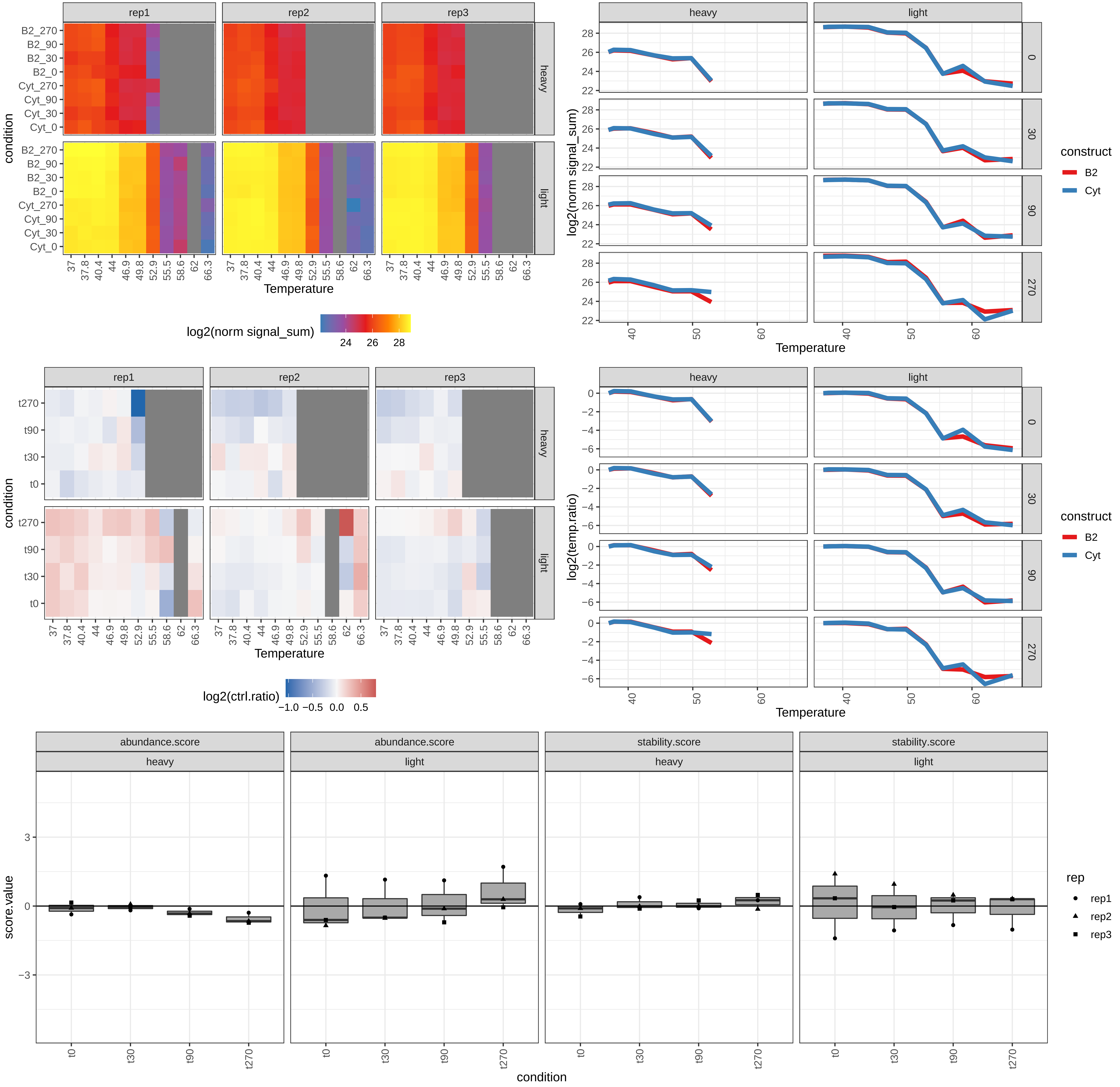

P33892 − eIF−2−alpha kinase activator GCN1 OS=Saccharomyces cerevisiae (strain ATCC 204508 / S288c) GN=GCN1 PE=1 SV=1

GCN2 − average.top3: 6.95108964285714 − max.qupm: 11

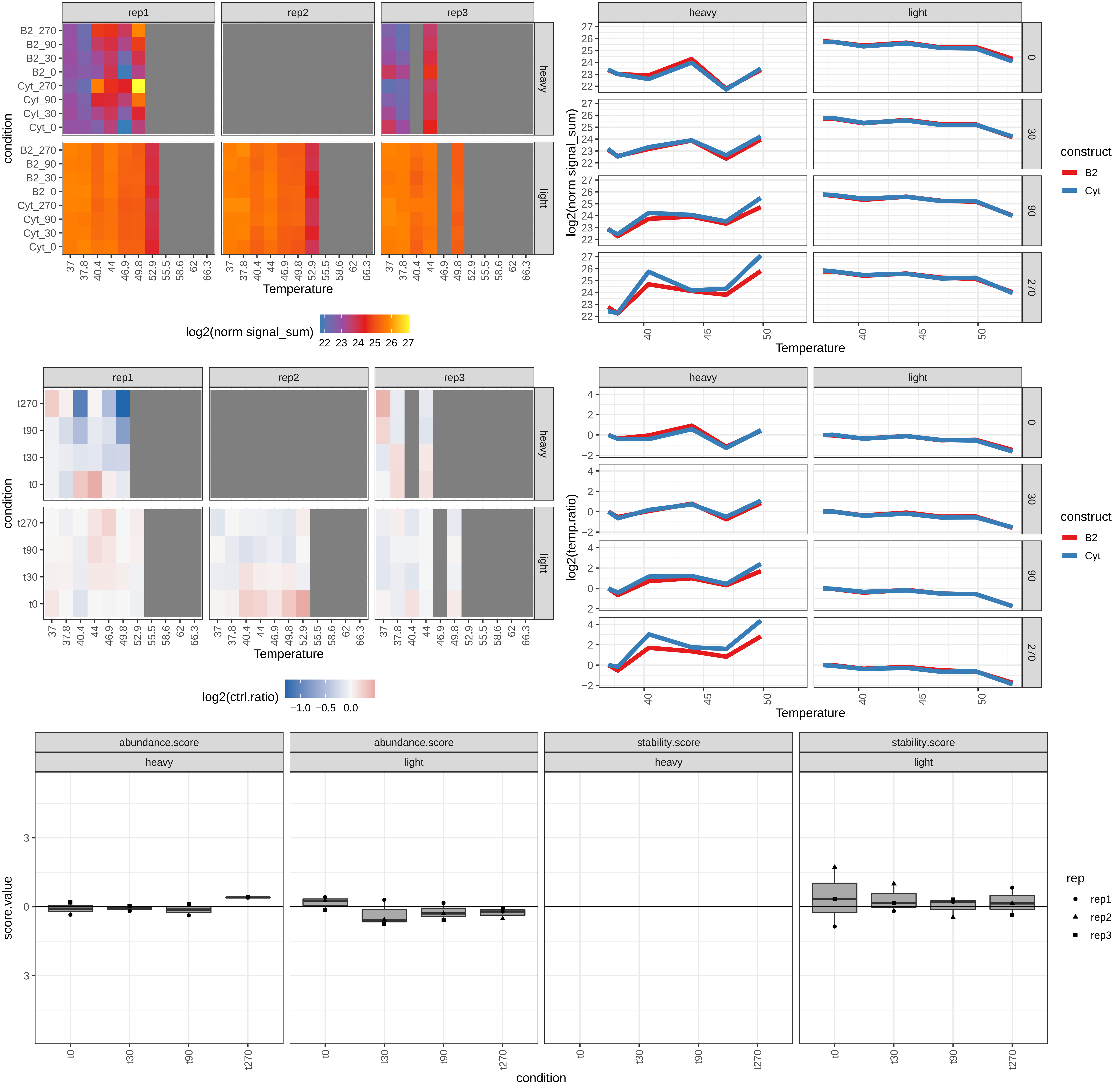

P15442 − eIF−2−alpha kinase GCN2 OS=Saccharomyces cerevisiae (strain ATCC 204508 / S288c) GN=GCN2 PE=1 SV=3

GCN20 − average.top3: 6.90687866666667 − max.qupm: 17

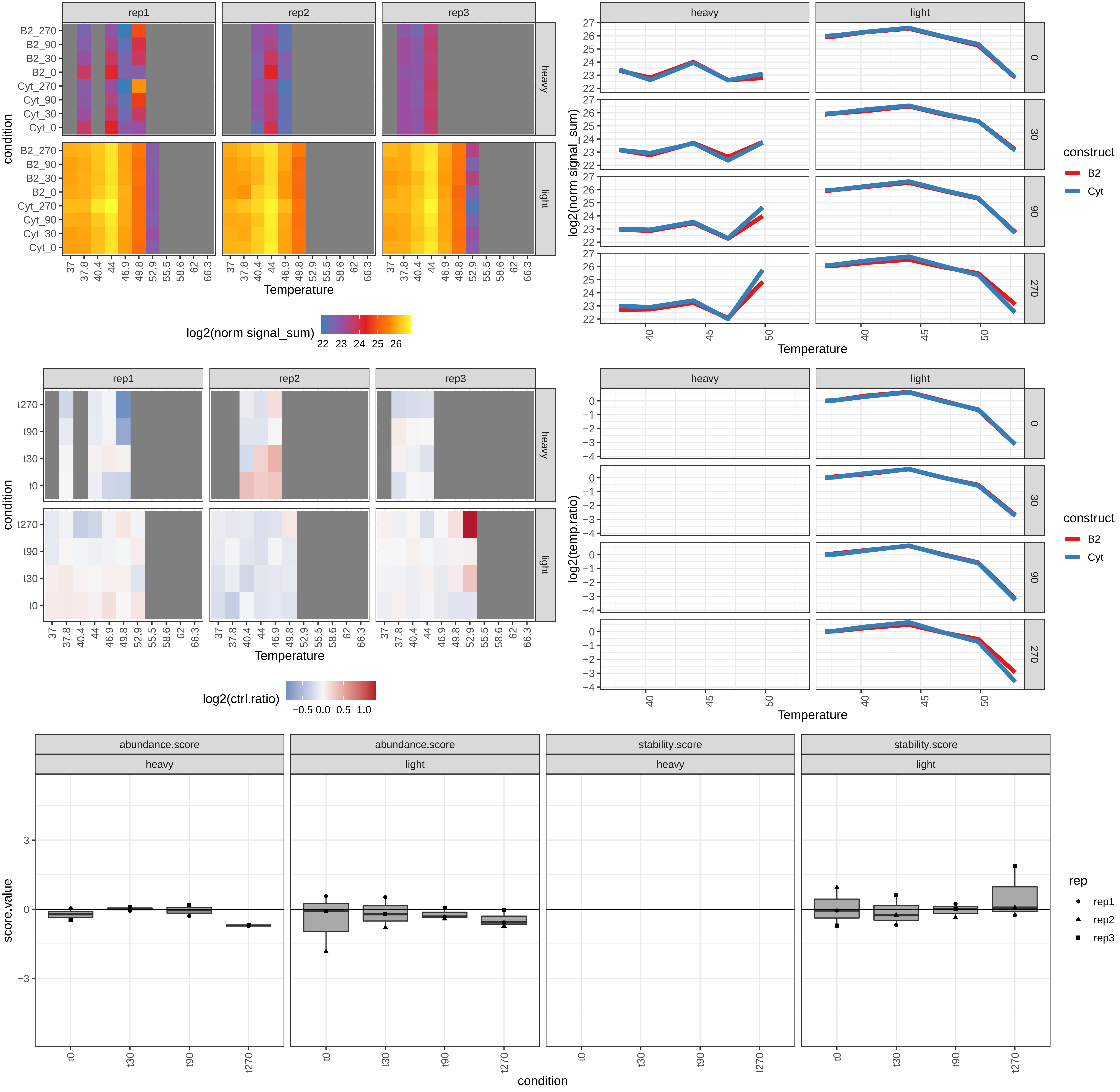

P43535 − Protein GCN20 OS=Saccharomyces cerevisiae (strain ATCC 204508 / S288c) GN=GCN20 PE=1 SV=1

GCN3 − average.top3: 7.02332055555556 − max.qupm: 7

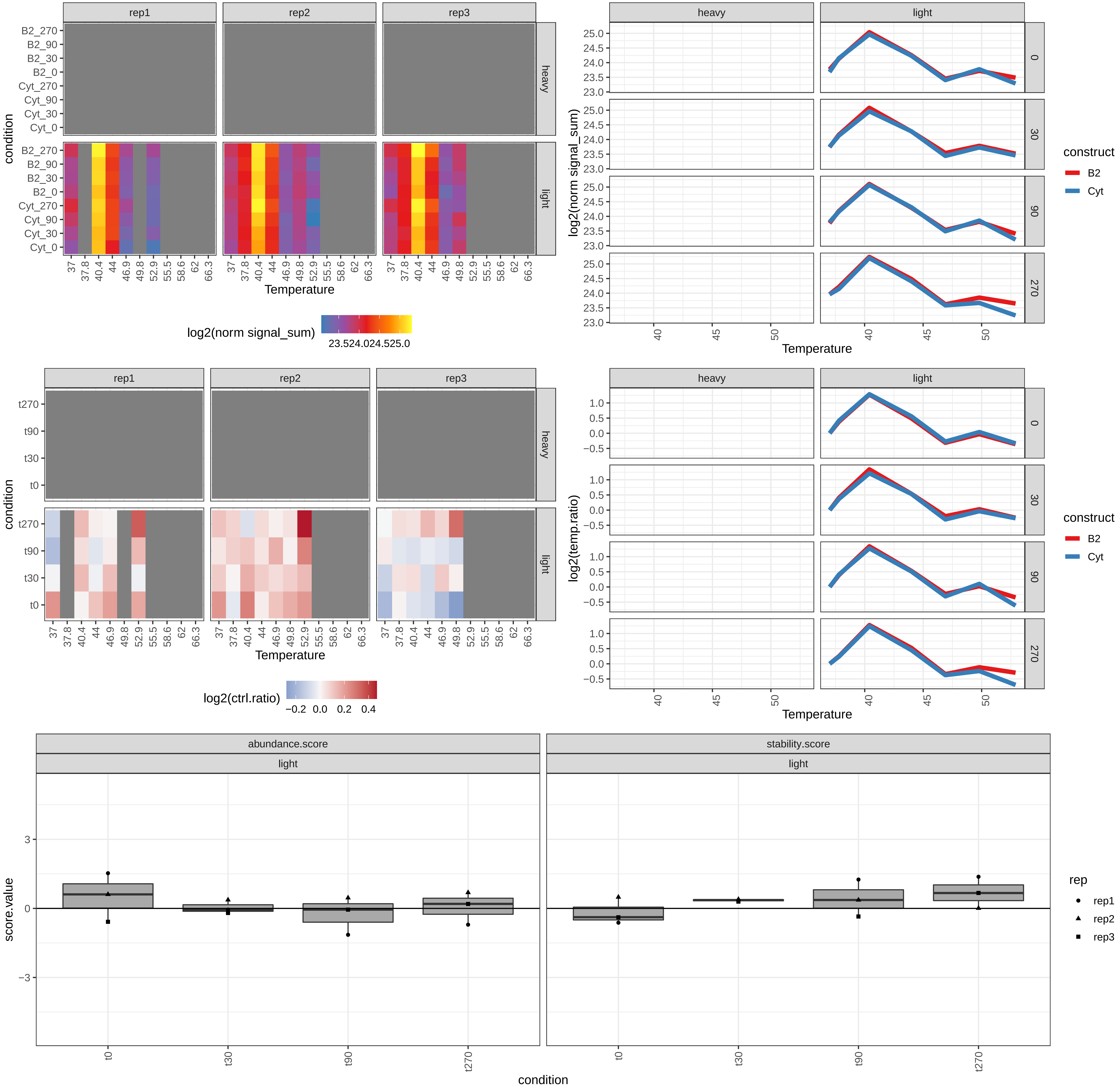

P14741 − Translation initiation factor eIF−2B subunit alpha OS=Saccharomyces cerevisiae (strain ATCC 204508 / S288c) GN=GCN3 PE=1 SV=1

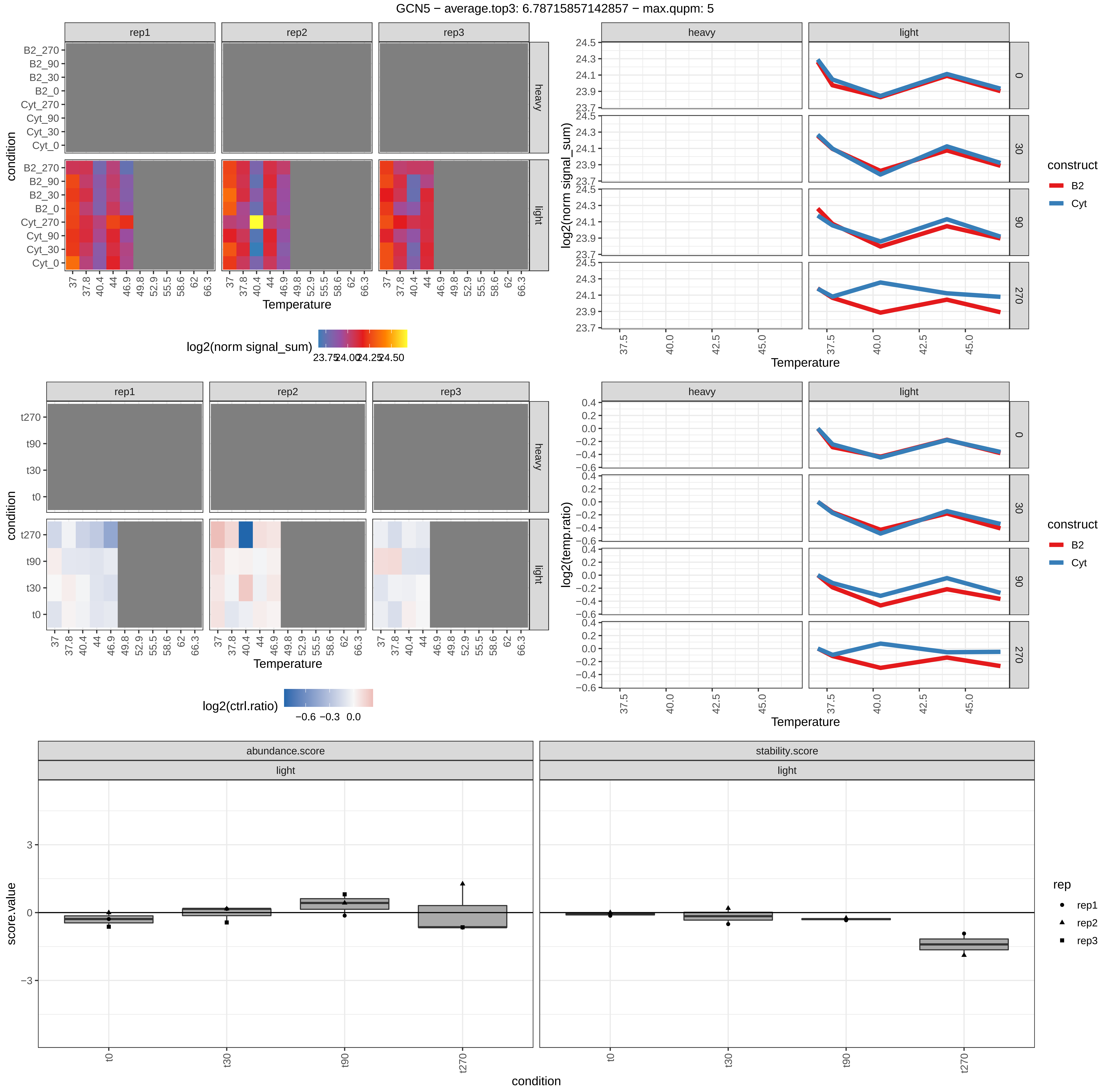

Q03330 − Histone acetyltransferase GCN5 OS=Saccharomyces cerevisiae (strain ATCC 204508 / S288c) GN=GCN5 PE=1 SV=1

GCS1 − average.top3: 7.02305818181818 − max.qupm: 11

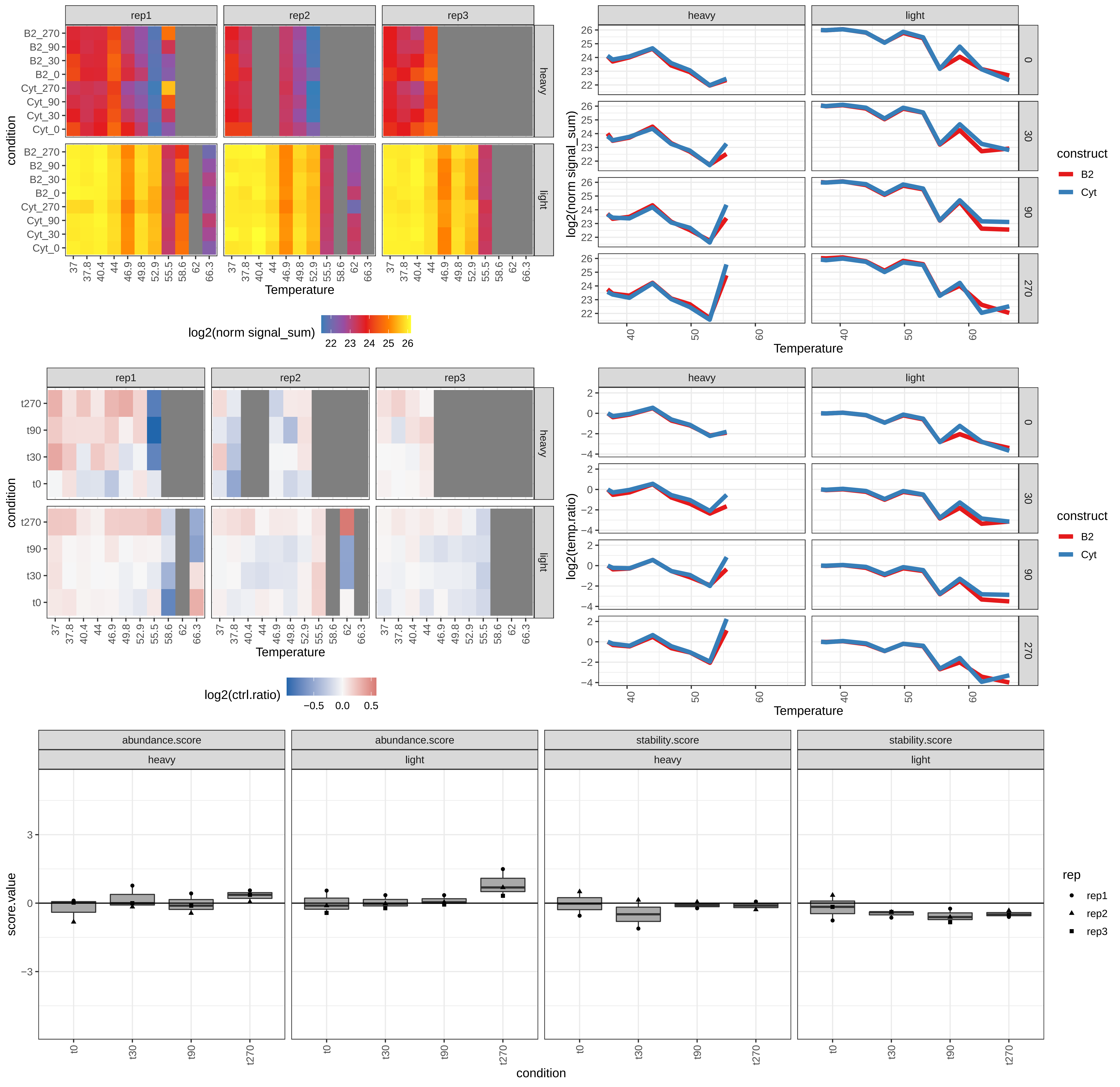

P35197 − ADP−ribosylation factor GTPase−activating protein GCS1 OS=Saccharomyces cerevisiae (strain ATCC 204508 / S288c) GN=GCS1 PE=1 SV=1

GCV1 − average.top3: 6.83790555555556 − max.qupm: 4

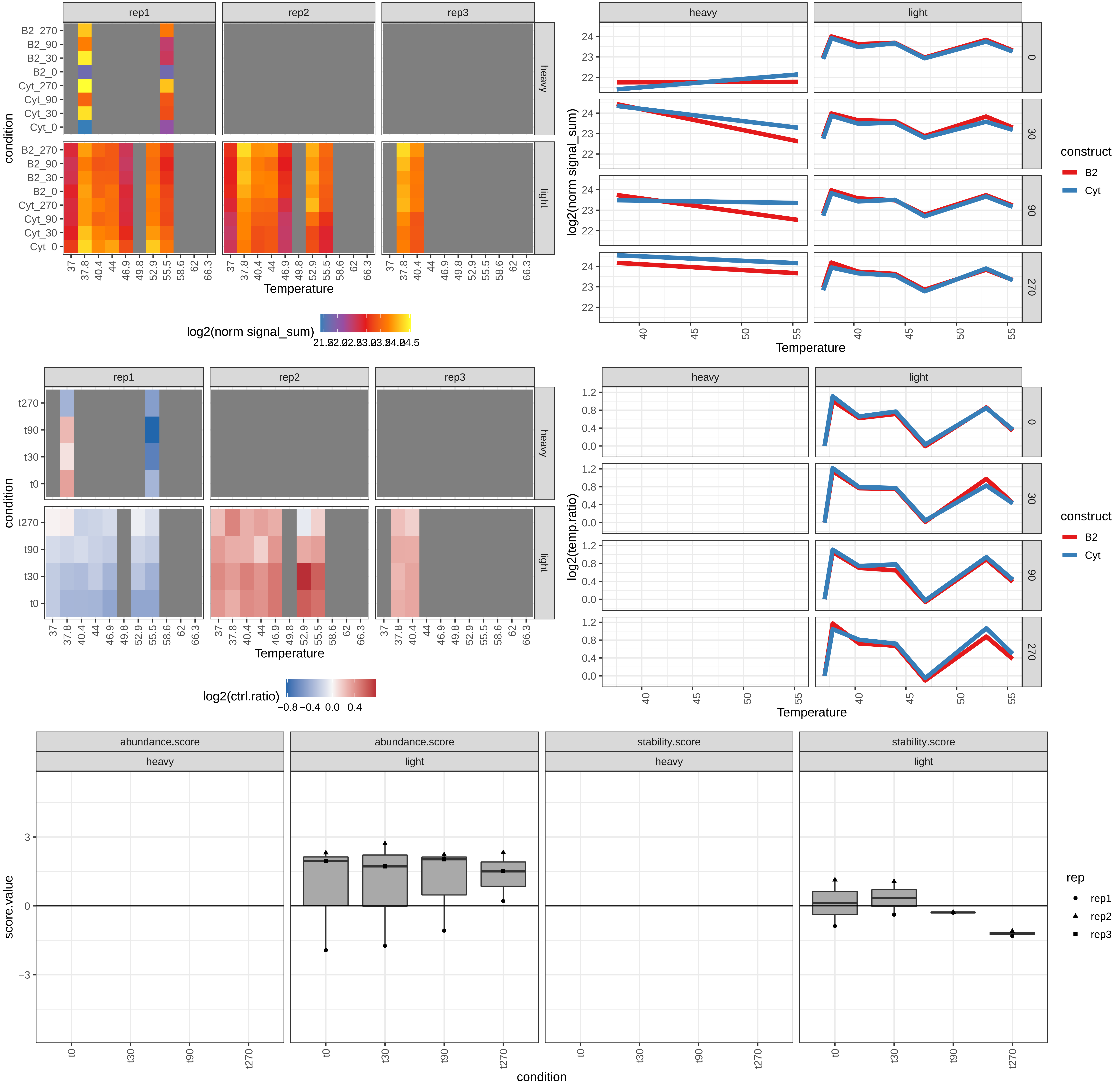

P48015 − Aminomethyltransferase, mitochondrial OS=Saccharomyces cerevisiae (strain ATCC 204508 / S288c) GN=GCV1 PE=1 SV=2

GCV2 − average.top3: 6.3848872 − max.qupm: 6

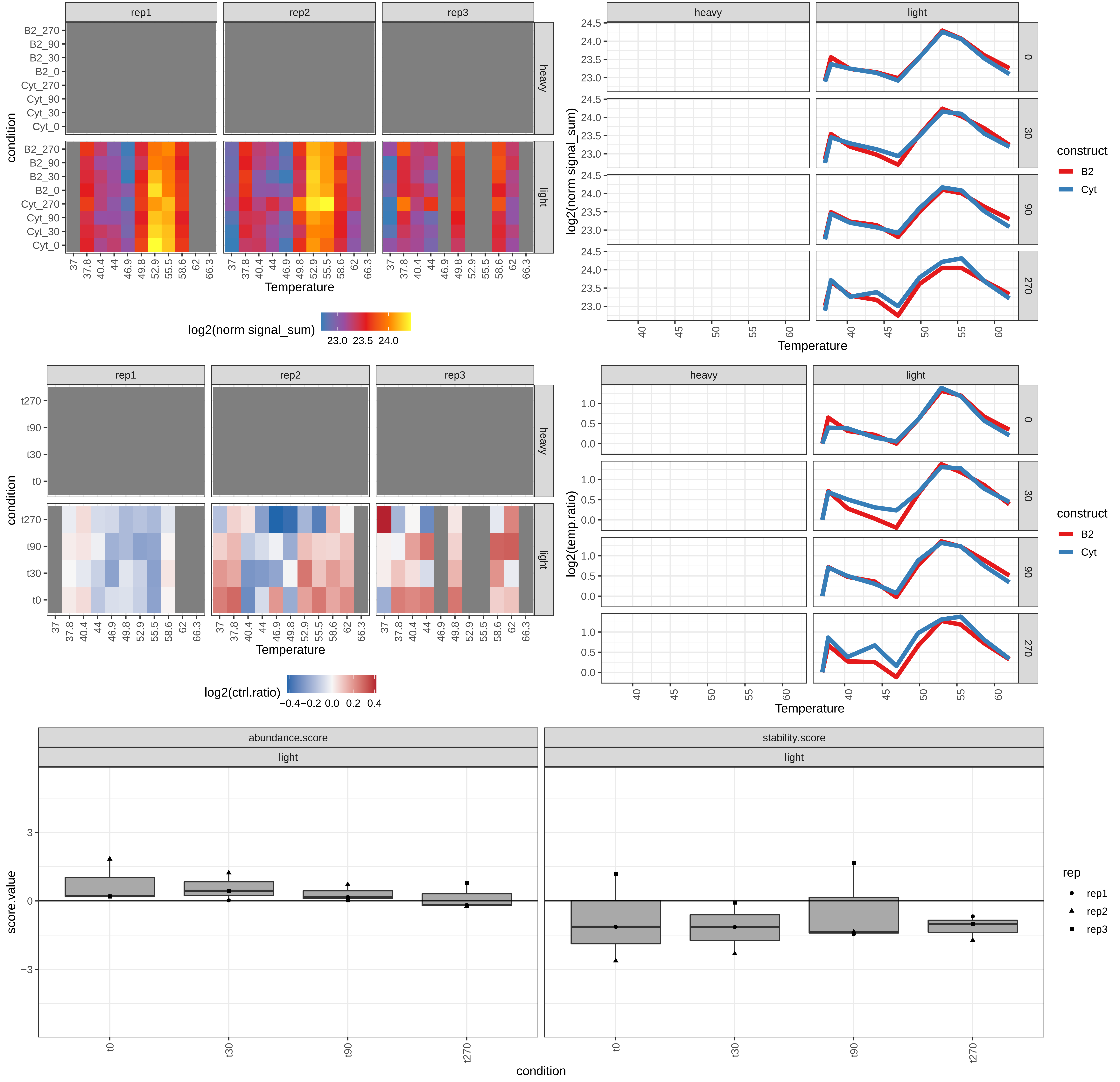

P49095 − Glycine dehydrogenase (decarboxylating), mitochondrial OS=Saccharomyces cerevisiae (strain ATCC 204508 / S288c) GN=GCV2 PE=1 SV=1

GCV3 − average.top3: 7.05680136363636 − max.qupm: 4

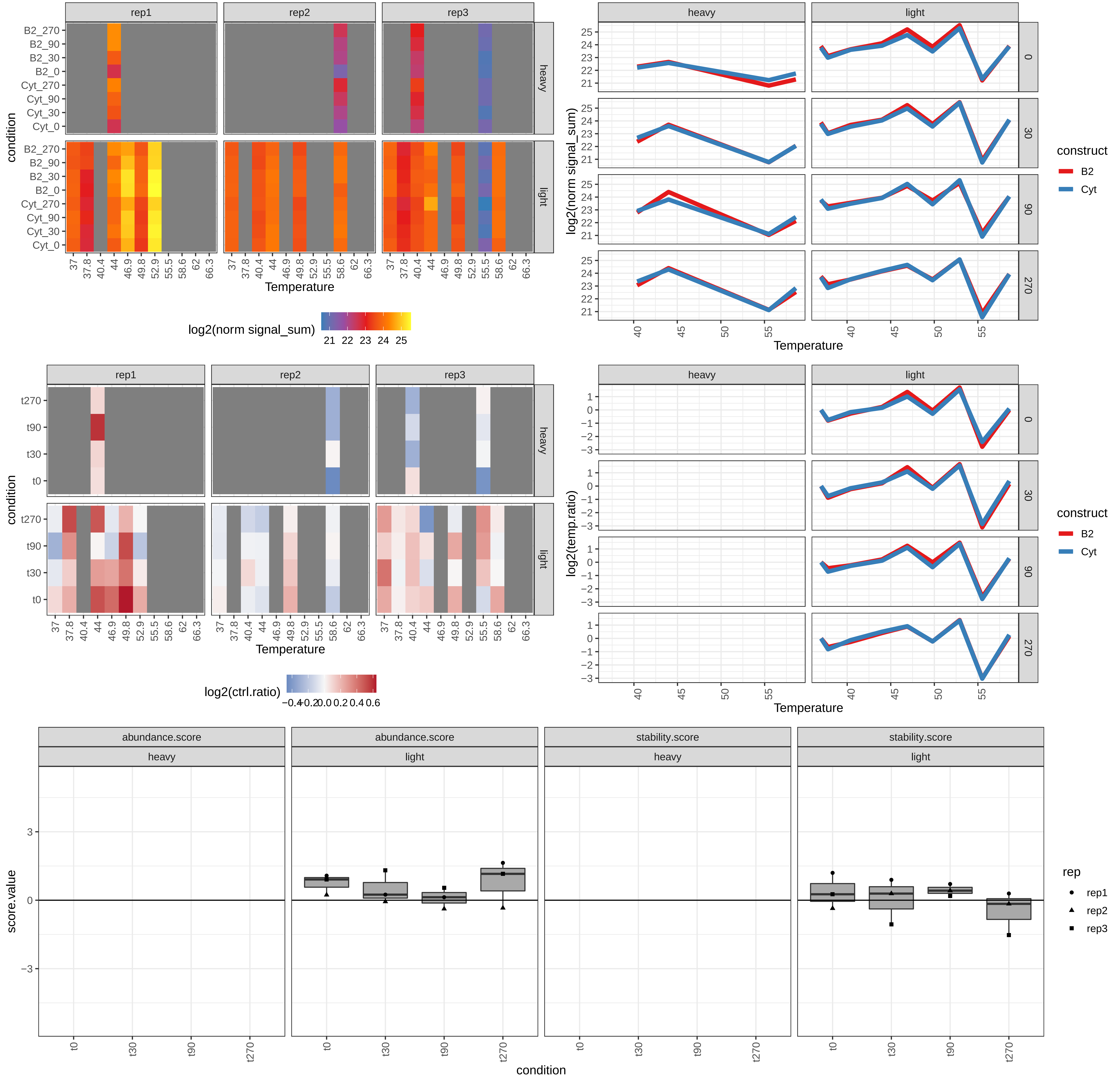

P39726 − Glycine cleavage system H protein, mitochondrial OS=Saccharomyces cerevisiae (strain ATCC 204508 / S288c) GN=GCV3 PE=1 SV=3

GCY1 − average.top3: 7.31440033333333 − max.qupm: 11

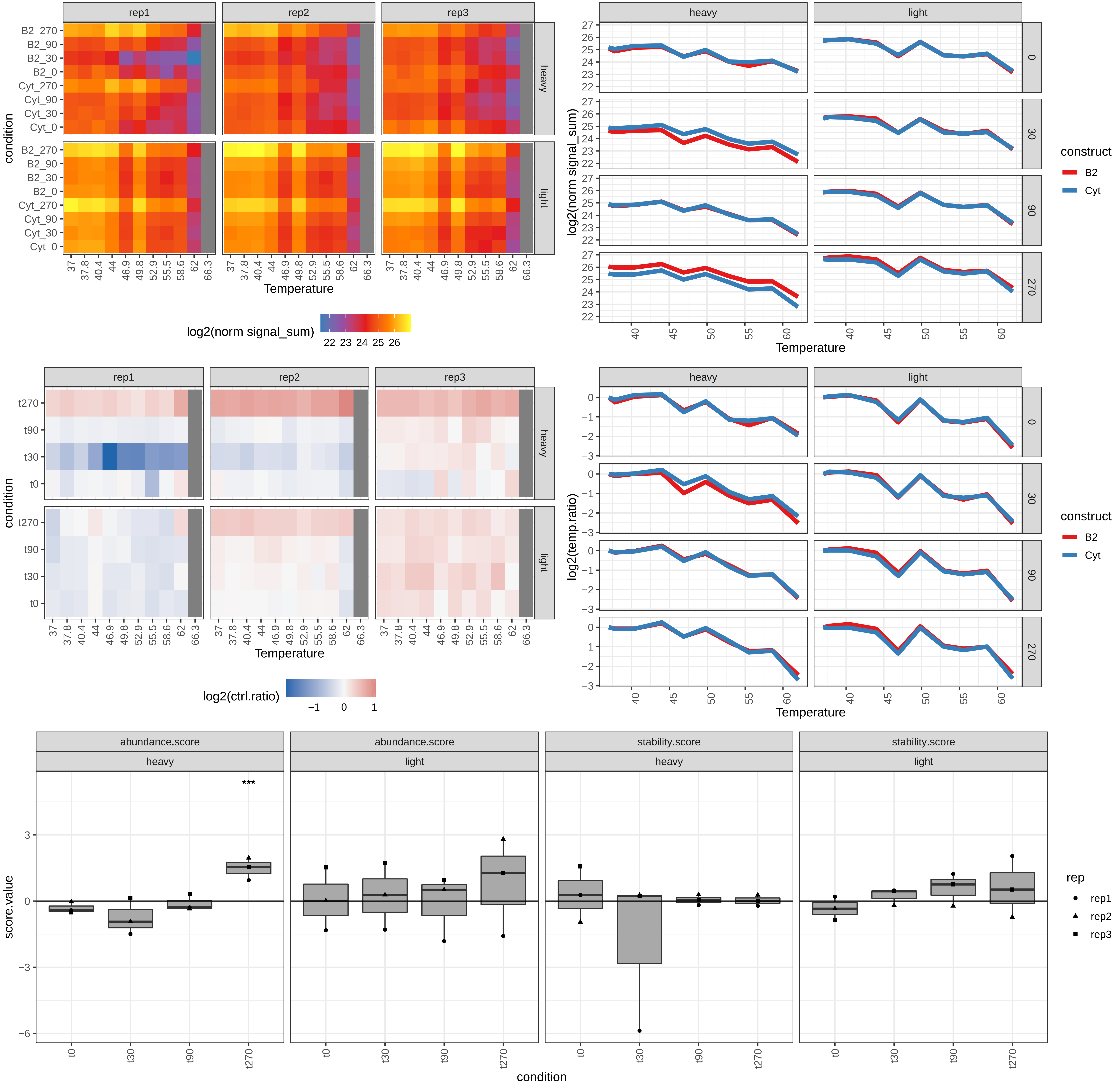

P14065 − Glycerol 2−dehydrogenase (NADP(+)) OS=Saccharomyces cerevisiae (strain ATCC 204508 / S288c) GN=GCY1 PE=1 SV=1

GDA1 − average.top3: 6.73159515151515 − max.qupm: 8

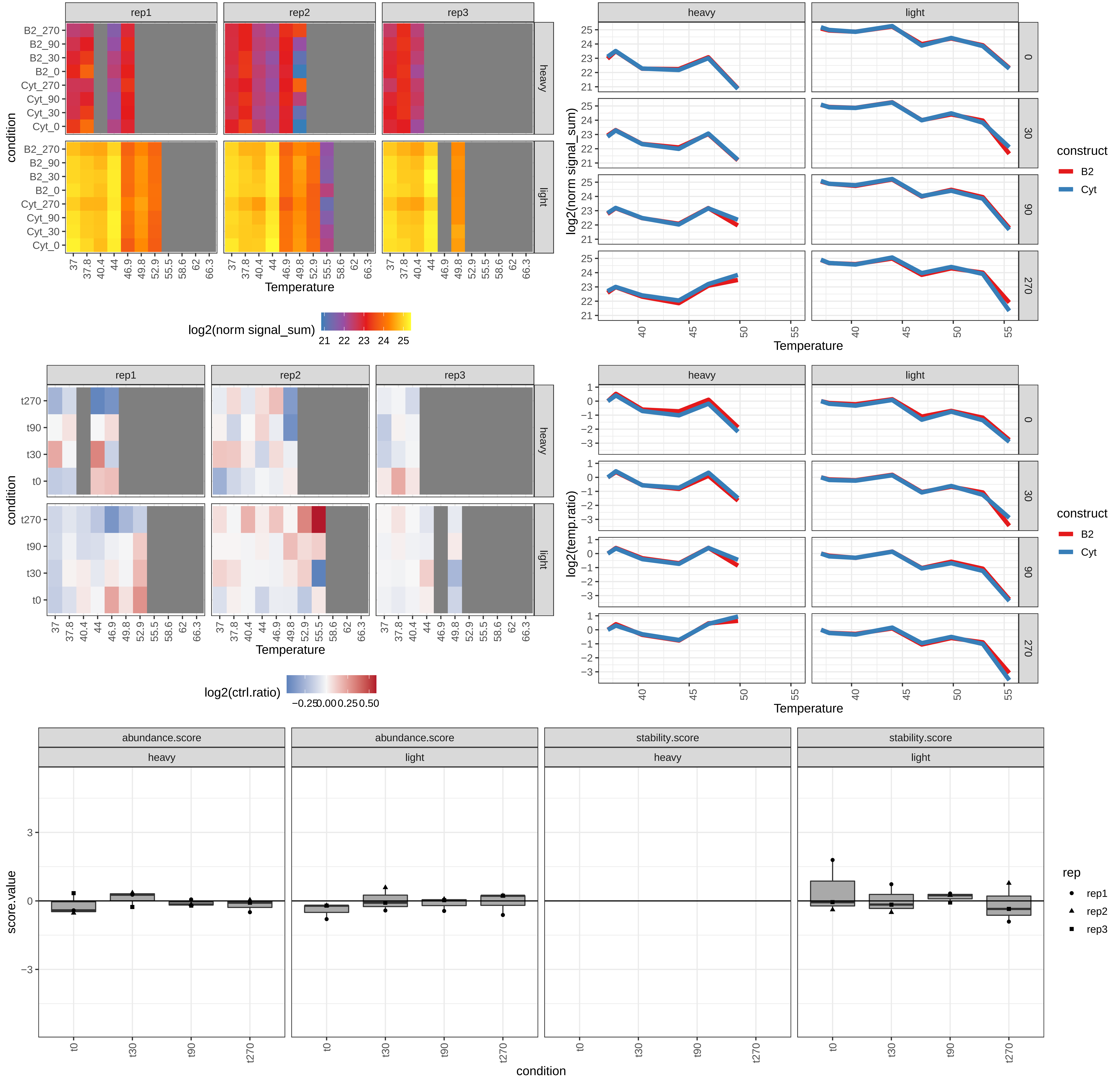

P32621 − Guanosine−diphosphatase OS=Saccharomyces cerevisiae (strain ATCC 204508 / S288c) GN=GDA1 PE=1 SV=1

GDB1 − average.top3: 7.36245725 − max.qupm: 33

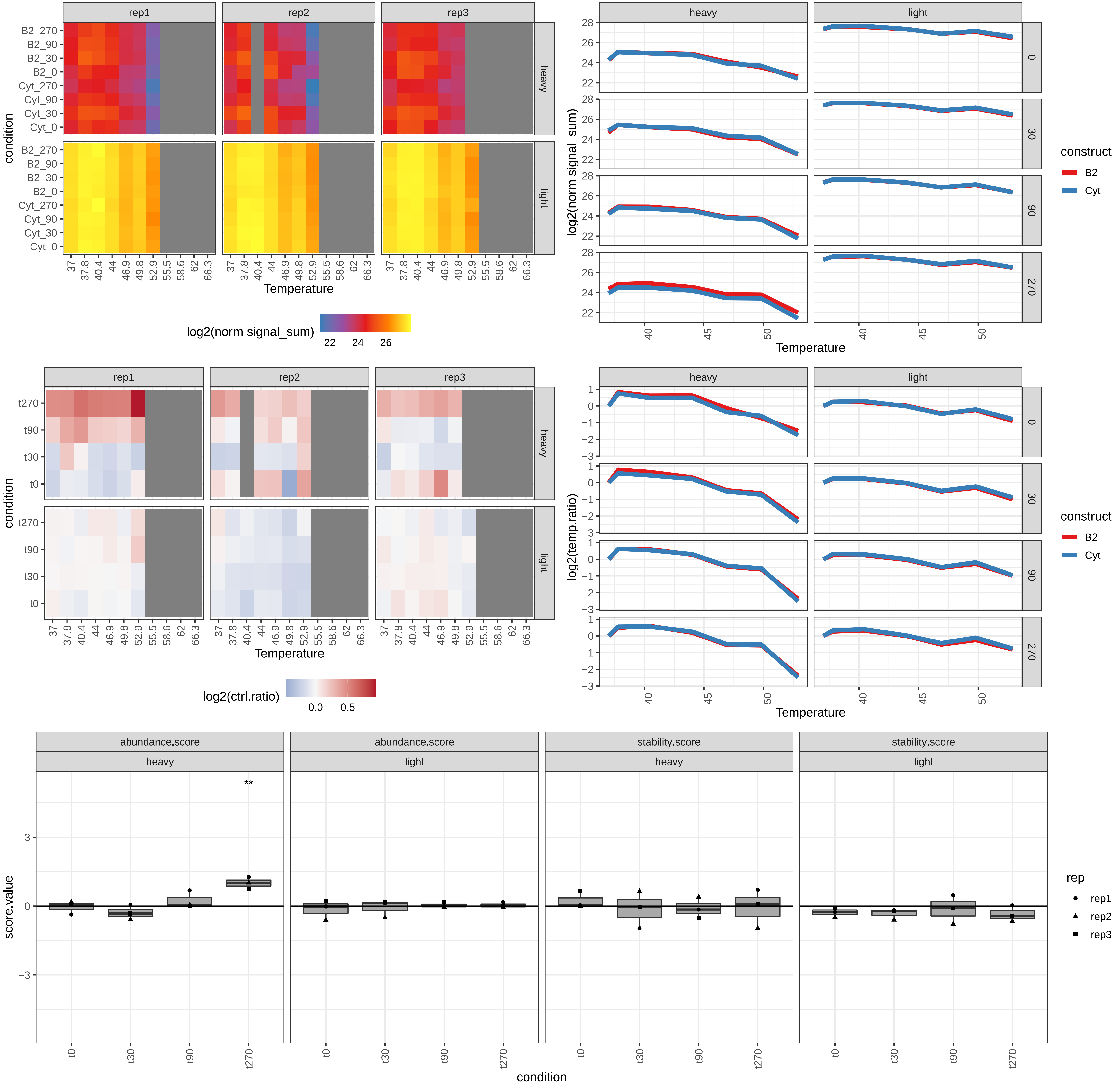

Q06625 − Glycogen debranching enzyme OS=Saccharomyces cerevisiae (strain ATCC 204508 / S288c) GN=GDB1 PE=1 SV=1

GDE1 − average.top3: 6.63992470588235 − max.qupm: 9

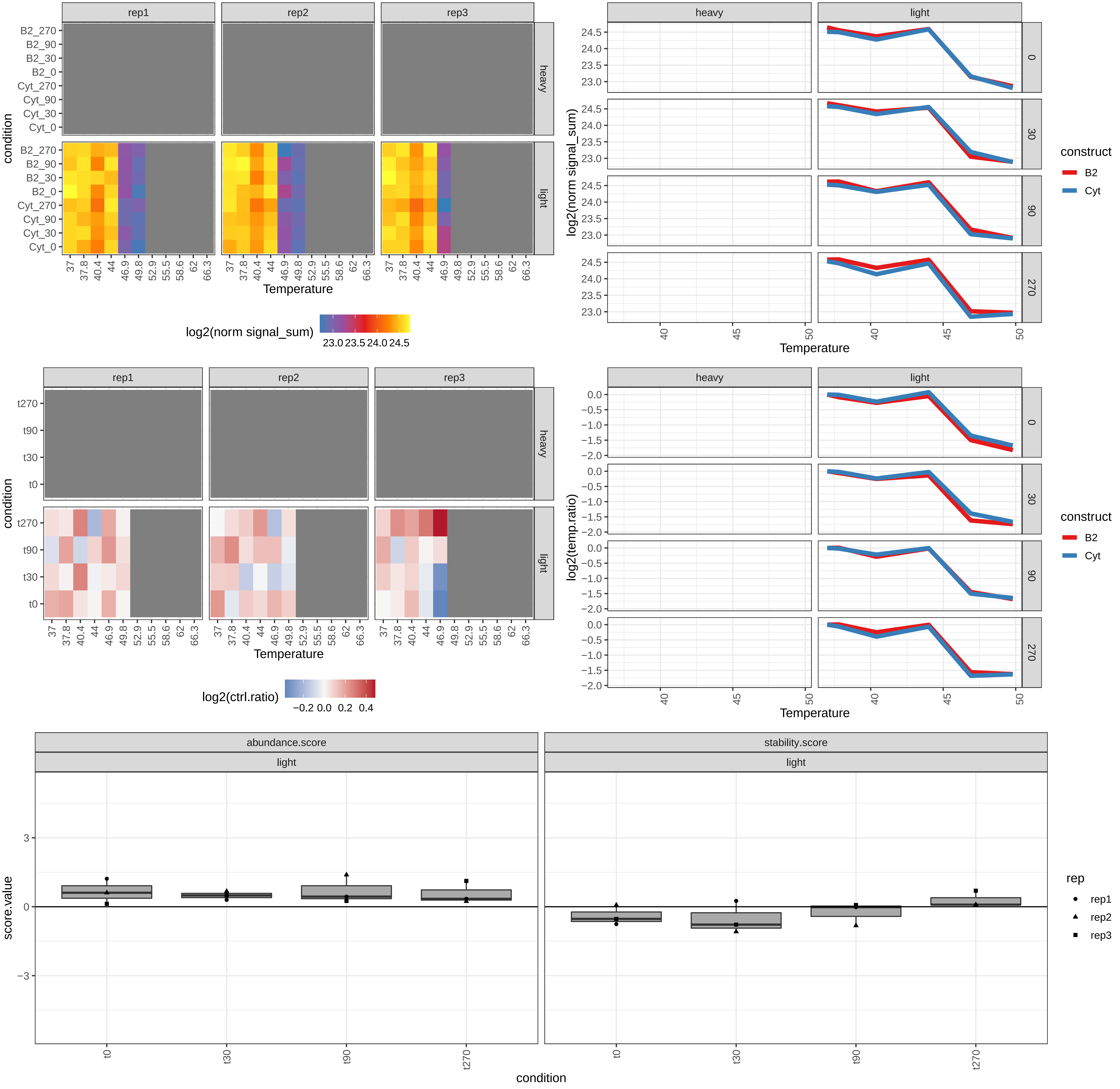

Q02979 − Glycerophosphodiester phosphodiesterase GDE1 OS=Saccharomyces cerevisiae (strain ATCC 204508 / S288c) GN=GDE1 PE=1 SV=1

GDH1 − average.top3: 8.18789590909091 − max.qupm: 18

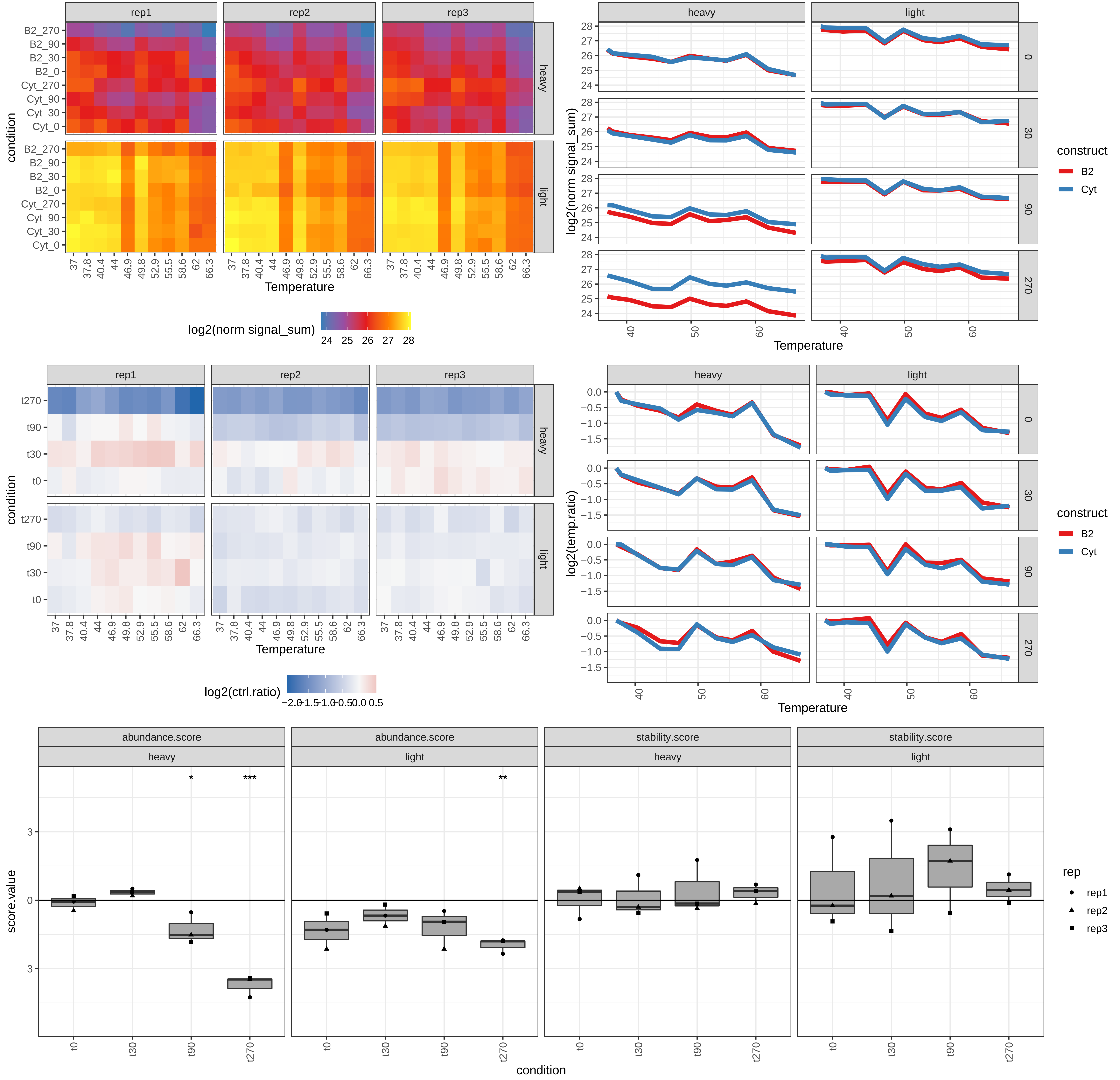

P07262 − NADP−specific glutamate dehydrogenase 1 OS=Saccharomyces cerevisiae (strain ATCC 204508 / S288c) GN=GDH1 PE=1 SV=2

GDH2 − average.top3: 7.89019852459016 − max.qupm: 38

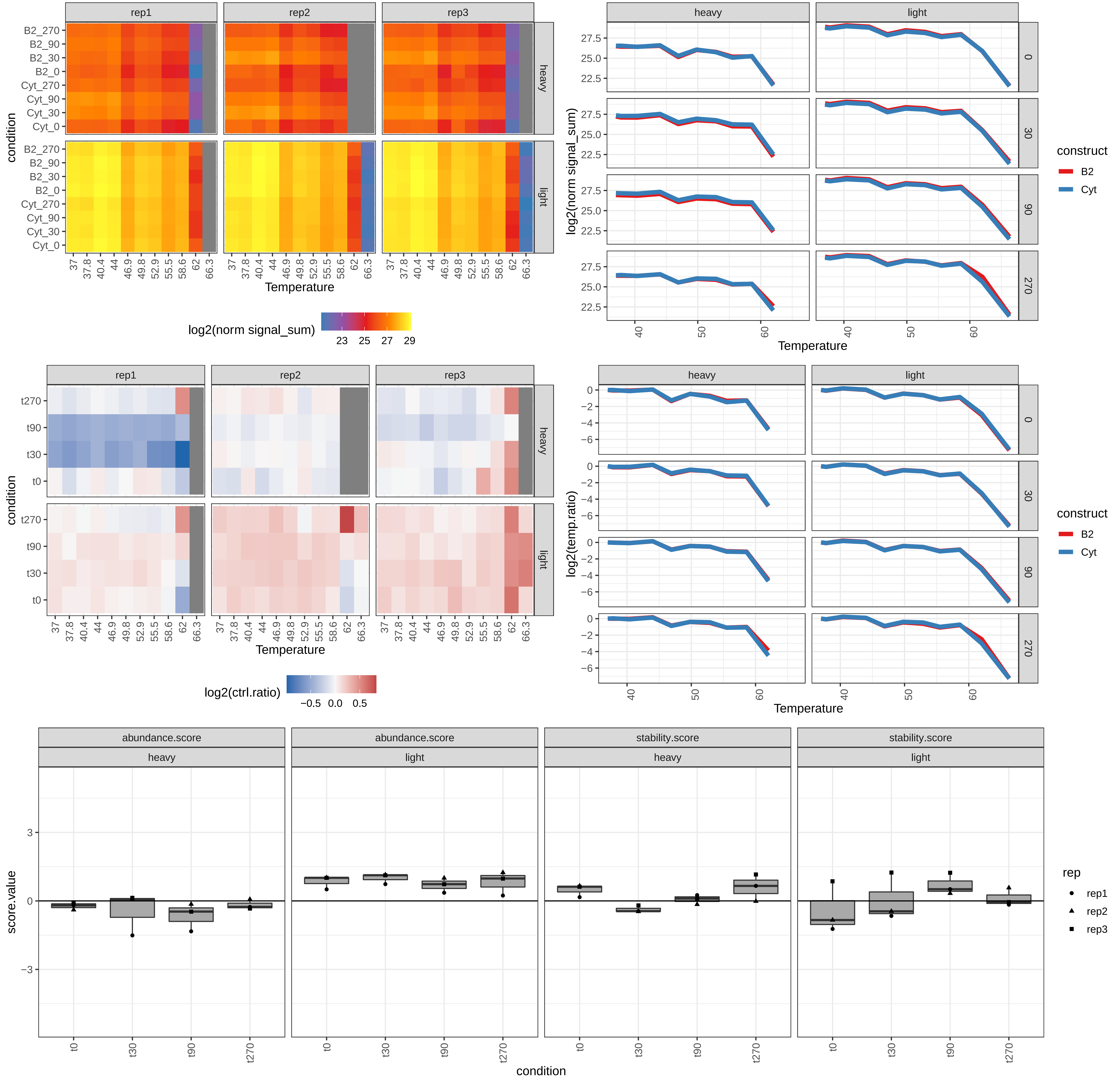

P33327 − NAD−specific glutamate dehydrogenase OS=Saccharomyces cerevisiae (strain ATCC 204508 / S288c) GN=GDH2 PE=1 SV=1

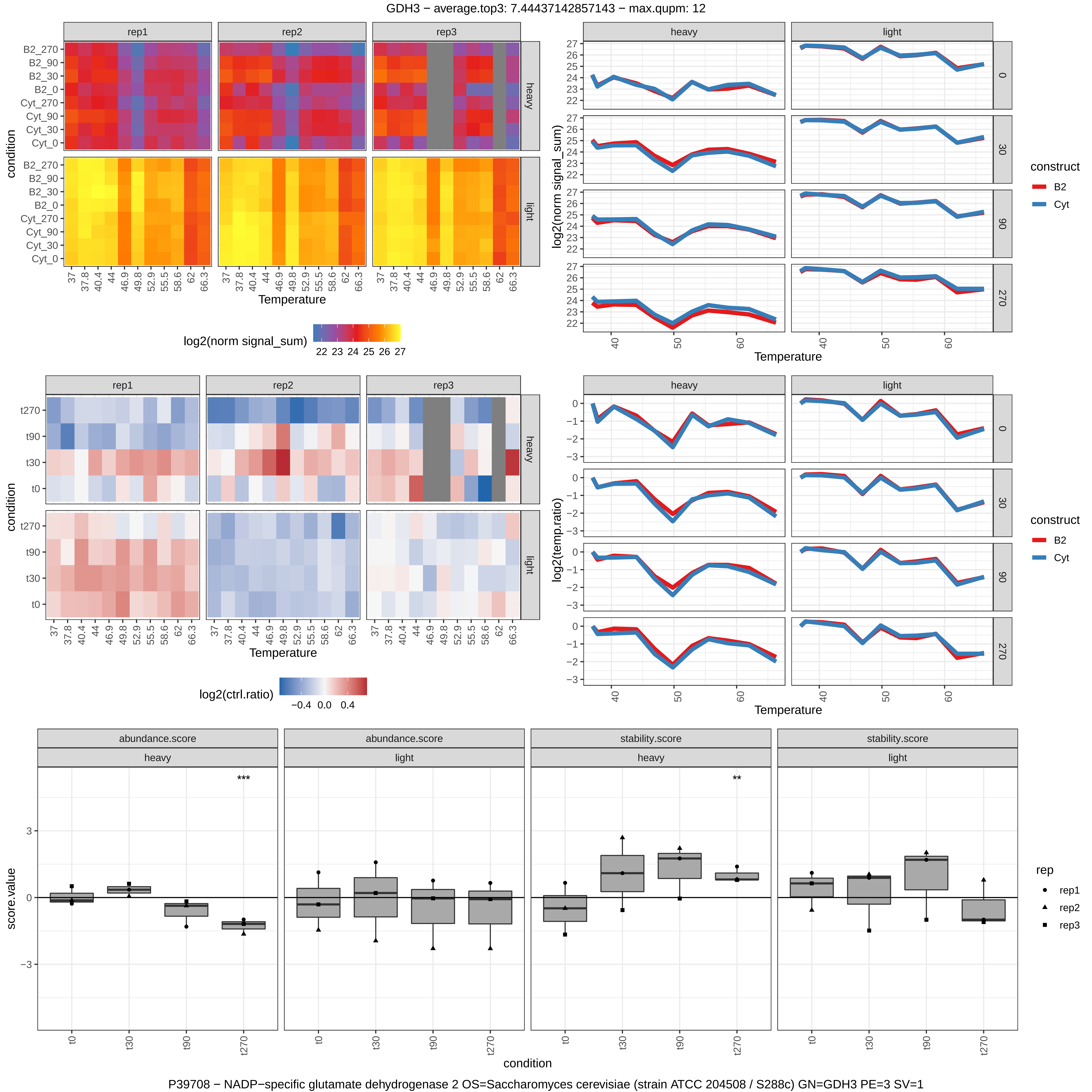
GDH3 − average.top3: 7.44437142857143 − max.qupm: 12
rep1
rep2
rep3
heavy
light
condition
Temperature
log2(norm signal_sum)
log2(temp.ratio)
log2(ctrl.ratio)
construct
B2
Cyt
abundance.score
stability.score
score.value
rep
rep1
rep2
rep3
***
**
P39708 − NADP−specific glutamate dehydrogenase 2 OS=Saccharomyces cerevisiae (strain ATCC 204508 / S288c) GN=GDH3 PE=3 SV=1

GDI1 − average.top3: 7.39801692307692 − max.qupm: 15

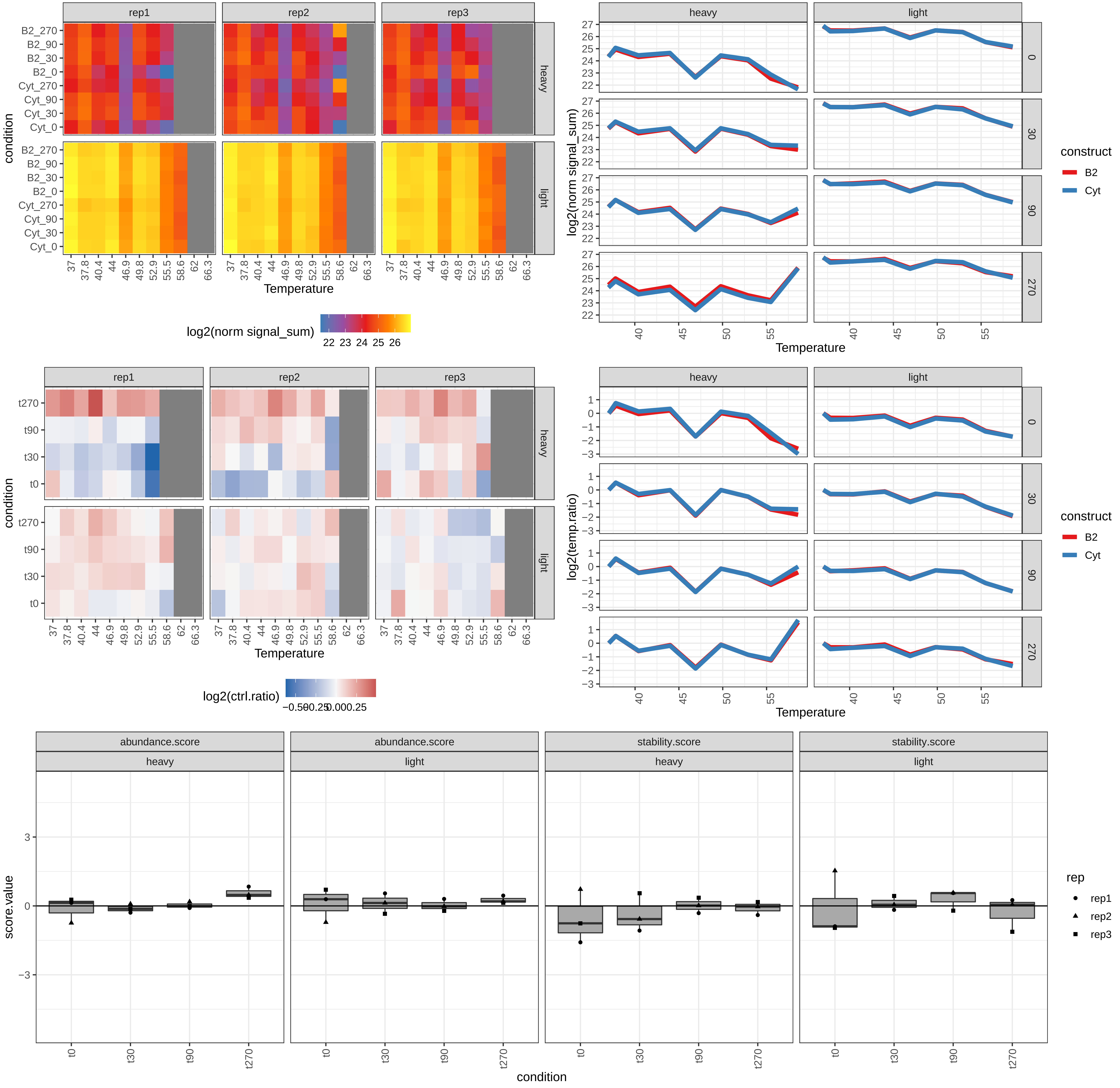

P39958 − Rab GDP−dissociation inhibitor OS=Saccharomyces cerevisiae (strain ATCC 204508 / S288c) GN=GDI1 PE=1 SV=1

GEA1 − average.top3: 6.81010875 − max.qupm: 8

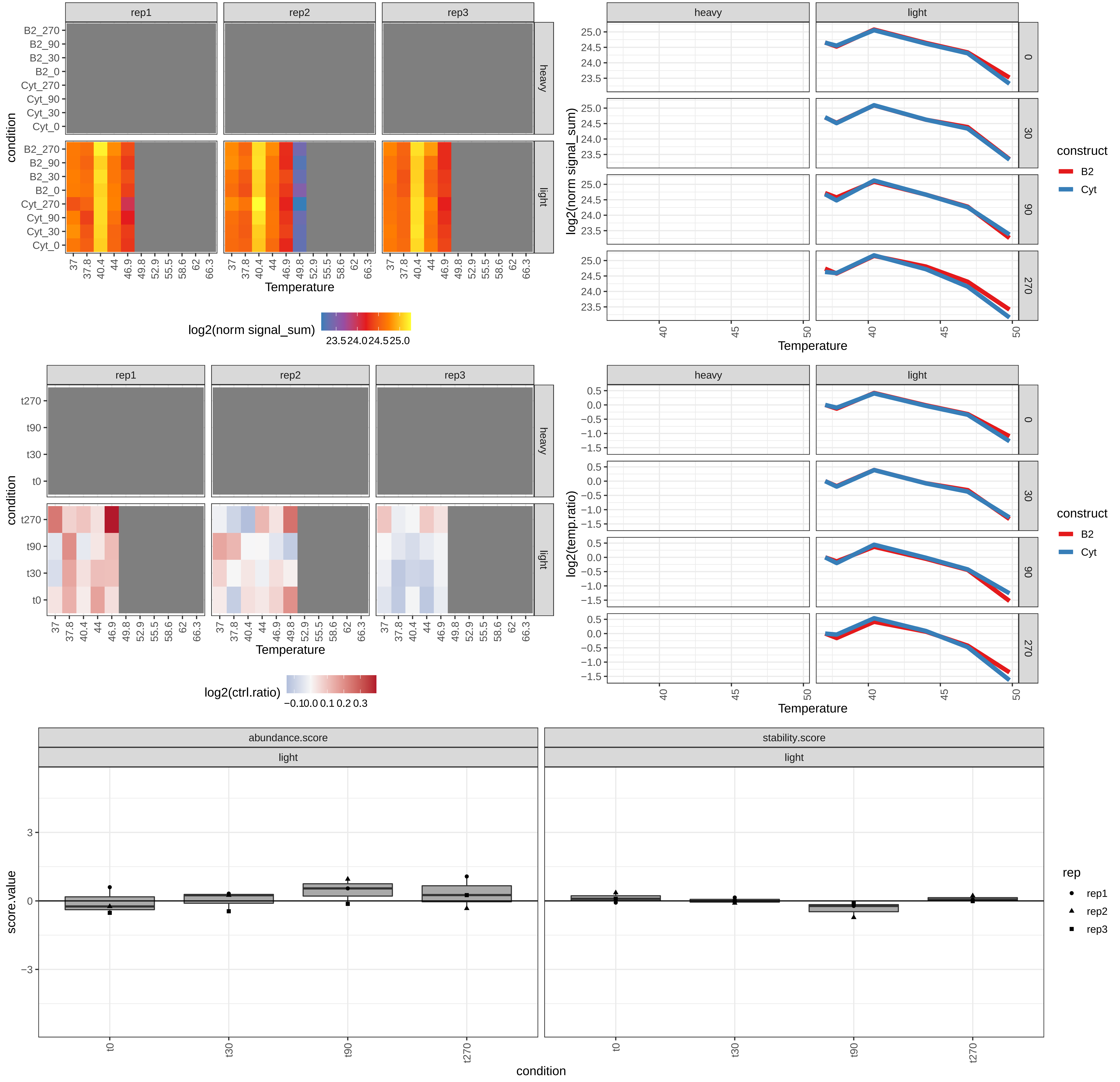

P47102 − ARF guanine−nucleotide exchange factor 1 OS=Saccharomyces cerevisiae (strain ATCC 204508 / S288c) GN=GEA1 PE=1 SV=1

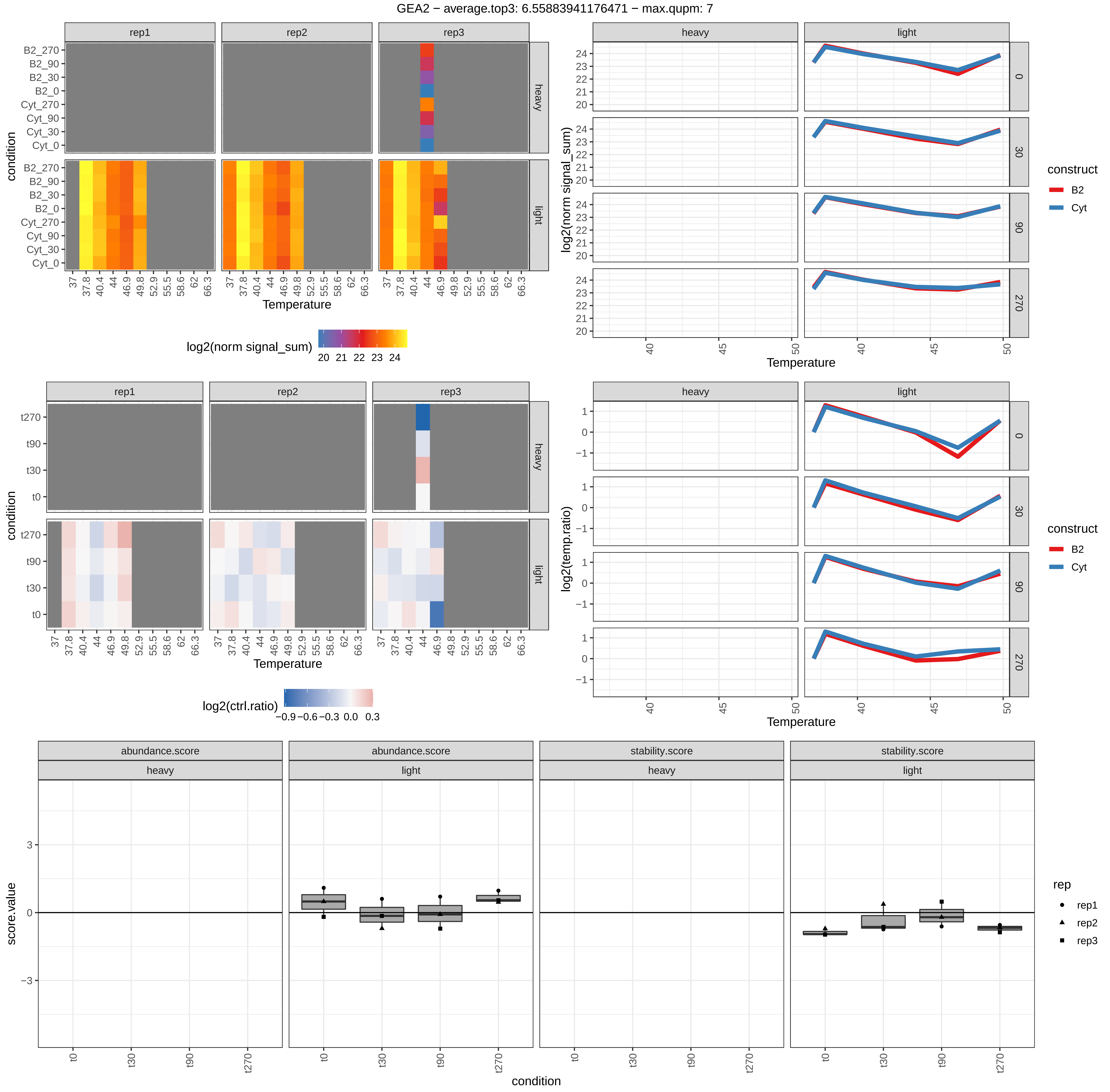

P39993 − ARF guanine−nucleotide exchange factor 2 OS=Saccharomyces cerevisiae (strain ATCC 204508 / S288c) GN=GEA2 PE=1 SV=1

GEF1 − average.top3: 6.66437894736842 − max.qupm: 8

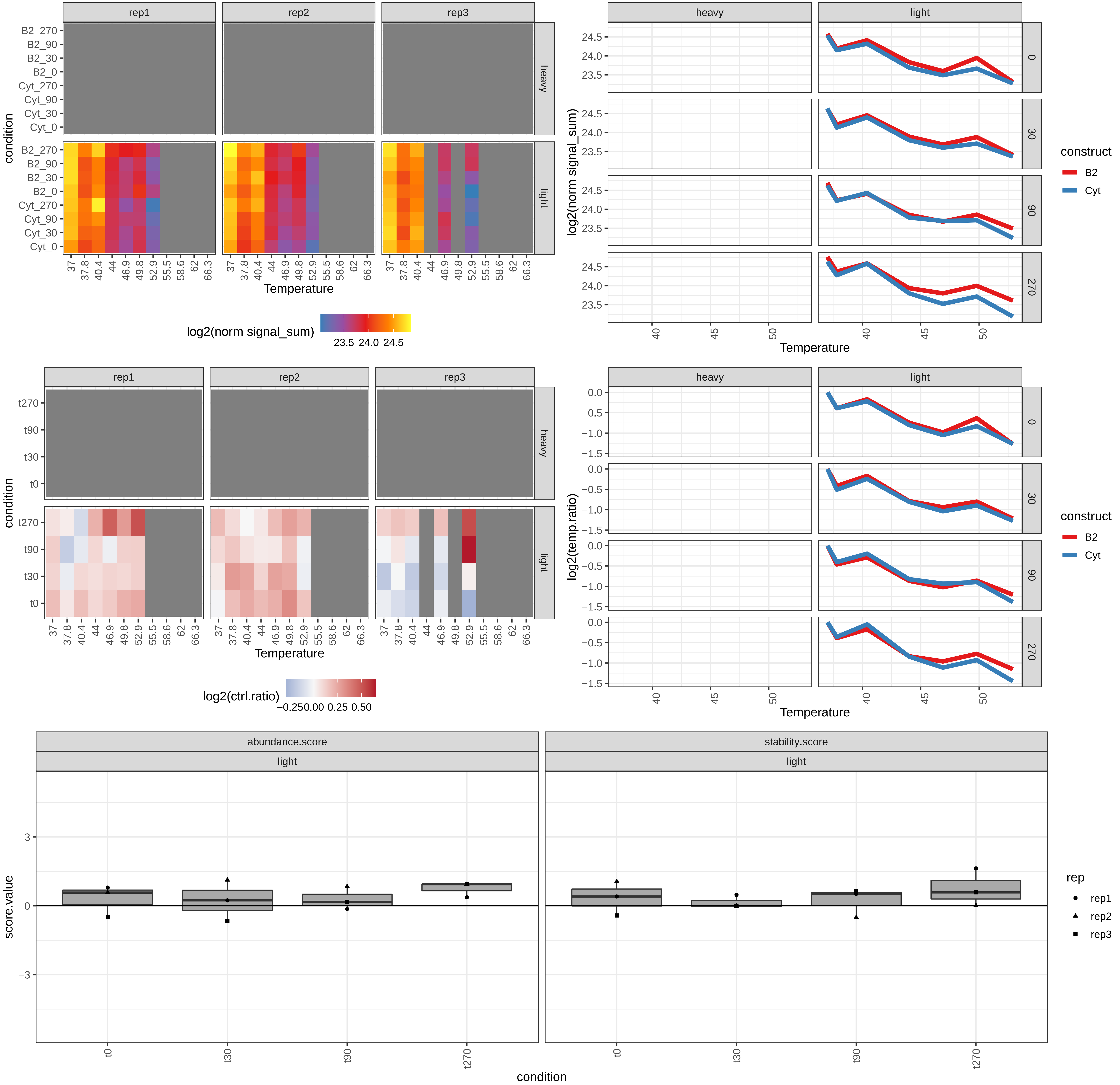

P37020 − Anion/proton exchange transporter GEF1 OS=Saccharomyces cerevisiae (strain ATCC 204508 / S288c) GN=GEF1 PE=1 SV=2

GEM1 − average.top3: 6.447840625 − max.qupm: 5

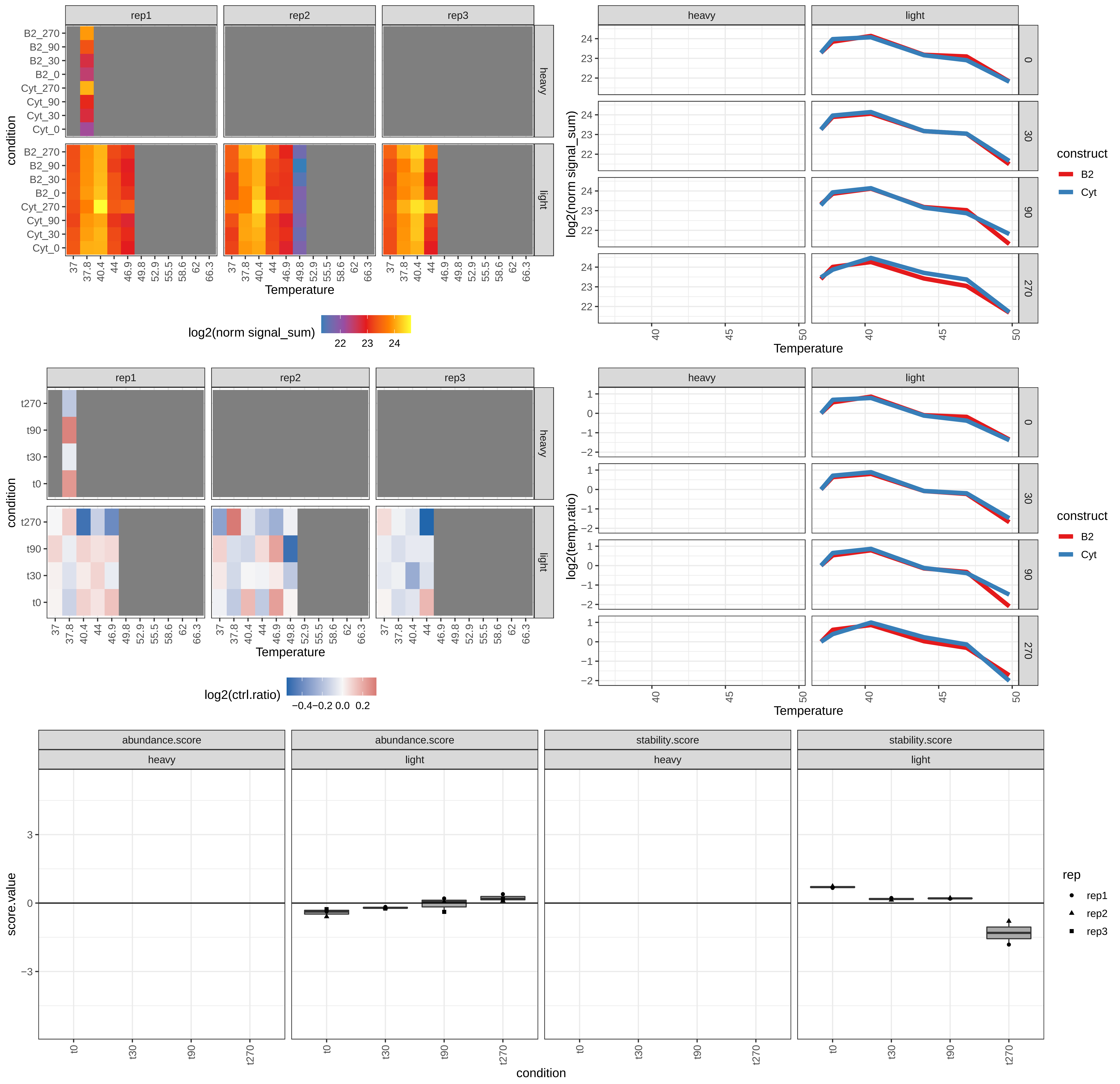

P39722 − Mitochondrial Rho GTPase 1 OS=Saccharomyces cerevisiae (strain ATCC 204508 / S288c) GN=GEM1 PE=1 SV=1

GEP3 − average.top3: 6.27816333333333 − max.qupm: 7

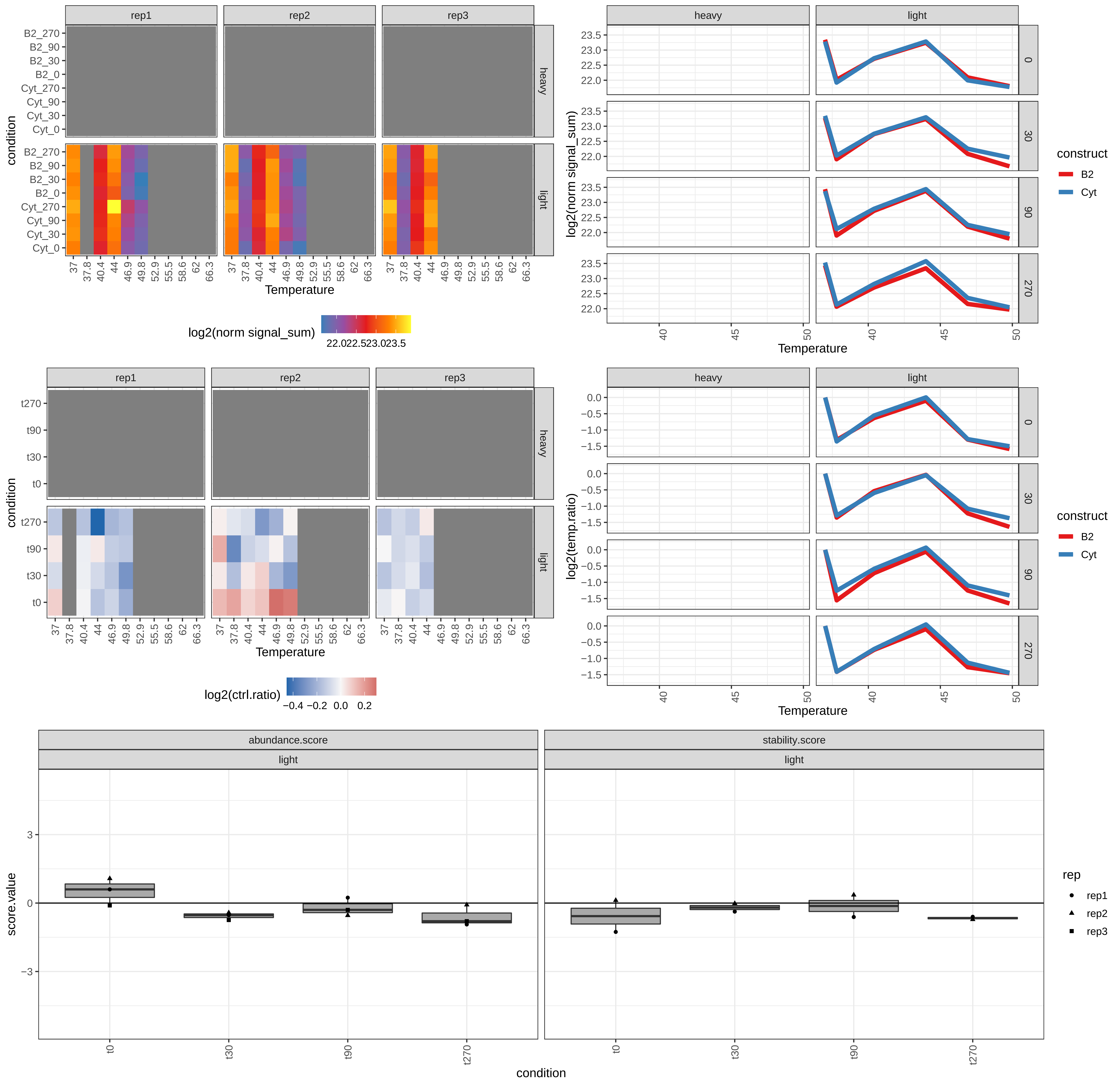

Q08622 − Genetic interactor of prohibitins 3, mitochondrial OS=Saccharomyces cerevisiae (strain ATCC 204508 / S288c) GN=GEP3 PE=1 SV=1

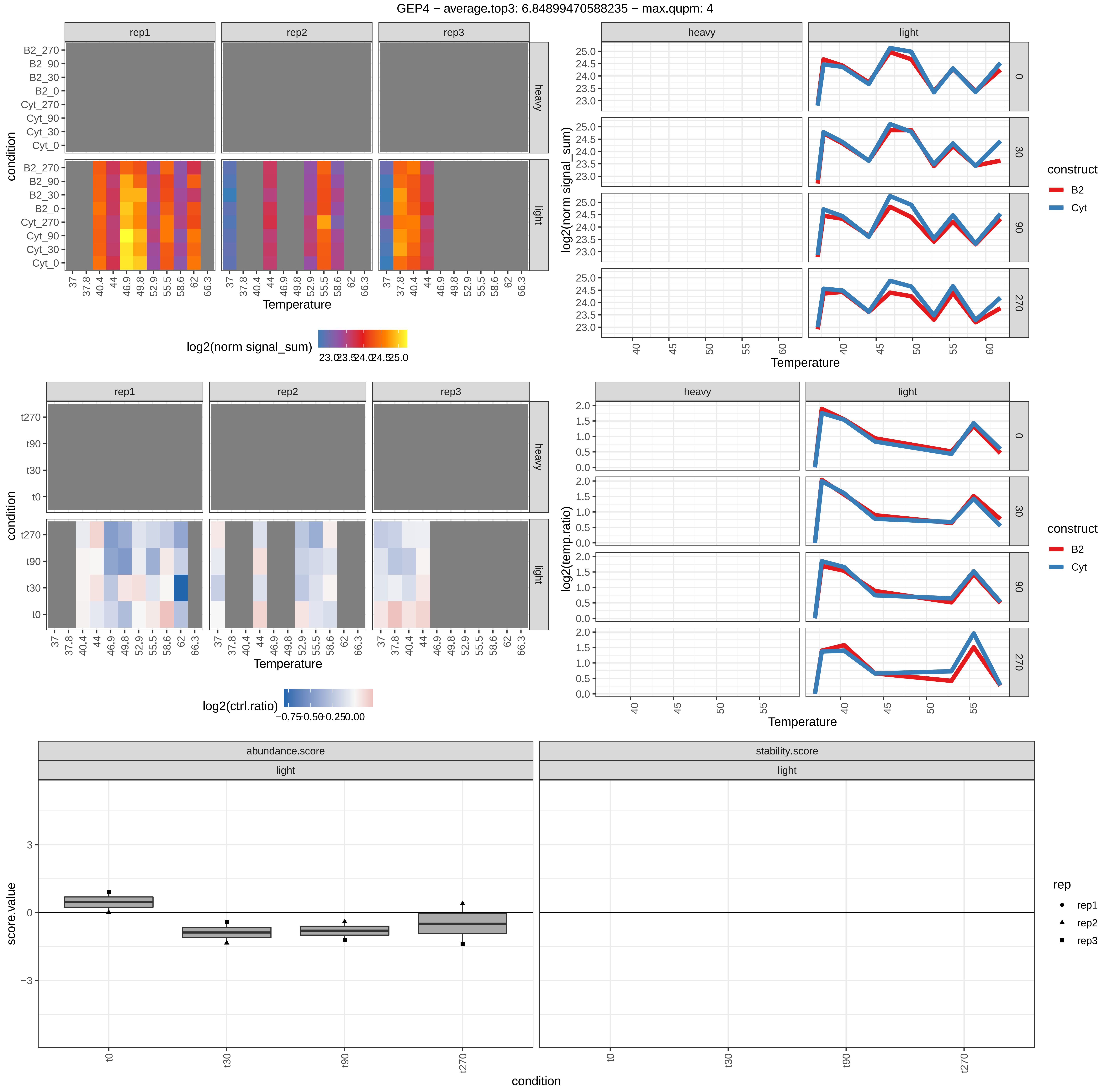

P38812 − Phosphatidylglycerophosphatase GEP4, mitochondrial OS=Saccharomyces cerevisiae (strain ATCC 204508 / S288c) GN=GEP4 PE=1 SV=1

GET1 − average.top3: 6.77710513513513 − max.qupm: 4

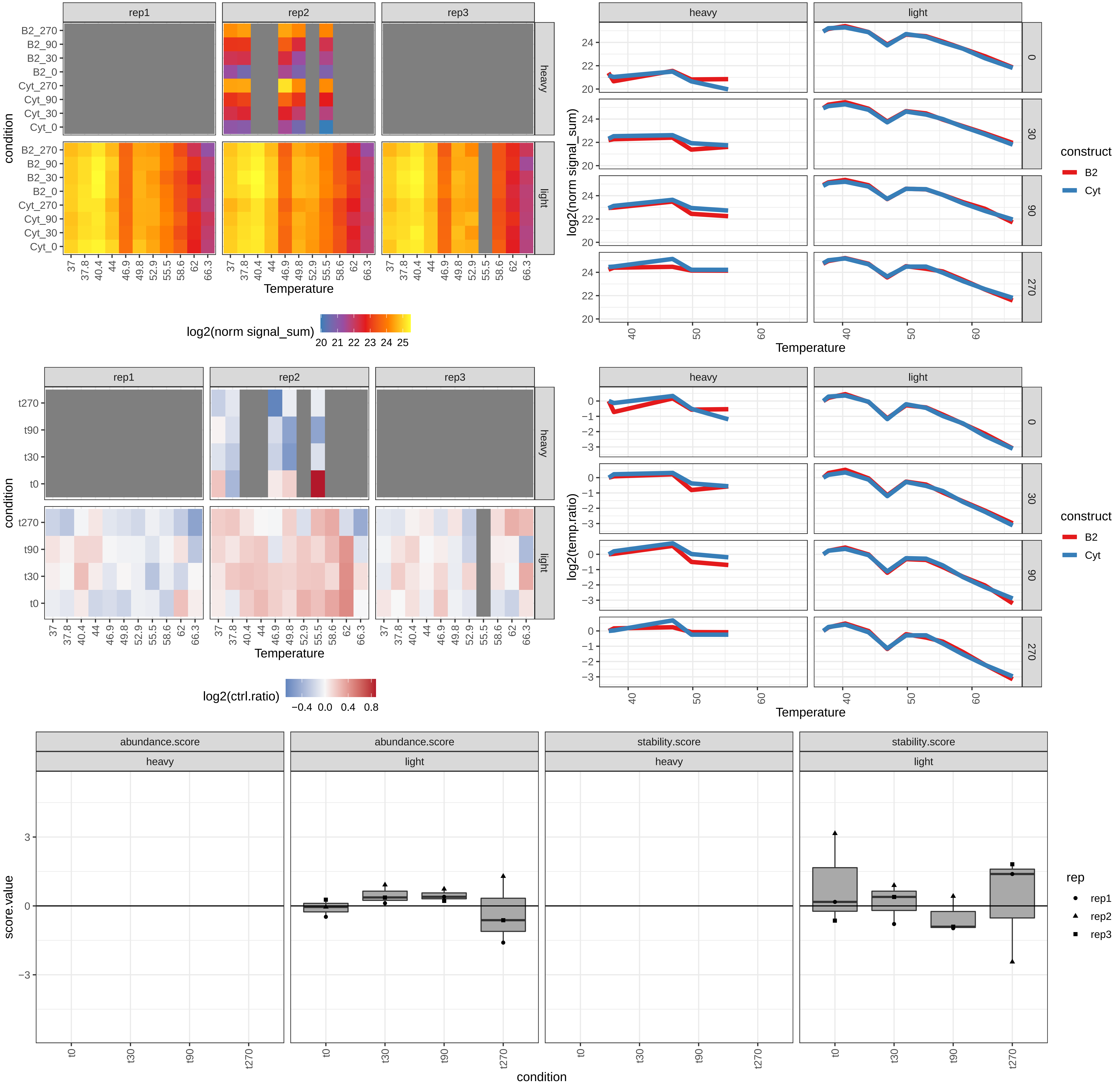

P53192 − Golgi to ER traffic protein 1 OS=Saccharomyces cerevisiae (strain ATCC 204508 / S288c) GN=GET1 PE=1 SV=1

GET3 − average.top3: 7.21681456521739 − max.qupm: 9

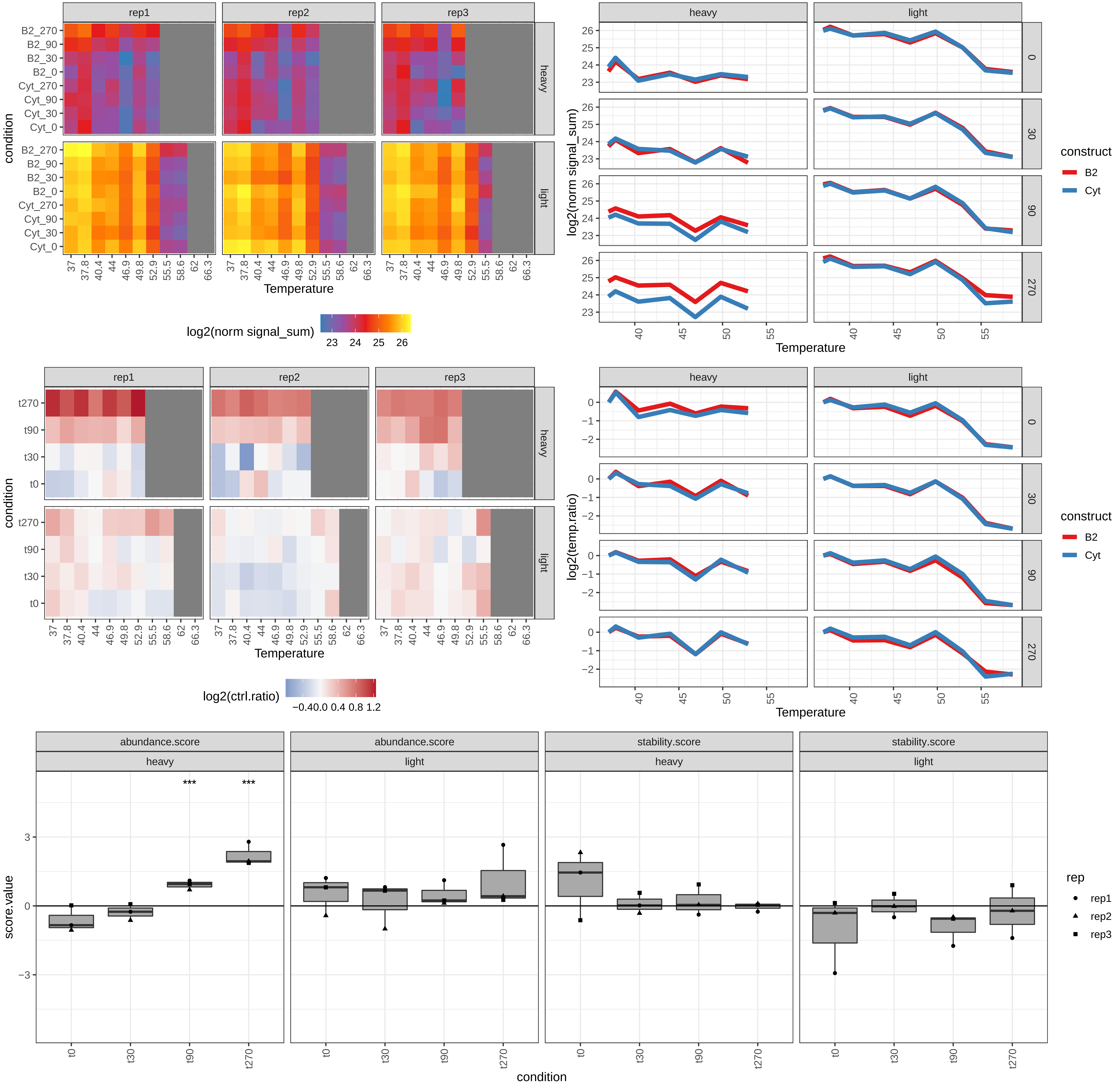

Q12154 − ATPase GET3 OS=Saccharomyces cerevisiae (strain ATCC 204508 / S288c) GN=GET3 PE=1 SV=1

GET4 − average.top3: 6.957643243243243 − max.qupm: 9

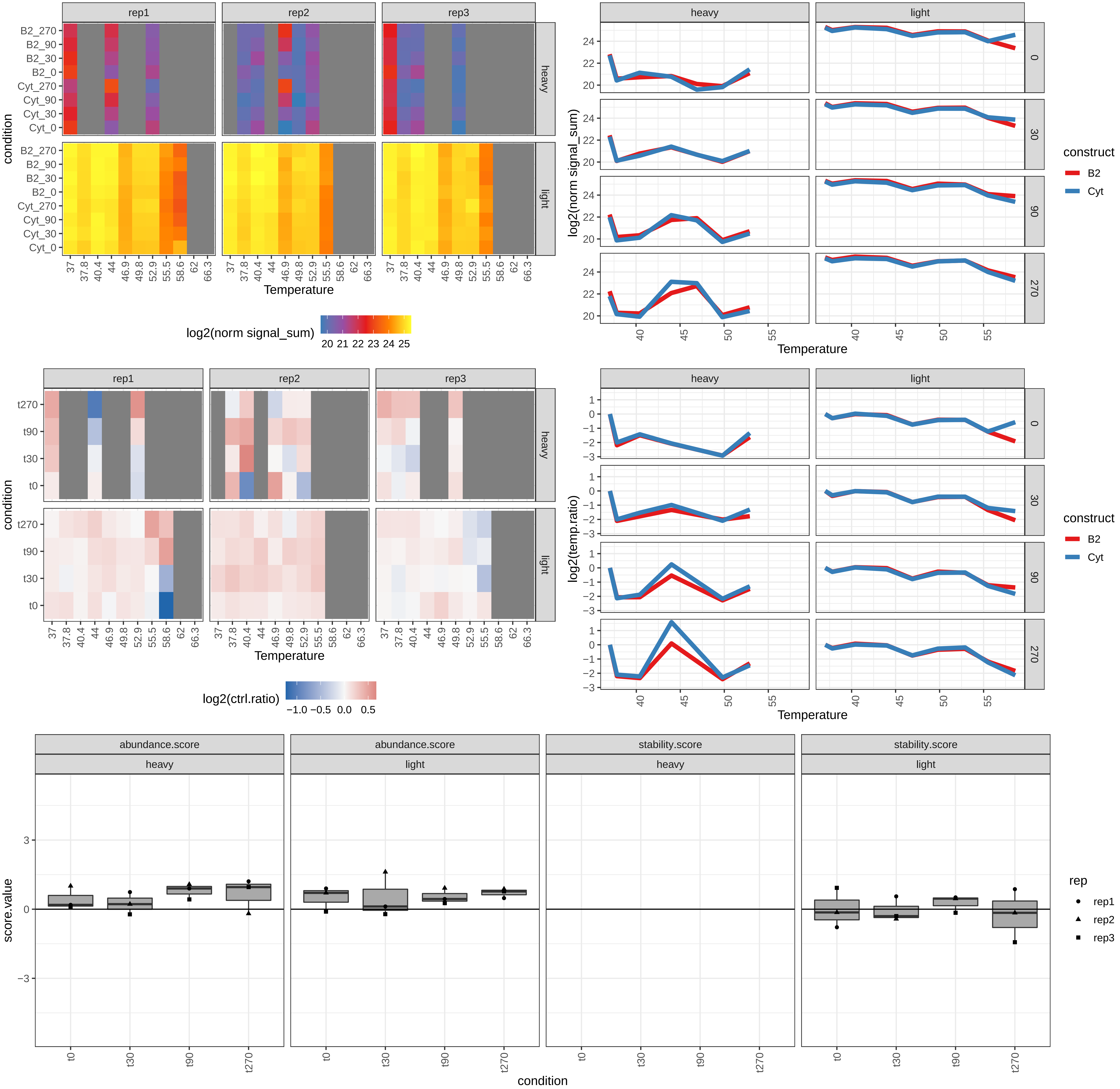

Q12125 − Golgi to ER traffic protein 4 OS=Saccharomyces cerevisiae (strain ATCC 204508 / S288c) GN=GET4 PE=1 SV=1

GFA1 − average.top3: 7.20396530612245 − max.qupm: 21

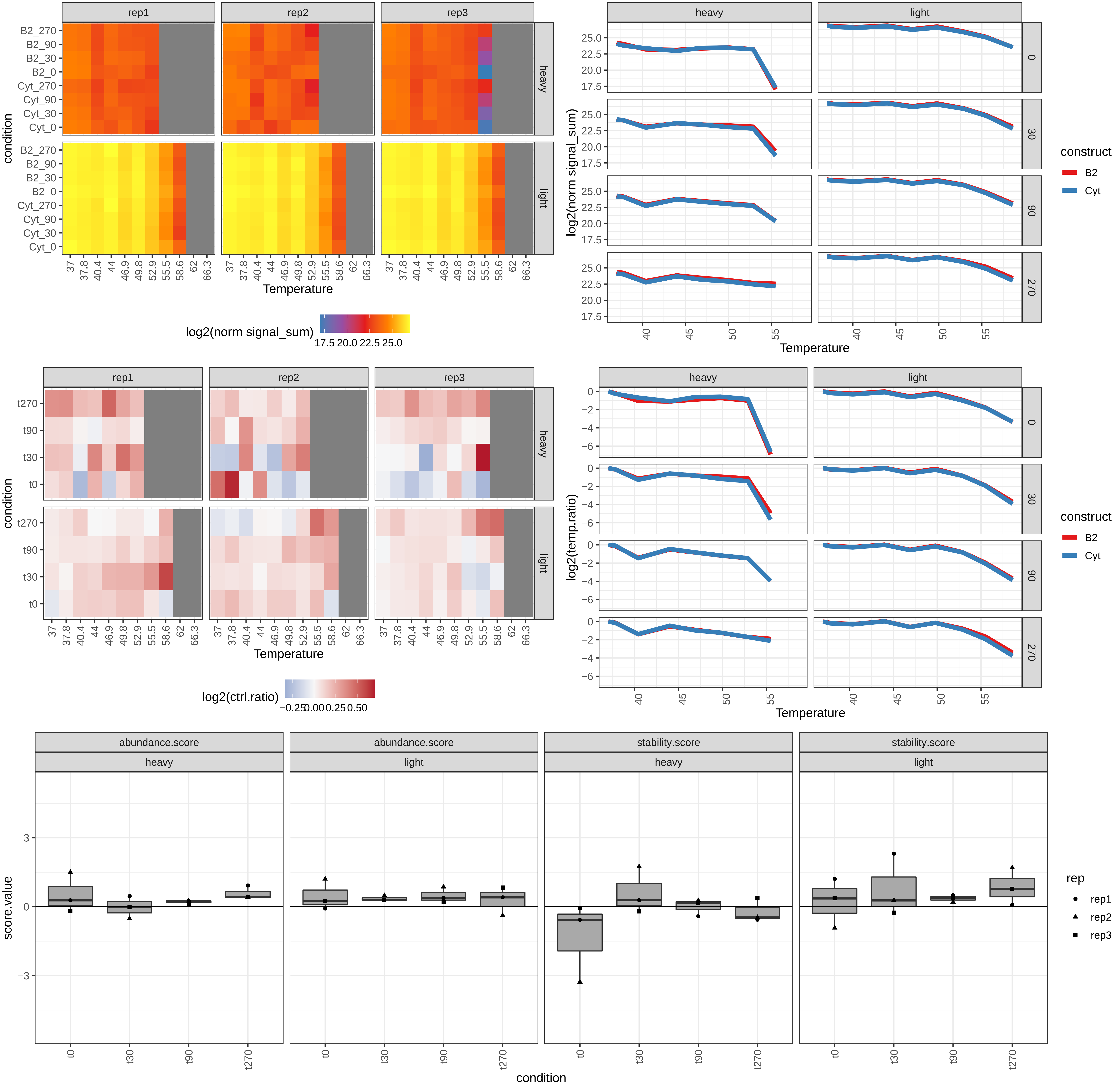

P14742 − Glutamine−−fructose−6−phosphate aminotransferase [isomerizing] OS=Saccharomyces cerevisiae (strain ATCC 204508 / S288c) GN=GFA1 PE=1 SV=4

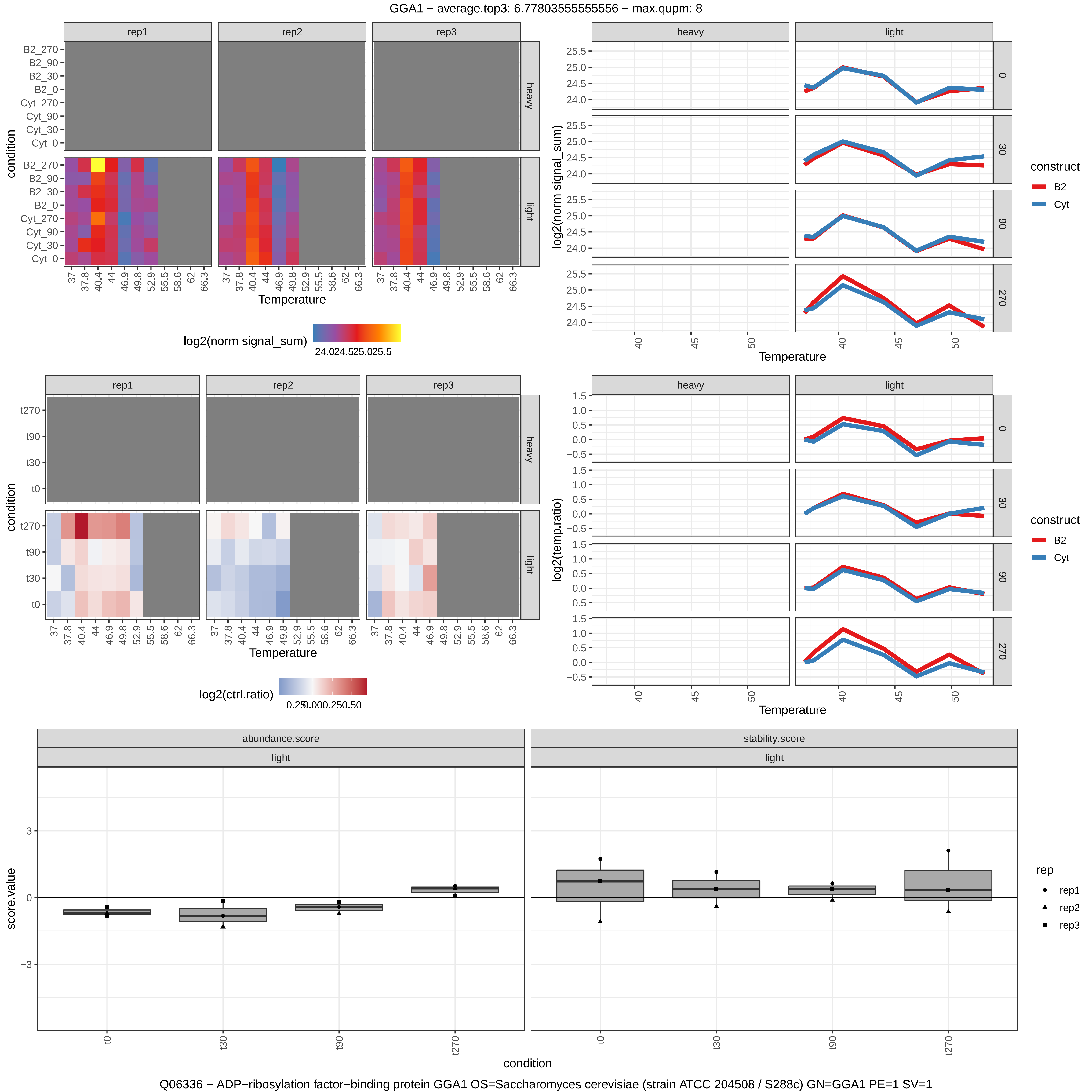
GGA1 − average.top3: 6.77803555555556 − max.qupm: 8
rep1
rep2
rep3
heavy
light
condition
Temperature
log2(norm signal_sum)
construct
B2
Cyt
log2(ctrl.ratio)
log2(temp.ratio)
abundance.score
stability.score
score.value
rep
condition
Q06336 − ADP−ribosylation factor−binding protein GGA1 OS=Saccharomyces cerevisiae (strain ATCC 204508 / S288c) GN=GGA1 PE=1 SV=1

GGA2 − average.top3: 6.88686884615385 − max.qupm: 7

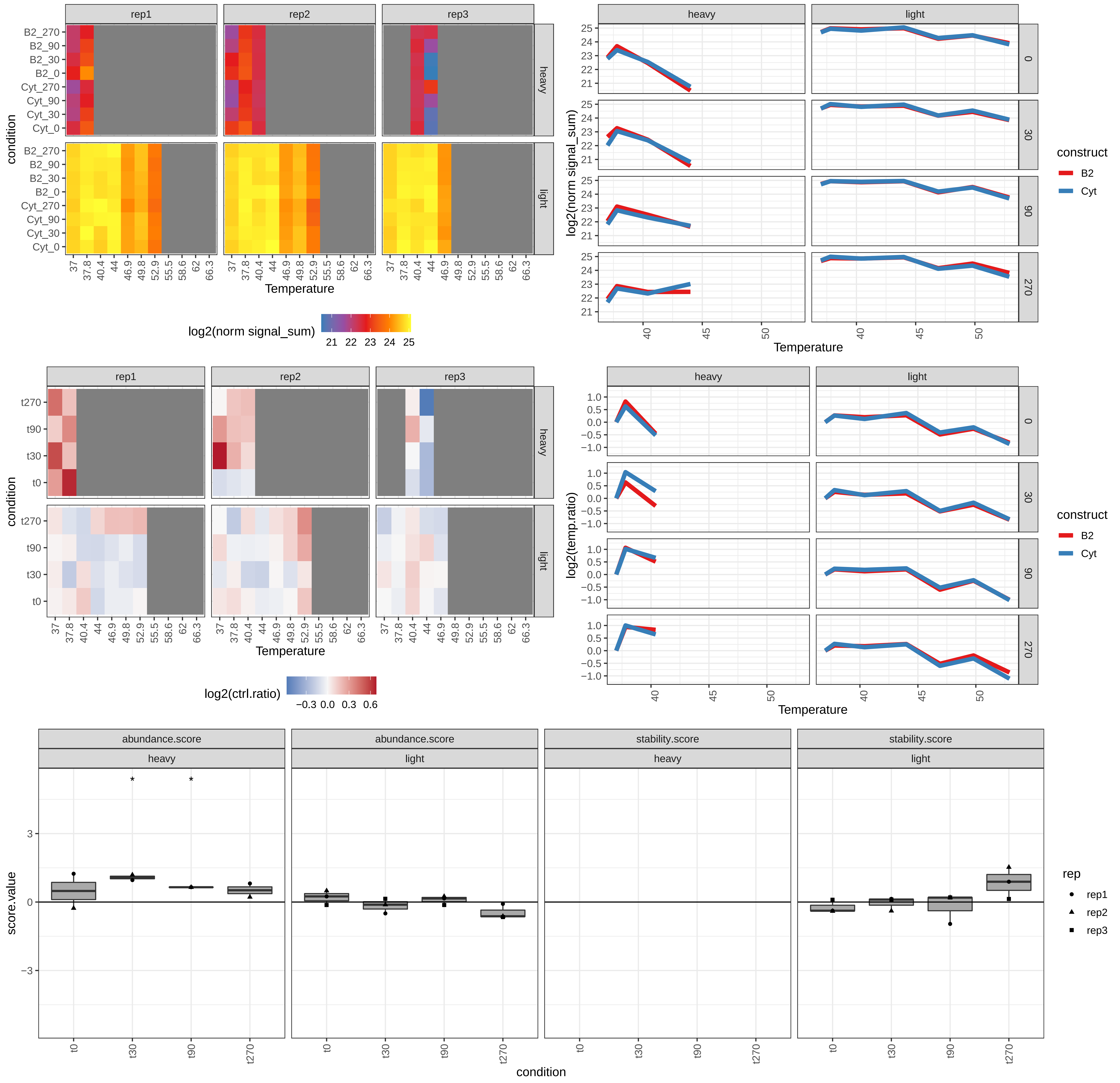

P38817 − ADP−ribosylation factor−binding protein GGA2 OS=Saccharomyces cerevisiae (strain ATCC 204508 / S288c) GN=GGA2 PE=1 SV=1

GGC1 − average.top3: 7.1970035 − max.qupm: 14

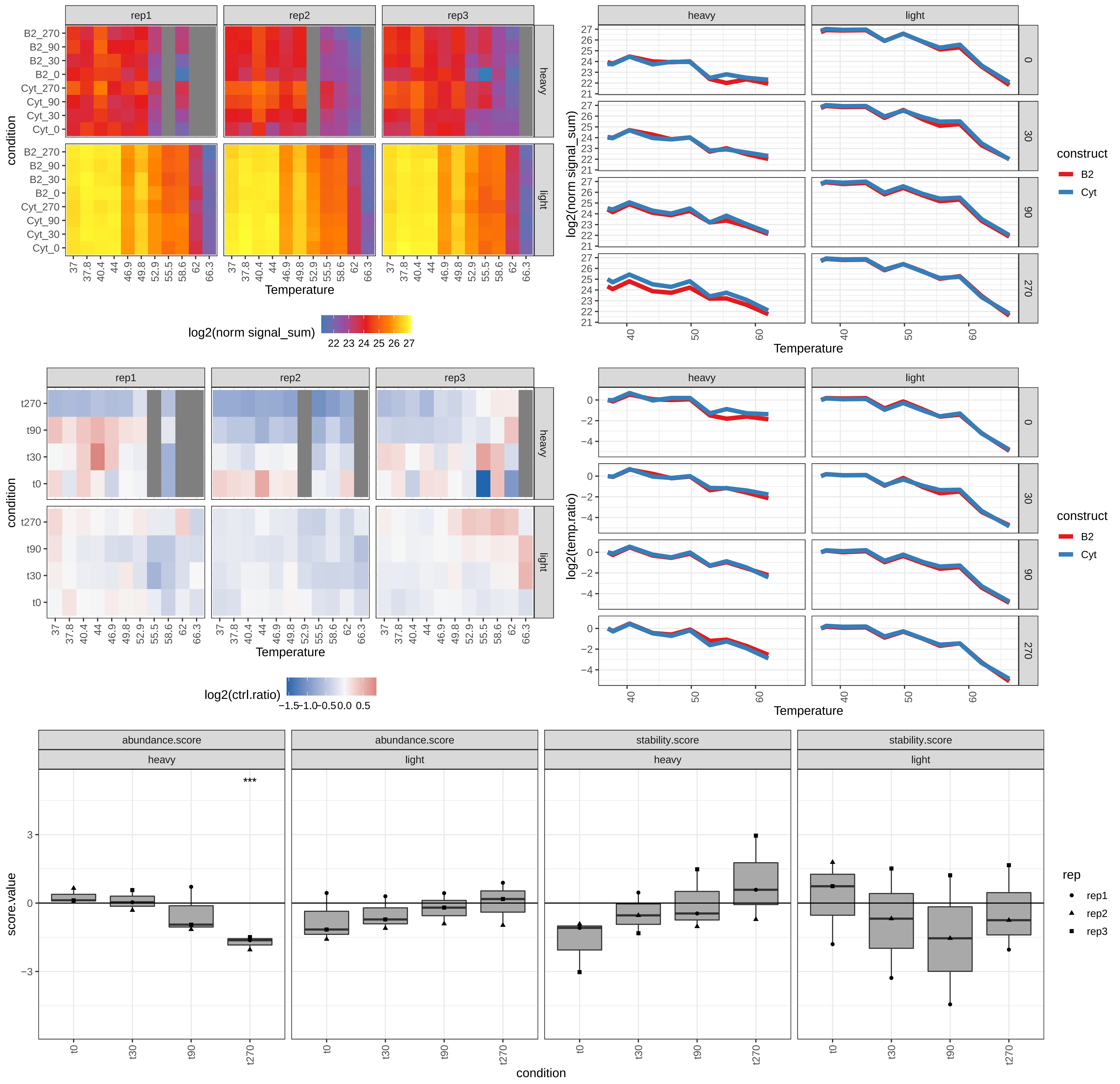

P38988 − Mitochondrial GTP/GDP carrier protein 1 OS=Saccharomyces cerevisiae (strain ATCC 204508 / S288c) GN=GGC1 PE=1 SV=1

GID7 − average.top3: 7.02404444444444 − max.qupm: 9

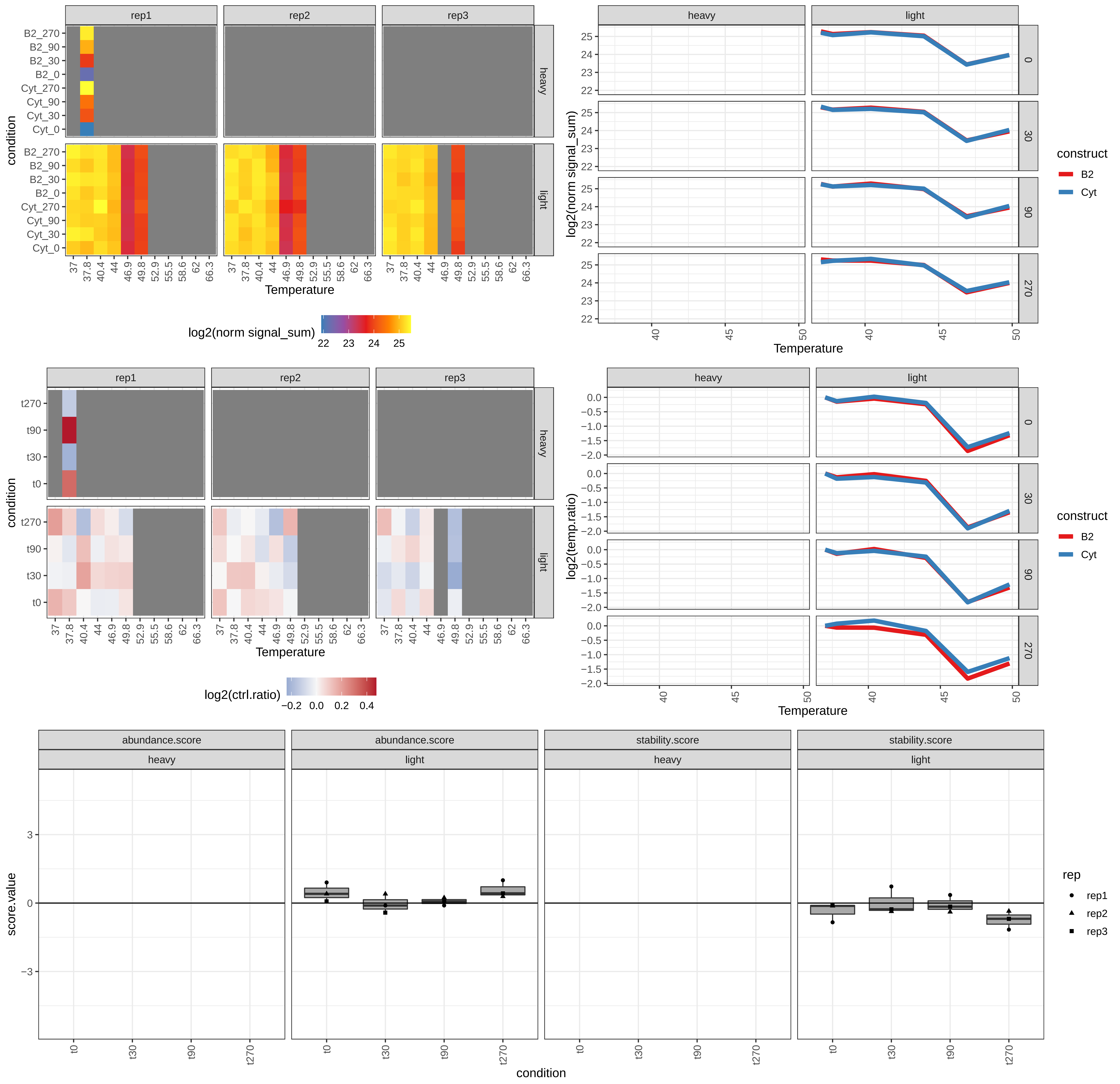

P25569 − Glucose−induced degradation protein 7 OS=Saccharomyces cerevisiae (strain ATCC 204508 / S288c) GN=GID7 PE=1 SV=2

GID8 − average.top3: 6.59257 − max.qupm: 5

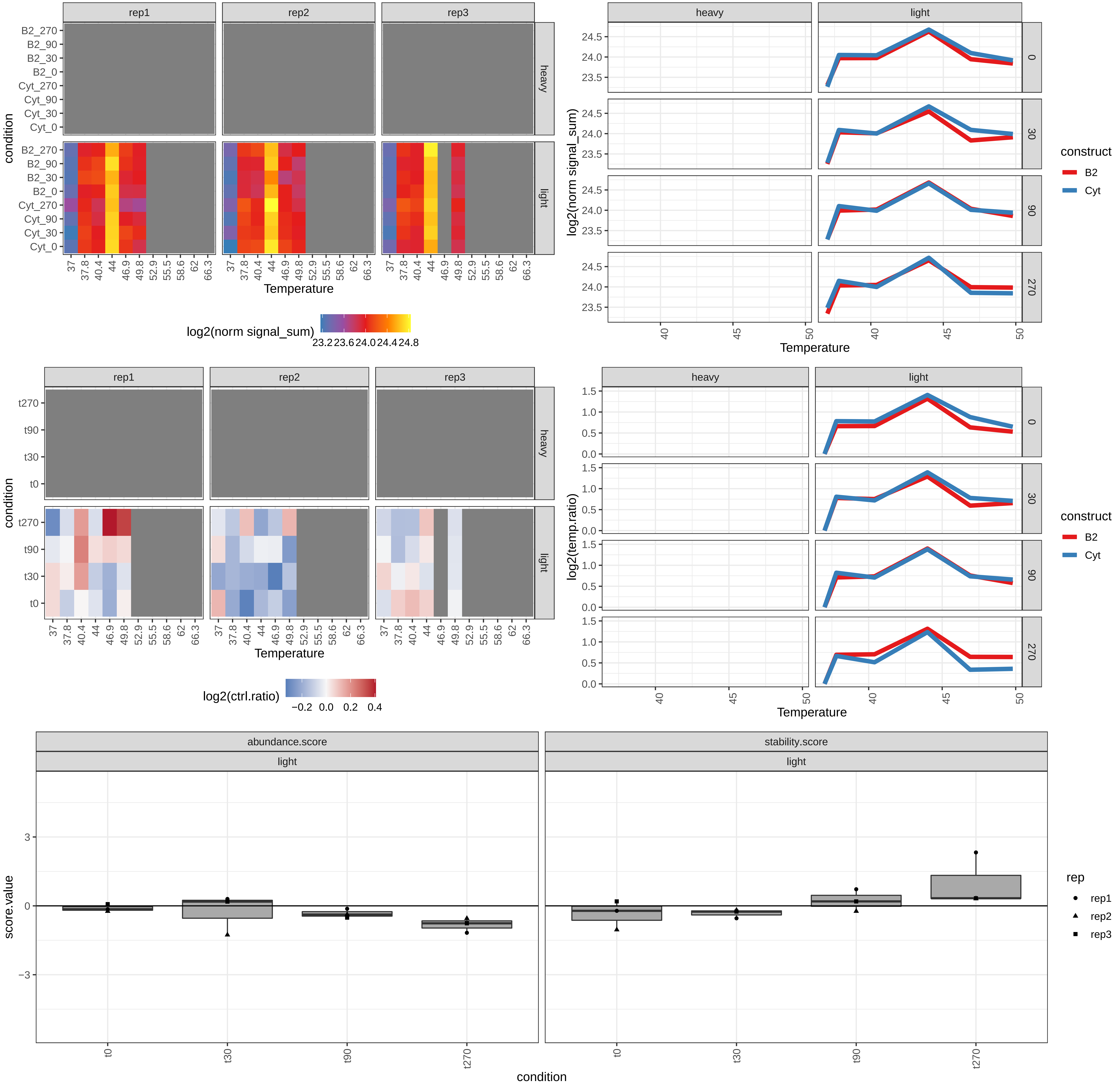

P40208 − Glucose−induced degradation protein 8 OS=Saccharomyces cerevisiae (strain ATCC 204508 / S288c) GN=GID8 PE=1 SV=1

GIM3 − average.top3: 7.128715 − max.qupm: 3

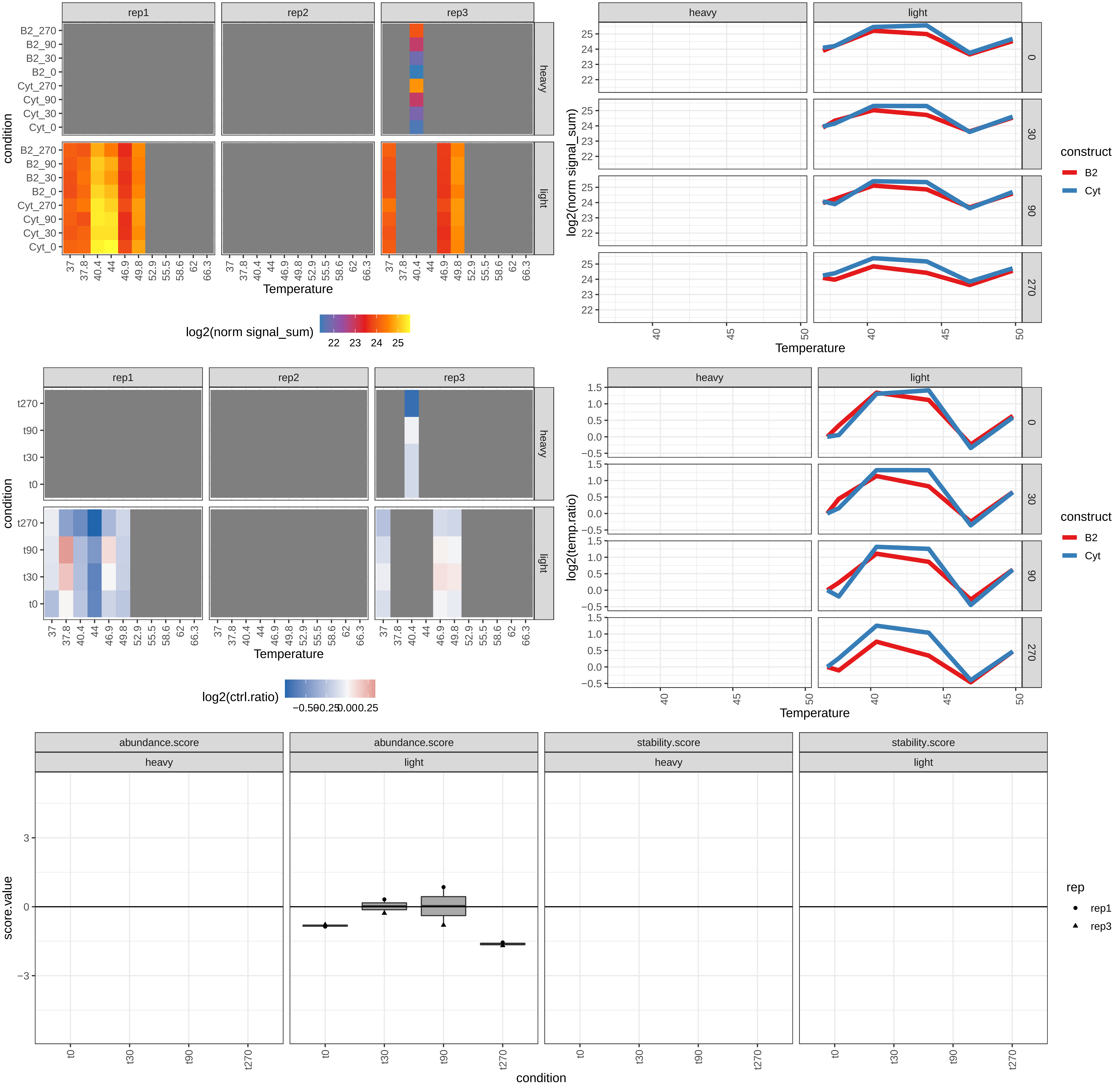

P53900 − Prefoldin subunit 4 OS=Saccharomyces cerevisiae (strain ATCC 204508 / S288c) GN=GIM3 PE=1 SV=1

GIM4 − average.top3: 7.06884380952381 − max.qupm: 5

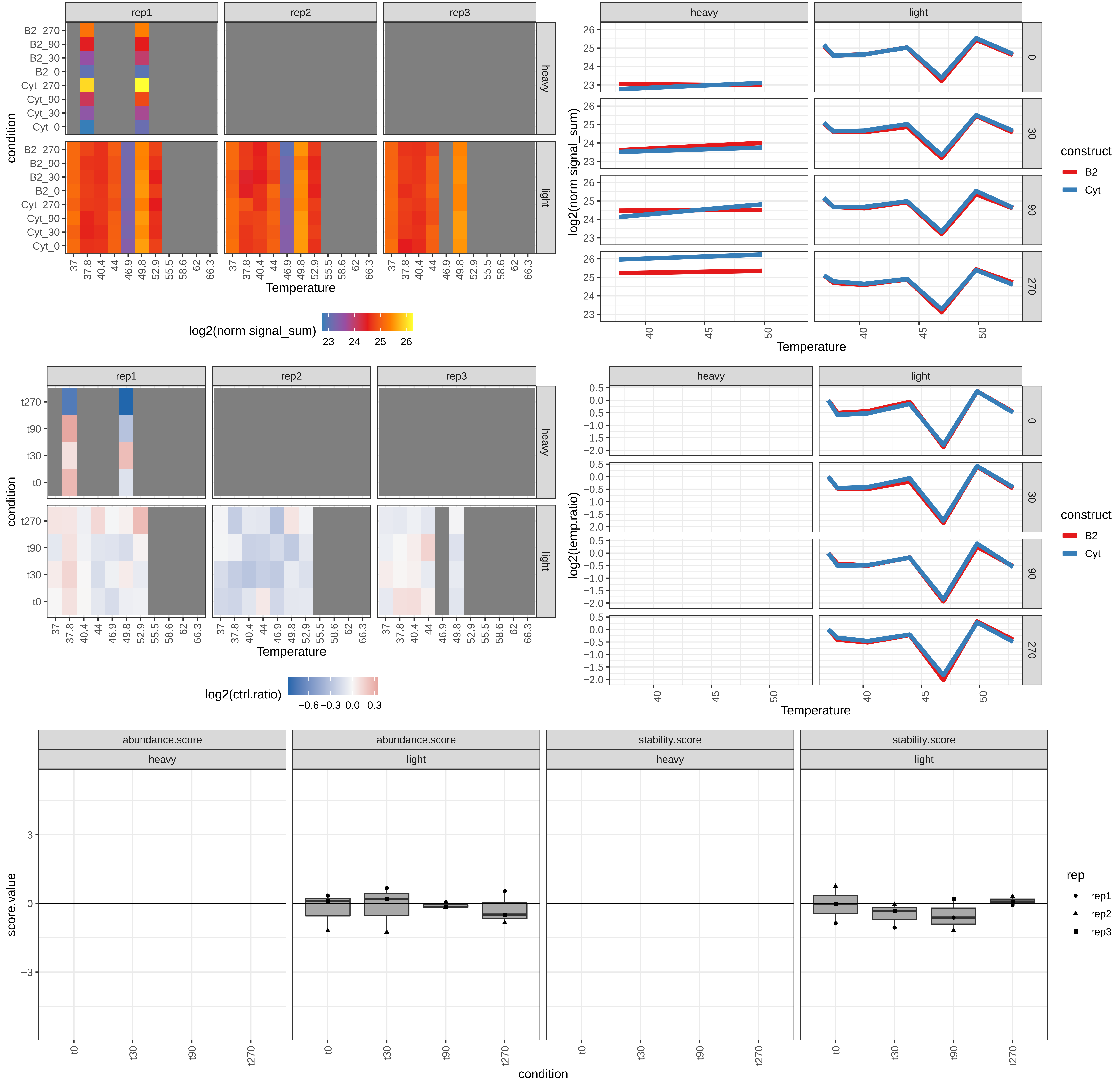

P40005 − Prefoldin subunit 2 OS=Saccharomyces cerevisiae (strain ATCC 204508 / S288c) GN=GIM4 PE=1 SV=2

GIN4 − average.top3: 6.58815756756757 − max.qupm: 10

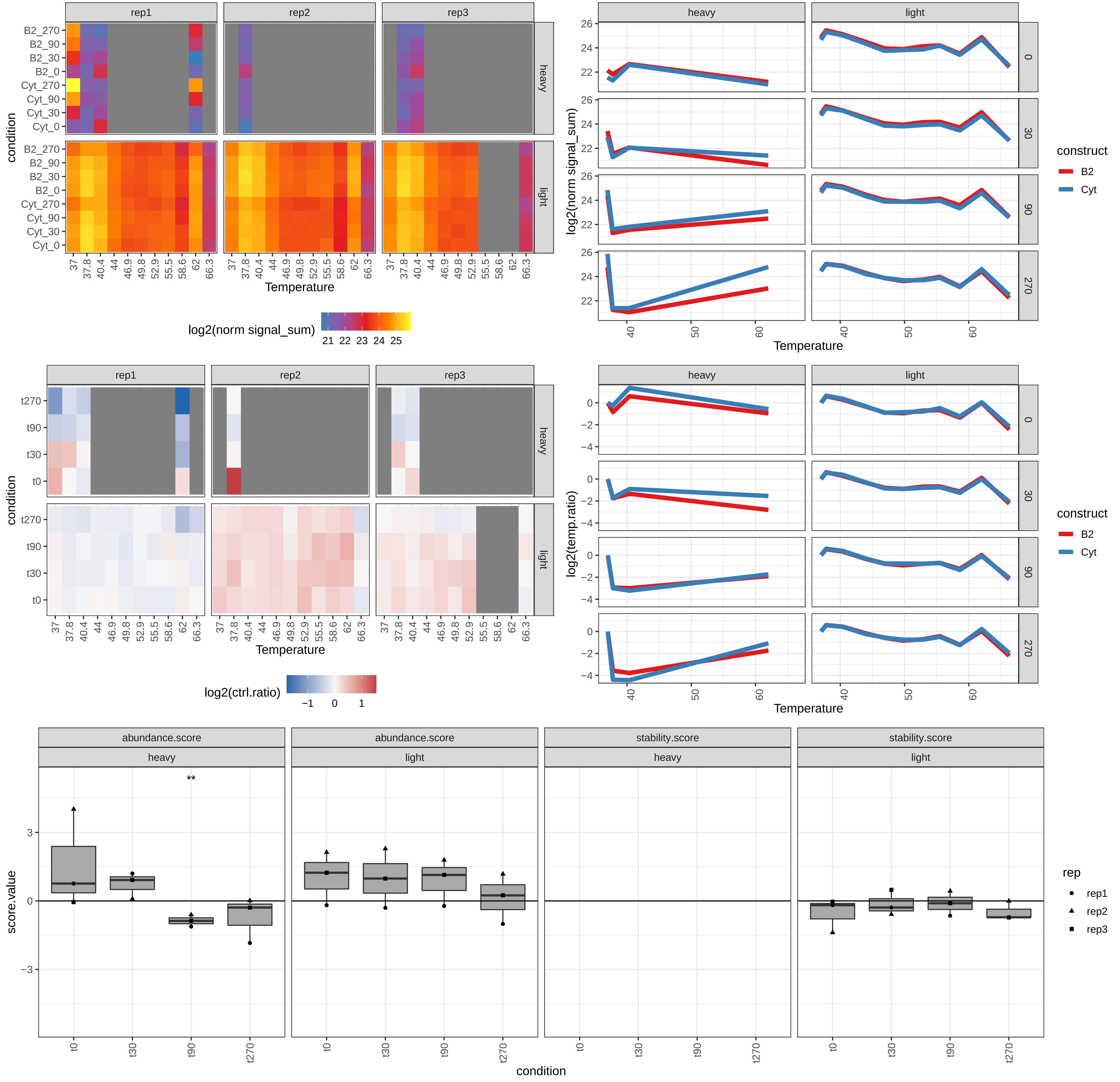

Q12263 − Serine/threonine−protein kinase GIN4 OS=Saccharomyces cerevisiae (strain ATCC 204508 / S288c) GN=GIN4 PE=1 SV=1

GIP4 − average.top3: 6.7197525 − max.qupm: 6

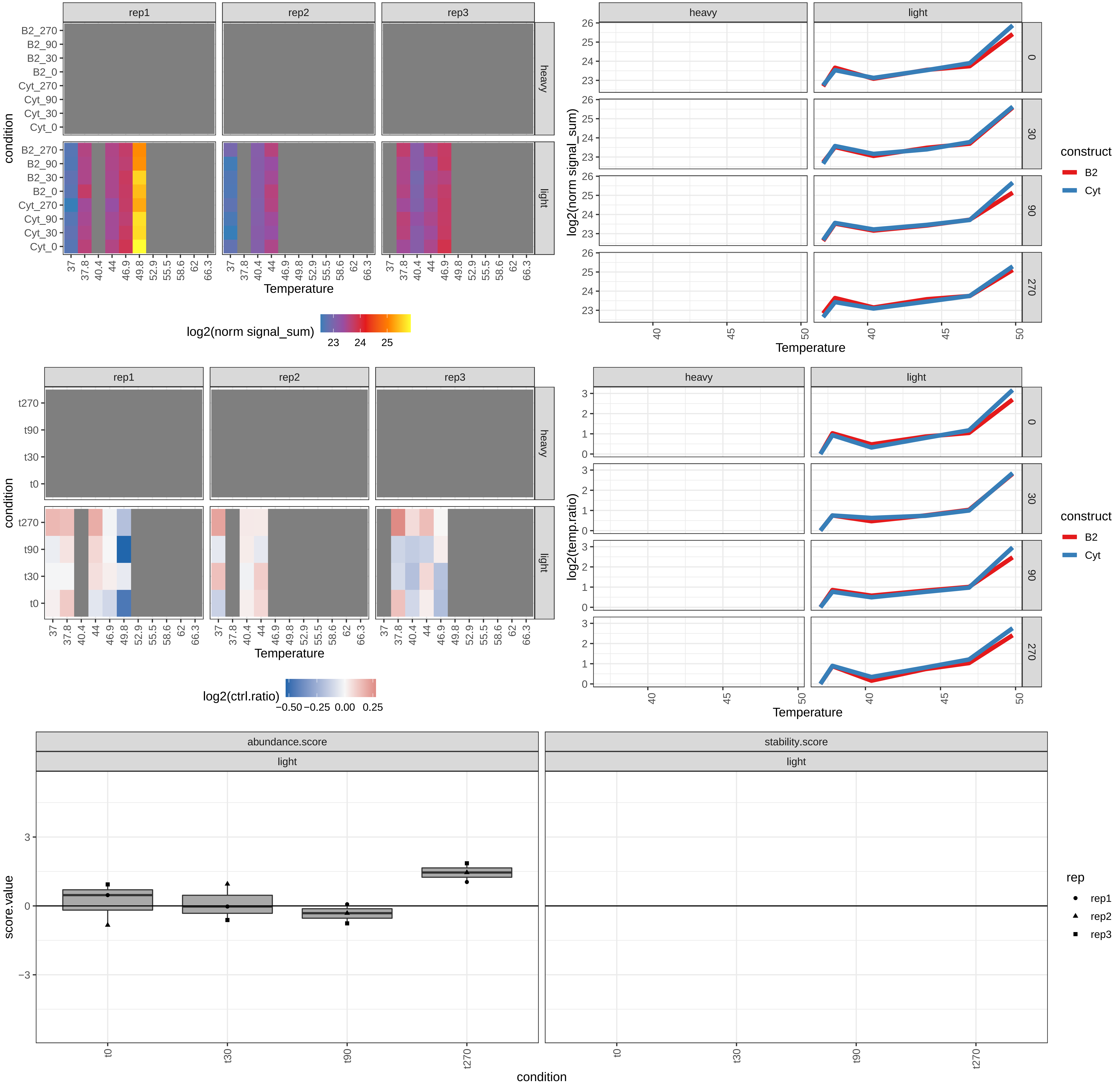

P39732 − GLC7−interacting protein 4 OS=Saccharomyces cerevisiae (strain ATCC 204508 / S288c) GN=GIP4 PE=1 SV=2

GIS1 − average.top3: 6.63153882352941 − max.qupm: 4

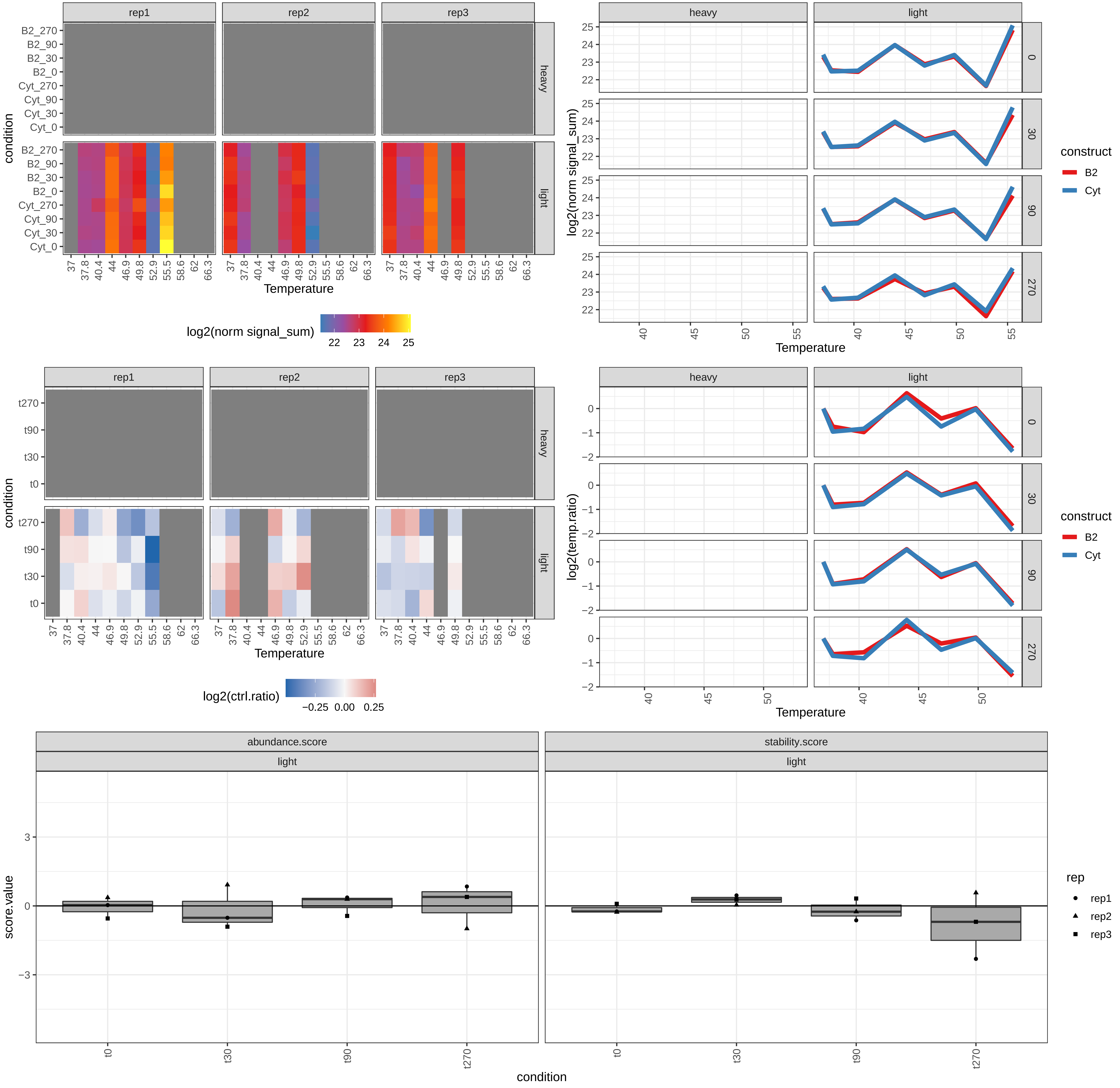

Q03833 − Transcriptional activator/repressor GIS1 OS=Saccharomyces cerevisiae (strain ATCC 204508 / S288c) GN=GIS1 PE=1 SV=1

GIS2 − average.top3: 7.44532769230769 − max.qupm: 13

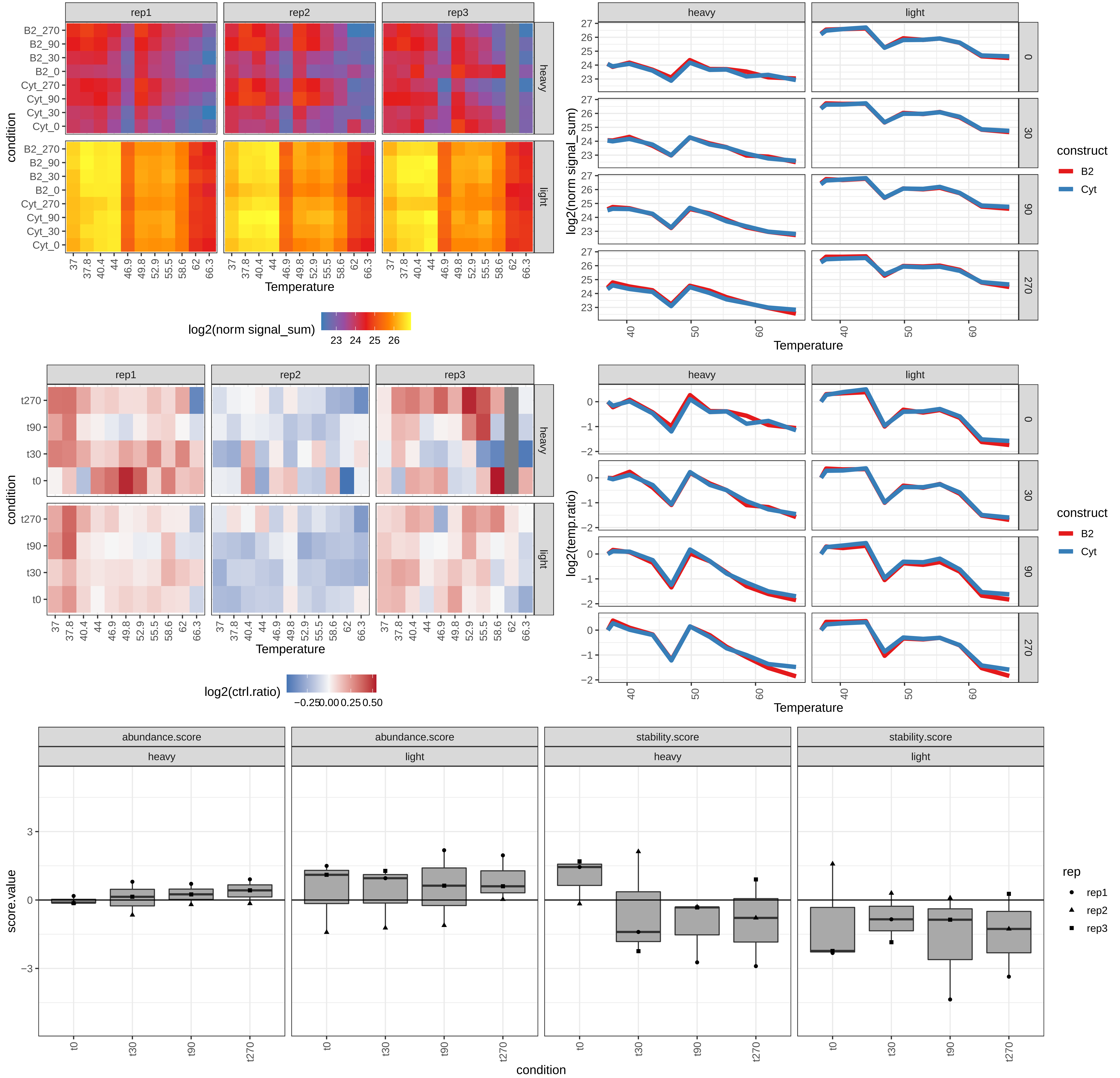

P53849 − Zinc finger protein GIS2 OS=Saccharomyces cerevisiae (strain ATCC 204508 / S288c) GN=GIS2 PE=1 SV=1

GLC3 − average.top3: 7.4670875862069 − max.qupm: 21

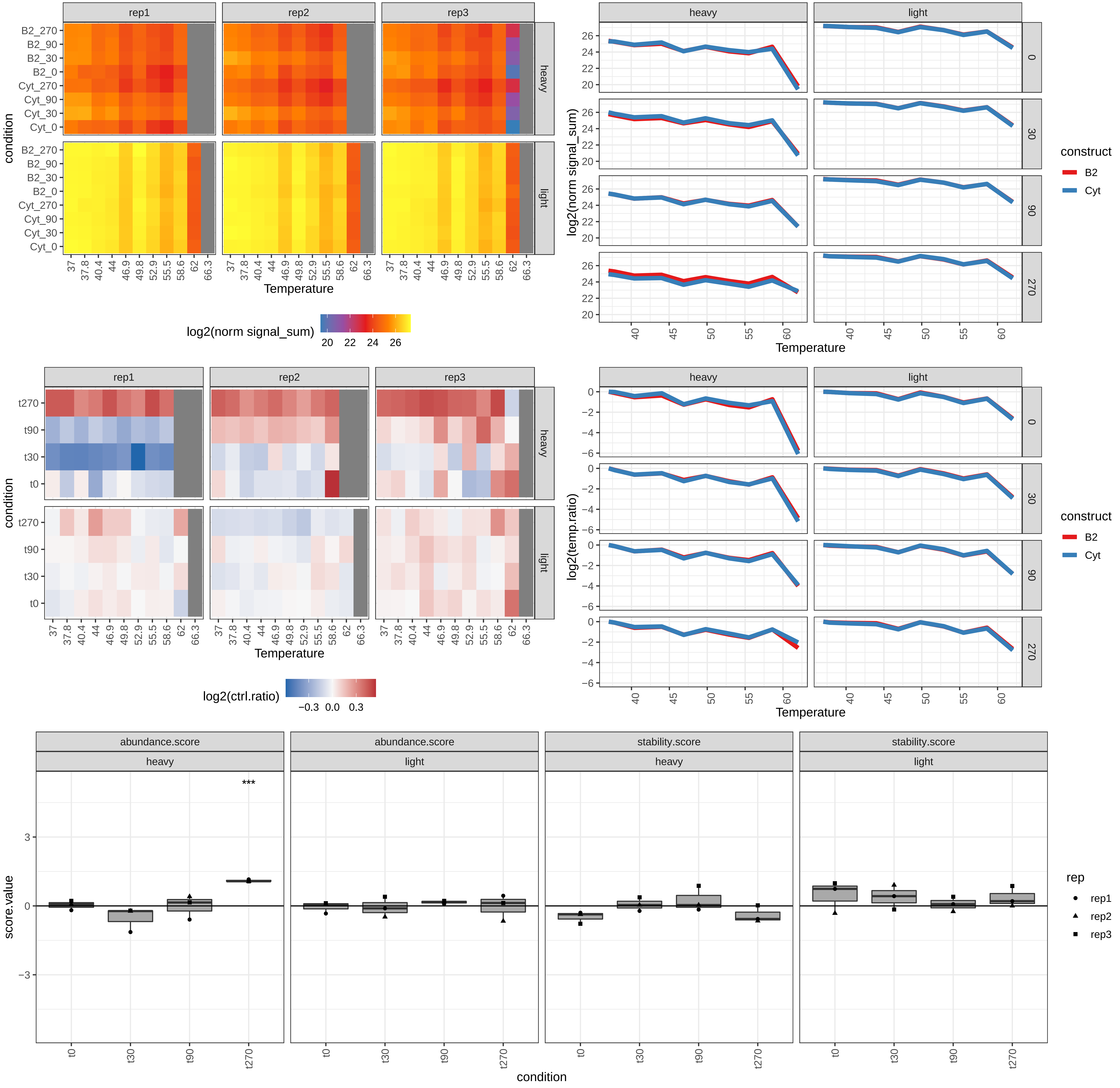

P32775 − 1,4−alpha−glucan−branching enzyme OS=Saccharomyces cerevisiae (strain ATCC 204508 / S288c) GN=GLC3 PE=1 SV=2

GLC7 − average.top3: 7.15339388888889 − max.qupm: 10

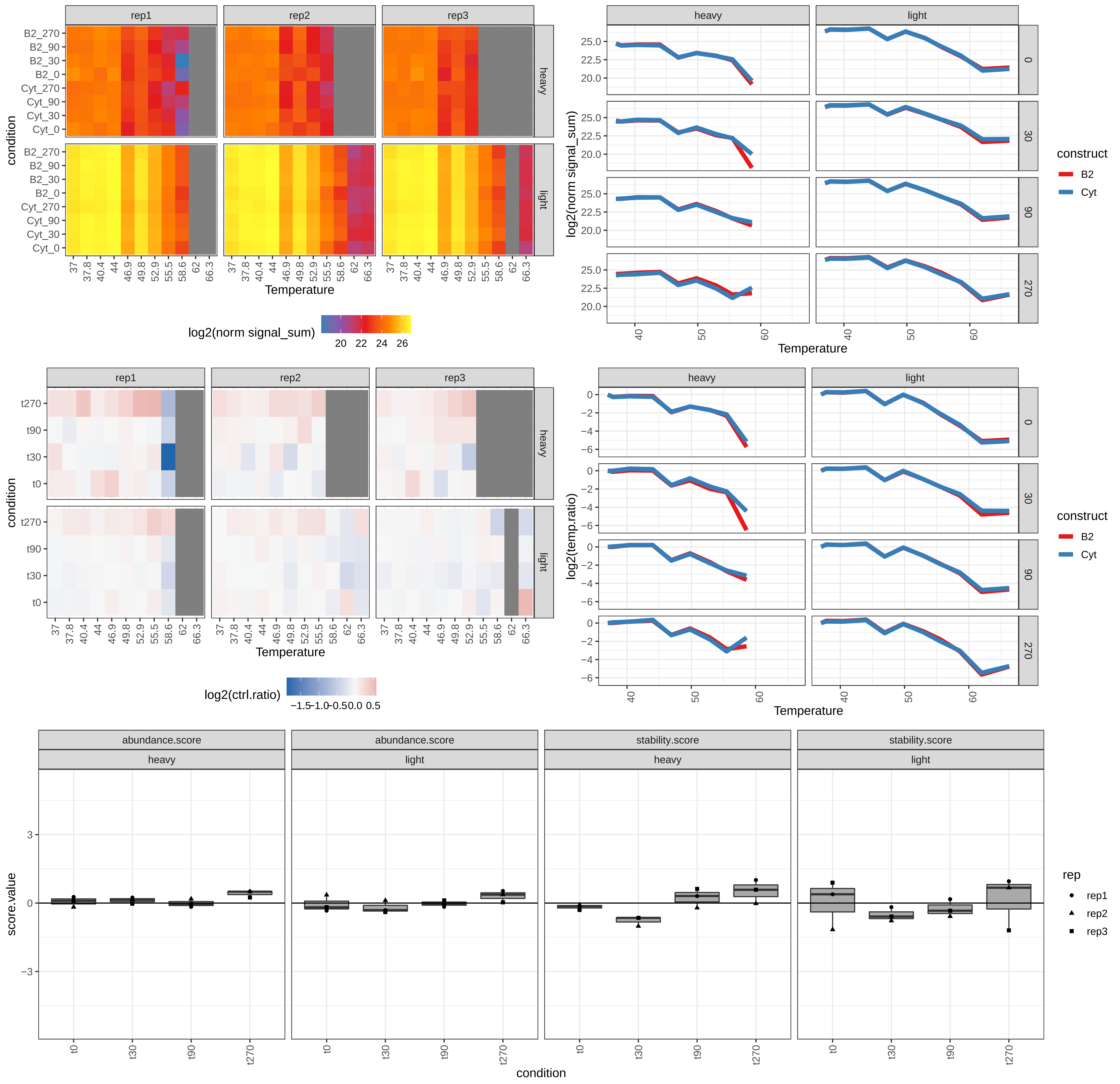

P32598 − Serine/threonine−protein phosphatase PP1−2 OS=Saccharomyces cerevisiae (strain ATCC 204508 / S288c) GN=GLC7 PE=1 SV=1

GLE1 − average.top3: 6.4730488 − max.qupm: 5

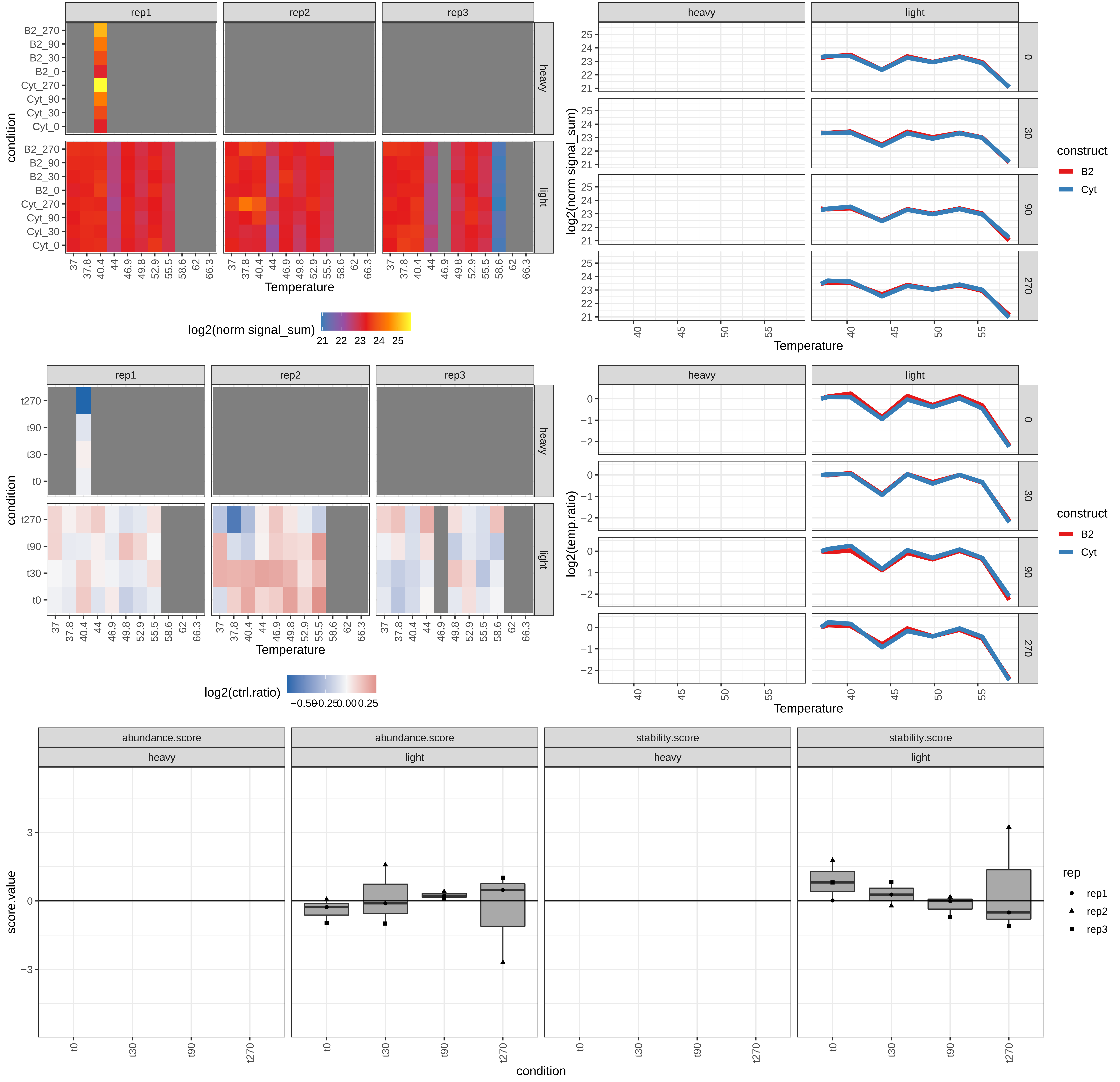

Q12315 − Nucleoporin GLE1 OS=Saccharomyces cerevisiae (strain ATCC 204508 / S288c) GN=GLE1 PE=1 SV=1

GLE2 − average.top3: 6.661674 − max.qupm: 3

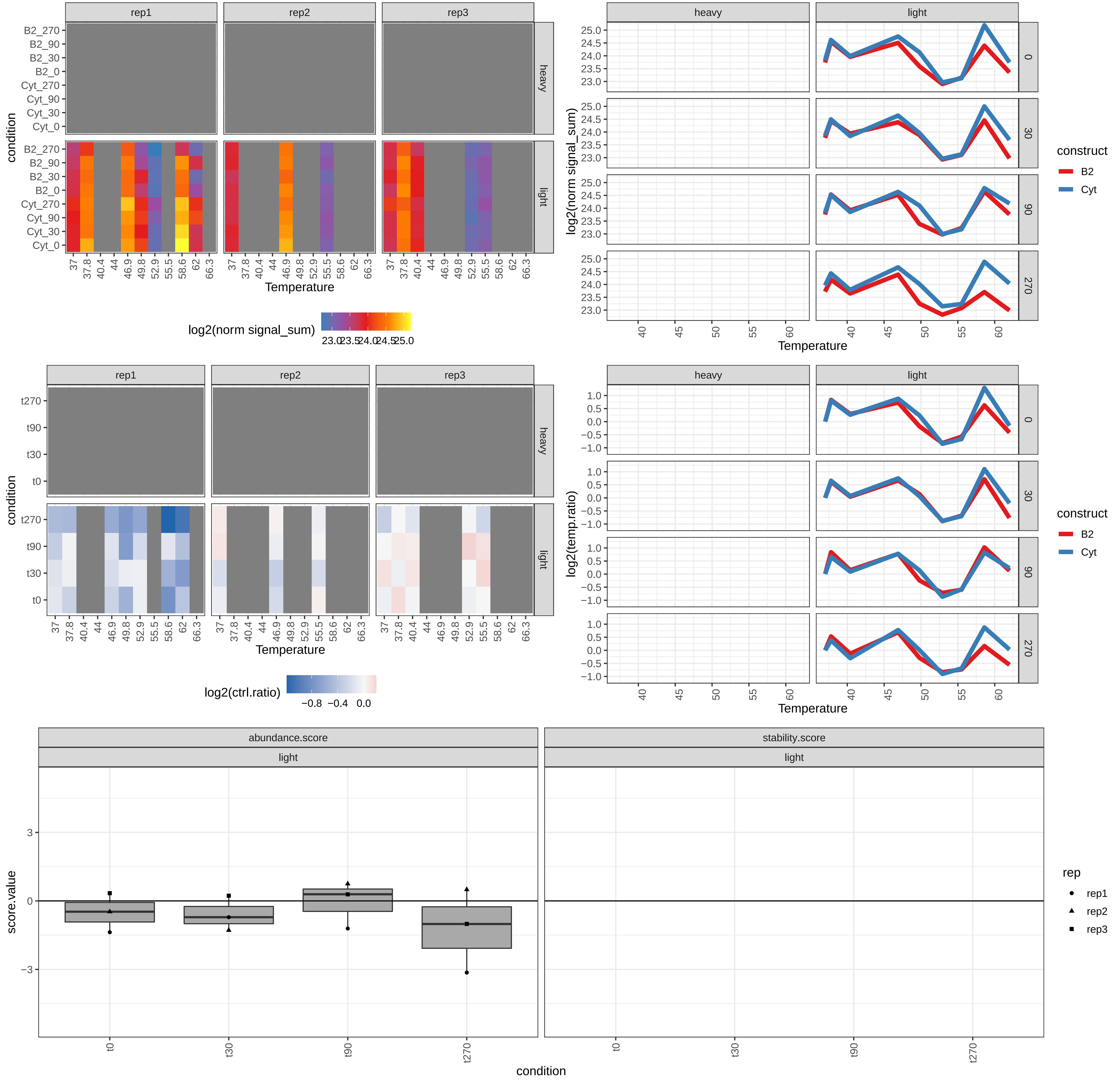

P40066 − Nucleoporin GLE2 OS=Saccharomyces cerevisiae (strain ATCC 204508 / S288c) GN=GLE2 PE=1 SV=1

GLG2 − average.top3: 6.86653914285714 − max.qupm: 6

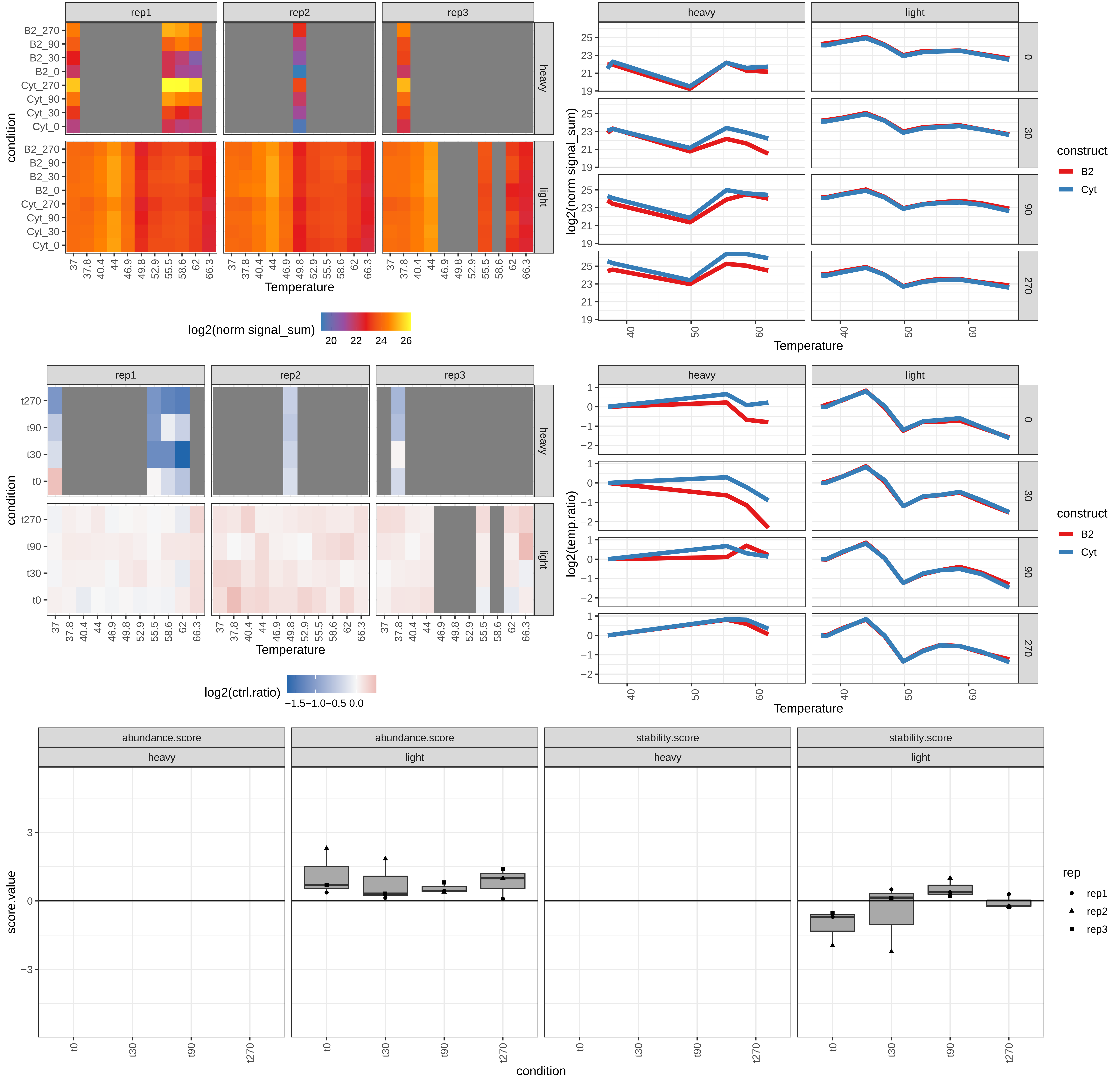

P47011 − Glycogenin−2 OS=Saccharomyces cerevisiae (strain ATCC 204508 / S288c) GN=GLG2 PE=1 SV=1

GLK1 − average.top3: 8.08157271186441 − max.qupm: 19

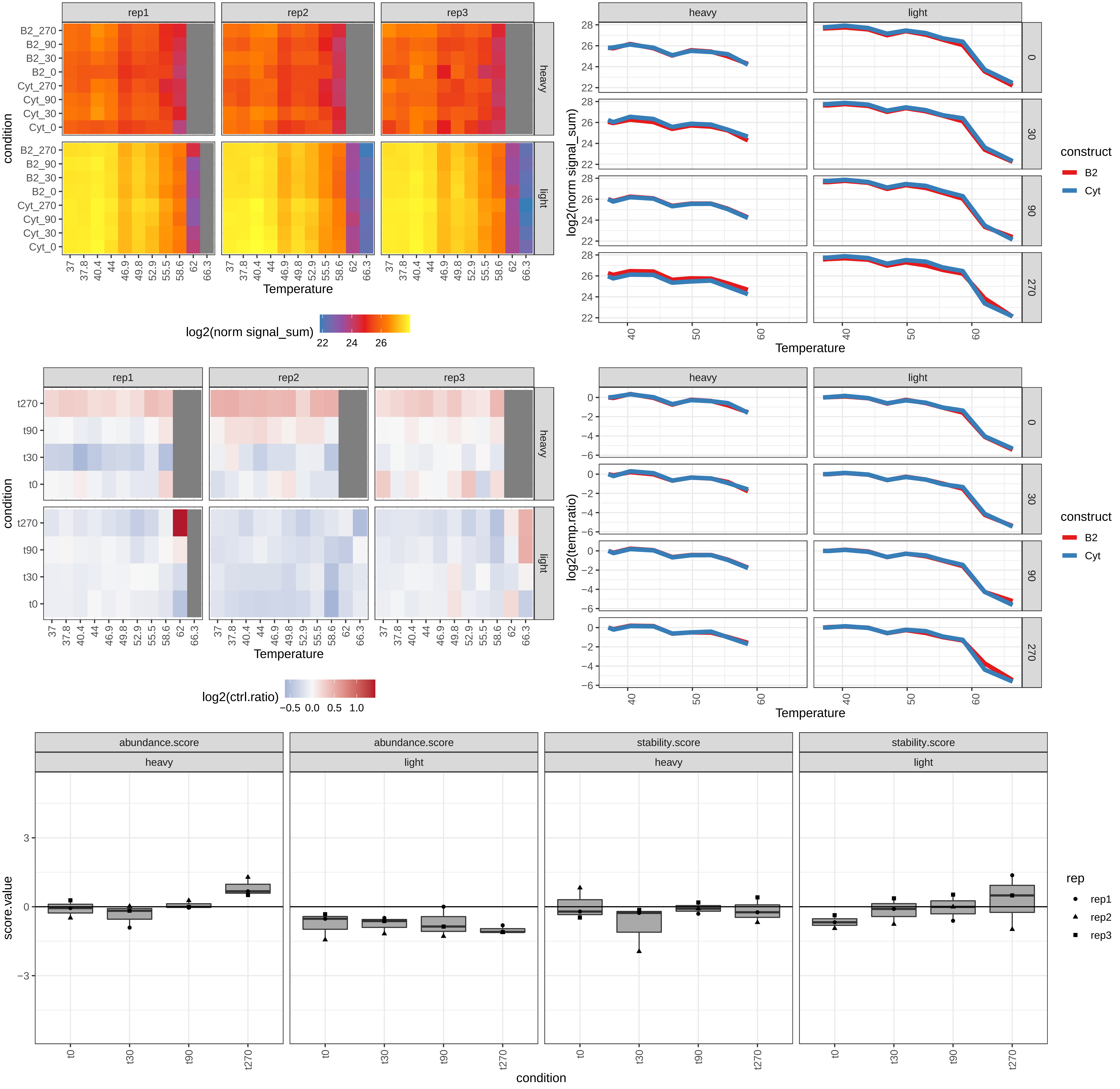

P17709 − Glucokinase−1 OS=Saccharomyces cerevisiae (strain ATCC 204508 / S288c) GN=GLK1 PE=1 SV=1

GLN1 − average.top3: 7.33461764705882 − max.qupm: 10

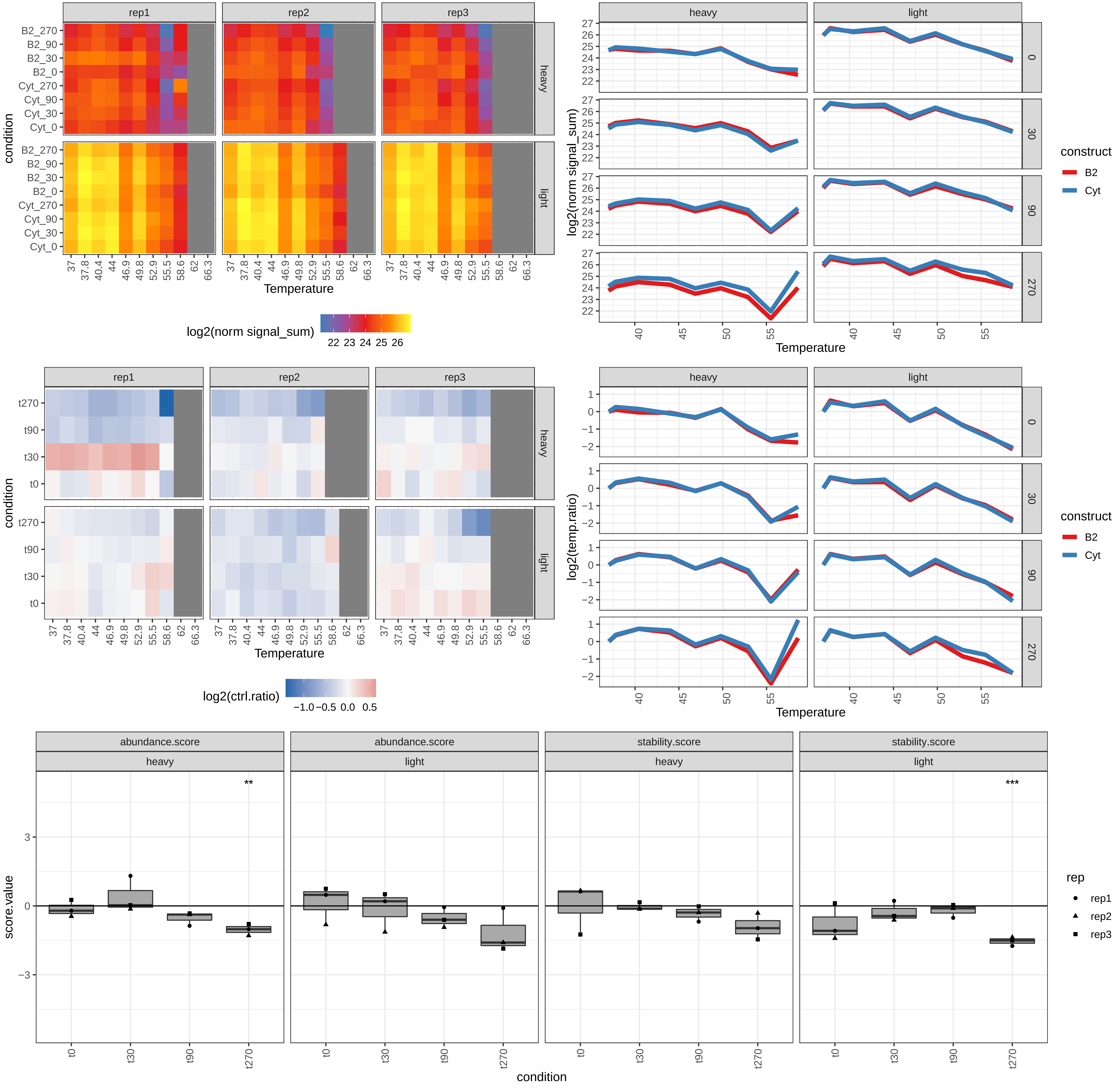

P32288 − Glutamine synthetase OS=Saccharomyces cerevisiae (strain ATCC 204508 / S288c) GN=GLN1 PE=1 SV=4

GLN4 − average.top3: 7.27717860465116 − max.qupm: 41

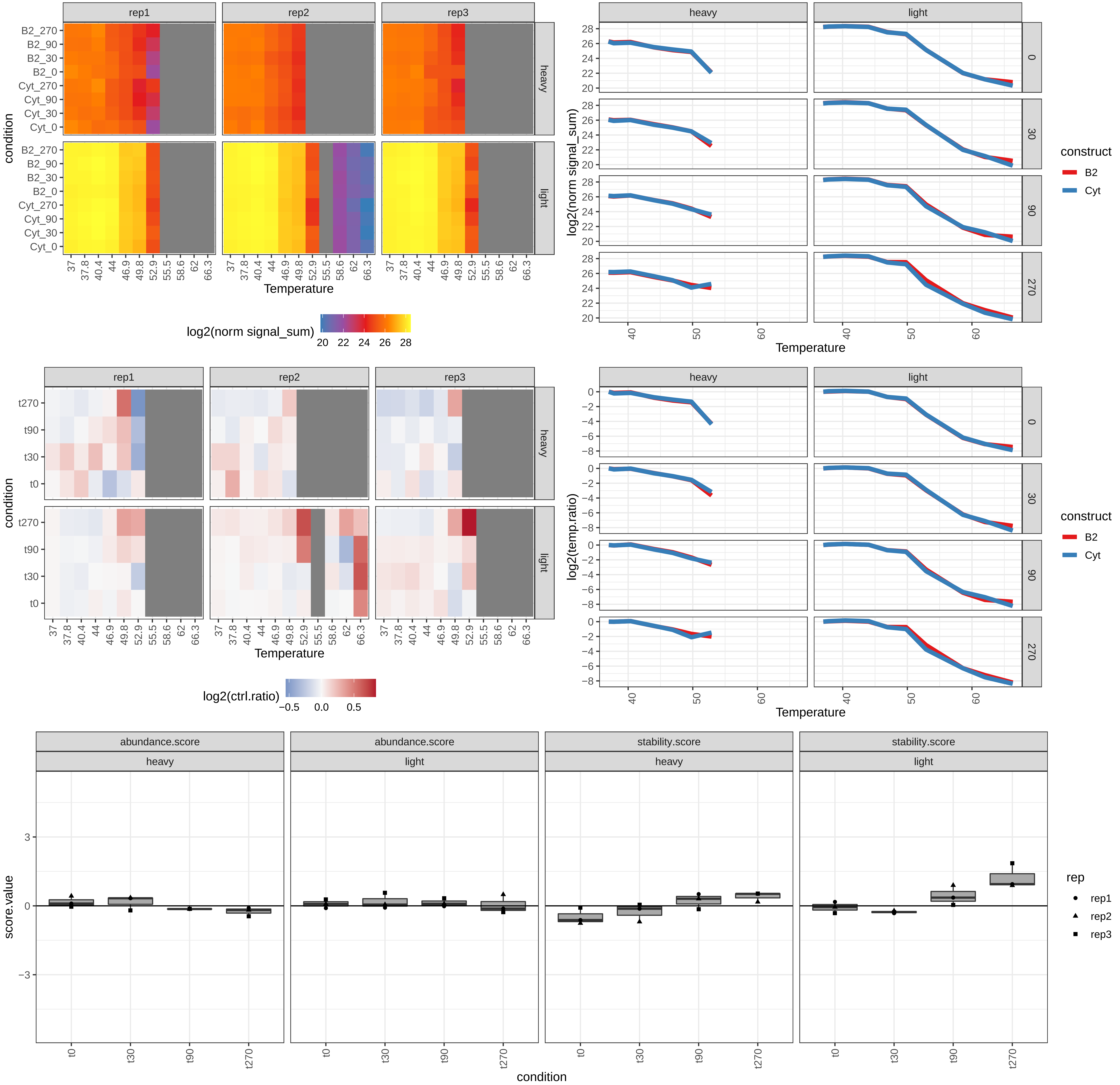

P13188 − Glutamine−−tRNA ligase OS=Saccharomyces cerevisiae (strain ATCC 204508 / S288c) GN=GLN4 PE=1 SV=2

GLO1 − average.top3: 7.08589255813953 − max.qupm: 9

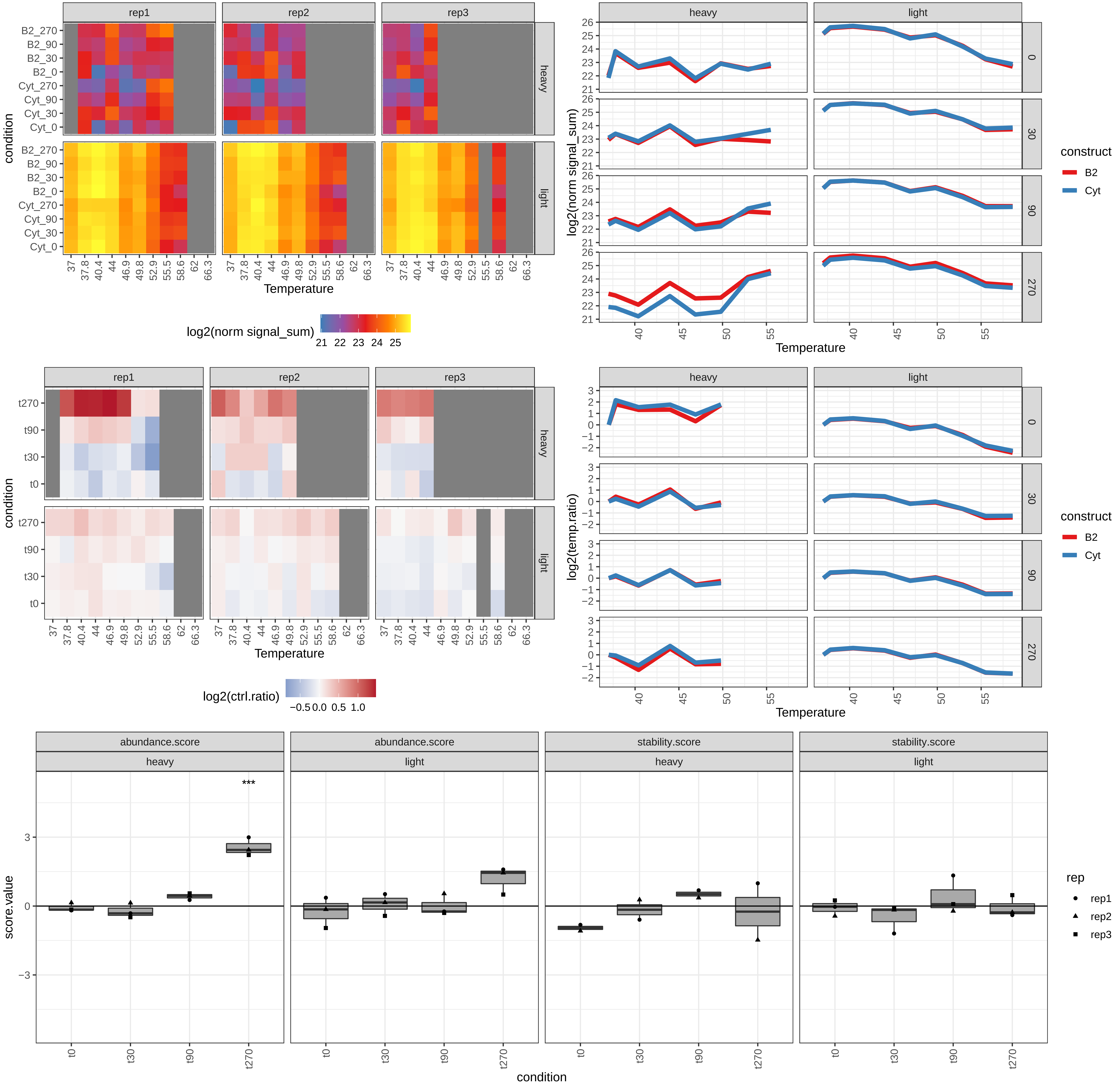

P50107 − Lactoylglutathione lyase OS=Saccharomyces cerevisiae (strain ATCC 204508 / S288c) GN=GLO1 PE=1 SV=1

GLO2 − average.top3: 6.99198619047619 − max.qupm: 11

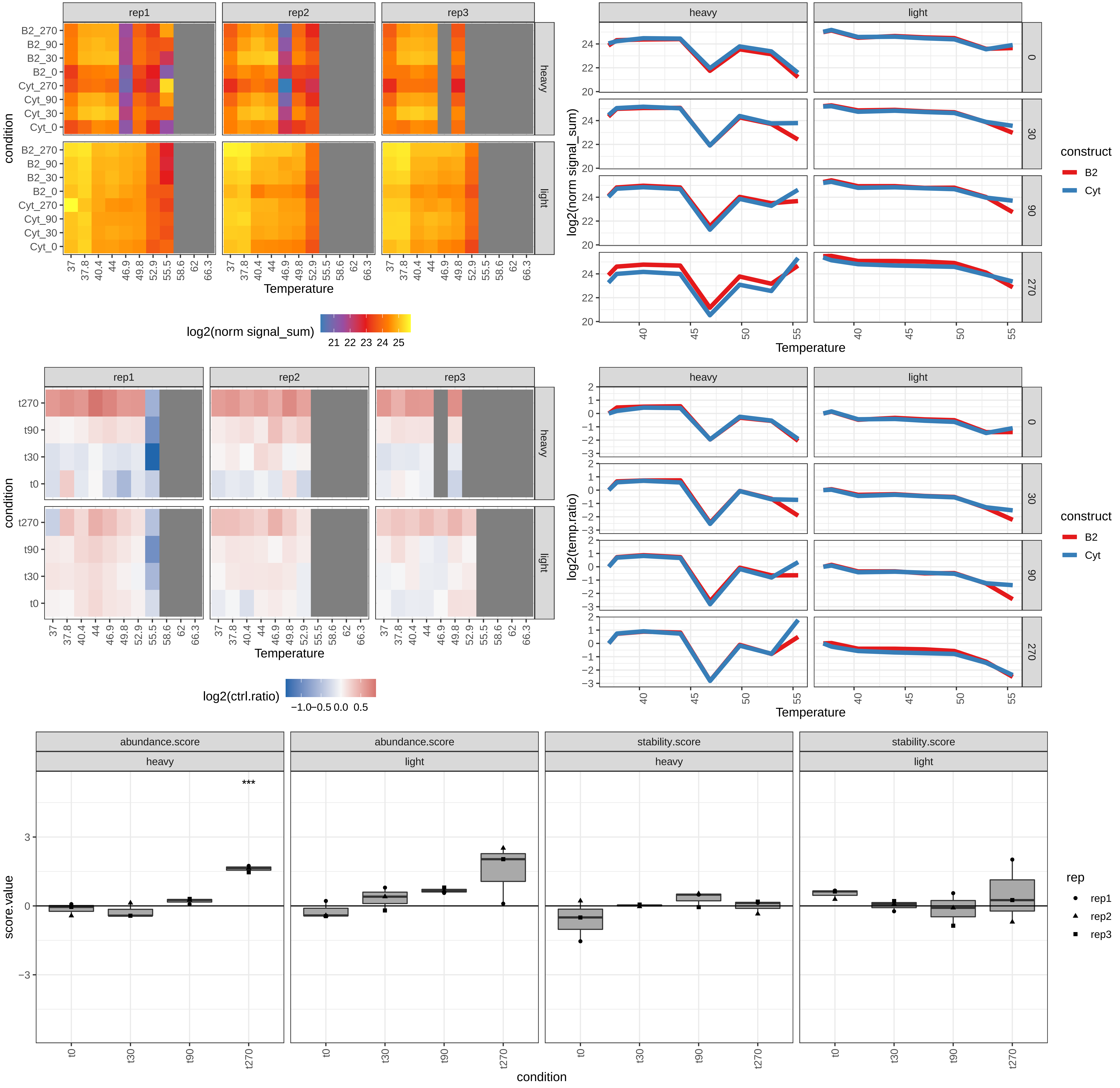

Q05584 − Hydroxyacylglutathione hydrolase, cytoplasmic isozyme OS=Saccharomyces cerevisiae (strain ATCC 204508 / S288c) GN=GLO2 PE=1 SV=1

GLO3 − average.top3: 7.20190235294118 − max.qupm: 5

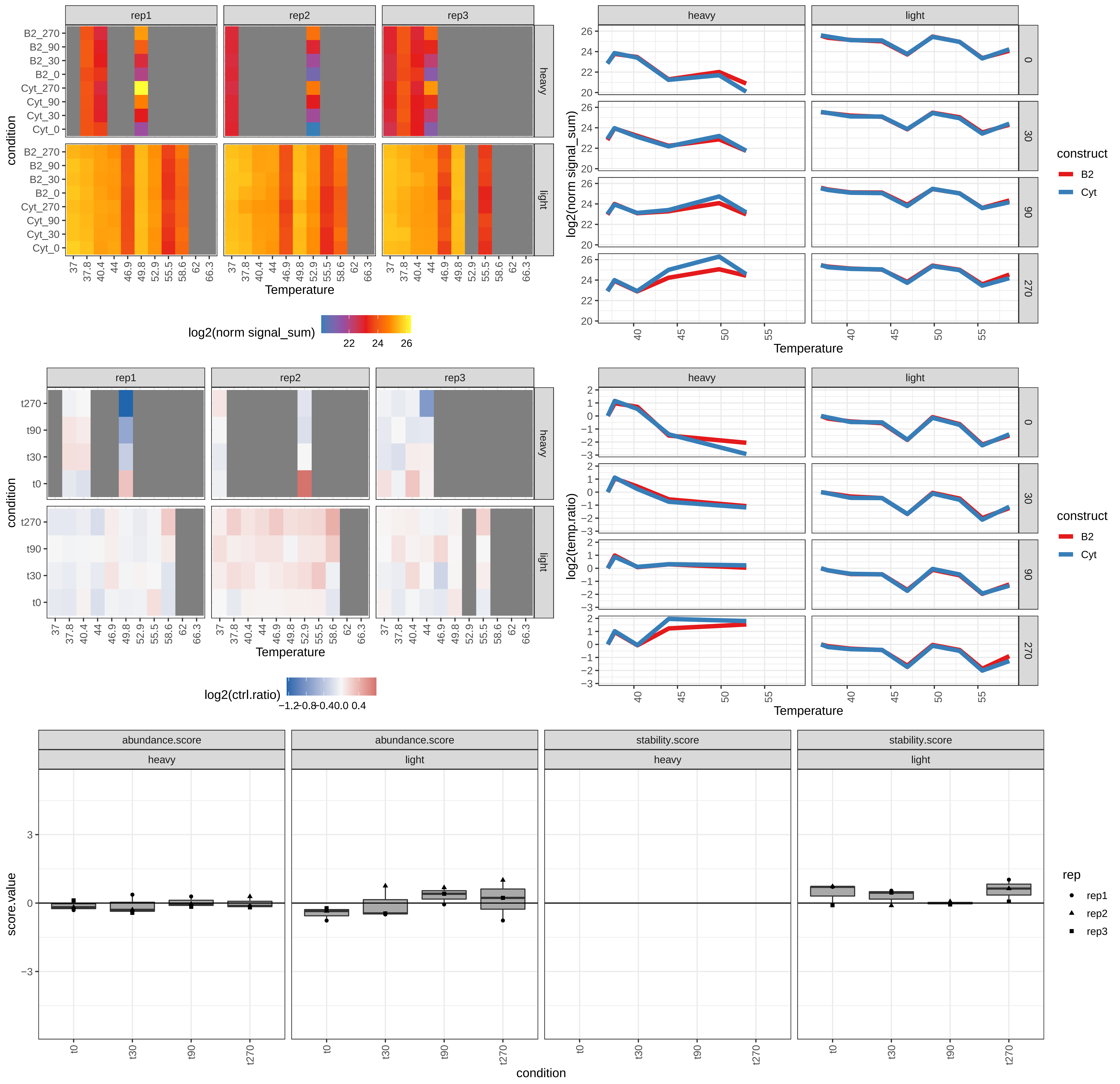

P38682 − ADP−ribosylation factor GTPase−activating protein GLO3 OS=Saccharomyces cerevisiae (strain ATCC 204508 / S288c) GN=GLO3 PE=1 SV=1

GLO4 − average.top3: 7.02464268292683 − max.qupm: 10

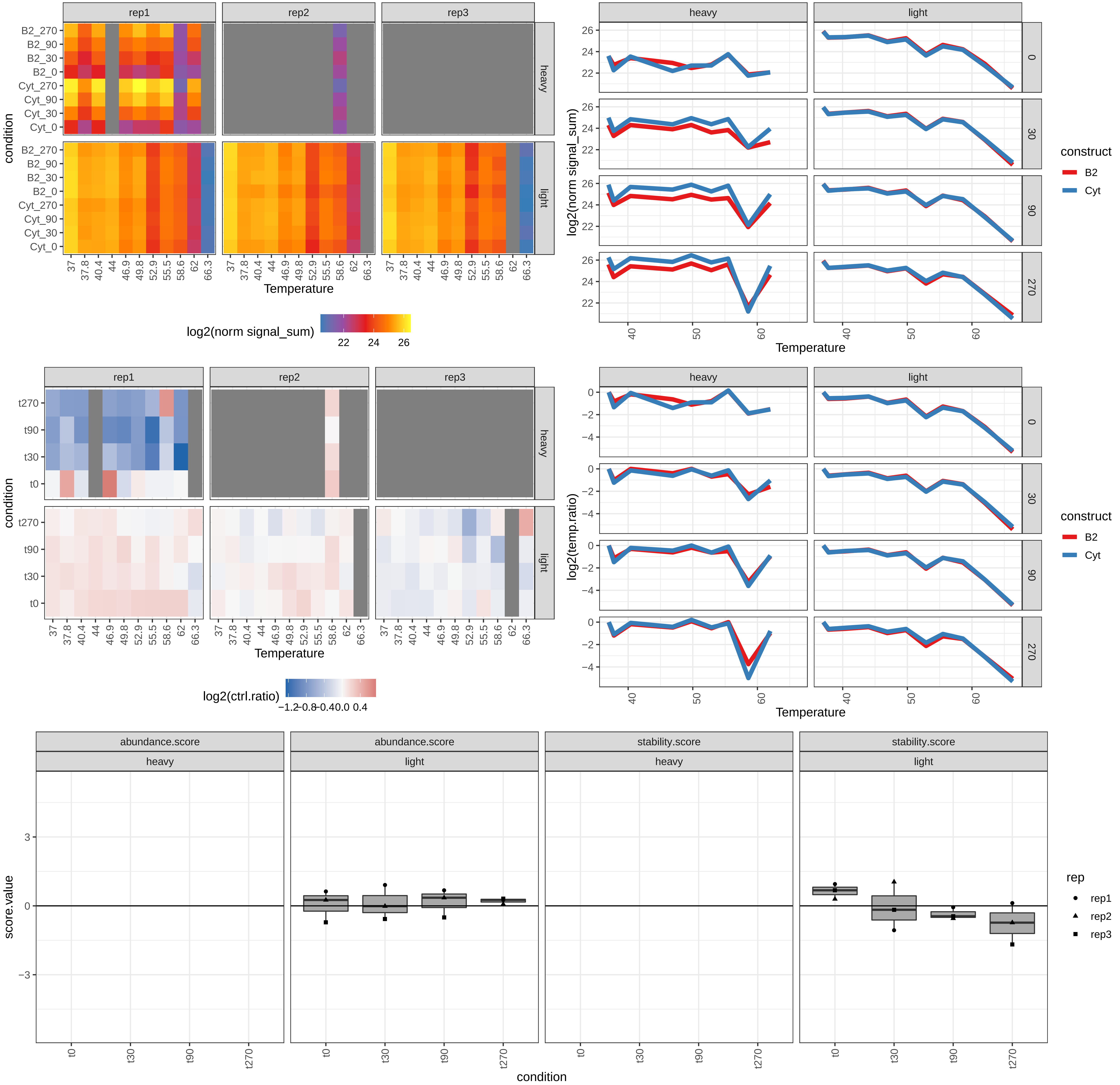

Q12320 − Hydroxyacylglutathione hydrolase, mitochondrial OS=Saccharomyces cerevisiae (strain ATCC 204508 / S288c) GN=GLO4 PE=3 SV=1

GLR1 − average.top3: 7.59547830508475 − max.qupm: 18

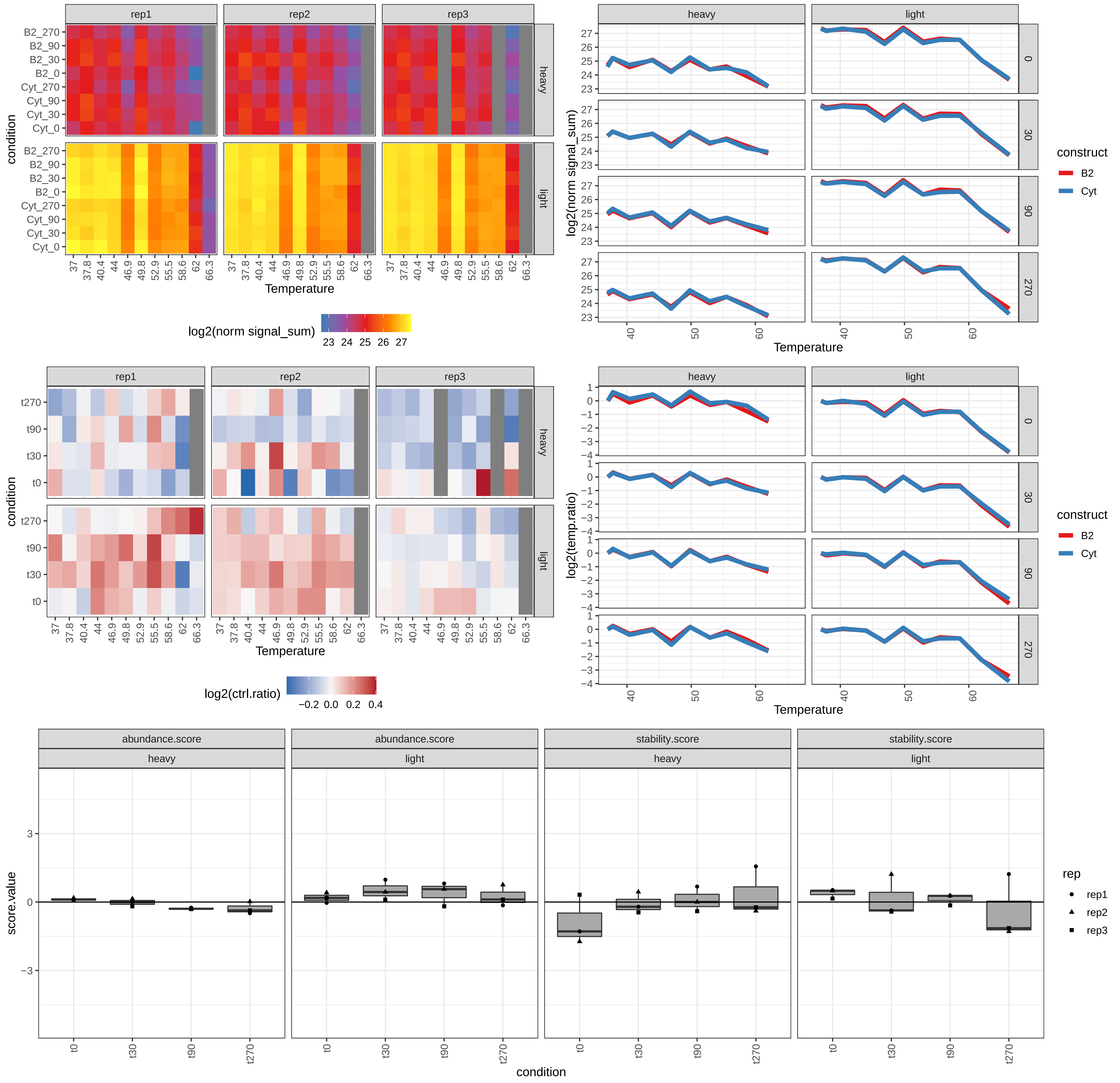

2 − Glutathione reductase OS=Saccharomyces cerevisiae (strain ATCC 204508 / S288c) GN=GLR1 PE=1 SV=2|Isoform 2 of Glutathione reductase OS=Saccharomyces cerevisiae (strain ATCC 204508 / S

GLT1 − average.top3: 7.29008117647059 − max.qupm: 53

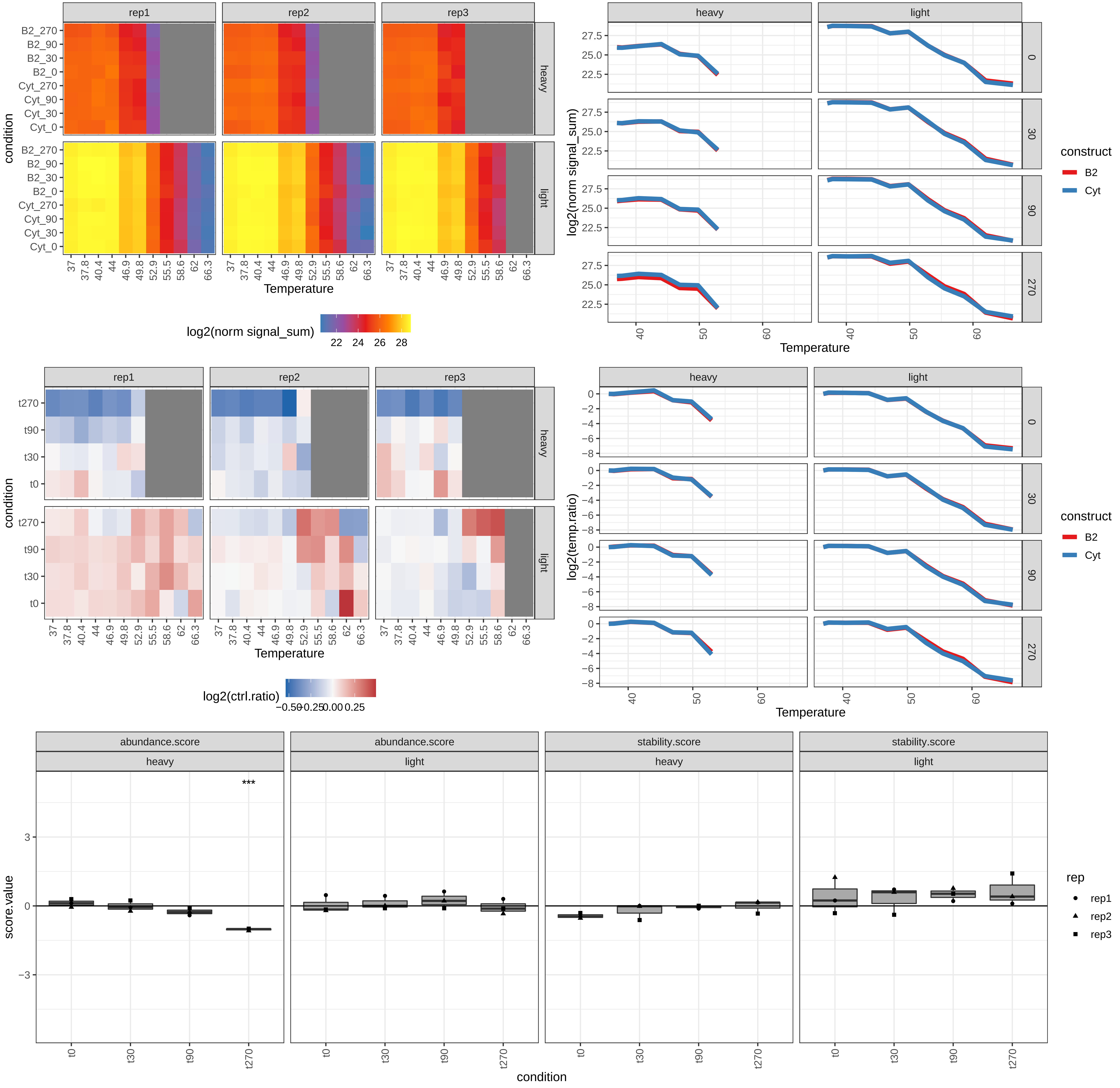

Q12680 − Glutamate synthase [NADH] OS=Saccharomyces cerevisiae (strain ATCC 204508 / S288c) GN=GLT1 PE=1 SV=2

GLY1 − average.top3: 6.97577857142857 − max.qupm: 7

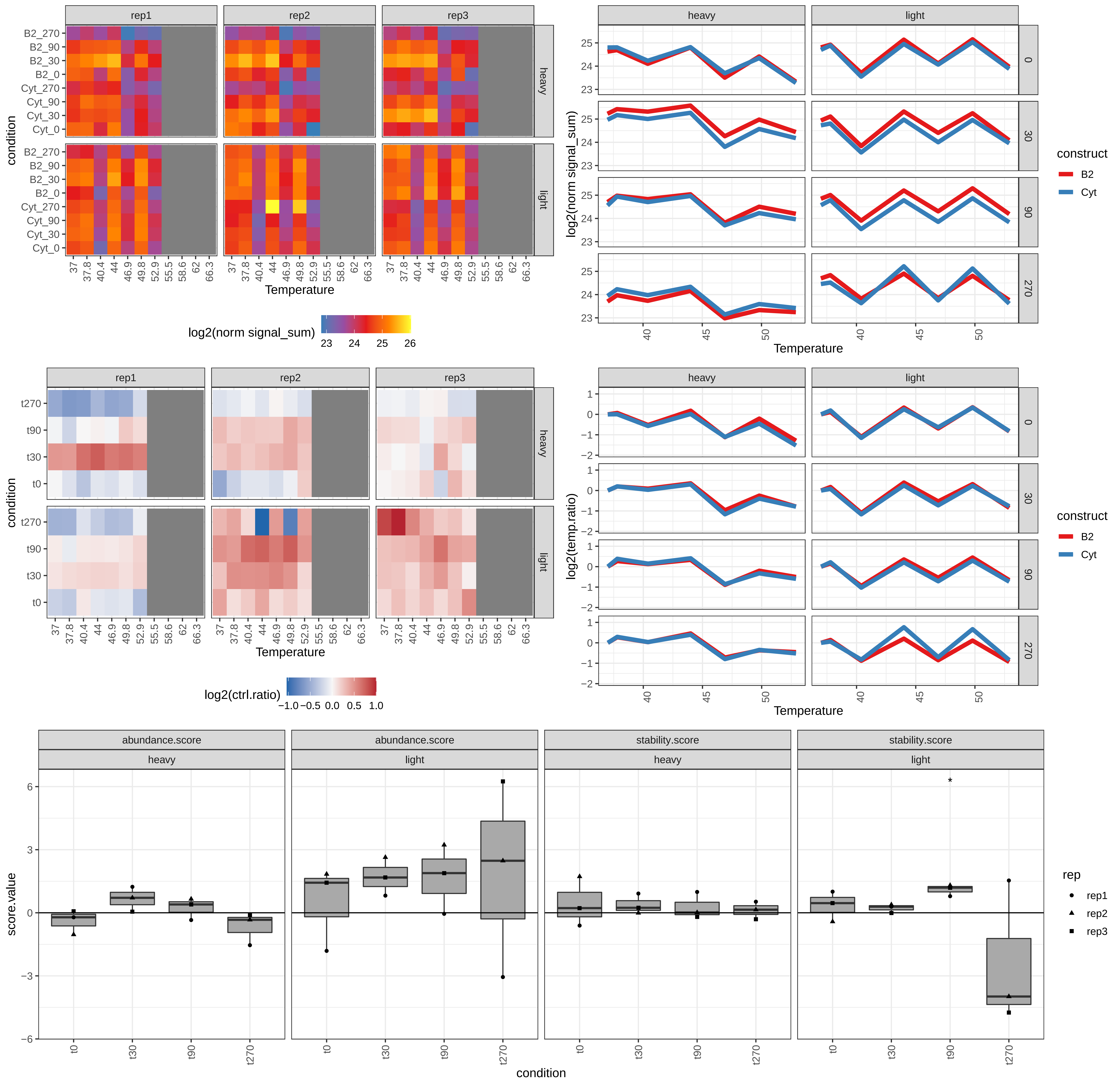

P37303 − Low specificity L−threonine aldolase OS=Saccharomyces cerevisiae (strain ATCC 204508 / S288c) GN=GLY1 PE=1 SV=2

GNA1 − average.top3: 6.97254404255319 − max.qupm: 7

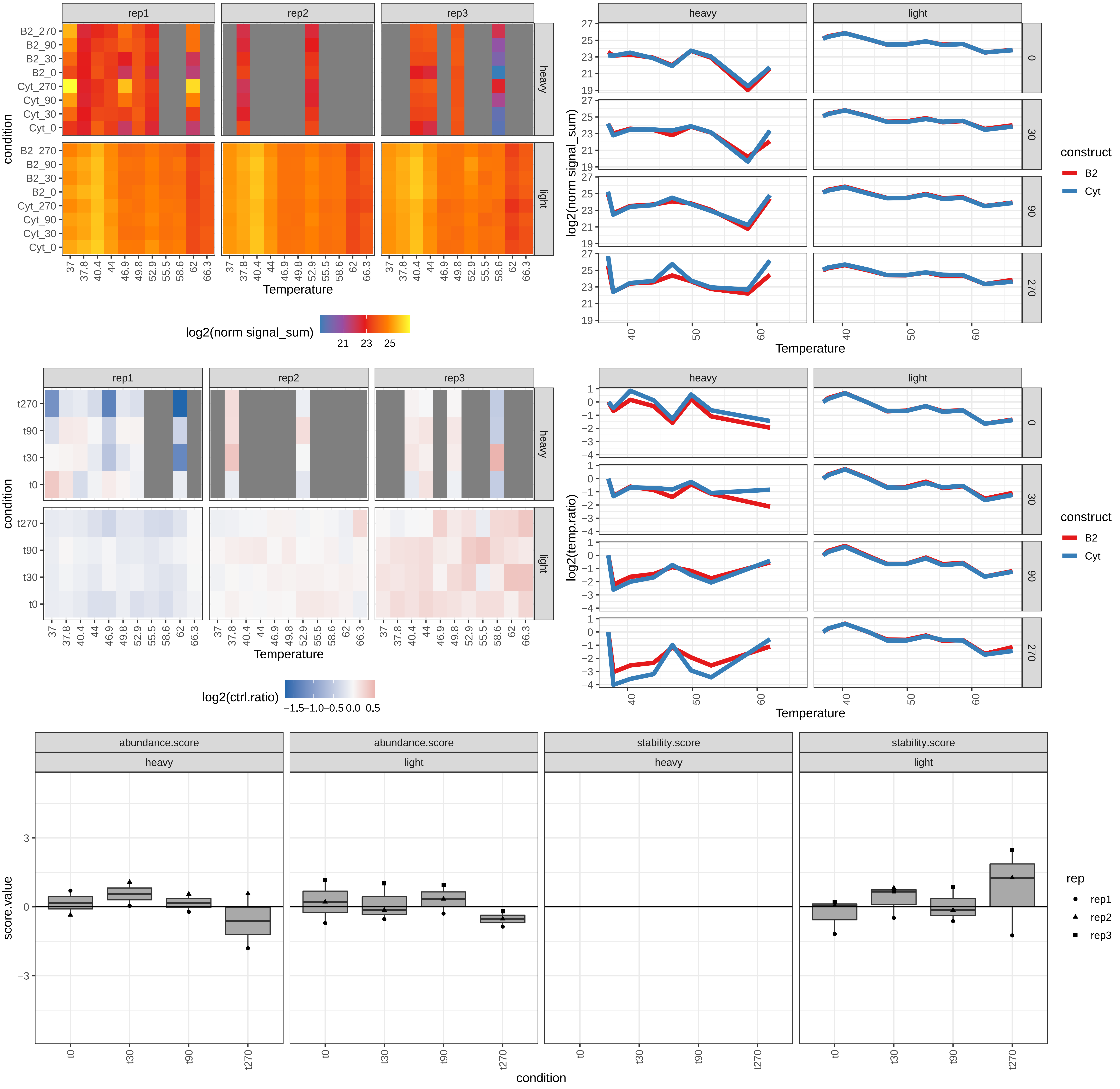

P43577 − Glucosamine 6−phosphate N−acetyltransferase OS=Saccharomyces cerevisiae (strain ATCC 204508 / S288c) GN=GNA1 PE=1 SV=1

GND1 − average.top3: 7.93280526315789 − max.qupm: 21

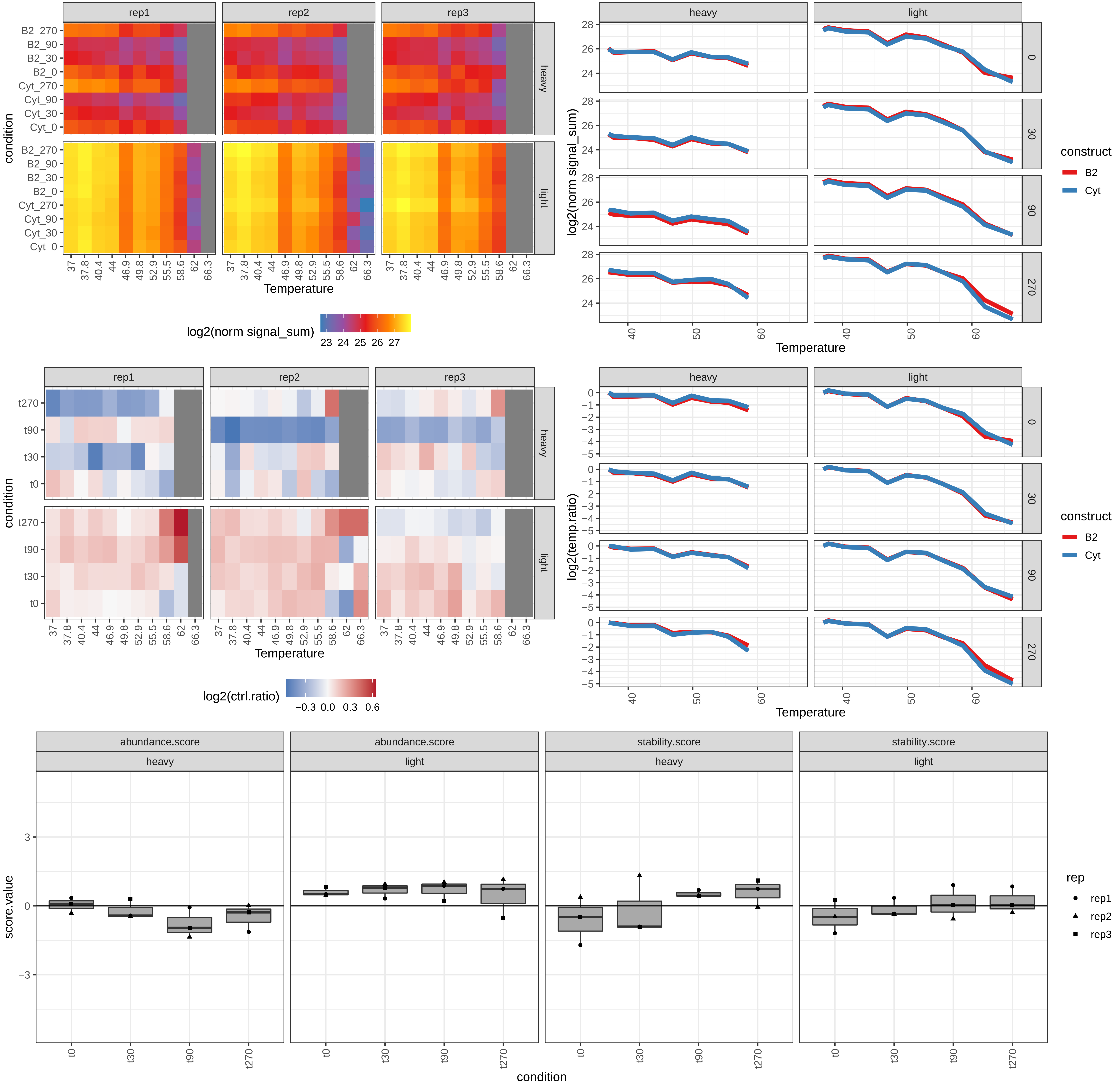

P38720 − 6−phosphogluconate dehydrogenase, decarboxylating 1 OS=Saccharomyces cerevisiae (strain ATCC 204508 / S288c) GN=GND1 PE=1 SV=1

GND2 − average.top3: 6.75760394736842 − max.qupm: 9

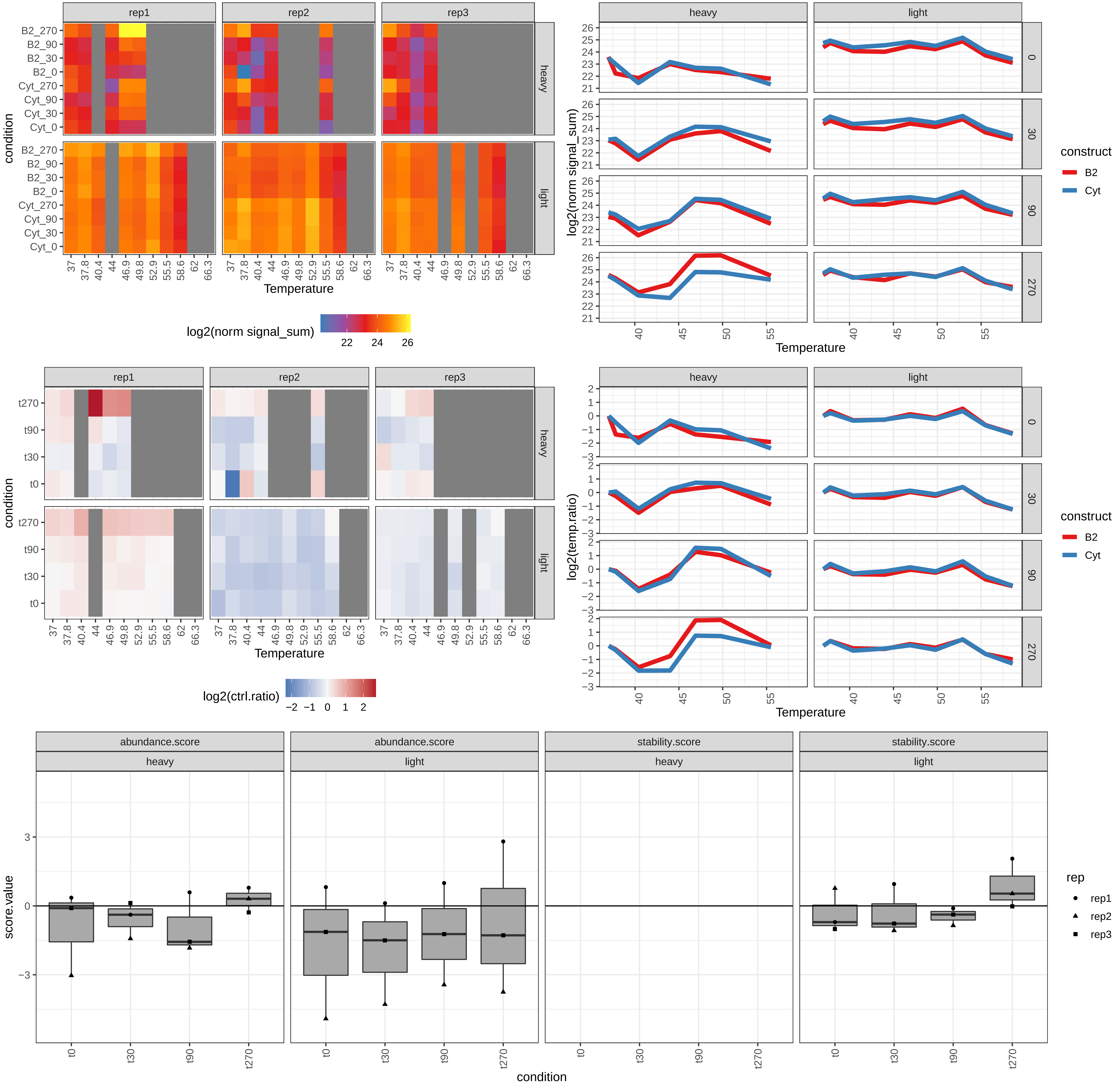

P53319 − 6−phosphogluconate dehydrogenase, decarboxylating 2 OS=Saccharomyces cerevisiae (strain ATCC 204508 / S288c) GN=GND2 PE=1 SV=1

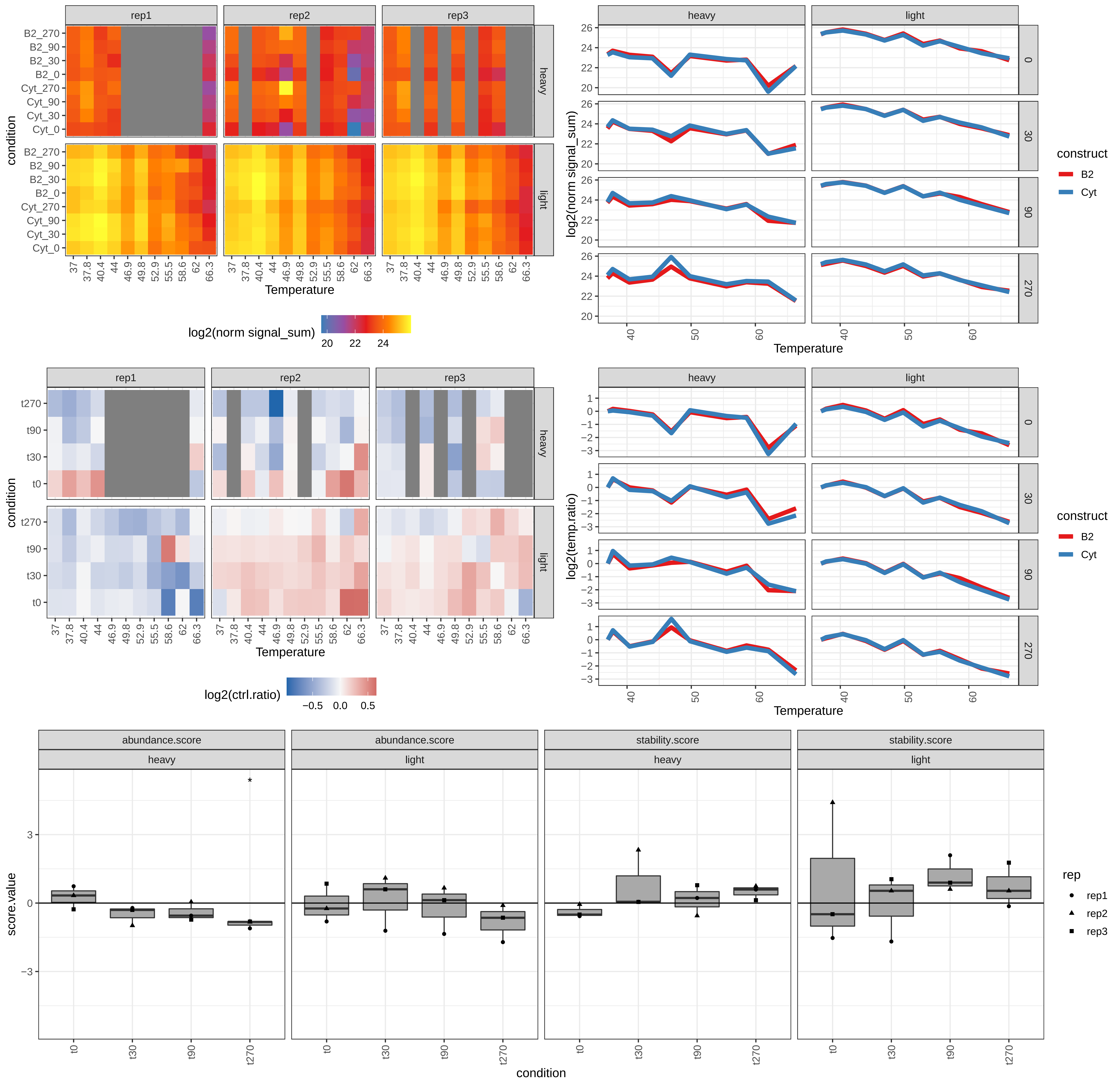

P48813 − High−affinity glutamine permease OS=Saccharomyces cerevisiae (strain ATCC 204508 / S288c) GN=GNP1 PE=1 SV=2

GNT1 − average.top3: 6.42277709677419 − max.qupm: 7

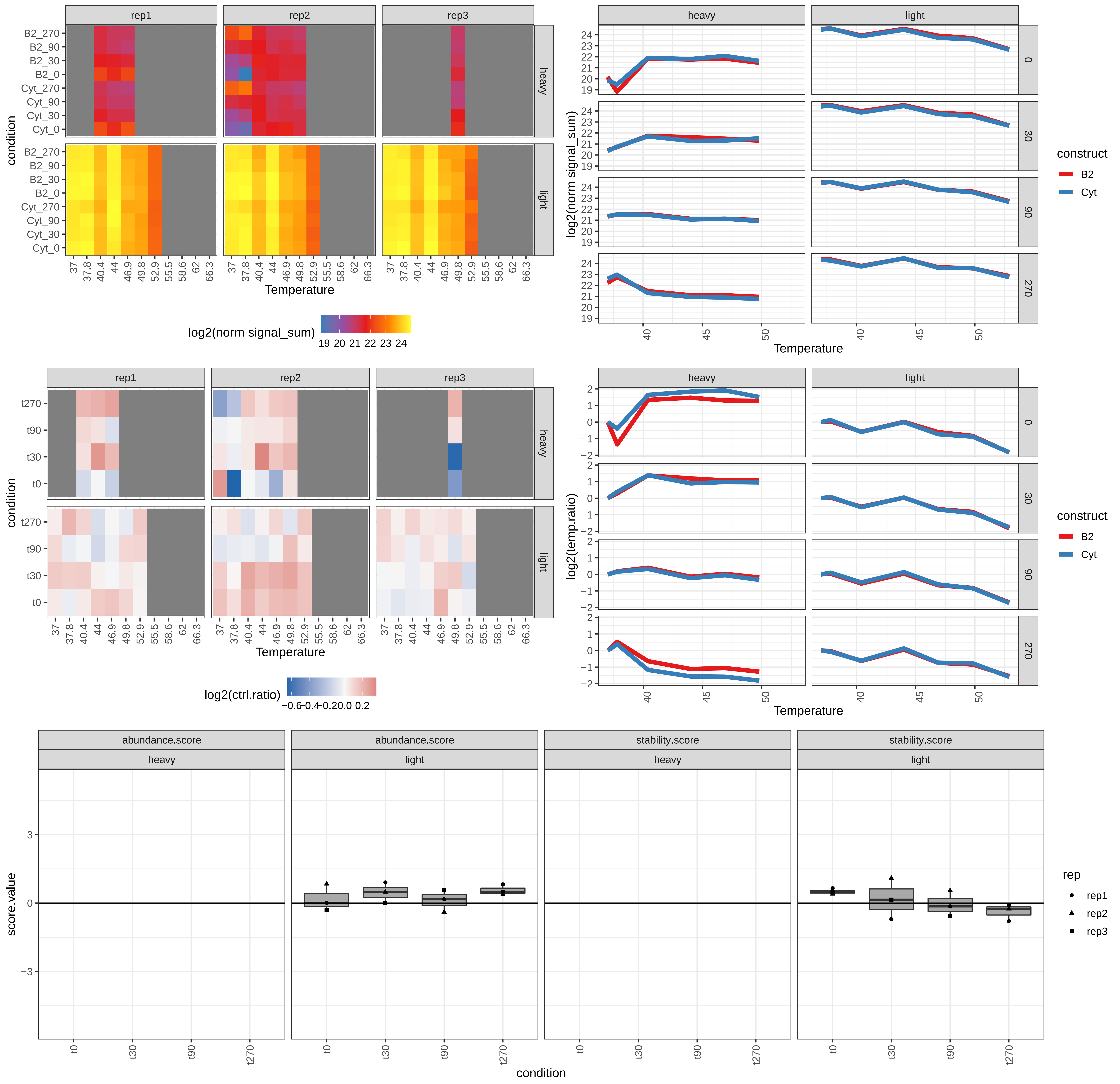

Q12096 − Glucose N−acetyltransferase 1 OS=Saccharomyces cerevisiae (strain ATCC 204508 / S288c) GN=GNT1 PE=1 SV=1

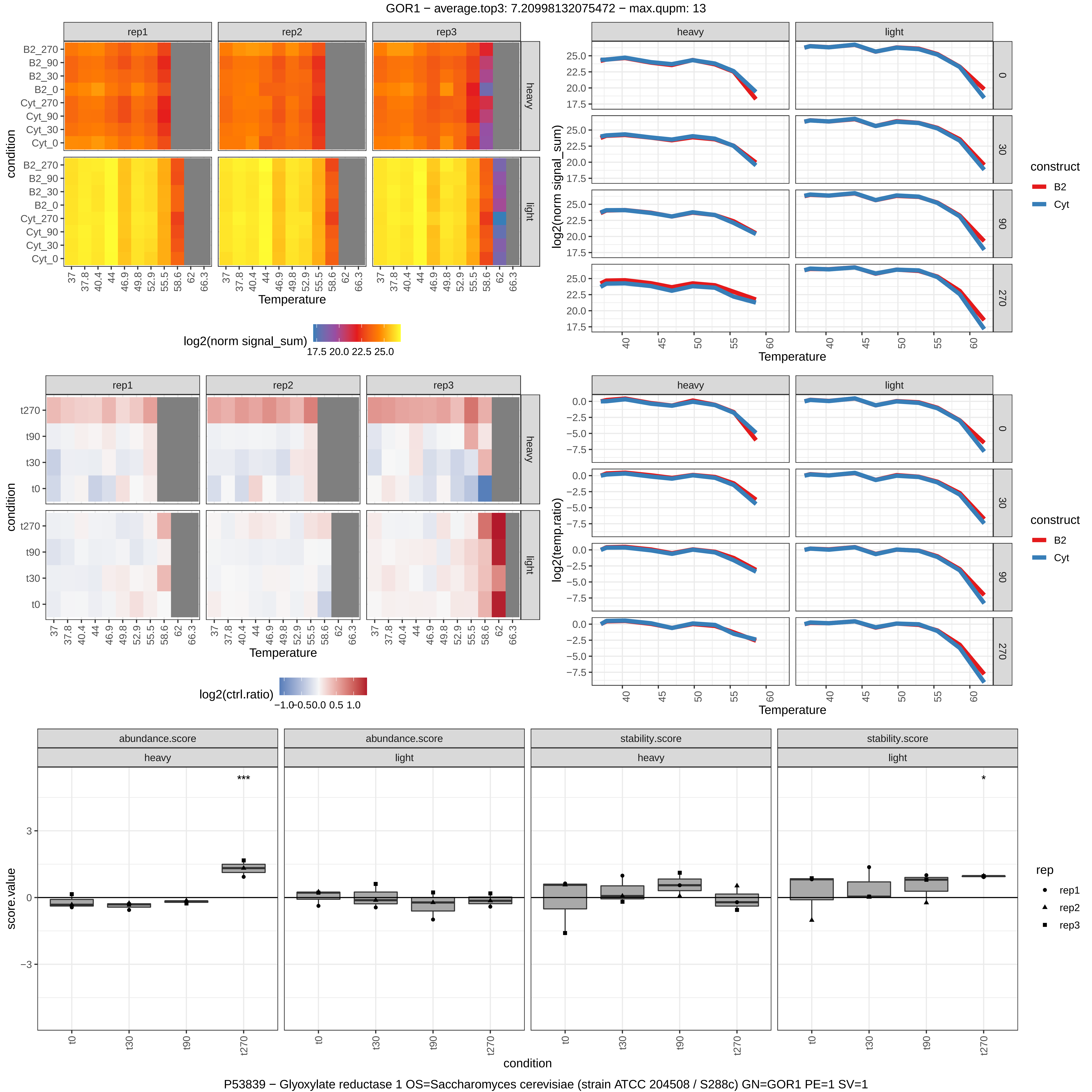

GOR1 − average.top3: 7.20998132075472 − max.qupm: 13
rep1
rep2
rep3
heavy
light
condition
Temperature
log2(norm signal_sum)
17.5 20.0 22.5 25.0
construct
B2
Cyt
log2(ctrl.ratio)
−1.0 −0.5 0.0 0.5 1.0
log2(temp.ratio)
abundance.score
stability.score
score.value
rep
***
*
P53839 − Glyoxylate reductase 1 OS=Saccharomyces cerevisiae (strain ATCC 204508 / S288c) GN=GOR1 PE=1 SV=1

GOS1 − average.top3: 6.54775608695652 − max.qupm: 7

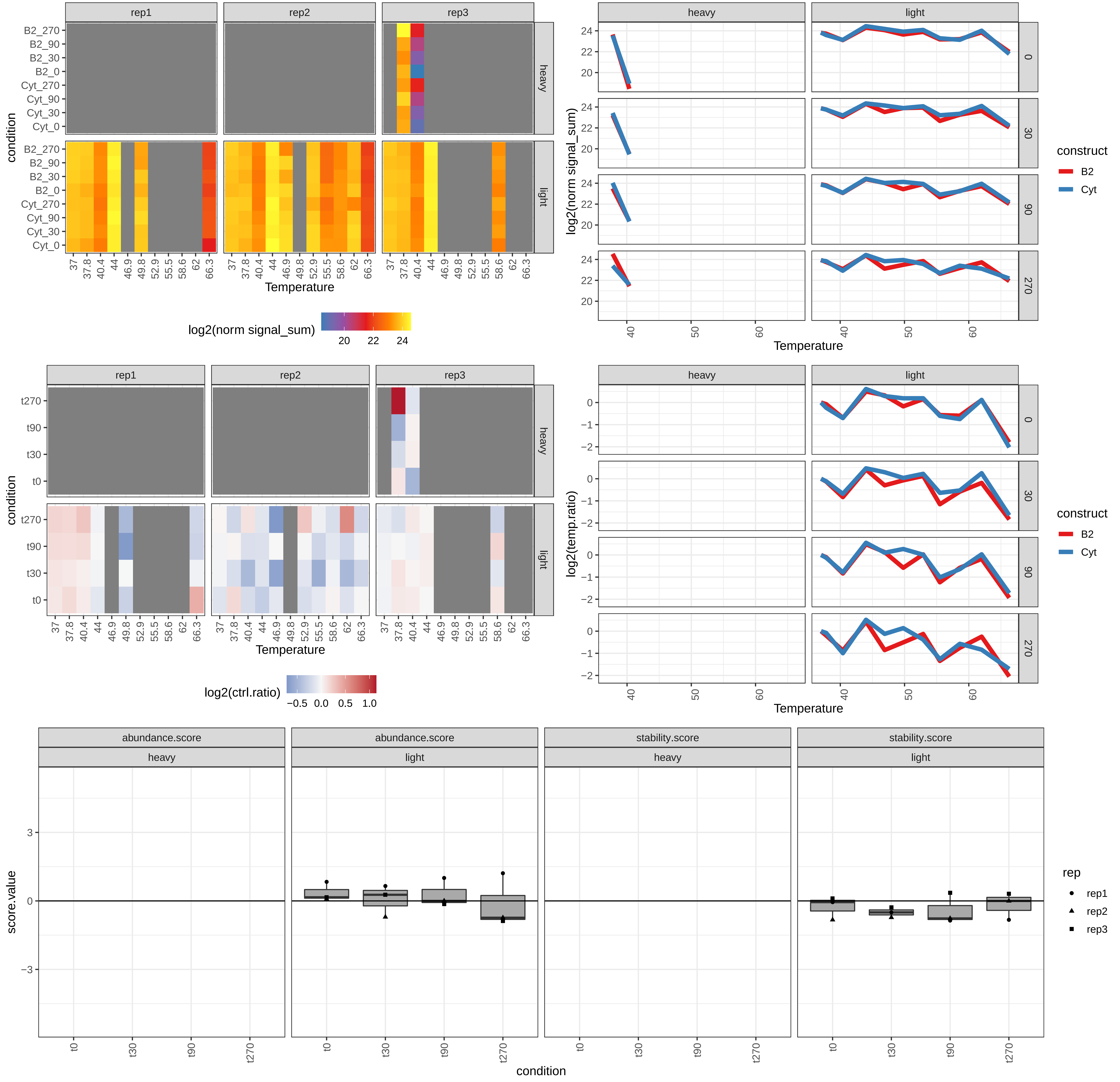

P38736 − Golgi SNAP receptor complex member 1 OS=Saccharomyces cerevisiae (strain ATCC 204508 / S288c) GN=GOS1 PE=1 SV=1

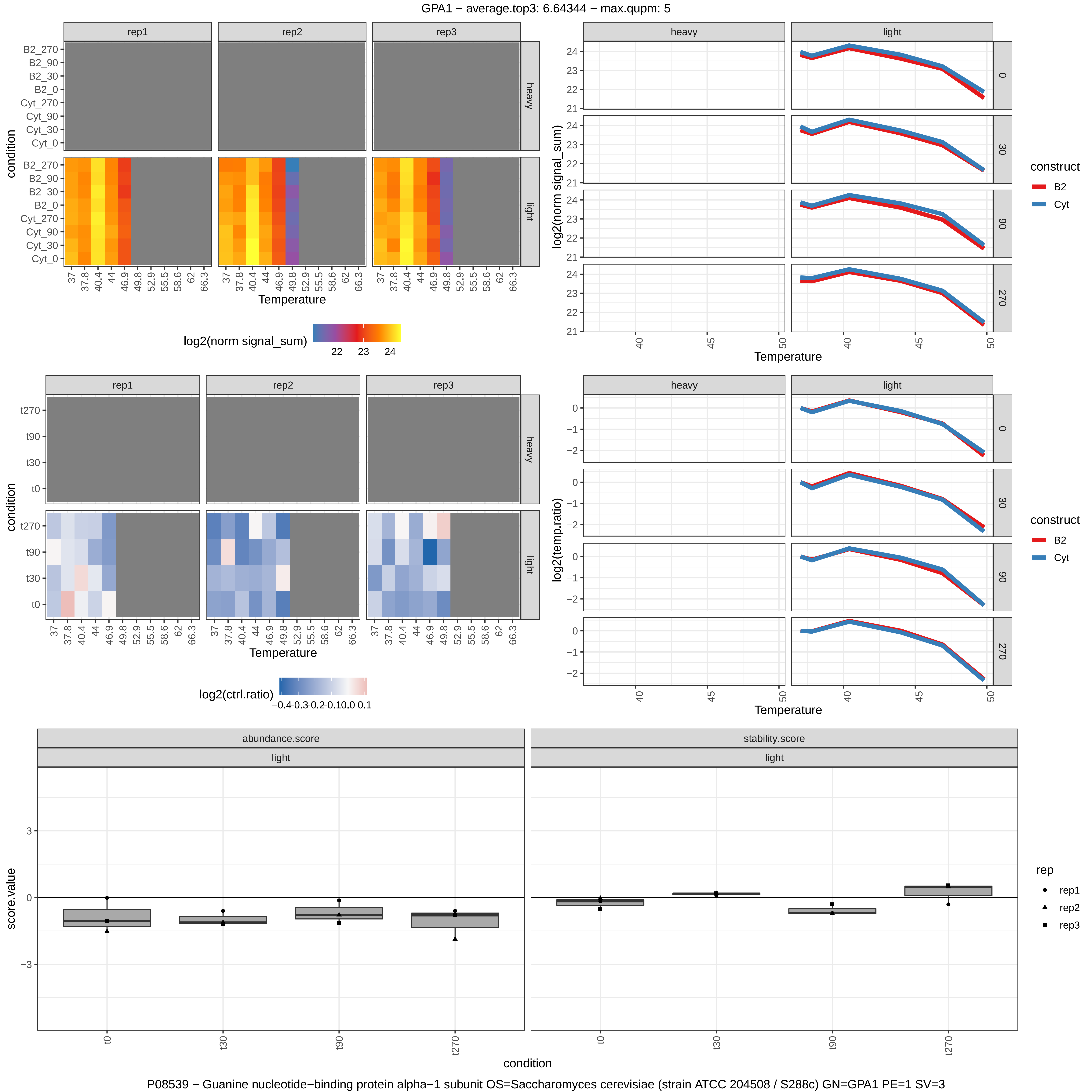
GPA1 − average.top3: 6.64344 − max.qupm: 5
rep1
rep2
rep3
heavy
light
condition
Temperature
log2(norm signal_sum)
construct
B2
Cyt
log2(ctrl.ratio)
log2(temp.ratio)
abundance.score
stability.score
score.value
rep
rep1
rep2
rep3
P08539 − Guanine nucleotide−binding protein alpha−1 subunit OS=Saccharomyces cerevisiae (strain ATCC 204508 / S288c) GN=GPA1 PE=1 SV=3

GPA2 − average.top3: 6.50120666666667 − max.qupm: 5

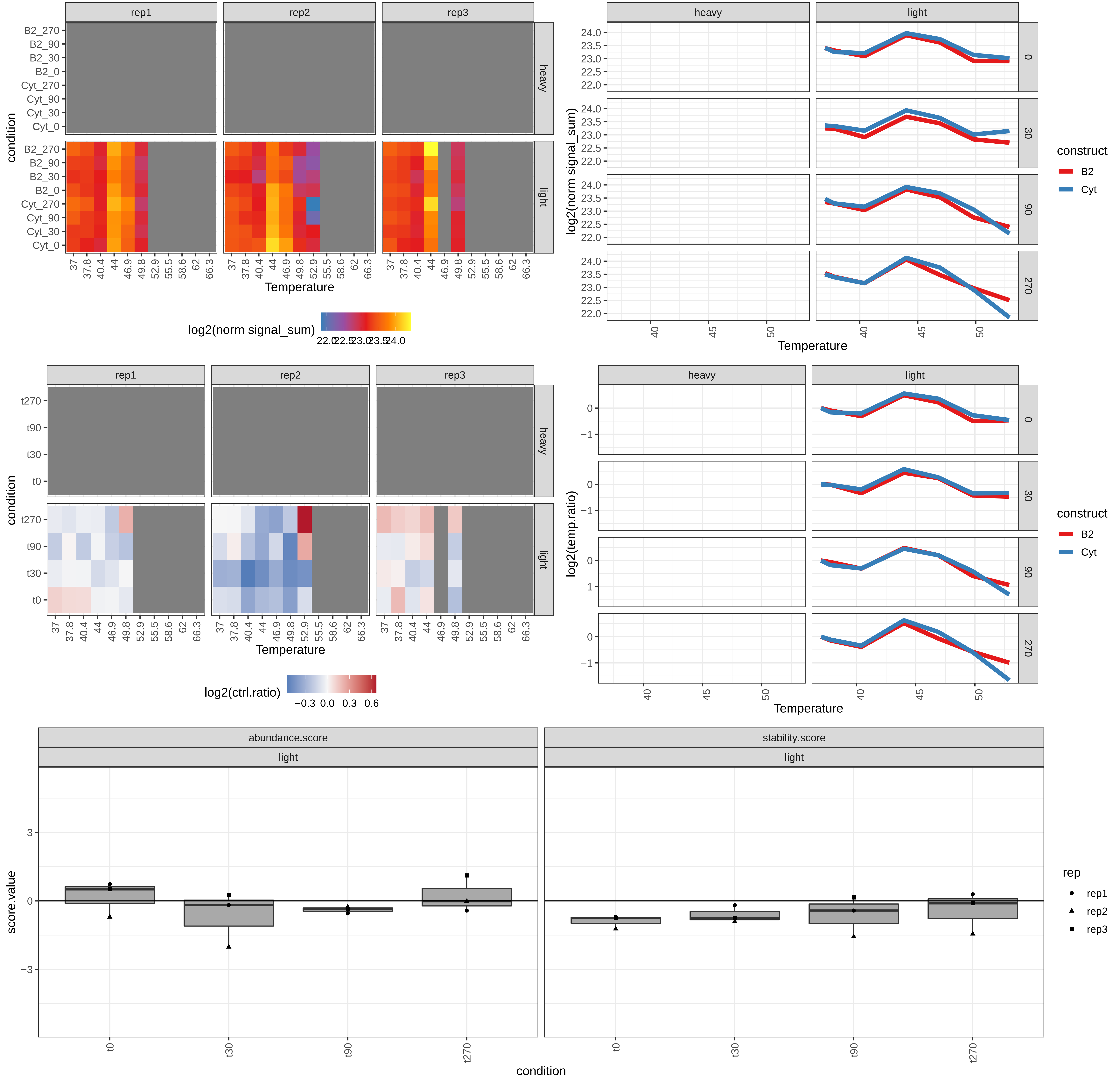

P10823 − Guanine nucleotide−binding protein alpha−2 subunit OS=Saccharomyces cerevisiae (strain ATCC 204508 / S288c) GN=GPA2 PE=1 SV=2

GPB1 − average.top3: 6.673345625 − max.qupm: 6

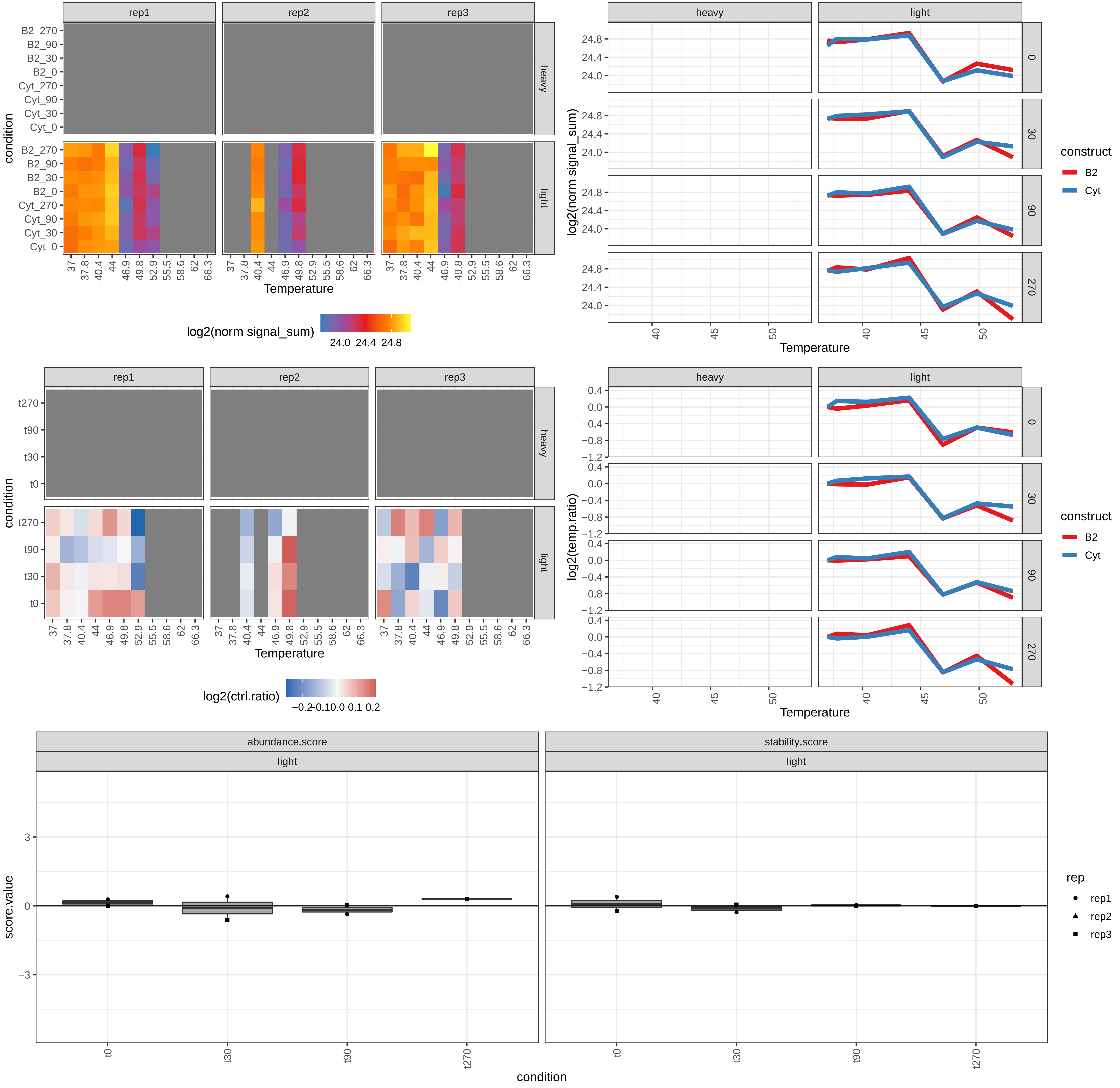

Q08886 − Guanine nucleotide−binding protein subunit beta 1 OS=Saccharomyces cerevisiae (strain ATCC 204508 / S288c) GN=GPB1 PE=1 SV=1

GPD1 − average.top3: 8.03334173913044 − max.qupm: 12

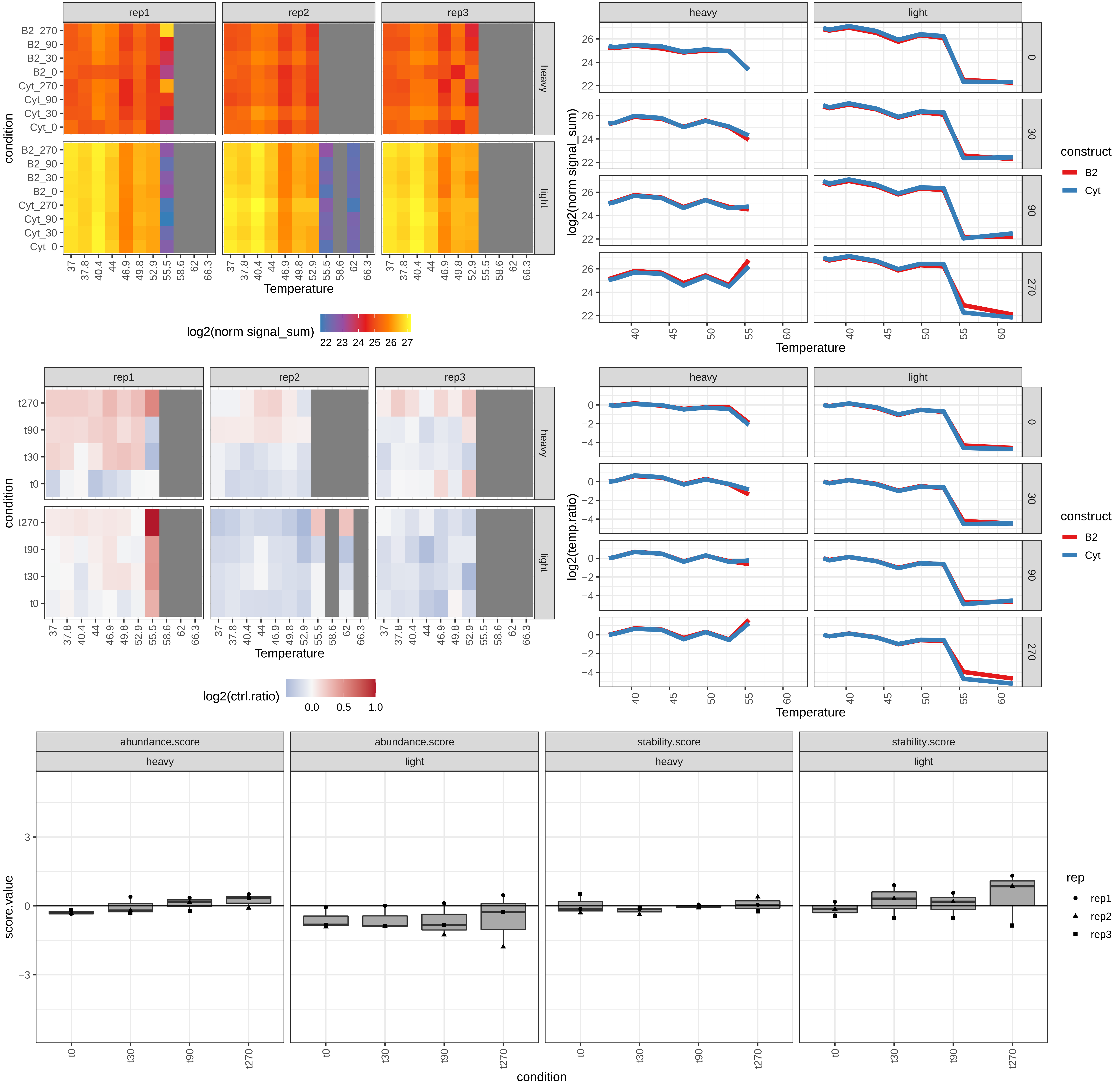

Q00055 − Glycerol−3−phosphate dehydrogenase [NAD(+)] 1 OS=Saccharomyces cerevisiae (strain ATCC 204508 / S288c) GN=GPD1 PE=1 SV=4

GPD2 − average.top3: 7.090608 − max.qupm: 9

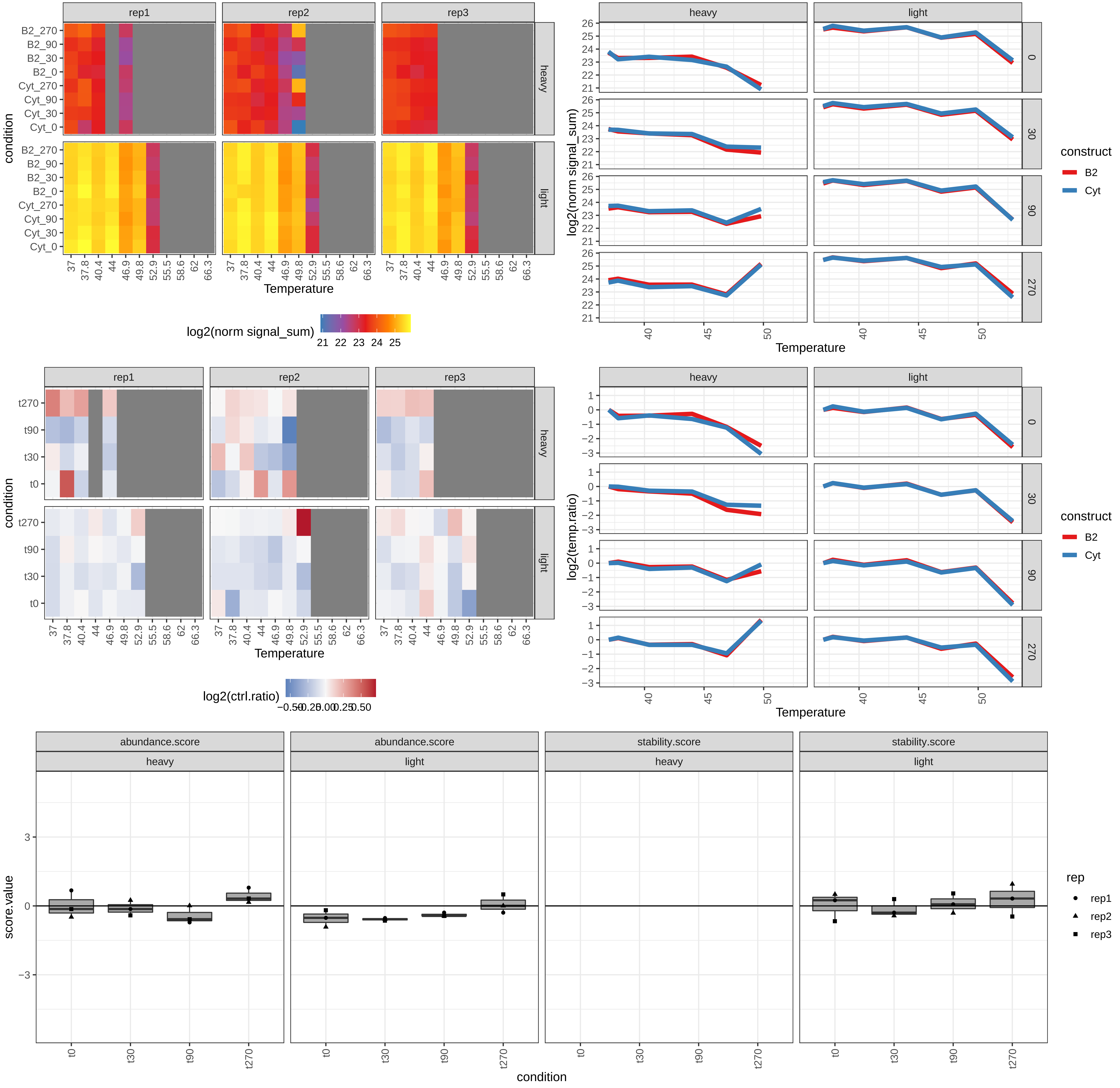

P41911 − Glycerol−3−phosphate dehydrogenase [NAD(+)] 2, mitochondrial OS=Saccharomyces cerevisiae (strain ATCC 204508 / S288c) GN=GPD2 PE=1 SV=2

GPH1 − average.top3: 7.47416282608696 − max.qupm: 30

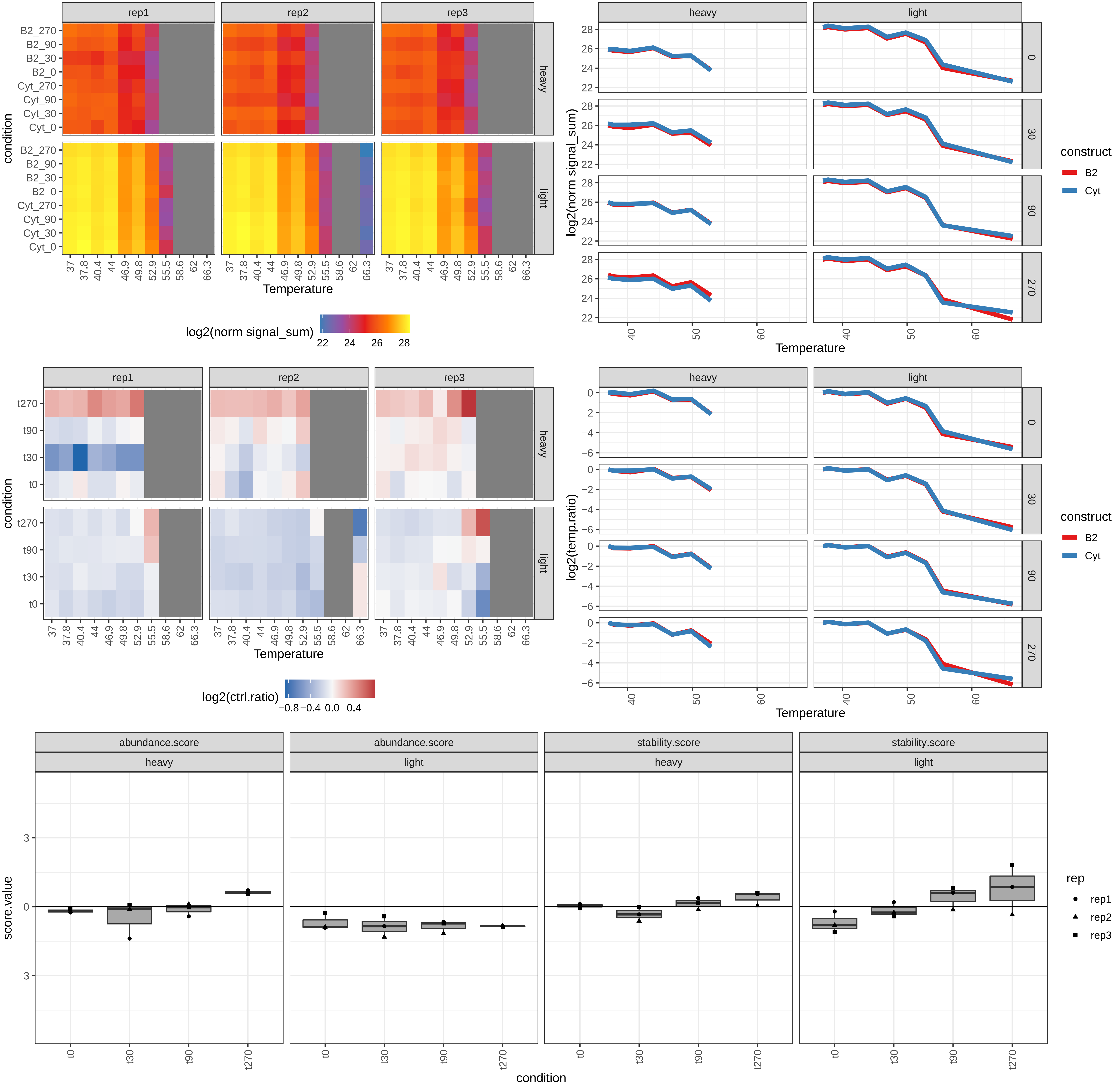

P06738 − Glycogen phosphorylase OS=Saccharomyces cerevisiae (strain ATCC 204508 / S288c) GN=GPH1 PE=1 SV=4

GPI10 − average.top3: 6.41587625 − max.qupm: 4

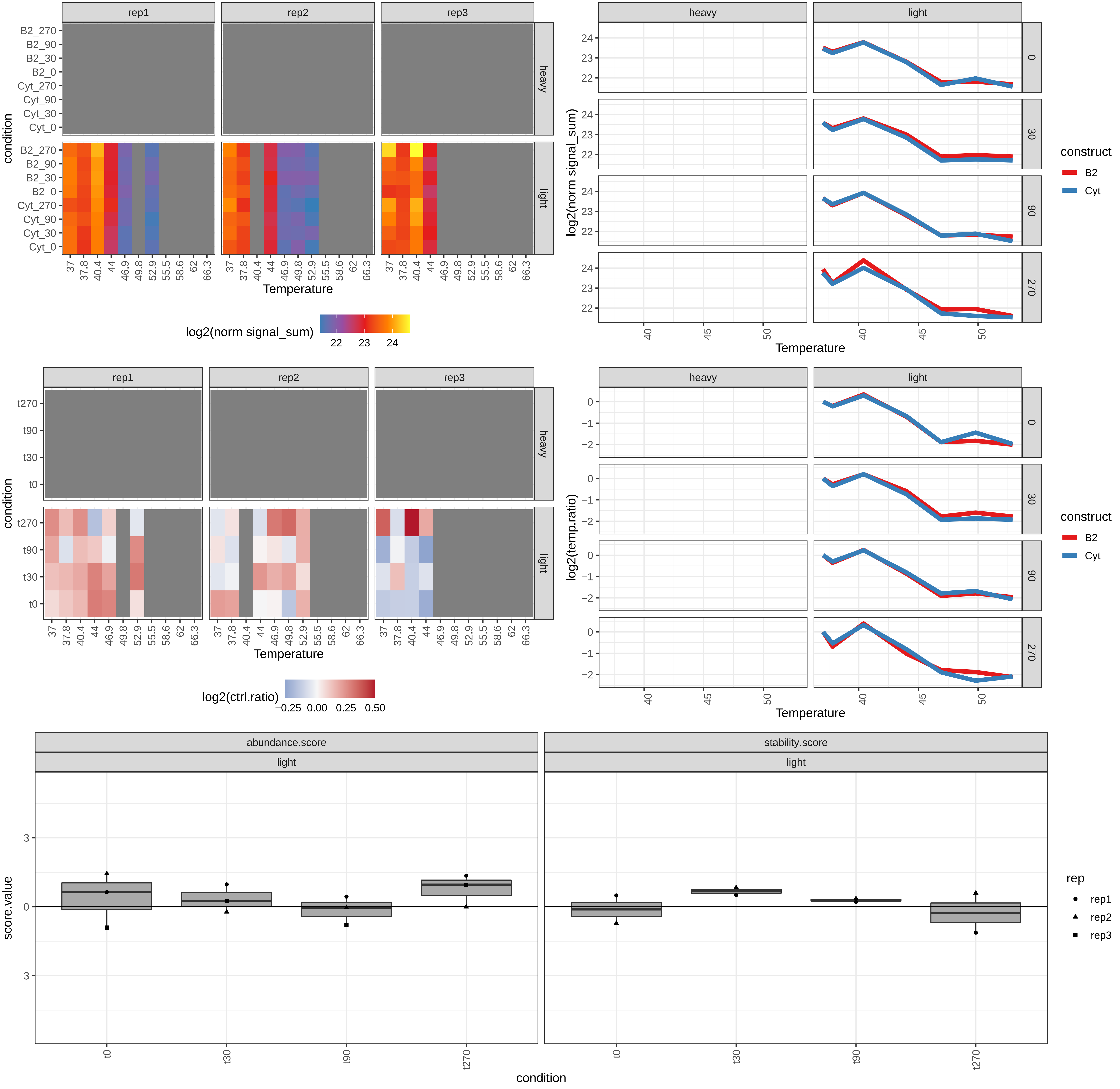

P30777 − GPI mannosyltransferase 3 OS=Saccharomyces cerevisiae (strain ATCC 204508 / S288c) GN=GPI10 PE=1 SV=2

GPI12 − average.top3: 6.93310761904762 − max.qupm: 5

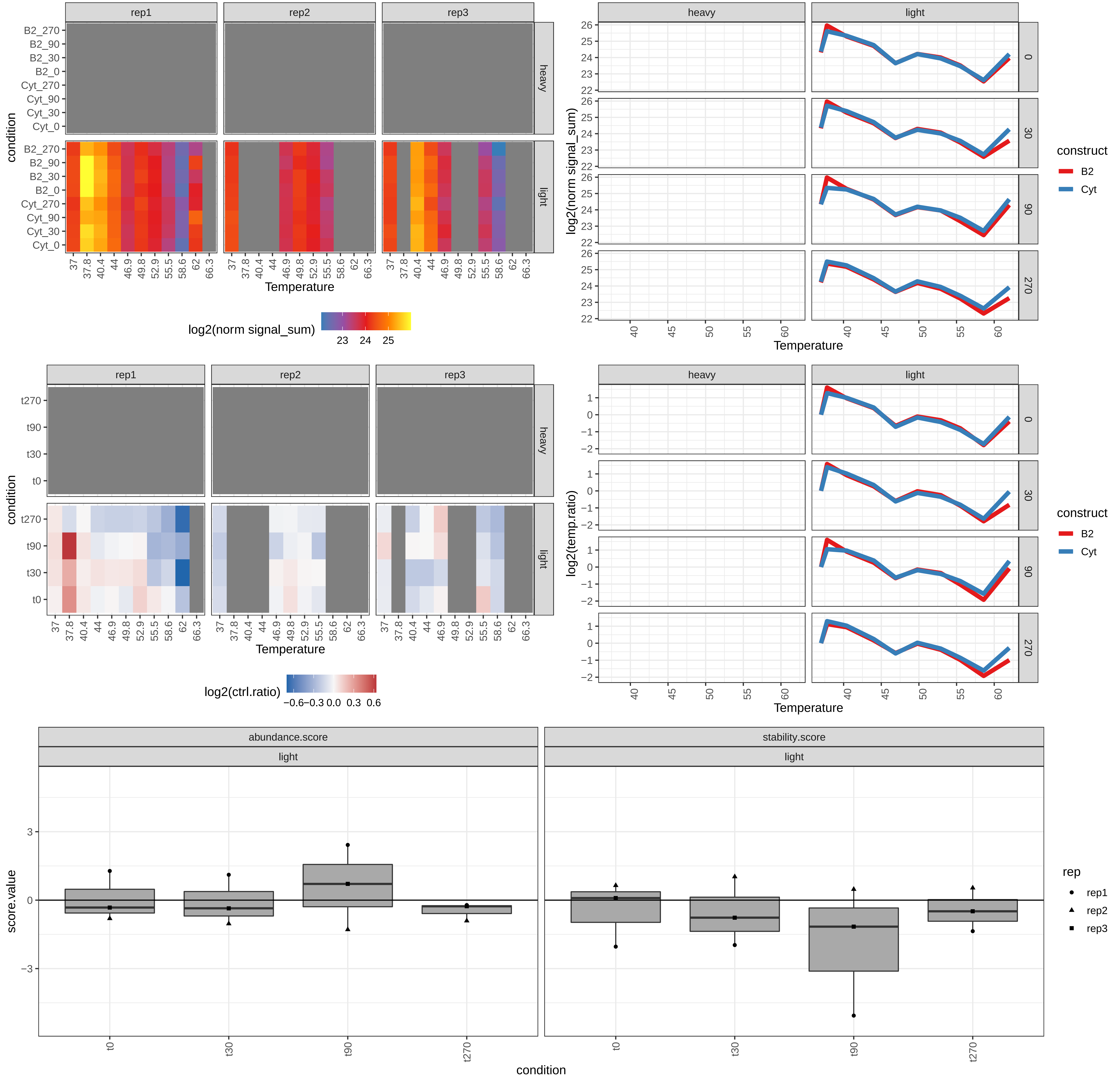

P23797 − N−acetylglucosaminyl−phosphatidylinositol de−N−acetylase OS=Saccharomyces cerevisiae (strain ATCC 204508 / S288c) GN=GPI12 PE=1 SV=2

GPI16 − average.top3: 7.049096 − max.qupm: 8

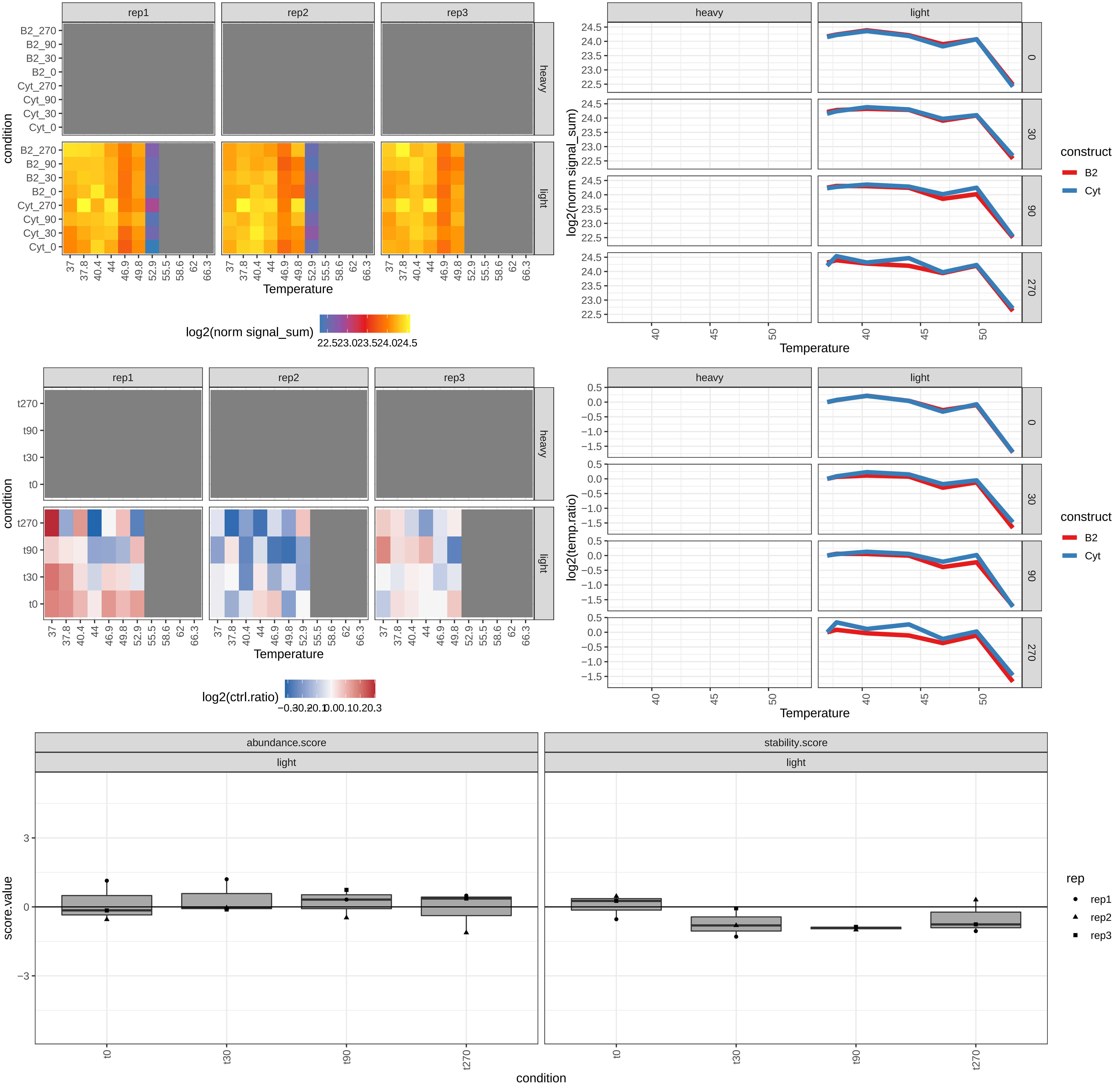

P38875 − GPI transamidase component GPI16 OS=Saccharomyces cerevisiae (strain ATCC 204508 / S288c) GN=GPI16 PE=1 SV=2

GPI17 − average.top3: 5.998655 − max.qupm: 4

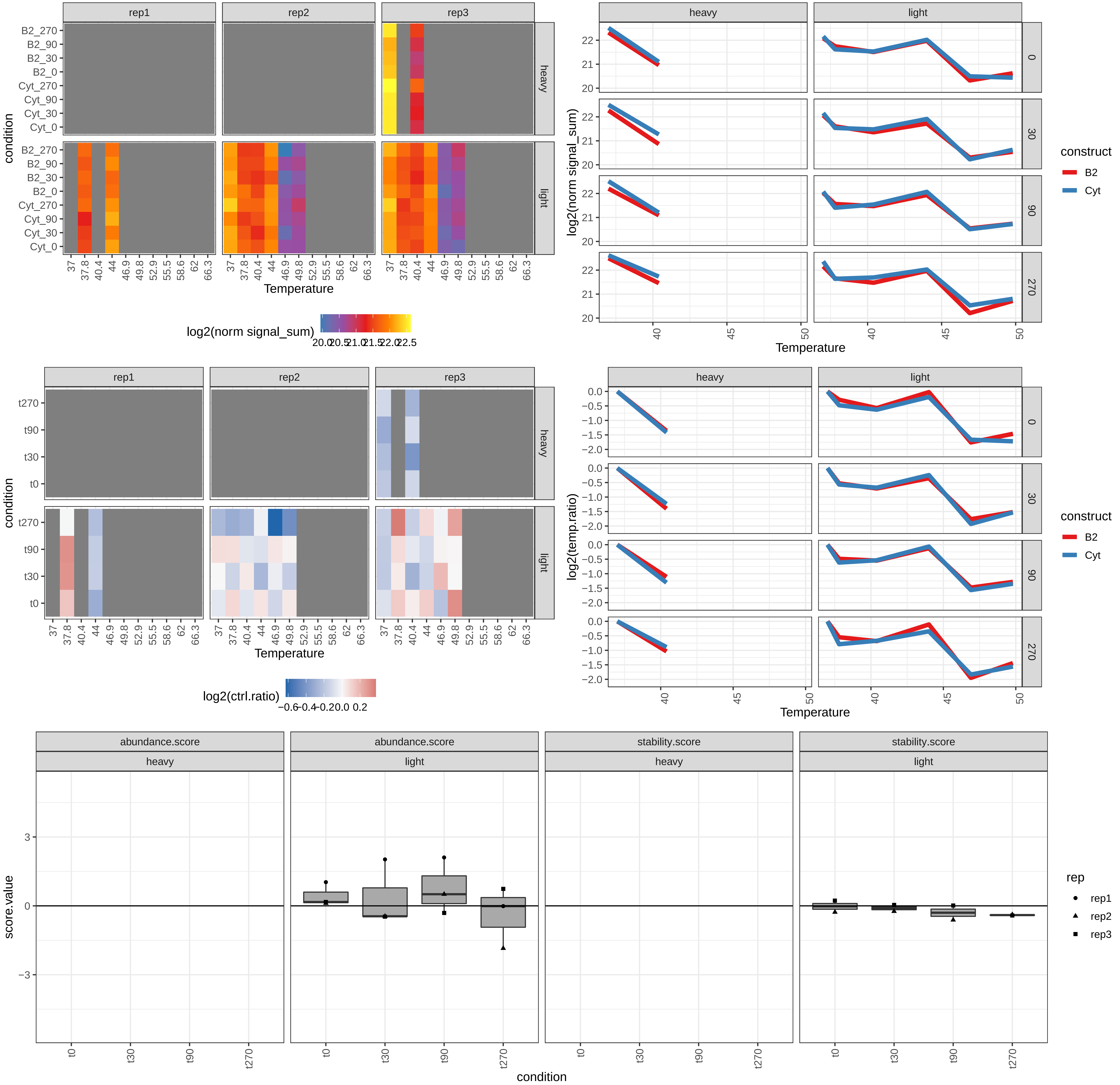

Q04080 − GPI transamidase component GPI17 OS=Saccharomyces cerevisiae (strain ATCC 204508 / S288c) GN=GPI17 PE=1 SV=1

GPM1 − average.top3: 8.21054818181818 − max.qupm: 13

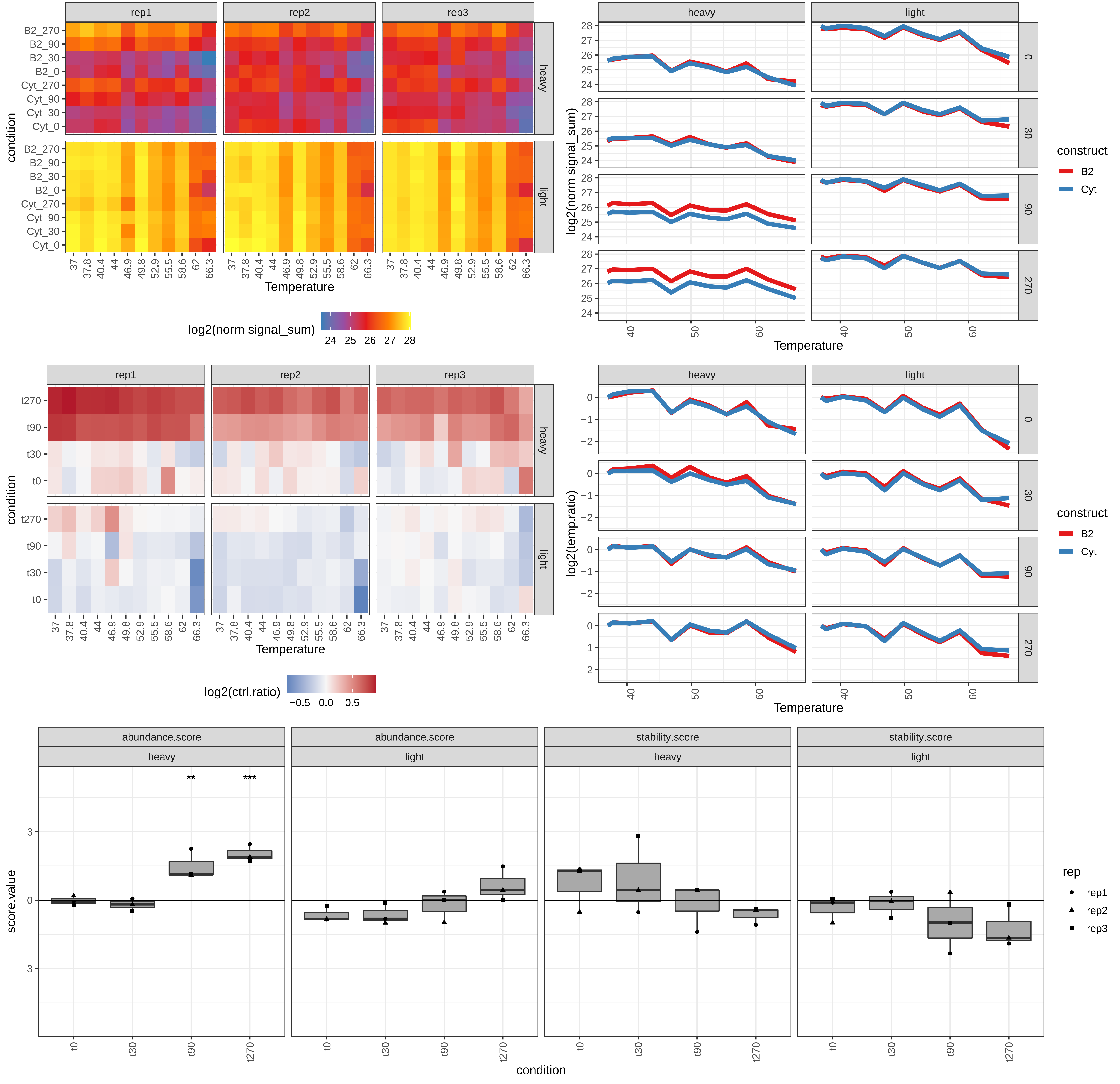

P00950 − Phosphoglycerate mutase 1 OS=Saccharomyces cerevisiae (strain ATCC 204508 / S288c) GN=GPM1 PE=1 SV=3

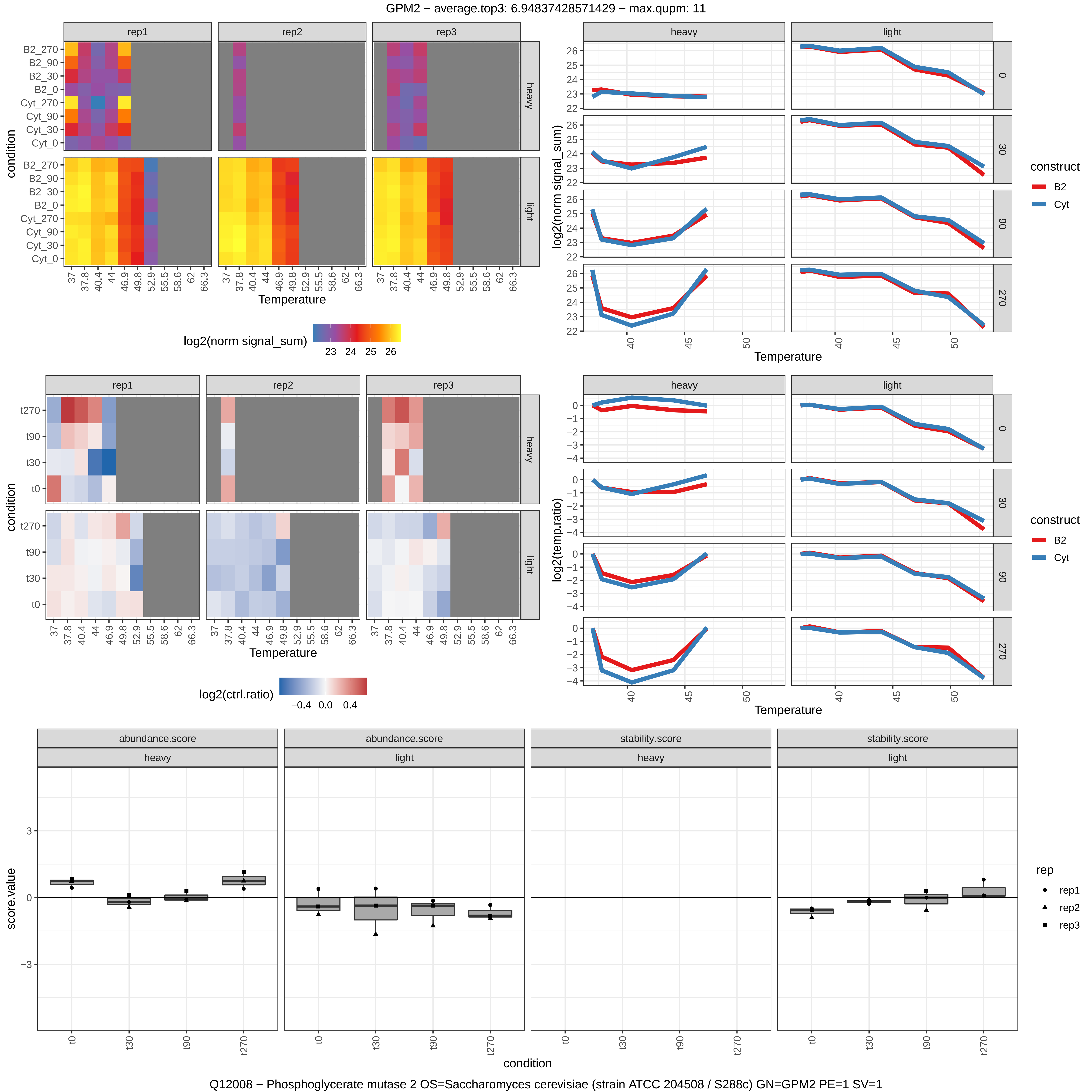
GPM2 − average.top3: 6.94837428571429 − max.qupm: 11
rep1
rep2
rep3
heavy
light
condition
Temperature
log2(norm signal_sum)
log2(norm signal_sum)
construct
B2
Cyt
log2(ctrl.ratio)
log2(temp.ratio)
abundance.score
stability.score
score.value
rep
rep1
rep2
rep3
condition
Q12008 − Phosphoglycerate mutase 2 OS=Saccharomyces cerevisiae (strain ATCC 204508 / S288c) GN=GPM2 PE=1 SV=1

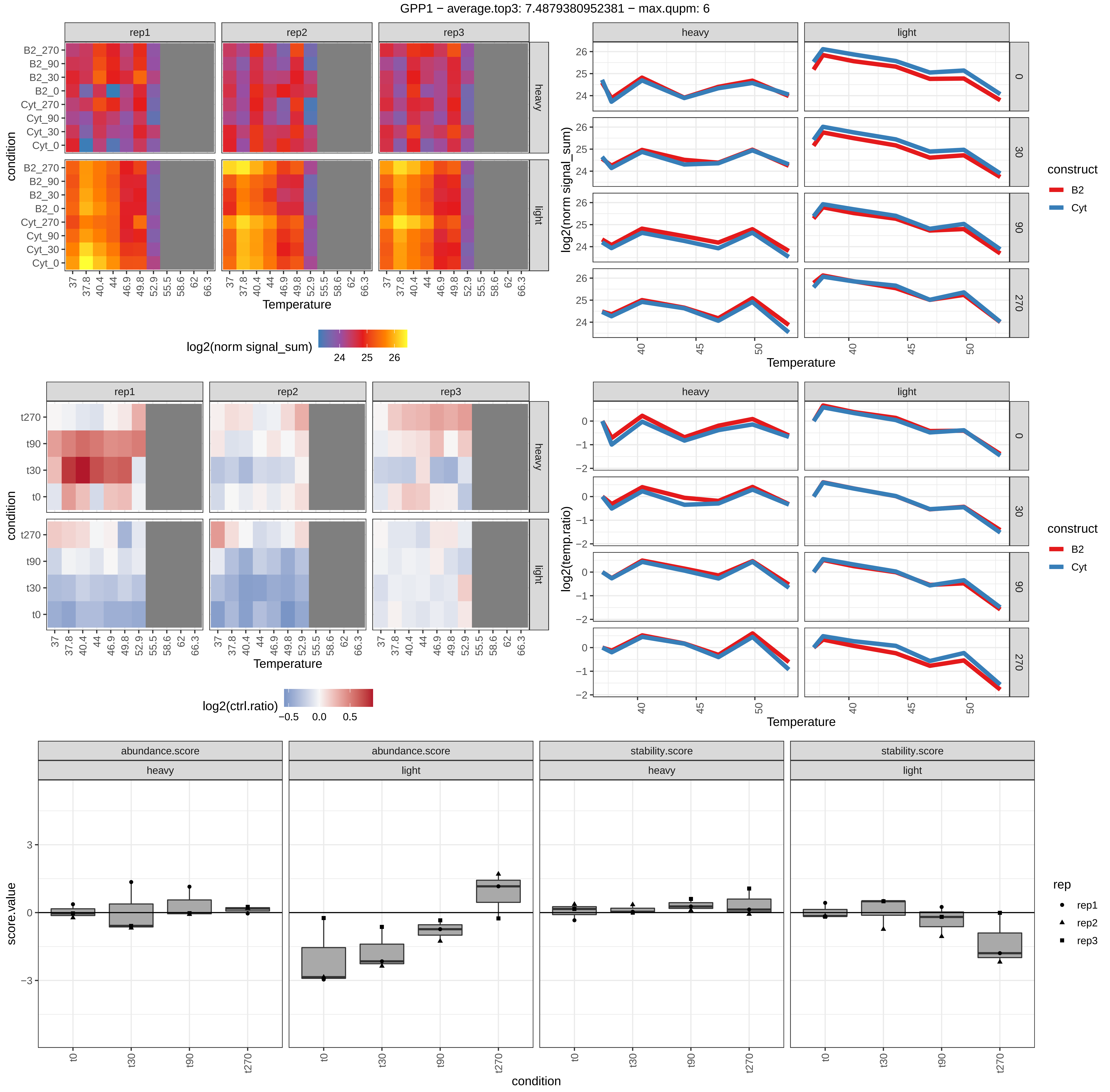

P41277 − Glycerol−1−phosphate phosphohydrolase 1 OS=Saccharomyces cerevisiae (strain ATCC 204508 / S288c) GN=GPP1 PE=1 SV=3

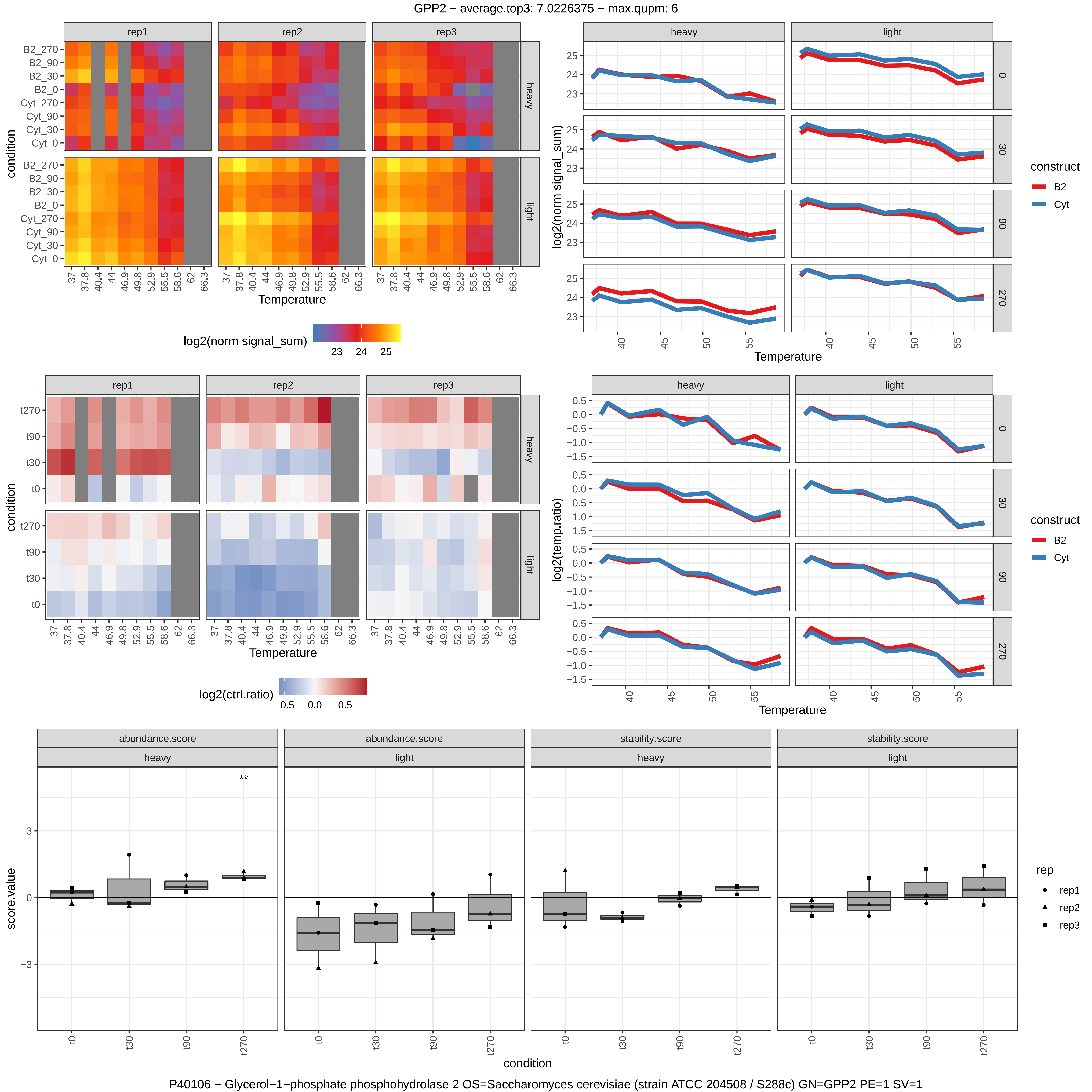
GPP2 − average.top3: 7.0226375 − max.qupm: 6
rep1
rep2
rep3
heavy
light
condition
Temperature
log2(norm signal_sum)
construct
B2
Cyt
t270
t90
t30
t0
log2(temp.ratio)
log2(ctrl.ratio)
abundance.score
stability.score
score.value
rep
rep1
rep2
rep3
P40106 − Glycerol−1−phosphate phosphohydrolase 2 OS=Saccharomyces cerevisiae (strain ATCC 204508 / S288c) GN=GPP2 PE=1 SV=1

GPT2 − average.top3: 6.85477147058824 − max.qupm: 15

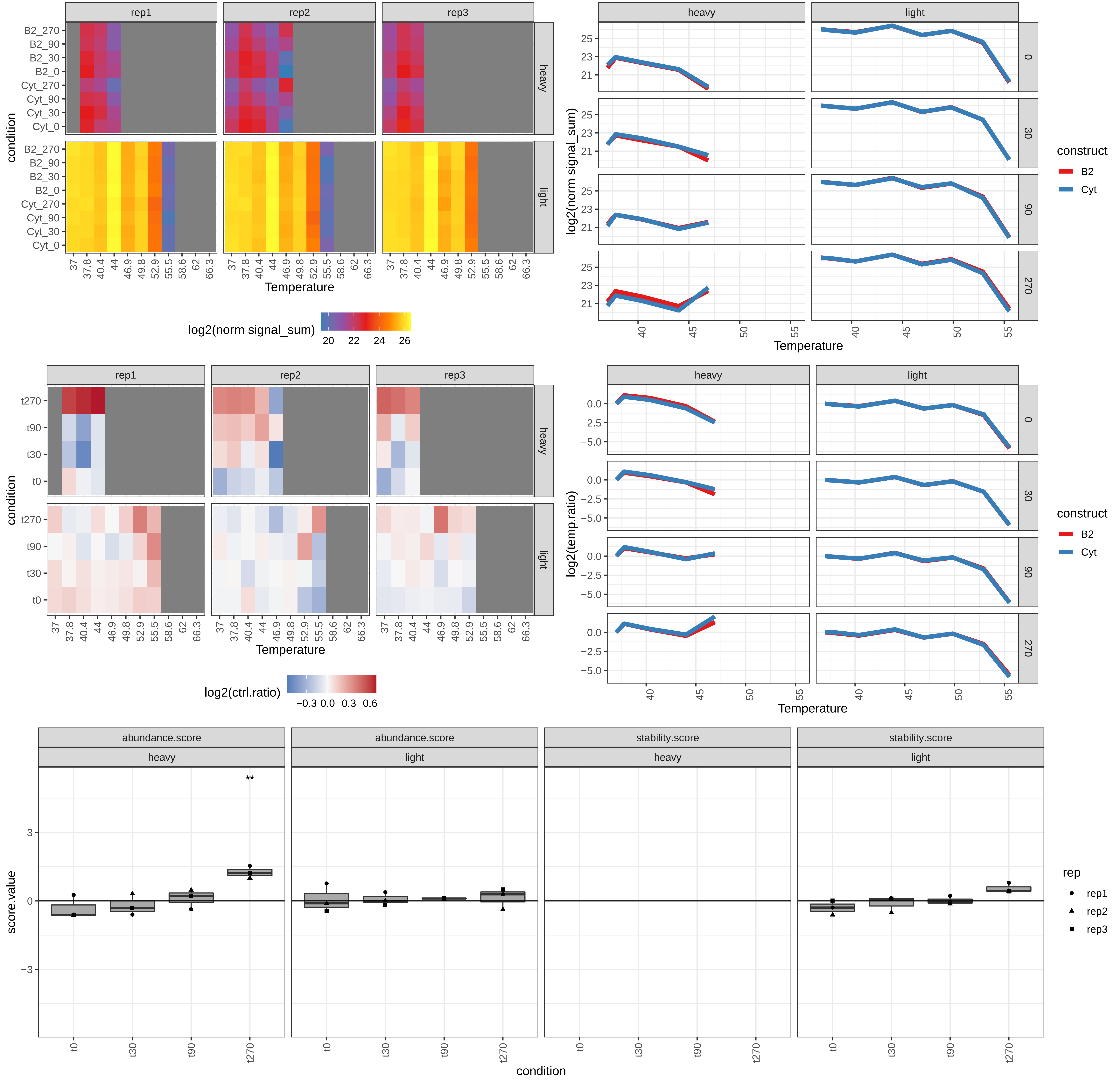

P36148 − Glycerol−3−phosphate O−acyltransferase 2 OS=Saccharomyces cerevisiae (strain ATCC 204508 / S288c) GN=GPT2 PE=1 SV=1

GPX1 − average.top3: 7.21987181818182 − max.qupm: 3

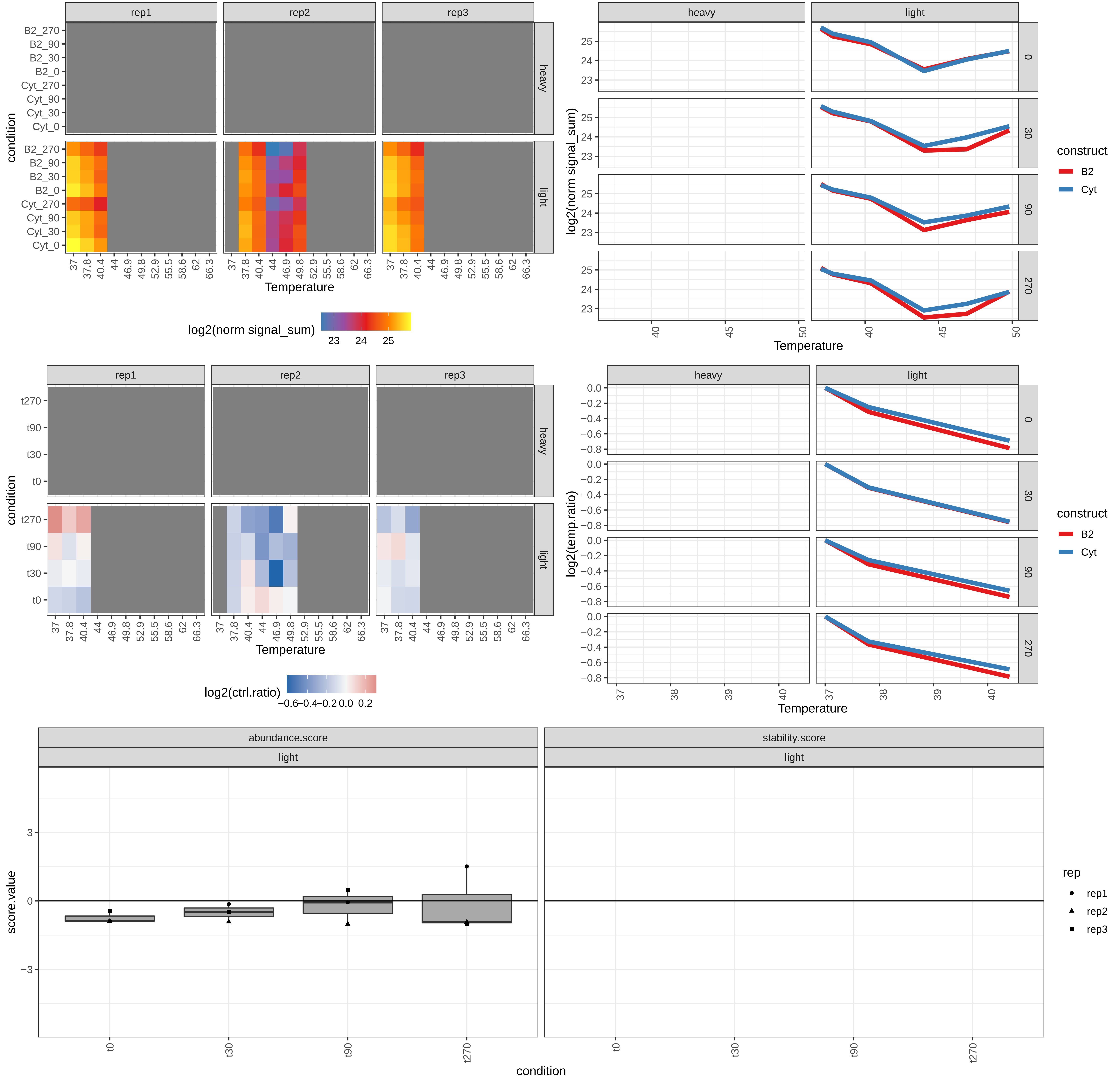

P36014 − Glutathione peroxidase 1 OS=Saccharomyces cerevisiae (strain ATCC 204508 / S288c) GN=GPX1 PE=3 SV=1

GPX2 − average.top3: 6.65698789473684 − max.qupm: 4

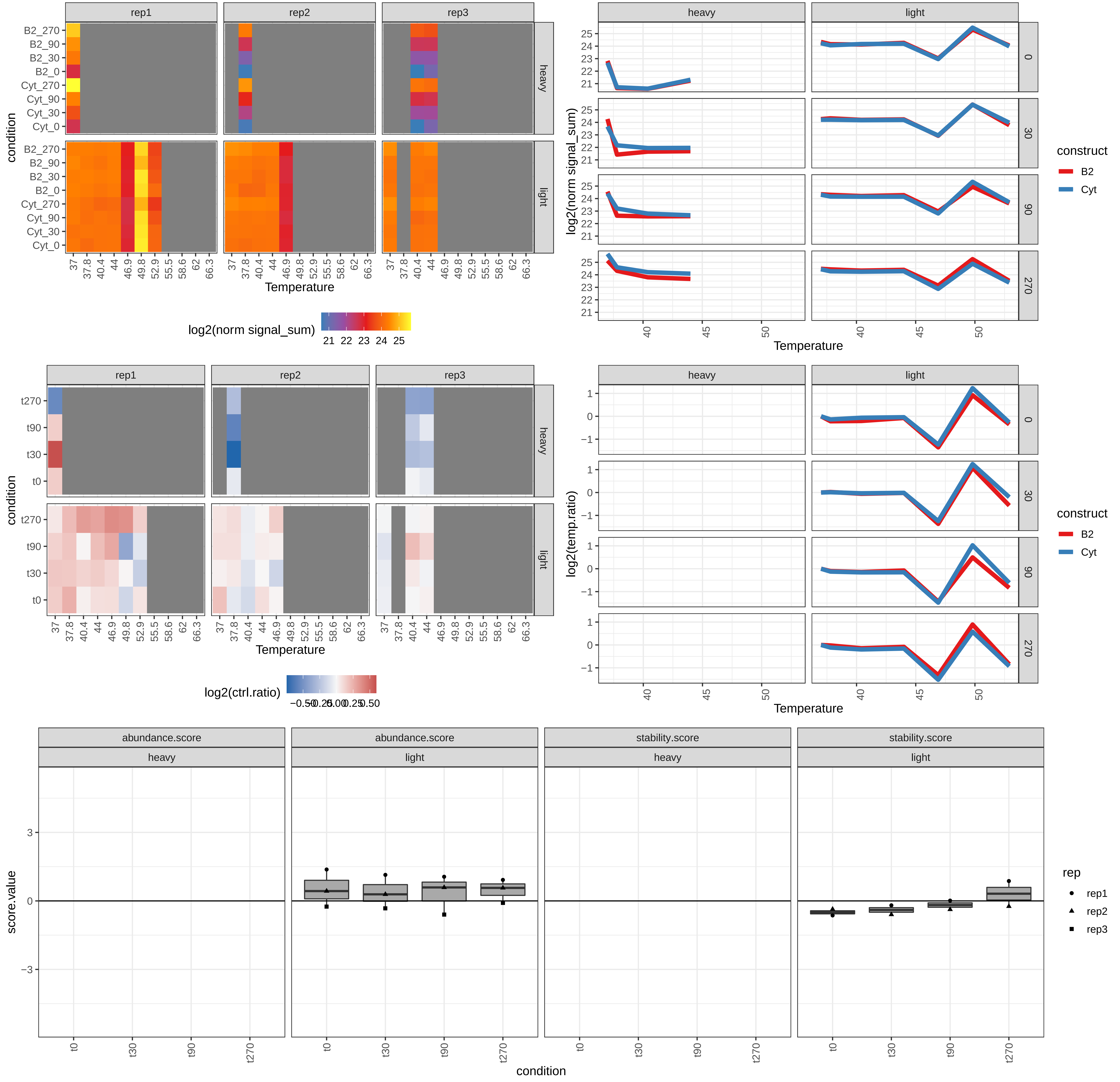

P38143 − Glutathione peroxidase 2 OS=Saccharomyces cerevisiae (strain ATCC 204508 / S288c) GN=GPX2 PE=1 SV=1

GRE1 − average.top3: 6.6501519047619 − max.qupm: 3

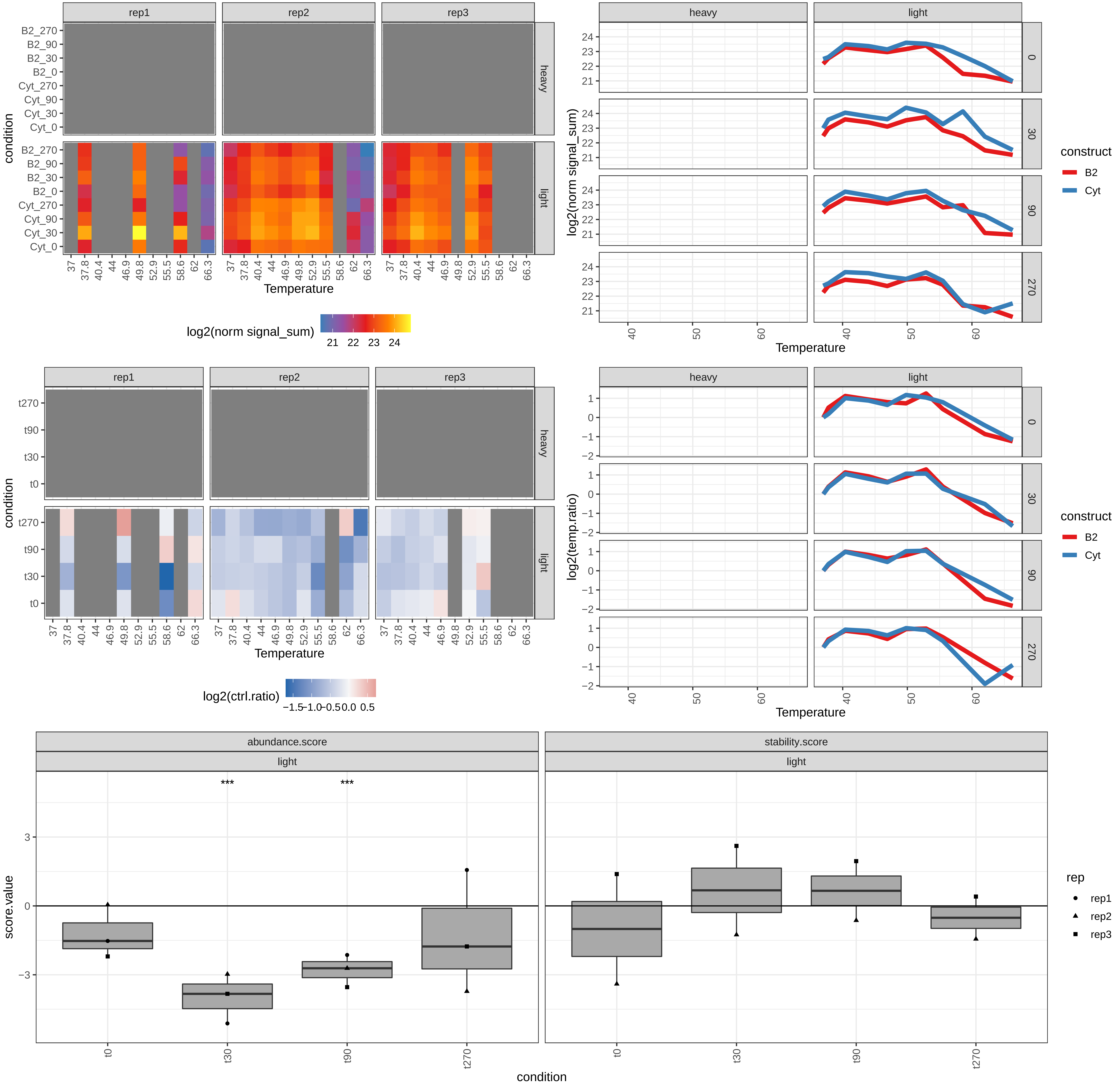

Q08969 − Protein GRE1 OS=Saccharomyces cerevisiae (strain ATCC 204508 / S288c) GN=GRE1 PE=1 SV=1

GRE2 − average.top3: 7.44694575 − max.qupm: 13

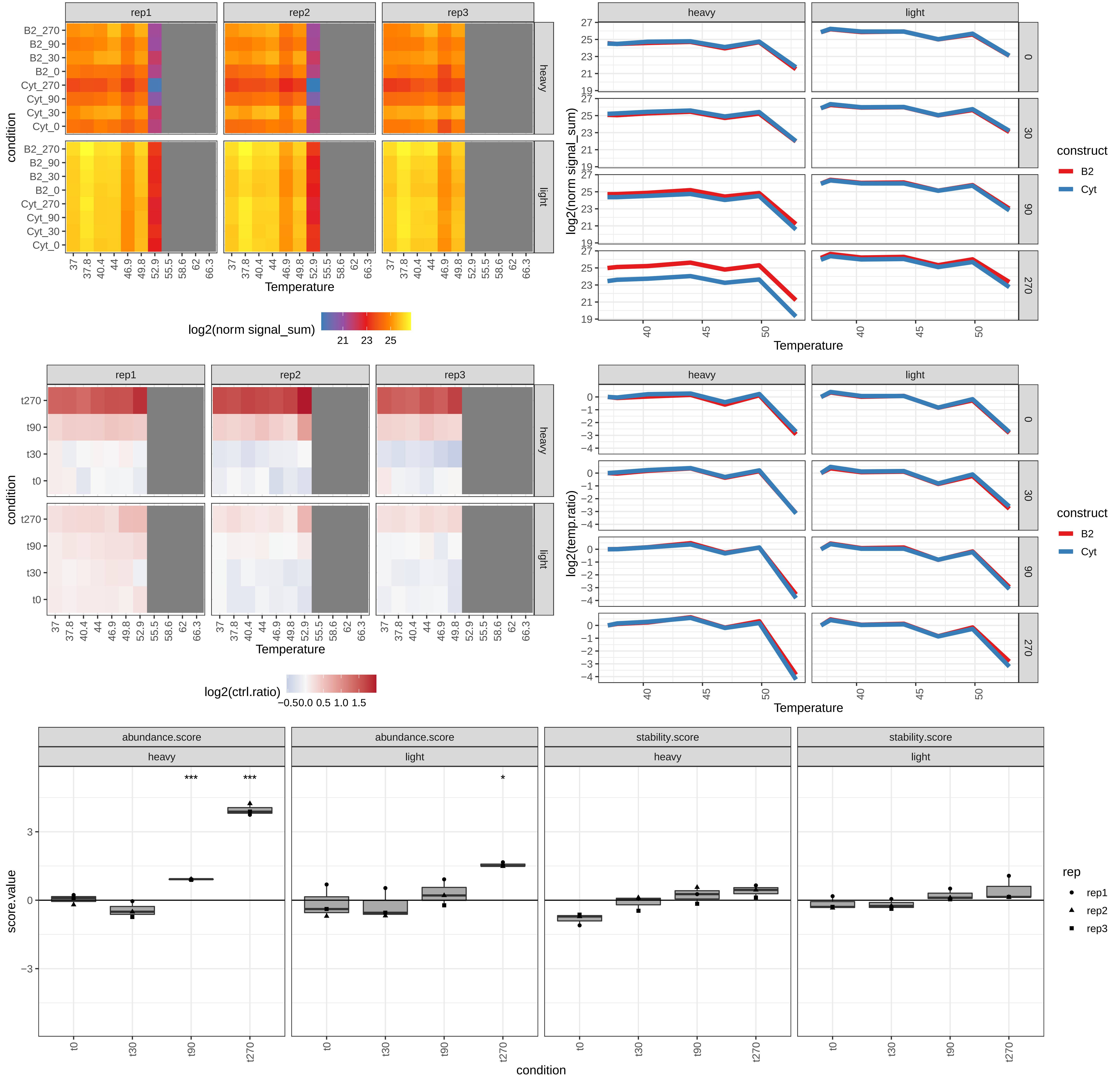

Q12068 − NADPH−dependent methylglyoxal reductase GRE2 OS=Saccharomyces cerevisiae (strain ATCC 204508 / S288c) GN=GRE2 PE=1 SV=1

GRE3 − average.top3: 7.92573806451613 − max.qupm: 21

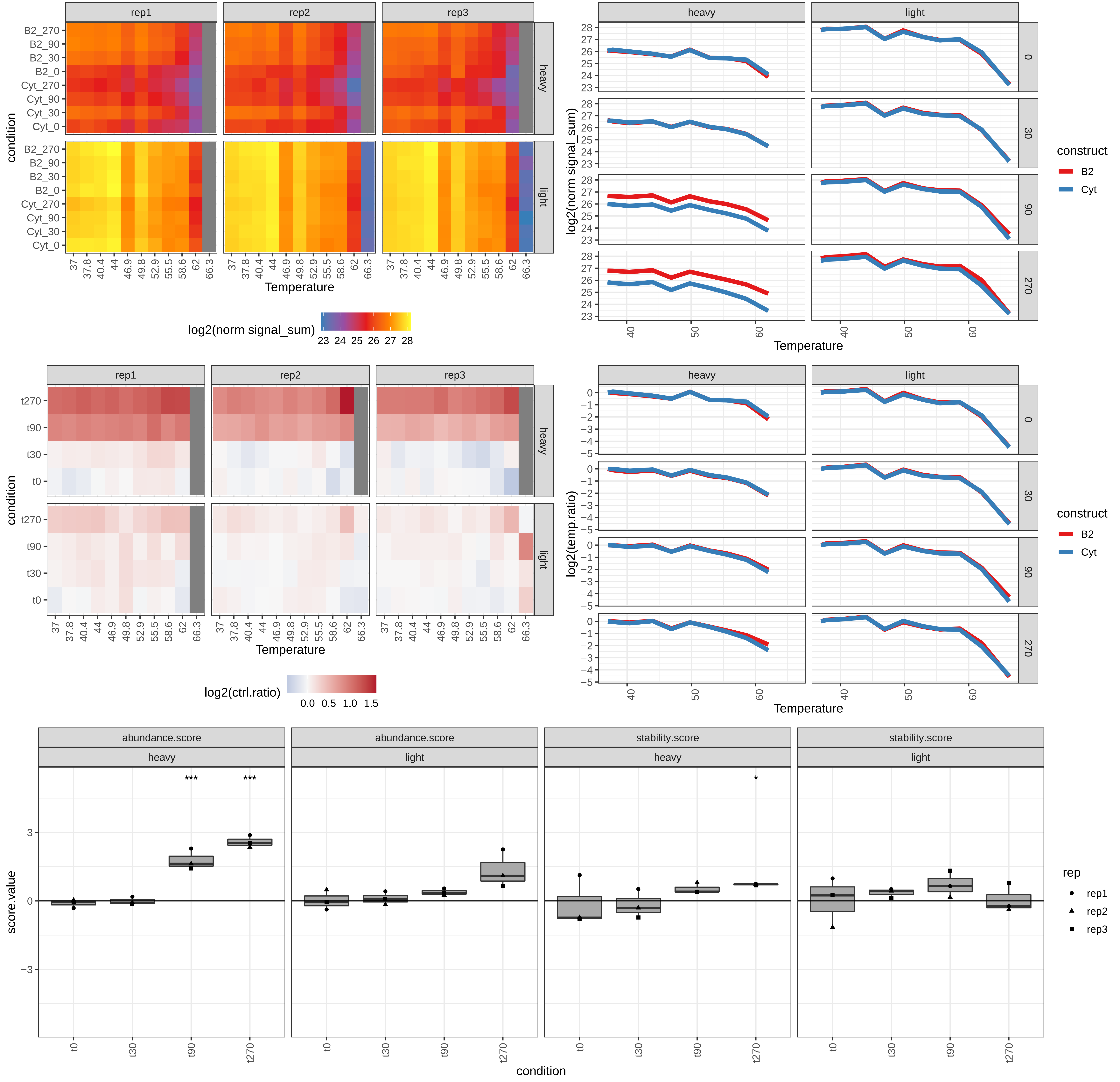

P38715 − NADPH−dependent aldose reductase GRE3 OS=Saccharomyces cerevisiae (strain ATCC 204508 / S288c) GN=GRE3 PE=1 SV=1

GRH1 − average.top3: 7.13300473684211 − max.qupm: 5

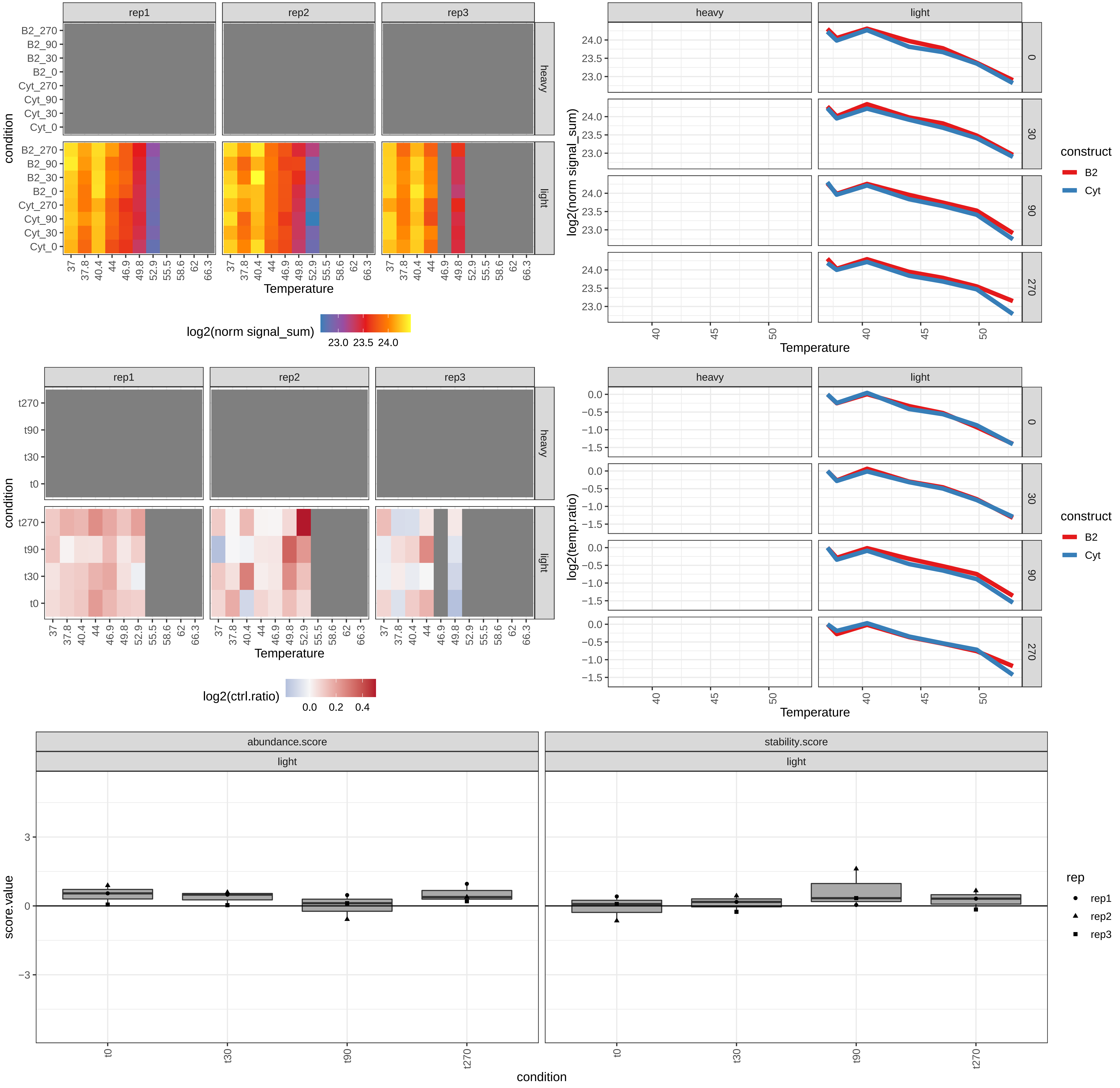

Q04410 − GRASP65 homolog protein 1 OS=Saccharomyces cerevisiae (strain ATCC 204508 / S288c) GN=GRH1 PE=1 SV=1

GRR1 − average.top3: 6.03535 − max.qupm: 4

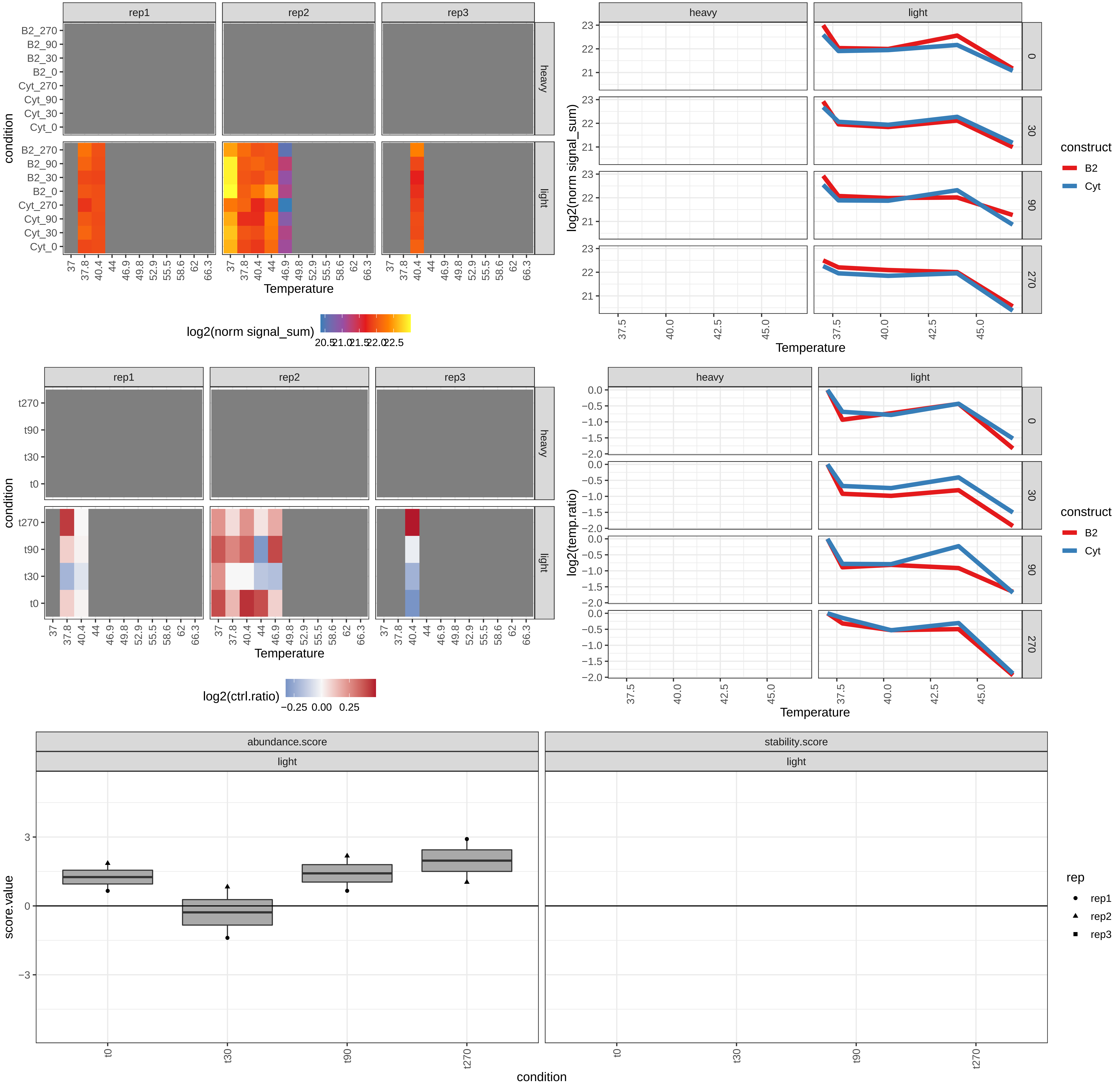

P24814 − SCF E3 ubiquitin ligase complex F−box protein GRR1 OS=Saccharomyces cerevisiae (strain ATCC 204508 / S288c) GN=GRR1 PE=1 SV=1

GRS1 − average.top3: 7.65370872727273 − max.qupm: 32

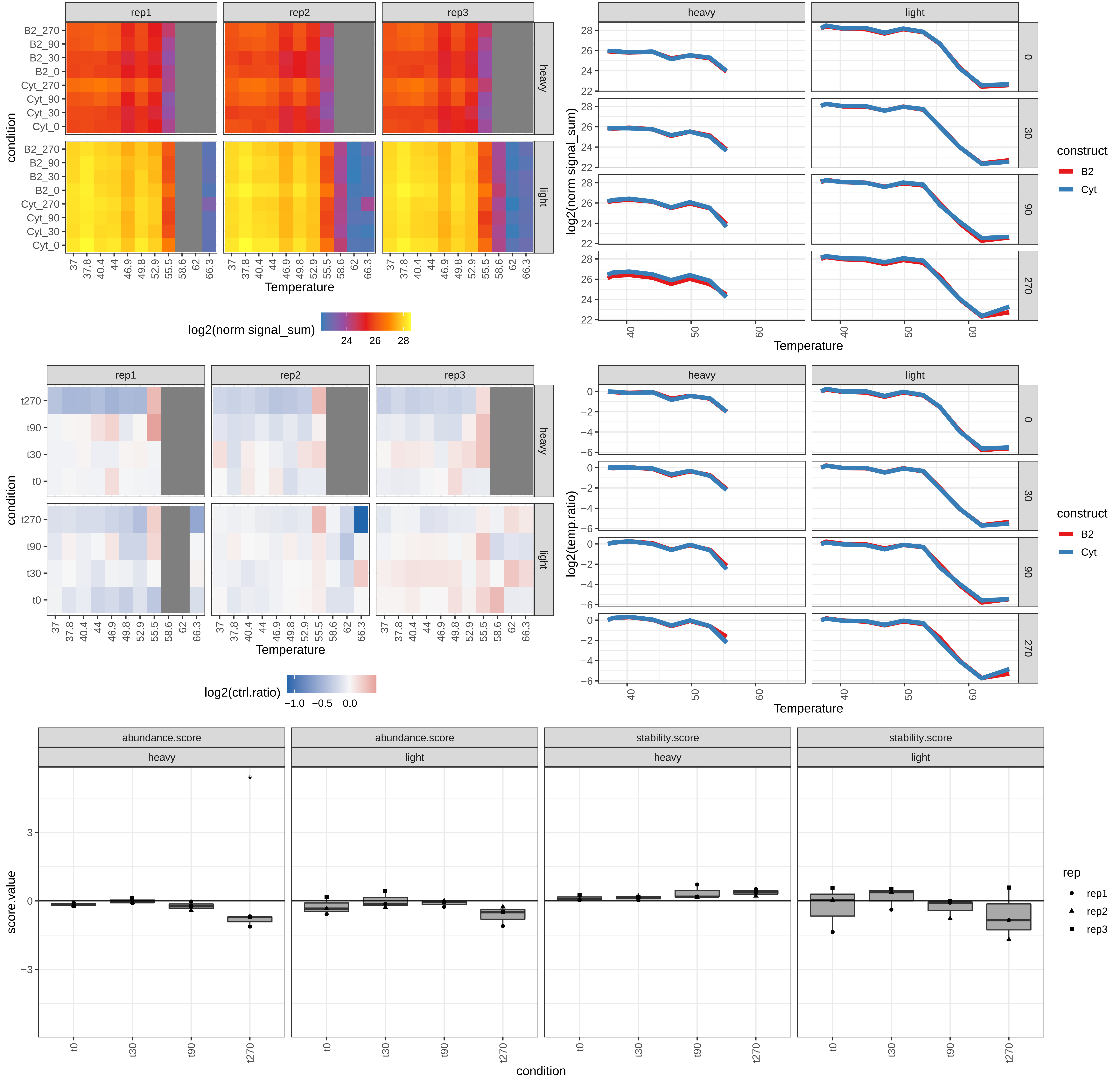

ase 1, mitochondrial OS=Saccharomyces cerevisiae (strain ATCC 204508 / S288c) GN=GRS1 PE=1 SV=3|Isoform Cytoplasmic of Glycine−−tRNA ligase 1, mitochondrial OS=Saccharomyces cerevisiae (s

GRS2 − average.top3: 6.83280615384615 − max.qupm: 11

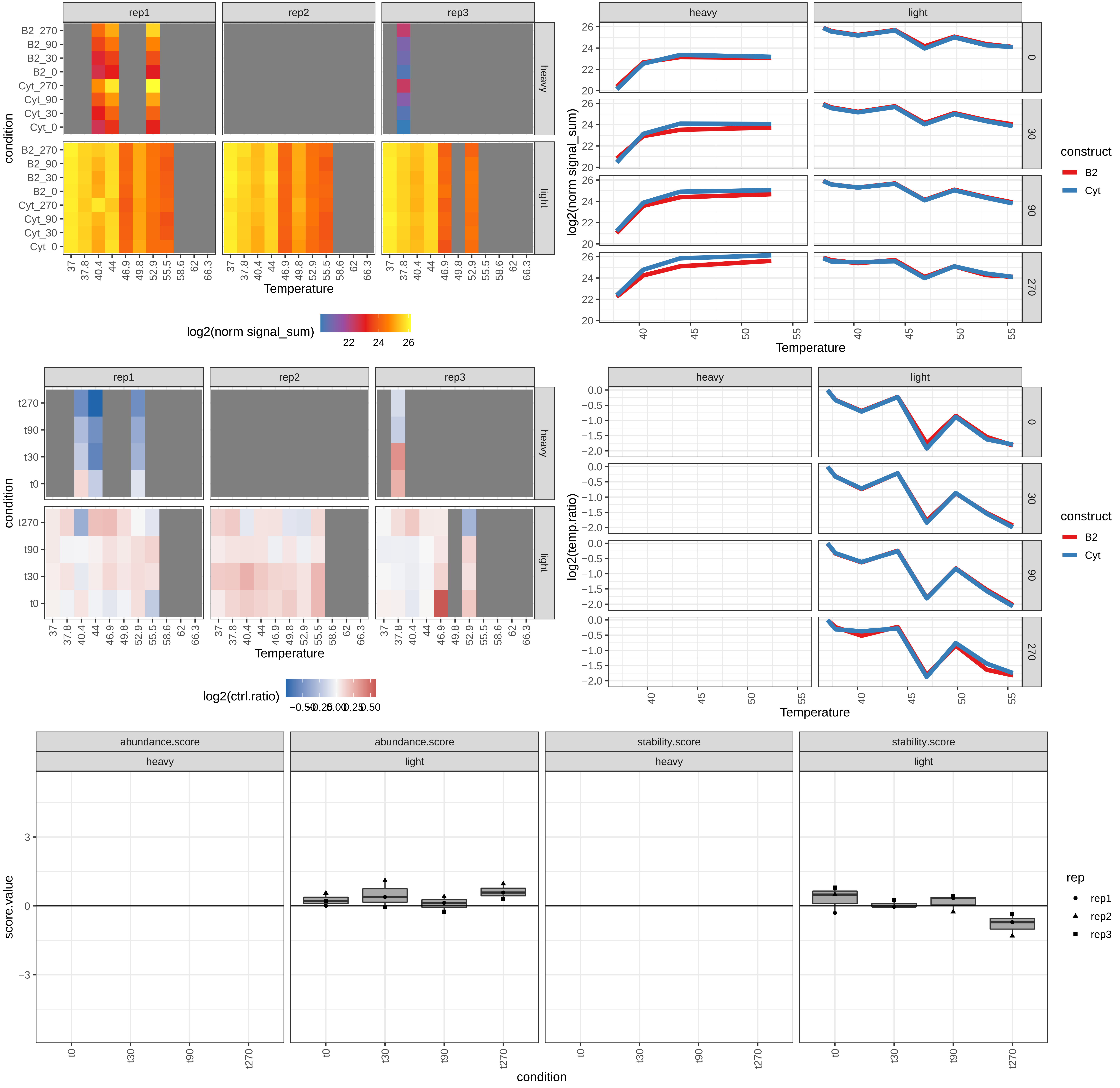

Q06817 − Glycine−−tRNA ligase 2 OS=Saccharomyces cerevisiae (strain ATCC 204508 / S288c) GN=GRS2 PE=1 SV=1

GRX1 − average.top3: 7.32209754385965 − max.qupm: 8

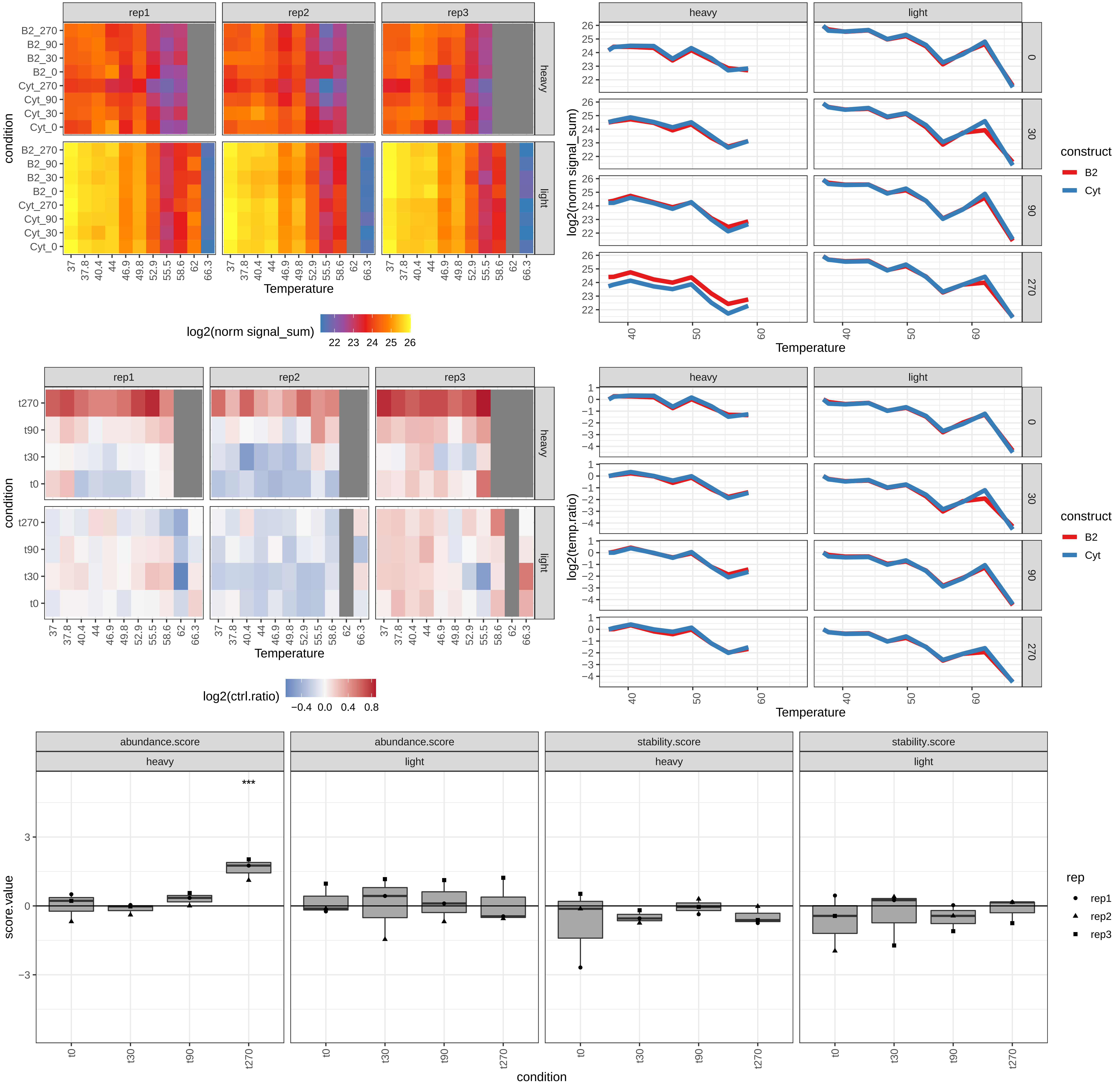

P25373 − Glutaredoxin−1 OS=Saccharomyces cerevisiae (strain ATCC 204508 / S288c) GN=GRX1 PE=1 SV=1

GRX2 − average.top3: 6.93212107142857 − max.qupm: 6

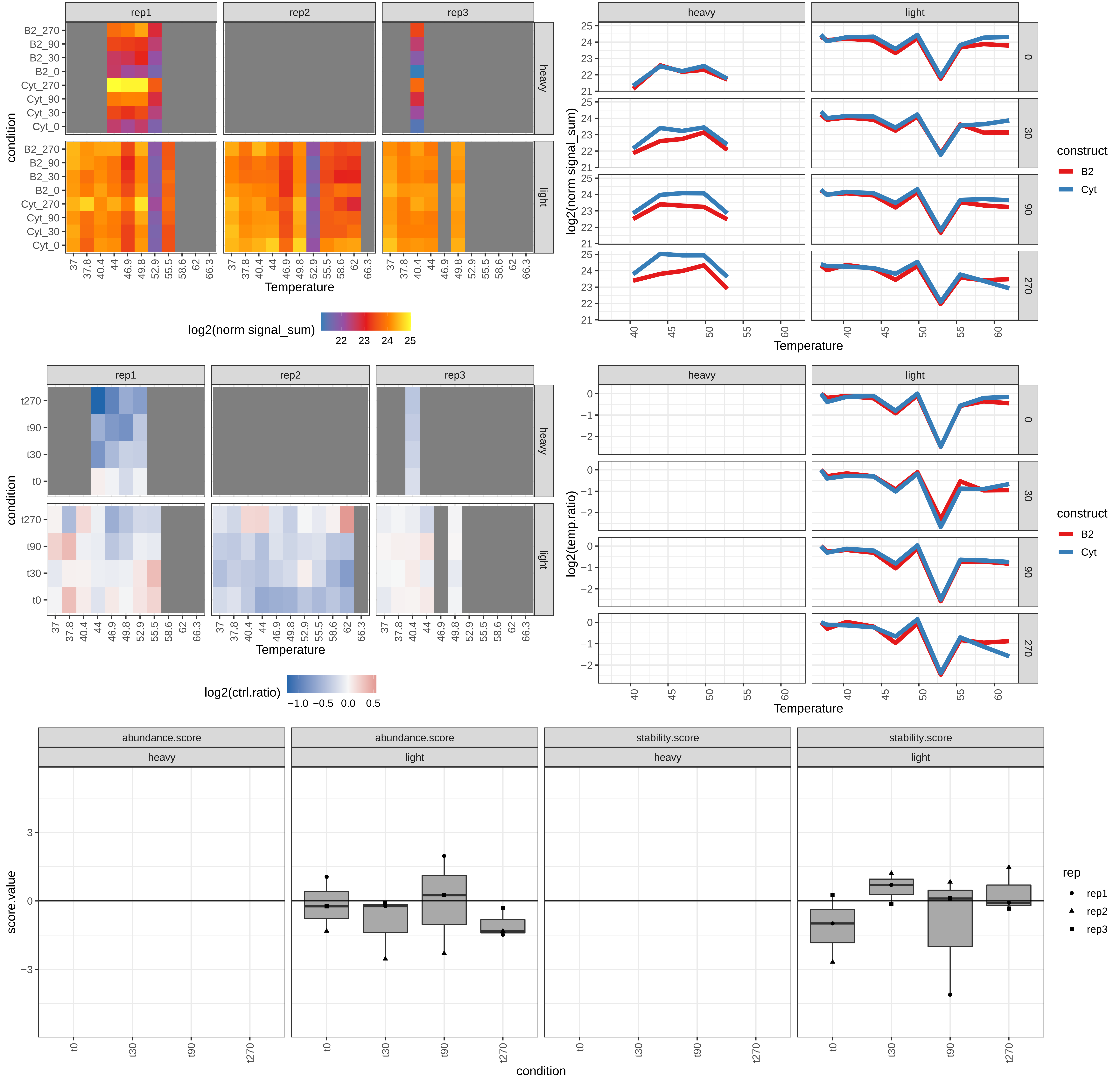

P17695 − Glutaredoxin−2, mitochondrial OS=Saccharomyces cerevisiae (strain ATCC 204508 / S288c) GN=GRX2 PE=1 SV=3

GRX3 − average.top3: 6.88816725 − max.qupm: 4

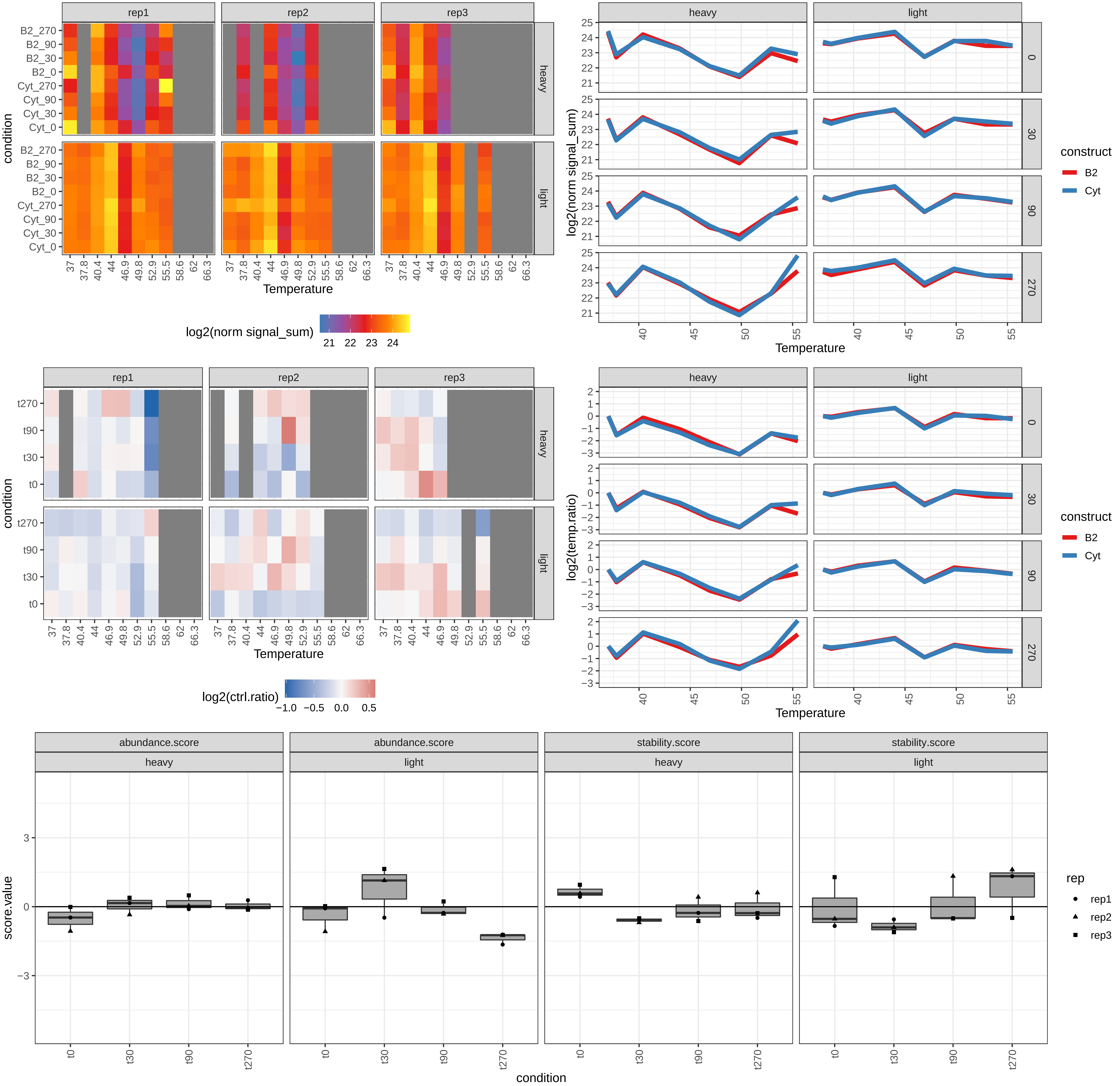

Q03835 − Monothiol glutaredoxin−3 OS=Saccharomyces cerevisiae (strain ATCC 204508 / S288c) GN=GRX3 PE=1 SV=2

GRX7 − average.top3: 6.76410266666667 − max.qupm: 7

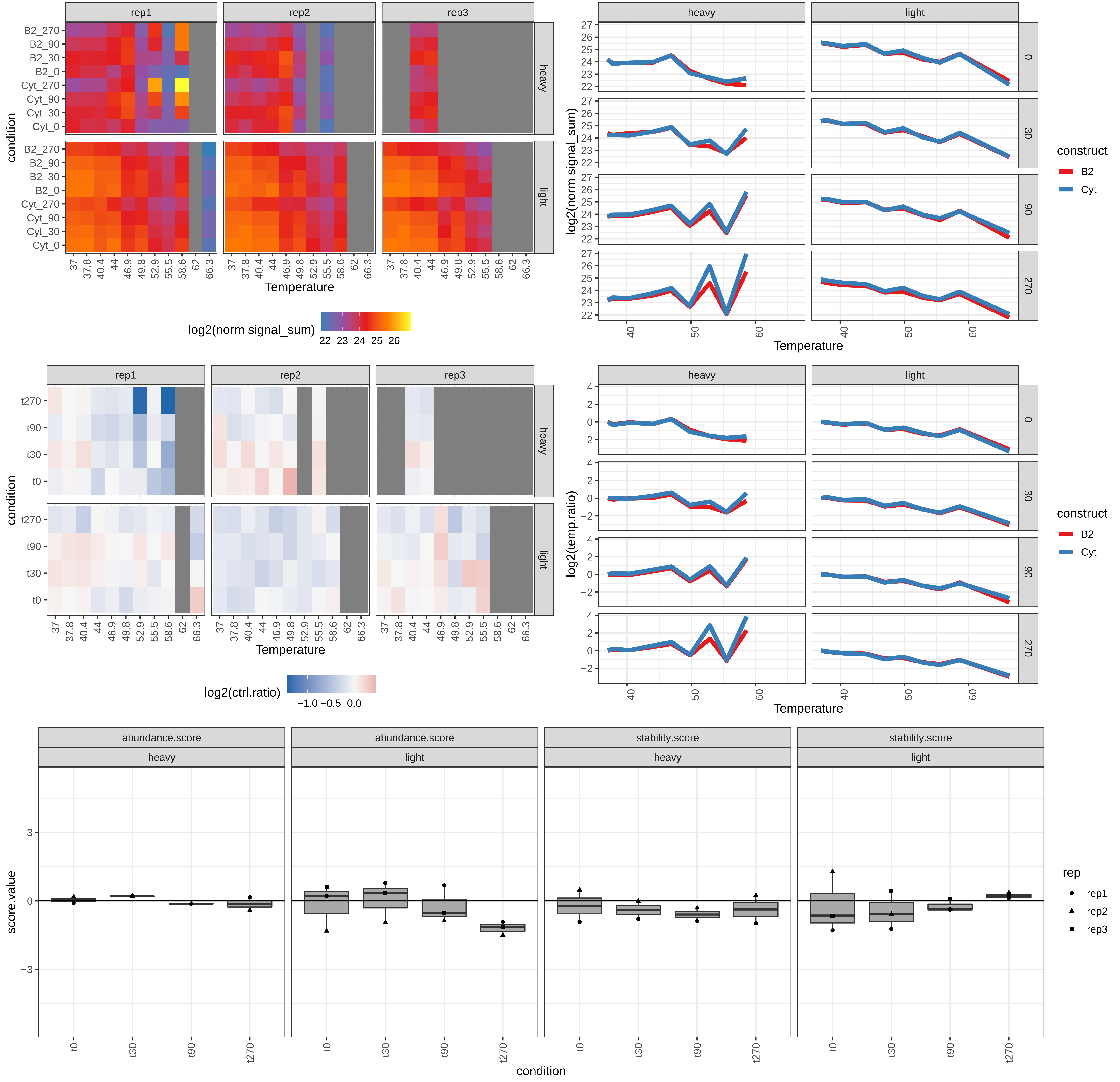

P38068 − Monothiol glutaredoxin−7 OS=Saccharomyces cerevisiae (strain ATCC 204508 / S288c) GN=GRX7 PE=1 SV=1

GRX8 − average.top3: 6.8960976 − max.qupm: 4

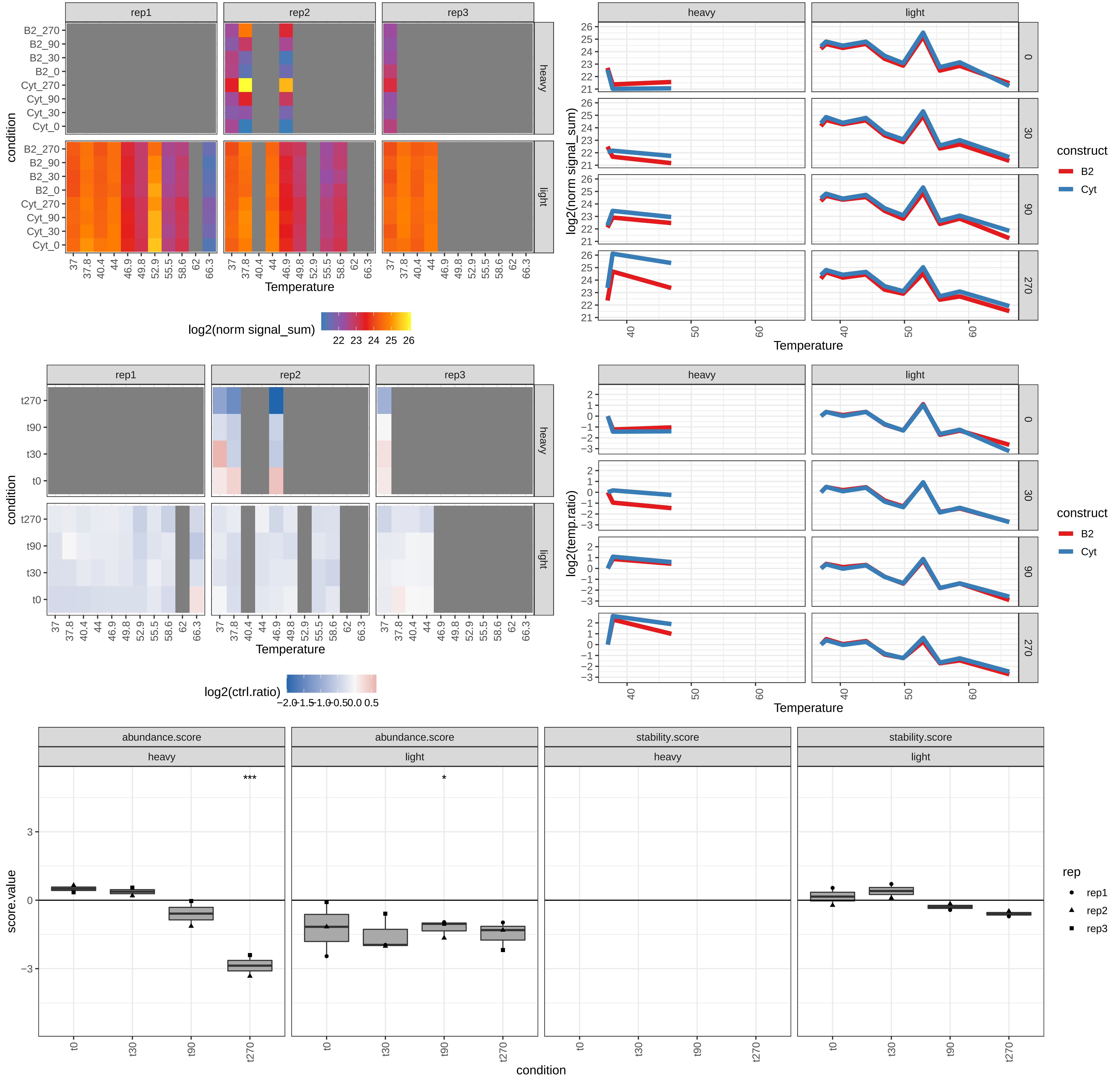

Q05926 − Glutaredoxin−8 OS=Saccharomyces cerevisiae (strain ATCC 204508 / S288c) GN=GRX8 PE=1 SV=1

GSC2 − average.top3: 7.09596720930233 − max.qupm: 13

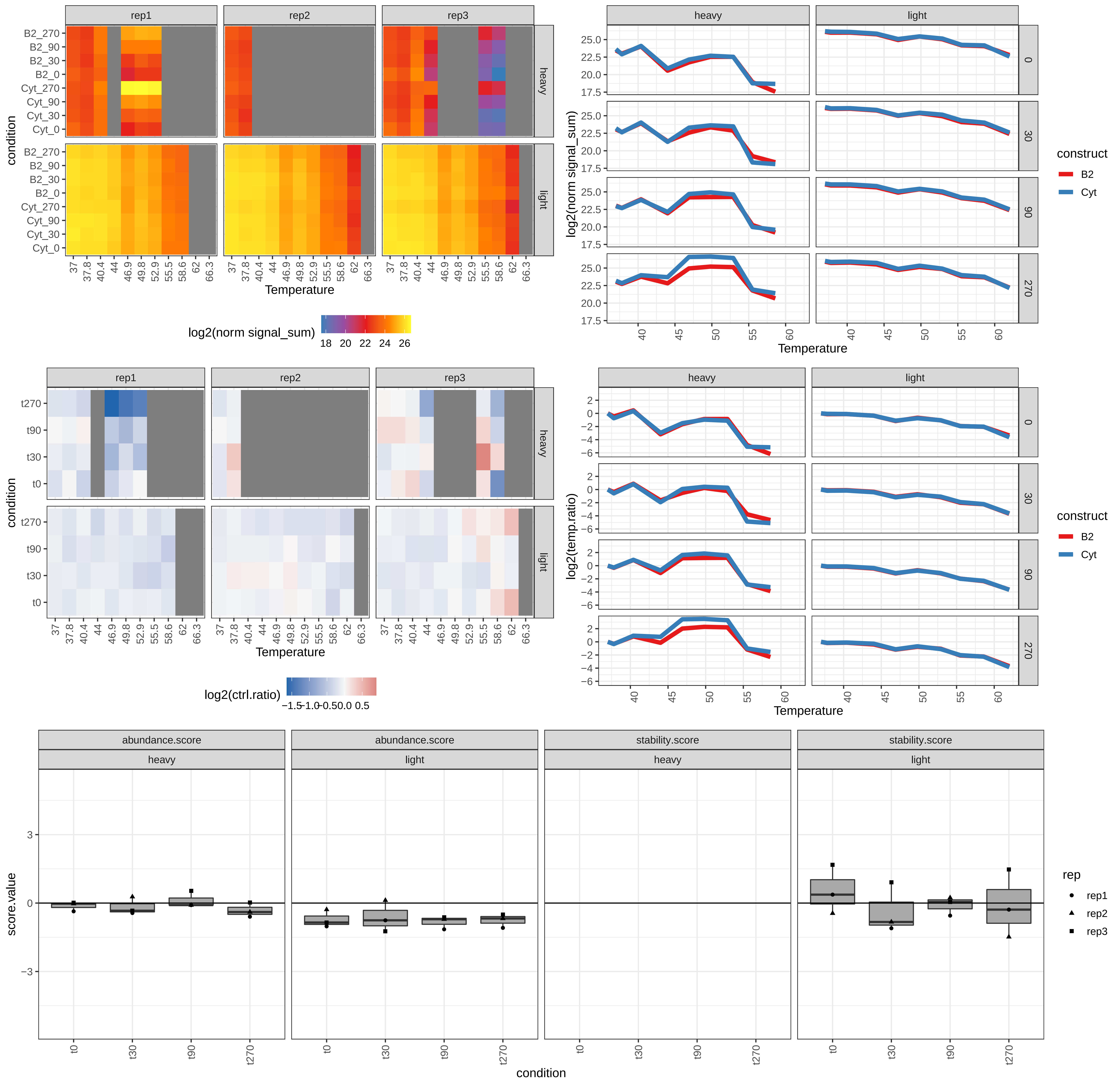

P40989 − 1,3−beta−glucan synthase component GSC2 OS=Saccharomyces cerevisiae (strain ATCC 204508 / S288c) GN=GSC2 PE=1 SV=2

GSF2 − average.top3: 7.19638941176471 − max.qupm: 9

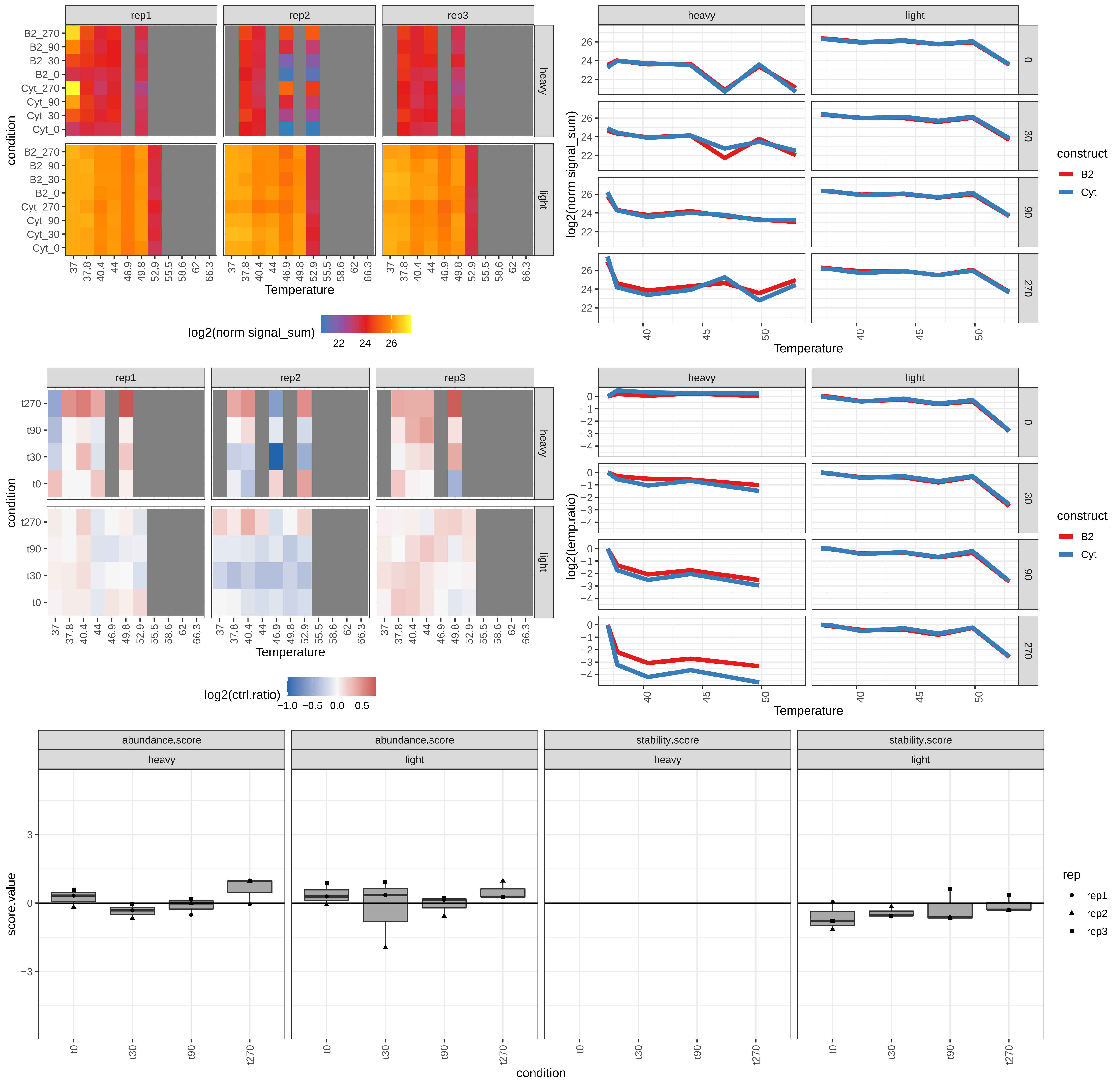

Q04697 − Glucose−signaling factor 2 OS=Saccharomyces cerevisiae (strain ATCC 204508 / S288c) GN=GSF2 PE=1 SV=1

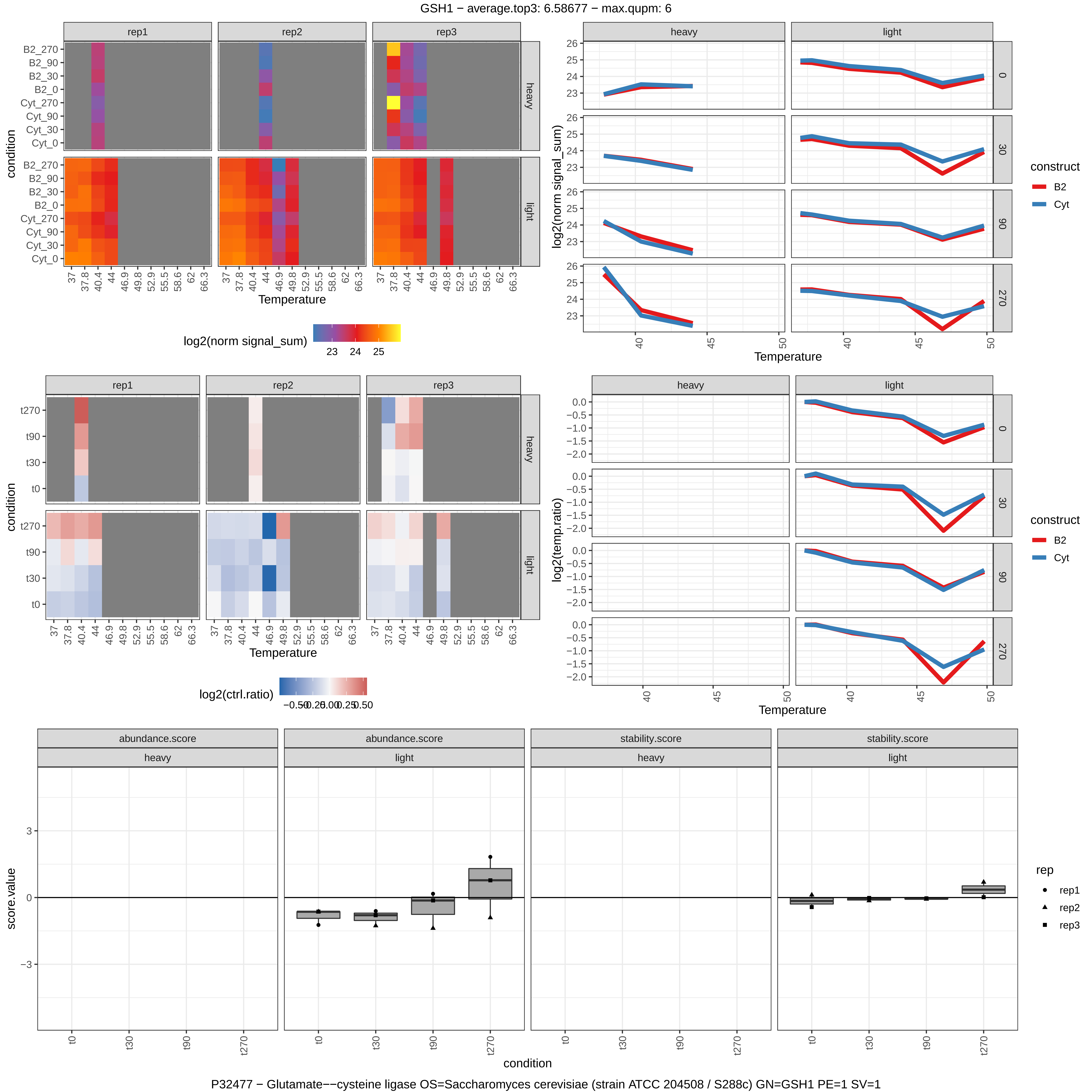

GSH1 − average.top3: 6.58677 − max.qupm: 6
rep1
rep2
rep3
heavy
light
condition
Temperature
log2(norm signal_sum)
construct
B2
Cyt
log2(ctrl.ratio)
log2(temp.ratio)
abundance.score
stability.score
score.value
rep
rep1
rep2
rep3
P32477 − Glutamate−−cysteine ligase OS=Saccharomyces cerevisiae (strain ATCC 204508 / S288c) GN=GSH1 PE=1 SV=1

GSH2 − average.top3: 7.16989571428571 − max.qupm: 16

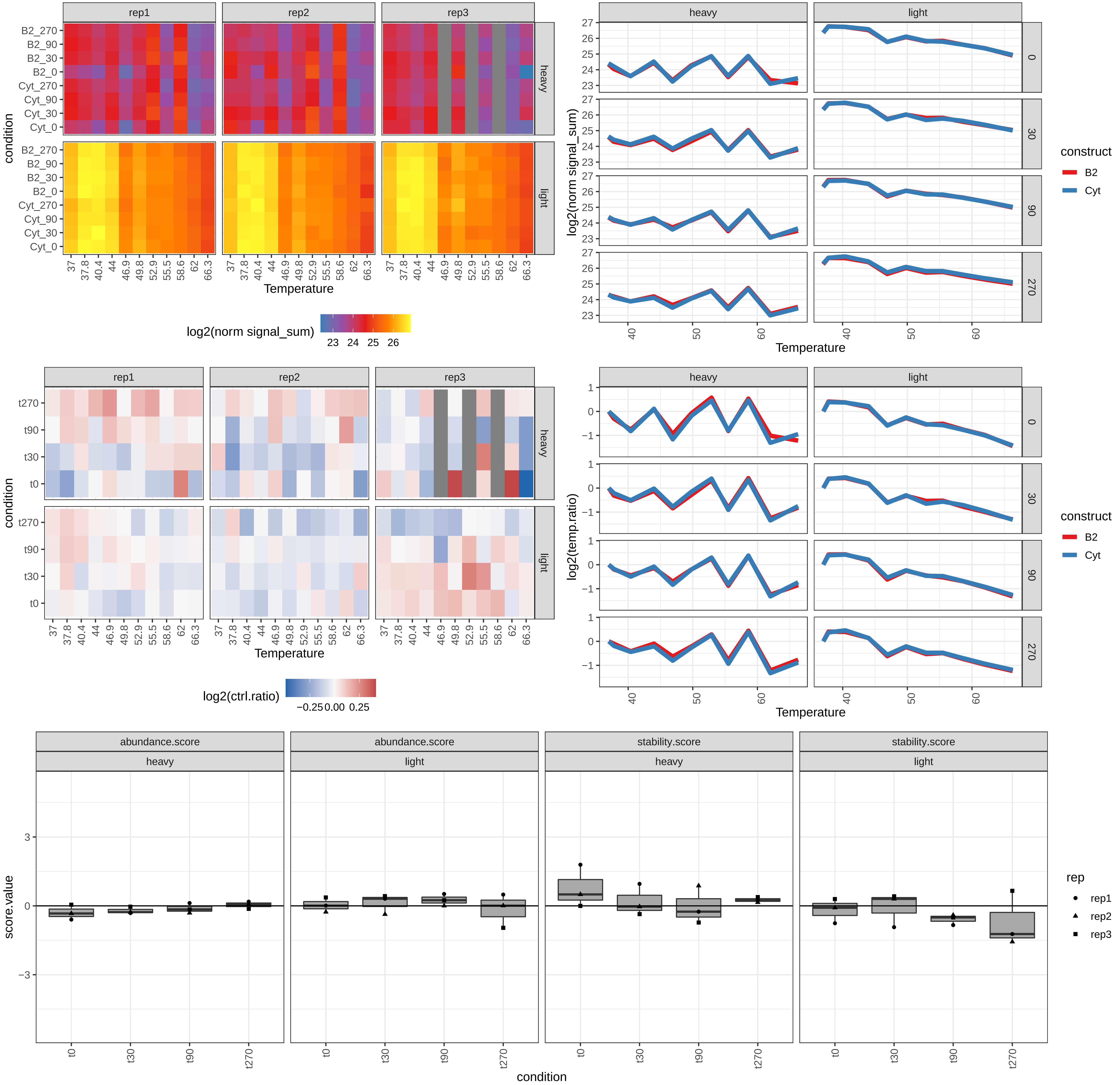

Q08220 − Glutathione synthetase OS=Saccharomyces cerevisiae (strain ATCC 204508 / S288c) GN=GSH2 PE=1 SV=1

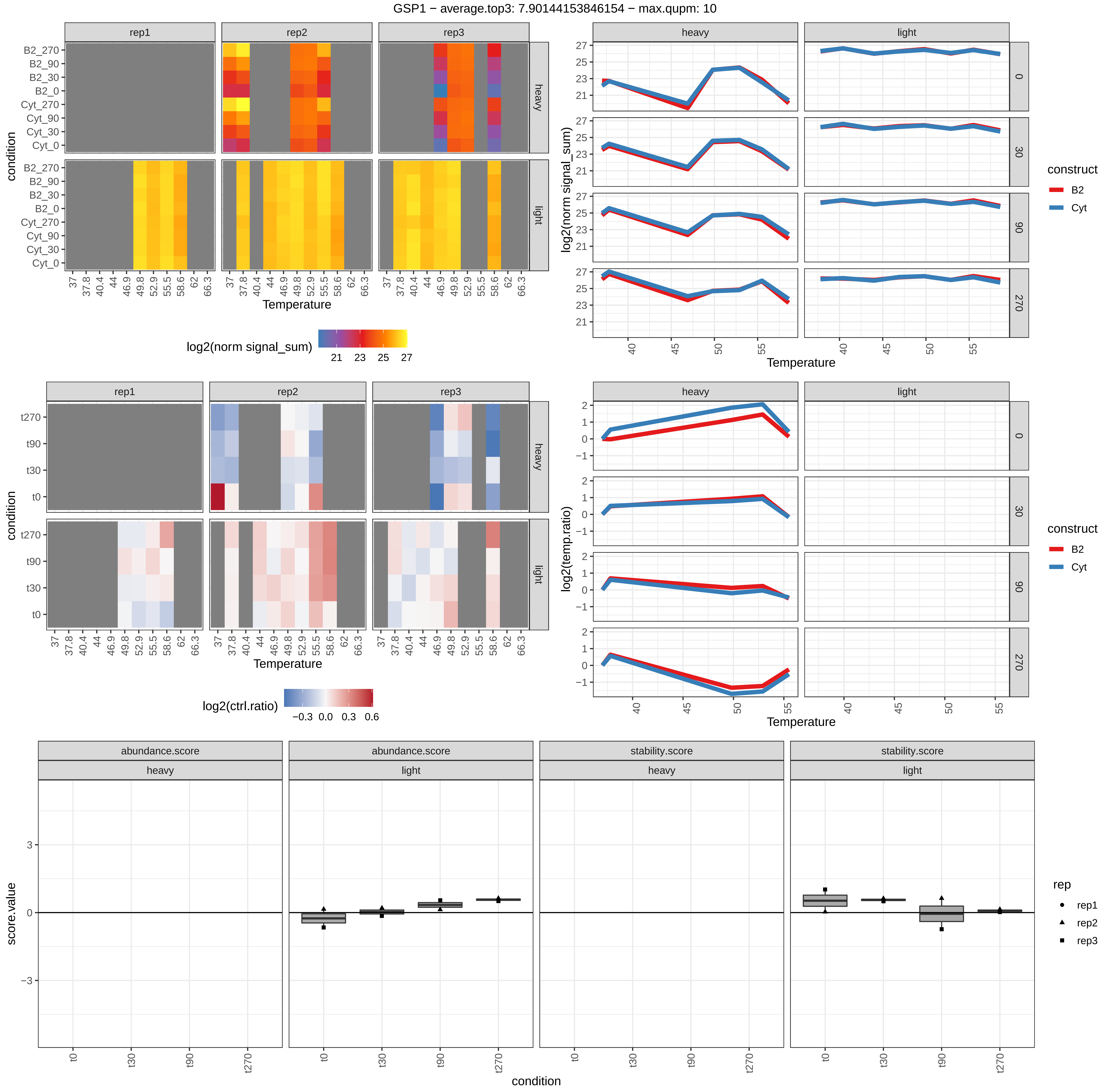

P32835 − GTP−binding nuclear protein GSP1/CNR1 OS=Saccharomyces cerevisiae (strain ATCC 204508 / S288c) GN=GSP1 PE=1 SV=1

GSP1|GSP2 − average.top3: 7.58862228571429 − max.qupm: 7

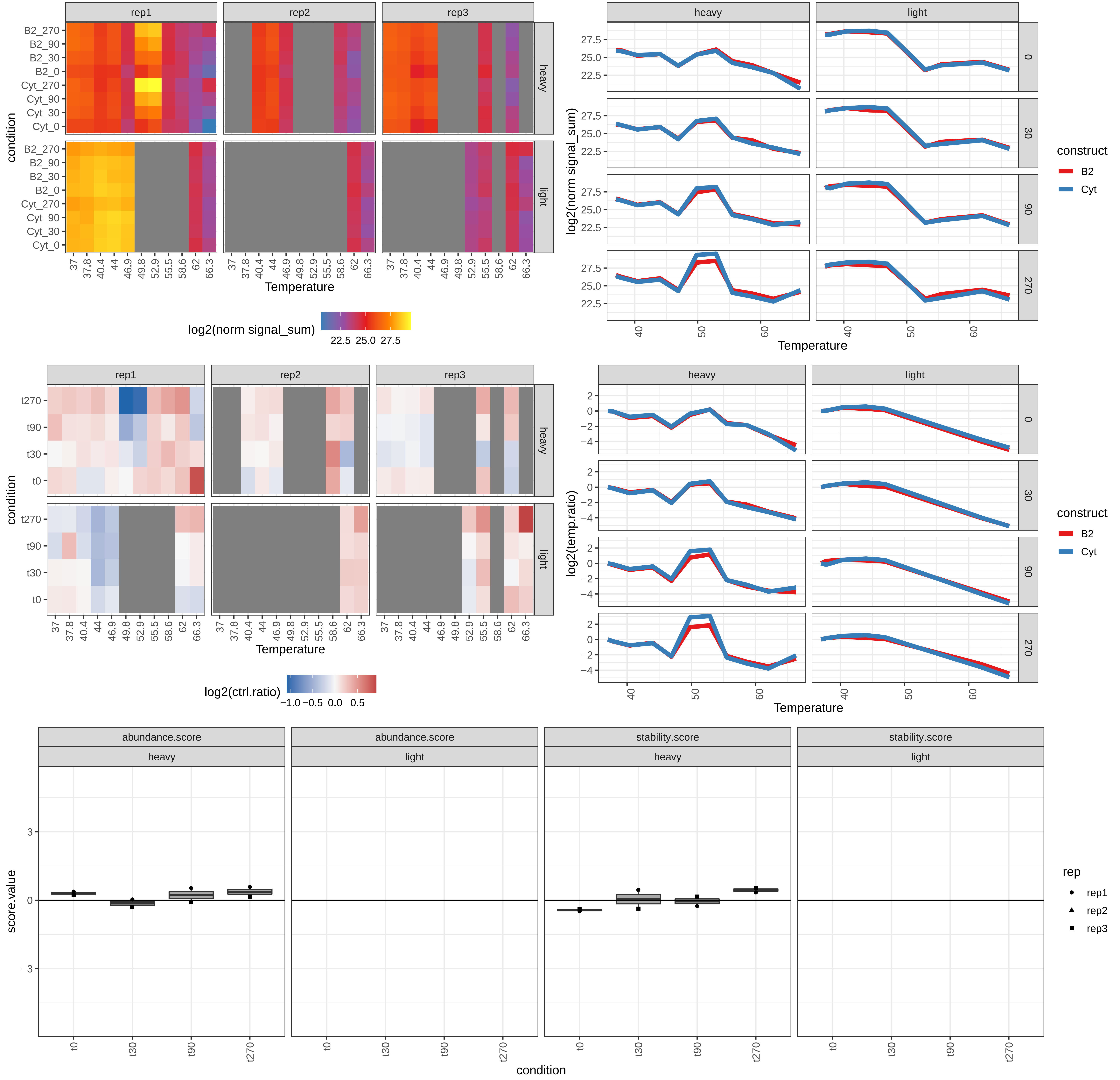

ear protein GSP1/CNR1 OS=Saccharomyces cerevisiae (strain ATCC 204508 / S288c) GN=GSP1 PE=1 SV=1|GTP−binding nuclear protein GSP2/CNR2 OS=Saccharomyces cerevisiae (strain ATCC 204

GSY1 − average.top3: 7.0705712195122 − max.qupm: 11

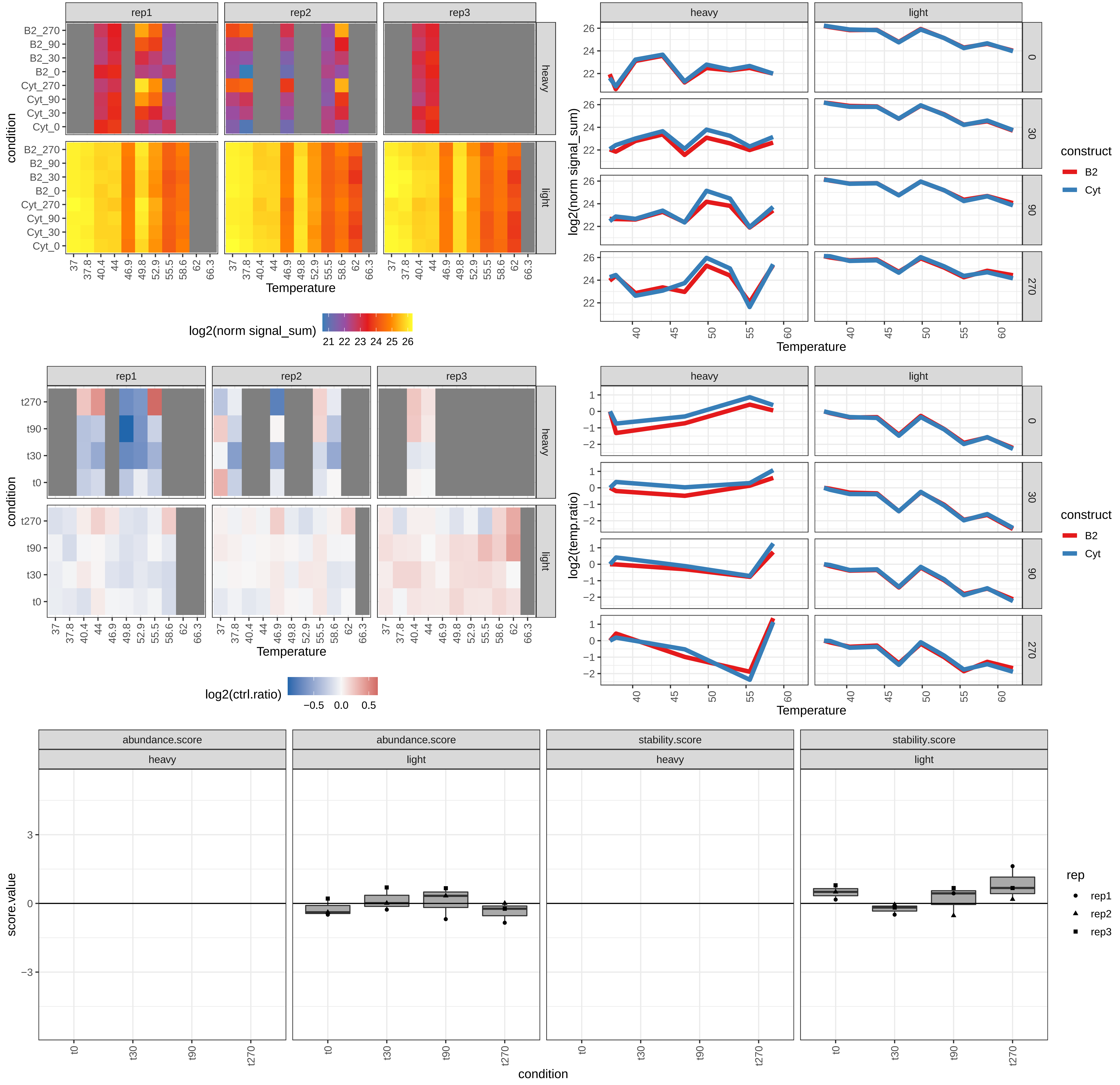

P23337 − Glycogen [starch] synthase isoform 1 OS=Saccharomyces cerevisiae (strain ATCC 204508 / S288c) GN=GSY1 PE=1 SV=3

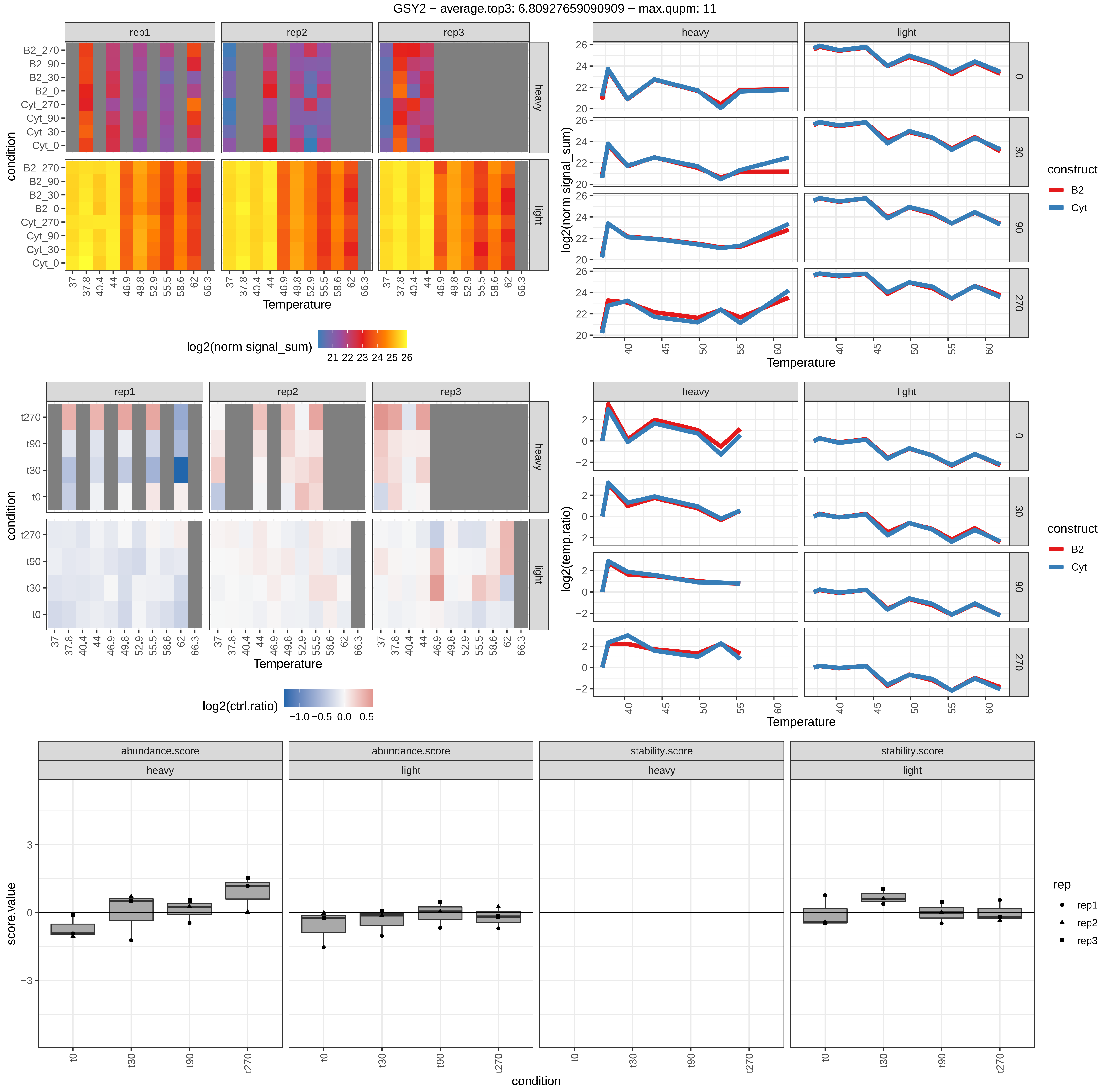

P27472 − Glycogen [starch] synthase isoform 2 OS=Saccharomyces cerevisiae (strain ATCC 204508 / S288c) GN=GSY2 PE=1 SV=3

GTB1 − average.top3: 6.86918875 − max.qupm: 8

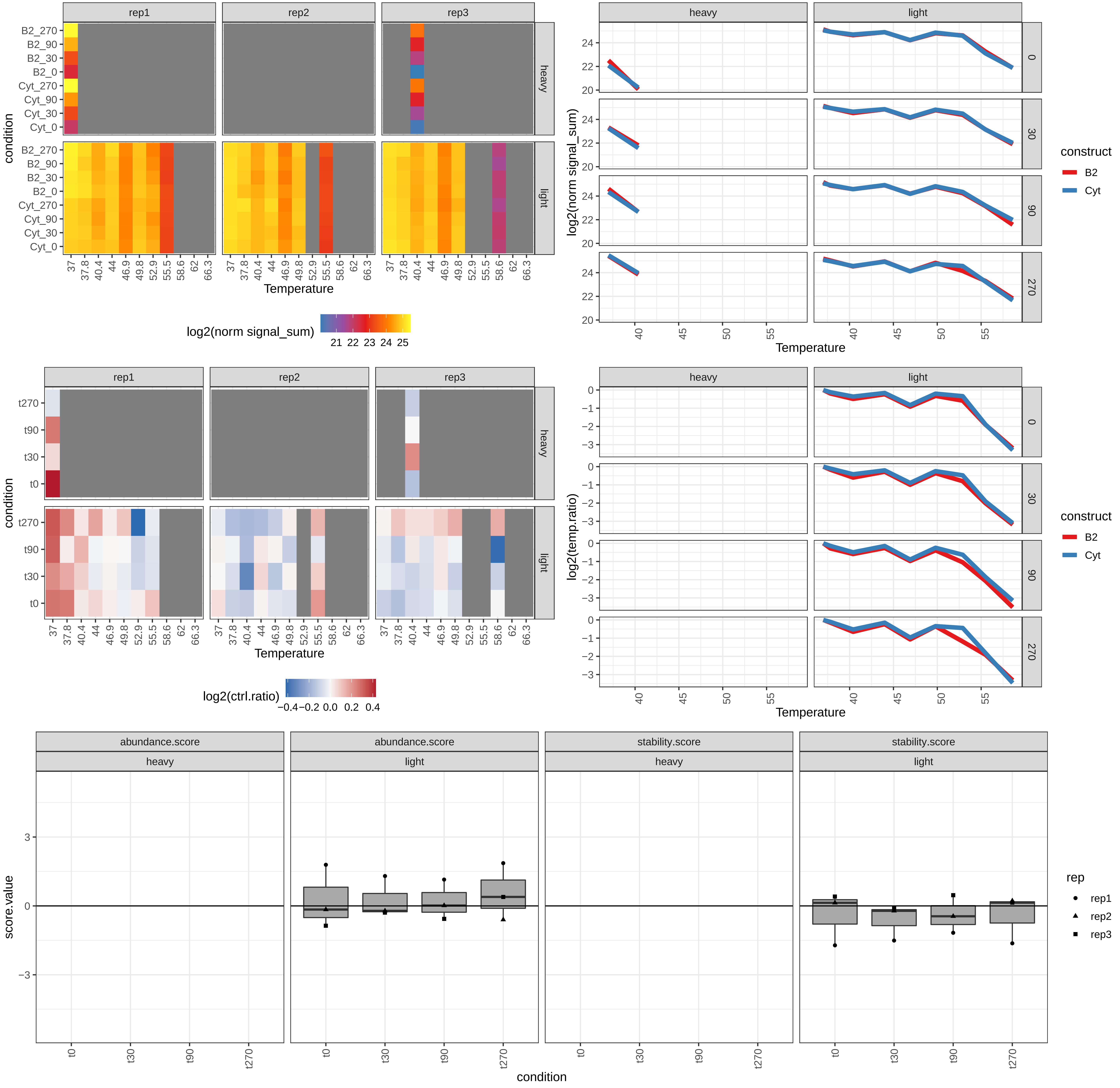

Q04924 − Glucosidase 2 subunit beta OS=Saccharomyces cerevisiae (strain ATCC 204508 / S288c) GN=GTB1 PE=1 SV=1

GTF1 − average.top3: 6.66291421052632 − max.qupm: 5

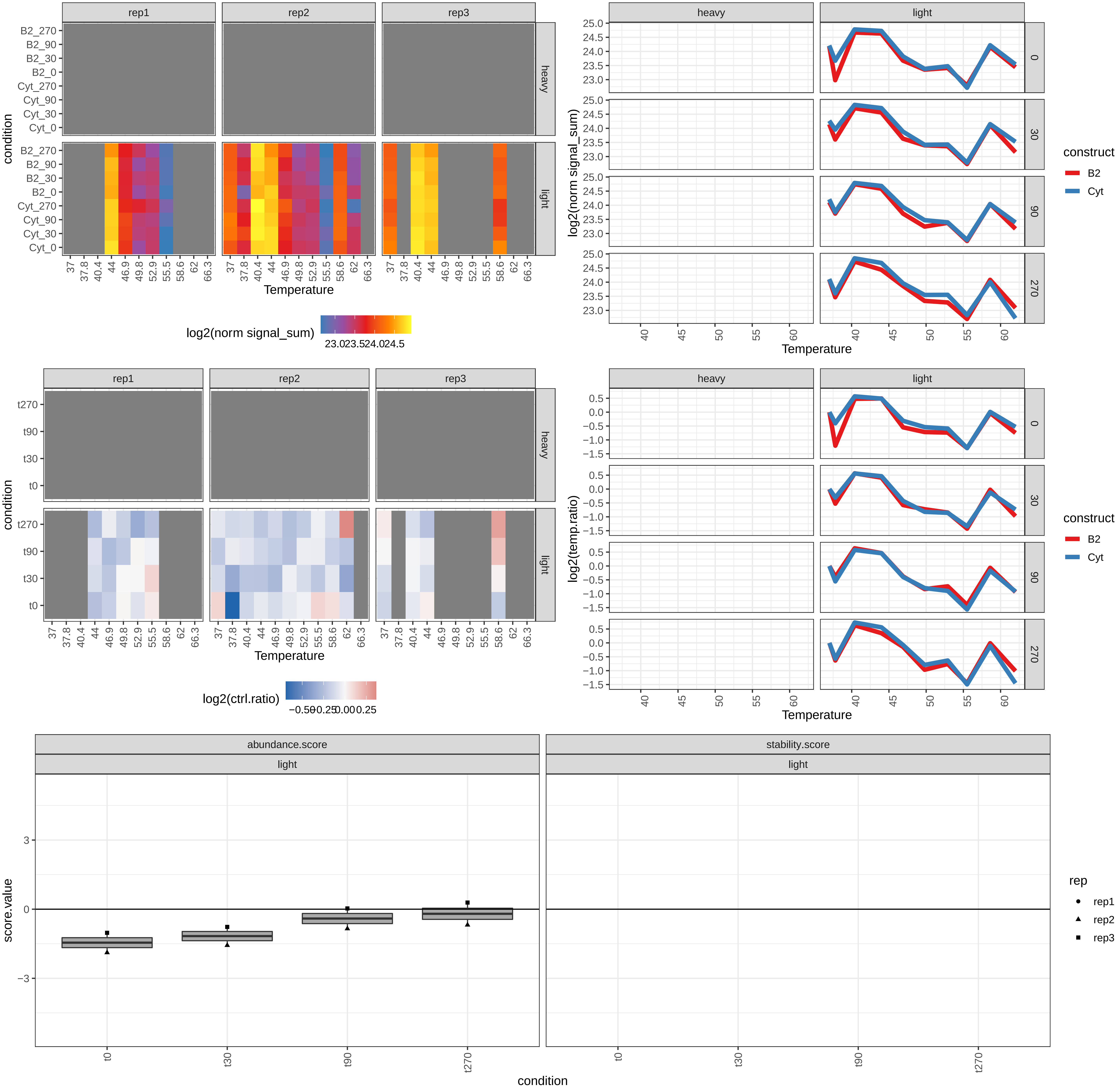

P53260 − Glutamyl−tRNA(Gln) amidotransferase subunit F, mitochondrial OS=Saccharomyces cerevisiae (strain ATCC 204508 / S288c) GN=GTF1 PE=1 SV=1

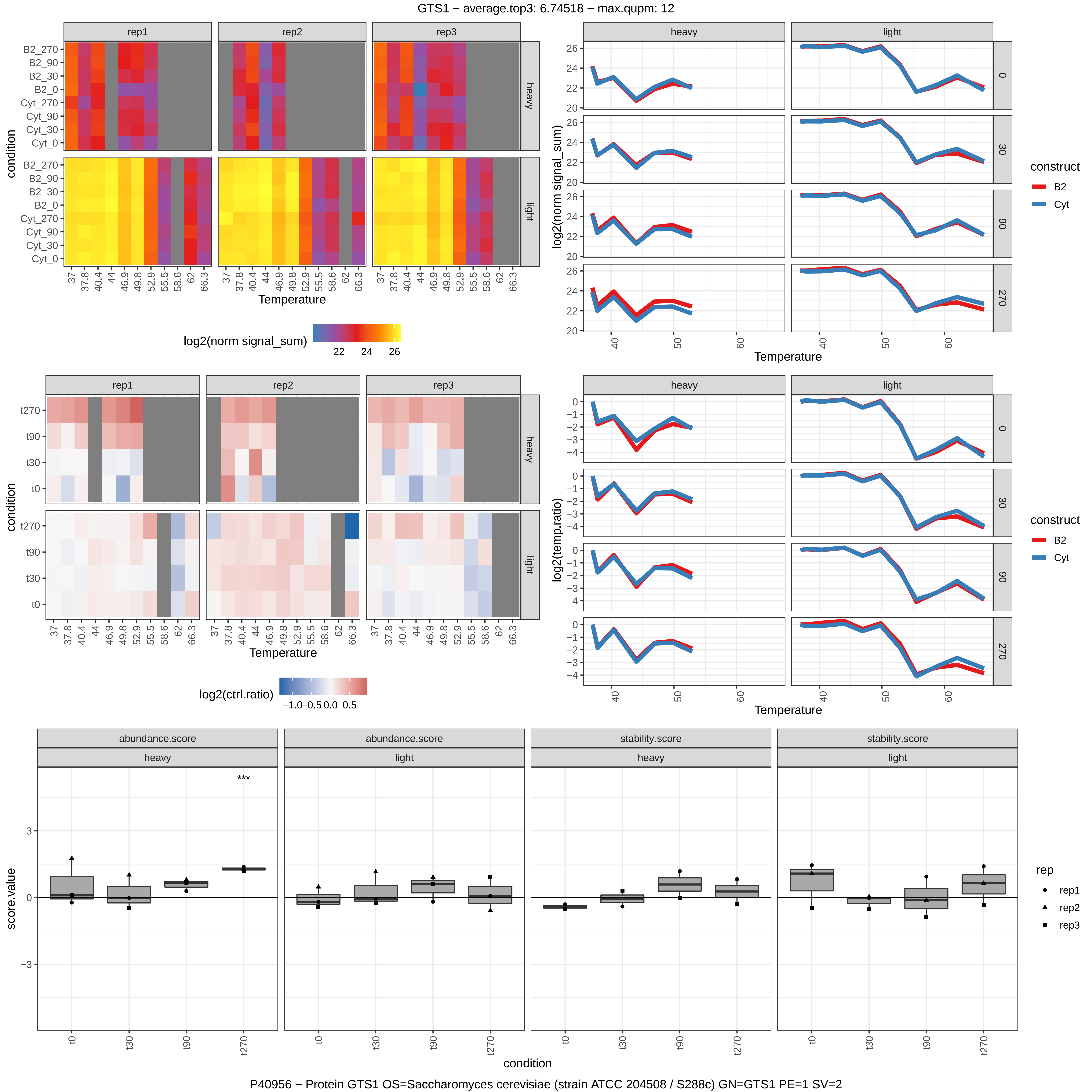

GTS1 − average.top3: 6.74518 − max.qupm: 12
rep1
rep2
rep3
heavy
light
condition
Temperature
log2(norm signal_sum)
construct
B2
Cyt
log2(temp.ratio)
log2(ctrl.ratio)
abundance.score
stability.score
score.value
rep
rep1
rep2
rep3
***
P40956 − Protein GTS1 OS=Saccharomyces cerevisiae (strain ATCC 204508 / S288c) GN=GTS1 PE=1 SV=2

GTT1 − average.top3: 7.49450378787879 − max.qupm: 11

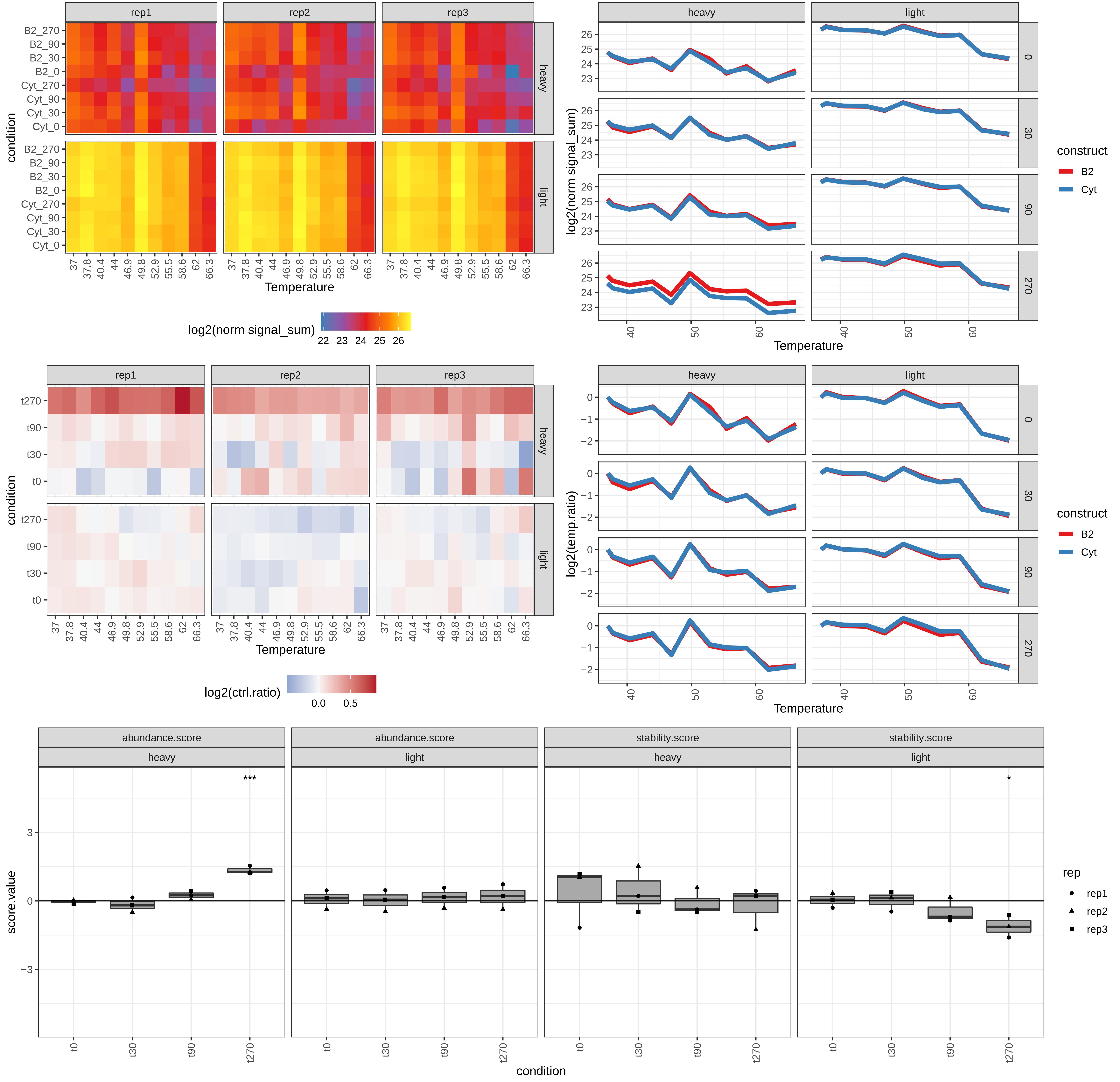

P40582 − Glutathione S−transferase 1 OS=Saccharomyces cerevisiae (strain ATCC 204508 / S288c) GN=GTT1 PE=1 SV=1

GTT3 − average.top3: 6.553533125 − max.qupm: 2

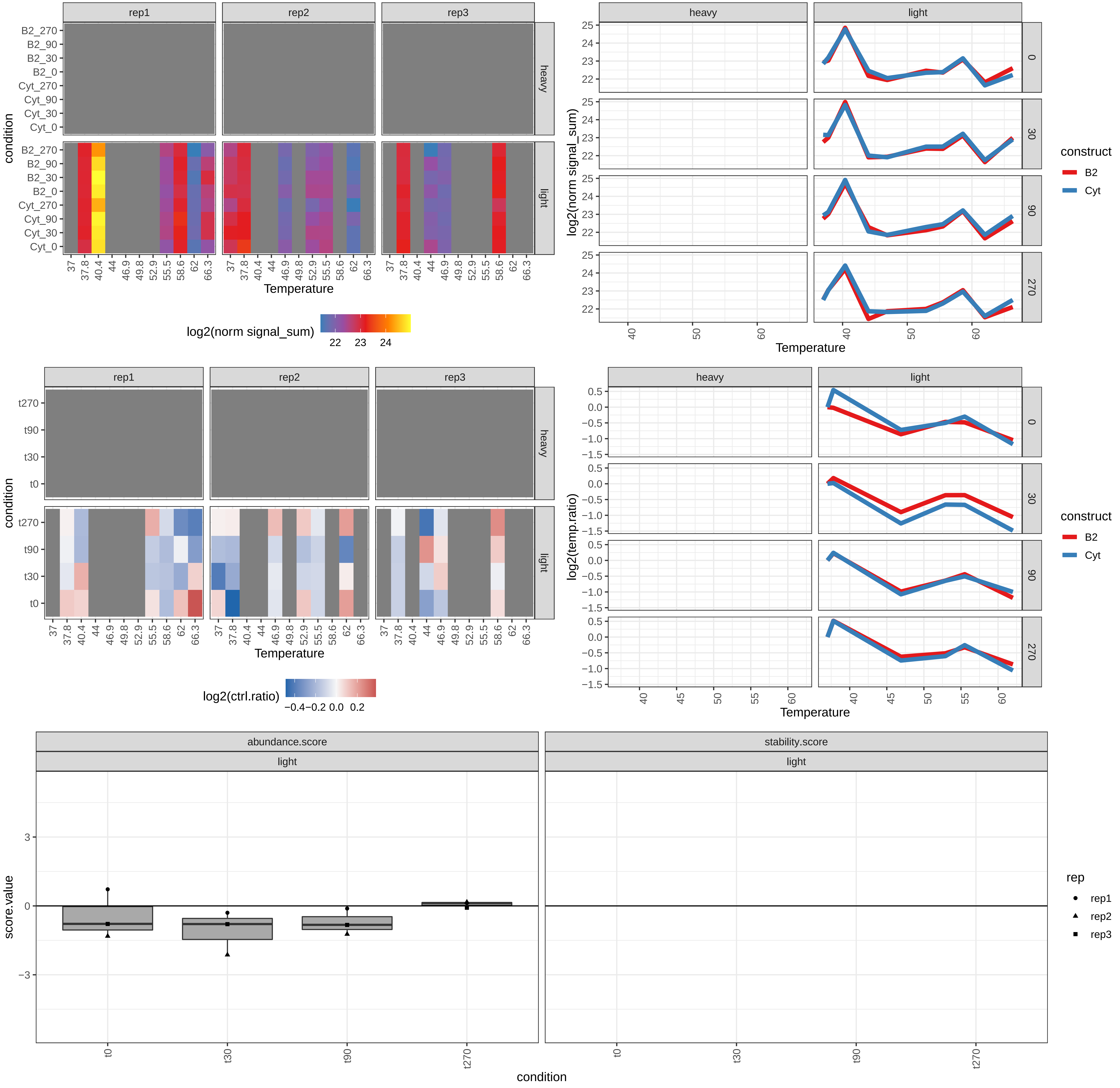

P39996 − Glutathione transferase 3 OS=Saccharomyces cerevisiae (strain ATCC 204508 / S288c) GN=GTT3 PE=1 SV=1

GUA1 − average.top3: 7.74213 − max.qupm: 23

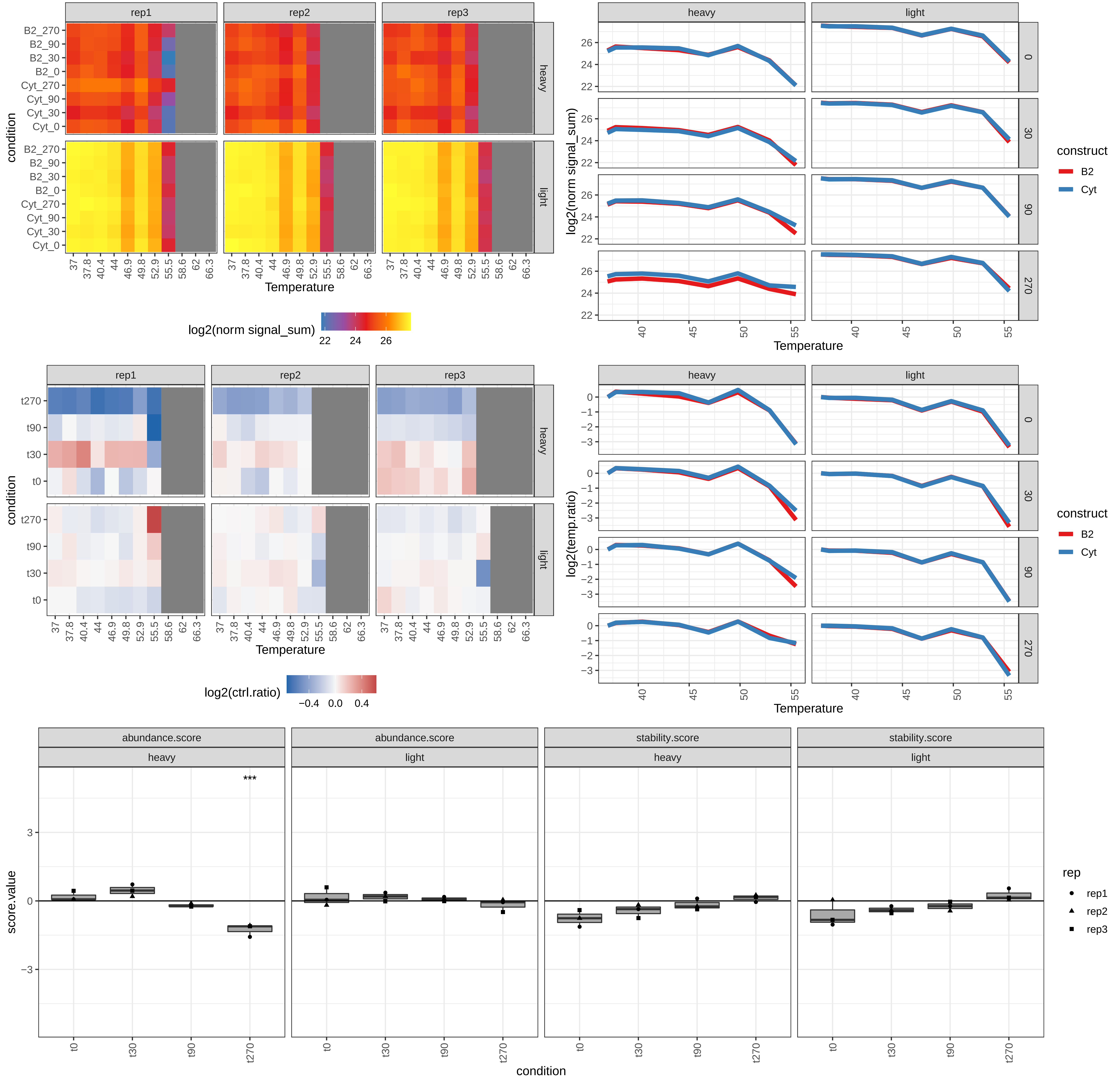

P38625 − GMP synthase [glutamine−hydrolyzing] OS=Saccharomyces cerevisiae (strain ATCC 204508 / S288c) GN=GUA1 PE=1 SV=4

GUD1 − average.top3: 6.41287904761905 − max.qupm: 5

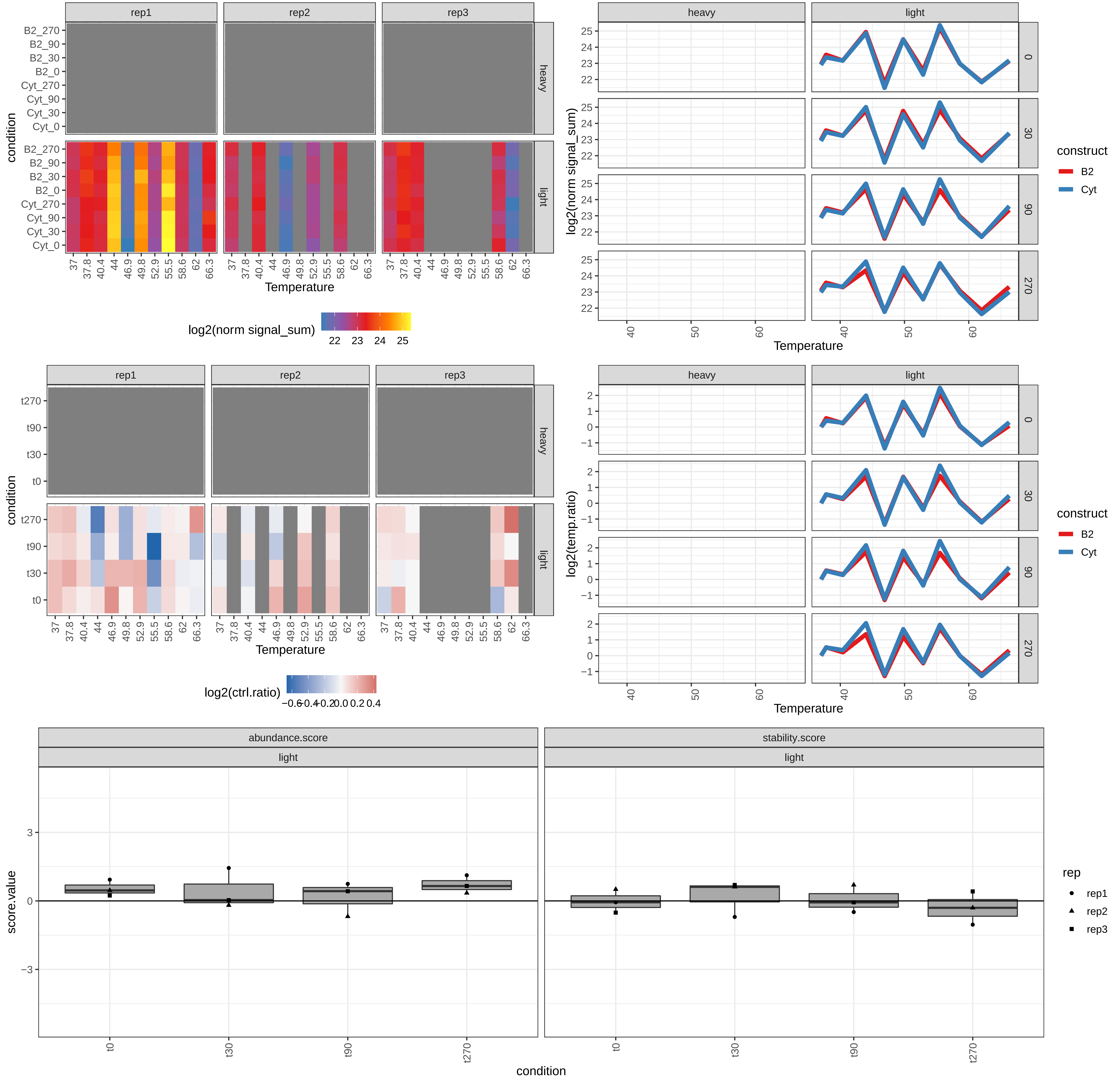

Q07729 − Probable guanine deaminase OS=Saccharomyces cerevisiae (strain ATCC 204508 / S288c) GN=GUD1 PE=1 SV=1

GUK1 − average.top3: 7.1860175 − max.qupm: 8

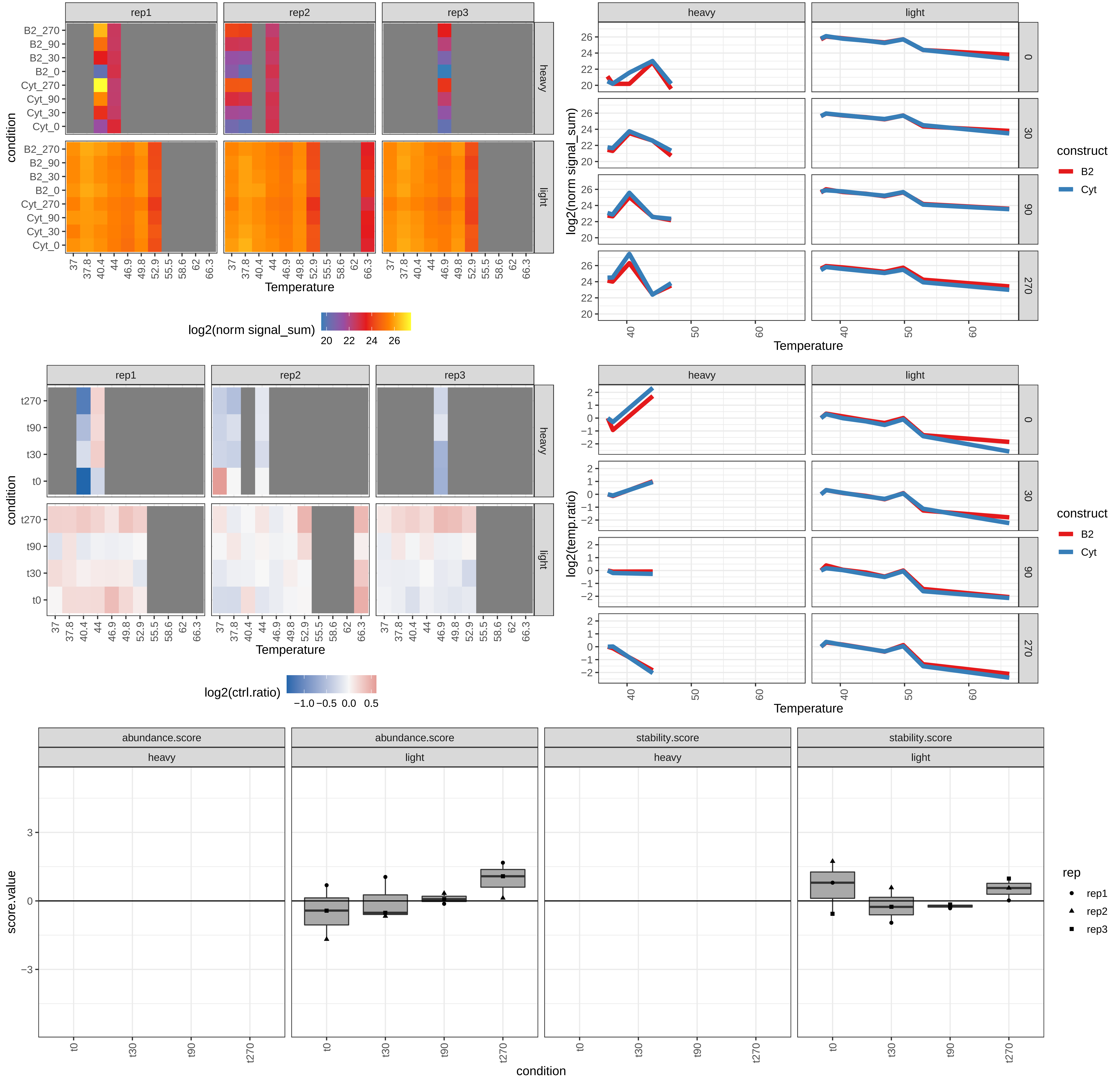

P15454 − Guanylate kinase OS=Saccharomyces cerevisiae (strain ATCC 204508 / S288c) GN=GUK1 PE=1 SV=2

GUP1 − average.top3: 6.90869785714286 − max.qupm: 7

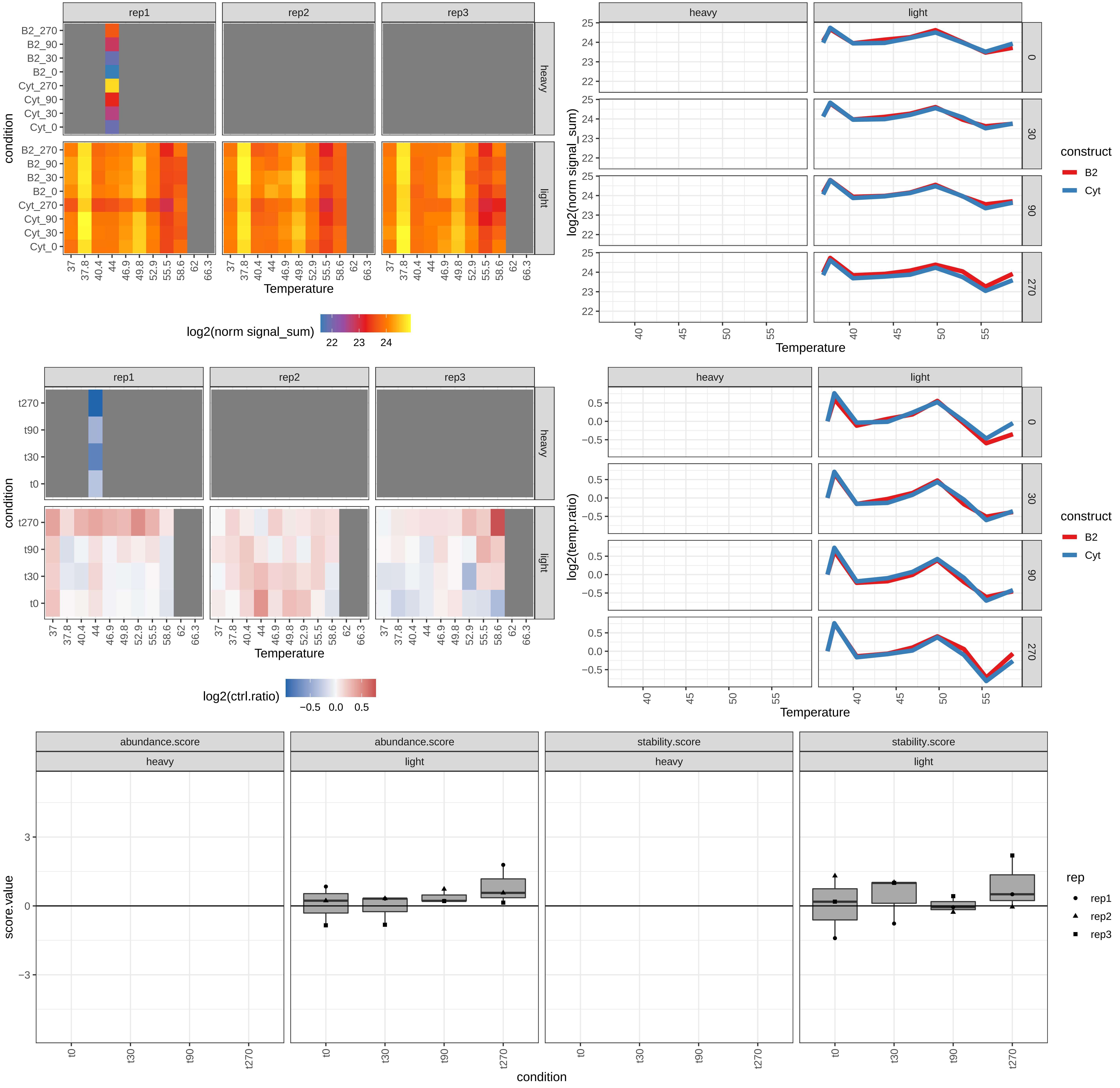

P53154 − Glycerol uptake protein 1 OS=Saccharomyces cerevisiae (strain ATCC 204508 / S288c) GN=GUP1 PE=1 SV=1

GUS1 − average.top3: 7.74729491525424 − max.qupm: 46

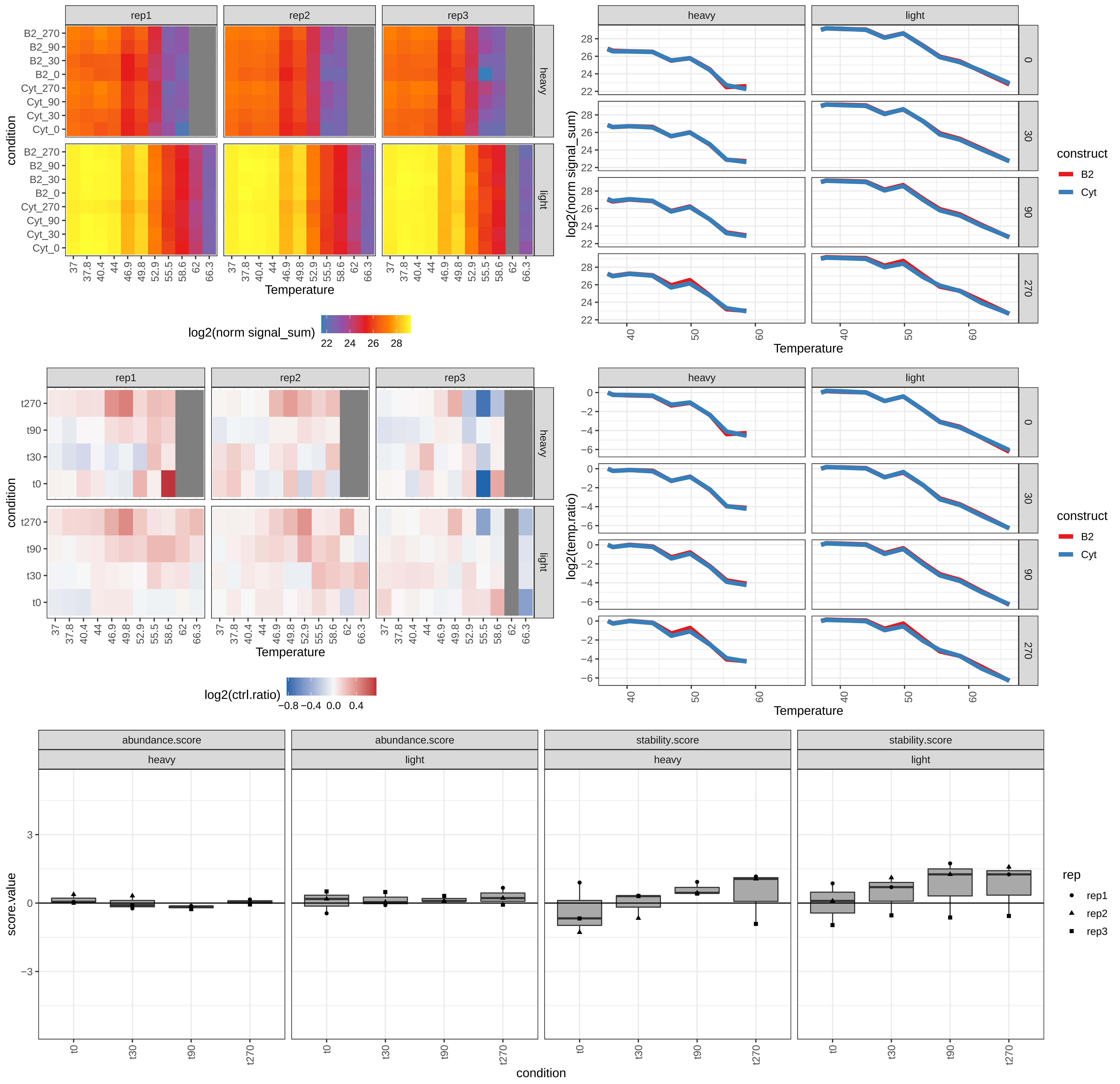

P46655 − Glutamate−−tRNA ligase, cytoplasmic OS=Saccharomyces cerevisiae (strain ATCC 204508 / S288c) GN=GUS1 PE=1 SV=3

GUT1 − average.top3: 7.30839442622951 − max.qupm: 26

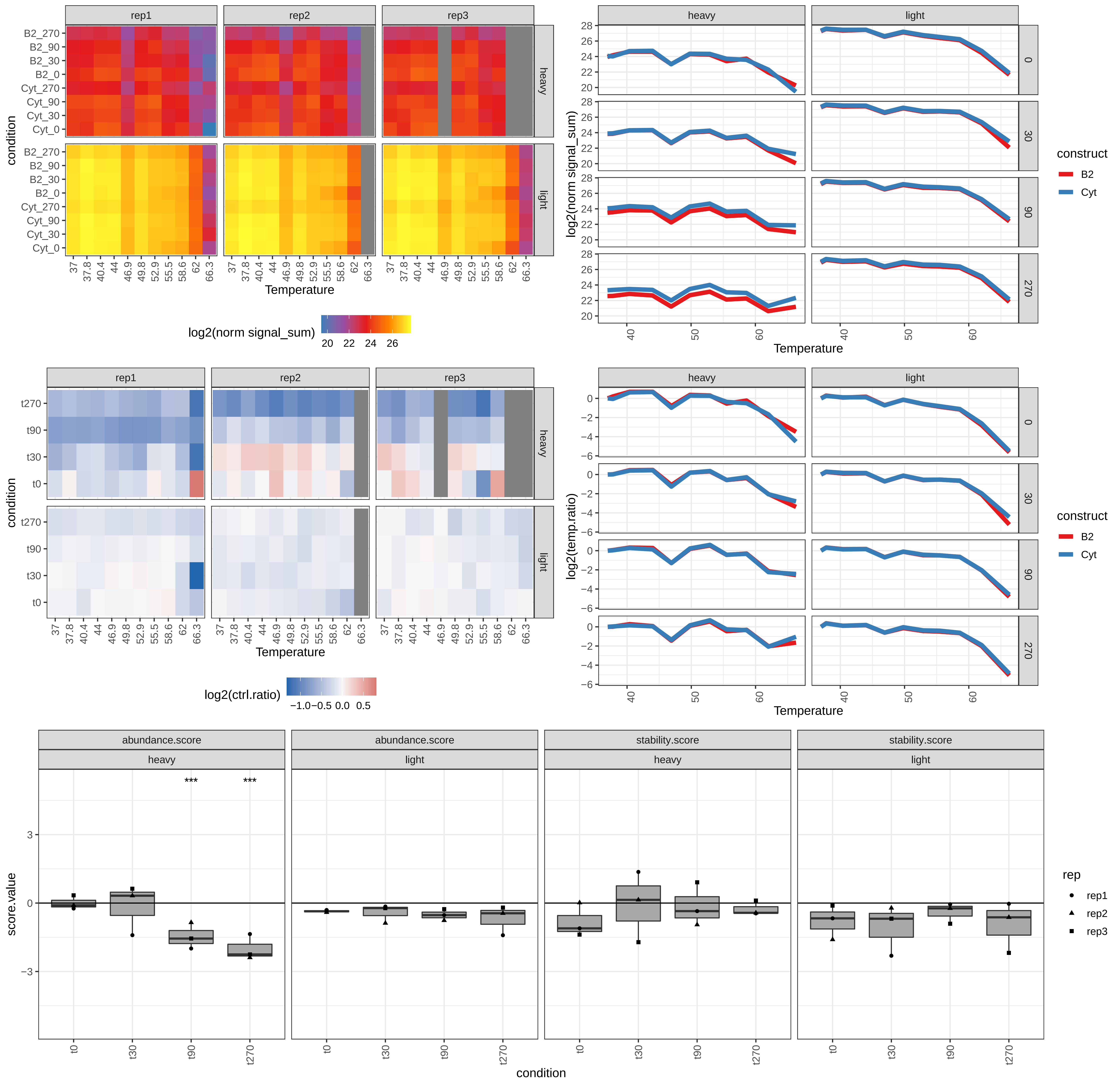

P32190 − Glycerol kinase OS=Saccharomyces cerevisiae (strain ATCC 204508 / S288c) GN=GUT1 PE=3 SV=1

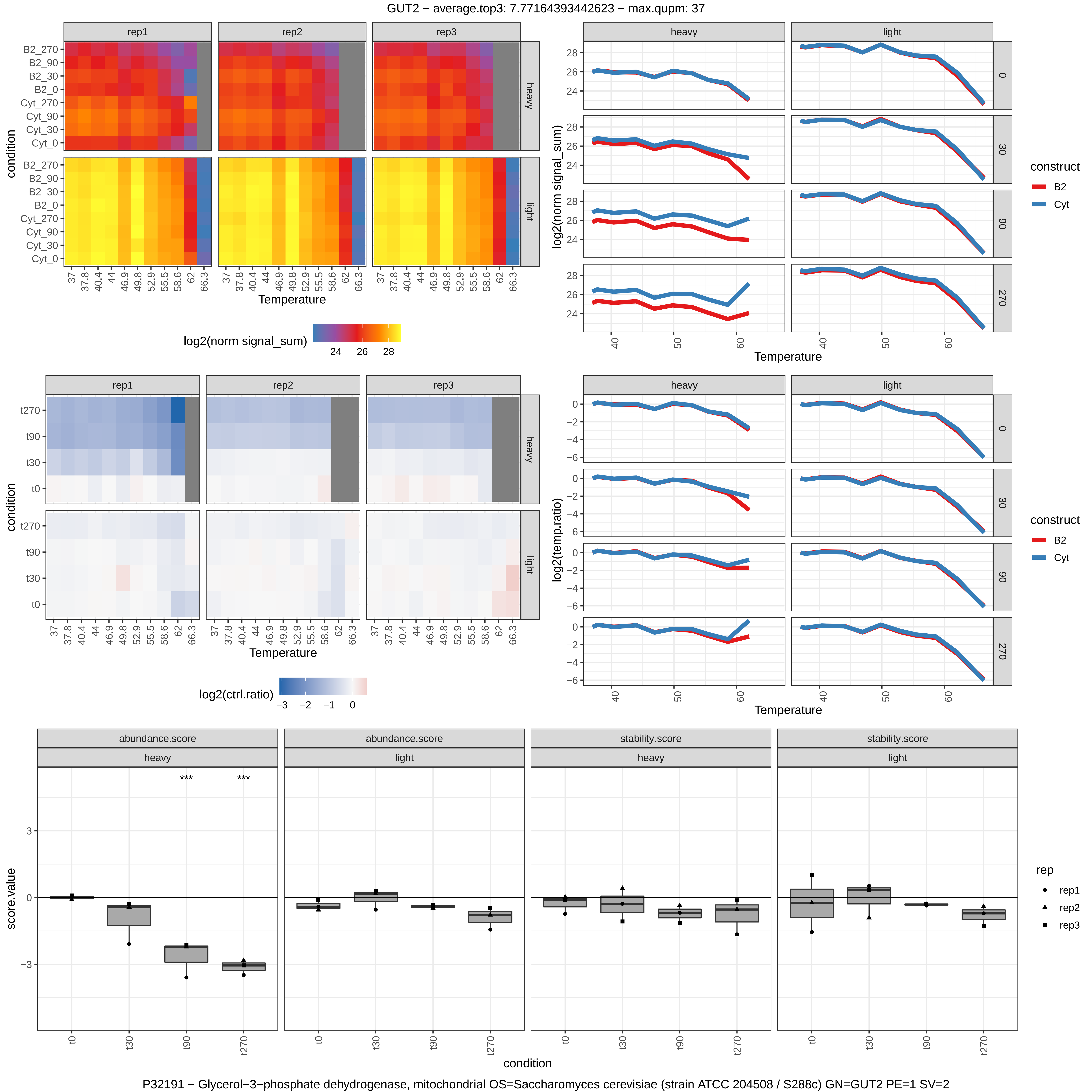

GUT2 − average.top3: 7.77164393442623 − max.qupm: 37
rep1
rep2
rep3
heavy
light
condition
Temperature
log2(norm signal_sum)
construct
B2
Cyt
log2(ctrl.ratio)
log2(temp.ratio)
abundance.score
stability.score
score.value
rep
***
P32191 − Glycerol−3−phosphate dehydrogenase, mitochondrial OS=Saccharomyces cerevisiae (strain ATCC 204508 / S288c) GN=GUT2 PE=1 SV=2

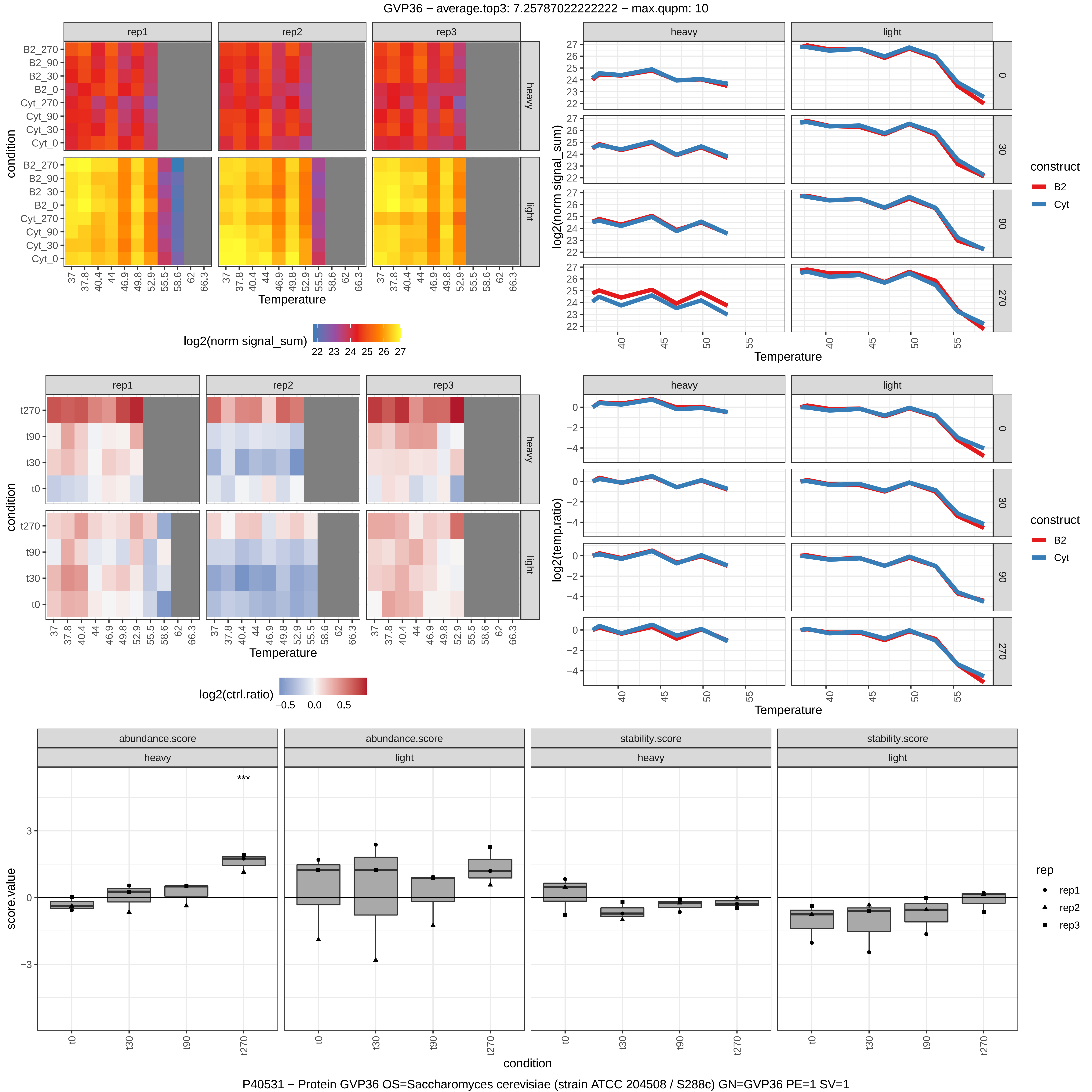

GVP36 − average.top3: 7.25787022222222 − max.qupm: 10
rep1
rep2
rep3
heavy
light
condition
Temperature
log2(norm signal_sum)
construct
B2
Cyt
log2(ctrl.ratio)
log2(temp.ratio)
abundance.score
stability.score
score.value
rep
***
P40531 − Protein GVP36 OS=Saccharomyces cerevisiae (strain ATCC 204508 / S288c) GN=GVP36 PE=1 SV=1

GYL1 − average.top3: 6.41947 − max.qupm: 8

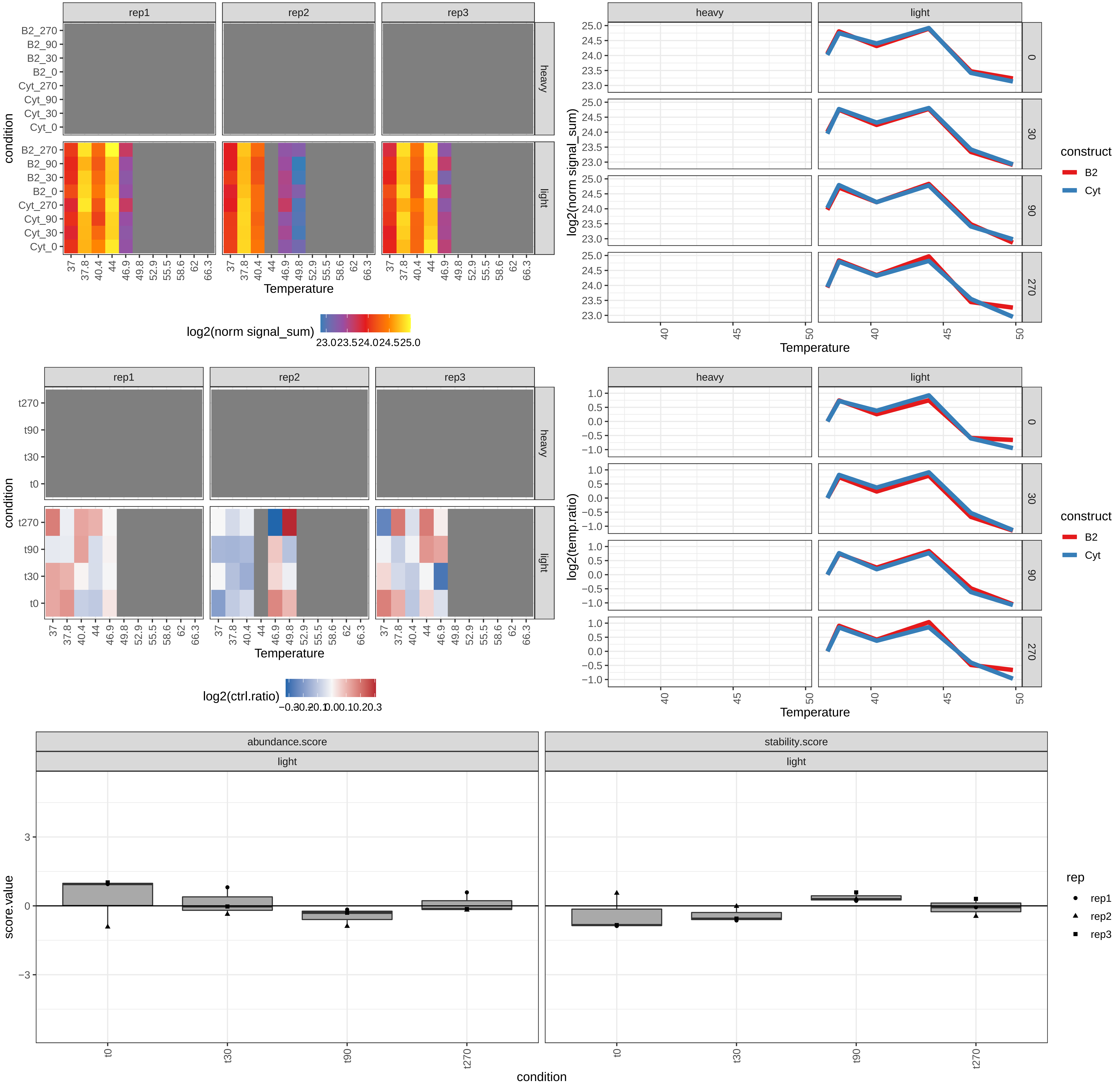

Q04322 − Probable GTPase−activating protein GYL1 OS=Saccharomyces cerevisiae (strain ATCC 204508 / S288c) GN=GYL1 PE=1 SV=1

GYP6 − average.top3: 5.93644818181818 − max.qupm: 3

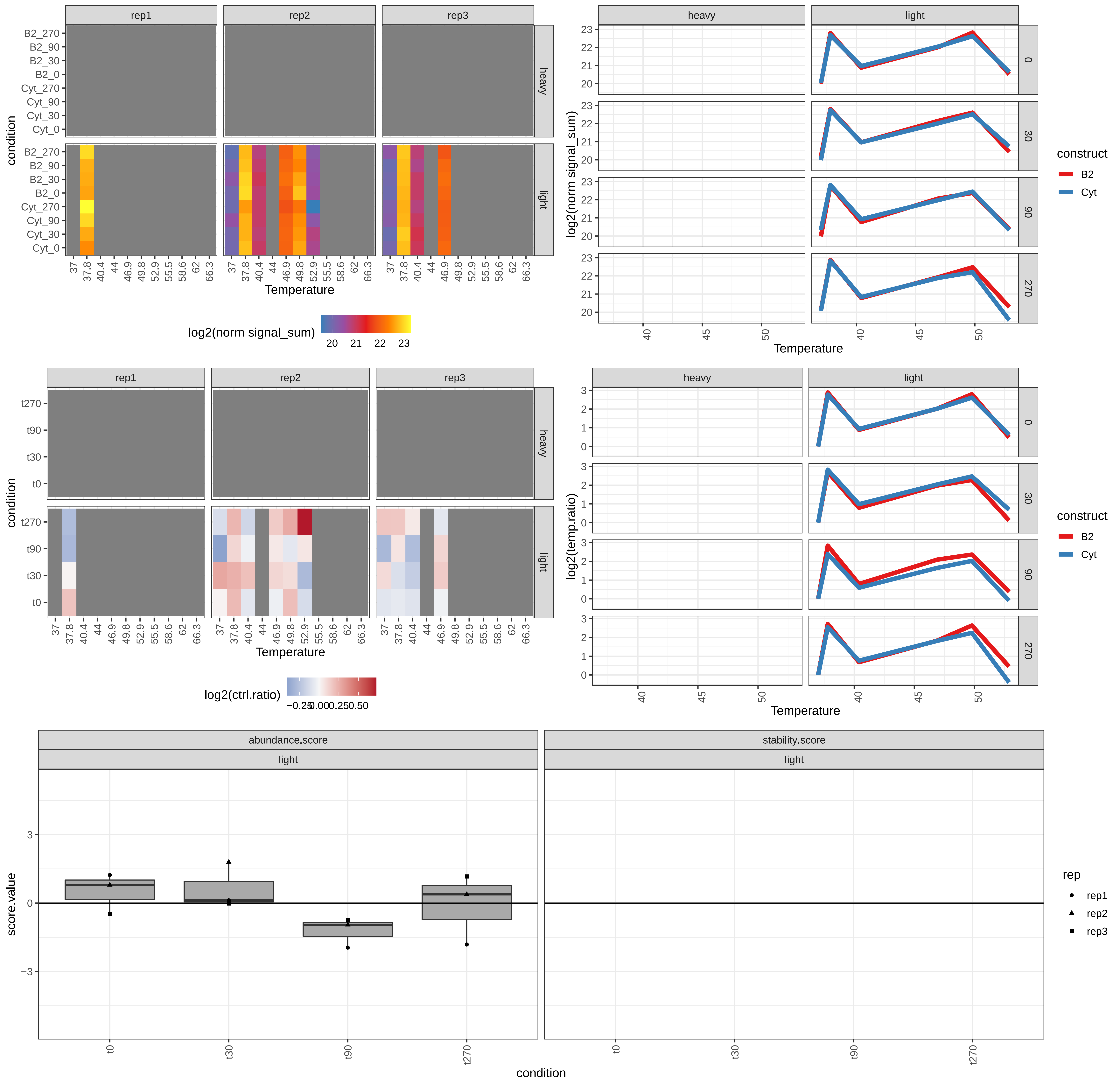

P32806 − GTPase−activating protein GYP6 OS=Saccharomyces cerevisiae (strain ATCC 204508 / S288c) GN=GYP6 PE=1 SV=2

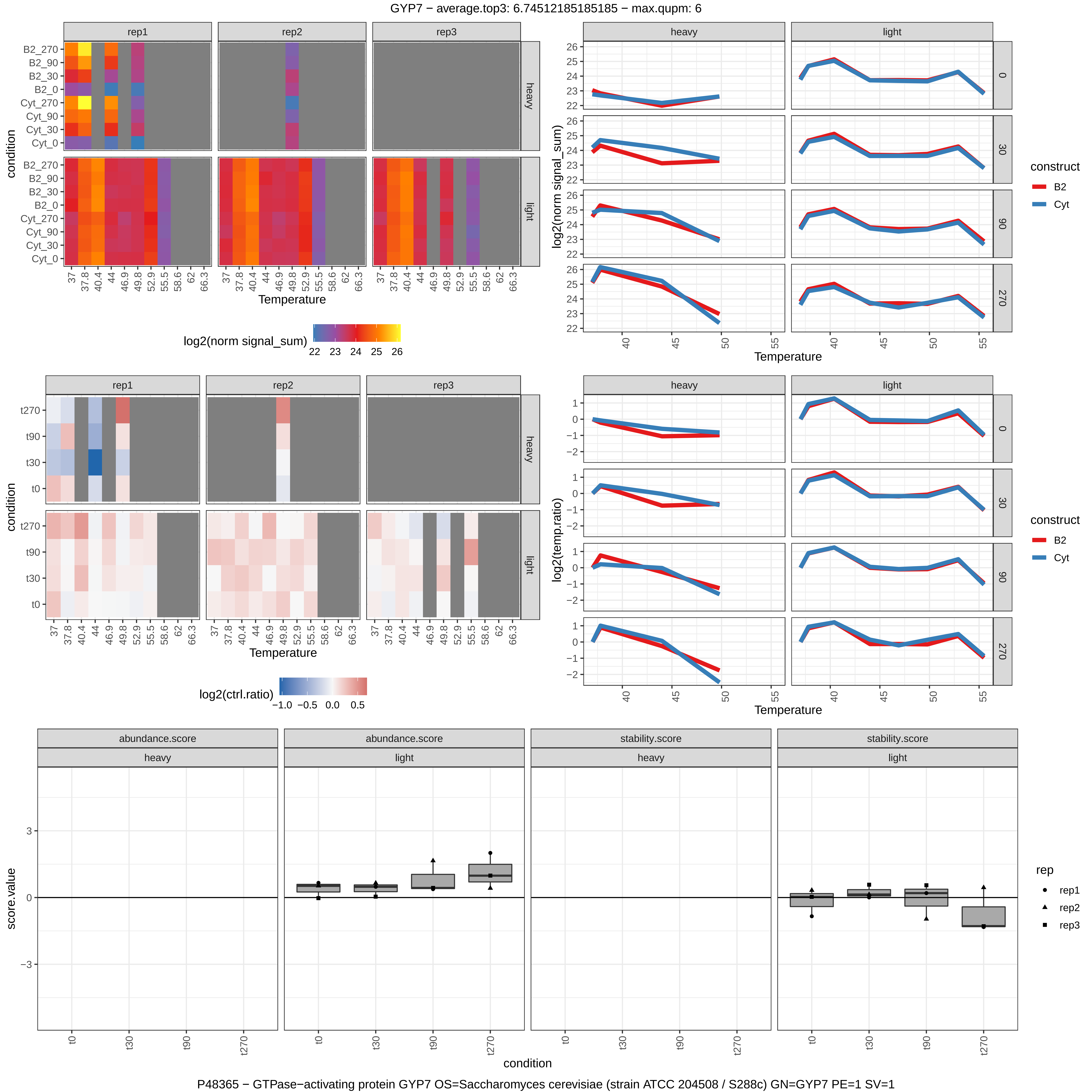
GYP7 − average.top3: 6.74512185185185 − max.qupm: 6
rep1
rep2
rep3
heavy
light
B2_270
B2_90
B2_30
B2_0
Cyt_270
Cyt_90
Cyt_30
Cyt_0
condition
37
37.8
40.4
44
46.9
49.8
52.9
55.5
58.6
62
66.3
Temperature
log2(norm signal_sum)
22
23
24
25
26
log2(norm signal_sum)
0
30
90
270
40
45
50
55
construct
B2
Cyt
t270
t90
t30
t0
log2(ctrl.ratio)
−1.0
−0.5
0.0
0.5
log2(temp.ratio)
abundance.score
stability.score
score.value
3
0
−3
rep
rep1
rep2
rep3
P48365 − GTPase−activating protein GYP7 OS=Saccharomyces cerevisiae (strain ATCC 204508 / S288c) GN=GYP7 PE=1 SV=1

HAL5 − average.top3: 6.52793 − max.qupm: 4

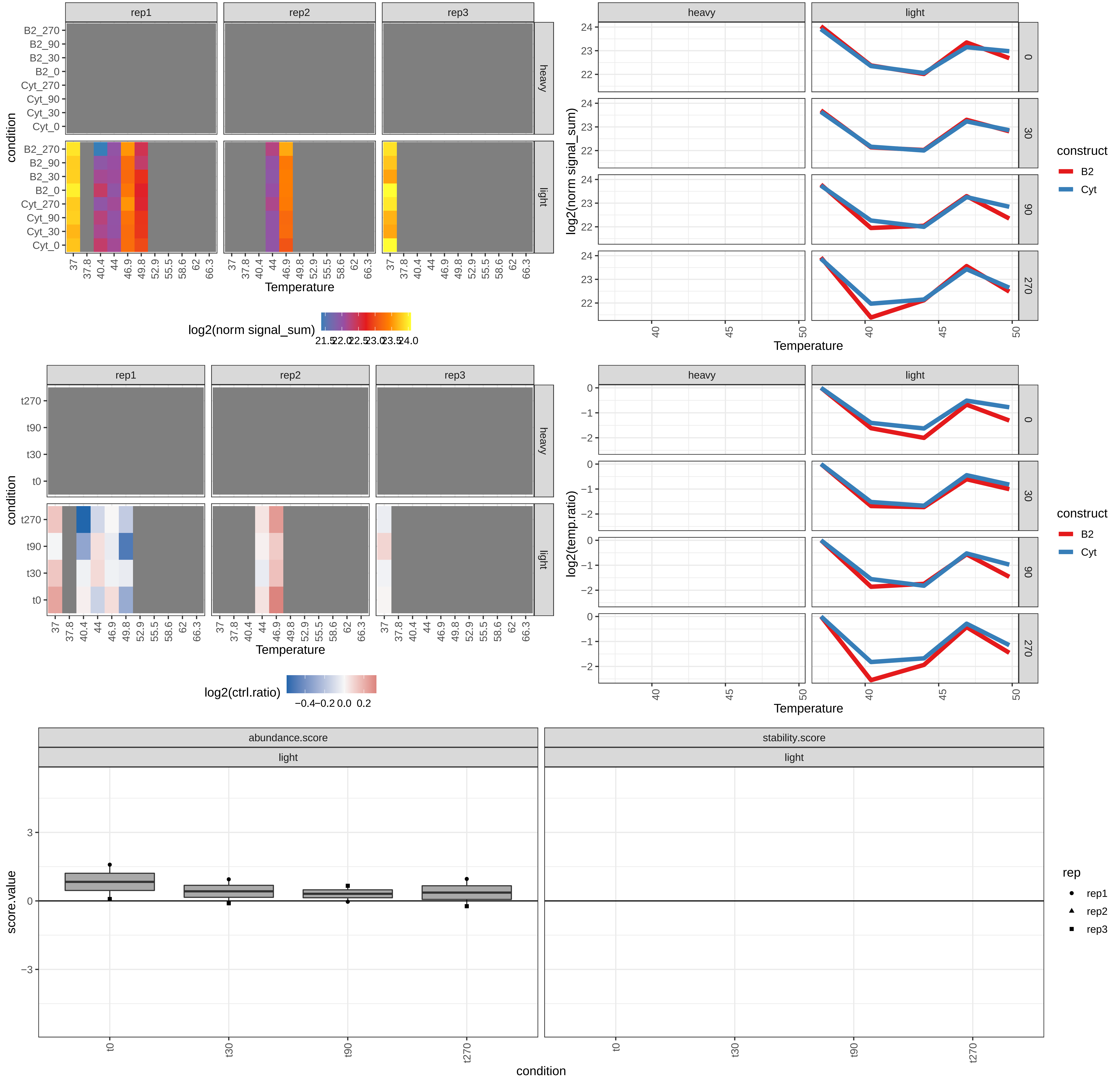

P38970 − Serine/threonine−protein kinase HAL5 OS=Saccharomyces cerevisiae (strain ATCC 204508 / S288c) GN=HAL5 PE=1 SV=2

HAL9 − average.top3: 6.29593066666667 − max.qupm: 4

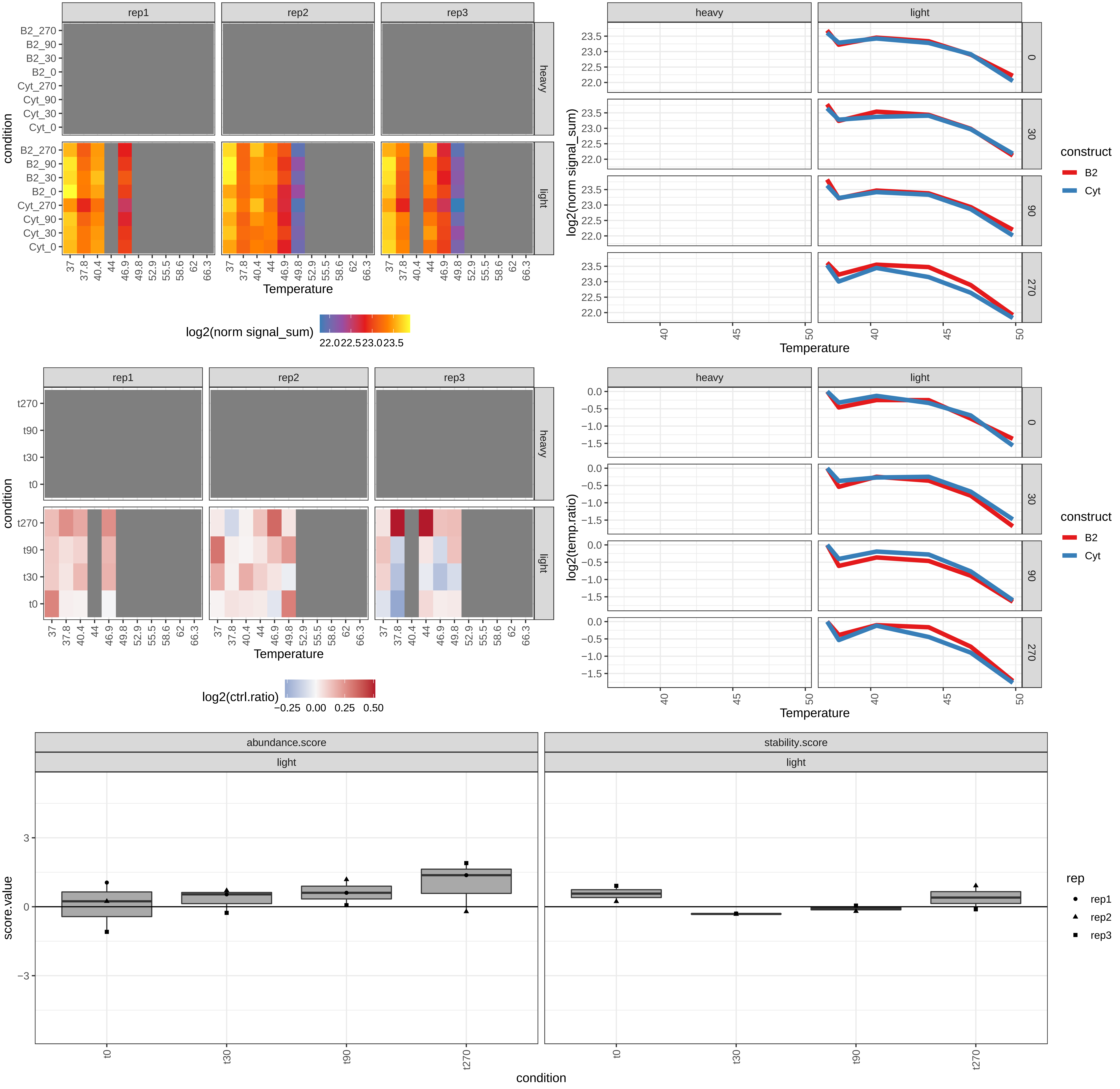

Q12180 − Halotolerance protein 9 OS=Saccharomyces cerevisiae (strain ATCC 204508 / S288c) GN=HAL9 PE=1 SV=1

HAM1 − average.top3: 7.05268066666667 − max.qupm: 8

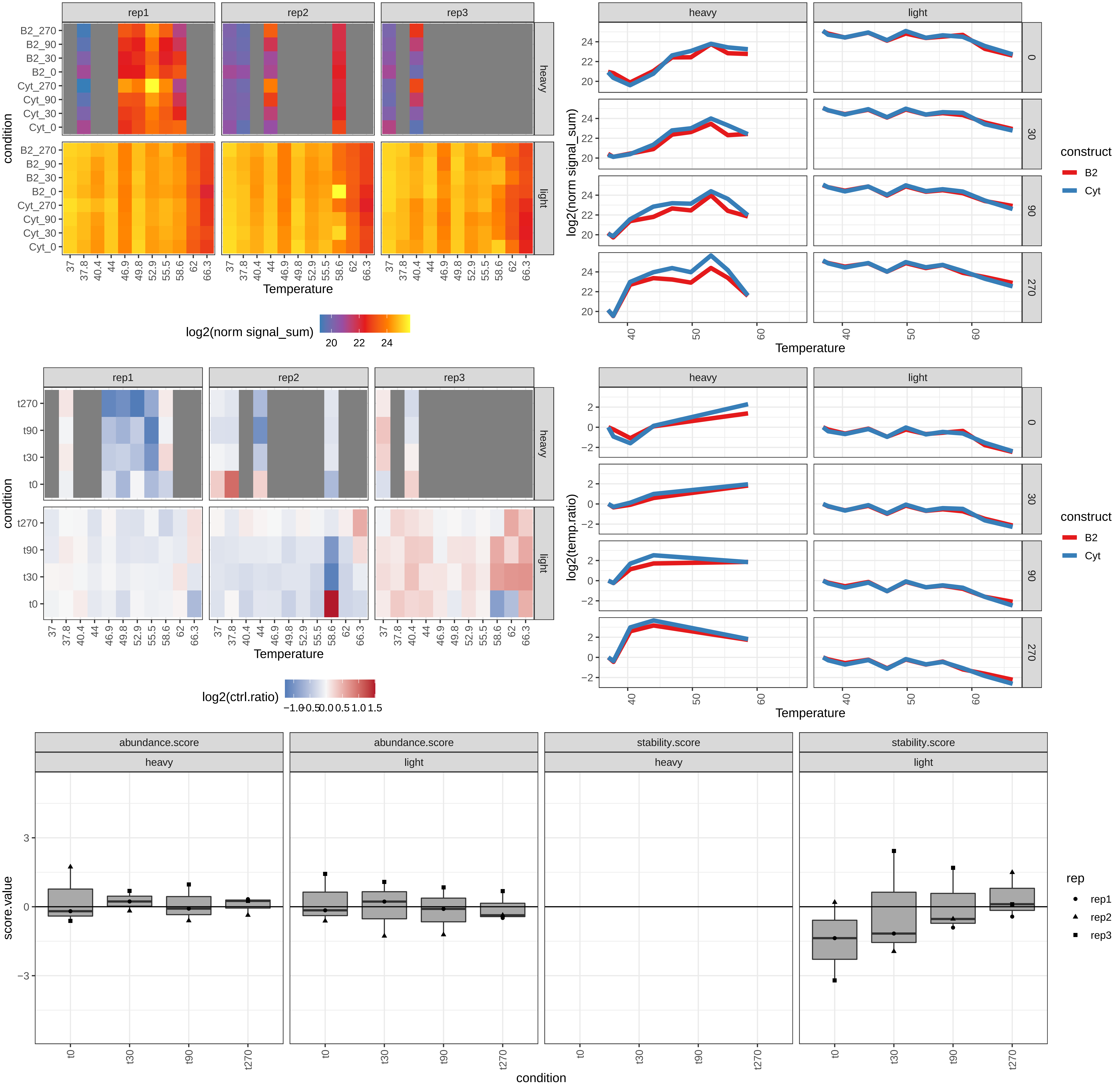

P47119 − Inosine triphosphate pyrophosphatase OS=Saccharomyces cerevisiae (strain ATCC 204508 / S288c) GN=HAM1 PE=1 SV=1

HAS1 − average.top3: 6.96076193548387 − max.qupm: 13

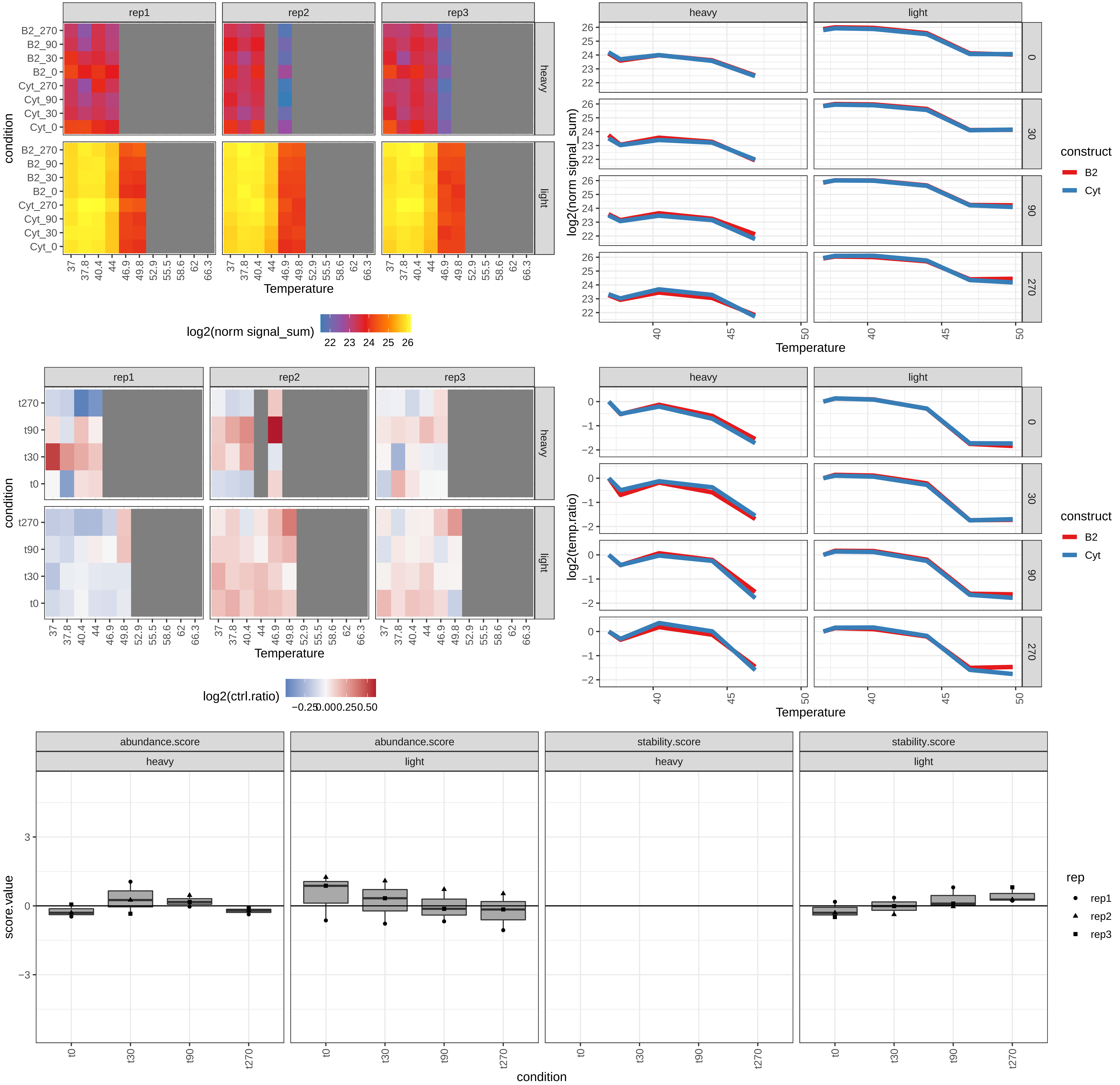

Q03532 − ATP−dependent RNA helicase HAS1 OS=Saccharomyces cerevisiae (strain ATCC 204508 / S288c) GN=HAS1 PE=1 SV=1

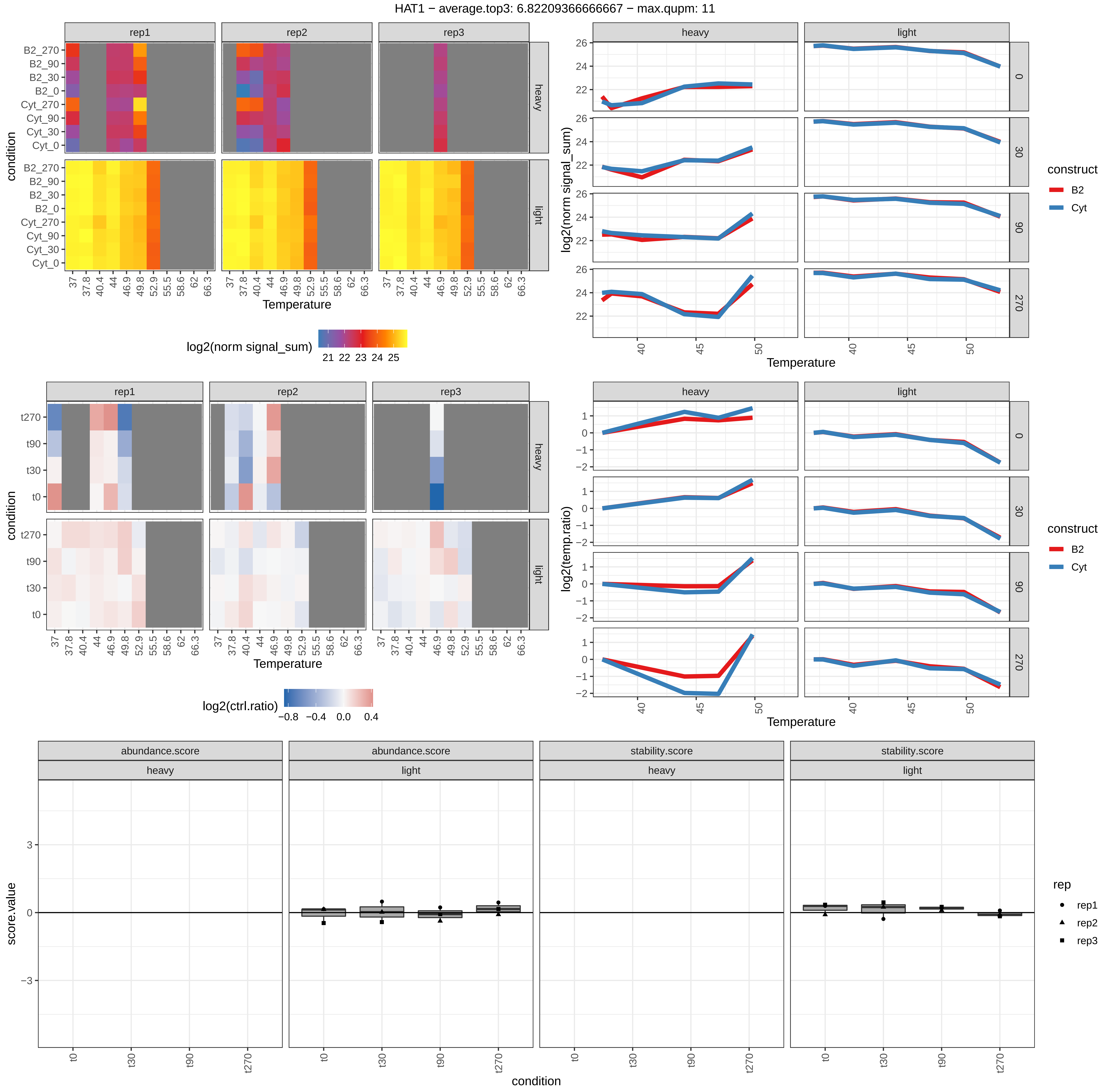

Q12341 − Histone acetyltransferase type B catalytic subunit OS=Saccharomyces cerevisiae (strain ATCC 204508 / S288c) GN=HAT1 PE=1 SV=1

HAT2 − average.top3: 6.68884095238095 − max.qupm: 6

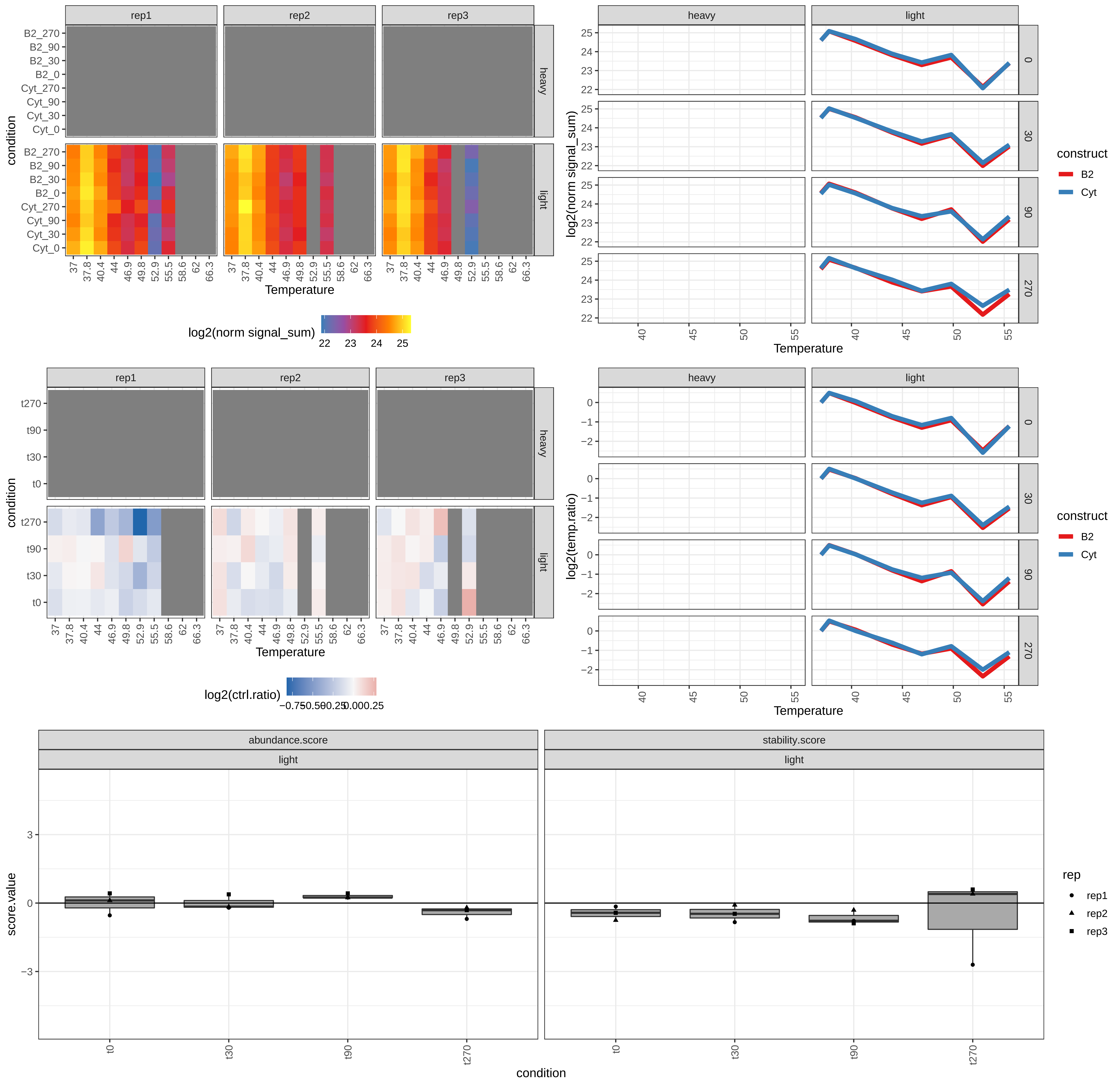

P39984 − Histone acetyltransferase type B subunit 2 OS=Saccharomyces cerevisiae (strain ATCC 204508 / S288c) GN=HAT2 PE=1 SV=1

HBN1 − average.top3: 7.12366018518519 − max.qupm: 5

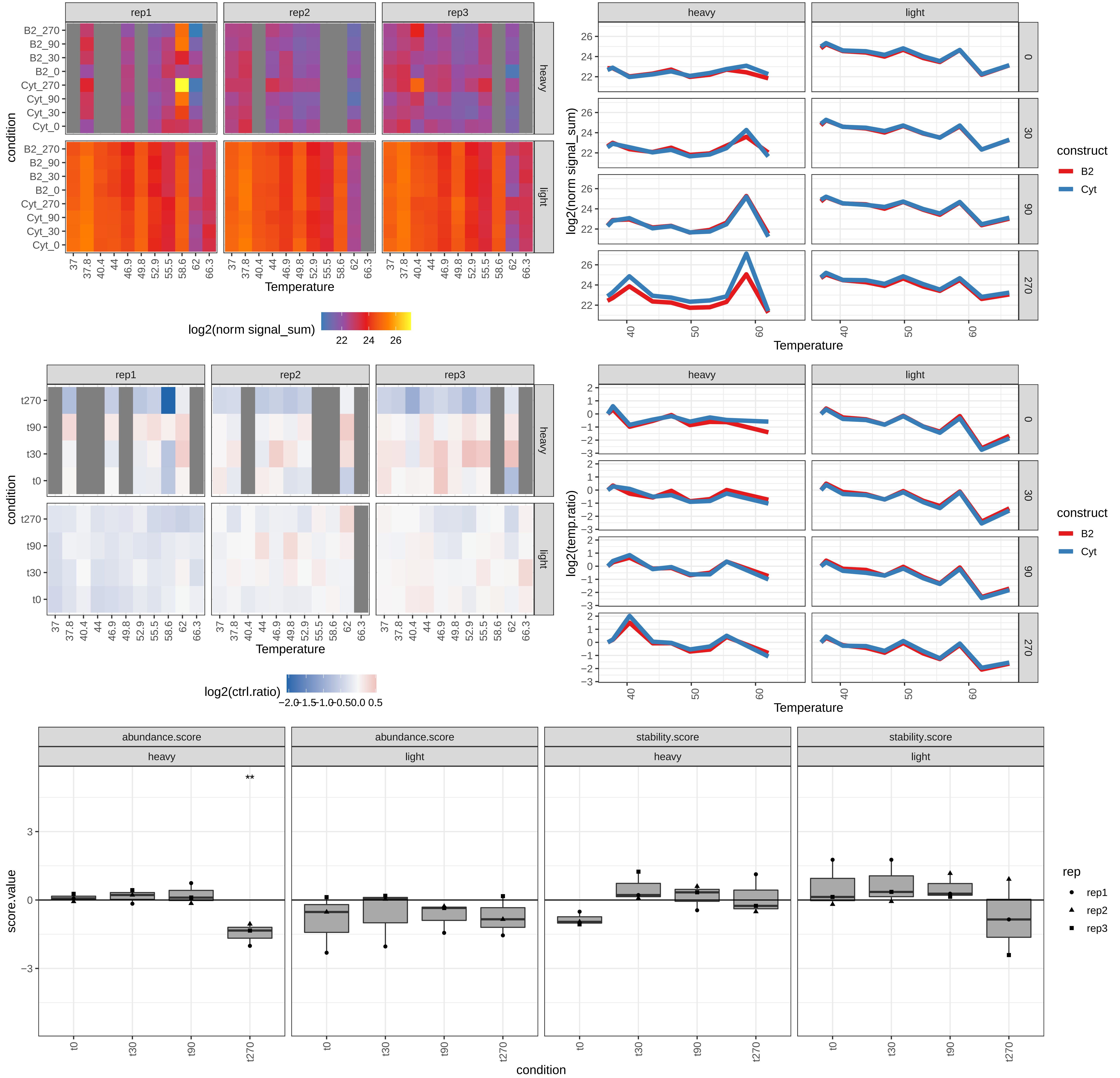

Q96VH4 − Putative nitroreductase HBN1 OS=Saccharomyces cerevisiae (strain ATCC 204508 / S288c) GN=HBN1 PE=1 SV=1

HBS1 − average.top3: 7.08117043478261 − max.qupm: 5

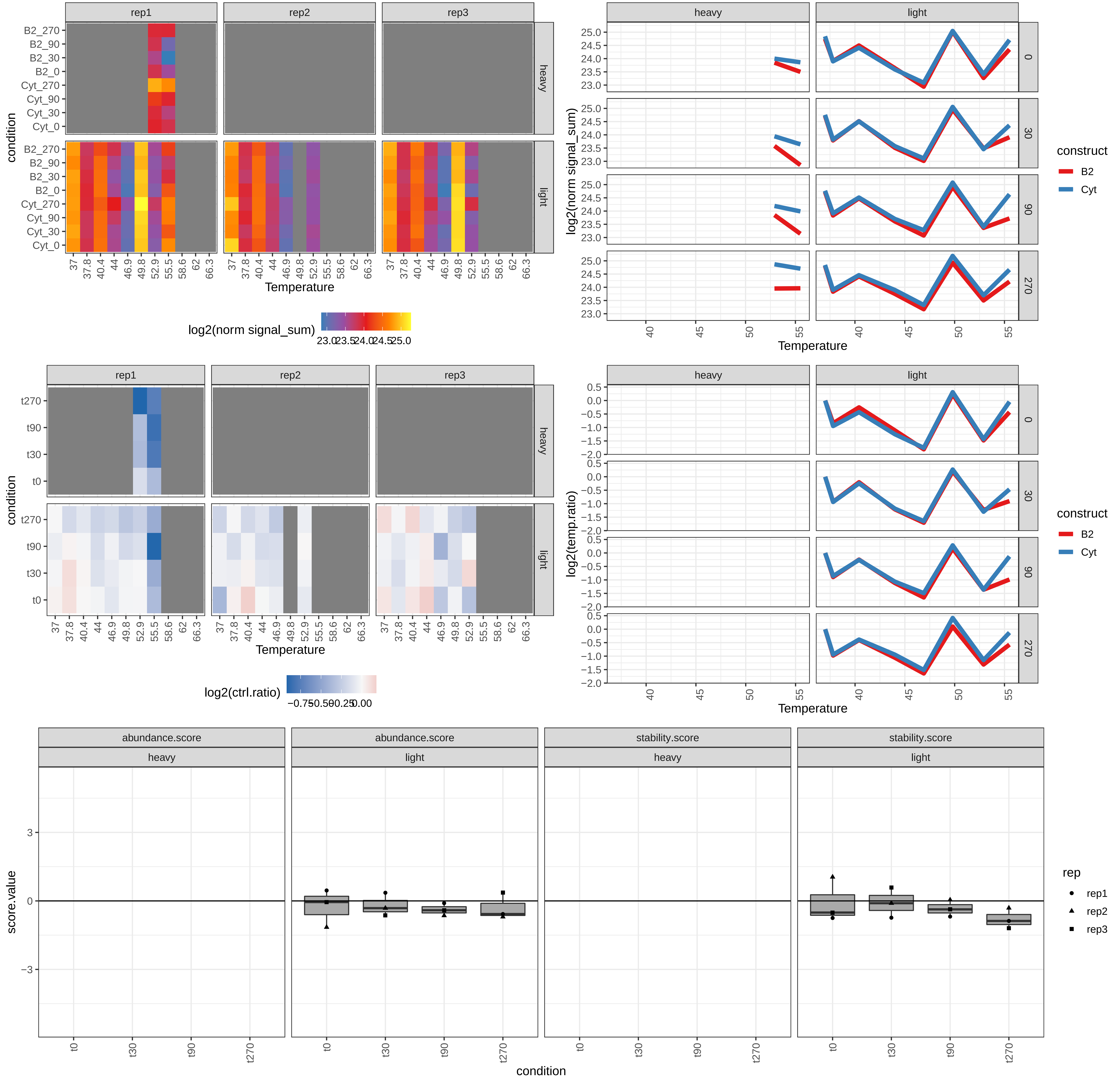

P32769 − Elongation factor 1 alpha−like protein OS=Saccharomyces cerevisiae (strain ATCC 204508 / S288c) GN=HBS1 PE=1 SV=2

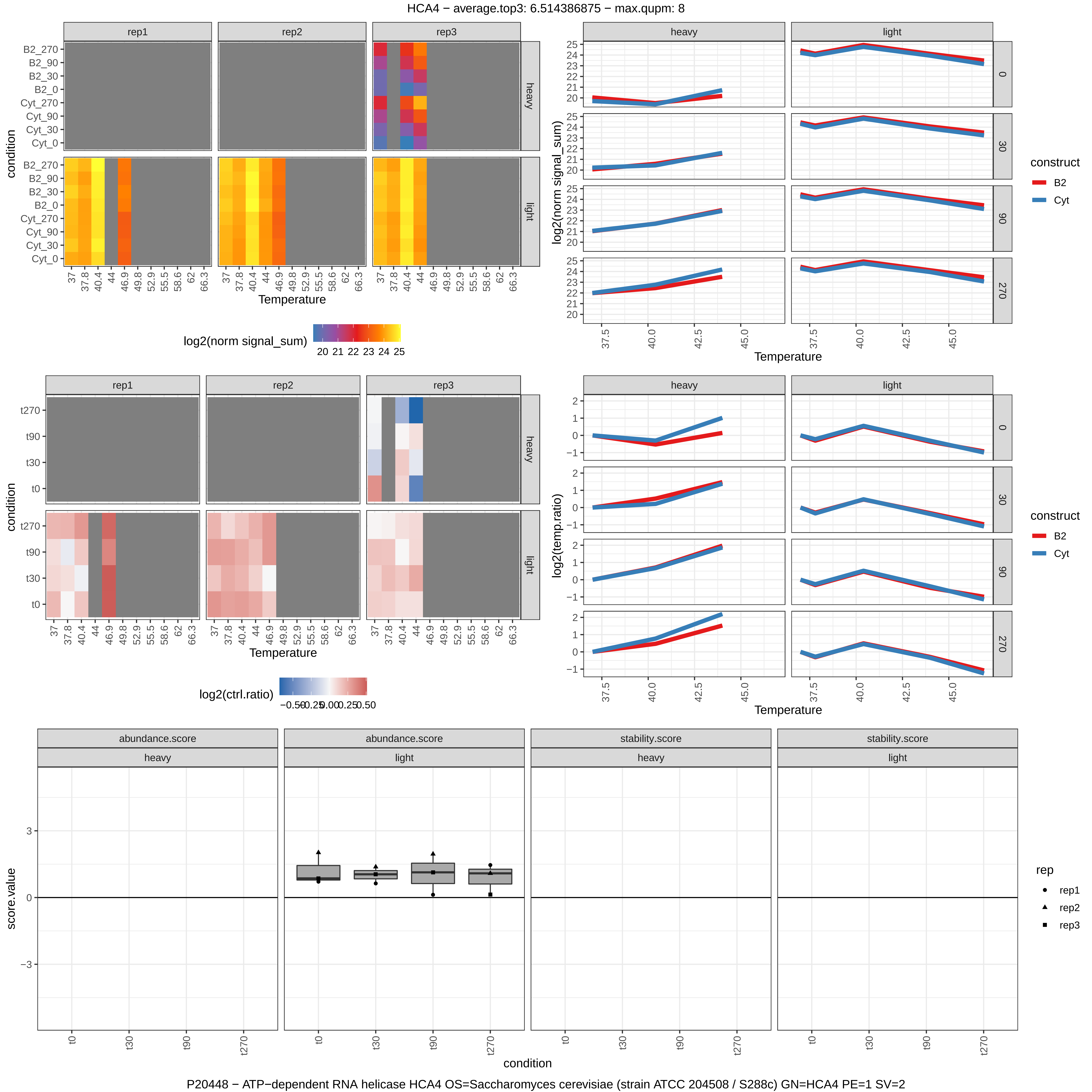

HCA4 − average.top3: 6.514386875 − max.qupm: 8
rep1
rep2
rep3
heavy
light
condition
Temperature
log2(norm signal_sum)
construct
B2
Cyt
log2(ctrl.ratio)
log2(temp.ratio)
abundance.score
stability.score
score.value
rep
rep1
rep2
rep3
P20448 − ATP−dependent RNA helicase HCA4 OS=Saccharomyces cerevisiae (strain ATCC 204508 / S288c) GN=HCA4 PE=1 SV=2

HCR1 − average.top3: 7.20800766666667 − max.qupm: 9

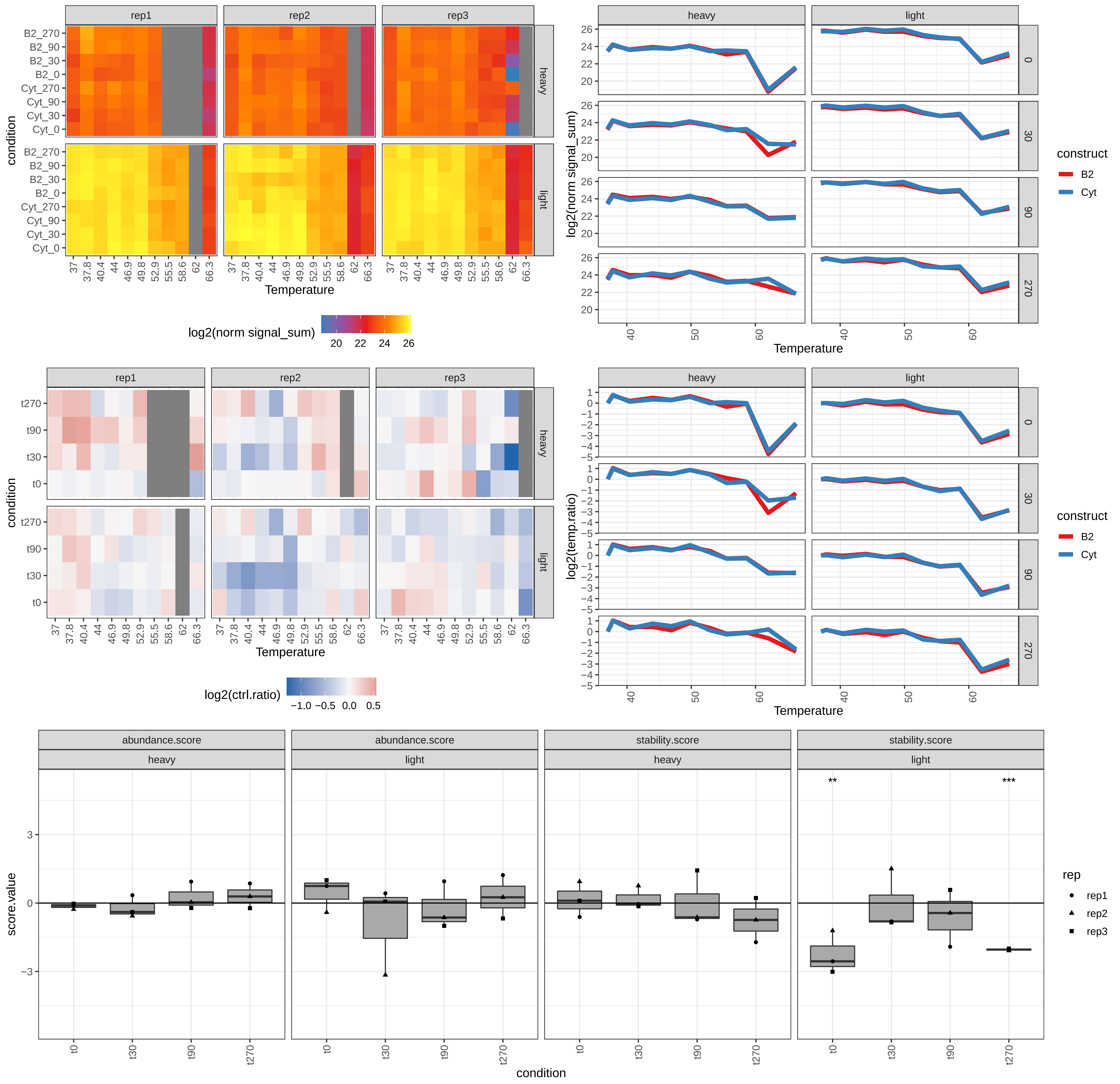

Q05775 − Eukaryotic translation initiation factor 3 subunit J OS=Saccharomyces cerevisiae (strain ATCC 204508 / S288c) GN=HCR1 PE=1 SV=1

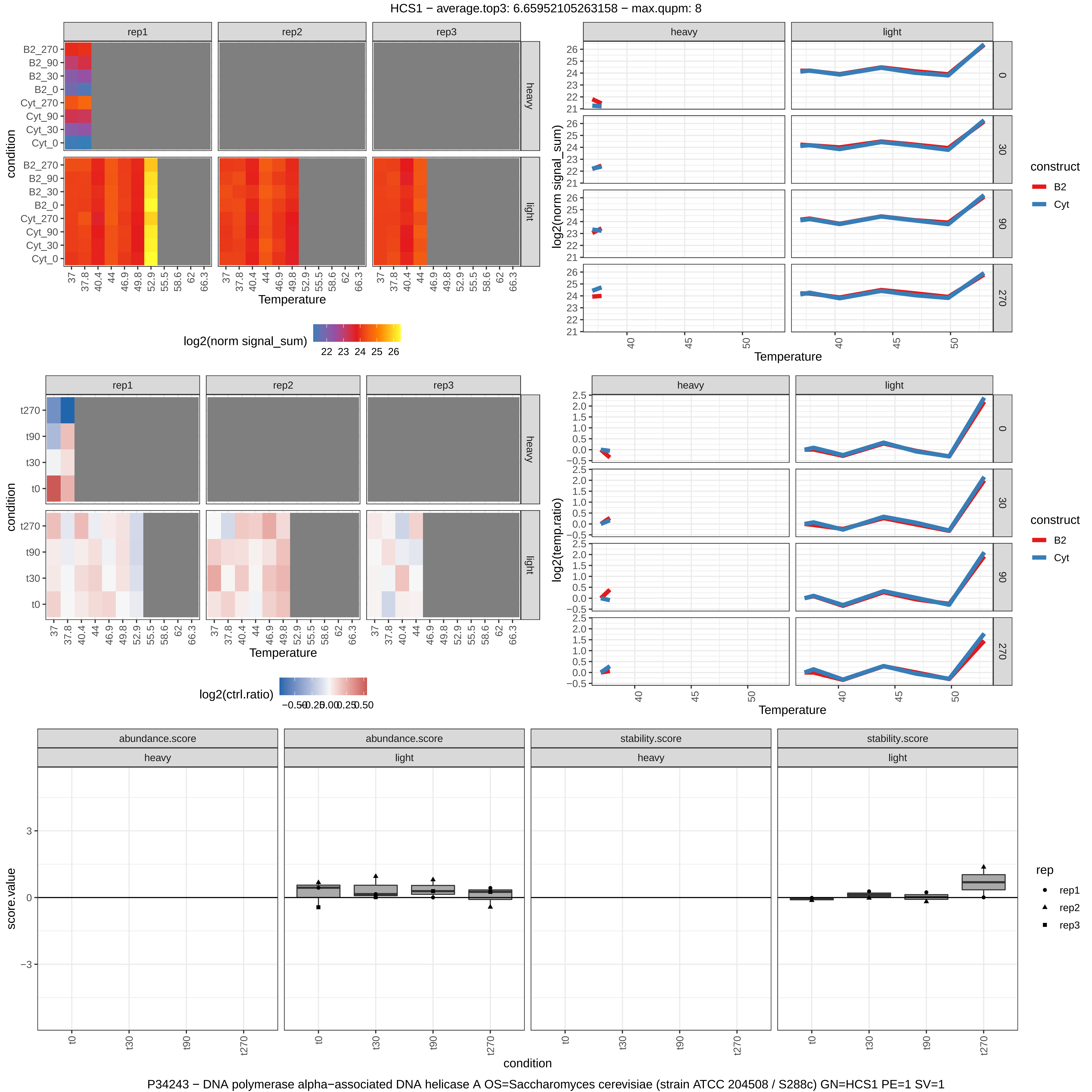
HCS1 − average.top3: 6.65952105263158 − max.qupm: 8
rep1
rep2
rep3
heavy
light
condition
Temperature
log2(norm signal_sum)
log2(ctrl.ratio)
log2(temp.ratio)
construct
B2
Cyt
abundance.score
stability.score
score.value
rep
rep1
rep2
rep3
P34243 − DNA polymerase alpha−associated DNA helicase A OS=Saccharomyces cerevisiae (strain ATCC 204508 / S288c) GN=HCS1 PE=1 SV=1

HDA1 − average.top3: 6.85420357142857 − max.qupm: 8

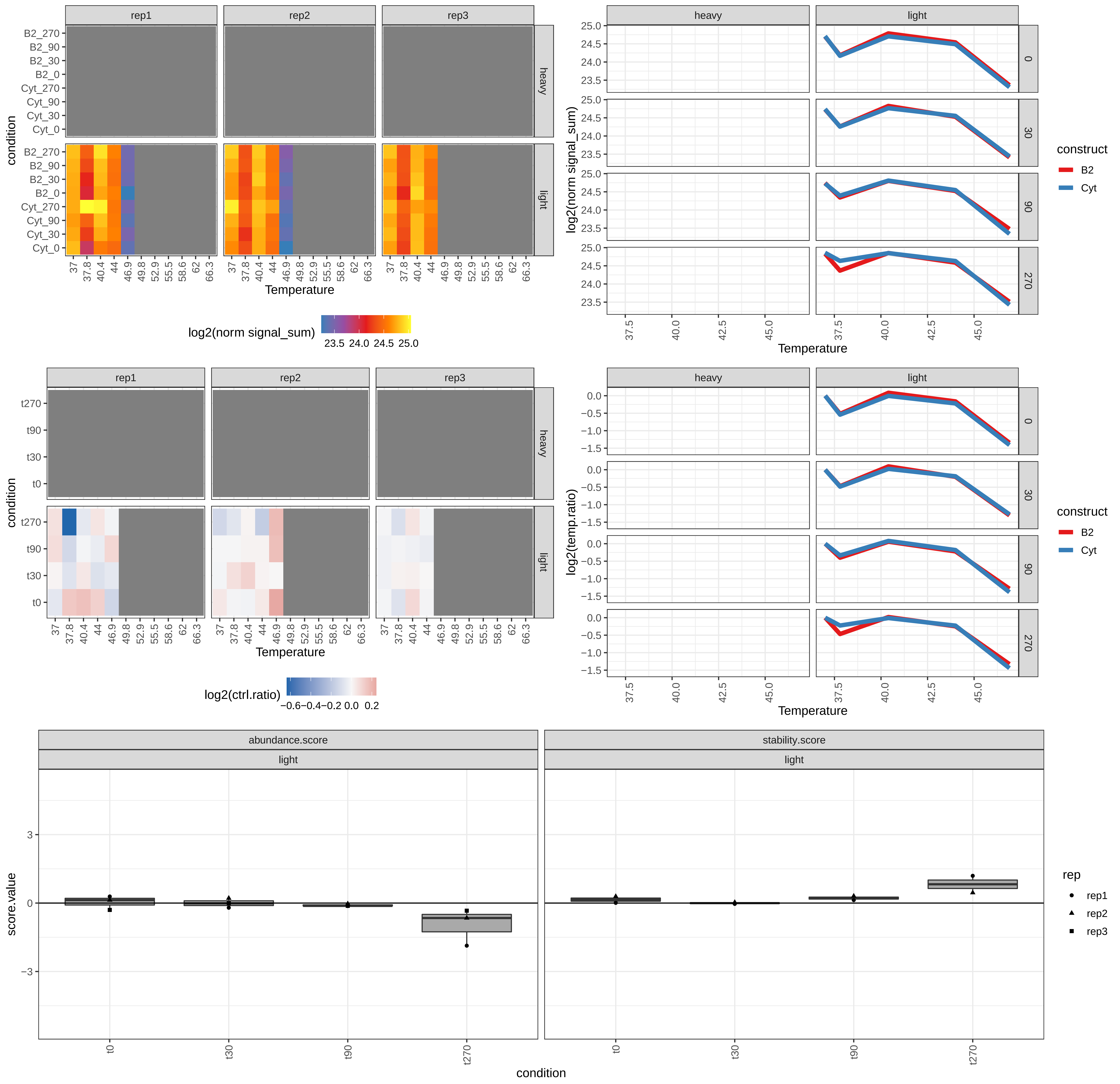

P53973 − Histone deacetylase HDA1 OS=Saccharomyces cerevisiae (strain ATCC 204508 / S288c) GN=HDA1 PE=1 SV=1

HDA2 − average.top3: 6.70242909090909 − max.qupm: 4

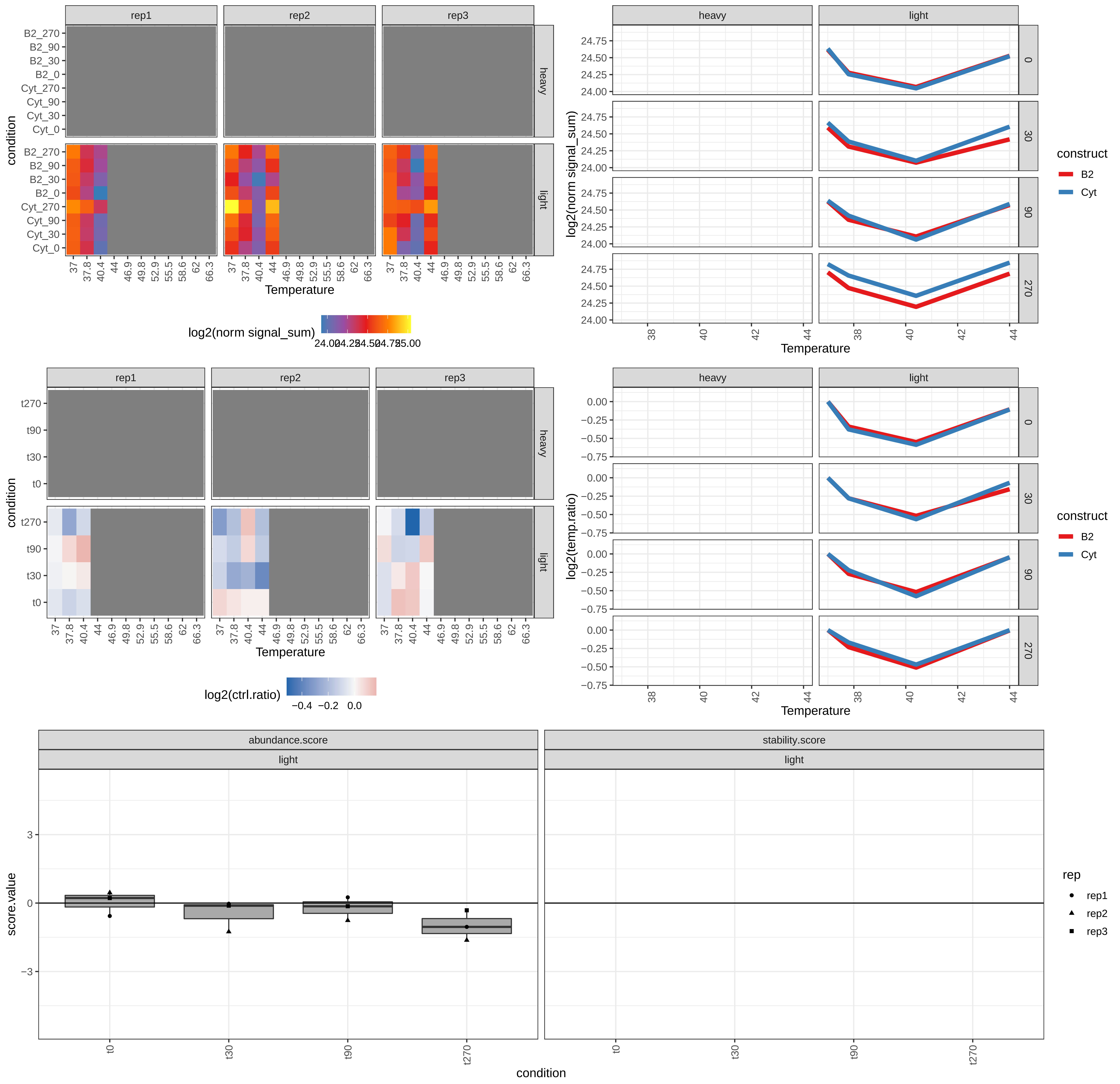

Q06629 − HDA1 complex subunit 2 OS=Saccharomyces cerevisiae (strain ATCC 204508 / S288c) GN=HDA2 PE=1 SV=1

HDA3 − average.top3: 6.52125454545455 − max.qupm: 5

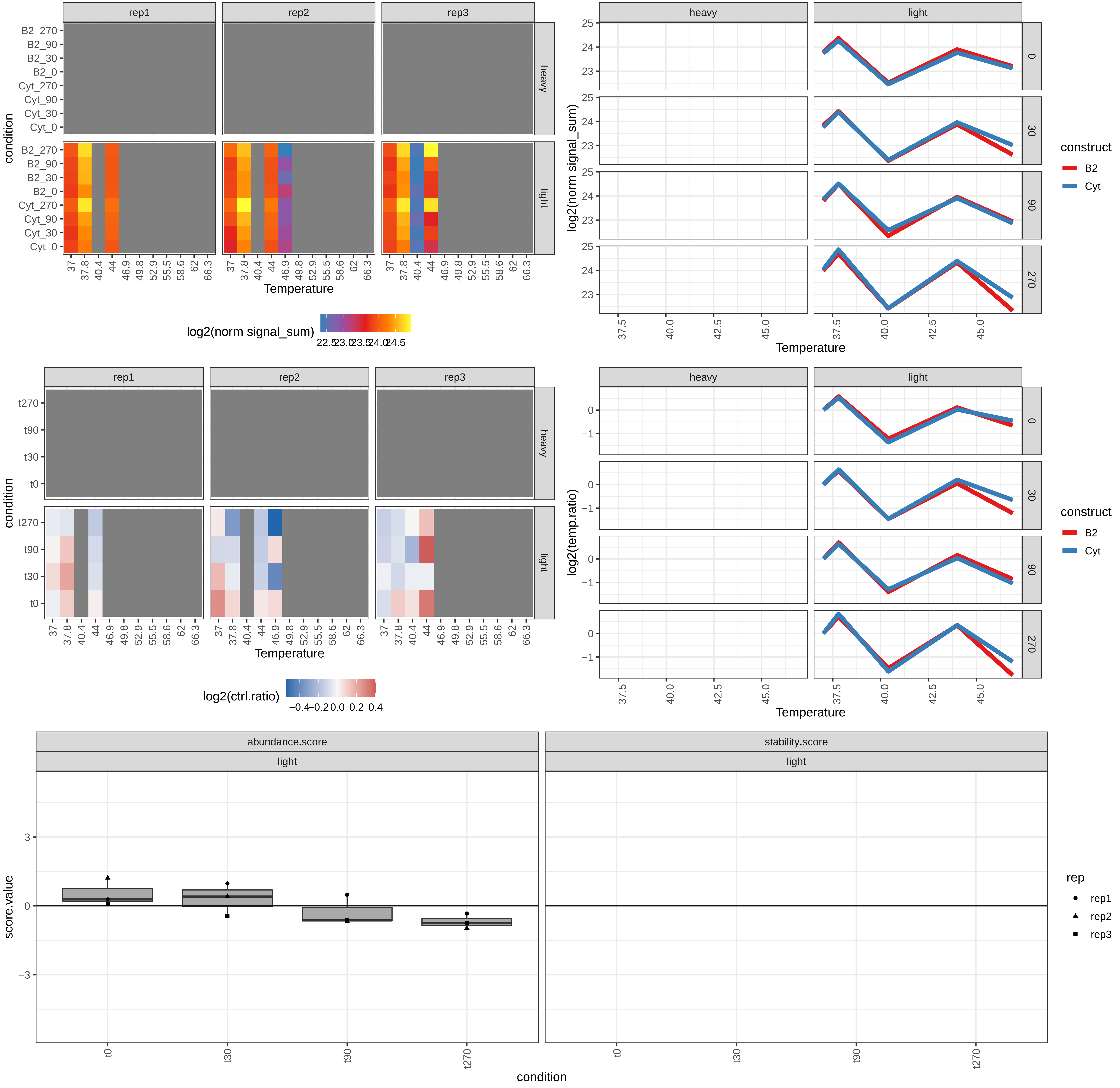

Q06623 − HDA1 complex subunit 3 OS=Saccharomyces cerevisiae (strain ATCC 204508 / S288c) GN=HDA3 PE=1 SV=1

HEF3 − average.top3: 6.8435225 − max.qupm: 12

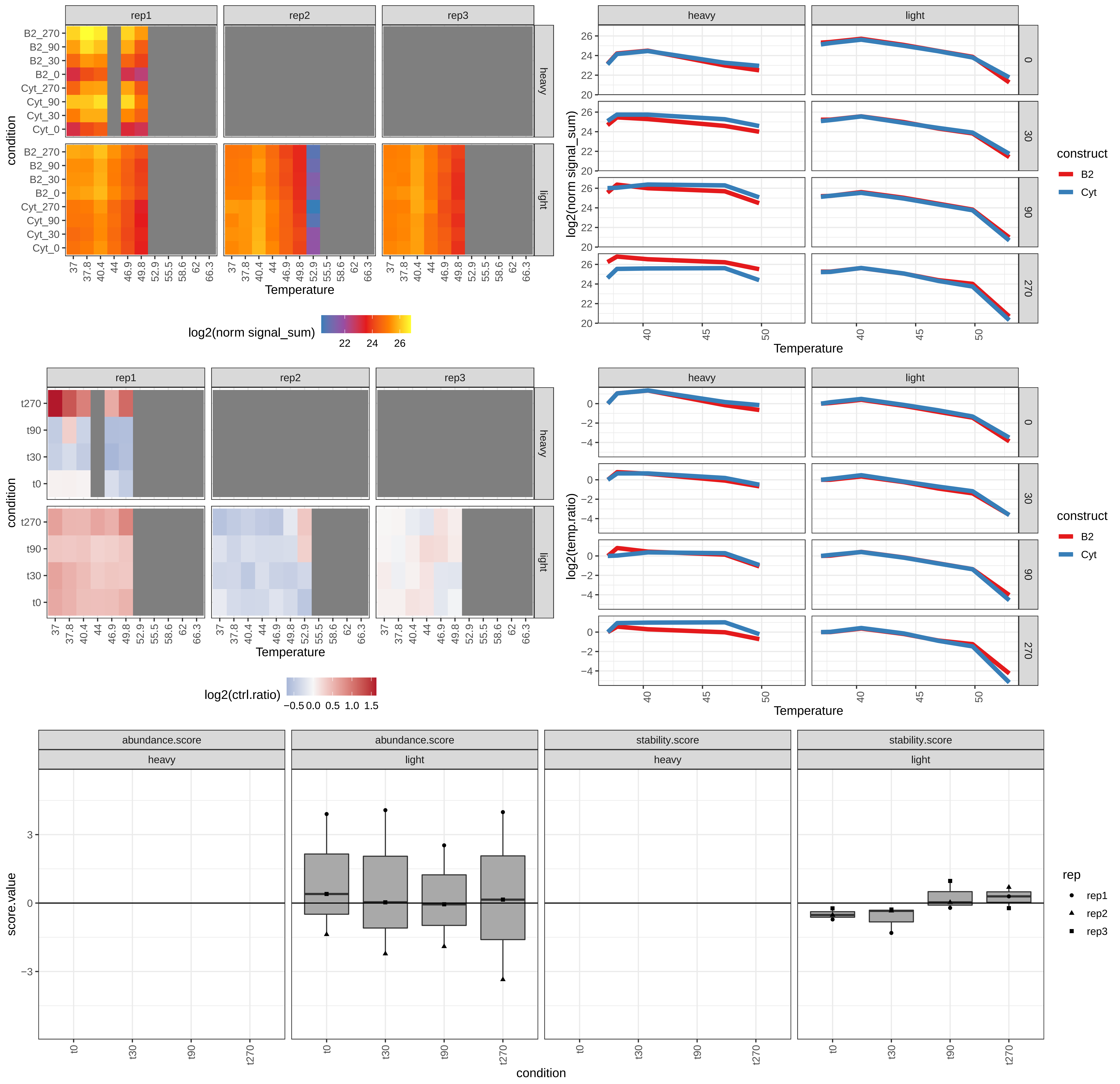

P53978 − Elongation factor 3B OS=Saccharomyces cerevisiae (strain ATCC 204508 / S288c) GN=HEF3 PE=1 SV=2

HEH2 − average.top3: 6.46243 − max.qupm: 5

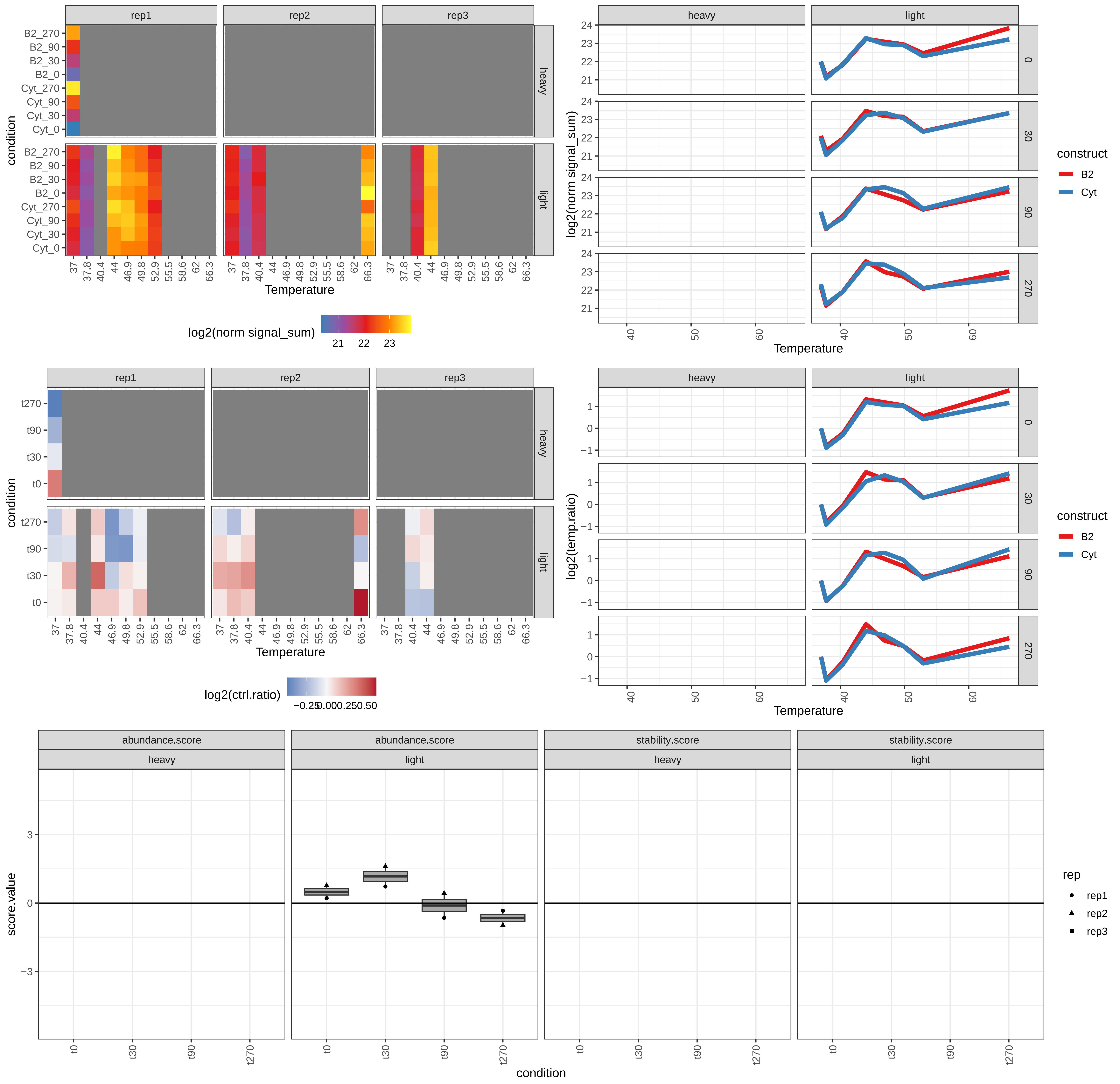

Q03281 − Inner nuclear membrane protein HEH2 OS=Saccharomyces cerevisiae (strain ATCC 204508 / S288c) GN=HEH2 PE=1 SV=1

HEK2 − average.top3: 6.94297458333333 − max.qupm: 5

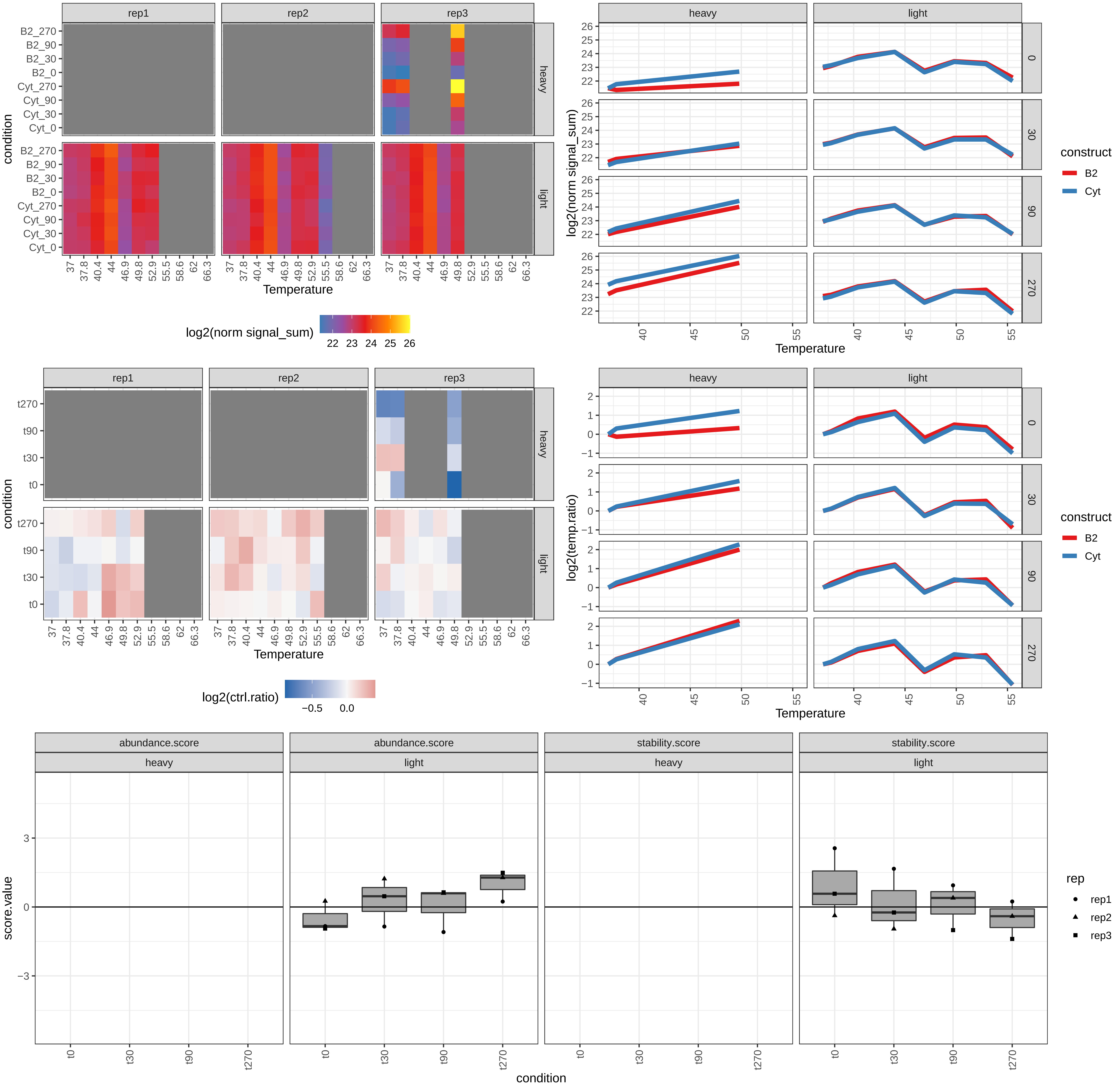

P38199 − Heterogeneous nuclear rnp K−like protein 2 OS=Saccharomyces cerevisiae (strain ATCC 204508 / S288c) GN=HEK2 PE=1 SV=1

HEL2 − average.top3: 6.67054291666667 − max.qupm: 6

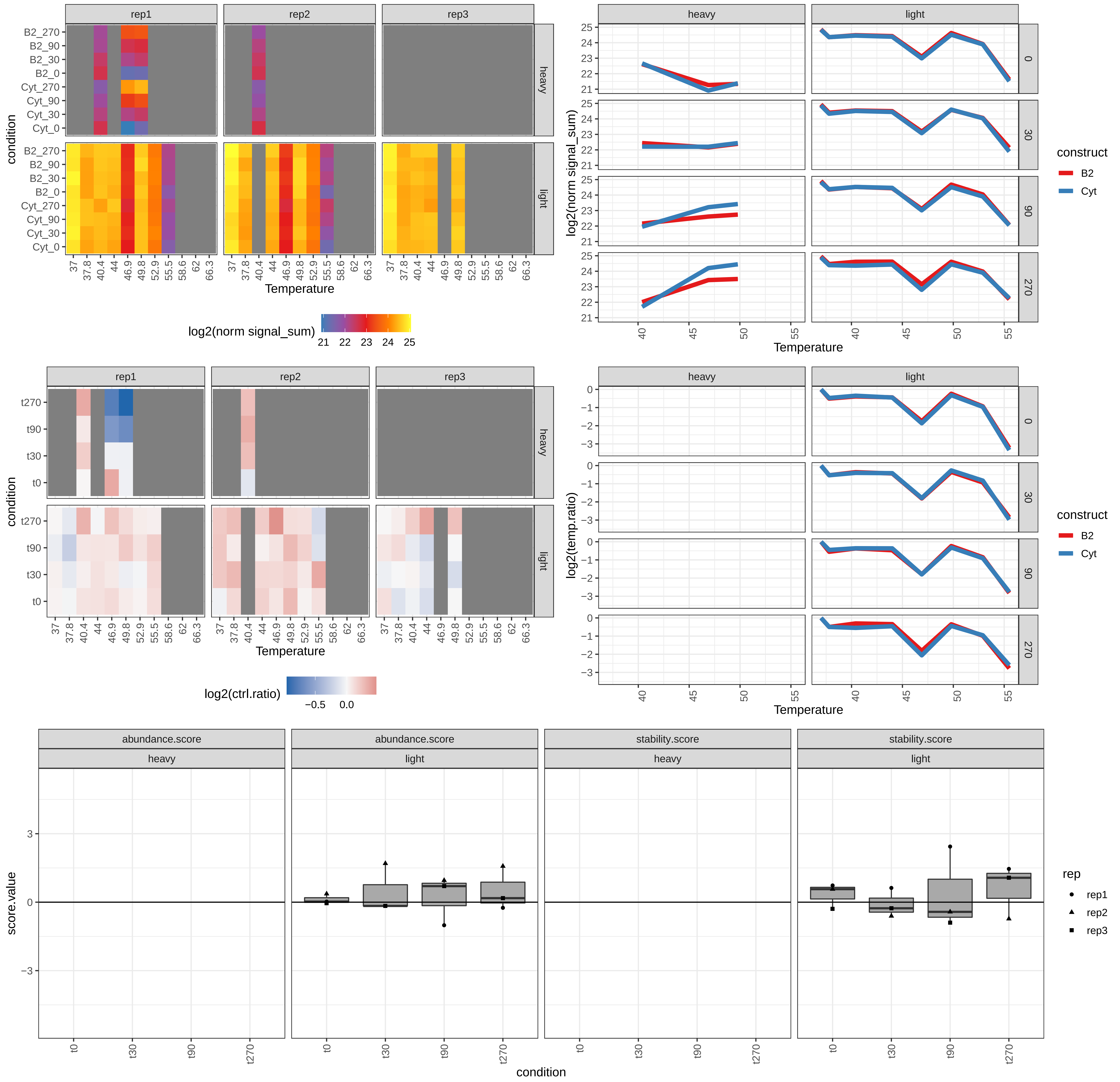

Q05580 − E3 ubiquitin−protein ligase HEL2 OS=Saccharomyces cerevisiae (strain ATCC 204508 / S288c) GN=HEL2 PE=1 SV=1

HEM1 − average.top3: 7.29814697674419 − max.qupm: 9

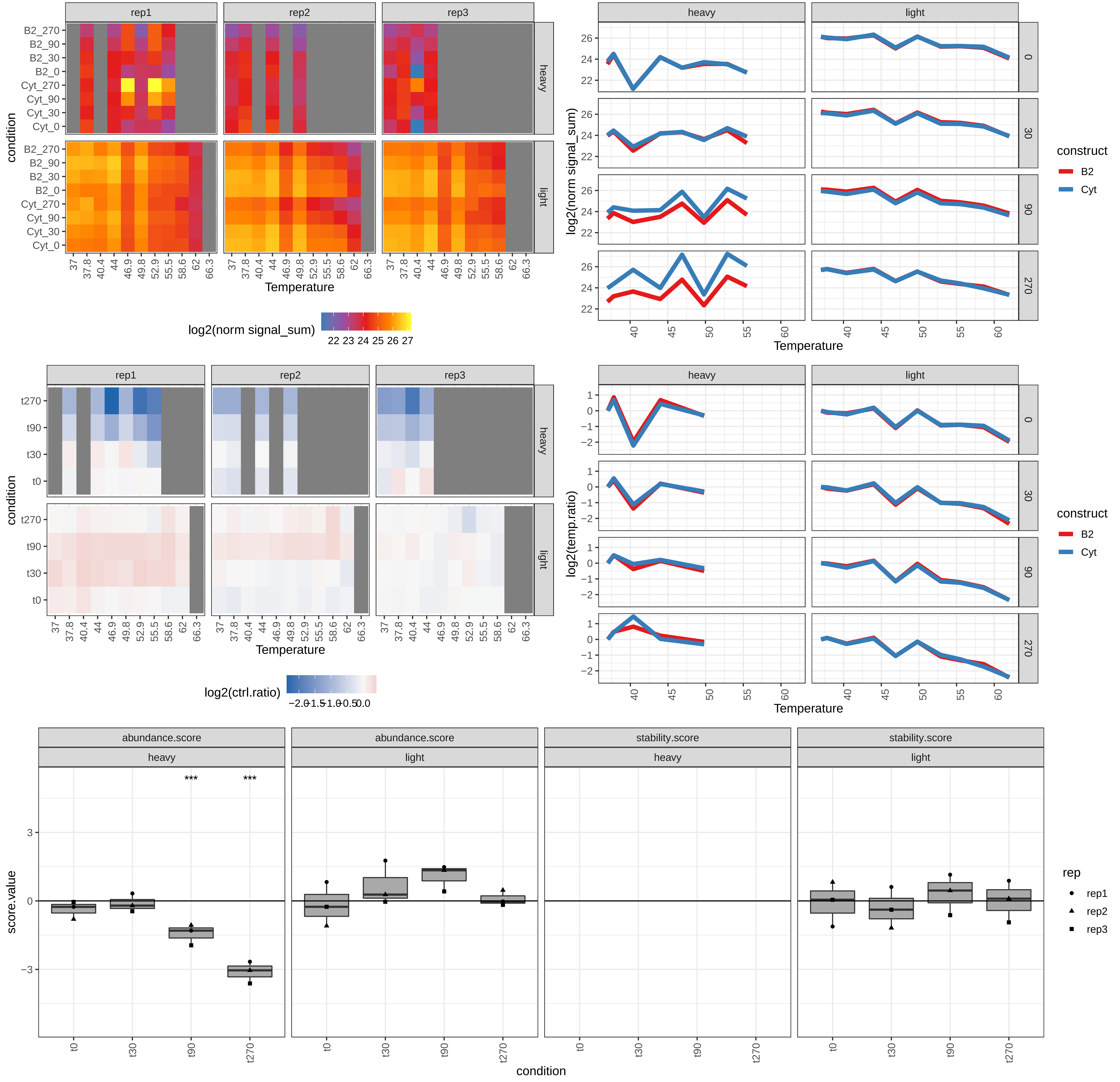

P09950 − 5−aminolevulinate synthase, mitochondrial OS=Saccharomyces cerevisiae (strain ATCC 204508 / S288c) GN=HEM1 PE=1 SV=1

HEM12 − average.top3: 6.97742018518518 − max.qupm: 12

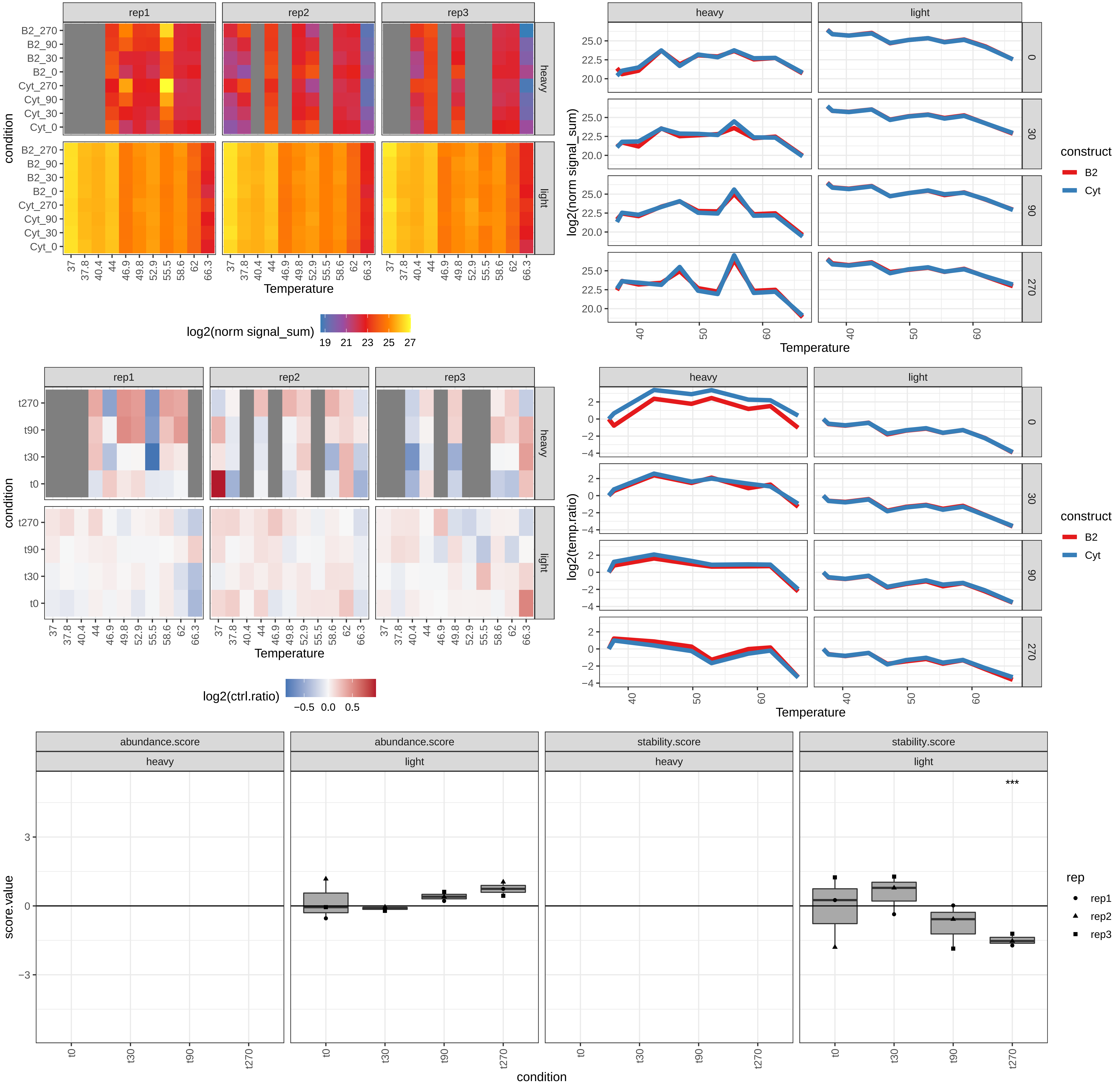

P32347 − Uroporphyrinogen decarboxylase OS=Saccharomyces cerevisiae (strain ATCC 204508 / S288c) GN=HEM12 PE=1 SV=1

HEM13 − average.top3: 7.18575775510204 − max.qupm: 13

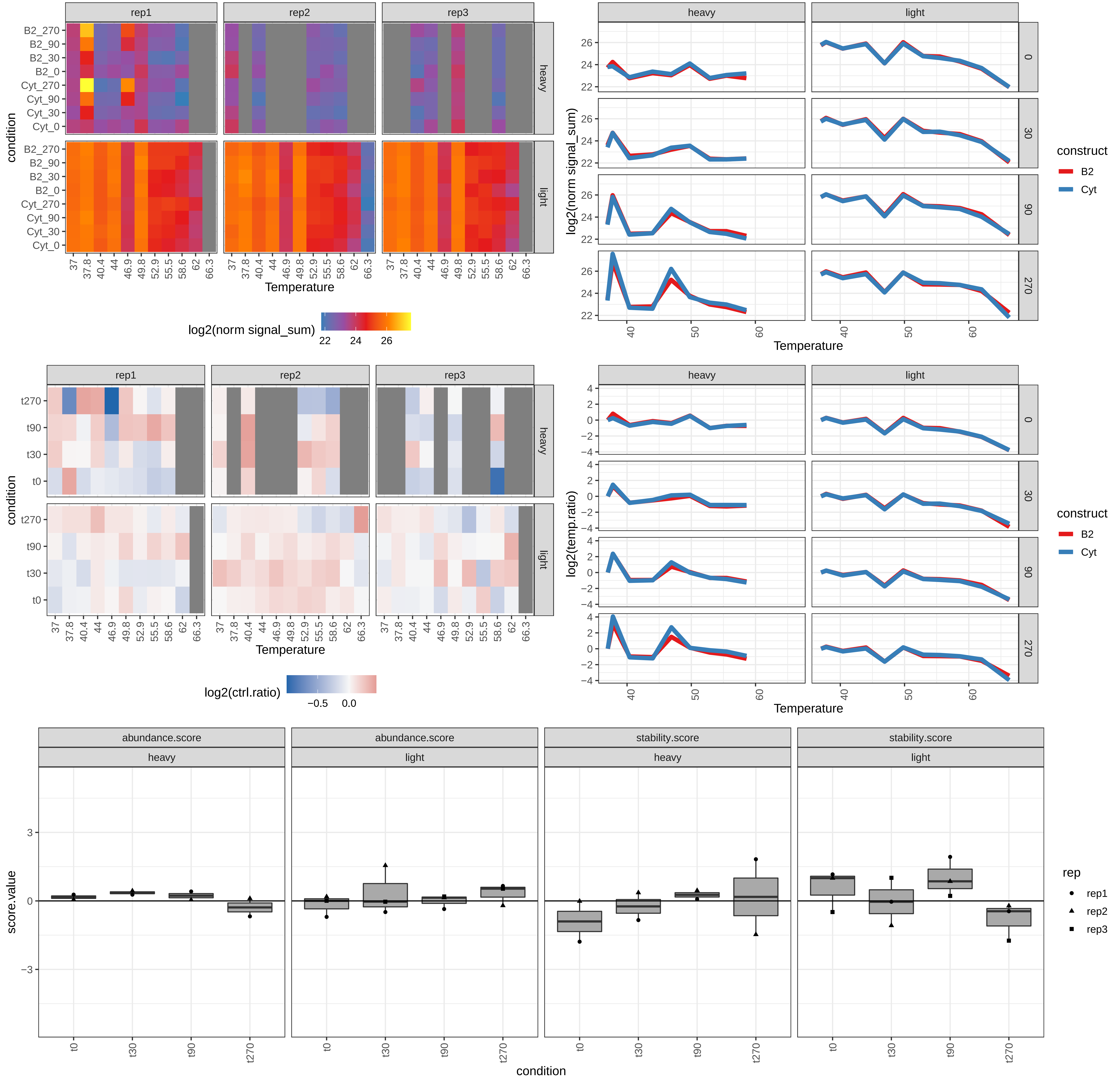

P11353 − Oxygen−dependent coproporphyrinogen−III oxidase OS=Saccharomyces cerevisiae (strain ATCC 204508 / S288c) GN=HEM13 PE=1 SV=2

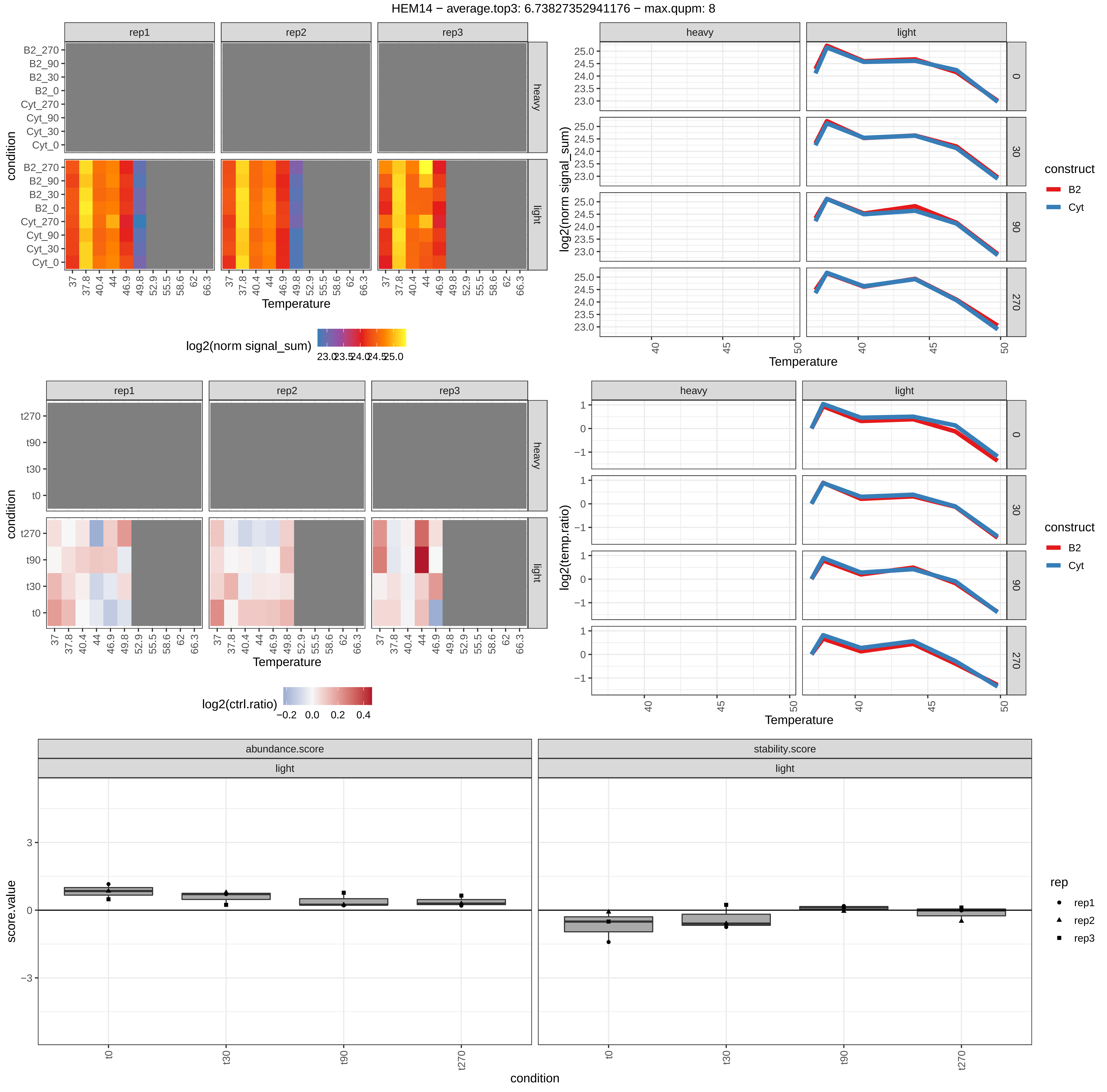

P40012 − Protoporphyrinogen oxidase OS=Saccharomyces cerevisiae (strain ATCC 204508 / S288c) GN=HEM14 PE=1 SV=1

HEM15 − average.top3: 7.426238 − max.qupm: 14

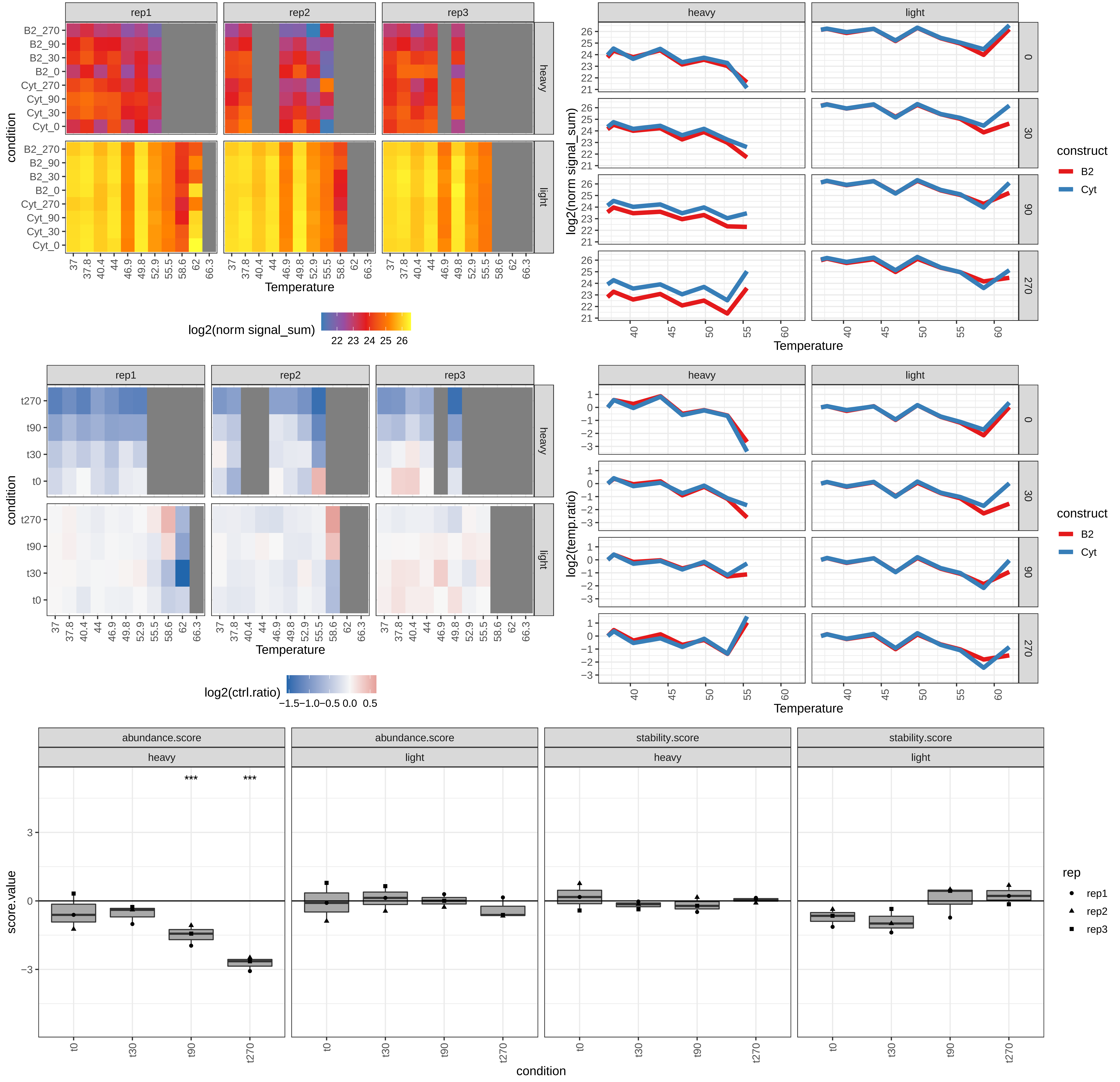

P16622 − Ferrochelatase, mitochondrial OS=Saccharomyces cerevisiae (strain ATCC 204508 / S288c) GN=HEM15 PE=1 SV=1

HEM2 − average.top3: 7.47835944444444 − max.qupm: 12

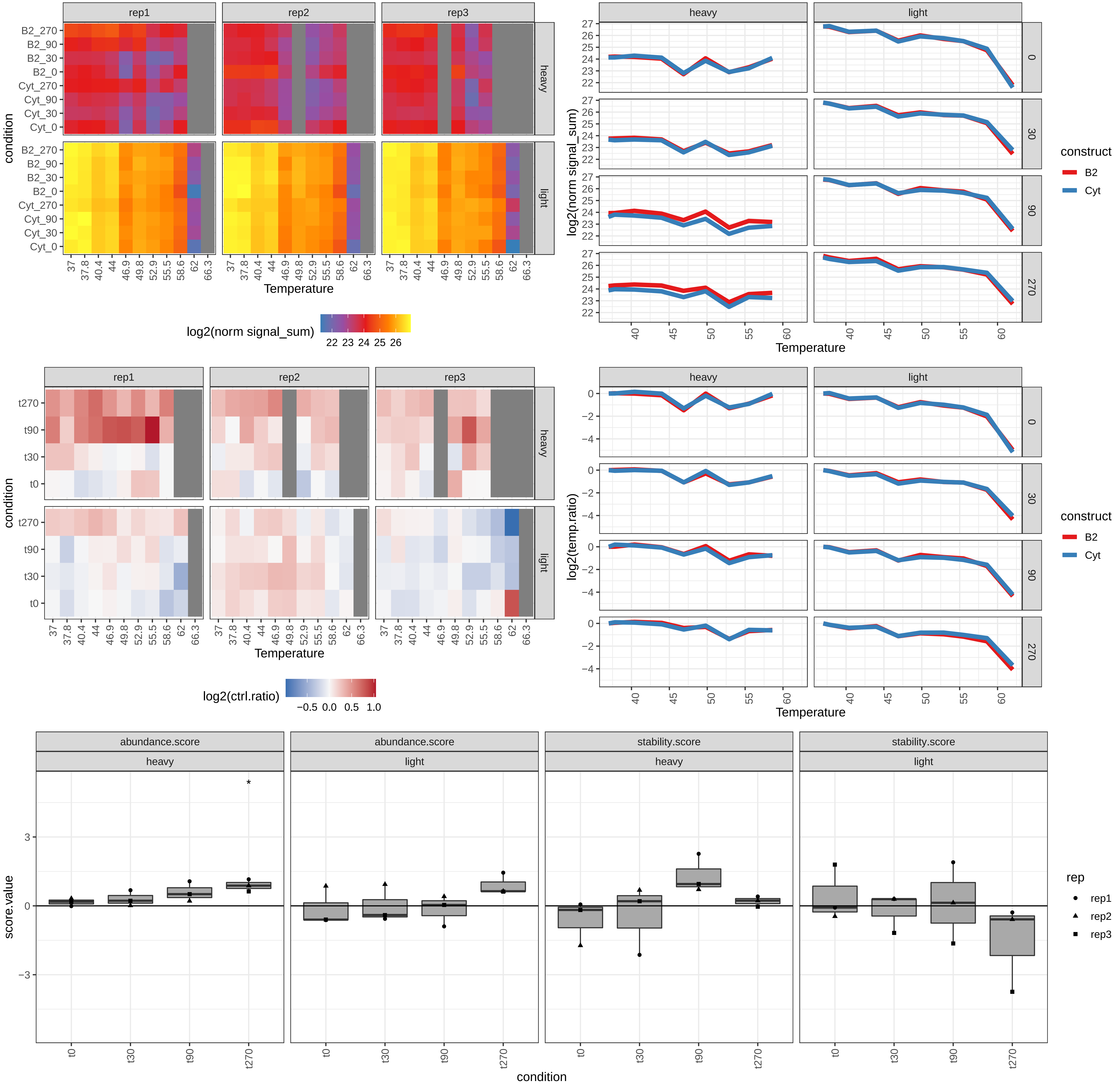

P05373 − Delta−aminolevulinic acid dehydratase OS=Saccharomyces cerevisiae (strain ATCC 204508 / S288c) GN=HEM2 PE=1 SV=2

HEM3 − average.top3: 6.68502909090909 − max.qupm: 8

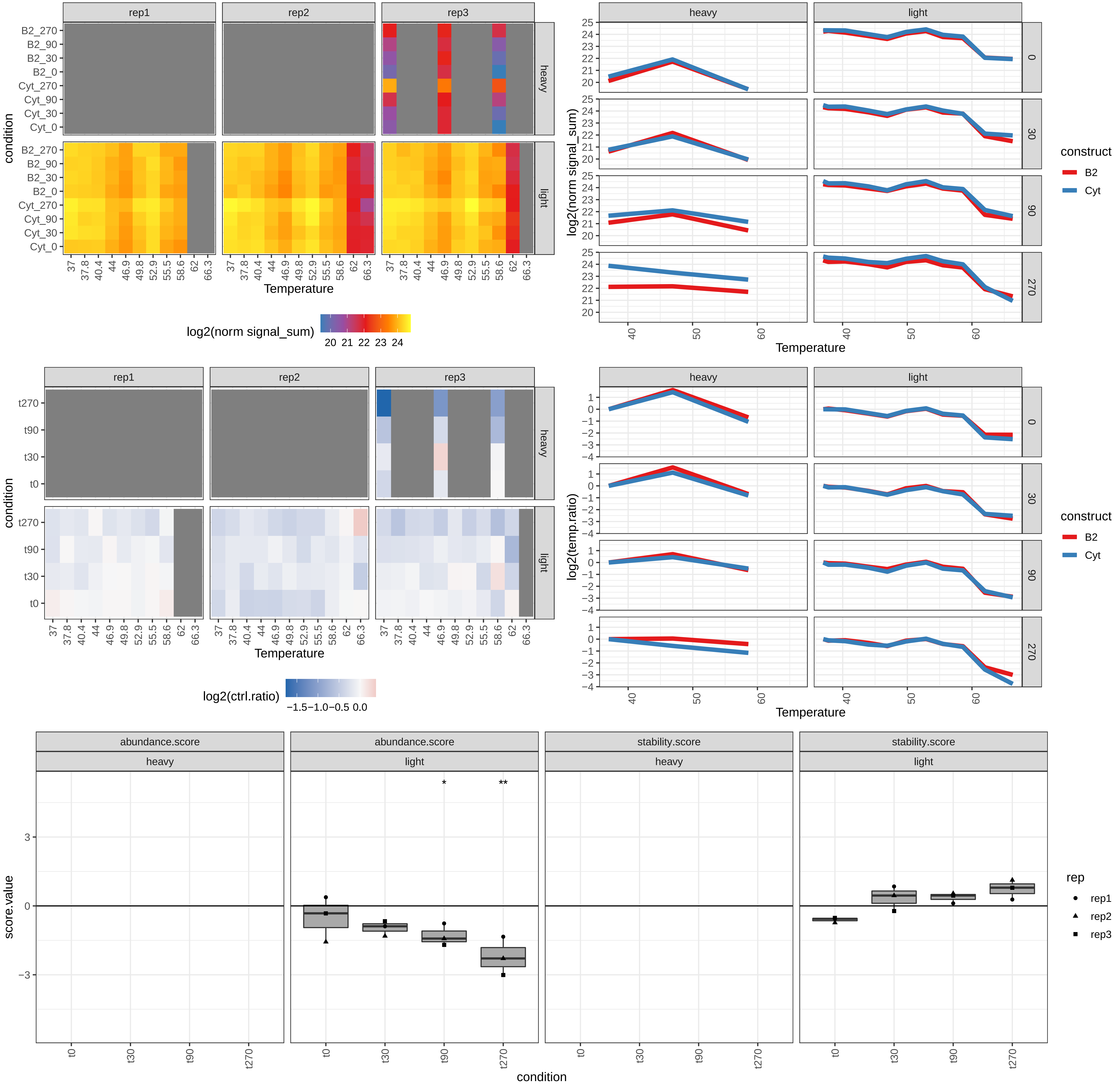

P28789 − Porphobilinogen deaminase OS=Saccharomyces cerevisiae (strain ATCC 204508 / S288c) GN=HEM3 PE=1 SV=1

HEM4 − average.top3: 6.77764 − max.qupm: 6

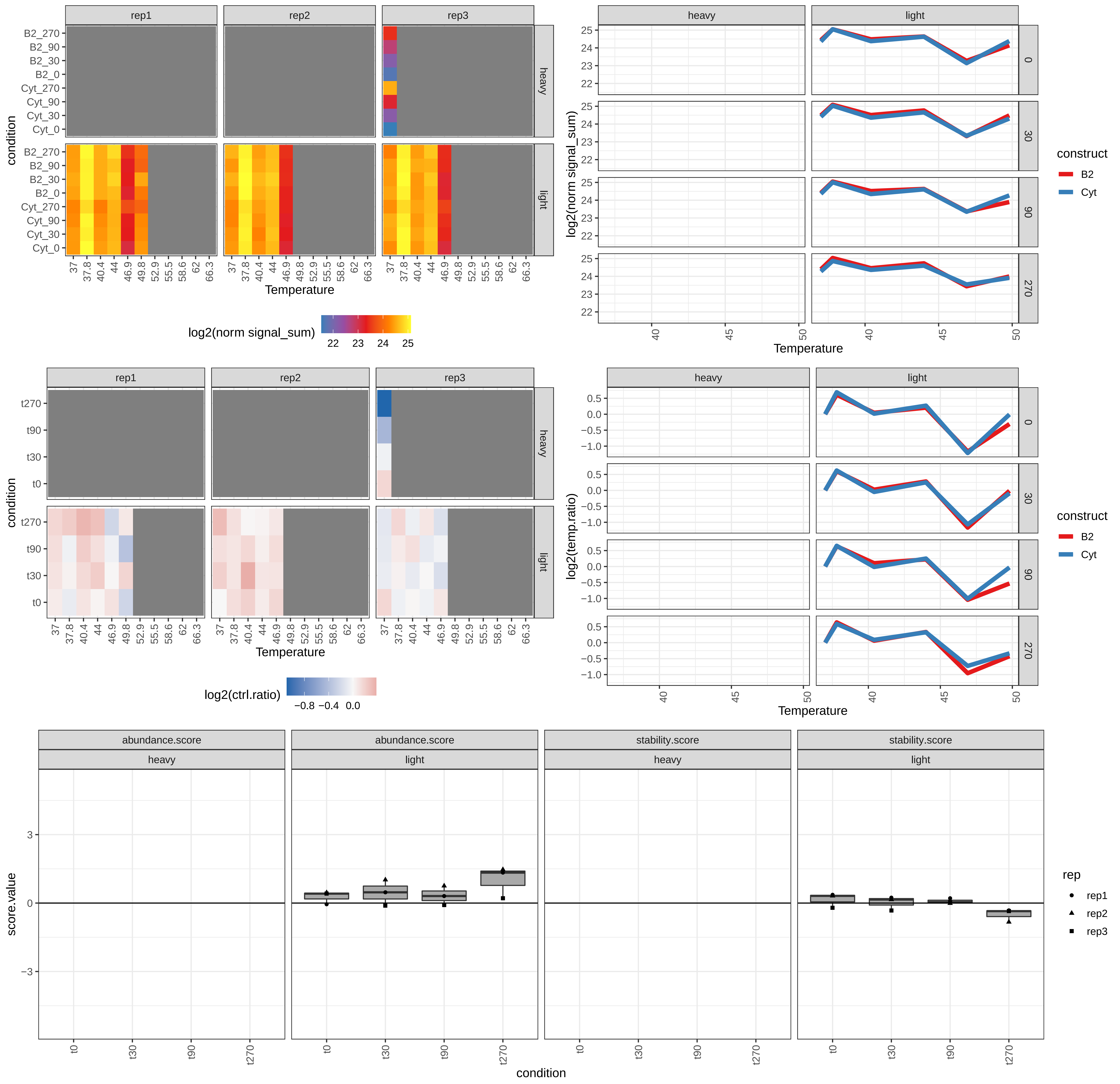

P06174 − Uroporphyrinogen−III synthase OS=Saccharomyces cerevisiae (strain ATCC 204508 / S288c) GN=HEM4 PE=1 SV=2

HER1 − average.top3: 6.28774545454545 − max.qupm: 6

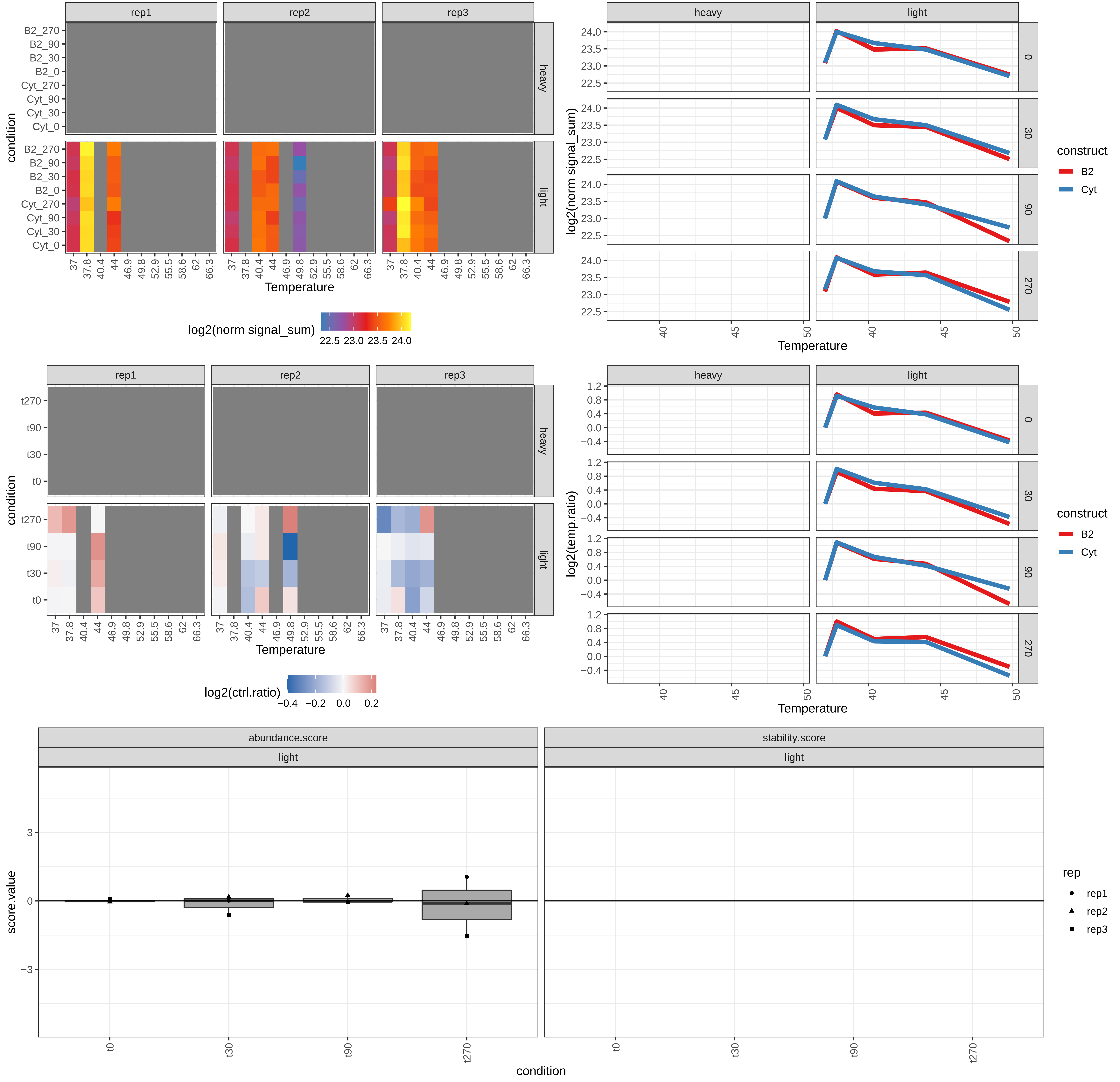

Q12276 − HMG2−induced ER−remodeling protein 1 OS=Saccharomyces cerevisiae (strain ATCC 204508 / S288c) GN=HER1 PE=1 SV=1

HFA1 − average.top3: 6.18363090909091 − max.qupm: 6

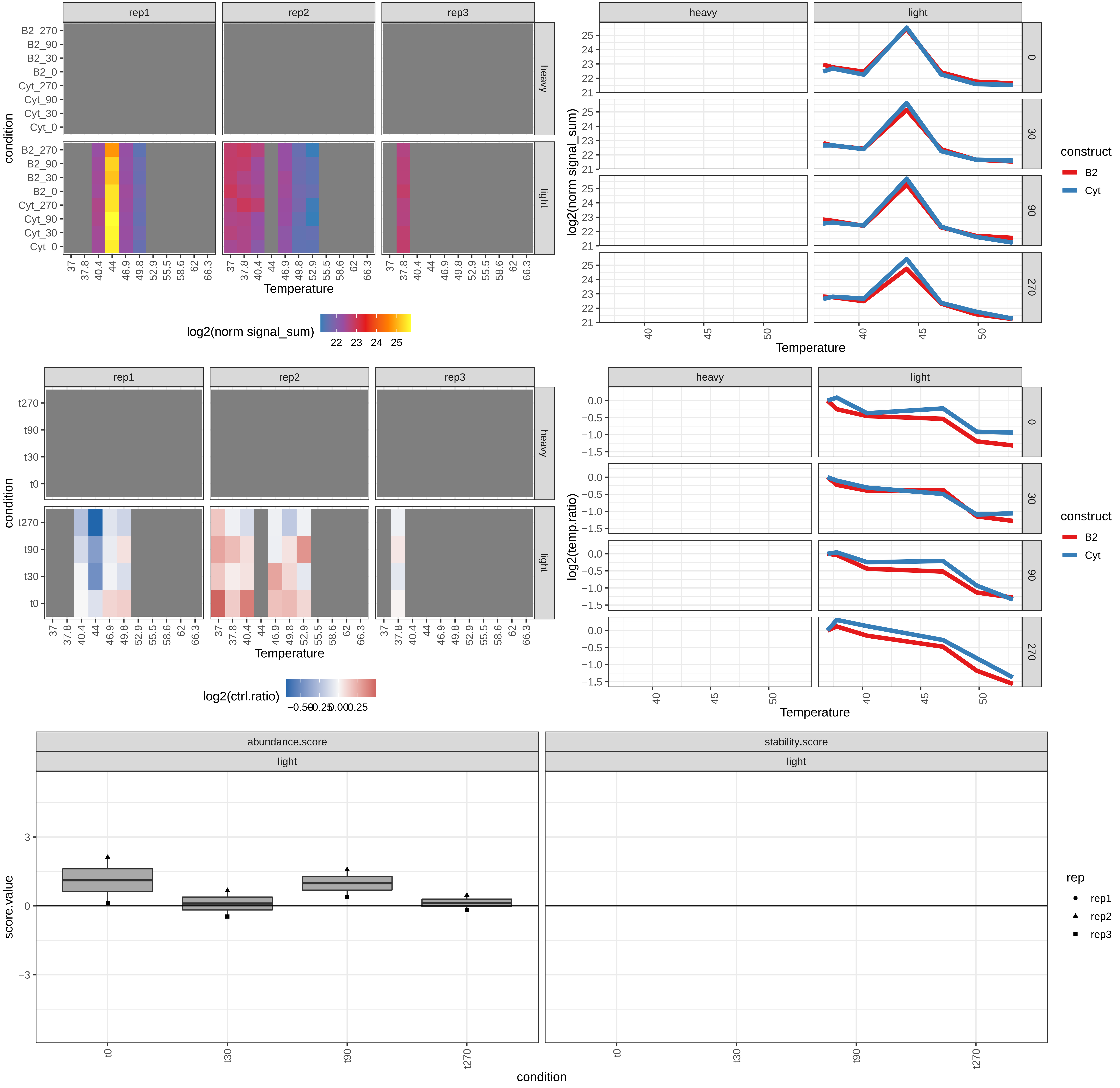

P32874 − Acetyl−CoA carboxylase, mitochondrial OS=Saccharomyces cerevisiae (strain ATCC 204508 / S288c) GN=HFA1 PE=1 SV=2

HFD1 − average.top3: 7.4346052 − max.qupm: 13

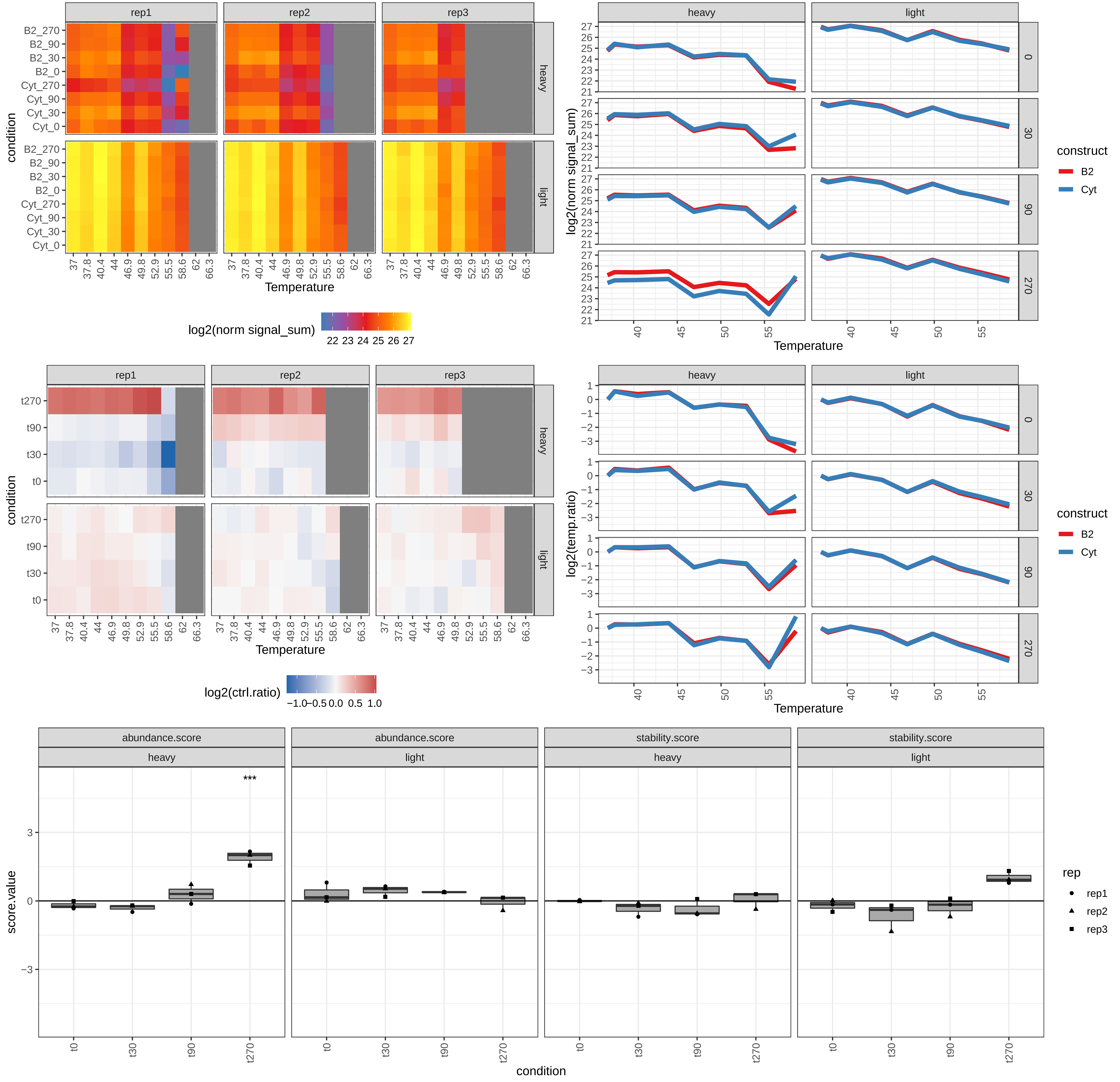

Q04458 − Fatty aldehyde dehydrogenase HFD1 OS=Saccharomyces cerevisiae (strain ATCC 204508 / S288c) GN=HFD1 PE=1 SV=1

HFI1 − average.top3: 6.01354625 − max.qupm: 3

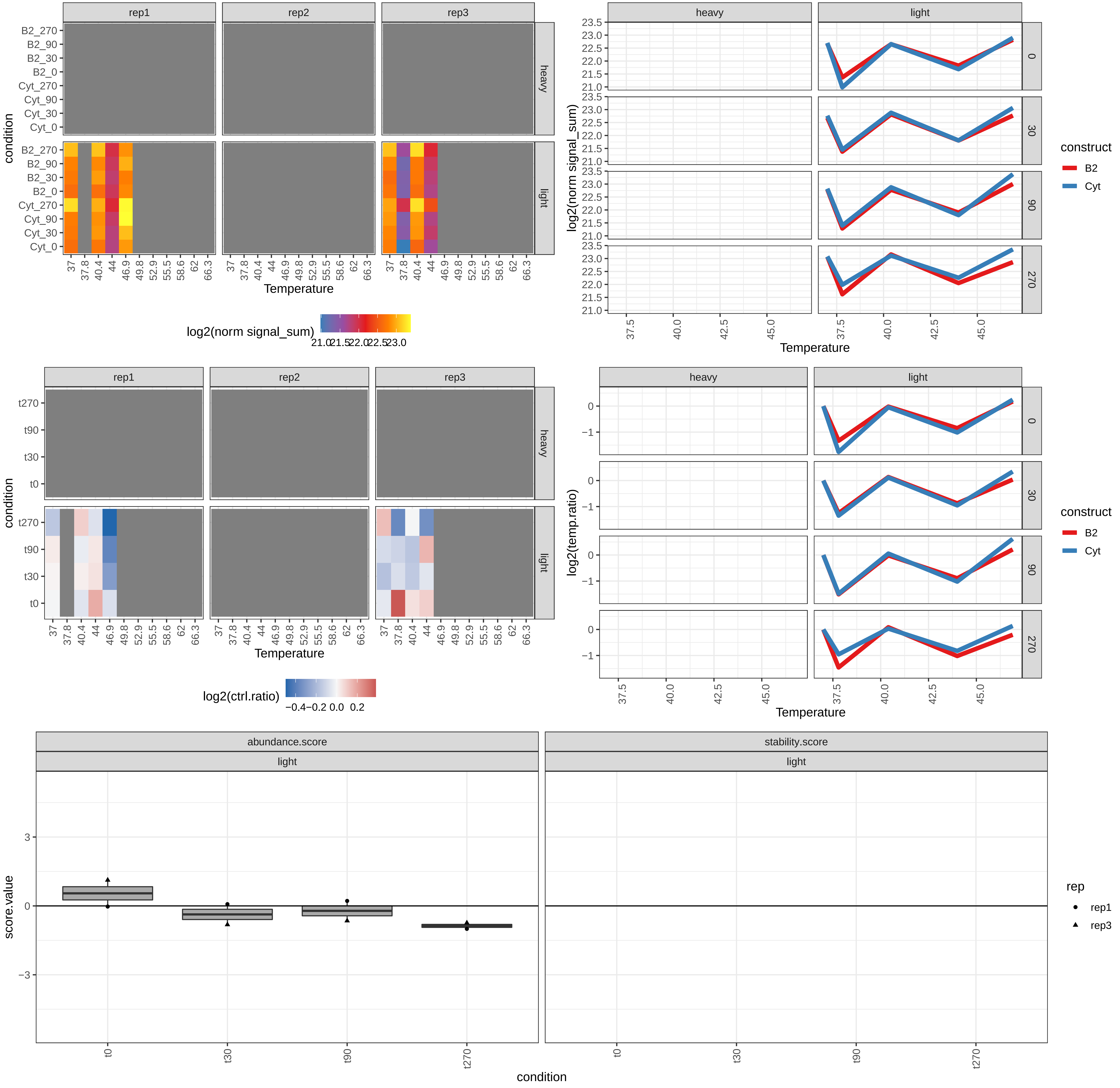

Q12060 − Transcriptional coactivator HFI1/ADA1 OS=Saccharomyces cerevisiae (strain ATCC 204508 / S288c) GN=HFI1 PE=1 SV=1

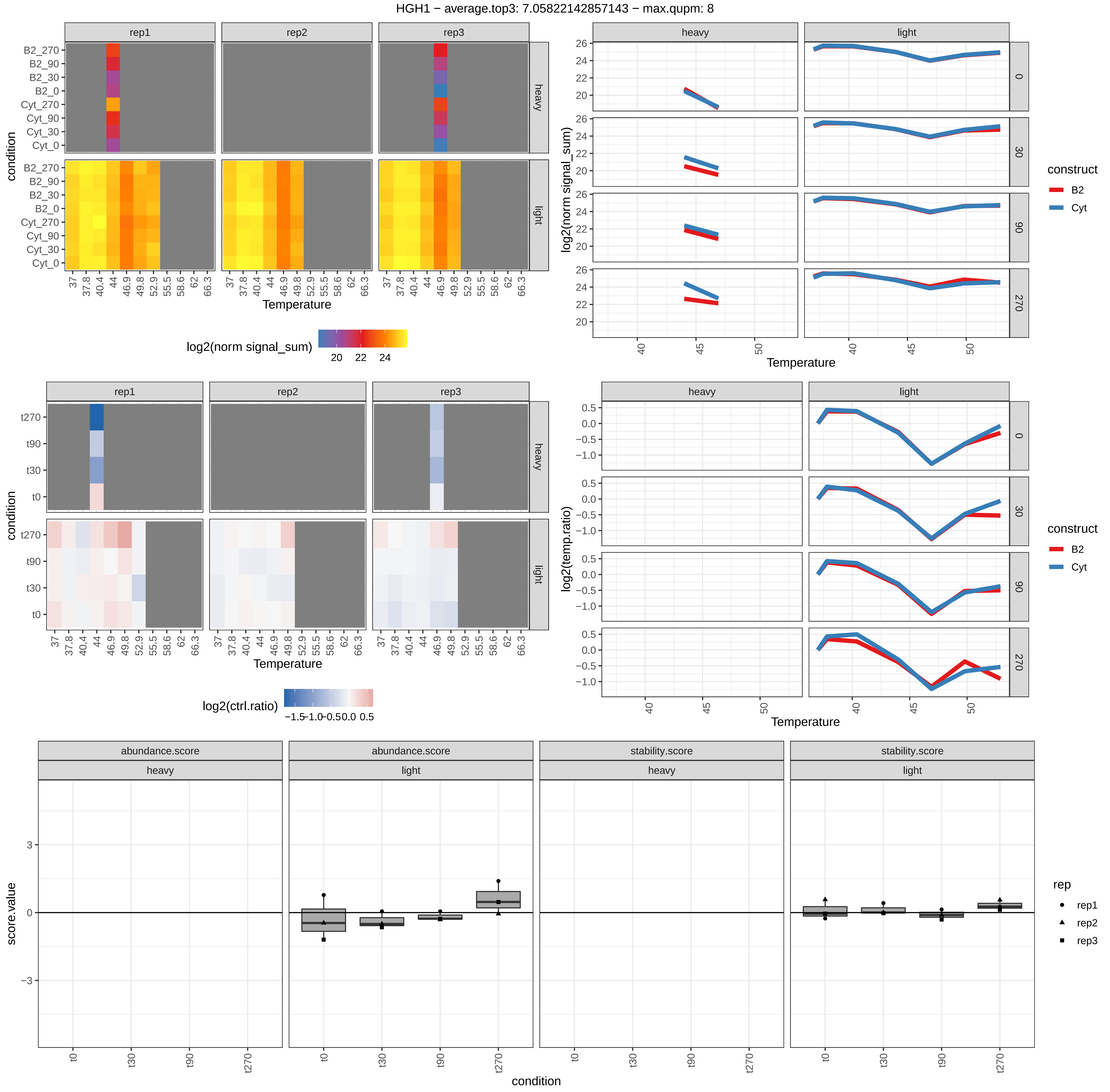

P48362 − Protein HGH1 OS=Saccharomyces cerevisiae (strain ATCC 204508 / S288c) GN=HGH1 PE=1 SV=2

HHF1 − average.top3: 7.89930563636364 − max.qupm: 6

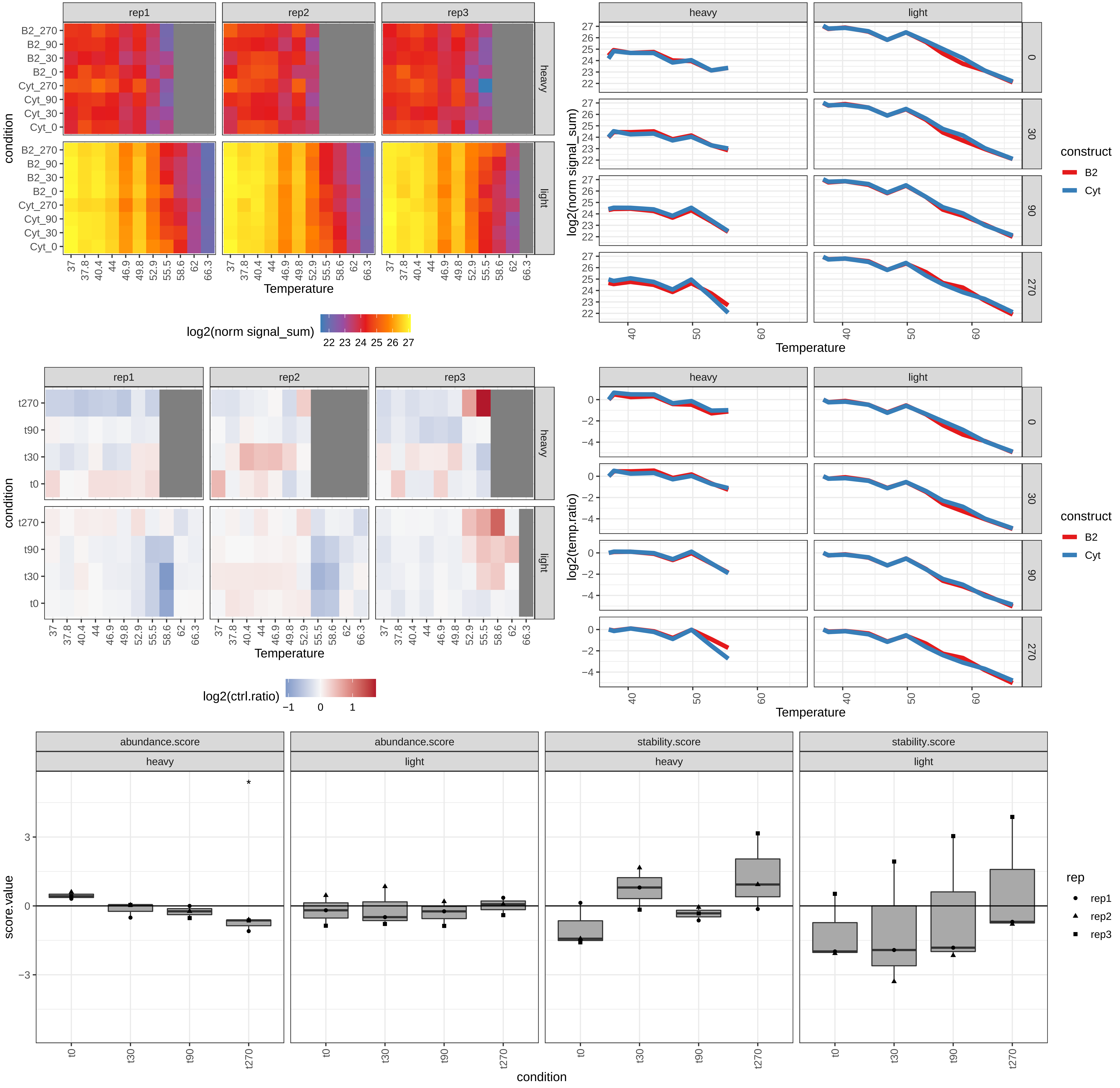

P02309 − Histone H4 OS=Saccharomyces cerevisiae (strain ATCC 204508 / S288c) GN=HHF1 PE=1 SV=2

HHT1 − average.top3: 7.80343254901961 − max.qupm: 7

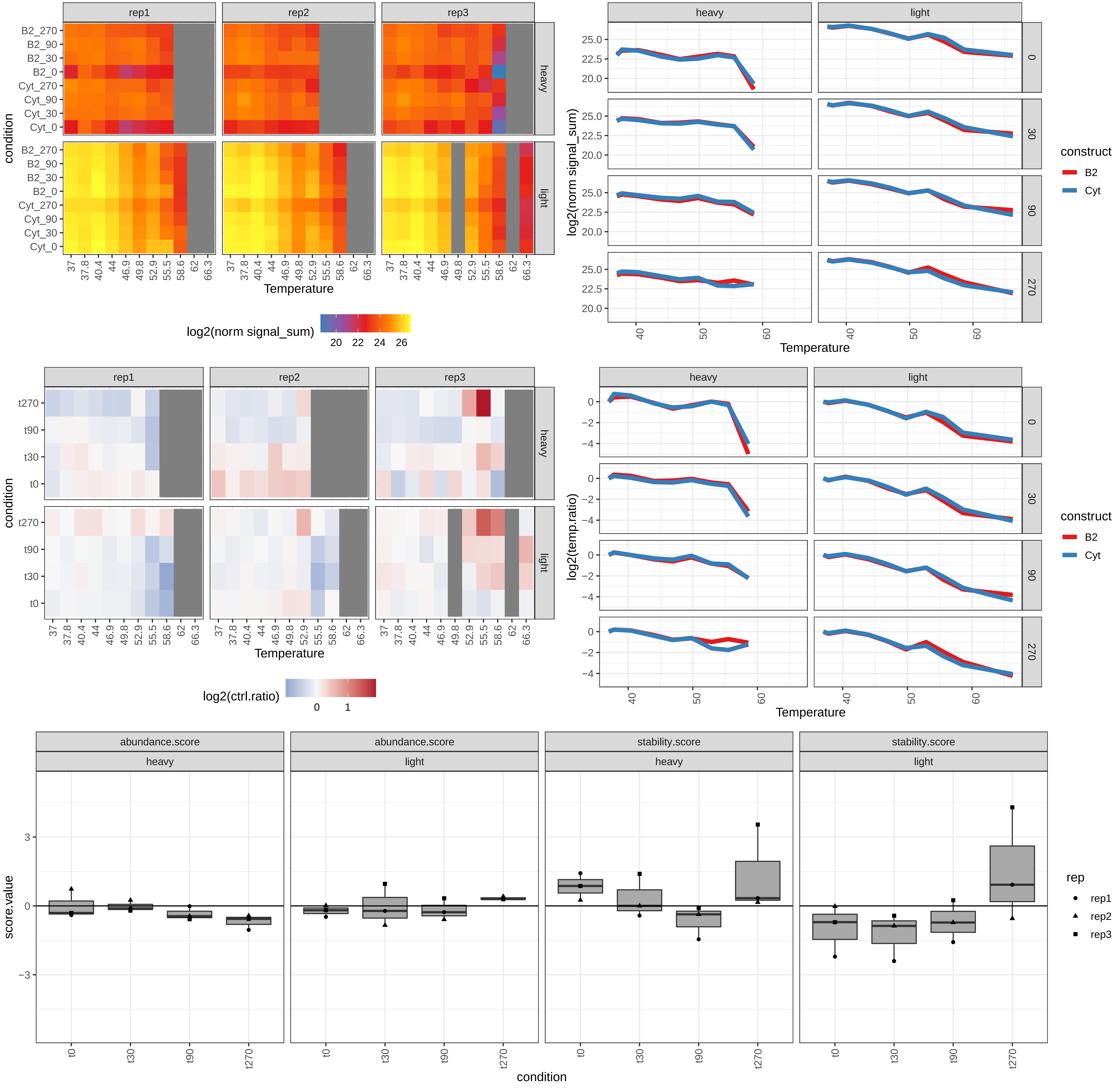

P61830 − Histone H3 OS=Saccharomyces cerevisiae (strain ATCC 204508 / S288c) GN=HHT1 PE=1 SV=2

HIR2 − average.top3: 6.40455823529412 − max.qupm: 7

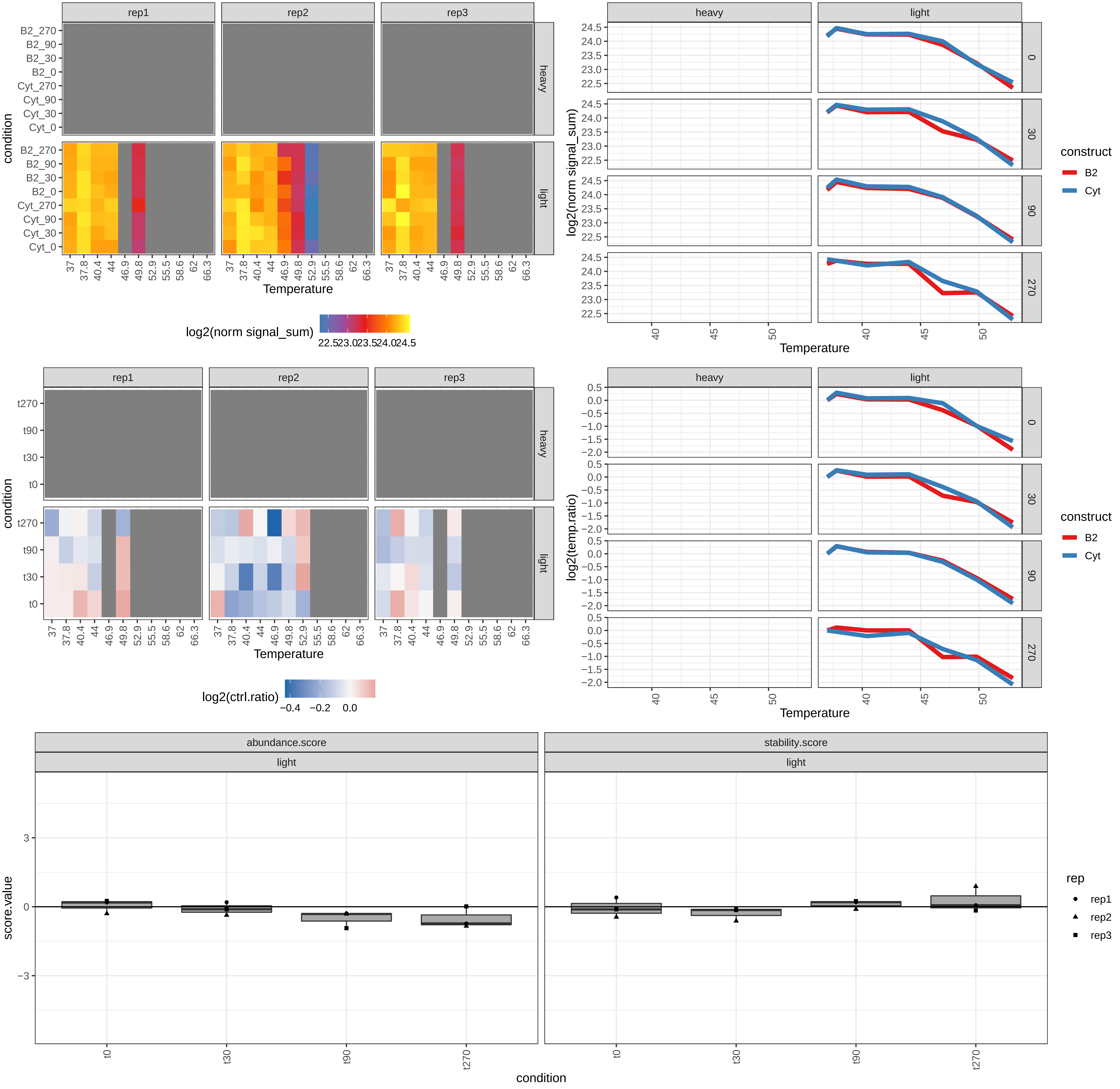

P32480 − Protein HIR2 OS=Saccharomyces cerevisiae (strain ATCC 204508 / S288c) GN=HIR2 PE=1 SV=2

HIR3 − average.top3: 6.24389625 − max.qupm: 3

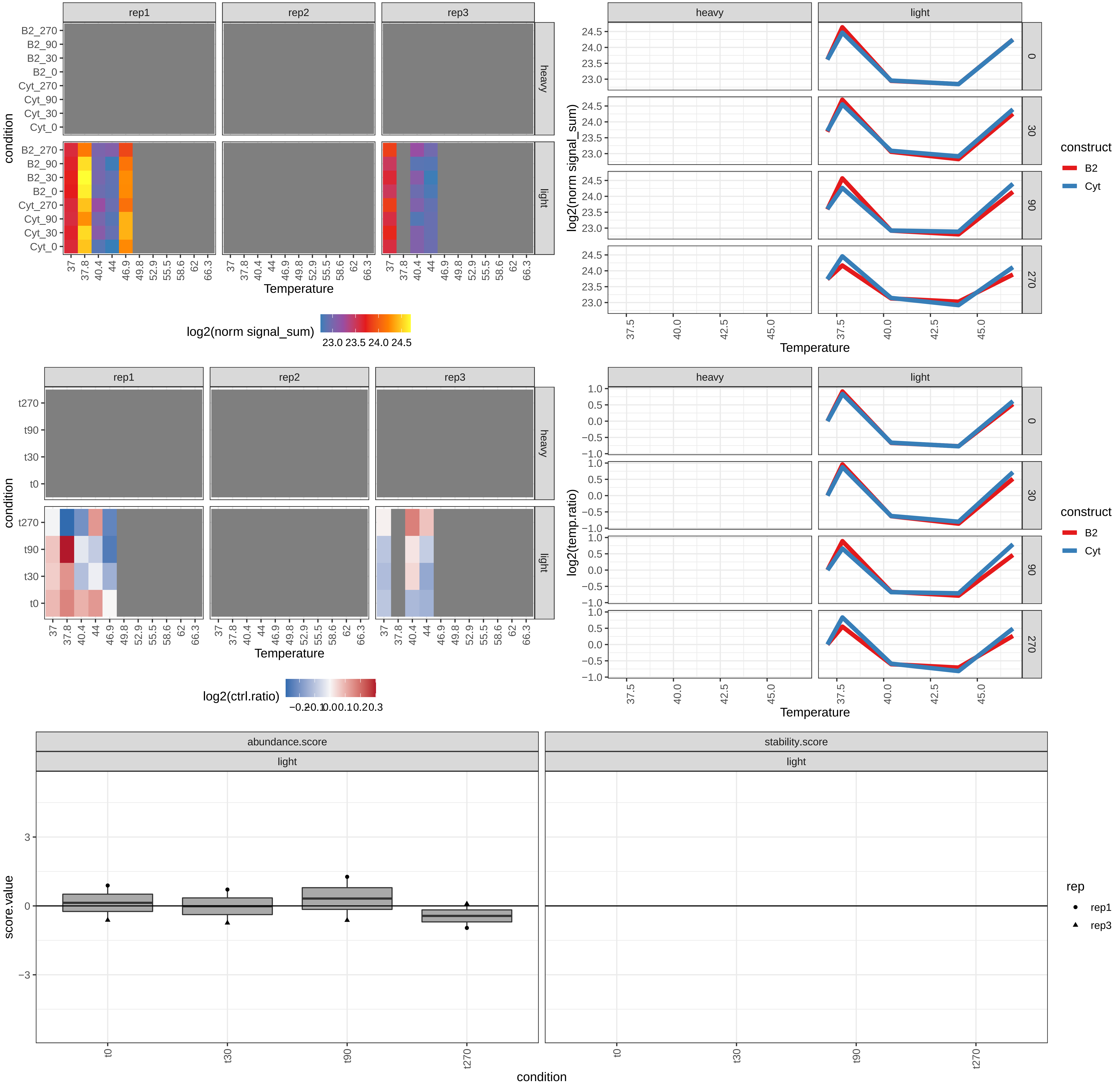

P47171 − Histone transcription regulator 3 OS=Saccharomyces cerevisiae (strain ATCC 204508 / S288c) GN=HIR3 PE=1 SV=1

HIS1 − average.top3: 6.9301415 − max.qupm: 10

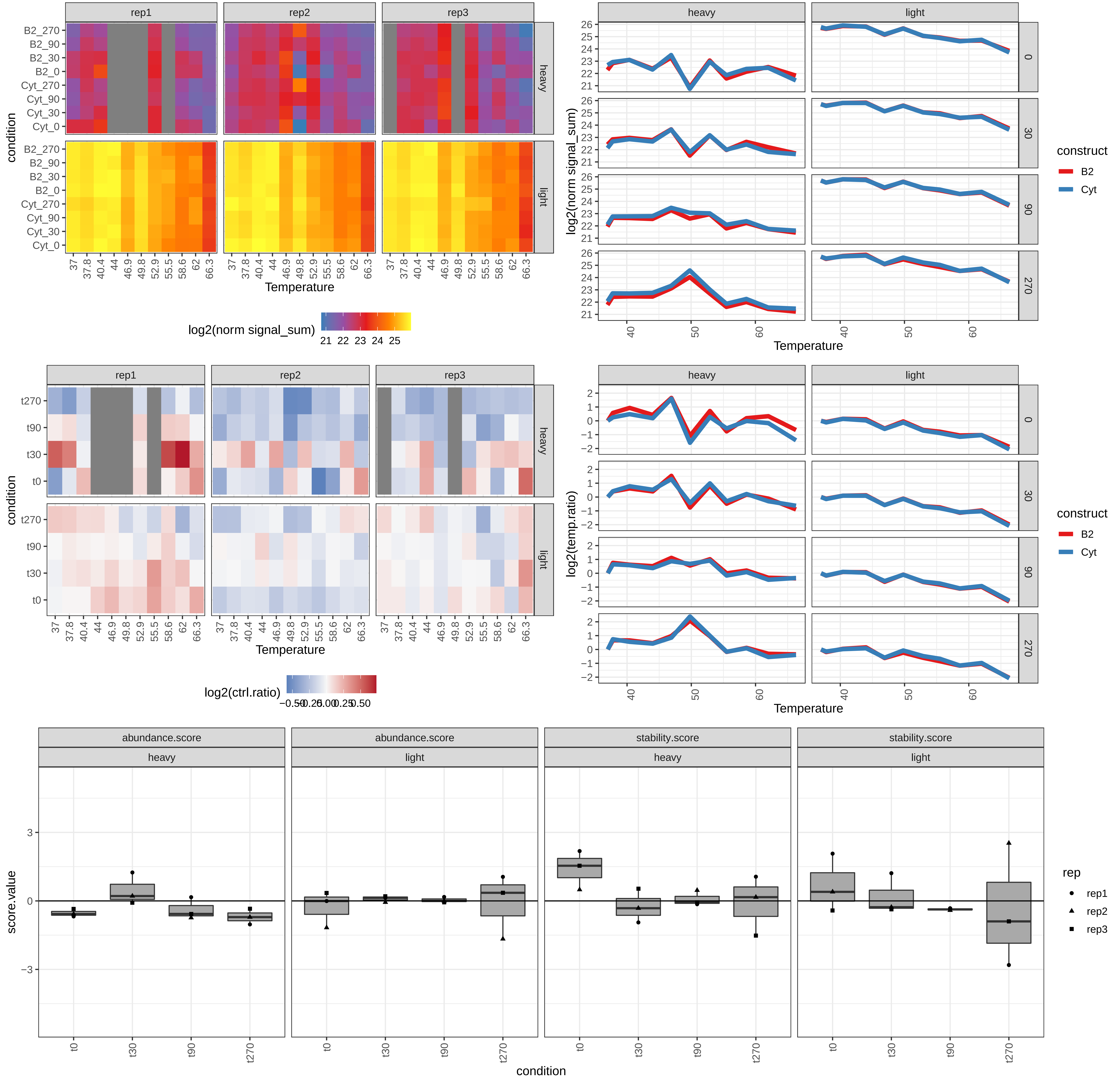

P00498 − ATP phosphoribosyltransferase OS=Saccharomyces cerevisiae (strain ATCC 204508 / S288c) GN=HIS1 PE=1 SV=1

HIS2 − average.top3: 7.13167704545455 − max.qupm: 9

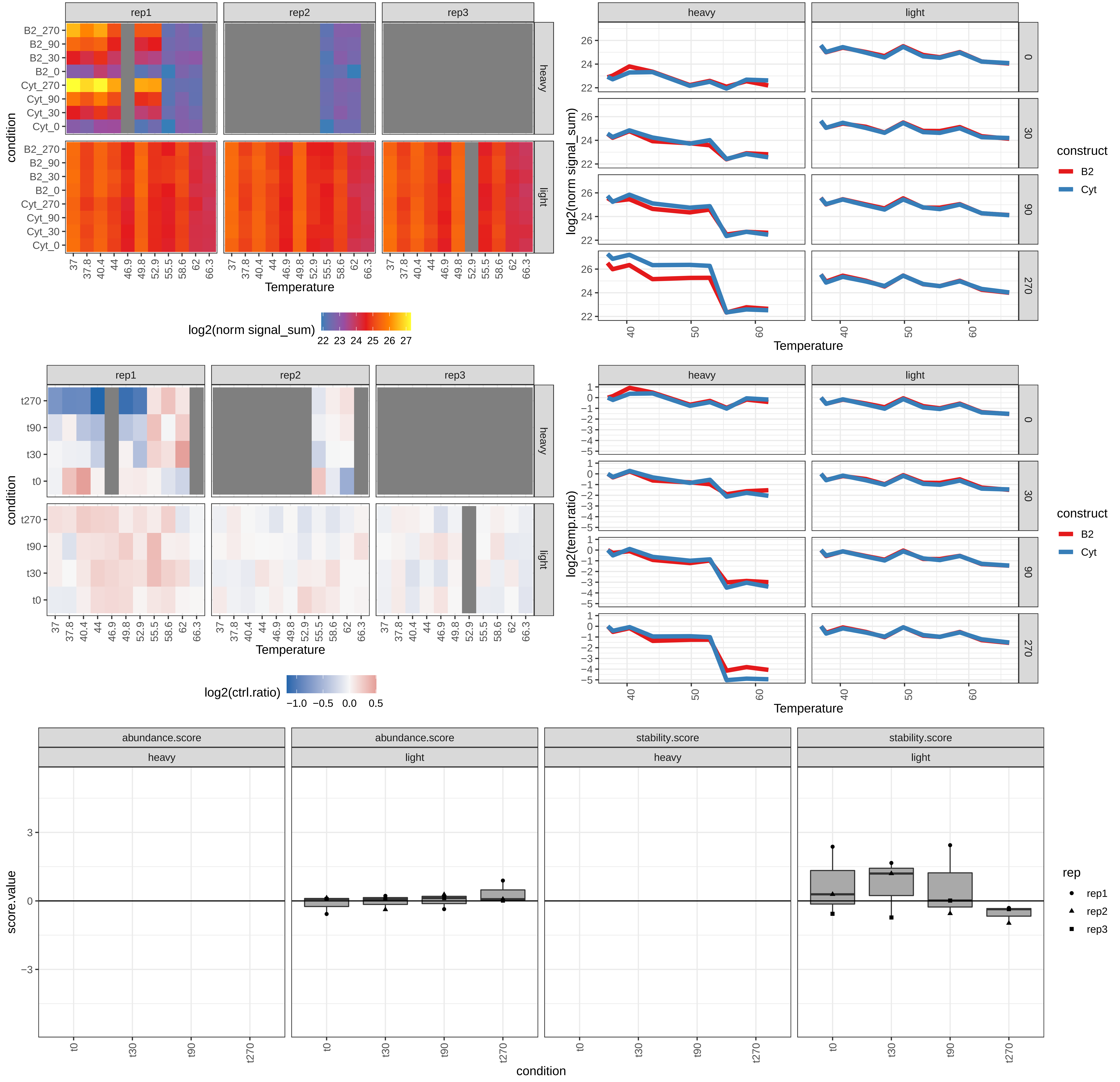

P38635 − Histidinol−phosphatase OS=Saccharomyces cerevisiae (strain ATCC 204508 / S288c) GN=HIS2 PE=1 SV=2

HIS4 − average.top3: 7.39335316666667 − max.qupm: 24

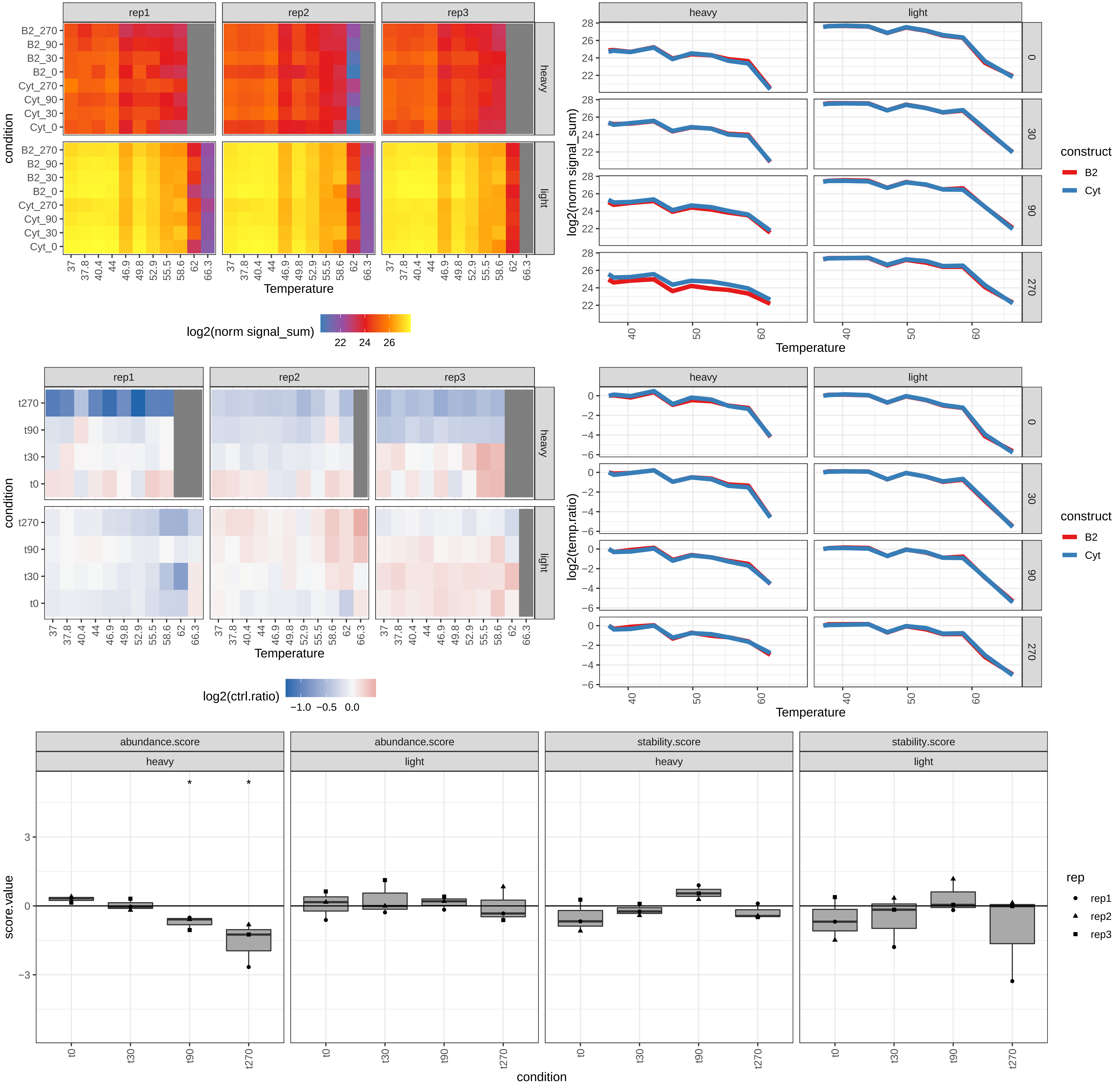

P00815 − Histidine biosynthesis trifunctional protein OS=Saccharomyces cerevisiae (strain ATCC 204508 / S288c) GN=HIS4 PE=1 SV=3

HIS5 − average.top3: 7.5008553125 − max.qupm: 8

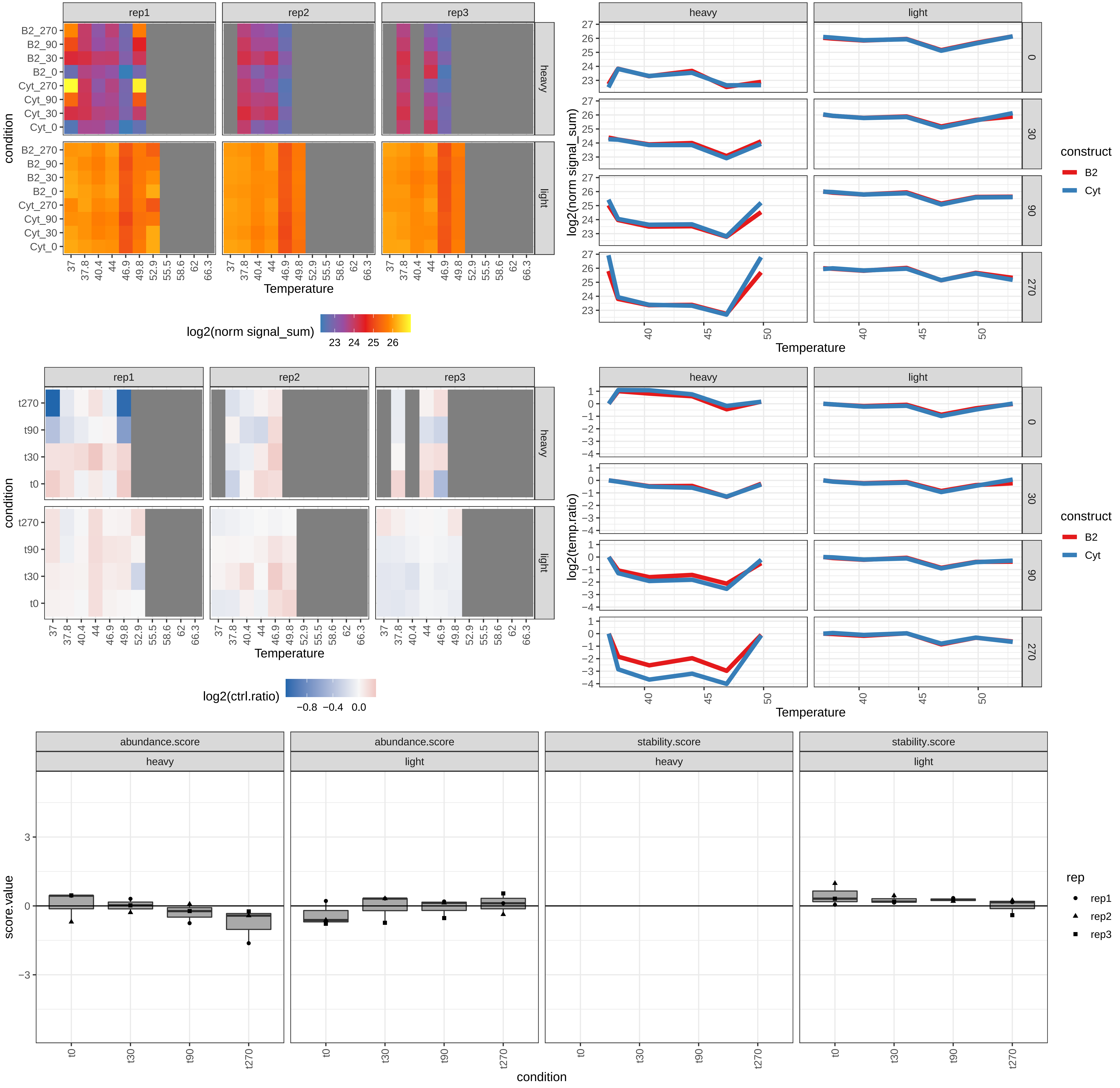

P07172 − Histidinol−phosphate aminotransferase OS=Saccharomyces cerevisiae (strain ATCC 204508 / S288c) GN=HIS5 PE=3 SV=2

HIS6 − average.top3: 7.265566 − max.qupm: 8

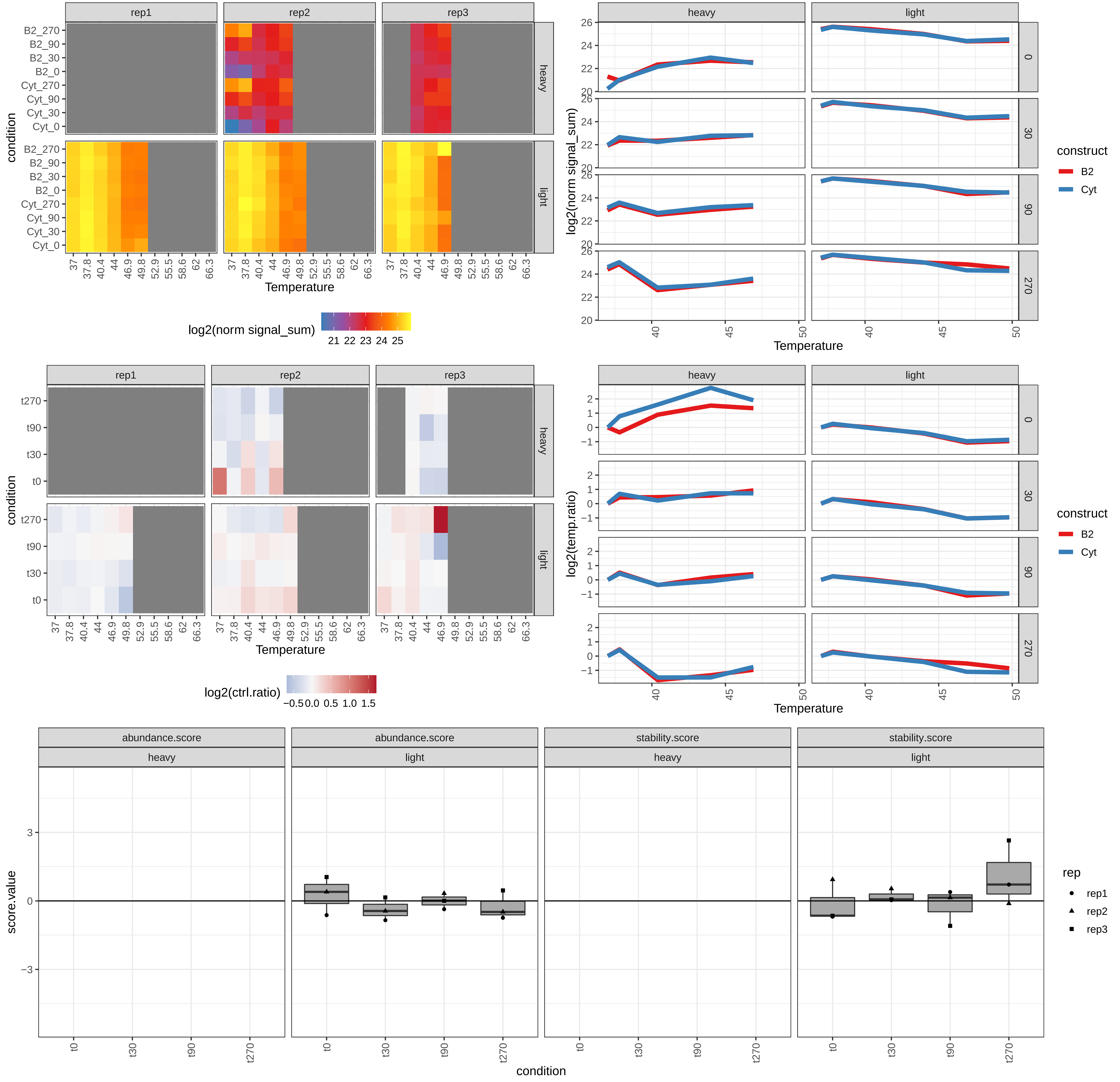

P40545 − 1−(5−phosphoribosyl)−5−[(5−phosphoribosylamino)methylideneamino] imidazole−4−carboxamide isomerase OS=Saccharomyces cerevisiae (strain ATCC 204508 / S288c) GN=HIS6 PE=1 SV=1

HIS7 − average.top3: 7.22707767857143 − max.qupm: 23

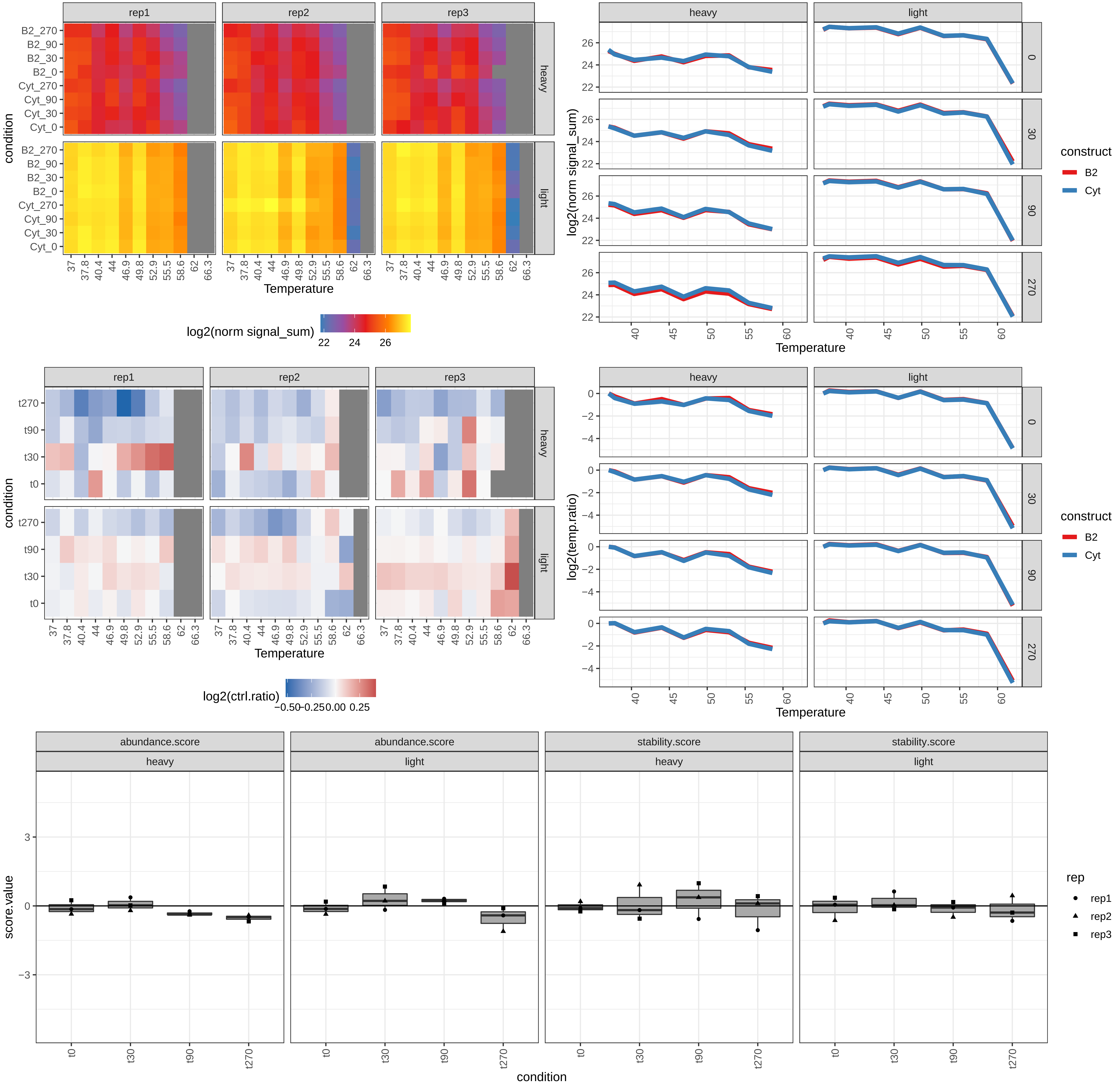

P33734 − Imidazole glycerol phosphate synthase hisHF OS=Saccharomyces cerevisiae (strain ATCC 204508 / S288c) GN=HIS7 PE=1 SV=2

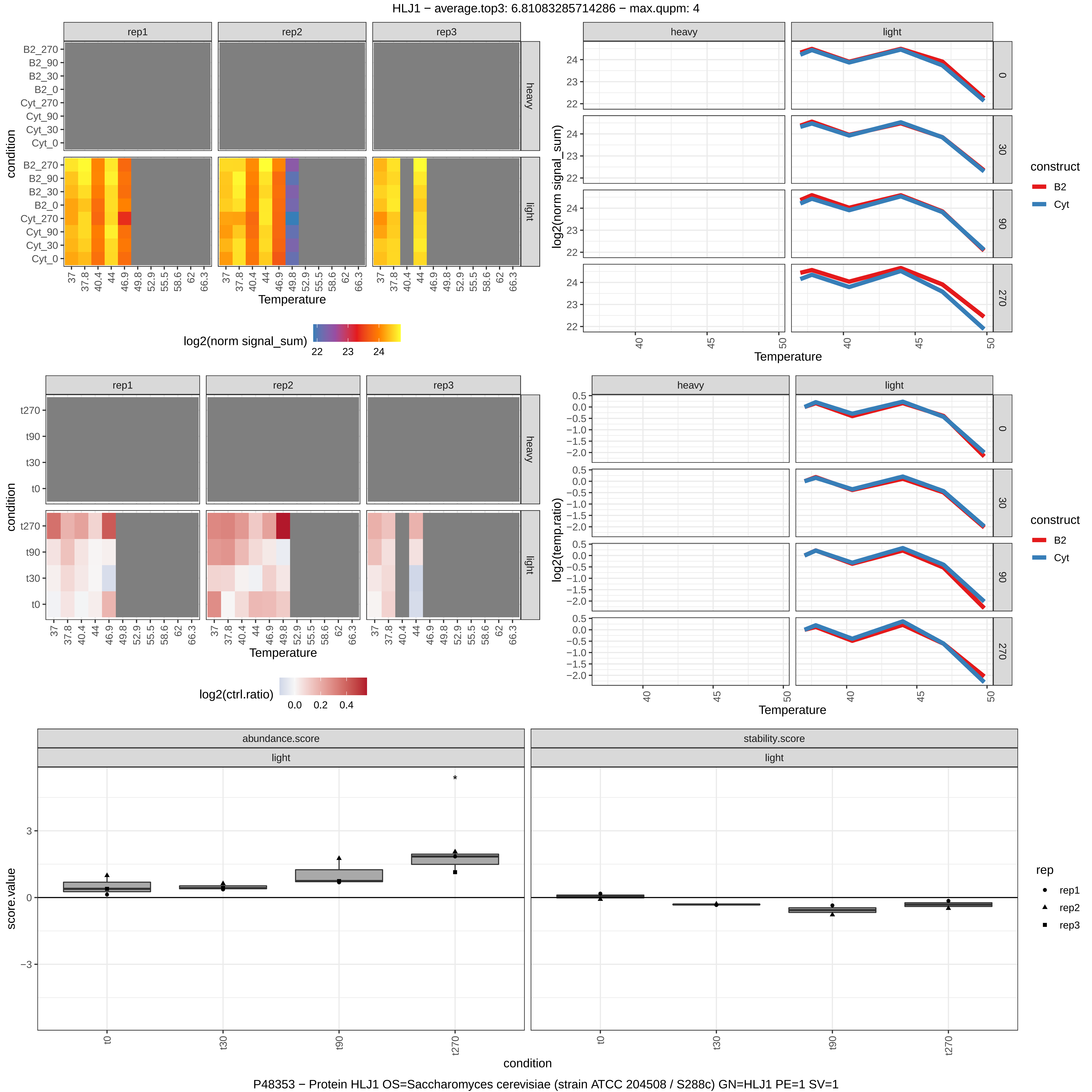
HLJ1 − average.top3: 6.81083285714286 − max.qupm: 4
rep1
rep2
rep3
heavy
light
condition
Temperature
log2(norm signal_sum)
log2(temp.ratio)
log2(ctrl.ratio)
construct
B2
Cyt
abundance.score
stability.score
score.value
rep
rep1
rep2
rep3
t0
t30
t90
t270
B2_270
B2_90
B2_30
B2_0
Cyt_270
Cyt_90
Cyt_30
Cyt_0
P48353 − Protein HLJ1 OS=Saccharomyces cerevisiae (strain ATCC 204508 / S288c) GN=HLJ1 PE=1 SV=1

HMF1 − average.top3: 7.60009616666667 − max.qupm: 5

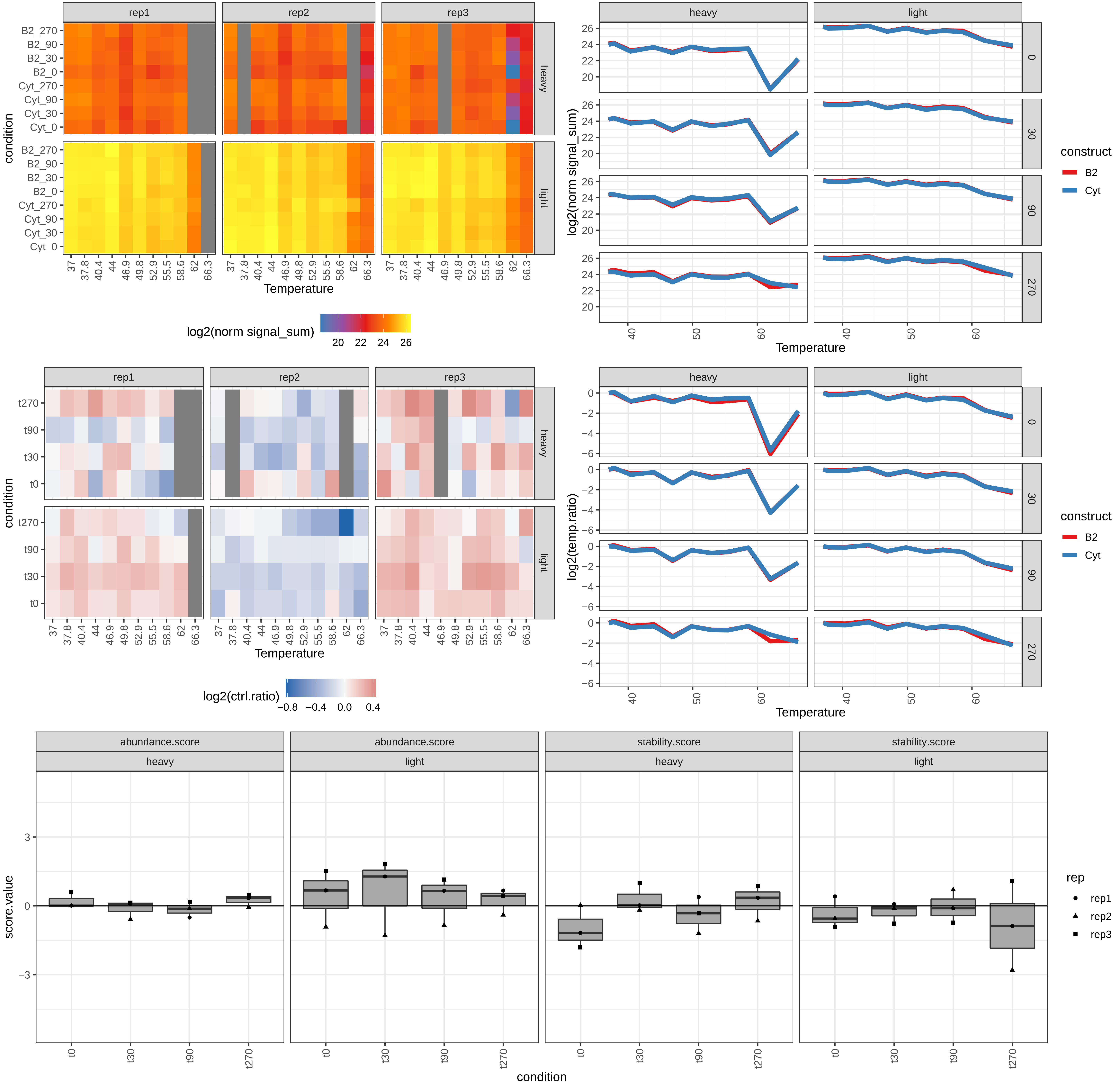

P40037 − Protein HMF1 OS=Saccharomyces cerevisiae (strain ATCC 204508 / S288c) GN=HMF1 PE=1 SV=1

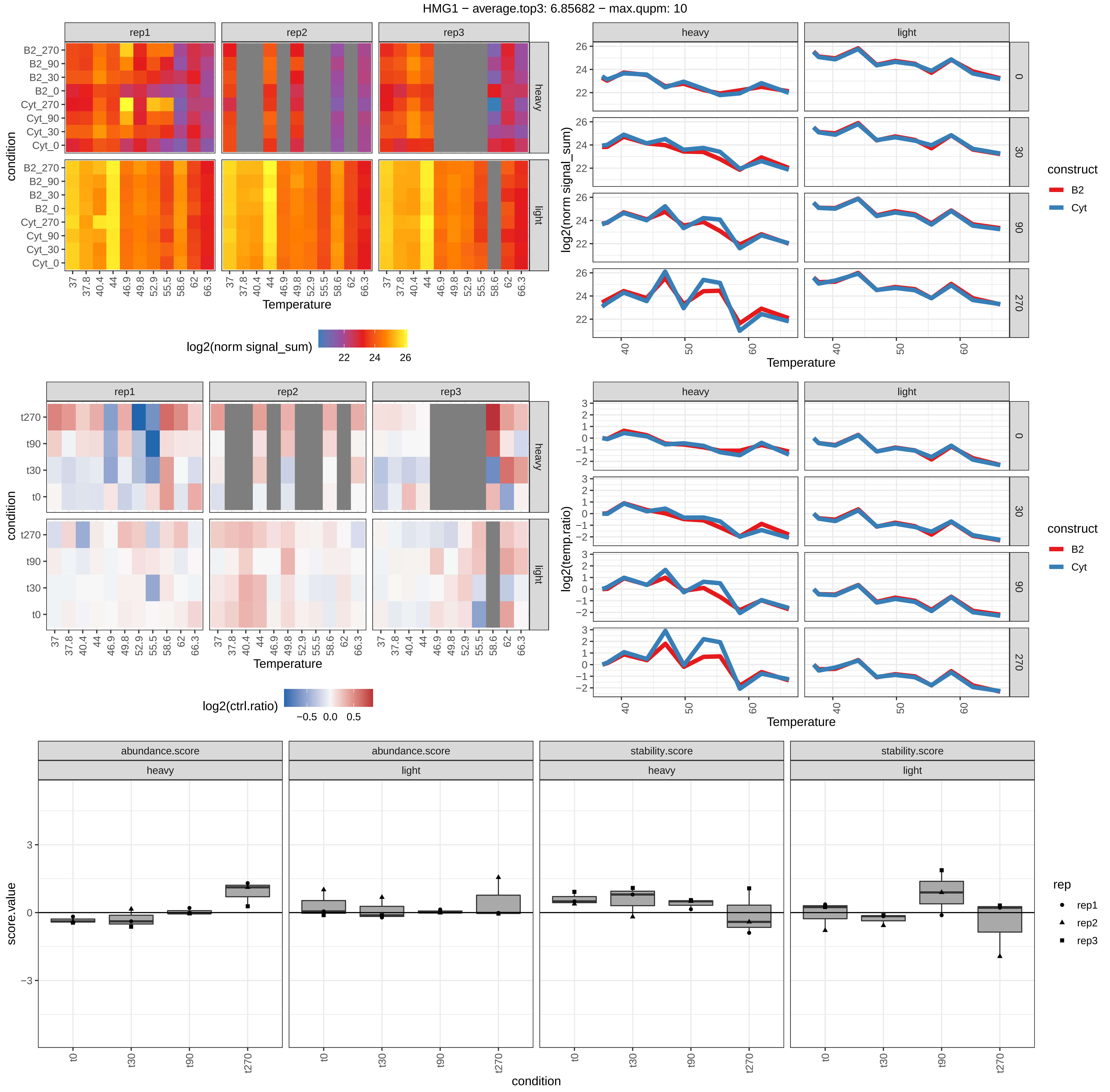

P12683 − 3−hydroxy−3−methylglutaryl−coenzyme A reductase 1 OS=Saccharomyces cerevisiae (strain ATCC 204508 / S288c) GN=HMG1 PE=1 SV=1

HMG2 − average.top3: 6.38505454545455 − max.qupm: 5

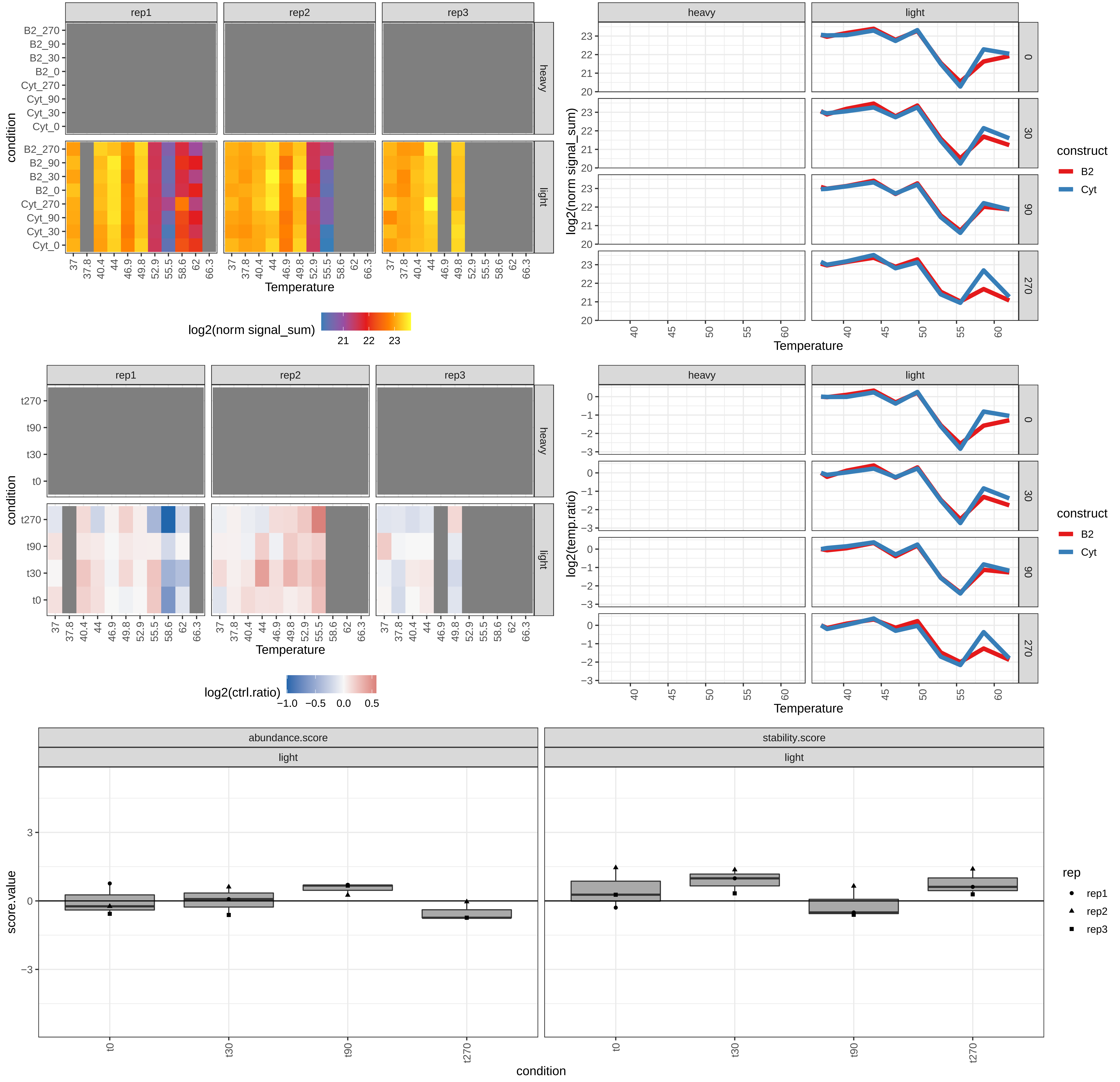

P12684 − 3−hydroxy−3−methylglutaryl−coenzyme A reductase 2 OS=Saccharomyces cerevisiae (strain ATCC 204508 / S288c) GN=HMG2 PE=1 SV=1

HMO1 − average.top3: 6.73617037037037 − max.qupm: 4

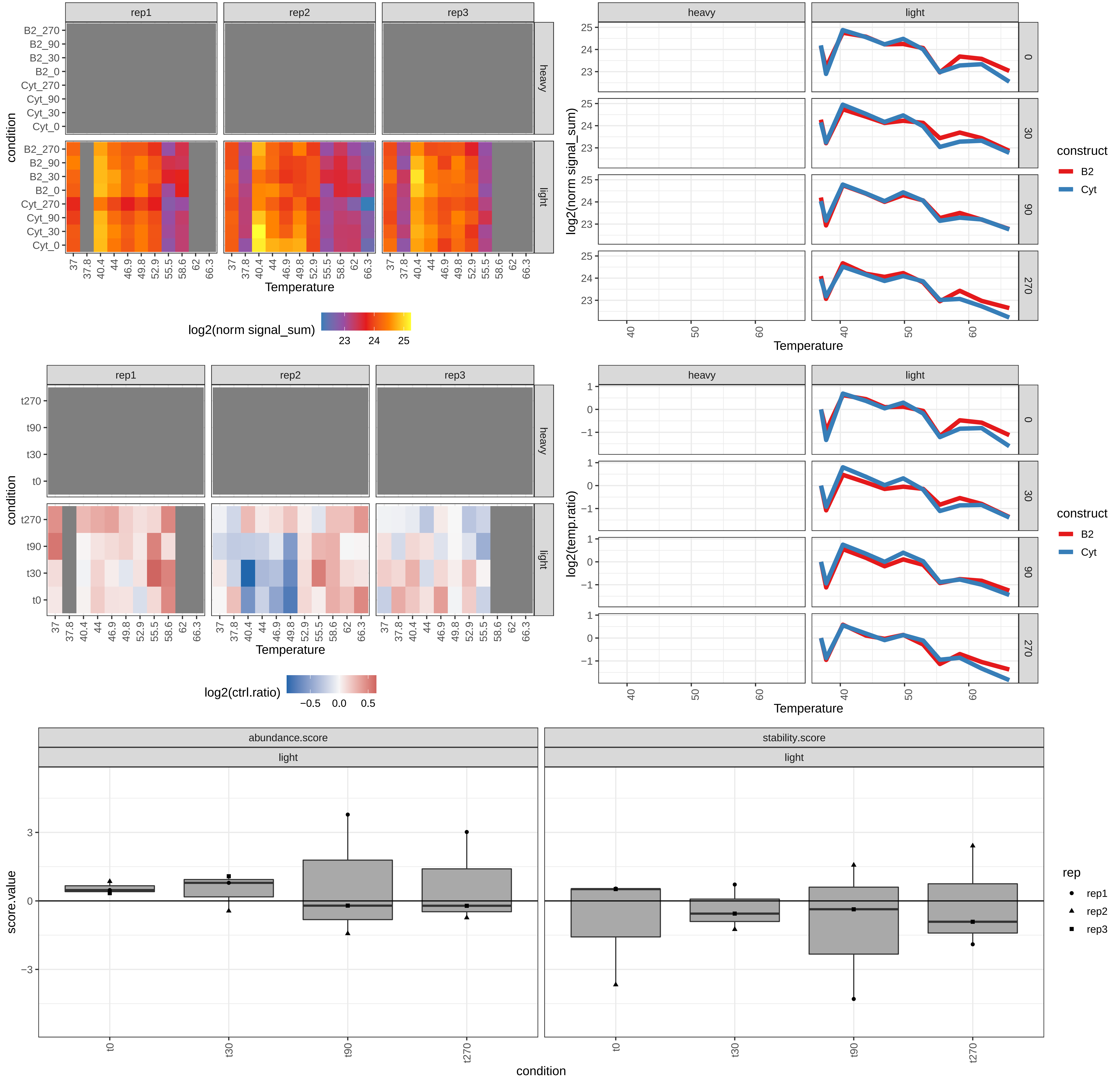

Q03973 − High mobility group protein 1 OS=Saccharomyces cerevisiae (strain ATCC 204508 / S288c) GN=HMO1 PE=1 SV=1

HMT1 − average.top3: 6.99468181818182 − max.qupm: 8

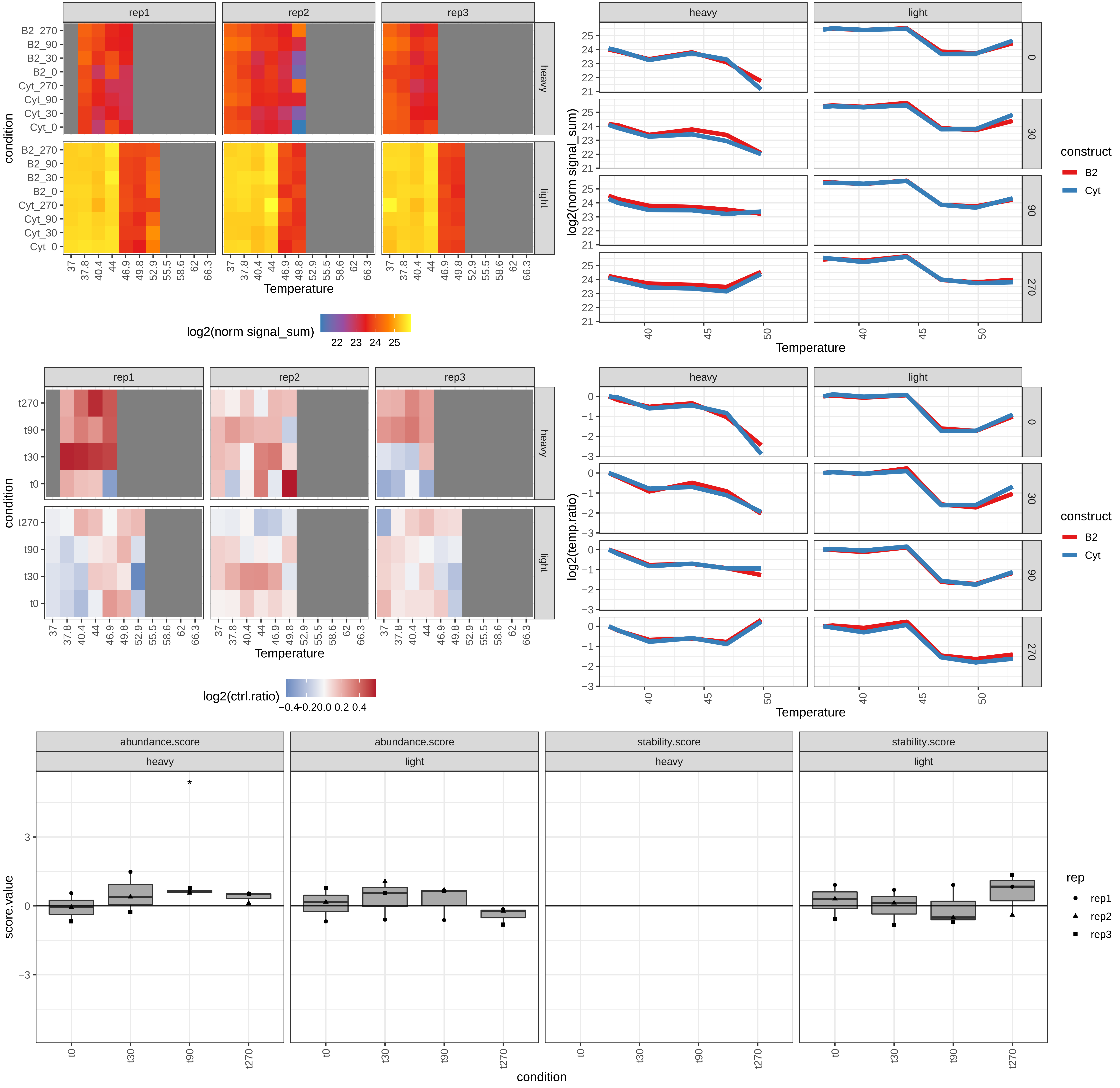

P38074 − Protein arginine N−methyltransferase 1 OS=Saccharomyces cerevisiae (strain ATCC 204508 / S288c) GN=HMT1 PE=1 SV=1

HNT1 − average.top3: 7.54948114754098 − max.qupm: 6

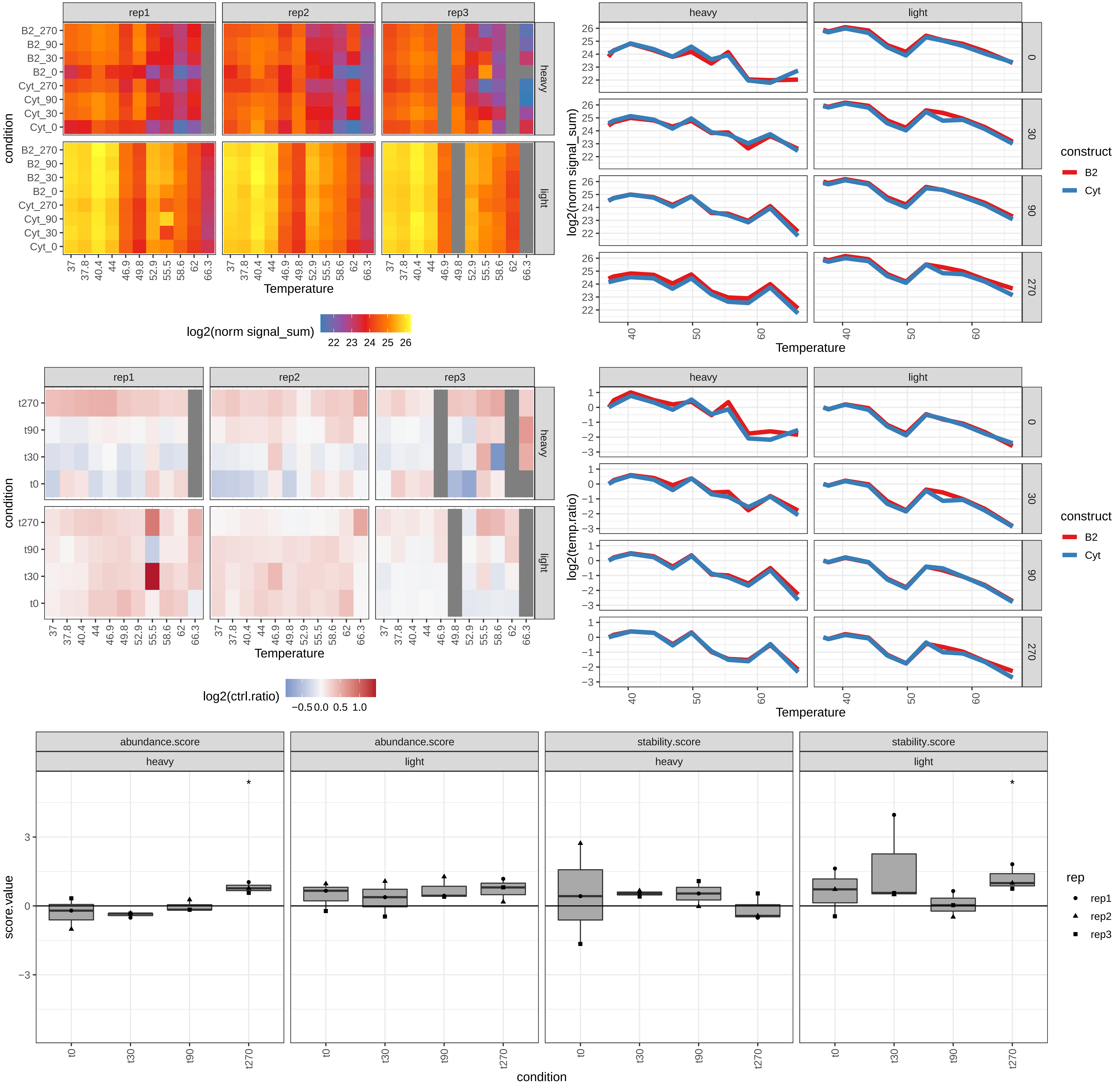

Q04344 − Hit family protein 1 OS=Saccharomyces cerevisiae (strain ATCC 204508 / S288c) GN=HNT1 PE=1 SV=2

HNT2 − average.top3: 6.534768 − max.qupm: 4

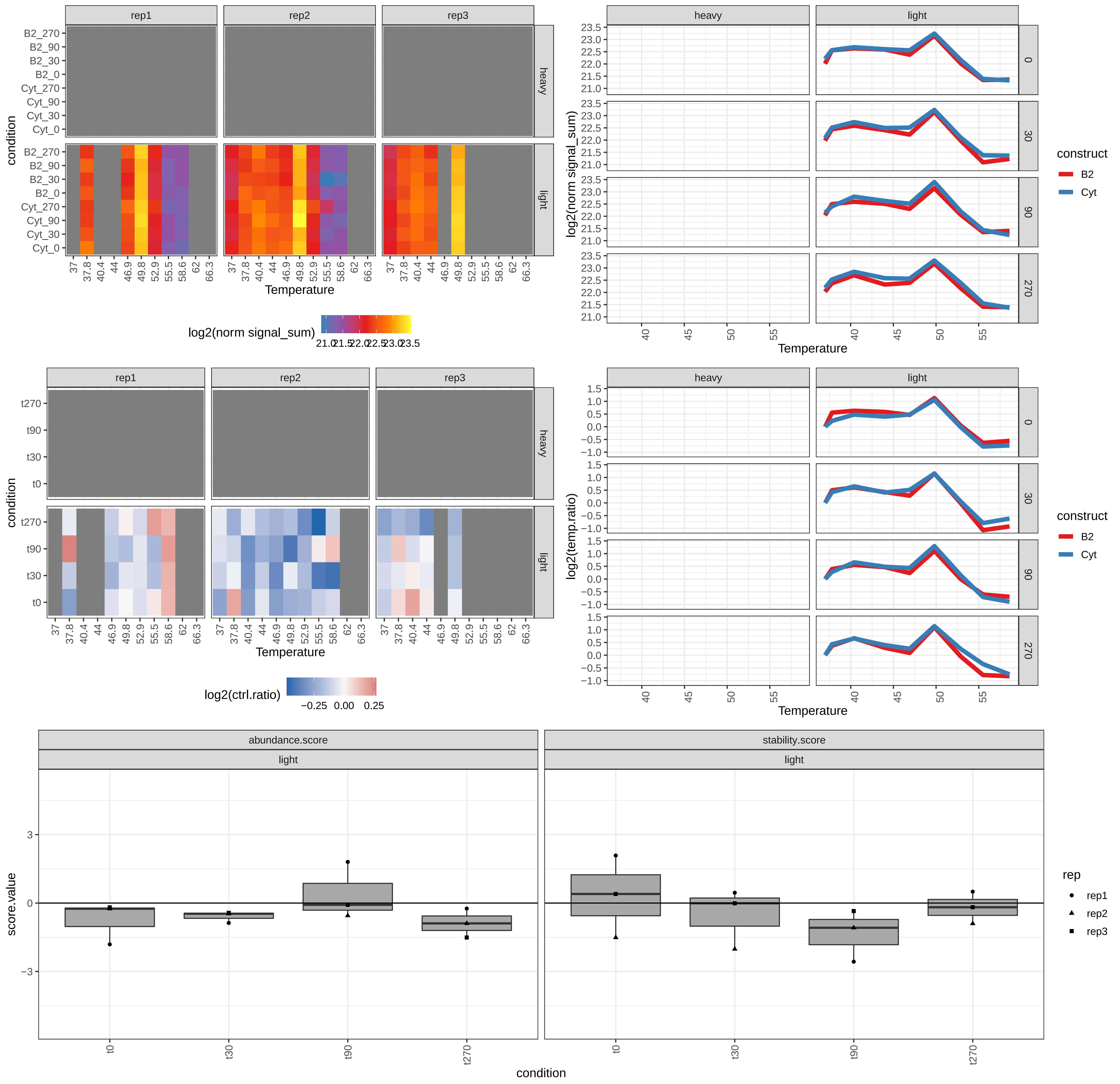

P49775 − Bis(5'−adenosyl)−triphosphatase OS=Saccharomyces cerevisiae (strain ATCC 204508 / S288c) GN=HNT2 PE=1 SV=2

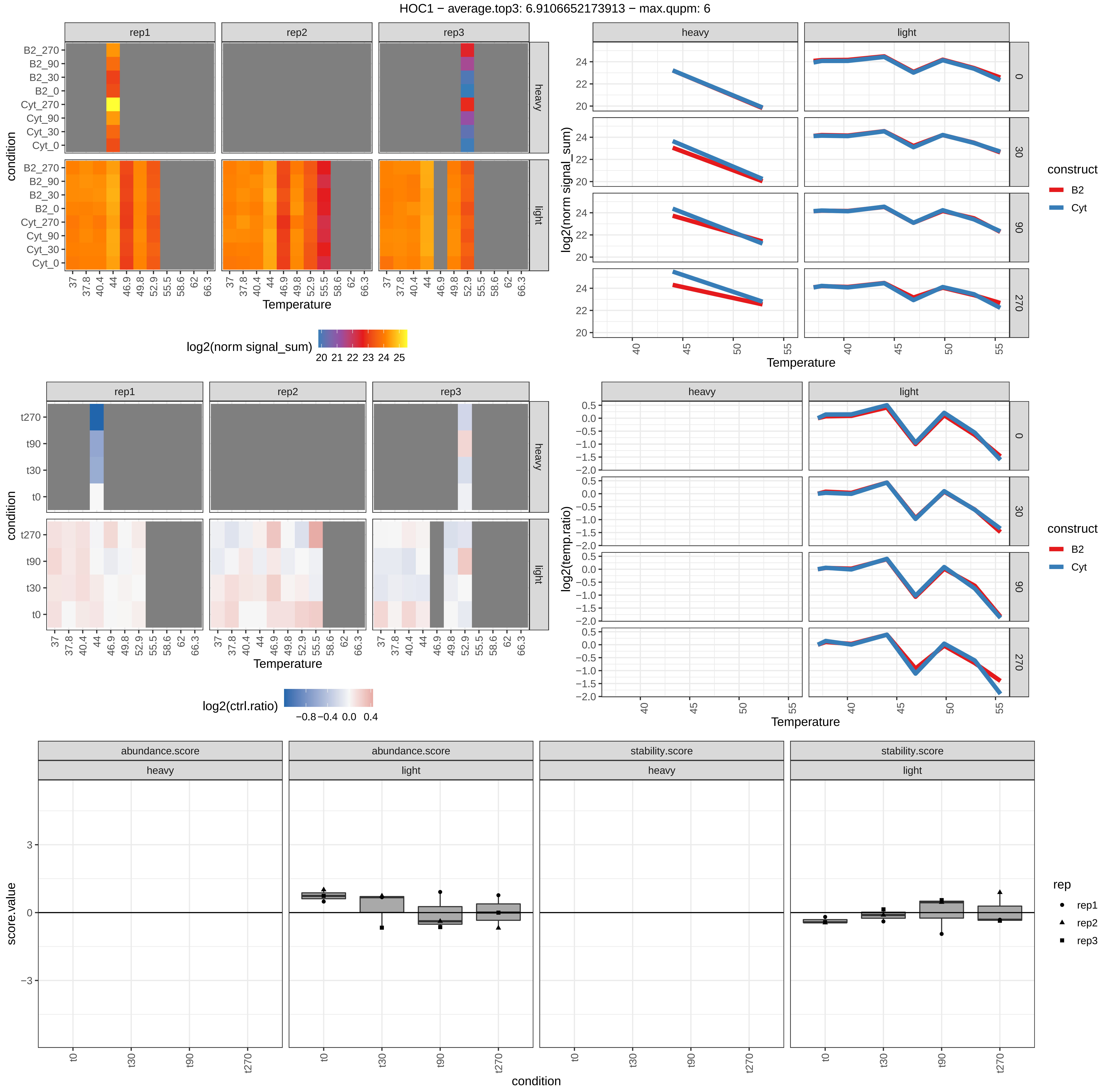

P47124 − Putative glycosyltransferase HOC1 OS=Saccharomyces cerevisiae (strain ATCC 204508 / S288c) GN=HOC1 PE=1 SV=3

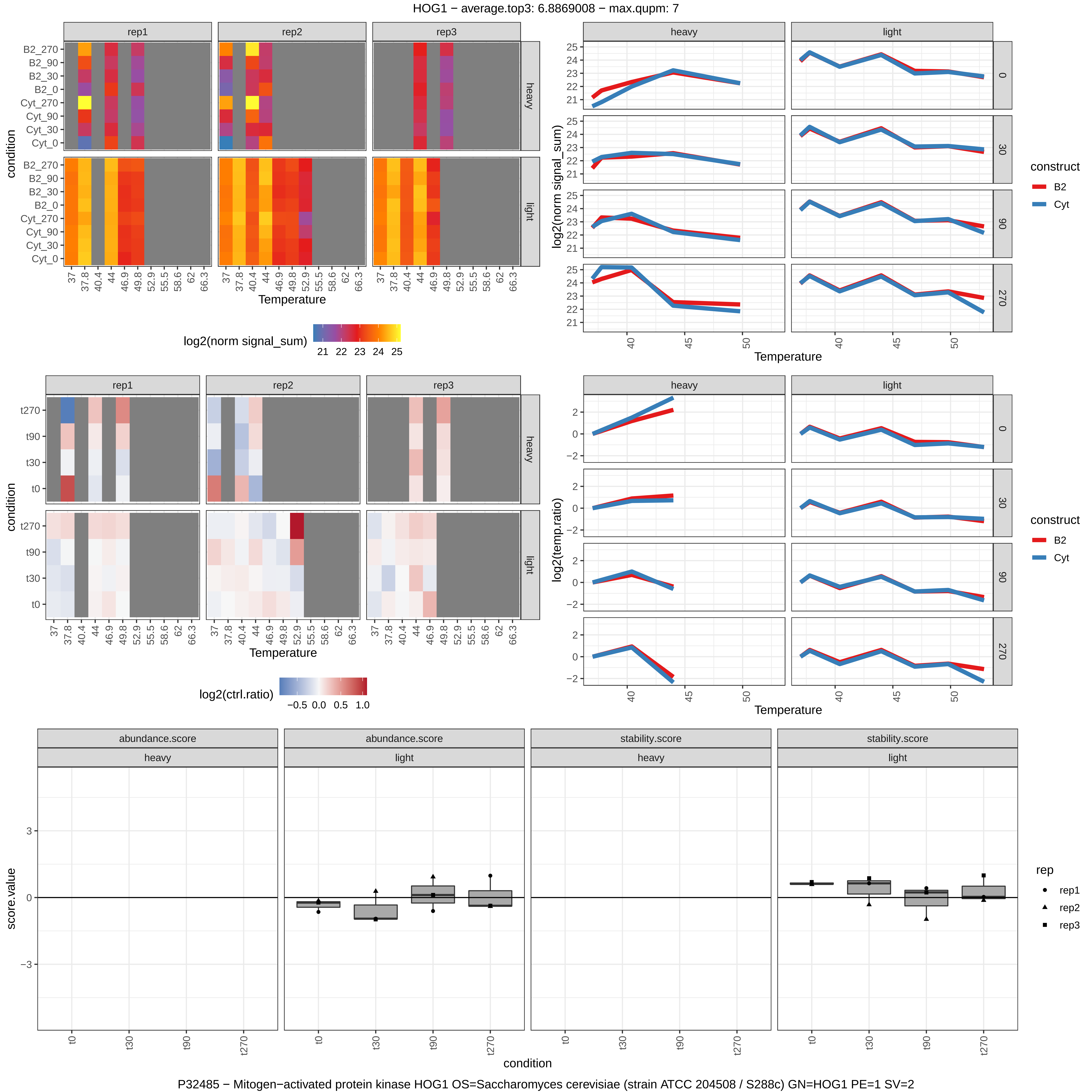
HOG1 − average.top3: 6.8869008 − max.qupm: 7
rep1
rep2
rep3
heavy
light
B2_270
B2_90
B2_30
B2_0
Cyt_270
Cyt_90
Cyt_30
Cyt_0
condition
37
37.8
40.4
44
46.9
49.8
52.9
55.5
58.6
62
66.3
Temperature
log2(norm signal_sum)
21 22 23 24 25
log2(norm signal_sum)
0
30
90
270
40
45
50
construct
B2
Cyt
t270
t90
t30
t0
log2(ctrl.ratio)
−0.5 0.0 0.5 1.0
log2(temp.ratio)
abundance.score
stability.score
score.value
3
0
−3
rep
rep1
rep2
rep3
condition
P32485 − Mitogen−activated protein kinase HOG1 OS=Saccharomyces cerevisiae (strain ATCC 204508 / S288c) GN=HOG1 PE=1 SV=2

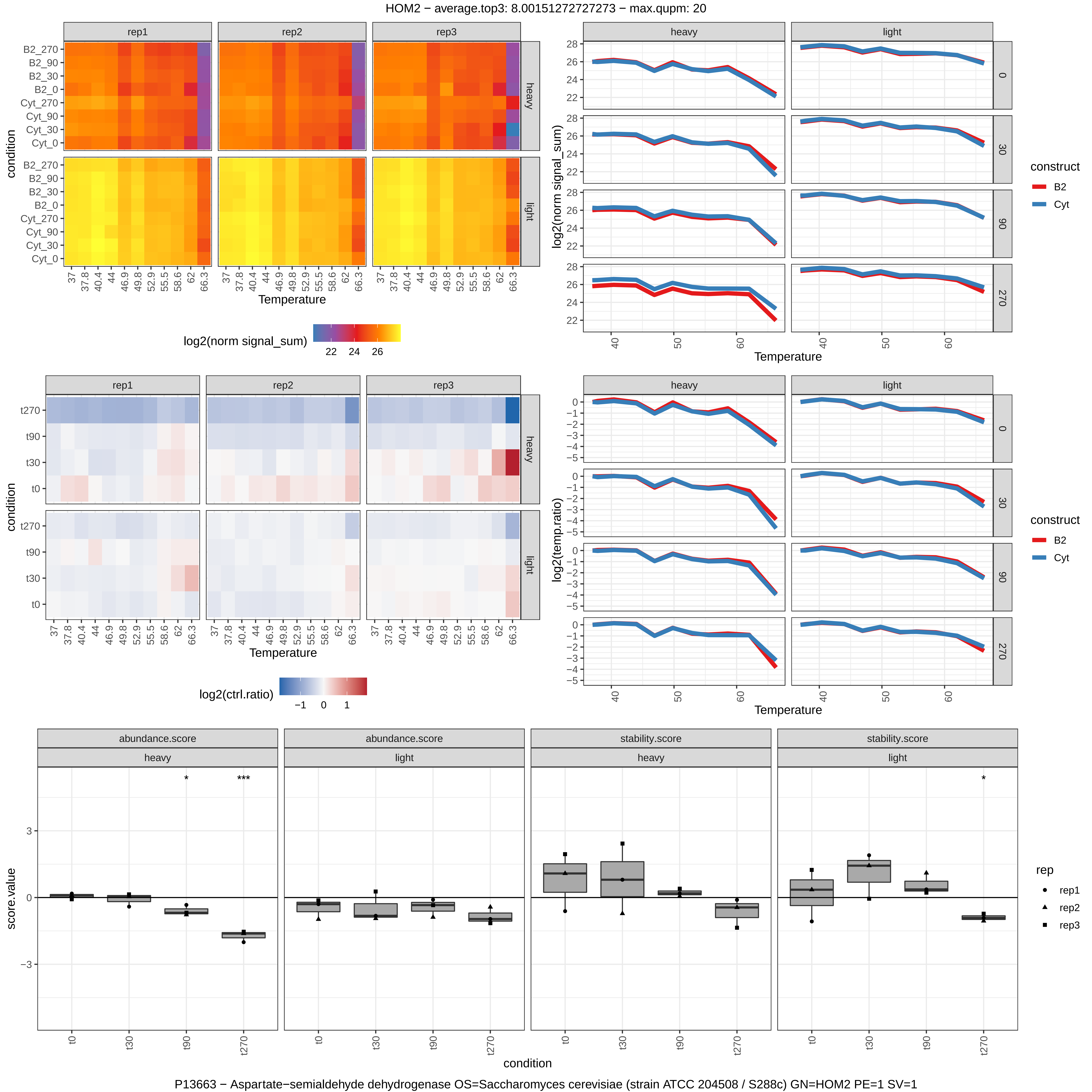

HOM2 − average.top3: 8.00151272727273 − max.qupm: 20
rep1
rep2
rep3
heavy
light
condition
Temperature
log2(norm signal_sum)
construct
B2
Cyt
log2(temp.ratio)
log2(ctrl.ratio)
abundance.score
stability.score
score.value
rep
rep1
rep2
rep3
P13663 − Aspartate−semialdehyde dehydrogenase OS=Saccharomyces cerevisiae (strain ATCC 204508 / S288c) GN=HOM2 PE=1 SV=1

HOM3 − average.top3: 7.37349525423729 − max.qupm: 17

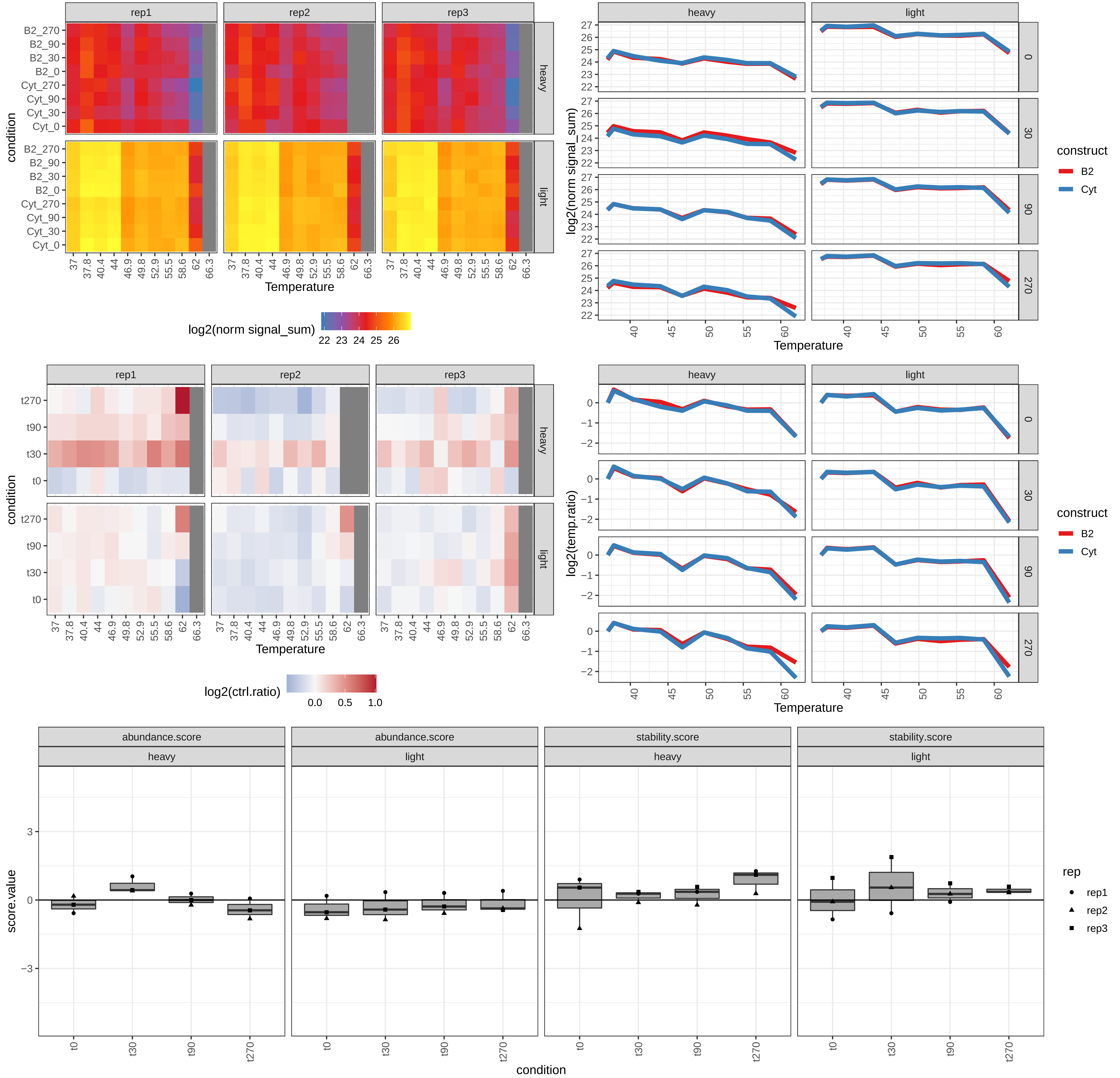

P10869 − Aspartokinase OS=Saccharomyces cerevisiae (strain ATCC 204508 / S288c) GN=HOM3 PE=1 SV=2

HOM6 − average.top3: 8.0200096969697 − max.qupm: 24

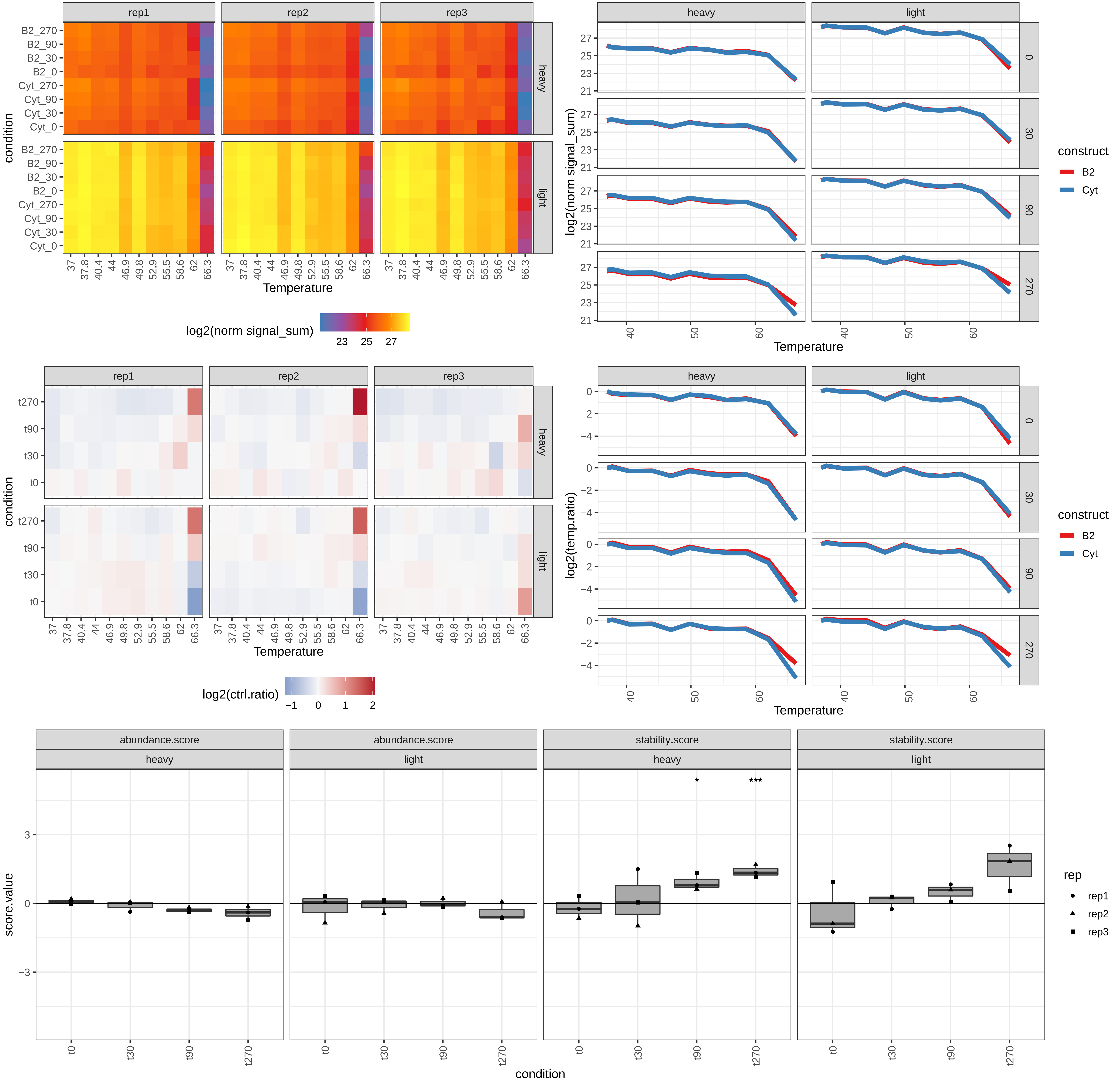

P31116 − Homoserine dehydrogenase OS=Saccharomyces cerevisiae (strain ATCC 204508 / S288c) GN=HOM6 PE=1 SV=1

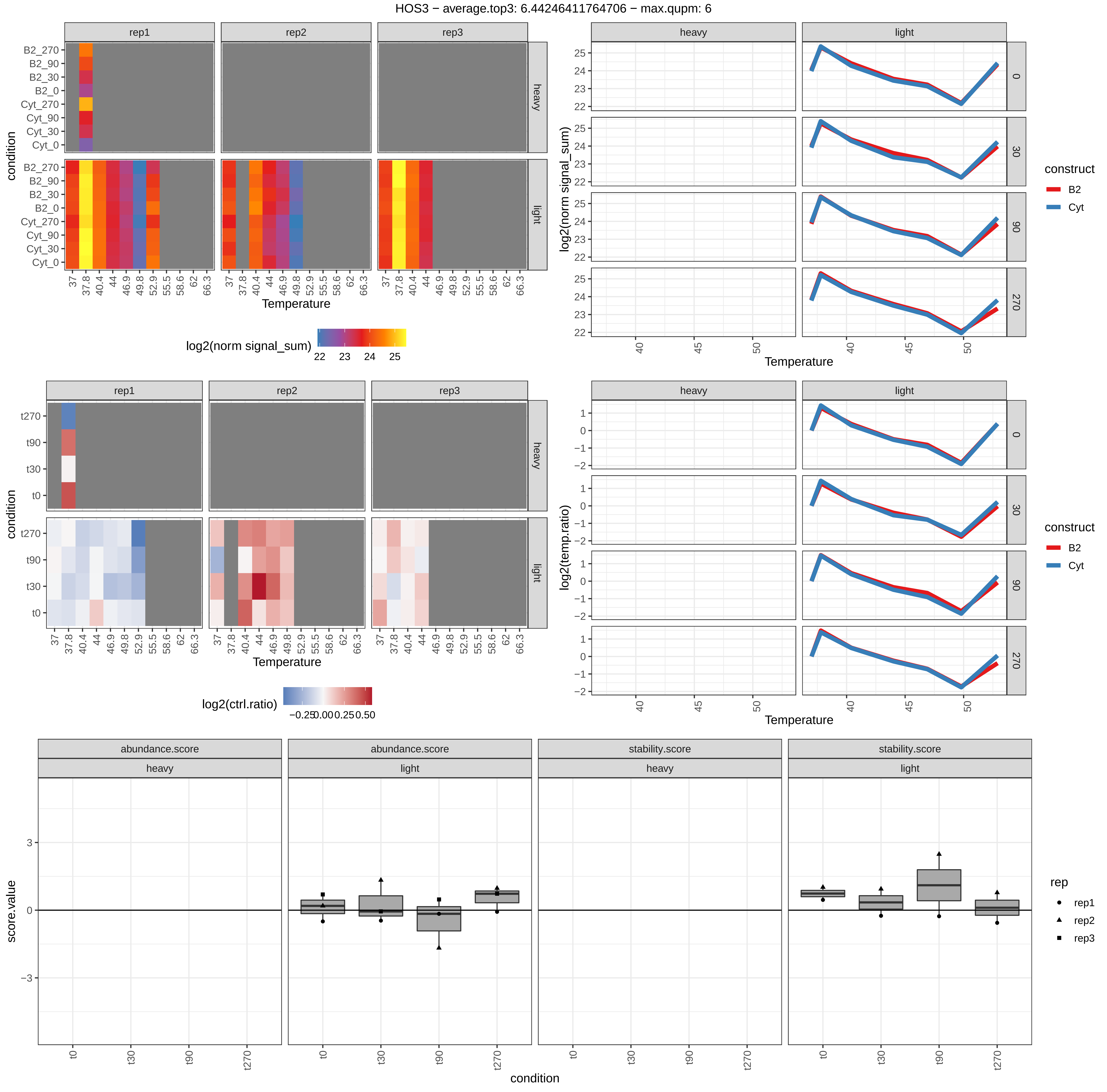

Q02959 − Histone deacetylase HOS3 OS=Saccharomyces cerevisiae (strain ATCC 204508 / S288c) GN=HOS3 PE=1 SV=1

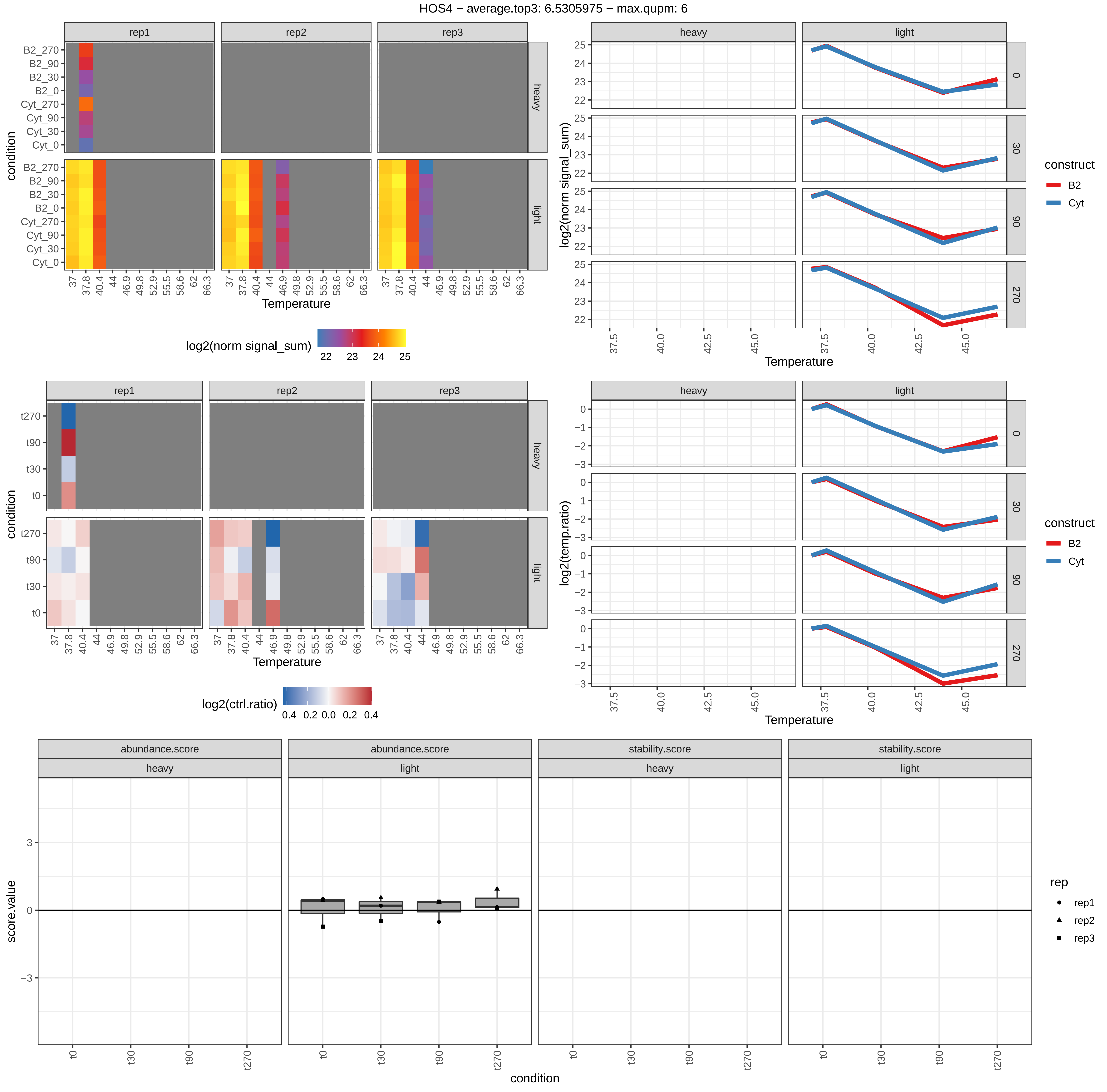

P40480 − Protein HOS4 OS=Saccharomyces cerevisiae (strain ATCC 204508 / S288c) GN=HOS4 PE=1 SV=1

HPA2 − average.top3: 6.73258941176471 − max.qupm: 4

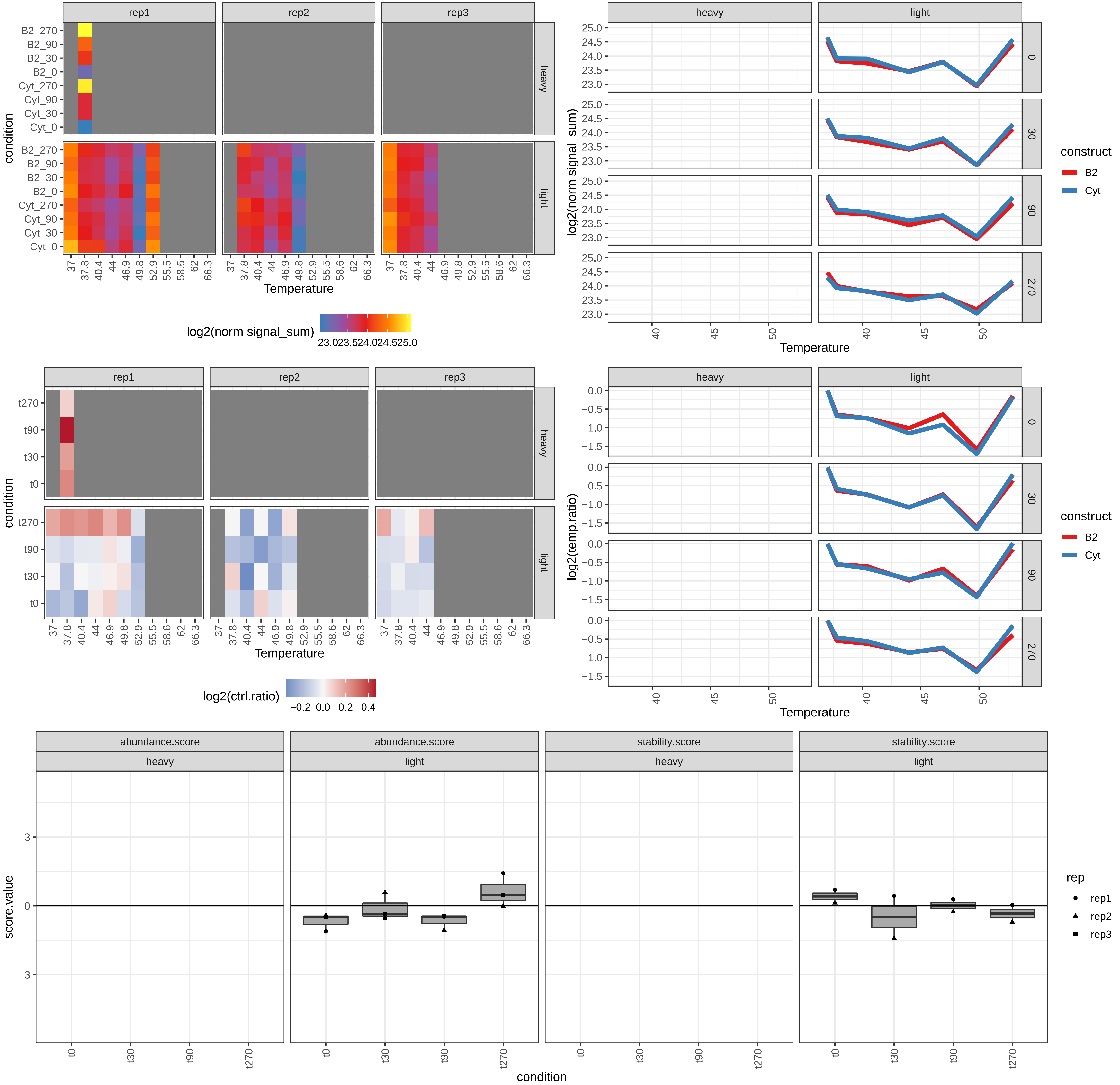

Q06592 − Histone acetyltransferase HPA2 OS=Saccharomyces cerevisiae (strain ATCC 204508 / S288c) GN=HPA2 PE=1 SV=1

HPA3 − average.top3: 7.37530511111111 − max.qupm: 7

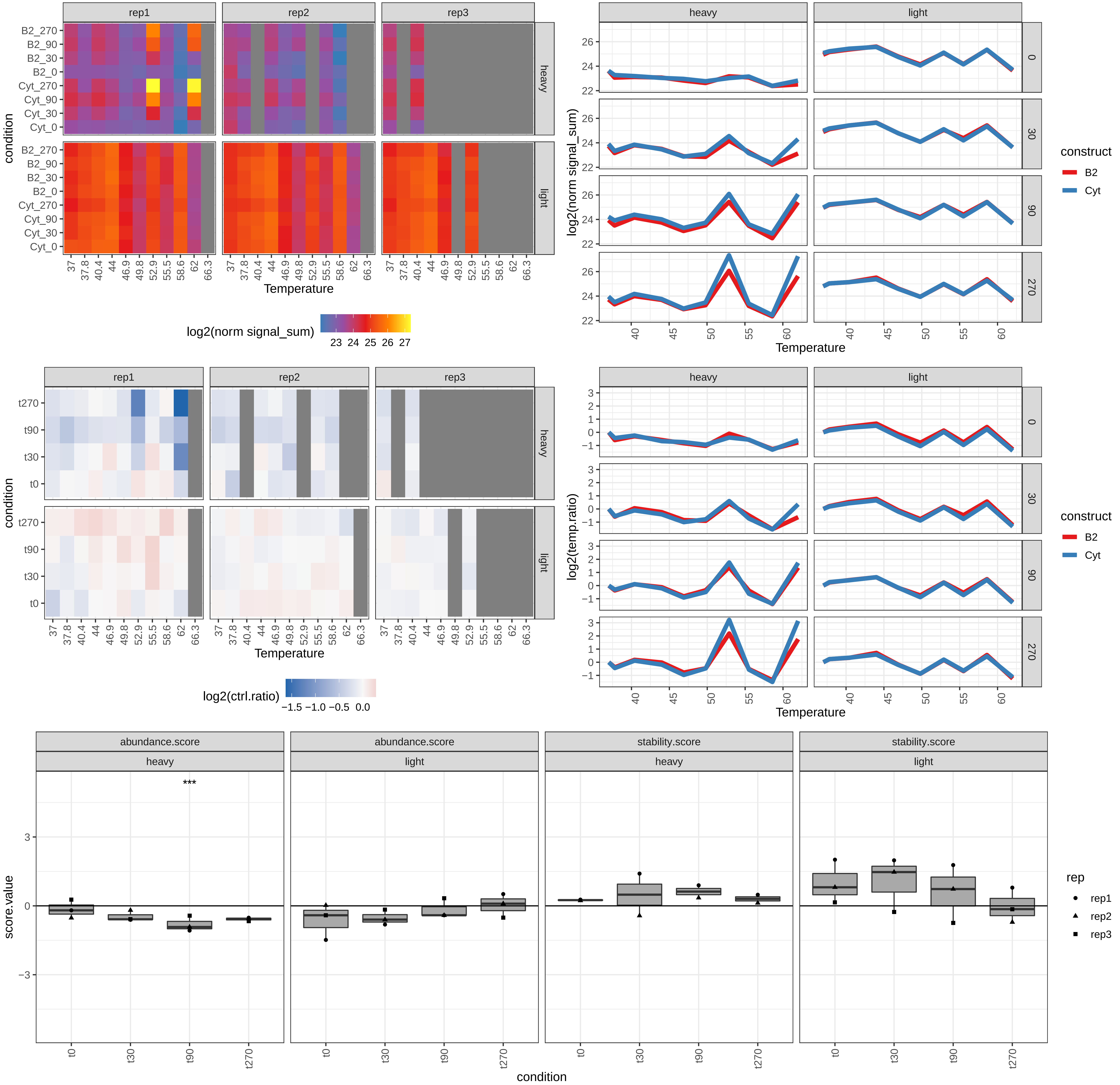

P39979 − D−amino−acid N−acetyltransferase HPA3 OS=Saccharomyces cerevisiae (strain ATCC 204508 / S288c) GN=HPA3 PE=1 SV=2

HPR1 − average.top3: 6.66590055555556 − max.qupm: 8

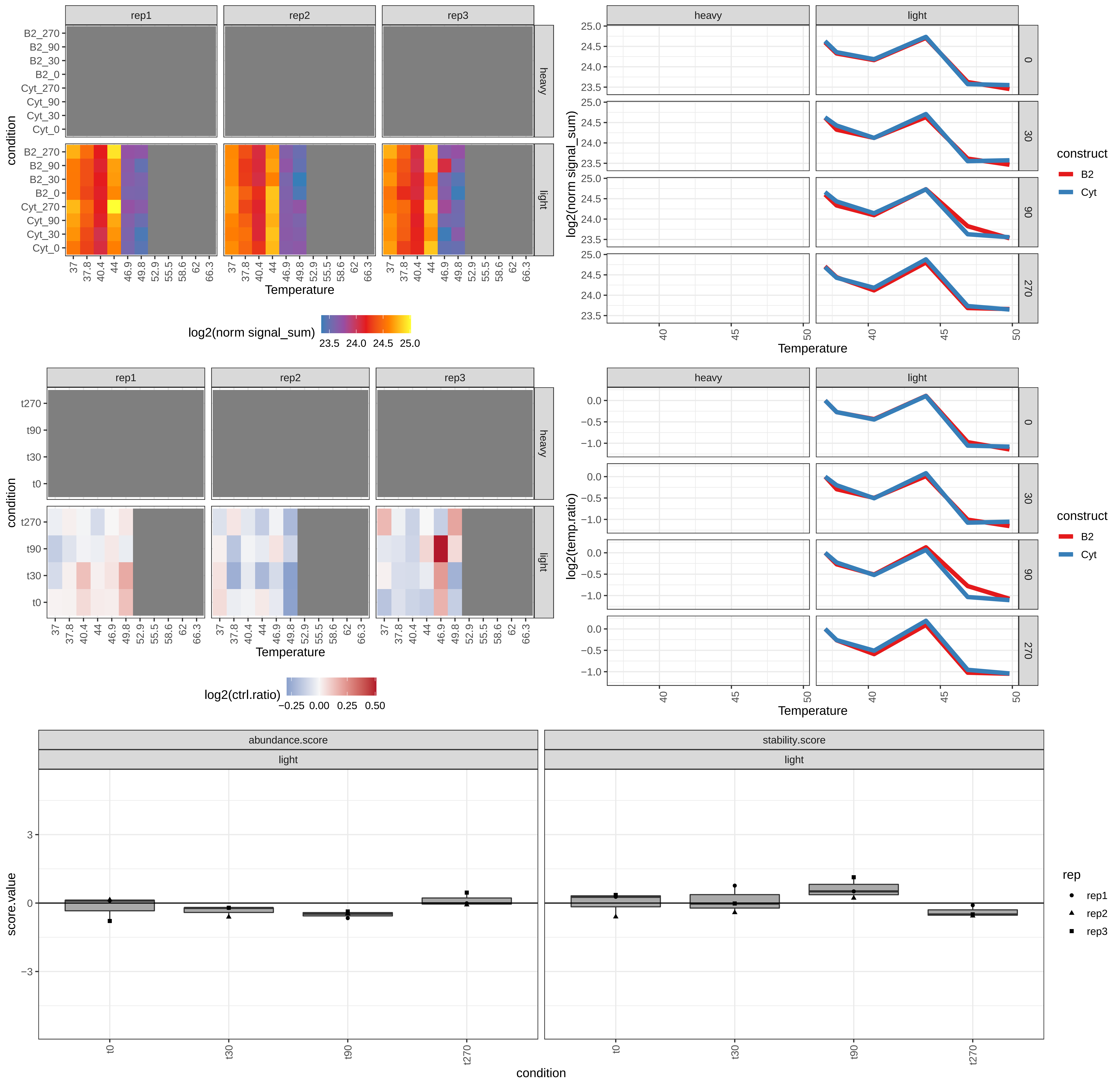

P17629 − THO complex subunit HPR1 OS=Saccharomyces cerevisiae (strain ATCC 204508 / S288c) GN=HPR1 PE=1 SV=2

HPT1 − average.top3: 6.97039166666667 − max.qupm: 8

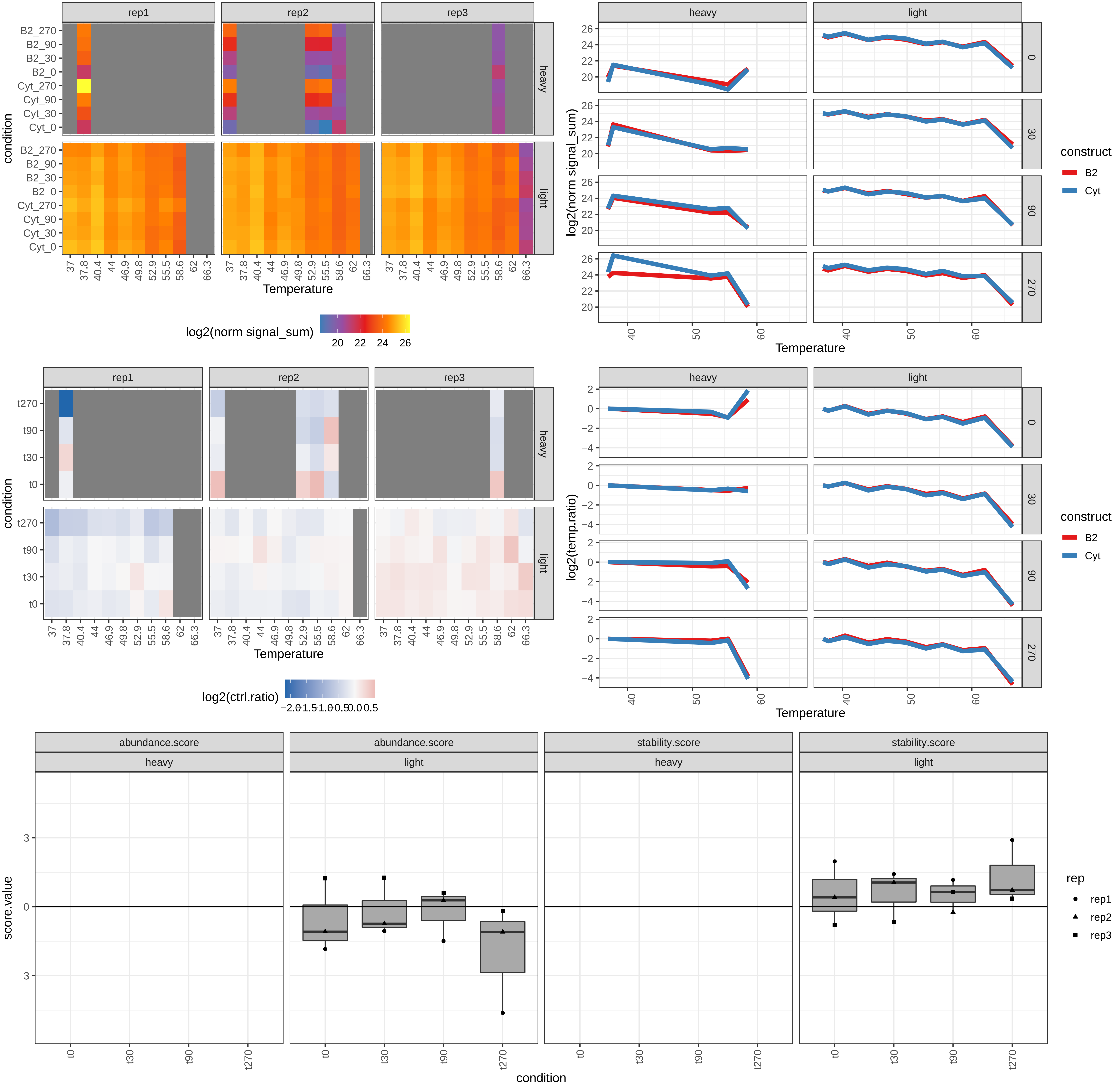

Q04178 − Hypoxanthine−guanine phosphoribosyltransferase OS=Saccharomyces cerevisiae (strain ATCC 204508 / S288c) GN=HPT1 PE=1 SV=1

HRB1 − average.top3: 6.75801916666667 − max.qupm: 8

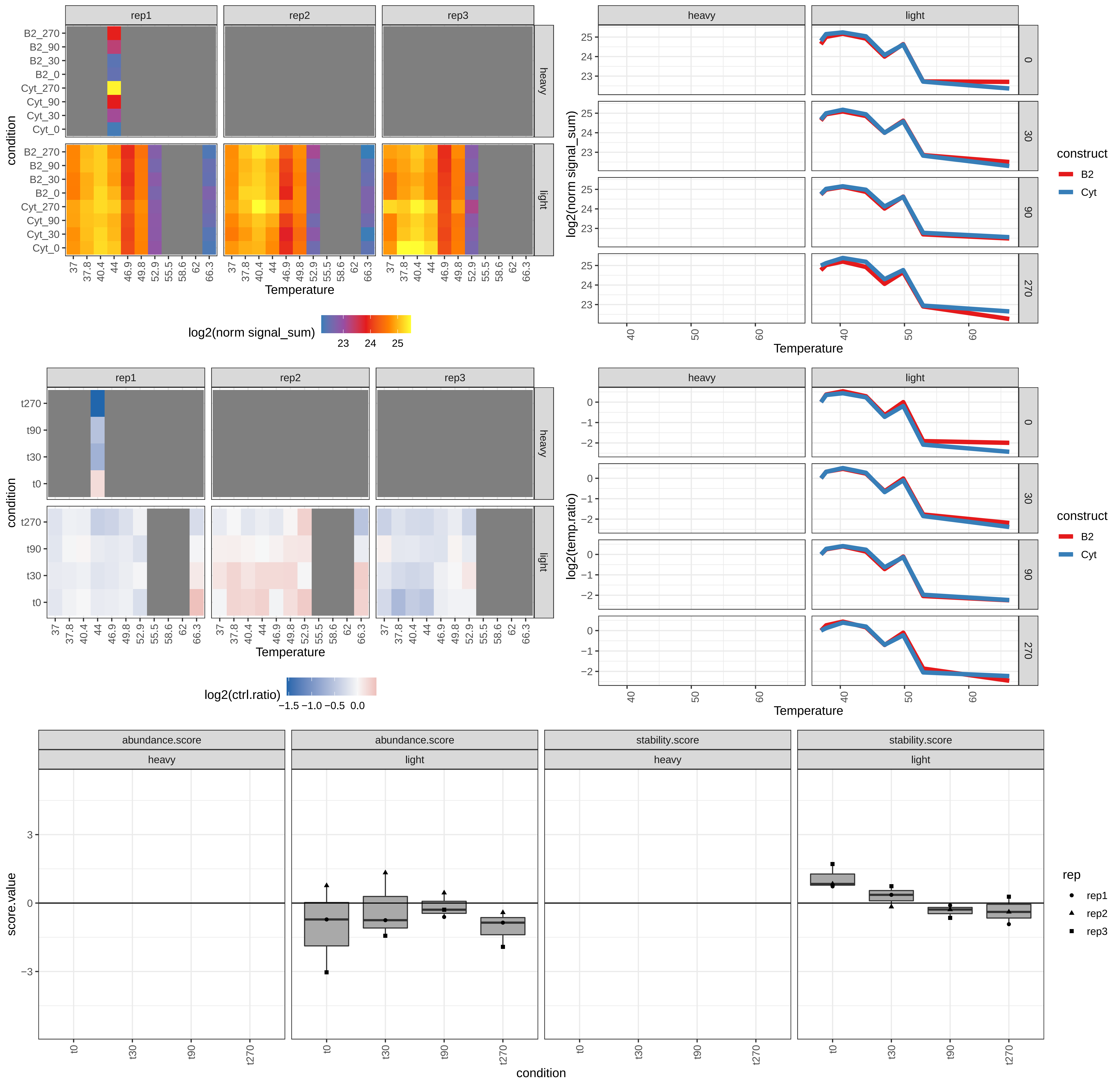

P38922 − Protein HRB1 OS=Saccharomyces cerevisiae (strain ATCC 204508 / S288c) GN=HRB1 PE=1 SV=2

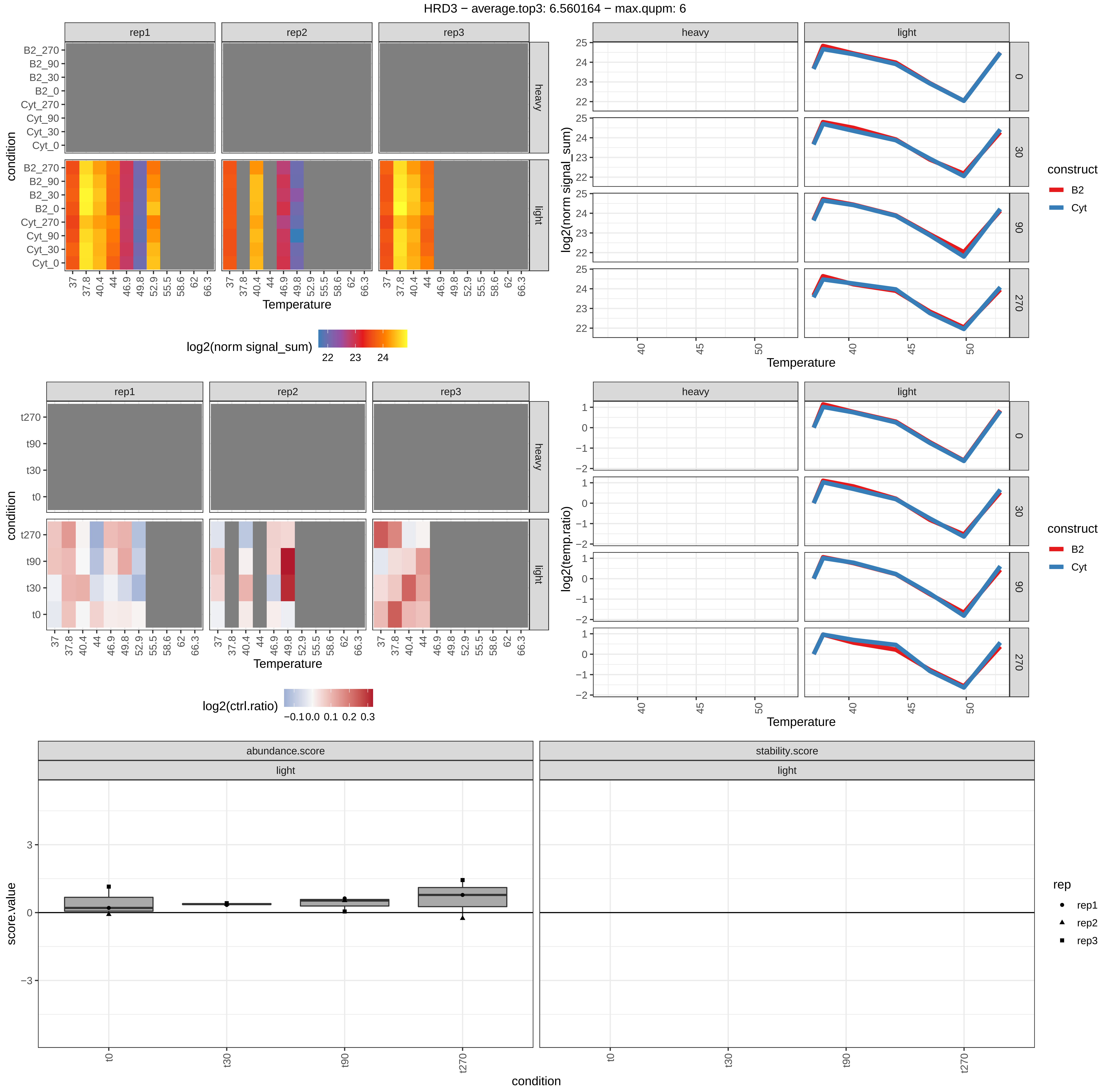

Q05787 − ERAD−associated E3 ubiquitin−protein ligase component HRD3 OS=Saccharomyces cerevisiae (strain ATCC 204508 / S288c) GN=HRD3 PE=1 SV=1

HRI1 − average.top3: 7.41672681818182 − max.qupm: 13

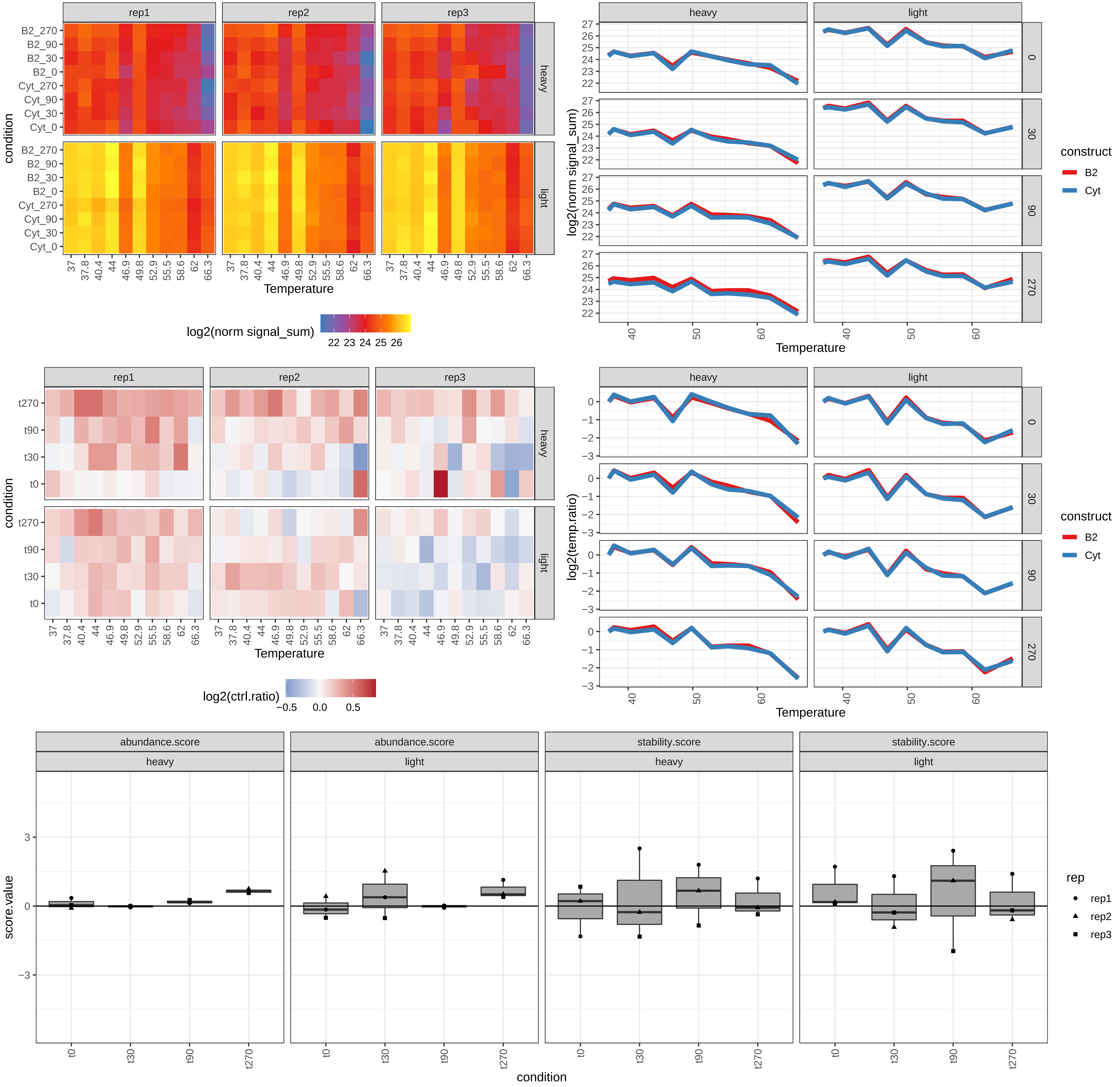

Q05905 − Protein HRI1 OS=Saccharomyces cerevisiae (strain ATCC 204508 / S288c) GN=HRI1 PE=1 SV=1

HRK1 − average.top3: 7.07430090909091 − max.qupm: 7

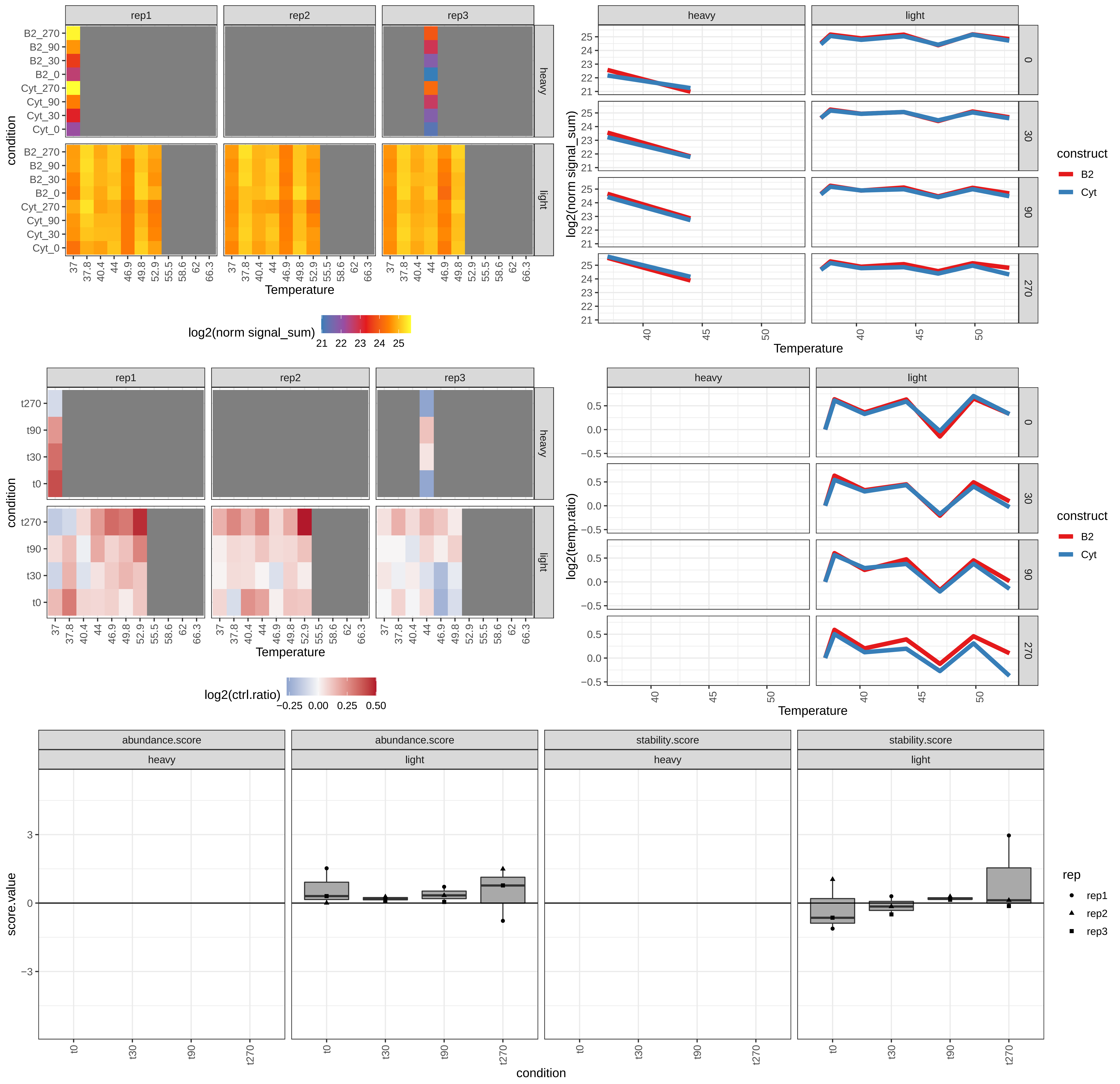

Q08732 − Serine/threonine−protein kinase HRK1 OS=Saccharomyces cerevisiae (strain ATCC 204508 / S288c) GN=HRK1 PE=1 SV=1

HRP1 − average.top3: 7.06098162790698 − max.qupm: 11

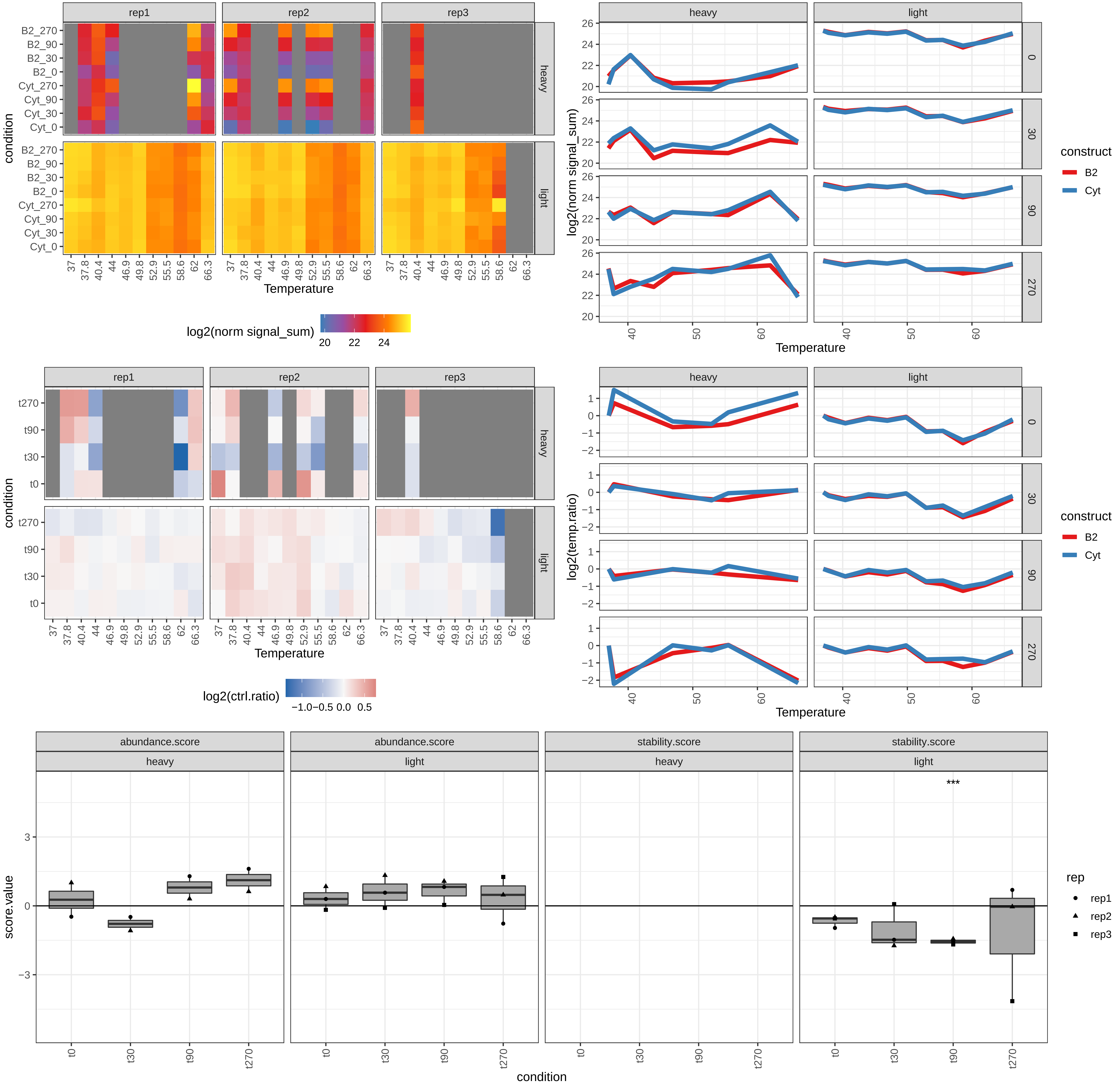

Q99383 − Nuclear polyadenylated RNA−binding protein 4 OS=Saccharomyces cerevisiae (strain ATCC 204508 / S288c) GN=HRP1 PE=1 SV=1

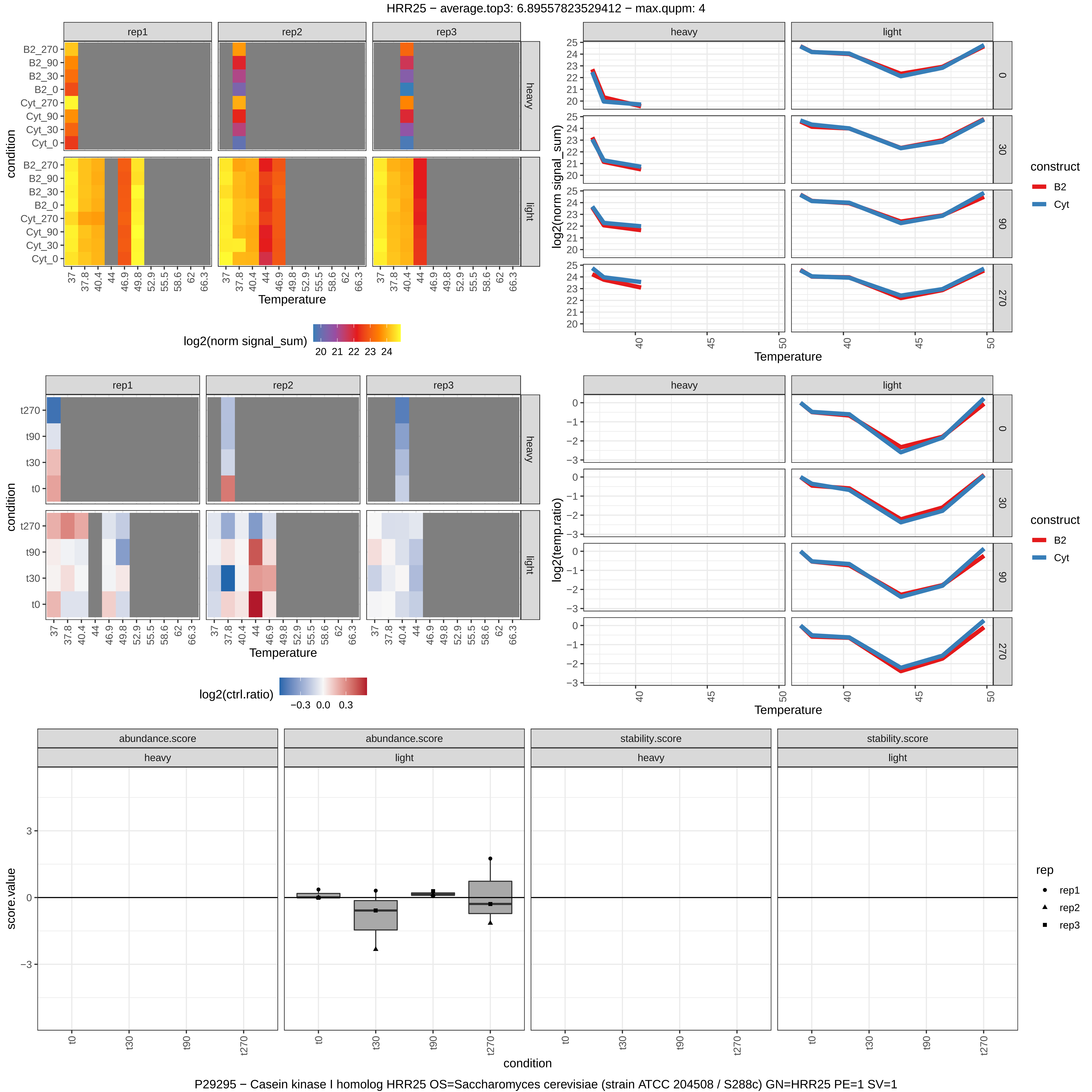
HRR25 − average.top3: 6.89557823529412 − max.qupm: 4
rep1
rep2
rep3
heavy
light
condition
Temperature
log2(norm signal_sum)
log2(ctrl.ratio)
log2(temp.ratio)
construct
B2
Cyt
abundance.score
stability.score
score.value
rep
rep1
rep2
rep3
P29295 − Casein kinase I homolog HRR25 OS=Saccharomyces cerevisiae (strain ATCC 204508 / S288c) GN=HRR25 PE=1 SV=1

HSC82 − average.top3: 7.29125612903226 − max.qupm: 10

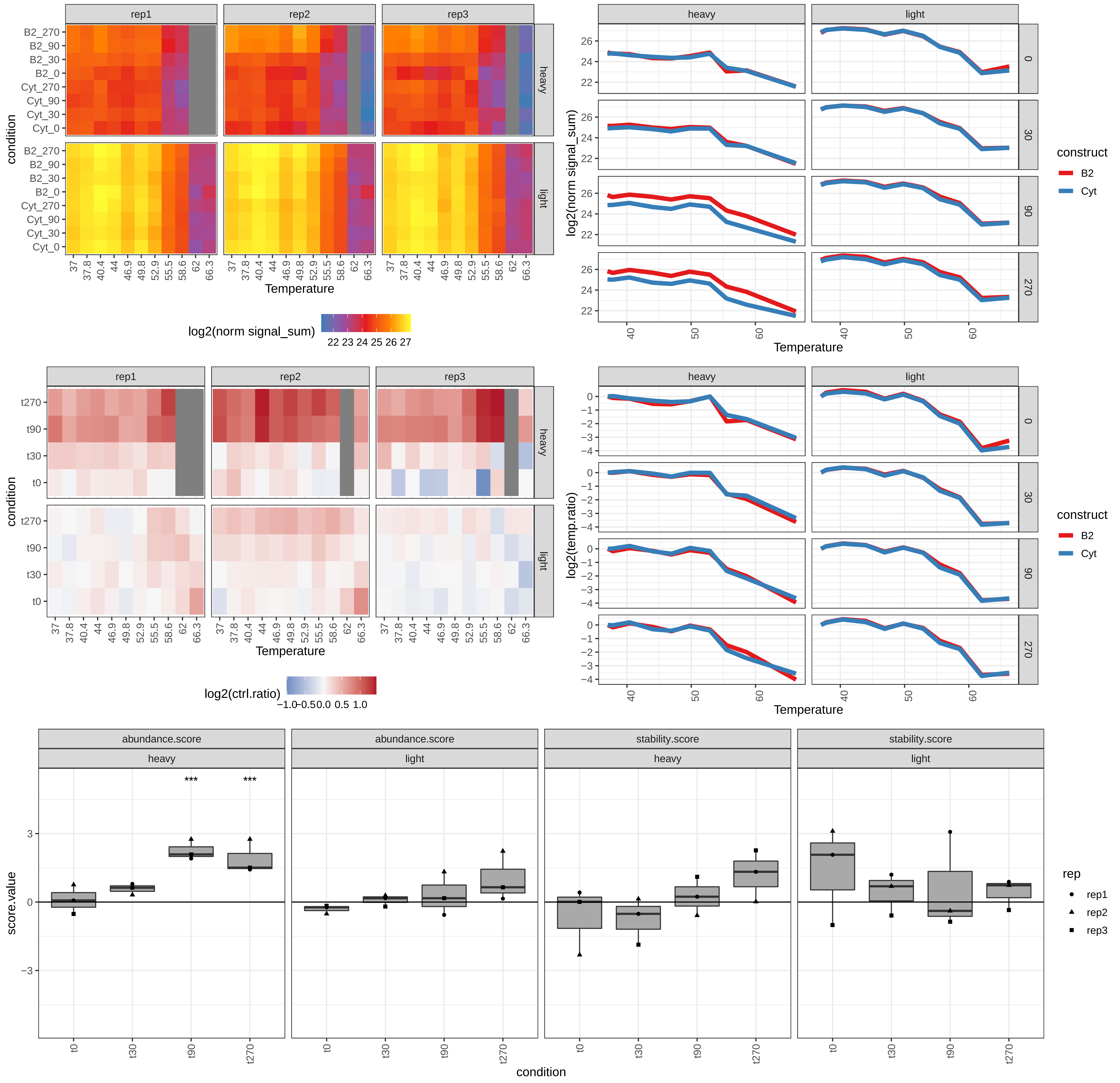

P15108 − ATP−dependent molecular chaperone HSC82 OS=Saccharomyces cerevisiae (strain ATCC 204508 / S288c) GN=HSC82 PE=1 SV=4

HSE1 − average.top3: 6.40394727272727 − max.qupm: 3

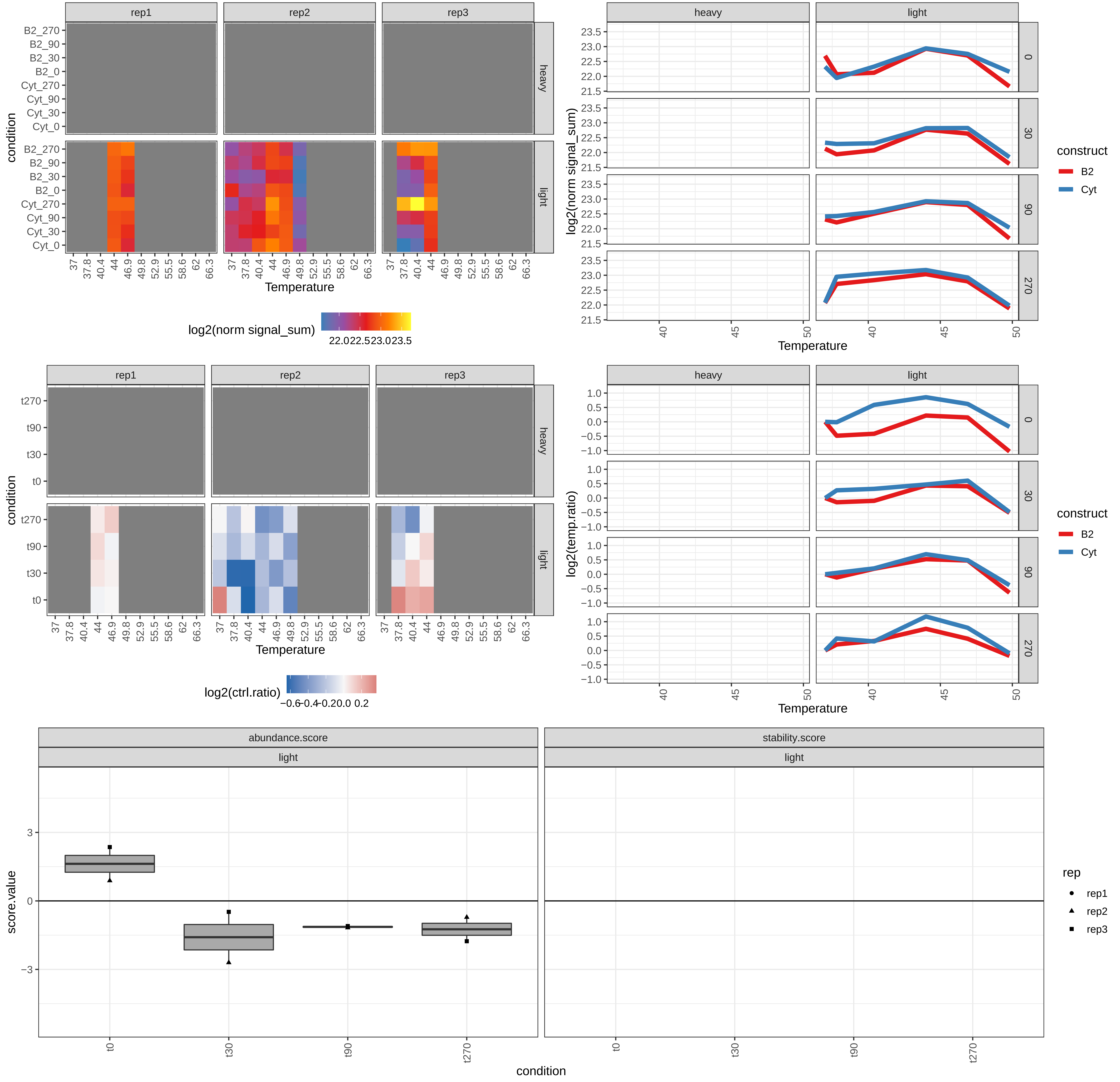

P38753 − Class E vacuolar protein−sorting machinery protein HSE1 OS=Saccharomyces cerevisiae (strain ATCC 204508 / S288c) GN=HSE1 PE=1 SV=1

HSM3 − average.top3: 7.1068704 − max.qupm: 7

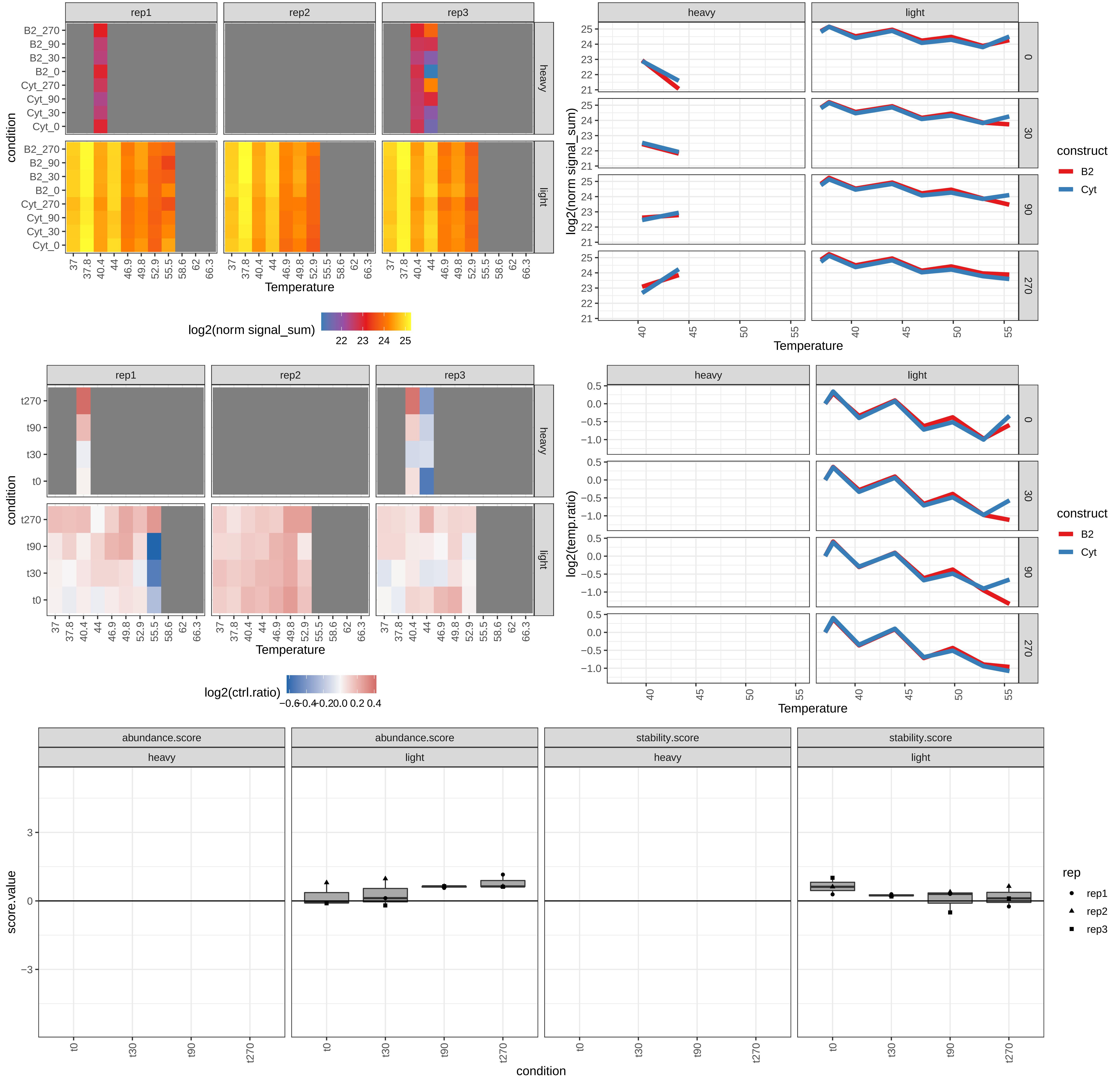

P38348 − DNA mismatch repair protein HSM3 OS=Saccharomyces cerevisiae (strain ATCC 204508 / S288c) GN=HSM3 PE=1 SV=1

HSP10 − average.top3: 6.44824051282051 − max.qupm: 5

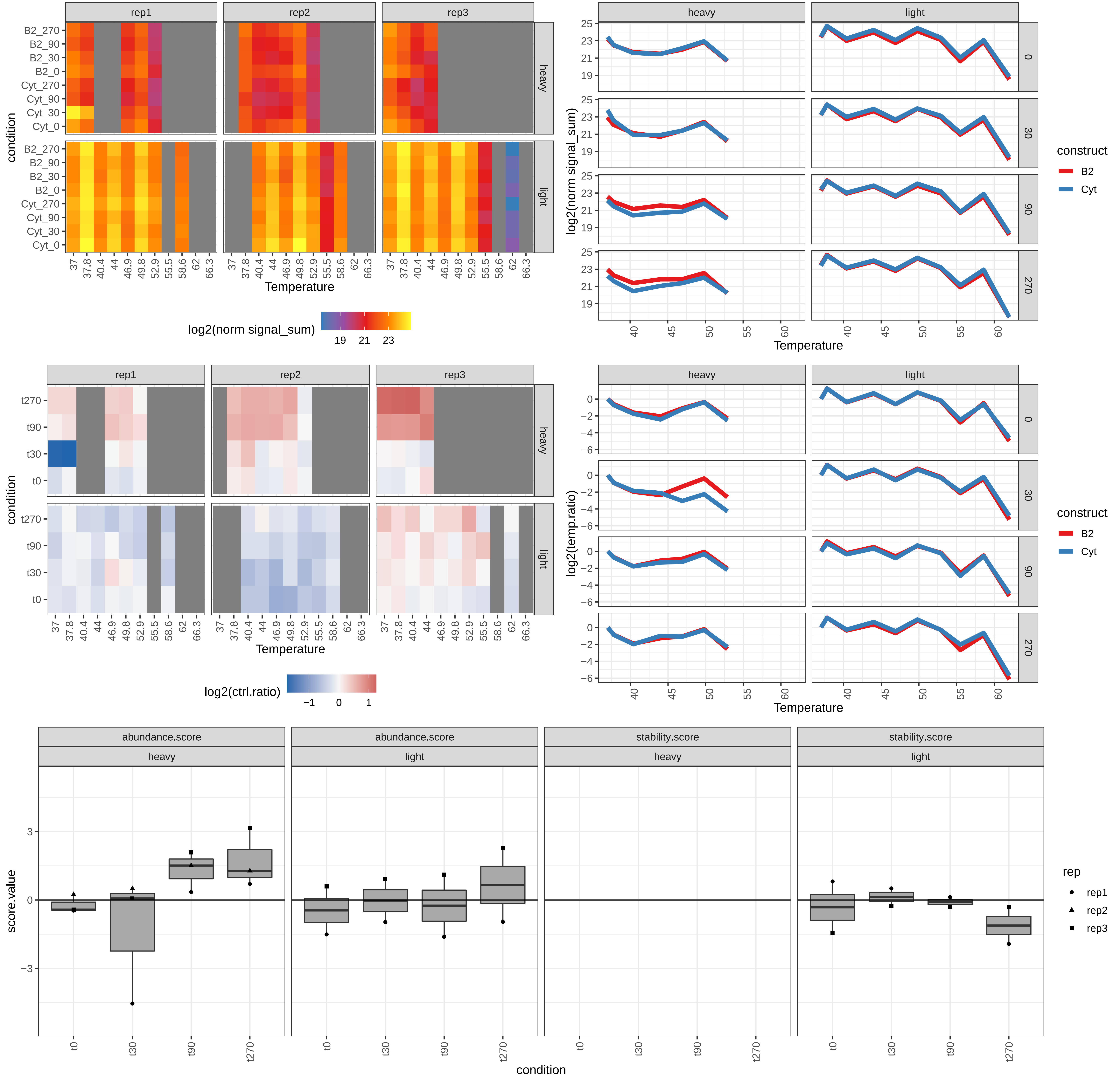

P38910 − 10 kDa heat shock protein, mitochondrial OS=Saccharomyces cerevisiae (strain ATCC 204508 / S288c) GN=HSP10 PE=1 SV=1

HSP104 − average.top3: 7.805215 − max.qupm: 56

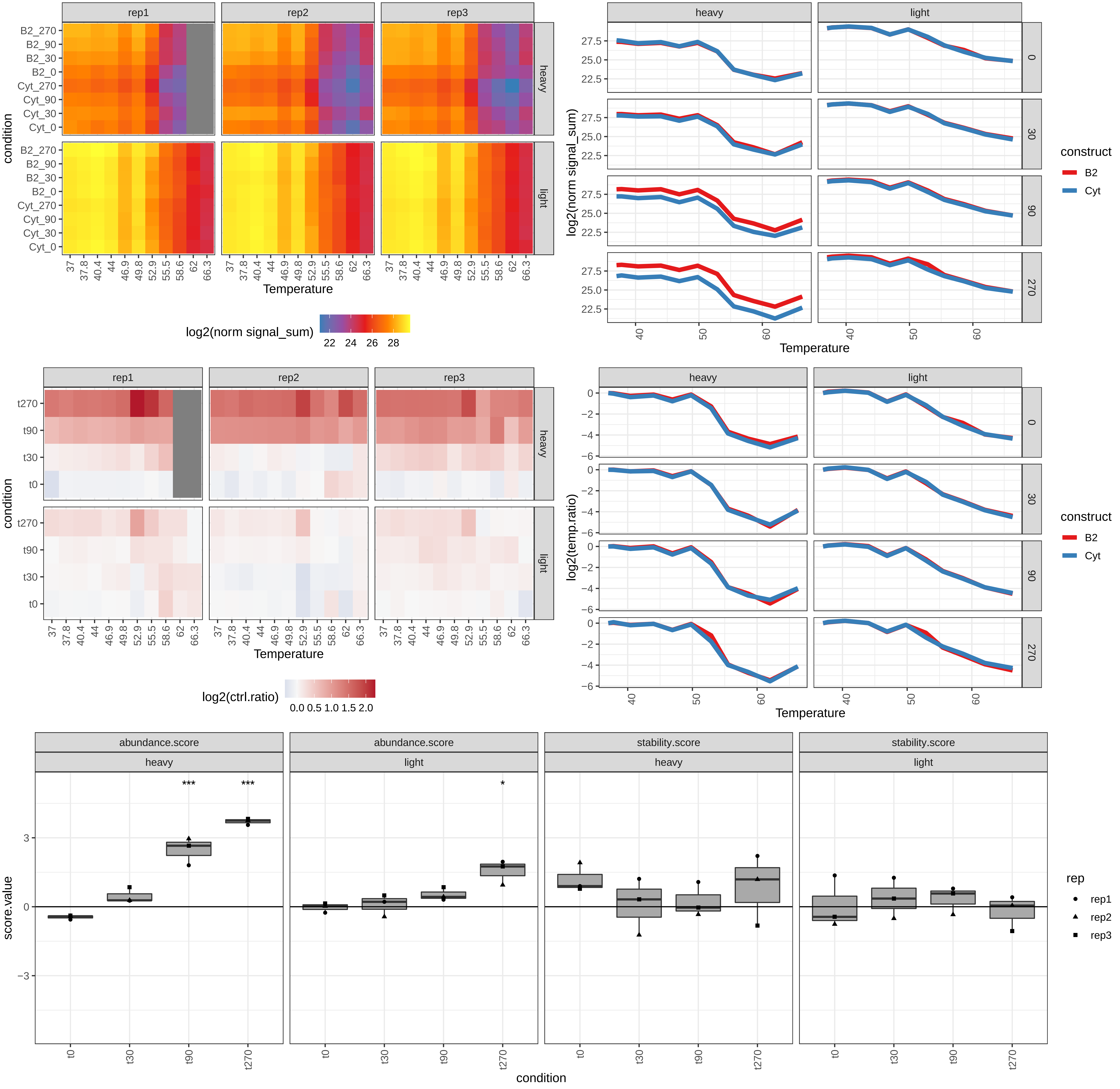

P31539 − Heat shock protein 104 OS=Saccharomyces cerevisiae (strain ATCC 204508 / S288c) GN=HSP104 PE=1 SV=2

HSP12 − average.top3: 6.94625593220339 − max.qupm: 6

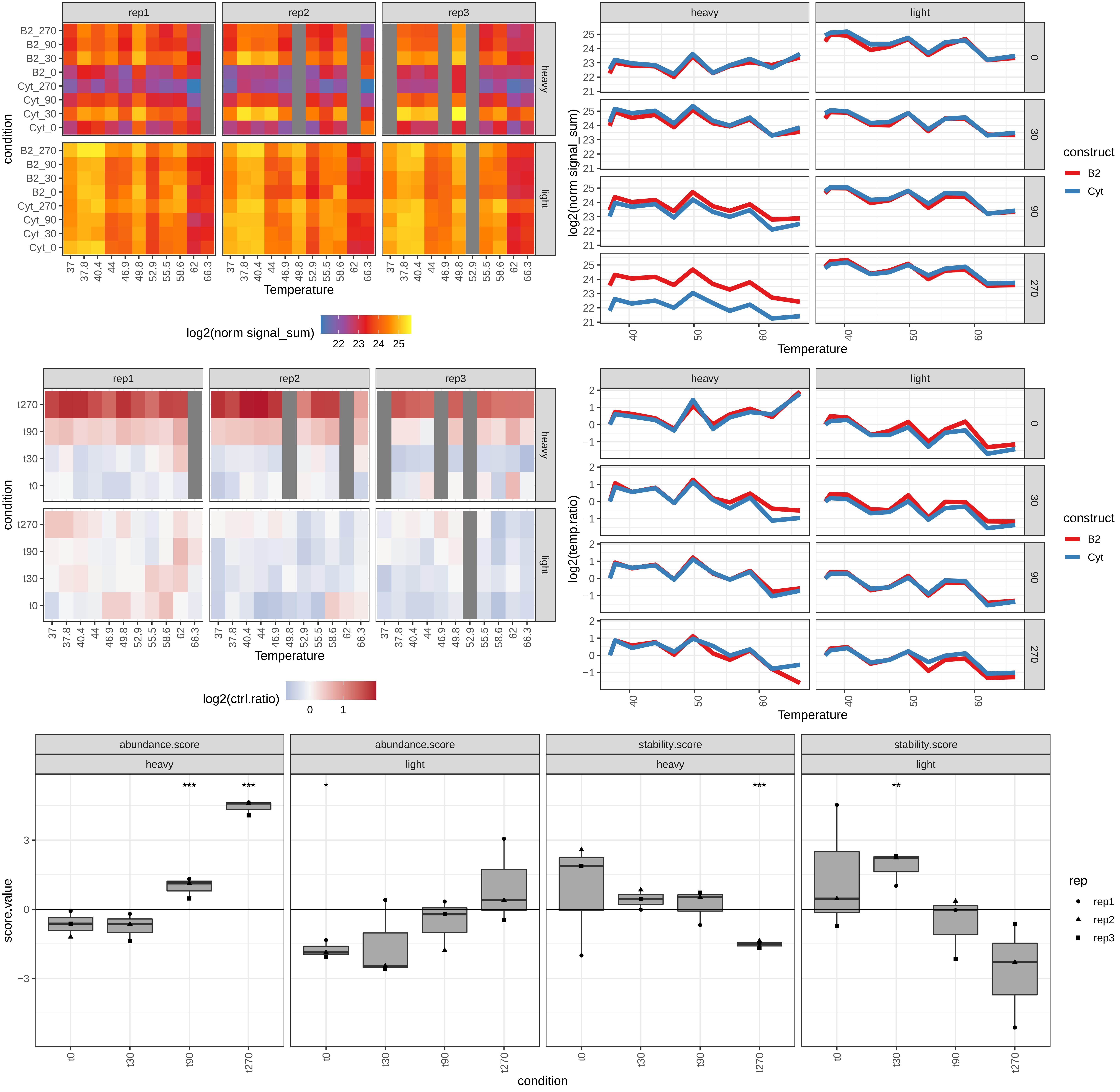

P22943 − 12 kDa heat shock protein OS=Saccharomyces cerevisiae (strain ATCC 204508 / S288c) GN=HSP12 PE=1 SV=1

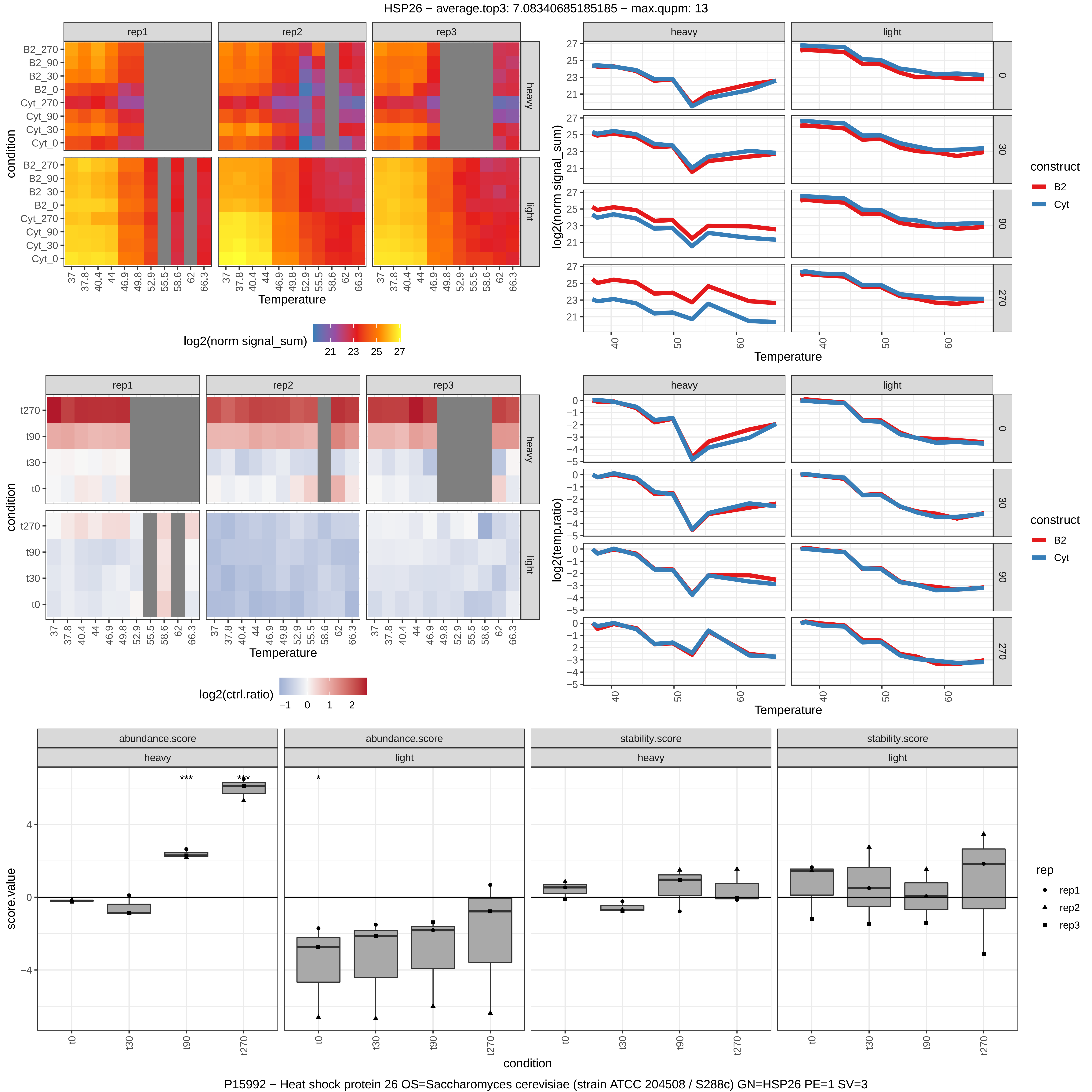
HSP26 − average.top3: 7.08340685185185 − max.qupm: 13
rep1
rep2
rep3
heavy
light
condition
Temperature
log2(norm signal_sum)
log2(ctrl.ratio)
construct
B2
Cyt
log2(temp.ratio)
abundance.score
stability.score
score.value
rep
rep1
rep2
rep3
condition
P15992 − Heat shock protein 26 OS=Saccharomyces cerevisiae (strain ATCC 204508 / S288c) GN=HSP26 PE=1 SV=3

HSP31 − average.top3: 7.106578125 − max.qupm: 7

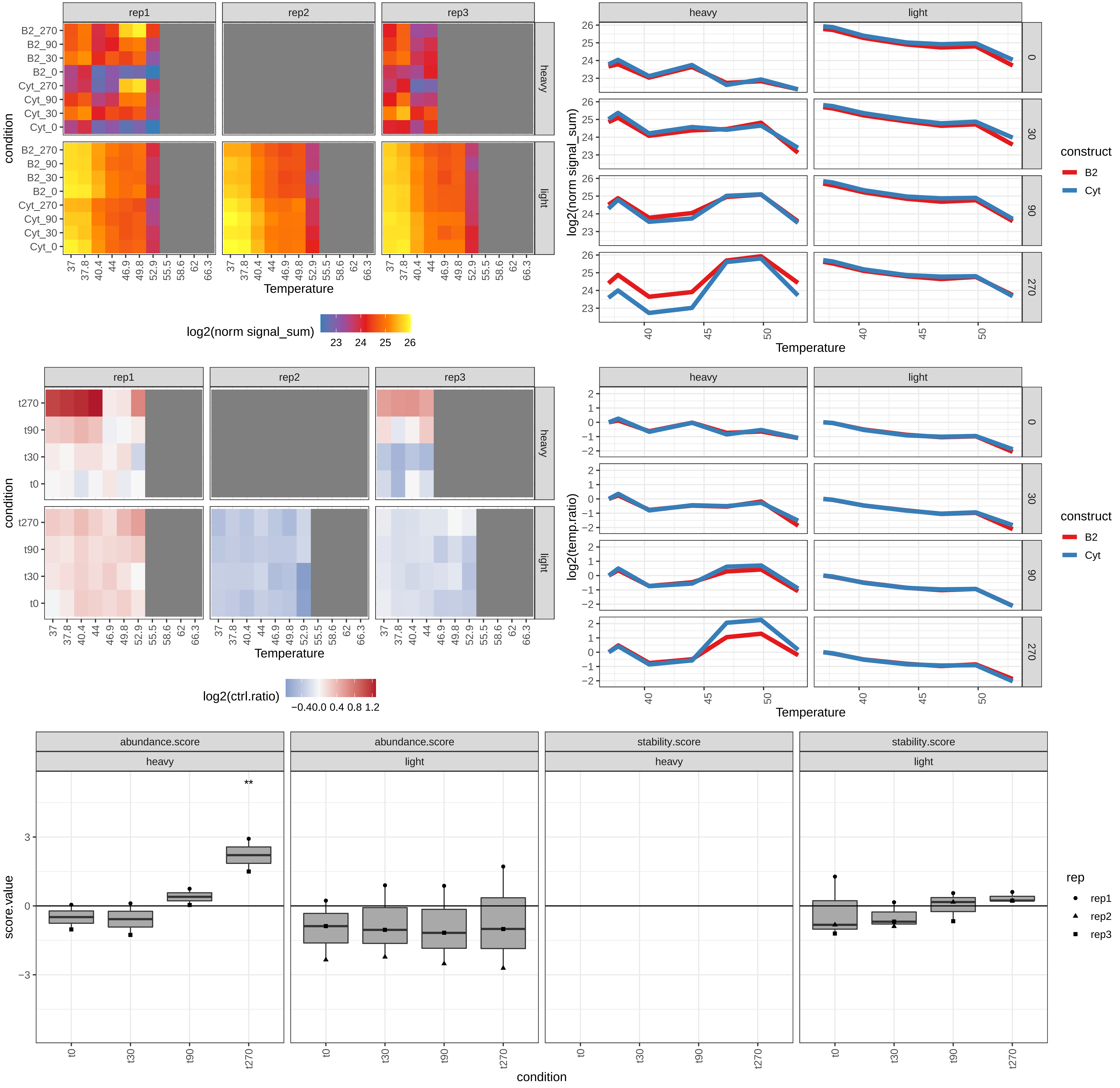

Q04432 − Glutathione−independent glyoxalase HSP31 OS=Saccharomyces cerevisiae (strain ATCC 204508 / S288c) GN=HSP31 PE=1 SV=1

HSP42 − average.top3: 7.32760192307692 − max.qupm: 7

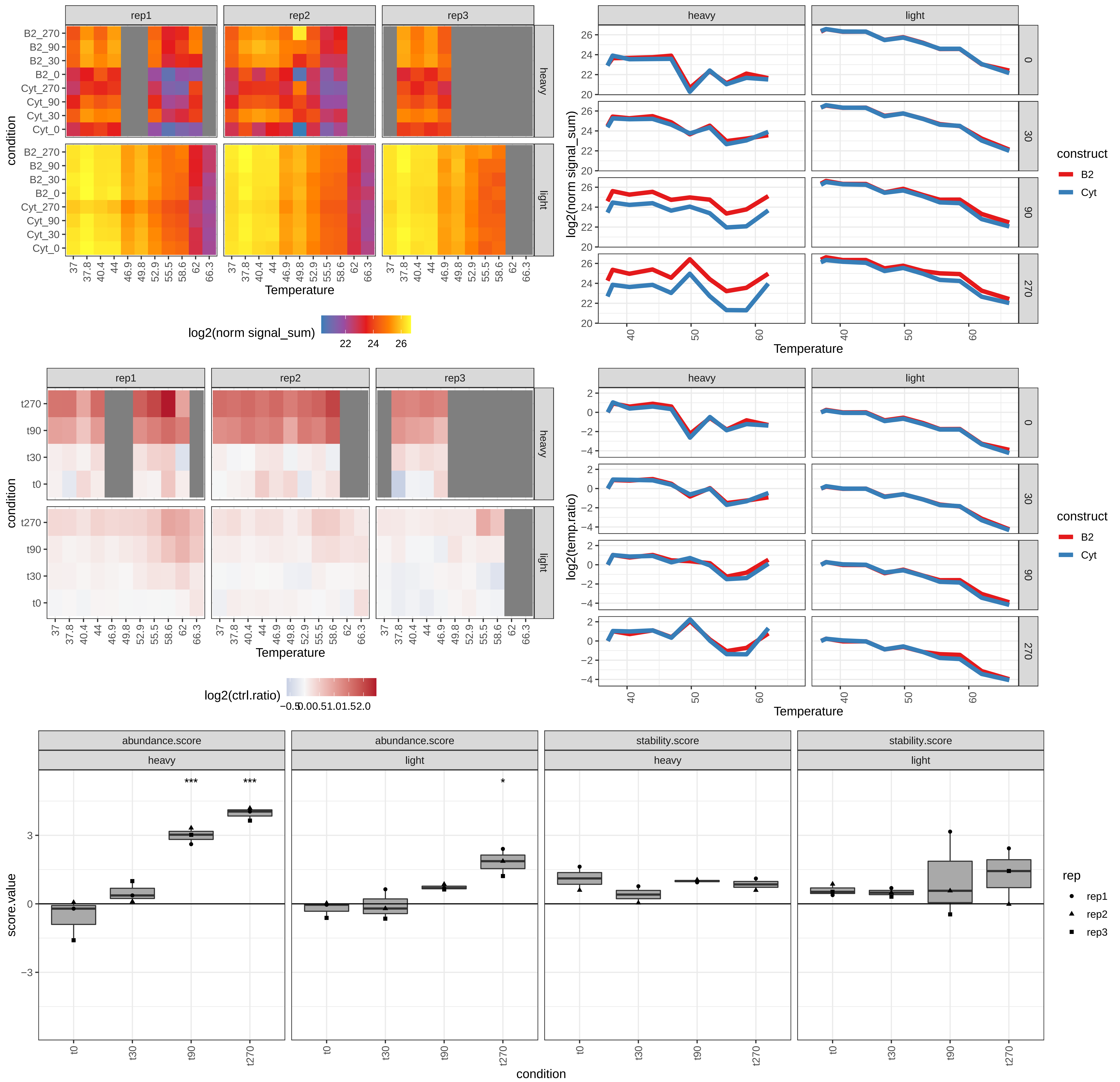

Q12329 − Heat shock protein 42 OS=Saccharomyces cerevisiae (strain ATCC 204508 / S288c) GN=HSP42 PE=1 SV=1

HSP60 − average.top3: 8.17536272727273 − max.qupm: 33

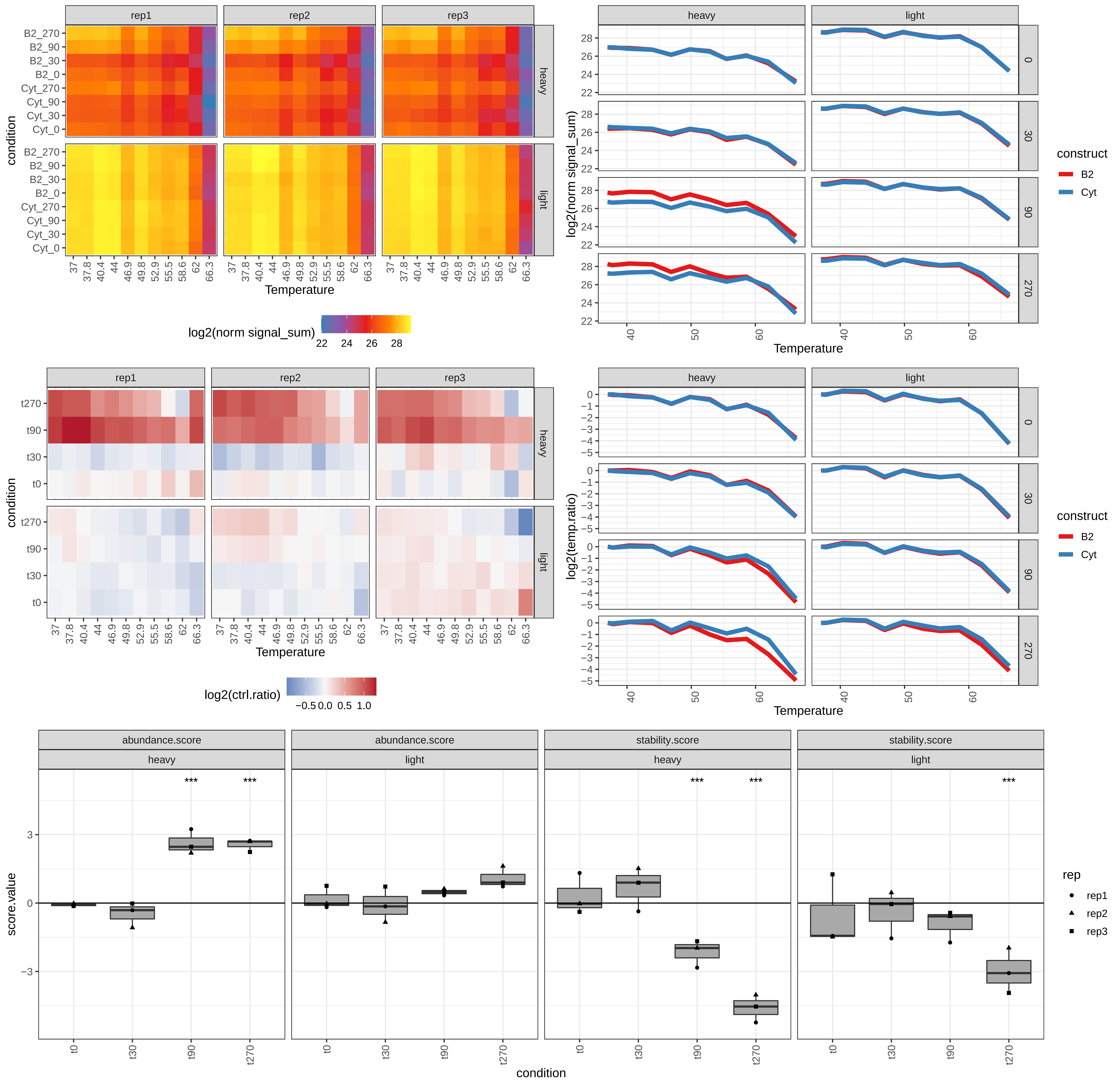

P19882 − Heat shock protein 60, mitochondrial OS=Saccharomyces cerevisiae (strain ATCC 204508 / S288c) GN=HSP60 PE=1 SV=1

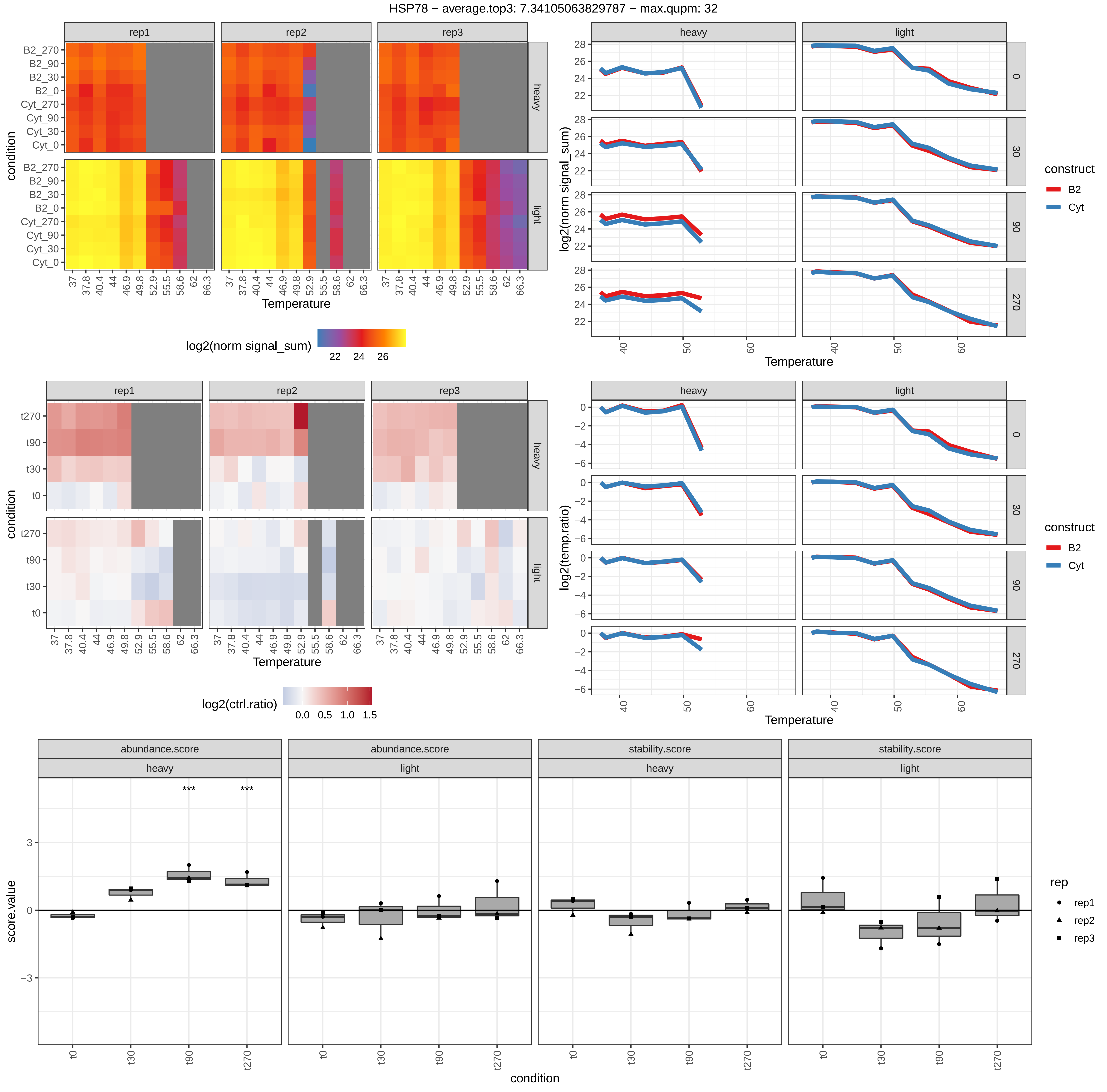

P33416 − Heat shock protein 78, mitochondrial OS=Saccharomyces cerevisiae (strain ATCC 204508 / S288c) GN=HSP78 PE=1 SV=2

HSP82 − average.top3: 7.4008406557377 − max.qupm: 8

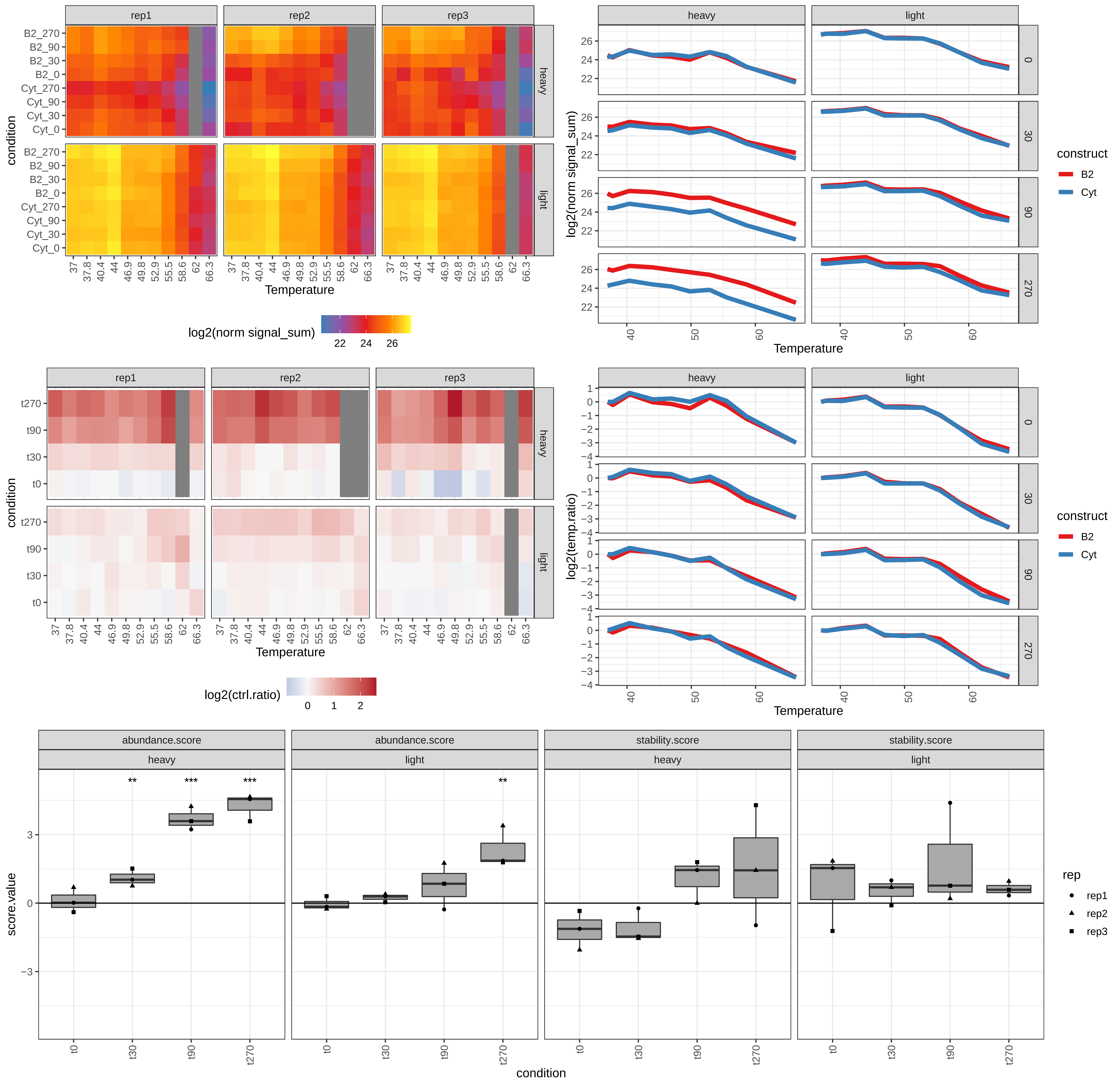

P02829 − ATP−dependent molecular chaperone HSP82 OS=Saccharomyces cerevisiae (strain ATCC 204508 / S288c) GN=HSP82 PE=1 SV=1

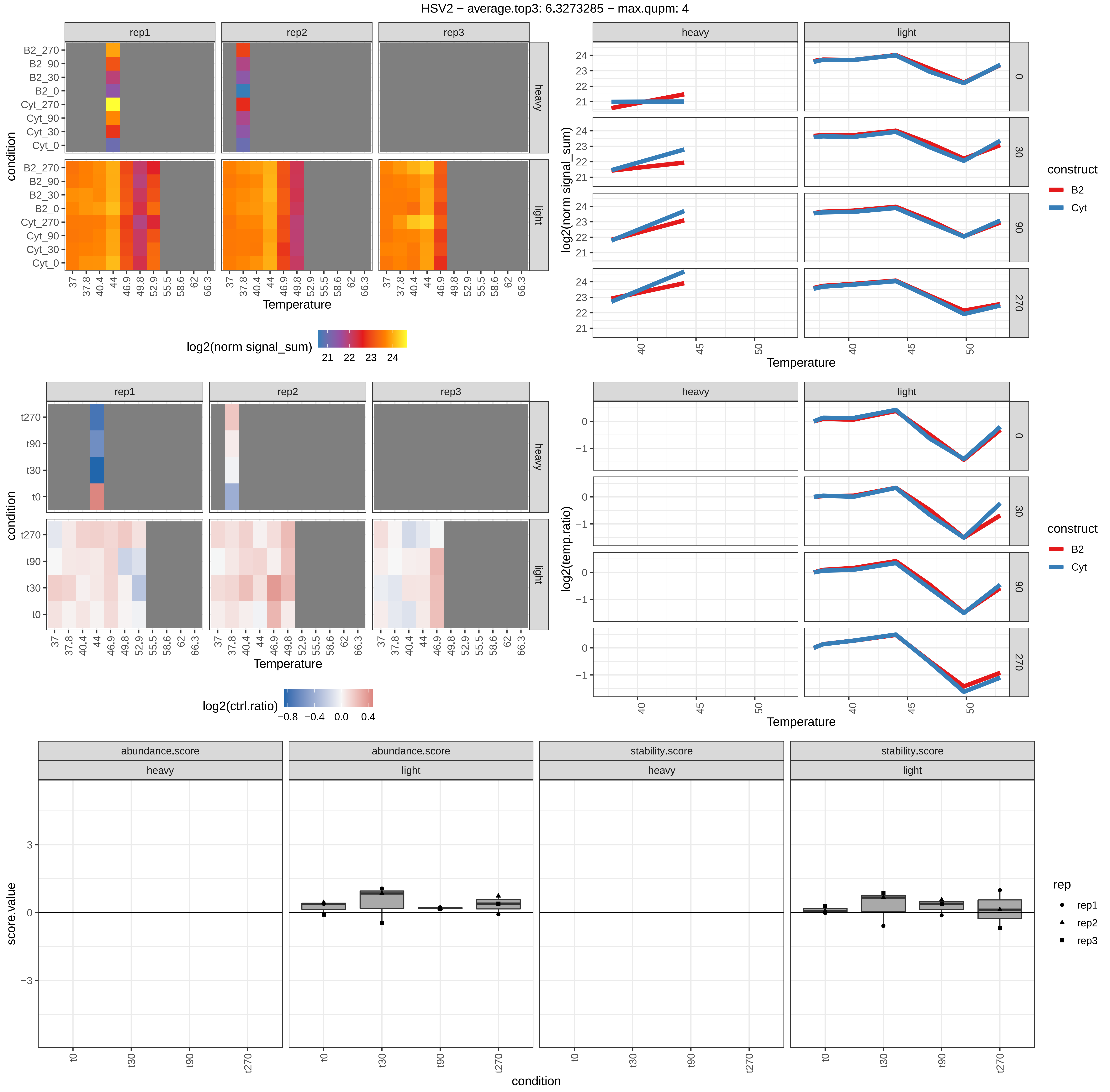

P50079 − SVP1−like protein 2 OS=Saccharomyces cerevisiae (strain ATCC 204508 / S288c) GN=HSV2 PE=1 SV=1

HTA1|HTA2 − average.top3: 7.82846675 − max.qupm: 3

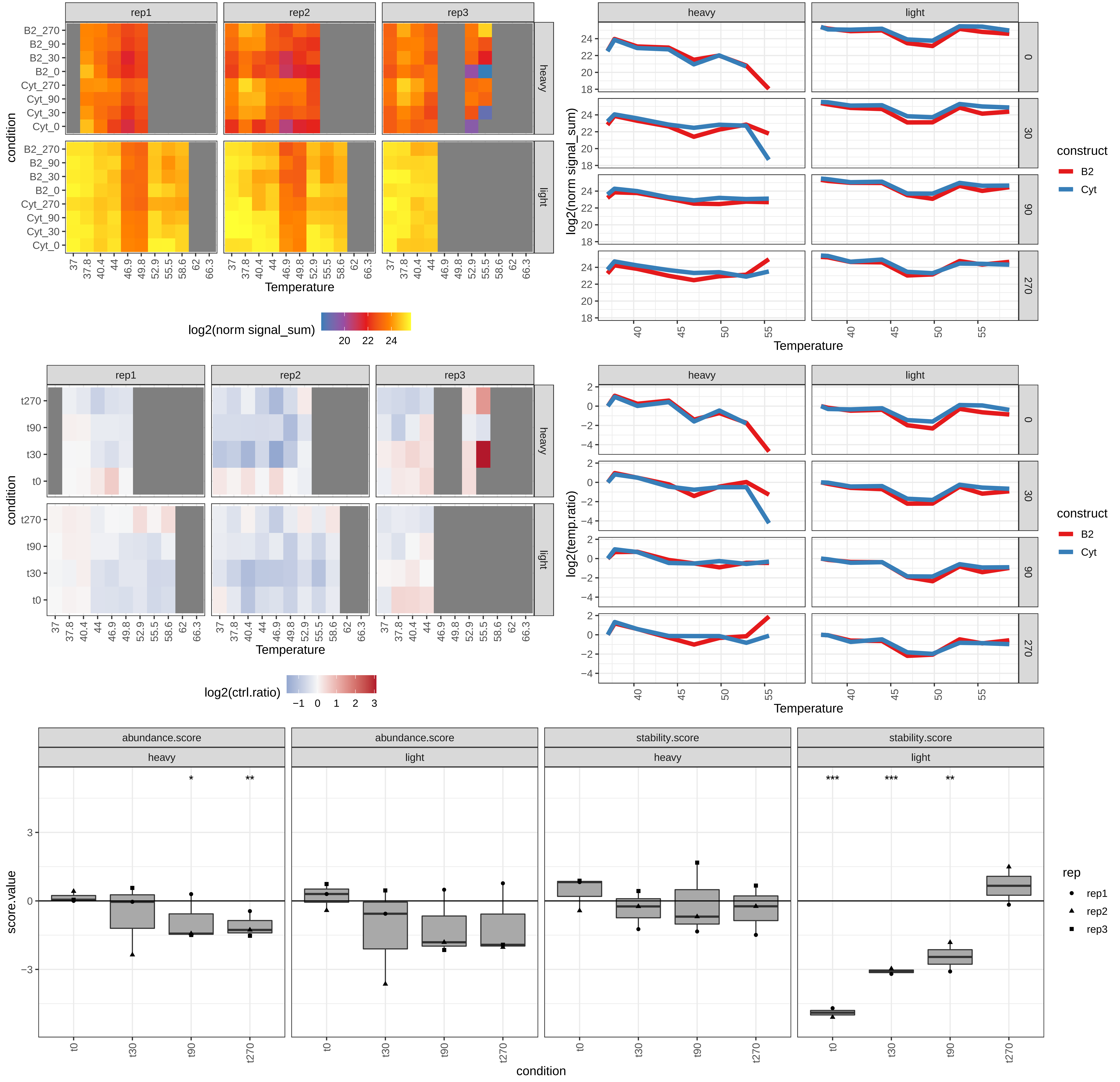

P04912 − Histone H2A.1 OS=Saccharomyces cerevisiae (strain ATCC 204508 / S288c) GN=HTA1 PE=1 SV=2|Histone H2A.2 OS=Saccharomyces cerevisiae (strain ATCC 204508 / S288c) GN=HTA2 PE=

HTB1 − average.top3: 7.66490142857143 − max.qupm: 10

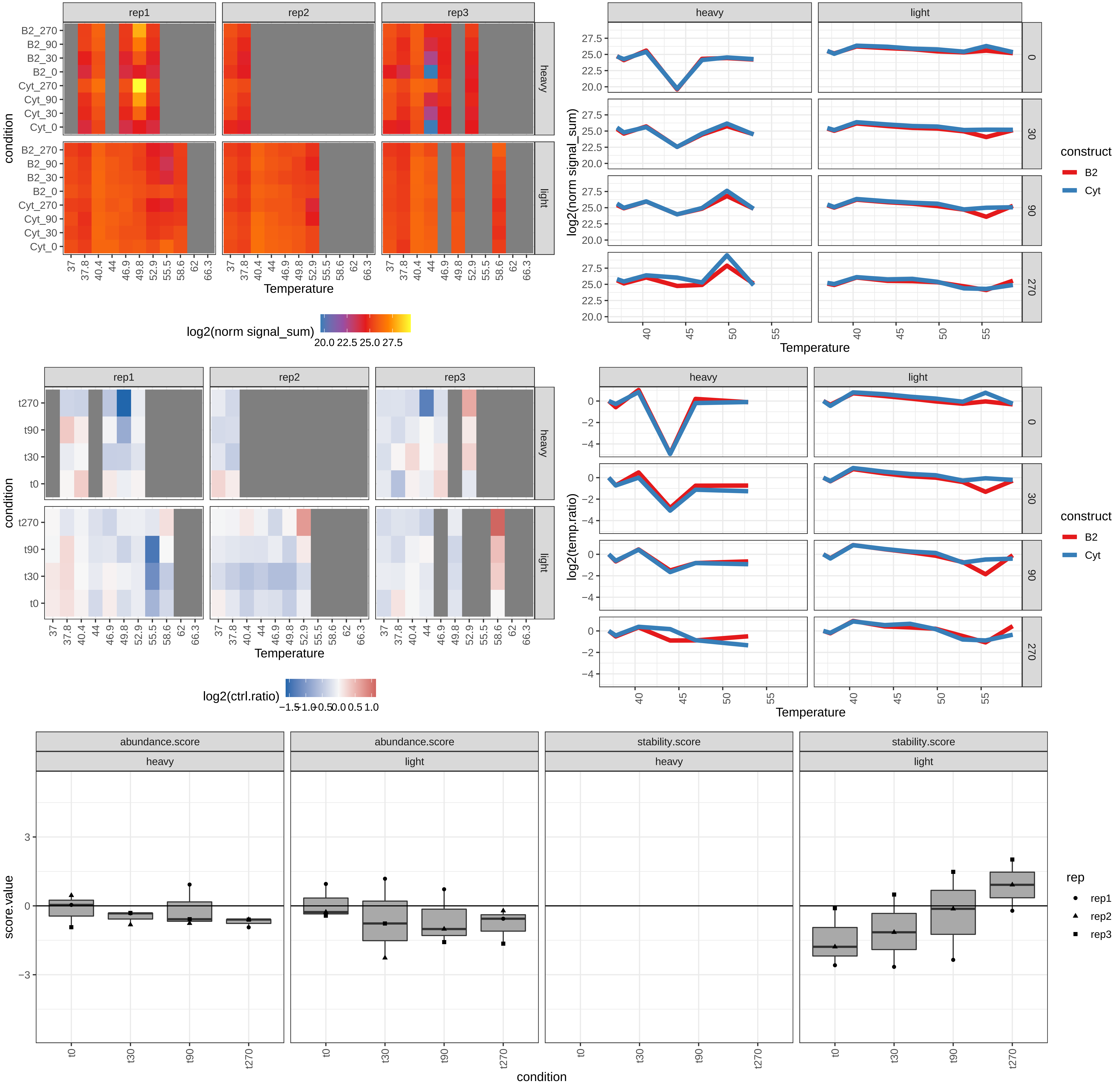

P02293 − Histone H2B.1 OS=Saccharomyces cerevisiae (strain ATCC 204508 / S288c) GN=HTB1 PE=1 SV=2

HTB2 − average.top3: 7.47973363636364 − max.qupm: 8

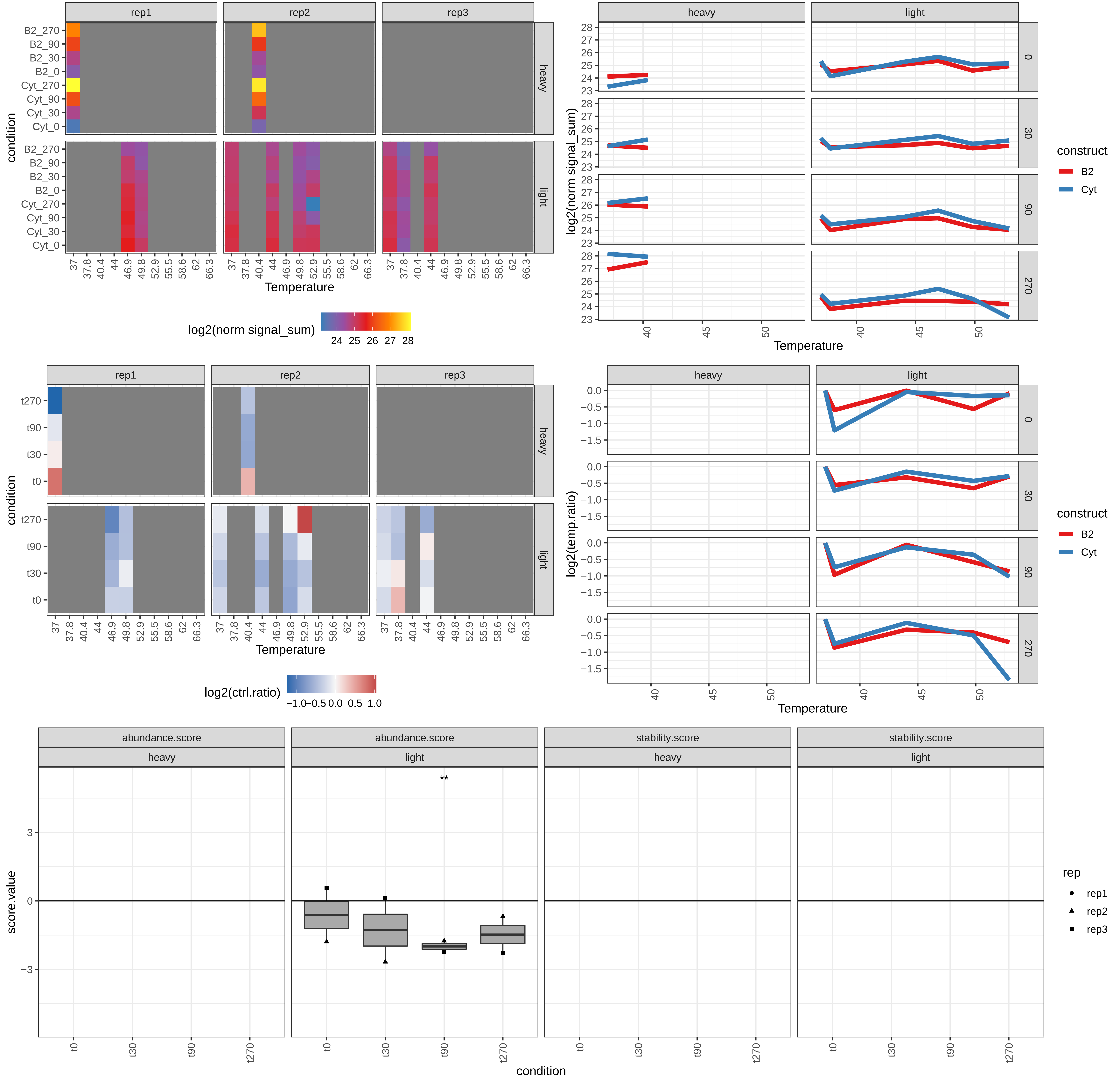

P02294 − Histone H2B.2 OS=Saccharomyces cerevisiae (strain ATCC 204508 / S288c) GN=HTB2 PE=1 SV=2

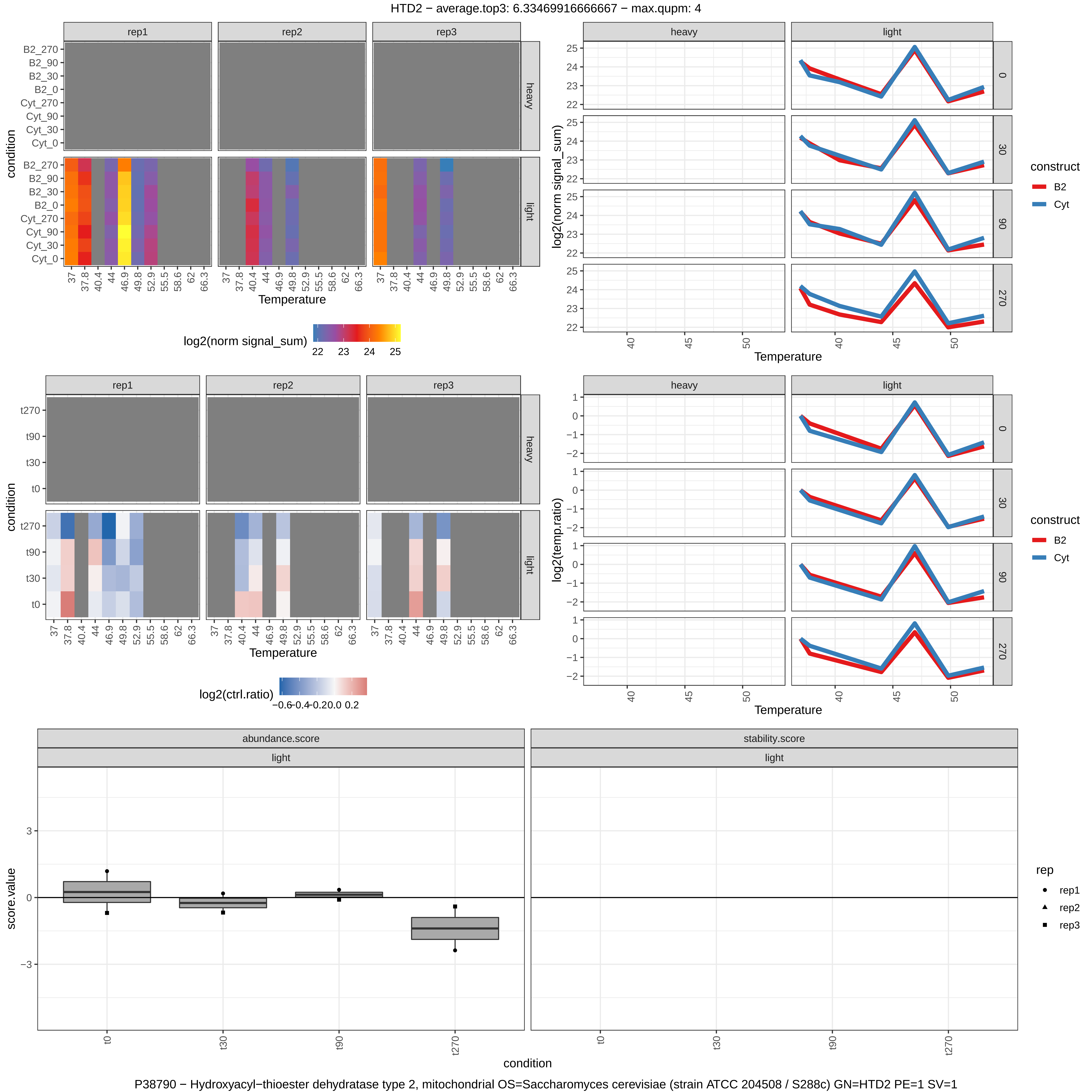
HTD2 − average.top3: 6.33469916666667 − max.qupm: 4
rep1
rep2
rep3
heavy
light
condition
Temperature
log2(norm signal_sum)
log2(ctrl.ratio)
log2(temp.ratio)
construct
B2
Cyt
abundance.score
stability.score
score.value
rep
rep1
rep2
rep3
P38790 − Hydroxyacyl−thioester dehydratase type 2, mitochondrial OS=Saccharomyces cerevisiae (strain ATCC 204508 / S288c) GN=HTD2 PE=1 SV=1

HTS1 − average.top3: 7.33776979166667 − max.qupm: 19

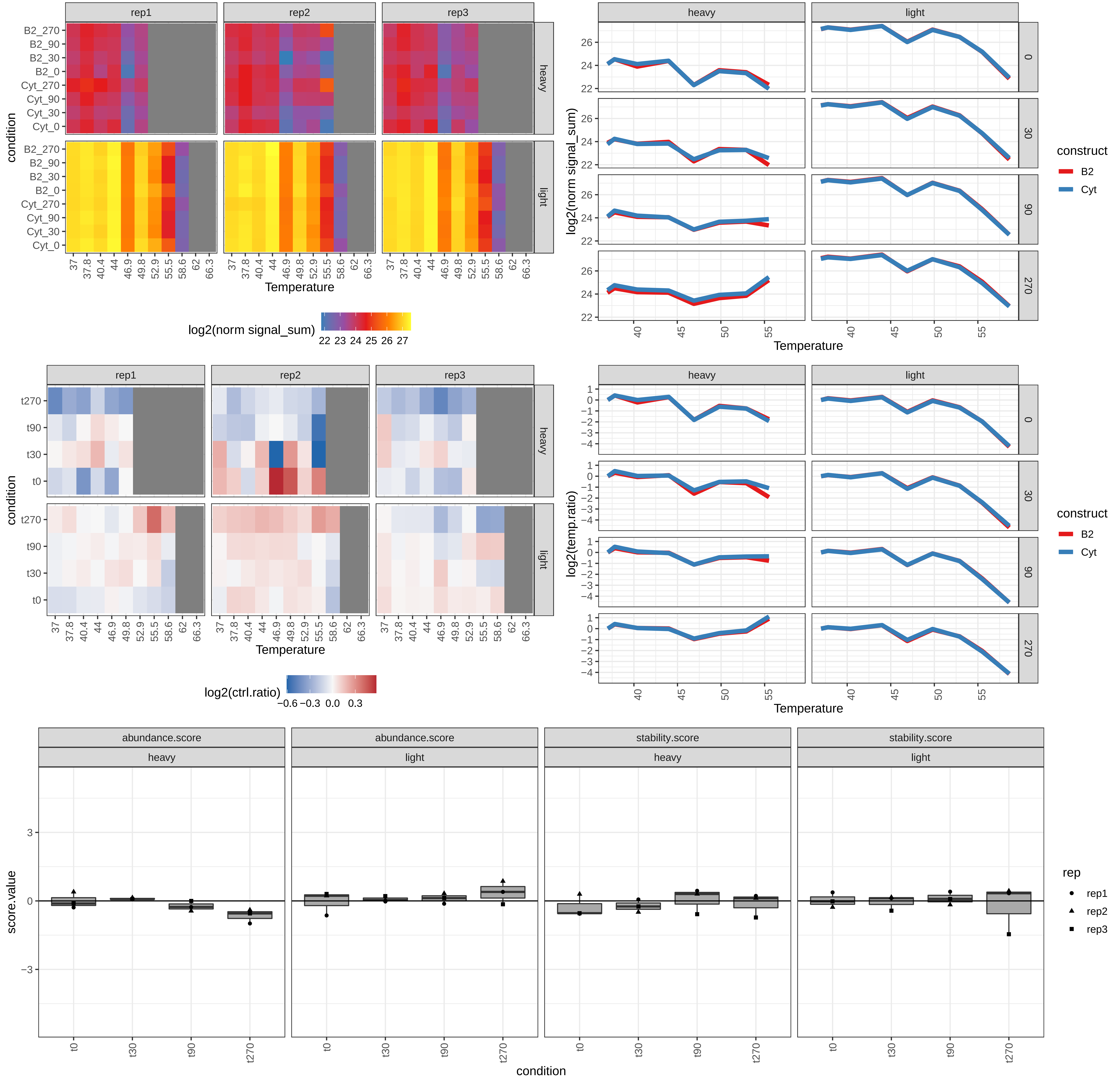

ligase, mitochondrial OS=Saccharomyces cerevisiae (strain ATCC 204508 / S288c) GN=HTS1 PE=1 SV=2|Isoform Cytoplasmic of Histidine−−tRNA ligase, mitochondrial OS=Saccharomyces cerevisiae (st

HTZ1 − average.top3: 7.4384980952381 − max.qupm: 2

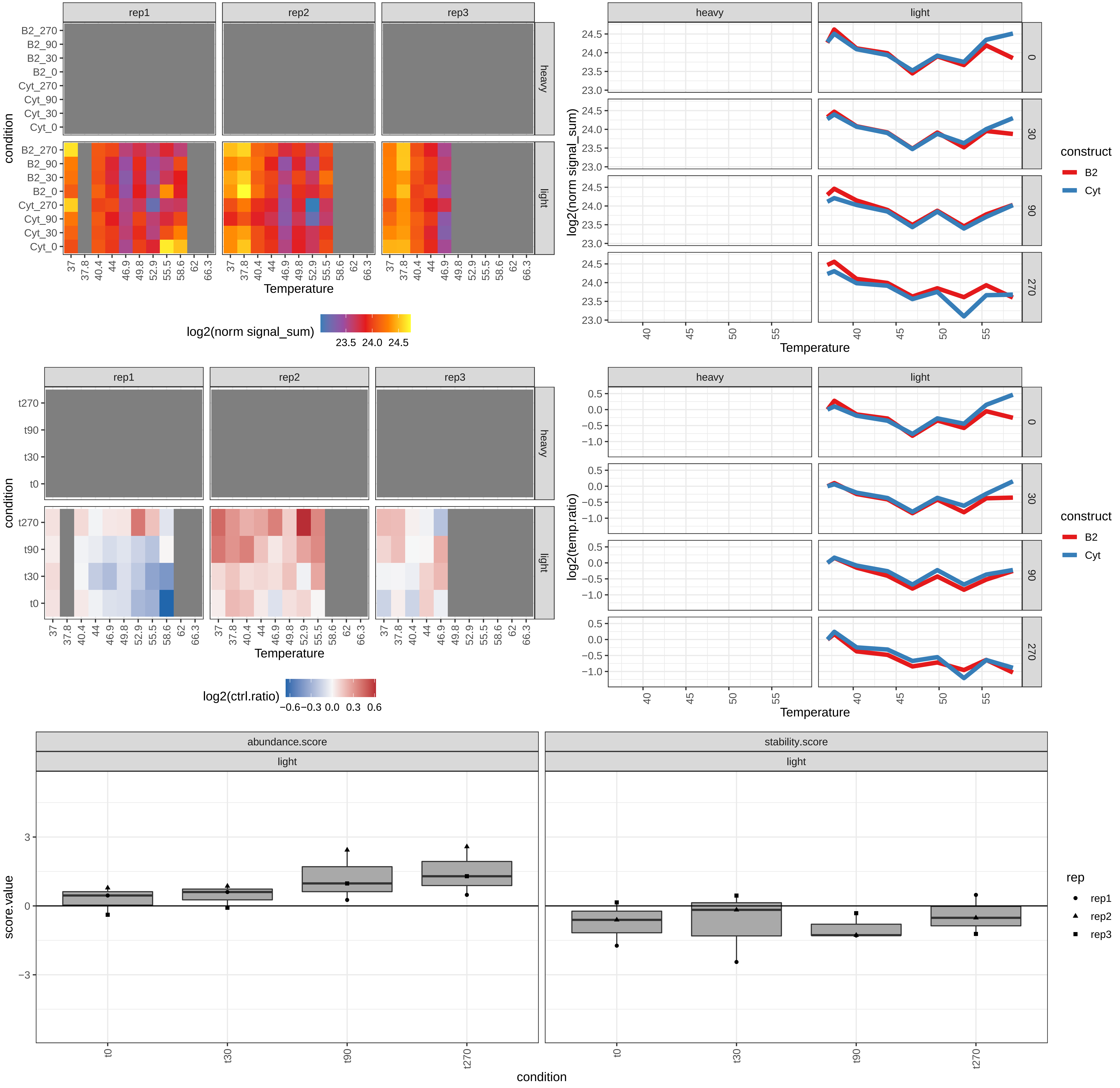

Q12692 − Histone H2A.Z OS=Saccharomyces cerevisiae (strain ATCC 204508 / S288c) GN=HTZ1 PE=1 SV=3

HUB1 − average.top3: 6.85937294117647 − max.qupm: 3

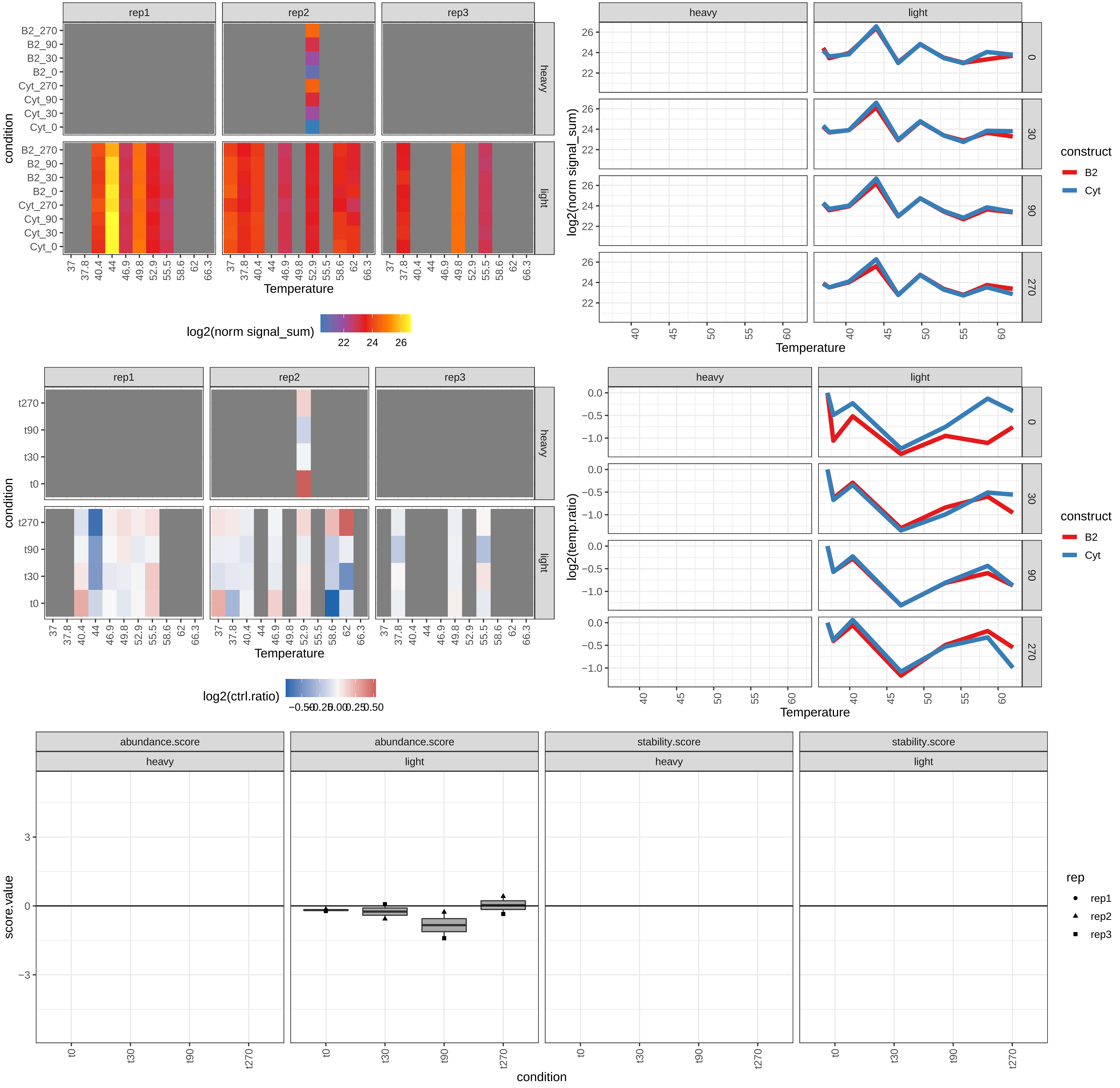

Q6Q546 − Ubiquitin−like modifier HUB1 OS=Saccharomyces cerevisiae (strain ATCC 204508 / S288c) GN=HUB1 PE=1 SV=2

HUL5 − average.top3: 6.32537222222222 − max.qupm: 3

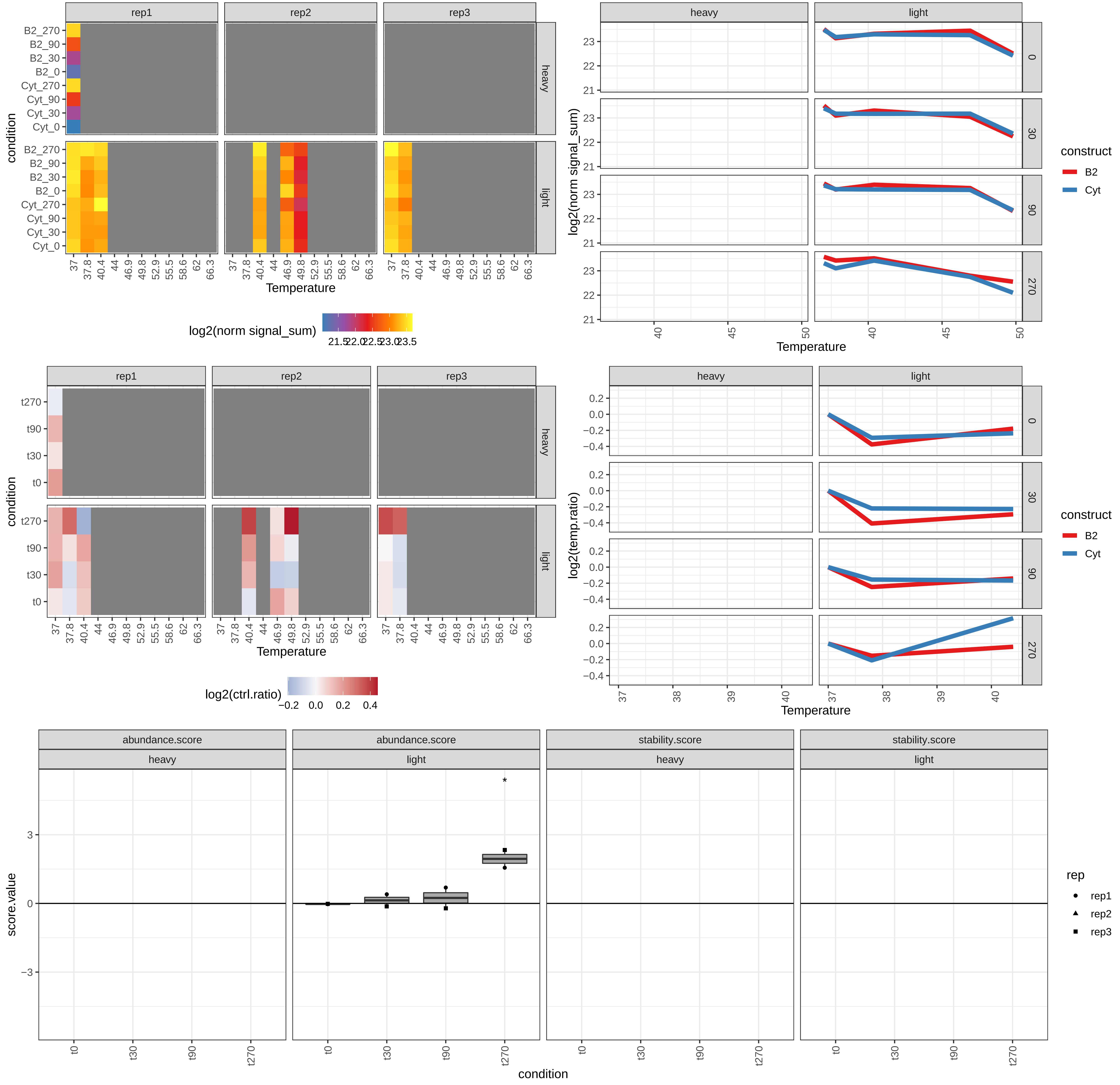

P53119 − Probable E3 ubiquitin−protein ligase HUL5 OS=Saccharomyces cerevisiae (strain ATCC 204508 / S288c) GN=HUL5 PE=1 SV=1

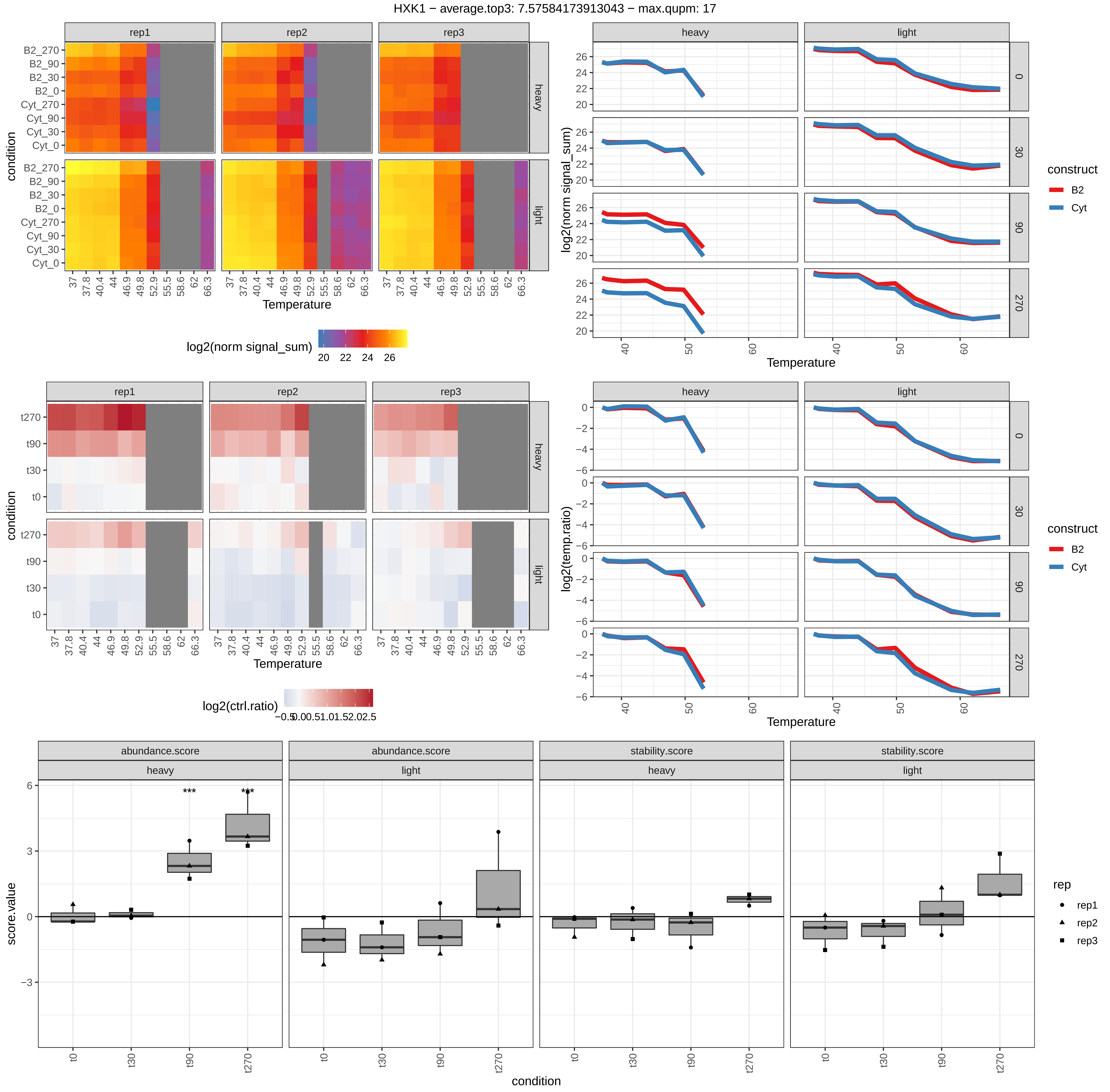

P04806 − Hexokinase−1 OS=Saccharomyces cerevisiae (strain ATCC 204508 / S288c) GN=HXK1 PE=1 SV=2

HXK2 − average.top3: 7.7182031372549 − max.qupm: 21

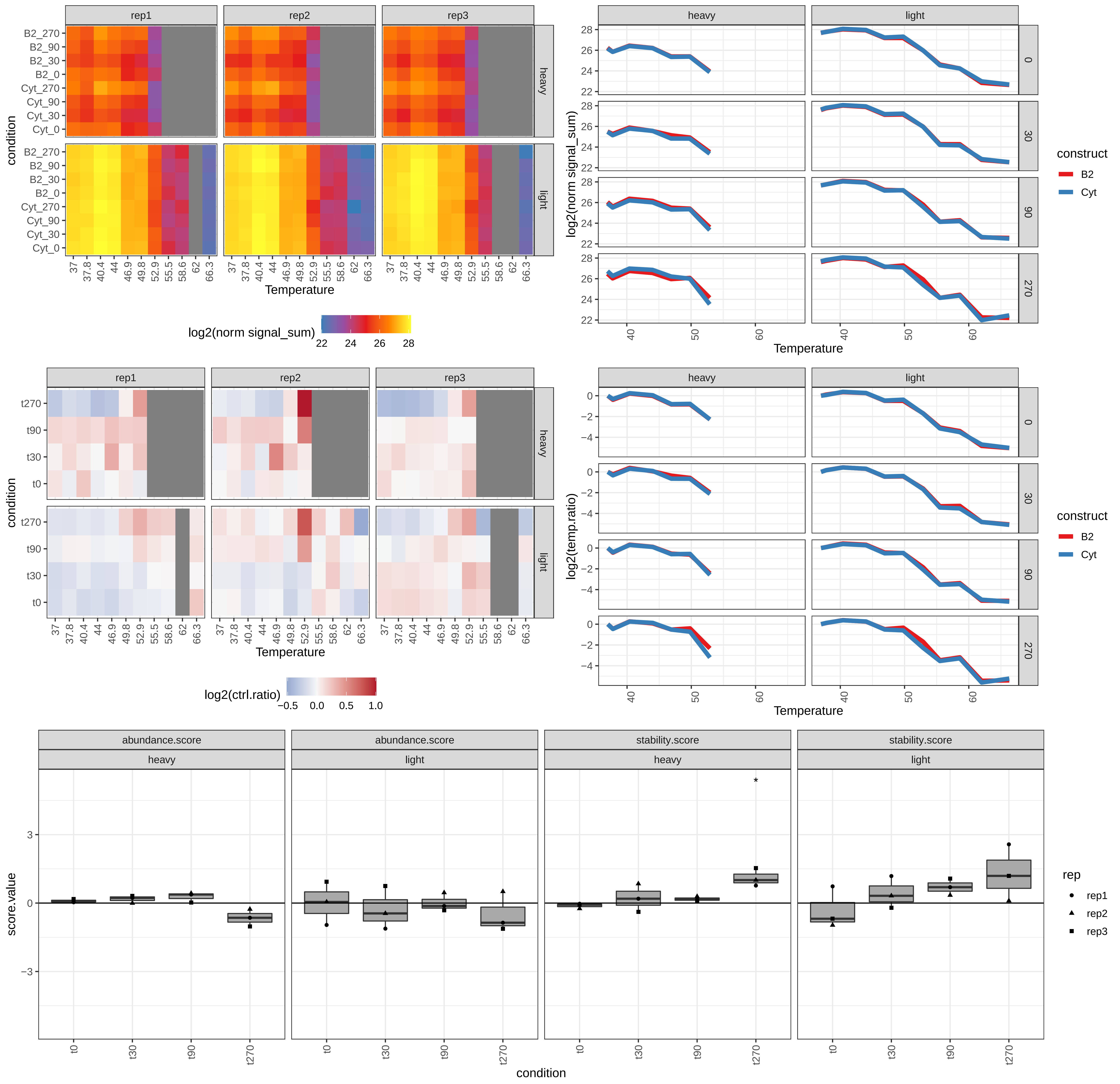

P04807 − Hexokinase−2 OS=Saccharomyces cerevisiae (strain ATCC 204508 / S288c) GN=HXK2 PE=1 SV=4

HXT5 − average.top3: 7.23617580645161 − max.qupm: 5

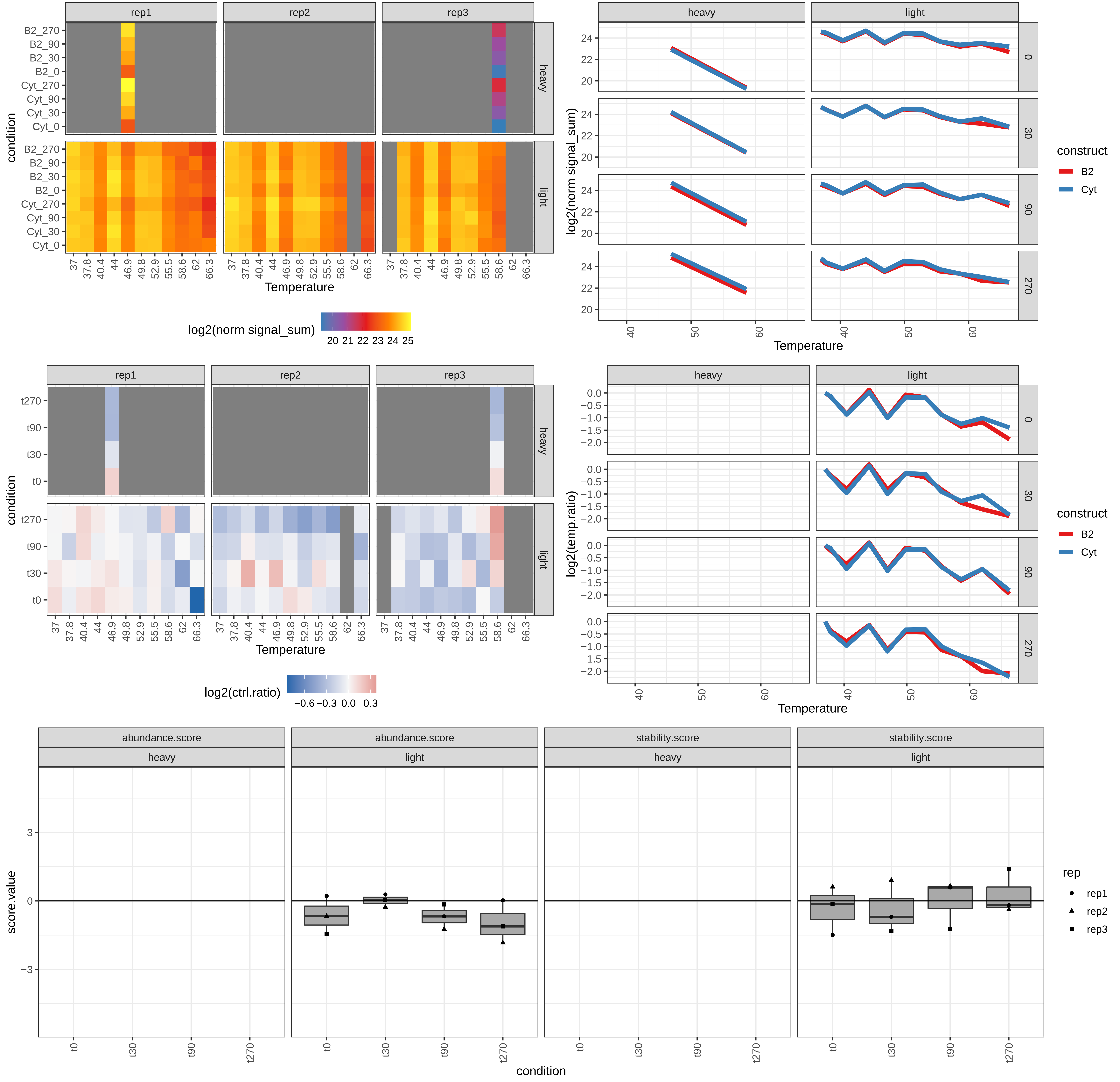

P38695 − Probable glucose transporter HXT5 OS=Saccharomyces cerevisiae (strain ATCC 204508 / S288c) GN=HXT5 PE=1 SV=1

HXT6|HXT7 − average.top3: 6.88263263157895 − max.qupm: 5

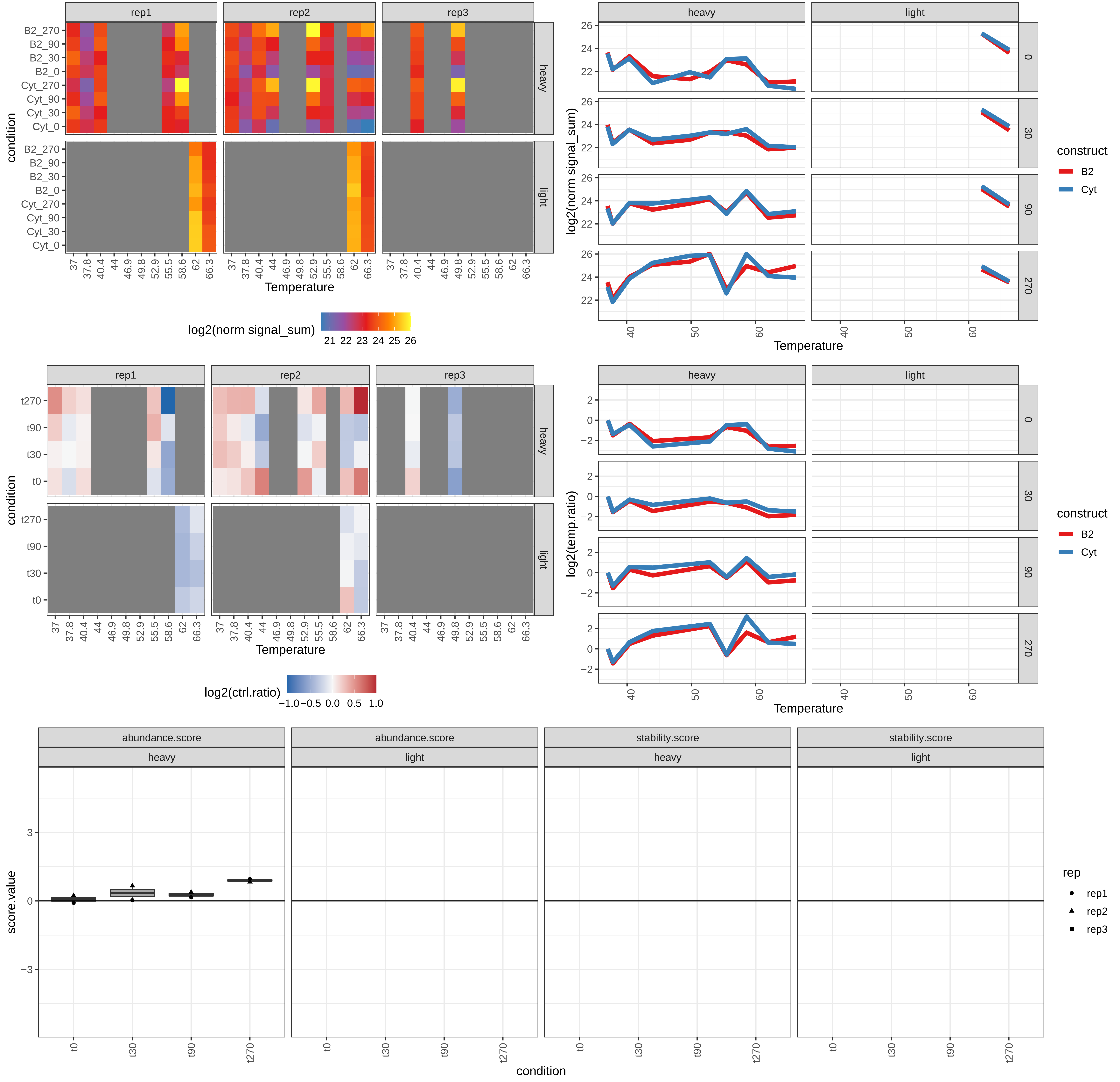

hexose transporter HXT6 OS=Saccharomyces cerevisiae (strain ATCC 204508 / S288c) GN=HXT6 PE=1 SV=2|High−affinity hexose transporter HXT6 OS=Saccharomyces cerevisiae (strain ATCC 204508

HXT7 − average.top3: 7.53267222222222 − max.qupm: 9

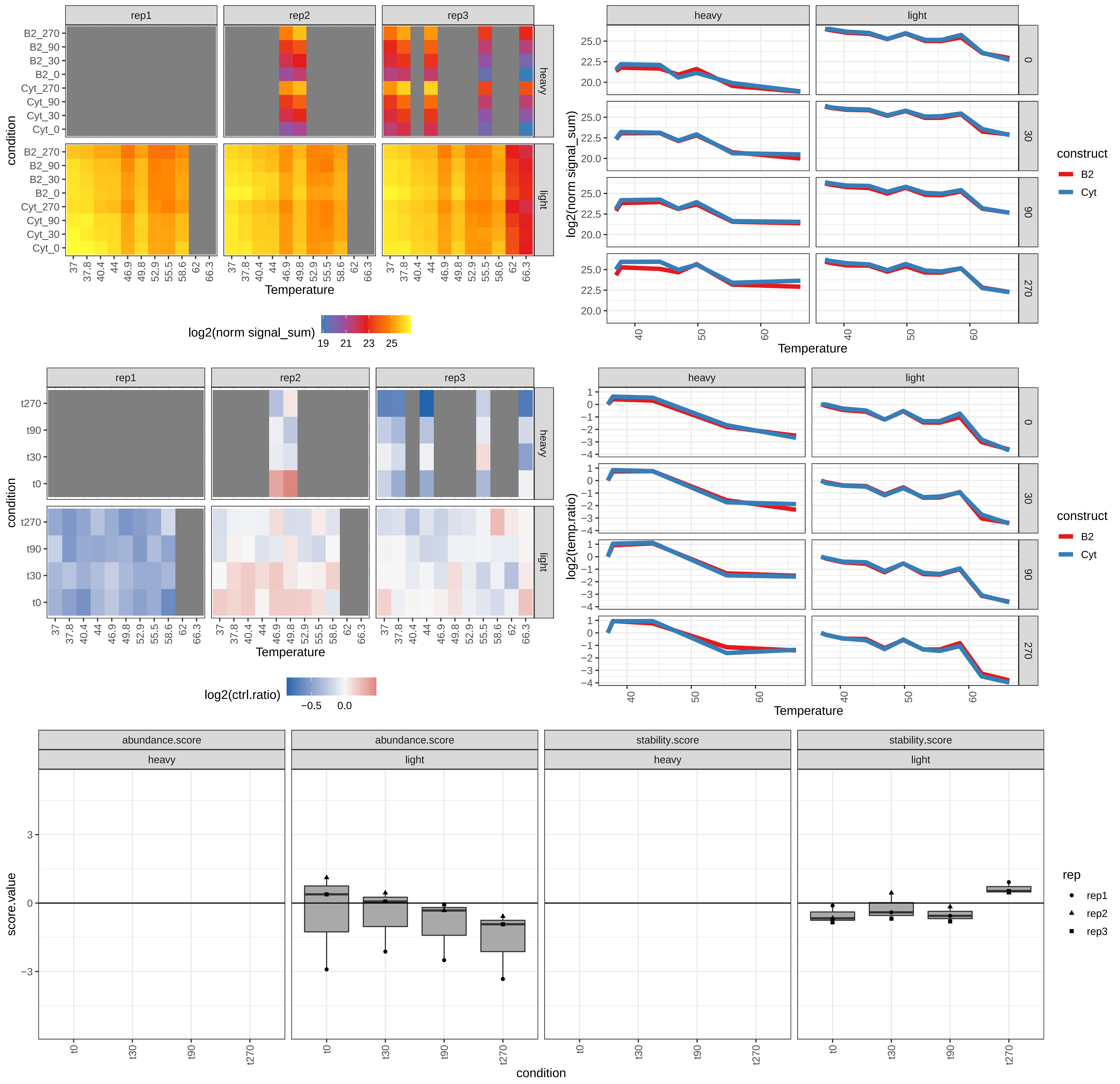

P39004 − High−affinity hexose transporter HXT6 OS=Saccharomyces cerevisiae (strain ATCC 204508 / S288c) GN=HXT7 PE=1 SV=1

HYM1 − average.top3: 5.97629117647059 − max.qupm: 3

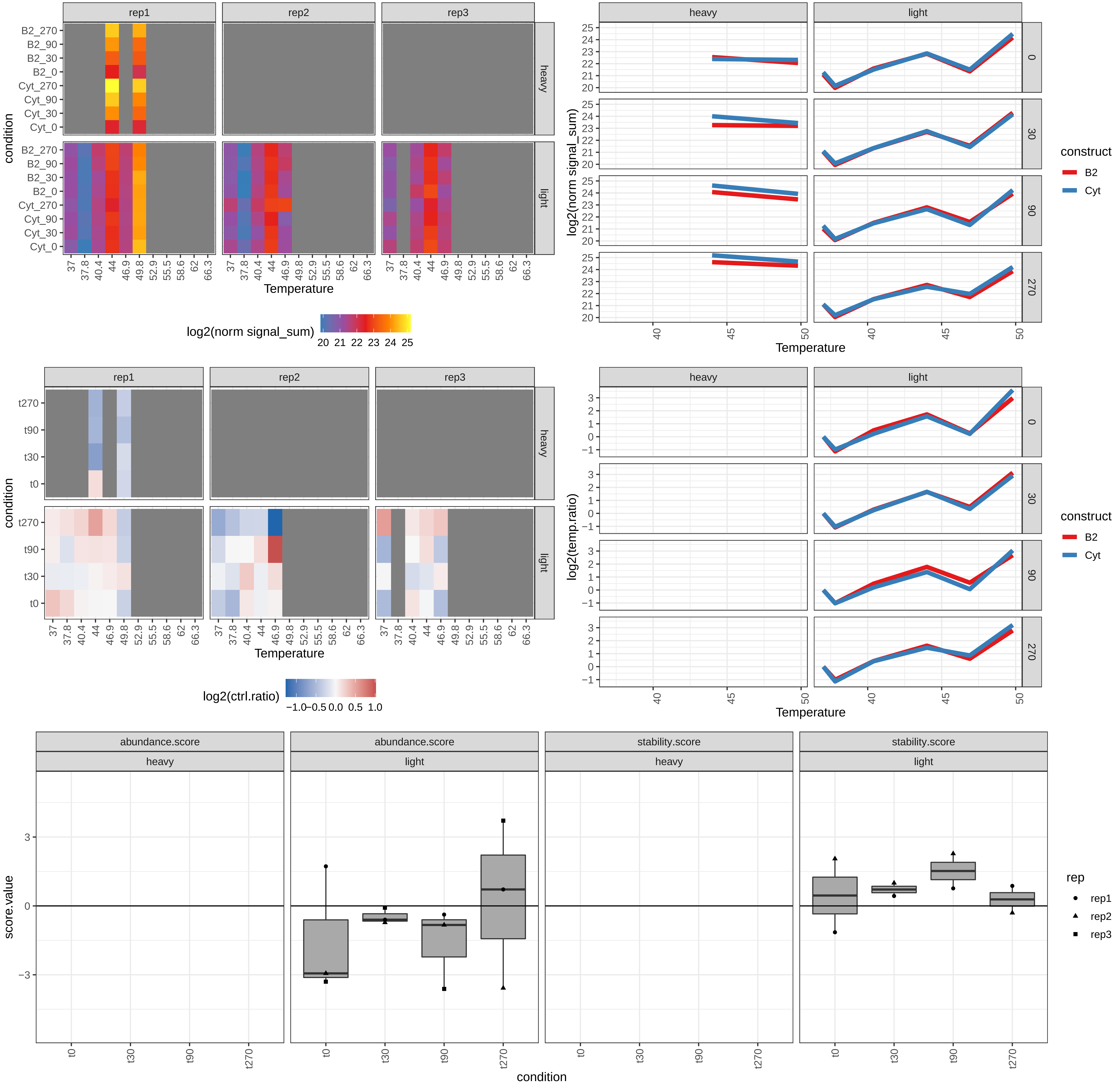

P32464 − Protein HYM1 OS=Saccharomyces cerevisiae (strain ATCC 204508 / S288c) GN=HYM1 PE=1 SV=1

HYP2 − average.top3: 7.61533723404255 − max.qupm: 7

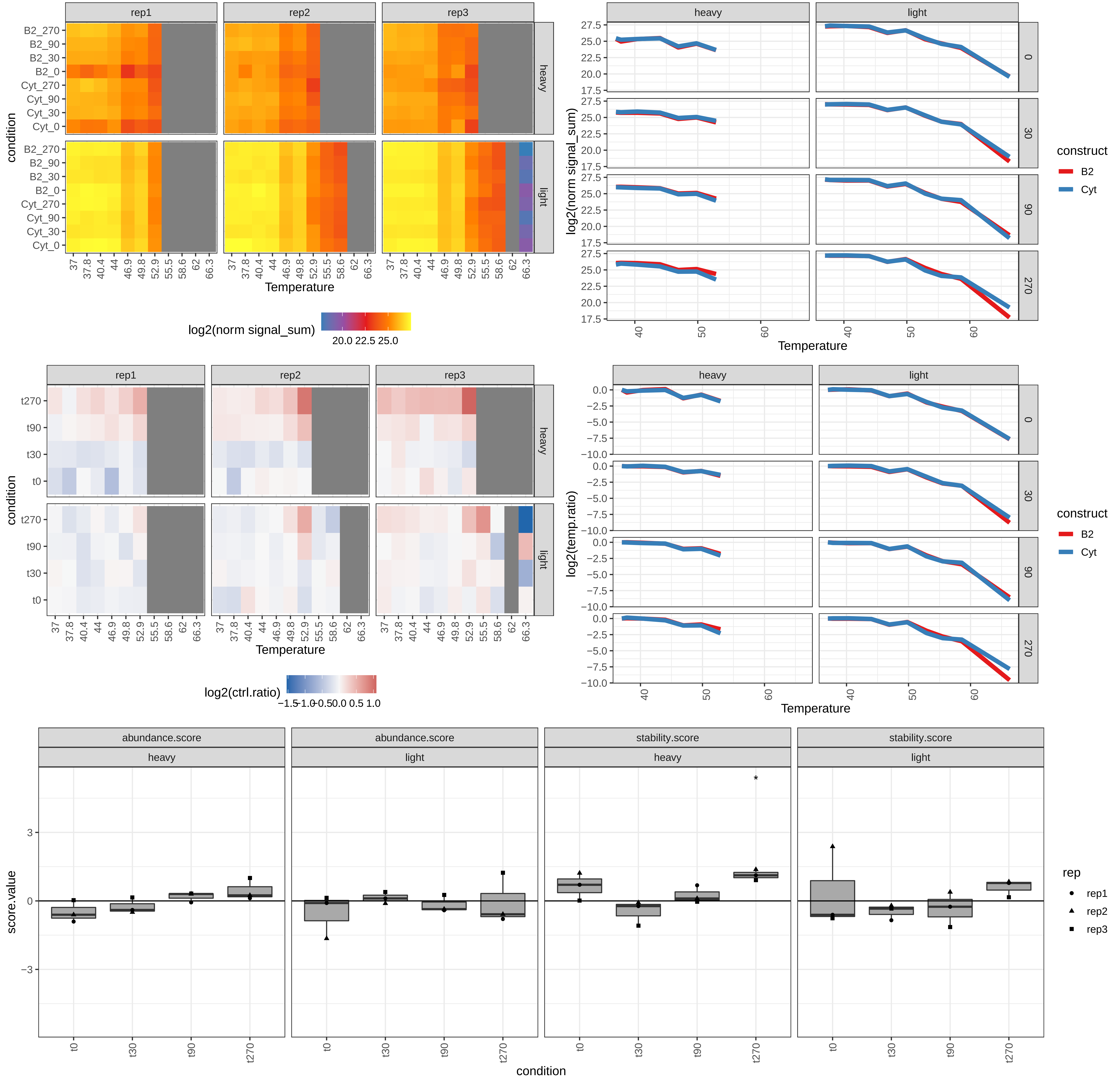

P23301 − Eukaryotic translation initiation factor 5A−1 OS=Saccharomyces cerevisiae (strain ATCC 204508 / S288c) GN=HYP2 PE=1 SV=3

HYR1 − average.top3: 7.37990886792453 − max.qupm: 8

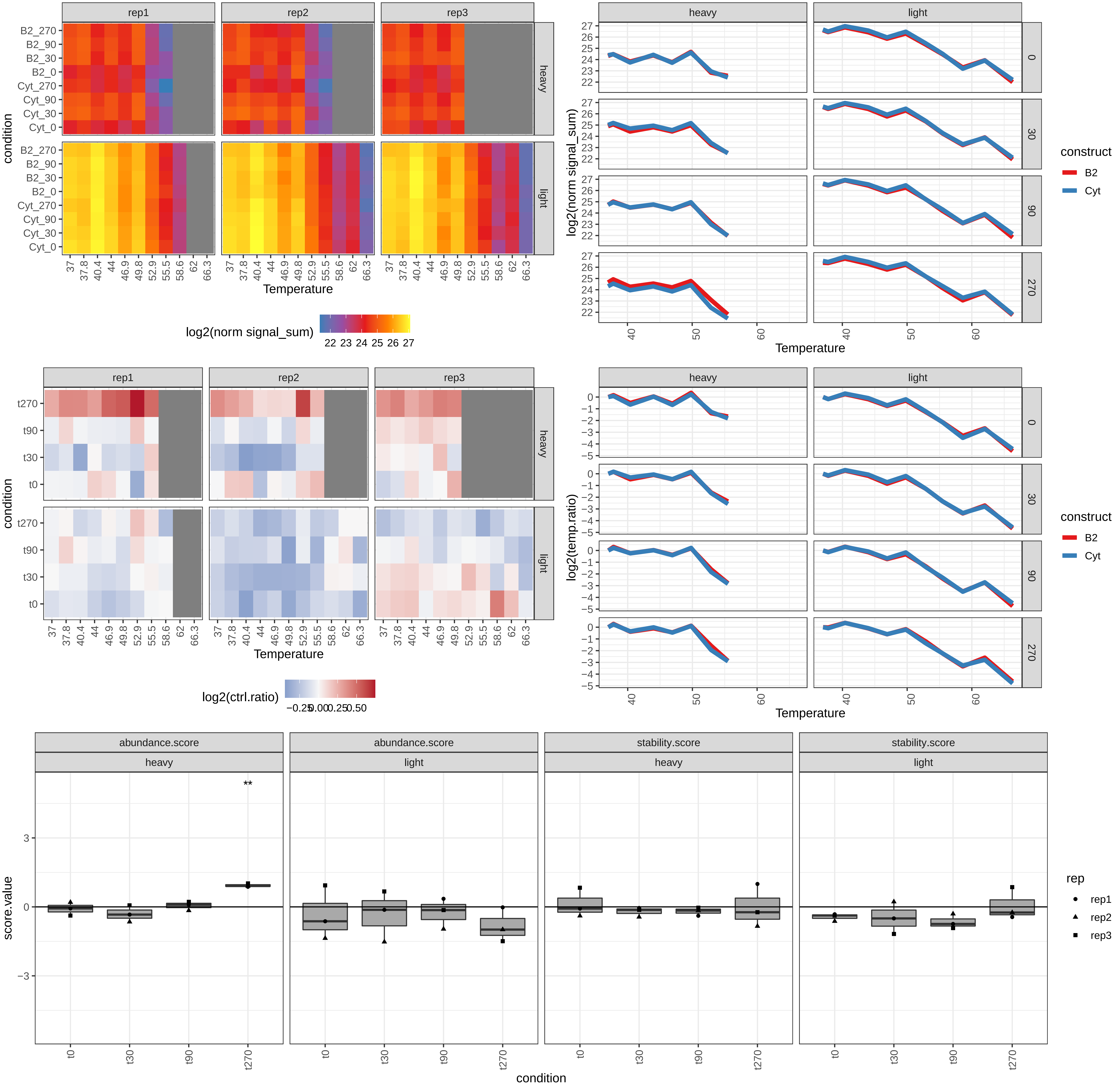

P40581 − Peroxiredoxin HYR1 OS=Saccharomyces cerevisiae (strain ATCC 204508 / S288c) GN=HYR1 PE=1 SV=1

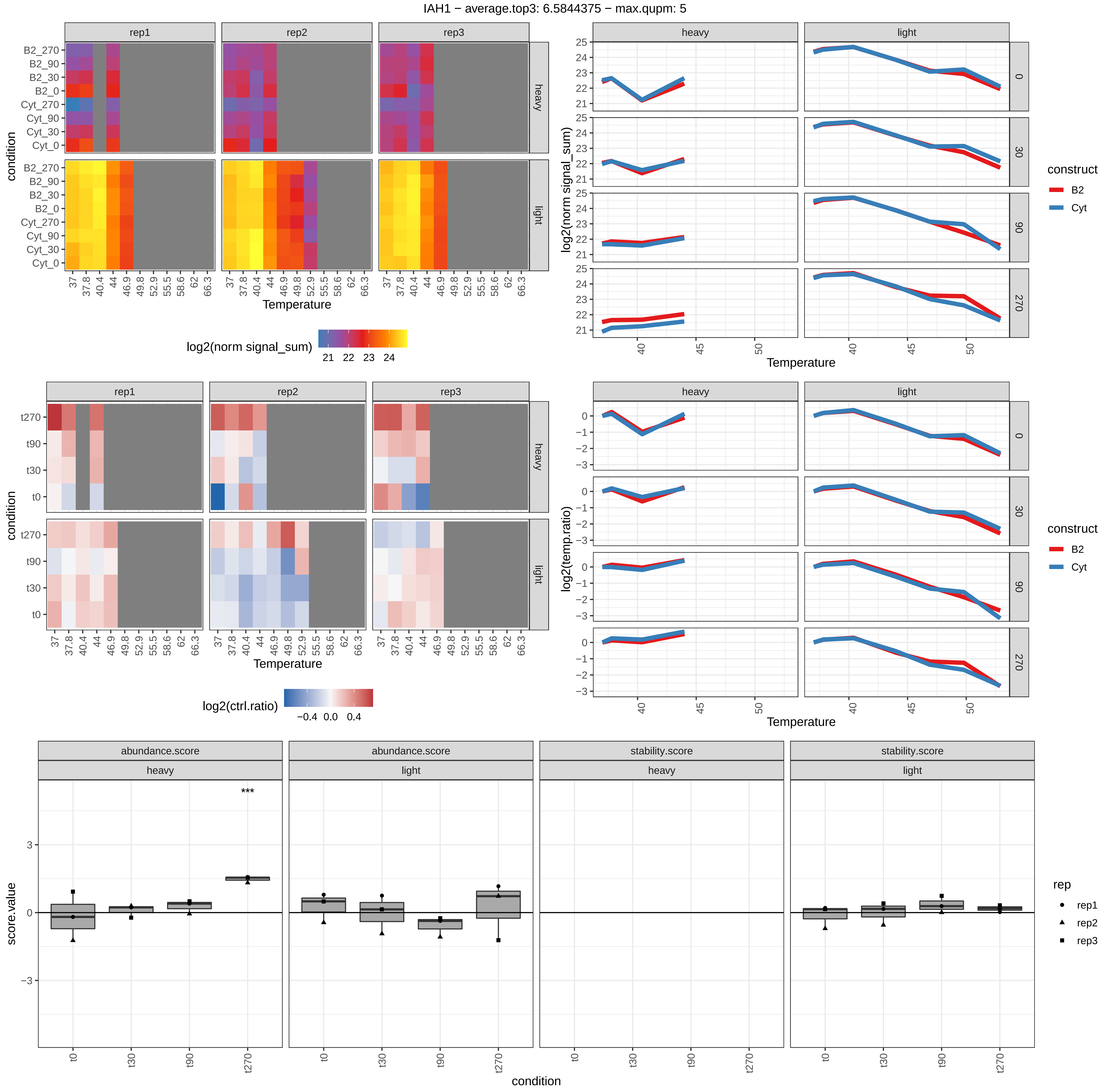

P41734 − Isoamyl acetate−hydrolyzing esterase OS=Saccharomyces cerevisiae (strain ATCC 204508 / S288c) GN=IAH1 PE=1 SV=1

IBA57 − average.top3: 6.31670764705882 − max.qupm: 5

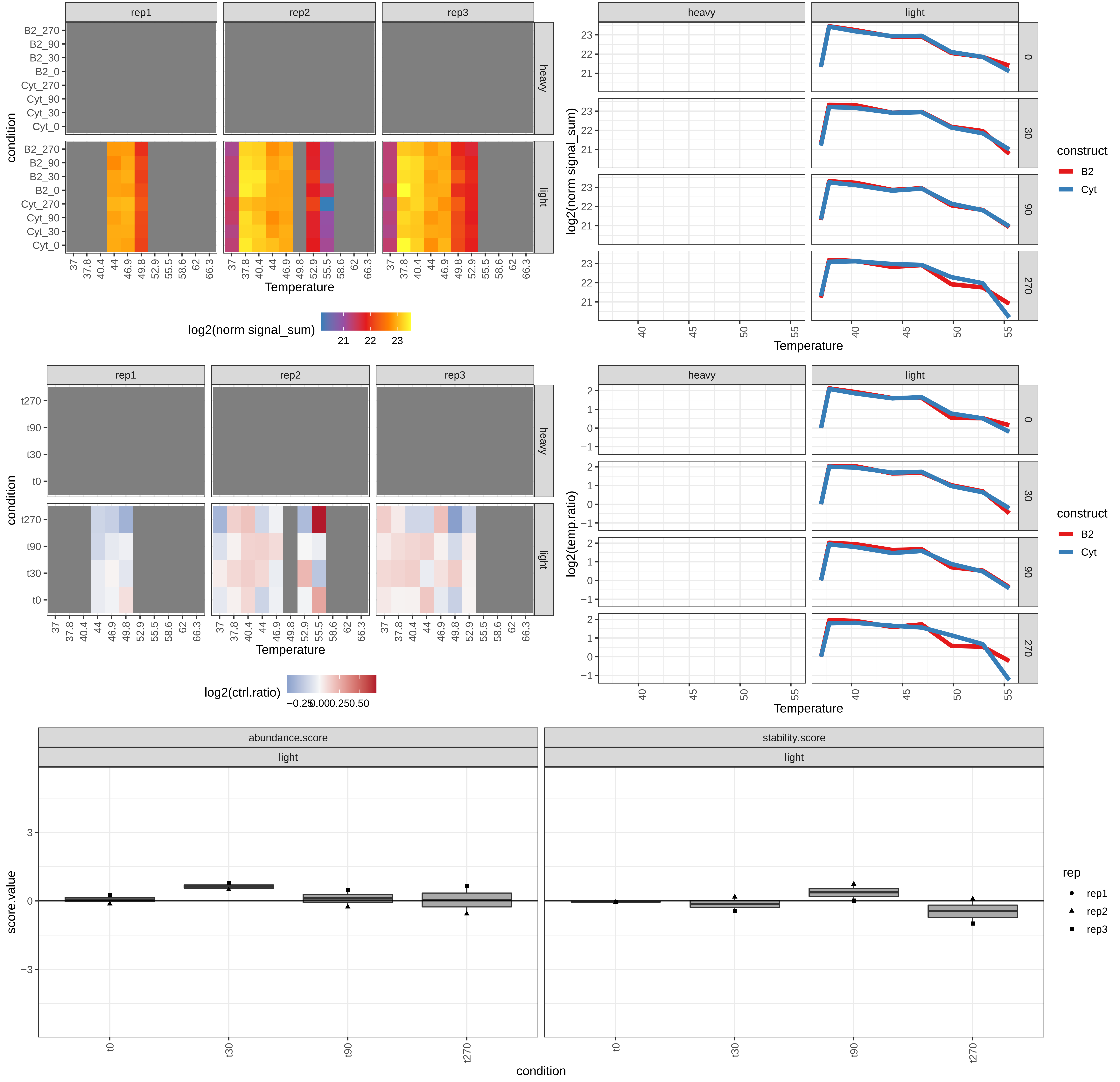

P47158 − Putative transferase CAF17, mitochondrial OS=Saccharomyces cerevisiae (strain ATCC 204508 / S288c) GN=IBA57 PE=1 SV=1

ICE2 − average.top3: 6.34343 − max.qupm: 3

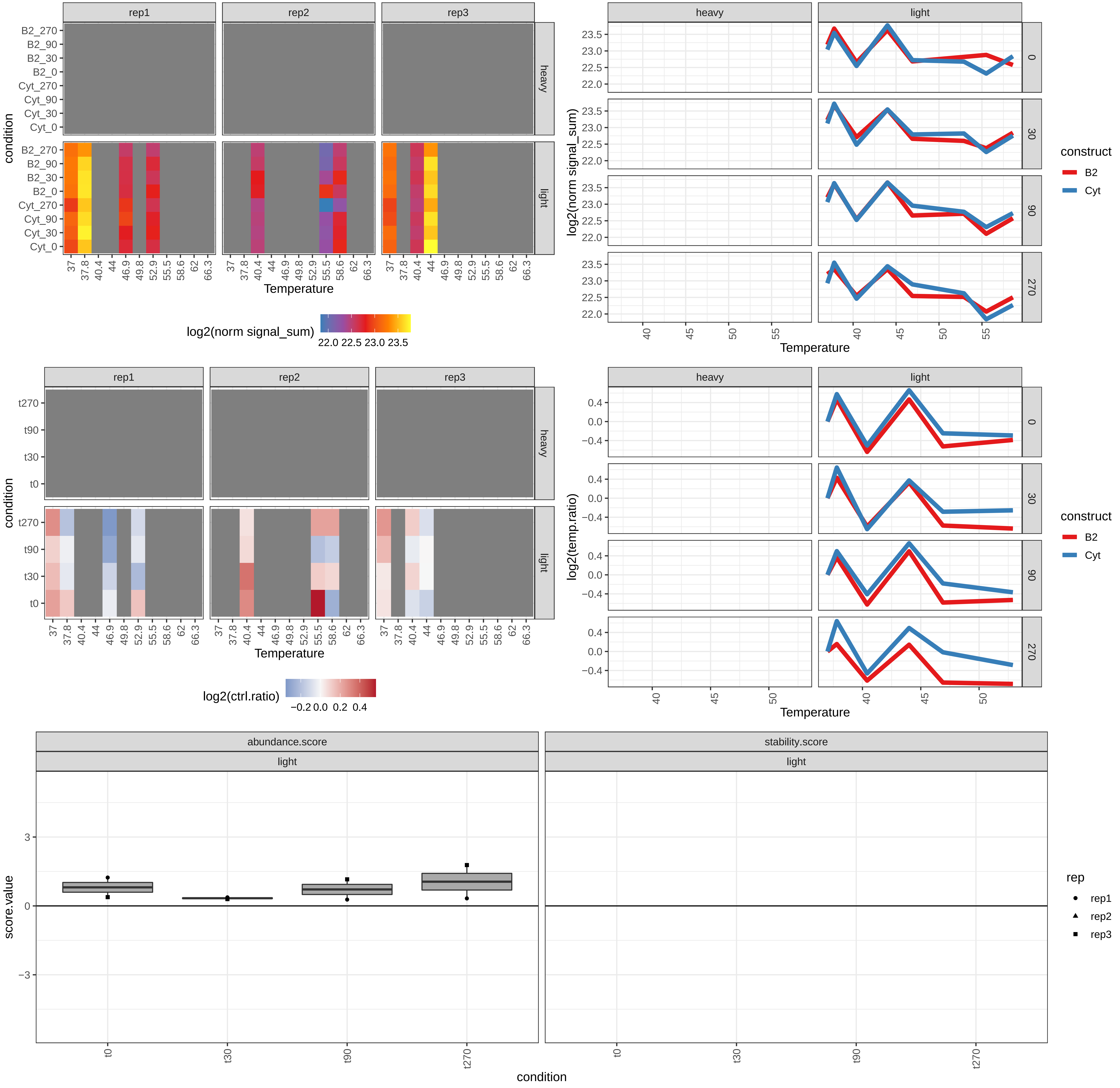

P40499 − Protein ICE2 OS=Saccharomyces cerevisiae (strain ATCC 204508 / S288c) GN=ICE2 PE=1 SV=1

ICL1 − average.top3: 8.12556423728814 − max.qupm: 23

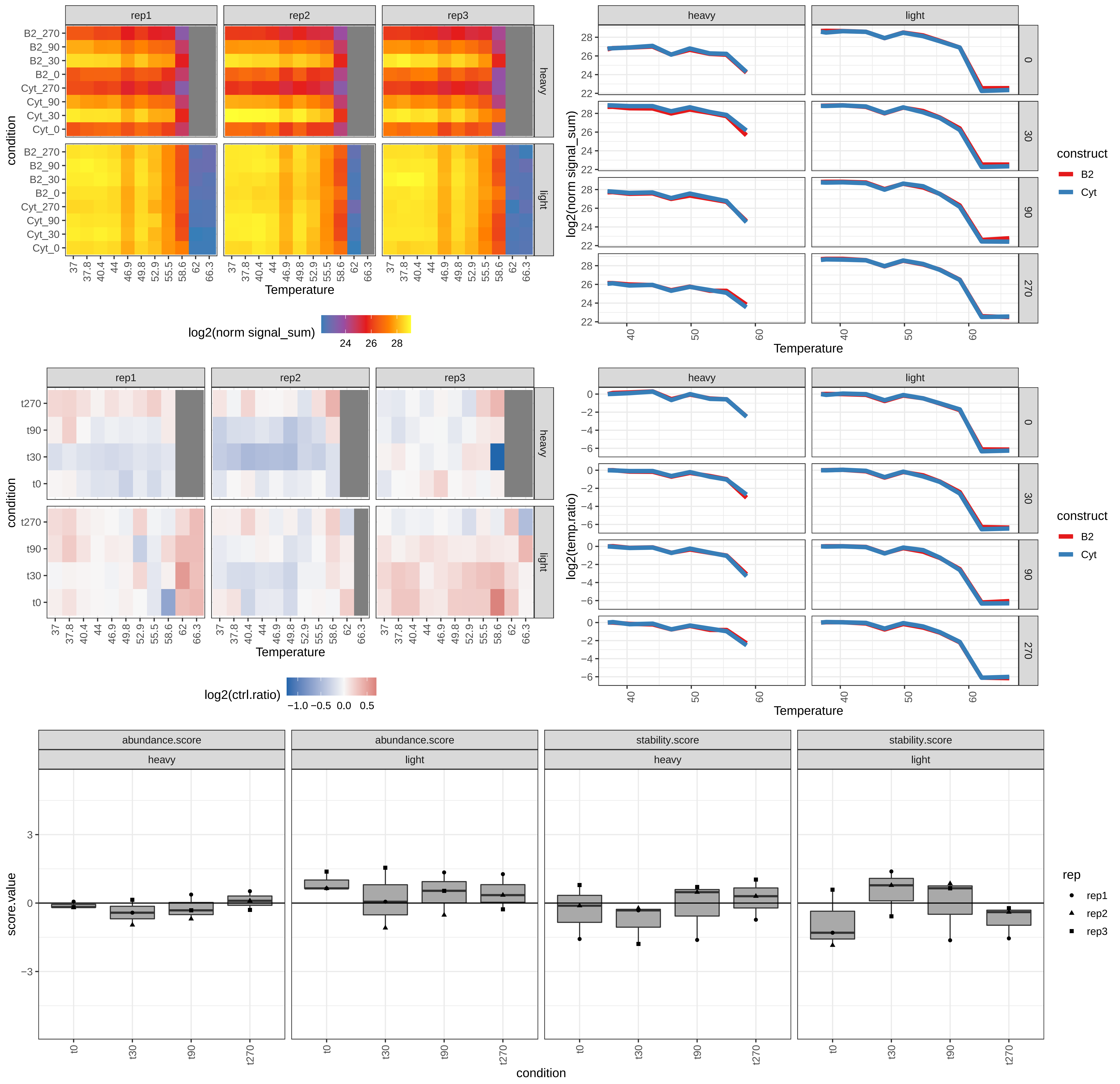

P28240 − Isocitrate lyase OS=Saccharomyces cerevisiae (strain ATCC 204508 / S288c) GN=ICL1 PE=1 SV=1

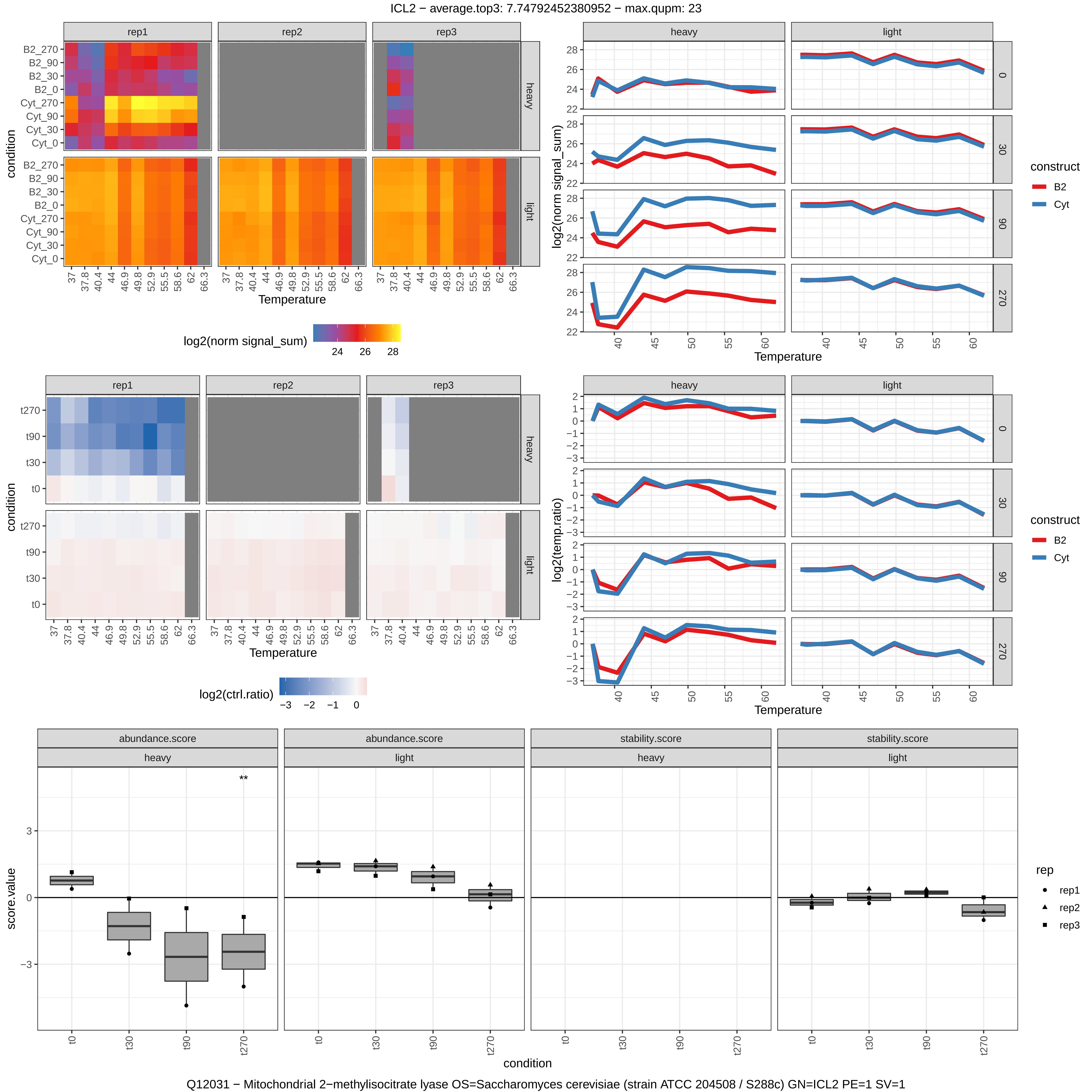
ICL2 − average.top3: 7.74792452380952 − max.qupm: 23
rep1
rep2
rep3
heavy
light
condition
Temperature
log2(norm signal_sum)
construct
B2
Cyt
log2(ctrl.ratio)
log2(temp.ratio)
abundance.score
stability.score
score.value
rep
rep1
rep2
rep3
**
Q12031 − Mitochondrial 2−methylisocitrate lyase OS=Saccharomyces cerevisiae (strain ATCC 204508 / S288c) GN=ICL2 PE=1 SV=1

ICP55 − average.top3: 7.03622235294118 − max.qupm: 7

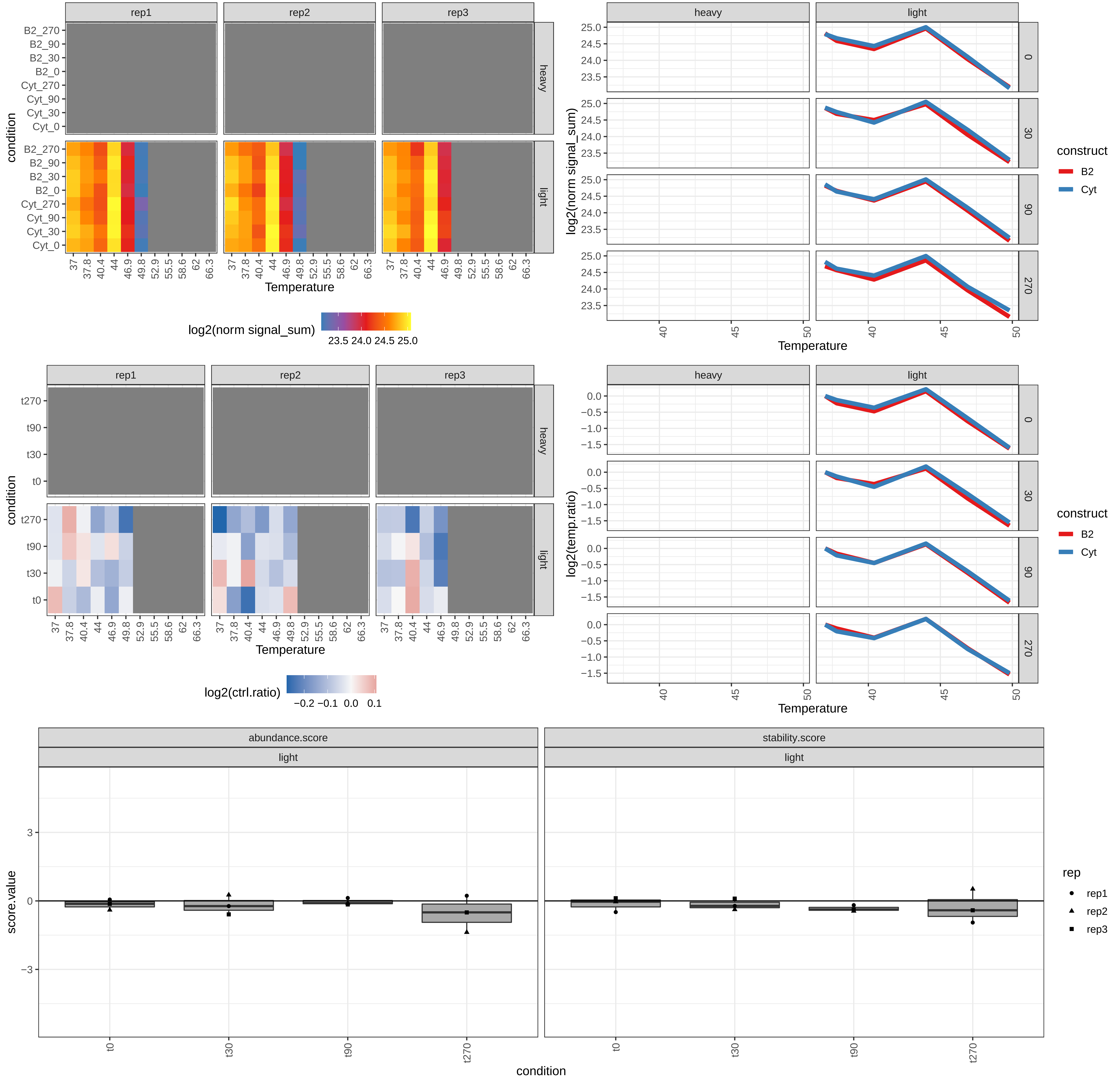

P40051 − Intermediate cleaving peptidase 55 OS=Saccharomyces cerevisiae (strain ATCC 204508 / S288c) GN=ICP55 PE=1 SV=1

IDH1 − average.top3: 8.03390640625 − max.qupm: 15

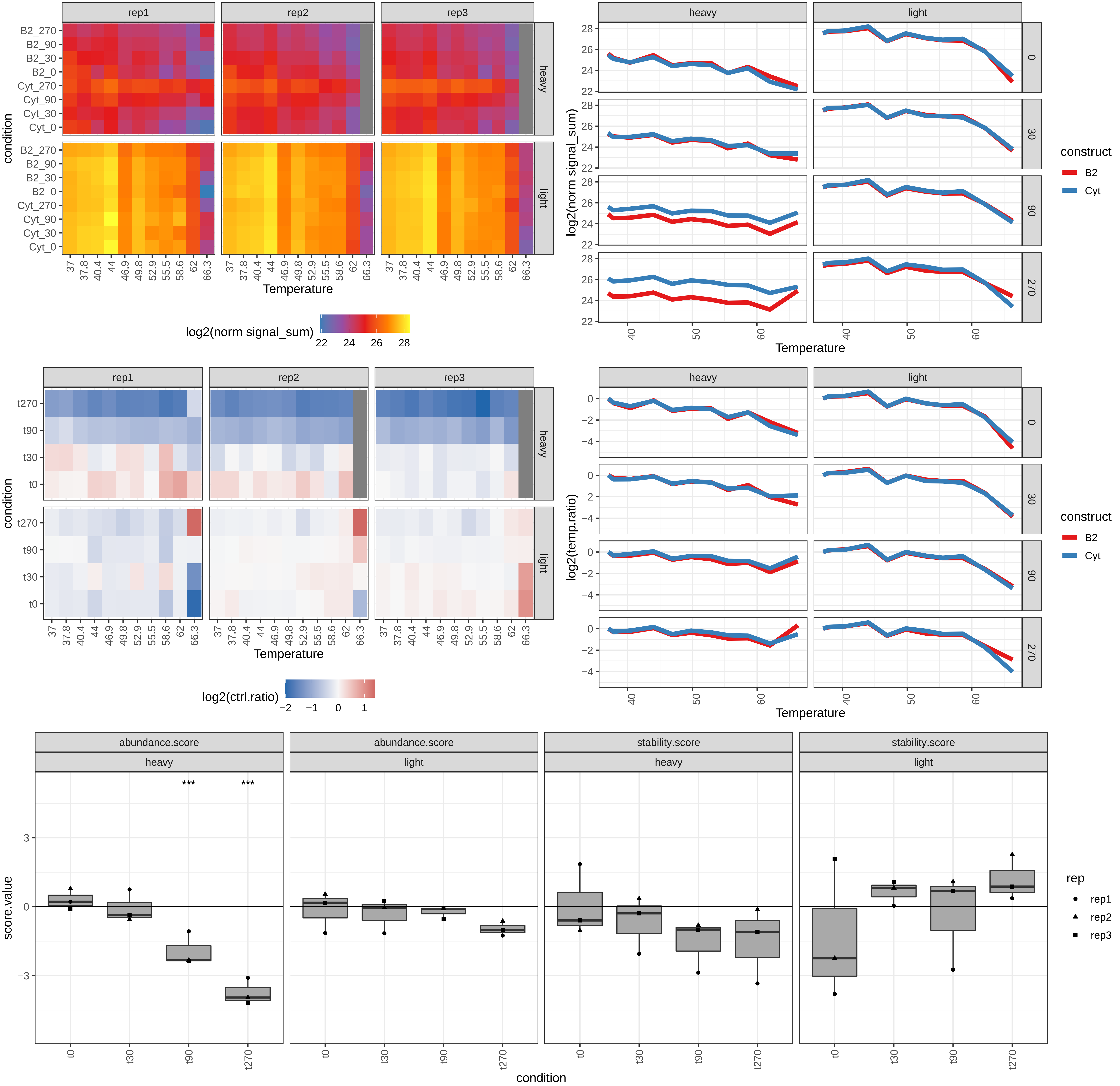

P28834 − Isocitrate dehydrogenase [NAD] subunit 1, mitochondrial OS=Saccharomyces cerevisiae (strain ATCC 204508 / S288c) GN=IDH1 PE=1 SV=2

IDH2 − average.top3: 7.73386555555556 − max.qupm: 13

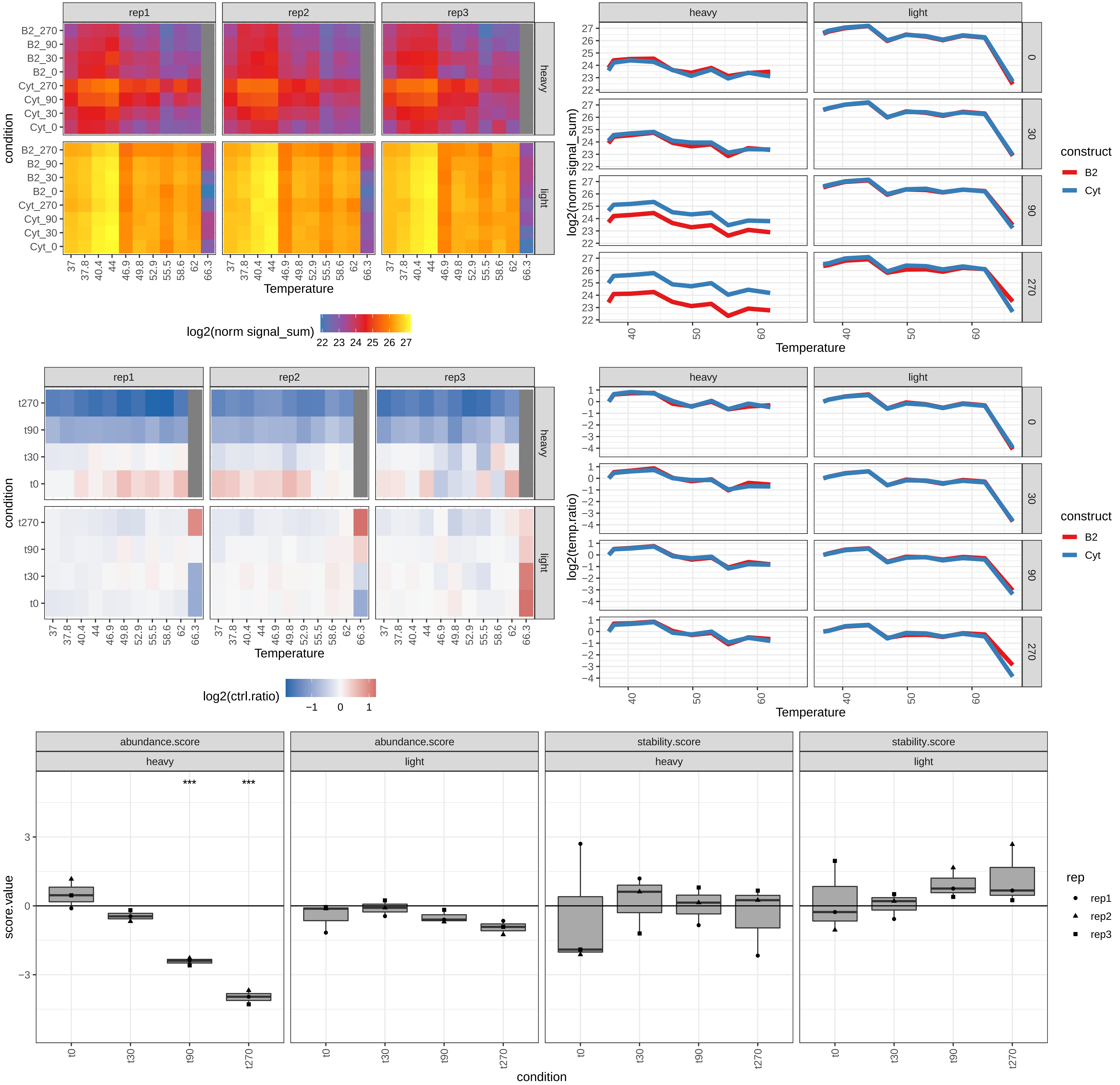

P28241 − Isocitrate dehydrogenase [NAD] subunit 2, mitochondrial OS=Saccharomyces cerevisiae (strain ATCC 204508 / S288c) GN=IDH2 PE=1 SV=1

IDI1 − average.top3: 7.1993806557377 − max.qupm: 9

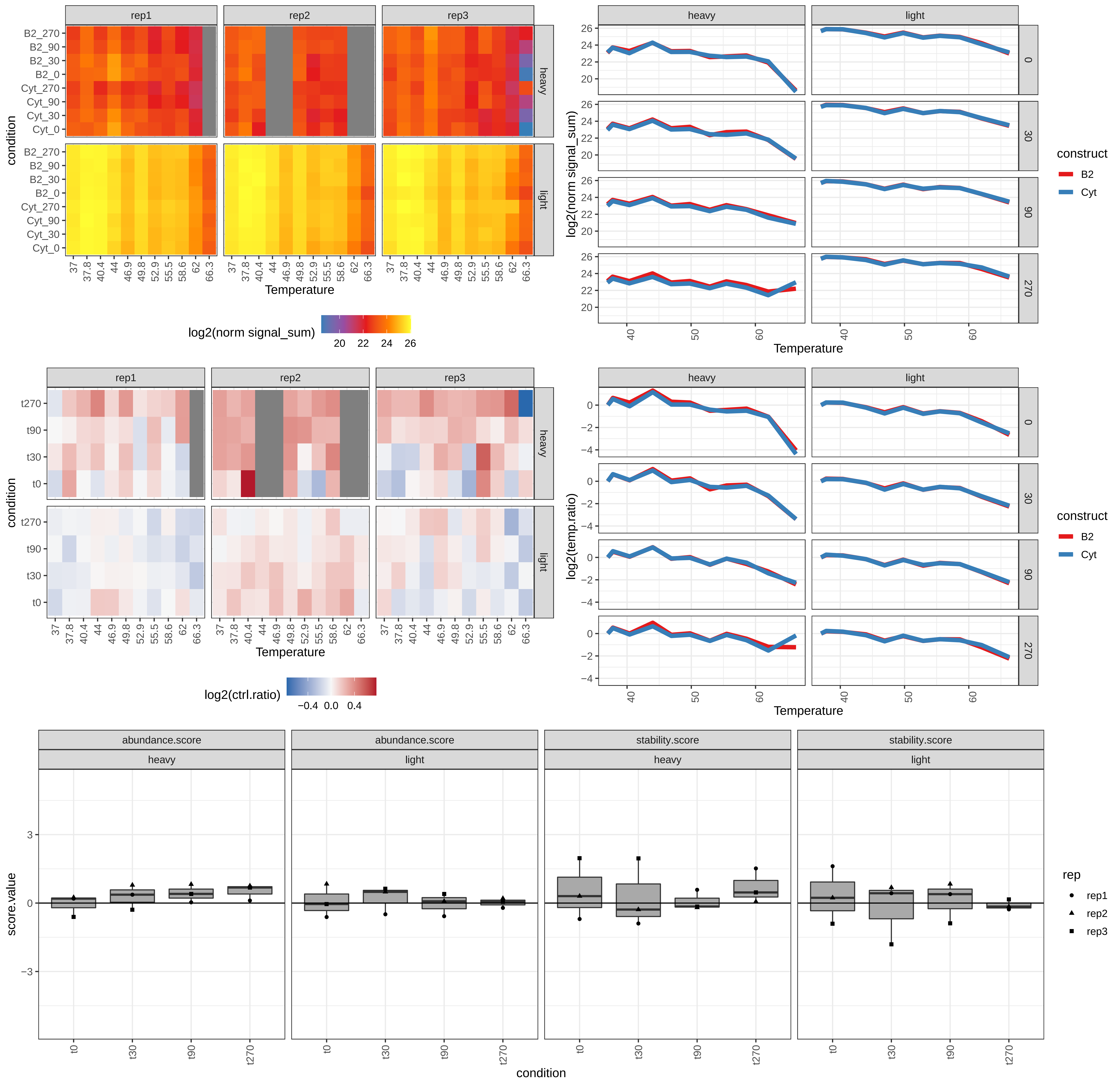

P15496 − Isopentenyl−diphosphate Delta−isomerase OS=Saccharomyces cerevisiae (strain ATCC 204508 / S288c) GN=IDI1 PE=1 SV=2

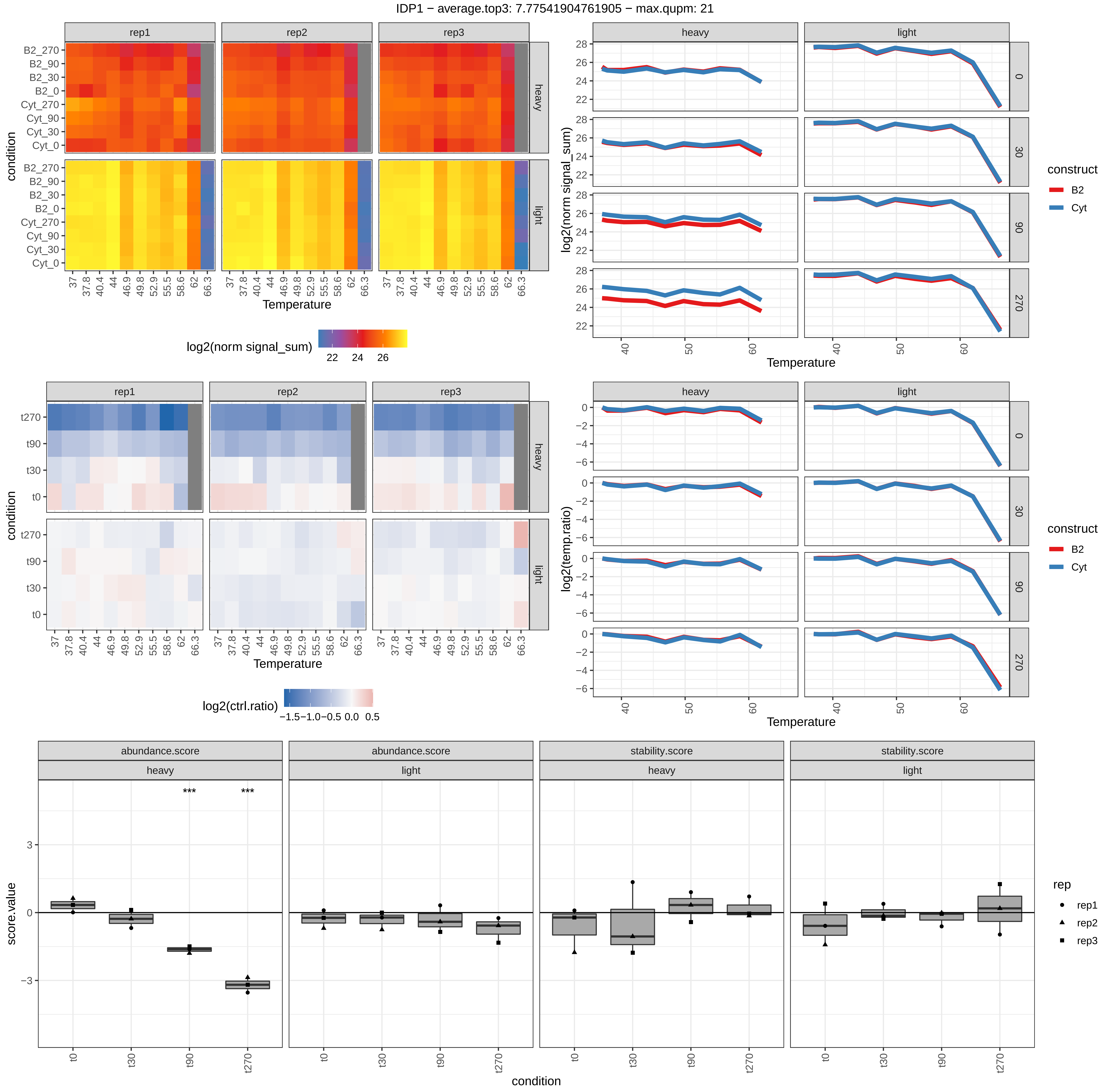

P21954 − Isocitrate dehydrogenase [NADP], mitochondrial OS=Saccharomyces cerevisiae (strain ATCC 204508 / S288c) GN=IDP1 PE=1 SV=1

IDP2 − average.top3: 7.67304016949153 − max.qupm: 22

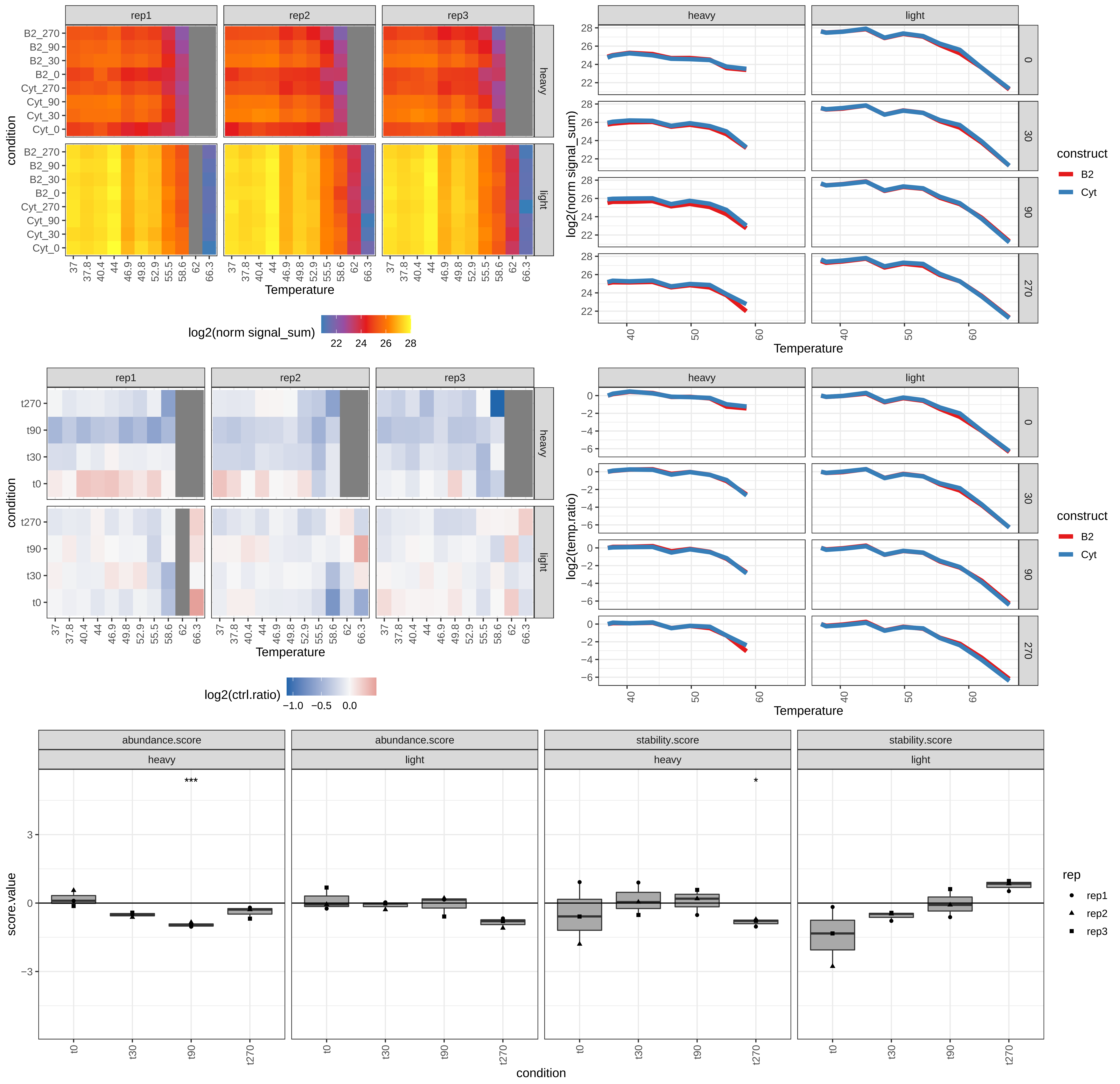

P41939 − Isocitrate dehydrogenase [NADP] cytoplasmic OS=Saccharomyces cerevisiae (strain ATCC 204508 / S288c) GN=IDP2 PE=1 SV=2

IDP3 − average.top3: 7.3965984375 − max.qupm: 17

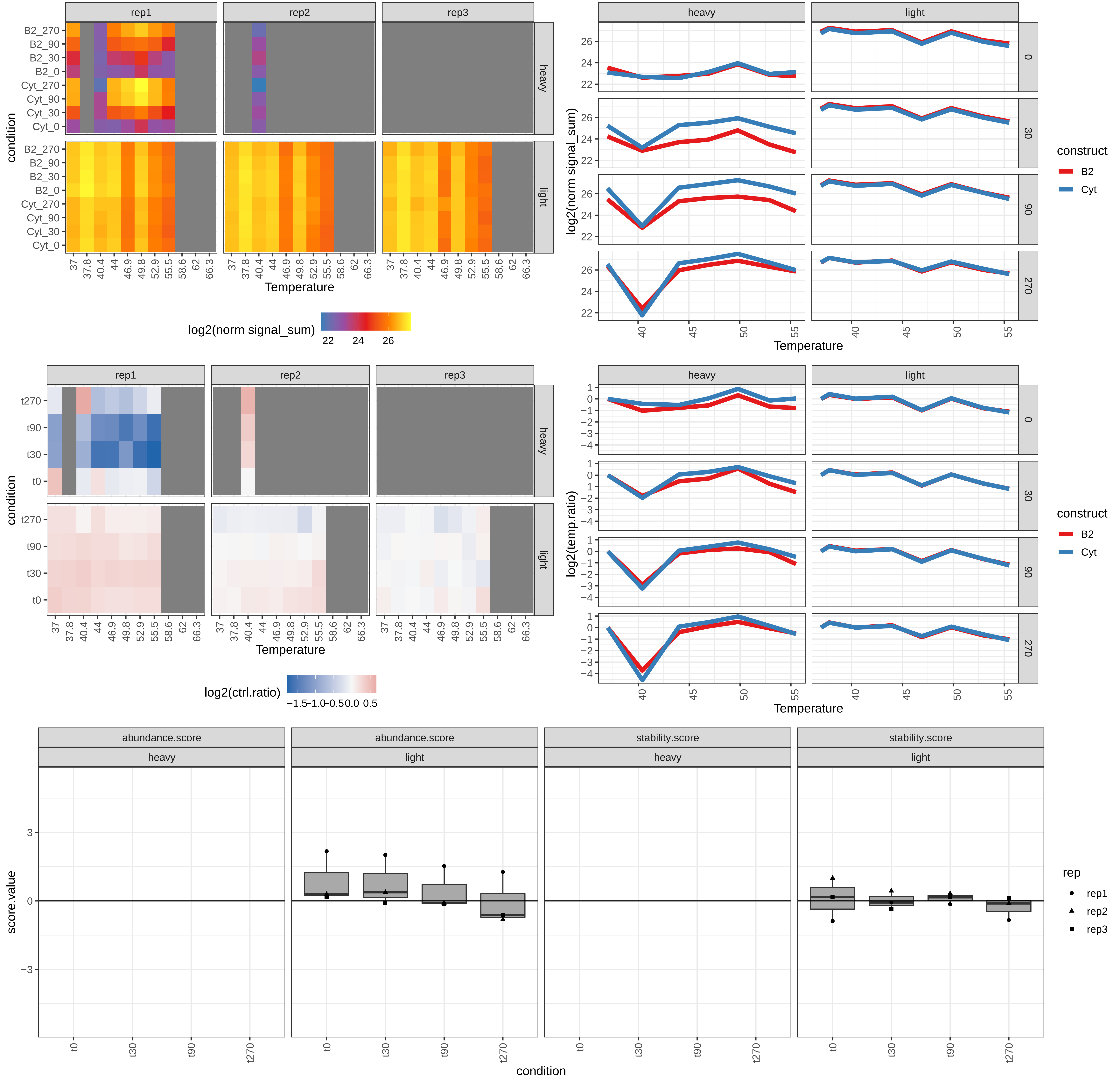

P53982 − Isocitrate dehydrogenase [NADP] OS=Saccharomyces cerevisiae (strain ATCC 204508 / S288c) GN=IDP3 PE=3 SV=1

IES1 − average.top3: 6.526561 − max.qupm: 4

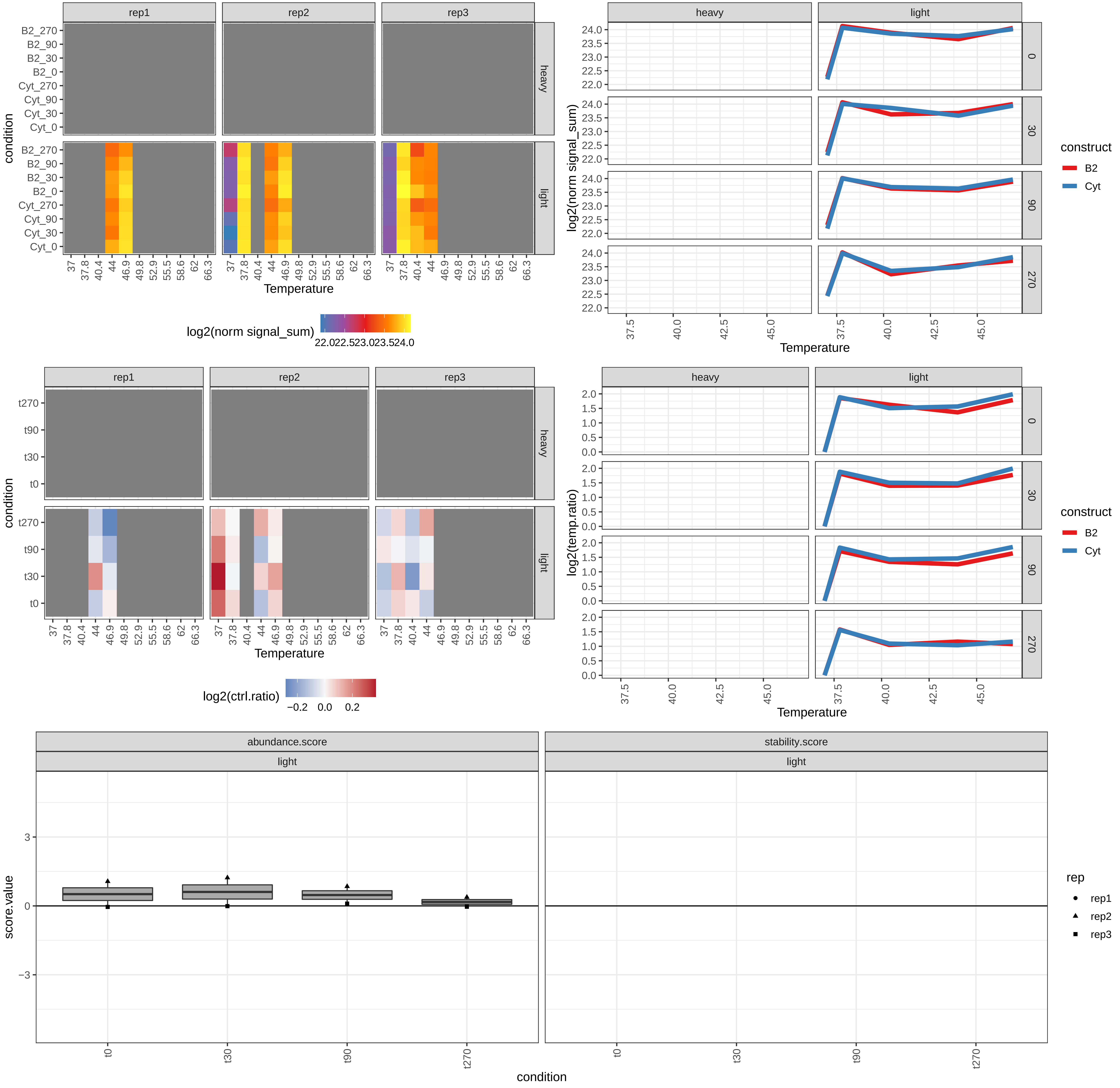

P43579 − Ino eighty subunit 1 OS=Saccharomyces cerevisiae (strain ATCC 204508 / S288c) GN=IES1 PE=1 SV=1

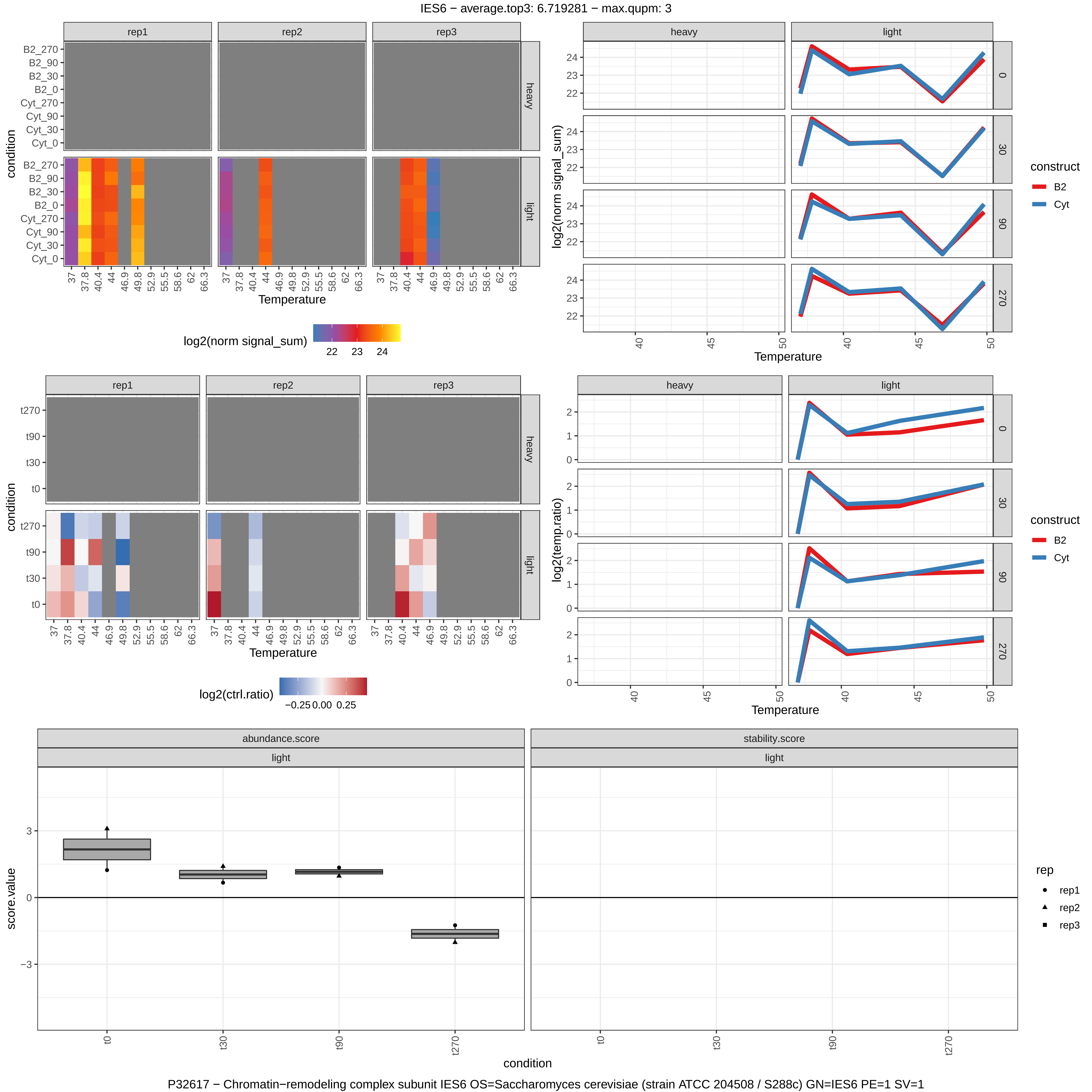
IES6 − average.top3: 6.719281 − max.qupm: 3
rep1
rep2
rep3
heavy
light
condition
Temperature
log2(norm signal_sum)
construct
B2
Cyt
log2(ctrl.ratio)
log2(temp.ratio)
abundance.score
stability.score
score.value
rep
rep1
rep2
rep3
P32617 − Chromatin−remodeling complex subunit IES6 OS=Saccharomyces cerevisiae (strain ATCC 204508 / S288c) GN=IES6 PE=1 SV=1

IFA38 − average.top3: 7.27419977272727 − max.qupm: 11

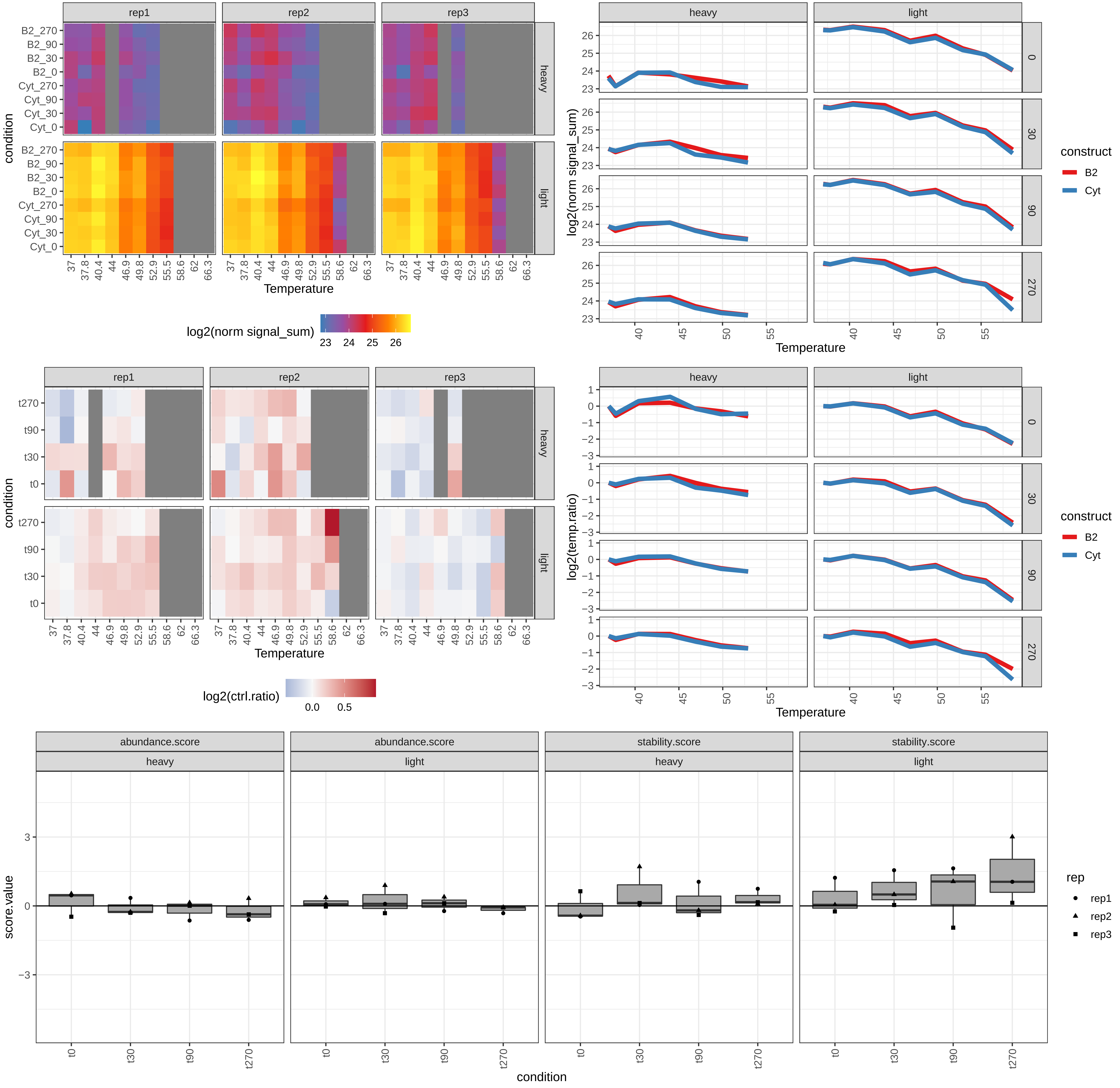

P38286 − Very−long−chain 3−oxoacyl−CoA reductase OS=Saccharomyces cerevisiae (strain ATCC 204508 / S288c) GN=IFA38 PE=1 SV=1

IFM1 − average.top3: 6.67699857142857 − max.qupm: 7

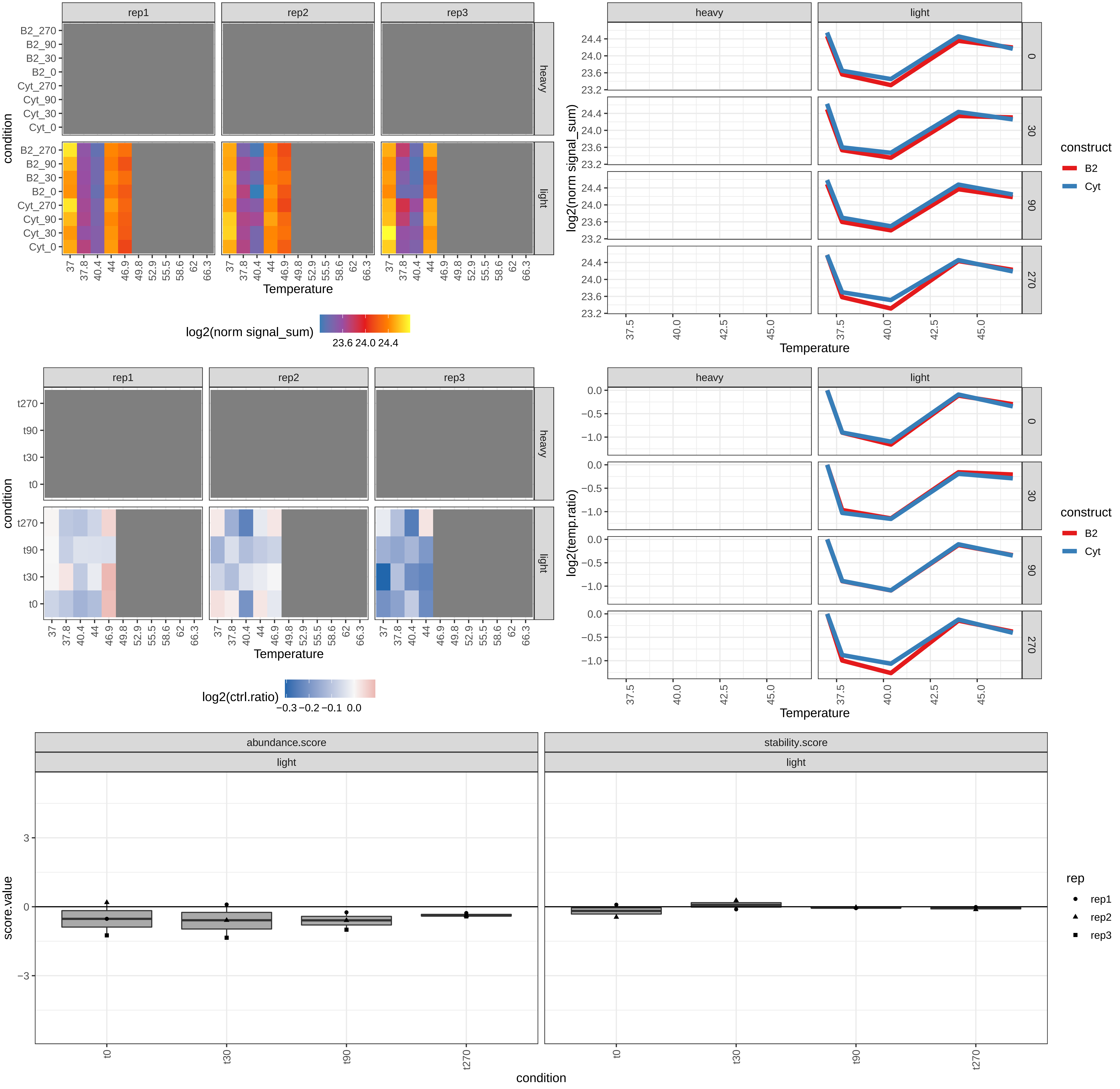

P25038 − Translation initiation factor IF−2, mitochondrial OS=Saccharomyces cerevisiae (strain ATCC 204508 / S288c) GN=IFM1 PE=1 SV=2

IKI3 − average.top3: 7.1423158974359 − max.qupm: 21

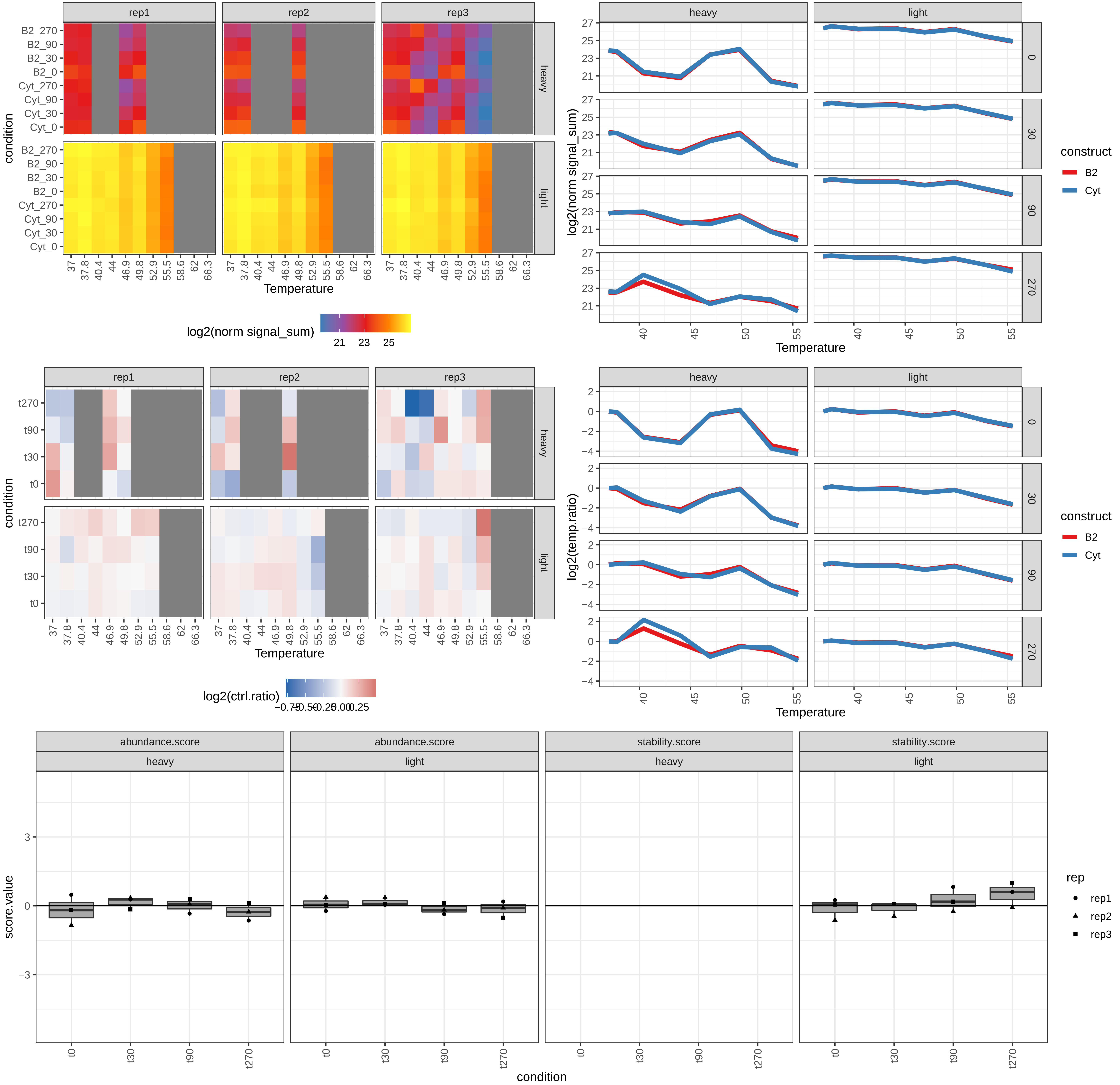

Q06706 − Elongator complex protein 1 OS=Saccharomyces cerevisiae (strain ATCC 204508 / S288c) GN=IKI3 PE=1 SV=1

ILS1 − average.top3: 7.43122527272727 − max.qupm: 41

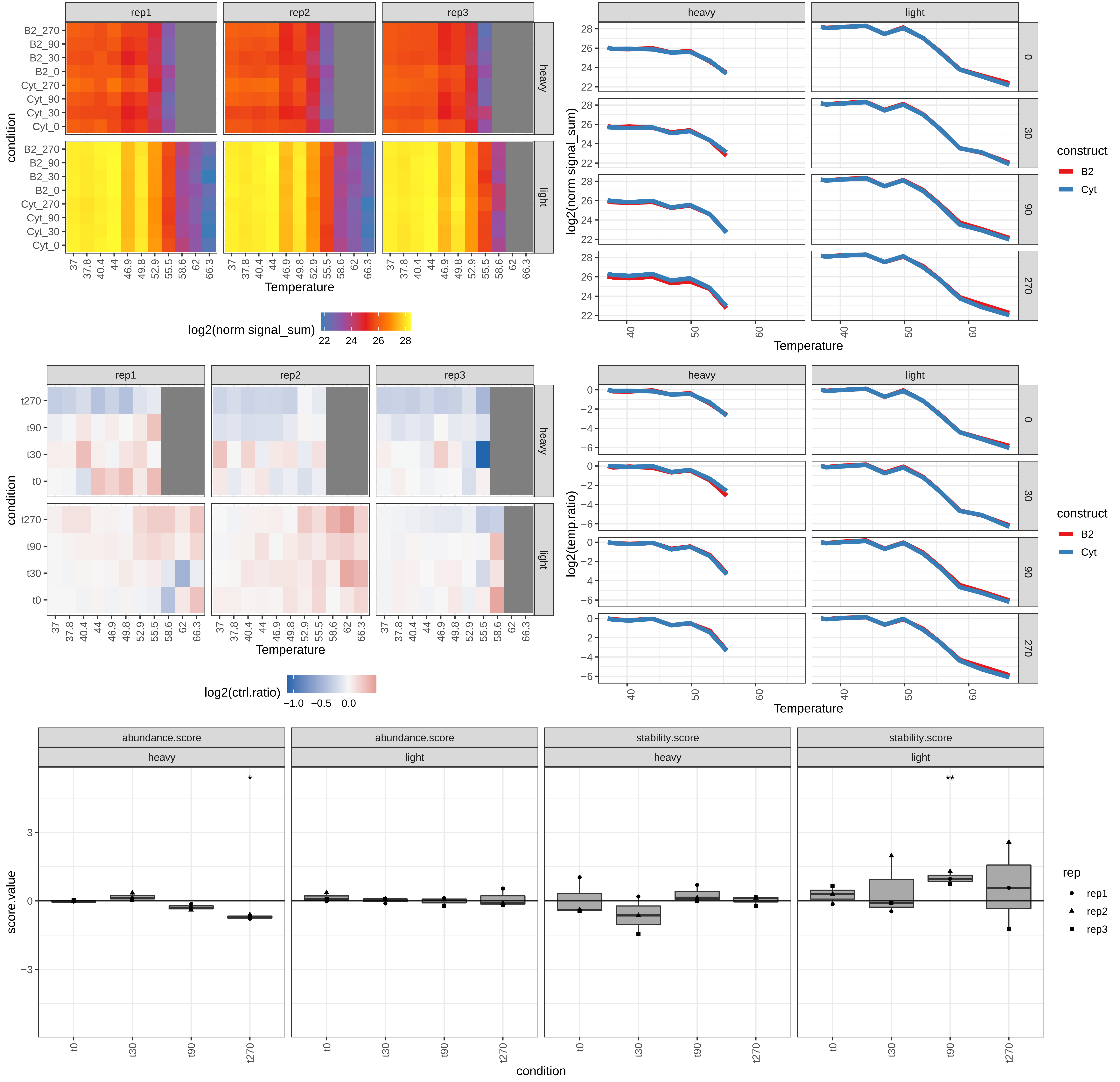

P09436 − Isoleucine−−tRNA ligase, cytoplasmic OS=Saccharomyces cerevisiae (strain ATCC 204508 / S288c) GN=ILS1 PE=1 SV=1

ILV1 − average.top3: 7.26214384615385 − max.qupm: 25

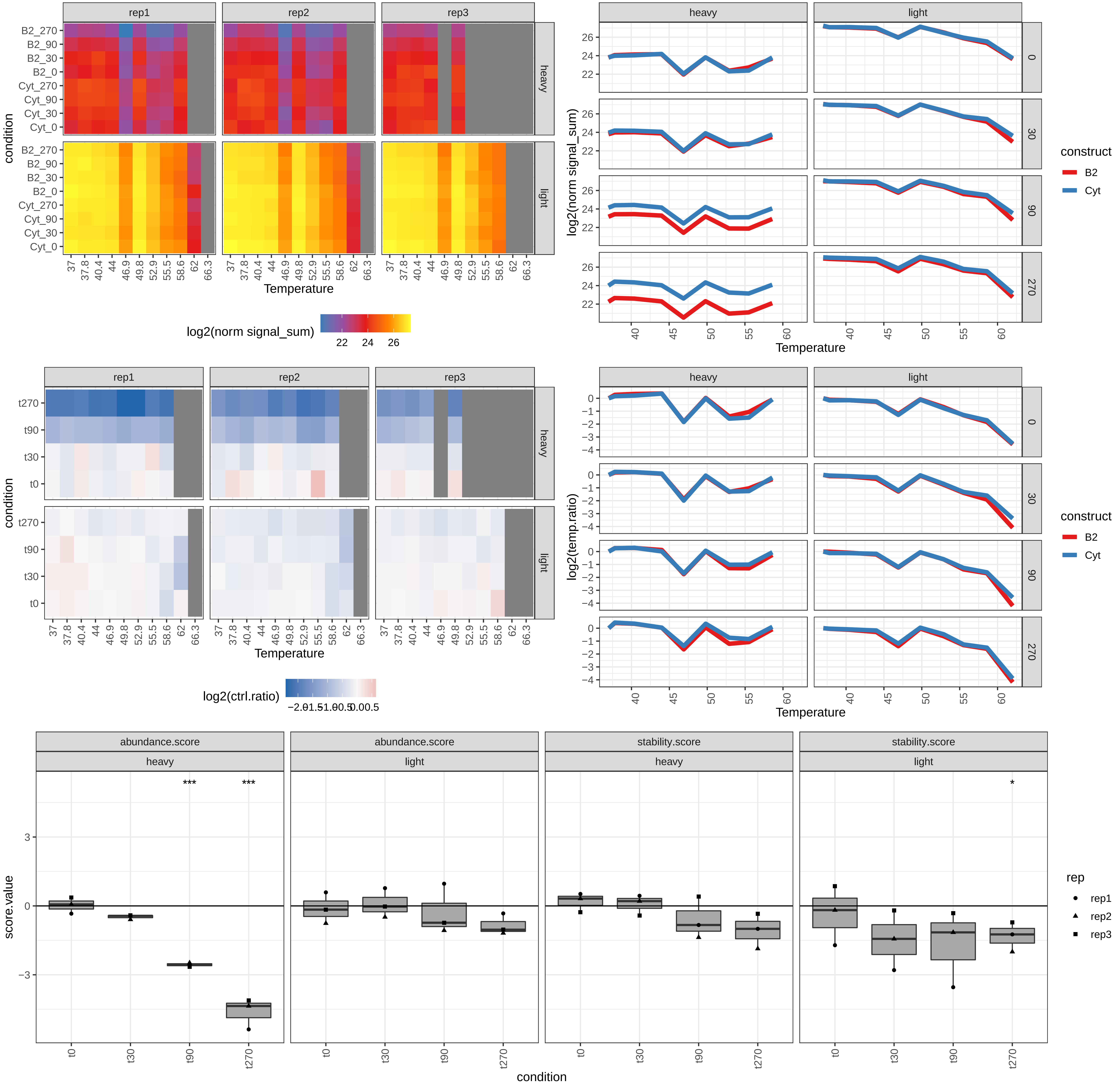

P00927 − Threonine dehydratase, mitochondrial OS=Saccharomyces cerevisiae (strain ATCC 204508 / S288c) GN=ILV1 PE=1 SV=2

ILV2 − average.top3: 7.45146127272727 − max.qupm: 18

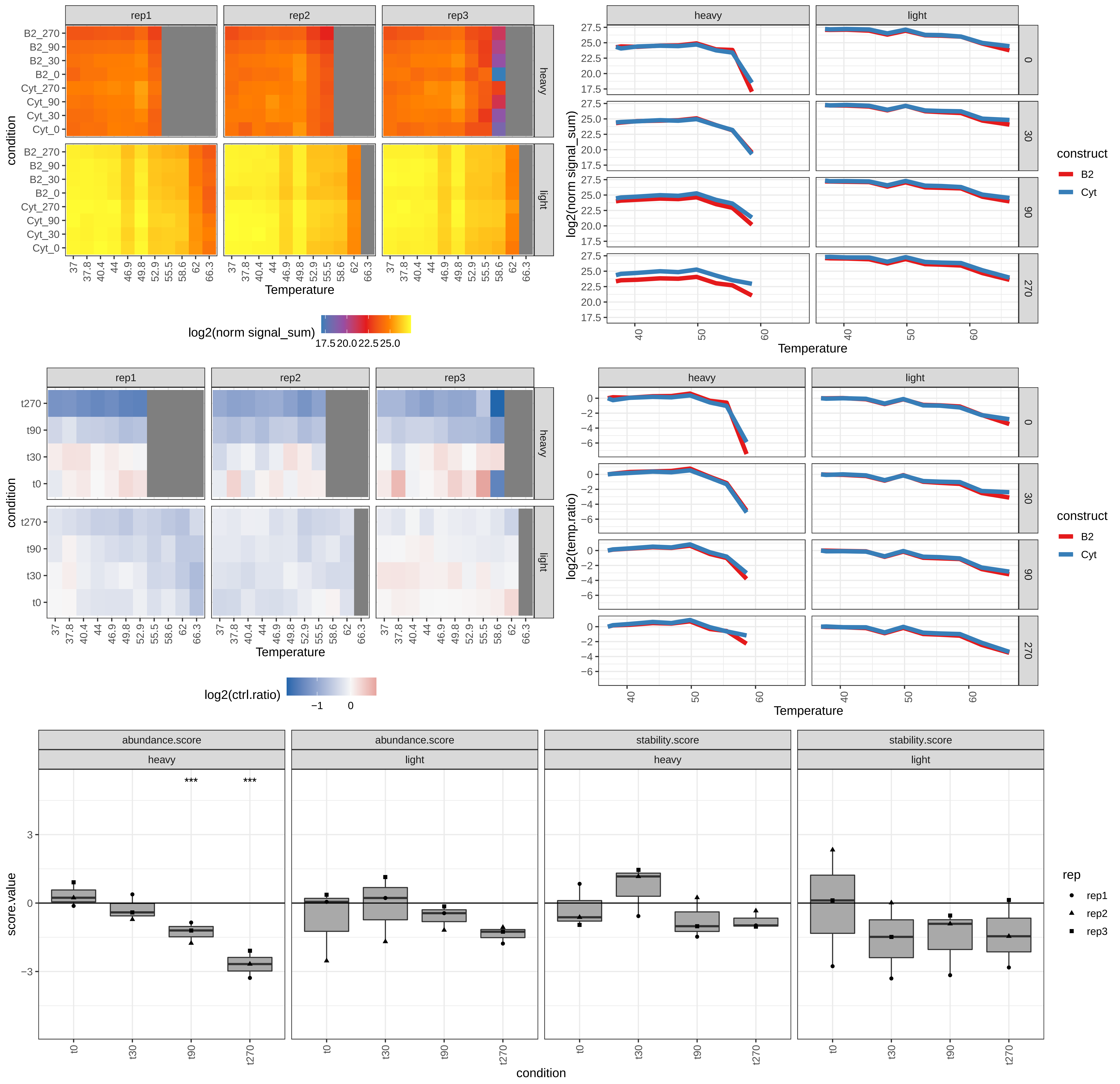

P07342 − Acetolactate synthase catalytic subunit, mitochondrial OS=Saccharomyces cerevisiae (strain ATCC 204508 / S288c) GN=ILV2 PE=1 SV=1

ILV3 − average.top3: 7.67322777777778 − max.qupm: 26

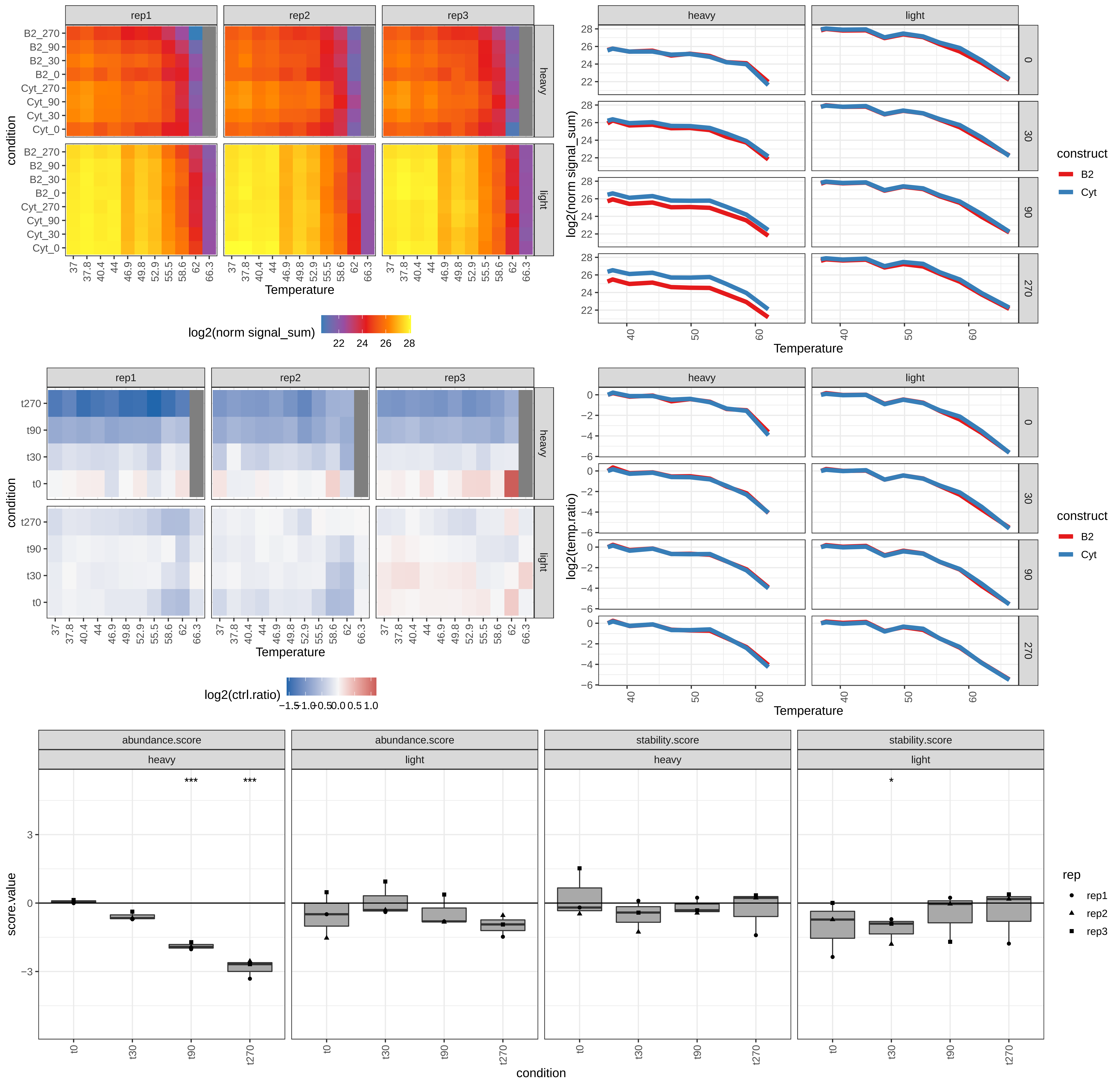

P39522 − Dihydroxy−acid dehydratase, mitochondrial OS=Saccharomyces cerevisiae (strain ATCC 204508 / S288c) GN=ILV3 PE=1 SV=2

ILV5 − average.top3: 7.87934737704918 − max.qupm: 20

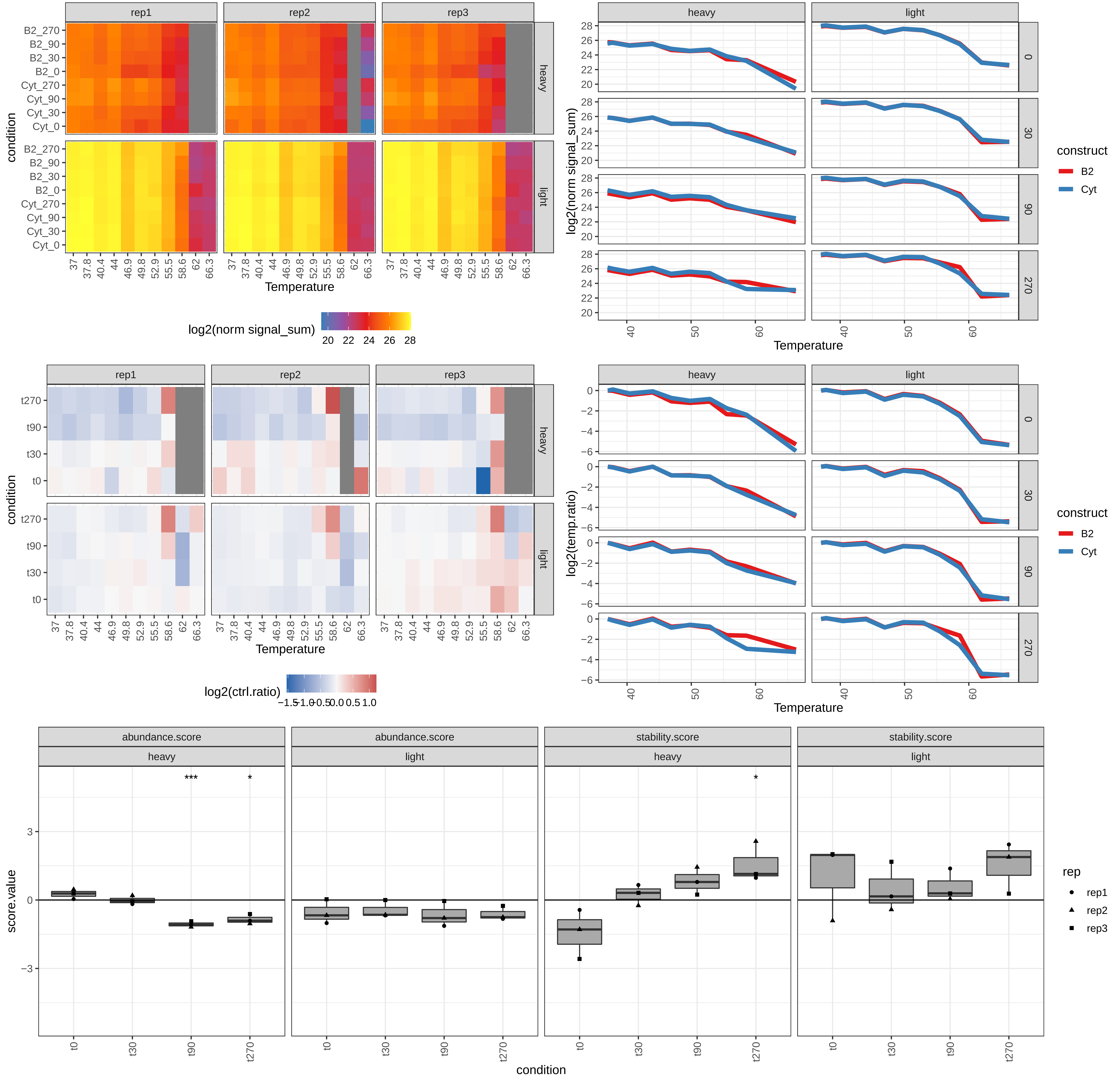

P06168 − Ketol−acid reductoisomerase, mitochondrial OS=Saccharomyces cerevisiae (strain ATCC 204508 / S288c) GN=ILV5 PE=1 SV=1

ILV6 − average.top3: 7.17844727272727 − max.qupm: 6

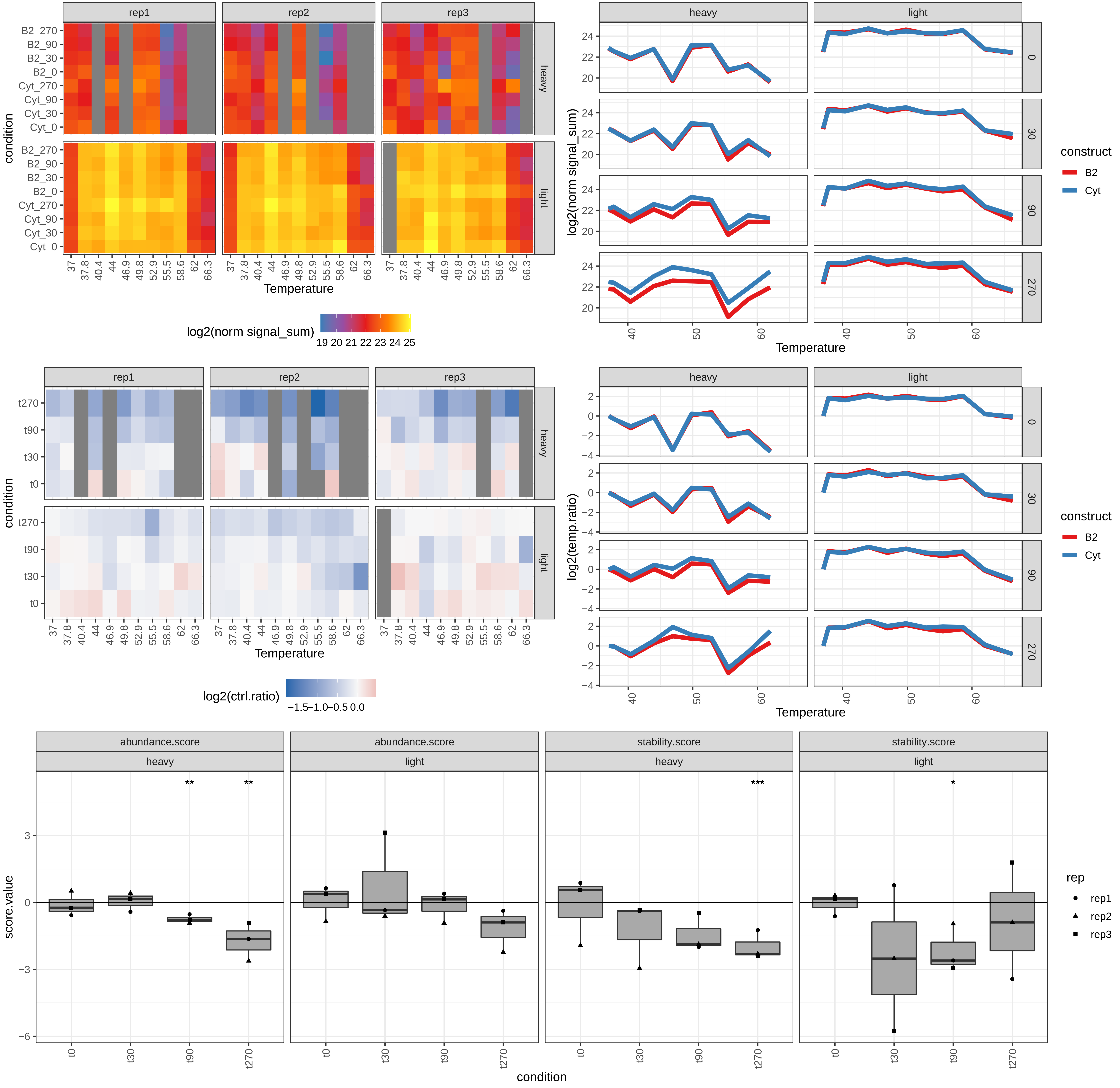

P25605 − Acetolactate synthase small subunit, mitochondrial OS=Saccharomyces cerevisiae (strain ATCC 204508 / S288c) GN=ILV6 PE=1 SV=2

IMA3|IMA4|IMA2 − average.top3: 6.76680555555556 − max.qupm: 9

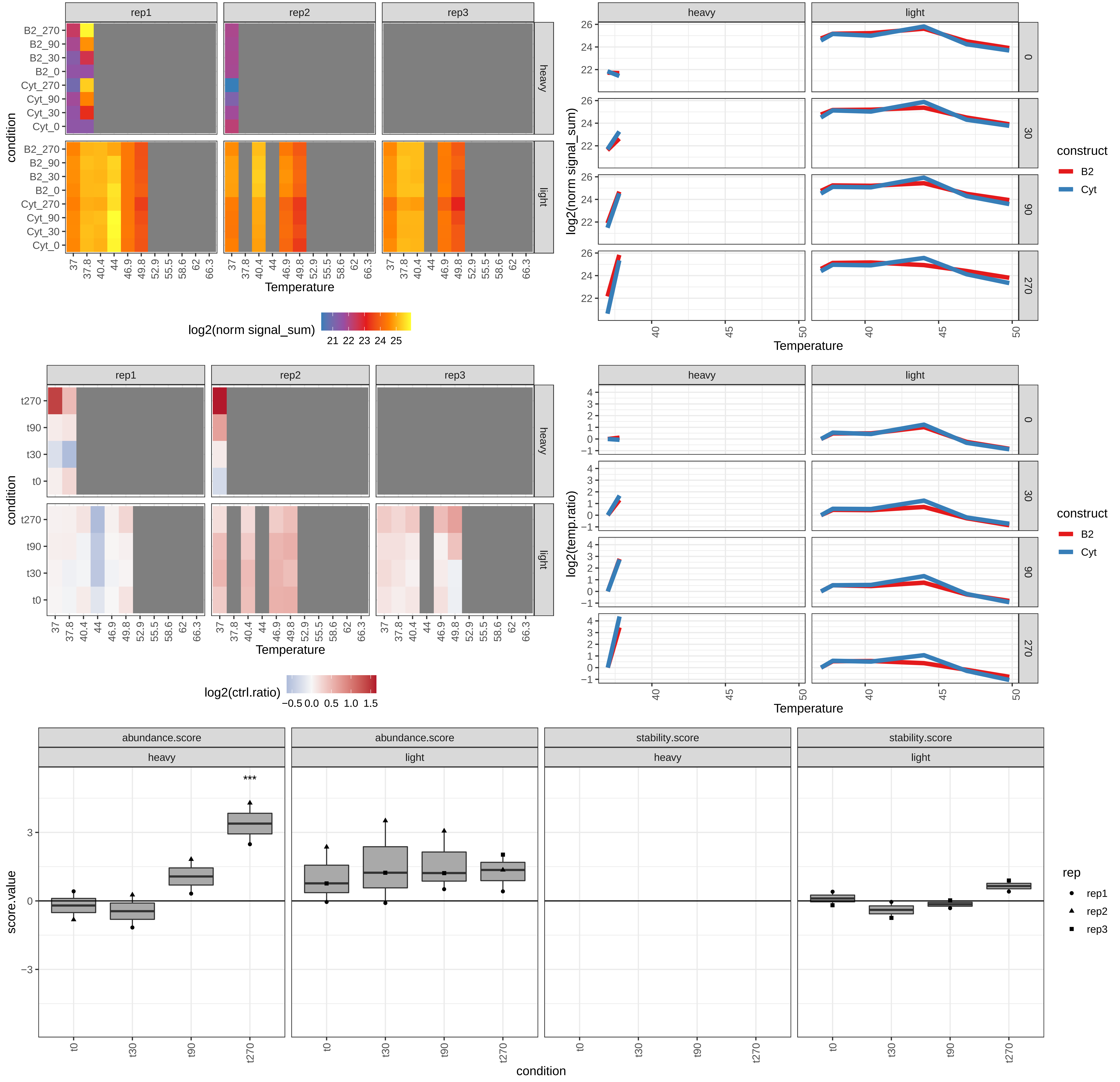

visiae (strain ATCC 204508 / S288c) GN=IMA3 PE=1 SV=1|Oligo−1,6−glucosidase IMA4 OS=Saccharomyces cerevisiae (strain ATCC 204508 / S288c) GN=IMA4 PE=3 SV=1|Oligo−1,6−glucosidase IMA2

IMD3 − average.top3: 7.33436416666667 − max.qupm: 10

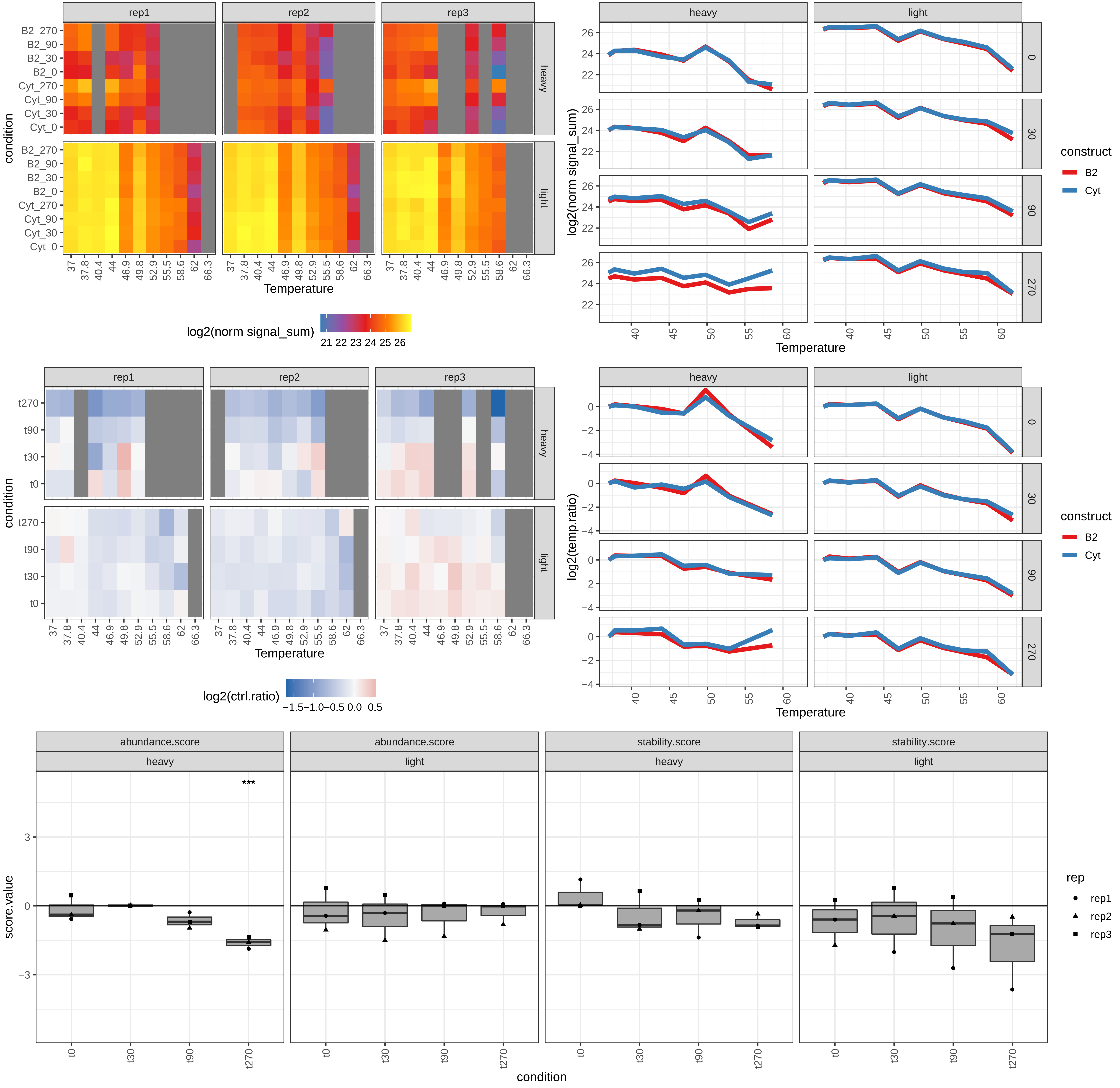

P50095 − Inosine−5'−monophosphate dehydrogenase 3 OS=Saccharomyces cerevisiae (strain ATCC 204508 / S288c) GN=IMD3 PE=1 SV=1

IMD4 − average.top3: 6.96099333333333 − max.qupm: 7

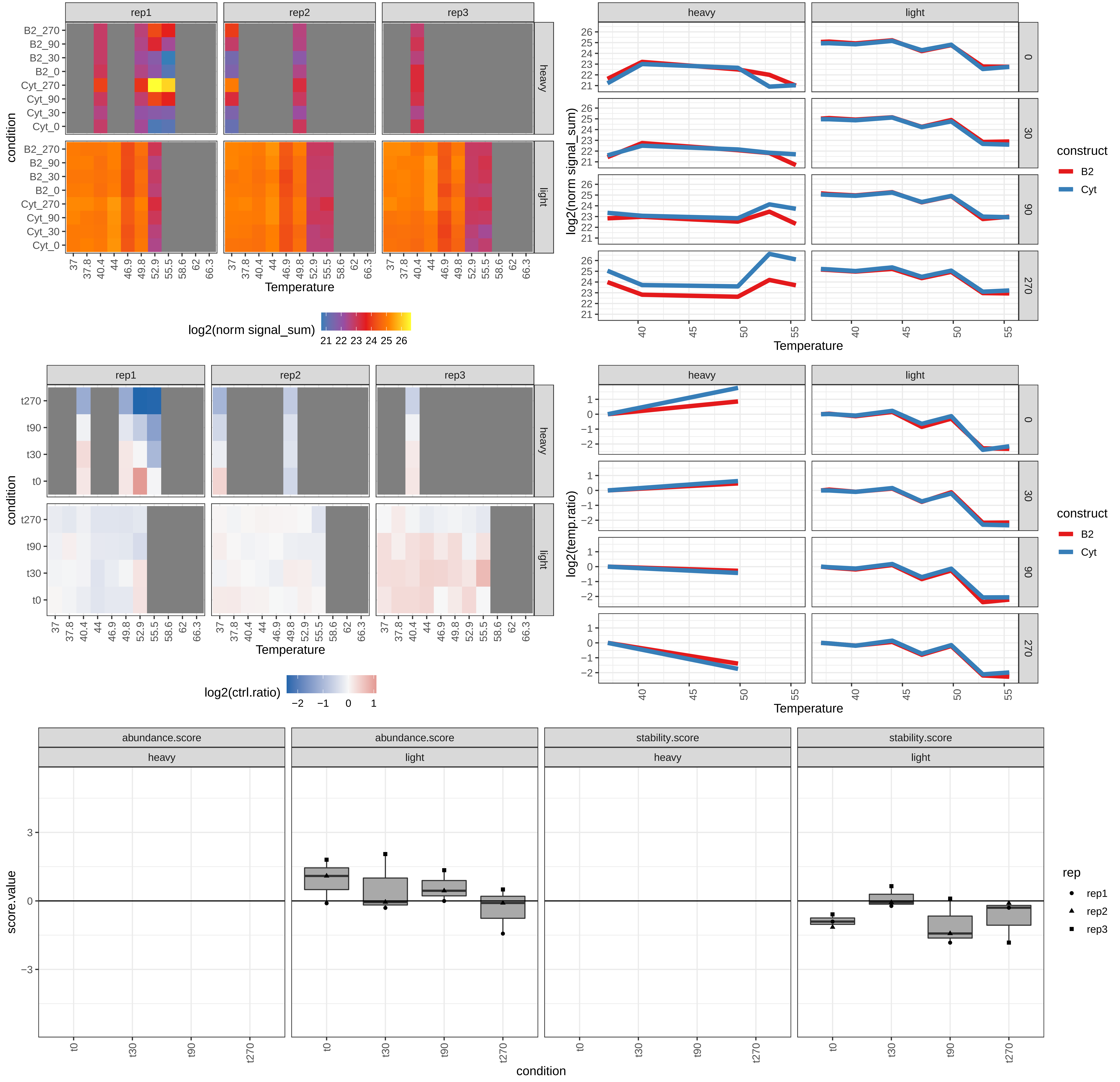

P50094 − Inosine−5'−monophosphate dehydrogenase 4 OS=Saccharomyces cerevisiae (strain ATCC 204508 / S288c) GN=IMD4 PE=1 SV=1

IMG1 − average.top3: 6.41278230769231 − max.qupm: 4

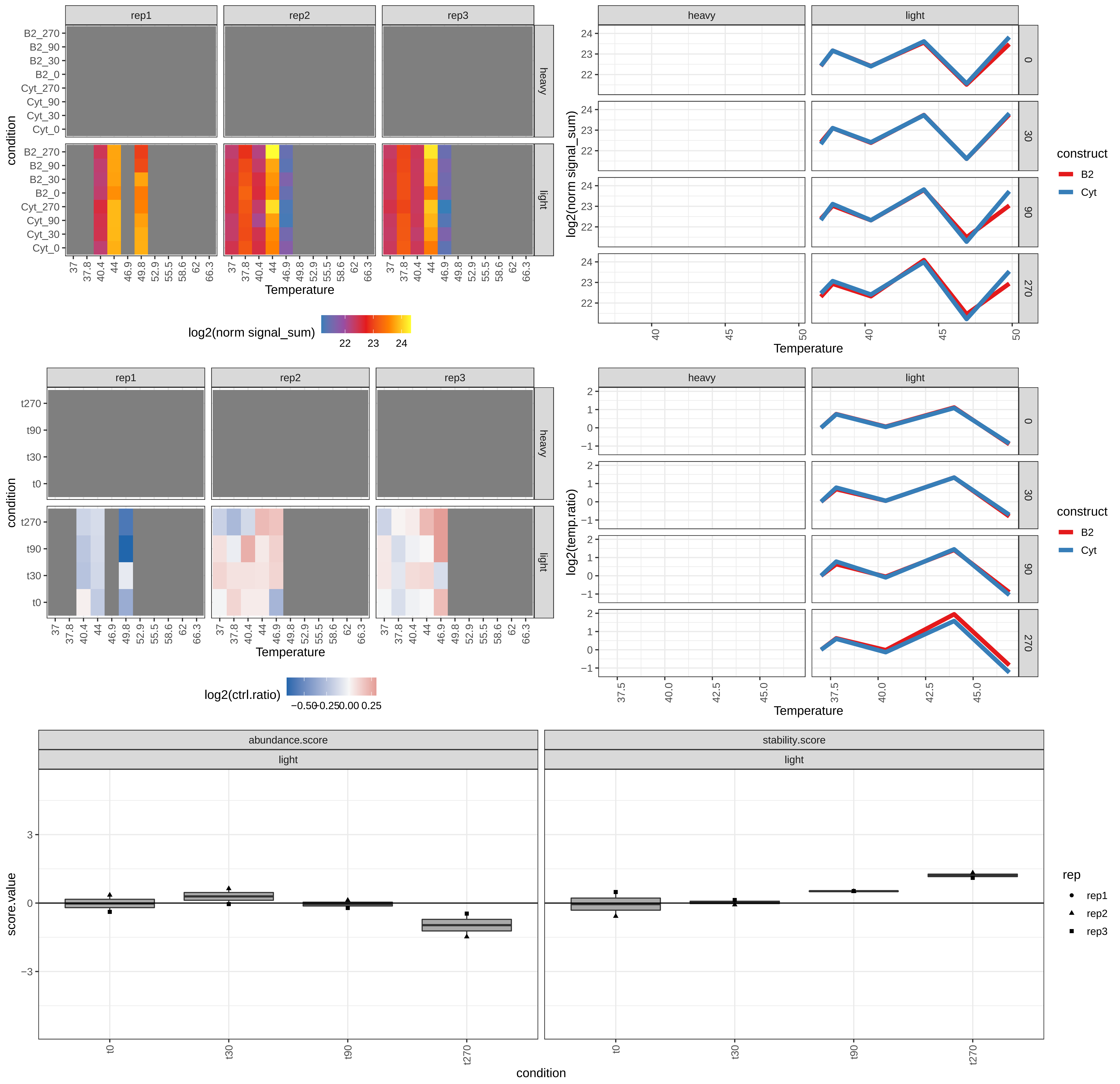

P25626 − 54S ribosomal protein IMG1, mitochondrial OS=Saccharomyces cerevisiae (strain ATCC 204508 / S288c) GN=IMG1 PE=1 SV=1

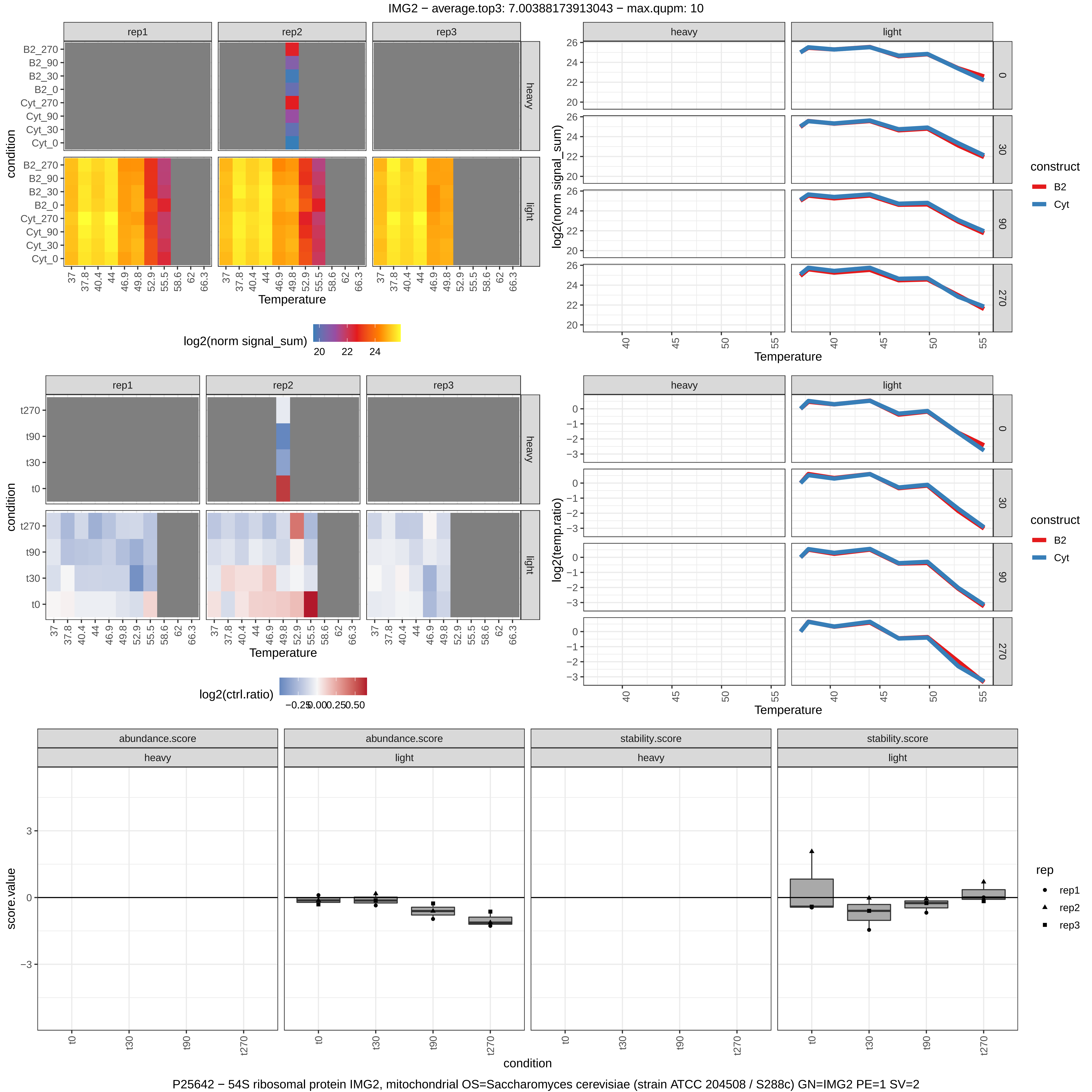
IMG2 − average.top3: 7.00388173913043 − max.qupm: 10
rep1
rep2
rep3
heavy
light
condition
B2_270
B2_90
B2_30
B2_0
Cyt_270
Cyt_90
Cyt_30
Cyt_0
Temperature
log2(norm signal_sum)
log2(ctrl.ratio)
log2(temp.ratio)
construct
B2
Cyt
t270
t90
t30
t0
abundance.score
stability.score
score.value
rep
P25642 − 54S ribosomal protein IMG2, mitochondrial OS=Saccharomyces cerevisiae (strain ATCC 204508 / S288c) GN=IMG2 PE=1 SV=2

IMH1 − average.top3: 6.44838115384615 − max.qupm: 14

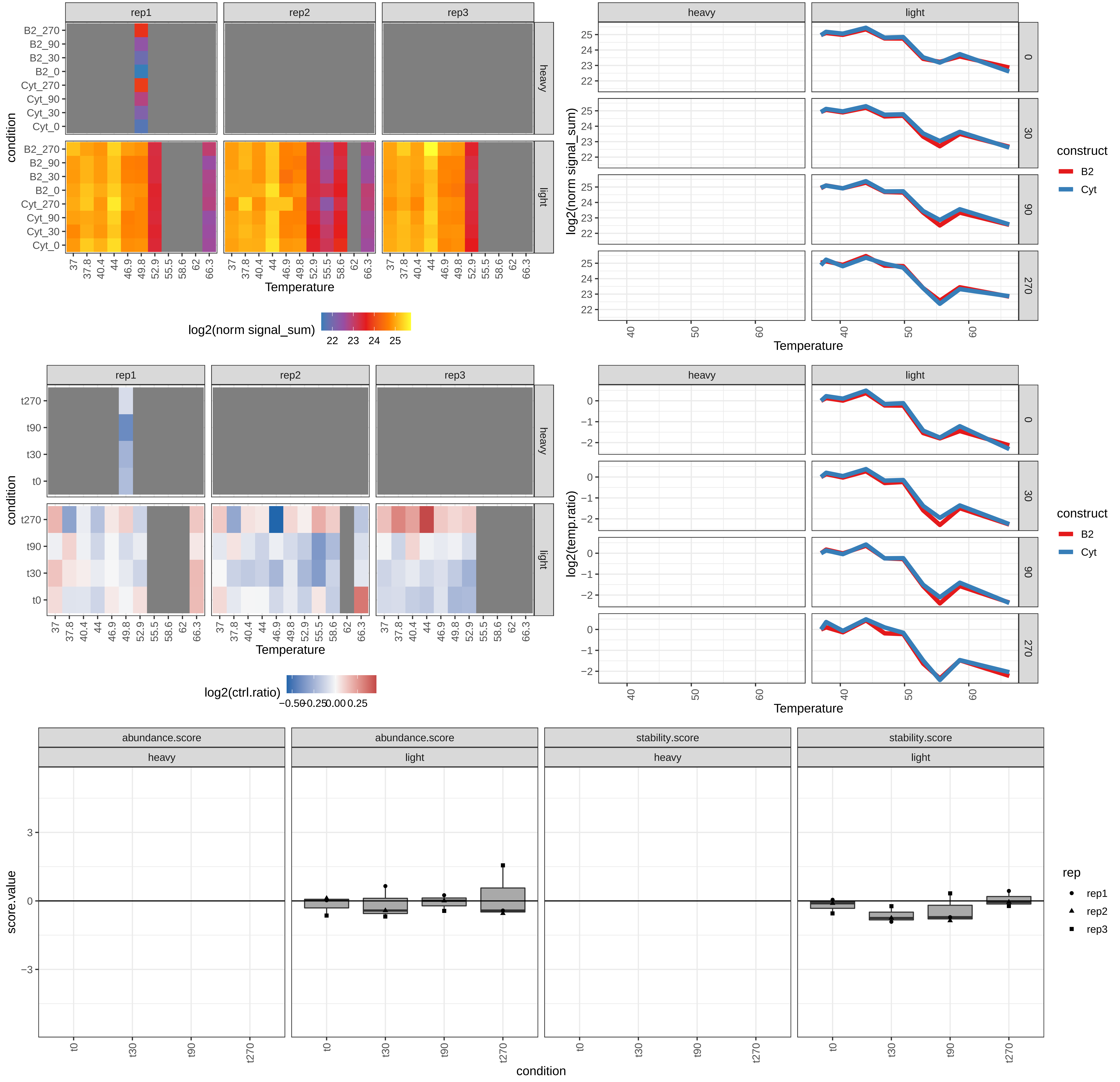

Q06704 − Golgin IMH1 OS=Saccharomyces cerevisiae (strain ATCC 204508 / S288c) GN=IMH1 PE=1 SV=1

IML1 − average.top3: 6.58177368421053 − max.qupm: 10

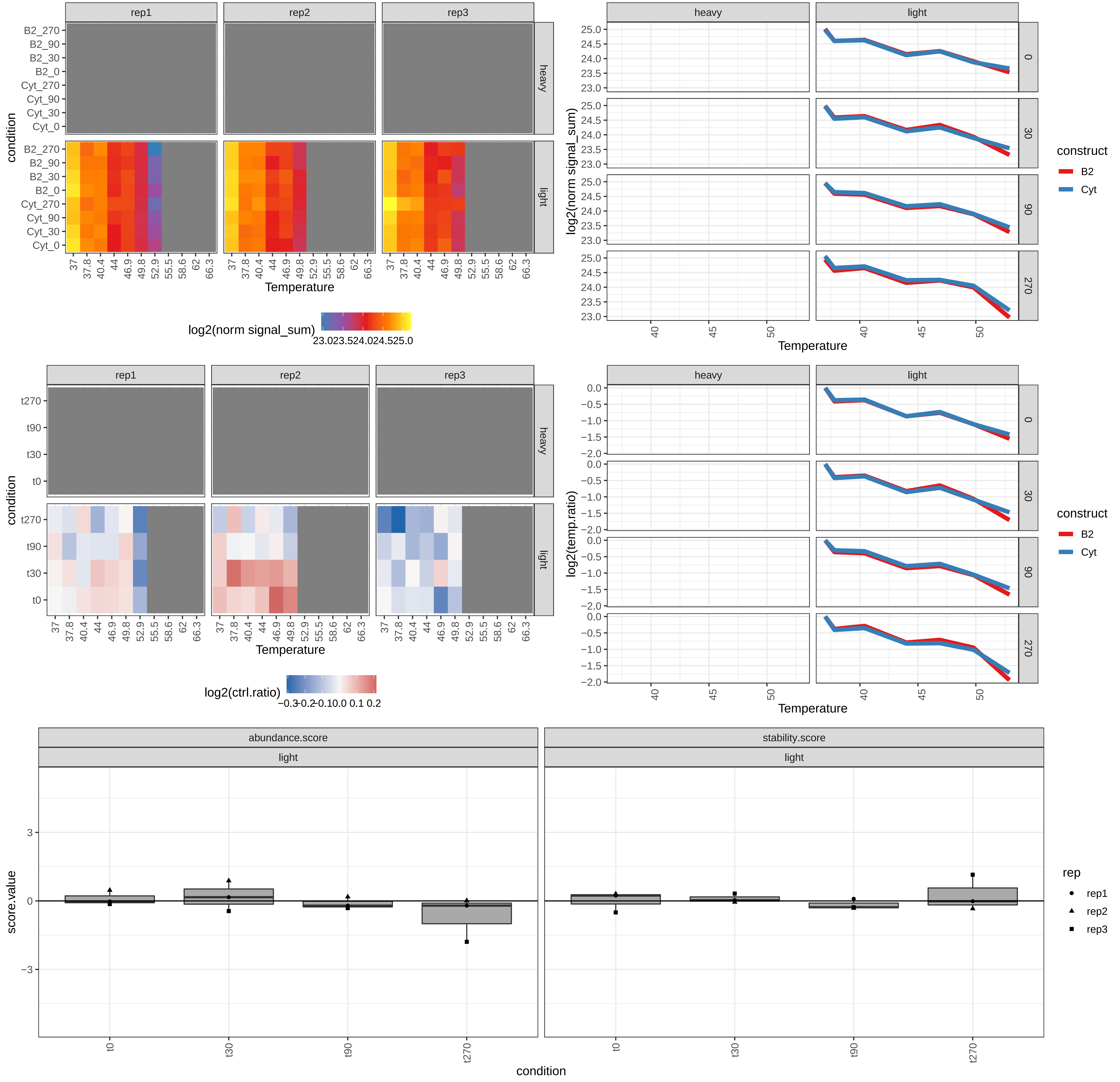

P47170 − Vacuolar membrane−associated protein IML1 OS=Saccharomyces cerevisiae (strain ATCC 204508 / S288c) GN=IML1 PE=1 SV=1

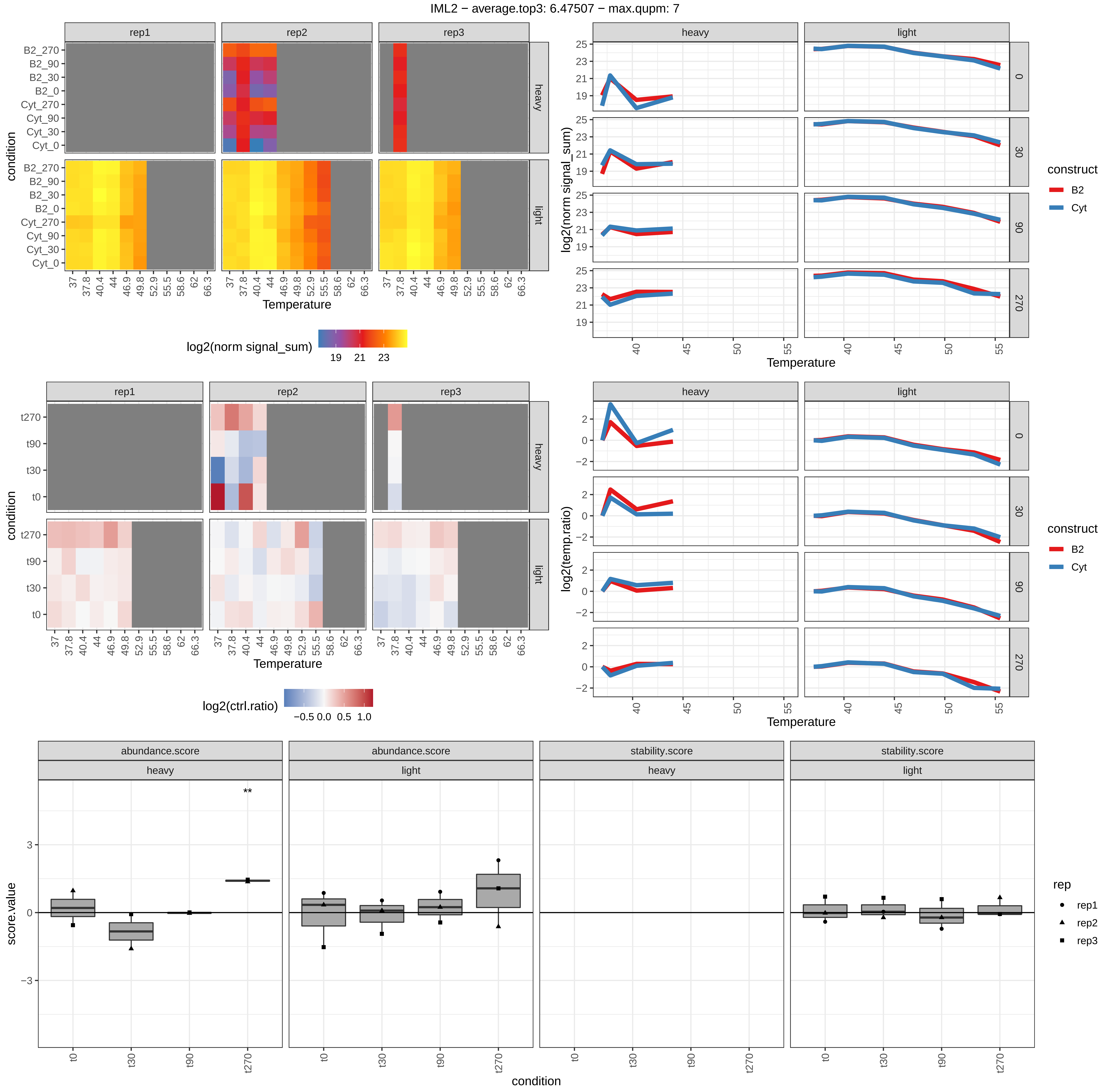

P47031 − Mitochondrial outer membrane protein IML2 OS=Saccharomyces cerevisiae (strain ATCC 204508 / S288c) GN=IML2 PE=1 SV=1

IMO32 − average.top3: 6.582385 − max.qupm: 5

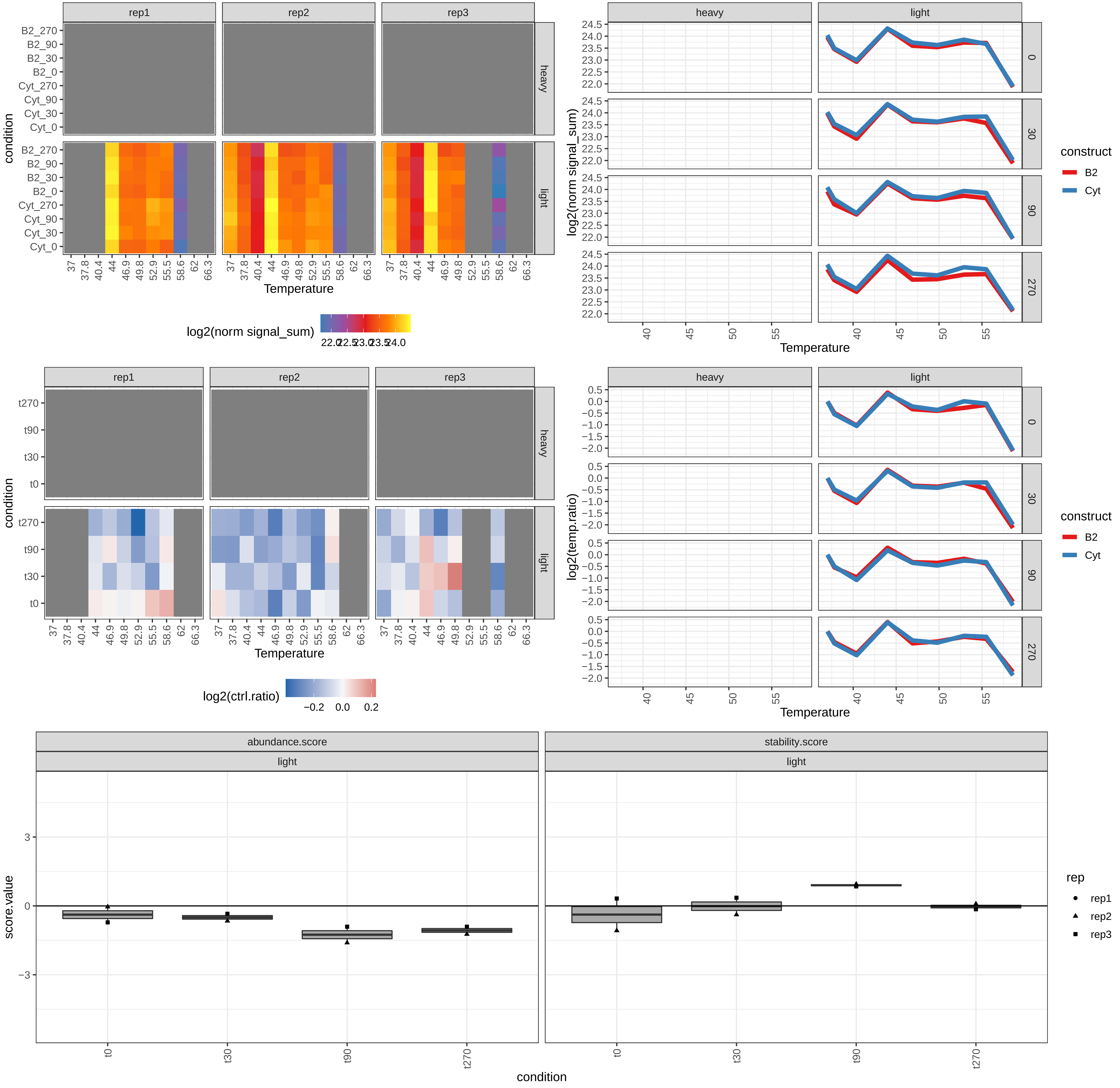

P53219 − Abhydrolase domain−containing protein IMO32 OS=Saccharomyces cerevisiae (strain ATCC 204508 / S288c) GN=IMO32 PE=1 SV=1

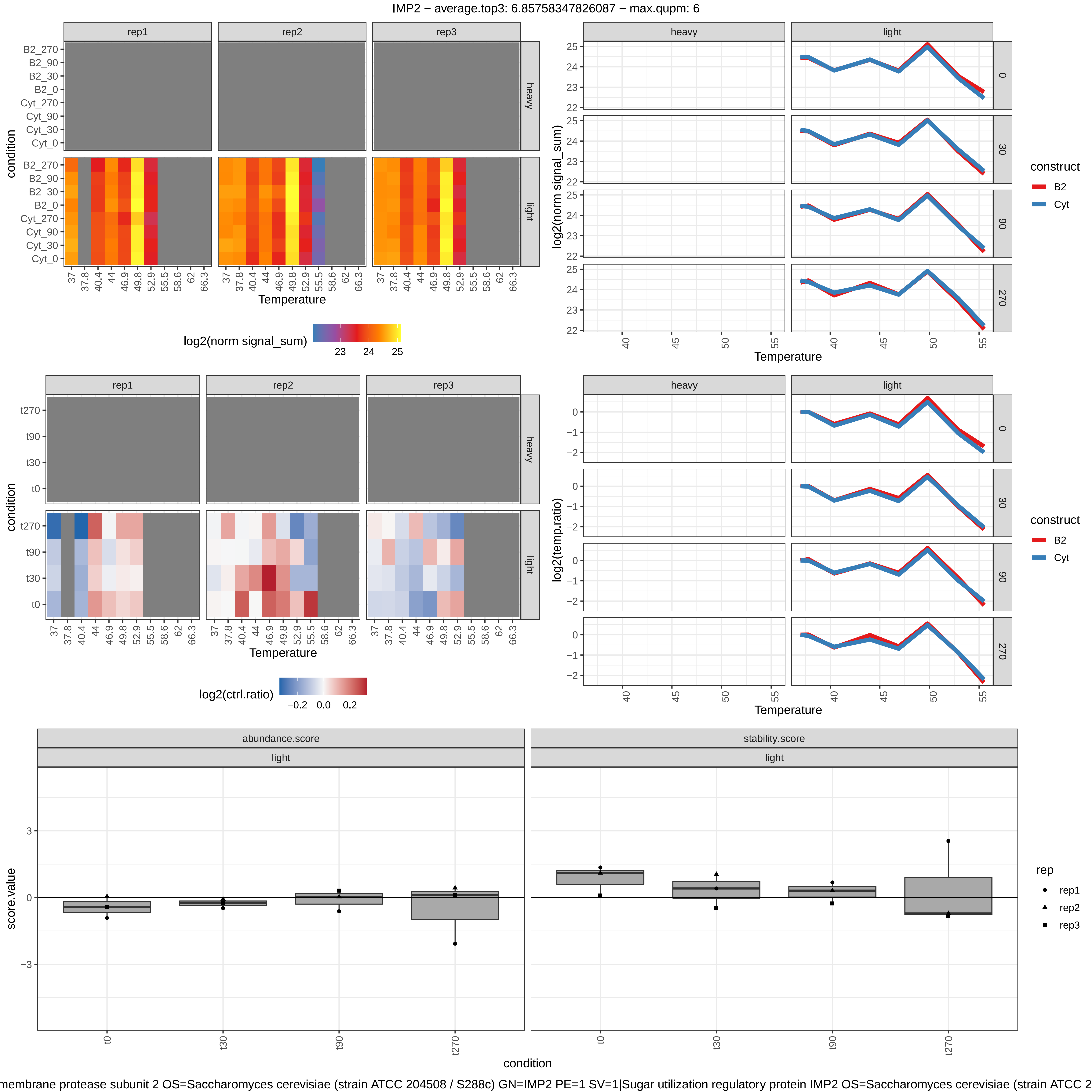
IMP2 − average.top3: 6.85758347826087 − max.qupm: 6
rep1
rep2
rep3
heavy
light
condition
Temperature
log2(norm signal_sum)
log2(temp.ratio)
log2(ctrl.ratio)
construct
B2
Cyt
abundance.score
stability.score
score.value
rep
condition
membrane protease subunit 2 OS=Saccharomyces cerevisiae (strain ATCC 204508 / S288c) GN=IMP2 PE=1 SV=1|Sugar utilization regulatory protein IMP2 OS=Saccharomyces cerevisiae (strain ATCC 2

IMP3 − average.top3: 6.43651615384615 − max.qupm: 6

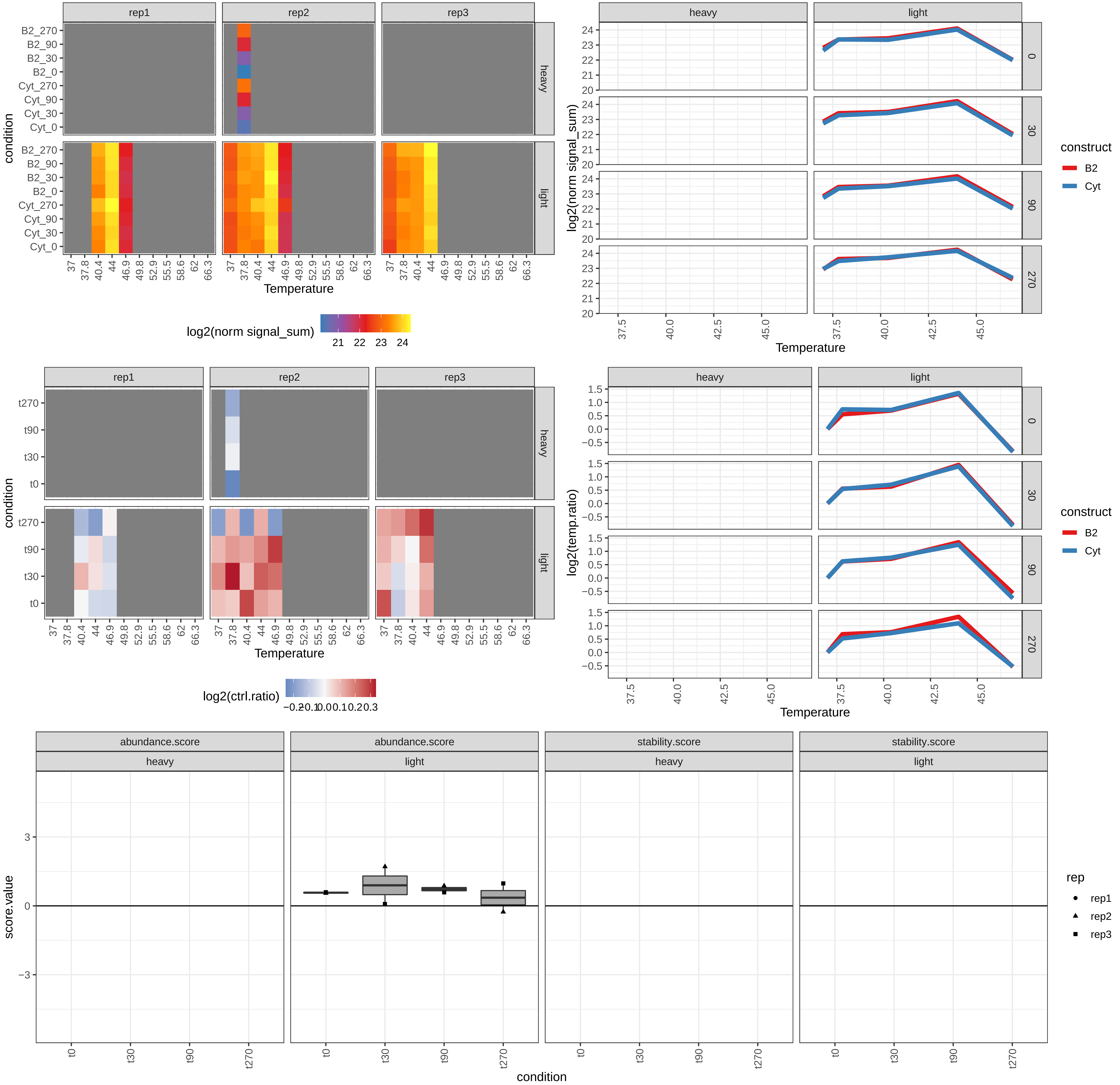

P32899 − U3 small nucleolar ribonucleoprotein protein IMP3 OS=Saccharomyces cerevisiae (strain ATCC 204508 / S288c) GN=IMP3 PE=1 SV=1

IMP4 − average.top3: 6.826589 − max.qupm: 5

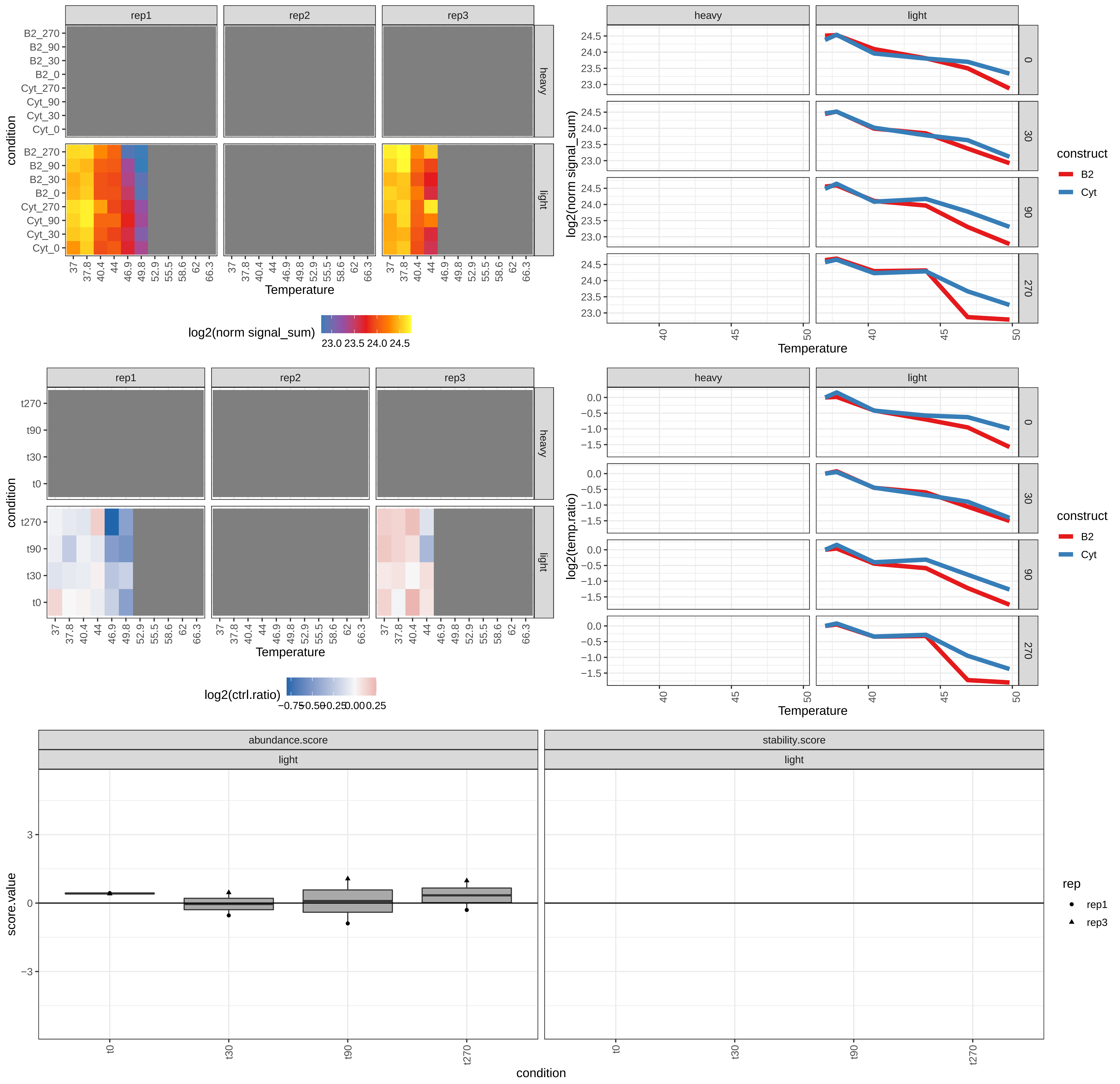

P53941 − U3 small nucleolar ribonucleoprotein protein IMP4 OS=Saccharomyces cerevisiae (strain ATCC 204508 / S288c) GN=IMP4 PE=1 SV=1

INH1 − average.top3: 7.60995423728814 − max.qupm: 6

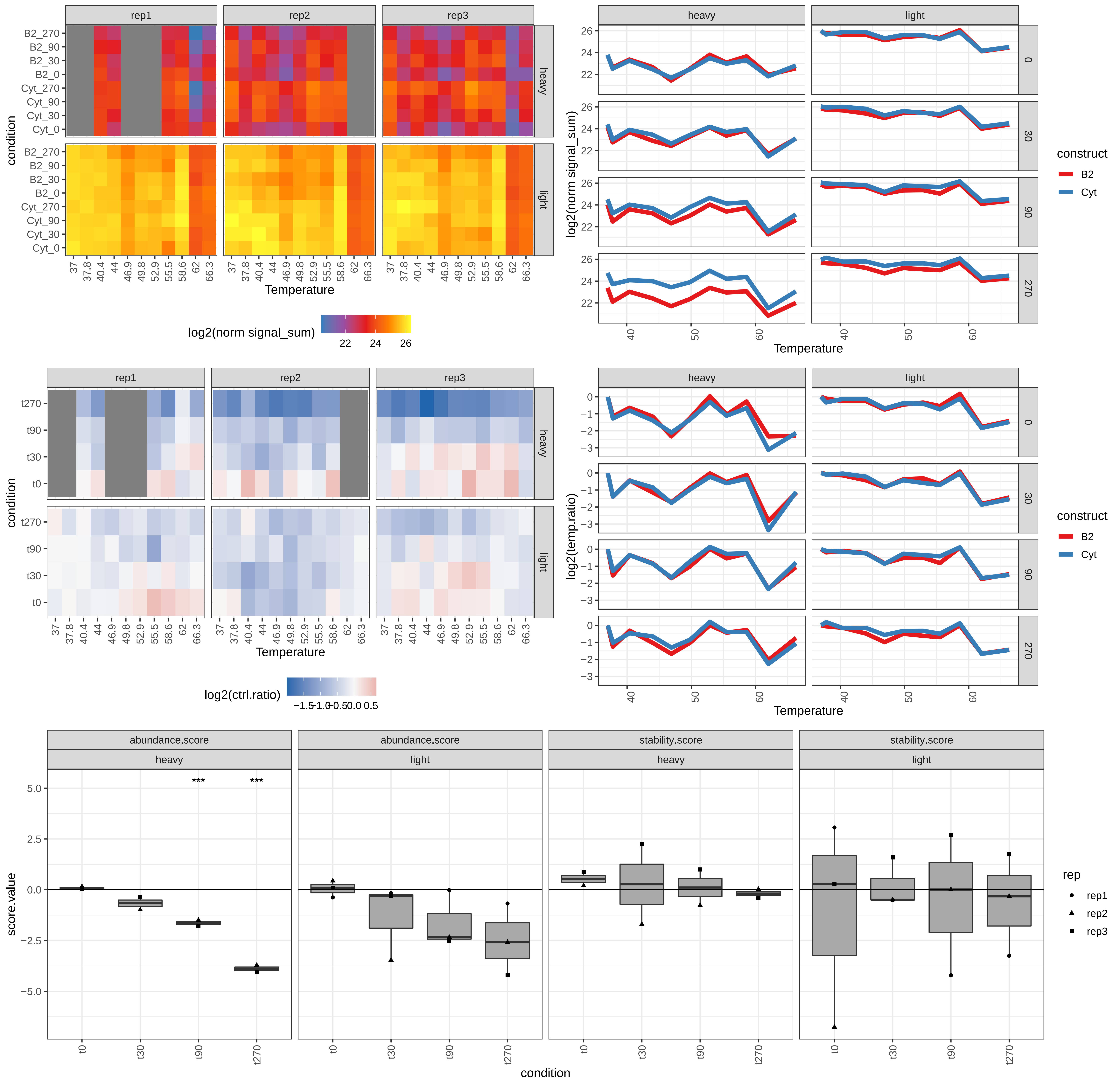

P01097 − ATPase inhibitor, mitochondrial OS=Saccharomyces cerevisiae (strain ATCC 204508 / S288c) GN=INH1 PE=1 SV=2

INM1 − average.top3: 6.67414810810811 − max.qupm: 6

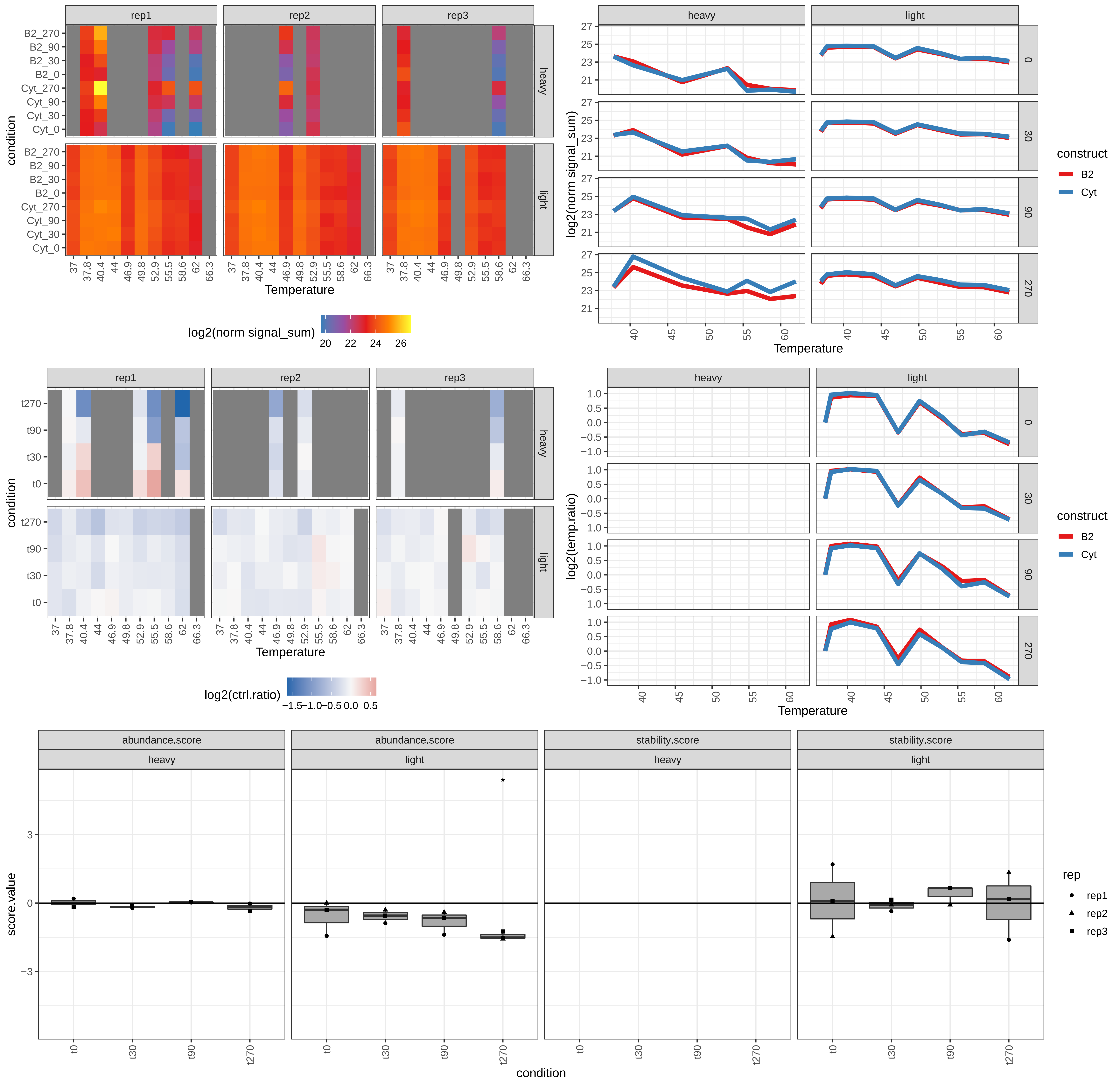

P38710 − Inositol monophosphatase 1 OS=Saccharomyces cerevisiae (strain ATCC 204508 / S288c) GN=INM1 PE=1 SV=1

INM2 − average.top3: 6.79630970588235 − max.qupm: 9

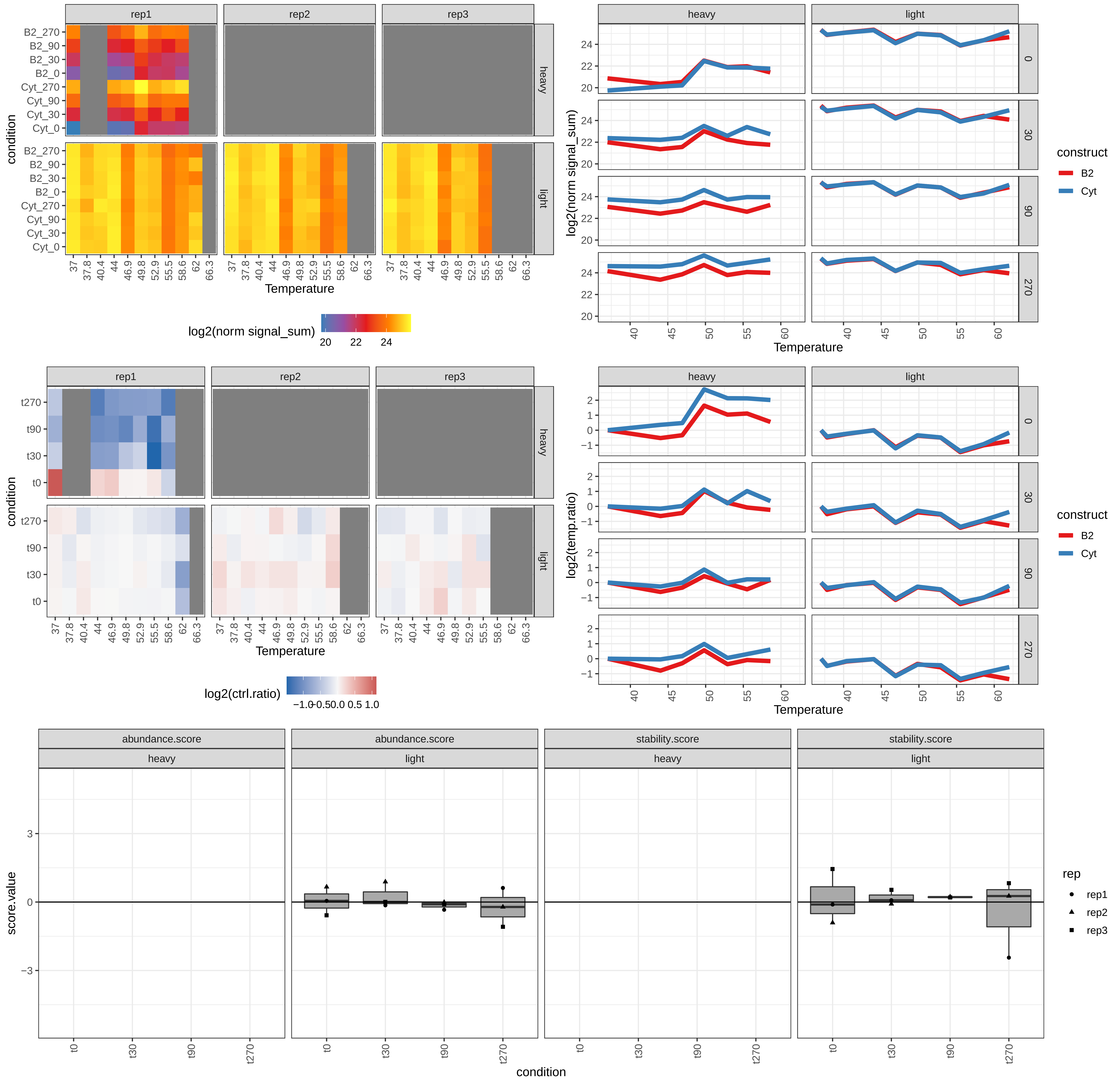

Q05533 − Inositol monophosphatase 2 OS=Saccharomyces cerevisiae (strain ATCC 204508 / S288c) GN=INM2 PE=1 SV=1

INO1 − average.top3: 8.34738772727273 − max.qupm: 30

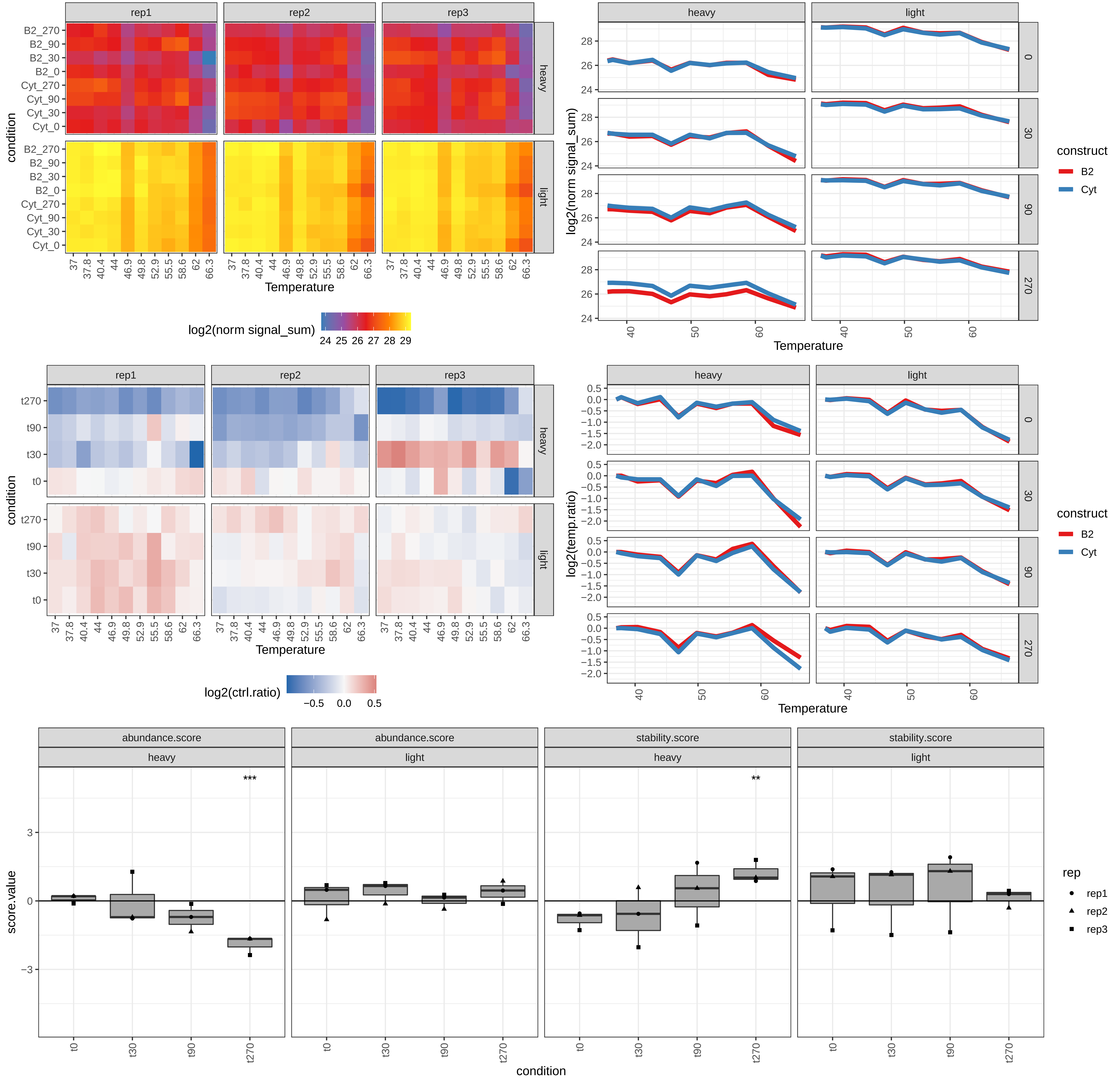

P11986 − Inositol−3−phosphate synthase OS=Saccharomyces cerevisiae (strain ATCC 204508 / S288c) GN=INO1 PE=1 SV=3

INO80 − average.top3: 6.78506666666667 − max.qupm: 8

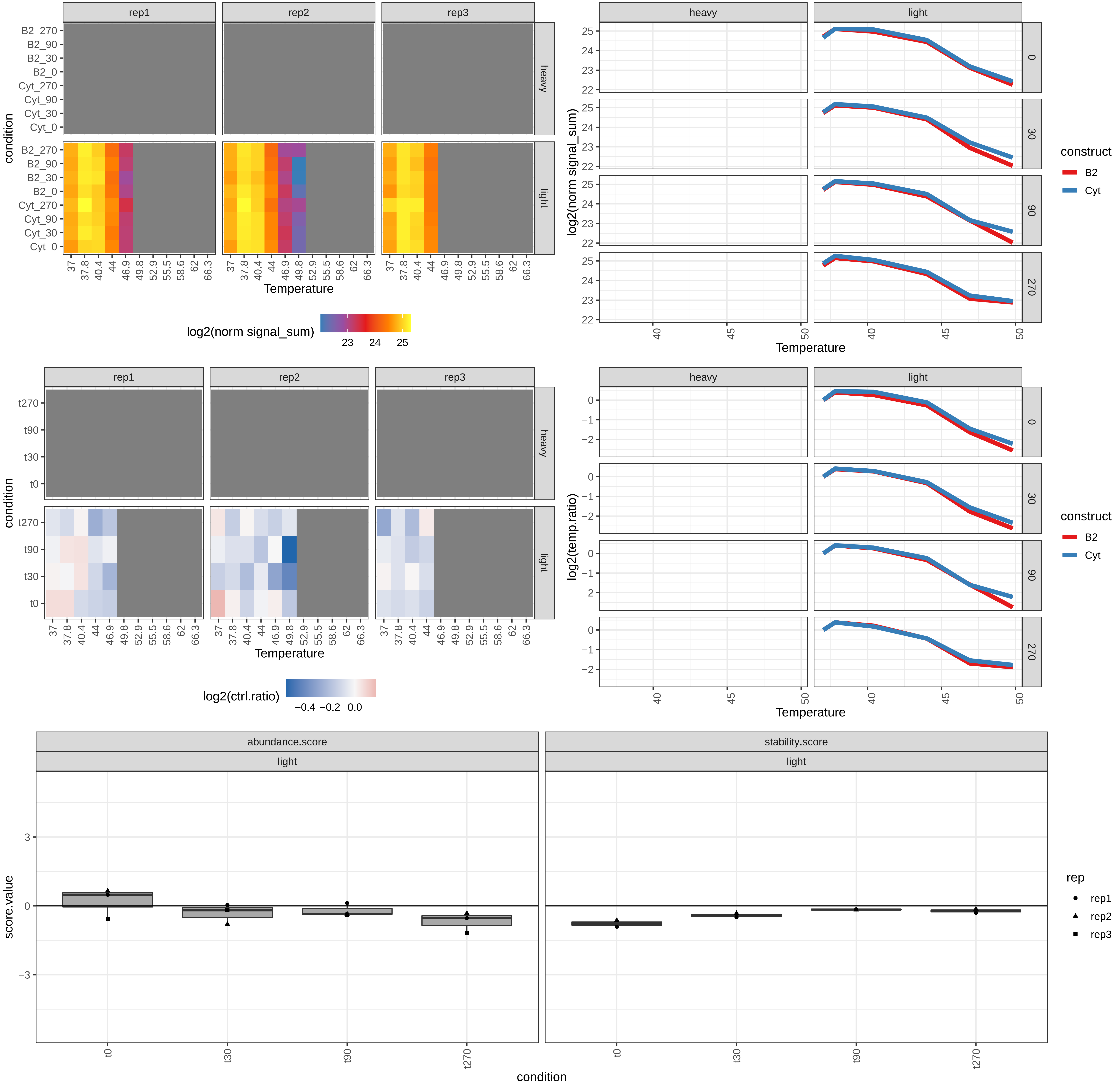

P53115 − Putative DNA helicase INO80 OS=Saccharomyces cerevisiae (strain ATCC 204508 / S288c) GN=INO80 PE=1 SV=1

INP51 − average.top3: 6.55471166666667 − max.qupm: 9

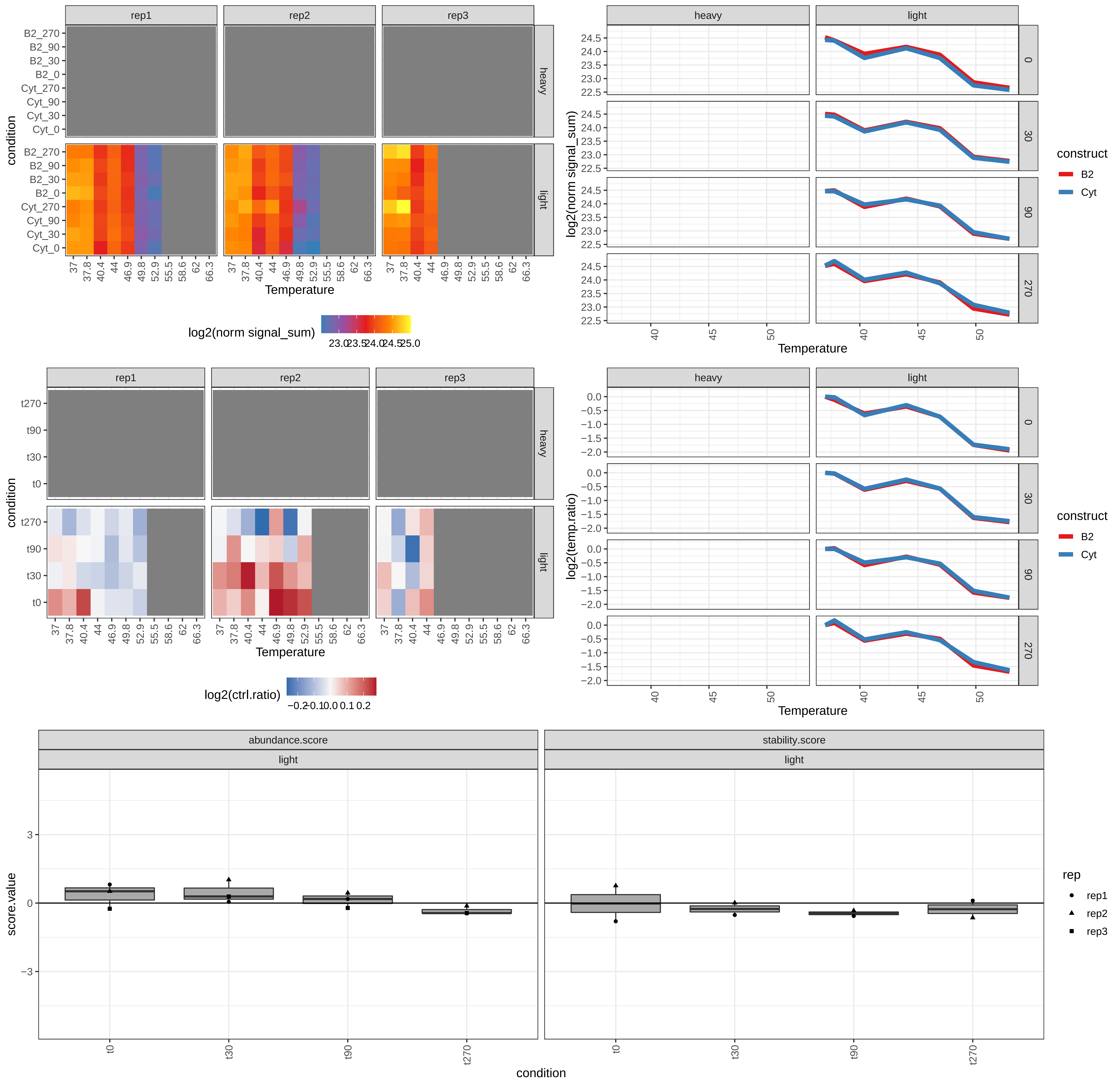

P40559 − Phosphatidylinositol 4,5−bisphosphate 5−phosphatase INP51 OS=Saccharomyces cerevisiae (strain ATCC 204508 / S288c) GN=INP51 PE=1 SV=1

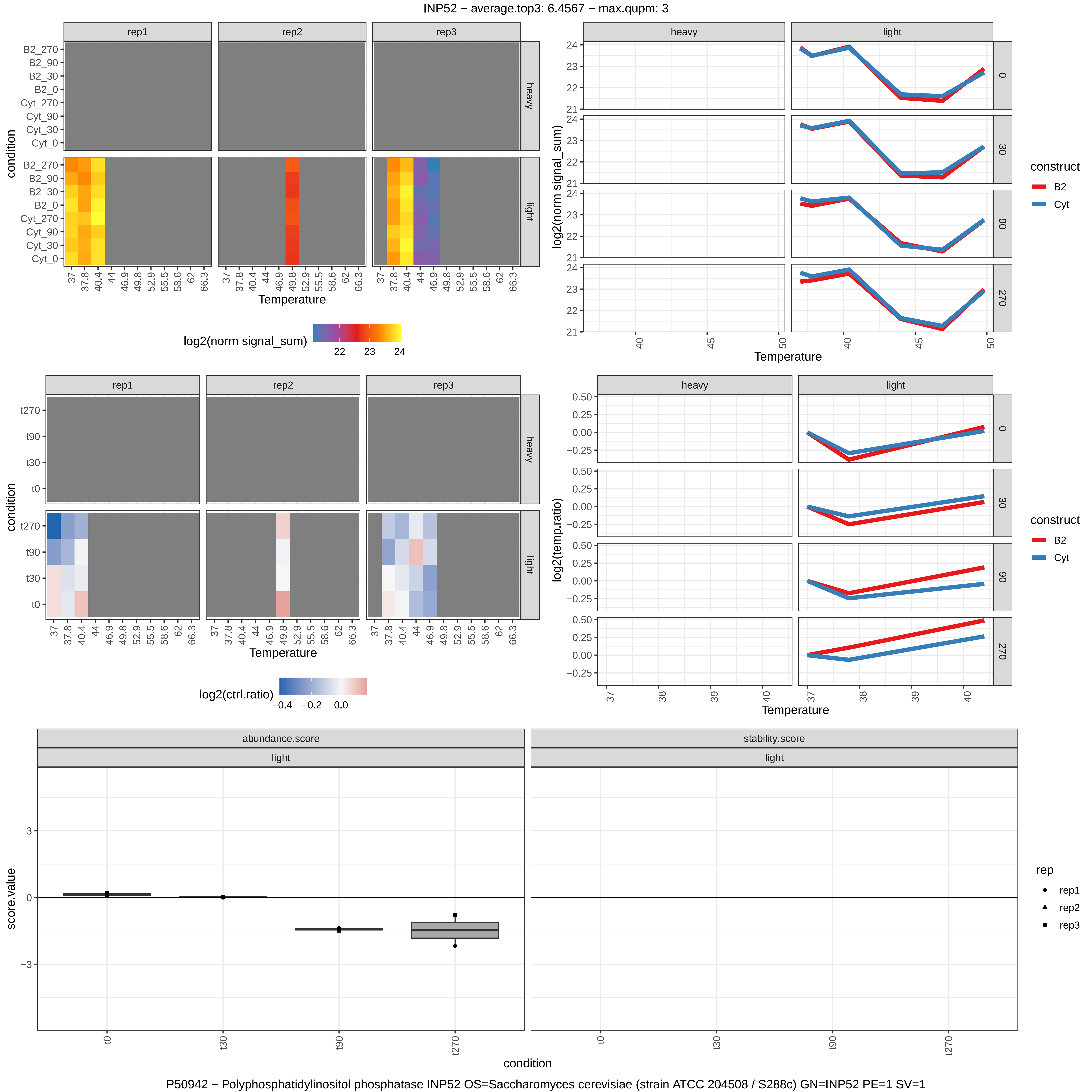
INP52 − average.top3: 6.4567 − max.qupm: 3
rep1
rep2
rep3
heavy
light
condition
Temperature
log2(norm signal_sum)
construct
B2
Cyt
log2(ctrl.ratio)
log2(temp.ratio)
abundance.score
stability.score
score.value
rep
rep1
rep2
rep3
P50942 − Polyphosphatidylinositol phosphatase INP52 OS=Saccharomyces cerevisiae (strain ATCC 204508 / S288c) GN=INP52 PE=1 SV=1

INP53 − average.top3: 6.83124128205128 − max.qupm: 12

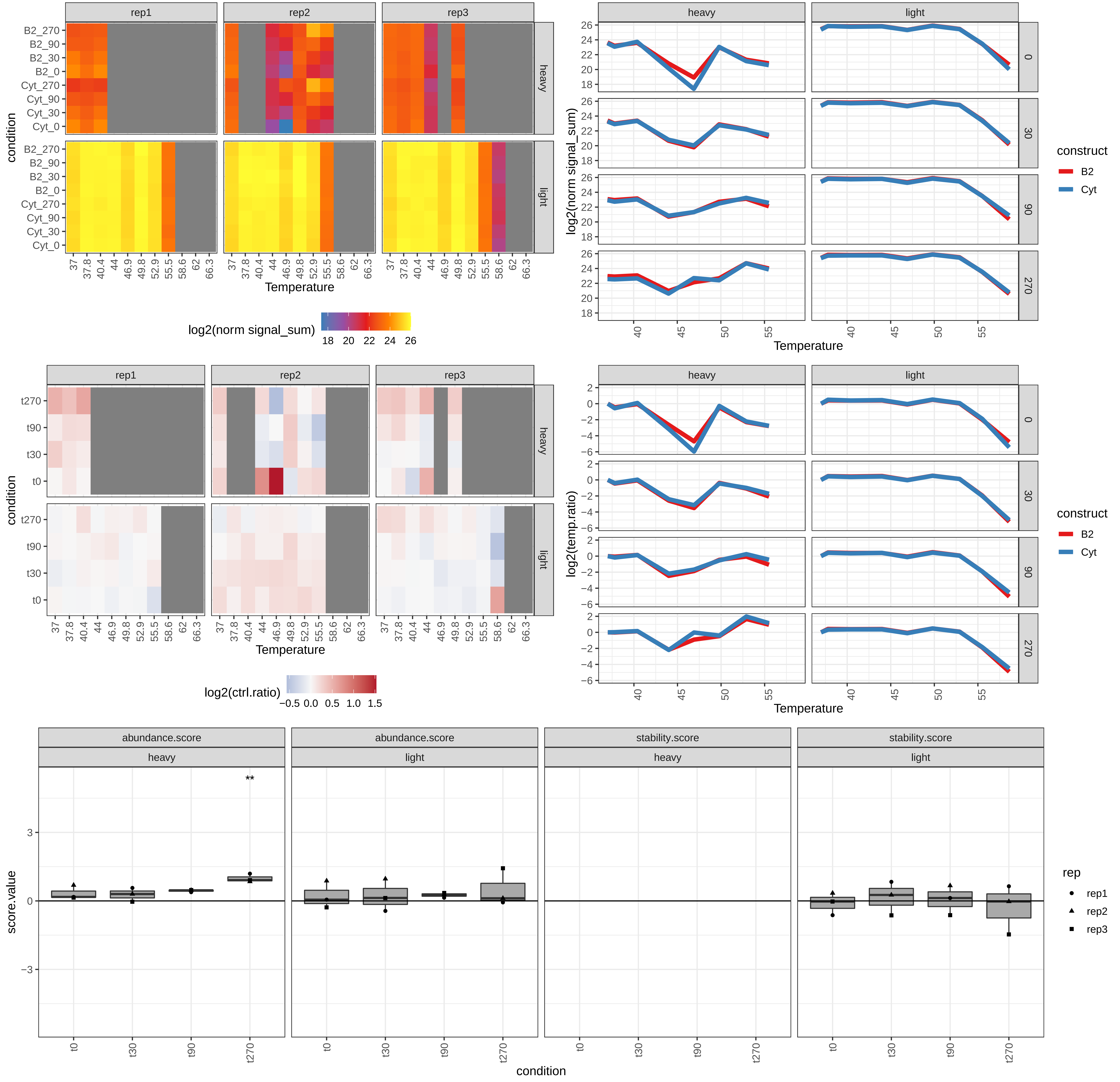

Q12271 − Polyphosphatidylinositol phosphatase INP53 OS=Saccharomyces cerevisiae (strain ATCC 204508 / S288c) GN=INP53 PE=1 SV=1

INP54 − average.top3: 6.73078923076923 − max.qupm: 5

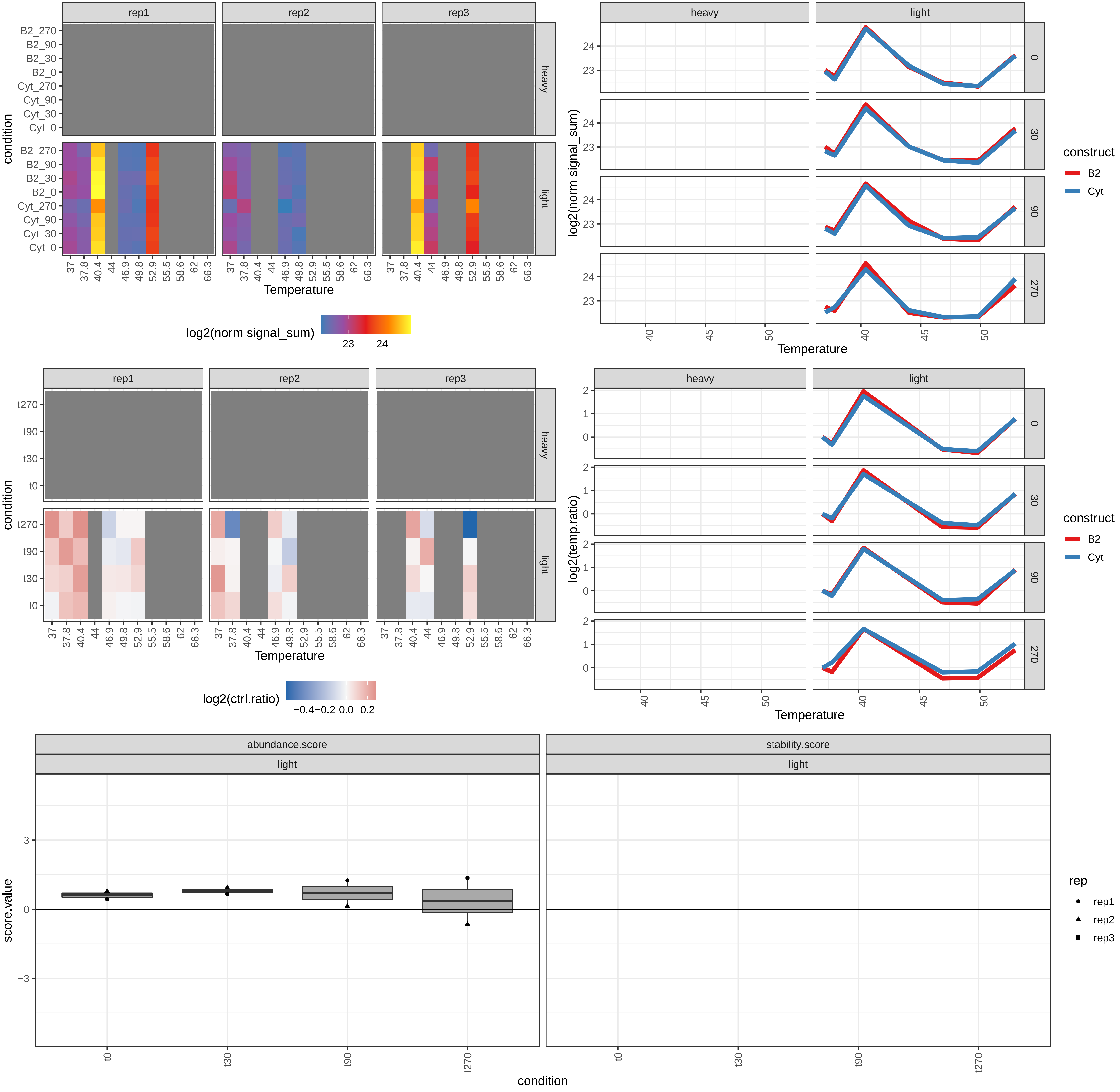

Q08227 − Phosphatidylinositol 4,5−bisphosphate 5−phosphatase INP54 OS=Saccharomyces cerevisiae (strain ATCC 204508 / S288c) GN=INP54 PE=1 SV=1

IOC2 − average.top3: 6.50935384615385 − max.qupm: 5

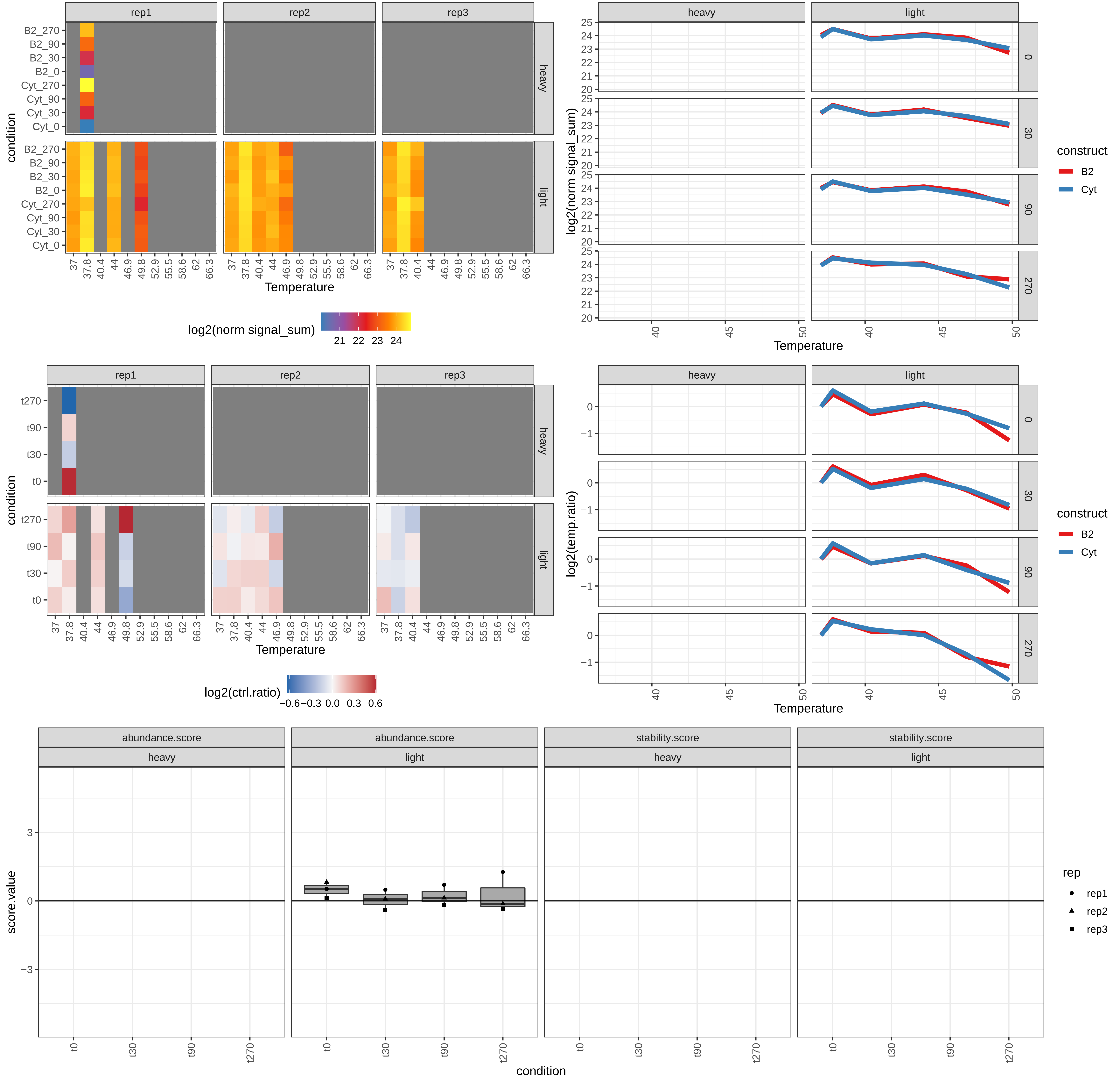

Q12072 − ISWI one complex protein 2 OS=Saccharomyces cerevisiae (strain ATCC 204508 / S288c) GN=IOC2 PE=1 SV=1

IOC3 − average.top3: 6.37226846153846 − max.qupm: 5

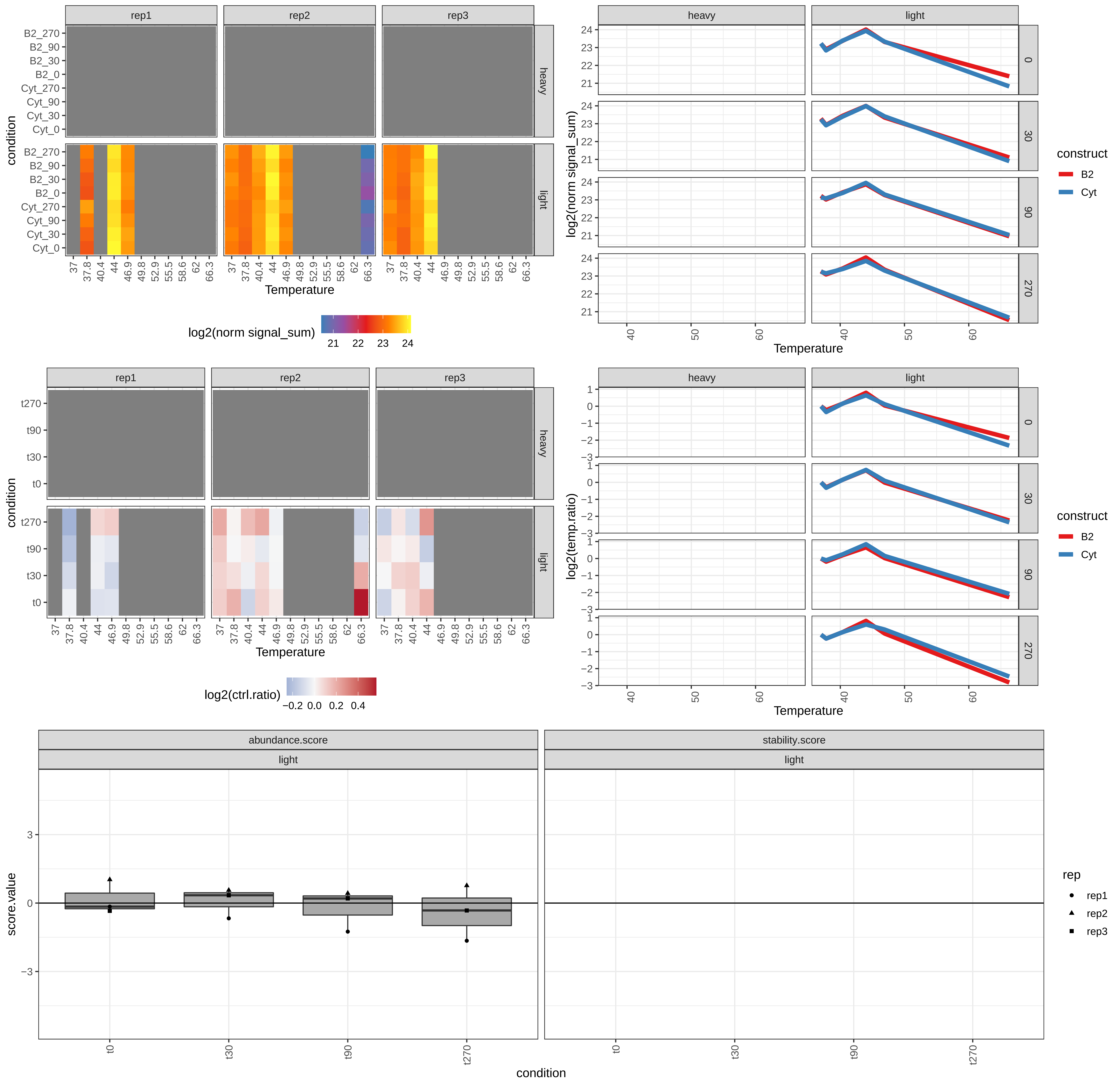

P43596 − ISWI one complex protein 3 OS=Saccharomyces cerevisiae (strain ATCC 204508 / S288c) GN=IOC3 PE=1 SV=1

IPI3 − average.top3: 6.80895588235294 − max.qupm: 5

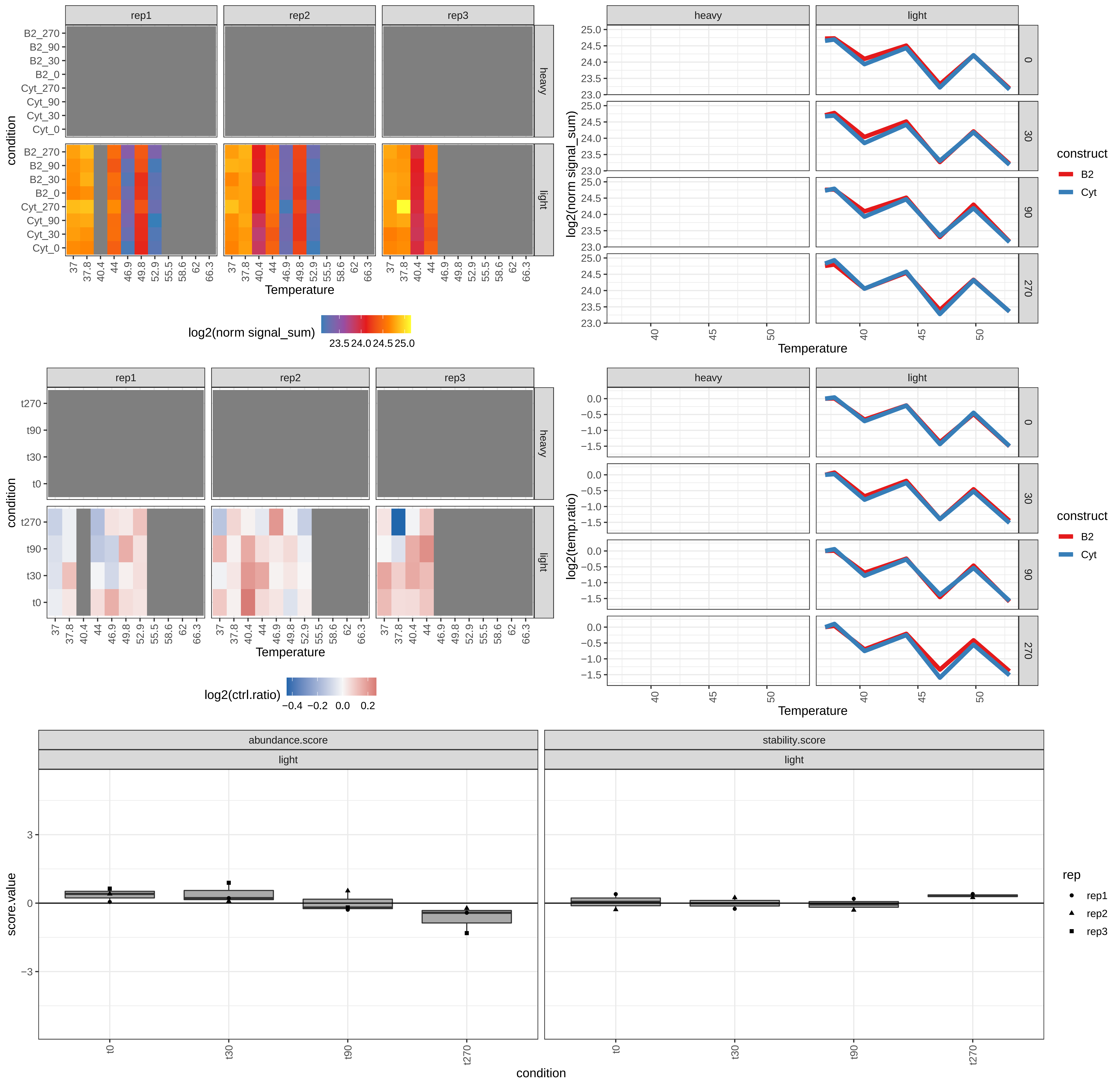

P53877 − Pre−rRNA−processing protein IPI3 OS=Saccharomyces cerevisiae (strain ATCC 204508 / S288c) GN=IPI3 PE=1 SV=1

IPP1 − average.top3: 7.95111457627119 − max.qupm: 17

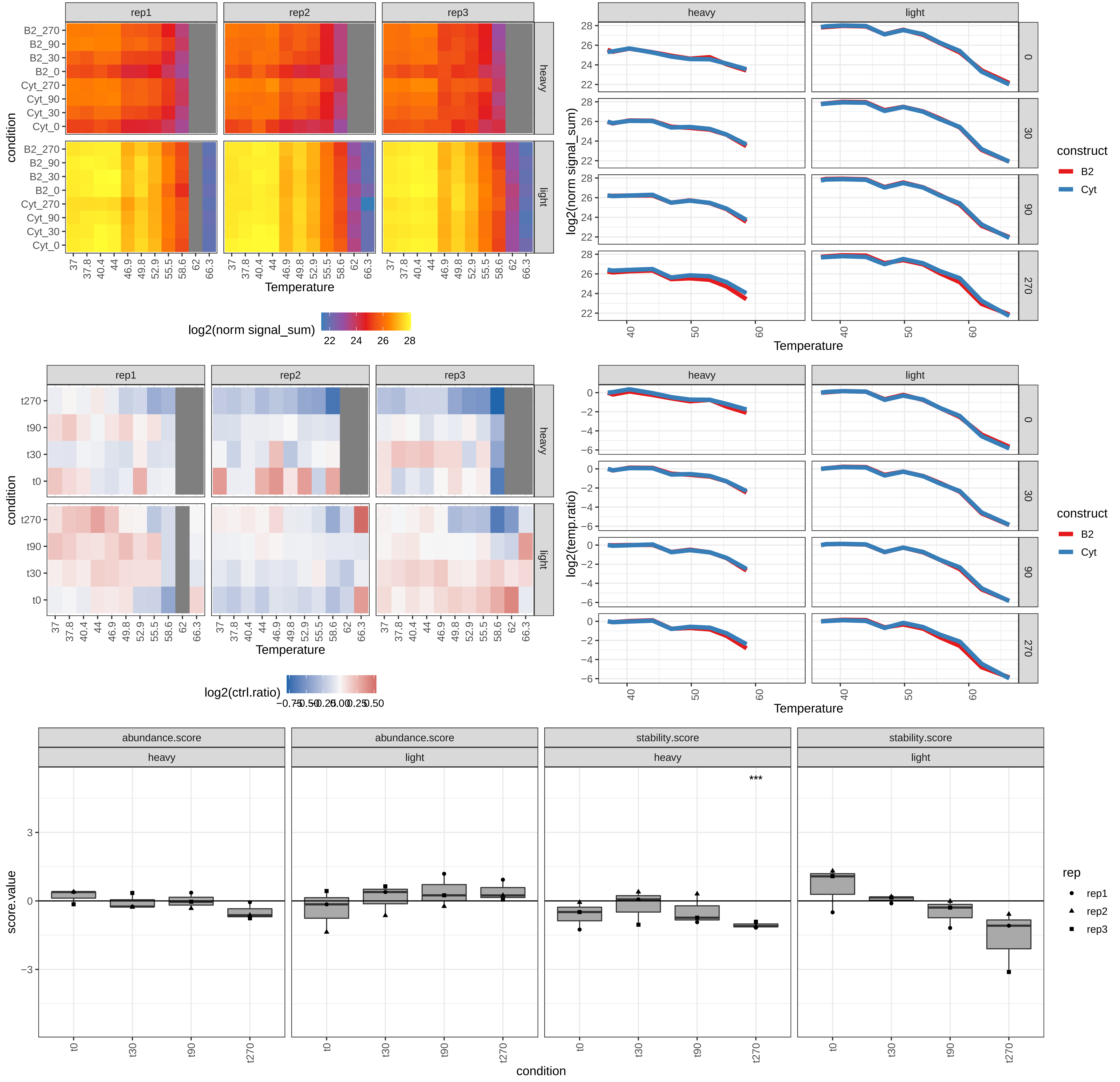

P00817 − Inorganic pyrophosphatase OS=Saccharomyces cerevisiae (strain ATCC 204508 / S288c) GN=IPP1 PE=1 SV=4

IRA1 − average.top3: 6.71990789473684 − max.qupm: 14

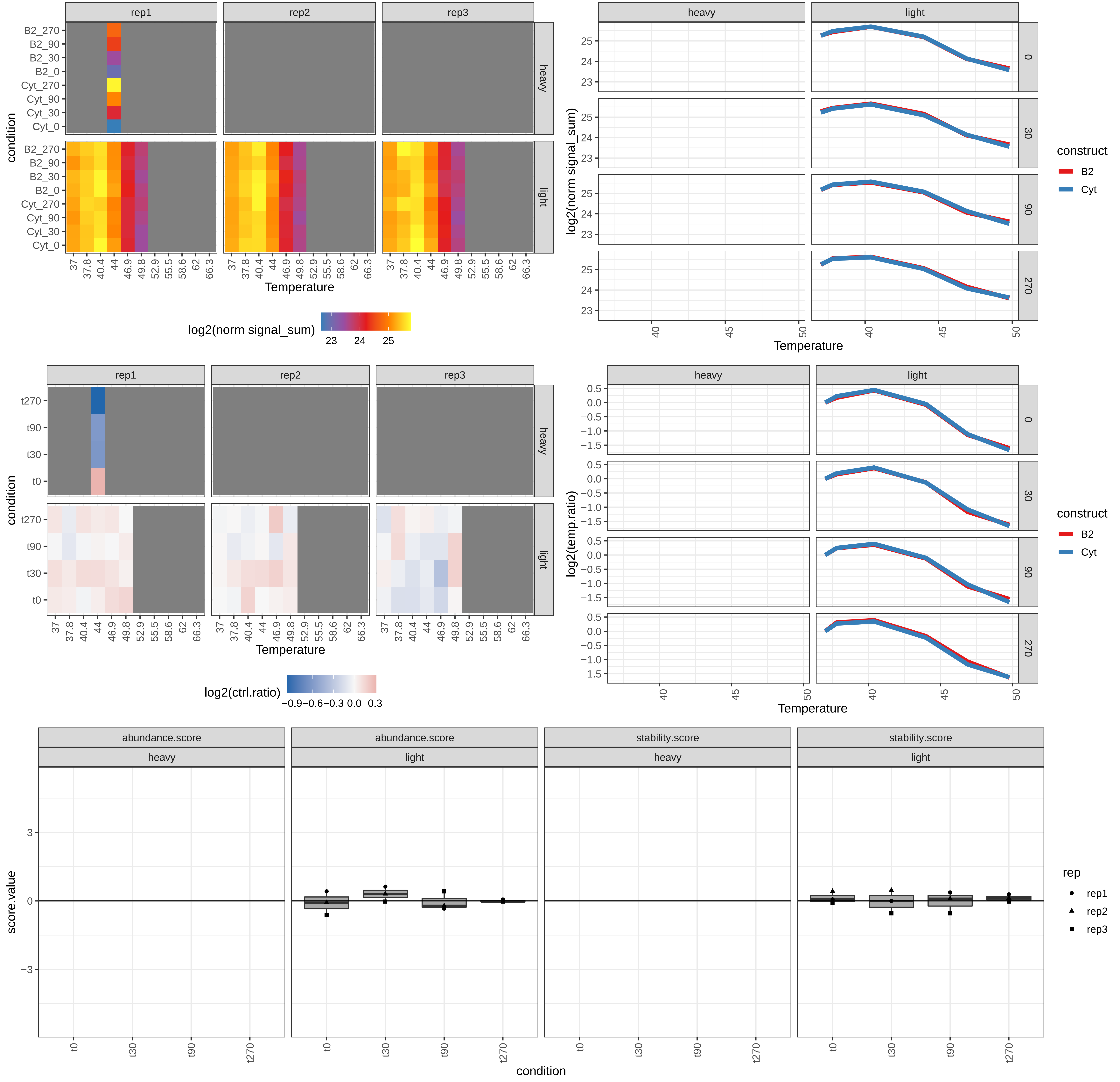

P18963 − Inhibitory regulator protein IRA1 OS=Saccharomyces cerevisiae (strain ATCC 204508 / S288c) GN=IRA1 PE=1 SV=2

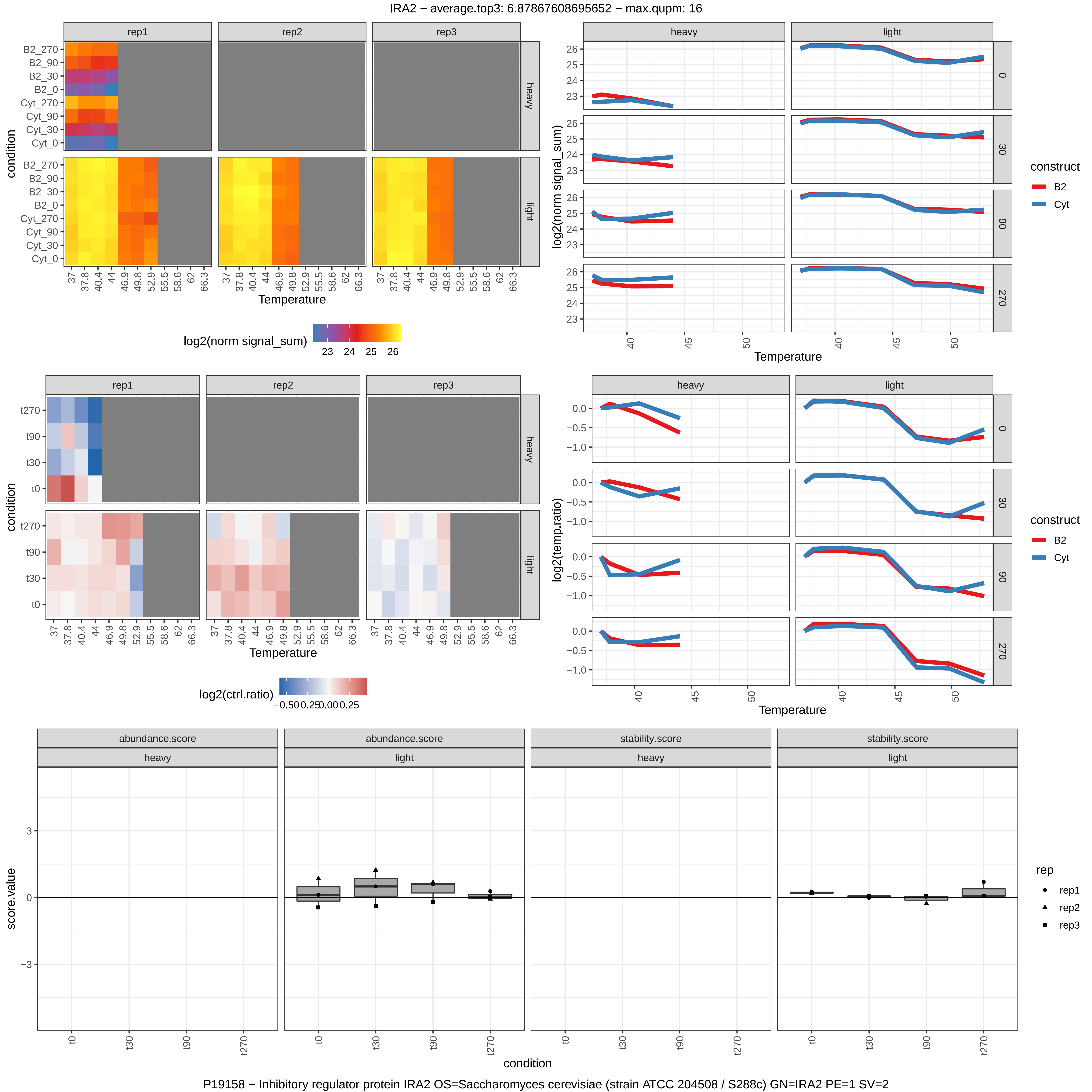

IRA2 − average.top3: 6.87867608695652 − max.qupm: 16
rep1
rep2
rep3
heavy
light
condition
Temperature
log2(norm signal_sum)
log2(temp.ratio)
log2(ctrl.ratio)
construct
B2
Cyt
abundance.score
stability.score
score.value
rep
rep1
rep2
rep3
P19158 − Inhibitory regulator protein IRA2 OS=Saccharomyces cerevisiae (strain ATCC 204508 / S288c) GN=IRA2 PE=1 SV=2

IRC15 − average.top3: 6.52299923076923 − max.qupm: 6

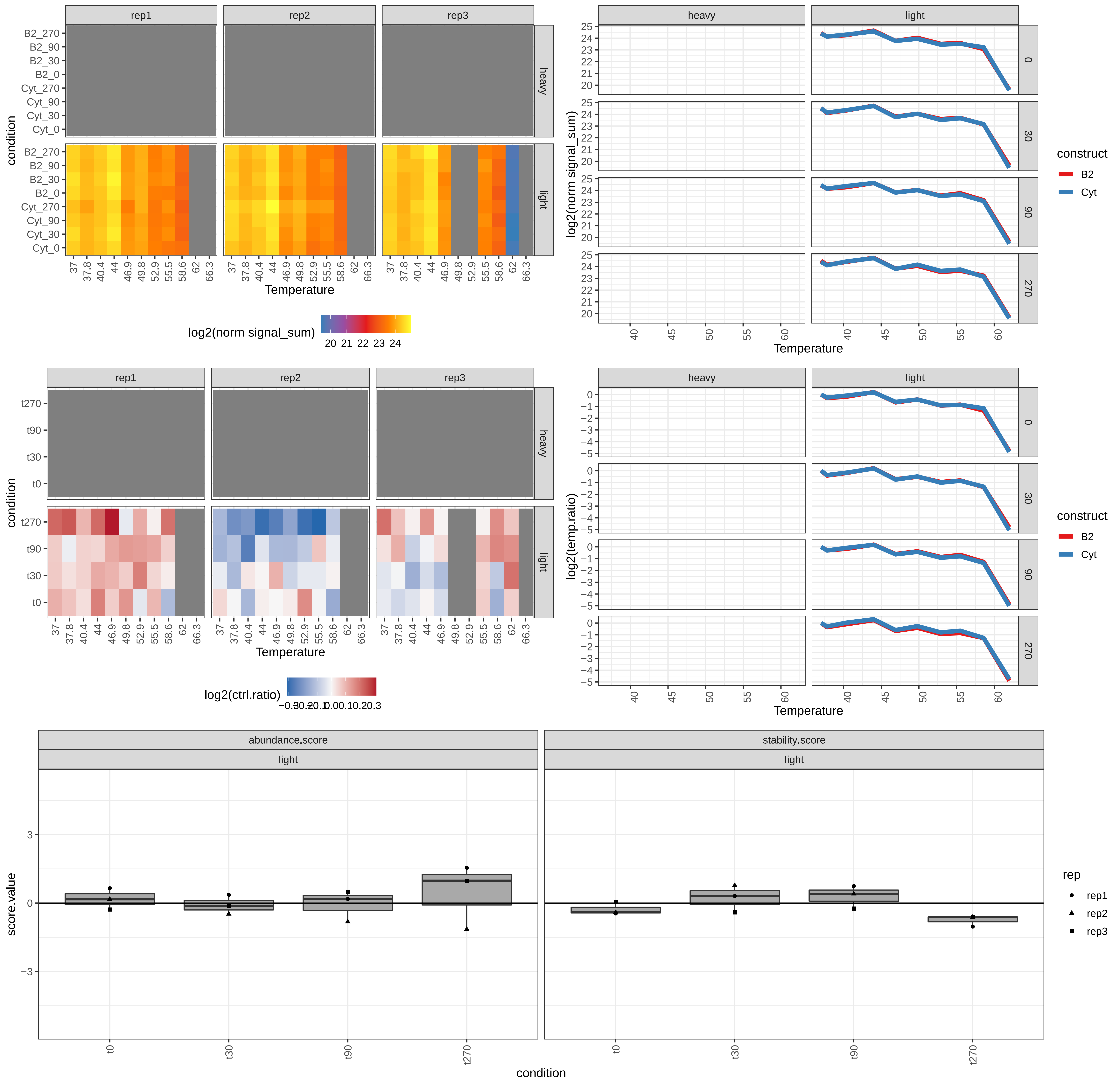

Q02733 − Increased recombination centers protein 15 OS=Saccharomyces cerevisiae (strain ATCC 204508 / S288c) GN=IRC15 PE=1 SV=1

IRC19 − average.top3: 6.26698333333333 − max.qupm: 5

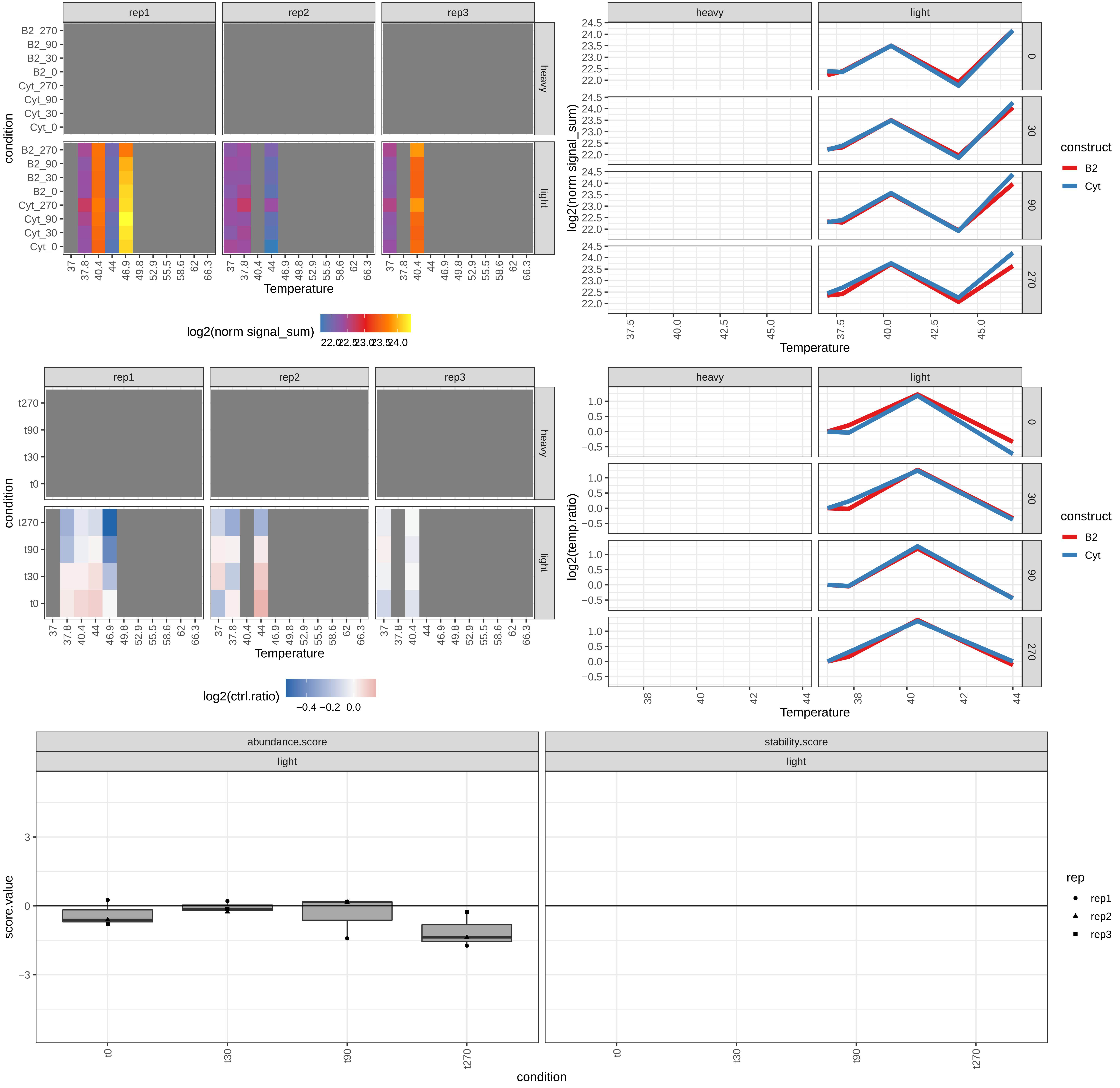

Q07843 − Increased recombination centers protein 19 OS=Saccharomyces cerevisiae (strain ATCC 204508 / S288c) GN=IRC19 PE=2 SV=1

IRC21 − average.top3: 6.78077212121212 − max.qupm: 6

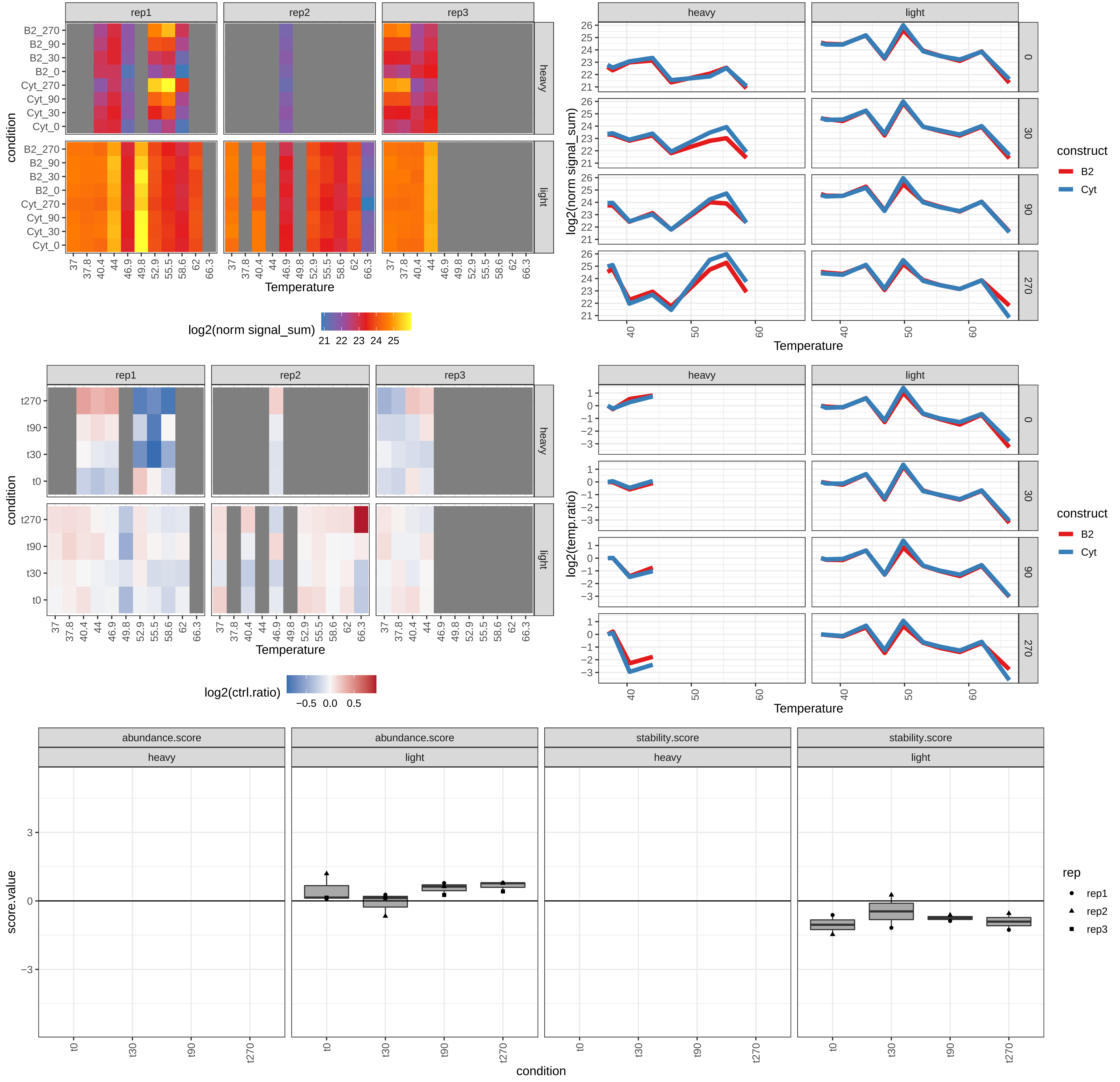

Q04772 − Increased recombination centers protein 21 OS=Saccharomyces cerevisiae (strain ATCC 204508 / S288c) GN=IRC21 PE=1 SV=1

IRC24 − average.top3: 7.17813333333333 − max.qupm: 11

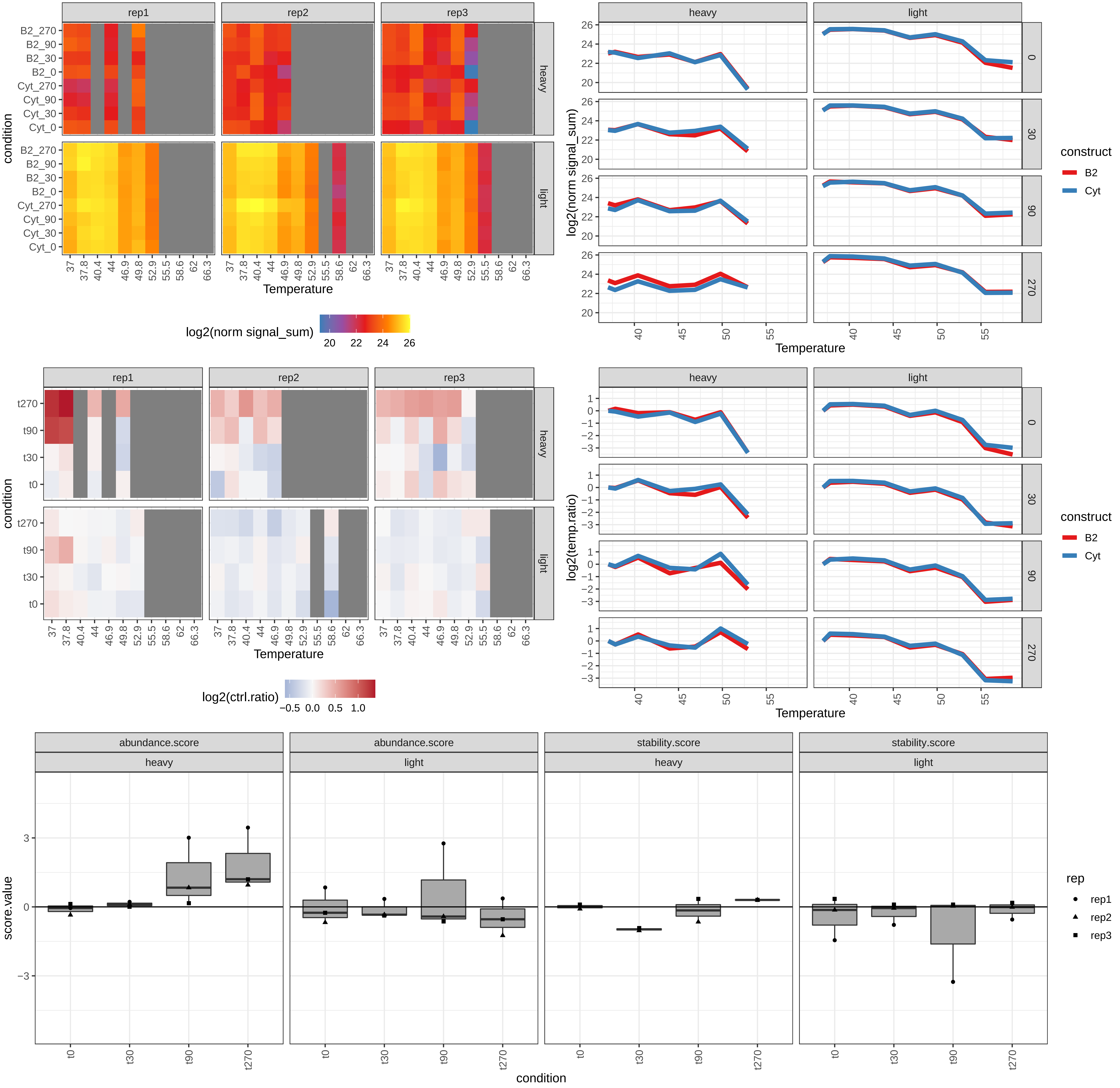

P40580 − Benzil reductase ((S)−benzoin forming) IRC24 OS=Saccharomyces cerevisiae (strain ATCC 204508 / S288c) GN=IRC24 PE=1 SV=1

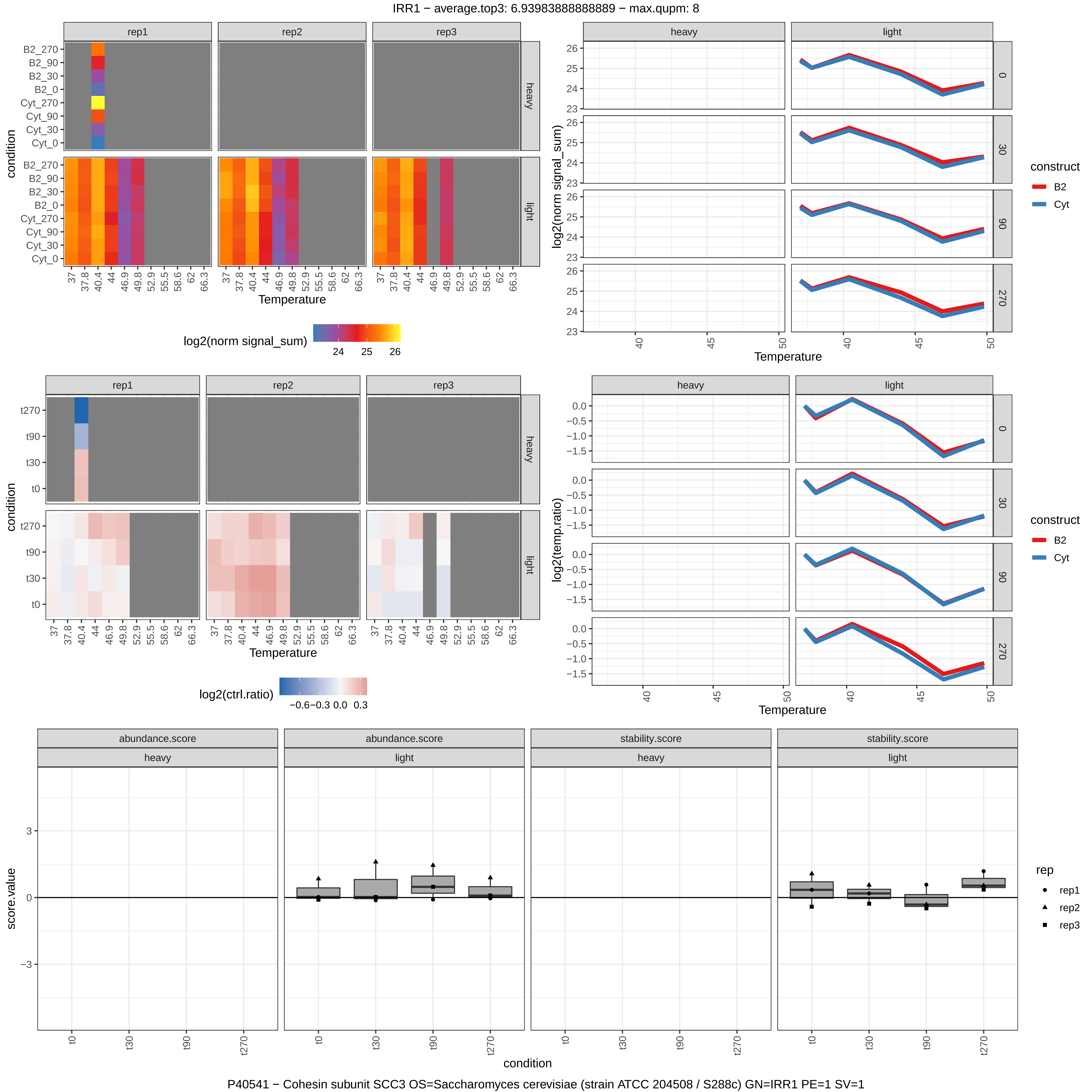
IRR1 − average.top3: 6.93983888888889 − max.qupm: 8
rep1
rep2
rep3
heavy
light
condition
Temperature
log2(norm signal_sum)
construct
B2
Cyt
log2(ctrl.ratio)
log2(temp.ratio)
abundance.score
stability.score
score.value
rep
rep1
rep2
rep3
P40541 − Cohesin subunit SCC3 OS=Saccharomyces cerevisiae (strain ATCC 204508 / S288c) GN=IRR1 PE=1 SV=1

ISD11 − average.top3: 7.24495536585366 − max.qupm: 8

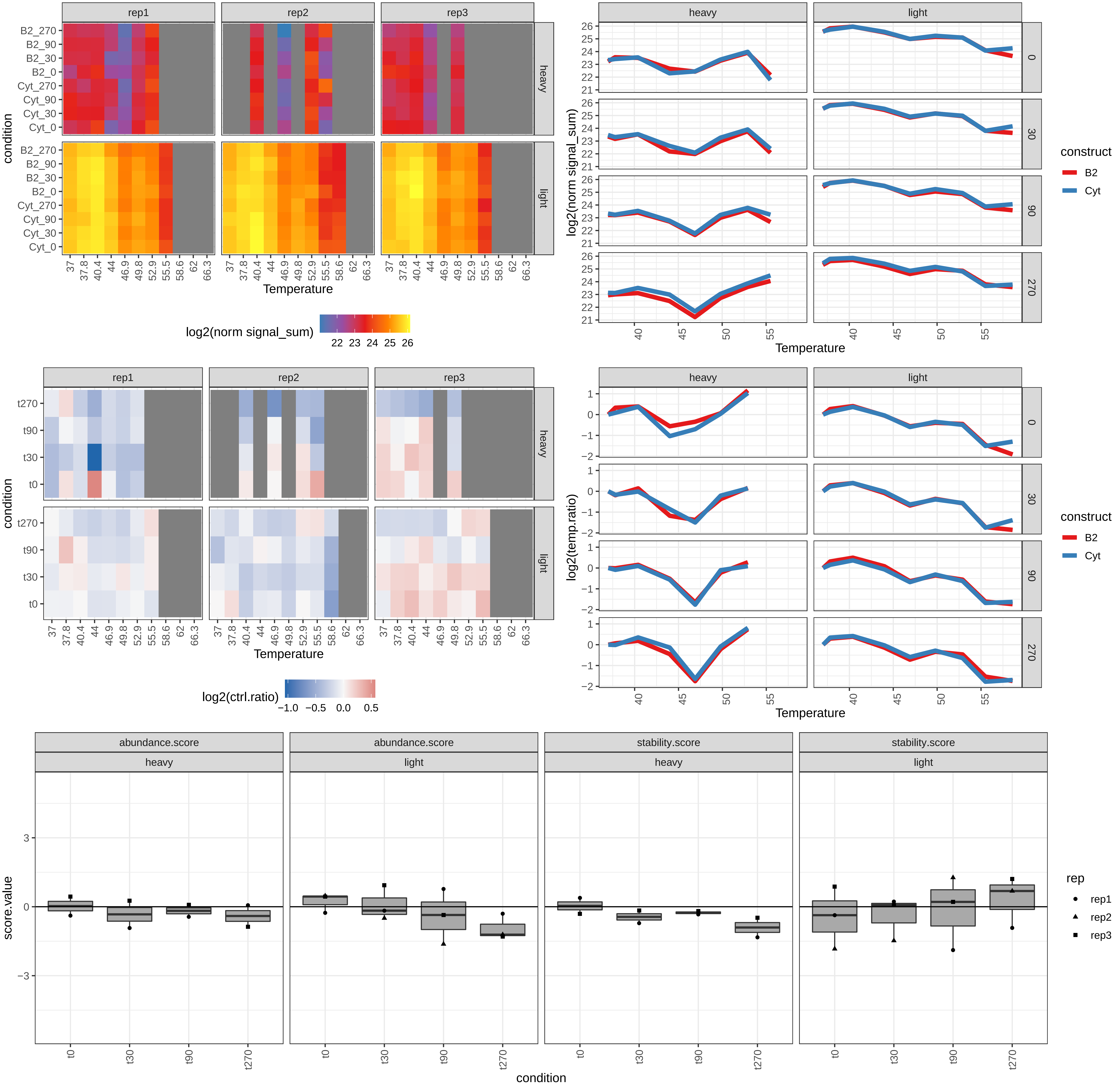

Q6Q560 − Protein ISD11 OS=Saccharomyces cerevisiae (strain ATCC 204508 / S288c) GN=ISD11 PE=1 SV=1

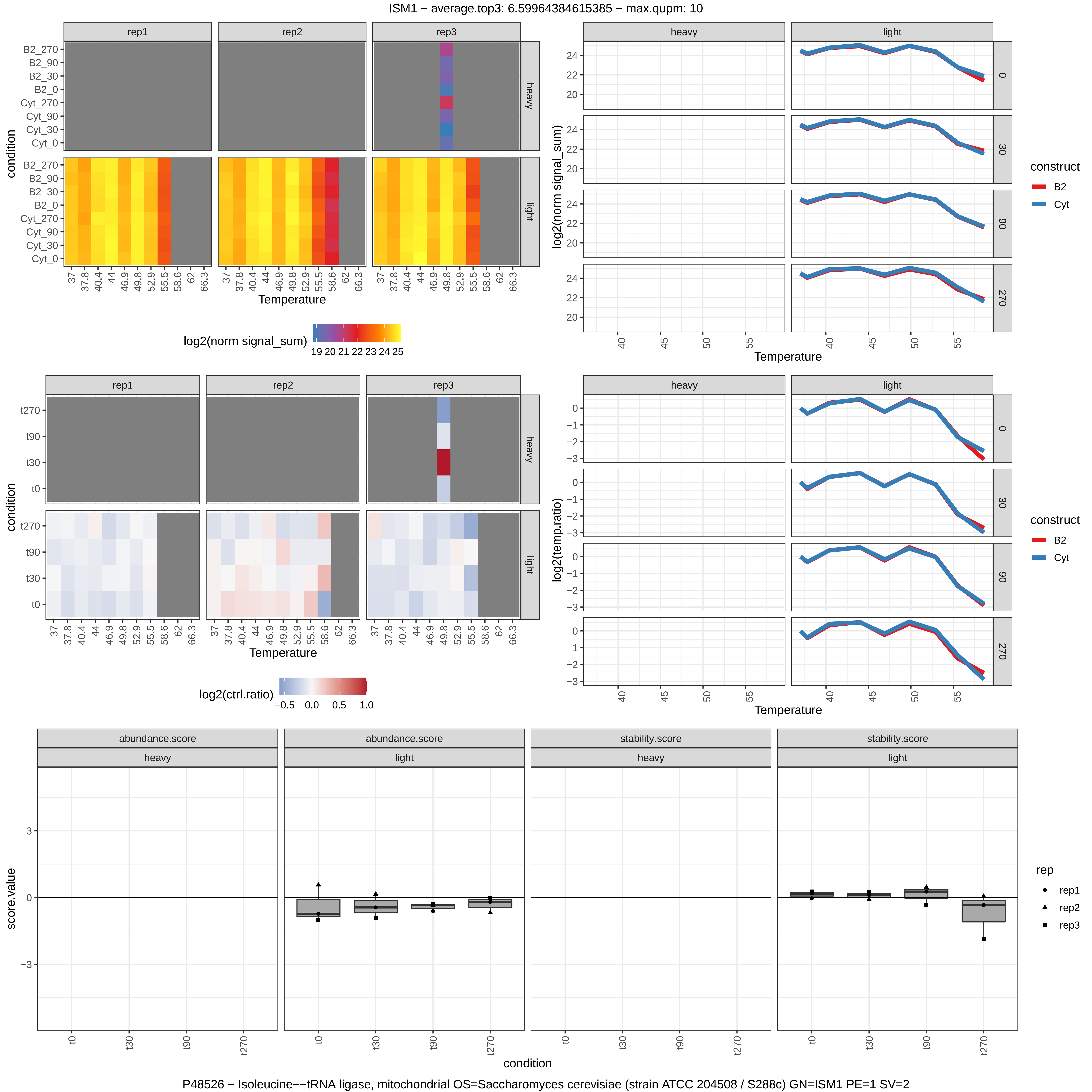

ISM1 − average.top3: 6.59964384615385 − max.qupm: 10
rep1
rep2
rep3
heavy
light
condition
Temperature
log2(norm signal_sum)
construct
B2
Cyt
log2(ctrl.ratio)
log2(temp.ratio)
abundance.score
stability.score
score.value
rep
rep1
rep2
rep3
P48526 − Isoleucine−−tRNA ligase, mitochondrial OS=Saccharomyces cerevisiae (strain ATCC 204508 / S288c) GN=ISM1 PE=1 SV=2

ISN1 − average.top3: 6.92386914893617 − max.qupm: 11

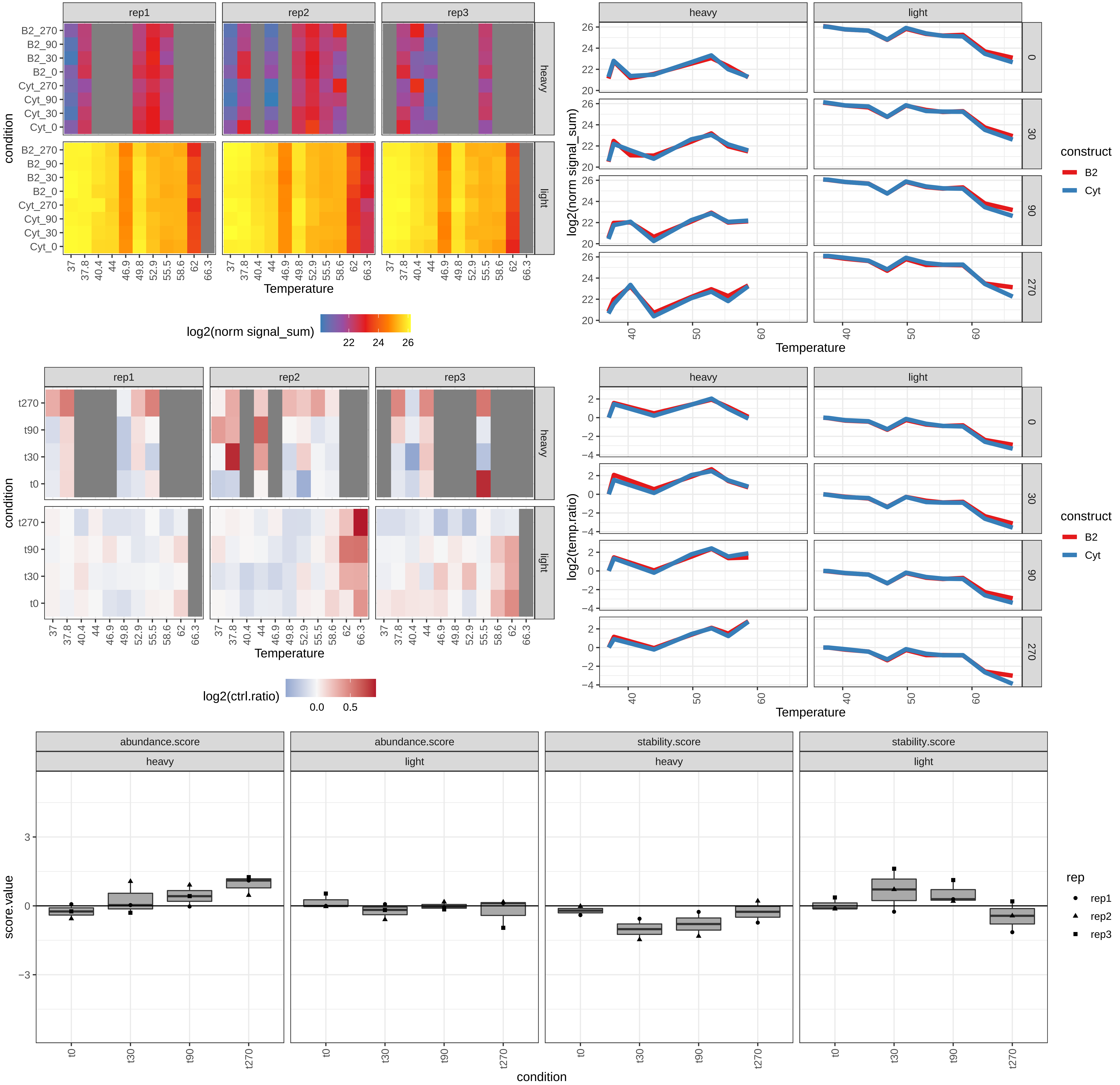

Q99312 − IMP−specific 5'−nucleotidase 1 OS=Saccharomyces cerevisiae (strain ATCC 204508 / S288c) GN=ISN1 PE=1 SV=1

IST1 − average.top3: 6.76564387096774 − max.qupm: 6

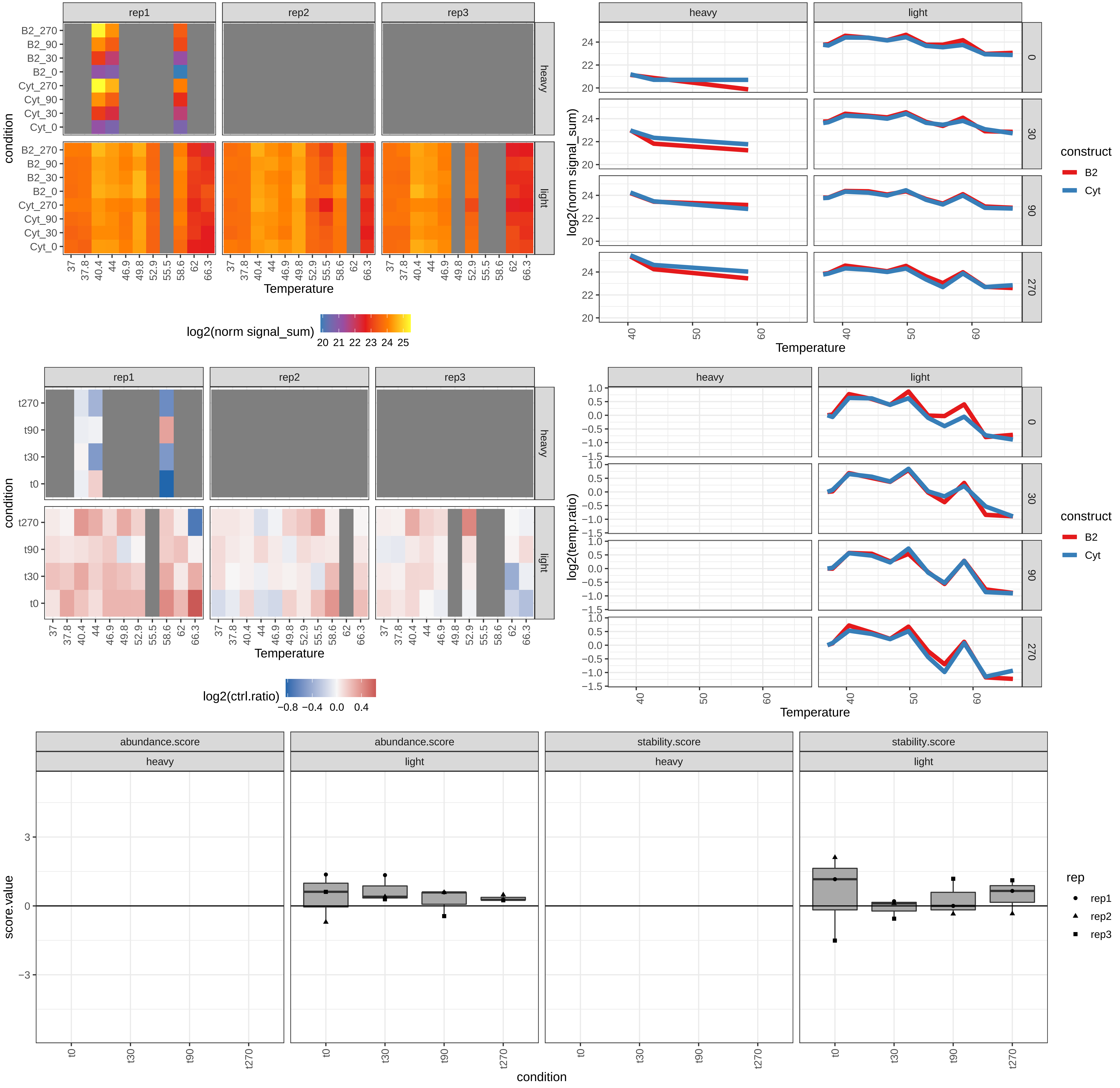

P53843 − Vacuolar protein sorting−associated protein IST1 OS=Saccharomyces cerevisiae (strain ATCC 204508 / S288c) GN=IST1 PE=1 SV=1

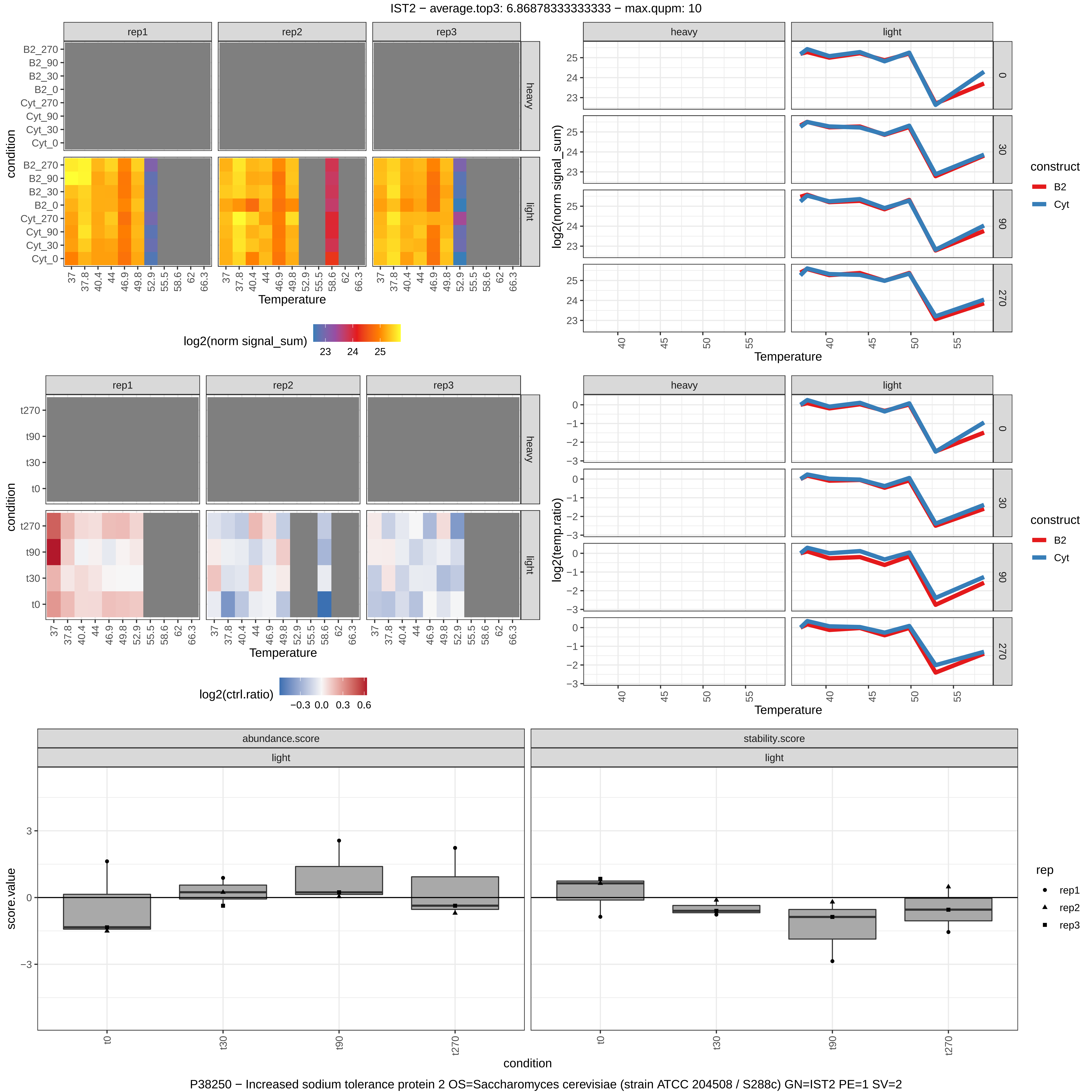
IST2 − average.top3: 6.86878333333333 − max.qupm: 10
rep1
rep2
rep3
heavy
light
condition
B2_270
B2_90
B2_30
B2_0
Cyt_270
Cyt_90
Cyt_30
Cyt_0
Temperature
37 37.8 40.4 44 46.9 49.8 52.9 55.5 58.6 62 66.3
log2(norm signal_sum)
23 24 25
log2(norm signal_sum)
0
30
90
270
construct
B2
Cyt
t270
t90
t30
t0
log2(ctrl.ratio)
−0.3 0.0 0.3 0.6
log2(temp.ratio)
abundance.score
stability.score
score.value
rep
rep1
rep2
rep3
condition
P38250 − Increased sodium tolerance protein 2 OS=Saccharomyces cerevisiae (strain ATCC 204508 / S288c) GN=IST2 PE=1 SV=2

ISU1 − average.top3: 7.01141 − max.qupm: 3

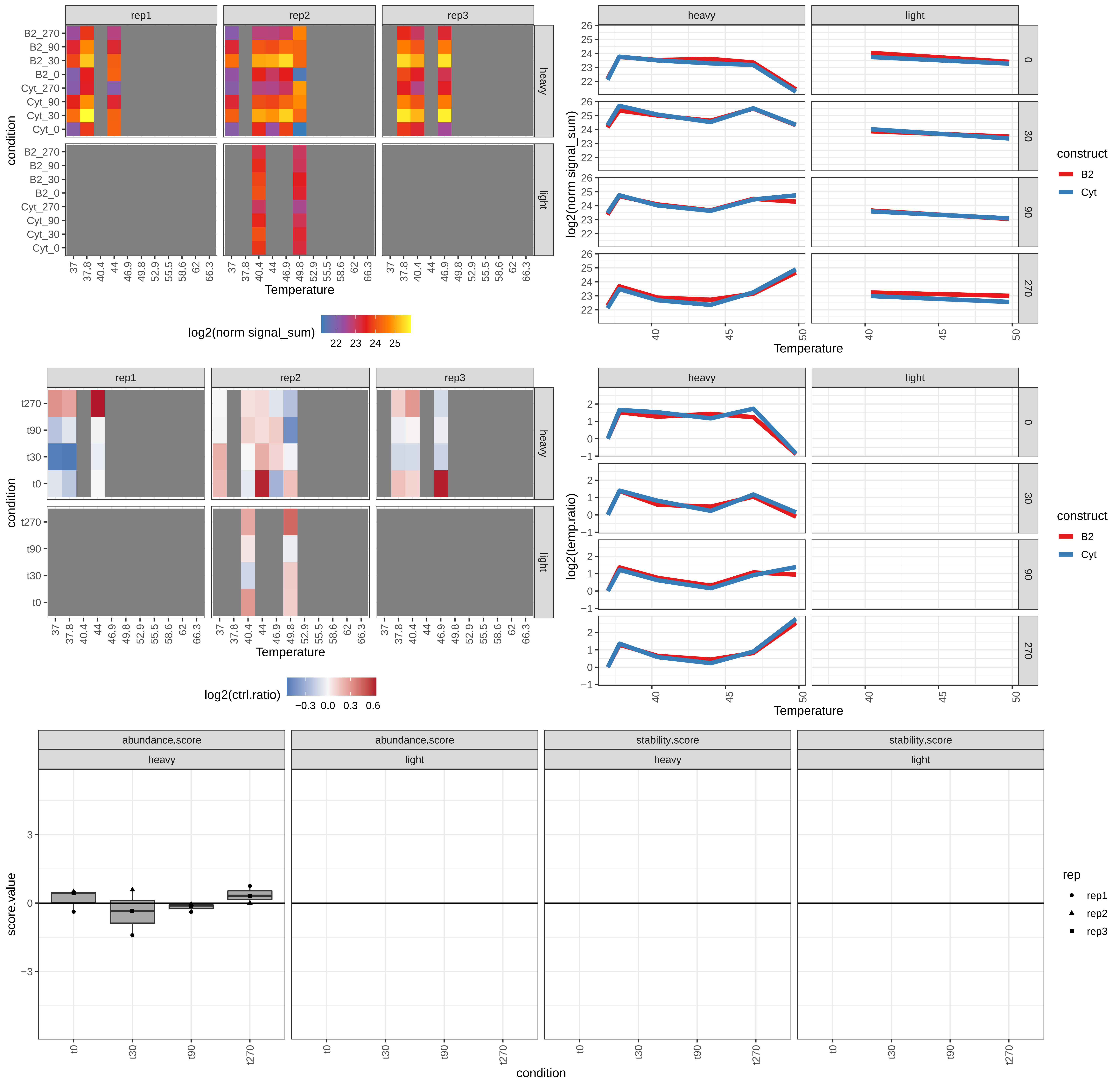

Q03020 − Iron sulfur cluster assembly protein 1, mitochondrial OS=Saccharomyces cerevisiae (strain ATCC 204508 / S288c) GN=ISU1 PE=1 SV=1

ISW1 − average.top3: 6.98027588235294 − max.qupm: 28

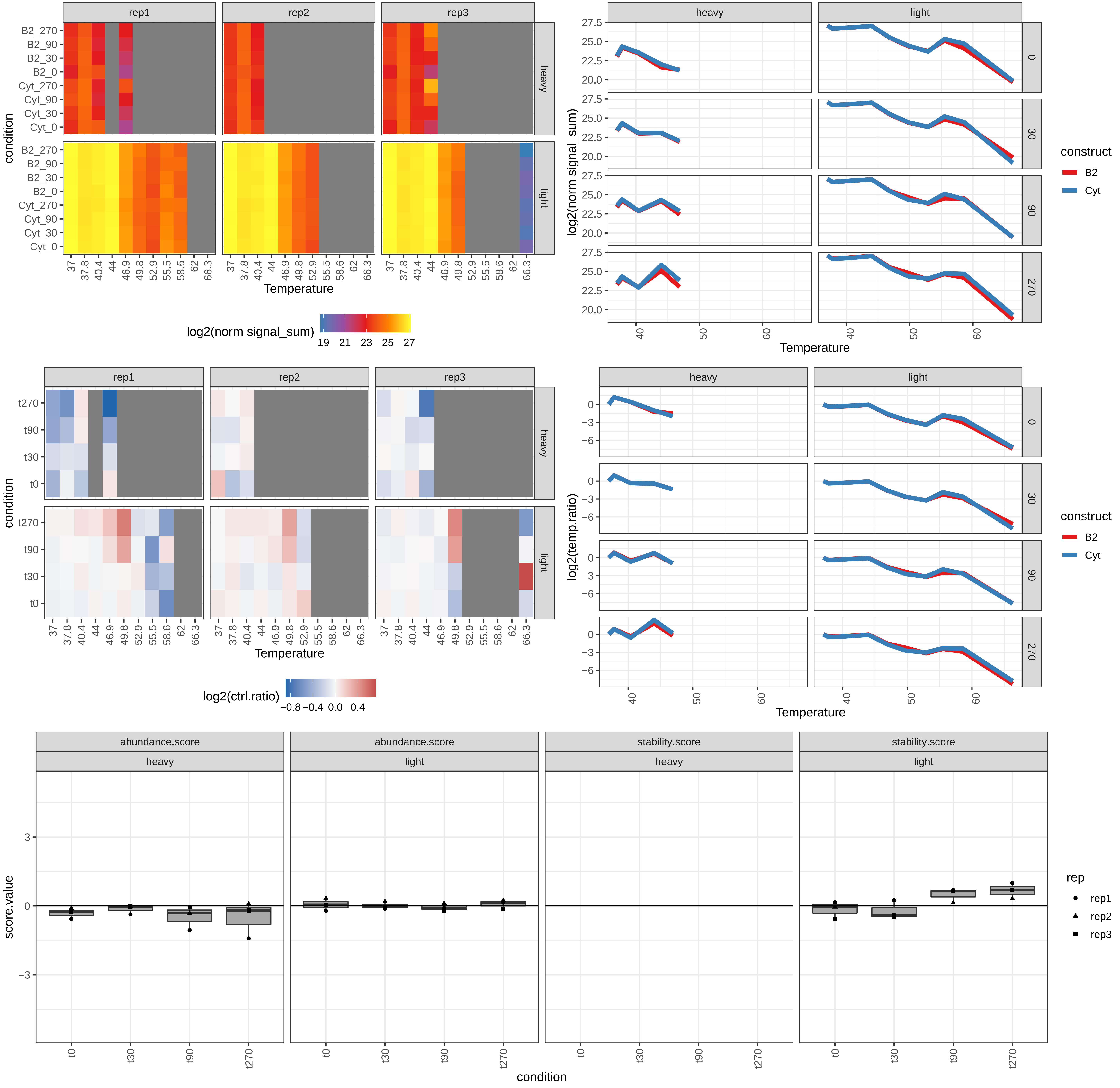

P38144 − ISWI chromatin−remodeling complex ATPase ISW1 OS=Saccharomyces cerevisiae (strain ATCC 204508 / S288c) GN=ISW1 PE=1 SV=2

ISW2 − average.top3: 6.57460923076923 − max.qupm: 6

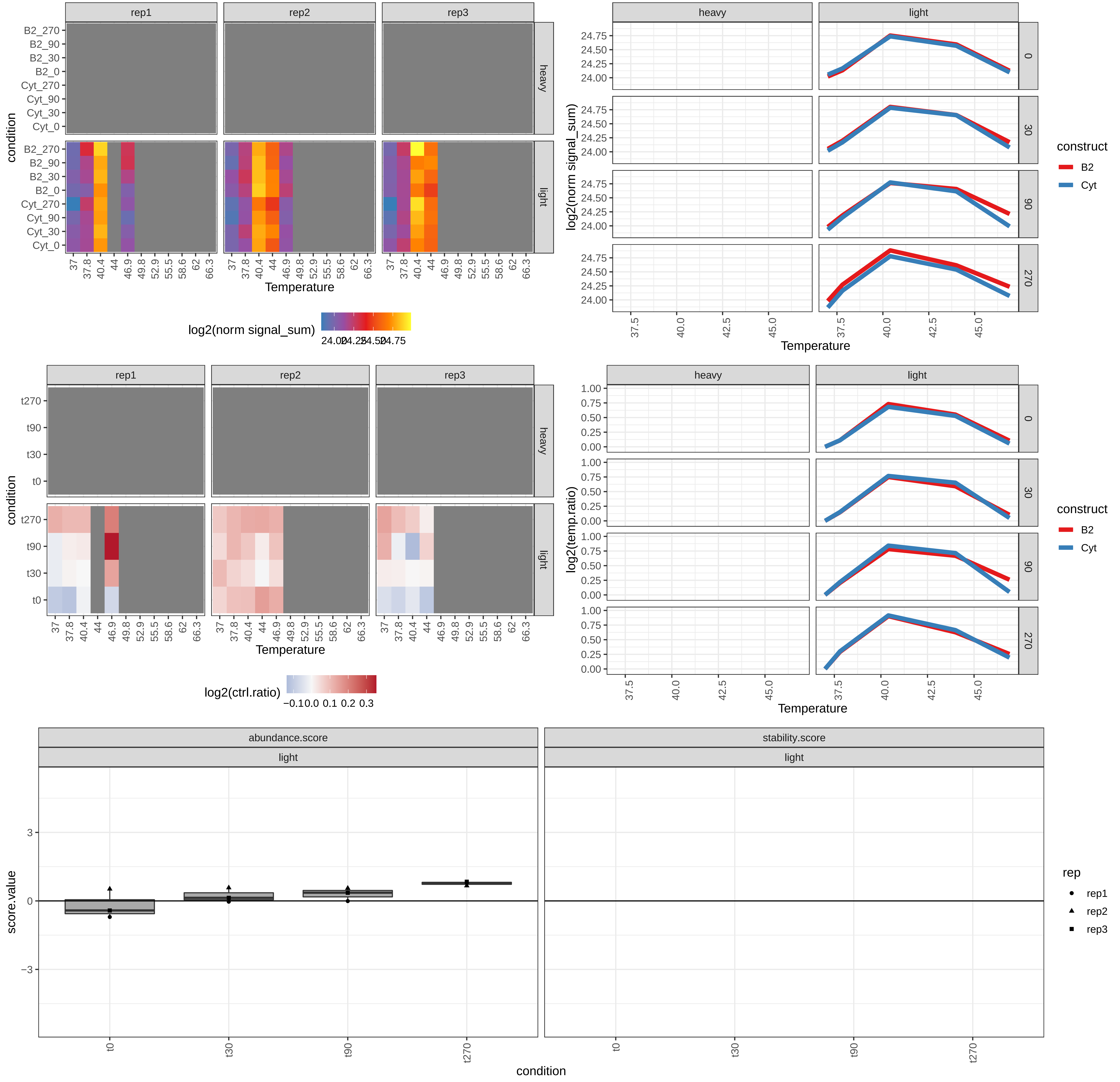

Q08773 − ISWI chromatin−remodeling complex ATPase ISW2 OS=Saccharomyces cerevisiae (strain ATCC 204508 / S288c) GN=ISW2 PE=1 SV=1

ITC1 − average.top3: 6.52640733333333 − max.qupm: 8

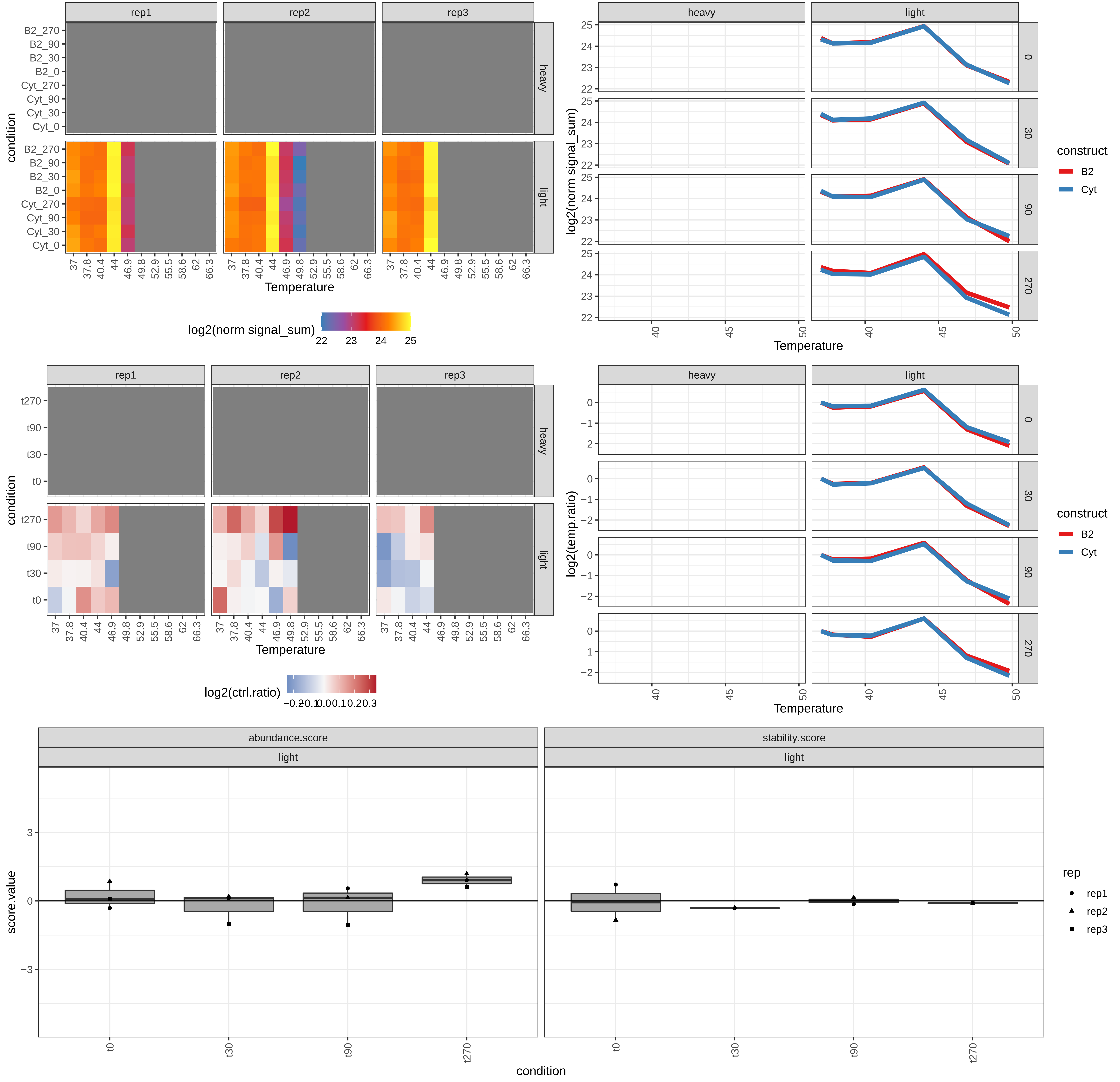

P53125 − Imitation switch two complex protein 1 OS=Saccharomyces cerevisiae (strain ATCC 204508 / S288c) GN=ITC1 PE=1 SV=1

ITR1 − average.top3: 7.18763206896552 − max.qupm: 6

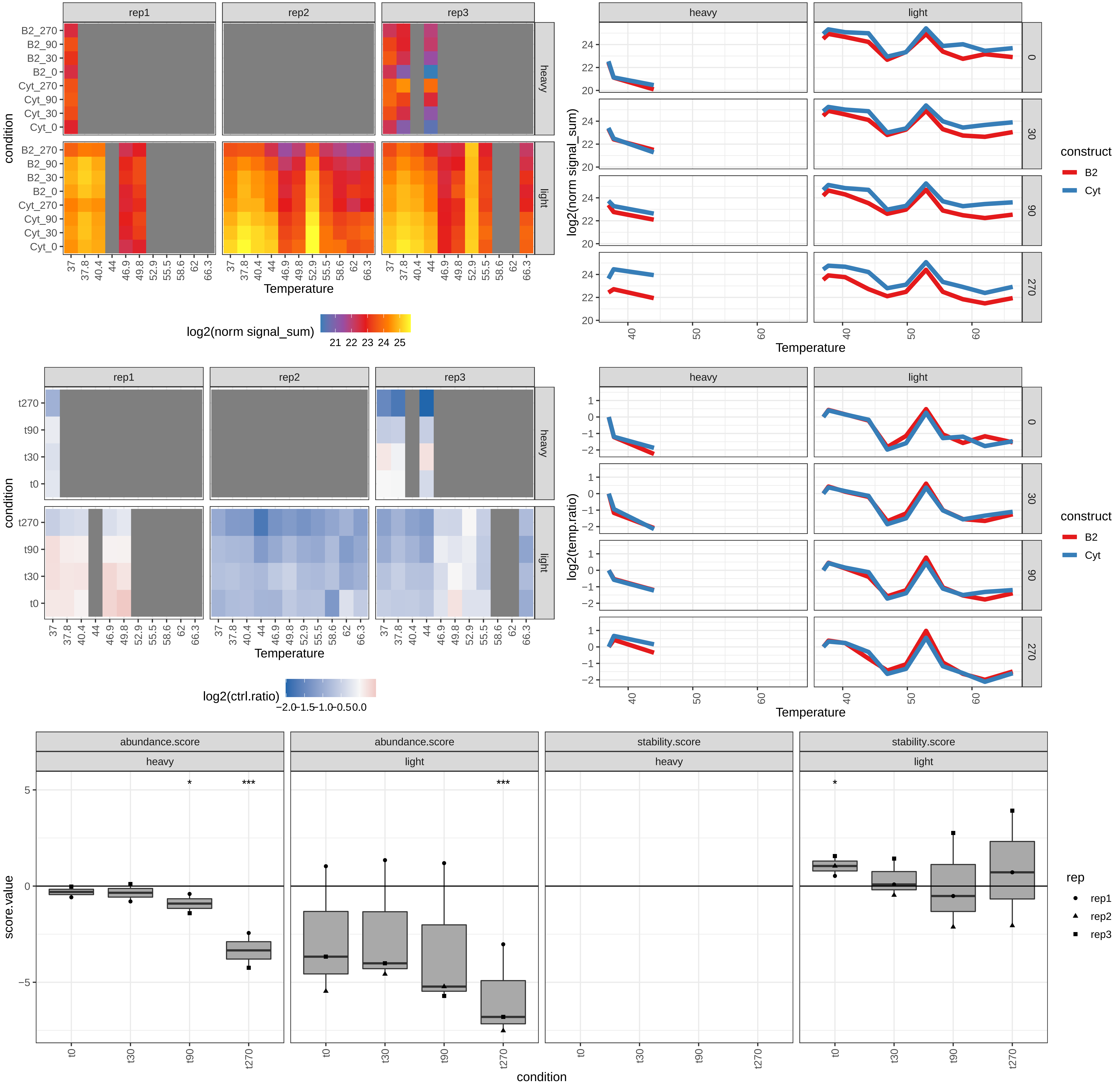

P30605 − Myo−inositol transporter 1 OS=Saccharomyces cerevisiae (strain ATCC 204508 / S288c) GN=ITR1 PE=1 SV=2

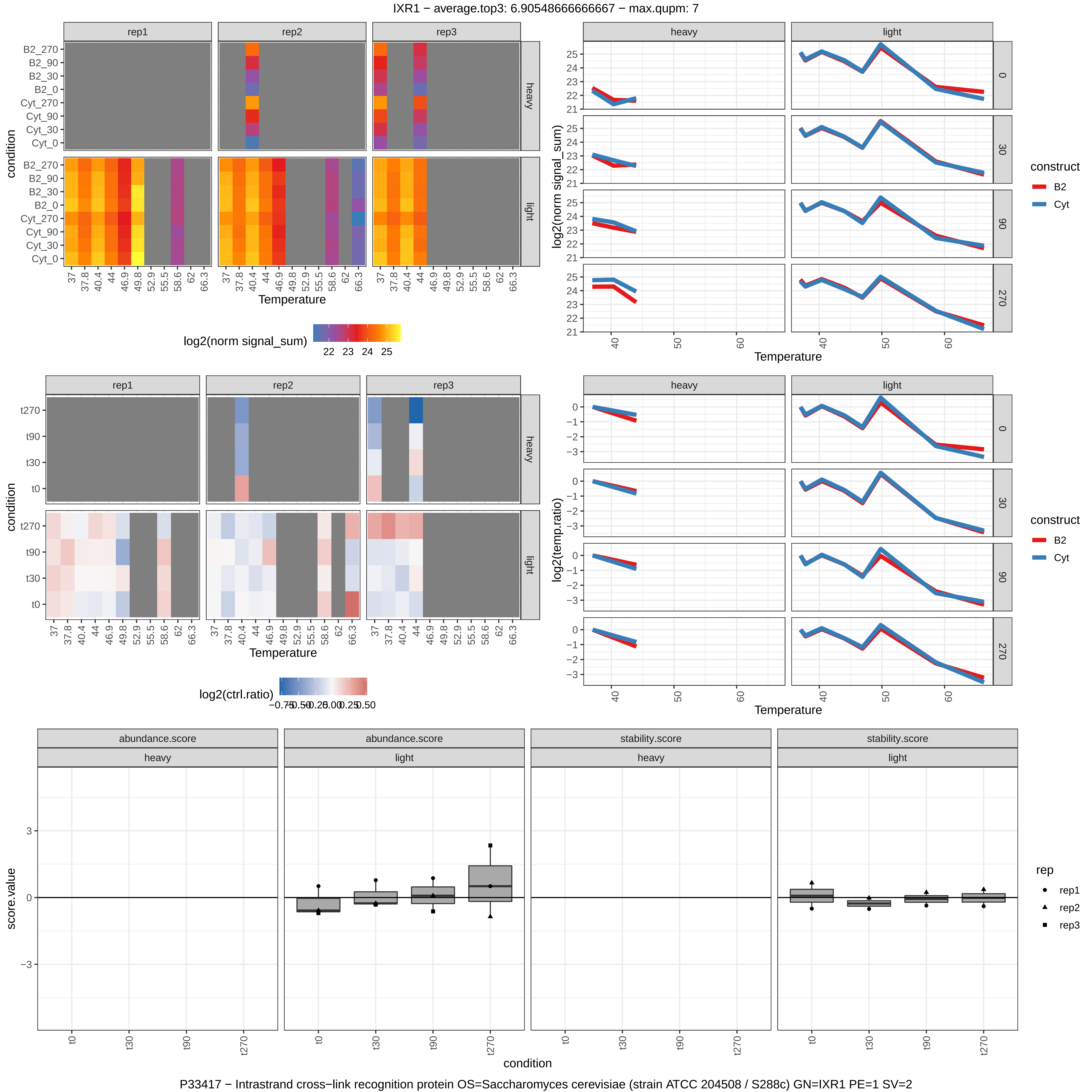
IXR1 − average.top3: 6.90548666666667 − max.qupm: 7
rep1
rep2
rep3
heavy
light
condition
Temperature
log2(norm signal_sum)
log2(ctrl.ratio)
log2(temp.ratio)
construct
B2
Cyt
abundance.score
stability.score
score.value
rep
condition
P33417 − Intrastrand cross−link recognition protein OS=Saccharomyces cerevisiae (strain ATCC 204508 / S288c) GN=IXR1 PE=1 SV=2

JEN1 − average.top3: 7.66910728813559 − max.qupm: 8

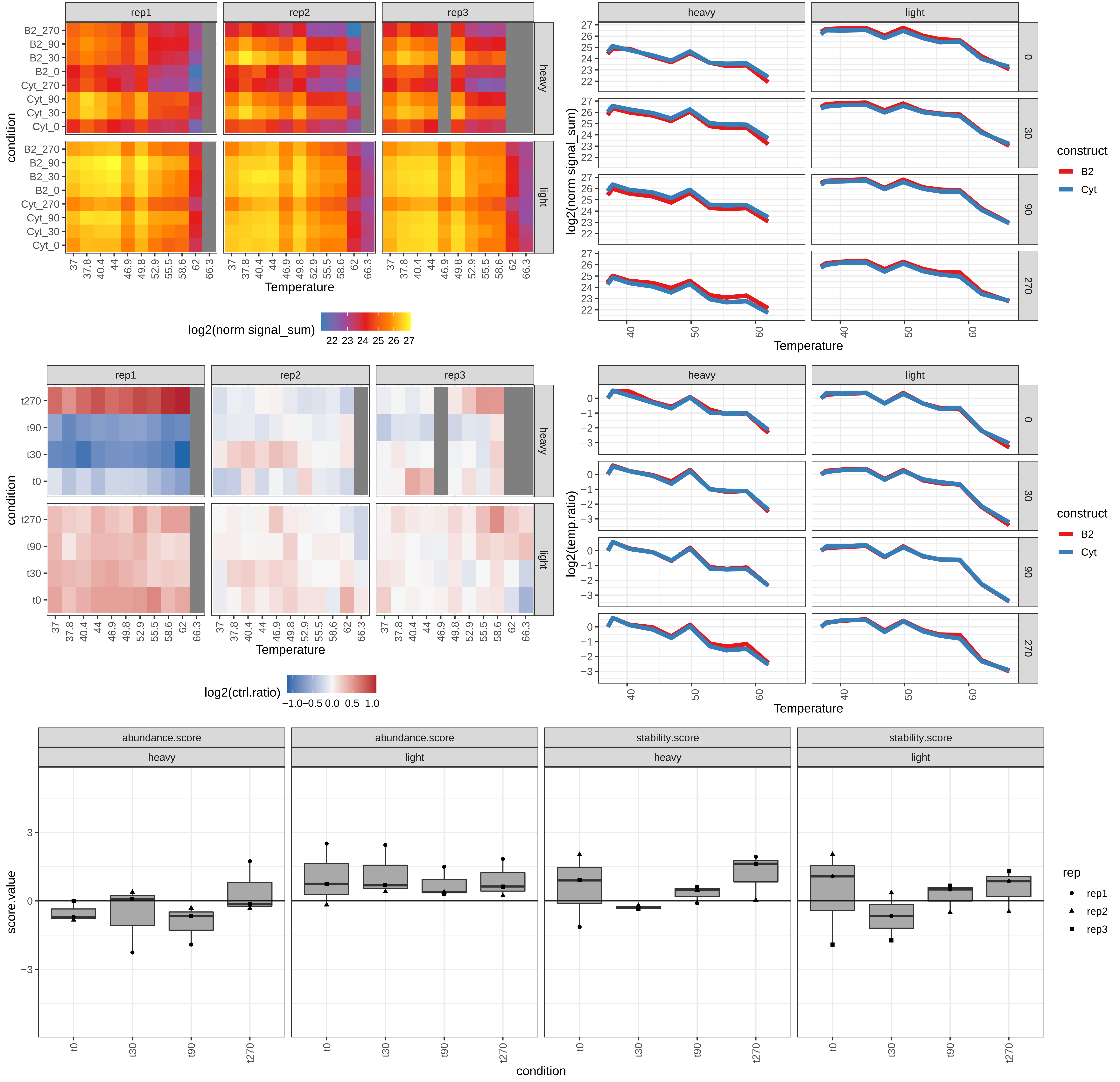

P36035 − Carboxylic acid transporter protein homolog OS=Saccharomyces cerevisiae (strain ATCC 204508 / S288c) GN=JEN1 PE=1 SV=1

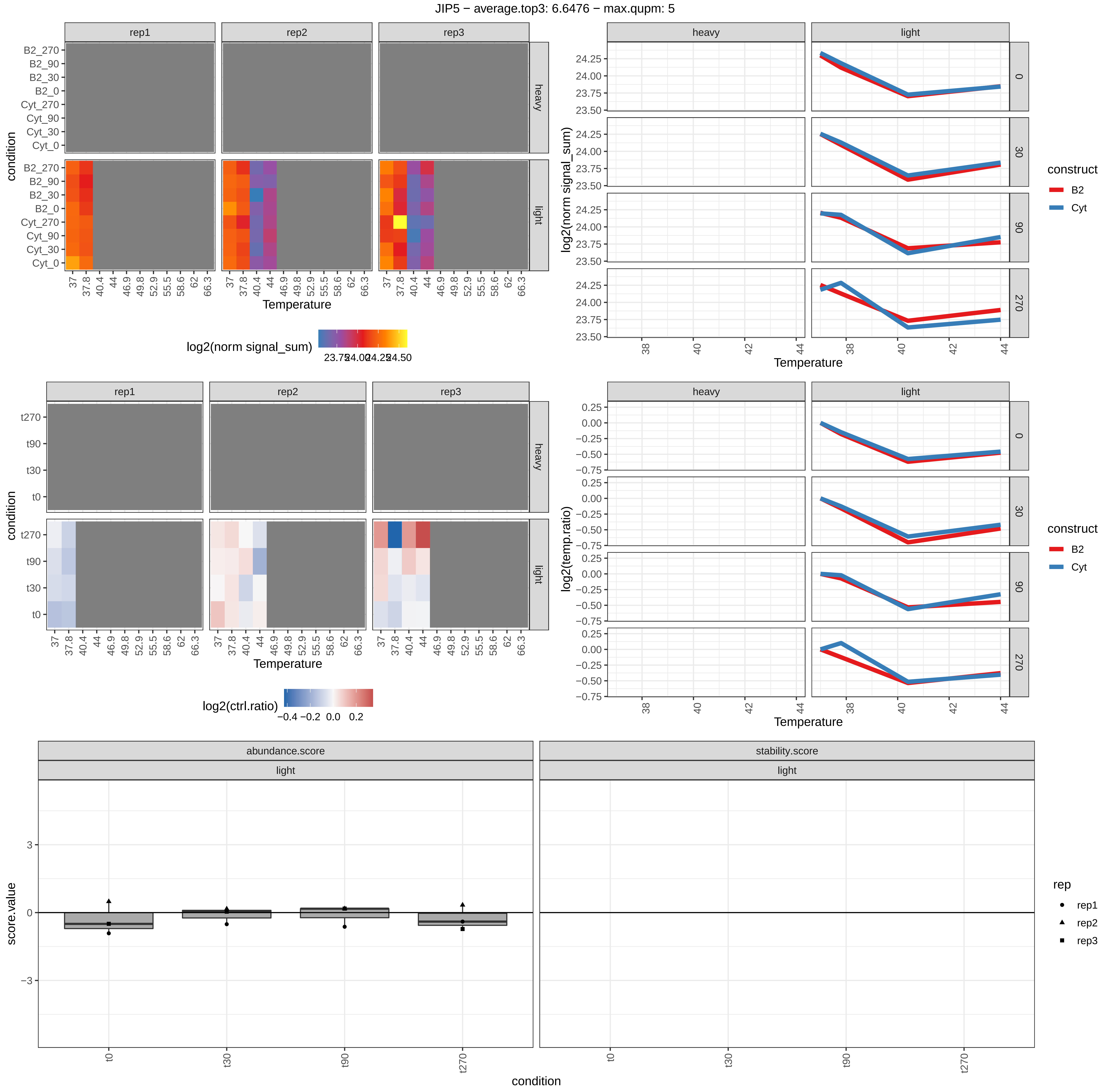

Q06214 − WD repeat−containing protein JIP5 OS=Saccharomyces cerevisiae (strain ATCC 204508 / S288c) GN=JIP5 PE=1 SV=2

JSN1 − average.top3: 6.58029285714286 − max.qupm: 6

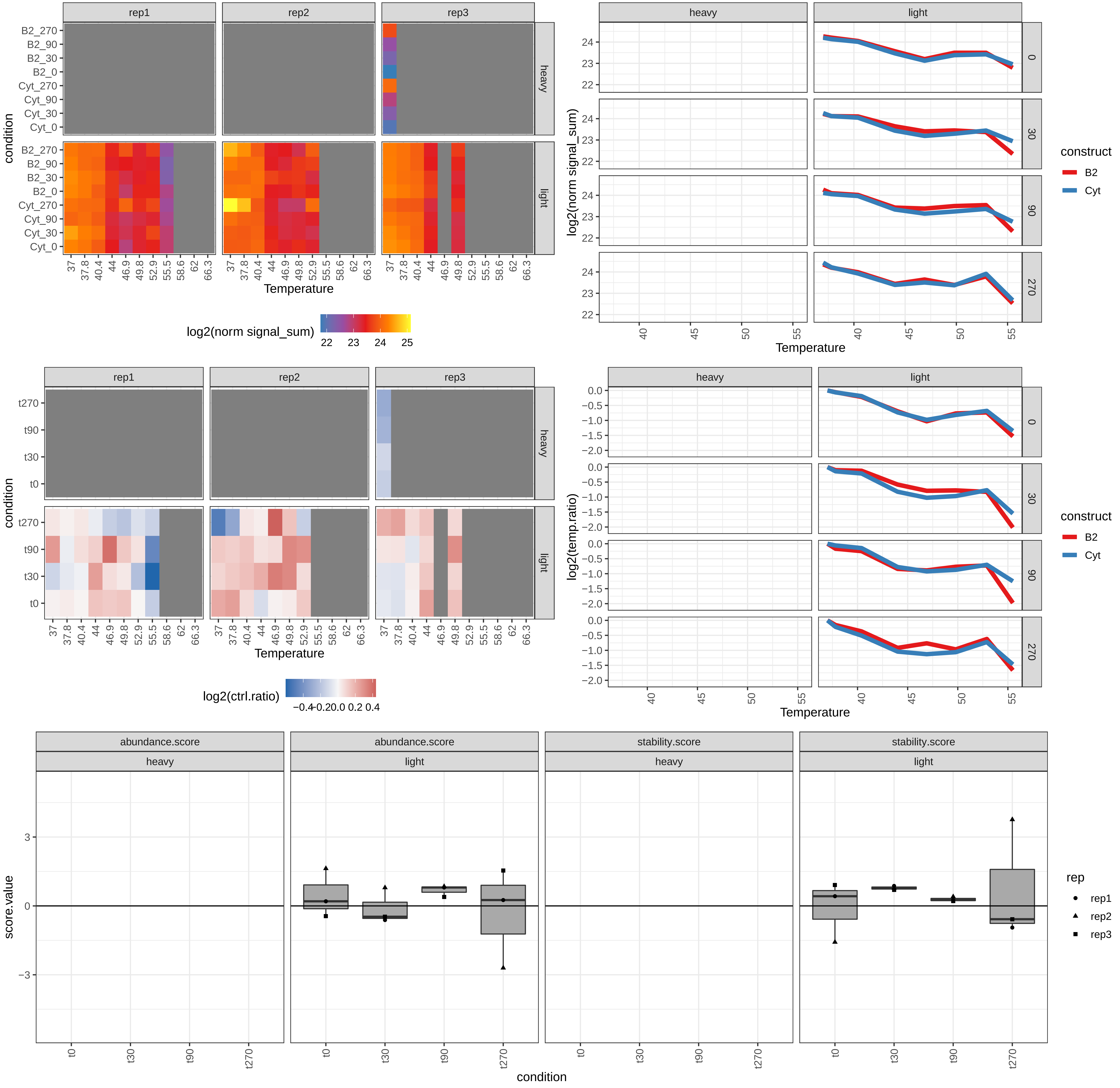

P47135 − Protein JSN1 OS=Saccharomyces cerevisiae (strain ATCC 204508 / S288c) GN=JSN1 PE=1 SV=1

KAE1 − average.top3: 6.72597038461538 − max.qupm: 6

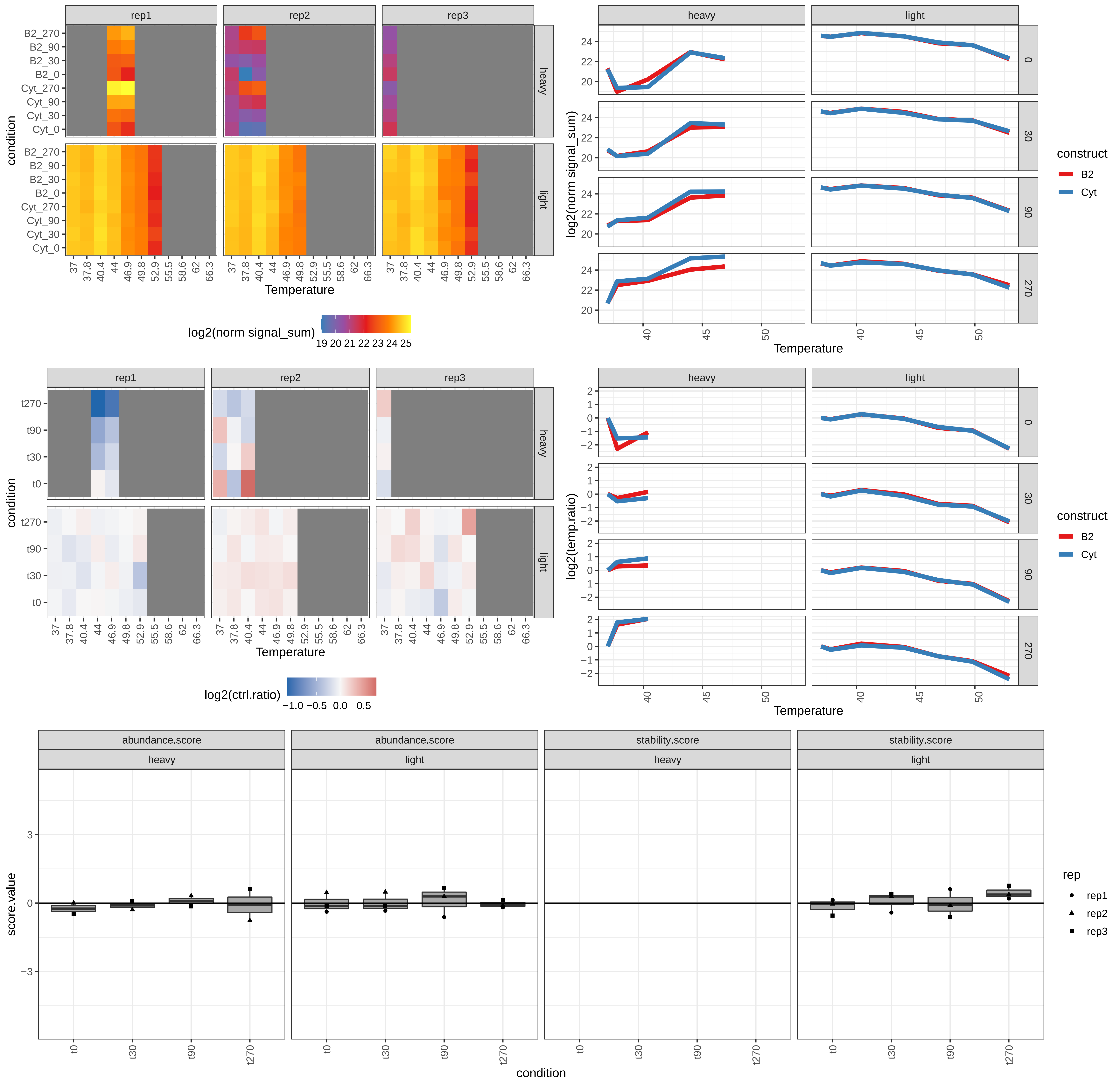

P36132 − tRNA N6−adenosine threonylcarbamoyltransferase OS=Saccharomyces cerevisiae (strain ATCC 204508 / S288c) GN=KAE1 PE=1 SV=2

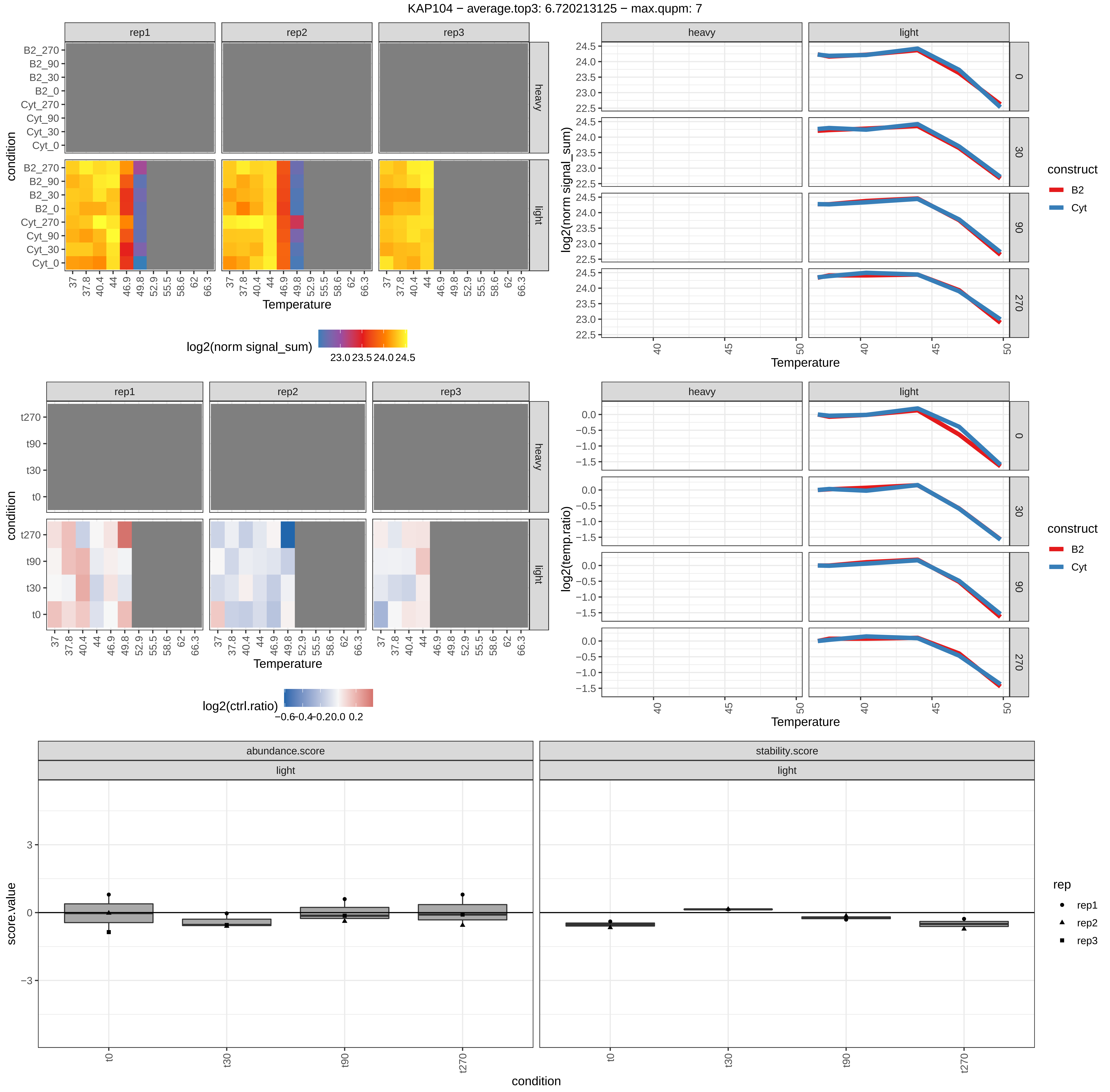

P38217 − Importin subunit beta−2 OS=Saccharomyces cerevisiae (strain ATCC 204508 / S288c) GN=KAP104 PE=1 SV=1

KAP114 − average.top3: 6.73431277777778 − max.qupm: 7

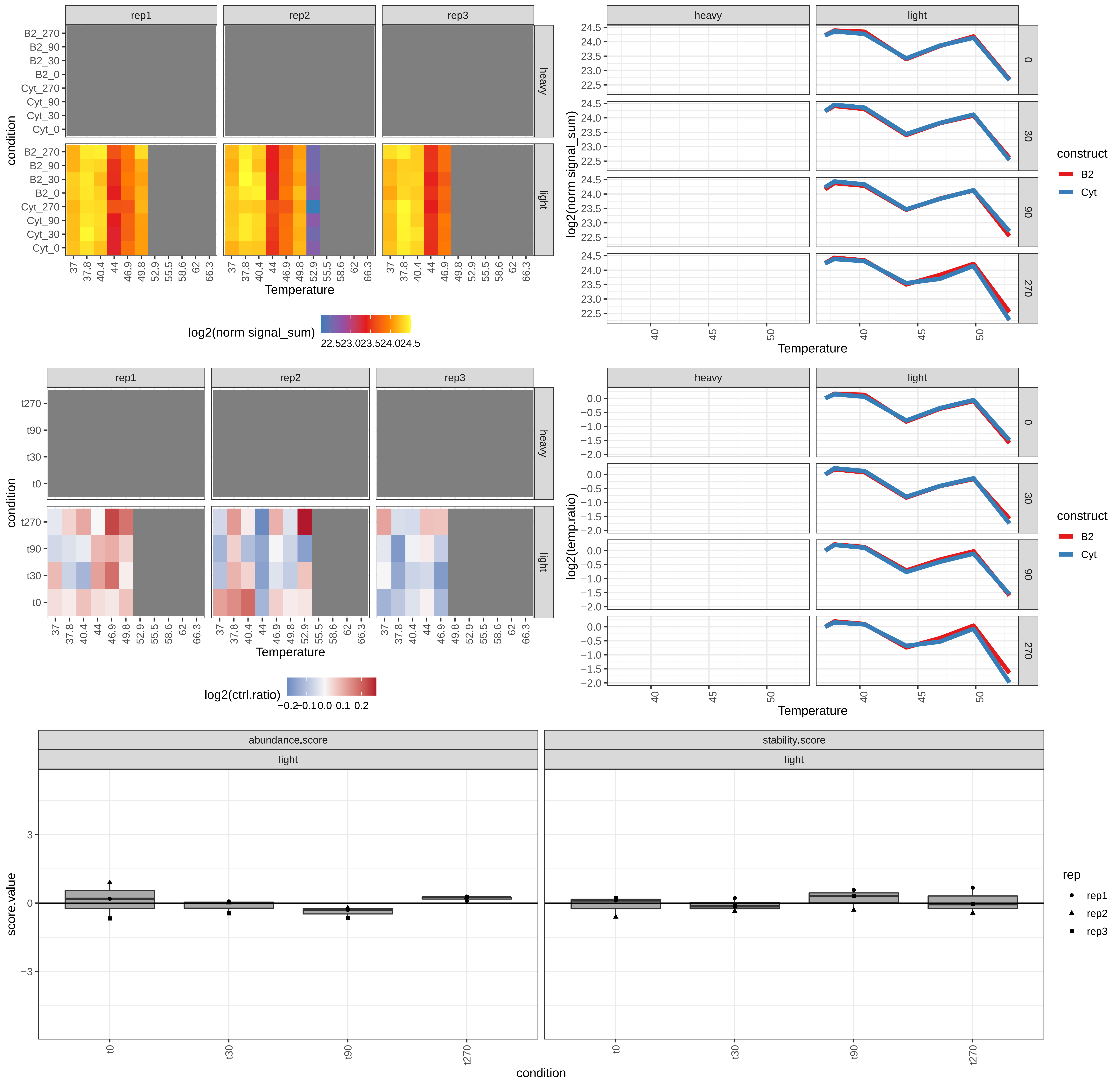

P53067 − Importin subunit beta−5 OS=Saccharomyces cerevisiae (strain ATCC 204508 / S288c) GN=KAP114 PE=1 SV=1

KAP120 − average.top3: 6.80358583333333 − max.qupm: 8

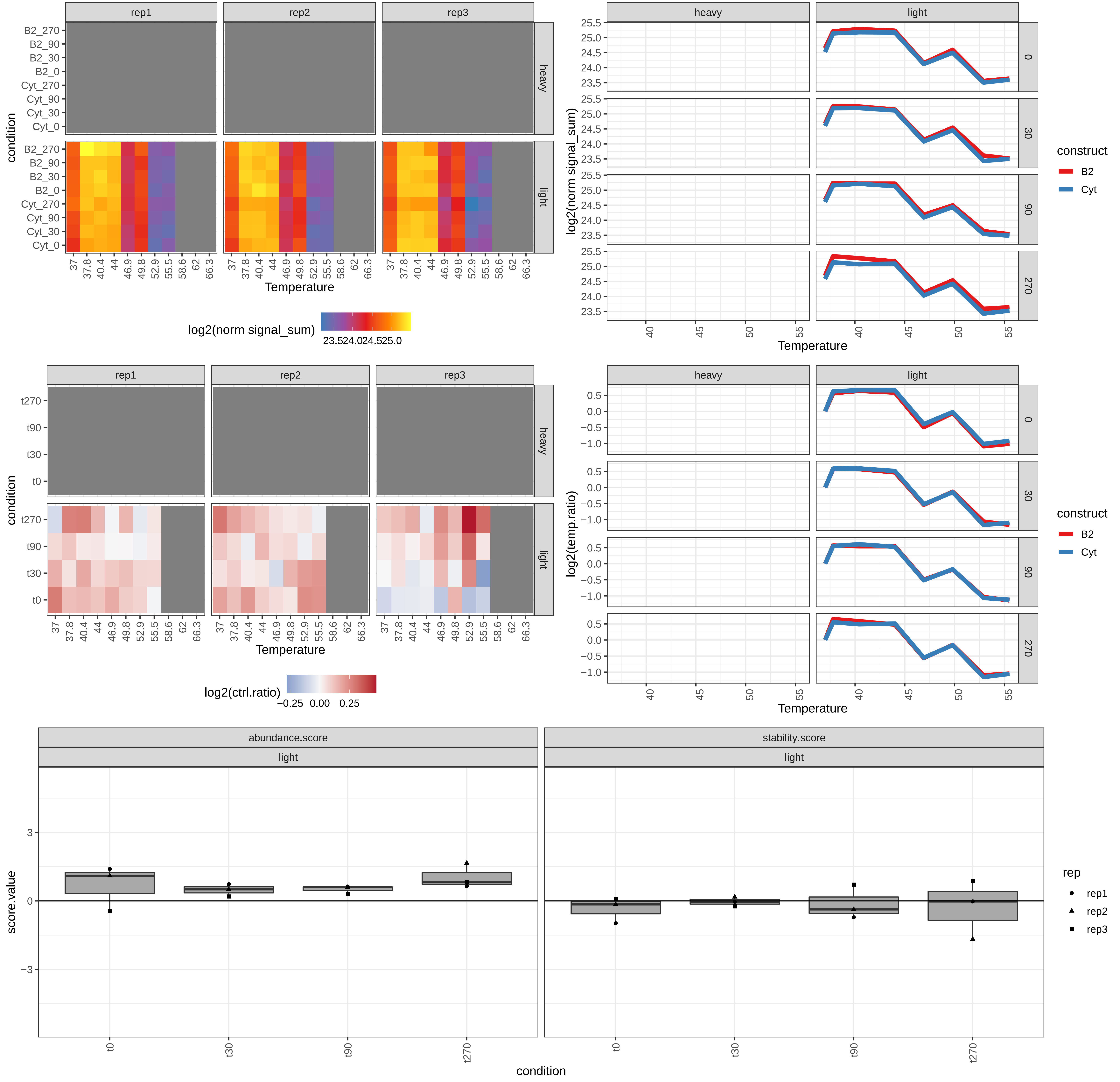

Q02932 − Importin beta−like protein KAP120 OS=Saccharomyces cerevisiae (strain ATCC 204508 / S288c) GN=KAP120 PE=1 SV=1

KAP122 − average.top3: 6.59722166666667 − max.qupm: 4

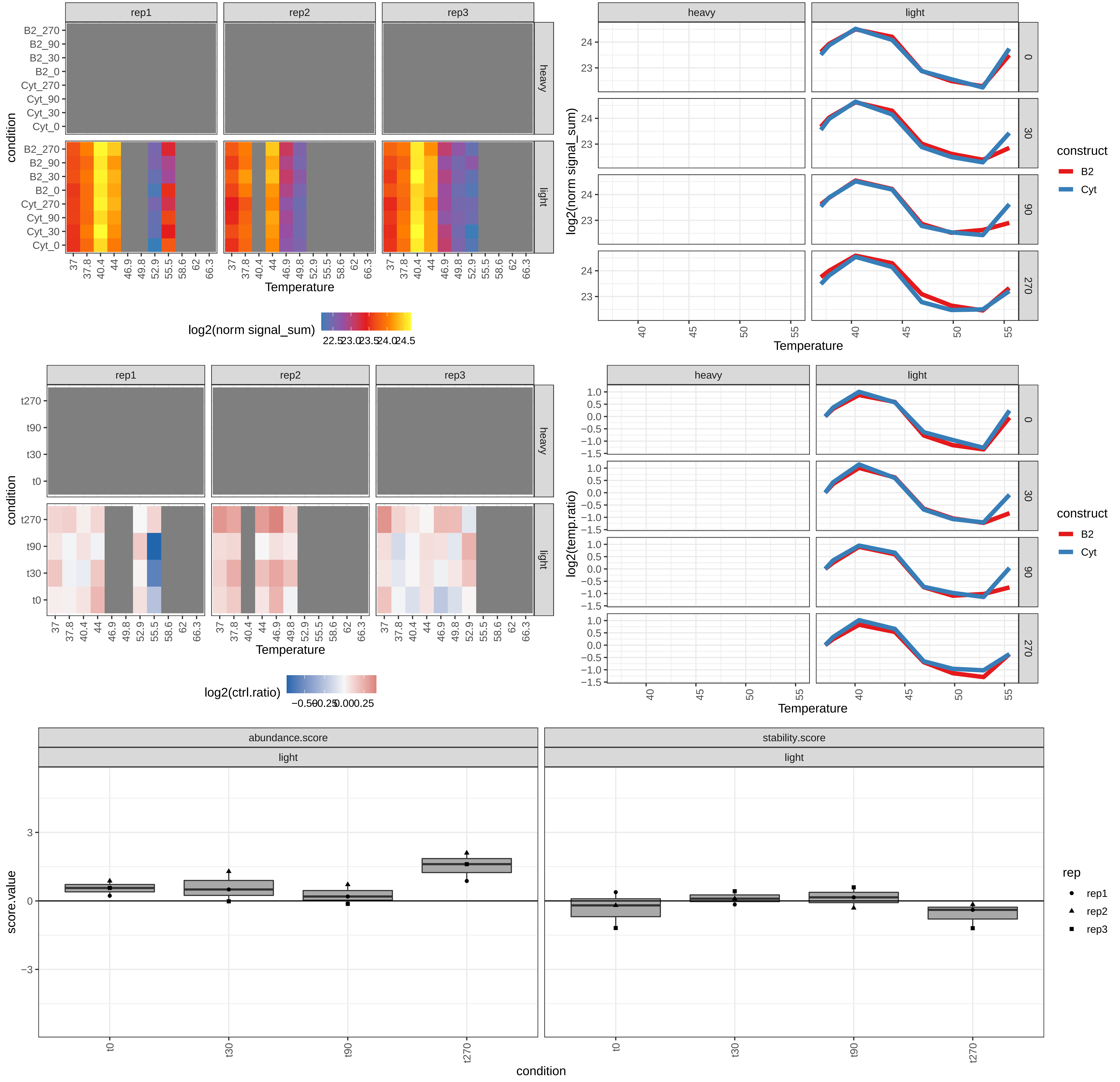

P32767 − Importin beta−like protein KAP122 OS=Saccharomyces cerevisiae (strain ATCC 204508 / S288c) GN=KAP122 PE=1 SV=1

KAP123 − average.top3: 7.01172260869565 − max.qupm: 15

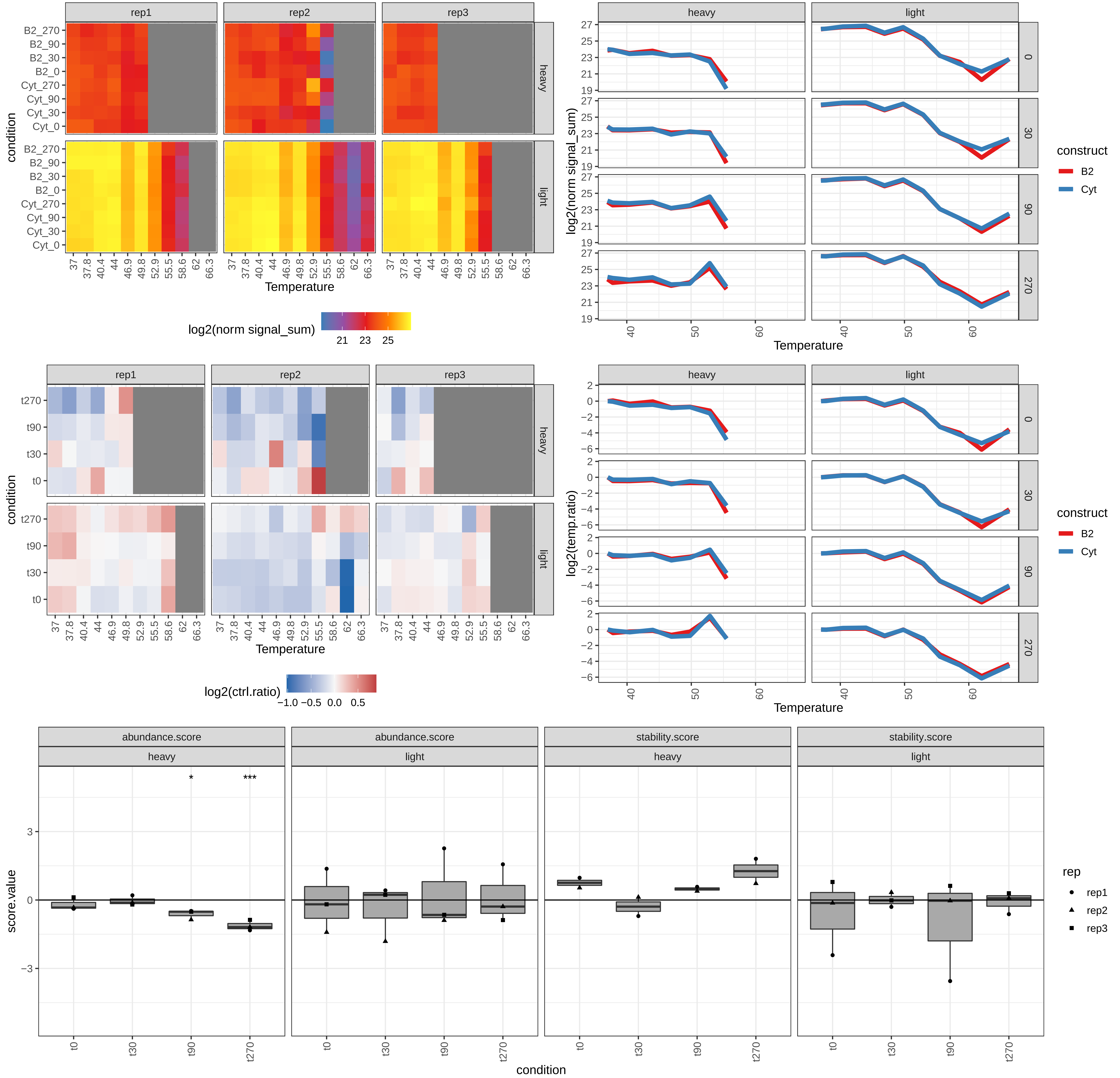

P40069 − Importin subunit beta−4 OS=Saccharomyces cerevisiae (strain ATCC 204508 / S288c) GN=KAP123 PE=1 SV=1

KAR2 − average.top3: 7.70240492307692 − max.qupm: 28

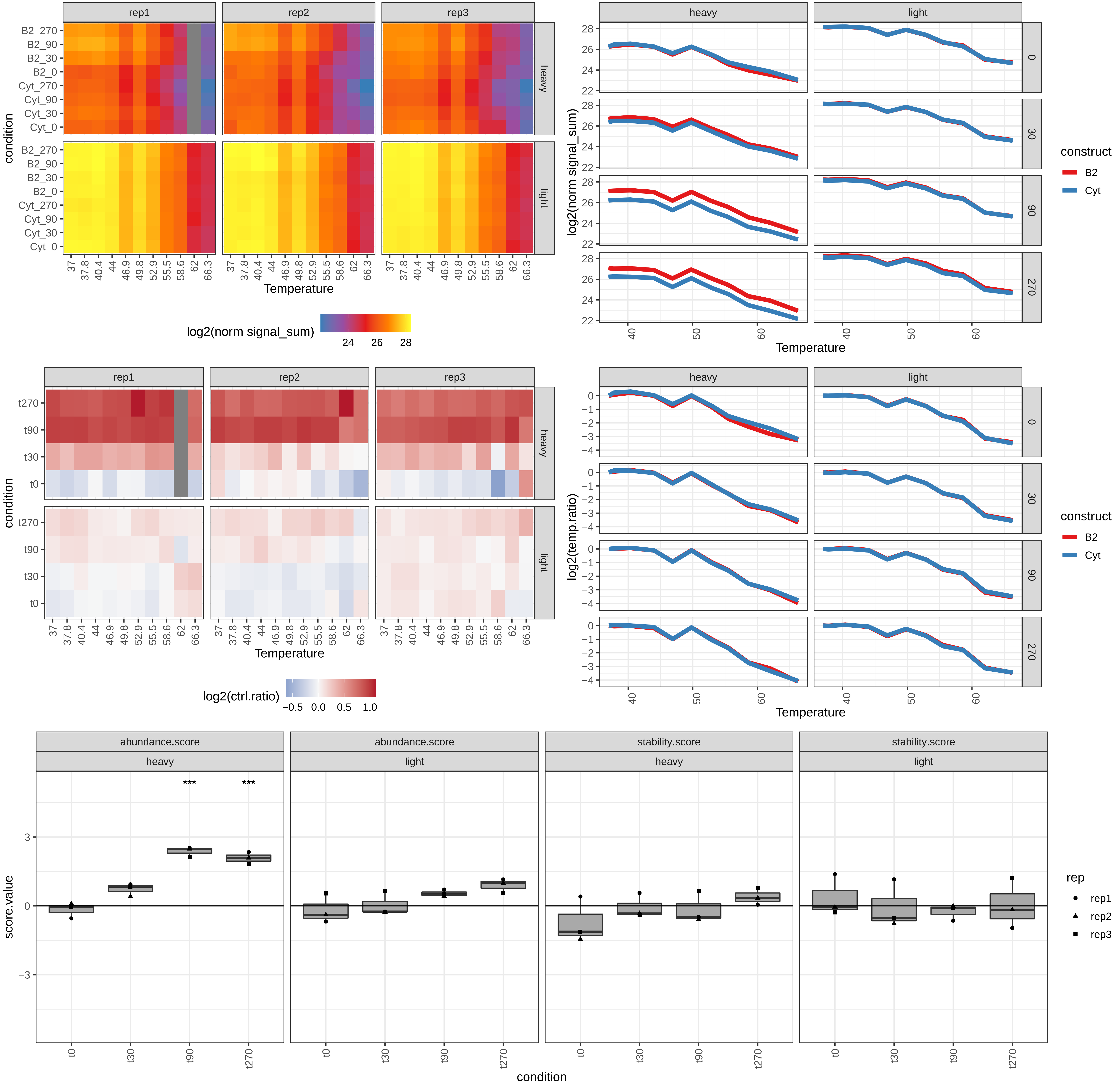

P16474 − 78 kDa glucose−regulated protein homolog OS=Saccharomyces cerevisiae (strain ATCC 204508 / S288c) GN=KAR2 PE=1 SV=1

KCS1 − average.top3: 6.90491111111111 − max.qupm: 6

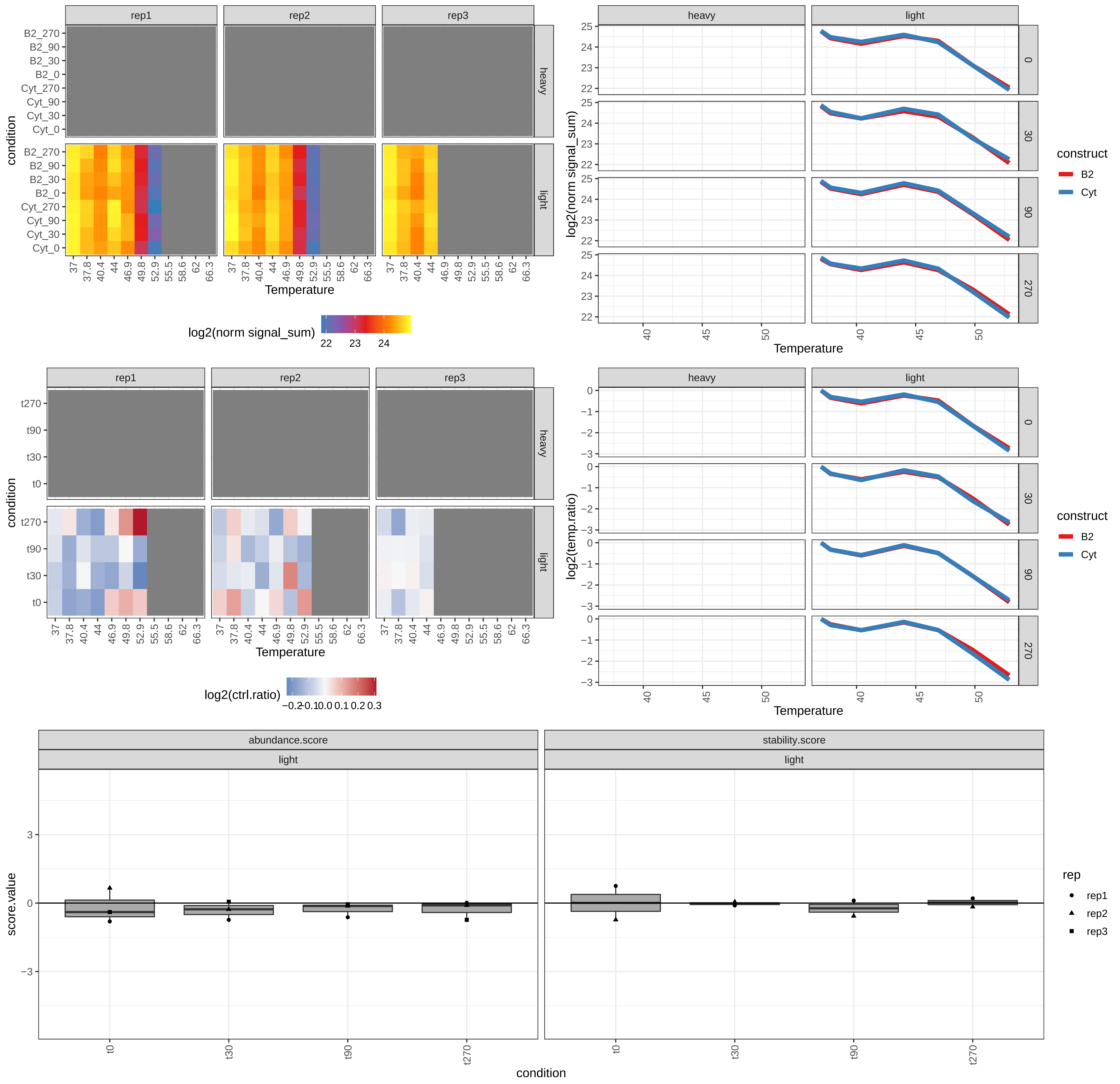

Q12494 − Inositol hexakisphosphate kinase 1 OS=Saccharomyces cerevisiae (strain ATCC 204508 / S288c) GN=KCS1 PE=1 SV=1

KEL1 − average.top3: 6.60971611111111 − max.qupm: 6

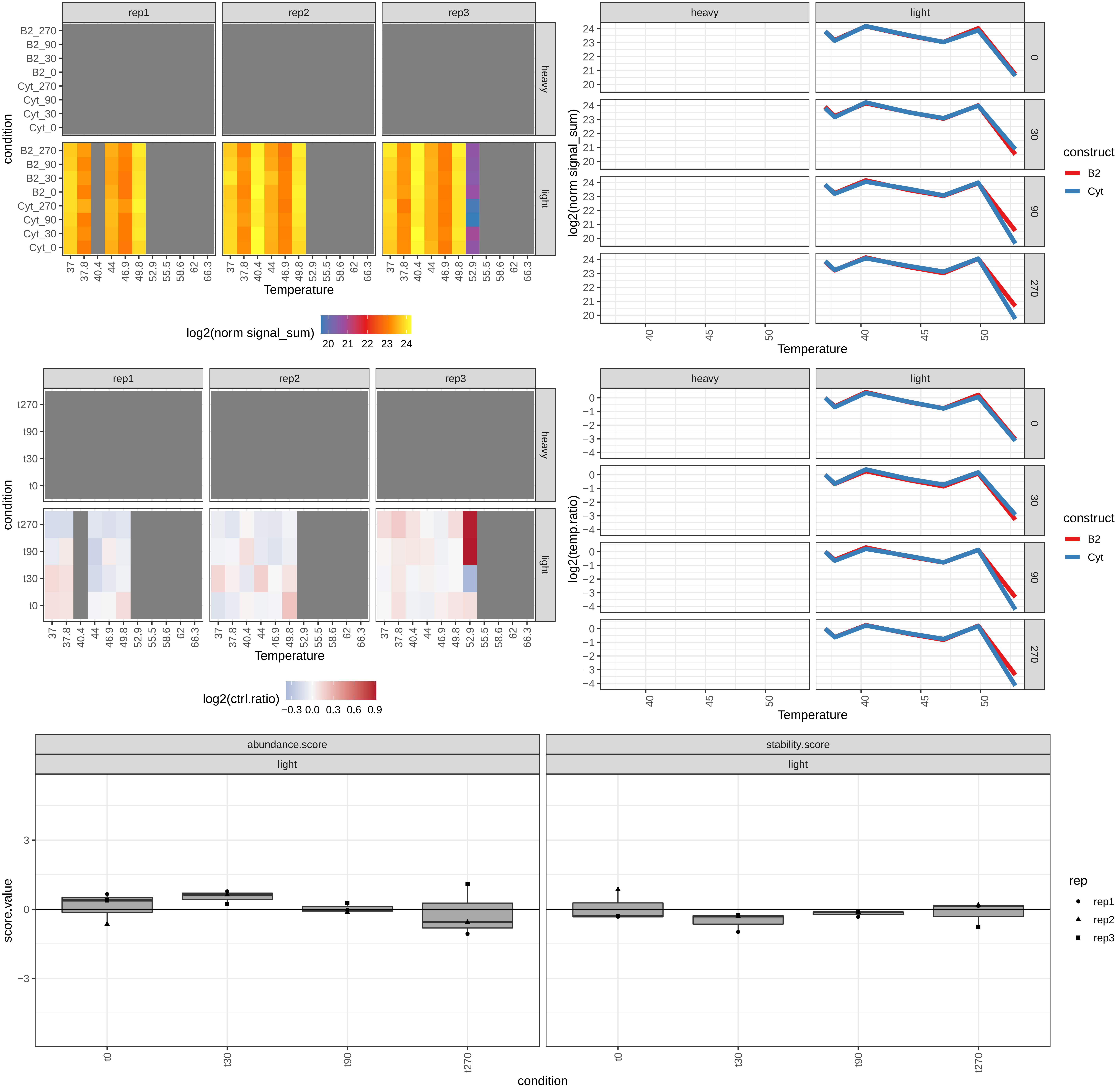

P38853 − Kelch repeat−containing protein 1 OS=Saccharomyces cerevisiae (strain ATCC 204508 / S288c) GN=KEL1 PE=1 SV=1

KES1 − average.top3: 7.34868780487805 − max.qupm: 18

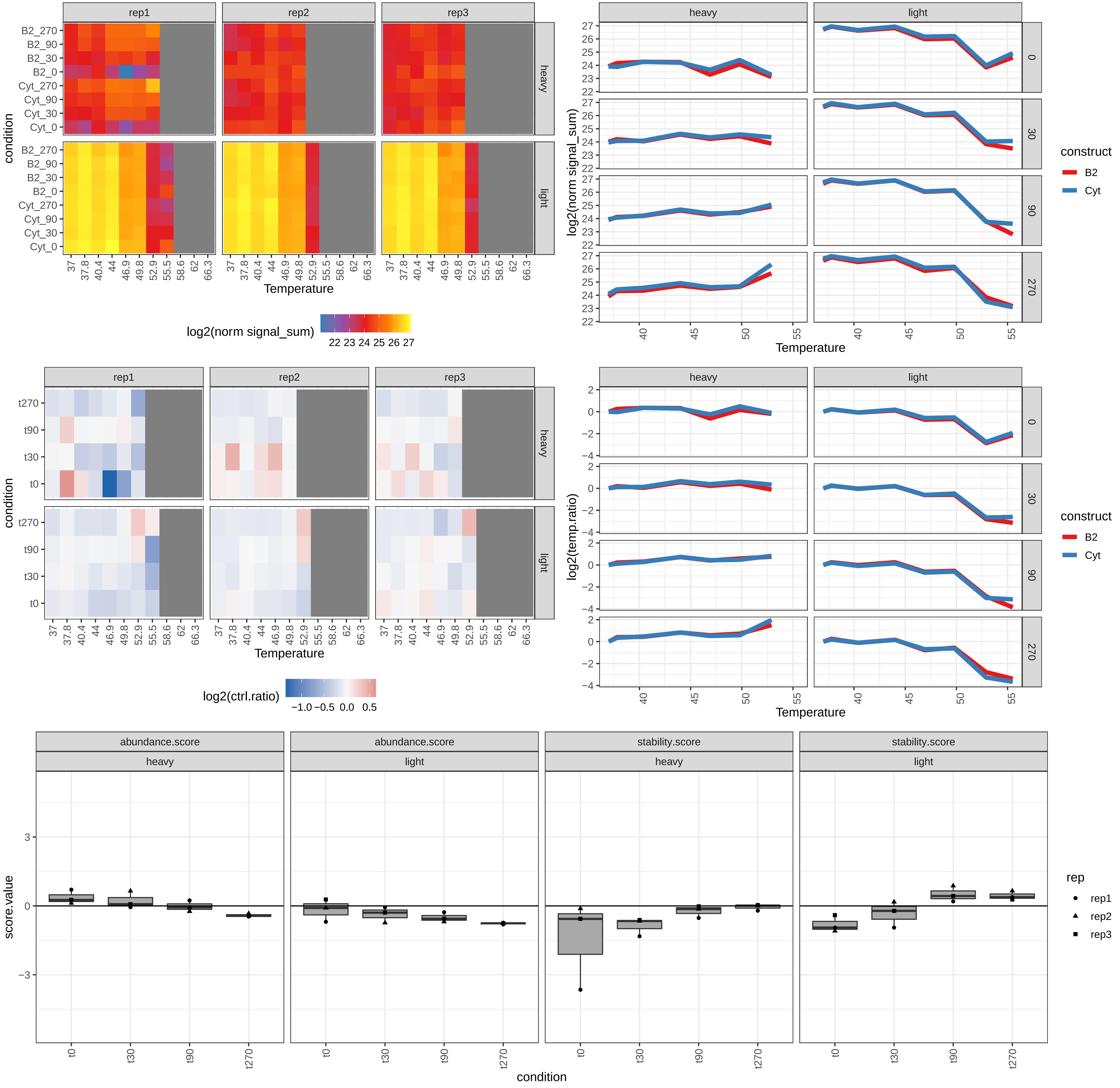

P35844 − Oxysterol−binding protein homolog 4 OS=Saccharomyces cerevisiae (strain ATCC 204508 / S288c) GN=KES1 PE=1 SV=1

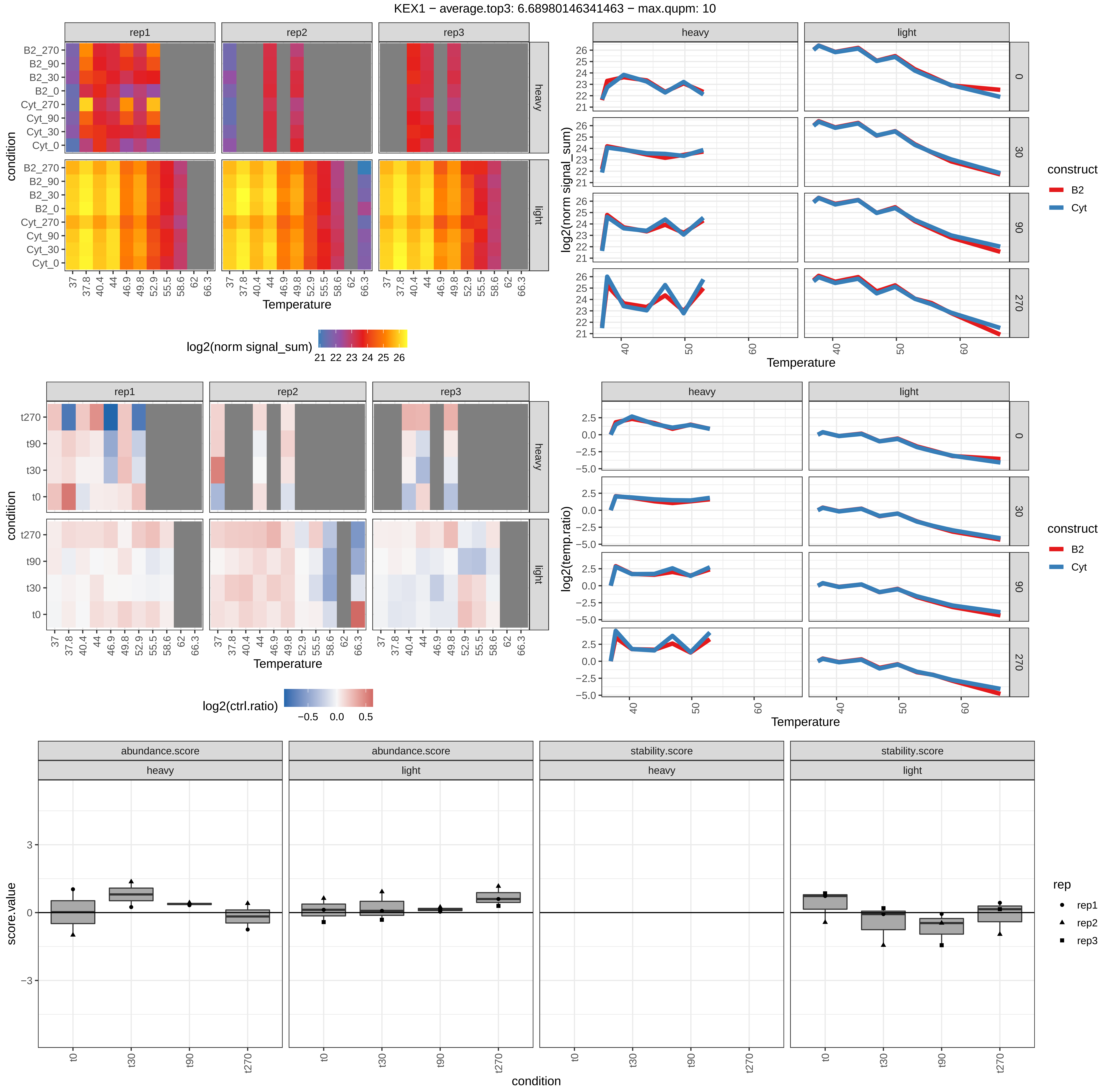

P09620 − Pheromone−processing carboxypeptidase KEX1 OS=Saccharomyces cerevisiae (strain ATCC 204508 / S288c) GN=KEX1 PE=1 SV=1

KGD1 − average.top3: 8.03792881355932 − max.qupm: 32

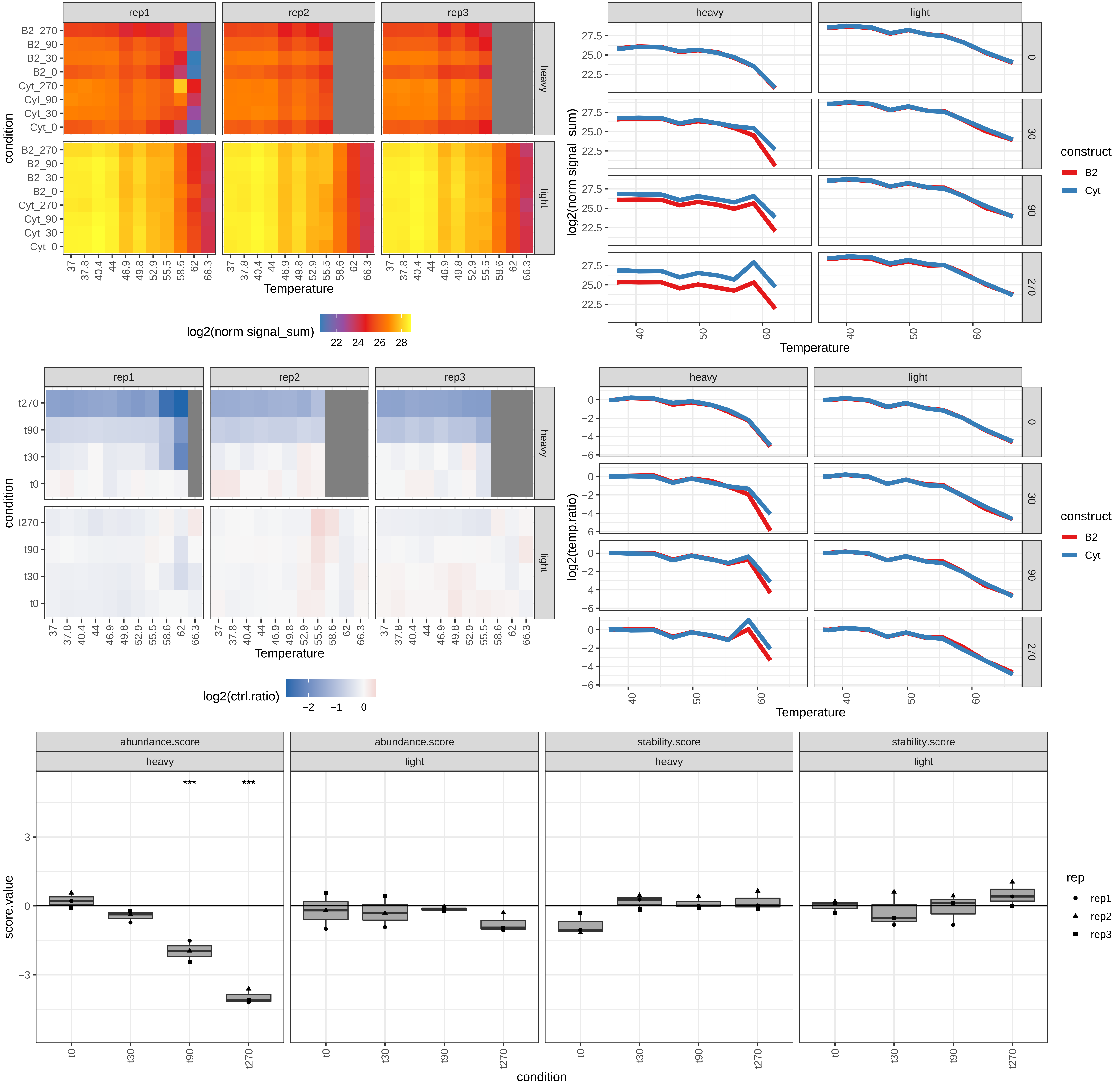

P20967 − 2−oxoglutarate dehydrogenase, mitochondrial OS=Saccharomyces cerevisiae (strain ATCC 204508 / S288c) GN=KGD1 PE=1 SV=2

KGD2 − average.top3: 7.23678210526316 − max.qupm: 10

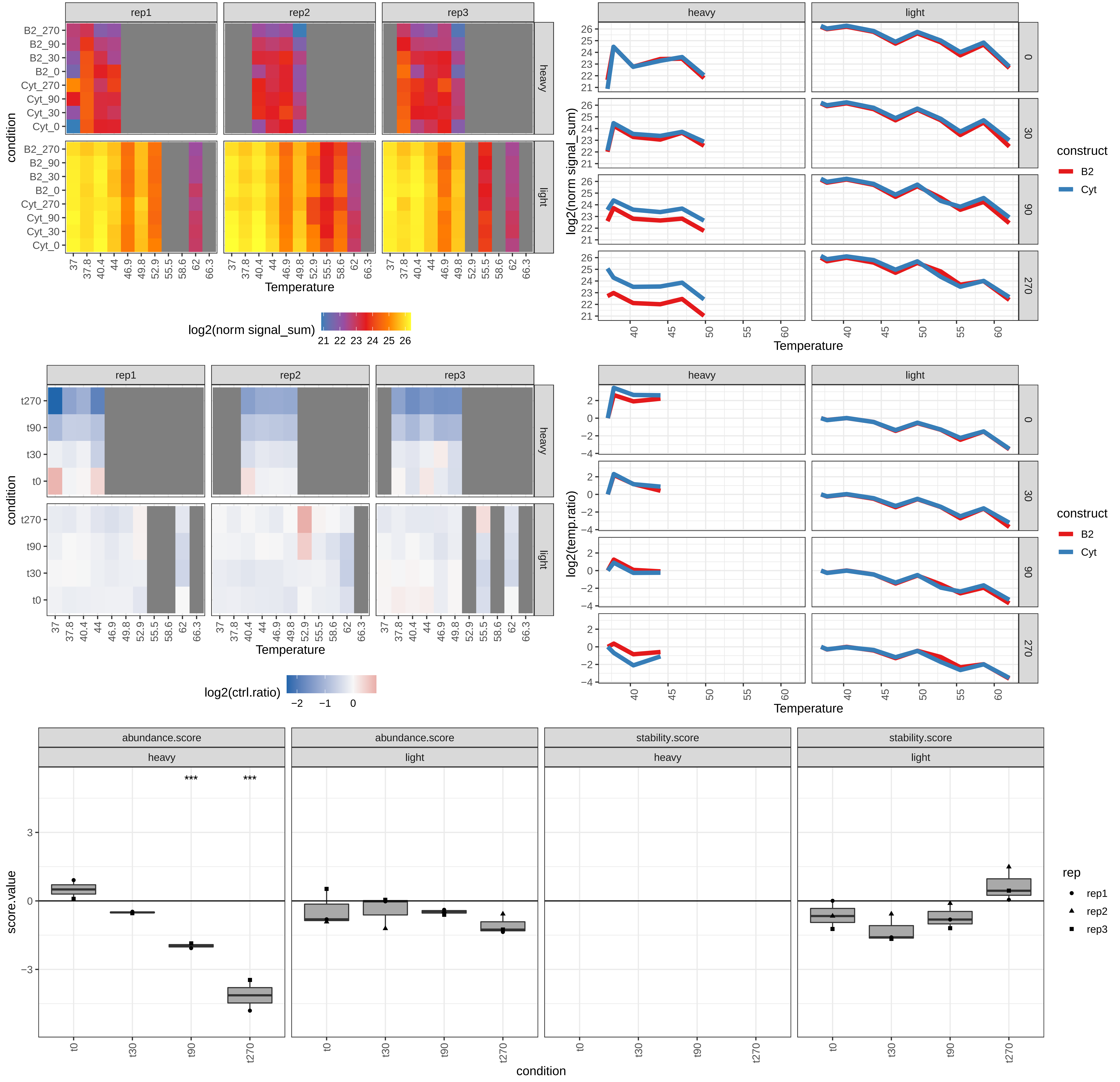

62 − Dihydrolipoyllysine−residue succinyltransferase component of 2−oxoglutarate dehydrogenase complex, mitochondrial OS=Saccharomyces cerevisiae (strain ATCC 204508 / S288c) GN=KGD2 PE=1 S

KIC1 − average.top3: 6.817695 − max.qupm: 2

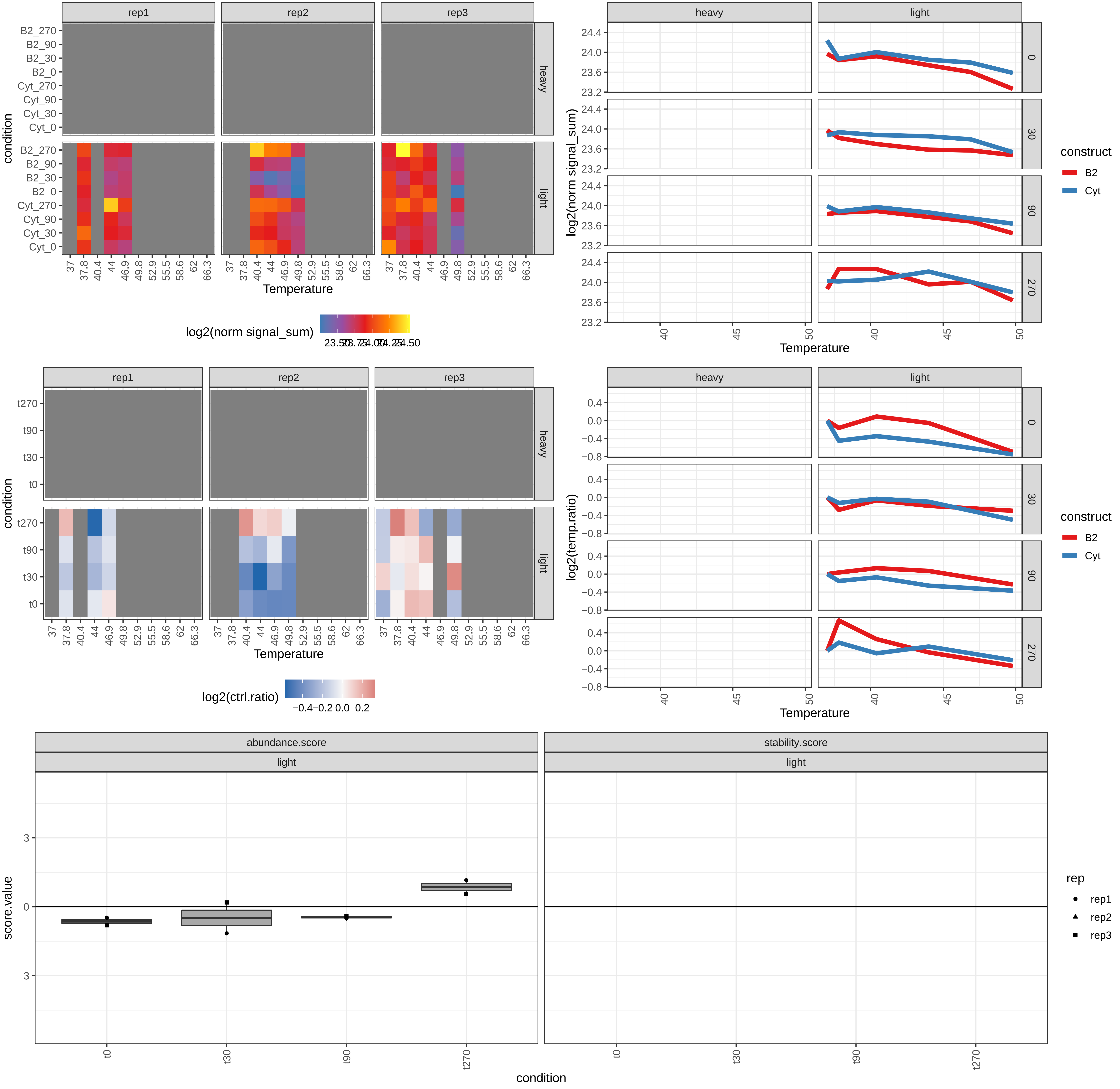

P38692 − Serine/threonine−protein kinase KIC1 OS=Saccharomyces cerevisiae (strain ATCC 204508 / S288c) GN=KIC1 PE=1 SV=1

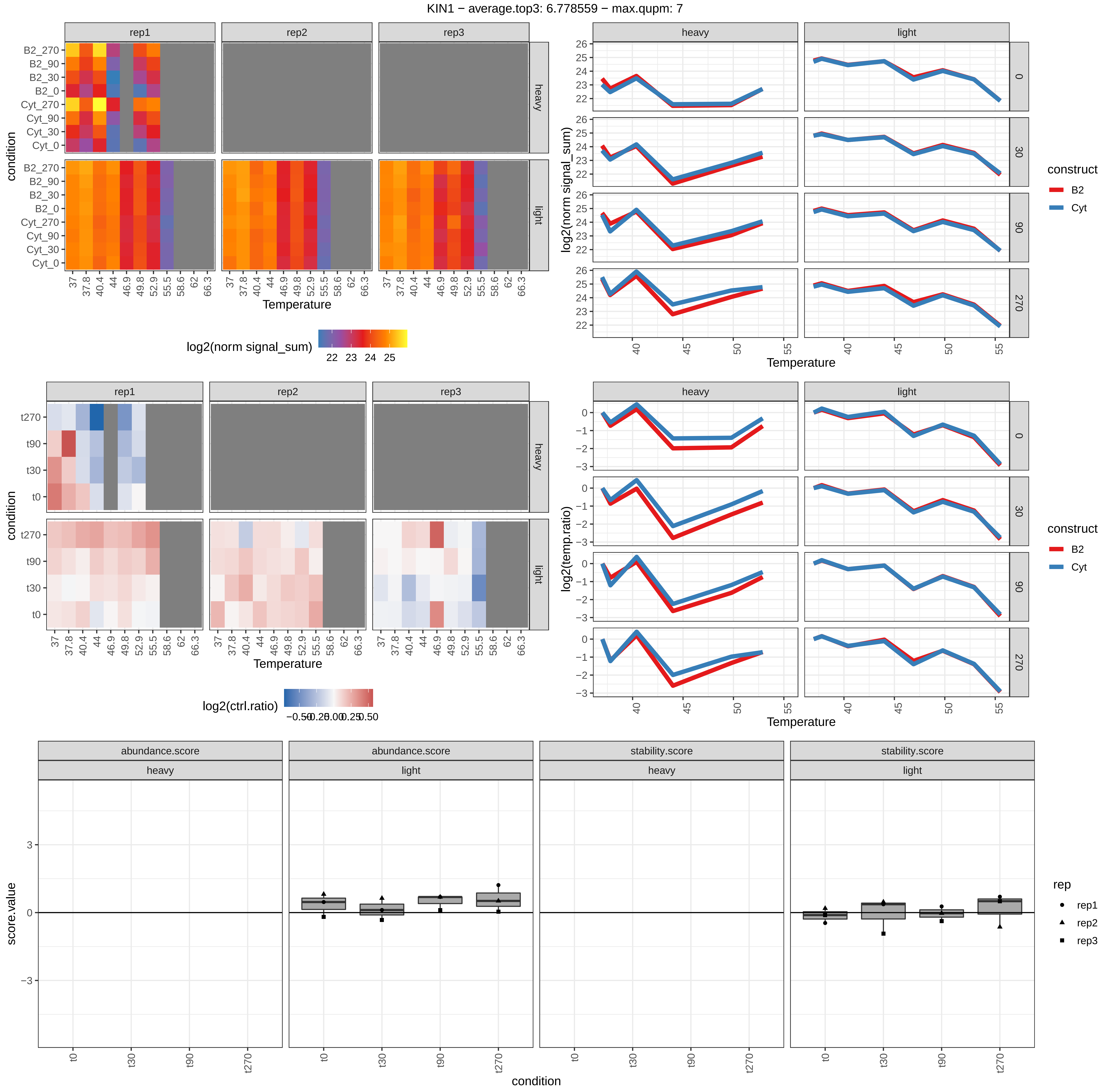

P13185 − Serine/threonine protein kinase KIN1 OS=Saccharomyces cerevisiae (strain ATCC 204508 / S288c) GN=KIN1 PE=1 SV=3

KIN28 − average.top3: 6.43303266666667 − max.qupm: 5

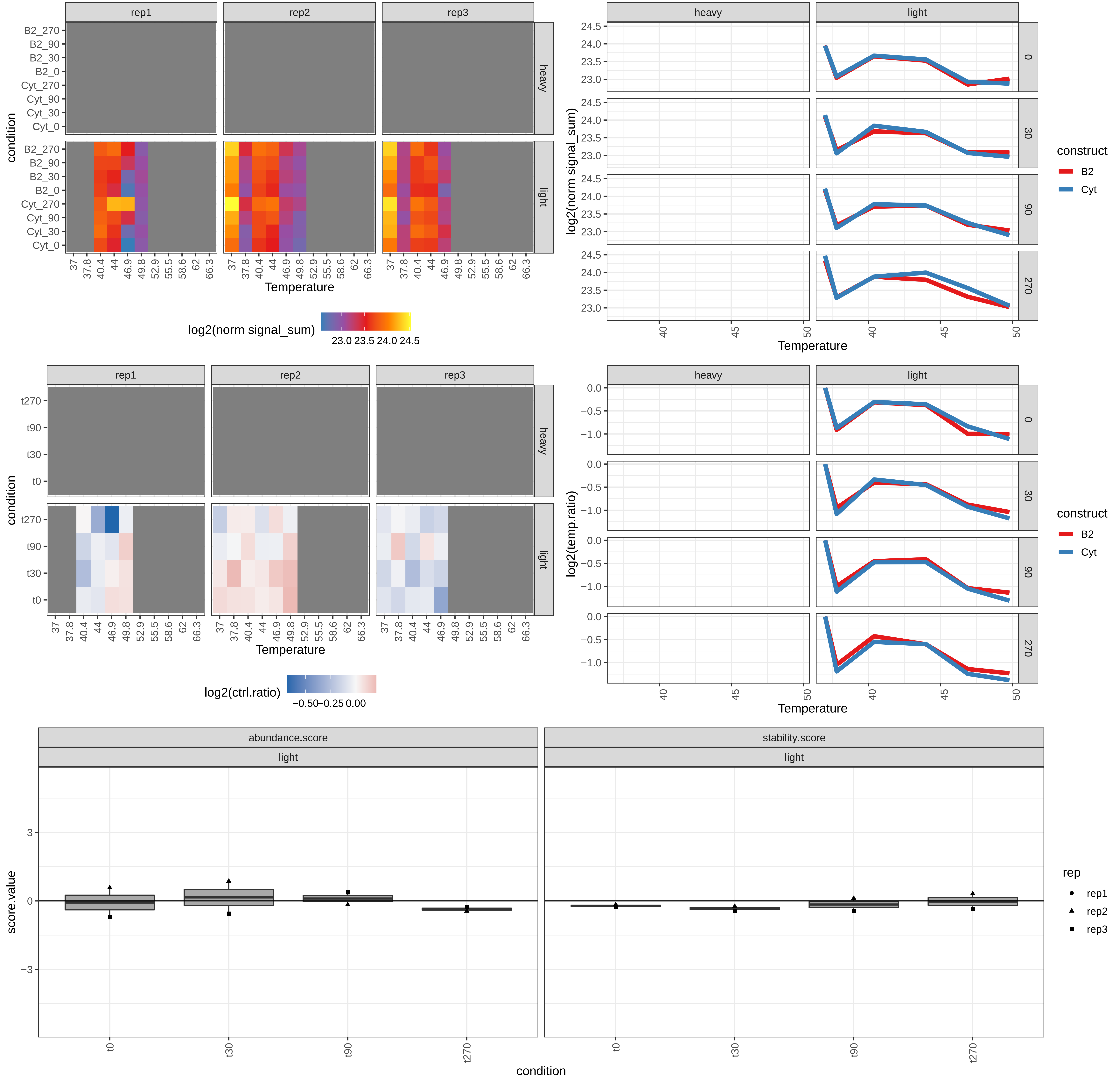

P06242 − Serine/threonine−protein kinase KIN28 OS=Saccharomyces cerevisiae (strain ATCC 204508 / S288c) GN=KIN28 PE=1 SV=1

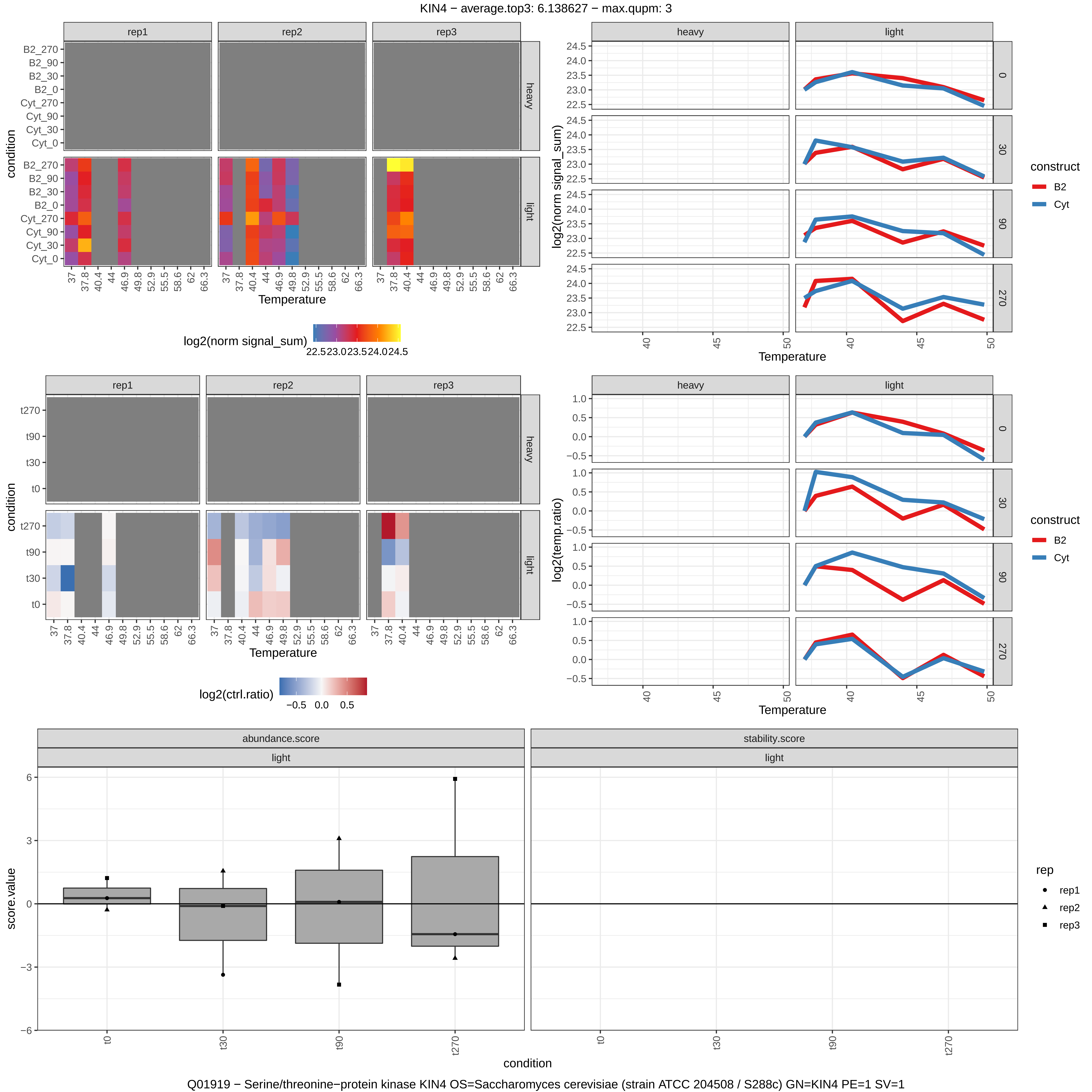

KIN4 − average.top3: 6.138627 − max.qupm: 3
rep1
rep2
rep3
heavy
light
condition
Temperature
log2(norm signal_sum)
log2(ctrl.ratio)
log2(temp.ratio)
construct
B2
Cyt
abundance.score
stability.score
score.value
rep
rep1
rep2
rep3
Q01919 − Serine/threonine−protein kinase KIN4 OS=Saccharomyces cerevisiae (strain ATCC 204508 / S288c) GN=KIN4 PE=1 SV=1

KOG1 − average.top3: 6.41153692307692 − max.qupm: 7

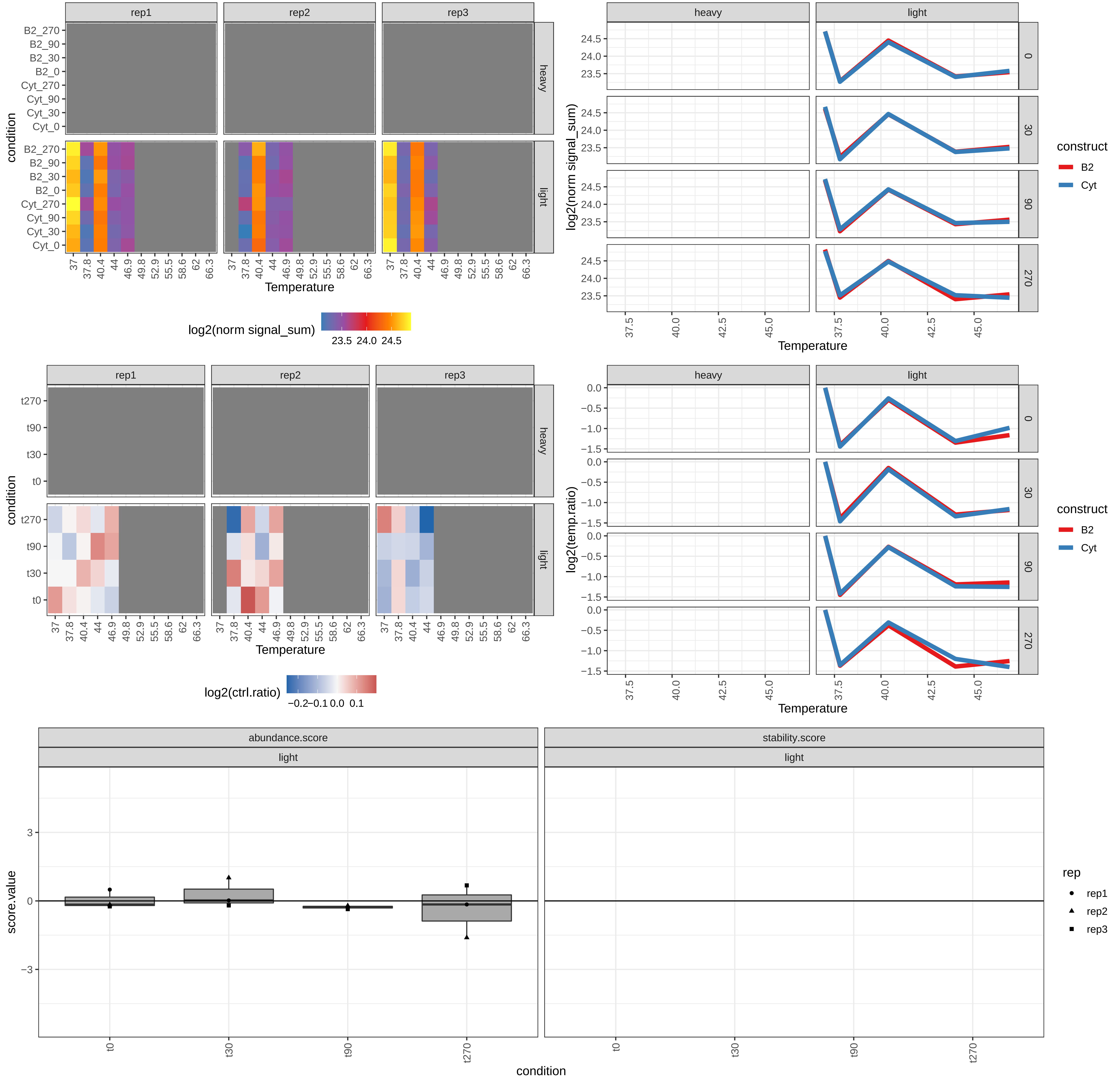

P38873 − Target of rapamycin complex 1 subunit KOG1 OS=Saccharomyces cerevisiae (strain ATCC 204508 / S288c) GN=KOG1 PE=1 SV=2

KRE2 − average.top3: 7.08869175 − max.qupm: 10

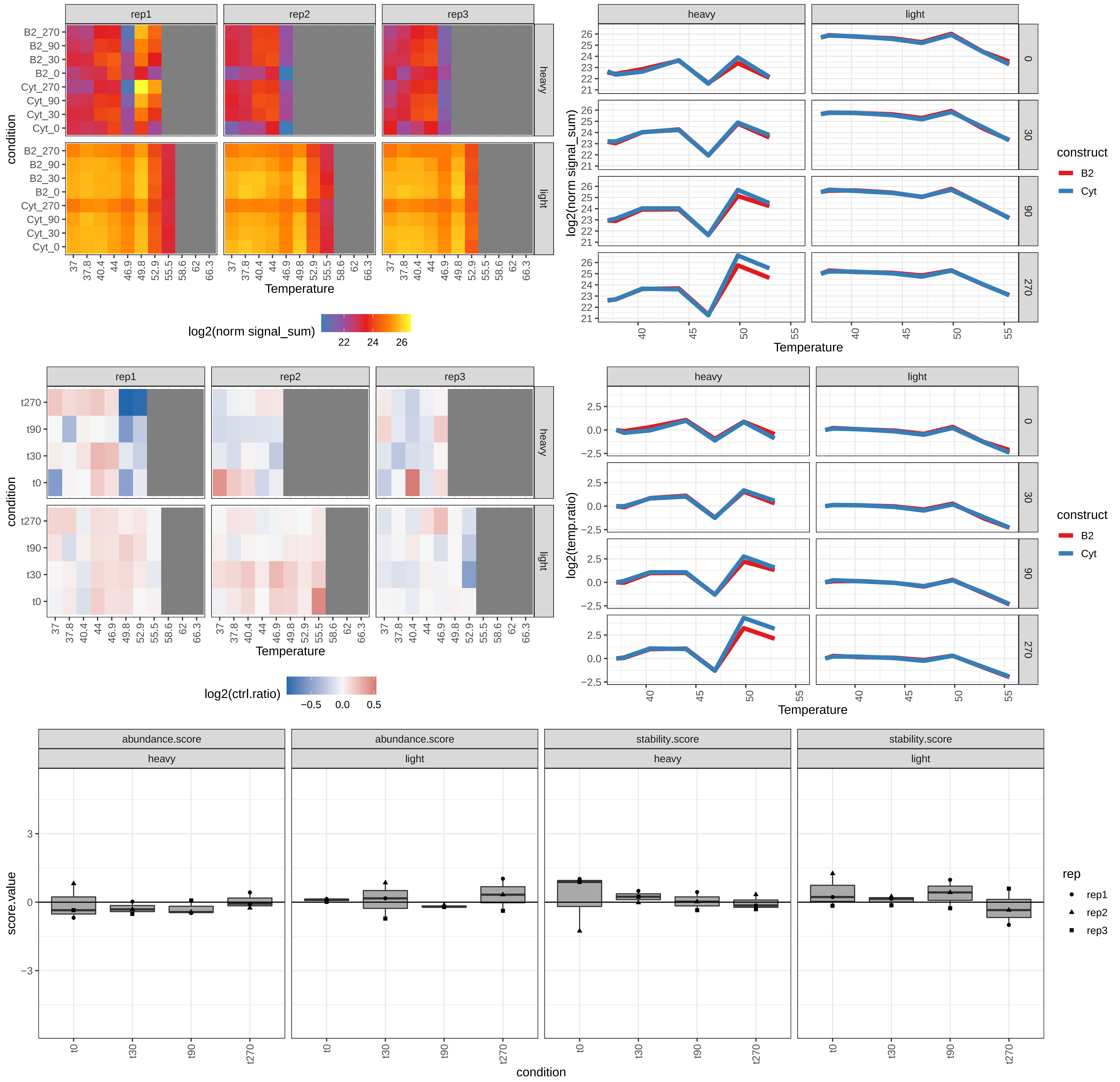

P27809 − Glycolipid 2−alpha−mannosyltransferase OS=Saccharomyces cerevisiae (strain ATCC 204508 / S288c) GN=KRE2 PE=1 SV=1

KRE5 − average.top3: 6.871539 − max.qupm: 15

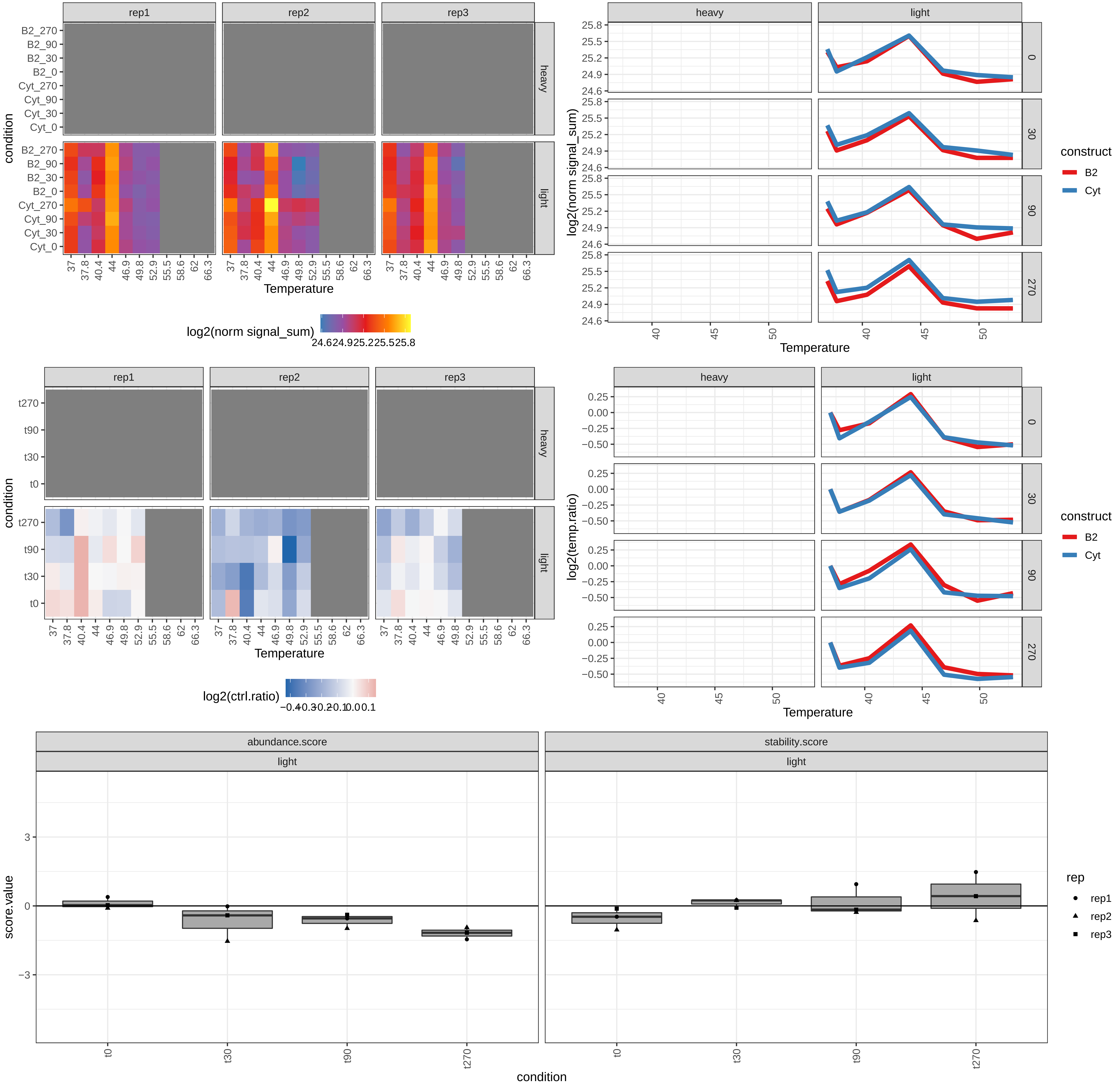

P22023 − Killer toxin−resistance protein 5 OS=Saccharomyces cerevisiae (strain ATCC 204508 / S288c) GN=KRE5 PE=1 SV=2

KRE6 − average.top3: 6.56699578947368 − max.qupm: 4

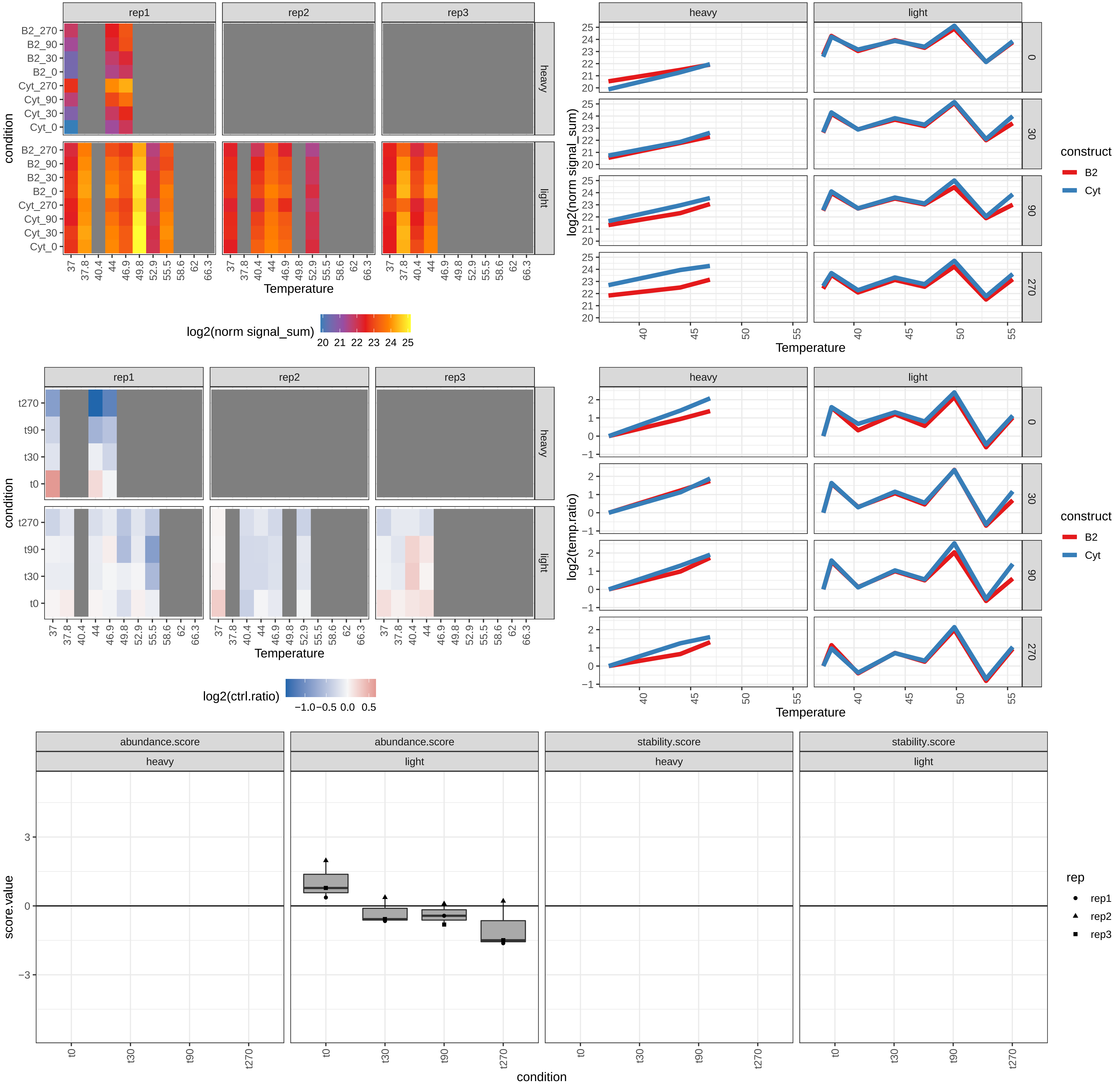

P32486 − Beta−glucan synthesis−associated protein KRE6 OS=Saccharomyces cerevisiae (strain ATCC 204508 / S288c) GN=KRE6 PE=1 SV=2

KRI1 − average.top3: 6.11044571428571 − max.qupm: 4

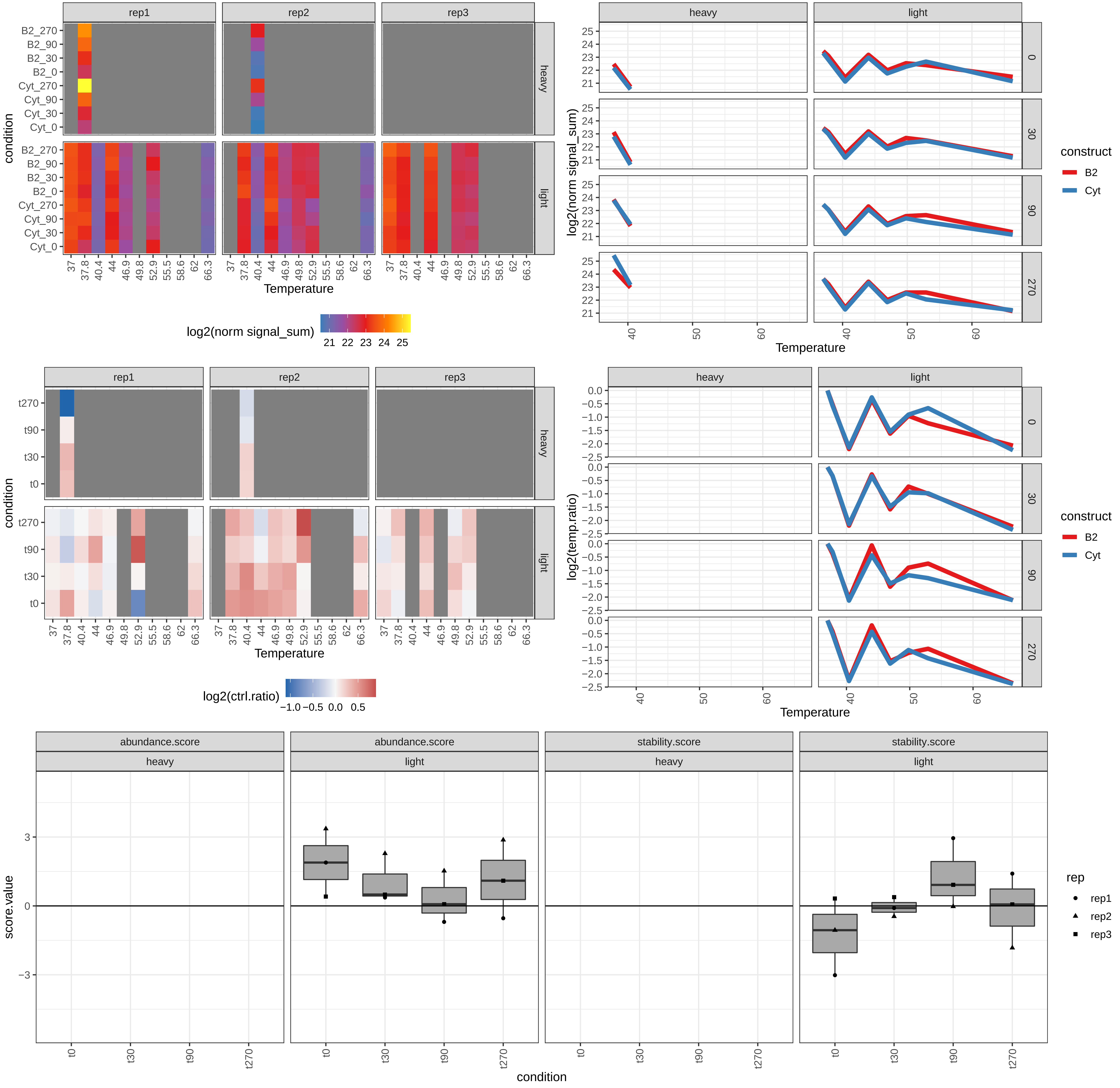

P42846 − Protein KRI1 OS=Saccharomyces cerevisiae (strain ATCC 204508 / S288c) GN=KRI1 PE=1 SV=1

KRR1 − average.top3: 6.81229535714286 − max.qupm: 8

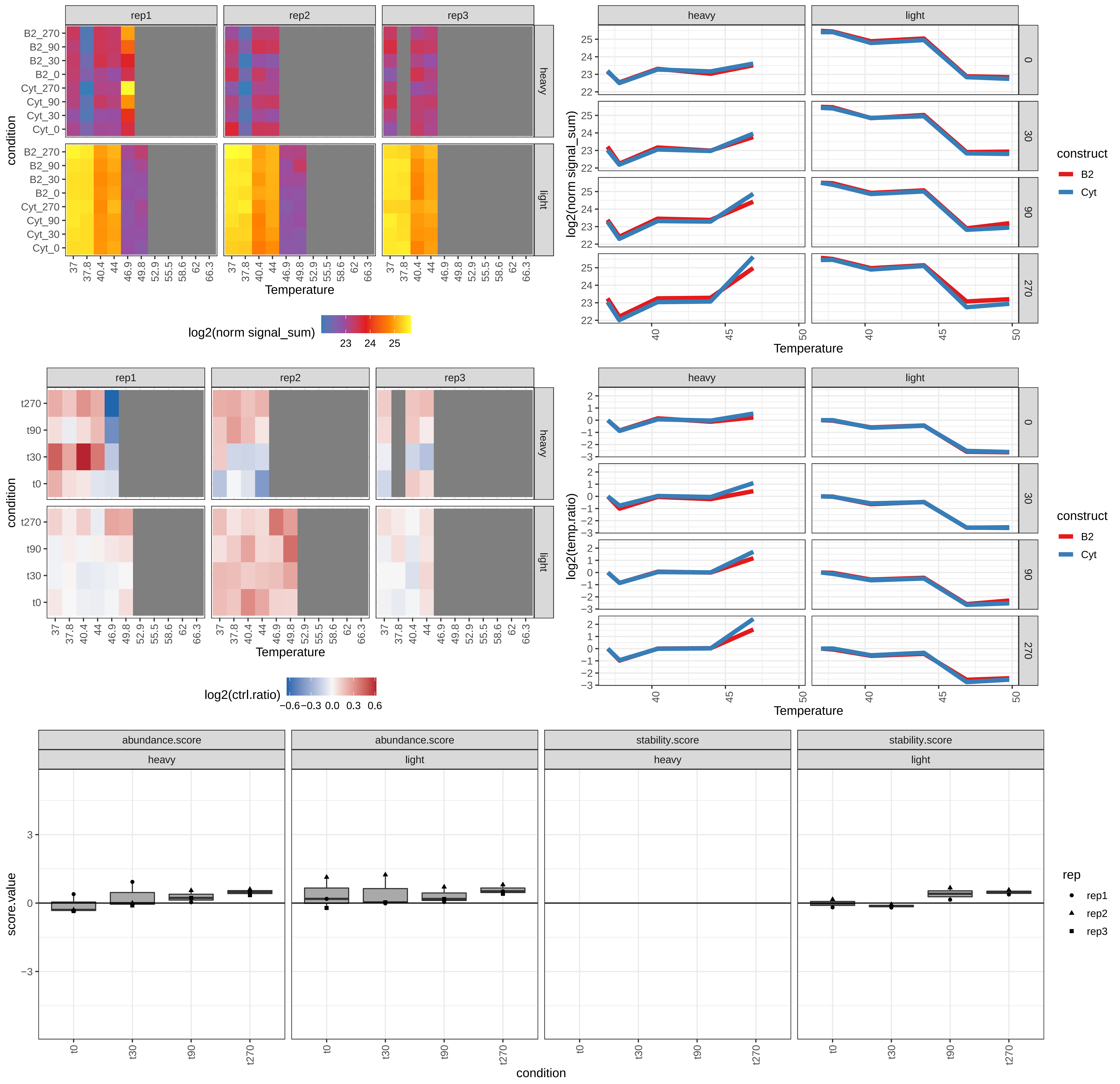

P25586 − KRR1 small subunit processome component OS=Saccharomyces cerevisiae (strain ATCC 204508 / S288c) GN=KRR1 PE=1 SV=1

KRS1 − average.top3: 7.67444230769231 − max.qupm: 28

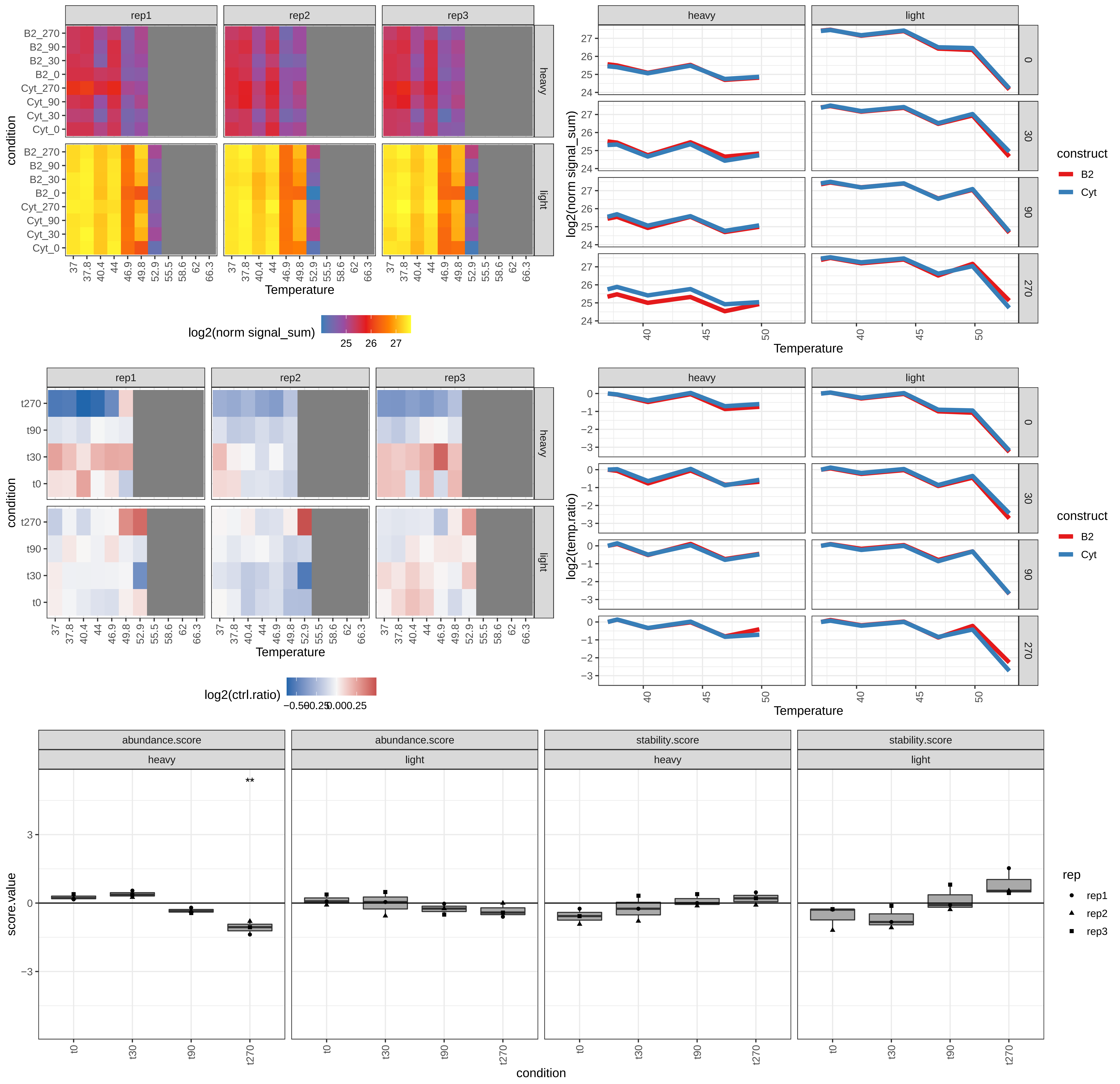

P15180 − Lysine−−tRNA ligase, cytoplasmic OS=Saccharomyces cerevisiae (strain ATCC 204508 / S288c) GN=KRS1 PE=1 SV=2

KTI12 − average.top3: 6.83558416666667 − max.qupm: 3

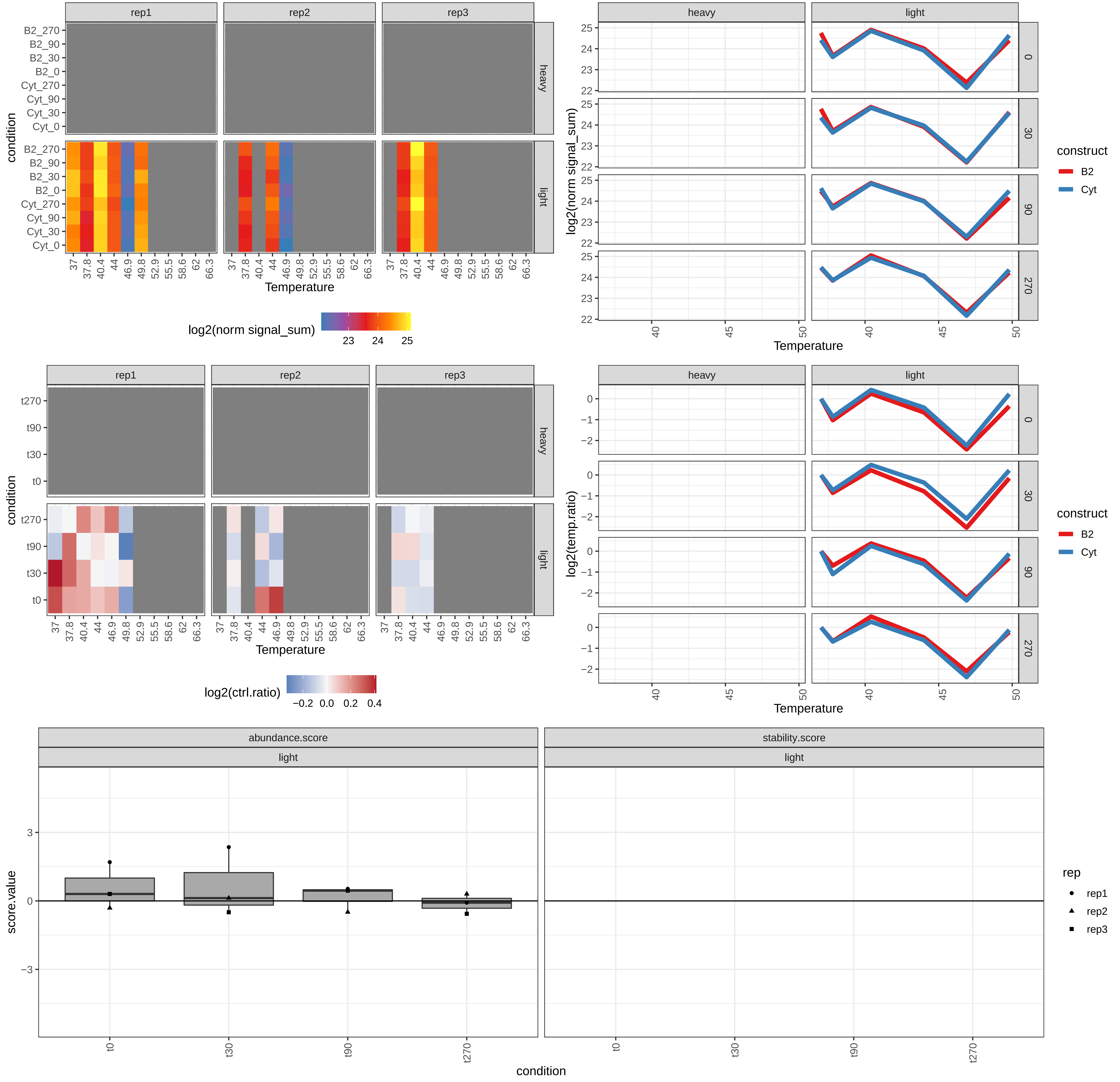

P34253 − Protein KTI12 OS=Saccharomyces cerevisiae (strain ATCC 204508 / S288c) GN=KTI12 PE=1 SV=2

KTR1 − average.top3: 7.13364084745763 − max.qupm: 9

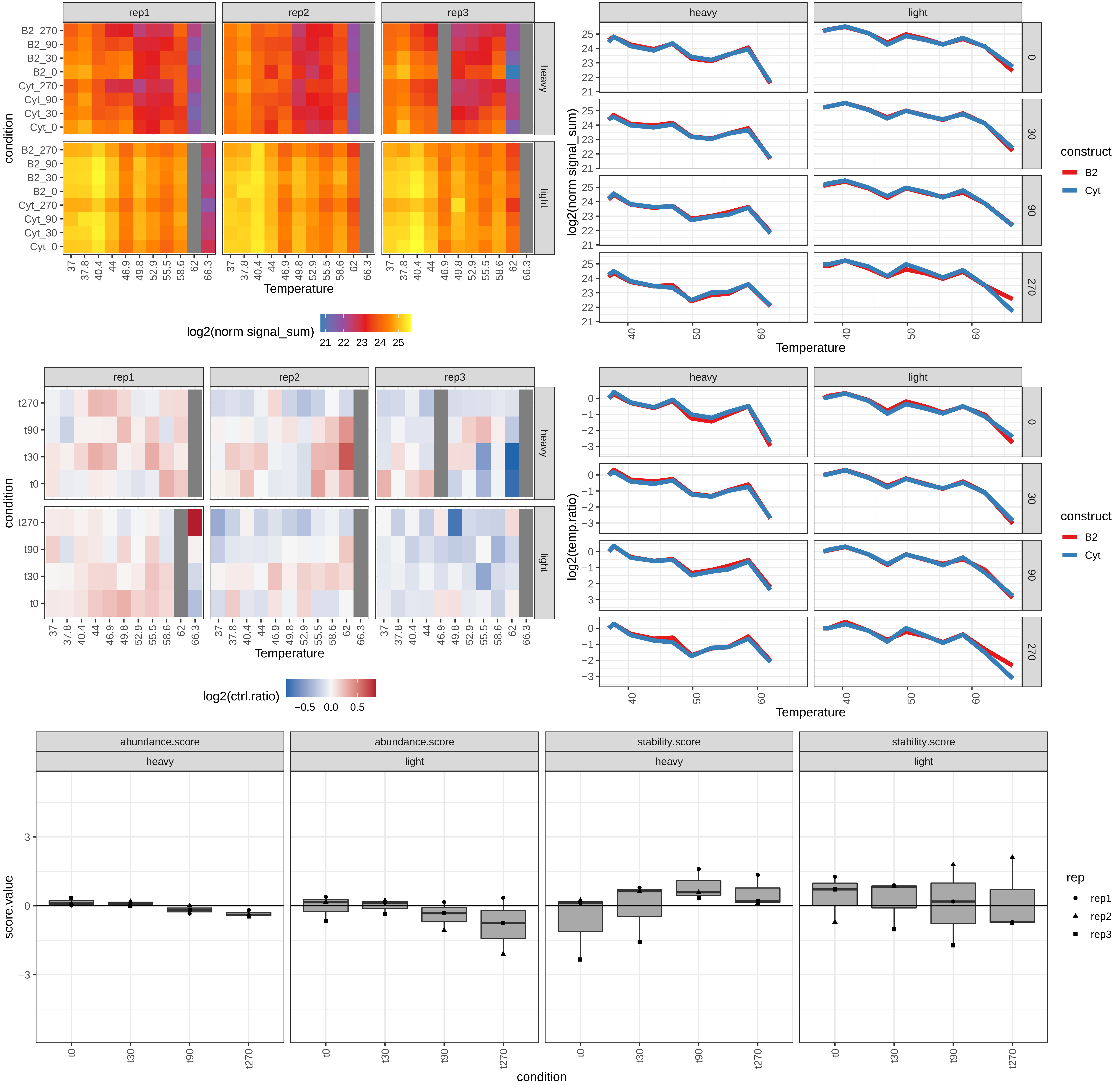

P27810 − Alpha−1,2 mannosyltransferase KTR1 OS=Saccharomyces cerevisiae (strain ATCC 204508 / S288c) GN=KTR1 PE=1 SV=1

KTR3 − average.top3: 6.97171135135135 − max.qupm: 11

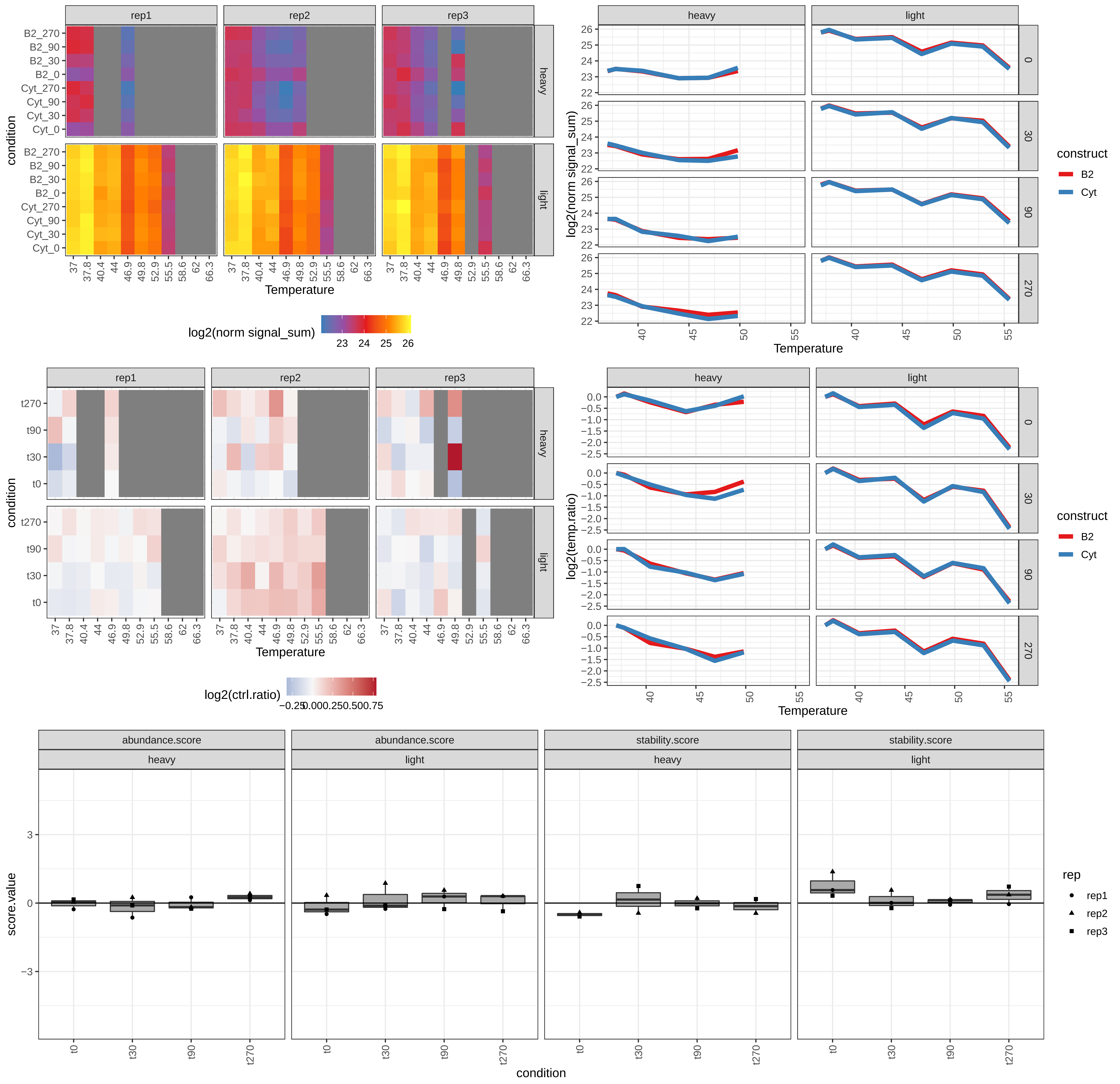

P38130 − Probable mannosyltransferase KTR3 OS=Saccharomyces cerevisiae (strain ATCC 204508 / S288c) GN=KTR3 PE=1 SV=1

KTR4 − average.top3: 6.69917133333333 − max.qupm: 7

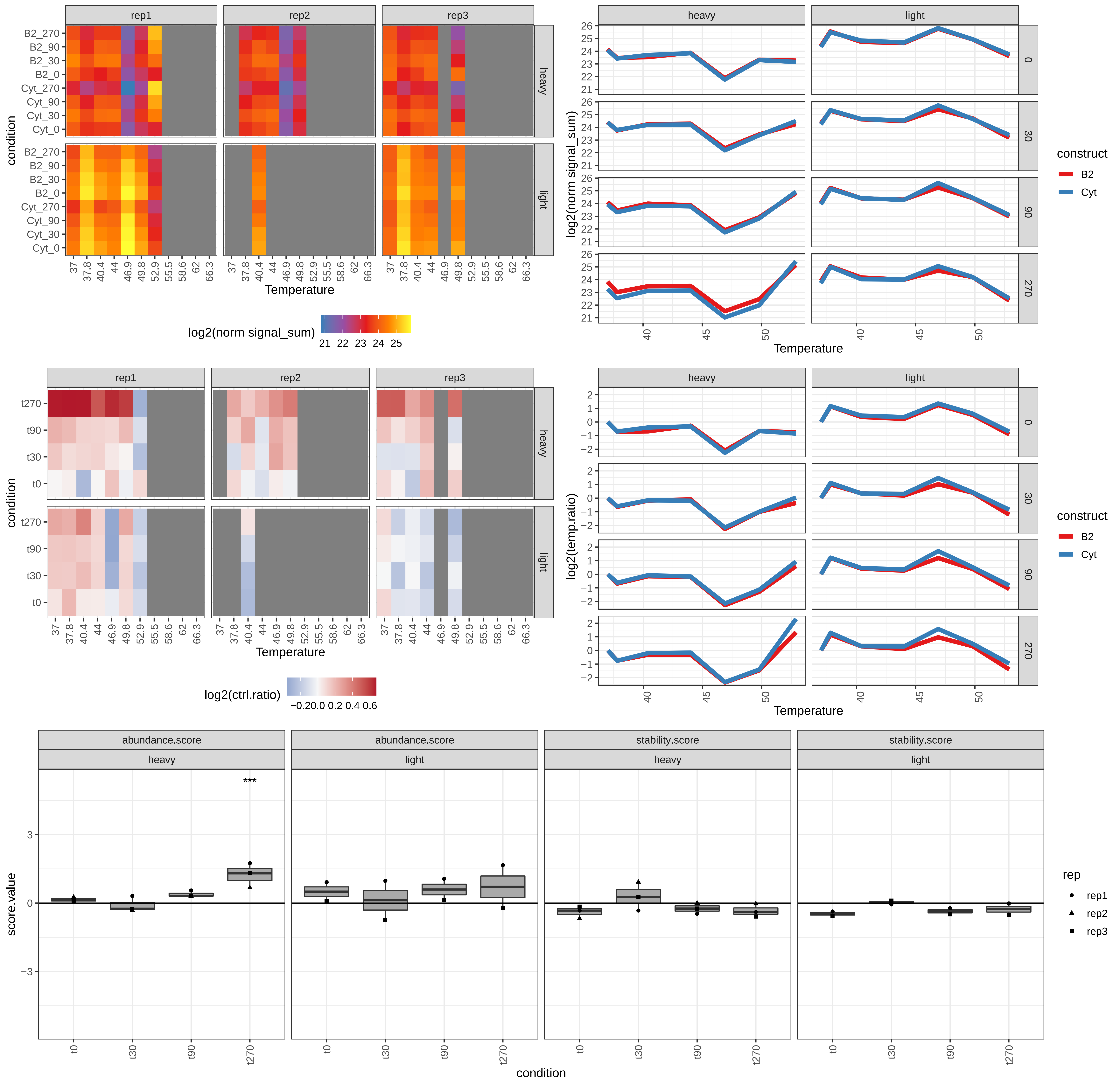

P38131 − Probable mannosyltransferase KTR4 OS=Saccharomyces cerevisiae (strain ATCC 204508 / S288c) GN=KTR4 PE=1 SV=1

KTR6 − average.top3: 6.85189727272727 − max.qupm: 4

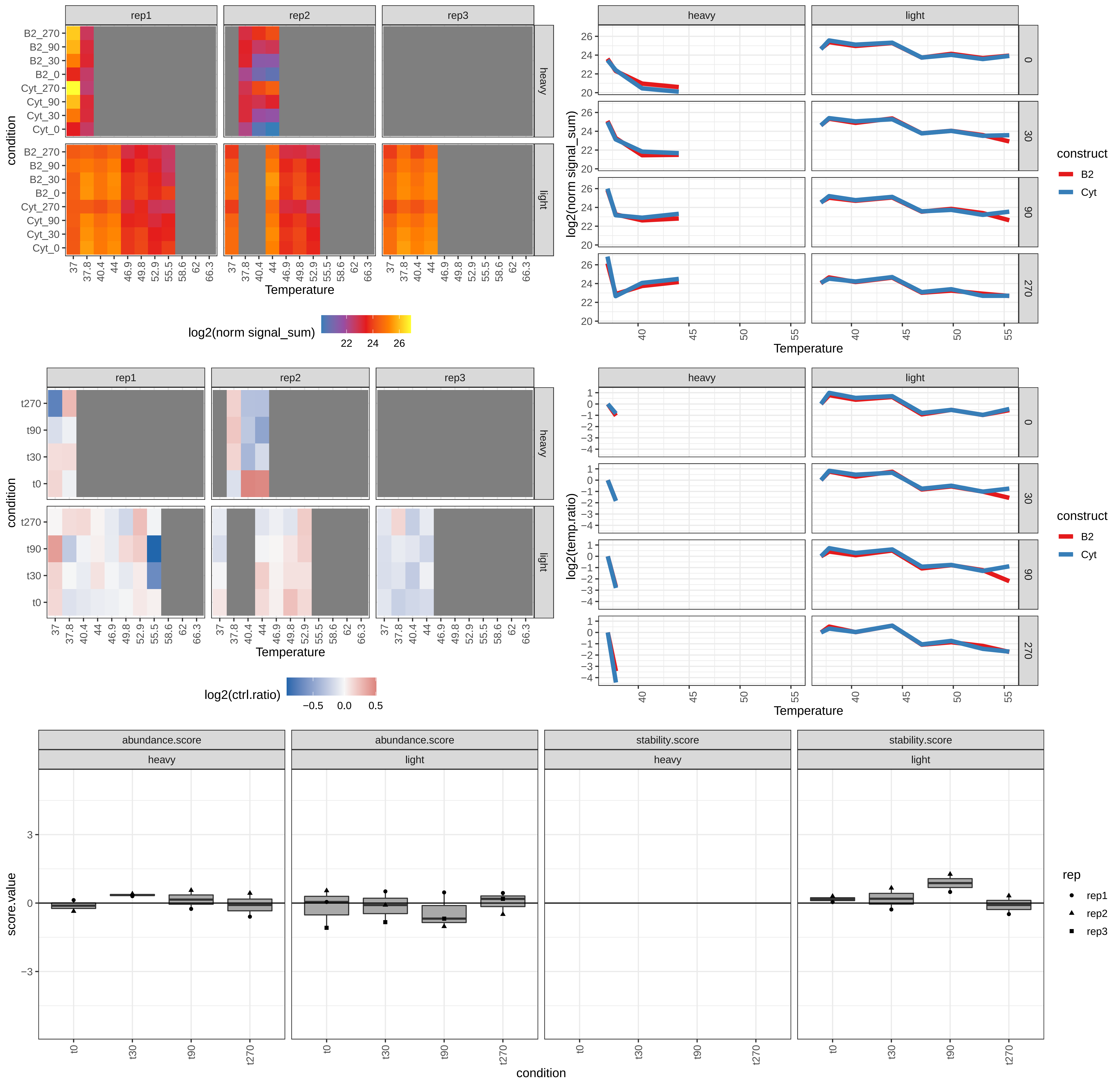

P54070 − Mannosyltransferase KTR6 OS=Saccharomyces cerevisiae (strain ATCC 204508 / S288c) GN=KTR6 PE=1 SV=1

KXD1 − average.top3: 6.56744647058824 − max.qupm: 4

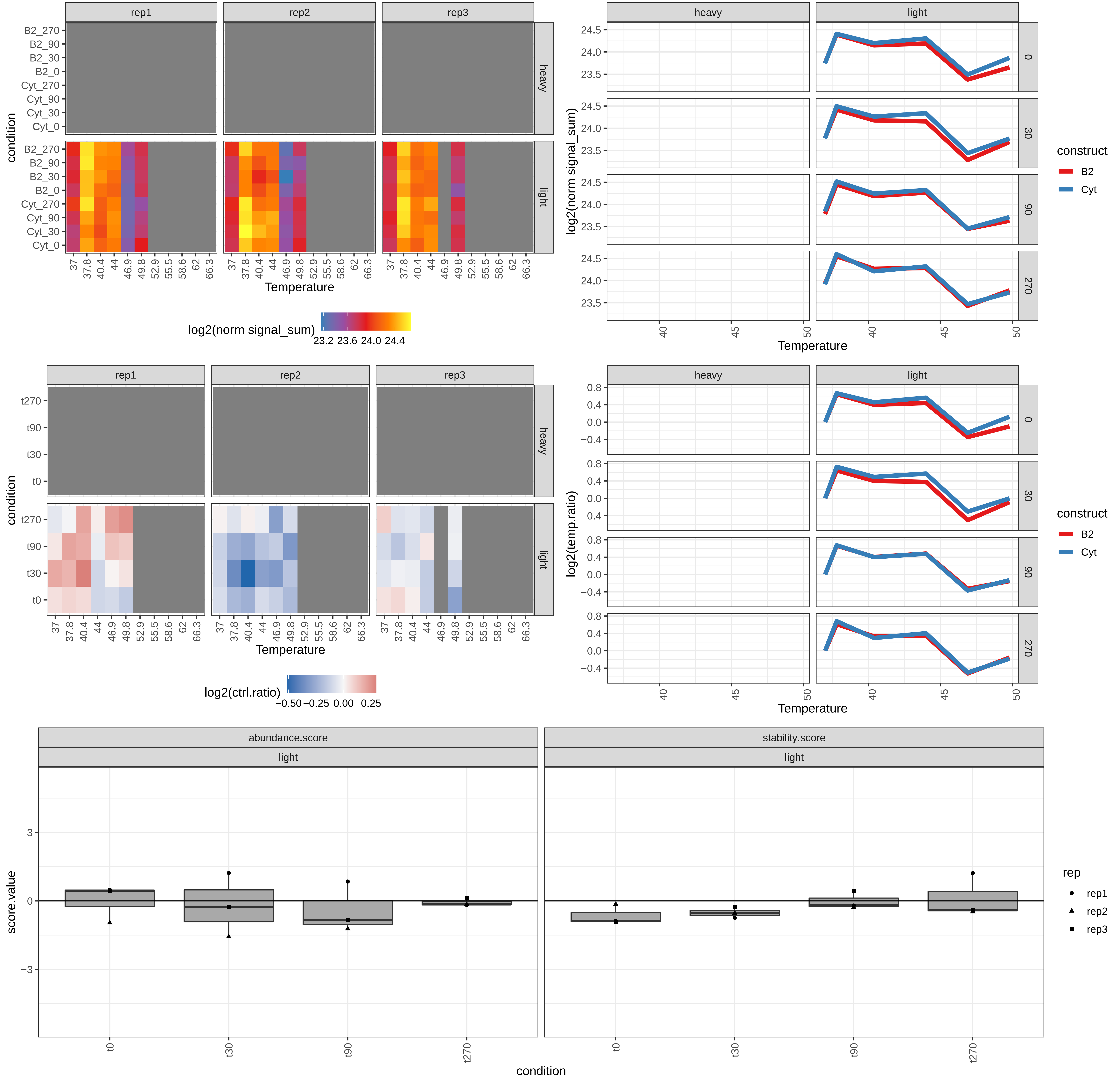

P53158 − Biogenesis of lysosome−related organelles complex 1 subunit KXD1 OS=Saccharomyces cerevisiae (strain ATCC 204508 / S288c) GN=KXD1 PE=1 SV=1

LAA1 − average.top3: 6.72134703703704 − max.qupm: 14

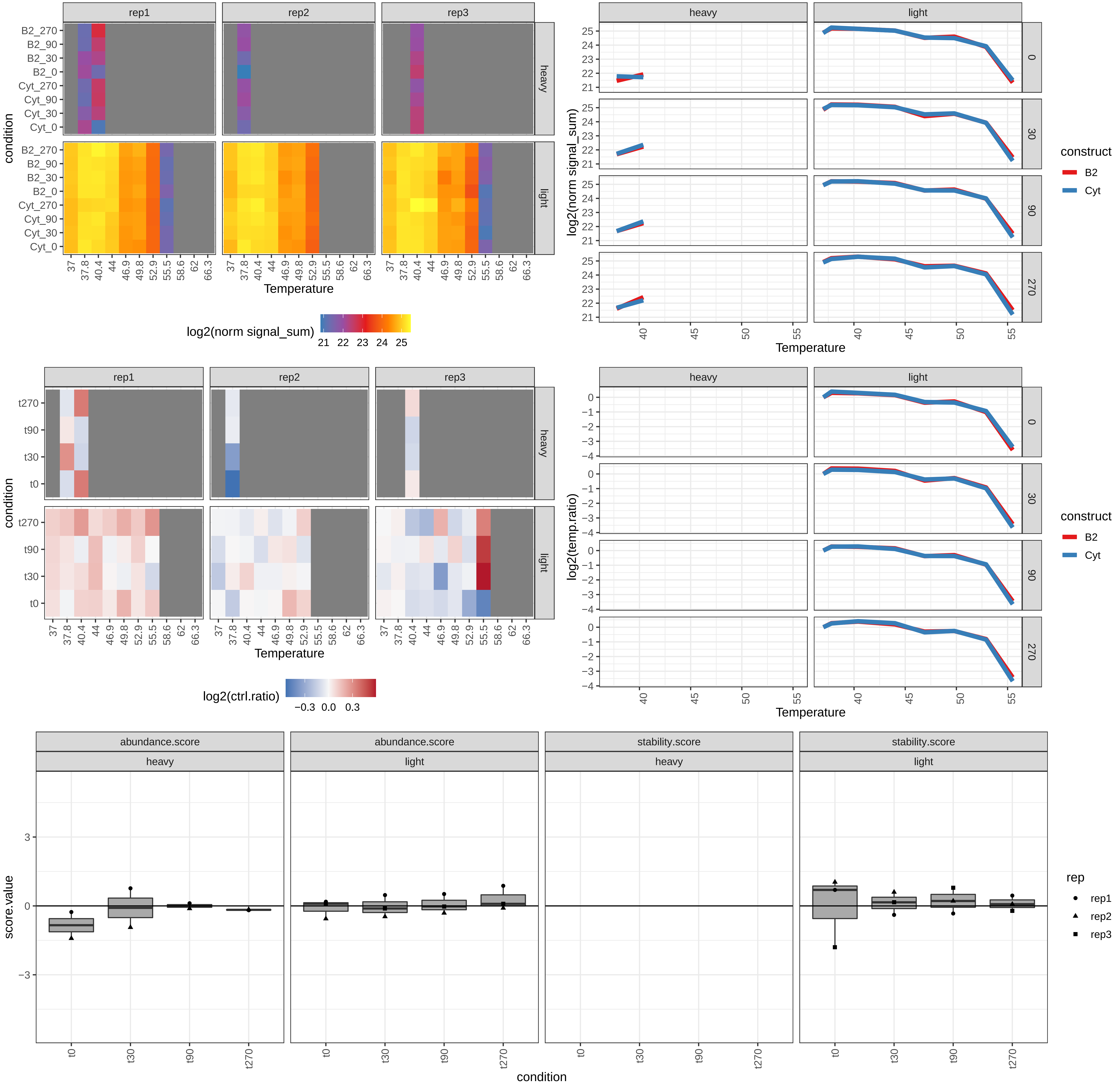

P39526 − AP−1 accessory protein LAA1 OS=Saccharomyces cerevisiae (strain ATCC 204508 / S288c) GN=LAA1 PE=1 SV=1

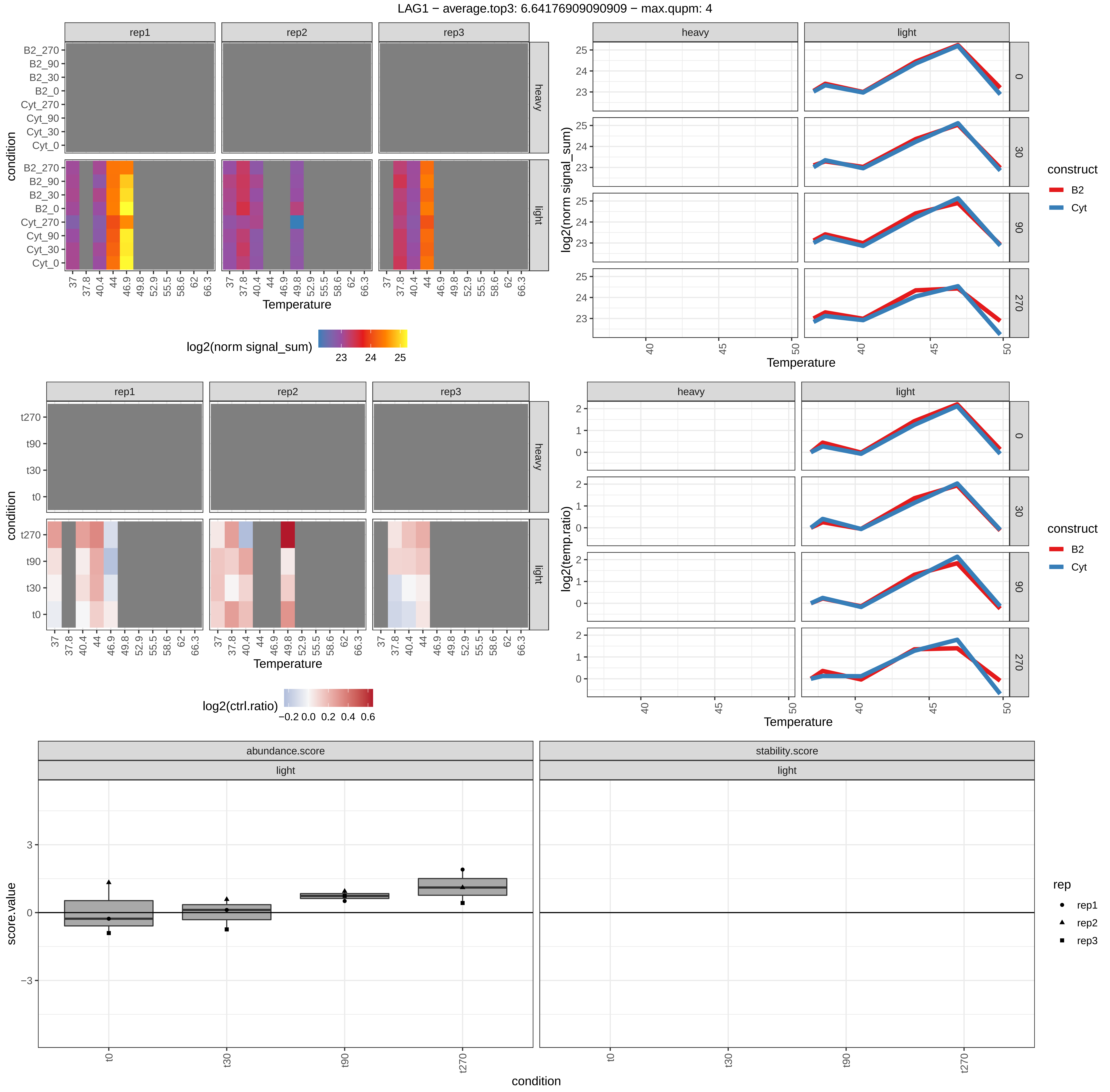

P38703 − Sphingosine N−acyltransferase LAG1 OS=Saccharomyces cerevisiae (strain ATCC 204508 / S288c) GN=LAG1 PE=1 SV=1

LAG2 − average.top3: 6.56642647058823 − max.qupm: 5

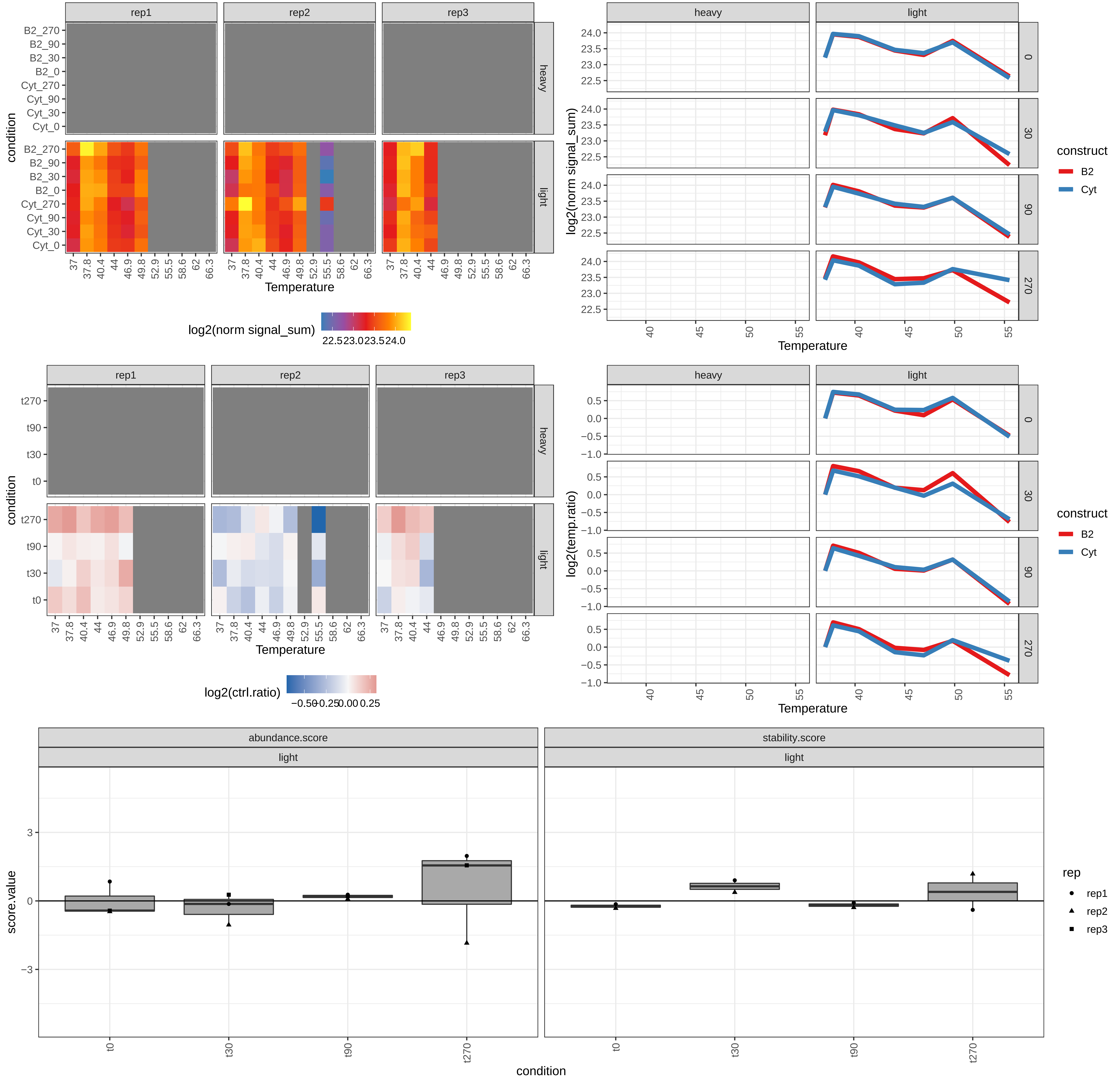

Q92325 − Cullin−associated NEDD8−dissociated protein 1 homolog OS=Saccharomyces cerevisiae (strain ATCC 204508 / S288c) GN=LAG2 PE=1 SV=2

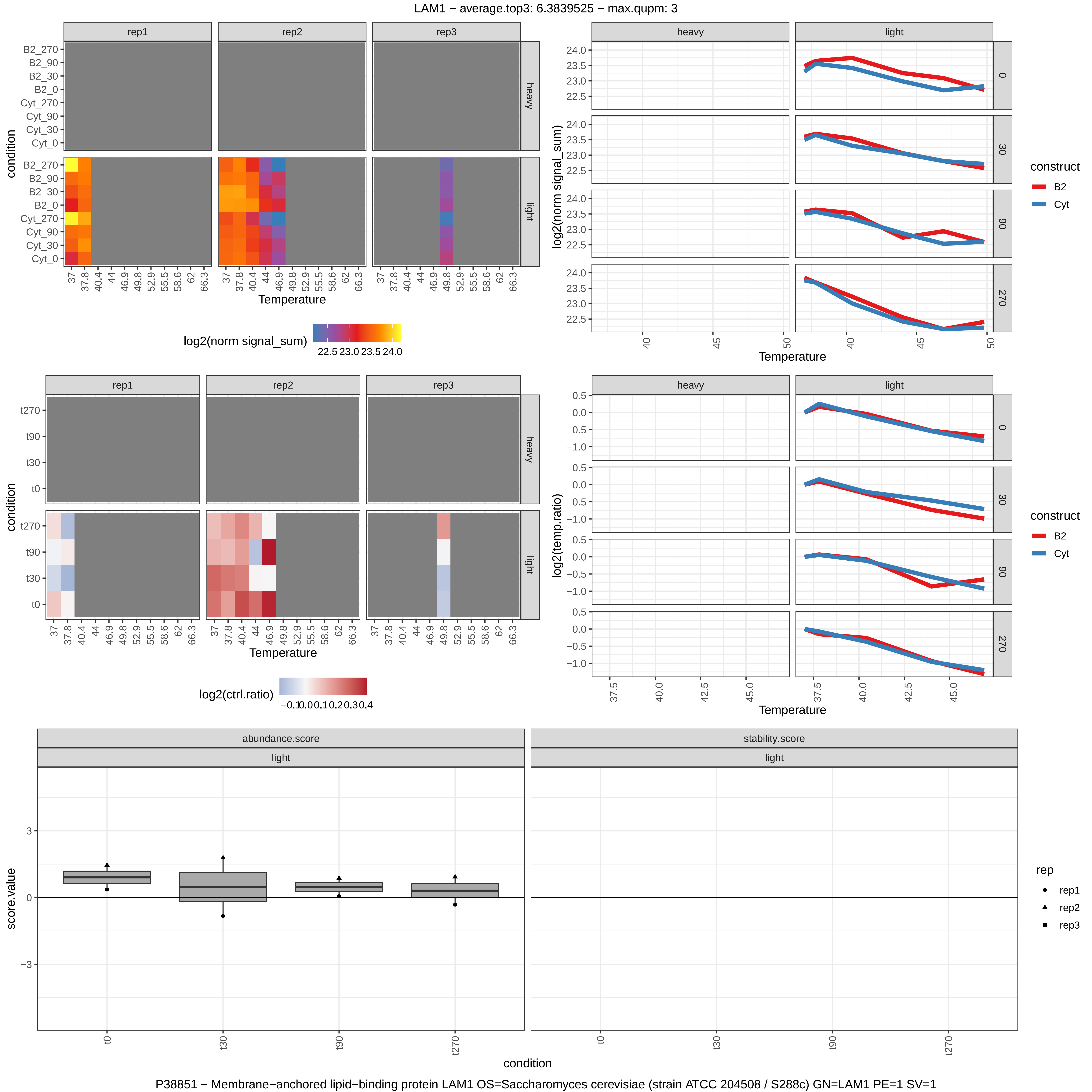

LAM1 − average.top3: 6.3839525 − max.qupm: 3
rep1
rep2
rep3
heavy
light
condition
B2_270
B2_90
B2_30
B2_0
Cyt_270
Cyt_90
Cyt_30
Cyt_0
37
37.8
40.4
44
46.9
49.8
52.9
55.5
58.6
62
66.3
Temperature
log2(norm signal_sum)
22.5 23.0 23.5 24.0
0
30
90
270
construct
B2
Cyt
t270
t90
t30
t0
log2(ctrl.ratio)
−0.1 0.0 0.1 0.2 0.3 0.4
log2(temp.ratio)
abundance.score
stability.score
score.value
rep
rep1
rep2
rep3
P38851 − Membrane−anchored lipid−binding protein LAM1 OS=Saccharomyces cerevisiae (strain ATCC 204508 / S288c) GN=LAM1 PE=1 SV=1

LAM5 − average.top3: 6.85448058823529 − max.qupm: 6

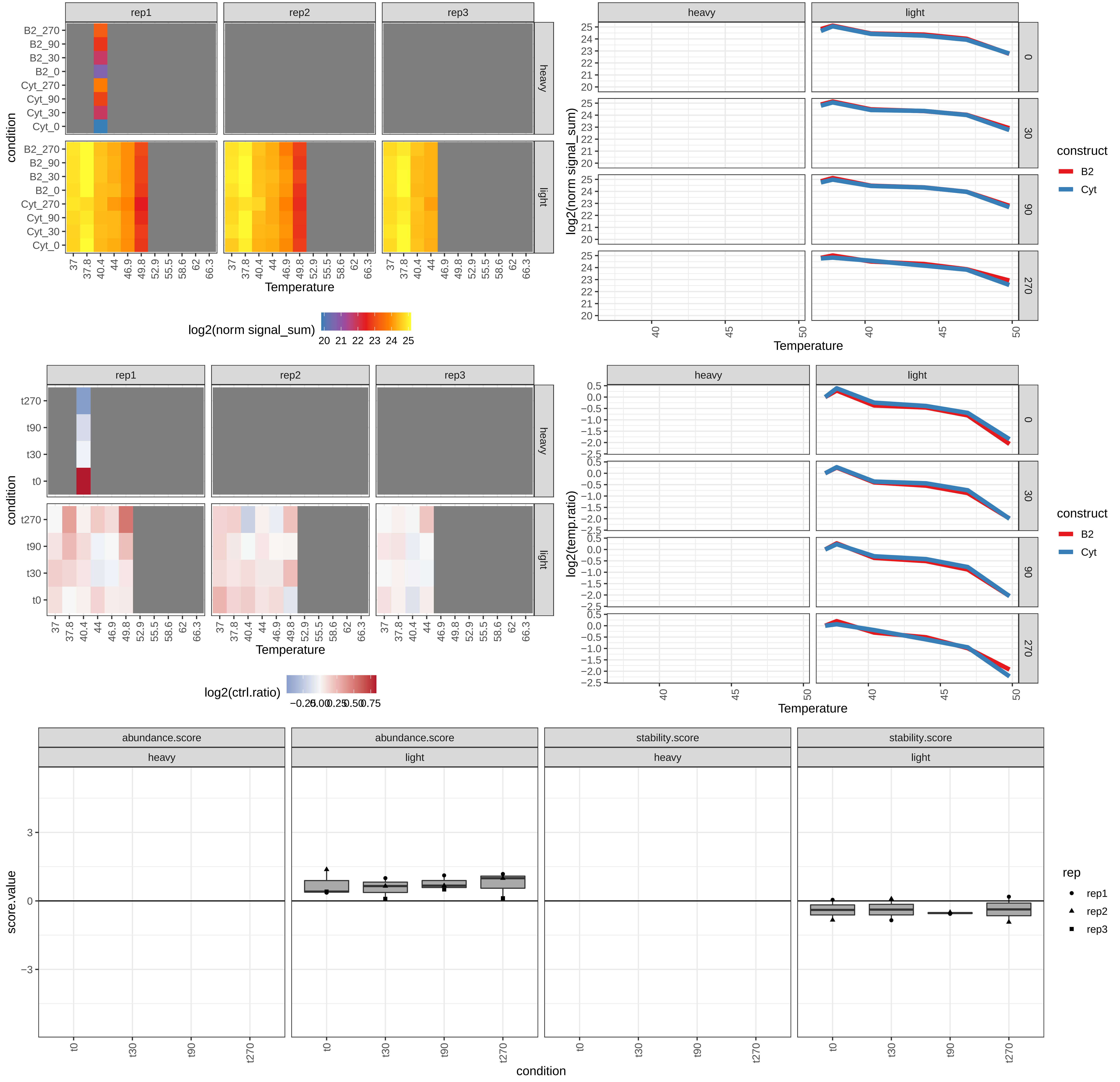

P43560 − Membrane−anchored lipid−binding protein LAM5 OS=Saccharomyces cerevisiae (strain ATCC 204508 / S288c) GN=LAM5 PE=1 SV=2

LAM6 − average.top3: 6.57597692307692 − max.qupm: 5

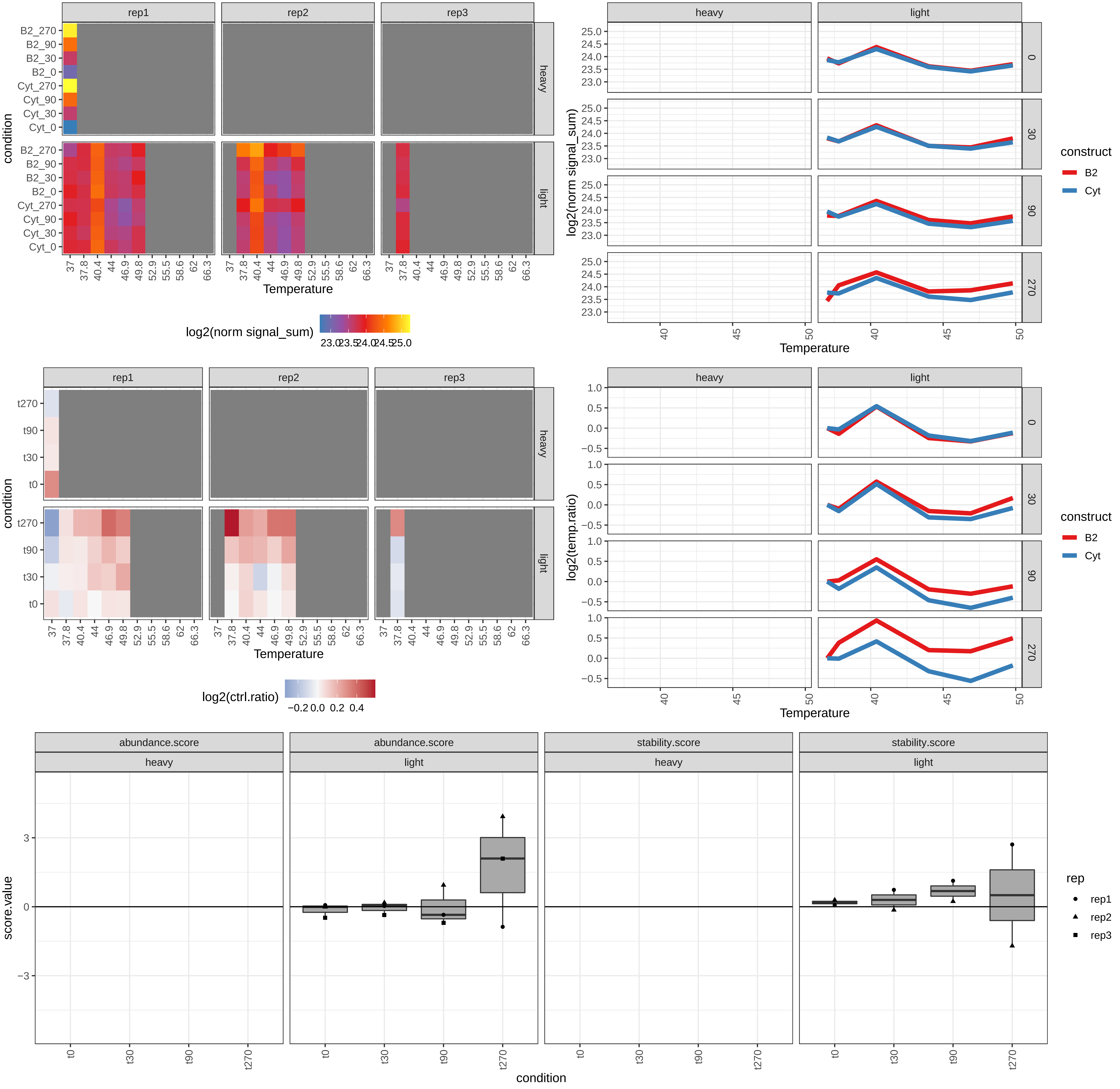

Q08001 − Membrane−anchored lipid−binding protein LAM6 OS=Saccharomyces cerevisiae (strain ATCC 204508 / S288c) GN=LAM6 PE=1 SV=1

LAP2 − average.top3: 7.20838254237288 − max.qupm: 19

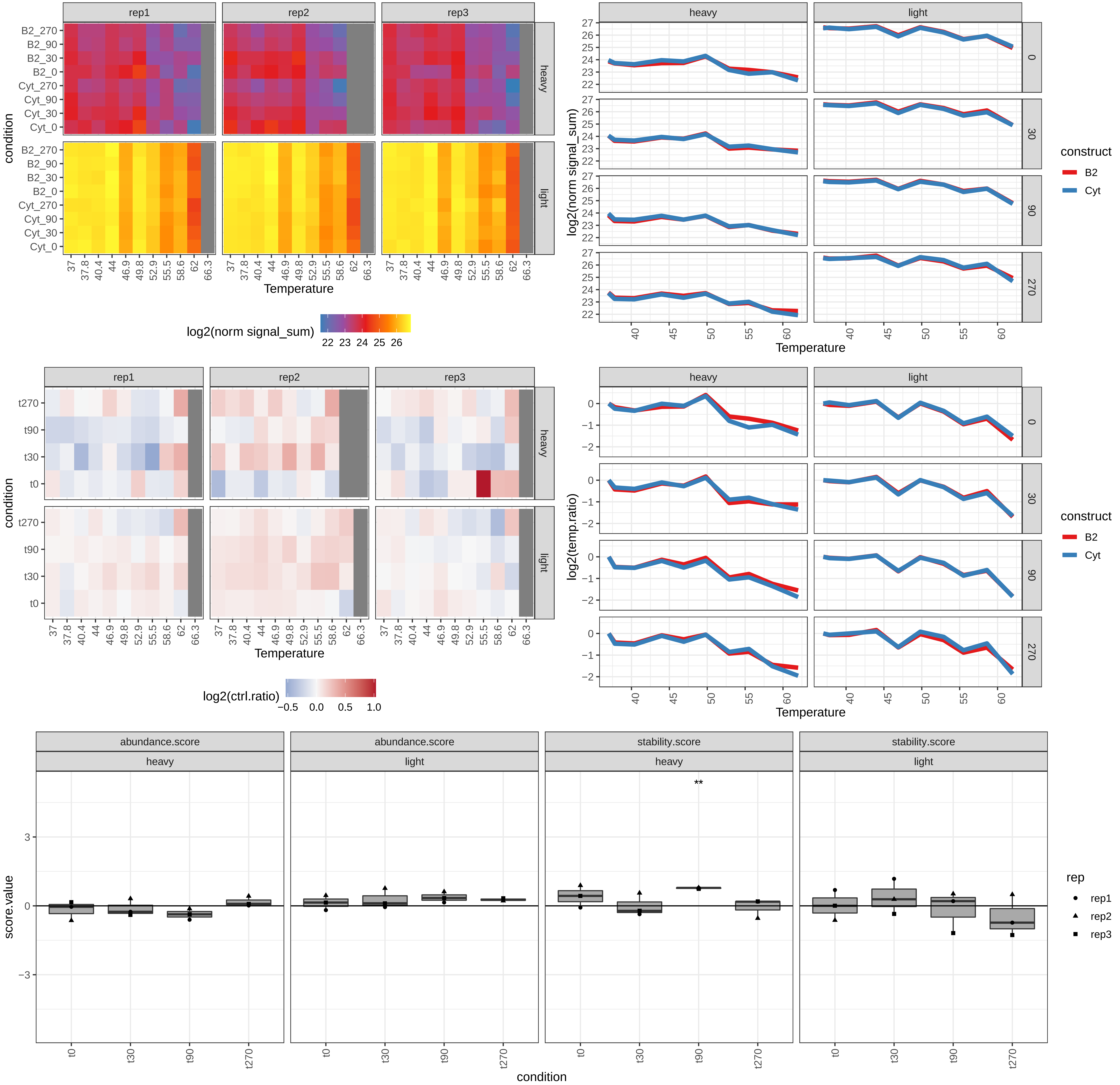

Q10740 − Leukotriene A−4 hydrolase homolog OS=Saccharomyces cerevisiae (strain ATCC 204508 / S288c) GN=LAP2 PE=1 SV=1

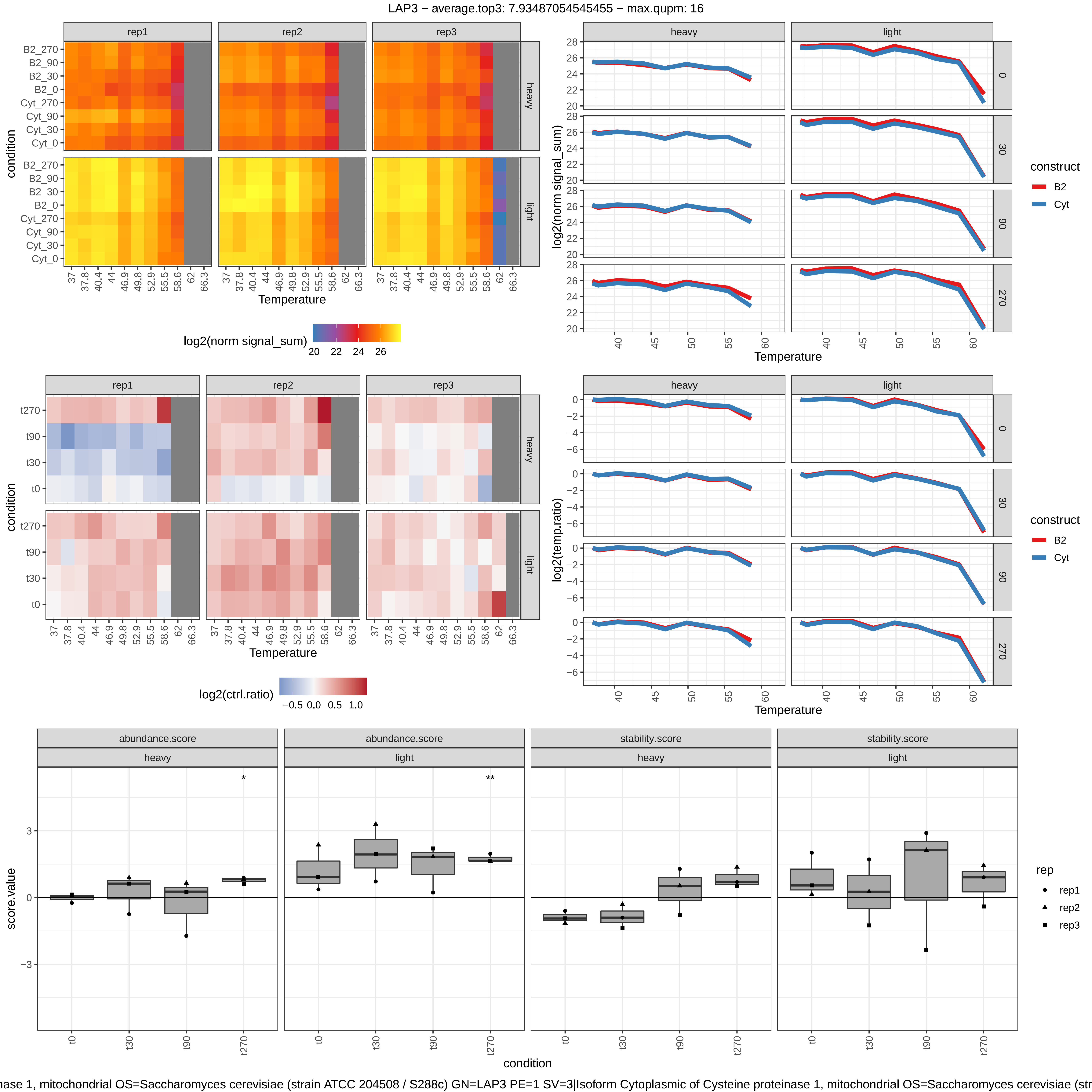

LAP3 − average.top3: 7.93487054545455 − max.qupm: 16
rep1
rep2
rep3
heavy
light
condition
Temperature
log2(norm signal_sum)
construct
B2
Cyt
log2(ctrl.ratio)
log2(temp.ratio)
abundance.score
stability.score
score.value
rep
rep1
rep2
rep3
ase 1, mitochondrial OS=Saccharomyces cerevisiae (strain ATCC 204508 / S288c) GN=LAP3 PE=1 SV=3|Isoform Cytoplasmic of Cysteine proteinase 1, mitochondrial OS=Saccharomyces cerevisiae (str

LAS17 − average.top3: 7.07712166666667 − max.qupm: 5

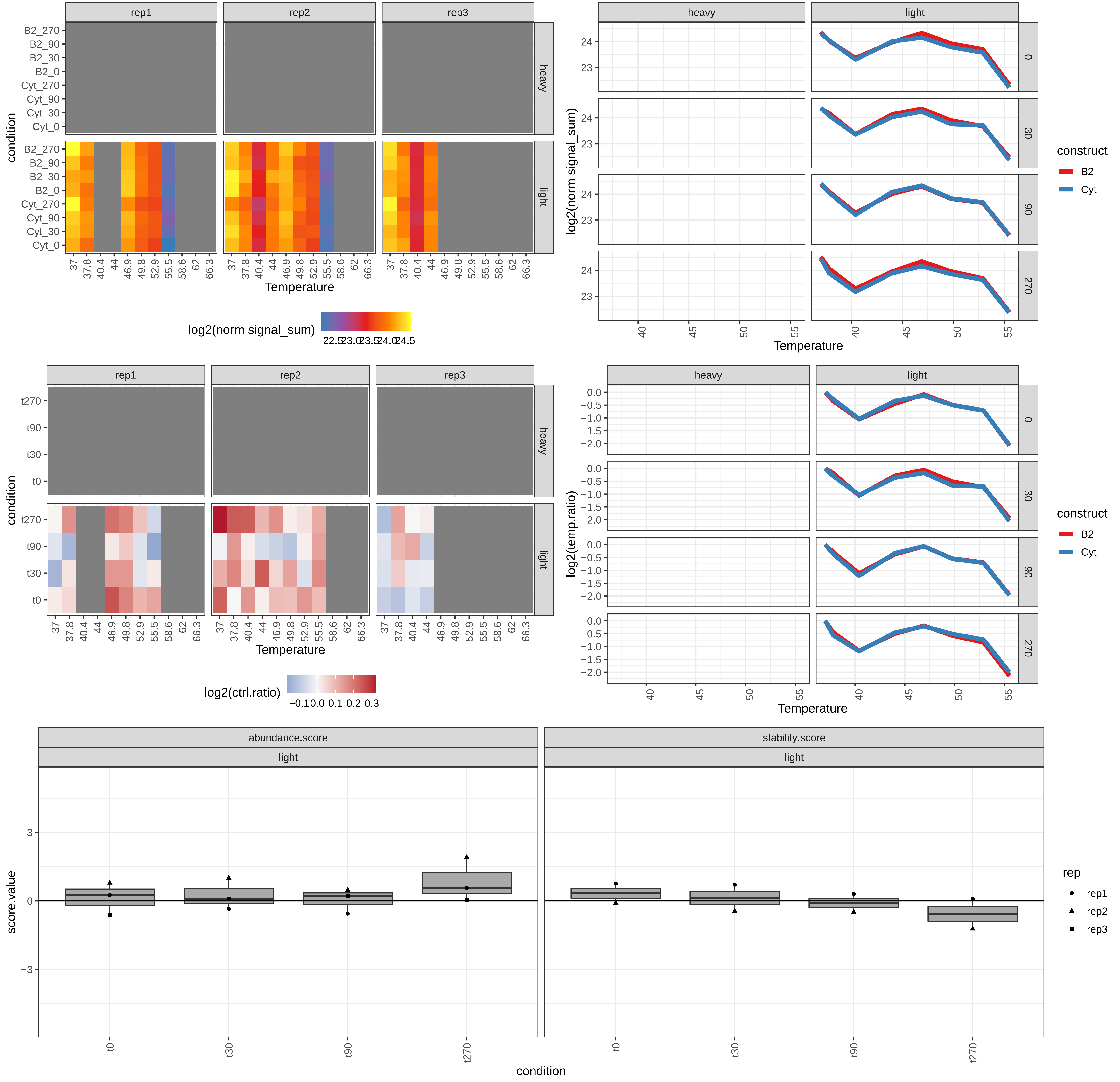

Q12446 − Proline−rich protein LAS17 OS=Saccharomyces cerevisiae (strain ATCC 204508 / S288c) GN=LAS17 PE=1 SV=1

LAS21 − average.top3: 6.55384222222222 − max.qupm: 3

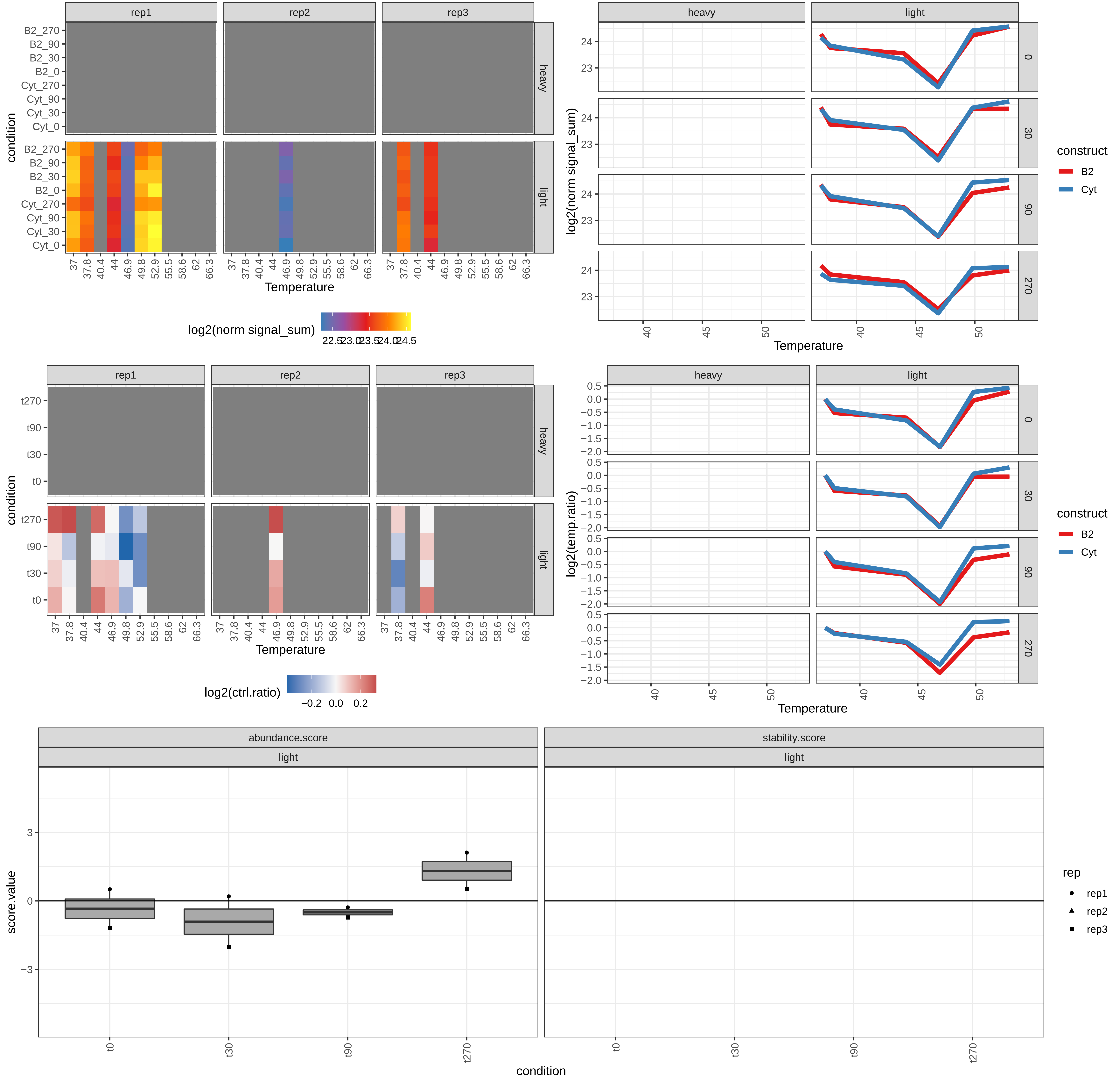

P40367 − GPI ethanolamine phosphate transferase 2 OS=Saccharomyces cerevisiae (strain ATCC 204508 / S288c) GN=LAS21 PE=1 SV=1

LAT1 − average.top3: 7.38810169491525 − max.qupm: 14

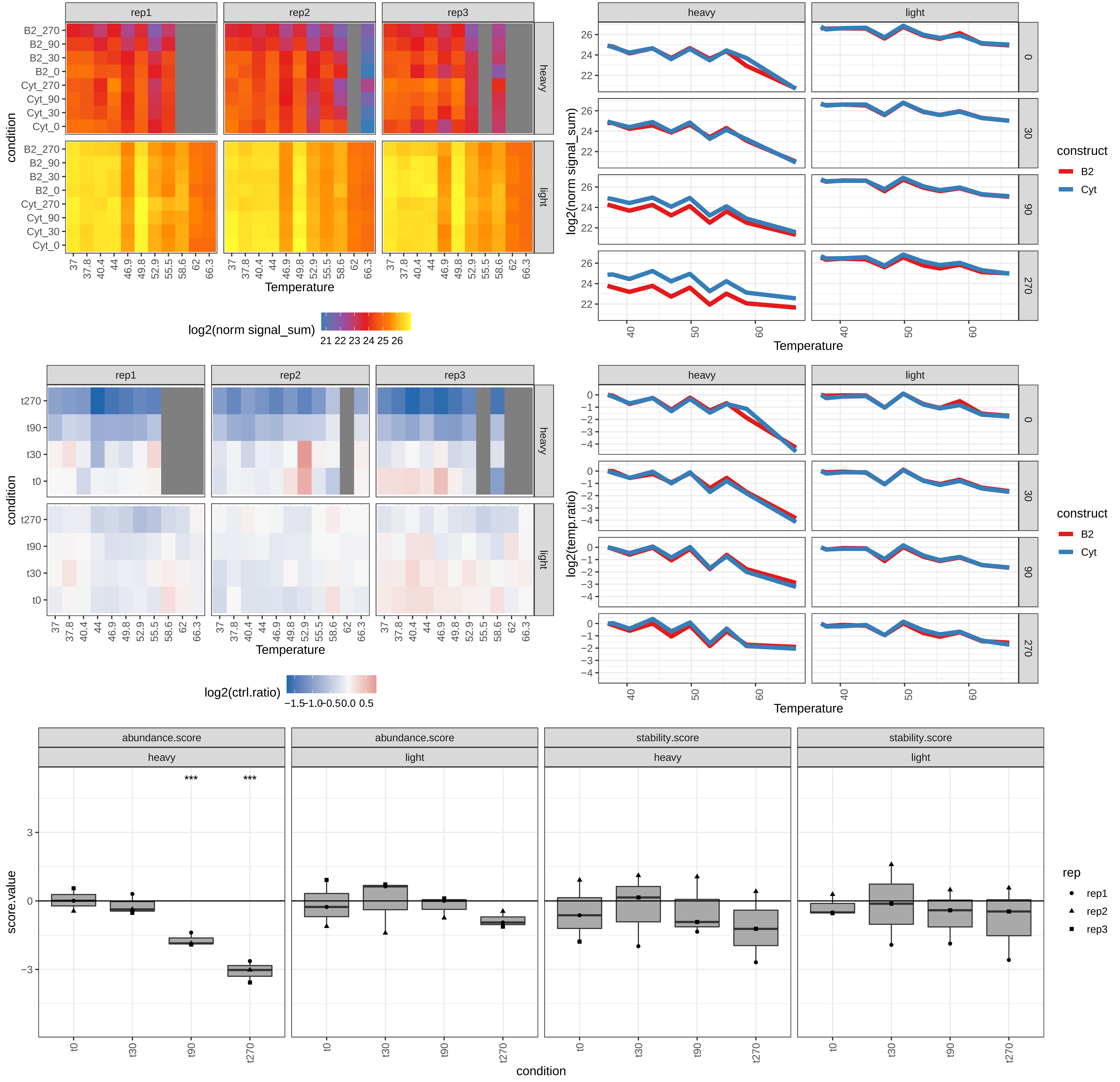

P12695 − Dihydrolipoyllysine−residue acetyltransferase component of pyruvate dehydrogenase complex, mitochondrial OS=Saccharomyces cerevisiae (strain ATCC 204508 / S288c) GN=LAT1 PE=1 SV=1

LCB1 − average.top3: 6.5749028 − max.qupm: 8

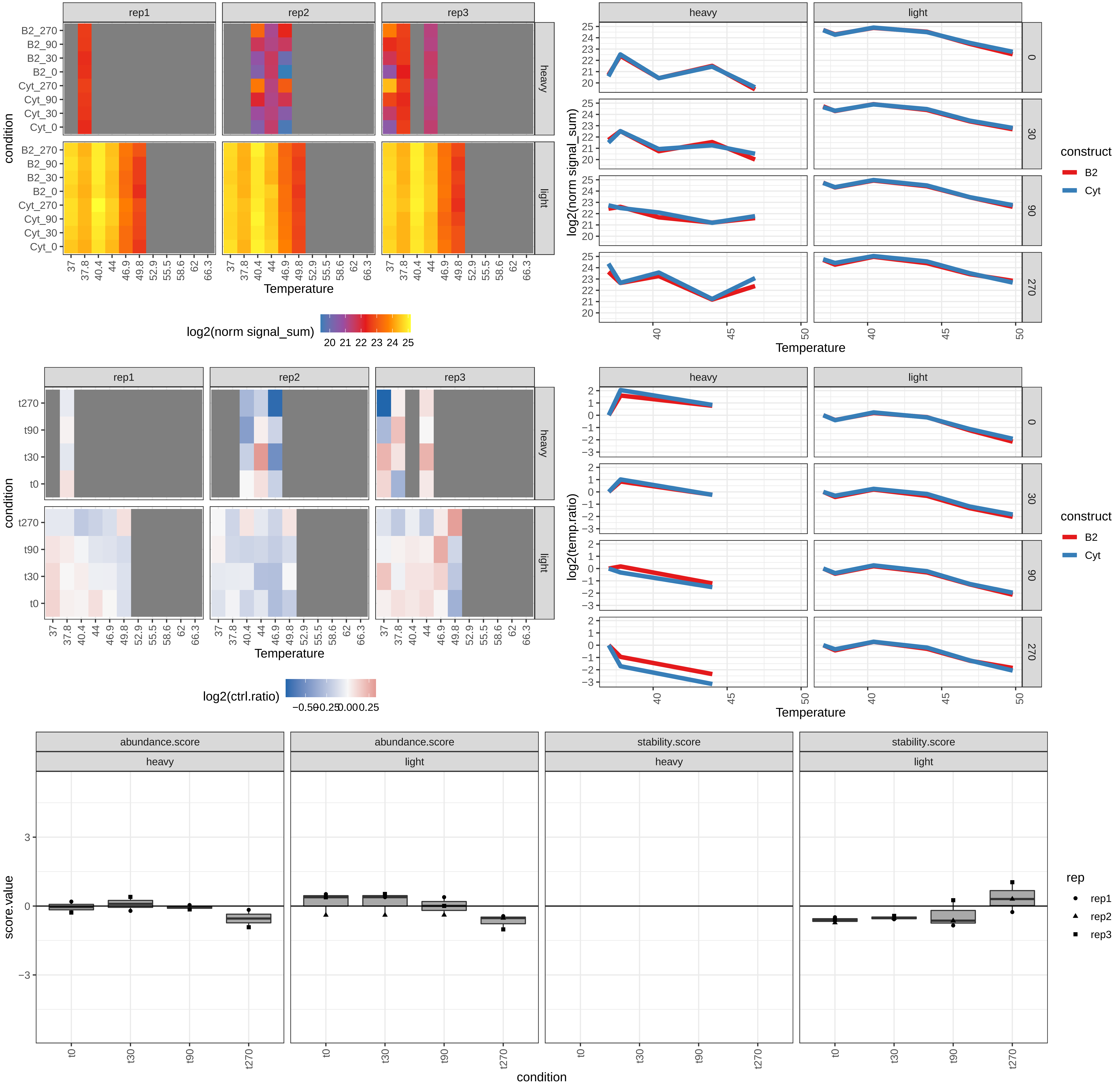

P25045 − Serine palmitoyltransferase 1 OS=Saccharomyces cerevisiae (strain ATCC 204508 / S288c) GN=LCB1 PE=1 SV=2

LCB2 − average.top3: 6.95189692307692 − max.qupm: 11

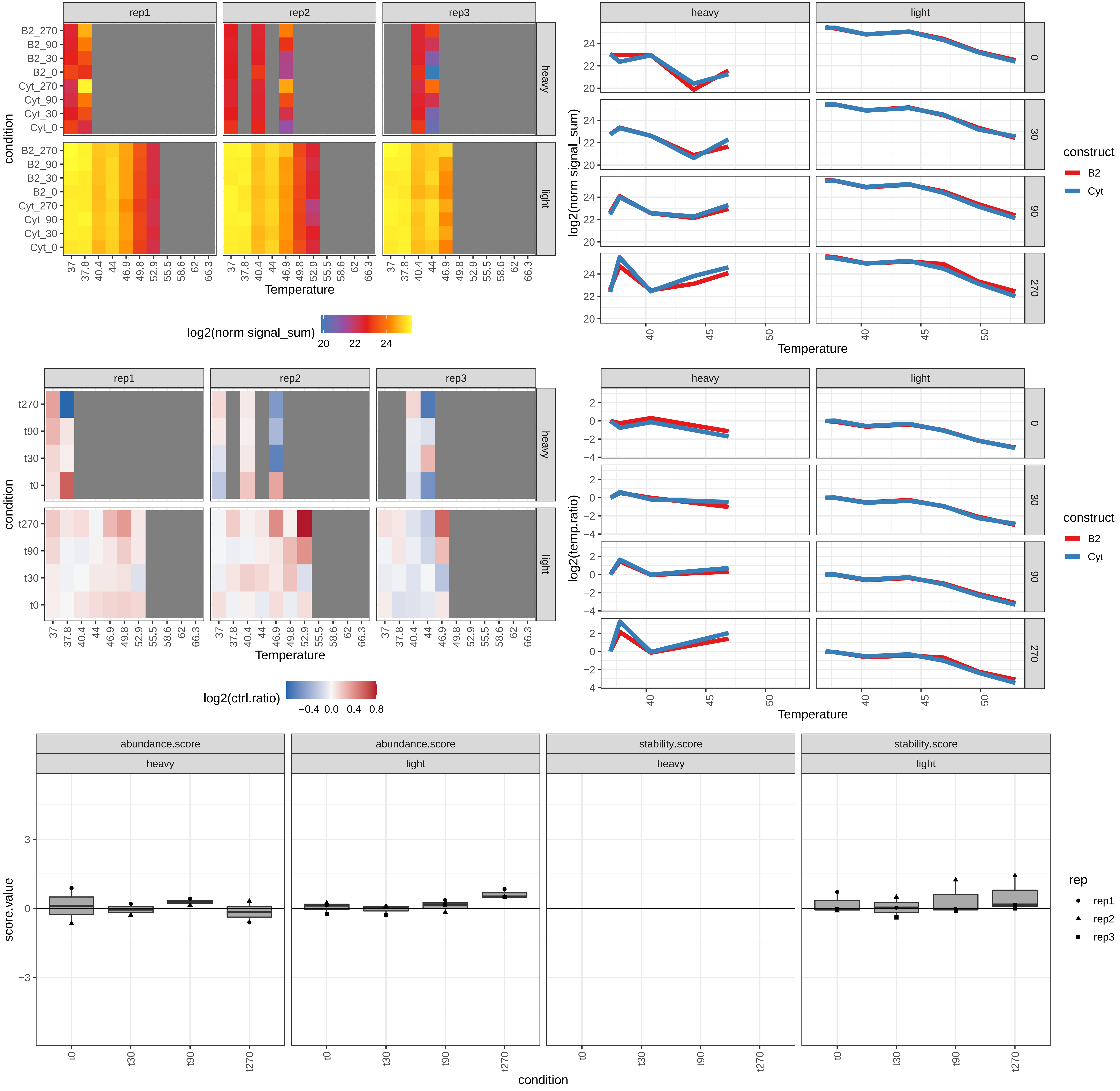

P40970 − Serine palmitoyltransferase 2 OS=Saccharomyces cerevisiae (strain ATCC 204508 / S288c) GN=LCB2 PE=1 SV=1

LDH1 − average.top3: 6.88504625 − max.qupm: 5

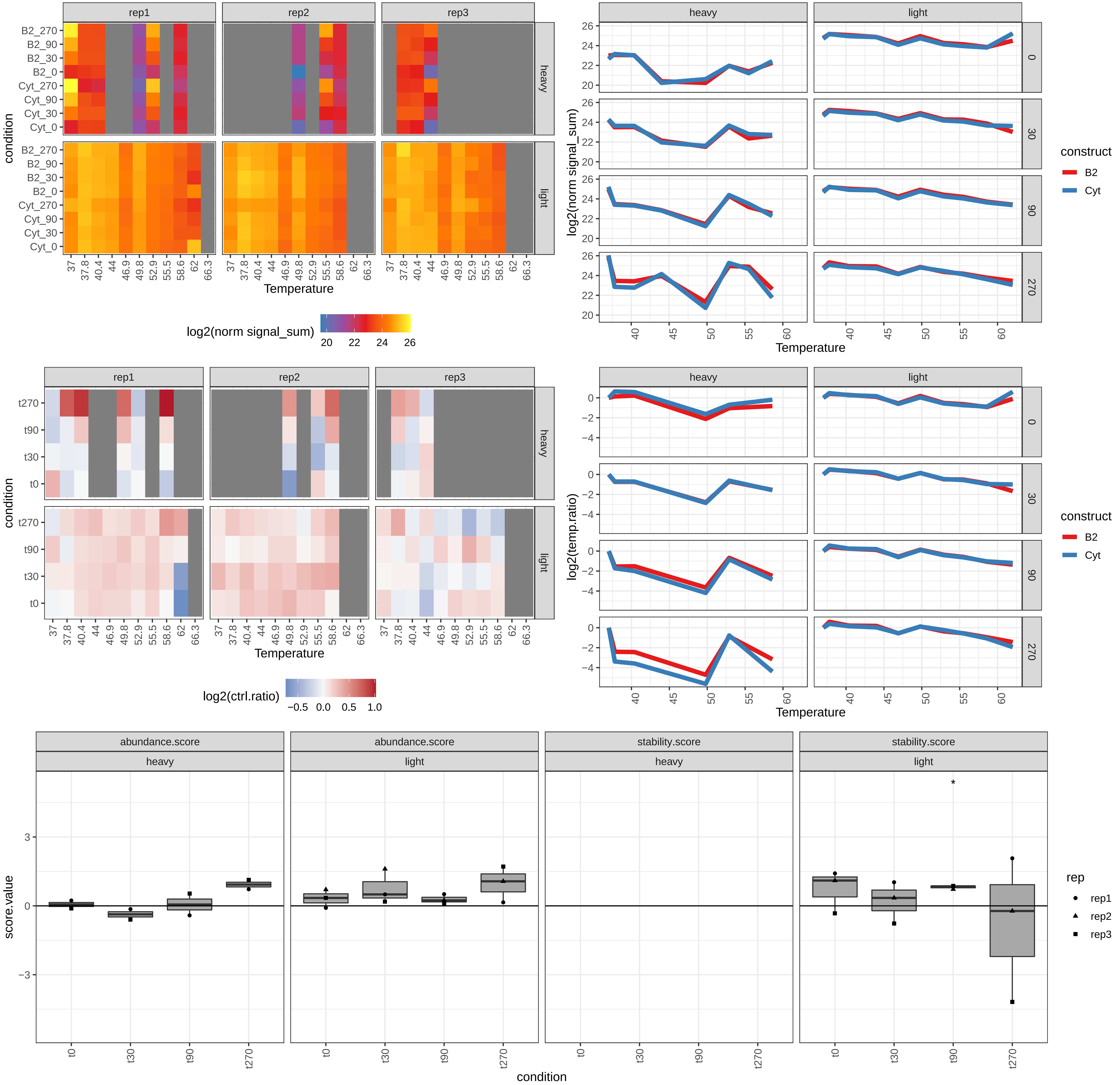

P38139 − Lipid droplet hydrolase 1 OS=Saccharomyces cerevisiae (strain ATCC 204508 / S288c) GN=LDH1 PE=1 SV=2

LEA1 − average.top3: 6.5632455 − max.qupm: 5

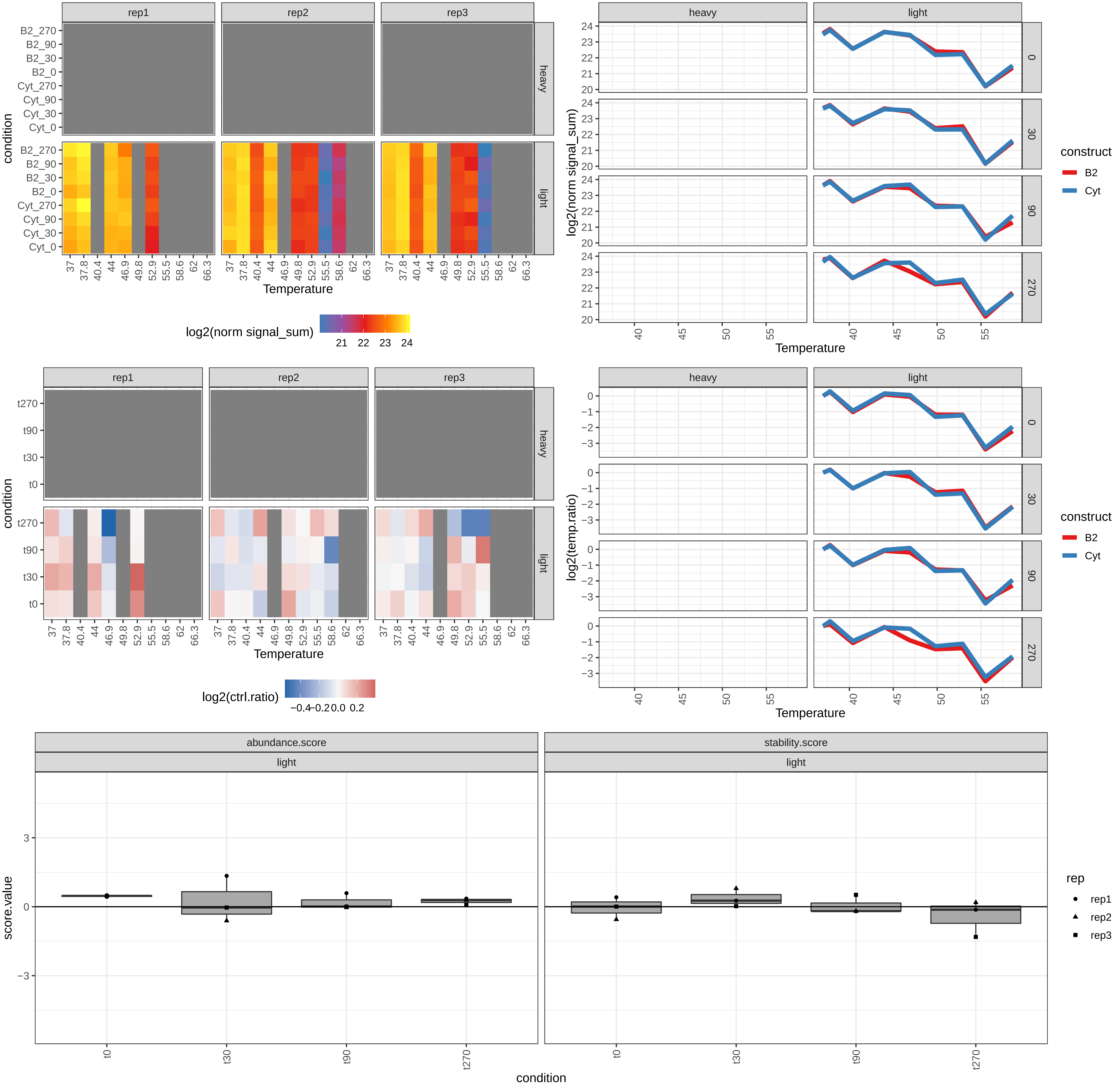

Q08963 − U2 small nuclear ribonucleoprotein A' OS=Saccharomyces cerevisiae (strain ATCC 204508 / S288c) GN=LEA1 PE=1 SV=1

LEM3 − average.top3: 6.88092975 − max.qupm: 5

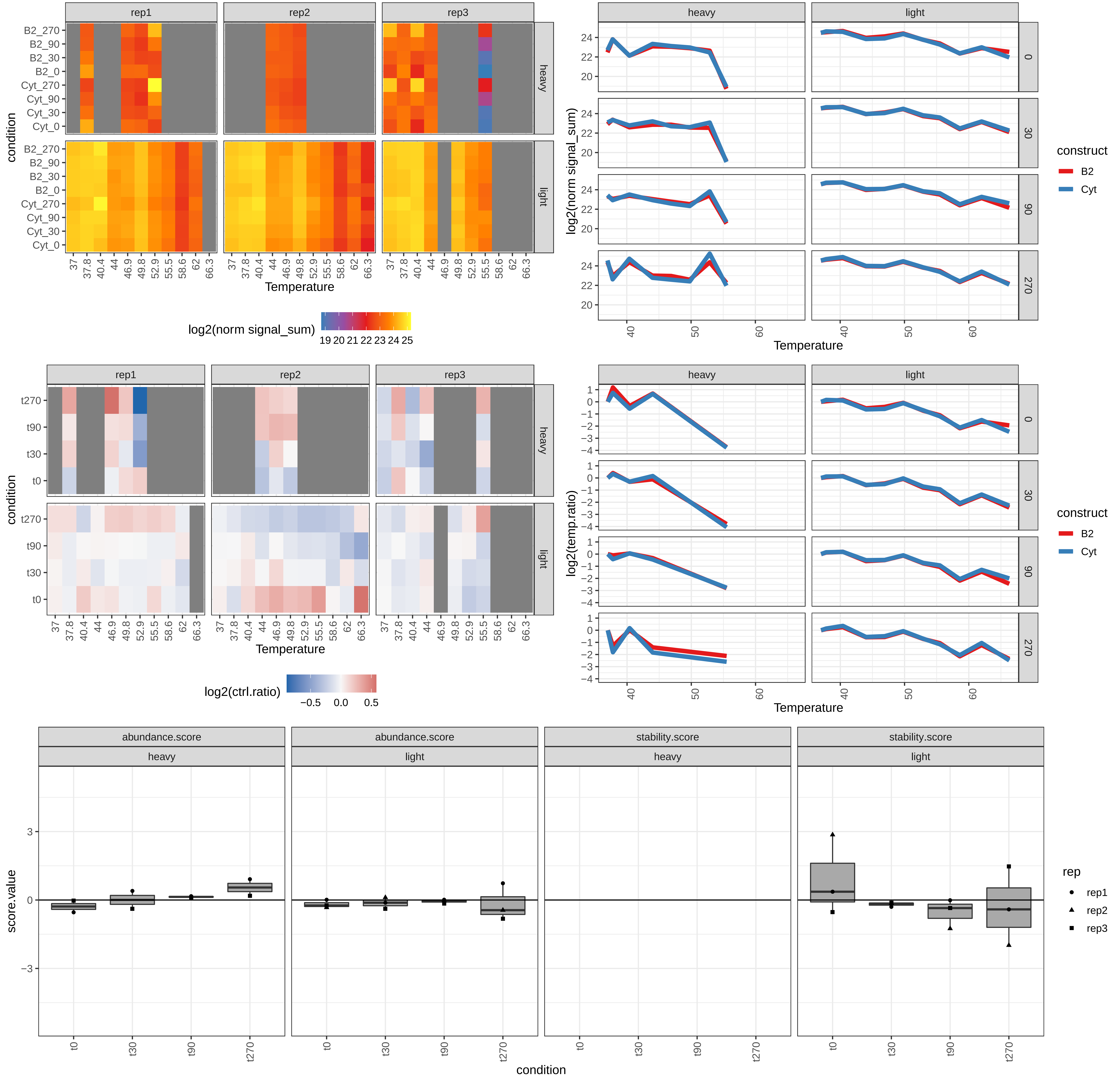

P42838 − Alkylphosphocholine resistance protein LEM3 OS=Saccharomyces cerevisiae (strain ATCC 204508 / S288c) GN=LEM3 PE=1 SV=1

LEO1 − average.top3: 6.99352923076923 − max.qupm: 4

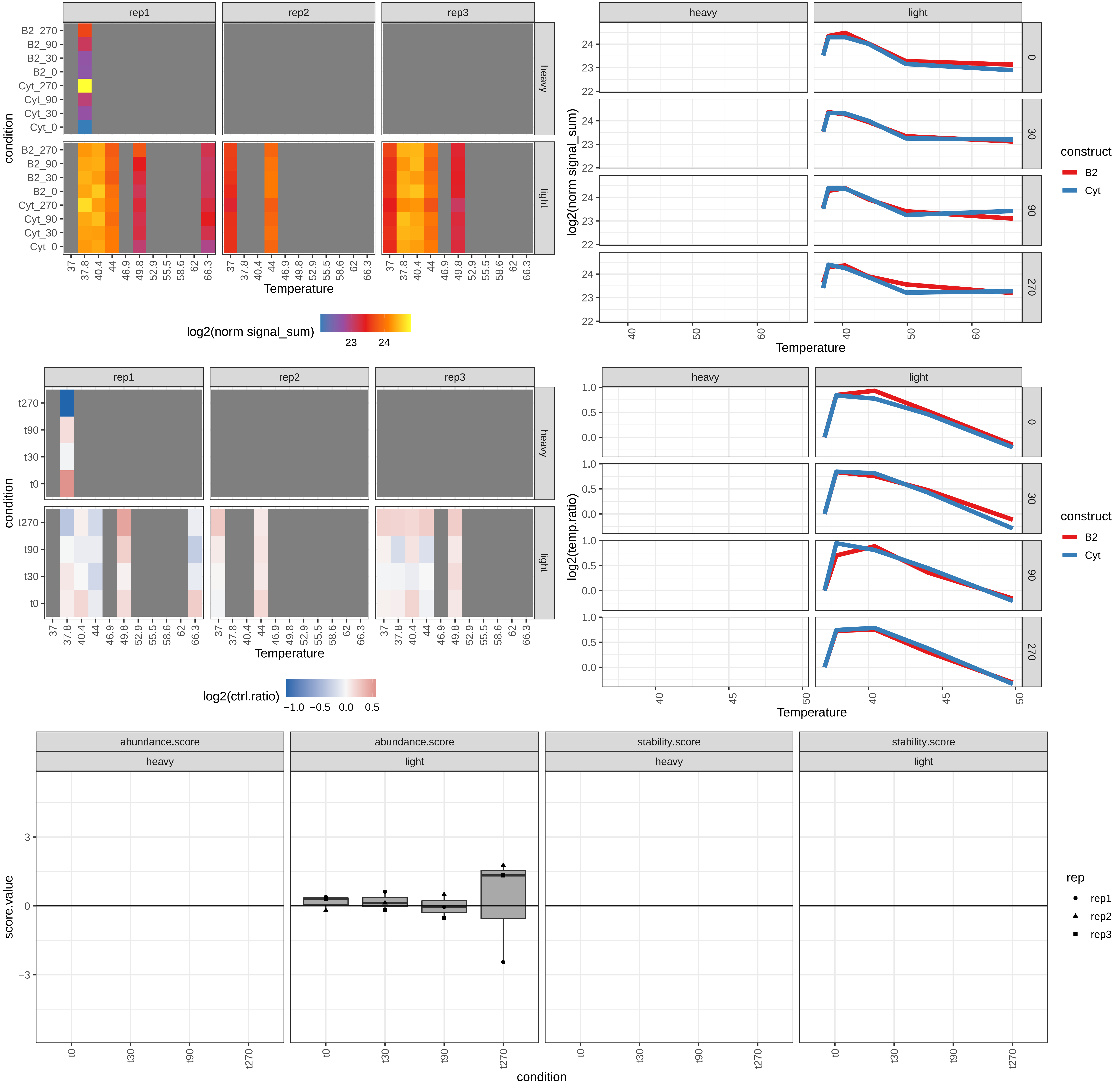

P38439 − RNA polymerase−associated protein LEO1 OS=Saccharomyces cerevisiae (strain ATCC 204508 / S288c) GN=LEO1 PE=1 SV=1

LEU1 − average.top3: 7.9080074 − max.qupm: 34

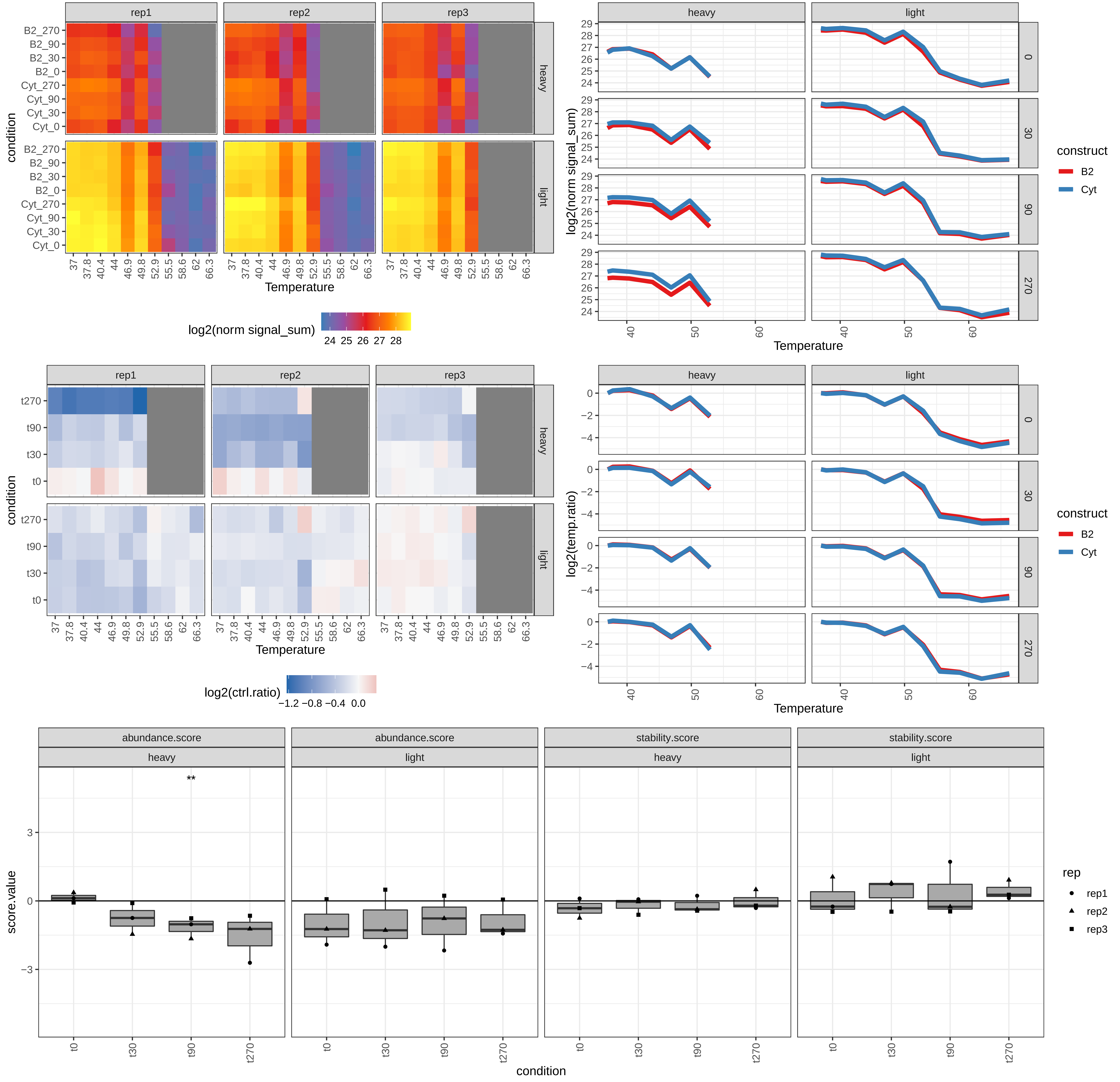

P07264 − 3−isopropylmalate dehydratase OS=Saccharomyces cerevisiae (strain ATCC 204508 / S288c) GN=LEU1 PE=1 SV=3

LEU4 − average.top3: 7.96838178571429 − max.qupm: 18

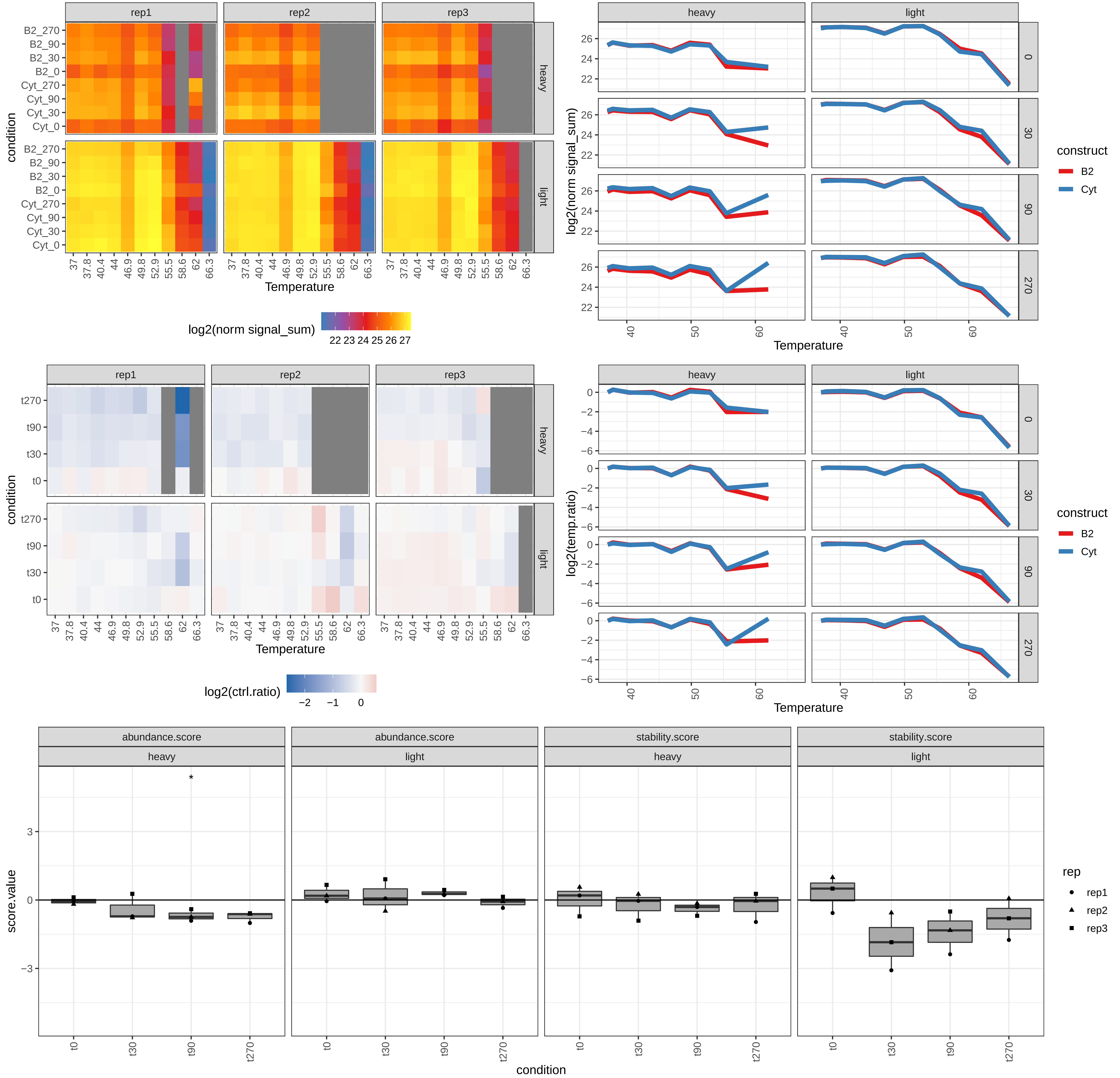

opylmalate synthase OS=Saccharomyces cerevisiae (strain ATCC 204508 / S288c) GN=LEU4 PE=1 SV=1|Isoform Cytoplasmic of 2−isopropylmalate synthase OS=Saccharomyces cerevisiae (strain ATCC

LEU9 − average.top3: 6.68012421052632 − max.qupm: 5

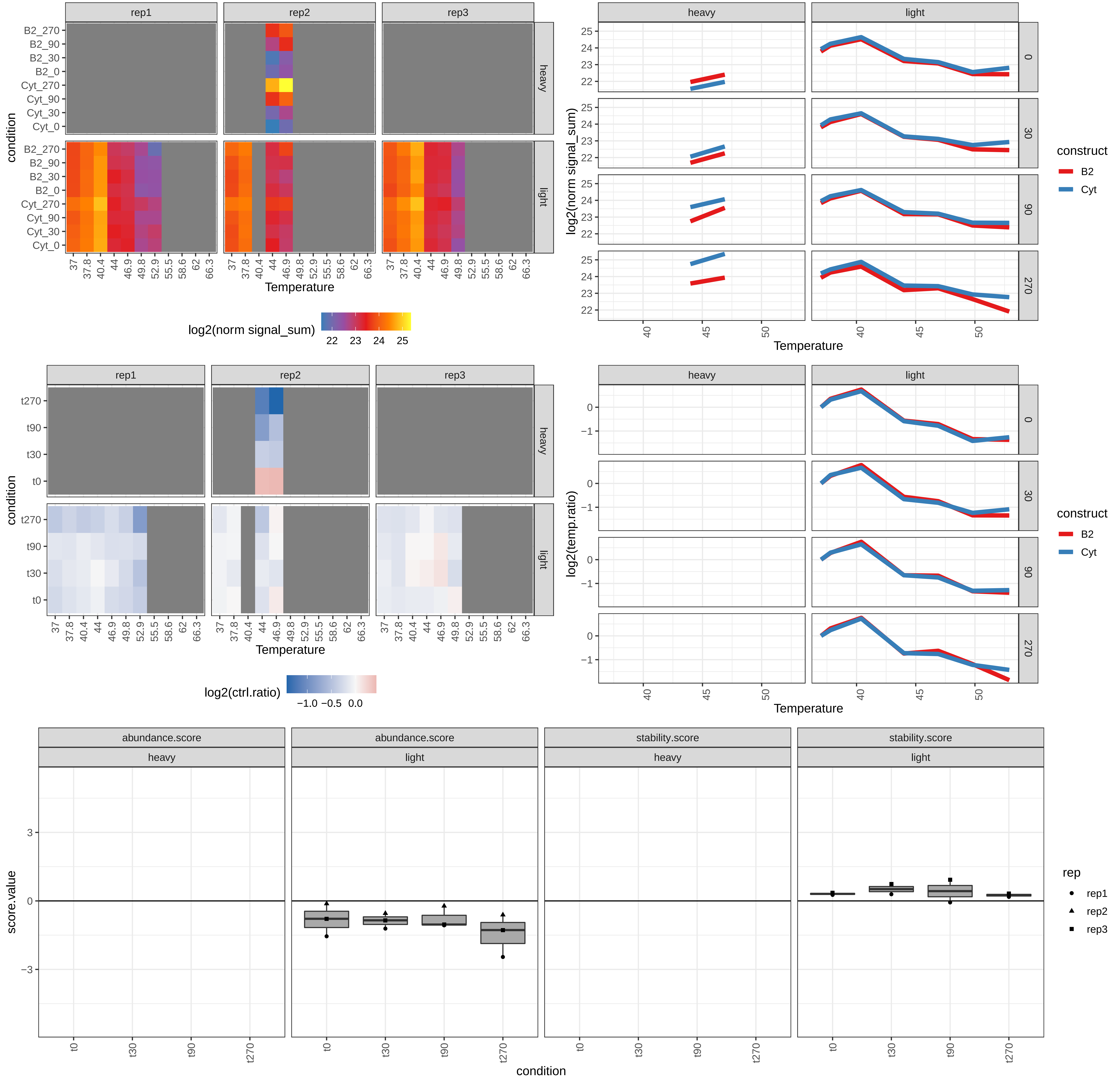

Q12166 − 2−isopropylmalate synthase 2, mitochondrial OS=Saccharomyces cerevisiae (strain ATCC 204508 / S288c) GN=LEU9 PE=1 SV=1

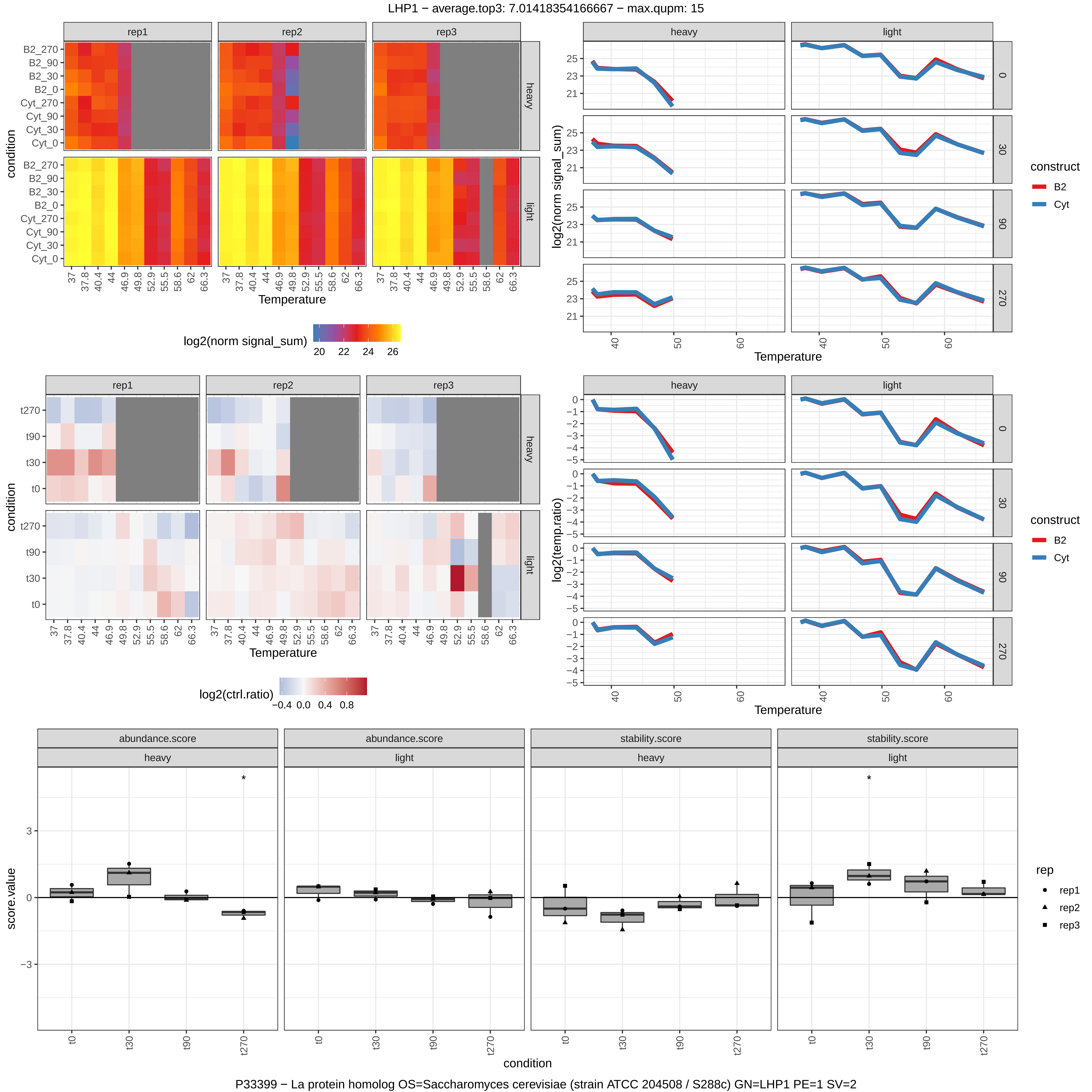
LHP1 − average.top3: 7.01418354166667 − max.qupm: 15
rep1
rep2
rep3
heavy
light
condition
Temperature
log2(norm signal_sum)
log2(ctrl.ratio)
construct
B2
Cyt
log2(temp.ratio)
abundance.score
stability.score
score.value
rep
rep1
rep2
rep3
P33399 − La protein homolog OS=Saccharomyces cerevisiae (strain ATCC 204508 / S288c) GN=LHP1 PE=1 SV=2

LHS1 − average.top3: 6.84302918918919 − max.qupm: 15

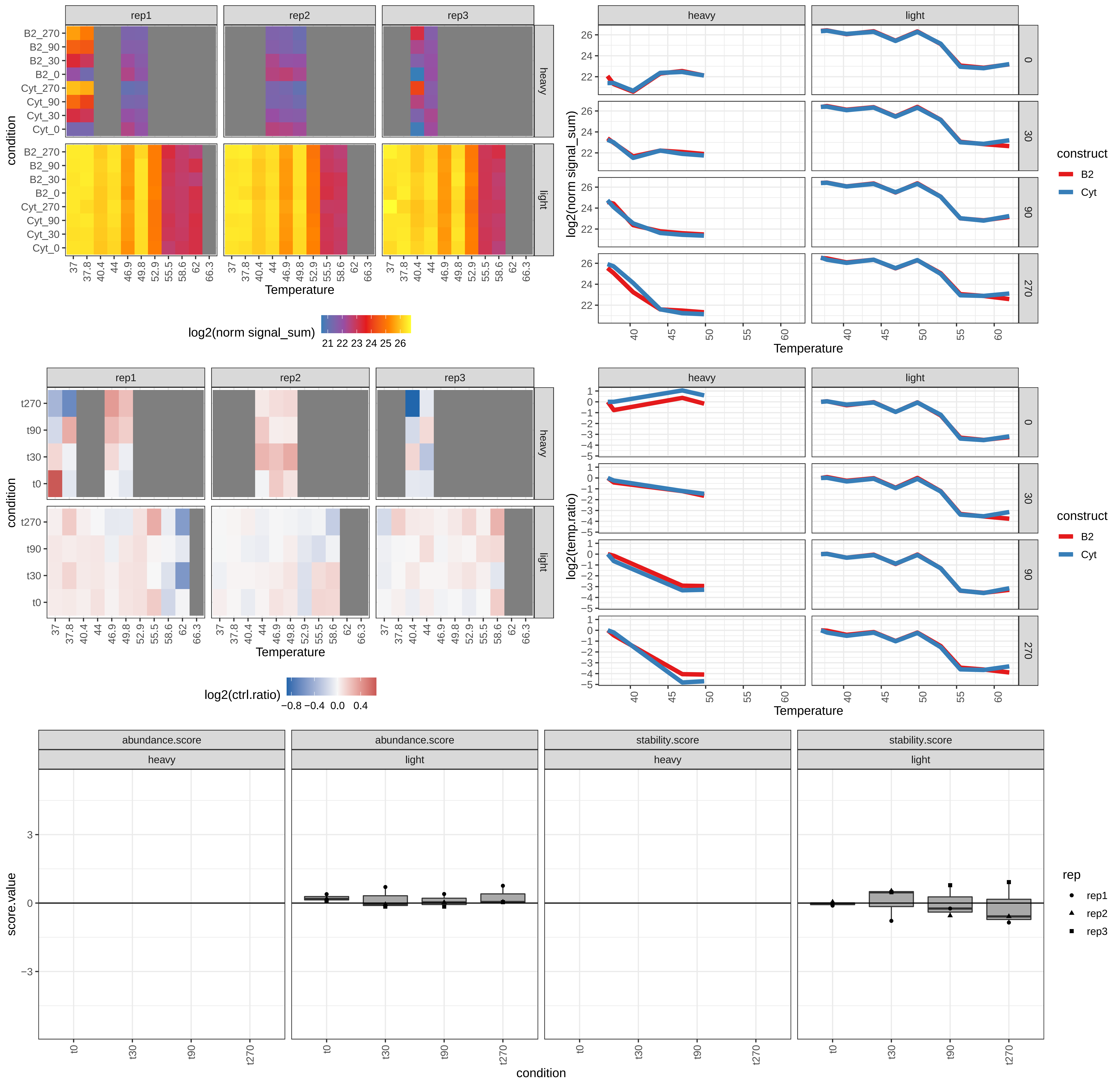

P36016 − Heat shock protein 70 homolog LHS1 OS=Saccharomyces cerevisiae (strain ATCC 204508 / S288c) GN=LHS1 PE=1 SV=1

LIA1 − average.top3: 7.0994196 − max.qupm: 15

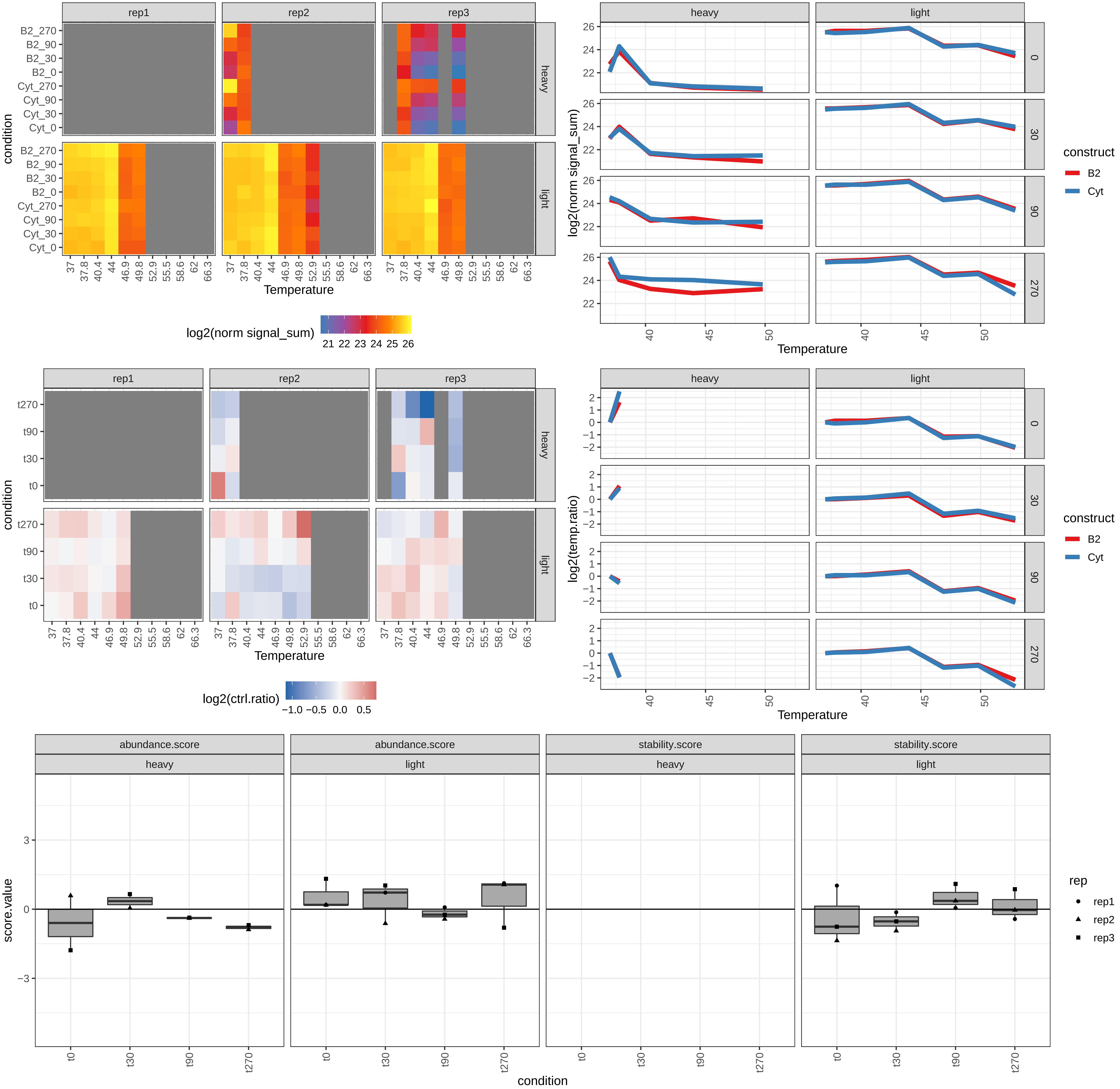

P47120 − Deoxyhypusine hydroxylase OS=Saccharomyces cerevisiae (strain ATCC 204508 / S288c) GN=LIA1 PE=1 SV=1

LIP1 − average.top3: 6.95077590909091 − max.qupm: 3

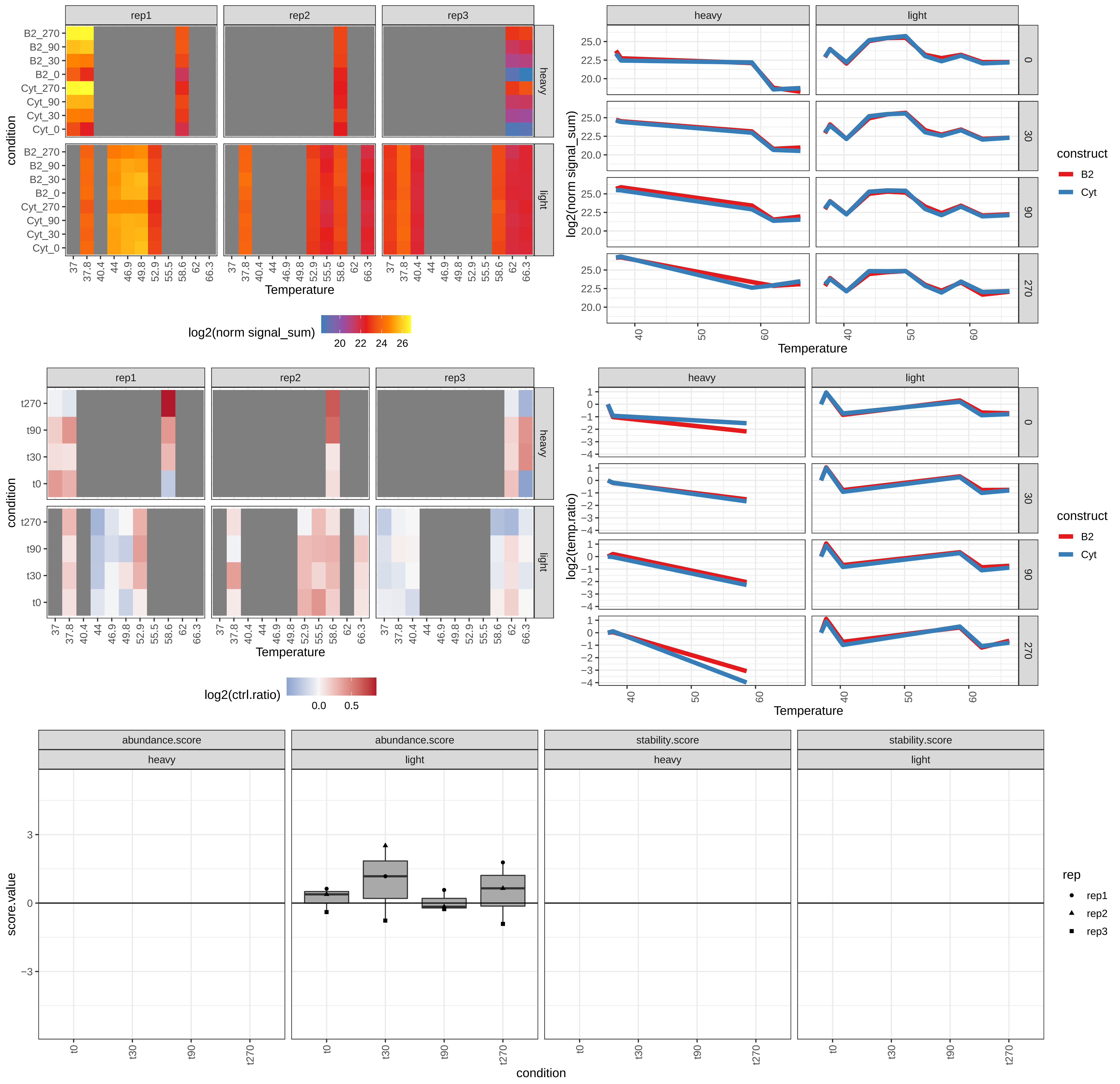

Q03579 − Ceramide synthase subunit LIP1 OS=Saccharomyces cerevisiae (strain ATCC 204508 / S288c) GN=LIP1 PE=1 SV=1

LIP2 − average.top3: 6.49598454545455 − max.qupm: 4

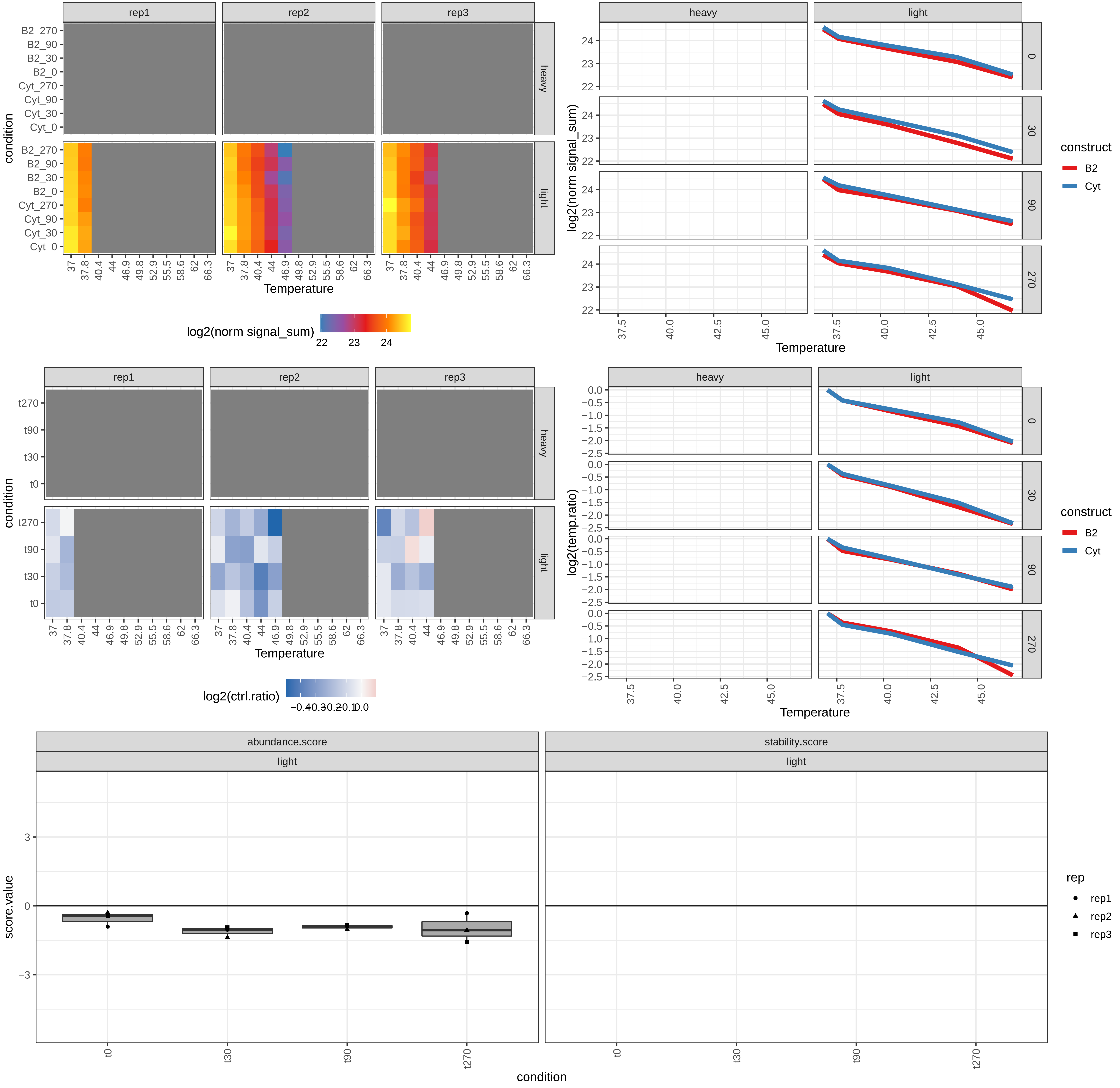

Q06005 − Octanoyltransferase, mitochondrial OS=Saccharomyces cerevisiae (strain ATCC 204508 / S288c) GN=LIP2 PE=1 SV=1

LIP5 − average.top3: 6.51689583333333 − max.qupm: 6

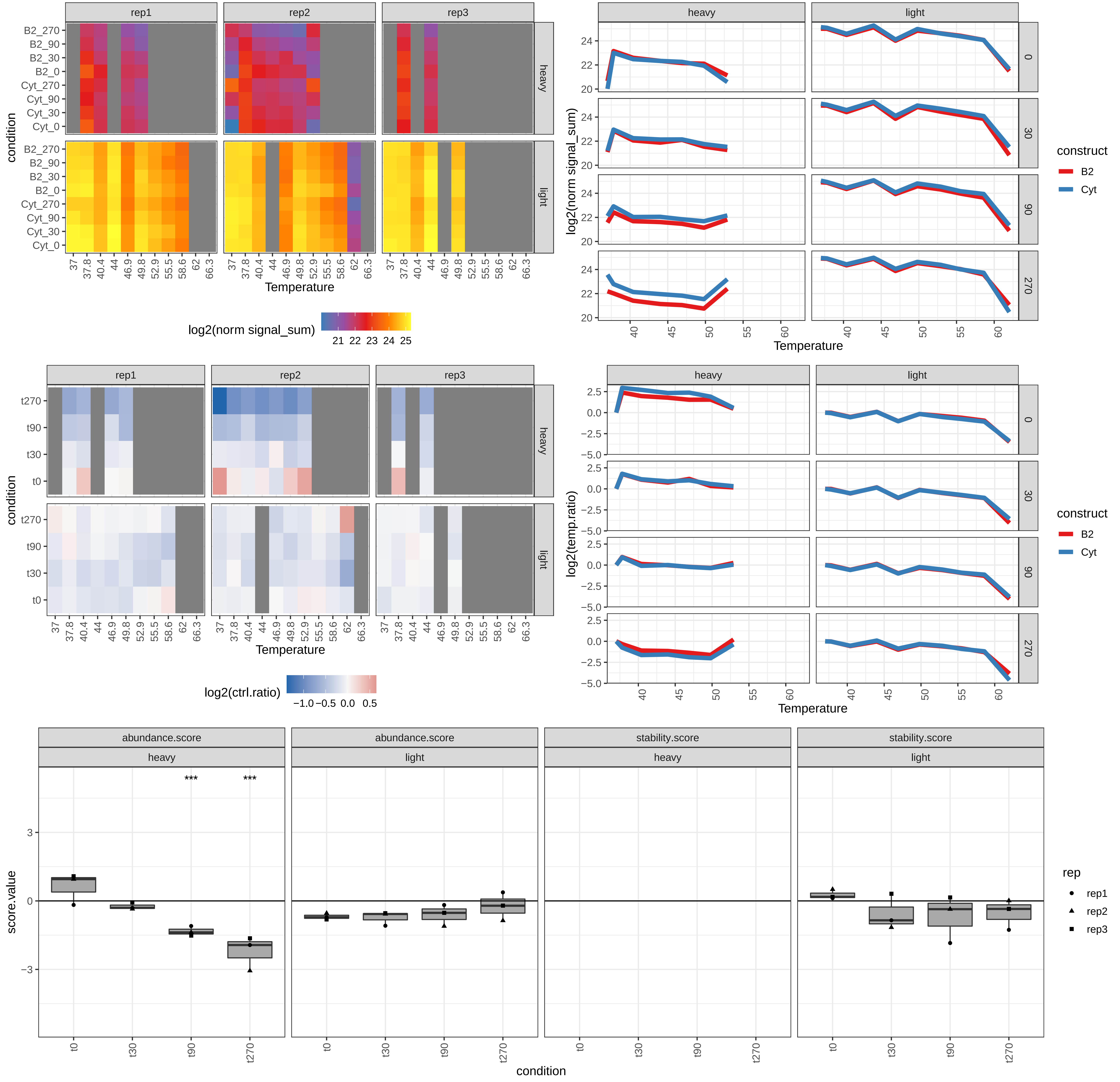

P32875 − Lipoyl synthase, mitochondrial OS=Saccharomyces cerevisiae (strain ATCC 204508 / S288c) GN=LIP5 PE=1 SV=1

LOC1 − average.top3: 6.52808 − max.qupm: 5

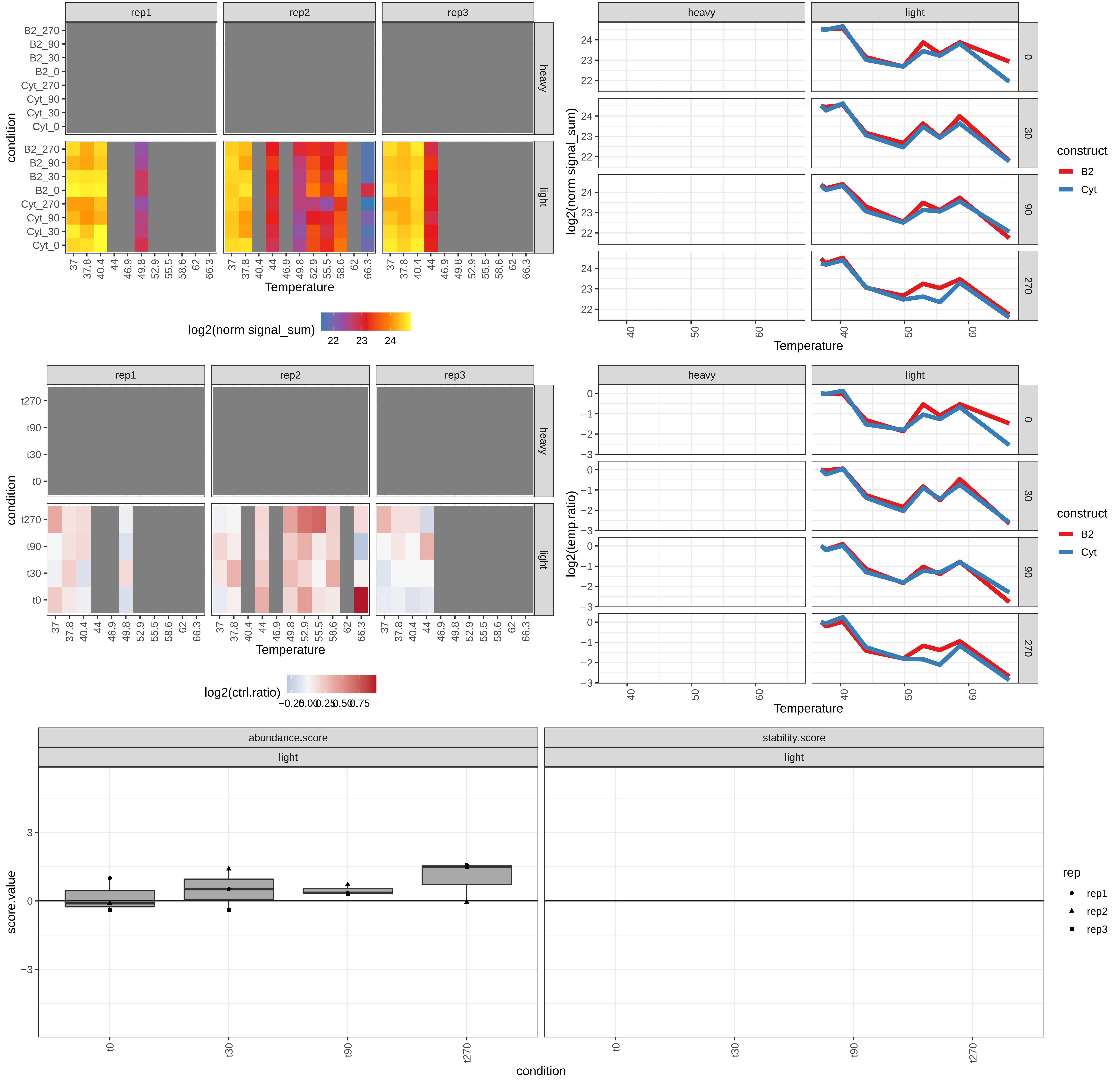

P43586 − 60S ribosomal subunit assembly/export protein LOC1 OS=Saccharomyces cerevisiae (strain ATCC 204508 / S288c) GN=LOC1 PE=1 SV=1

LOS1 − average.top3: 6.50091375 − max.qupm: 5

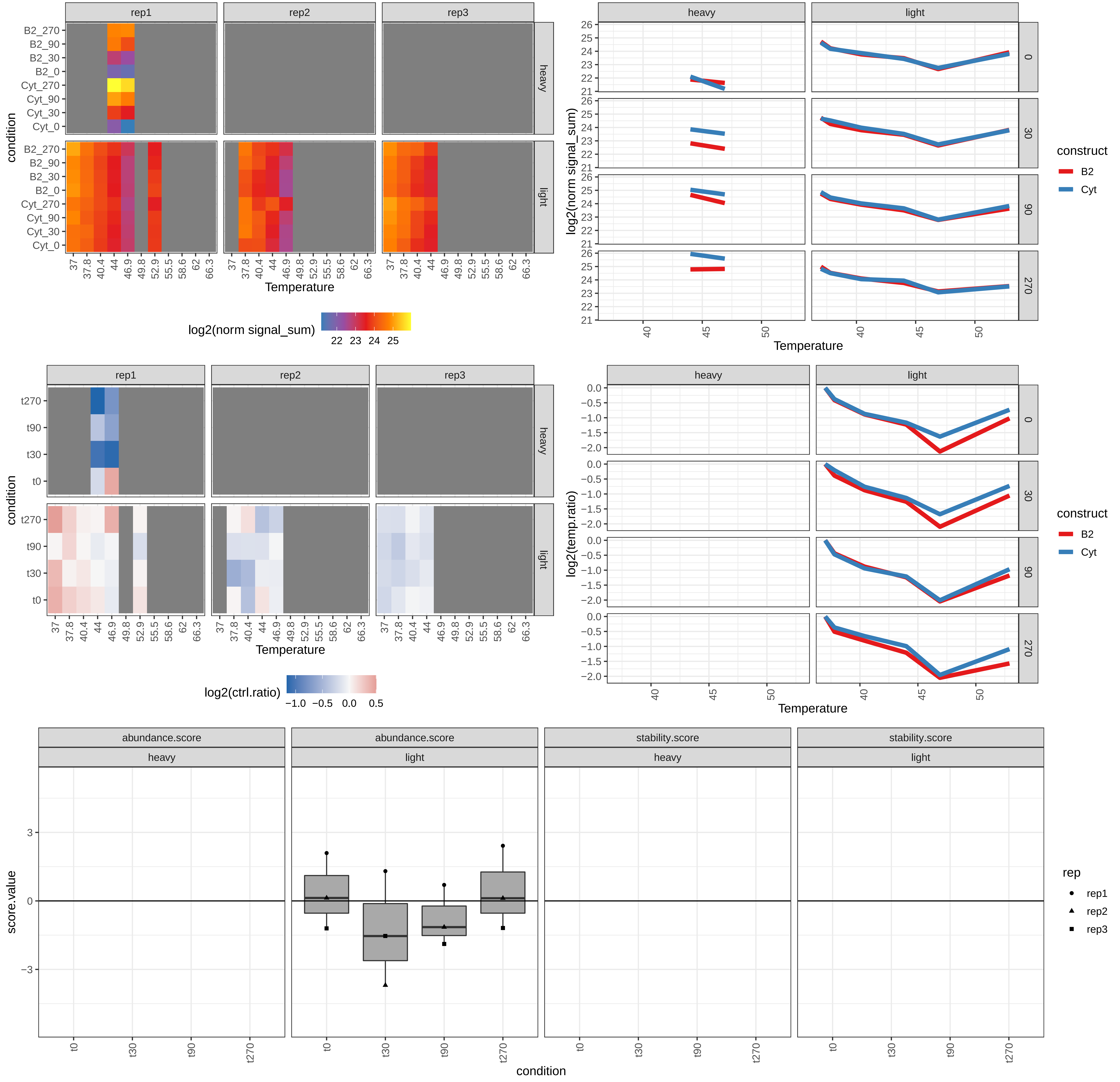

P33418 − Exportin−T OS=Saccharomyces cerevisiae (strain ATCC 204508 / S288c) GN=LOS1 PE=1 SV=1

LOT6 − average.top3: 6.54268384615385 − max.qupm: 6

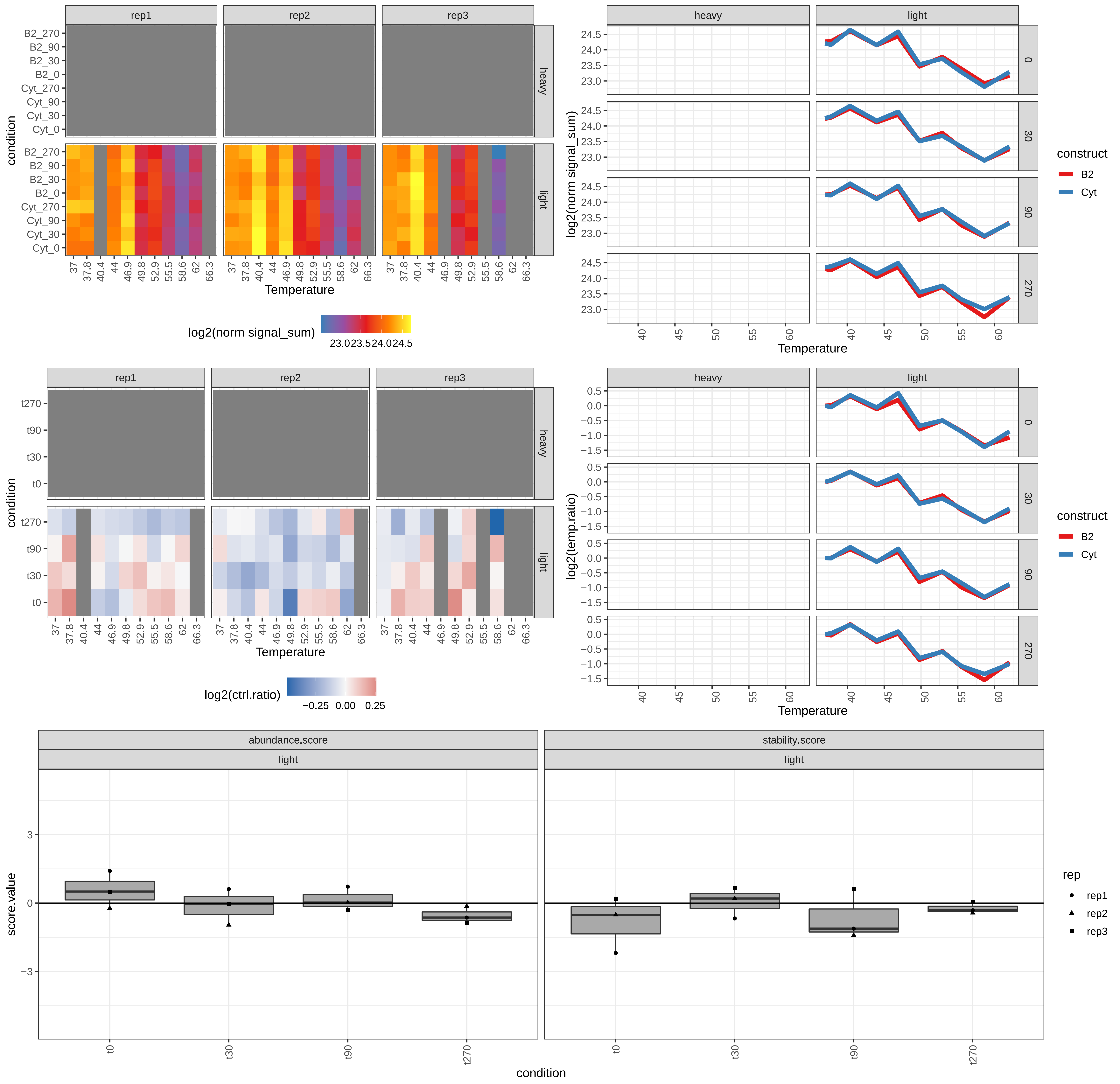

Q07923 − NAD(P)H−dependent FMN reductase LOT6 OS=Saccharomyces cerevisiae (strain ATCC 204508 / S288c) GN=LOT6 PE=1 SV=1

LPD1 − average.top3: 8.28009787878788 − max.qupm: 28

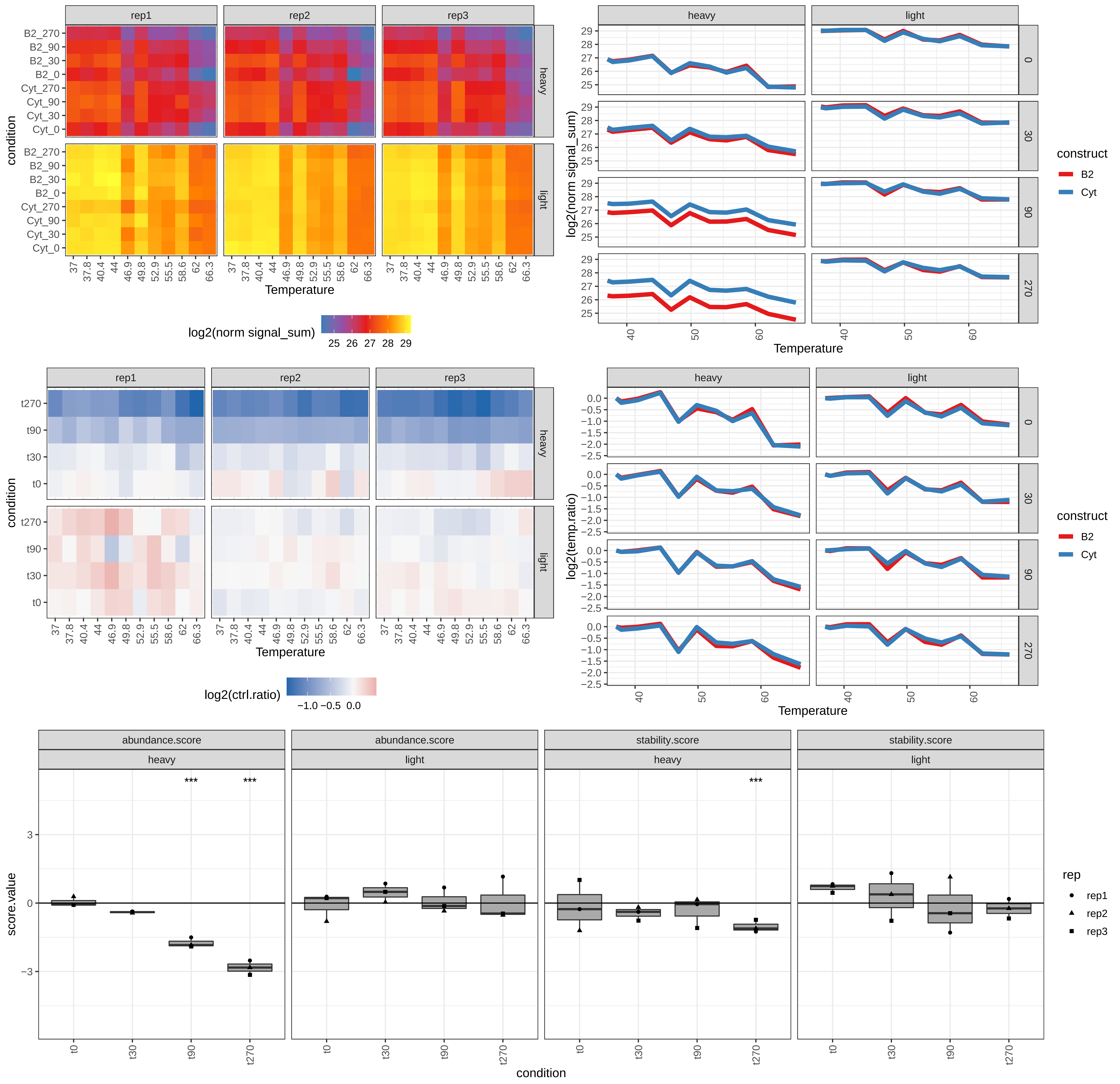

P09624 − Dihydrolipoyl dehydrogenase, mitochondrial OS=Saccharomyces cerevisiae (strain ATCC 204508 / S288c) GN=LPD1 PE=1 SV=1

LPX1 − average.top3: 7.31107771428571 − max.qupm: 13

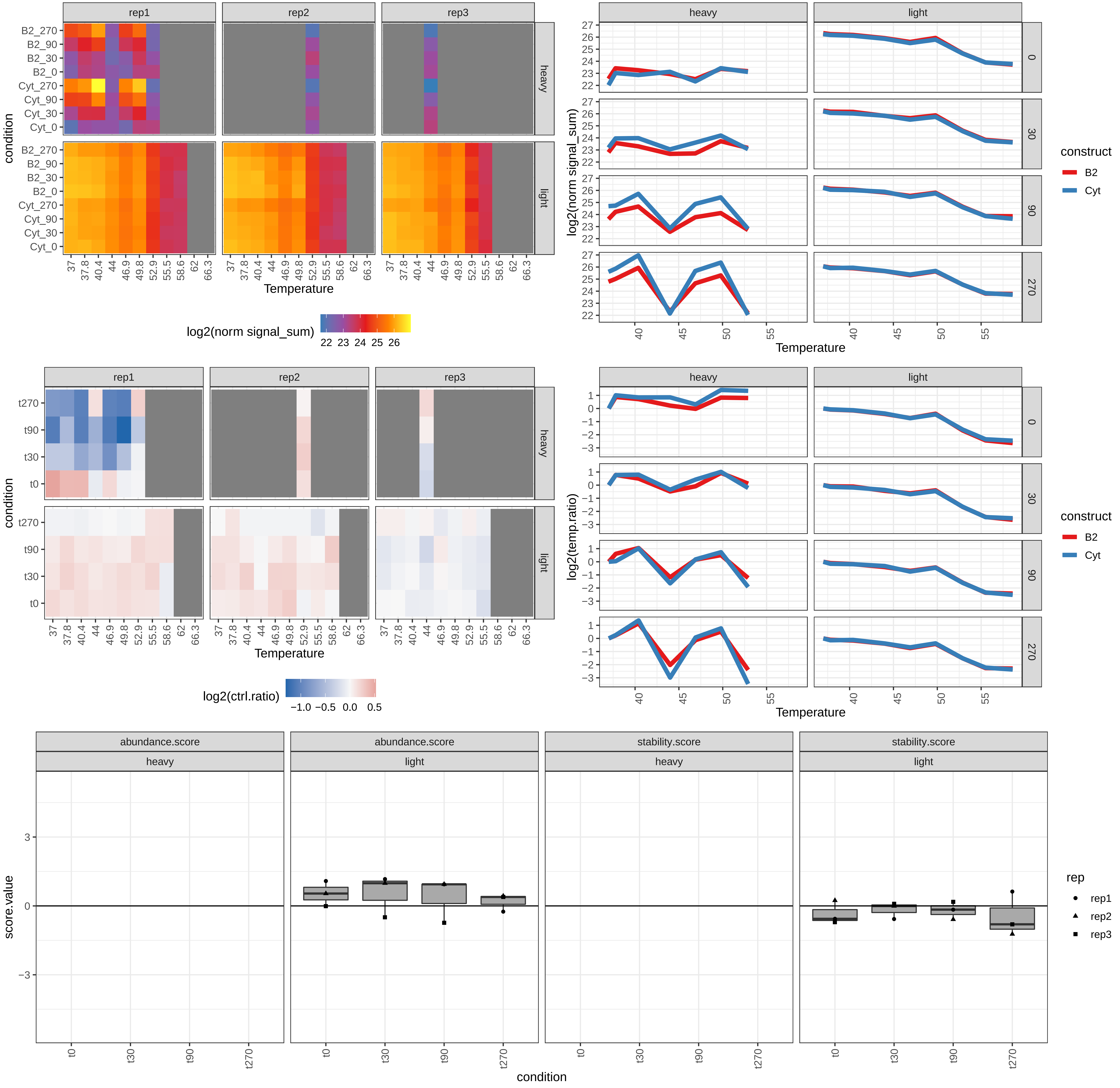

Q12405 − Peroxisomal membrane protein LPX1 OS=Saccharomyces cerevisiae (strain ATCC 204508 / S288c) GN=LPX1 PE=1 SV=1

LRO1 − average.top3: 6.54426363636364 − max.qupm: 6

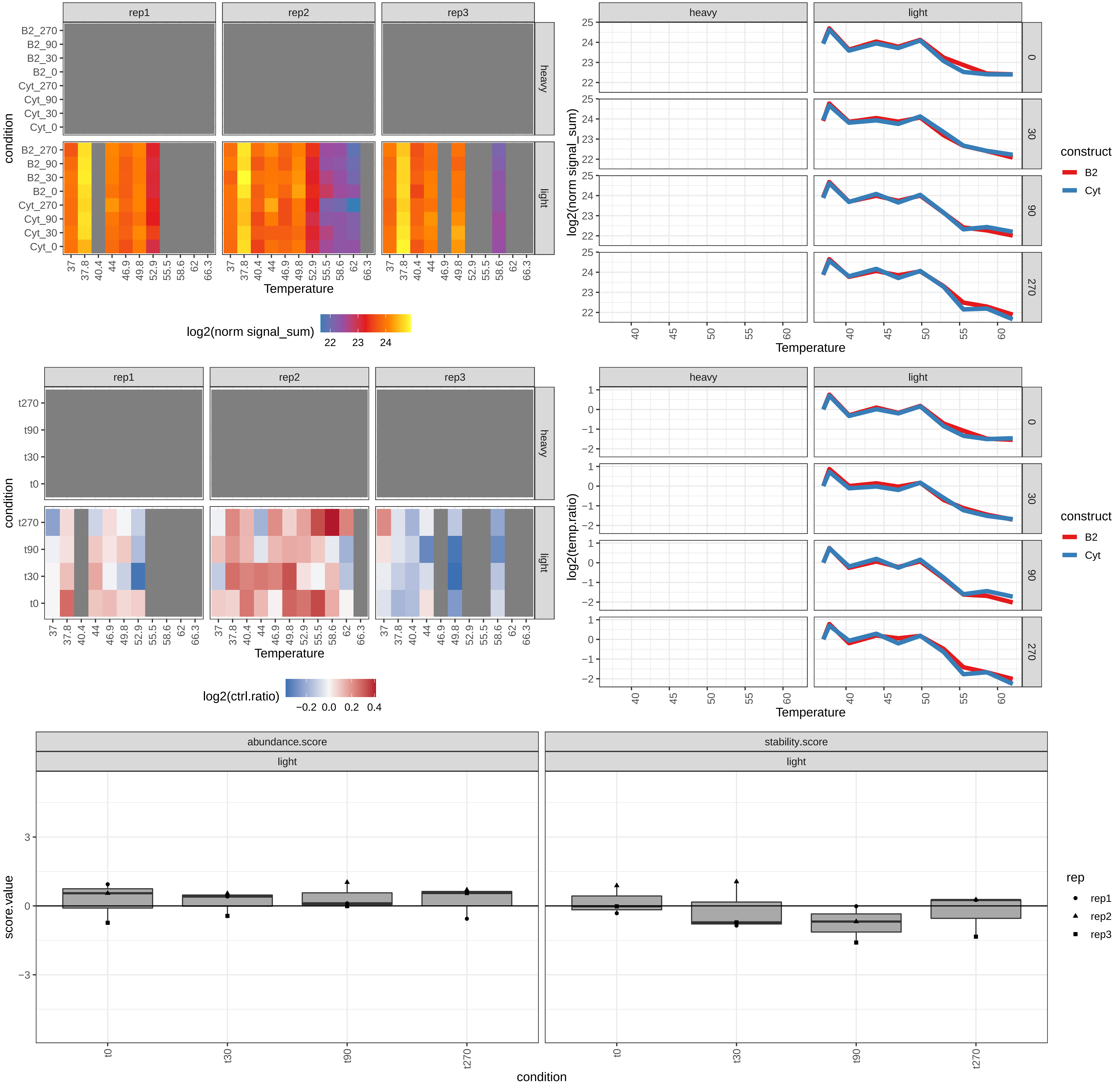

P40345 − Phospholipid:diacylglycerol acyltransferase OS=Saccharomyces cerevisiae (strain ATCC 204508 / S288c) GN=LRO1 PE=1 SV=1

LSB3 − average.top3: 7.0681428 − max.qupm: 8

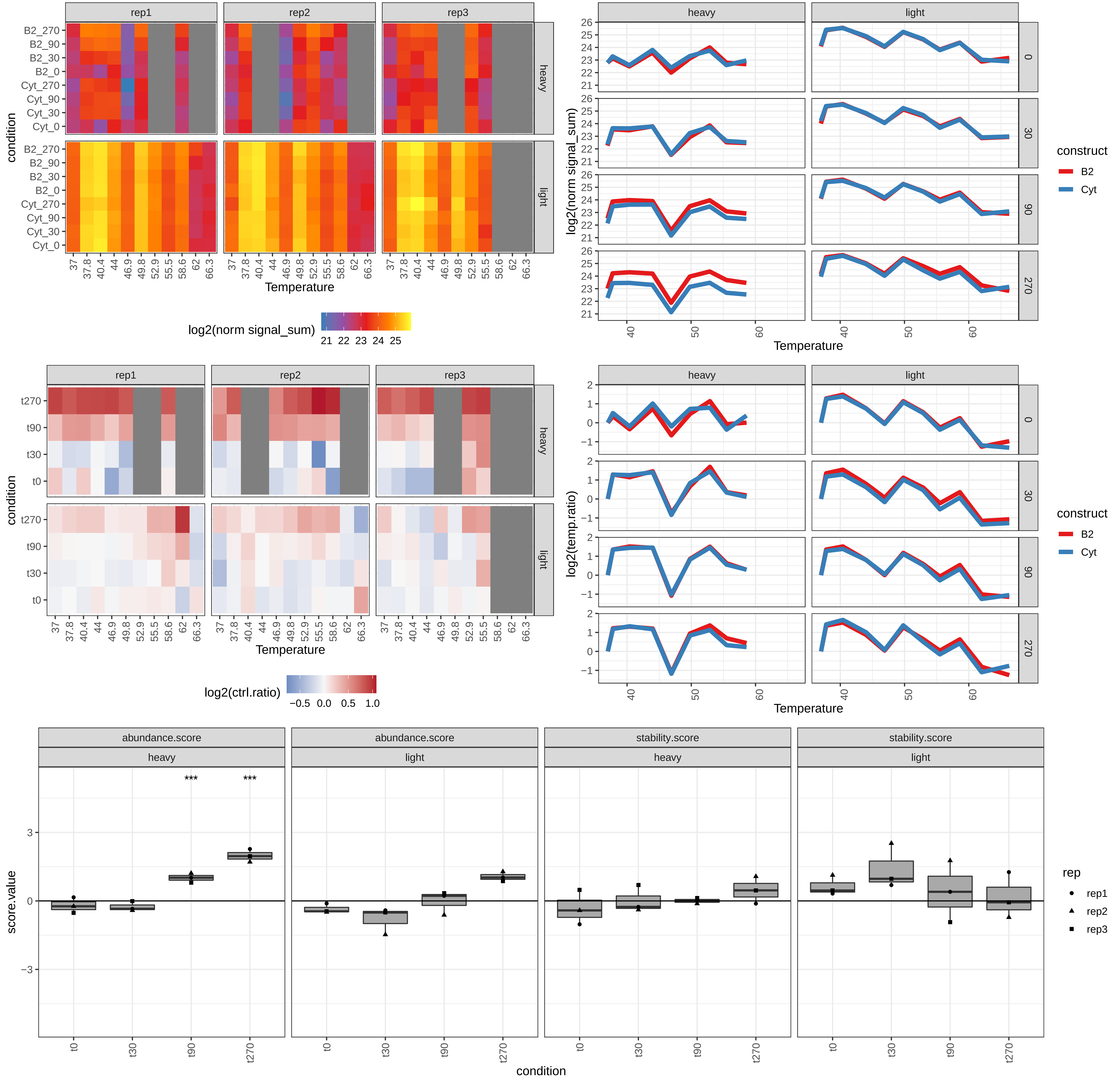

P43603 − LAS seventeen−binding protein 3 OS=Saccharomyces cerevisiae (strain ATCC 204508 / S288c) GN=LSB3 PE=1 SV=3

LSB5 − average.top3: 6.60116318181818 − max.qupm: 4

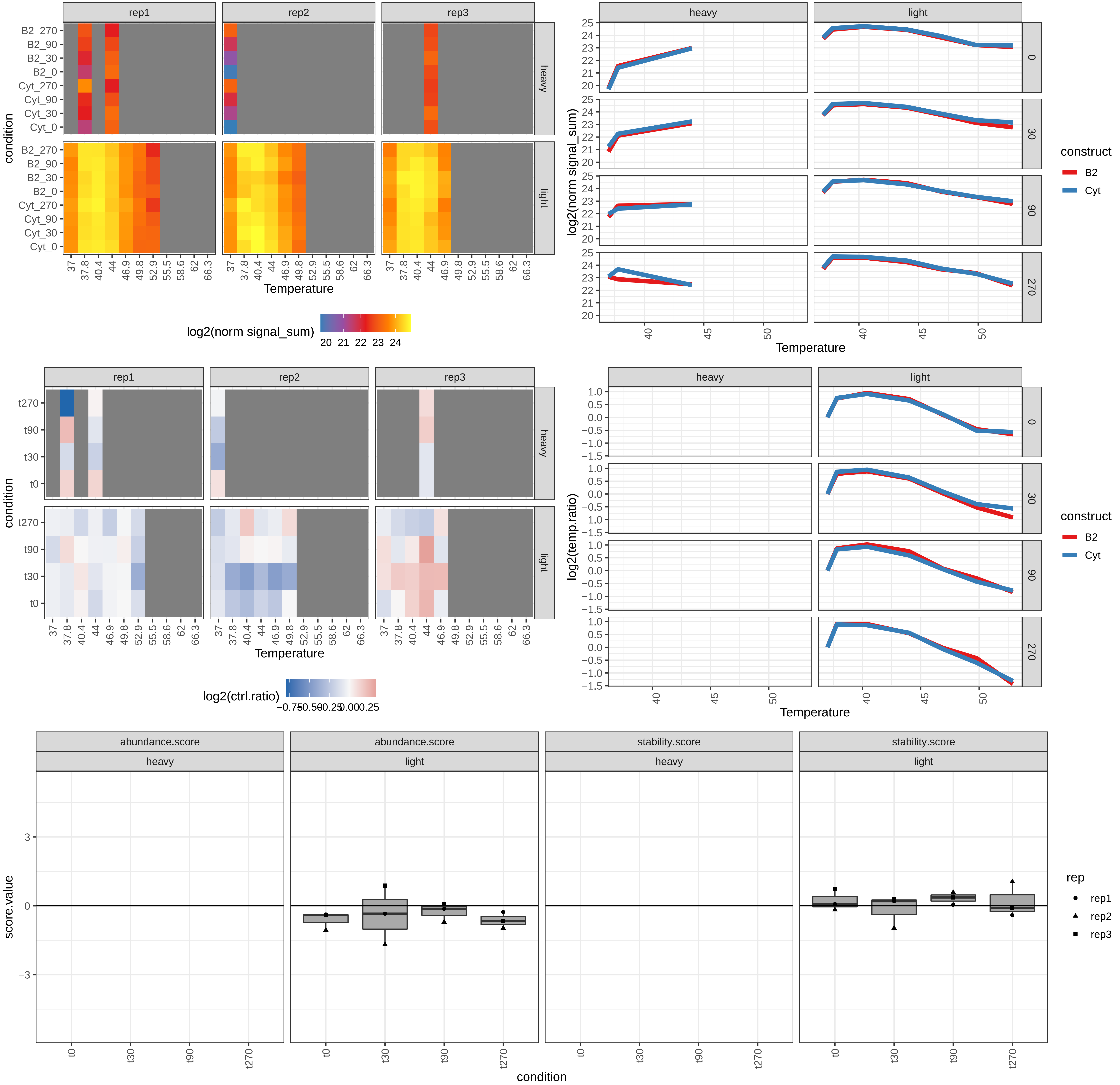

P25369 − LAS seventeen−binding protein 5 OS=Saccharomyces cerevisiae (strain ATCC 204508 / S288c) GN=LSB5 PE=1 SV=3

LSC1 − average.top3: 7.50765814814815 − max.qupm: 10

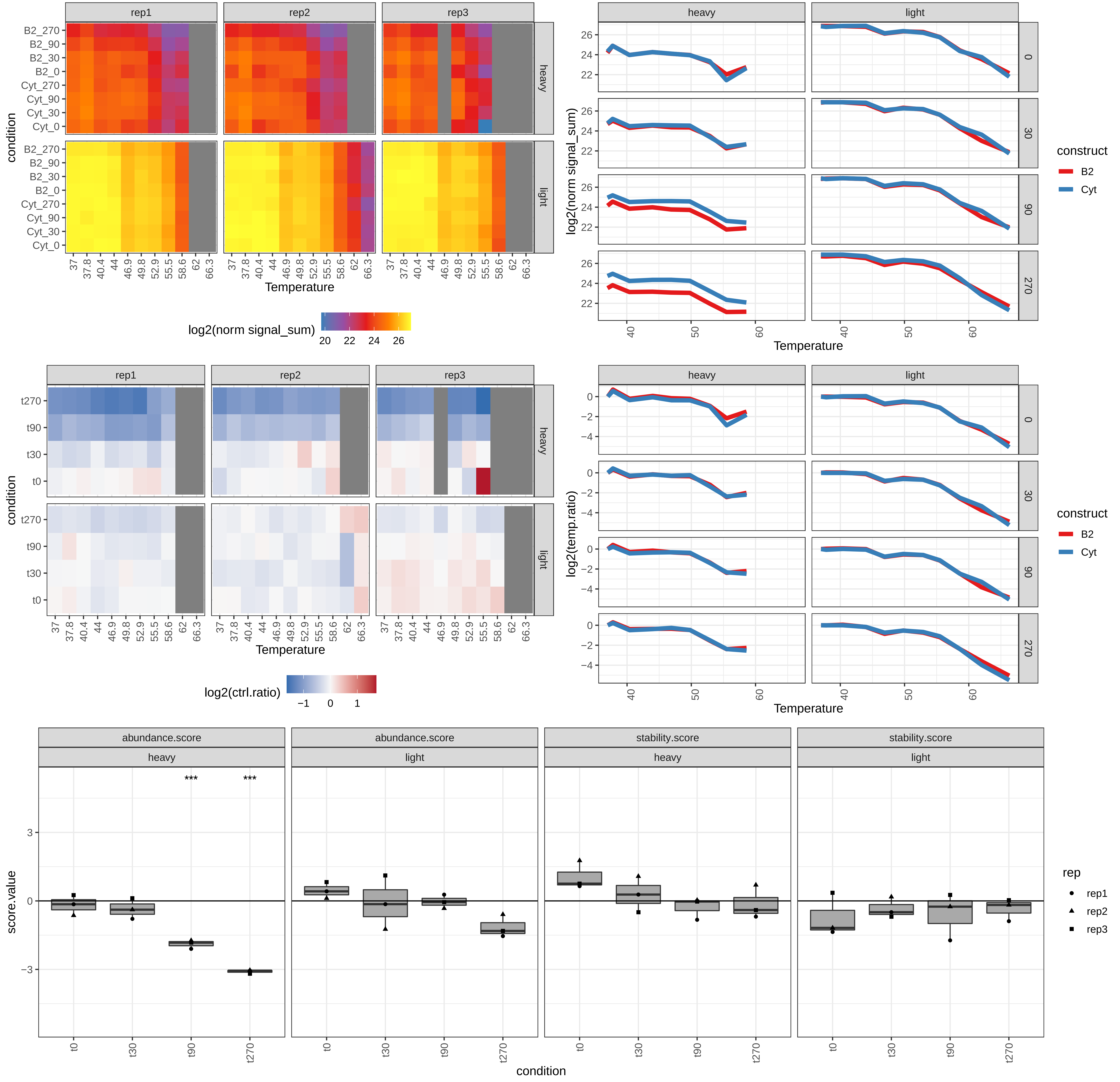

P53598 − Succinyl−CoA ligase [ADP−forming] subunit alpha, mitochondrial OS=Saccharomyces cerevisiae (strain ATCC 204508 / S288c) GN=LSC1 PE=1 SV=1

LSC2 − average.top3: 7.75333862745098 − max.qupm: 26

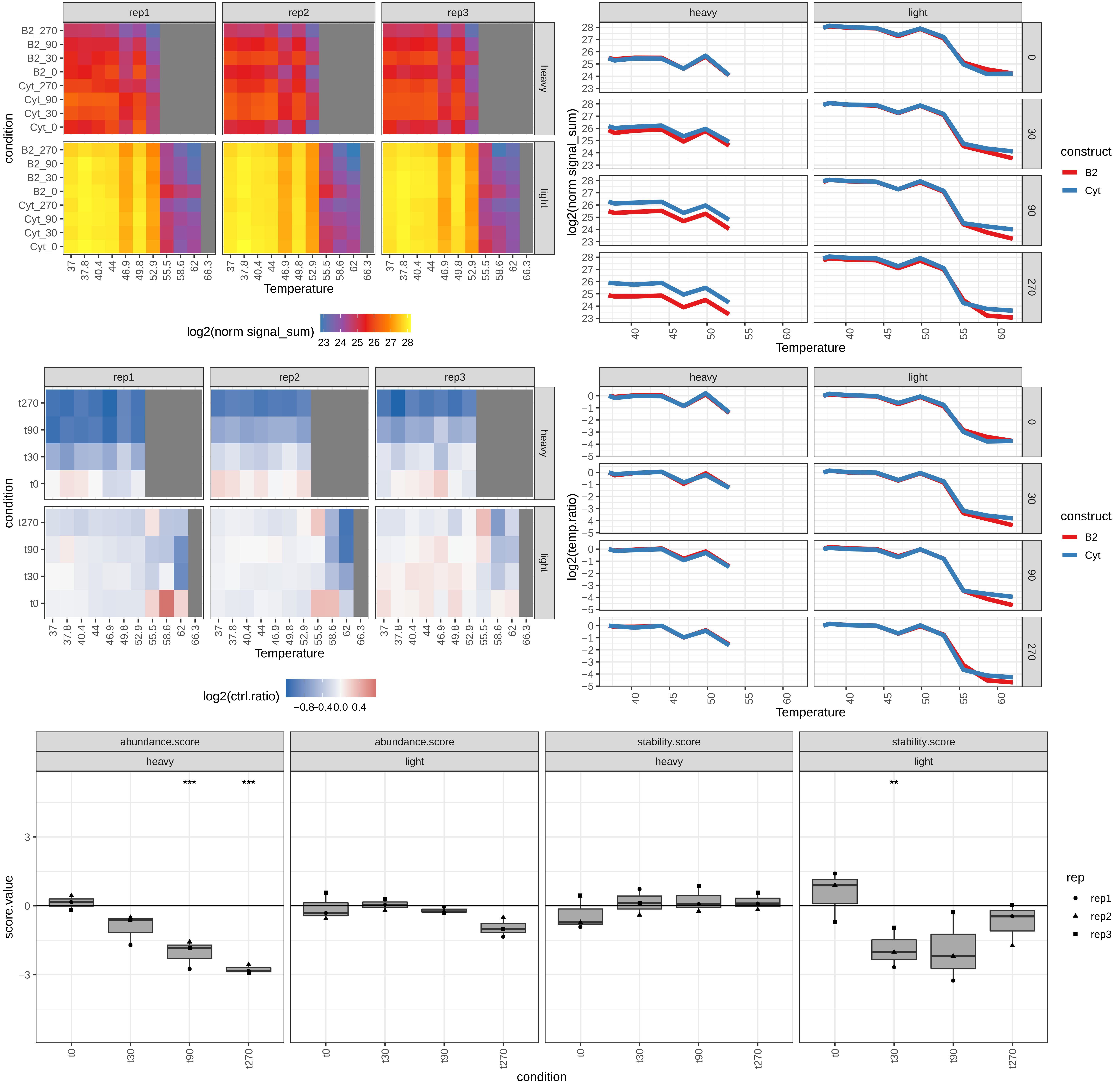

P53312 − Succinyl−CoA ligase [ADP−forming] subunit beta, mitochondrial OS=Saccharomyces cerevisiae (strain ATCC 204508 / S288c) GN=LSC2 PE=1 SV=1

LSG1 − average.top3: 6.7878445 − max.qupm: 15

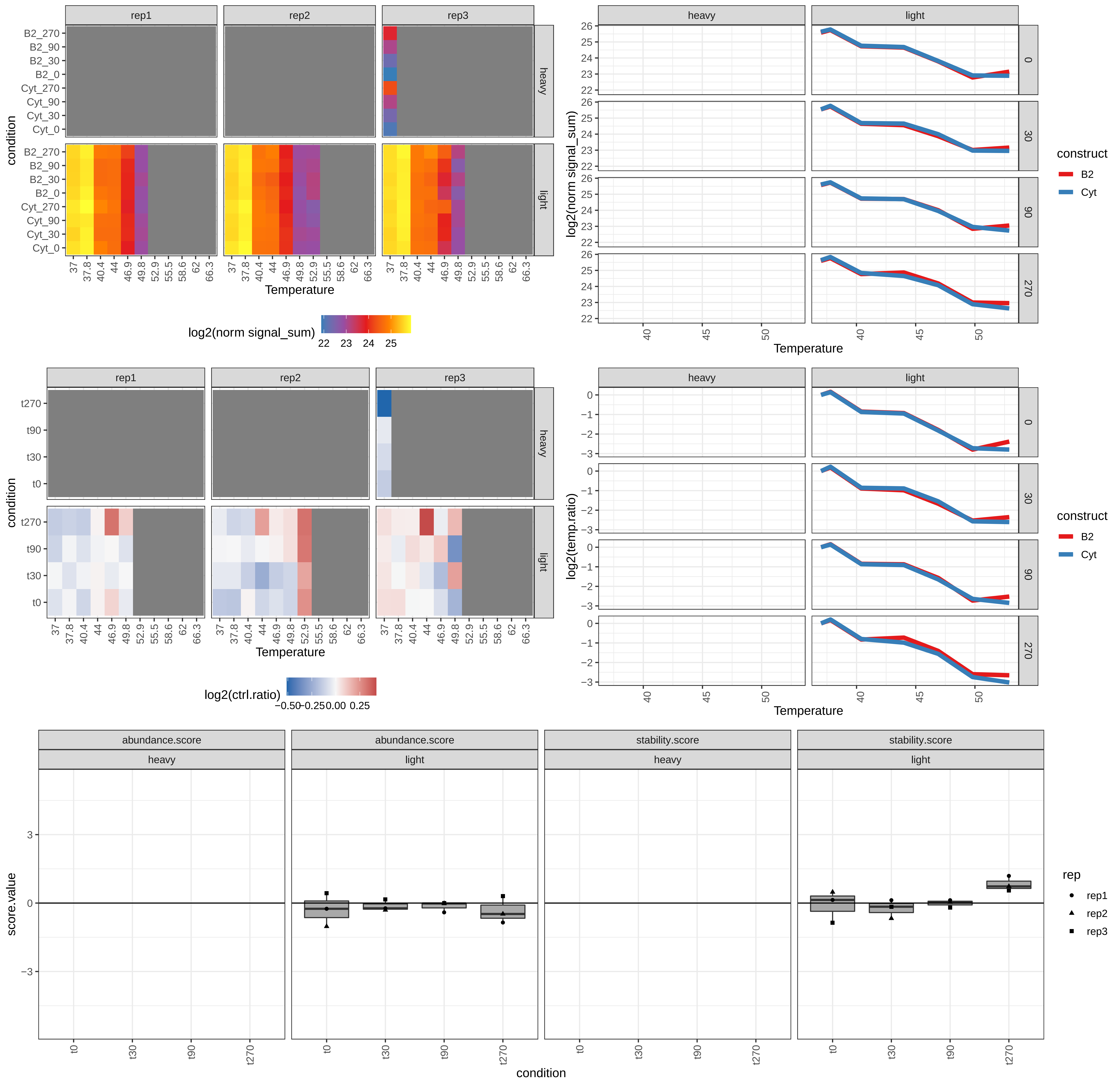

P53145 − Large subunit GTPase 1 OS=Saccharomyces cerevisiae (strain ATCC 204508 / S288c) GN=LSG1 PE=1 SV=1

LSM1 − average.top3: 7.09391161290323 − max.qupm: 5

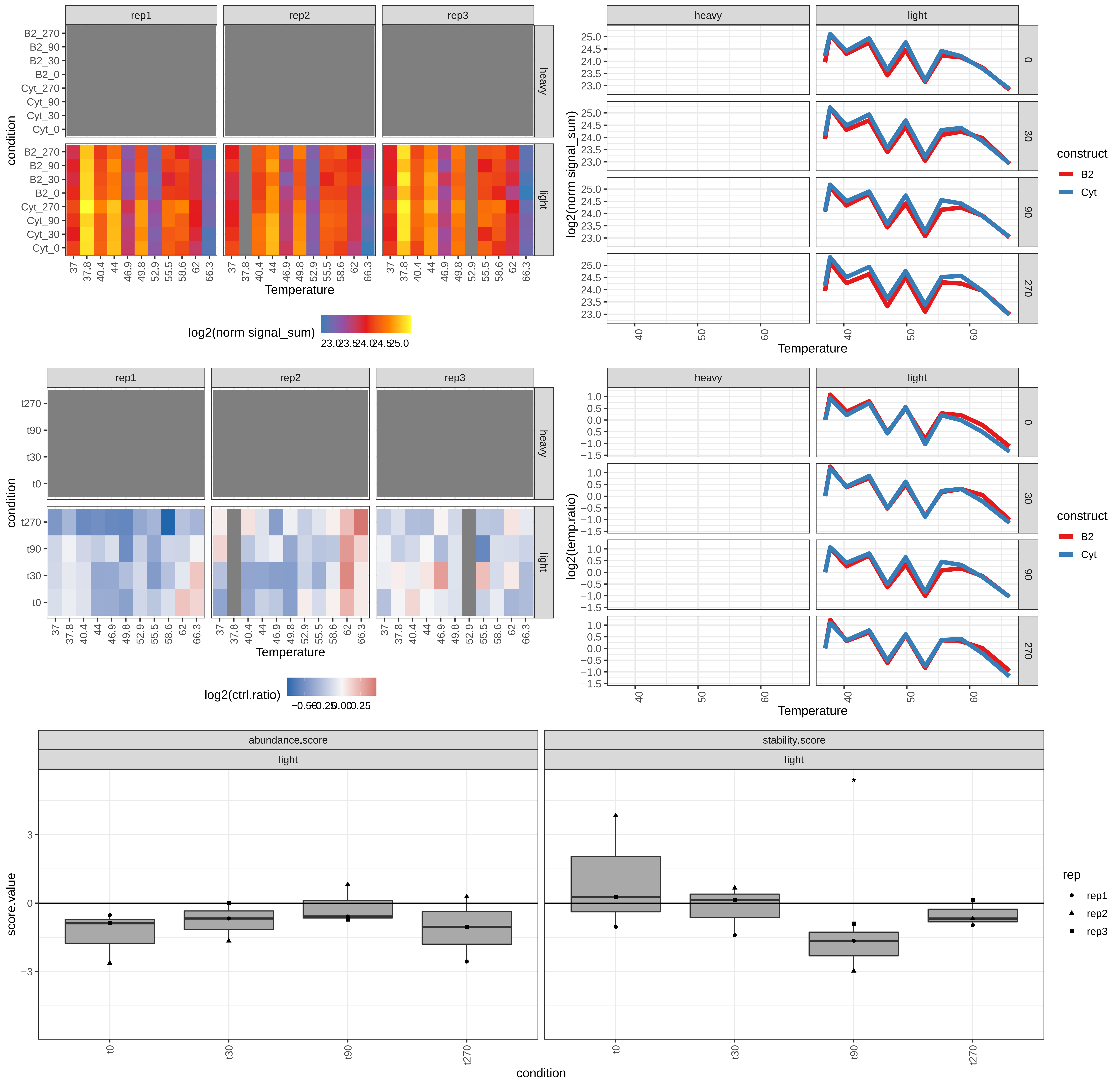

P47017 − Sm−like protein LSm1 OS=Saccharomyces cerevisiae (strain ATCC 204508 / S288c) GN=LSM1 PE=1 SV=1

LSM12 − average.top3: 6.7954404 − max.qupm: 7

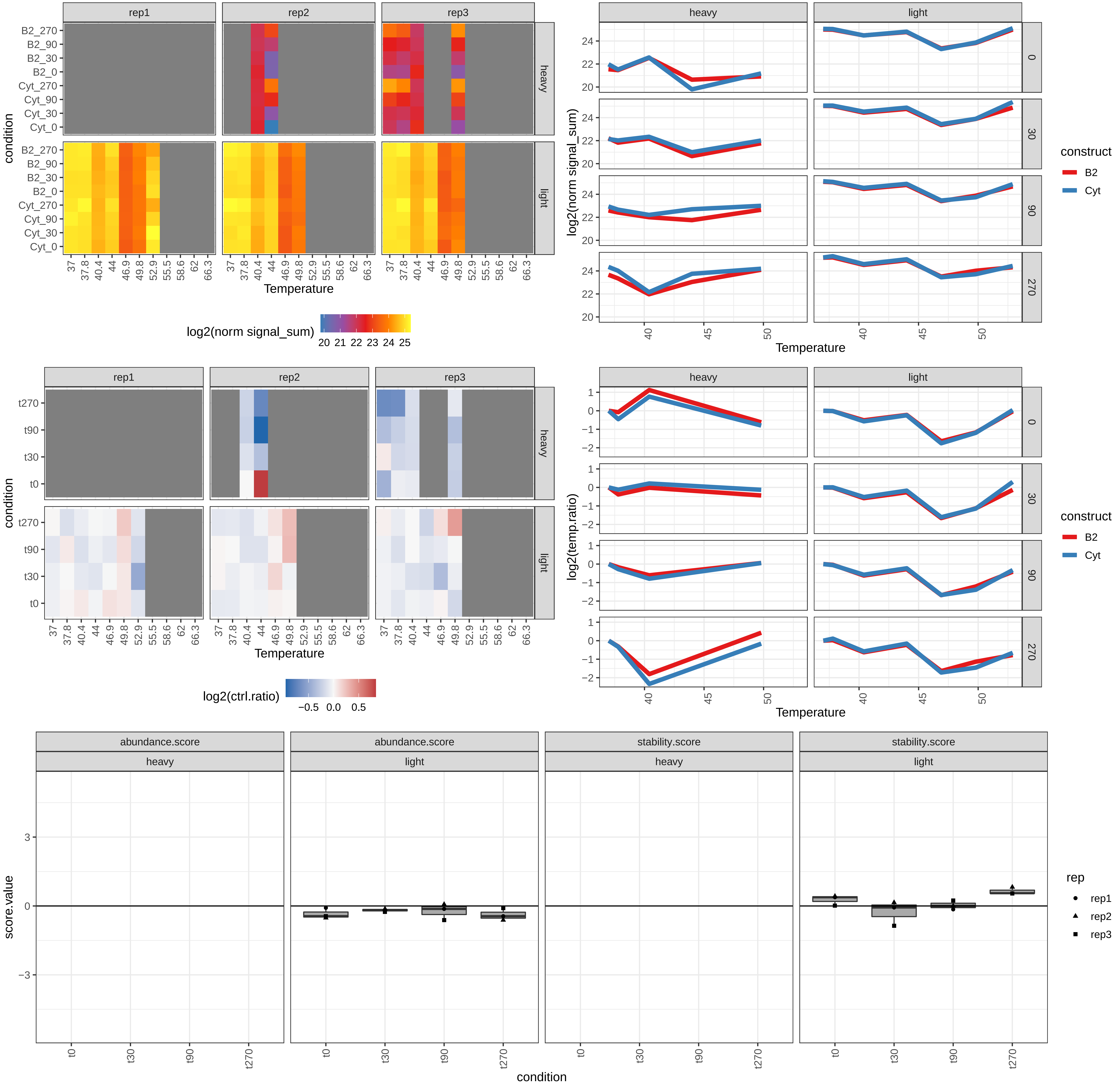

P38828 − Protein LSM12 OS=Saccharomyces cerevisiae (strain ATCC 204508 / S288c) GN=LSM12 PE=1 SV=1

LSM2 − average.top3: 6.9936465 − max.qupm: 6

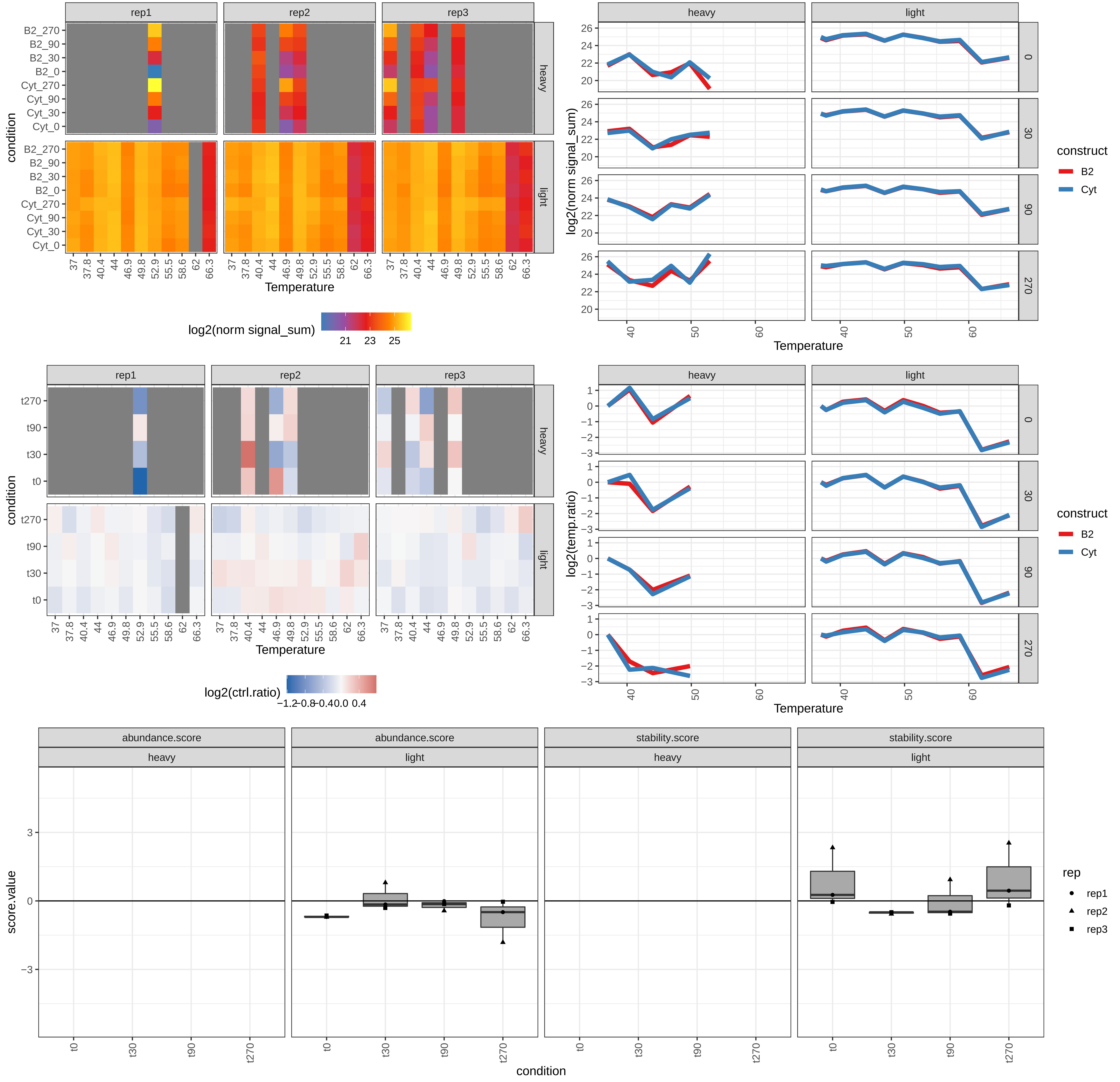

P38203 − U6 snRNA−associated Sm−like protein LSm2 OS=Saccharomyces cerevisiae (strain ATCC 204508 / S288c) GN=LSM2 PE=1 SV=1

LSM4 − average.top3: 6.7037652173913 − max.qupm: 3

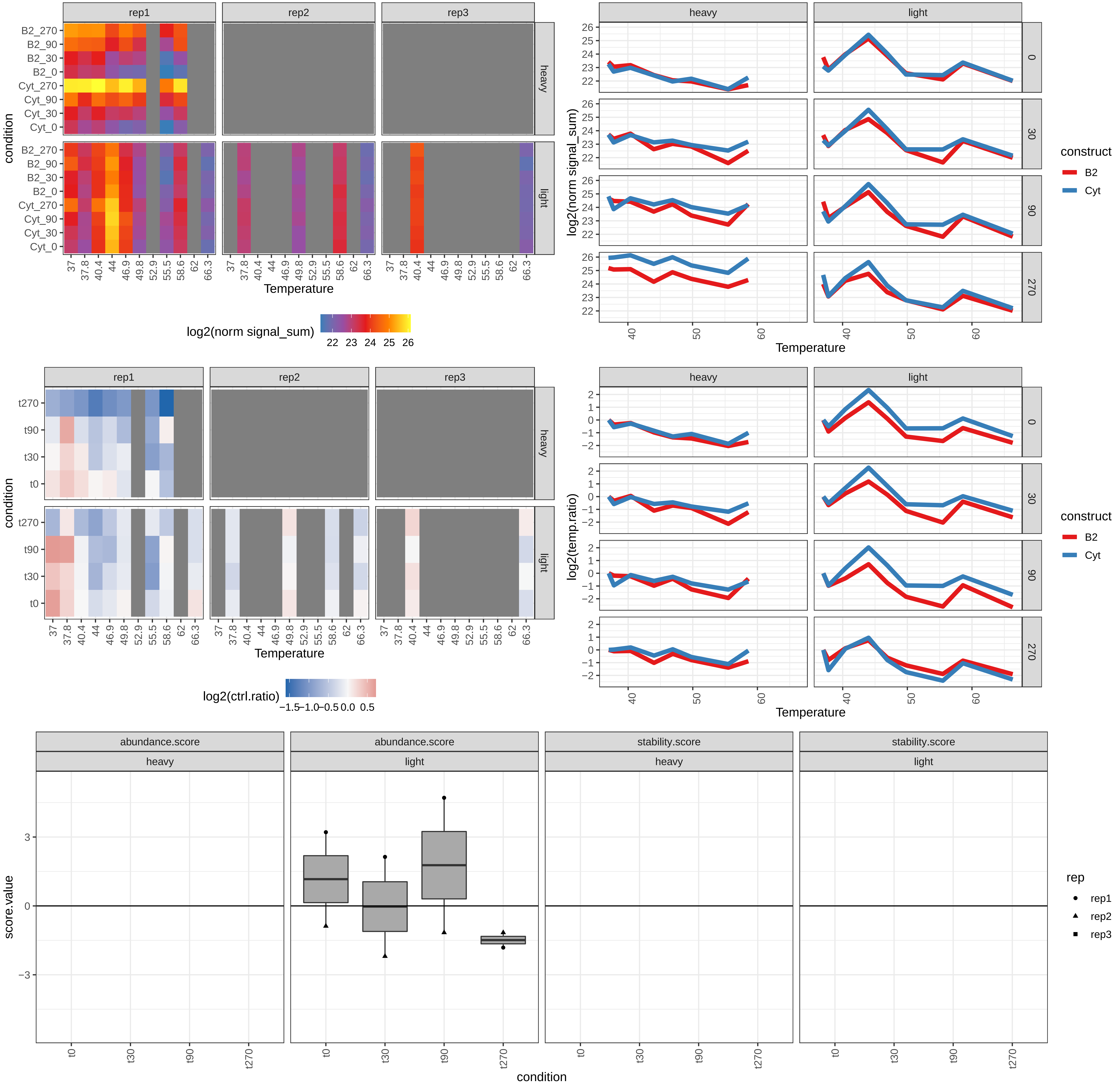

P40070 − U6 snRNA−associated Sm−like protein LSm4 OS=Saccharomyces cerevisiae (strain ATCC 204508 / S288c) GN=LSM4 PE=1 SV=1

LSM6 − average.top3: 7.03600083333333 − max.qupm: 2

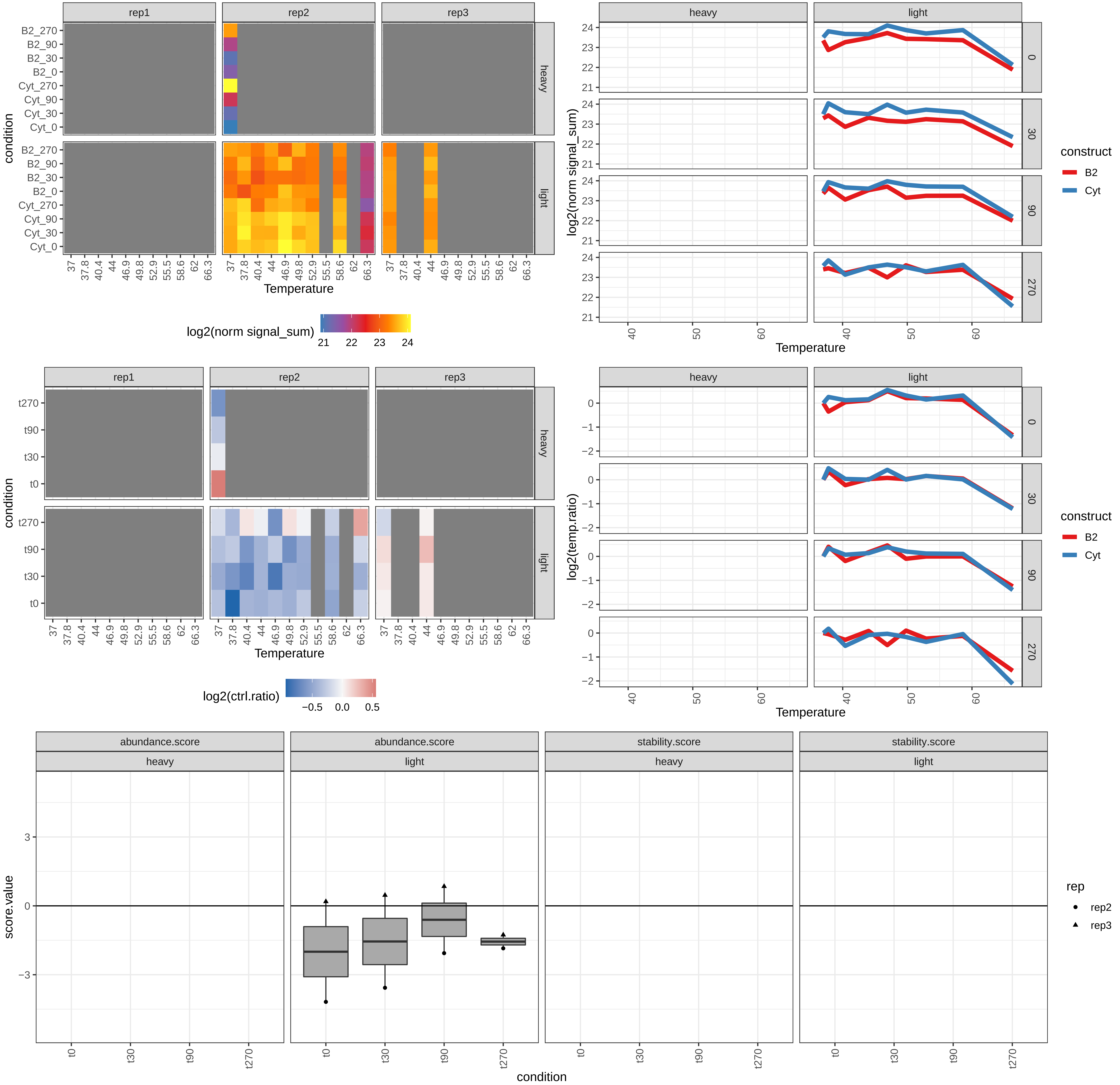

Q06406 − U6 snRNA−associated Sm−like protein LSm6 OS=Saccharomyces cerevisiae (strain ATCC 204508 / S288c) GN=LSM6 PE=1 SV=2

LSM7 − average.top3: 6.905579375 − max.qupm: 5

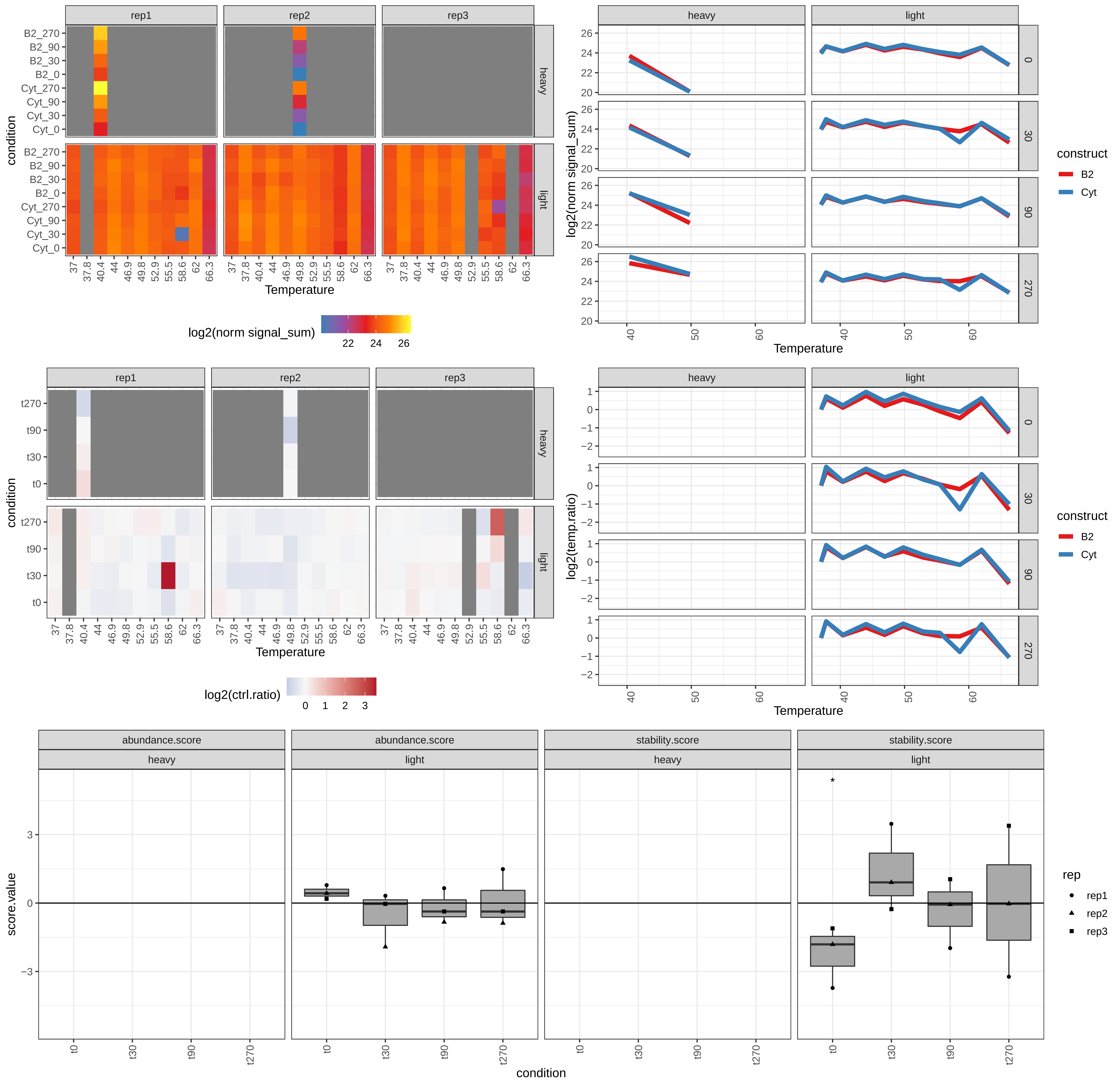

P53905 − U6 snRNA−associated Sm−like protein LSm7 OS=Saccharomyces cerevisiae (strain ATCC 204508 / S288c) GN=LSM7 PE=1 SV=2

LSP1 − average.top3: 7.50187581818182 − max.qupm: 13

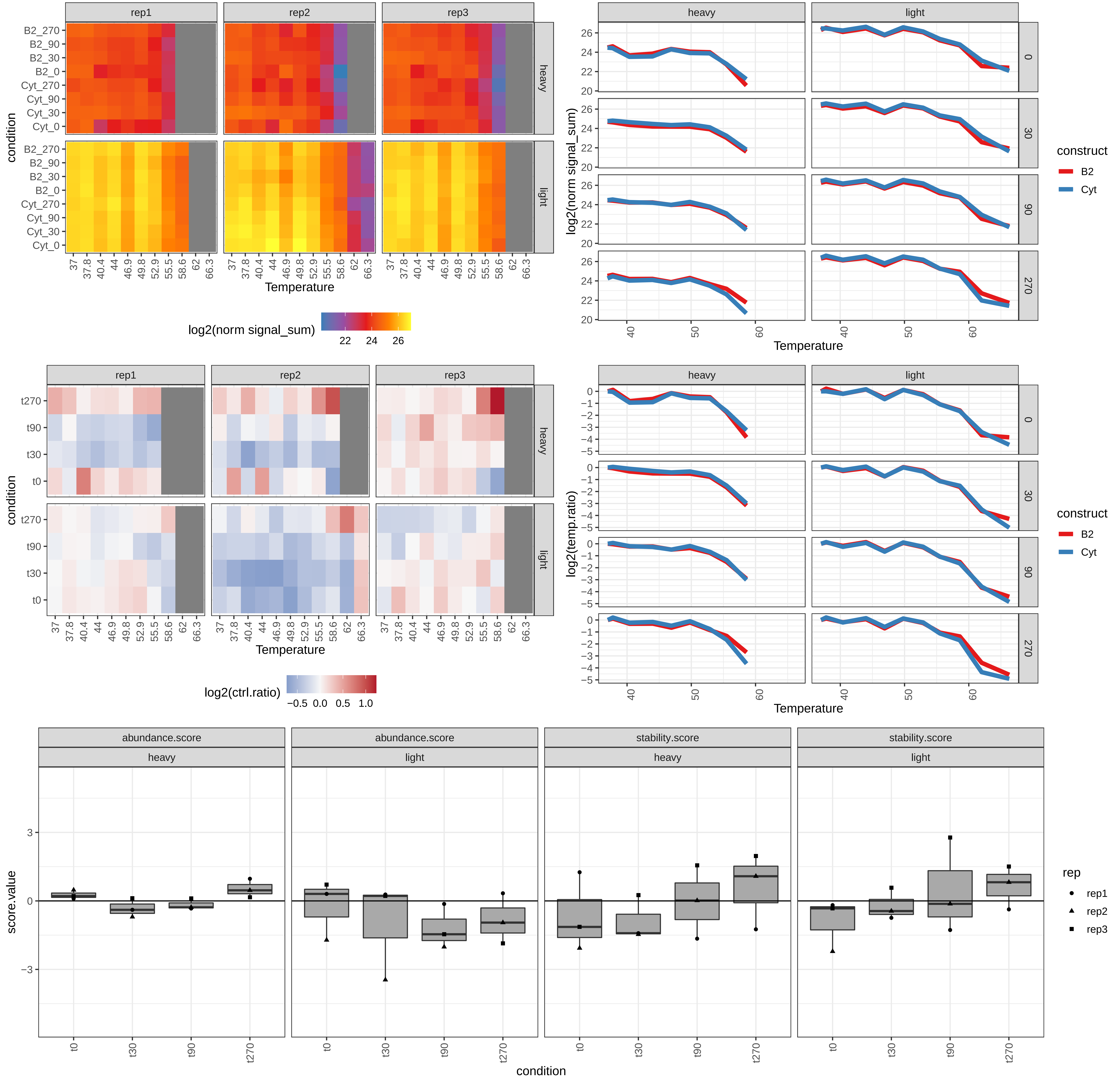

Q12230 − Sphingolipid long chain base−responsive protein LSP1 OS=Saccharomyces cerevisiae (strain ATCC 204508 / S288c) GN=LSP1 PE=1 SV=1

LTE1 − average.top3: 6.35926 − max.qupm: 5

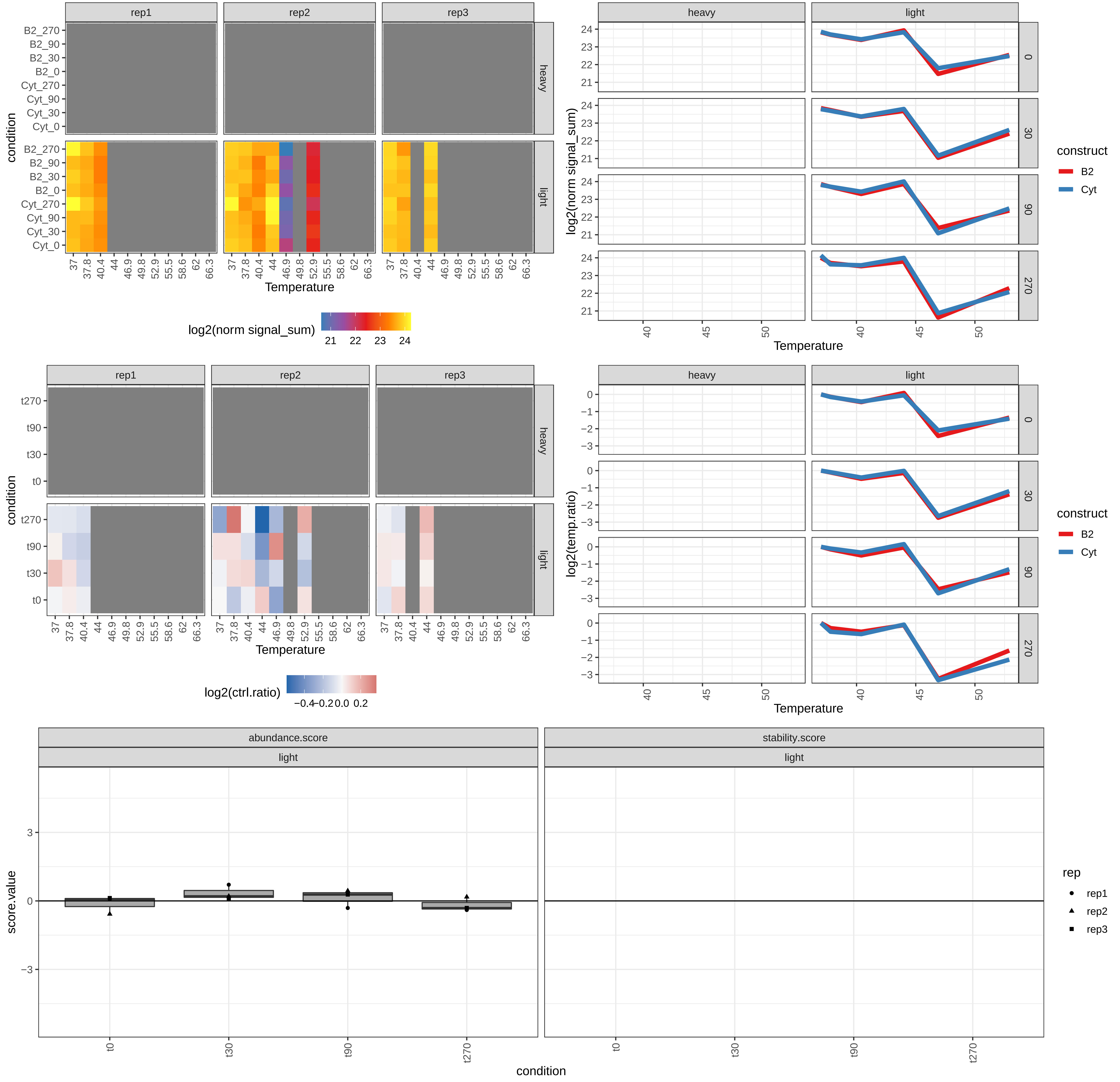

P07866 − Guanine nucleotide exchange factor LTE1 OS=Saccharomyces cerevisiae (strain ATCC 204508 / S288c) GN=LTE1 PE=1 SV=2

LTP1 − average.top3: 7.06506608695652 − max.qupm: 4

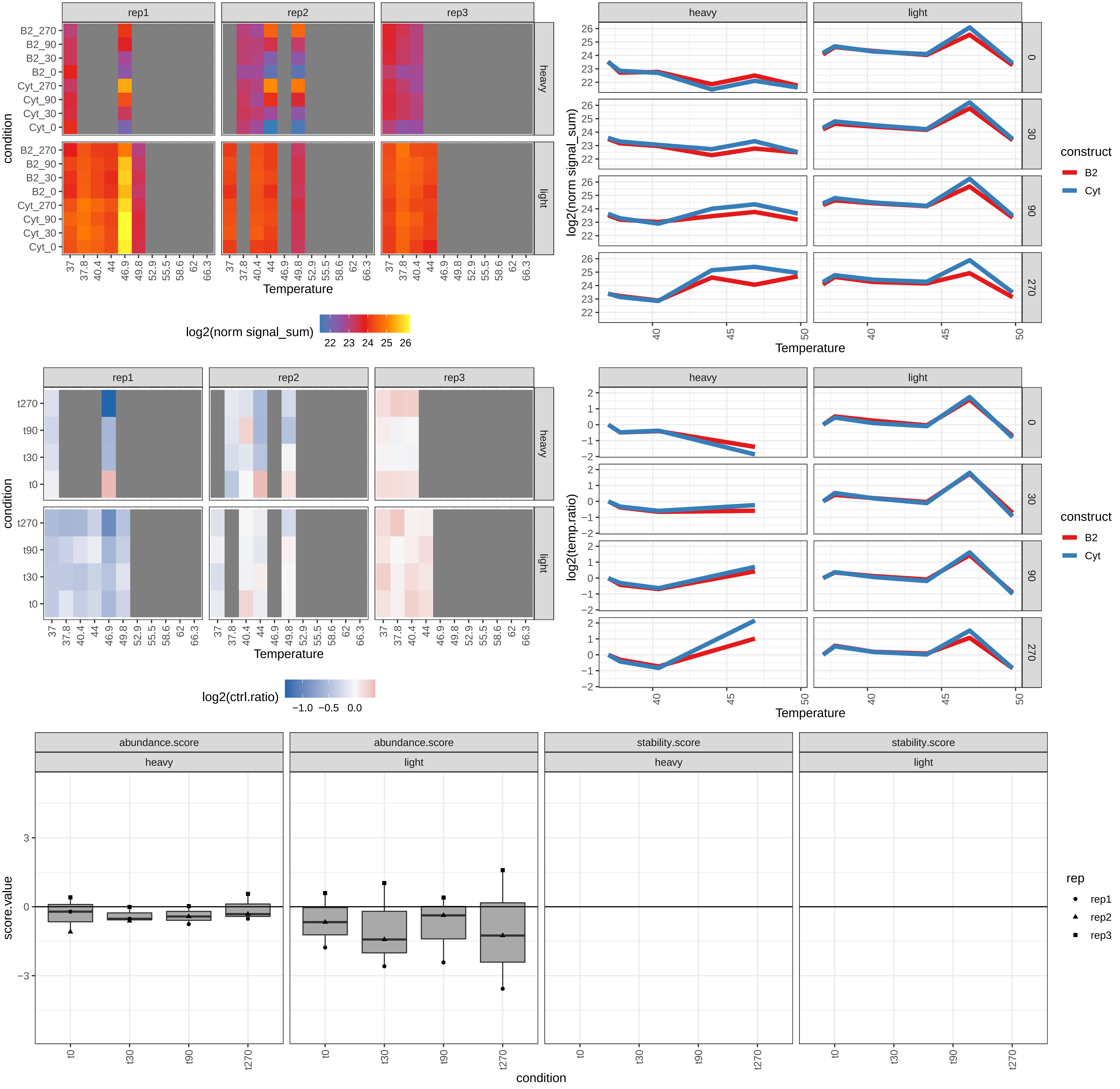

P40347 − Low molecular weight phosphotyrosine protein phosphatase OS=Saccharomyces cerevisiae (strain ATCC 204508 / S288c) GN=LTP1 PE=1 SV=1

LYP1 − average.top3: 6.64757095238095 − max.qupm: 4

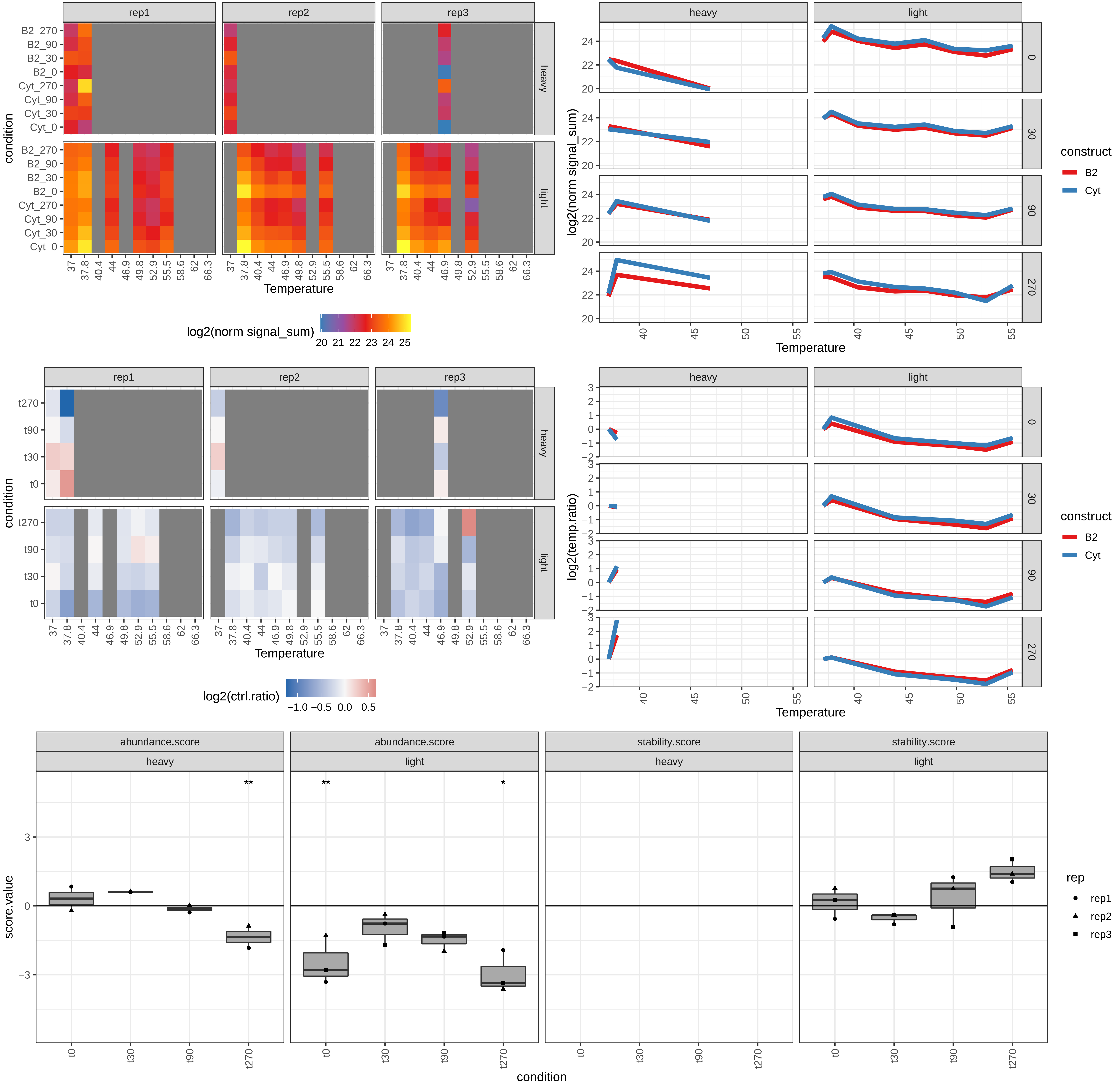

P32487 − Lysine−specific permease OS=Saccharomyces cerevisiae (strain ATCC 204508 / S288c) GN=LYP1 PE=1 SV=2

LYS1 − average.top3: 7.44782319148936 − max.qupm: 12

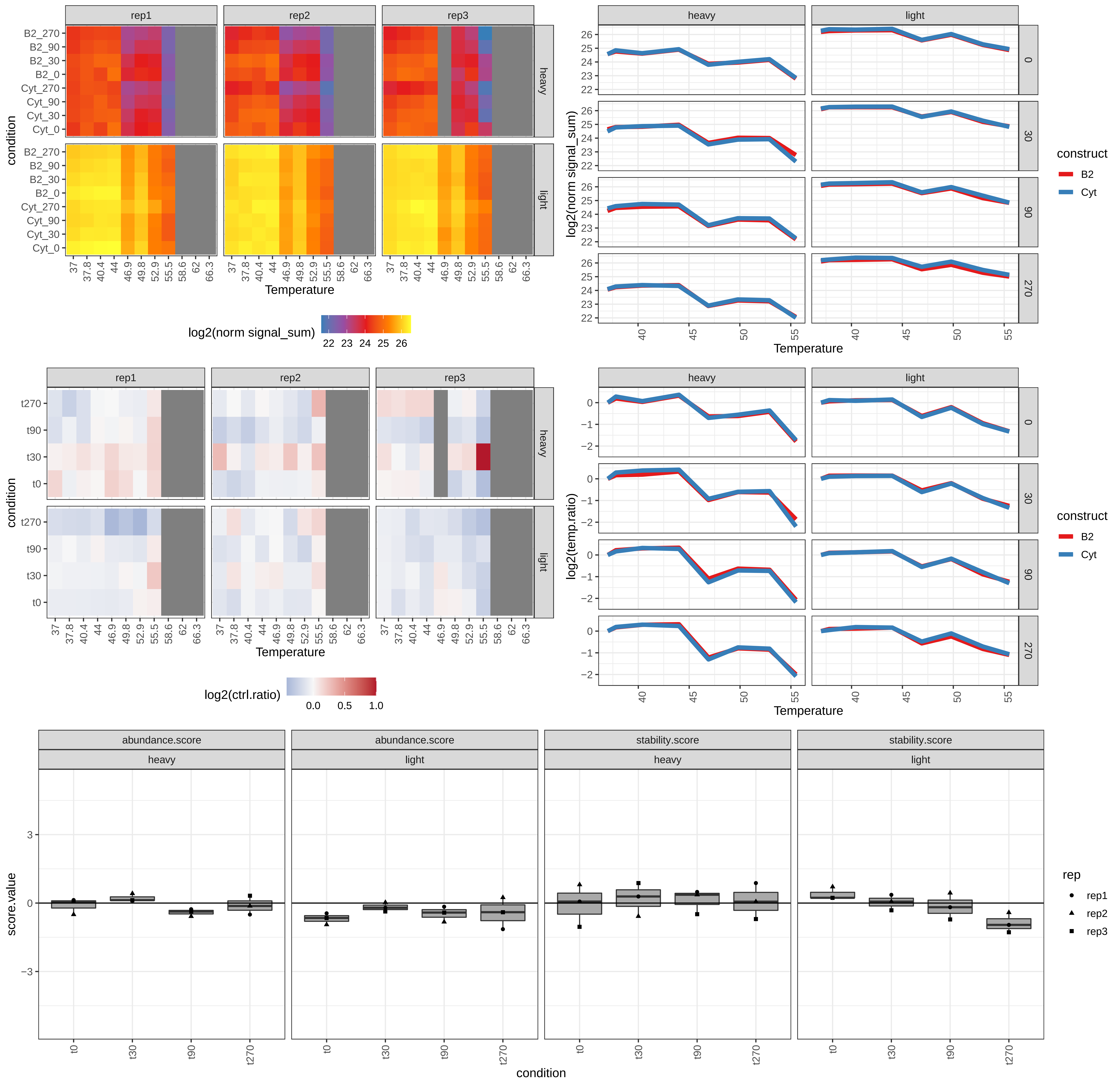

P38998 − Saccharopine dehydrogenase [NAD(+), L−lysine−forming] OS=Saccharomyces cerevisiae (strain ATCC 204508 / S288c) GN=LYS1 PE=1 SV=3

LYS12 − average.top3: 7.52522107692308 − max.qupm: 17

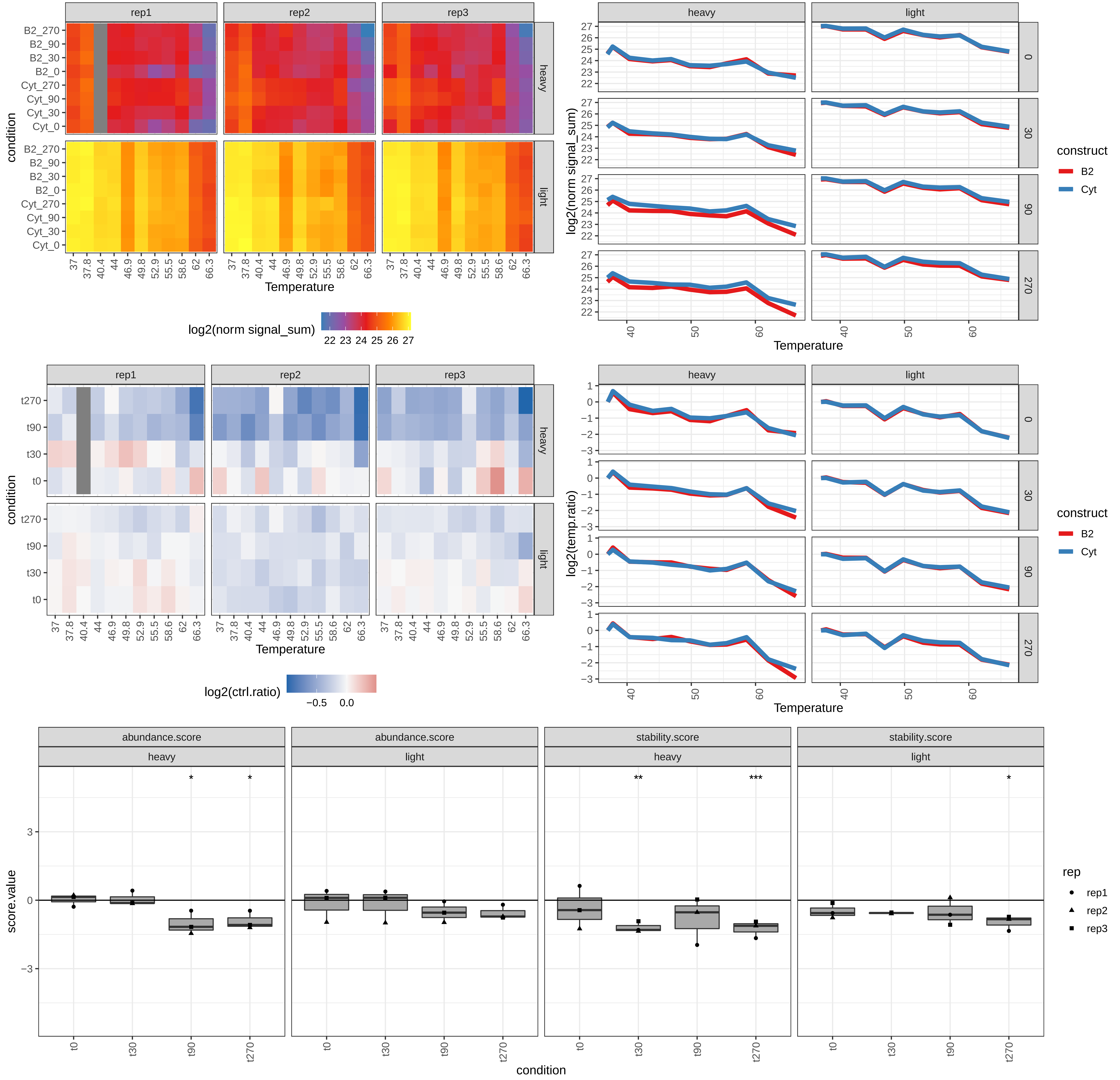

P40495 − Homoisocitrate dehydrogenase, mitochondrial OS=Saccharomyces cerevisiae (strain ATCC 204508 / S288c) GN=LYS12 PE=1 SV=1

LYS20 − average.top3: 6.95477367346939 − max.qupm: 12

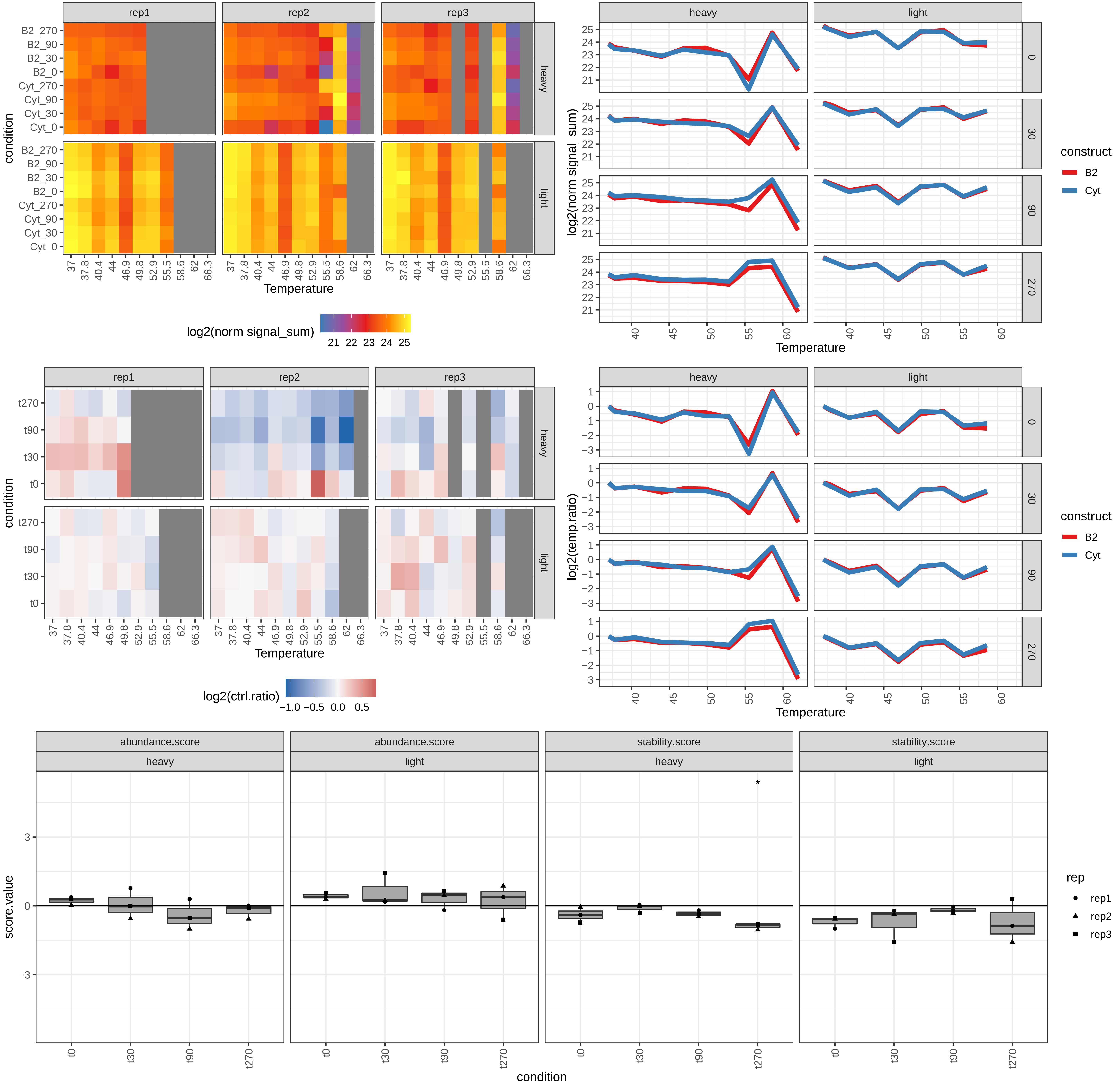

P48570 − Homocitrate synthase, cytosolic isozyme OS=Saccharomyces cerevisiae (strain ATCC 204508 / S288c) GN=LYS20 PE=1 SV=1

LYS21 − average.top3: 6.82833081081081 − max.qupm: 5

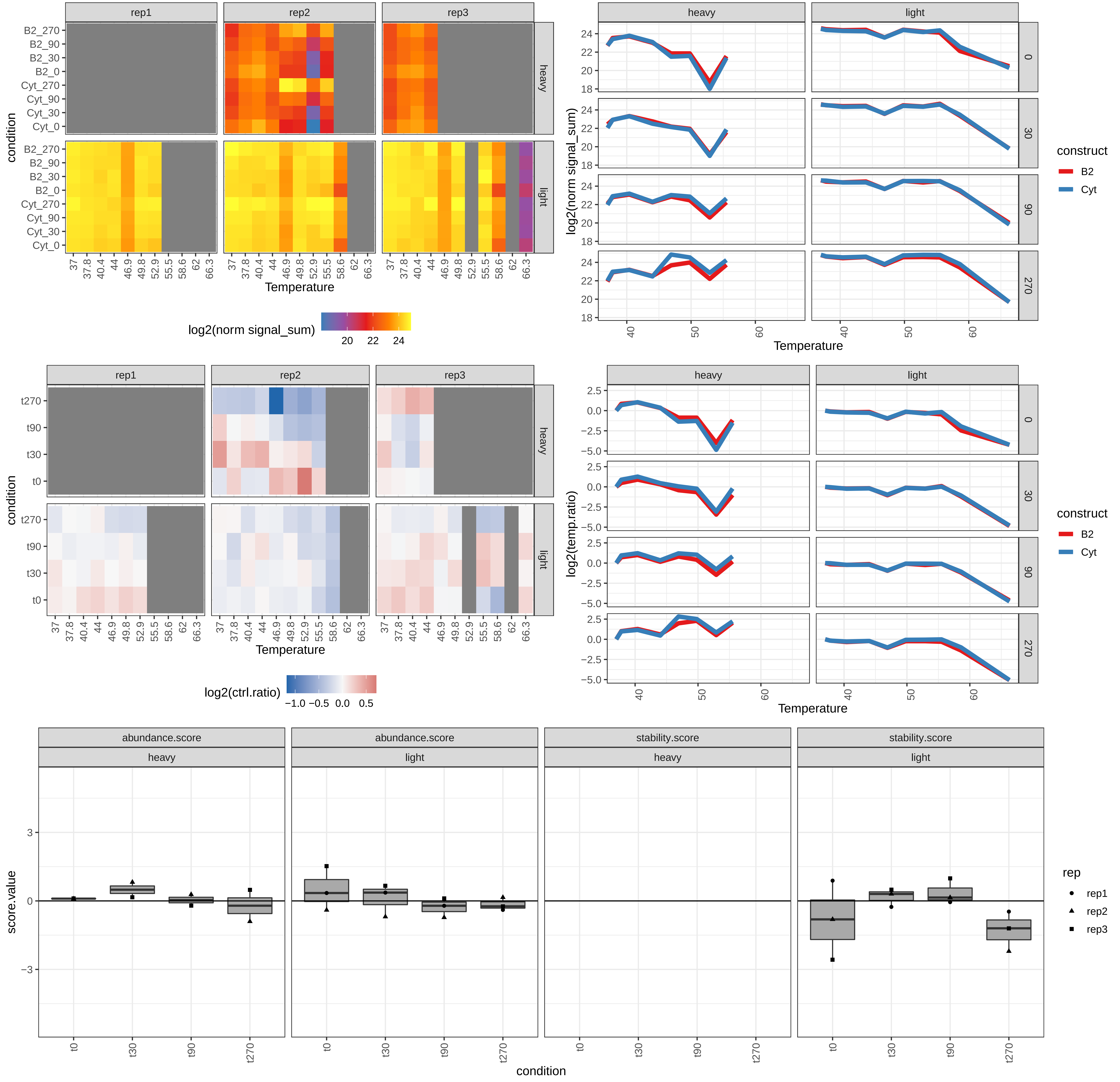

Q12122 − Homocitrate synthase, mitochondrial OS=Saccharomyces cerevisiae (strain ATCC 204508 / S288c) GN=LYS21 PE=1 SV=1

LYS4 − average.top3: 6.81756461538462 − max.qupm: 8

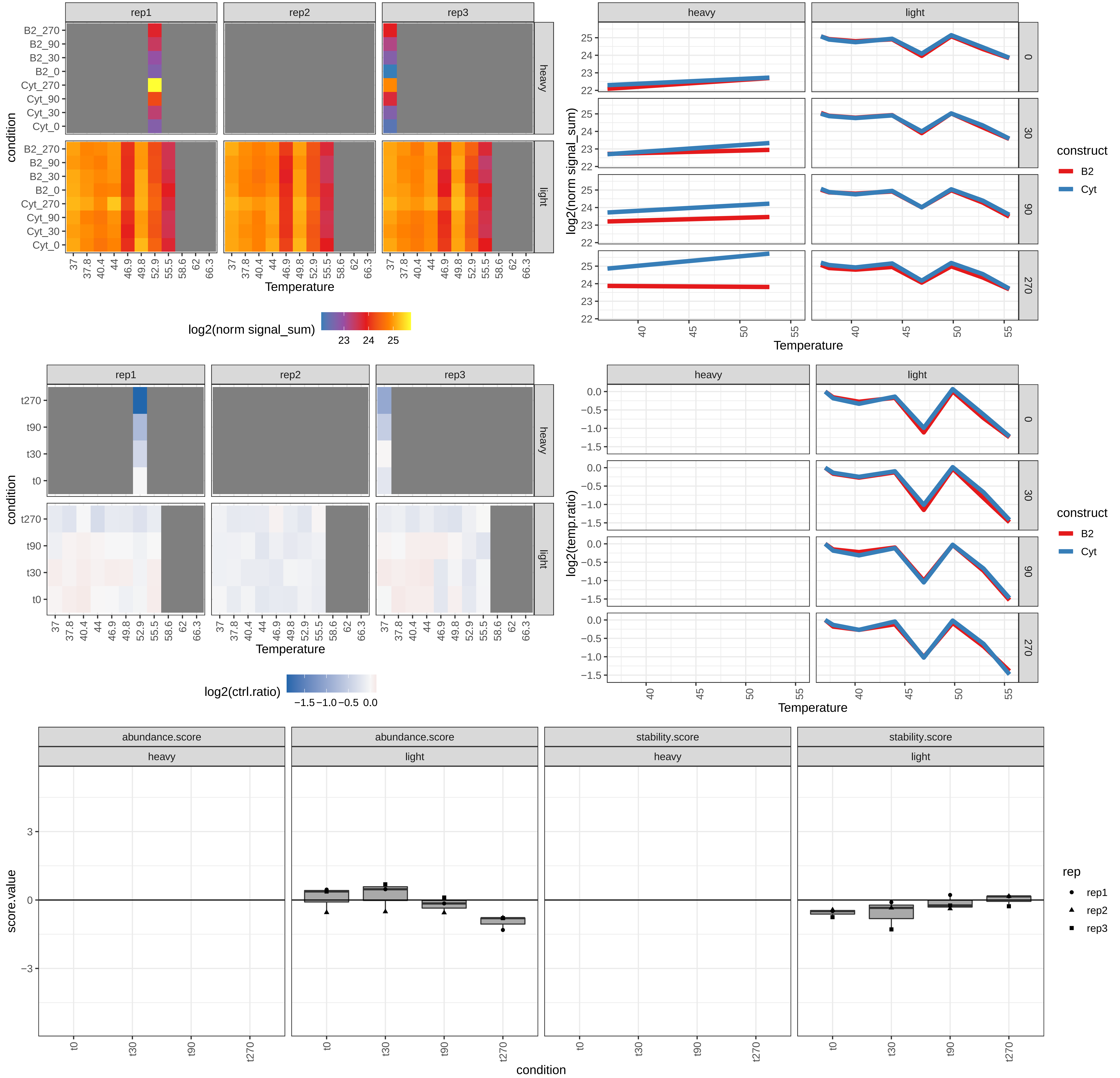

P49367 − Homoaconitase, mitochondrial OS=Saccharomyces cerevisiae (strain ATCC 204508 / S288c) GN=LYS4 PE=1 SV=1

LYS9 − average.top3: 6.81309 − max.qupm: 16

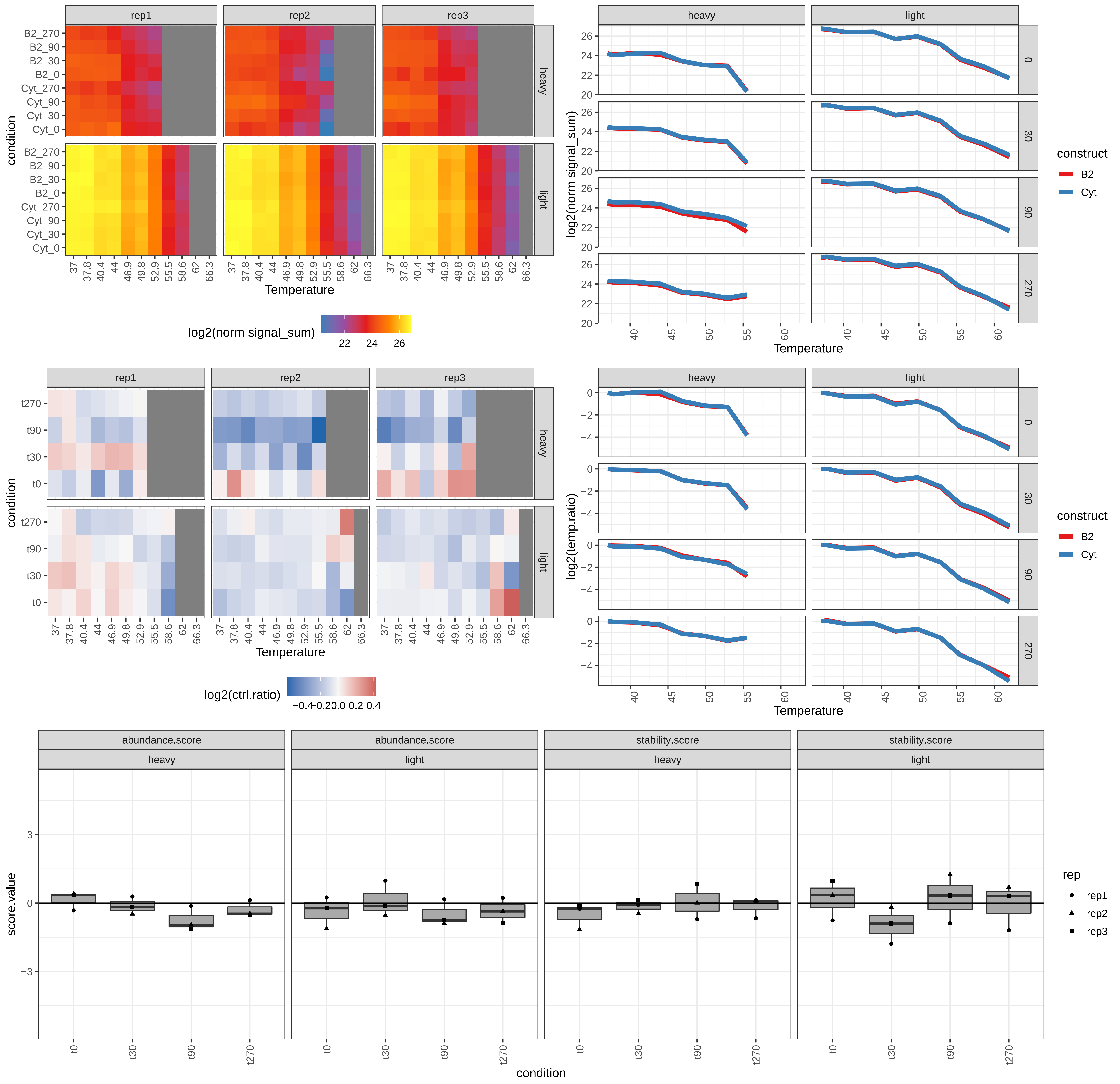

P38999 − Saccharopine dehydrogenase [NADP(+), L−glutamate−forming] OS=Saccharomyces cerevisiae (strain ATCC 204508 / S288c) GN=LYS9 PE=1 SV=1

MAD1 − average.top3: 6.04263444444444 − max.qupm: 4

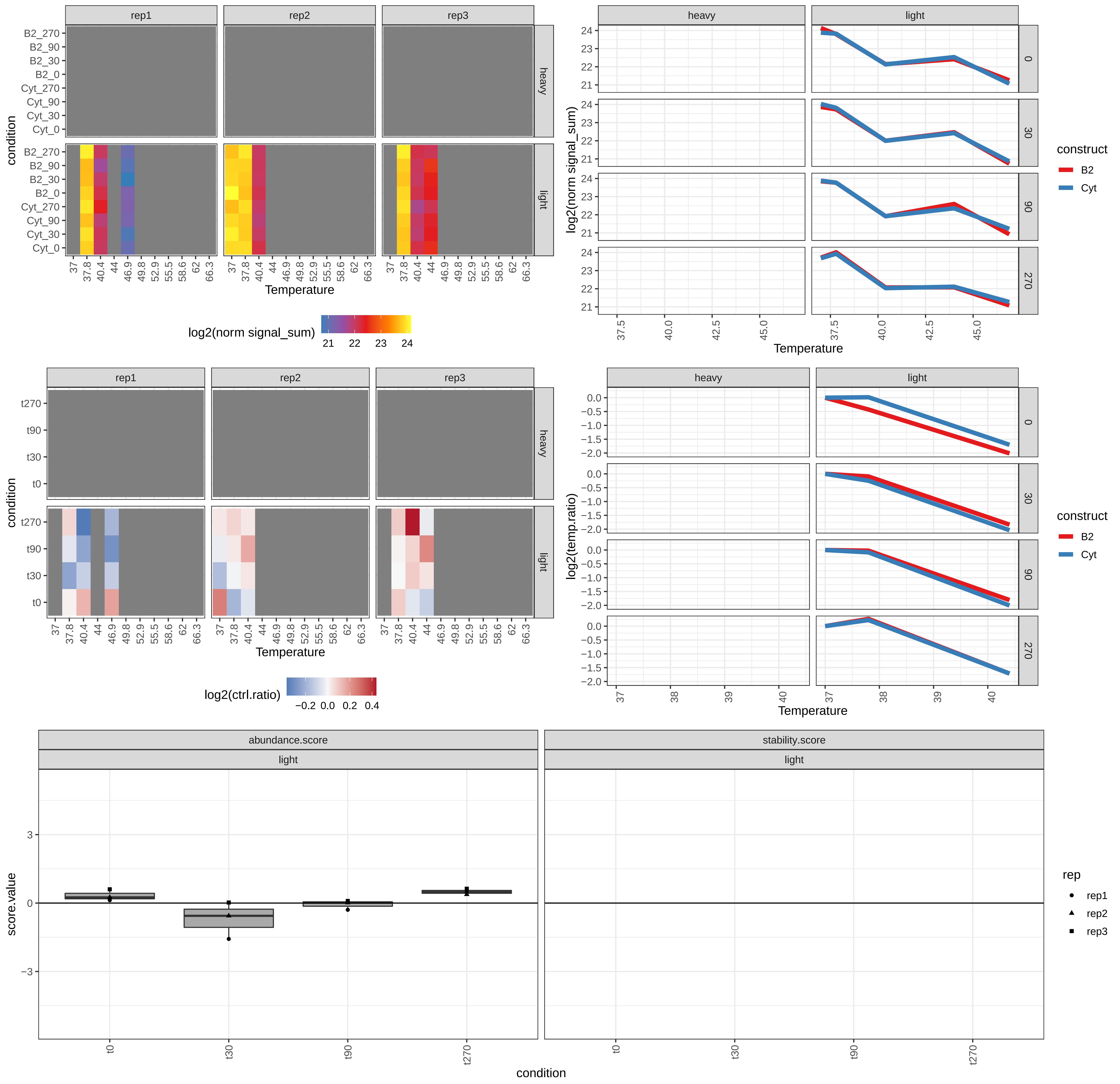

P40957 − Spindle assembly checkpoint component MAD1 OS=Saccharomyces cerevisiae (strain ATCC 204508 / S288c) GN=MAD1 PE=1 SV=1

MAE1 − average.top3: 6.78714473684211 − max.qupm: 5

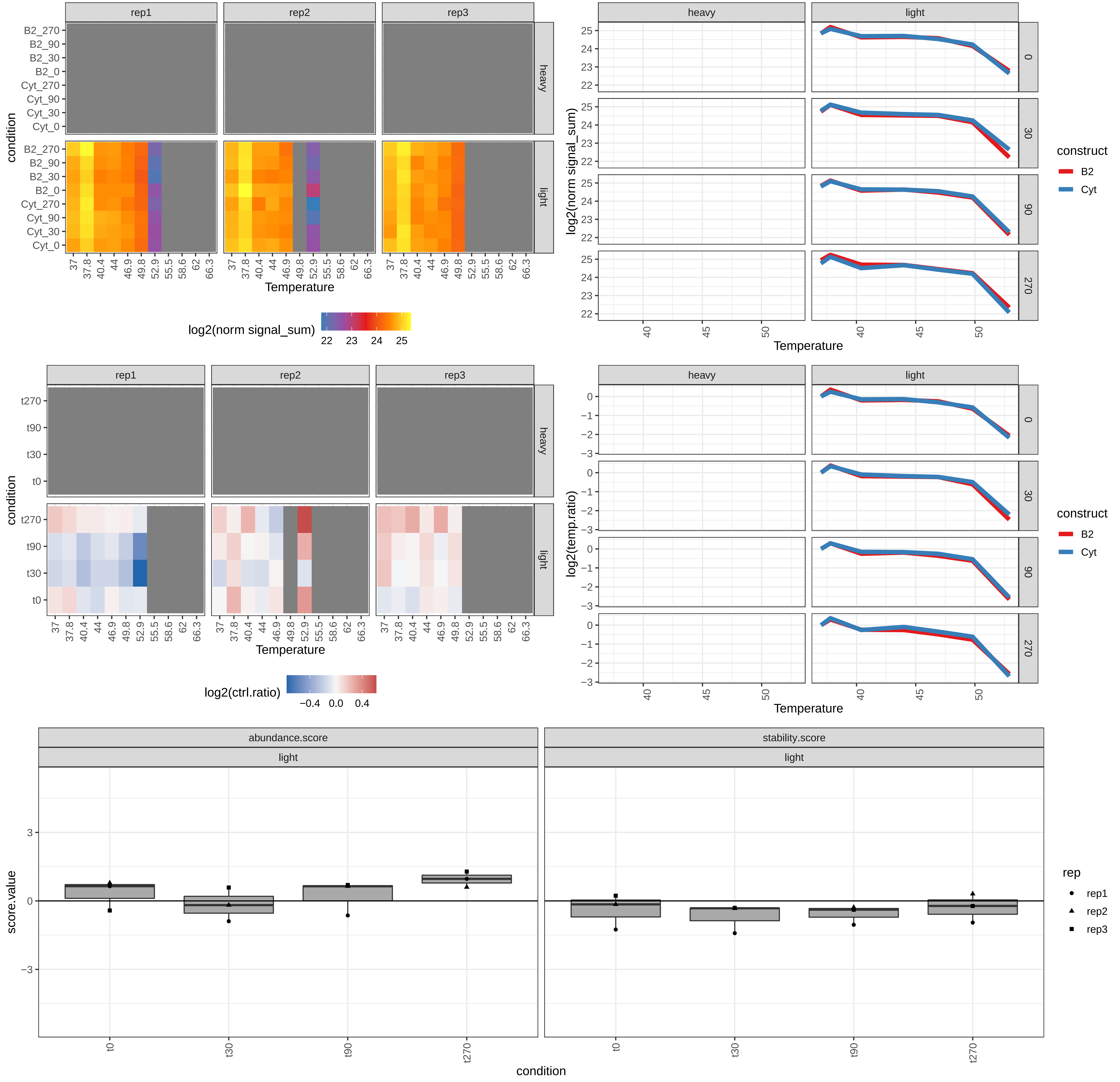

P36013 − NAD−dependent malic enzyme, mitochondrial OS=Saccharomyces cerevisiae (strain ATCC 204508 / S288c) GN=MAE1 PE=1 SV=1

MAF1 − average.top3: 6.41480333333333 − max.qupm: 3

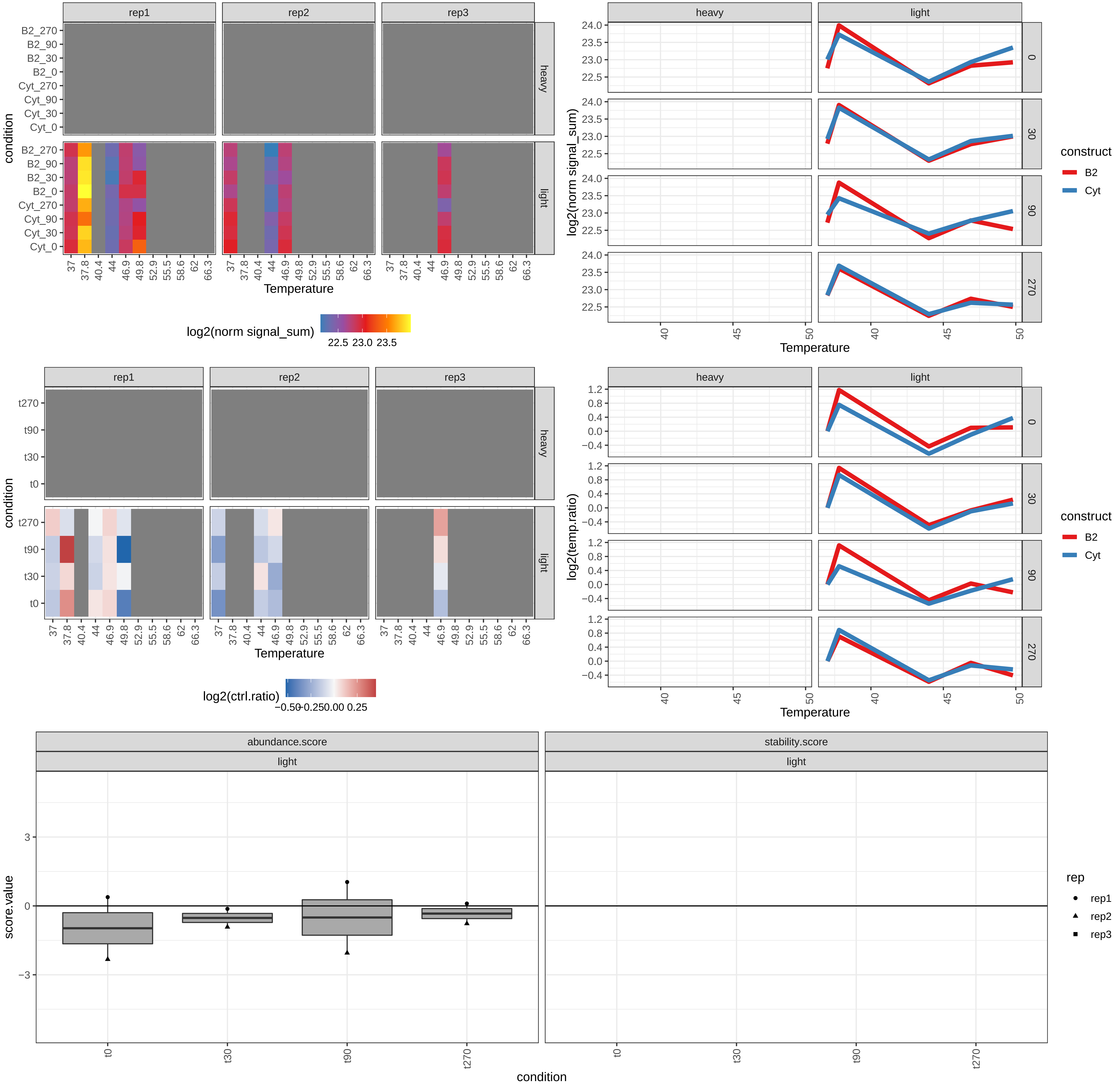

P41910 − Repressor of RNA polymerase III transcription MAF1 OS=Saccharomyces cerevisiae (strain ATCC 204508 / S288c) GN=MAF1 PE=1 SV=1

MAK10 − average.top3: 6.6039188 − max.qupm: 7

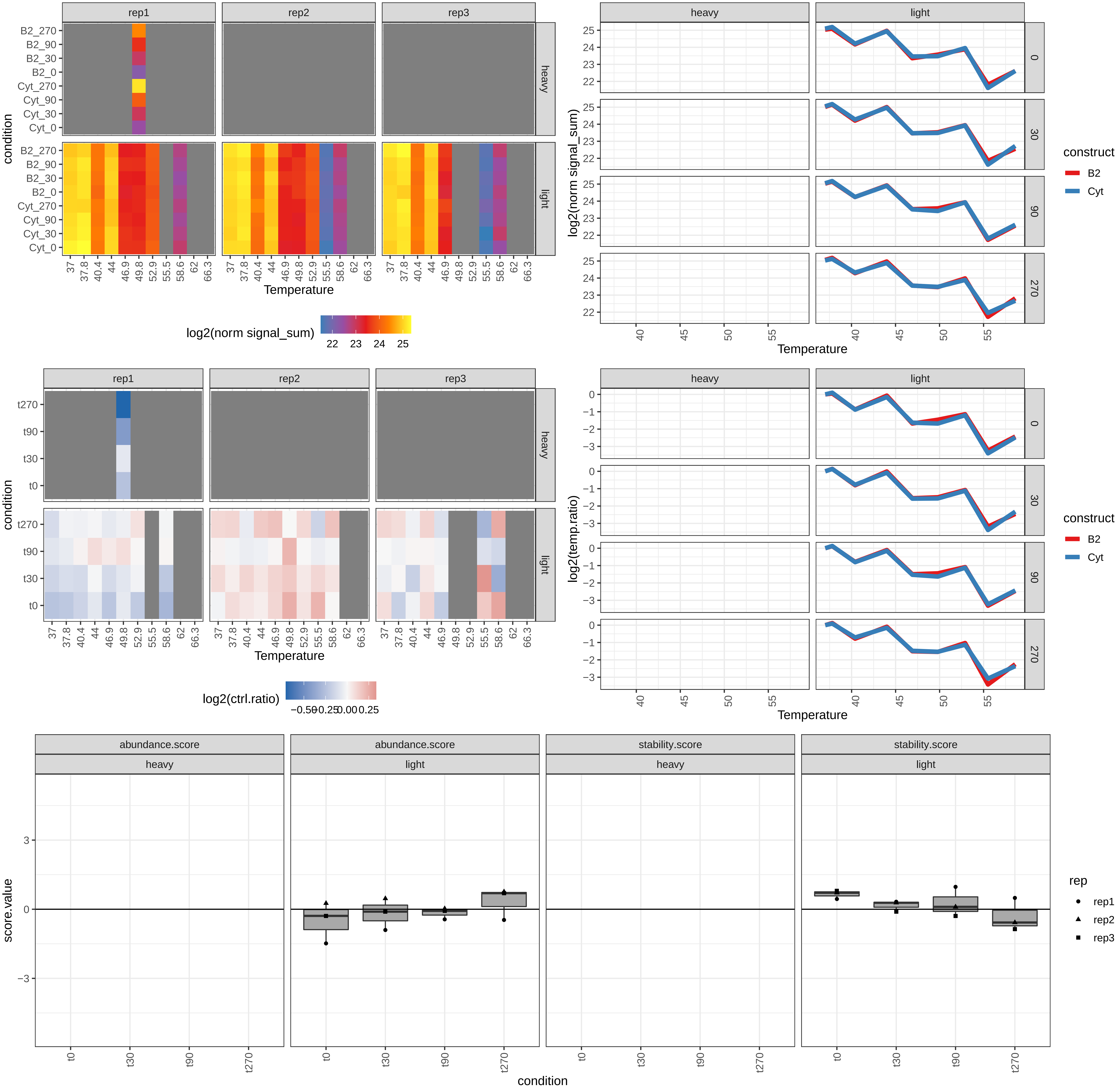

Q02197 − N−alpha−acetyltransferase, 35 NatC auxiliary subunit OS=Saccharomyces cerevisiae (strain ATCC 204508 / S288c) GN=MAK10 PE=1 SV=1

MAK11 − average.top3: 7.06314822222222 − max.qupm: 10

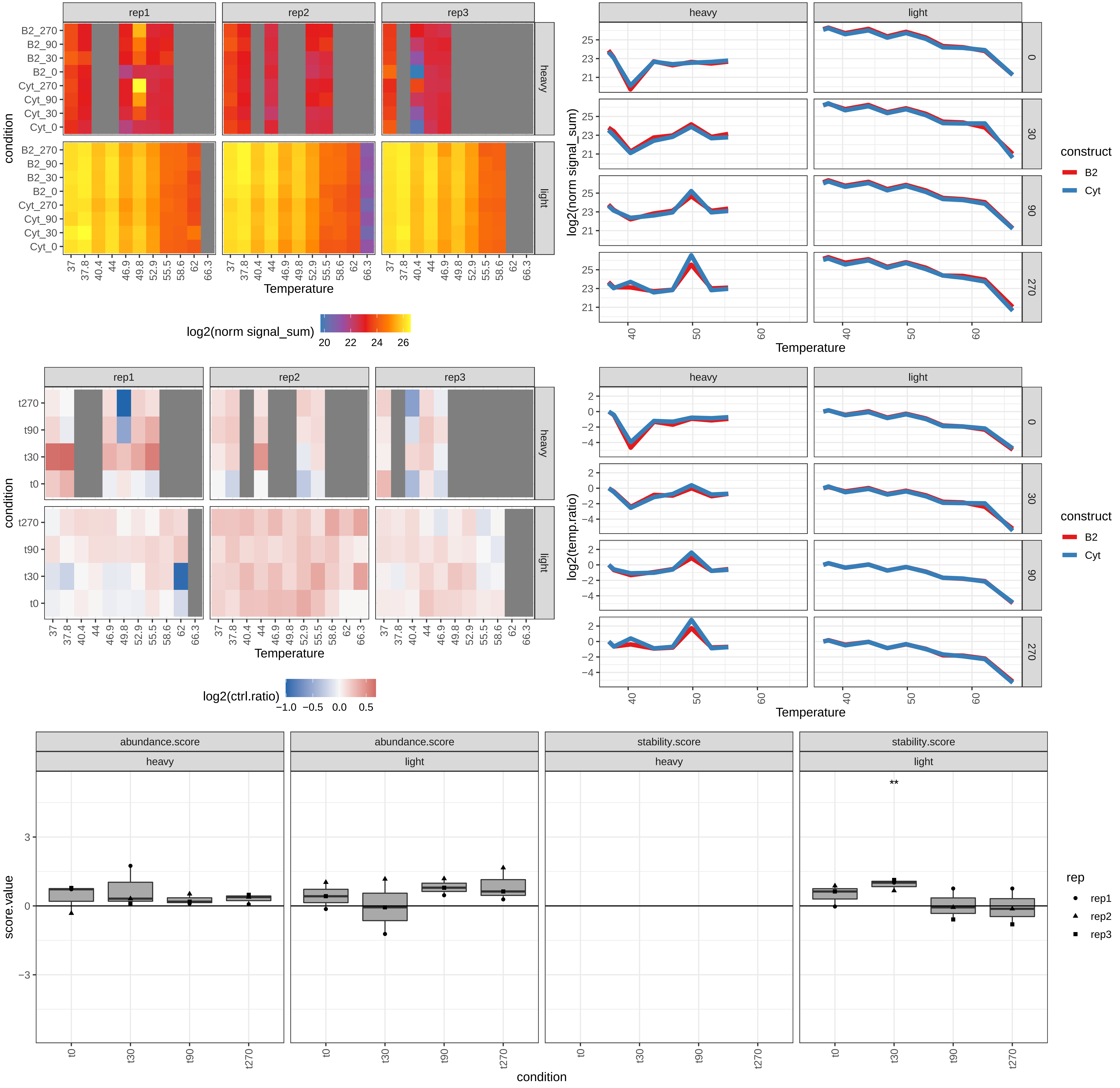

P20484 − Protein MAK11 OS=Saccharomyces cerevisiae (strain ATCC 204508 / S288c) GN=MAK11 PE=1 SV=2

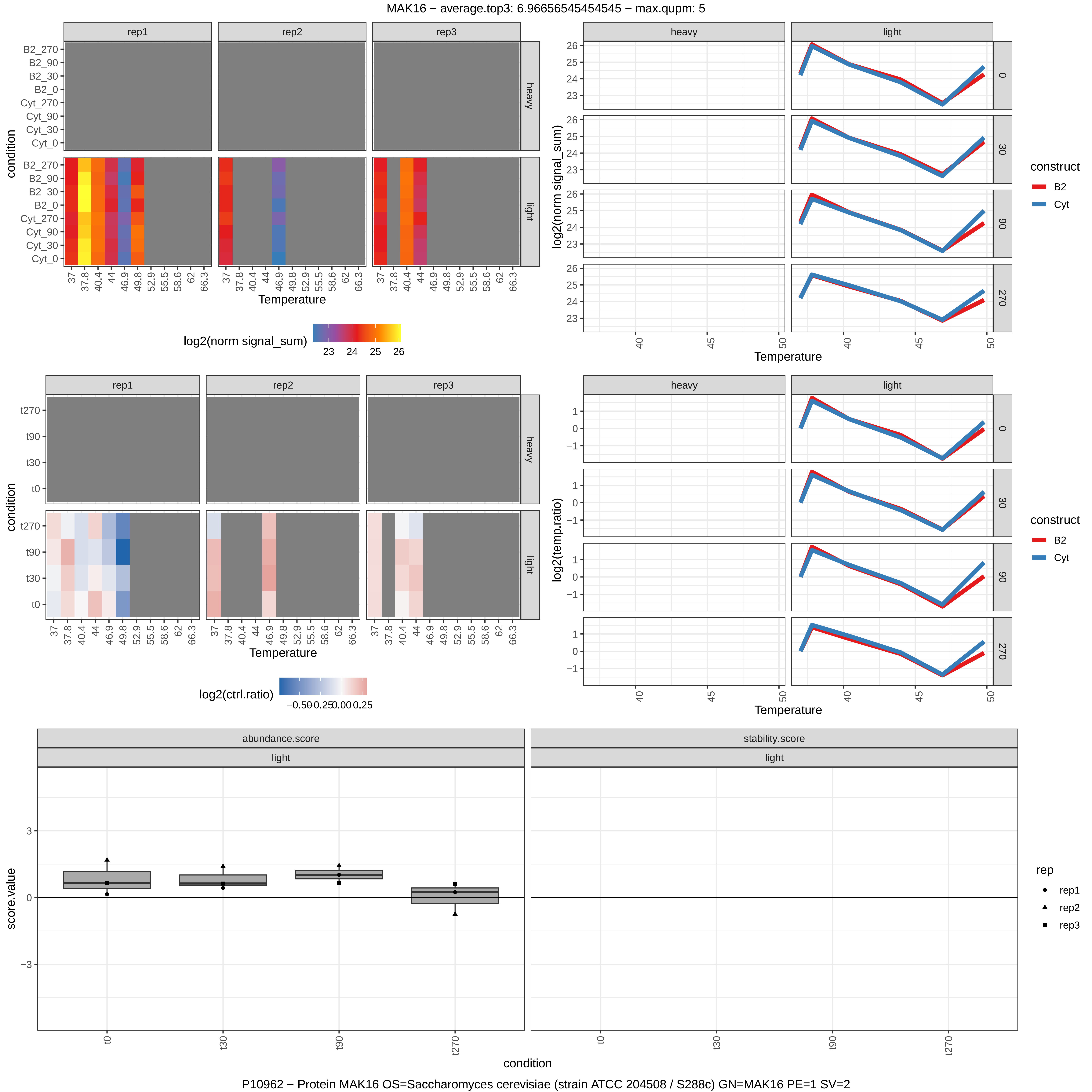

MAK16 − average.top3: 6.96656545454545 − max.qupm: 5
rep1
rep2
rep3
heavy
light
condition
Temperature
log2(norm signal_sum)
log2(ctrl.ratio)
log2(temp.ratio)
construct
B2
Cyt
abundance.score
stability.score
score.value
rep
rep1
rep2
rep3
P10962 − Protein MAK16 OS=Saccharomyces cerevisiae (strain ATCC 204508 / S288c) GN=MAK16 PE=1 SV=2

MAK21 − average.top3: 6.96200266666667 − max.qupm: 10

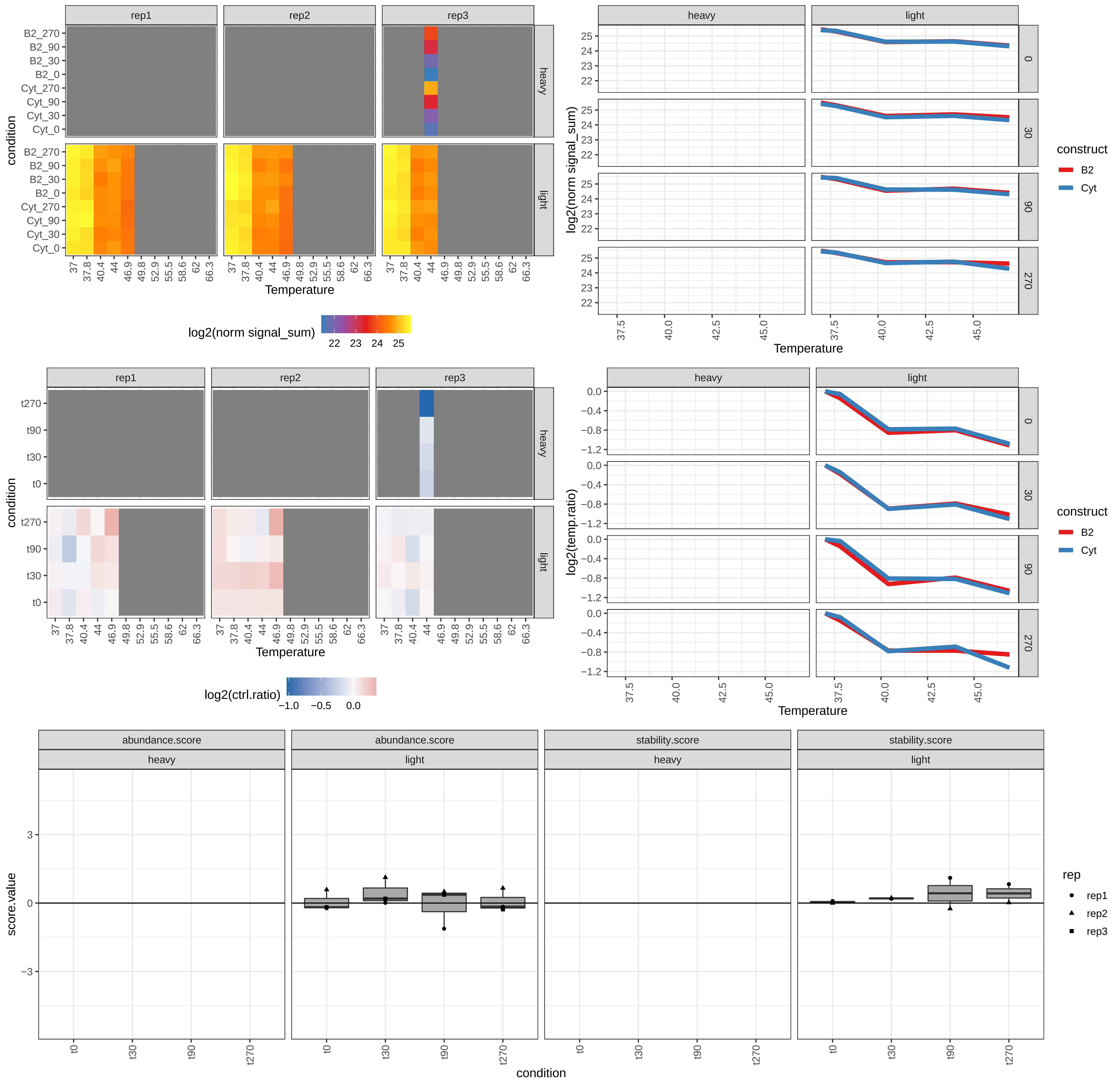

Q12176 − Ribosome biogenesis protein MAK21 OS=Saccharomyces cerevisiae (strain ATCC 204508 / S288c) GN=MAK21 PE=1 SV=1

MAK5 − average.top3: 6.6170885 − max.qupm: 6

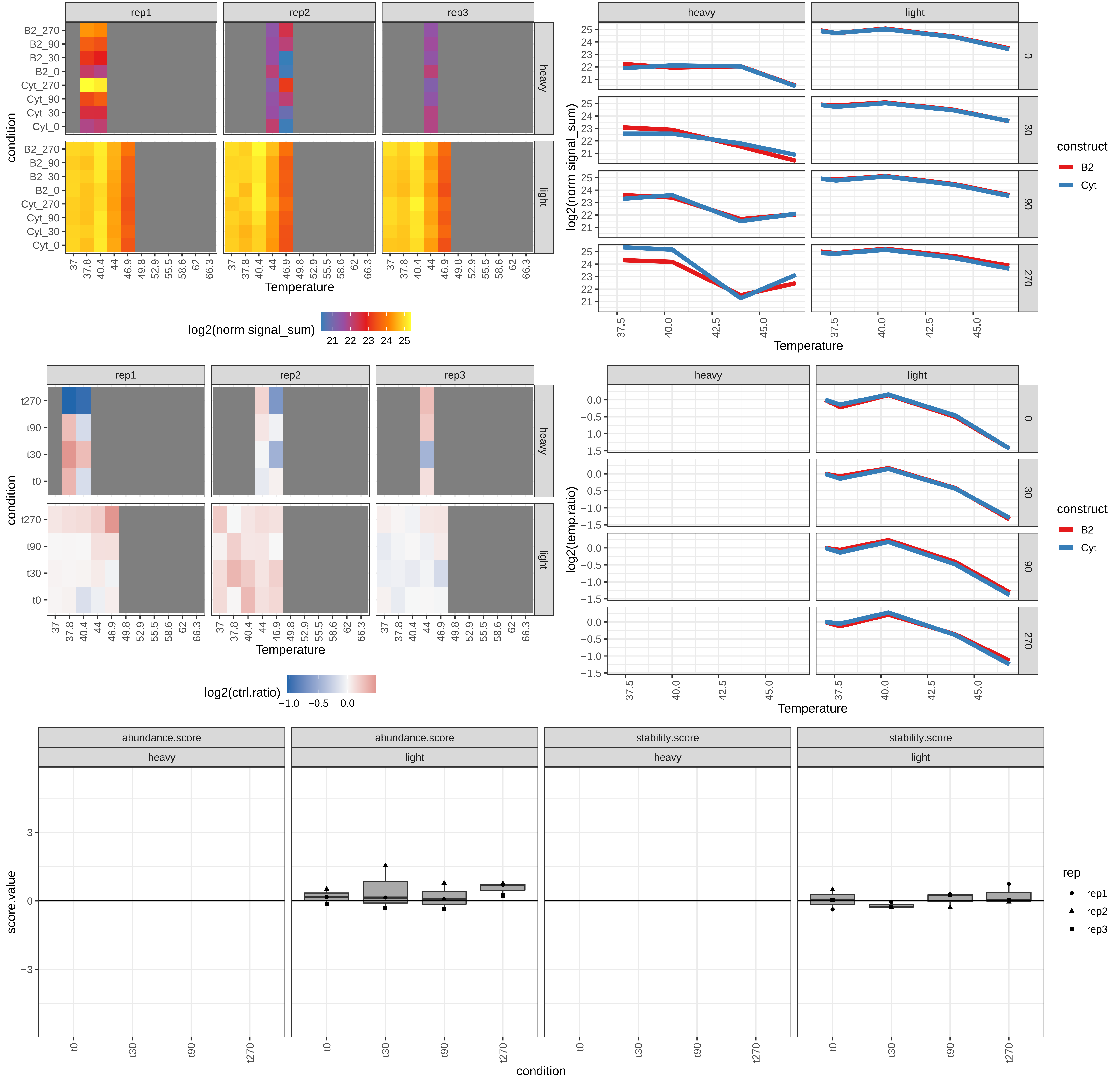

P38112 − ATP−dependent RNA helicase MAK5 OS=Saccharomyces cerevisiae (strain ATCC 204508 / S288c) GN=MAK5 PE=1 SV=1

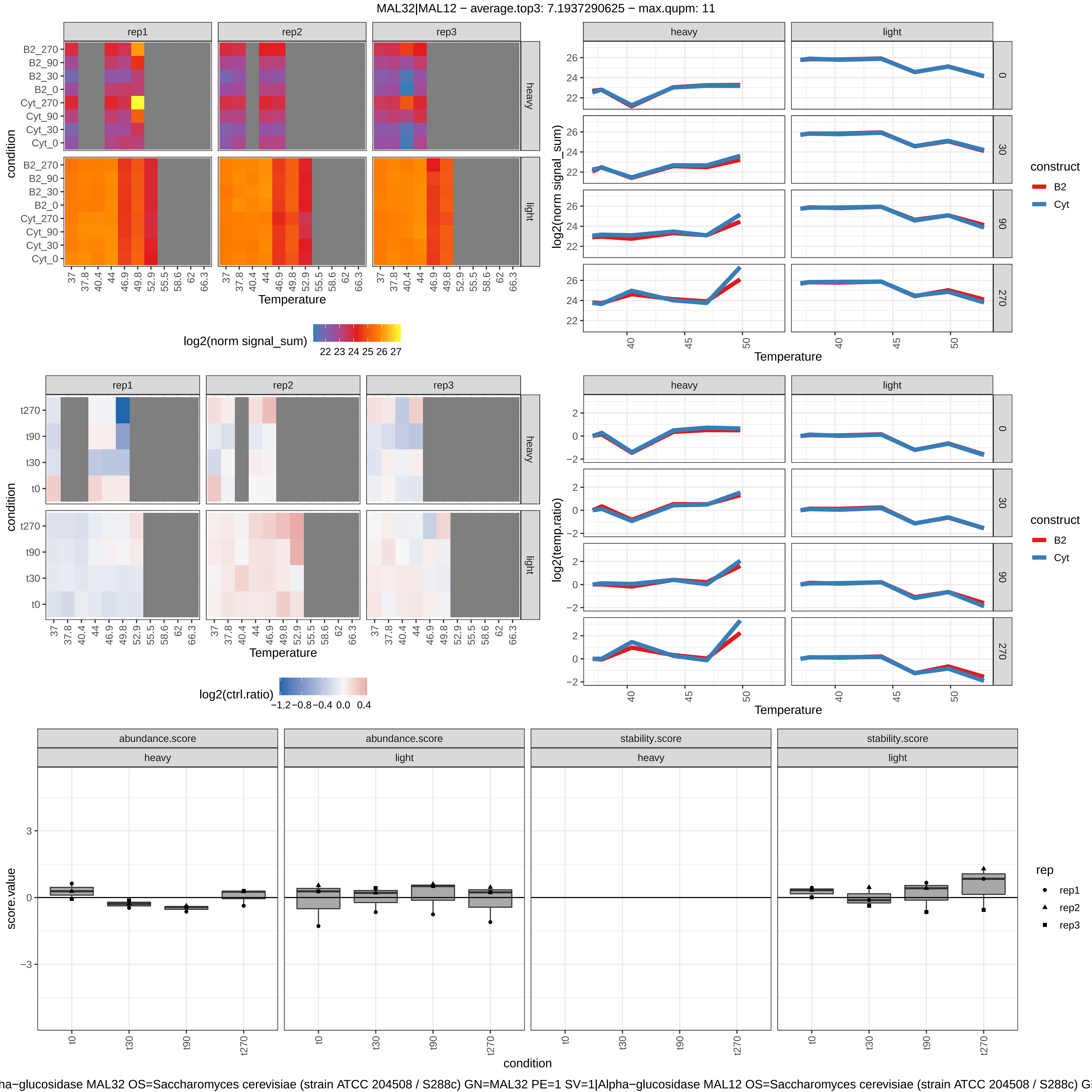
MAL32|MAL12 − average.top3: 7.1937290625 − max.qupm: 11
rep1
rep2
rep3
heavy
light
B2_270
B2_90
B2_30
B2_0
Cyt_270
Cyt_90
Cyt_30
Cyt_0
condition
37
37.8
40.4
44
46.9
49.8
52.9
55.5
58.6
62
66.3
Temperature
log2(norm signal_sum)
22 23 24 25 26 27
log2(norm signal_sum)
0
30
90
270
construct
B2
Cyt
t270
t90
t30
t0
log2(ctrl.ratio)
−1.2 −0.8 −0.4 0.0 0.4
log2(temp.ratio)
abundance.score
stability.score
score.value
rep
rep1
rep2
rep3
ha−glucosidase MAL32 OS=Saccharomyces cerevisiae (strain ATCC 204508 / S288c) GN=MAL32 PE=1 SV=1|Alpha−glucosidase MAL12 OS=Saccharomyces cerevisiae (strain ATCC 204508 / S288c) G

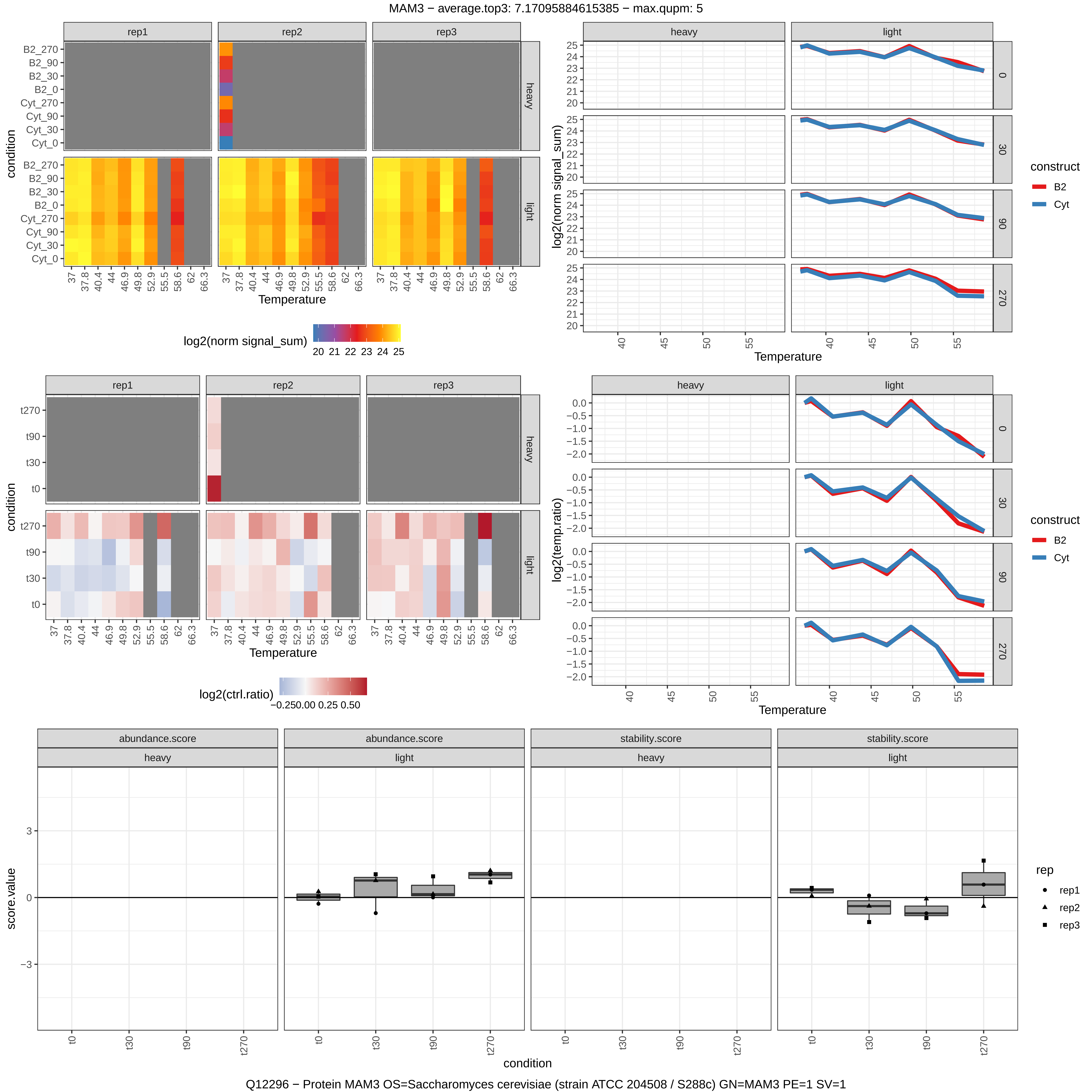
MAM3 − average.top3: 7.17095884615385 − max.qupm: 5
rep1
rep2
rep3
heavy
light
condition
B2_270
B2_90
B2_30
B2_0
Cyt_270
Cyt_90
Cyt_30
Cyt_0
Temperature
log2(norm signal_sum)
log2(norm signal_sum)
construct
B2
Cyt
t270
t90
t30
t0
log2(ctrl.ratio)
log2(temp.ratio)
abundance.score
stability.score
score.value
rep
condition
Q12296 − Protein MAM3 OS=Saccharomyces cerevisiae (strain ATCC 204508 / S288c) GN=MAM3 PE=1 SV=1

MAM33 − average.top3: 7.09824826086956 − max.qupm: 5

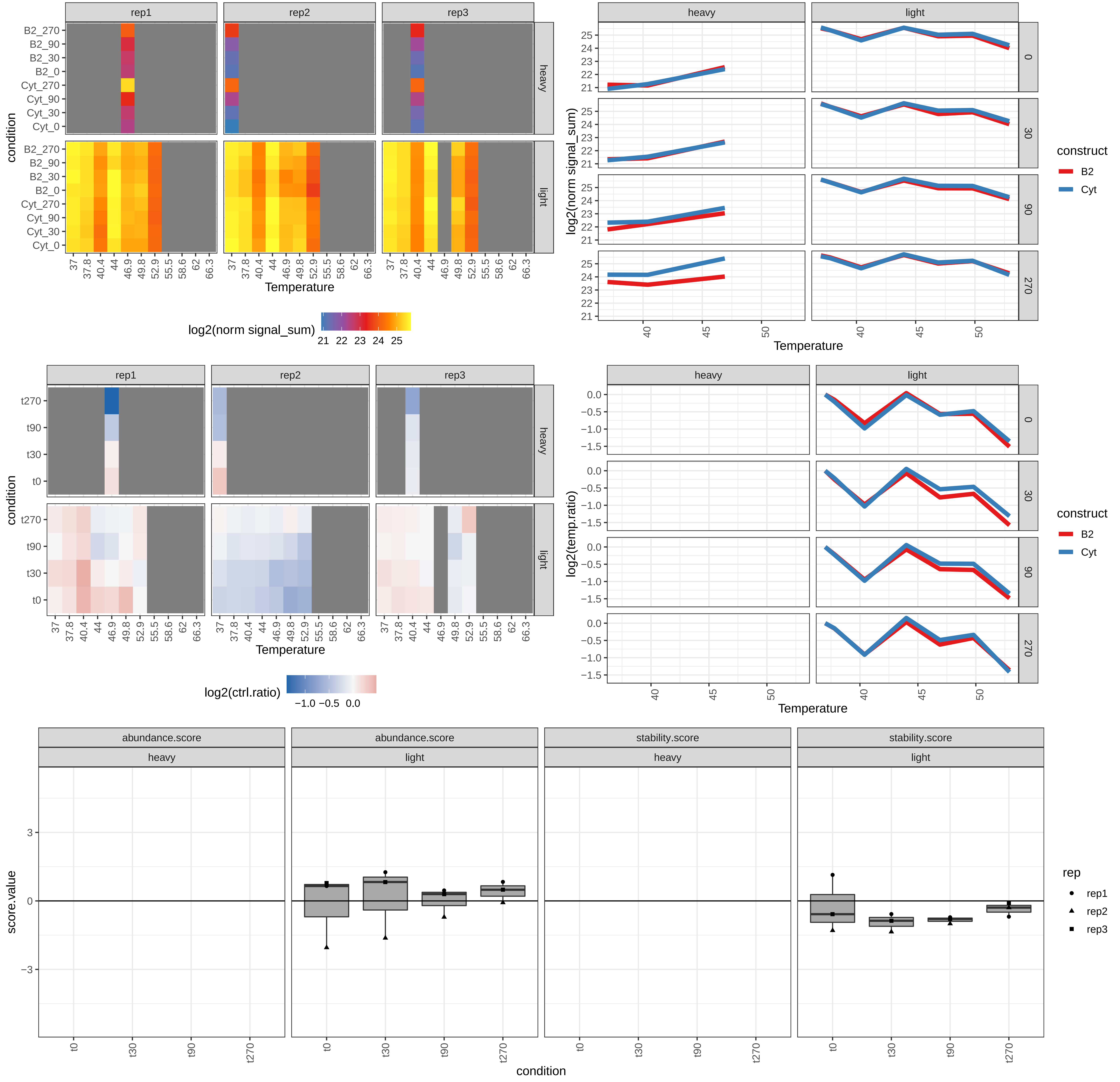

P40513 − Mitochondrial acidic protein MAM33 OS=Saccharomyces cerevisiae (strain ATCC 204508 / S288c) GN=MAM33 PE=1 SV=1

MAP1 − average.top3: 6.92421863636364 − max.qupm: 7

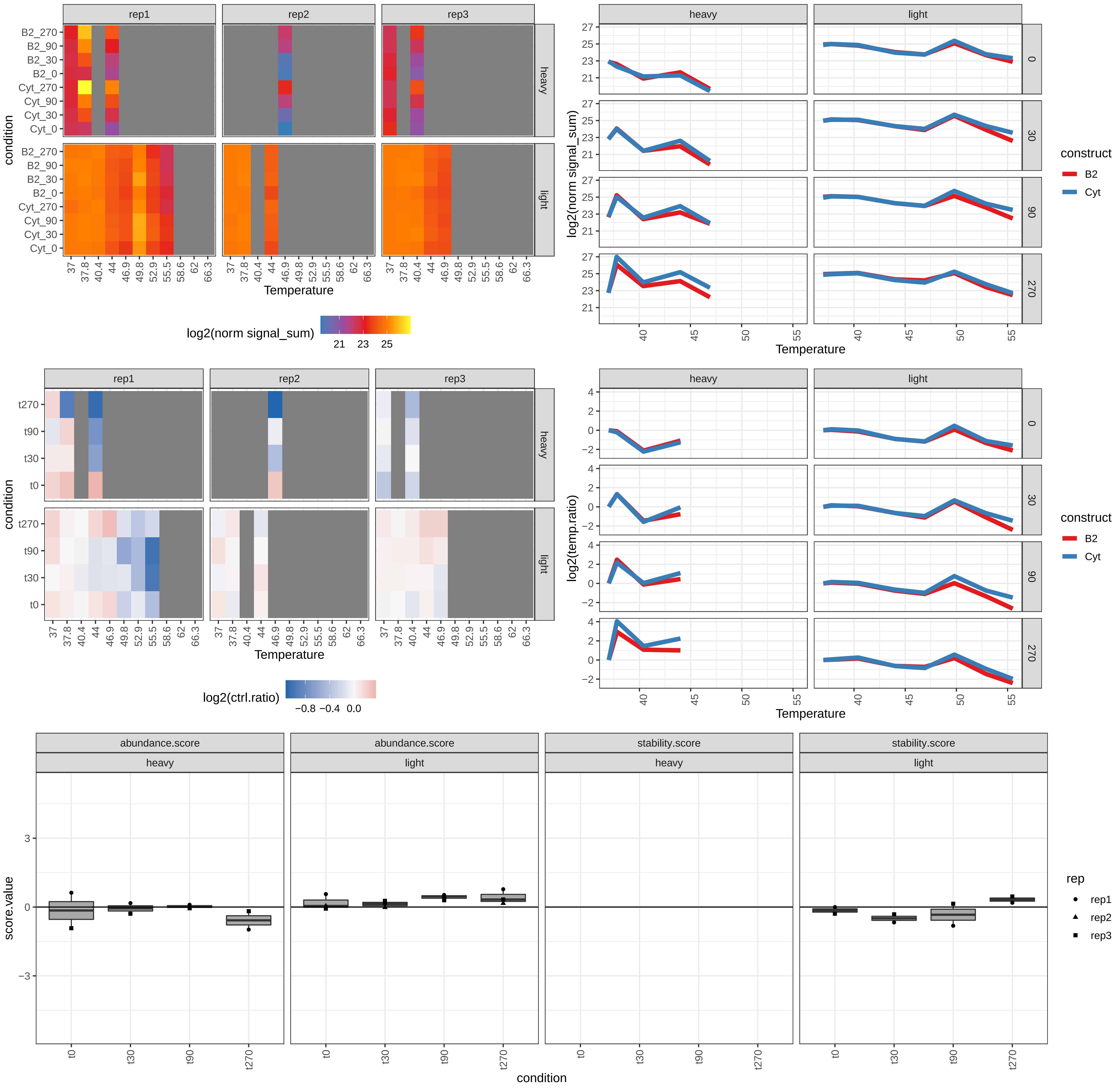

Q01662 − Methionine aminopeptidase 1 OS=Saccharomyces cerevisiae (strain ATCC 204508 / S288c) GN=MAP1 PE=1 SV=2

MAP2 − average.top3: 7.00366239130435 − max.qupm: 12

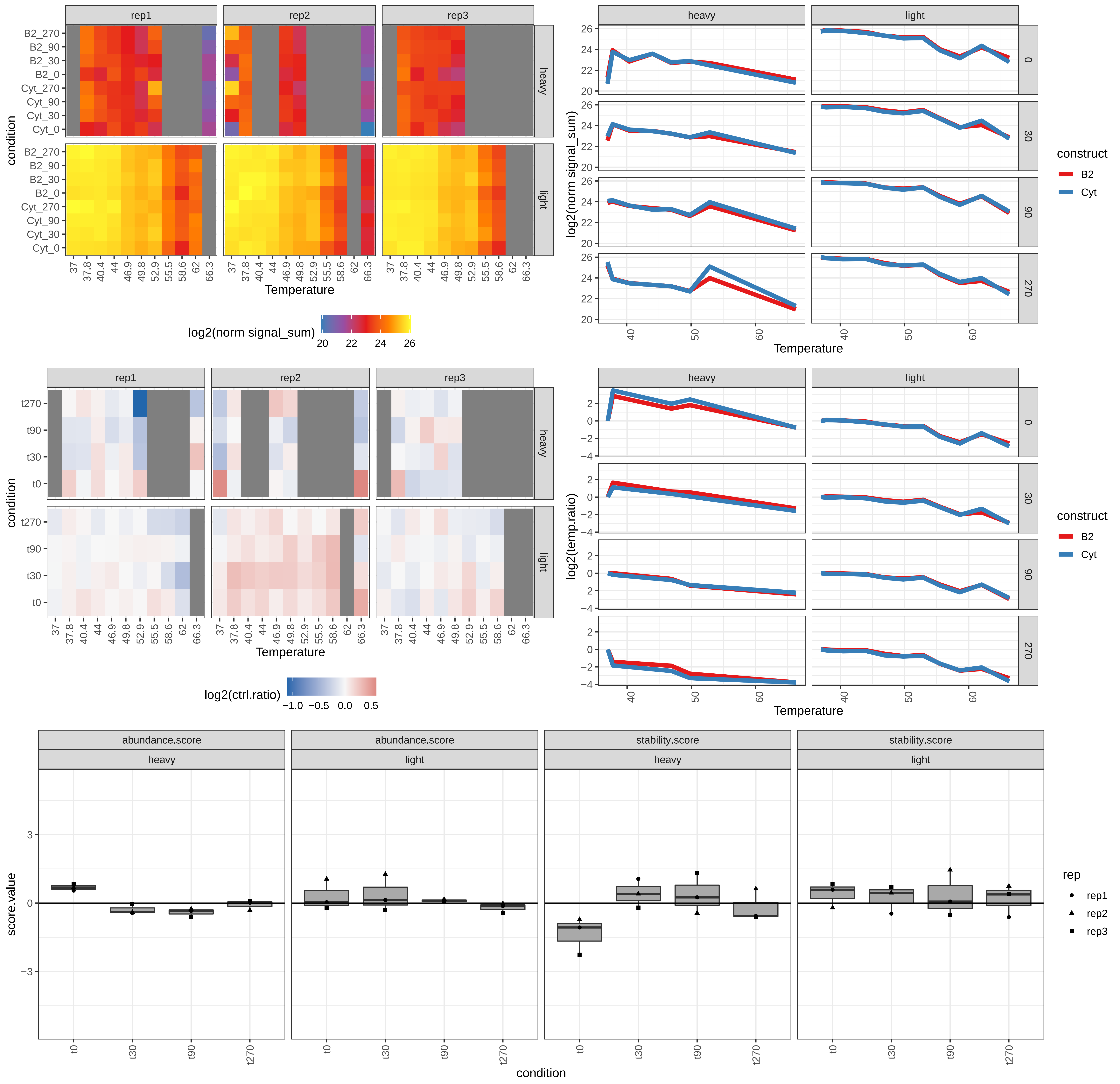

P38174 − Methionine aminopeptidase 2 OS=Saccharomyces cerevisiae (strain ATCC 204508 / S288c) GN=MAP2 PE=1 SV=4

MAS1 − average.top3: 7.0732259375 − max.qupm: 11

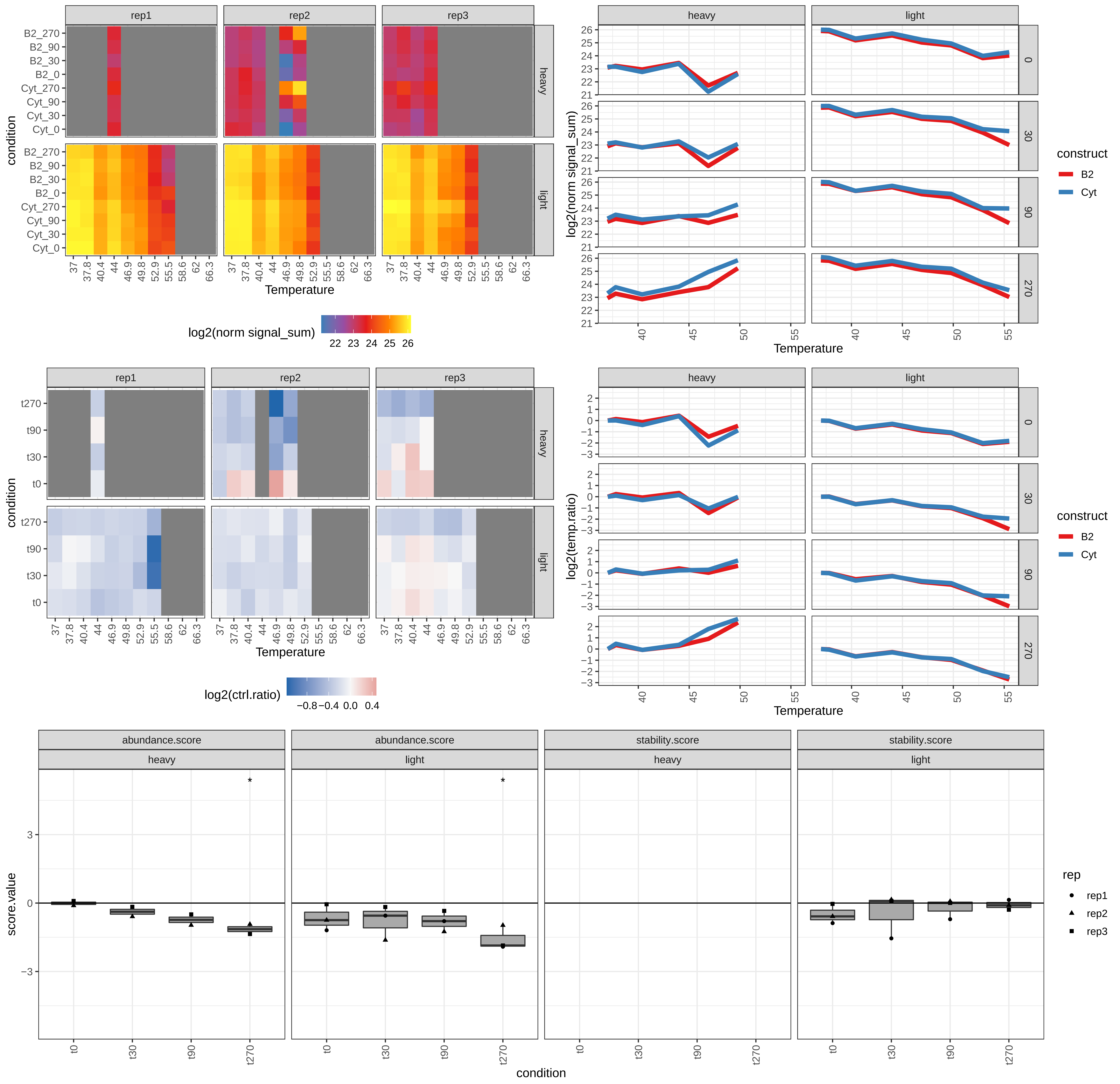

P10507 − Mitochondrial−processing peptidase subunit beta OS=Saccharomyces cerevisiae (strain ATCC 204508 / S288c) GN=MAS1 PE=1 SV=1

MAS2 − average.top3: 6.93598029411765 − max.qupm: 13

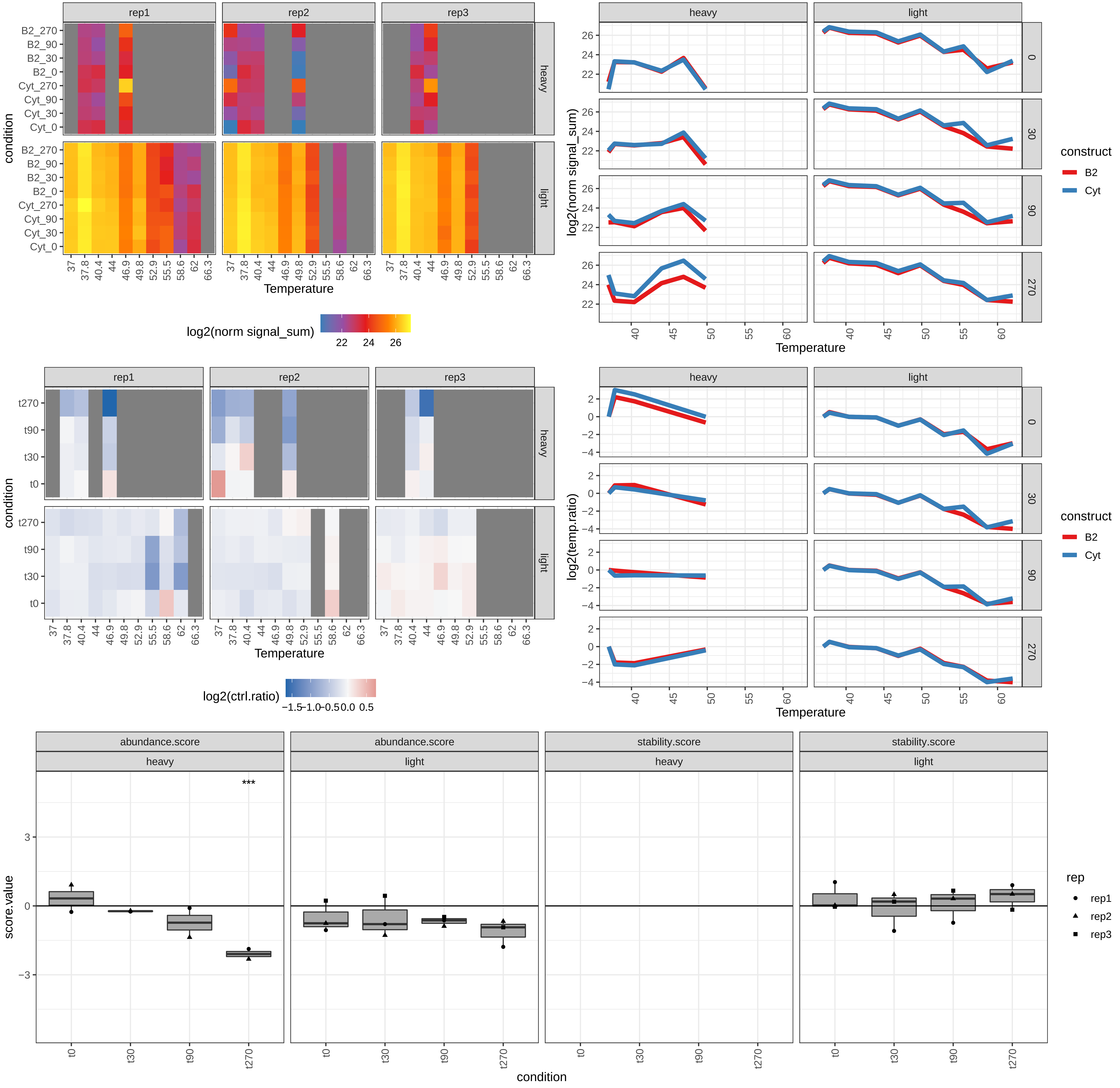

P11914 − Mitochondrial−processing peptidase subunit alpha OS=Saccharomyces cerevisiae (strain ATCC 204508 / S288c) GN=MAS2 PE=1 SV=1

MBF1 − average.top3: 6.69830047619048 − max.qupm: 5

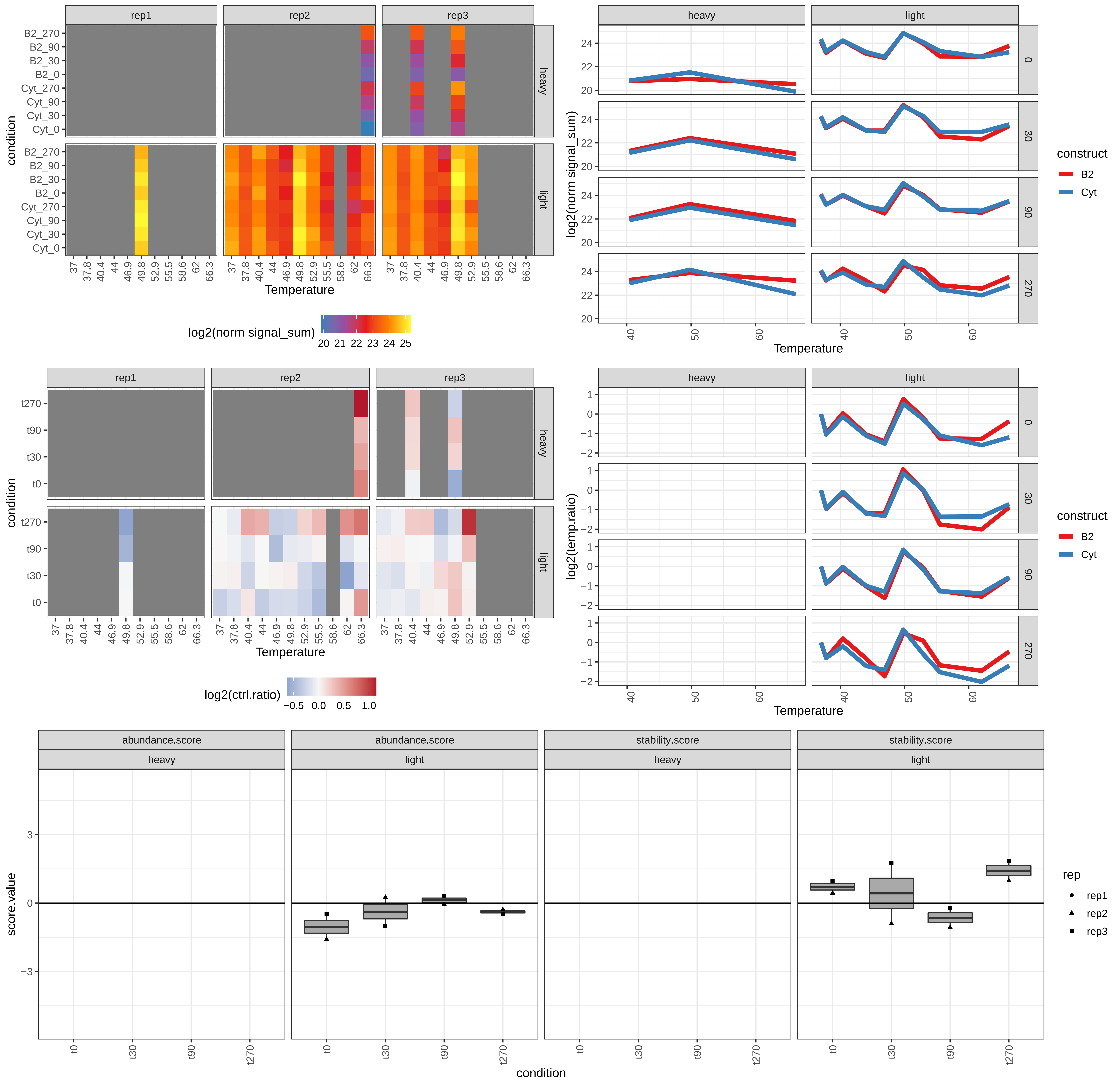

O14467 − Multiprotein−bridging factor 1 OS=Saccharomyces cerevisiae (strain ATCC 204508 / S288c) GN=MBF1 PE=1 SV=2

MCA1 − average.top3: 6.20185777777778 − max.qupm: 3

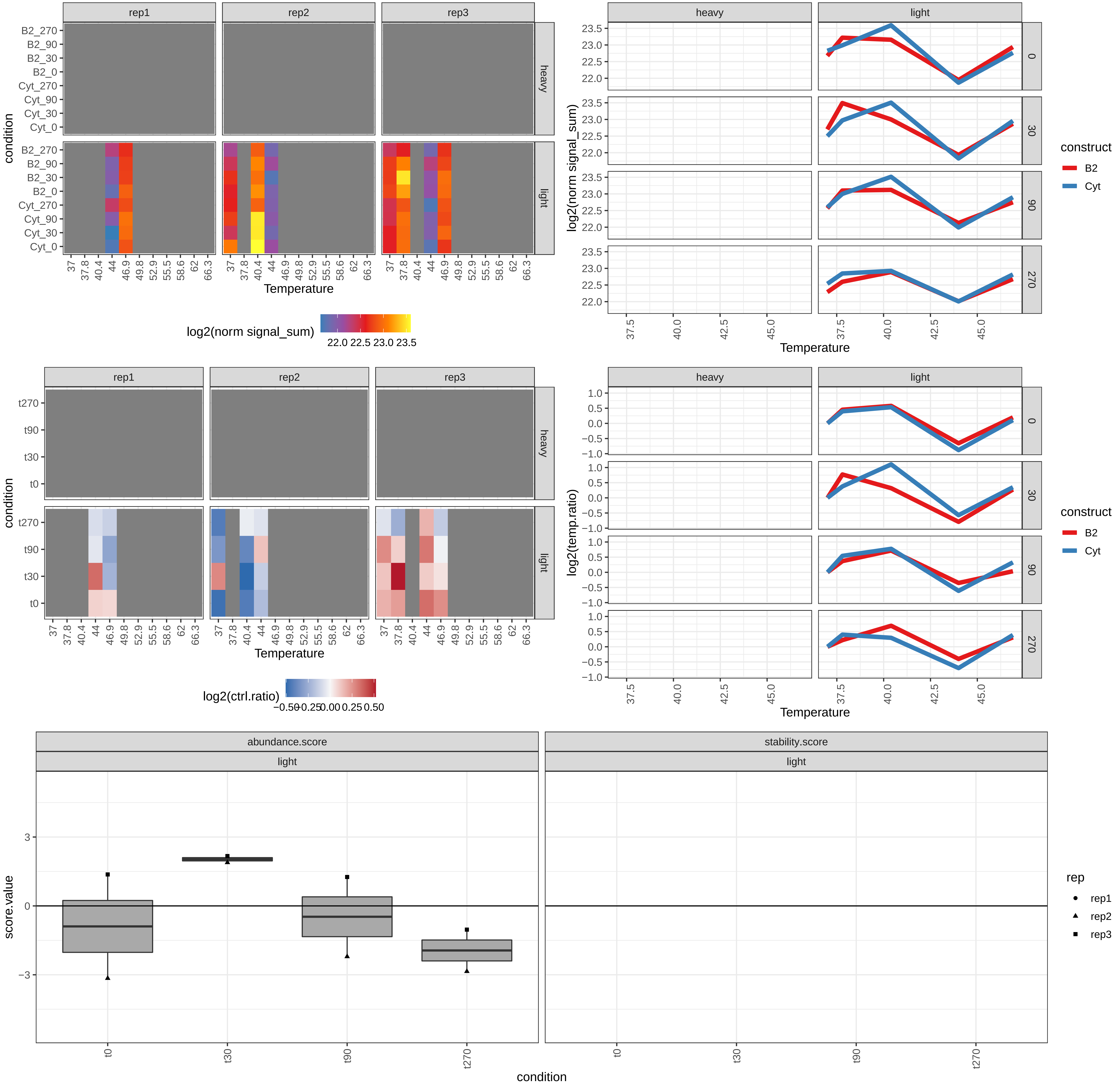

Q08601 − Metacaspase−1 OS=Saccharomyces cerevisiae (strain ATCC 204508 / S288c) GN=MCA1 PE=1 SV=2

MCD4 − average.top3: 6.26736227272727 − max.qupm: 8

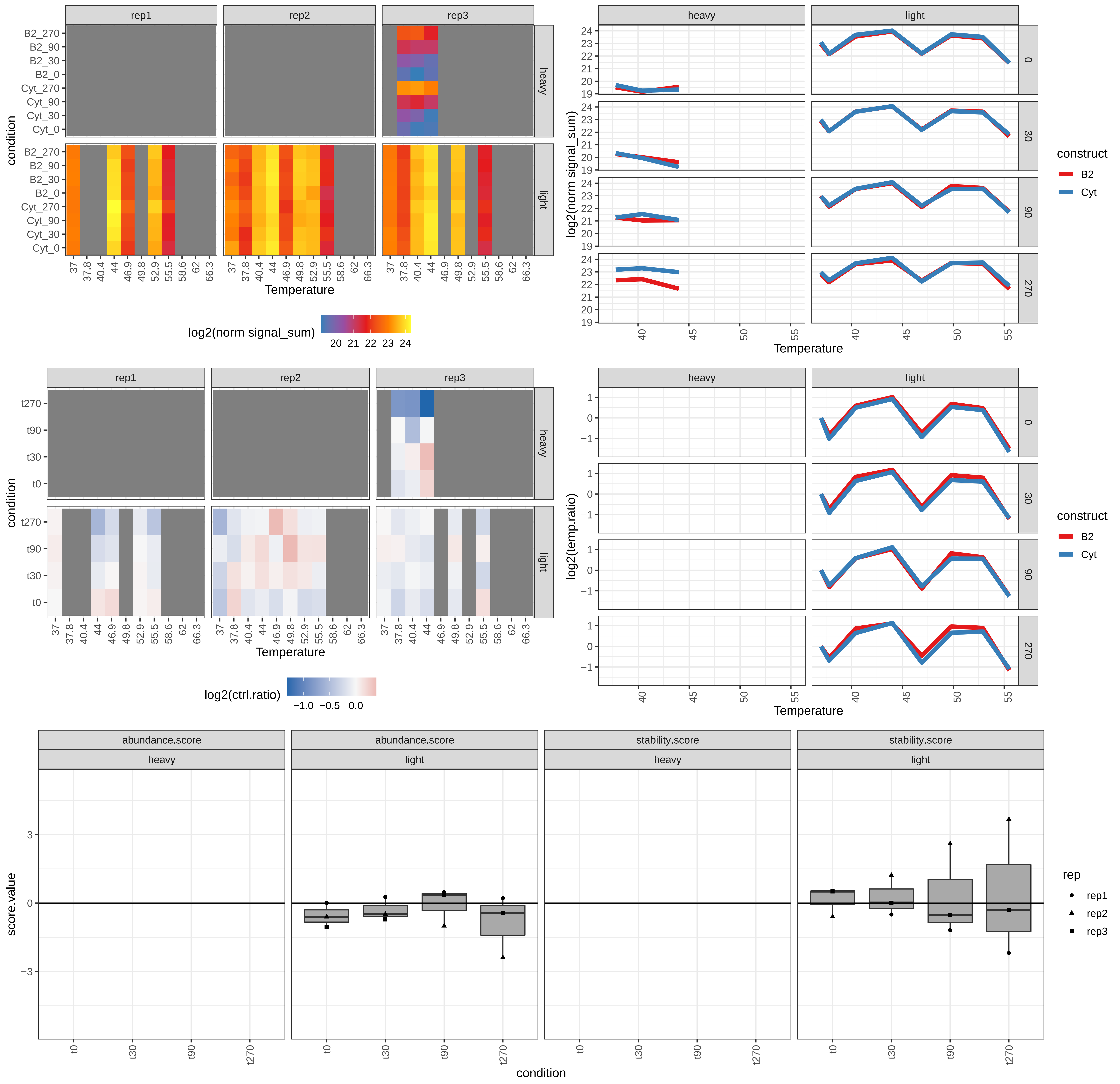

P36051 − GPI ethanolamine phosphate transferase 1 OS=Saccharomyces cerevisiae (strain ATCC 204508 / S288c) GN=MCD4 PE=1 SV=1

MCK1 − average.top3: 7.370527 − max.qupm: 14

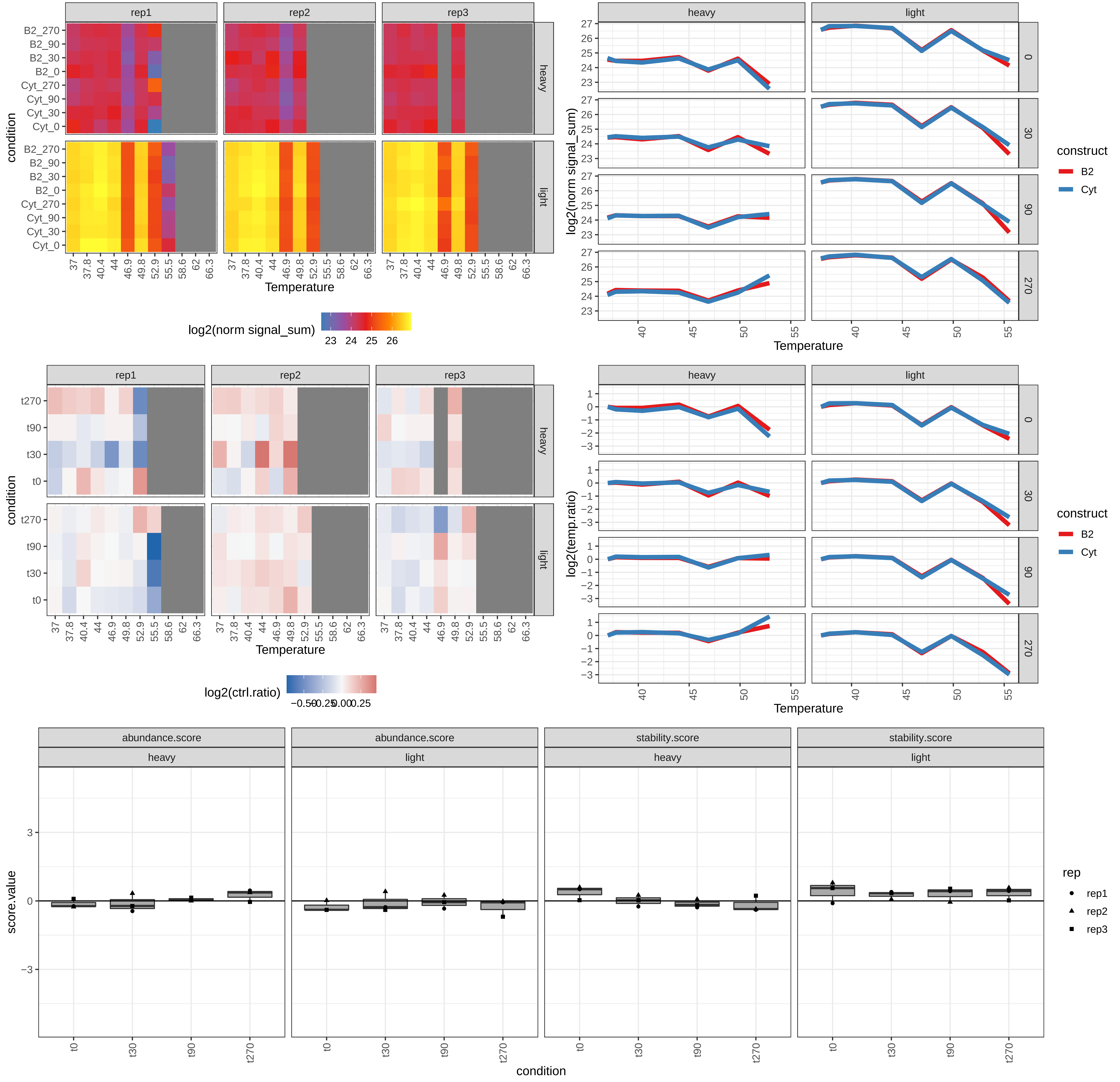

P21965 − Protein kinase MCK1 OS=Saccharomyces cerevisiae (strain ATCC 204508 / S288c) GN=MCK1 PE=1 SV=1

MCM2 − average.top3: 6.48348277777778 − max.qupm: 9

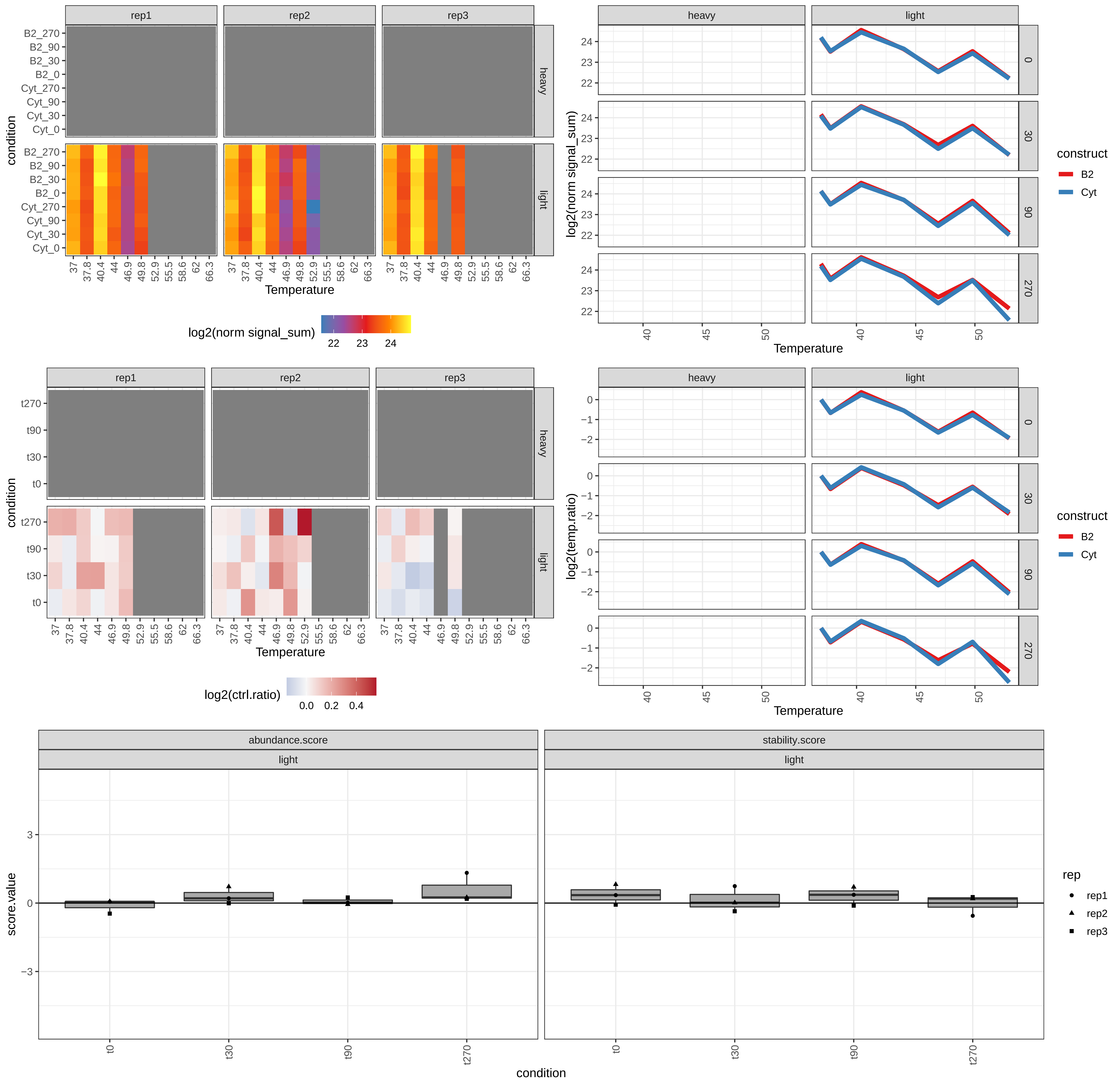

P29469 − DNA replication licensing factor MCM2 OS=Saccharomyces cerevisiae (strain ATCC 204508 / S288c) GN=MCM2 PE=1 SV=2

MCM3 − average.top3: 6.28940818181818 − max.qupm: 6

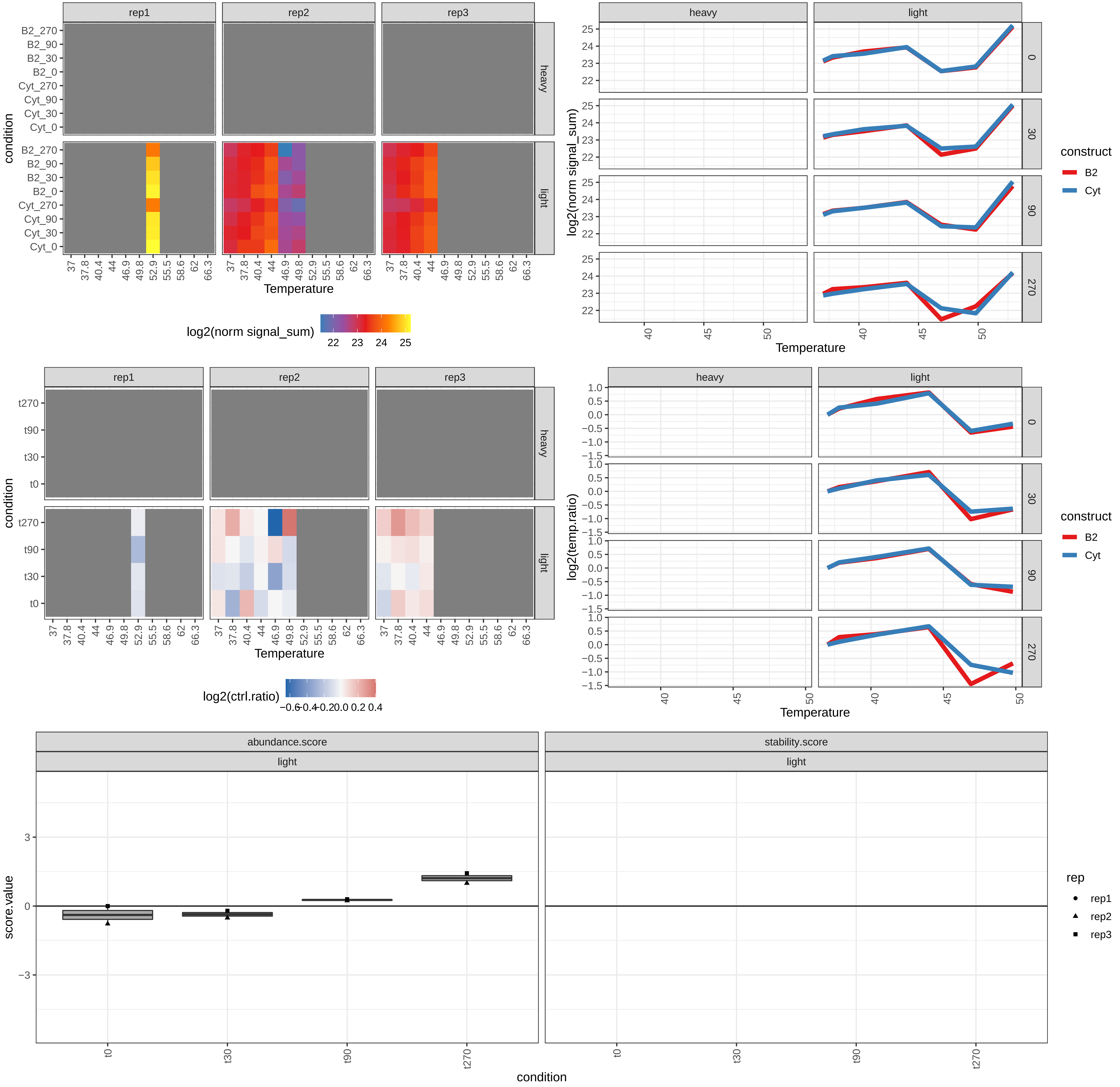

P24279 − DNA replication licensing factor MCM3 OS=Saccharomyces cerevisiae (strain ATCC 204508 / S288c) GN=MCM3 PE=1 SV=1

MCM4 − average.top3: 6.61966483870968 − max.qupm: 10

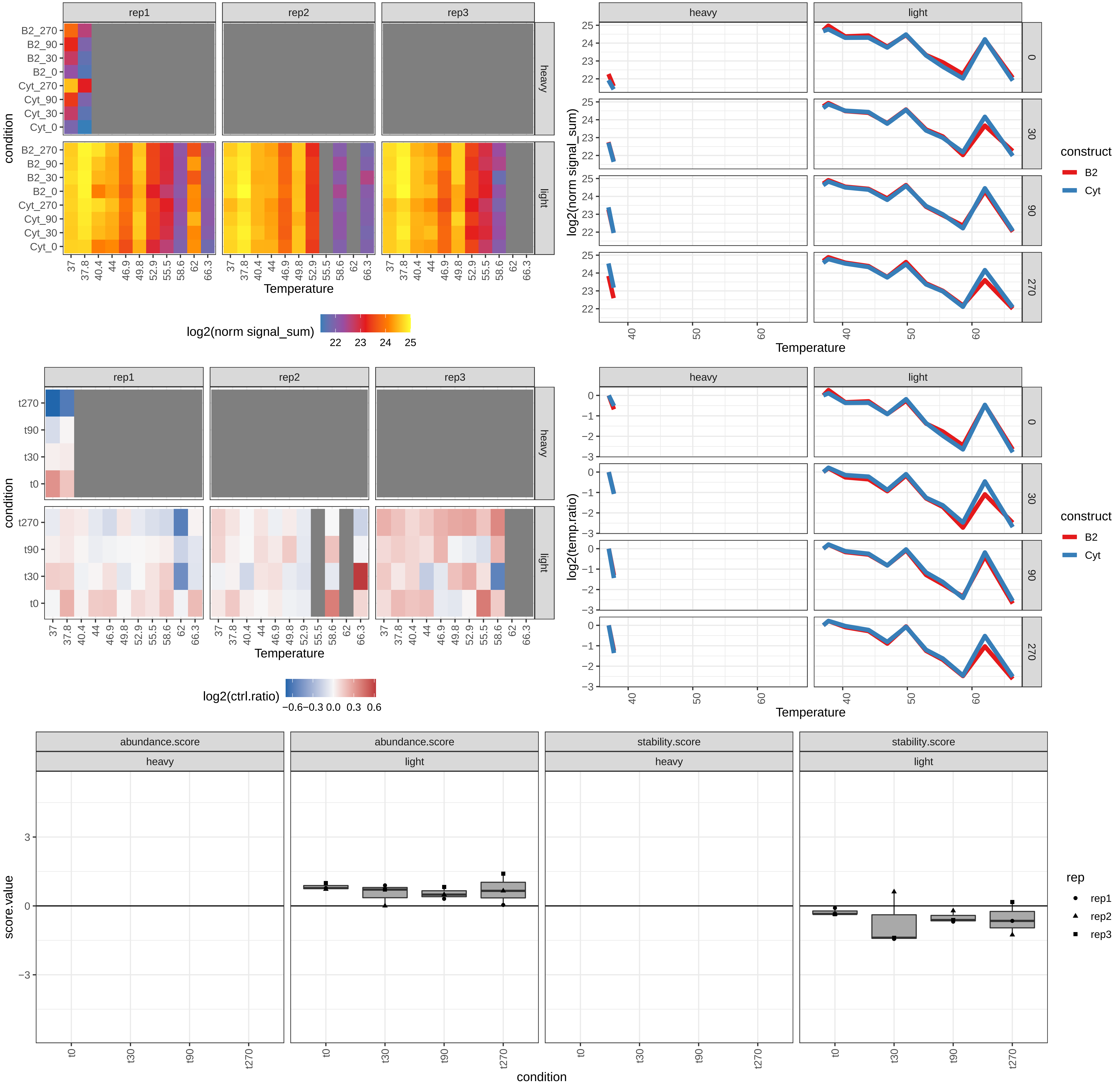

P30665 − DNA replication licensing factor MCM4 OS=Saccharomyces cerevisiae (strain ATCC 204508 / S288c) GN=MCM4 PE=1 SV=2

MCM5 − average.top3: 6.45716666666667 − max.qupm: 5

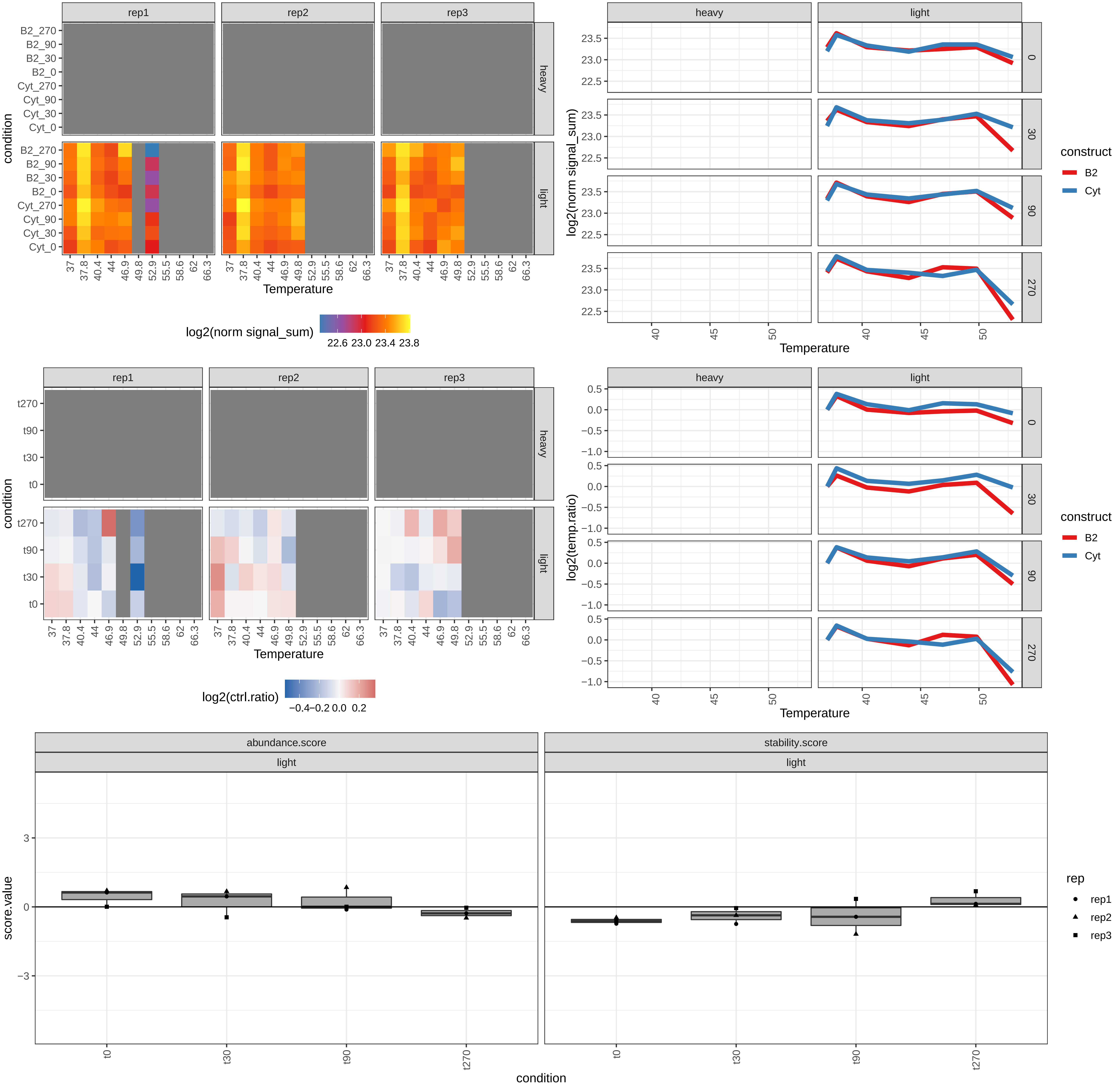

P29496 − Minichromosome maintenance protein 5 OS=Saccharomyces cerevisiae (strain ATCC 204508 / S288c) GN=MCM5 PE=1 SV=1

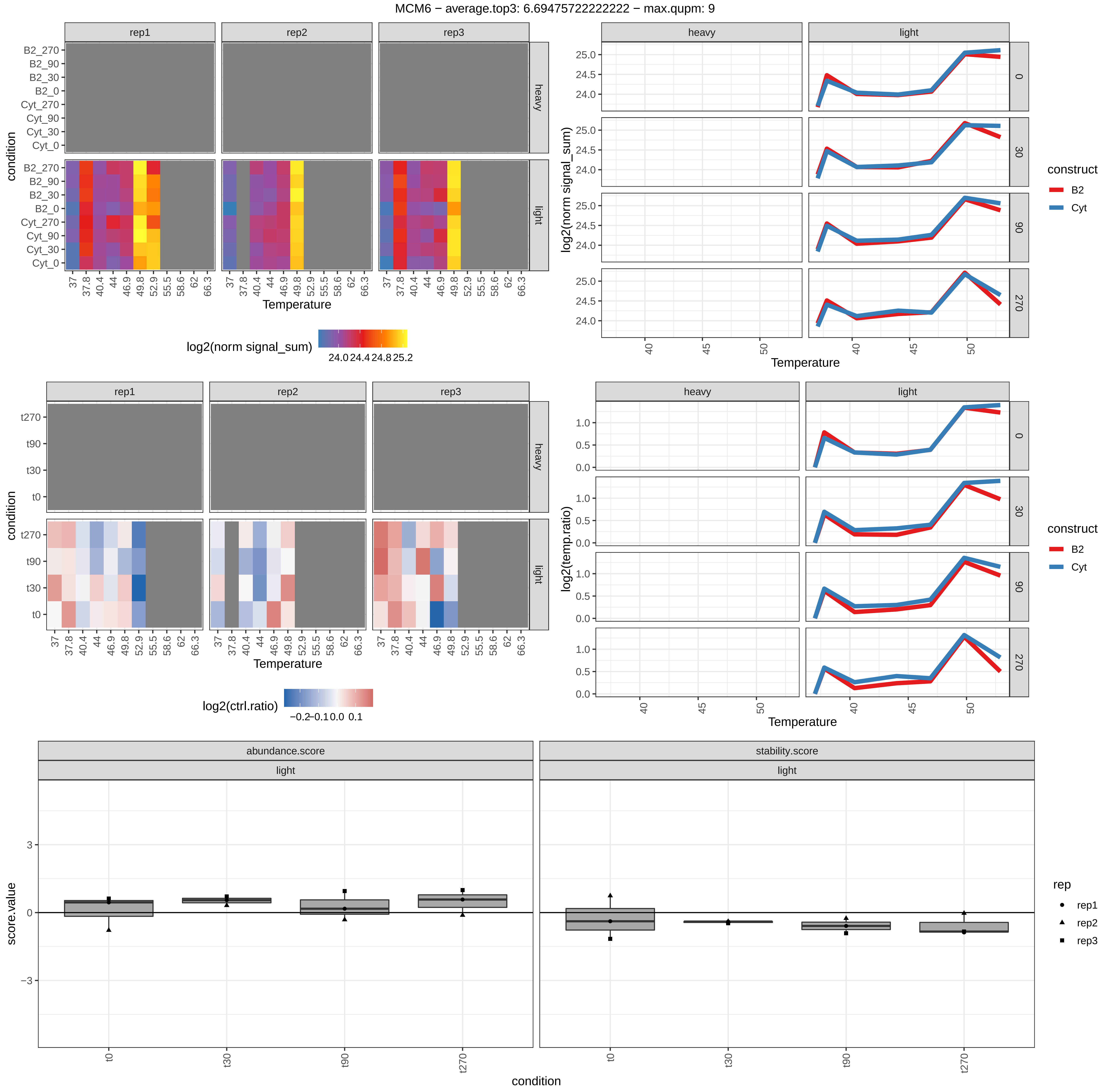

P53091 − DNA replication licensing factor MCM6 OS=Saccharomyces cerevisiae (strain ATCC 204508 / S288c) GN=MCM6 PE=1 SV=2

MCM7 − average.top3: 6.06741888888889 − max.qupm: 4

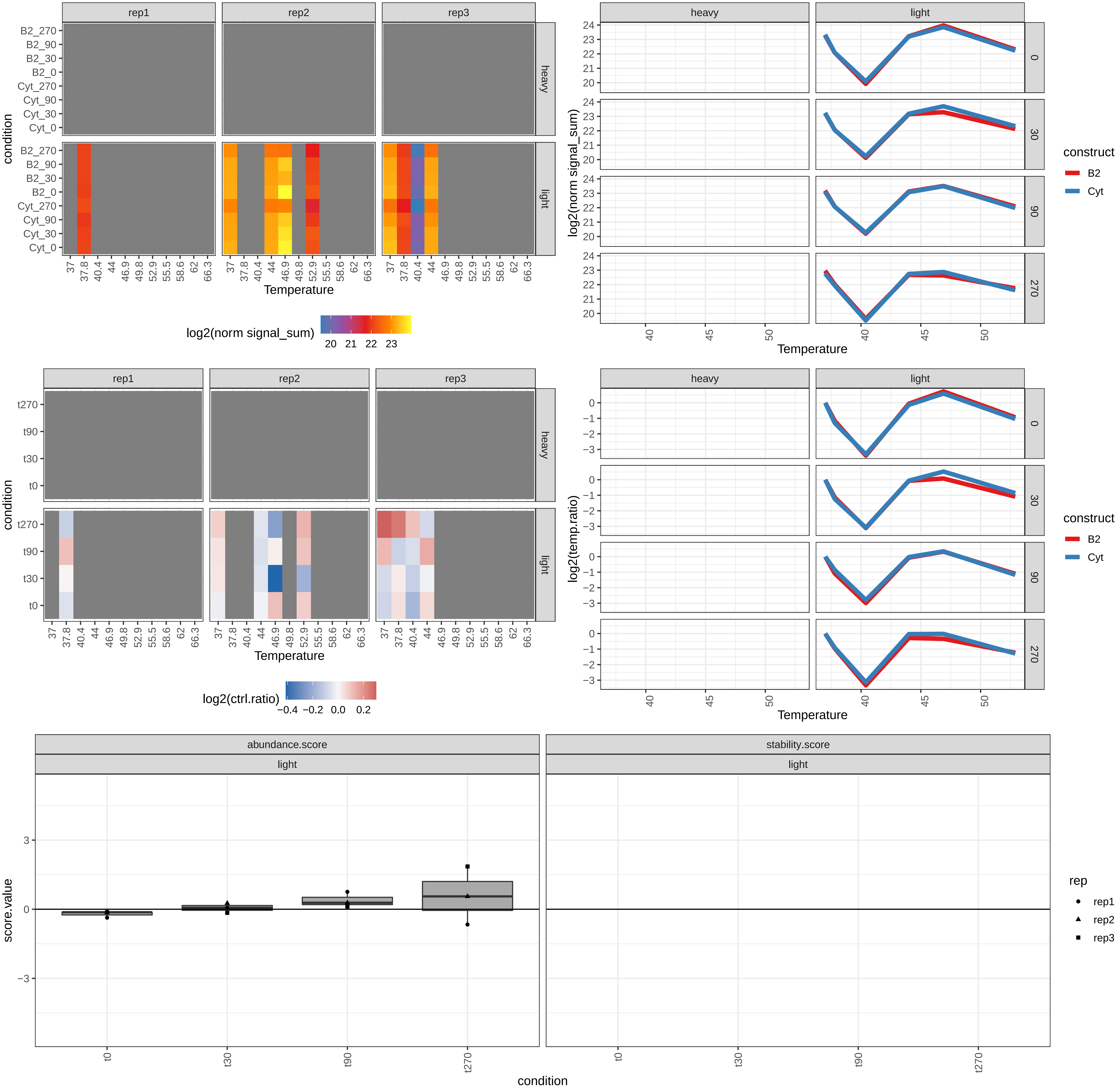

P38132 − DNA replication licensing factor MCM7 OS=Saccharomyces cerevisiae (strain ATCC 204508 / S288c) GN=MCM7 PE=1 SV=4

MCP2 − average.top3: 6.57984681818182 − max.qupm: 5

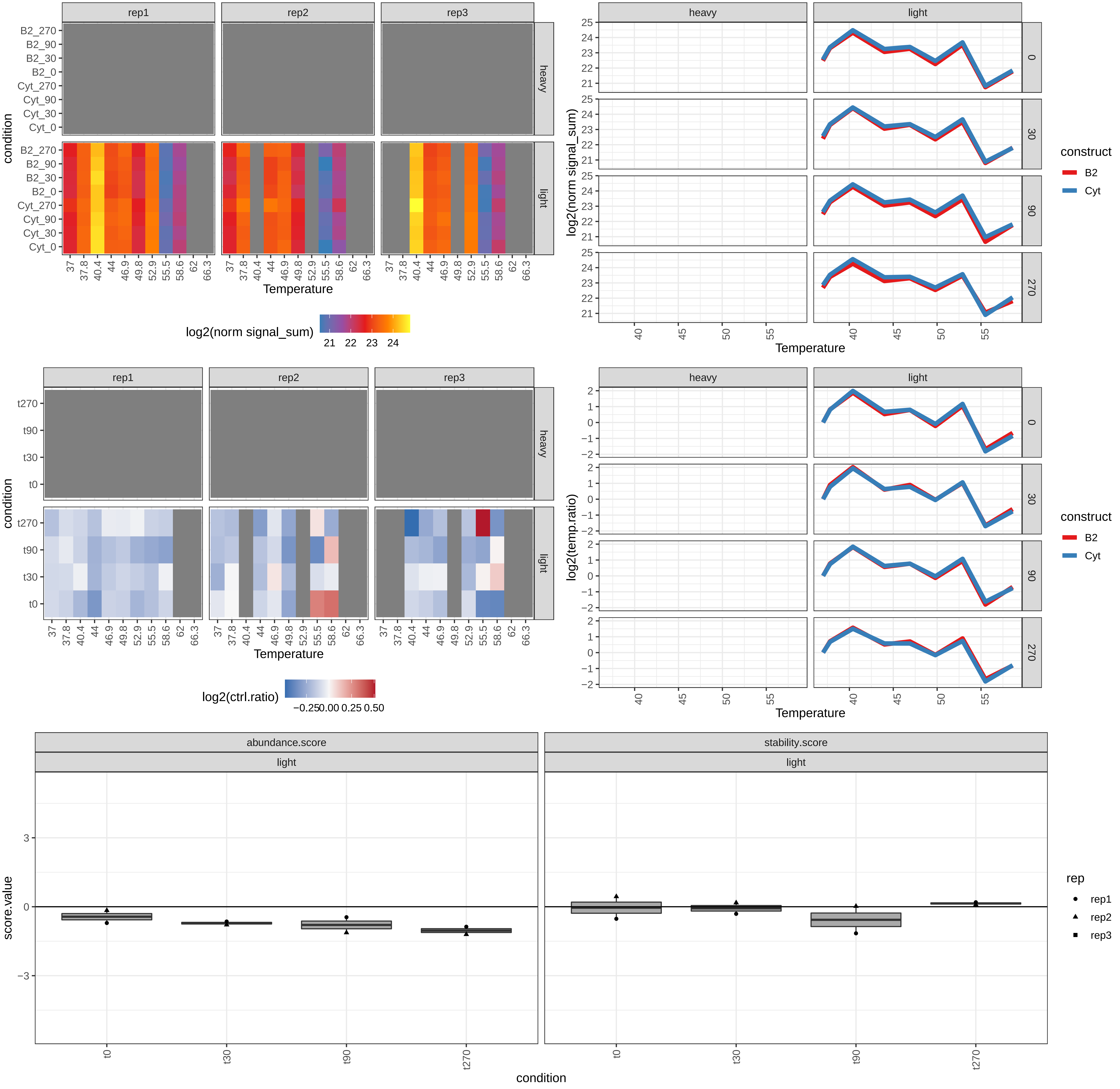

Q06567 − ABC1 family protein MCP2 OS=Saccharomyces cerevisiae (strain ATCC 204508 / S288c) GN=MCP2 PE=1 SV=1

MCR1 − average.top3: 7.41765022727273 − max.qupm: 19

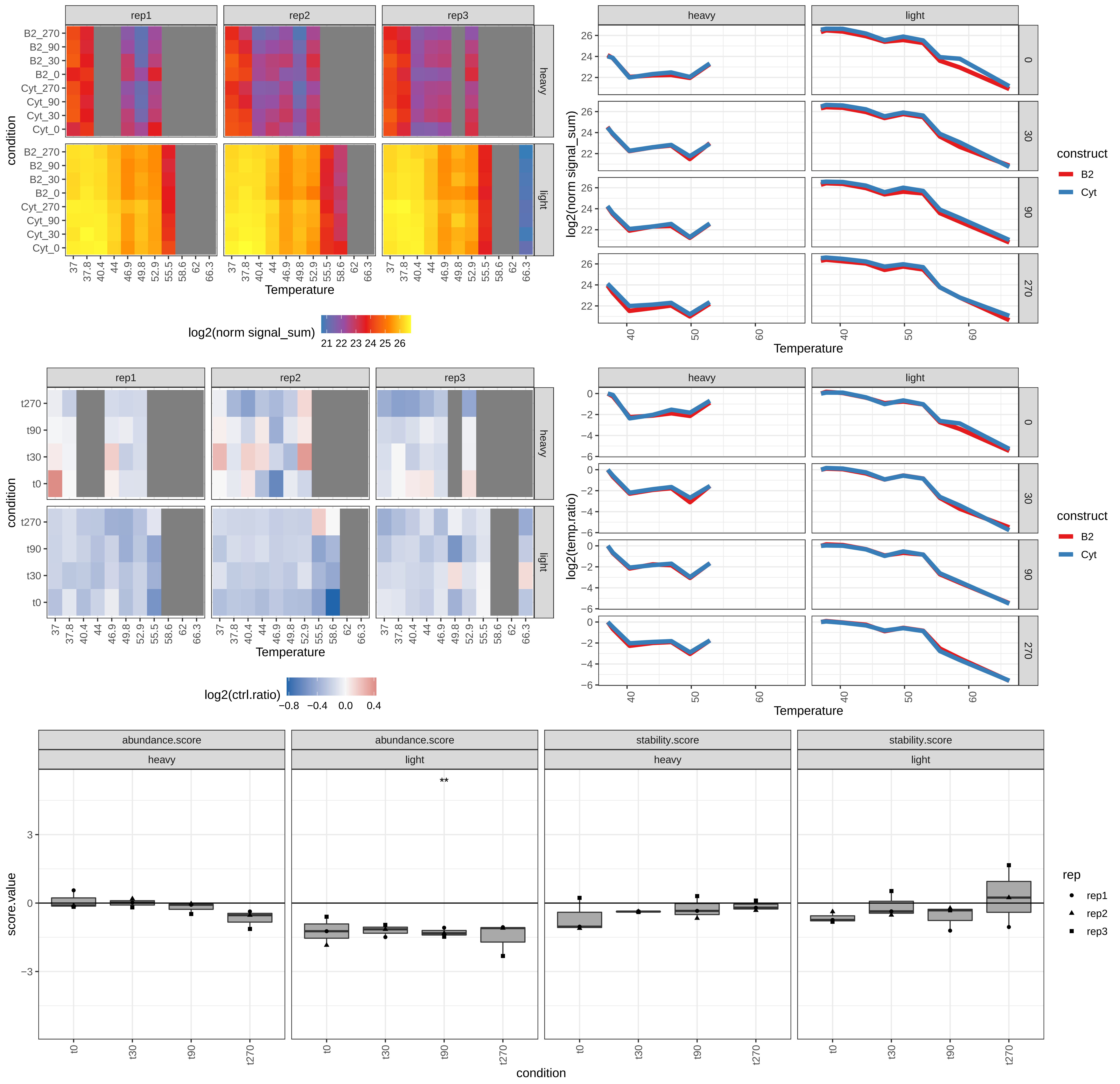

P36060 − NADH−cytochrome b5 reductase 2 OS=Saccharomyces cerevisiae (strain ATCC 204508 / S288c) GN=MCR1 PE=1 SV=1

MCX1 − average.top3: 6.23756727272727 − max.qupm: 3

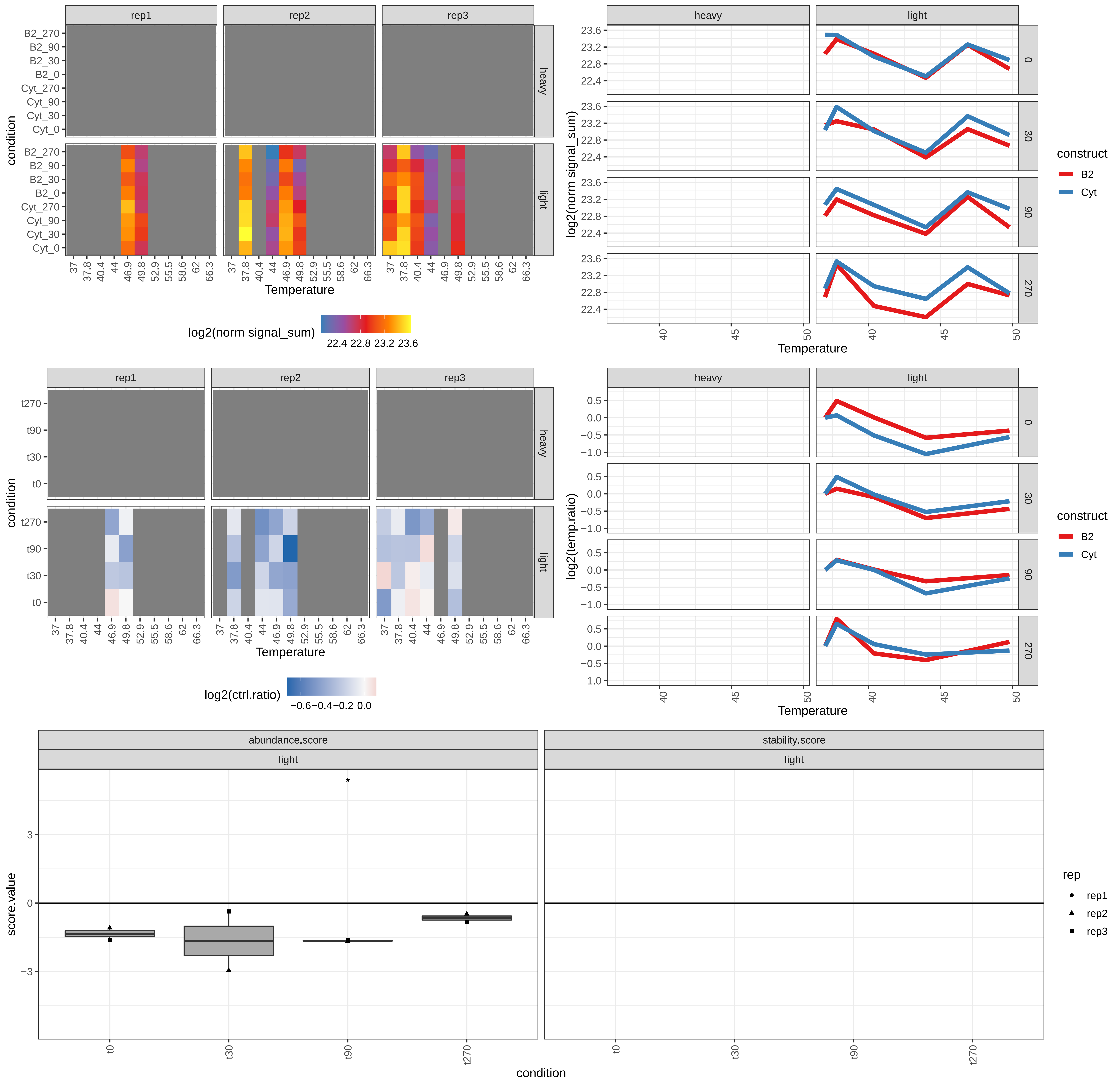

P38323 − ATP−dependent clpX−like chaperone, mitochondrial OS=Saccharomyces cerevisiae (strain ATCC 204508 / S288c) GN=MCX1 PE=1 SV=1

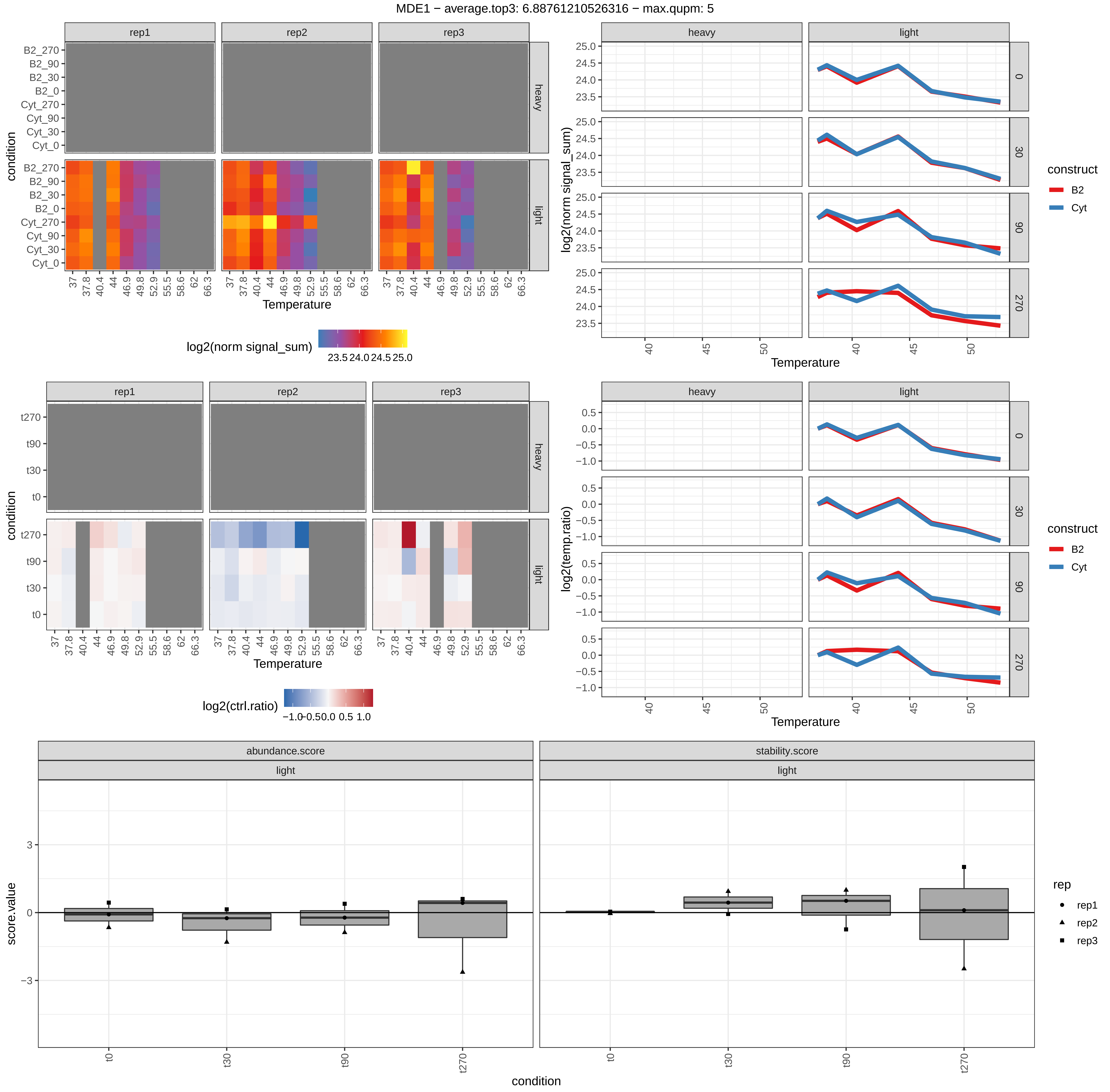

P47095 − Methylthioribulose−1−phosphate dehydratase OS=Saccharomyces cerevisiae (strain ATCC 204508 / S288c) GN=MDE1 PE=1 SV=1

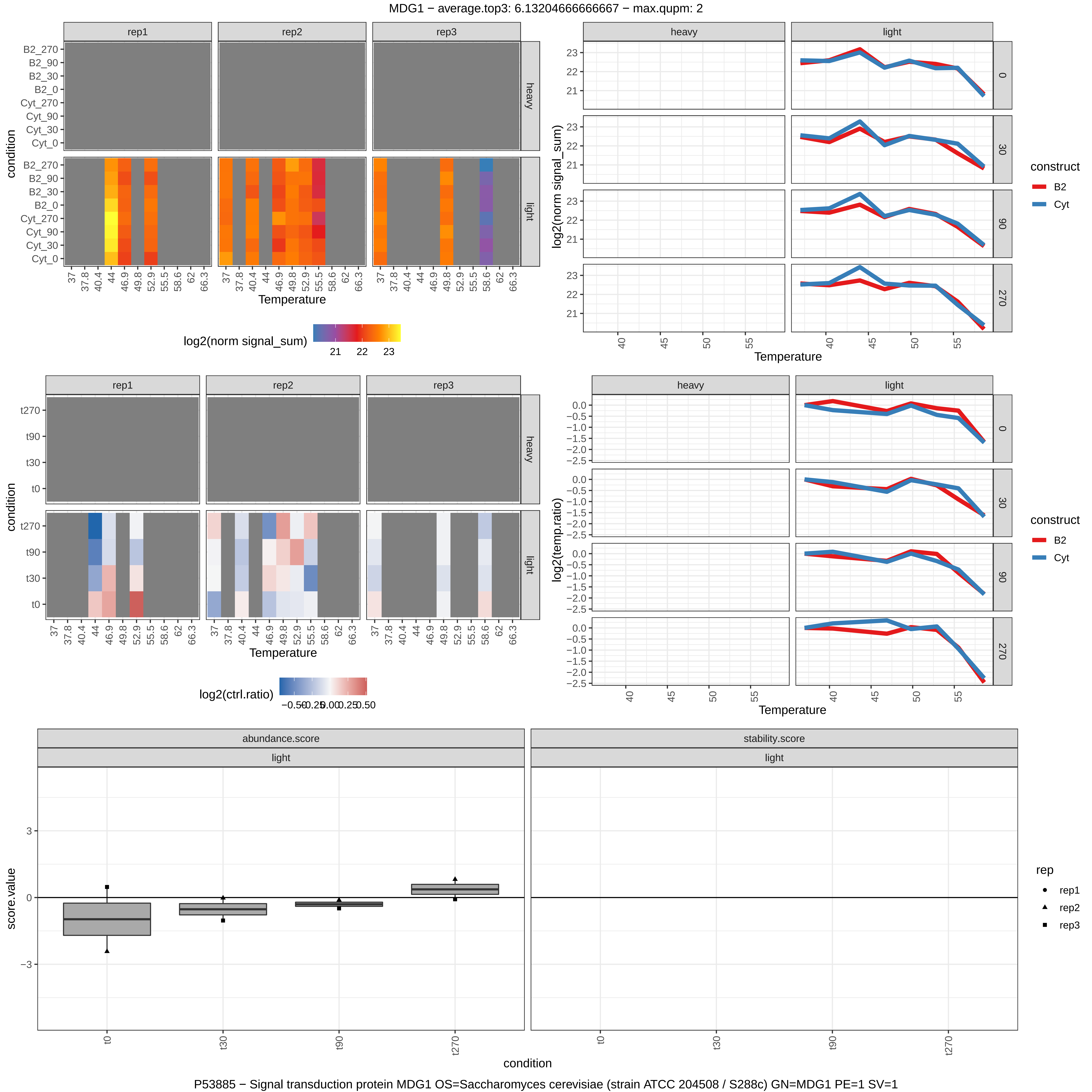
MDG1 − average.top3: 6.13204666666667 − max.qupm: 2
P53885 − Signal transduction protein MDG1 OS=Saccharomyces cerevisiae (strain ATCC 204508 / S288c) GN=MDG1 PE=1 SV=1

MDH1 − average.top3: 7.81222157894737 − max.qupm: 15

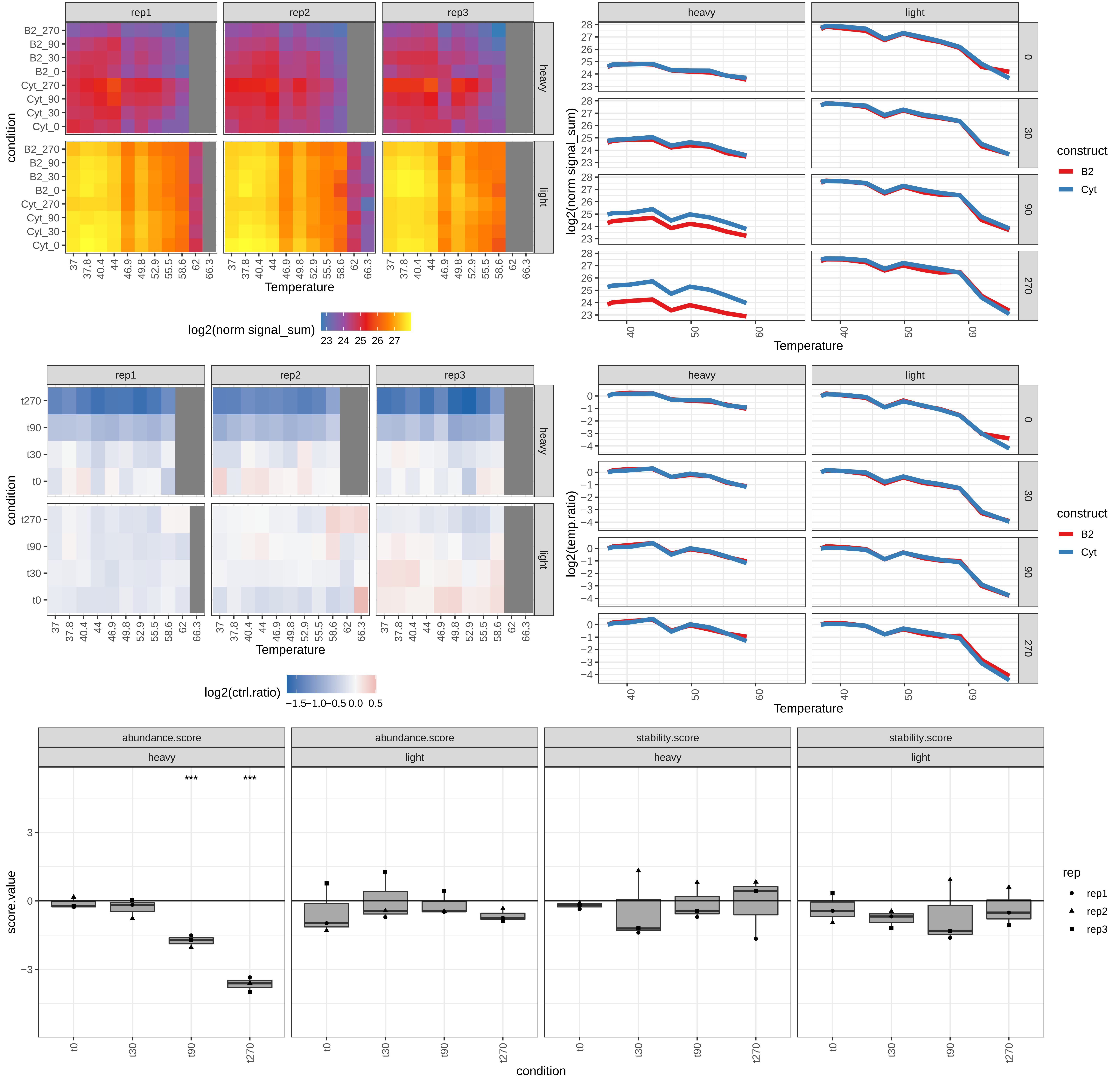

P17505 − Malate dehydrogenase, mitochondrial OS=Saccharomyces cerevisiae (strain ATCC 204508 / S288c) GN=MDH1 PE=1 SV=2

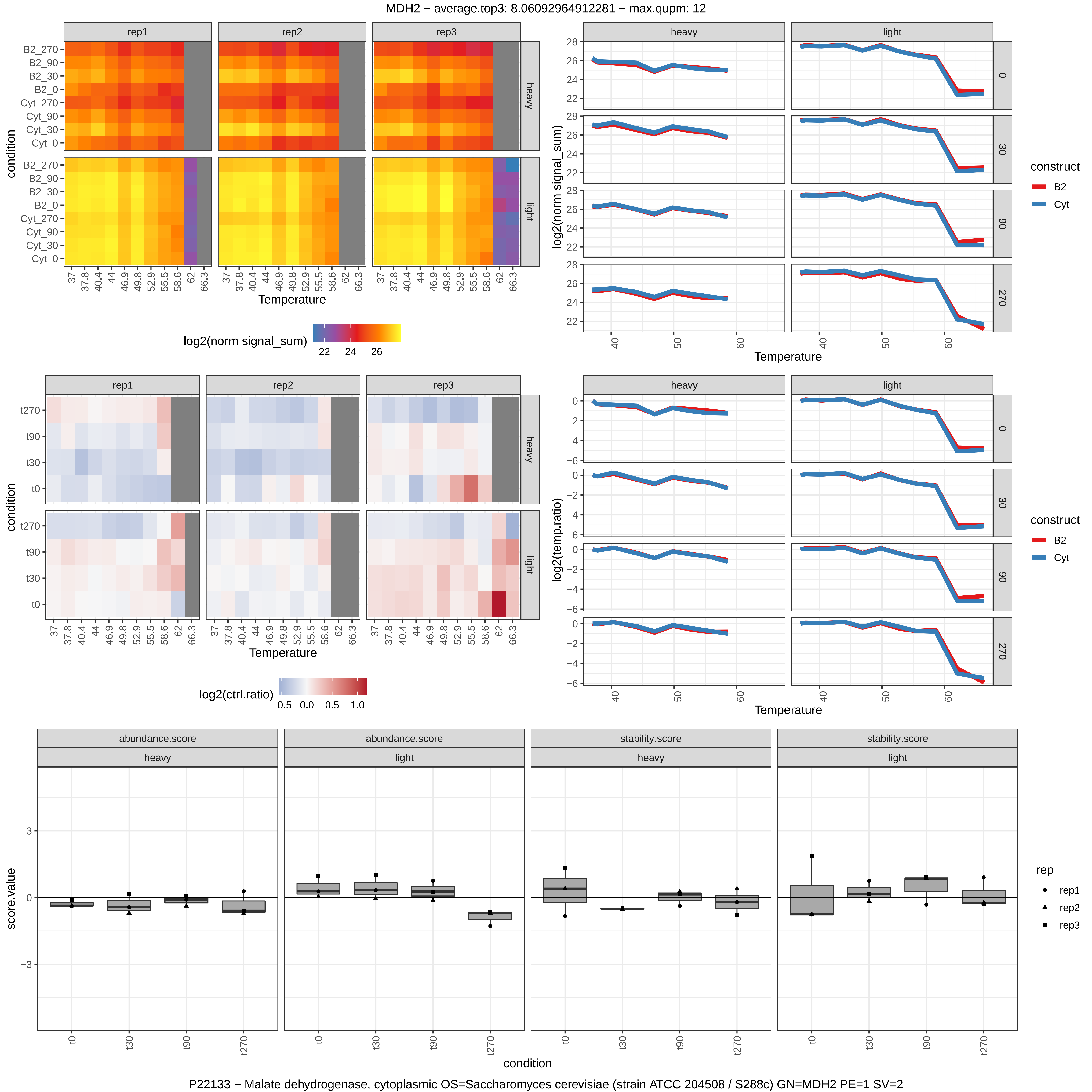
MDH2 − average.top3: 8.06092964912281 − max.qupm: 12
rep1
rep2
rep3
heavy
light
condition
Temperature
log2(norm signal_sum)
log2(ctrl.ratio)
log2(temp.ratio)
construct
B2
Cyt
abundance.score
stability.score
score.value
rep
rep1
rep2
rep3
P22133 − Malate dehydrogenase, cytoplasmic OS=Saccharomyces cerevisiae (strain ATCC 204508 / S288c) GN=MDH2 PE=1 SV=2

MDH3 − average.top3: 7.68477559322034 − max.qupm: 15

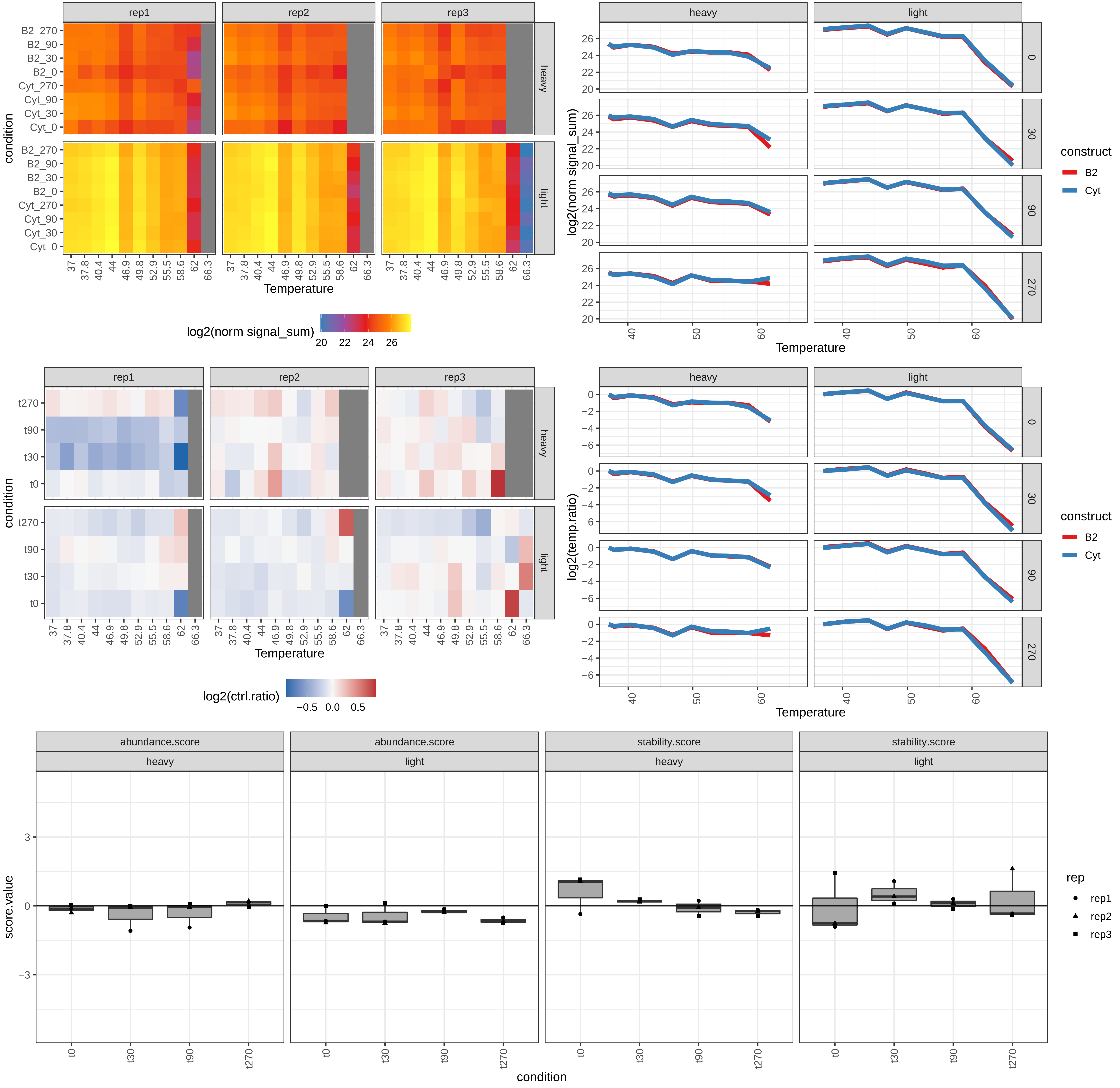

P32419 − Malate dehydrogenase, peroxisomal OS=Saccharomyces cerevisiae (strain ATCC 204508 / S288c) GN=MDH3 PE=1 SV=3

MDJ1 − average.top3: 7.1376796 − max.qupm: 7

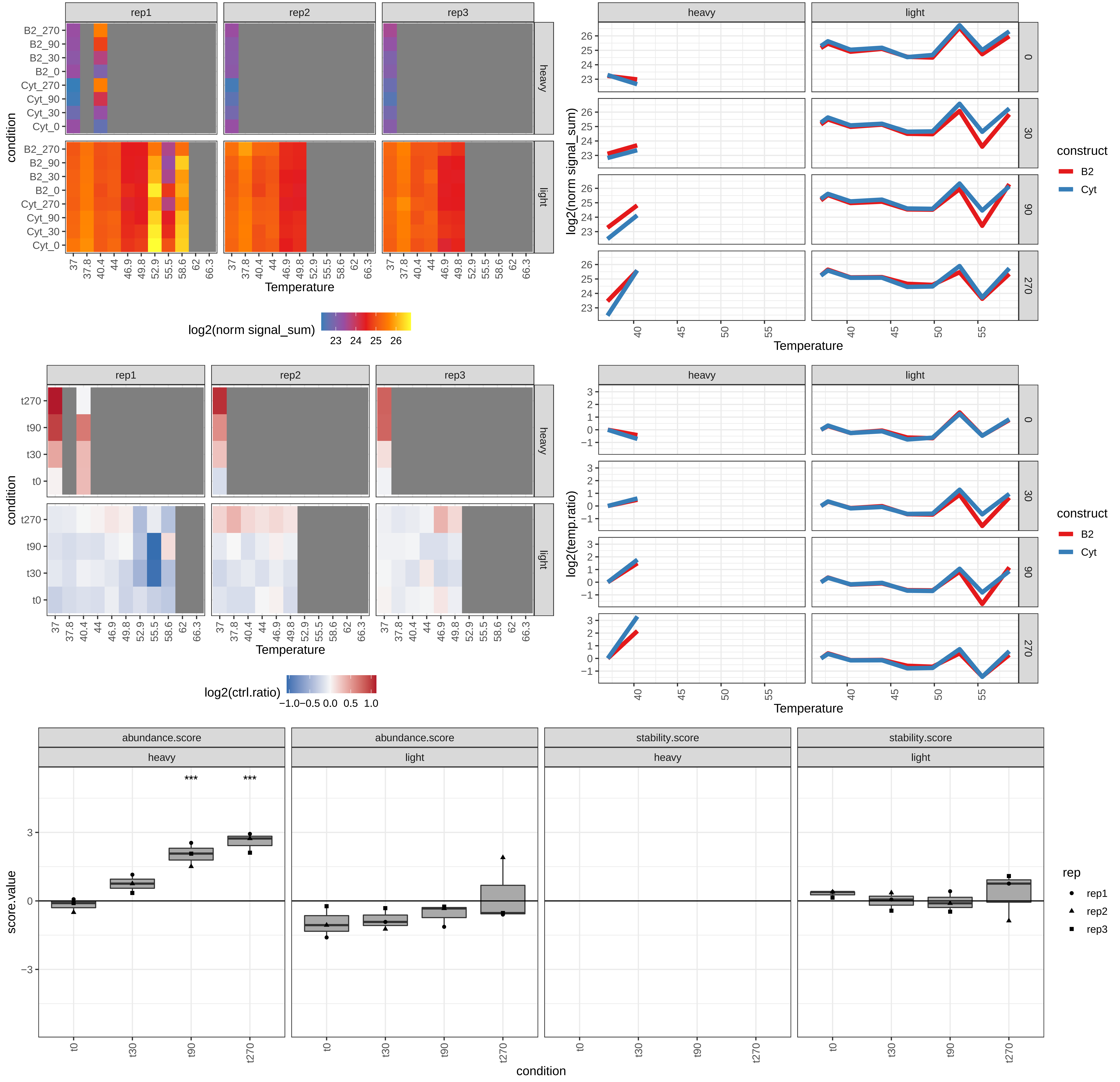

P35191 − DnaJ homolog 1, mitochondrial OS=Saccharomyces cerevisiae (strain ATCC 204508 / S288c) GN=MDJ1 PE=1 SV=1

MDL1 − average.top3: 6.59386238095238 − max.qupm: 6

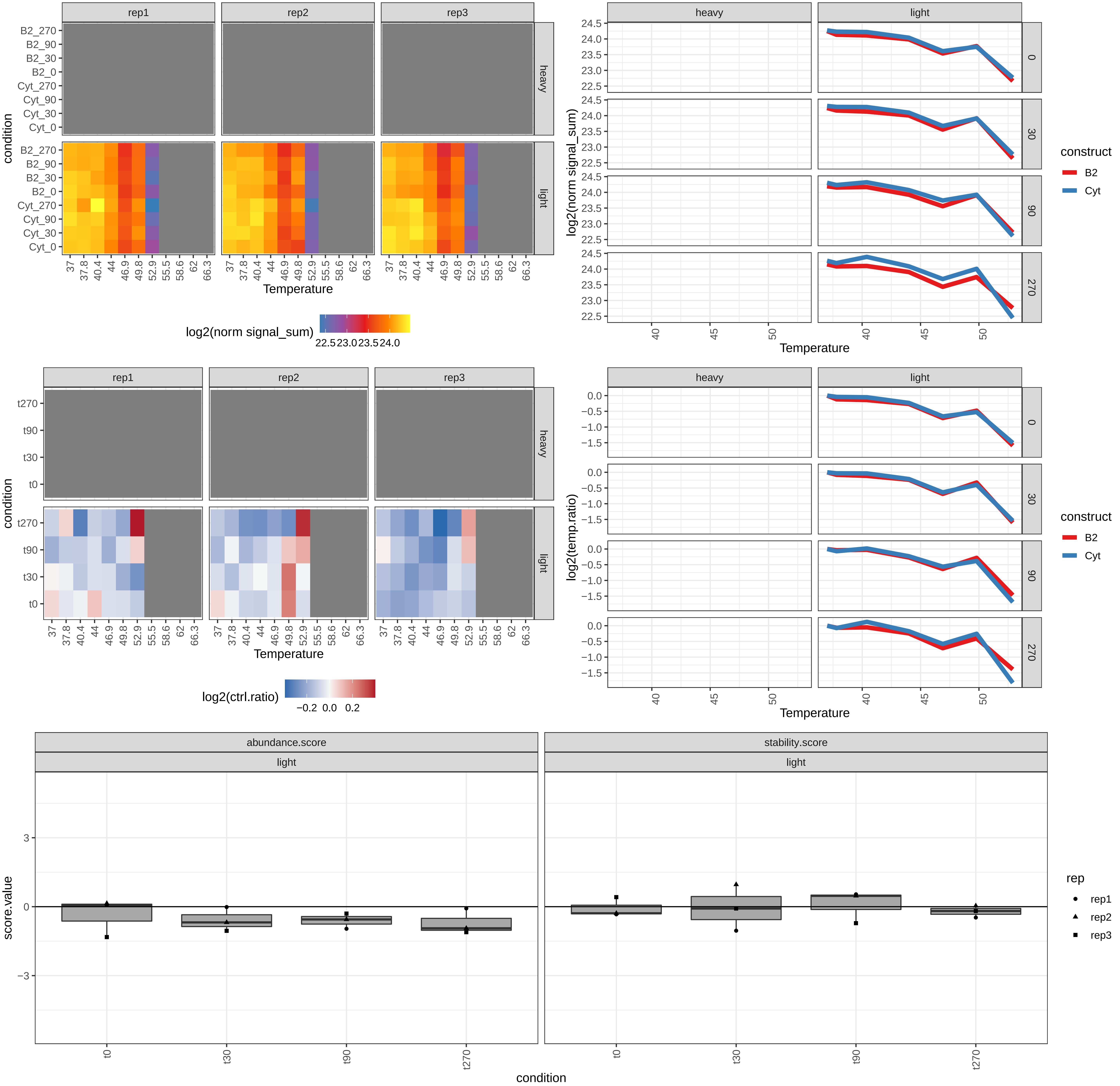

P33310 − ATP−dependent permease MDL1, mitochondrial OS=Saccharomyces cerevisiae (strain ATCC 204508 / S288c) GN=MDL1 PE=1 SV=2

MDL2 − average.top3: 6.74082952380952 − max.qupm: 7

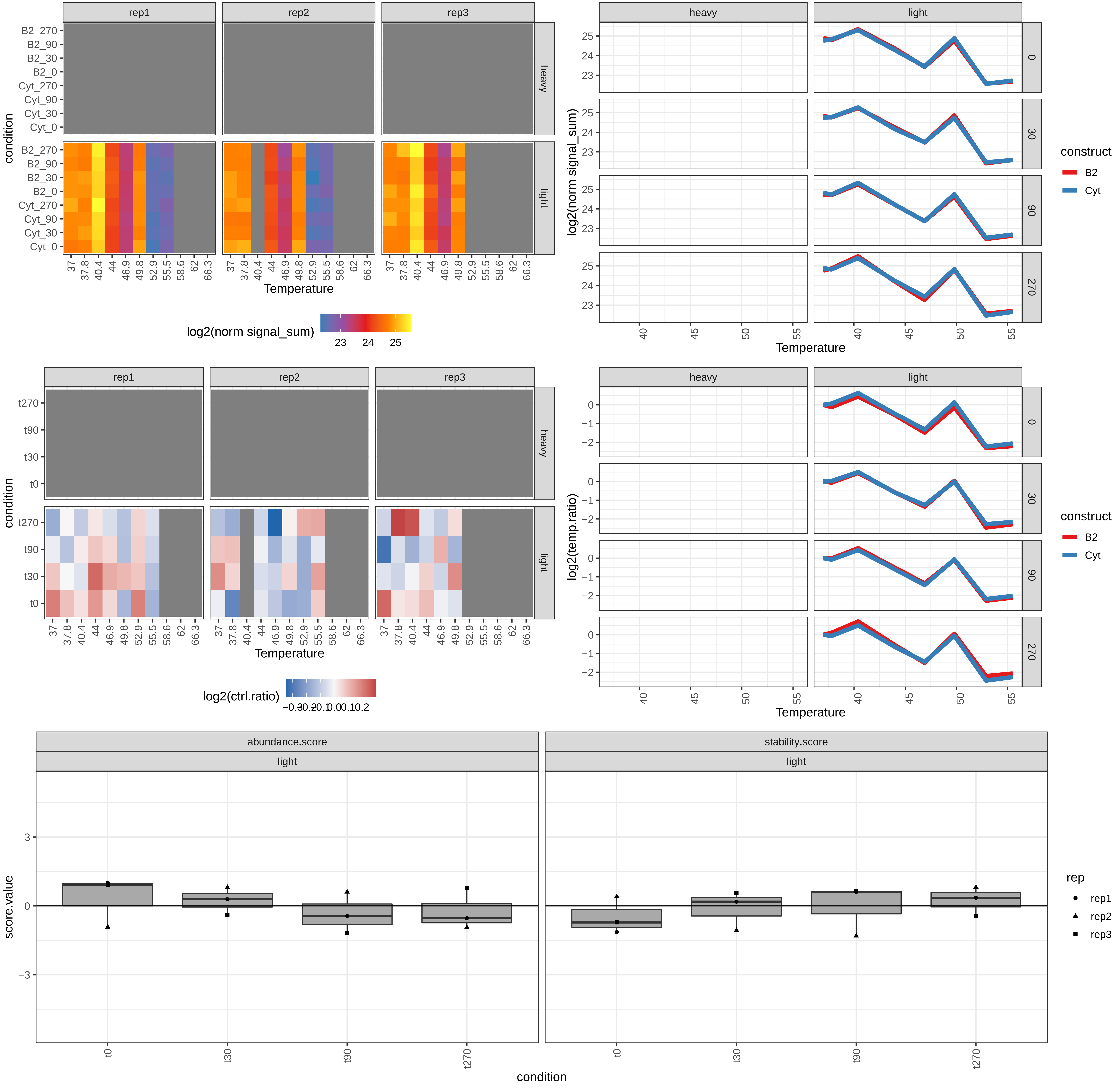

P33311 − ATP−dependent permease MDL2, mitochondrial OS=Saccharomyces cerevisiae (strain ATCC 204508 / S288c) GN=MDL2 PE=1 SV=3

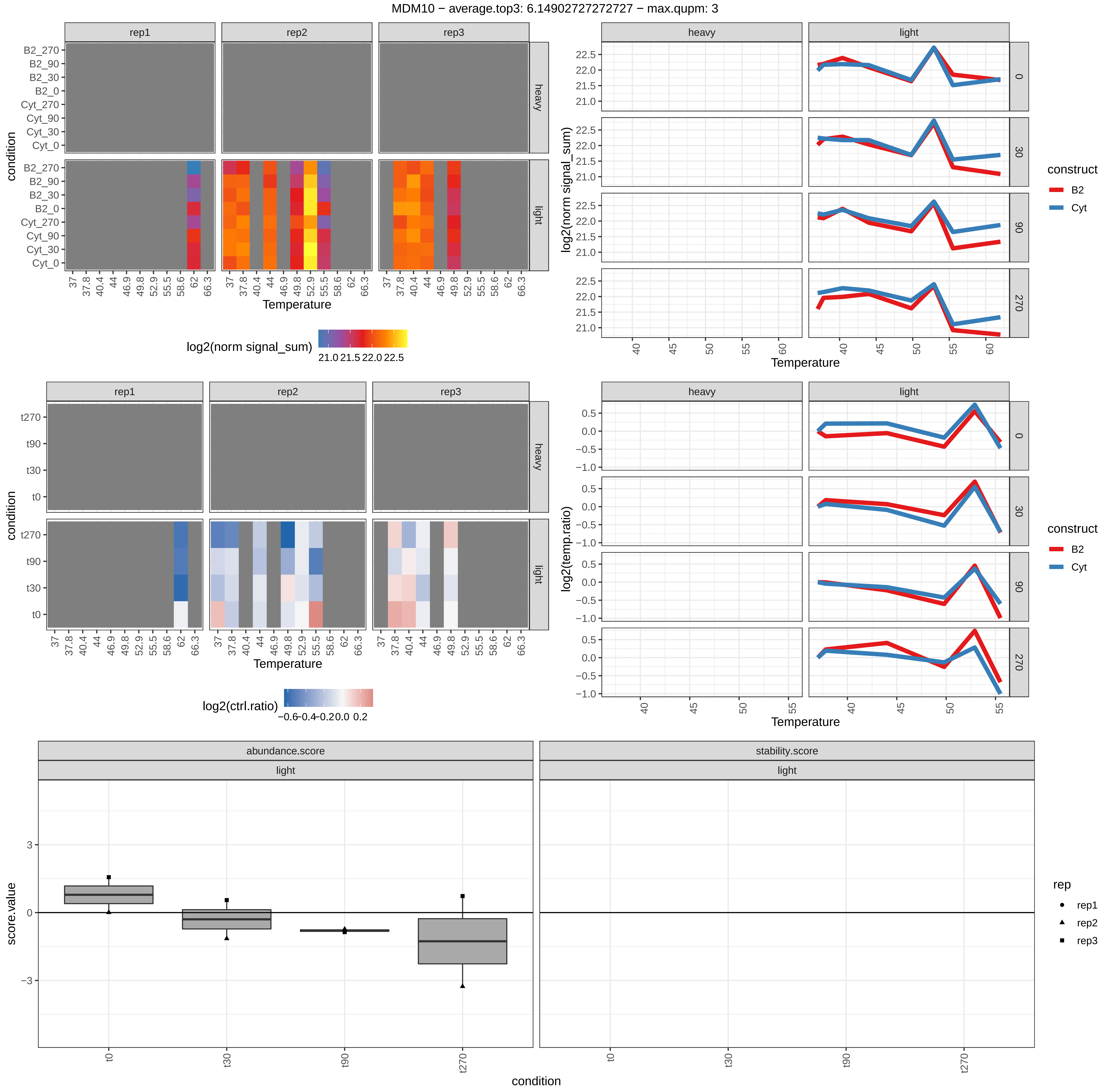

P18409 − Mitochondrial distribution and morphology protein 10 OS=Saccharomyces cerevisiae (strain ATCC 204508 / S288c) GN=MDM10 PE=1 SV=5

MDM20 − average.top3: 7.0444052173913 − max.qupm: 16

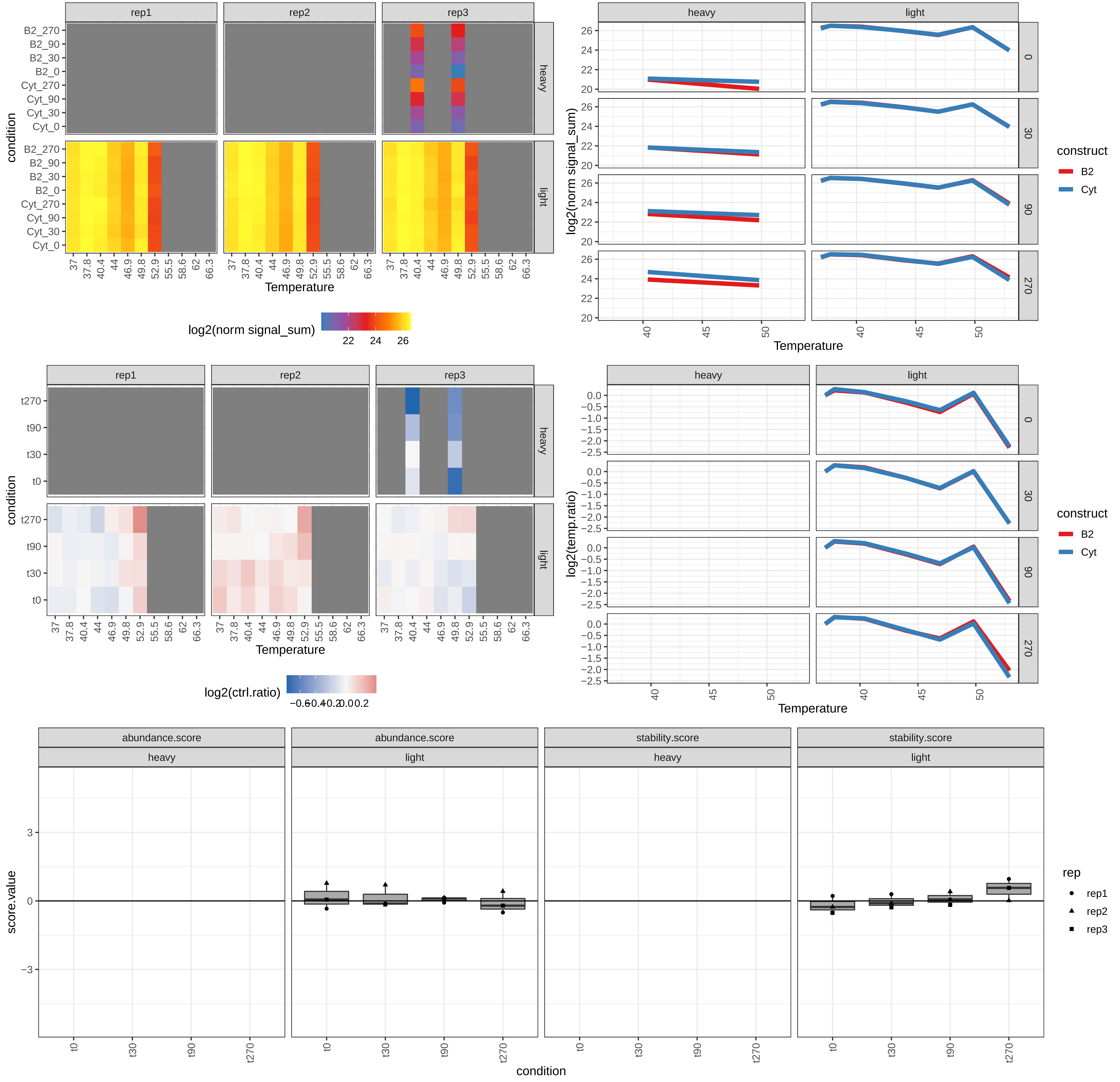

Q12387 − N−terminal acetyltransferase B complex subunit MDM20 OS=Saccharomyces cerevisiae (strain ATCC 204508 / S288c) GN=MDM20 PE=1 SV=1

MDM38 − average.top3: 7.30096586206897 − max.qupm: 7

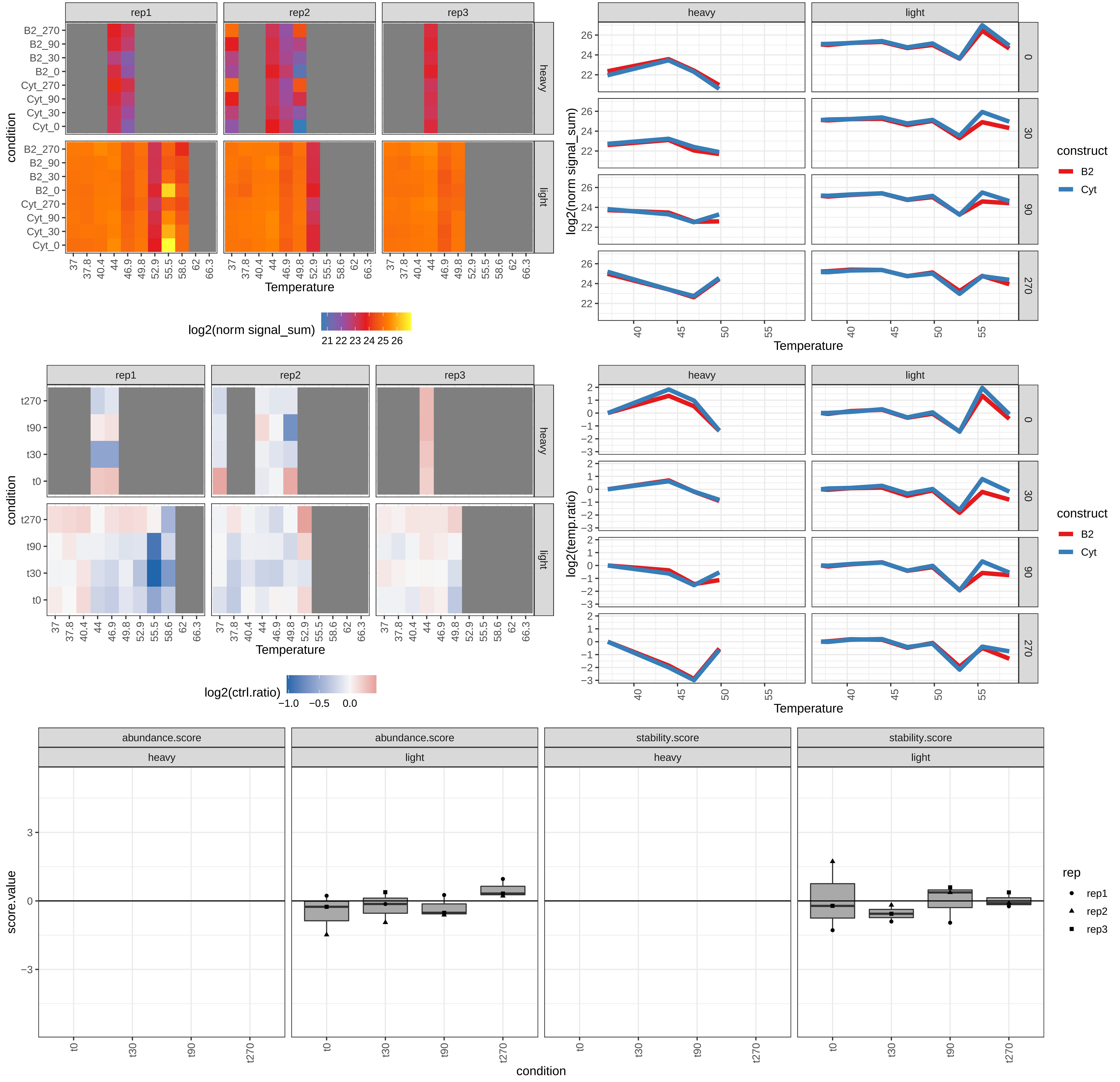

Q08179 − Mitochondrial distribution and morphology protein 38 OS=Saccharomyces cerevisiae (strain ATCC 204508 / S288c) GN=MDM38 PE=1 SV=1

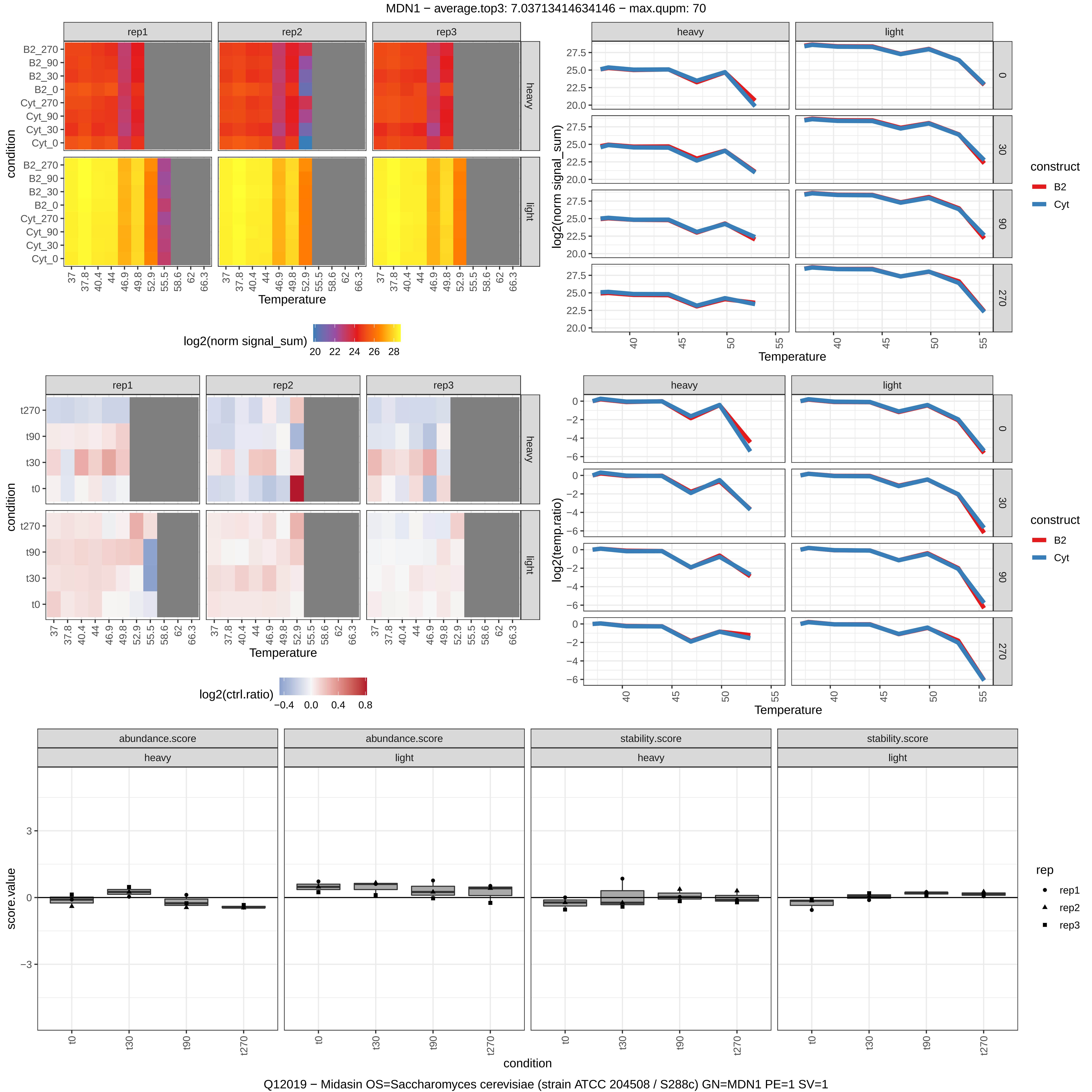

MDN1 − average.top3: 7.03713414634146 − max.qupm: 70
Q12019 − Midasin OS=Saccharomyces cerevisiae (strain ATCC 204508 / S288c) GN=MDN1 PE=1 SV=1

MDR1 − average.top3: 6.8192284 − max.qupm: 9

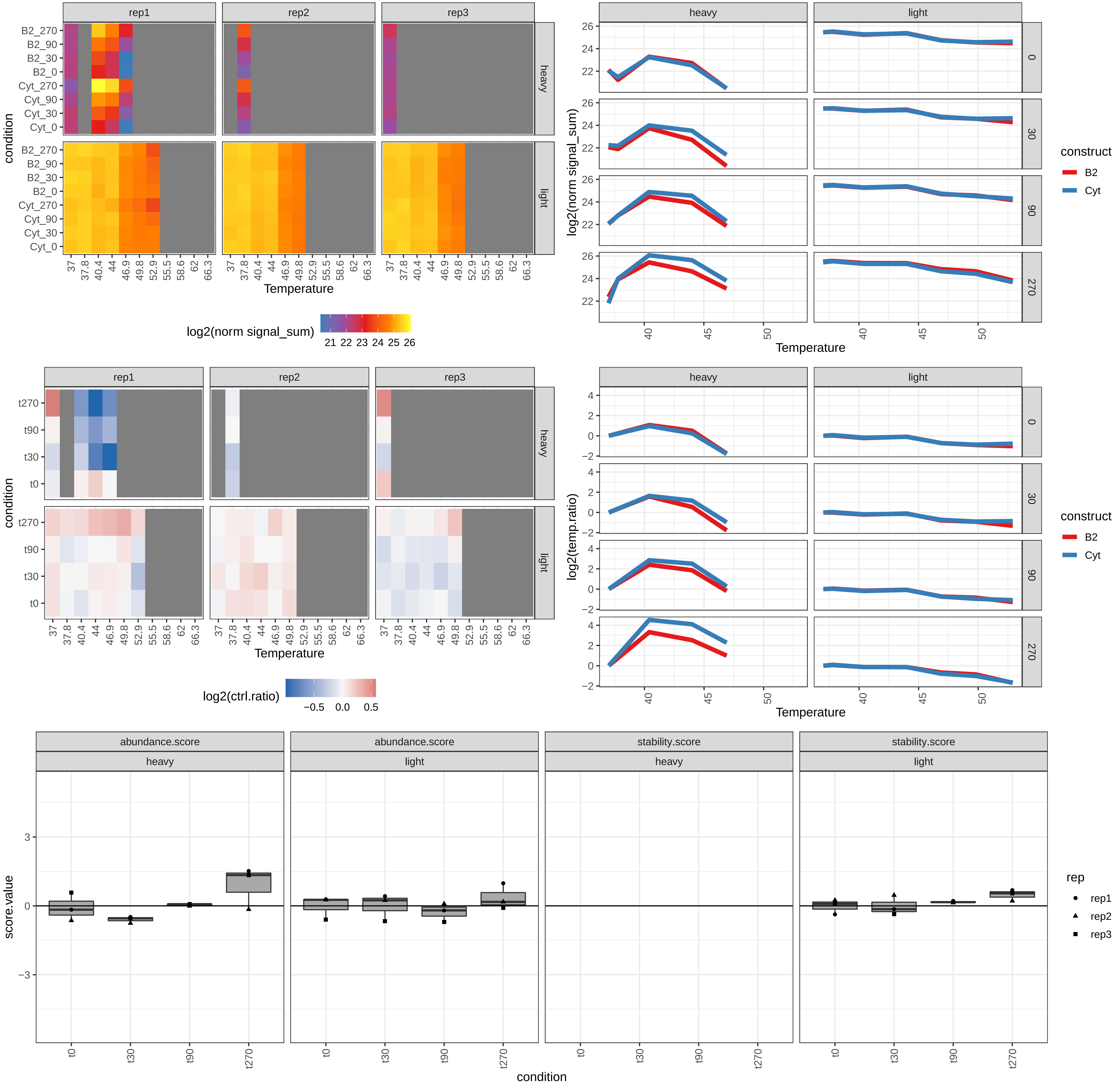

P53258 − GTPase−activating protein GYP2 OS=Saccharomyces cerevisiae (strain ATCC 204508 / S288c) GN=MDR1 PE=1 SV=1

MDS3 − average.top3: 6.40315526315789 − max.qupm: 5

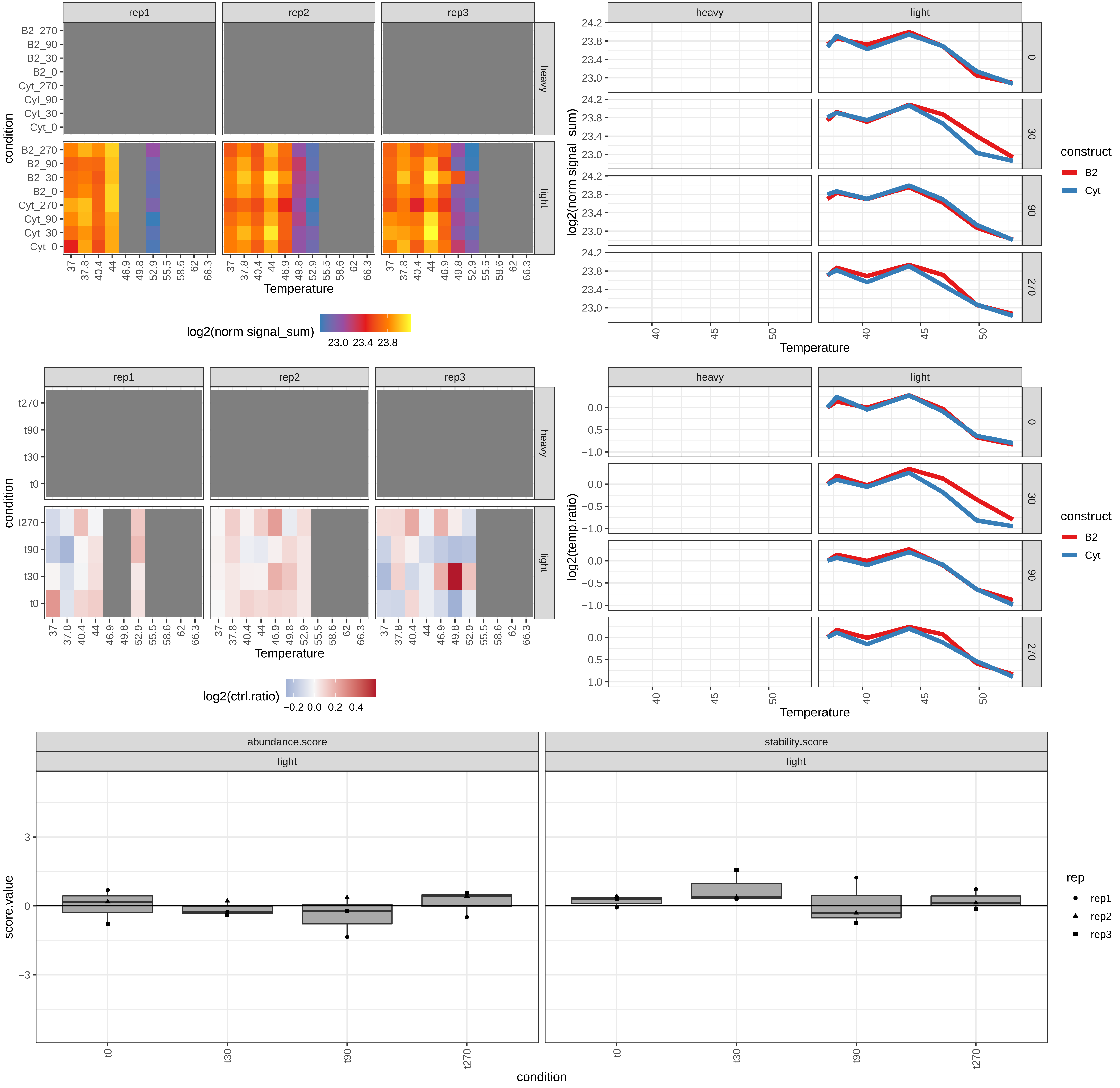

P53094 − Negative regulator of sporulation MDS3 OS=Saccharomyces cerevisiae (strain ATCC 204508 / S288c) GN=MDS3 PE=1 SV=2

MDV1 − average.top3: 6.84985227272727 − max.qupm: 4

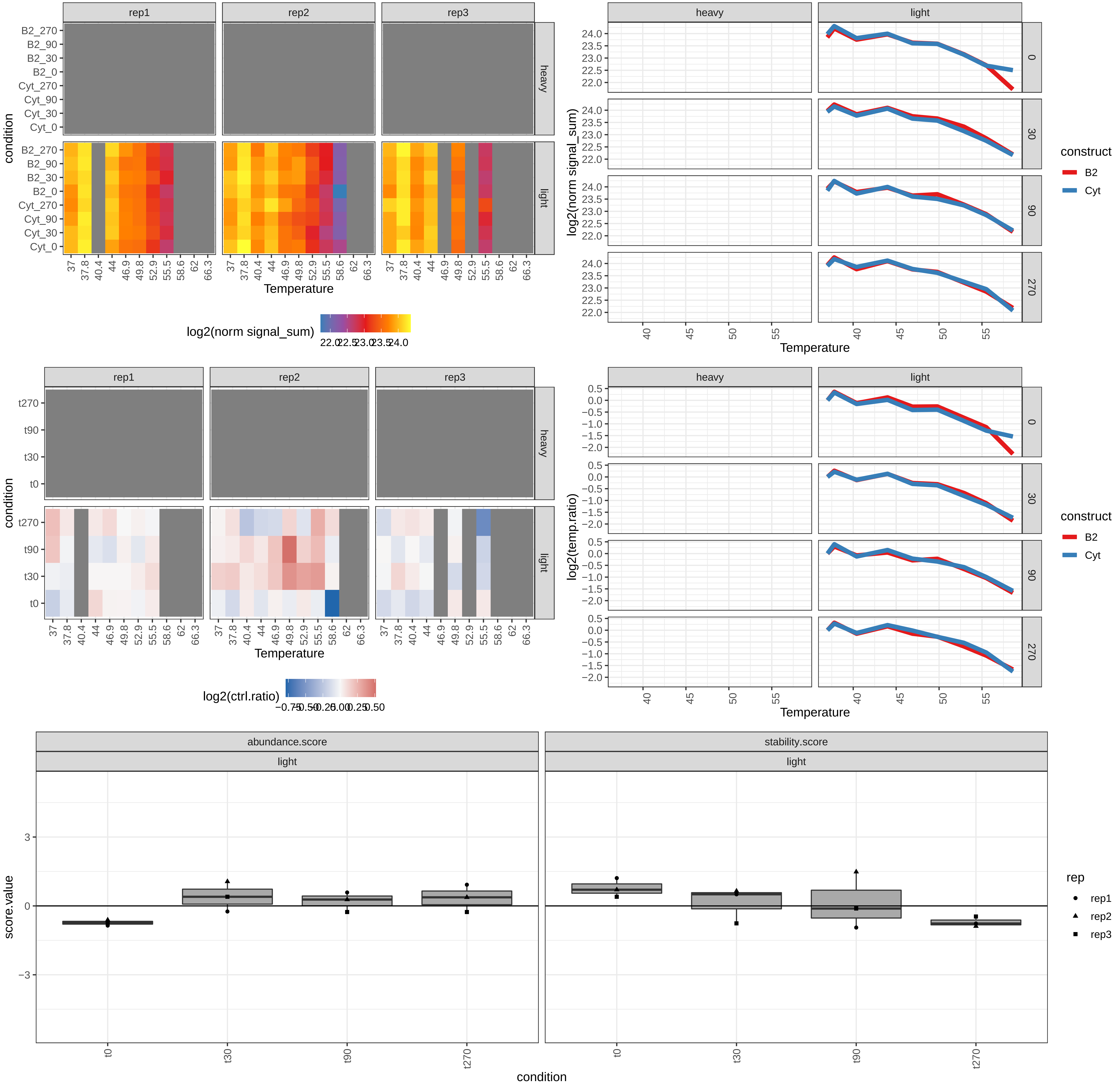

P47025 − Mitochondrial division protein 1 OS=Saccharomyces cerevisiae (strain ATCC 204508 / S288c) GN=MDV1 PE=1 SV=1

MDY2 − average.top3: 6.62884411764706 − max.qupm: 3

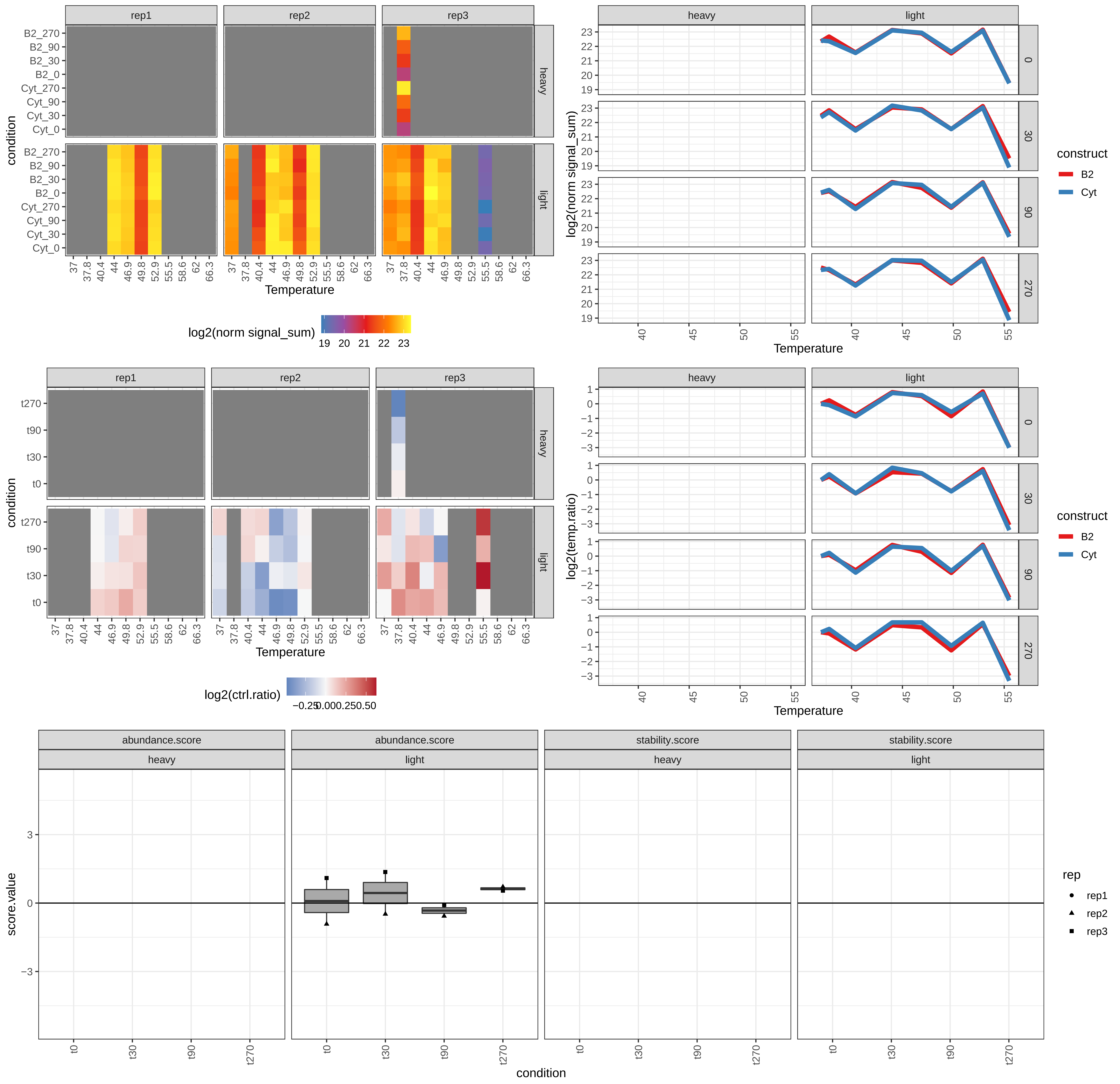

Q12285 − Ubiquitin−like protein MDY2 OS=Saccharomyces cerevisiae (strain ATCC 204508 / S288c) GN=MDY2 PE=1 SV=1

MEC1 − average.top3: 6.28431777777778 − max.qupm: 5

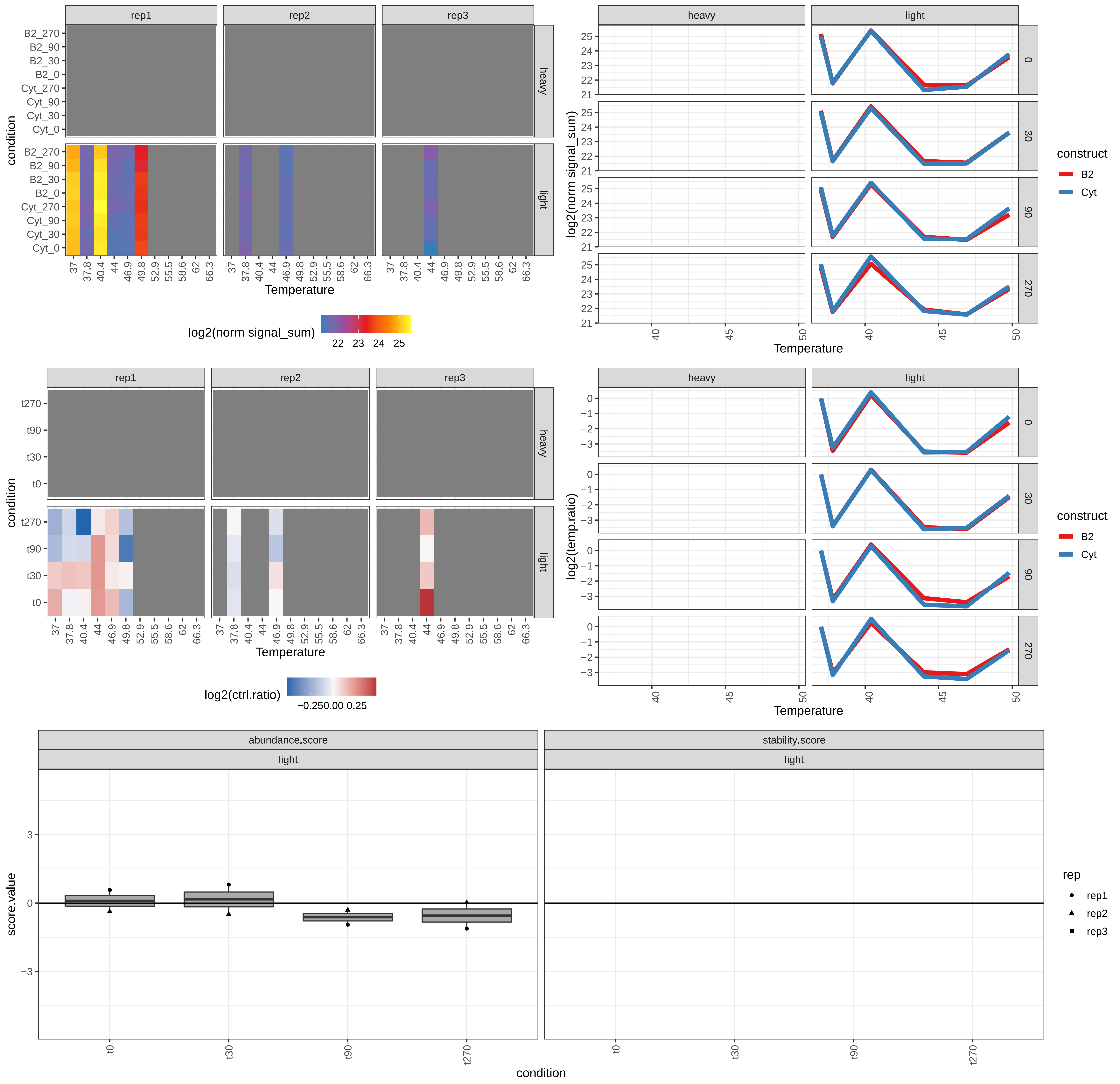

P38111 − Serine/threonine−protein kinase MEC1 OS=Saccharomyces cerevisiae (strain ATCC 204508 / S288c) GN=MEC1 PE=1 SV=1

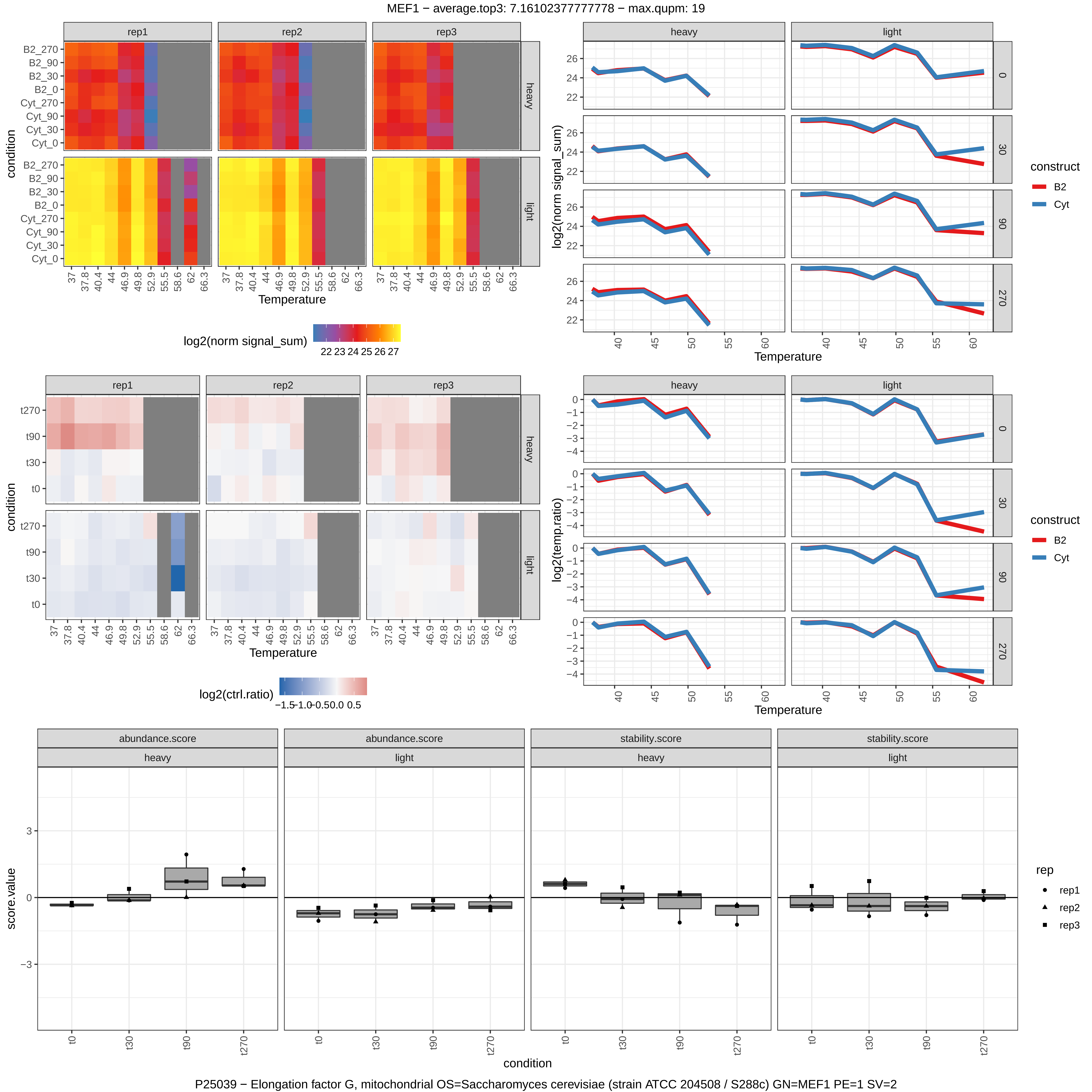
MEF1 − average.top3: 7.16102377777778 − max.qupm: 19
rep1
rep2
rep3
heavy
light
condition
Temperature
log2(norm signal_sum)
log2(ctrl.ratio)
log2(temp.ratio)
construct
B2
Cyt
abundance.score
stability.score
score.value
rep
rep1
rep2
rep3
P25039 − Elongation factor G, mitochondrial OS=Saccharomyces cerevisiae (strain ATCC 204508 / S288c) GN=MEF1 PE=1 SV=2

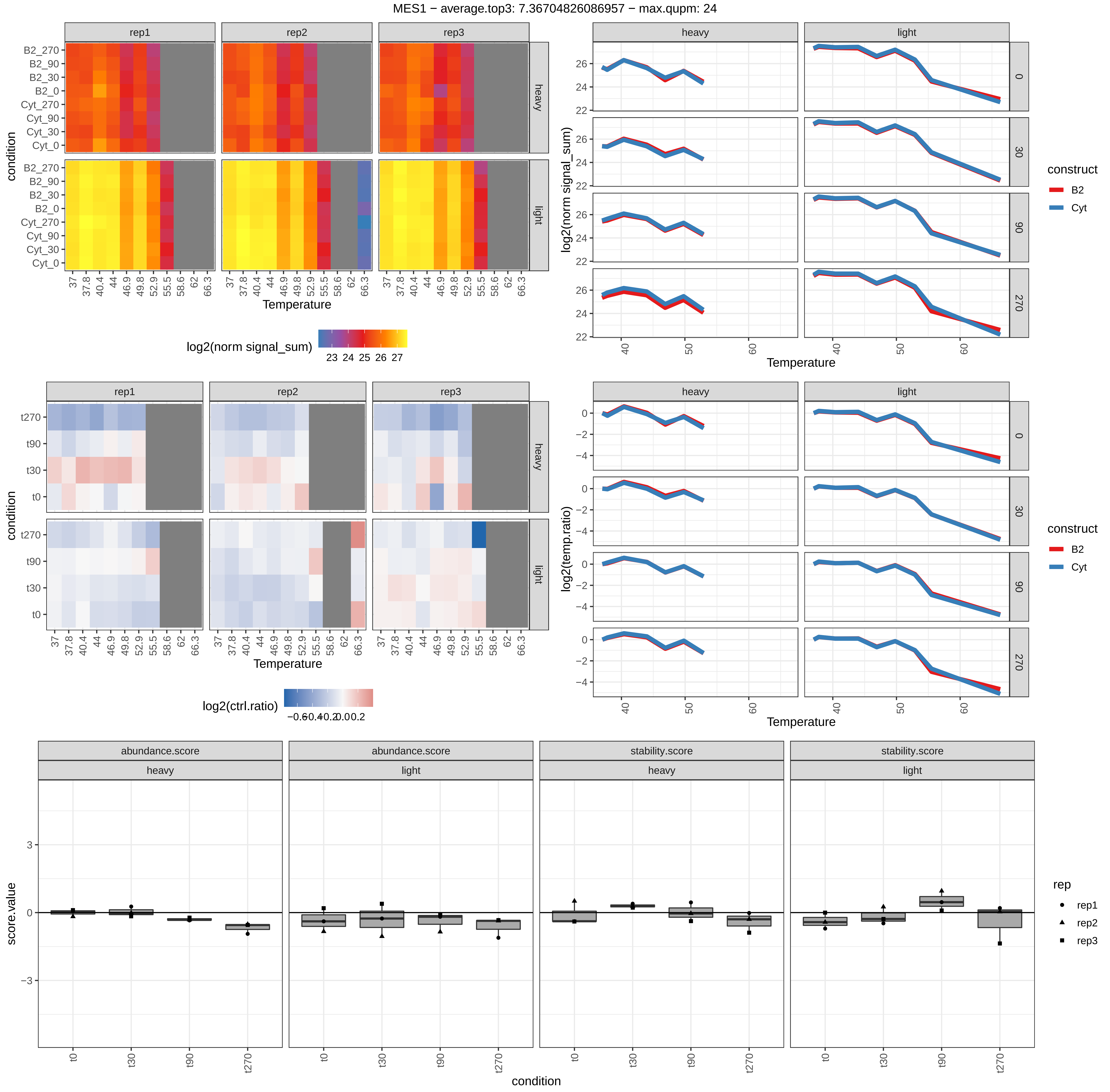

P00958 − Methionine−−tRNA ligase, cytoplasmic OS=Saccharomyces cerevisiae (strain ATCC 204508 / S288c) GN=MES1 PE=1 SV=4

MET1 − average.top3: 6.52192444444444 − max.qupm: 6

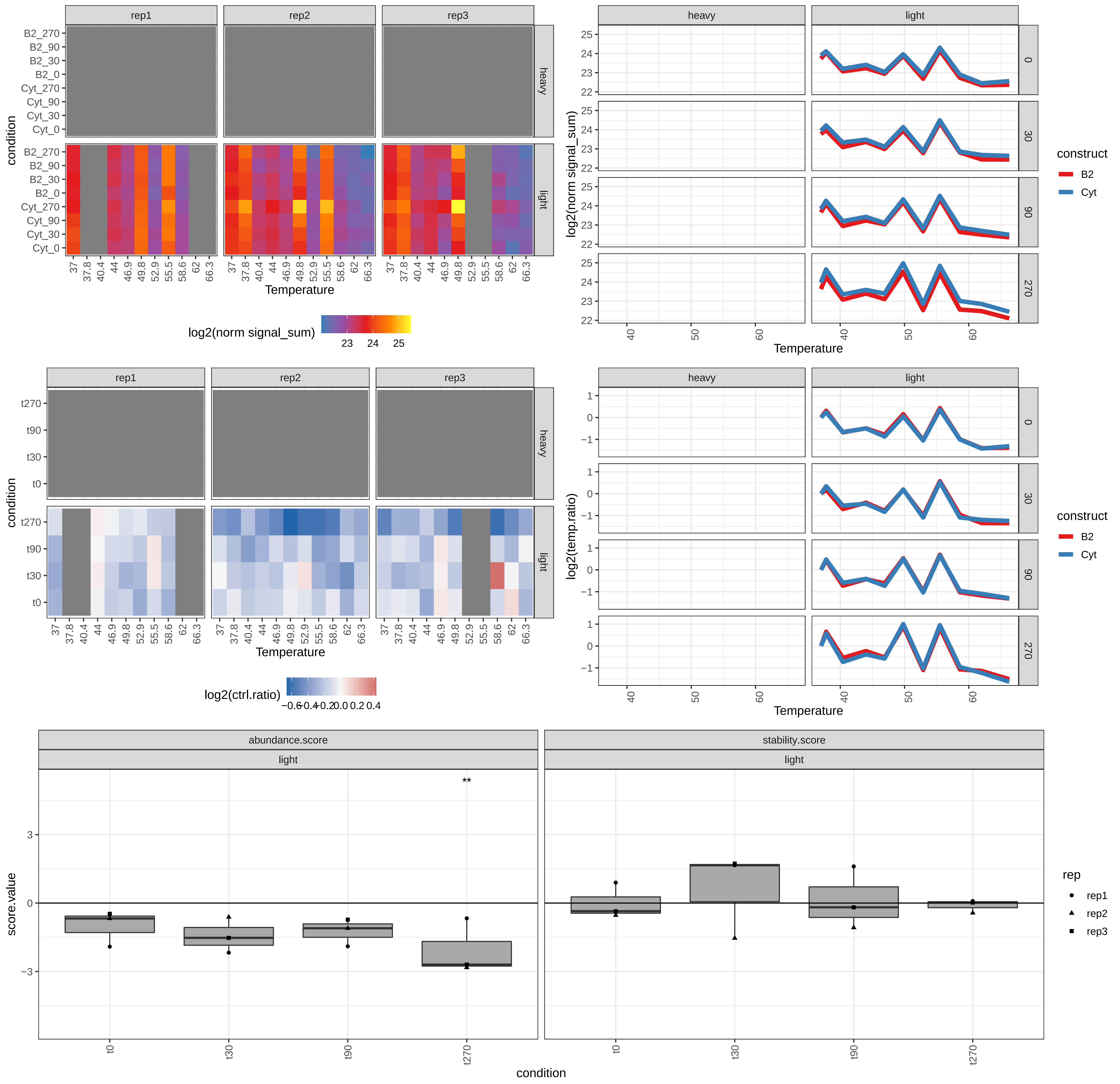

P36150 − Uroporphyrinogen−III C−methyltransferase OS=Saccharomyces cerevisiae (strain ATCC 204508 / S288c) GN=MET1 PE=1 SV=1

MET10 − average.top3: 6.82439 − max.qupm: 21

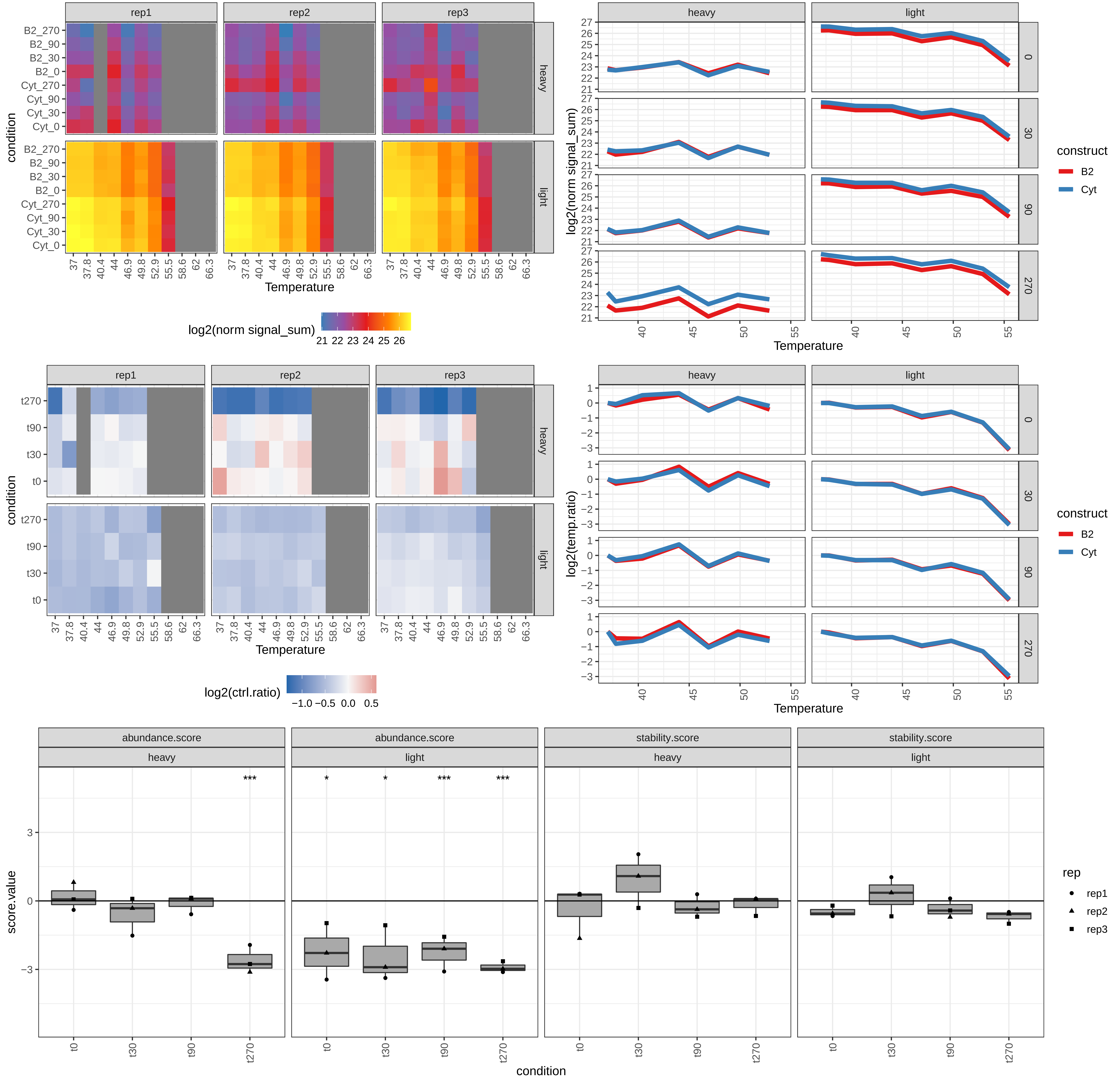

P39692 − Sulfite reductase [NADPH] flavoprotein component OS=Saccharomyces cerevisiae (strain ATCC 204508 / S288c) GN=MET10 PE=1 SV=2

MET12 − average.top3: 6.87562529411765 − max.qupm: 11

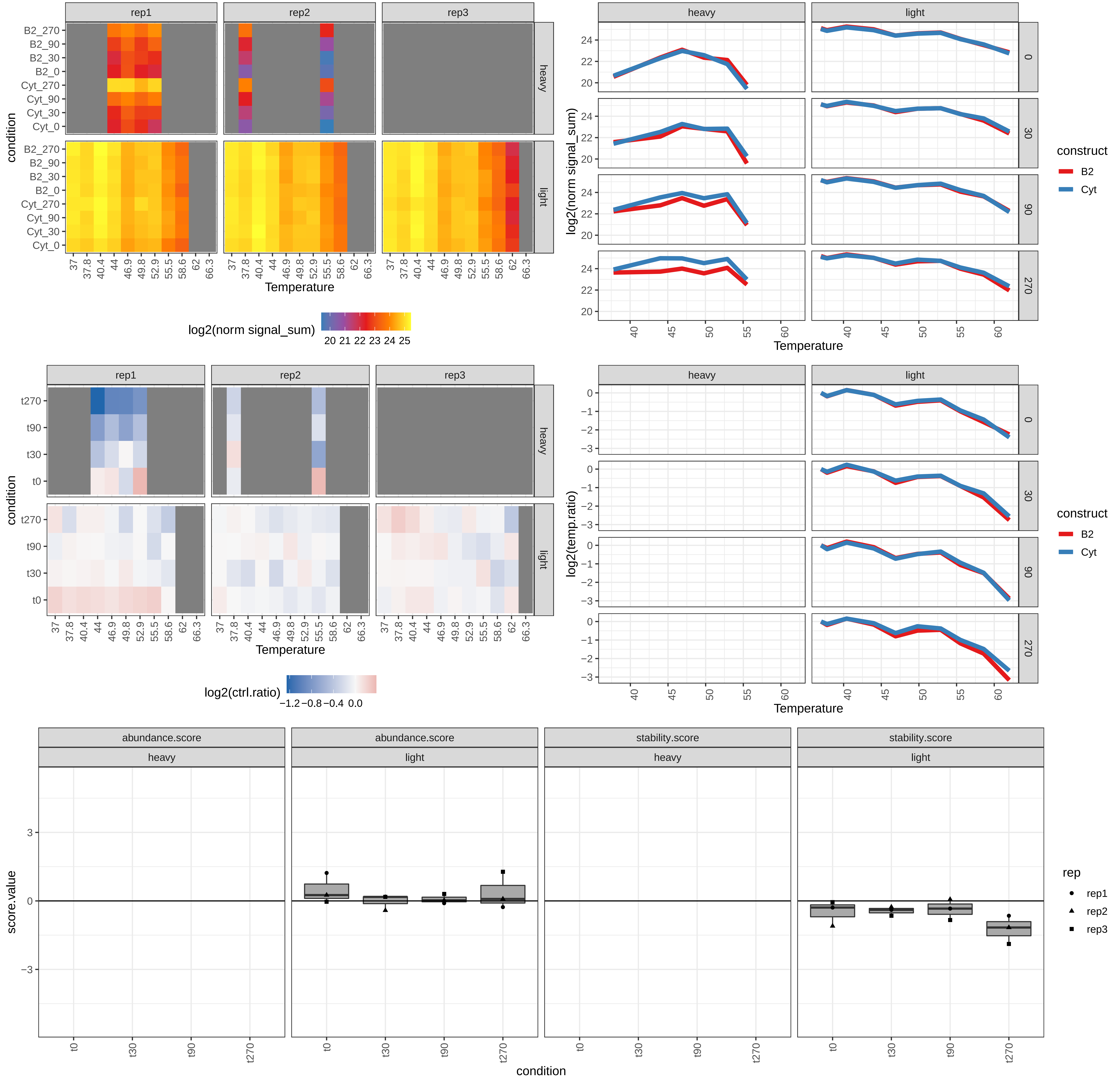

P46151 − Methylenetetrahydrofolate reductase 1 OS=Saccharomyces cerevisiae (strain ATCC 204508 / S288c) GN=MET12 PE=1 SV=2

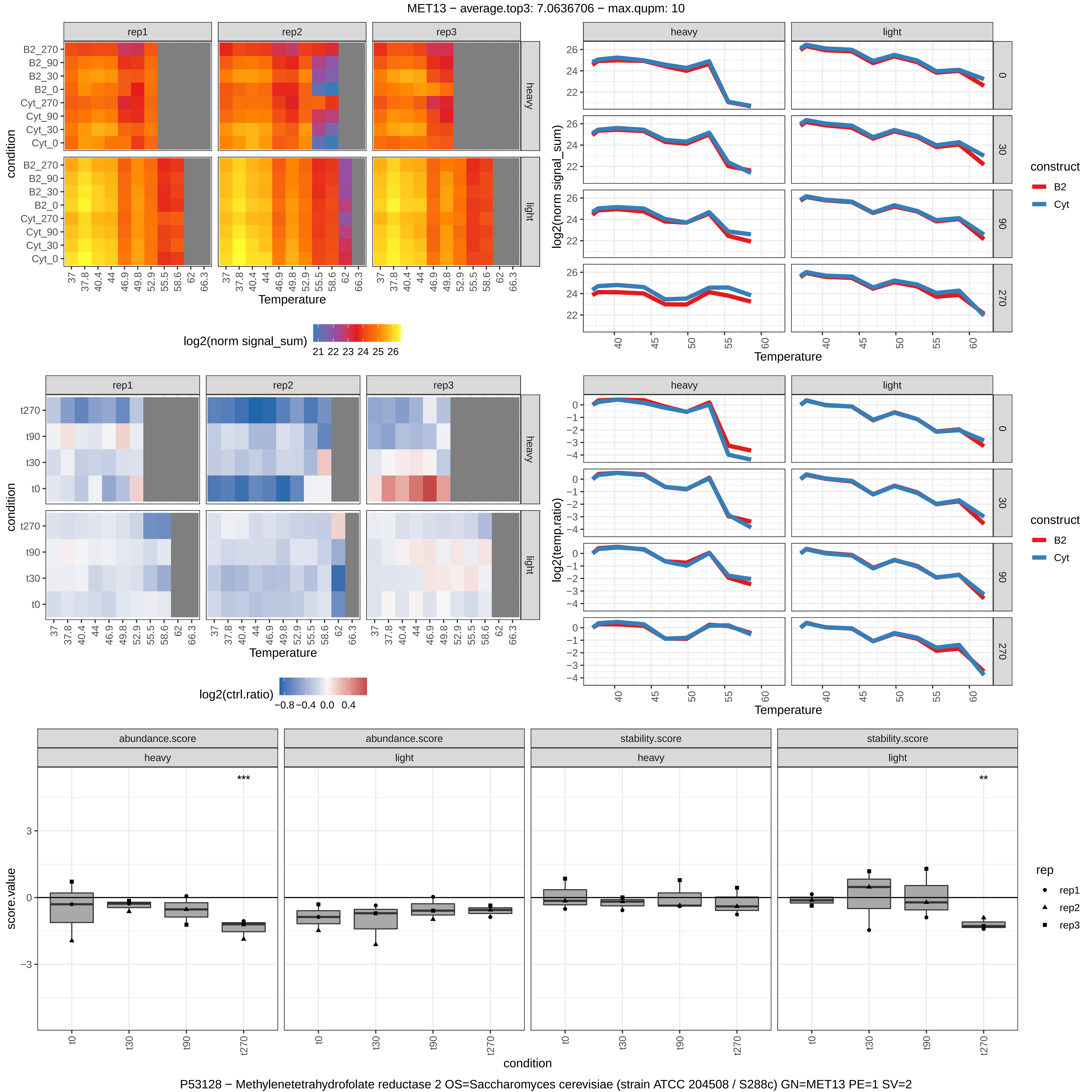
MET13 − average.top3: 7.0636706 − max.qupm: 10
rep1
rep2
rep3
heavy
light
condition
B2_270
B2_90
B2_30
B2_0
Cyt_270
Cyt_90
Cyt_30
Cyt_0
37 37.8 40.4 44 46.9 49.8 52.9 55.5 58.6 62 66.3
Temperature
log2(norm signal_sum)
21 22 23 24 25 26
log2(norm signal_sum)
0
30
90
270
construct
B2
Cyt
t270
t90
t30
t0
log2(ctrl.ratio)
−0.8 −0.4 0.0 0.4
log2(temp.ratio)
abundance.score
stability.score
score.value
***
**
rep
rep1
rep2
rep3
P53128 − Methylenetetrahydrofolate reductase 2 OS=Saccharomyces cerevisiae (strain ATCC 204508 / S288c) GN=MET13 PE=1 SV=2

MET14 − average.top3: 7.48511677419355 − max.qupm: 6

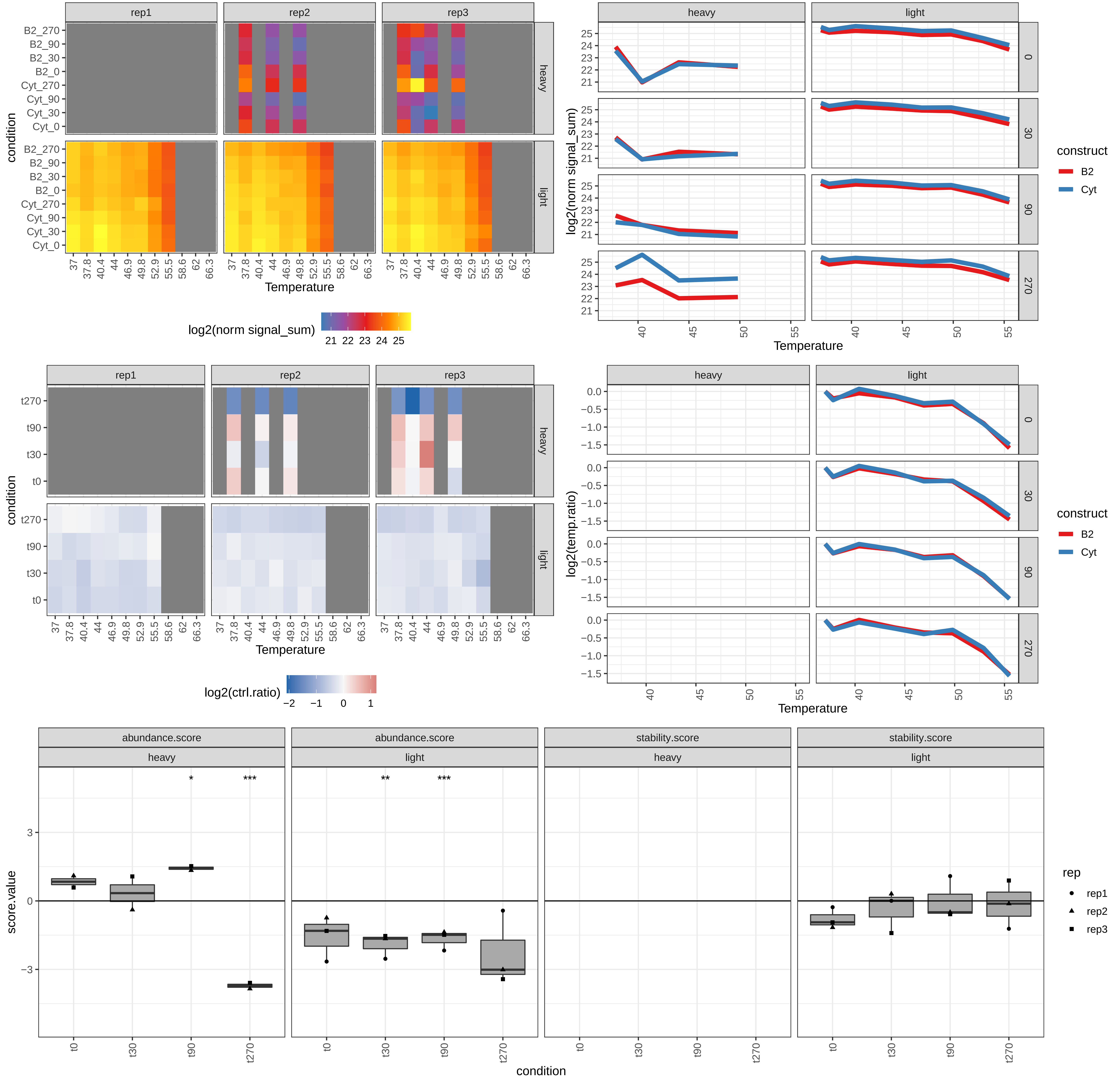

Q02196 − Adenylyl−sulfate kinase OS=Saccharomyces cerevisiae (strain ATCC 204508 / S288c) GN=MET14 PE=1 SV=1

MET17 − average.top3: 8.32033875 − max.qupm: 18

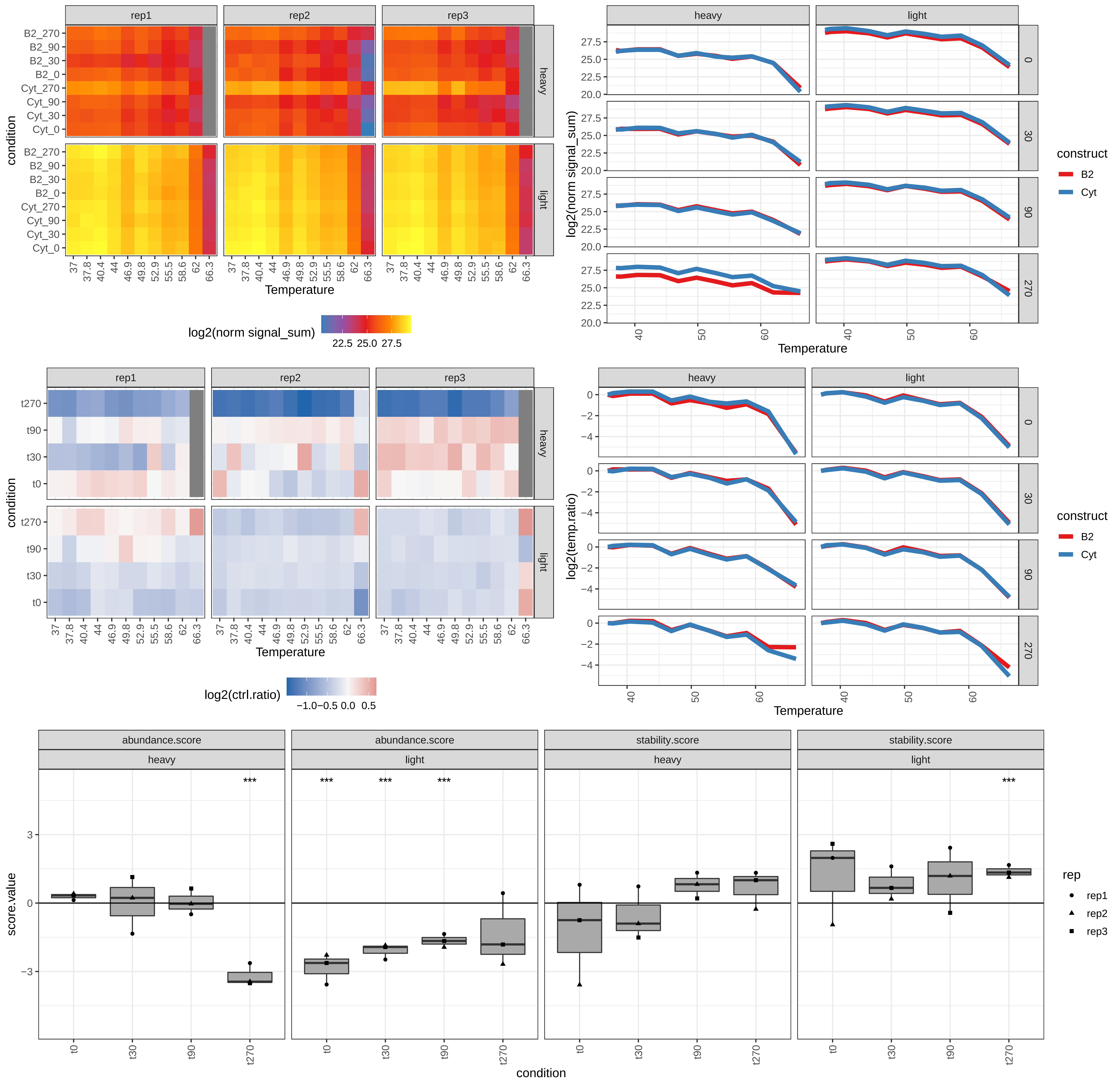

P06106 − Homocysteine/cysteine synthase OS=Saccharomyces cerevisiae (strain ATCC 204508 / S288c) GN=MET17 PE=1 SV=3

MET18 − average.top3: 6.48262875 − max.qupm: 7

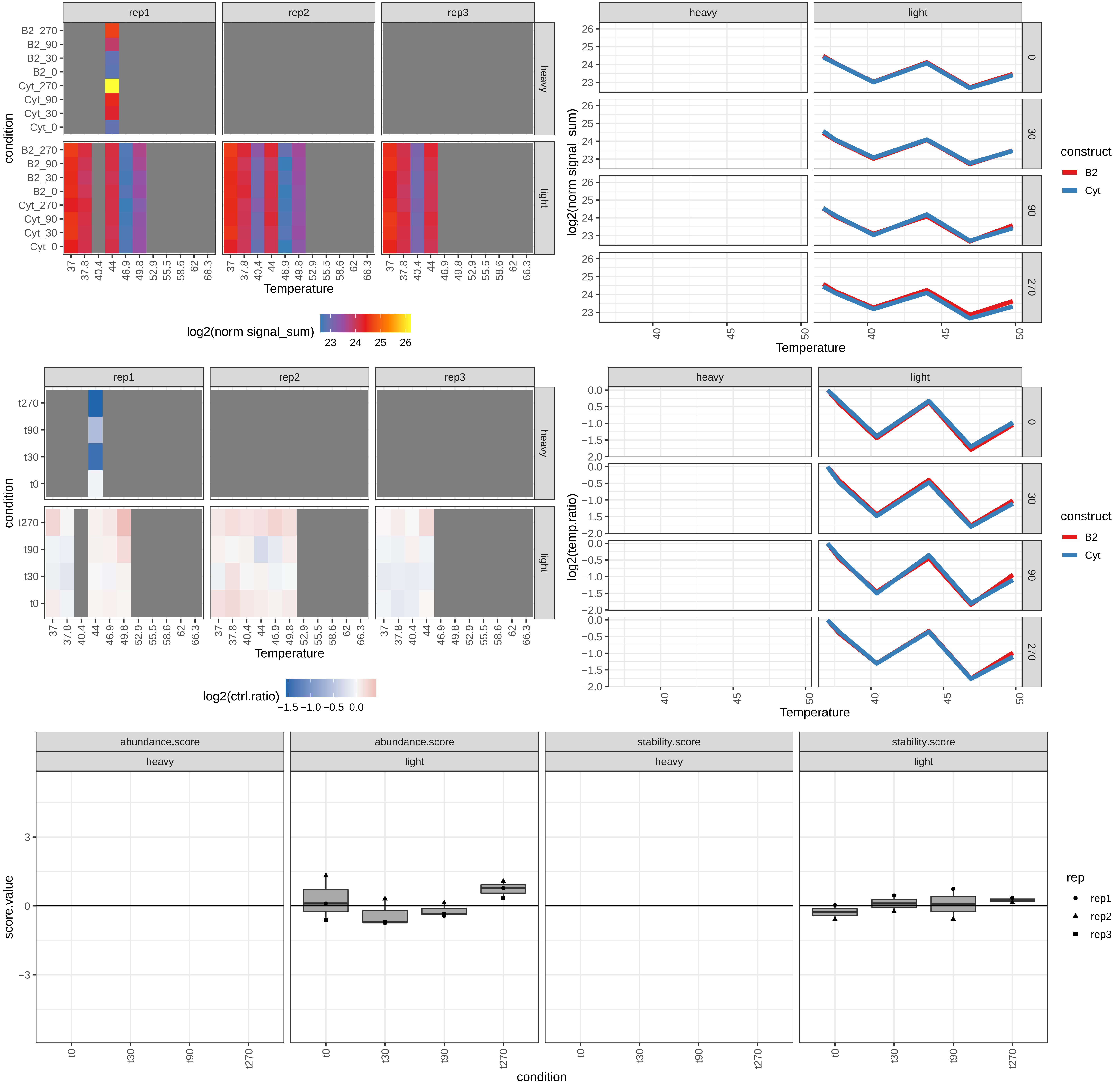

P40469 − DNA repair/transcription protein MET18/MMS19 OS=Saccharomyces cerevisiae (strain ATCC 204508 / S288c) GN=MET18 PE=1 SV=1

MET2 − average.top3: 6.471653 − max.qupm: 4

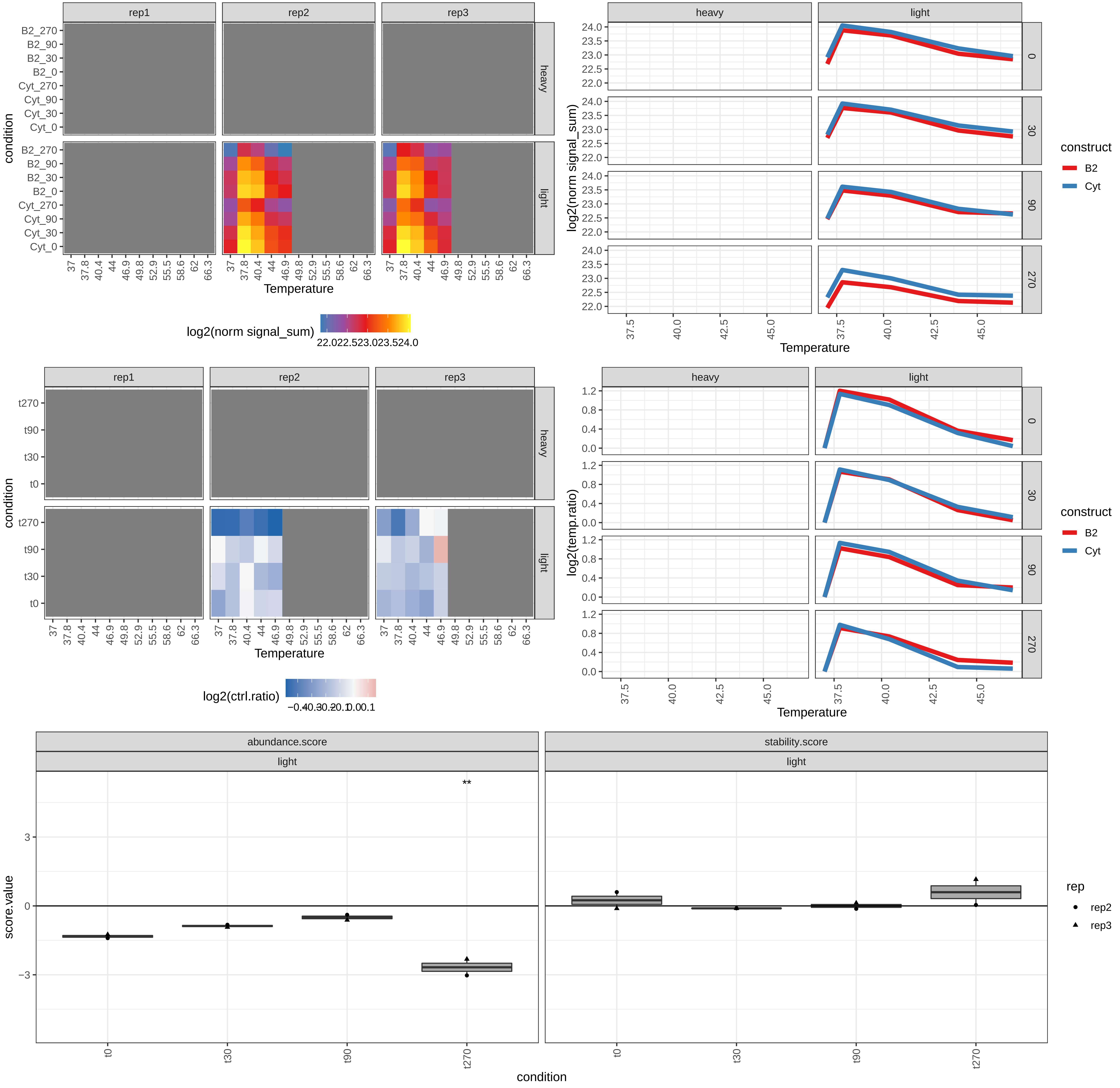

P08465 − Homoserine O−acetyltransferase OS=Saccharomyces cerevisiae (strain ATCC 204508 / S288c) GN=MET2 PE=1 SV=2

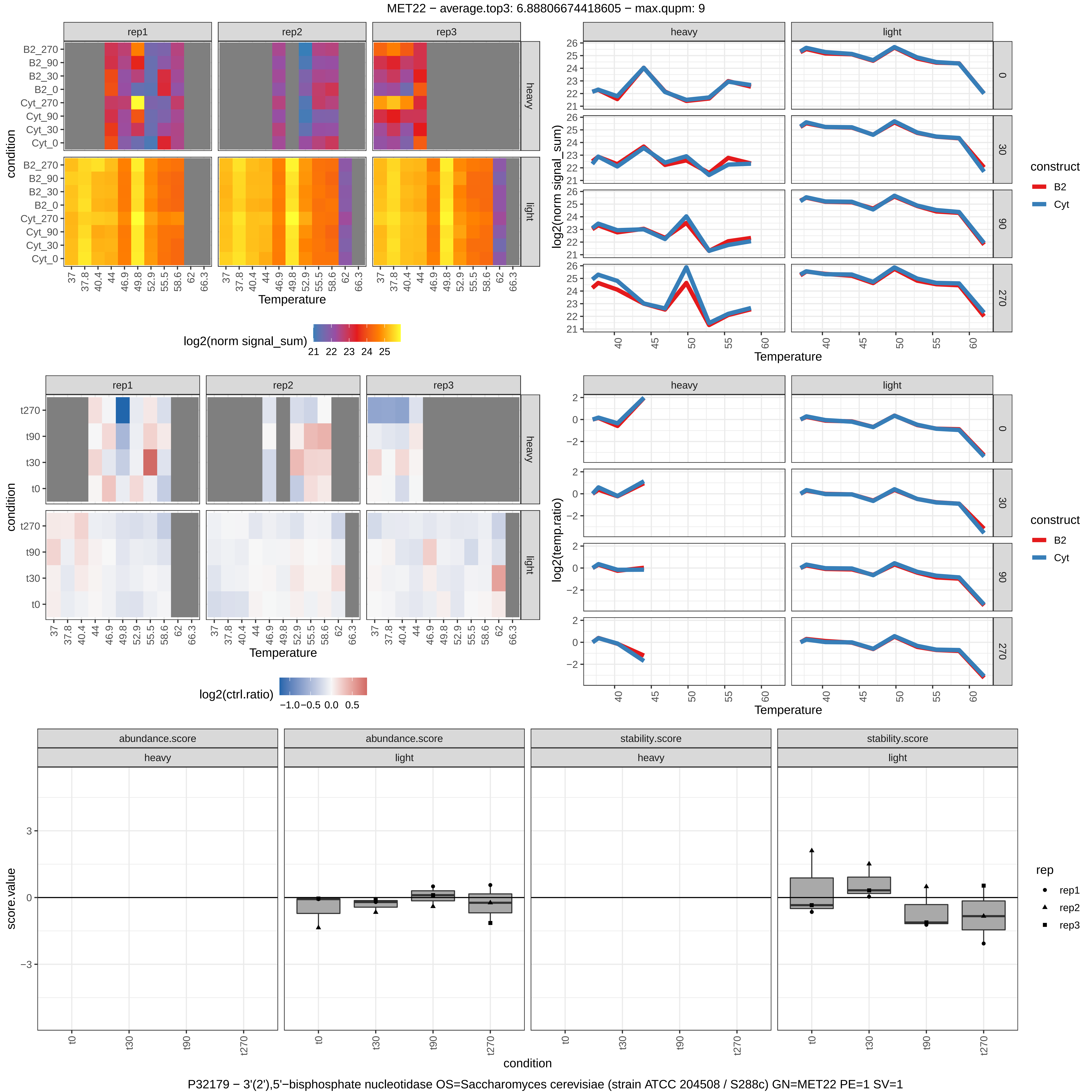
MET22 − average.top3: 6.88806674418605 − max.qupm: 9
rep1
rep2
rep3
heavy
light
condition
Temperature
log2(norm signal_sum)
construct
B2
Cyt
log2(ctrl.ratio)
log2(temp.ratio)
abundance.score
stability.score
score.value
rep
rep1
rep2
rep3
P32179 − 3'(2'),5'−bisphosphate nucleotidase OS=Saccharomyces cerevisiae (strain ATCC 204508 / S288c) GN=MET22 PE=1 SV=1

MET3 − average.top3: 7.43145340909091 − max.qupm: 13

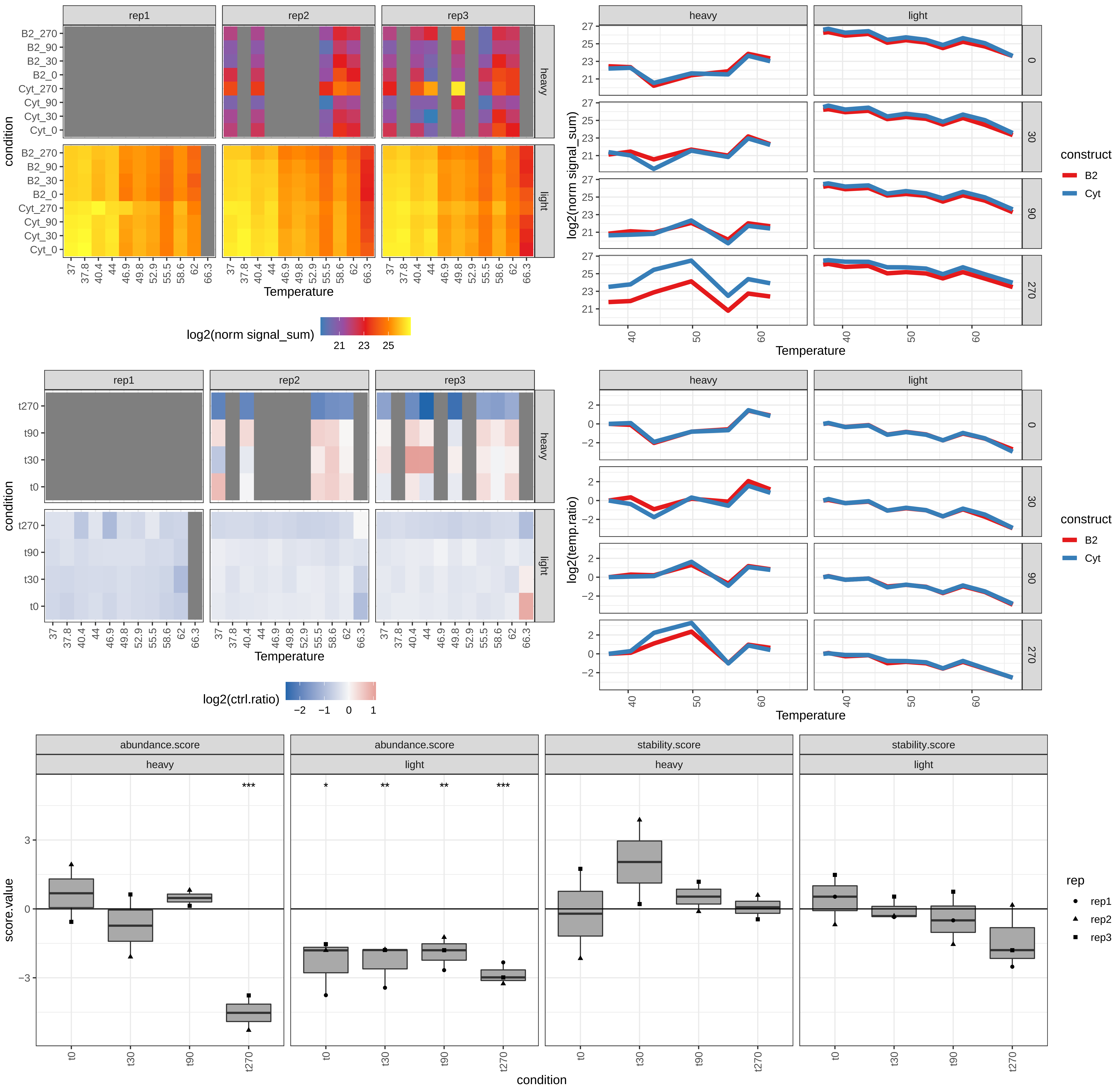

P08536 − Sulfate adenylyltransferase OS=Saccharomyces cerevisiae (strain ATCC 204508 / S288c) GN=MET3 PE=1 SV=2

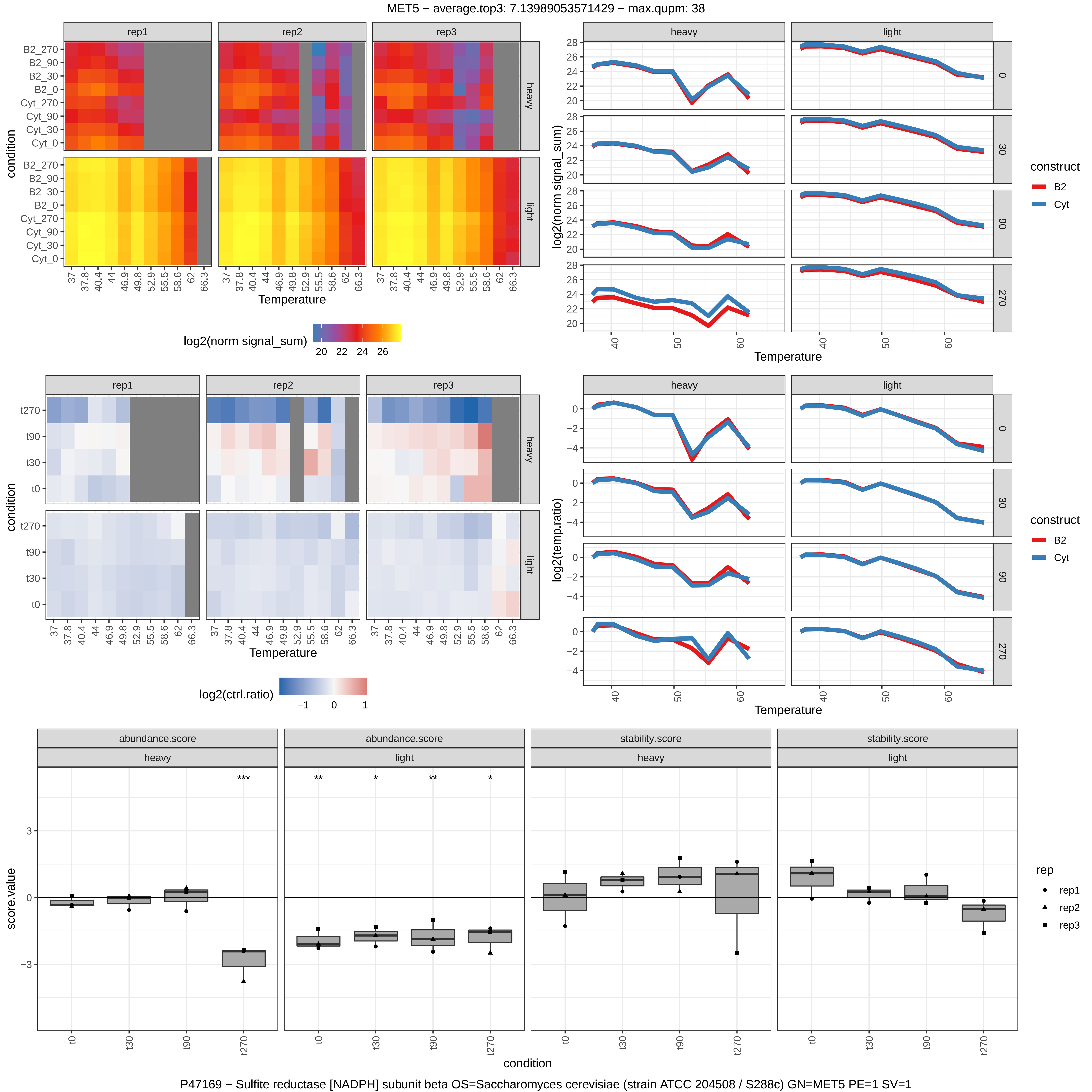
MET5 − average.top3: 7.13989053571429 − max.qupm: 38
rep1
rep2
rep3
heavy
light
condition
B2_270
B2_90
B2_30
B2_0
Cyt_270
Cyt_90
Cyt_30
Cyt_0
Temperature
log2(norm signal_sum)
20
22
24
26
log2(norm signal_sum)
construct
B2
Cyt
0
30
90
270
t270
t90
t30
t0
log2(ctrl.ratio)
−1
0
1
log2(temp.ratio)
abundance.score
stability.score
score.value
rep
rep1
rep2
rep3
***
**
*
P47169 − Sulfite reductase [NADPH] subunit beta OS=Saccharomyces cerevisiae (strain ATCC 204508 / S288c) GN=MET5 PE=1 SV=1

MET6 − average.top3: 8.39270106060606 − max.qupm: 44

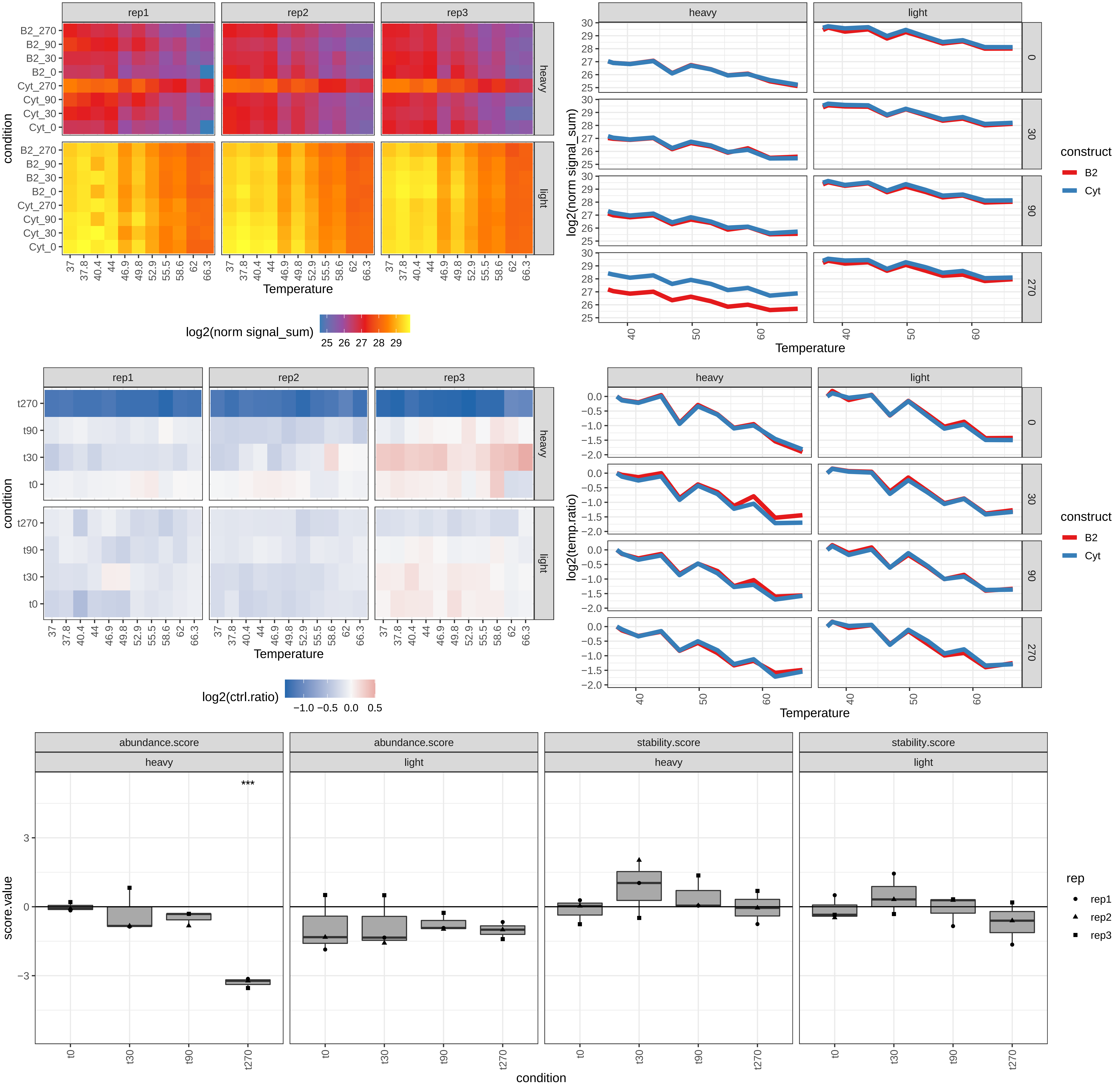

P05694 − 5−methyltetrahydropteroyltriglutamate−−homocysteine methyltransferase OS=Saccharomyces cerevisiae (strain ATCC 204508 / S288c) GN=MET6 PE=1 SV=4

MET7 − average.top3: 7.05319952380952 − max.qupm: 7

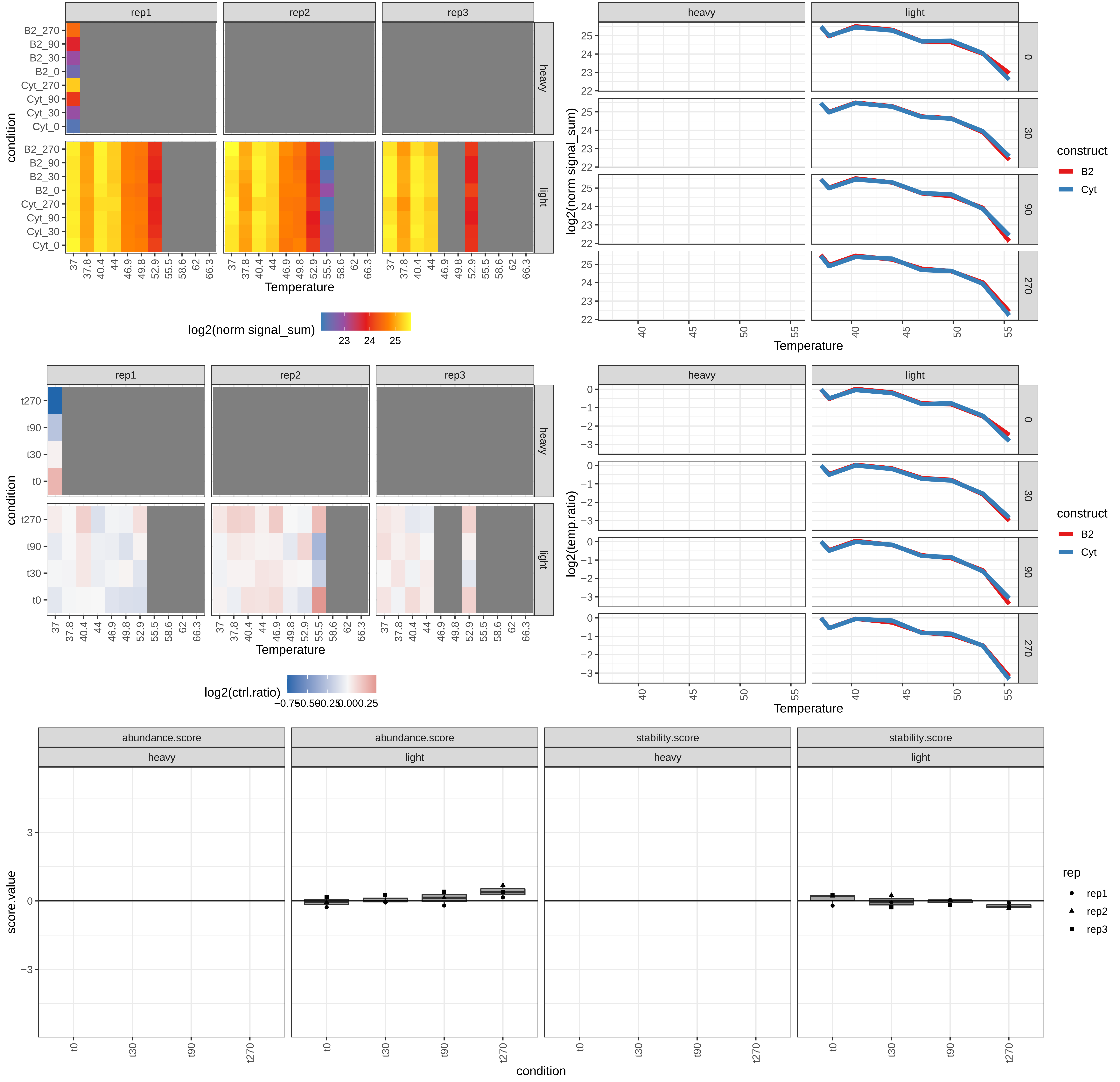

Q08645 − Folylpolyglutamate synthase OS=Saccharomyces cerevisiae (strain ATCC 204508 / S288c) GN=MET7 PE=1 SV=1

MET8 − average.top3: 6.488388 − max.qupm: 4

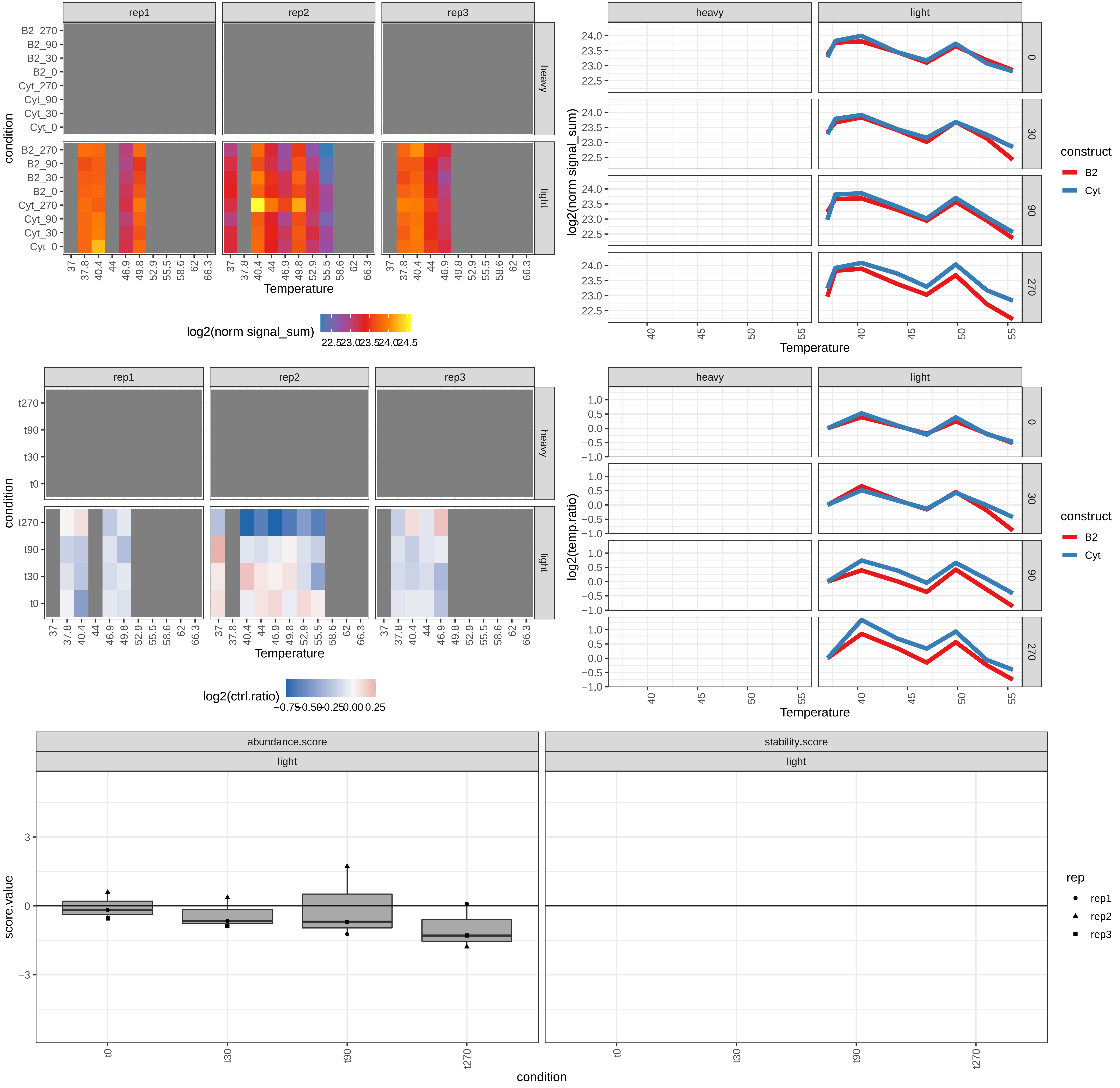

P15807 − Siroheme biosynthesis protein MET8 OS=Saccharomyces cerevisiae (strain ATCC 204508 / S288c) GN=MET8 PE=1 SV=1

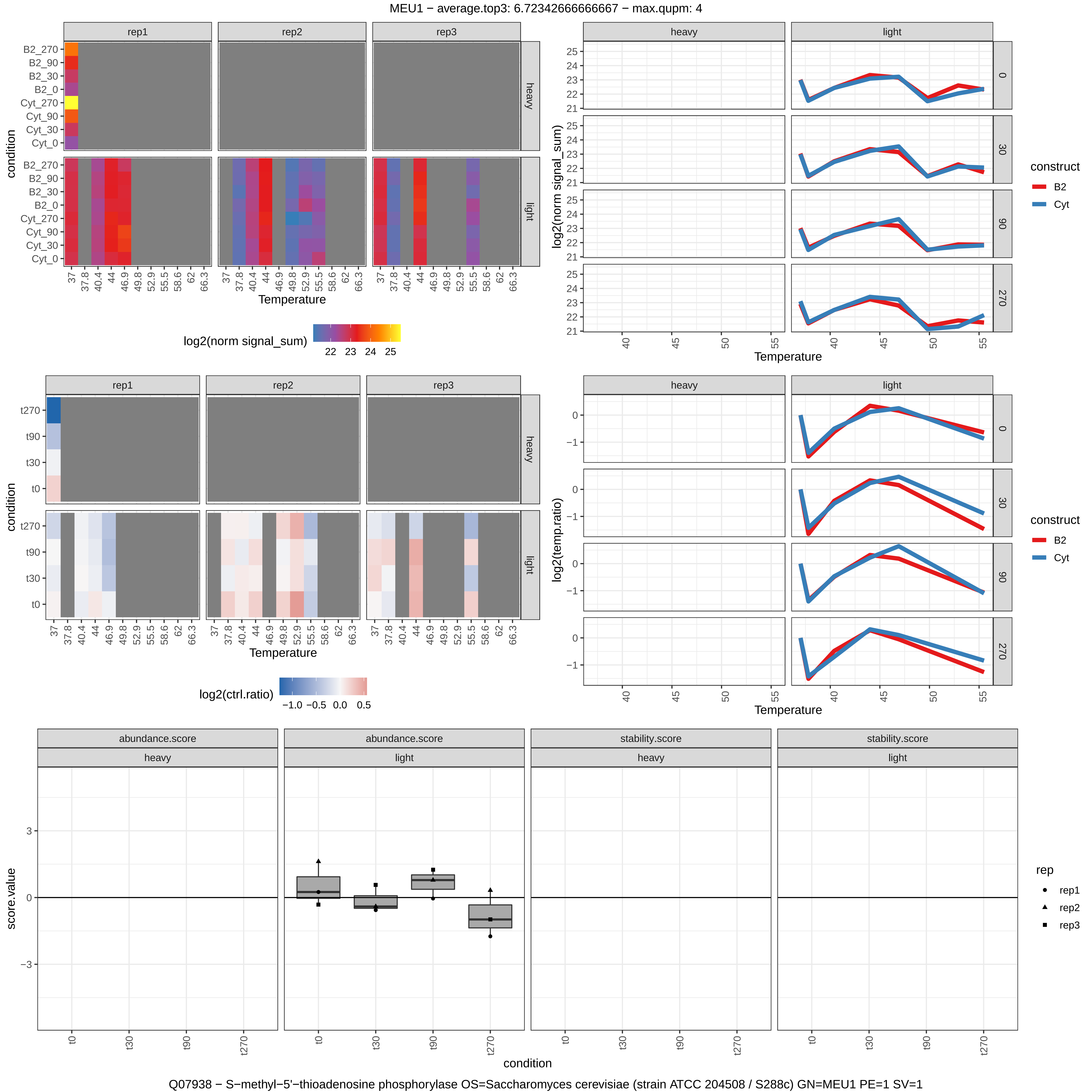

MEU1 − average.top3: 6.72342666666667 − max.qupm: 4
rep1
rep2
rep3
heavy
light
condition
Temperature
log2(norm signal_sum)
log2(ctrl.ratio)
log2(temp.ratio)
construct
B2
Cyt
abundance.score
stability.score
score.value
rep
rep1
rep2
rep3
Q07938 − S−methyl−5'−thioadenosine phosphorylase OS=Saccharomyces cerevisiae (strain ATCC 204508 / S288c) GN=MEU1 PE=1 SV=1

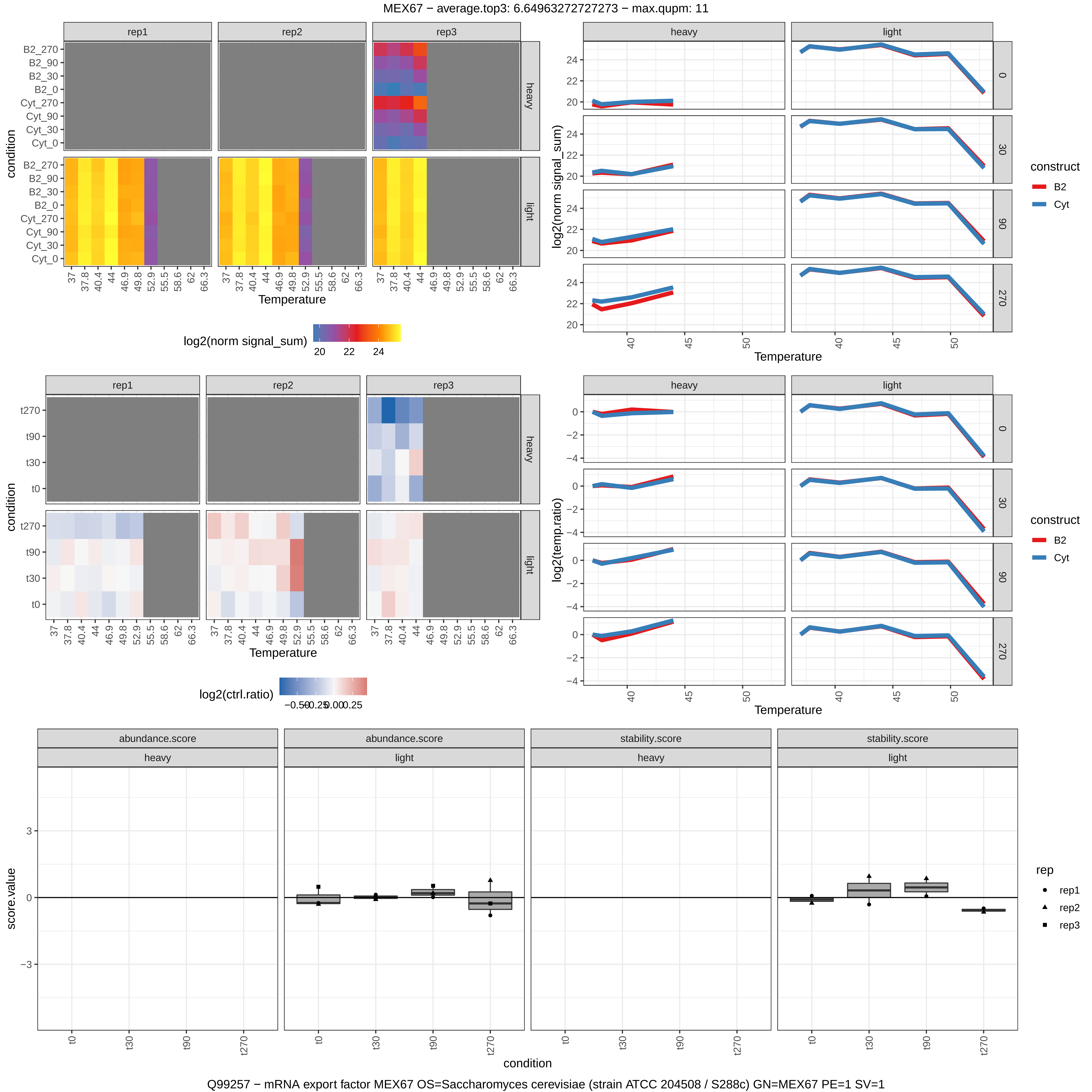
MEX67 − average.top3: 6.64963272727273 − max.qupm: 11
rep1
rep2
rep3
heavy
light
condition
Temperature
log2(norm signal_sum)
log2(ctrl.ratio)
log2(temp.ratio)
construct
B2
Cyt
abundance.score
stability.score
score.value
rep
rep1
rep2
rep3
Q99257 − mRNA export factor MEX67 OS=Saccharomyces cerevisiae (strain ATCC 204508 / S288c) GN=MEX67 PE=1 SV=1

MFT1 − average.top3: 6.74144222222222 − max.qupm: 8

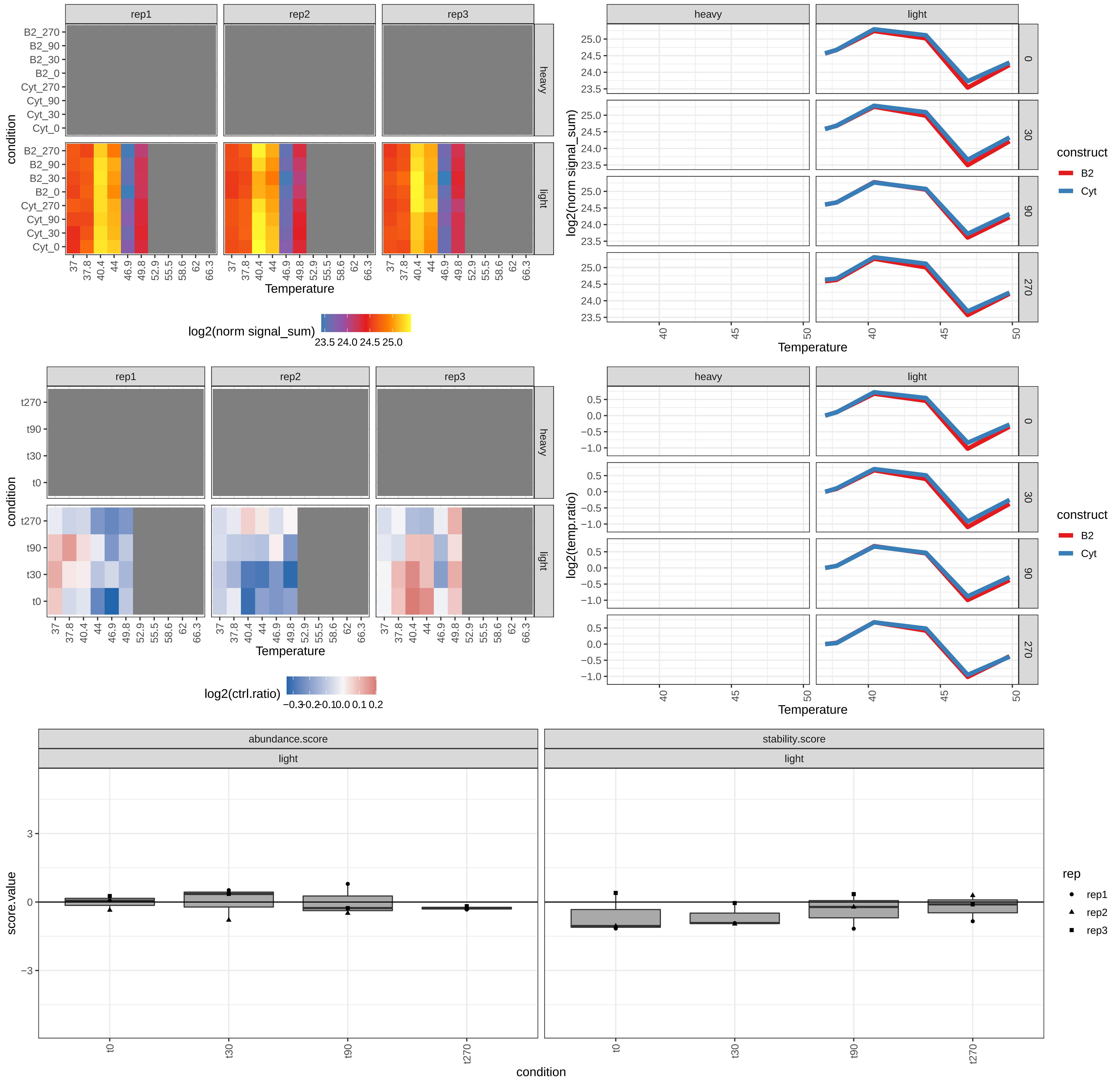

P33441 − THO complex subunit MFT1 OS=Saccharomyces cerevisiae (strain ATCC 204508 / S288c) GN=MFT1 PE=1 SV=1

MGE1 − average.top3: 6.84197652173913 − max.qupm: 5

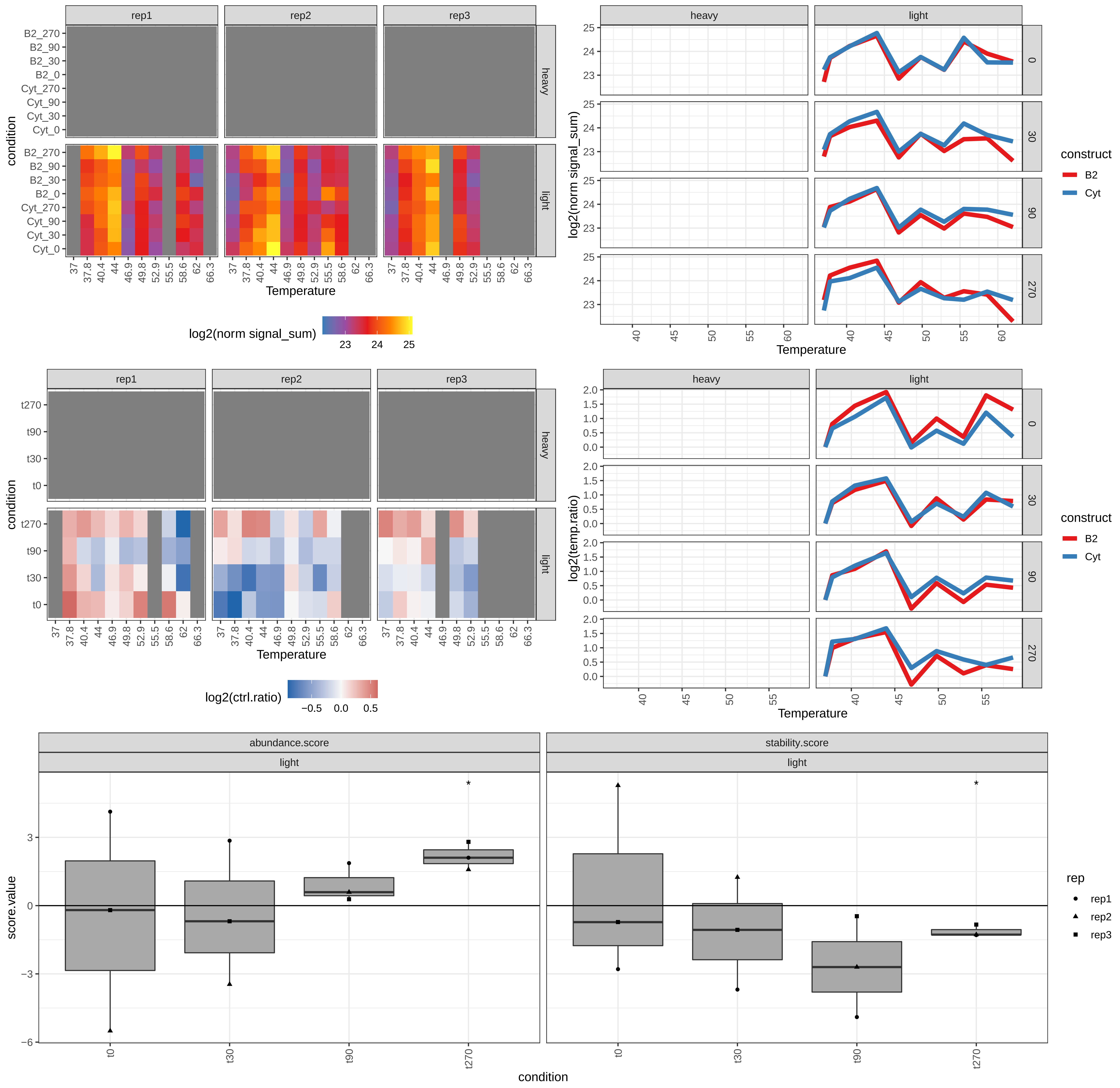

P38523 − GrpE protein homolog, mitochondrial OS=Saccharomyces cerevisiae (strain ATCC 204508 / S288c) GN=MGE1 PE=1 SV=1

MGM1 − average.top3: 6.74171764705882 − max.qupm: 9

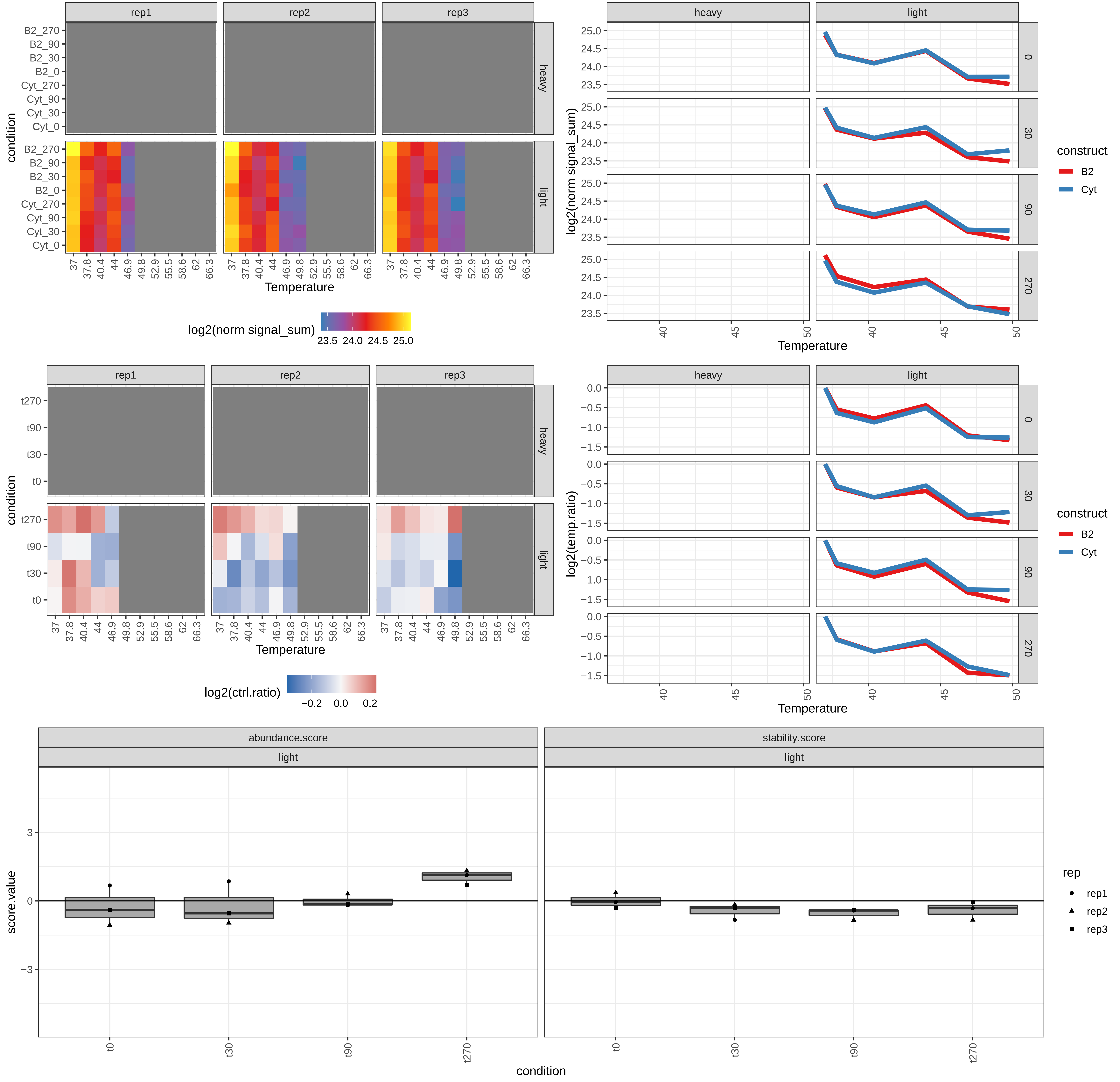

P32266 − Dynamin−like GTPase MGM1, mitochondrial OS=Saccharomyces cerevisiae (strain ATCC 204508 / S288c) GN=MGM1 PE=1 SV=3

MGM101 − average.top3: 6.99584071428571 − max.qupm: 9

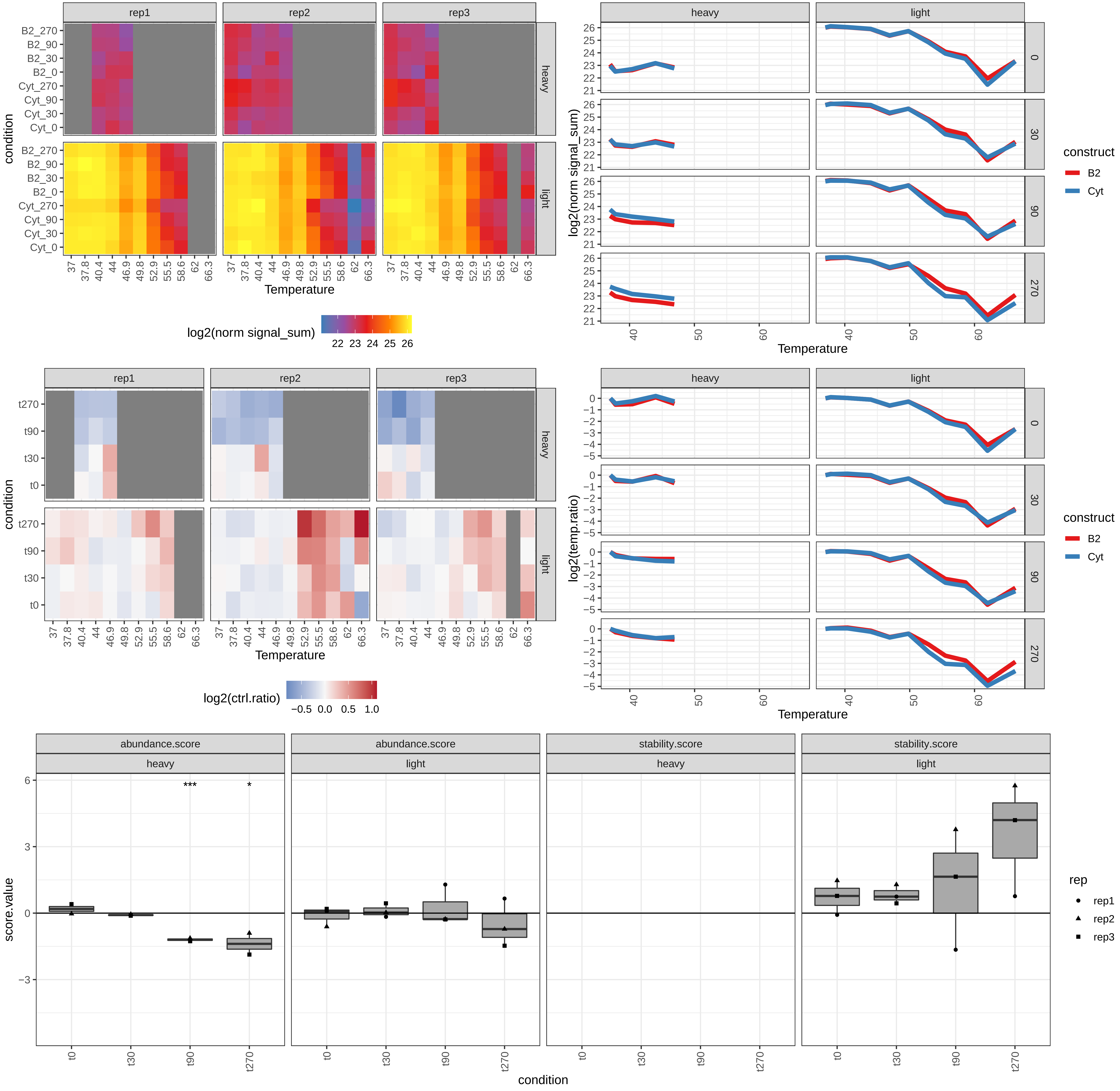

P32787 − Mitochondrial genome maintenance protein MGM101 OS=Saccharomyces cerevisiae (strain ATCC 204508 / S288c) GN=MGM101 PE=1 SV=1

MGR1 − average.top3: 6.6851 − max.qupm: 4

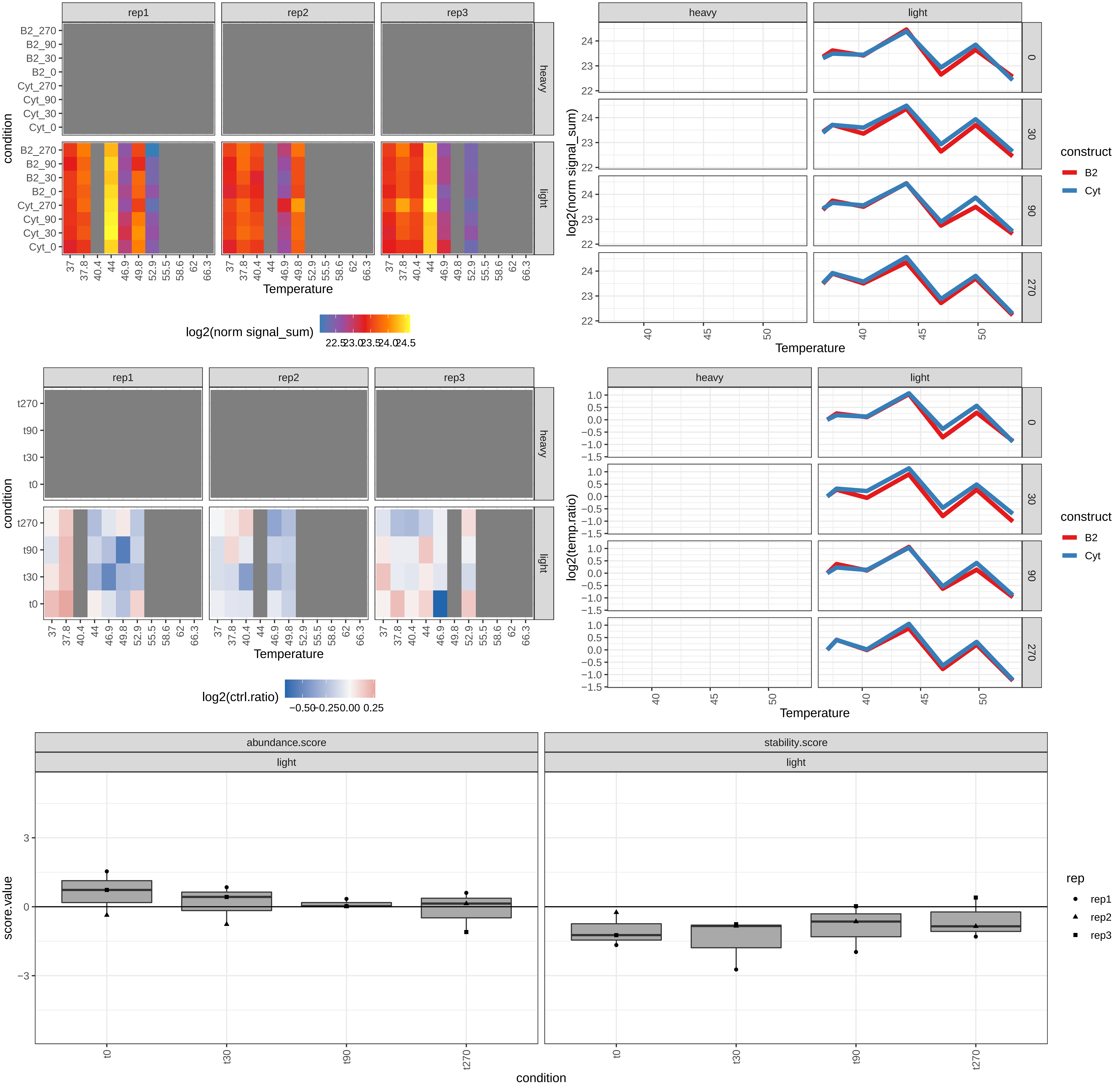

P25573 − Mitochondrial inner membrane i−AAA protease supercomplex subunit MGR1 OS=Saccharomyces cerevisiae (strain ATCC 204508 / S288c) GN=MGR1 PE=1 SV=1

MGR3 − average.top3: 6.94093952380952 − max.qupm: 8

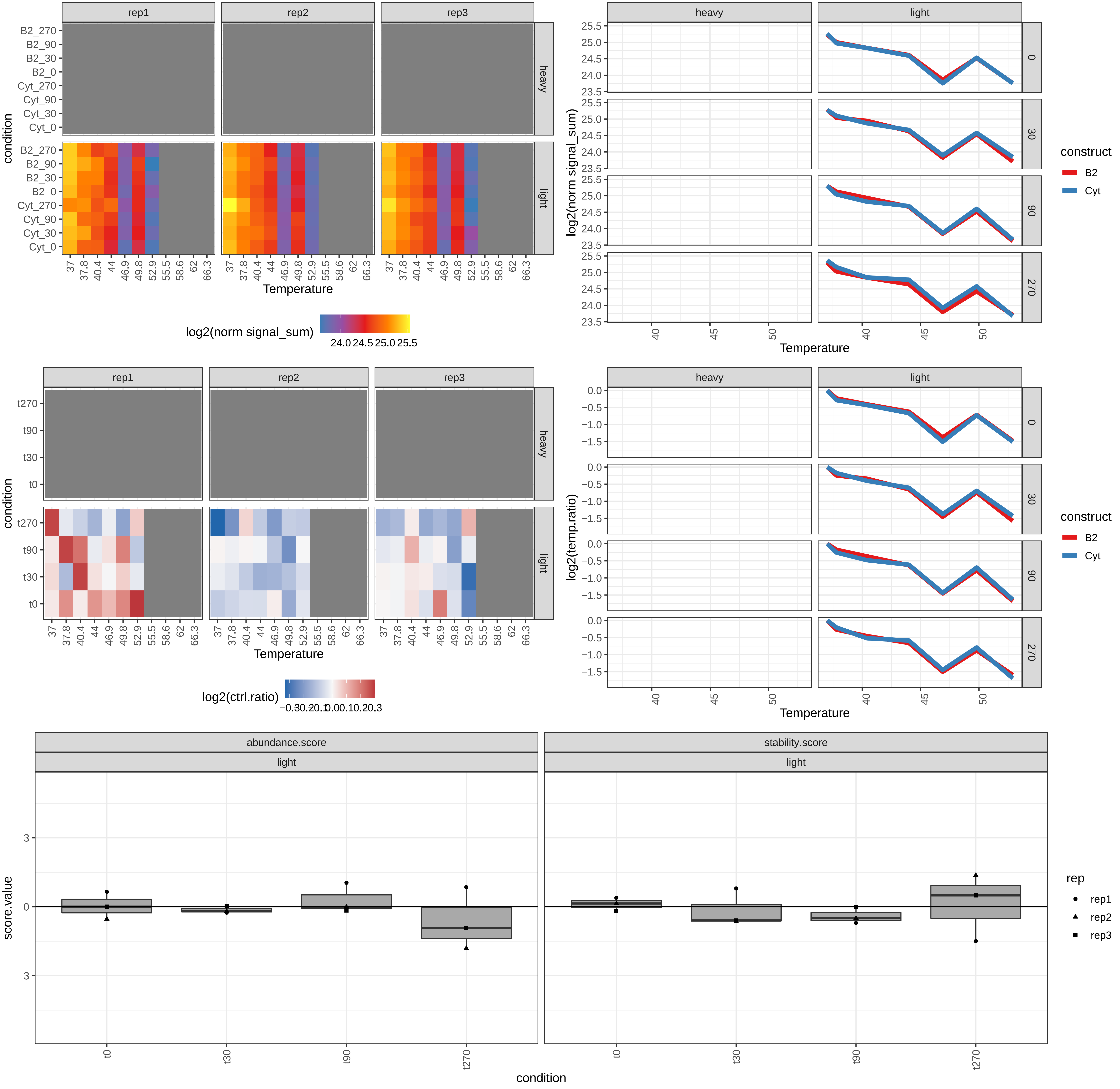

Q04472 − Mitochondrial inner membrane i−AAA protease supercomplex subunit MGR3 OS=Saccharomyces cerevisiae (strain ATCC 204508 / S288c) GN=MGR3 PE=1 SV=1

MHP1 − average.top3: 6.46078275862069 − max.qupm: 12

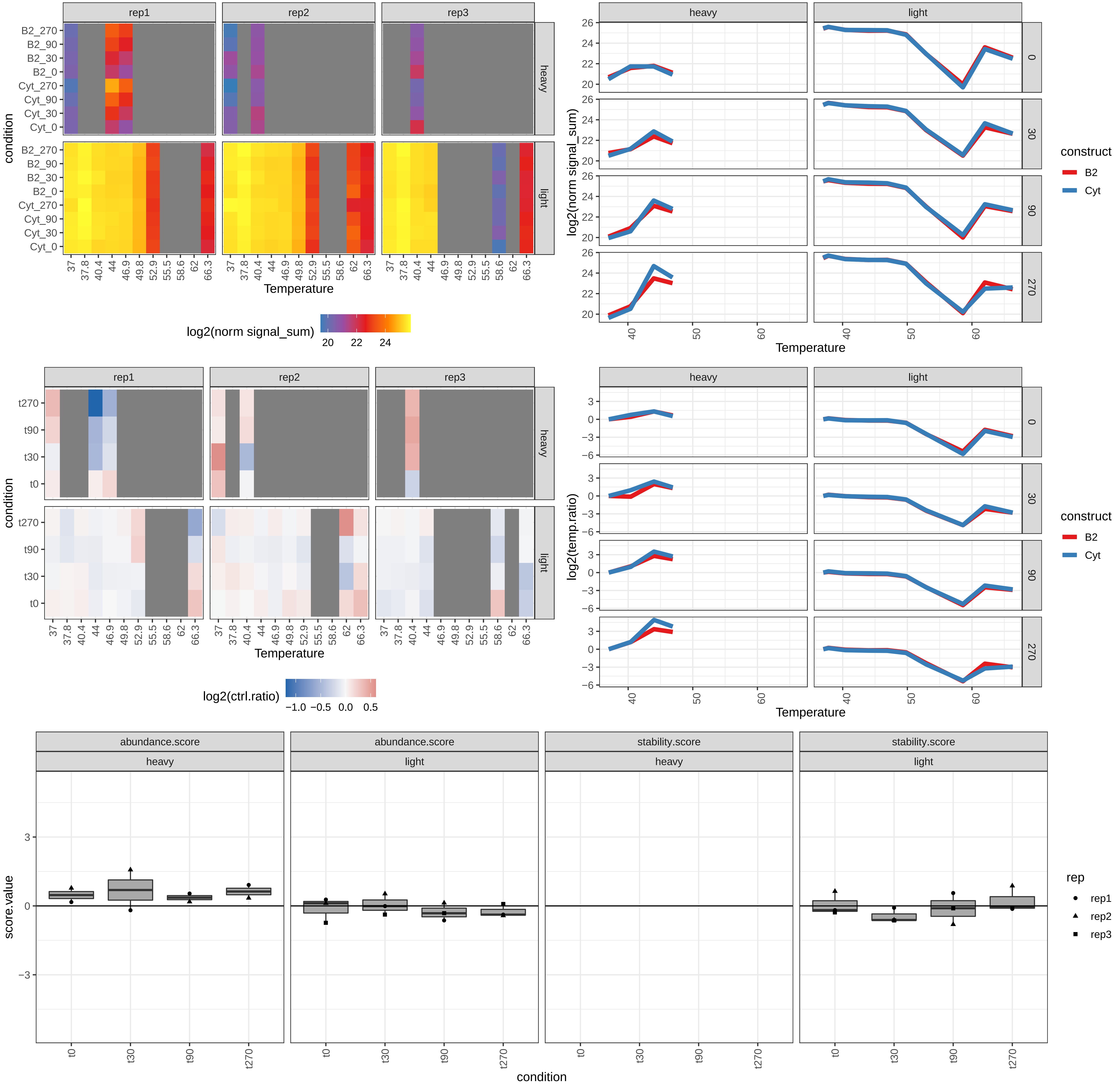

P43638 − MAP−homologous protein 1 OS=Saccharomyces cerevisiae (strain ATCC 204508 / S288c) GN=MHP1 PE=1 SV=1

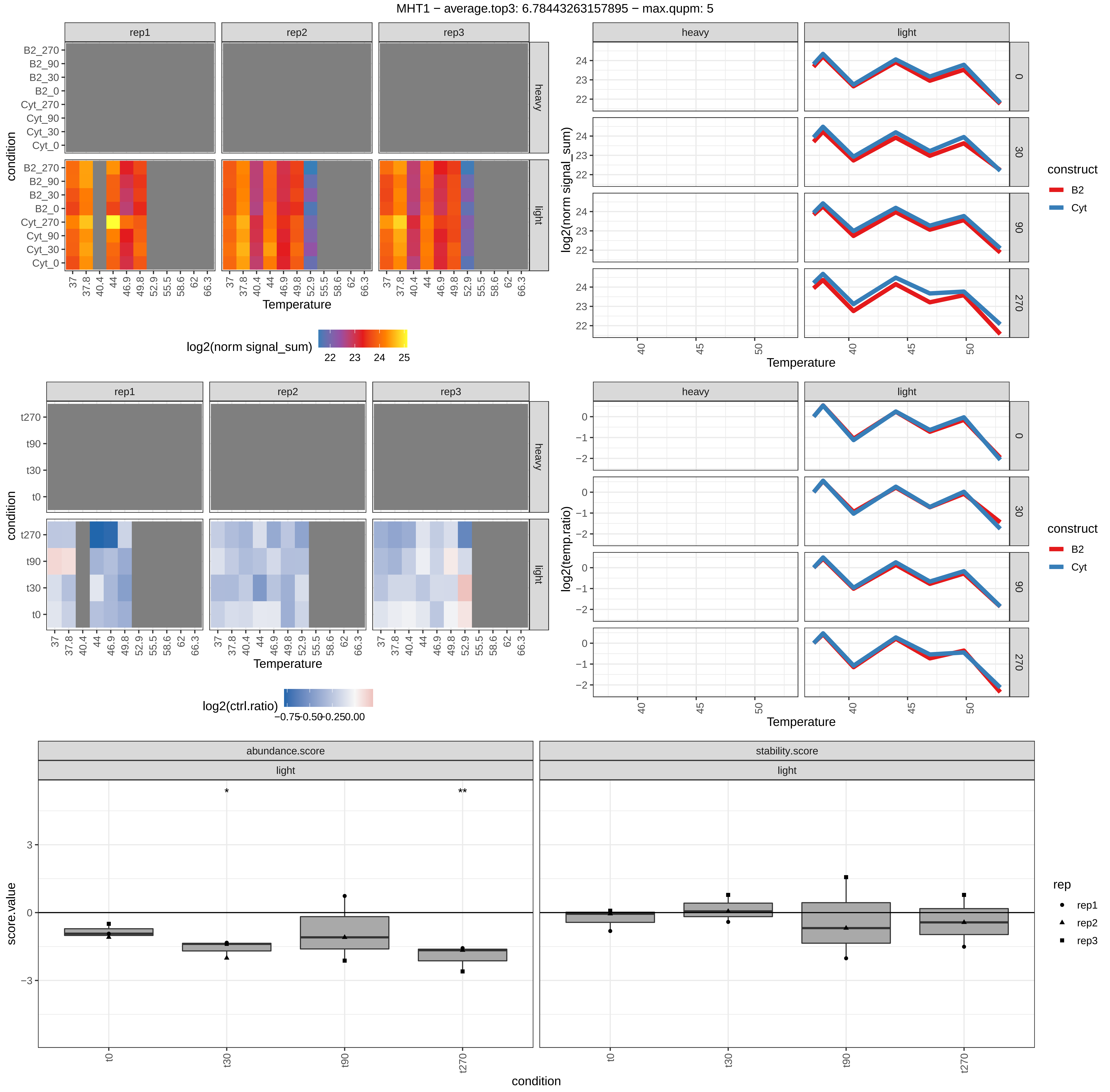

Q12525 − Homocysteine S−methyltransferase 1 OS=Saccharomyces cerevisiae (strain ATCC 204508 / S288c) GN=MHT1 PE=1 SV=1

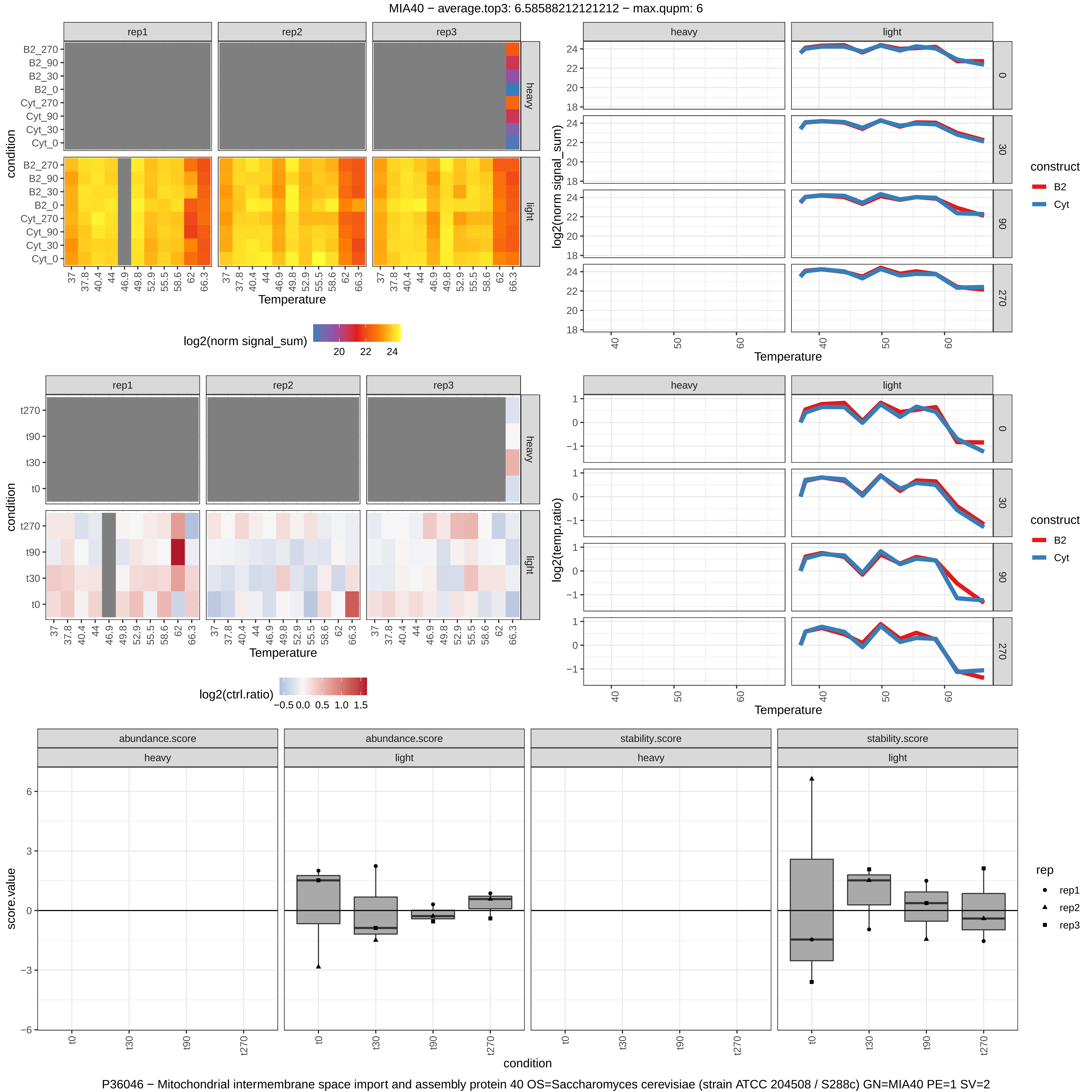
MIA40 − average.top3: 6.58588212121212 − max.qupm: 6
rep1
rep2
rep3
heavy
light
condition
Temperature
log2(norm signal_sum)
log2(temp.ratio)
log2(ctrl.ratio)
construct
B2
Cyt
abundance.score
stability.score
score.value
rep
rep1
rep2
rep3
P36046 − Mitochondrial intermembrane space import and assembly protein 40 OS=Saccharomyces cerevisiae (strain ATCC 204508 / S288c) GN=MIA40 PE=1 SV=2

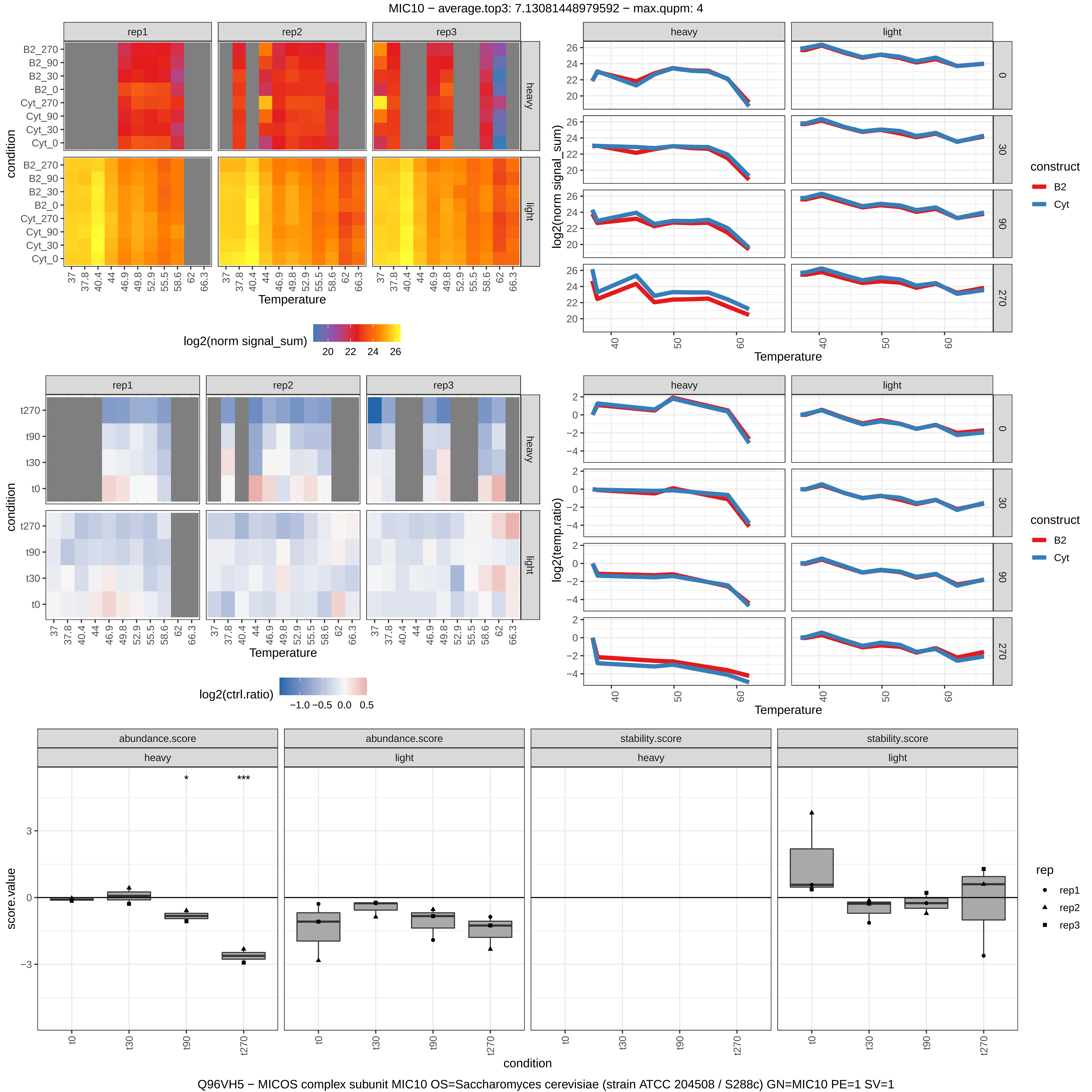
MIC10 − average.top3: 7.13081448979592 − max.qupm: 4
rep1
rep2
rep3
heavy
light
condition
B2_270
B2_90
B2_30
B2_0
Cyt_270
Cyt_90
Cyt_30
Cyt_0
Temperature
log2(norm signal_sum)
20 22 24 26
log2(norm signal_sum)
construct
B2
Cyt
0
30
90
270
t270
t90
t30
t0
log2(ctrl.ratio)
−1.0 −0.5 0.0 0.5
log2(temp.ratio)
abundance.score
stability.score
score.value
rep
rep1
rep2
rep3
*
***
condition
Q96VH5 − MICOS complex subunit MIC10 OS=Saccharomyces cerevisiae (strain ATCC 204508 / S288c) GN=MIC10 PE=1 SV=1

MIC12 − average.top3: 7.06147863636364 − max.qupm: 6

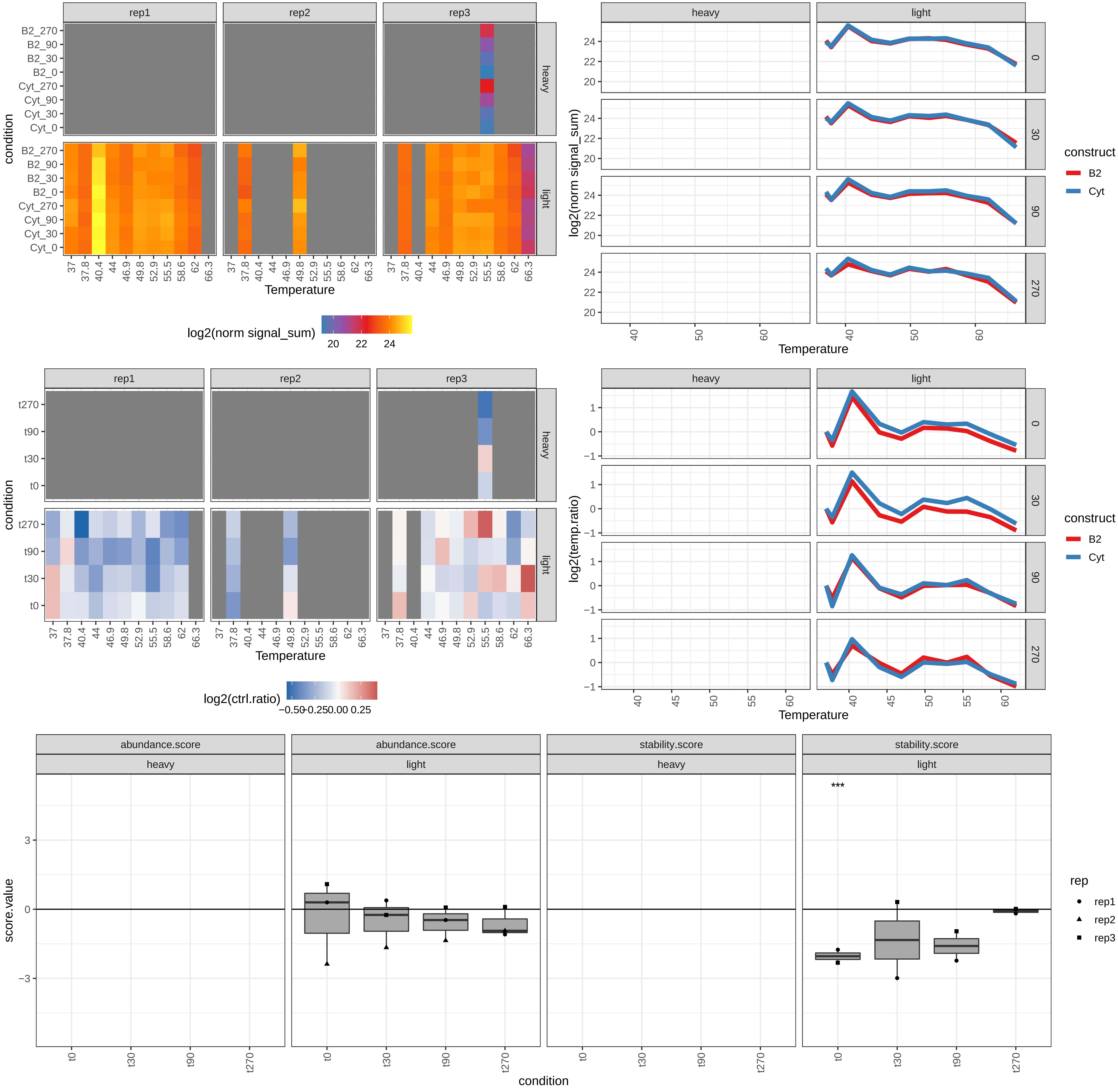

P38341 − MICOS complex subunit MIC12 OS=Saccharomyces cerevisiae (strain ATCC 204508 / S288c) GN=MIC12 PE=1 SV=1

MIC26 − average.top3: 6.68794111111111 − max.qupm: 3

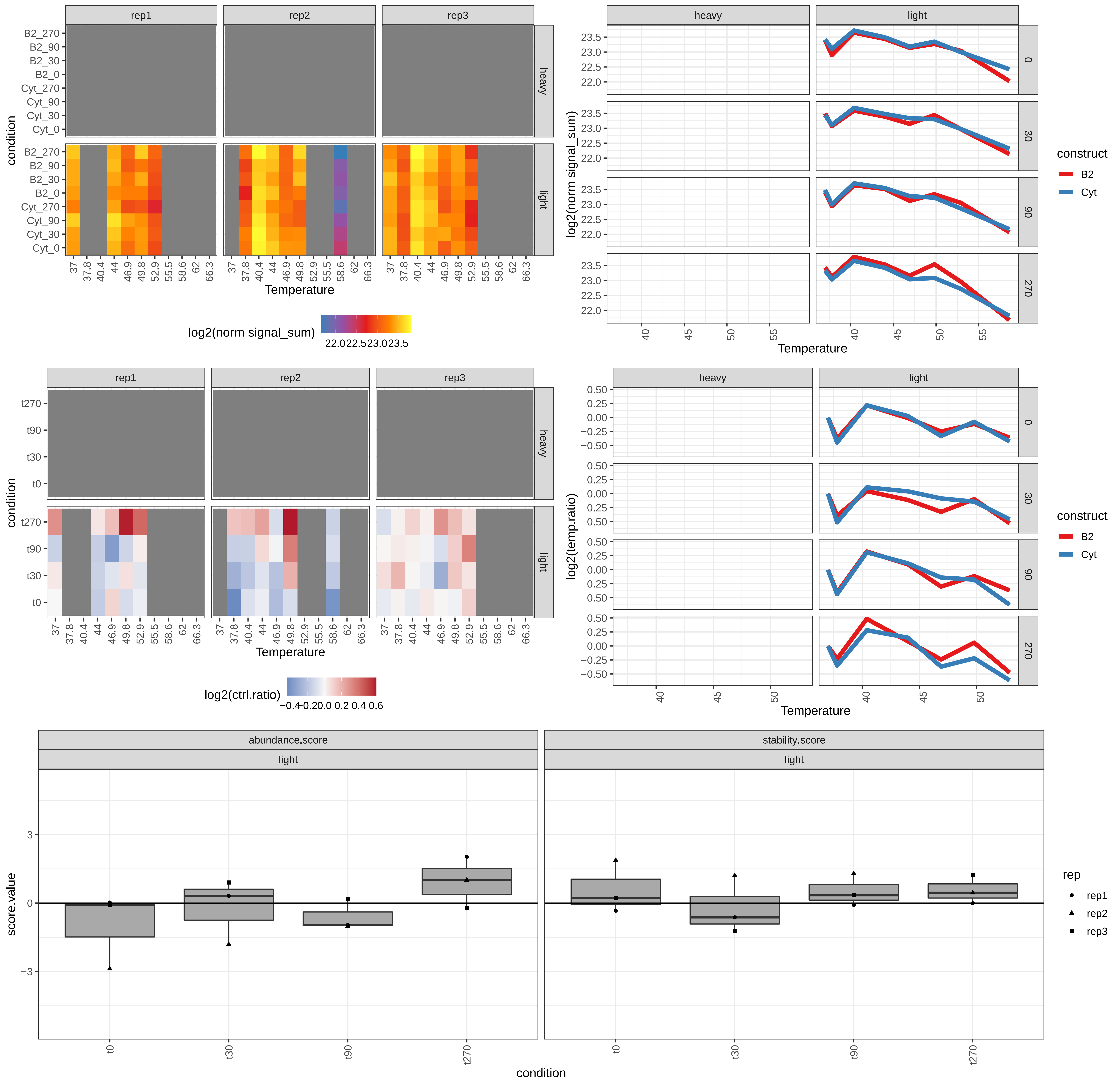

P50087 − MICOS subunit MIC26 OS=Saccharomyces cerevisiae (strain ATCC 204508 / S288c) GN=MIC26 PE=1 SV=1

MIC27 − average.top3: 7.1078065625 − max.qupm: 6

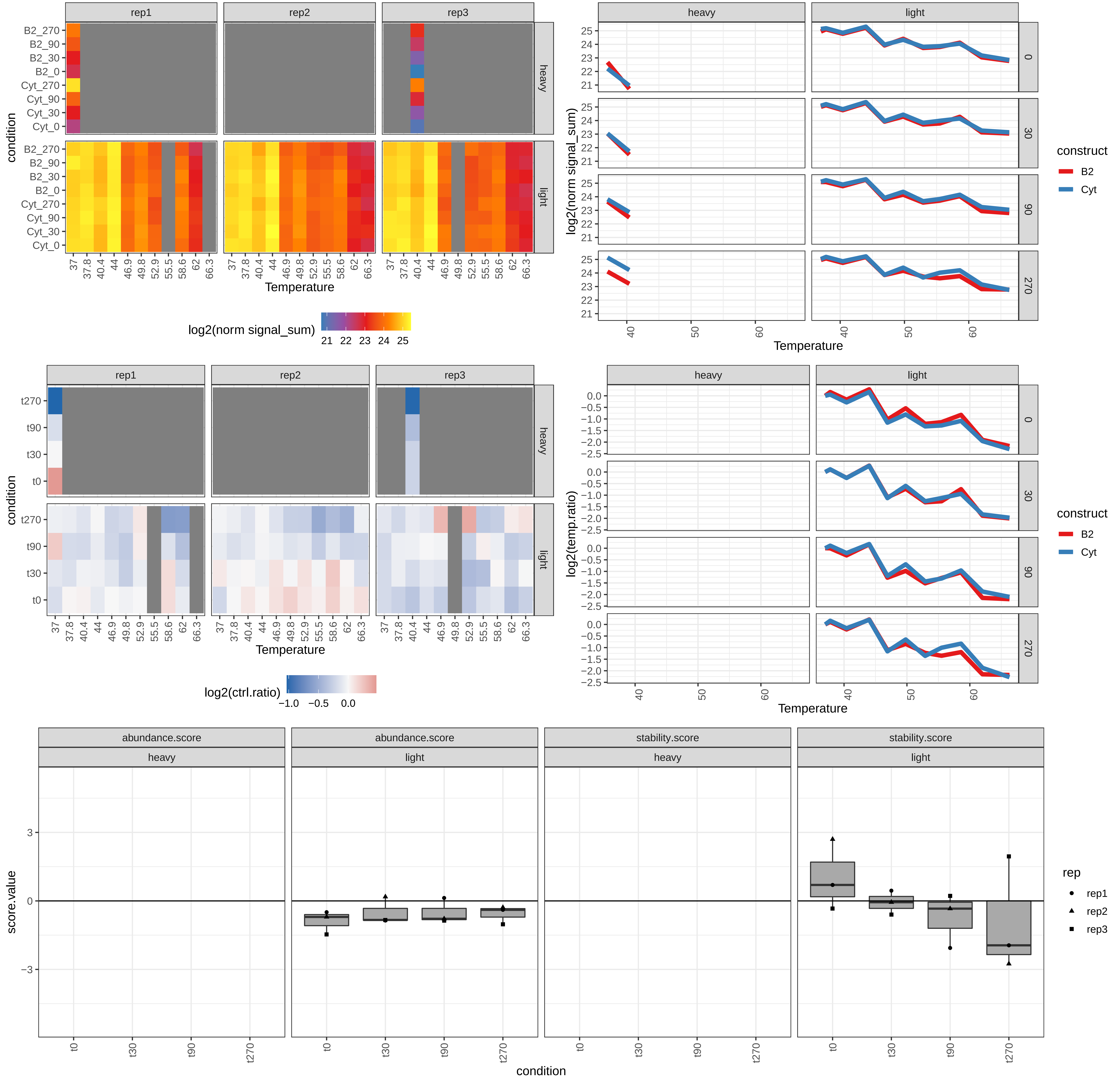

P50945 − MICOS complex subunit MIC27 OS=Saccharomyces cerevisiae (strain ATCC 204508 / S288c) GN=MIC27 PE=1 SV=1

MIC60 − average.top3: 6.536434 − max.qupm: 9

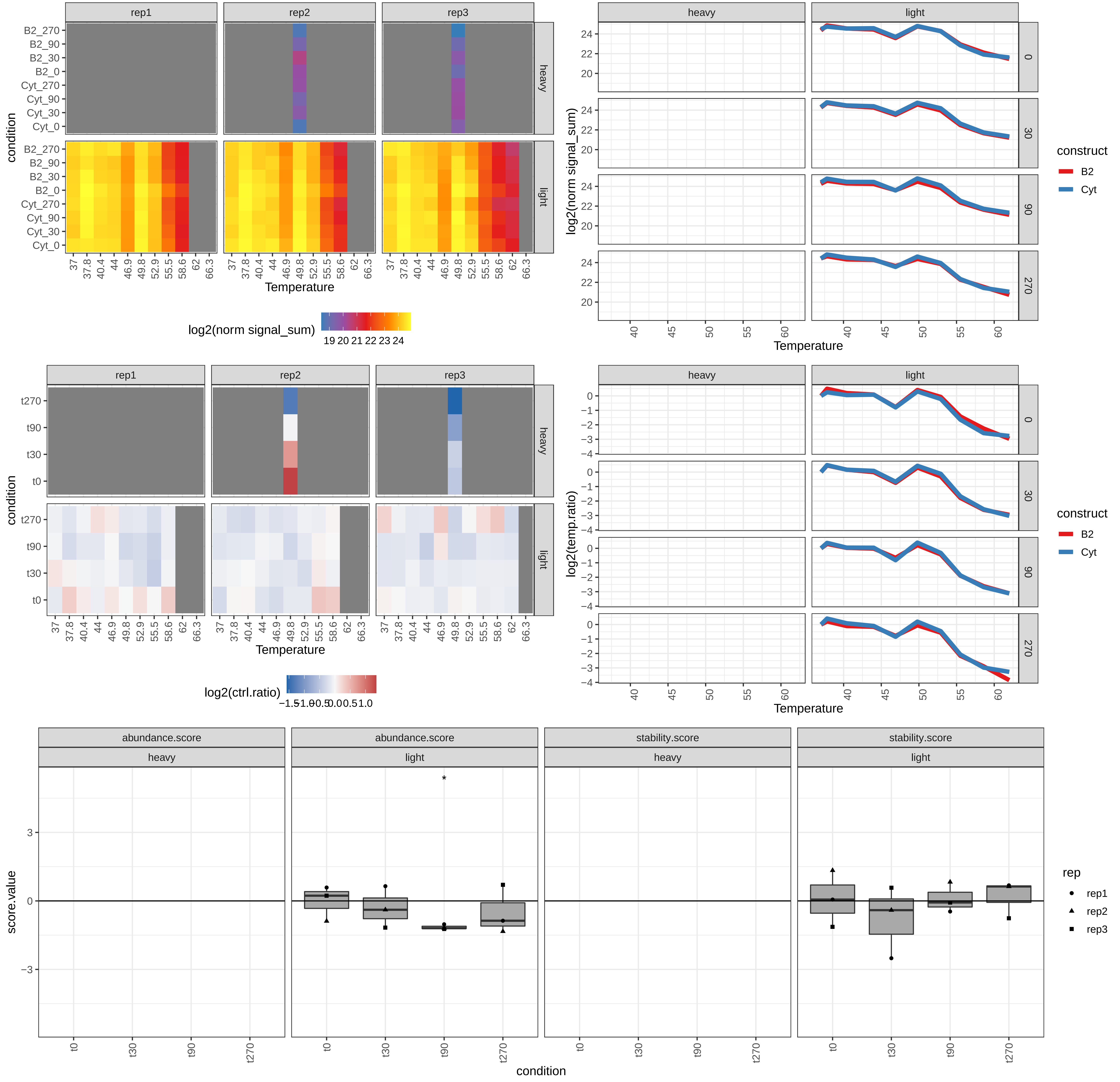

P36112 − MICOS complex subunit MIC60 OS=Saccharomyces cerevisiae (strain ATCC 204508 / S288c) GN=MIC60 PE=1 SV=1

MIR1 − average.top3: 7.614038 − max.qupm: 10

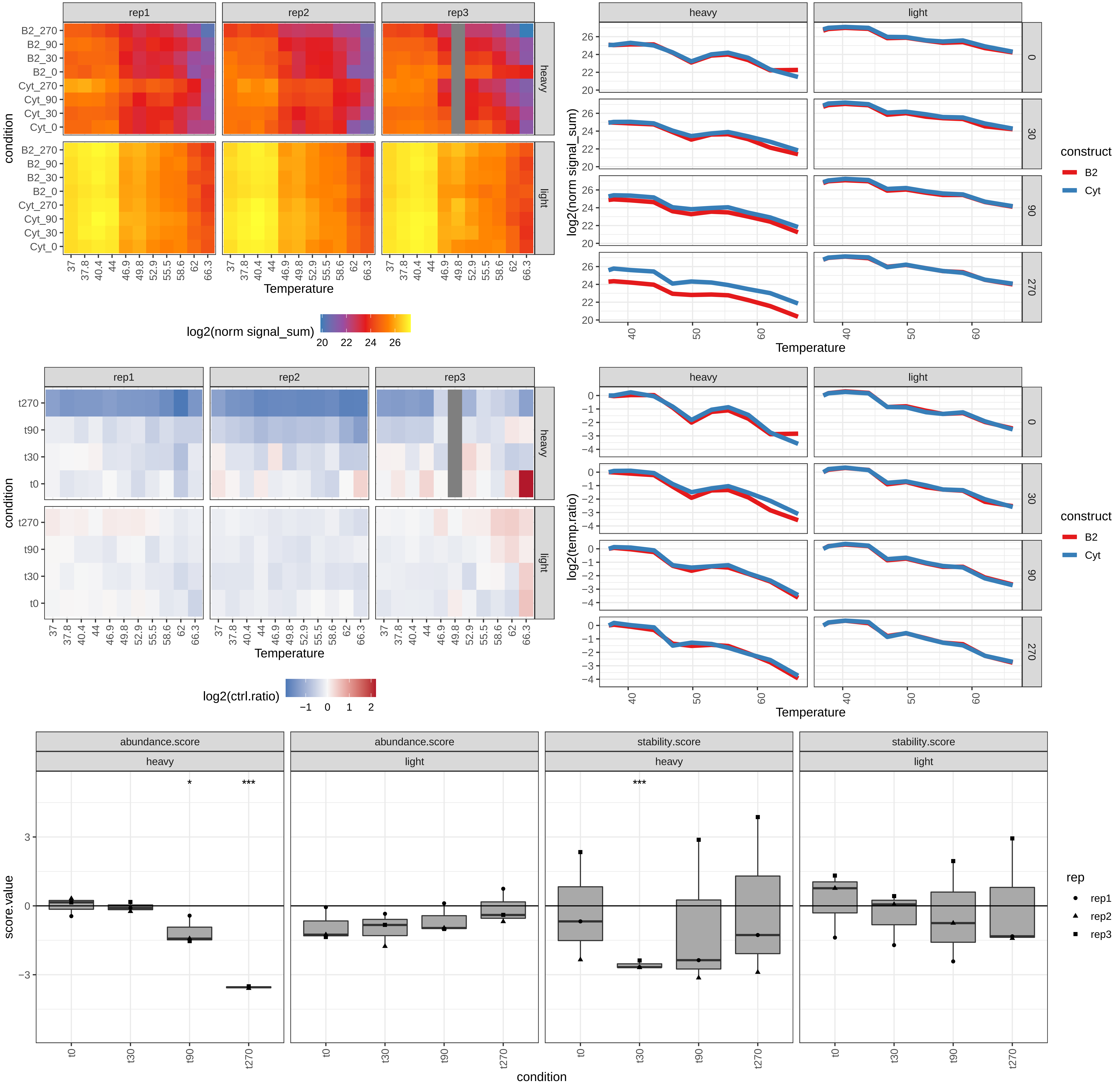

P23641 − Mitochondrial phosphate carrier protein OS=Saccharomyces cerevisiae (strain ATCC 204508 / S288c) GN=MIR1 PE=1 SV=1

MIS1 − average.top3: 6.99100276595745 − max.qupm: 18

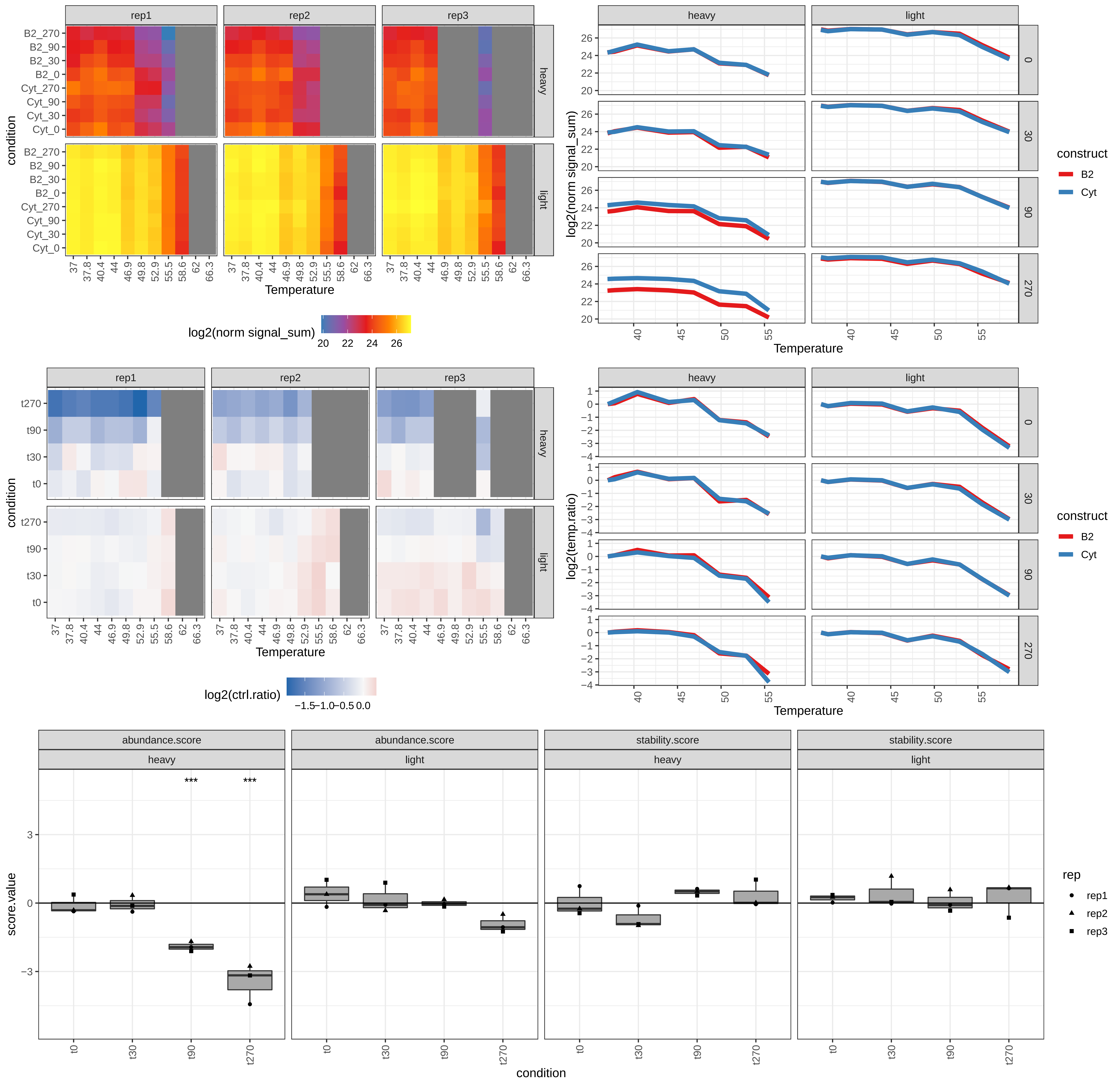

P09440 − C−1−tetrahydrofolate synthase, mitochondrial OS=Saccharomyces cerevisiae (strain ATCC 204508 / S288c) GN=MIS1 PE=1 SV=1

MIX23 − average.top3: 6.65602695652174 − max.qupm: 5

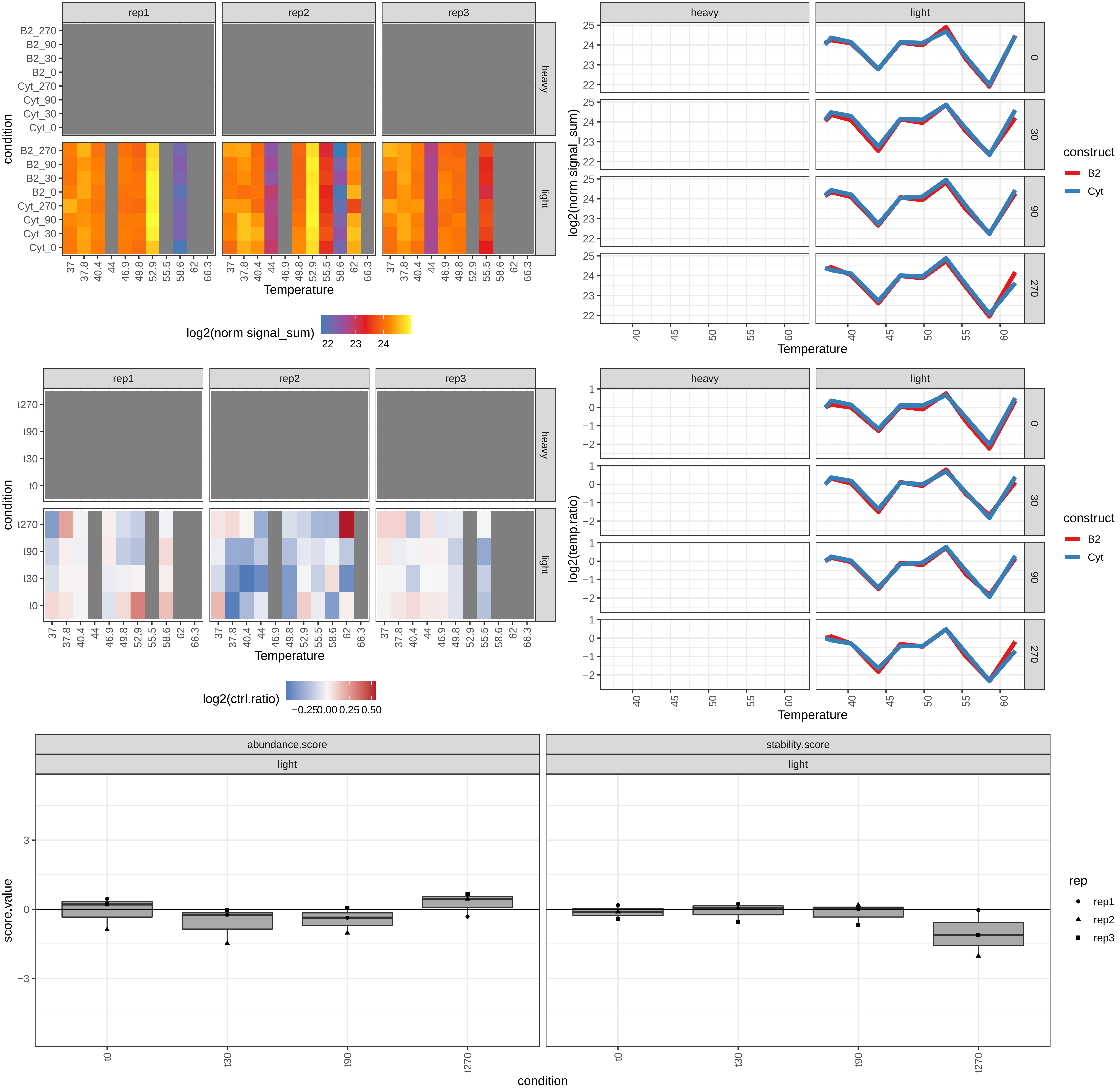

P38162 − Mitochondrial intermembrane space cysteine motif−containing protein MIX23 OS=Saccharomyces cerevisiae (strain ATCC 204508 / S288c) GN=MIX23 PE=1 SV=1

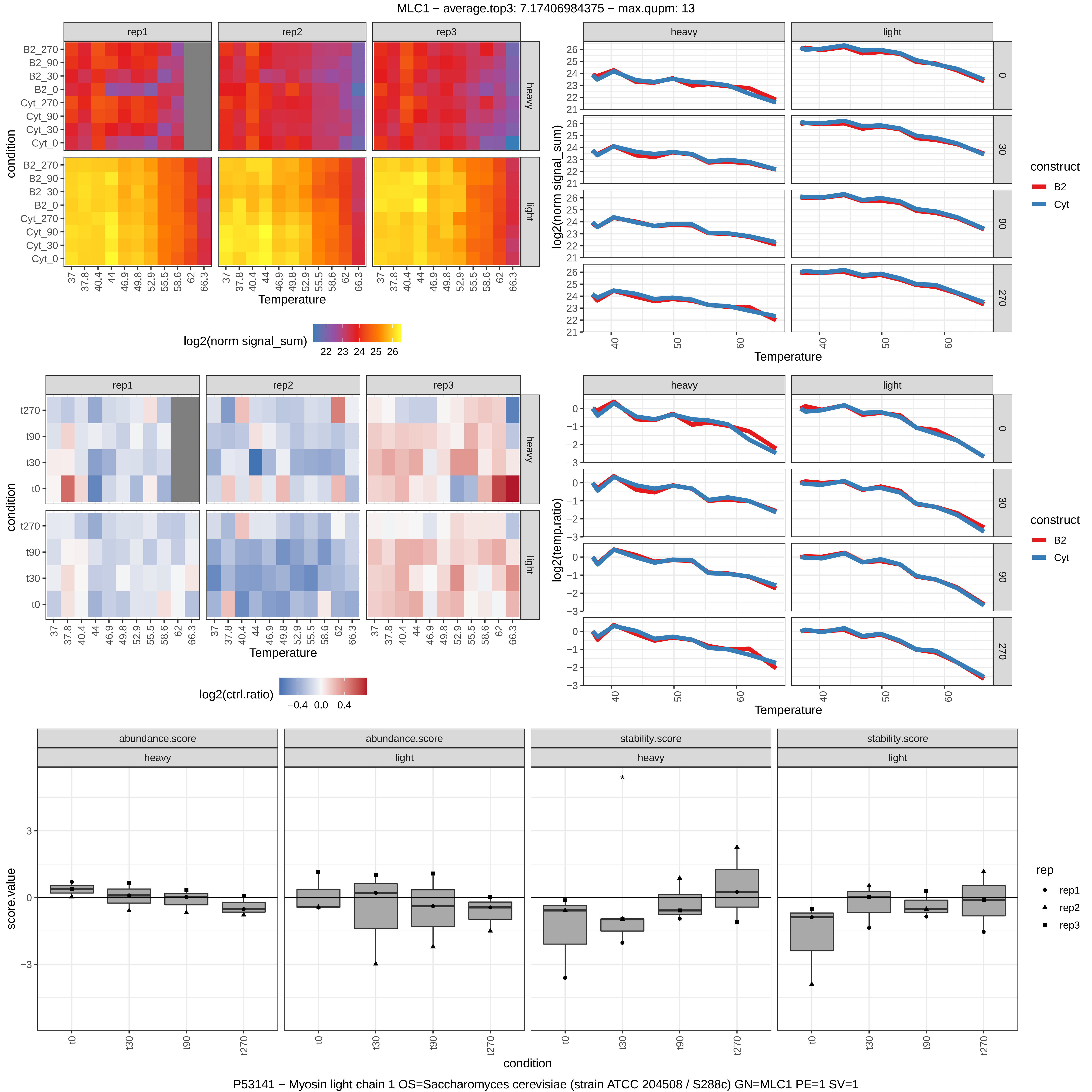
MLC1 − average.top3: 7.17406984375 − max.qupm: 13
rep1
rep2
rep3
heavy
light
B2_270
B2_90
B2_30
B2_0
Cyt_270
Cyt_90
Cyt_30
Cyt_0
condition
Temperature
log2(norm signal_sum)
22 23 24 25 26
log2(norm signal_sum)
construct
B2
Cyt
t270
t90
t30
t0
log2(ctrl.ratio)
−0.4 0.0 0.4
log2(temp.ratio)
abundance.score
stability.score
score.value
rep
rep1
rep2
rep3
condition
P53141 − Myosin light chain 1 OS=Saccharomyces cerevisiae (strain ATCC 204508 / S288c) GN=MLC1 PE=1 SV=1

MLP1 − average.top3: 6.667783125 − max.qupm: 19

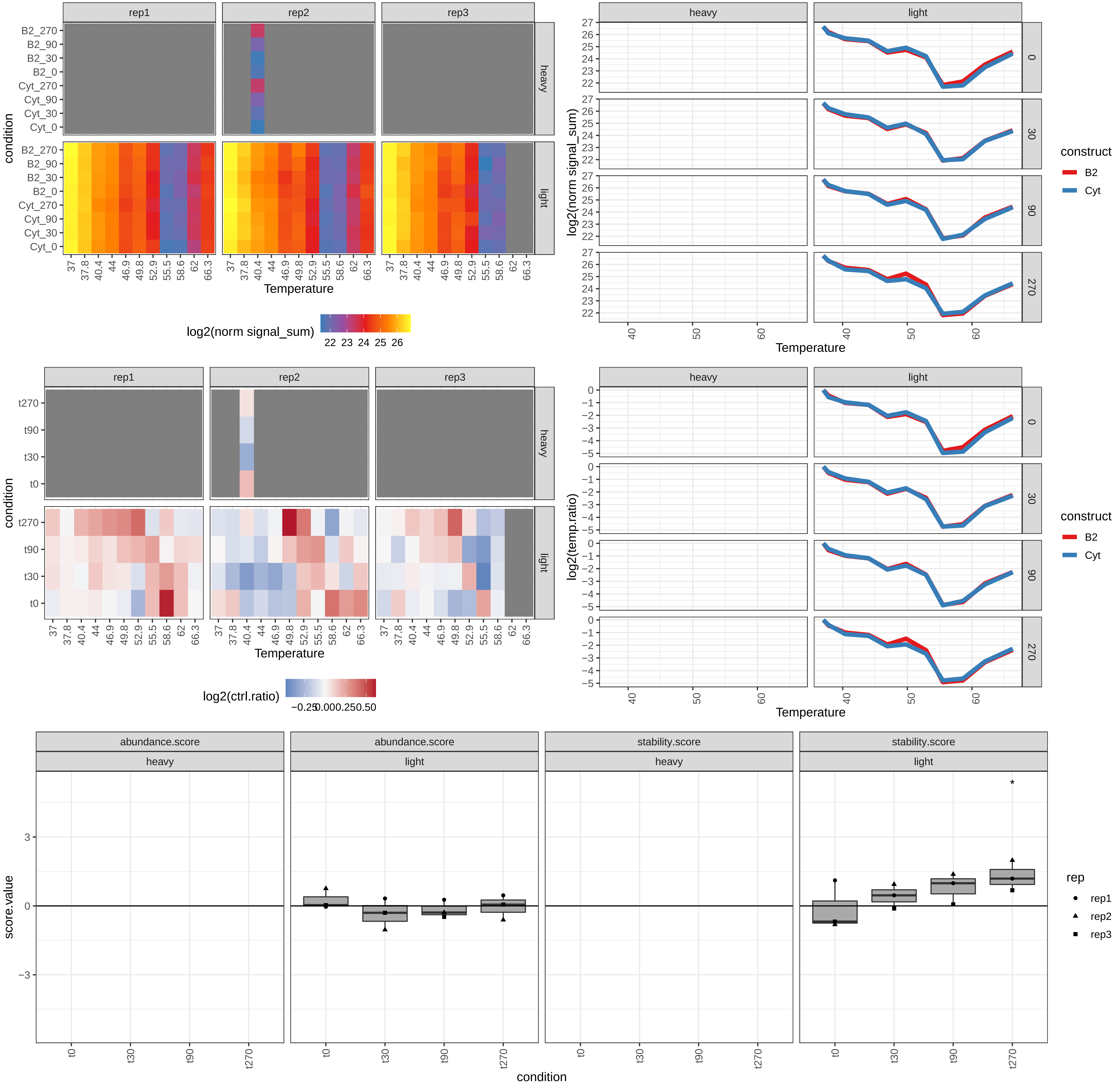

Q02455 − Protein MLP1 OS=Saccharomyces cerevisiae (strain ATCC 204508 / S288c) GN=MLP1 PE=1 SV=2

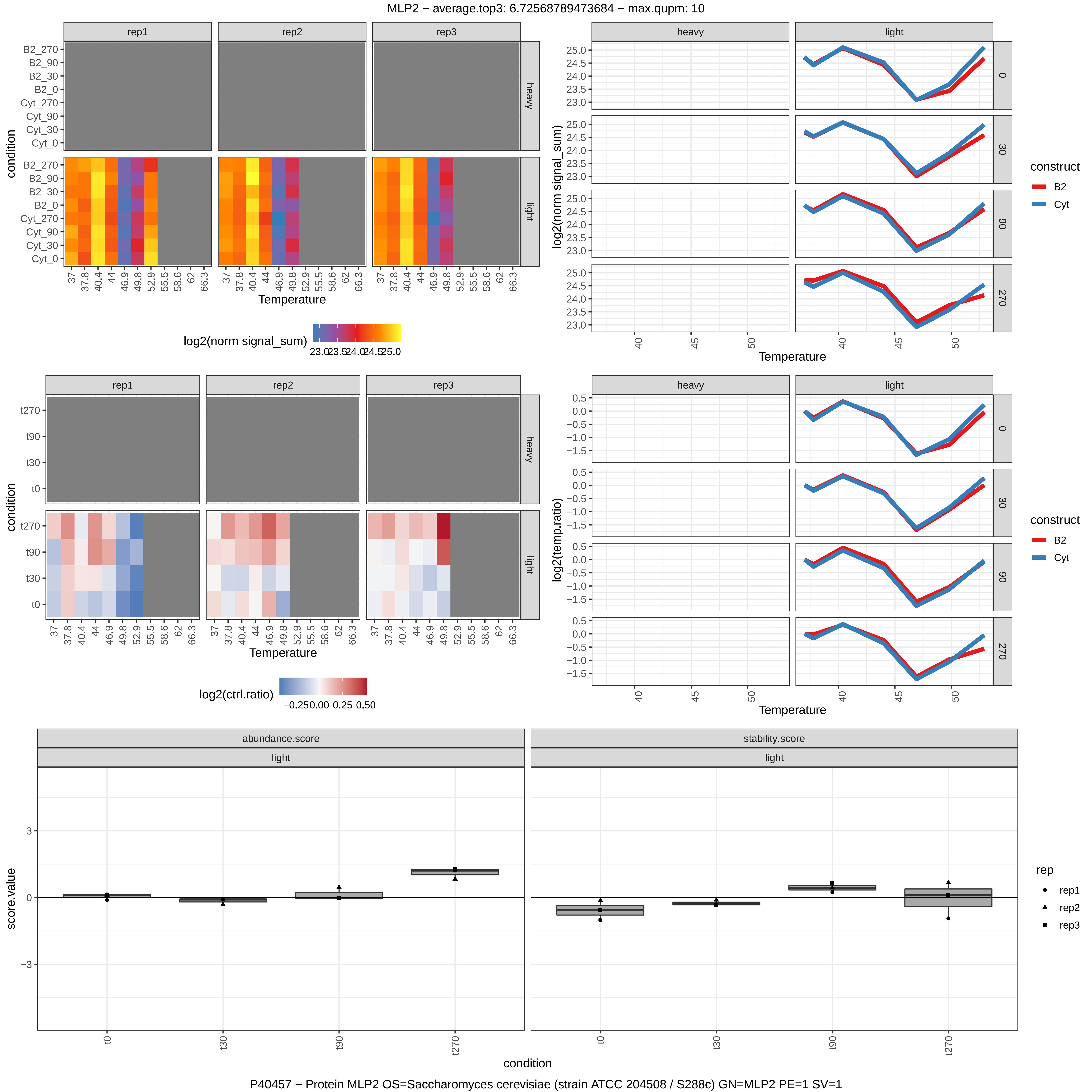

MLP2 − average.top3: 6.72568789473684 − max.qupm: 10
rep1
rep2
rep3
heavy
light
condition
Temperature
log2(norm signal_sum)
construct
B2
Cyt
log2(ctrl.ratio)
log2(temp.ratio)
abundance.score
stability.score
score.value
rep
rep1
rep2
rep3
P40457 − Protein MLP2 OS=Saccharomyces cerevisiae (strain ATCC 204508 / S288c) GN=MLP2 PE=1 SV=1

MLS1 − average.top3: 8.02489564516129 − max.qupm: 27

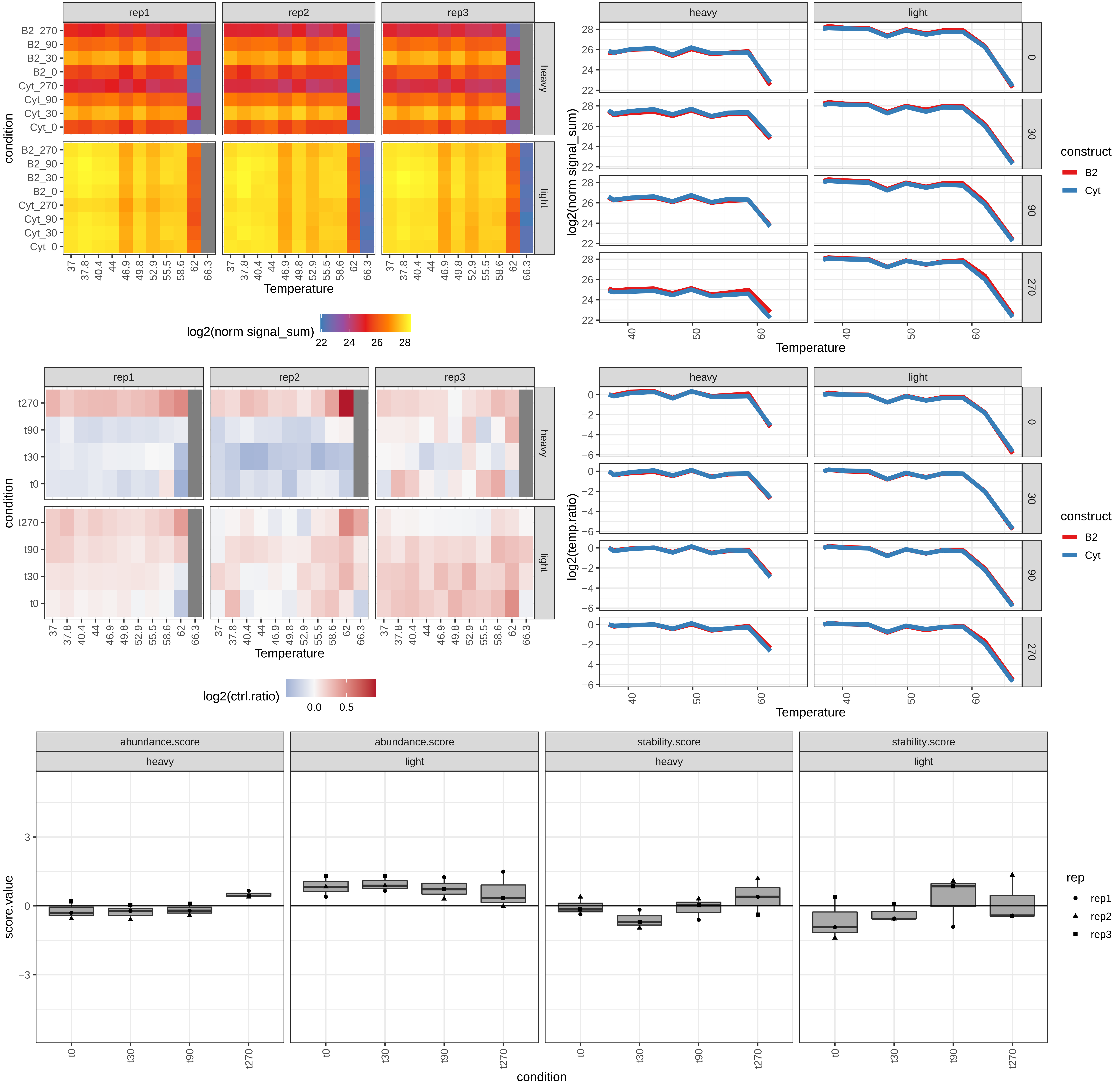

P30952 − Malate synthase 1, glyoxysomal OS=Saccharomyces cerevisiae (strain ATCC 204508 / S288c) GN=MLS1 PE=1 SV=1

MMF1 − average.top3: 7.14308126984127 − max.qupm: 5

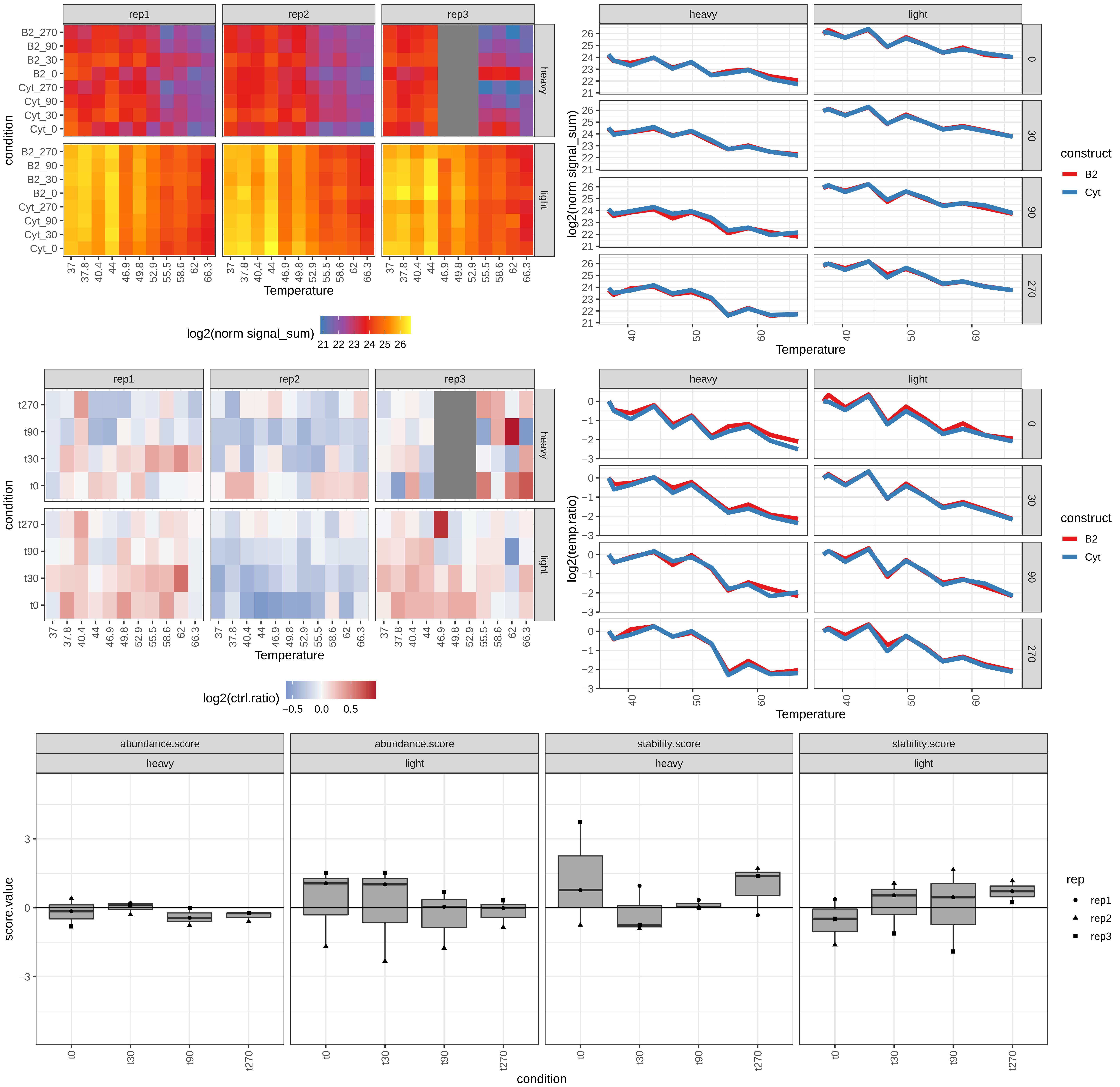

P40185 − Protein MMF1, mitochondrial OS=Saccharomyces cerevisiae (strain ATCC 204508 / S288c) GN=MMF1 PE=1 SV=1

MMM1 − average.top3: 6.273611 − max.qupm: 7

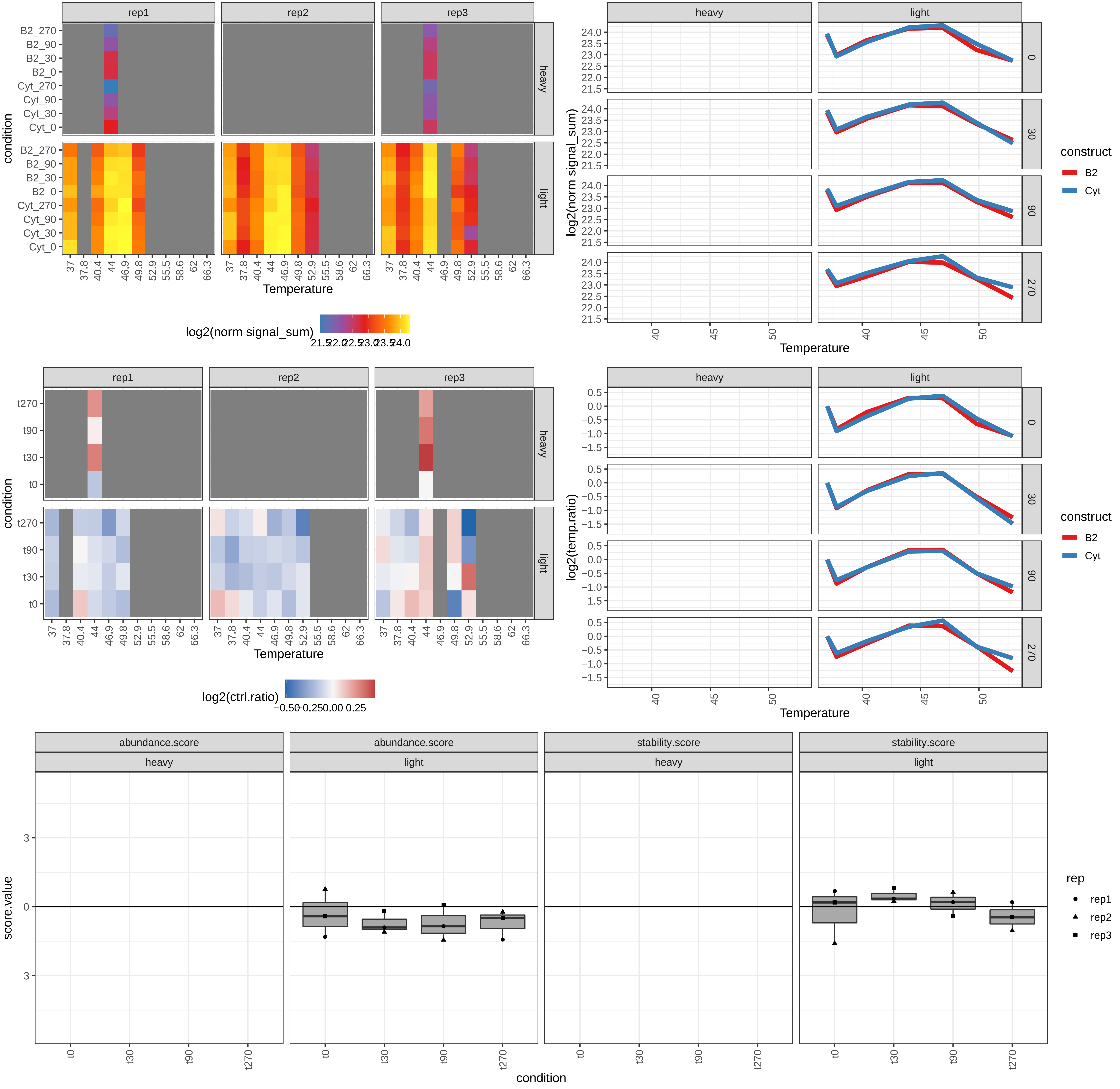

P41800 − Maintenance of mitochondrial morphology protein 1 OS=Saccharomyces cerevisiae (strain ATCC 204508 / S288c) GN=MMM1 PE=1 SV=2

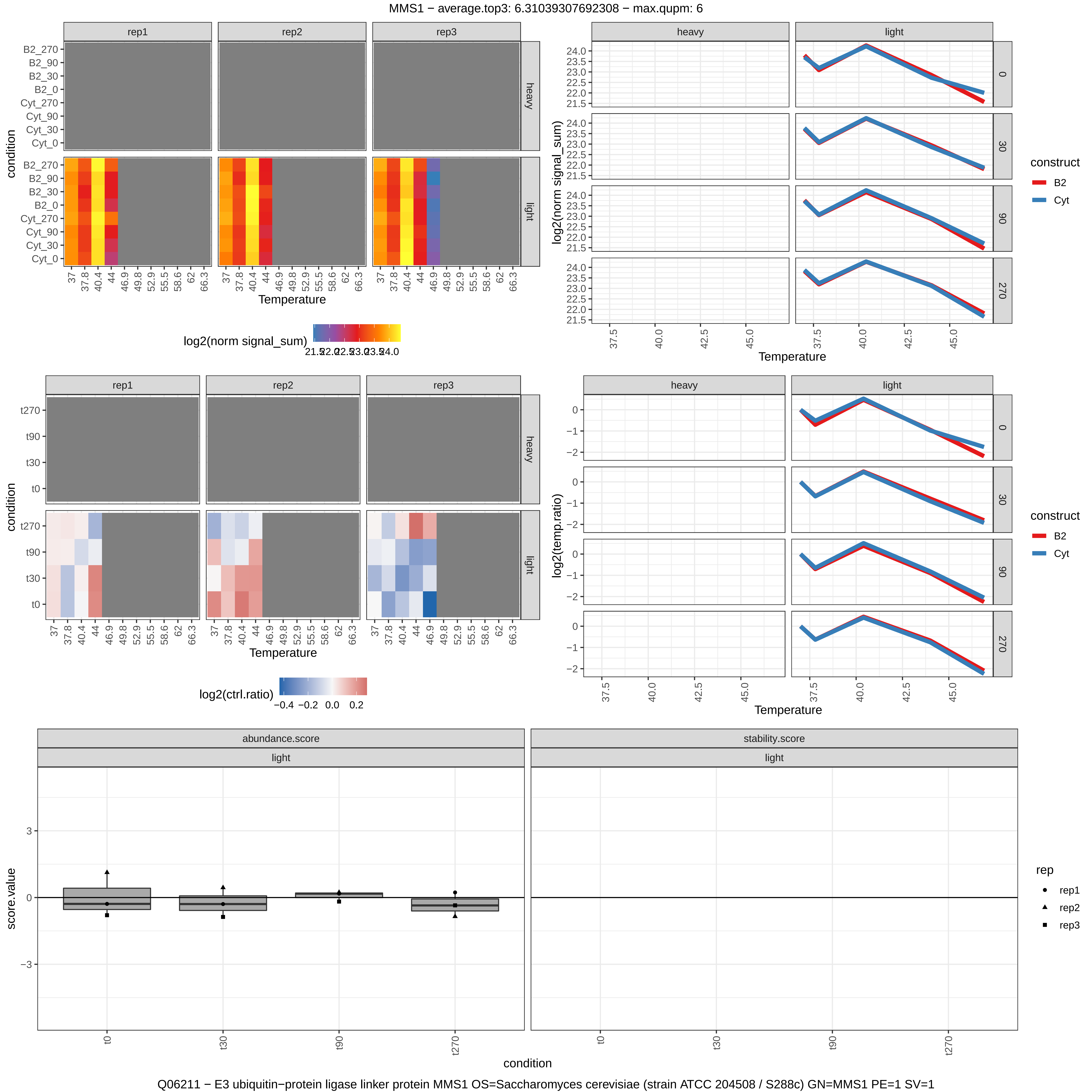
MMS1 − average.top3: 6.31039307692308 − max.qupm: 6
rep1
rep2
rep3
heavy
light
condition
Temperature
log2(norm signal_sum)
log2(ctrl.ratio)
log2(temp.ratio)
construct
B2
Cyt
abundance.score
stability.score
score.value
rep
rep1
rep2
rep3
Q06211 − E3 ubiquitin−protein ligase linker protein MMS1 OS=Saccharomyces cerevisiae (strain ATCC 204508 / S288c) GN=MMS1 PE=1 SV=1

MNE1 − average.top3: 5.840557 − max.qupm: 3

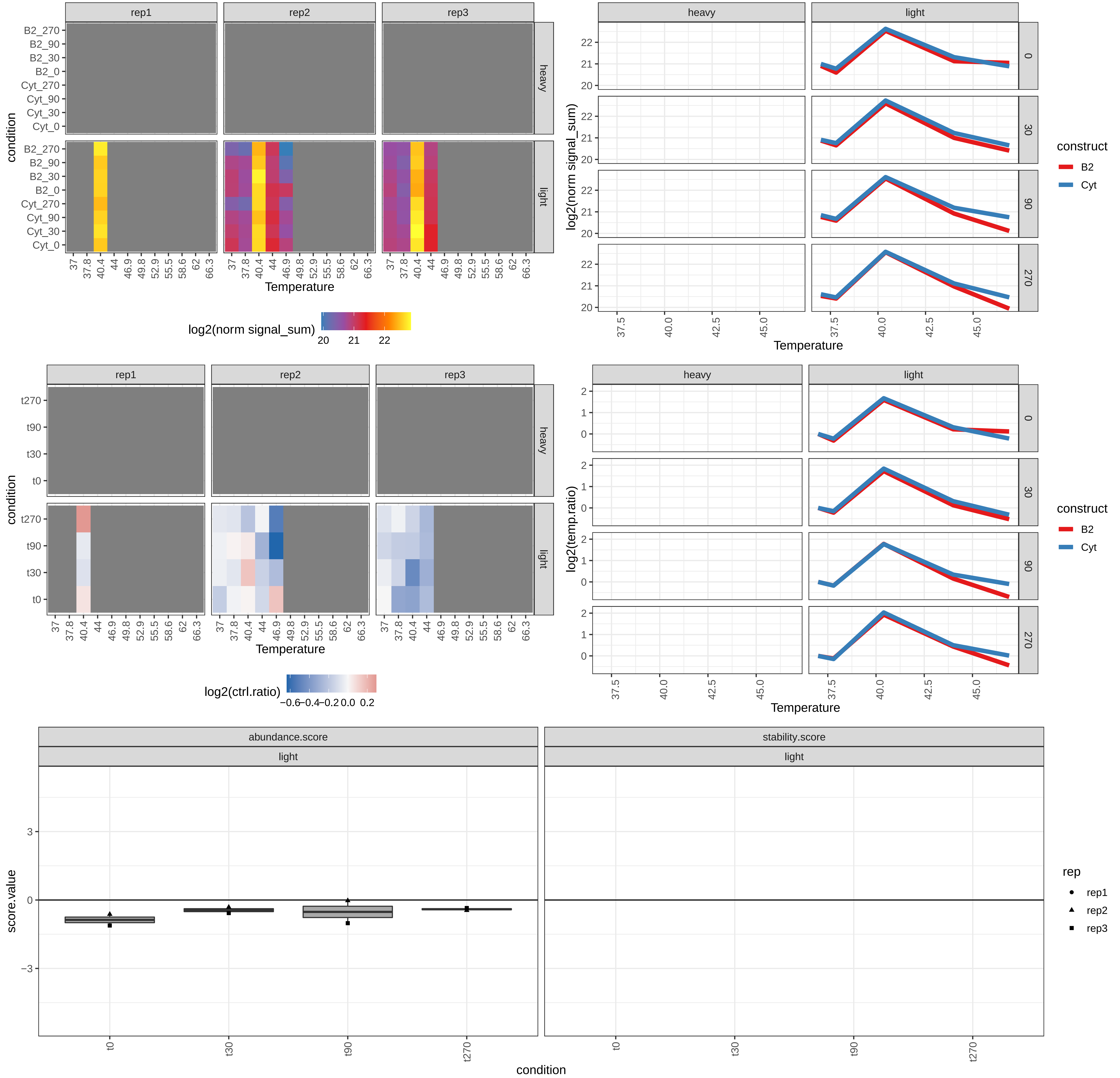

P24720 − Protein MNE1 OS=Saccharomyces cerevisiae (strain ATCC 204508 / S288c) GN=MNE1 PE=1 SV=2

MNL2 − average.top3: 6.896004 − max.qupm: 4

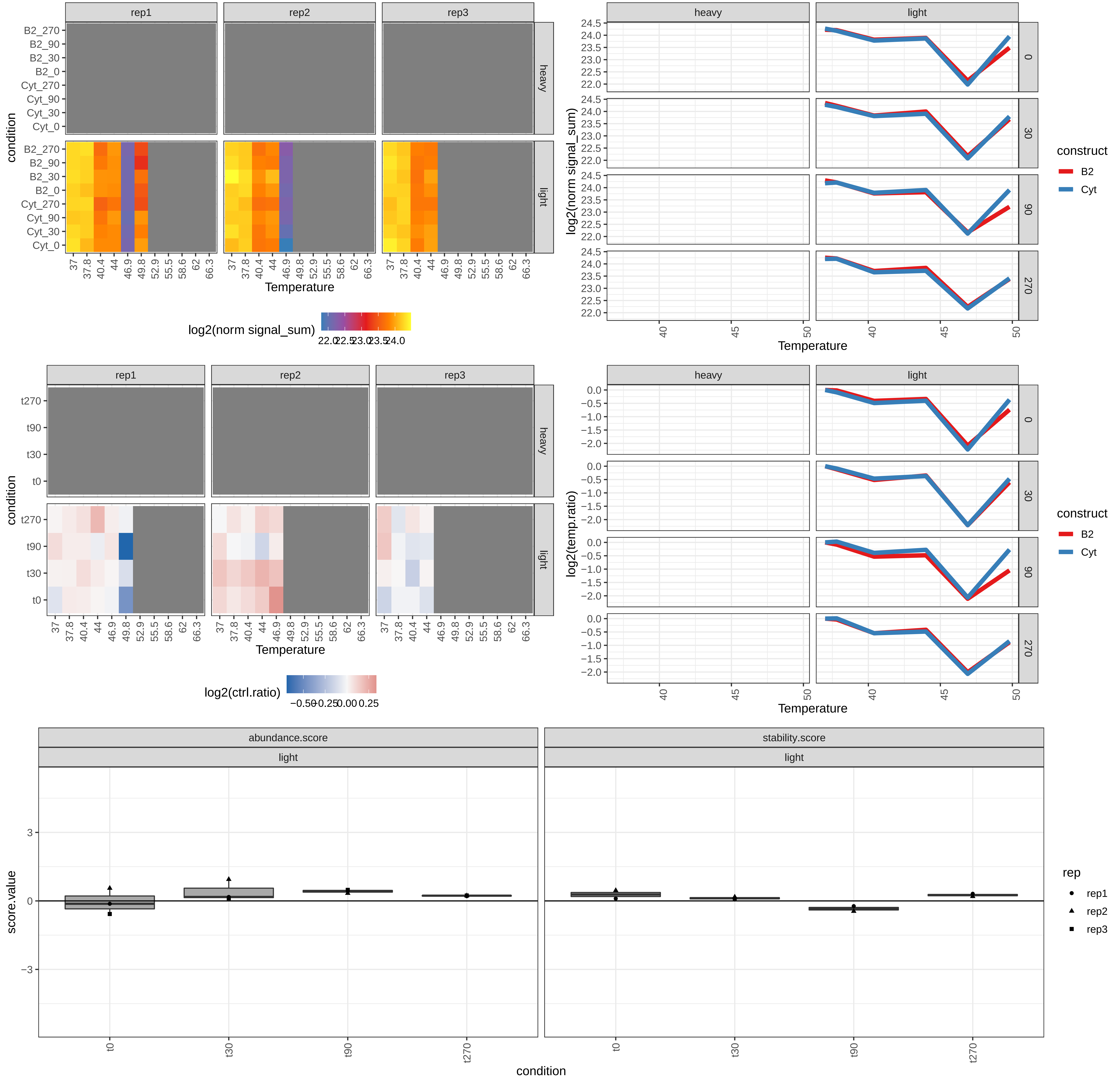

Q12205 − Putative endoplasmic reticulum mannosidase MNL2 OS=Saccharomyces cerevisiae (strain ATCC 204508 / S288c) GN=MNL2 PE=1 SV=1

MNN1 − average.top3: 6.50755125 − max.qupm: 7

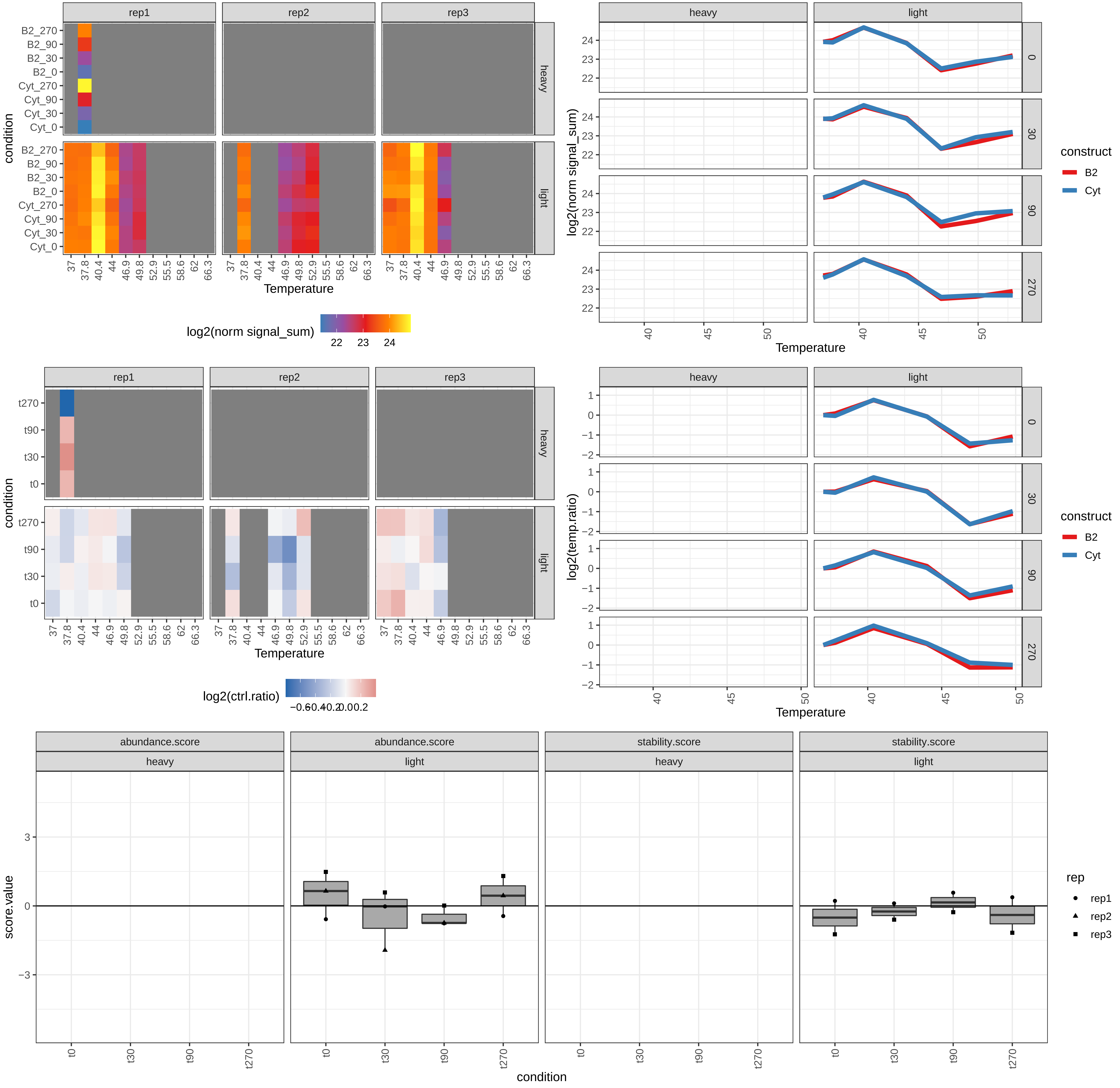

P39106 − Alpha−1,3−mannosyltransferase MNN1 OS=Saccharomyces cerevisiae (strain ATCC 204508 / S288c) GN=MNN1 PE=1 SV=1

MNN10 − average.top3: 7.0180425 − max.qupm: 6

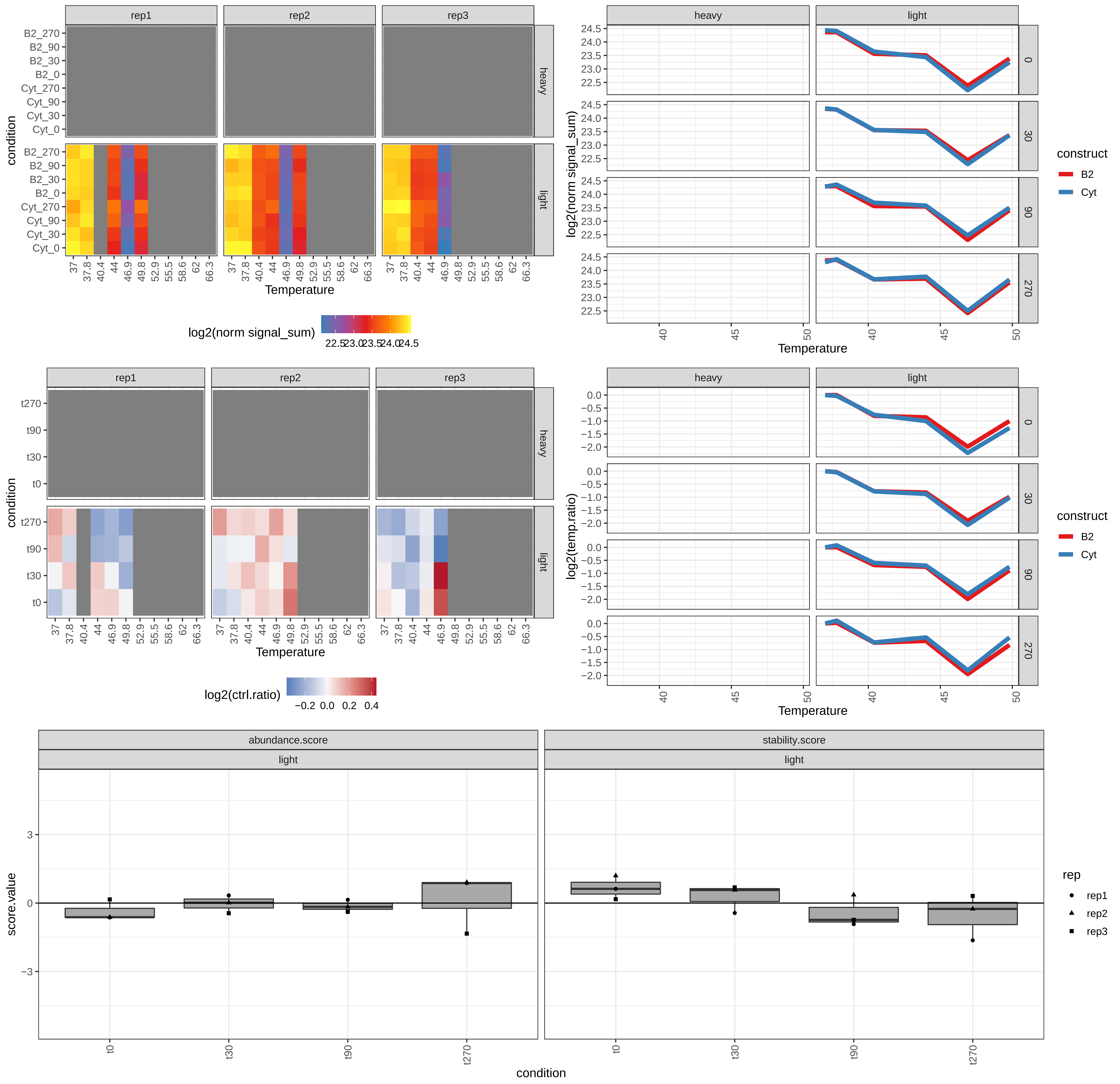

P50108 − Probable alpha−1,6−mannosyltransferase MNN10 OS=Saccharomyces cerevisiae (strain ATCC 204508 / S288c) GN=MNN10 PE=1 SV=1

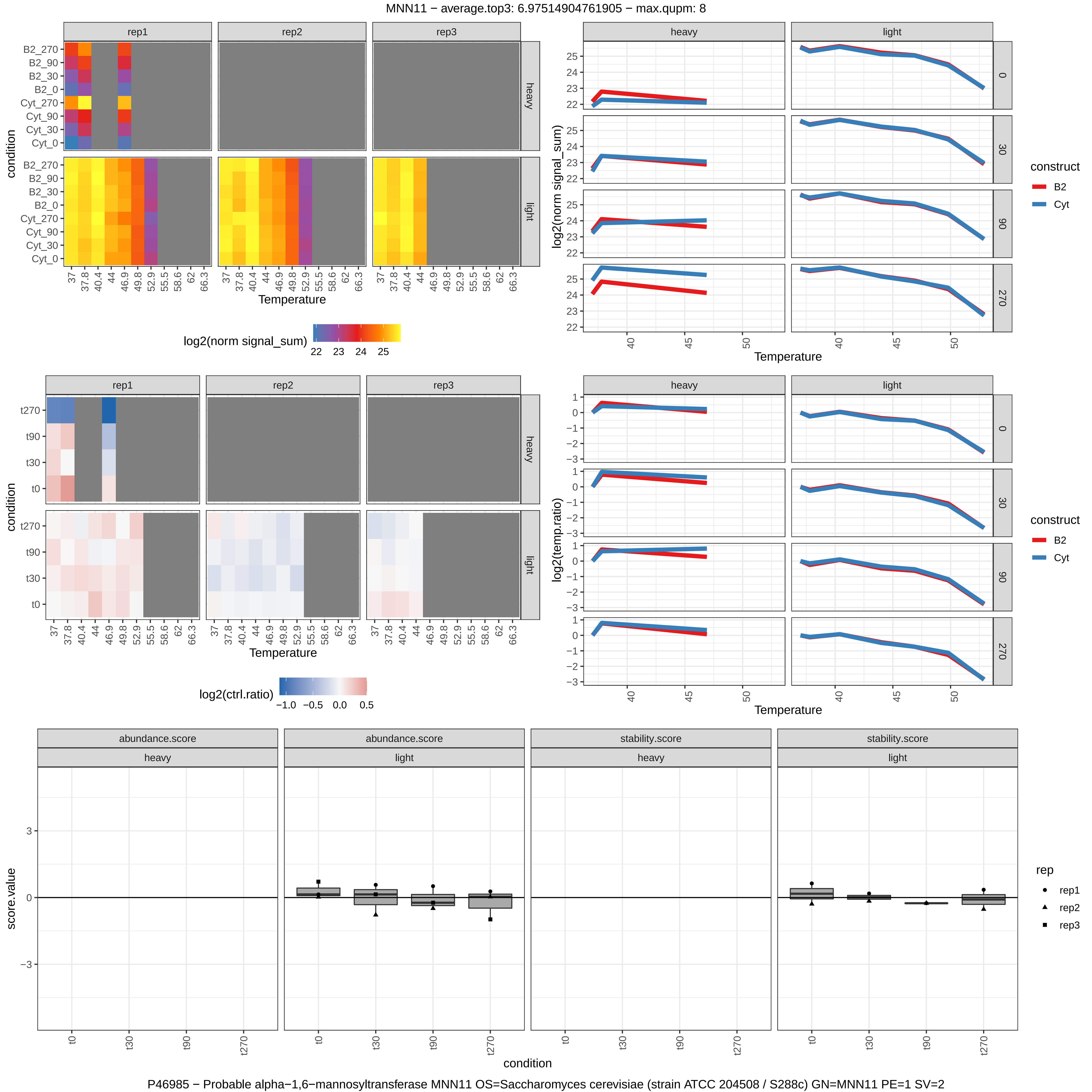
MNN11 − average.top3: 6.97514904761905 − max.qupm: 8
rep1
rep2
rep3
heavy
light
condition
Temperature
log2(norm signal_sum)
construct
B2
Cyt
log2(temp.ratio)
log2(ctrl.ratio)
abundance.score
stability.score
score.value
rep
rep1
rep2
rep3
P46985 − Probable alpha−1,6−mannosyltransferase MNN11 OS=Saccharomyces cerevisiae (strain ATCC 204508 / S288c) GN=MNN11 PE=1 SV=2

MNN2 − average.top3: 6.70514 − max.qupm: 14

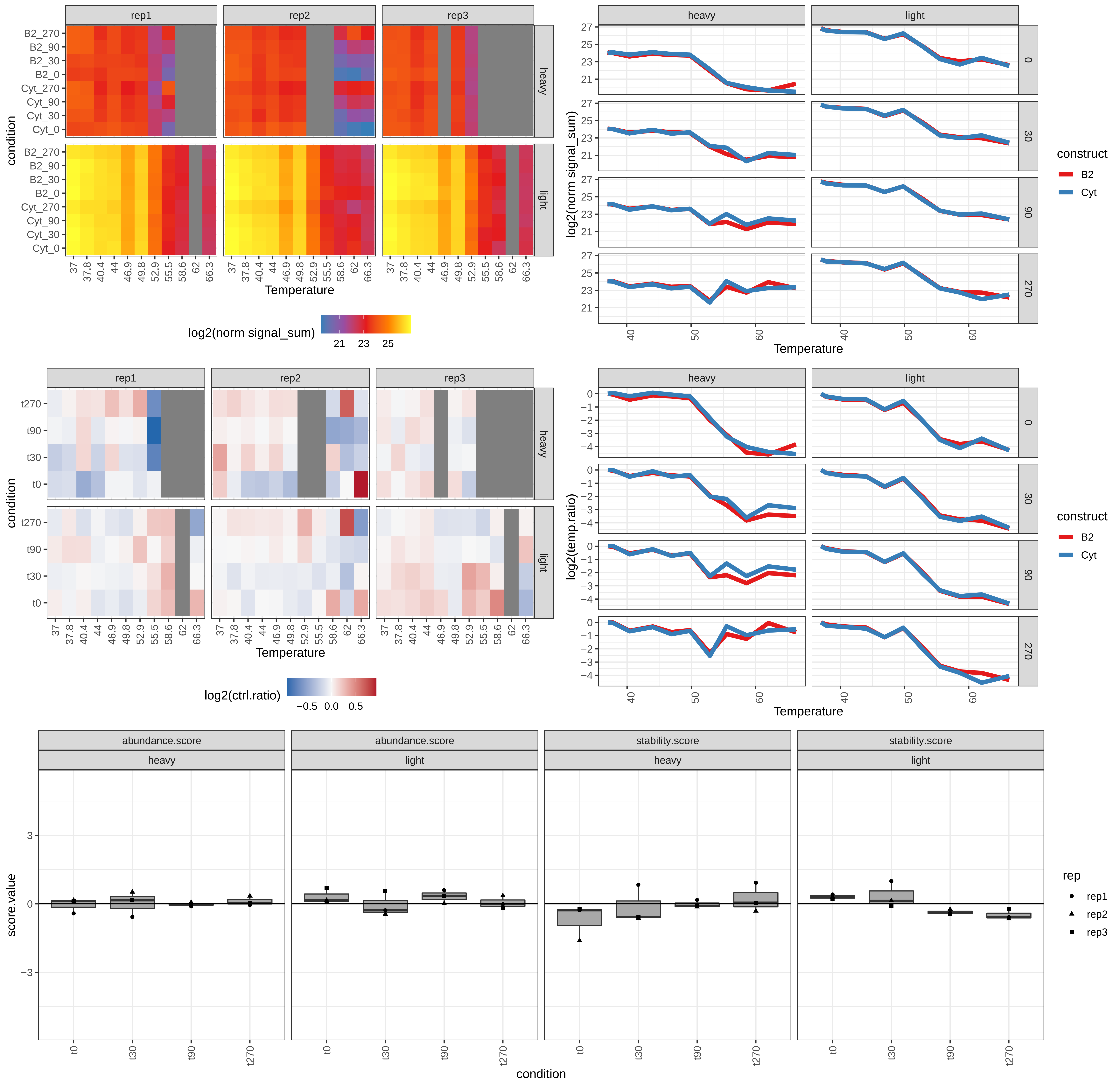

P38069 − Alpha−1,2−mannosyltransferase MNN2 OS=Saccharomyces cerevisiae (strain ATCC 204508 / S288c) GN=MNN2 PE=1 SV=1

MNN4 − average.top3: 6.68807411764706 − max.qupm: 6

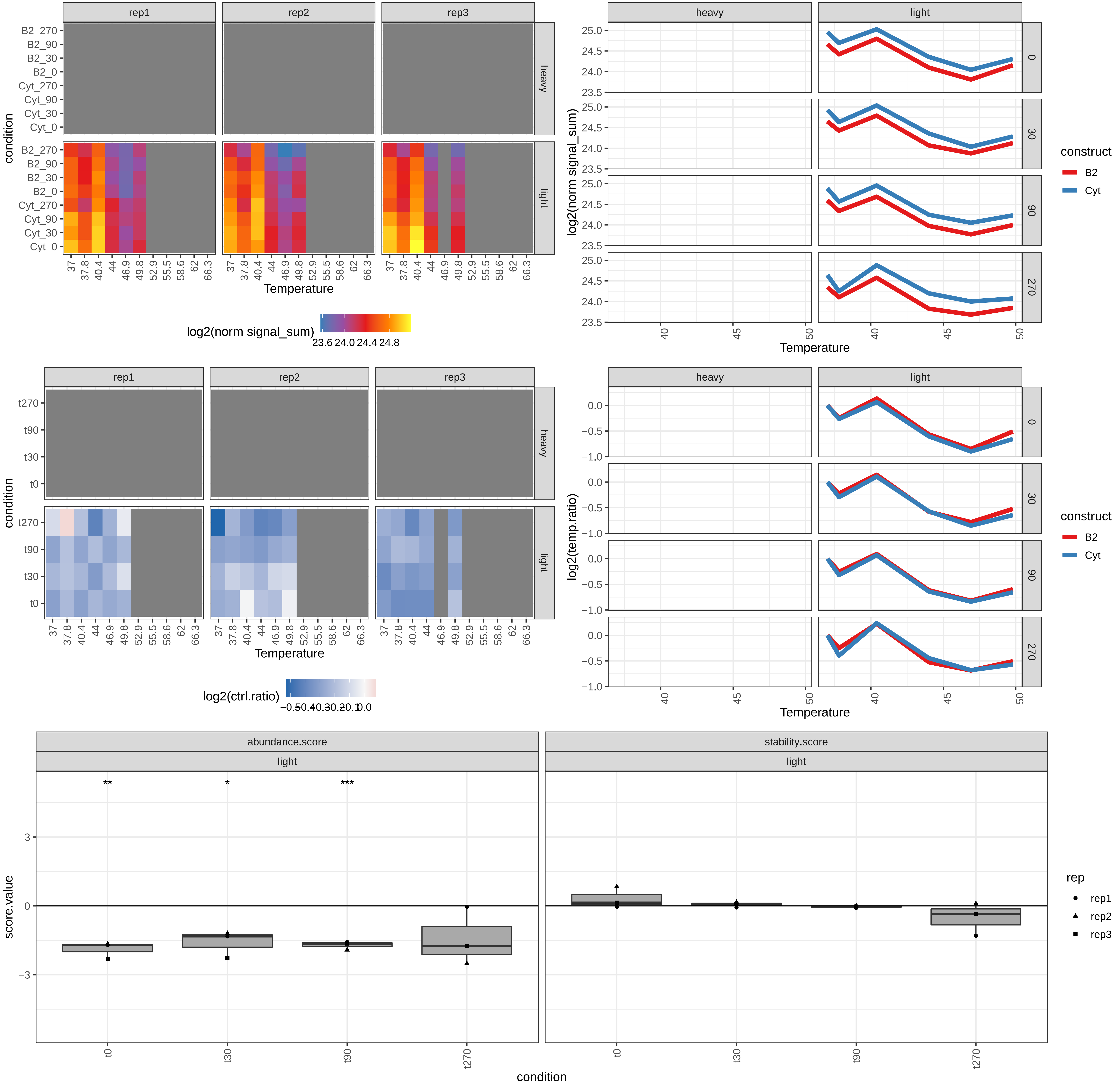

P36044 − Protein MNN4 OS=Saccharomyces cerevisiae (strain ATCC 204508 / S288c) GN=MNN4 PE=4 SV=2

MNN5 − average.top3: 7.07304463414634 − max.qupm: 10

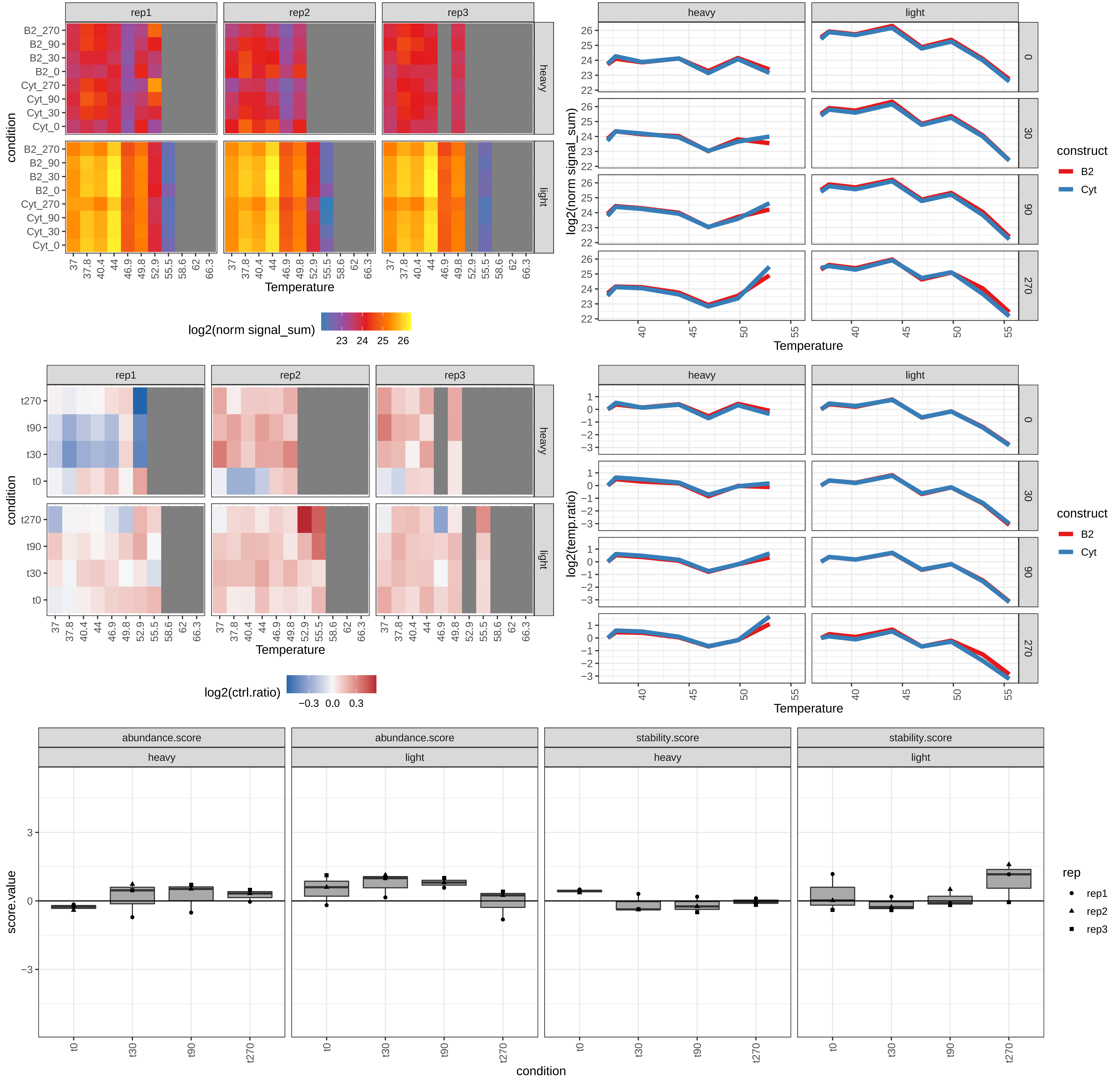

P46982 − Alpha−1,2−mannosyltransferase MNN5 OS=Saccharomyces cerevisiae (strain ATCC 204508 / S288c) GN=MNN5 PE=1 SV=2

MNN9 − average.top3: 6.950786875 − max.qupm: 7

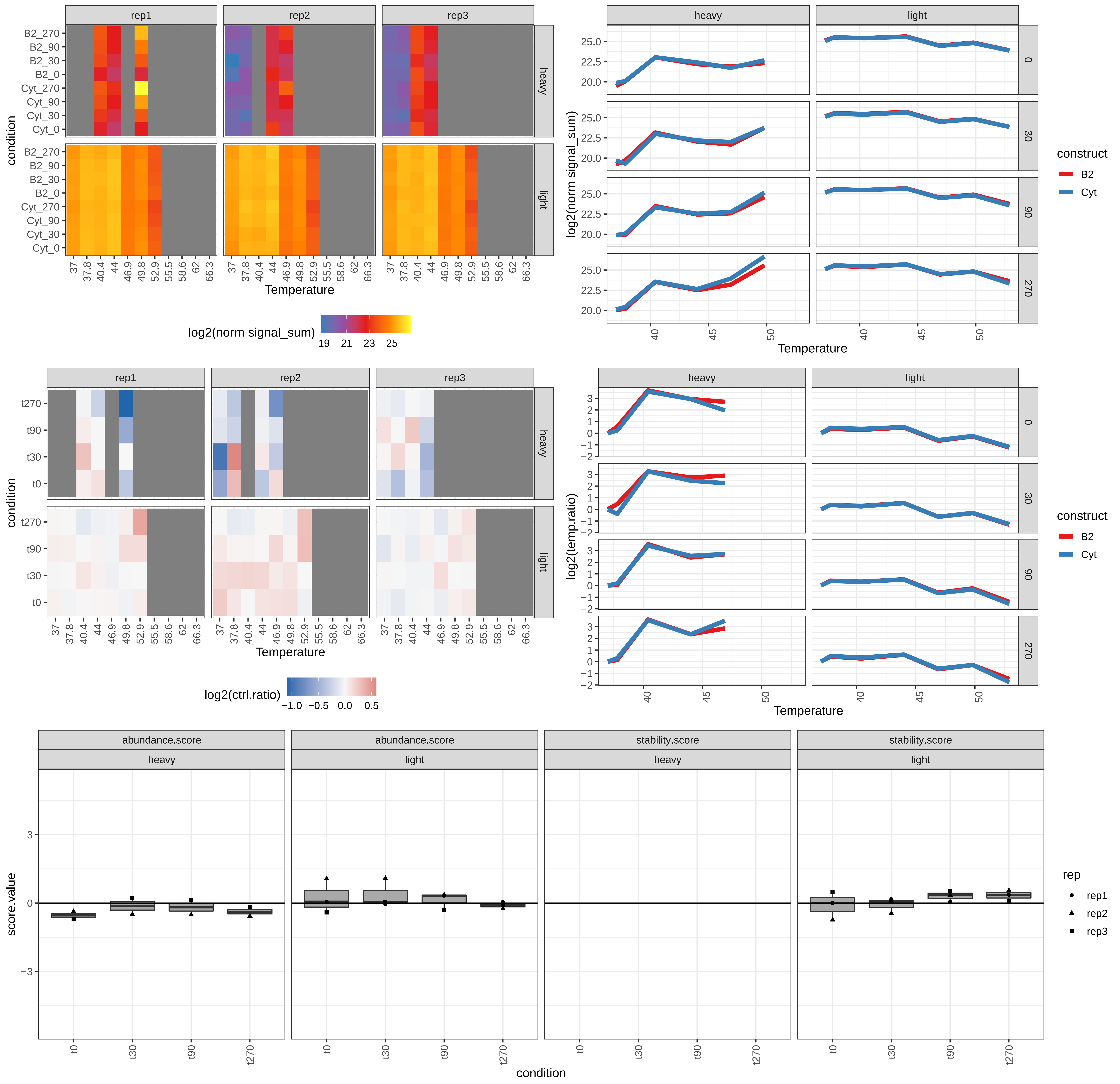

P39107 − Mannan polymerase complexes subunit MNN9 OS=Saccharomyces cerevisiae (strain ATCC 204508 / S288c) GN=MNN9 PE=1 SV=3

MNP1 − average.top3: 7.22433538461538 − max.qupm: 5

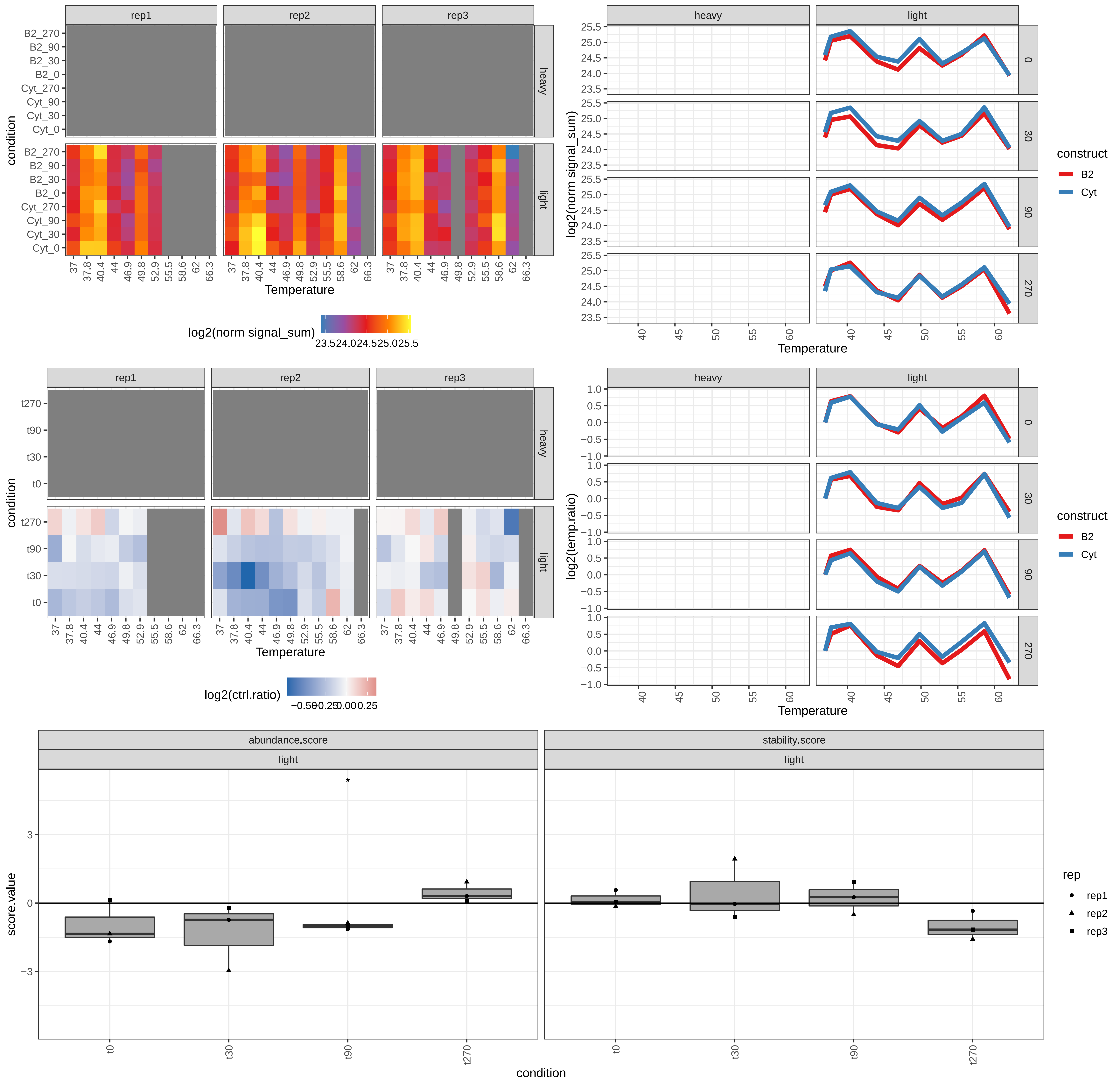

P53163 − 54S ribosomal protein L12, mitochondrial OS=Saccharomyces cerevisiae (strain ATCC 204508 / S288c) GN=MNP1 PE=1 SV=1

MNS1 − average.top3: 6.95761052631579 − max.qupm: 12

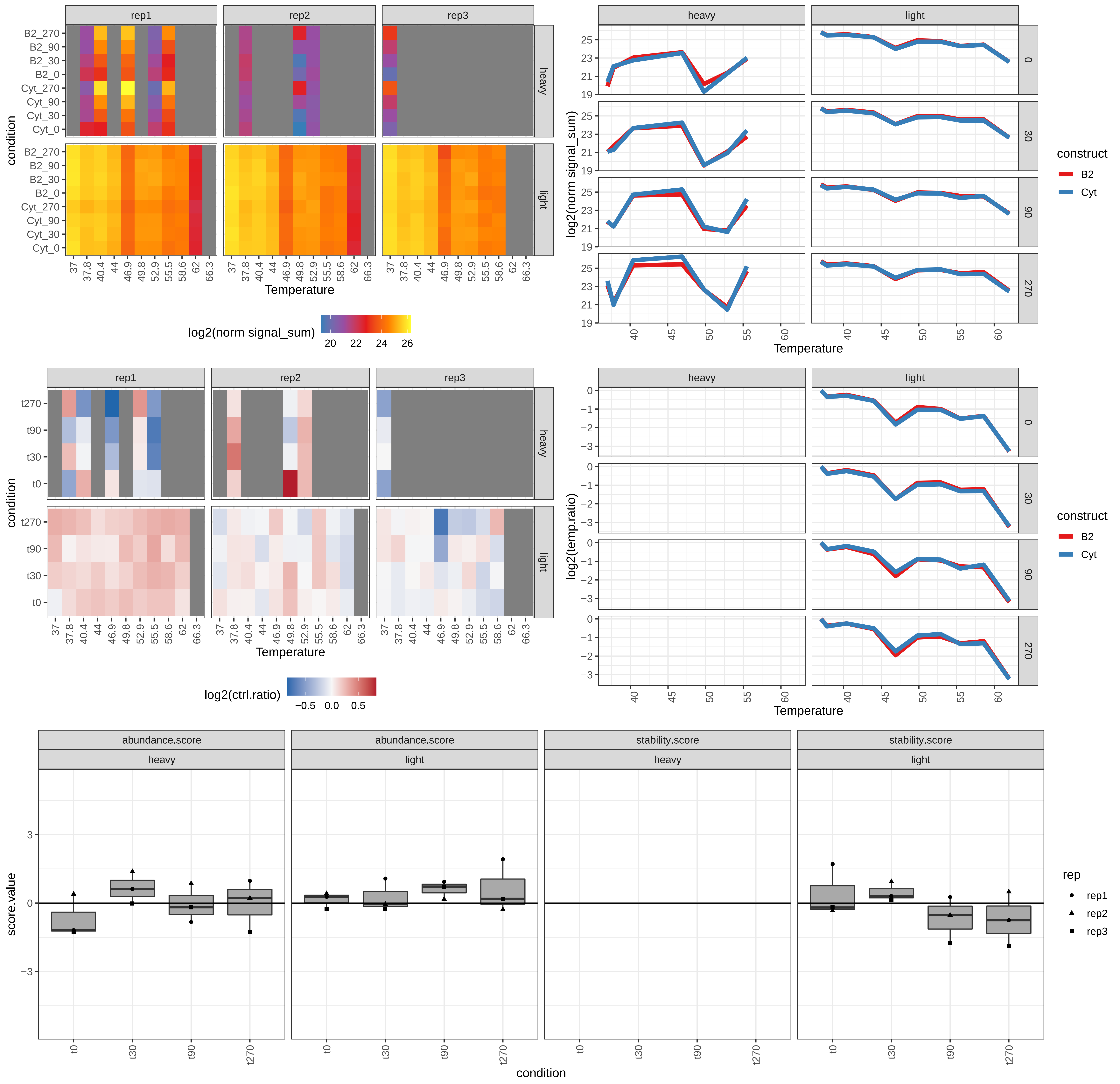

P32906 − Endoplasmic reticulum mannosyl−oligosaccharide 1,2−alpha−mannosidase OS=Saccharomyces cerevisiae (strain ATCC 204508 / S288c) GN=MNS1 PE=1 SV=1

MNT3 − average.top3: 6.715035625 − max.qupm: 4

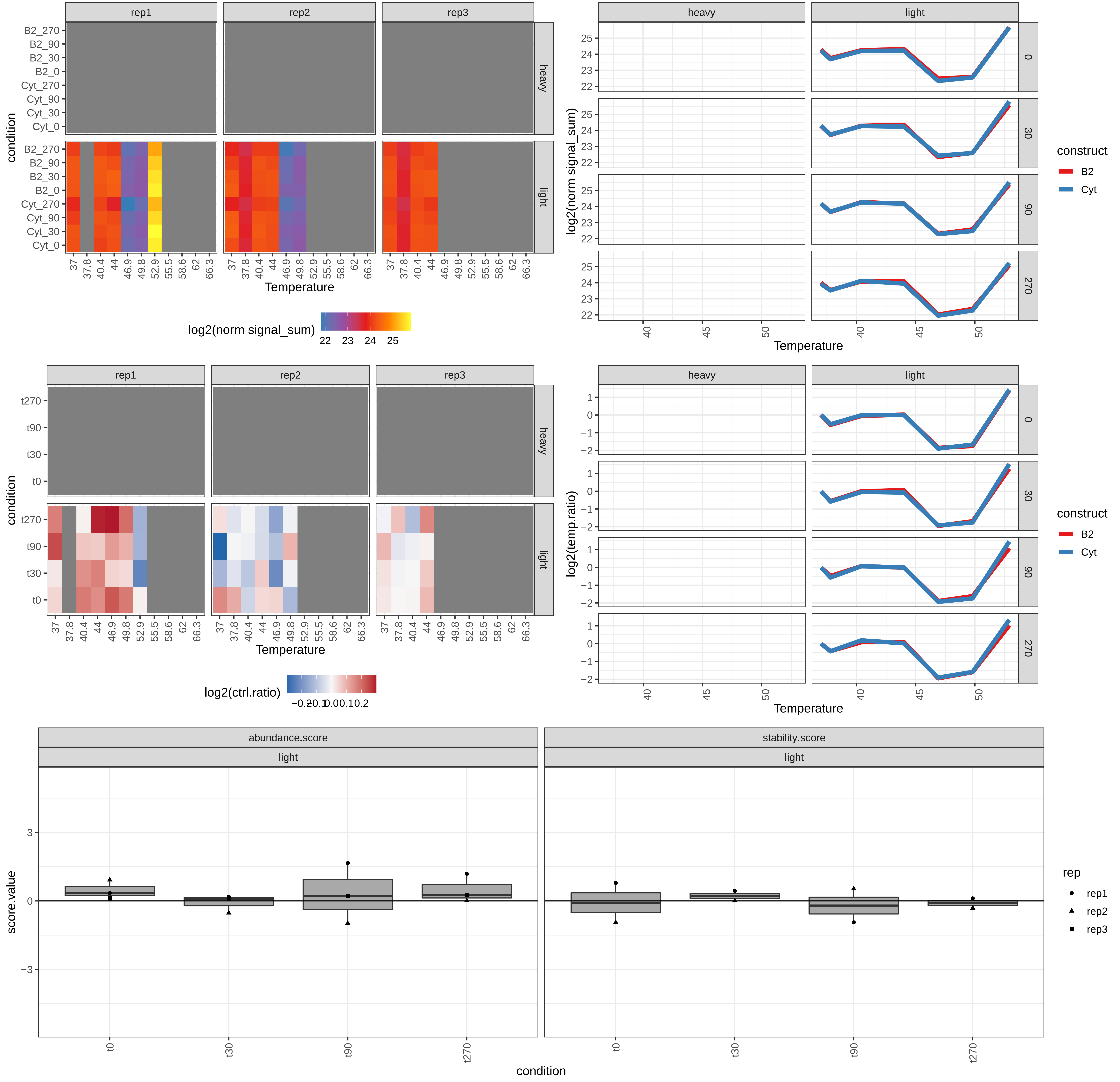

P40549 − Alpha−1,3−mannosyltransferase MNT3 OS=Saccharomyces cerevisiae (strain ATCC 204508 / S288c) GN=MNT3 PE=1 SV=1

MOB1 − average.top3: 6.567142 − max.qupm: 3

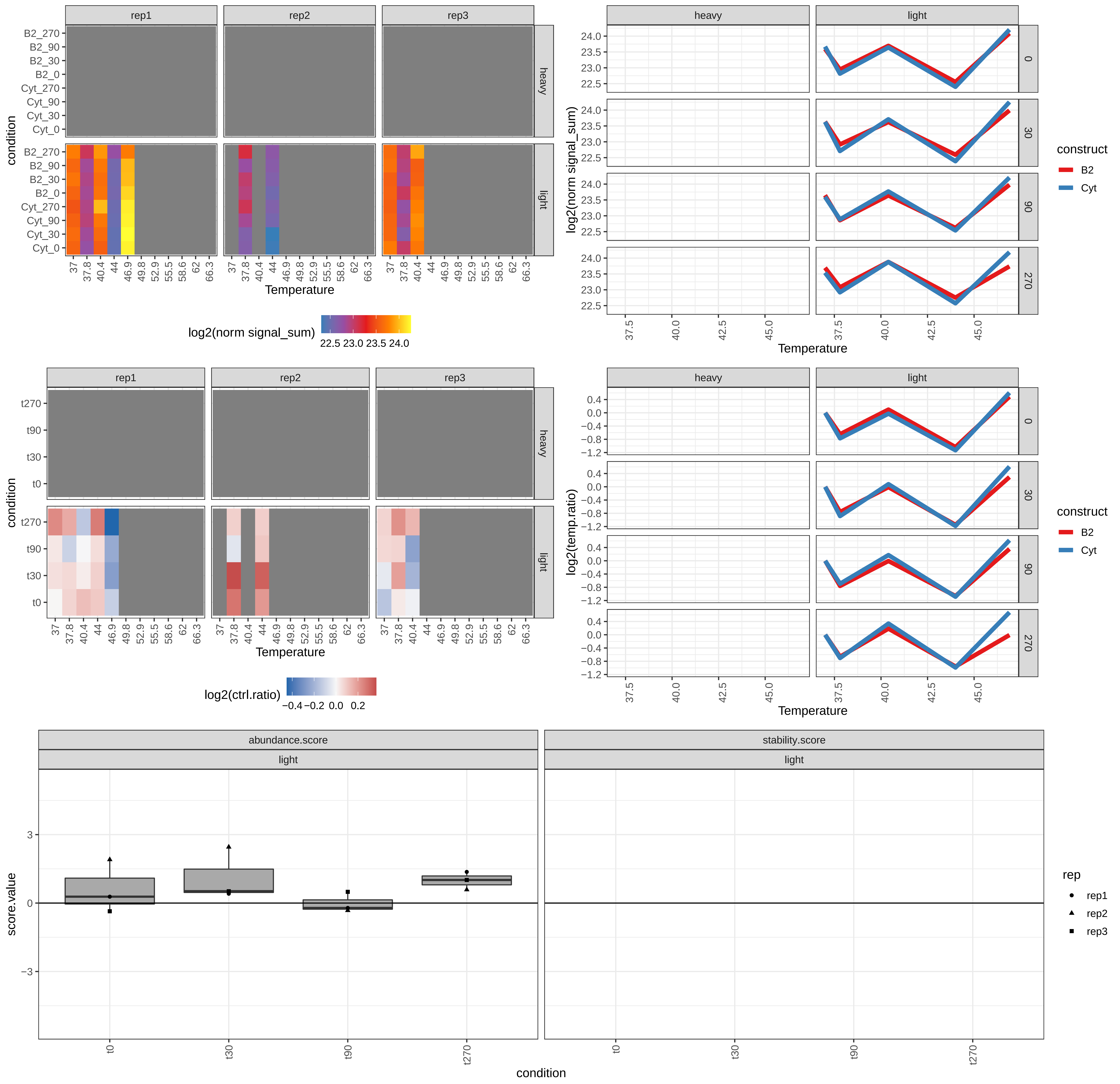

P40484 − DBF2 kinase activator protein MOB1 OS=Saccharomyces cerevisiae (strain ATCC 204508 / S288c) GN=MOB1 PE=1 SV=2

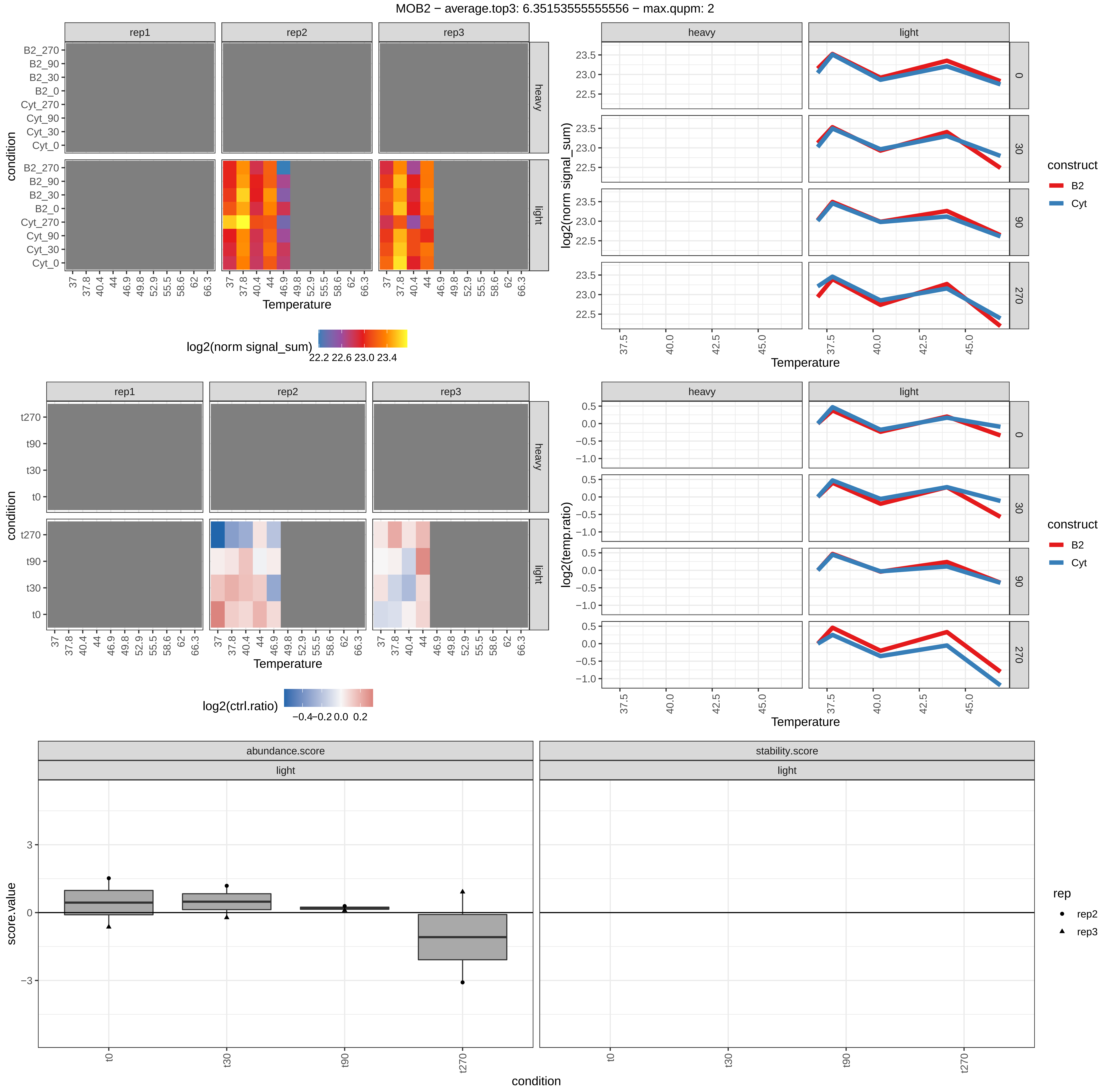

P43563 − CBK1 kinase activator protein MOB2 OS=Saccharomyces cerevisiae (strain ATCC 204508 / S288c) GN=MOB2 PE=1 SV=2

MOG1 − average.top3: 6.70271071428571 − max.qupm: 2

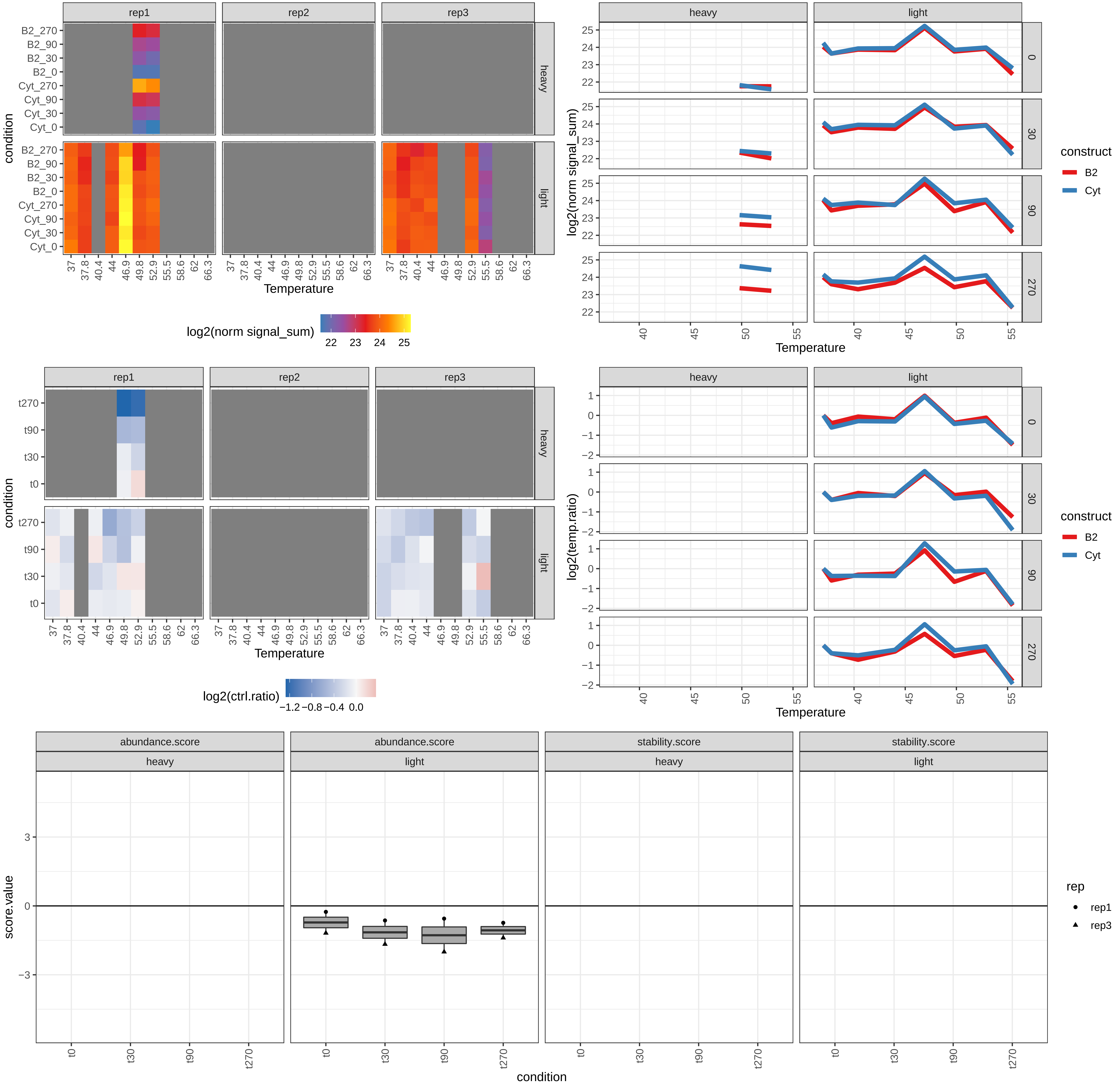

P47123 − Nuclear import protein MOG1 OS=Saccharomyces cerevisiae (strain ATCC 204508 / S288c) GN=MOG1 PE=1 SV=1

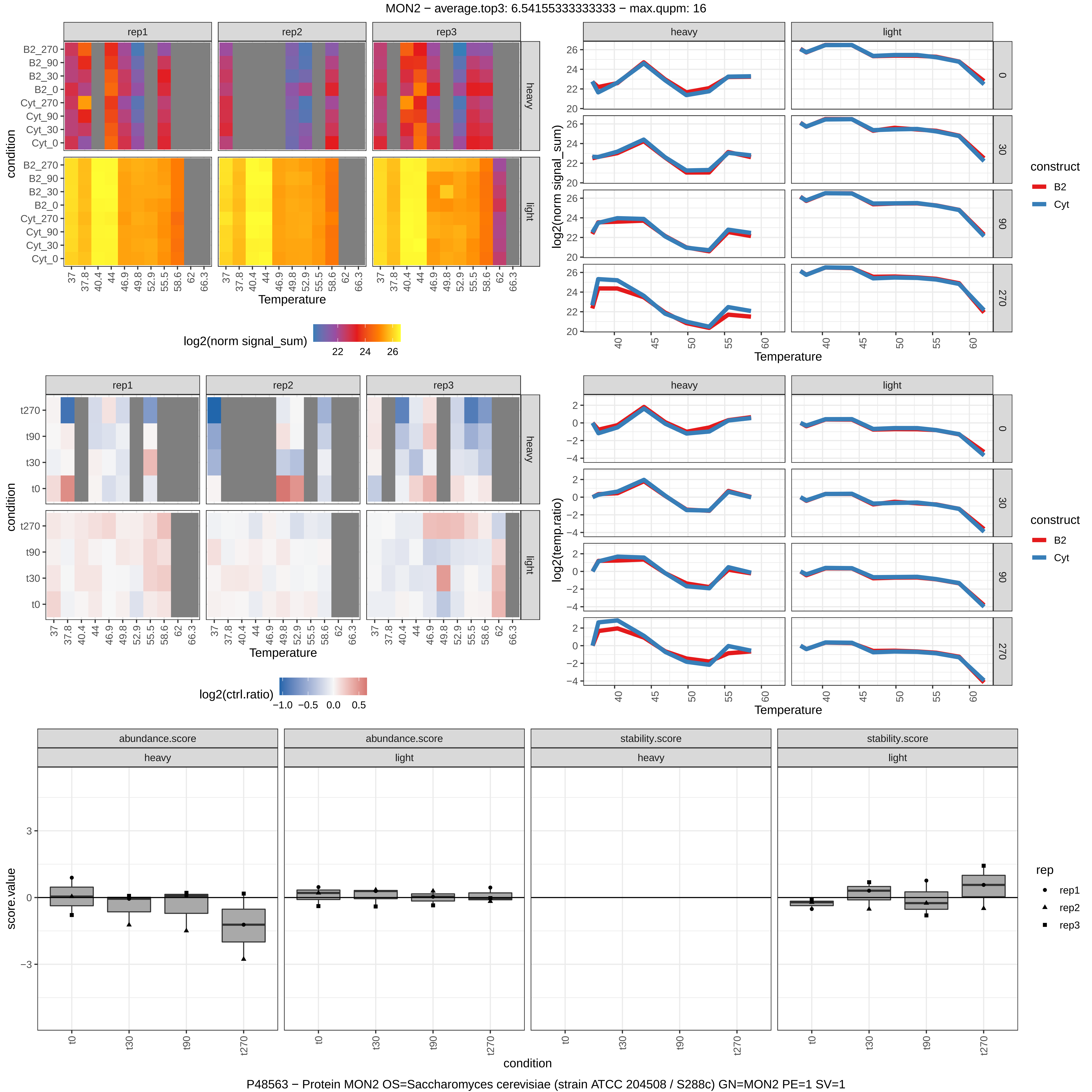
MON2 − average.top3: 6.54155333333333 − max.qupm: 16
rep1
rep2
rep3
heavy
light
condition
Temperature
log2(norm signal_sum)
22
24
26
construct
B2
Cyt
log2(ctrl.ratio)
−1.0
−0.5
0.0
0.5
log2(temp.ratio)
abundance.score
stability.score
score.value
rep
condition
P48563 − Protein MON2 OS=Saccharomyces cerevisiae (strain ATCC 204508 / S288c) GN=MON2 PE=1 SV=1

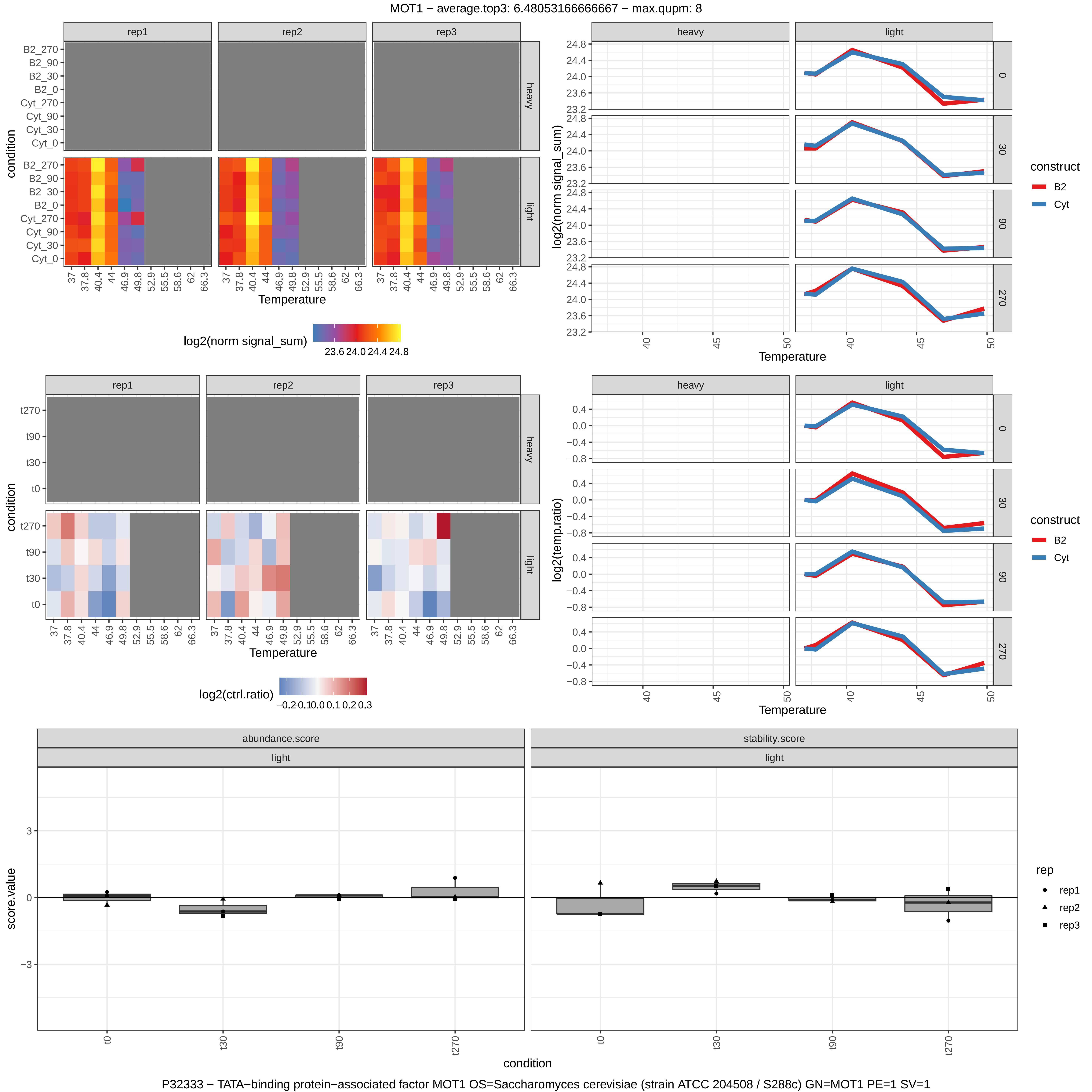
MOT1 − average.top3: 6.48053166666667 − max.qupm: 8
rep1
rep2
rep3
heavy
light
condition
Temperature
log2(norm signal_sum)
log2(ctrl.ratio)
log2(temp.ratio)
construct
B2
Cyt
abundance.score
stability.score
score.value
rep
rep1
rep2
rep3
condition
P32333 − TATA−binding protein−associated factor MOT1 OS=Saccharomyces cerevisiae (strain ATCC 204508 / S288c) GN=MOT1 PE=1 SV=1

MOT2 − average.top3: 7.0397552631579 − max.qupm: 8

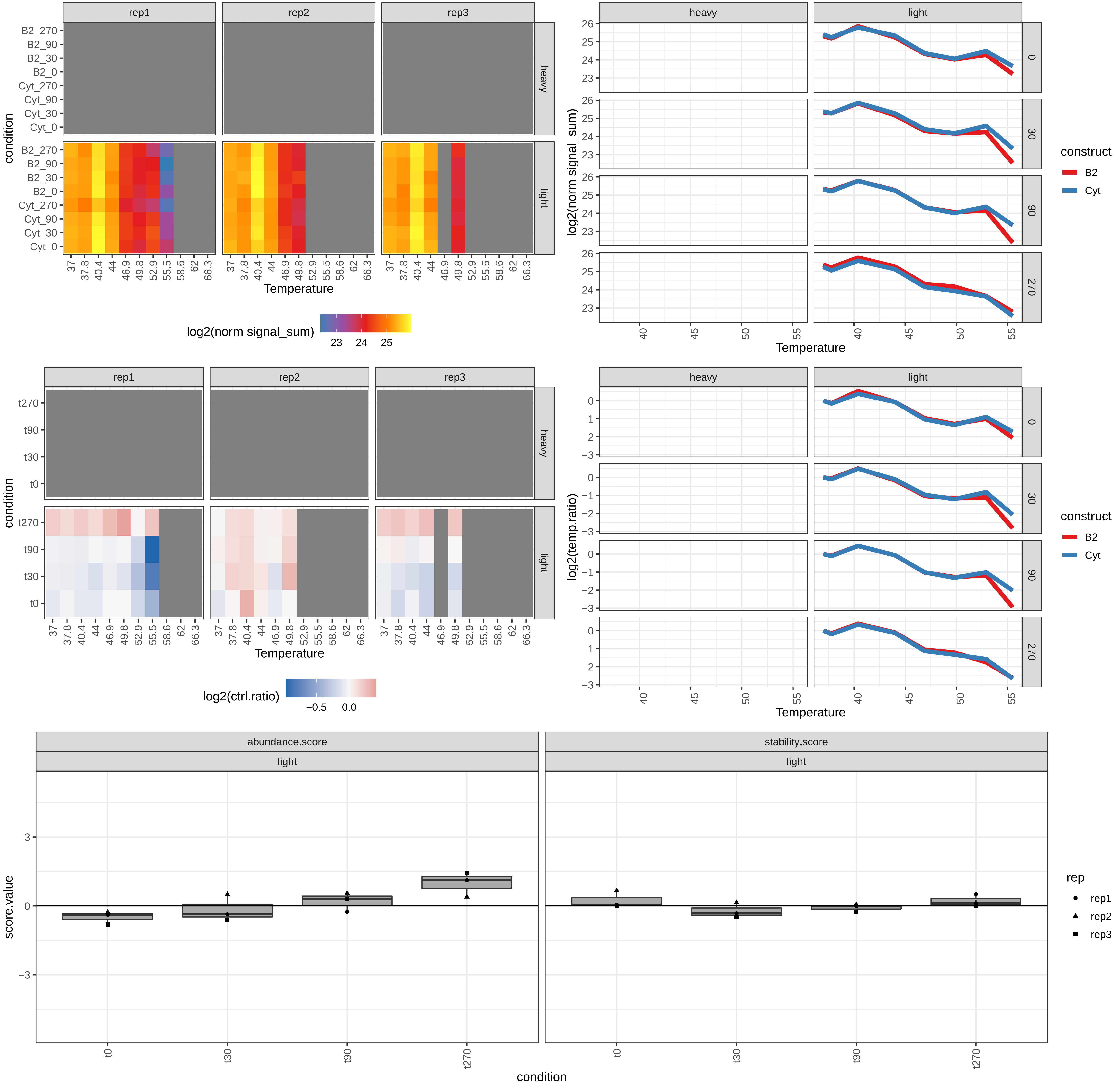

P34909 − General negative regulator of transcription subunit 4 OS=Saccharomyces cerevisiae (strain ATCC 204508 / S288c) GN=MOT2 PE=1 SV=1

MPD1 − average.top3: 6.60439192307692 − max.qupm: 10

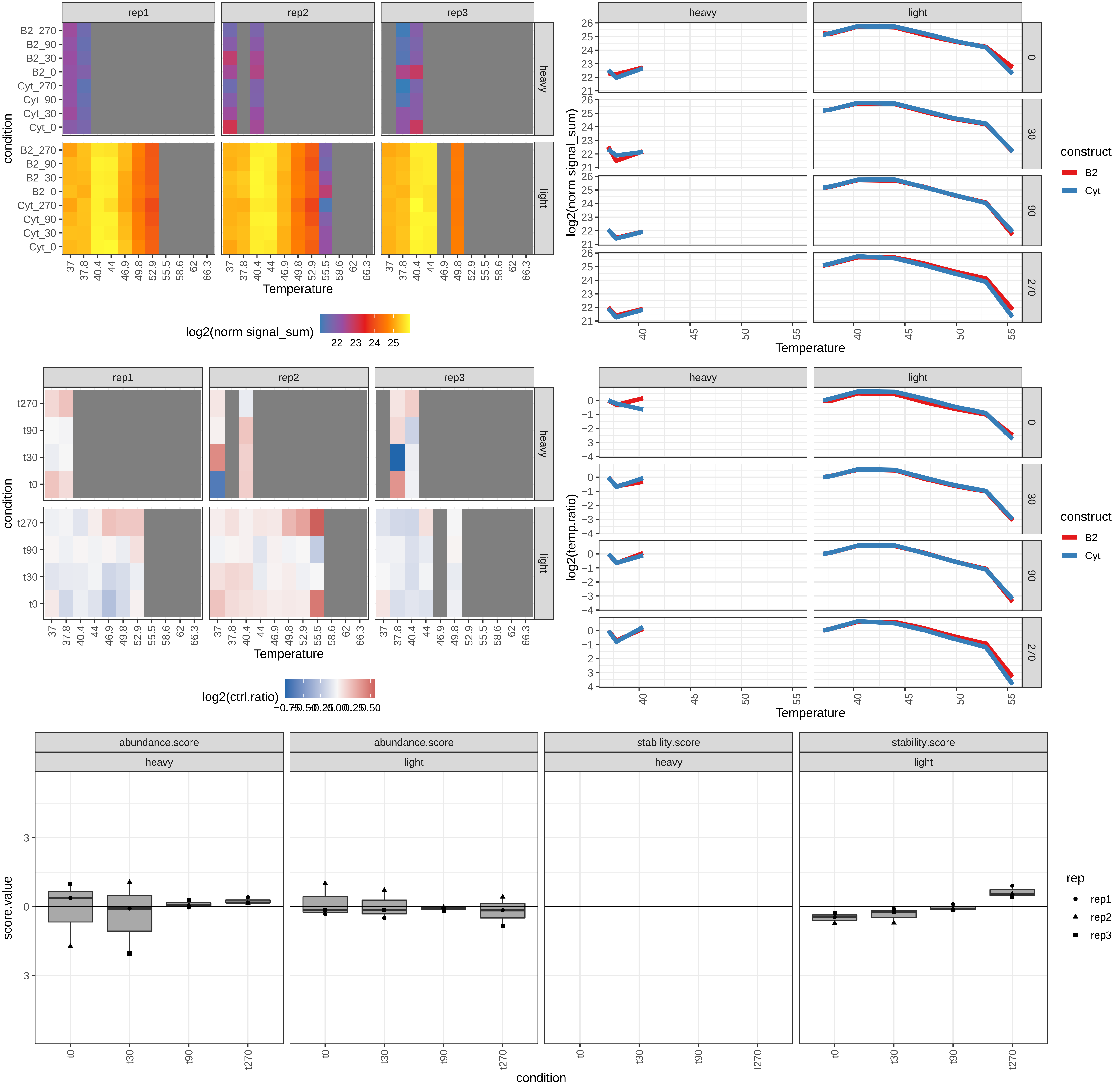

Q12404 − Protein disulfide−isomerase MPD1 OS=Saccharomyces cerevisiae (strain ATCC 204508 / S288c) GN=MPD1 PE=1 SV=1

MPD2 − average.top3: 7.03156303571429 − max.qupm: 10

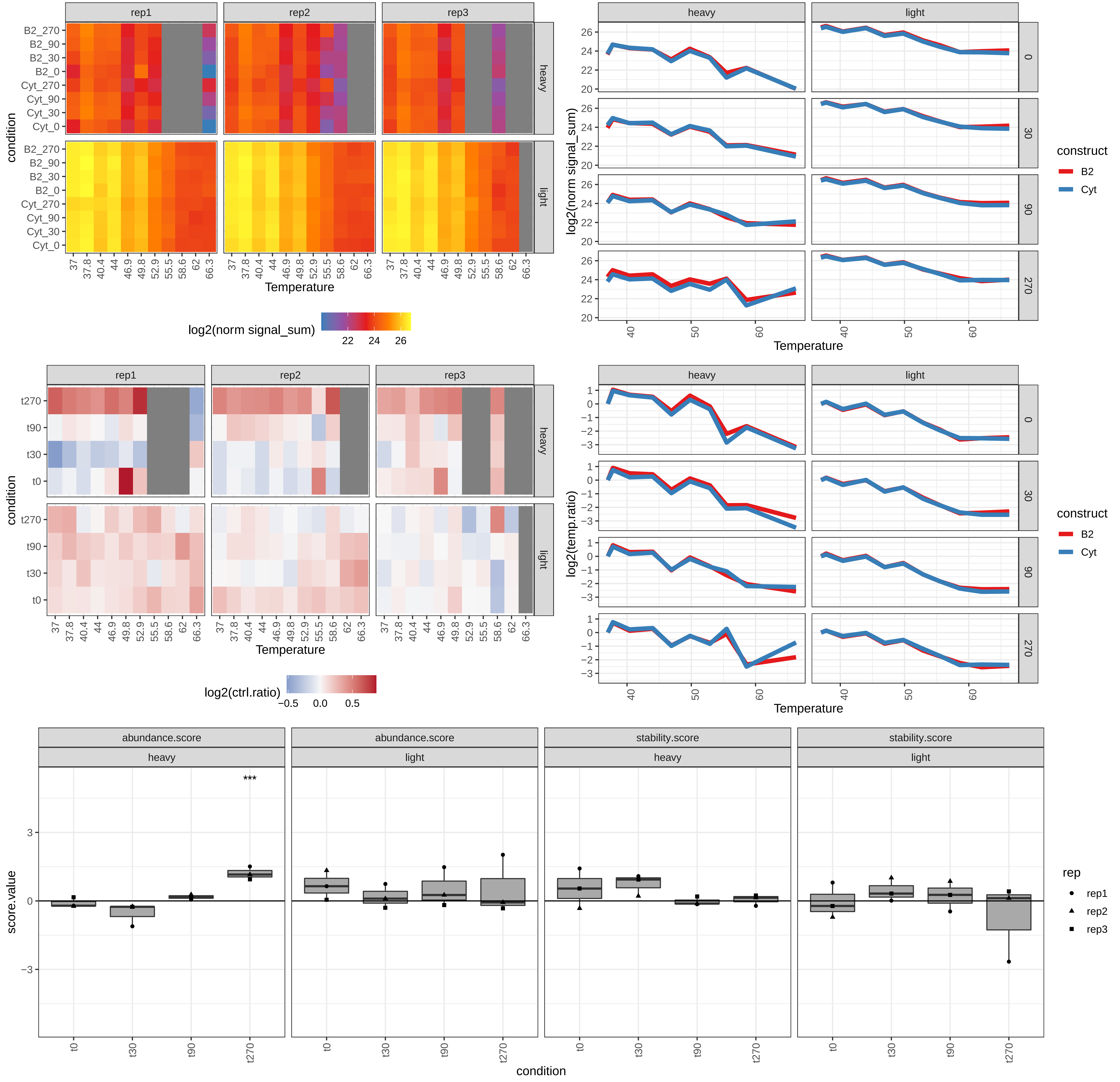

Q99316 − Protein disulfide isomerase MPD2 OS=Saccharomyces cerevisiae (strain ATCC 204508 / S288c) GN=MPD2 PE=1 SV=1

MPE1 − average.top3: 6.0701225 − max.qupm: 3

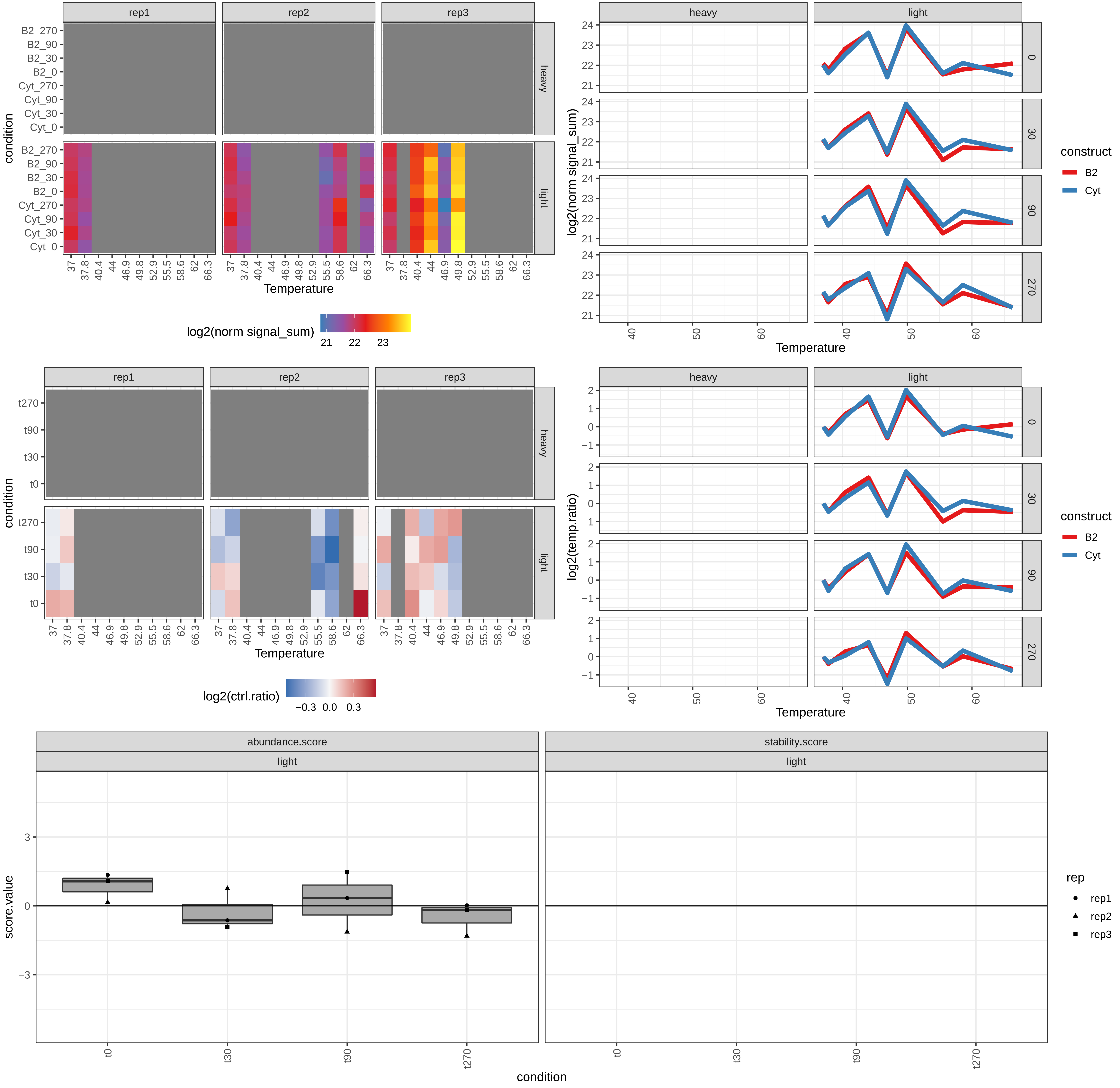

P35728 − Protein MPE1 OS=Saccharomyces cerevisiae (strain ATCC 204508 / S288c) GN=MPE1 PE=1 SV=1

MPM1 − average.top3: 6.4827725 − max.qupm: 3

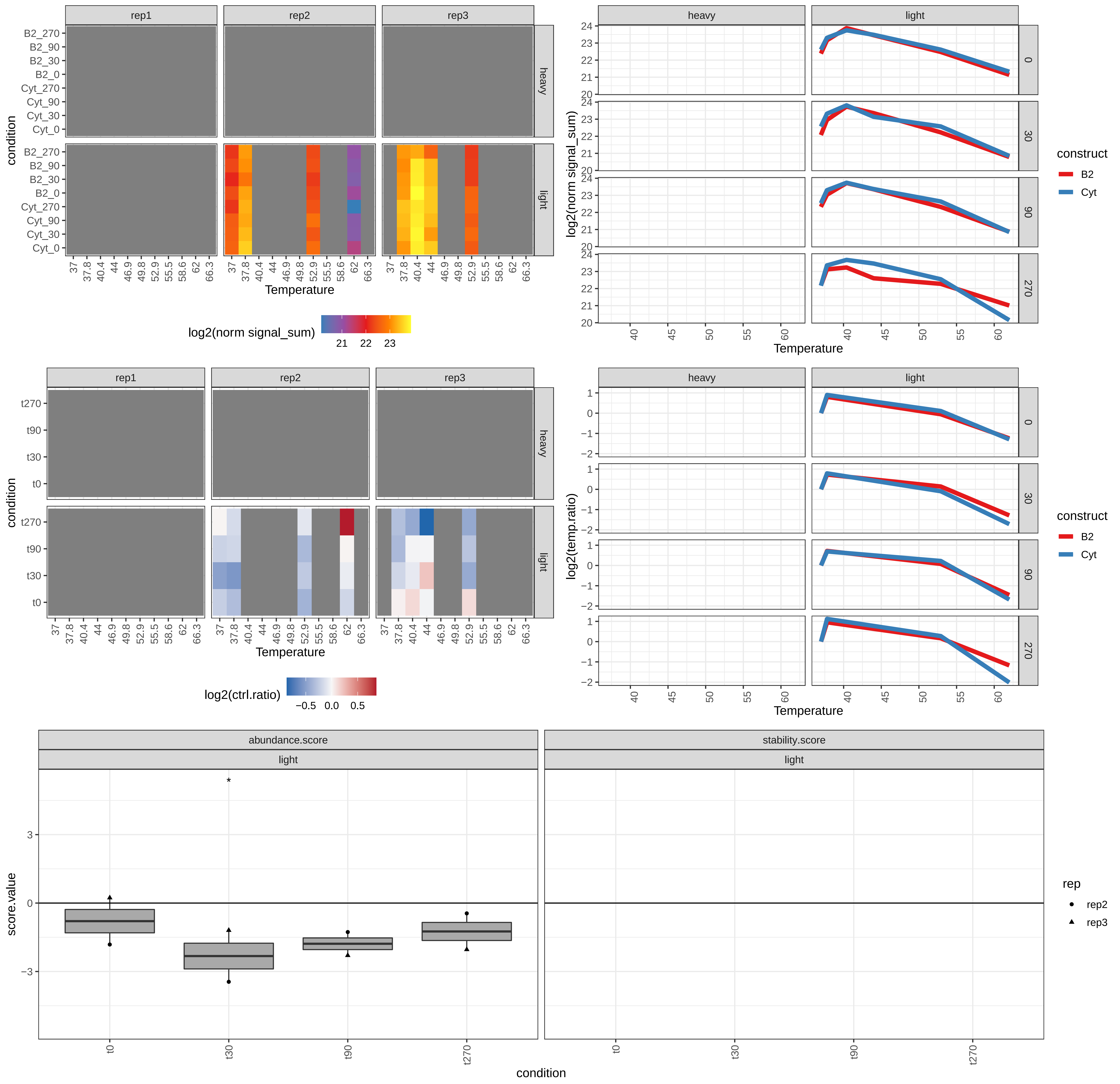

P40364 − Mitochondrial peculiar membrane protein 1 OS=Saccharomyces cerevisiae (strain ATCC 204508 / S288c) GN=MPM1 PE=1 SV=1

MRD1 − average.top3: 6.70232866666667 − max.qupm: 8

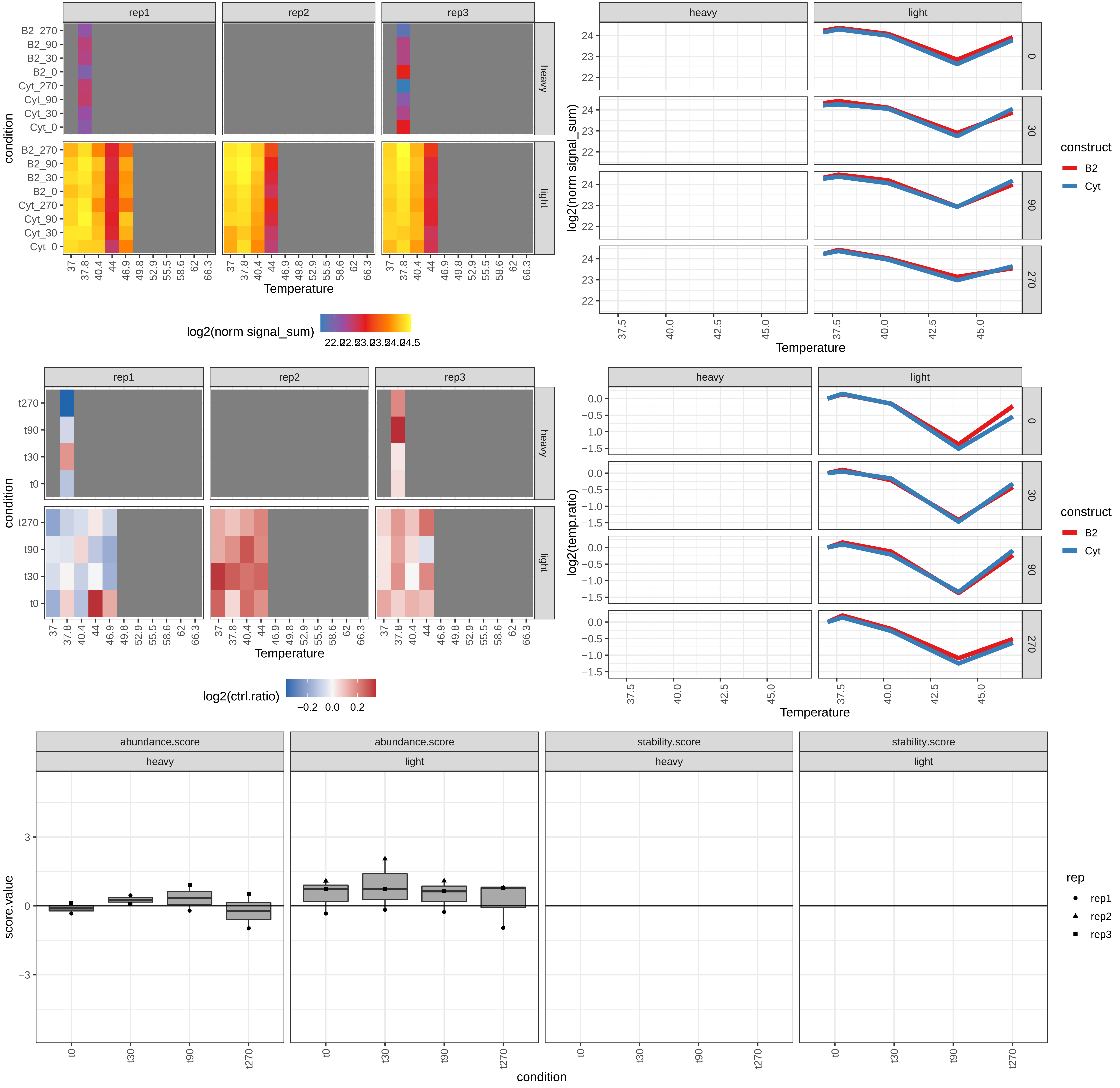

Q06106 − Multiple RNA−binding domain−containing protein 1 OS=Saccharomyces cerevisiae (strain ATCC 204508 / S288c) GN=MRD1 PE=1 SV=1

MRI1 − average.top3: 7.22998344827586 − max.qupm: 8

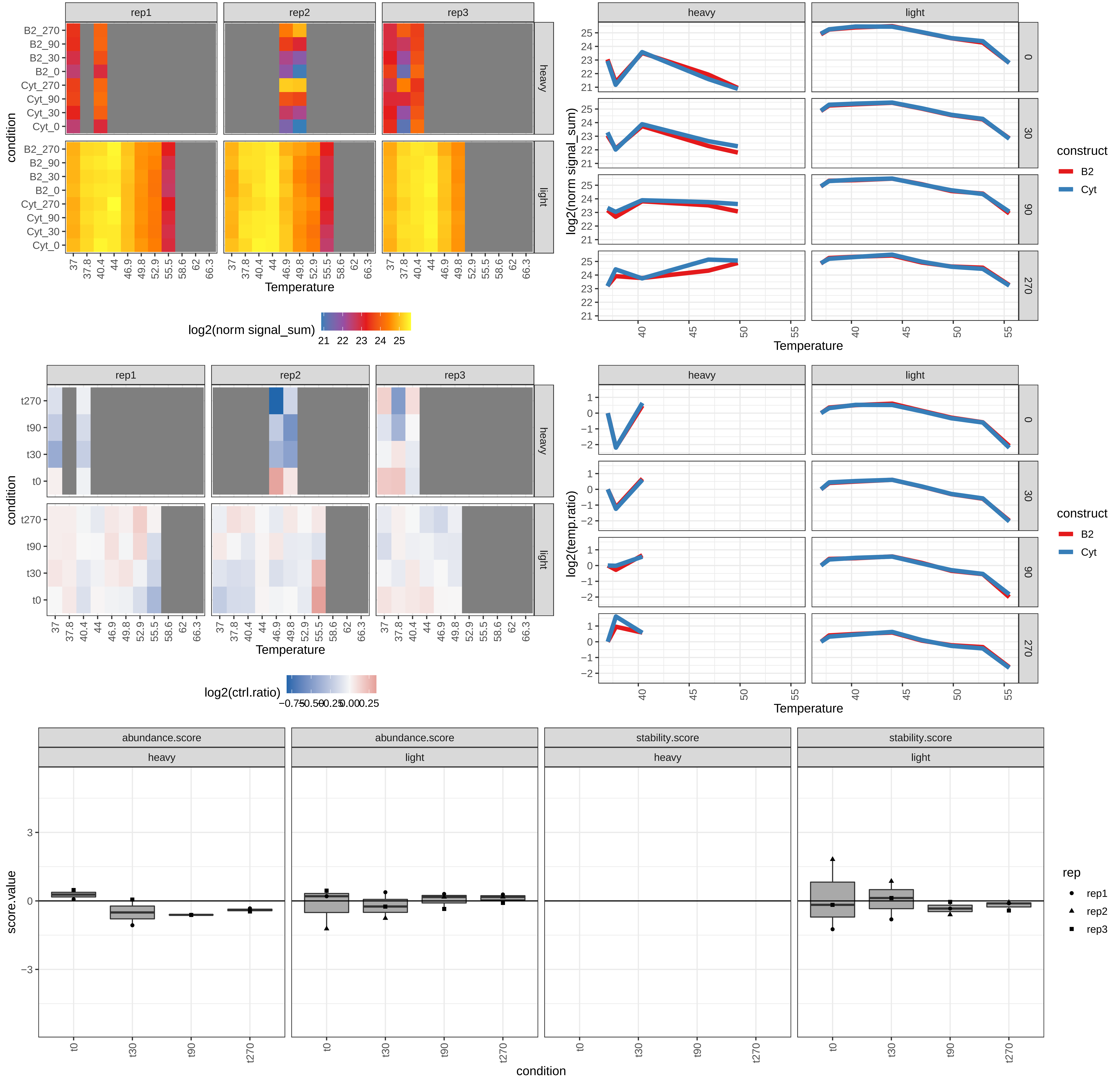

Q06489 − Methylthioribose−1−phosphate isomerase OS=Saccharomyces cerevisiae (strain ATCC 204508 / S288c) GN=MRI1 PE=1 SV=1

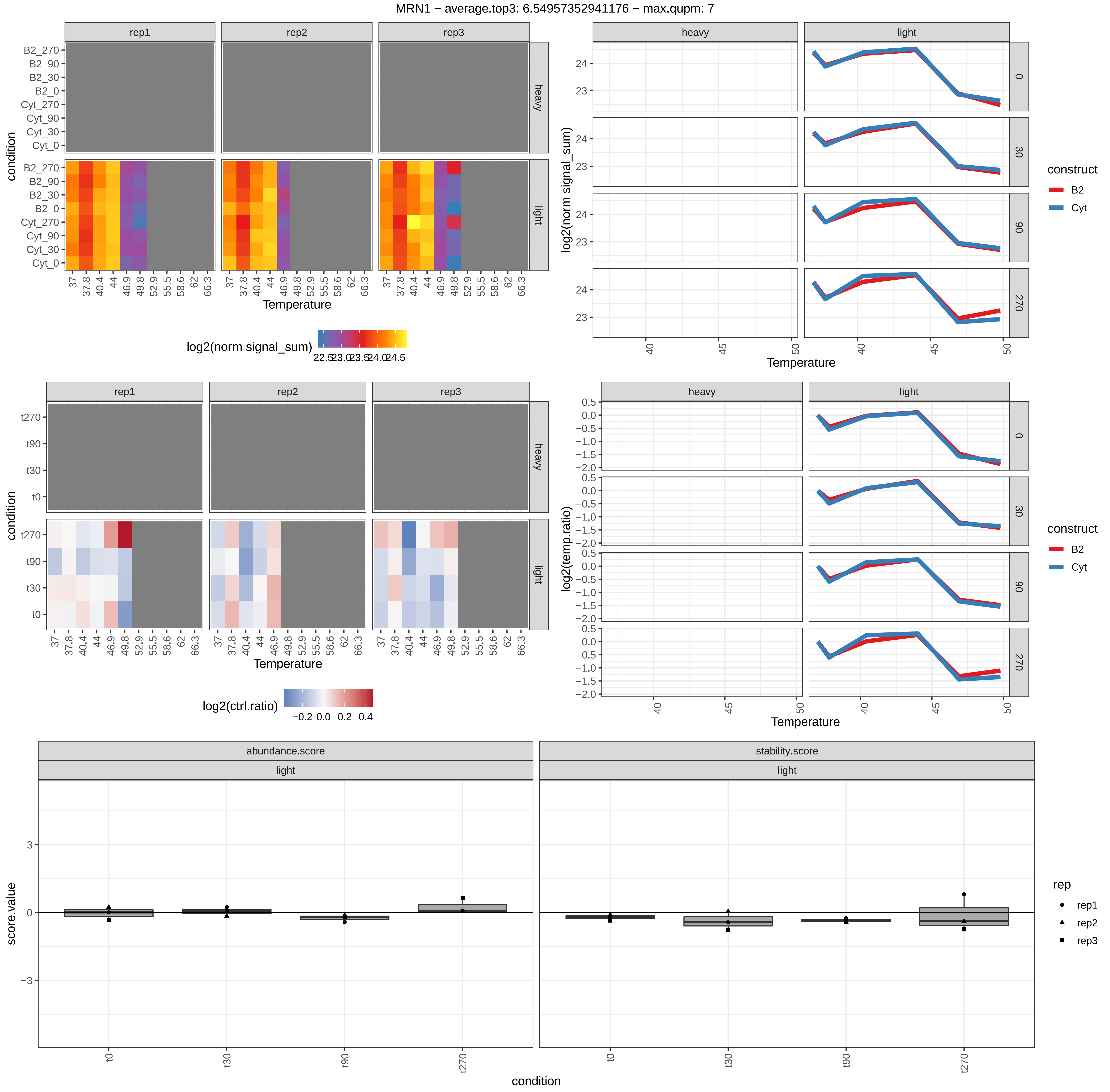

Q08925 − RNA−binding protein MRN1 OS=Saccharomyces cerevisiae (strain ATCC 204508 / S288c) GN=MRN1 PE=1 SV=1

MRP1 − average.top3: 6.91749333333333 − max.qupm: 7

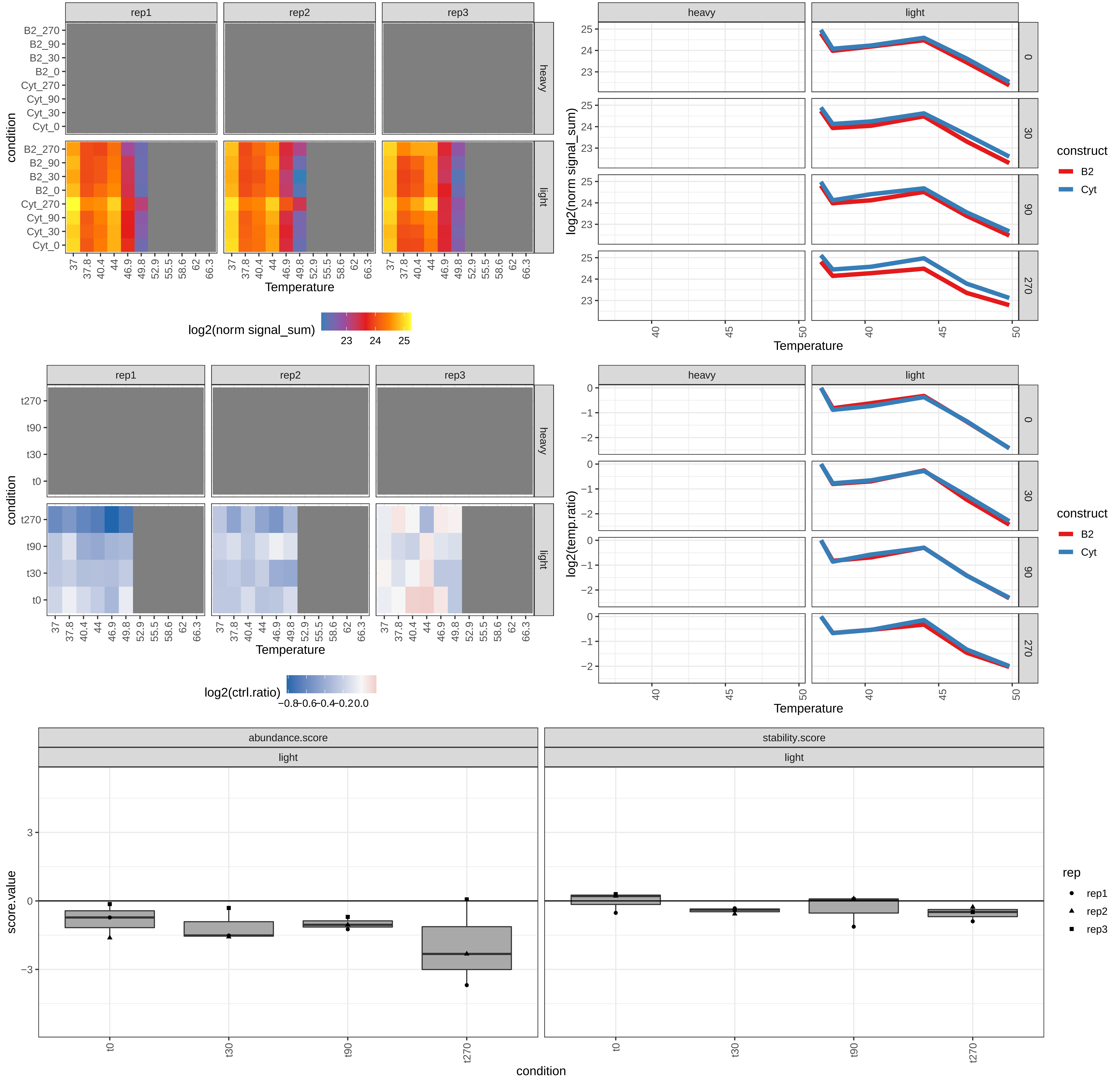

P10662 − 37S ribosomal protein MRP1, mitochondrial OS=Saccharomyces cerevisiae (strain ATCC 204508 / S288c) GN=MRP1 PE=1 SV=2

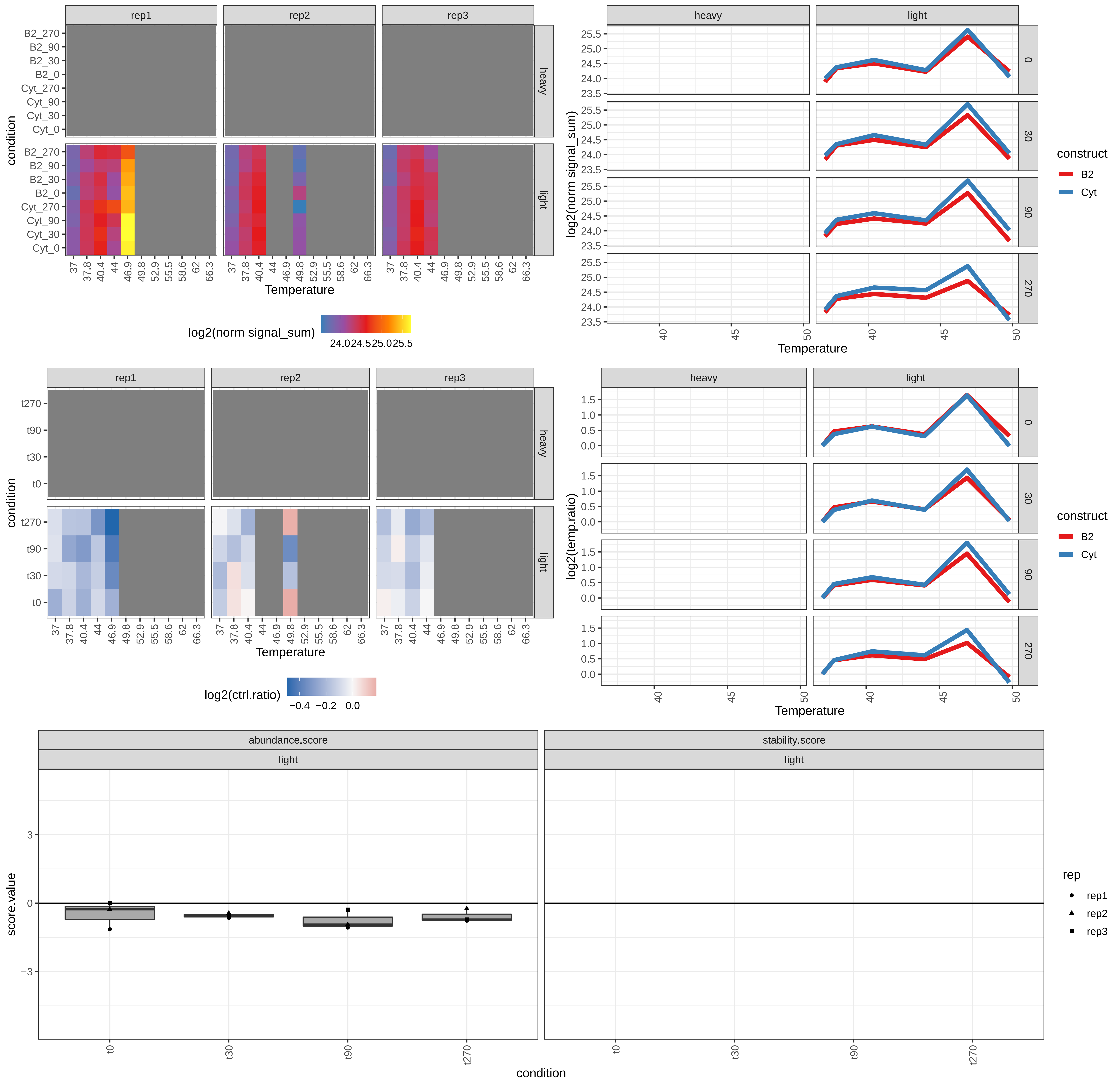

P28778 − 37S ribosomal protein MRP17, mitochondrial OS=Saccharomyces cerevisiae (strain ATCC 204508 / S288c) GN=MRP17 PE=1 SV=1

MRP2 − average.top3: 7.18469071428571 − max.qupm: 5

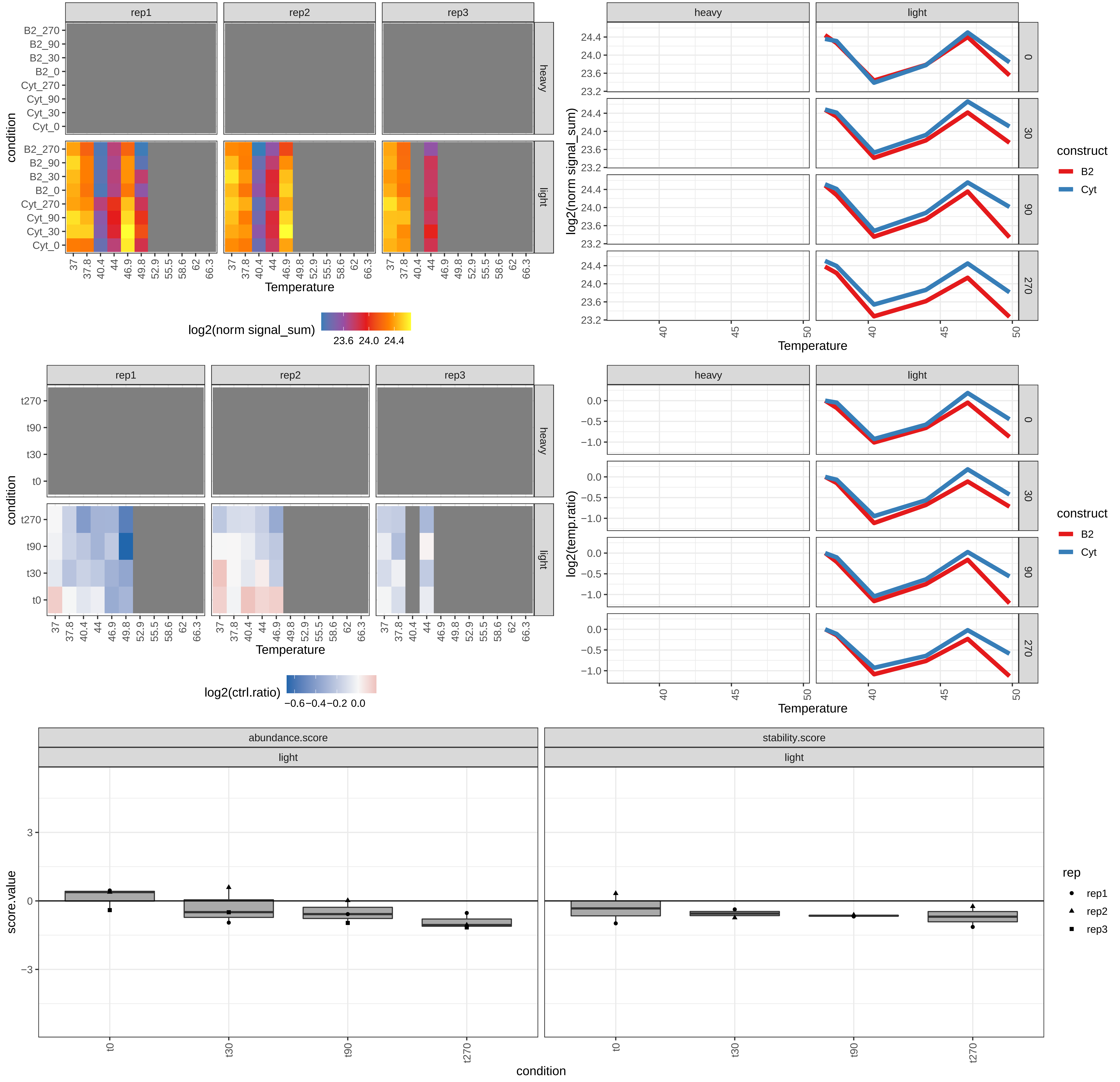

P10663 − 37S ribosomal protein MRP2, mitochondrial OS=Saccharomyces cerevisiae (strain ATCC 204508 / S288c) GN=MRP2 PE=1 SV=1

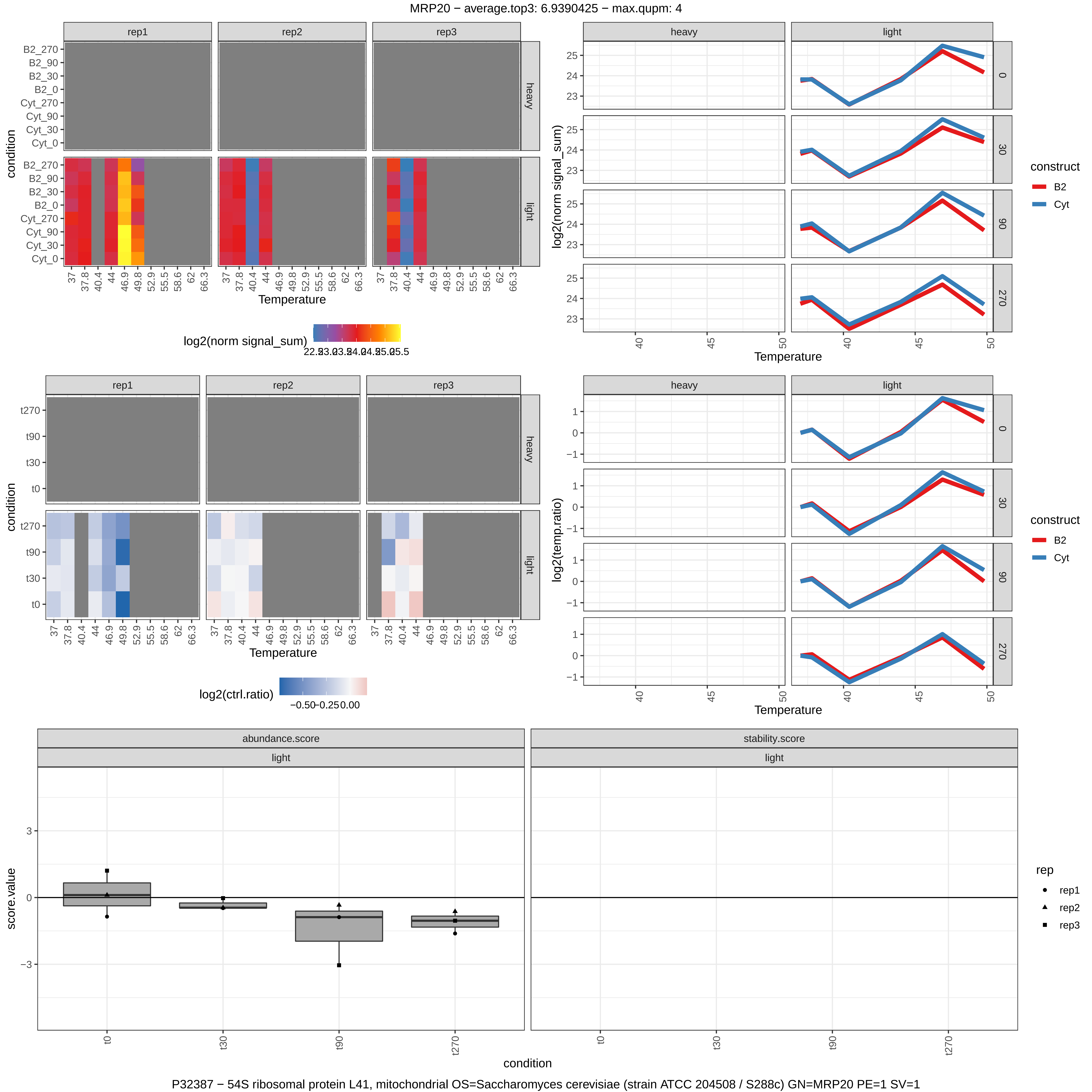

MRP20 − average.top3: 6.9390425 − max.qupm: 4
rep1
rep2
rep3
heavy
light
condition
Temperature
log2(norm signal_sum)
construct
B2
Cyt
log2(ctrl.ratio)
log2(temp.ratio)
abundance.score
stability.score
score.value
rep
rep1
rep2
rep3
P32387 − 54S ribosomal protein L41, mitochondrial OS=Saccharomyces cerevisiae (strain ATCC 204508 / S288c) GN=MRP20 PE=1 SV=1

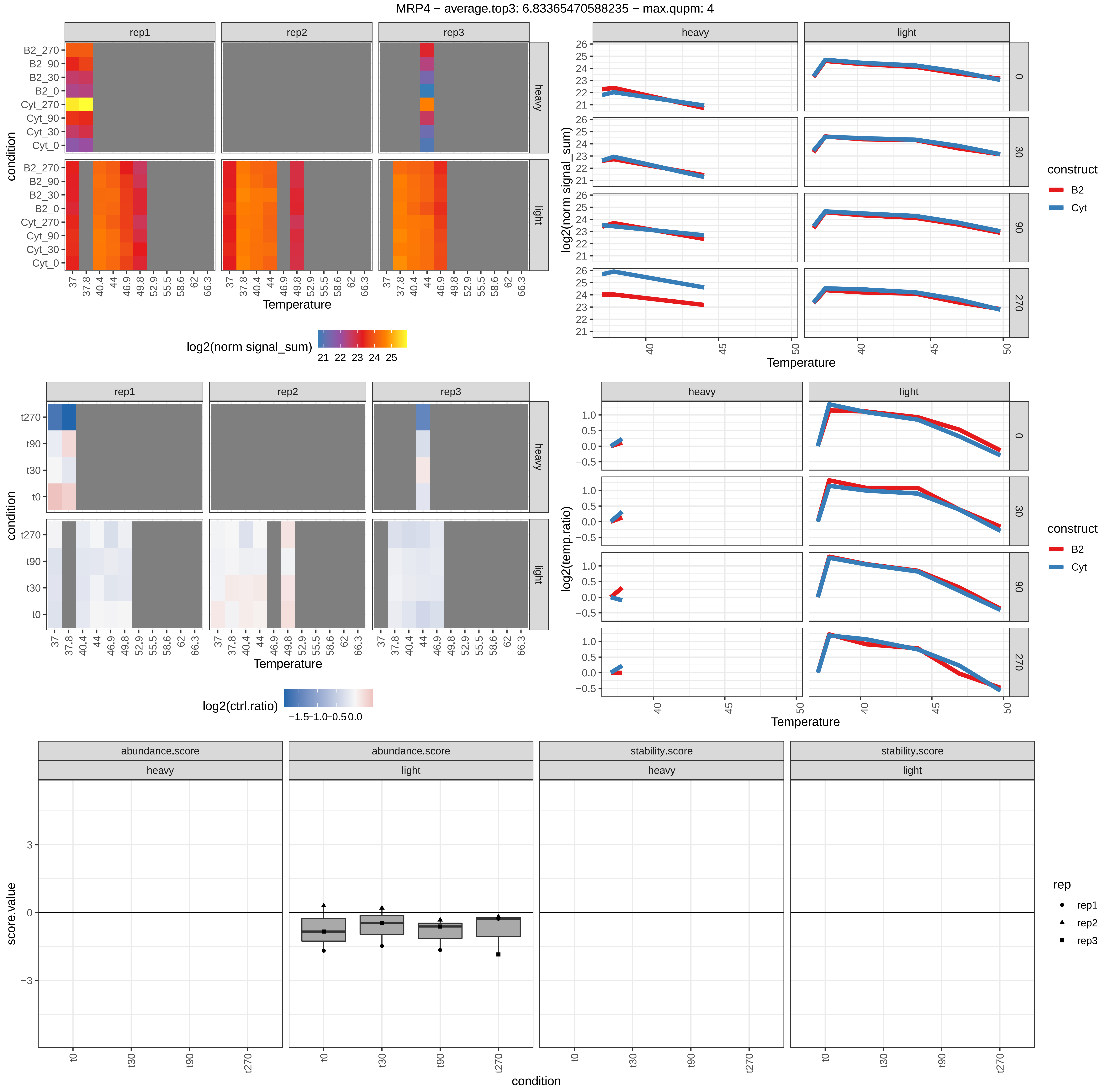

P32902 − 37S ribosomal protein MRP4, mitochondrial OS=Saccharomyces cerevisiae (strain ATCC 204508 / S288c) GN=MRP4 PE=1 SV=1

MRP51 − average.top3: 6.97344666666667 − max.qupm: 9

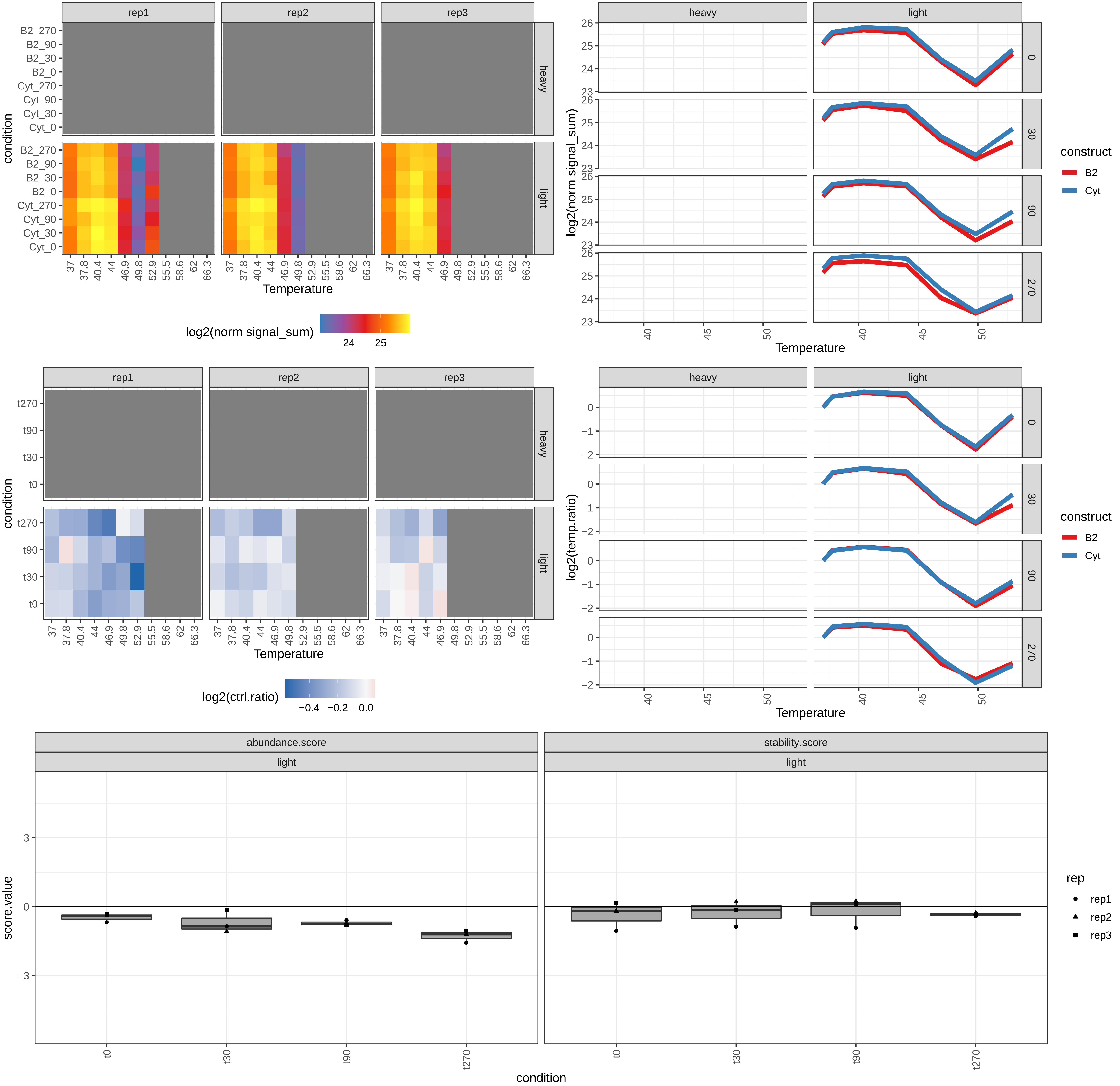

Q02950 − 37S ribosomal protein MRP51, mitochondrial OS=Saccharomyces cerevisiae (strain ATCC 204508 / S288c) GN=MRP51 PE=1 SV=1

MRP7 − average.top3: 6.96030103448276 − max.qupm: 15

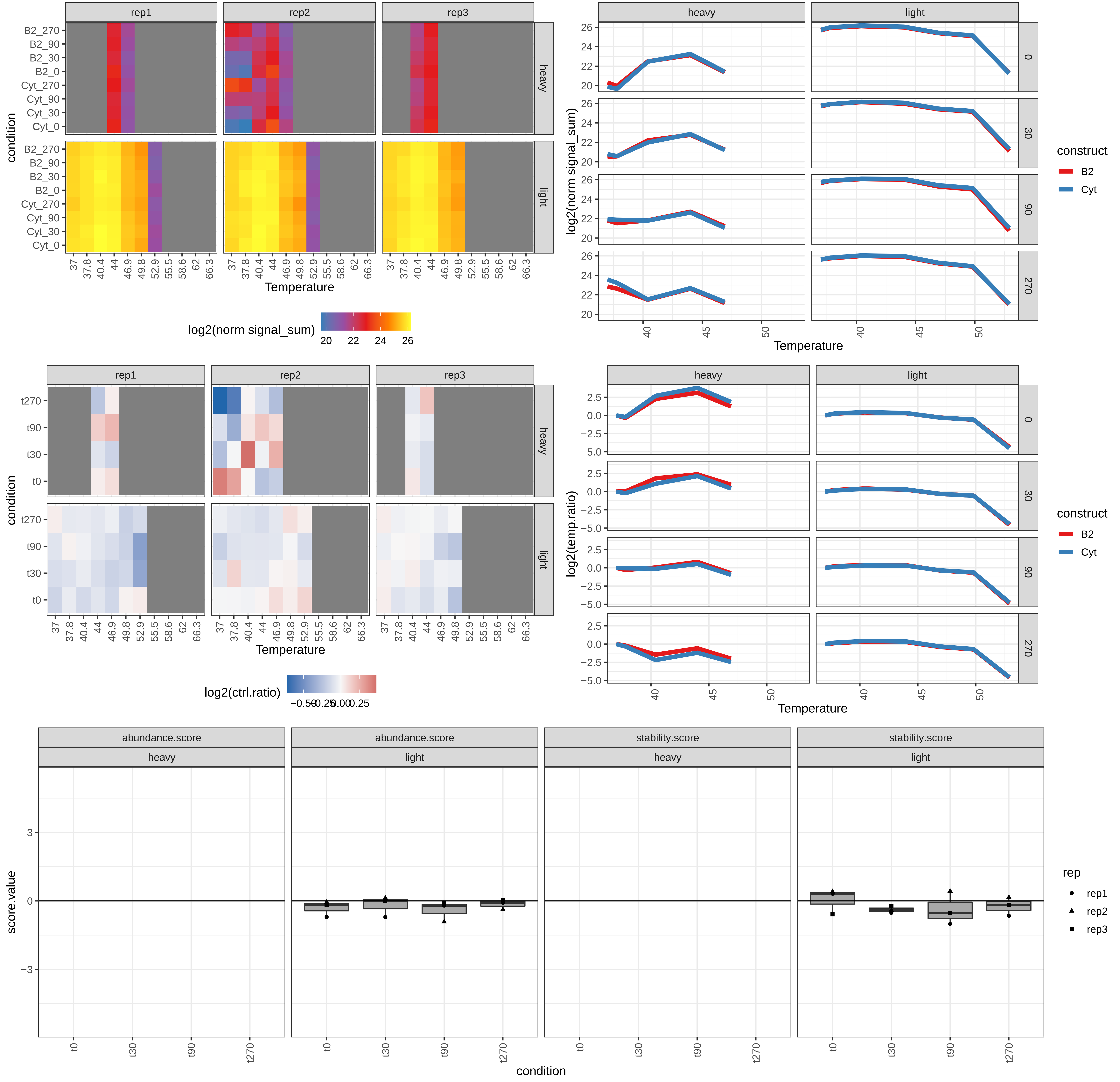

P12687 − 54S ribosomal protein L2, mitochondrial OS=Saccharomyces cerevisiae (strain ATCC 204508 / S288c) GN=MRP7 PE=1 SV=1

MRP8 − average.top3: 7.27012212765957 − max.qupm: 15

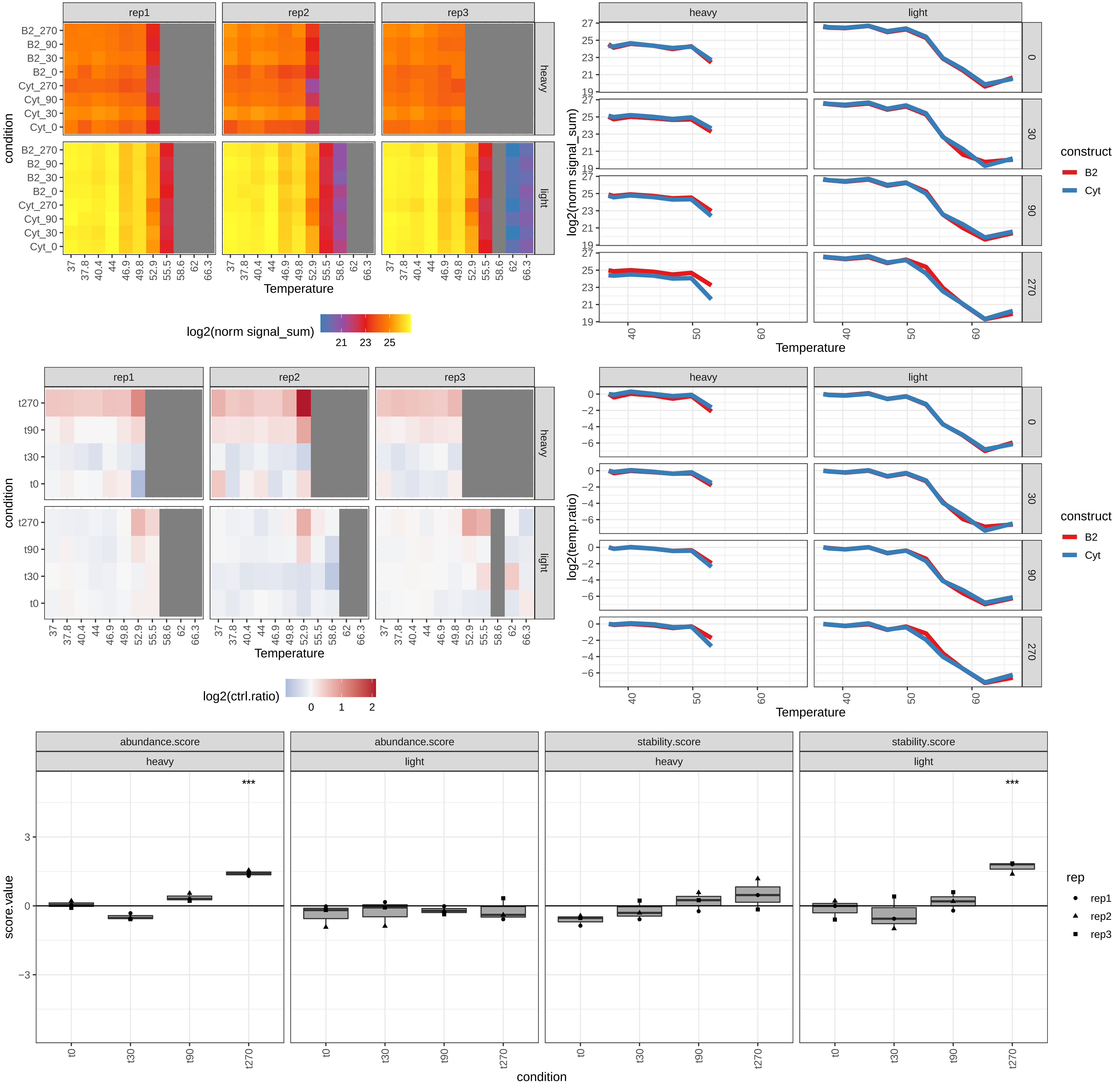

P35719 − Uncharacterized protein MRP8 OS=Saccharomyces cerevisiae (strain ATCC 204508 / S288c) GN=MRP8 PE=1 SV=1

MRPL1 − average.top3: 6.95140578947368 − max.qupm: 7

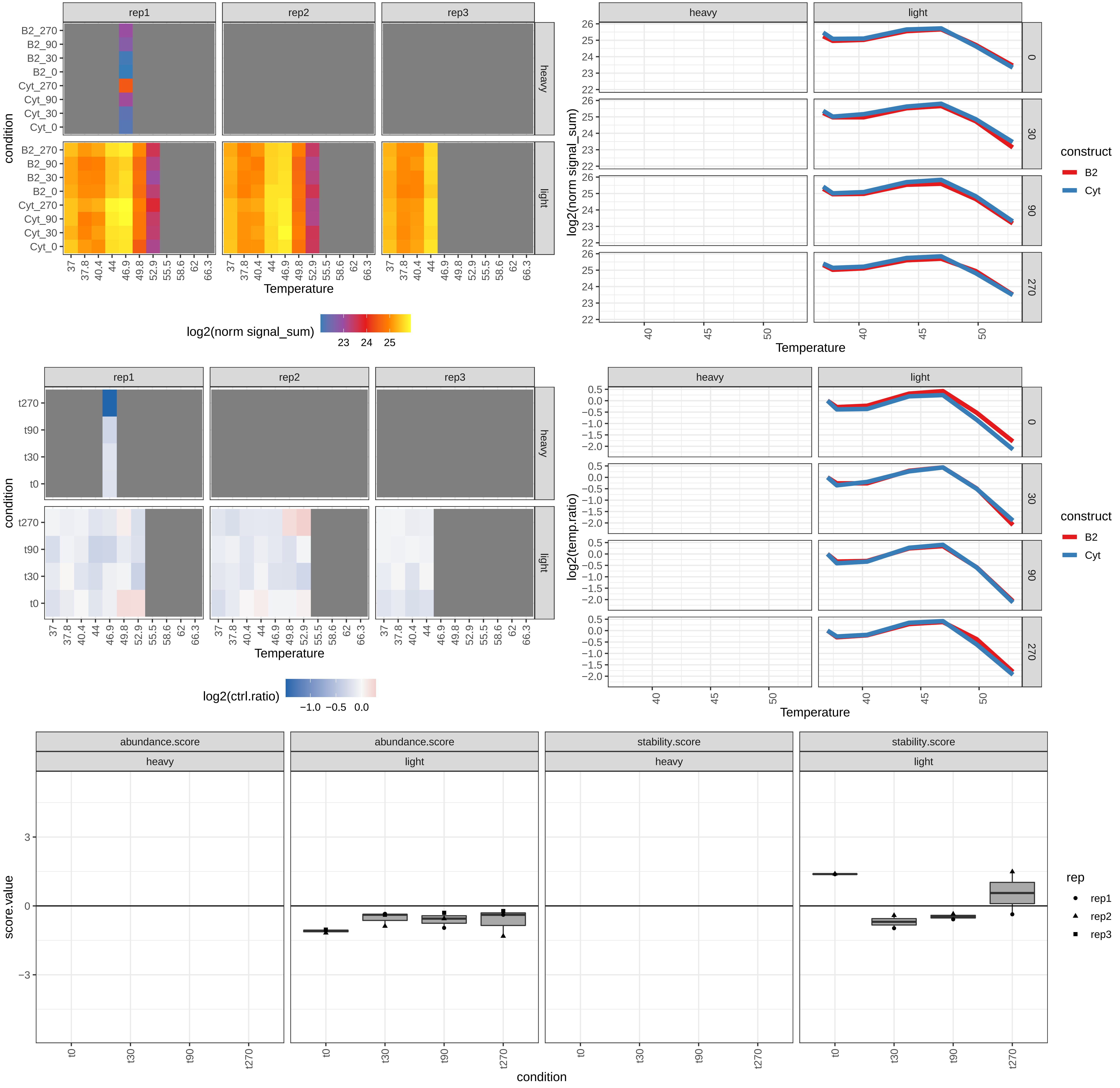

Q04599 − 54S ribosomal protein L1, mitochondrial OS=Saccharomyces cerevisiae (strain ATCC 204508 / S288c) GN=MRPL1 PE=1 SV=1

MRPL10 − average.top3: 7.226818 − max.qupm: 7

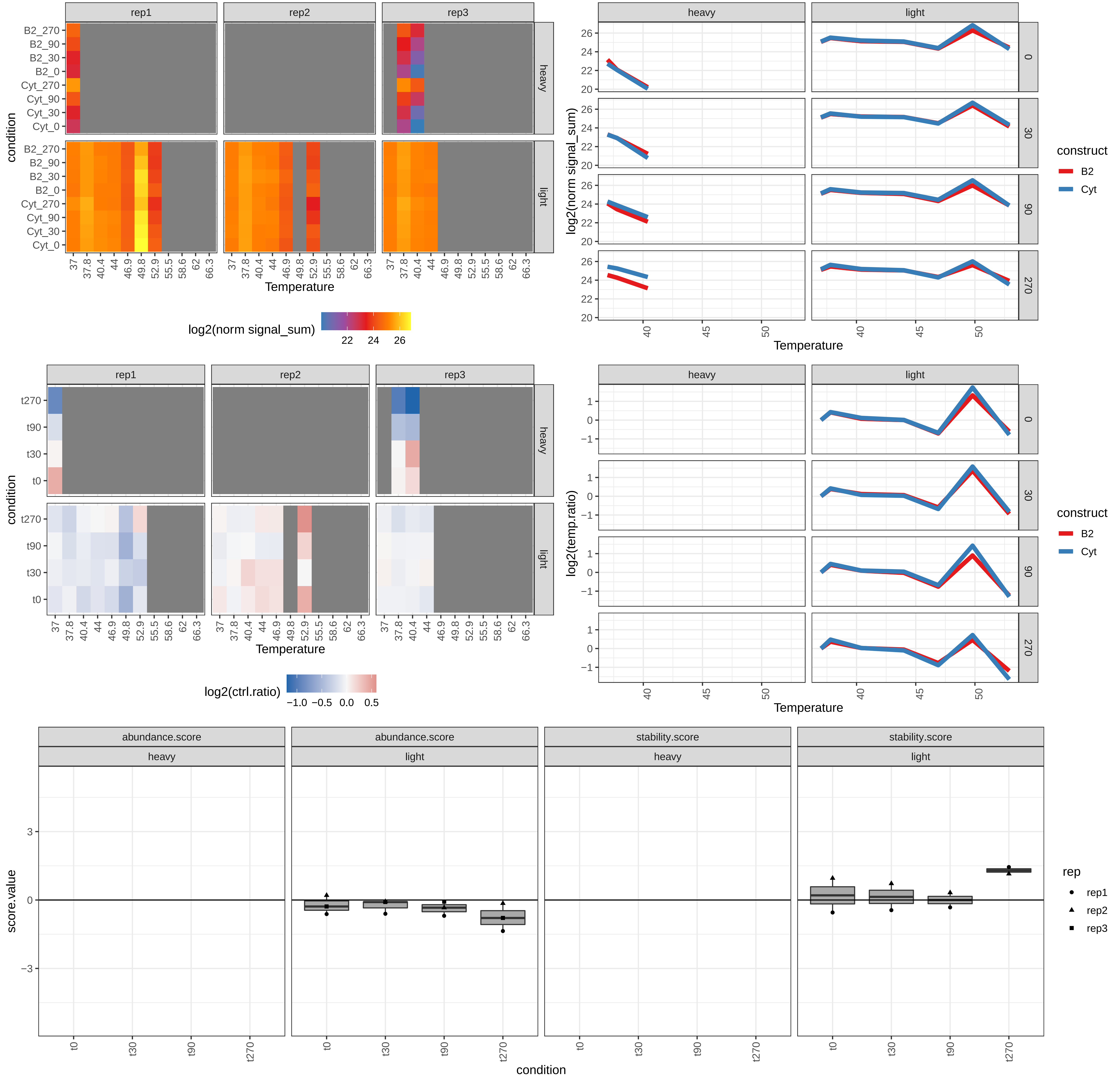

P36520 − 54S ribosomal protein L10, mitochondrial OS=Saccharomyces cerevisiae (strain ATCC 204508 / S288c) GN=MRPL10 PE=1 SV=2

MRPL11 − average.top3: 6.44546 − max.qupm: 3

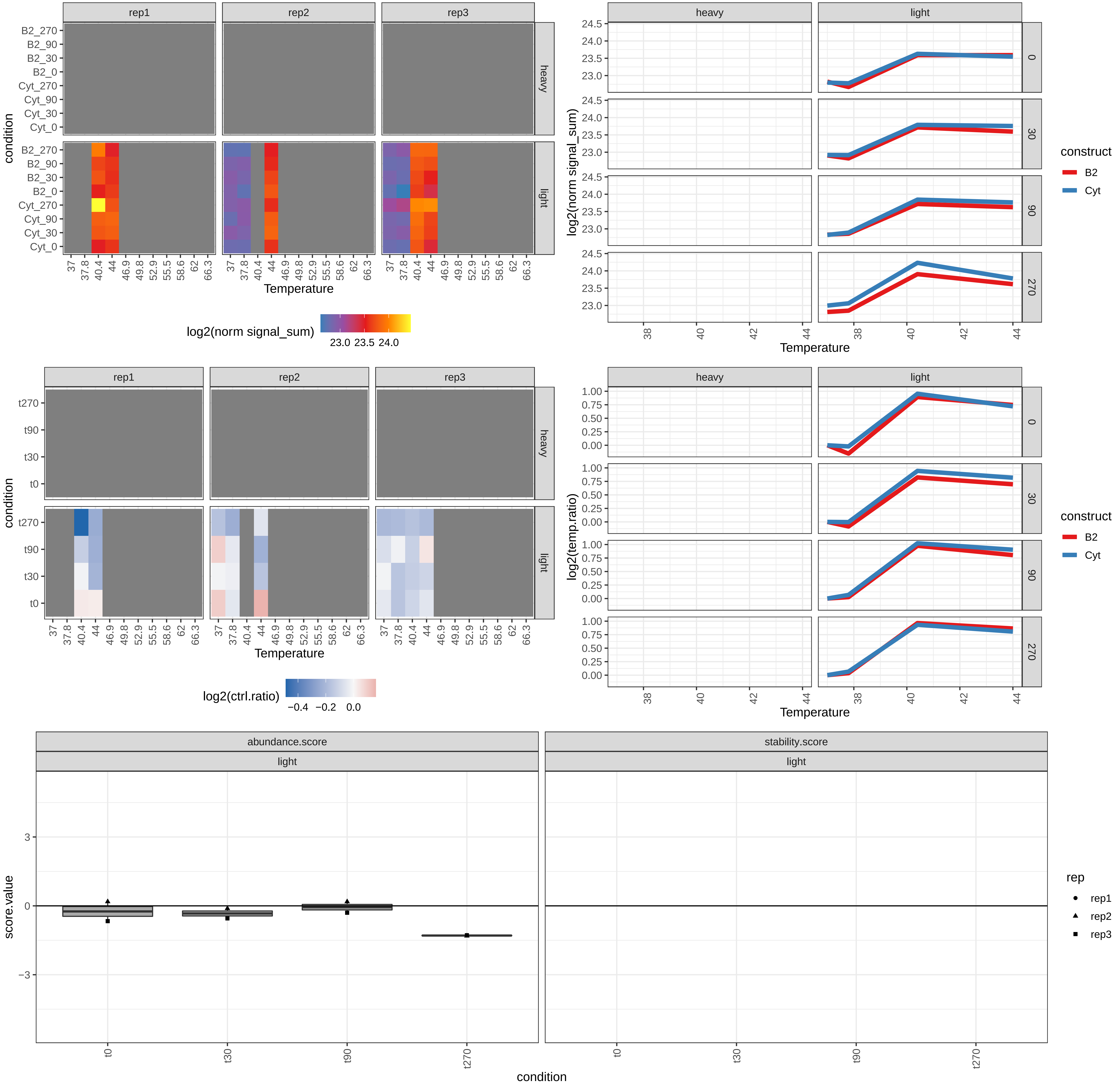

P36521 − 54S ribosomal protein L11, mitochondrial OS=Saccharomyces cerevisiae (strain ATCC 204508 / S288c) GN=MRPL11 PE=1 SV=2

MRPL15 − average.top3: 6.8041 − max.qupm: 5

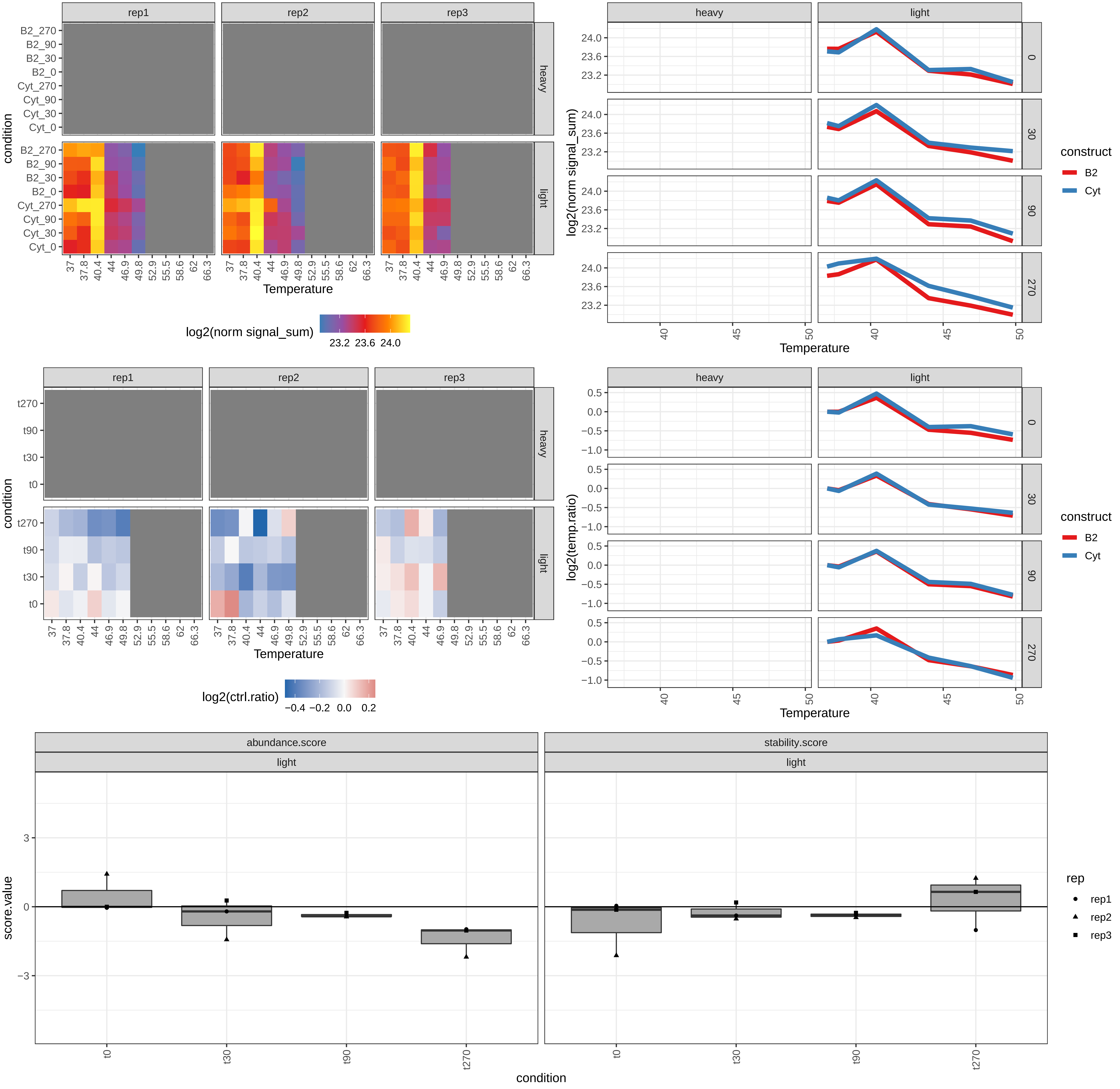

P36523 − 54S ribosomal protein L15, mitochondrial OS=Saccharomyces cerevisiae (strain ATCC 204508 / S288c) GN=MRPL15 PE=1 SV=3

MRPL16 − average.top3: 6.89504 − max.qupm: 5

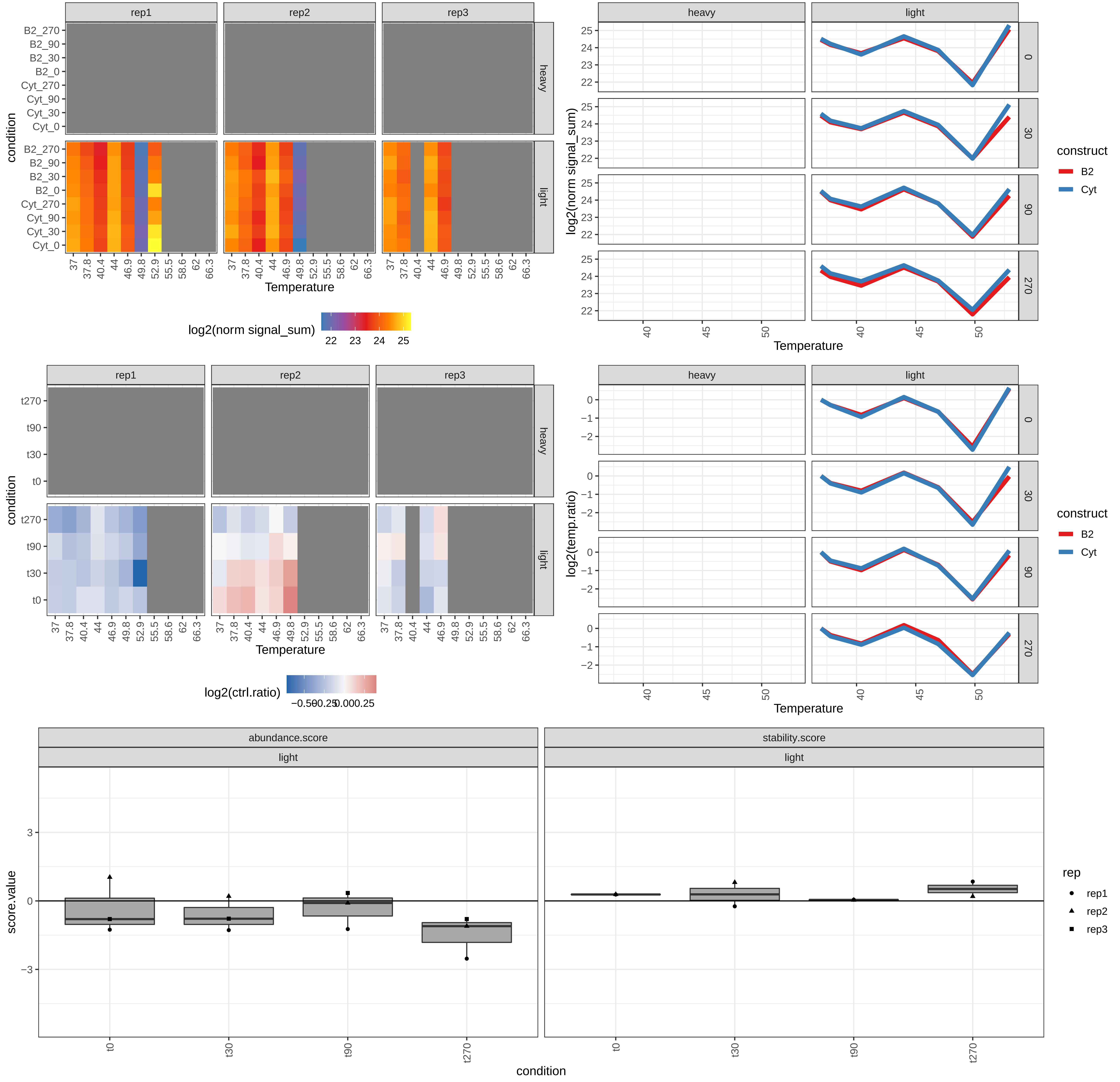

P38064 − 54S ribosomal protein L16, mitochondrial OS=Saccharomyces cerevisiae (strain ATCC 204508 / S288c) GN=MRPL16 PE=1 SV=1

MRPL17 − average.top3: 6.952798 − max.qupm: 8

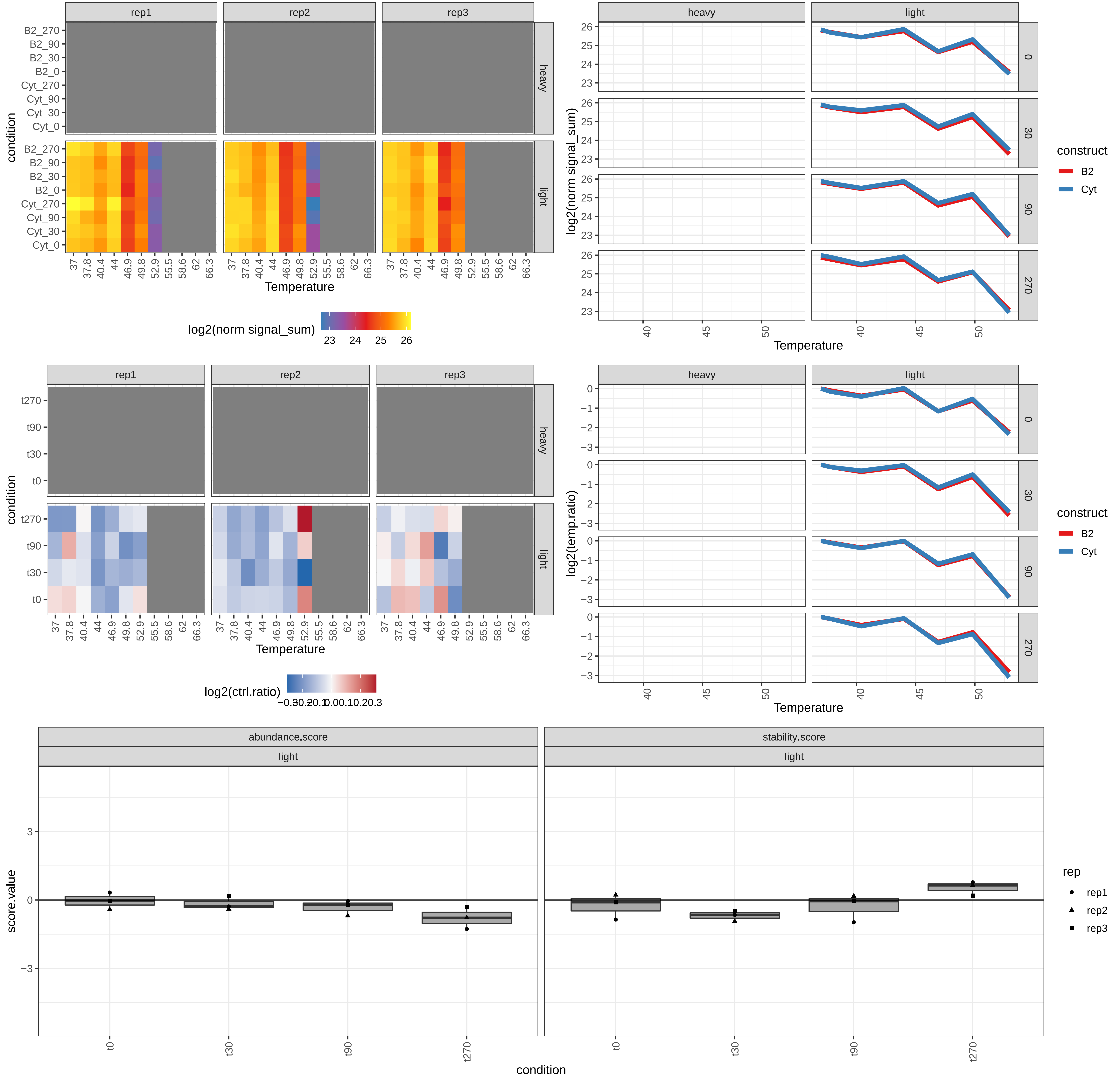

P36528 − 54S ribosomal protein L17, mitochondrial OS=Saccharomyces cerevisiae (strain ATCC 204508 / S288c) GN=MRPL17 PE=1 SV=2

MRPL20 − average.top3: 6.62956428571429 − max.qupm: 4

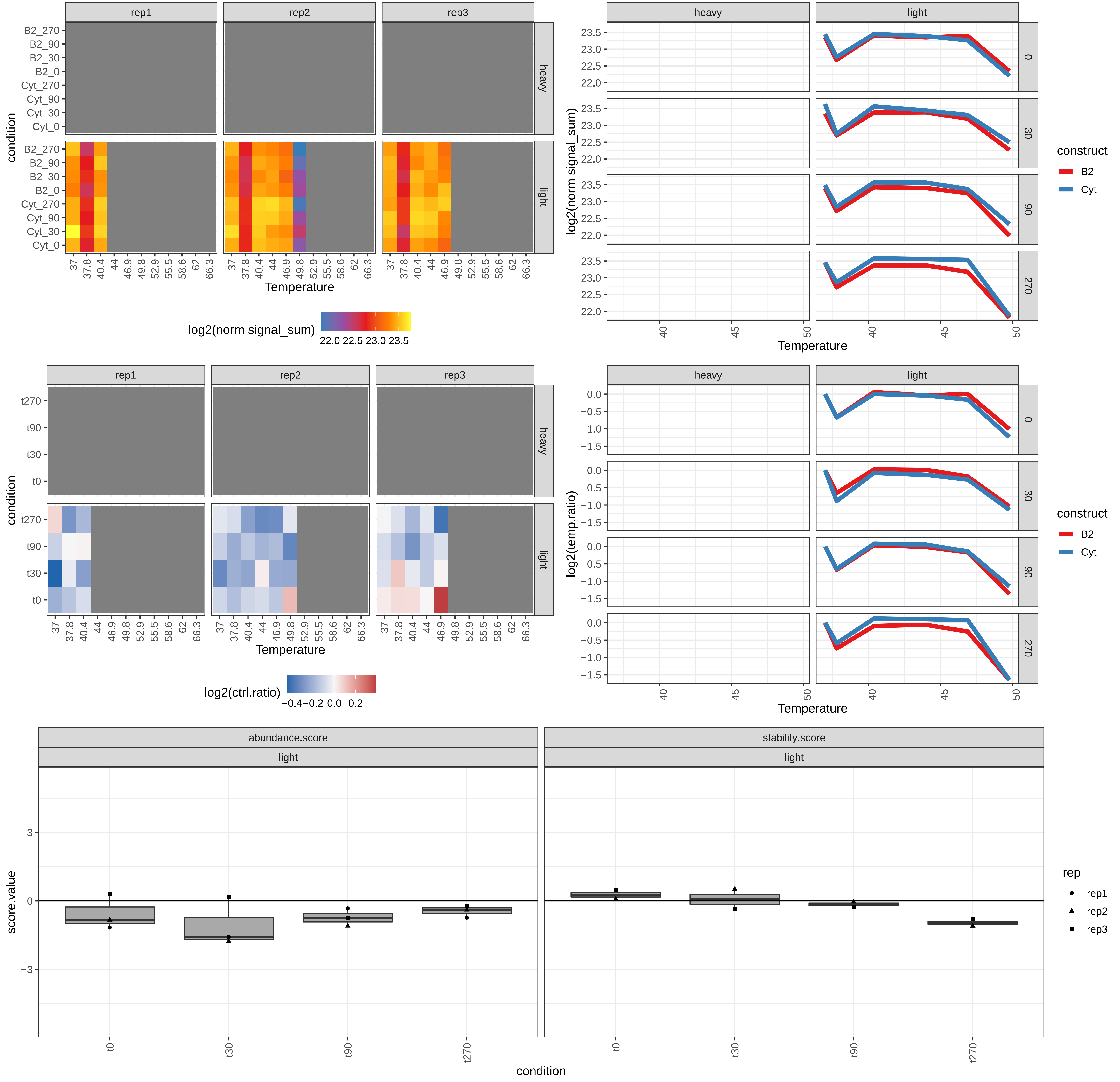

P22354 − 54S ribosomal protein L20, mitochondrial OS=Saccharomyces cerevisiae (strain ATCC 204508 / S288c) GN=MRPL20 PE=1 SV=2

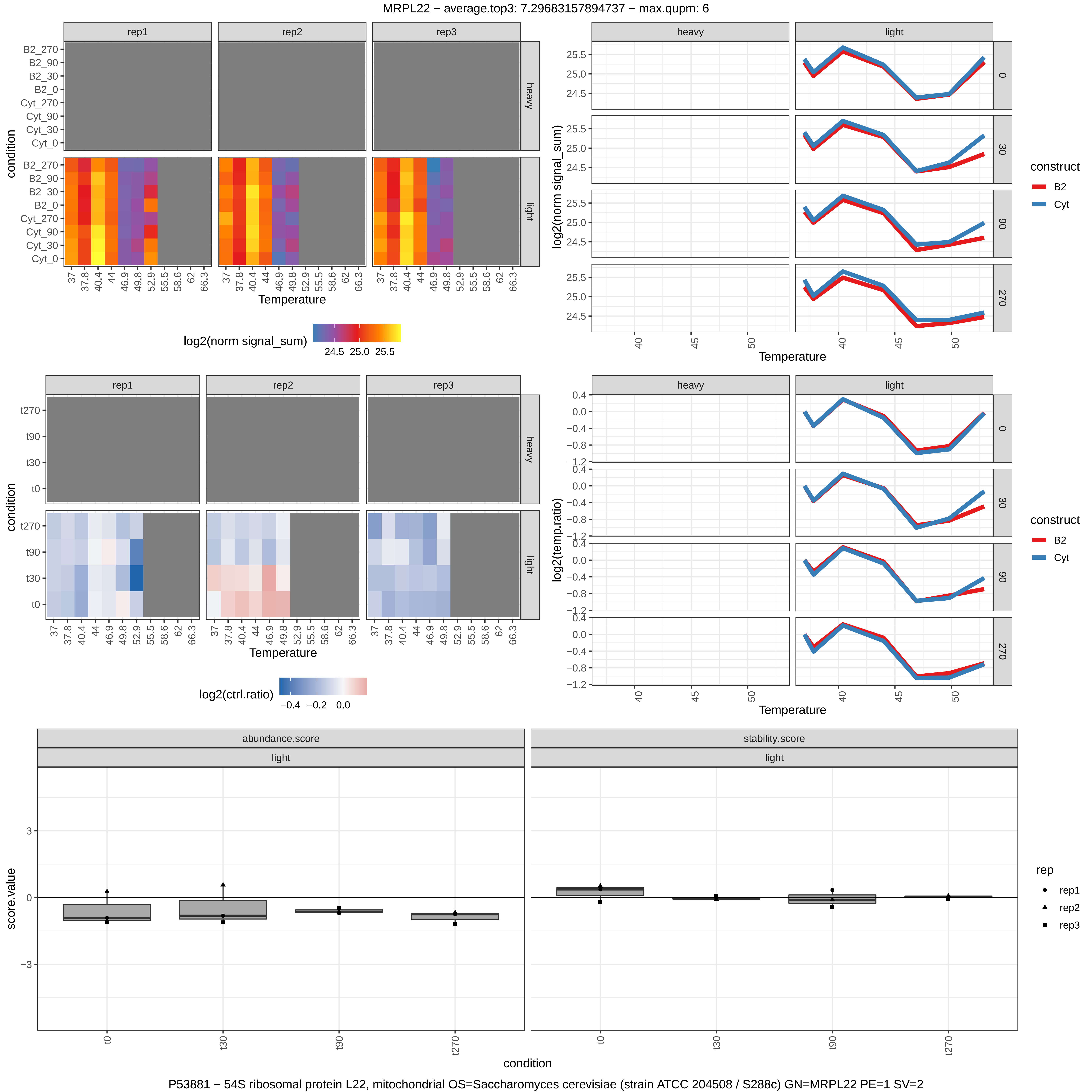

MRPL22 − average.top3: 7.29683157894737 − max.qupm: 6
rep1
rep2
rep3
heavy
light
condition
Temperature
log2(norm signal_sum)
construct
B2
Cyt
log2(temp.ratio)
log2(ctrl.ratio)
abundance.score
stability.score
score.value
rep
rep1
rep2
rep3
P53881 − 54S ribosomal protein L22, mitochondrial OS=Saccharomyces cerevisiae (strain ATCC 204508 / S288c) GN=MRPL22 PE=1 SV=2

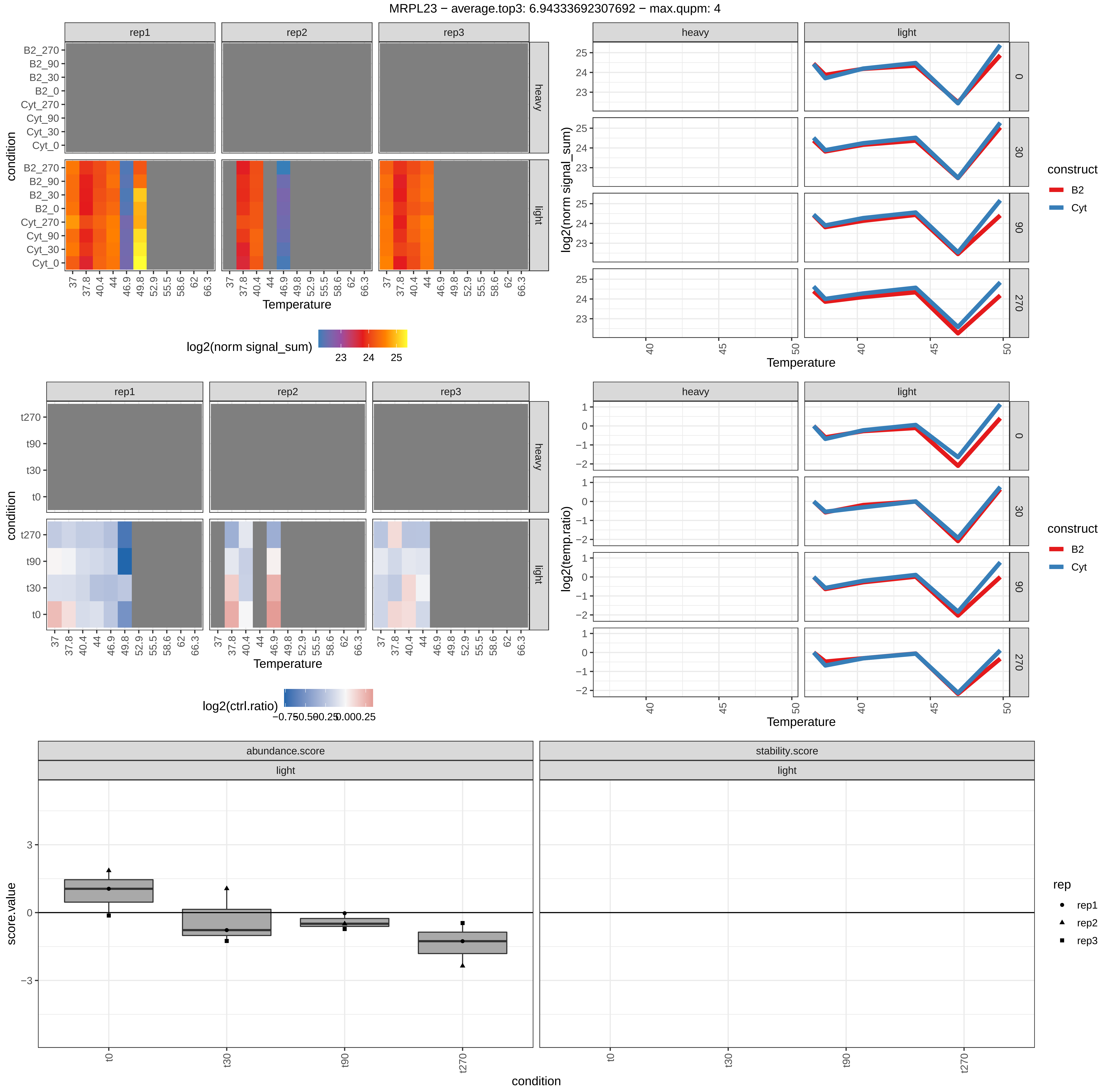

Q12487 − 54S ribosomal protein L23, mitochondrial OS=Saccharomyces cerevisiae (strain ATCC 204508 / S288c) GN=MRPL23 PE=1 SV=1

MRPL24 − average.top3: 6.85570722222222 − max.qupm: 6

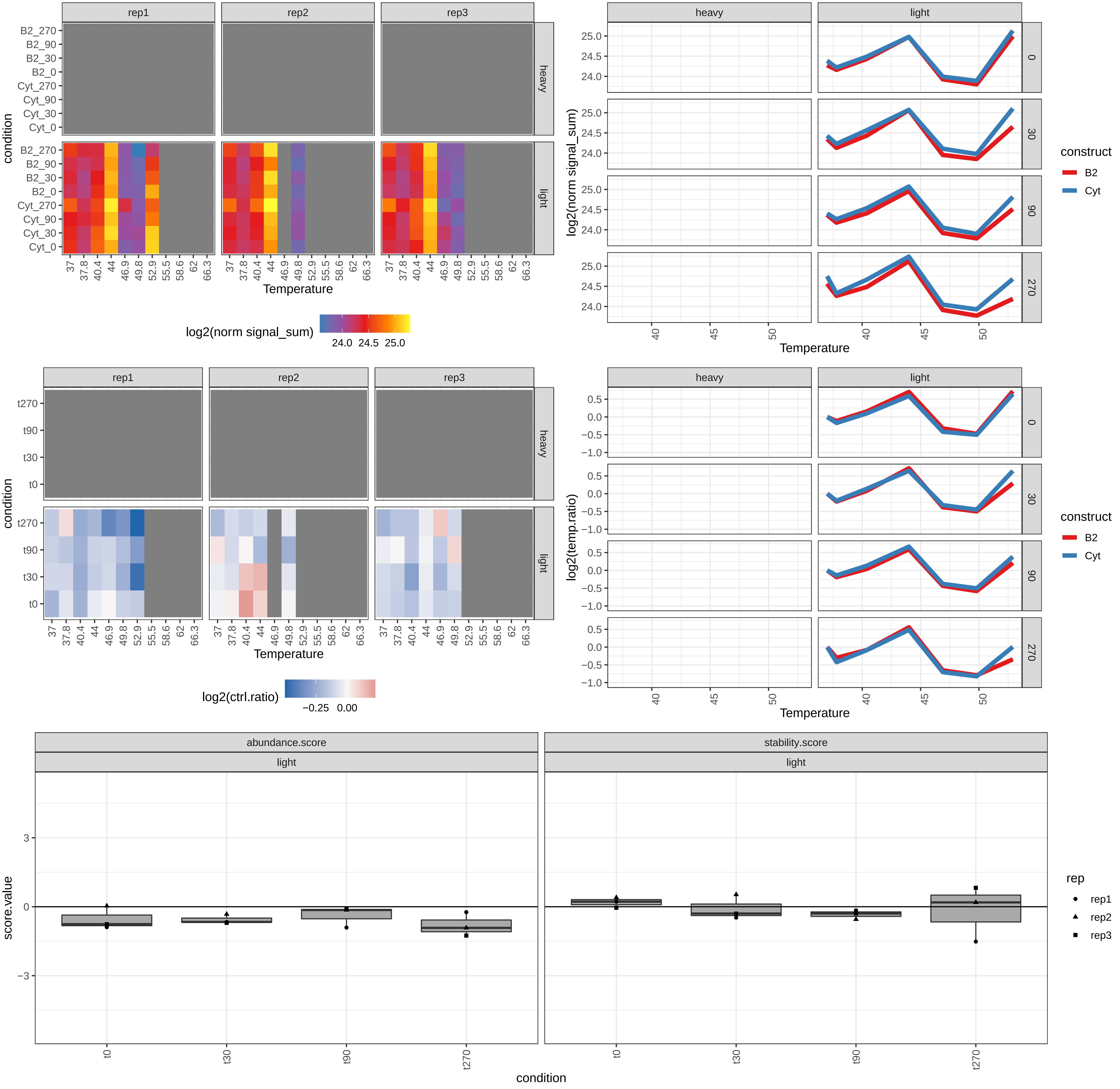

P36525 − 54S ribosomal protein L24, mitochondrial OS=Saccharomyces cerevisiae (strain ATCC 204508 / S288c) GN=MRPL24 PE=1 SV=2

MRPL25 − average.top3: 6.923965 − max.qupm: 4

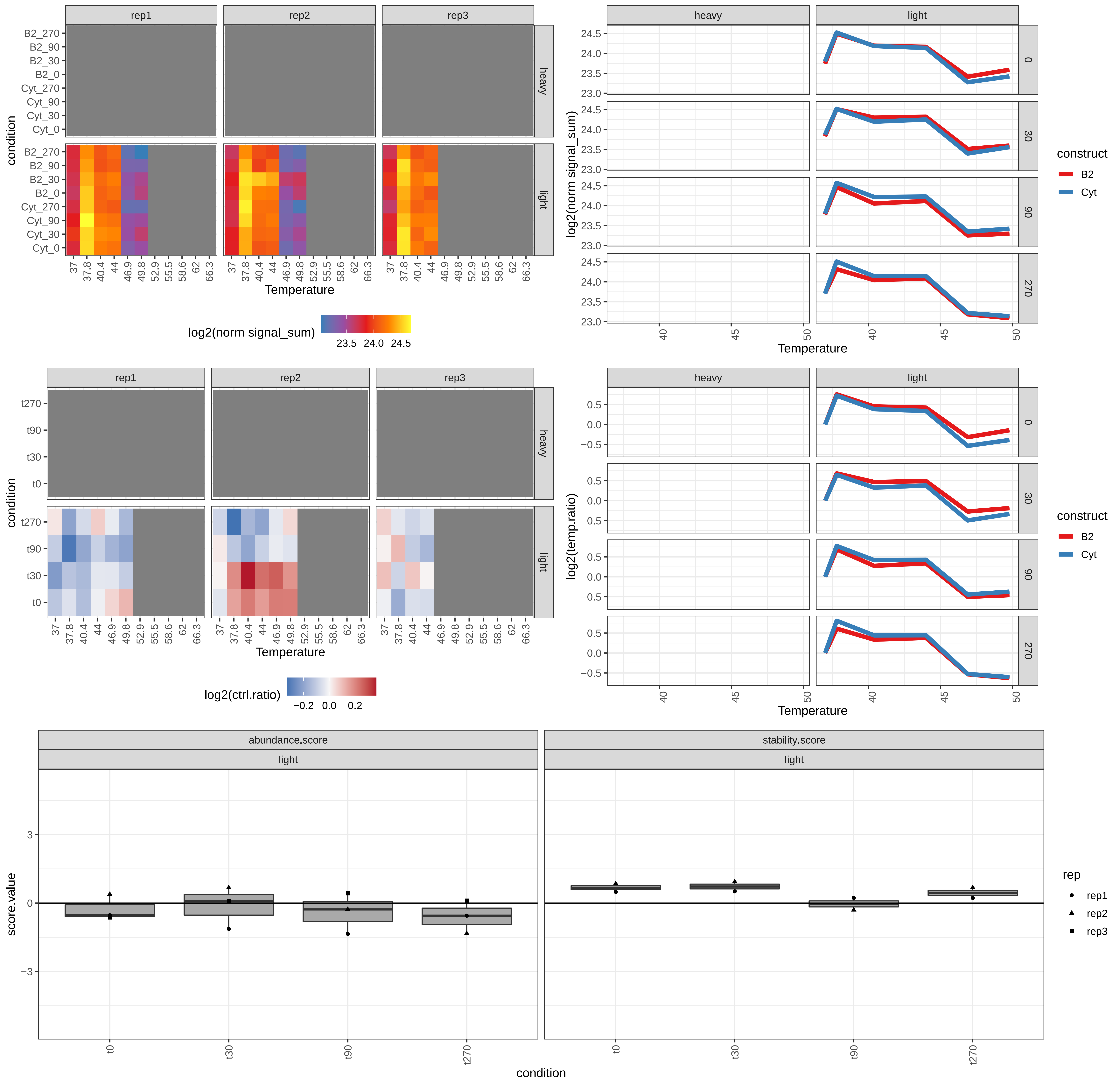

P23369 − 54S ribosomal protein L25, mitochondrial OS=Saccharomyces cerevisiae (strain ATCC 204508 / S288c) GN=MRPL25 PE=1 SV=2

MRPL27 − average.top3: 6.856164 − max.qupm: 4

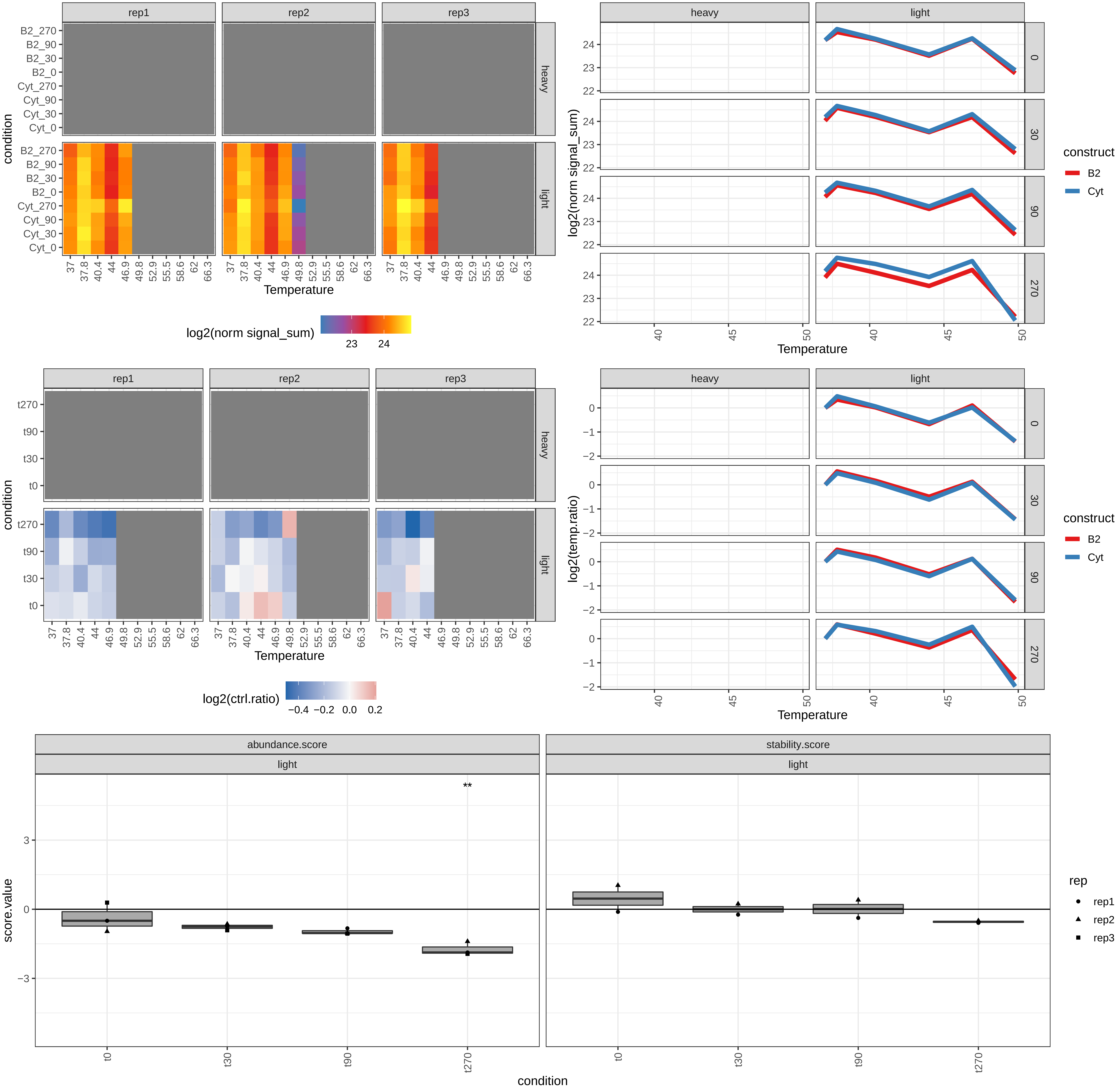

P36526 − 54S ribosomal protein L27, mitochondrial OS=Saccharomyces cerevisiae (strain ATCC 204508 / S288c) GN=MRPL27 PE=1 SV=2

MRPL3 − average.top3: 7.2315535 − max.qupm: 12

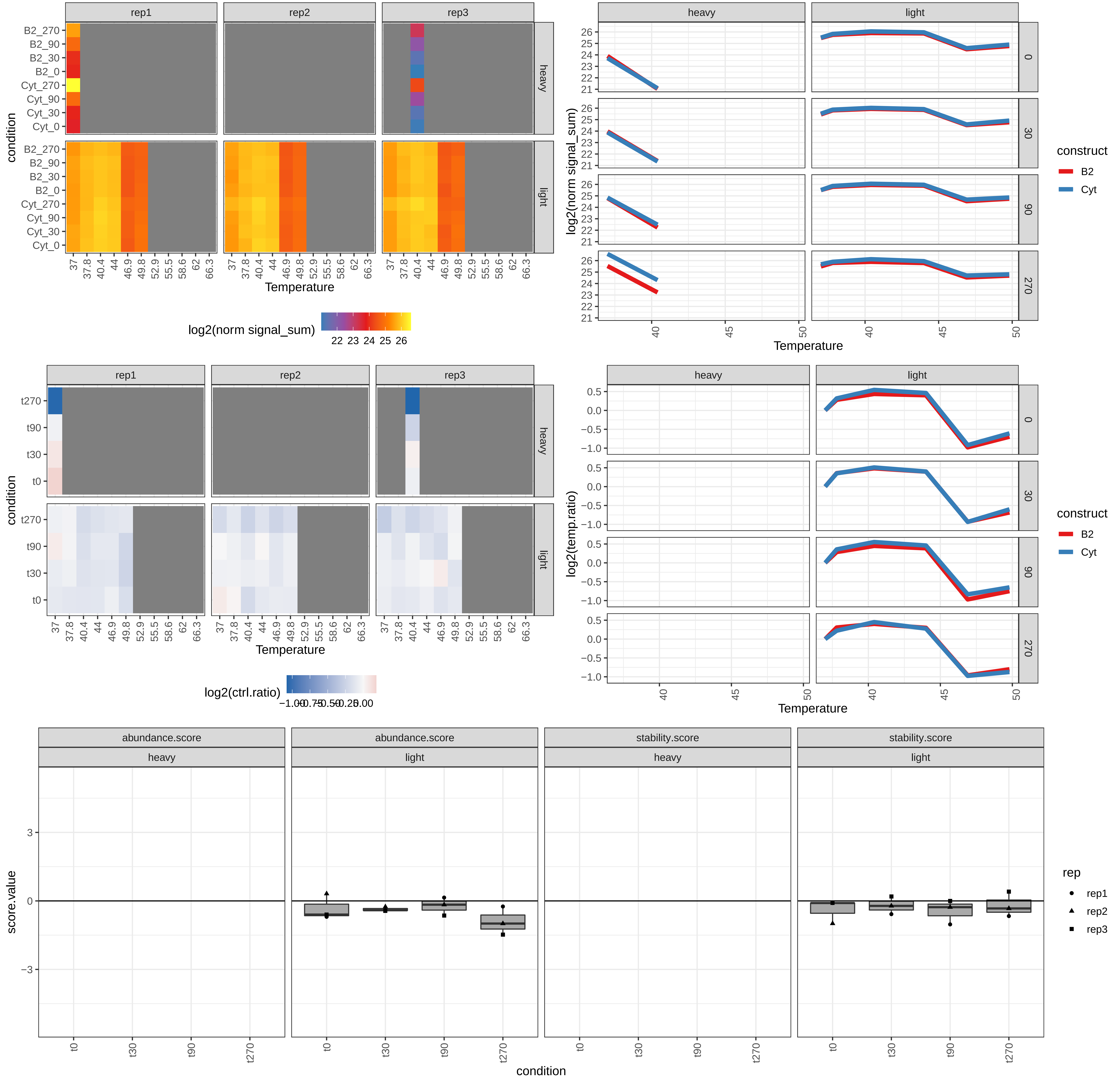

P36516 − 54S ribosomal protein L3, mitochondrial OS=Saccharomyces cerevisiae (strain ATCC 204508 / S288c) GN=MRPL3 PE=1 SV=2

MRPL32 − average.top3: 6.72935666666667 − max.qupm: 3

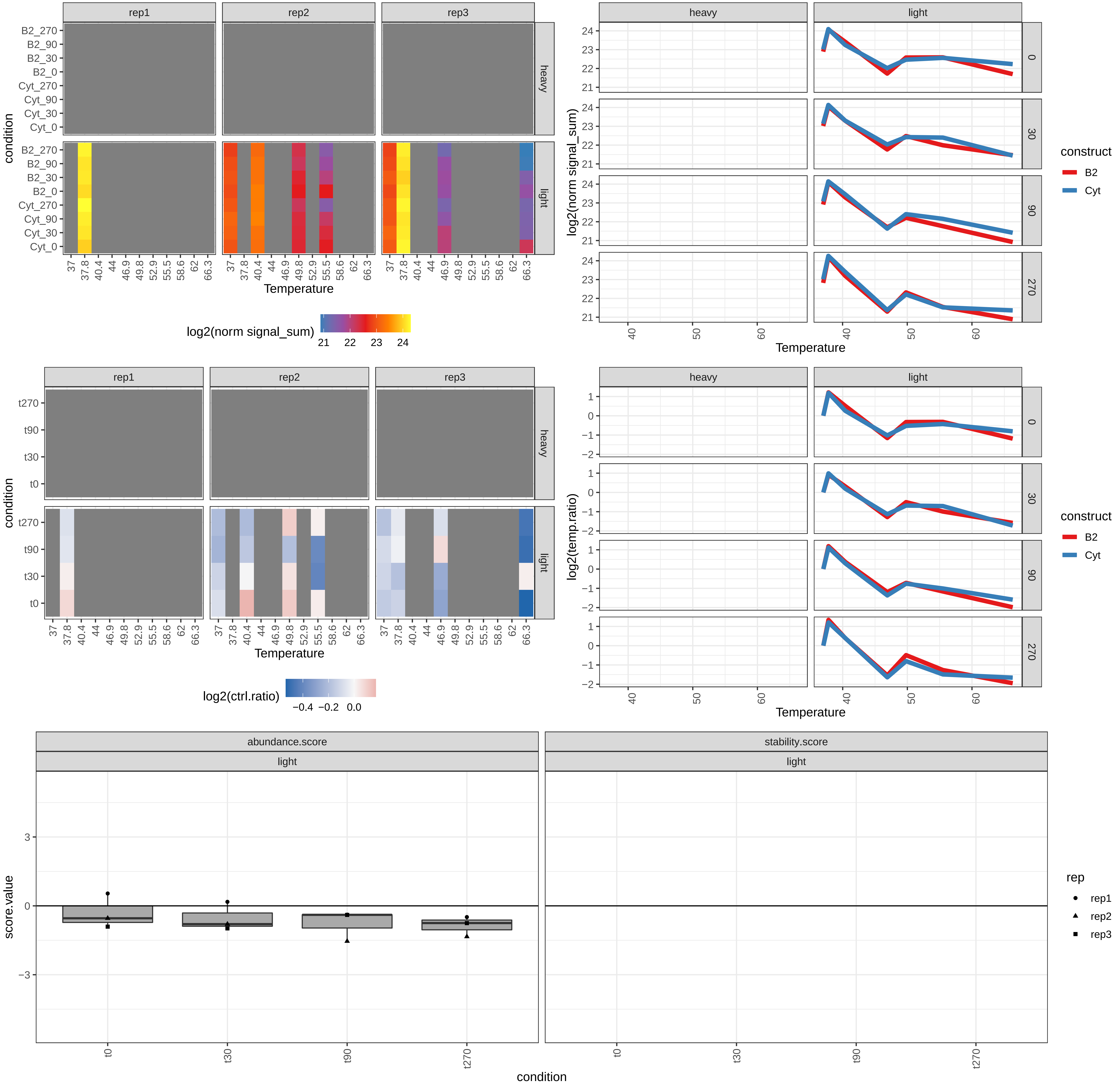

P25348 − 54S ribosomal protein L32, mitochondrial OS=Saccharomyces cerevisiae (strain ATCC 204508 / S288c) GN=MRPL32 PE=1 SV=1

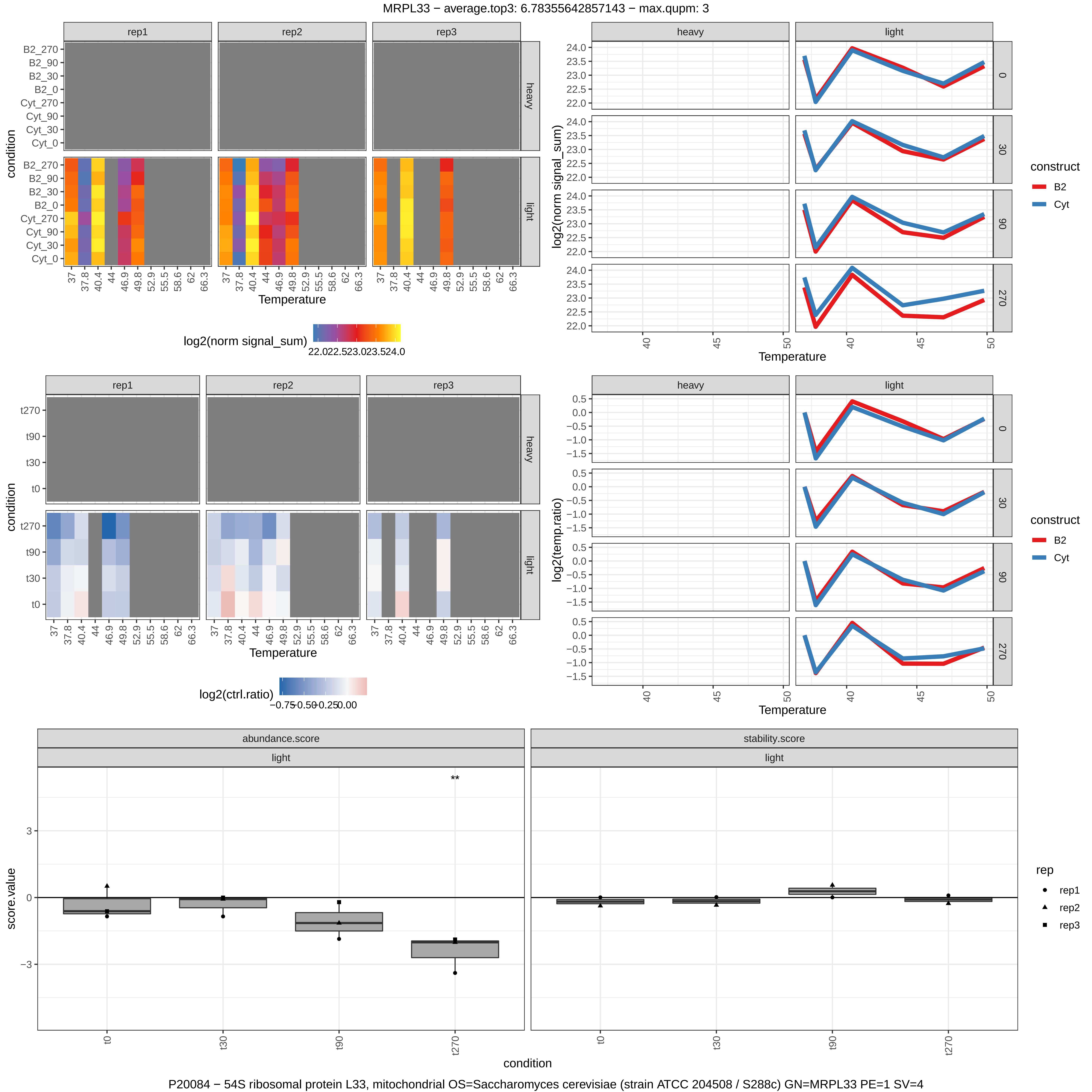

MRPL33 − average.top3: 6.78355642857143 − max.qupm: 3
rep1
rep2
rep3
heavy
light
condition
Temperature
log2(norm signal_sum)
construct
B2
Cyt
log2(temp.ratio)
log2(ctrl.ratio)
abundance.score
stability.score
score.value
rep
**
P20084 − 54S ribosomal protein L33, mitochondrial OS=Saccharomyces cerevisiae (strain ATCC 204508 / S288c) GN=MRPL33 PE=1 SV=4

MRPL35 − average.top3: 7.13258125 − max.qupm: 10

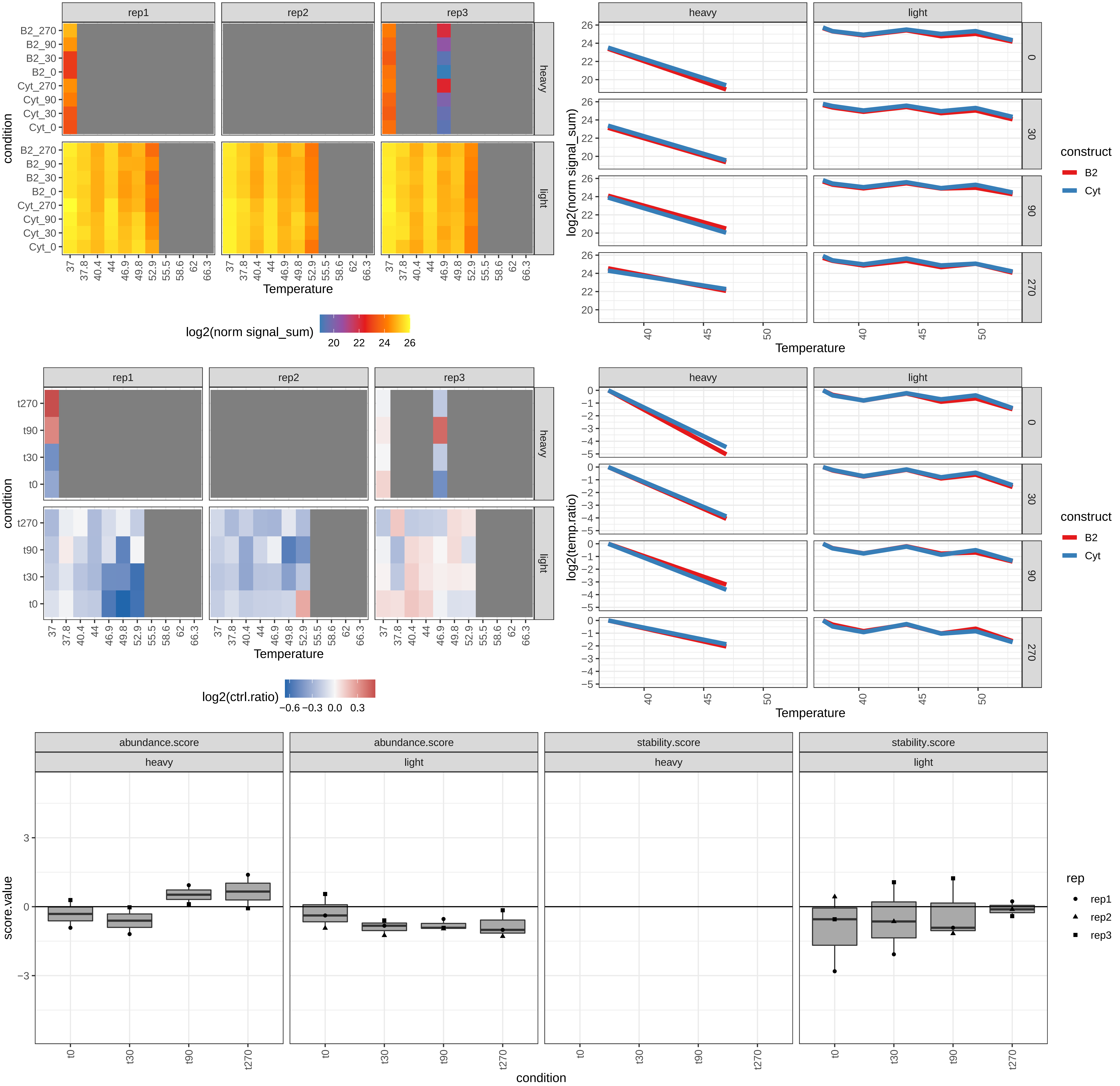

Q06678 − 54S ribosomal protein L35, mitochondrial OS=Saccharomyces cerevisiae (strain ATCC 204508 / S288c) GN=MRPL35 PE=1 SV=1

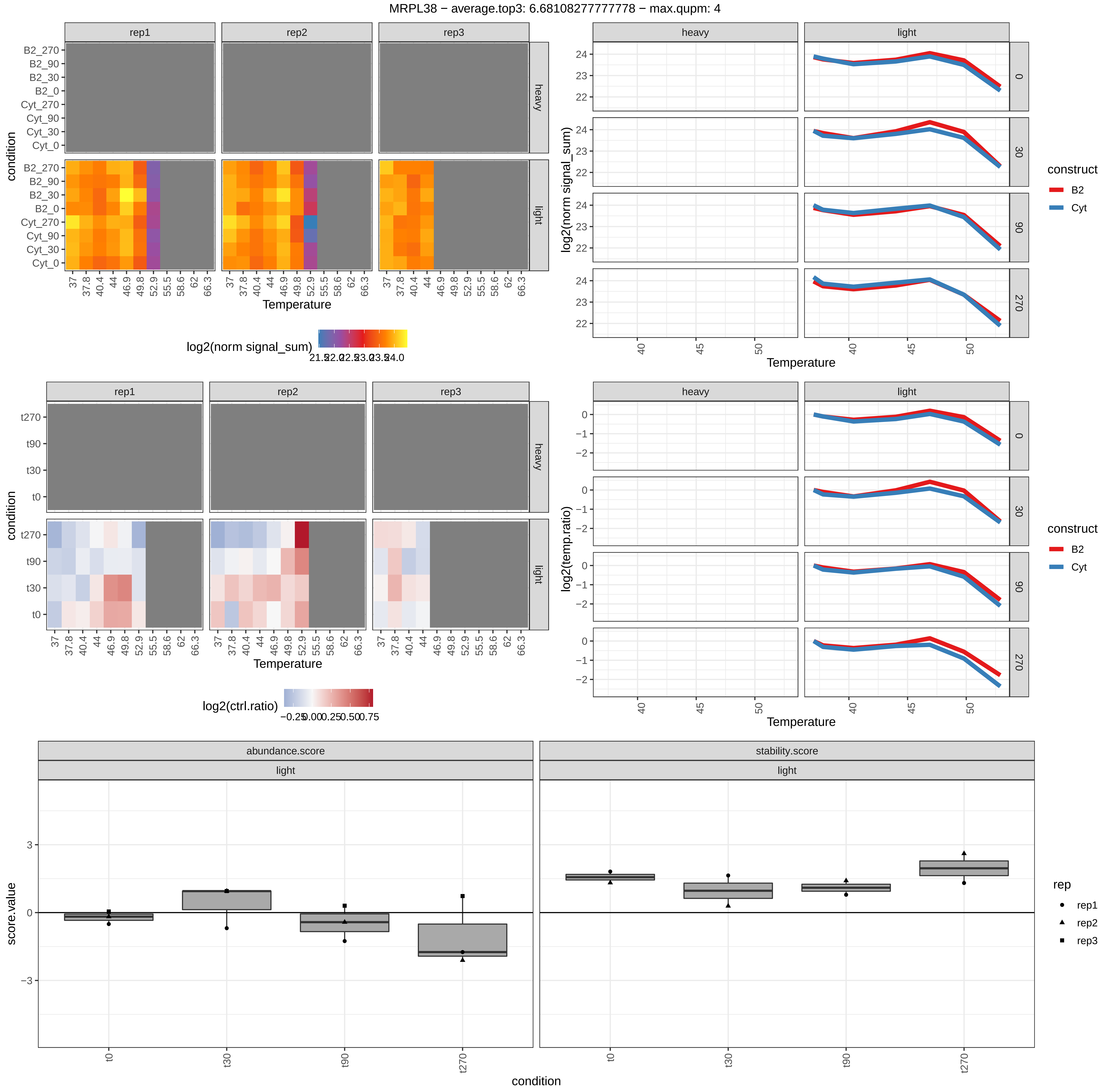

P35996 − 54S ribosomal protein L38, mitochondrial OS=Saccharomyces cerevisiae (strain ATCC 204508 / S288c) GN=MRPL38 PE=1 SV=1

MRPL4 − average.top3: 6.92682714285714 − max.qupm: 6

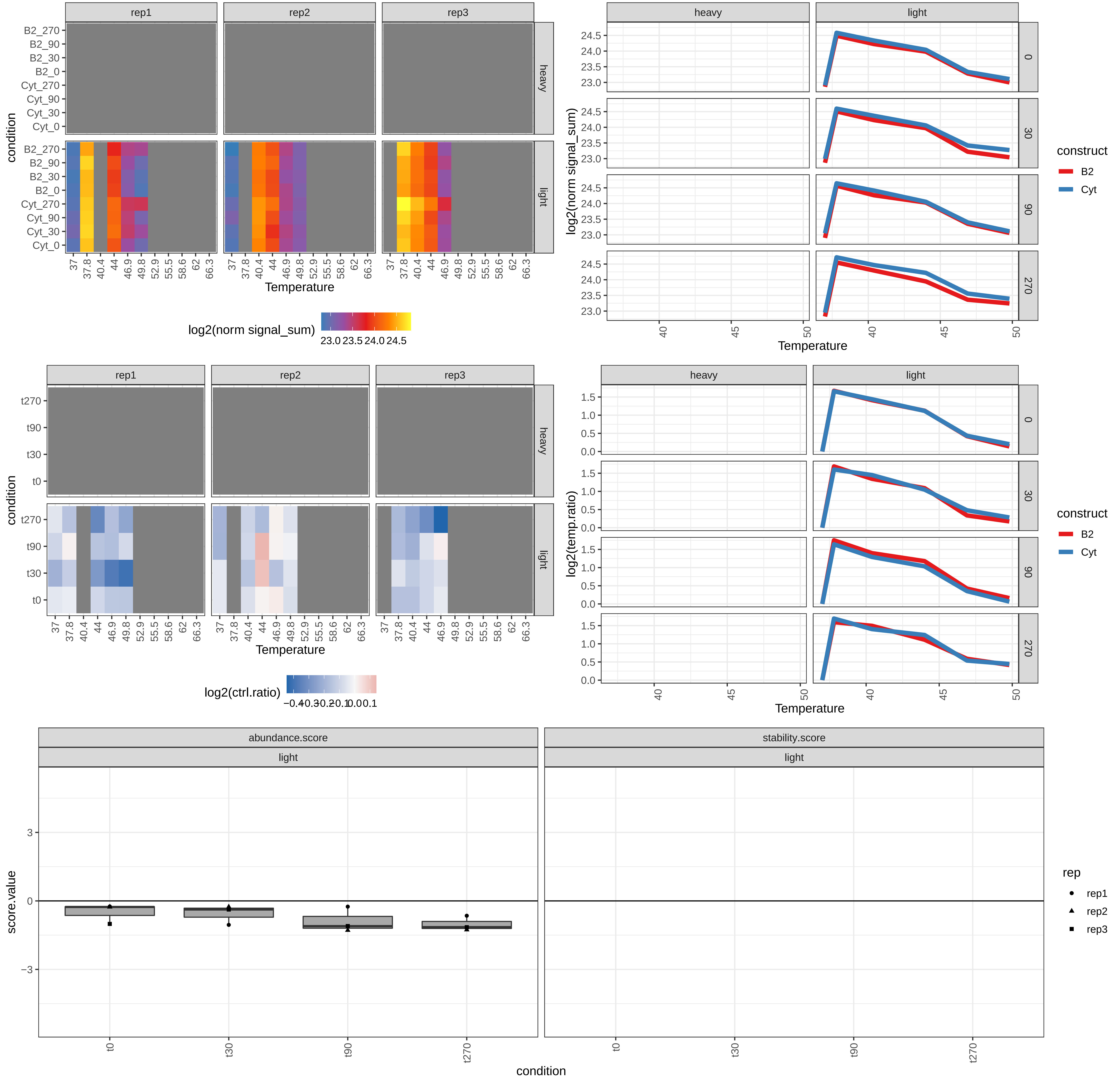

P36517 − 54S ribosomal protein L4, mitochondrial OS=Saccharomyces cerevisiae (strain ATCC 204508 / S288c) GN=MRPL4 PE=1 SV=2

MRPL40 − average.top3: 6.80114611111111 − max.qupm: 7

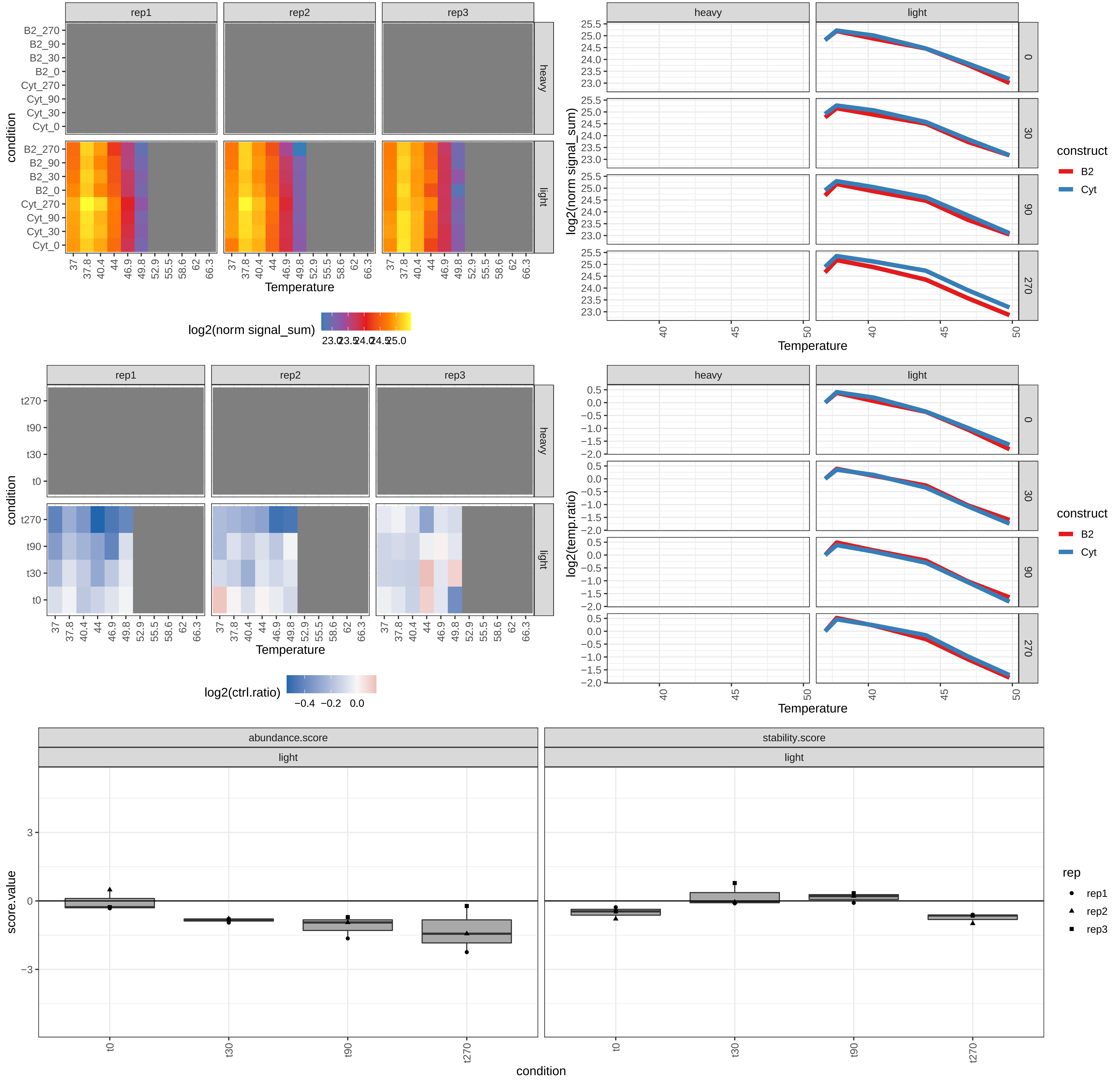

P36534 − 54S ribosomal protein L40, mitochondrial OS=Saccharomyces cerevisiae (strain ATCC 204508 / S288c) GN=MRPL40 PE=1 SV=2

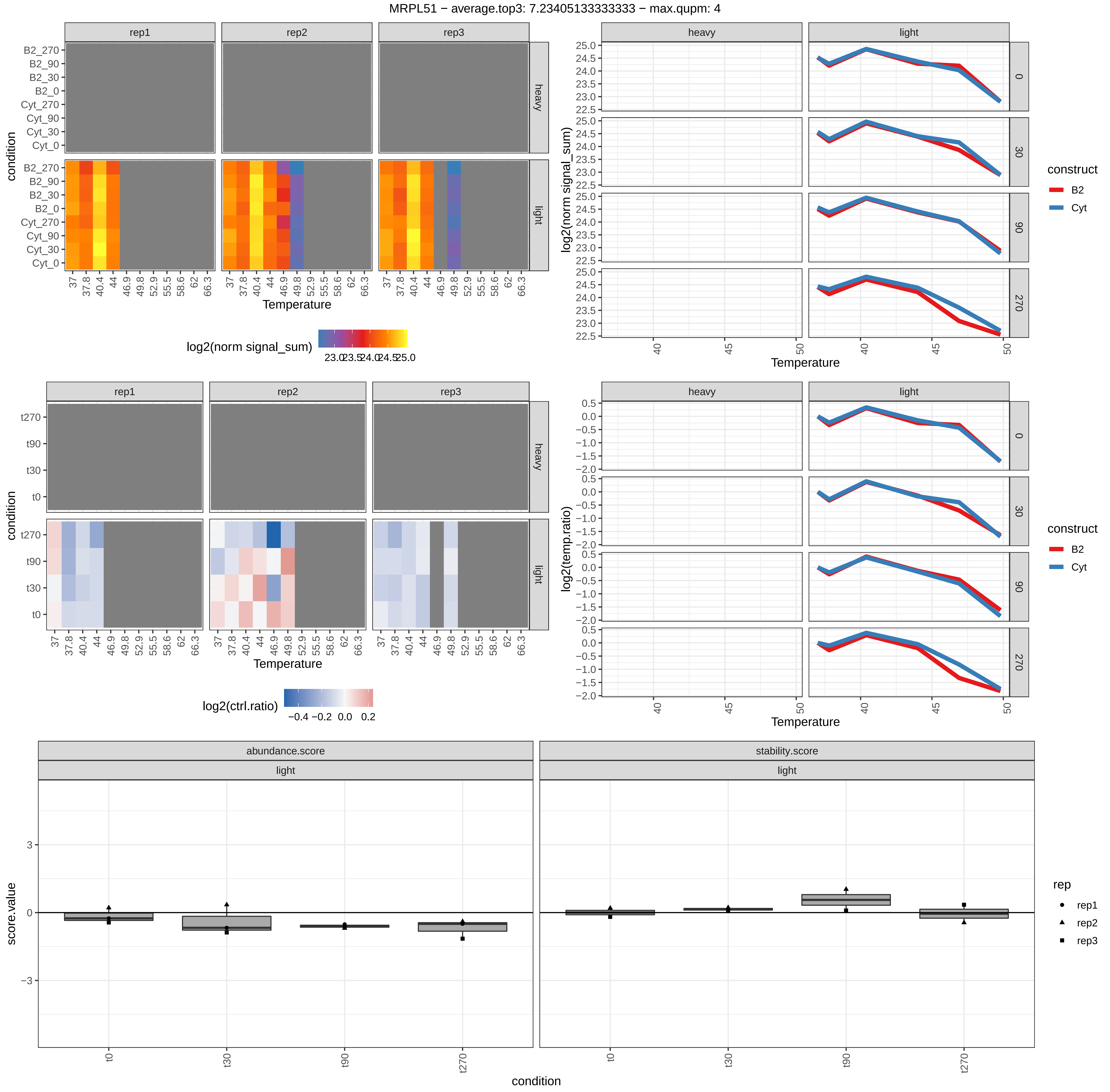

Q06090 − 54S ribosomal protein L51, mitochondrial OS=Saccharomyces cerevisiae (strain ATCC 204508 / S288c) GN=MRPL51 PE=1 SV=1

MRPL6 − average.top3: 6.75115833333333 − max.qupm: 5

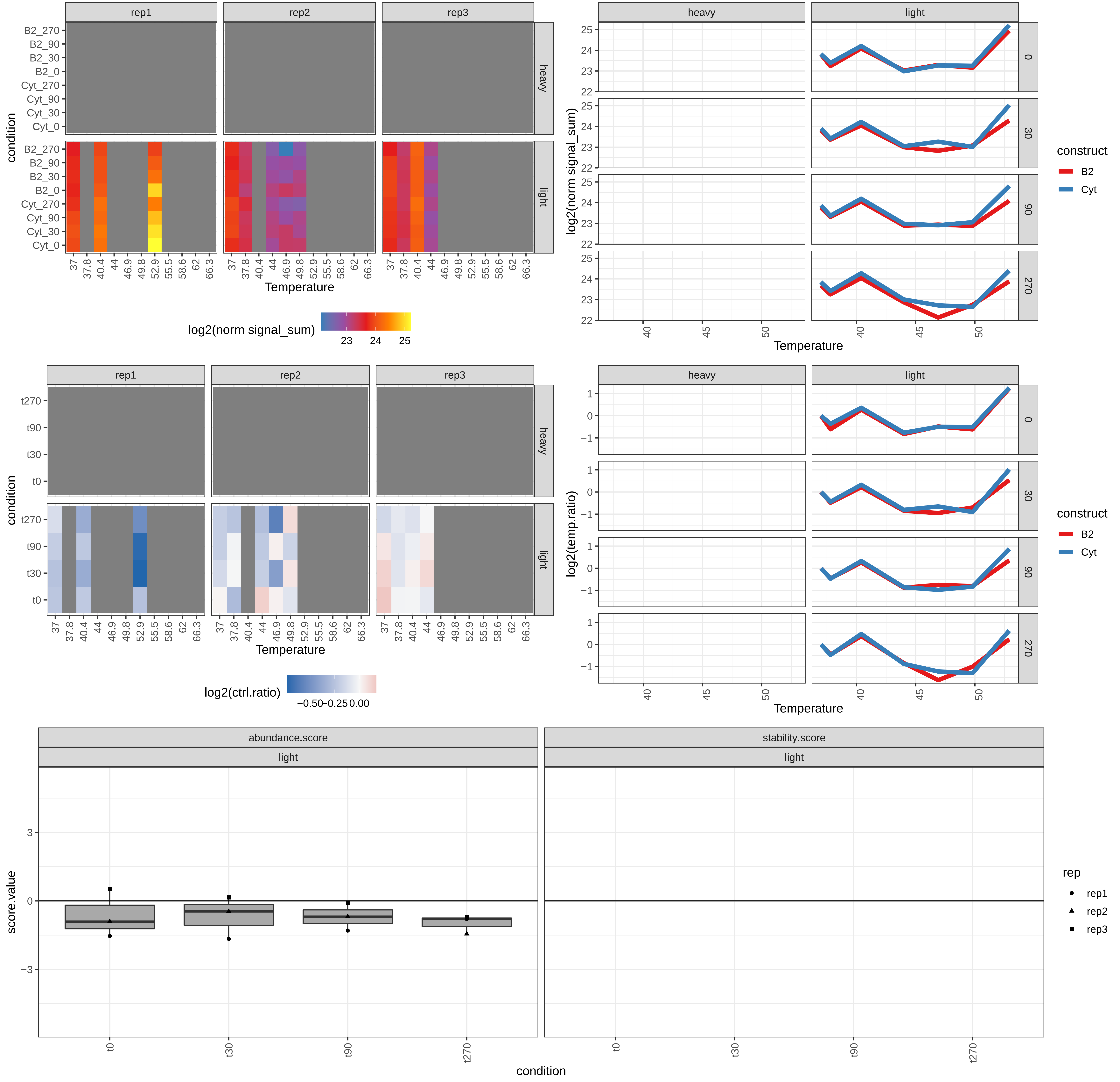

P32904 − 54S ribosomal protein L6, mitochondrial OS=Saccharomyces cerevisiae (strain ATCC 204508 / S288c) GN=MRPL6 PE=1 SV=2

MRPL7 − average.top3: 6.75432 − max.qupm: 4

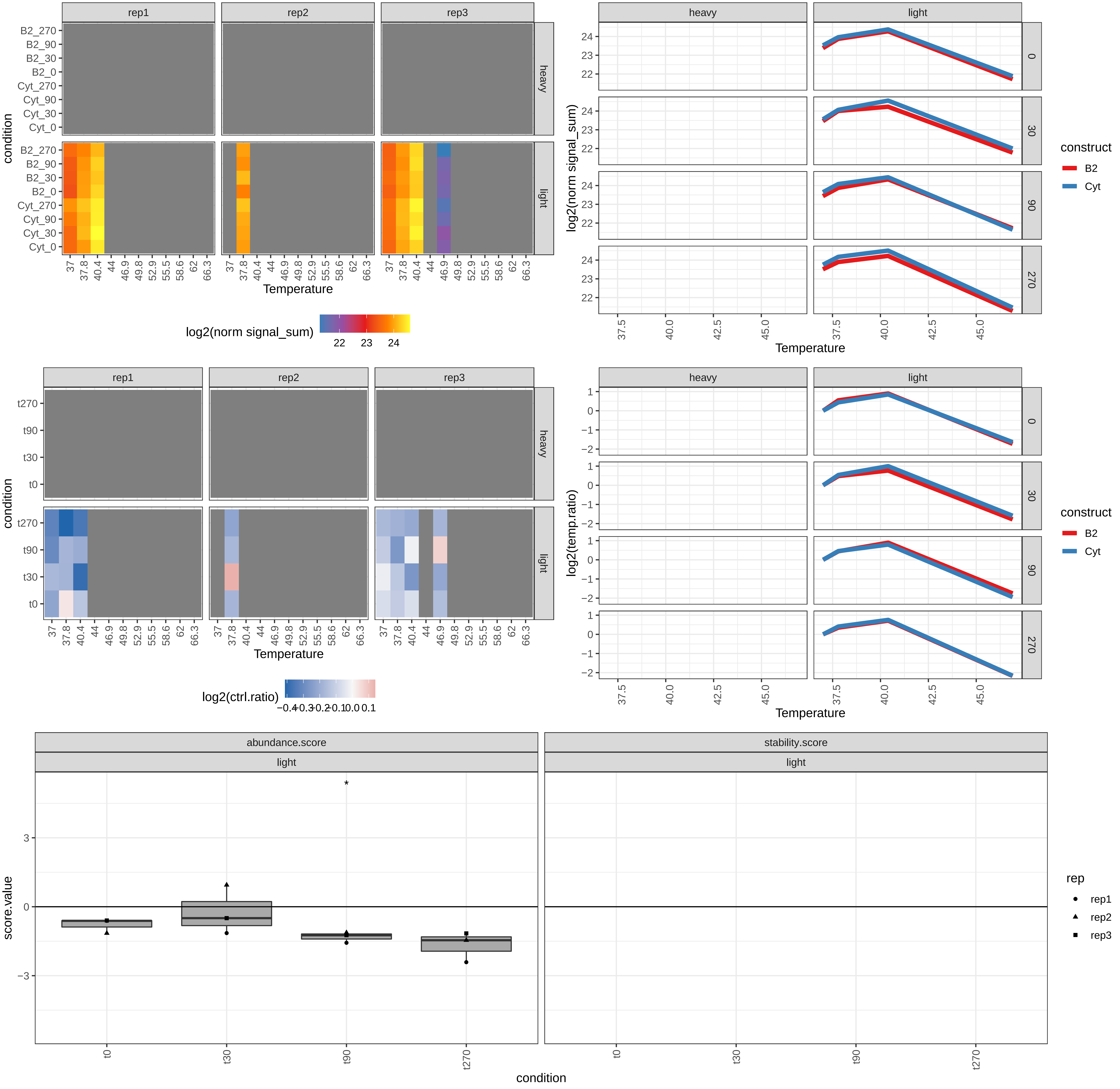

P36519 − 54S ribosomal protein L7, mitochondrial OS=Saccharomyces cerevisiae (strain ATCC 204508 / S288c) GN=MRPL7 PE=1 SV=2

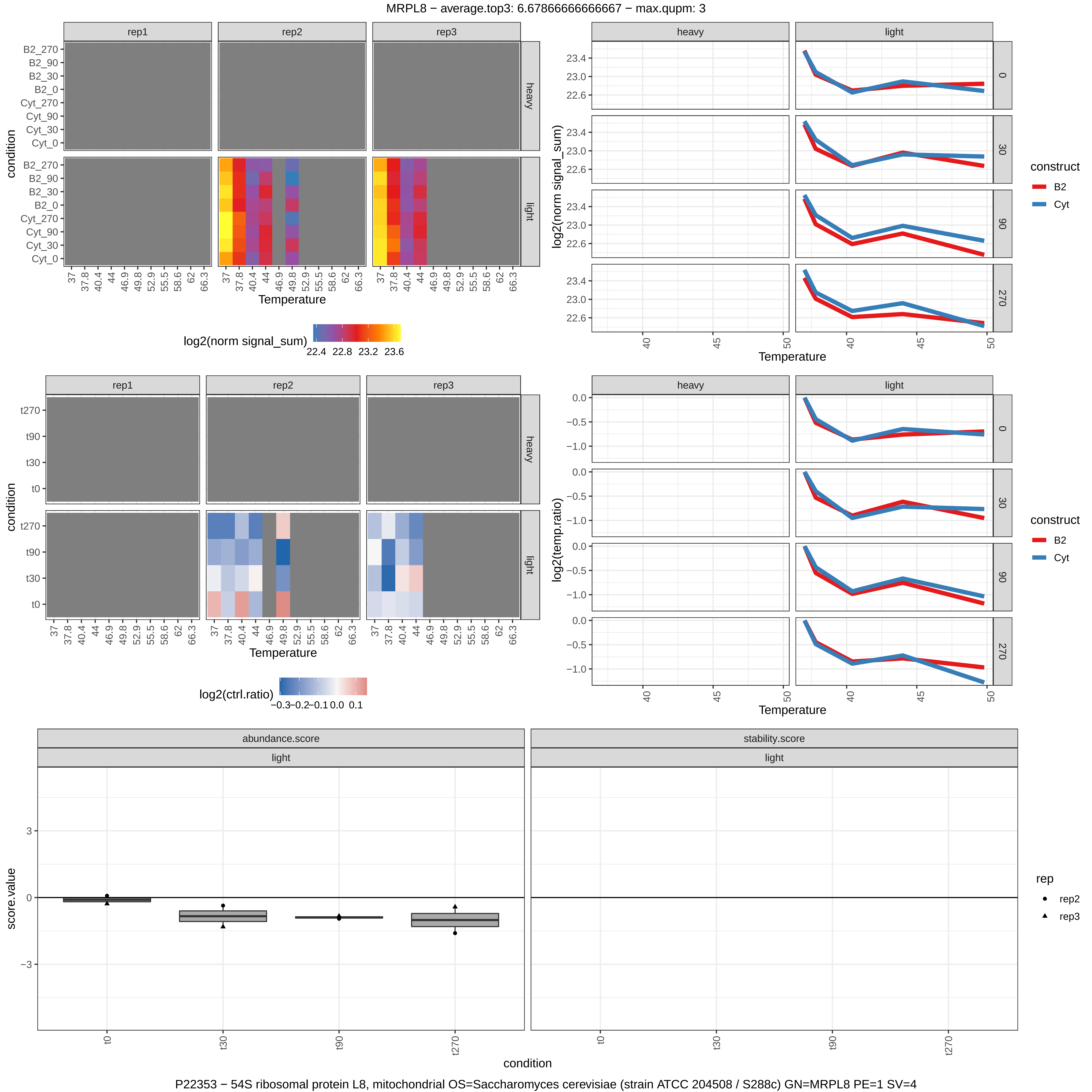
MRPL8 − average.top3: 6.67866666666667 − max.qupm: 3
rep1
rep2
rep3
heavy
light
condition
Temperature
log2(norm signal_sum)
22.4 22.8 23.2 23.6
construct
B2
Cyt
log2(ctrl.ratio)
−0.3 −0.2 −0.1 0.0 0.1
log2(temp.ratio)
abundance.score
stability.score
score.value
rep
rep2
rep3
P22353 − 54S ribosomal protein L8, mitochondrial OS=Saccharomyces cerevisiae (strain ATCC 204508 / S288c) GN=MRPL8 PE=1 SV=4

MRPL9 − average.top3: 6.79760526315789 − max.qupm: 4

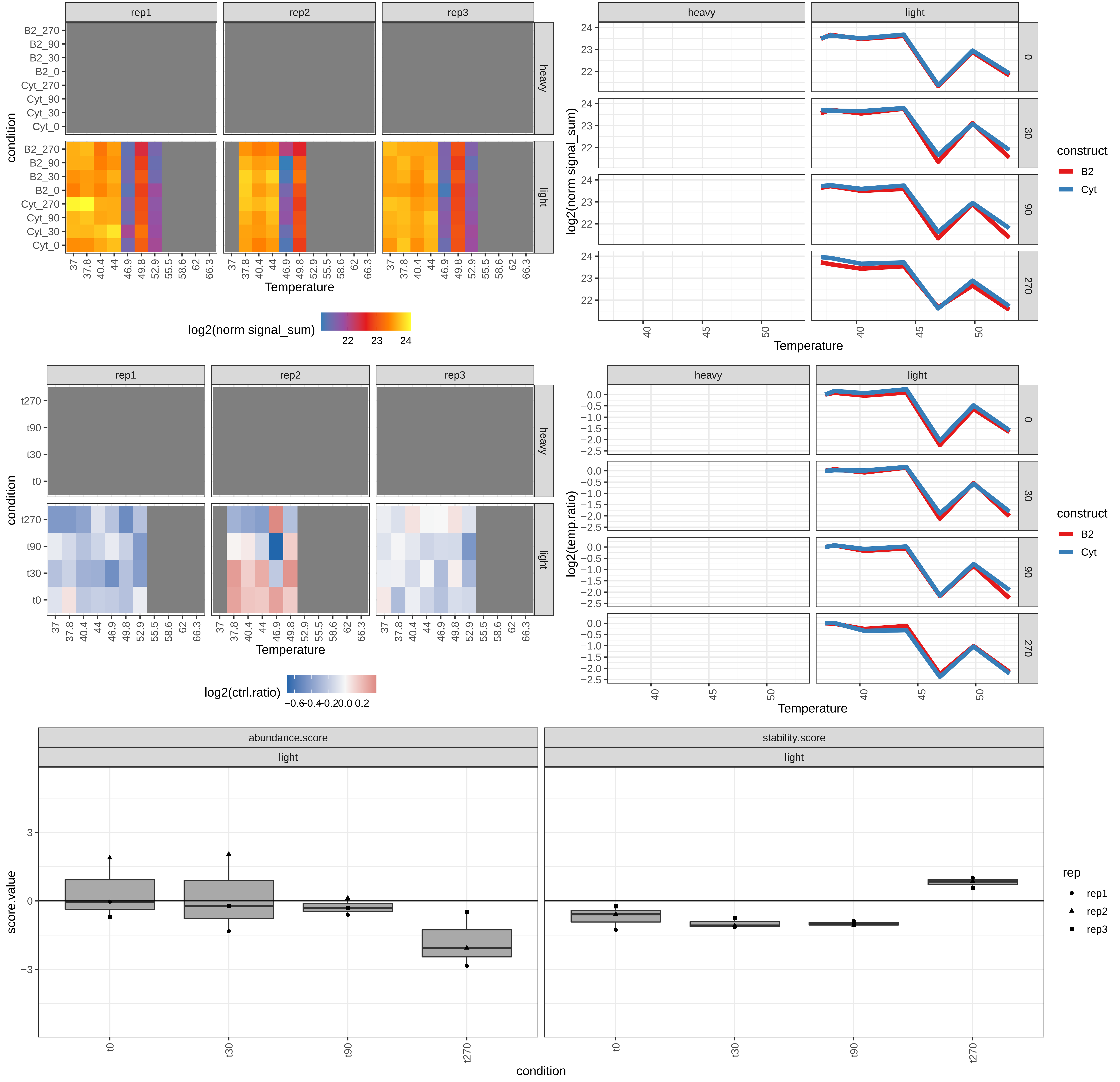

P31334 − 54S ribosomal protein L9, mitochondrial OS=Saccharomyces cerevisiae (strain ATCC 204508 / S288c) GN=MRPL9 PE=1 SV=3

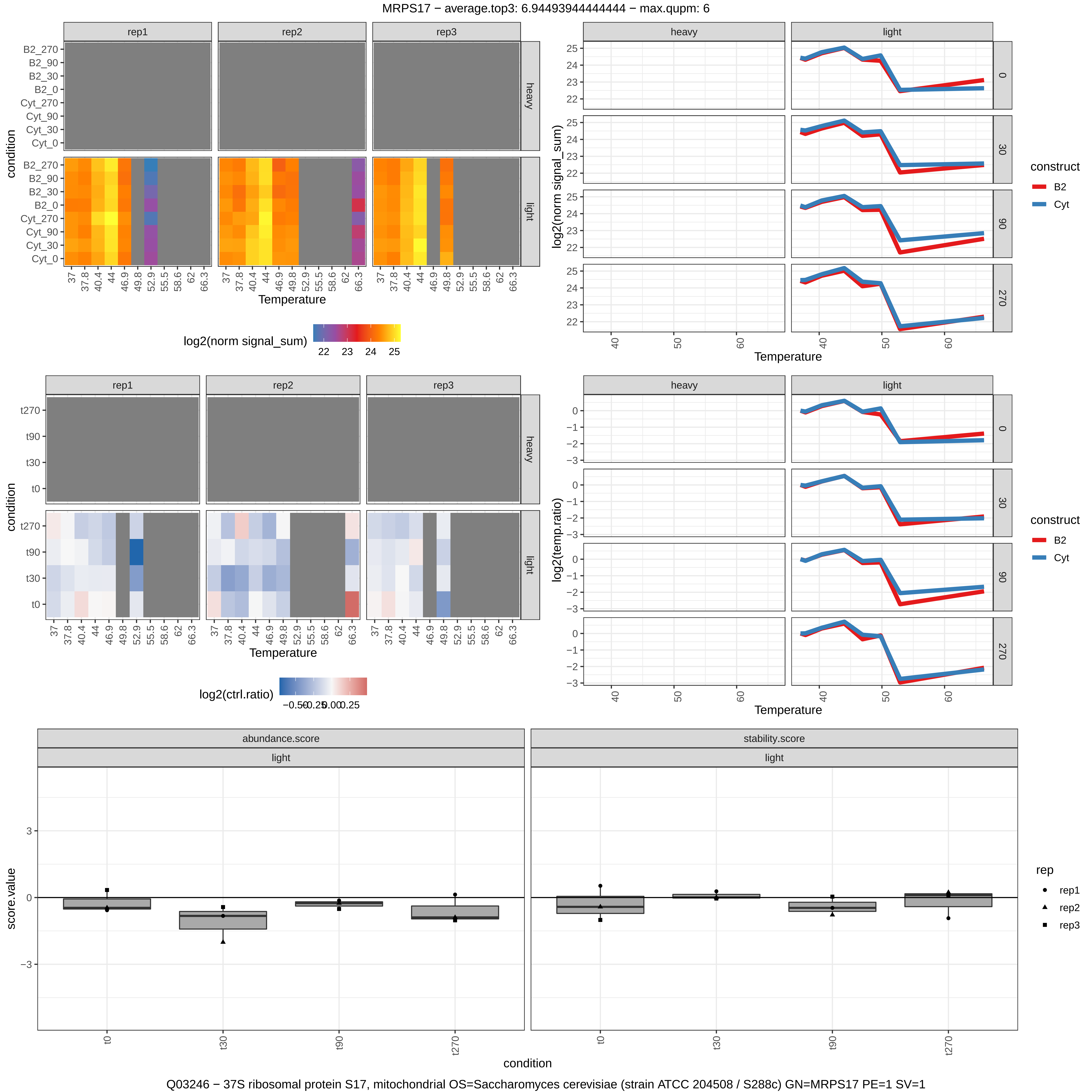
MRPS17 − average.top3: 6.94493944444444 − max.qupm: 6
rep1
rep2
rep3
heavy
light
condition
B2_270
B2_90
B2_30
B2_0
Cyt_270
Cyt_90
Cyt_30
Cyt_0
Temperature
log2(norm signal_sum)
log2(norm signal_sum)
construct
B2
Cyt
t270
t90
t30
t0
log2(ctrl.ratio)
log2(temp.ratio)
abundance.score
stability.score
score.value
rep
condition
Q03246 − 37S ribosomal protein S17, mitochondrial OS=Saccharomyces cerevisiae (strain ATCC 204508 / S288c) GN=MRPS17 PE=1 SV=1

MRPS28 − average.top3: 6.14041705882353 − max.qupm: 3

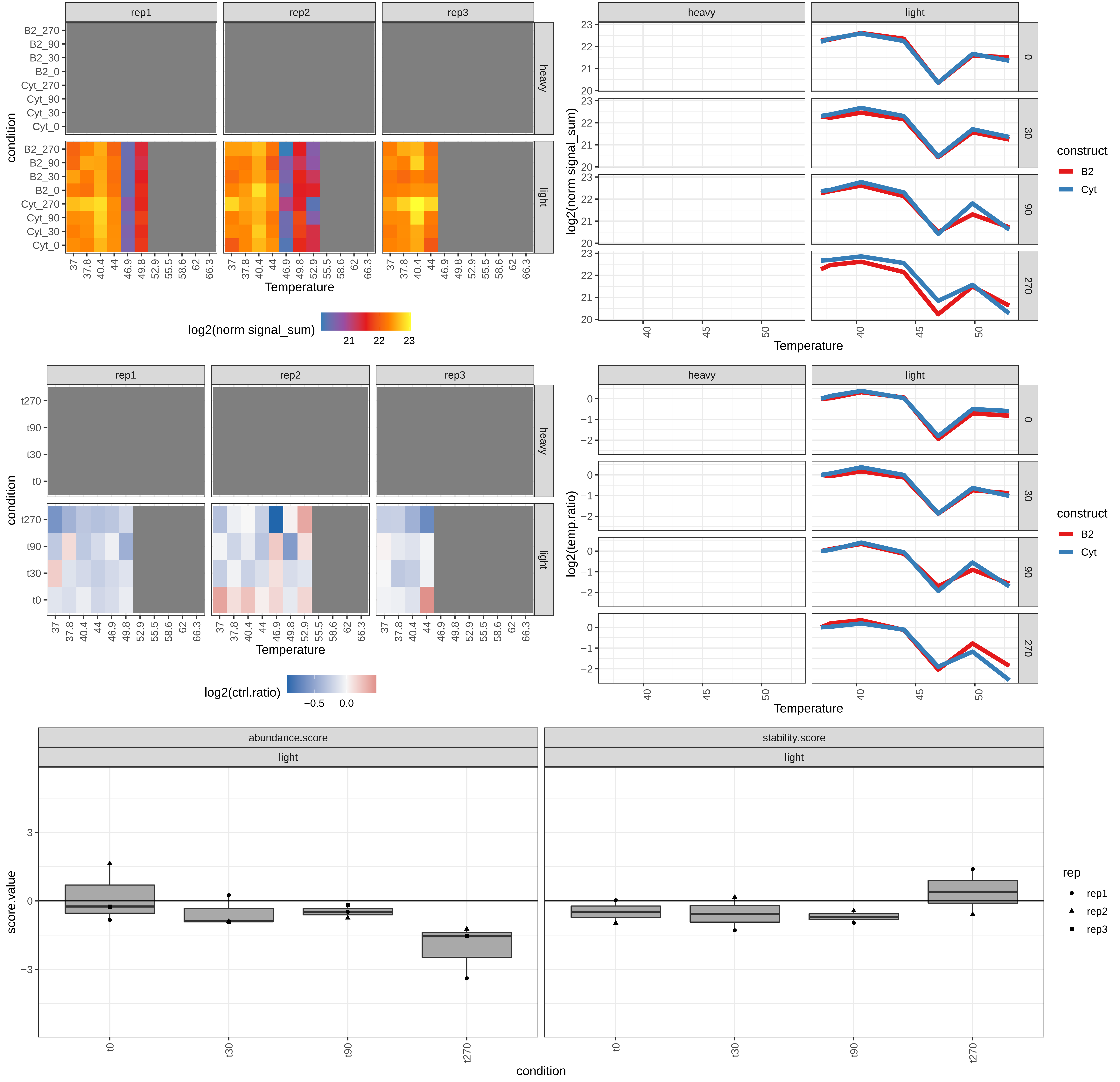

P21771 − 37S ribosomal protein S28, mitochondrial OS=Saccharomyces cerevisiae (strain ATCC 204508 / S288c) GN=MRPS28 PE=1 SV=1

MRPS35 − average.top3: 6.67792 − max.qupm: 6

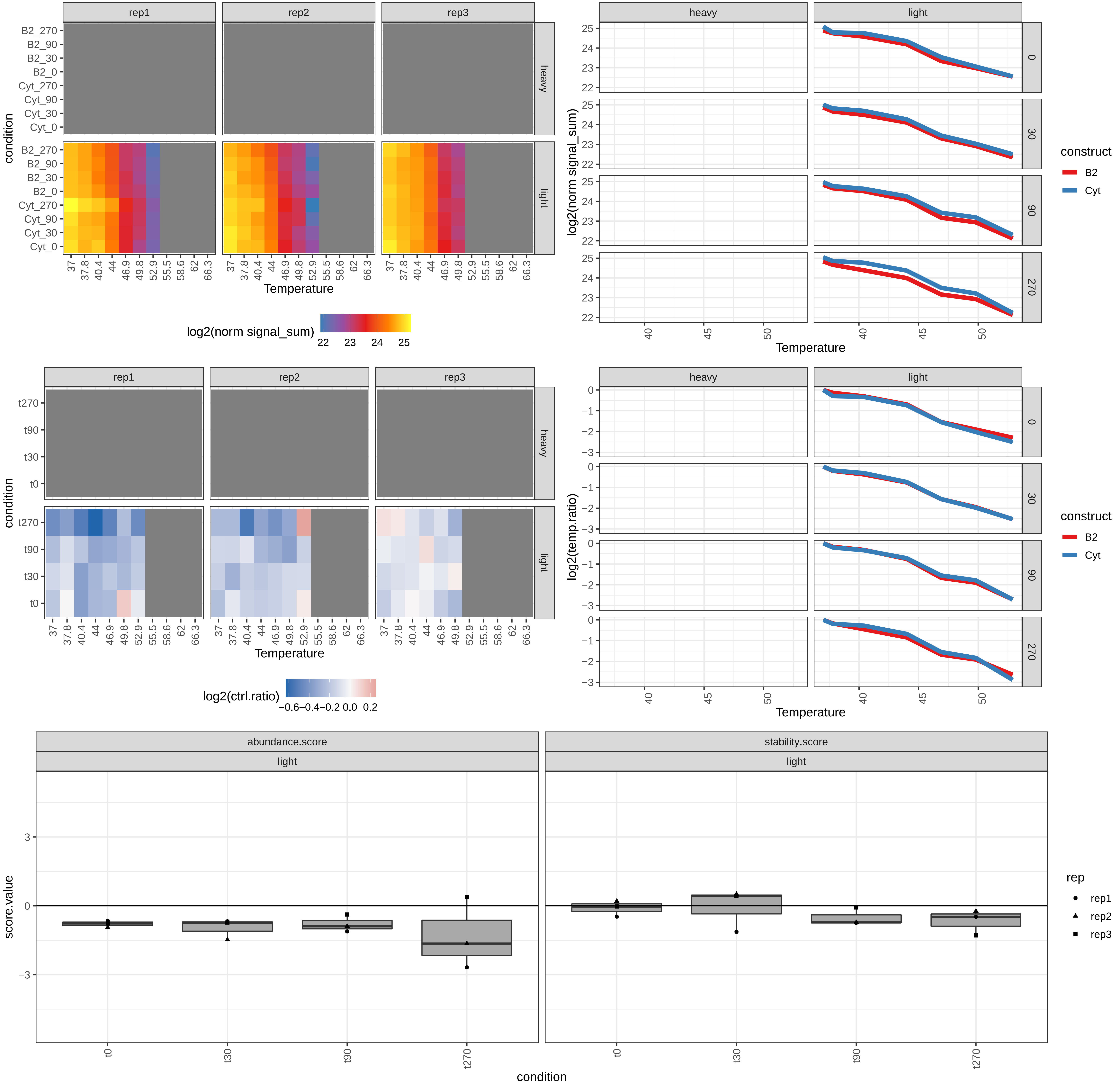

P53292 − 37S ribosomal protein S35, mitochondrial OS=Saccharomyces cerevisiae (strain ATCC 204508 / S288c) GN=MRPS35 PE=1 SV=1

MRPS5 − average.top3: 6.92935954545455 − max.qupm: 9

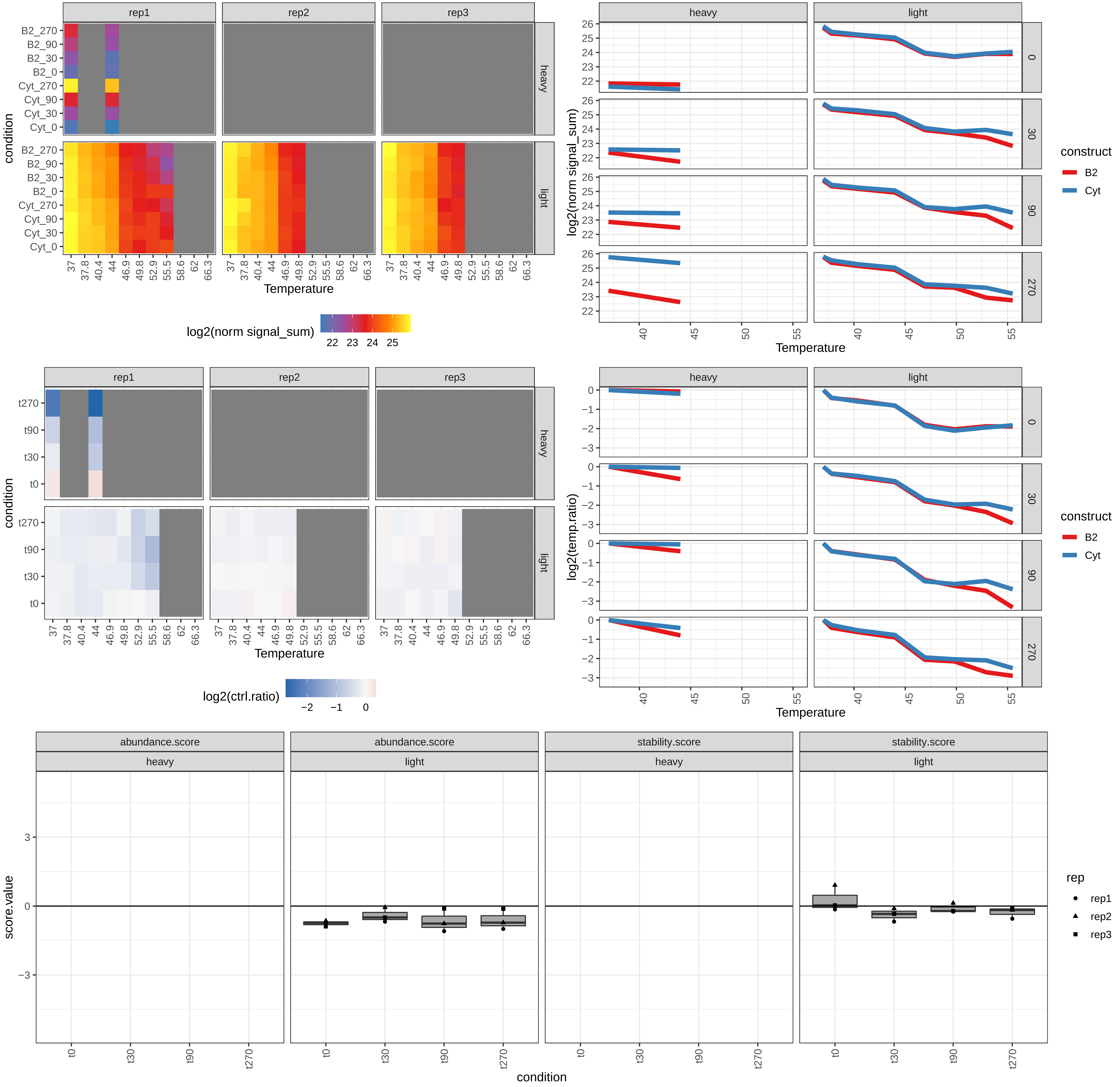

P33759 − 37S ribosomal protein S5, mitochondrial OS=Saccharomyces cerevisiae (strain ATCC 204508 / S288c) GN=MRPS5 PE=1 SV=1

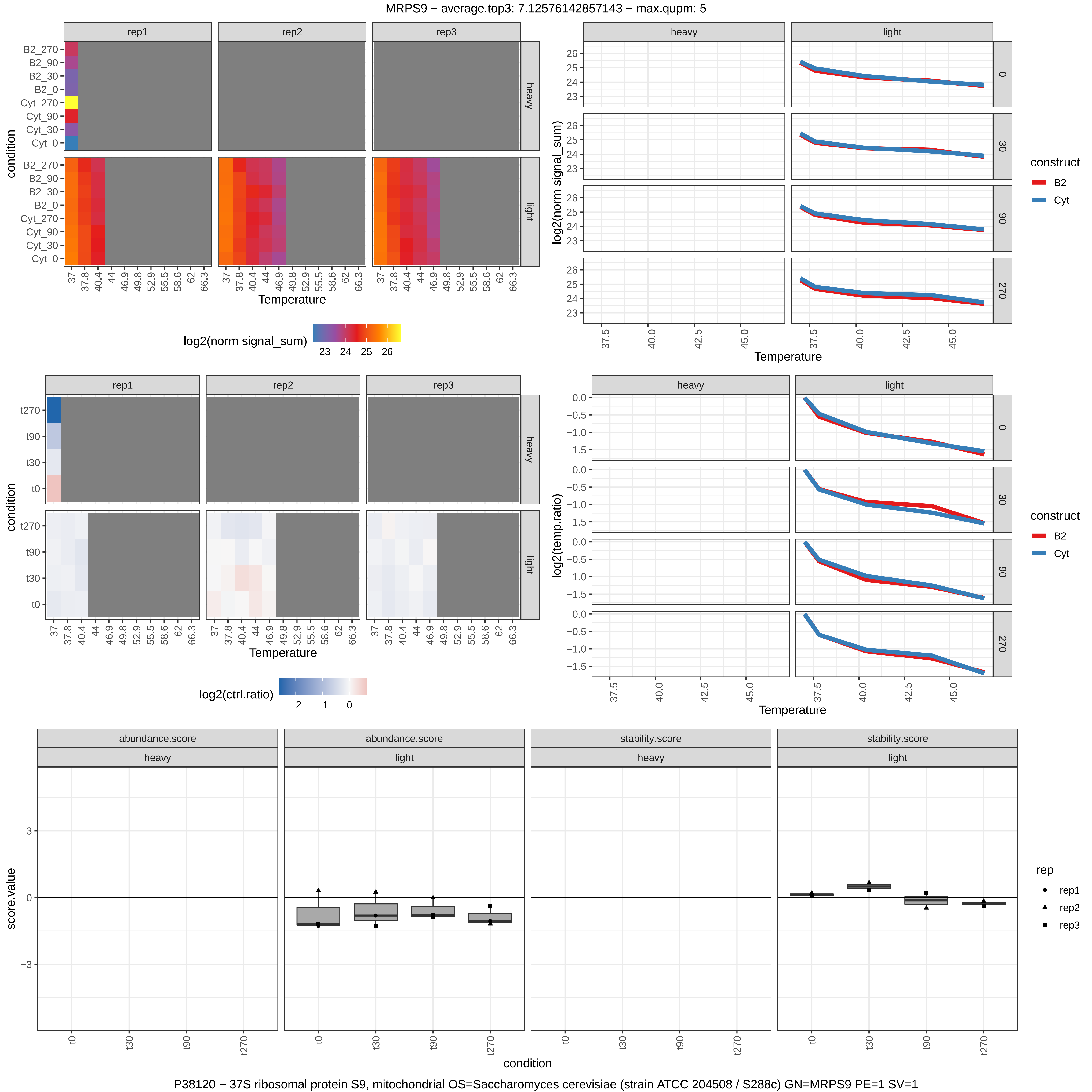
MRPS9 − average.top3: 7.12576142857143 − max.qupm: 5
rep1
rep2
rep3
heavy
light
condition
Temperature
log2(norm signal_sum)
construct
B2
Cyt
log2(ctrl.ratio)
log2(temp.ratio)
abundance.score
stability.score
score.value
rep
rep1
rep2
rep3
P38120 − 37S ribosomal protein S9, mitochondrial OS=Saccharomyces cerevisiae (strain ATCC 204508 / S288c) GN=MRPS9 PE=1 SV=1

MRS1 − average.top3: 6.55499625 − max.qupm: 6

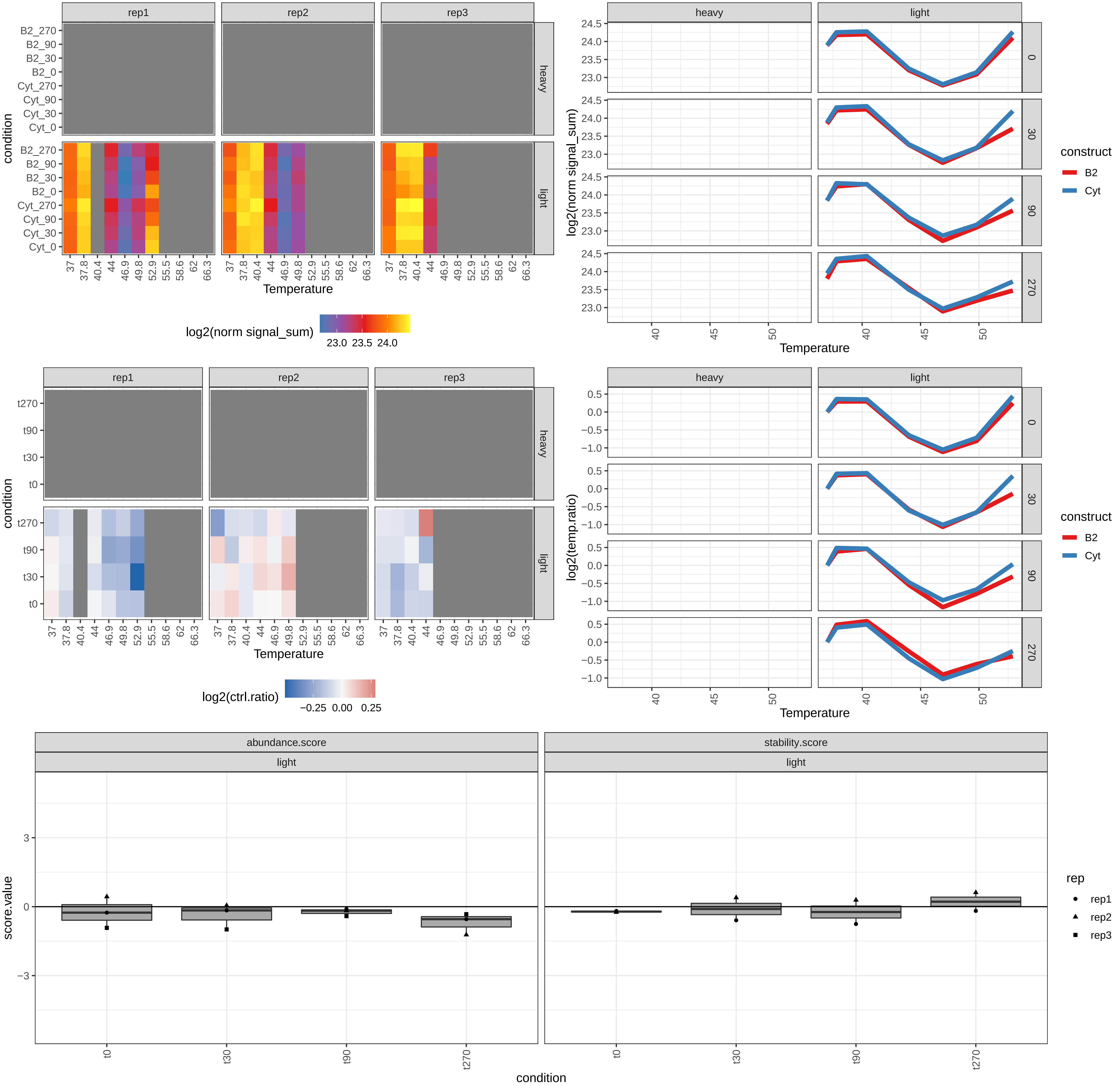

P07266 − Mitochondrial RNA−splicing protein MRS1 OS=Saccharomyces cerevisiae (strain ATCC 204508 / S288c) GN=MRS1 PE=1 SV=2

MRS6 − average.top3: 6.77463476190476 − max.qupm: 6

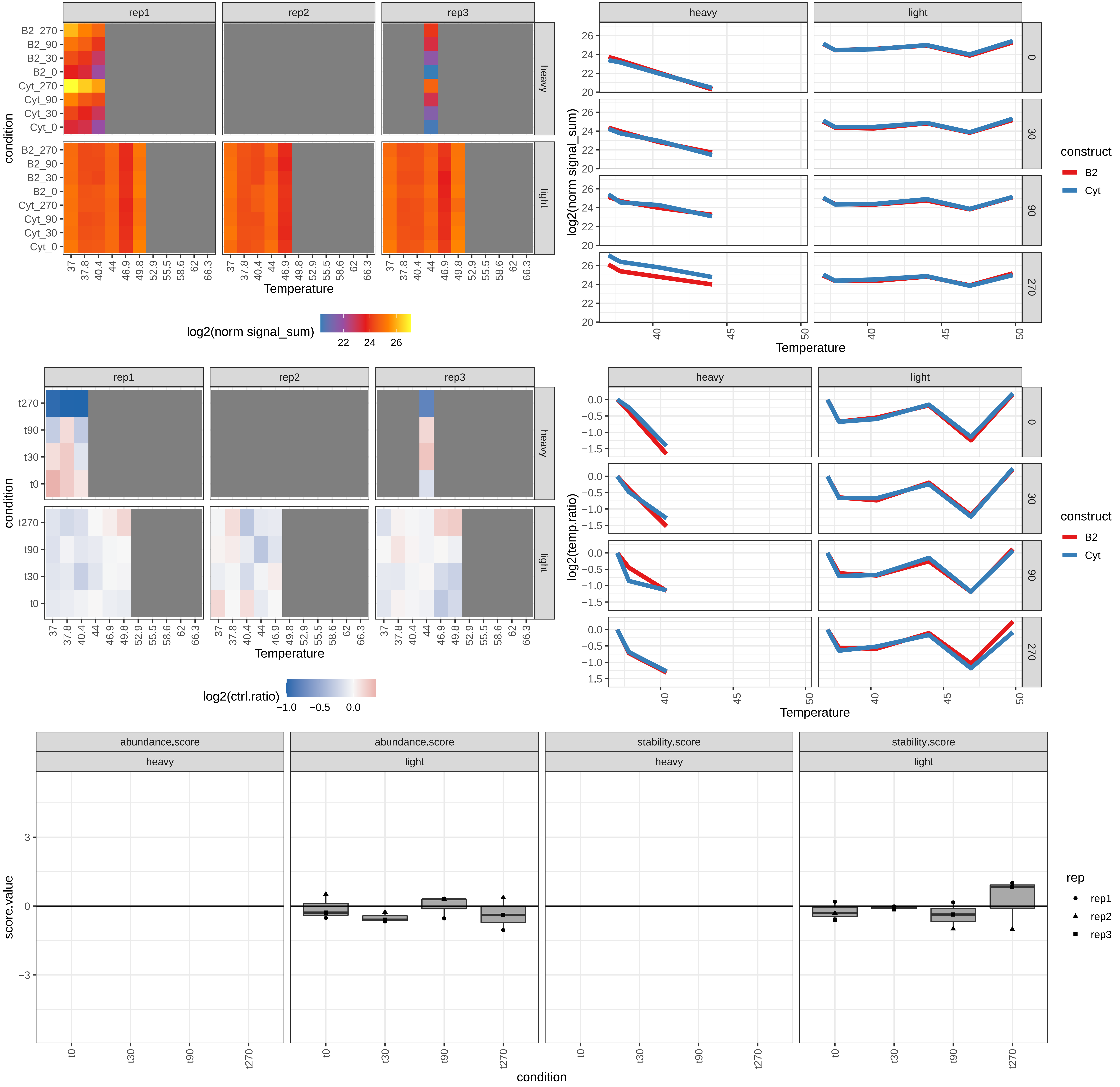

P32864 − Rab proteins geranylgeranyltransferase component A OS=Saccharomyces cerevisiae (strain ATCC 204508 / S288c) GN=MRS6 PE=1 SV=2

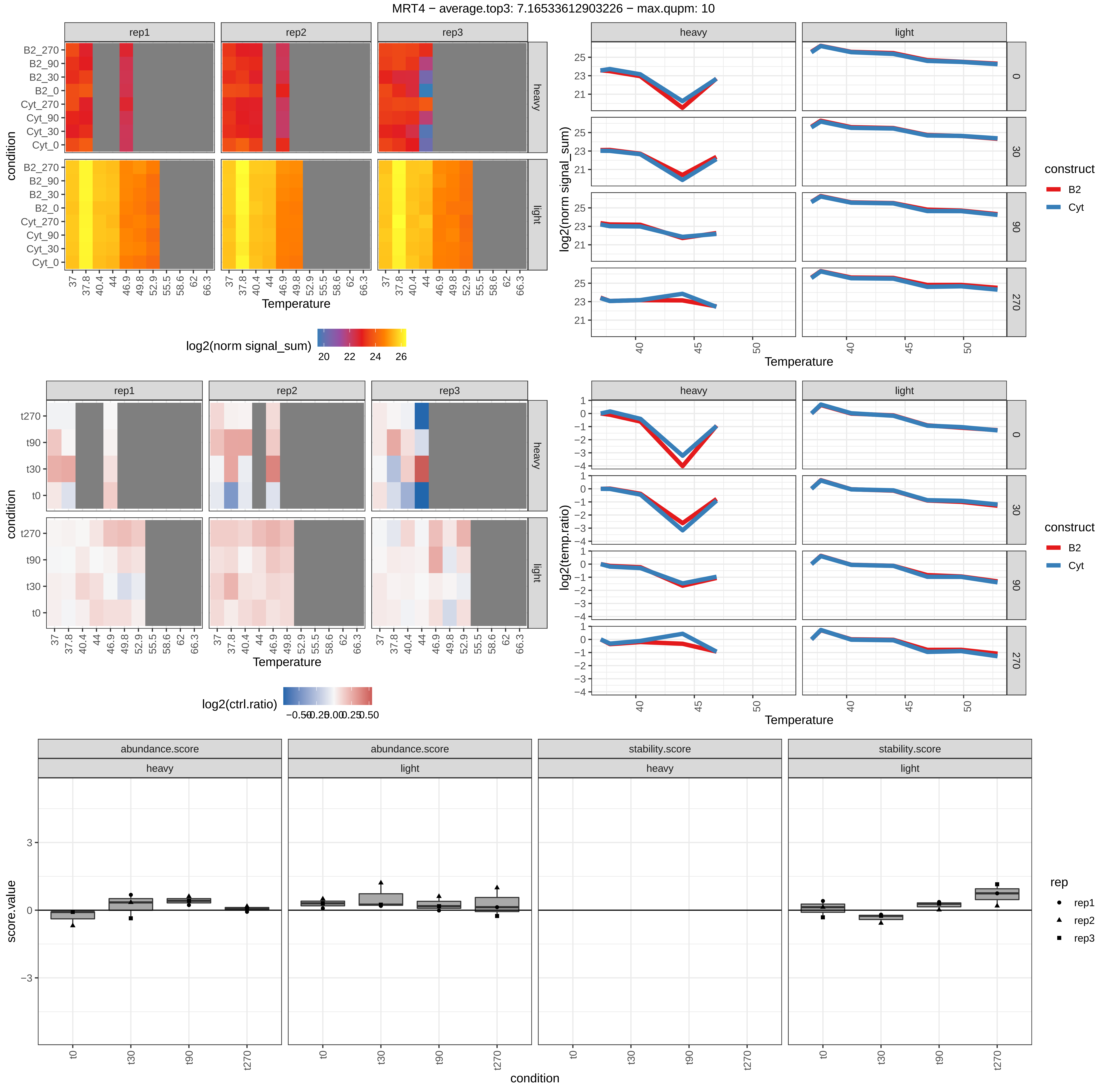

P33201 − Ribosome assembly factor MRT4 OS=Saccharomyces cerevisiae (strain ATCC 204508 / S288c) GN=MRT4 PE=1 SV=1

MRX1 − average.top3: 6.45102846153846 − max.qupm: 6

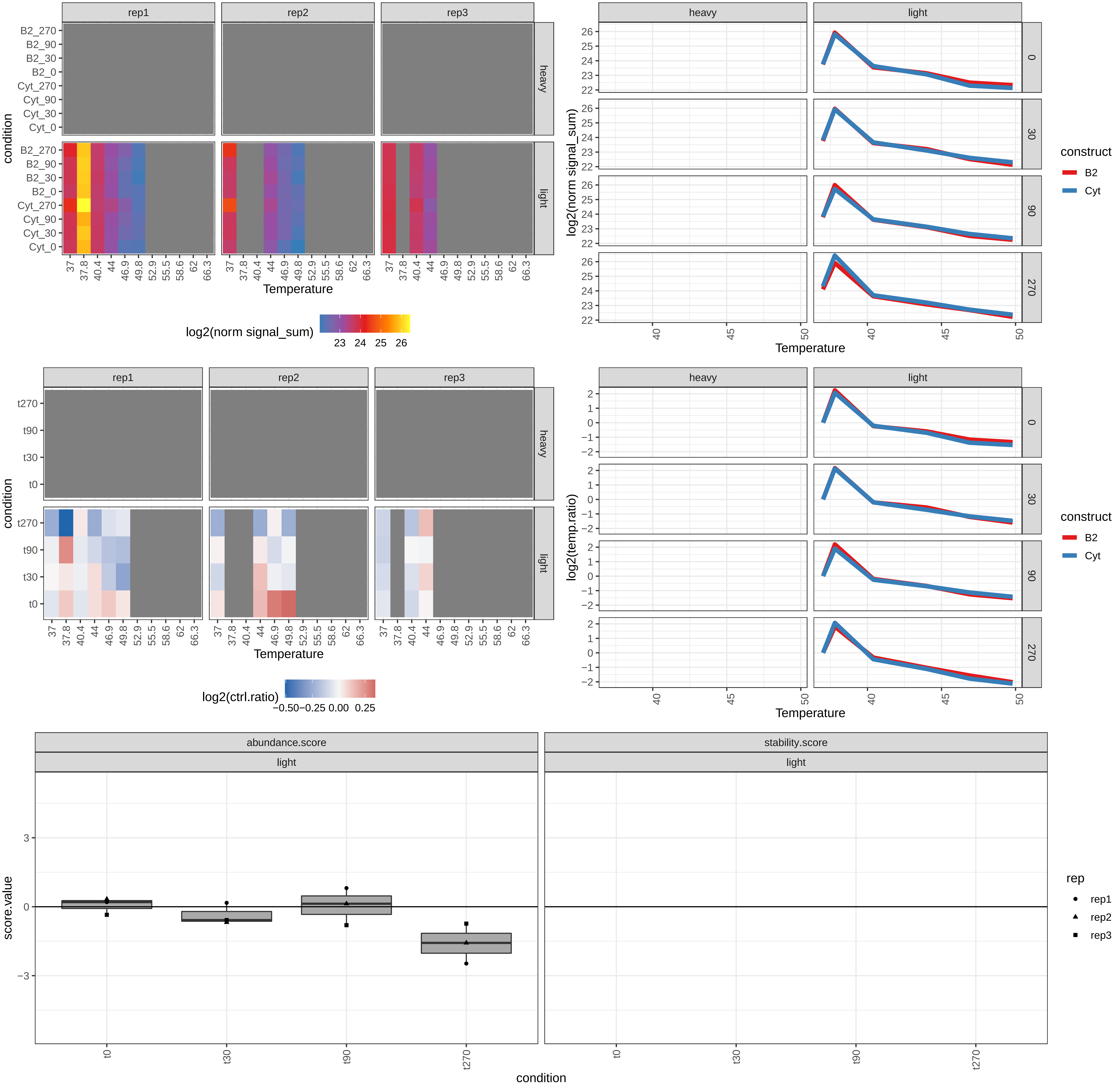

P40050 − MIOREX complex component 1 OS=Saccharomyces cerevisiae (strain ATCC 204508 / S288c) GN=MRX1 PE=1 SV=1

MRX10 − average.top3: 6.777515 − max.qupm: 5

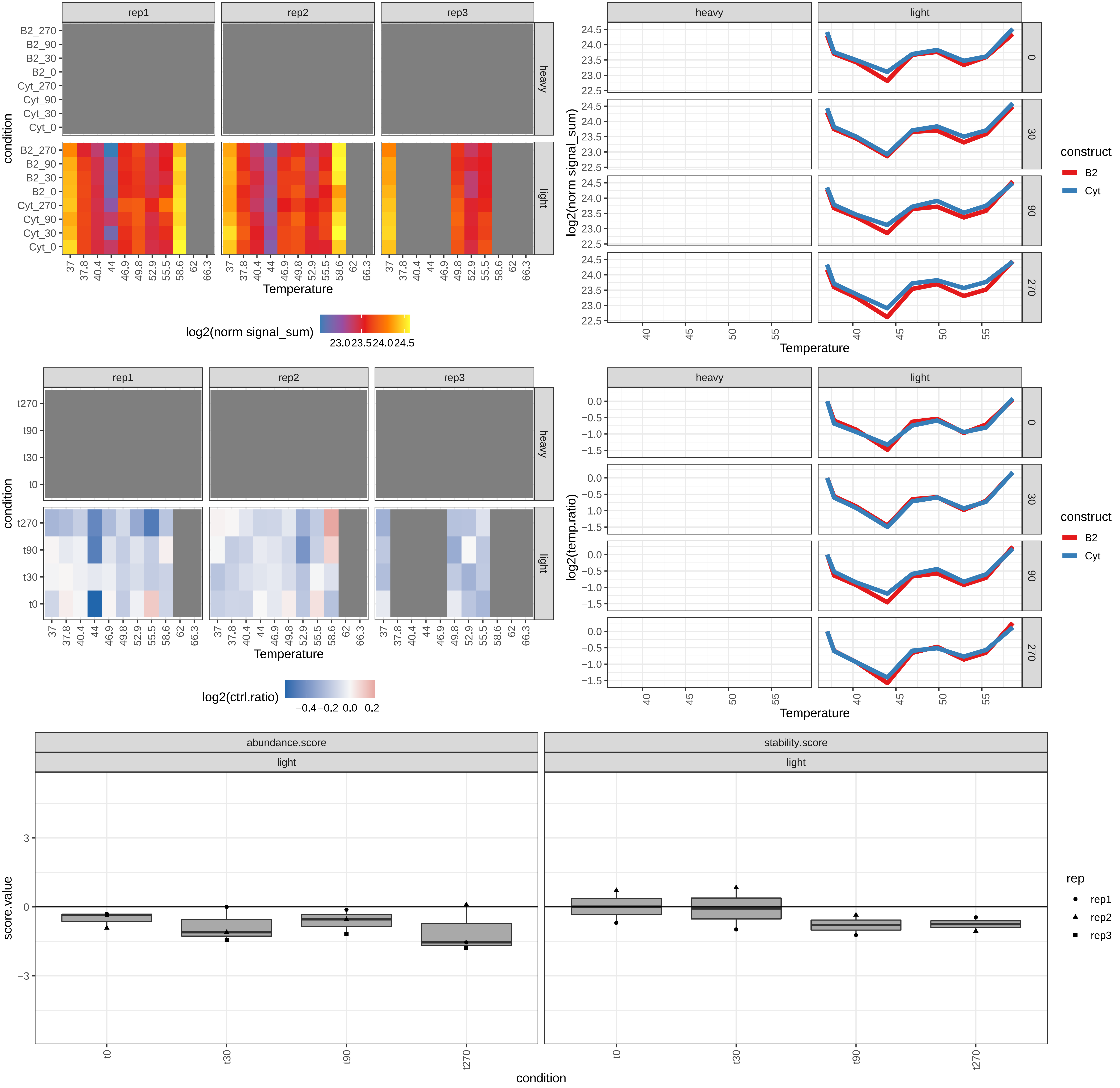

Q05648 − MIOREX complex component 10 OS=Saccharomyces cerevisiae (strain ATCC 204508 / S288c) GN=MRX10 PE=1 SV=1

MRX12 − average.top3: 6.29646461538462 − max.qupm: 5

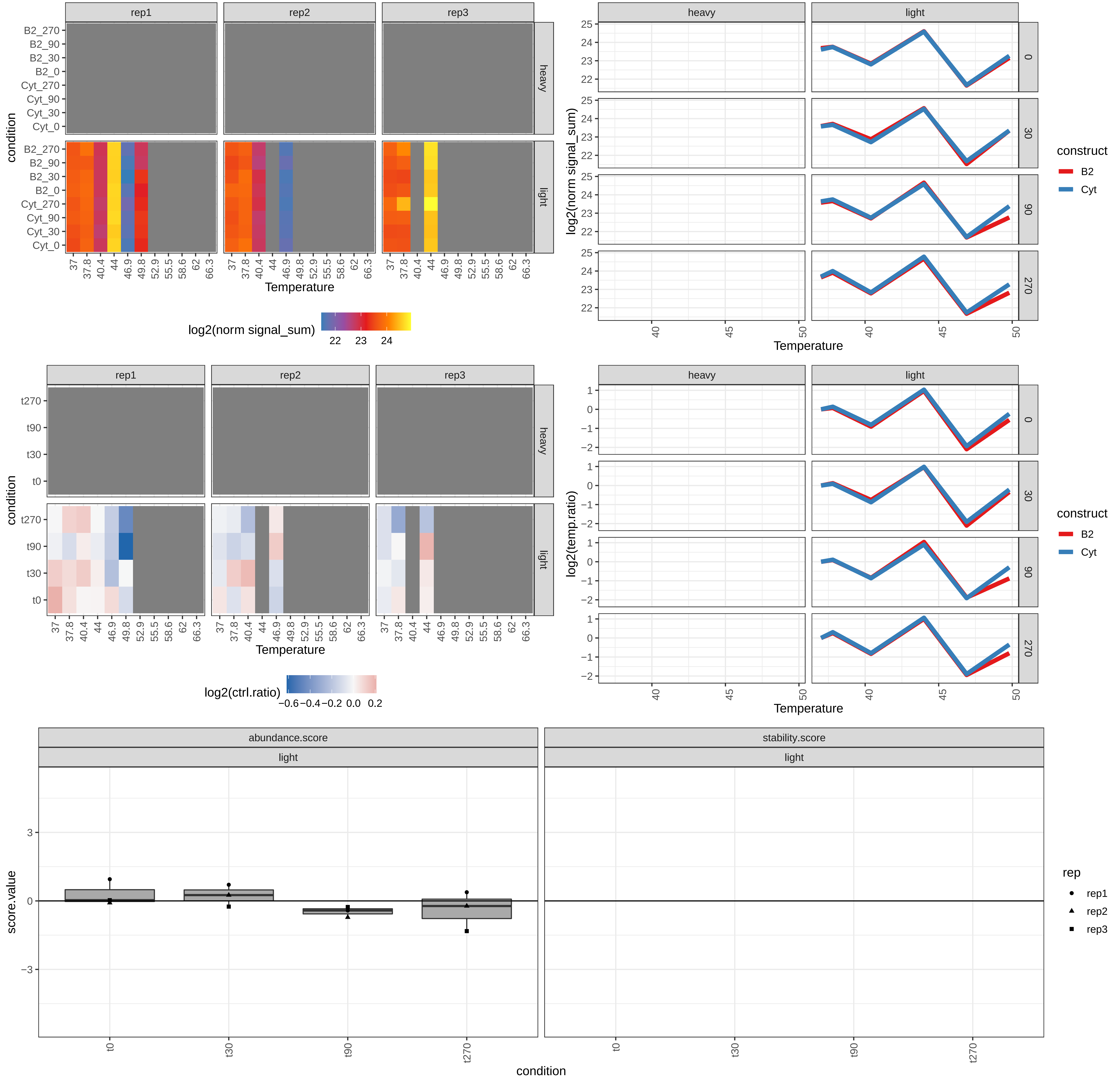

P47084 − MIOREX complex component 12 OS=Saccharomyces cerevisiae (strain ATCC 204508 / S288c) GN=MRX12 PE=1 SV=2

MRX3 − average.top3: 6.7279 − max.qupm: 4

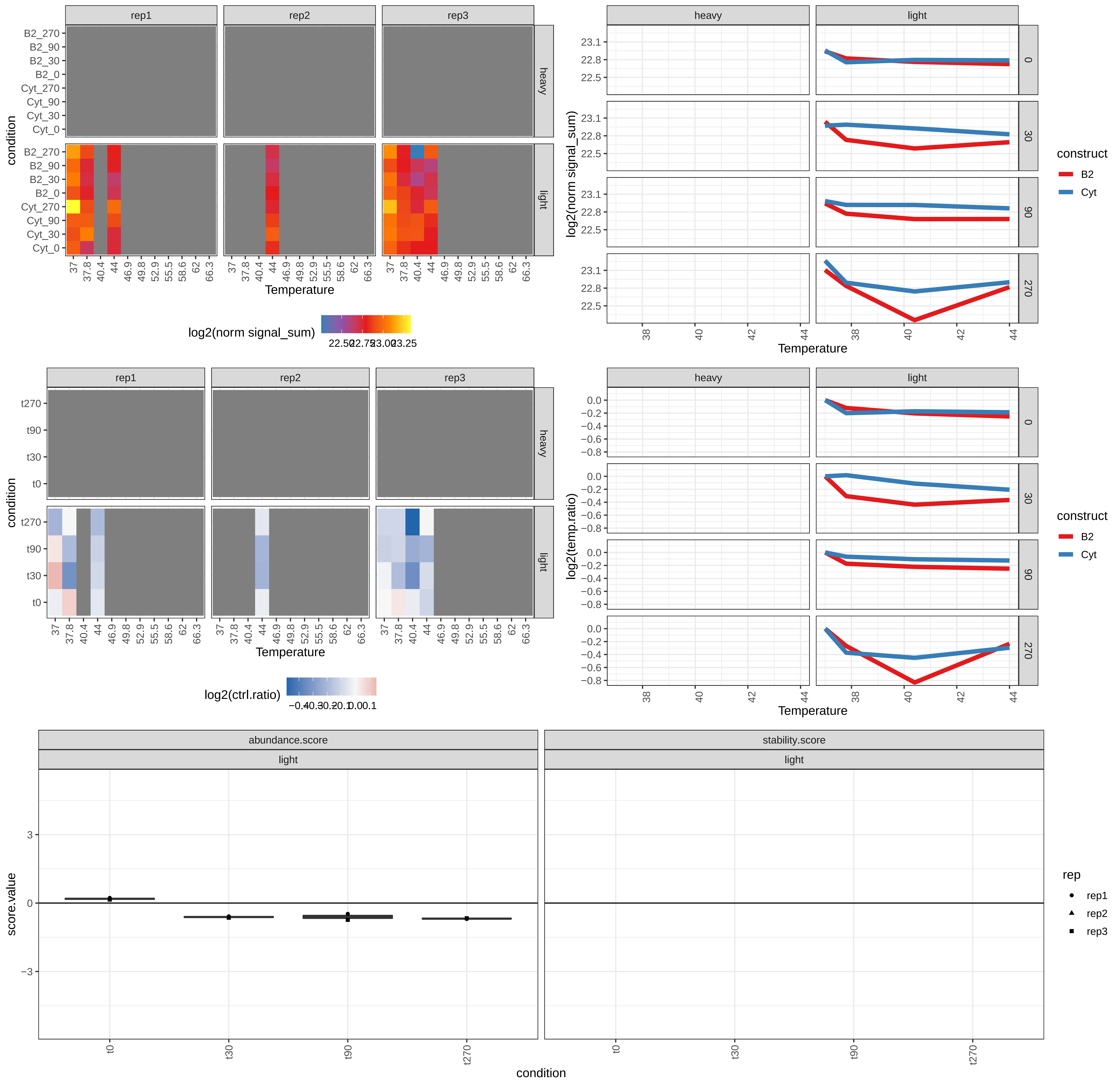

P38172 − MIOREX complex component 3 OS=Saccharomyces cerevisiae (strain ATCC 204508 / S288c) GN=MRX3 PE=1 SV=1

MRX9 − average.top3: 7.2745262962963 − max.qupm: 8

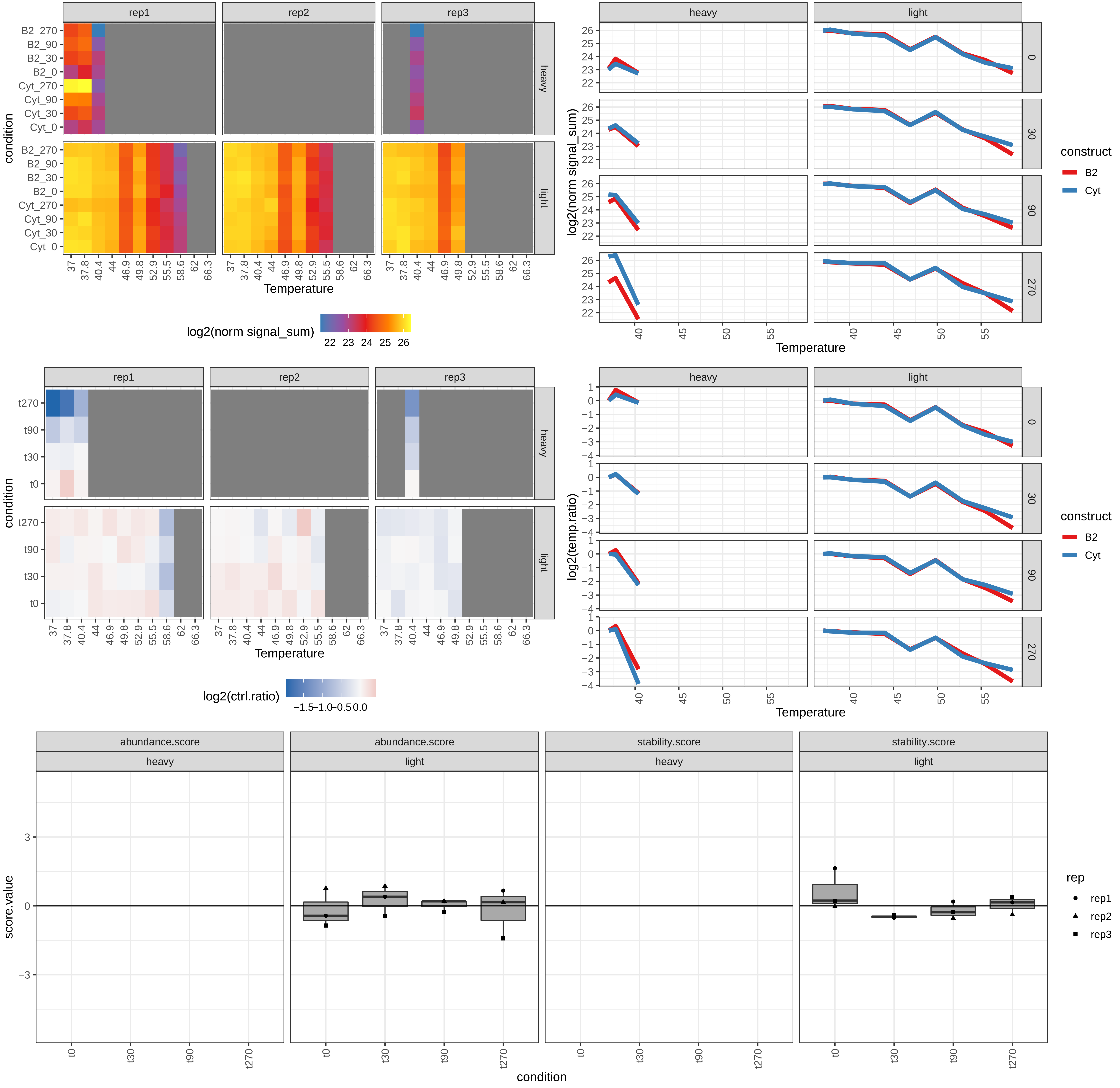

Q07349 − MIOREX complex component 9 OS=Saccharomyces cerevisiae (strain ATCC 204508 / S288c) GN=MRX9 PE=1 SV=1

MSB3 − average.top3: 6.36754 − max.qupm: 4

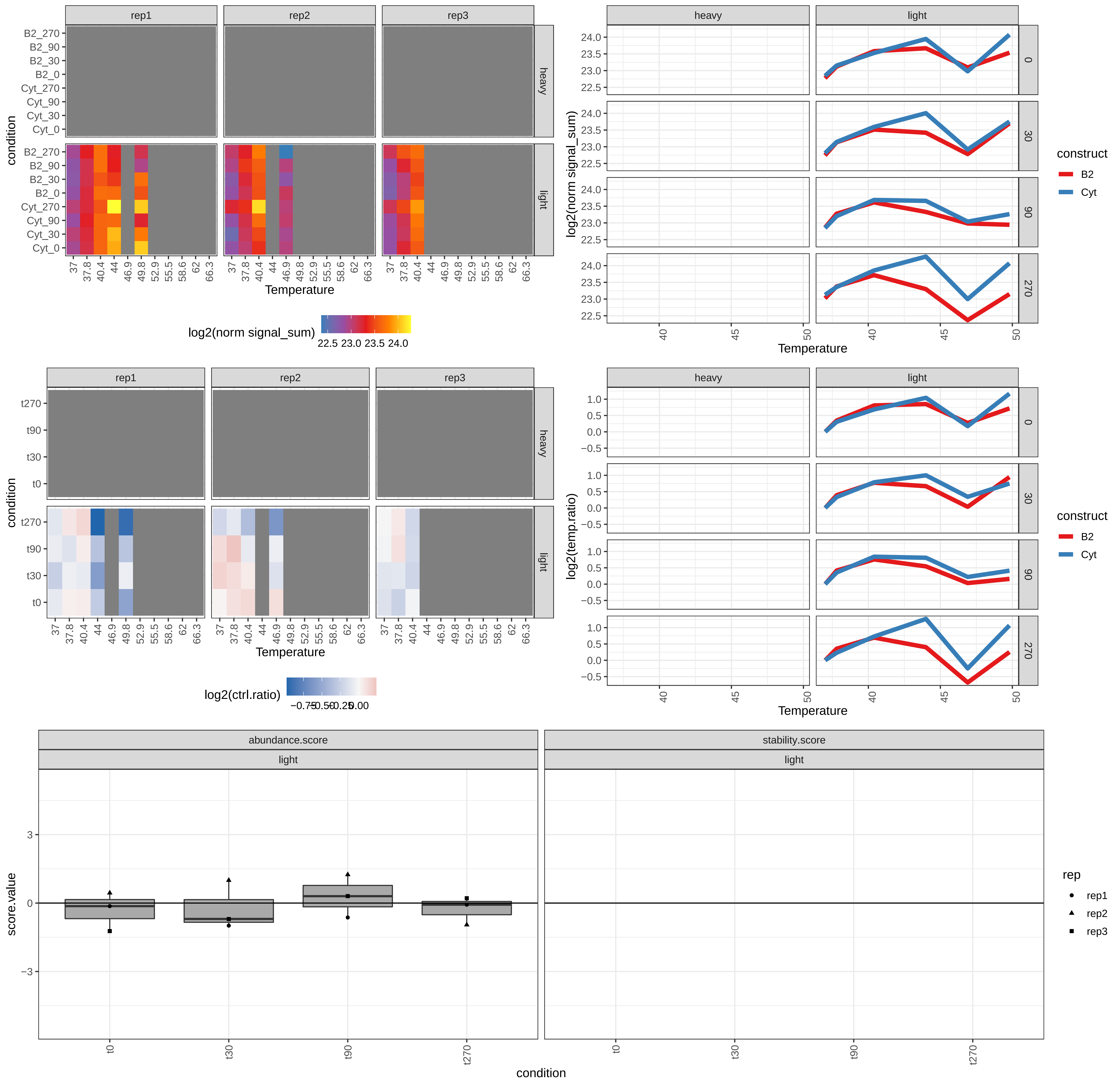

P48566 − GTPase−activating protein GYP3 OS=Saccharomyces cerevisiae (strain ATCC 204508 / S288c) GN=MSB3 PE=1 SV=1

MSC1 − average.top3: 7.22639833333333 − max.qupm: 14

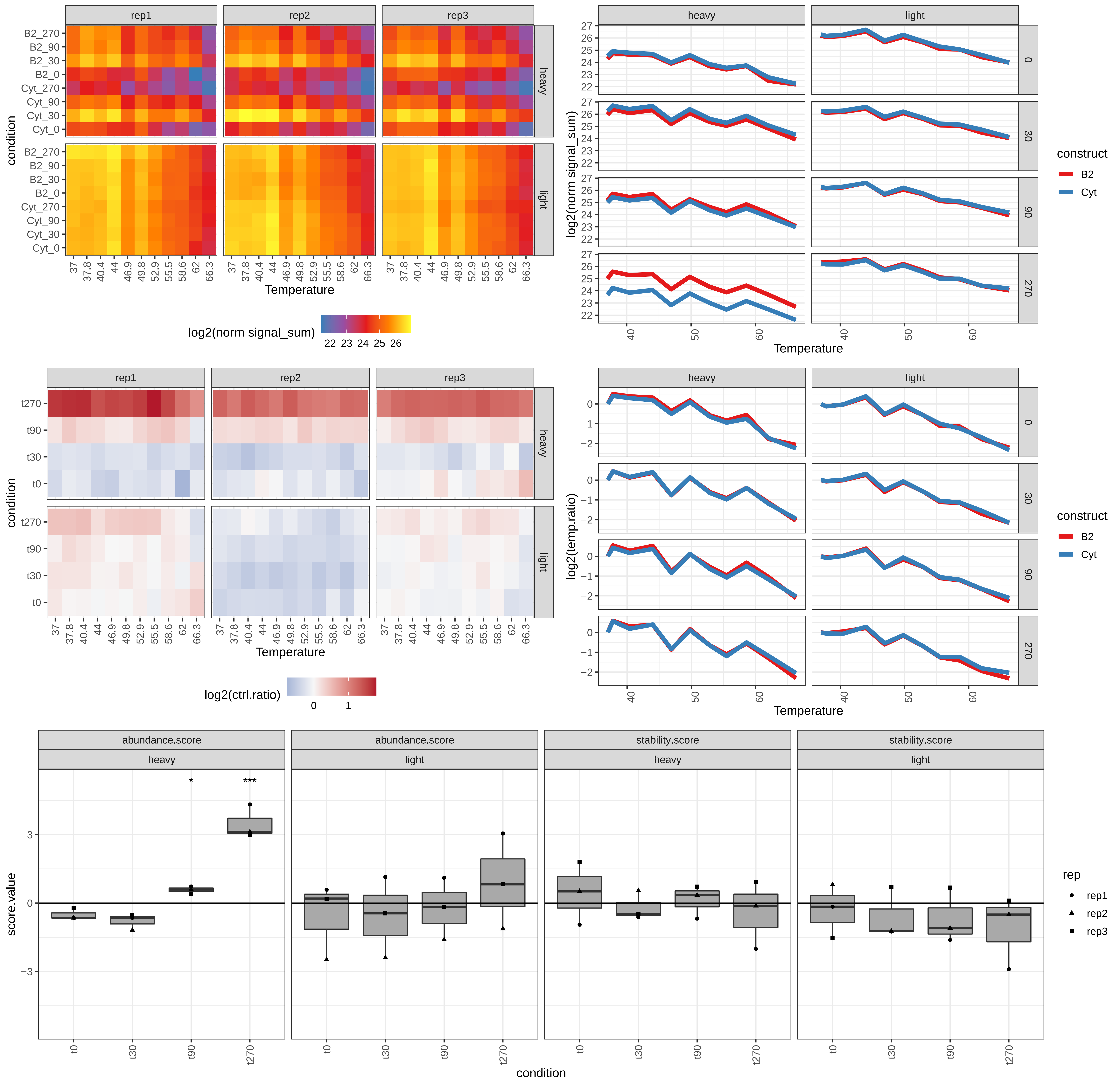

Q03104 − Meiotic sister chromatid recombination protein 1 OS=Saccharomyces cerevisiae (strain ATCC 204508 / S288c) GN=MSC1 PE=1 SV=1

MSC2 − average.top3: 6.59335052631579 − max.qupm: 3

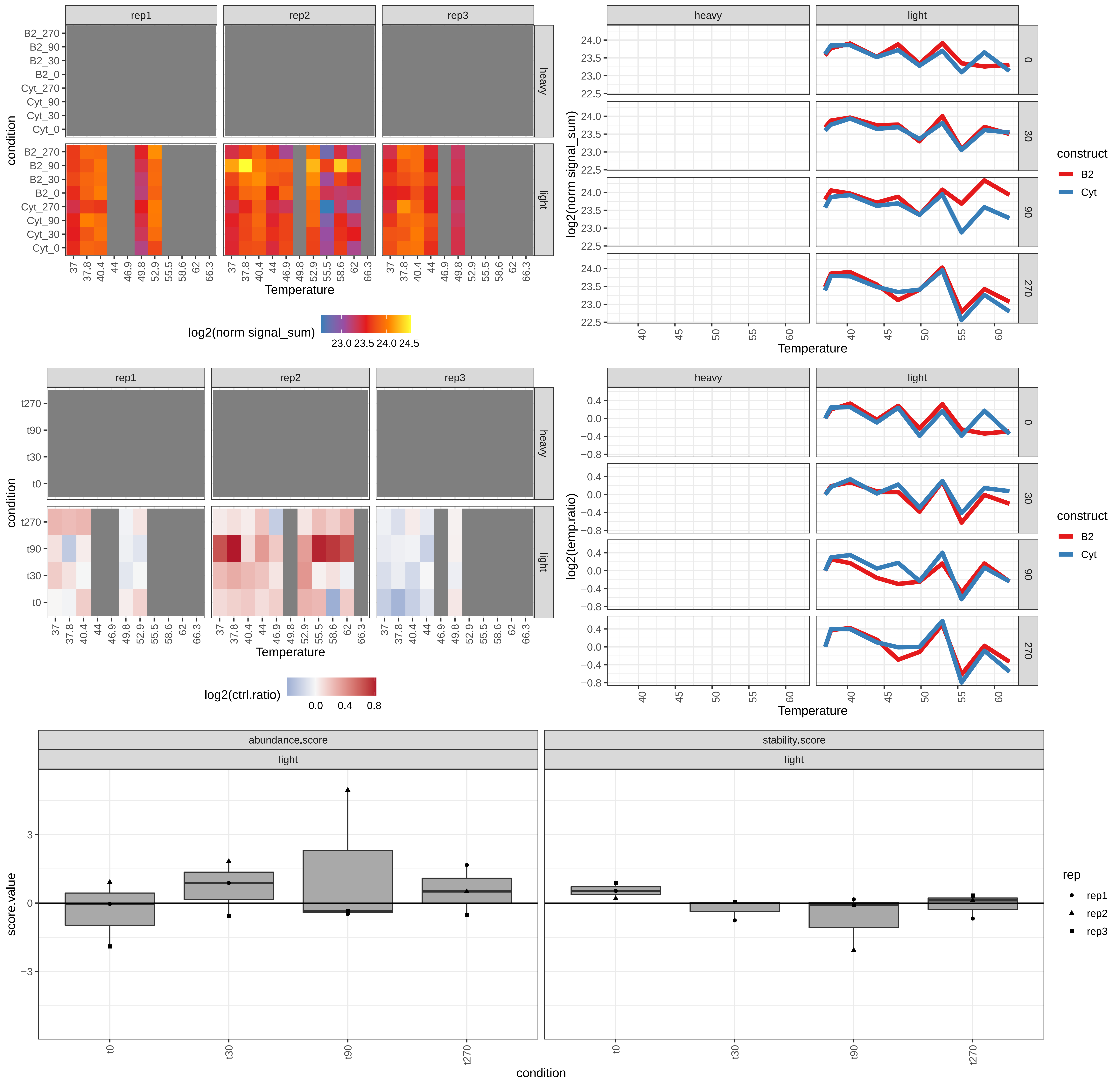

Q03455 − Probable zinc transporter MSC2 OS=Saccharomyces cerevisiae (strain ATCC 204508 / S288c) GN=MSC2 PE=1 SV=2

MSC6 − average.top3: 7.25573 − max.qupm: 14

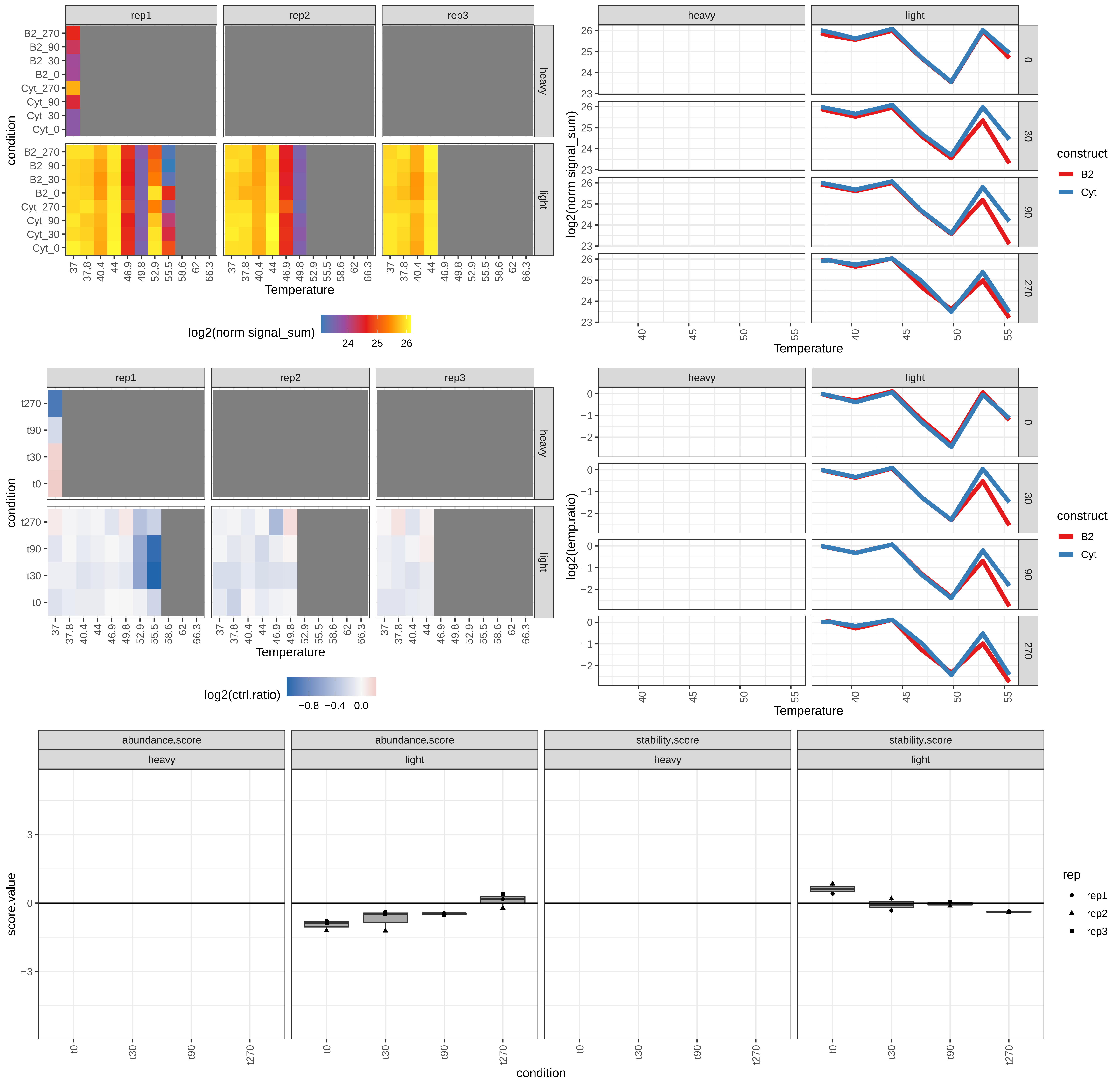

Q08818 − Meiotic sister−chromatid recombination protein 6, mitochondrial OS=Saccharomyces cerevisiae (strain ATCC 204508 / S288c) GN=MSC6 PE=1 SV=1

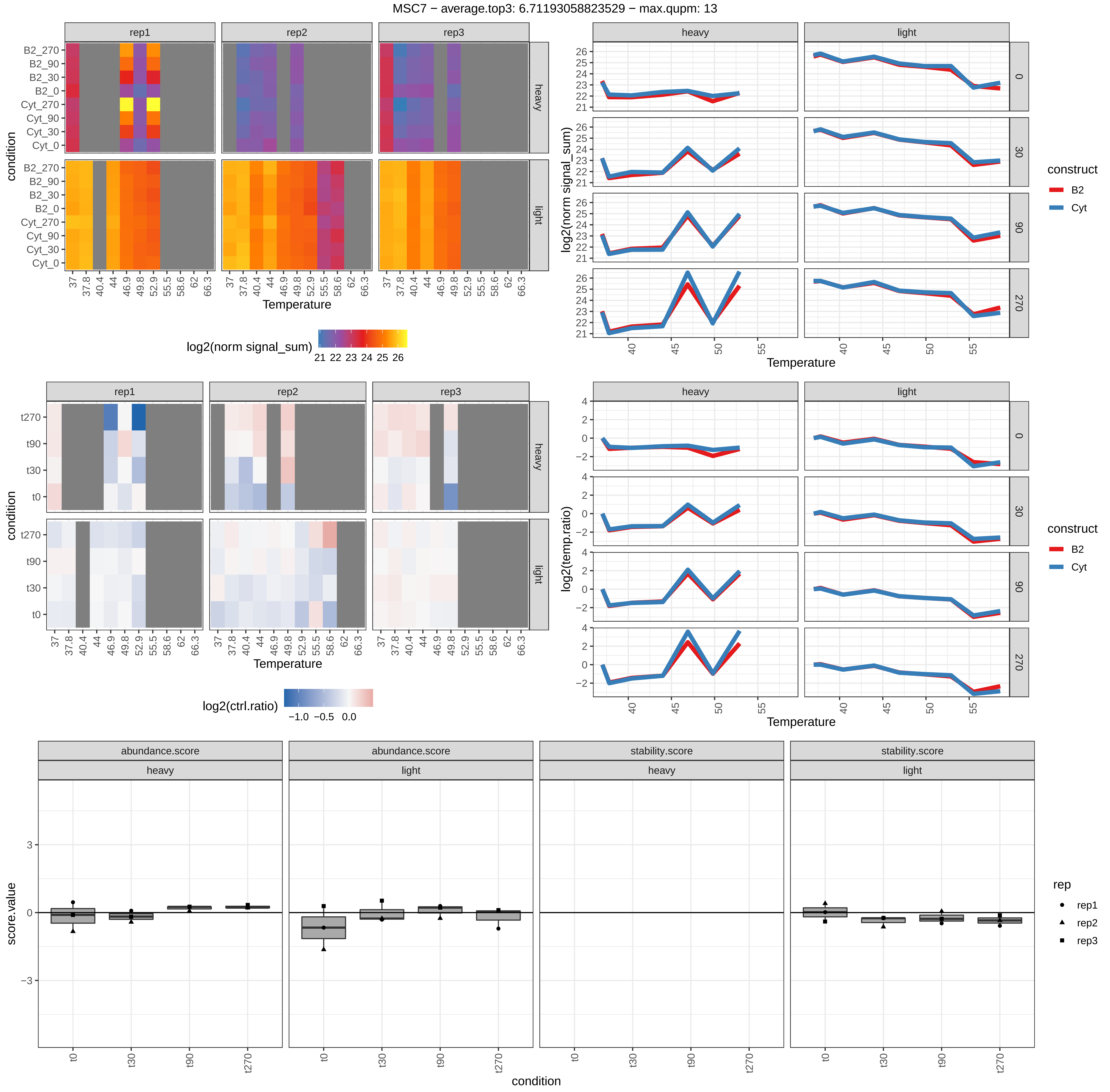

P38694 − Putative aldehyde dehydrogenase−like protein YHR039C OS=Saccharomyces cerevisiae (strain ATCC 204508 / S288c) GN=MSC7 PE=1 SV=1

MSD1 − average.top3: 6.87567333333333 − max.qupm: 10

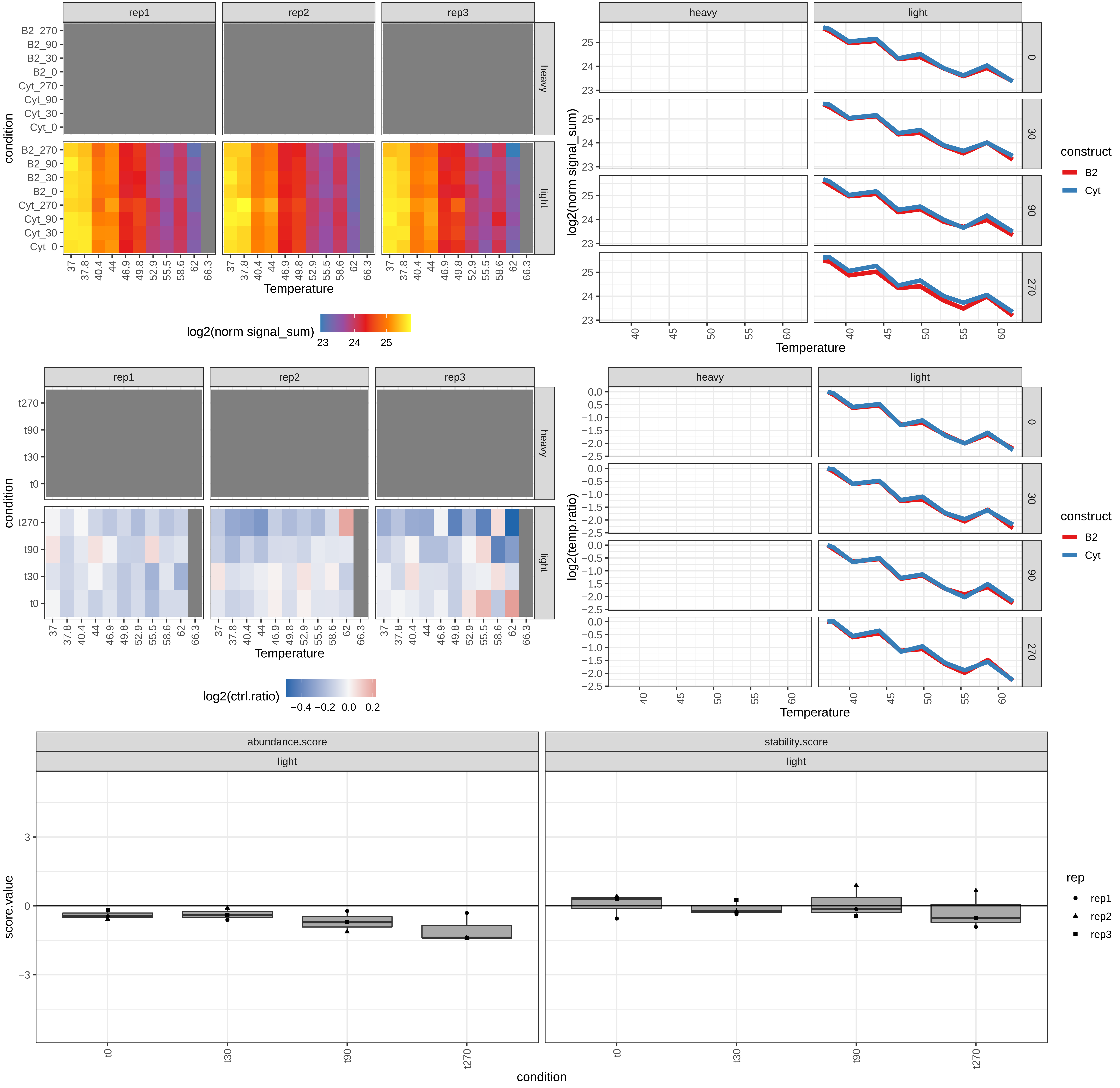

P15179 − Aspartate−−tRNA ligase, mitochondrial OS=Saccharomyces cerevisiae (strain ATCC 204508 / S288c) GN=MSD1 PE=1 SV=1

MSE1 − average.top3: 6.7443225 − max.qupm: 4

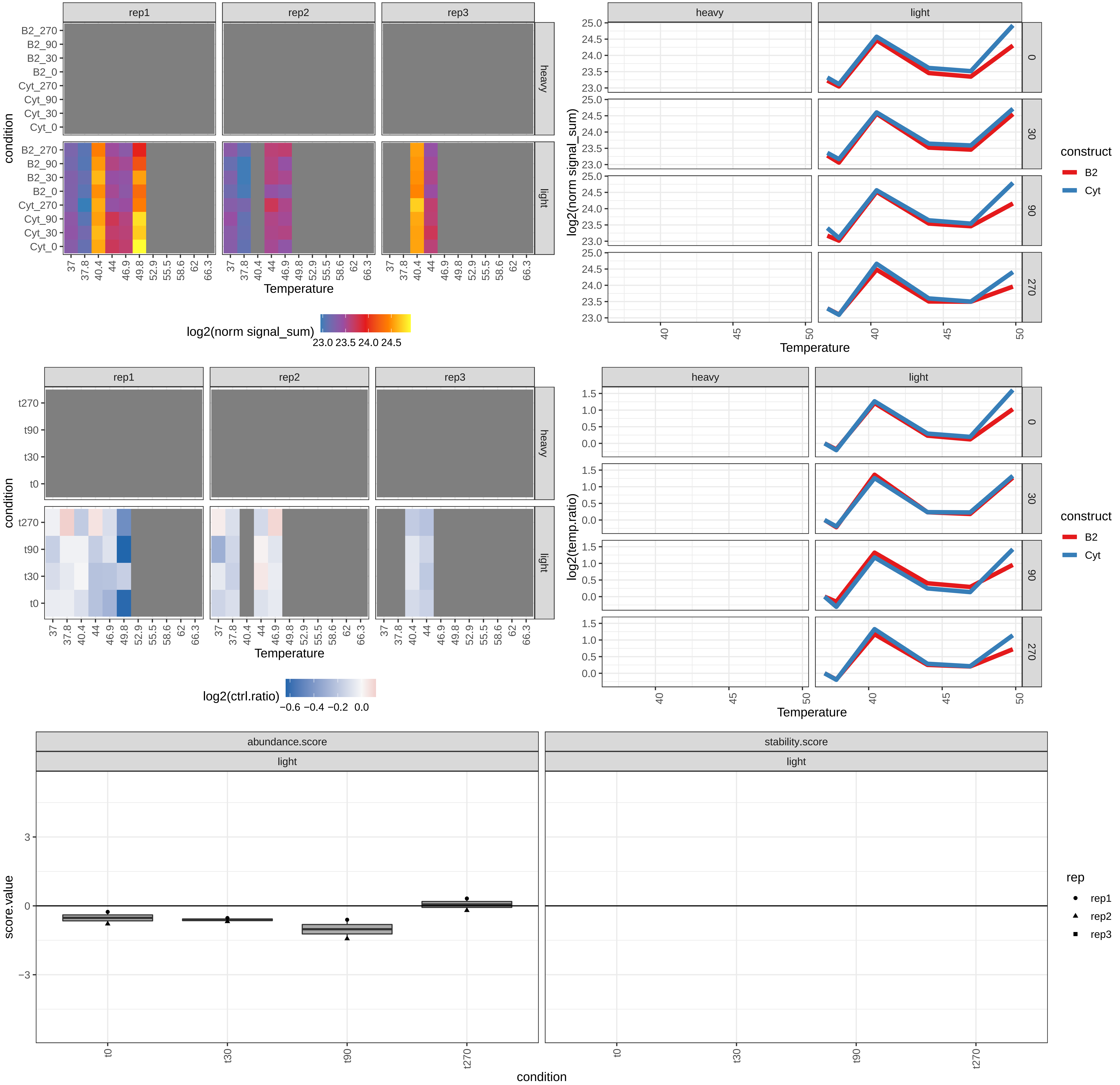

P48525 − Glutamate−−tRNA ligase, mitochondrial OS=Saccharomyces cerevisiae (strain ATCC 204508 / S288c) GN=MSE1 PE=1 SV=2

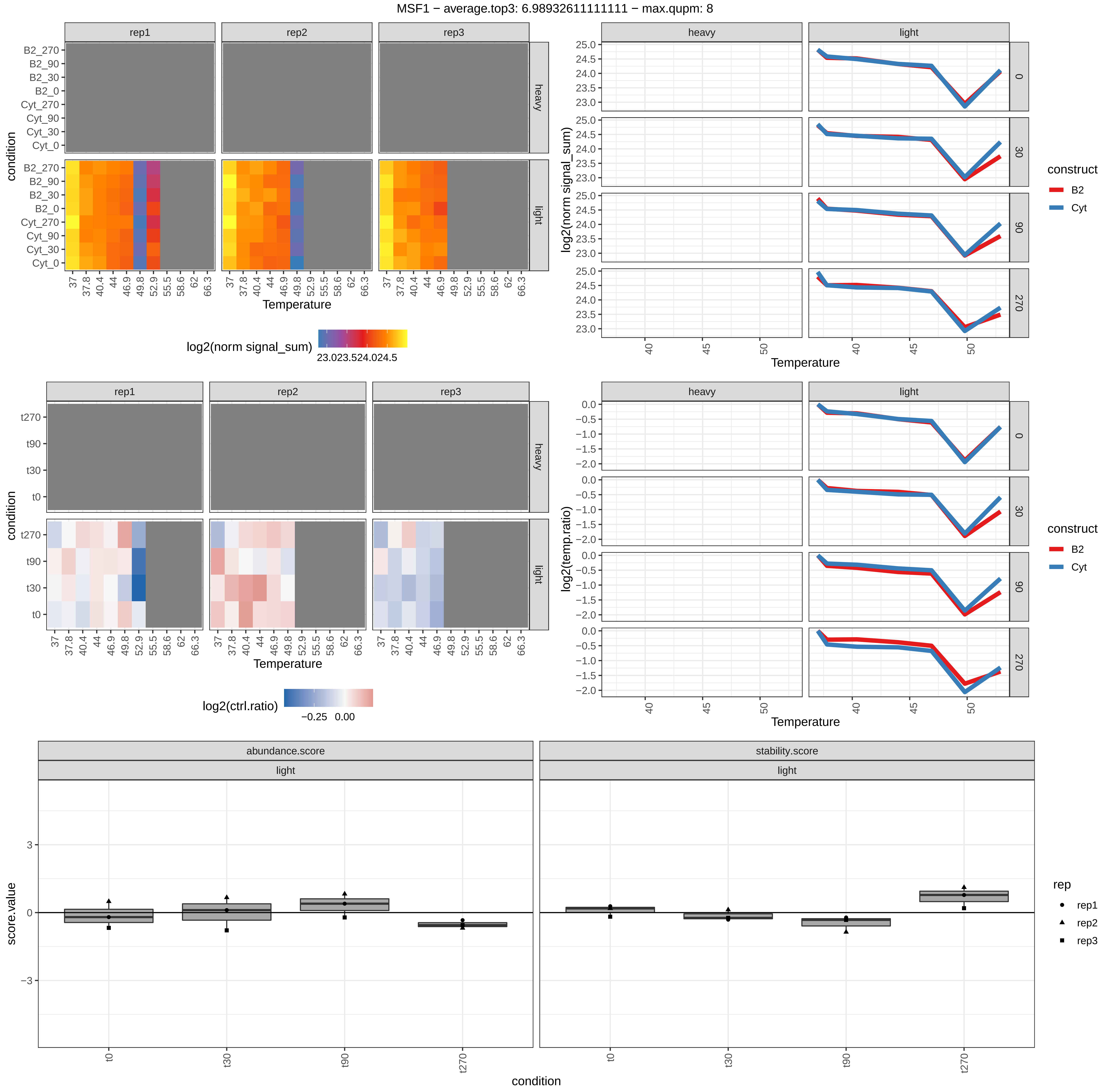

P08425 − Phenylalanine−−tRNA ligase, mitochondrial OS=Saccharomyces cerevisiae (strain ATCC 204508 / S288c) GN=MSF1 PE=1 SV=3

MSH2 − average.top3: 6.69258272727273 − max.qupm: 12

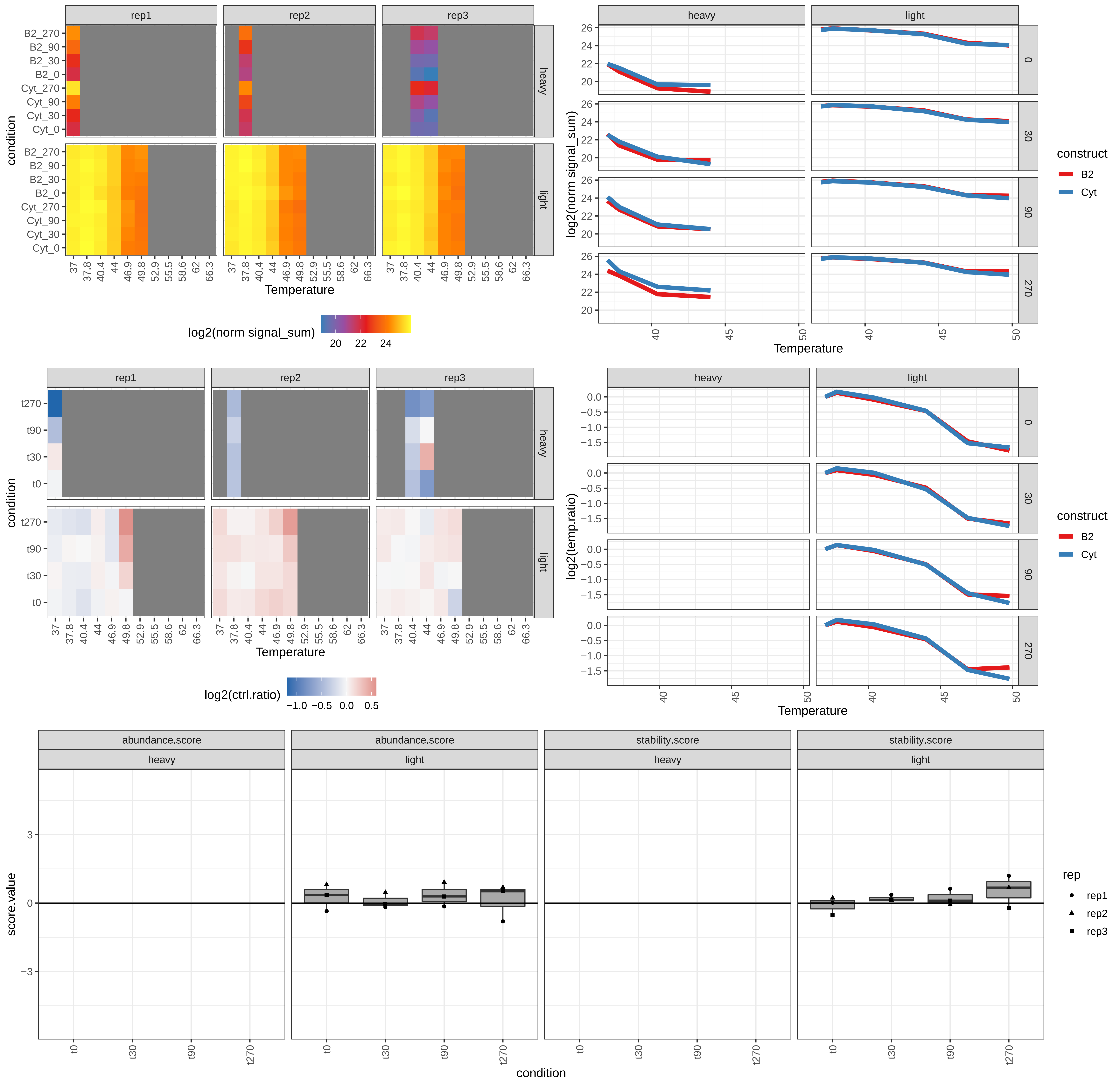

P25847 − DNA mismatch repair protein MSH2 OS=Saccharomyces cerevisiae (strain ATCC 204508 / S288c) GN=MSH2 PE=1 SV=2

MSH3 − average.top3: 6.34468545454545 − max.qupm: 5

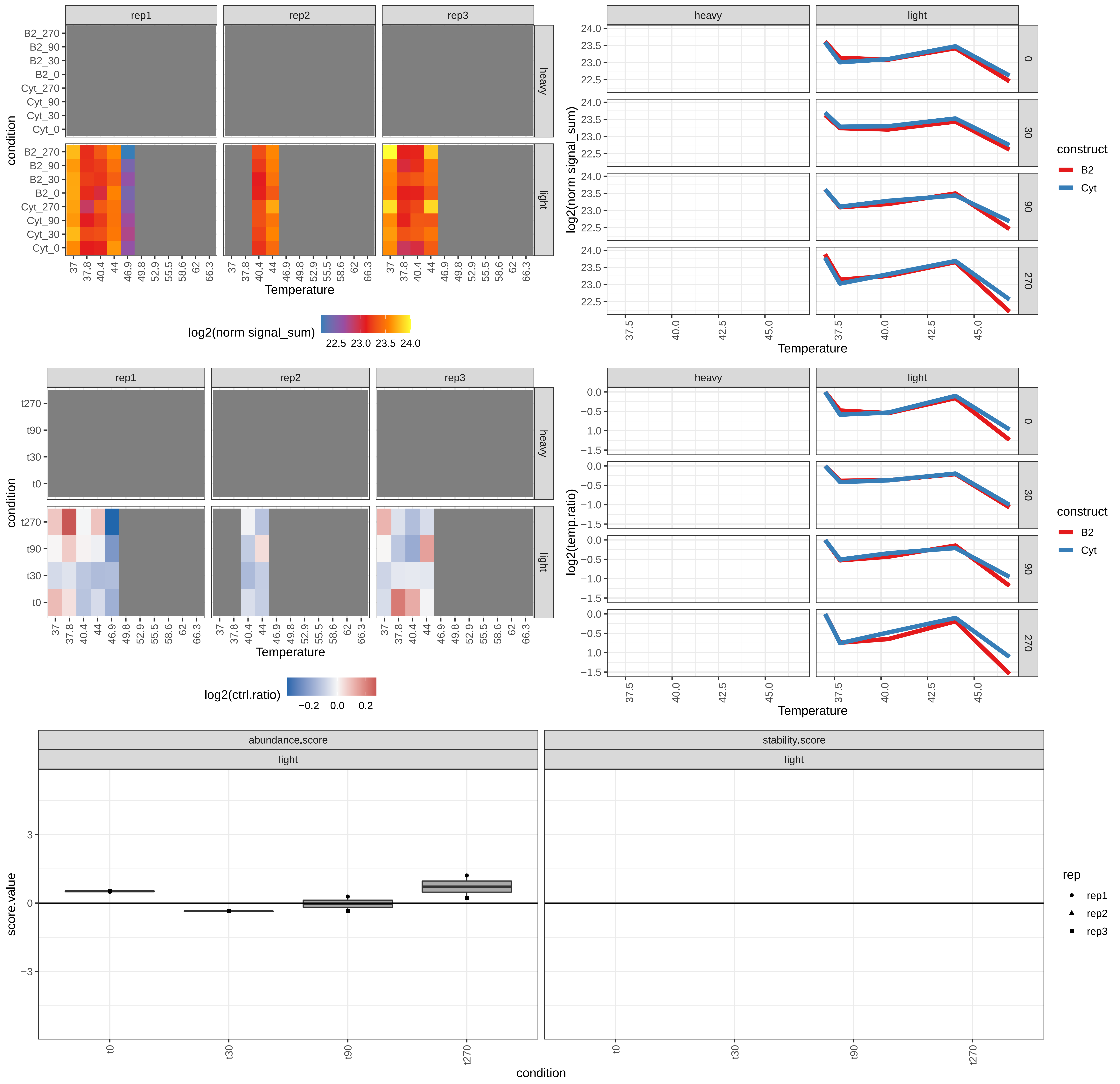

P25336 − DNA mismatch repair protein MSH3 OS=Saccharomyces cerevisiae (strain ATCC 204508 / S288c) GN=MSH3 PE=1 SV=2

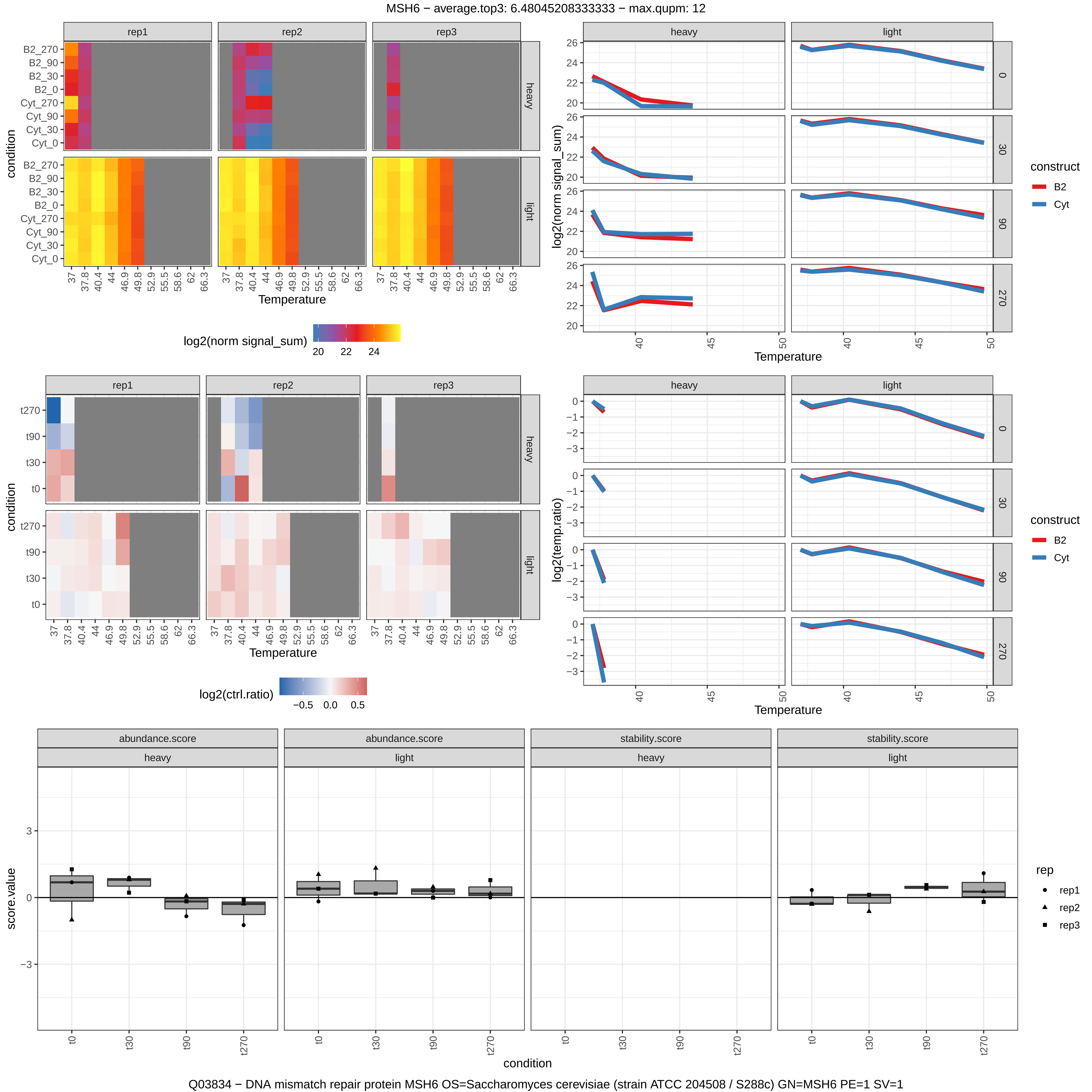

MSH6 − average.top3: 6.48045208333333 − max.qupm: 12
rep1
rep2
rep3
heavy
light
condition
Temperature
log2(norm signal_sum)
log2(norm signal_sum)
construct
B2
Cyt
log2(ctrl.ratio)
log2(temp.ratio)
abundance.score
stability.score
score.value
rep
rep1
rep2
rep3
Q03834 − DNA mismatch repair protein MSH6 OS=Saccharomyces cerevisiae (strain ATCC 204508 / S288c) GN=MSH6 PE=1 SV=1

MSK1 − average.top3: 6.50764230769231 − max.qupm: 5

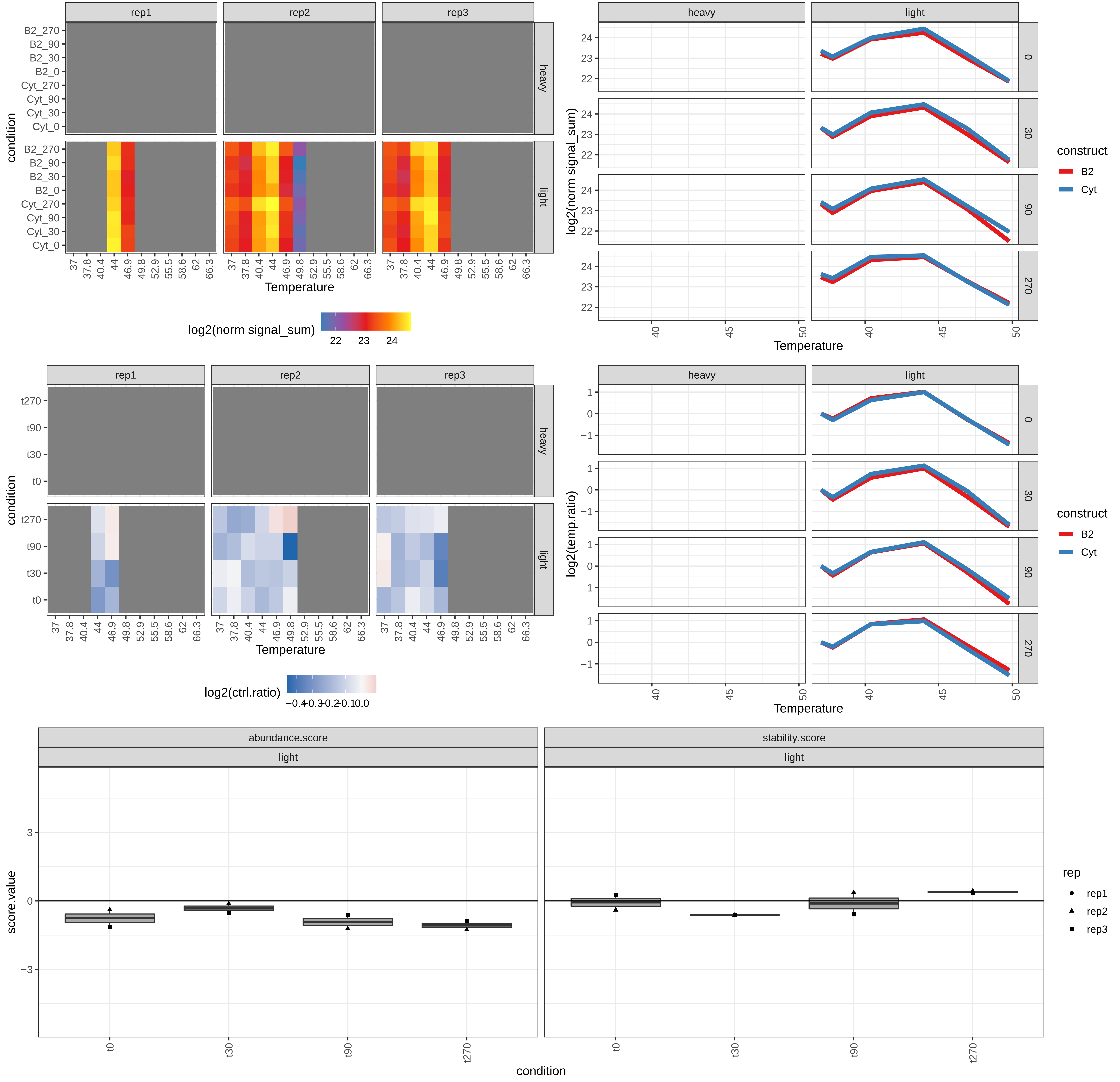

P32048 − Lysine−−tRNA ligase, mitochondrial OS=Saccharomyces cerevisiae (strain ATCC 204508 / S288c) GN=MSK1 PE=1 SV=3

MSM1 − average.top3: 6.63474740740741 − max.qupm: 8

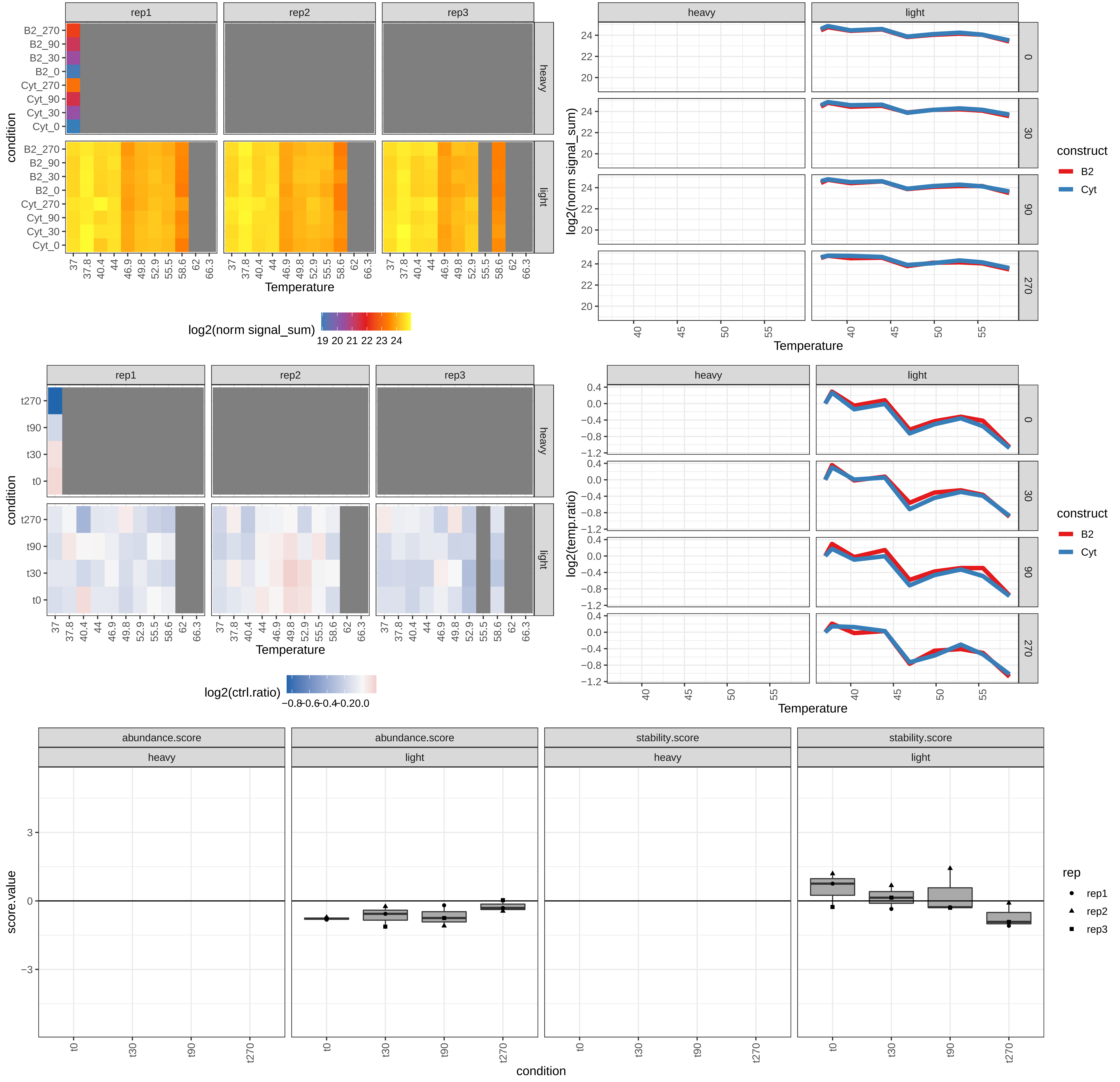

P22438 − Methionine−−tRNA ligase, mitochondrial OS=Saccharomyces cerevisiae (strain ATCC 204508 / S288c) GN=MSM1 PE=1 SV=2

MSN5 − average.top3: 6.92590233333333 − max.qupm: 18

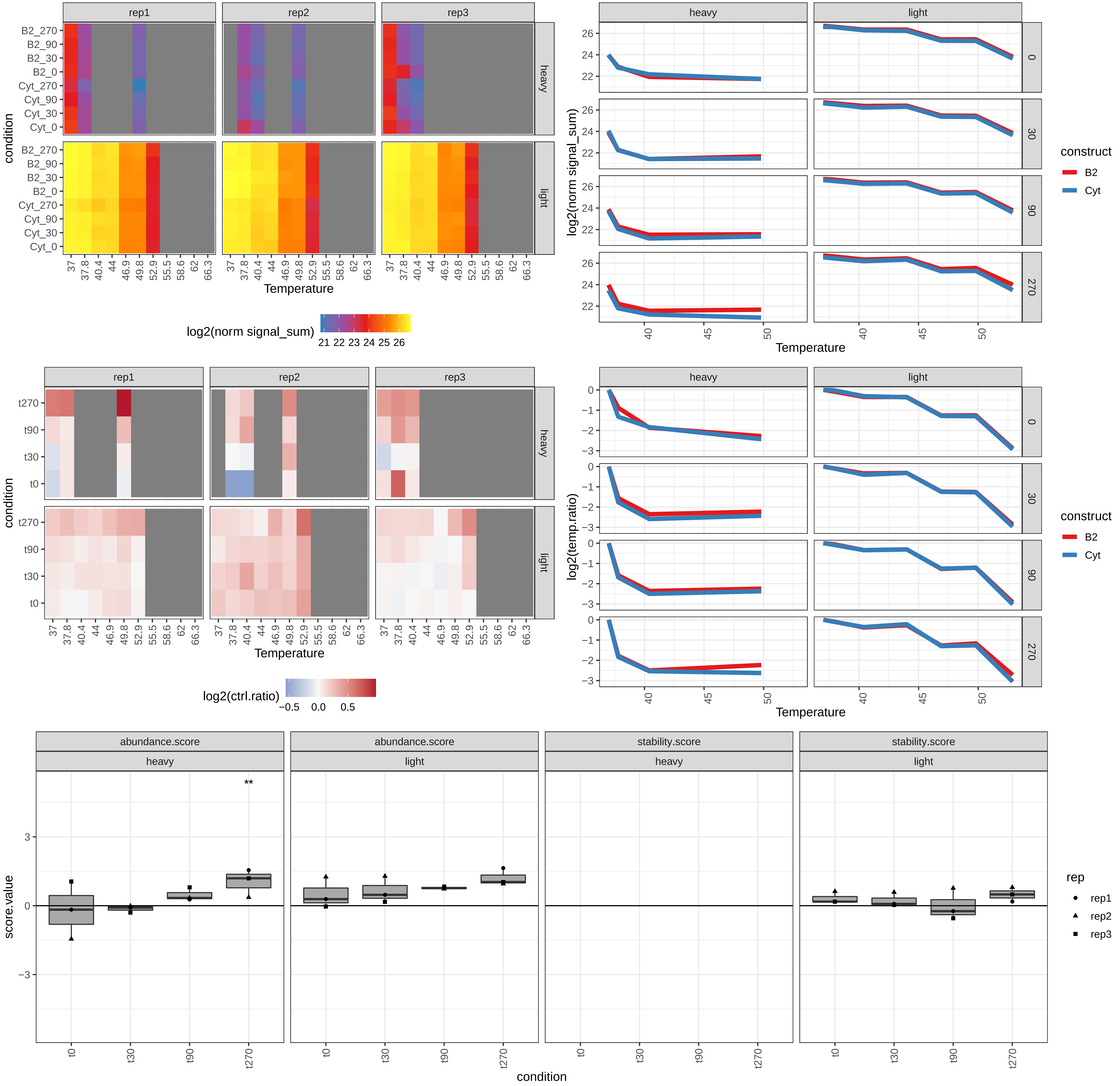

P52918 − Protein MSN5 OS=Saccharomyces cerevisiae (strain ATCC 204508 / S288c) GN=MSN5 PE=1 SV=1

MSP1 − average.top3: 6.82992523809524 − max.qupm: 6

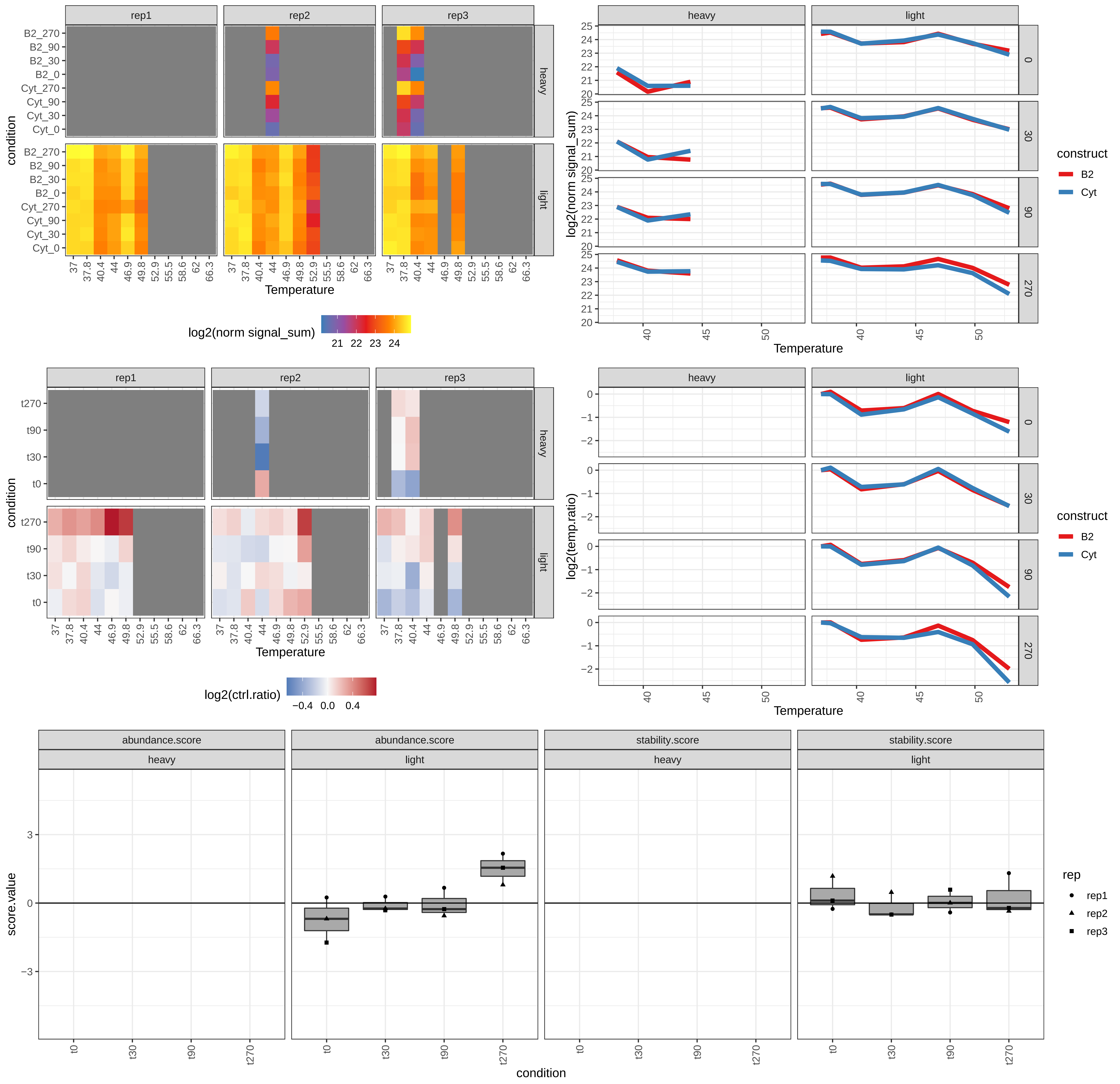

P28737 − Protein MSP1 OS=Saccharomyces cerevisiae (strain ATCC 204508 / S288c) GN=MSP1 PE=1 SV=2

MSR1 − average.top3: 6.48528869565217 − max.qupm: 7

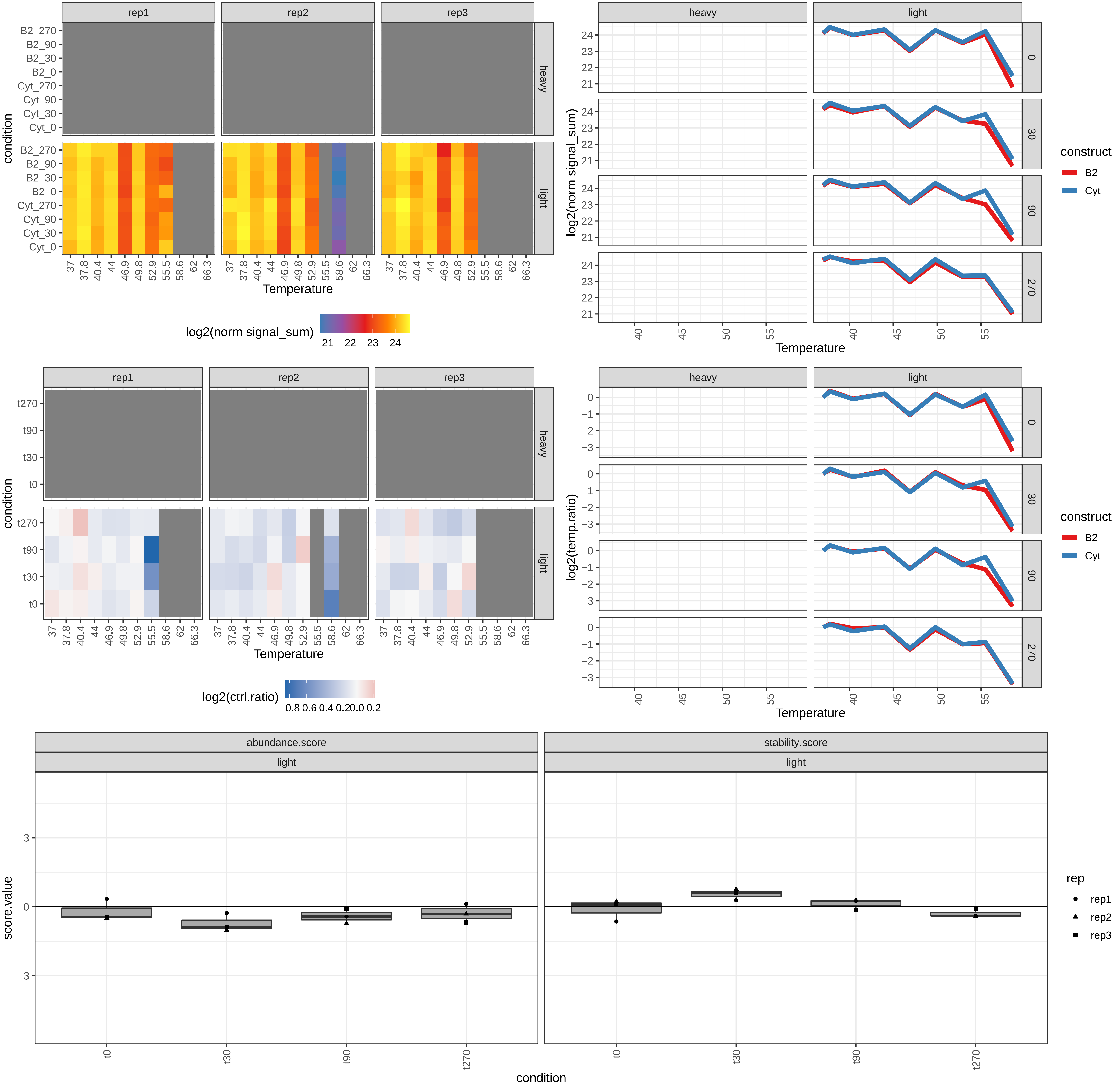

P38714 − Arginine−−tRNA ligase, mitochondrial OS=Saccharomyces cerevisiae (strain ATCC 204508 / S288c) GN=MSR1 PE=3 SV=3

MSS116 − average.top3: 6.81451575 − max.qupm: 16

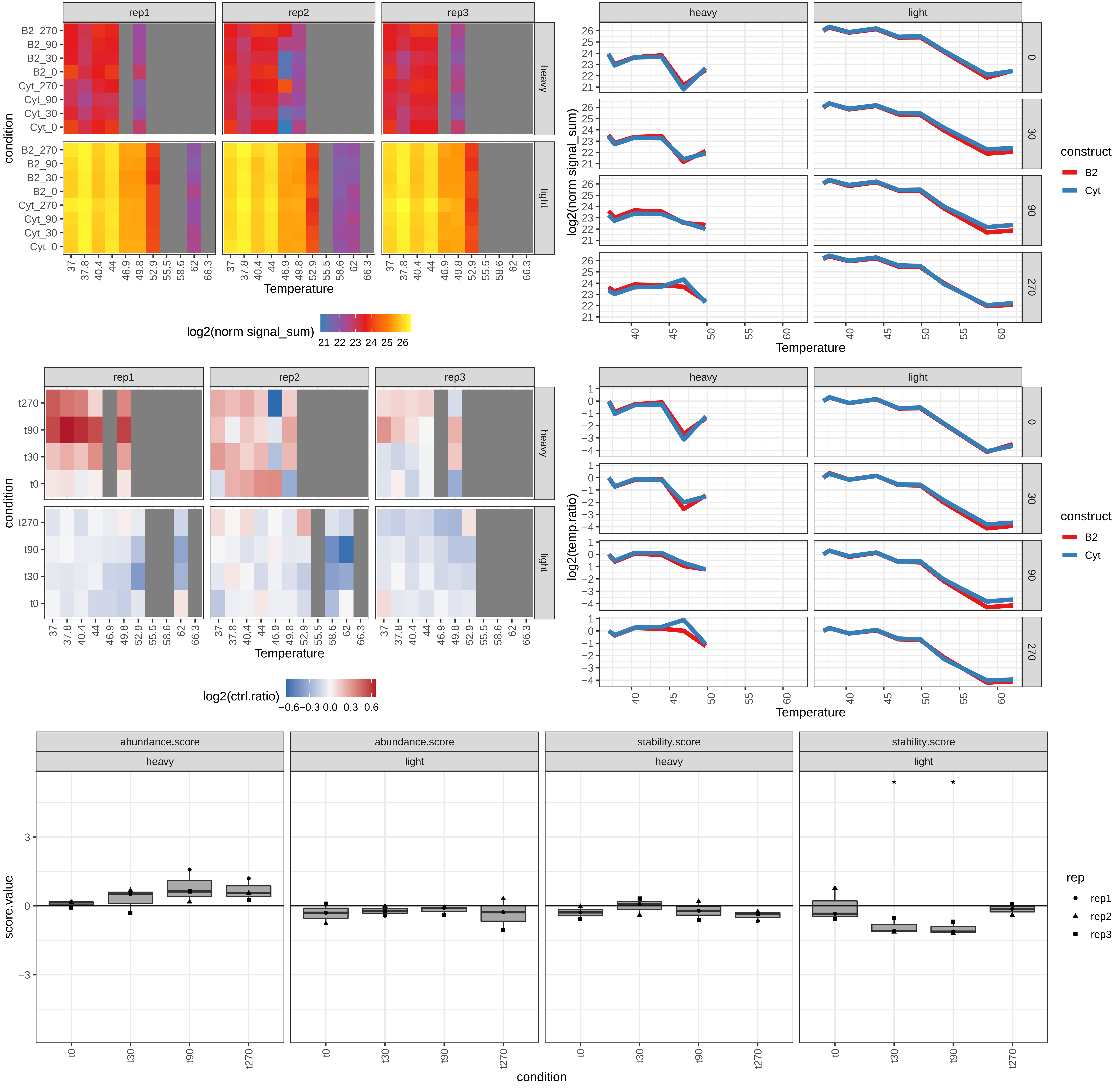

P15424 − ATP−dependent RNA helicase MSS116, mitochondrial OS=Saccharomyces cerevisiae (strain ATCC 204508 / S288c) GN=MSS116 PE=1 SV=1

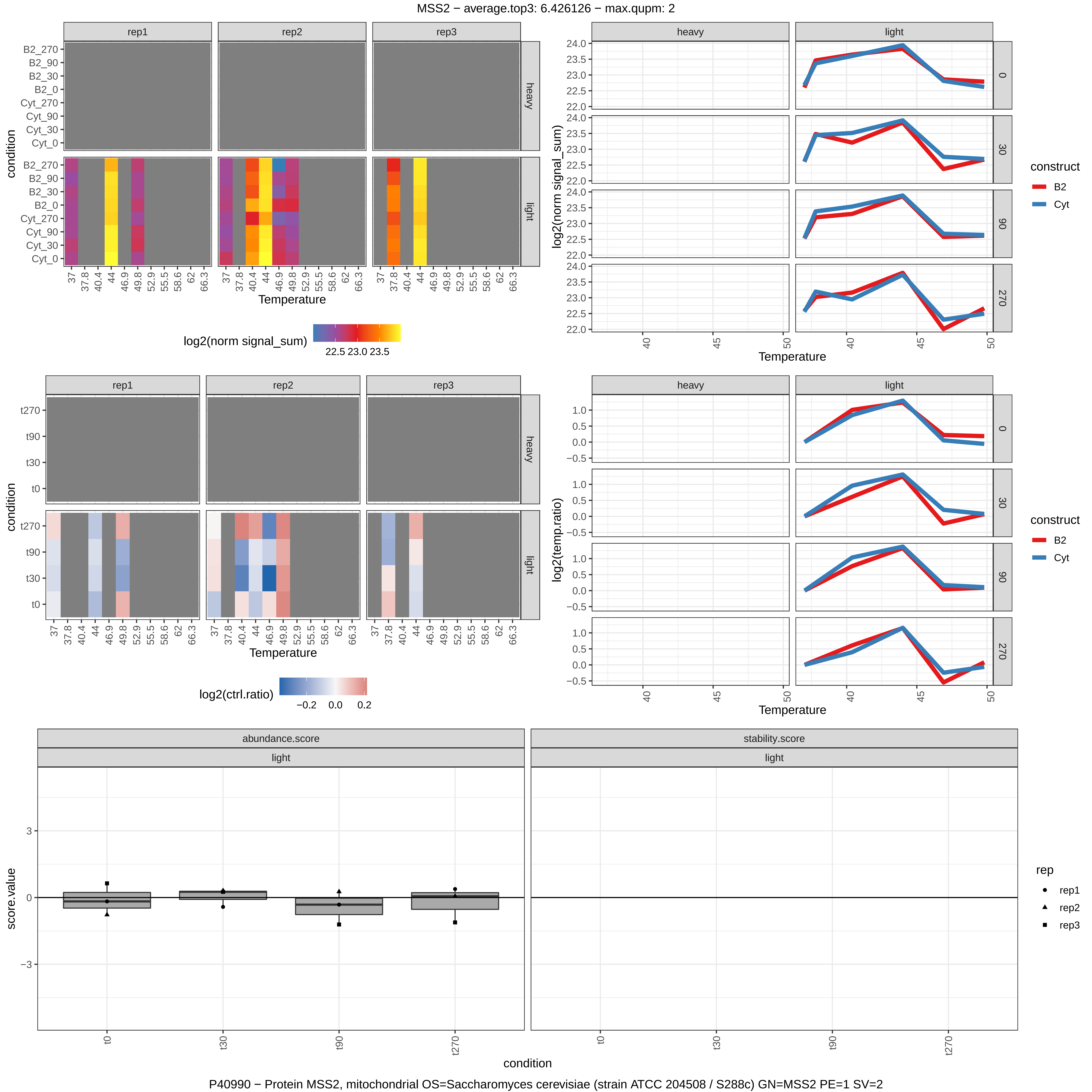
MSS2 − average.top3: 6.426126 − max.qupm: 2
rep1
rep2
rep3
heavy
light
condition
B2_270
B2_90
B2_30
B2_0
Cyt_270
Cyt_90
Cyt_30
Cyt_0
Temperature
log2(norm signal_sum)
22.5 23.0 23.5
log2(norm signal_sum)
construct
B2
Cyt
t270
t90
t30
t0
log2(ctrl.ratio)
−0.2 0.0 0.2
log2(temp.ratio)
abundance.score
stability.score
score.value
rep
rep1
rep2
rep3
condition
P40990 − Protein MSS2, mitochondrial OS=Saccharomyces cerevisiae (strain ATCC 204508 / S288c) GN=MSS2 PE=1 SV=2

MSS51 − average.top3: 7.10425755102041 − max.qupm: 15

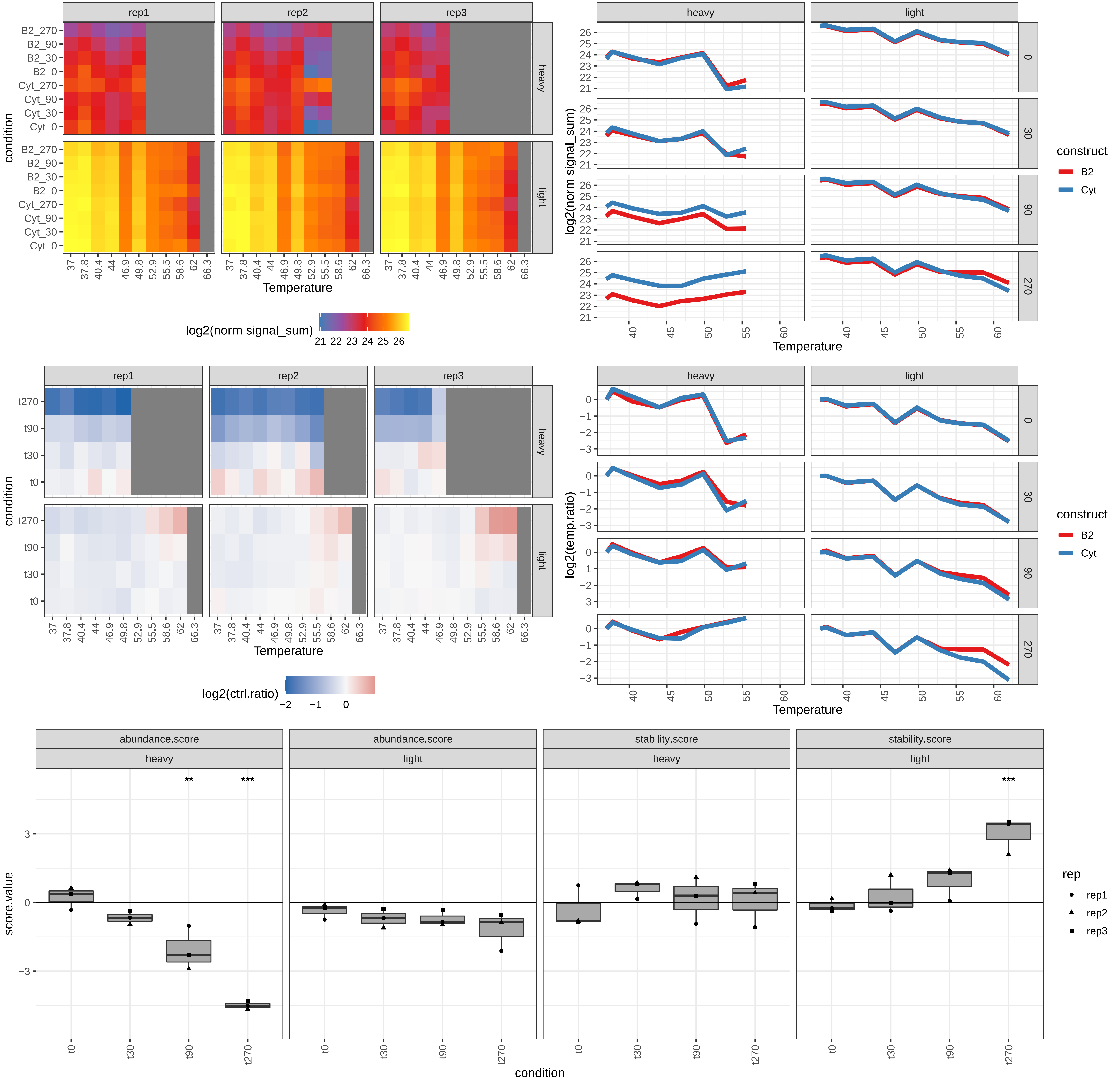

P32335 − Protein MSS51, mitochondrial OS=Saccharomyces cerevisiae (strain ATCC 204508 / S288c) GN=MSS51 PE=1 SV=2

MST1 − average.top3: 6.775234 − max.qupm: 9

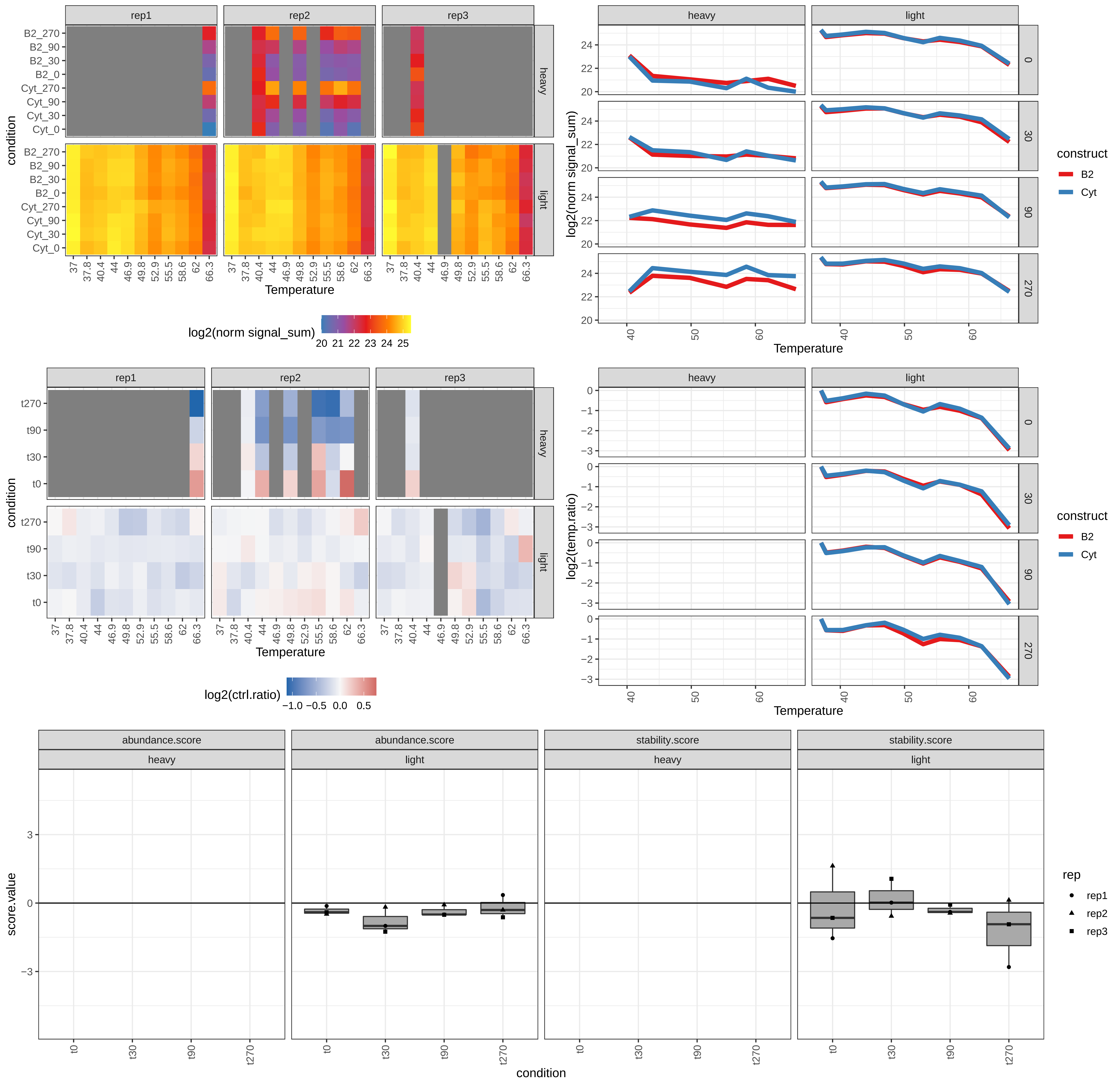

P07236 − Threonine−−tRNA ligase, mitochondrial OS=Saccharomyces cerevisiae (strain ATCC 204508 / S288c) GN=MST1 PE=1 SV=2

MSW1 − average.top3: 6.5557724 − max.qupm: 8

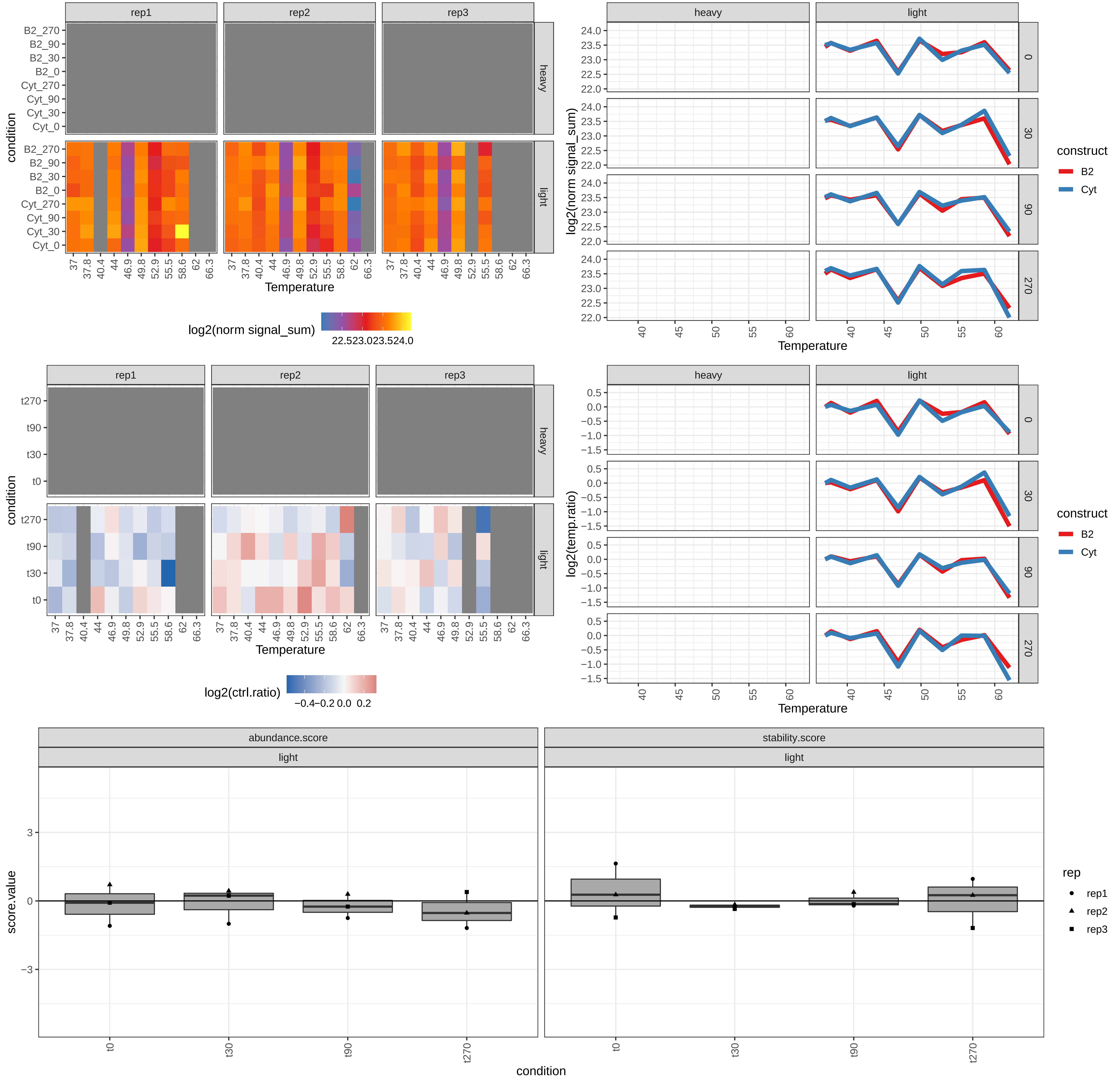

P04803 − Tryptophan−−tRNA ligase, mitochondrial OS=Saccharomyces cerevisiae (strain ATCC 204508 / S288c) GN=MSW1 PE=1 SV=2

MSY1 − average.top3: 6.800272 − max.qupm: 8

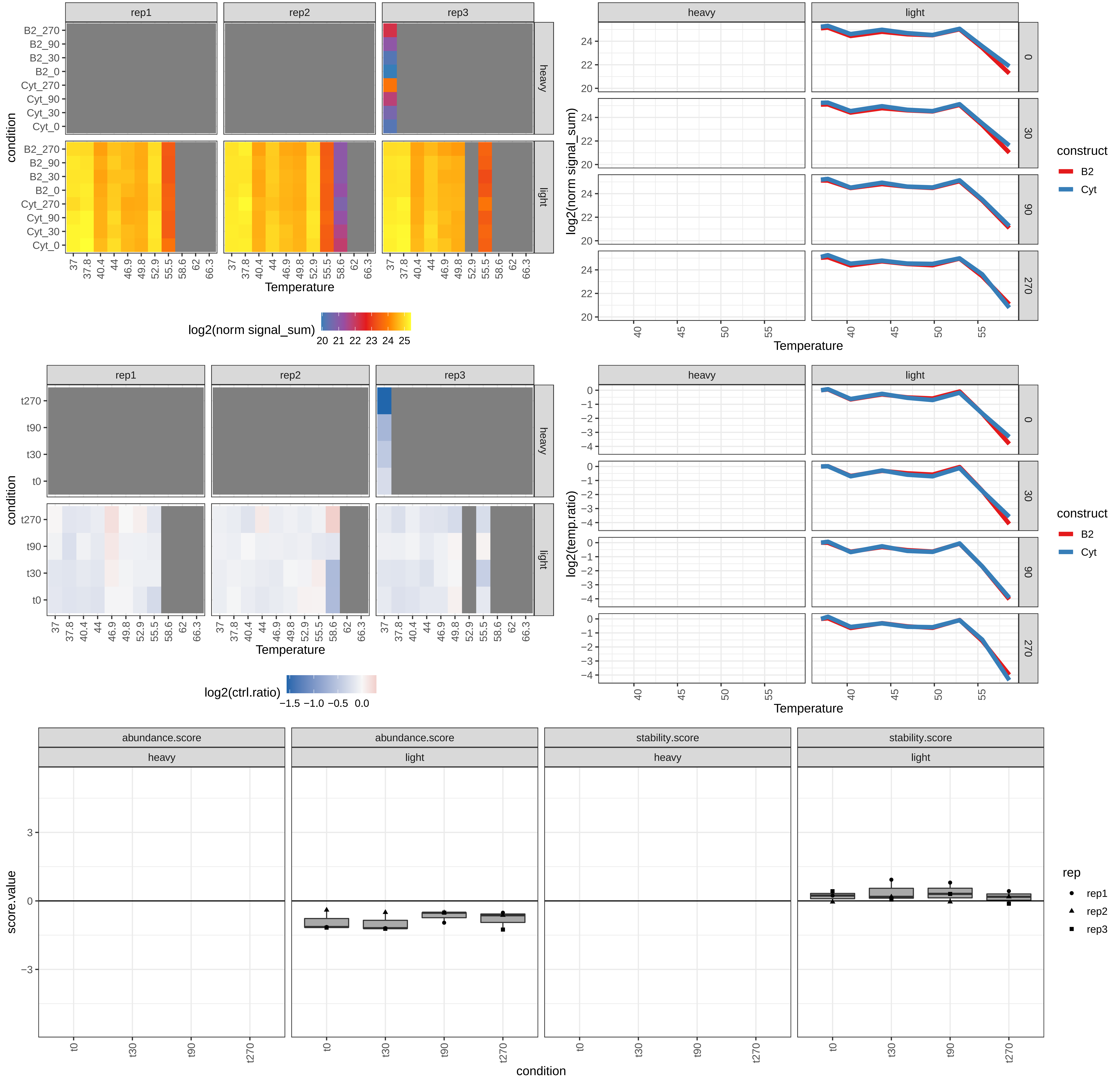

P48527 − Tyrosine−−tRNA ligase, mitochondrial OS=Saccharomyces cerevisiae (strain ATCC 204508 / S288c) GN=MSY1 PE=1 SV=1

MTC1 − average.top3: 7.01494403508772 − max.qupm: 10

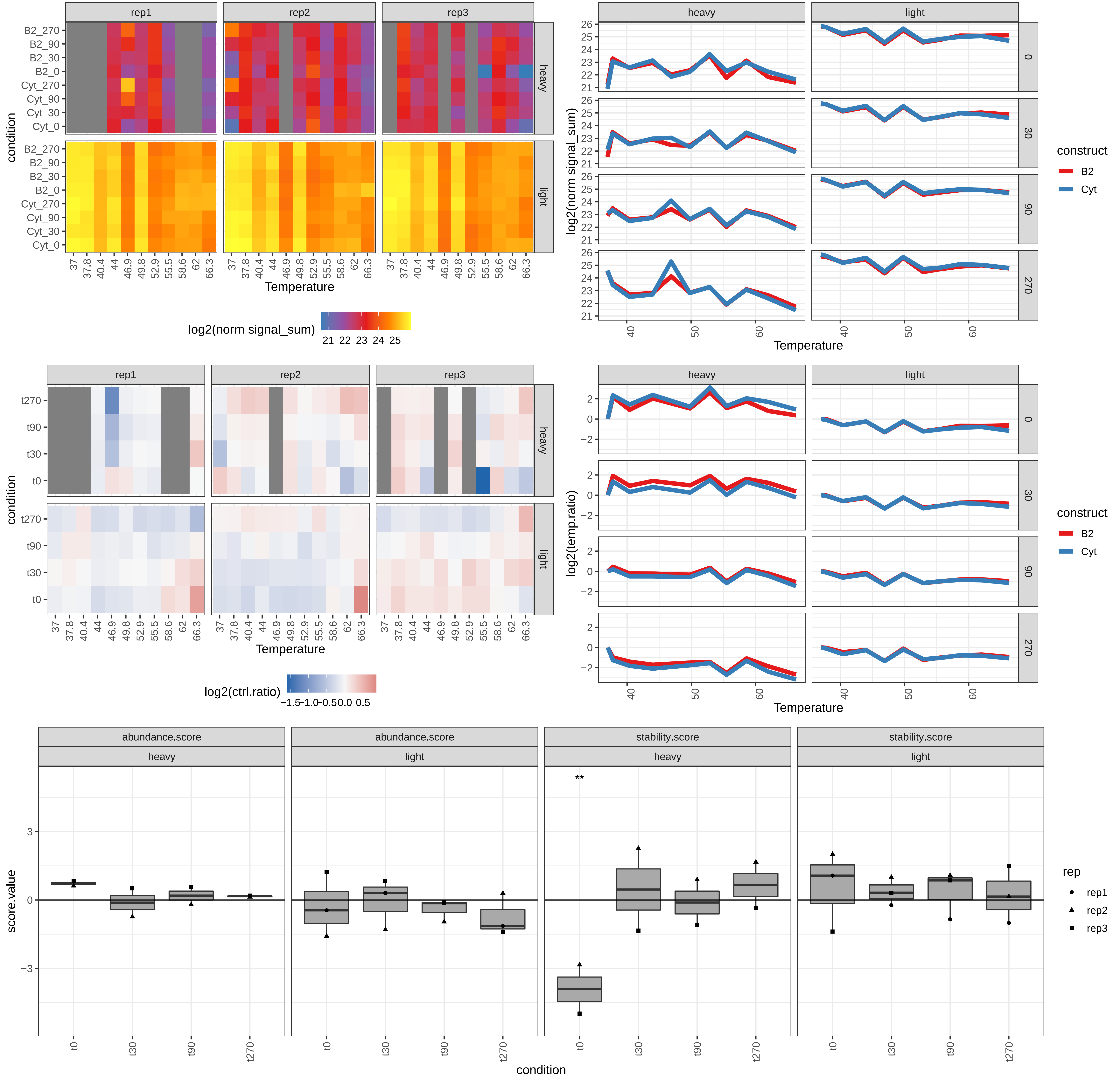

P47018 − Maintenance of telomere capping protein 1 OS=Saccharomyces cerevisiae (strain ATCC 204508 / S288c) GN=MTC1 PE=1 SV=1

MTD1 − average.top3: 6.76067142857143 − max.qupm: 4

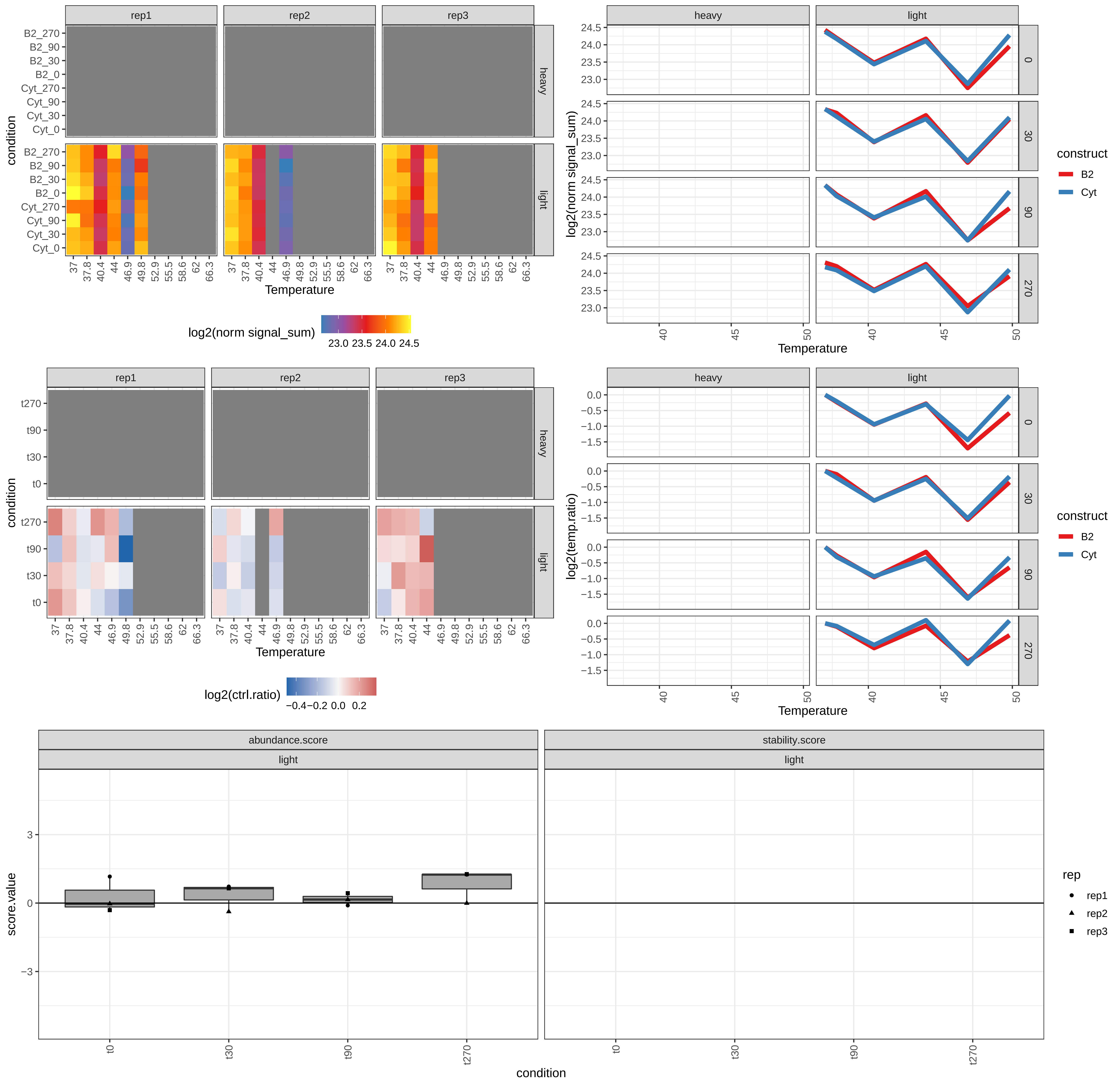

Q02046 − Methylenetetrahydrofolate dehydrogenase [NAD(+)] OS=Saccharomyces cerevisiae (strain ATCC 204508 / S288c) GN=MTD1 PE=1 SV=1

MTF1 − average.top3: 6.78274611111111 − max.qupm: 4

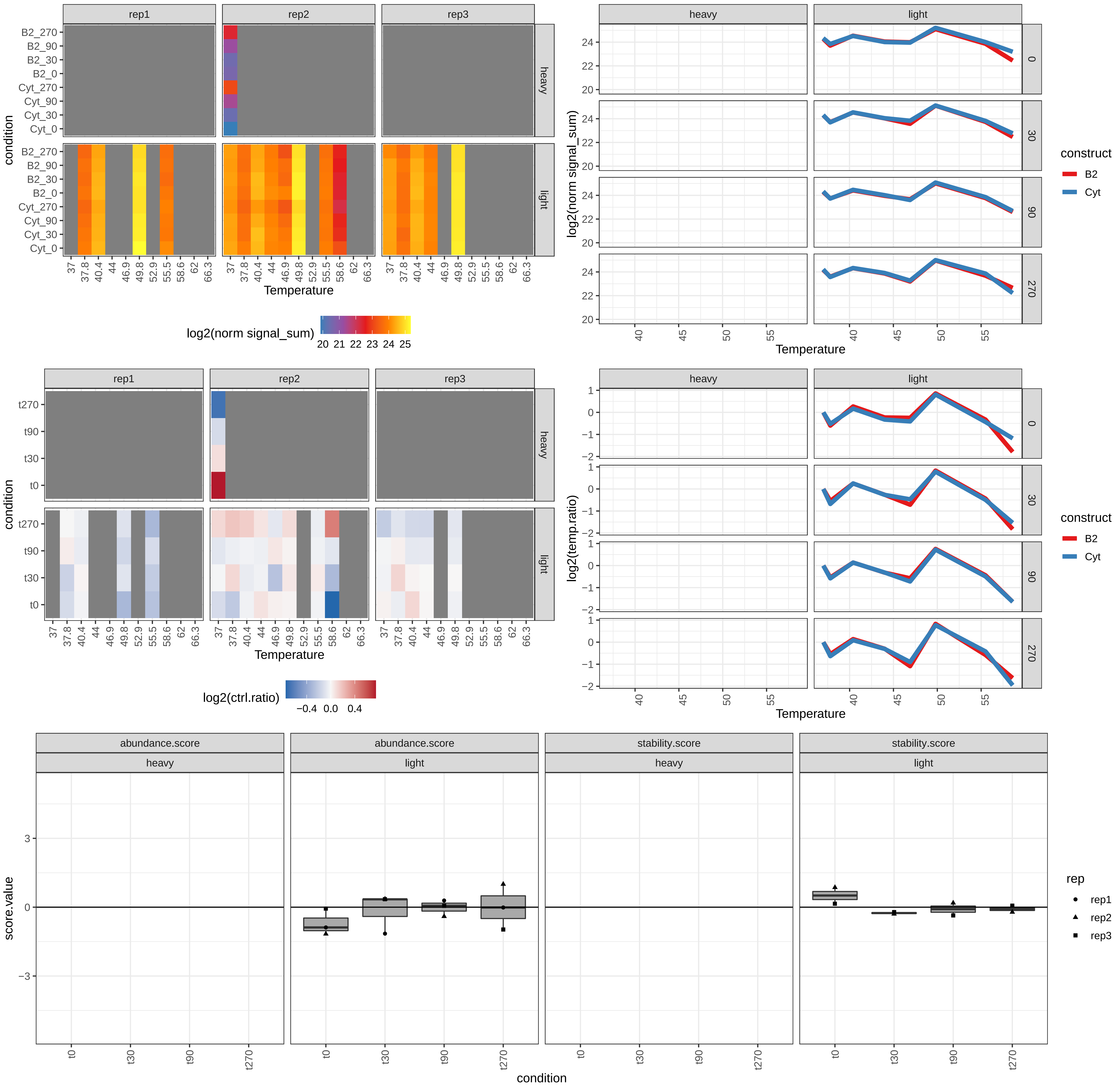

P14908 − Mitochondrial transcription factor 1 OS=Saccharomyces cerevisiae (strain ATCC 204508 / S288c) GN=MTF1 PE=1 SV=4

MTF2 − average.top3: 6.388334 − max.qupm: 4

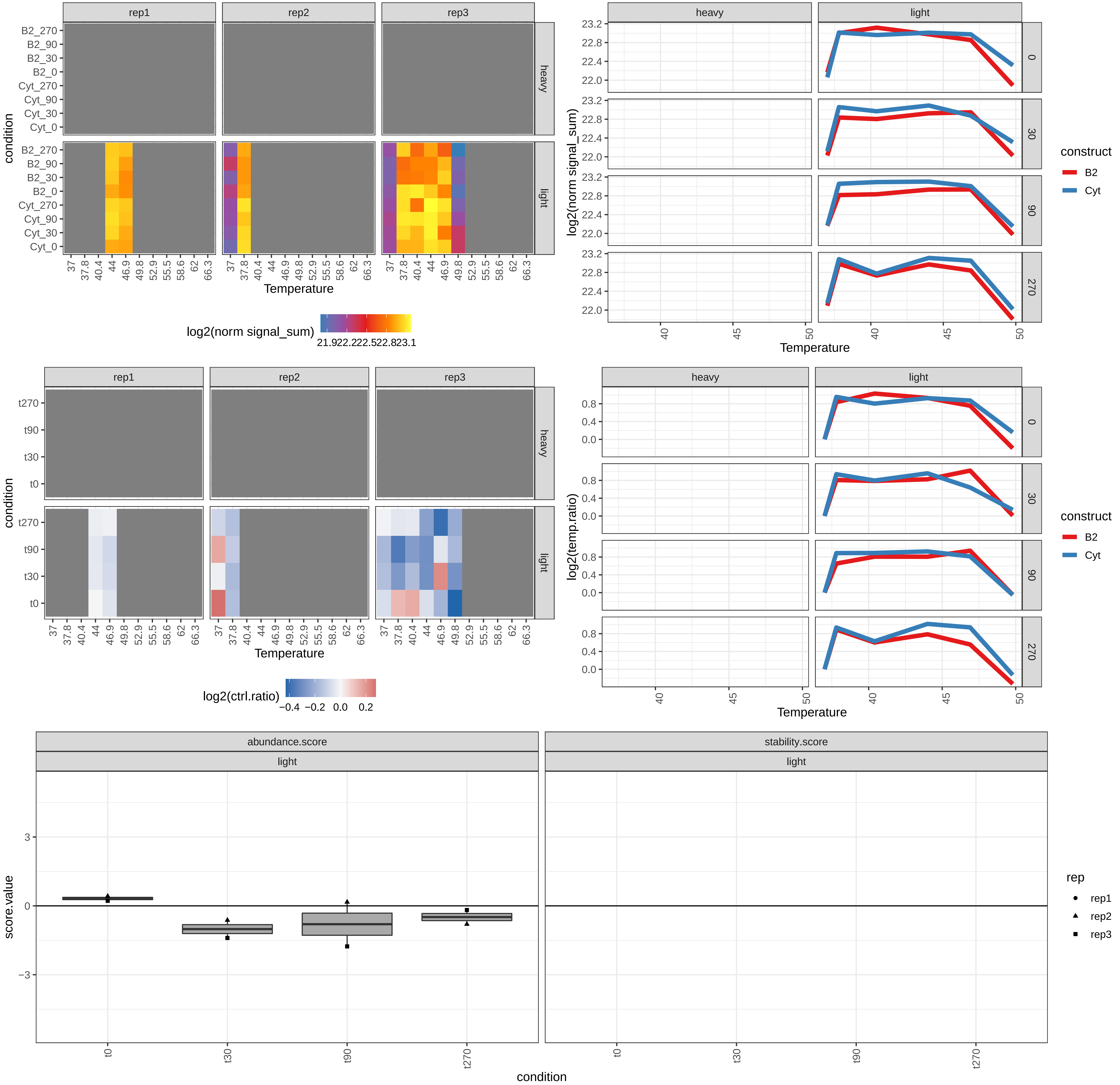

P10849 − Mitochondrial transcription factor 2 OS=Saccharomyces cerevisiae (strain ATCC 204508 / S288c) GN=MTF2 PE=1 SV=1

MTO1 − average.top3: 6.48435736842105 − max.qupm: 6

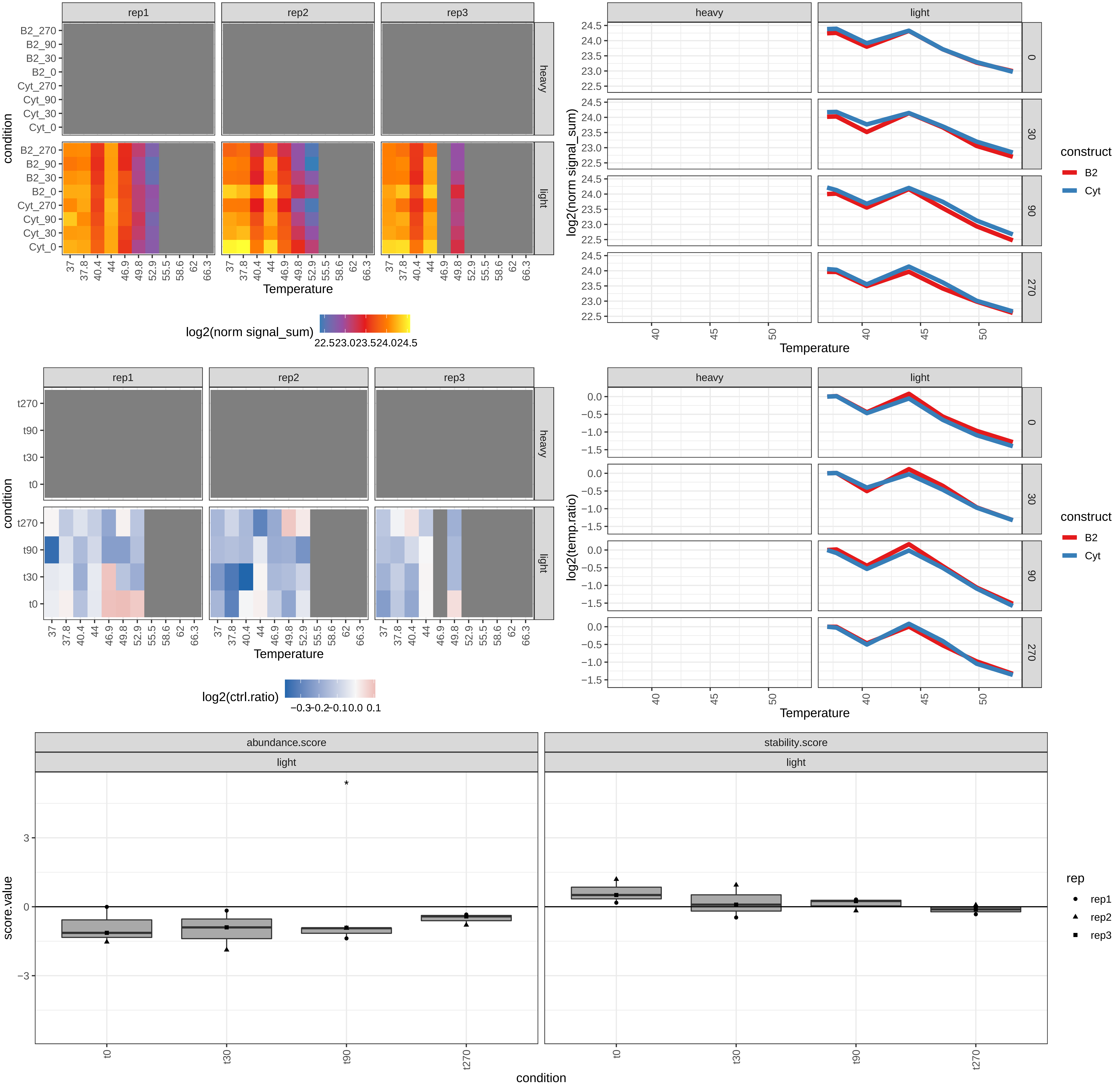

P53070 − Mitochondrial translation optimization protein 1 OS=Saccharomyces cerevisiae (strain ATCC 204508 / S288c) GN=MTO1 PE=1 SV=2

MTR10 − average.top3: 6.67061909090909 − max.qupm: 6

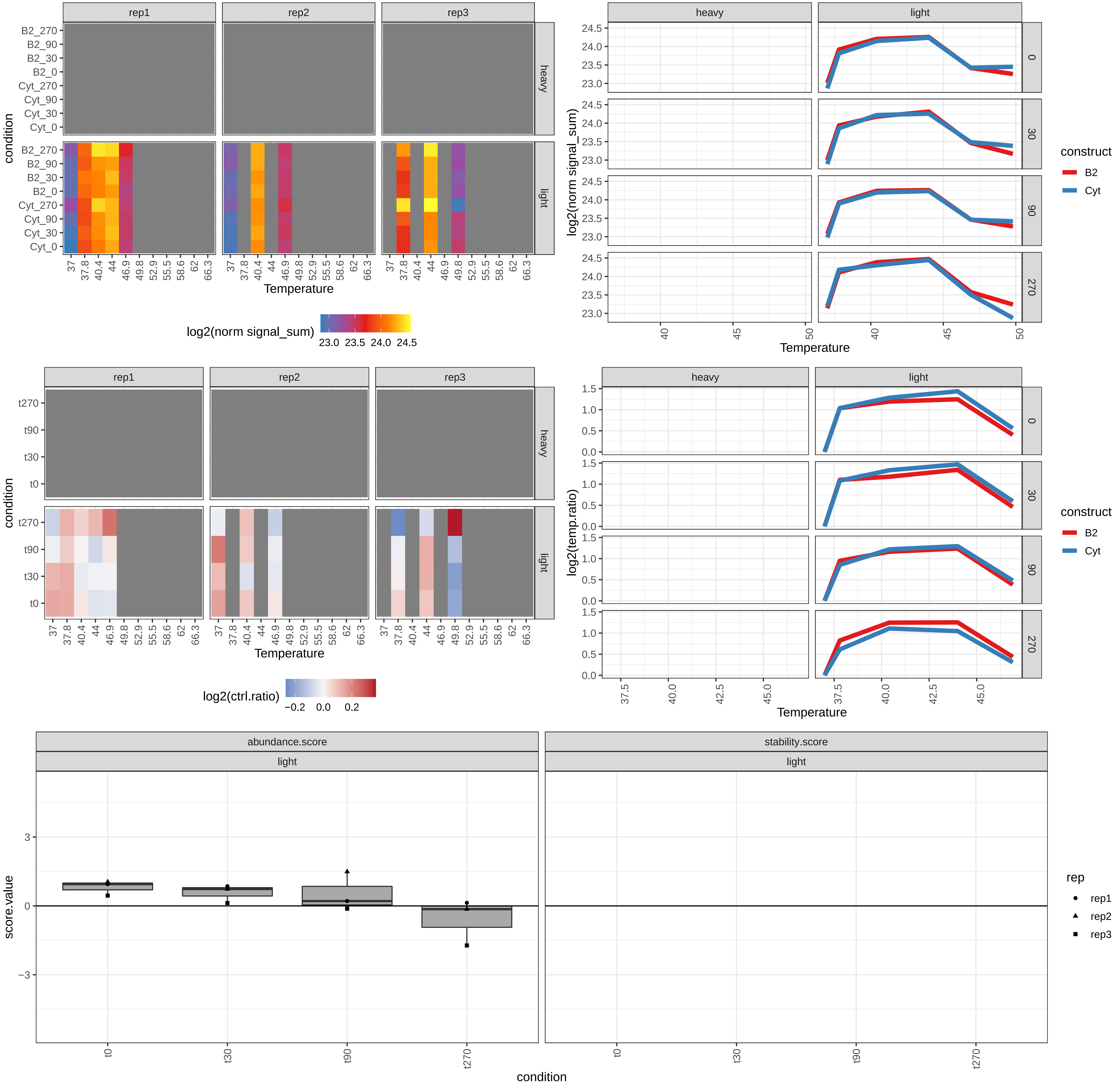

Q99189 − mRNA transport regulator MTR10 OS=Saccharomyces cerevisiae (strain ATCC 204508 / S288c) GN=MTR10 PE=1 SV=1

MTR3 − average.top3: 6.74053090909091 − max.qupm: 3

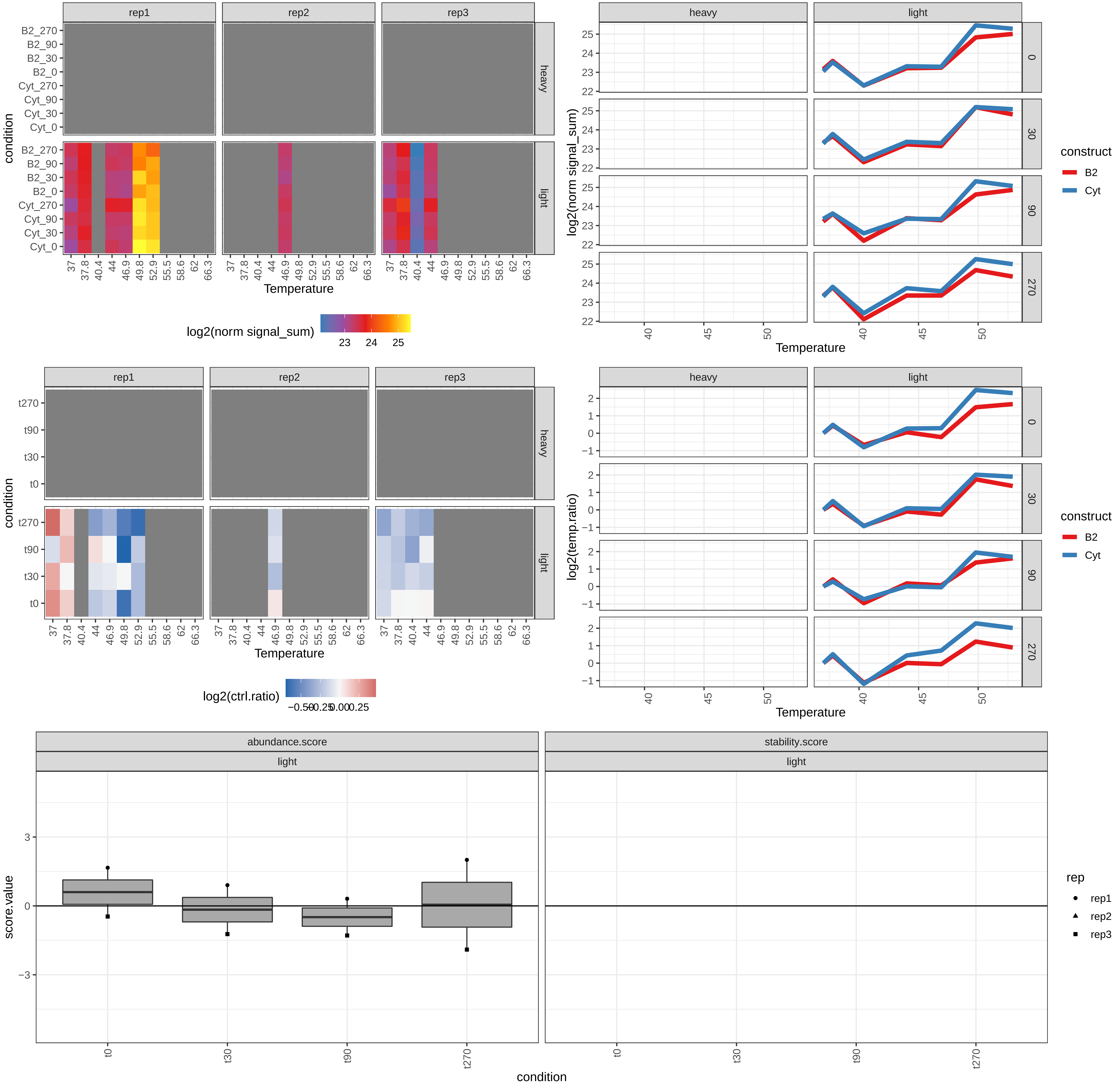

P48240 − Exosome complex component MTR3 OS=Saccharomyces cerevisiae (strain ATCC 204508 / S288c) GN=MTR3 PE=1 SV=1

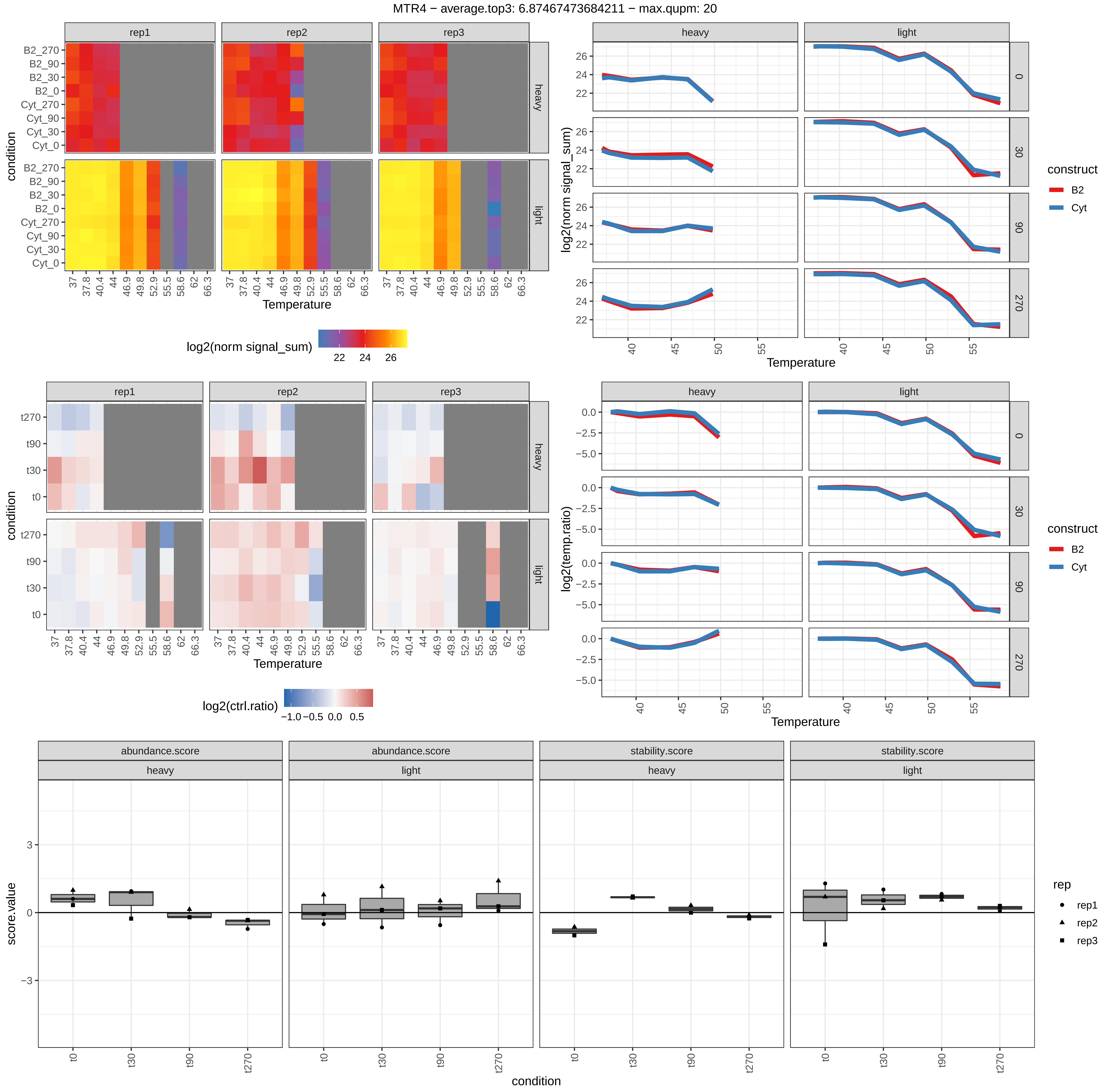

P47047 − ATP−dependent RNA helicase DOB1 OS=Saccharomyces cerevisiae (strain ATCC 204508 / S288c) GN=MTR4 PE=1 SV=1

MTW1 − average.top3: 5.879354 − max.qupm: 3

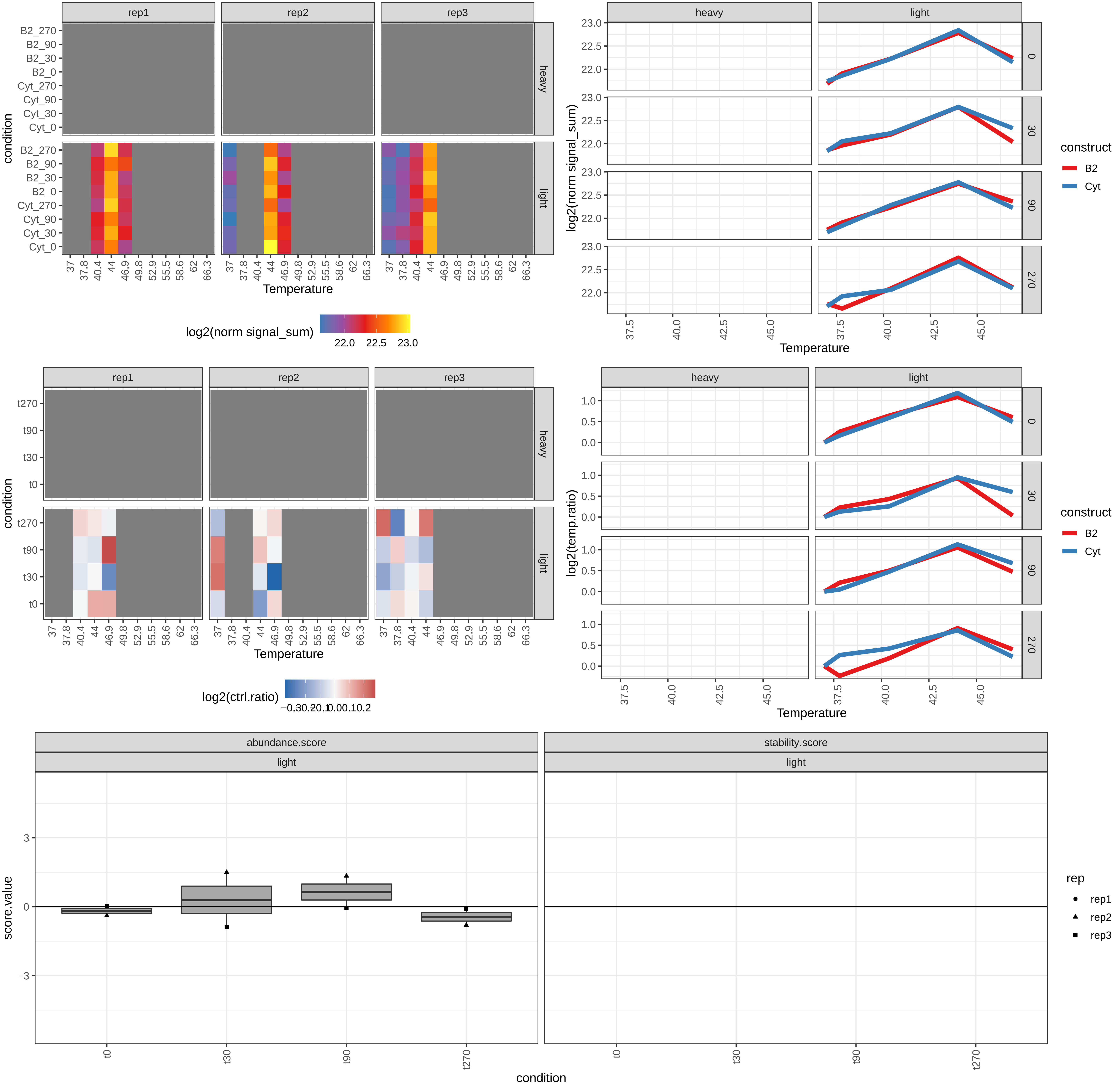

P39731 − Kinetochore−associated protein MTW1 OS=Saccharomyces cerevisiae (strain ATCC 204508 / S288c) GN=MTW1 PE=1 SV=2

MUK1 − average.top3: 6.35629727272727 − max.qupm: 4

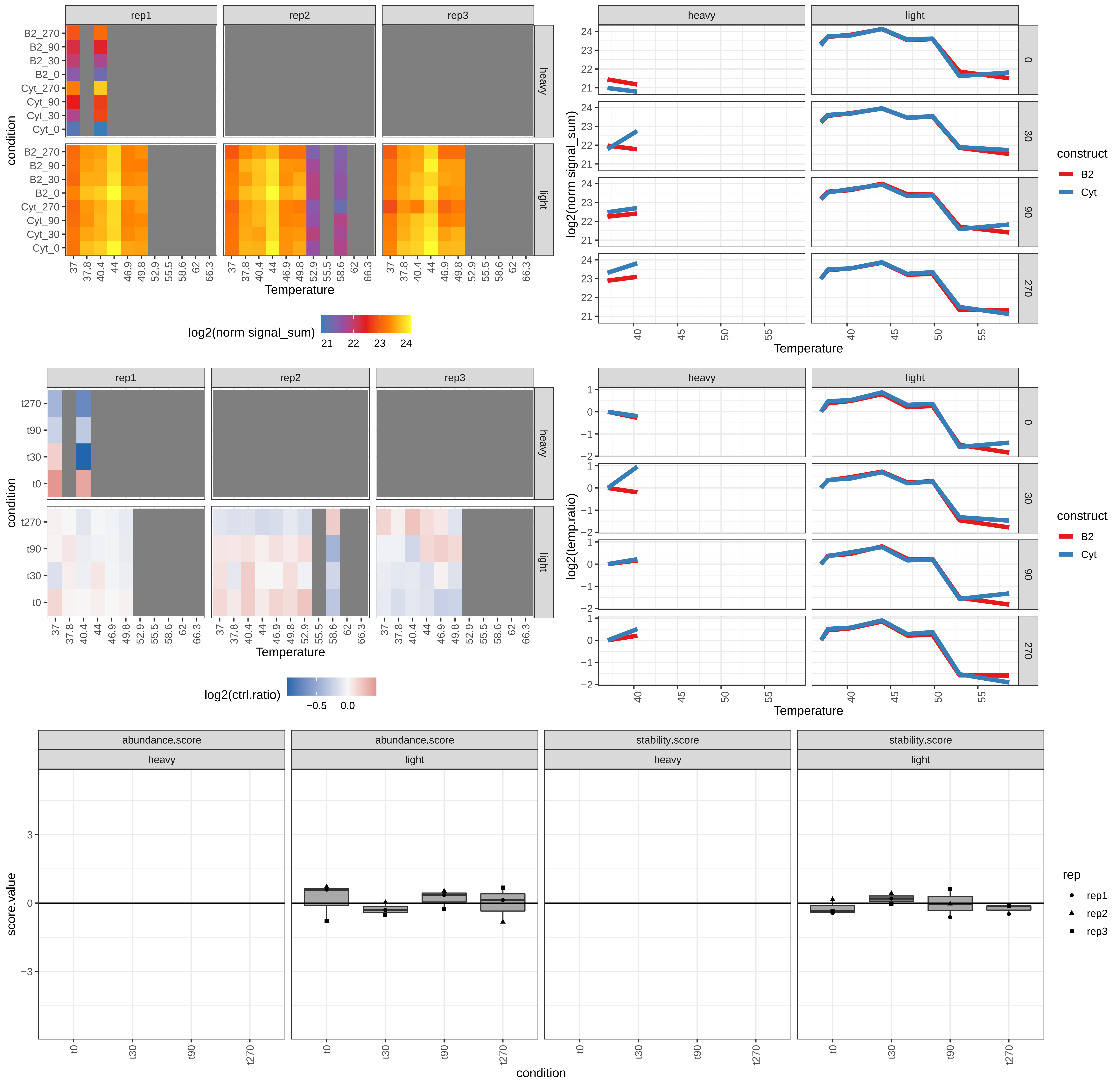

Q02866 − Protein MUK1 OS=Saccharomyces cerevisiae (strain ATCC 204508 / S288c) GN=MUK1 PE=1 SV=1

MVD1 − average.top3: 7.040671 − max.qupm: 9

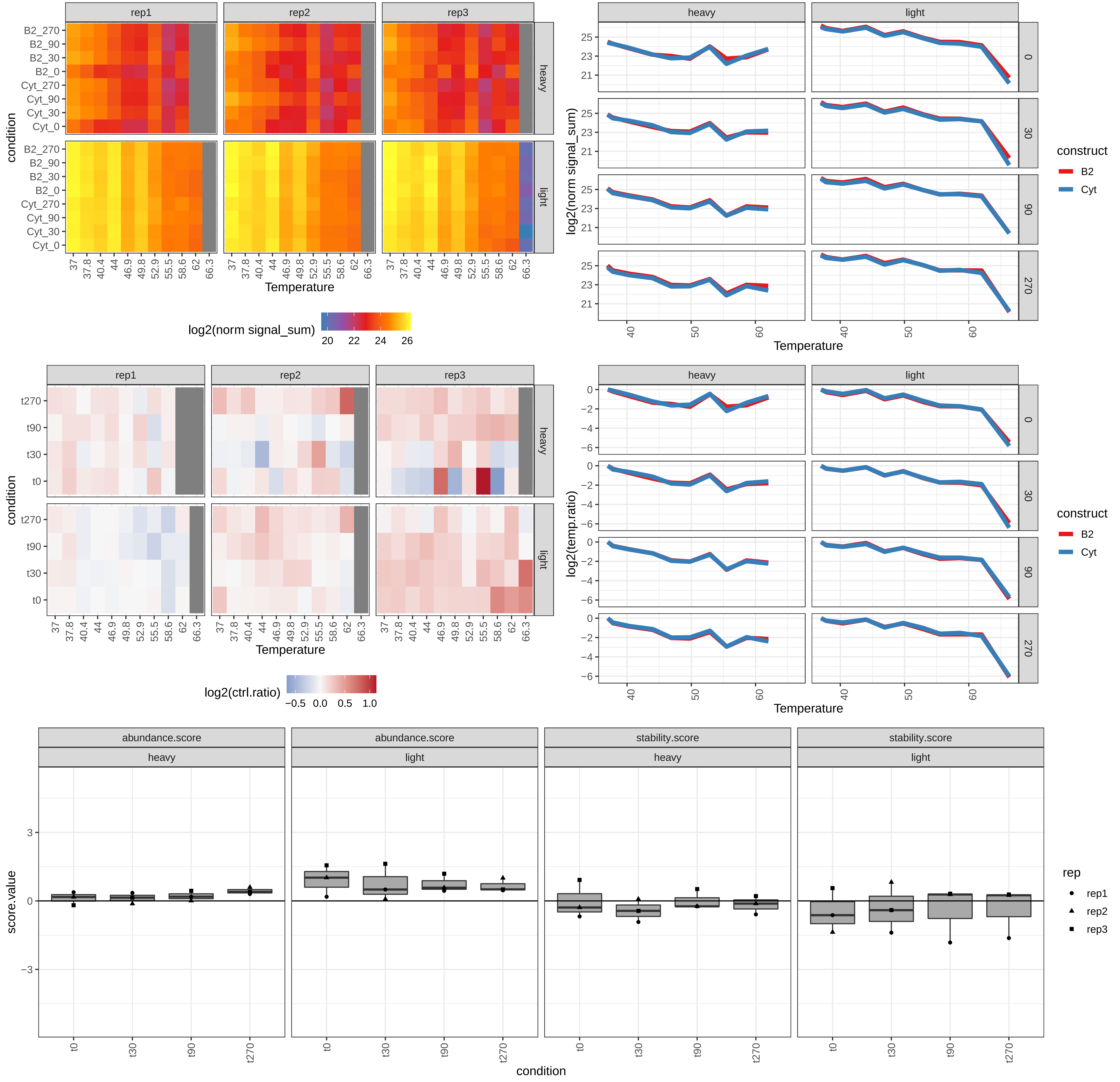

P32377 − Diphosphomevalonate decarboxylase OS=Saccharomyces cerevisiae (strain ATCC 204508 / S288c) GN=MVD1 PE=1 SV=2

MVP1 − average.top3: 6.72617962962963 − max.qupm: 9

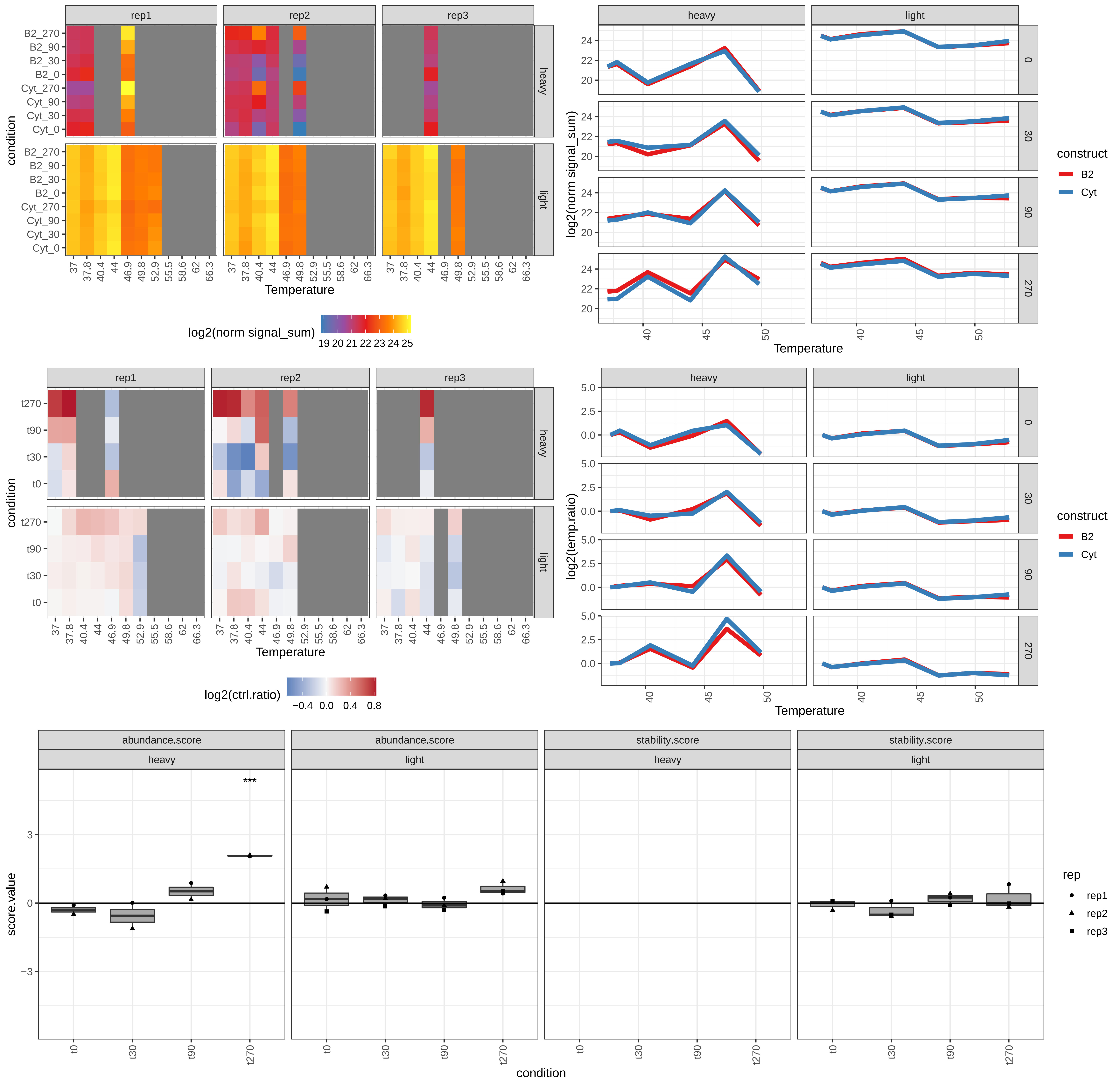

P40959 − Sorting nexin MVP1 OS=Saccharomyces cerevisiae (strain ATCC 204508 / S288c) GN=MVP1 PE=1 SV=2

MXR1 − average.top3: 6.93296466666667 − max.qupm: 9

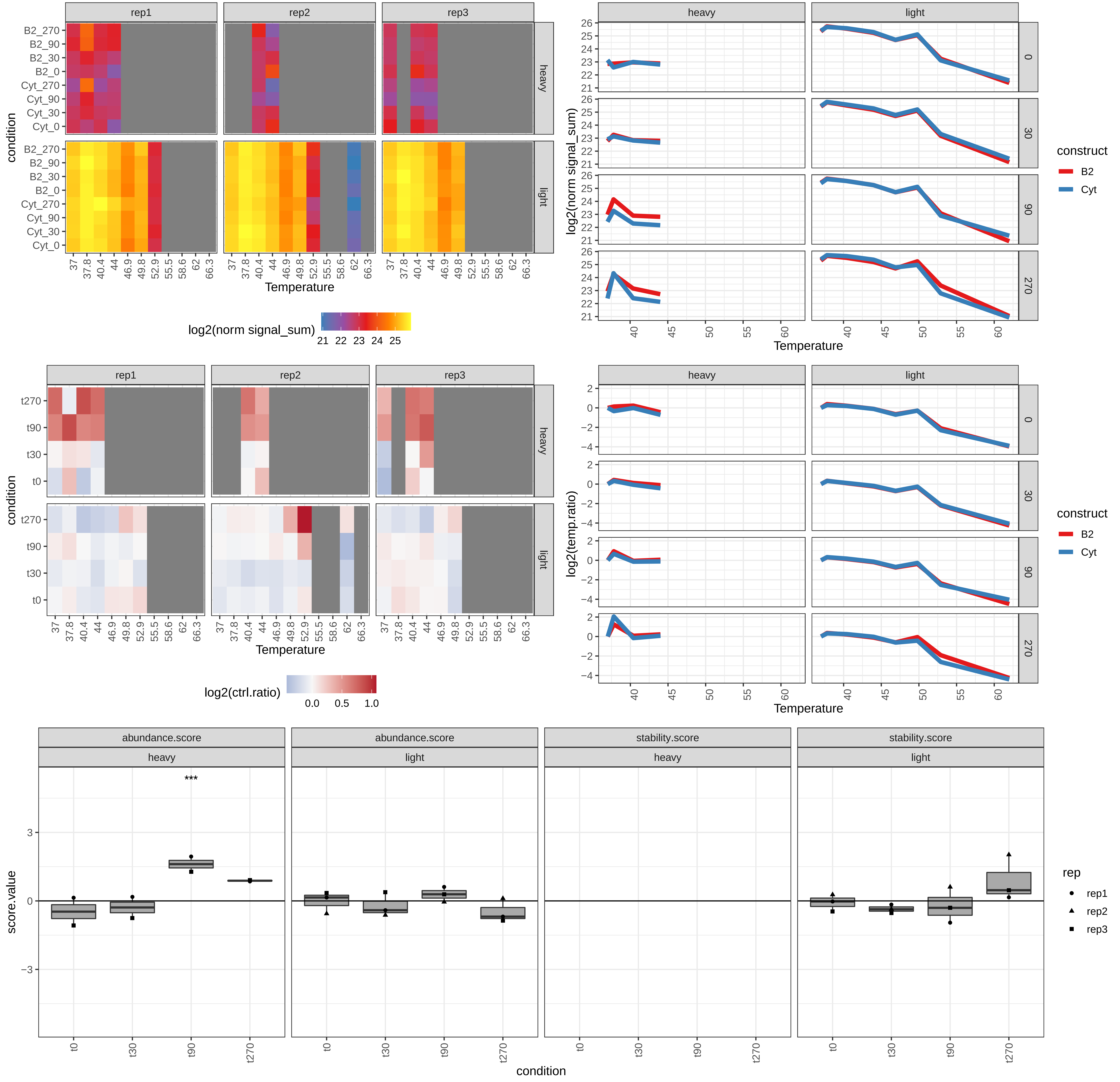

P40029 − Peptide methionine sulfoxide reductase OS=Saccharomyces cerevisiae (strain ATCC 204508 / S288c) GN=MXR1 PE=1 SV=1

MYO1 − average.top3: 6.33121375 − max.qupm: 7

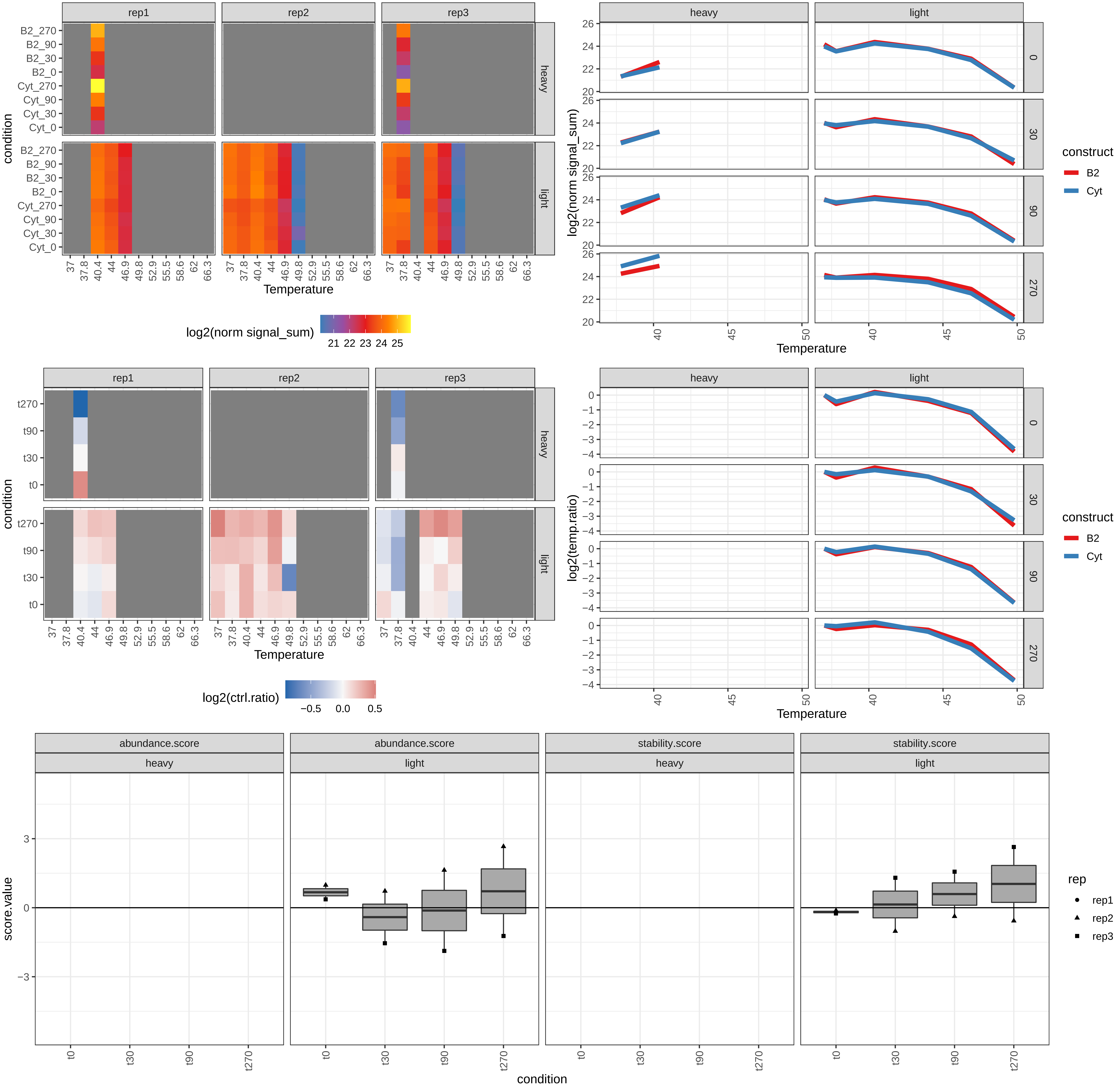

P08964 − Myosin−1 OS=Saccharomyces cerevisiae (strain ATCC 204508 / S288c) GN=MYO1 PE=1 SV=3

MYO2 − average.top3: 6.8511221875 − max.qupm: 22

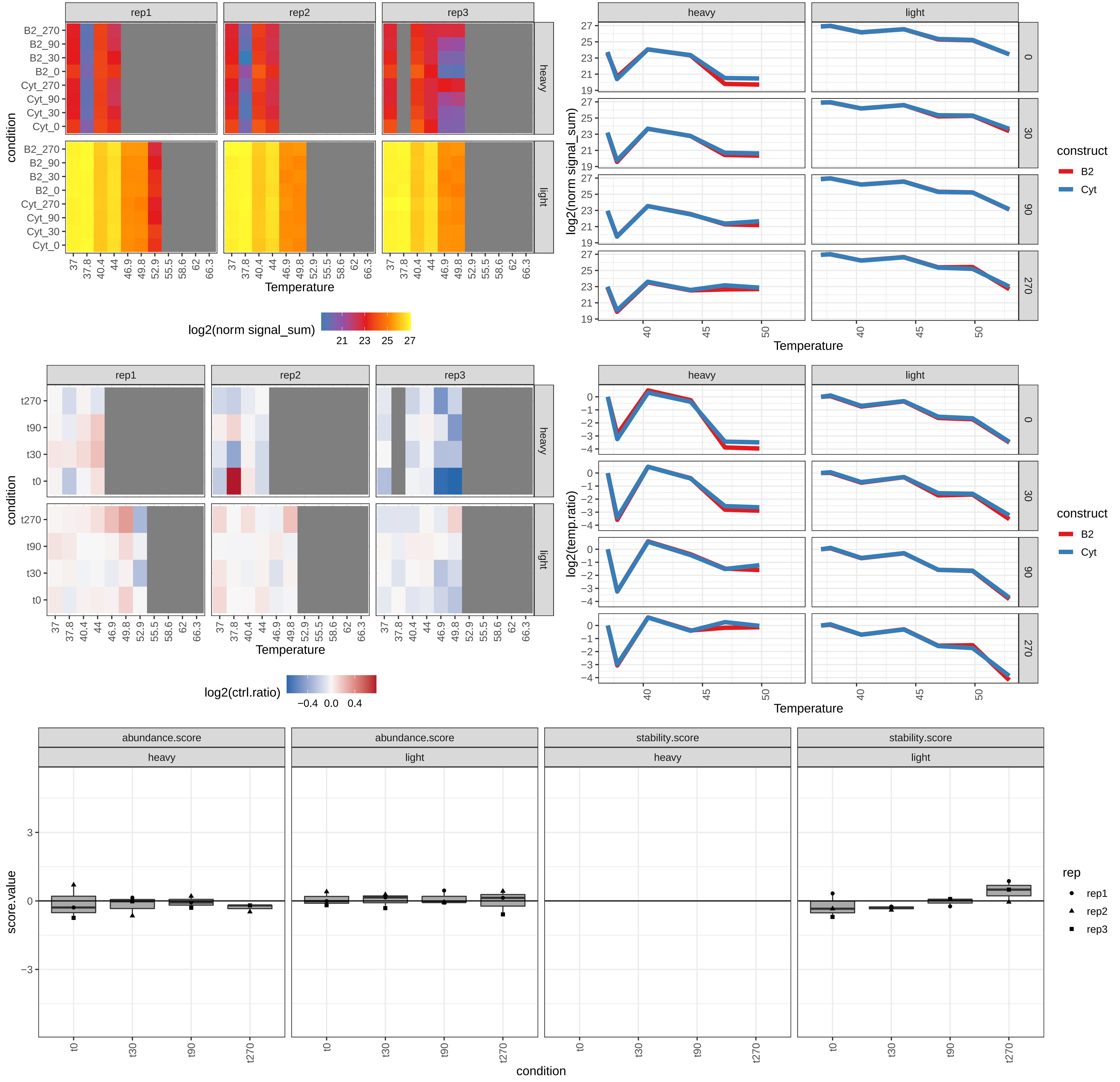

P19524 − Myosin−2 OS=Saccharomyces cerevisiae (strain ATCC 204508 / S288c) GN=MYO2 PE=1 SV=1

MYO3 − average.top3: 6.9780740625 − max.qupm: 15

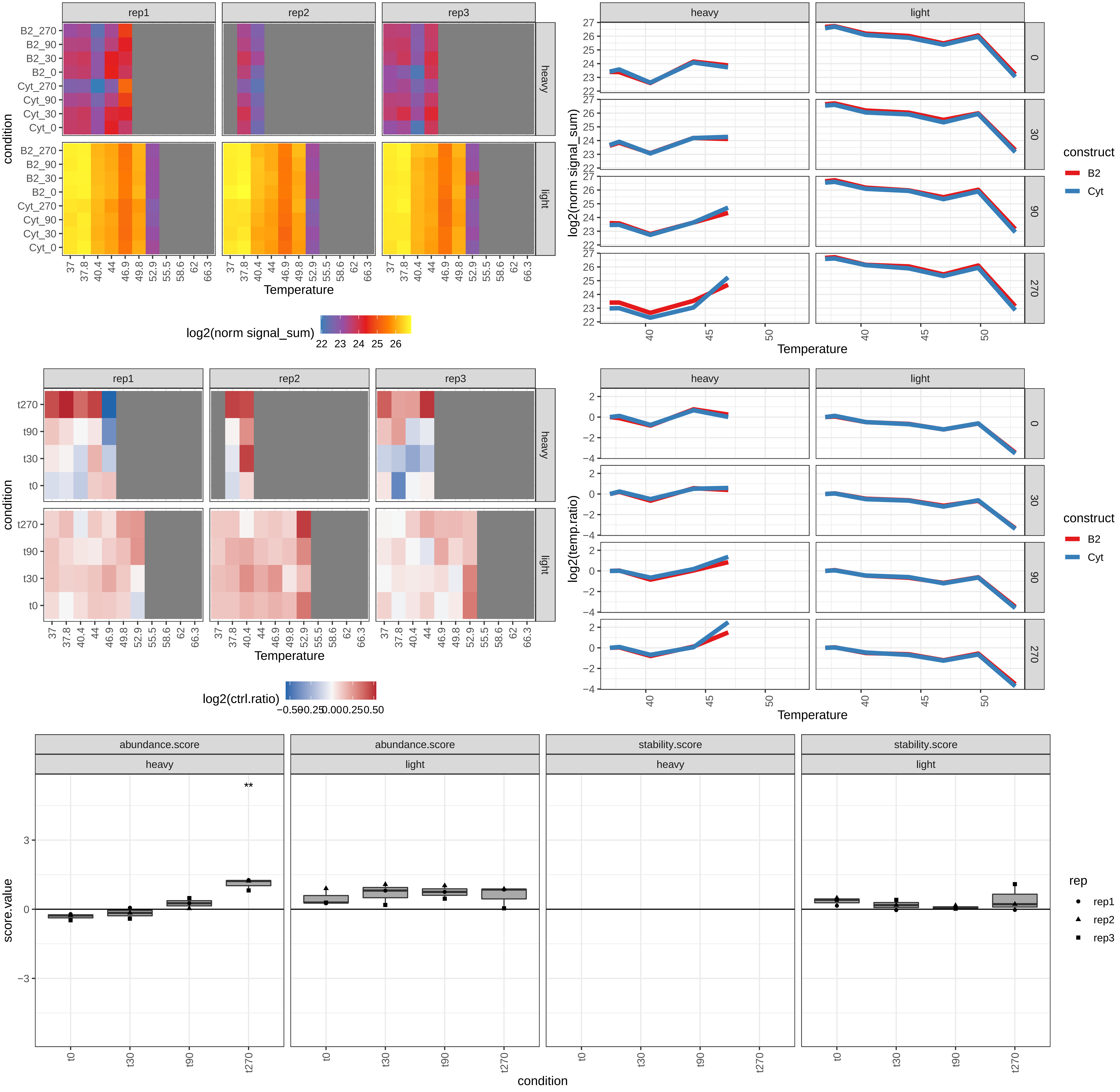

P36006 − Myosin−3 OS=Saccharomyces cerevisiae (strain ATCC 204508 / S288c) GN=MYO3 PE=1 SV=4

MYO4 − average.top3: 6.84651133333333 − max.qupm: 8

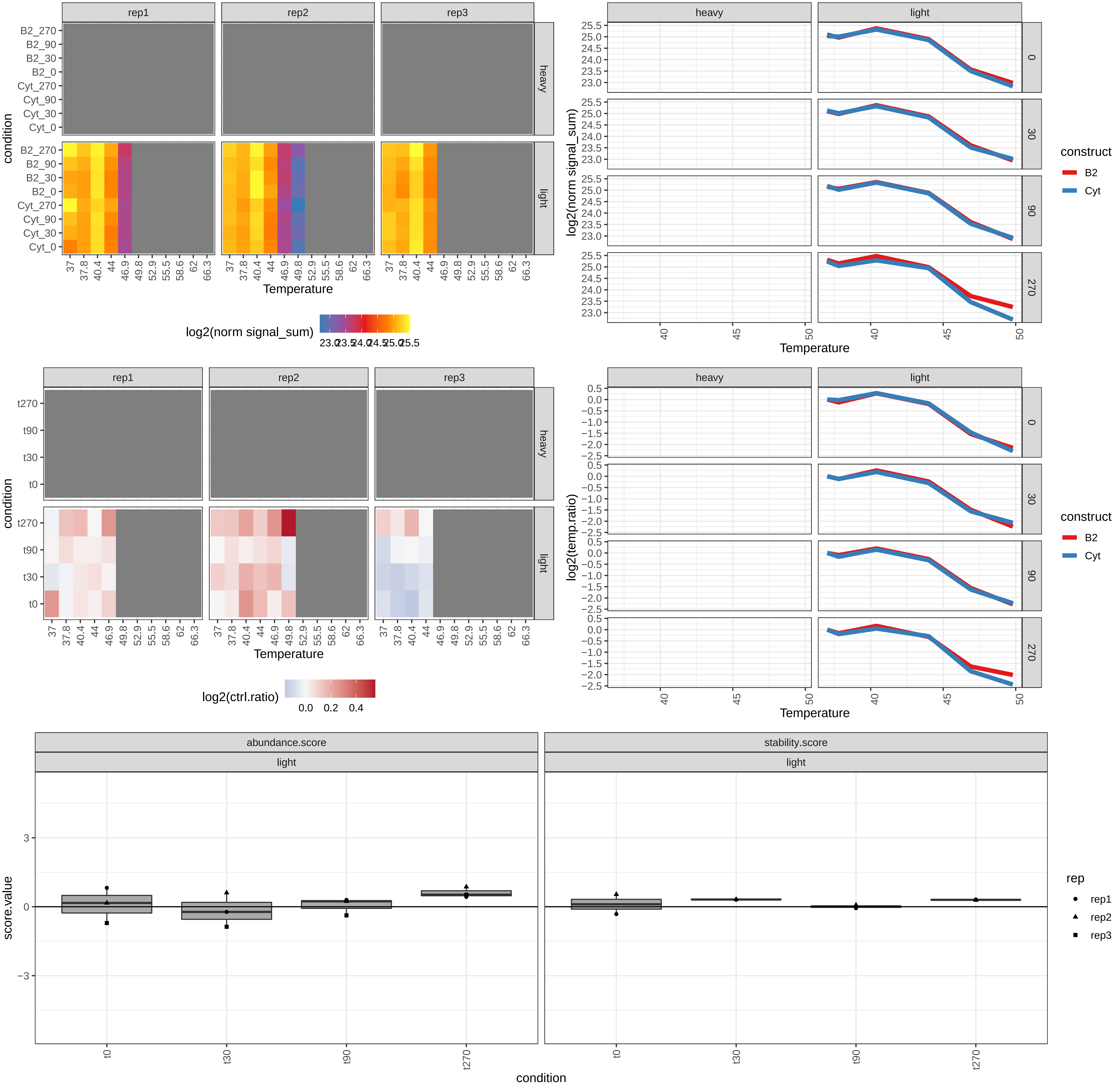

P32492 − Myosin−4 OS=Saccharomyces cerevisiae (strain ATCC 204508 / S288c) GN=MYO4 PE=1 SV=1

MYO5 − average.top3: 6.96177121212121 − max.qupm: 17

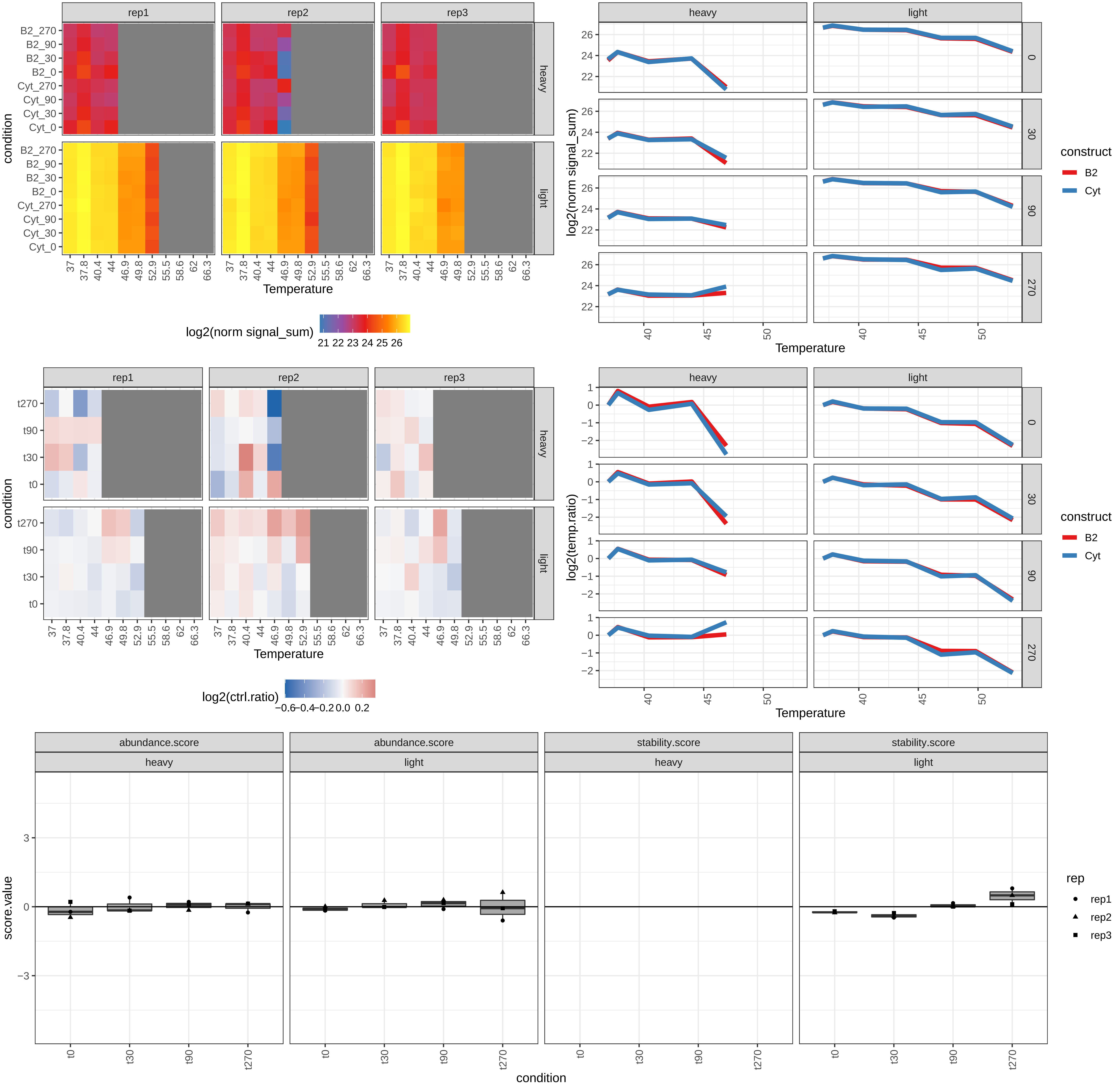

Q04439 − Myosin−5 OS=Saccharomyces cerevisiae (strain ATCC 204508 / S288c) GN=MYO5 PE=1 SV=1

NAB2 − average.top3: 6.98987918918919 − max.qupm: 8

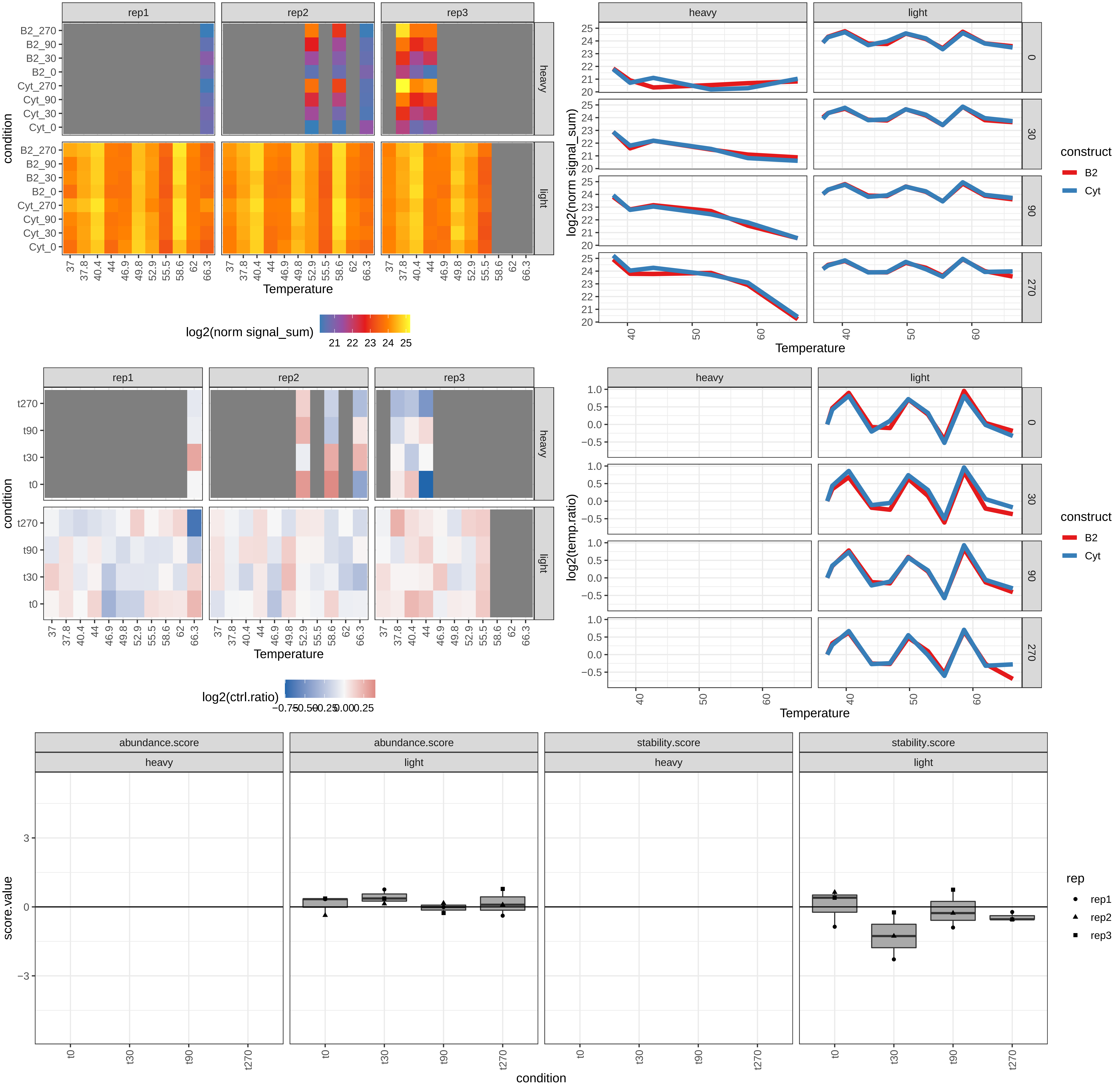

P32505 − Nuclear polyadenylated RNA−binding protein NAB2 OS=Saccharomyces cerevisiae (strain ATCC 204508 / S288c) GN=NAB2 PE=1 SV=1

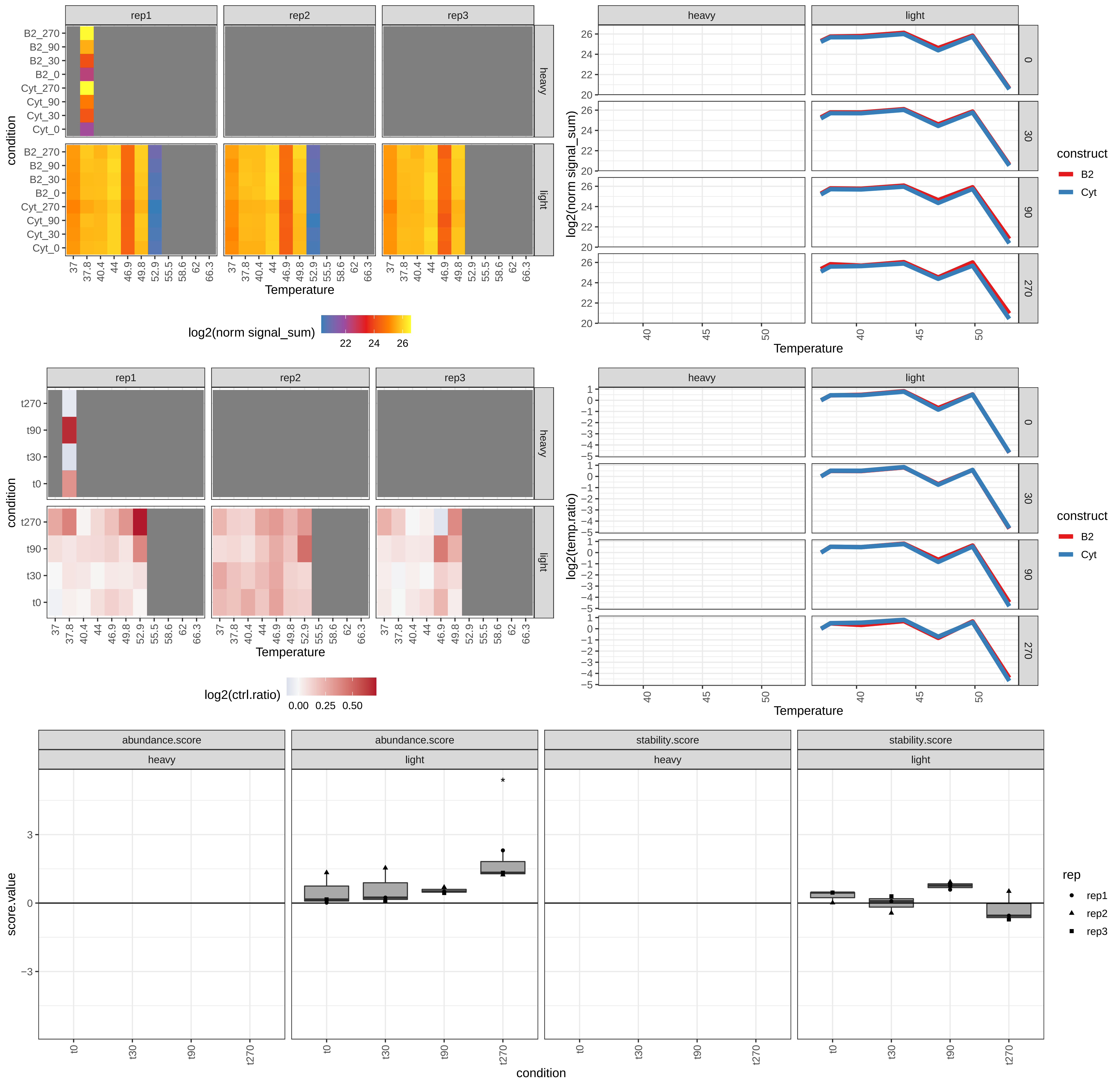

P38996 − Nuclear polyadenylated RNA−binding protein 3 OS=Saccharomyces cerevisiae (strain ATCC 204508 / S288c) GN=NAB3 PE=1 SV=1

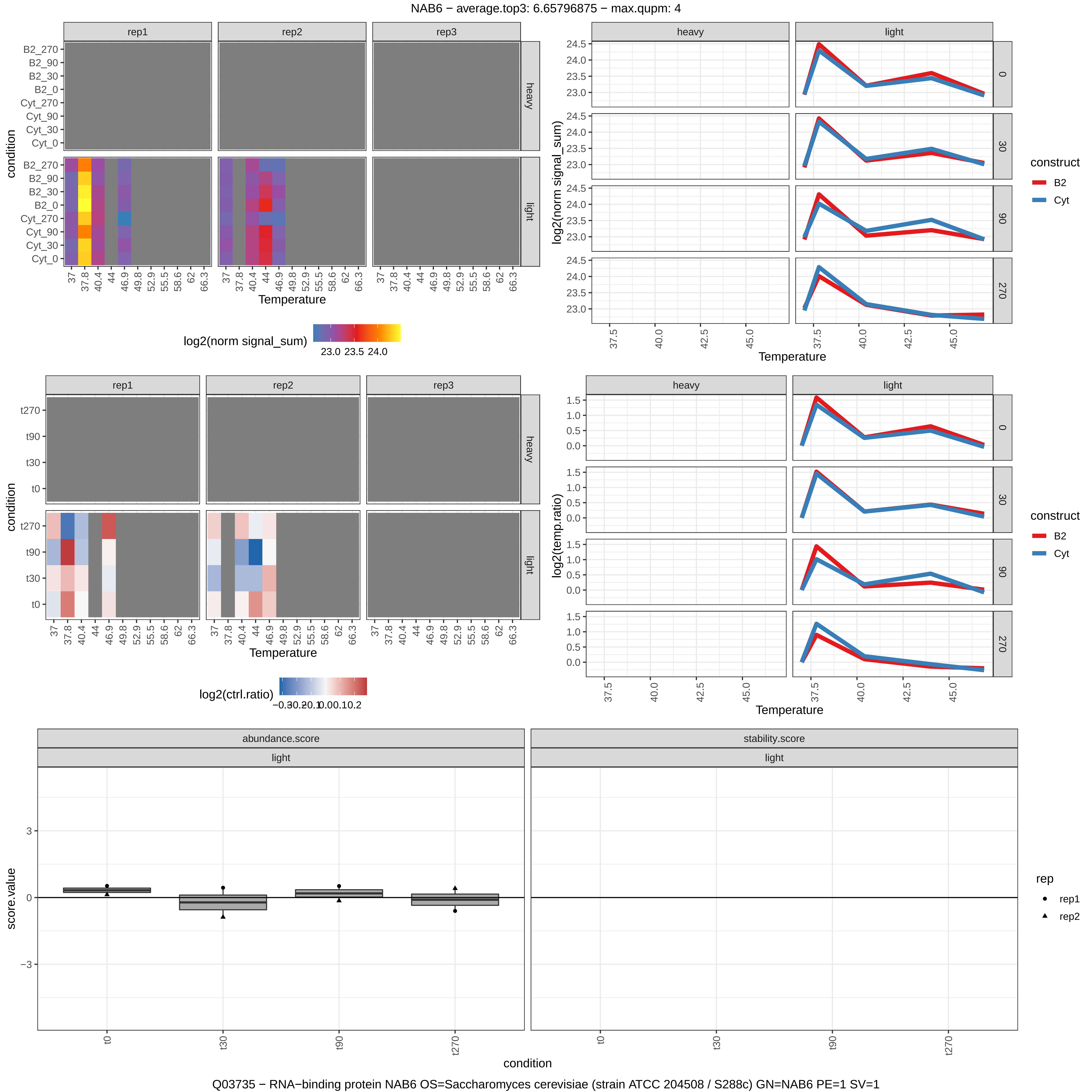

NAB6 − average.top3: 6.65796875 − max.qupm: 4
rep1
rep2
rep3
heavy
light
condition
B2_270
B2_90
B2_30
B2_0
Cyt_270
Cyt_90
Cyt_30
Cyt_0
37
37.8
40.4
44
46.9
49.8
52.9
55.5
58.6
62
66.3
Temperature
log2(norm signal_sum)
23.0 23.5 24.0
log2(norm signal_sum)
0
30
90
270
construct
B2
Cyt
t270
t90
t30
t0
log2(ctrl.ratio)
−0.3 −0.2 −0.1 0.0 0.1 0.2
log2(temp.ratio)
abundance.score
stability.score
score.value
condition
rep
rep1
rep2
Q03735 − RNA−binding protein NAB6 OS=Saccharomyces cerevisiae (strain ATCC 204508 / S288c) GN=NAB6 PE=1 SV=1

NAM2 − average.top3: 6.476856875 − max.qupm: 8

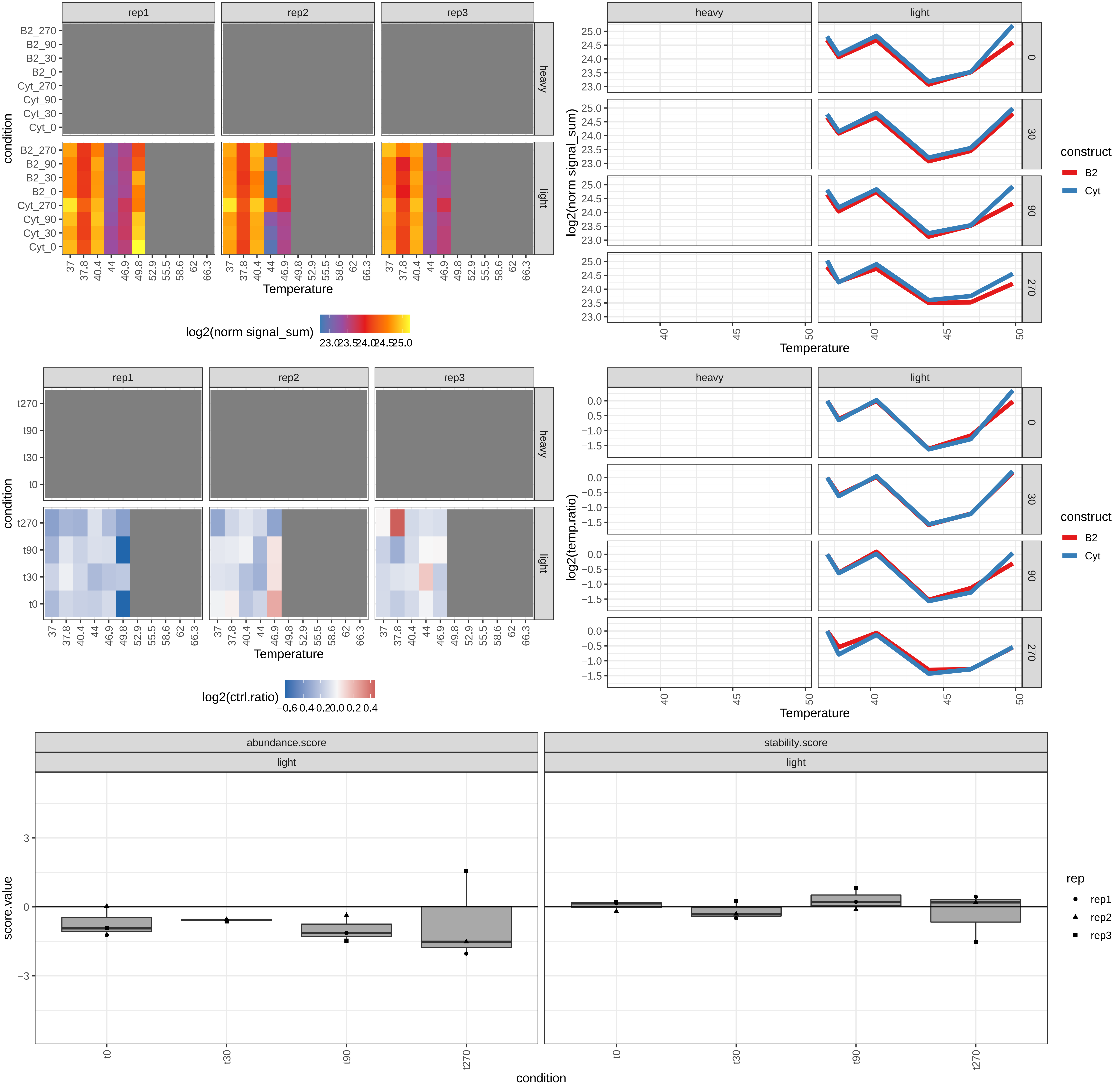

P11325 − Leucine−−tRNA ligase, mitochondrial OS=Saccharomyces cerevisiae (strain ATCC 204508 / S288c) GN=NAM2 PE=1 SV=1

NAM7 − average.top3: 6.91864142857143 − max.qupm: 13

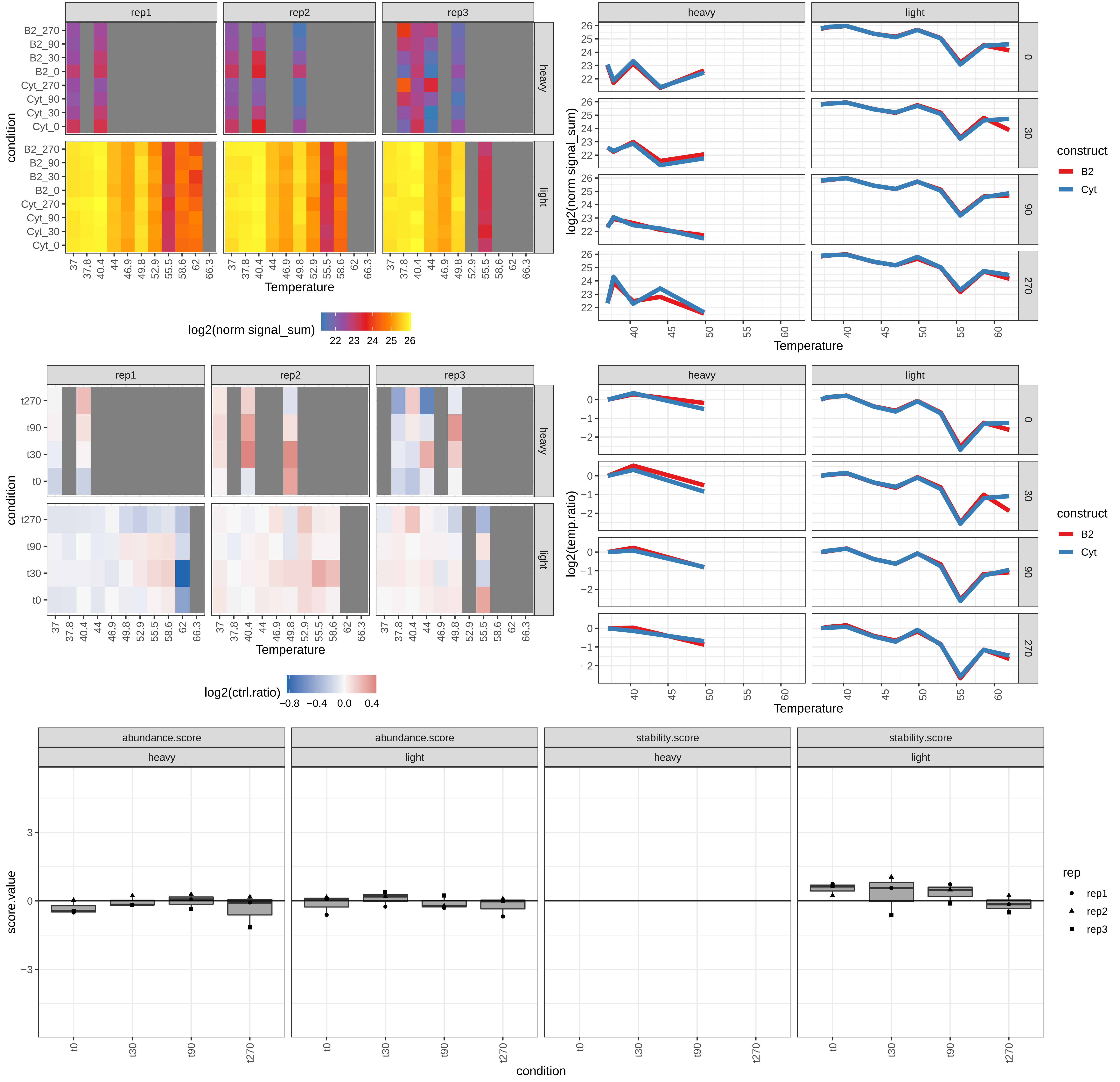

P30771 − ATP−dependent helicase NAM7 OS=Saccharomyces cerevisiae (strain ATCC 204508 / S288c) GN=NAM7 PE=1 SV=1

NAM9 − average.top3: 6.981737 − max.qupm: 15

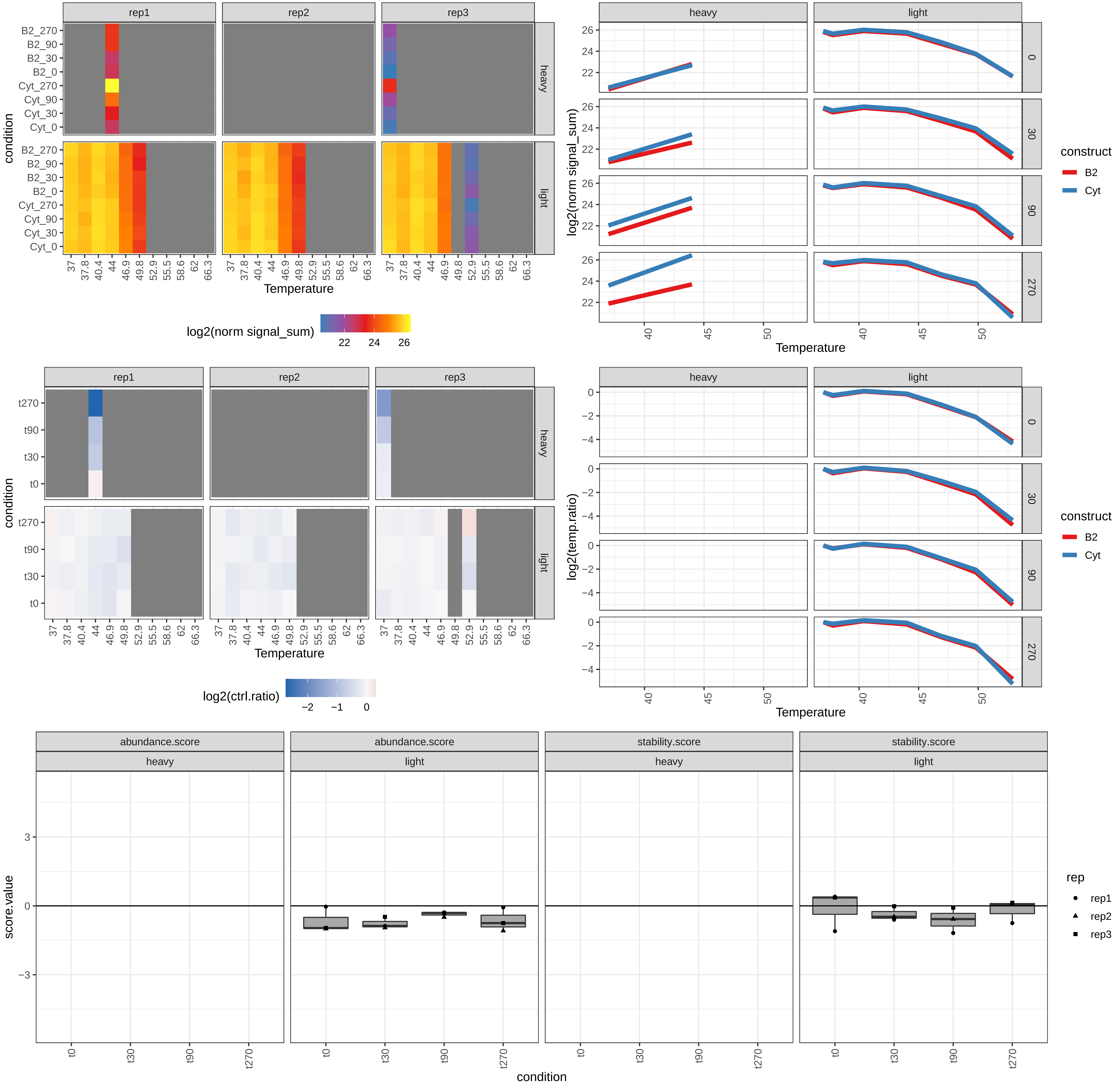

P27929 − 37S ribosomal protein NAM9, mitochondrial OS=Saccharomyces cerevisiae (strain ATCC 204508 / S288c) GN=NAM9 PE=1 SV=2

NAN1 − average.top3: 6.77373315789474 − max.qupm: 10

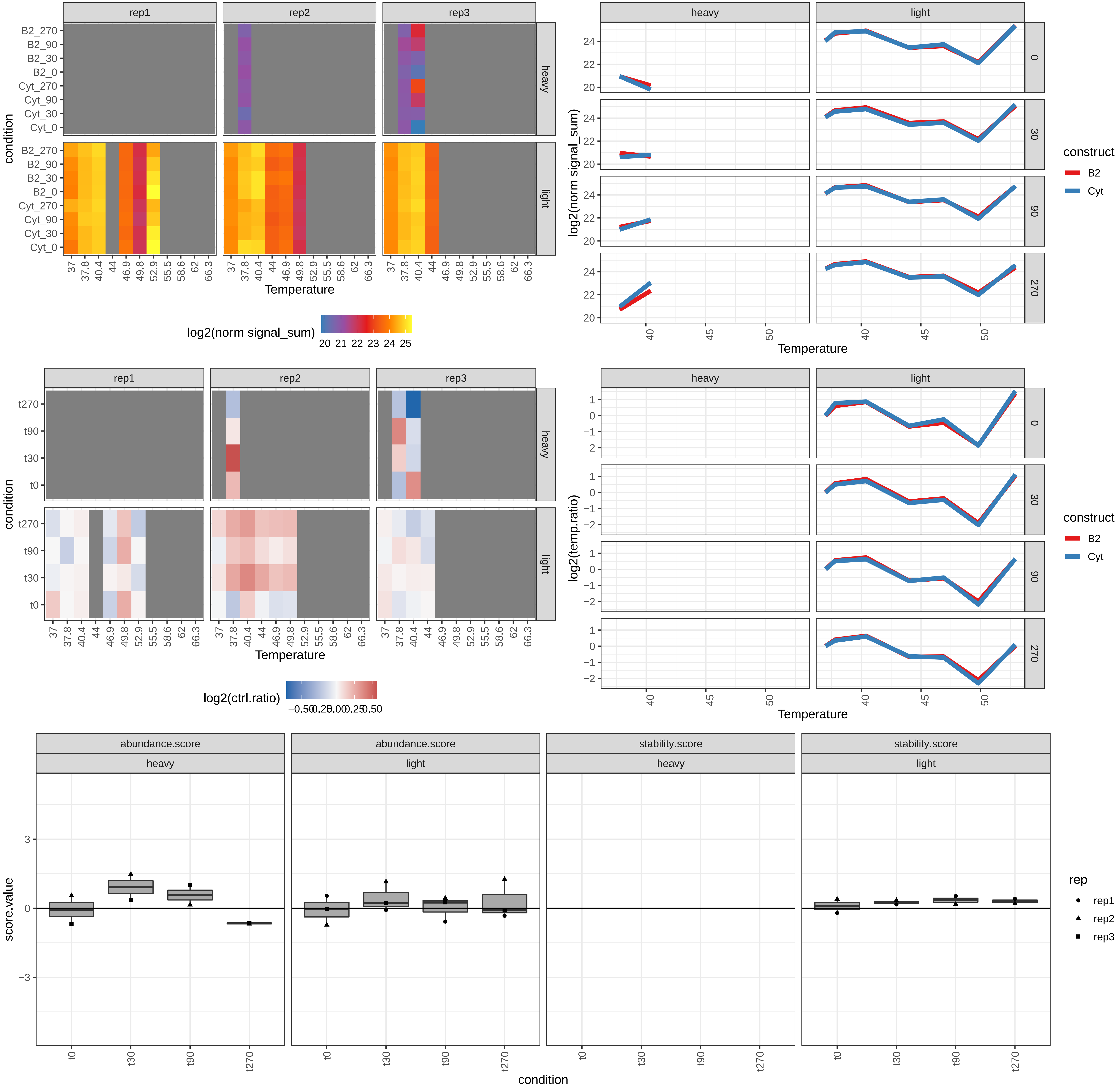

Q02931 − NET1−associated nuclear protein 1 OS=Saccharomyces cerevisiae (strain ATCC 204508 / S288c) GN=NAN1 PE=1 SV=1

NAP1 − average.top3: 6.74924228571429 − max.qupm: 9

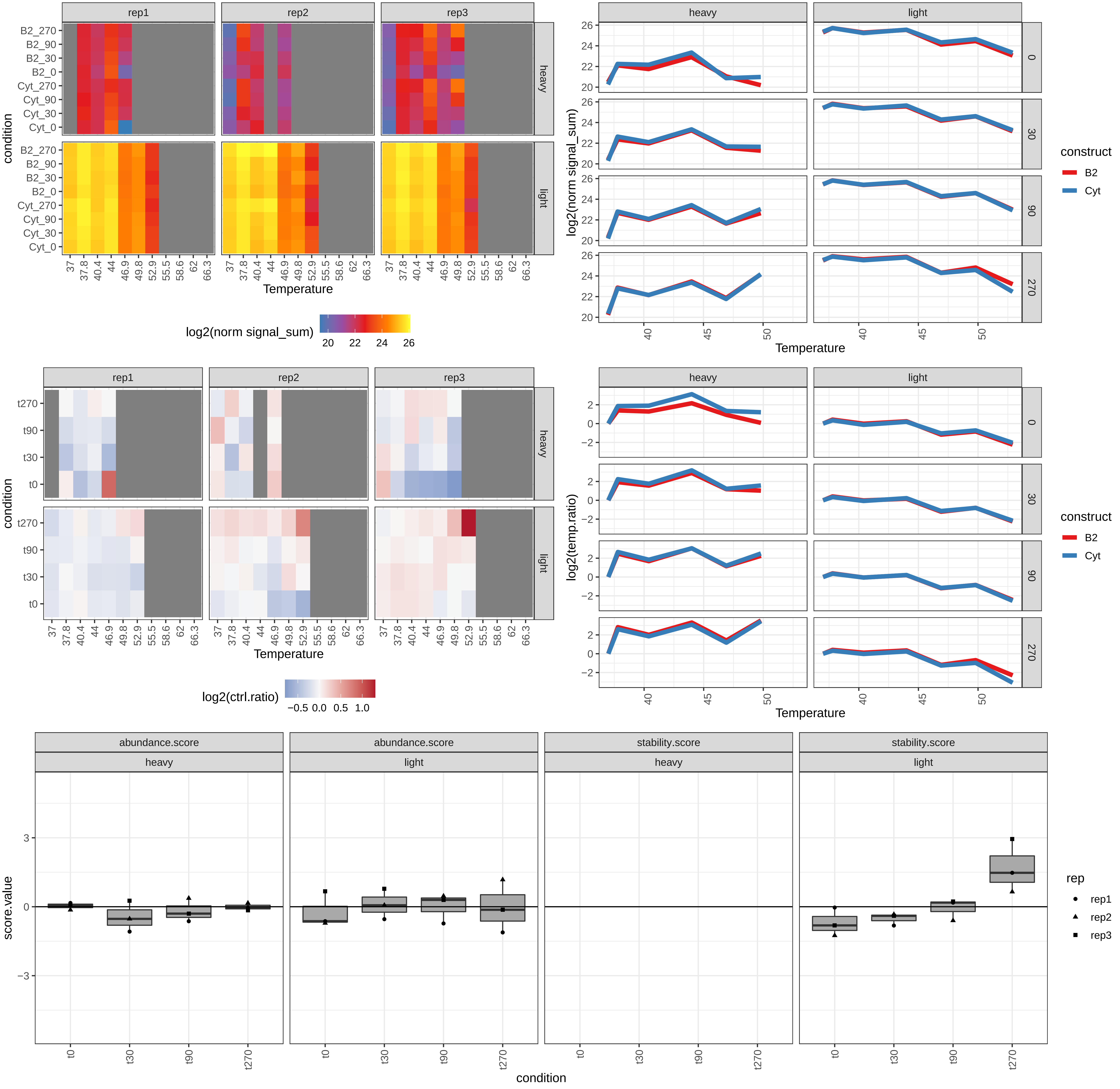

P25293 − Nucleosome assembly protein OS=Saccharomyces cerevisiae (strain ATCC 204508 / S288c) GN=NAP1 PE=1 SV=2

NAR1 − average.top3: 6.83472942857143 − max.qupm: 9

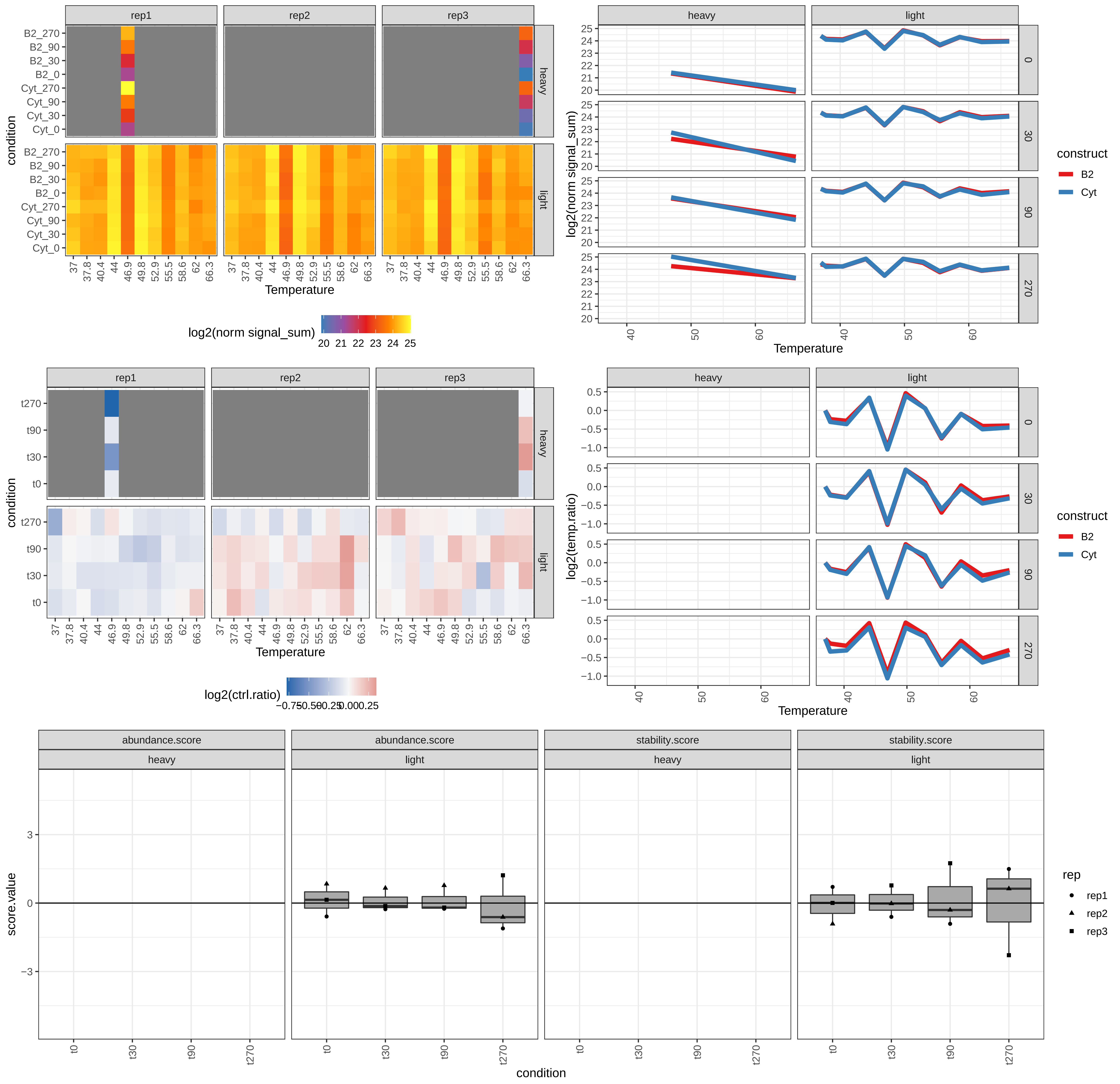

P23503 − Cytosolic Fe−S cluster assembly factor NAR1 OS=Saccharomyces cerevisiae (strain ATCC 204508 / S288c) GN=NAR1 PE=1 SV=2

NAS6 − average.top3: 7.27503368421053 − max.qupm: 8

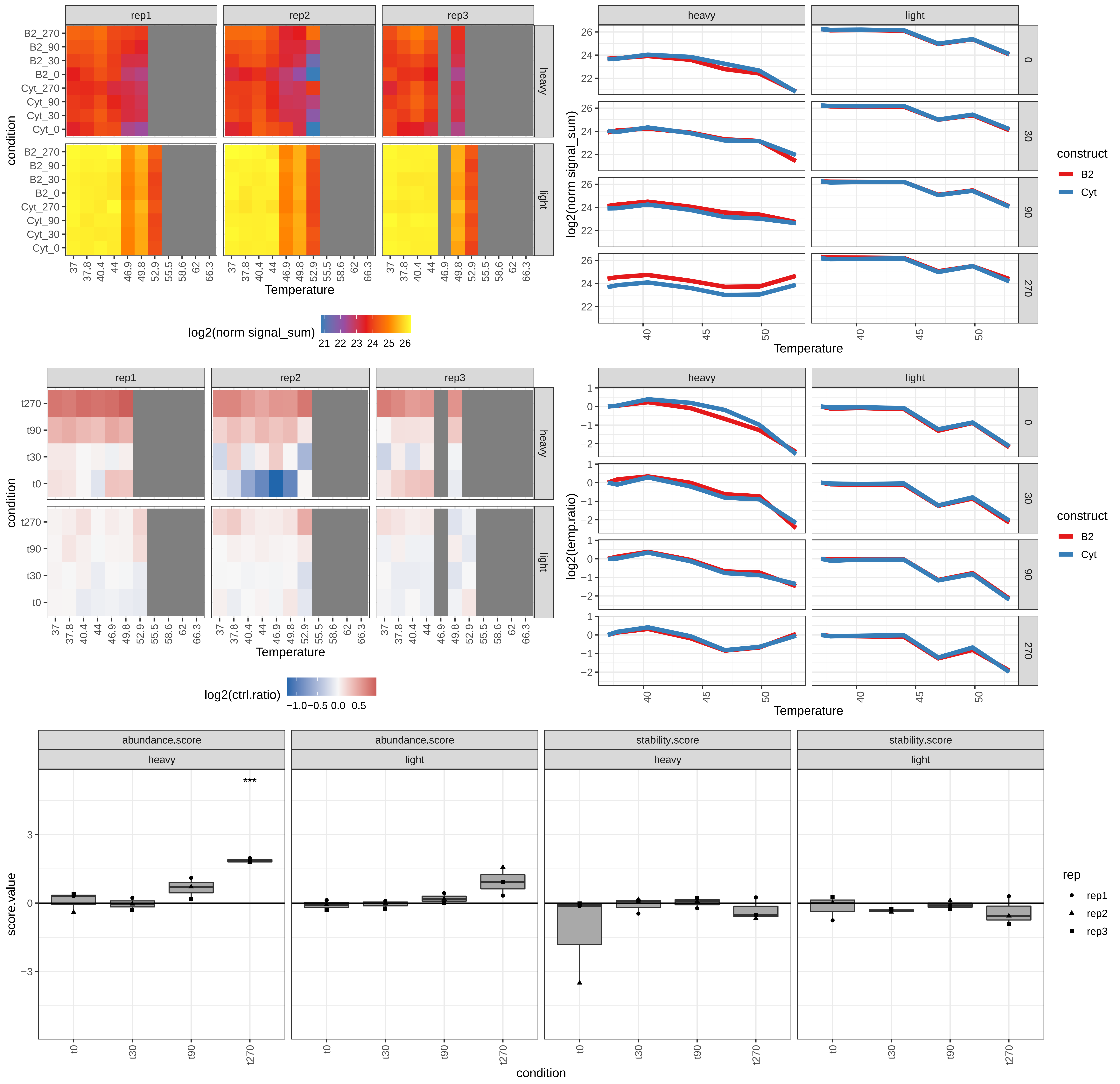

P50086 − Probable 26S proteasome regulatory subunit p28 OS=Saccharomyces cerevisiae (strain ATCC 204508 / S288c) GN=NAS6 PE=1 SV=1

NAT1 − average.top3: 7.16146177777778 − max.qupm: 33

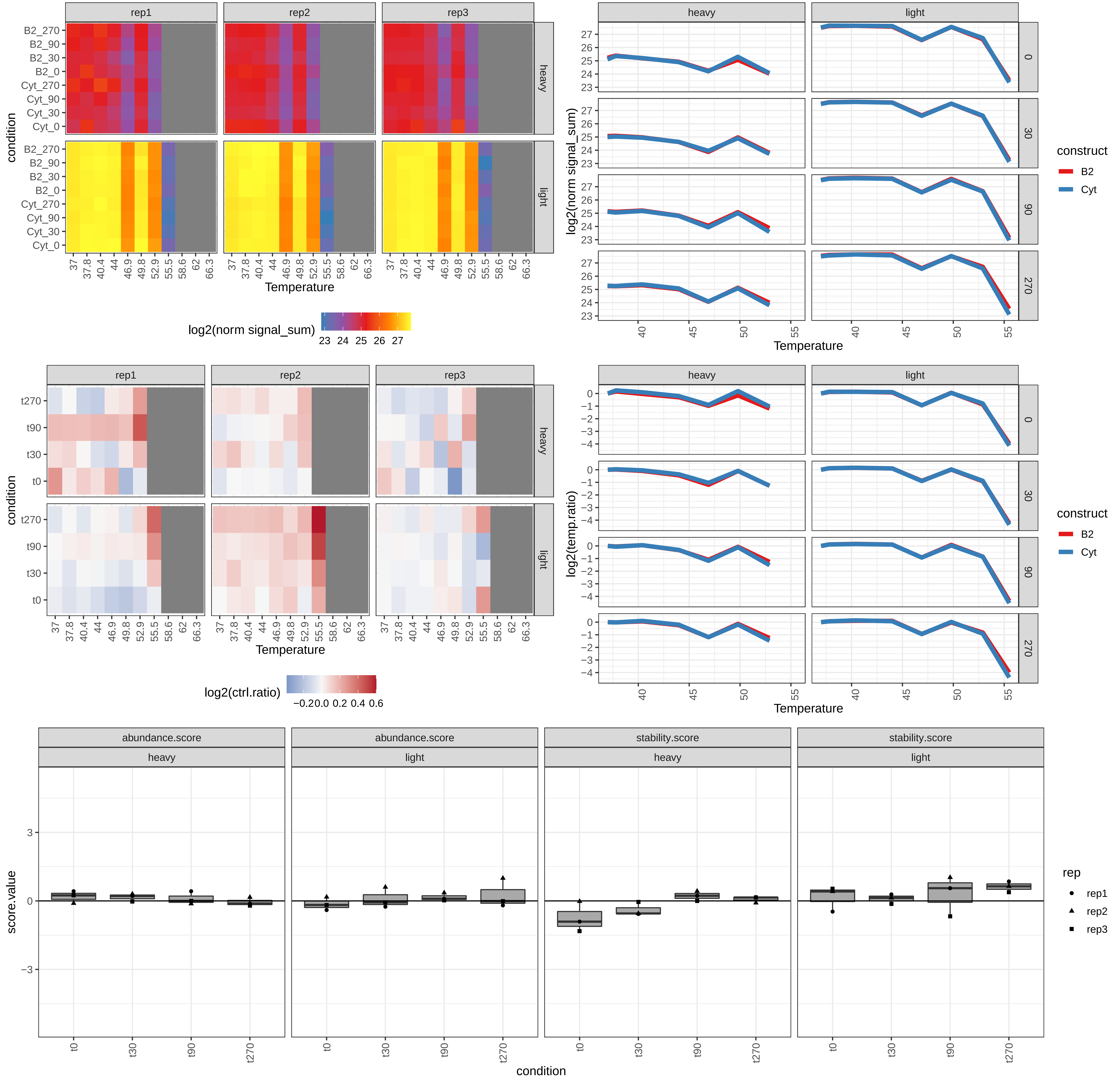

P12945 − N−terminal acetyltransferase A complex subunit NAT1 OS=Saccharomyces cerevisiae (strain ATCC 204508 / S288c) GN=NAT1 PE=1 SV=2

NAT10 − average.top3: 6.82462238095238 − max.qupm: 11

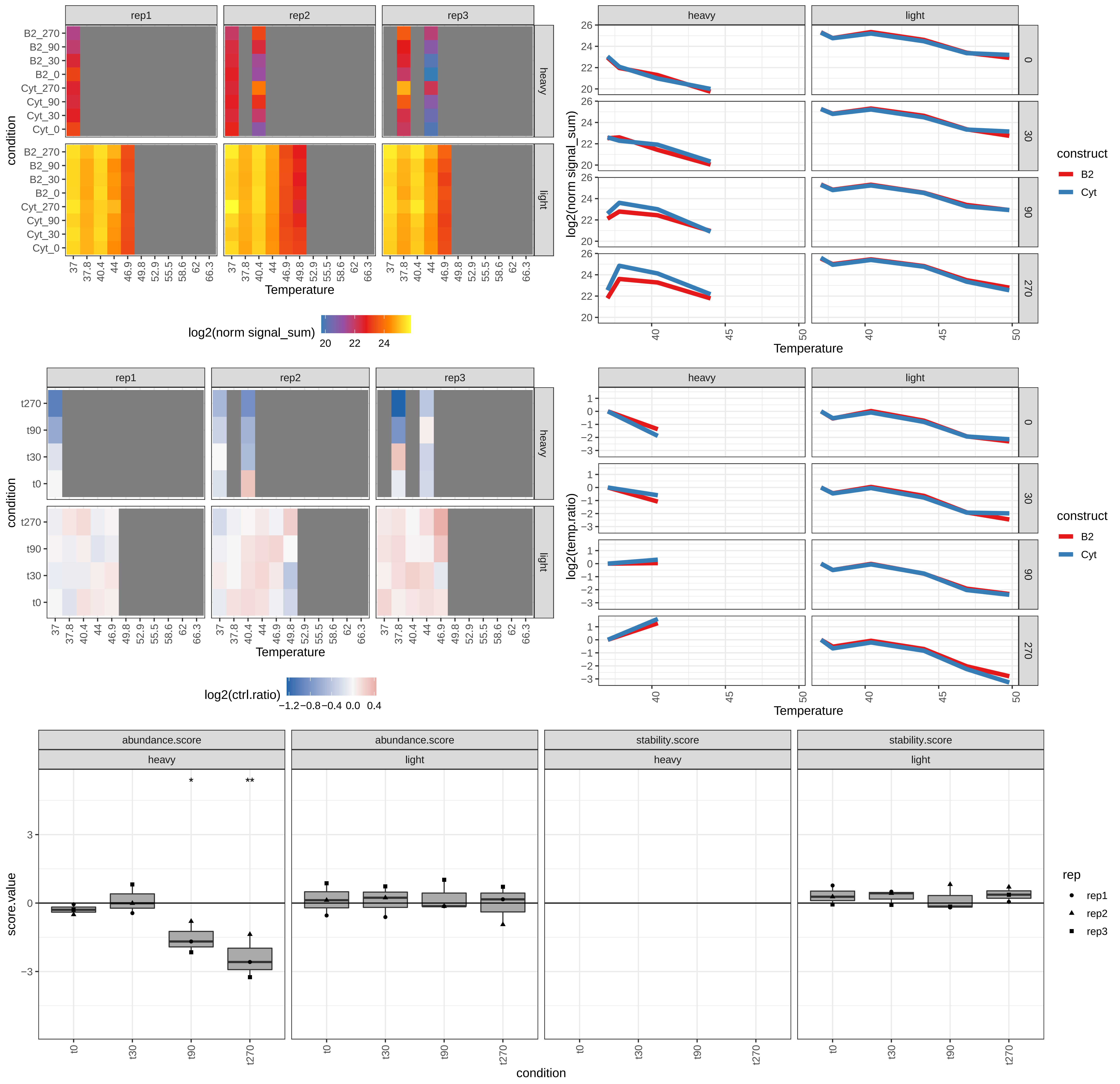

P53914 − RNA cytidine acetyltransferase OS=Saccharomyces cerevisiae (strain ATCC 204508 / S288c) GN=NAT10 PE=1 SV=1

NAT2 − average.top3: 6.83083333333333 − max.qupm: 6

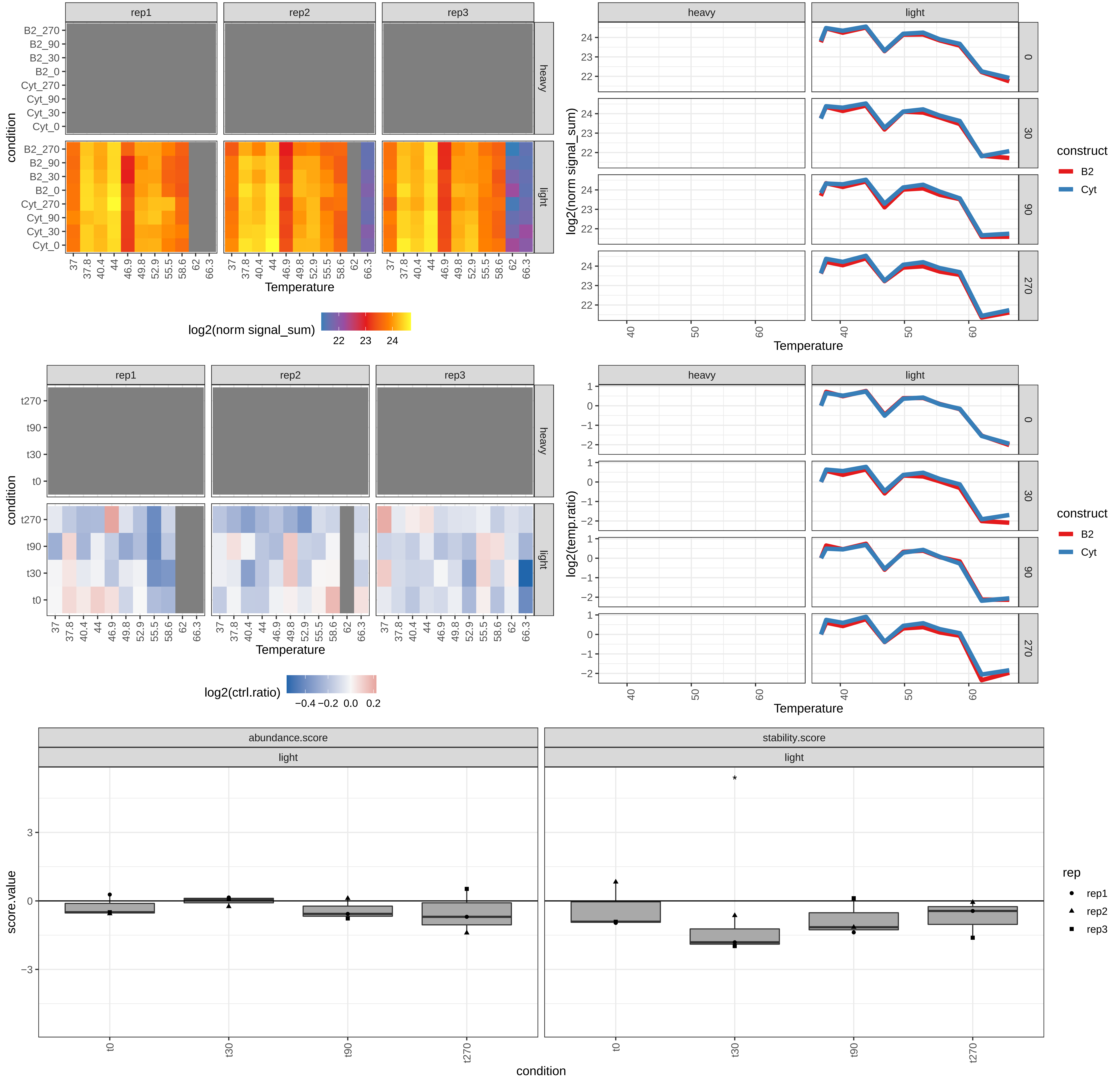

P37293 − N−terminal acetyltransferase 2 OS=Saccharomyces cerevisiae (strain ATCC 204508 / S288c) GN=NAT2 PE=1 SV=2

NAT3 − average.top3: 6.25543857142857 − max.qupm: 4

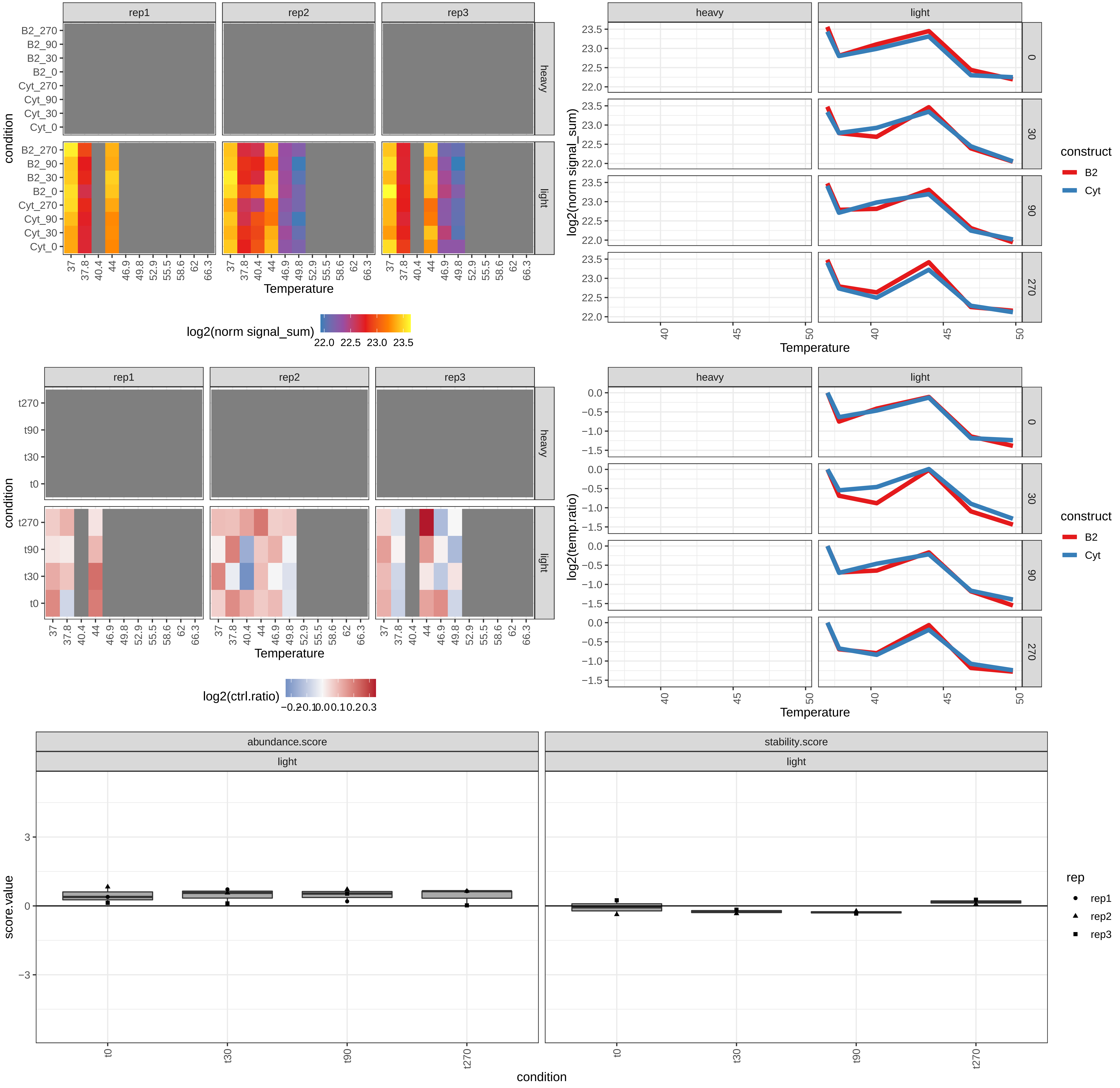

Q06504 − N−terminal acetyltransferase B complex catalytic subunit NAT3 OS=Saccharomyces cerevisiae (strain ATCC 204508 / S288c) GN=NAT3 PE=1 SV=2

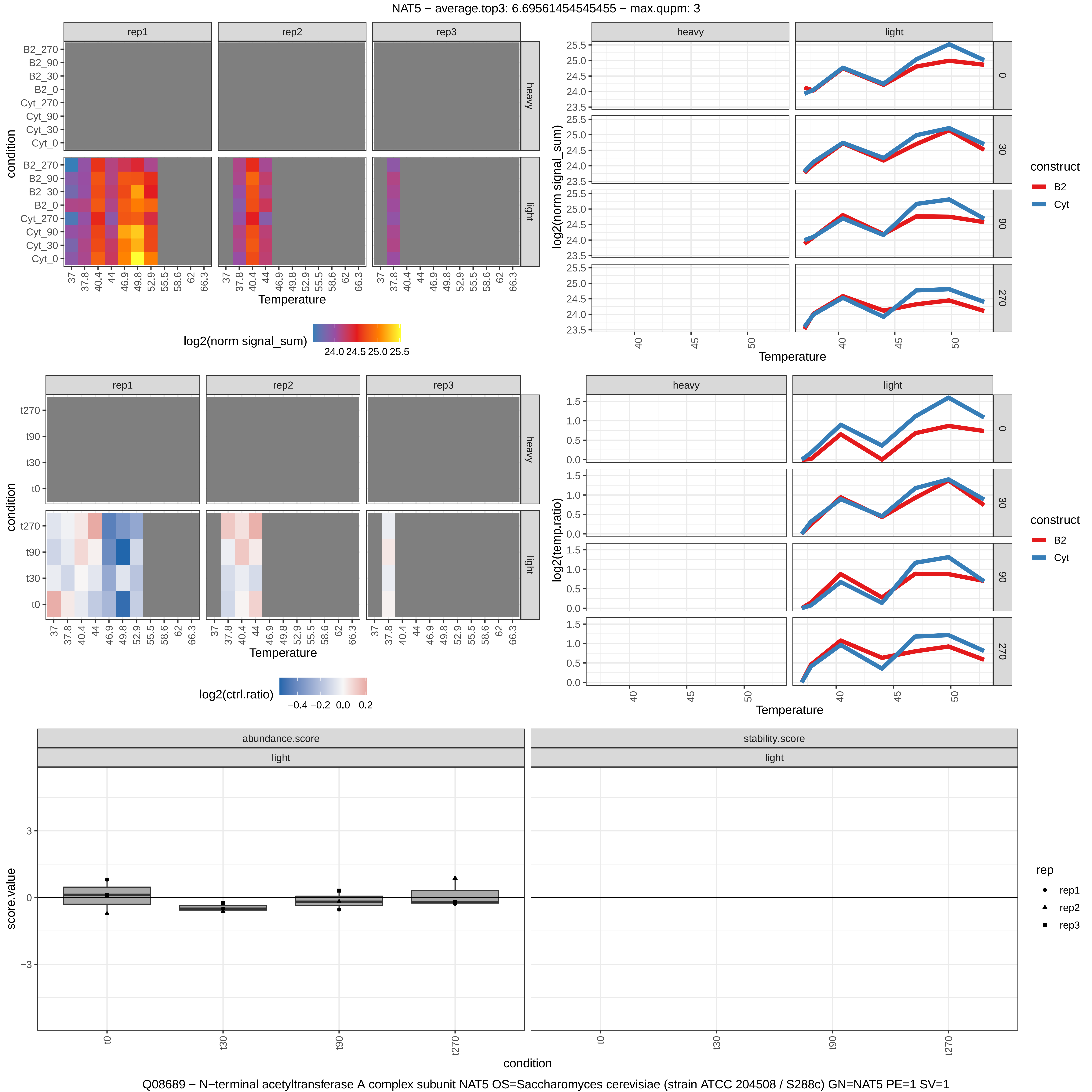
NAT5 − average.top3: 6.69561454545455 − max.qupm: 3
rep1
rep2
rep3
heavy
light
condition
Temperature
log2(norm signal_sum)
construct
B2
Cyt
log2(ctrl.ratio)
log2(temp.ratio)
abundance.score
stability.score
score.value
rep
rep1
rep2
rep3
Q08689 − N−terminal acetyltransferase A complex subunit NAT5 OS=Saccharomyces cerevisiae (strain ATCC 204508 / S288c) GN=NAT5 PE=1 SV=1

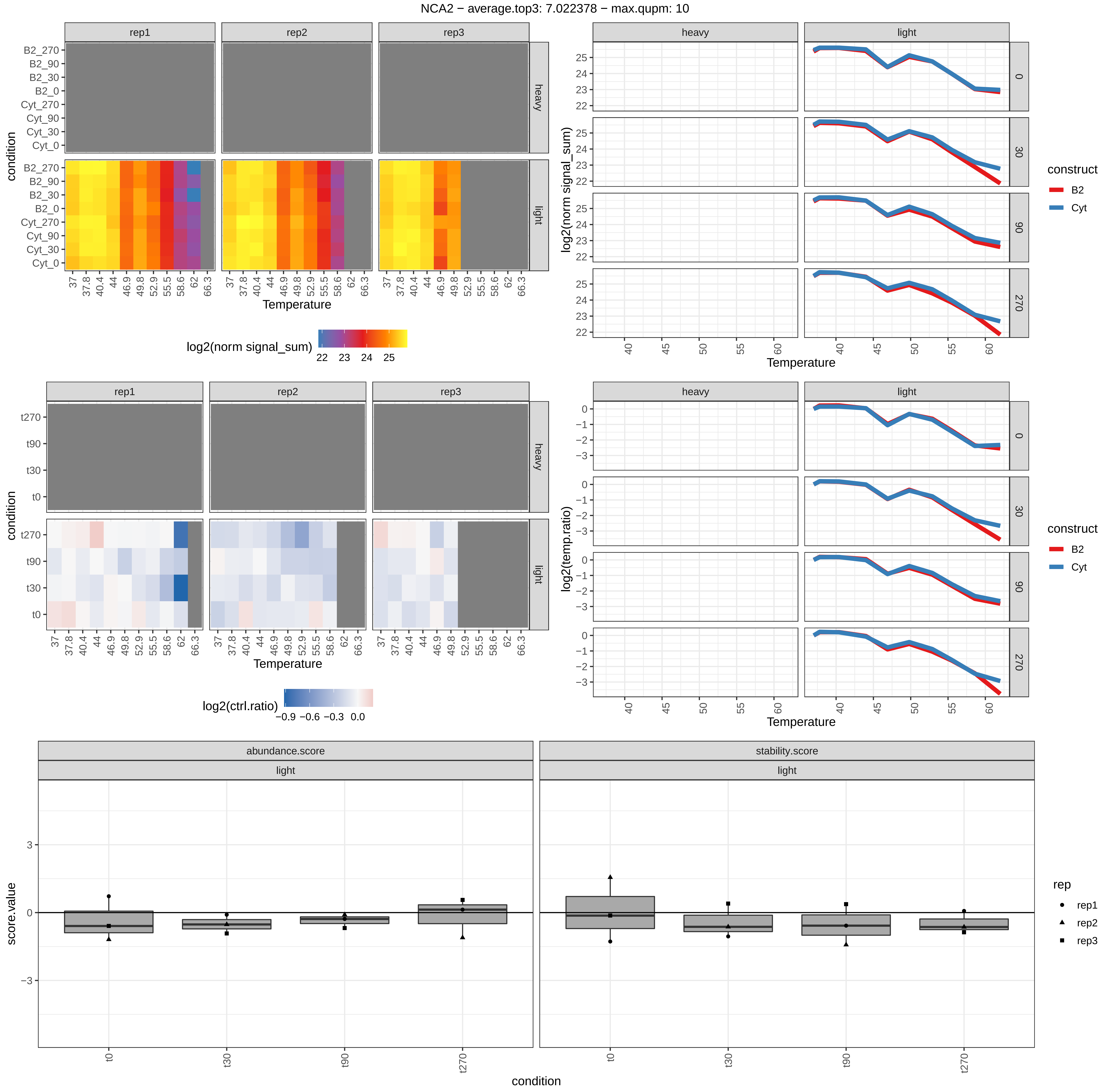

Q12374 − Nuclear control of ATPase protein 2 OS=Saccharomyces cerevisiae (strain ATCC 204508 / S288c) GN=NCA2 PE=1 SV=1

NCB2 − average.top3: 7.01183676470588 − max.qupm: 4

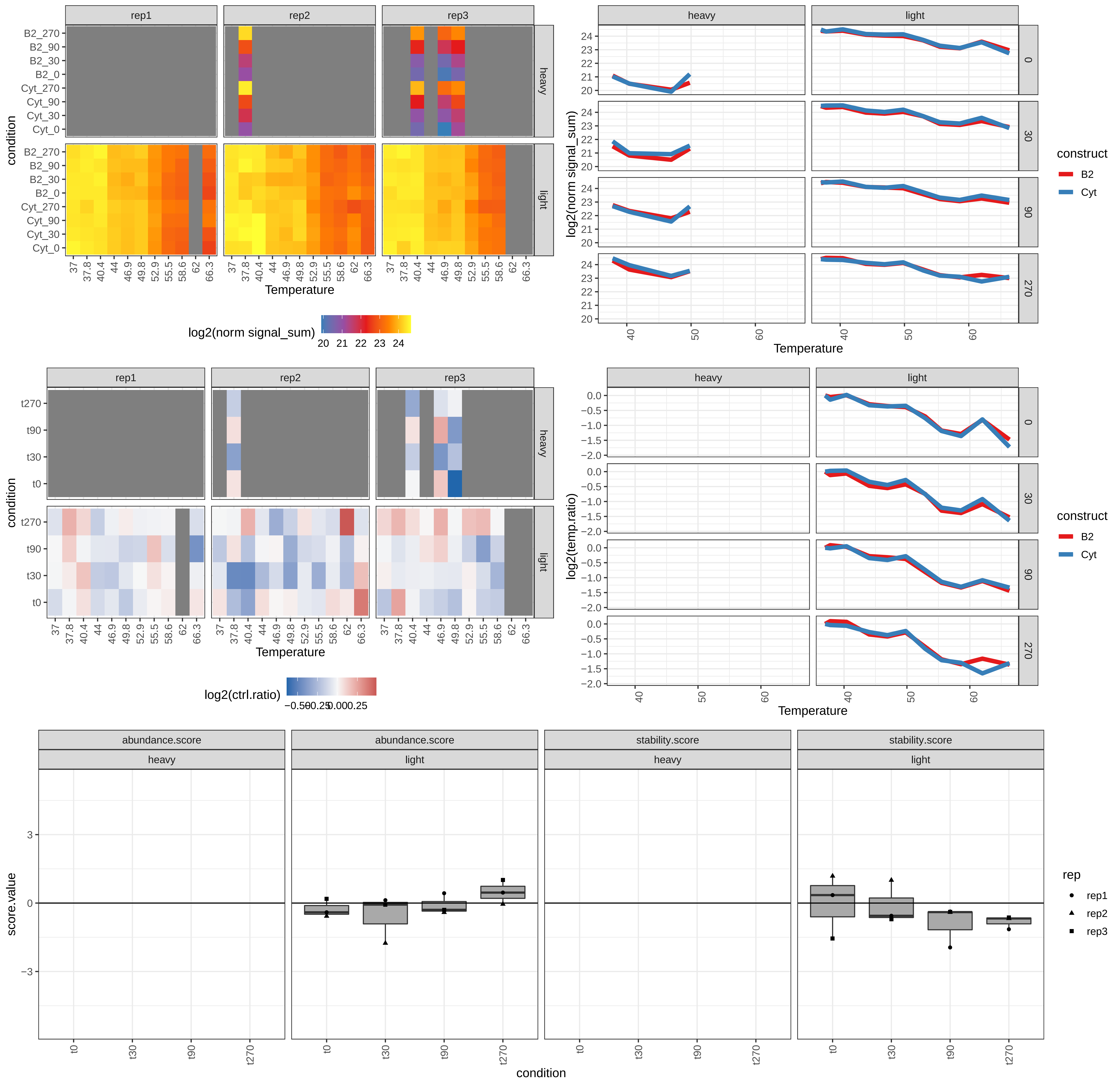

Q92317 − Negative cofactor 2 complex subunit beta OS=Saccharomyces cerevisiae (strain ATCC 204508 / S288c) GN=NCB2 PE=1 SV=1

NCE102 − average.top3: 7.52646661290323 − max.qupm: 4

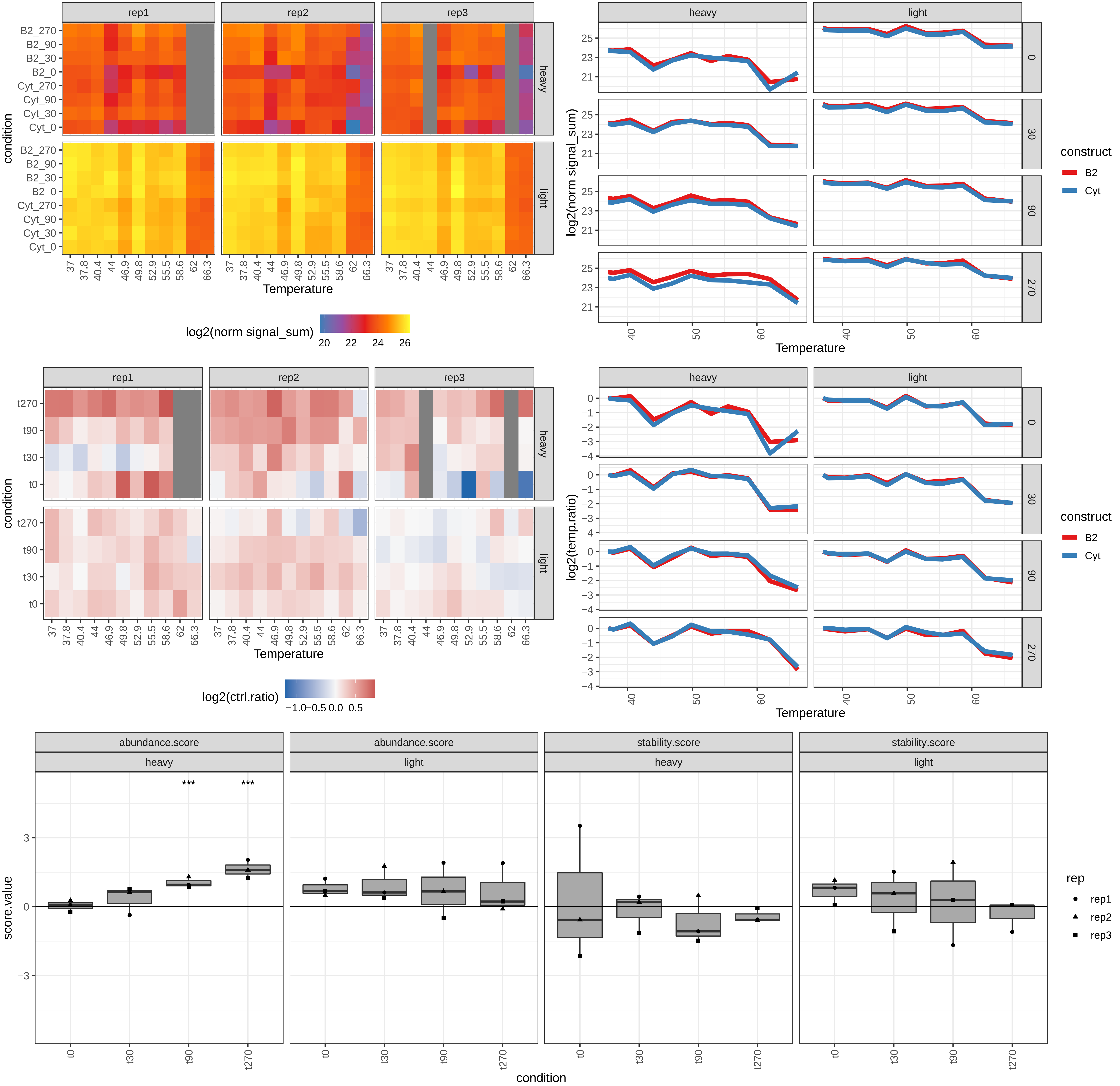

Q12207 − Non−classical export protein 2 OS=Saccharomyces cerevisiae (strain ATCC 204508 / S288c) GN=NCE102 PE=1 SV=1

NCE103 − average.top3: 7.59009842105263 − max.qupm: 5

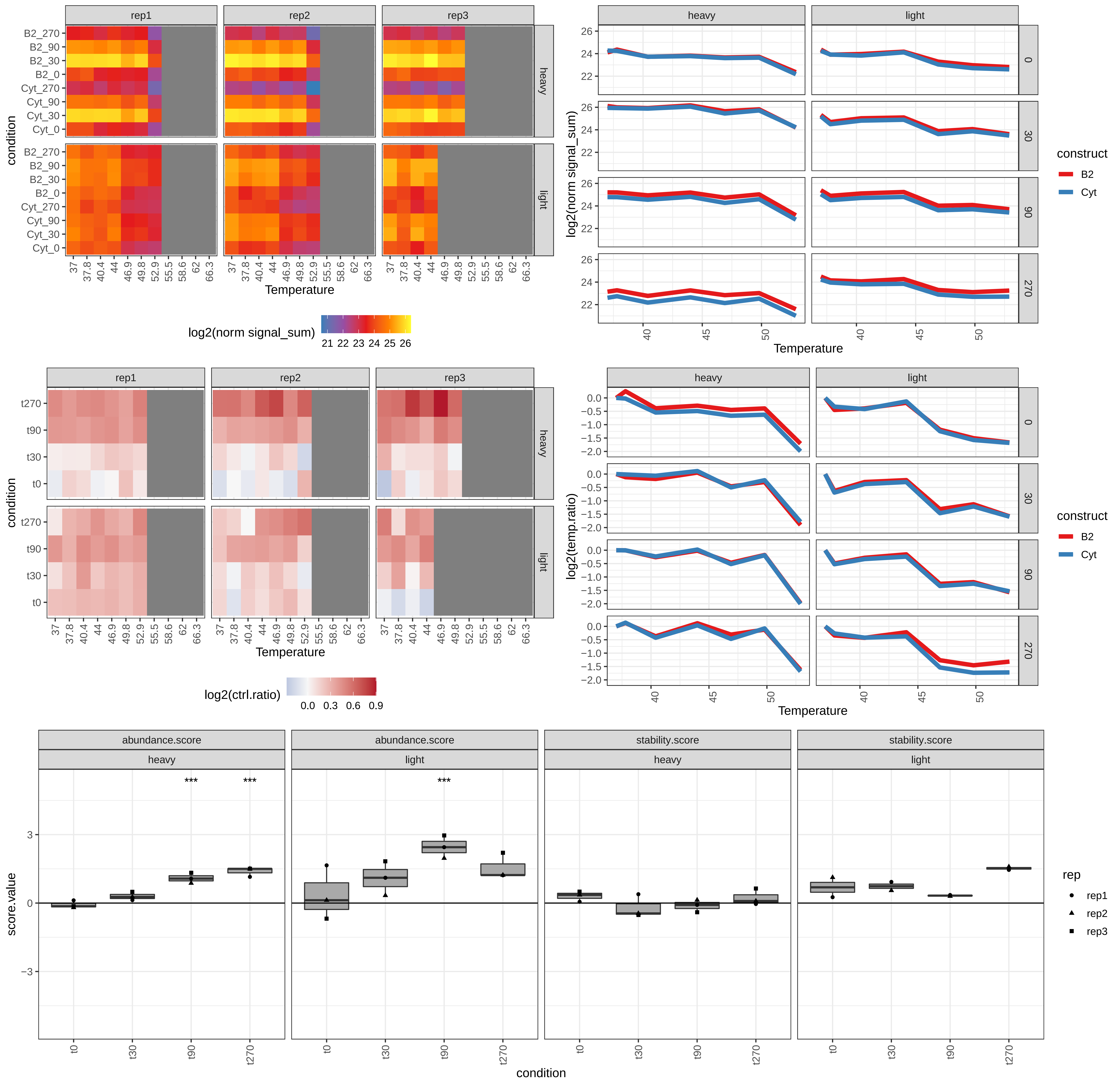

P53615 − Carbonic anhydrase OS=Saccharomyces cerevisiae (strain ATCC 204508 / S288c) GN=NCE103 PE=1 SV=1

NCL1 − average.top3: 7.10042431372549 − max.qupm: 20

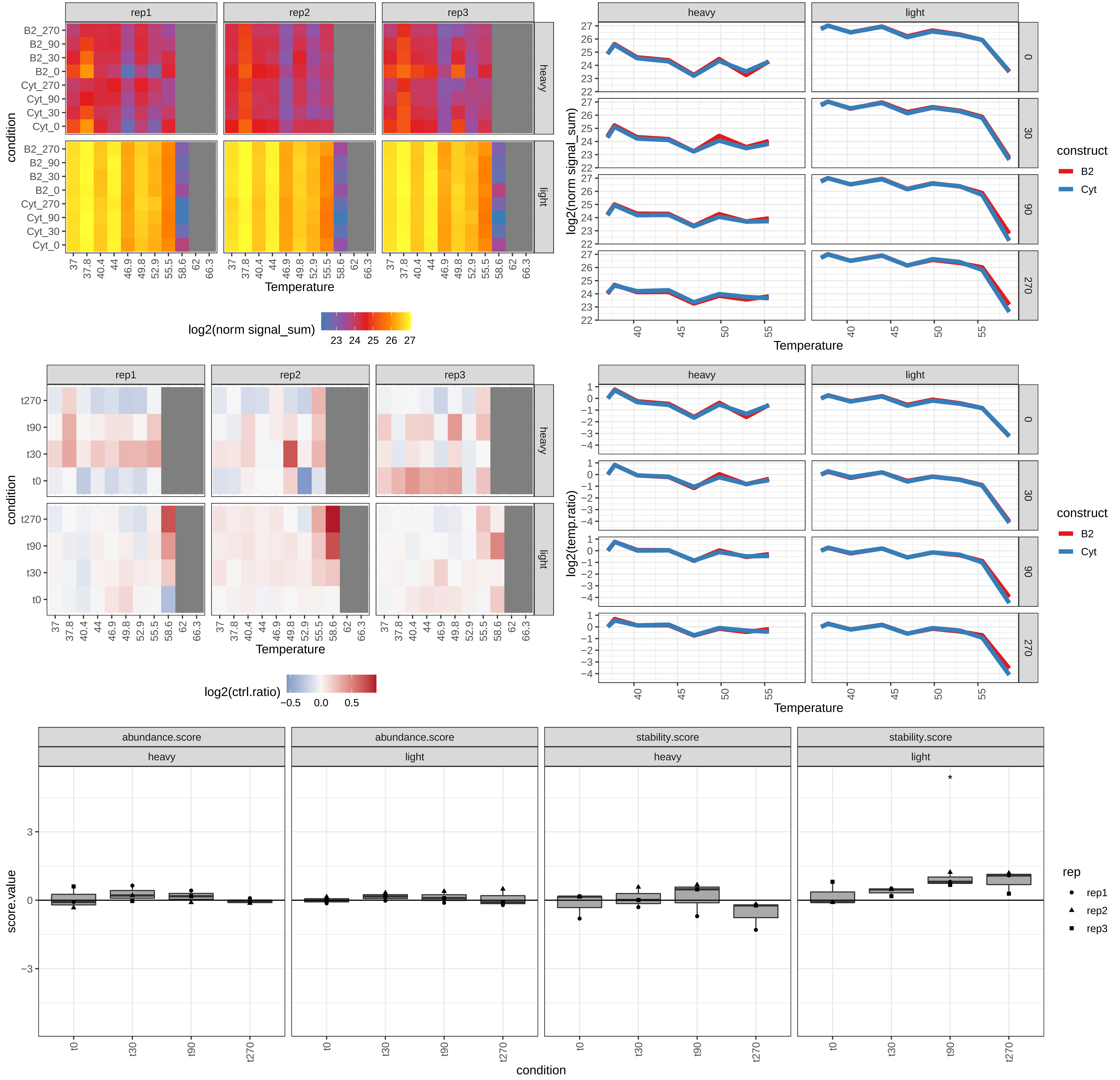

P38205 − Multisite−specific tRNA:(cytosine−C(5))−methyltransferase OS=Saccharomyces cerevisiae (strain ATCC 204508 / S288c) GN=NCL1 PE=1 SV=1

NCP1 − average.top3: 7.31440769230769 − max.qupm: 21

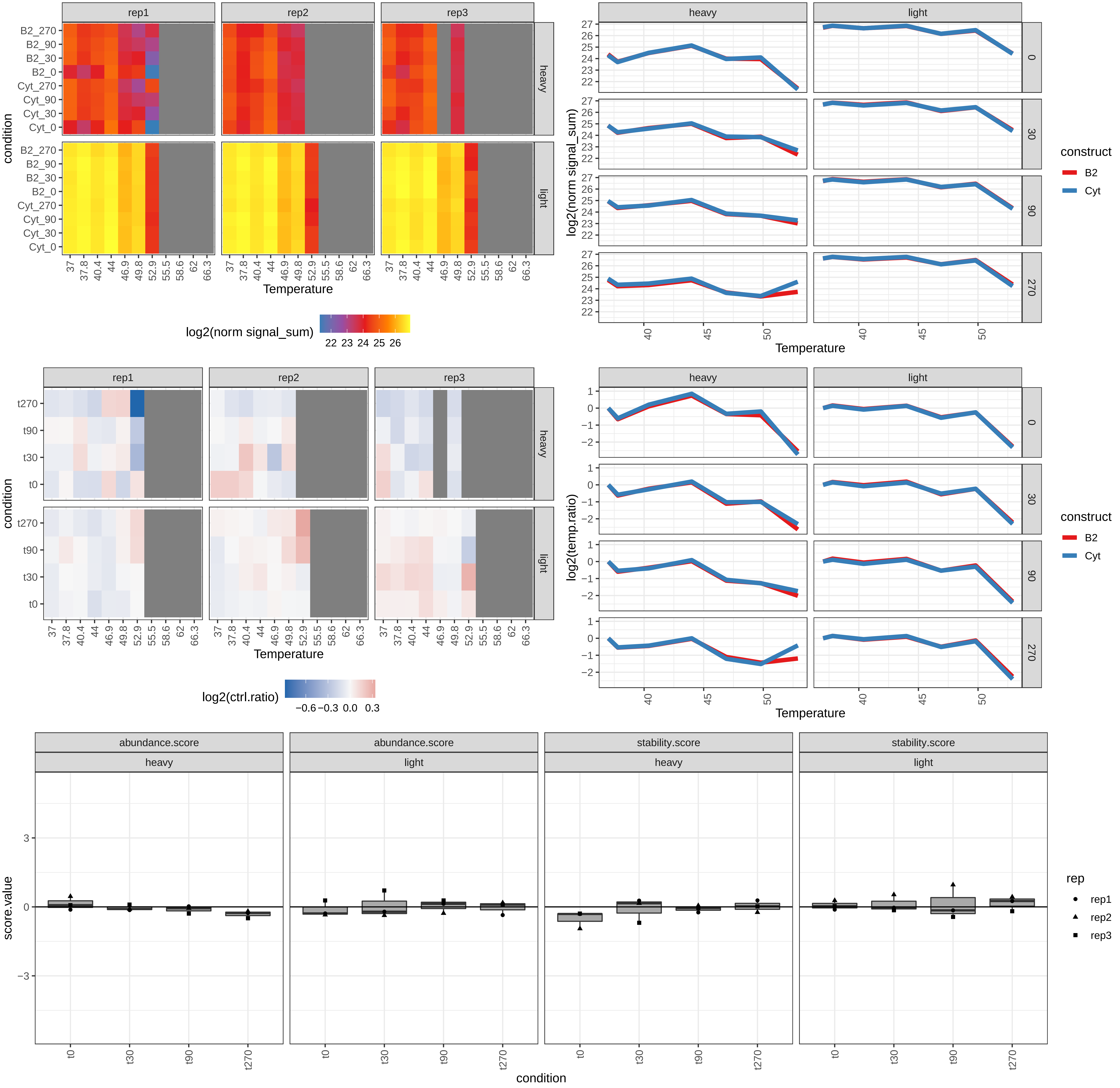

P16603 − NADPH−−cytochrome P450 reductase OS=Saccharomyces cerevisiae (strain ATCC 204508 / S288c) GN=NCP1 PE=1 SV=3

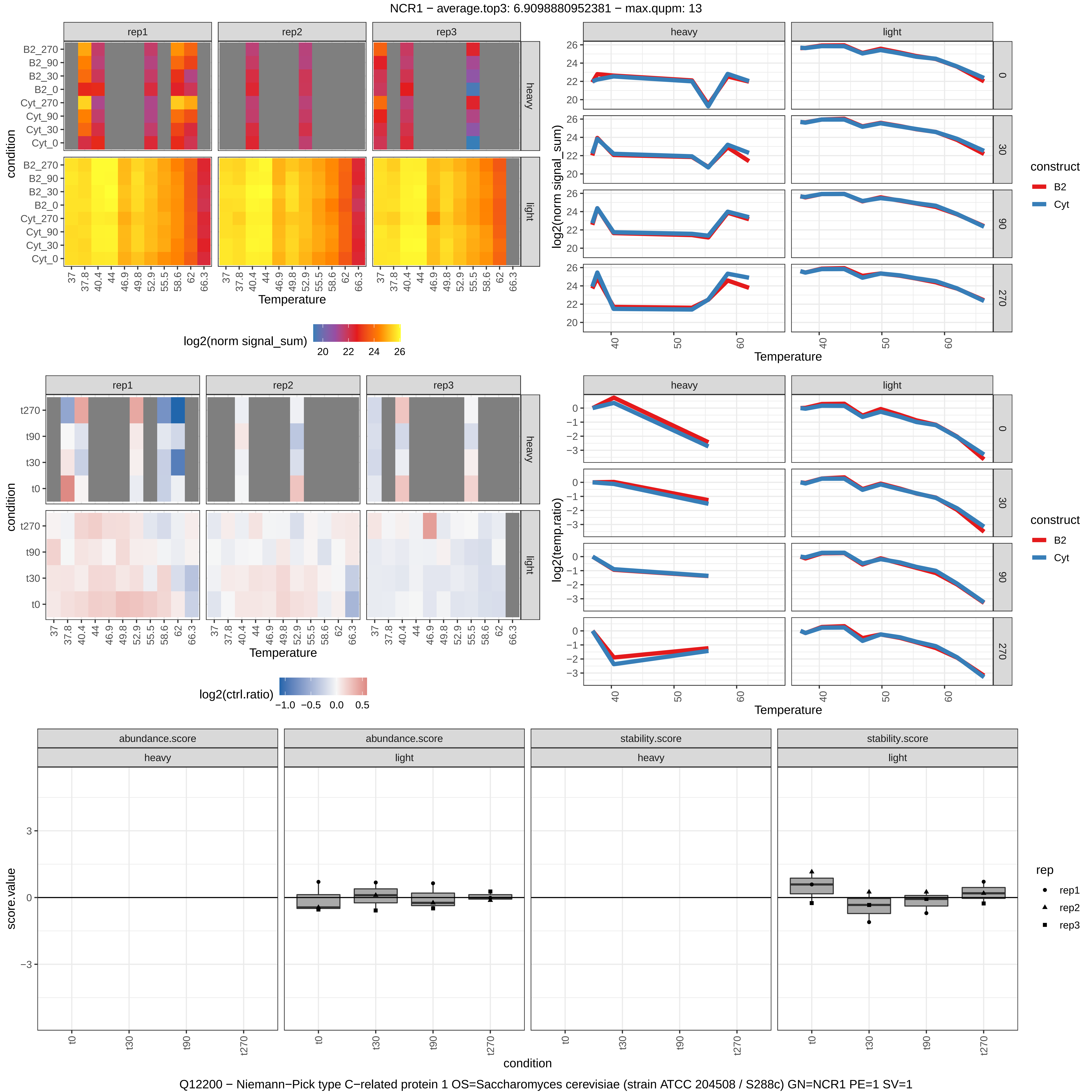
NCR1 − average.top3: 6.9098880952381 − max.qupm: 13
rep1
rep2
rep3
heavy
light
condition
B2_270
B2_90
B2_30
B2_0
Cyt_270
Cyt_90
Cyt_30
Cyt_0
37
37.8
40.4
44
46.9
49.8
52.9
55.5
58.6
62
66.3
Temperature
log2(norm signal_sum)
20
22
24
26
log2(norm signal_sum)
0
30
90
270
40
50
60
construct
B2
Cyt
t270
t90
t30
t0
log2(ctrl.ratio)
−1.0
−0.5
0.0
0.5
log2(temp.ratio)
0
−1
−2
−3
abundance.score
stability.score
score.value
3
0
−3
rep
rep1
rep2
rep3
Q12200 − Niemann−Pick type C−related protein 1 OS=Saccharomyces cerevisiae (strain ATCC 204508 / S288c) GN=NCR1 PE=1 SV=1

NCS2 − average.top3: 6.64618071428571 − max.qupm: 6

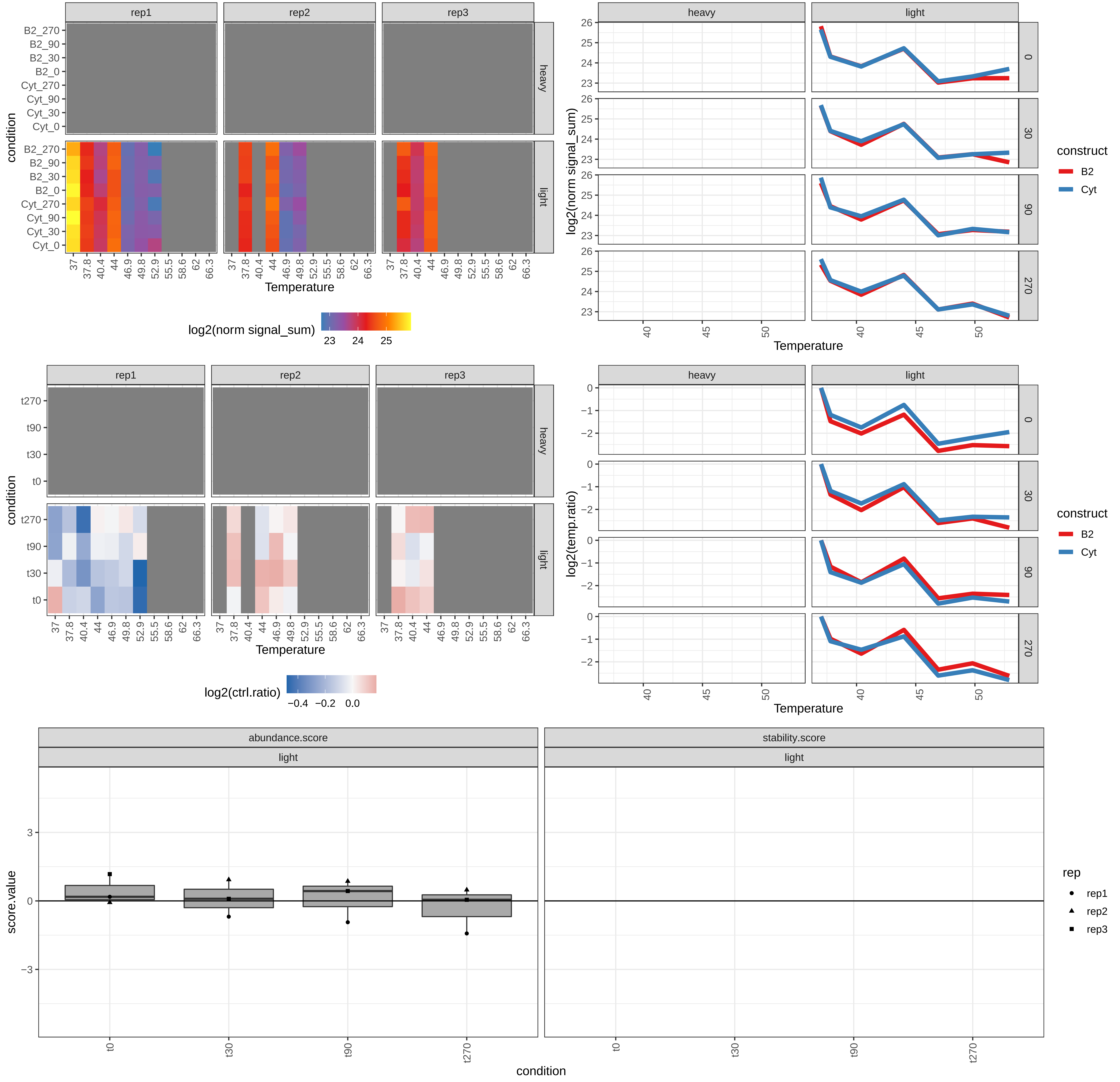

P53923 − Cytoplasmic tRNA 2−thiolation protein 2 OS=Saccharomyces cerevisiae (strain ATCC 204508 / S288c) GN=NCS2 PE=1 SV=1

NDC1 − average.top3: 6.73131666666667 − max.qupm: 5

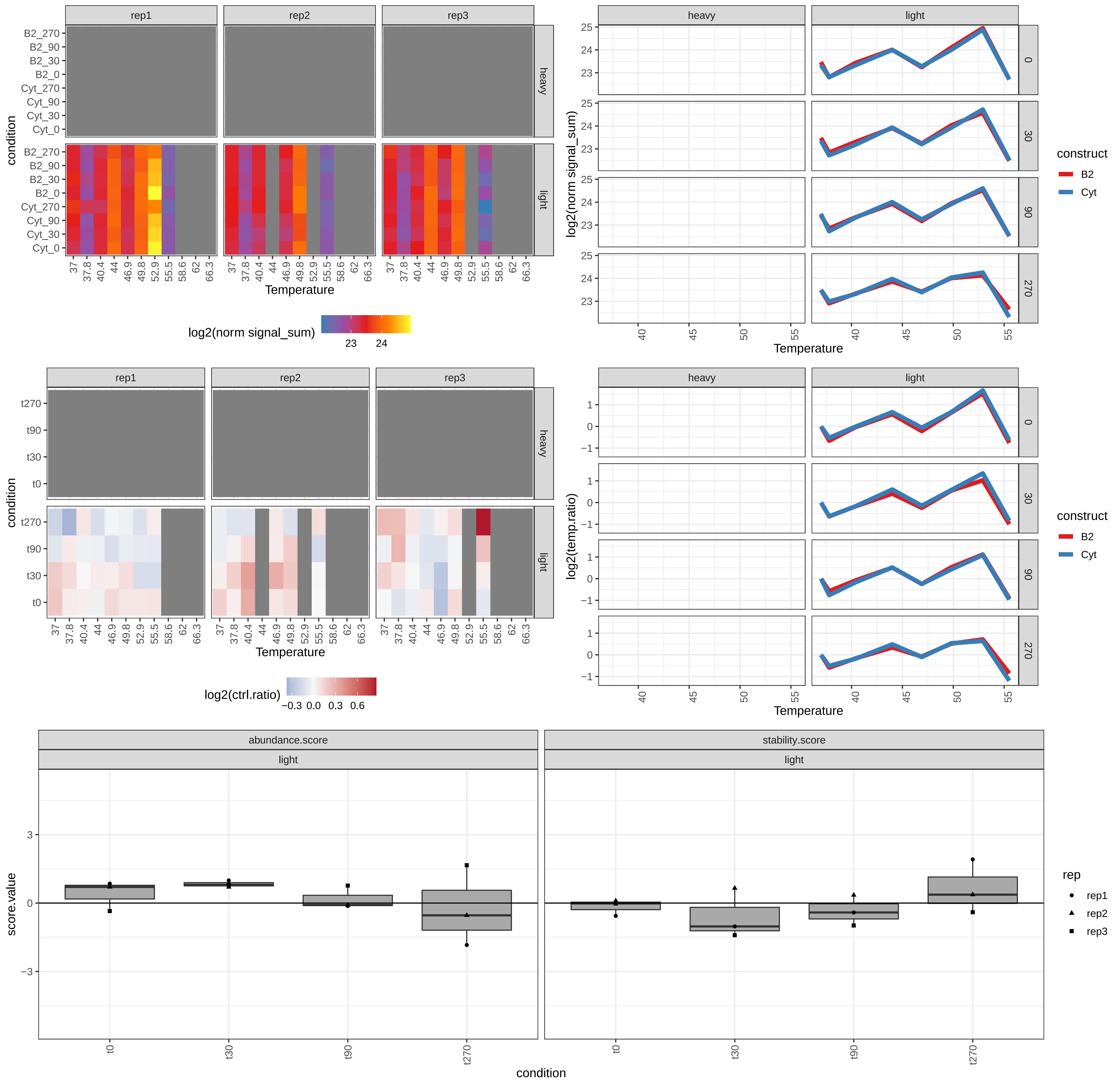

P32500 − Nucleoporin NDC1 OS=Saccharomyces cerevisiae (strain ATCC 204508 / S288c) GN=NDC1 PE=1 SV=1

NDC80 − average.top3: 6.264712 − max.qupm: 5

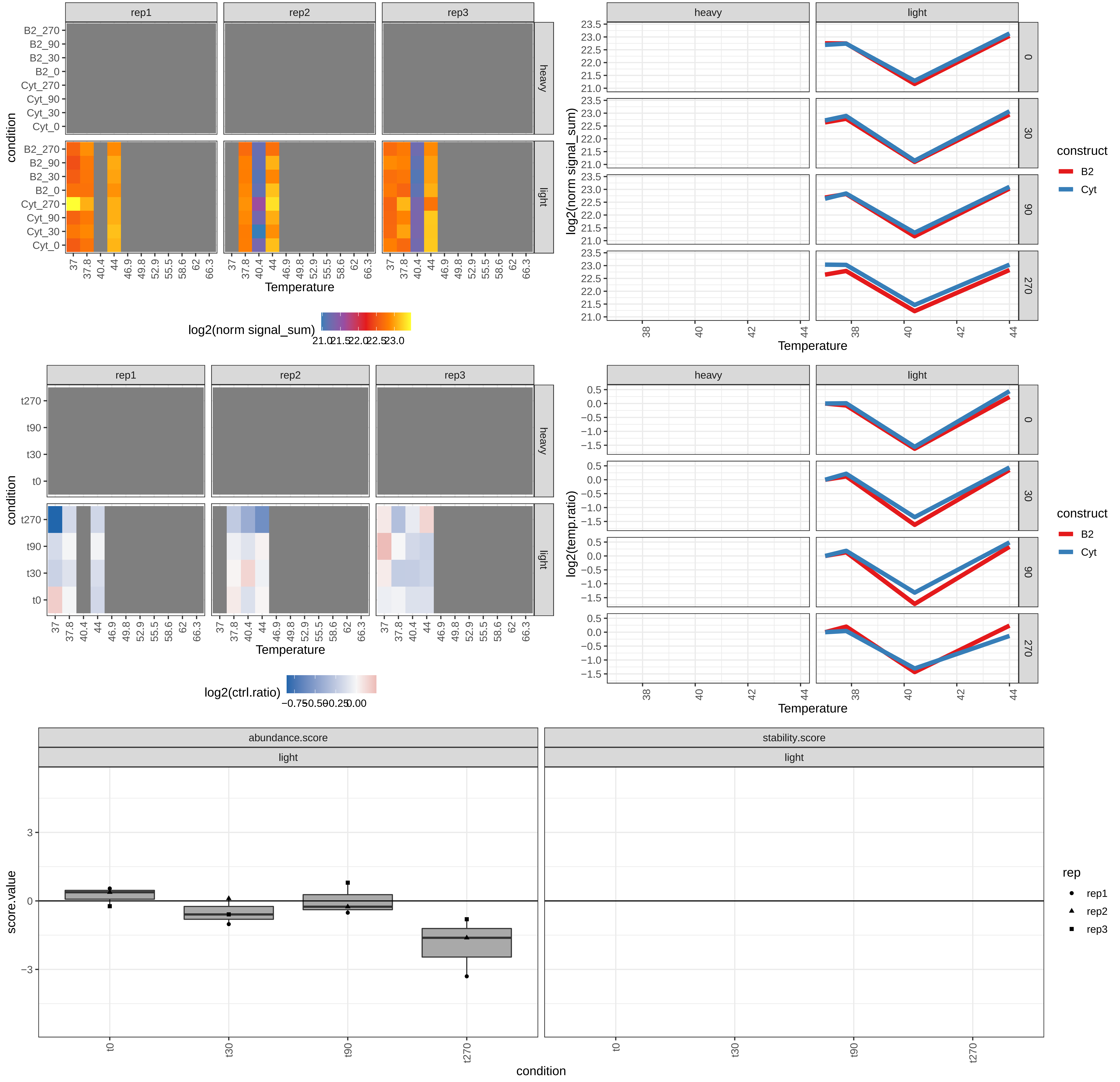

P40460 − Kinetochore protein NDC80 OS=Saccharomyces cerevisiae (strain ATCC 204508 / S288c) GN=NDC80 PE=1 SV=1

NDE1 − average.top3: 7.40848636363636 − max.qupm: 19

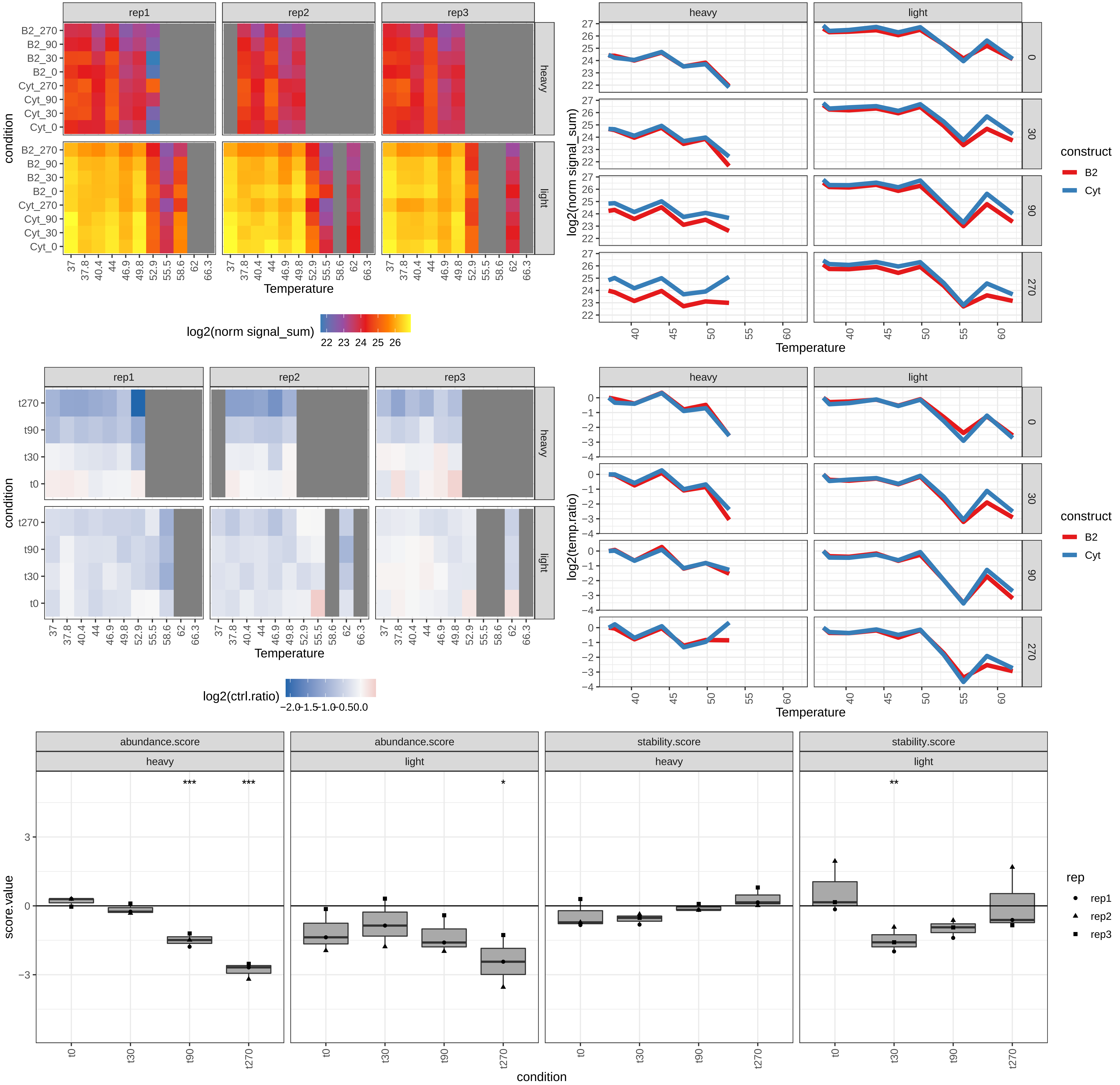

P40215 − External NADH−ubiquinone oxidoreductase 1, mitochondrial OS=Saccharomyces cerevisiae (strain ATCC 204508 / S288c) GN=NDE1 PE=1 SV=1

NDE2 − average.top3: 7.168874 − max.qupm: 8

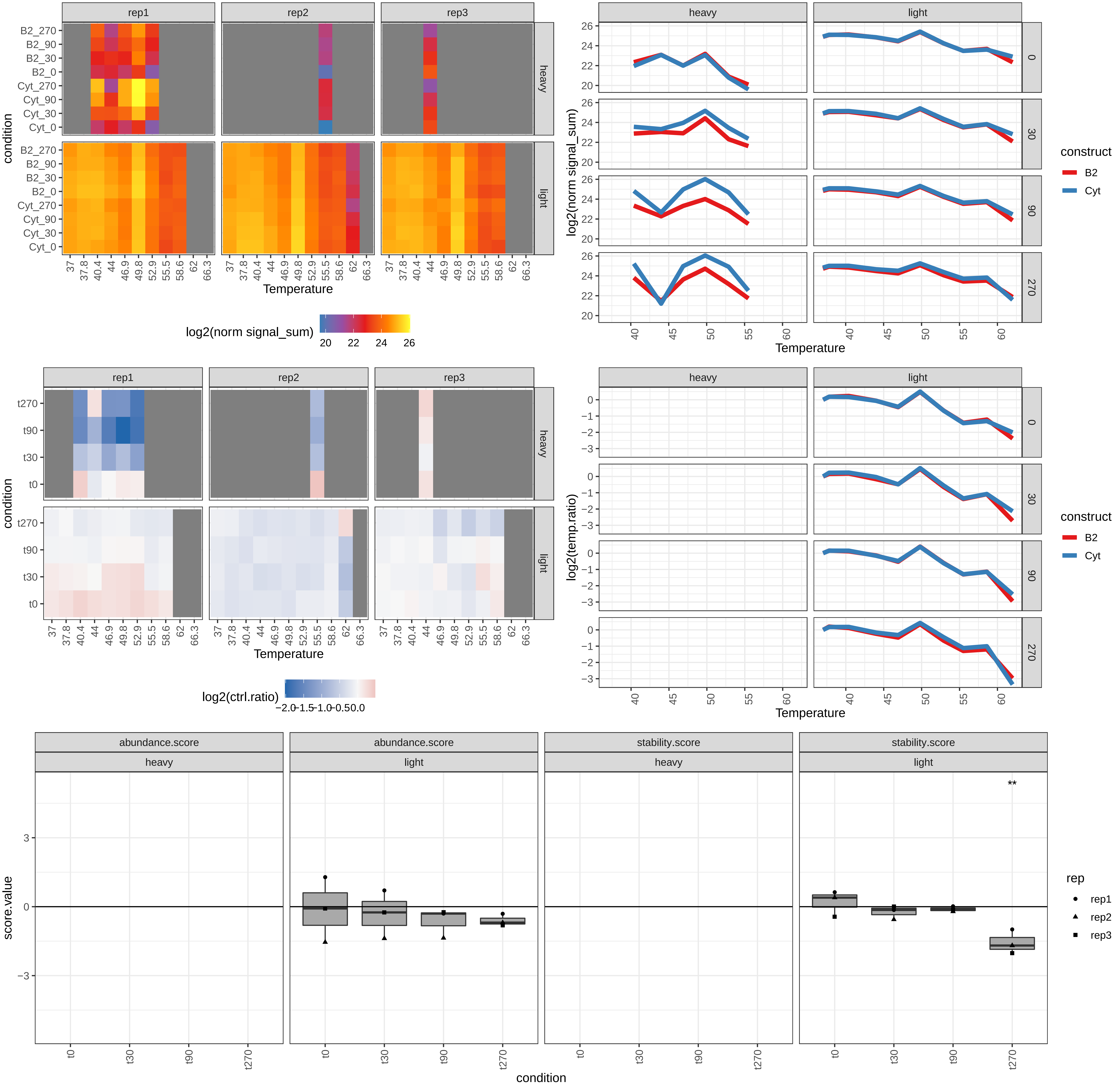

Q07500 − External NADH−ubiquinone oxidoreductase 2, mitochondrial OS=Saccharomyces cerevisiae (strain ATCC 204508 / S288c) GN=NDE2 PE=1 SV=1

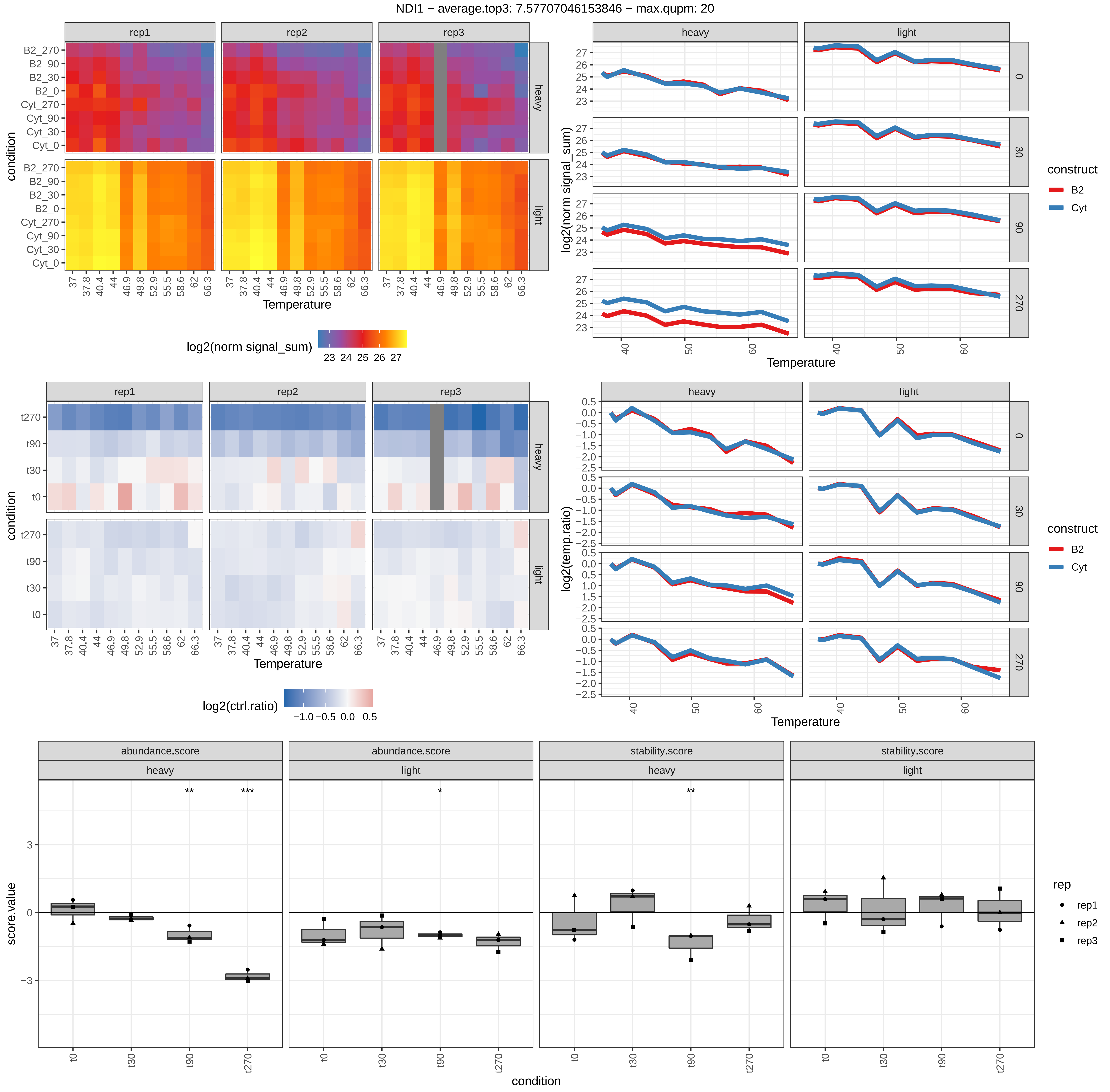

P32340 − Rotenone−insensitive NADH−ubiquinone oxidoreductase, mitochondrial OS=Saccharomyces cerevisiae (strain ATCC 204508 / S288c) GN=NDI1 PE=1 SV=1

NEO1 − average.top3: 6.74769105263158 − max.qupm: 8

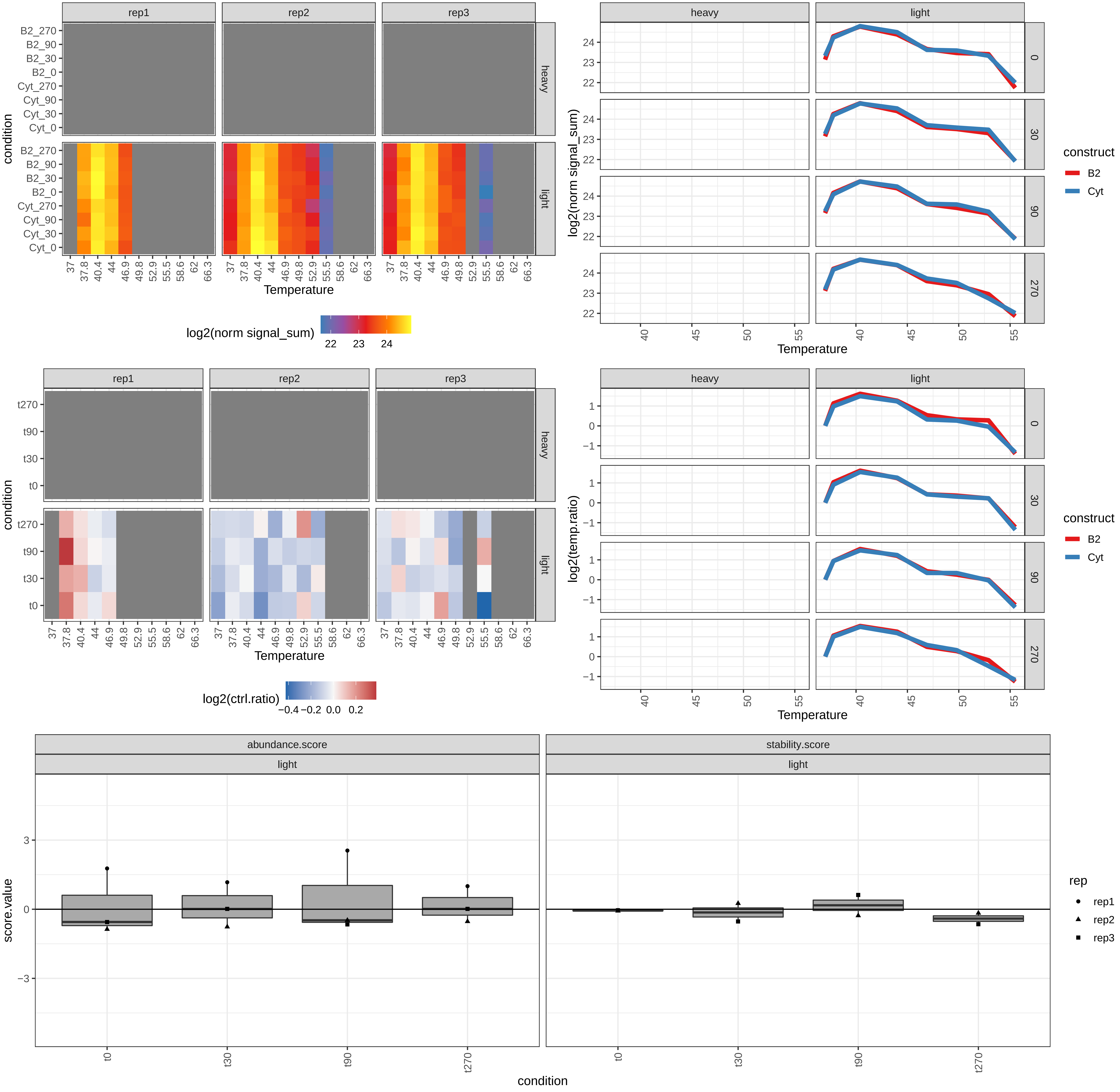

P40527 − Probable phospholipid−transporting ATPase NEO1 OS=Saccharomyces cerevisiae (strain ATCC 204508 / S288c) GN=NEO1 PE=1 SV=1

NEW1 − average.top3: 7.12256473684211 − max.qupm: 24

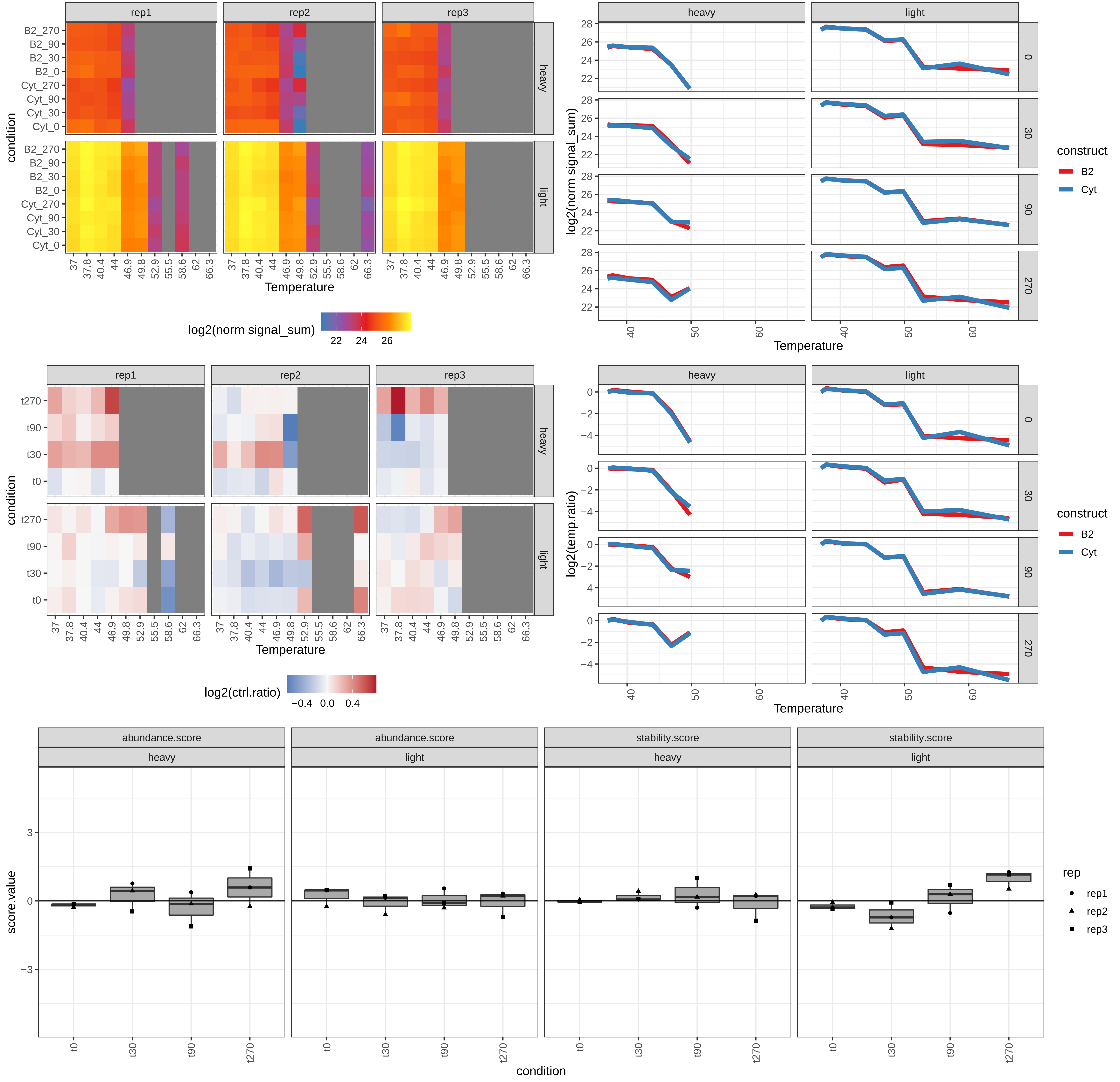

Q08972 − [NU+] prion formation protein 1 OS=Saccharomyces cerevisiae (strain ATCC 204508 / S288c) GN=NEW1 PE=1 SV=1

NFS1 − average.top3: 7.26557479166667 − max.qupm: 10

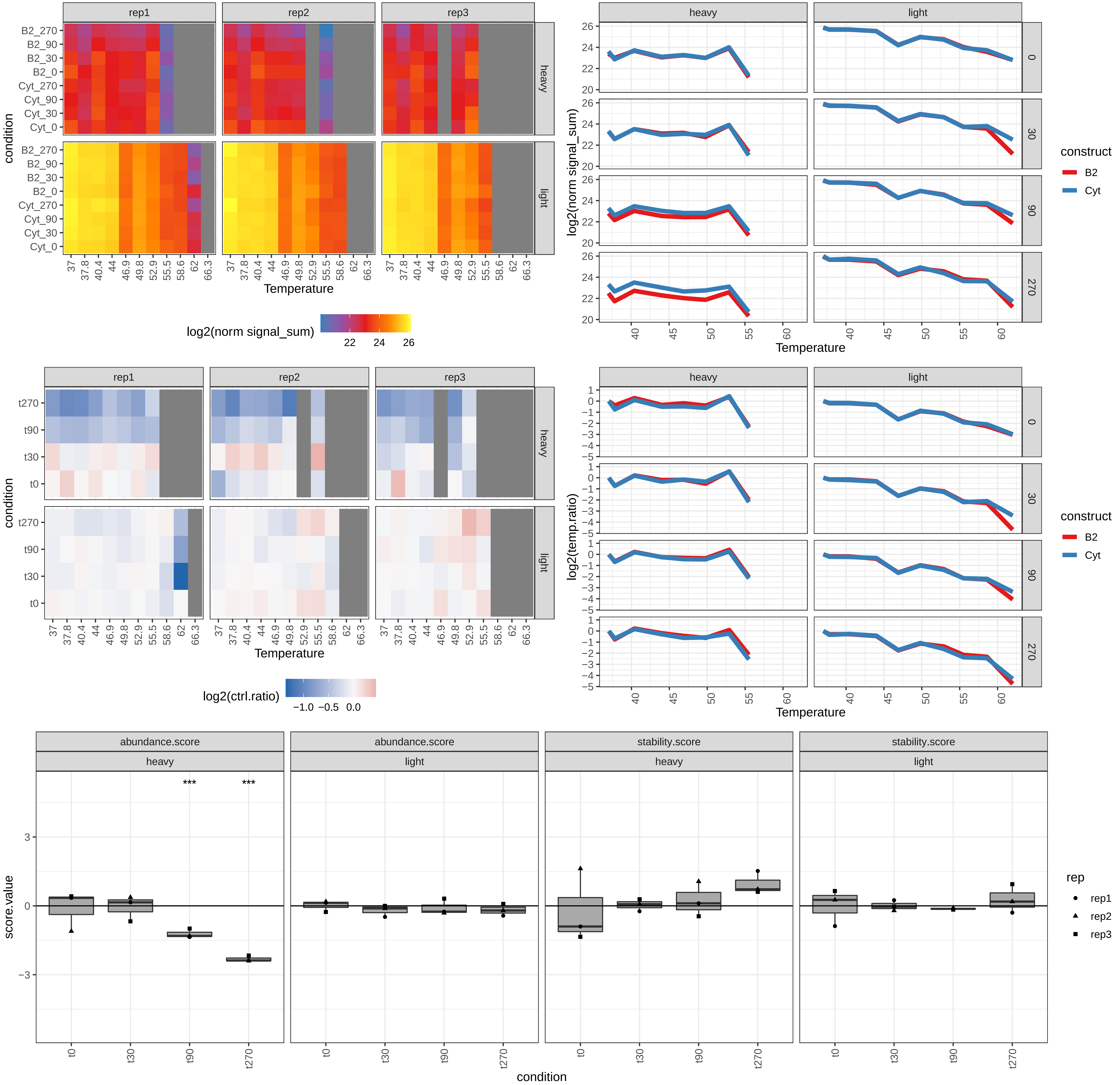

P25374 − Cysteine desulfurase, mitochondrial OS=Saccharomyces cerevisiae (strain ATCC 204508 / S288c) GN=NFS1 PE=1 SV=2

NFU1 − average.top3: 6.98523 − max.qupm: 8

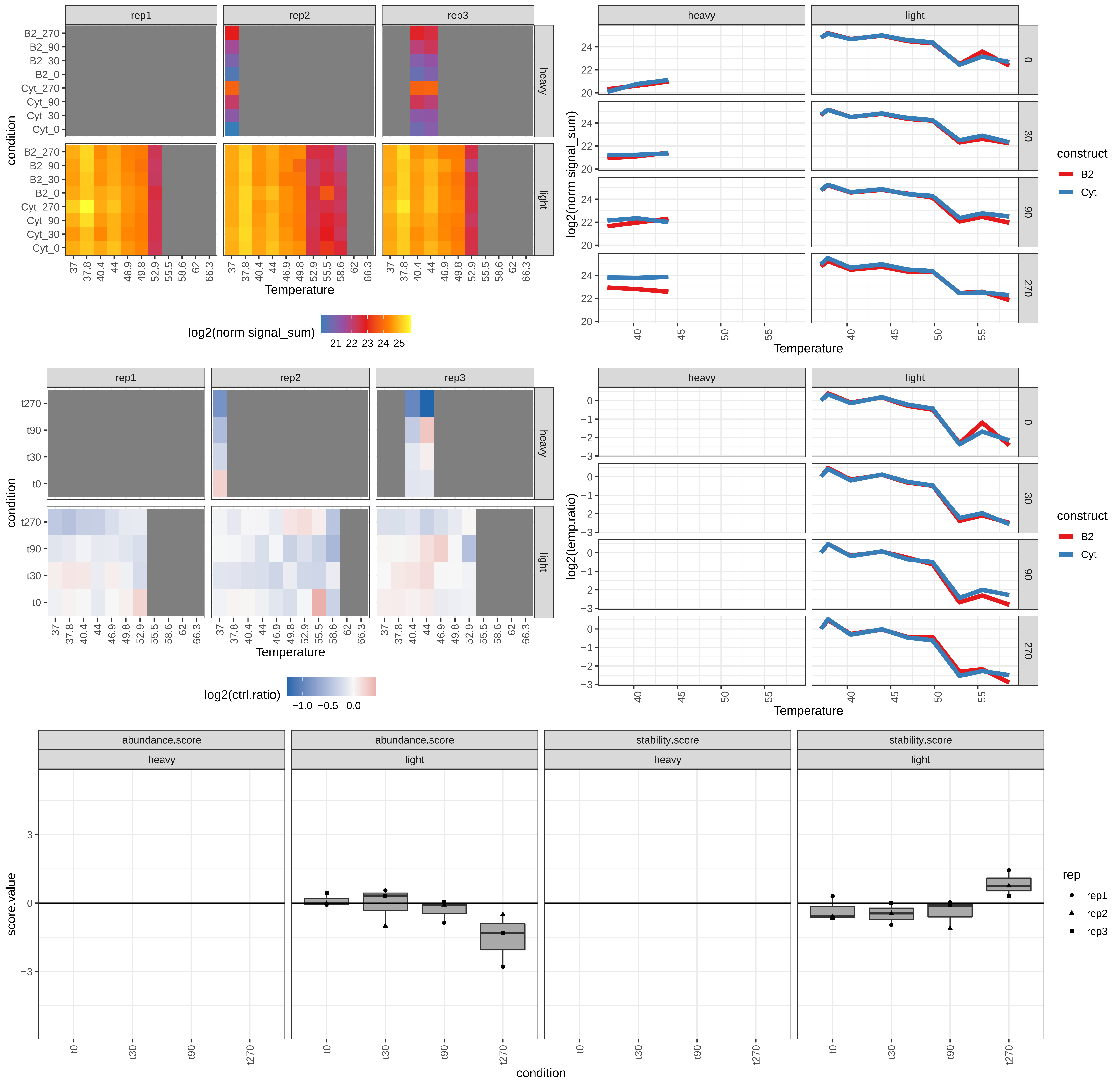

P32860 − NifU−like protein, mitochondrial OS=Saccharomyces cerevisiae (strain ATCC 204508 / S288c) GN=NFU1 PE=1 SV=2

NGR1 − average.top3: 7.04106052631579 − max.qupm: 4

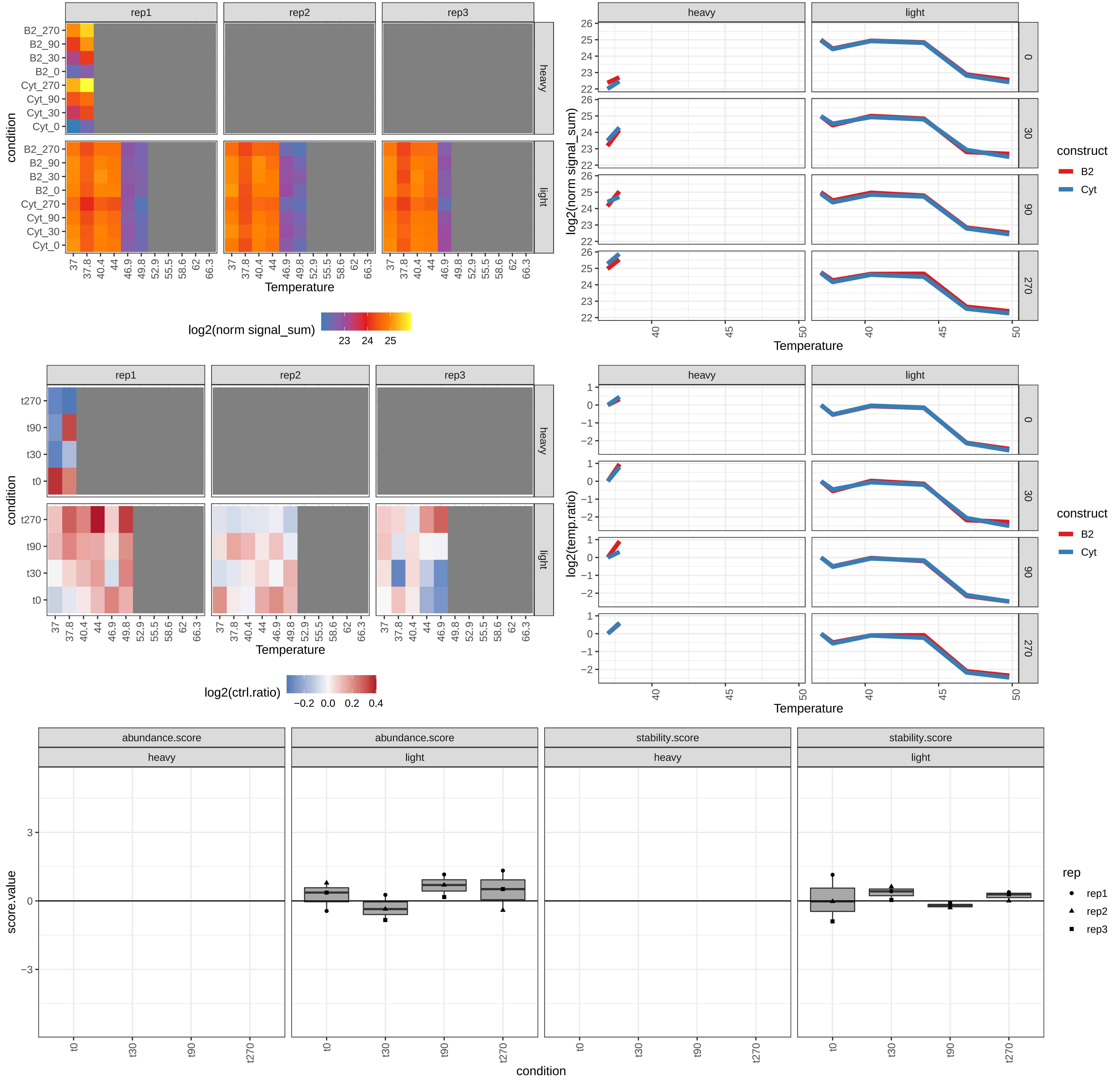

n regulatory protein NGR1 OS=Saccharomyces cerevisiae (strain ATCC 204508 / S288c) GN=NGR1 PE=1 SV=2|Isoform 2 of Negative growth regulatory protein NGR1 OS=Saccharomyces cerevisiae (stra

NHP10 − average.top3: 6.8516325 − max.qupm: 5

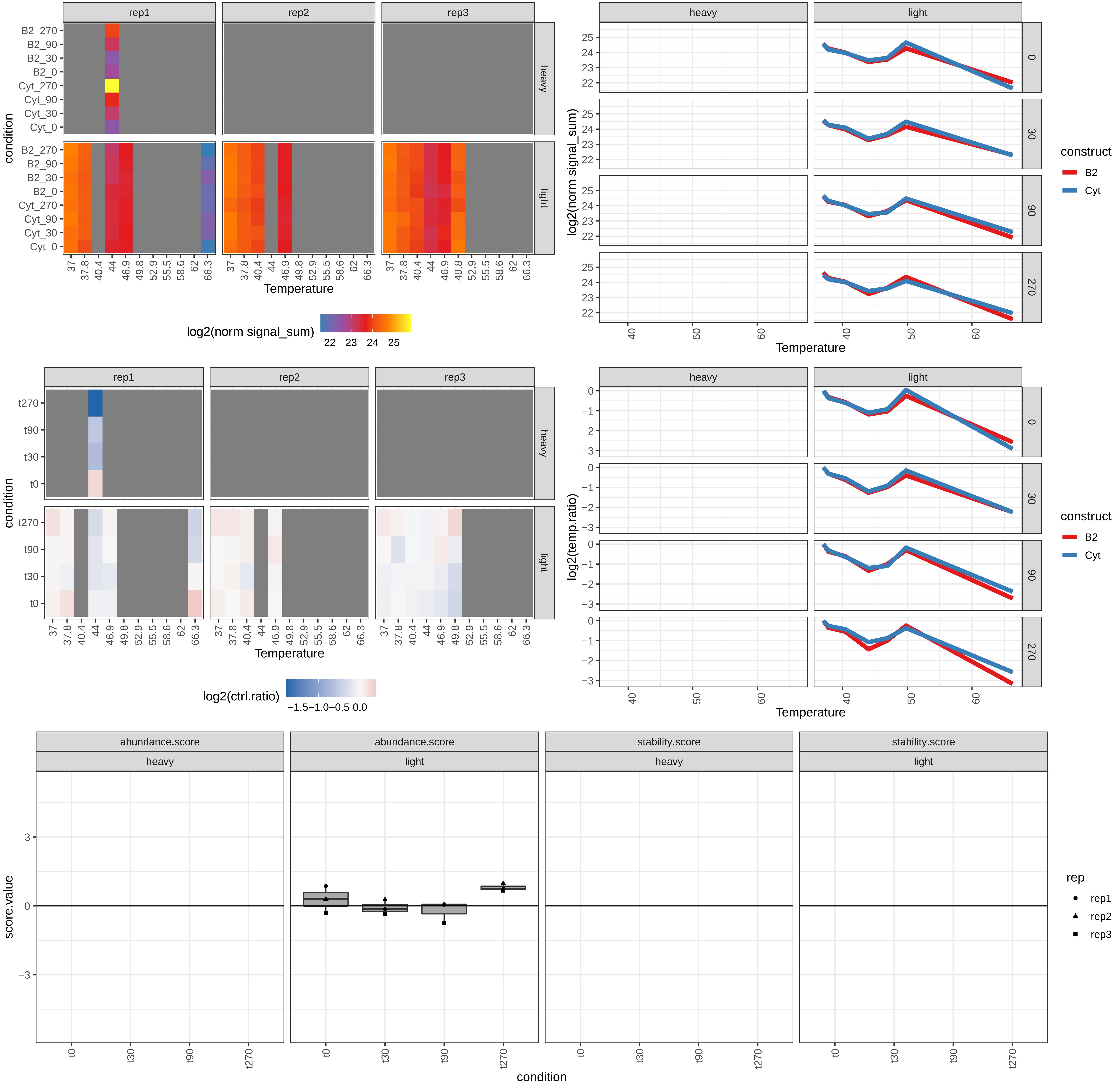

Q03435 − Non−histone protein 10 OS=Saccharomyces cerevisiae (strain ATCC 204508 / S288c) GN=NHP10 PE=1 SV=1

NHP2 − average.top3: 7.0850355 − max.qupm: 4

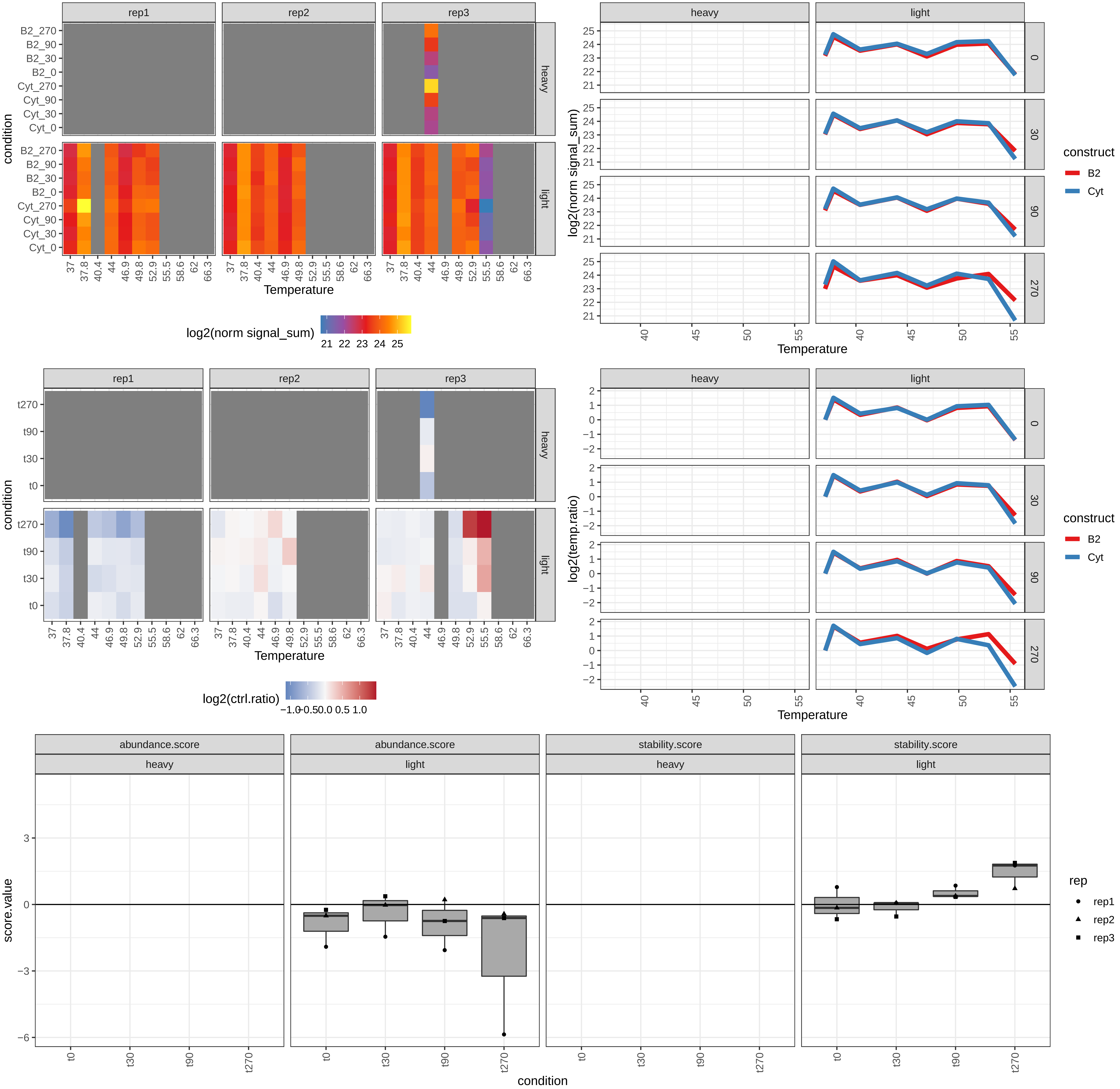

P32495 − H/ACA ribonucleoprotein complex subunit 2 OS=Saccharomyces cerevisiae (strain ATCC 204508 / S288c) GN=NHP2 PE=1 SV=2

NHP6A − average.top3: 7.02254676470588 − max.qupm: 4

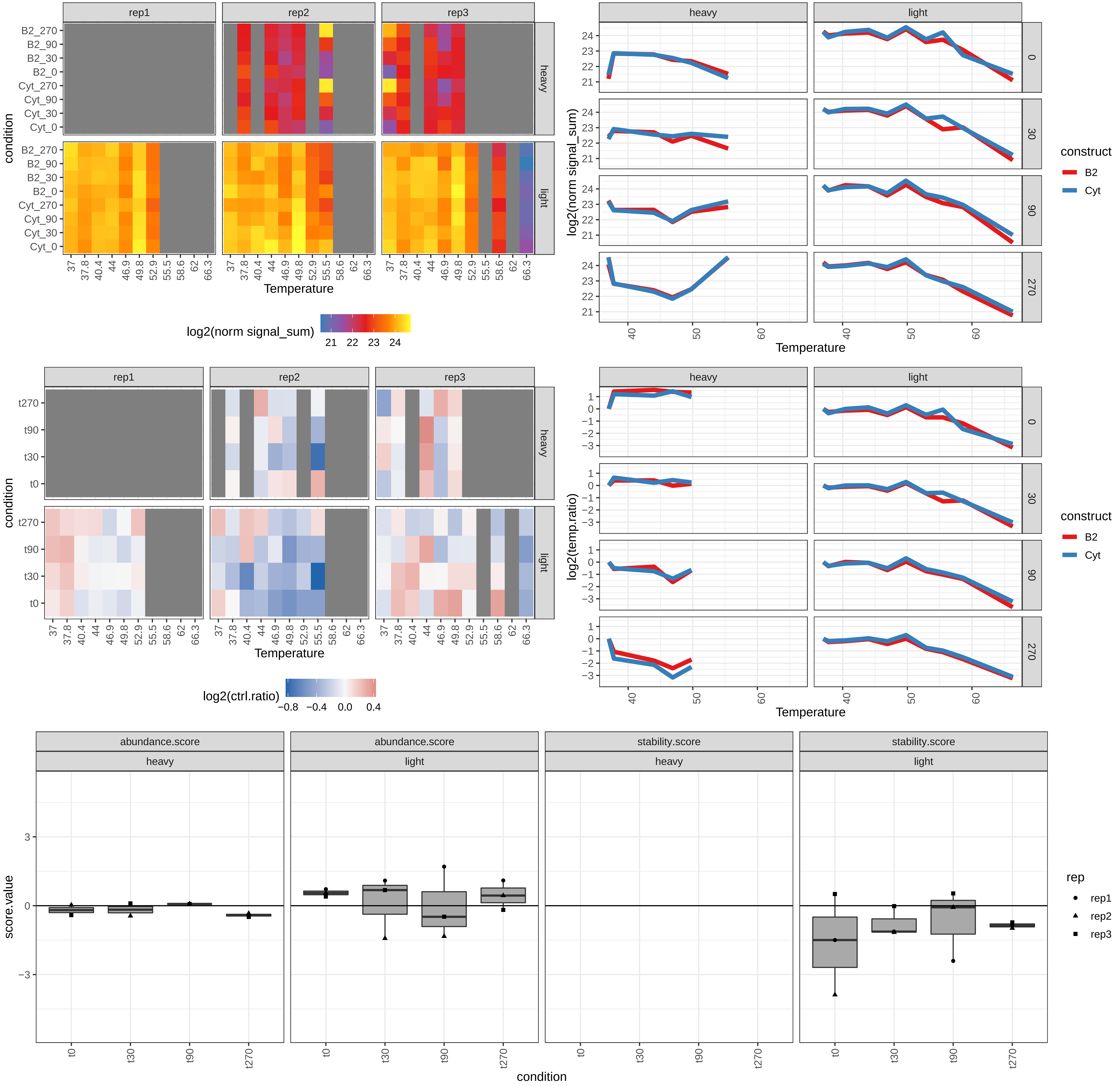

P11632 − Non−histone chromosomal protein 6A OS=Saccharomyces cerevisiae (strain ATCC 204508 / S288c) GN=NHP6A PE=1 SV=1

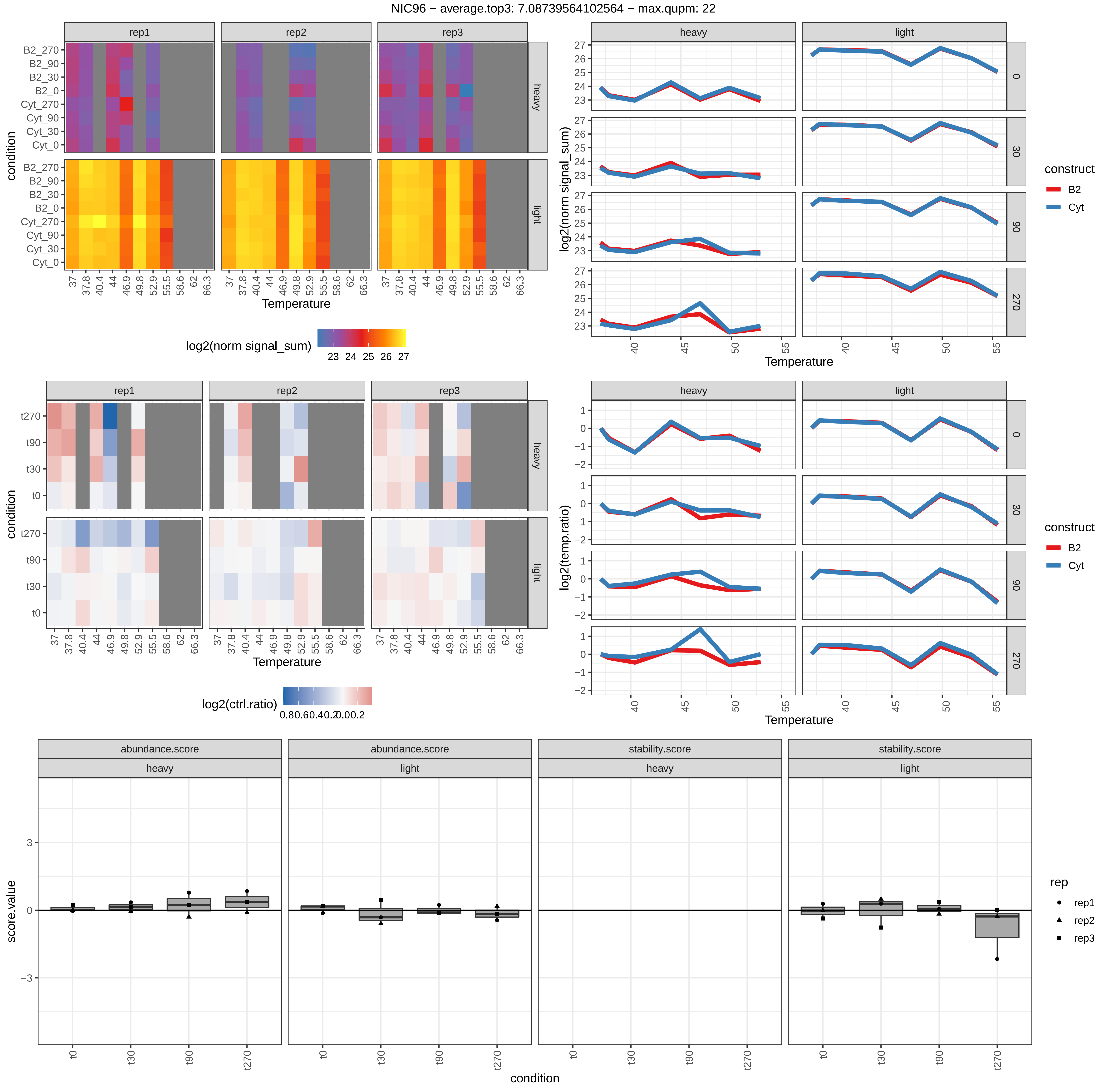

P34077 − Nucleoporin NIC96 OS=Saccharomyces cerevisiae (strain ATCC 204508 / S288c) GN=NIC96 PE=1 SV=2

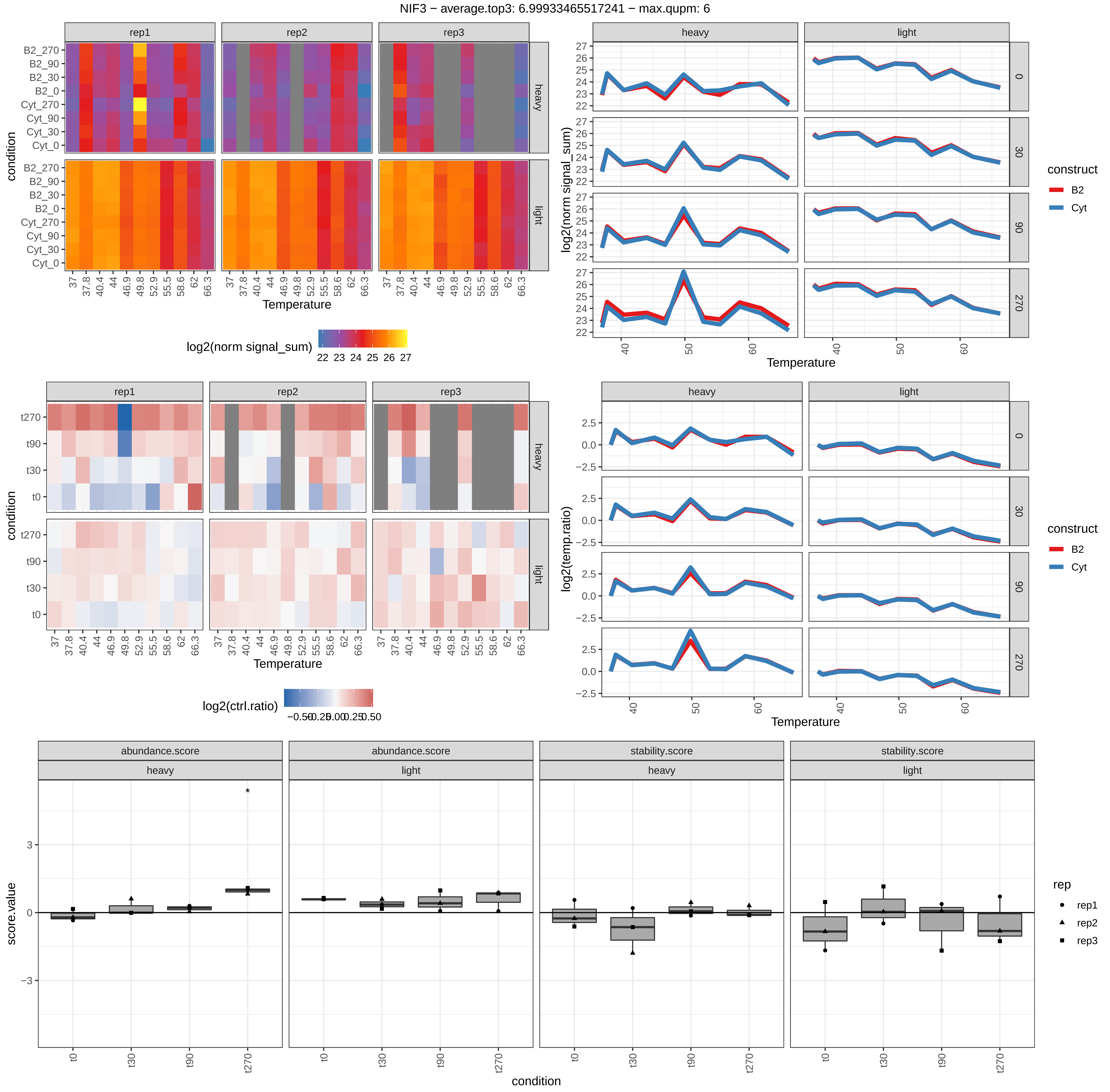

P53081 − NGG1−interacting factor 3 OS=Saccharomyces cerevisiae (strain ATCC 204508 / S288c) GN=NIF3 PE=1 SV=1

NIP1 − average.top3: 7.39365264150943 − max.qupm: 23

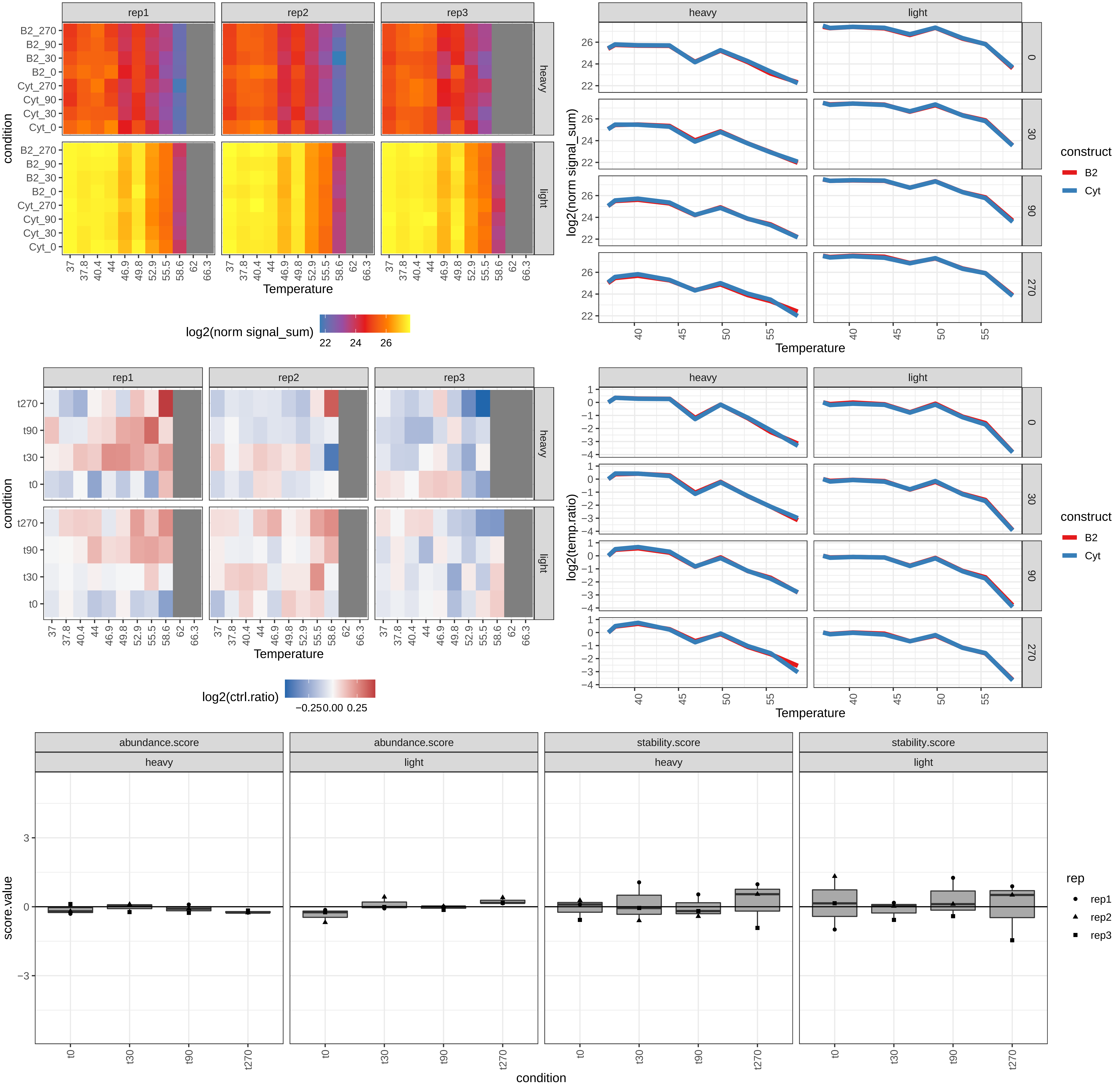

P32497 − Eukaryotic translation initiation factor 3 subunit C OS=Saccharomyces cerevisiae (strain ATCC 204508 / S288c) GN=NIP1 PE=1 SV=2

NIP7 − average.top3: 7.26928761904762 − max.qupm: 6

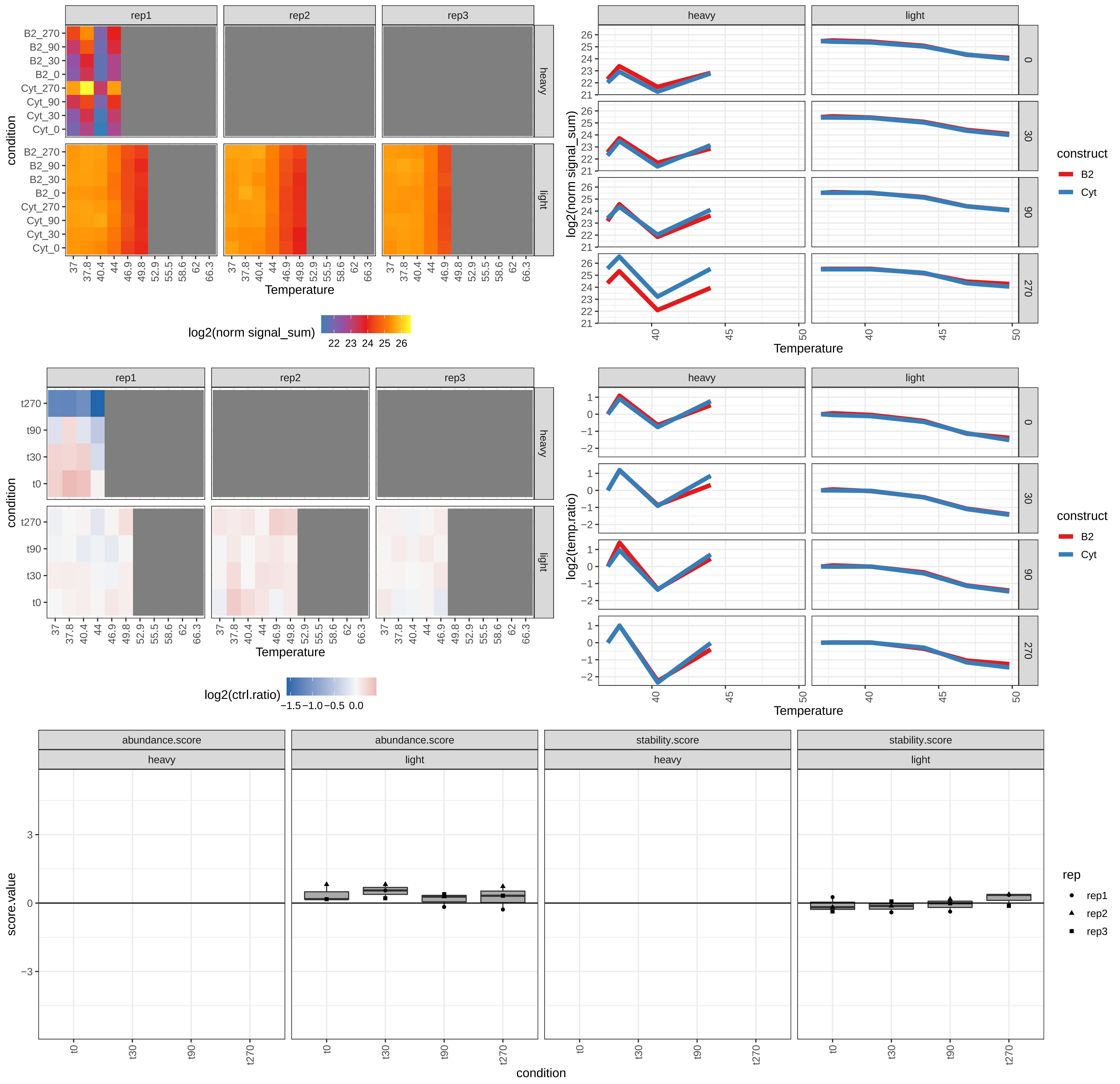

Q08962 − 60S ribosome subunit biogenesis protein NIP7 OS=Saccharomyces cerevisiae (strain ATCC 204508 / S288c) GN=NIP7 PE=1 SV=1

NIT2 − average.top3: 6.408476 − max.qupm: 6

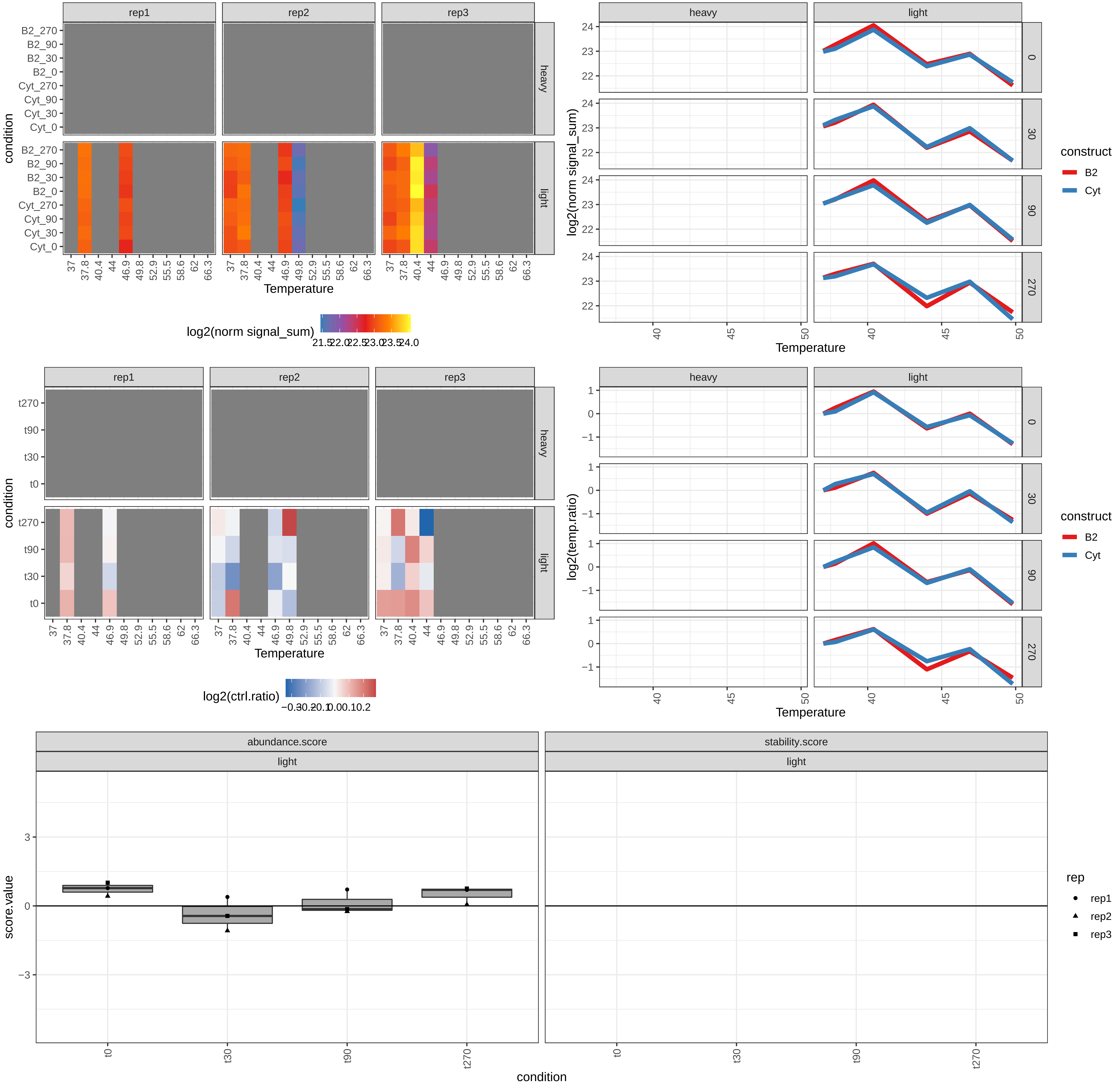

P47016 − Probable hydrolase NIT2 OS=Saccharomyces cerevisiae (strain ATCC 204508 / S288c) GN=NIT2 PE=1 SV=1

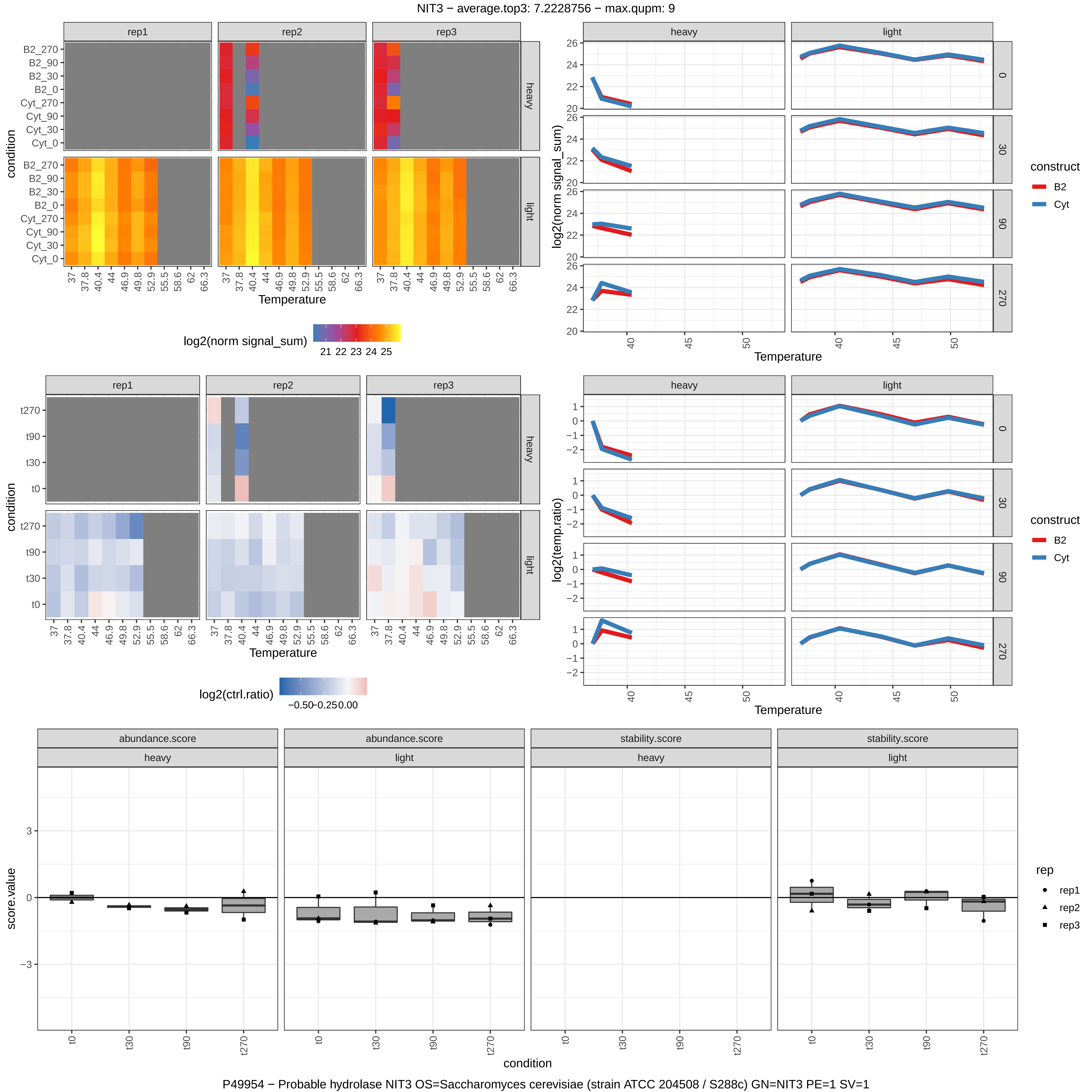

NIT3 − average.top3: 7.2228756 − max.qupm: 9
rep1
rep2
rep3
heavy
light
condition
Temperature
log2(norm signal_sum)
log2(ctrl.ratio)
log2(temp.ratio)
construct
B2
Cyt
abundance.score
stability.score
score.value
rep
P49954 − Probable hydrolase NIT3 OS=Saccharomyces cerevisiae (strain ATCC 204508 / S288c) GN=NIT3 PE=1 SV=1

NMA1 − average.top3: 6.5786352 − max.qupm: 5

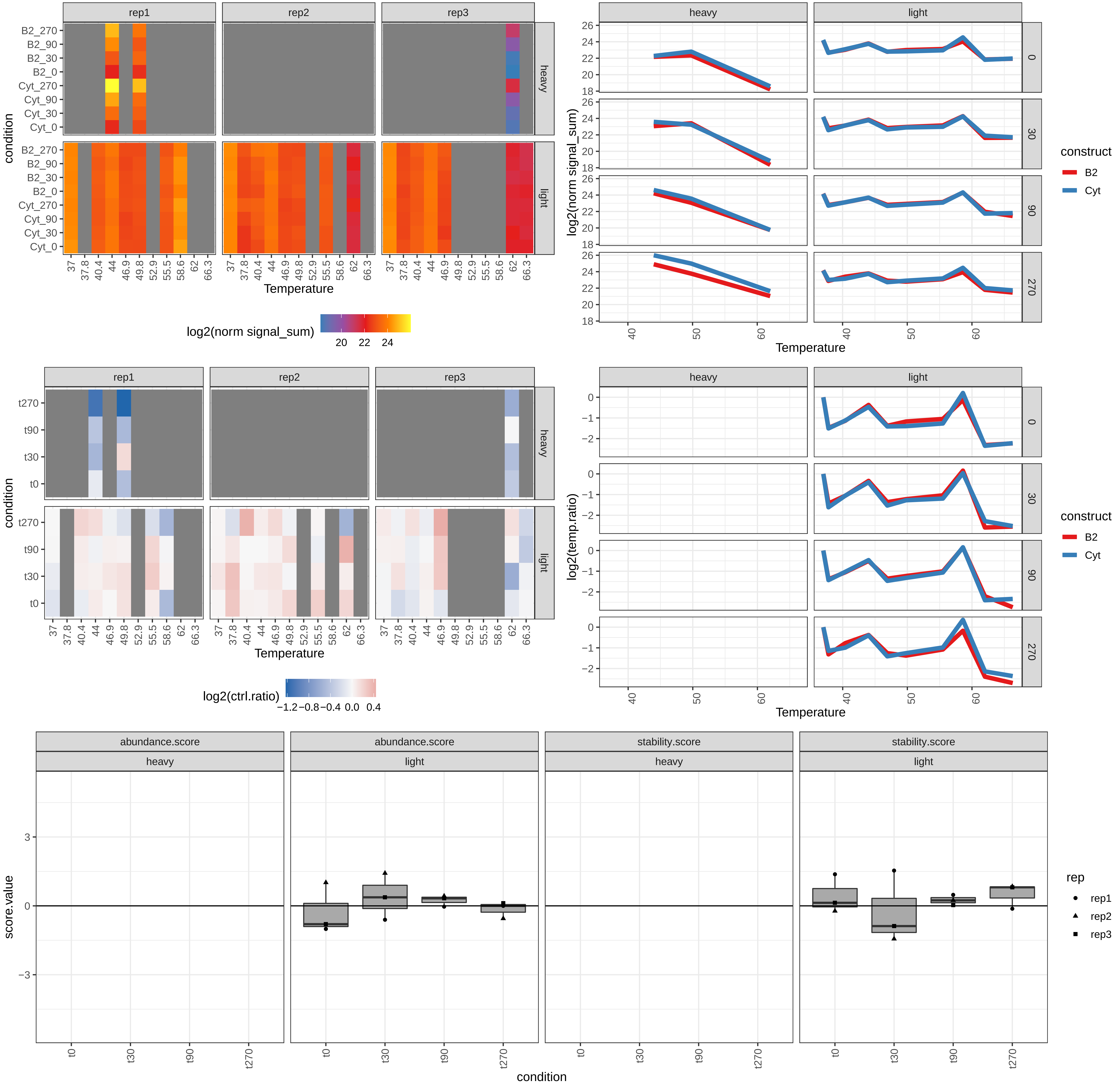

Q06178 − Nicotinamide/nicotinic acid mononucleotide adenylyltransferase 1 OS=Saccharomyces cerevisiae (strain ATCC 204508 / S288c) GN=NMA1 PE=1 SV=1

NMA111 − average.top3: 6.9267305 − max.qupm: 14

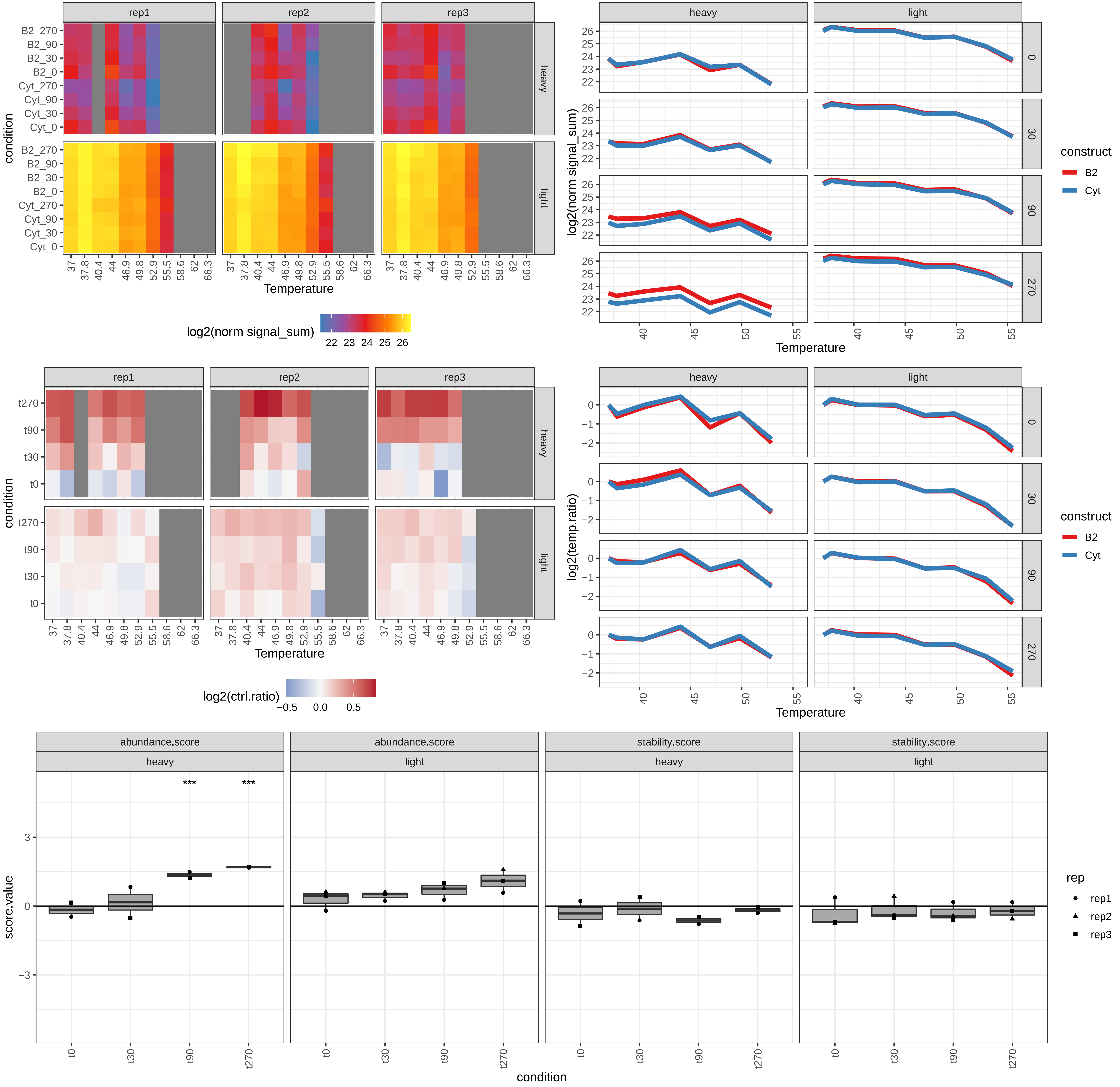

P53920 − Pro−apoptotic serine protease NMA111 OS=Saccharomyces cerevisiae (strain ATCC 204508 / S288c) GN=NMA111 PE=1 SV=1

NMA2 − average.top3: 6.563414375 − max.qupm: 4

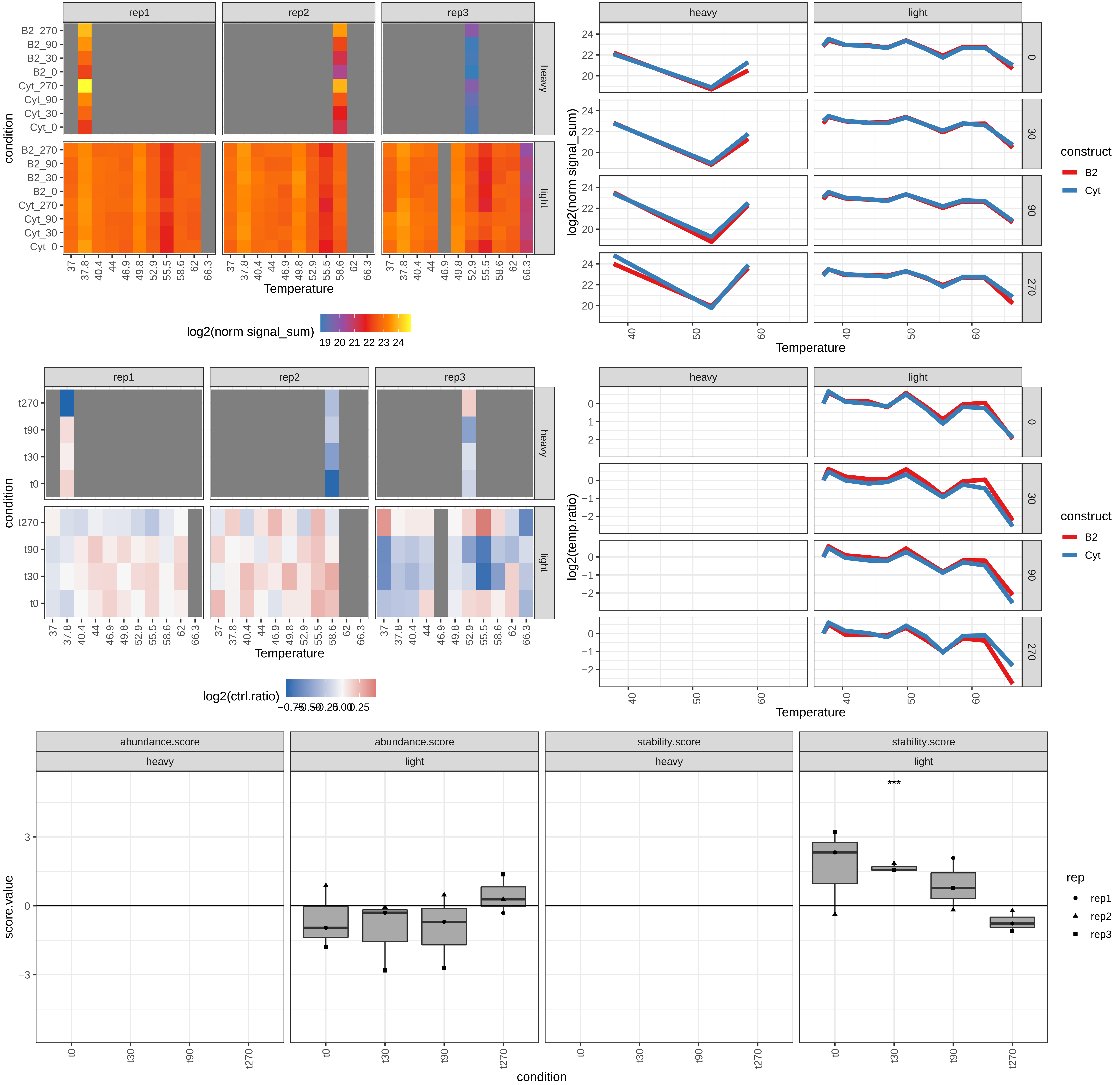

P53204 − Nicotinamide/nicotinic acid mononucleotide adenylyltransferase 2 OS=Saccharomyces cerevisiae (strain ATCC 204508 / S288c) GN=NMA2 PE=1 SV=1

NMD2 − average.top3: 6.83200857142857 − max.qupm: 11

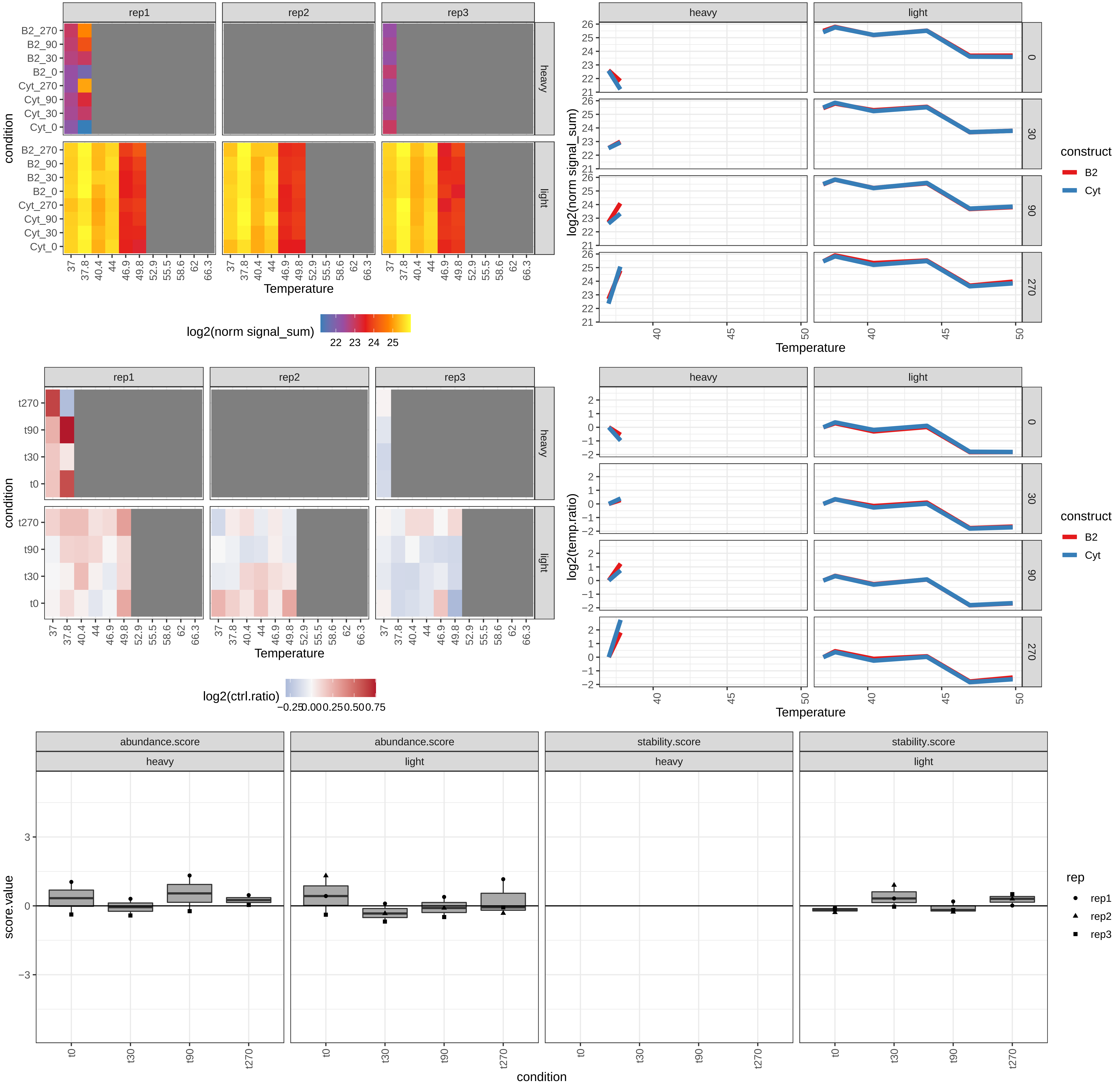

P38798 − Nonsense−mediated mRNA decay protein 2 OS=Saccharomyces cerevisiae (strain ATCC 204508 / S288c) GN=NMD2 PE=1 SV=2

NMD3 − average.top3: 6.92200481481481 − max.qupm: 10

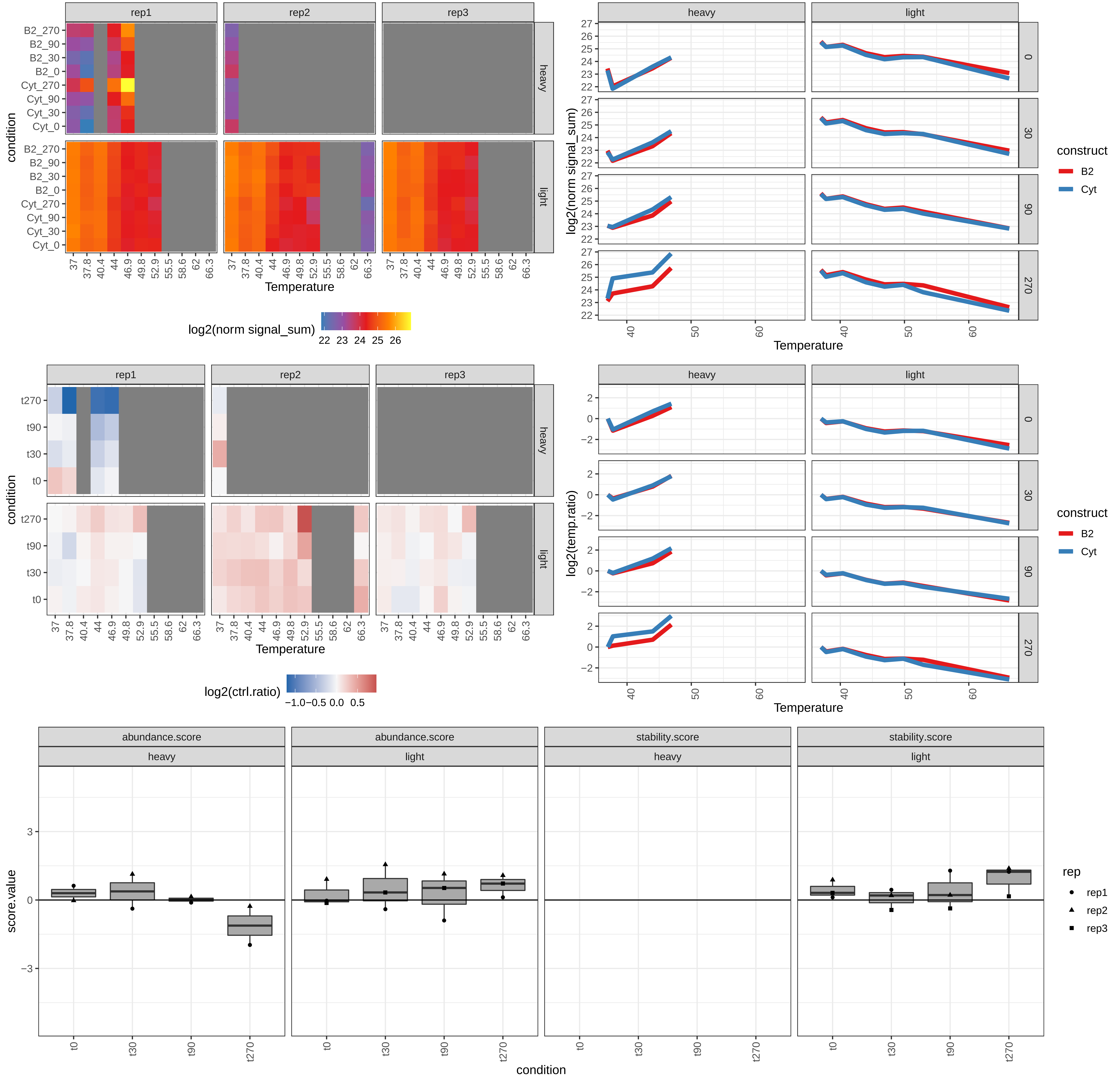

P38861 − 60S ribosomal export protein NMD3 OS=Saccharomyces cerevisiae (strain ATCC 204508 / S288c) GN=NMD3 PE=1 SV=3

NMD5 − average.top3: 6.64484 − max.qupm: 7

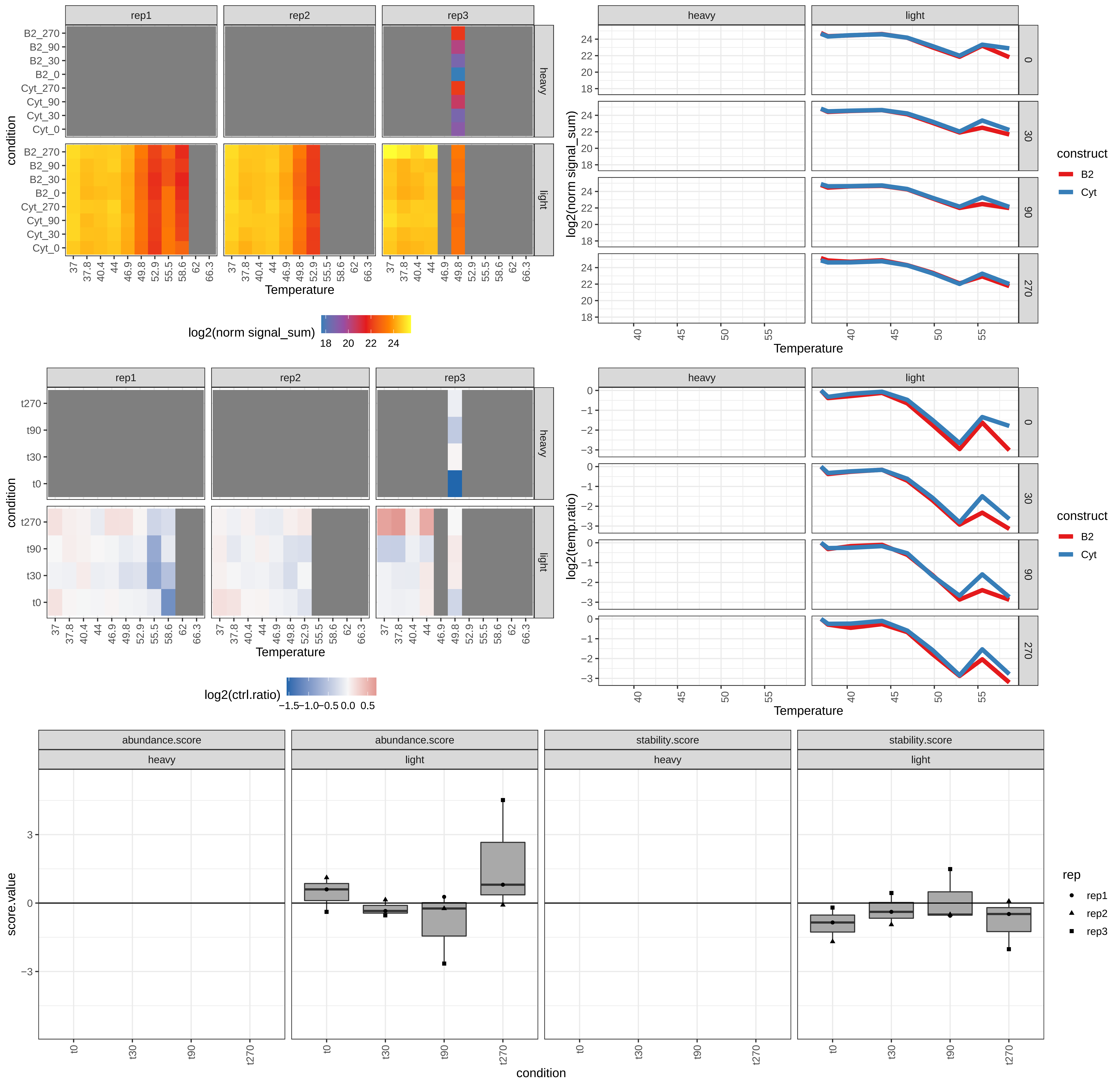

P46970 − Nonsense−mediated mRNA decay protein 5 OS=Saccharomyces cerevisiae (strain ATCC 204508 / S288c) GN=NMD5 PE=1 SV=2

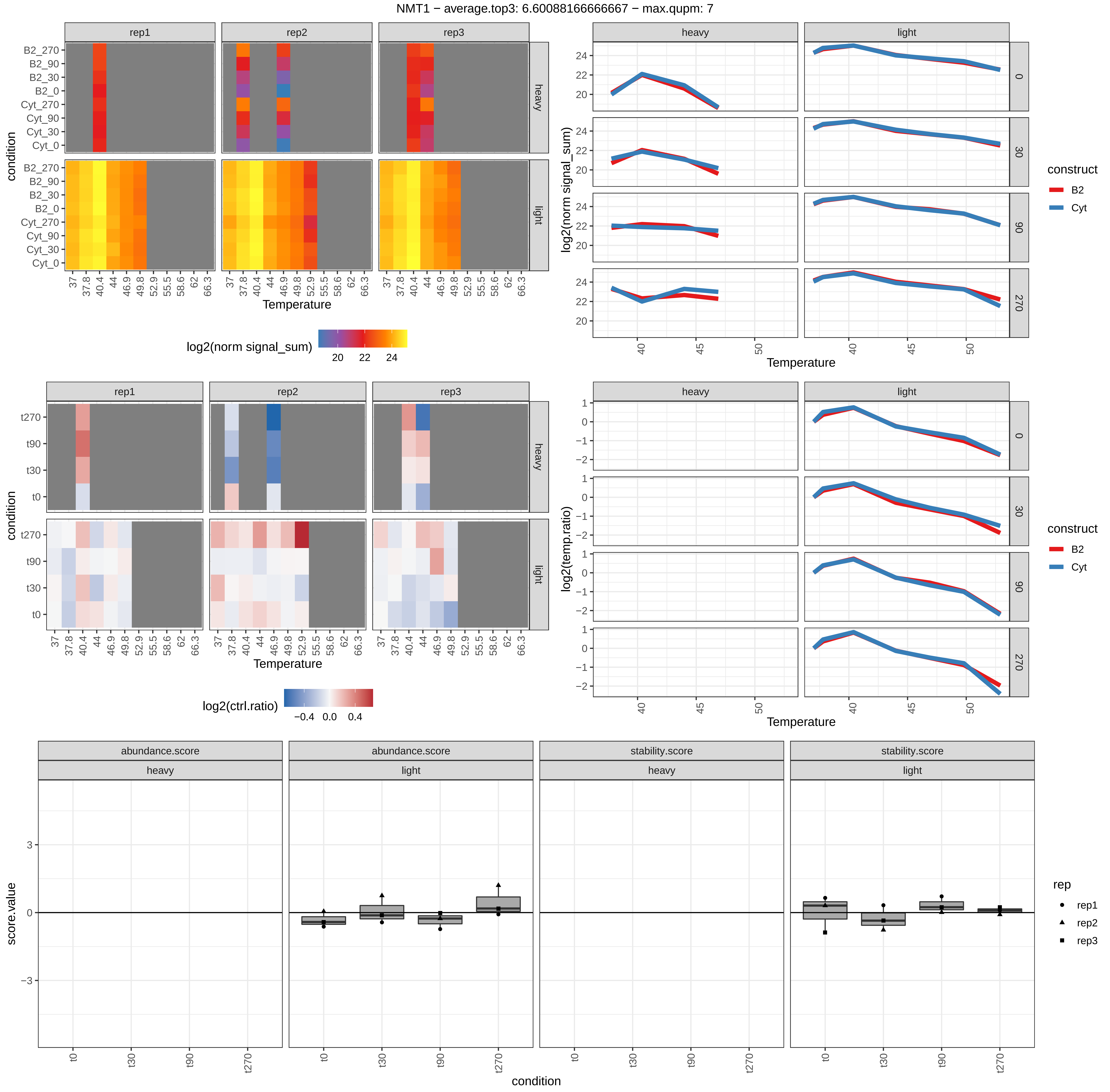

P14743 − Glycylpeptide N−tetradecanoyltransferase OS=Saccharomyces cerevisiae (strain ATCC 204508 / S288c) GN=NMT1 PE=1 SV=1

NNT1 − average.top3: 7.2060225 − max.qupm: 5

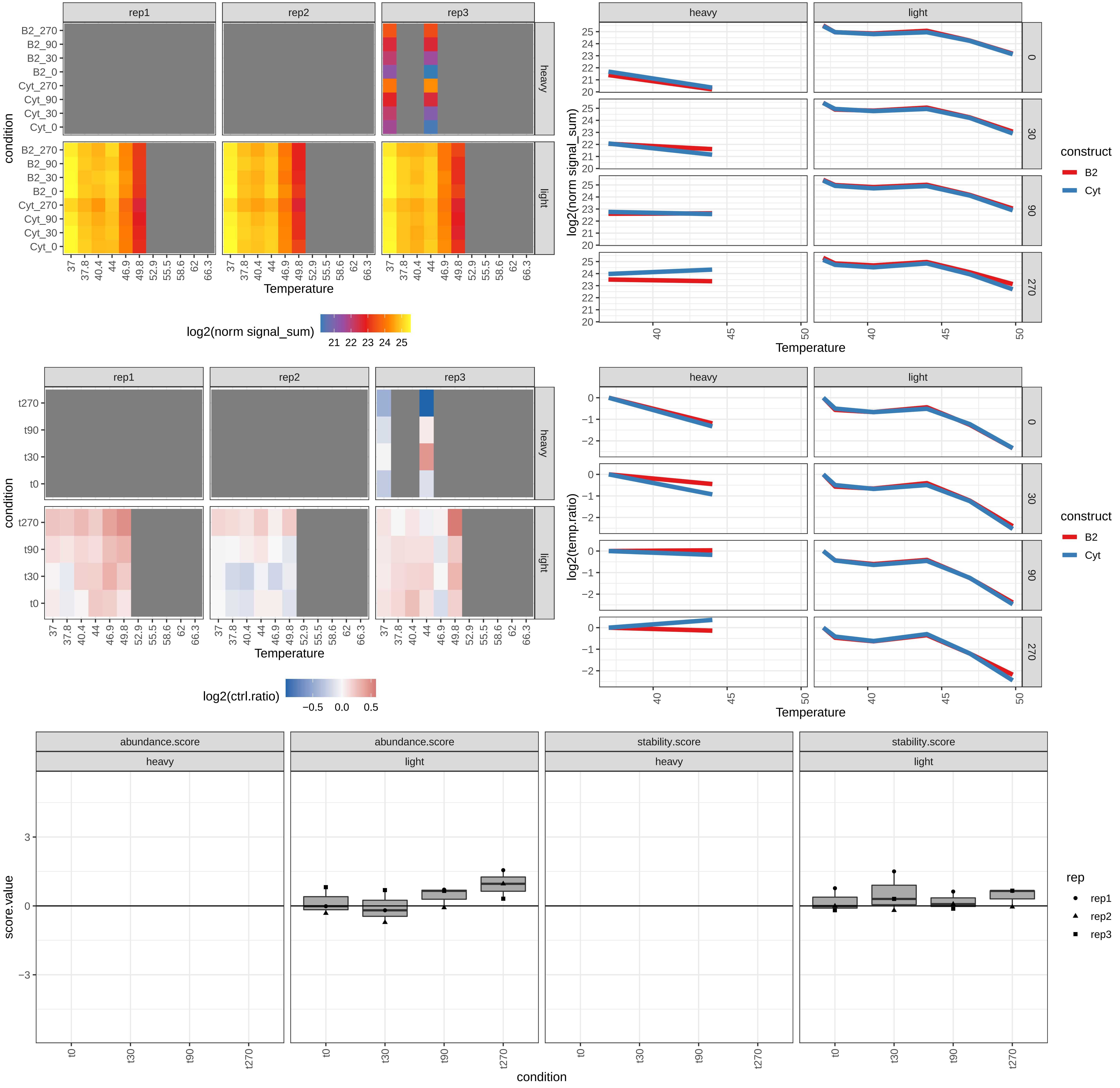

Q05874 − Protein N−methyltransferase NNT1 OS=Saccharomyces cerevisiae (strain ATCC 204508 / S288c) GN=NNT1 PE=1 SV=1

NOB1 − average.top3: 6.83397285714286 − max.qupm: 4

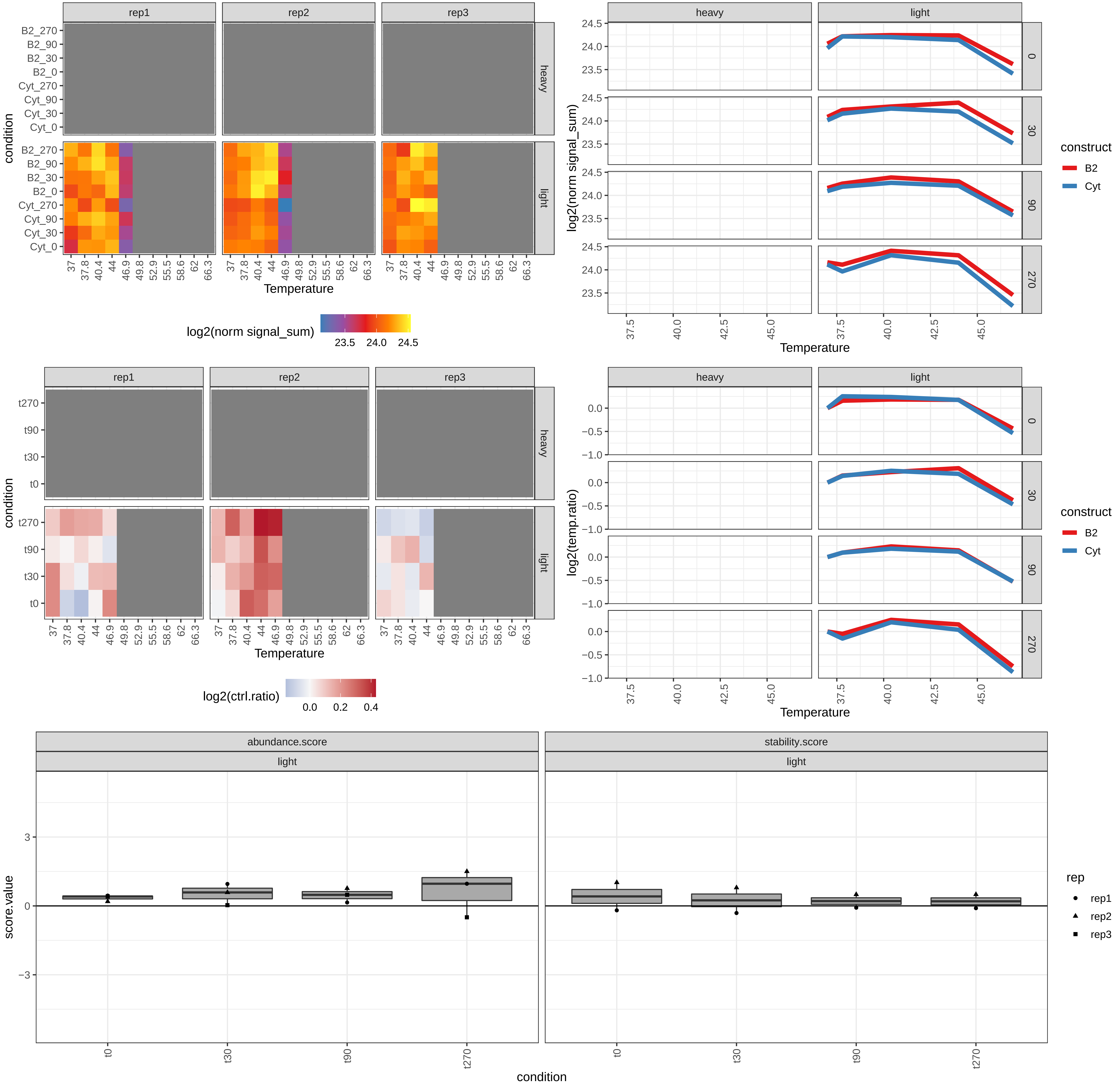

Q08444 − 20S−pre−rRNA D−site endonuclease NOB1 OS=Saccharomyces cerevisiae (strain ATCC 204508 / S288c) GN=NOB1 PE=1 SV=1

NOC2 − average.top3: 7.15395741935484 − max.qupm: 18

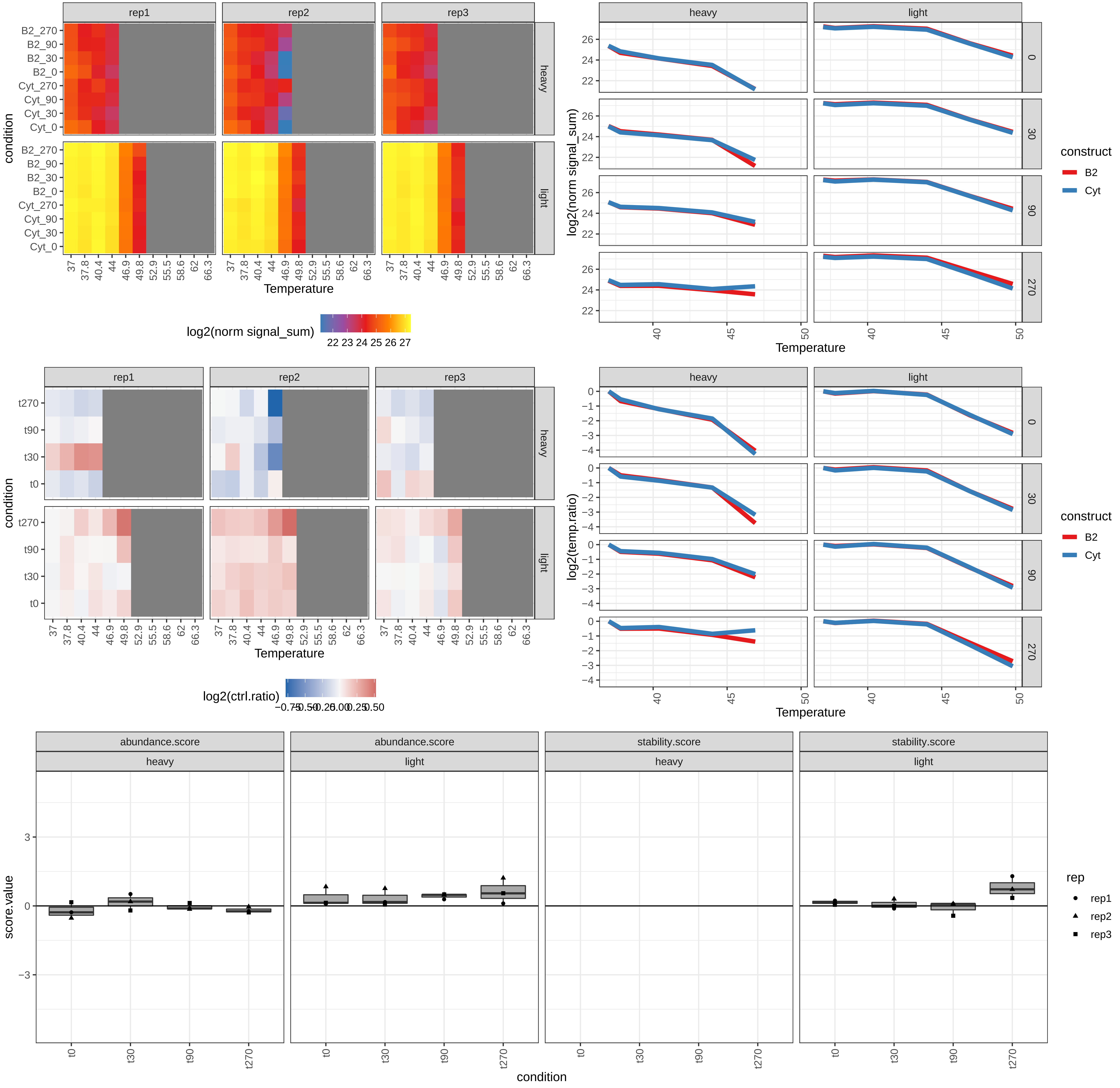

P39744 − Nucleolar complex protein 2 OS=Saccharomyces cerevisiae (strain ATCC 204508 / S288c) GN=NOC2 PE=1 SV=2

NOC4 − average.top3: 6.32201454545455 − max.qupm: 4

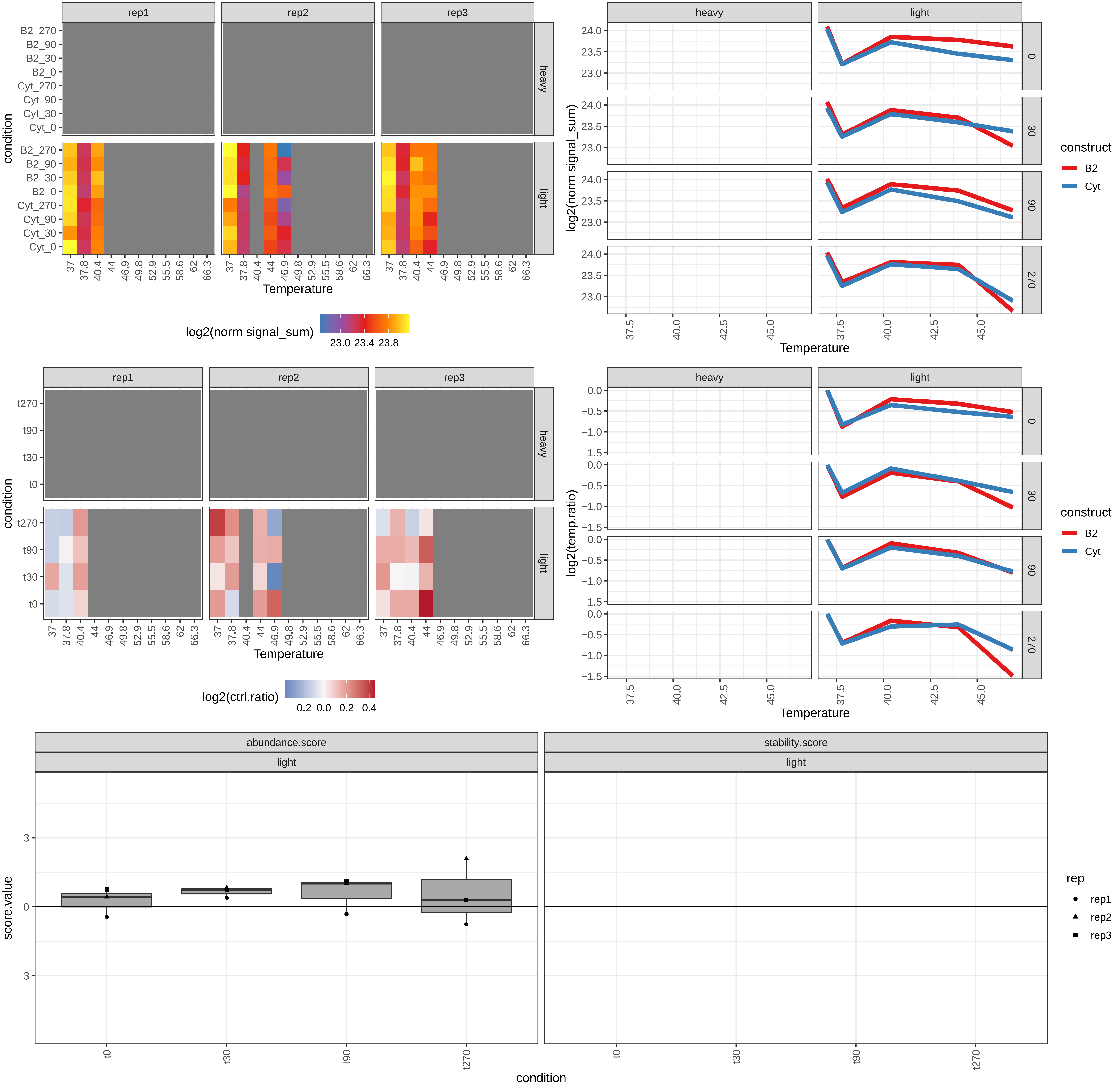

Q06512 − Nucleolar complex protein 4 OS=Saccharomyces cerevisiae (strain ATCC 204508 / S288c) GN=NOC4 PE=1 SV=1

NOG1 − average.top3: 6.79058352941176 − max.qupm: 14

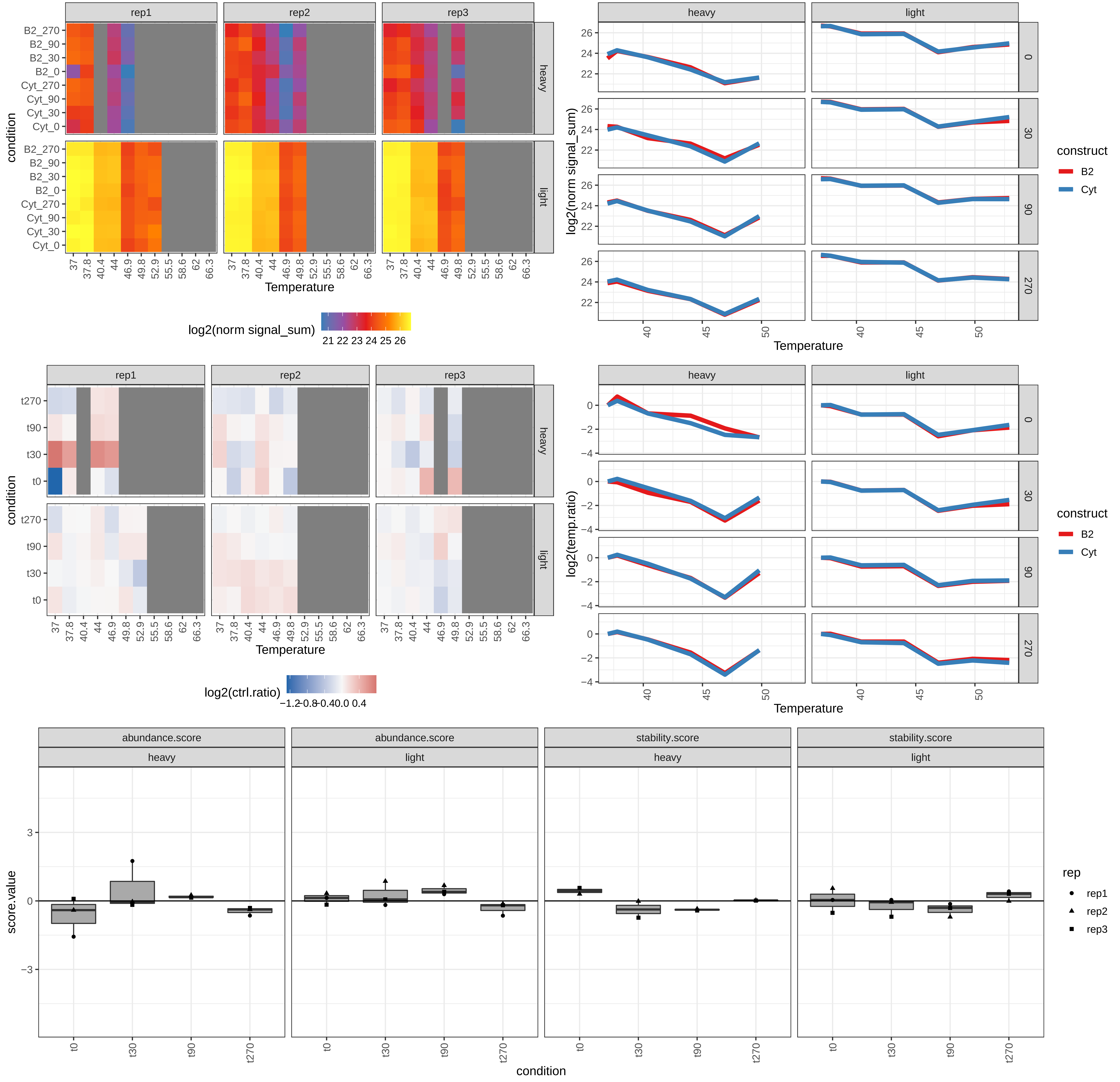

Q02892 − Nucleolar GTP−binding protein 1 OS=Saccharomyces cerevisiae (strain ATCC 204508 / S288c) GN=NOG1 PE=1 SV=1

NOG2 − average.top3: 6.4878 − max.qupm: 6

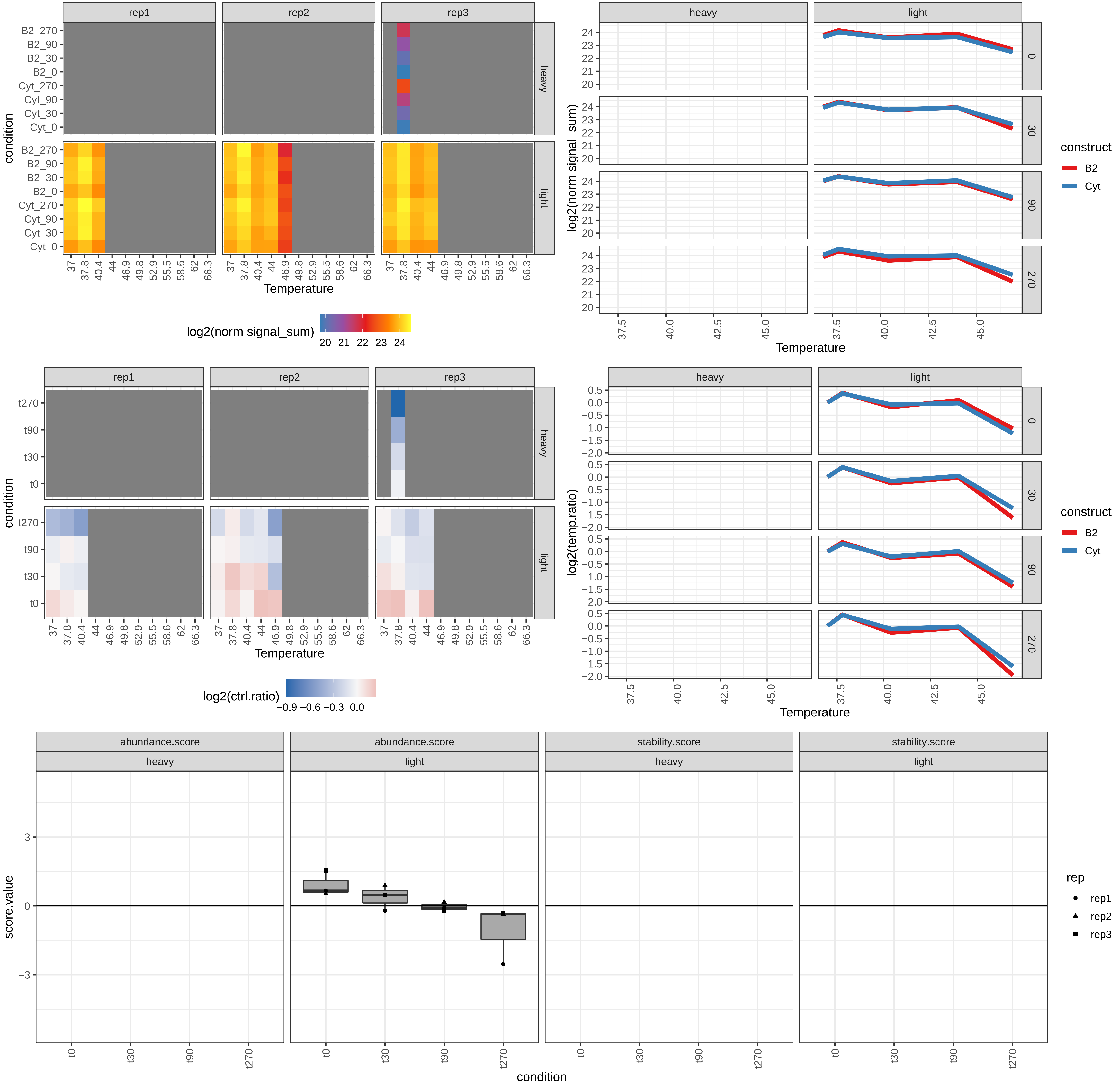

P53742 − Nucleolar GTP−binding protein 2 OS=Saccharomyces cerevisiae (strain ATCC 204508 / S288c) GN=NOG2 PE=1 SV=1

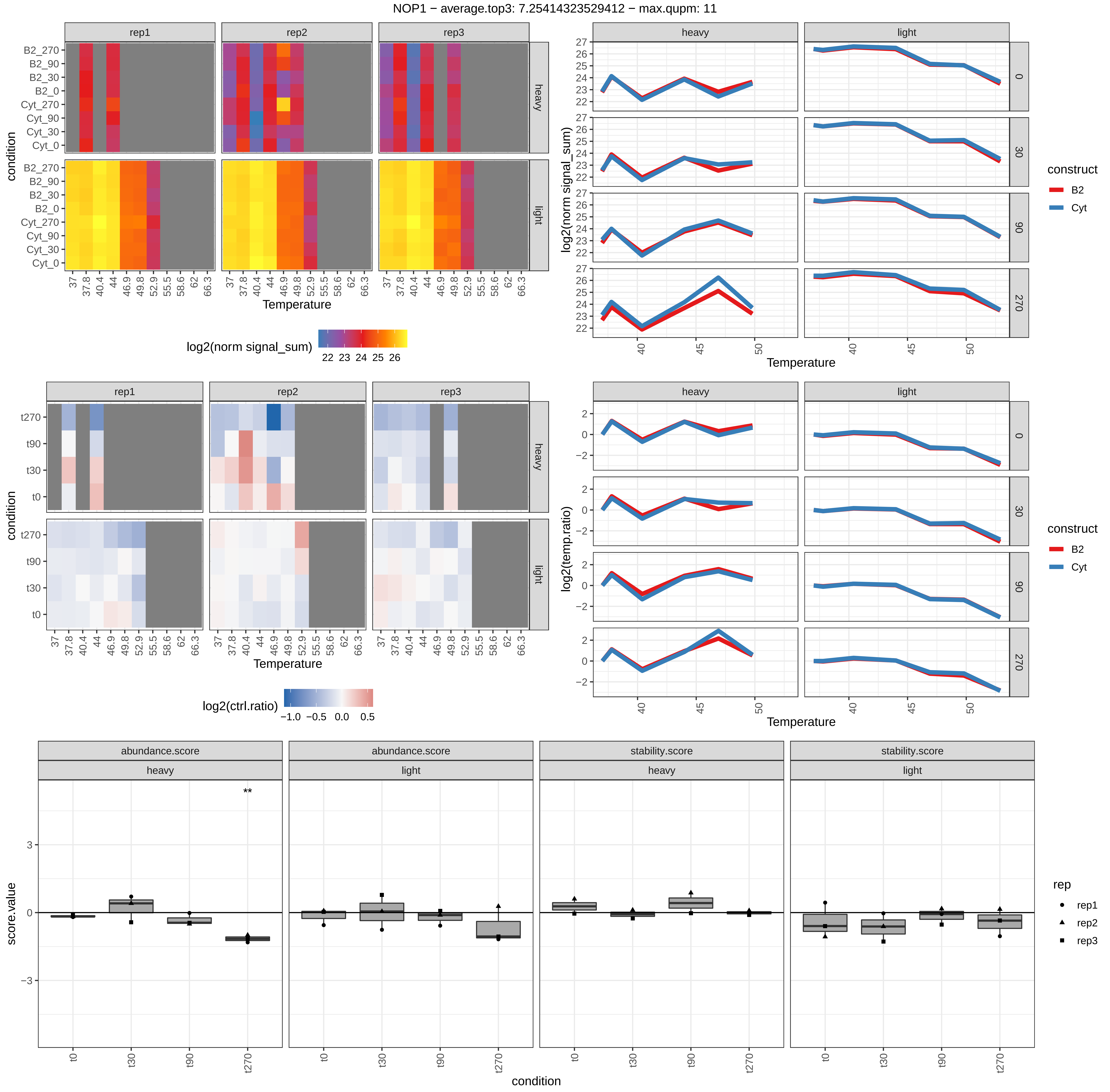

P15646 − rRNA 2'−O−methyltransferase fibrillarin OS=Saccharomyces cerevisiae (strain ATCC 204508 / S288c) GN=NOP1 PE=1 SV=1

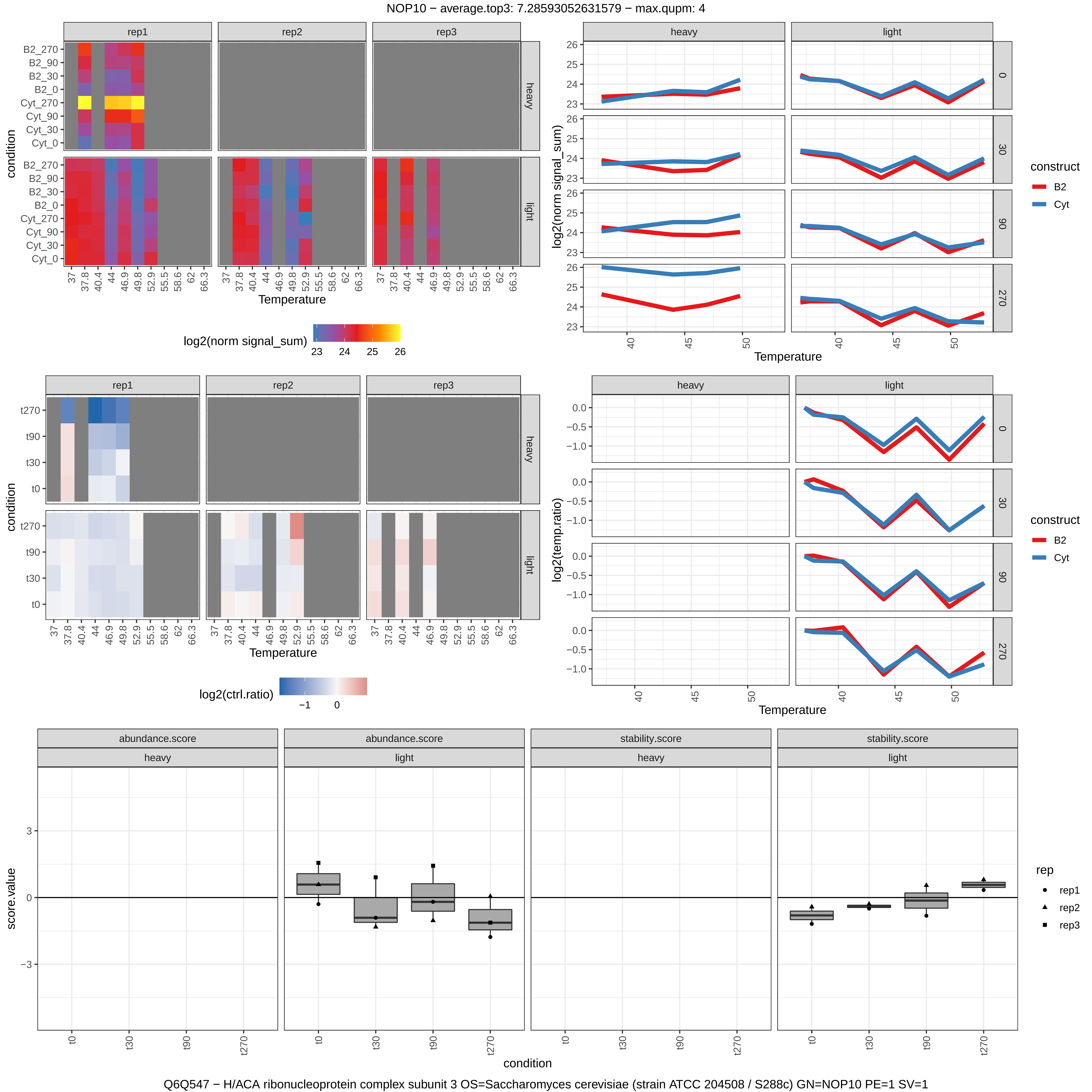

NOP10 − average.top3: 7.28593052631579 − max.qupm: 4
rep1
rep2
rep3
heavy
light
condition
Temperature
log2(norm signal_sum)
log2(ctrl.ratio)
log2(temp.ratio)
construct
B2
Cyt
abundance.score
stability.score
score.value
rep
rep1
rep2
rep3
Q6Q547 − H/ACA ribonucleoprotein complex subunit 3 OS=Saccharomyces cerevisiae (strain ATCC 204508 / S288c) GN=NOP10 PE=1 SV=1

NOP12 − average.top3: 6.86700166666667 − max.qupm: 9

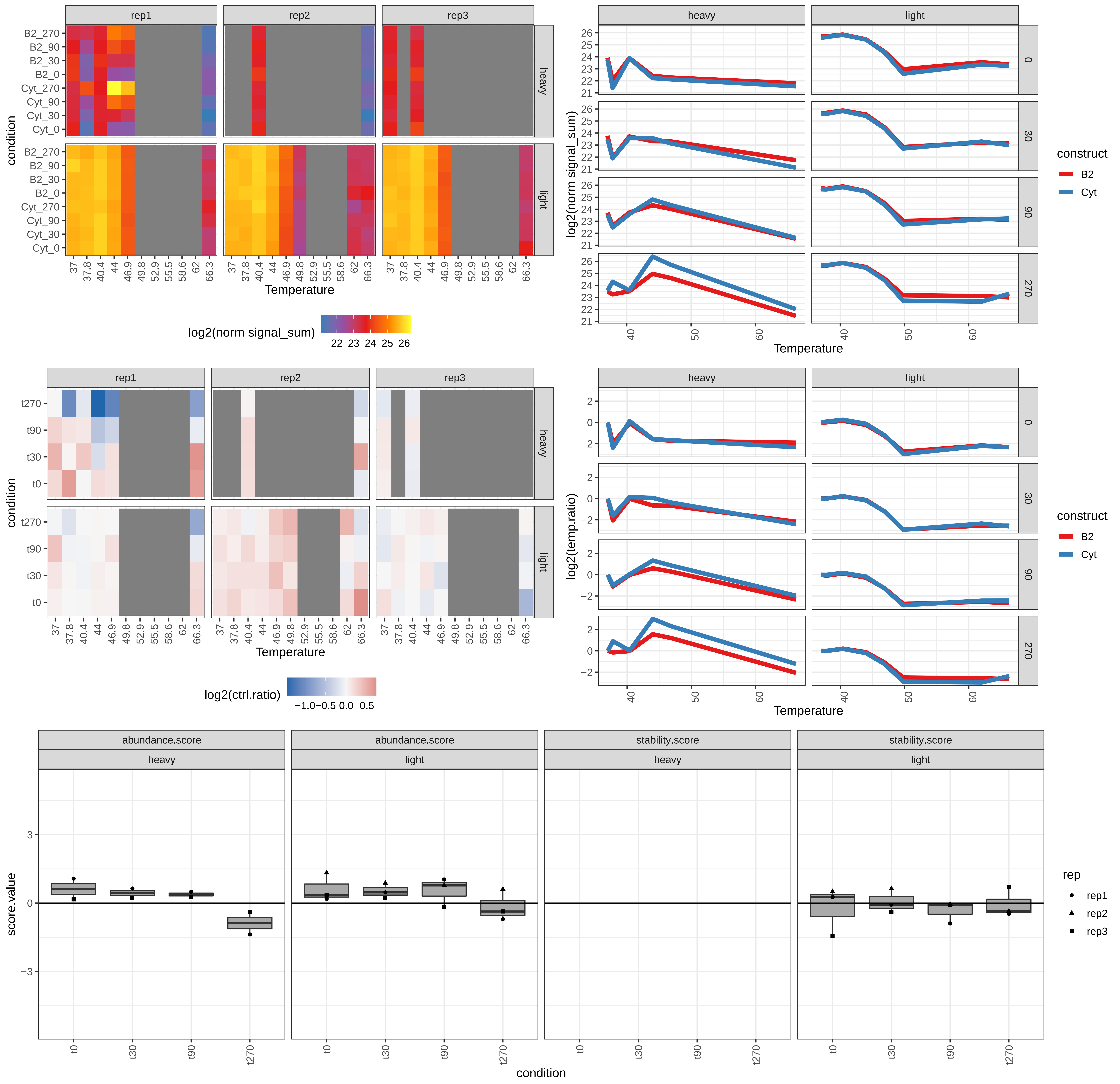

Q08208 − Nucleolar protein 12 OS=Saccharomyces cerevisiae (strain ATCC 204508 / S288c) GN=NOP12 PE=1 SV=1

NOP13 − average.top3: 6.86058457142857 − max.qupm: 14

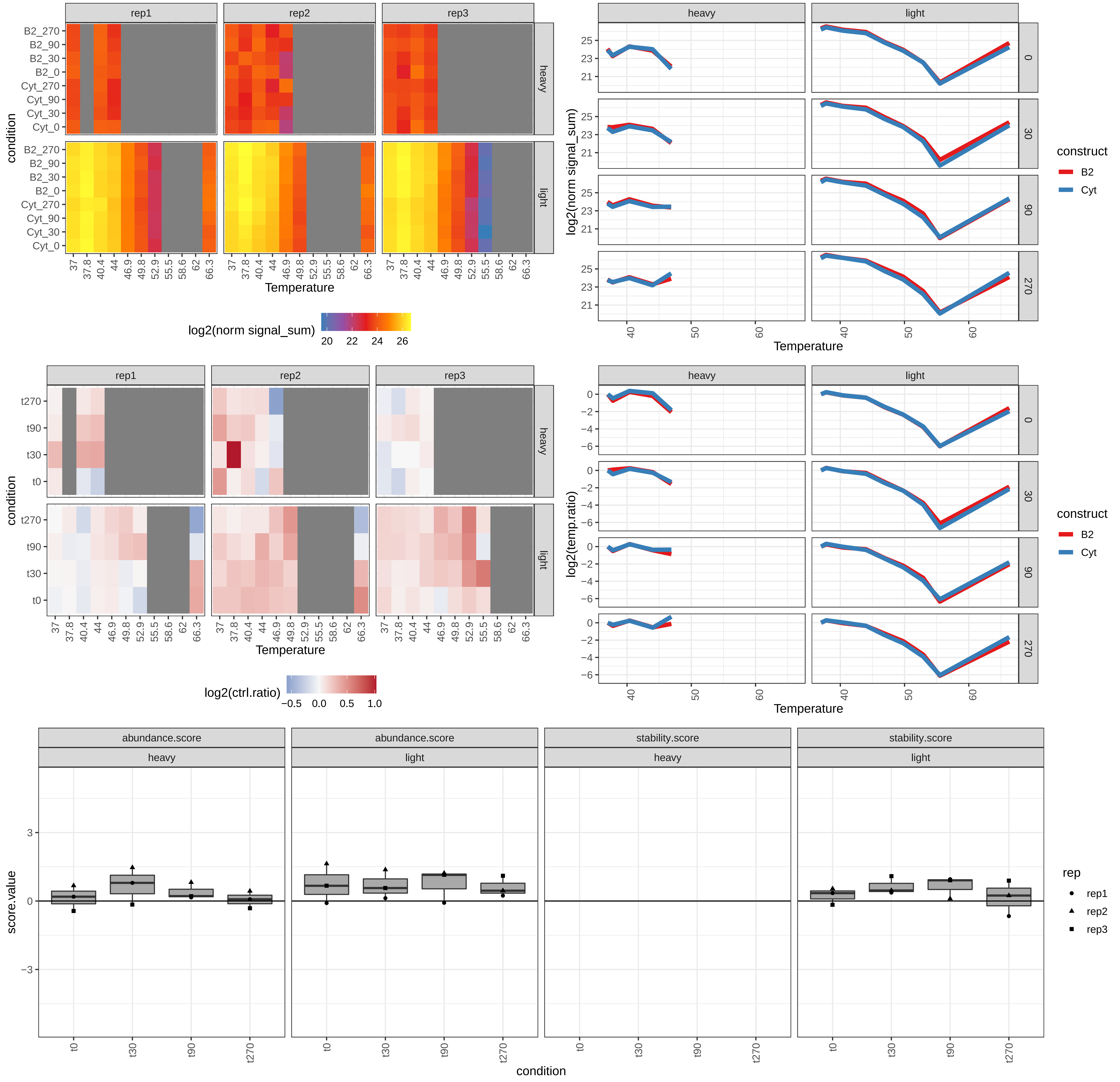

P53883 − Nucleolar protein 13 OS=Saccharomyces cerevisiae (strain ATCC 204508 / S288c) GN=NOP13 PE=1 SV=2

NOP14 − average.top3: 6.82660586206897 − max.qupm: 9

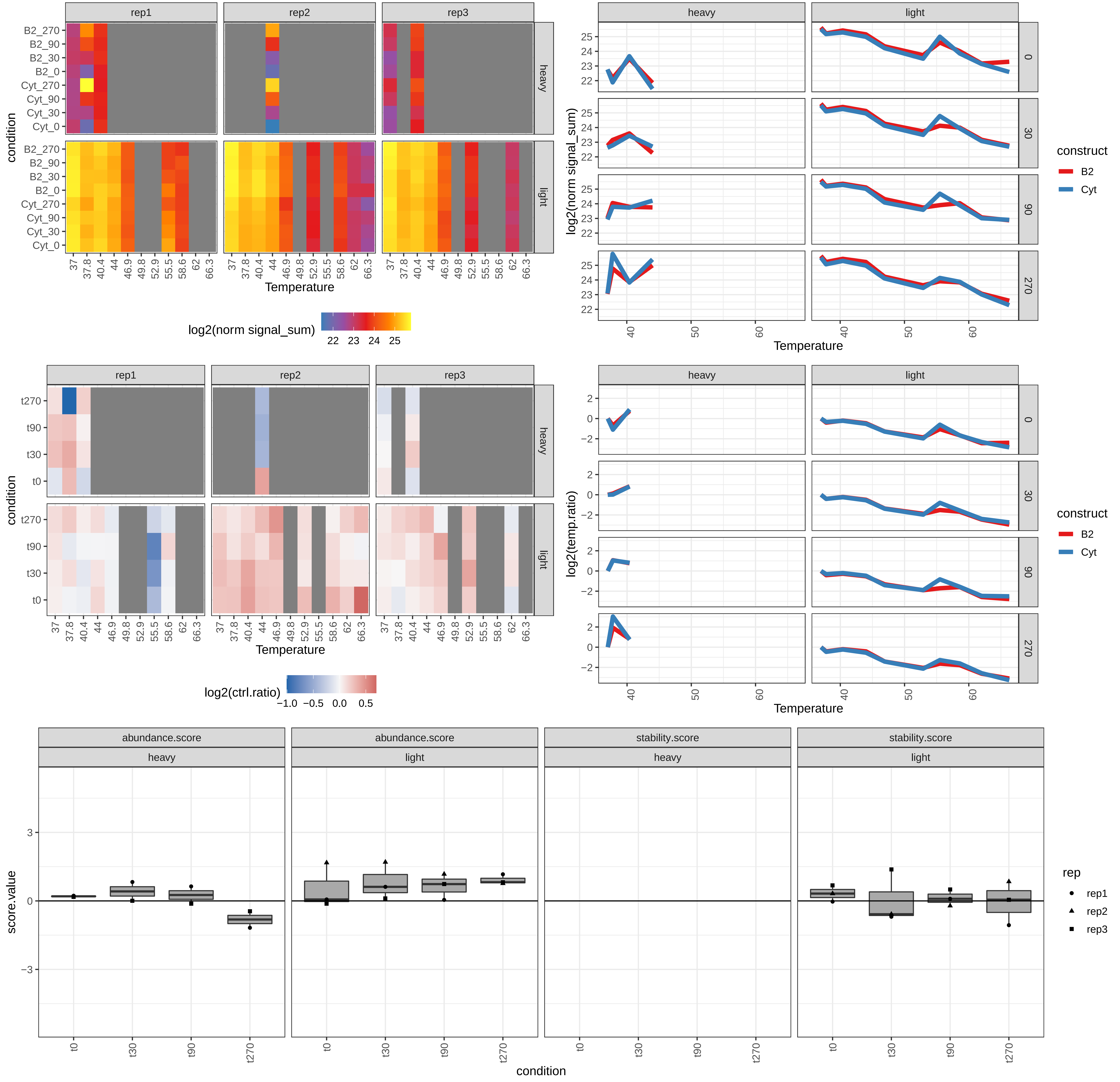

Q99207 − Nucleolar complex protein 14 OS=Saccharomyces cerevisiae (strain ATCC 204508 / S288c) GN=NOP14 PE=1 SV=1

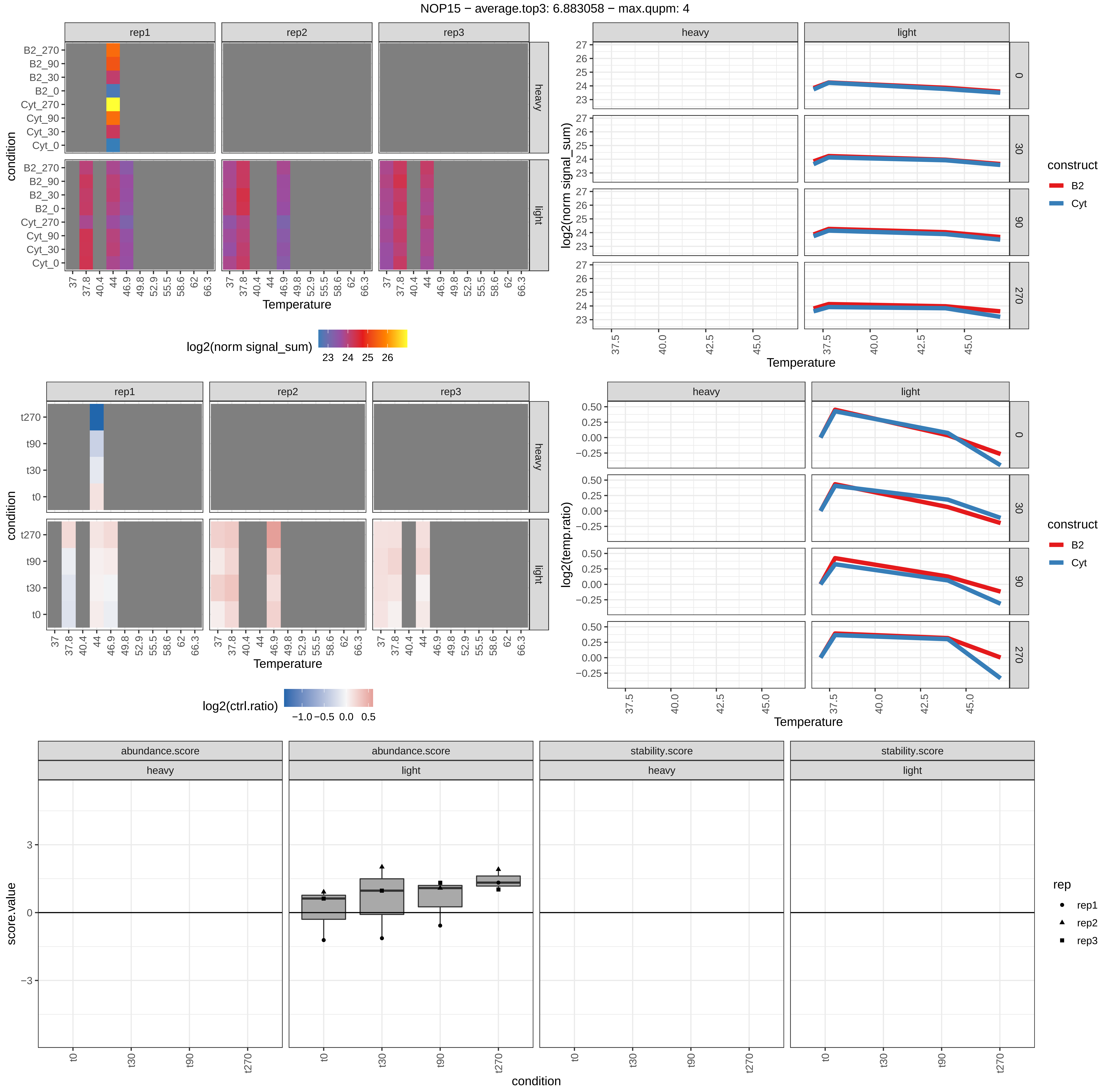

P53927 − Ribosome biogenesis protein 15 OS=Saccharomyces cerevisiae (strain ATCC 204508 / S288c) GN=NOP15 PE=1 SV=1

NOP16 − average.top3: 6.7532212 − max.qupm: 5

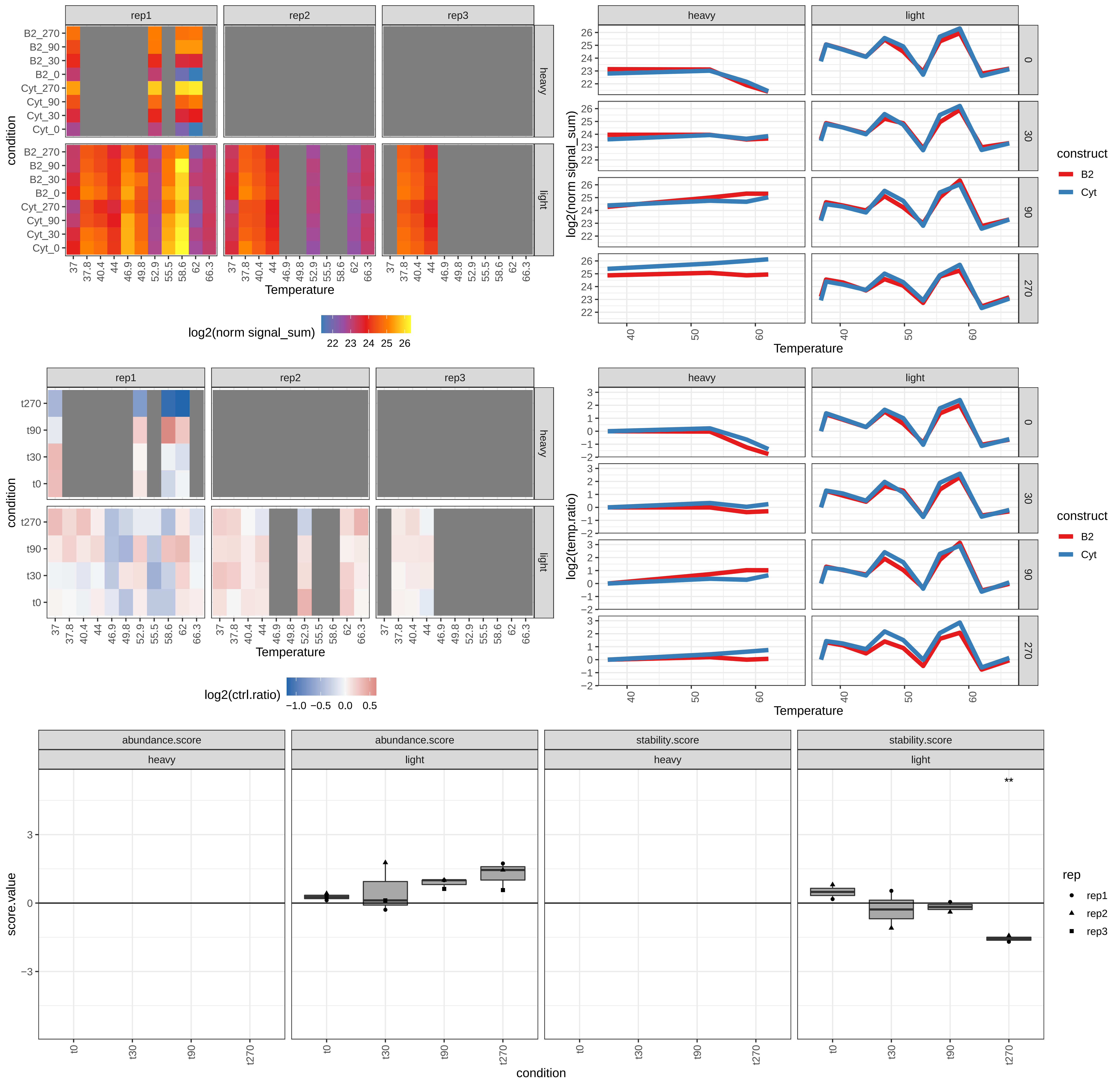

P40007 − Nucleolar protein 16 OS=Saccharomyces cerevisiae (strain ATCC 204508 / S288c) GN=NOP16 PE=1 SV=1

NOP2 − average.top3: 6.88766171428571 − max.qupm: 13

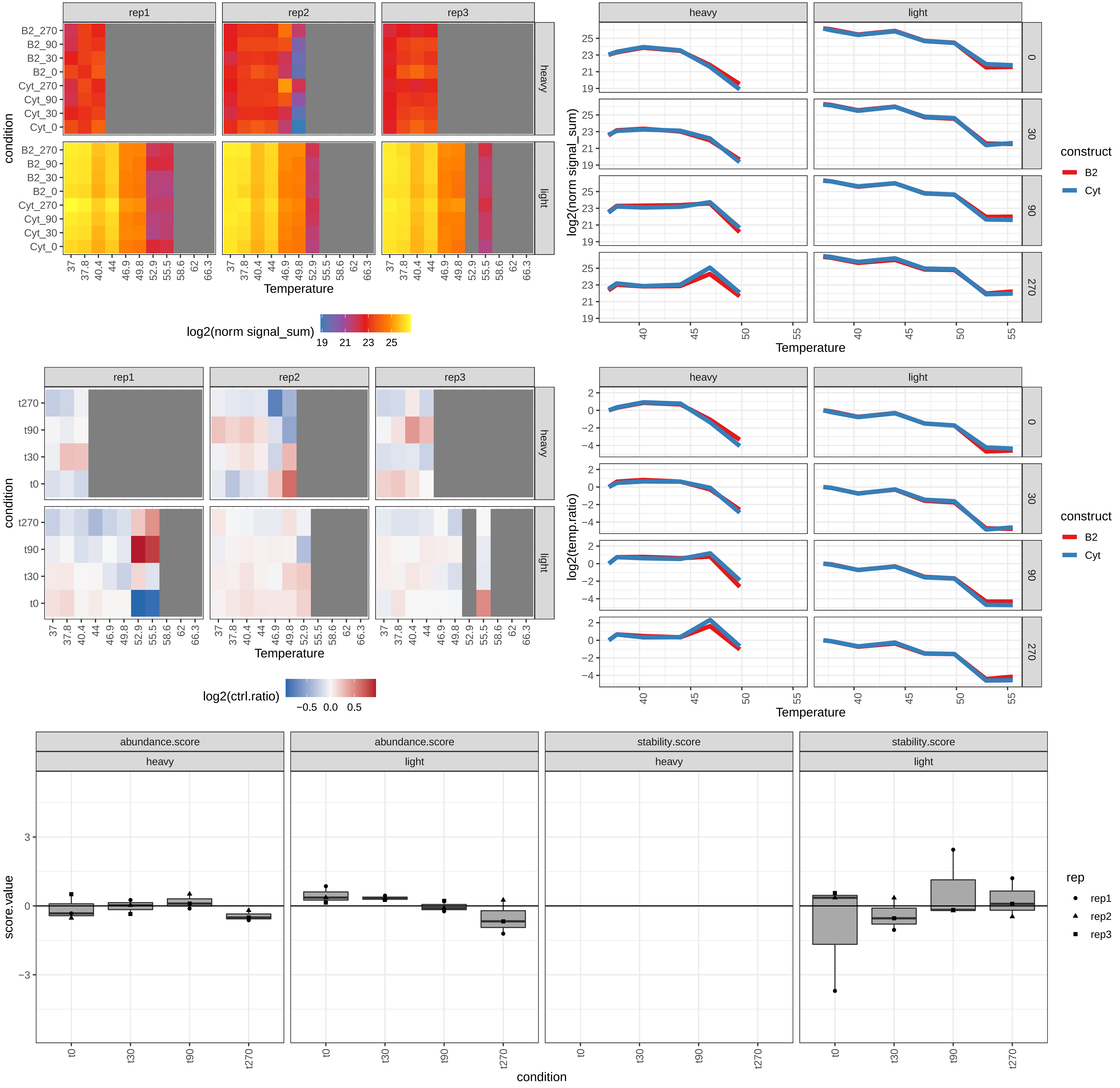

P40991 − 25S rRNA (cytosine(2870)−C(5))−methyltransferase OS=Saccharomyces cerevisiae (strain ATCC 204508 / S288c) GN=NOP2 PE=1 SV=1

NOP4 − average.top3: 6.572355625 − max.qupm: 5

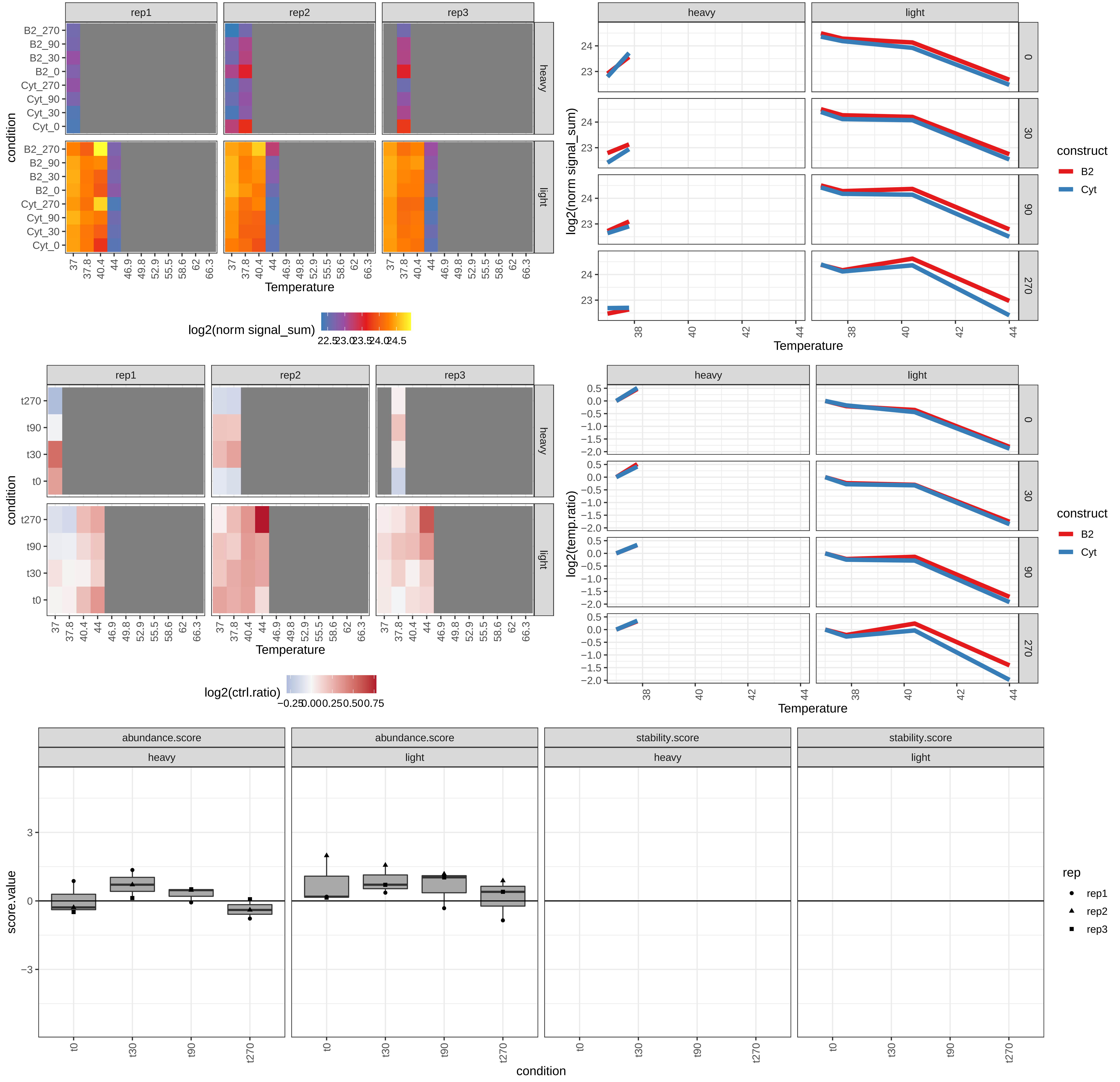

P37838 − Nucleolar protein 4 OS=Saccharomyces cerevisiae (strain ATCC 204508 / S288c) GN=NOP4 PE=1 SV=1

NOP53 − average.top3: 6.76766833333333 − max.qupm: 5

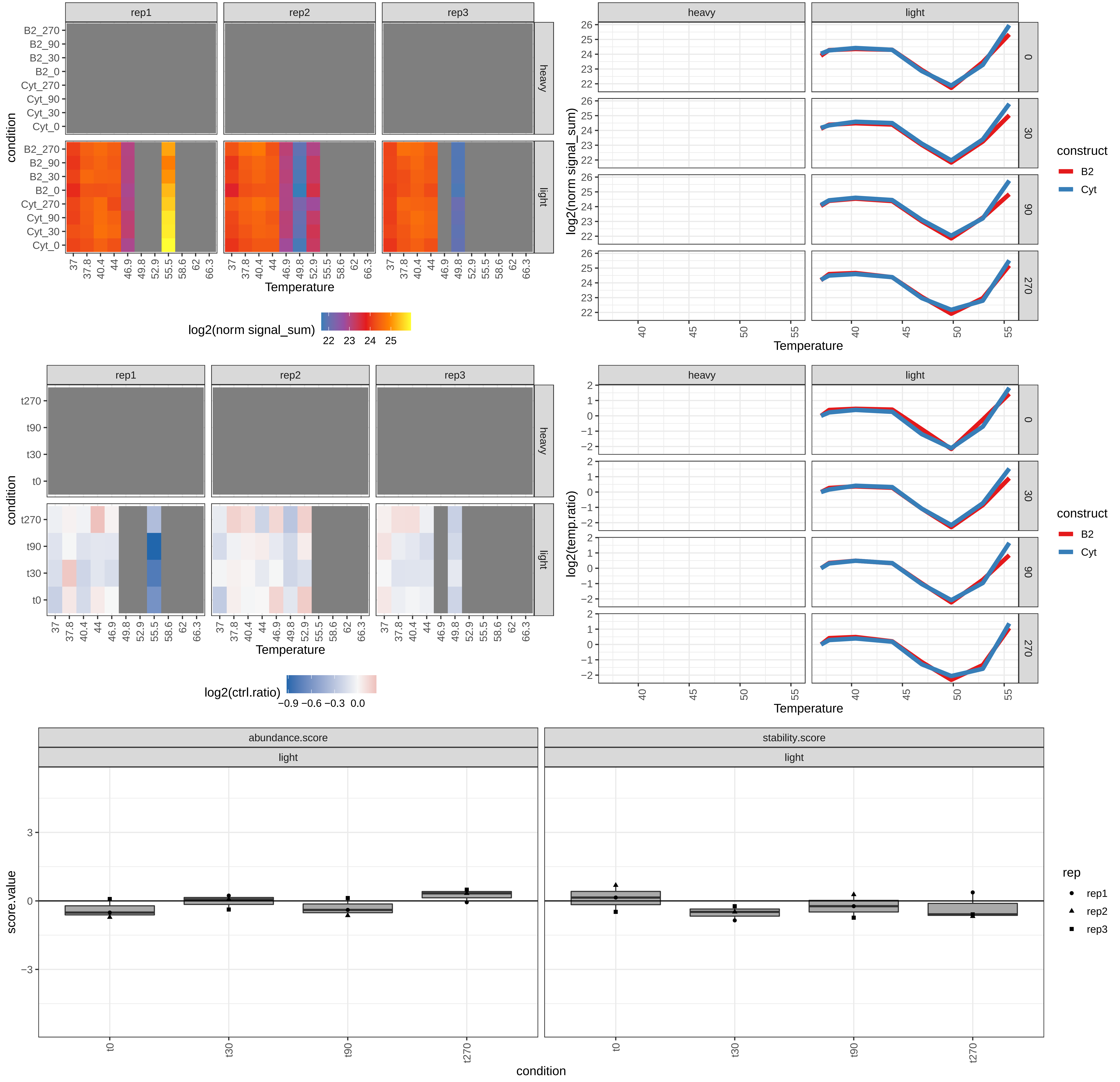

Q12080 − Ribosome biogenesis protein NOP53 OS=Saccharomyces cerevisiae (strain ATCC 204508 / S288c) GN=NOP53 PE=1 SV=1

NOP56 − average.top3: 7.24514160714286 − max.qupm: 17

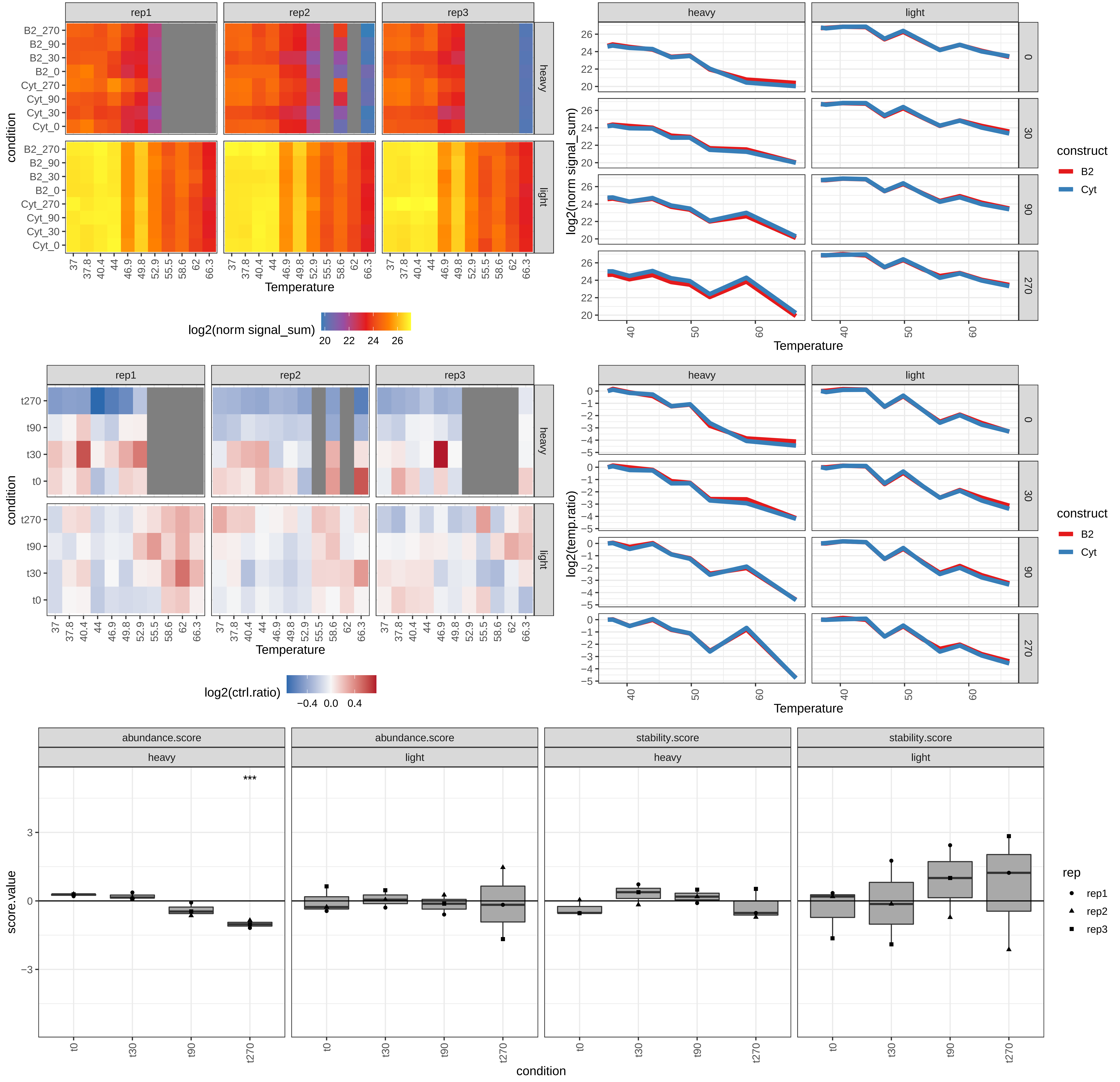

Q12460 − Nucleolar protein 56 OS=Saccharomyces cerevisiae (strain ATCC 204508 / S288c) GN=NOP56 PE=1 SV=1

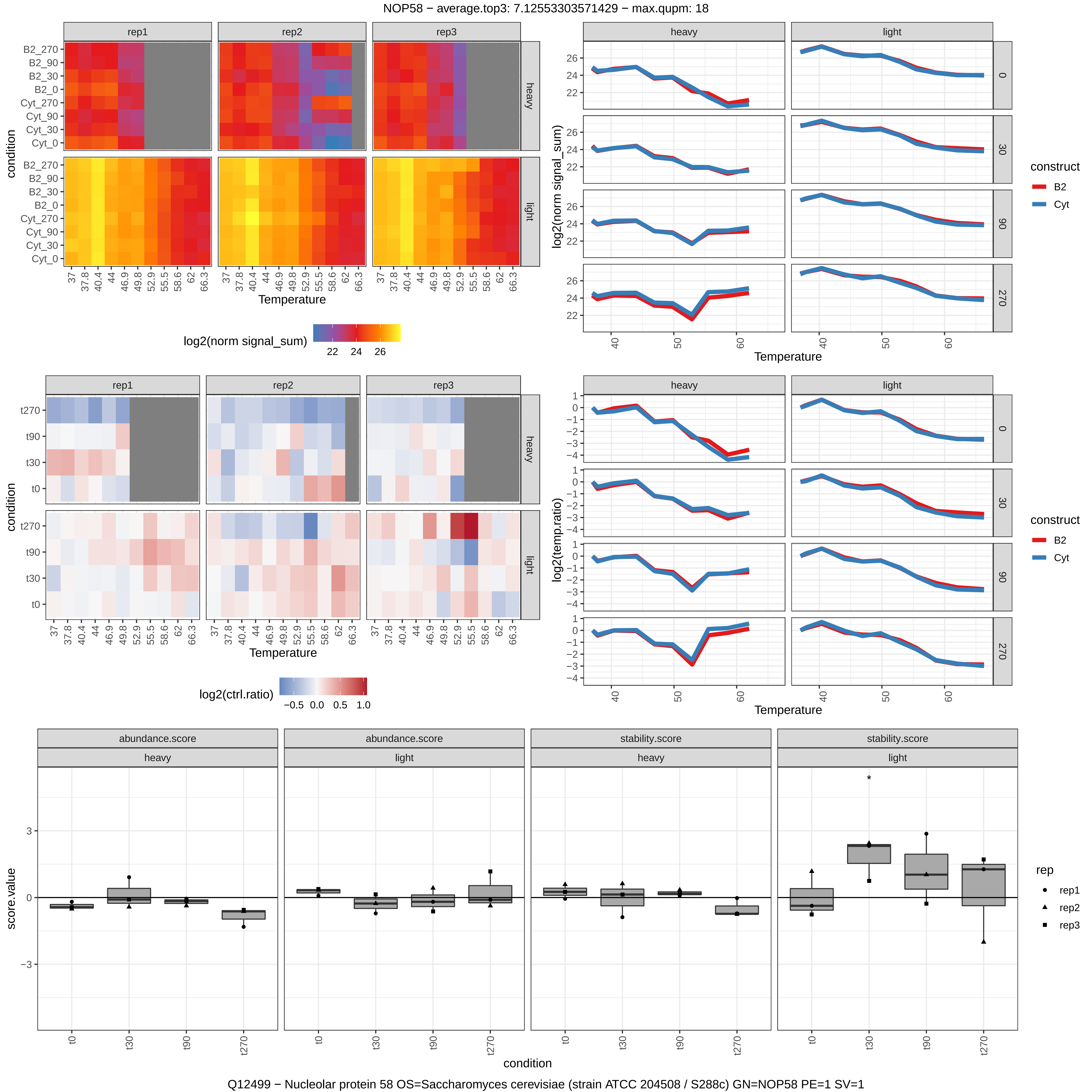
NOP58 − average.top3: 7.12553303571429 − max.qupm: 18
rep1
rep2
rep3
heavy
light
condition
Temperature
log2(norm signal_sum)
log2(ctrl.ratio)
log2(temp.ratio)
construct
B2
Cyt
abundance.score
stability.score
score.value
rep
rep1
rep2
rep3
Q12499 − Nucleolar protein 58 OS=Saccharomyces cerevisiae (strain ATCC 204508 / S288c) GN=NOP58 PE=1 SV=1

NOP6 − average.top3: 6.94939977777778 − max.qupm: 8

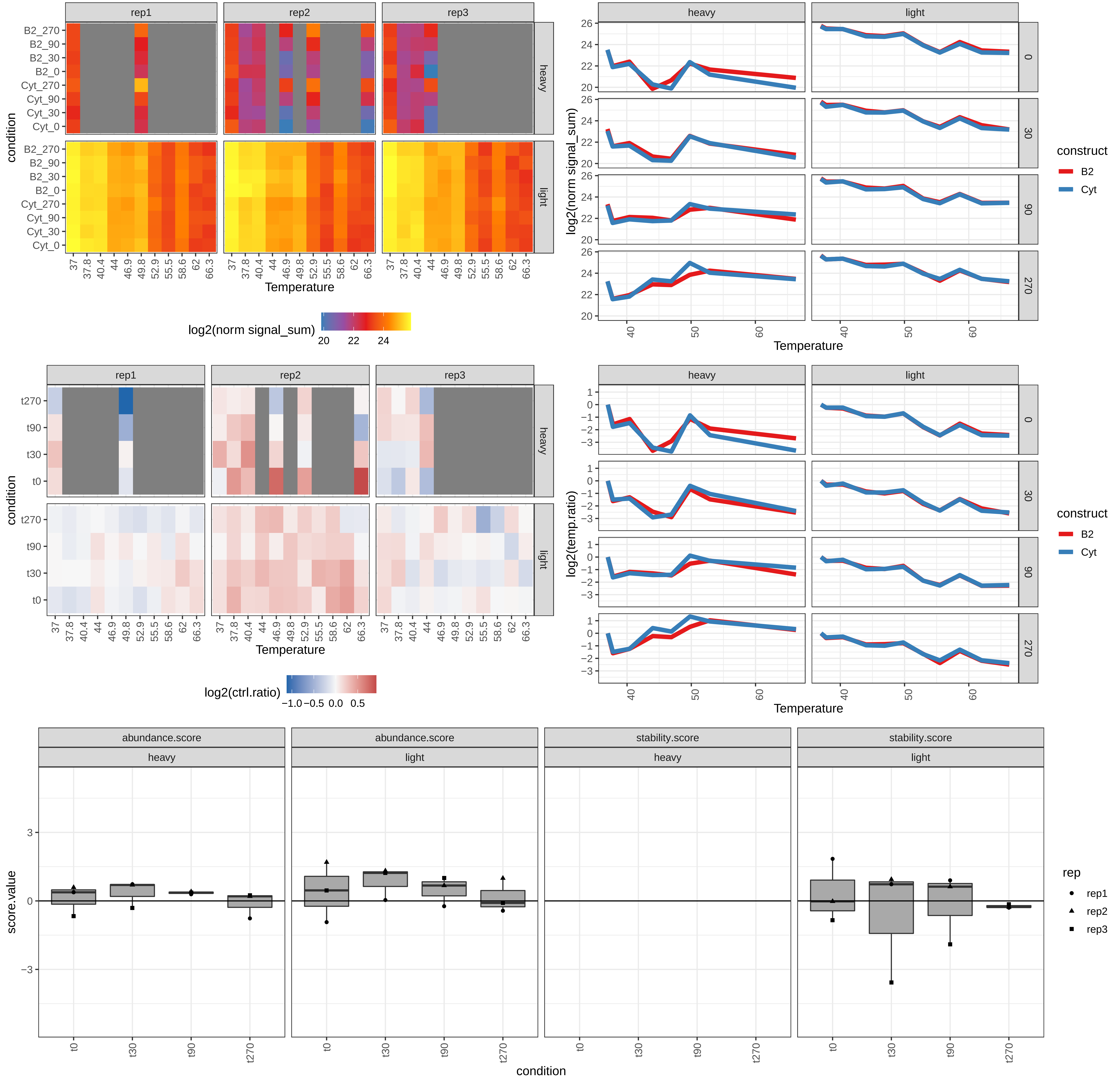

Q07623 − Nucleolar protein 6 OS=Saccharomyces cerevisiae (strain ATCC 204508 / S288c) GN=NOP6 PE=1 SV=1

NOP7 − average.top3: 7.04005464285714 − max.qupm: 12

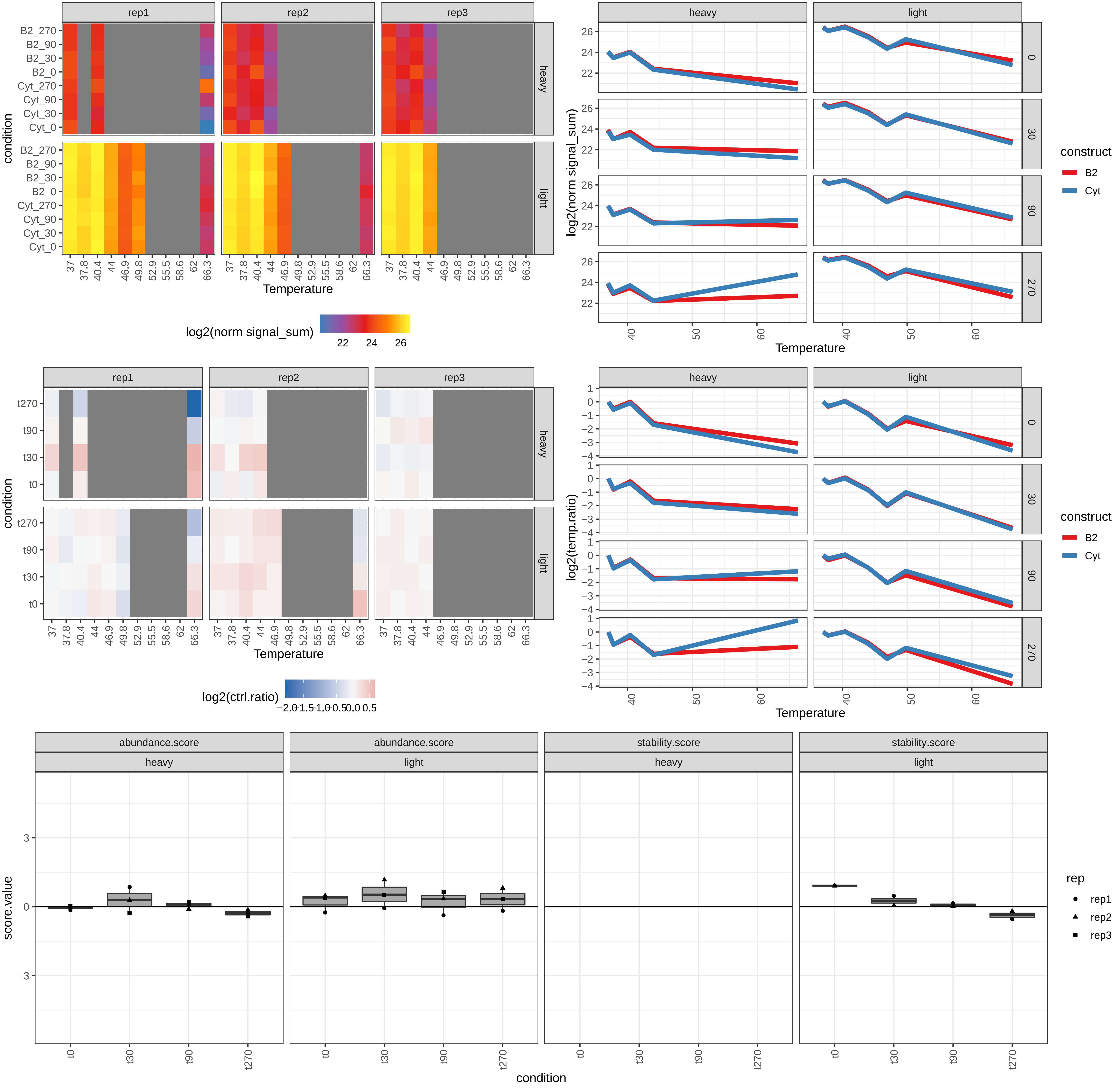

P53261 − Pescadillo homolog OS=Saccharomyces cerevisiae (strain ATCC 204508 / S288c) GN=NOP7 PE=1 SV=1

NOP9 − average.top3: 6.743674 − max.qupm: 12

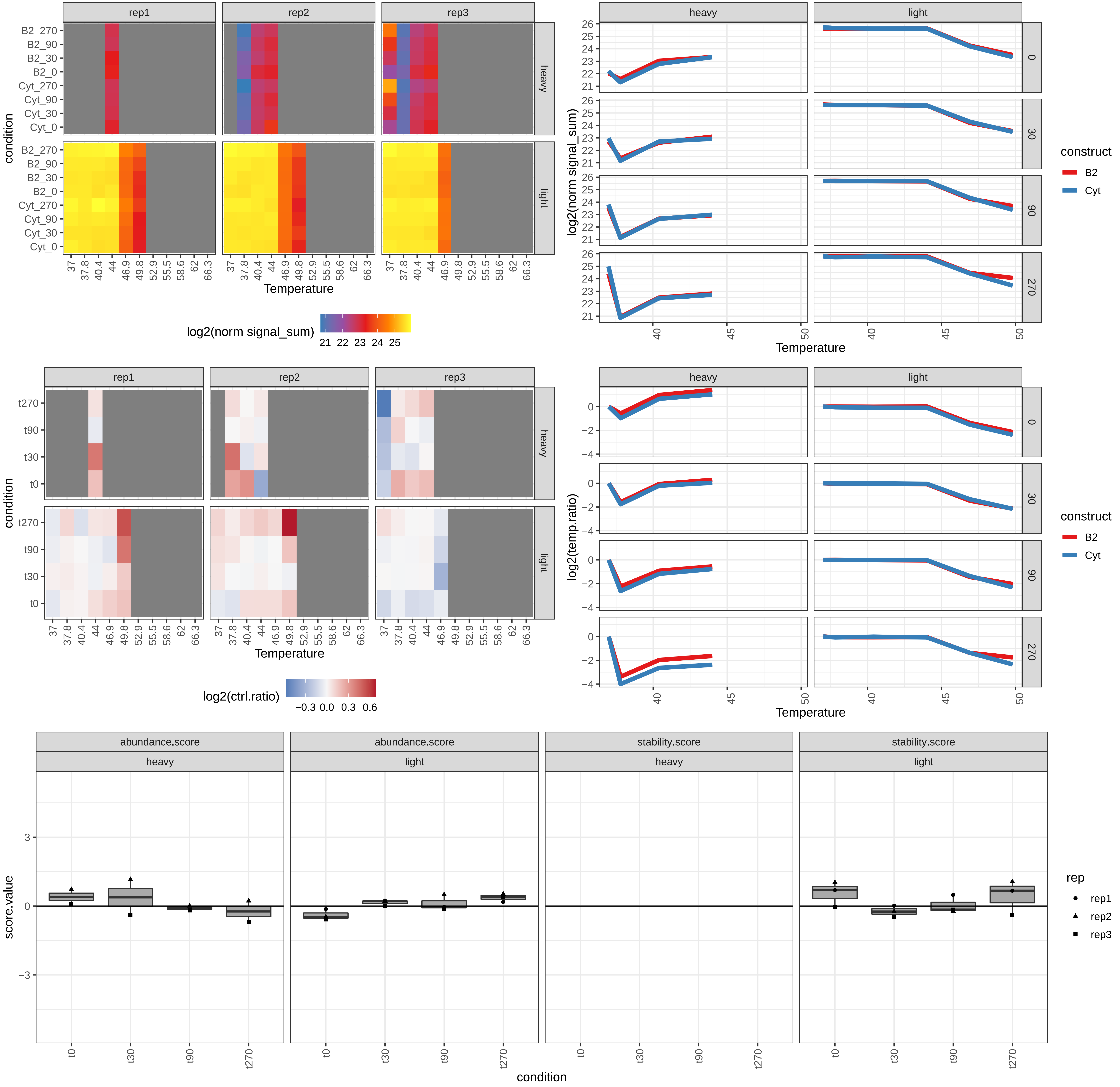

P47077 − Nucleolar protein 9 OS=Saccharomyces cerevisiae (strain ATCC 204508 / S288c) GN=NOP9 PE=1 SV=1

NOT3 − average.top3: 6.87750481481481 − max.qupm: 13

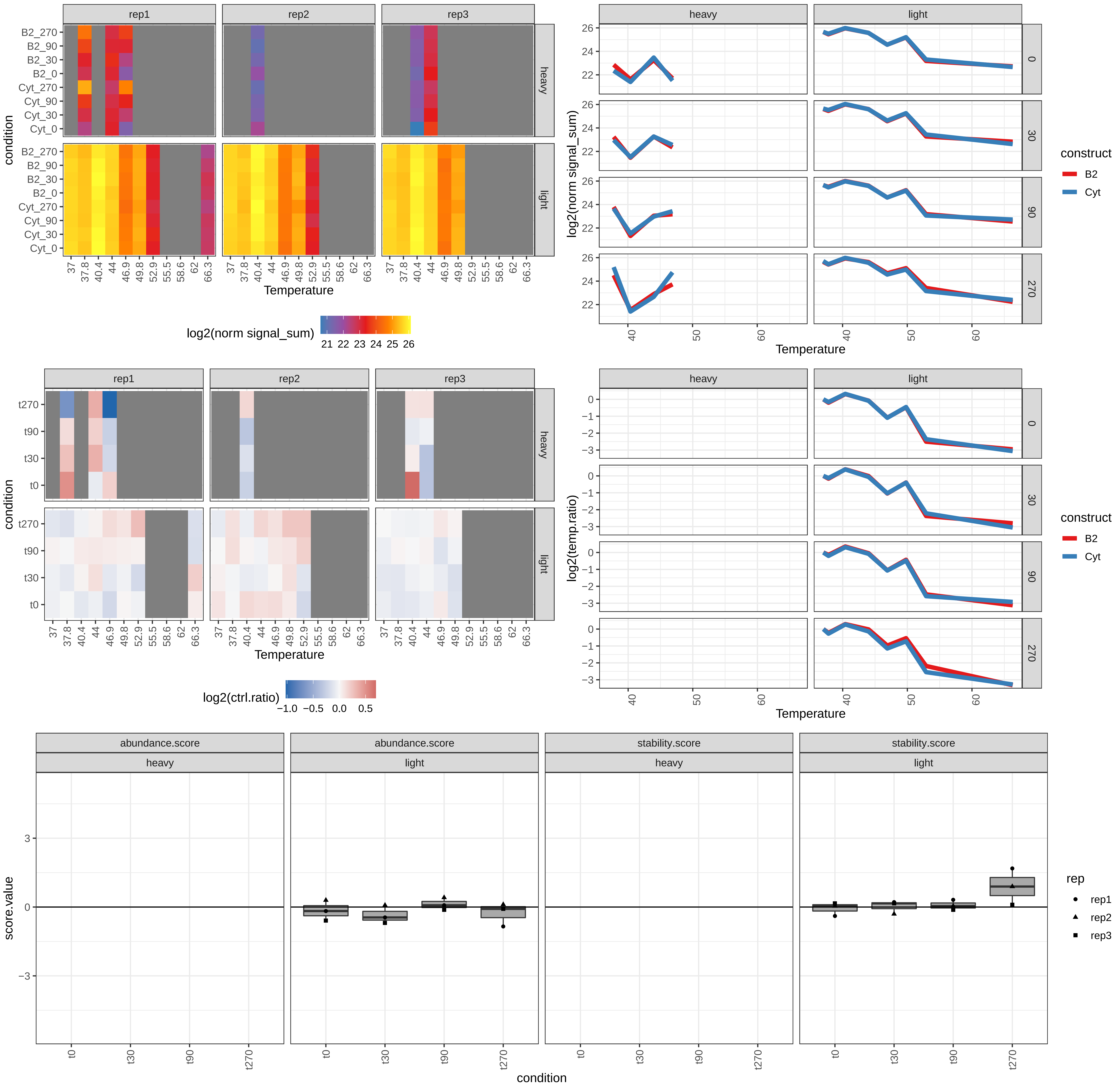

P06102 − General negative regulator of transcription subunit 3 OS=Saccharomyces cerevisiae (strain ATCC 204508 / S288c) GN=NOT3 PE=1 SV=2

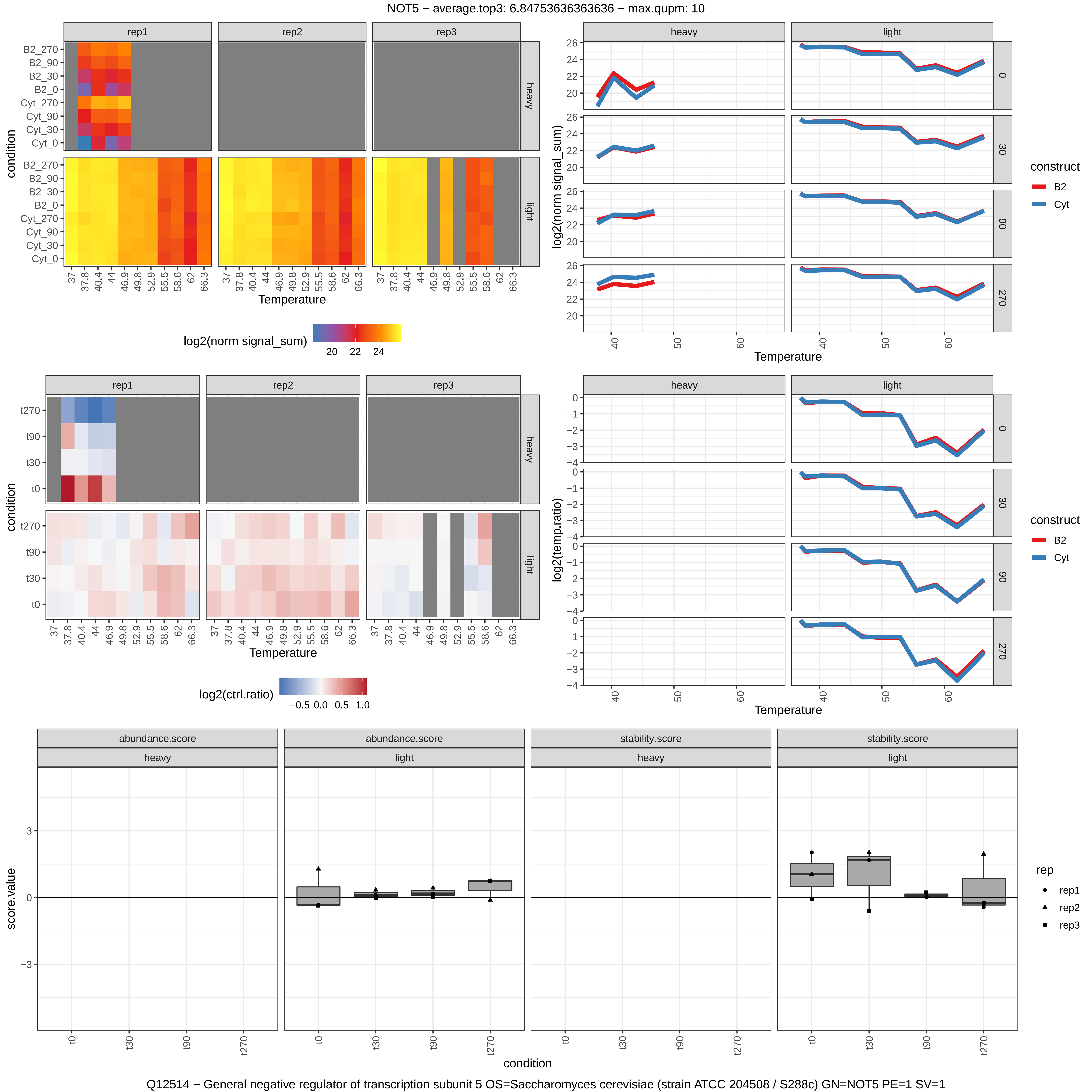

NOT5 − average.top3: 6.84753636363636 − max.qupm: 10
rep1
rep2
rep3
heavy
light
condition
B2_270
B2_90
B2_30
B2_0
Cyt_270
Cyt_90
Cyt_30
Cyt_0
37
37.8
40.4
44
46.9
49.8
52.9
55.5
58.6
62
66.3
Temperature
log2(norm signal_sum)
20
22
24
log2(norm signal_sum)
26
0
30
90
270
40
50
60
construct
B2
Cyt
t270
t90
t30
t0
log2(ctrl.ratio)
−0.5
0.0
0.5
1.0
log2(temp.ratio)
−1
−2
−3
−4
abundance.score
stability.score
score.value
3
−3
condition
rep
rep1
rep2
rep3
Q12514 − General negative regulator of transcription subunit 5 OS=Saccharomyces cerevisiae (strain ATCC 204508 / S288c) GN=NOT5 PE=1 SV=1

NPC2 − average.top3: 7.17286463414634 − max.qupm: 6

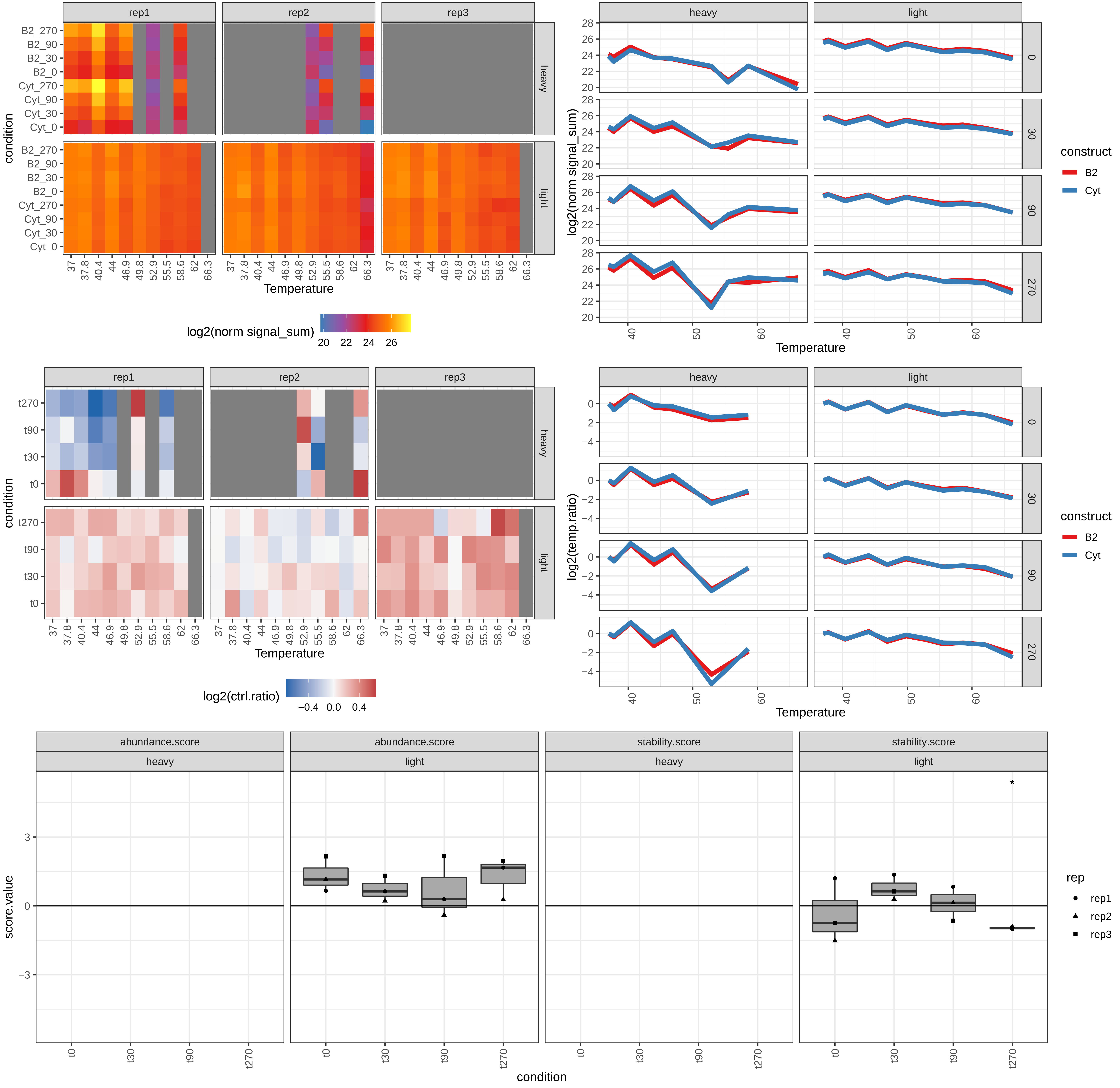

Q12408 − Phosphatidylglycerol/phosphatidylinositol transfer protein OS=Saccharomyces cerevisiae (strain ATCC 204508 / S288c) GN=NPC2 PE=1 SV=1

NPL3 − average.top3: 7.33033607843137 − max.qupm: 10

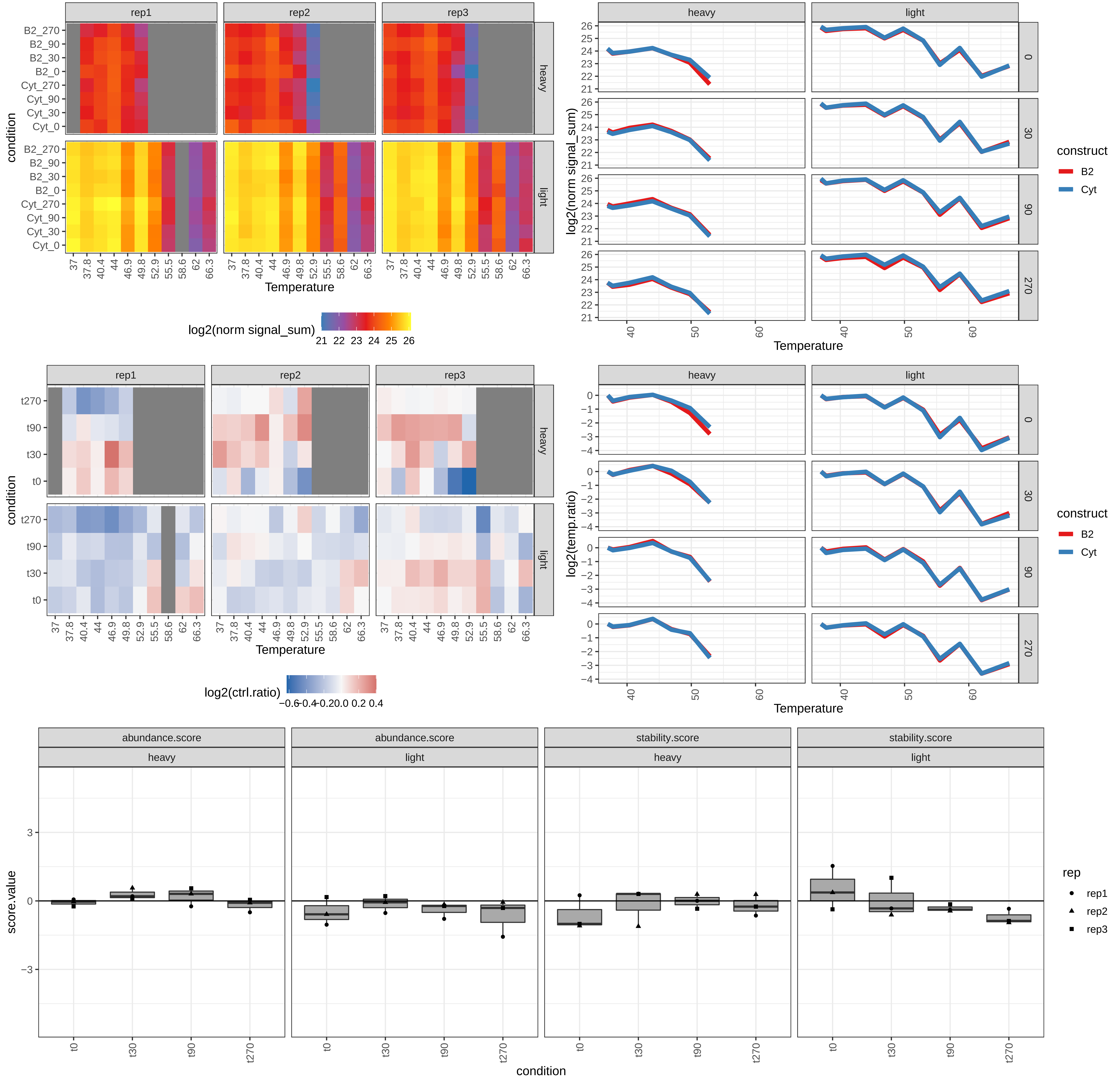

Q01560 − Nucleolar protein 3 OS=Saccharomyces cerevisiae (strain ATCC 204508 / S288c) GN=NPL3 PE=1 SV=1

NPL4 − average.top3: 6.69466382352941 − max.qupm: 8

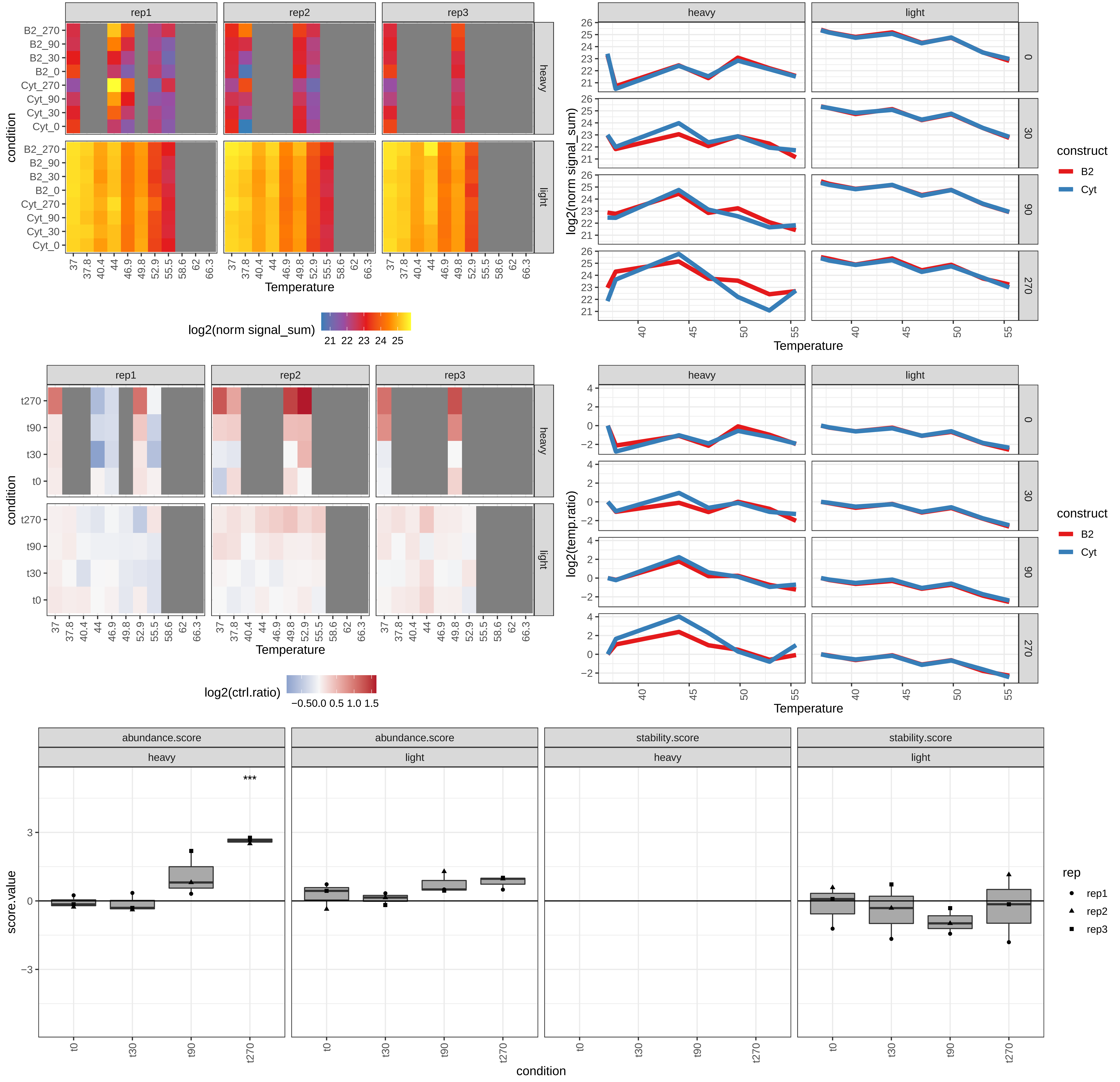

P33755 − Nuclear protein localization protein 4 OS=Saccharomyces cerevisiae (strain ATCC 204508 / S288c) GN=NPL4 PE=1 SV=1

NPL6 − average.top3: 7.17535157894737 − max.qupm: 7

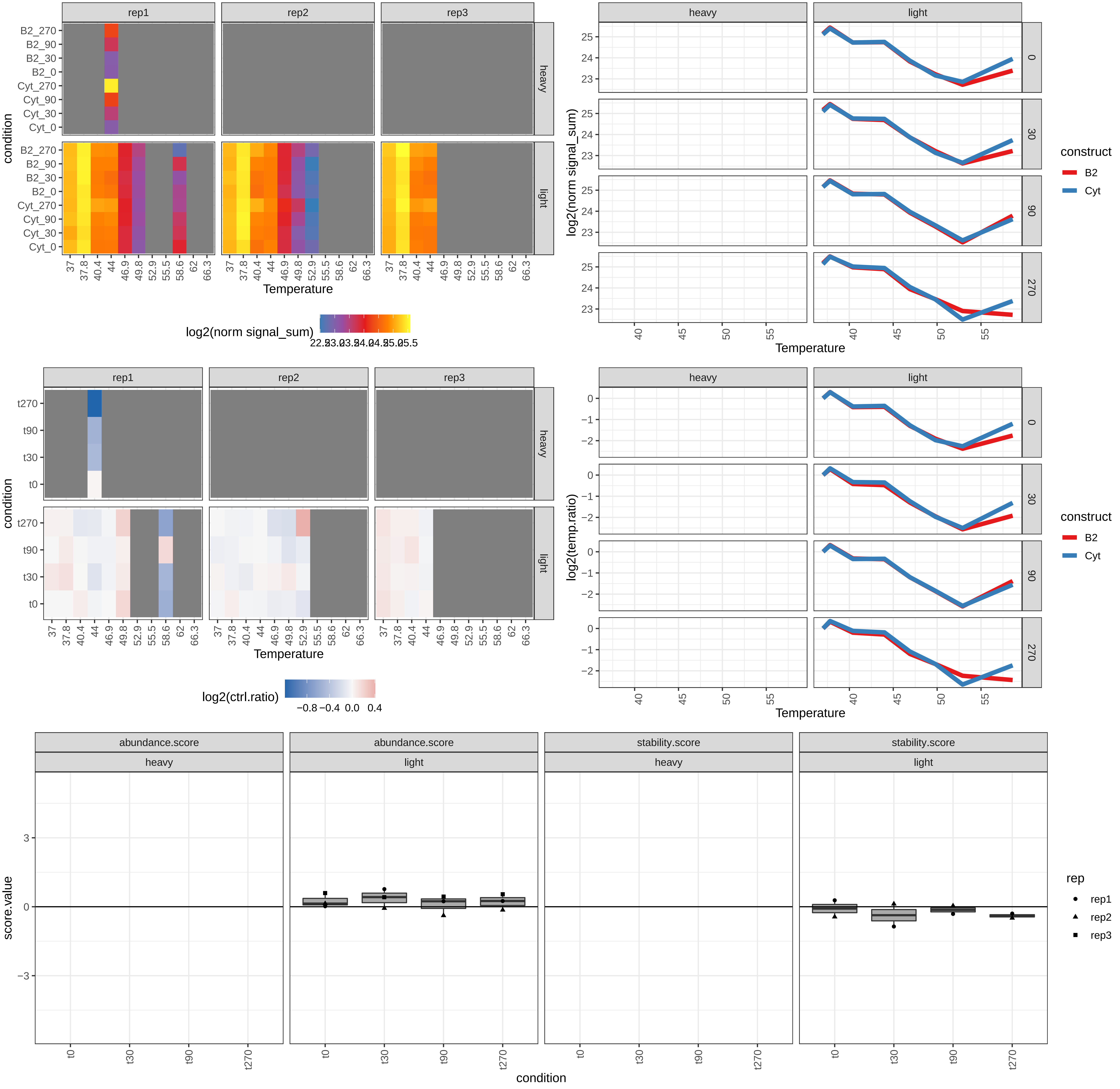

P32832 − Chromatin structure−remodeling complex subunit RSC7 OS=Saccharomyces cerevisiae (strain ATCC 204508 / S288c) GN=NPL6 PE=1 SV=1

NPP1 − average.top3: 6.44275 − max.qupm: 5

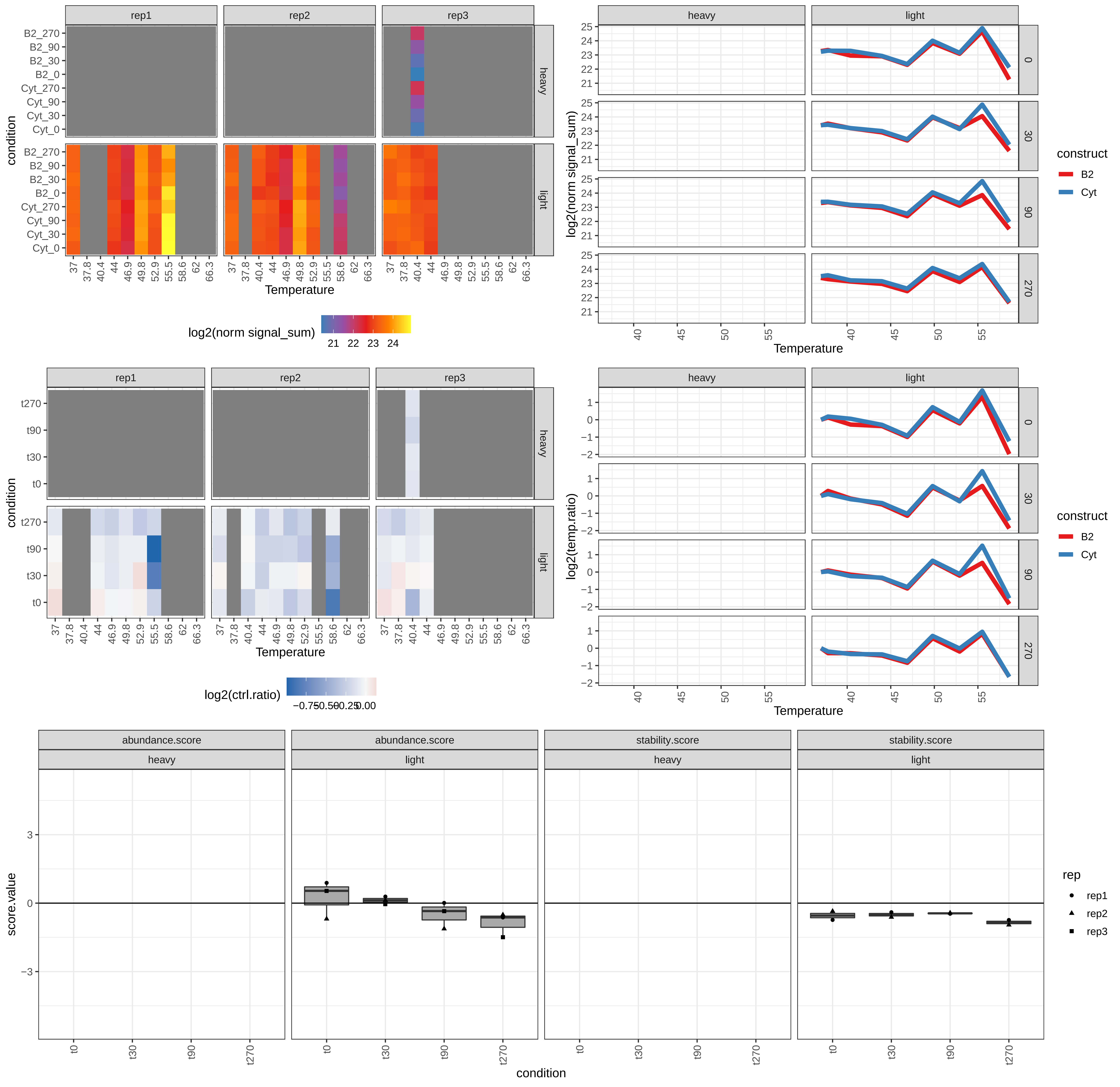

P25353 − Ectonucleotide pyrophosphatase/phosphodiesterase 1 OS=Saccharomyces cerevisiae (strain ATCC 204508 / S288c) GN=NPP1 PE=1 SV=2

NPR2 − average.top3: 6.49574230769231 − max.qupm: 5

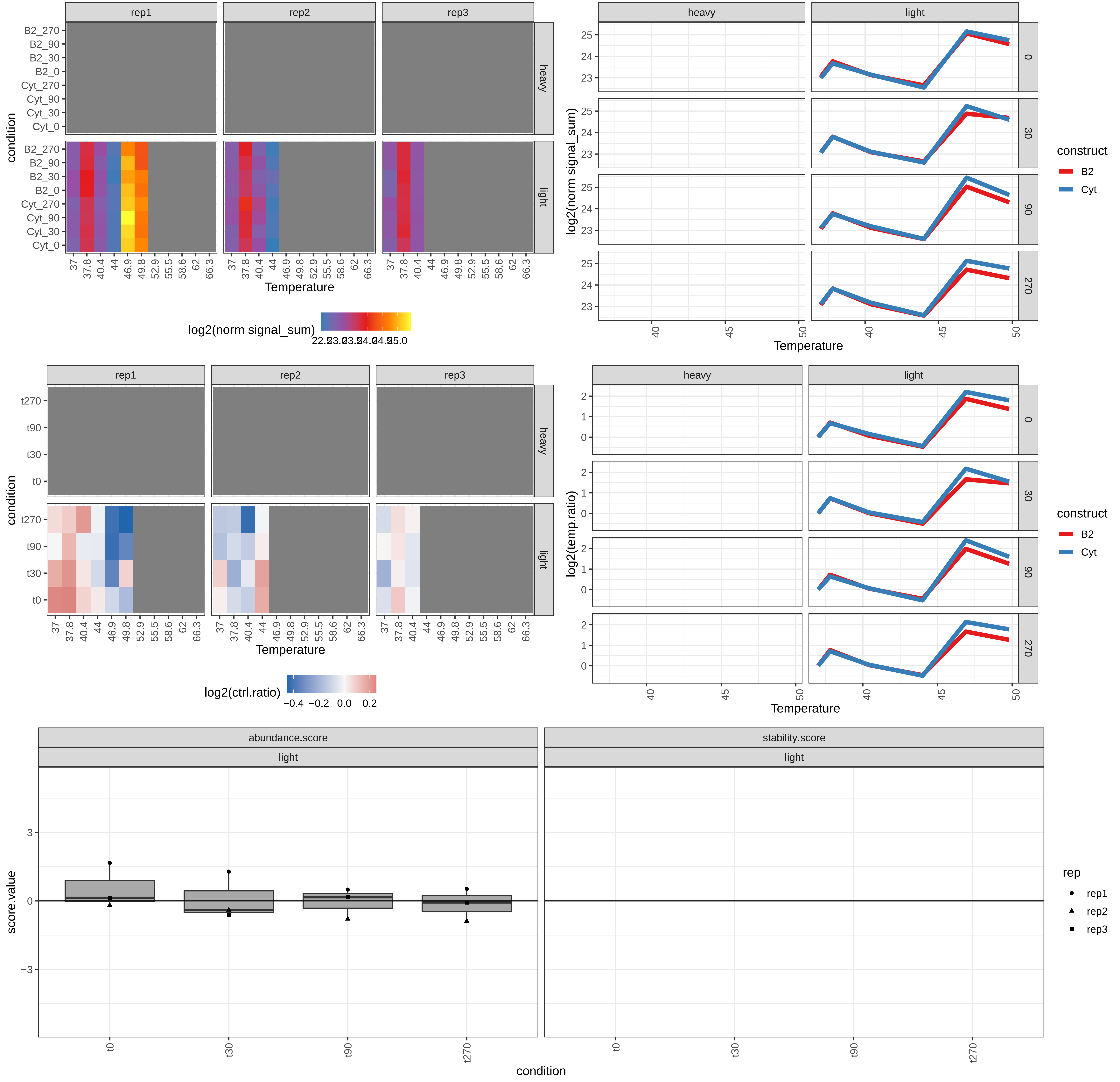

P39923 − Nitrogen permease regulator 2 OS=Saccharomyces cerevisiae (strain ATCC 204508 / S288c) GN=NPR2 PE=1 SV=2

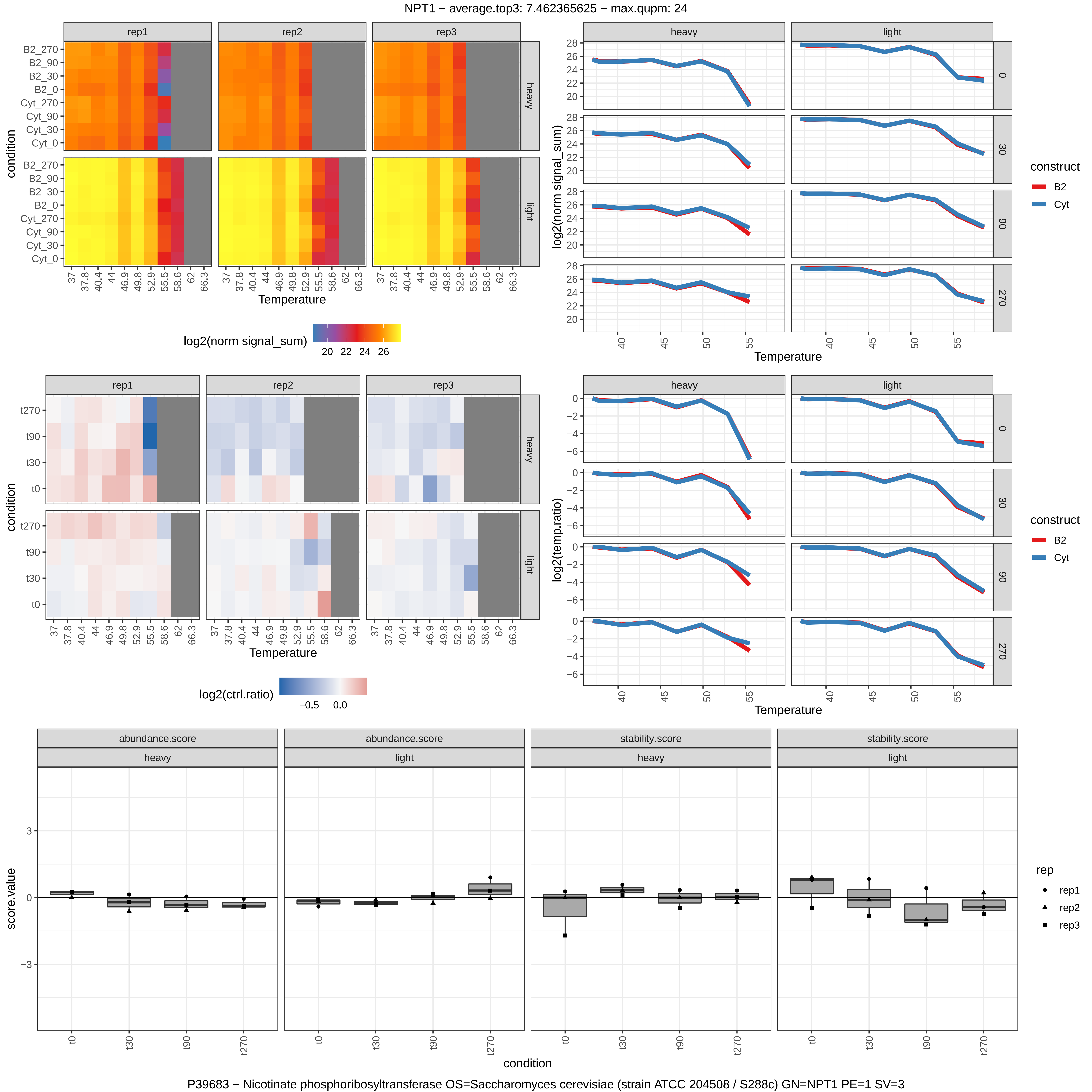

NPT1 − average.top3: 7.462365625 − max.qupm: 24
rep1
rep2
rep3
heavy
light
condition
Temperature
log2(norm signal_sum)
construct
B2
Cyt
log2(ctrl.ratio)
log2(temp.ratio)
abundance.score
stability.score
score.value
rep
rep1
rep2
rep3
P39683 − Nicotinate phosphoribosyltransferase OS=Saccharomyces cerevisiae (strain ATCC 204508 / S288c) GN=NPT1 PE=1 SV=3

NPY1 − average.top3: 6.88093529411765 − max.qupm: 6

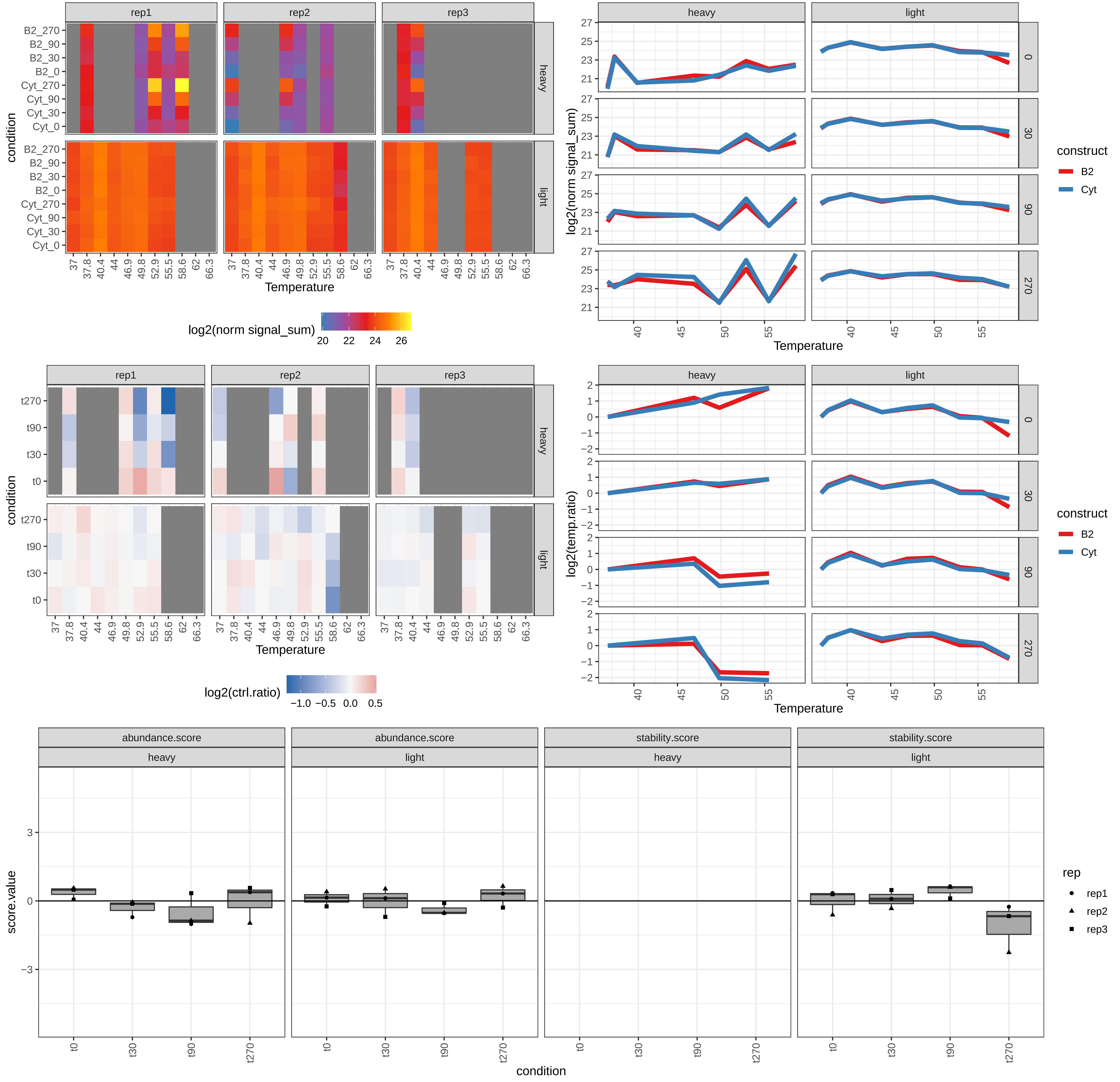

P53164 − NADH pyrophosphatase OS=Saccharomyces cerevisiae (strain ATCC 204508 / S288c) GN=NPY1 PE=1 SV=1

NQM1 − average.top3: 7.01274133333333 − max.qupm: 11

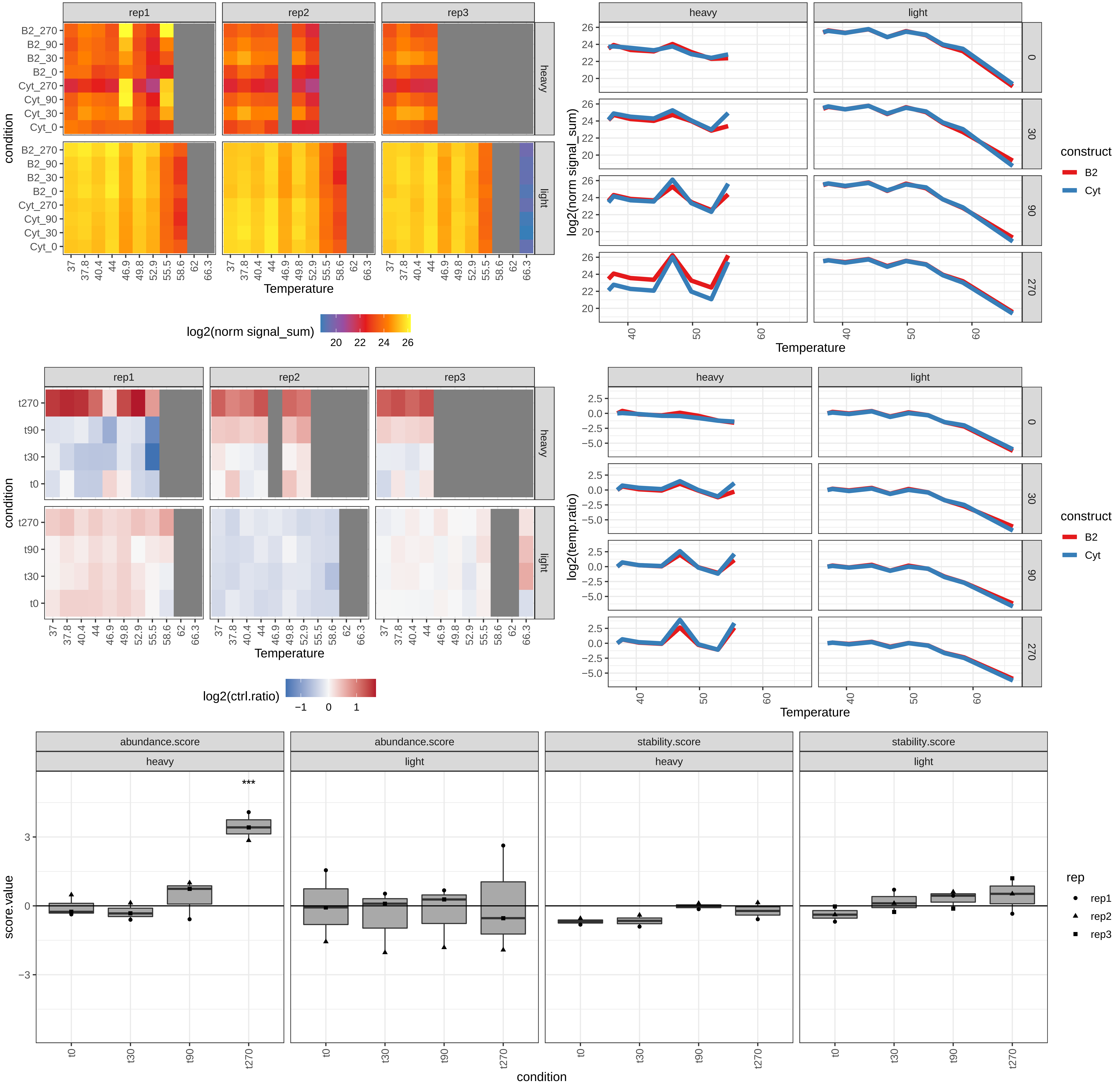

P53228 − Transaldolase NQM1 OS=Saccharomyces cerevisiae (strain ATCC 204508 / S288c) GN=NQM1 PE=1 SV=1

NRD1 − average.top3: 6.79525642857143 − max.qupm: 5

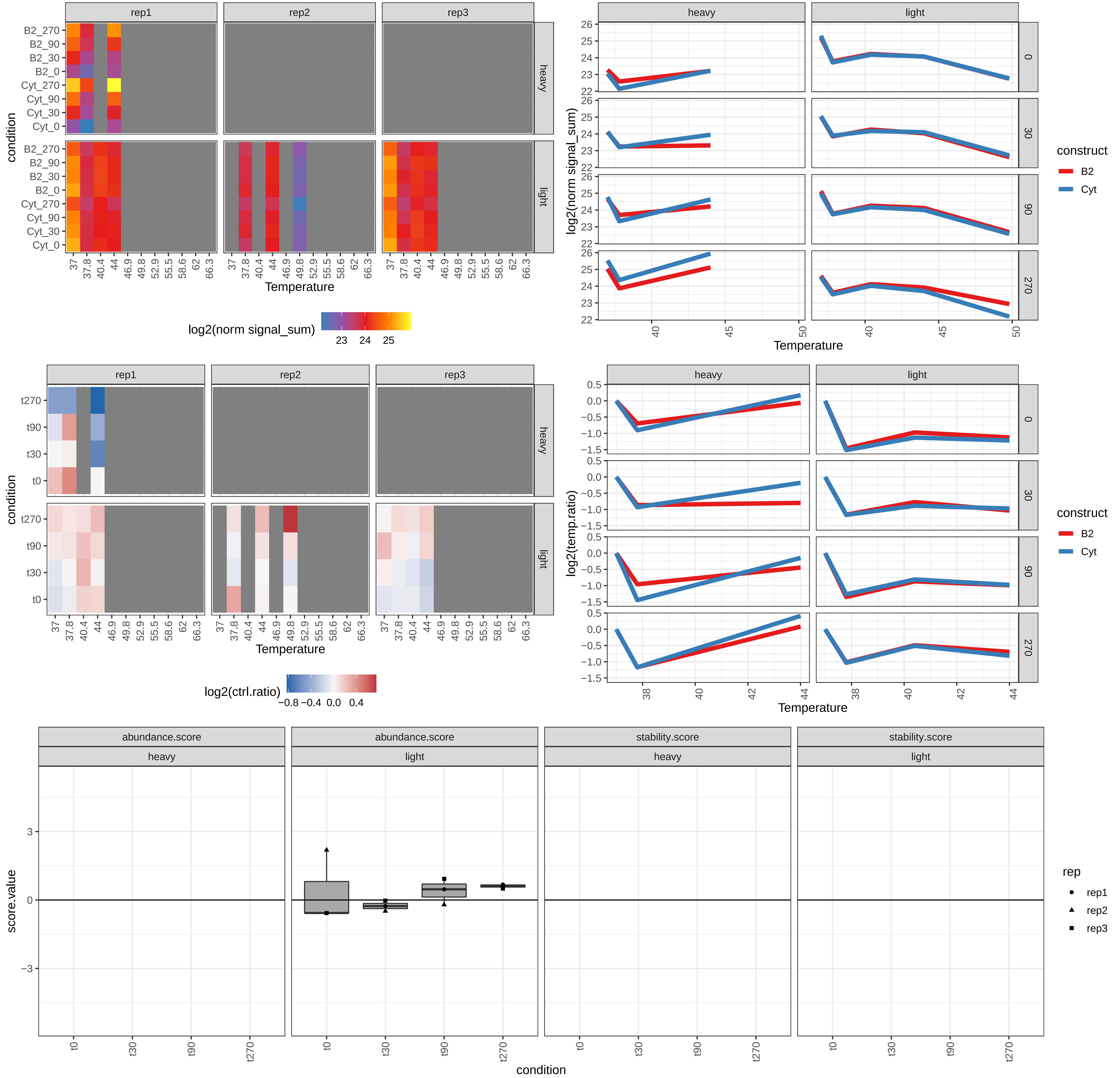

P53617 − Protein NRD1 OS=Saccharomyces cerevisiae (strain ATCC 204508 / S288c) GN=NRD1 PE=1 SV=1

NSA1 − average.top3: 6.79912344827586 − max.qupm: 12

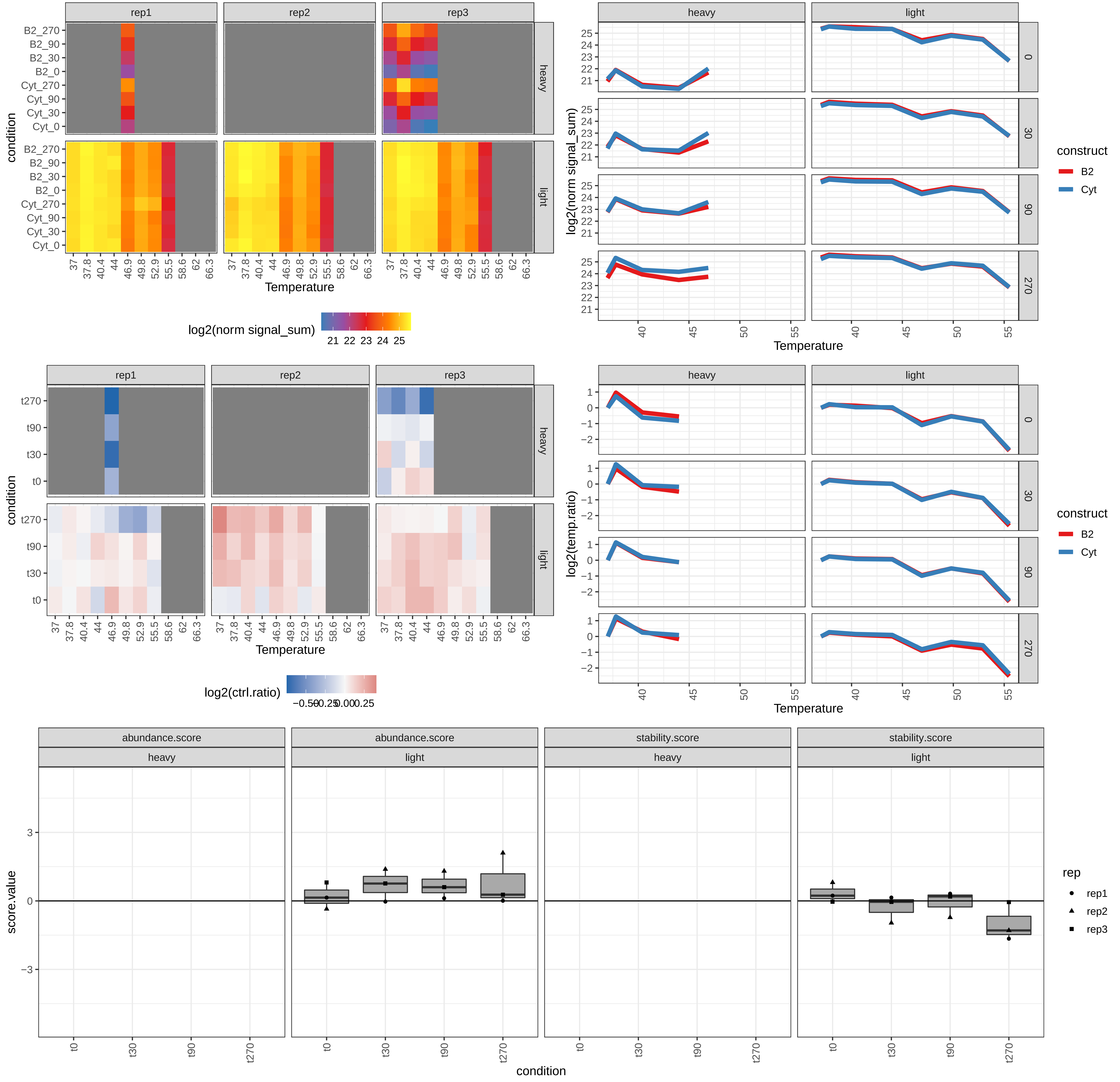

P53136 − Ribosome biogenesis protein NSA1 OS=Saccharomyces cerevisiae (strain ATCC 204508 / S288c) GN=NSA1 PE=1 SV=1

NSA2 − average.top3: 6.65615 − max.qupm: 4

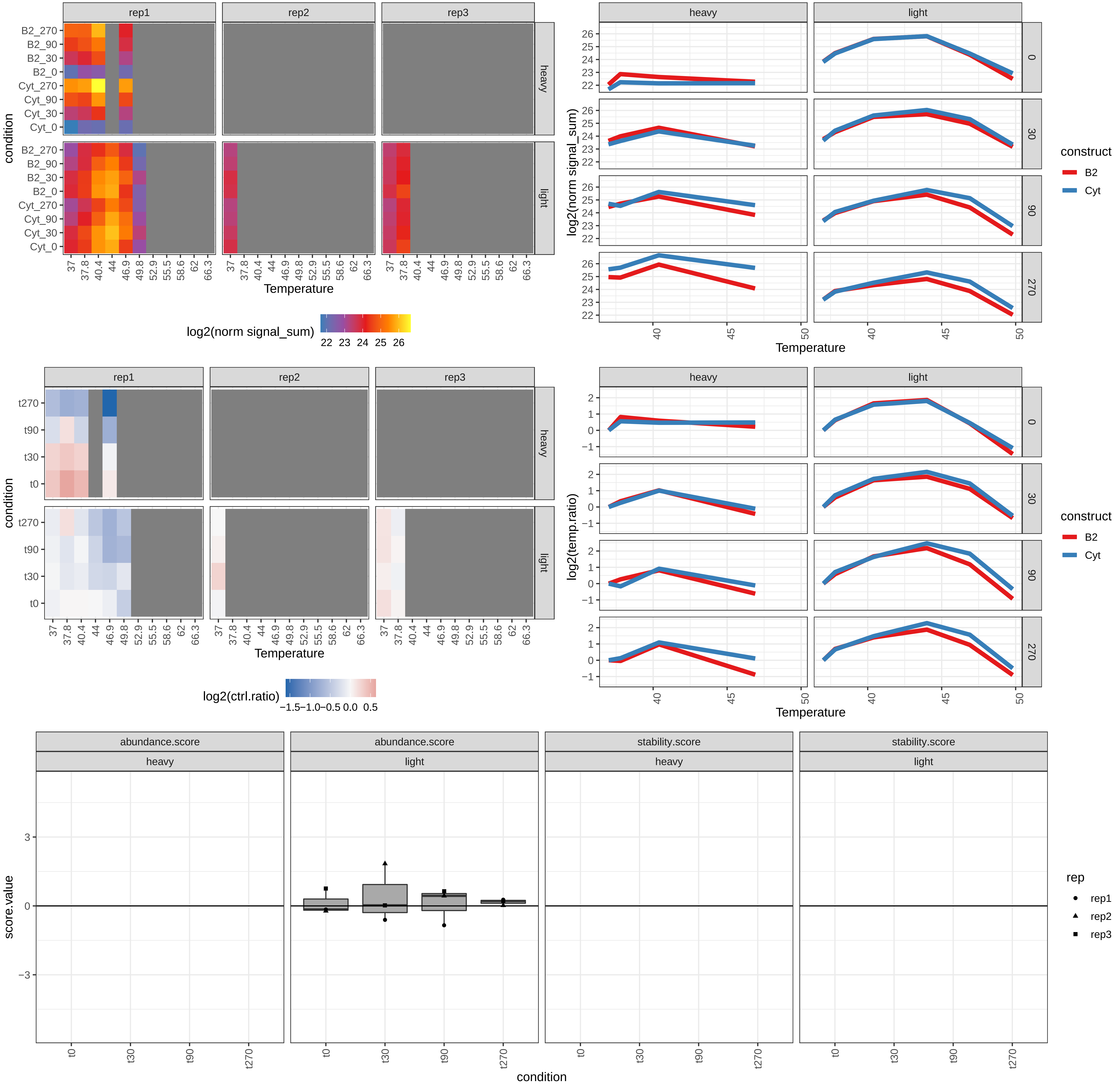

P40078 − Ribosome biogenesis protein NSA2 OS=Saccharomyces cerevisiae (strain ATCC 204508 / S288c) GN=NSA2 PE=1 SV=1

NSP1 − average.top3: 6.41871 − max.qupm: 4

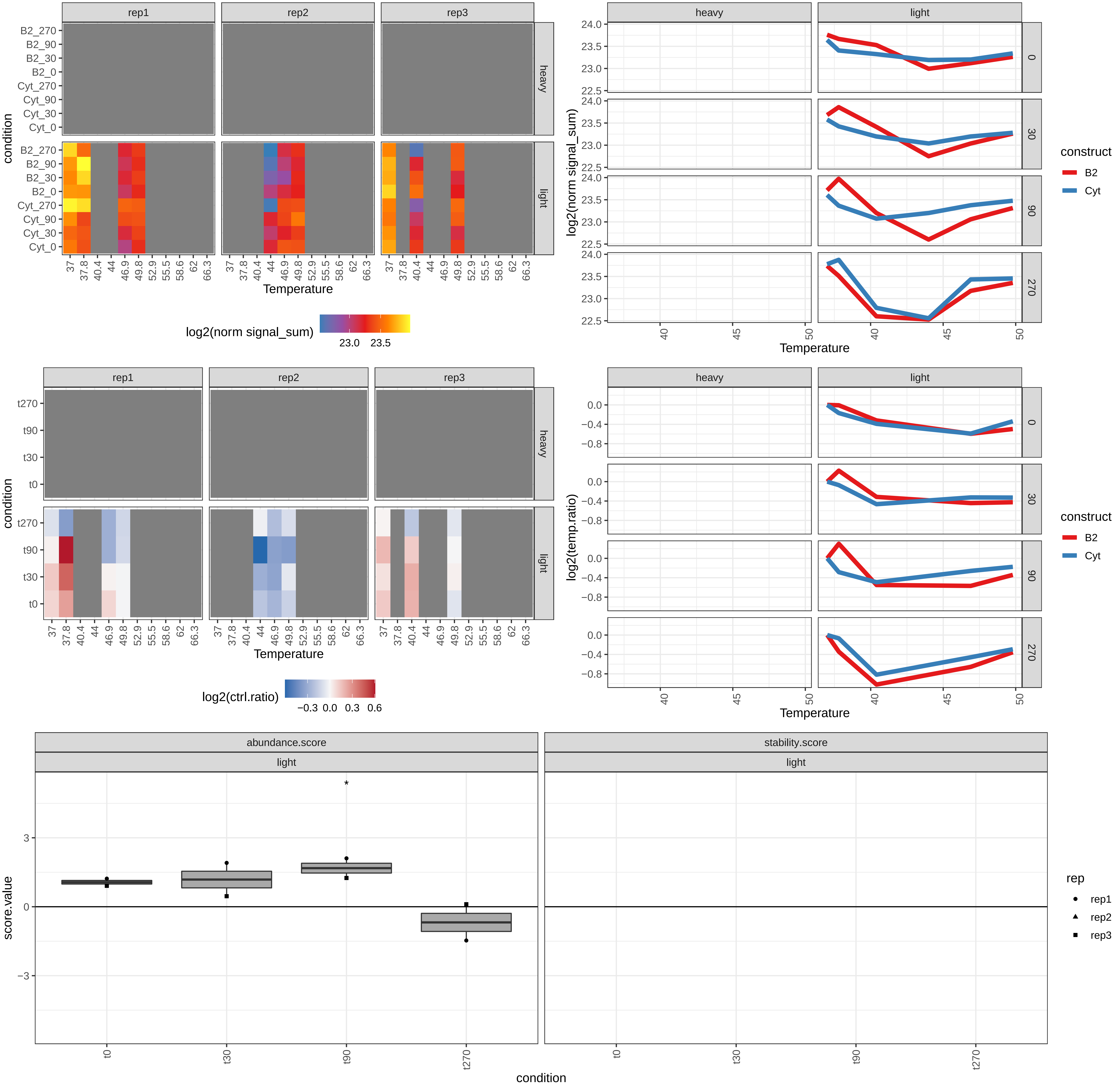

P14907 − Nucleoporin NSP1 OS=Saccharomyces cerevisiae (strain ATCC 204508 / S288c) GN=NSP1 PE=1 SV=1

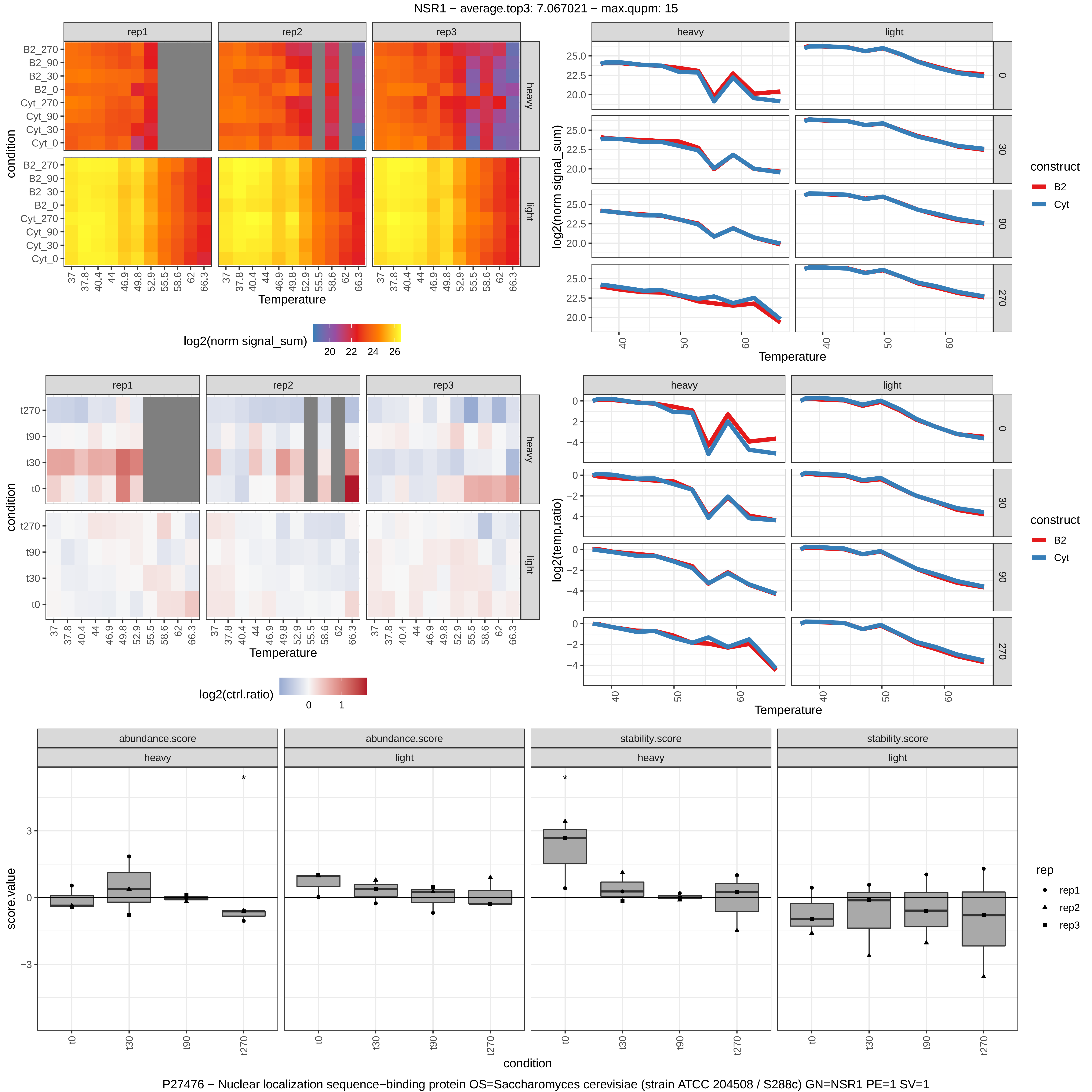
NSR1 − average.top3: 7.067021 − max.qupm: 15
rep1
rep2
rep3
heavy
light
condition
Temperature
log2(norm signal_sum)
construct
B2
Cyt
log2(temp.ratio)
log2(ctrl.ratio)
abundance.score
stability.score
score.value
rep
rep1
rep2
rep3
P27476 − Nuclear localization sequence−binding protein OS=Saccharomyces cerevisiae (strain ATCC 204508 / S288c) GN=NSR1 PE=1 SV=1

NST1 − average.top3: 6.22971434782609 − max.qupm: 5

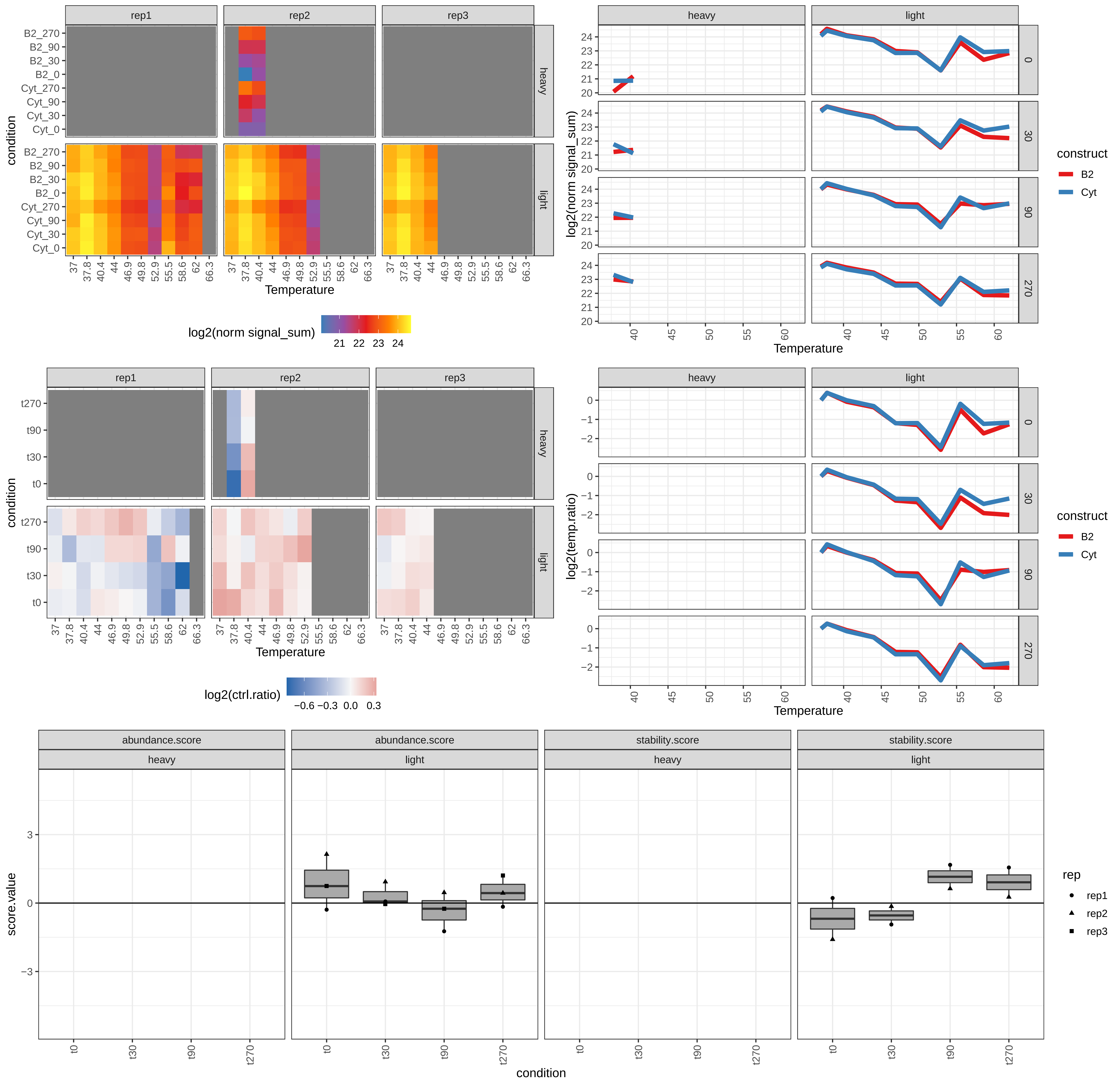

P53935 − Stress response protein NST1 OS=Saccharomyces cerevisiae (strain ATCC 204508 / S288c) GN=NST1 PE=1 SV=1

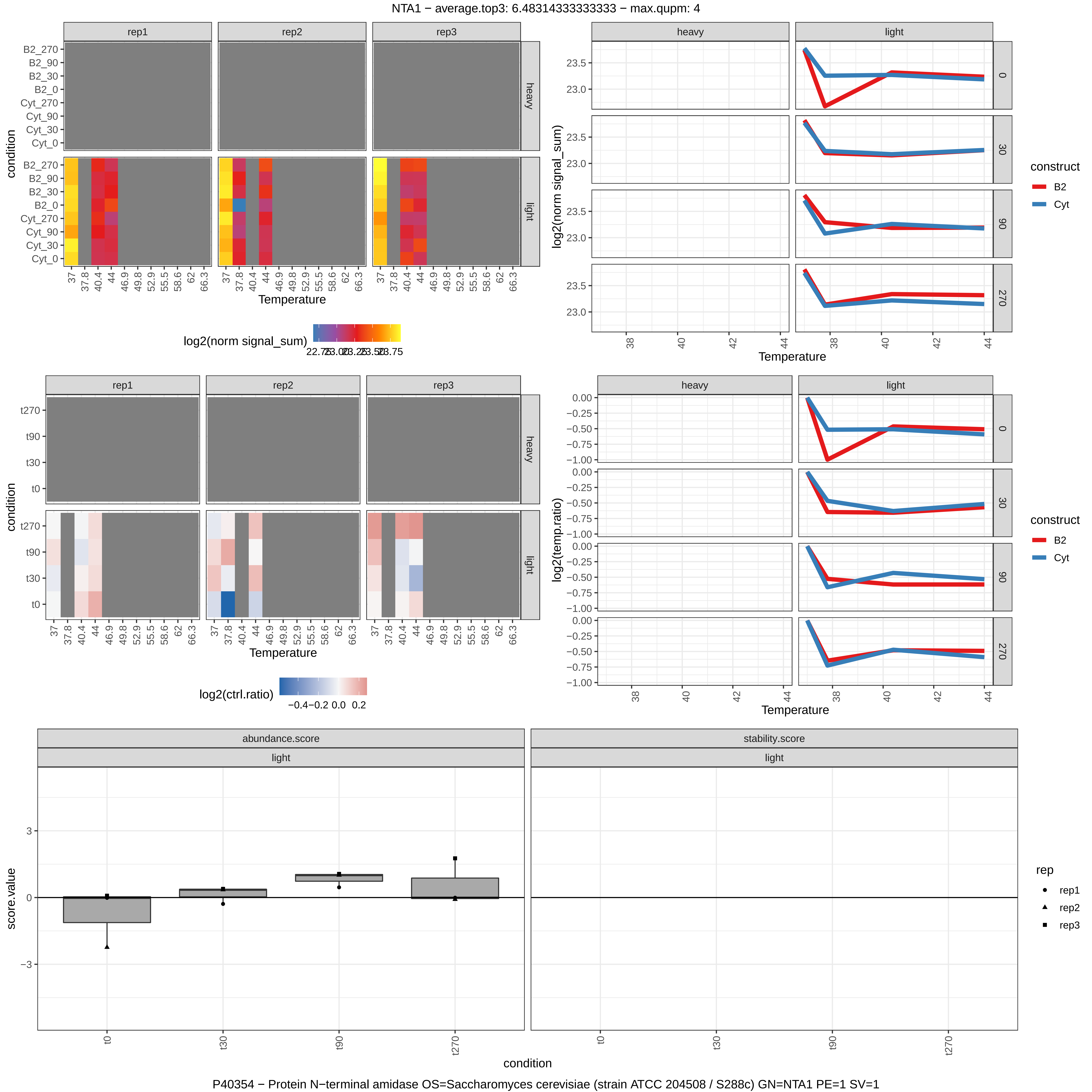

NTA1 − average.top3: 6.48314333333333 − max.qupm: 4
rep1
rep2
rep3
heavy
light
condition
B2_270
B2_90
B2_30
B2_0
Cyt_270
Cyt_90
Cyt_30
Cyt_0
37
37.8
40.4
44
46.9
49.8
52.9
55.5
58.6
62
66.3
Temperature
log2(norm signal_sum)
22.75
23.00
23.25
23.50
23.75
log2(norm signal_sum)
23.5
23.0
0
30
90
270
38
40
42
44
construct
B2
Cyt
t270
t90
t30
t0
log2(ctrl.ratio)
−0.4
−0.2
0.0
0.2
log2(temp.ratio)
0.00
−0.25
−0.50
−0.75
−1.00
abundance.score
stability.score
score.value
3
0
−3
condition
rep
rep1
rep2
rep3
P40354 − Protein N−terminal amidase OS=Saccharomyces cerevisiae (strain ATCC 204508 / S288c) GN=NTA1 PE=1 SV=1

NTE1 − average.top3: 6.97049 − max.qupm: 7

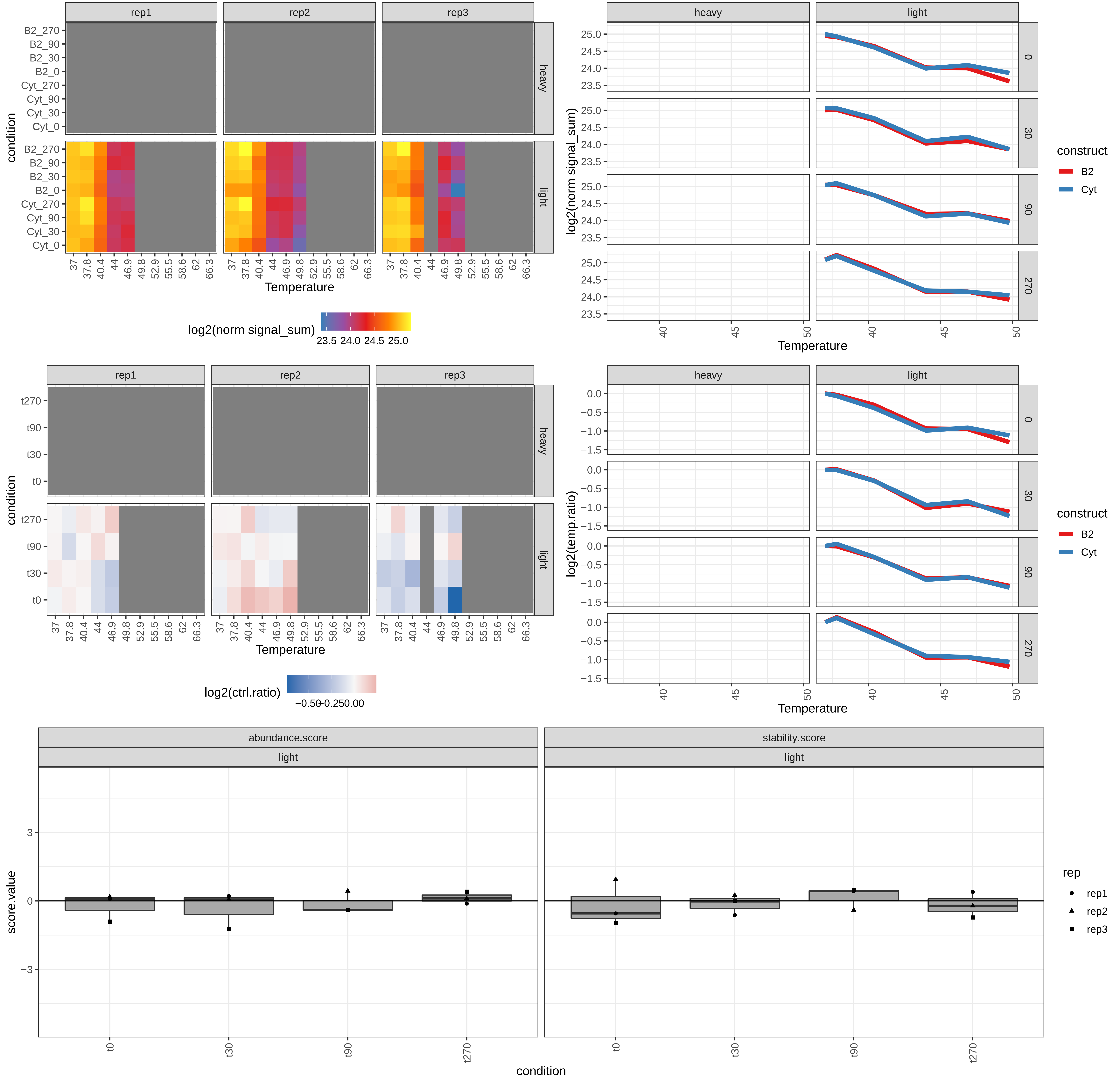

Q04958 − Lysophospholipase NTE1 OS=Saccharomyces cerevisiae (strain ATCC 204508 / S288c) GN=NTE1 PE=1 SV=1

NTH1 − average.top3: 7.1174048 − max.qupm: 16

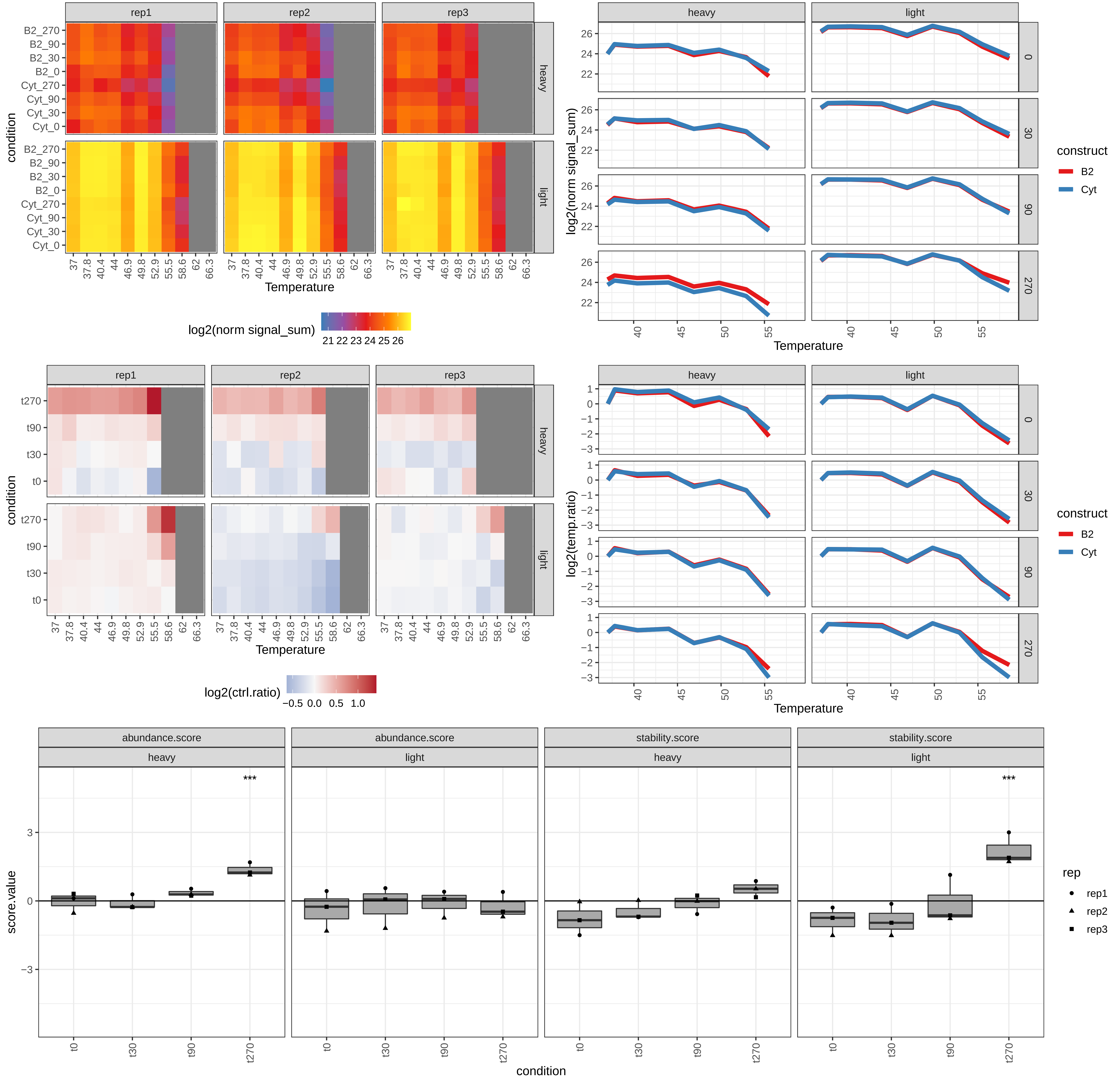

P32356 − Neutral trehalase OS=Saccharomyces cerevisiae (strain ATCC 204508 / S288c) GN=NTH1 PE=1 SV=3

NTH2 − average.top3: 6.5654 − max.qupm: 6

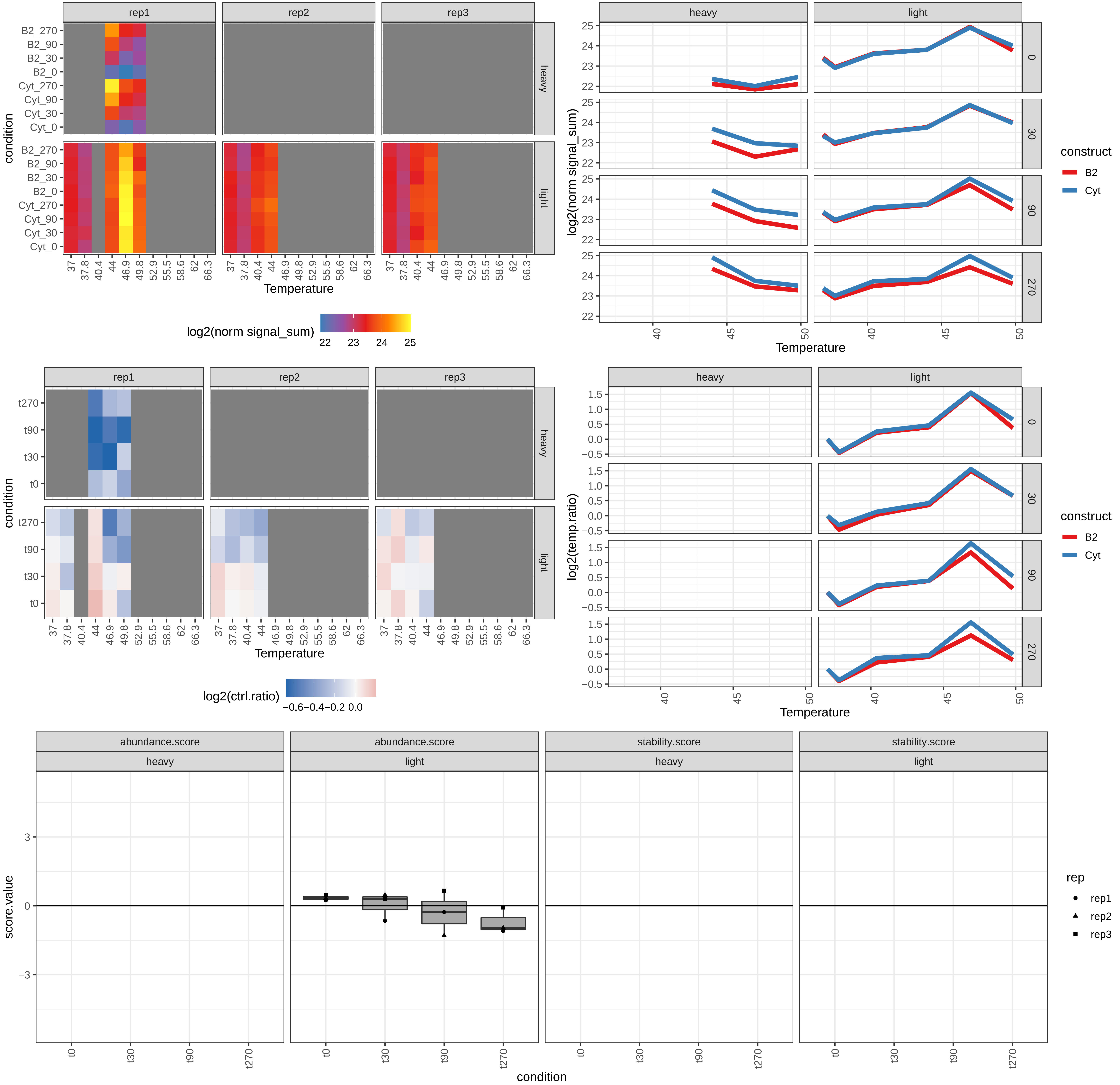

P35172 − Probable trehalase OS=Saccharomyces cerevisiae (strain ATCC 204508 / S288c) GN=NTH2 PE=1 SV=1

NUC1 − average.top3: 6.42470230769231 − max.qupm: 6

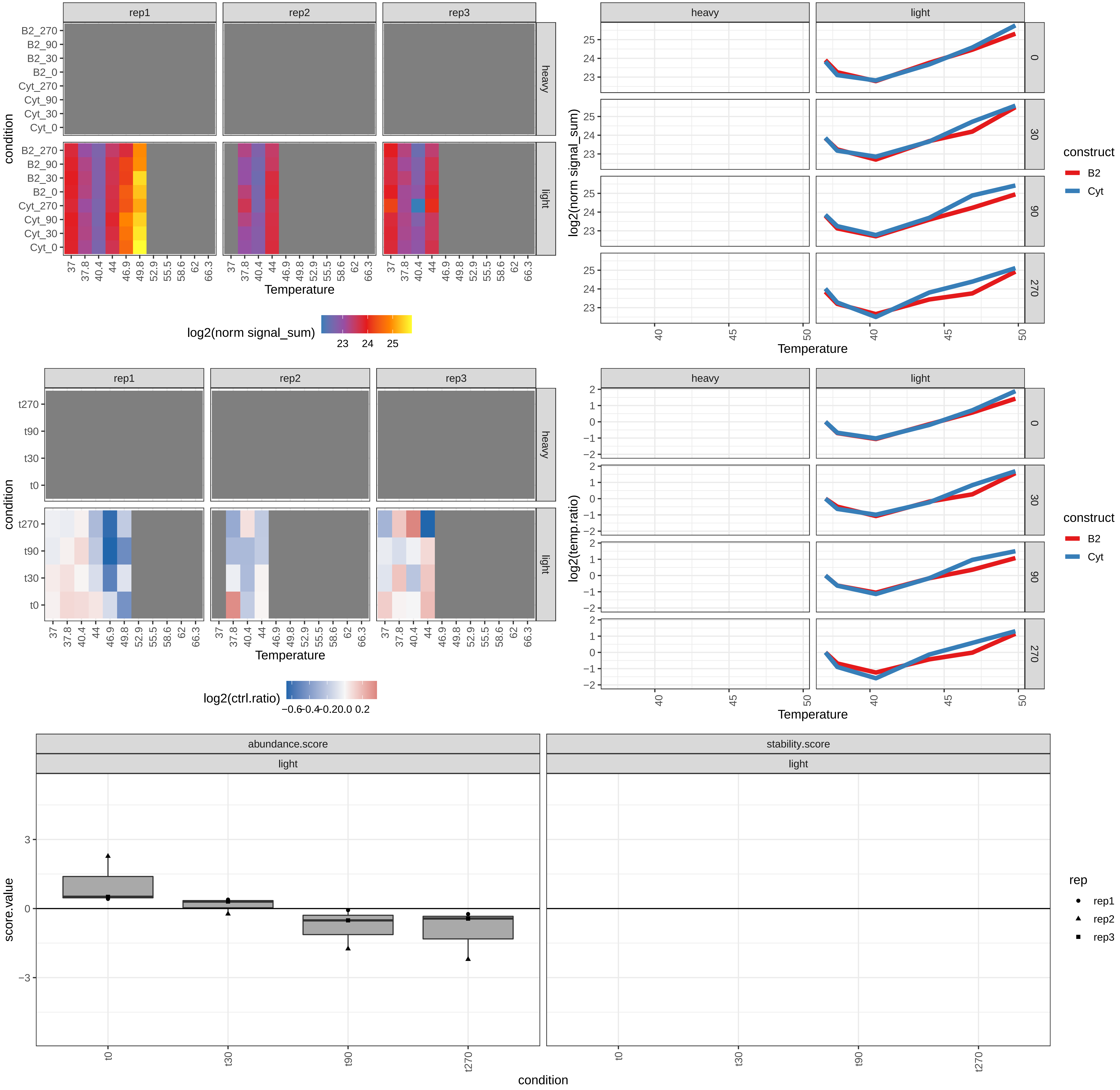

P08466 − Mitochondrial nuclease OS=Saccharomyces cerevisiae (strain ATCC 204508 / S288c) GN=NUC1 PE=1 SV=1

NUM1 − average.top3: 6.59497571428571 − max.qupm: 29

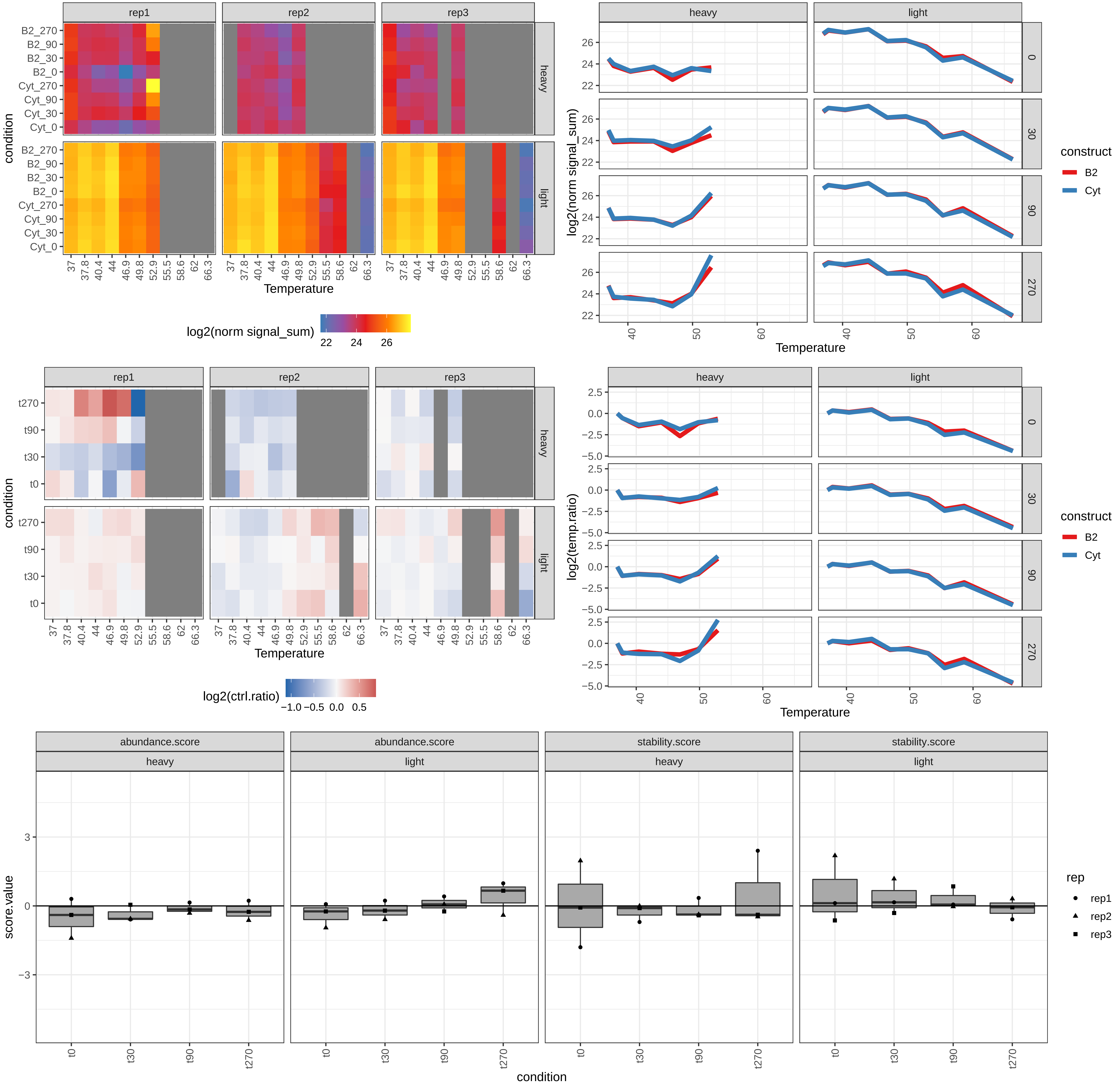

Q00402 − Nuclear migration protein NUM1 OS=Saccharomyces cerevisiae (strain ATCC 204508 / S288c) GN=NUM1 PE=1 SV=2

NUP116 − average.top3: 6.91893266666667 − max.qupm: 4

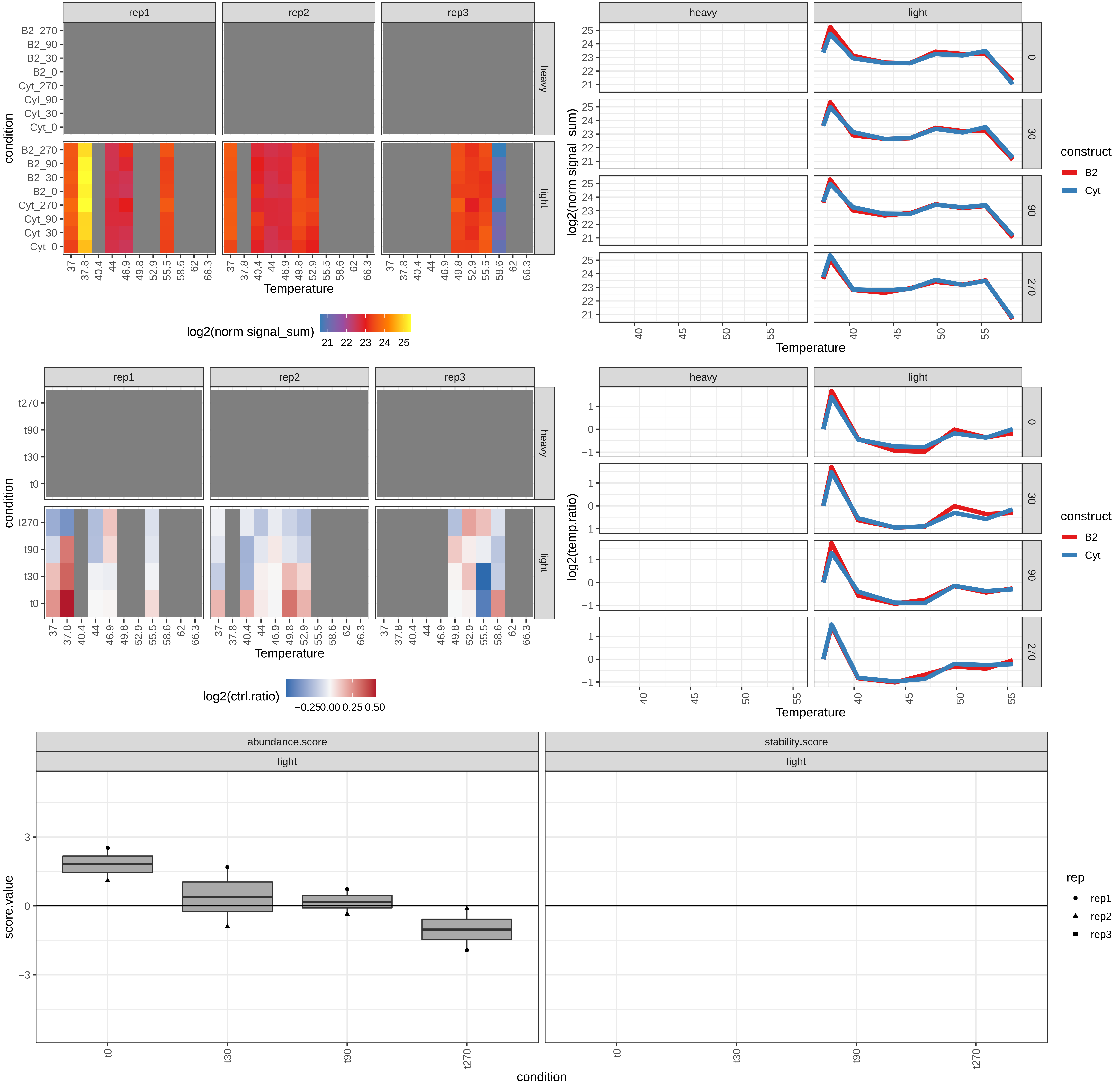

Q02630 − Nucleoporin NUP116/NSP116 OS=Saccharomyces cerevisiae (strain ATCC 204508 / S288c) GN=NUP116 PE=1 SV=2

NUP120 − average.top3: 6.85858166666667 − max.qupm: 18

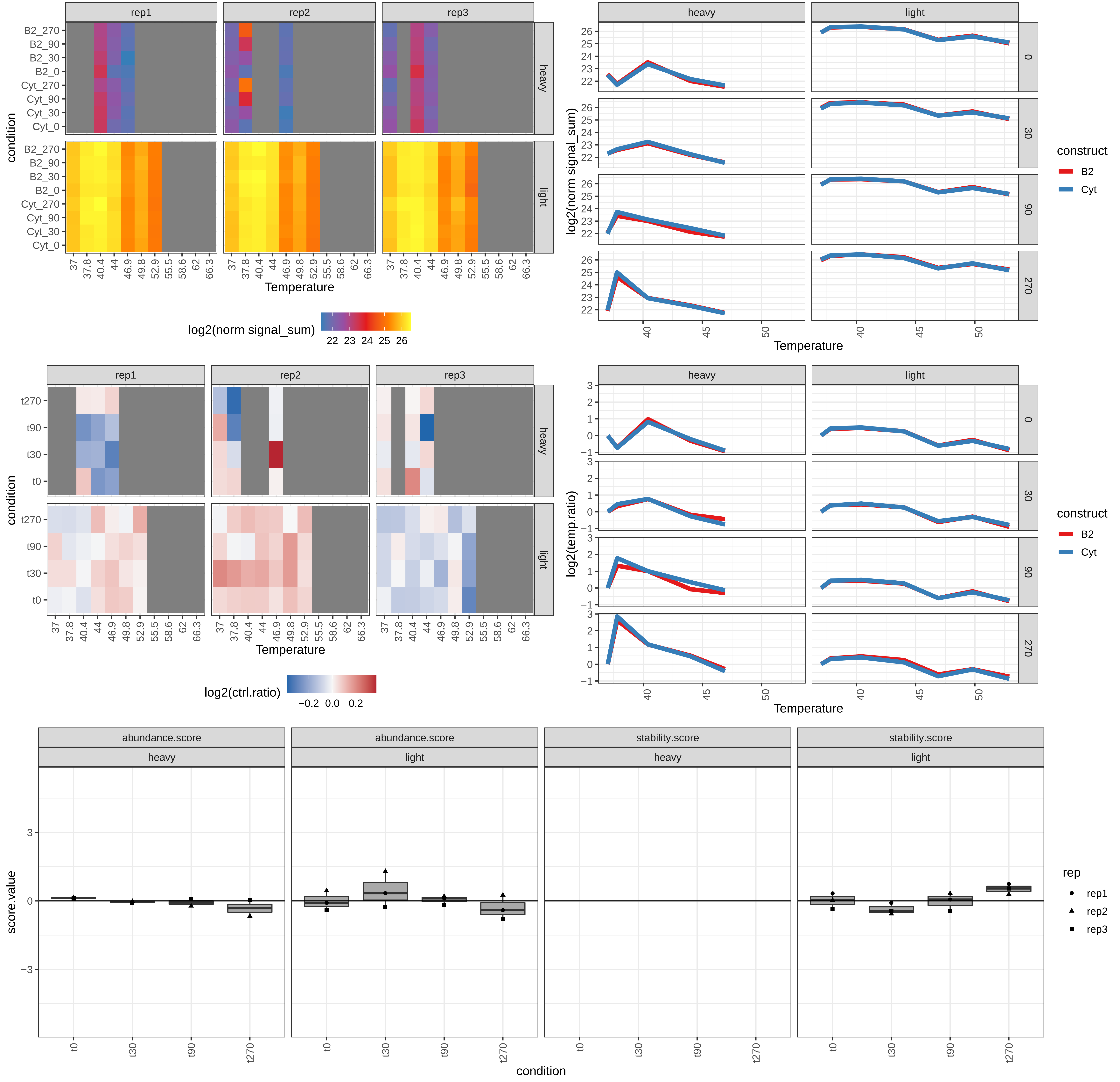

P35729 − Nucleoporin NUP120 OS=Saccharomyces cerevisiae (strain ATCC 204508 / S288c) GN=NUP120 PE=1 SV=1

NUP133 − average.top3: 6.70971558139535 − max.qupm: 17

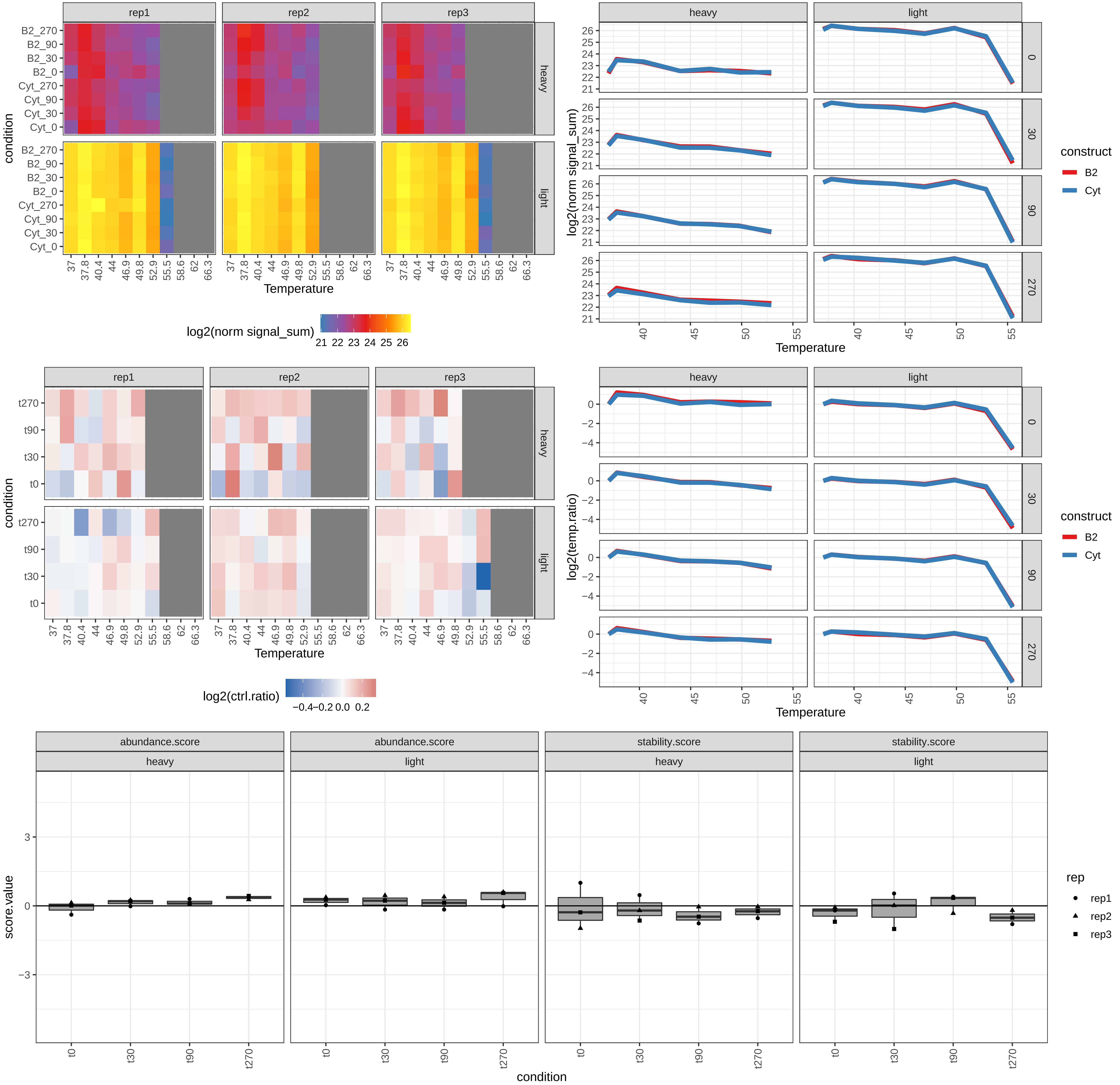

P36161 − Nucleoporin NUP133 OS=Saccharomyces cerevisiae (strain ATCC 204508 / S288c) GN=NUP133 PE=1 SV=1

NUP145 − average.top3: 7.06504181818182 − max.qupm: 16

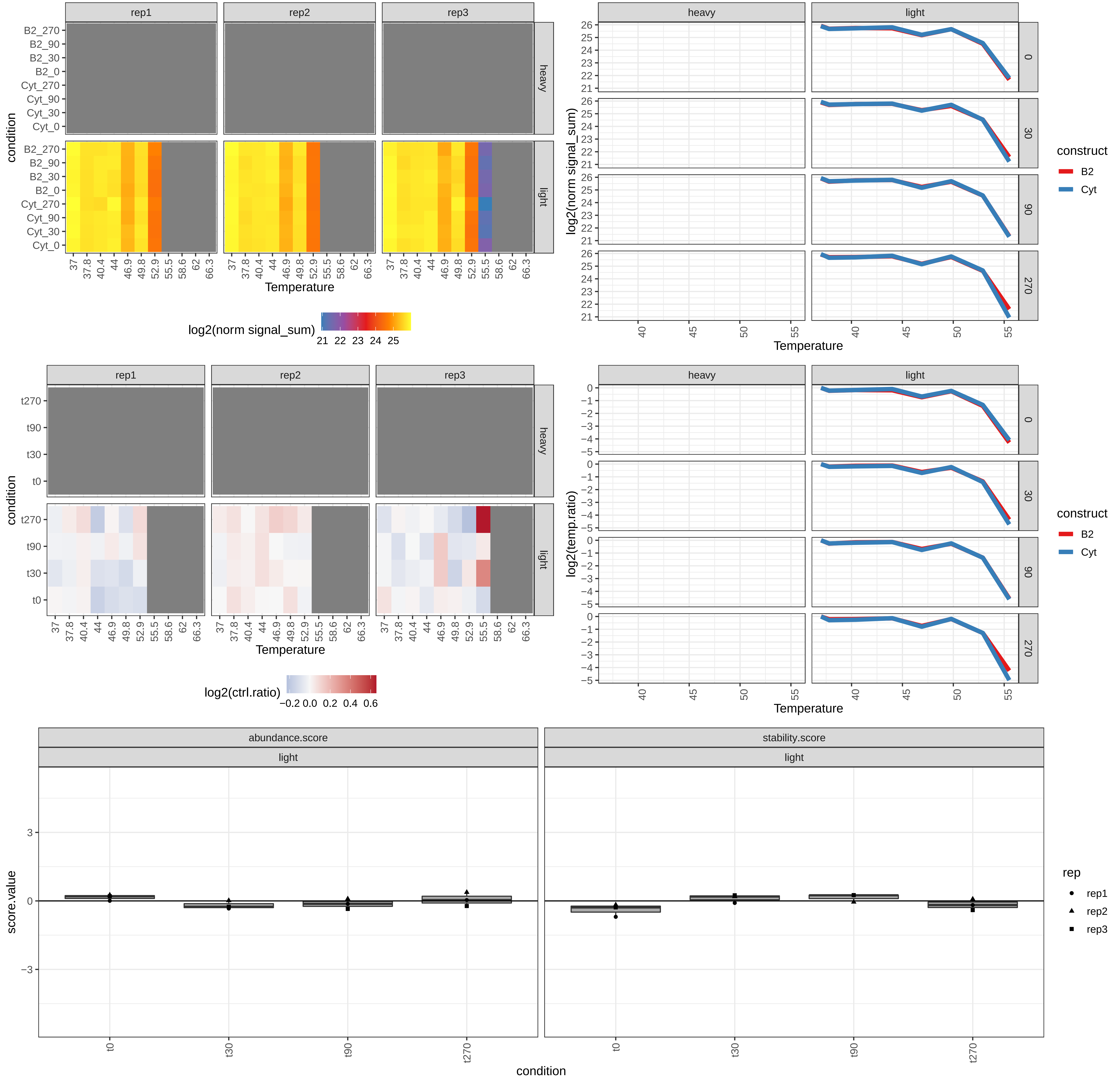

P49687 − Nucleoporin NUP145 OS=Saccharomyces cerevisiae (strain ATCC 204508 / S288c) GN=NUP145 PE=1 SV=1

NUP157 − average.top3: 6.81218482758621 − max.qupm: 14

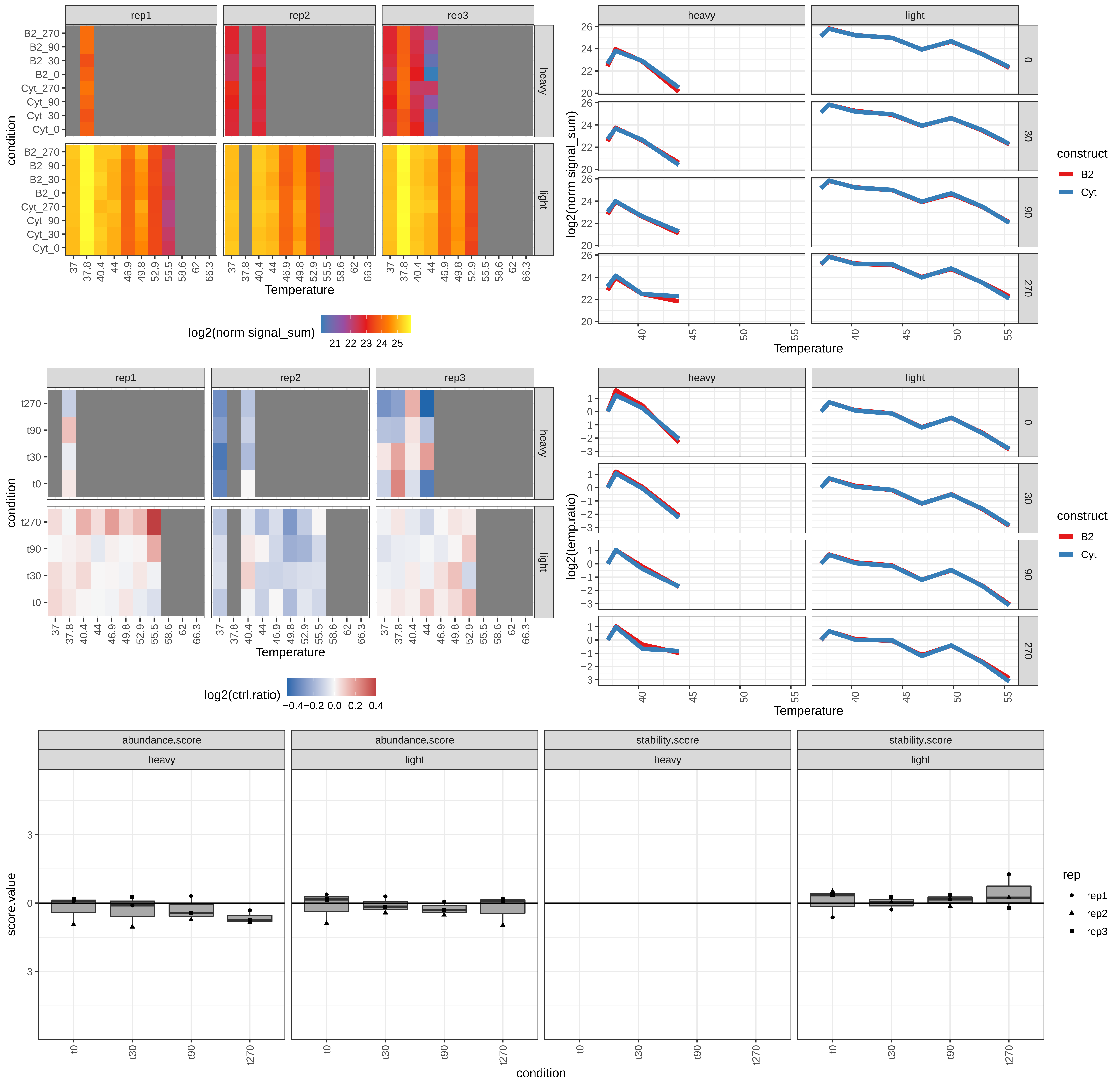

P40064 − Nucleoporin NUP157 OS=Saccharomyces cerevisiae (strain ATCC 204508 / S288c) GN=NUP157 PE=1 SV=1

NUP159 − average.top3: 6.93833583333333 − max.qupm: 6

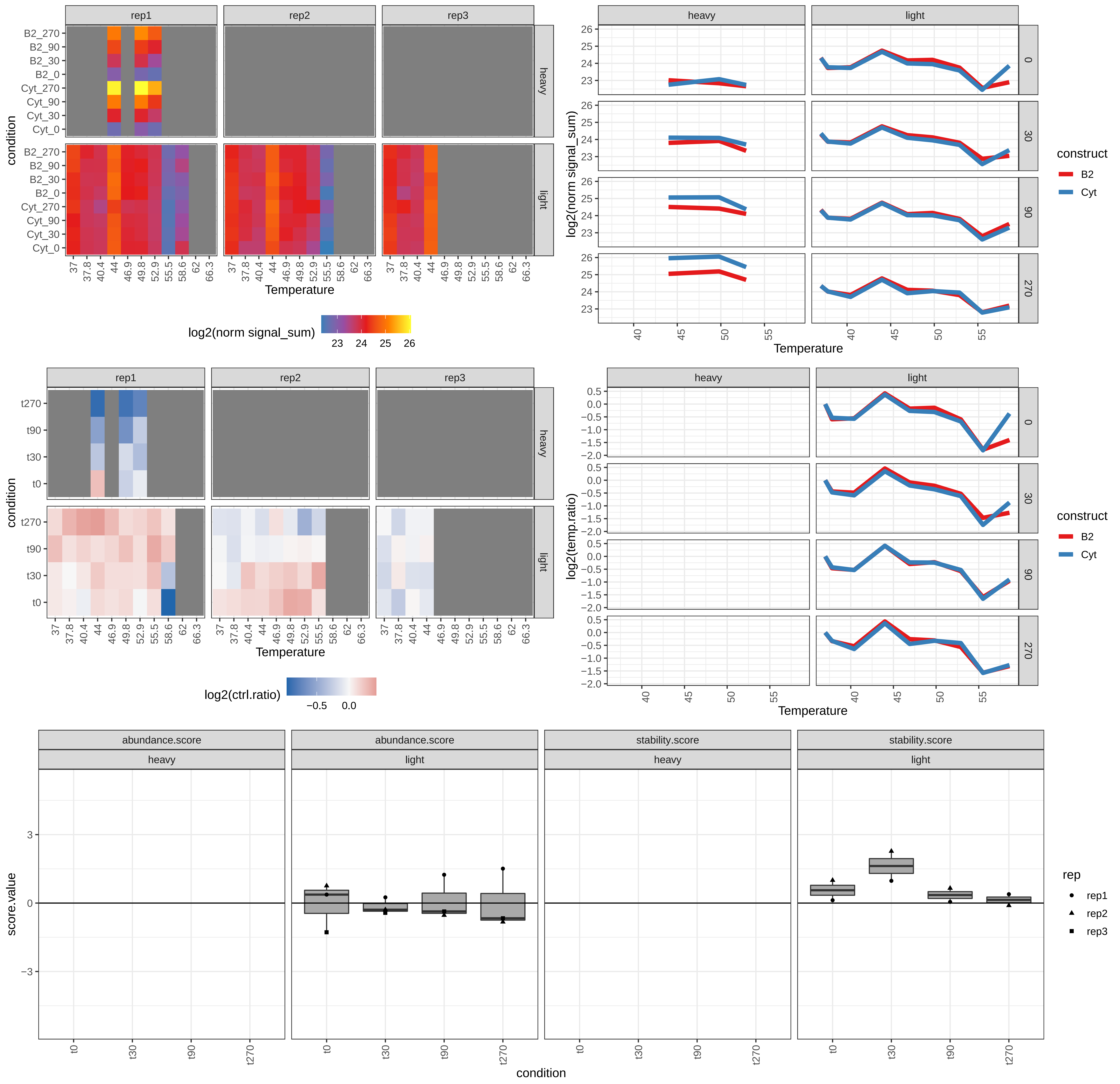

P40477 − Nucleoporin NUP159 OS=Saccharomyces cerevisiae (strain ATCC 204508 / S288c) GN=NUP159 PE=1 SV=1

NUP170 − average.top3: 6.84939208333333 − max.qupm: 13

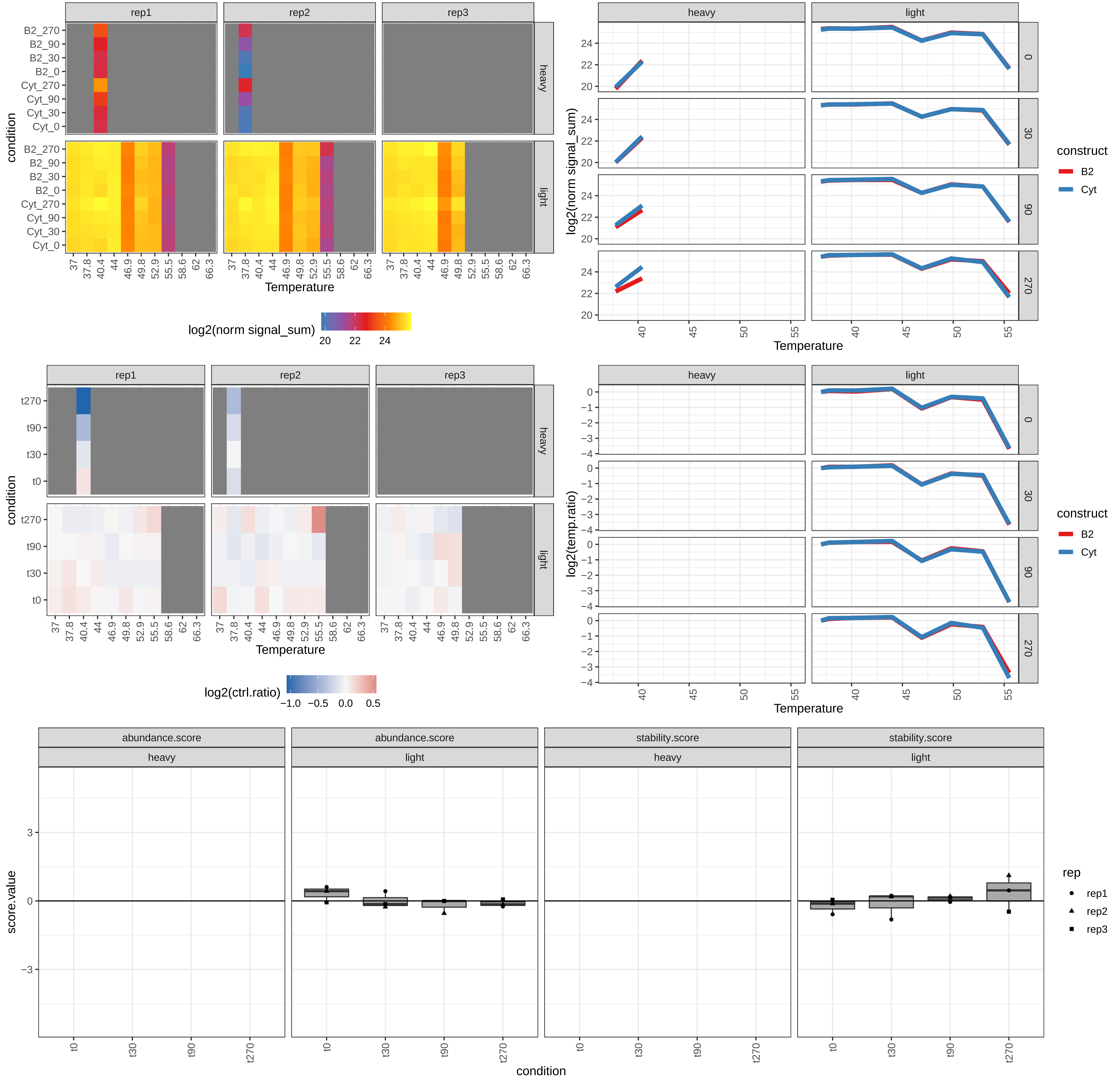

P38181 − Nucleoporin NUP170 OS=Saccharomyces cerevisiae (strain ATCC 204508 / S288c) GN=NUP170 PE=1 SV=1

NUP188 − average.top3: 6.826441 − max.qupm: 15

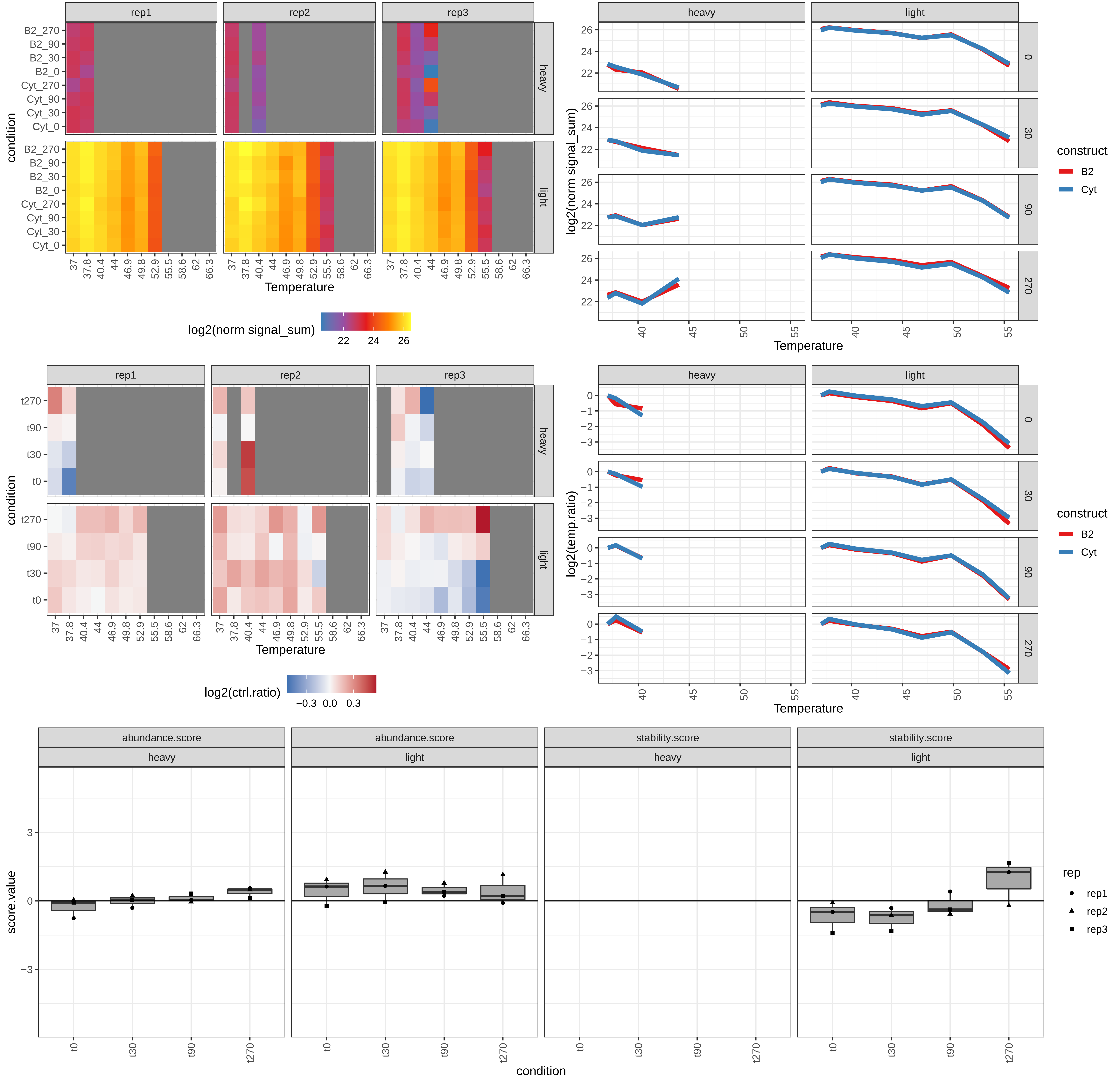

P52593 − Nucleoporin NUP188 OS=Saccharomyces cerevisiae (strain ATCC 204508 / S288c) GN=NUP188 PE=1 SV=1

NUP192 − average.top3: 6.87478178571429 − max.qupm: 13

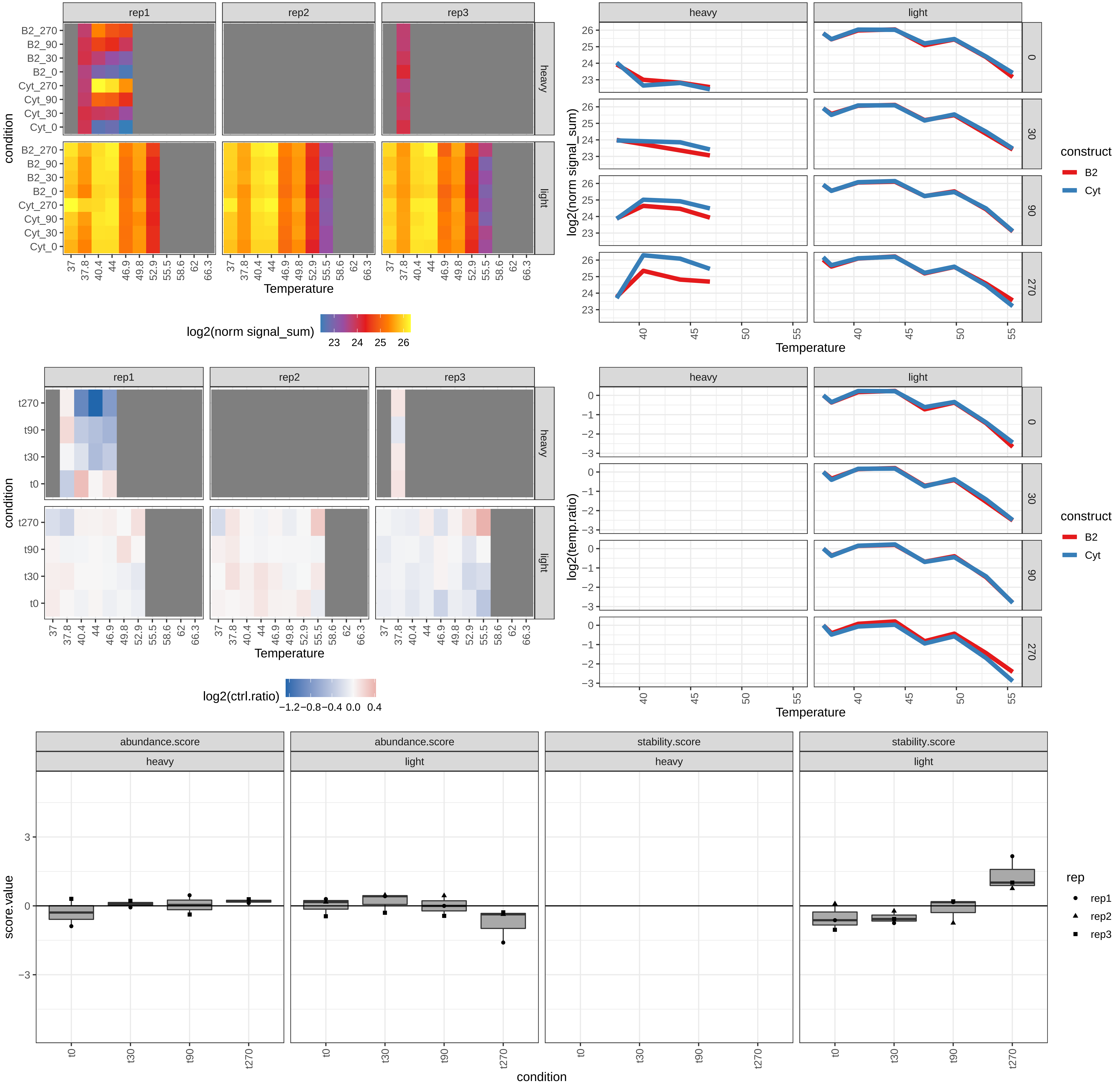

P47054 − Nucleoporin NUP192 OS=Saccharomyces cerevisiae (strain ATCC 204508 / S288c) GN=NUP192 PE=1 SV=1

NUP57 − average.top3: 6.96642285714286 − max.qupm: 8

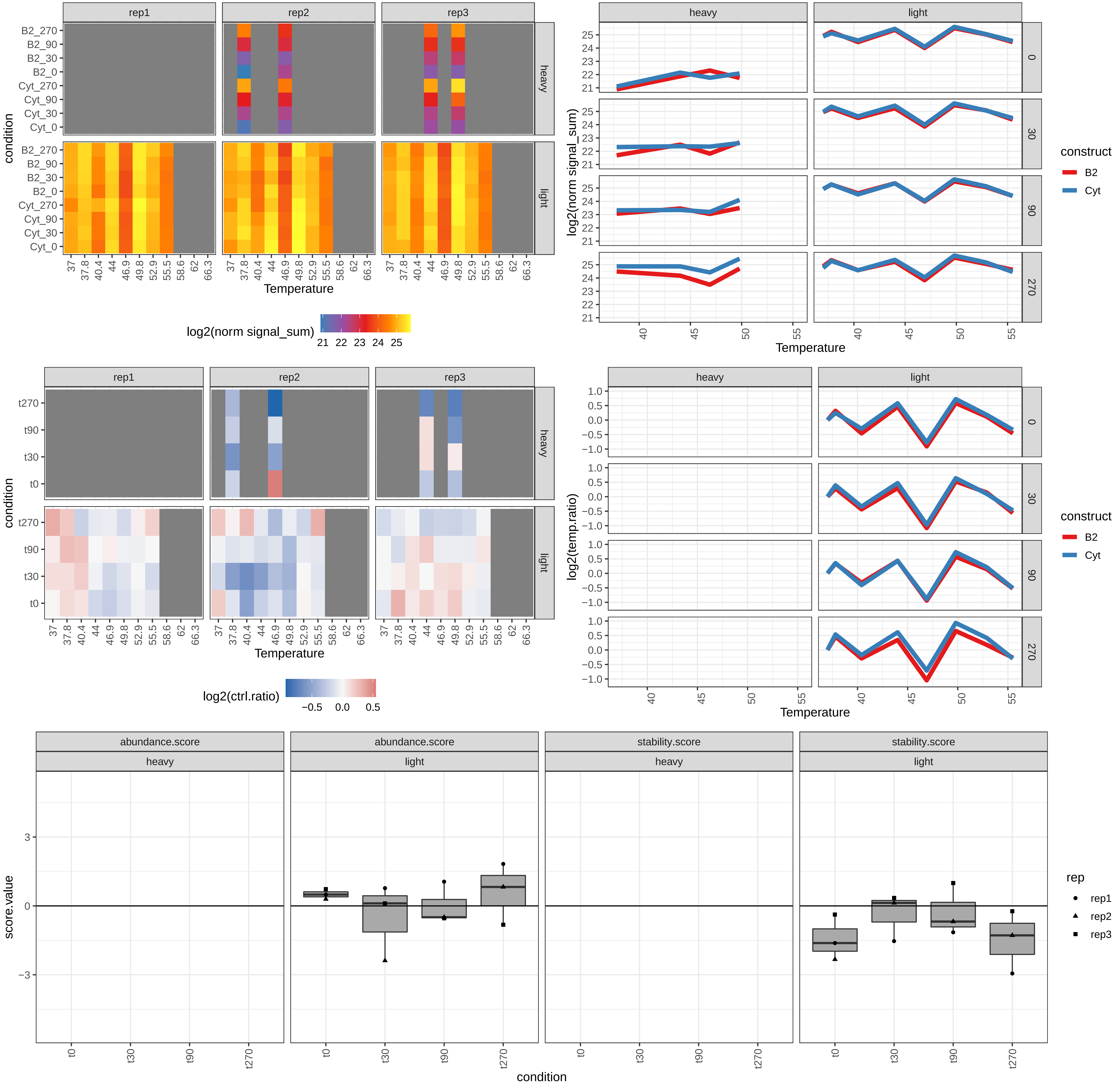

P48837 − Nucleoporin NUP57 OS=Saccharomyces cerevisiae (strain ATCC 204508 / S288c) GN=NUP57 PE=1 SV=1

NUP82 − average.top3: 6.50777617647059 − max.qupm: 10

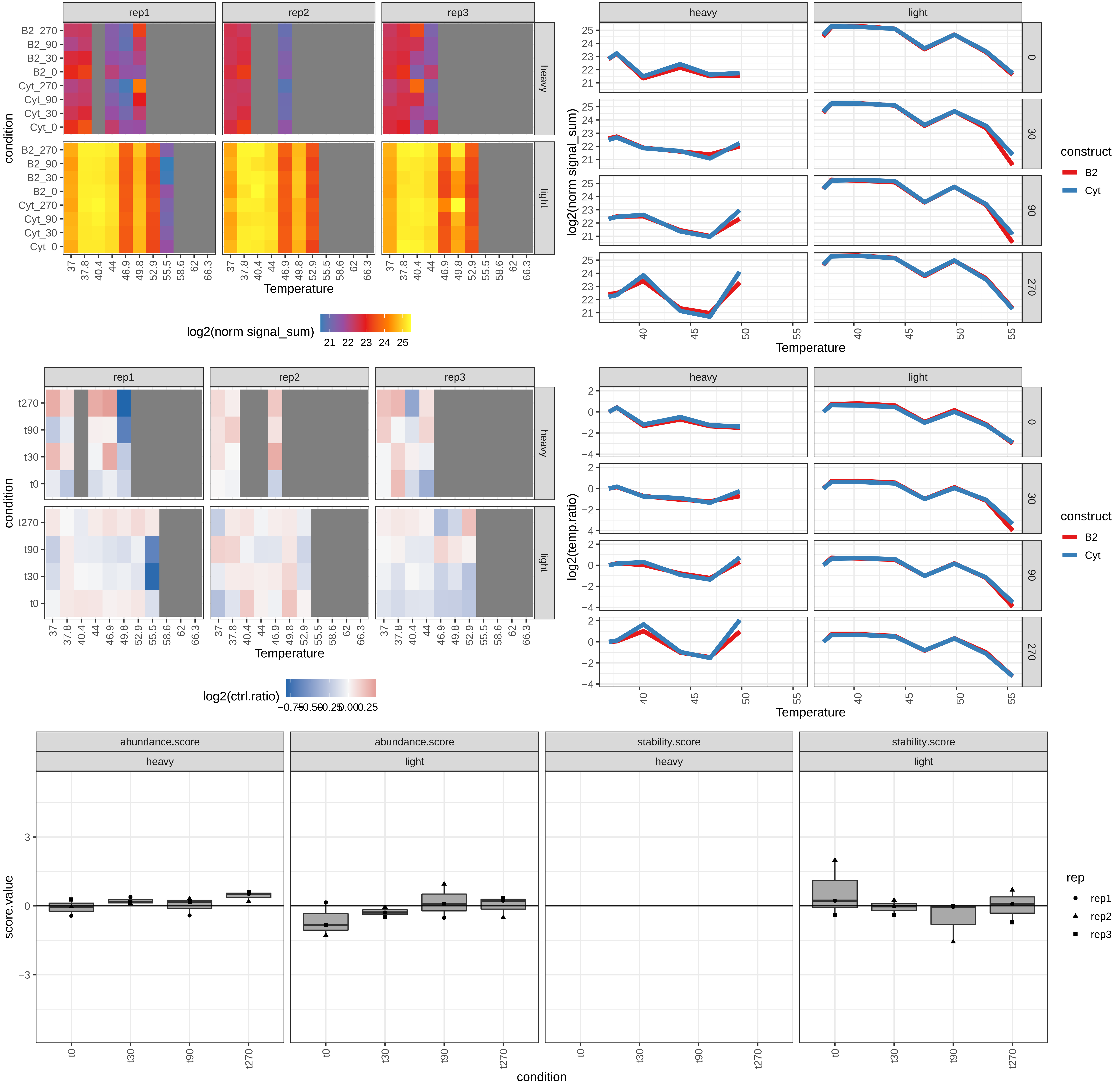

P40368 − Nucleoporin NUP82 OS=Saccharomyces cerevisiae (strain ATCC 204508 / S288c) GN=NUP82 PE=1 SV=2

NUP84 − average.top3: 7.14777181818182 − max.qupm: 11

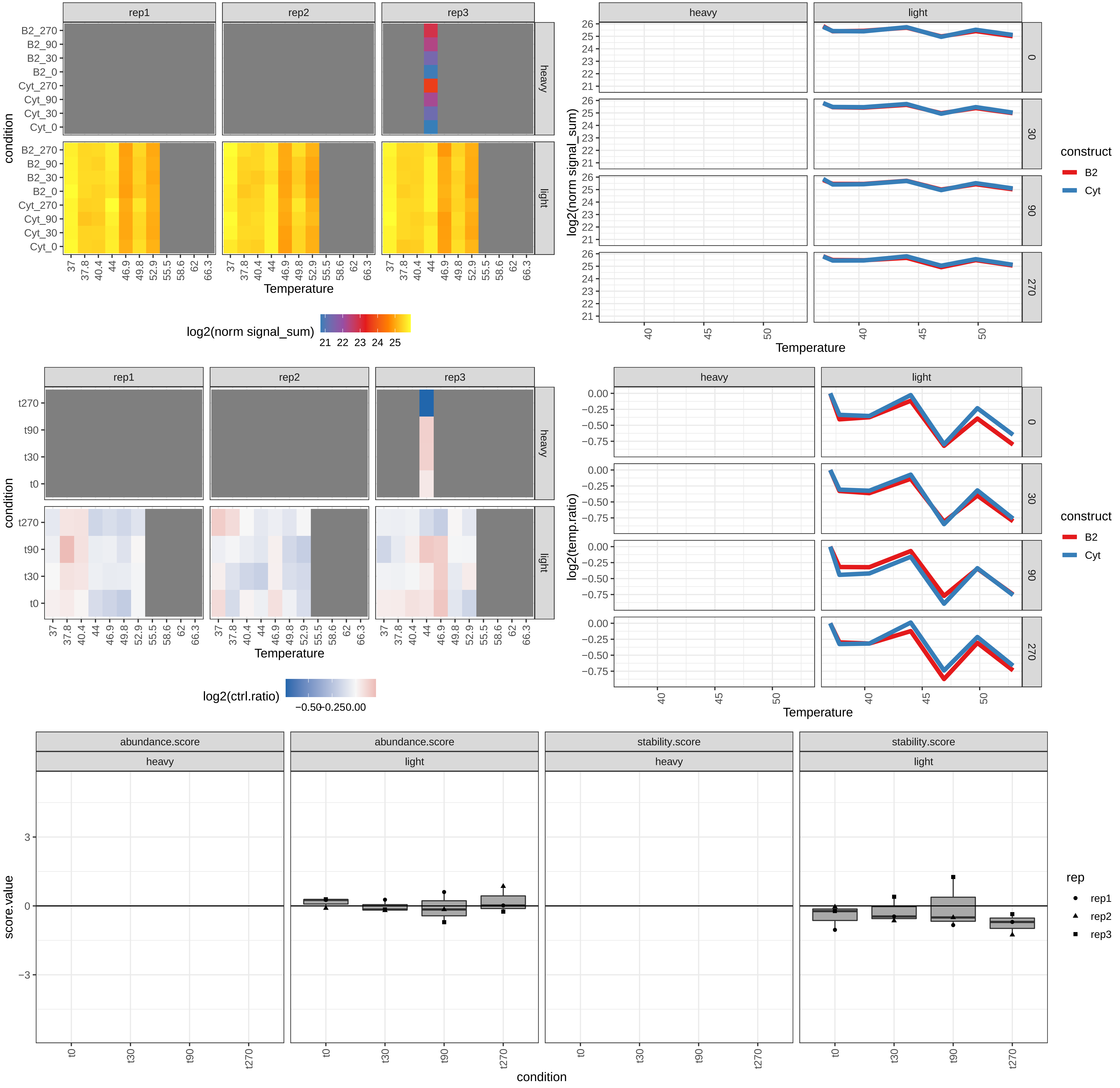

P52891 − Nucleoporin NUP84 OS=Saccharomyces cerevisiae (strain ATCC 204508 / S288c) GN=NUP84 PE=1 SV=1

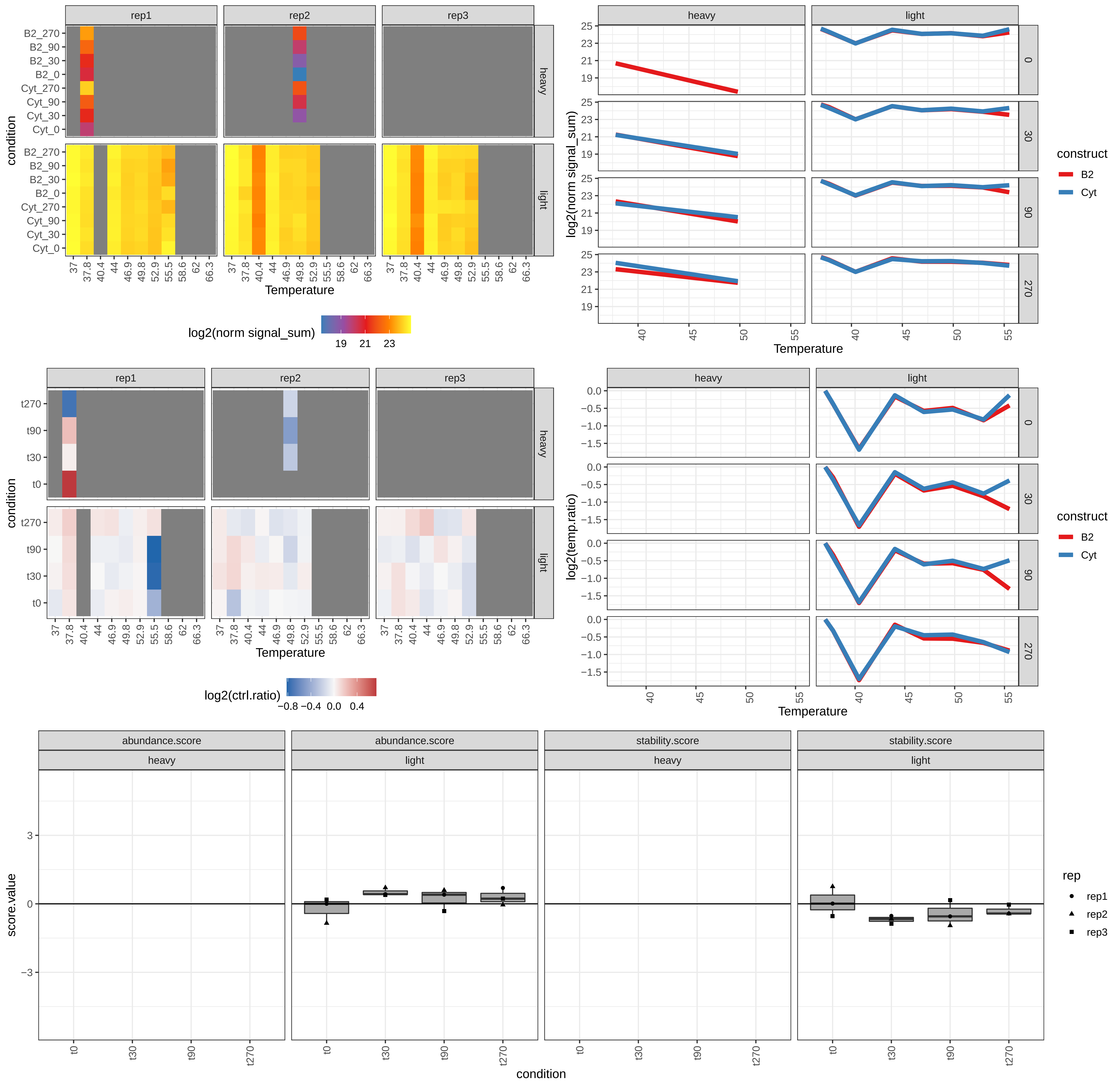

P46673 − Nucleoporin NUP85 OS=Saccharomyces cerevisiae (strain ATCC 204508 / S288c) GN=NUP85 PE=1 SV=1

NUS1 − average.top3: 6.49768615384615 − max.qupm: 4

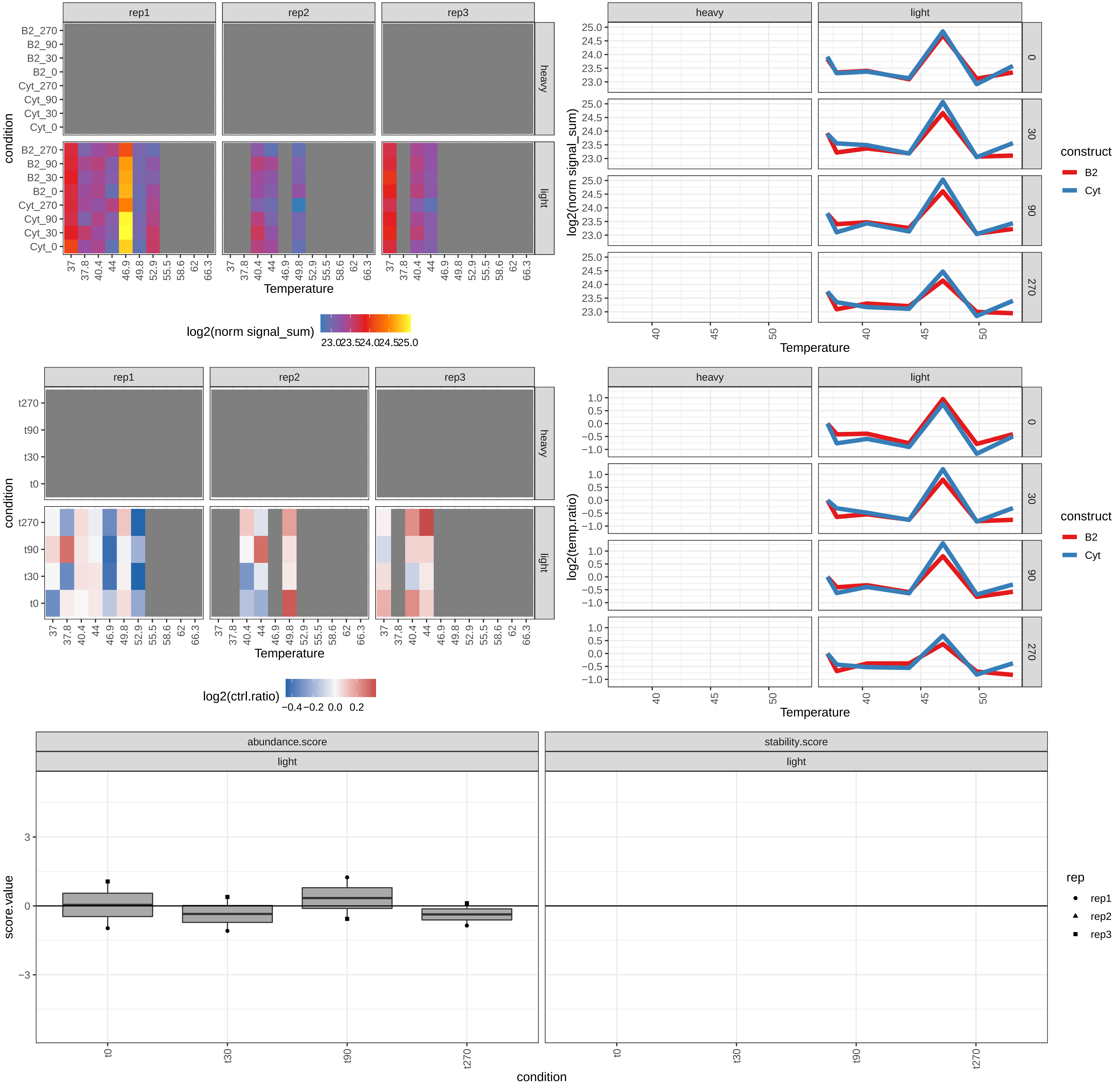

Q12063 − Dehydrodolichyl diphosphate synthase complex subunit NUS1 OS=Saccharomyces cerevisiae (strain ATCC 204508 / S288c) GN=NUS1 PE=1 SV=1

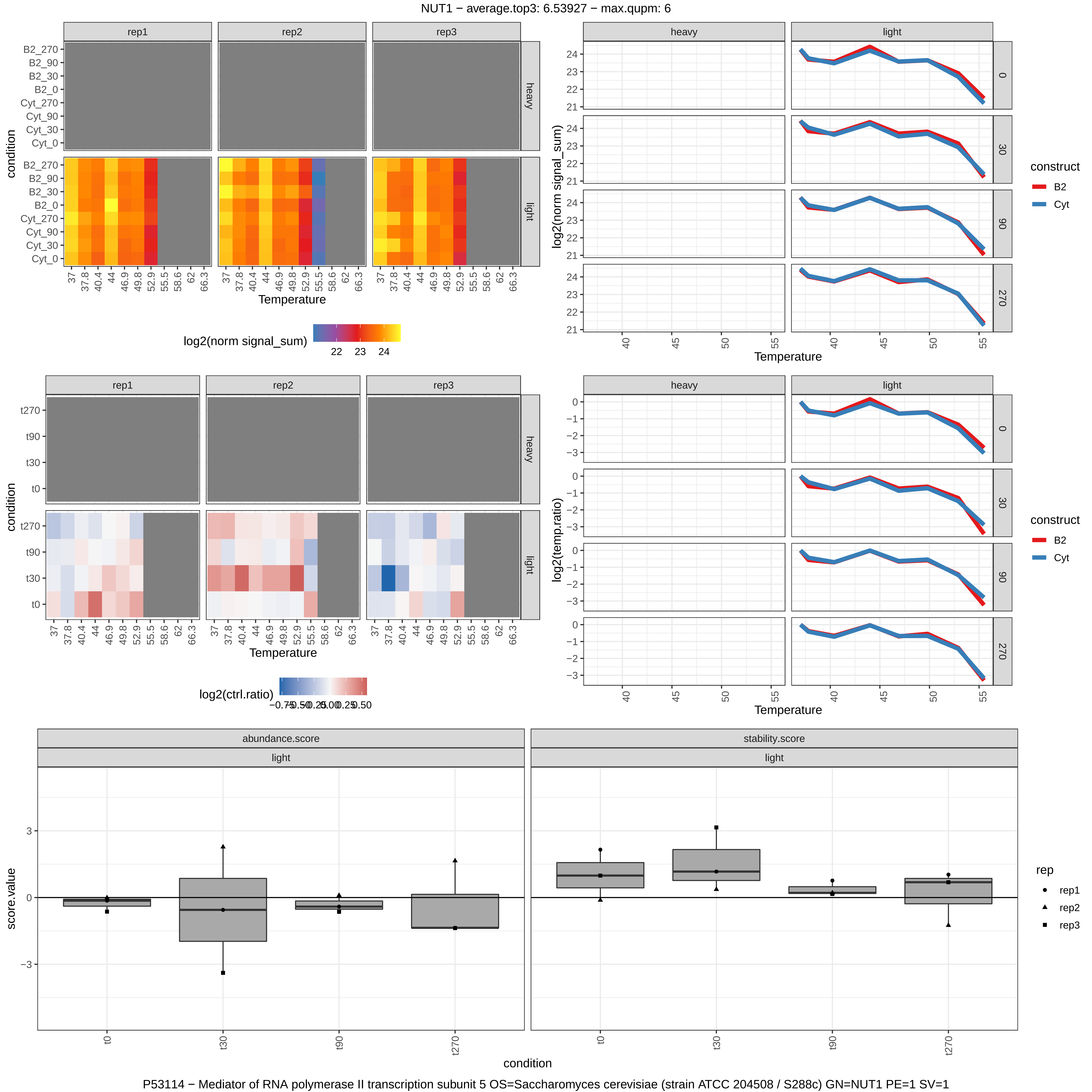

NUT1 − average.top3: 6.53927 − max.qupm: 6
rep1
rep2
rep3
heavy
light
condition
Temperature
log2(norm signal_sum)
log2(temp.ratio)
log2(ctrl.ratio)
construct
B2
Cyt
abundance.score
stability.score
score.value
rep
rep1
rep2
rep3
P53114 − Mediator of RNA polymerase II transcription subunit 5 OS=Saccharomyces cerevisiae (strain ATCC 204508 / S288c) GN=NUT1 PE=1 SV=1

OAC1 − average.top3: 7.07692 − max.qupm: 9

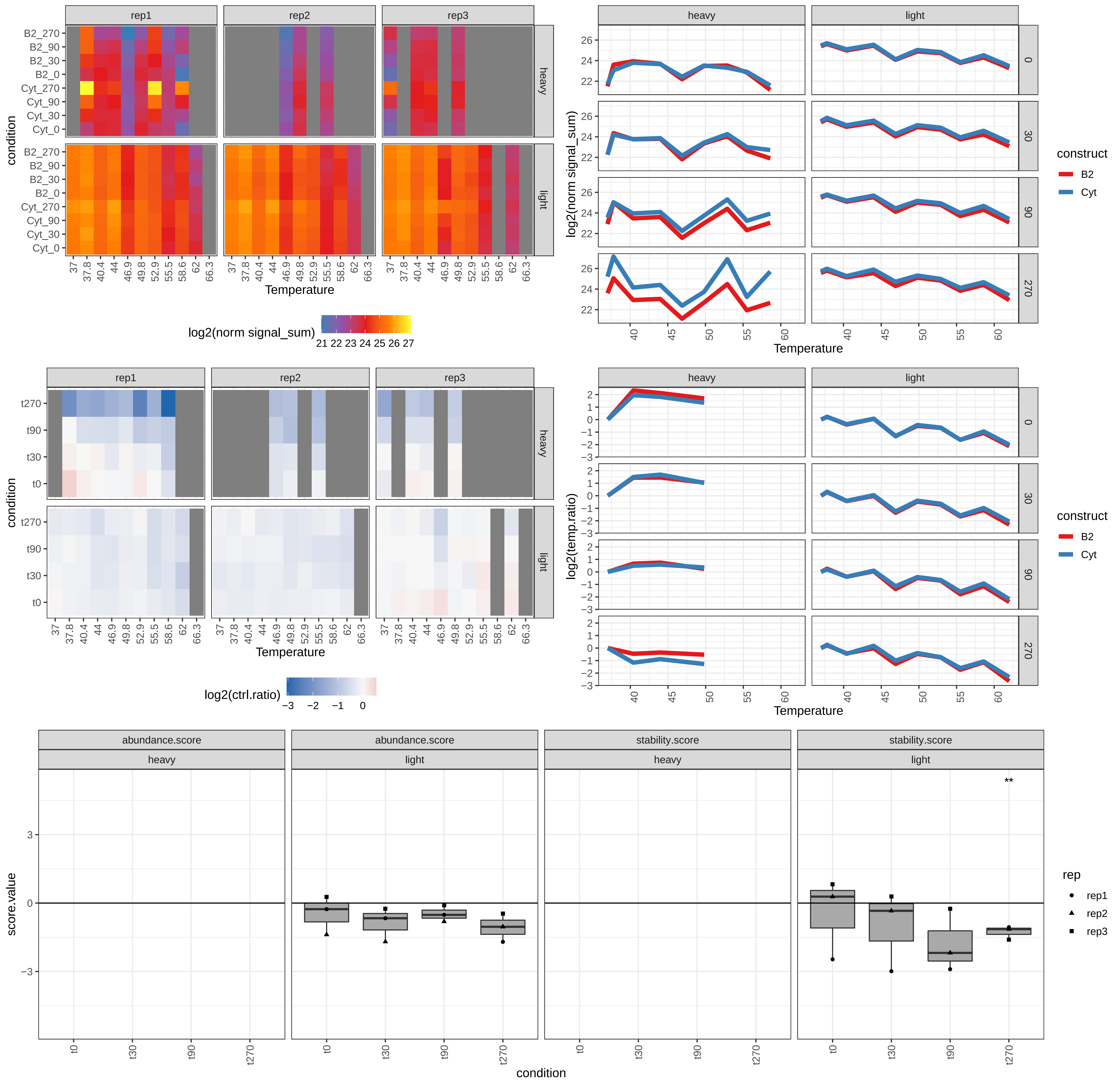

P32332 − Mitochondrial oxaloacetate transport protein OS=Saccharomyces cerevisiae (strain ATCC 204508 / S288c) GN=OAC1 PE=1 SV=1

OAF1 − average.top3: 6.54210866666667 − max.qupm: 4

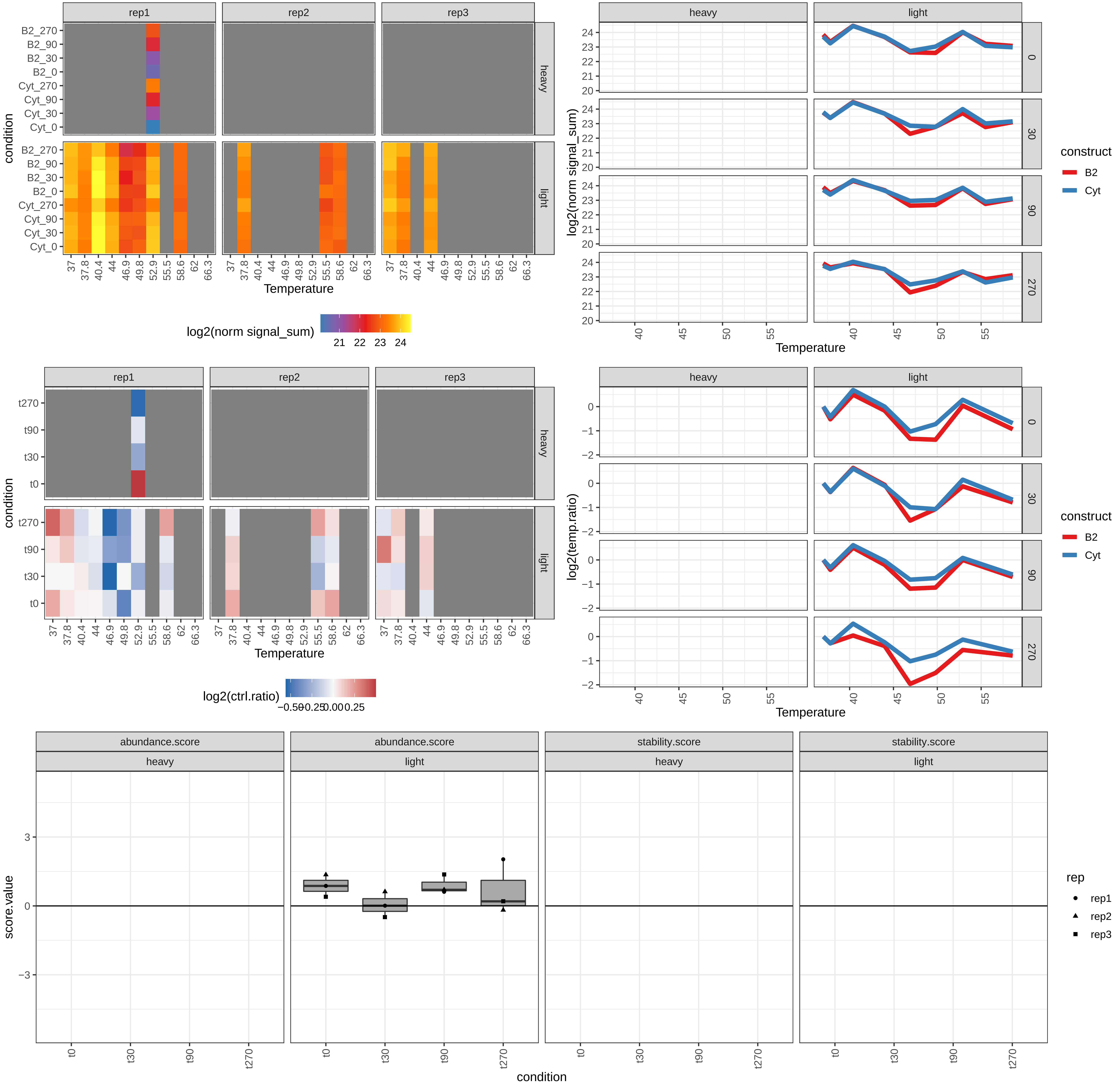

P39720 − Oleate−activated transcription factor 1 OS=Saccharomyces cerevisiae (strain ATCC 204508 / S288c) GN=OAF1 PE=1 SV=3

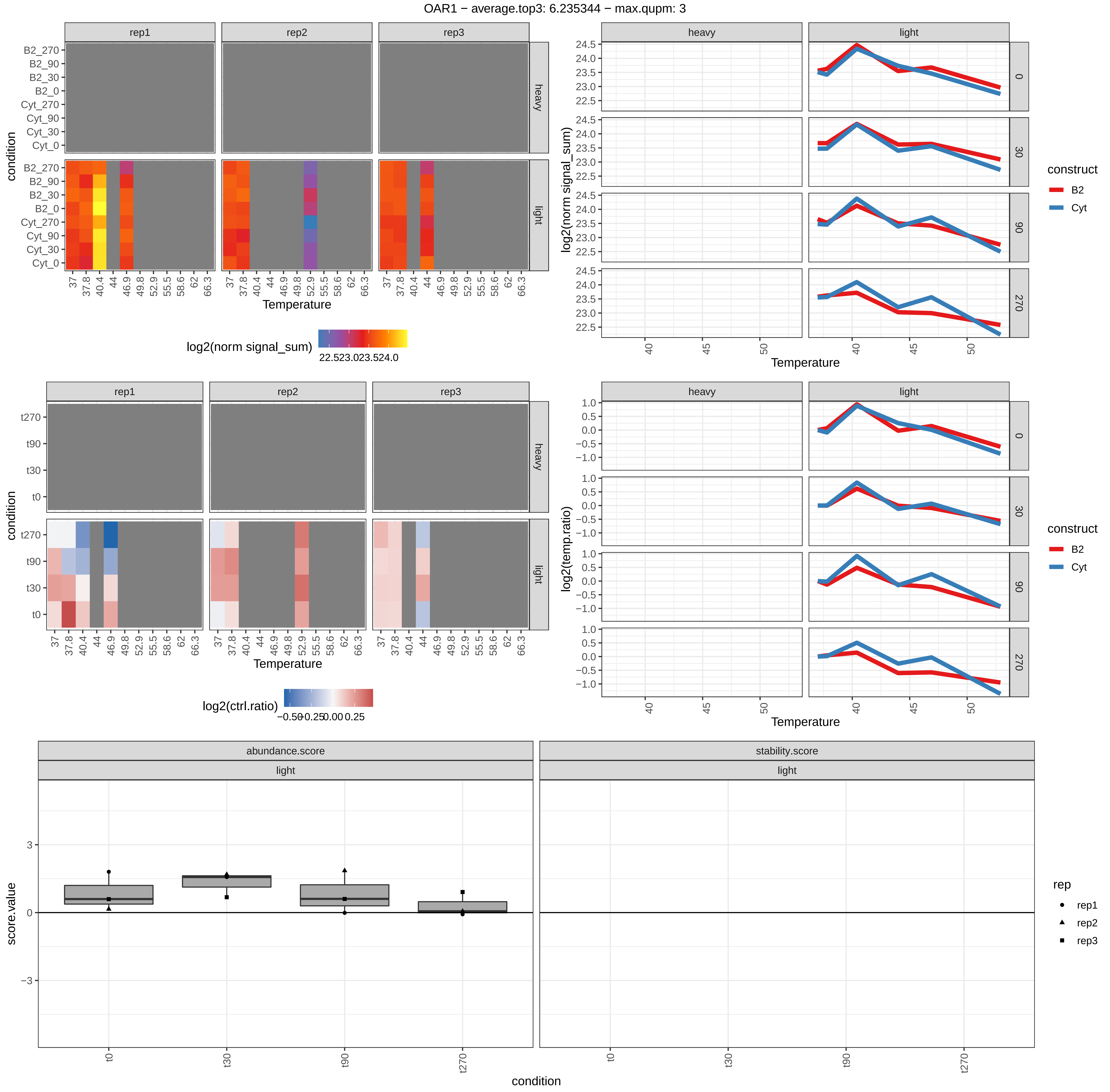

P35731 − 3−oxoacyl−[acyl−carrier−protein] reductase OS=Saccharomyces cerevisiae (strain ATCC 204508 / S288c) GN=OAR1 PE=1 SV=1

OCH1 − average.top3: 6.45365894736842 − max.qupm: 5

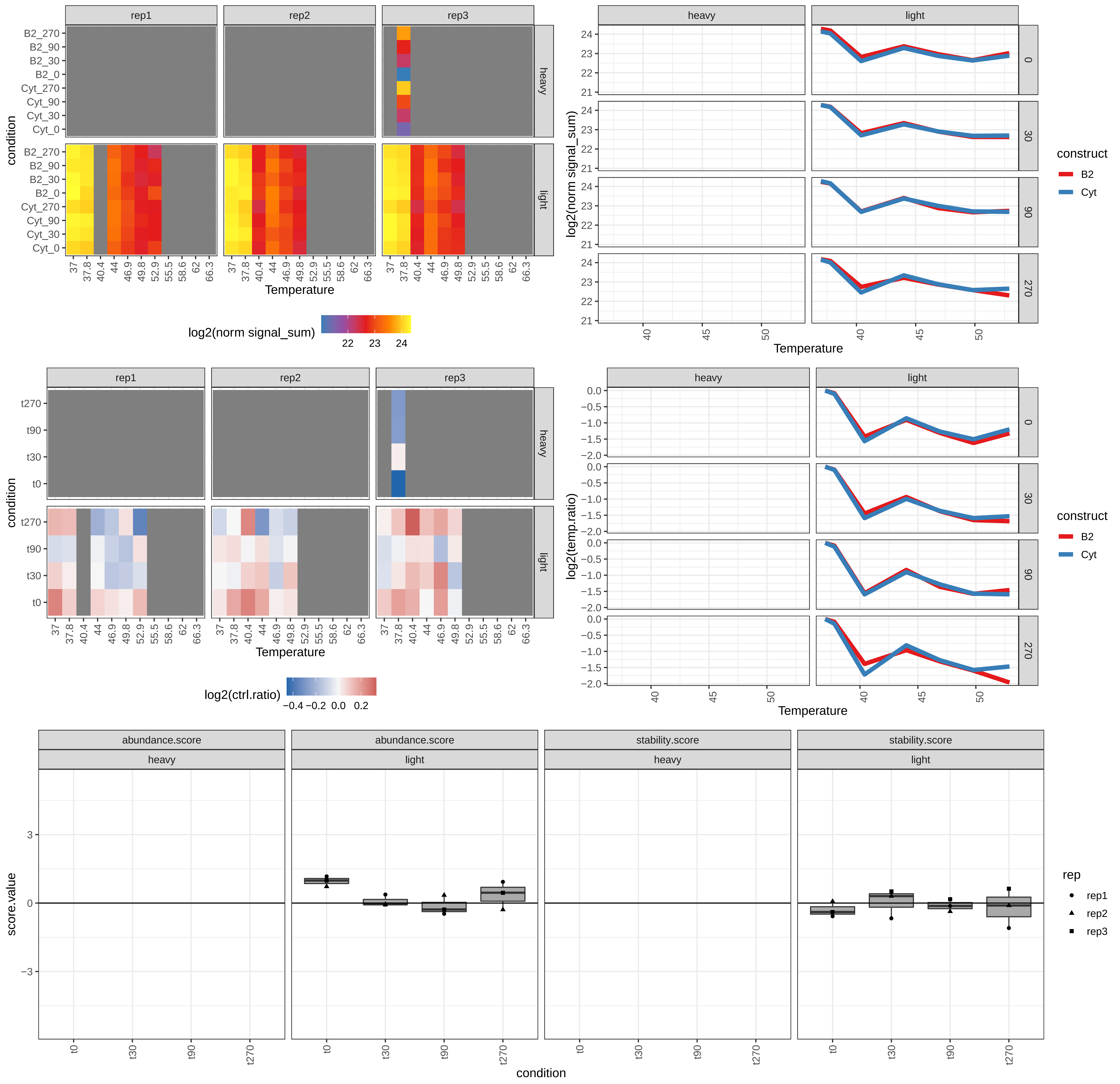

P31755 − Initiation−specific alpha−1,6−mannosyltransferase OS=Saccharomyces cerevisiae (strain ATCC 204508 / S288c) GN=OCH1 PE=1 SV=1

OCT1 − average.top3: 6.42227523809524 − max.qupm: 7

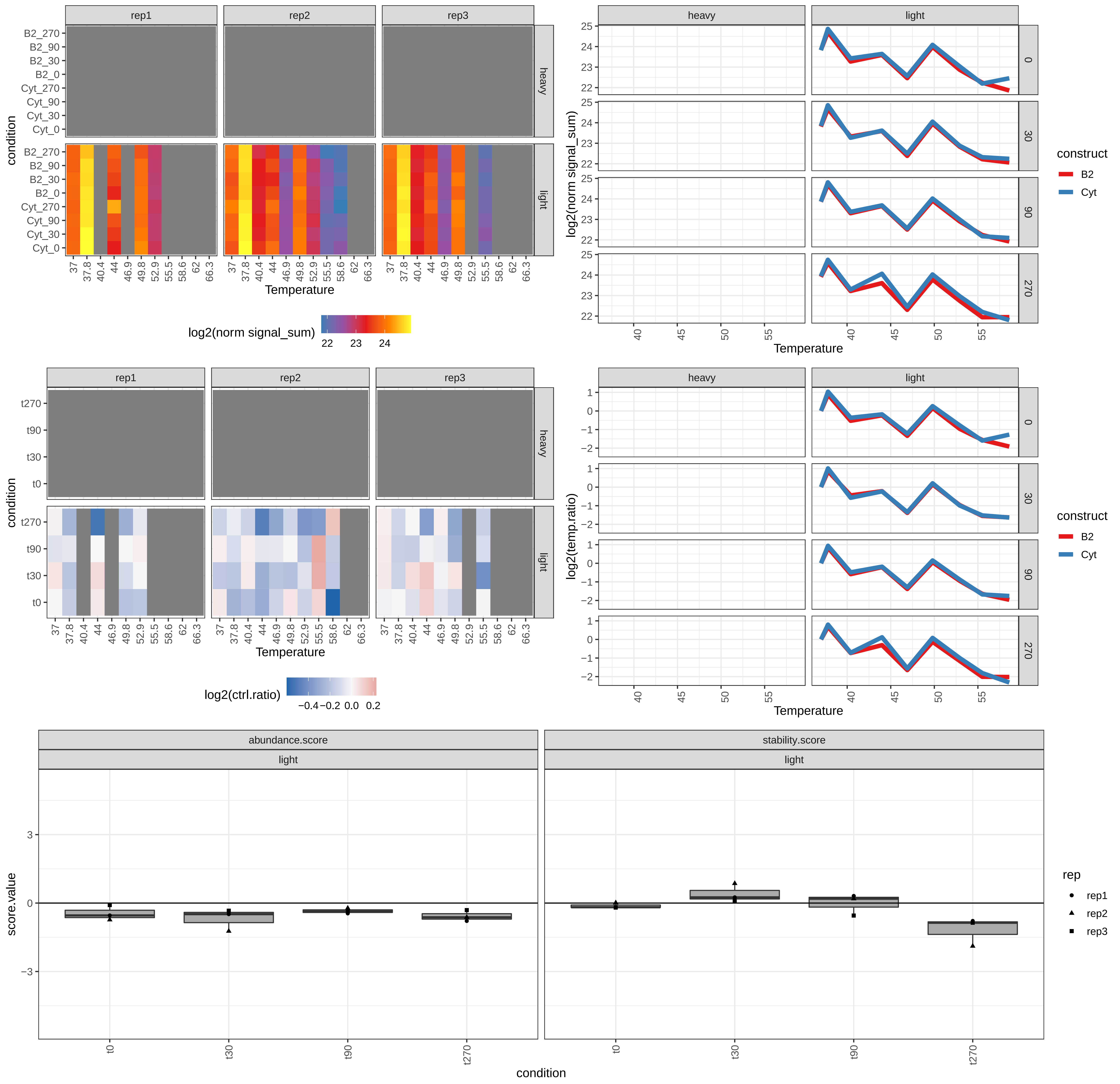

P35999 − Mitochondrial intermediate peptidase OS=Saccharomyces cerevisiae (strain ATCC 204508 / S288c) GN=OCT1 PE=1 SV=2

ODC1 − average.top3: 7.21705555555556 − max.qupm: 7

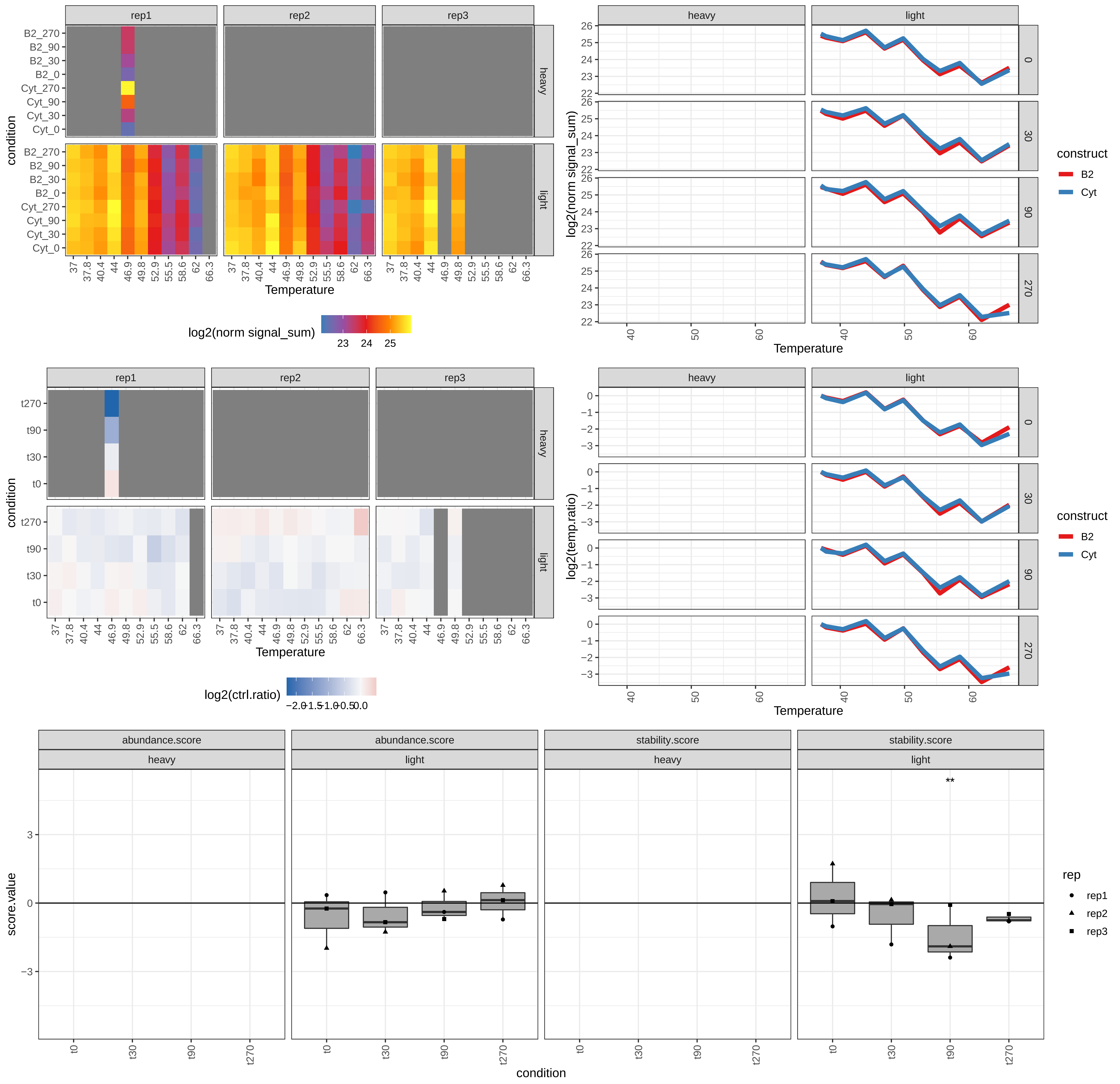

Q03028 − Mitochondrial 2−oxodicarboxylate carrier 1 OS=Saccharomyces cerevisiae (strain ATCC 204508 / S288c) GN=ODC1 PE=1 SV=1

OLA1 − average.top3: 7.5638595 − max.qupm: 19

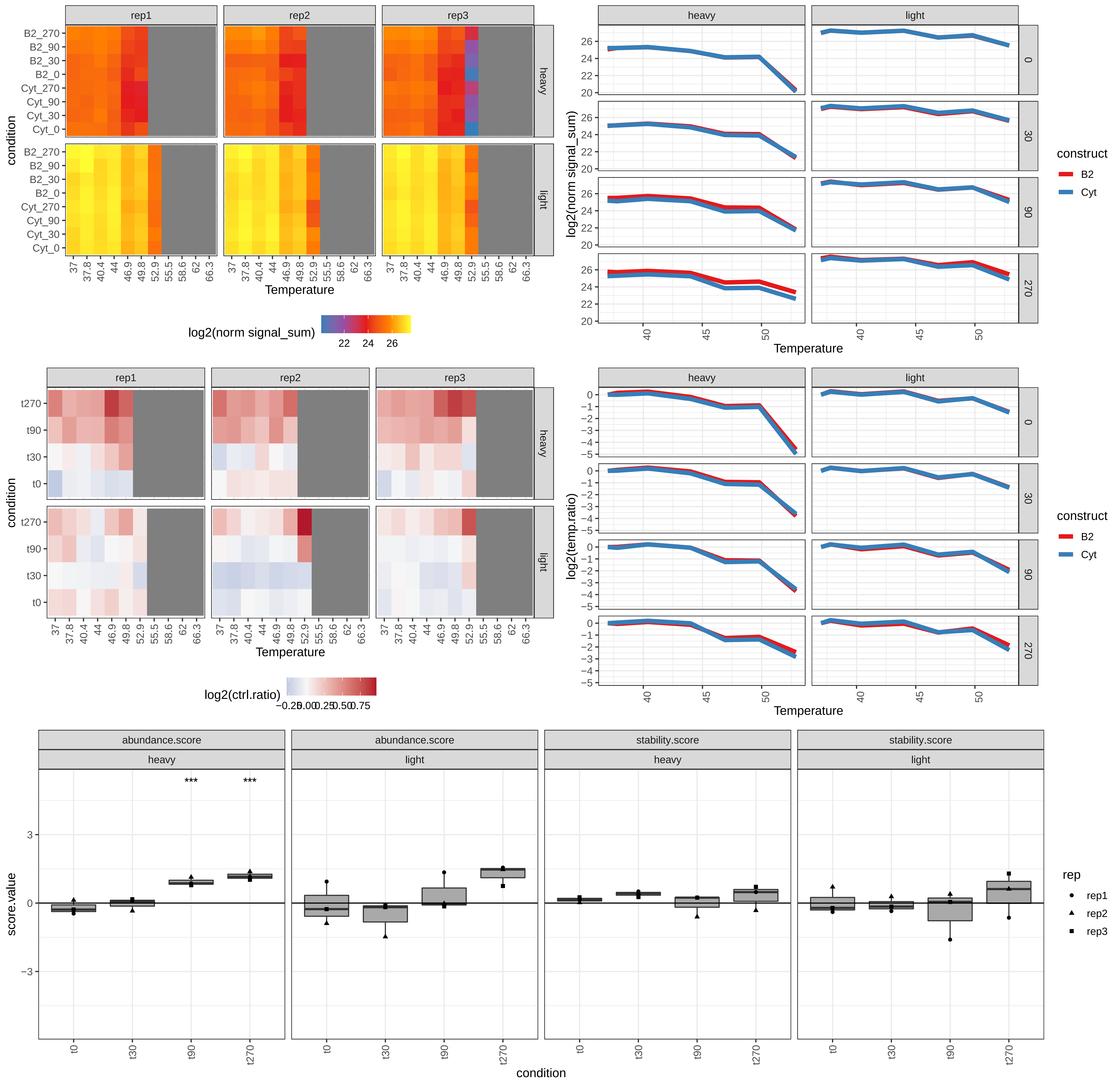

P38219 − Obg−like ATPase 1 OS=Saccharomyces cerevisiae (strain ATCC 204508 / S288c) GN=OLA1 PE=1 SV=1

OLE1 − average.top3: 6.83612578947368 − max.qupm: 7

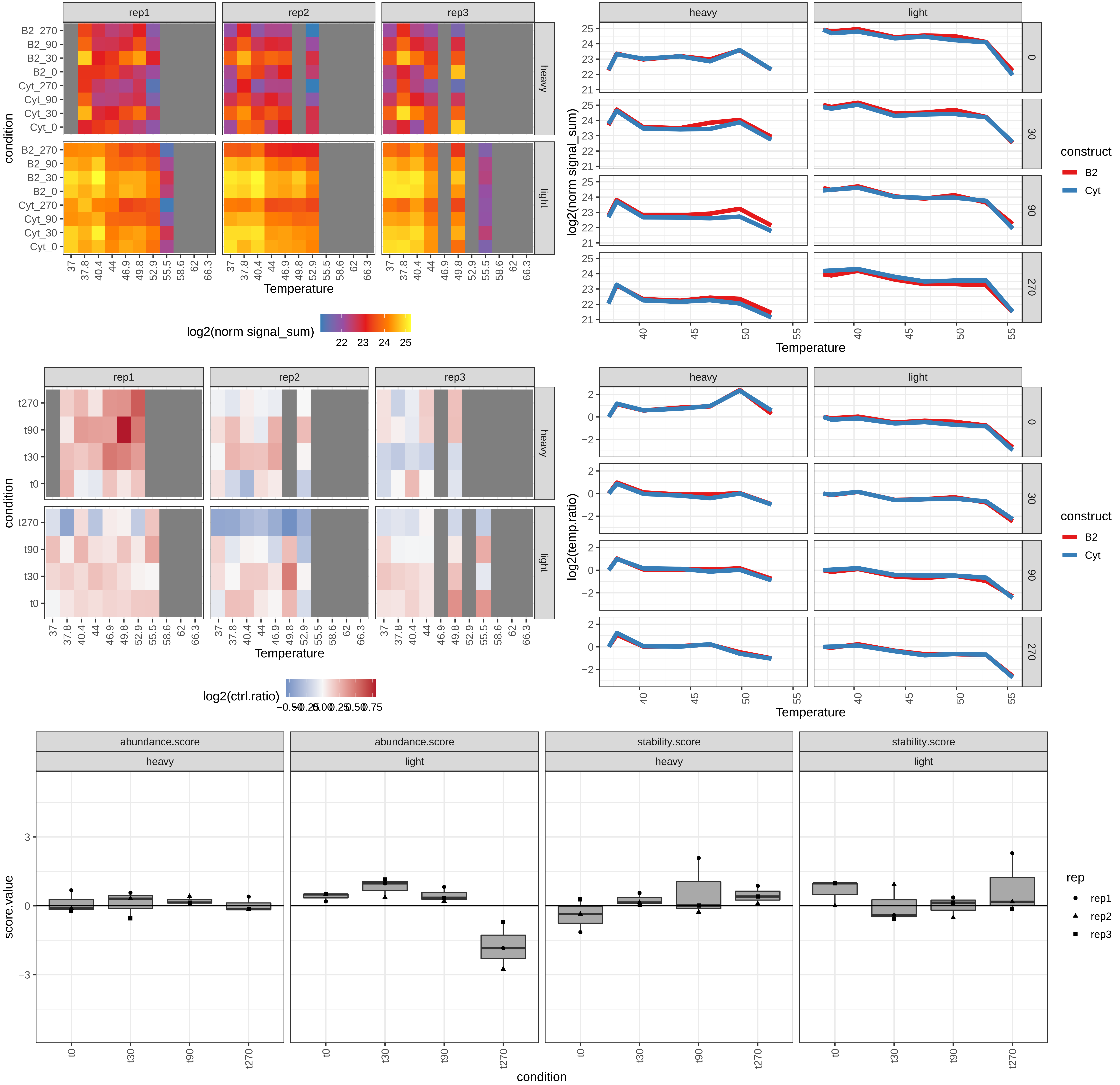

P21147 − Acyl−CoA desaturase 1 OS=Saccharomyces cerevisiae (strain ATCC 204508 / S288c) GN=OLE1 PE=1 SV=2

OM14 − average.top3: 6.88083846153846 − max.qupm: 5

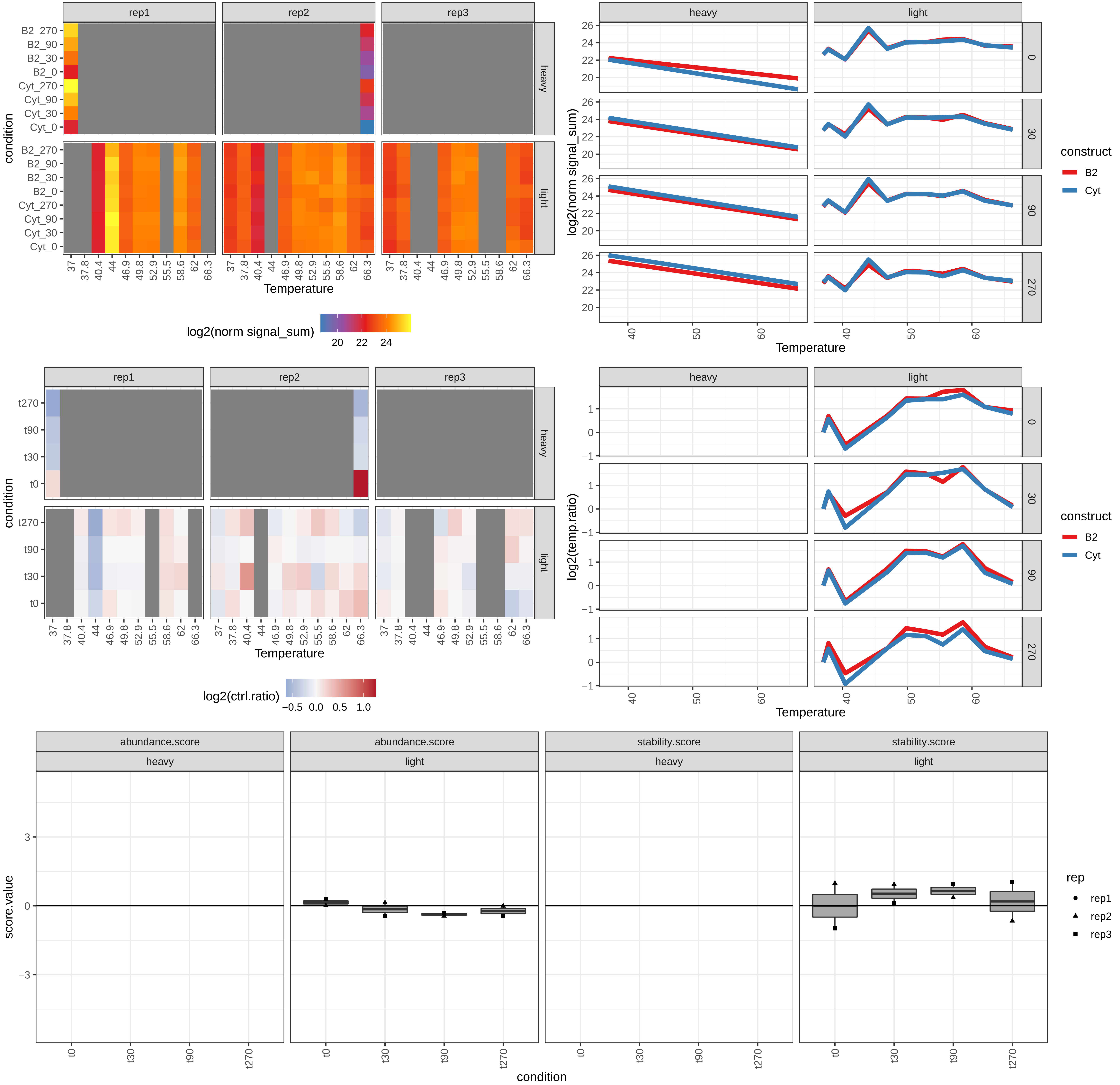

P38325 − Mitochondrial outer membrane protein OM14 OS=Saccharomyces cerevisiae (strain ATCC 204508 / S288c) GN=OM14 PE=1 SV=1

OM45 − average.top3: 7.83837954545455 − max.qupm: 31

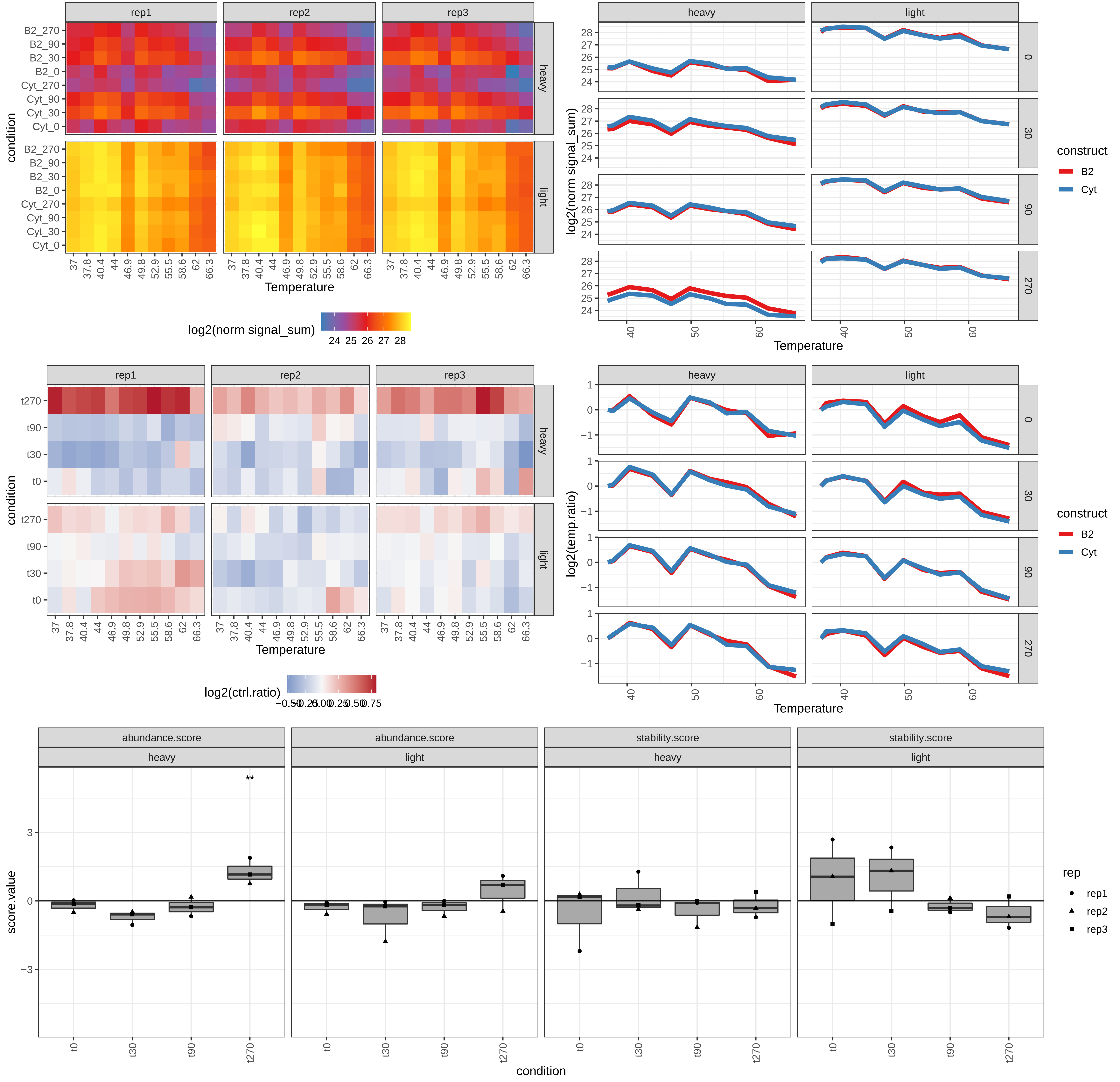

P16547 − Mitochondrial outer membrane protein OM45 OS=Saccharomyces cerevisiae (strain ATCC 204508 / S288c) GN=OM45 PE=1 SV=2

OMA1 − average.top3: 6.4633725 − max.qupm: 6

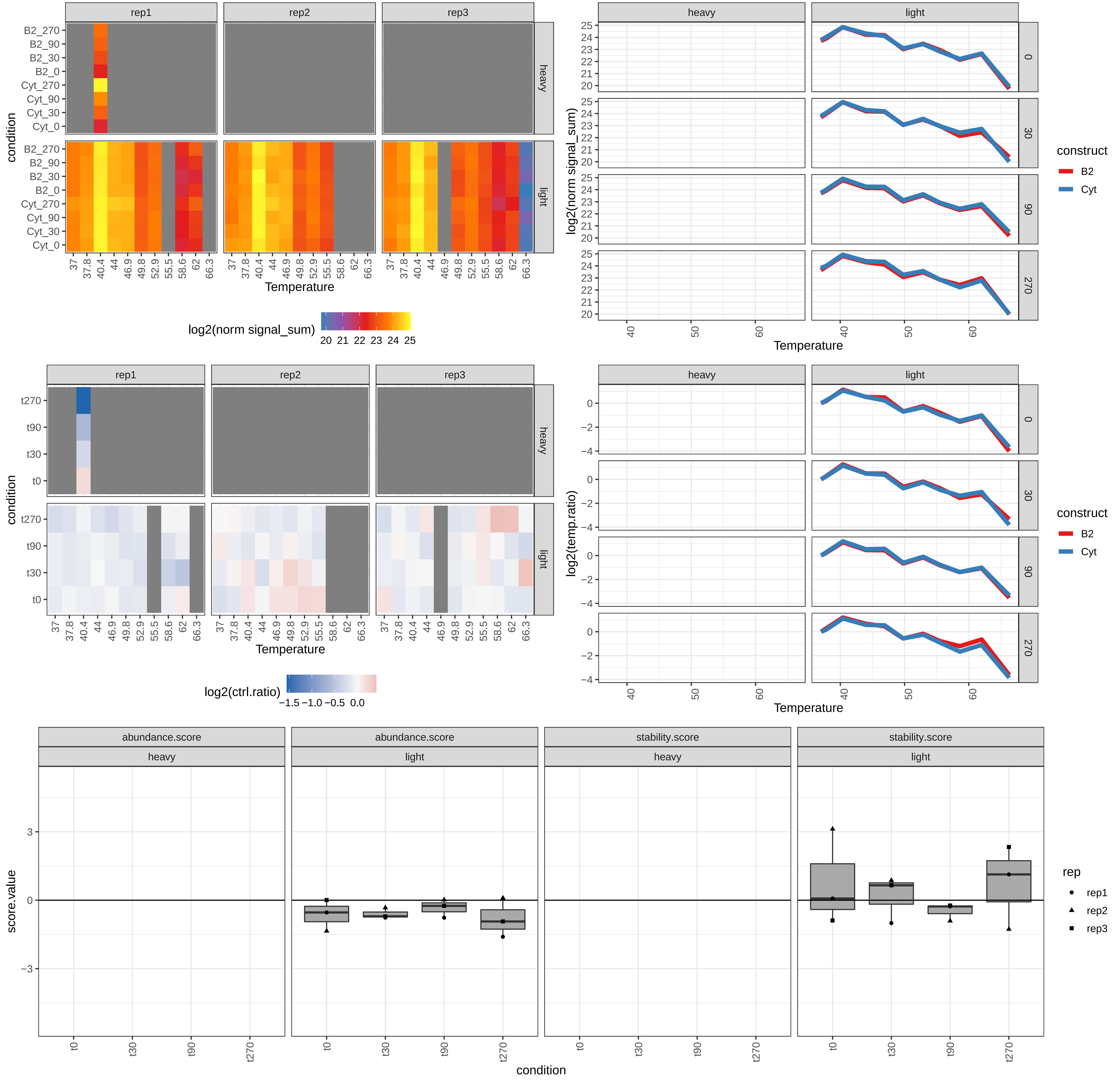

P36163 − Mitochondrial metalloendopeptidase OMA1 OS=Saccharomyces cerevisiae (strain ATCC 204508 / S288c) GN=OMA1 PE=1 SV=2

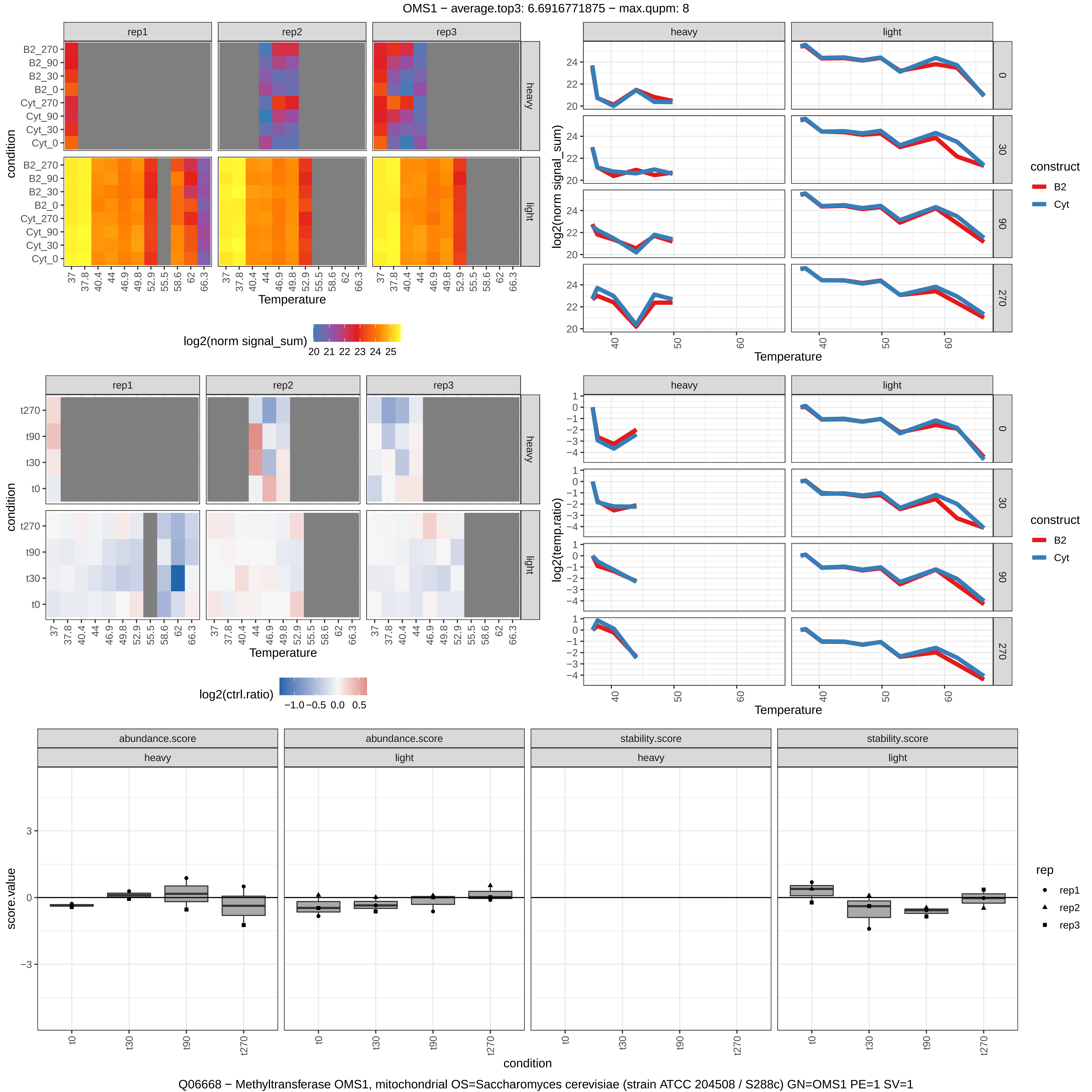
OMS1 − average.top3: 6.6916771875 − max.qupm: 8
rep1
rep2
rep3
heavy
light
condition
Temperature
log2(norm signal_sum)
log2(temp.ratio)
log2(ctrl.ratio)
construct
B2
Cyt
abundance.score
stability.score
score.value
rep
rep1
rep2
rep3
Q06668 − Methyltransferase OMS1, mitochondrial OS=Saccharomyces cerevisiae (strain ATCC 204508 / S288c) GN=OMS1 PE=1 SV=1

OPI10 − average.top3: 6.73017769230769 − max.qupm: 4

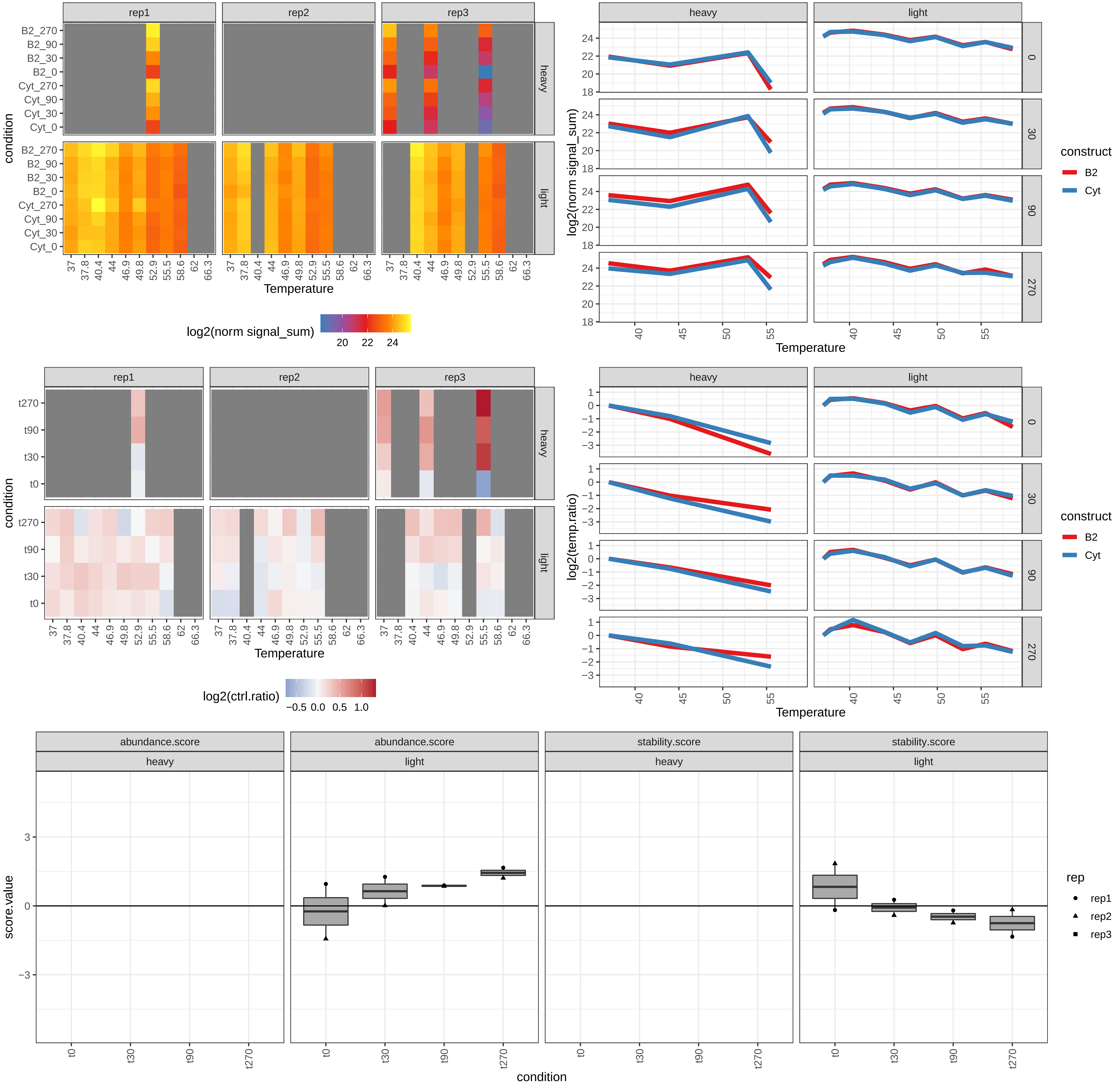

Q08202 − Protein OPI10 OS=Saccharomyces cerevisiae (strain ATCC 204508 / S288c) GN=OPI10 PE=1 SV=1

OPY1 − average.top3: 6.46042 − max.qupm: 4

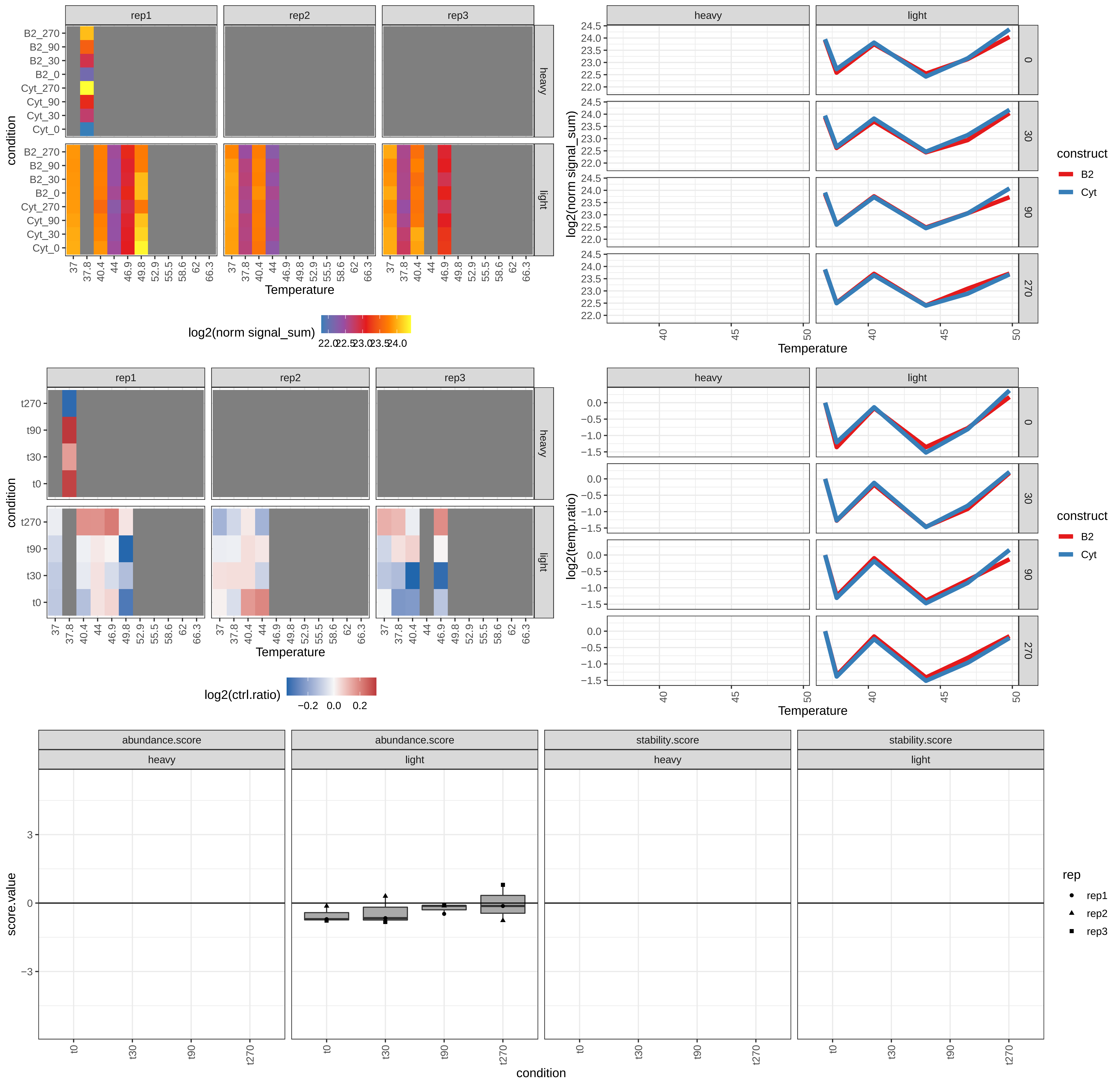

P38271 − Protein OPY1 OS=Saccharomyces cerevisiae (strain ATCC 204508 / S288c) GN=OPY1 PE=1 SV=1

ORC1 − average.top3: 6.36588222222222 − max.qupm: 5

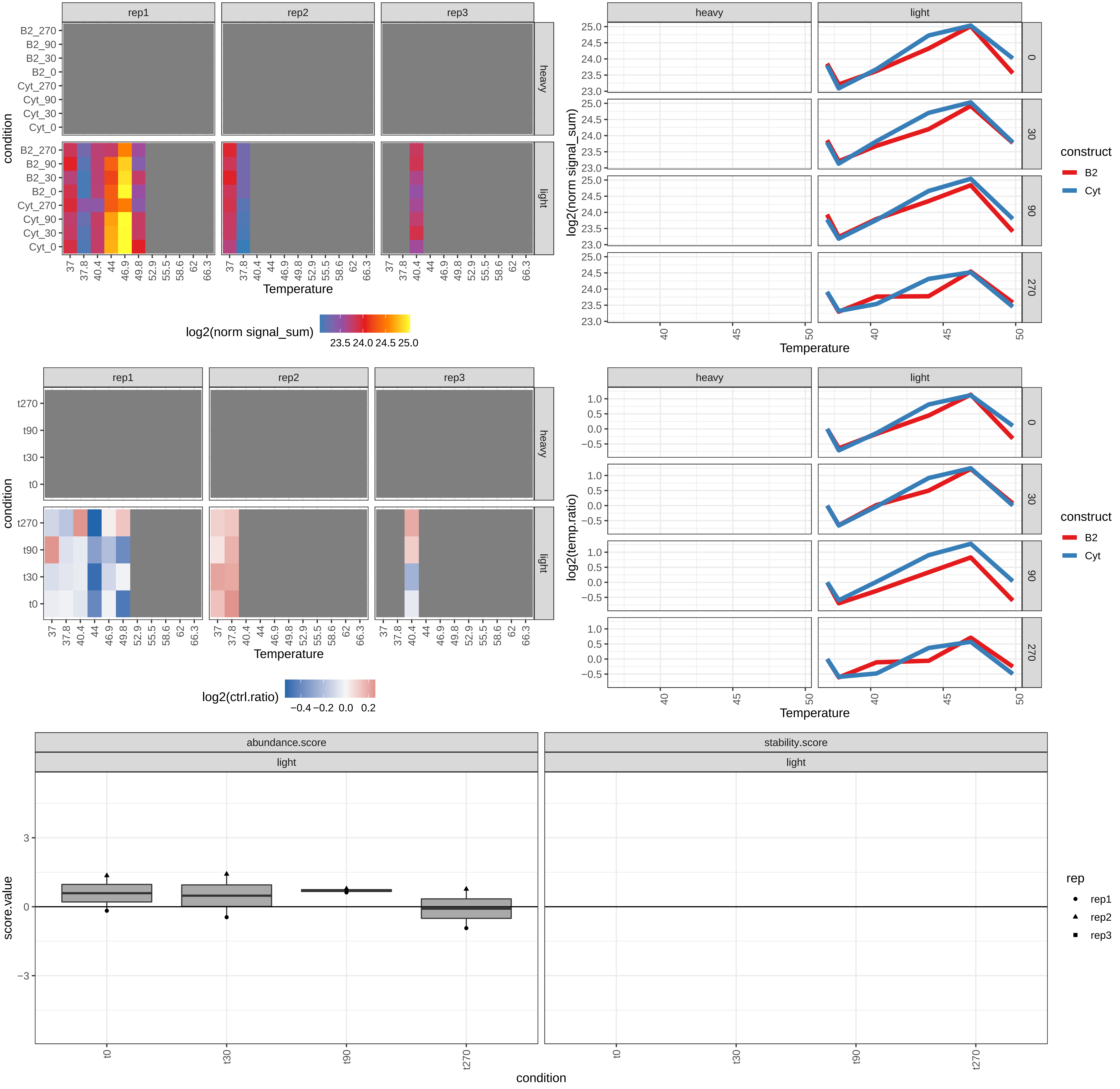

P54784 − Origin recognition complex subunit 1 OS=Saccharomyces cerevisiae (strain ATCC 204508 / S288c) GN=ORC1 PE=1 SV=1

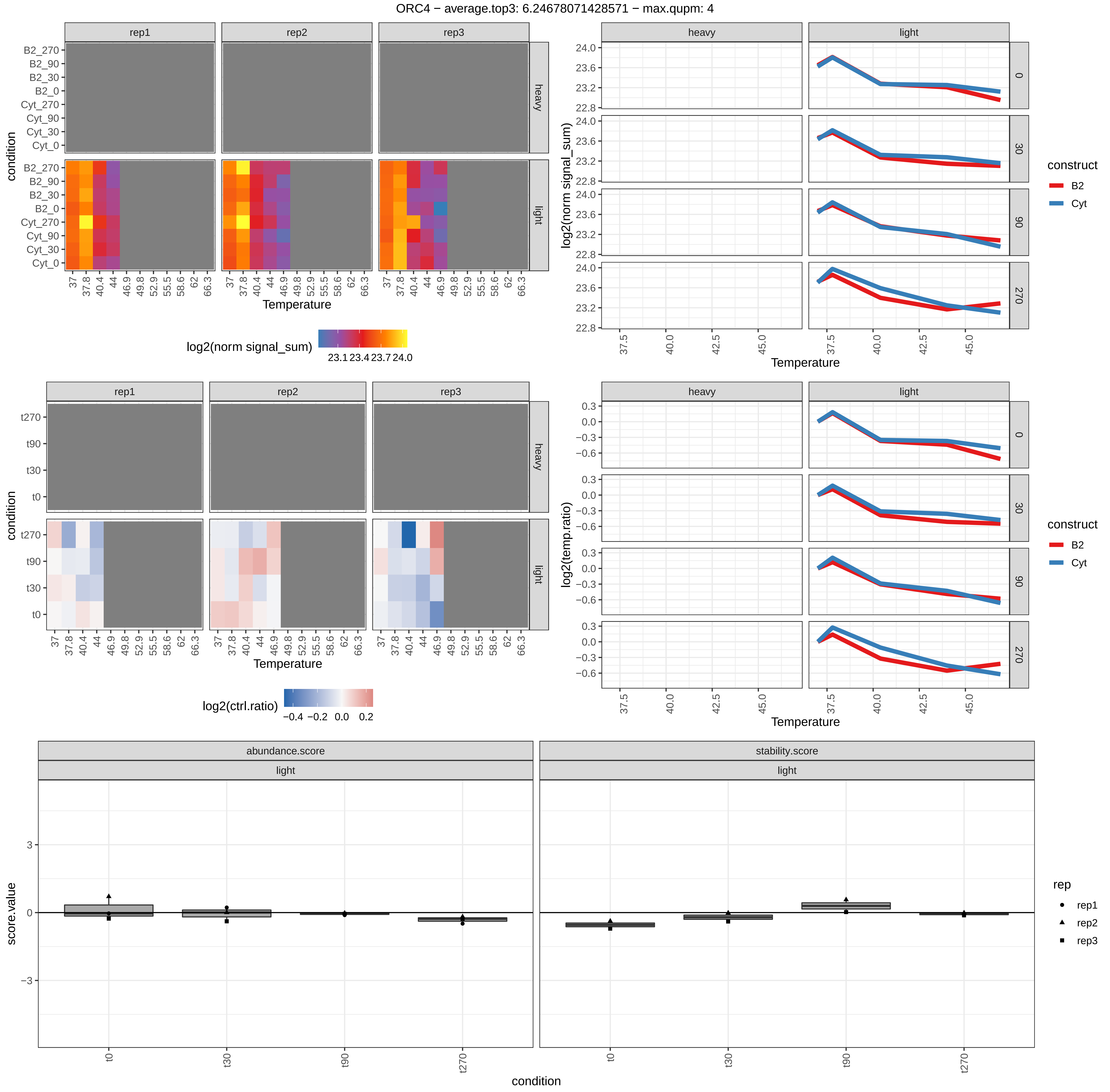

P54791 − Origin recognition complex subunit 4 OS=Saccharomyces cerevisiae (strain ATCC 204508 / S288c) GN=ORC4 PE=1 SV=1

ORT1 − average.top3: 6.570394 − max.qupm: 3

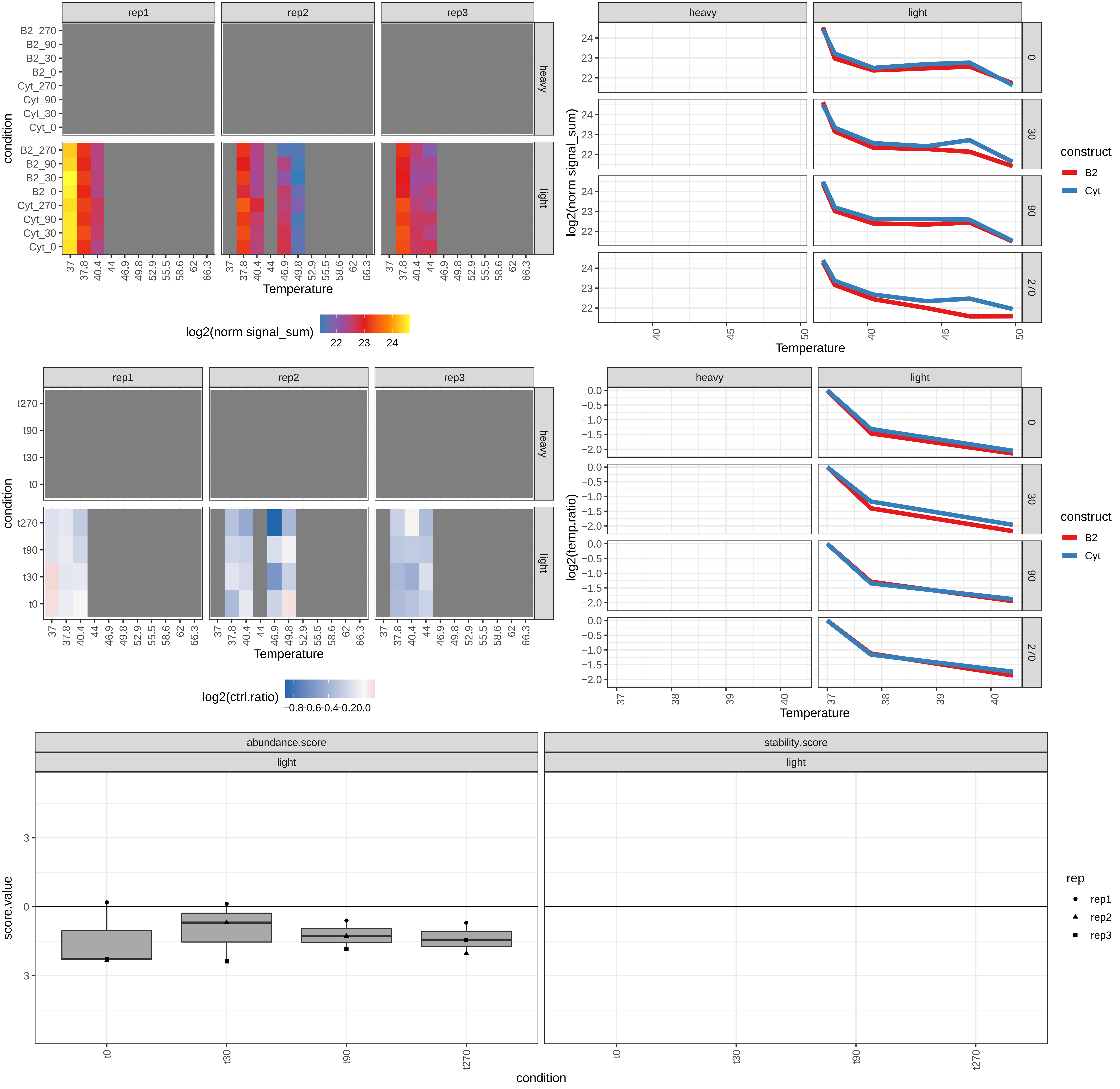

Q12375 − Mitochondrial ornithine transporter 1 OS=Saccharomyces cerevisiae (strain ATCC 204508 / S288c) GN=ORT1 PE=1 SV=2

OSH2 − average.top3: 6.91546590909091 − max.qupm: 9

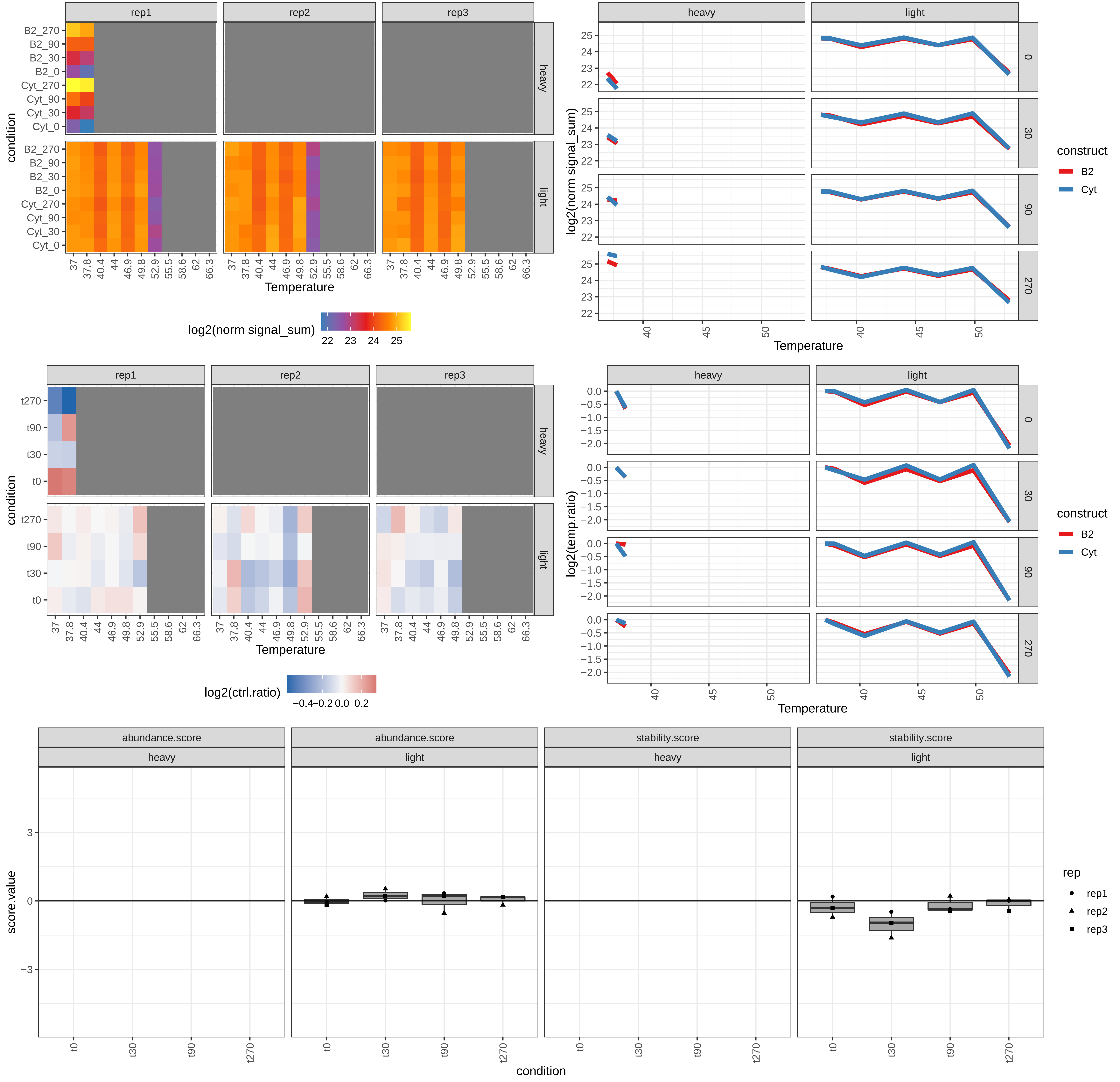

Q12451 − Oxysterol−binding protein homolog 2 OS=Saccharomyces cerevisiae (strain ATCC 204508 / S288c) GN=OSH2 PE=1 SV=1

OSH3 − average.top3: 6.61353444444444 − max.qupm: 4

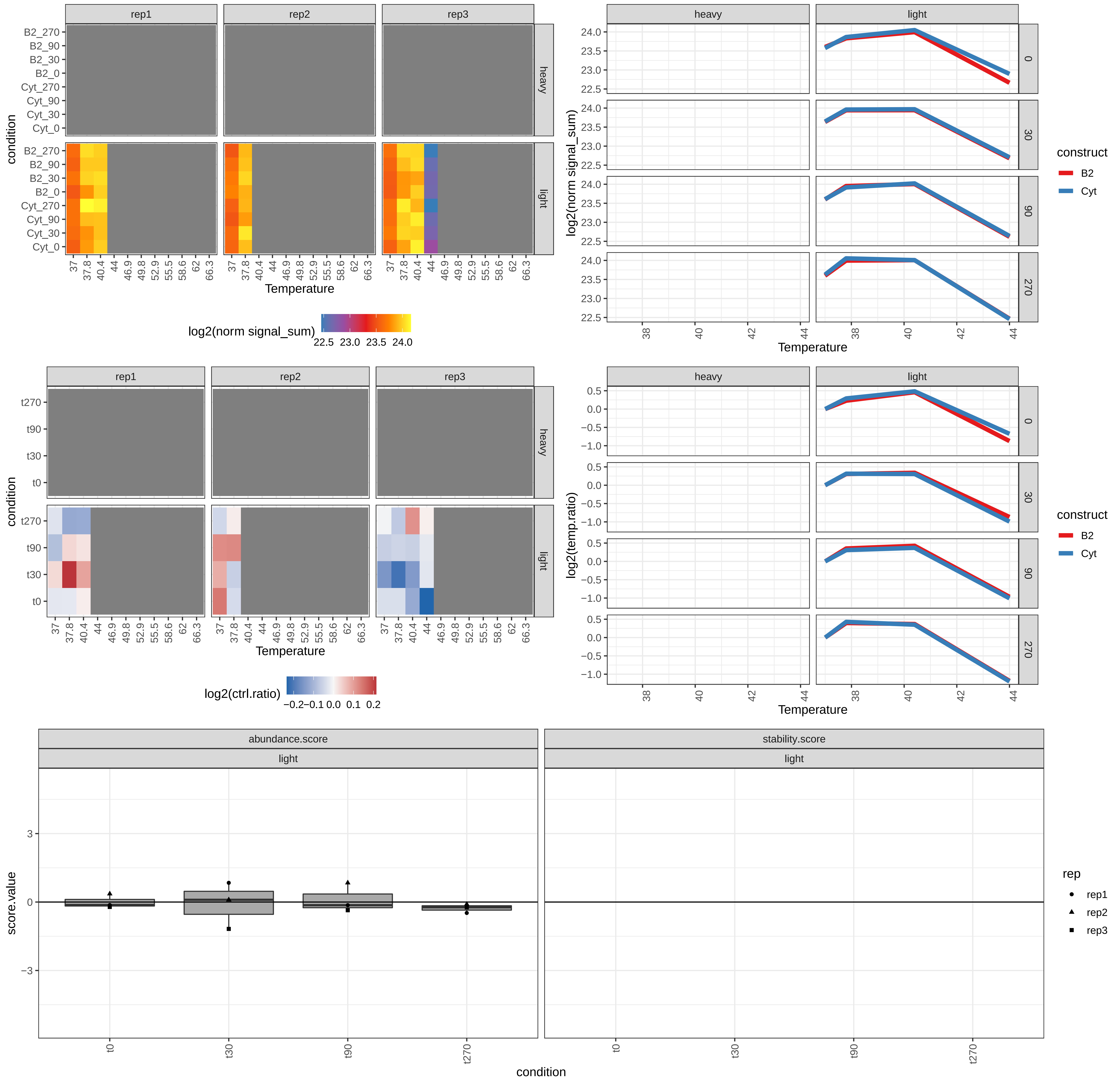

P38713 − Oxysterol−binding protein homolog 3 OS=Saccharomyces cerevisiae (strain ATCC 204508 / S288c) GN=OSH3 PE=1 SV=1

OSH6 − average.top3: 6.93966210526316 − max.qupm: 8

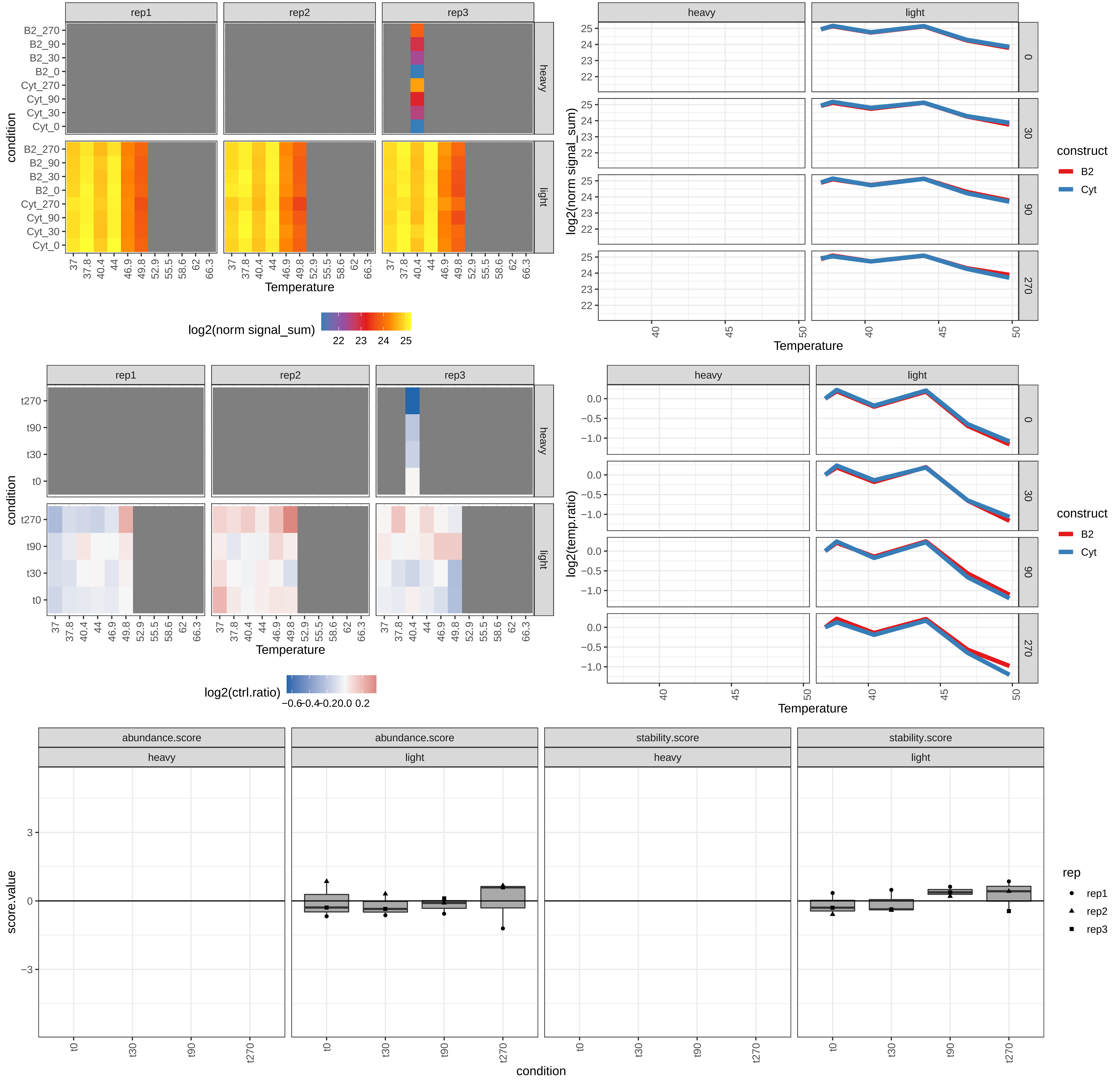

Q02201 − Oxysterol−binding protein homolog 6 OS=Saccharomyces cerevisiae (strain ATCC 204508 / S288c) GN=OSH6 PE=1 SV=1

OSH7 − average.top3: 6.90998766666667 − max.qupm: 14

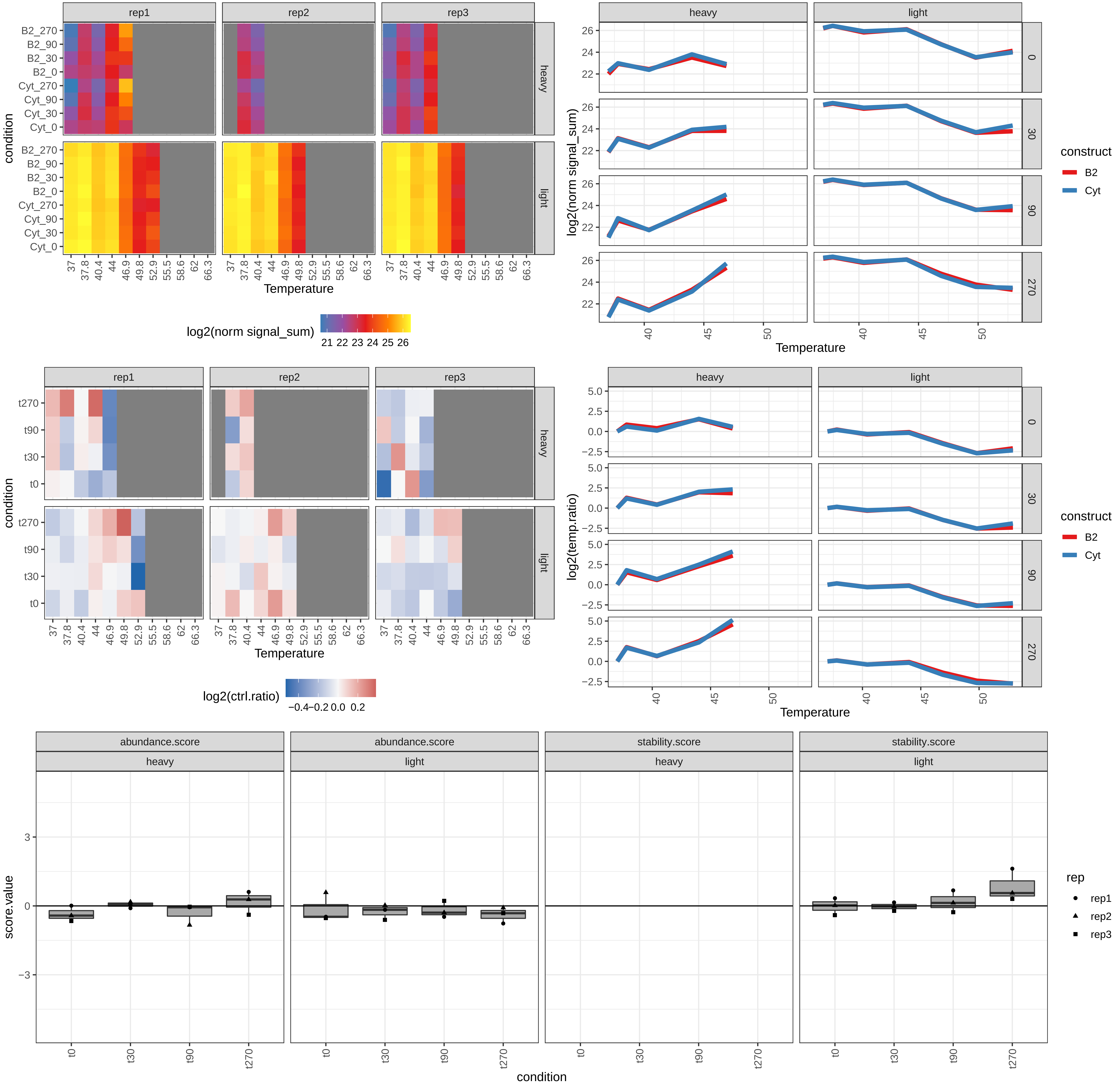

P38755 − Oxysterol−binding protein homolog 7 OS=Saccharomyces cerevisiae (strain ATCC 204508 / S288c) GN=OSH7 PE=1 SV=1

OSM1 − average.top3: 6.86441756756757 − max.qupm: 9

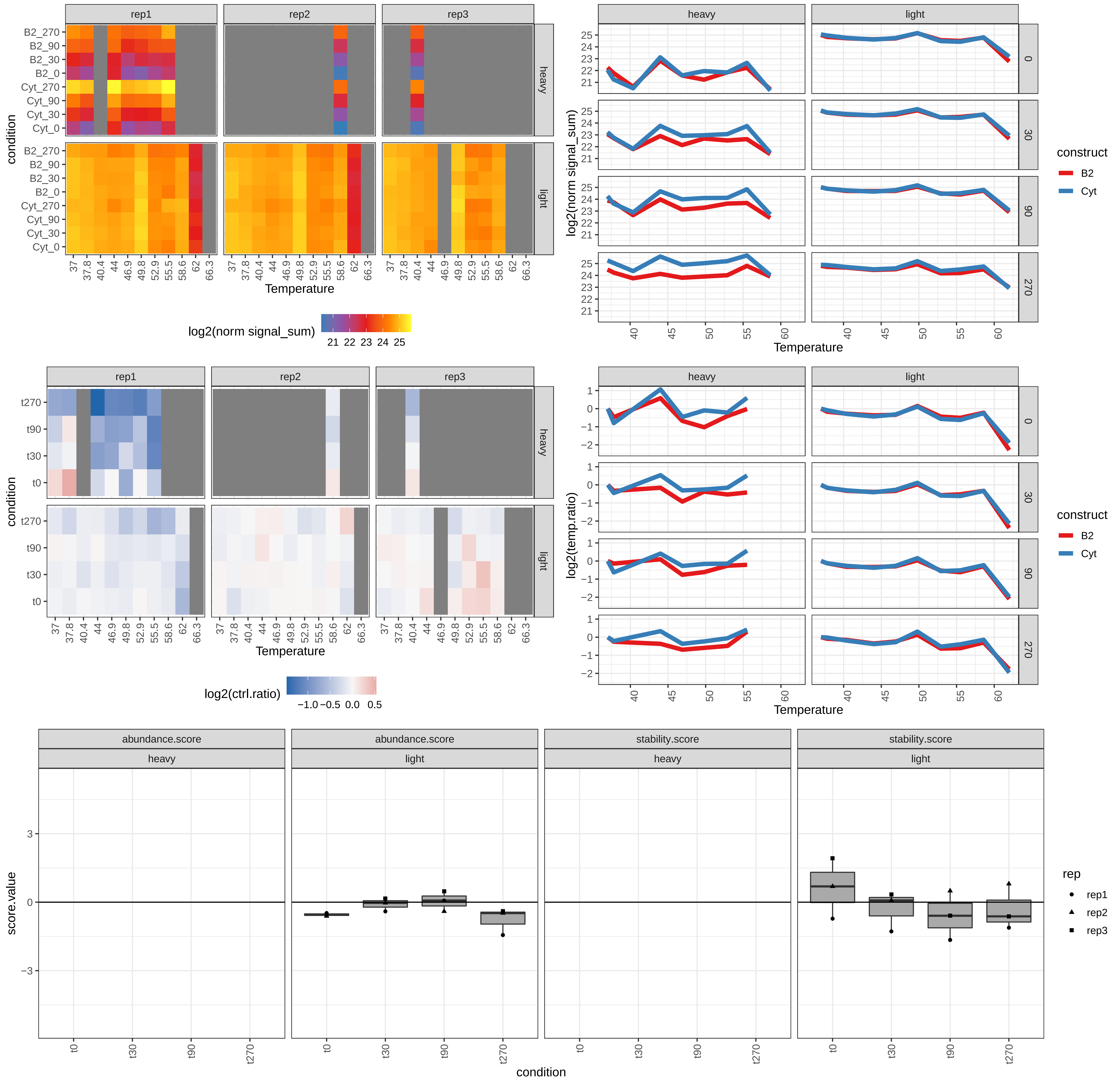

P21375 − Fumarate reductase 2 OS=Saccharomyces cerevisiae (strain ATCC 204508 / S288c) GN=OSM1 PE=1 SV=3

OST1 − average.top3: 7.01435142857143 − max.qupm: 6

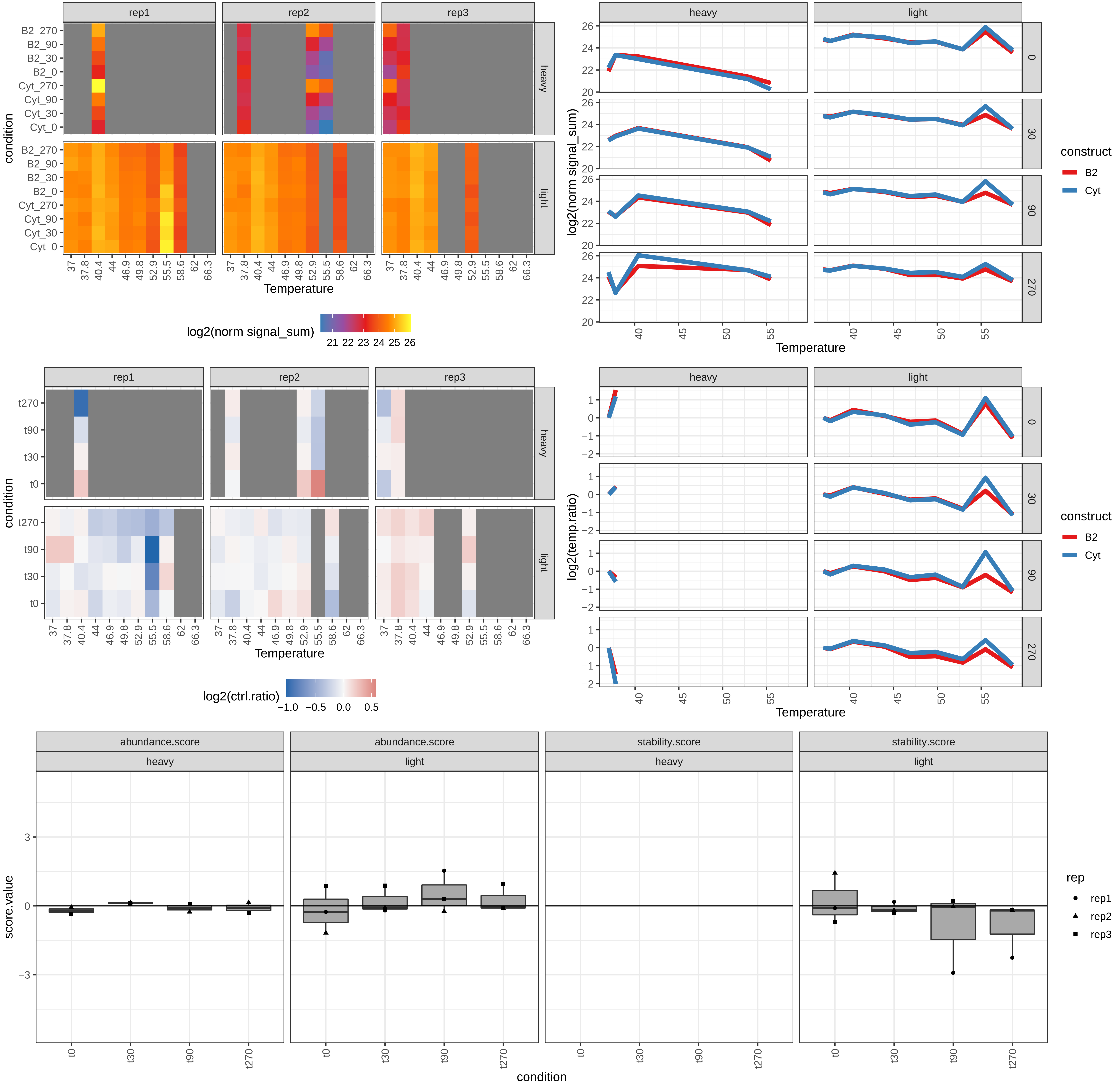

P41543 − Dolichyl−diphosphooligosaccharide−−protein glycosyltransferase subunit 1 OS=Saccharomyces cerevisiae (strain ATCC 204508 / S288c) GN=OST1 PE=1 SV=1

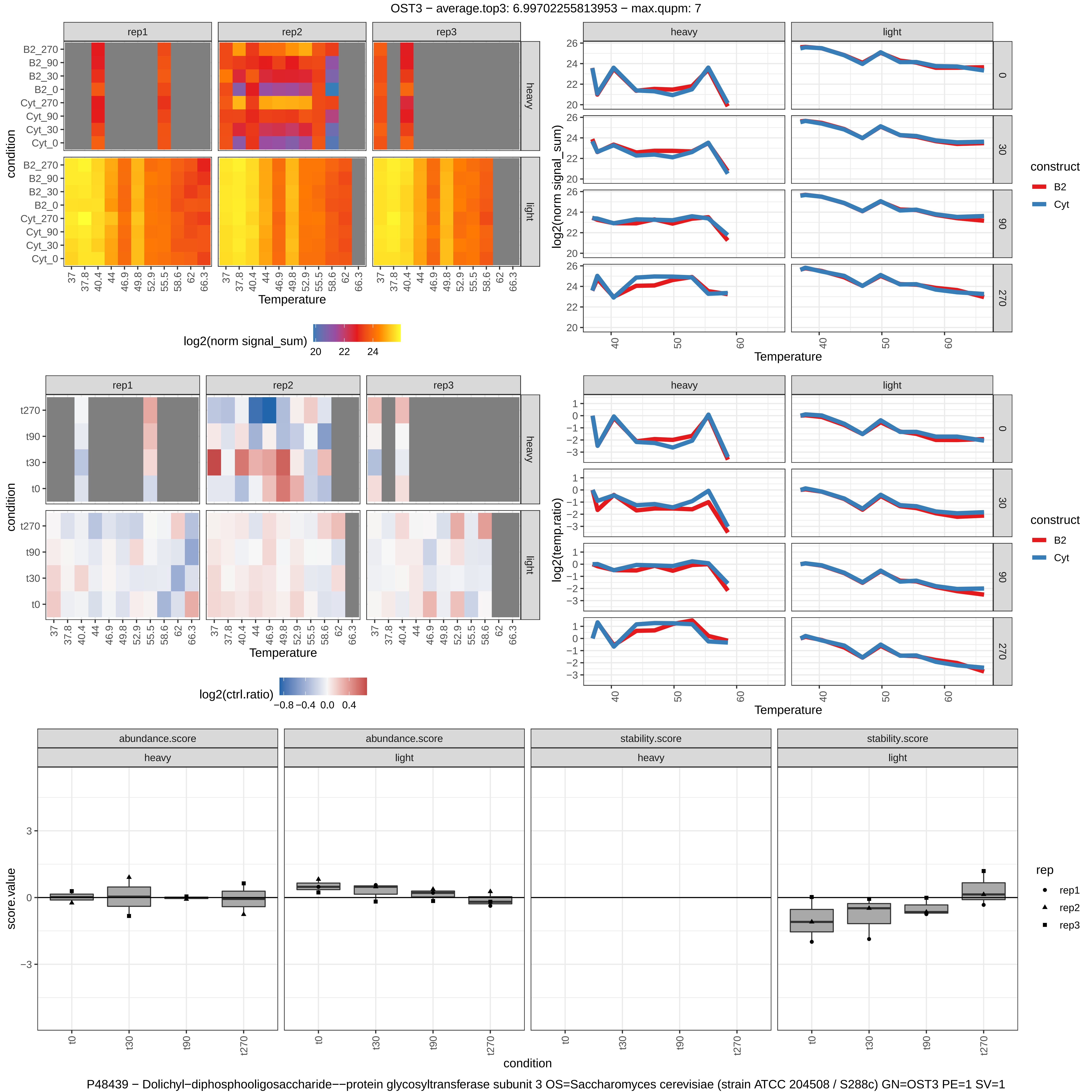
OST3 − average.top3: 6.99702255813953 − max.qupm: 7
rep1
rep2
rep3
heavy
light
condition
Temperature
log2(norm signal_sum)
log2(ctrl.ratio)
log2(temp.ratio)
construct
B2
Cyt
abundance.score
stability.score
score.value
rep
rep1
rep2
rep3
P48439 − Dolichyl−diphosphooligosaccharide−−protein glycosyltransferase subunit 3 OS=Saccharomyces cerevisiae (strain ATCC 204508 / S288c) GN=OST3 PE=1 SV=1

OST5 − average.top3: 6.83298068965517 − max.qupm: 4

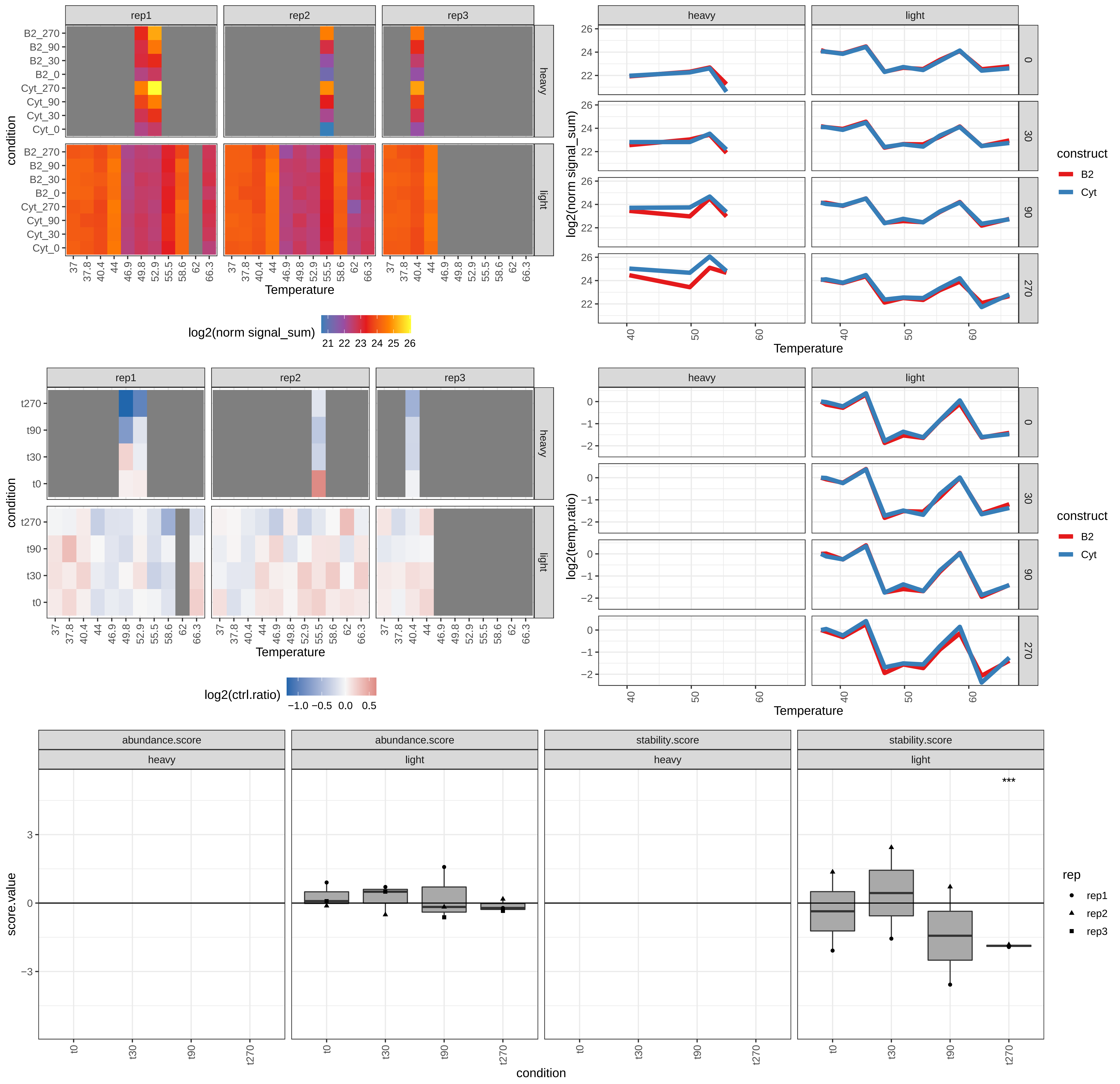

Q92316 − Dolichyl−diphosphooligosaccharide−−protein glycosyltransferase subunit OST5 OS=Saccharomyces cerevisiae (strain ATCC 204508 / S288c) GN=OST5 PE=1 SV=2

OSW7 − average.top3: 6.21732368421053 − max.qupm: 4

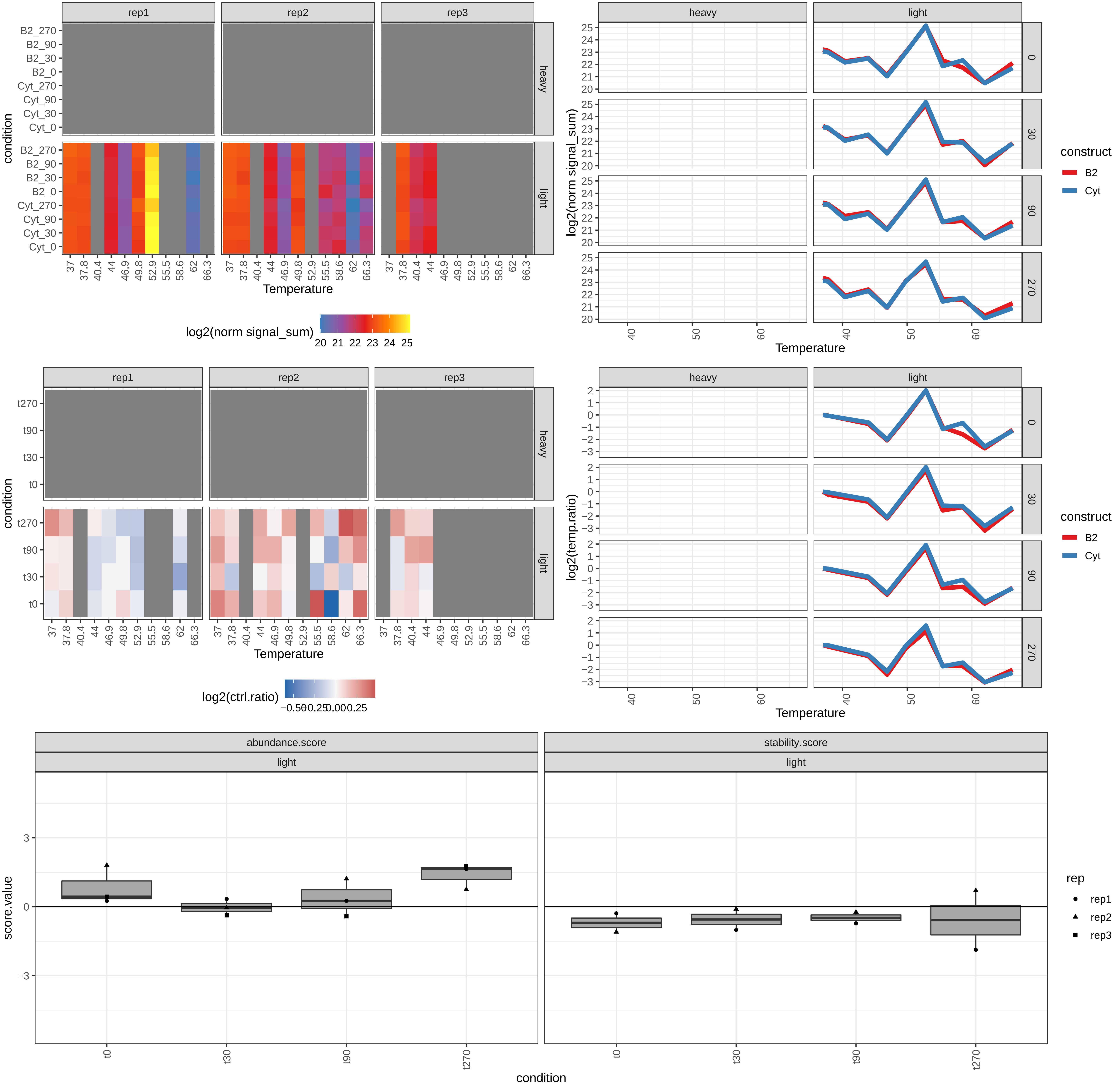

P43611 − Outer spore wall protein 7 OS=Saccharomyces cerevisiae (strain ATCC 204508 / S288c) GN=OSW7 PE=1 SV=1

OTU1 − average.top3: 6.93930631578947 − max.qupm: 6

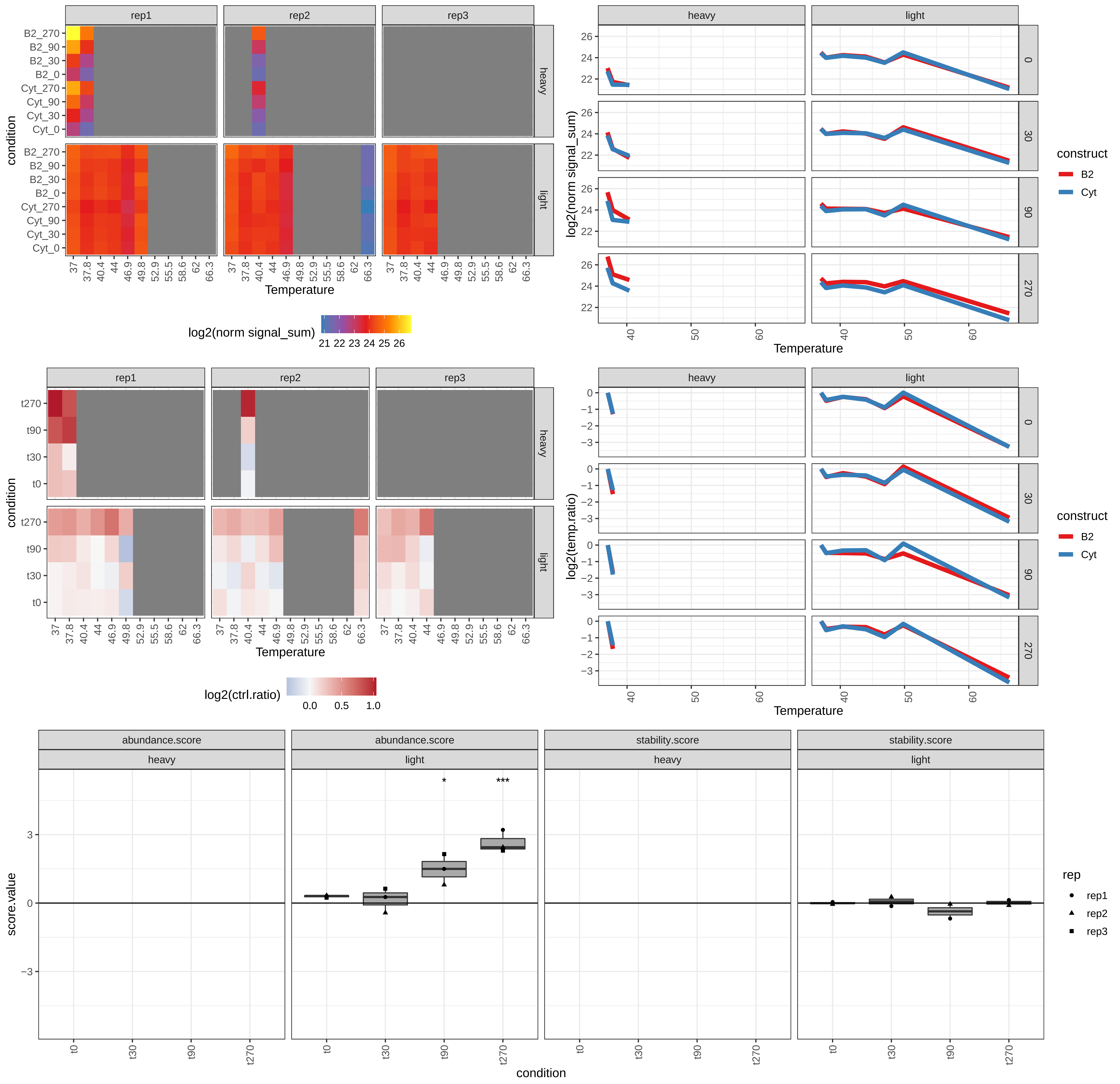

P43558 − Ubiquitin thioesterase OTU1 OS=Saccharomyces cerevisiae (strain ATCC 204508 / S288c) GN=OTU1 PE=1 SV=1

OXP1 − average.top3: 7.11133649122807 − max.qupm: 39

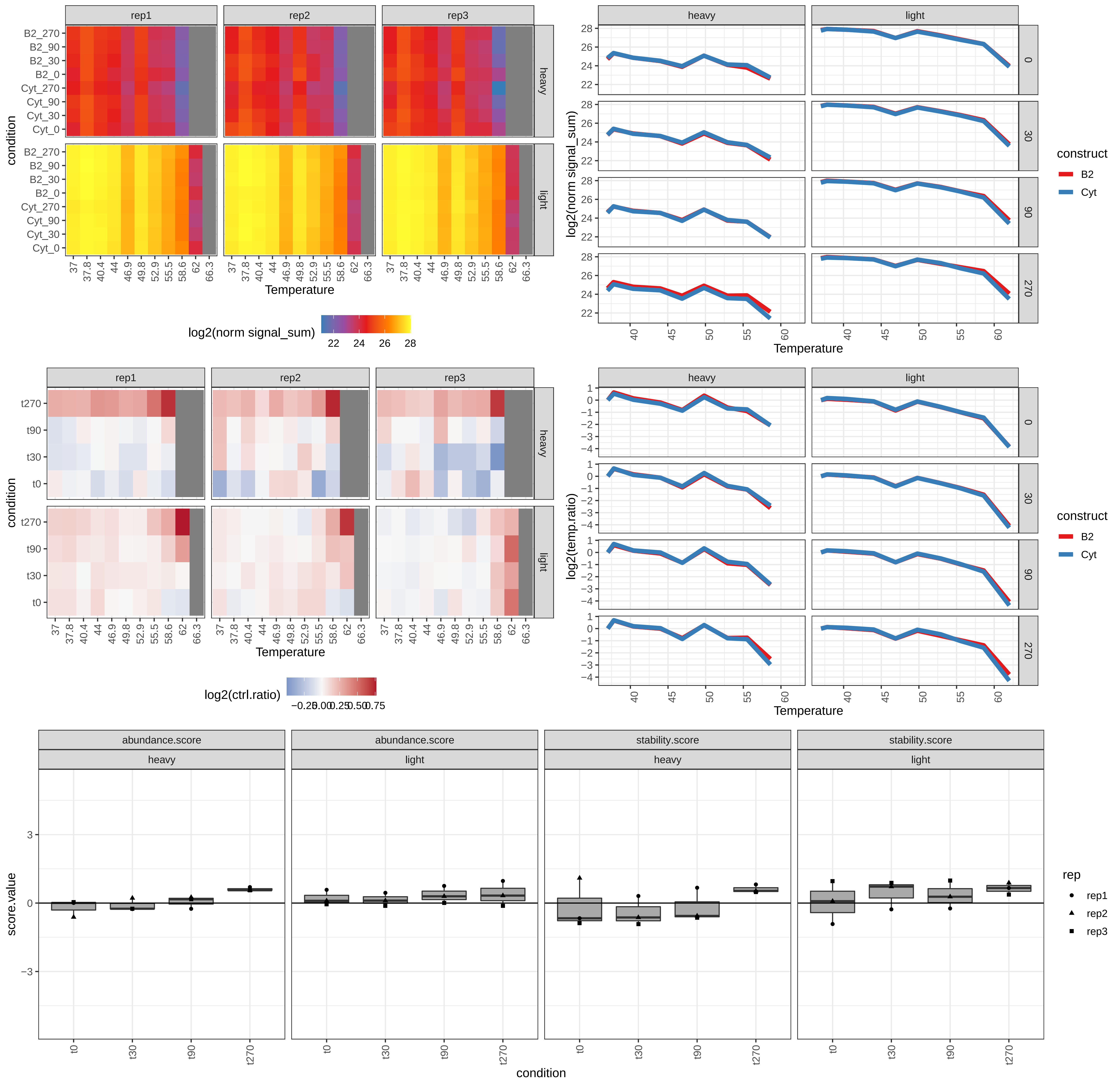

P28273 − 5−oxoprolinase OS=Saccharomyces cerevisiae (strain ATCC 204508 / S288c) GN=OXP1 PE=1 SV=2

OXR1 − average.top3: 6.47289333333333 − max.qupm: 3

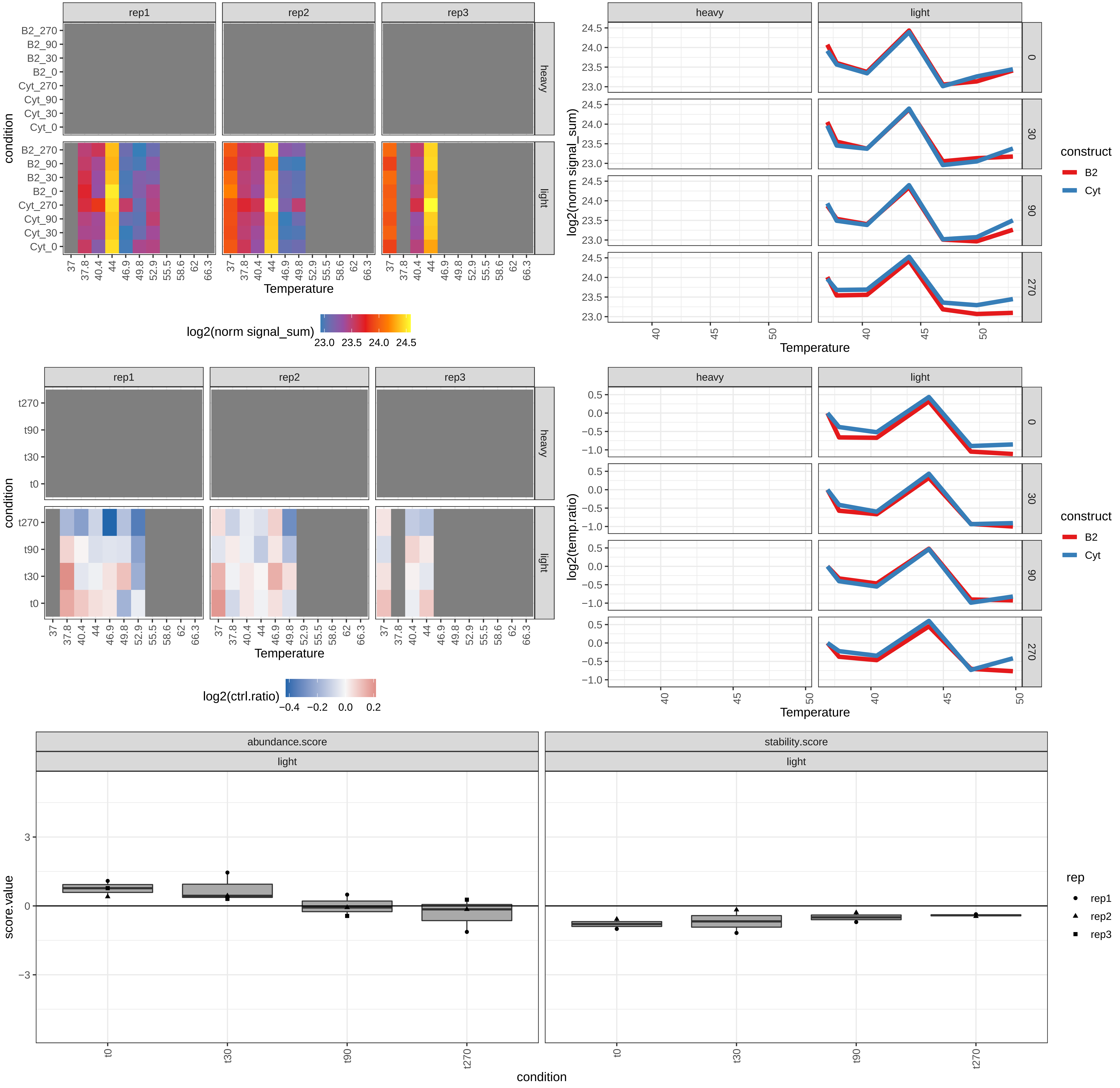

Q08952 − Oxidation resistance protein 1 OS=Saccharomyces cerevisiae (strain ATCC 204508 / S288c) GN=OXR1 PE=1 SV=1

OYE2 − average.top3: 7.44743734693878 − max.qupm: 17

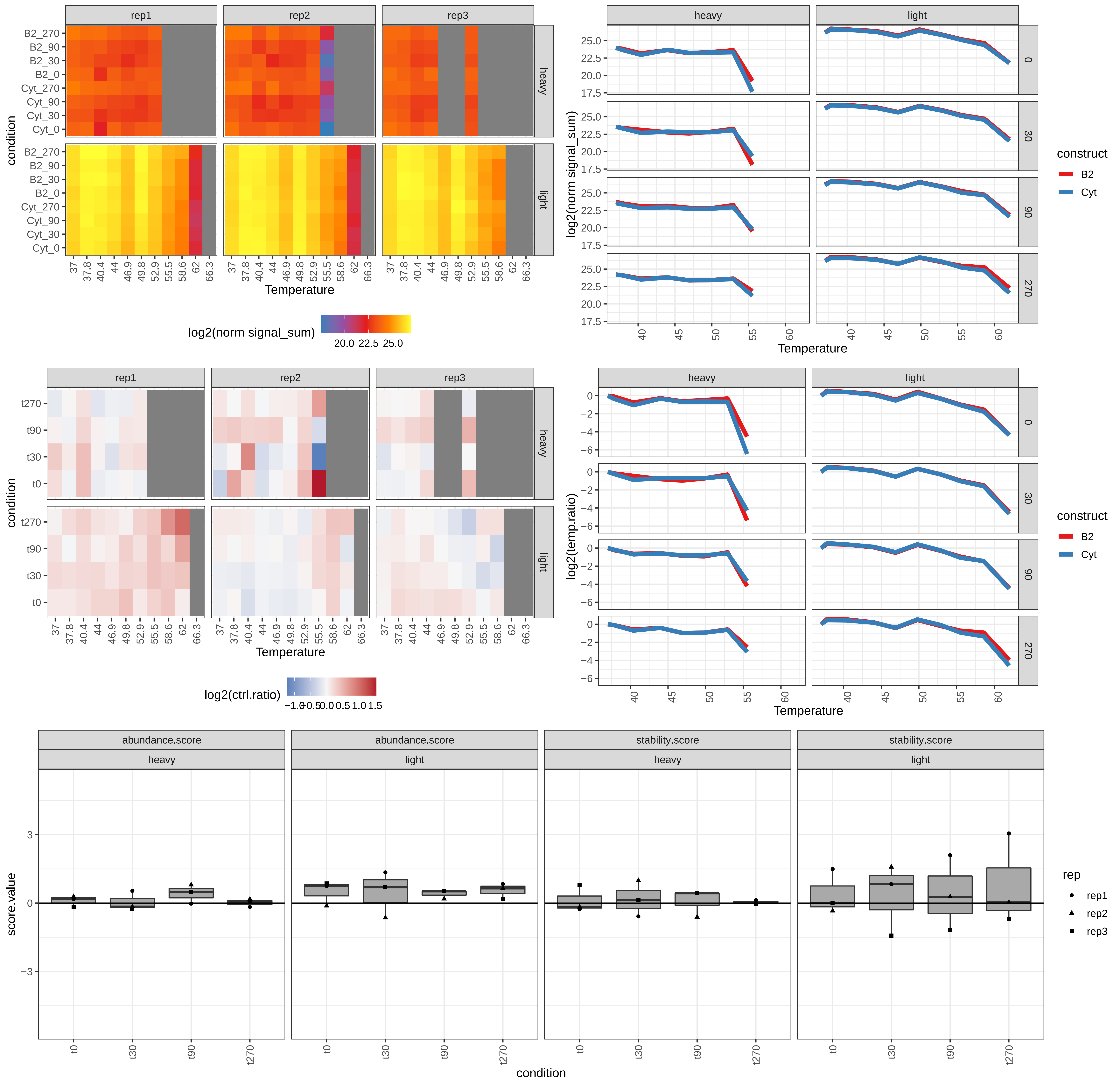

Q03558 − NADPH dehydrogenase 2 OS=Saccharomyces cerevisiae (strain ATCC 204508 / S288c) GN=OYE2 PE=1 SV=3

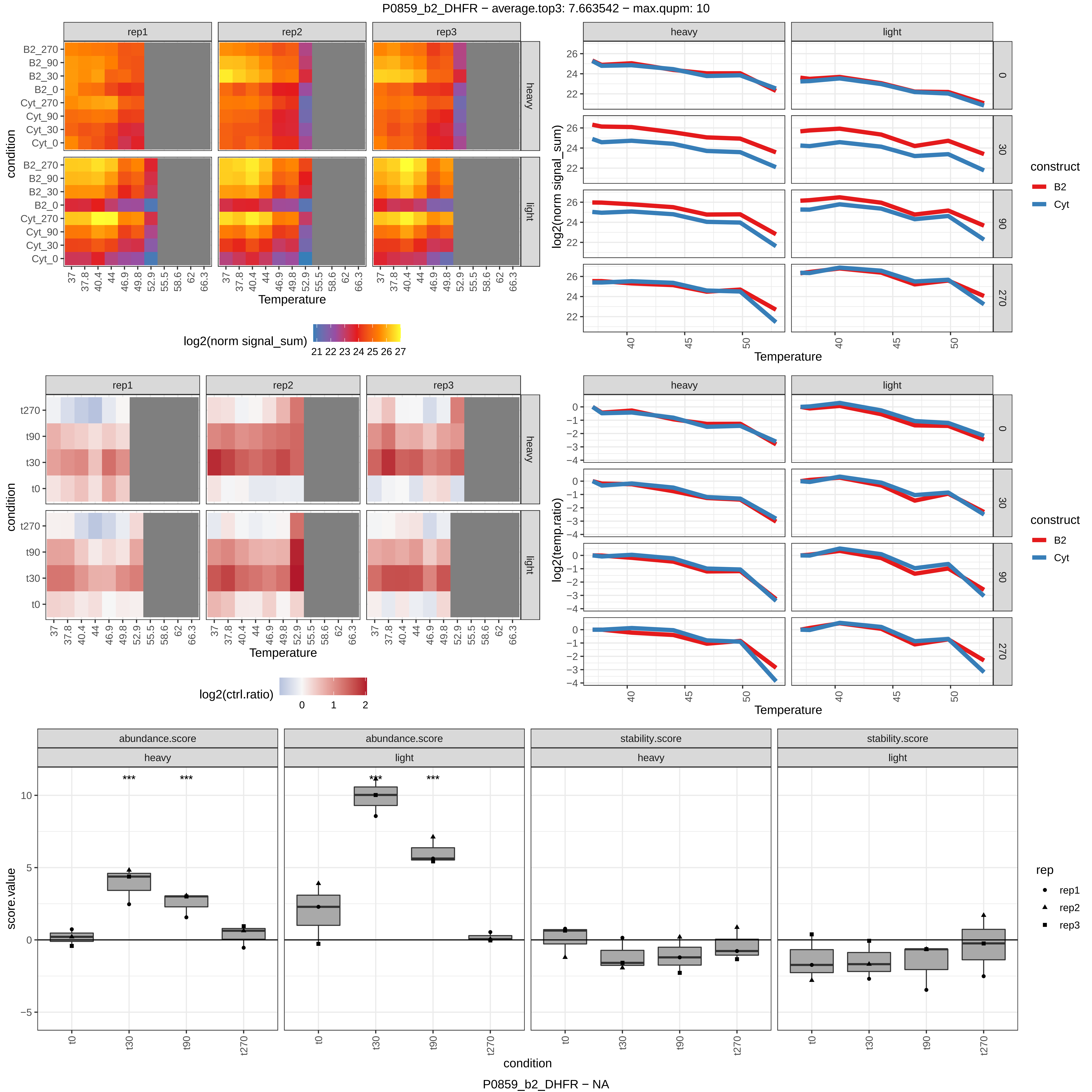
P0859_b2_DHFR − average.top3: 7.663542 − max.qupm: 10
rep1
rep2
rep3
heavy
light
condition
B2_270
B2_90
B2_30
B2_0
Cyt_270
Cyt_90
Cyt_30
Cyt_0
Temperature
log2(norm signal_sum)
21 22 23 24 25 26 27
log2(norm signal_sum)
construct
B2
Cyt
t270
t90
t30
t0
log2(ctrl.ratio)
log2(temp.ratio)
abundance.score
stability.score
score.value
rep
condition
P0859_b2_DHFR − NA

PAA1 − average.top3: 7.41108490566038 − max.qupm: 10

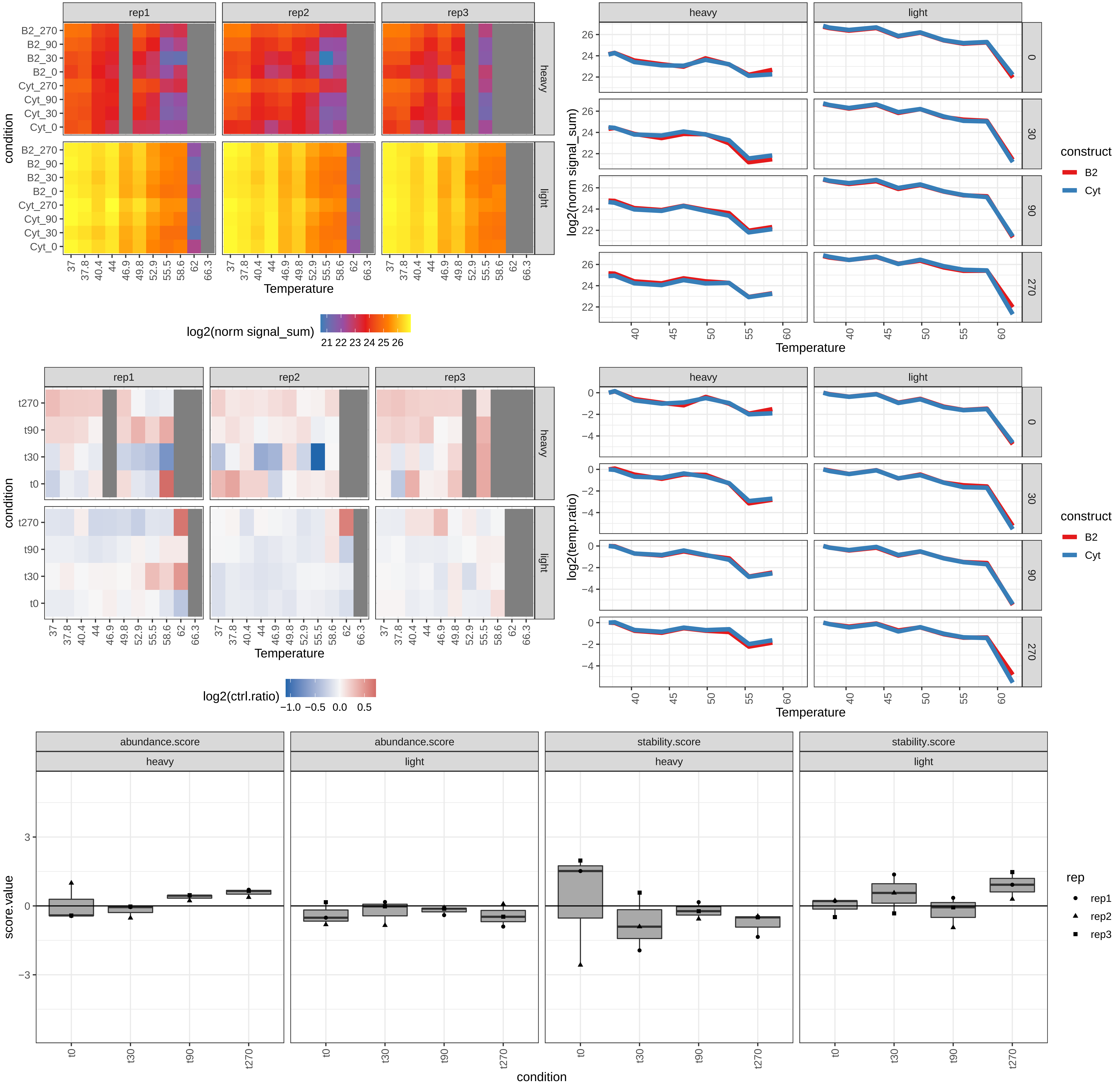

Q12447 − Polyamine N−acetyltransferase 1 OS=Saccharomyces cerevisiae (strain ATCC 204508 / S288c) GN=PAA1 PE=1 SV=1

PAB1 − average.top3: 7.63304890909091 − max.qupm: 20

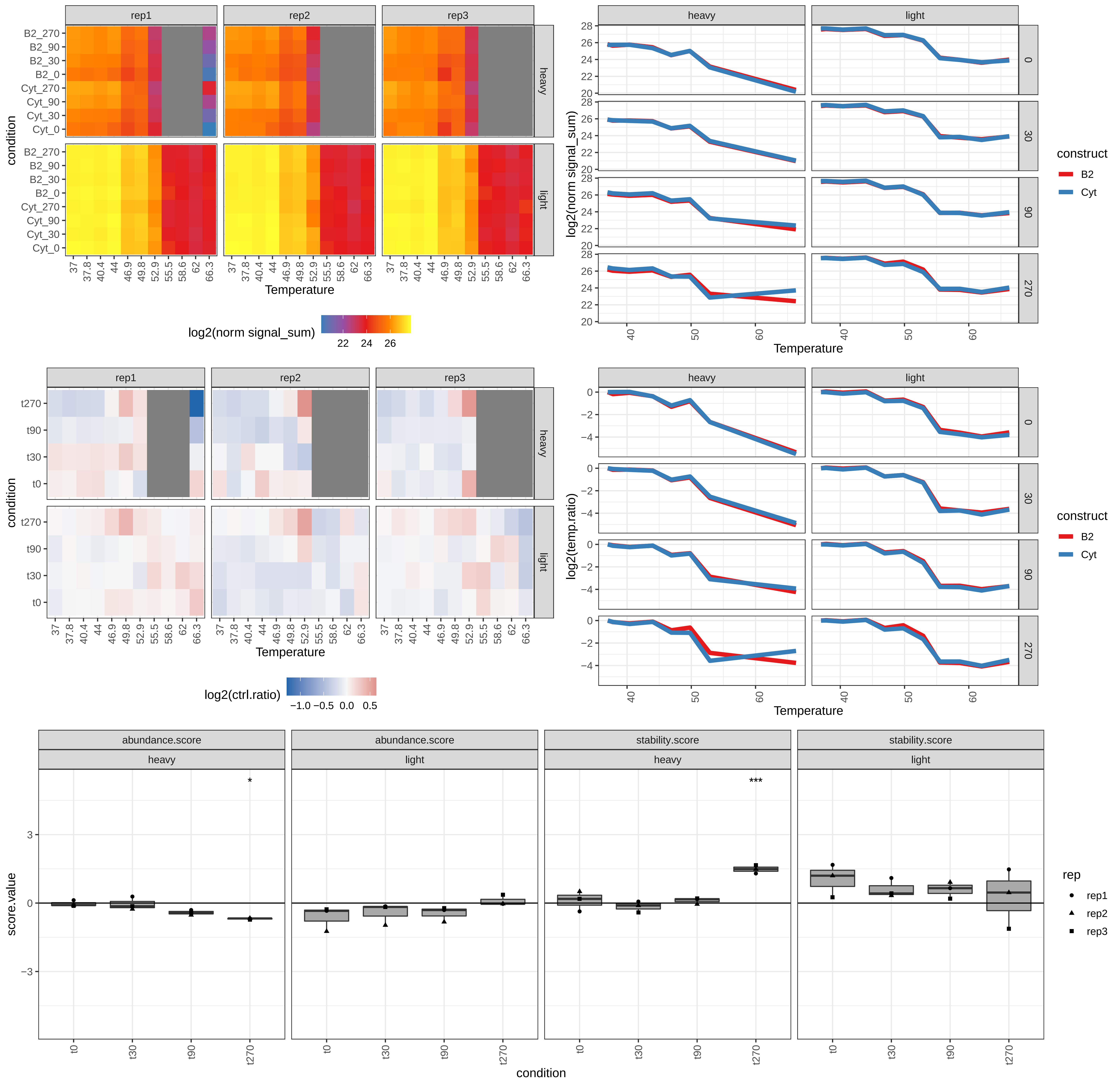

P04147 − Polyadenylate−binding protein, cytoplasmic and nuclear OS=Saccharomyces cerevisiae (strain ATCC 204508 / S288c) GN=PAB1 PE=1 SV=4

PAC10 − average.top3: 6.75084555555556 − max.qupm: 3

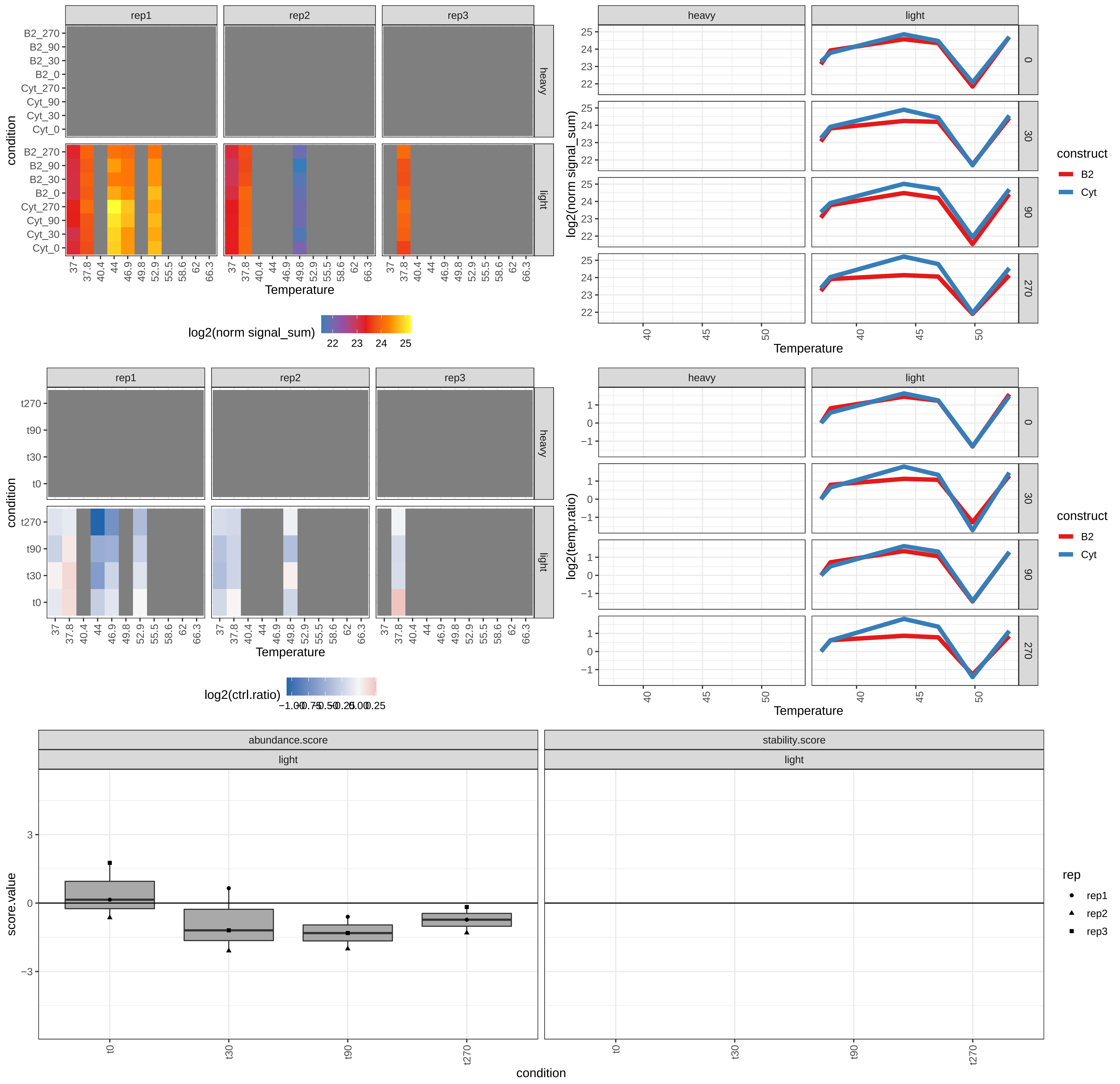

P48363 − Prefoldin subunit 3 OS=Saccharomyces cerevisiae (strain ATCC 204508 / S288c) GN=PAC10 PE=1 SV=1

PAF1 − average.top3: 6.8668545 − max.qupm: 11

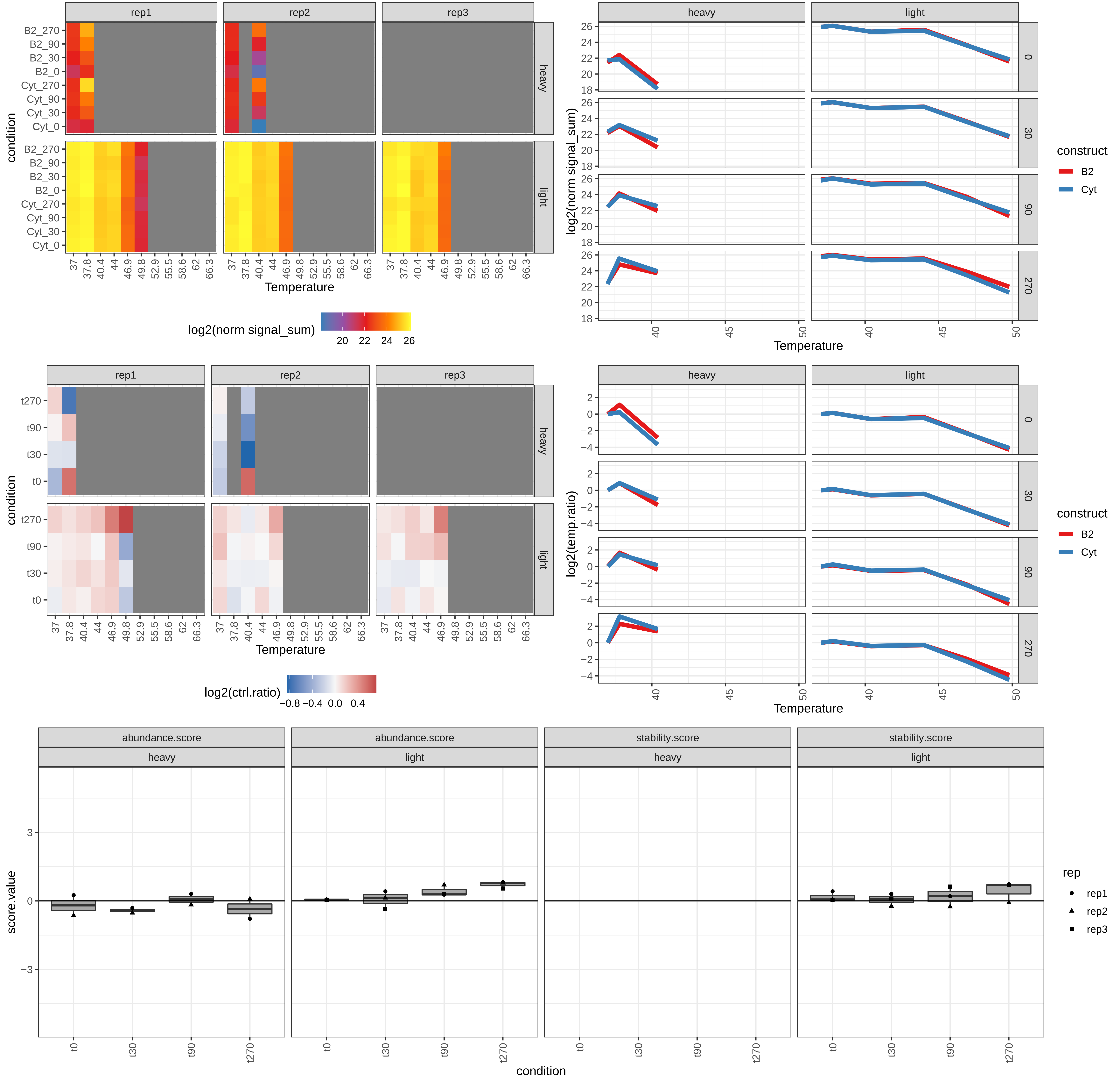

P38351 − RNA polymerase II−associated protein 1 OS=Saccharomyces cerevisiae (strain ATCC 204508 / S288c) GN=PAF1 PE=1 SV=1

PAH1 − average.top3: 6.766856 − max.qupm: 8

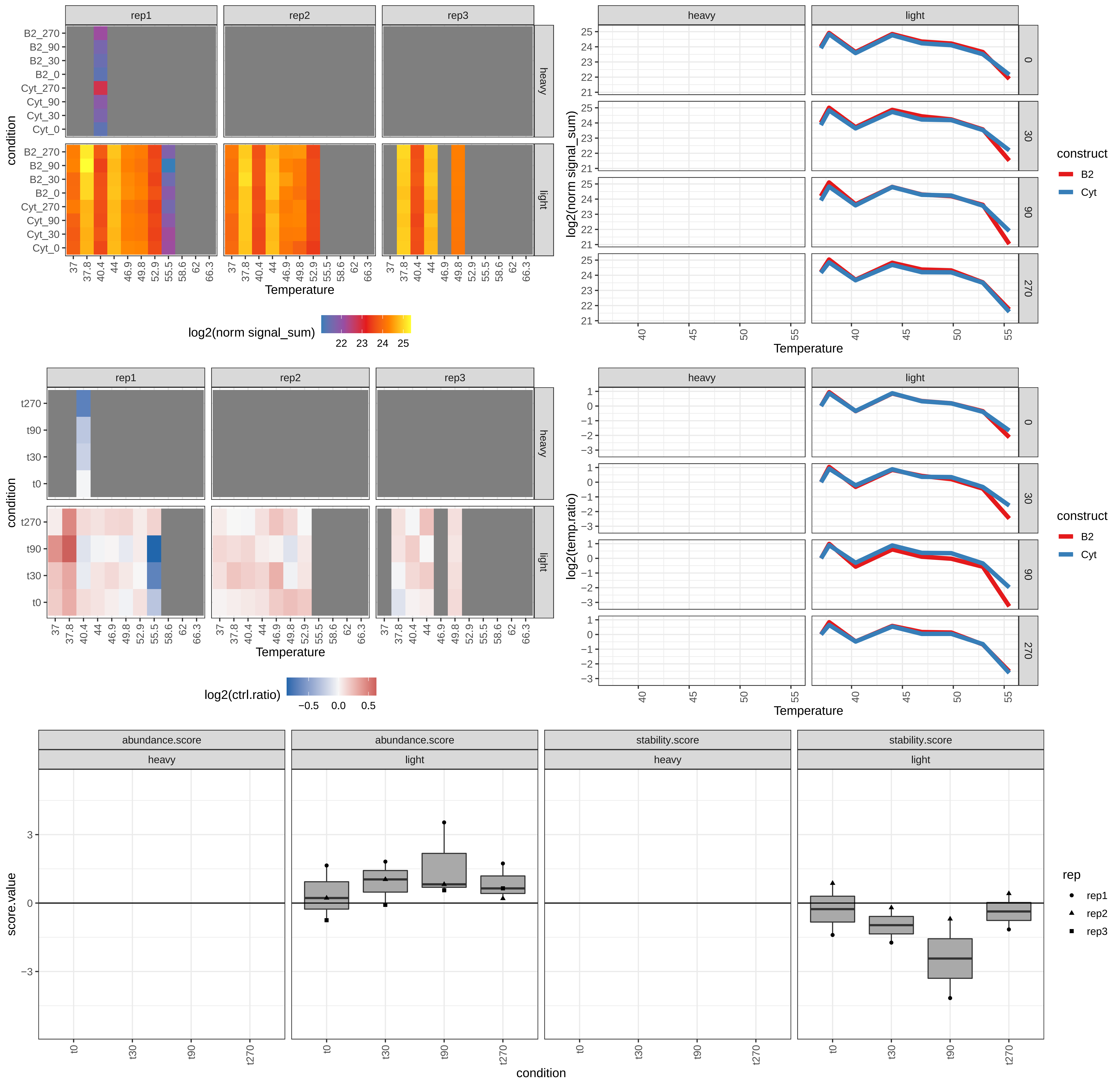

P32567 − Phosphatidic acid phosphohydrolase 1 OS=Saccharomyces cerevisiae (strain ATCC 204508 / S288c) GN=PAH1 PE=1 SV=1

PAM1 − average.top3: 6.56325 − max.qupm: 3

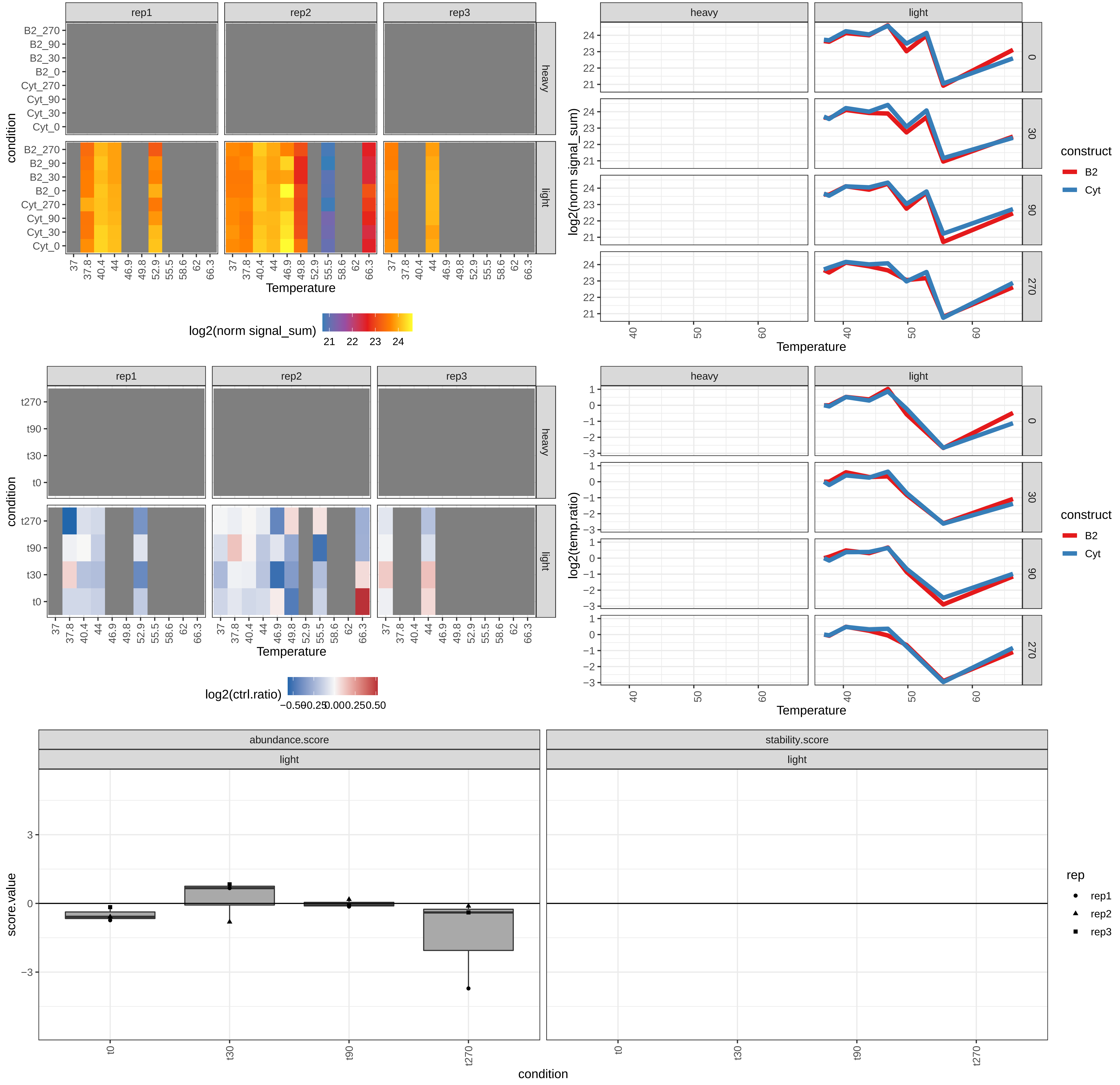

P37304 − Protein PAM1 OS=Saccharomyces cerevisiae (strain ATCC 204508 / S288c) GN=PAM1 PE=1 SV=2

PAM16 − average.top3: 6.91461928571429 − max.qupm: 6

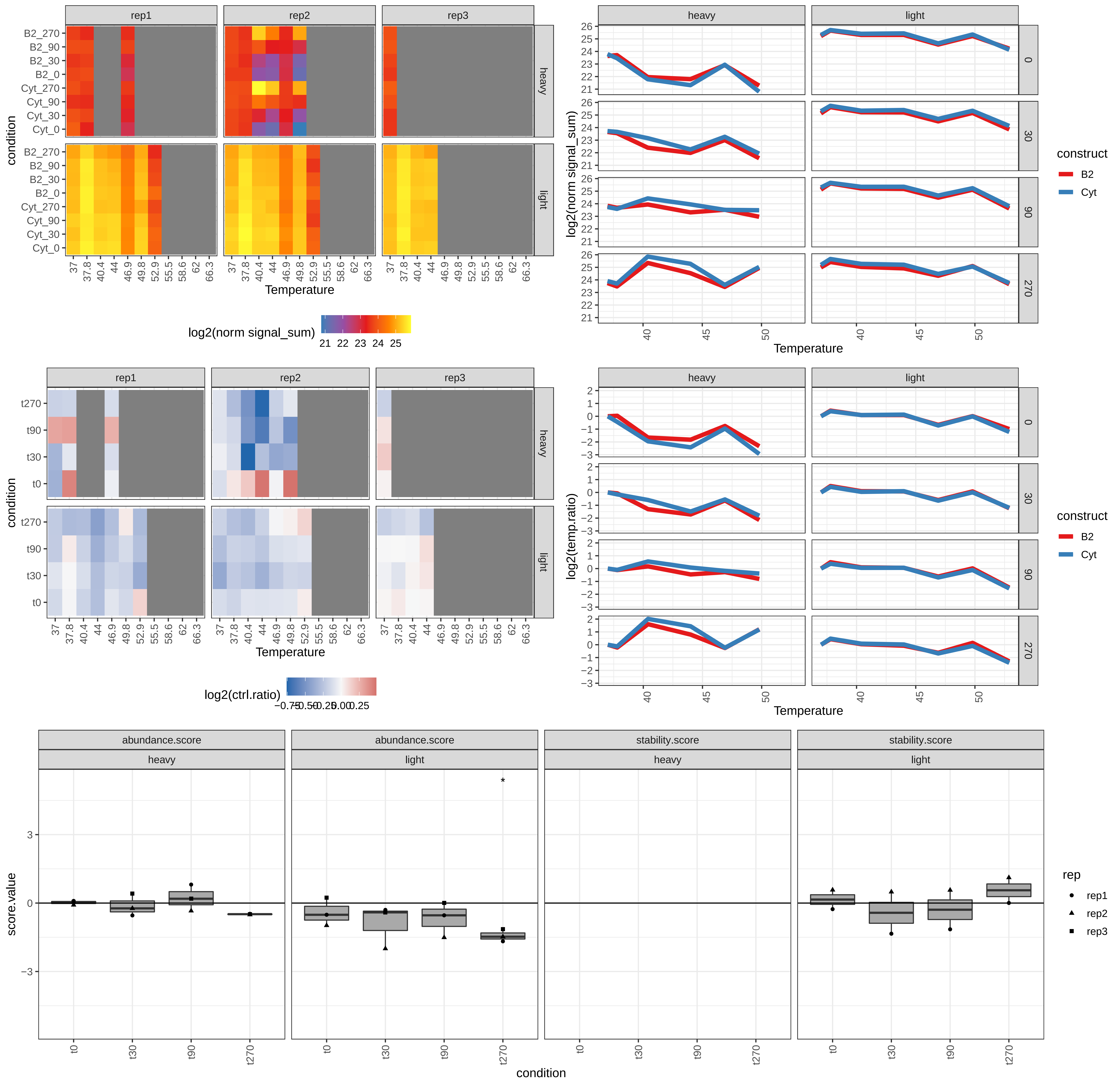

P42949 − Mitochondrial import inner membrane translocase subunit TIM16 OS=Saccharomyces cerevisiae (strain ATCC 204508 / S288c) GN=PAM16 PE=1 SV=1

PAM17 − average.top3: 6.96124166666667 − max.qupm: 3

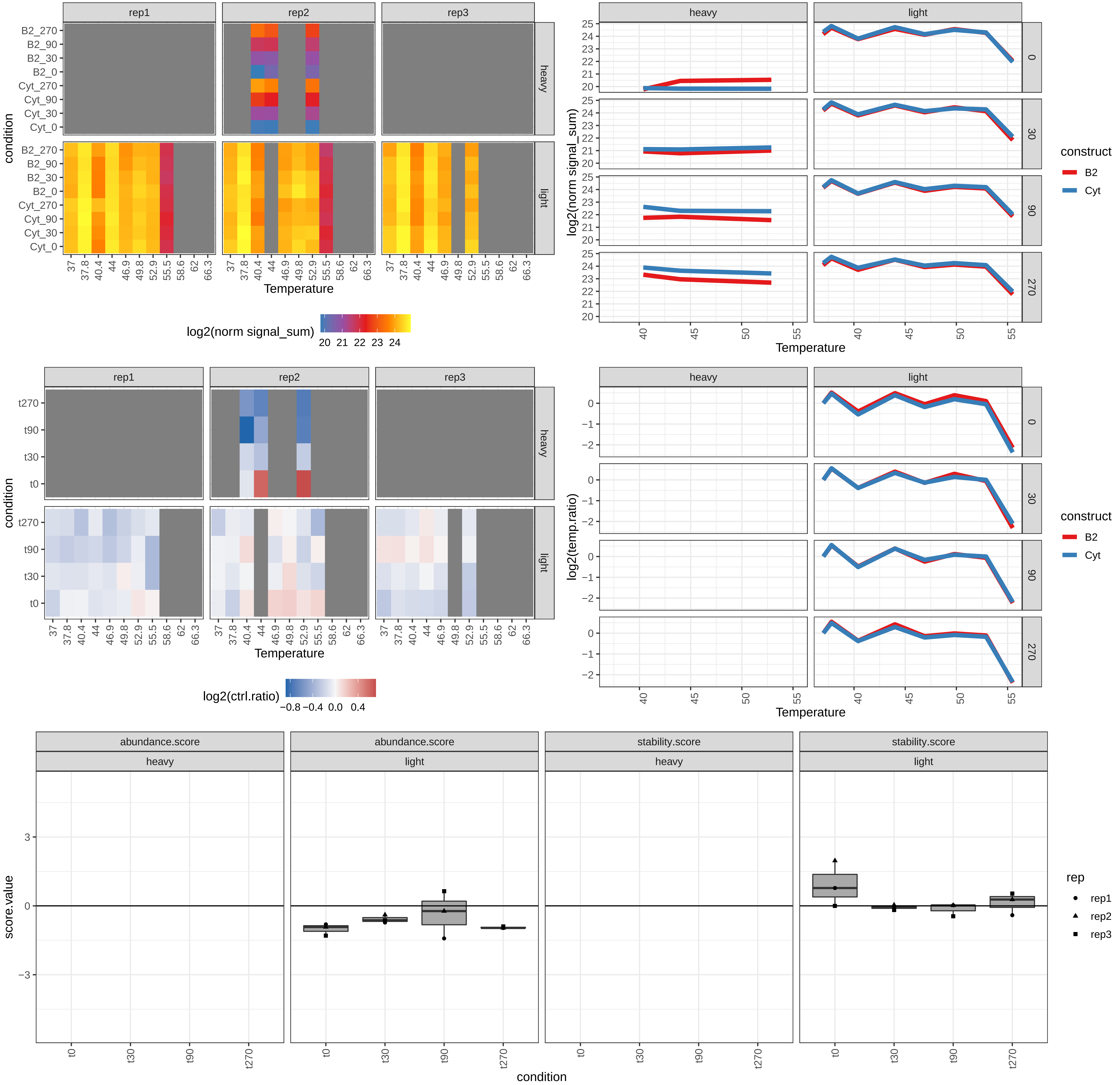

P36147 − Presequence translocated−associated motor subunit PAM17, mitochondrial OS=Saccharomyces cerevisiae (strain ATCC 204508 / S288c) GN=PAM17 PE=1 SV=1

PAM18 − average.top3: 6.73578538461538 − max.qupm: 4

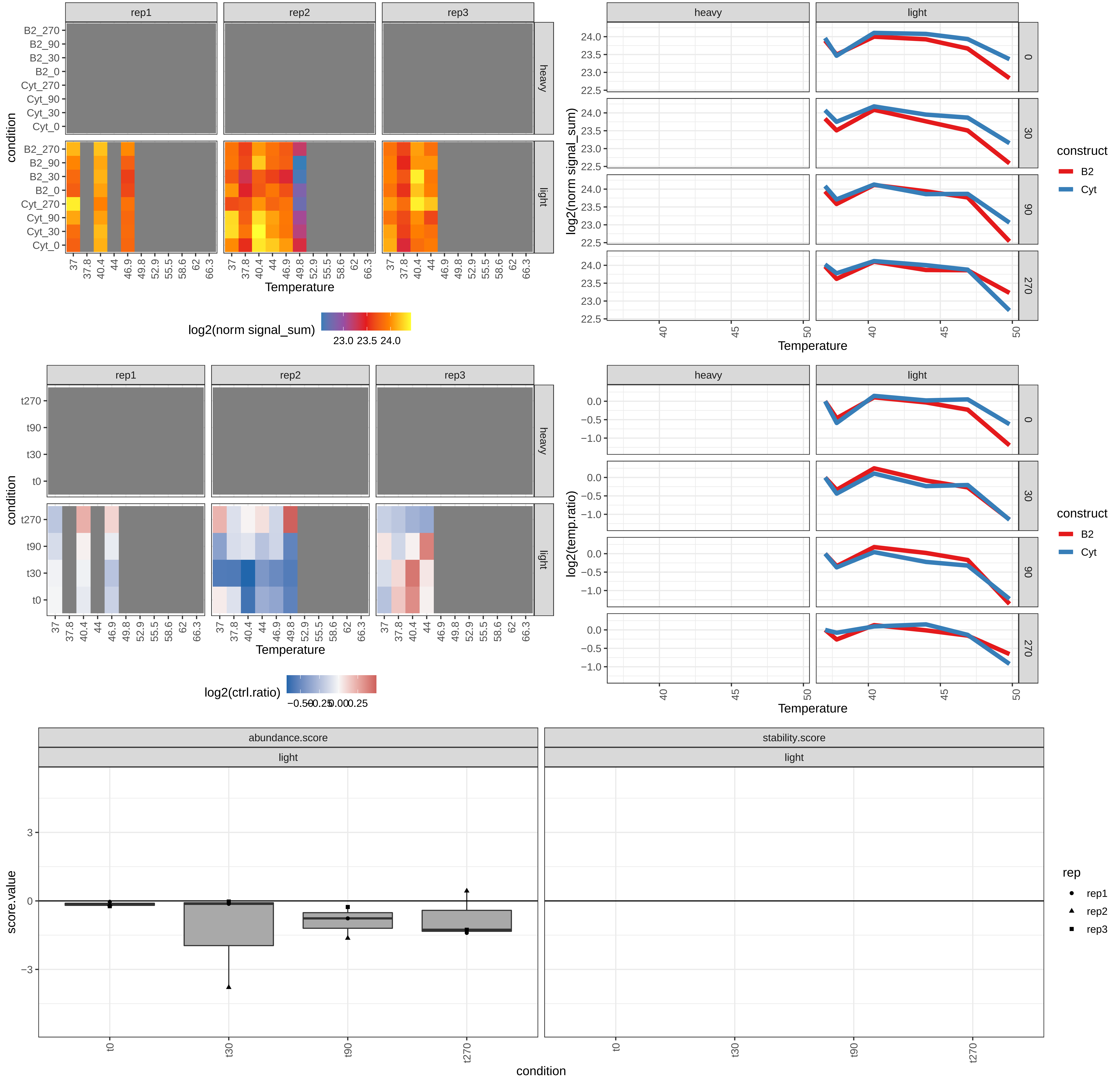

Q07914 − Mitochondrial import inner membrane translocase subunit TIM14 OS=Saccharomyces cerevisiae (strain ATCC 204508 / S288c) GN=PAM18 PE=1 SV=1

PAN1 − average.top3: 6.61630956521739 − max.qupm: 11

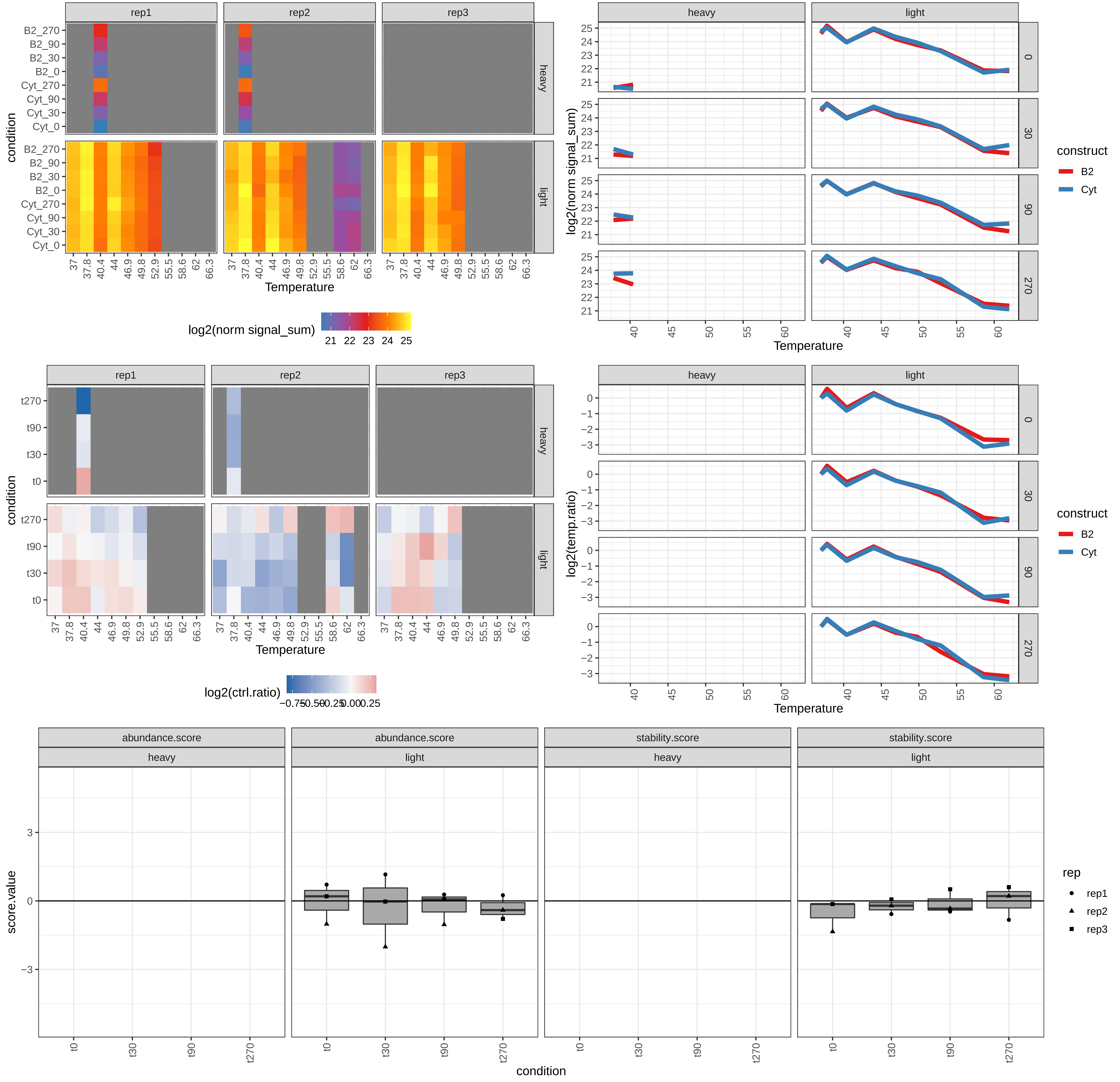

P32521 − Actin cytoskeleton−regulatory complex protein PAN1 OS=Saccharomyces cerevisiae (strain ATCC 204508 / S288c) GN=PAN1 PE=1 SV=2

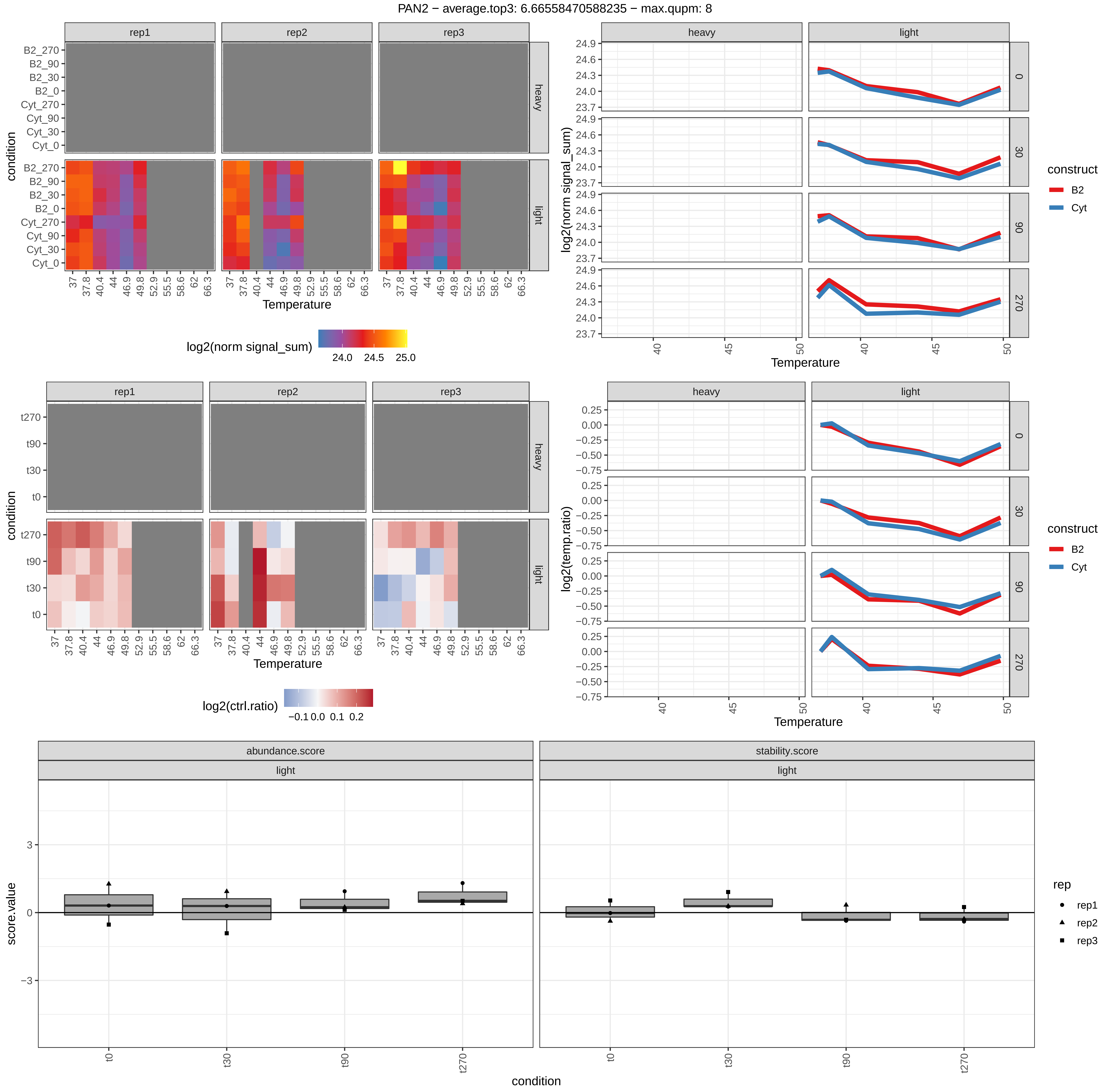

P53010 − PAB−dependent poly(A)−specific ribonuclease subunit PAN2 OS=Saccharomyces cerevisiae (strain ATCC 204508 / S288c) GN=PAN2 PE=1 SV=1

PAN3 − average.top3: 7.15050315789474 − max.qupm: 9

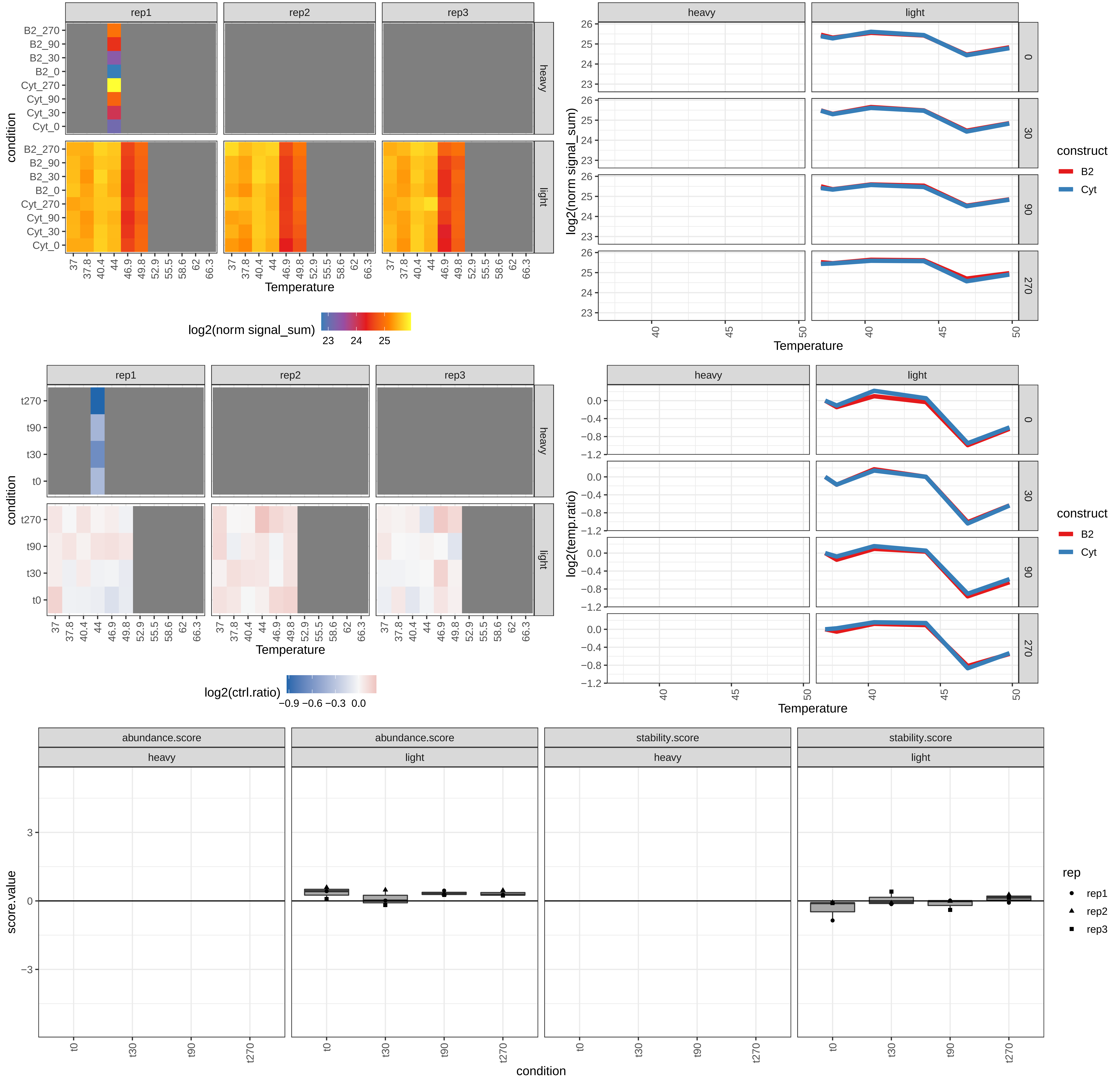

P36102 − PAB−dependent poly(A)−specific ribonuclease subunit PAN3 OS=Saccharomyces cerevisiae (strain ATCC 204508 / S288c) GN=PAN3 PE=1 SV=1

PAN5 − average.top3: 6.9379959375 − max.qupm: 12

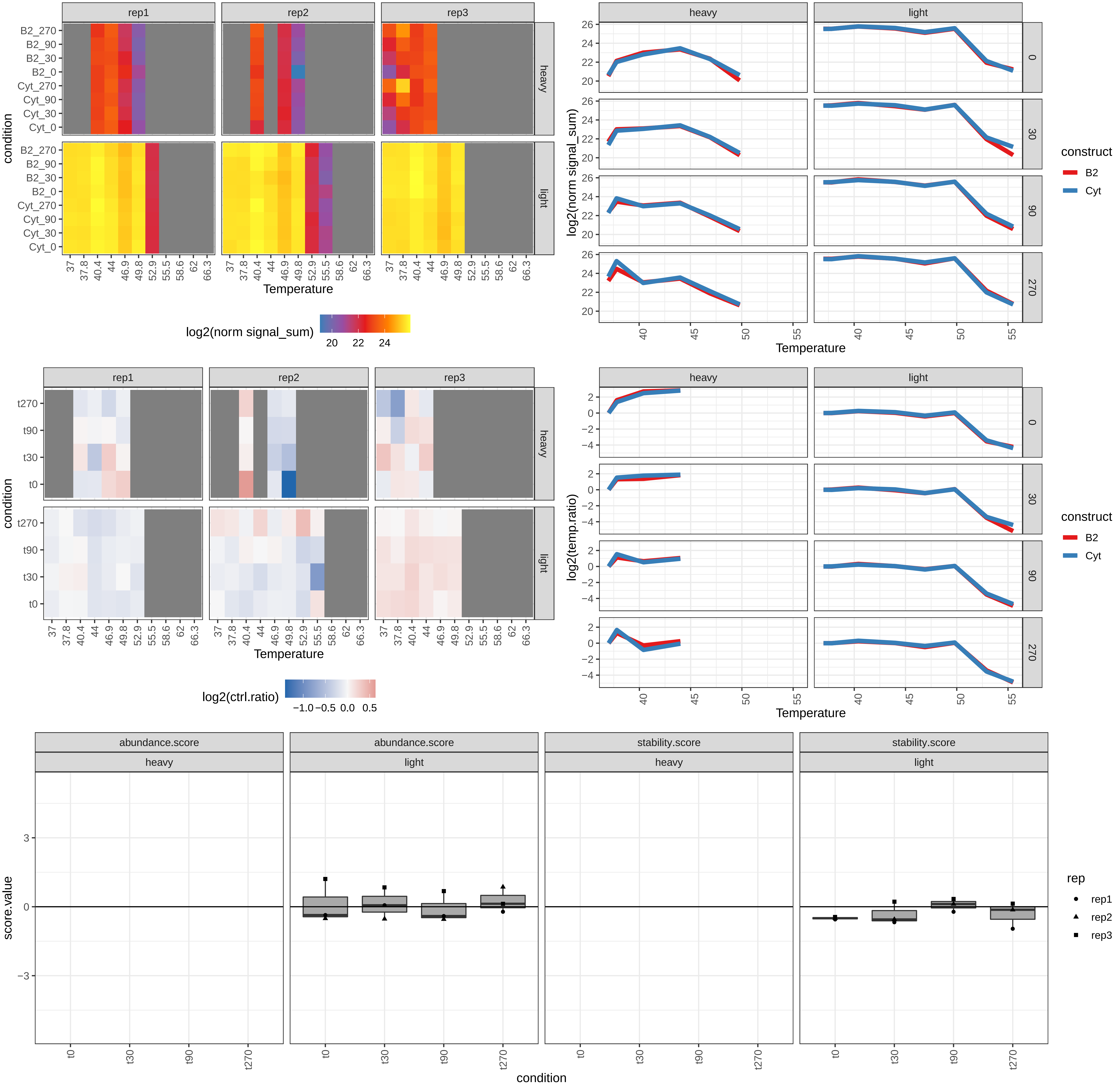

P38787 − 2−dehydropantoate 2−reductase OS=Saccharomyces cerevisiae (strain ATCC 204508 / S288c) GN=PAN5 PE=1 SV=1

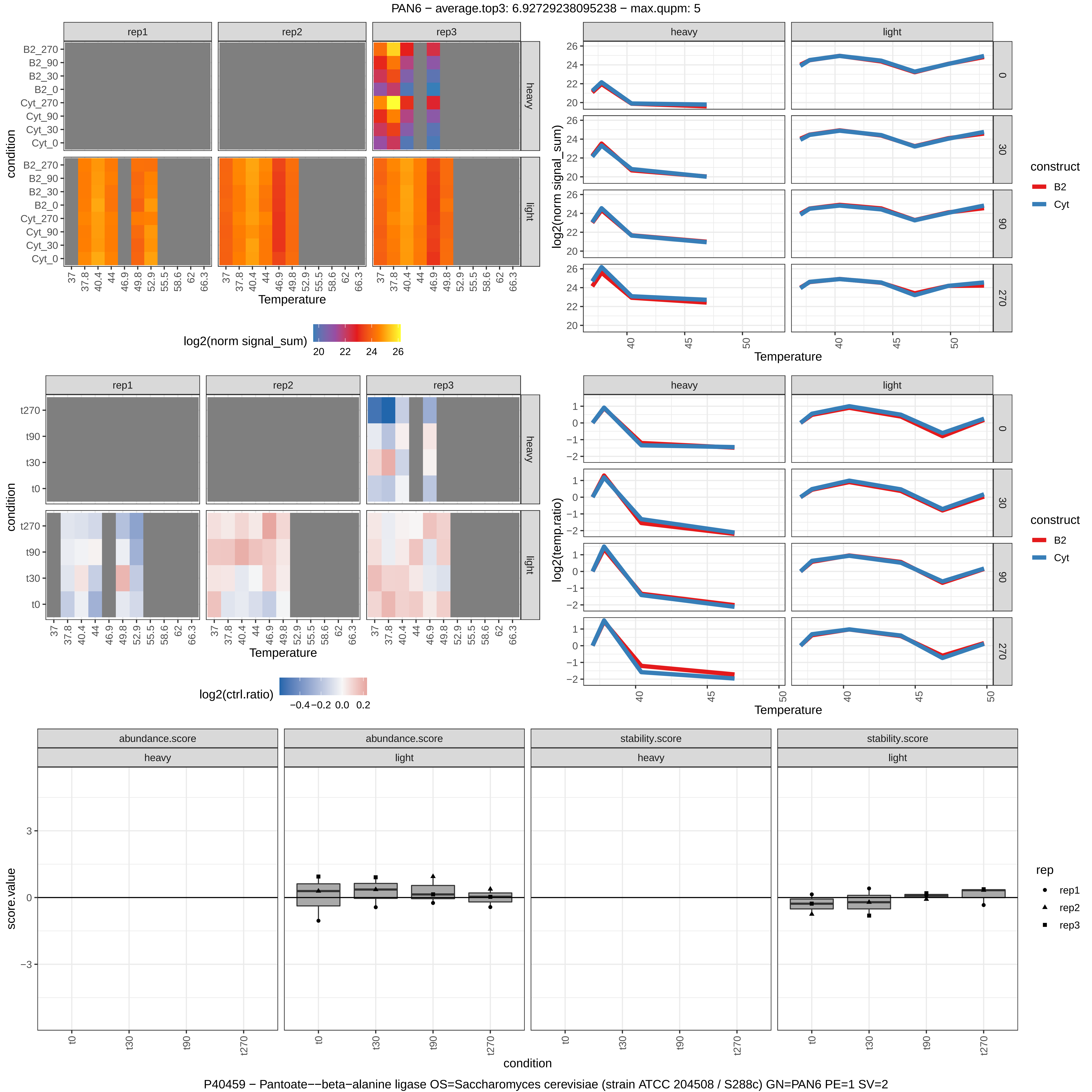
PAN6 − average.top3: 6.92729238095238 − max.qupm: 5
rep1
rep2
rep3
heavy
light
condition
Temperature
log2(norm signal_sum)
construct
B2
Cyt
log2(ctrl.ratio)
log2(temp.ratio)
abundance.score
stability.score
score.value
rep
rep1
rep2
rep3
P40459 − Pantoate−−beta−alanine ligase OS=Saccharomyces cerevisiae (strain ATCC 204508 / S288c) GN=PAN6 PE=1 SV=2

PAP1 − average.top3: 6.65979 − max.qupm: 15

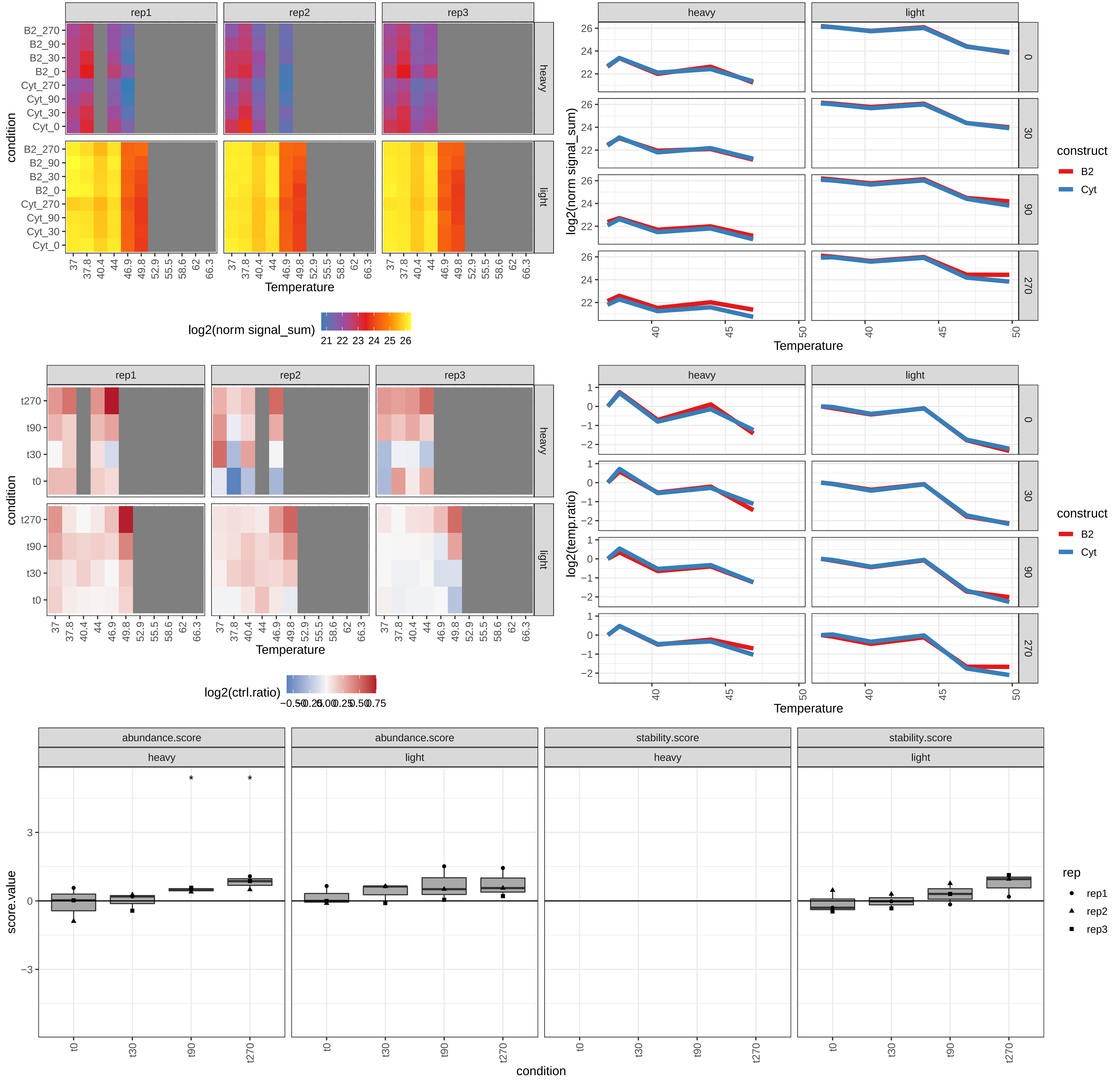

P29468 − Poly(A) polymerase OS=Saccharomyces cerevisiae (strain ATCC 204508 / S288c) GN=PAP1 PE=1 SV=1

PAT1 − average.top3: 6.79372368421053 − max.qupm: 5

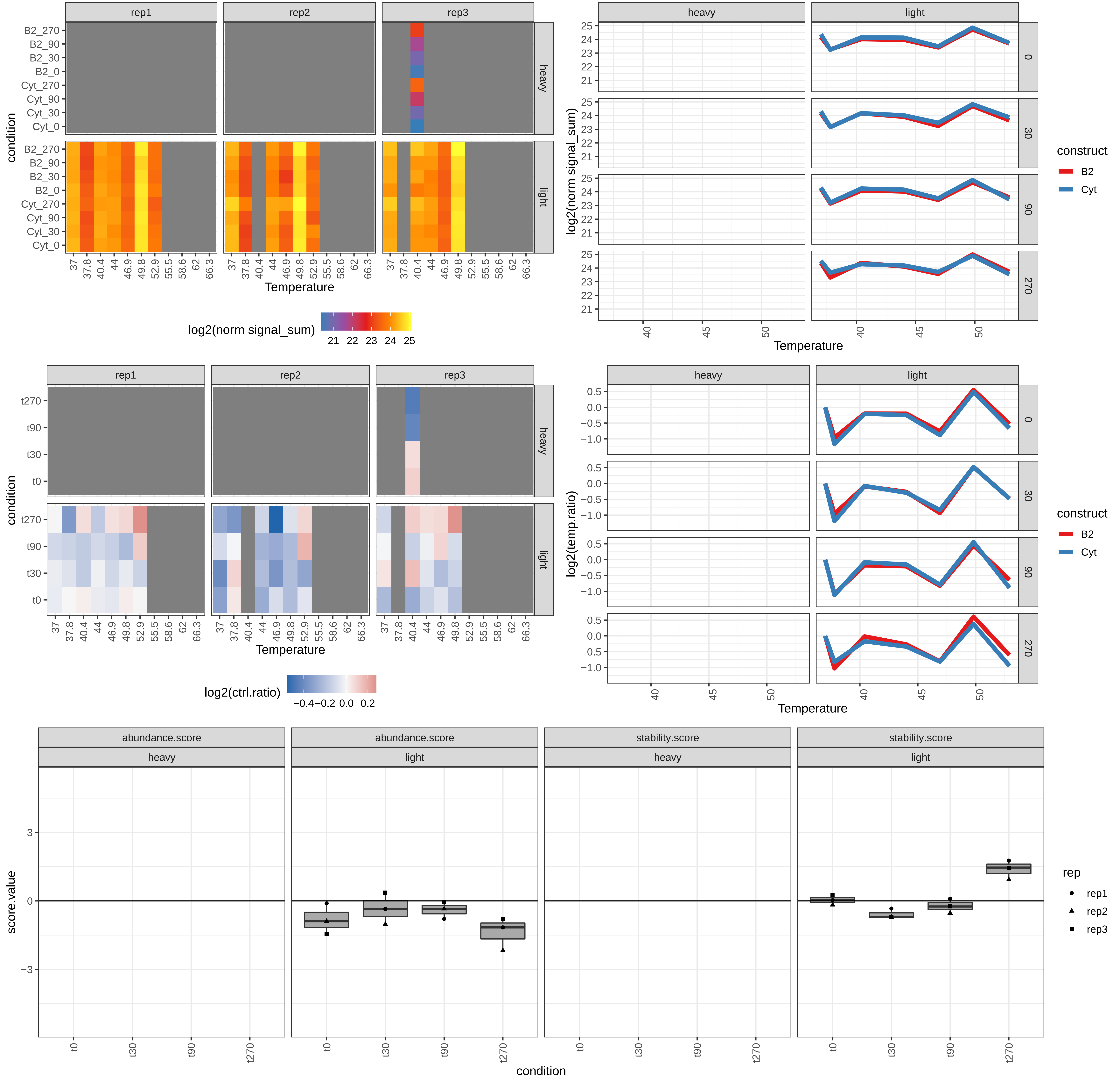

P25644 − DNA topoisomerase 2−associated protein PAT1 OS=Saccharomyces cerevisiae (strain ATCC 204508 / S288c) GN=PAT1 PE=1 SV=4

PBA1 − average.top3: 6.31915333333333 − max.qupm: 3

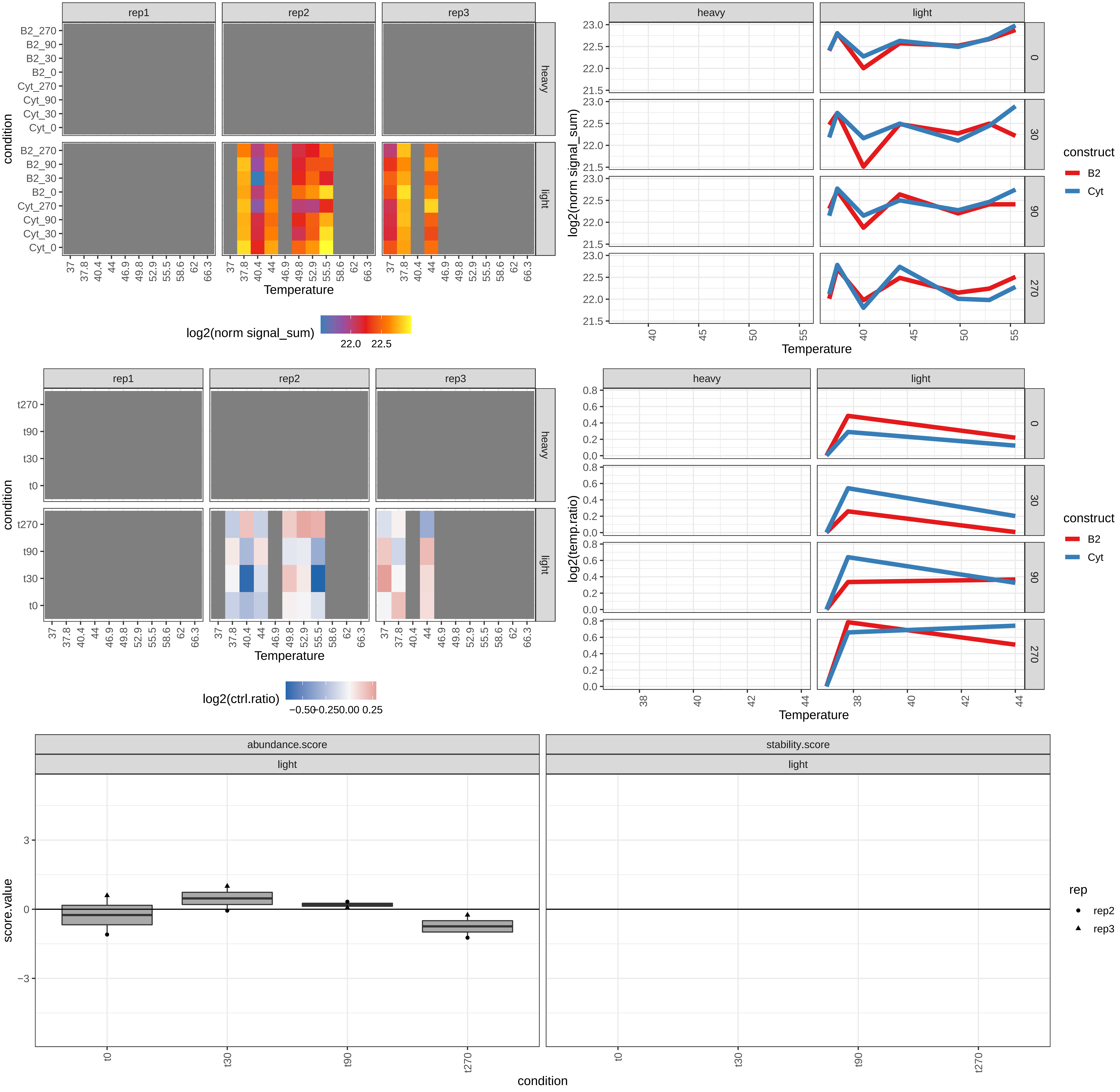

Q05778 − Proteasome chaperone 1 OS=Saccharomyces cerevisiae (strain ATCC 204508 / S288c) GN=PBA1 PE=1 SV=2

PBI2 − average.top3: 7.88808606060606 − max.qupm: 9

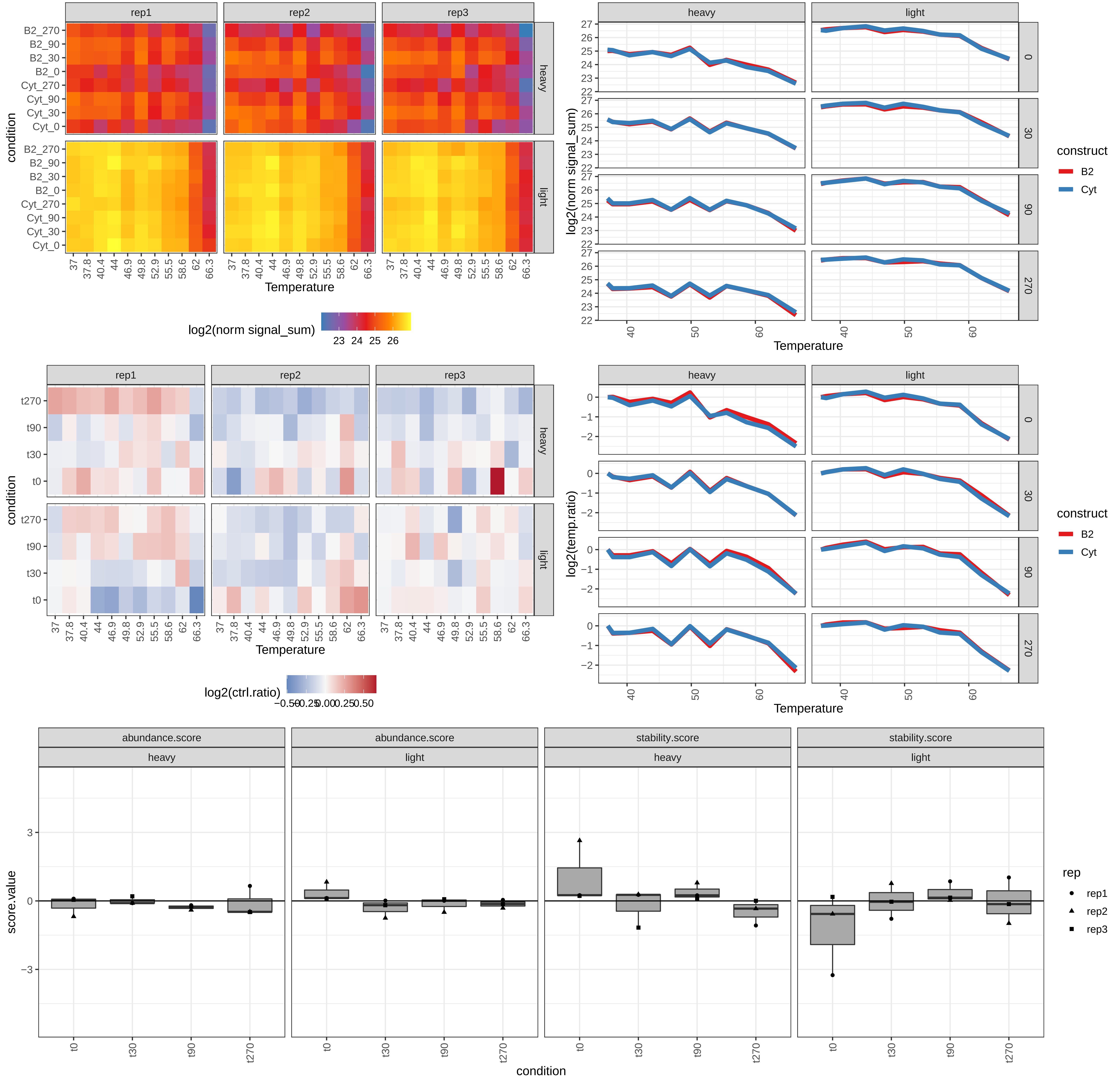

P0CT04 − Protease B inhibitor 2 OS=Saccharomyces cerevisiae (strain ATCC 204508 / S288c) GN=PBI2 PE=1 SV=1

PBN1 − average.top3: 6.9014495 − max.qupm: 8

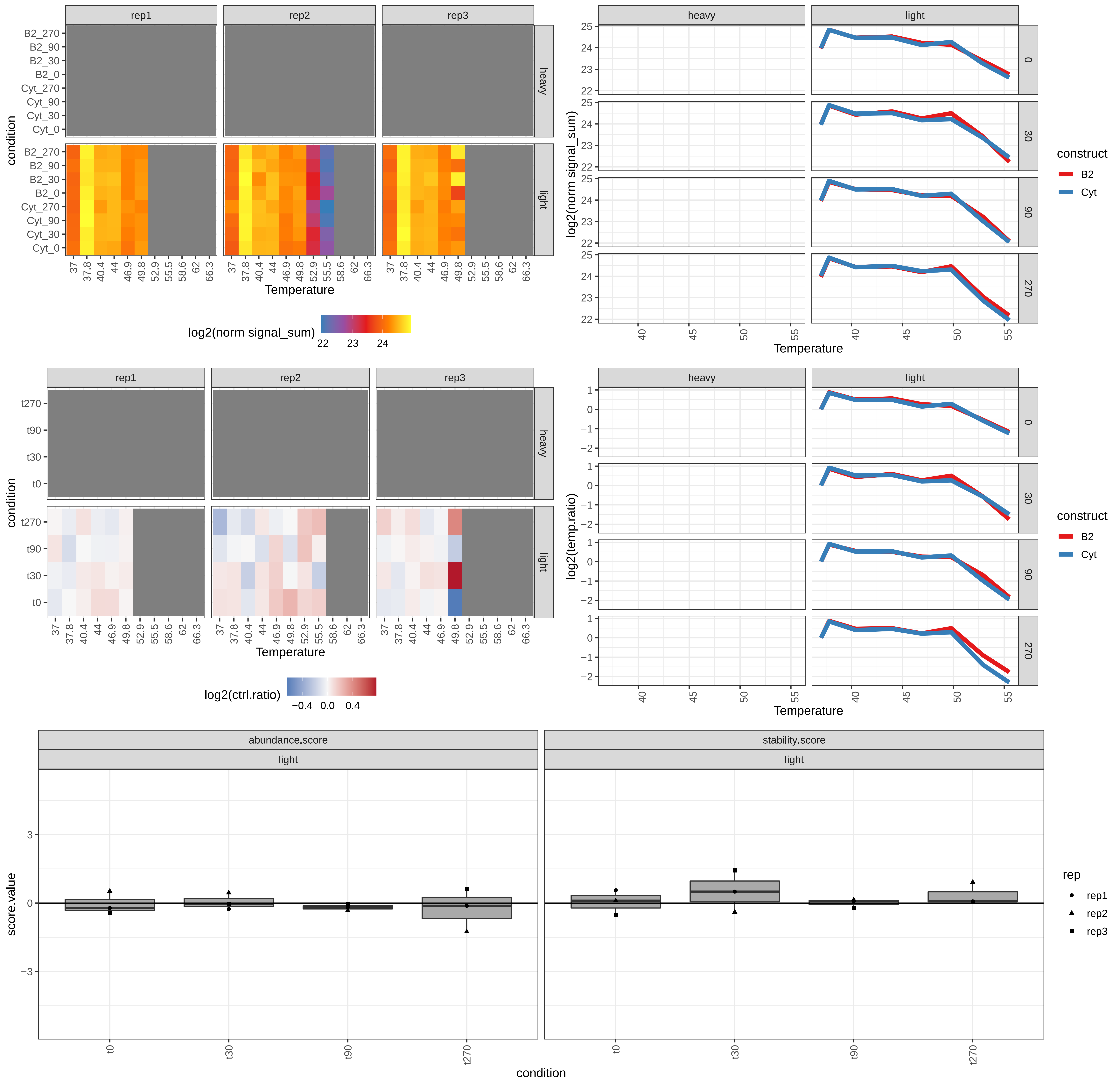

P25580 − Protein PBN1 OS=Saccharomyces cerevisiae (strain ATCC 204508 / S288c) GN=PBN1 PE=1 SV=1

PBY1 − average.top3: 7.20810136363636 − max.qupm: 12

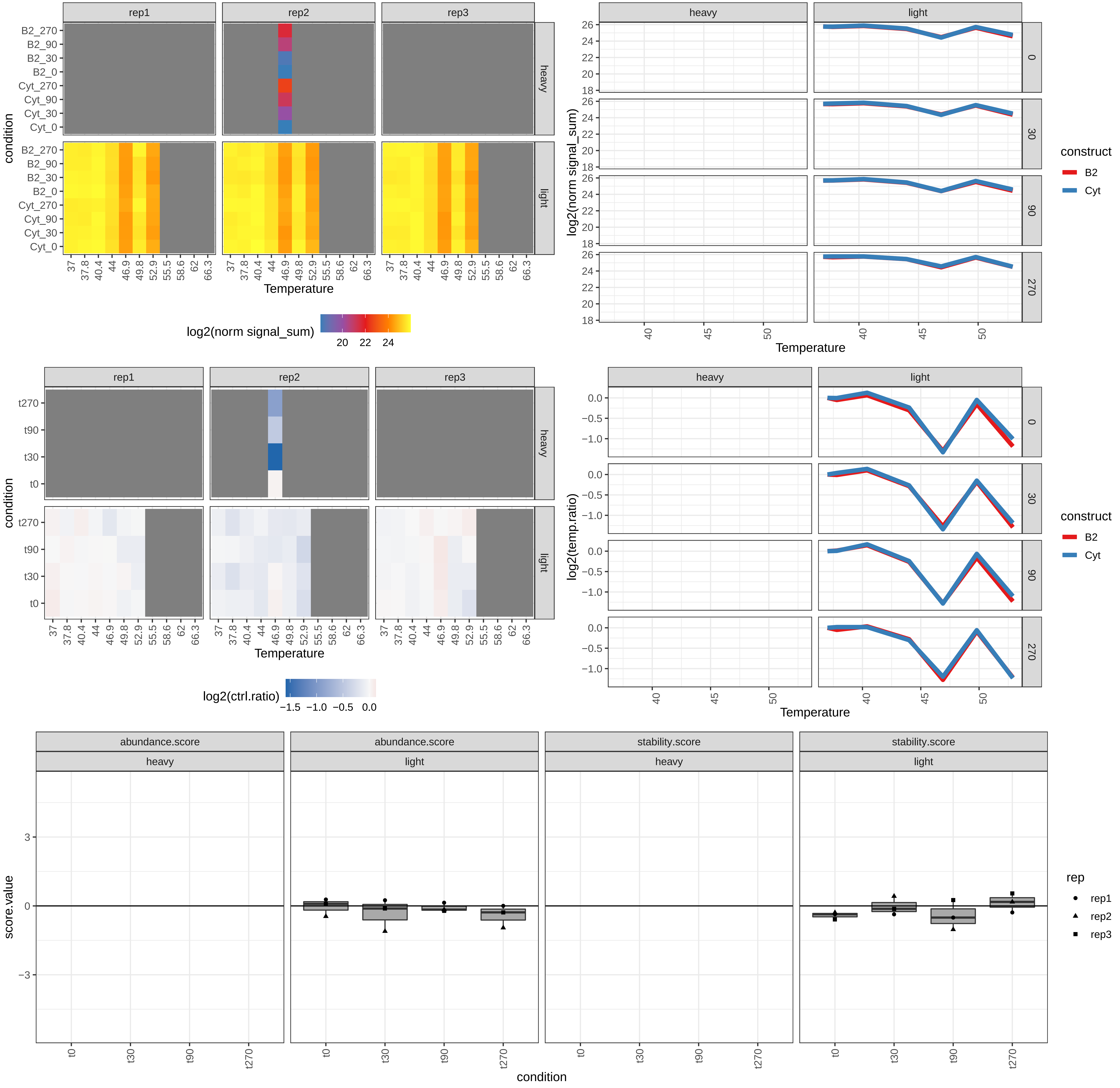

P38254 − Probable tubulin−−tyrosine ligase PBY1 OS=Saccharomyces cerevisiae (strain ATCC 204508 / S288c) GN=PBY1 PE=1 SV=2

PCC1 − average.top3: 6.74575555555556 − max.qupm: 3

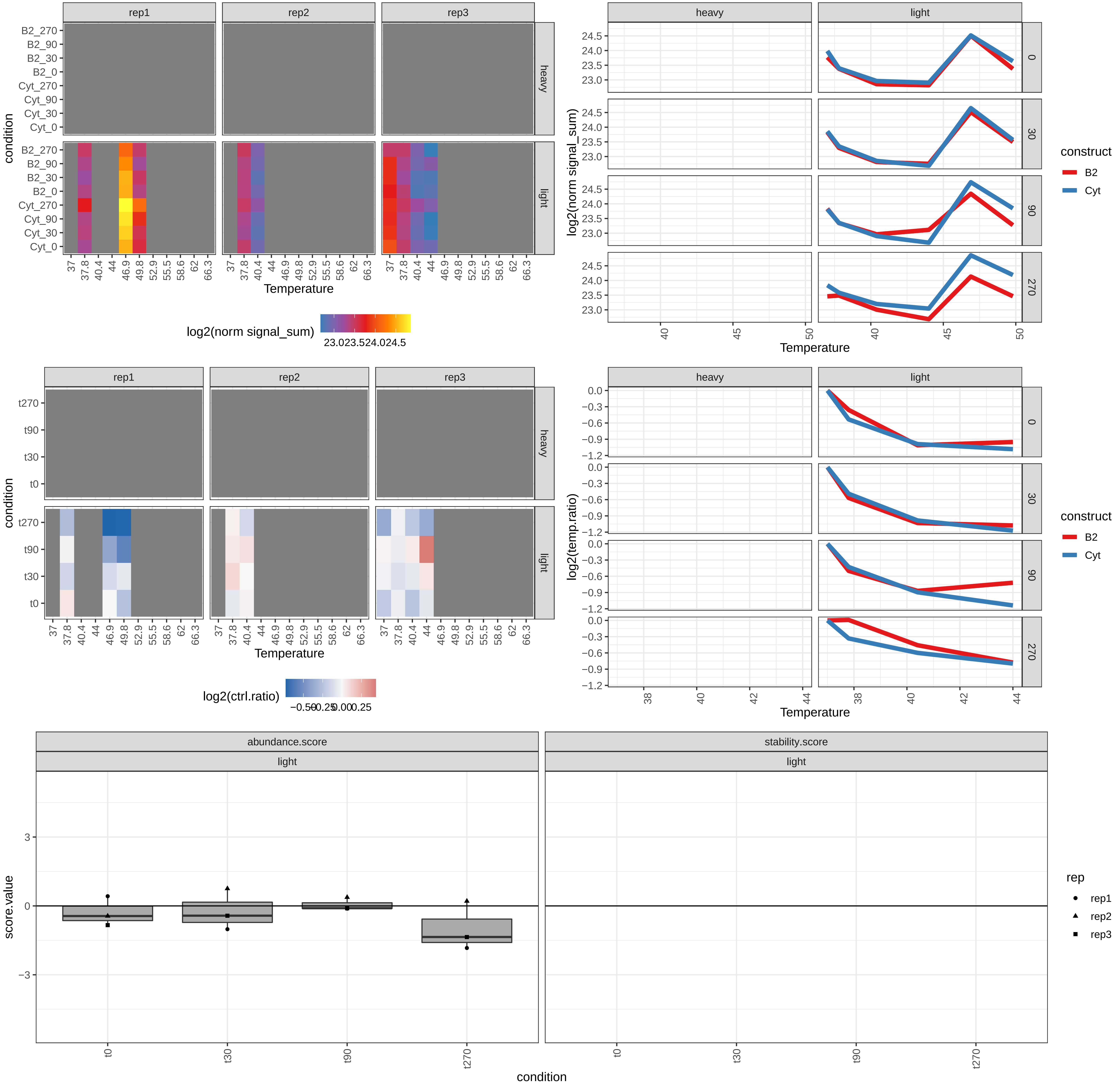

Q3E833 − EKC/KEOPS complex subunit PCC1 OS=Saccharomyces cerevisiae (strain ATCC 204508 / S288c) GN=PCC1 PE=1 SV=1

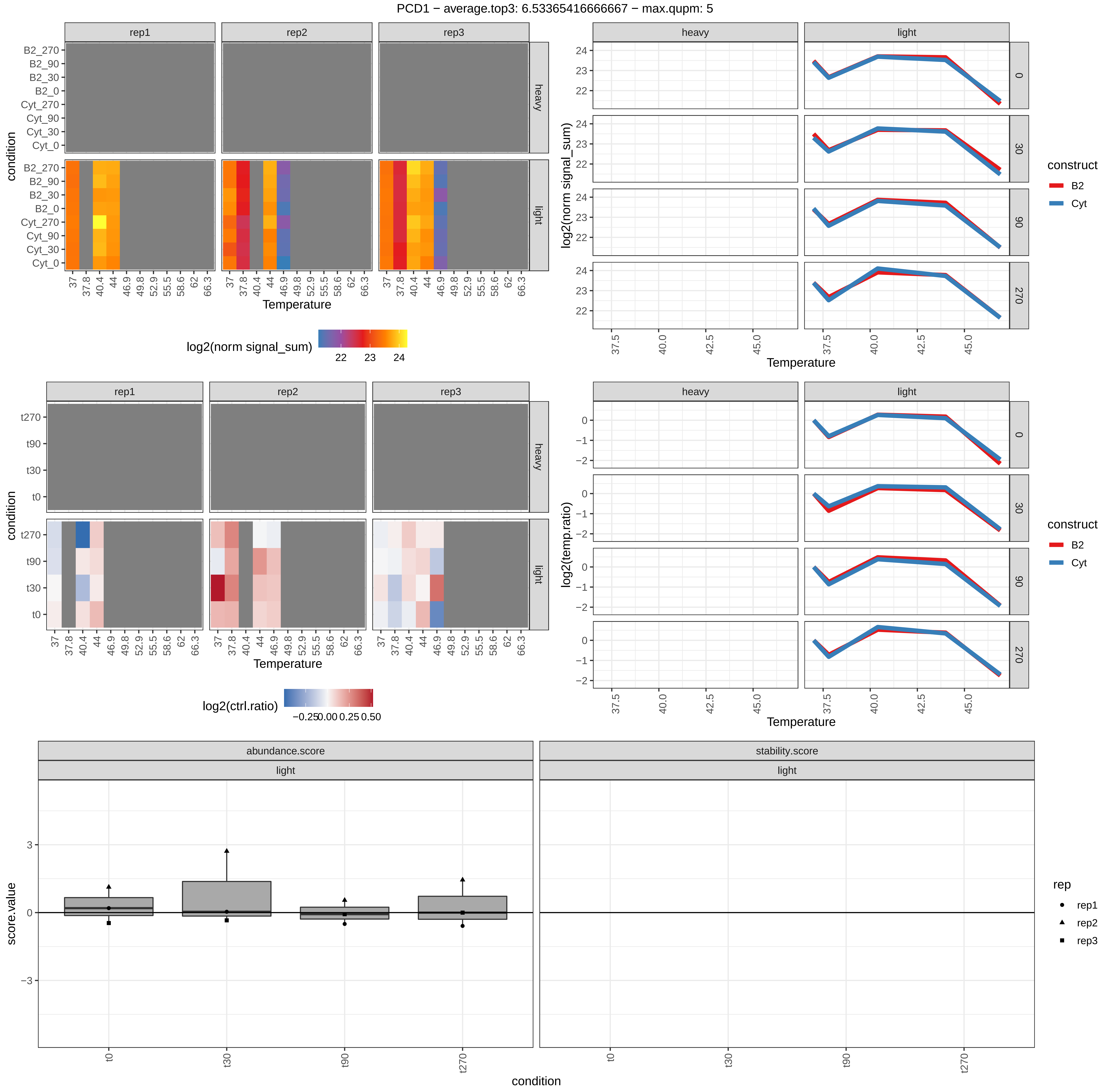

Q12524 − Peroxisomal coenzyme A diphosphatase 1, peroxisomal OS=Saccharomyces cerevisiae (strain ATCC 204508 / S288c) GN=PCD1 PE=1 SV=1

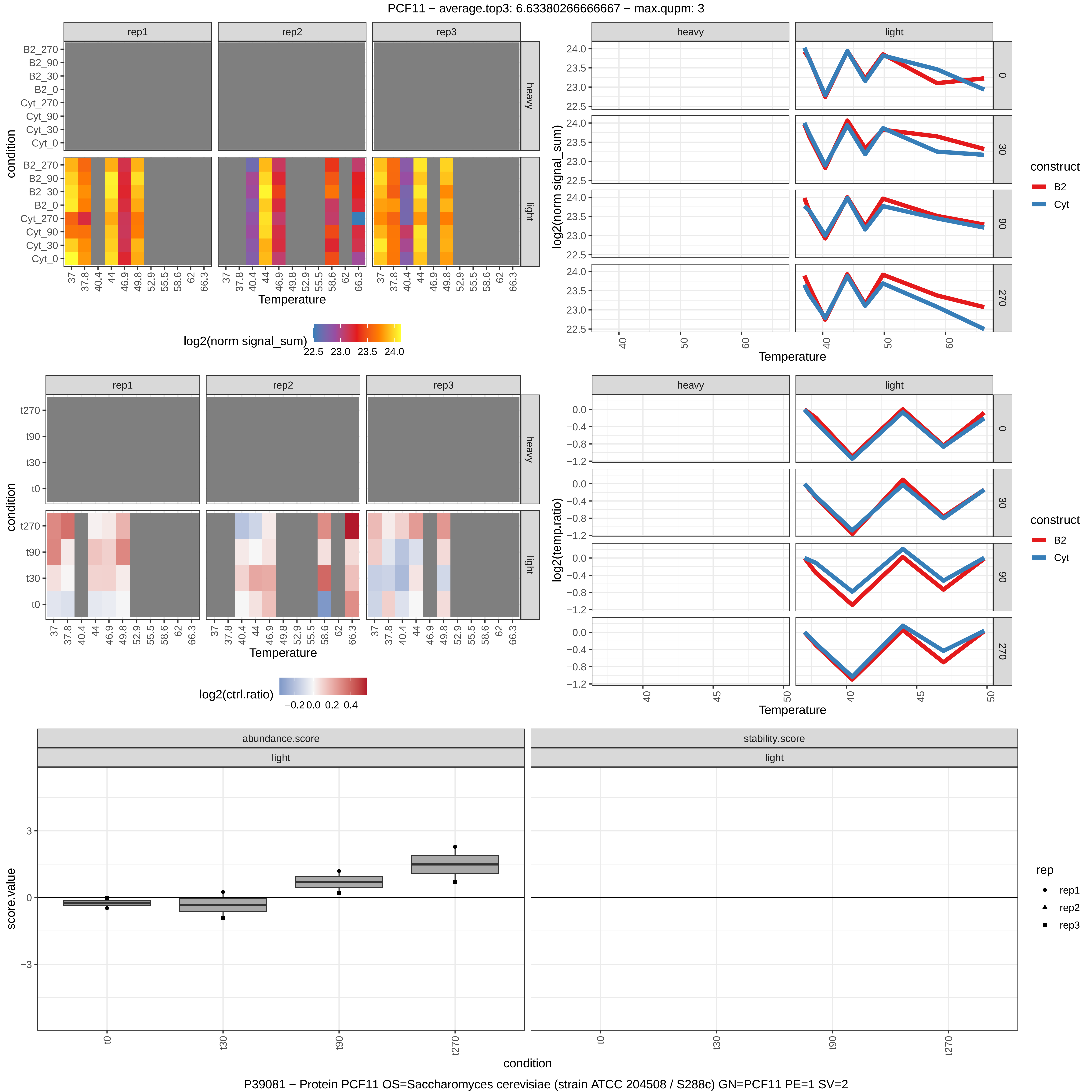
PCF11 − average.top3: 6.63380266666667 − max.qupm: 3
rep1
rep2
rep3
heavy
light
condition
B2_270
B2_90
B2_30
B2_0
Cyt_270
Cyt_90
Cyt_30
Cyt_0
37
37.8
40.4
44
46.9
49.8
52.9
55.5
58.6
62
66.3
Temperature
log2(norm signal_sum)
22.5
23.0
23.5
24.0
0
30
90
270
40
50
60
construct
B2
Cyt
t270
t90
t30
t0
log2(ctrl.ratio)
−0.2
0.0
0.2
0.4
log2(temp.ratio)
−1.2
−0.8
−0.4
45
abundance.score
stability.score
score.value
3
−3
condition
rep
rep1
rep2
rep3
P39081 − Protein PCF11 OS=Saccharomyces cerevisiae (strain ATCC 204508 / S288c) GN=PCF11 PE=1 SV=2

PCK1 − average.top3: 7.833644 − max.qupm: 21

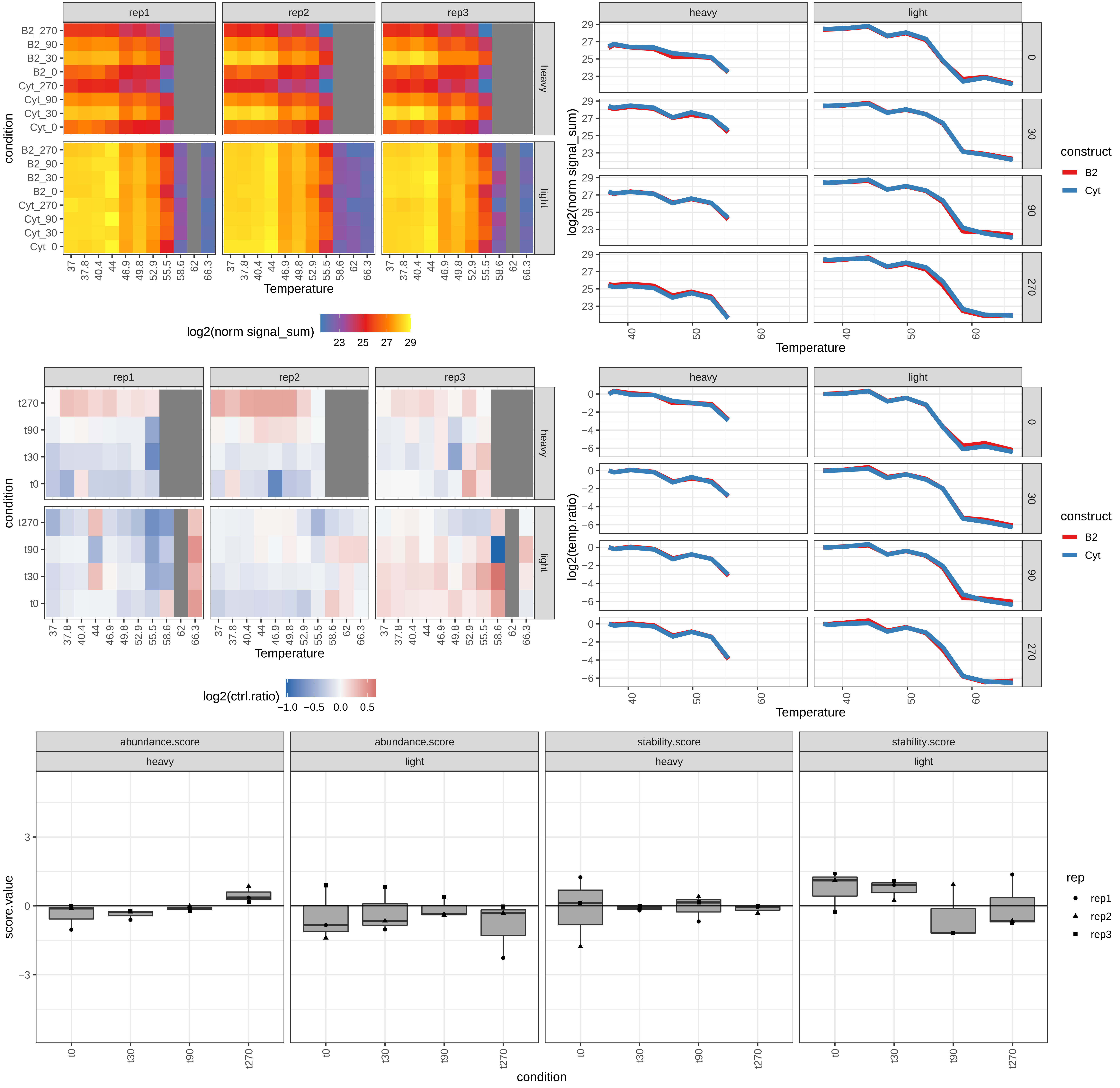

P10963 − Phosphoenolpyruvate carboxykinase [ATP] OS=Saccharomyces cerevisiae (strain ATCC 204508 / S288c) GN=PCK1 PE=1 SV=2

PCM1 − average.top3: 7.39453763157895 − max.qupm: 19

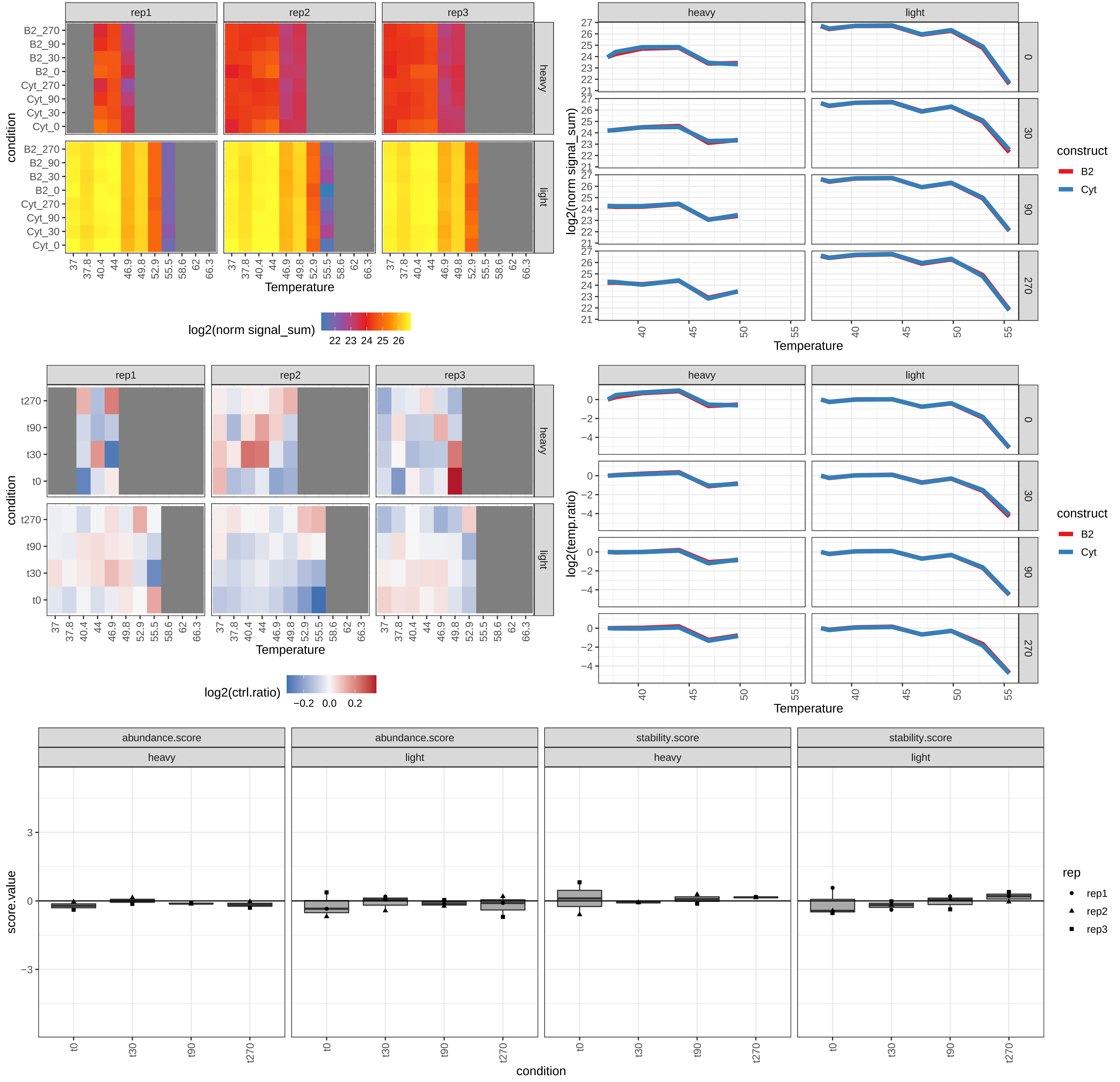

P38628 − Phosphoacetylglucosamine mutase OS=Saccharomyces cerevisiae (strain ATCC 204508 / S288c) GN=PCM1 PE=1 SV=2

PCS60 − average.top3: 7.37743581818182 − max.qupm: 19

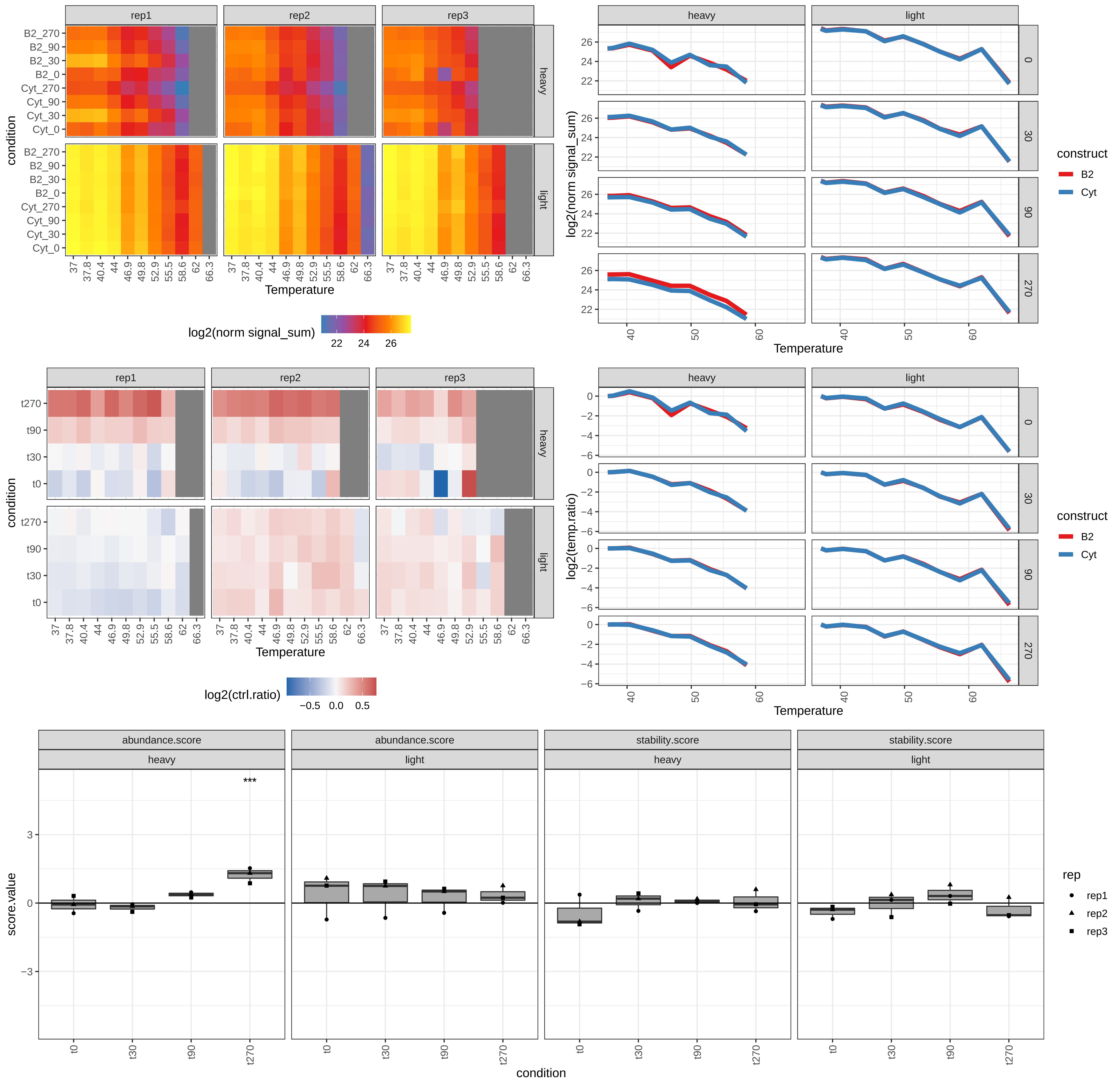

P38137 − Peroxisomal−coenzyme A synthetase OS=Saccharomyces cerevisiae (strain ATCC 204508 / S288c) GN=PCS60 PE=1 SV=1

PCT1 − average.top3: 6.58982666666667 − max.qupm: 8

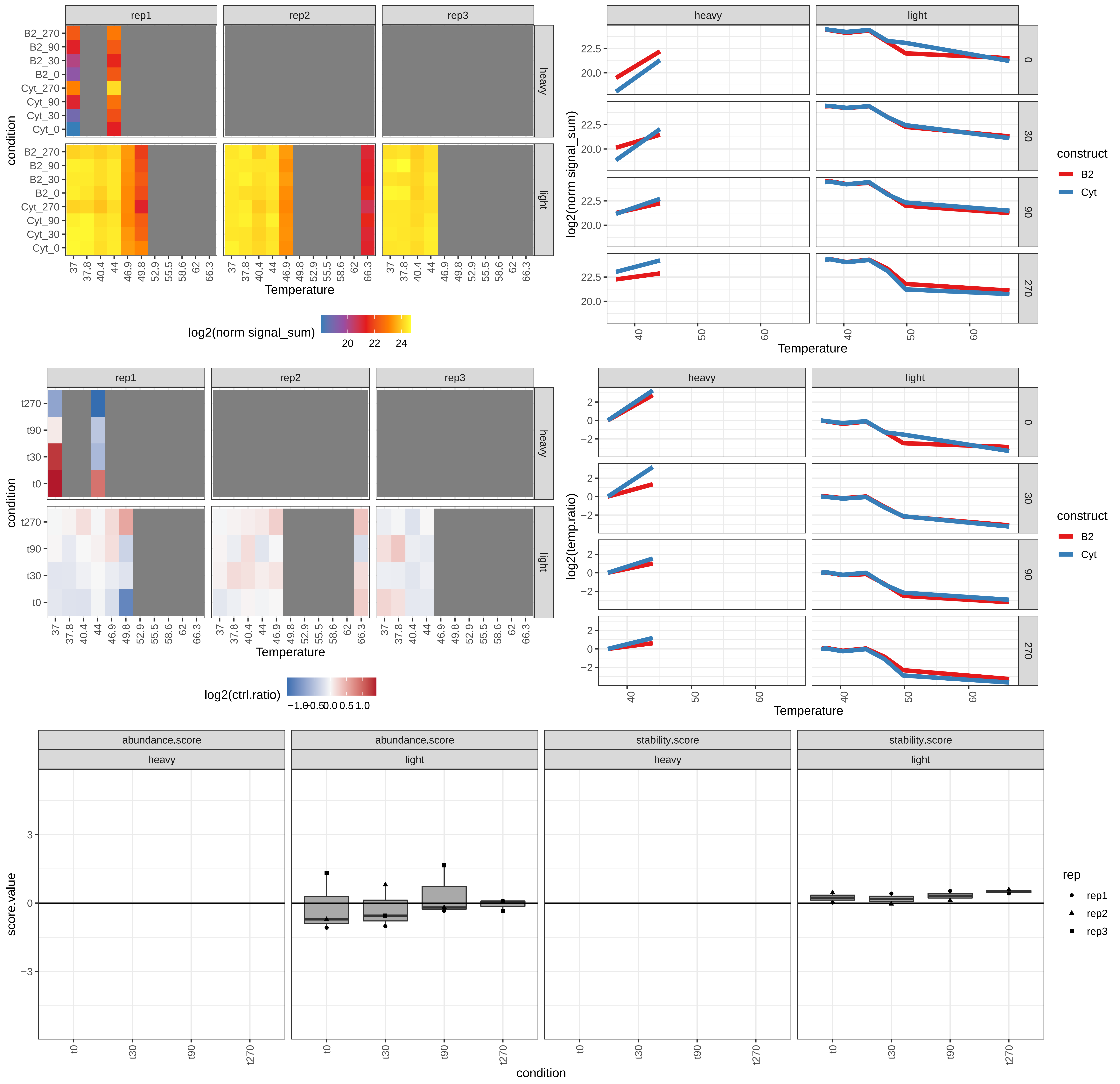

P13259 − Choline−phosphate cytidylyltransferase OS=Saccharomyces cerevisiae (strain ATCC 204508 / S288c) GN=PCT1 PE=1 SV=2

PDA1 − average.top3: 7.76093813559322 − max.qupm: 26

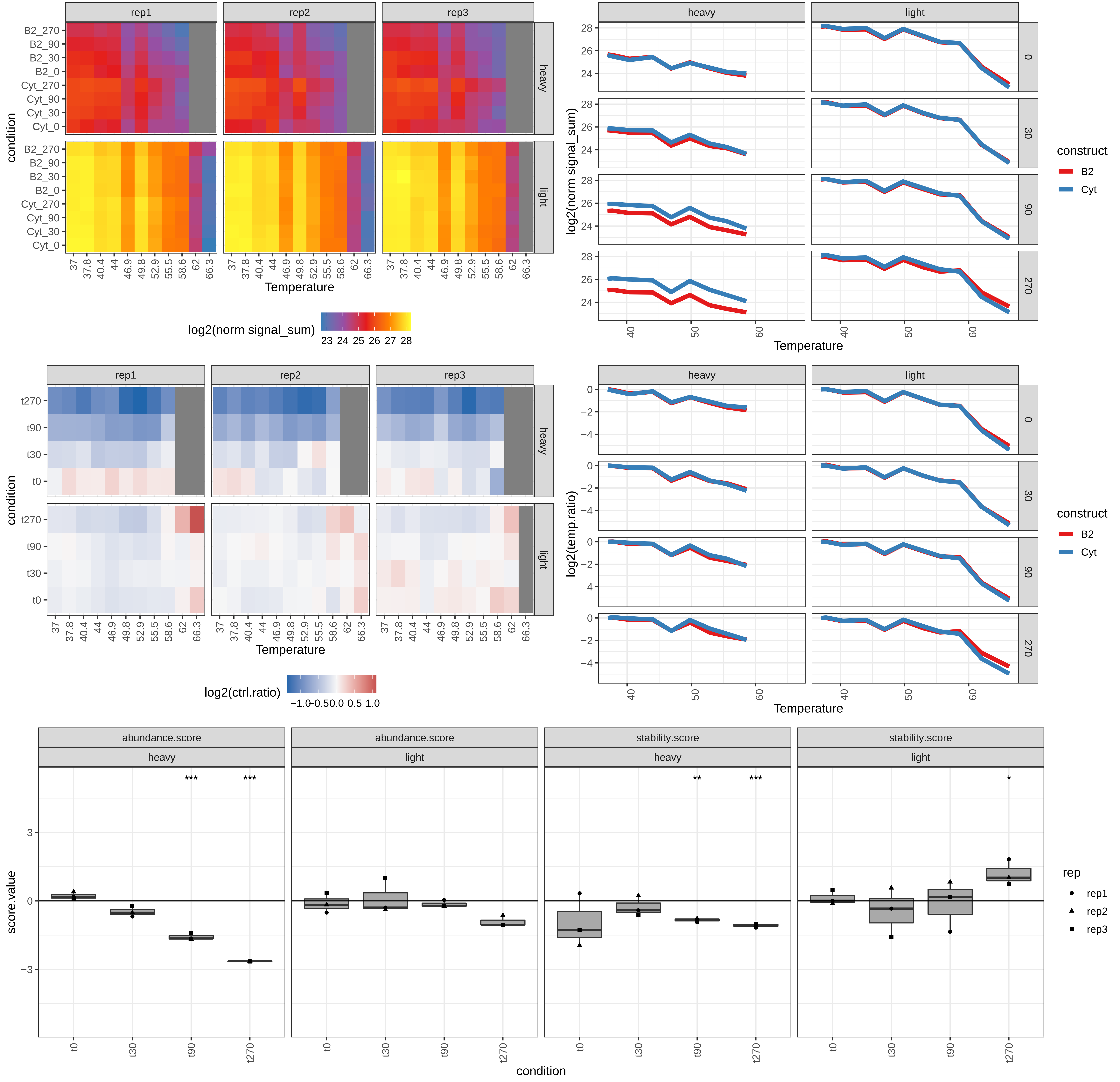

P16387 − Pyruvate dehydrogenase E1 component subunit alpha, mitochondrial OS=Saccharomyces cerevisiae (strain ATCC 204508 / S288c) GN=PDA1 PE=1 SV=2

PDB1 − average.top3: 7.29973275862069 − max.qupm: 9

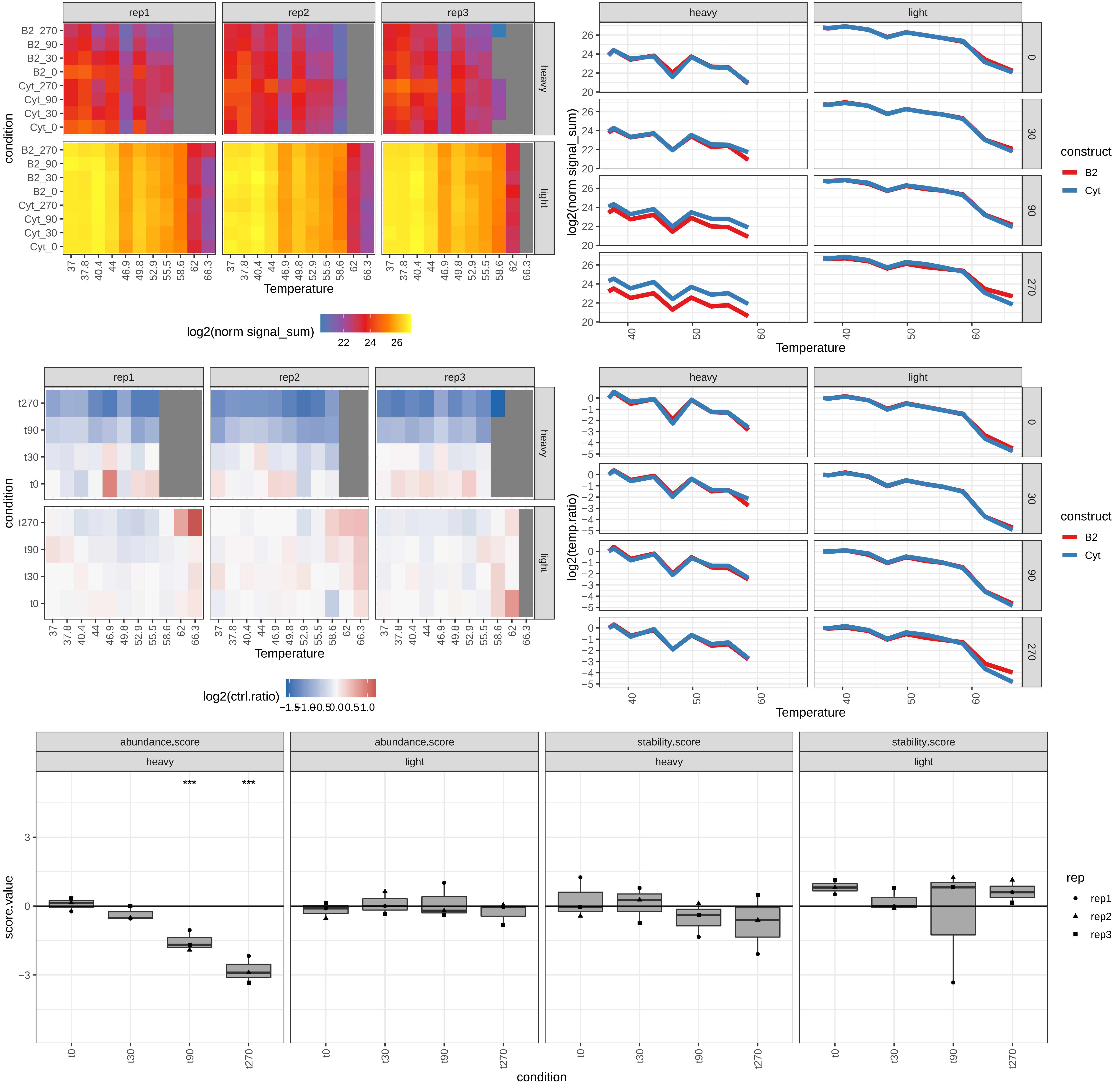

P32473 − Pyruvate dehydrogenase E1 component subunit beta, mitochondrial OS=Saccharomyces cerevisiae (strain ATCC 204508 / S288c) GN=PDB1 PE=1 SV=2

PDC1 − average.top3: 8.44248106060606 − max.qupm: 17

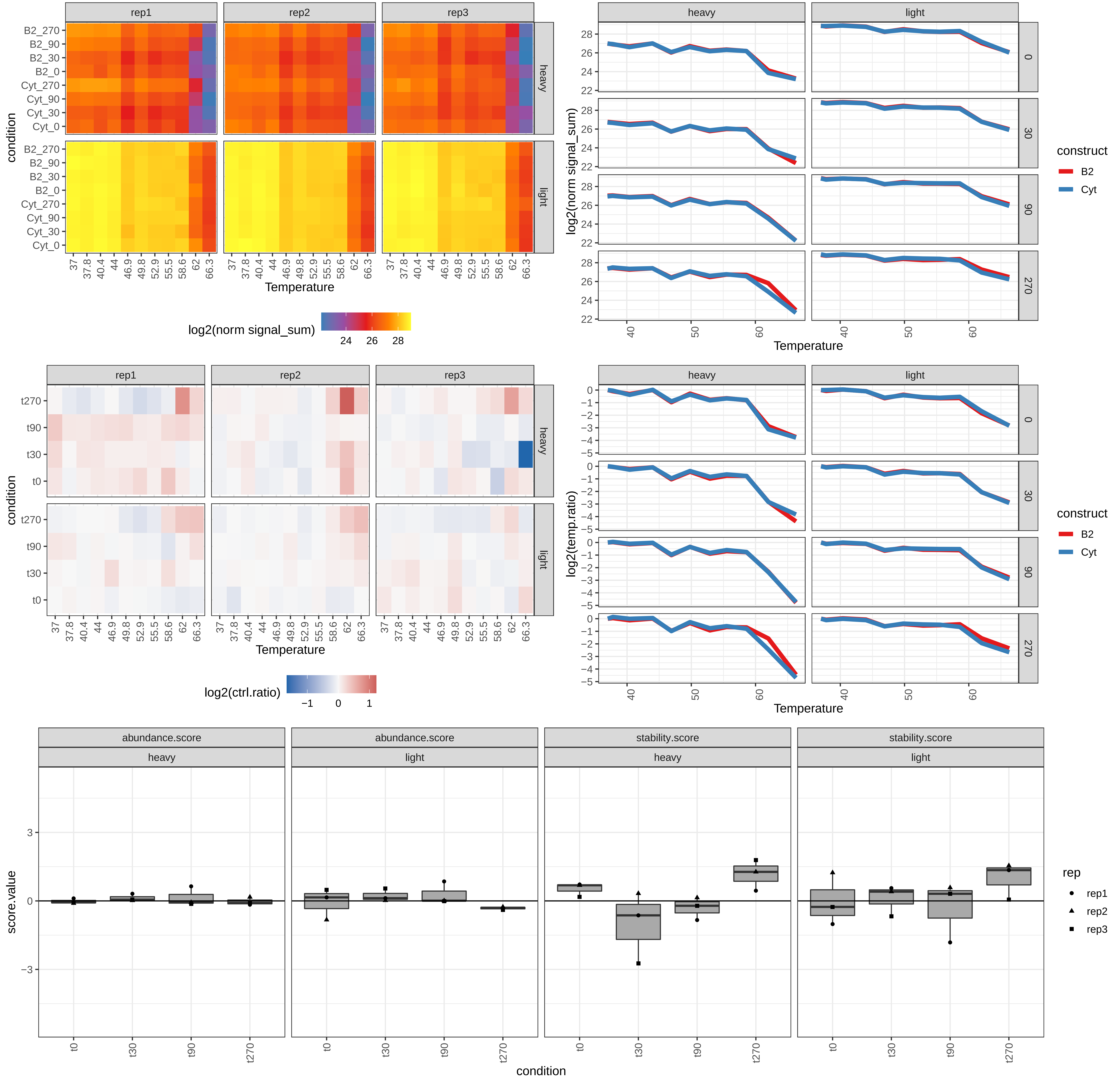

P06169 − Pyruvate decarboxylase isozyme 1 OS=Saccharomyces cerevisiae (strain ATCC 204508 / S288c) GN=PDC1 PE=1 SV=7

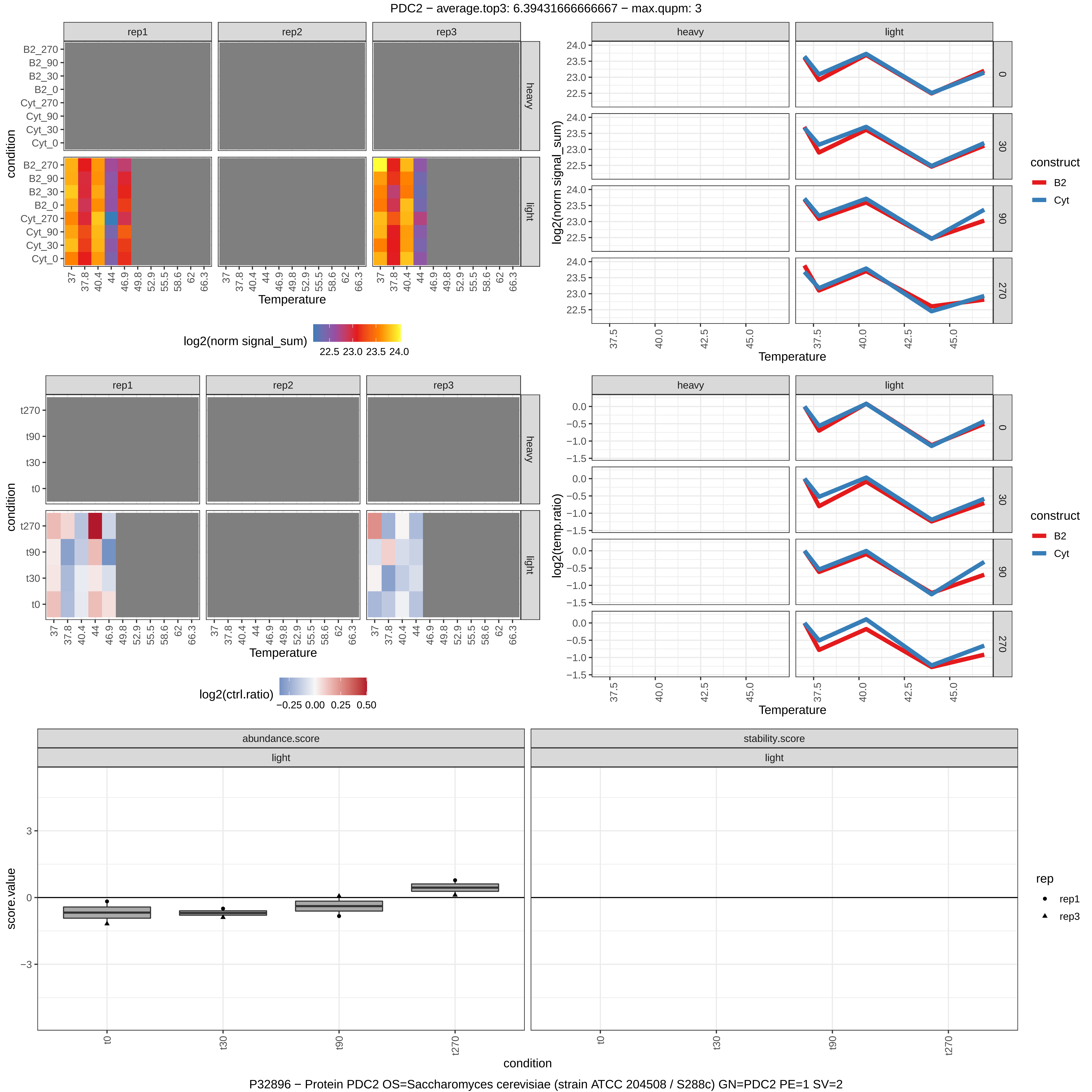

PDC2 − average.top3: 6.39431666666667 − max.qupm: 3
rep1
rep2
rep3
heavy
light
condition
Temperature
log2(norm signal_sum)
construct
B2
Cyt
log2(ctrl.ratio)
log2(temp.ratio)
abundance.score
stability.score
score.value
rep
rep1
rep3
P32896 − Protein PDC2 OS=Saccharomyces cerevisiae (strain ATCC 204508 / S288c) GN=PDC2 PE=1 SV=2

PDC6 − average.top3: 6.4139 − max.qupm: 3

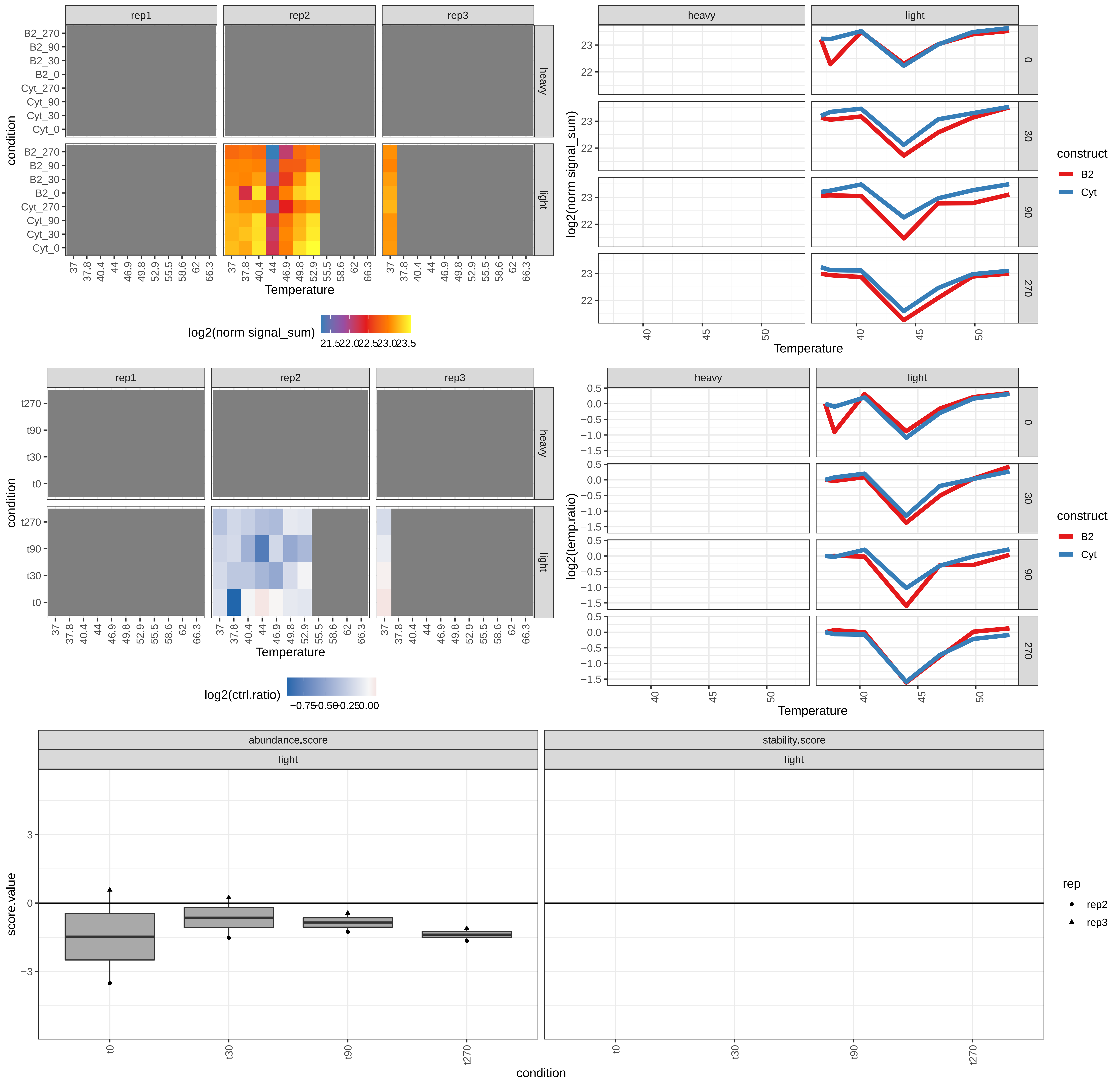

P26263 − Pyruvate decarboxylase isozyme 3 OS=Saccharomyces cerevisiae (strain ATCC 204508 / S288c) GN=PDC6 PE=1 SV=3

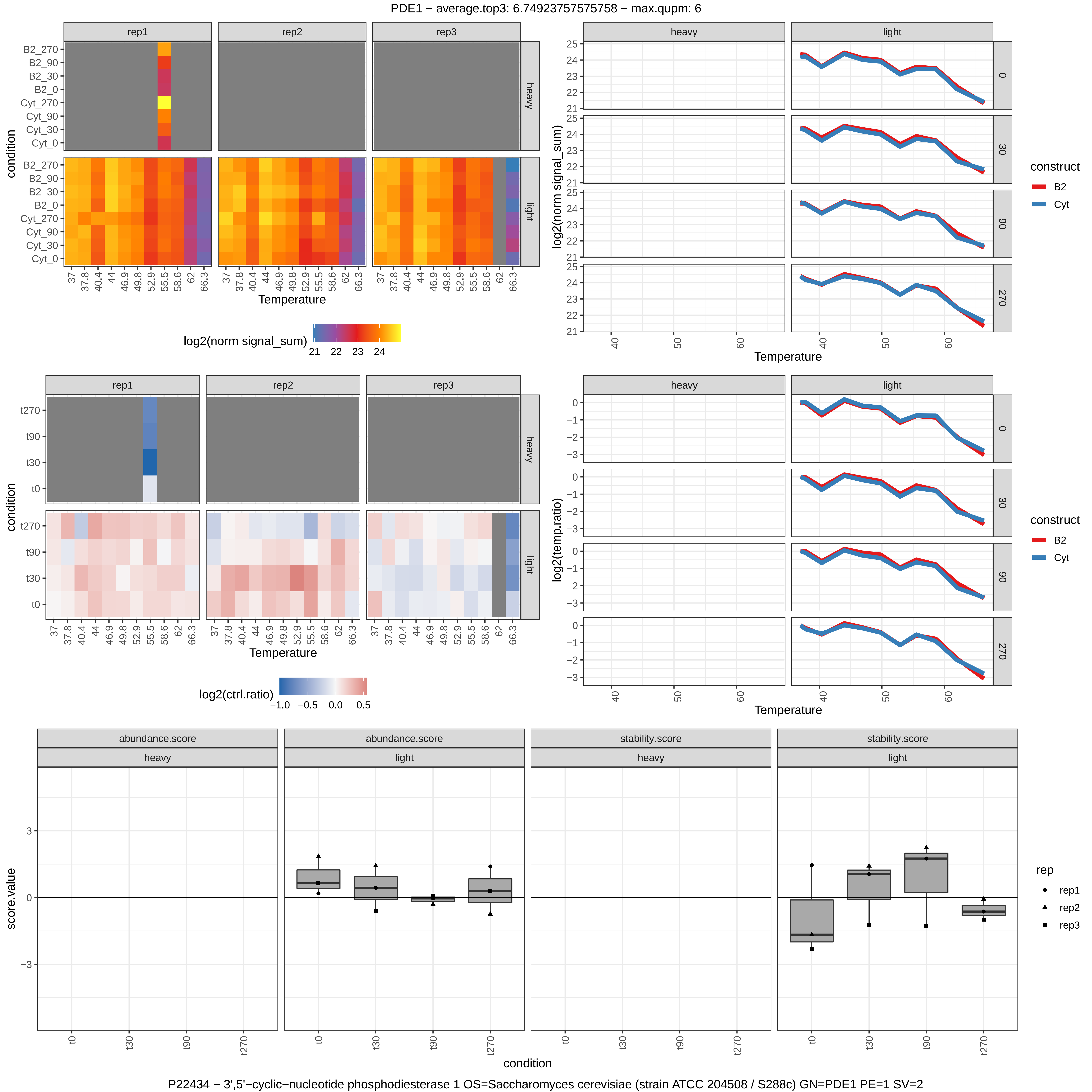

PDE1 − average.top3: 6.74923757575758 − max.qupm: 6
rep1
rep2
rep3
heavy
light
condition
B2_270
B2_90
B2_30
B2_0
Cyt_270
Cyt_90
Cyt_30
Cyt_0
Temperature
log2(norm signal_sum)
log2(ctrl.ratio)
log2(temp.ratio)
construct
B2
Cyt
t270
t90
t30
t0
abundance.score
stability.score
score.value
rep
P22434 − 3',5'−cyclic−nucleotide phosphodiesterase 1 OS=Saccharomyces cerevisiae (strain ATCC 204508 / S288c) GN=PDE1 PE=1 SV=2

PDE2 − average.top3: 6.8275637037037 − max.qupm: 6

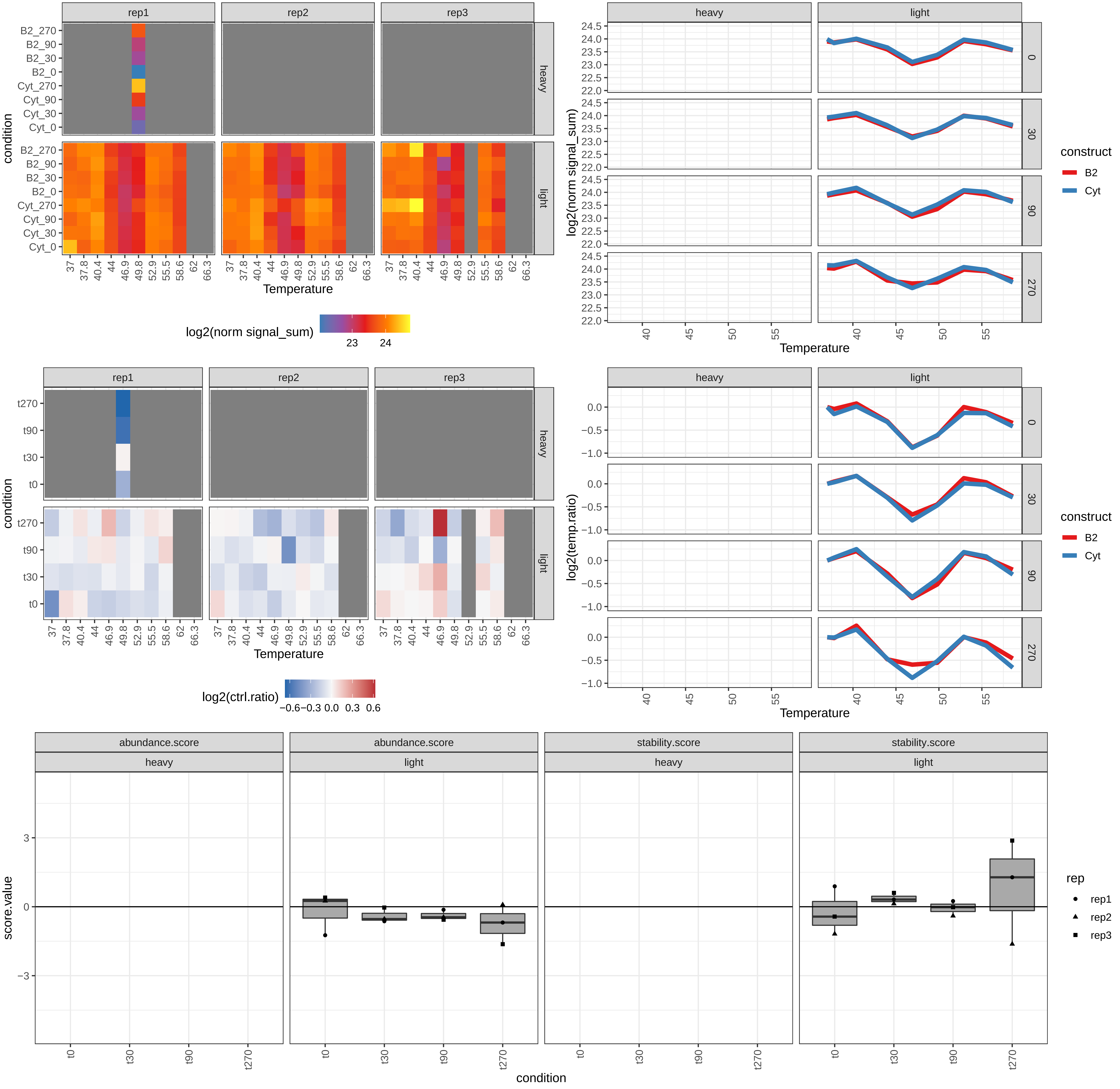

P06776 − 3',5'−cyclic−nucleotide phosphodiesterase 2 OS=Saccharomyces cerevisiae (strain ATCC 204508 / S288c) GN=PDE2 PE=1 SV=2

PDH1 − average.top3: 7.75825981481481 − max.qupm: 29

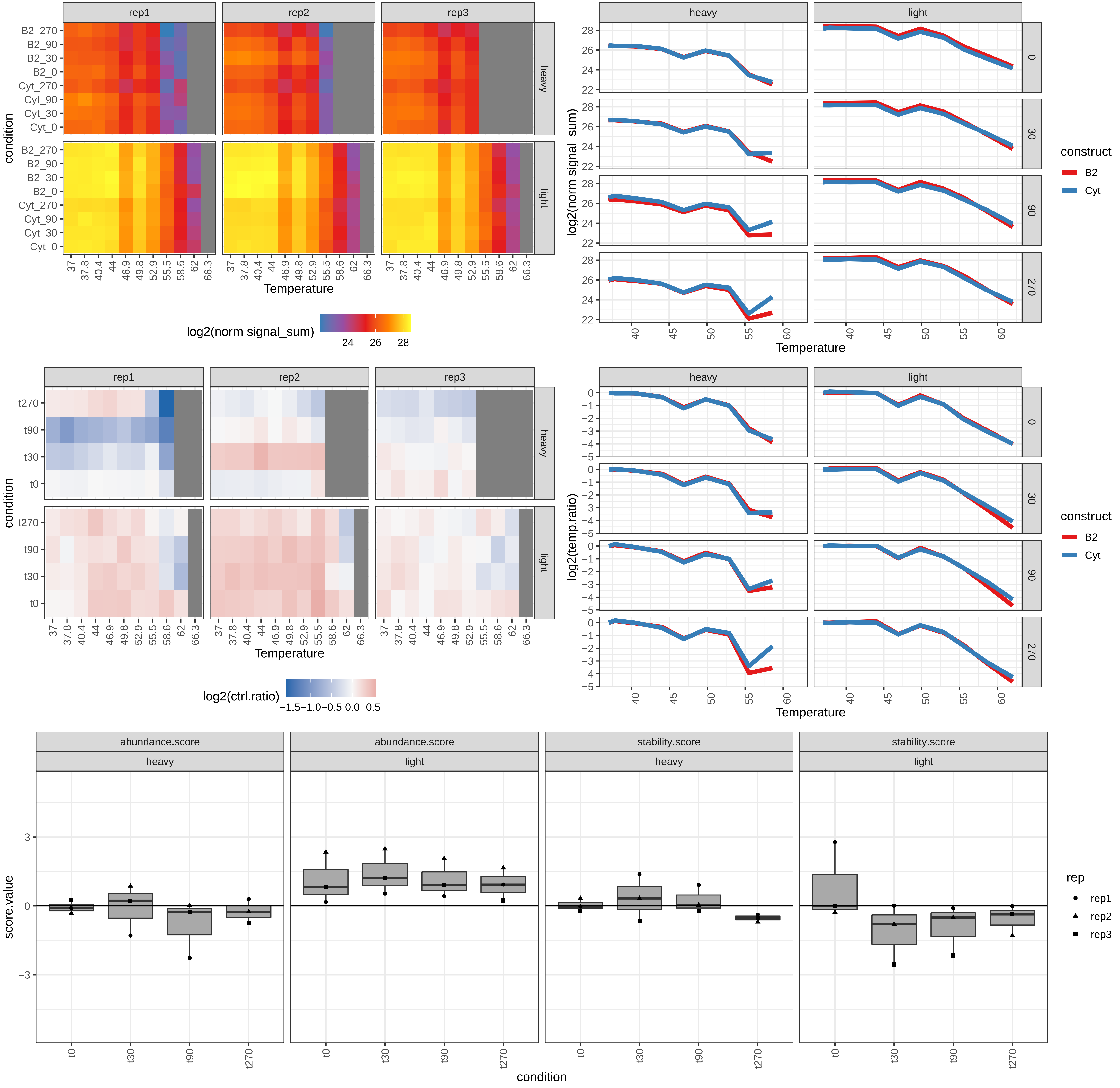

Q12428 − Probable 2−methylcitrate dehydratase OS=Saccharomyces cerevisiae (strain ATCC 204508 / S288c) GN=PDH1 PE=1 SV=1

PDI1 − average.top3: 7.54490692307692 − max.qupm: 22

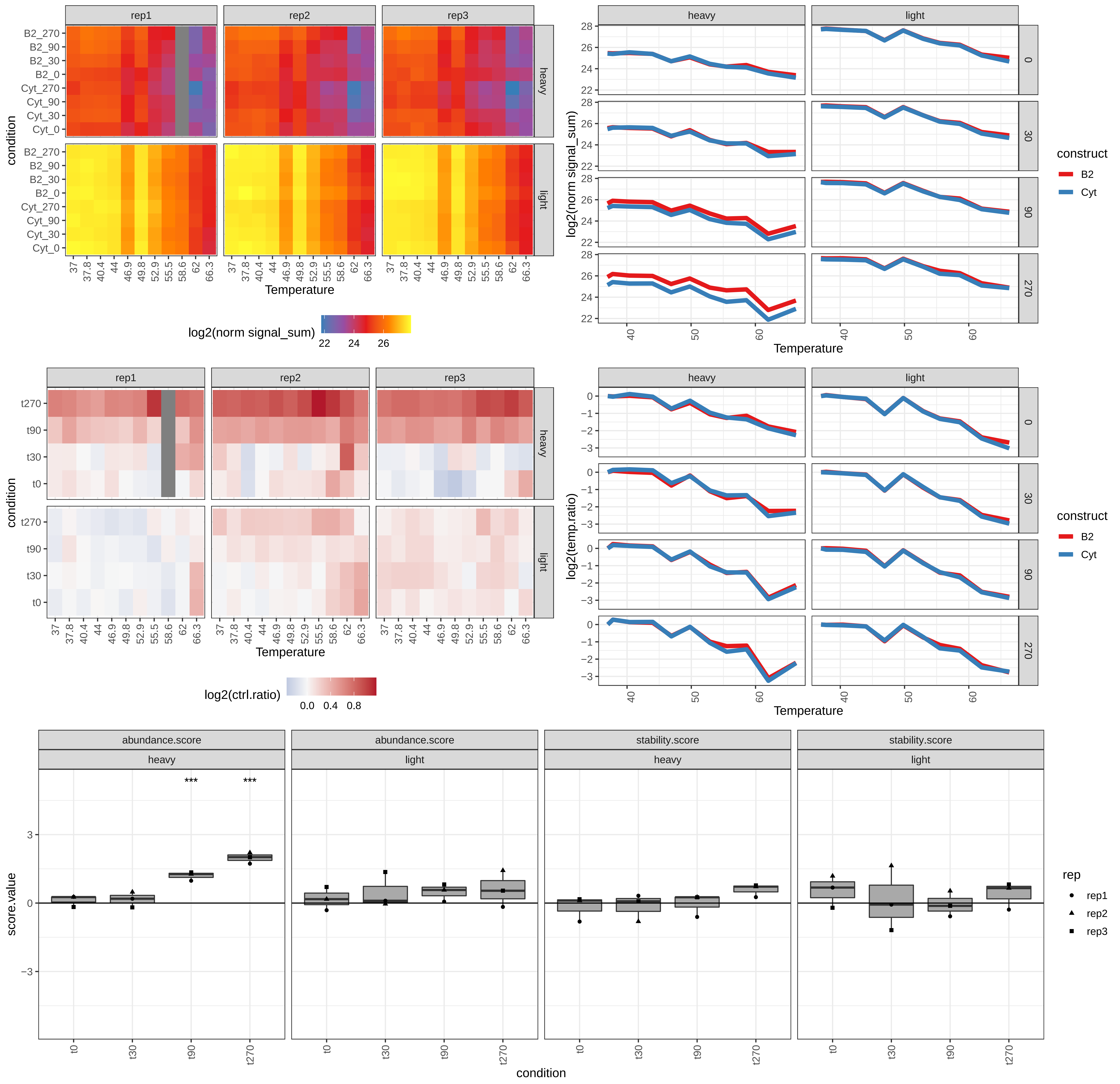

P17967 − Protein disulfide−isomerase OS=Saccharomyces cerevisiae (strain ATCC 204508 / S288c) GN=PDI1 PE=1 SV=2

PDR1 − average.top3: 6.66812028571429 − max.qupm: 7

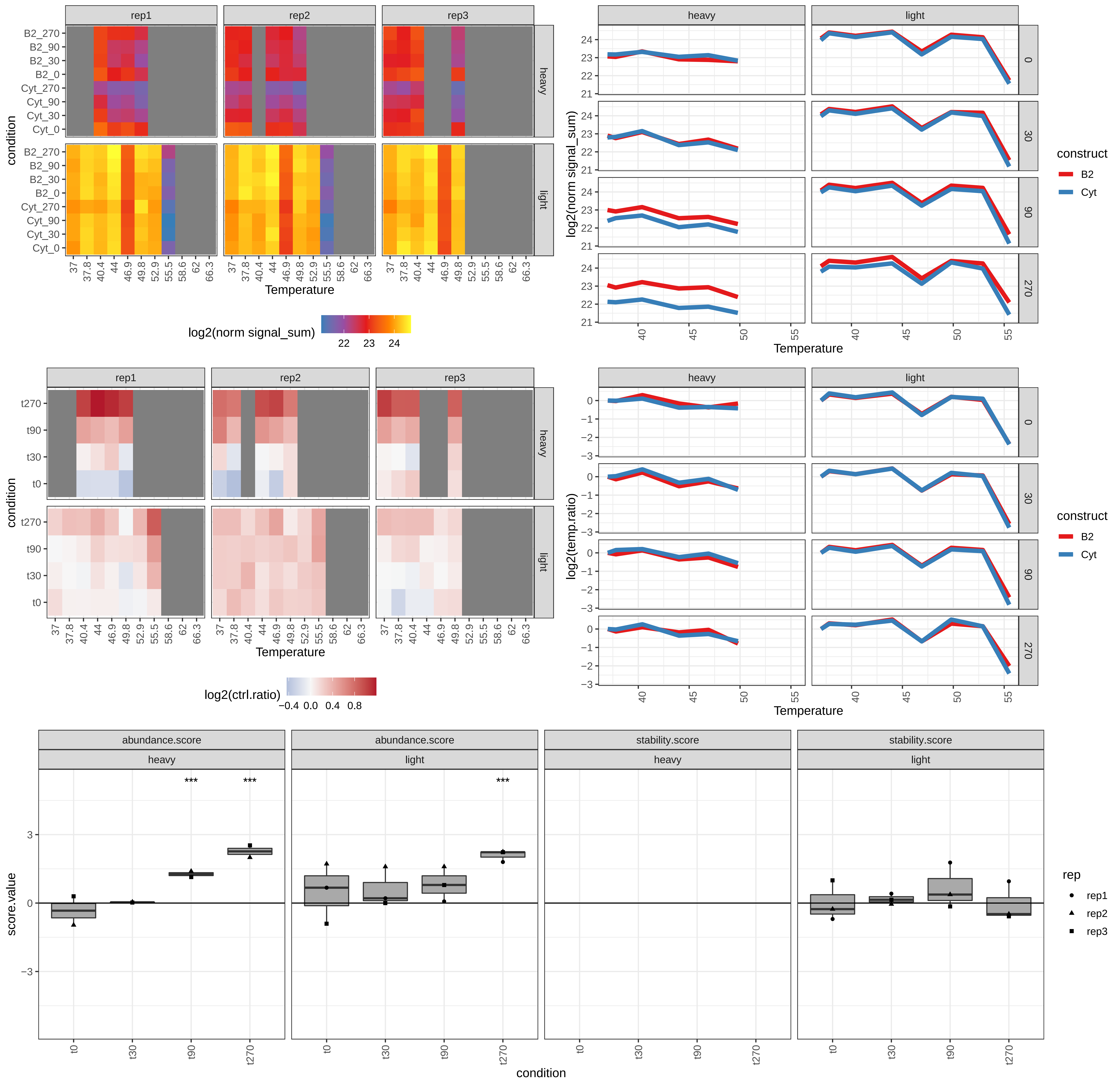

P12383 − Transcription factor PDR1 OS=Saccharomyces cerevisiae (strain ATCC 204508 / S288c) GN=PDR1 PE=1 SV=2

PDR12 − average.top3: 7.06056803921569 − max.qupm: 25

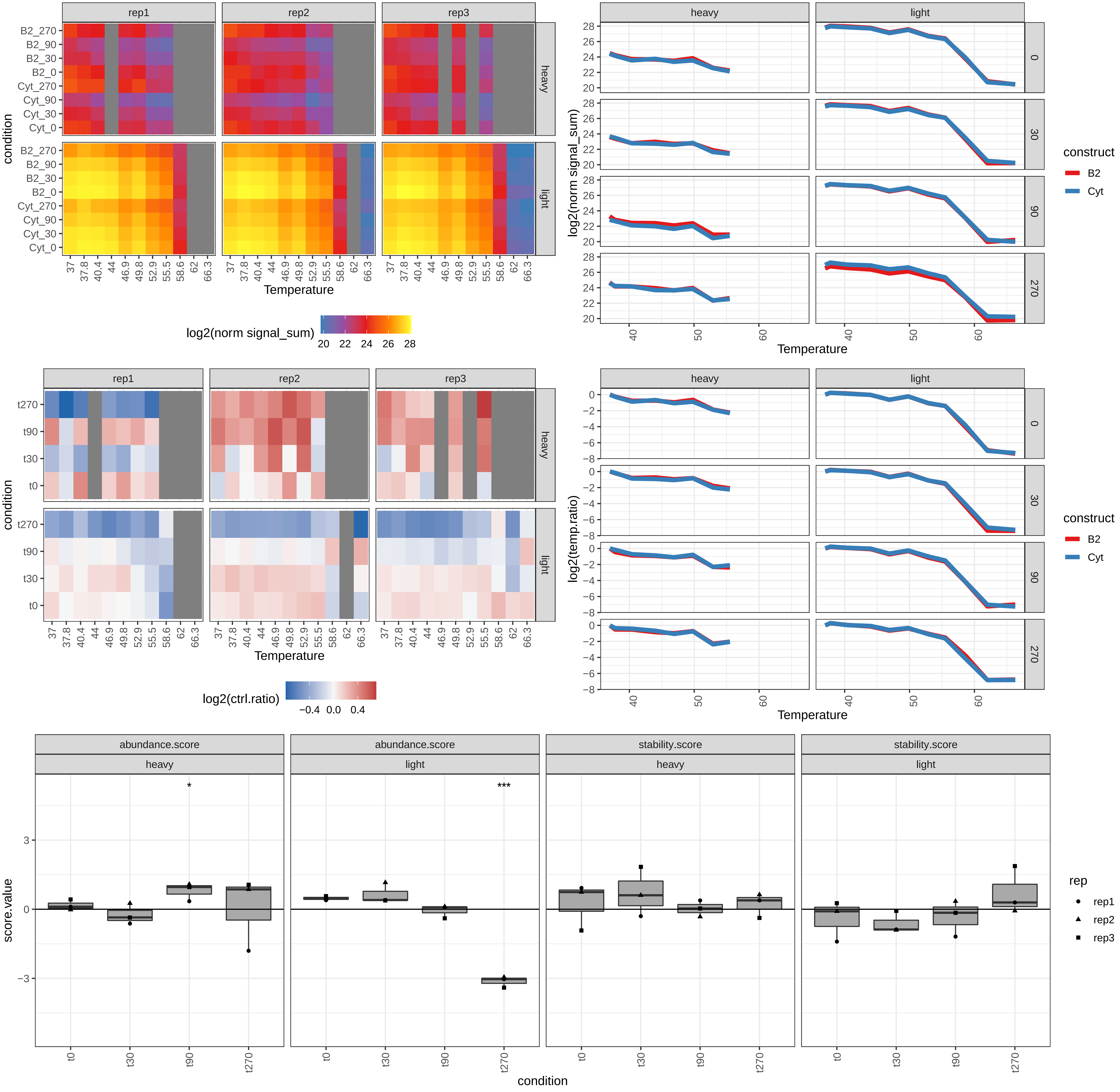

Q02785 − ATP−dependent permease PDR12 OS=Saccharomyces cerevisiae (strain ATCC 204508 / S288c) GN=PDR12 PE=1 SV=1

PDR16 − average.top3: 6.94817976744186 − max.qupm: 10

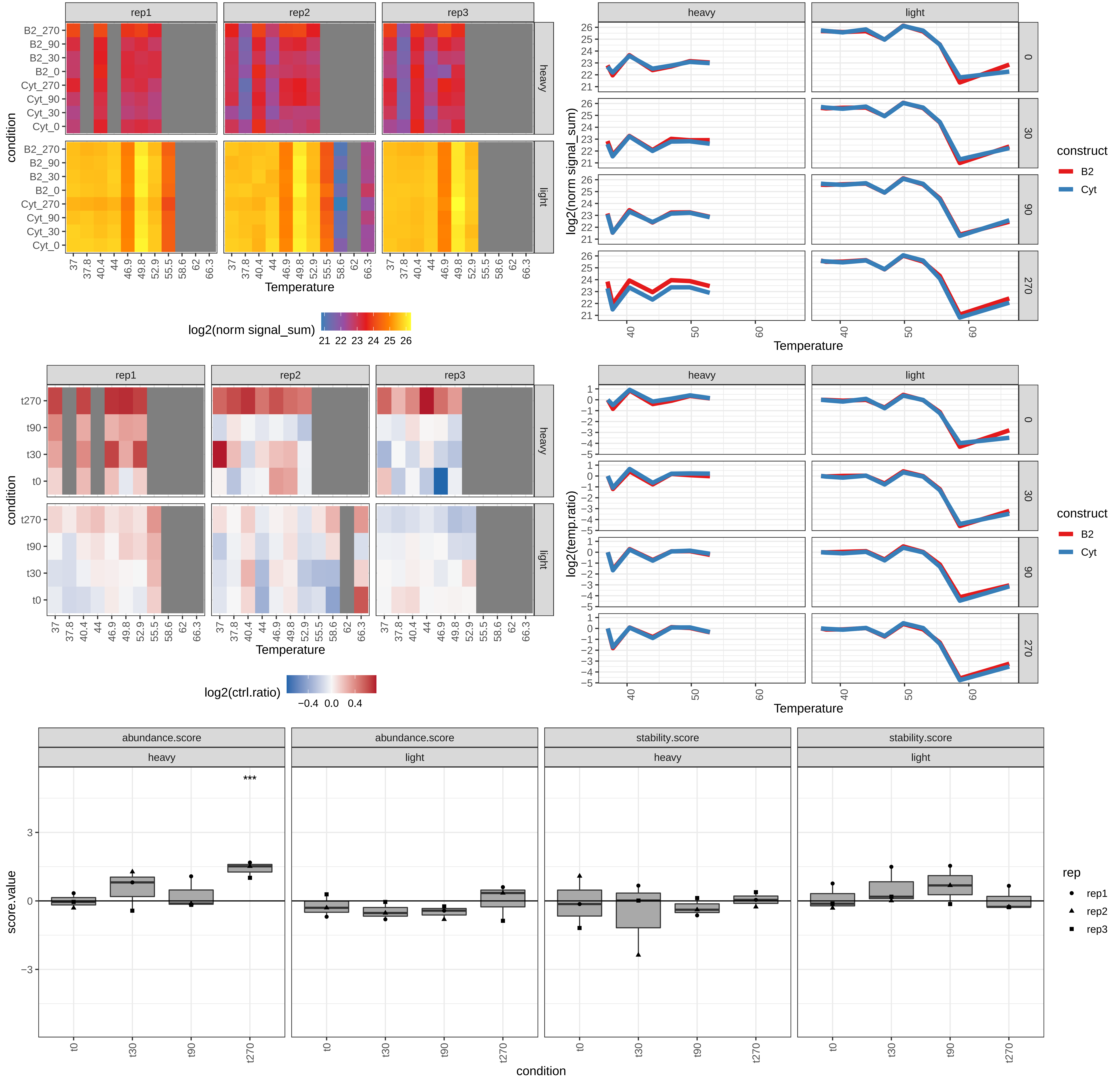

P53860 − Phosphatidylinositol transfer protein PDR16 OS=Saccharomyces cerevisiae (strain ATCC 204508 / S288c) GN=PDR16 PE=1 SV=1

PDR17 − average.top3: 6.918553 − max.qupm: 6

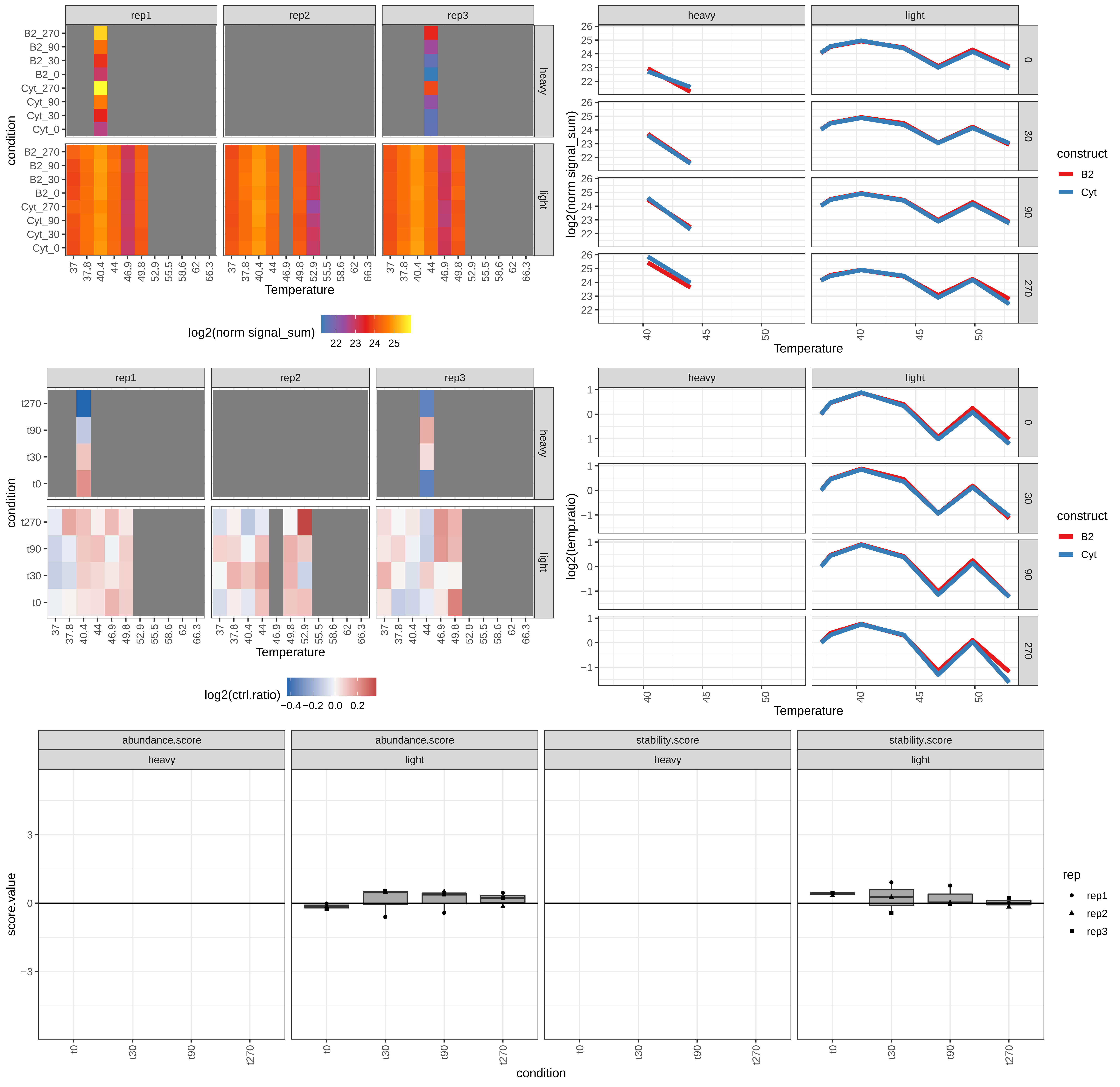

P53844 − Phosphatidylinositol transfer protein PDR17 OS=Saccharomyces cerevisiae (strain ATCC 204508 / S288c) GN=PDR17 PE=1 SV=1

PDR5 − average.top3: 7.17442444444444 − max.qupm: 14

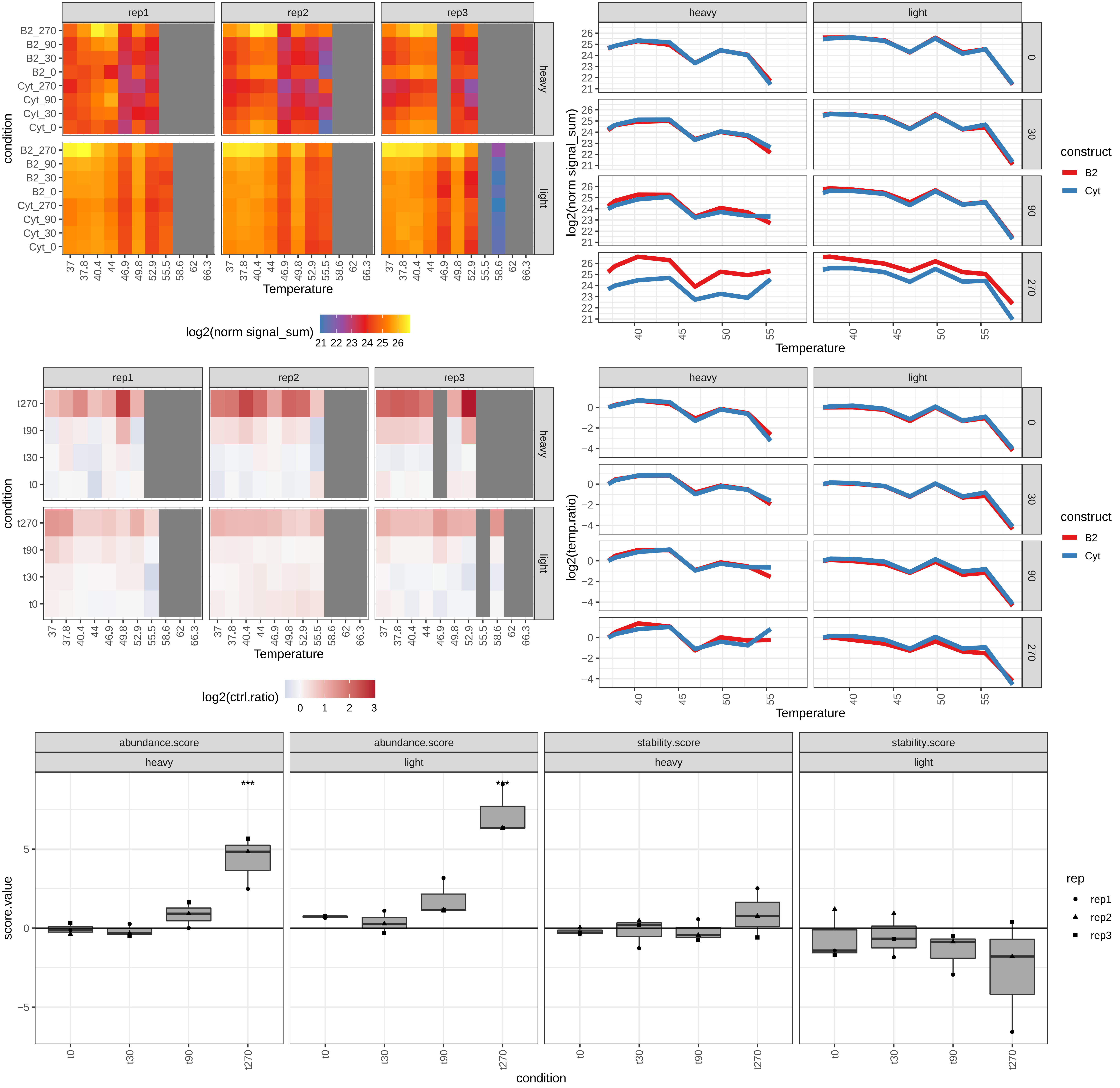

P33302 − Pleiotropic ABC efflux transporter of multiple drugs OS=Saccharomyces cerevisiae (strain ATCC 204508 / S288c) GN=PDR5 PE=1 SV=1

PDS5 − average.top3: 6.6579685 − max.qupm: 10

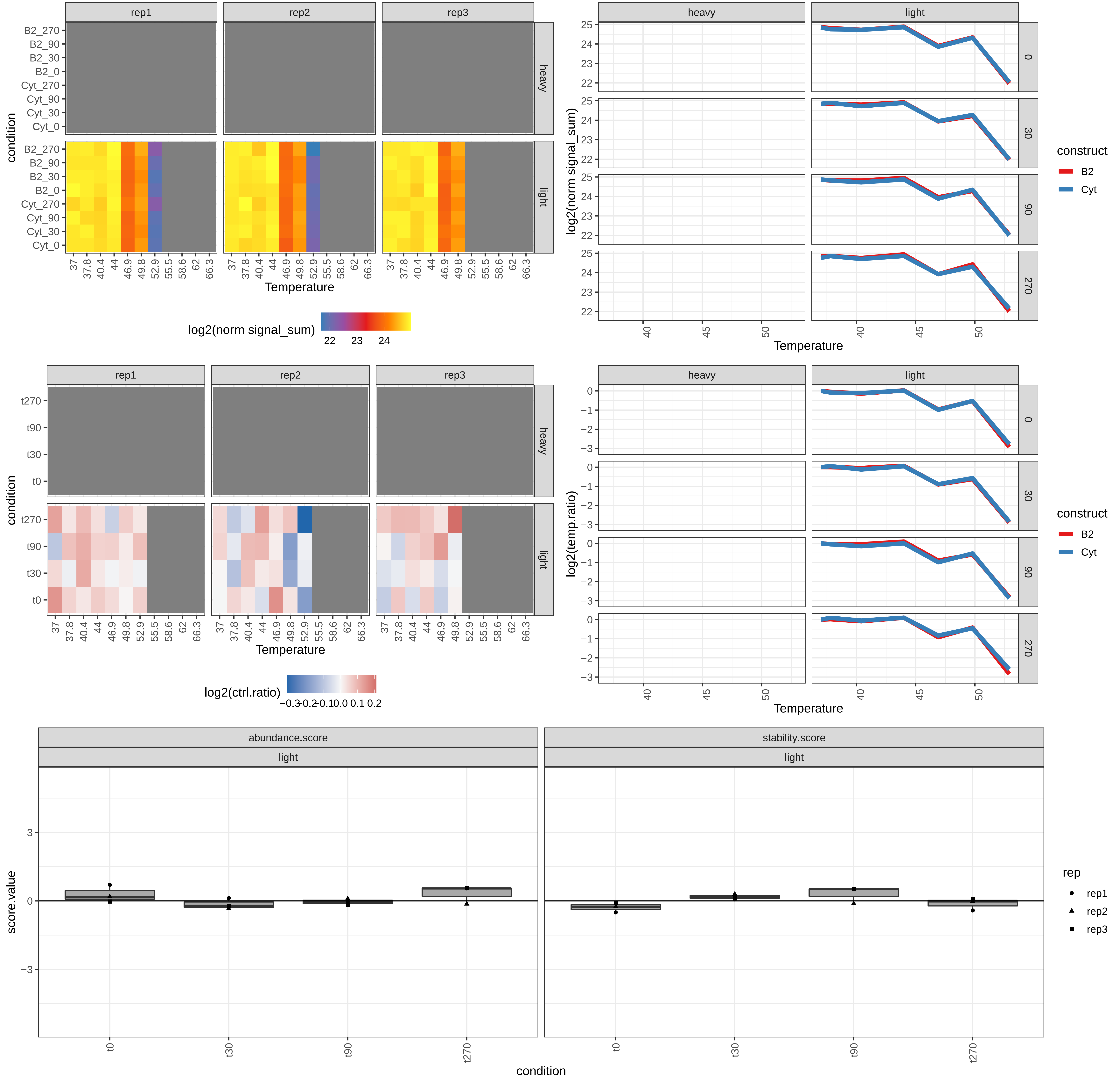

Q04264 − Sister chromatid cohesion protein PDS5 OS=Saccharomyces cerevisiae (strain ATCC 204508 / S288c) GN=PDS5 PE=1 SV=1

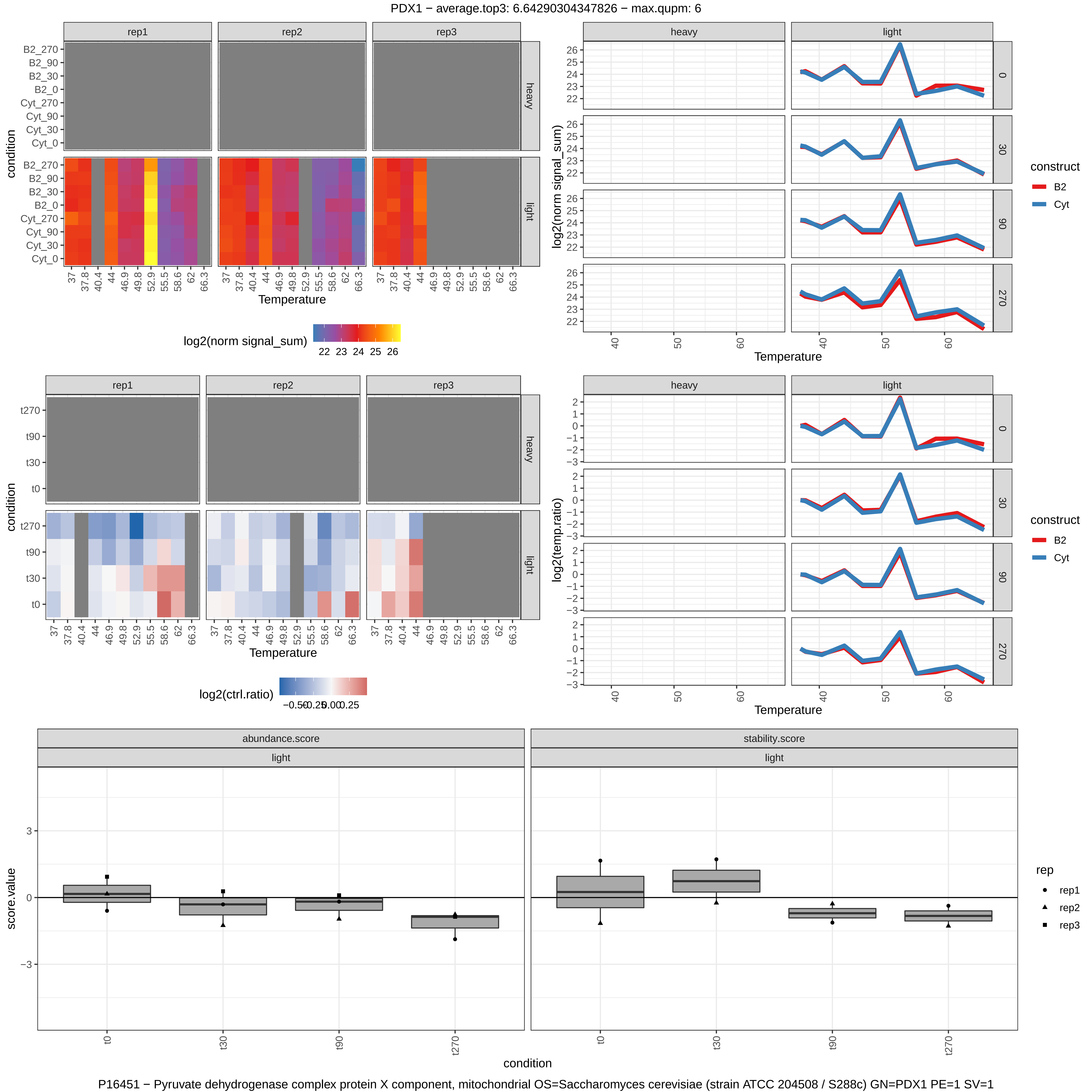
PDX1 − average.top3: 6.64290304347826 − max.qupm: 6
rep1
rep2
rep3
heavy
light
condition
Temperature
log2(norm signal_sum)
construct
B2
Cyt
log2(temp.ratio)
log2(ctrl.ratio)
abundance.score
stability.score
score.value
rep
rep1
rep2
rep3
P16451 − Pyruvate dehydrogenase complex protein X component, mitochondrial OS=Saccharomyces cerevisiae (strain ATCC 204508 / S288c) GN=PDX1 PE=1 SV=1

PDX3 − average.top3: 7.25745259259259 − max.qupm: 10

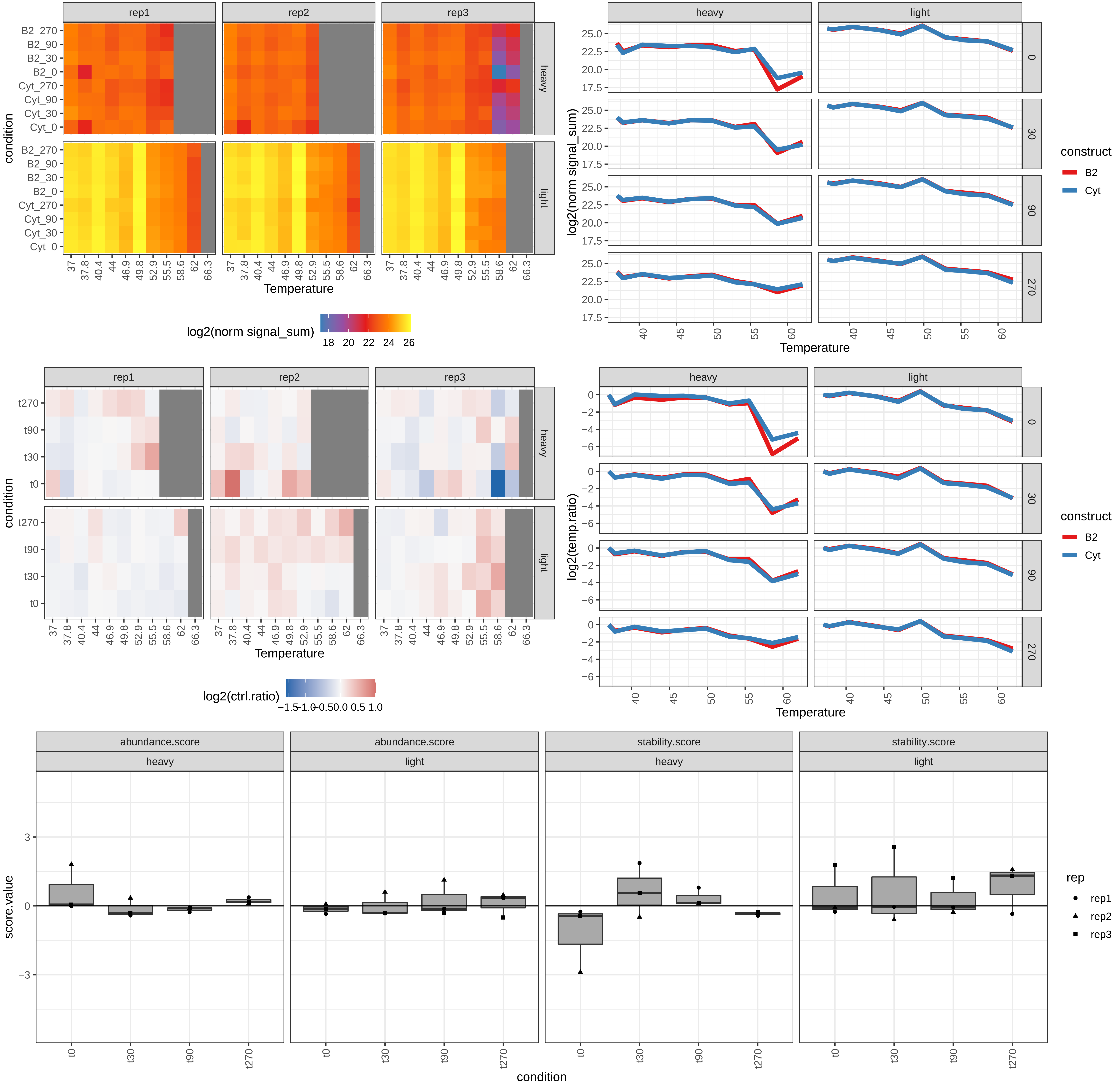

P38075 − Pyridoxamine 5'−phosphate oxidase OS=Saccharomyces cerevisiae (strain ATCC 204508 / S288c) GN=PDX3 PE=1 SV=1

PEP1 − average.top3: 7.01783362068965 − max.qupm: 25

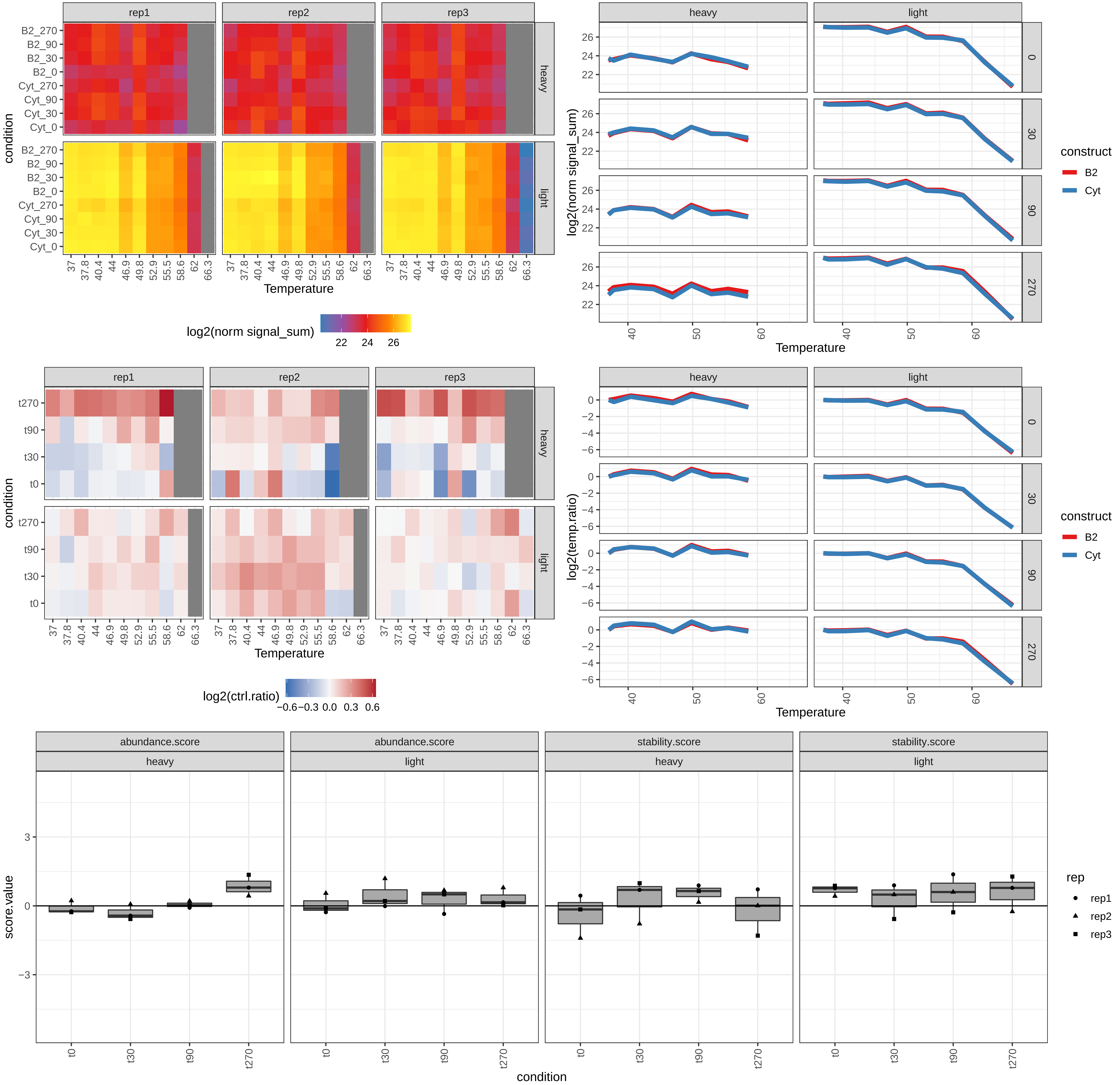

P32319 − Vacuolar protein sorting/targeting protein VPS10 OS=Saccharomyces cerevisiae (strain ATCC 204508 / S288c) GN=PEP1 PE=1 SV=1

PEP3 − average.top3: 6.92956565217391 − max.qupm: 15

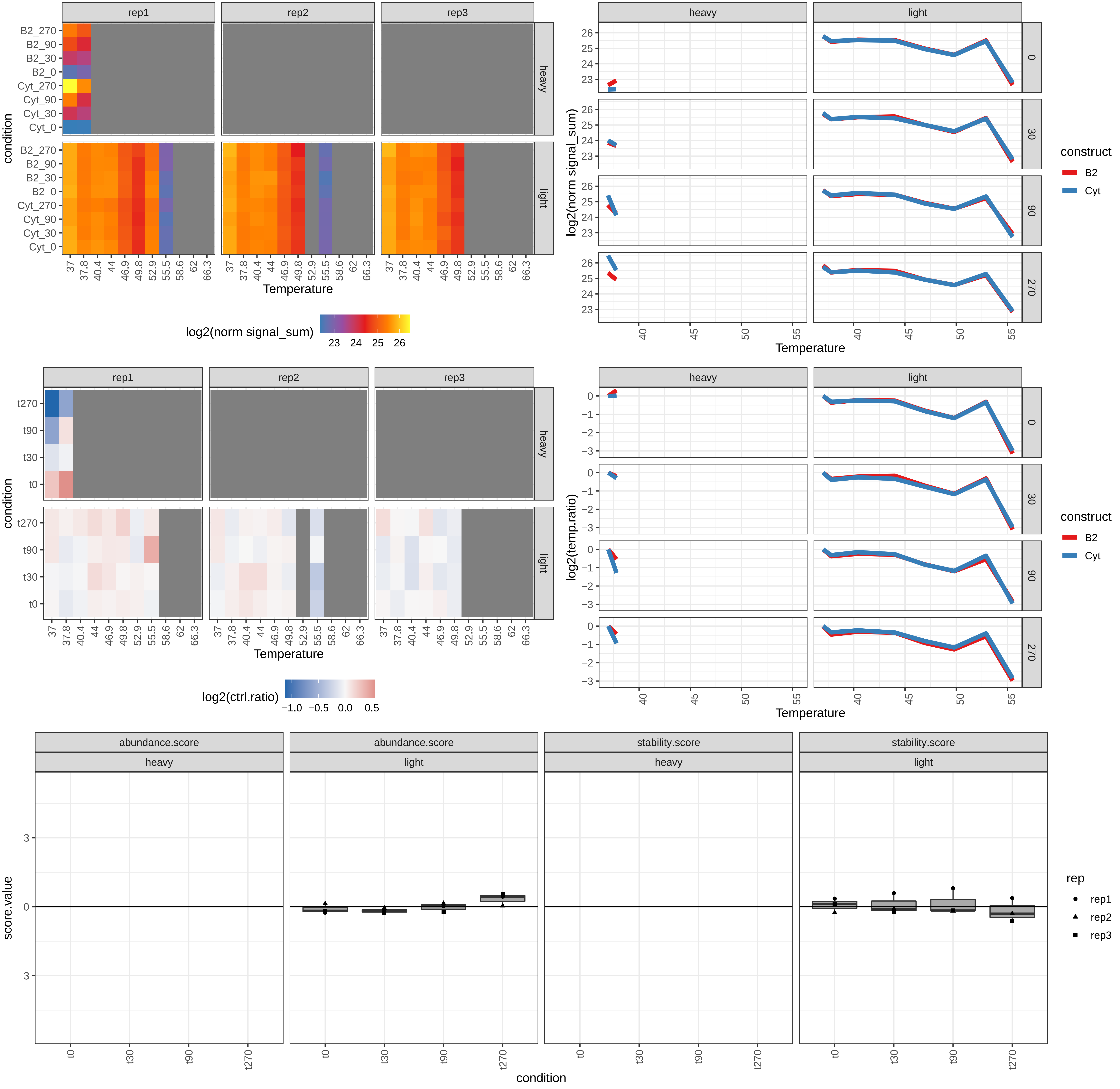

P27801 − Vacuolar membrane protein PEP3 OS=Saccharomyces cerevisiae (strain ATCC 204508 / S288c) GN=PEP3 PE=1 SV=1

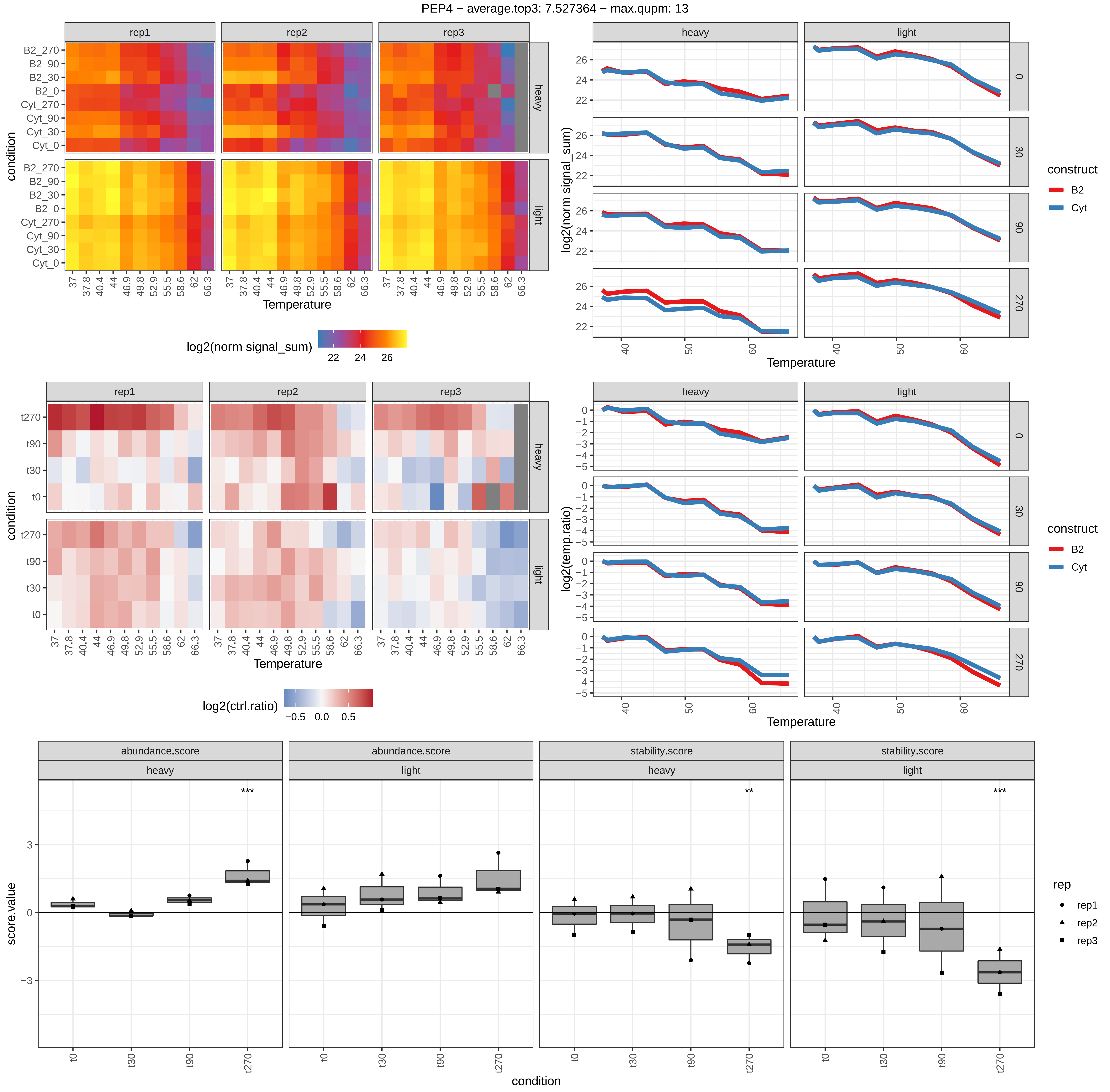

P07267 − Saccharopepsin OS=Saccharomyces cerevisiae (strain ATCC 204508 / S288c) GN=PEP4 PE=1 SV=1

PEP5 − average.top3: 6.70942533333333 − max.qupm: 7

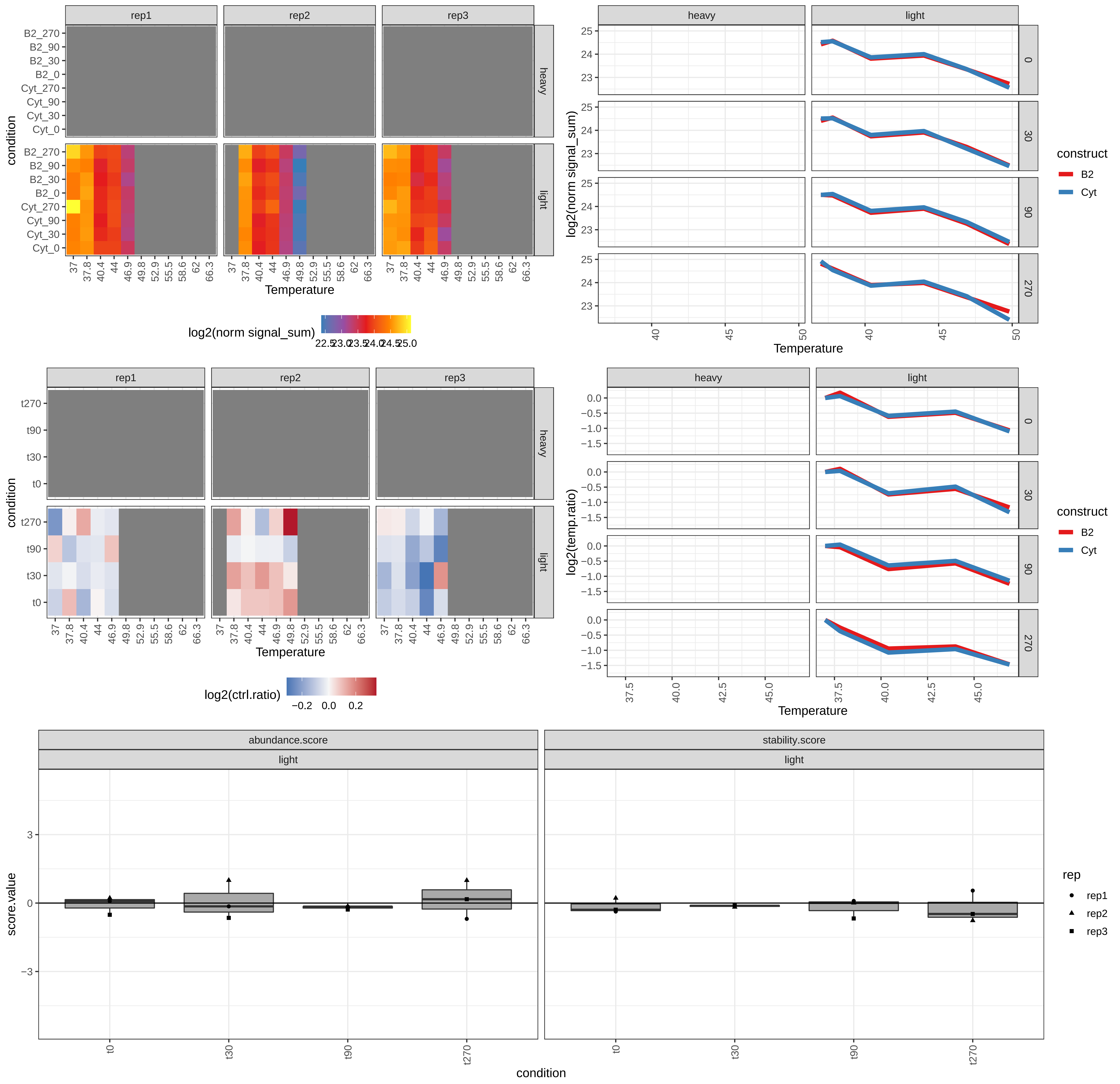

P12868 − E3 ubiquitin−protein ligase PEP5 OS=Saccharomyces cerevisiae (strain ATCC 204508 / S288c) GN=PEP5 PE=1 SV=2

PEP8 − average.top3: 6.77580578947368 − max.qupm: 7

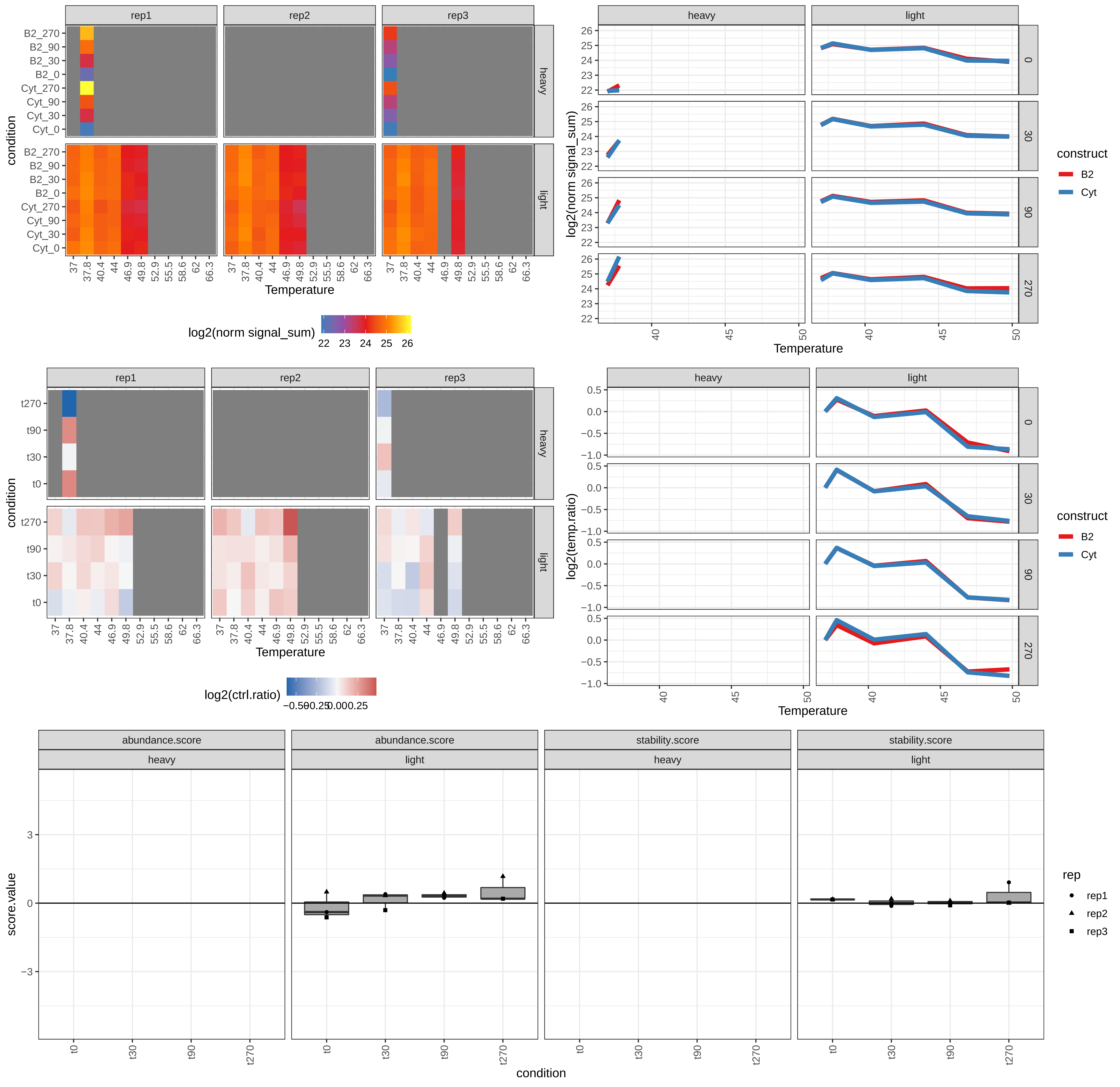

P40335 − Carboxypeptidase Y−deficient protein 8 OS=Saccharomyces cerevisiae (strain ATCC 204508 / S288c) GN=PEP8 PE=1 SV=2

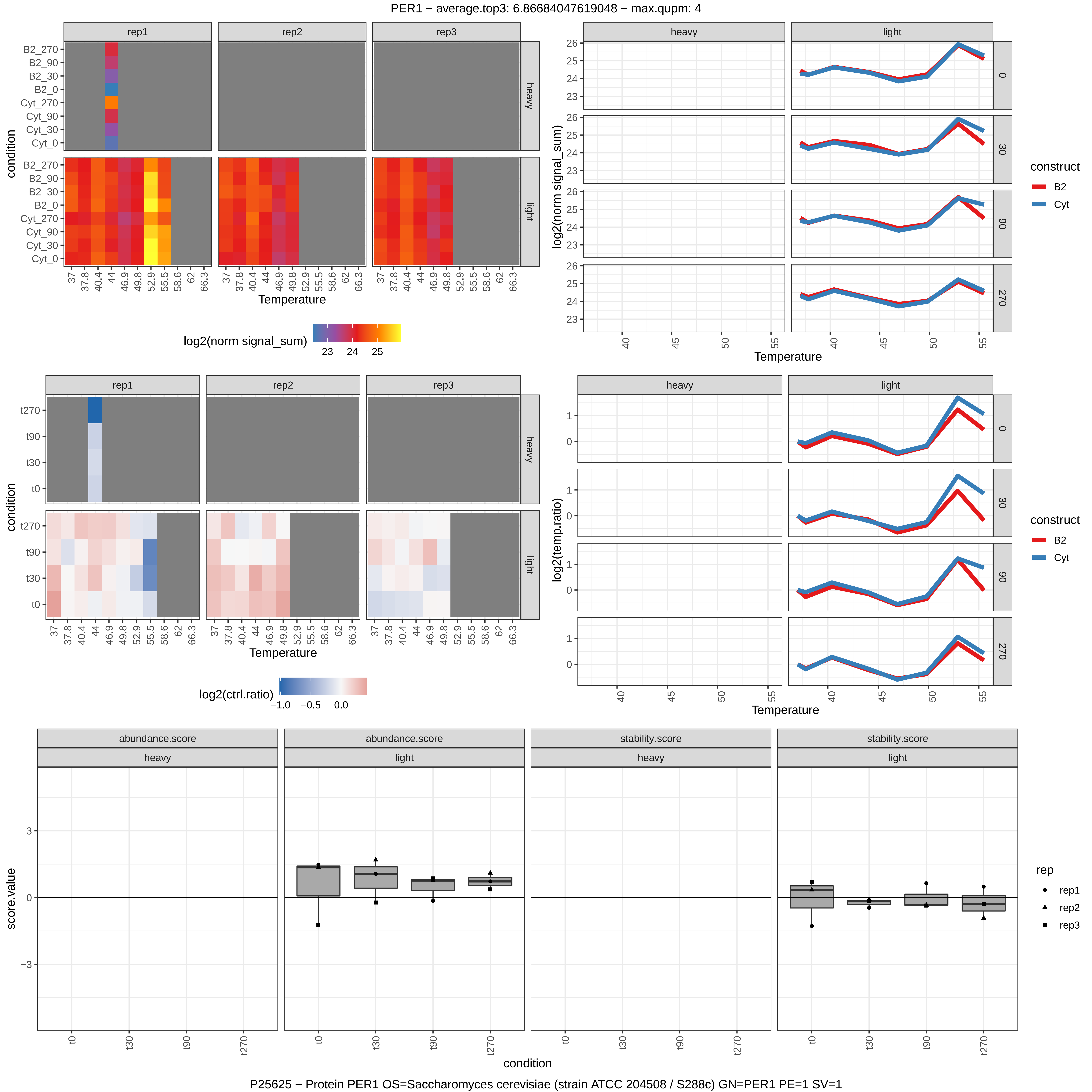
PER1 − average.top3: 6.86684047619048 − max.qupm: 4
rep1
rep2
rep3
heavy
light
condition
Temperature
log2(norm signal_sum)
log2(ctrl.ratio)
log2(temp.ratio)
construct
B2
Cyt
abundance.score
stability.score
score.value
rep
rep1
rep2
rep3
P25625 − Protein PER1 OS=Saccharomyces cerevisiae (strain ATCC 204508 / S288c) GN=PER1 PE=1 SV=1

PET123 − average.top3: 6.74248076923077 − max.qupm: 4

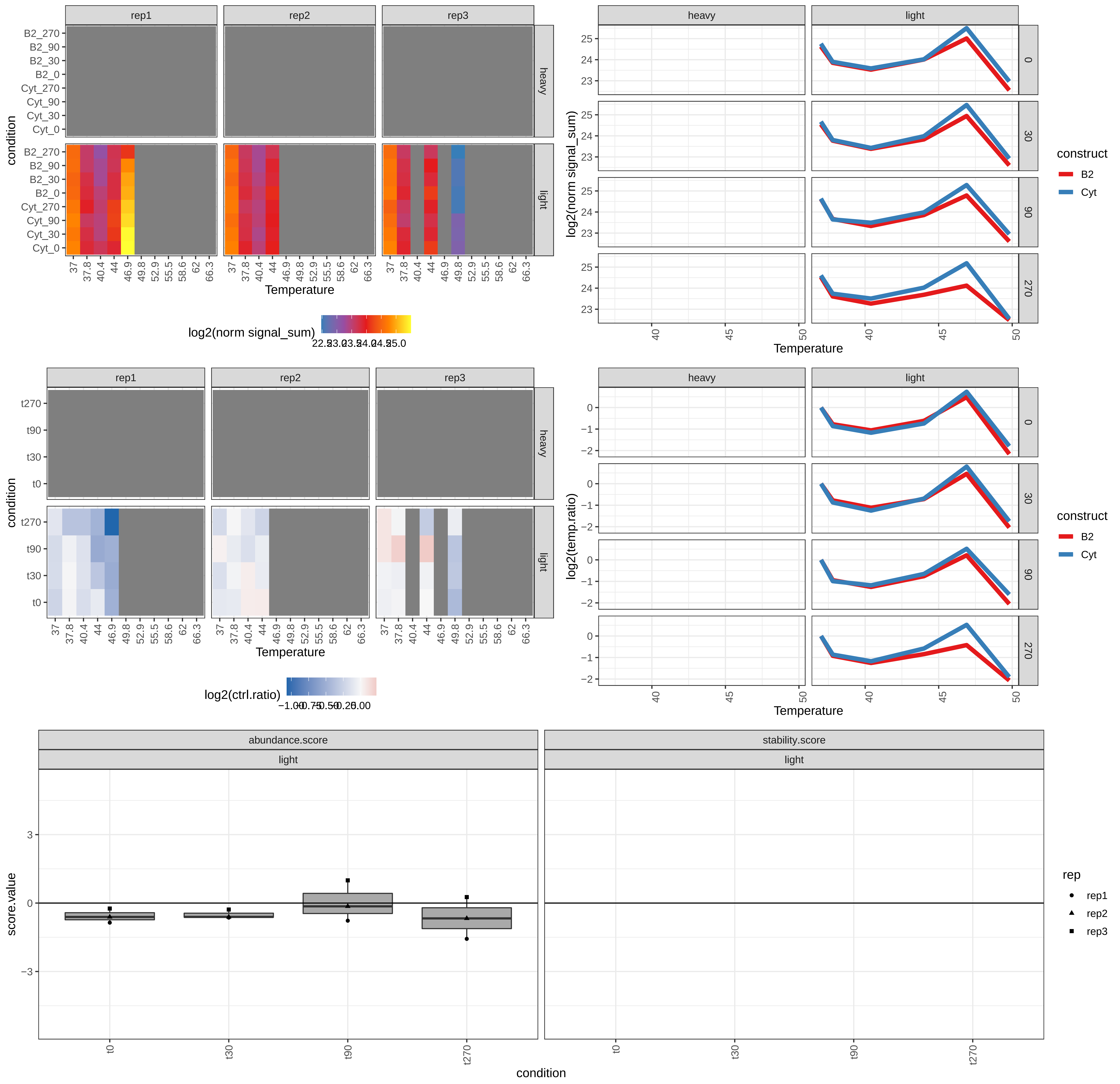

P17558 − 37S ribosomal protein PET123, mitochondrial OS=Saccharomyces cerevisiae (strain ATCC 204508 / S288c) GN=PET123 PE=1 SV=1

PET54 − average.top3: 6.72535388888889 − max.qupm: 6

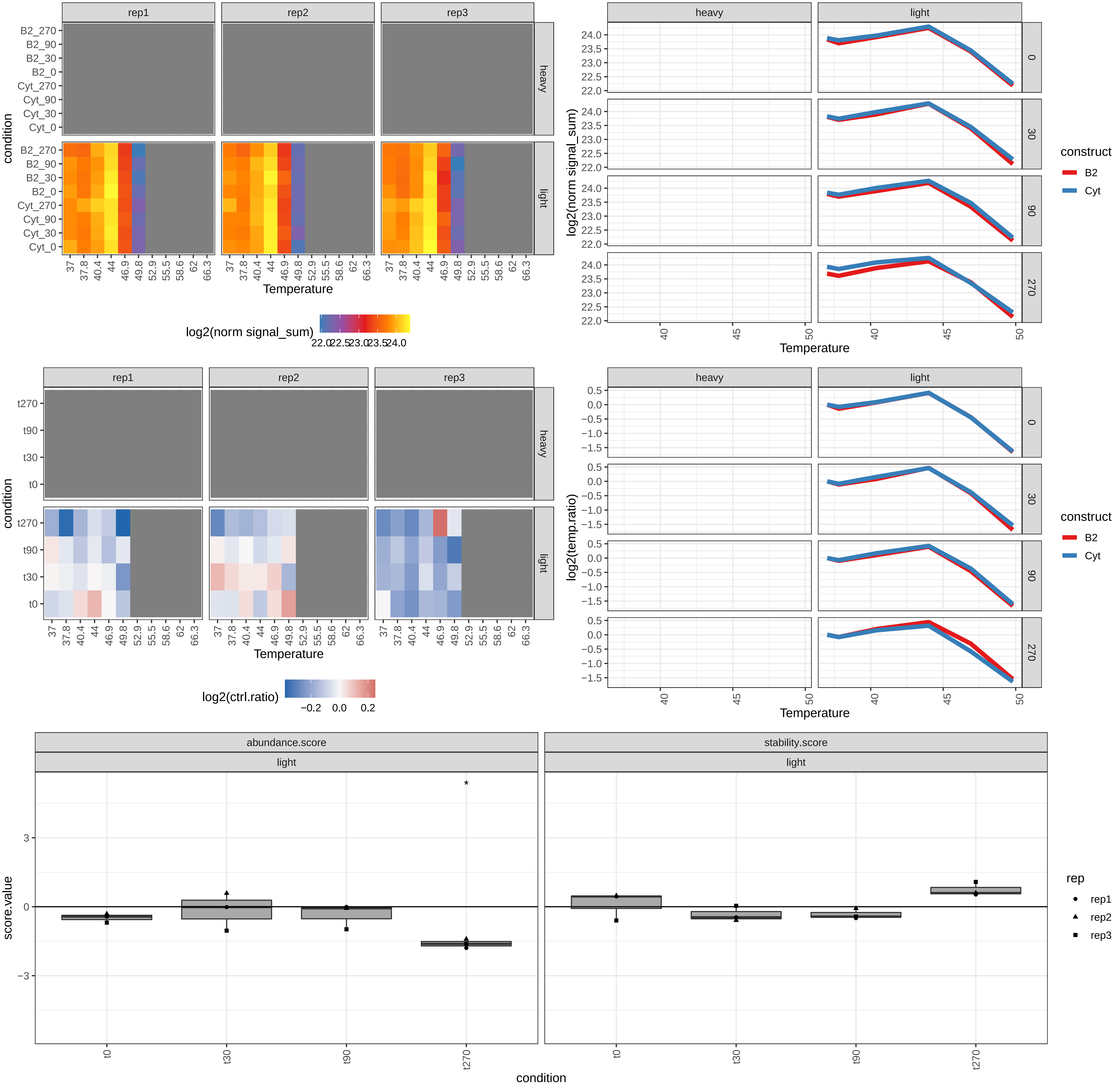

P10834 − Protein PET54 OS=Saccharomyces cerevisiae (strain ATCC 204508 / S288c) GN=PET54 PE=1 SV=1

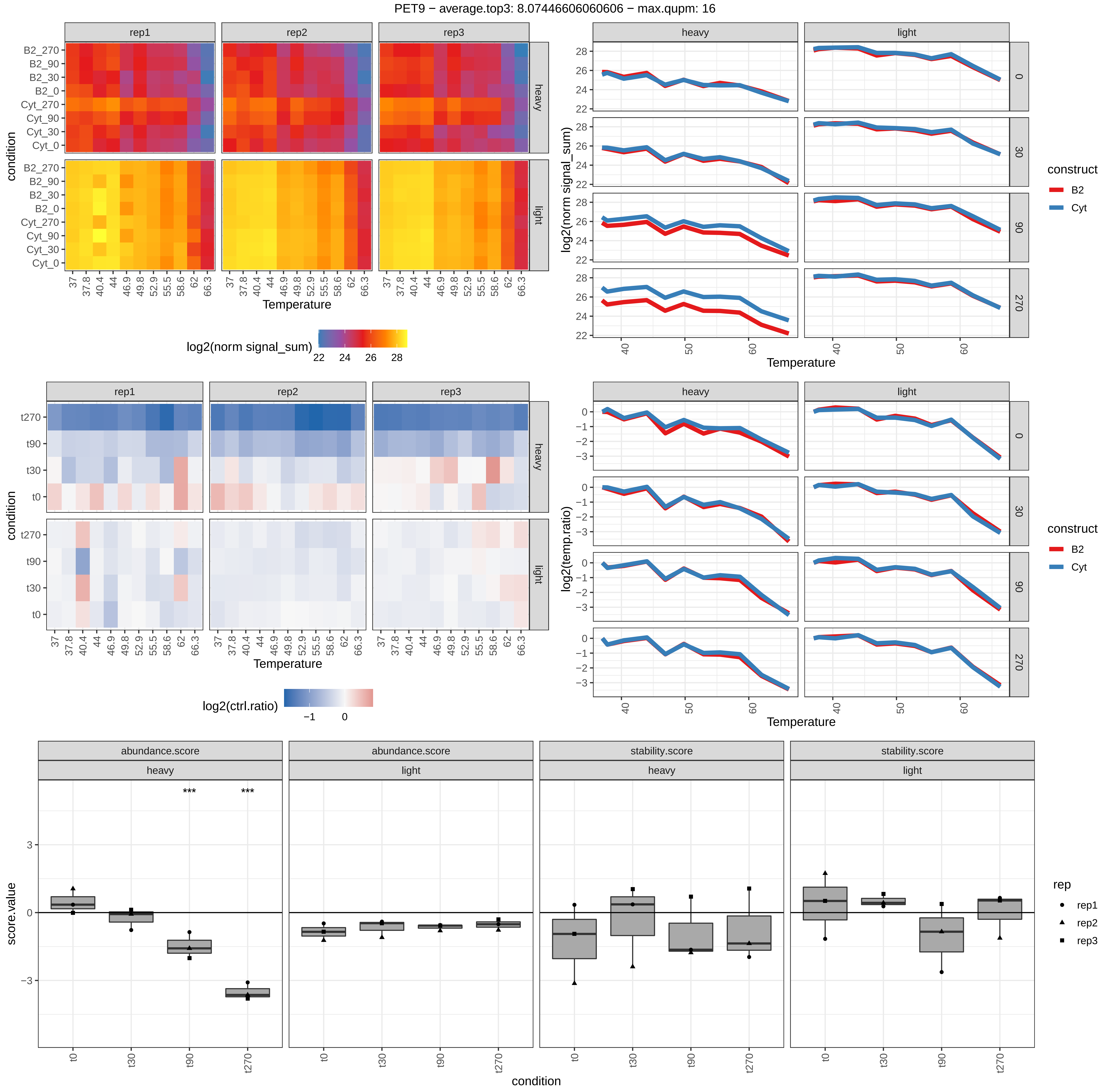

P18239 − ADP,ATP carrier protein 2 OS=Saccharomyces cerevisiae (strain ATCC 204508 / S288c) GN=PET9 PE=1 SV=2

PEX1 − average.top3: 6.3565925 − max.qupm: 6

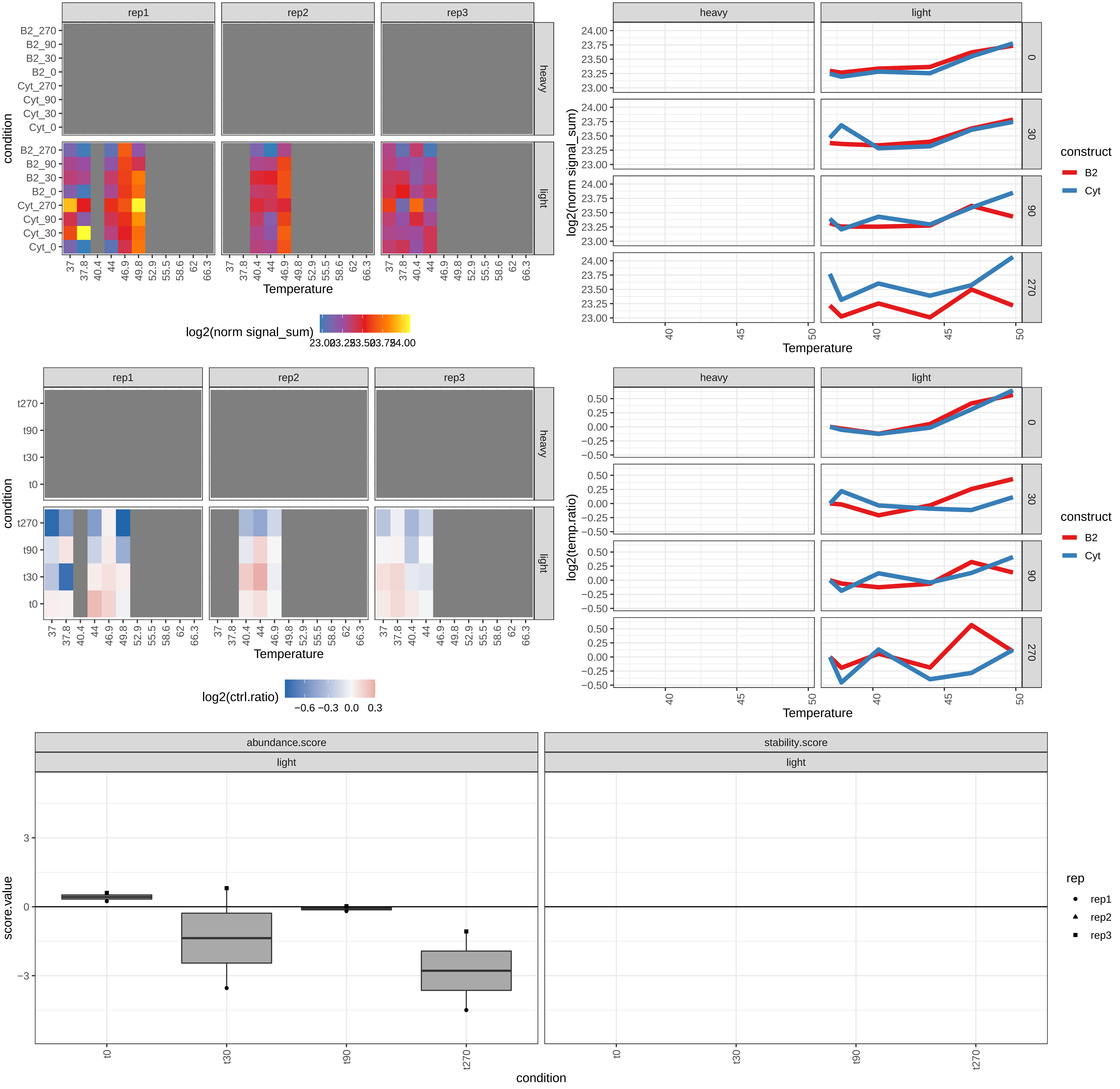

P24004 − Peroxisomal ATPase PEX1 OS=Saccharomyces cerevisiae (strain ATCC 204508 / S288c) GN=PEX1 PE=1 SV=2

PEX11 − average.top3: 6.94874259259259 − max.qupm: 5

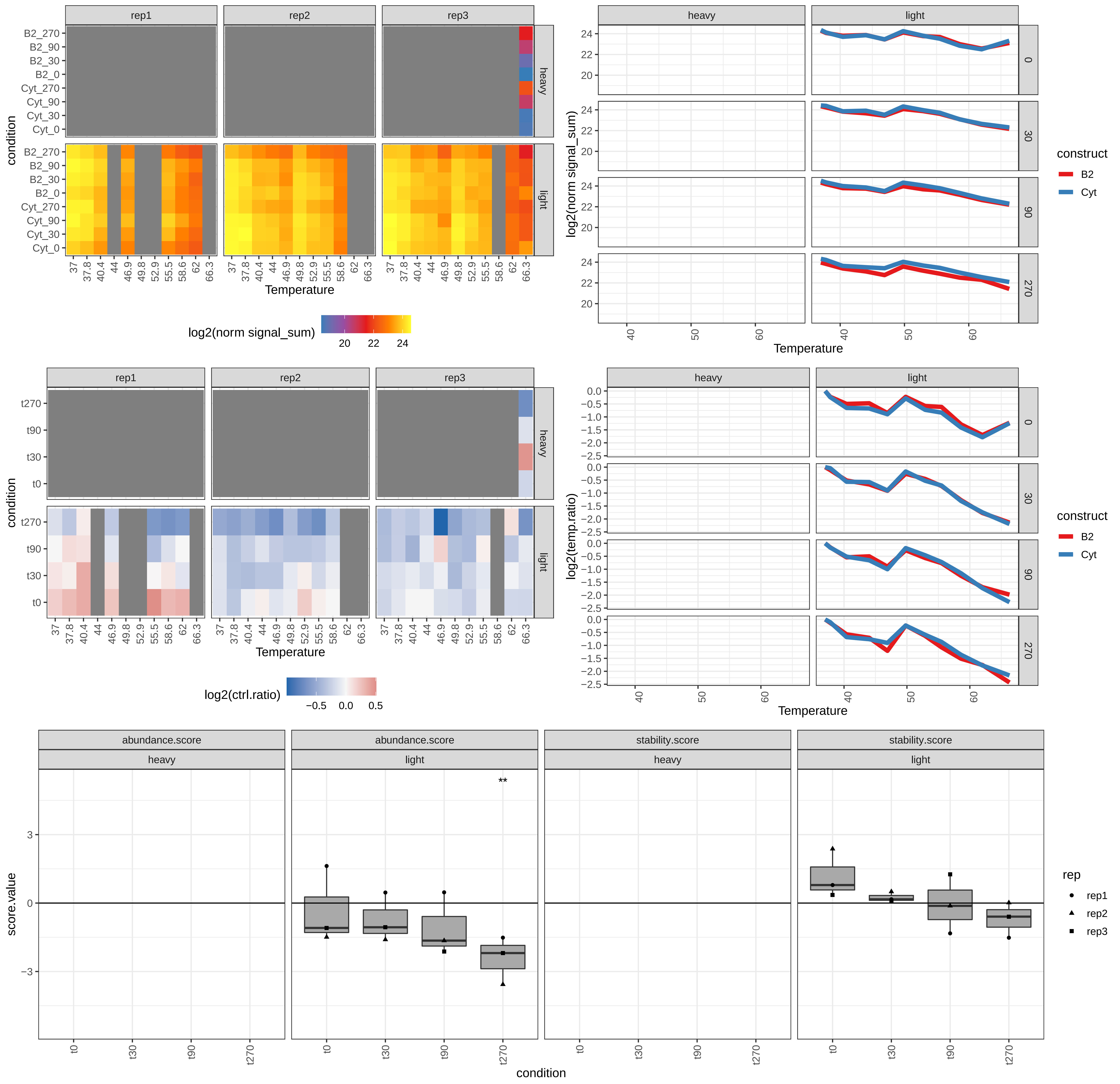

Q12462 − Peroxisomal membrane protein PMP27 OS=Saccharomyces cerevisiae (strain ATCC 204508 / S288c) GN=PEX11 PE=1 SV=2

PEX13 − average.top3: 6.58306363636364 − max.qupm: 3

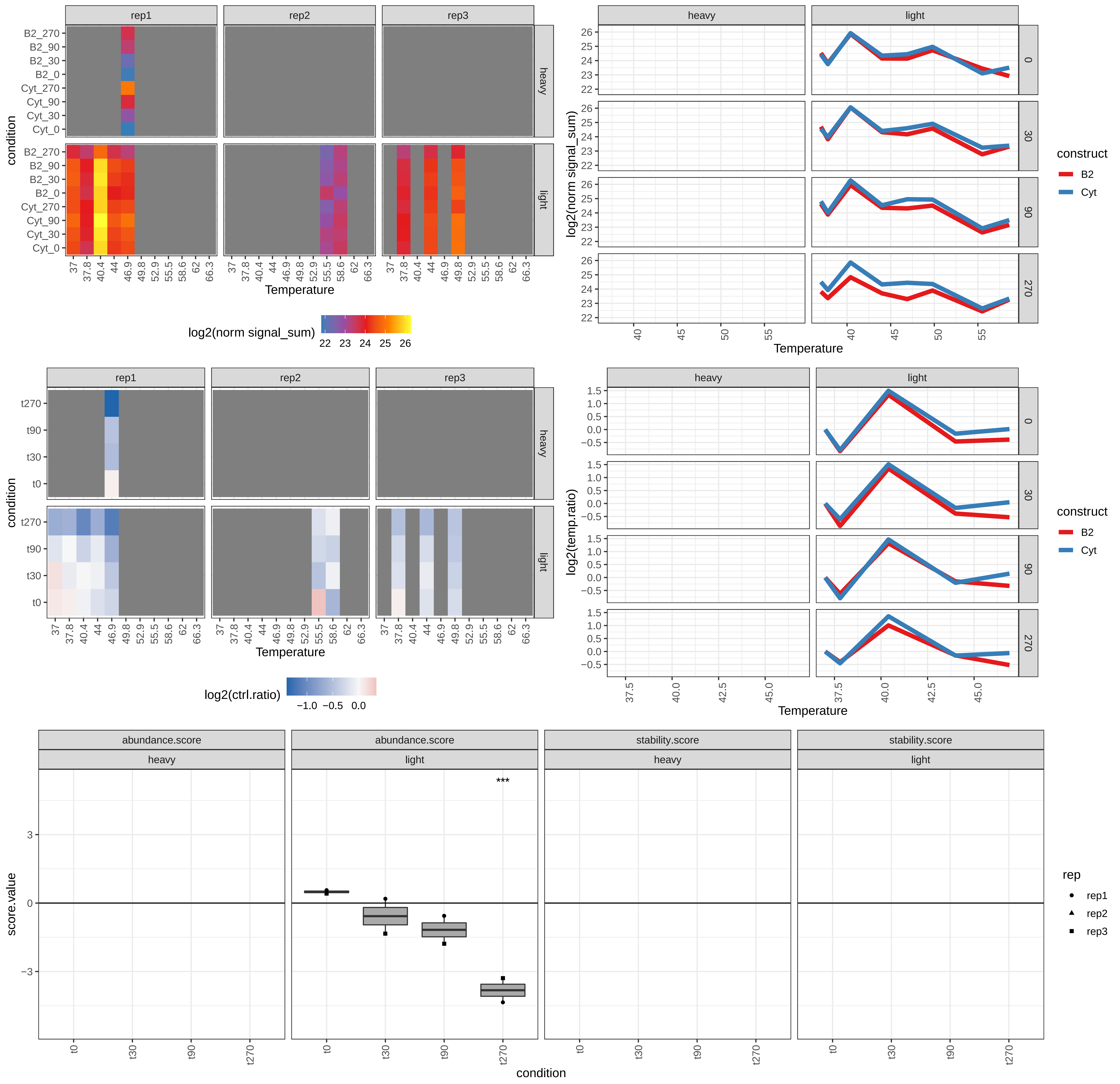

P80667 − Peroxisomal membrane protein PAS20 OS=Saccharomyces cerevisiae (strain ATCC 204508 / S288c) GN=PEX13 PE=1 SV=1

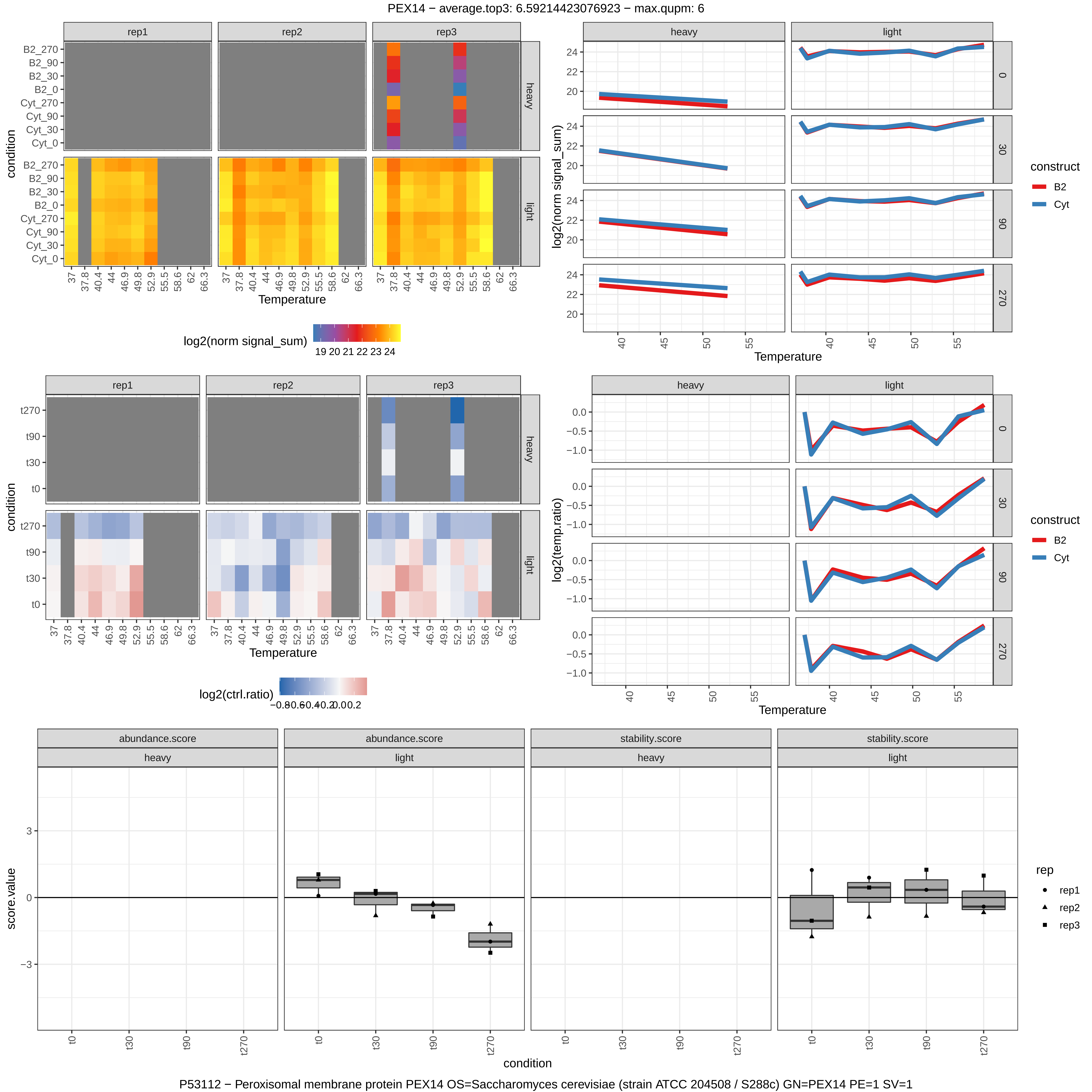

PEX14 − average.top3: 6.59214423076923 − max.qupm: 6
rep1
rep2
rep3
heavy
light
condition
Temperature
log2(norm signal_sum)
construct
B2
Cyt
log2(ctrl.ratio)
log2(temp.ratio)
abundance.score
stability.score
score.value
rep
rep1
rep2
rep3
P53112 − Peroxisomal membrane protein PEX14 OS=Saccharomyces cerevisiae (strain ATCC 204508 / S288c) GN=PEX14 PE=1 SV=1

PEX19 − average.top3: 6.66533444444444 − max.qupm: 5

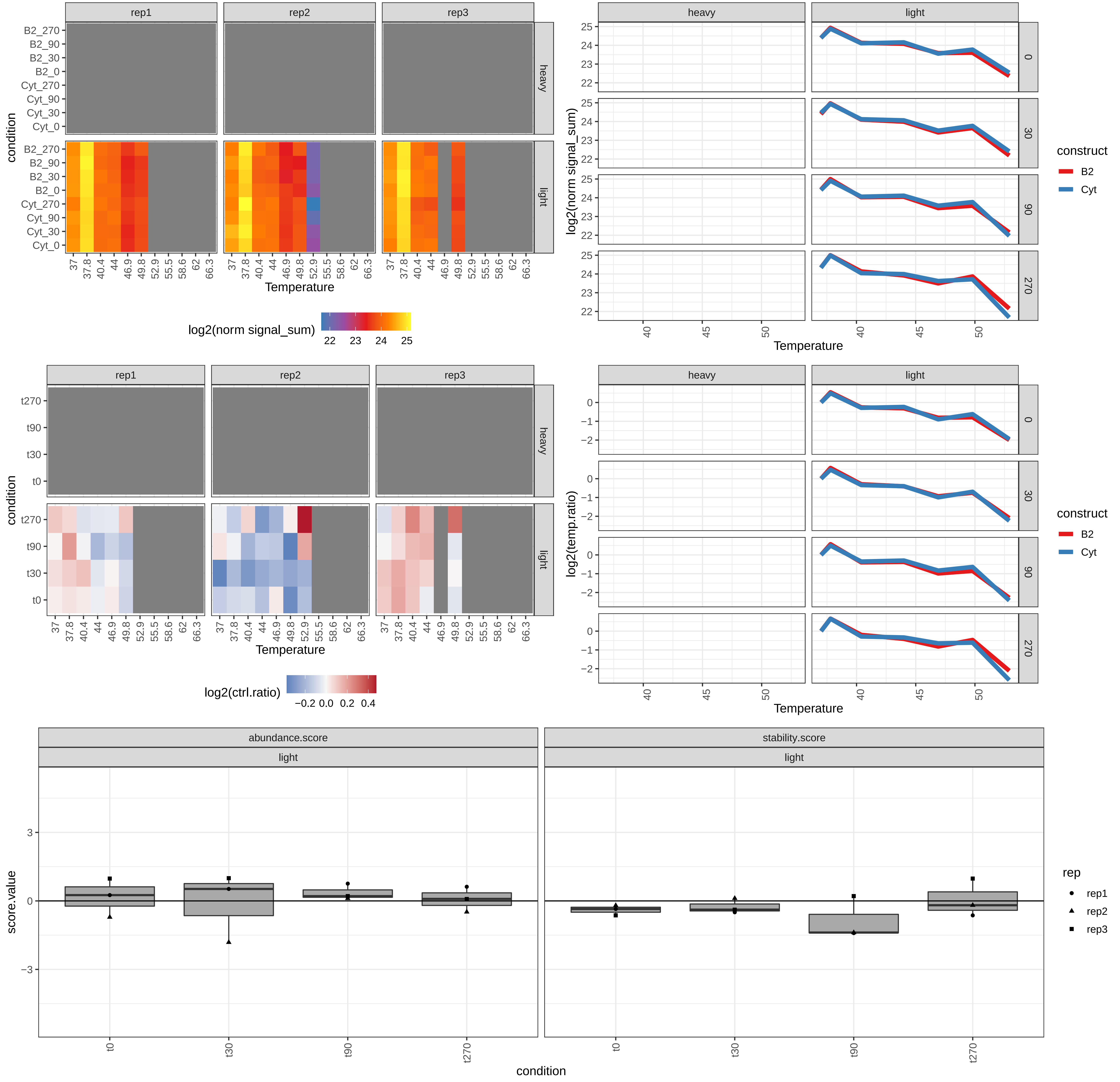

Q07418 − Peroxisomal membrane protein import receptor PEX19 OS=Saccharomyces cerevisiae (strain ATCC 204508 / S288c) GN=PEX19 PE=1 SV=2

PEX22 − average.top3: 6.45844181818182 − max.qupm: 4

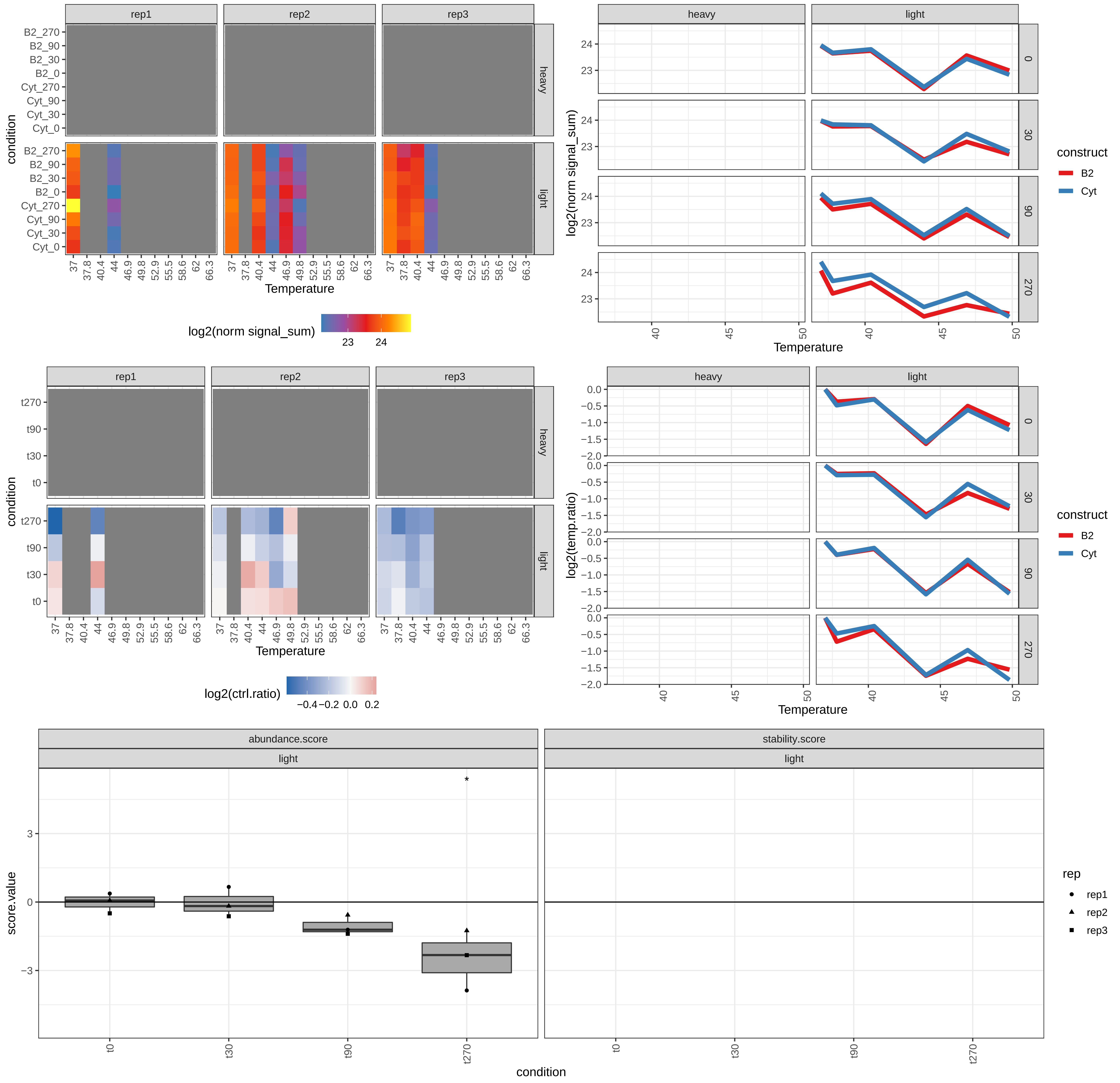

P39718 − Peroxisome assembly protein 22 OS=Saccharomyces cerevisiae (strain ATCC 204508 / S288c) GN=PEX22 PE=1 SV=1

PEX25 − average.top3: 6.16411041666667 − max.qupm: 6

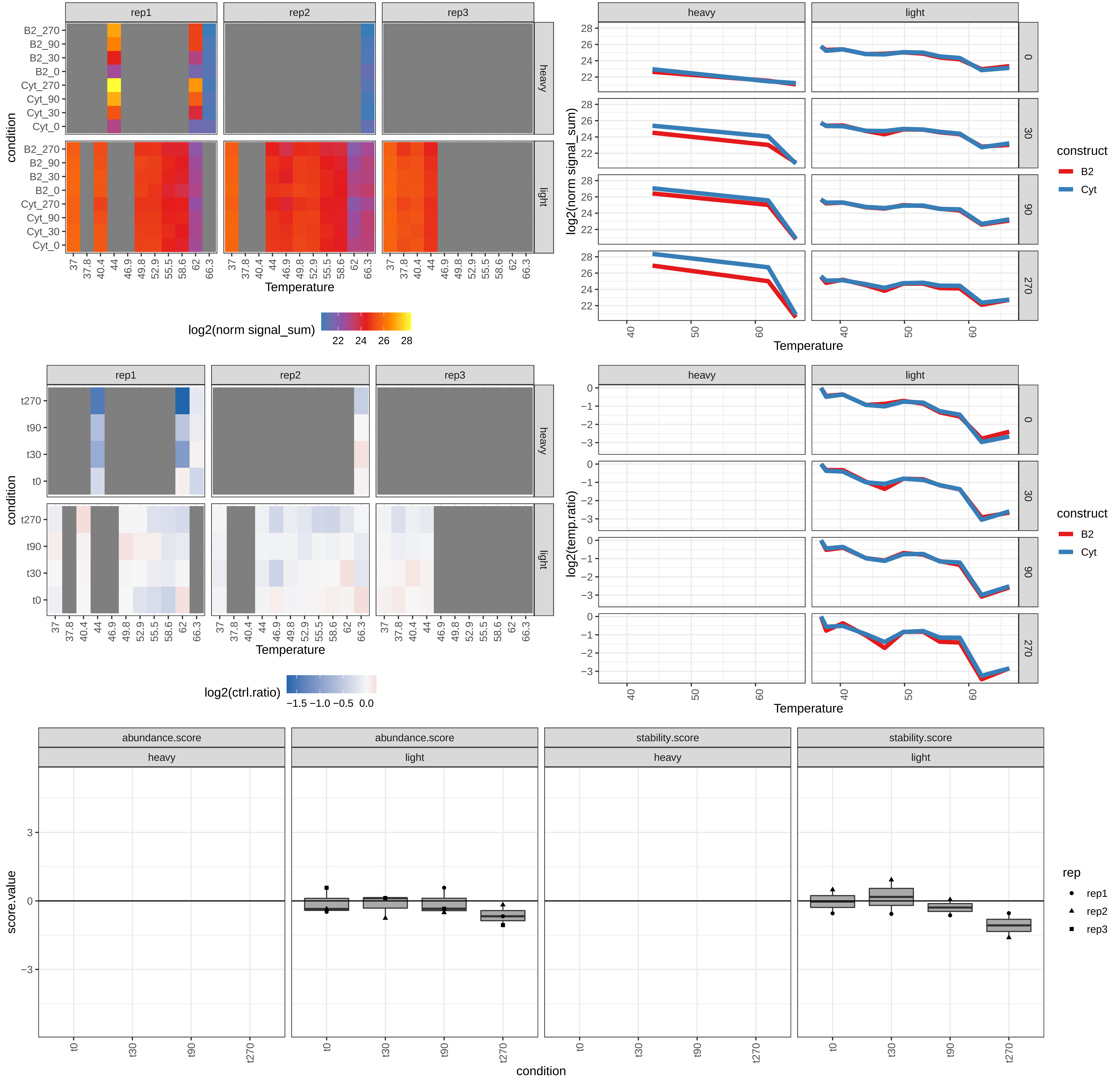

Q02969 − Peroxisomal membrane protein PEX25 OS=Saccharomyces cerevisiae (strain ATCC 204508 / S288c) GN=PEX25 PE=1 SV=1

PEX30 − average.top3: 6.718448 − max.qupm: 2

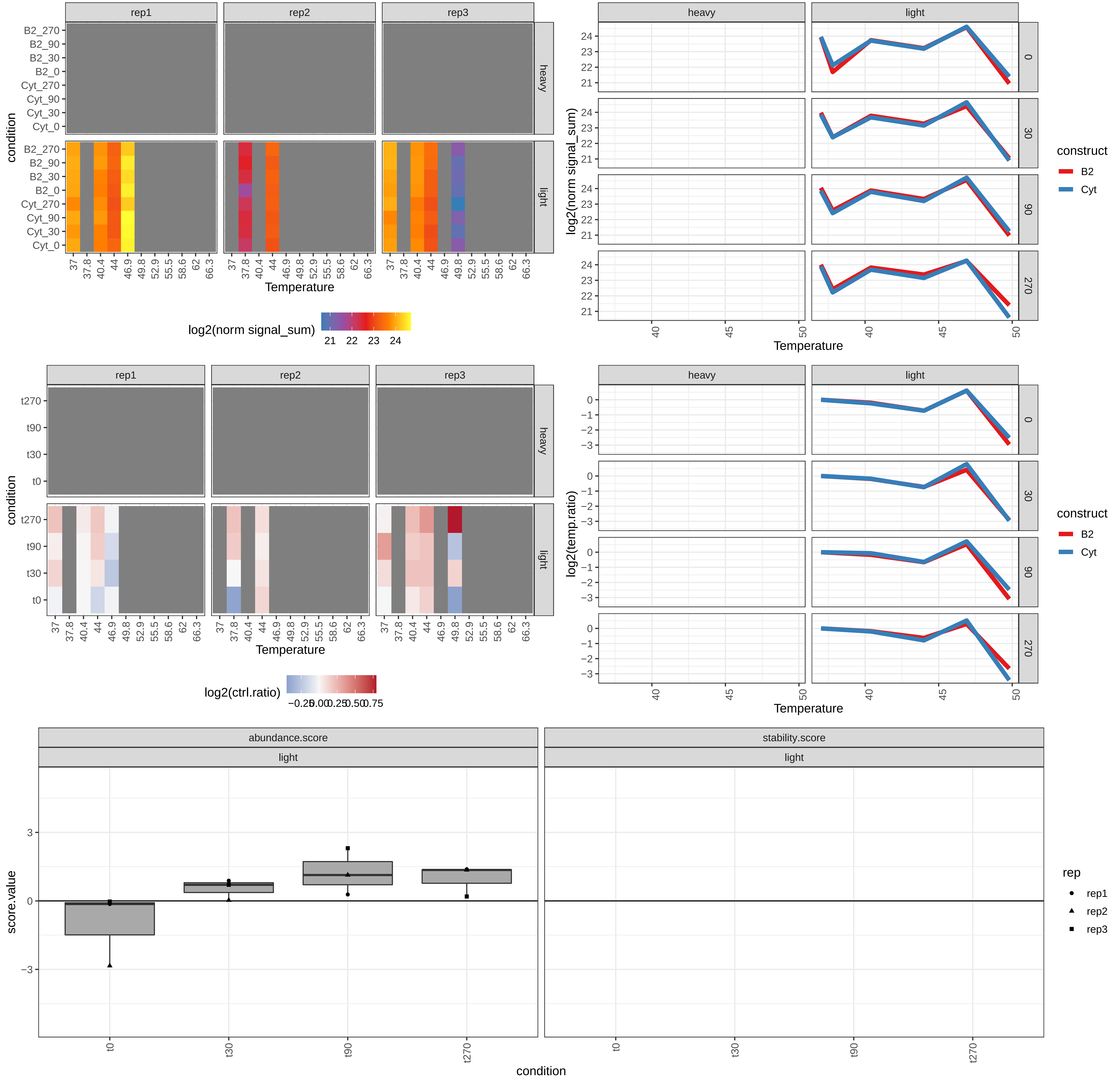

Q06169 − Peroxisomal membrane protein PEX30 OS=Saccharomyces cerevisiae (strain ATCC 204508 / S288c) GN=PEX30 PE=1 SV=1

PEX7 − average.top3: 6.33706818181818 − max.qupm: 3

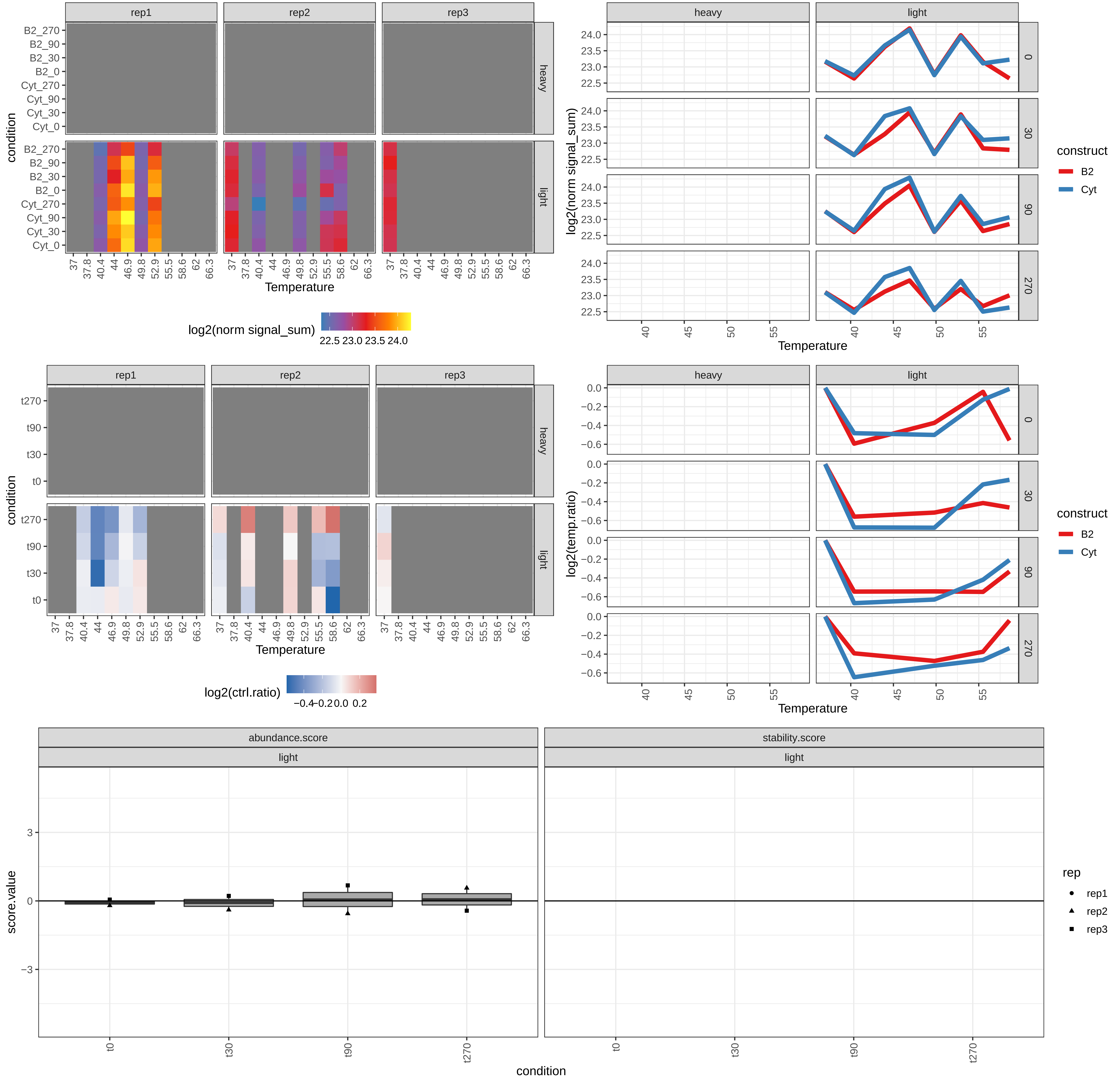

P39108 − Peroxisomal targeting signal 2 receptor OS=Saccharomyces cerevisiae (strain ATCC 204508 / S288c) GN=PEX7 PE=1 SV=1

PFD1 − average.top3: 6.81456736842105 − max.qupm: 4

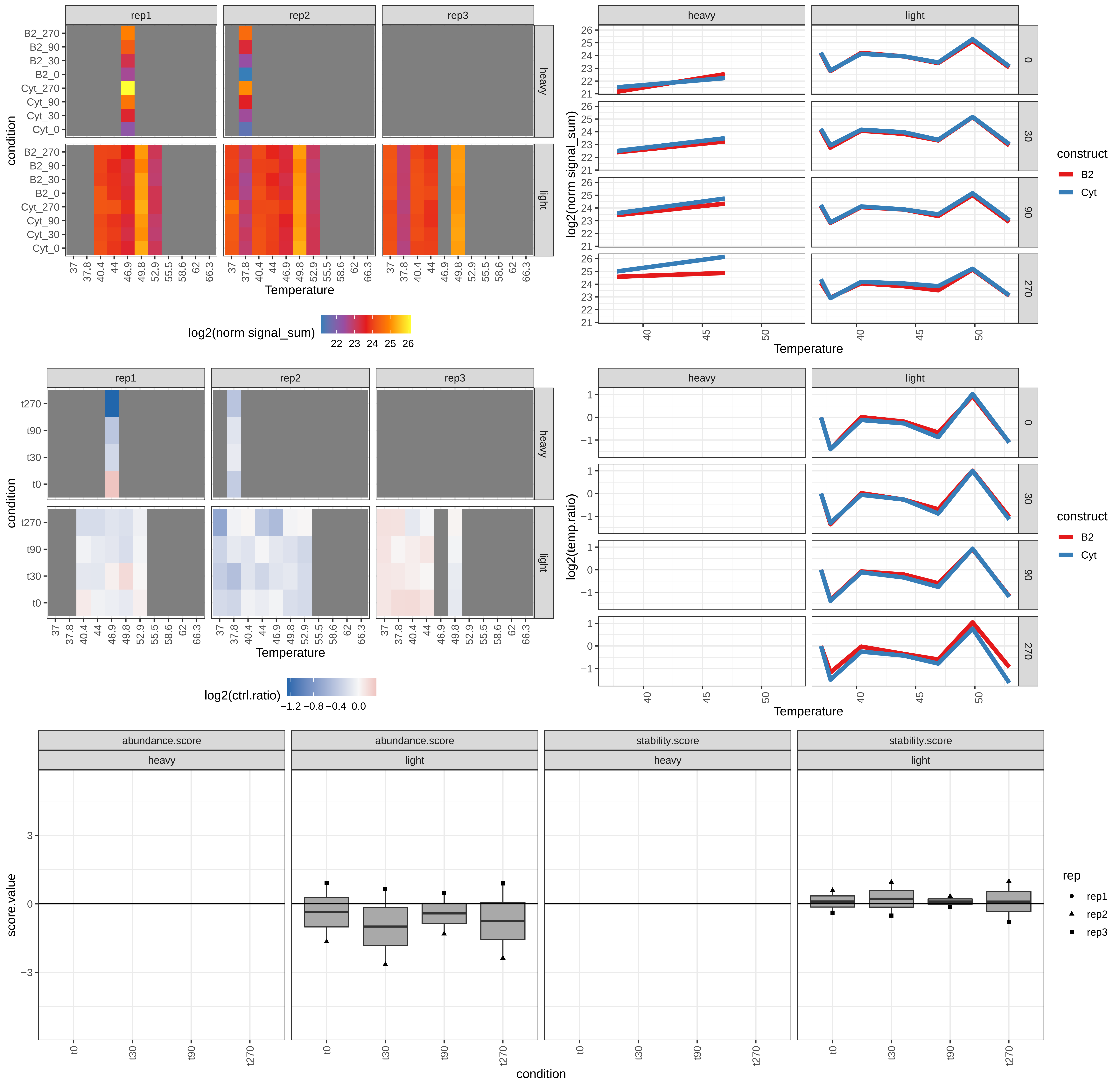

P46988 − Prefoldin subunit 1 OS=Saccharomyces cerevisiae (strain ATCC 204508 / S288c) GN=PFD1 PE=1 SV=2

PFF1 − average.top3: 6.95701452380952 − max.qupm: 13

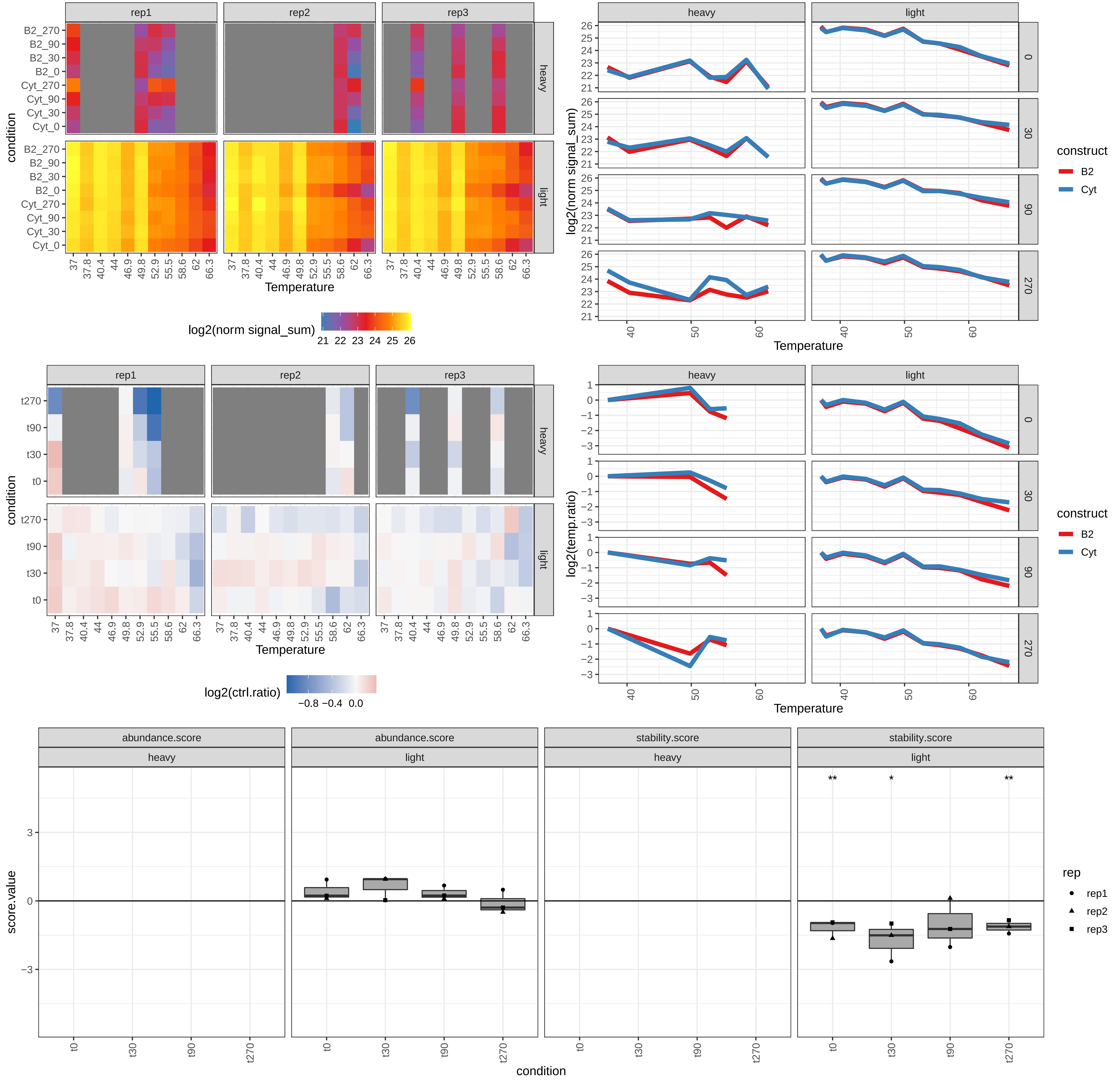

P38244 − Vacuolar membrane protease OS=Saccharomyces cerevisiae (strain ATCC 204508 / S288c) GN=PFF1 PE=1 SV=3

PFK1 − average.top3: 7.81556173076923 − max.qupm: 37

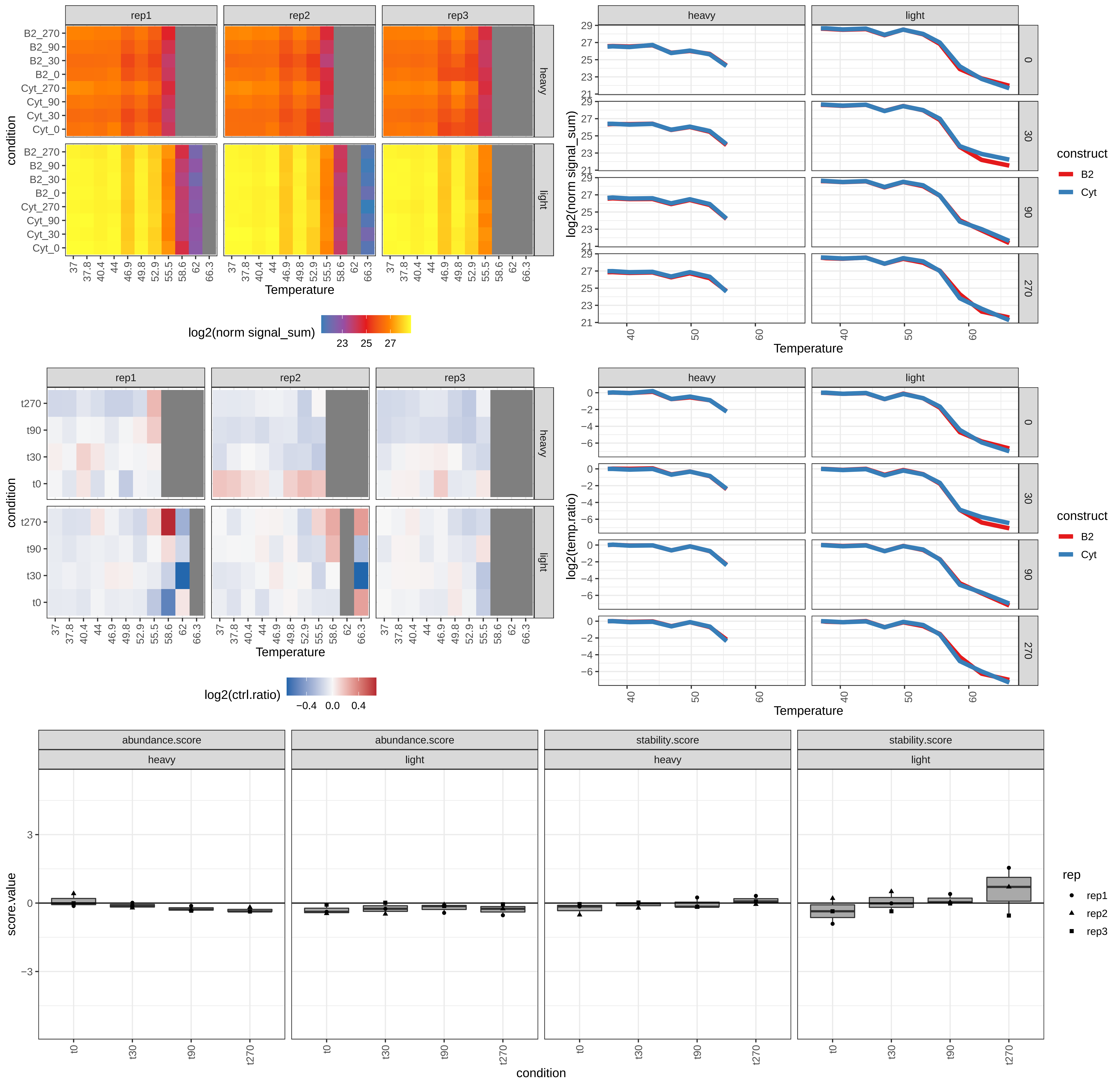

P16861 − ATP−dependent 6−phosphofructokinase subunit alpha OS=Saccharomyces cerevisiae (strain ATCC 204508 / S288c) GN=PFK1 PE=1 SV=1

PFK2 − average.top3: 7.75658725490196 − max.qupm: 28

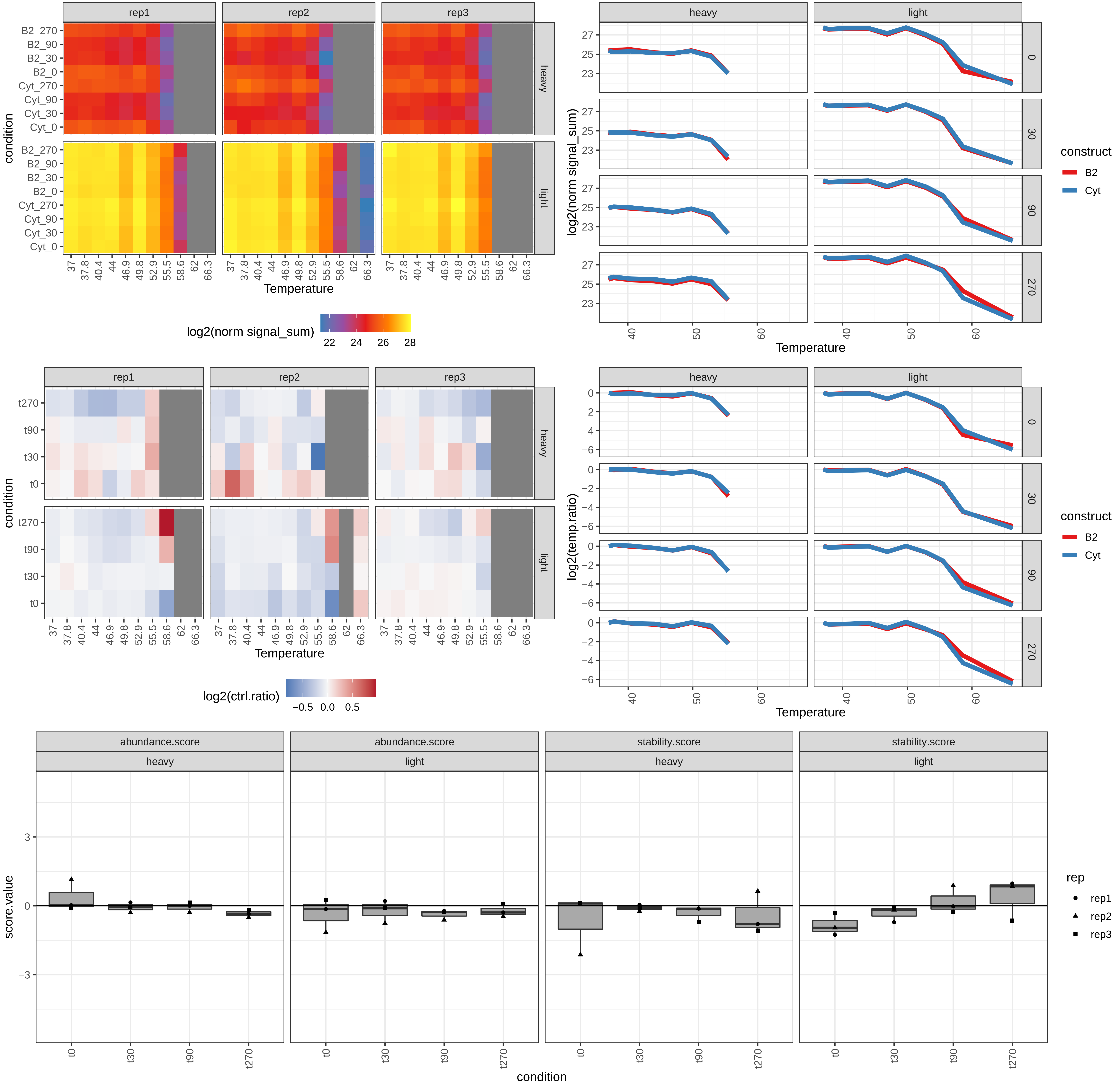

P16862 − ATP−dependent 6−phosphofructokinase subunit beta OS=Saccharomyces cerevisiae (strain ATCC 204508 / S288c) GN=PFK2 PE=1 SV=4

PFK26 − average.top3: 6.90262481481482 − max.qupm: 12

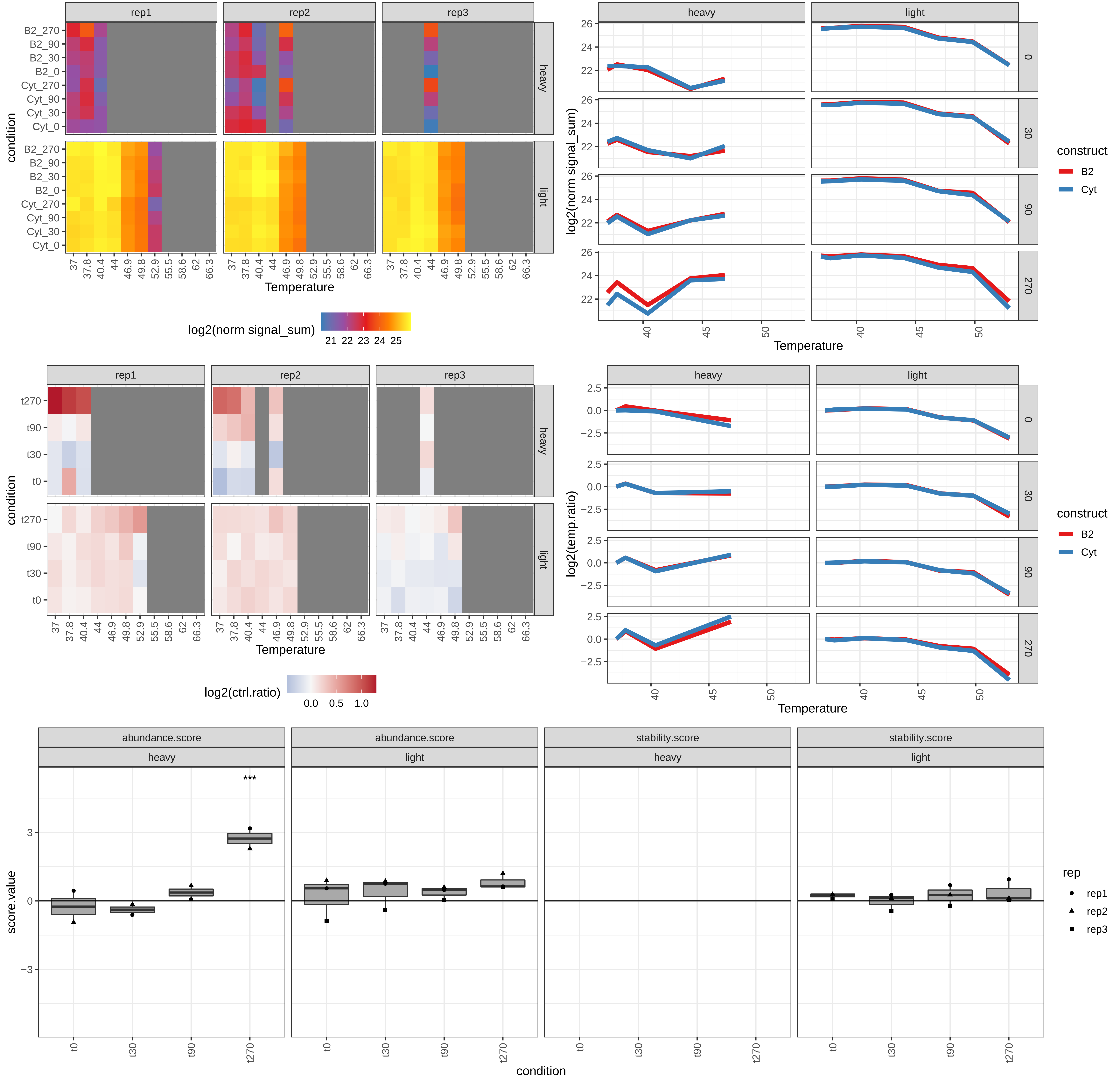

P40433 − 6−phosphofructo−2−kinase 1 OS=Saccharomyces cerevisiae (strain ATCC 204508 / S288c) GN=PFK26 PE=1 SV=1

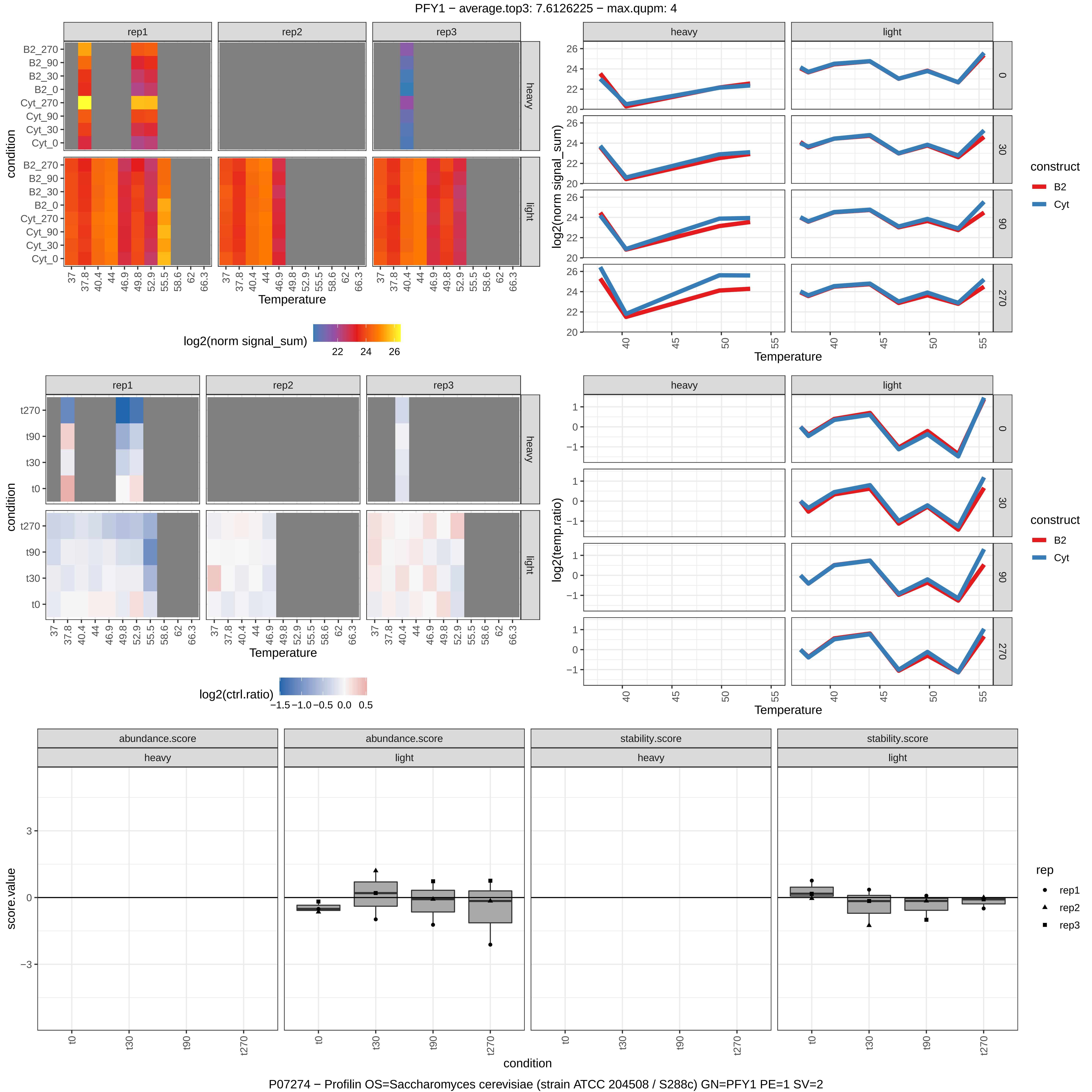
PFY1 − average.top3: 7.6126225 − max.qupm: 4
rep1
rep2
rep3
heavy
light
condition
Temperature
log2(norm signal_sum)
construct
B2
Cyt
log2(ctrl.ratio)
log2(temp.ratio)
abundance.score
stability.score
score.value
rep
rep1
rep2
rep3
P07274 − Profilin OS=Saccharomyces cerevisiae (strain ATCC 204508 / S288c) GN=PFY1 PE=1 SV=2

PGA2 − average.top3: 6.52264875 − max.qupm: 4

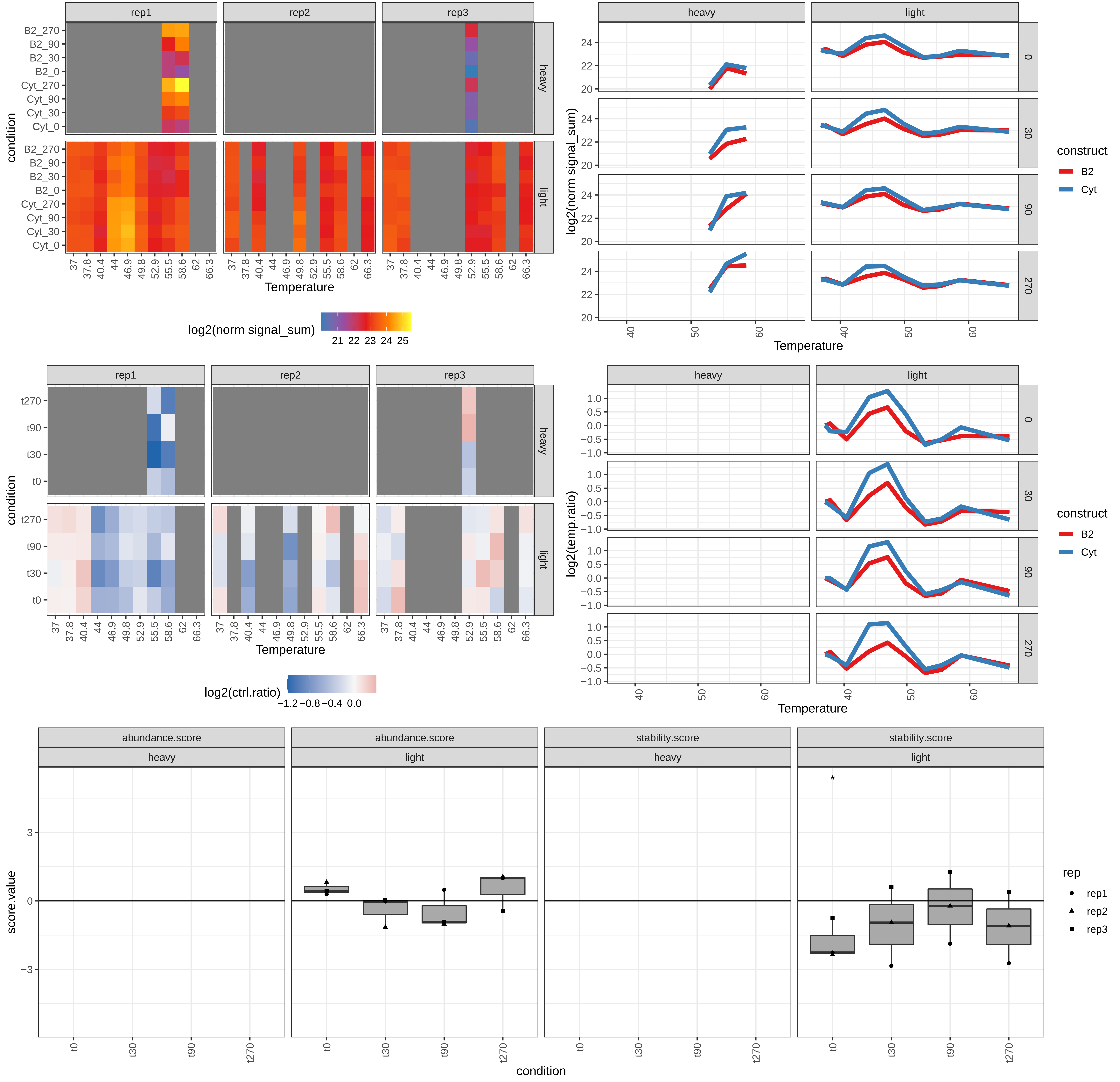

P53903 − Processing of GAS1 and ALP protein 2 OS=Saccharomyces cerevisiae (strain ATCC 204508 / S288c) GN=PGA2 PE=1 SV=3

PGA3 − average.top3: 6.802282 − max.qupm: 6

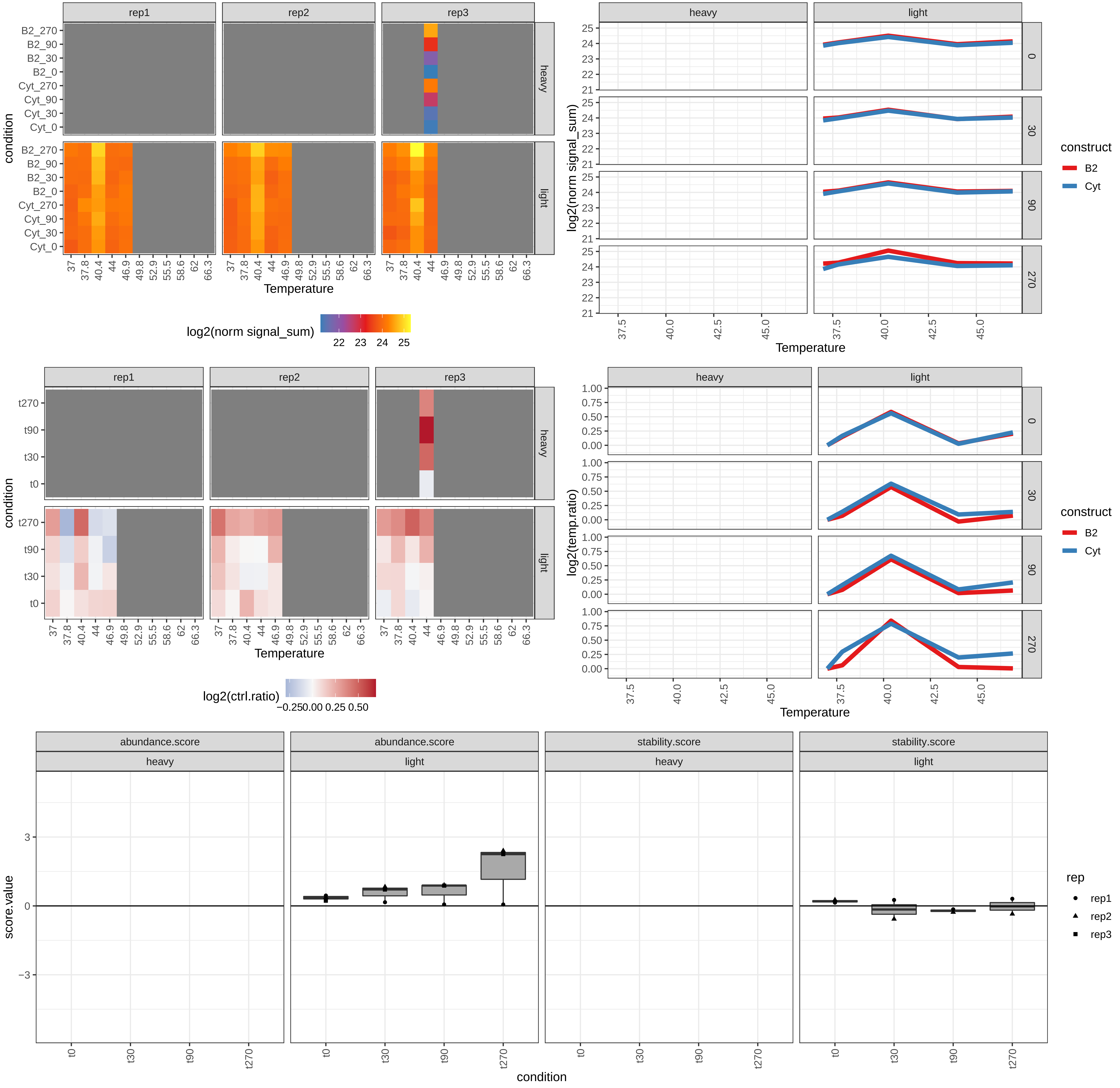

Q12746 − Plasma membrane−associated coenzyme Q6 reductase PGA3 OS=Saccharomyces cerevisiae (strain ATCC 204508 / S288c) GN=PGA3 PE=1 SV=1

PGC1 − average.top3: 6.689905 − max.qupm: 3

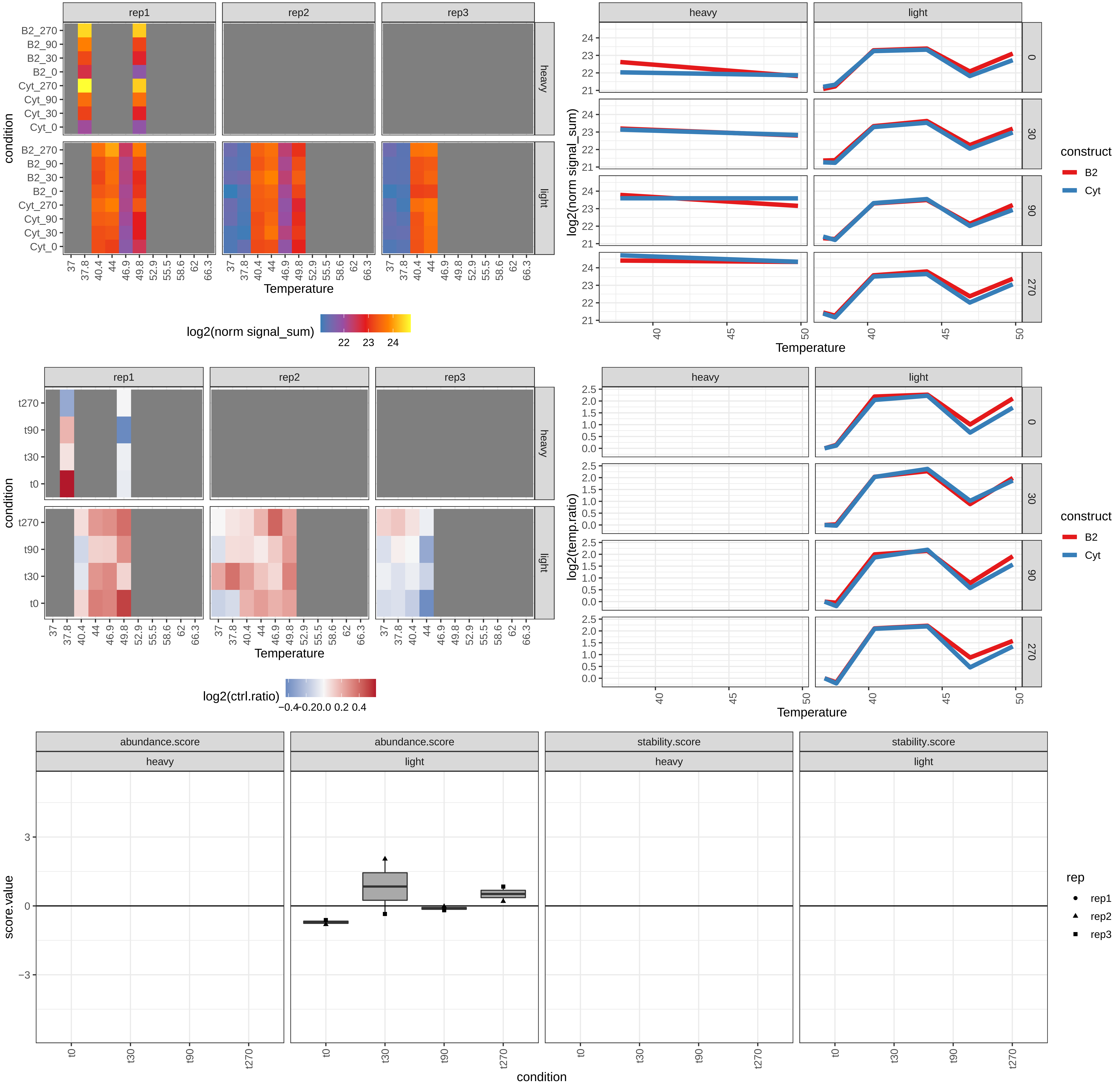

Q08959 − Phosphatidylglycerol phospholipase C OS=Saccharomyces cerevisiae (strain ATCC 204508 / S288c) GN=PGC1 PE=1 SV=1

PGD1 − average.top3: 5.67281375 − max.qupm: 3

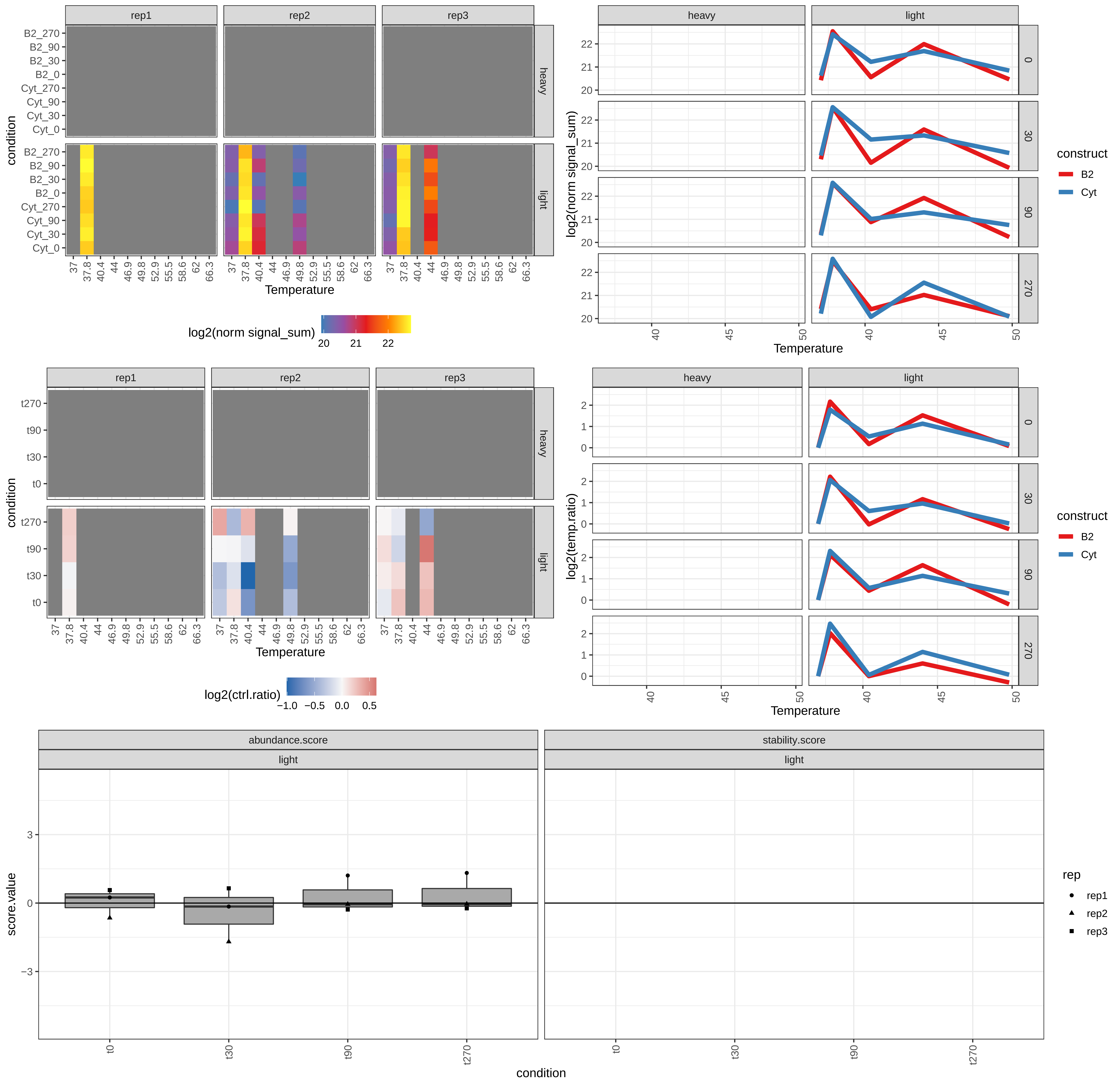

P40356 − Mediator of RNA polymerase II transcription subunit 3 OS=Saccharomyces cerevisiae (strain ATCC 204508 / S288c) GN=PGD1 PE=1 SV=2

PGI1 − average.top3: 8.462116 − max.qupm: 22

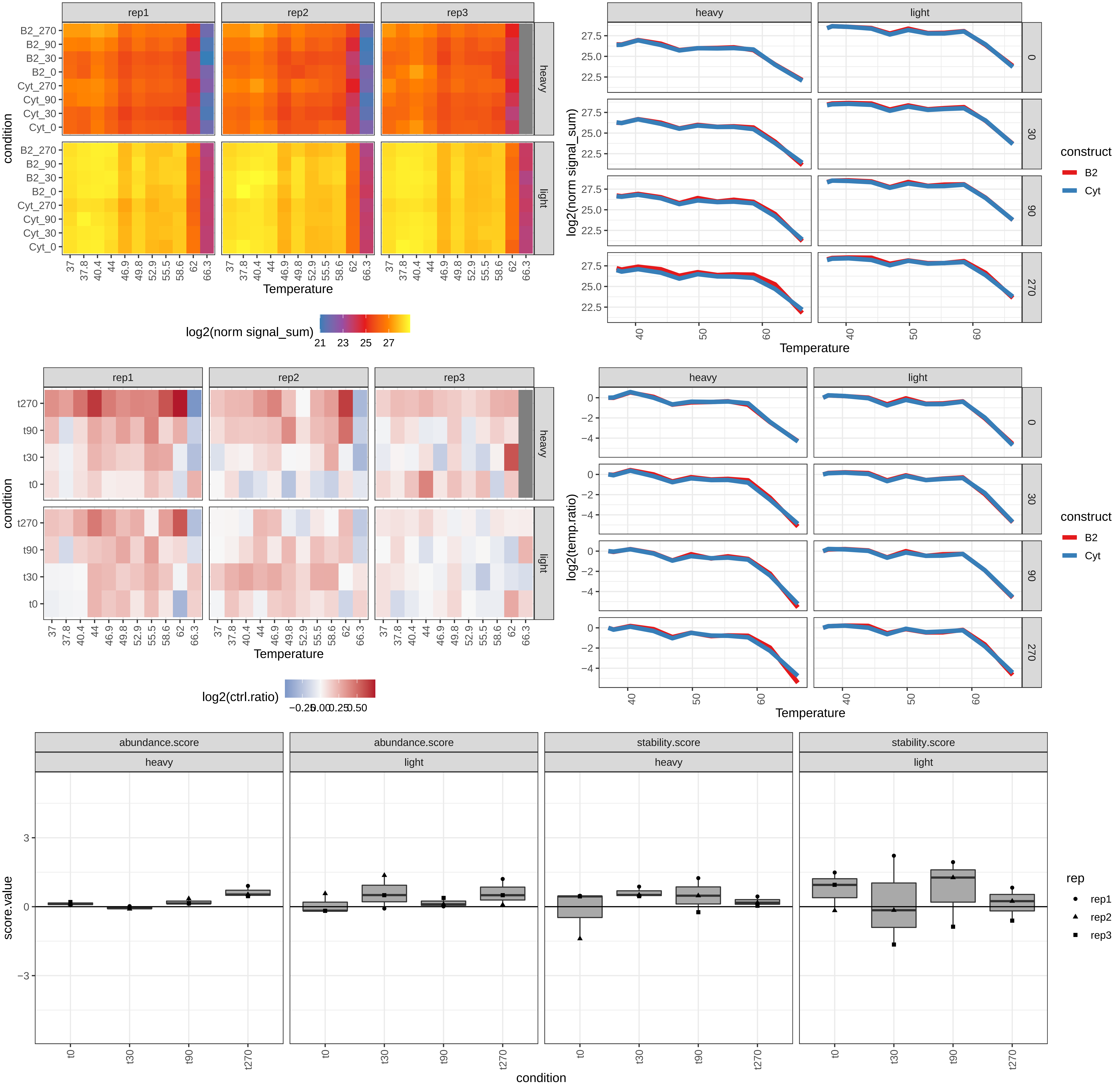

P12709 − Glucose−6−phosphate isomerase OS=Saccharomyces cerevisiae (strain ATCC 204508 / S288c) GN=PGI1 PE=1 SV=3

PGK1 − average.top3: 8.45813606060606 − max.qupm: 34

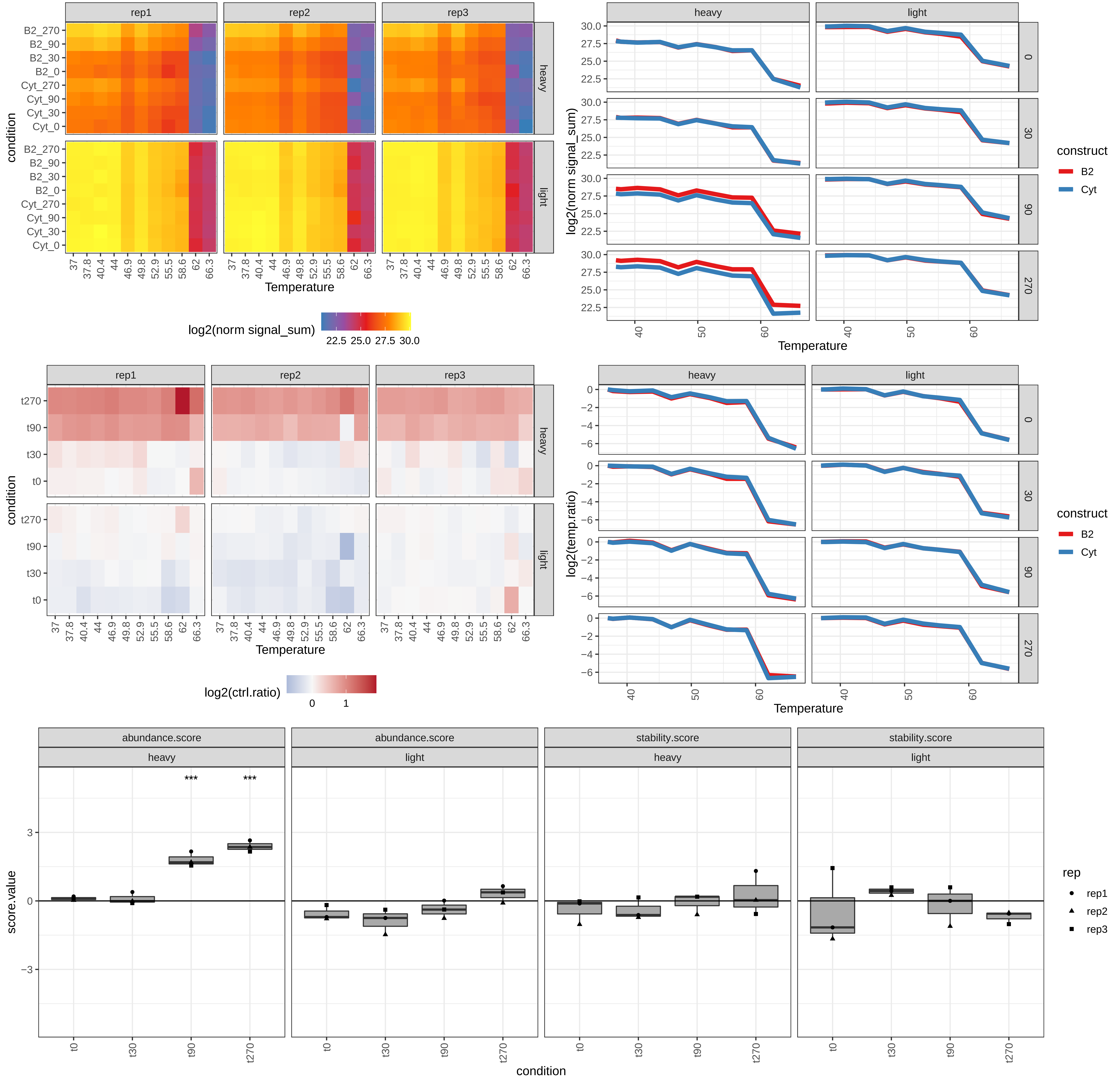

P00560 − Phosphoglycerate kinase OS=Saccharomyces cerevisiae (strain ATCC 204508 / S288c) GN=PGK1 PE=1 SV=2

PGM1 − average.top3: 6.78486519230769 − max.qupm: 10

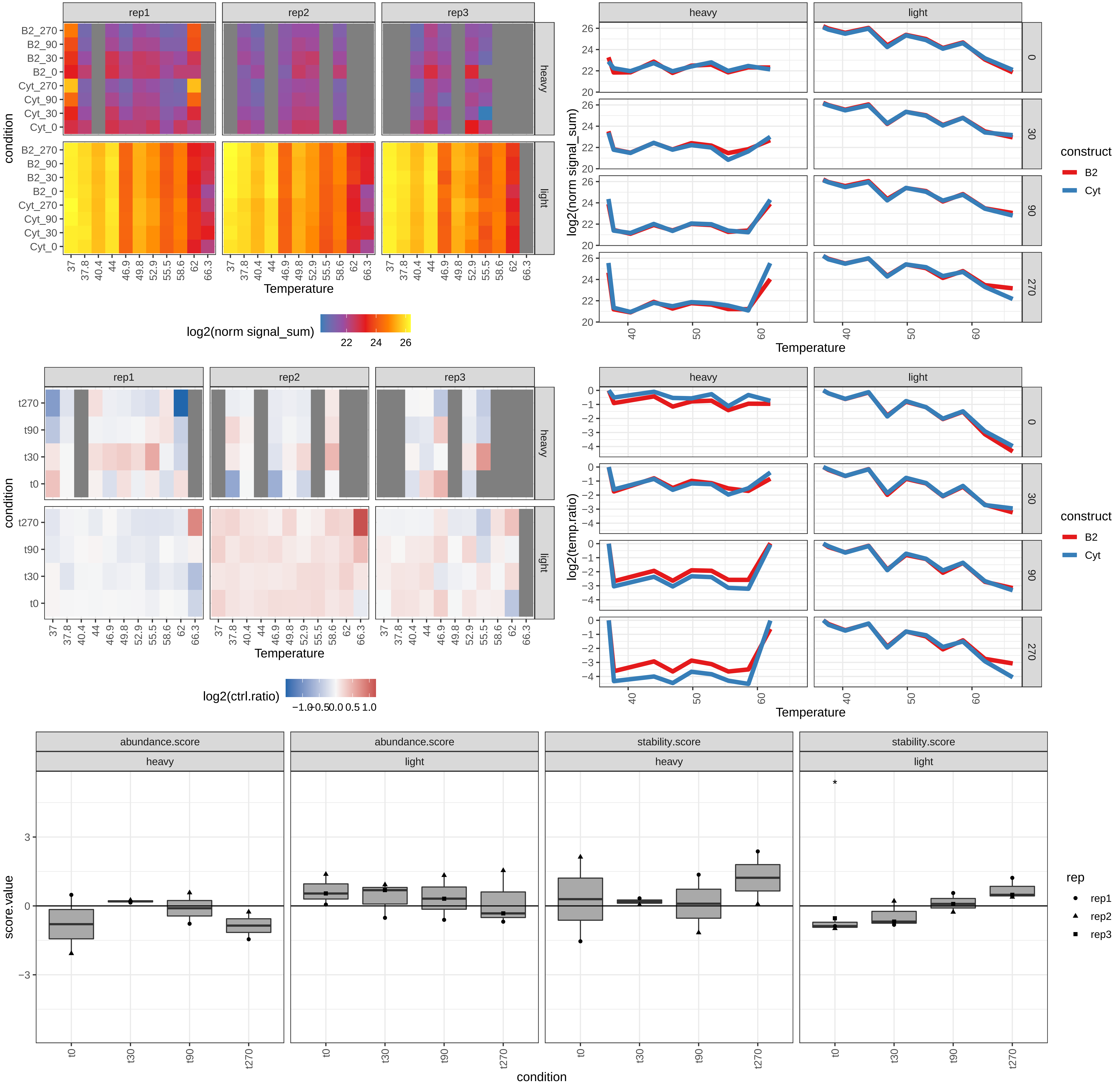

P33401 − Phosphoglucomutase 1 OS=Saccharomyces cerevisiae (strain ATCC 204508 / S288c) GN=PGM1 PE=1 SV=1

PGM2 − average.top3: 7.7146 − max.qupm: 18

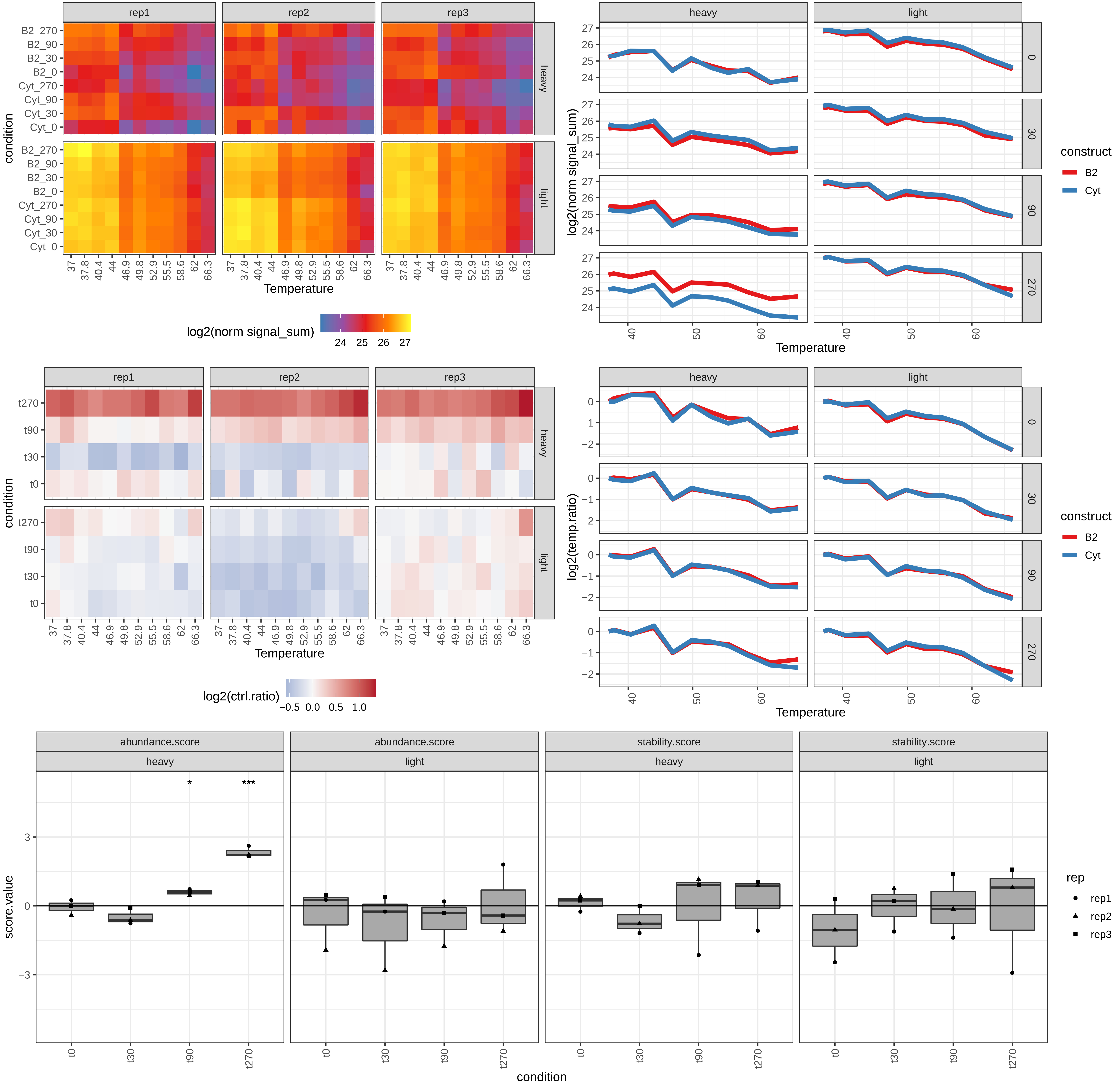

P37012 − Phosphoglucomutase 2 OS=Saccharomyces cerevisiae (strain ATCC 204508 / S288c) GN=PGM2 PE=1 SV=1

PHA2 − average.top3: 6.88150444444444 − max.qupm: 4

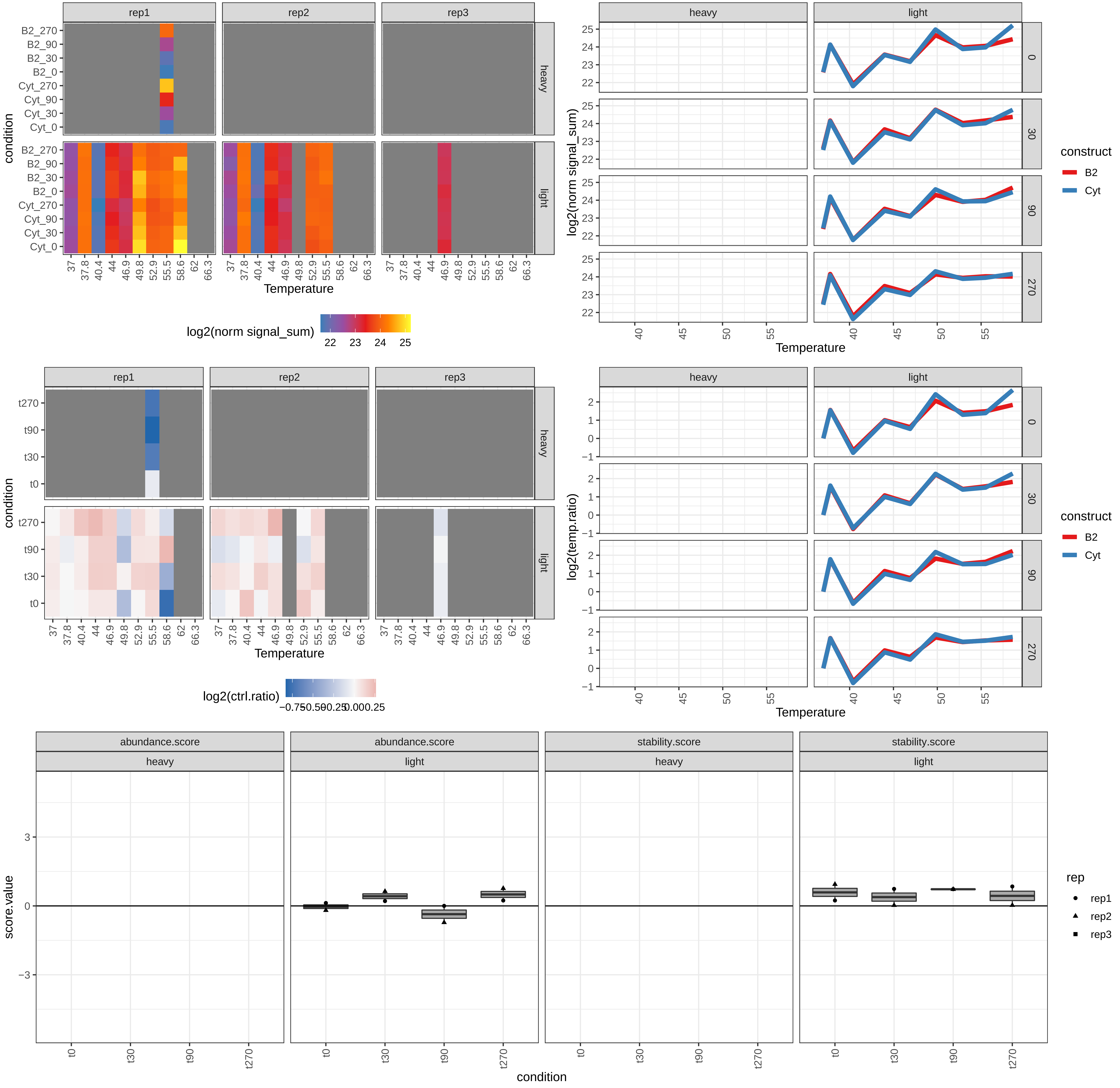

P32452 − Putative prephenate dehydratase OS=Saccharomyces cerevisiae (strain ATCC 204508 / S288c) GN=PHA2 PE=1 SV=3

PHB1 − average.top3: 7.37436589285714 − max.qupm: 10

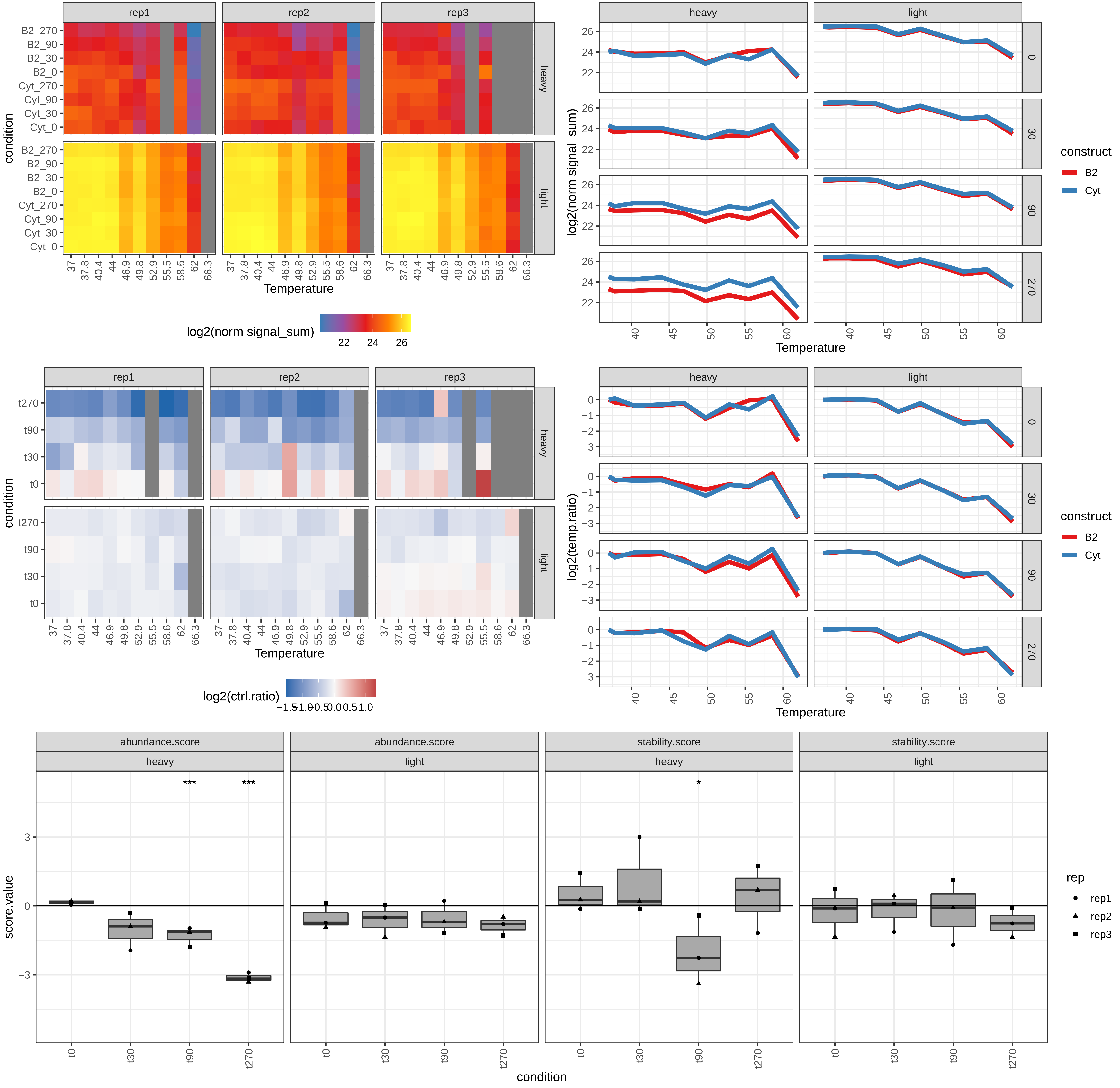

P40961 − Prohibitin−1 OS=Saccharomyces cerevisiae (strain ATCC 204508 / S288c) GN=PHB1 PE=1 SV=2

PHB2 − average.top3: 7.38504297297297 − max.qupm: 11

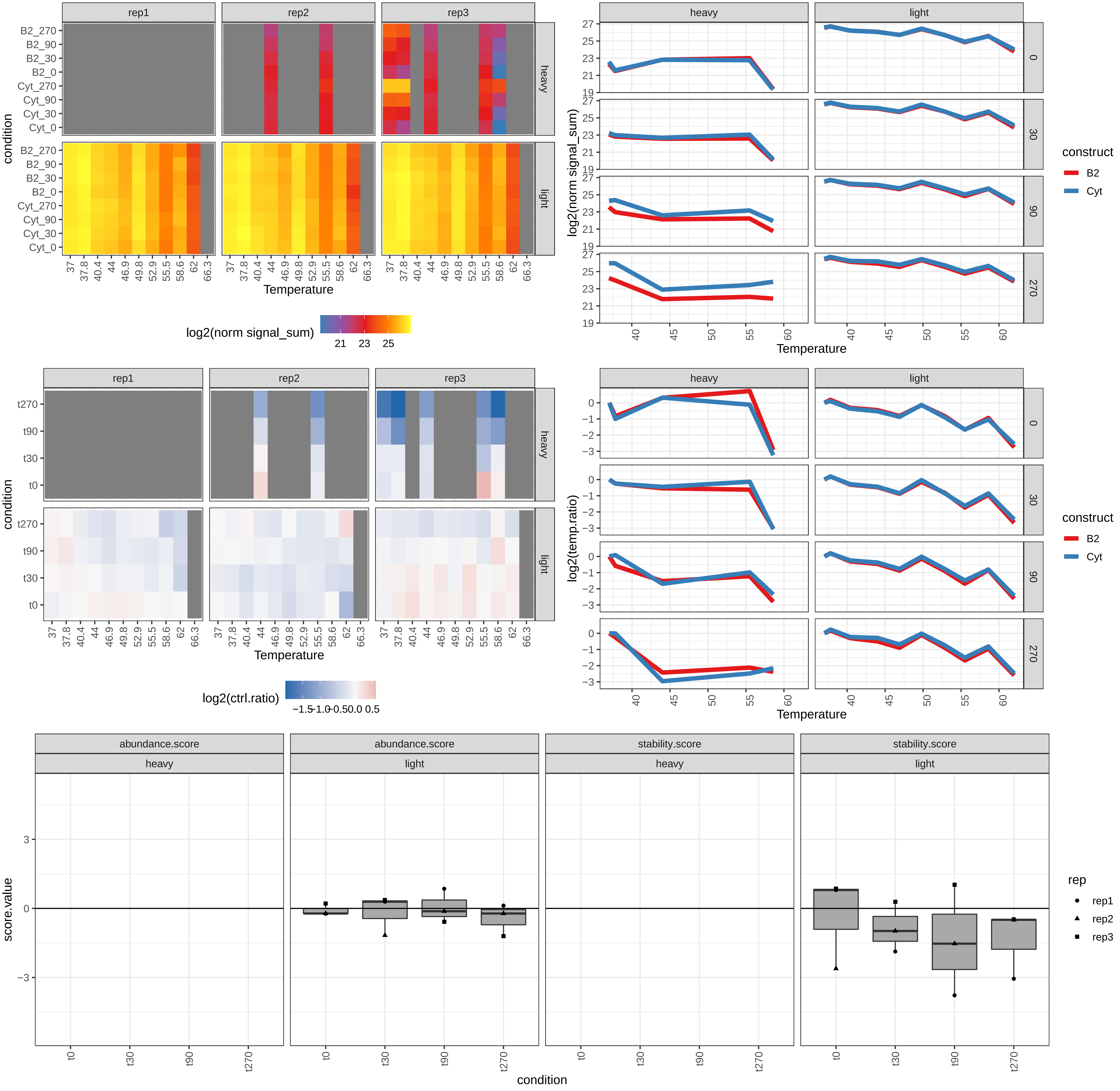

P50085 − Prohibitin−2 OS=Saccharomyces cerevisiae (strain ATCC 204508 / S288c) GN=PHB2 PE=1 SV=2

PHO13 − average.top3: 7.02108929824561 − max.qupm: 9

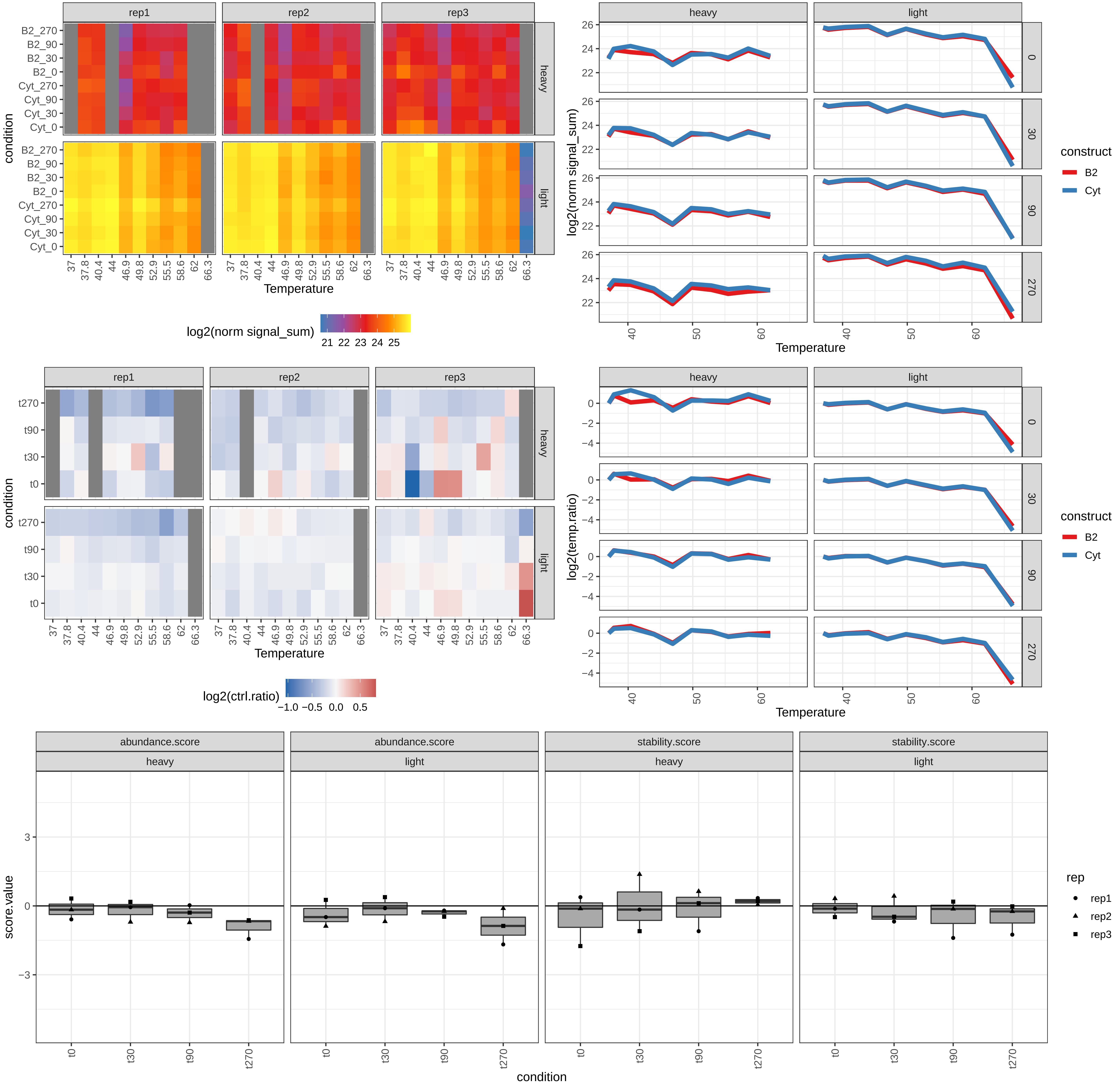

P19881 − 4−nitrophenylphosphatase OS=Saccharomyces cerevisiae (strain ATCC 204508 / S288c) GN=PHO13 PE=1 SV=2

PHO8 − average.top3: 7.10115411764706 − max.qupm: 14

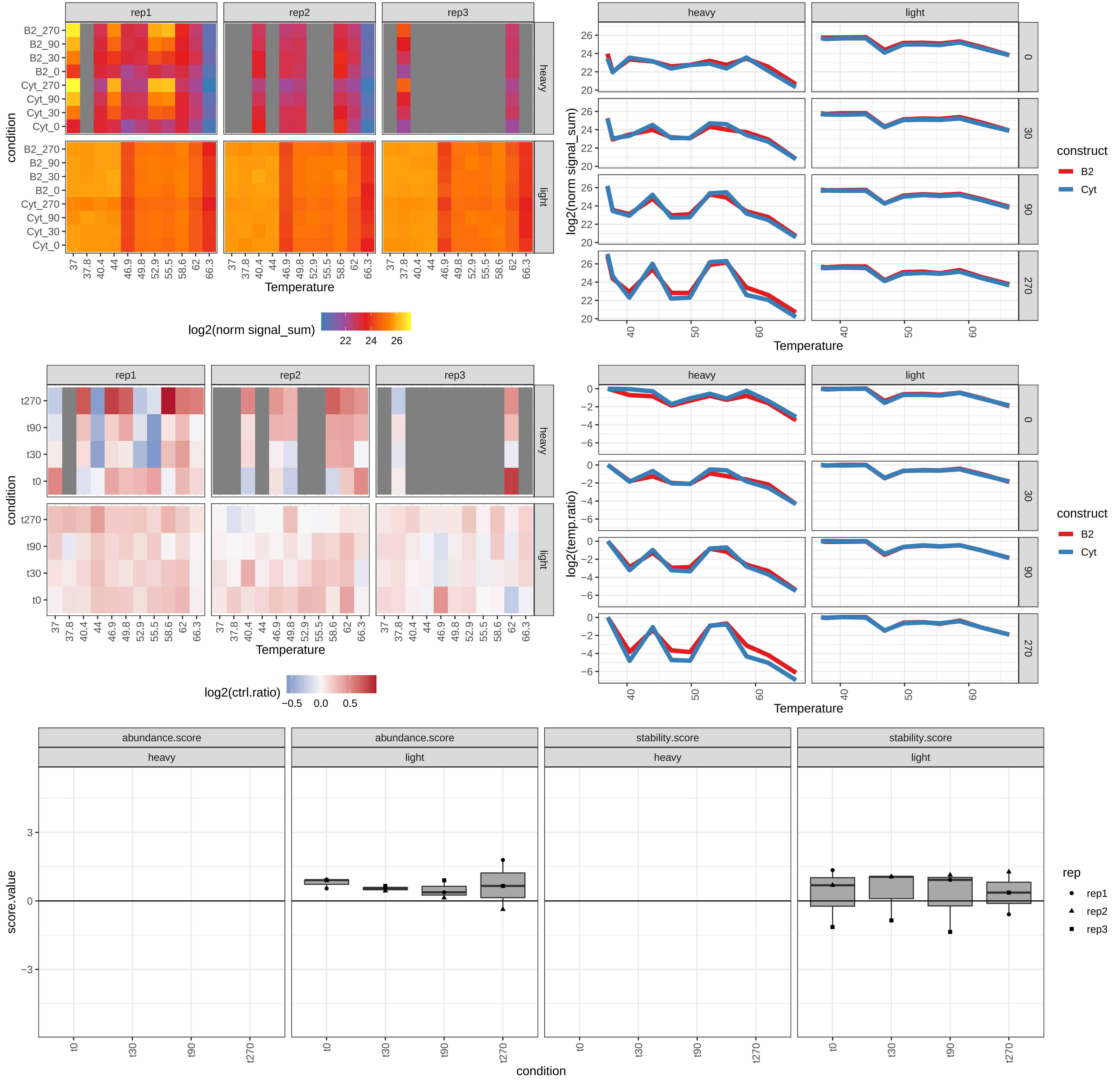

P11491 − Repressible alkaline phosphatase OS=Saccharomyces cerevisiae (strain ATCC 204508 / S288c) GN=PHO8 PE=1 SV=2

PHO81 − average.top3: 6.54905136363636 − max.qupm: 10

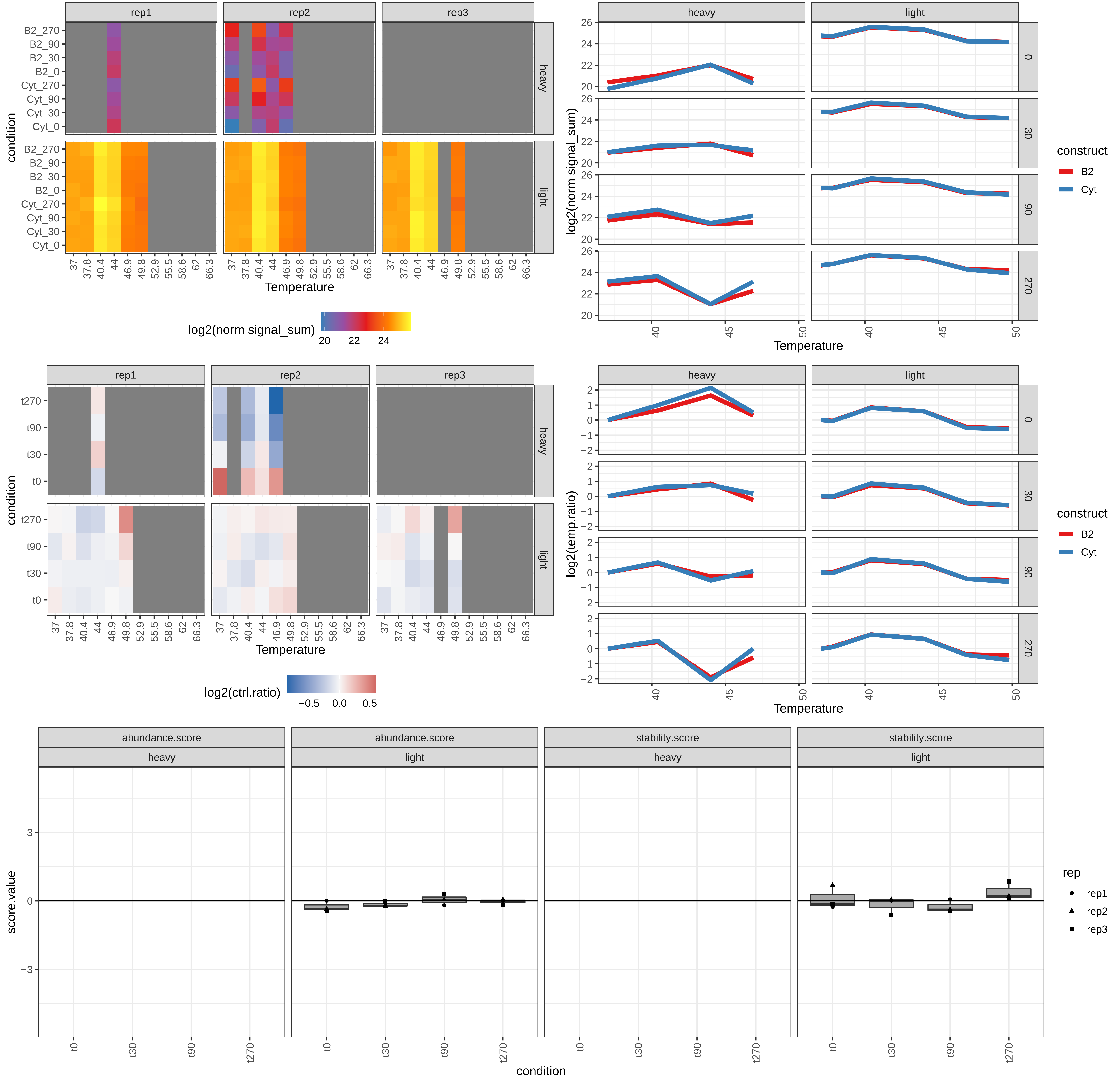

P17442 − Phosphate system positive regulatory protein PHO81 OS=Saccharomyces cerevisiae (strain ATCC 204508 / S288c) GN=PHO81 PE=1 SV=2

PHO85 − average.top3: 7.03813607142857 − max.qupm: 6

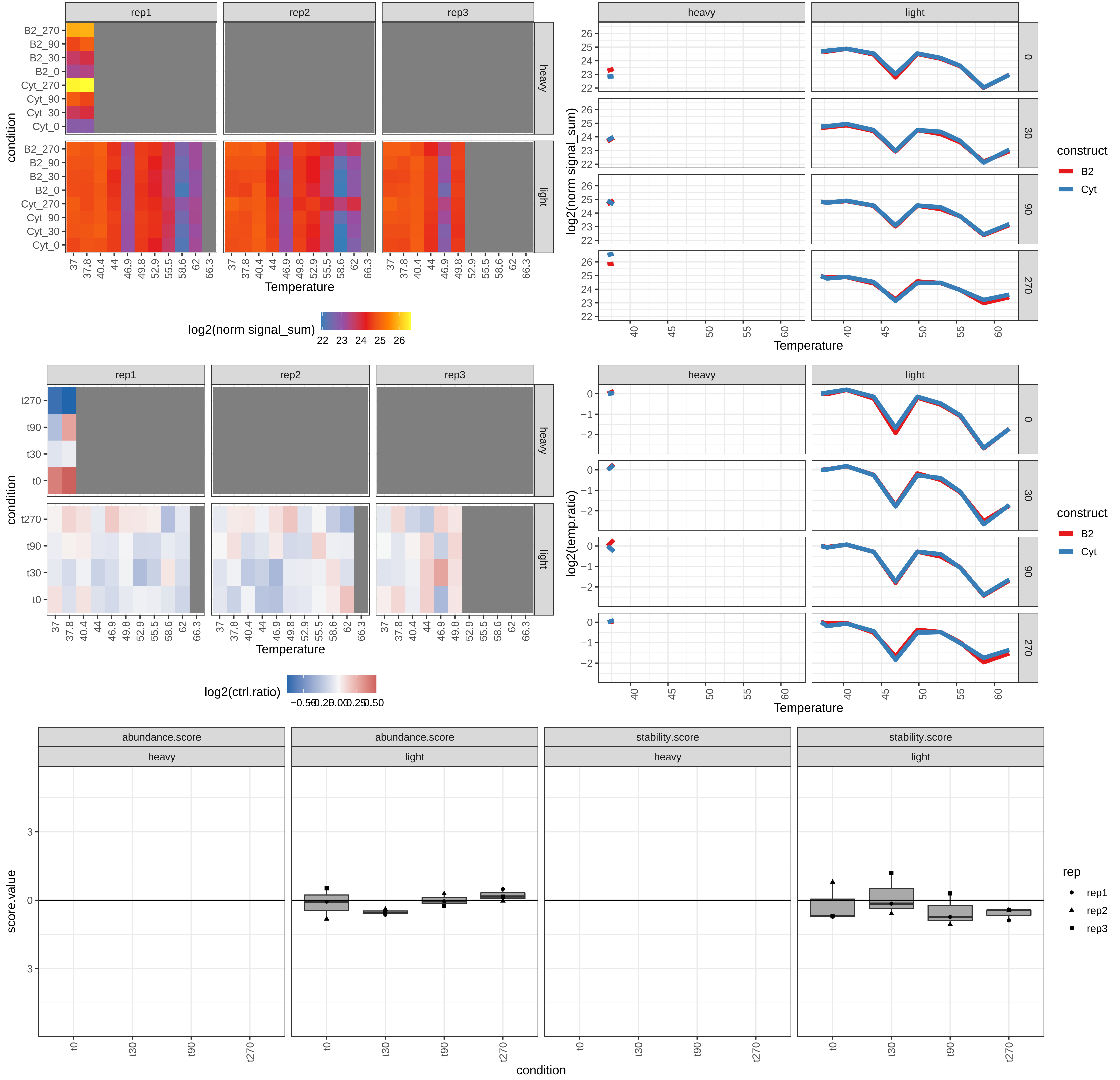

P17157 − Cyclin−dependent protein kinase PHO85 OS=Saccharomyces cerevisiae (strain ATCC 204508 / S288c) GN=PHO85 PE=1 SV=2

PHO86 − average.top3: 7.081333125 − max.qupm: 6

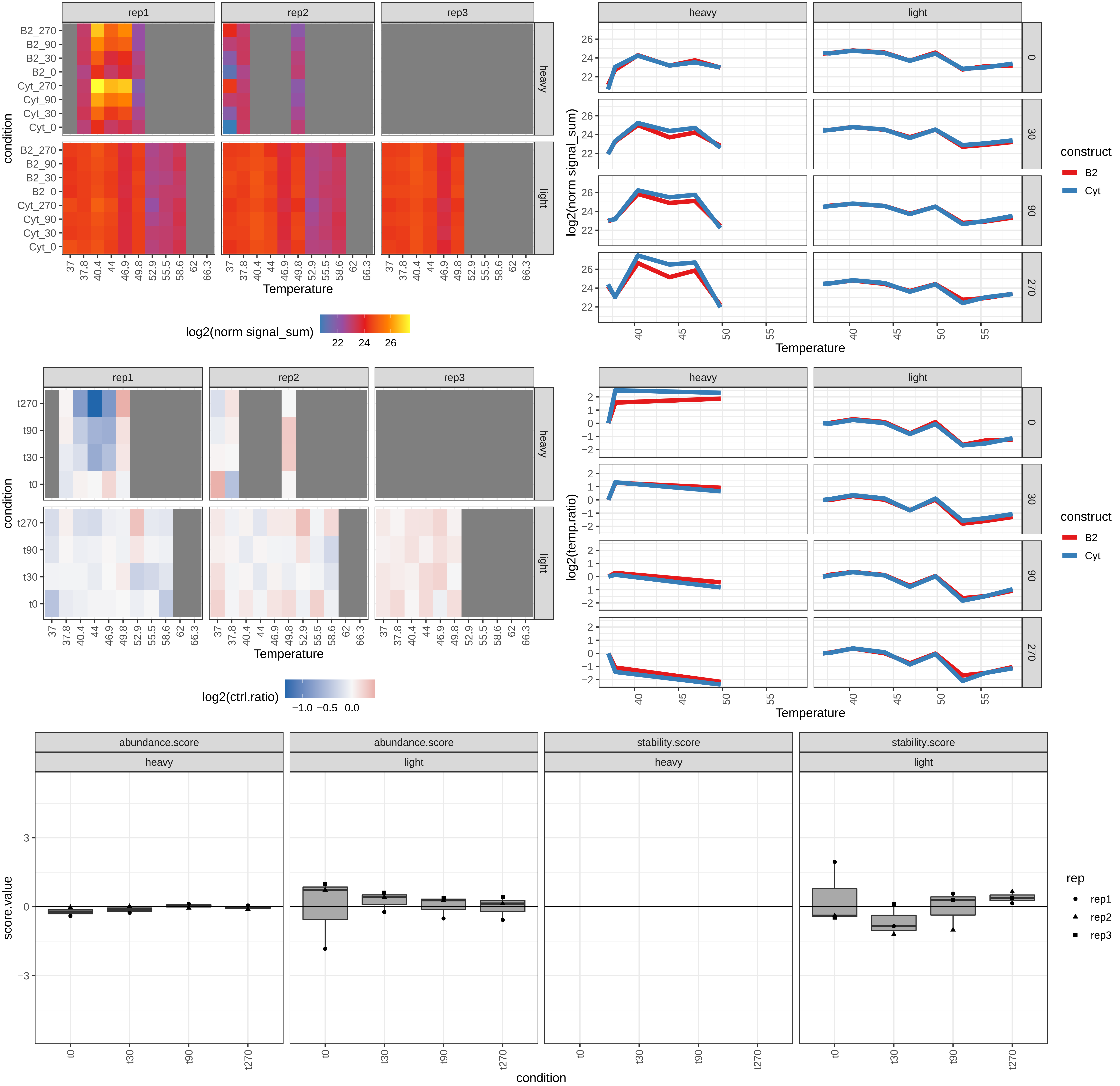

P46956 − Inorganic phosphate transporter PHO86 OS=Saccharomyces cerevisiae (strain ATCC 204508 / S288c) GN=PHO86 PE=1 SV=1

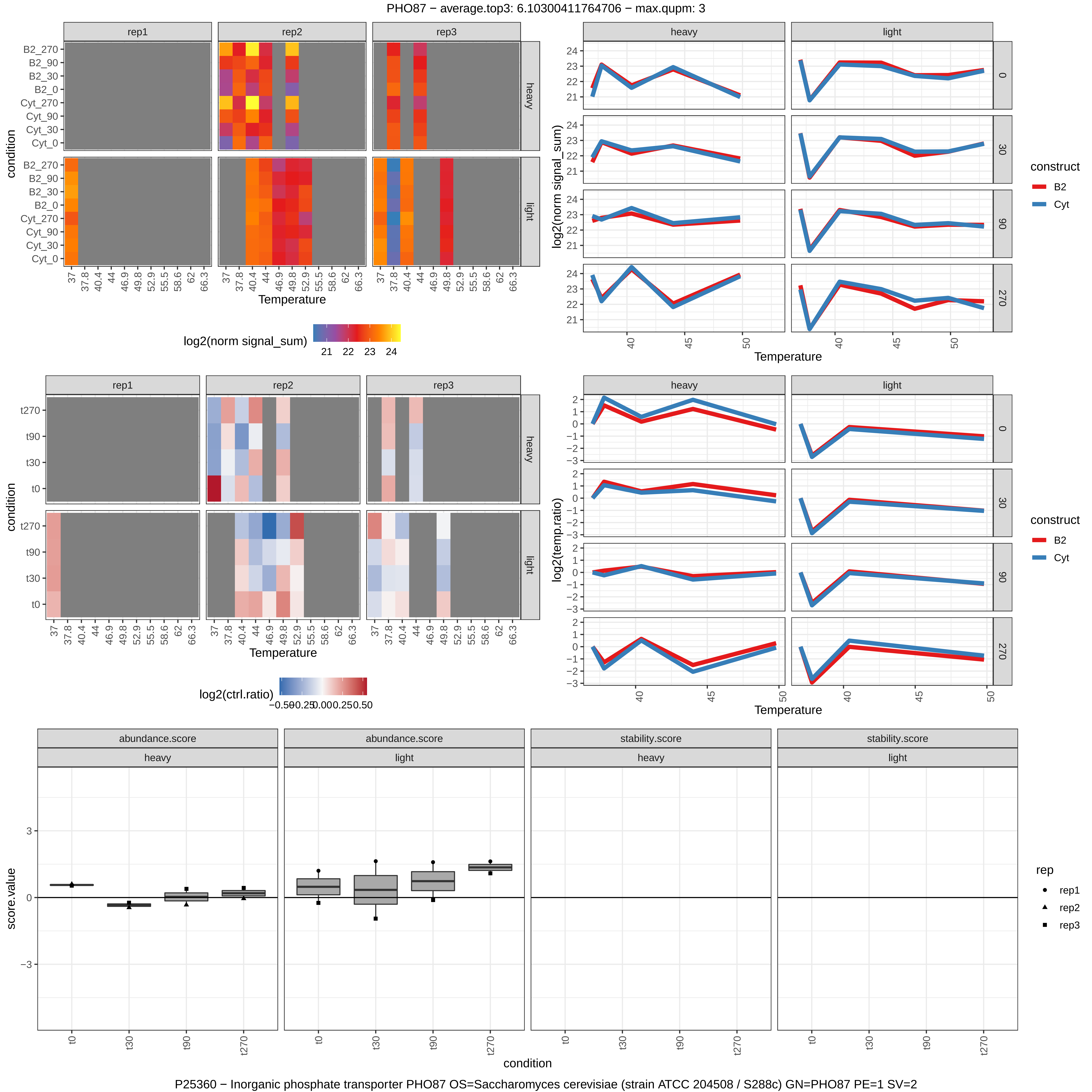
PHO87 − average.top3: 6.10300411764706 − max.qupm: 3
rep1
rep2
rep3
heavy
light
condition
Temperature
log2(norm signal_sum)
log2(ctrl.ratio)
construct
B2
Cyt
log2(temp.ratio)
abundance.score
stability.score
score.value
rep
rep1
rep2
rep3
P25360 − Inorganic phosphate transporter PHO87 OS=Saccharomyces cerevisiae (strain ATCC 204508 / S288c) GN=PHO87 PE=1 SV=2

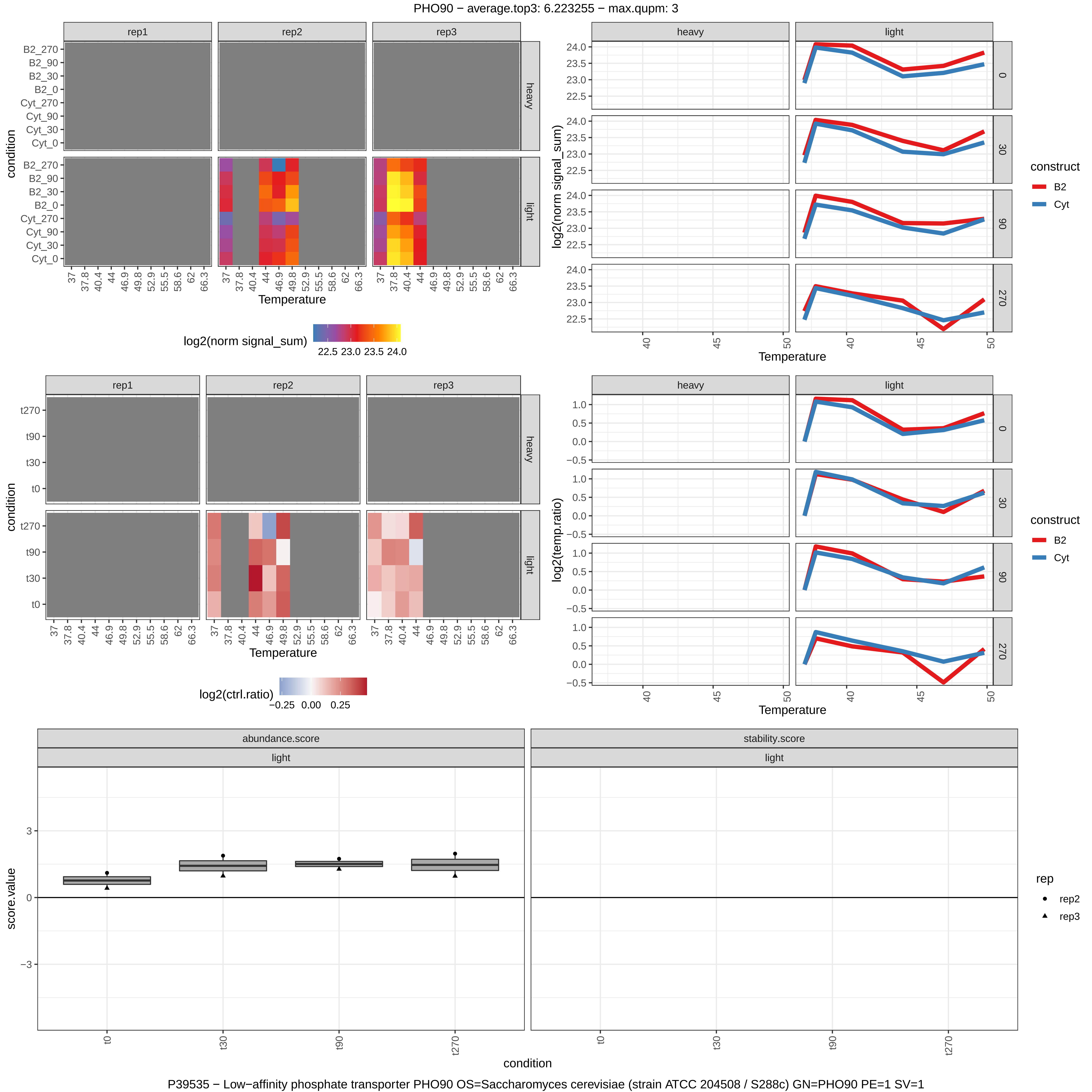
PHO90 − average.top3: 6.223255 − max.qupm: 3
rep1
rep2
rep3
heavy
light
B2_270
B2_90
B2_30
B2_0
Cyt_270
Cyt_90
Cyt_30
Cyt_0
condition
37 37.8 40.4 44 46.9 49.8 52.9 55.5 58.6 62 66.3
Temperature
log2(norm signal_sum)
22.5 23.0 23.5 24.0
log2(norm signal_sum)
0
30
90
270
40 45 50
construct
B2
Cyt
t270
t90
t30
t0
log2(ctrl.ratio)
−0.25 0.00 0.25
log2(temp.ratio)
1.0 0.5 0.0 −0.5
abundance.score
stability.score
score.value
3
0
−3
condition
rep
rep2
rep3
P39535 − Low−affinity phosphate transporter PHO90 OS=Saccharomyces cerevisiae (strain ATCC 204508 / S288c) GN=PHO90 PE=1 SV=1

PHO91 − average.top3: 6.601979 − max.qupm: 3

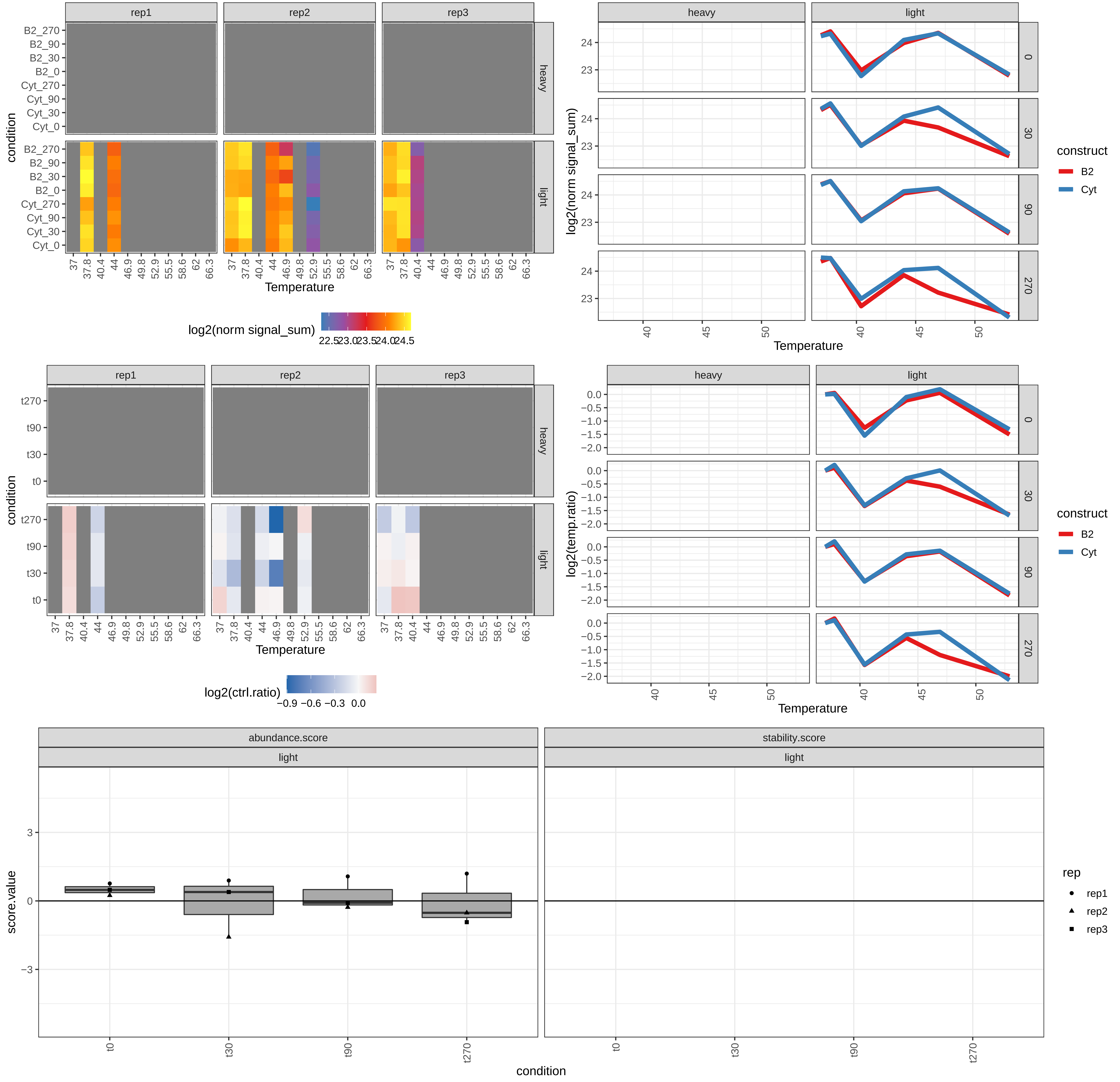

P27514 − Low−affinity phosphate transporter PHO91 OS=Saccharomyces cerevisiae (strain ATCC 204508 / S288c) GN=PHO91 PE=1 SV=2

PHR1 − average.top3: 6.432435625 − max.qupm: 5

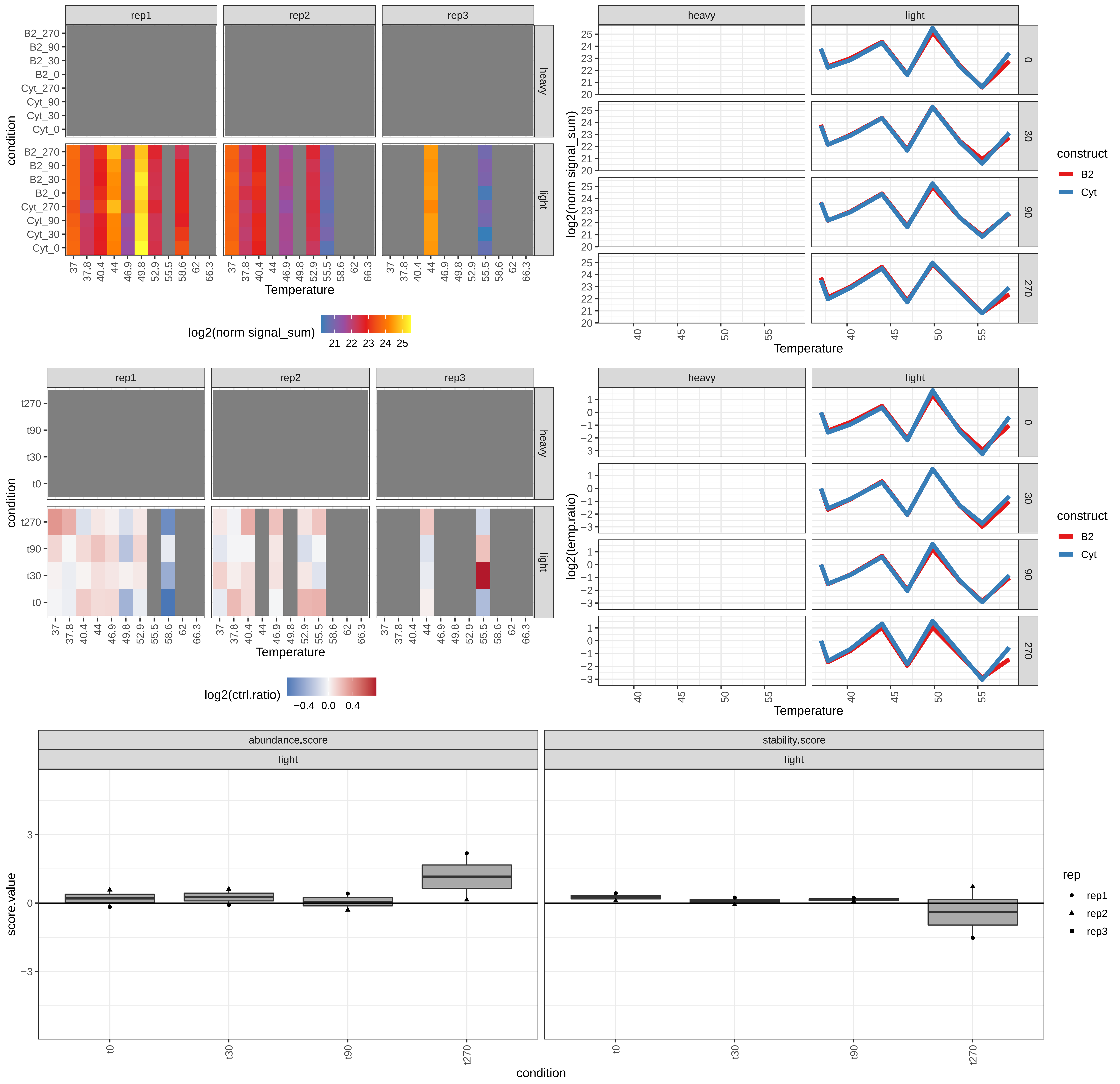

P05066 − Deoxyribodipyrimidine photo−lyase, mitochondrial OS=Saccharomyces cerevisiae (strain ATCC 204508 / S288c) GN=PHR1 PE=1 SV=1

PIB1 − average.top3: 6.49242333333333 − max.qupm: 5

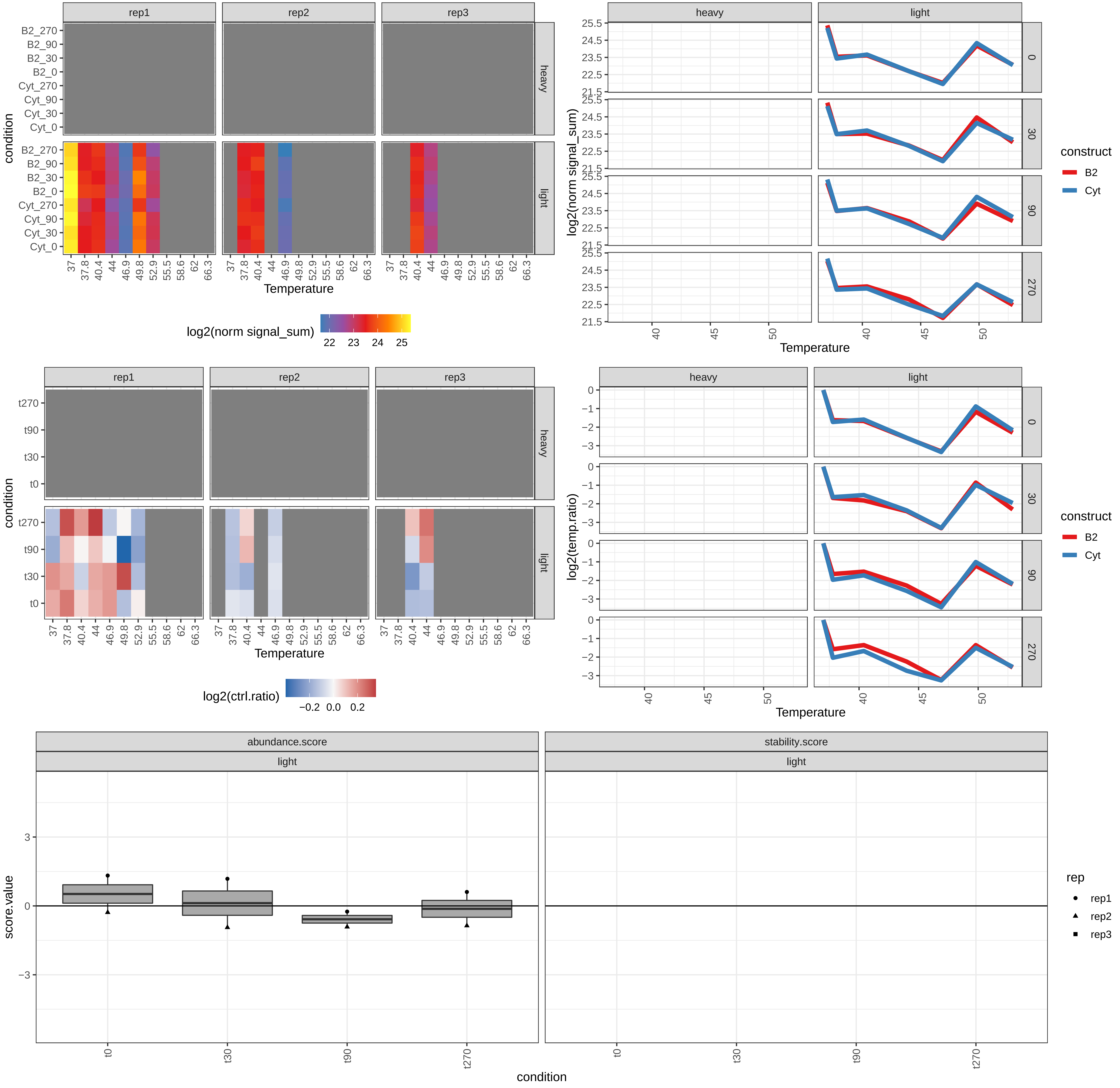

Q06651 − E3 ubiquitin−protein ligase PIB1 OS=Saccharomyces cerevisiae (strain ATCC 204508 / S288c) GN=PIB1 PE=1 SV=1

PIC2 − average.top3: 7.0149756 − max.qupm: 6

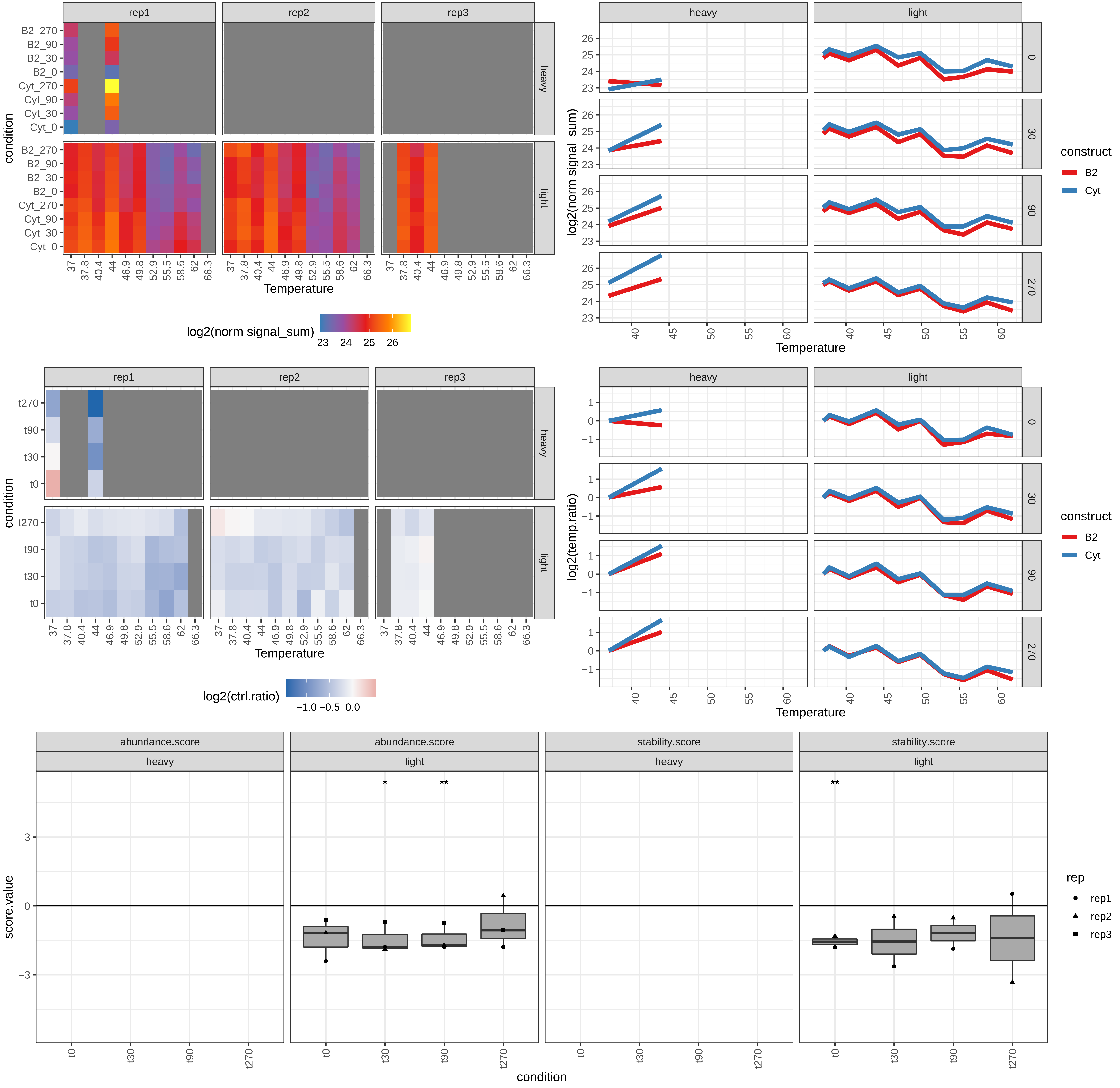

P40035 − Mitochondrial phosphate carrier protein 2 OS=Saccharomyces cerevisiae (strain ATCC 204508 / S288c) GN=PIC2 PE=1 SV=1

PIH1 − average.top3: 6.74579684210526 − max.qupm: 5

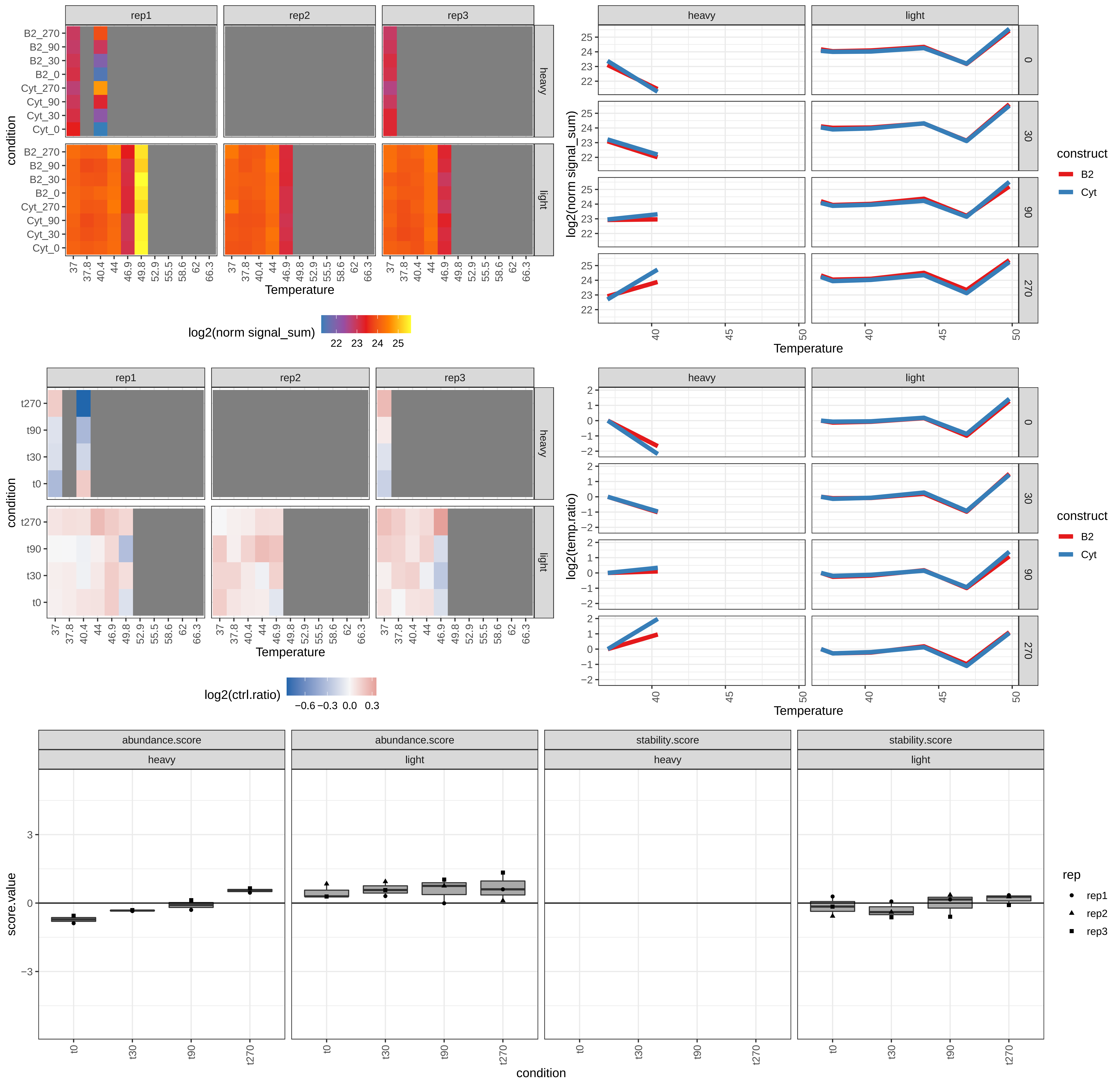

P38768 − Protein interacting with Hsp90 1 OS=Saccharomyces cerevisiae (strain ATCC 204508 / S288c) GN=PIH1 PE=1 SV=1

PIK1 − average.top3: 6.62339 − max.qupm: 4

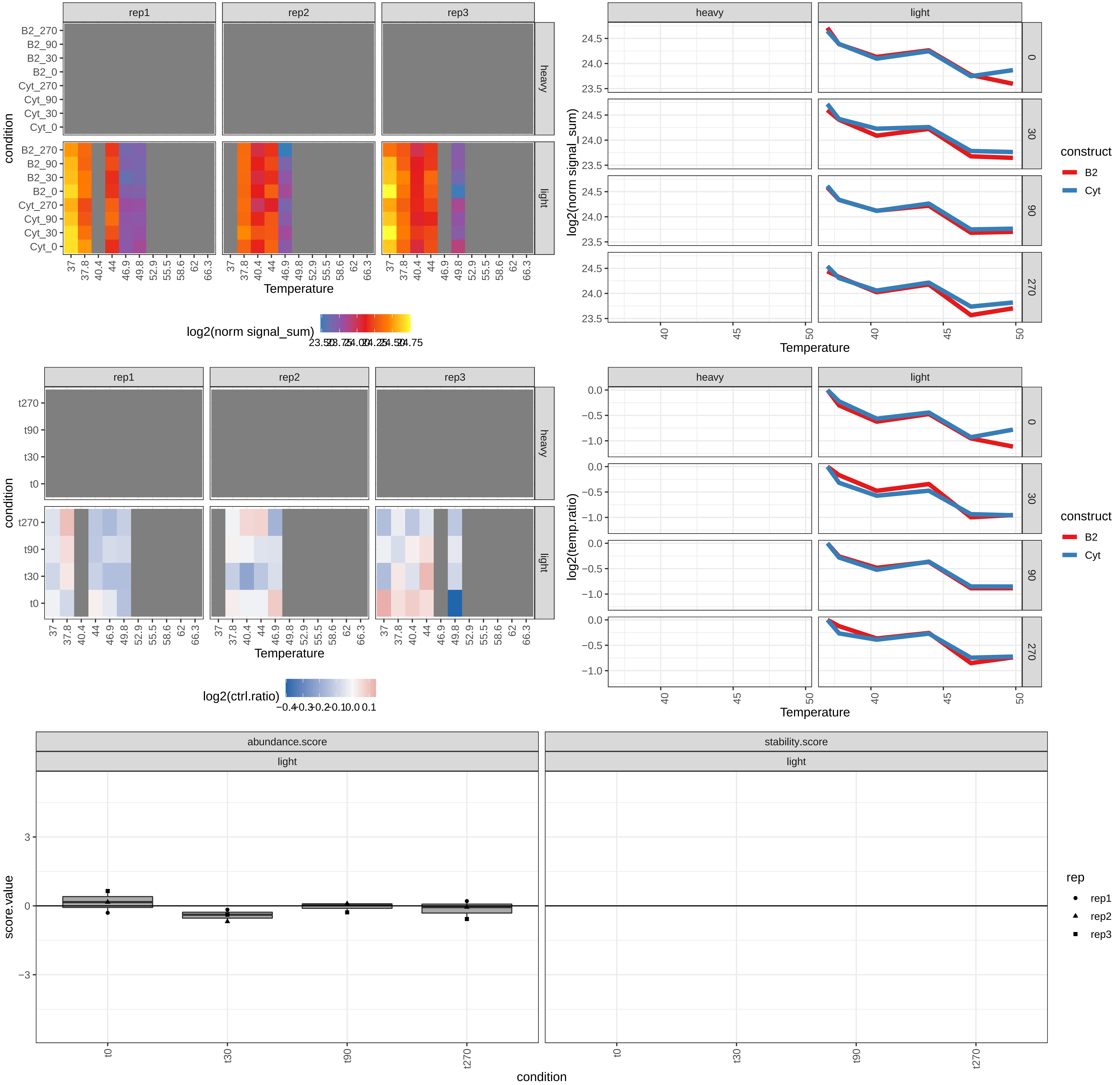

P39104 − Phosphatidylinositol 4−kinase PIK1 OS=Saccharomyces cerevisiae (strain ATCC 204508 / S288c) GN=PIK1 PE=1 SV=1

PIL1 − average.top3: 7.88230727272727 − max.qupm: 11

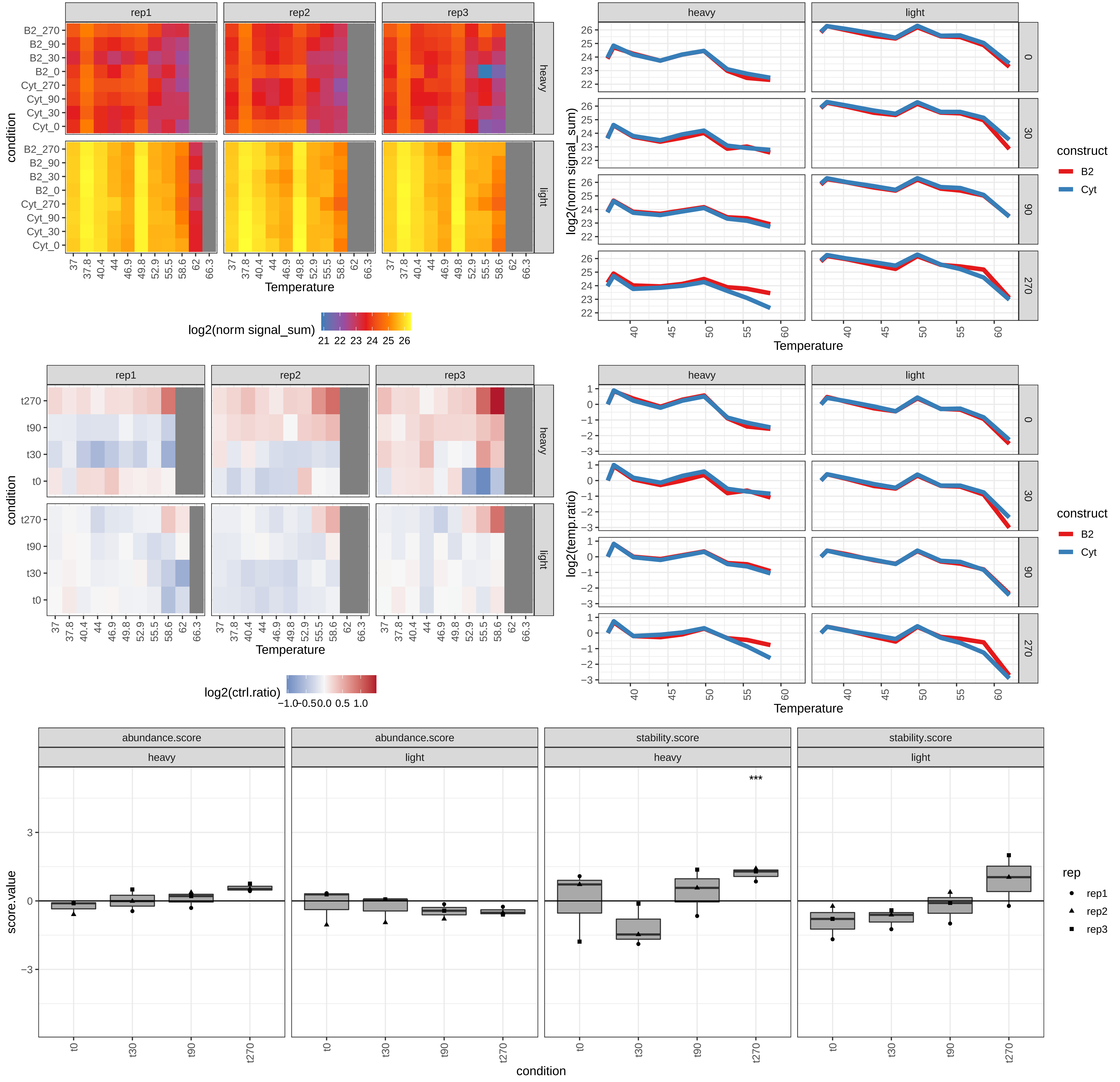

P53252 − Sphingolipid long chain base−responsive protein PIL1 OS=Saccharomyces cerevisiae (strain ATCC 204508 / S288c) GN=PIL1 PE=1 SV=1

PIM1 − average.top3: 6.89555441860465 − max.qupm: 22

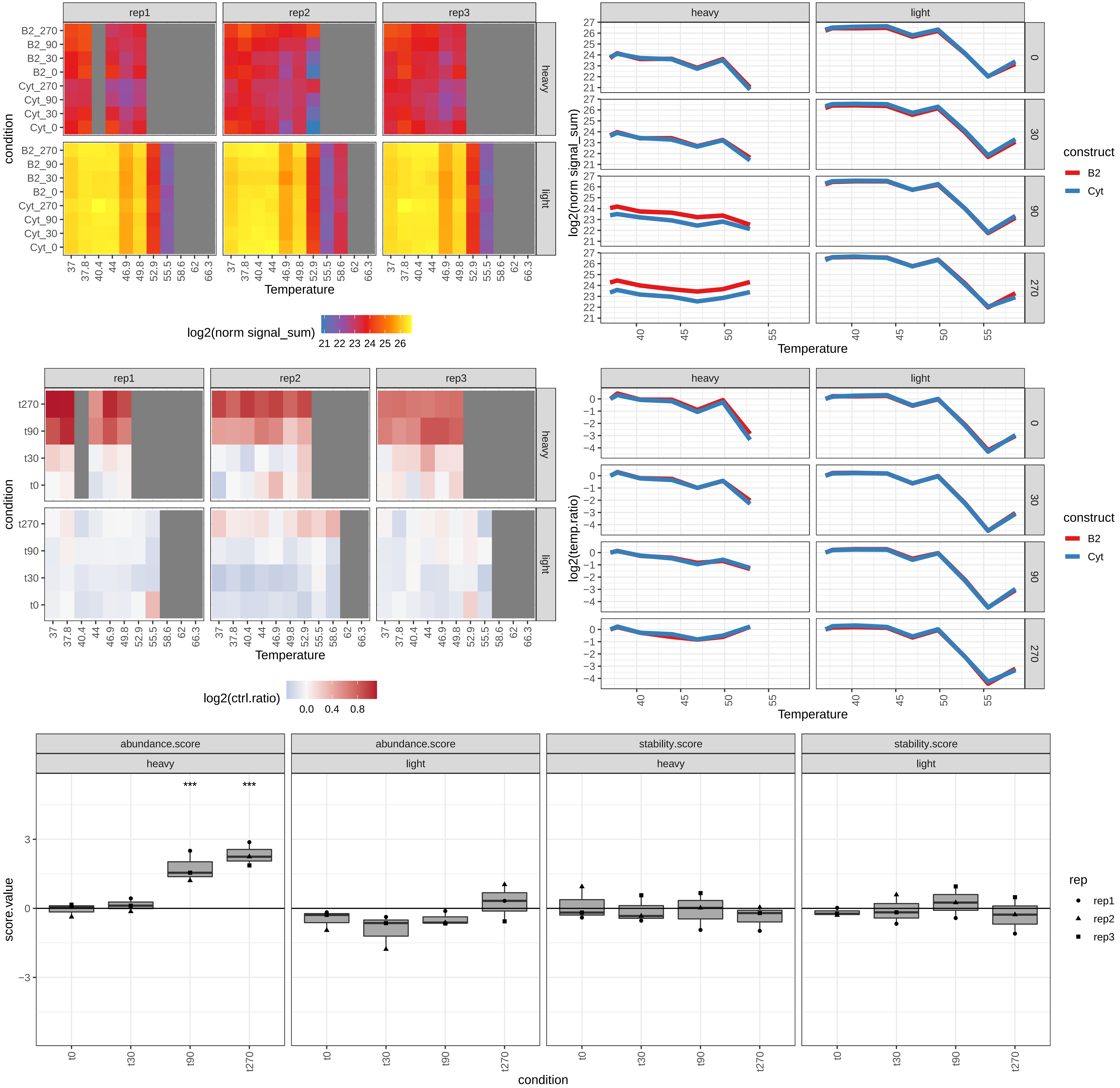

P36775 − Lon protease homolog, mitochondrial OS=Saccharomyces cerevisiae (strain ATCC 204508 / S288c) GN=PIM1 PE=1 SV=2

PIP2 − average.top3: 6.25445642857143 − max.qupm: 3

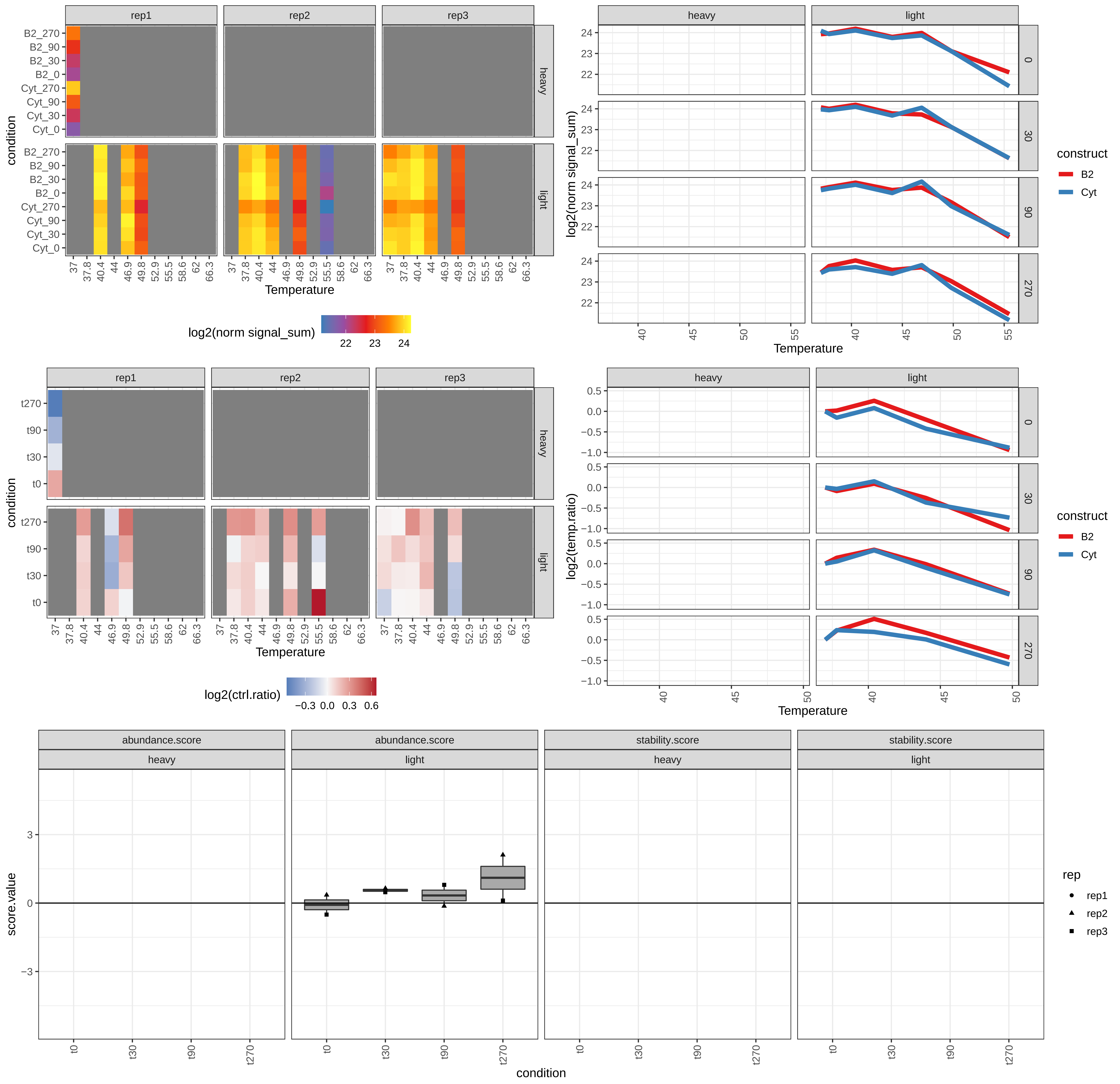

P52960 − Peroxisome proliferation transcriptional regulator OS=Saccharomyces cerevisiae (strain ATCC 204508 / S288c) GN=PIP2 PE=1 SV=1

PIS1 − average.top3: 7.06947290322581 − max.qupm: 2

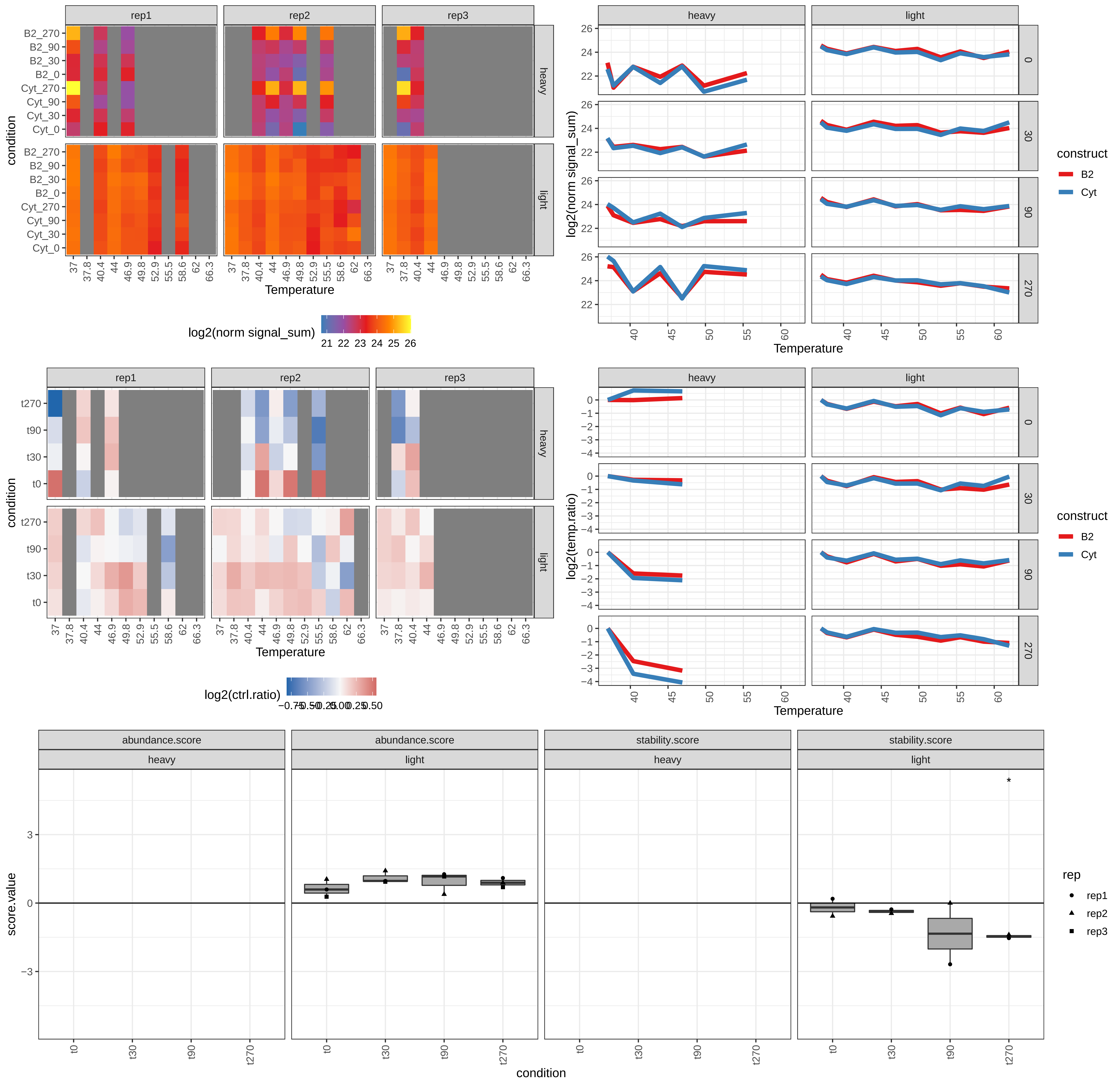

P06197 − CDP−diacylglycerol−−inositol 3−phosphatidyltransferase OS=Saccharomyces cerevisiae (strain ATCC 204508 / S288c) GN=PIS1 PE=1 SV=1

PKC1 − average.top3: 6.71173727272727 − max.qupm: 8

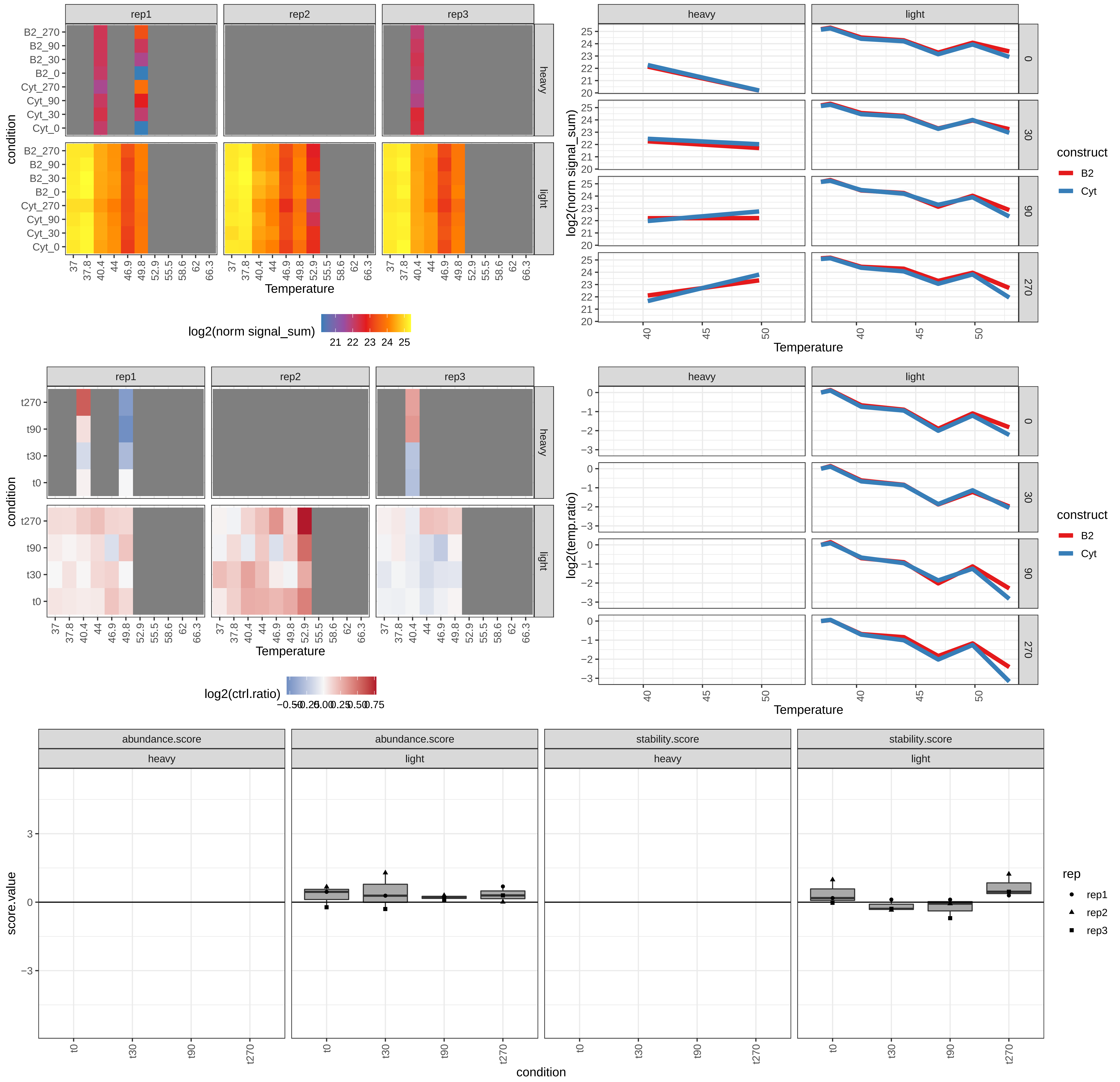

P24583 − Protein kinase C−like 1 OS=Saccharomyces cerevisiae (strain ATCC 204508 / S288c) GN=PKC1 PE=1 SV=3

PKH1 − average.top3: 6.708645 − max.qupm: 6

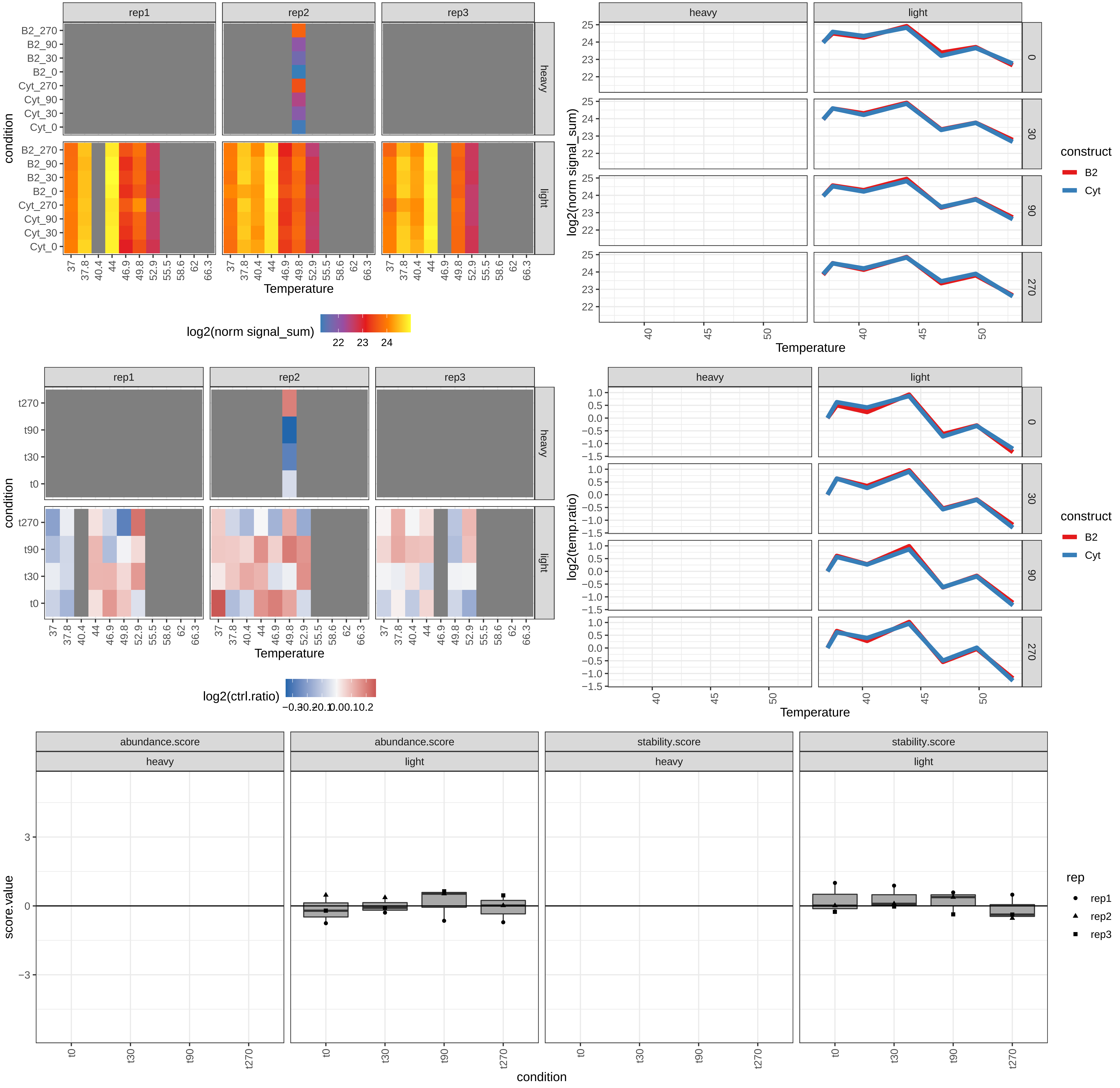

Q03407 − Serine/threonine−protein kinase PKH1 OS=Saccharomyces cerevisiae (strain ATCC 204508 / S288c) GN=PKH1 PE=1 SV=1

PKP1 − average.top3: 6.815982 − max.qupm: 4

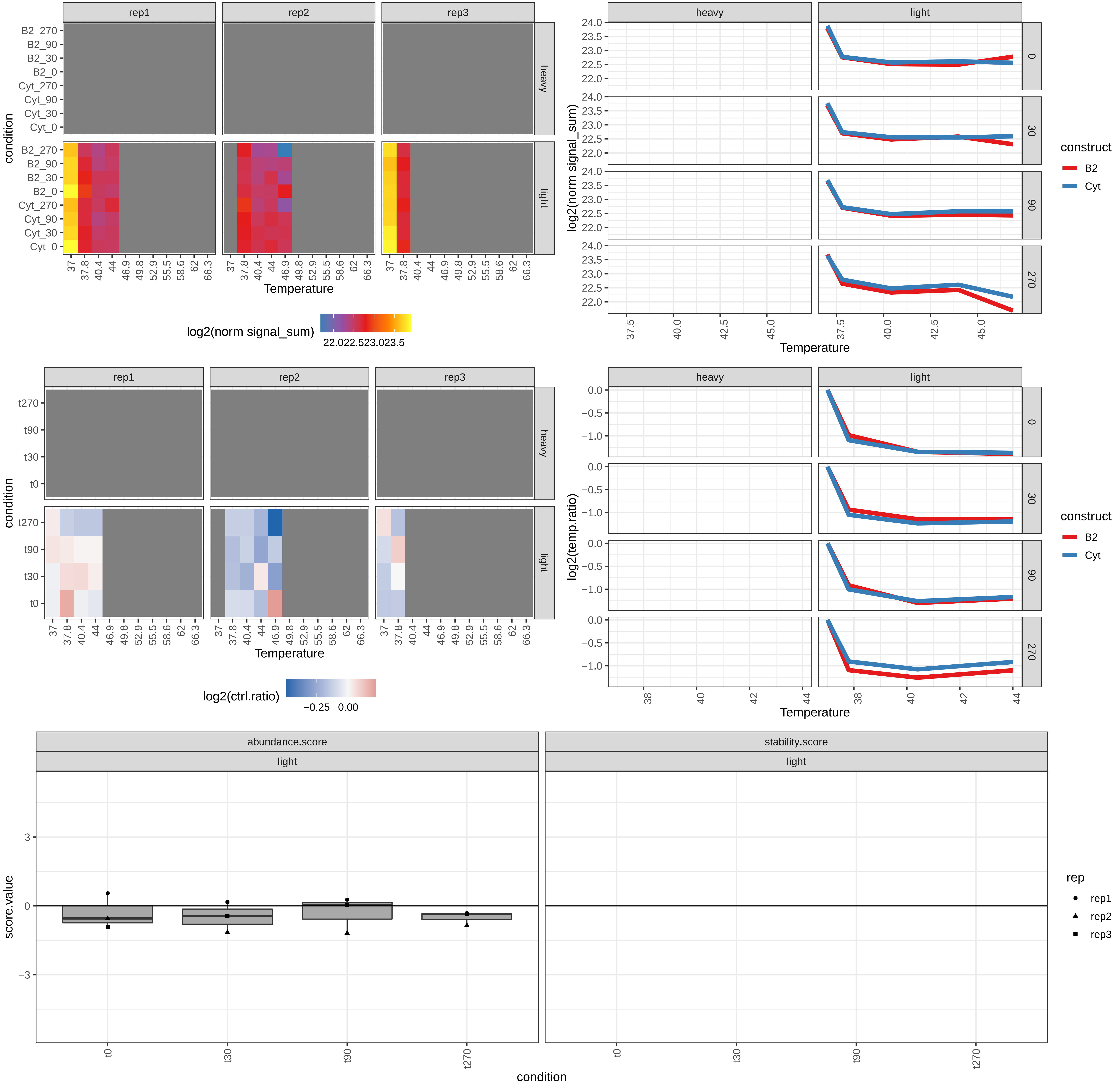

P40530 − [Pyruvate dehydrogenase (acetyl−transferring)] kinase 1, mitochondrial OS=Saccharomyces cerevisiae (strain ATCC 204508 / S288c) GN=PKP1 PE=1 SV=1

PKP2 − average.top3: 6.29242611111111 − max.qupm: 6

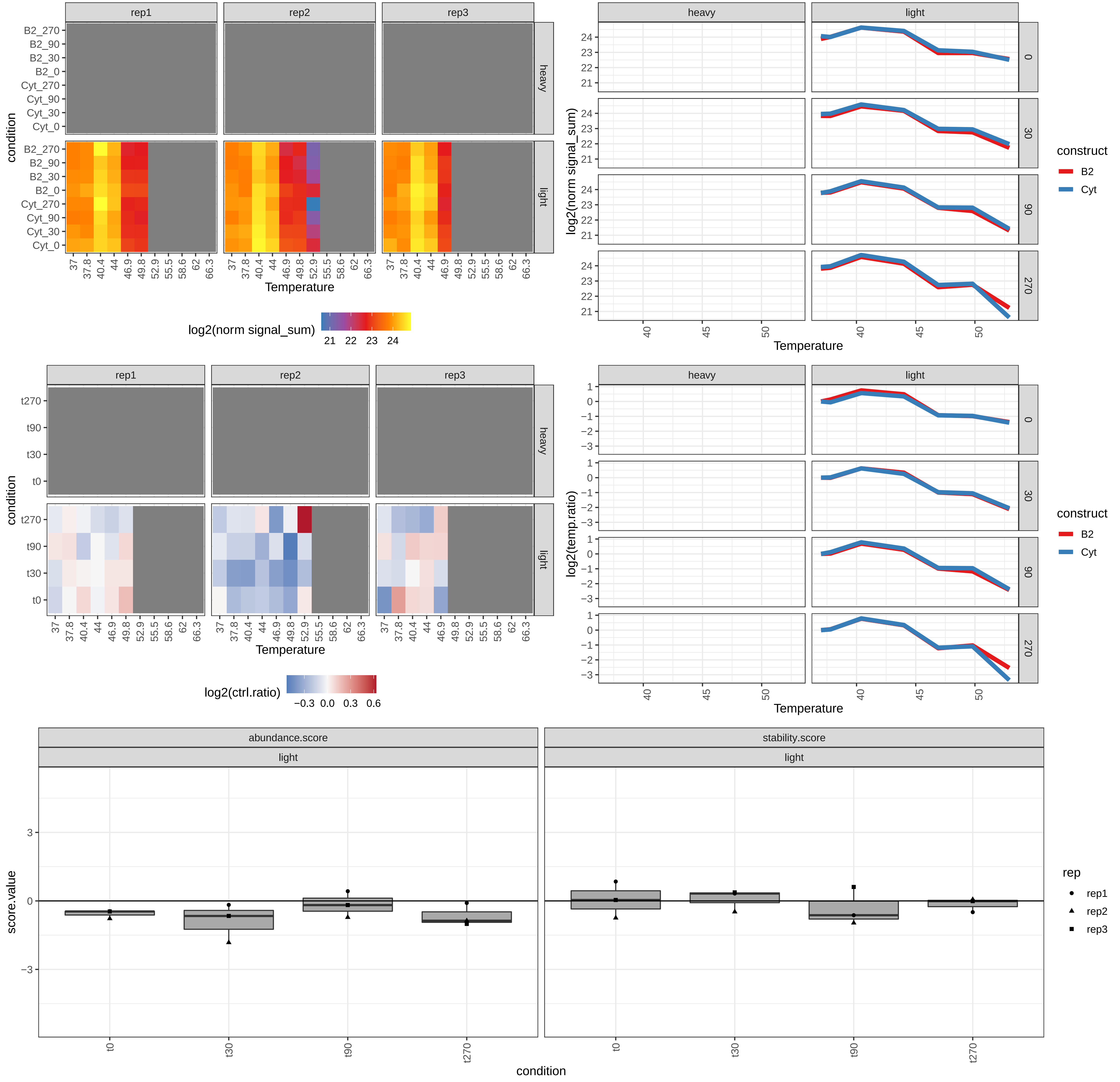

P53170 − [Pyruvate dehydrogenase (acetyl−transferring)] kinase 2, mitochondrial OS=Saccharomyces cerevisiae (strain ATCC 204508 / S288c) GN=PKP2 PE=1 SV=3

PKR1 − average.top3: 7.09790333333333 − max.qupm: 7

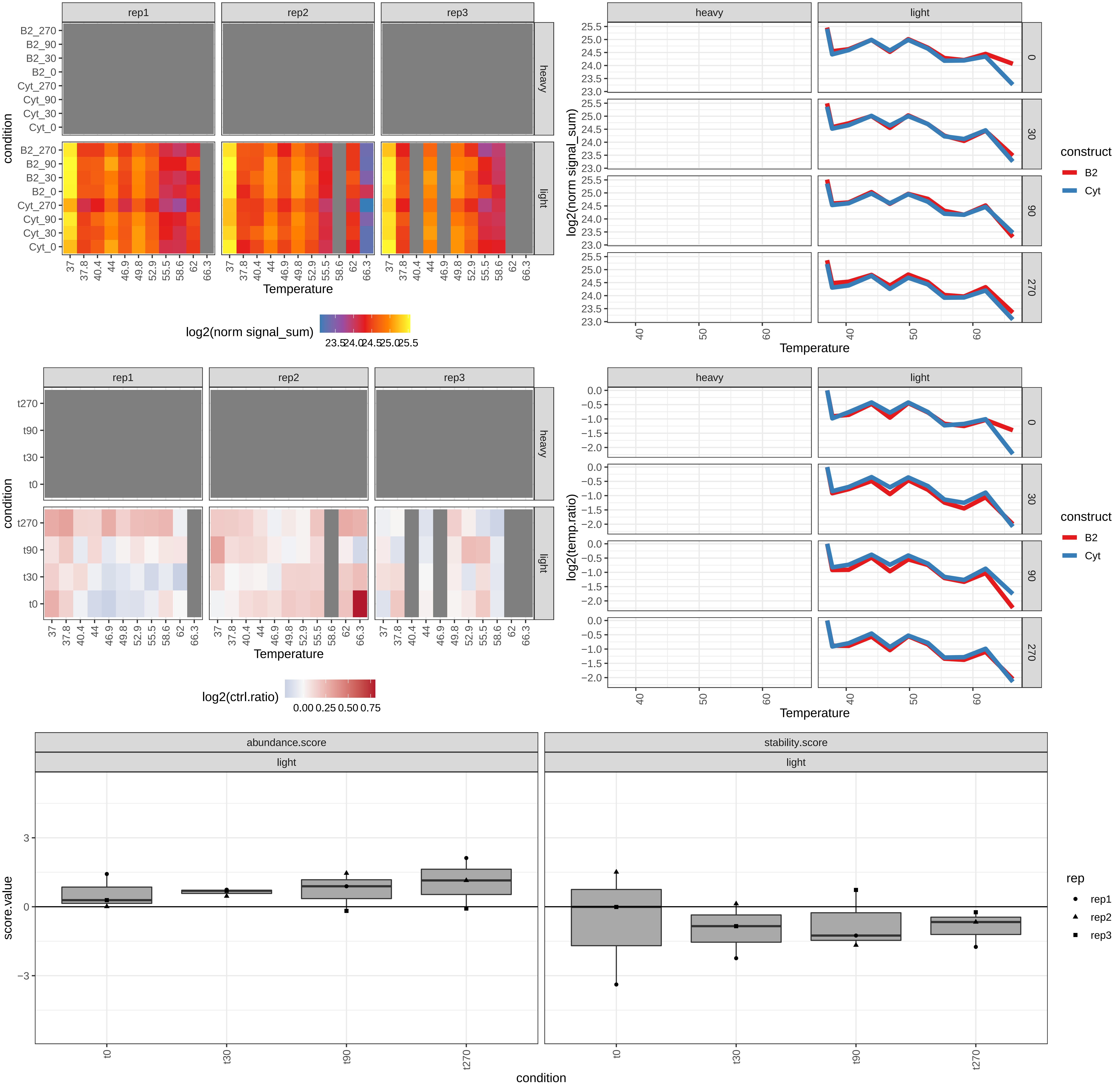

Q03880 − V−type ATPase assembly factor PKR1 OS=Saccharomyces cerevisiae (strain ATCC 204508 / S288c) GN=PKR1 PE=1 SV=1

PLB1 − average.top3: 7.11784621621622 − max.qupm: 9

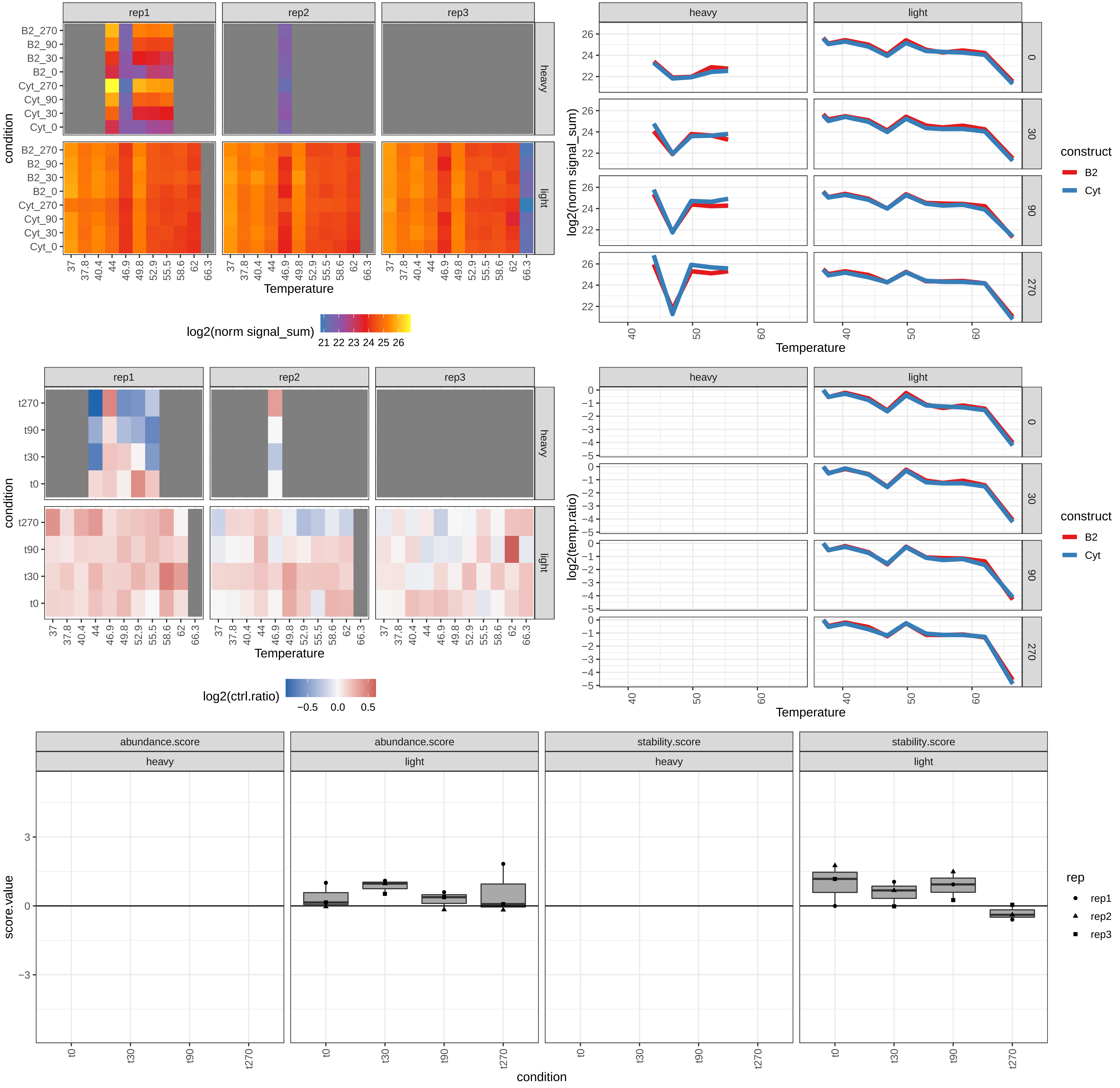

P39105 − Lysophospholipase 1 OS=Saccharomyces cerevisiae (strain ATCC 204508 / S288c) GN=PLB1 PE=1 SV=2

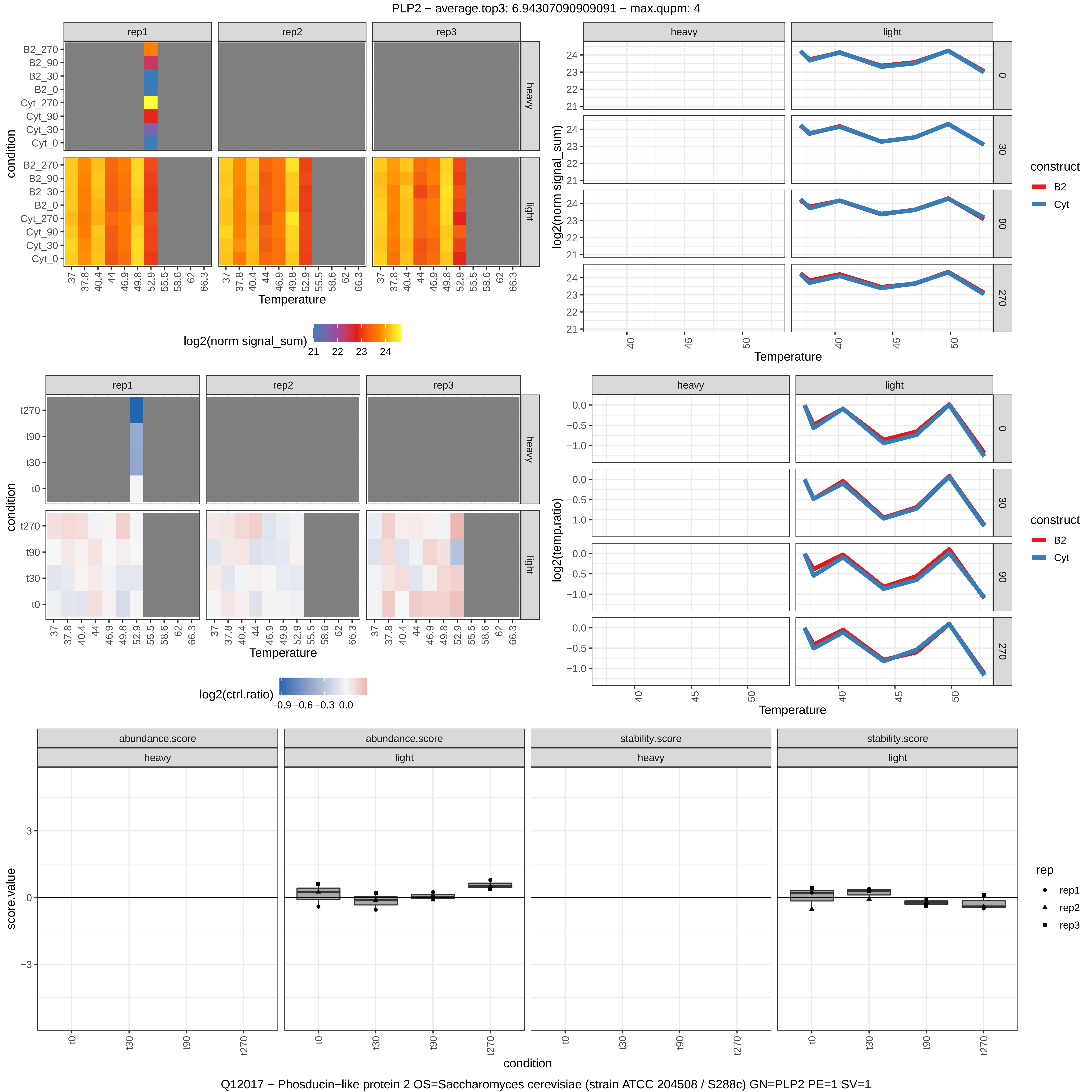
PLP2 − average.top3: 6.94307090909091 − max.qupm: 4
rep1
rep2
rep3
heavy
light
condition
Temperature
log2(norm signal_sum)
construct
B2
Cyt
log2(temp.ratio)
log2(ctrl.ratio)
abundance.score
stability.score
score.value
rep
rep1
rep2
rep3
Q12017 − Phosducin−like protein 2 OS=Saccharomyces cerevisiae (strain ATCC 204508 / S288c) GN=PLP2 PE=1 SV=1

PMA1 − average.top3: 7.89342403225806 − max.qupm: 25

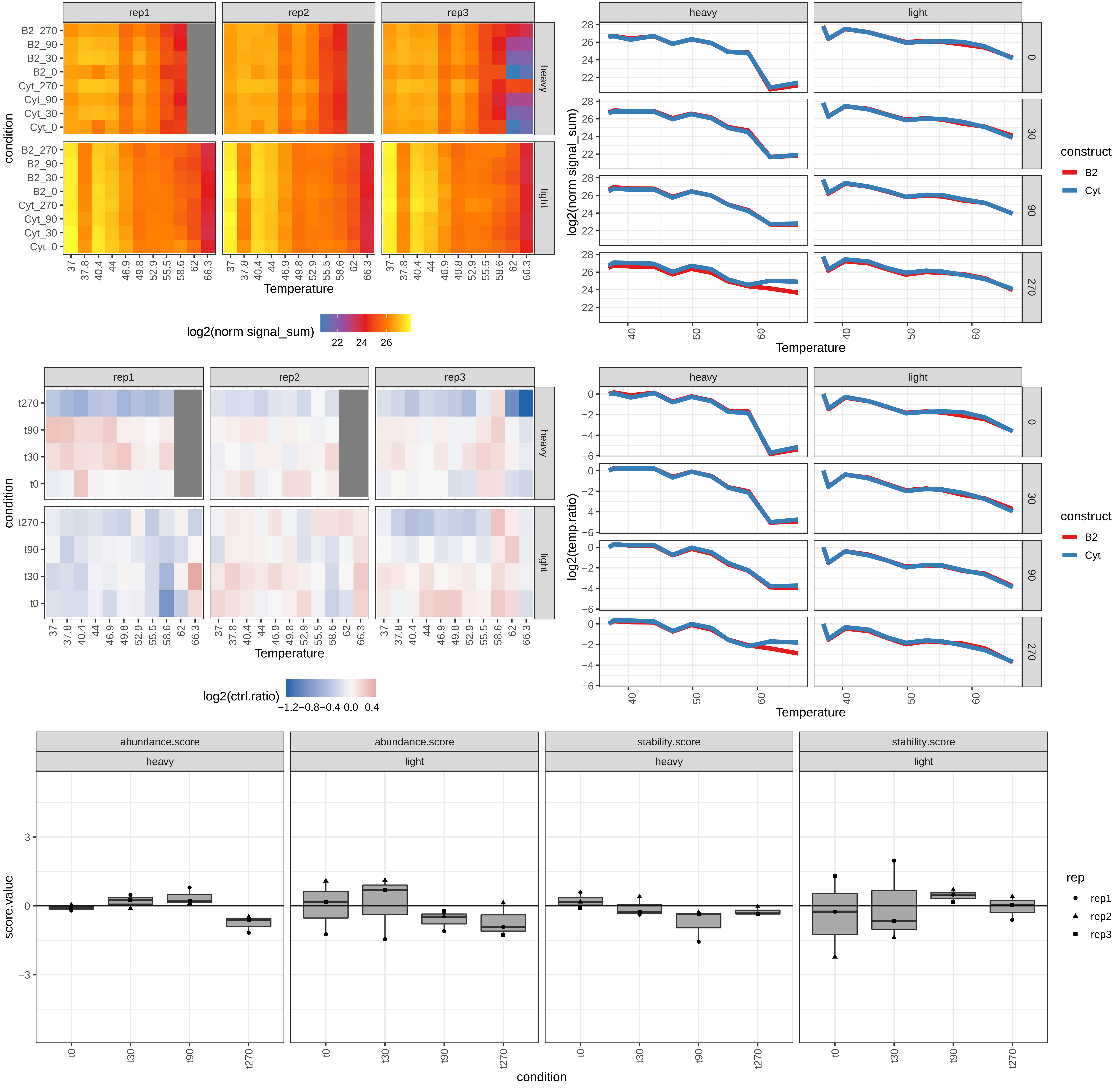

P05030 − Plasma membrane ATPase 1 OS=Saccharomyces cerevisiae (strain ATCC 204508 / S288c) GN=PMA1 PE=1 SV=2

PMC1 − average.top3: 7.06173451612903 − max.qupm: 21

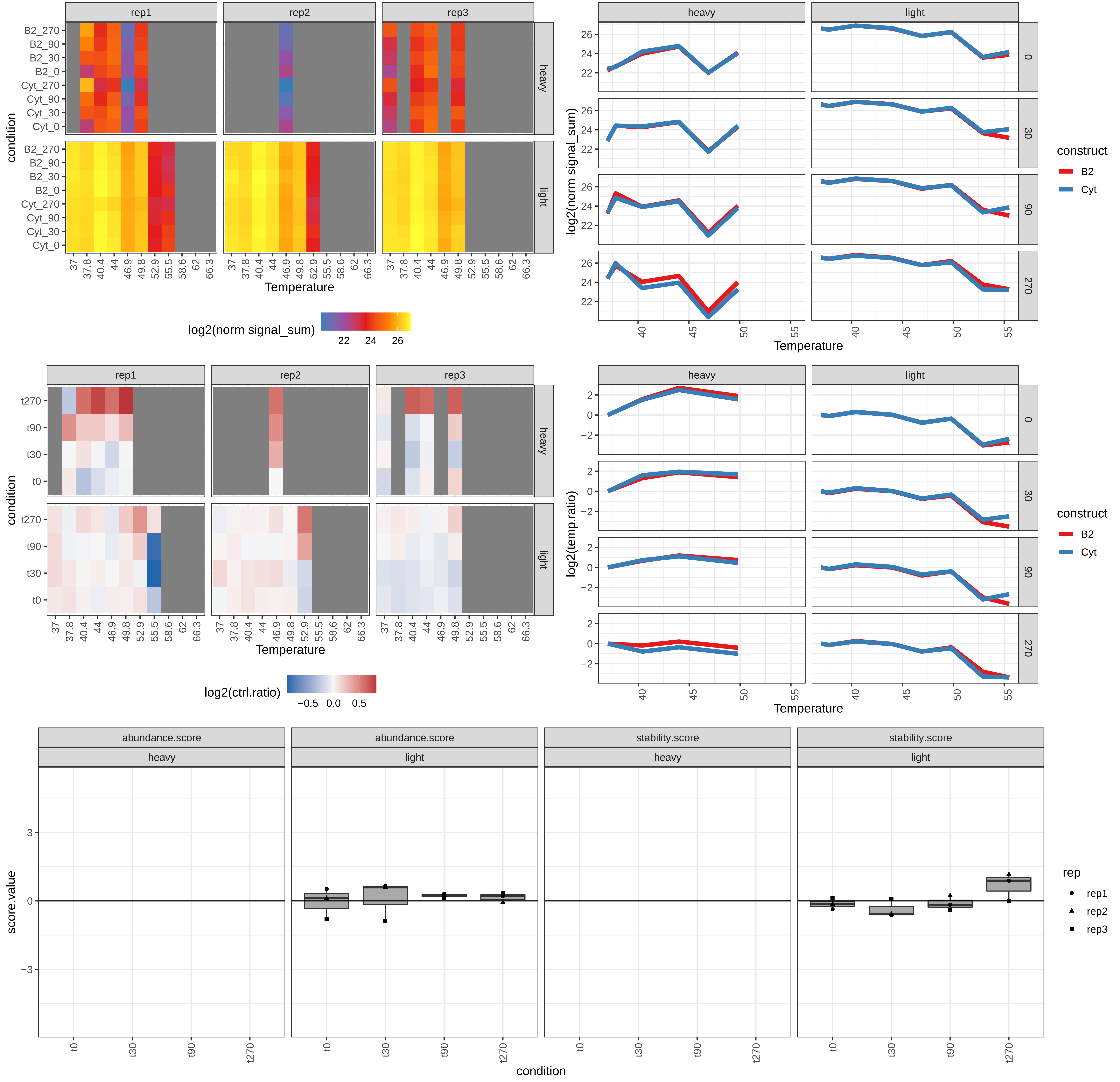

P38929 − Calcium−transporting ATPase 2 OS=Saccharomyces cerevisiae (strain ATCC 204508 / S288c) GN=PMC1 PE=1 SV=1

PMI40 − average.top3: 7.2587547826087 − max.qupm: 16

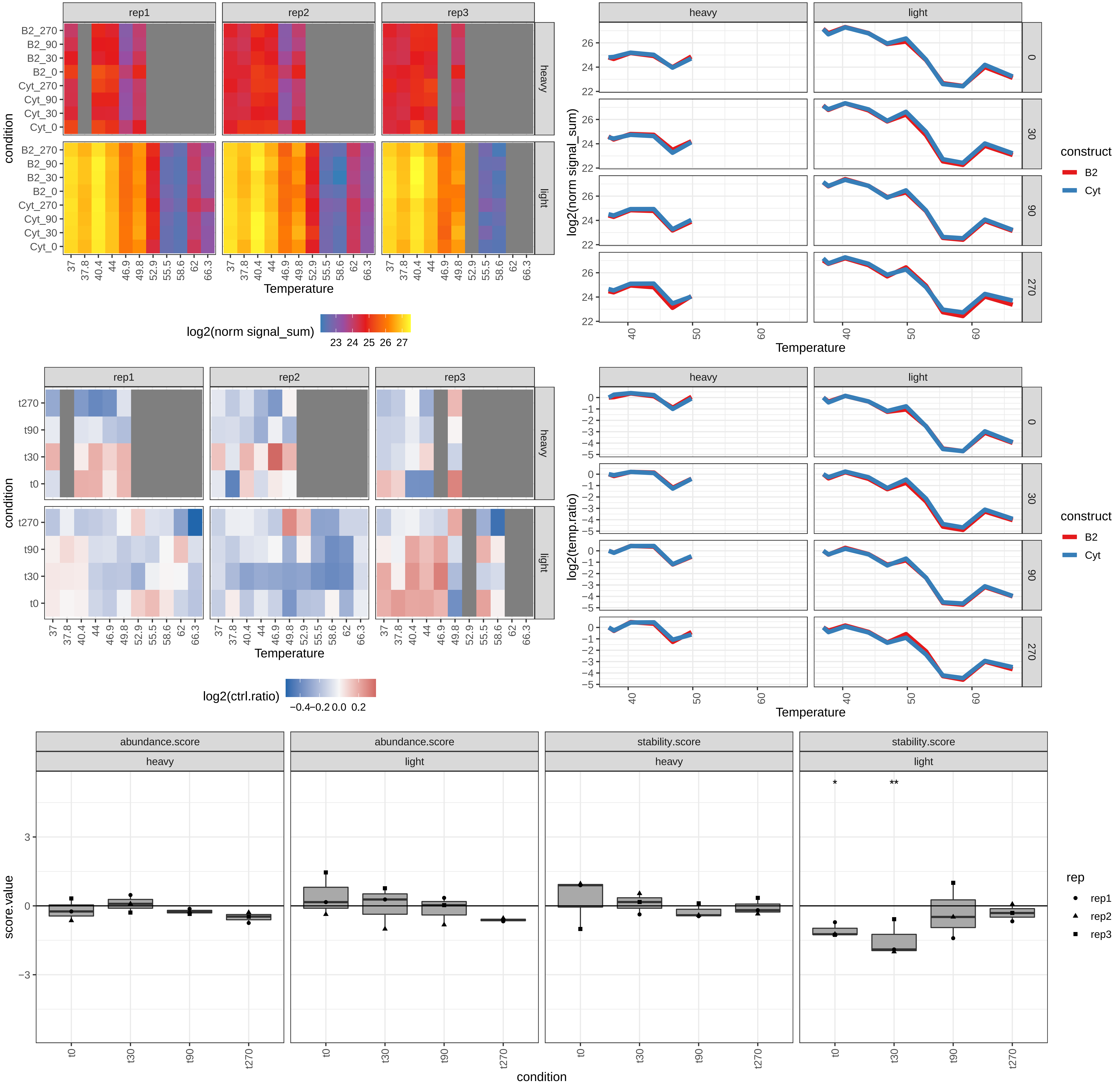

P29952 − Mannose−6−phosphate isomerase OS=Saccharomyces cerevisiae (strain ATCC 204508 / S288c) GN=PMI40 PE=1 SV=4

PMR1 − average.top3: 6.65387074074074 − max.qupm: 9

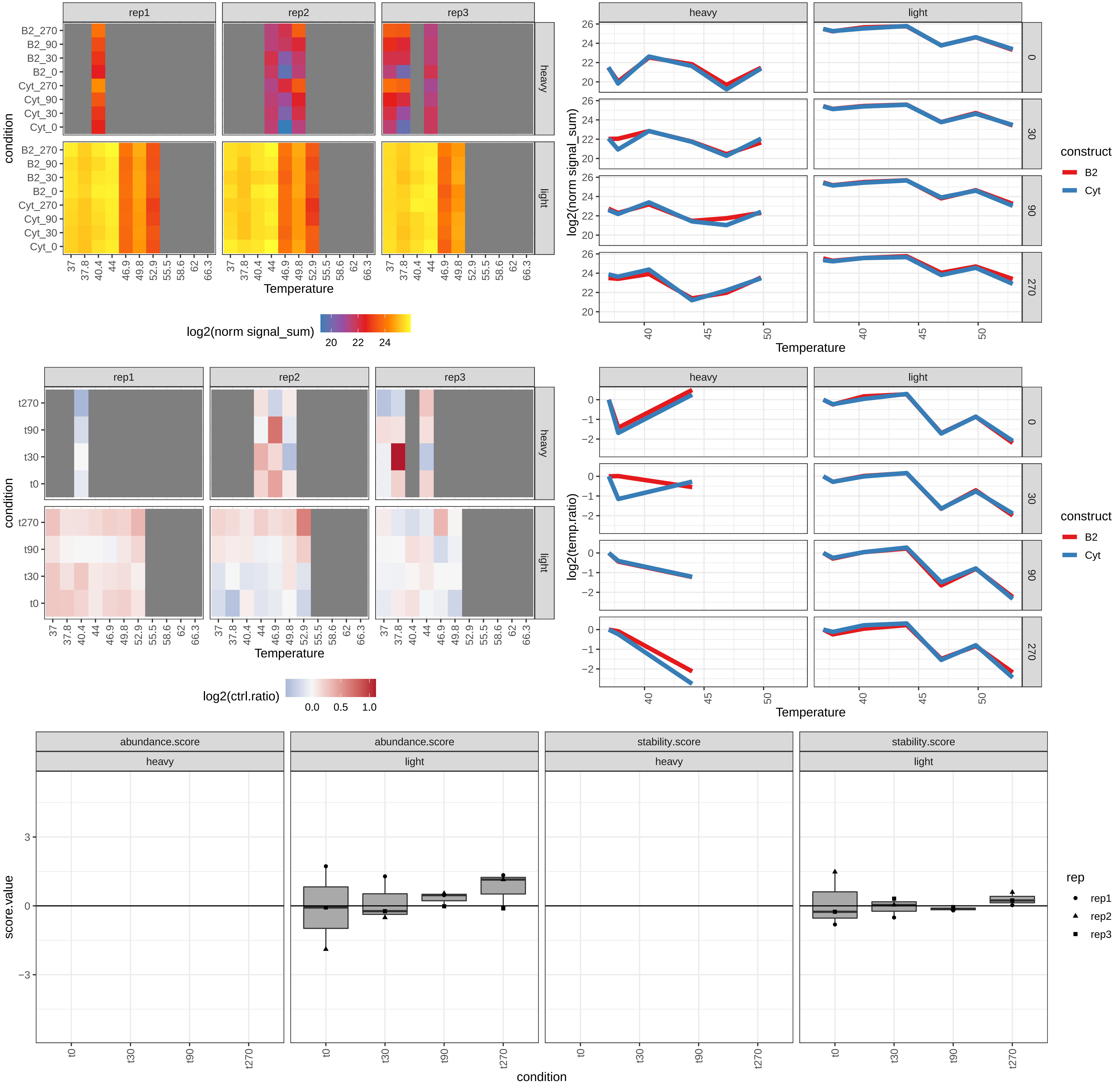

P13586 − Calcium−transporting ATPase 1 OS=Saccharomyces cerevisiae (strain ATCC 204508 / S288c) GN=PMR1 PE=1 SV=1

PMT1 − average.top3: 7.07636405405405 − max.qupm: 8

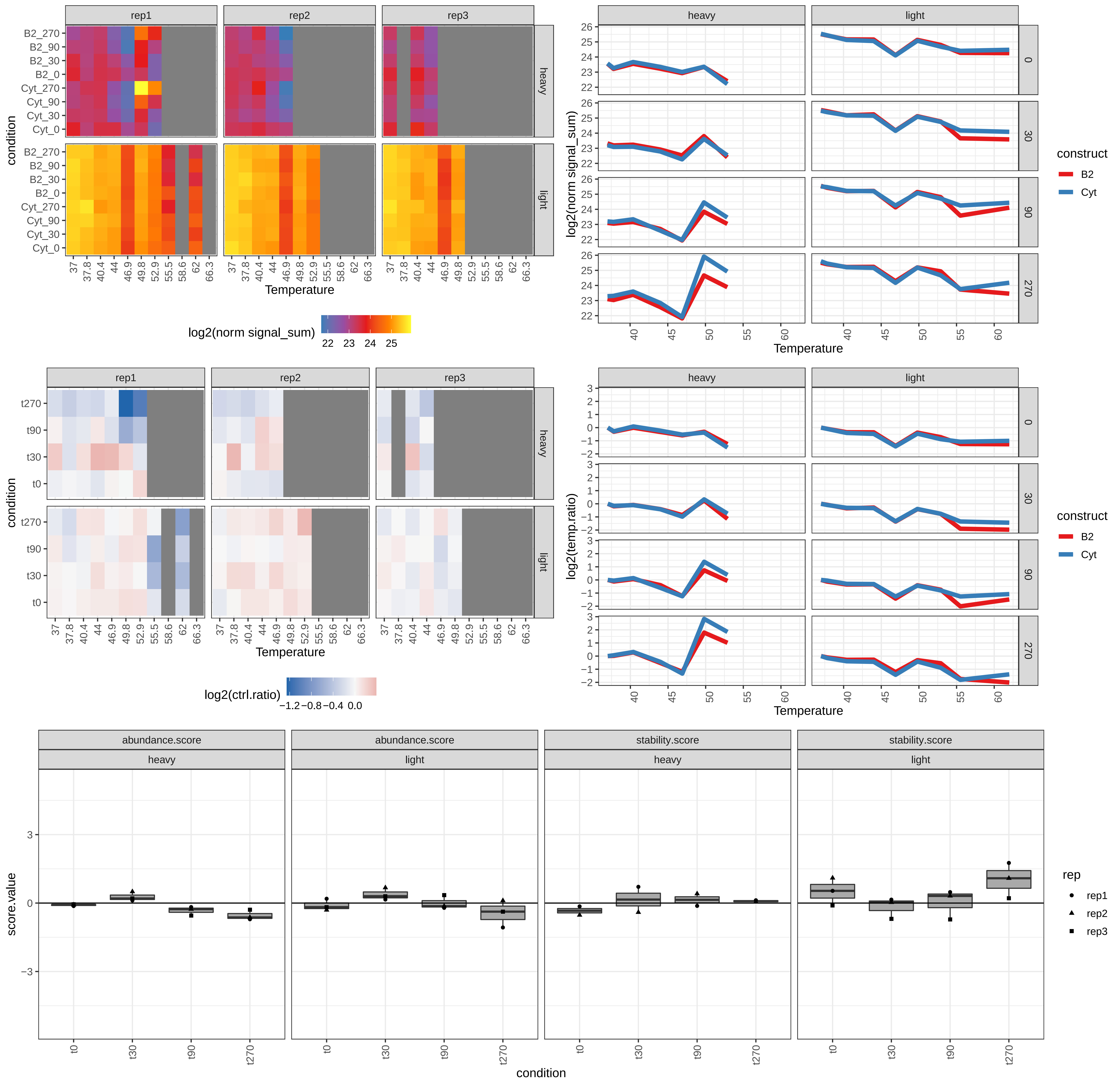

P33775 − Dolichyl−phosphate−mannose−−protein mannosyltransferase 1 OS=Saccharomyces cerevisiae (strain ATCC 204508 / S288c) GN=PMT1 PE=1 SV=1

PMT2 − average.top3: 7.13752222222222 − max.qupm: 11

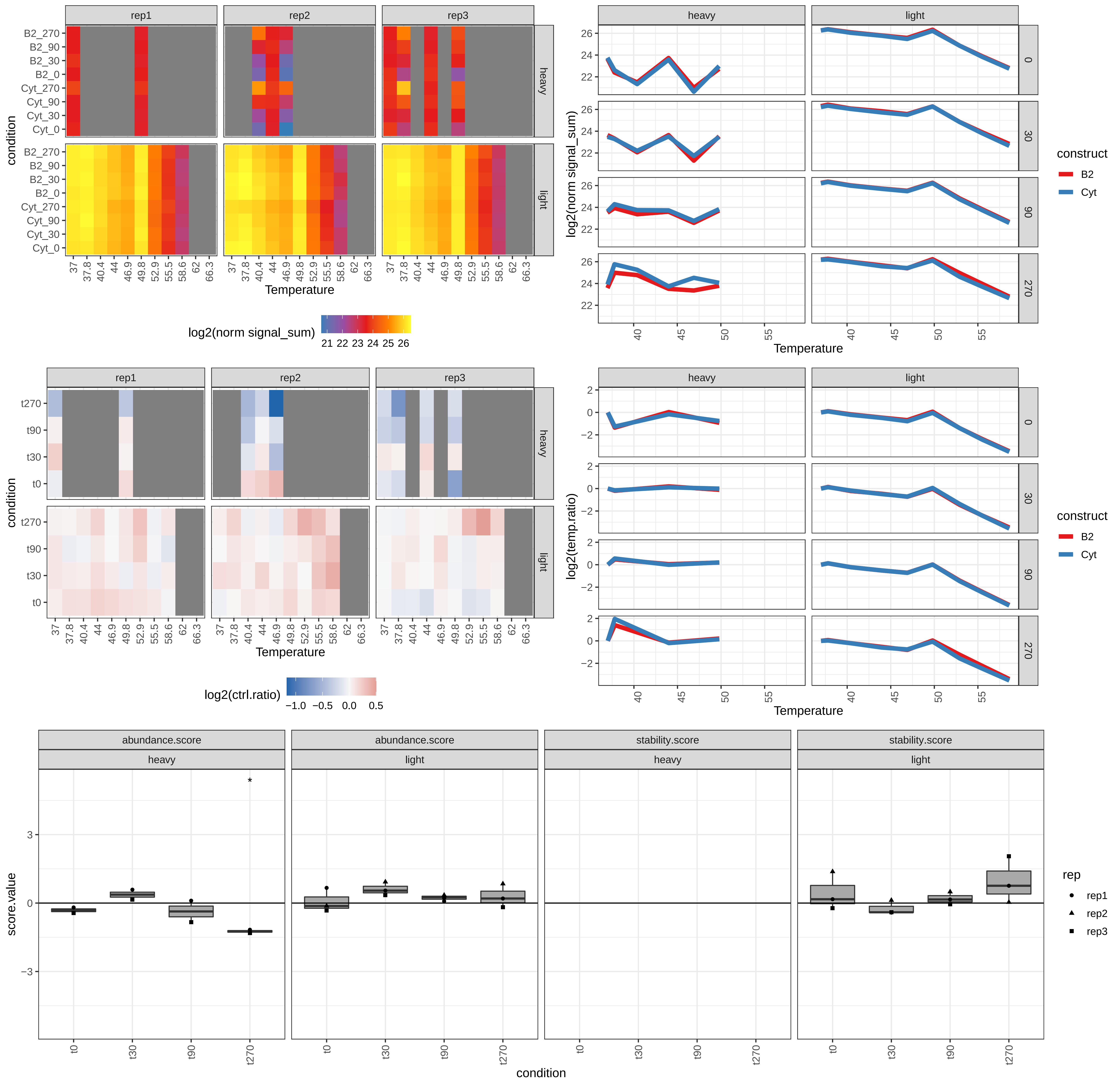

P31382 − Dolichyl−phosphate−mannose−−protein mannosyltransferase 2 OS=Saccharomyces cerevisiae (strain ATCC 204508 / S288c) GN=PMT2 PE=1 SV=2

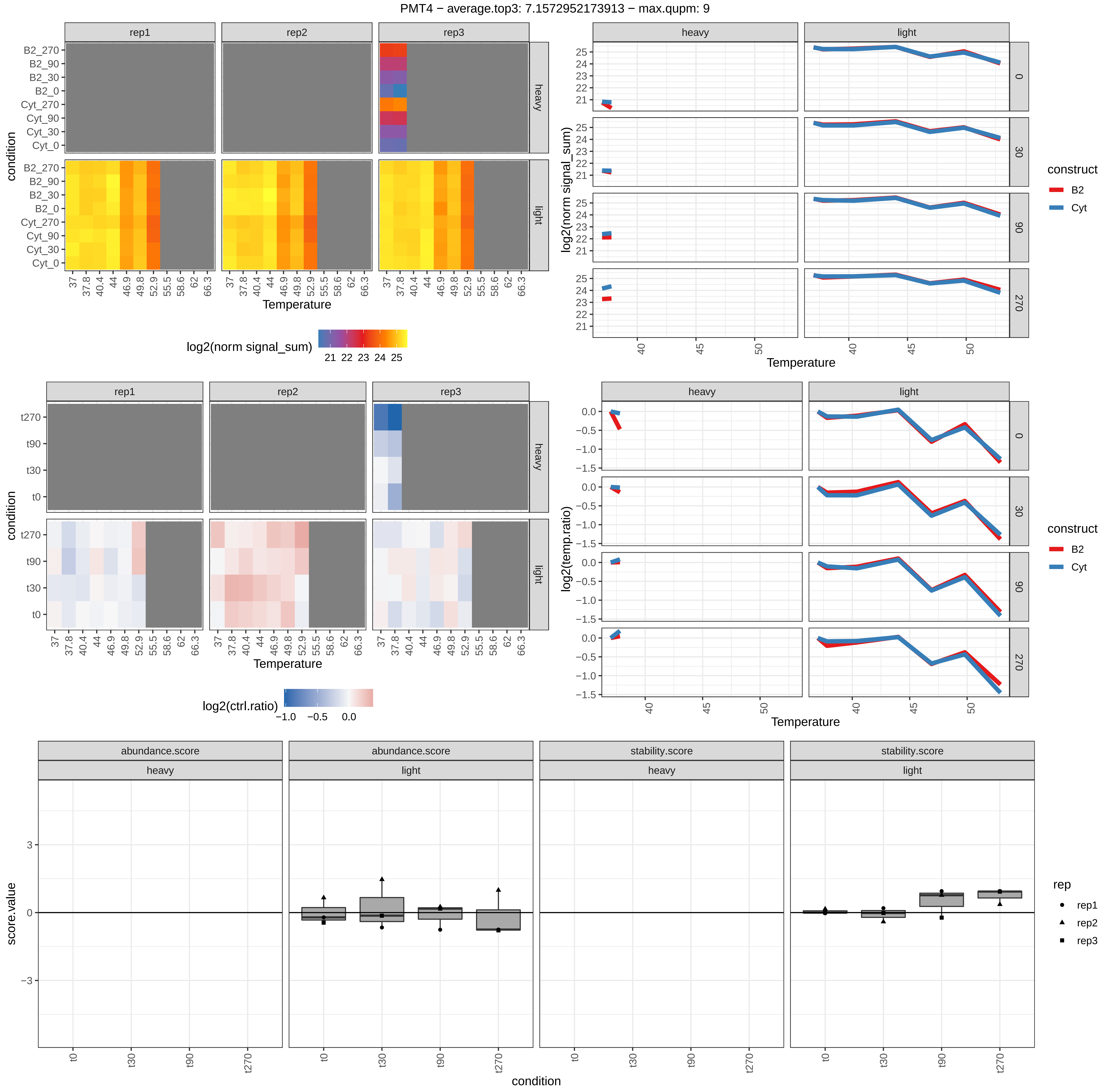

P46971 − Dolichyl−phosphate−mannose−−protein mannosyltransferase 4 OS=Saccharomyces cerevisiae (strain ATCC 204508 / S288c) GN=PMT4 PE=1 SV=1

PMT6 − average.top3: 6.70865076923077 − max.qupm: 4

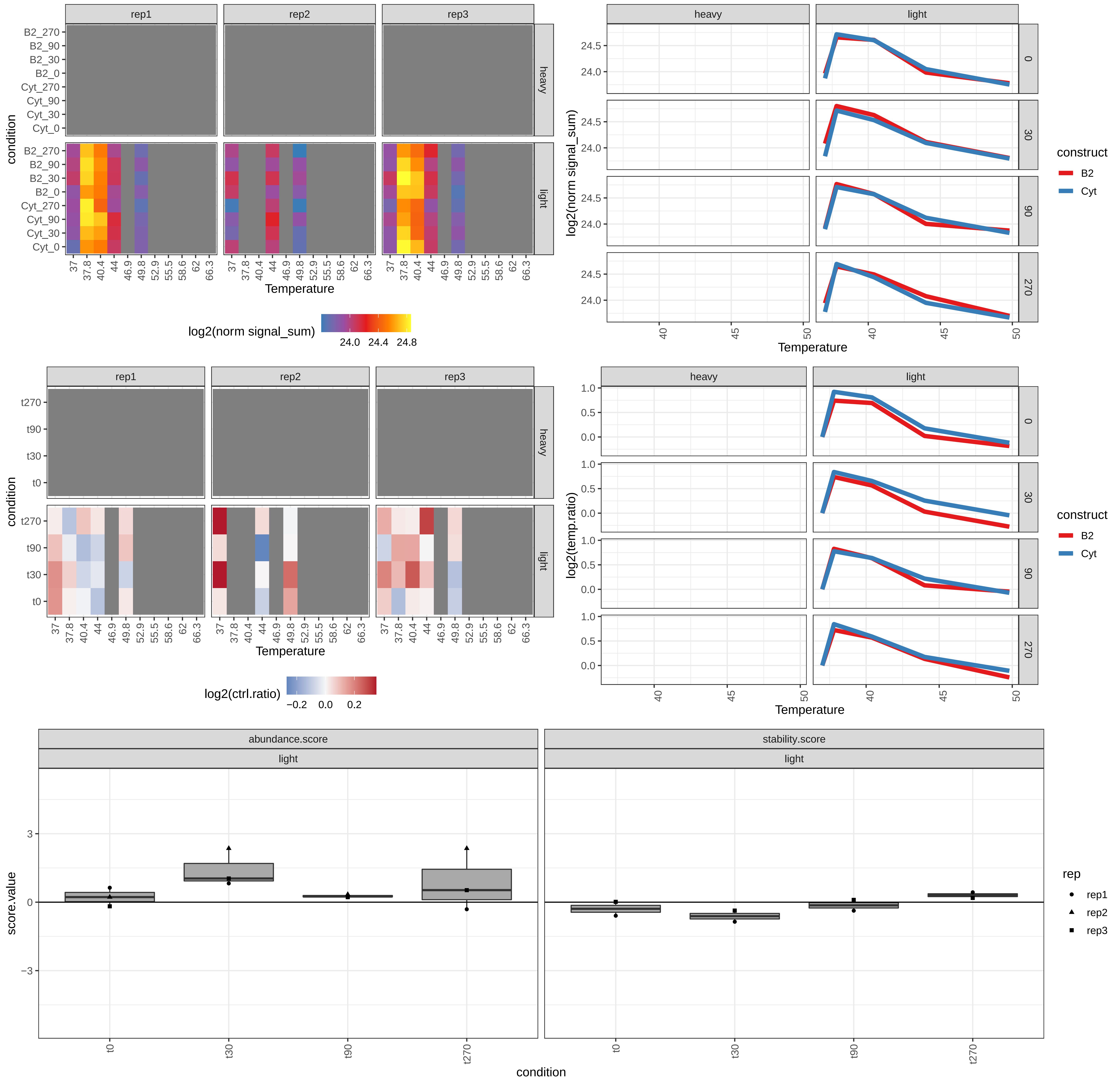

P42934 − Dolichyl−phosphate−mannose−−protein mannosyltransferase 6 OS=Saccharomyces cerevisiae (strain ATCC 204508 / S288c) GN=PMT6 PE=1 SV=1

PMT7 − average.top3: 6.75423411764706 − max.qupm: 7

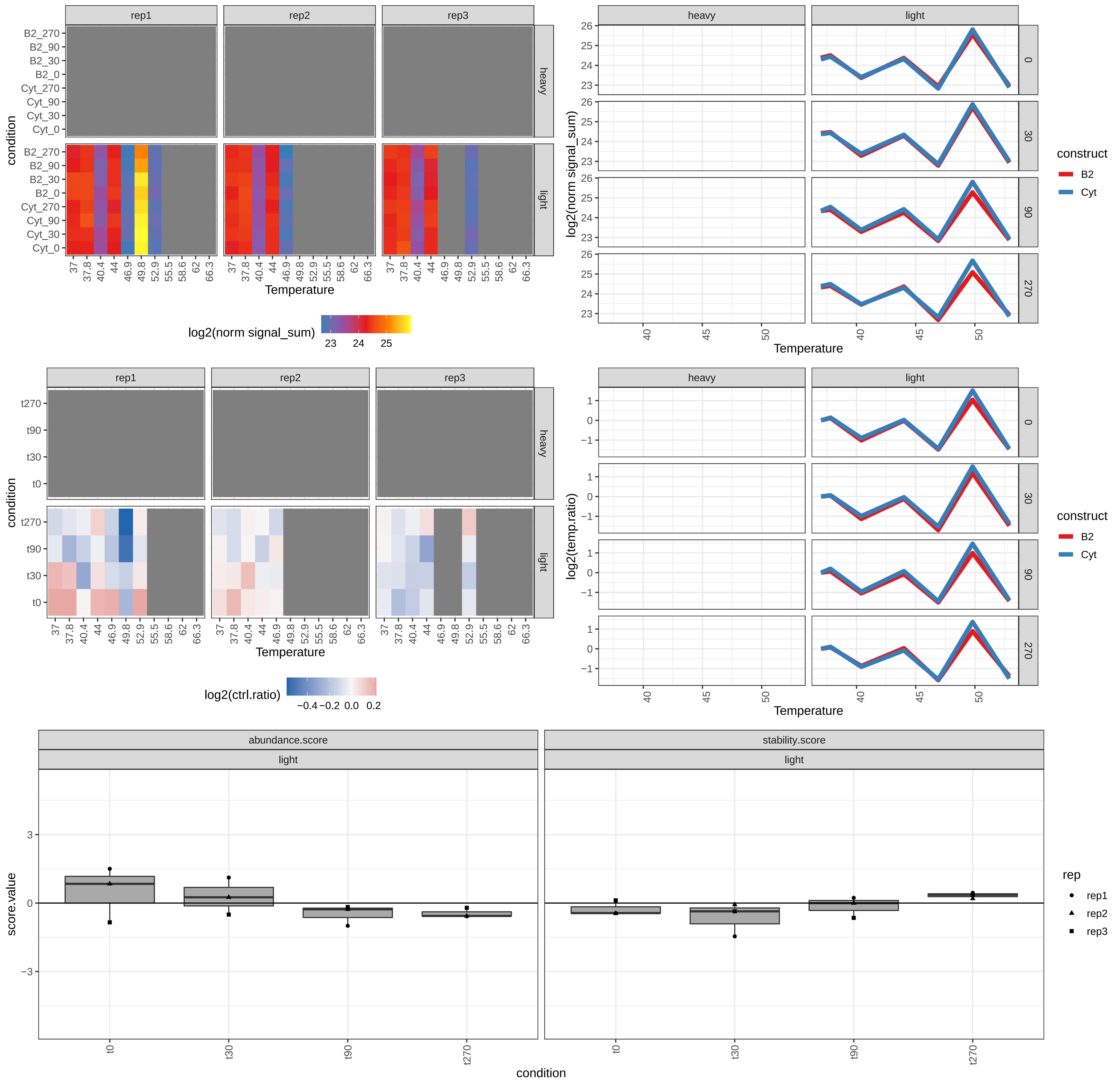

Q06644 − Probable dolichyl−phosphate−mannose−−protein mannosyltransferase 7 OS=Saccharomyces cerevisiae (strain ATCC 204508 / S288c) GN=PMT7 PE=1 SV=1

PMU1 − average.top3: 6.841735625 − max.qupm: 5

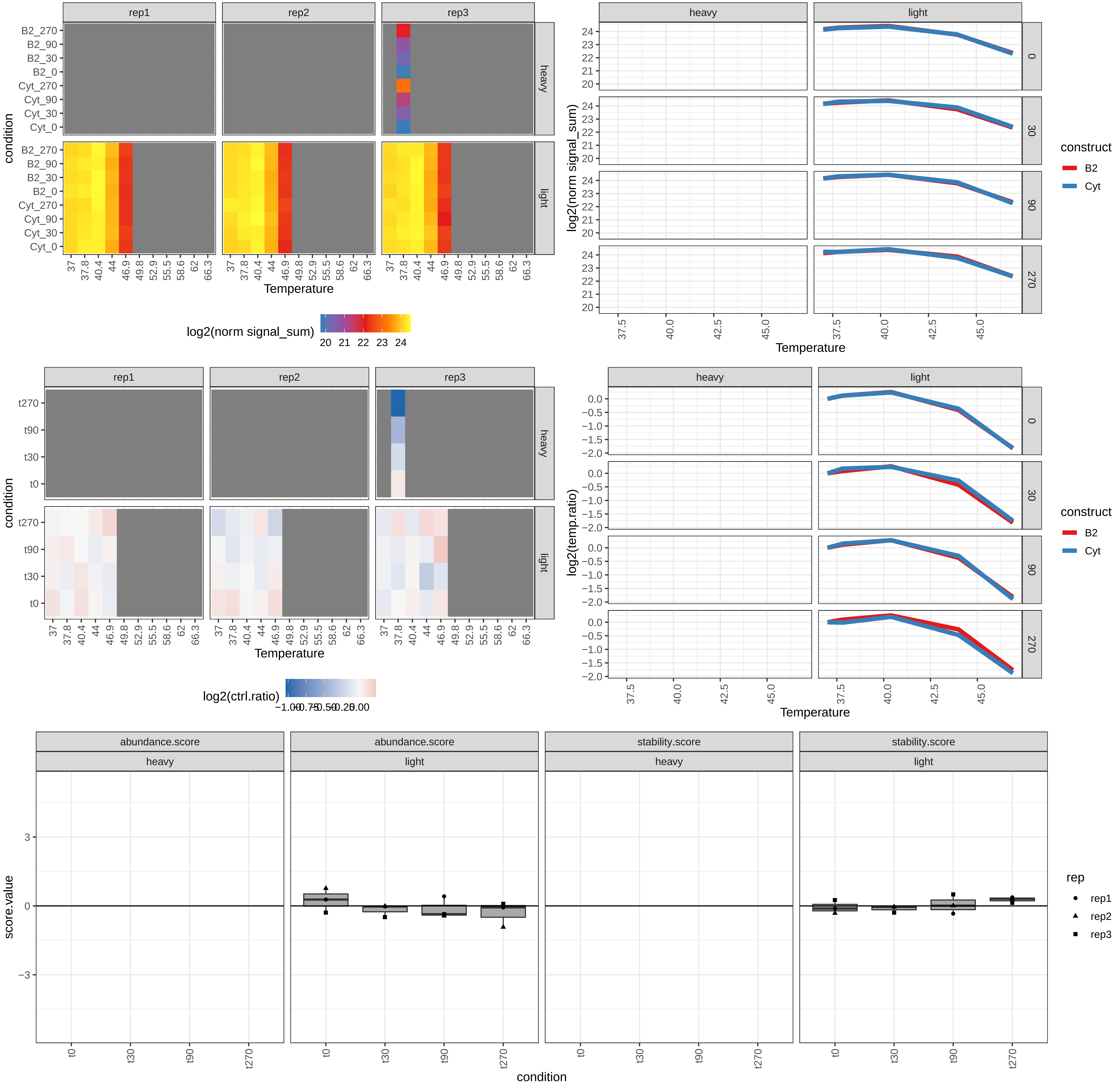

P36069 − Probable phosphoglycerate mutase PMU1 OS=Saccharomyces cerevisiae (strain ATCC 204508 / S288c) GN=PMU1 PE=1 SV=1

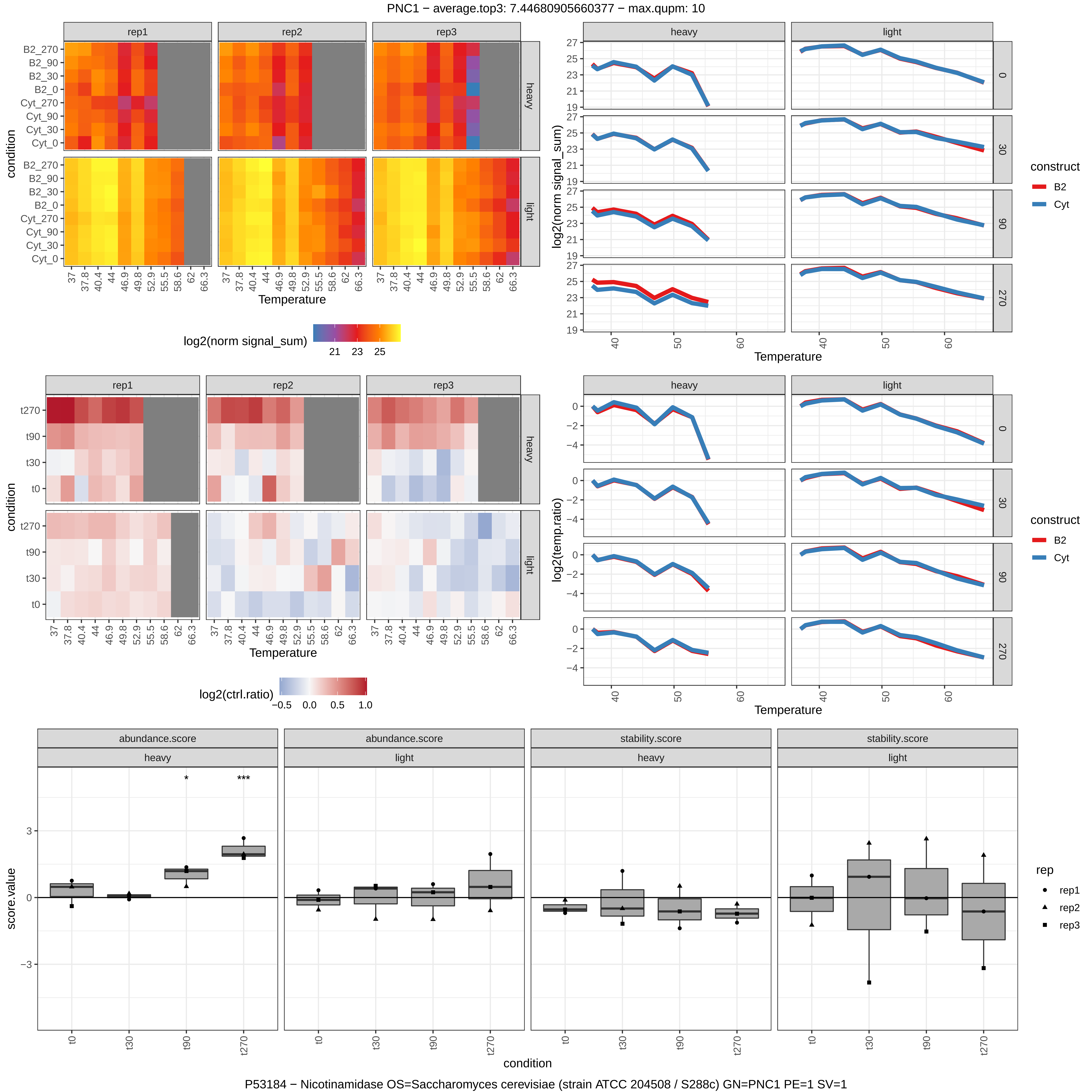

PNC1 − average.top3: 7.44680905660377 − max.qupm: 10
rep1
rep2
rep3
heavy
light
condition
Temperature
log2(norm signal_sum)
log2(norm signal_sum)
construct
B2
Cyt
log2(ctrl.ratio)
log2(temp.ratio)
abundance.score
stability.score
score.value
rep
rep1
rep2
rep3
P53184 − Nicotinamidase OS=Saccharomyces cerevisiae (strain ATCC 204508 / S288c) GN=PNC1 PE=1 SV=1

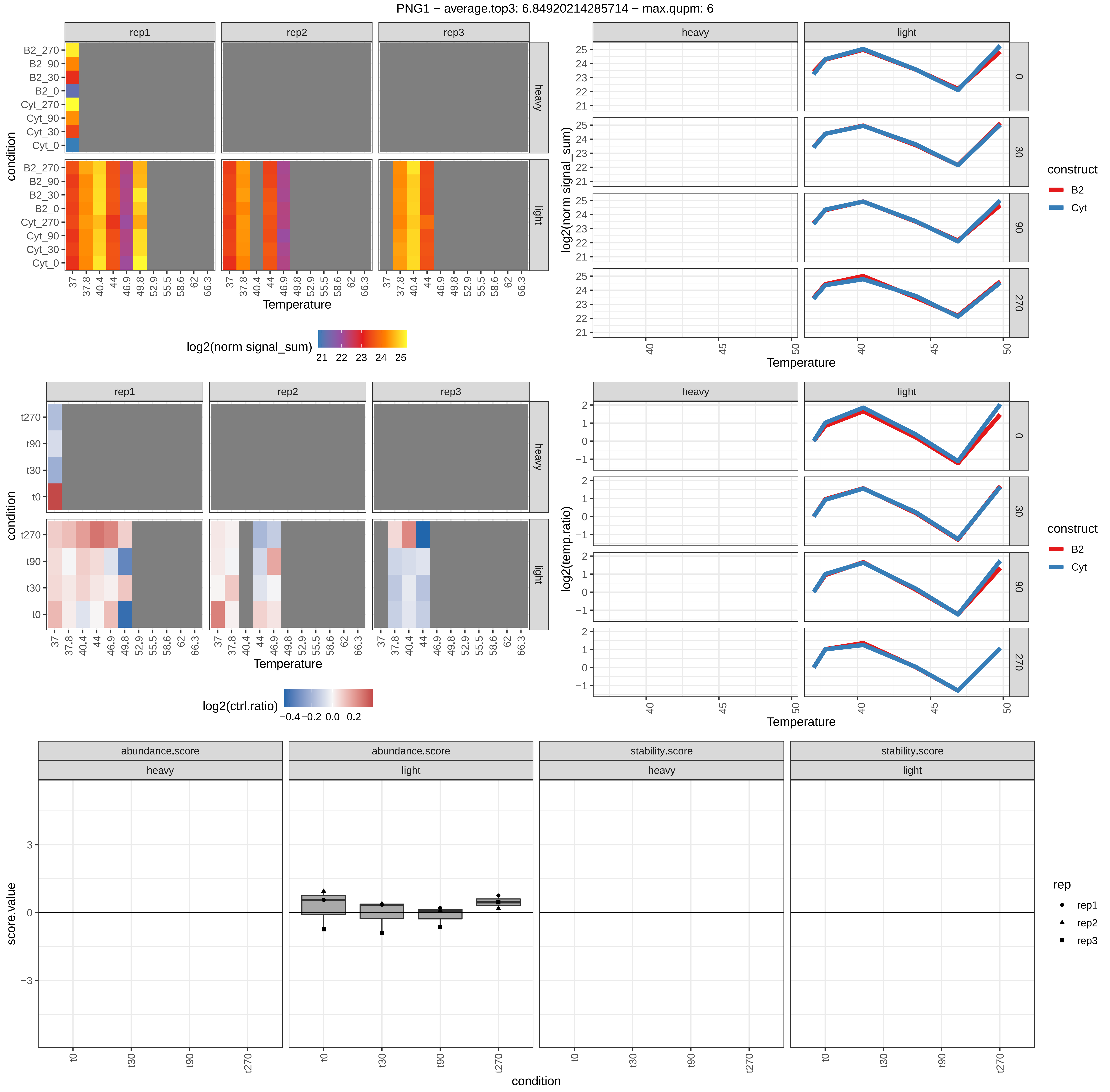

Q02890 − Peptide−N(4)−(N−acetyl−beta−glucosaminyl)asparagine amidase OS=Saccharomyces cerevisiae (strain ATCC 204508 / S288c) GN=PNG1 PE=1 SV=1

PNO1 − average.top3: 6.689407 − max.qupm: 4

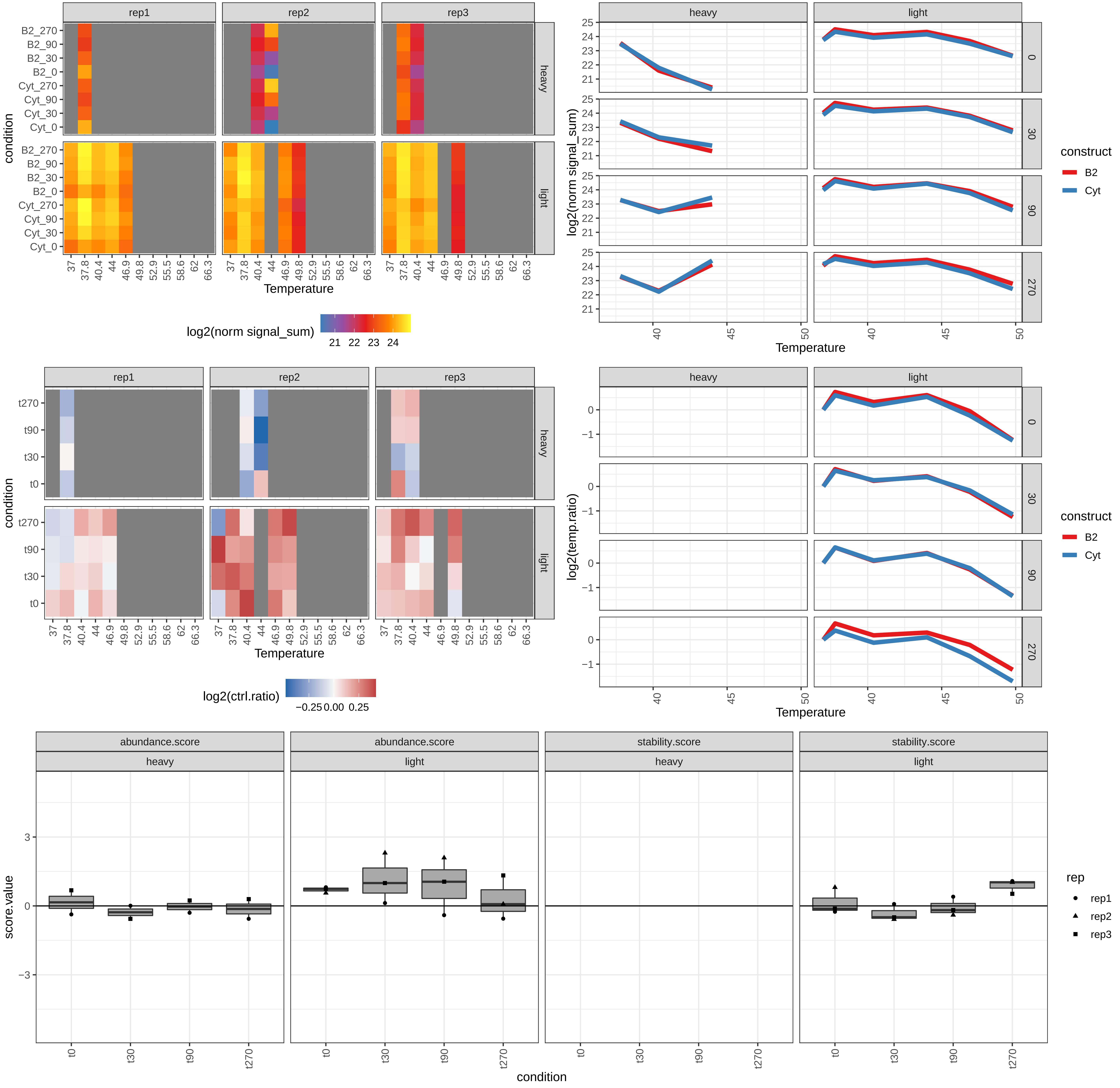

Q99216 − Pre−rRNA−processing protein PNO1 OS=Saccharomyces cerevisiae (strain ATCC 204508 / S288c) GN=PNO1 PE=1 SV=1

PNP1 − average.top3: 6.88409222222222 − max.qupm: 7

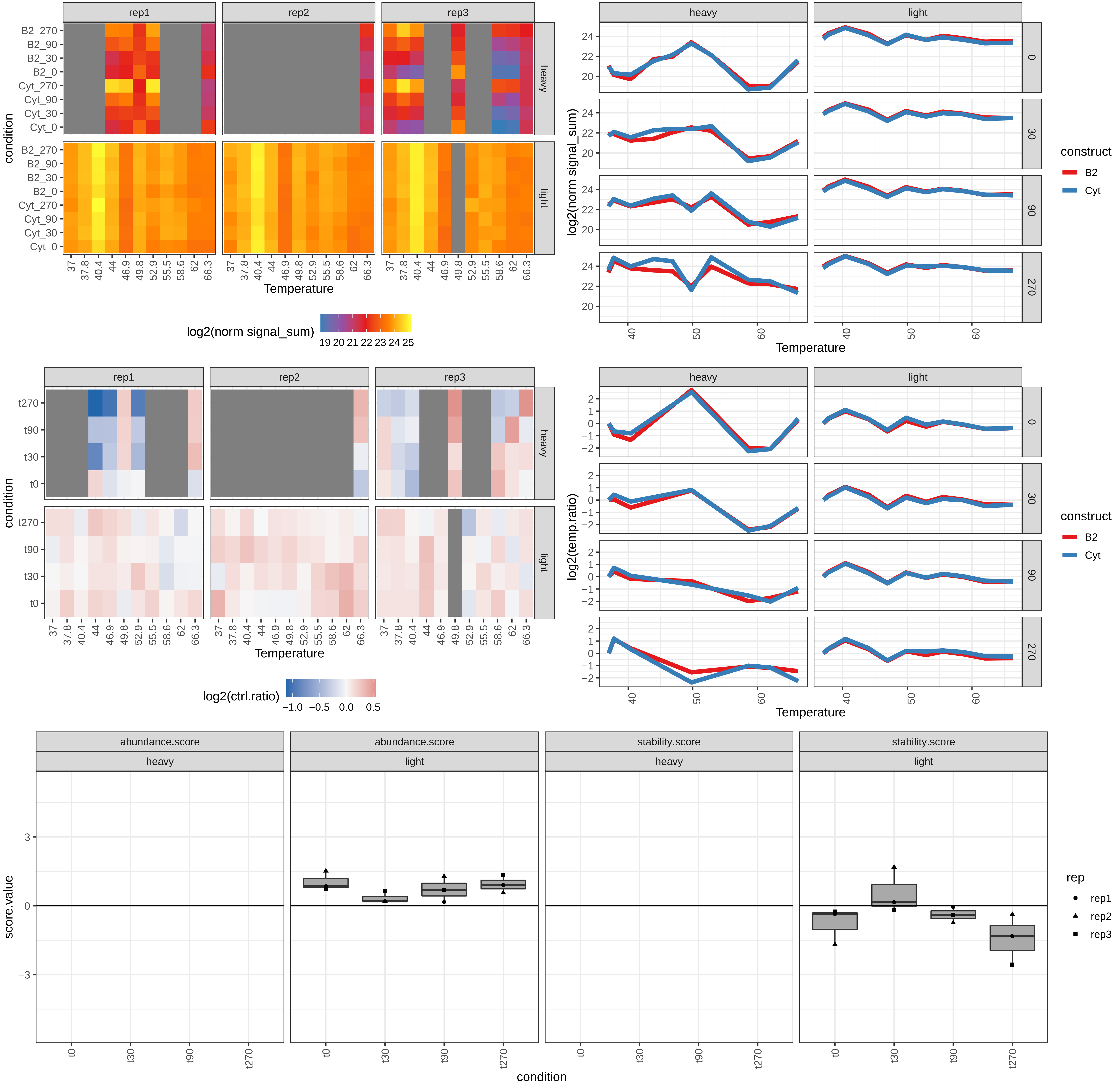

Q05788 − Purine nucleoside phosphorylase OS=Saccharomyces cerevisiae (strain ATCC 204508 / S288c) GN=PNP1 PE=1 SV=1

PNS1 − average.top3: 6.84541516129032 − max.qupm: 6

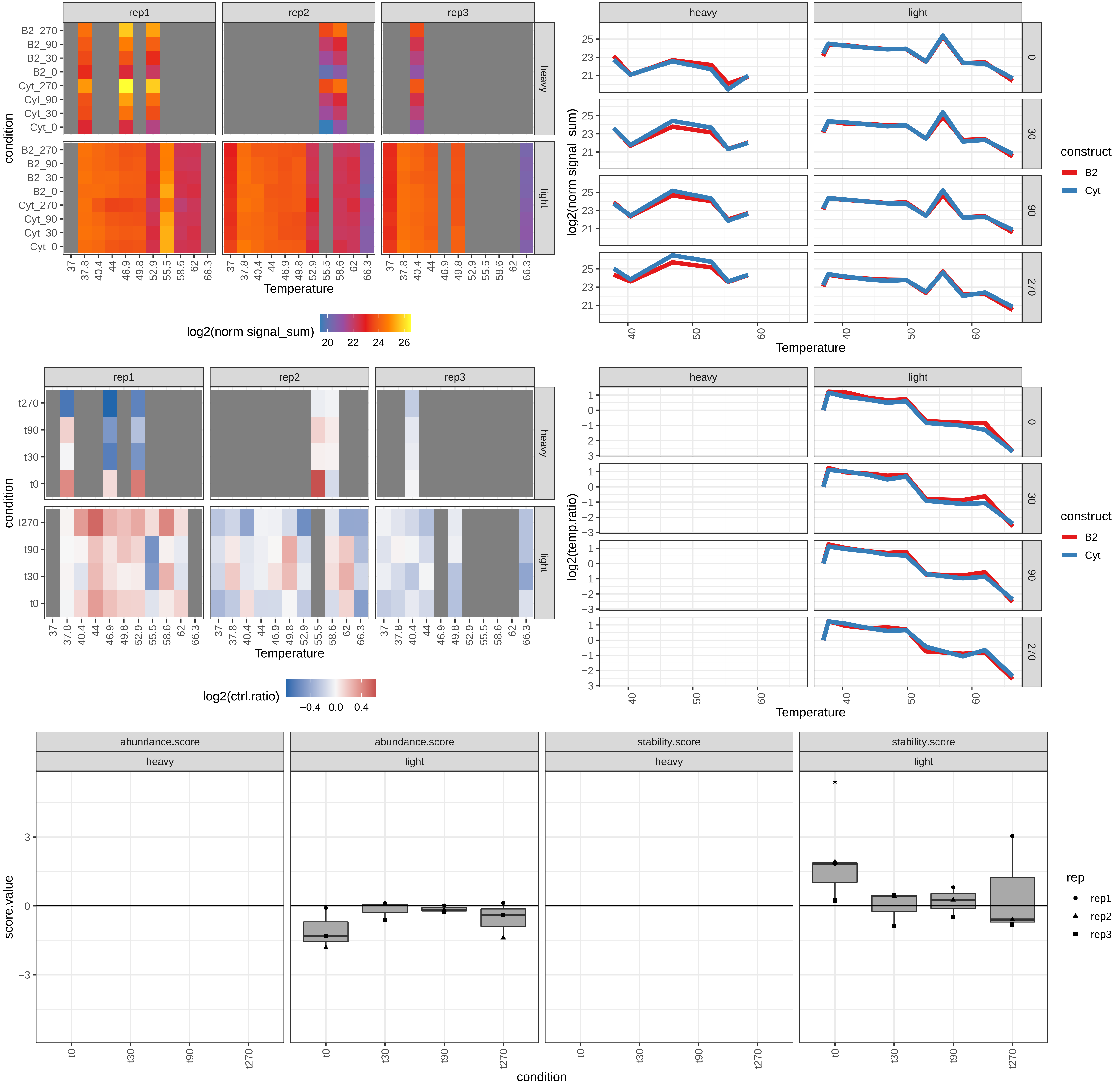

Q12412 − Protein PNS1 OS=Saccharomyces cerevisiae (strain ATCC 204508 / S288c) GN=PNS1 PE=1 SV=1

PNT1 − average.top3: 6.383324 − max.qupm: 3

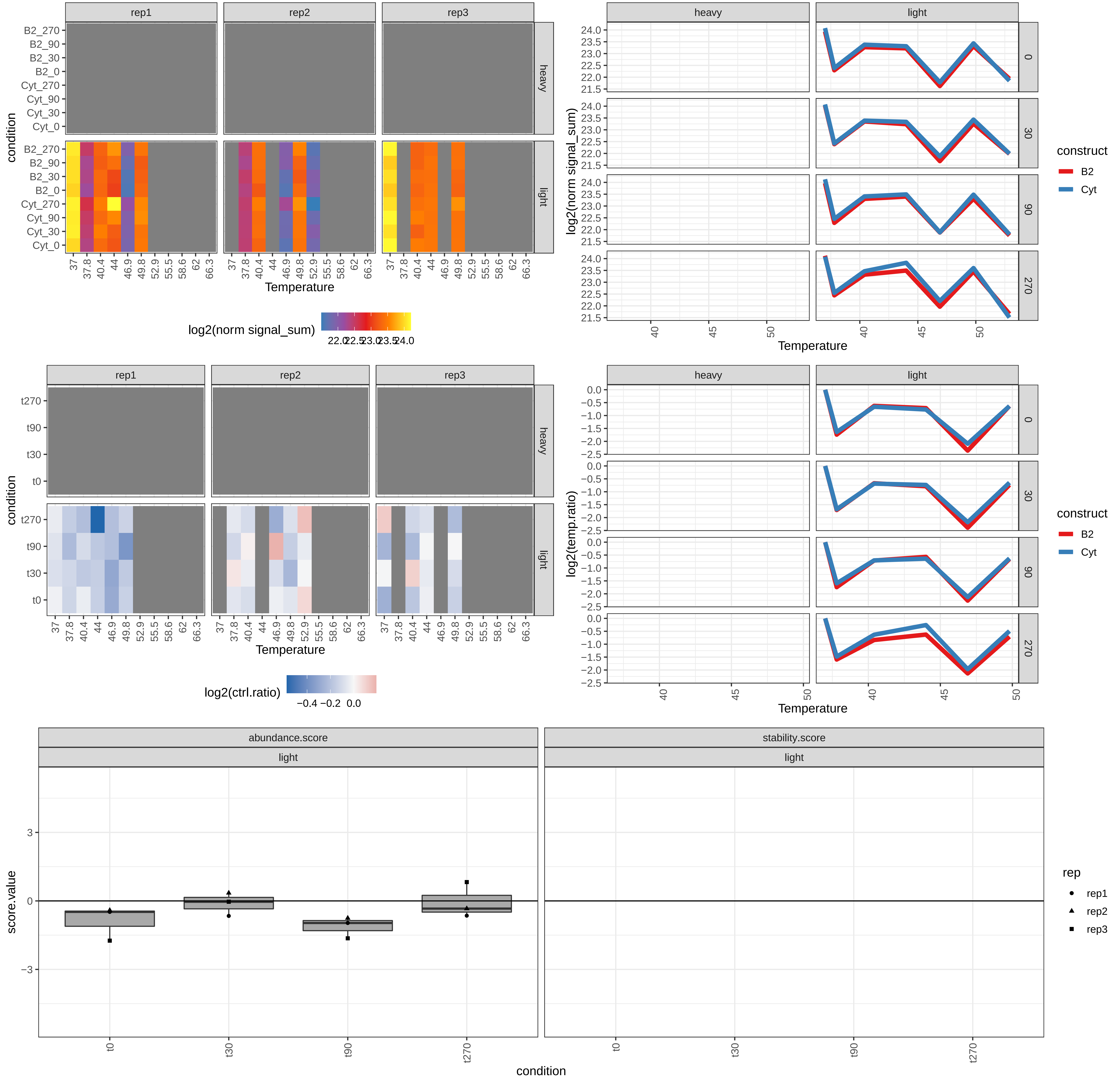

P38969 − Pentamidine resistance factor, mitochondrial OS=Saccharomyces cerevisiae (strain ATCC 204508 / S288c) GN=PNT1 PE=1 SV=2

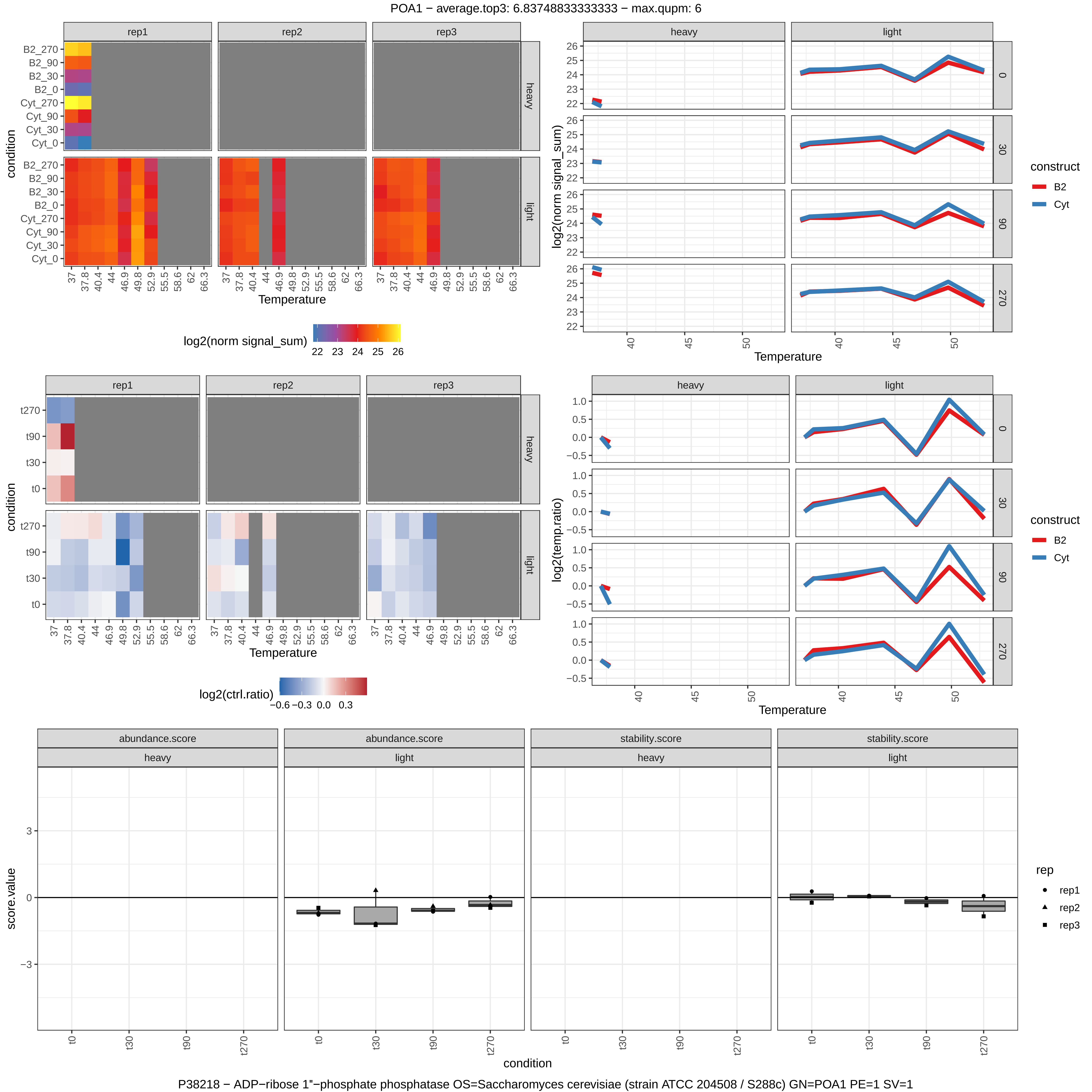

POA1 − average.top3: 6.83748833333333 − max.qupm: 6
rep1
rep2
rep3
heavy
light
condition
Temperature
log2(norm signal_sum)
log2(ctrl.ratio)
log2(temp.ratio)
construct
B2
Cyt
abundance.score
stability.score
score.value
rep
rep1
rep2
rep3
P38218 − ADP−ribose 1''−phosphate phosphatase OS=Saccharomyces cerevisiae (strain ATCC 204508 / S288c) GN=POA1 PE=1 SV=1

POB3 − average.top3: 6.87861627906977 − max.qupm: 9

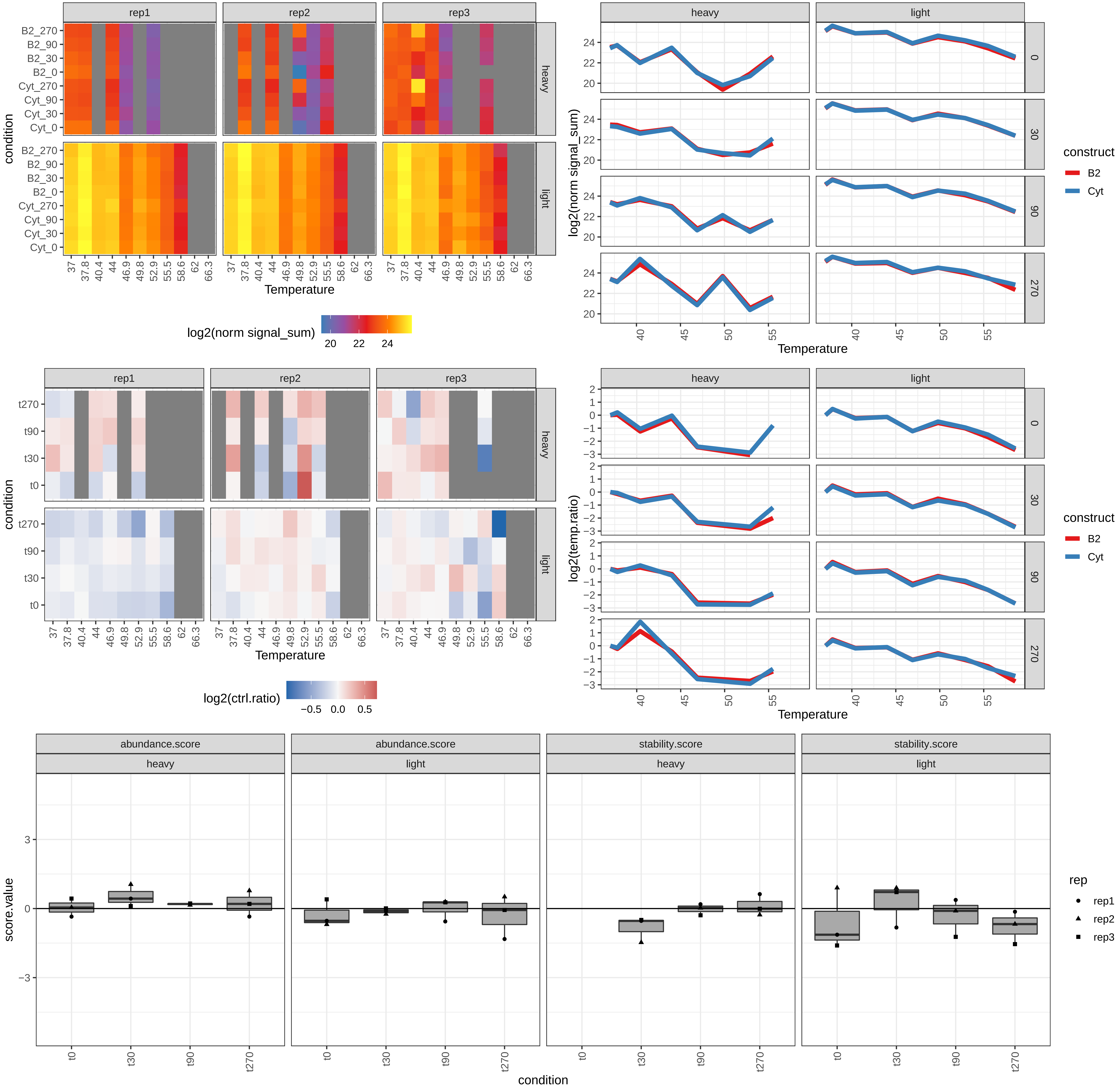

Q04636 − FACT complex subunit POB3 OS=Saccharomyces cerevisiae (strain ATCC 204508 / S288c) GN=POB3 PE=1 SV=1

POC4 − average.top3: 6.72817333333333 − max.qupm: 2

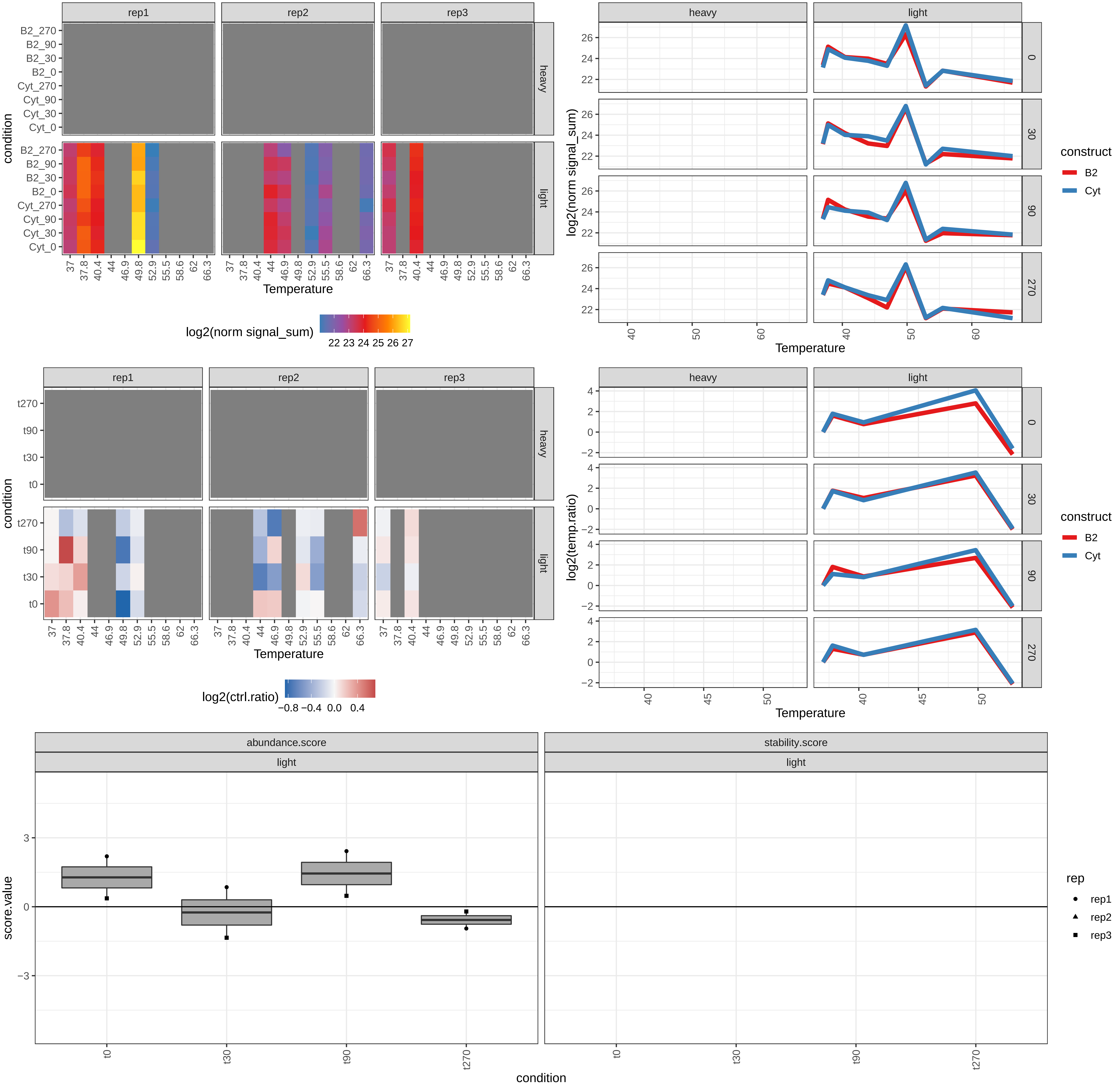

Q12245 − Proteasome chaperone 4 OS=Saccharomyces cerevisiae (strain ATCC 204508 / S288c) GN=POC4 PE=1 SV=1

POF1 − average.top3: 7.1400325 − max.qupm: 5

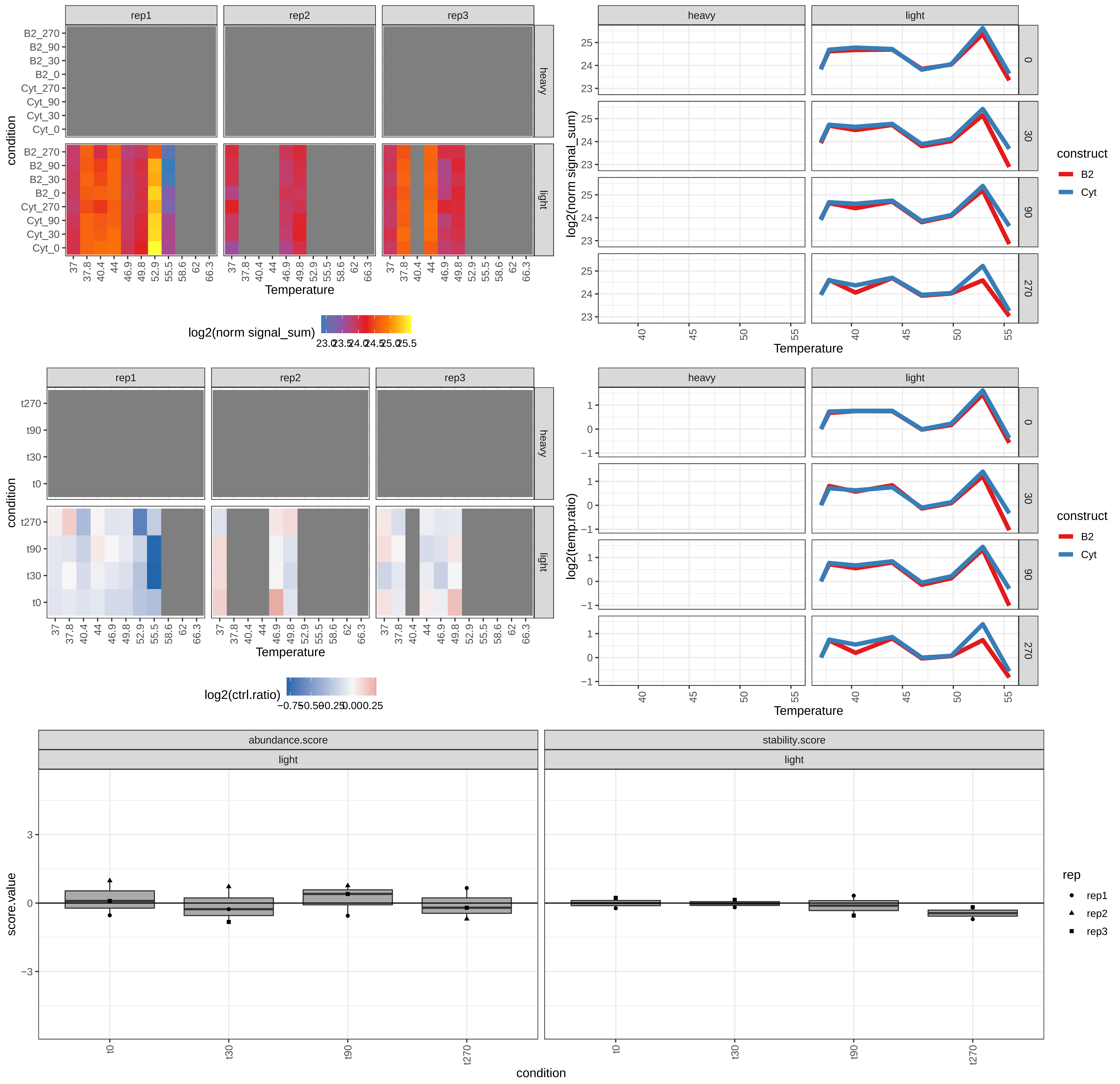

P25576 − Nicotinamide mononucleotide adenylyltransferase OS=Saccharomyces cerevisiae (strain ATCC 204508 / S288c) GN=POF1 PE=1 SV=1

POL1 − average.top3: 6.700454 − max.qupm: 11

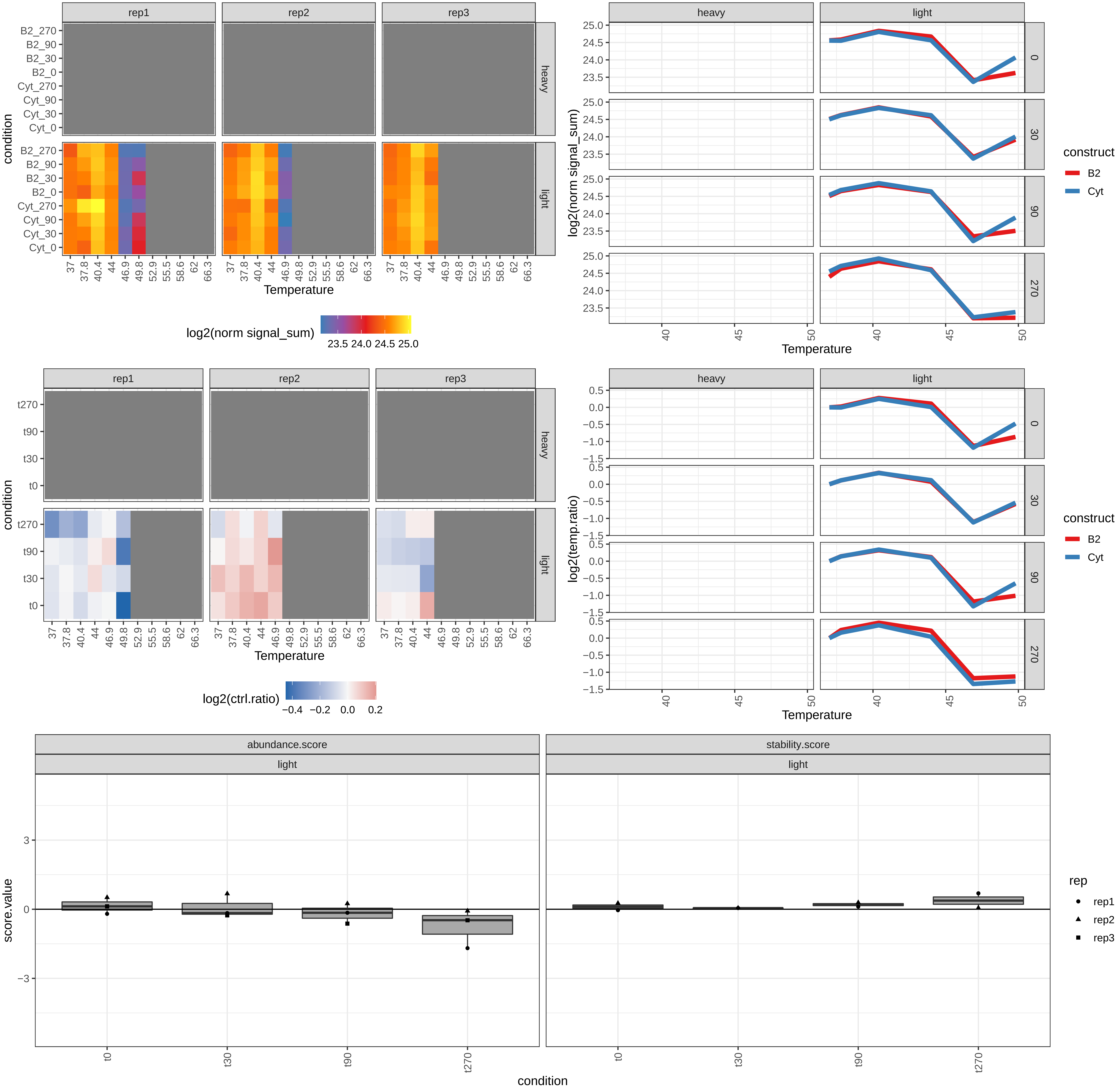

P13382 − DNA polymerase alpha catalytic subunit A OS=Saccharomyces cerevisiae (strain ATCC 204508 / S288c) GN=POL1 PE=1 SV=2

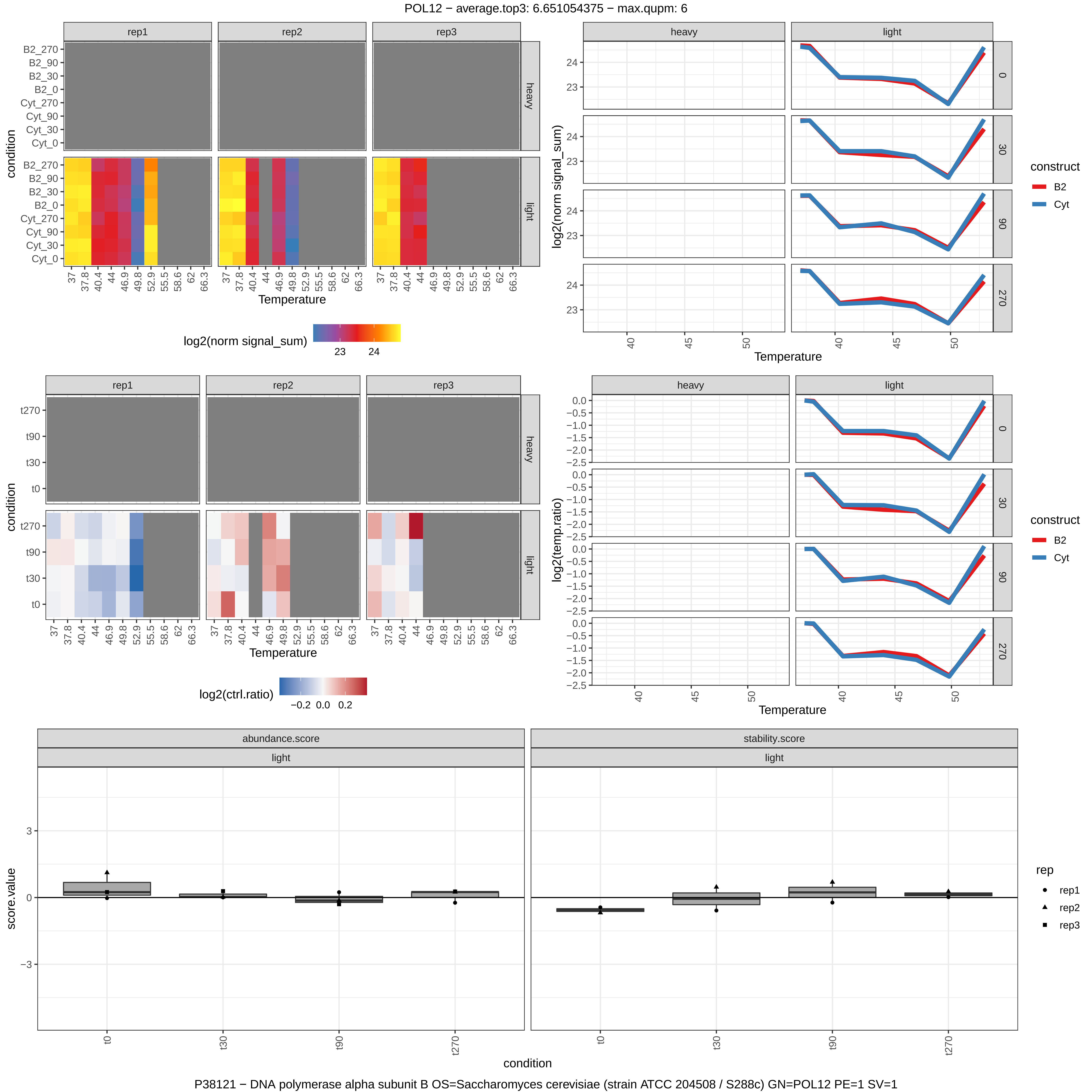
POL12 − average.top3: 6.651054375 − max.qupm: 6
rep1
rep2
rep3
heavy
light
B2_270
B2_90
B2_30
B2_0
Cyt_270
Cyt_90
Cyt_30
Cyt_0
condition
37
37.8
40.4
44
46.9
49.8
52.9
55.5
58.6
62
66.3
Temperature
log2(norm signal_sum)
23
24
0
30
90
270
40
45
50
construct
B2
Cyt
t270
t90
t30
t0
log2(temp.ratio)
log2(ctrl.ratio)
−0.2
0.0
0.2
0.0
−0.5
−1.0
−1.5
−2.0
−2.5
abundance.score
stability.score
score.value
3
0
−3
rep
rep1
rep2
rep3
P38121 − DNA polymerase alpha subunit B OS=Saccharomyces cerevisiae (strain ATCC 204508 / S288c) GN=POL12 PE=1 SV=1

POL2 − average.top3: 6.62101896551724 − max.qupm: 19

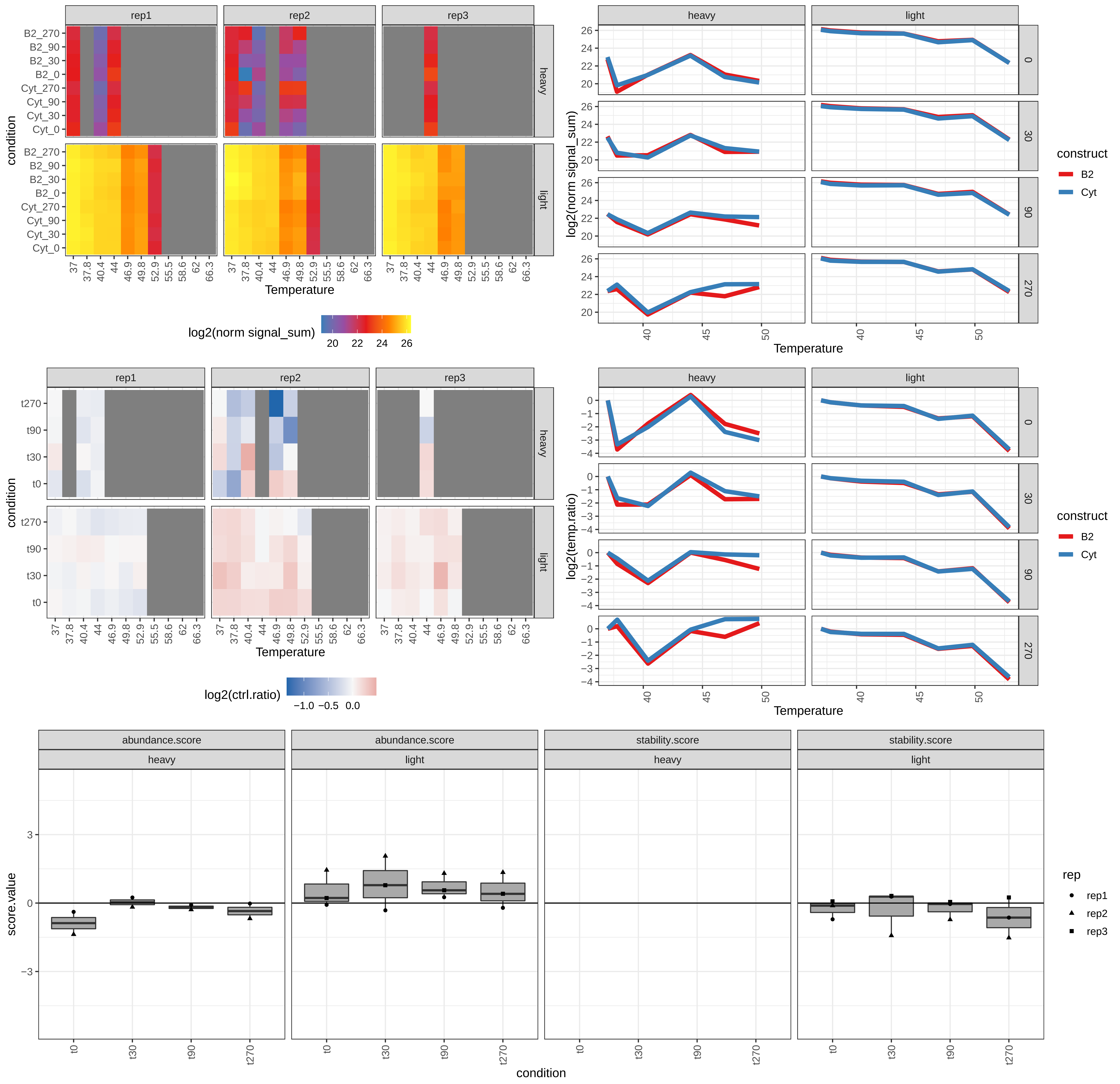

P21951 − DNA polymerase epsilon catalytic subunit A OS=Saccharomyces cerevisiae (strain ATCC 204508 / S288c) GN=POL2 PE=1 SV=1

POL3 − average.top3: 6.7770025 − max.qupm: 9

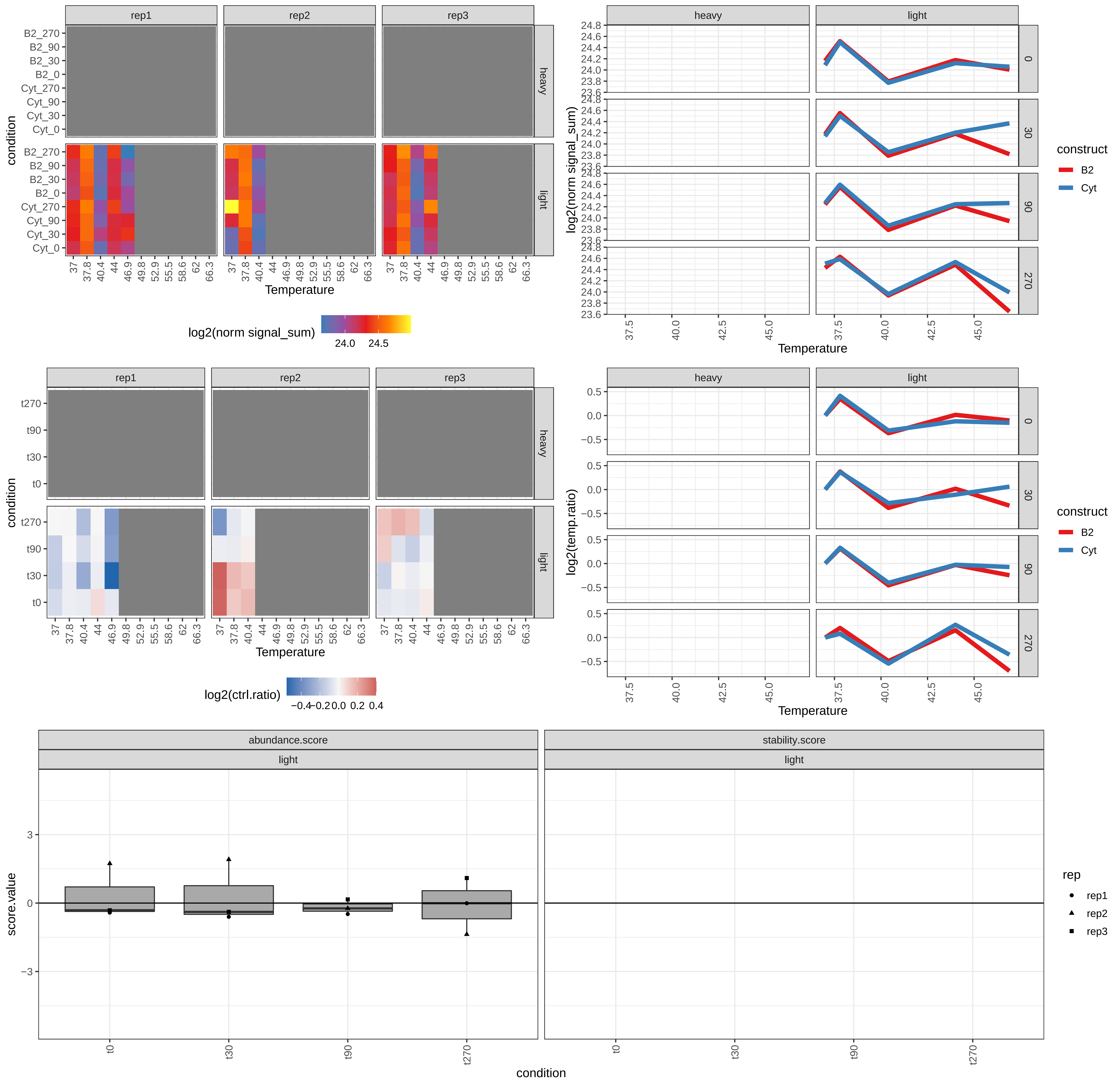

P15436 − DNA polymerase delta catalytic subunit OS=Saccharomyces cerevisiae (strain ATCC 204508 / S288c) GN=POL3 PE=1 SV=4

POL30 − average.top3: 7.19886615384615 − max.qupm: 11

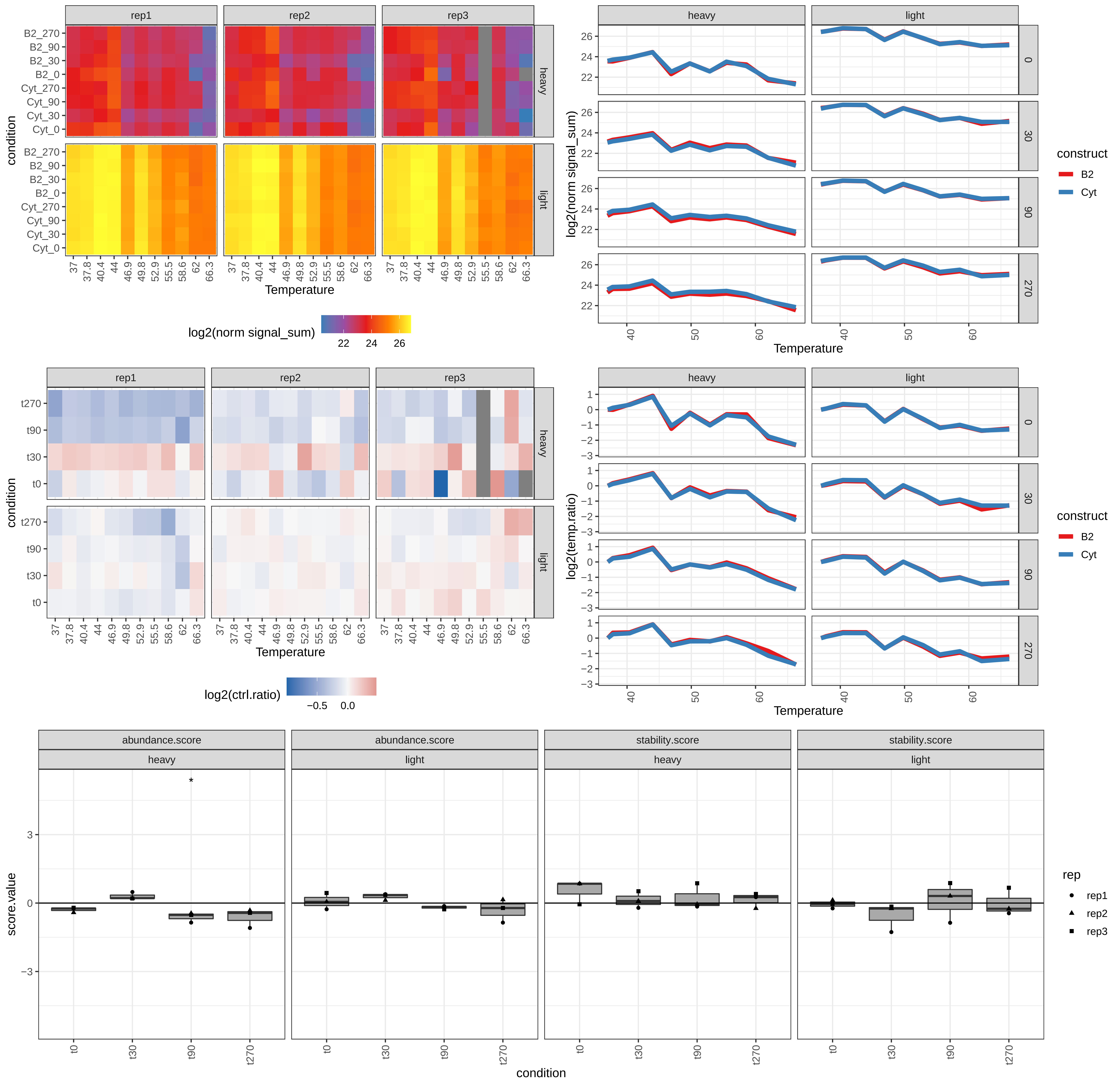

P15873 − Proliferating cell nuclear antigen OS=Saccharomyces cerevisiae (strain ATCC 204508 / S288c) GN=POL30 PE=1 SV=1

POL31 − average.top3: 6.84786692307692 − max.qupm: 6

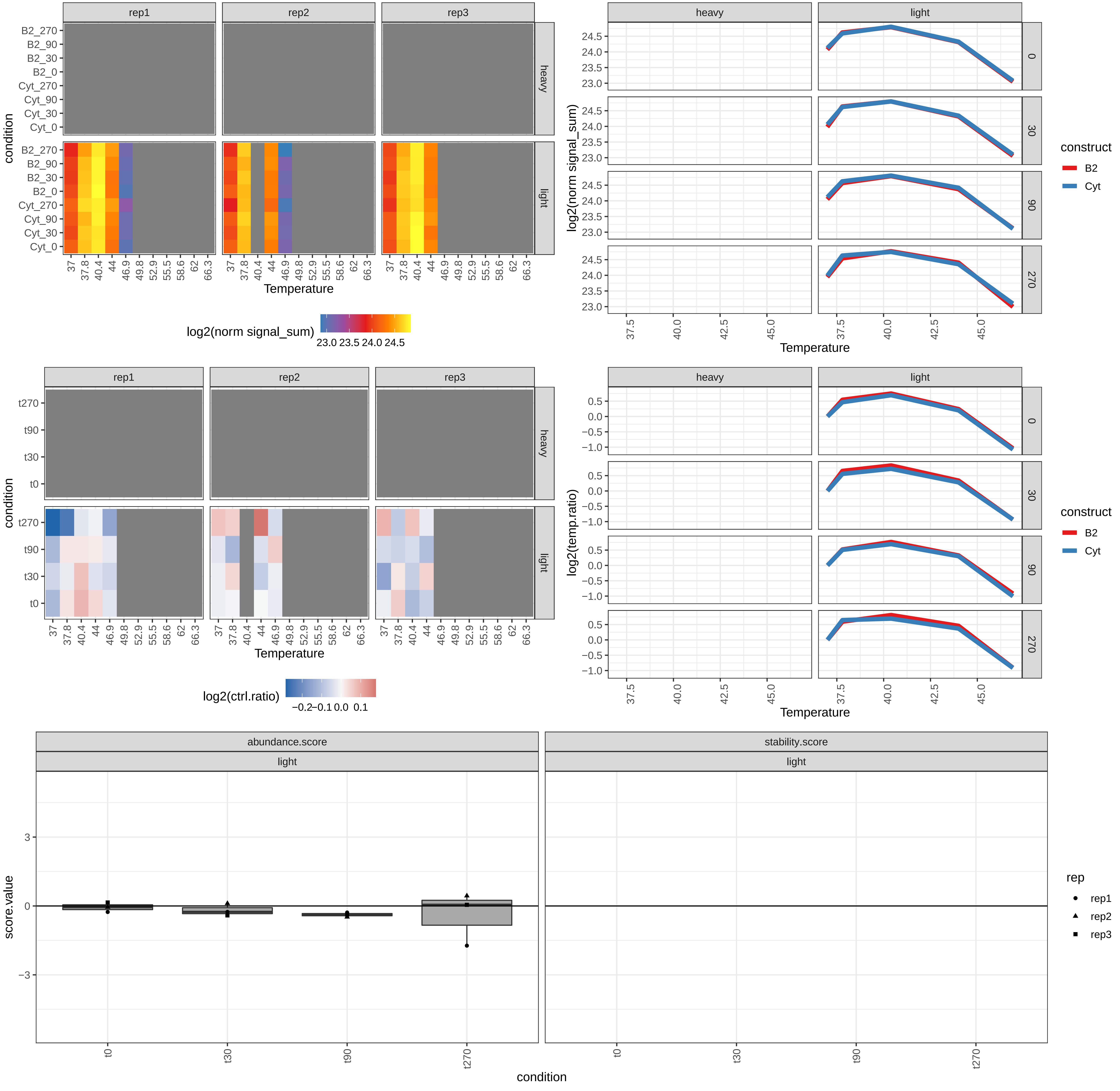

P46957 − DNA polymerase delta small subunit OS=Saccharomyces cerevisiae (strain ATCC 204508 / S288c) GN=POL31 PE=1 SV=1

POL5 − average.top3: 6.493295 − max.qupm: 9

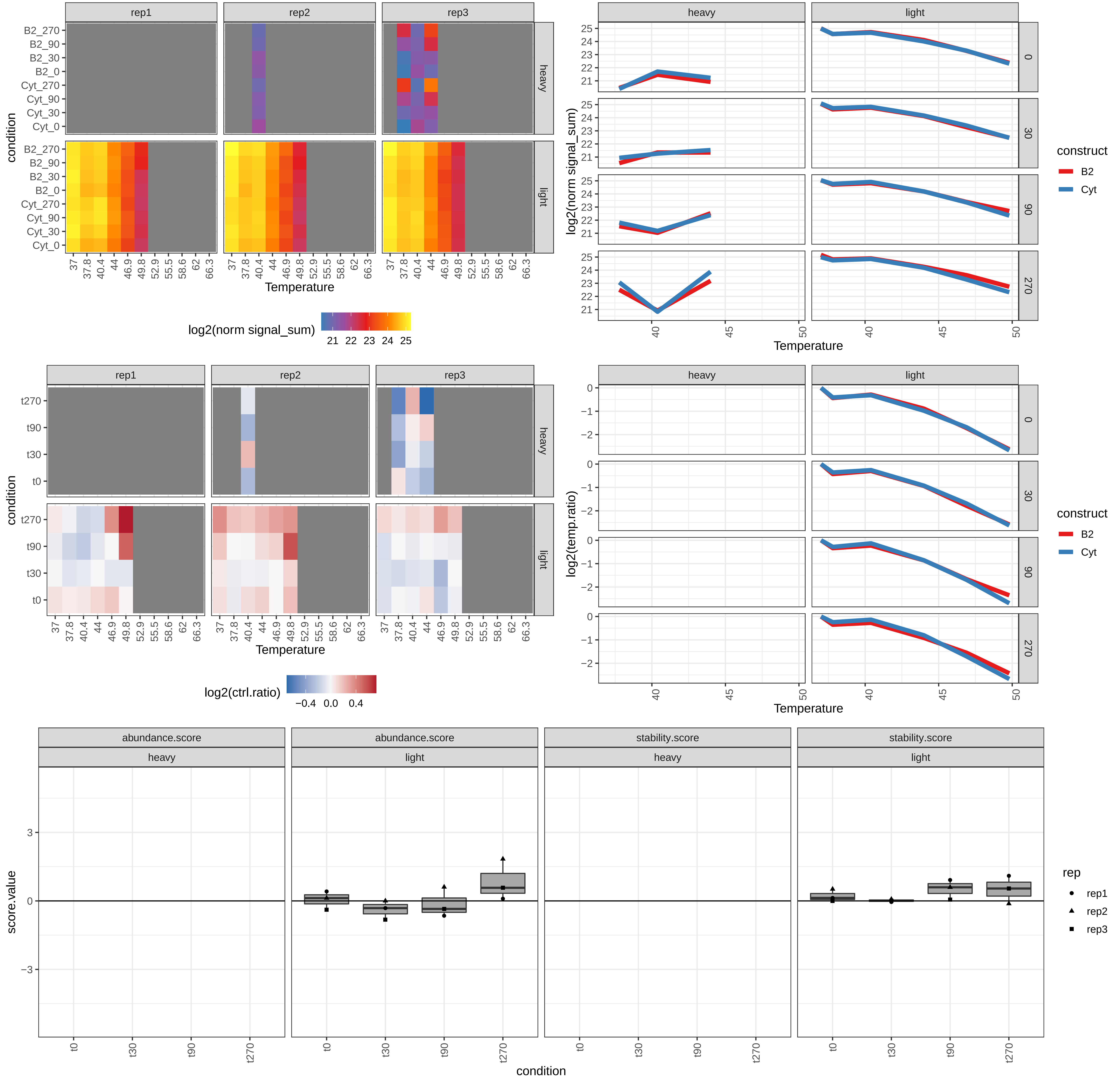

P39985 − DNA polymerase V OS=Saccharomyces cerevisiae (strain ATCC 204508 / S288c) GN=POL5 PE=1 SV=1

POM152 − average.top3: 6.87189897959184 − max.qupm: 23

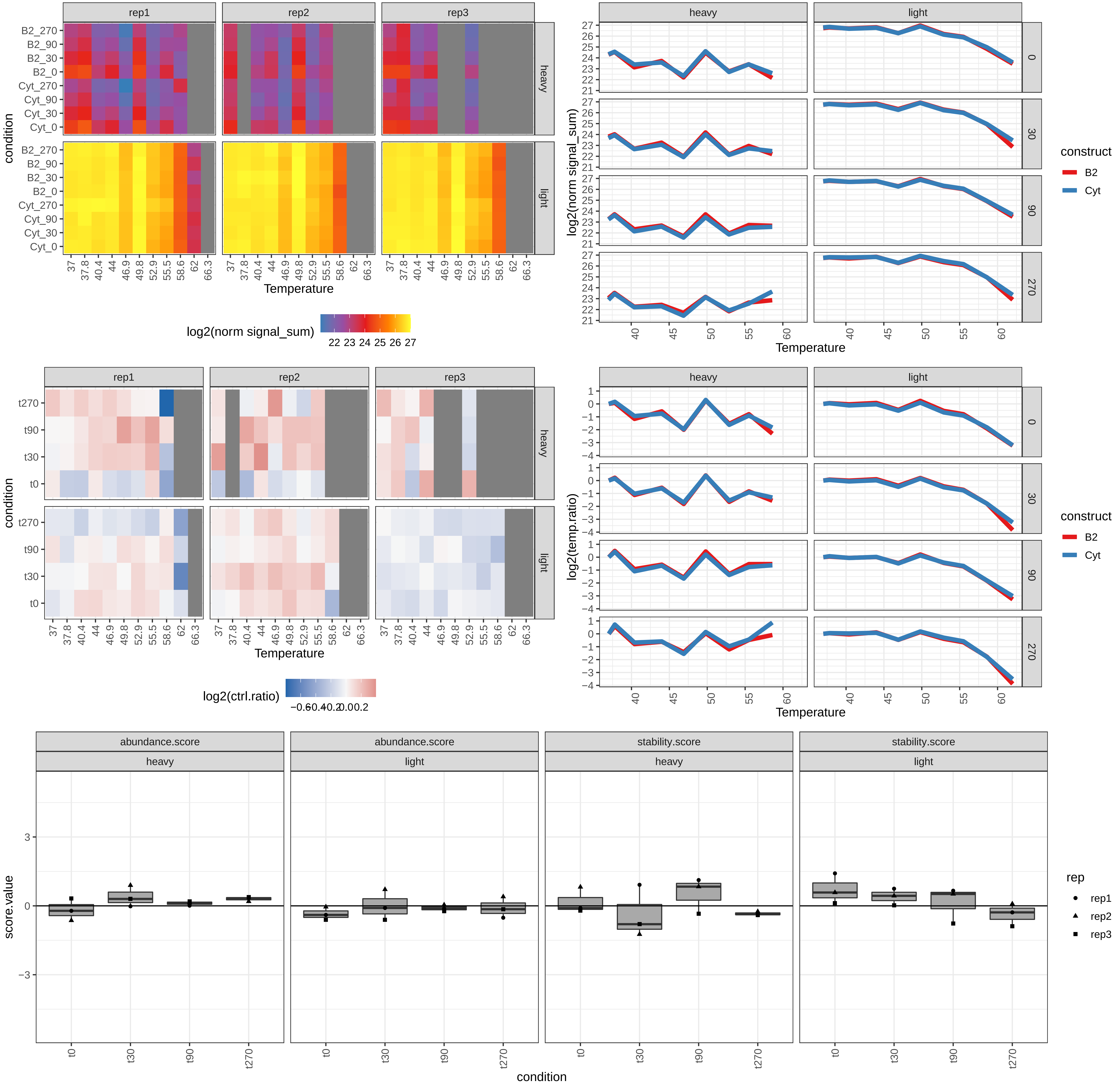

P39685 − Nucleoporin POM152 OS=Saccharomyces cerevisiae (strain ATCC 204508 / S288c) GN=POM152 PE=1 SV=1

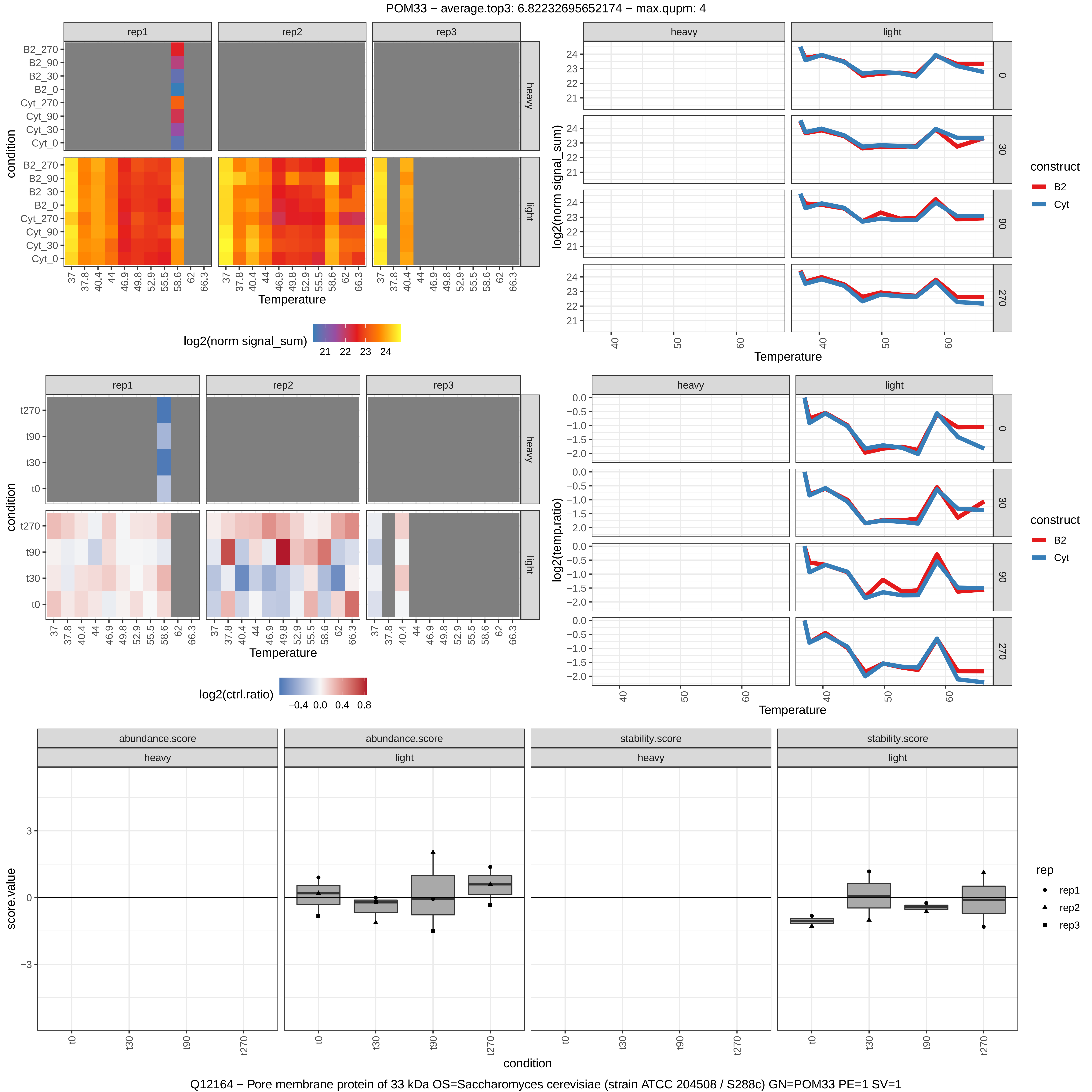
POM33 − average.top3: 6.82232695652174 − max.qupm: 4
rep1
rep2
rep3
heavy
light
condition
Temperature
log2(norm signal_sum)
construct
B2
Cyt
log2(ctrl.ratio)
log2(temp.ratio)
abundance.score
stability.score
score.value
rep
rep1
rep2
rep3
Q12164 − Pore membrane protein of 33 kDa OS=Saccharomyces cerevisiae (strain ATCC 204508 / S288c) GN=POM33 PE=1 SV=1

POP1 − average.top3: 6.75566823529412 − max.qupm: 7

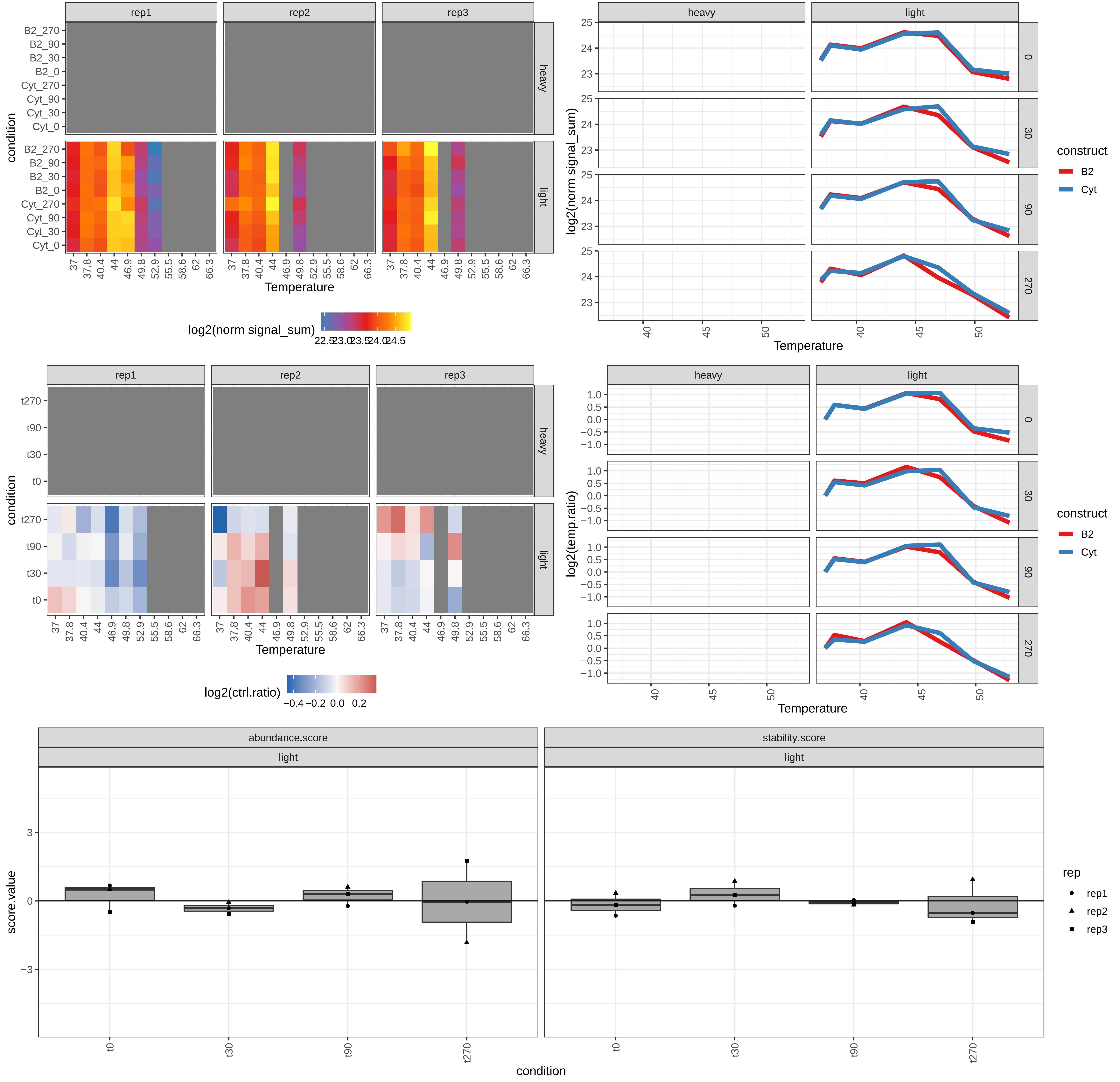

P41812 − Ribonucleases P/MRP protein subunit POP1 OS=Saccharomyces cerevisiae (strain ATCC 204508 / S288c) GN=POP1 PE=1 SV=1

POP2 − average.top3: 6.59451125 − max.qupm: 2

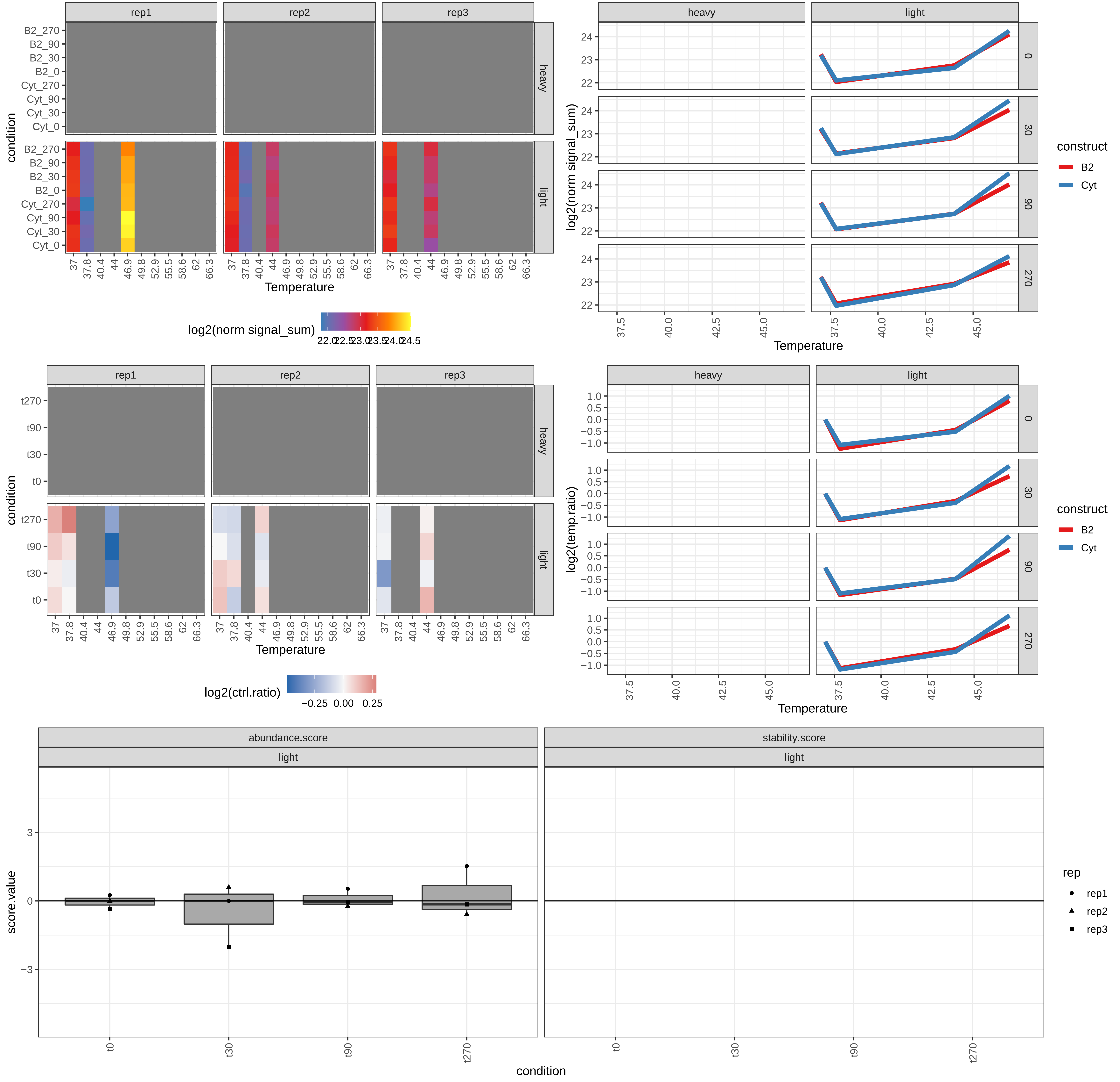

P39008 − Poly(A) ribonuclease POP2 OS=Saccharomyces cerevisiae (strain ATCC 204508 / S288c) GN=POP2 PE=1 SV=2

POP4 − average.top3: 6.46879454545455 − max.qupm: 3

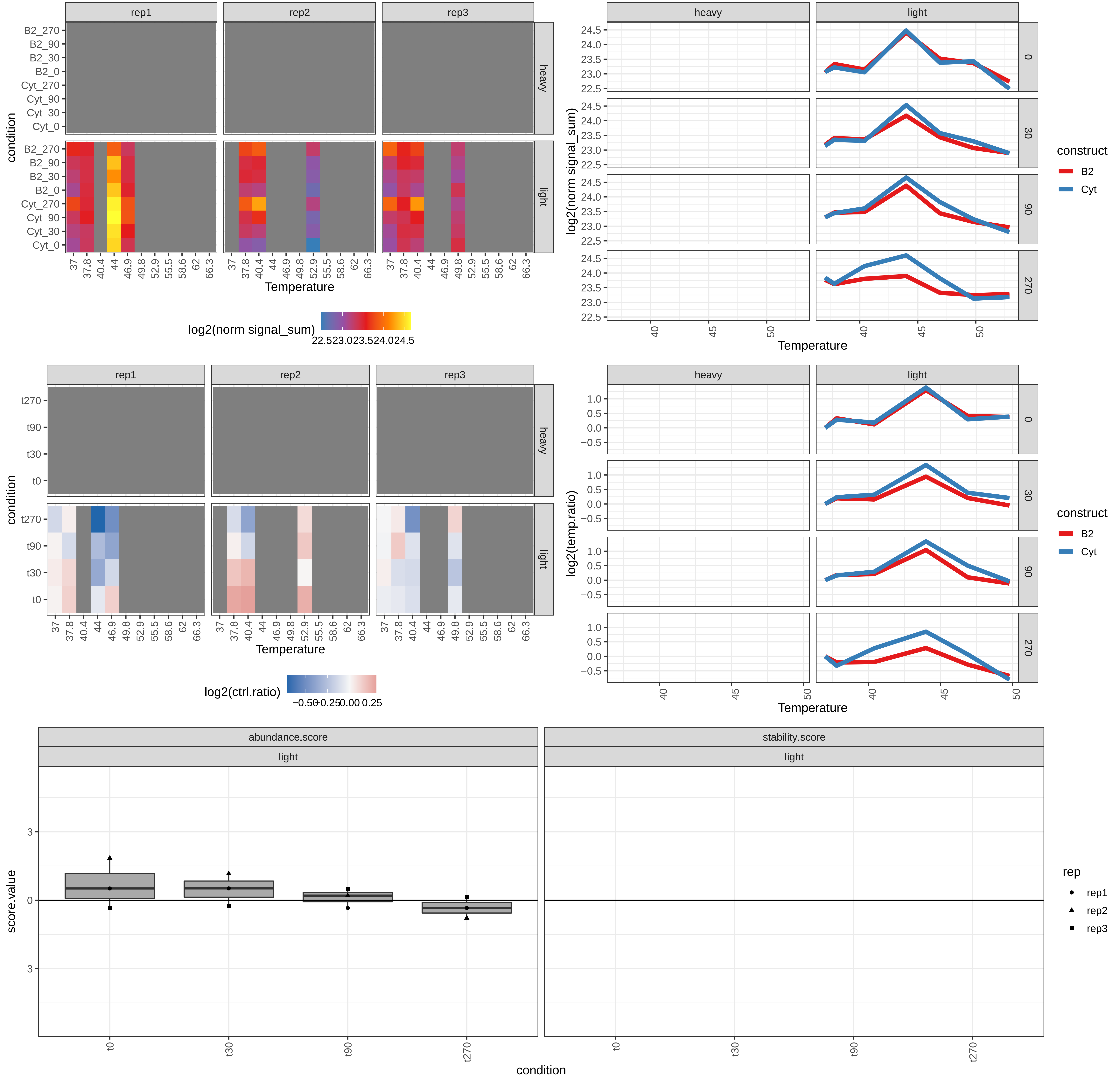

P38336 − RNases MRP/P 32.9 kDa subunit OS=Saccharomyces cerevisiae (strain ATCC 204508 / S288c) GN=POP4 PE=1 SV=1

POP6 − average.top3: 6.76665461538462 − max.qupm: 3

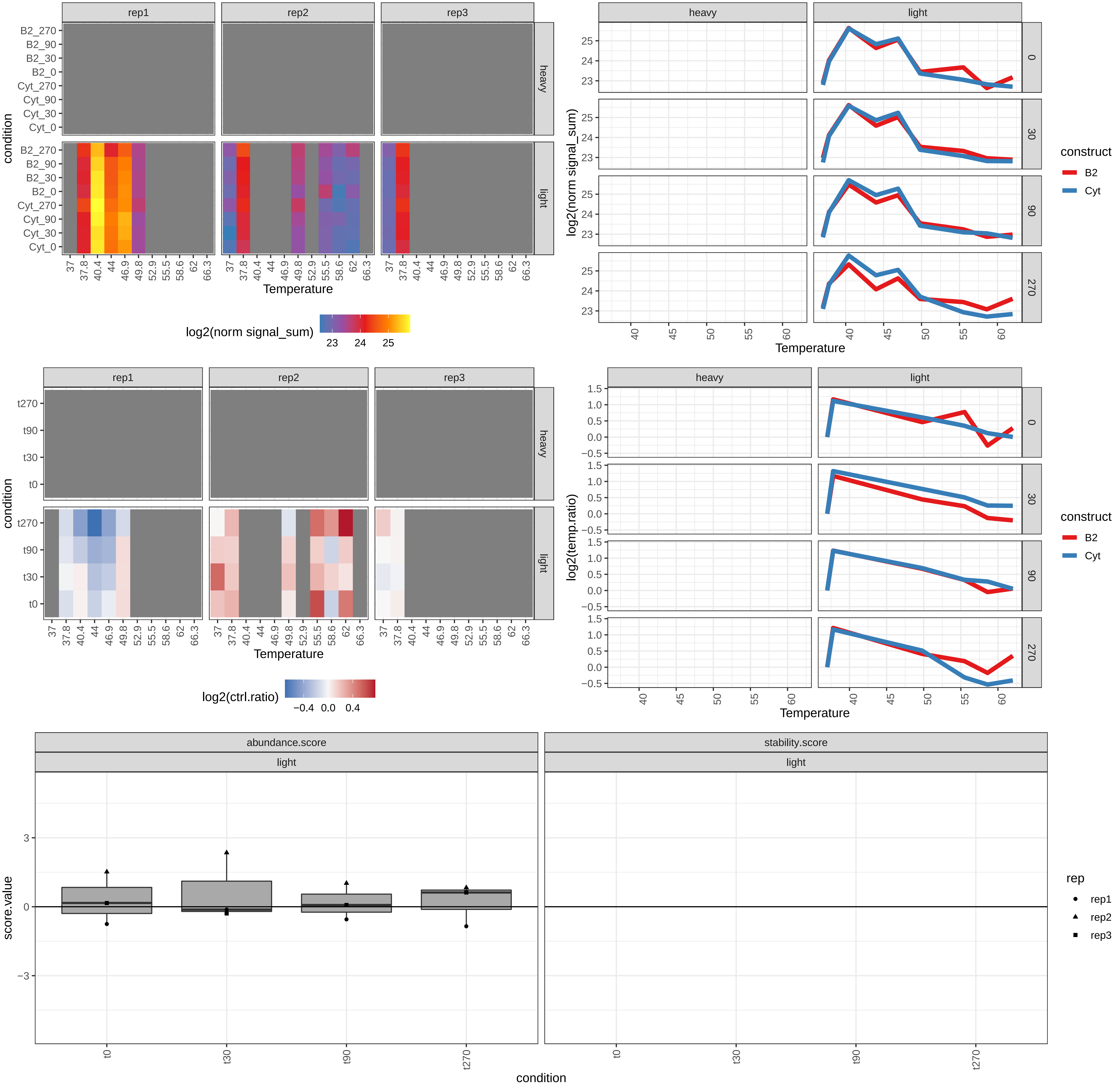

P53218 − Ribonucleases P/MRP protein subunit POP6 OS=Saccharomyces cerevisiae (strain ATCC 204508 / S288c) GN=POP6 PE=1 SV=1

POR1 − average.top3: 8.17838348484849 − max.qupm: 15

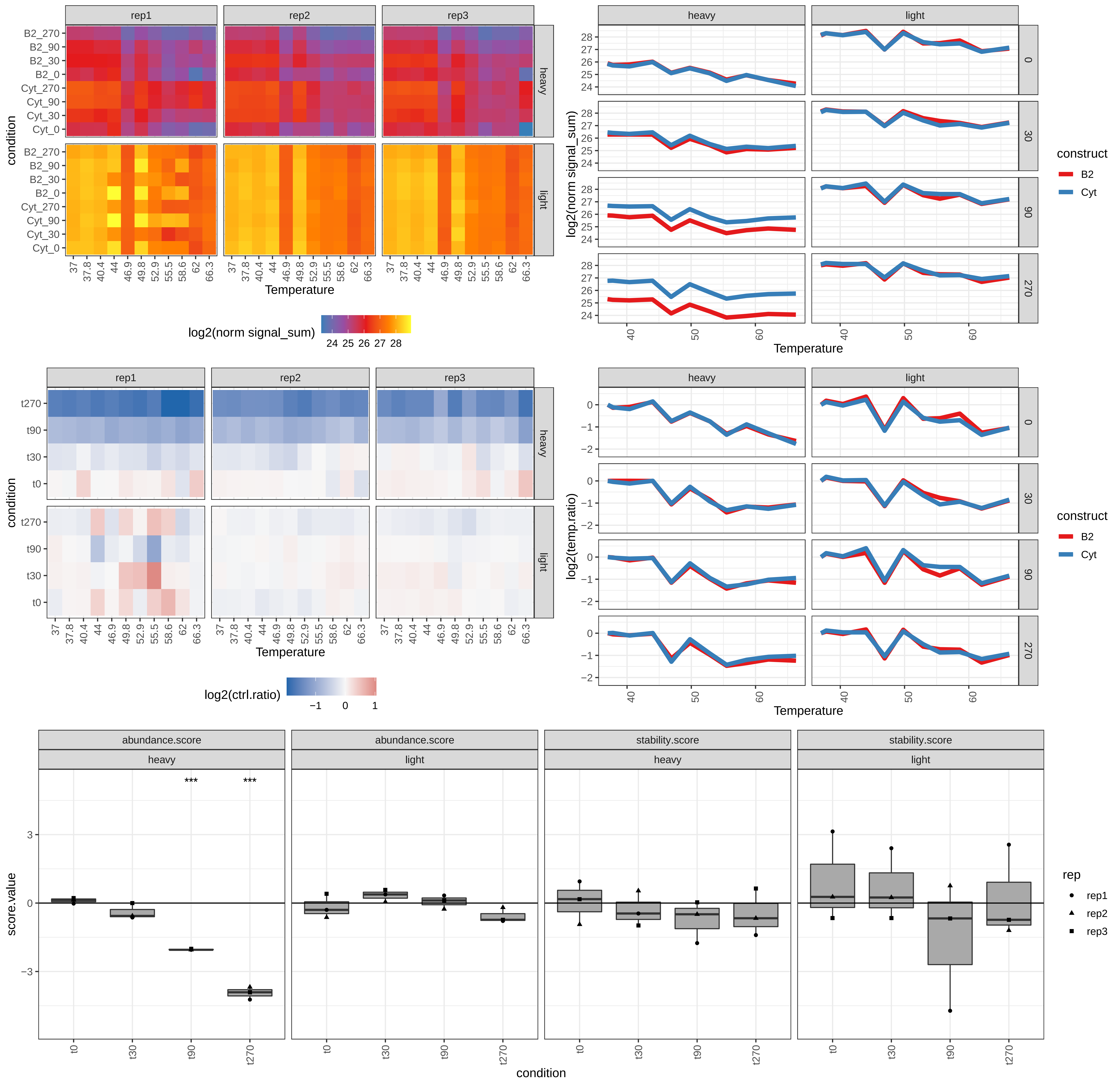

P04840 − Mitochondrial outer membrane protein porin 1 OS=Saccharomyces cerevisiae (strain ATCC 204508 / S288c) GN=POR1 PE=1 SV=4

POT1 − average.top3: 7.51203524590164 − max.qupm: 14

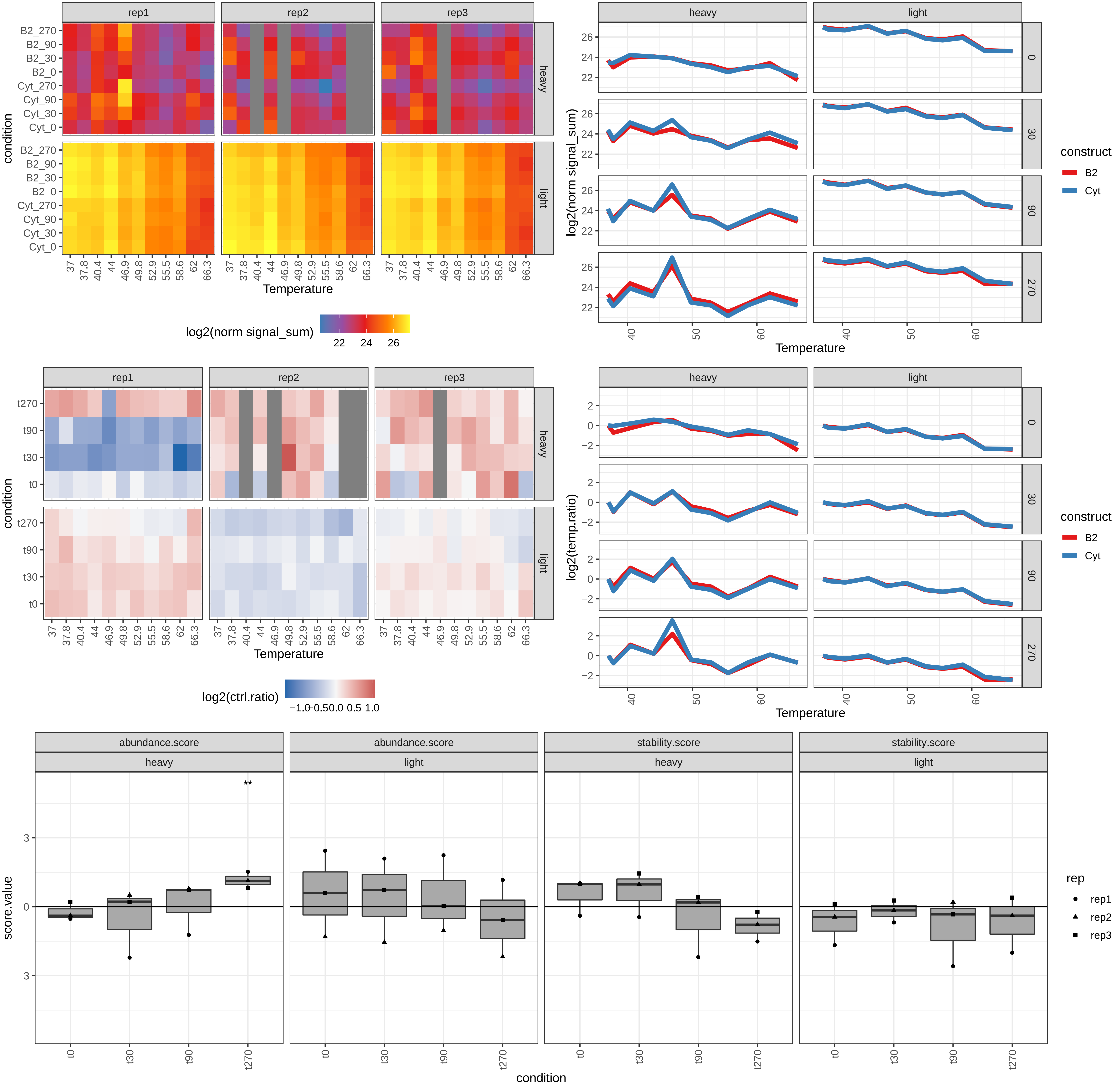

P27796 − 3−ketoacyl−CoA thiolase, peroxisomal OS=Saccharomyces cerevisiae (strain ATCC 204508 / S288c) GN=POT1 PE=1 SV=1

POX1 − average.top3: 7.24024622222222 − max.qupm: 22

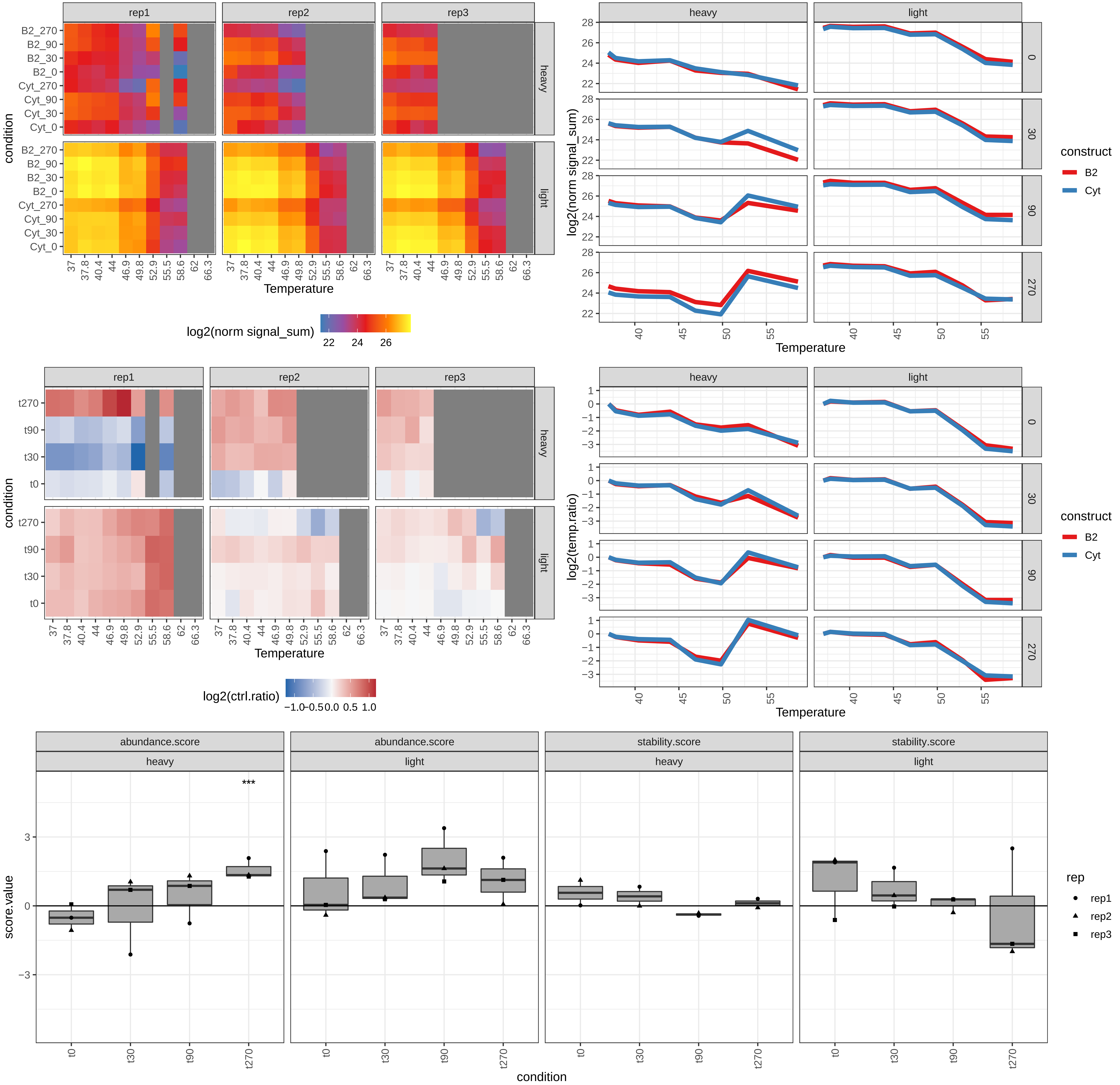

P13711 − Acyl−coenzyme A oxidase OS=Saccharomyces cerevisiae (strain ATCC 204508 / S288c) GN=POX1 PE=3 SV=2

PPA2 − average.top3: 6.82991117647059 − max.qupm: 5

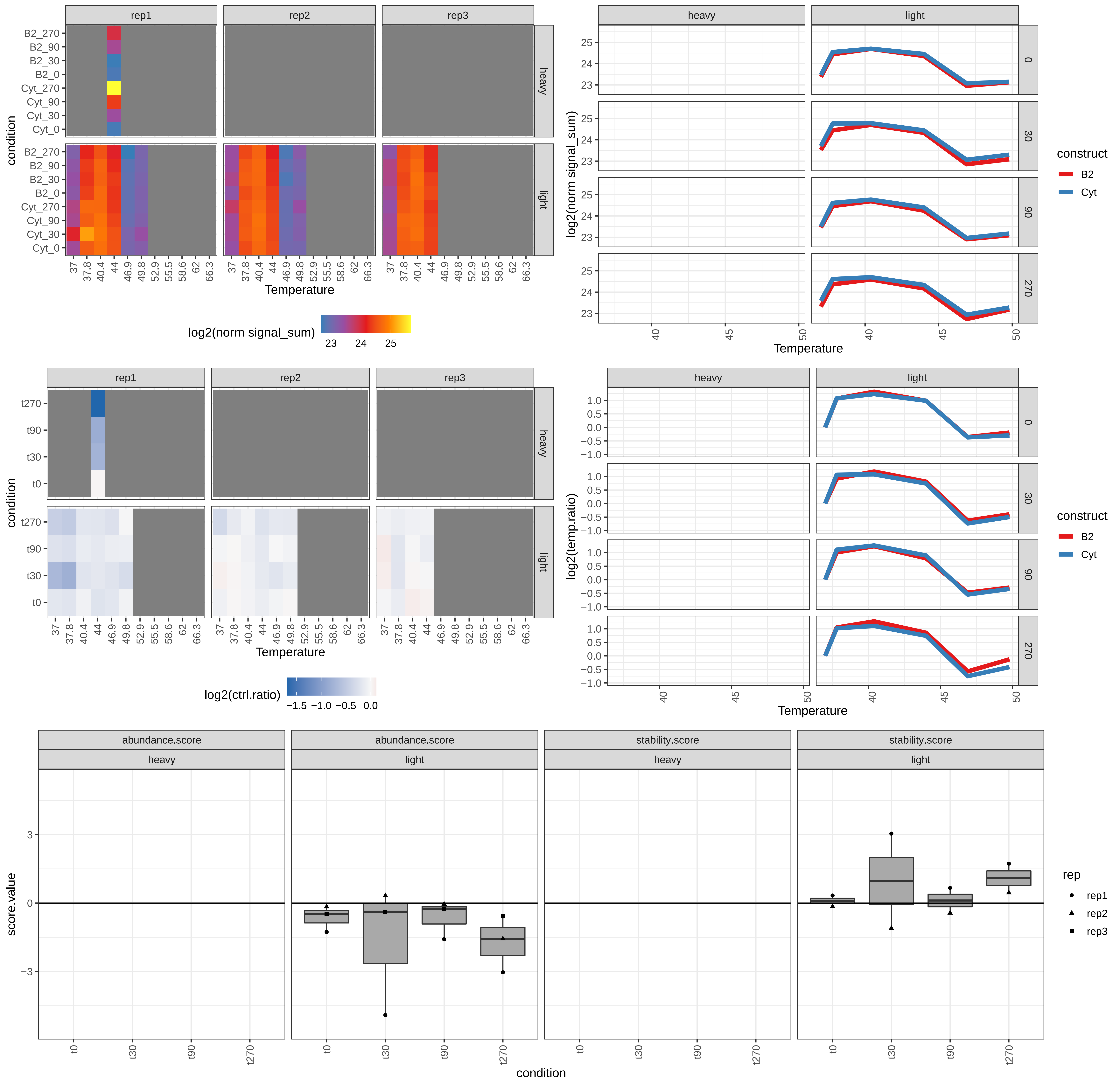

P28239 − Inorganic pyrophosphatase, mitochondrial OS=Saccharomyces cerevisiae (strain ATCC 204508 / S288c) GN=PPA2 PE=1 SV=1

PPE1 − average.top3: 6.78386333333333 − max.qupm: 4

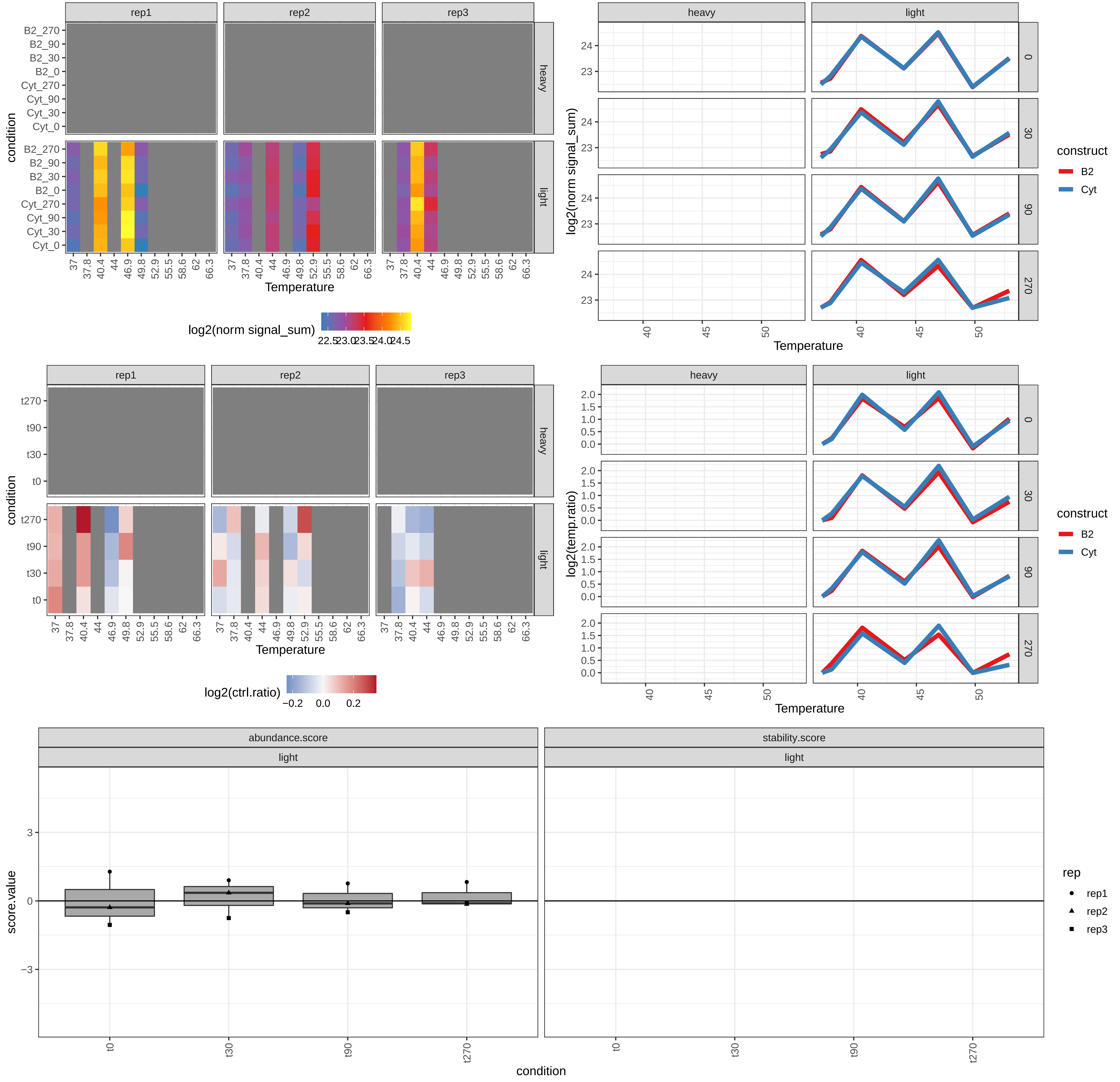

P38796 − Protein phosphatase methylesterase 1 OS=Saccharomyces cerevisiae (strain ATCC 204508 / S288c) GN=PPE1 PE=1 SV=1

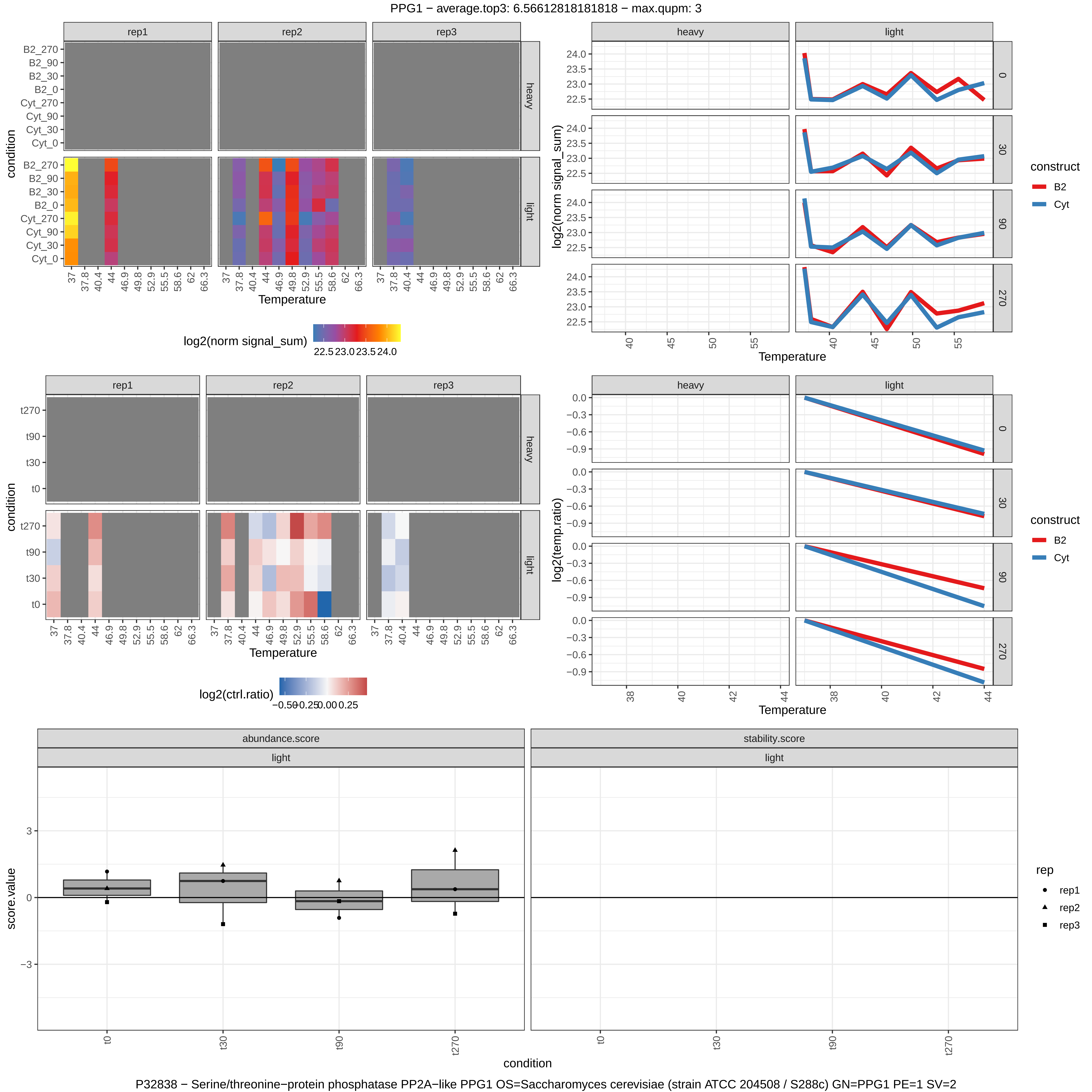

PPG1 − average.top3: 6.56612818181818 − max.qupm: 3
rep1
rep2
rep3
heavy
light
condition
Temperature
log2(norm signal_sum)
construct
B2
Cyt
log2(temp.ratio)
log2(ctrl.ratio)
abundance.score
stability.score
score.value
rep
rep1
rep2
rep3
P32838 − Serine/threonine−protein phosphatase PP2A−like PPG1 OS=Saccharomyces cerevisiae (strain ATCC 204508 / S288c) GN=PPG1 PE=1 SV=2

PPH21|PPH22 − average.top3: 6.58378733333333 − max.qupm: 7

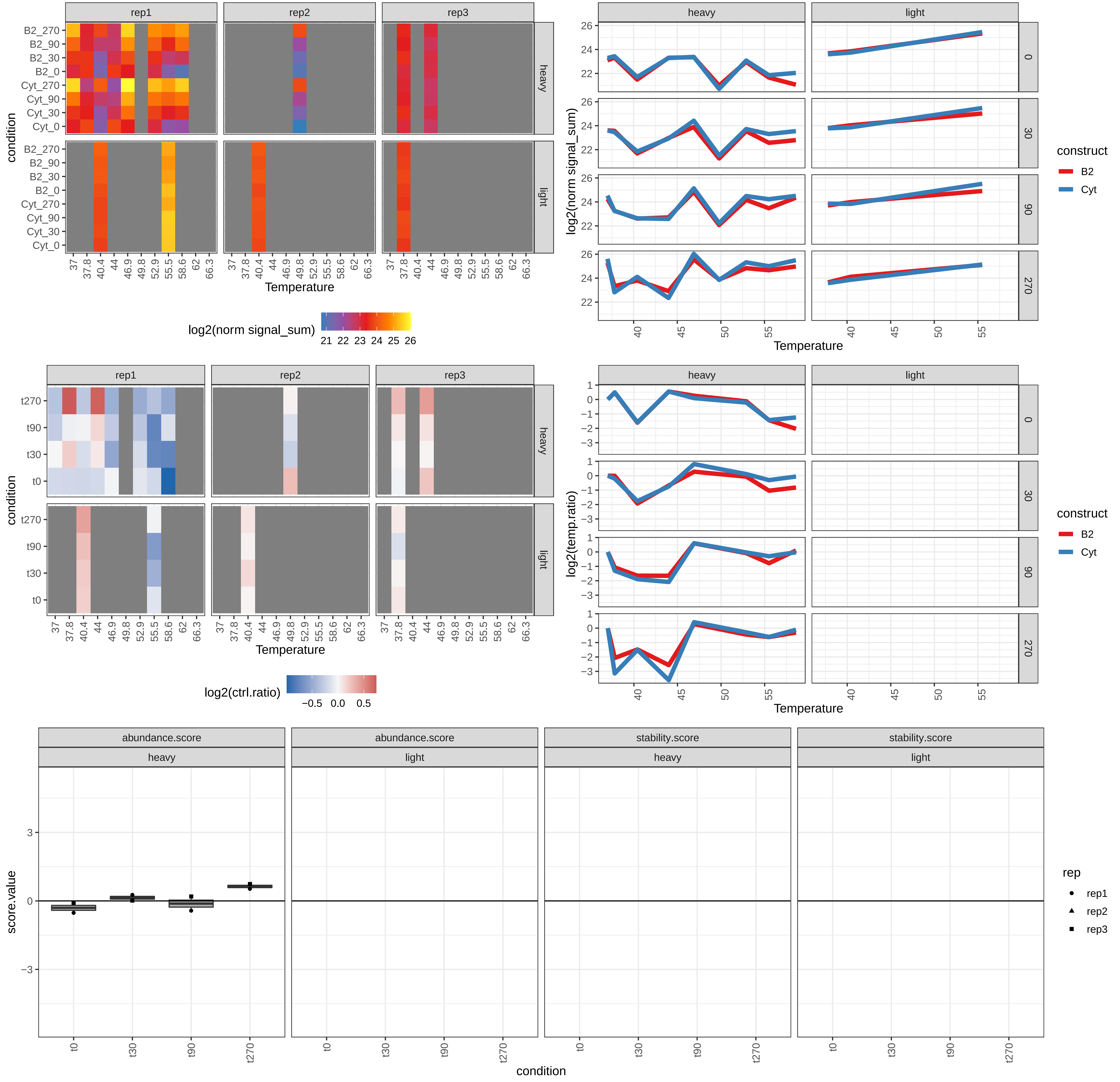

P2A−1 catalytic subunit OS=Saccharomyces cerevisiae (strain ATCC 204508 / S288c) GN=PPH21 PE=1 SV=1|Serine/threonine−protein phosphatase PP2A−2 catalytic subunit OS=Saccharomyces cerevi

PPH3 − average.top3: 6.348727 − max.qupm: 3

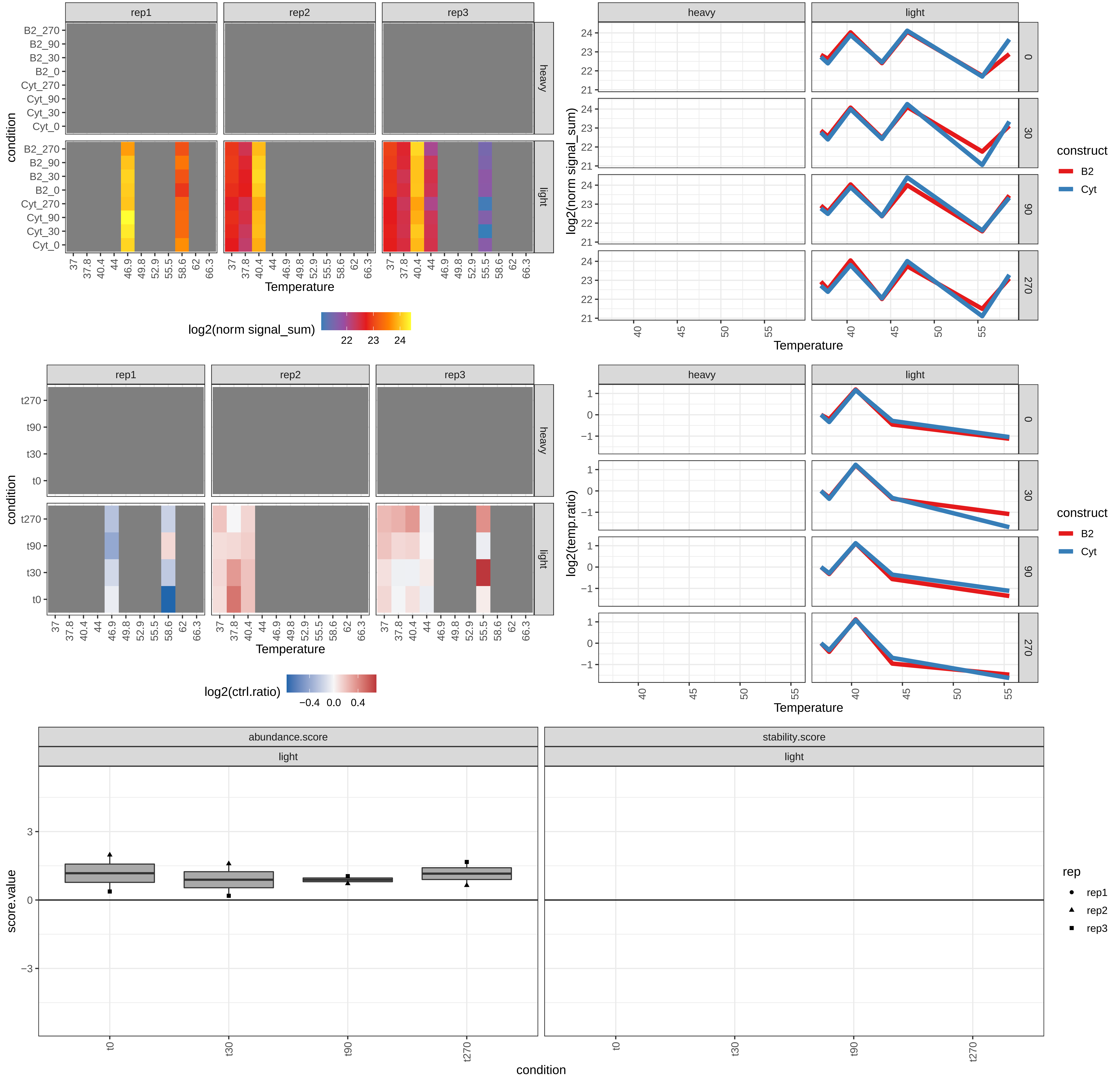

P32345 − Serine/threonine−protein phosphatase 4 catalytic subunit OS=Saccharomyces cerevisiae (strain ATCC 204508 / S288c) GN=PPH3 PE=1 SV=2

PPM1 − average.top3: 6.85409590909091 − max.qupm: 8

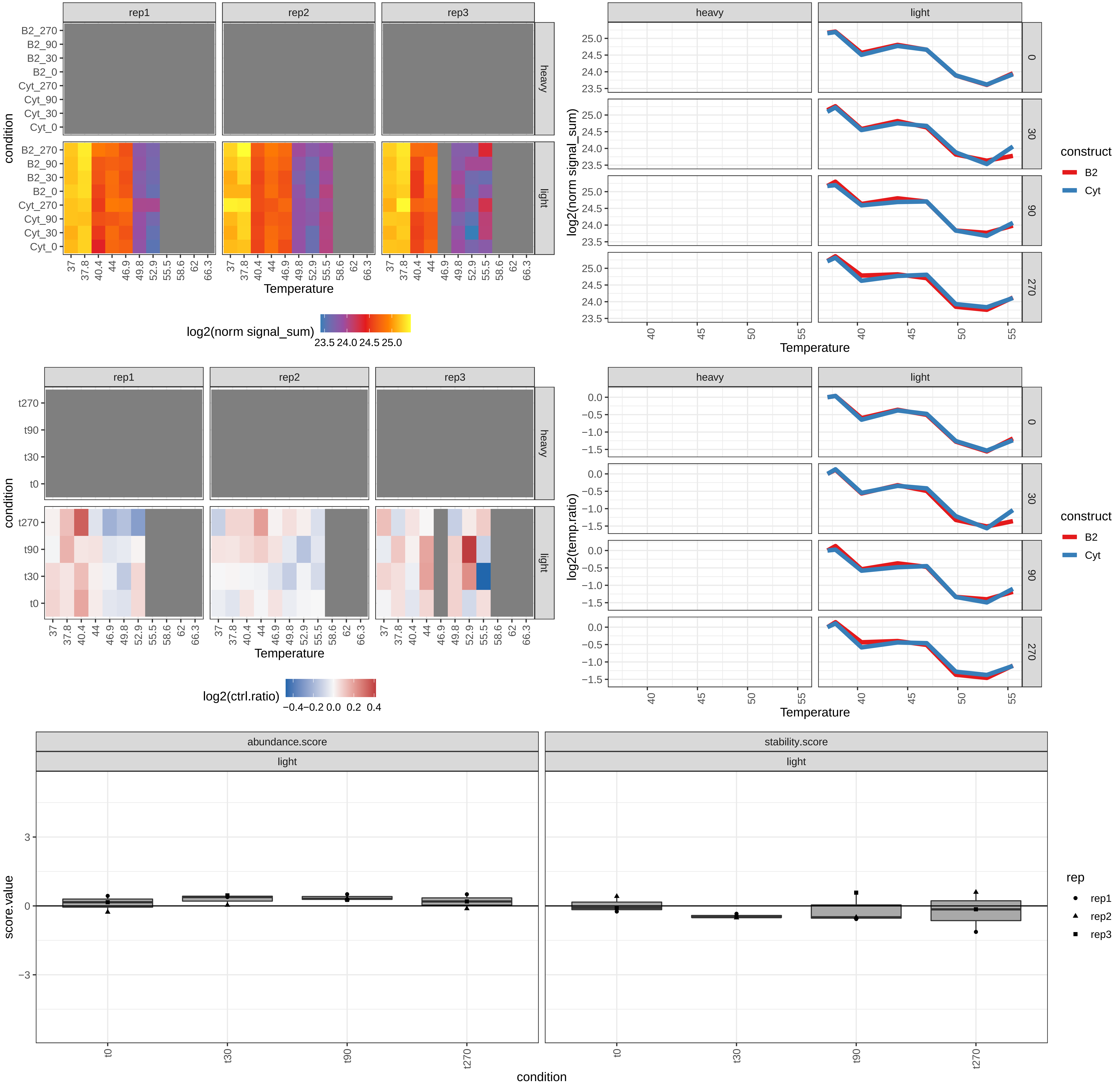

Q04081 − Leucine carboxyl methyltransferase 1 OS=Saccharomyces cerevisiae (strain ATCC 204508 / S288c) GN=PPM1 PE=1 SV=1

PPM2 − average.top3: 6.37933222222222 − max.qupm: 3

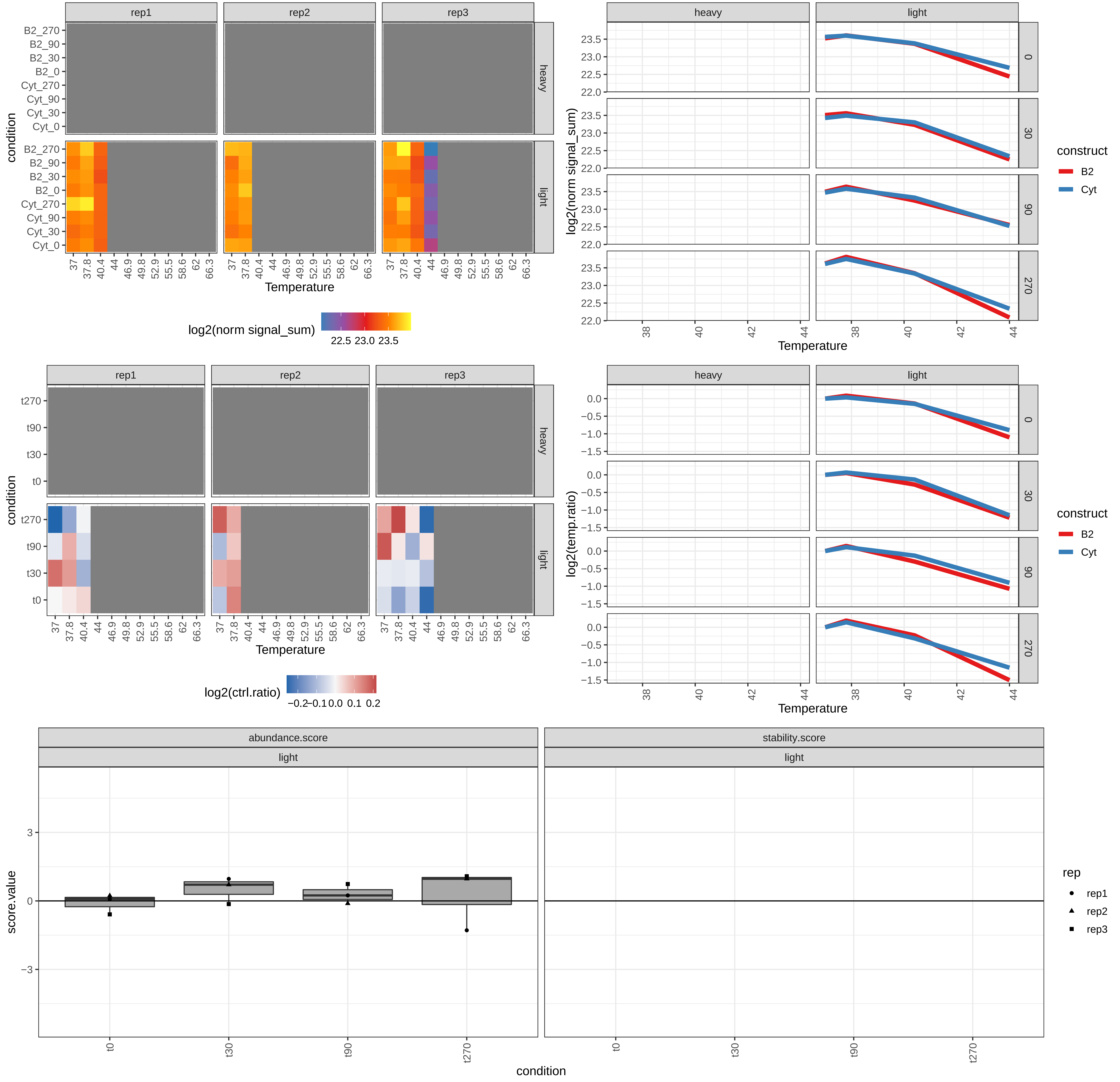

Q08282 − tRNA wybutosine−synthesizing protein 4 OS=Saccharomyces cerevisiae (strain ATCC 204508 / S288c) GN=PPM2 PE=1 SV=2

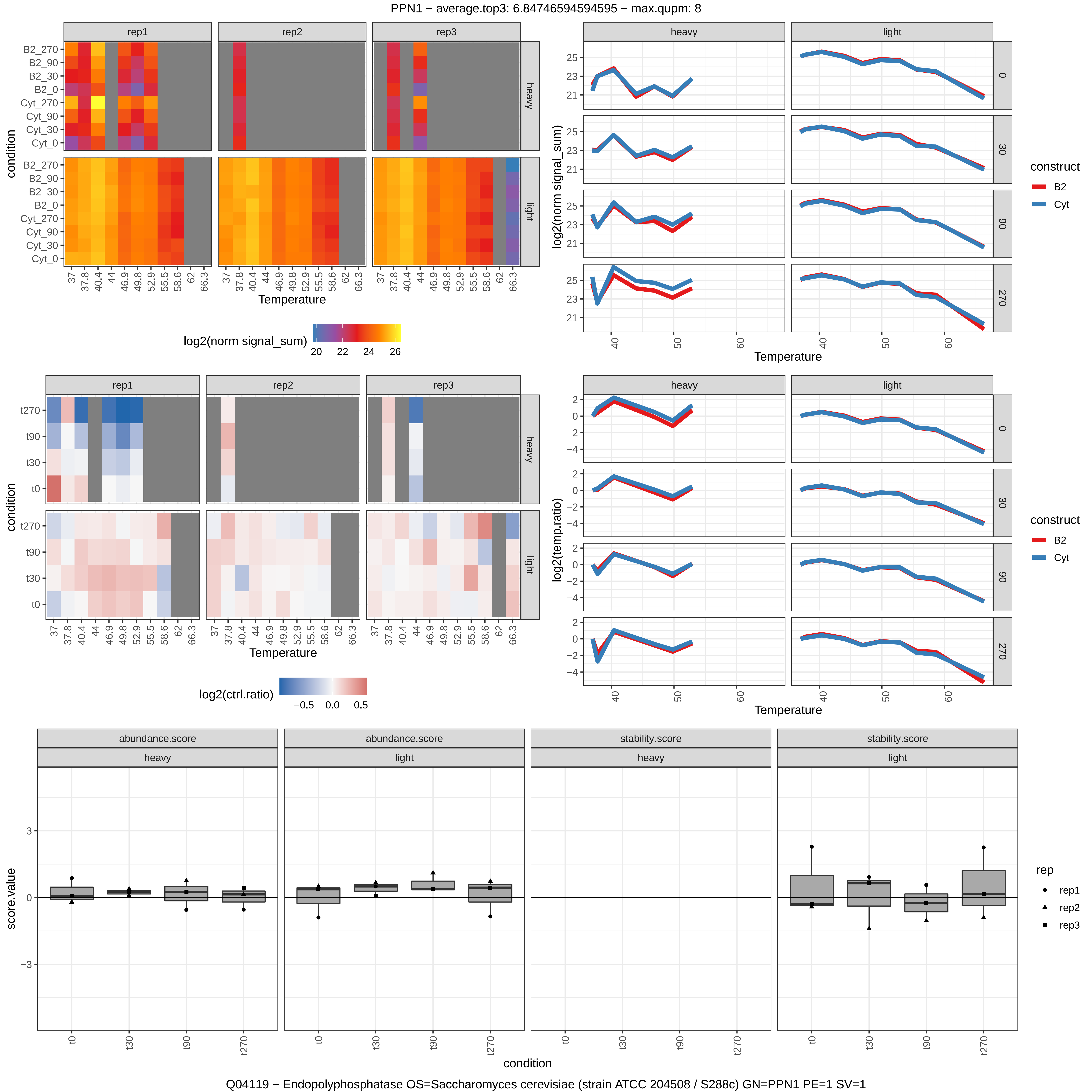

PPN1 − average.top3: 6.84746594594595 − max.qupm: 8
rep1
rep2
rep3
heavy
light
condition
Temperature
log2(norm signal_sum)
log2(temp.ratio)
log2(ctrl.ratio)
construct
B2
Cyt
abundance.score
stability.score
score.value
rep
rep1
rep2
rep3
Q04119 − Endopolyphosphatase OS=Saccharomyces cerevisiae (strain ATCC 204508 / S288c) GN=PPN1 PE=1 SV=1

PPS1 − average.top3: 6.41929071428571 − max.qupm: 4

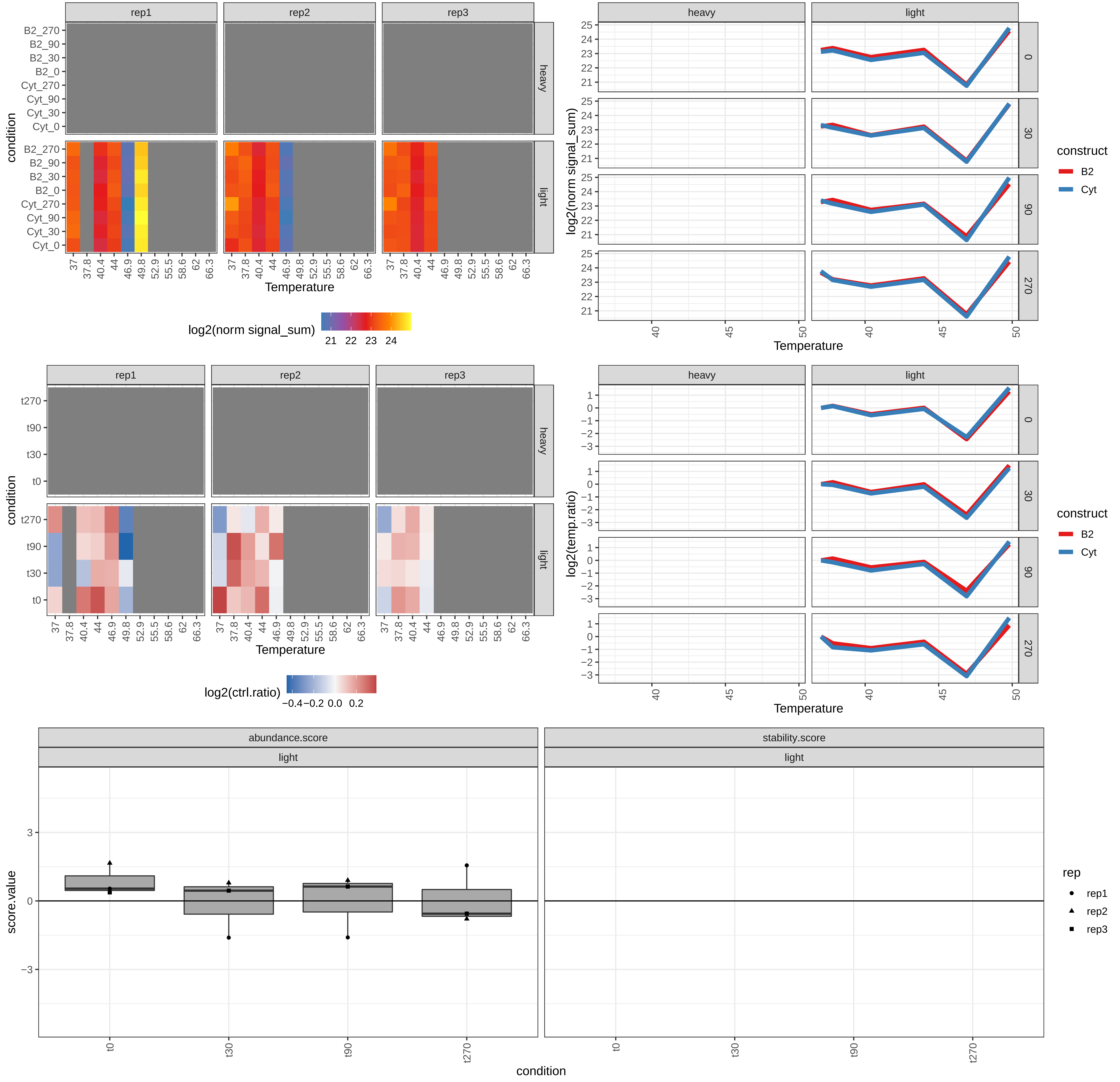

P38148 − Dual specificity protein phosphatase PPS1 OS=Saccharomyces cerevisiae (strain ATCC 204508 / S288c) GN=PPS1 PE=3 SV=1

PPT1 − average.top3: 6.68854071428571 − max.qupm: 12

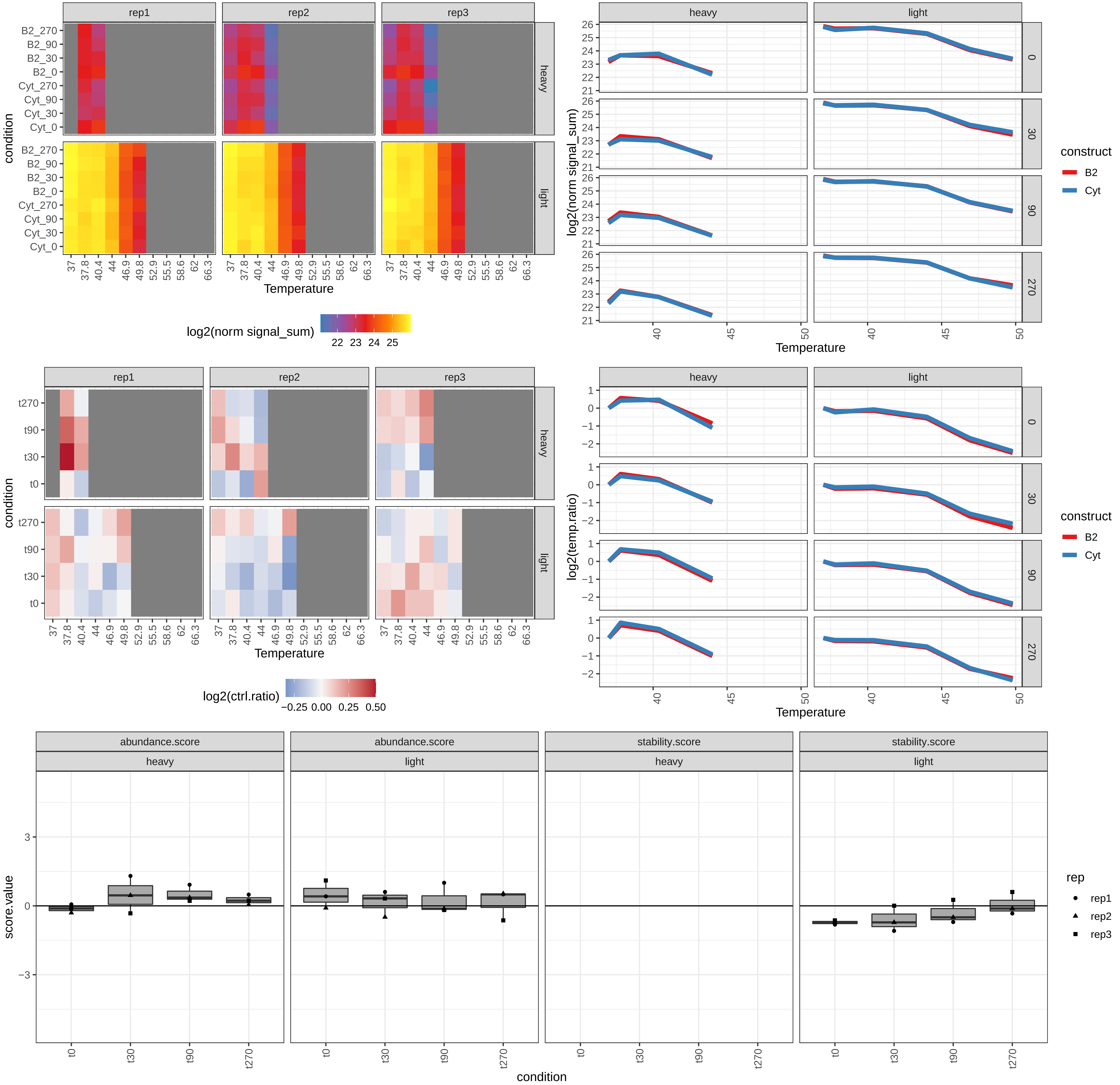

P53043 − Serine/threonine−protein phosphatase T OS=Saccharomyces cerevisiae (strain ATCC 204508 / S288c) GN=PPT1 PE=1 SV=1

PPX1 − average.top3: 7.0488 − max.qupm: 10

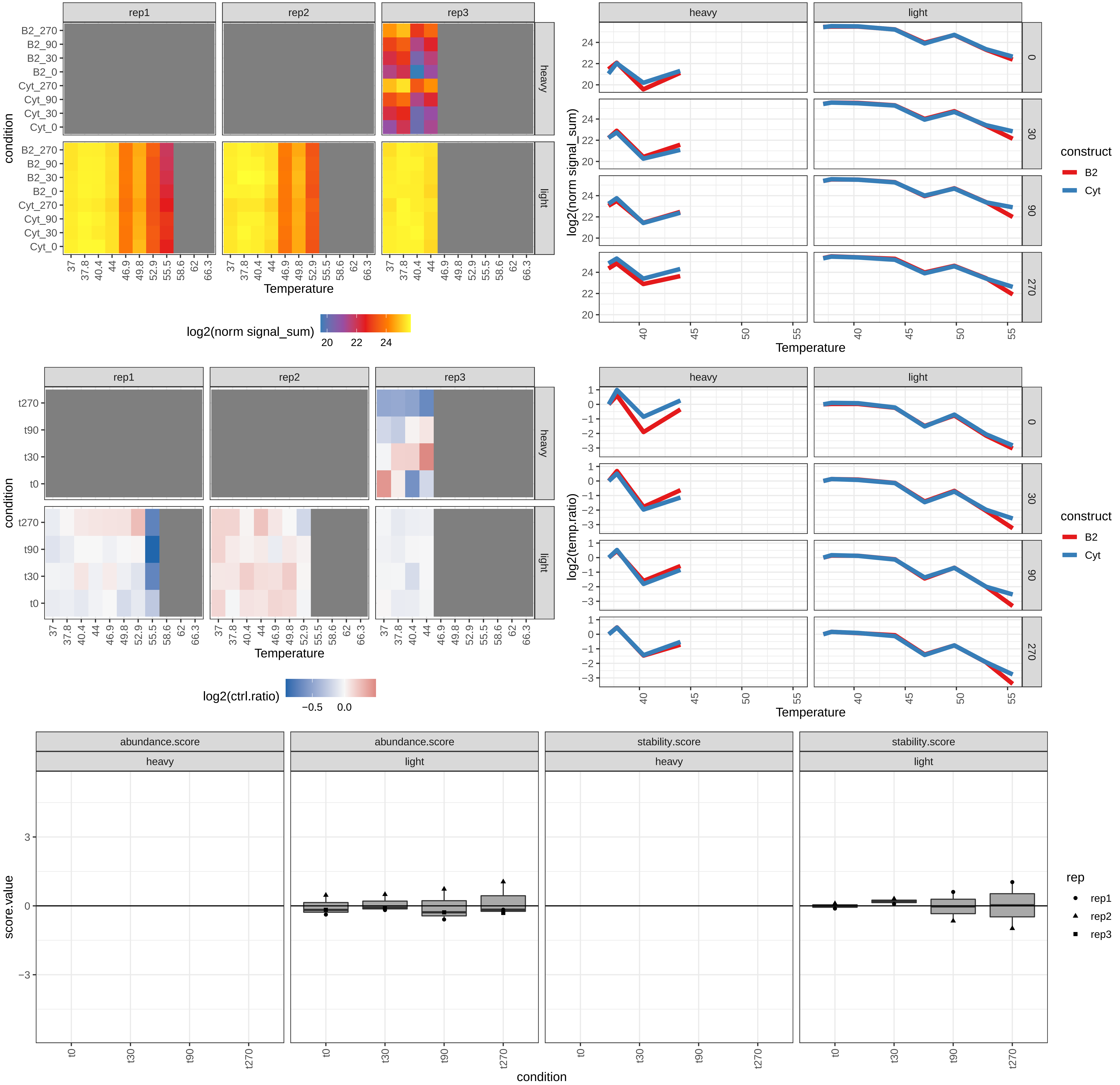

P38698 − Exopolyphosphatase OS=Saccharomyces cerevisiae (strain ATCC 204508 / S288c) GN=PPX1 PE=1 SV=1

PPZ1 − average.top3: 6.86185095238095 − max.qupm: 6

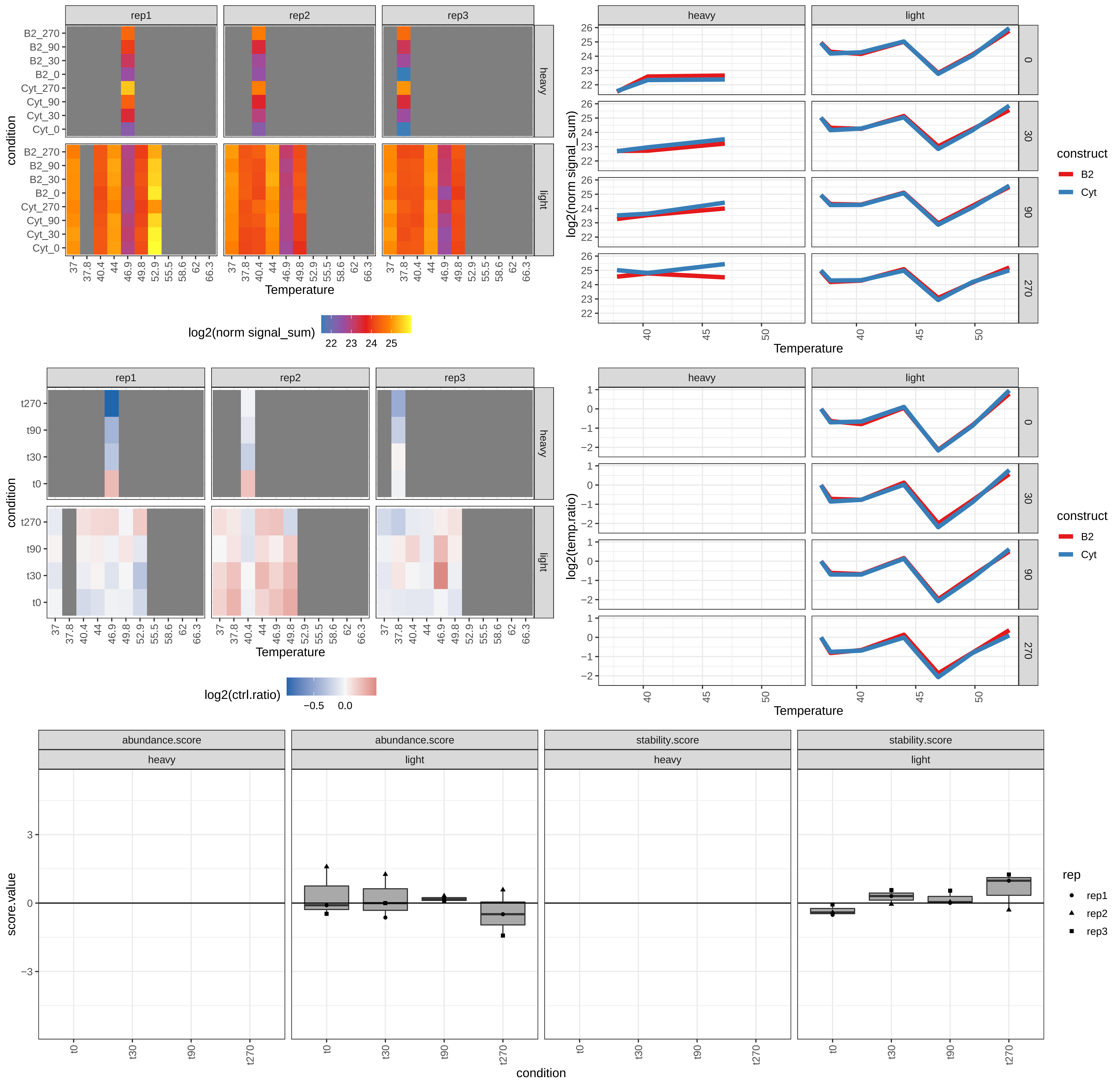

P26570 − Serine/threonine−protein phosphatase PP−Z1 OS=Saccharomyces cerevisiae (strain ATCC 204508 / S288c) GN=PPZ1 PE=1 SV=5

PRB1 − average.top3: 7.9519659375 − max.qupm: 12

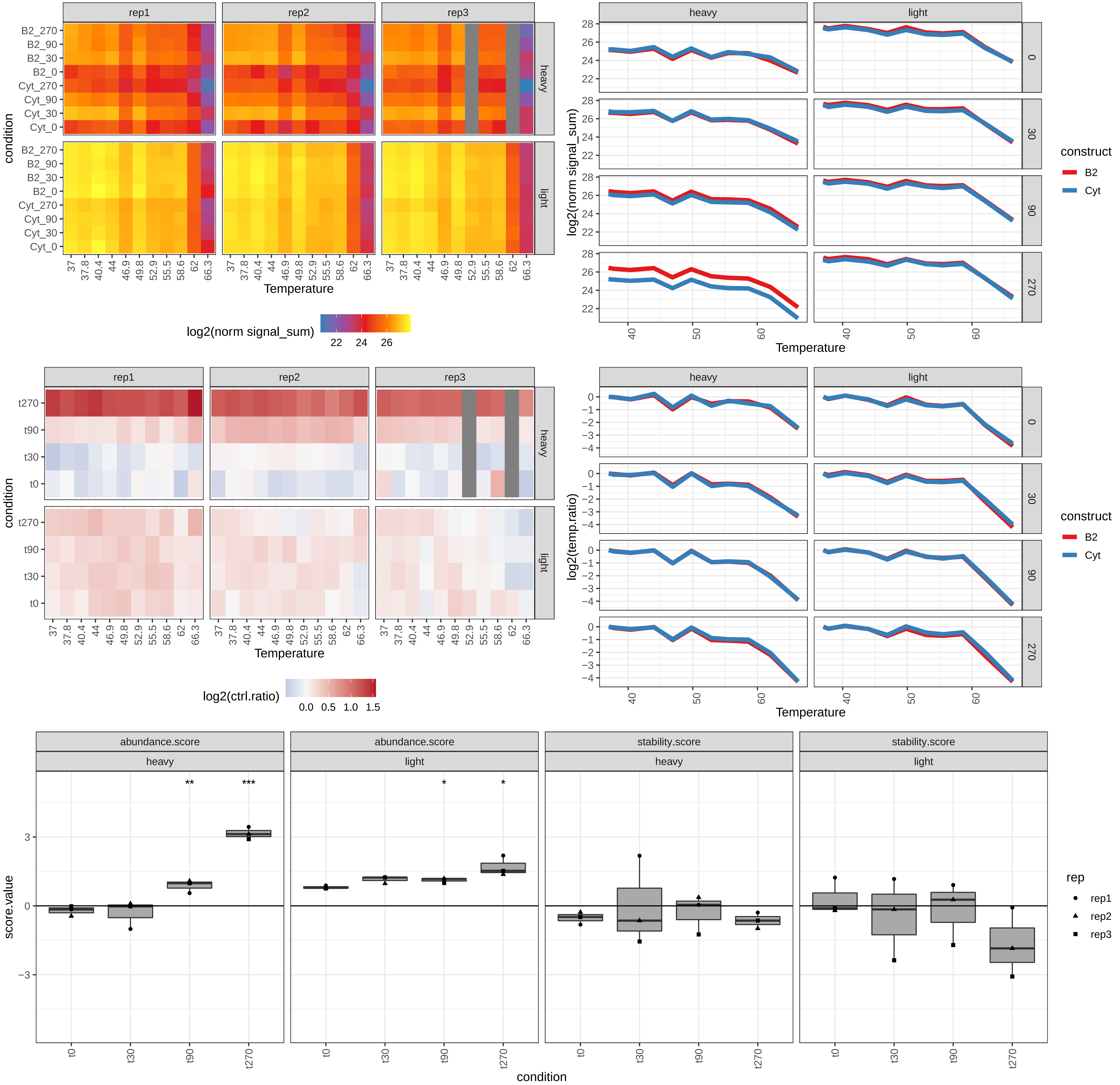

P09232 − Cerevisin OS=Saccharomyces cerevisiae (strain ATCC 204508 / S288c) GN=PRB1 PE=1 SV=1

PRC1 − average.top3: 7.55564134615385 − max.qupm: 6

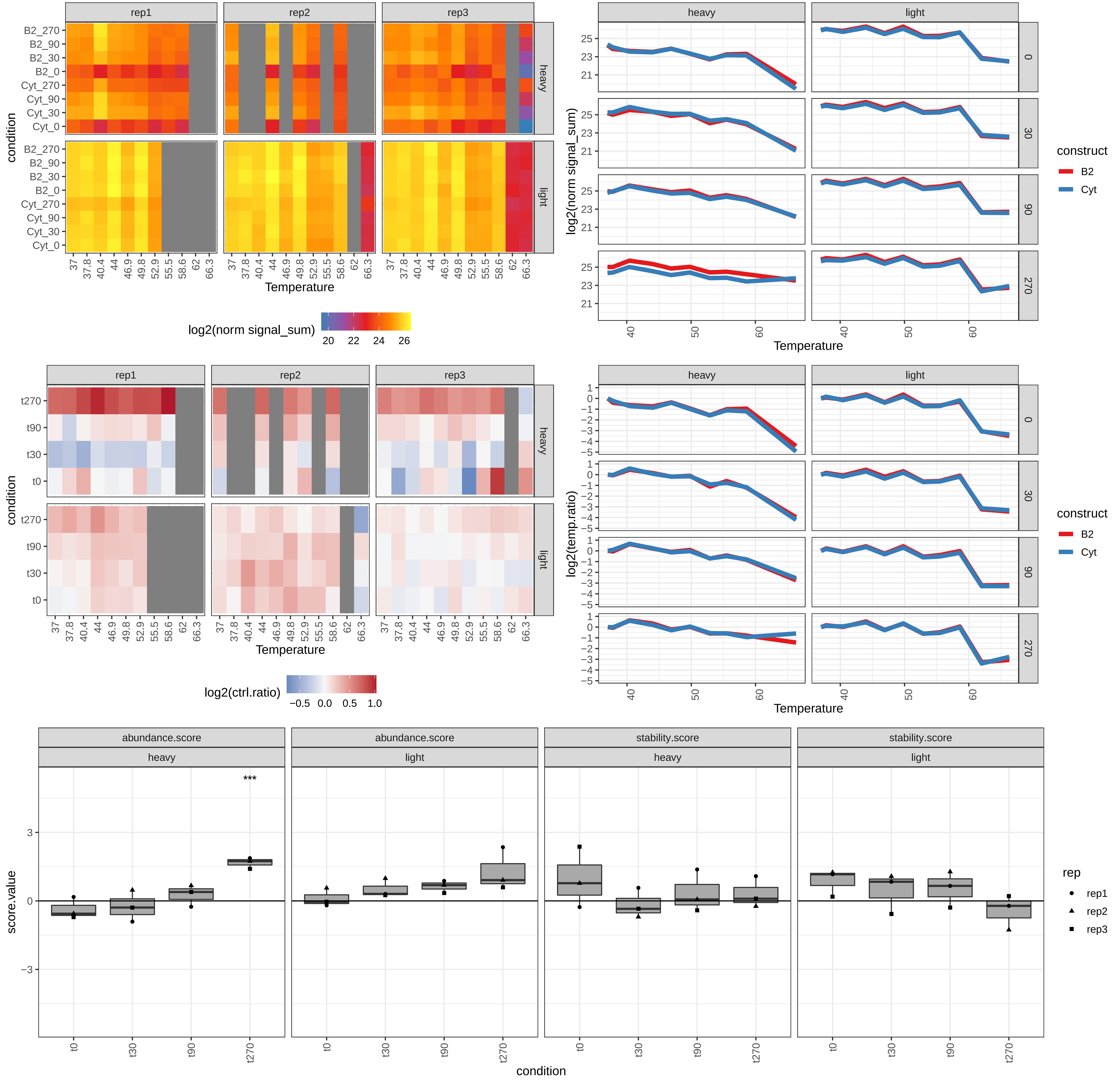

P00729 − Carboxypeptidase Y OS=Saccharomyces cerevisiae (strain ATCC 204508 / S288c) GN=PRC1 PE=1 SV=1

PRD1 − average.top3: 7.12930714285714 − max.qupm: 23

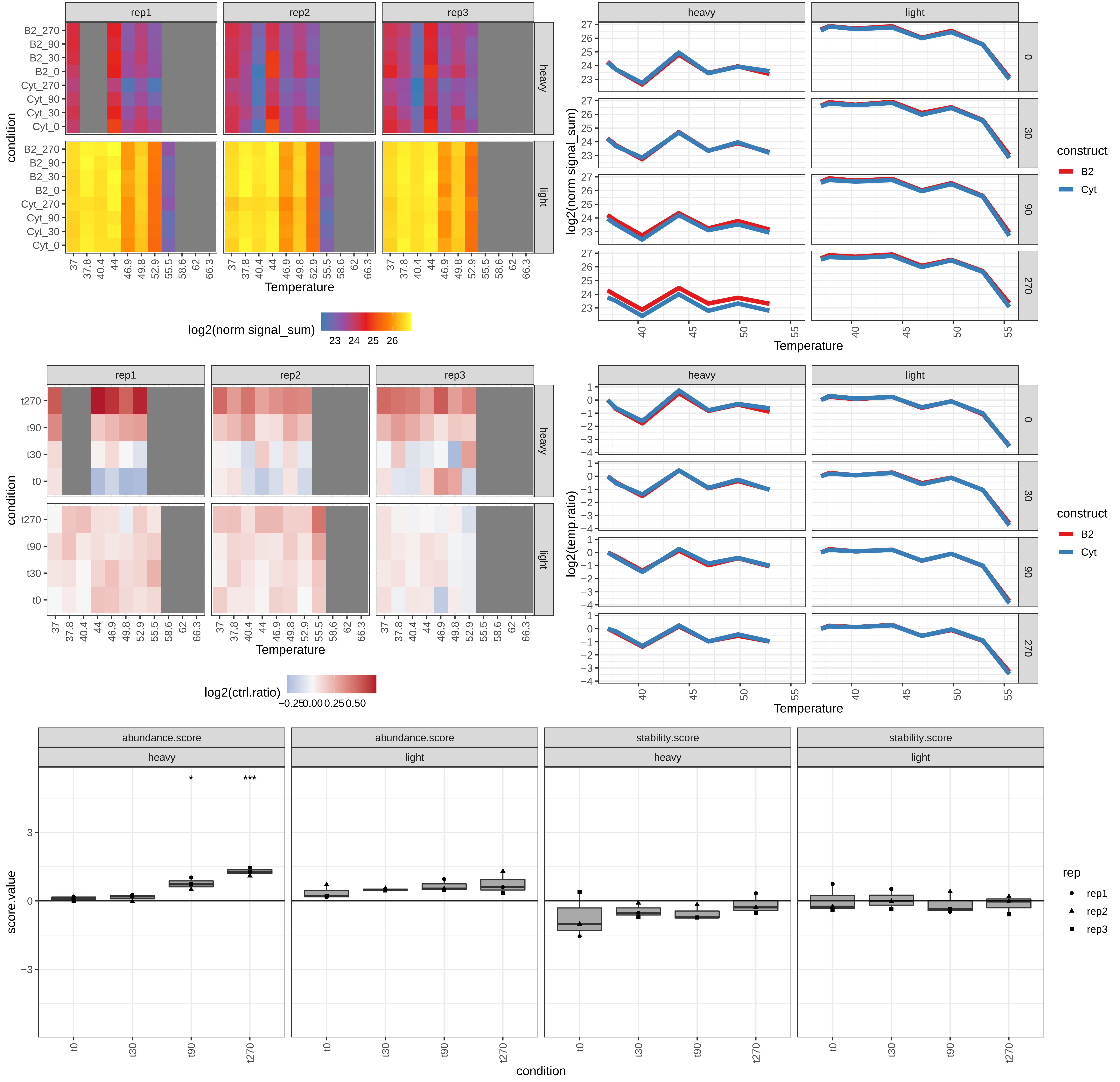

P25375 − Saccharolysin OS=Saccharomyces cerevisiae (strain ATCC 204508 / S288c) GN=PRD1 PE=1 SV=1

PRE1 − average.top3: 7.17174254545455 − max.qupm: 6

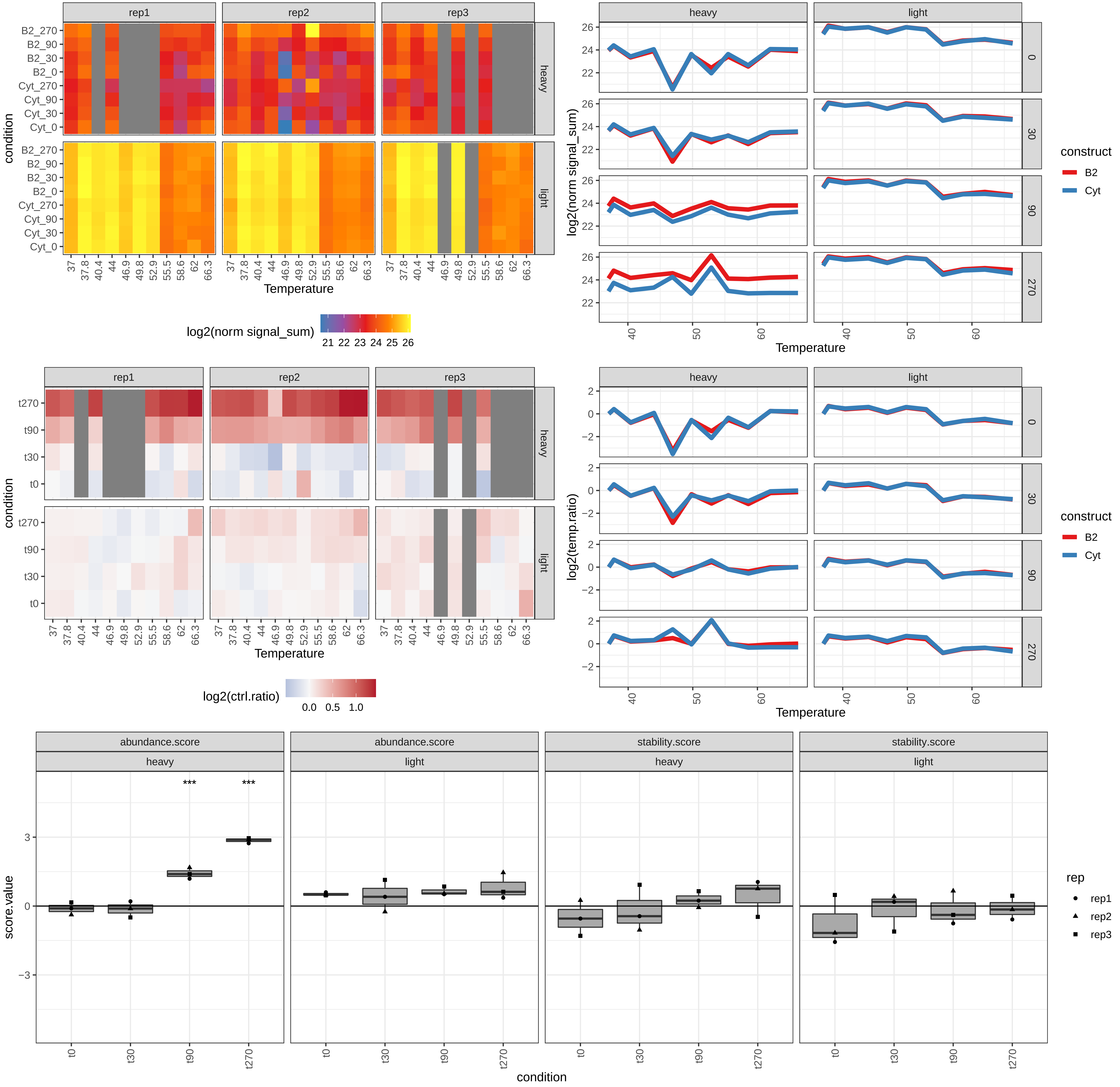

P22141 − Proteasome subunit beta type−4 OS=Saccharomyces cerevisiae (strain ATCC 204508 / S288c) GN=PRE1 PE=1 SV=2

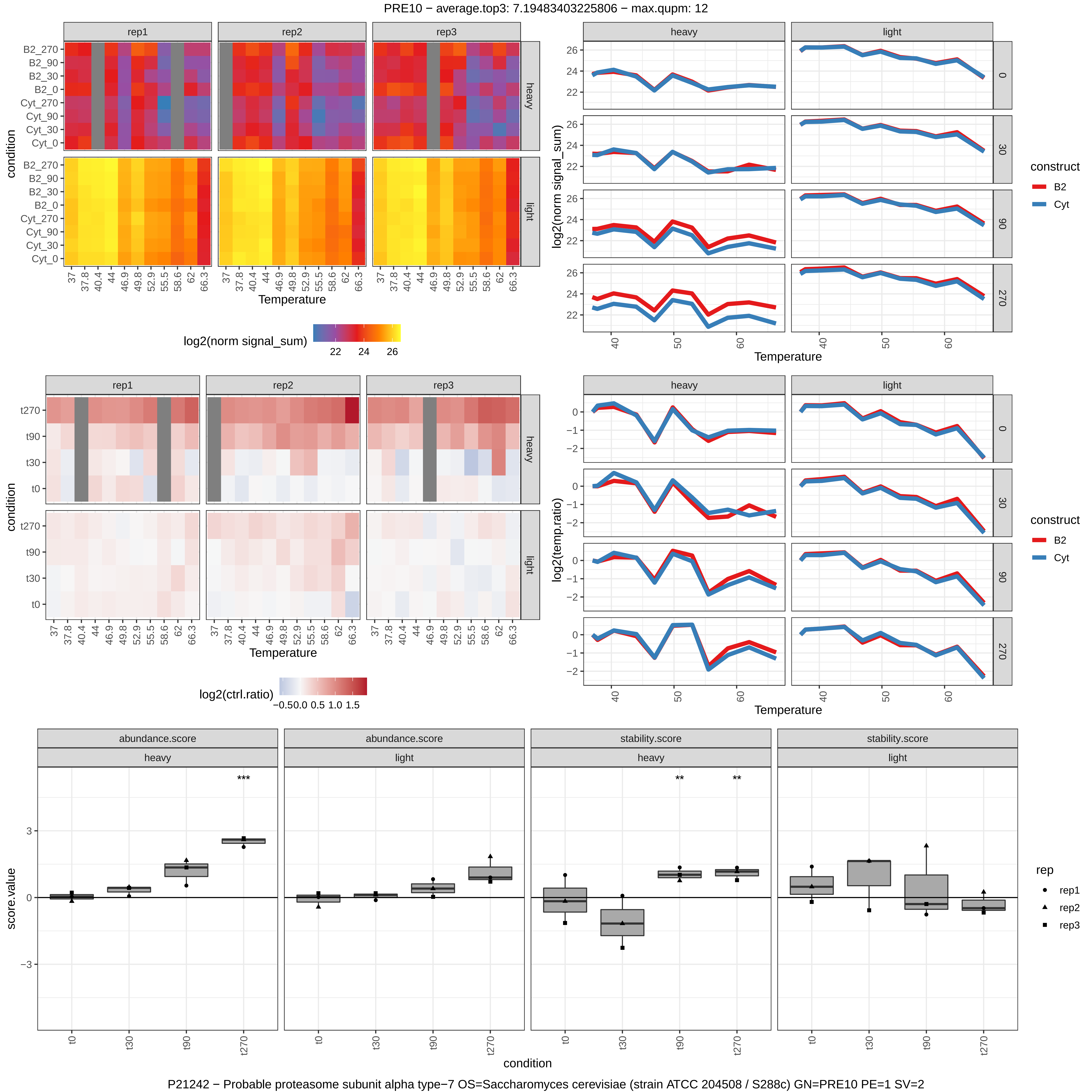

PRE10 − average.top3: 7.19483403225806 − max.qupm: 12
rep1
rep2
rep3
heavy
light
condition
Temperature
log2(norm signal_sum)
construct
B2
Cyt
log2(ctrl.ratio)
log2(temp.ratio)
abundance.score
stability.score
score.value
rep
rep1
rep2
rep3
***
**
**
P21242 − Probable proteasome subunit alpha type−7 OS=Saccharomyces cerevisiae (strain ATCC 204508 / S288c) GN=PRE10 PE=1 SV=2

PRE2 − average.top3: 7.09042719298246 − max.qupm: 6

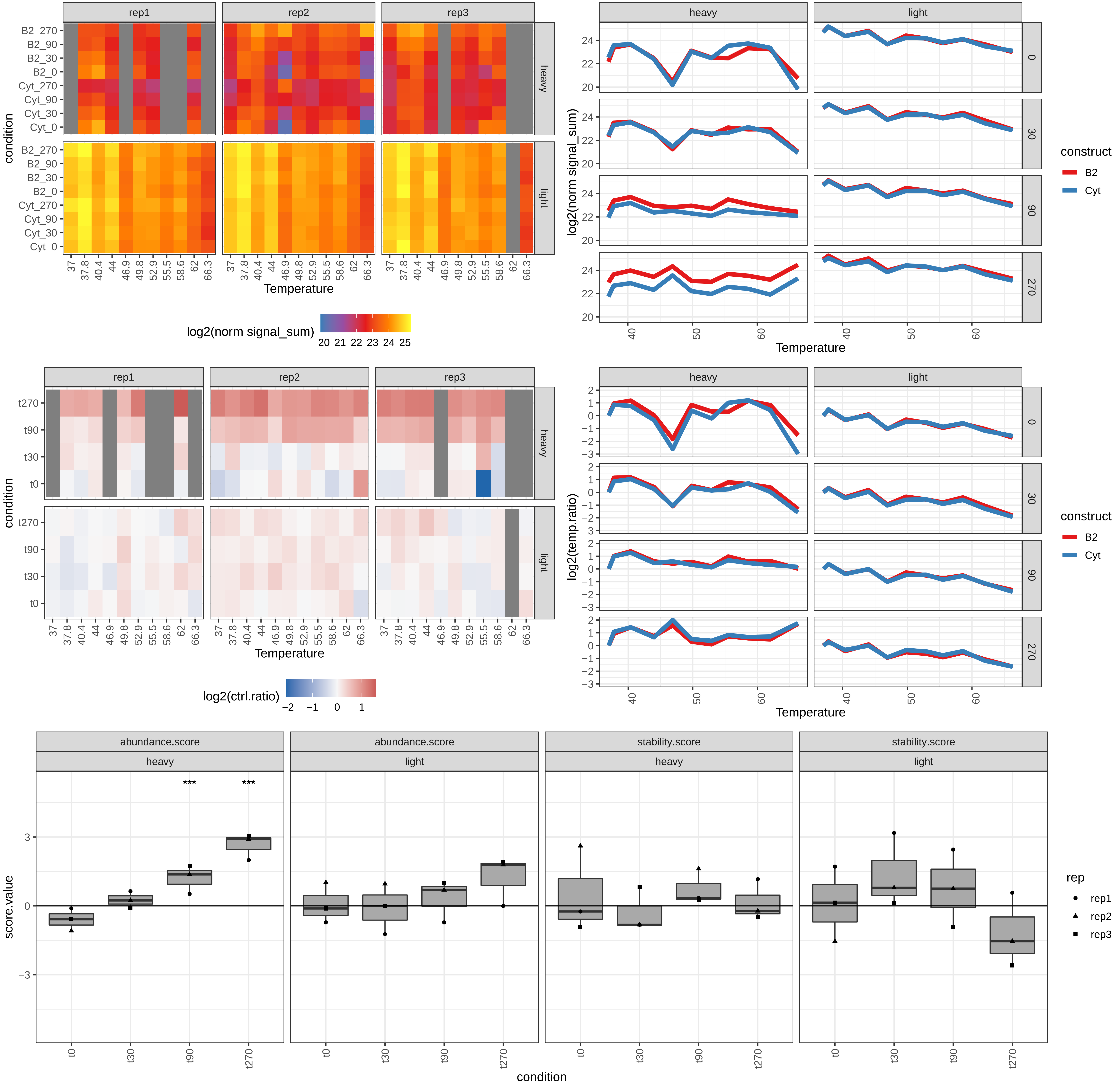

P30656 − Proteasome subunit beta type−5 OS=Saccharomyces cerevisiae (strain ATCC 204508 / S288c) GN=PRE2 PE=1 SV=3

PRE3 − average.top3: 7.36874359375 − max.qupm: 7

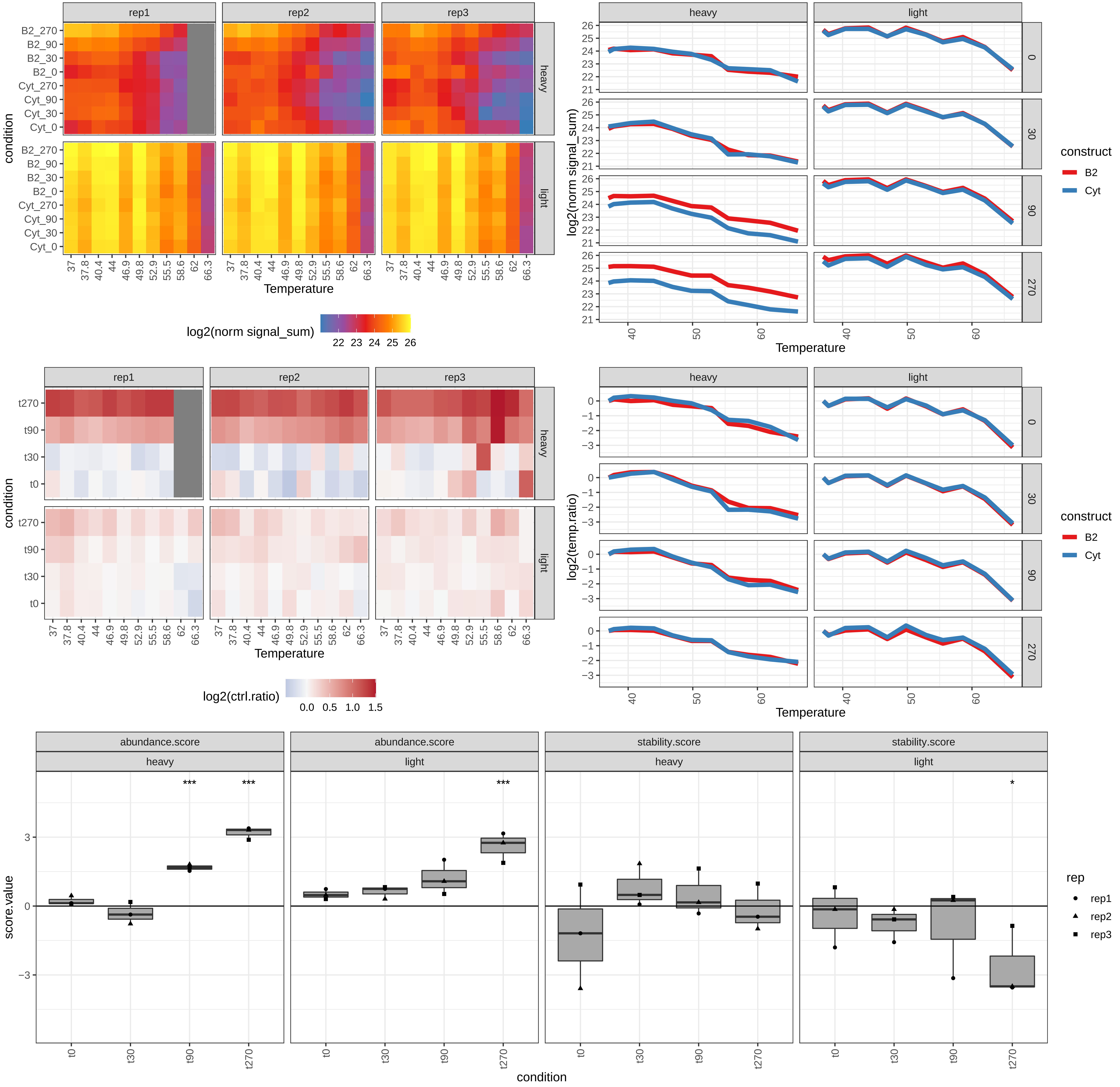

P38624 − Proteasome subunit beta type−1 OS=Saccharomyces cerevisiae (strain ATCC 204508 / S288c) GN=PRE3 PE=1 SV=2

PRE4 − average.top3: 7.31728428571429 − max.qupm: 6

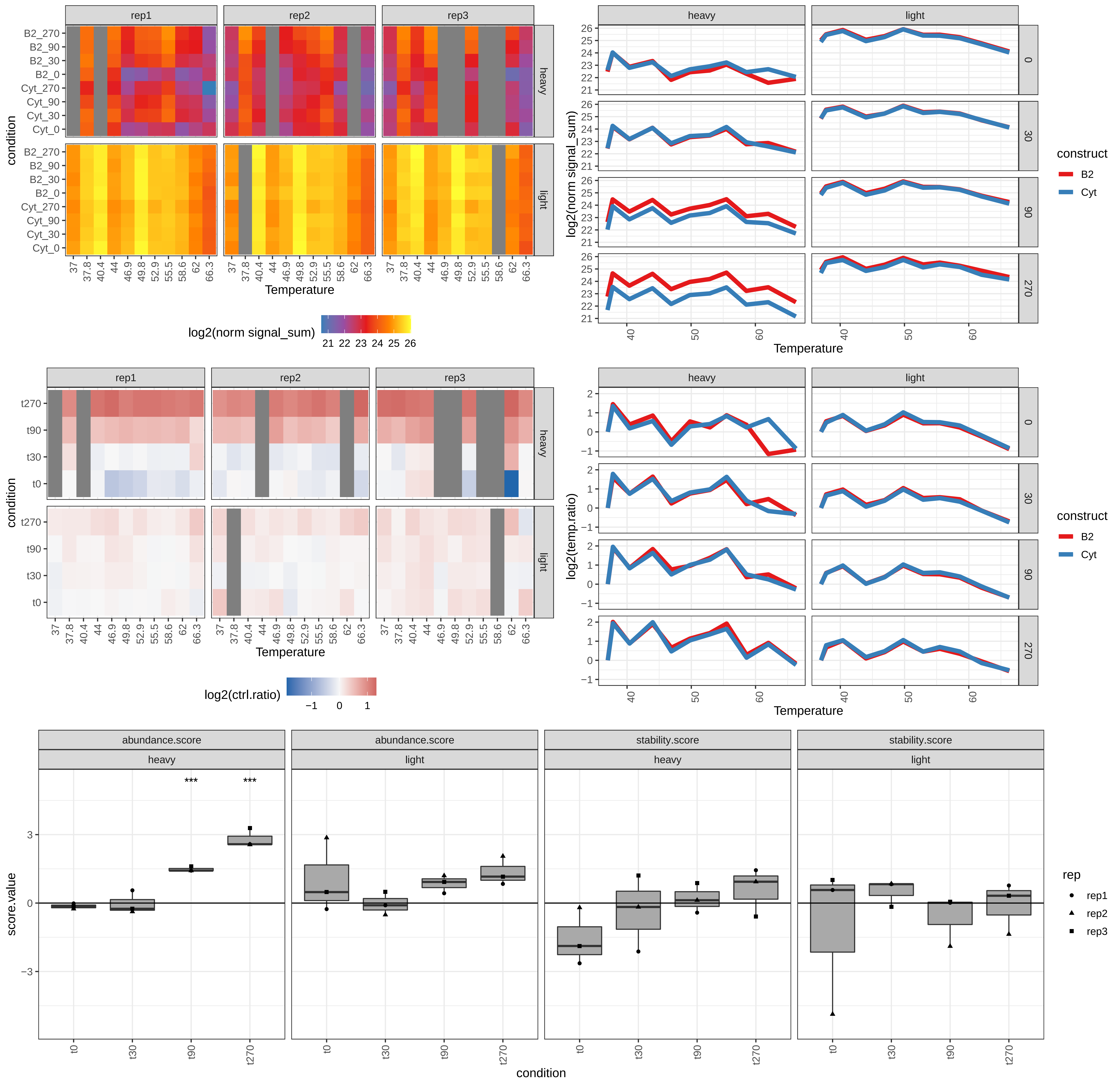

P30657 − Proteasome subunit beta type−7 OS=Saccharomyces cerevisiae (strain ATCC 204508 / S288c) GN=PRE4 PE=1 SV=1

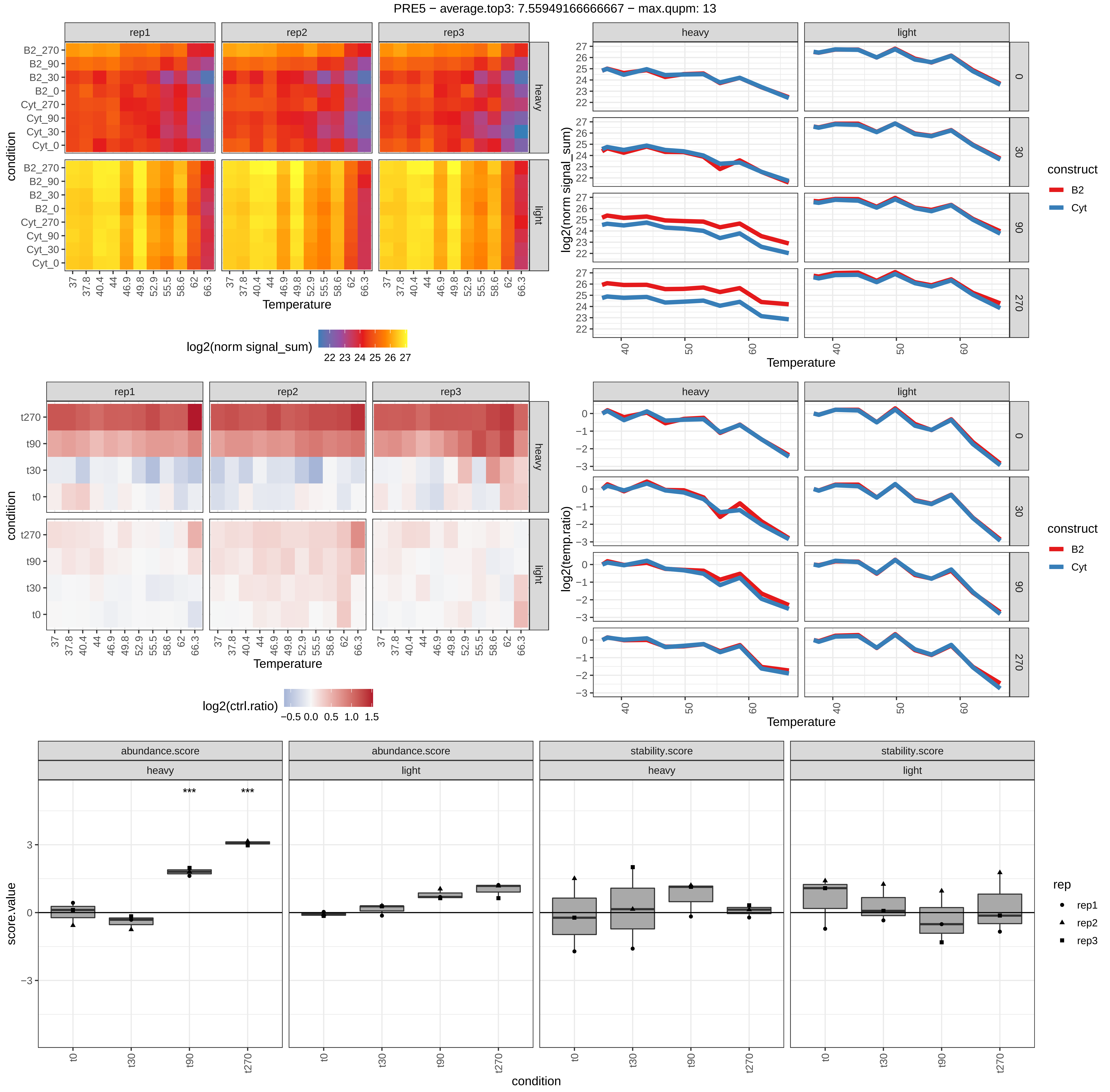

P40302 − Proteasome subunit alpha type−6 OS=Saccharomyces cerevisiae (strain ATCC 204508 / S288c) GN=PRE5 PE=1 SV=1

PRE6 − average.top3: 7.09497016129032 − max.qupm: 9

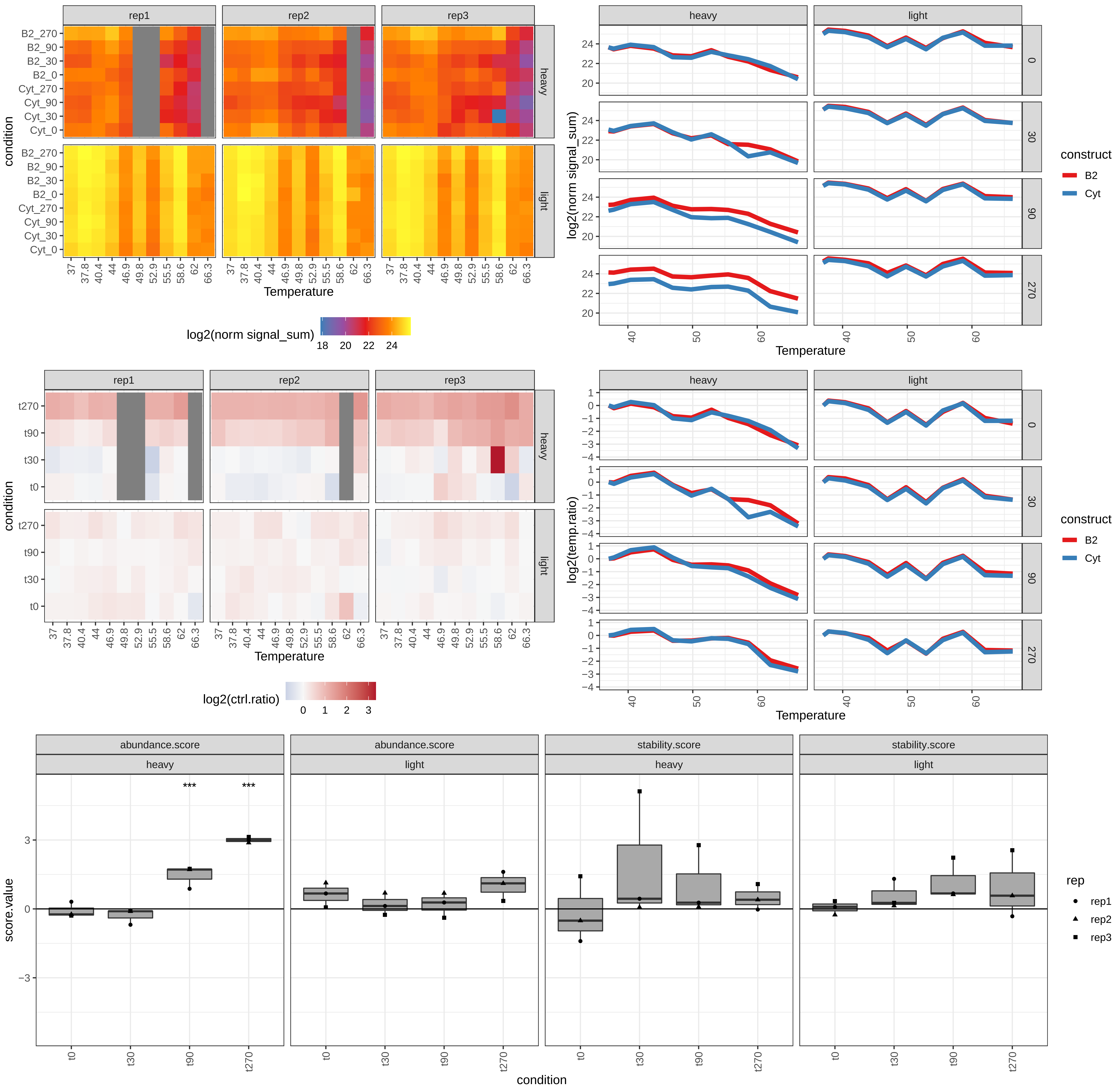

P40303 − Proteasome subunit alpha type−4 OS=Saccharomyces cerevisiae (strain ATCC 204508 / S288c) GN=PRE6 PE=1 SV=1

PRE7 − average.top3: 7.48546893939394 − max.qupm: 8

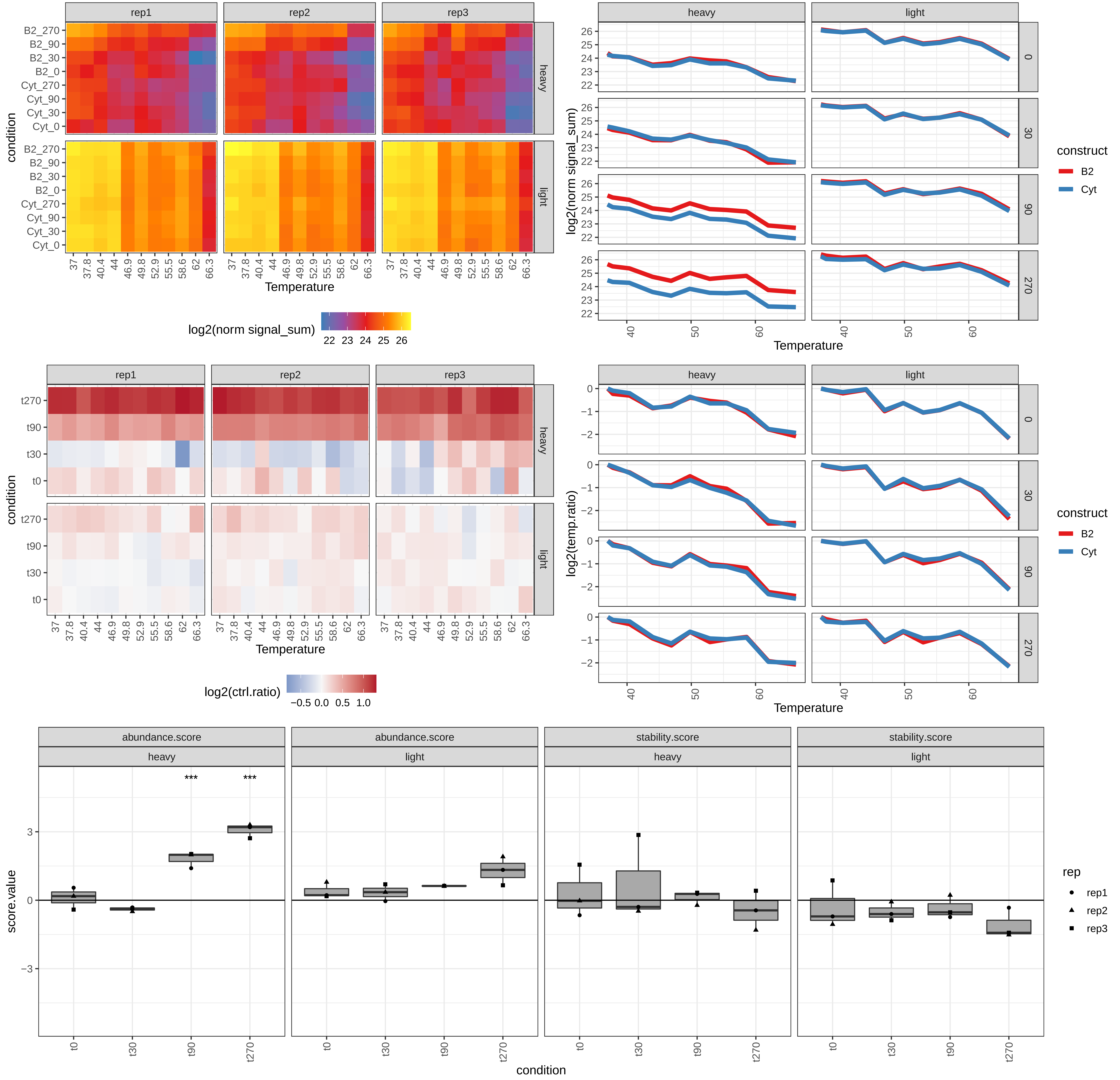

P23724 − Proteasome subunit beta type−6 OS=Saccharomyces cerevisiae (strain ATCC 204508 / S288c) GN=PRE7 PE=1 SV=1

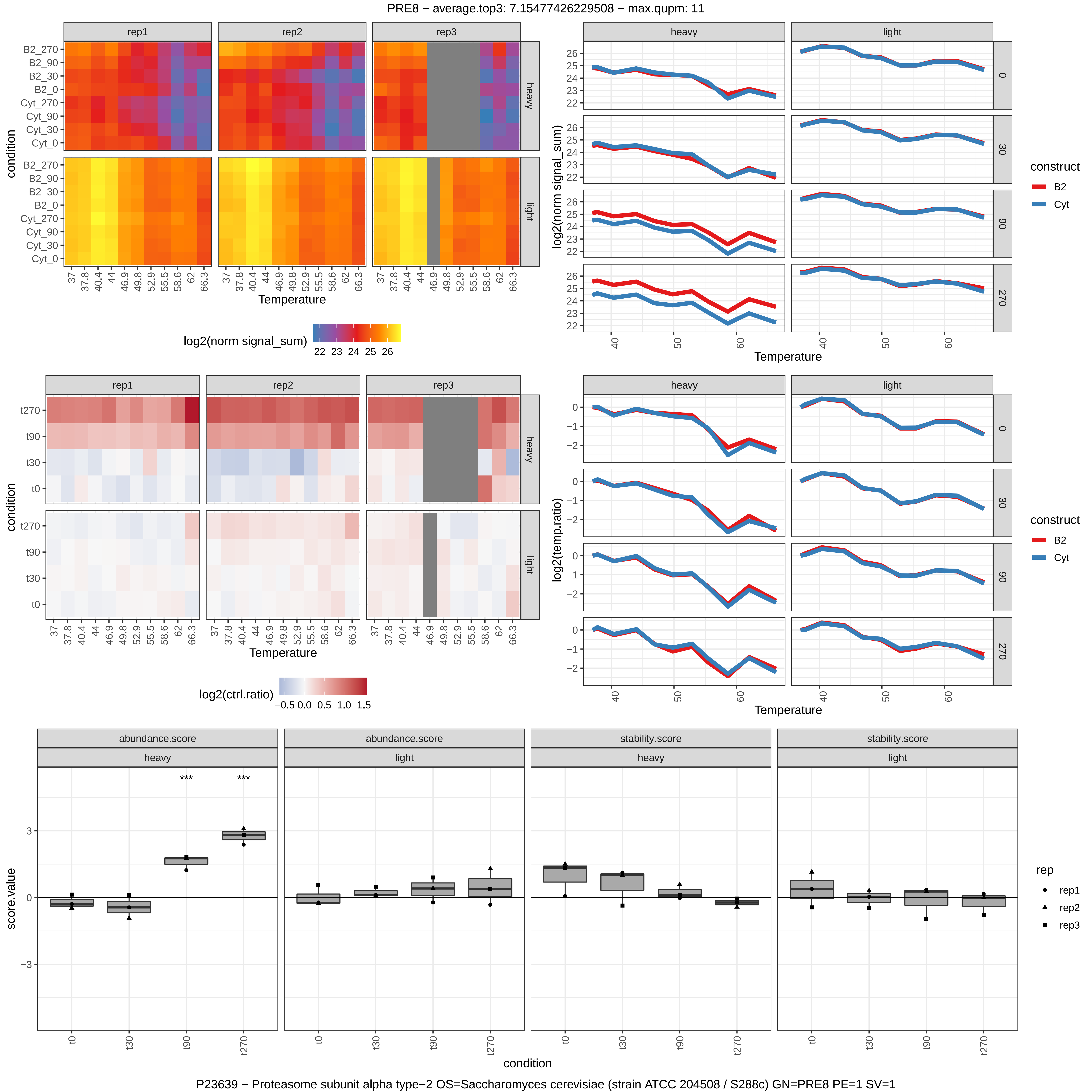

PRE8 − average.top3: 7.15477426229508 − max.qupm: 11
rep1
rep2
rep3
heavy
light
condition
Temperature
log2(norm signal_sum)
log2(norm signal_sum)
construct
B2
Cyt
t270
t90
t30
t0
log2(ctrl.ratio)
log2(temp.ratio)
abundance.score
stability.score
score.value
rep
***
P23639 − Proteasome subunit alpha type−2 OS=Saccharomyces cerevisiae (strain ATCC 204508 / S288c) GN=PRE8 PE=1 SV=1

PRE9 − average.top3: 6.99188507692308 − max.qupm: 11

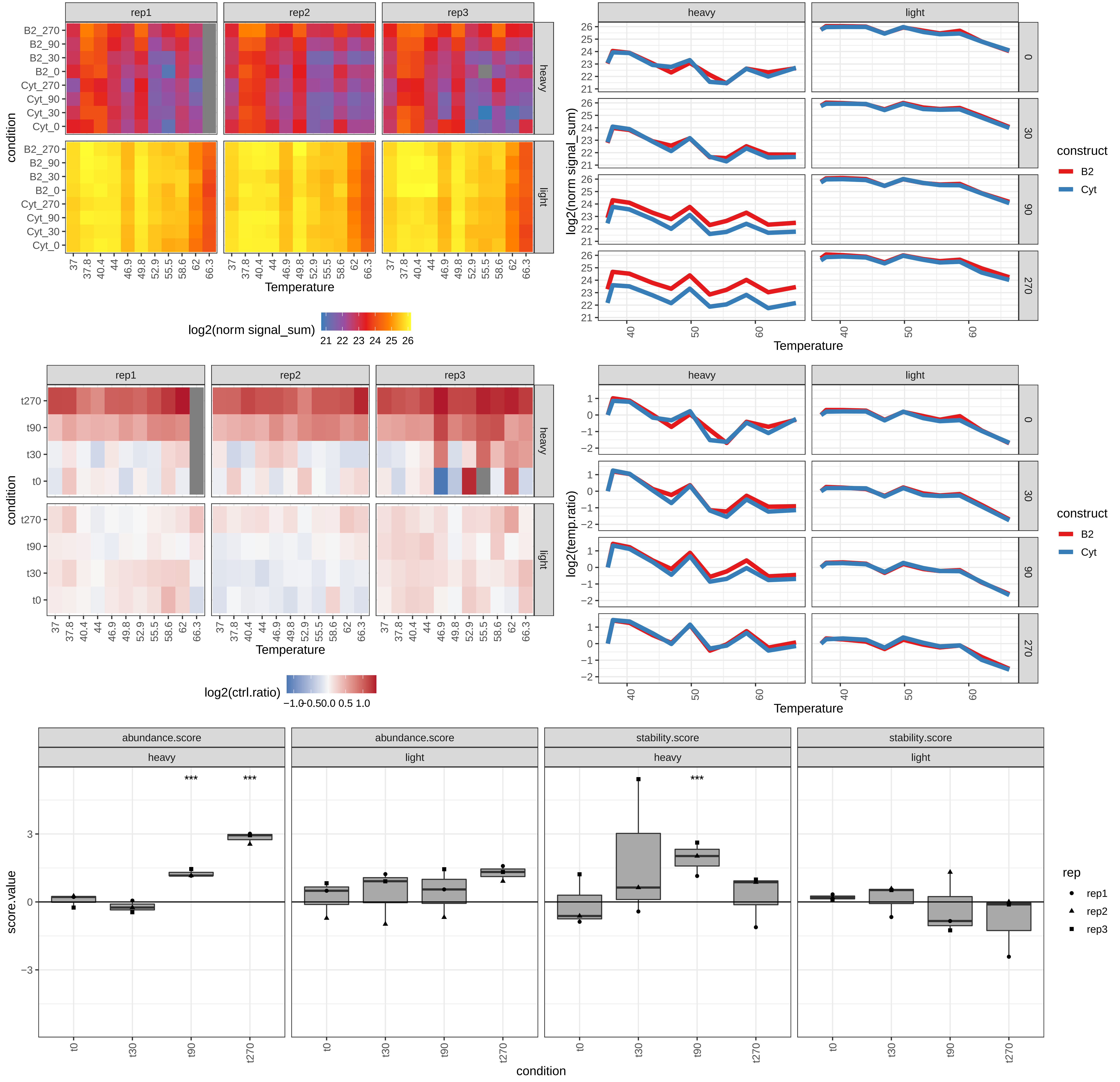

P23638 − Proteasome subunit alpha type−3 OS=Saccharomyces cerevisiae (strain ATCC 204508 / S288c) GN=PRE9 PE=1 SV=1

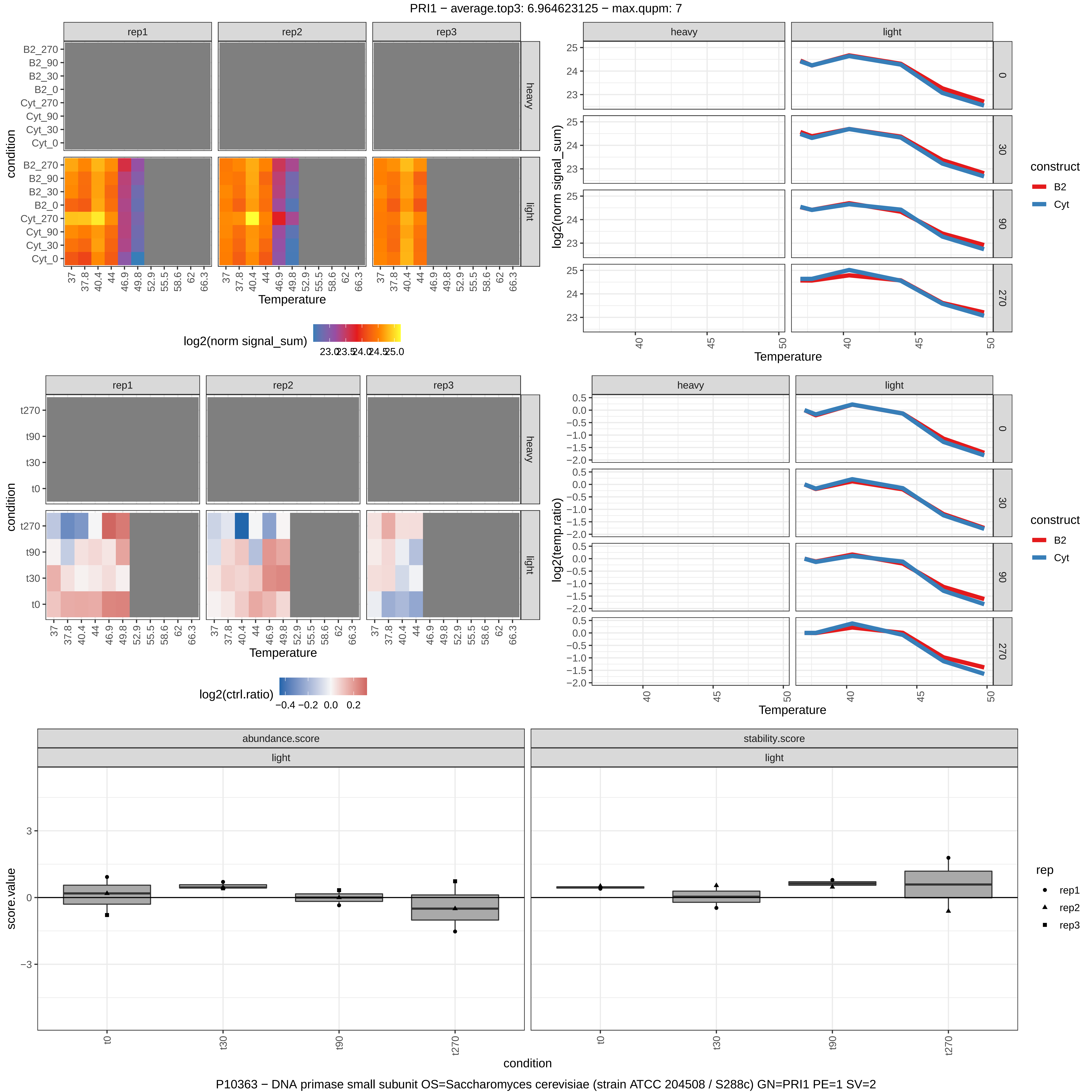
PRI1 − average.top3: 6.964623125 − max.qupm: 7
rep1
rep2
rep3
heavy
light
condition
Temperature
log2(norm signal_sum)
log2(ctrl.ratio)
log2(temp.ratio)
construct
B2
Cyt
abundance.score
stability.score
score.value
rep
rep1
rep2
rep3
P10363 − DNA primase small subunit OS=Saccharomyces cerevisiae (strain ATCC 204508 / S288c) GN=PRI1 PE=1 SV=2

PRI2 − average.top3: 6.411551 − max.qupm: 7

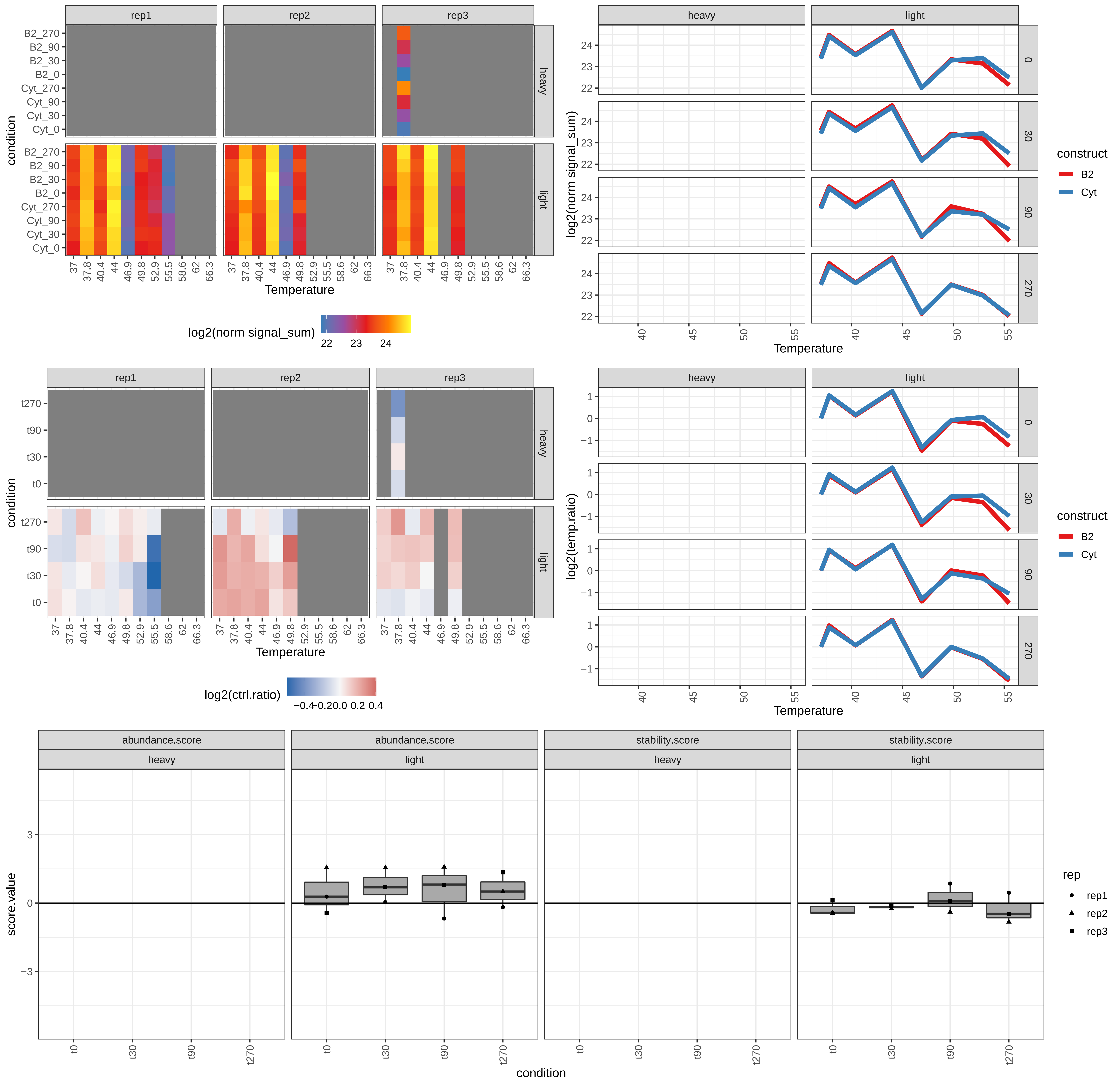

P20457 − DNA primase large subunit OS=Saccharomyces cerevisiae (strain ATCC 204508 / S288c) GN=PRI2 PE=1 SV=1

PRM15 − average.top3: 6.916884 − max.qupm: 11

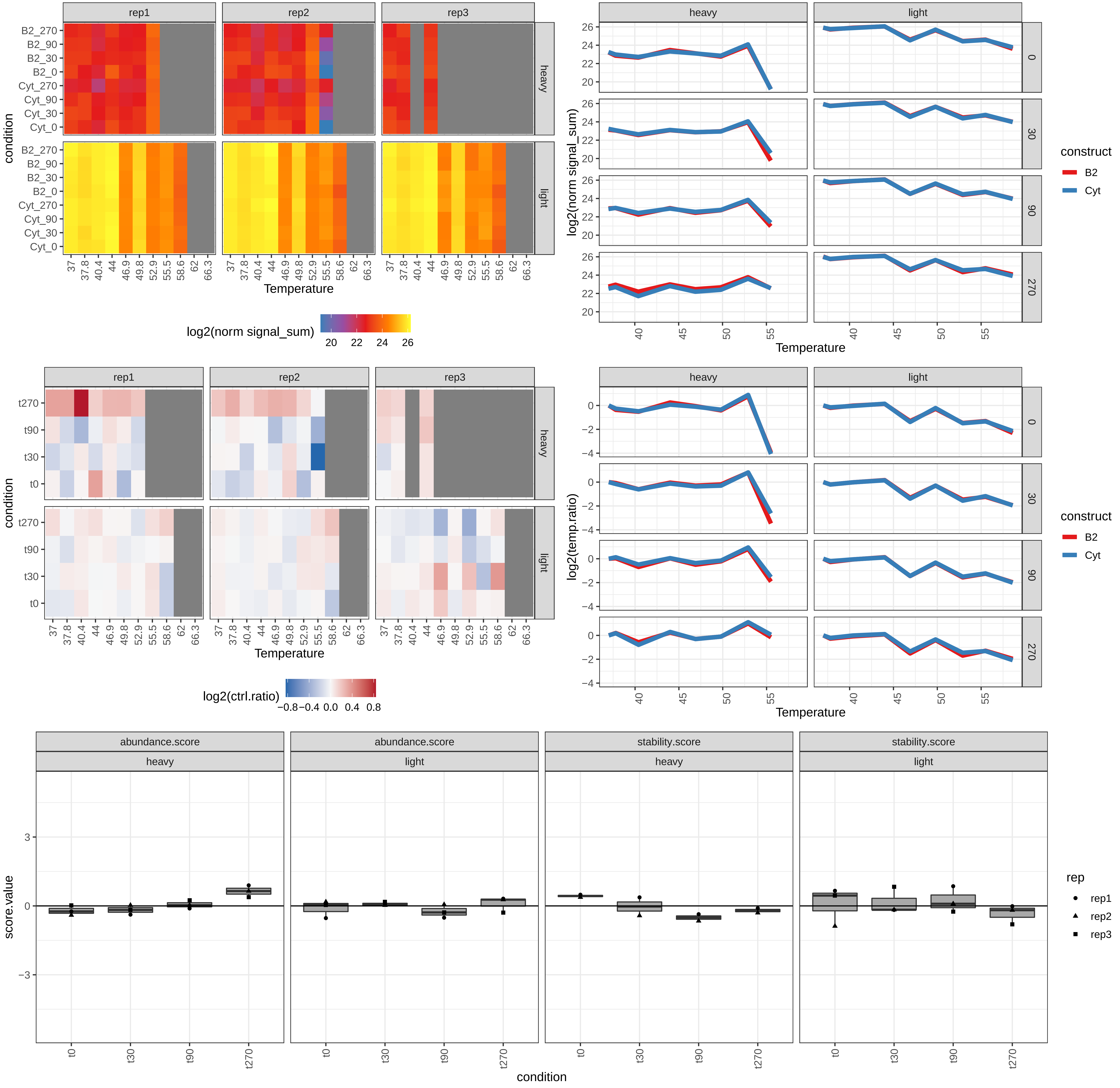

Q03262 − Phosphoribomutase OS=Saccharomyces cerevisiae (strain ATCC 204508 / S288c) GN=PRM15 PE=1 SV=1

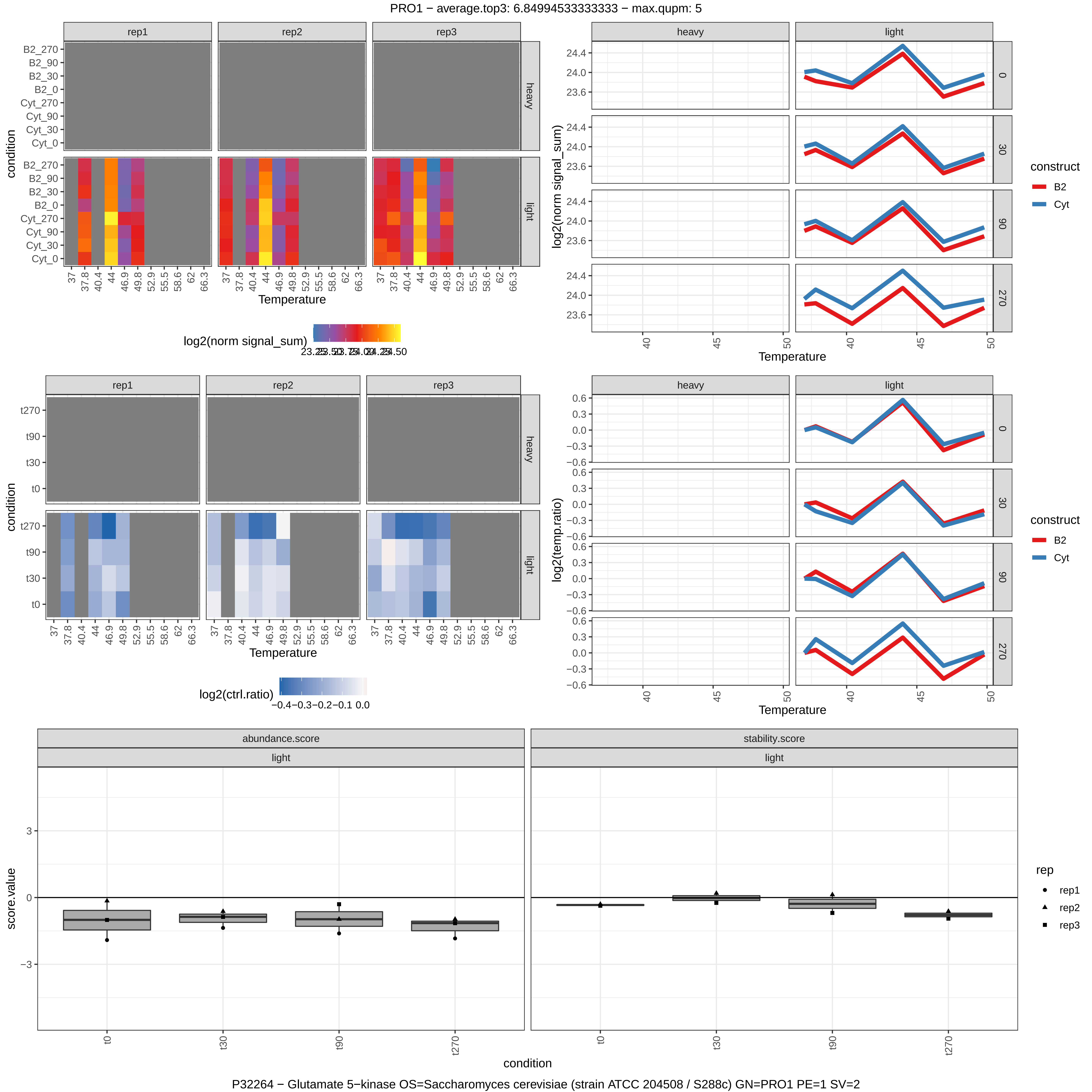

PRO1 − average.top3: 6.84994533333333 − max.qupm: 5
rep1
rep2
rep3
heavy
light
condition
Temperature
log2(norm signal_sum)
log2(ctrl.ratio)
log2(temp.ratio)
construct
B2
Cyt
abundance.score
stability.score
score.value
rep
rep1
rep2
rep3
P32264 − Glutamate 5−kinase OS=Saccharomyces cerevisiae (strain ATCC 204508 / S288c) GN=PRO1 PE=1 SV=2

PRO2 − average.top3: 7.38096033898305 − max.qupm: 18

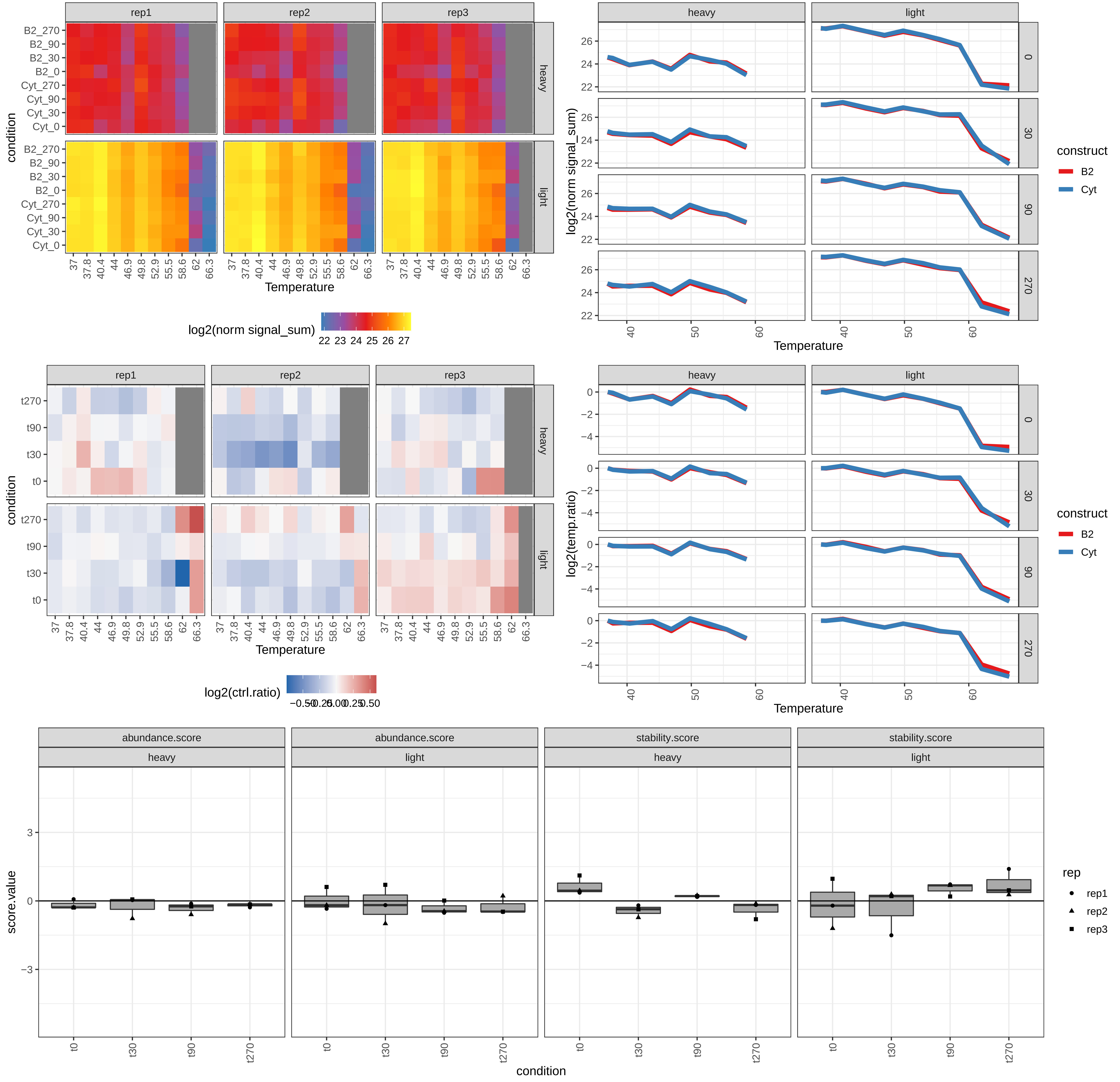

P54885 − Gamma−glutamyl phosphate reductase OS=Saccharomyces cerevisiae (strain ATCC 204508 / S288c) GN=PRO2 PE=1 SV=1

PRO3 − average.top3: 7.03958058823529 − max.qupm: 7

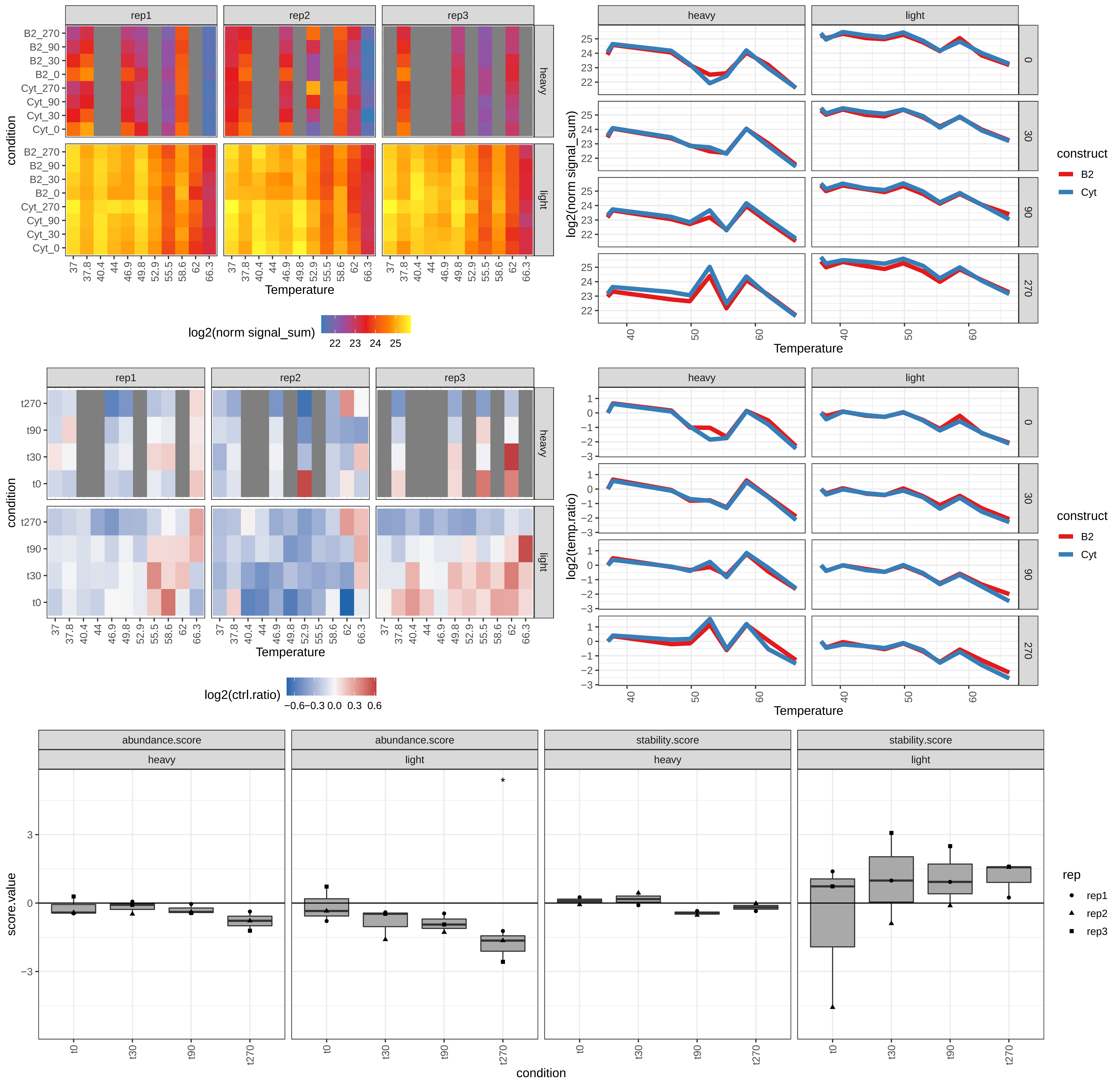

P32263 − Pyrroline−5−carboxylate reductase OS=Saccharomyces cerevisiae (strain ATCC 204508 / S288c) GN=PRO3 PE=1 SV=1

PRP19 − average.top3: 6.78296476190476 − max.qupm: 5

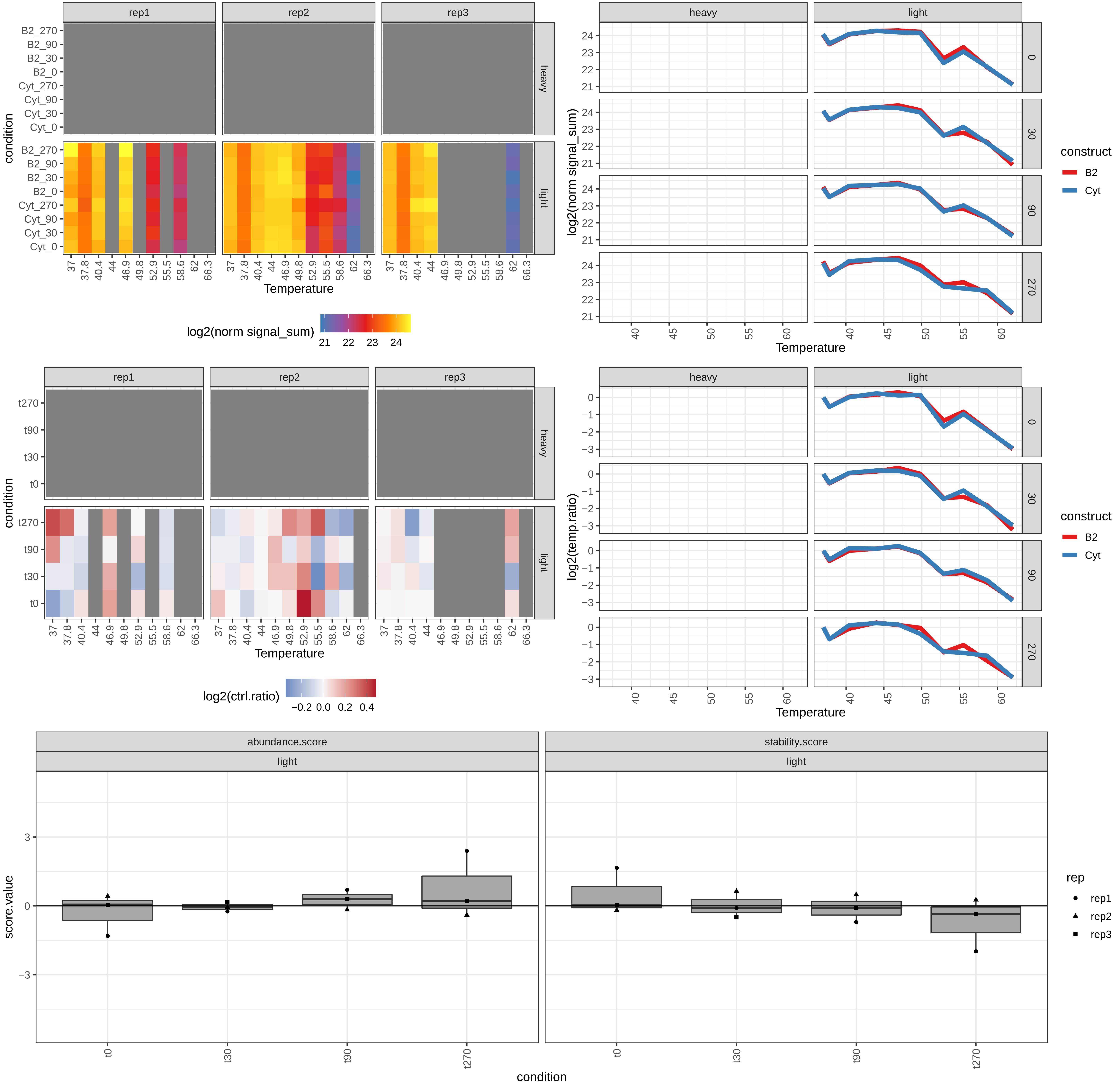

P32523 − Pre−mRNA−processing factor 19 OS=Saccharomyces cerevisiae (strain ATCC 204508 / S288c) GN=PRP19 PE=1 SV=2

PRP28 − average.top3: 6.75891384615385 − max.qupm: 6

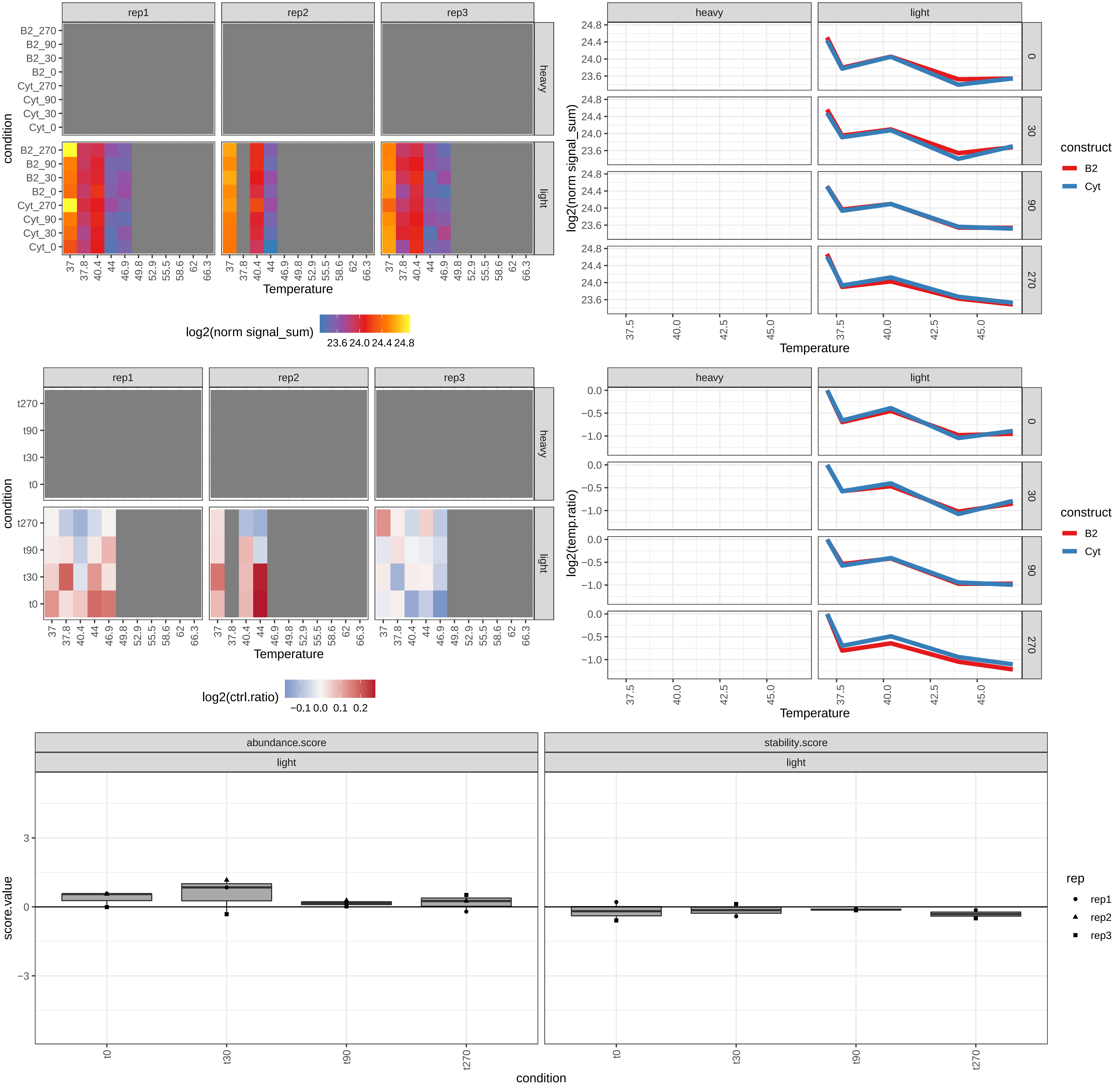

P23394 − Pre−mRNA−splicing ATP−dependent RNA helicase PRP28 OS=Saccharomyces cerevisiae (strain ATCC 204508 / S288c) GN=PRP28 PE=1 SV=2

PRP4 − average.top3: 6.74722923076923 − max.qupm: 4

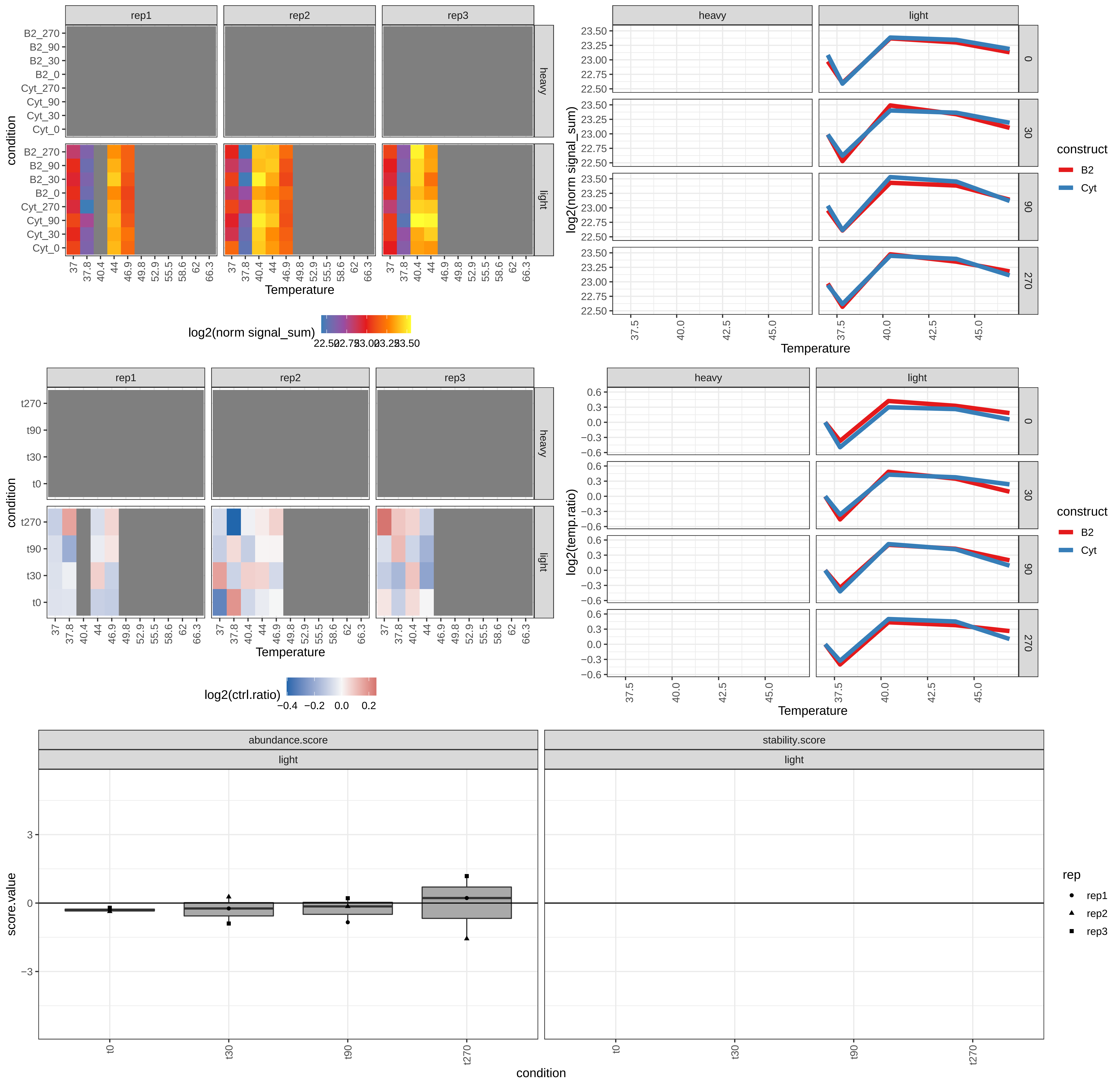

P20053 − U4/U6 small nuclear ribonucleoprotein PRP4 OS=Saccharomyces cerevisiae (strain ATCC 204508 / S288c) GN=PRP4 PE=1 SV=1

PRP42 − average.top3: 6.955115 − max.qupm: 3

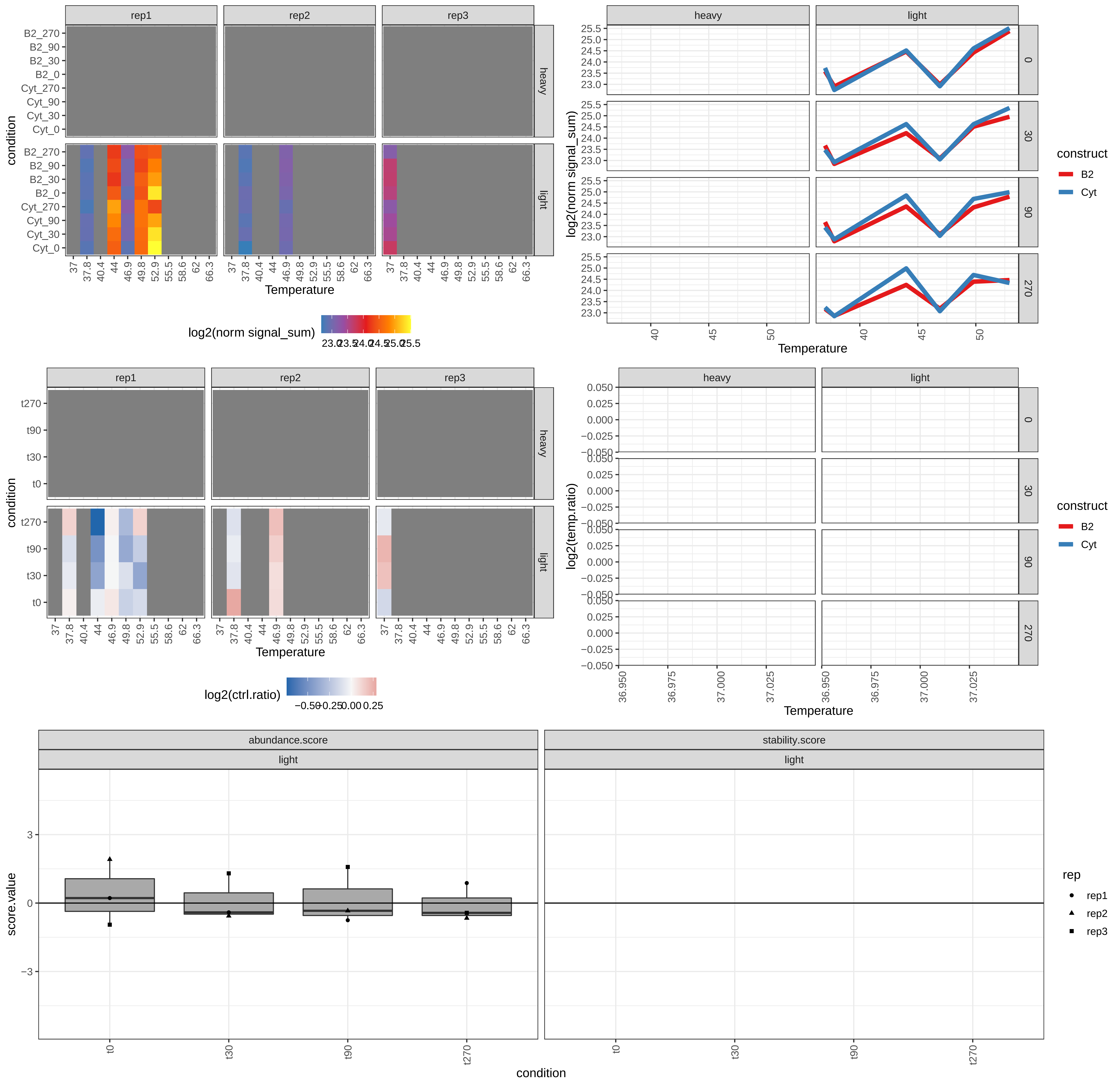

Q03776 − U1 small nuclear ribonucleoprotein component PRP42 OS=Saccharomyces cerevisiae (strain ATCC 204508 / S288c) GN=PRP42 PE=1 SV=1

PRP43 − average.top3: 6.9984025 − max.qupm: 16

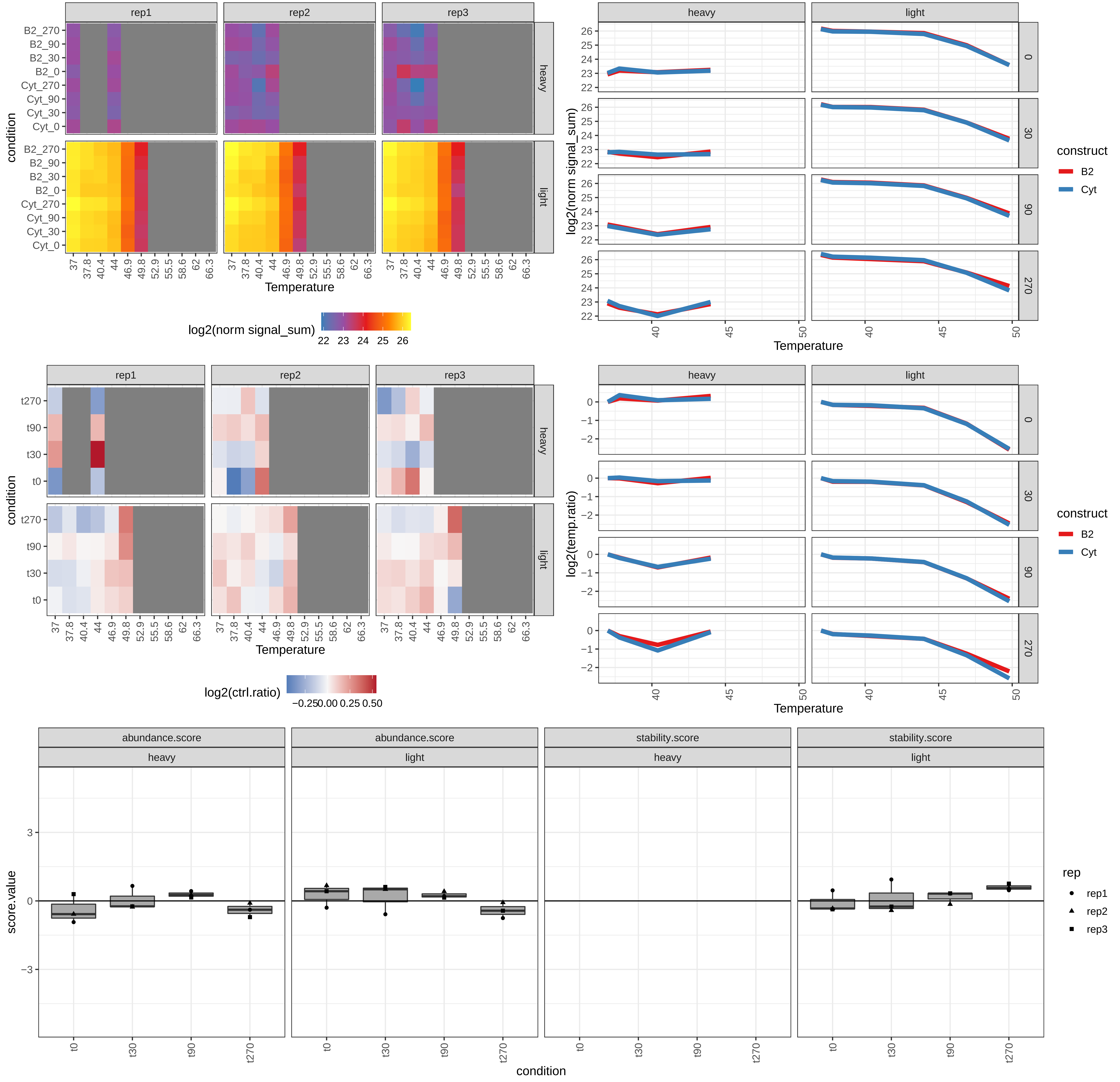

P53131 − Pre−mRNA−splicing factor ATP−dependent RNA helicase PRP43 OS=Saccharomyces cerevisiae (strain ATCC 204508 / S288c) GN=PRP43 PE=1 SV=1

PRP6 − average.top3: 6.77677375 − max.qupm: 4

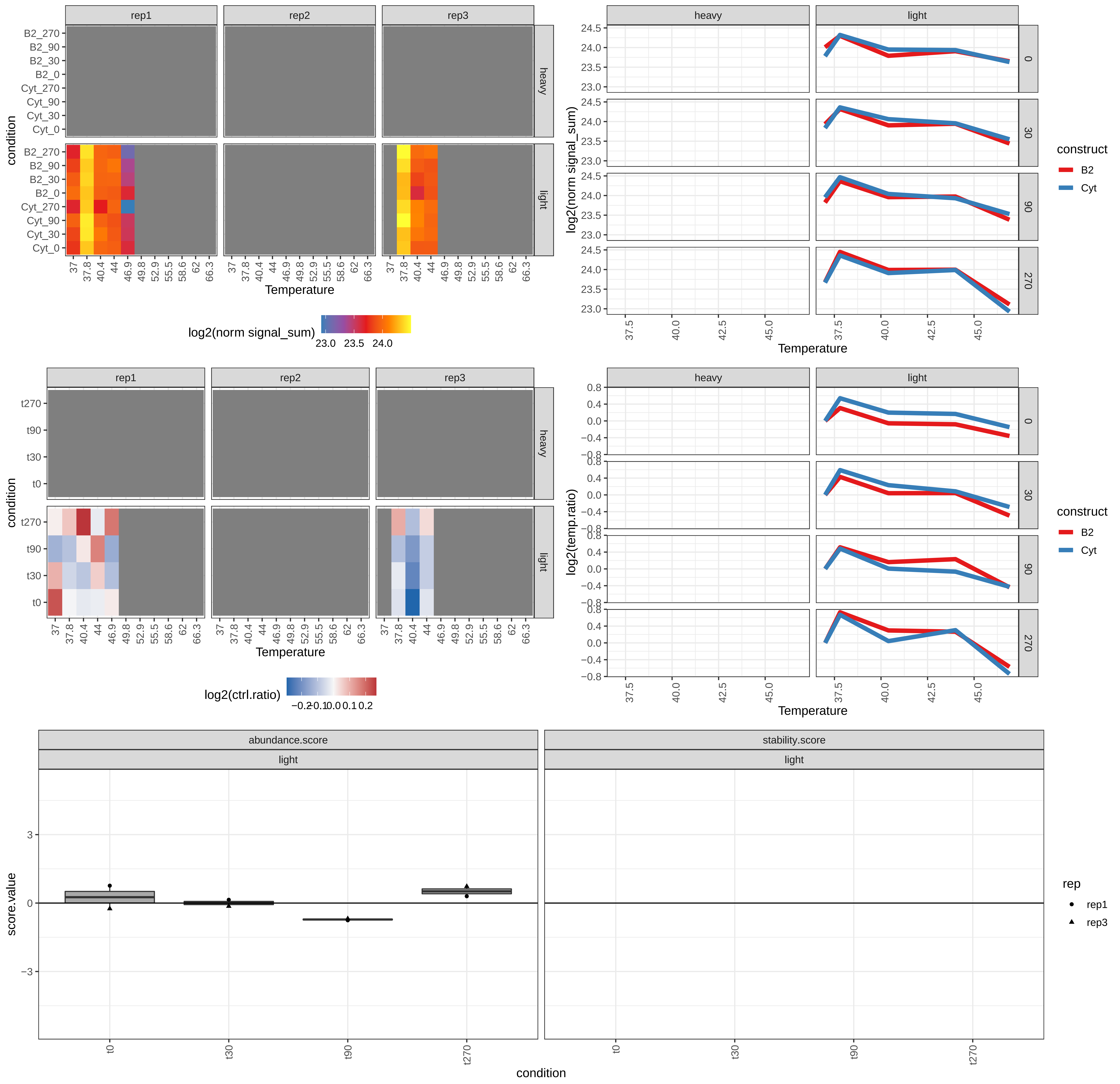

P19735 − Pre−mRNA−splicing factor 6 OS=Saccharomyces cerevisiae (strain ATCC 204508 / S288c) GN=PRP6 PE=1 SV=1

PRP8 − average.top3: 6.740469375 − max.qupm: 14

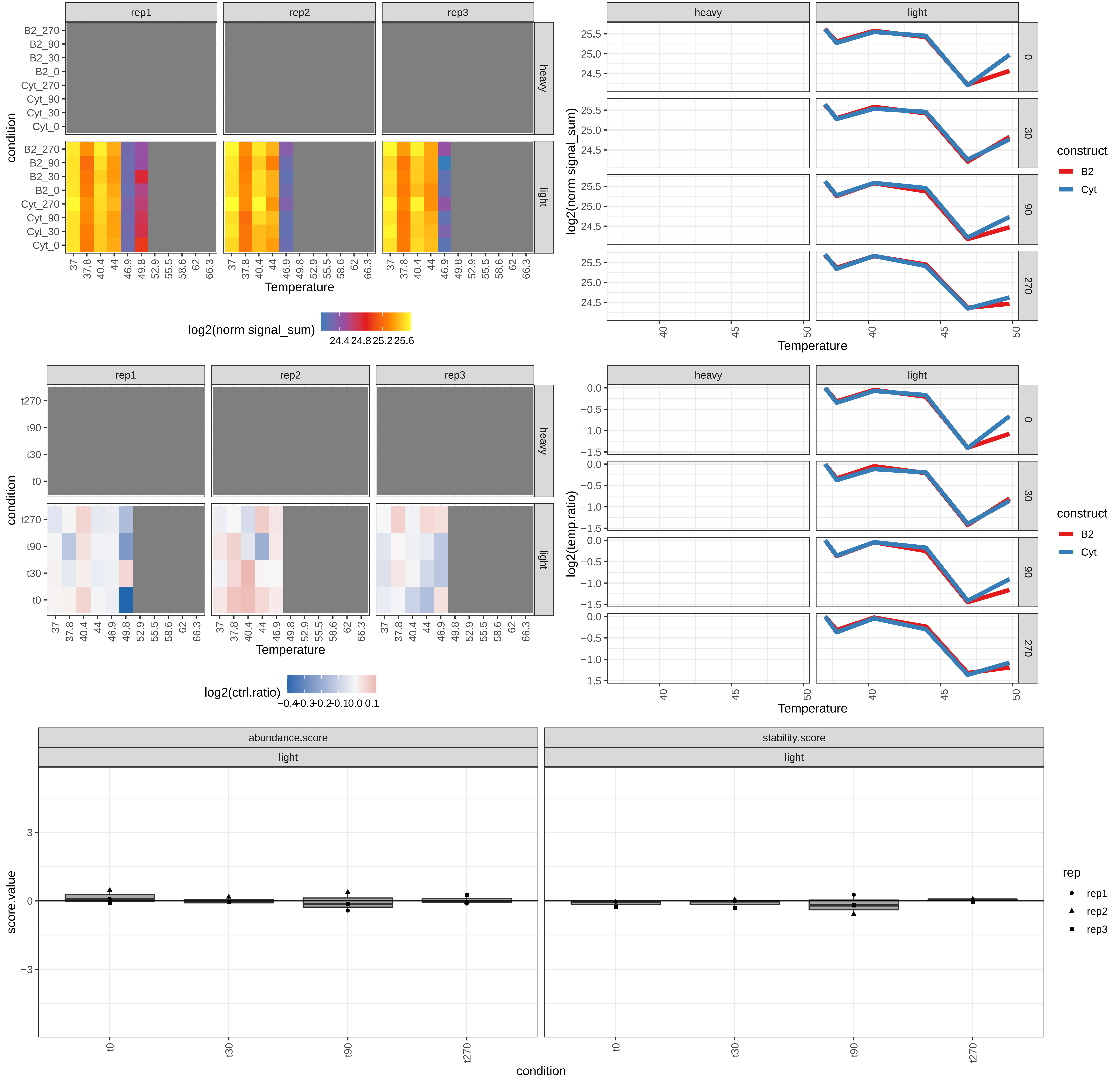

P33334 − Pre−mRNA−splicing factor 8 OS=Saccharomyces cerevisiae (strain ATCC 204508 / S288c) GN=PRP8 PE=1 SV=1

PRP9 − average.top3: 6.44119 − max.qupm: 4

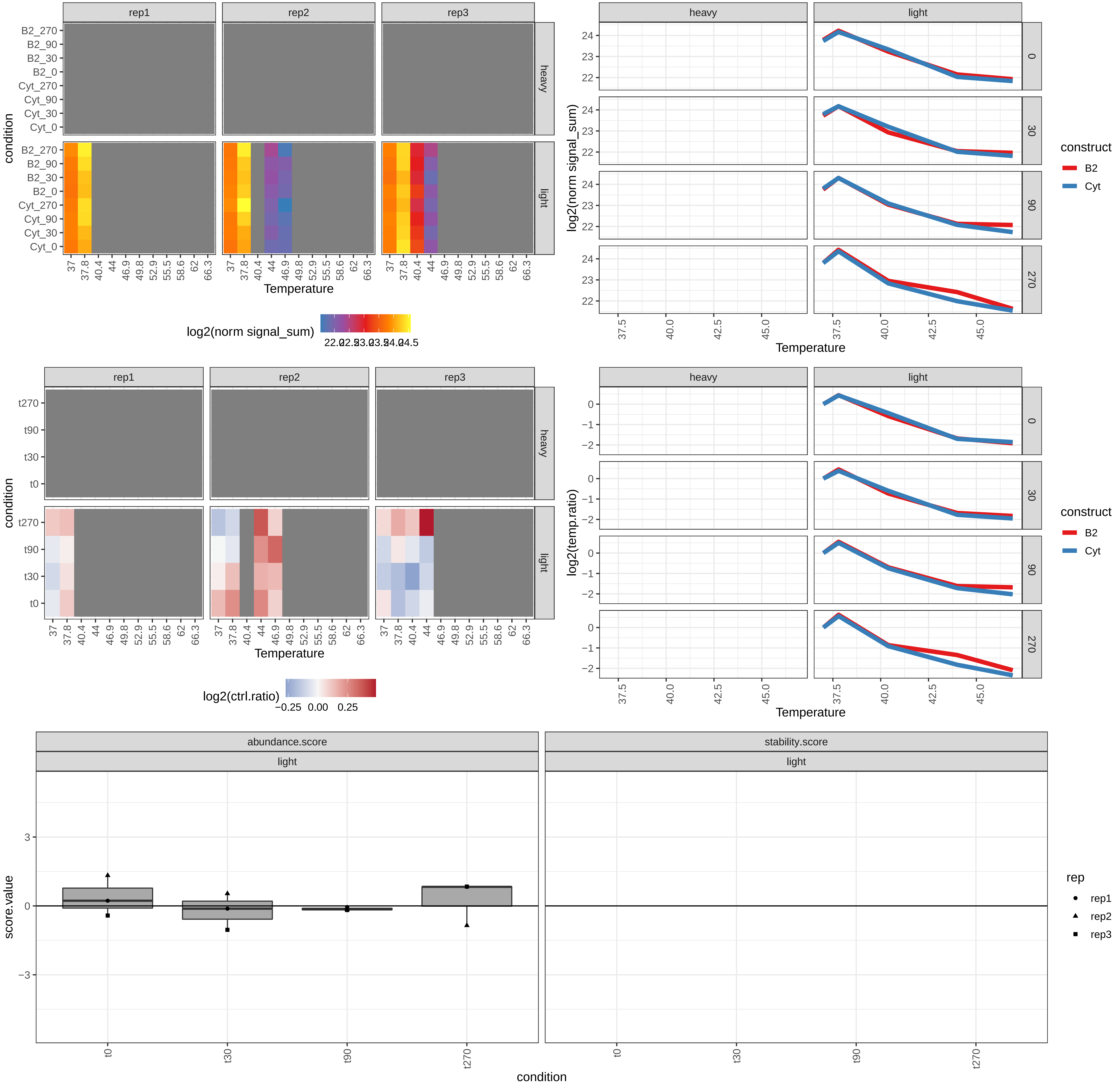

P19736 − Pre−mRNA−splicing factor PRP9 OS=Saccharomyces cerevisiae (strain ATCC 204508 / S288c) GN=PRP9 PE=1 SV=1

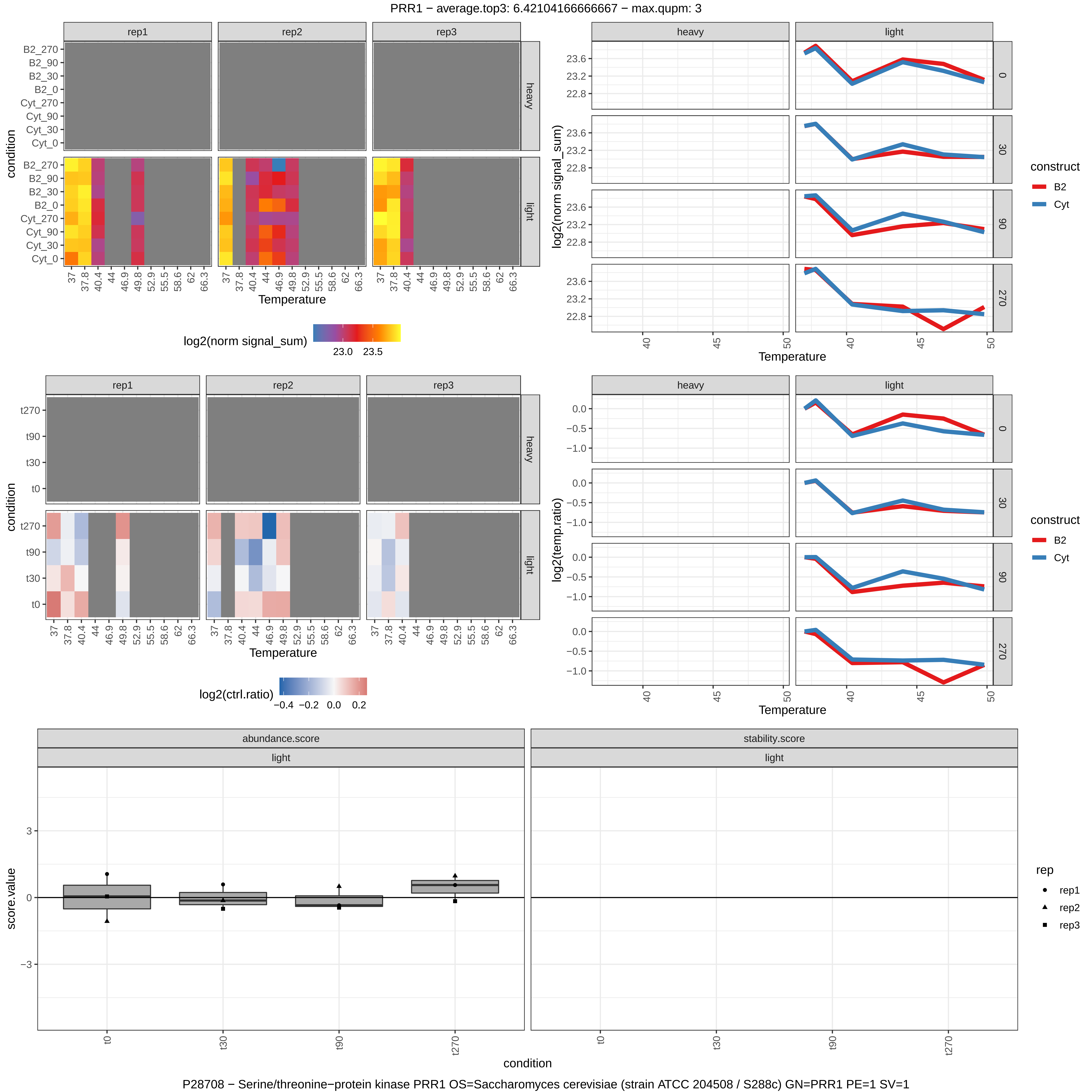

PRR1 − average.top3: 6.42104166666667 − max.qupm: 3
rep1
rep2
rep3
heavy
light
condition
B2_270
B2_90
B2_30
B2_0
Cyt_270
Cyt_90
Cyt_30
Cyt_0
Temperature
log2(norm signal_sum)
23.0
23.5
log2(norm signal_sum)
construct
B2
Cyt
t270
t90
t30
t0
log2(ctrl.ratio)
−0.4
−0.2
0.0
0.2
log2(temp.ratio)
abundance.score
stability.score
score.value
rep
condition
P28708 − Serine/threonine−protein kinase PRR1 OS=Saccharomyces cerevisiae (strain ATCC 204508 / S288c) GN=PRR1 PE=1 SV=1

PRS1 − average.top3: 6.91725133333333 − max.qupm: 10

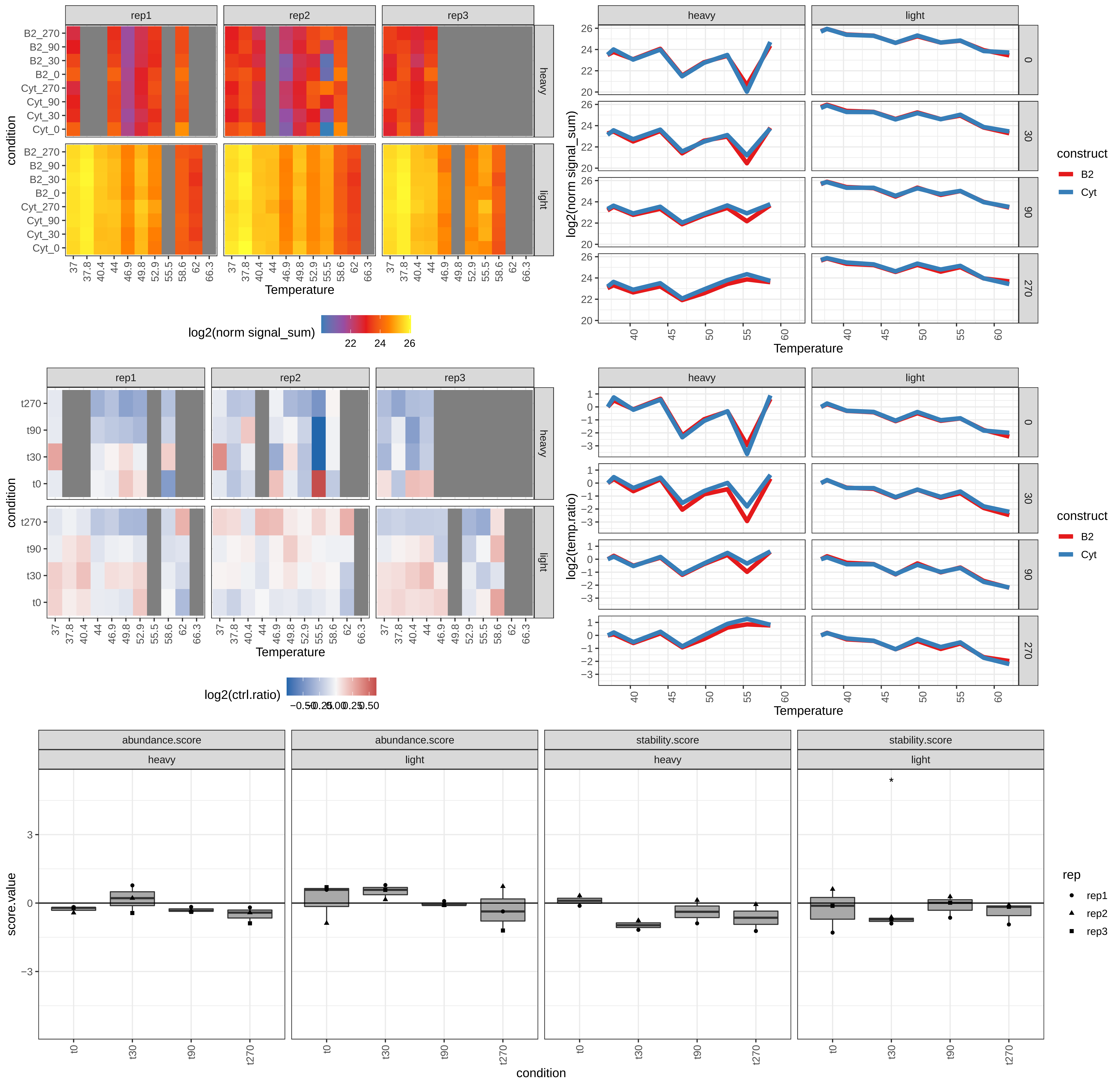

P32895 − Ribose−phosphate pyrophosphokinase 1 OS=Saccharomyces cerevisiae (strain ATCC 204508 / S288c) GN=PRS1 PE=1 SV=1

PRS2 − average.top3: 6.53975333333333 − max.qupm: 4

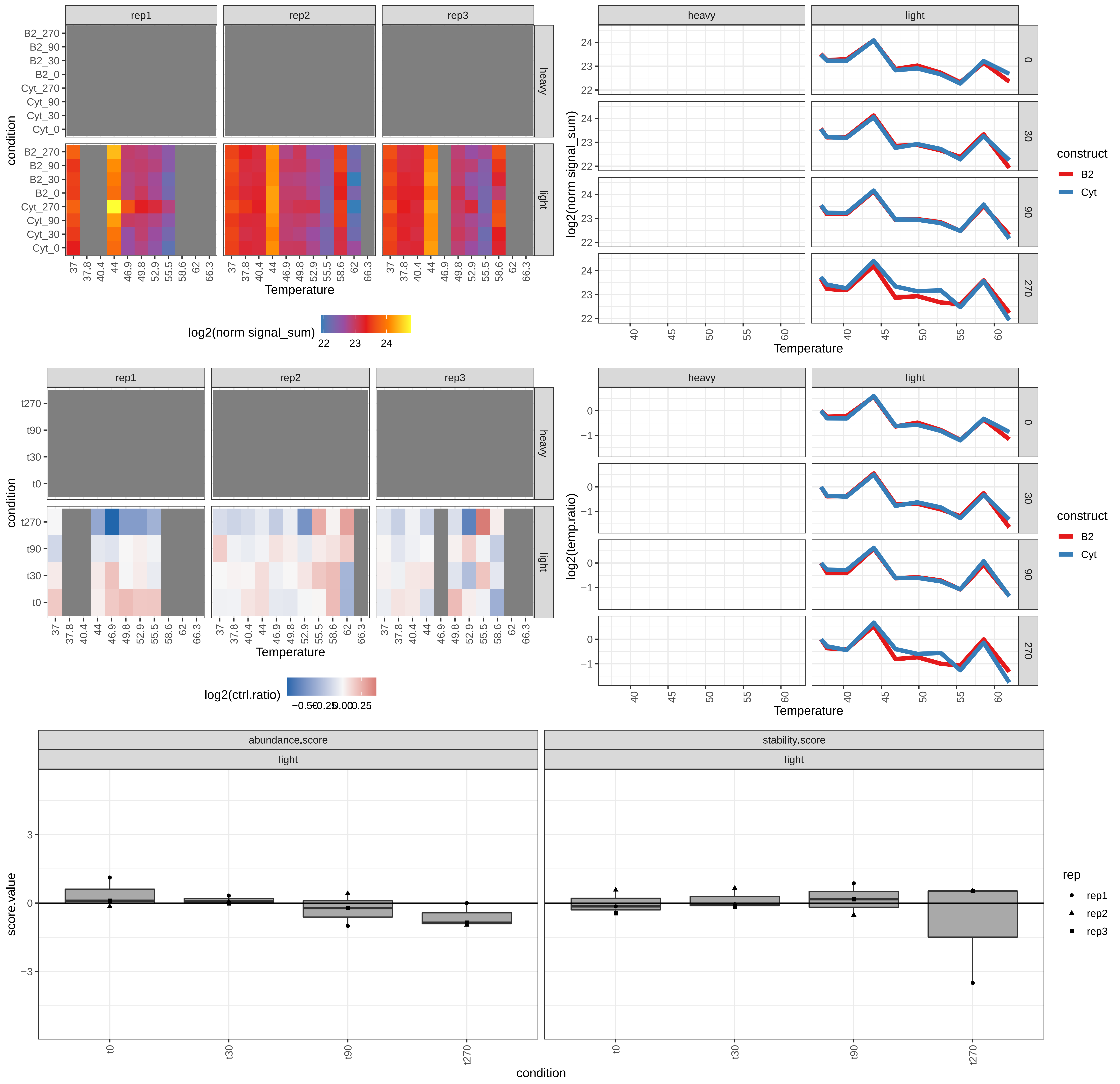

P38620 − Ribose−phosphate pyrophosphokinase 2 OS=Saccharomyces cerevisiae (strain ATCC 204508 / S288c) GN=PRS2 PE=1 SV=1

PRS3 − average.top3: 7.37731 − max.qupm: 9

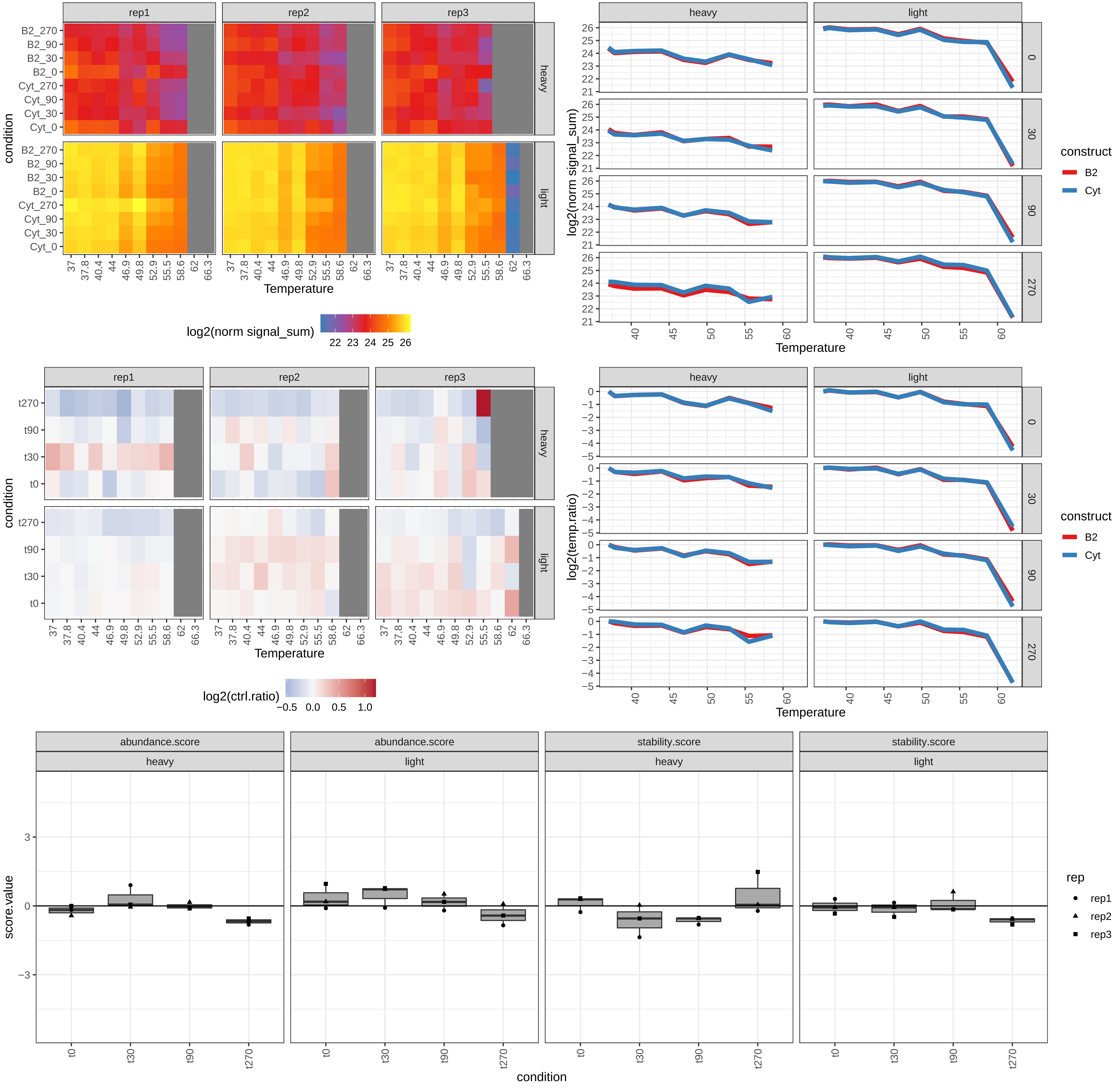

P38689 − Ribose−phosphate pyrophosphokinase 3 OS=Saccharomyces cerevisiae (strain ATCC 204508 / S288c) GN=PRS3 PE=1 SV=1

PRS4 − average.top3: 6.58459217391304 − max.qupm: 5

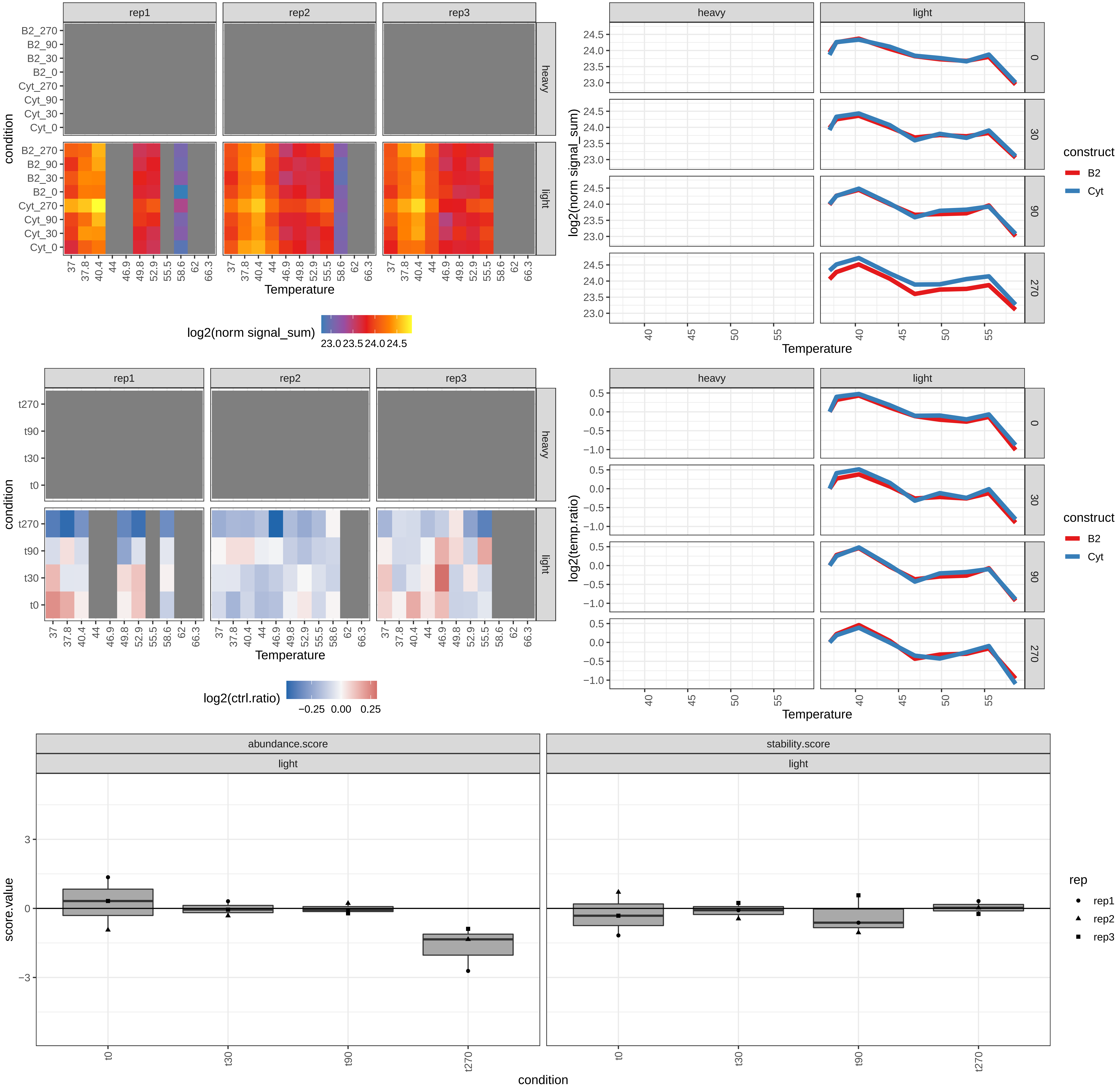

P38063 − Ribose−phosphate pyrophosphokinase 4 OS=Saccharomyces cerevisiae (strain ATCC 204508 / S288c) GN=PRS4 PE=1 SV=3

PRS5 − average.top3: 7.34085409090909 − max.qupm: 7

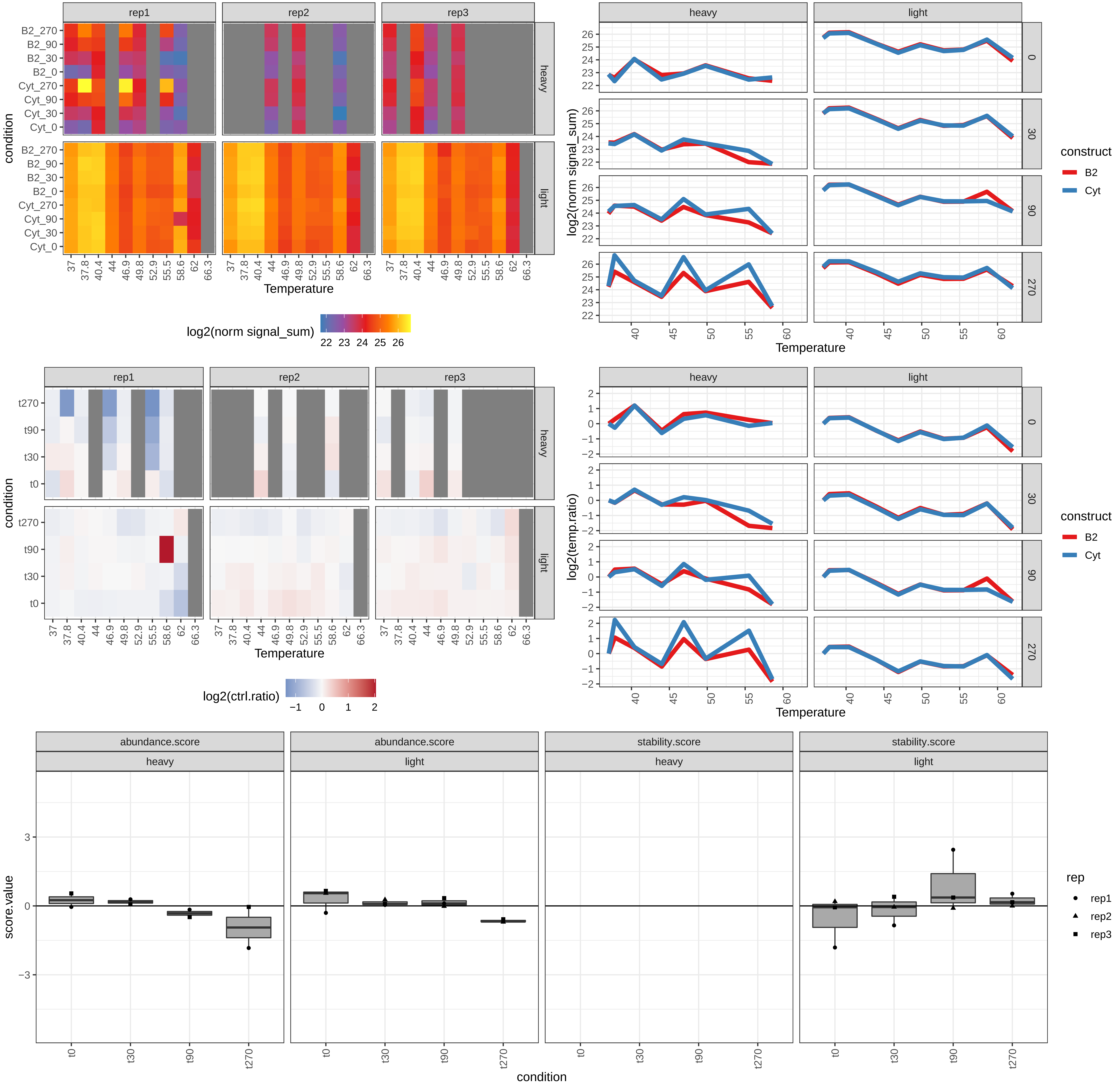

Q12265 − Ribose−phosphate pyrophosphokinase 5 OS=Saccharomyces cerevisiae (strain ATCC 204508 / S288c) GN=PRS5 PE=1 SV=1

PRT1 − average.top3: 7.27957674418605 − max.qupm: 22

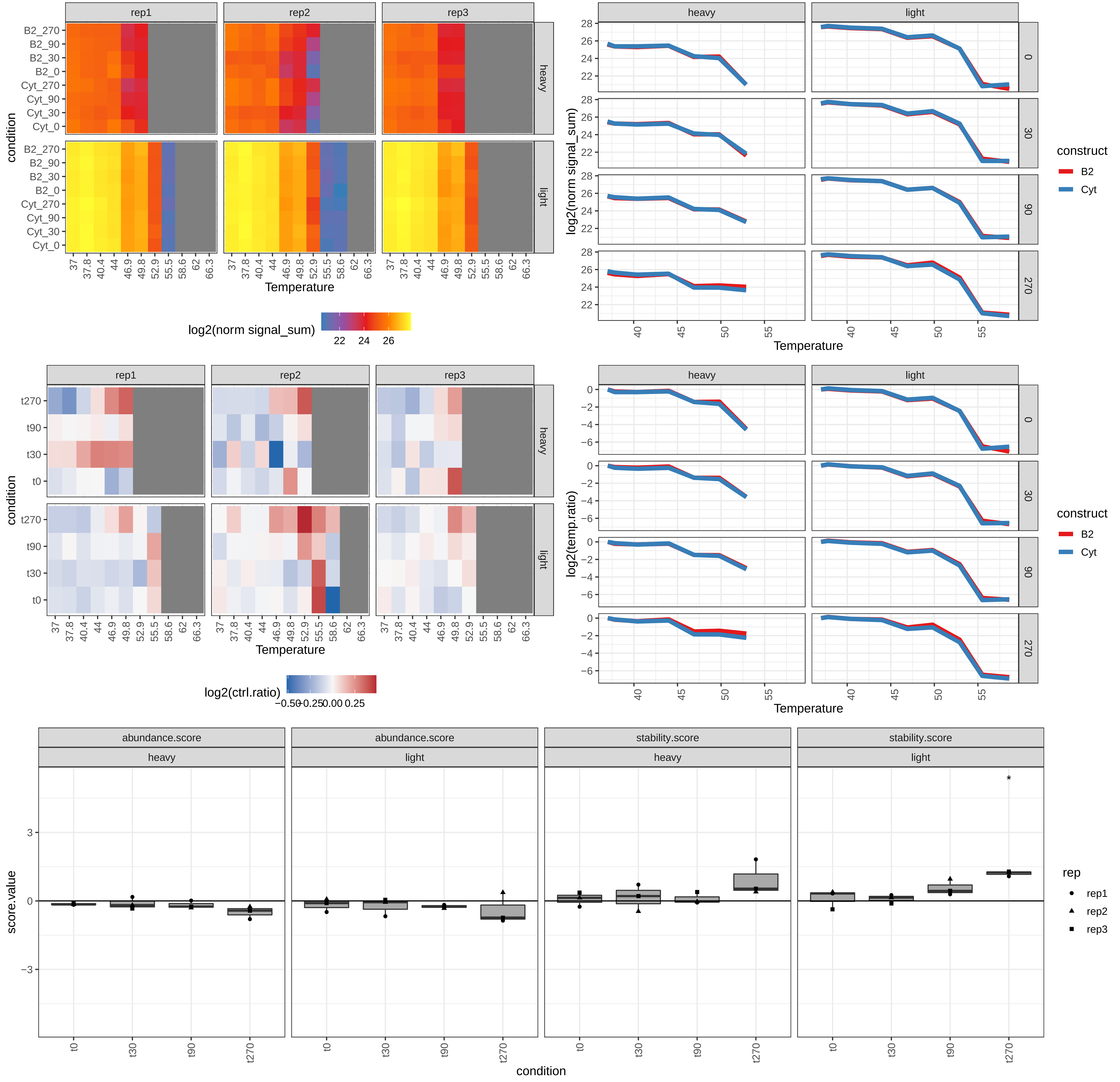

P06103 − Eukaryotic translation initiation factor 3 subunit B OS=Saccharomyces cerevisiae (strain ATCC 204508 / S288c) GN=PRT1 PE=1 SV=1

PRX1 − average.top3: 7.77330140625 − max.qupm: 10

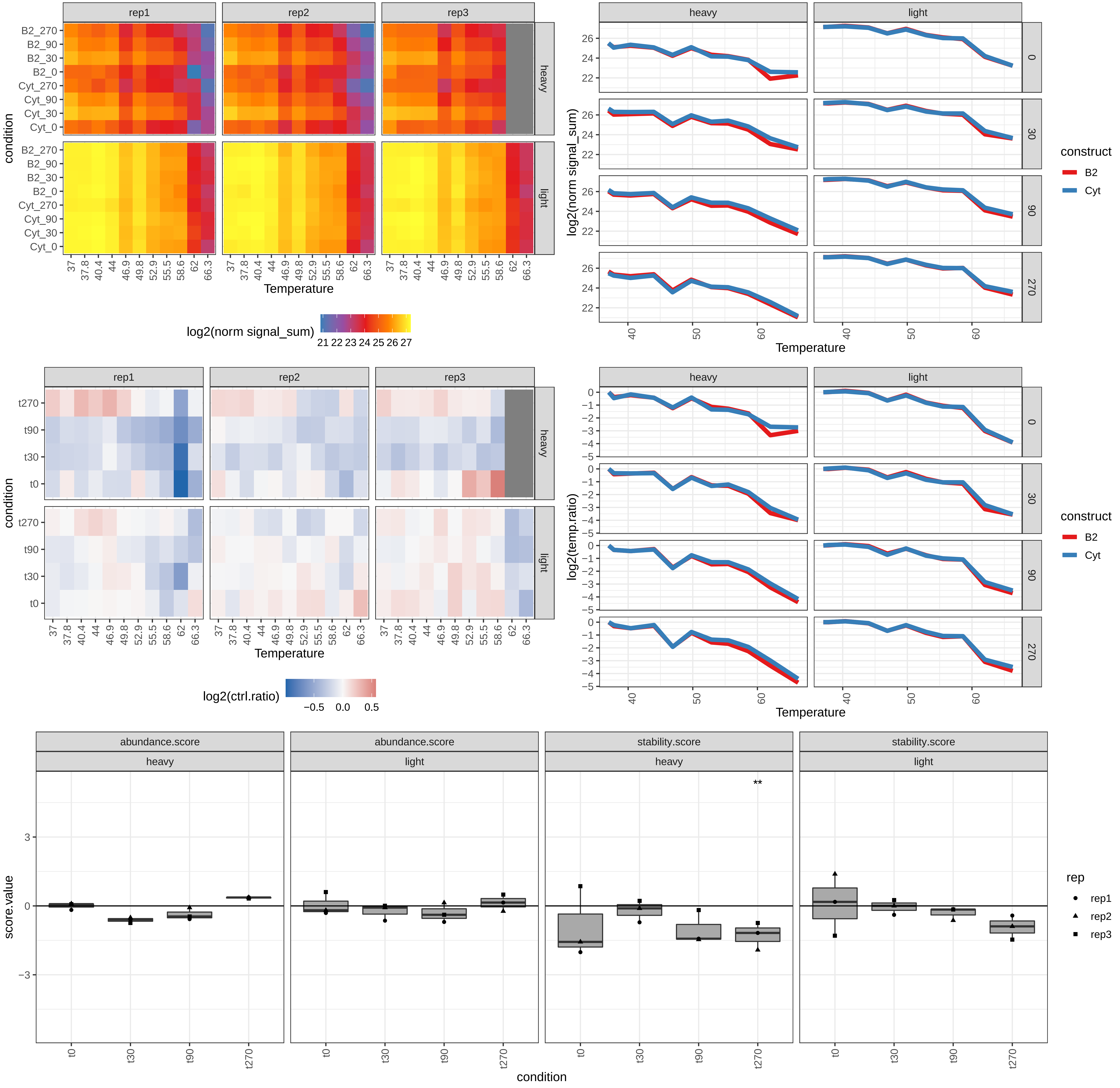

P34227 − Mitochondrial peroxiredoxin PRX1 OS=Saccharomyces cerevisiae (strain ATCC 204508 / S288c) GN=PRX1 PE=1 SV=1

PSA1 − average.top3: 7.519644 − max.qupm: 14

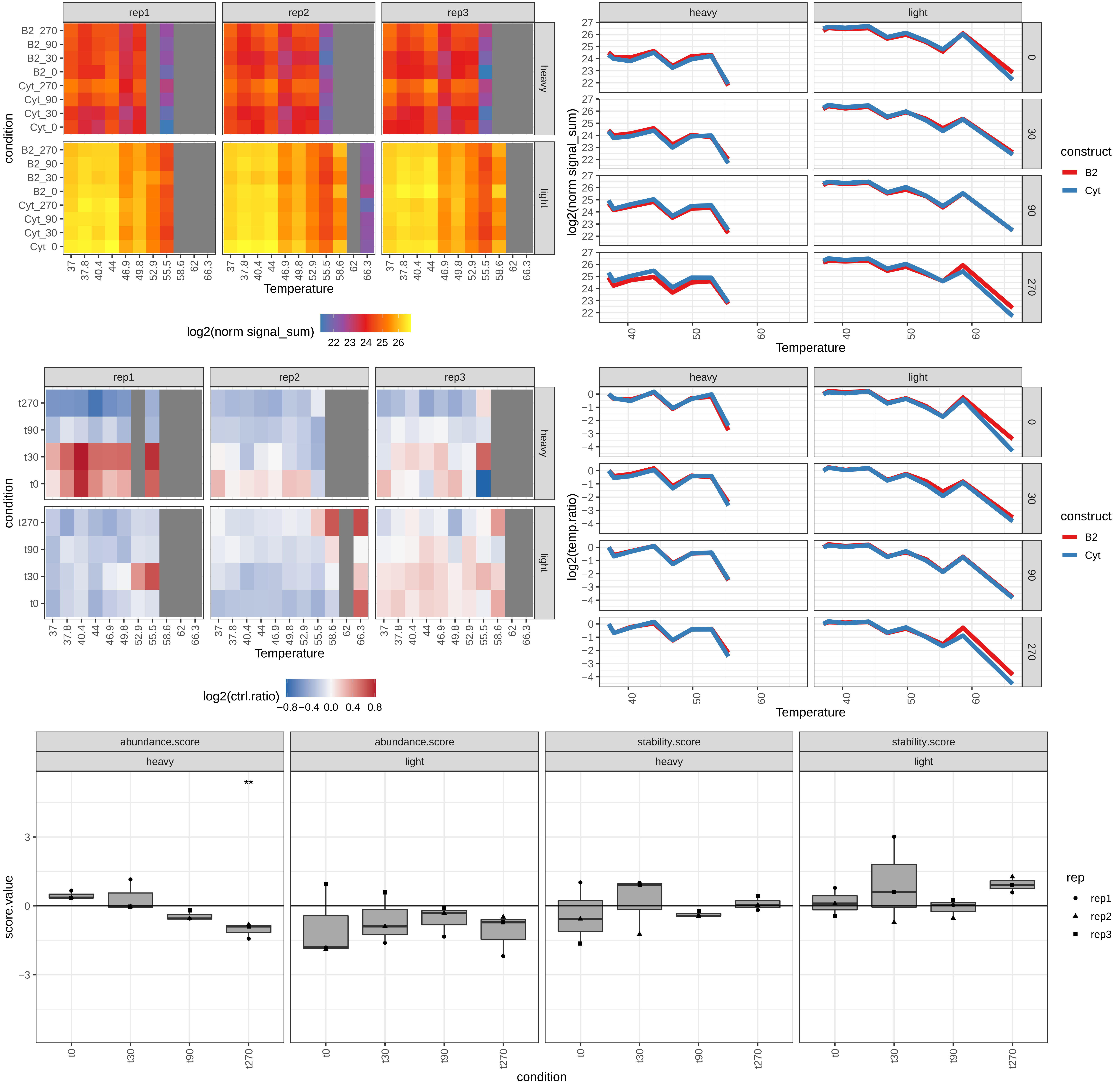

P41940 − Mannose−1−phosphate guanyltransferase OS=Saccharomyces cerevisiae (strain ATCC 204508 / S288c) GN=PSA1 PE=1 SV=2

PSD1 − average.top3: 6.63702714285714 − max.qupm: 3

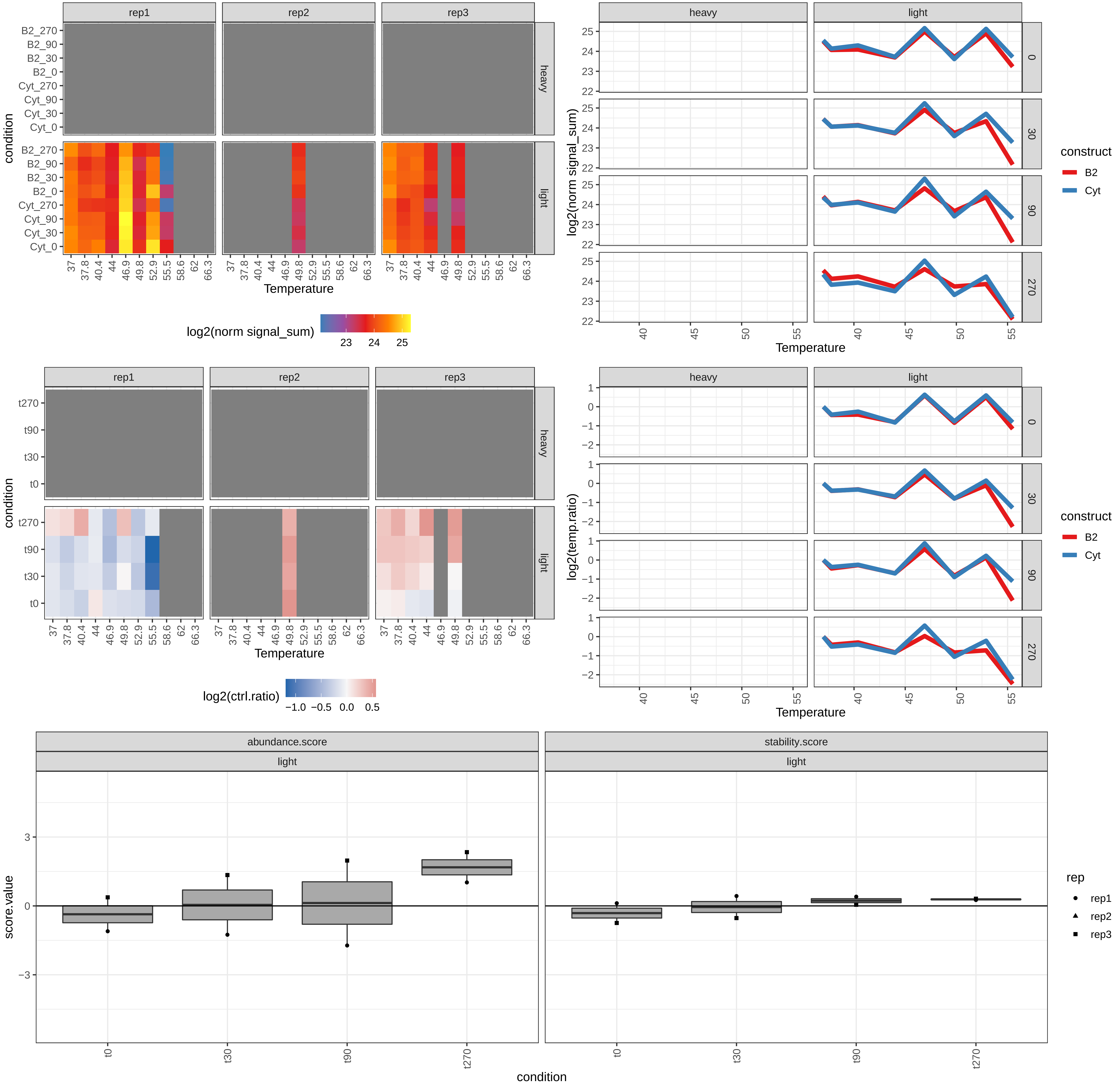

P39006 − Phosphatidylserine decarboxylase proenzyme 1, mitochondrial OS=Saccharomyces cerevisiae (strain ATCC 204508 / S288c) GN=PSD1 PE=1 SV=1

PSD2 − average.top3: 6.38643333333333 − max.qupm: 4

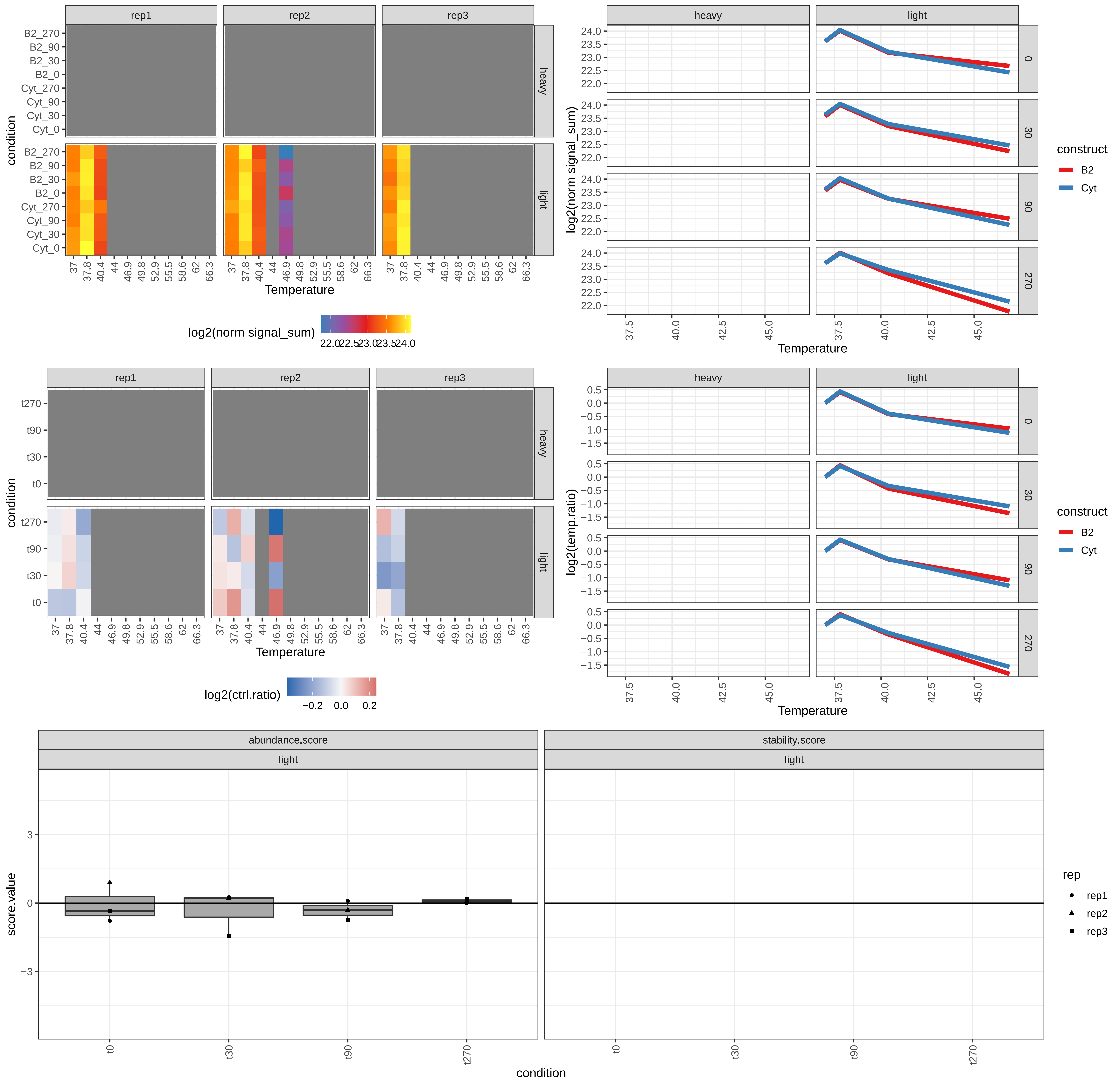

P53037 − Phosphatidylserine decarboxylase proenzyme 2 OS=Saccharomyces cerevisiae (strain ATCC 204508 / S288c) GN=PSD2 PE=1 SV=1

PSE1 − average.top3: 6.7722819047619 − max.qupm: 8

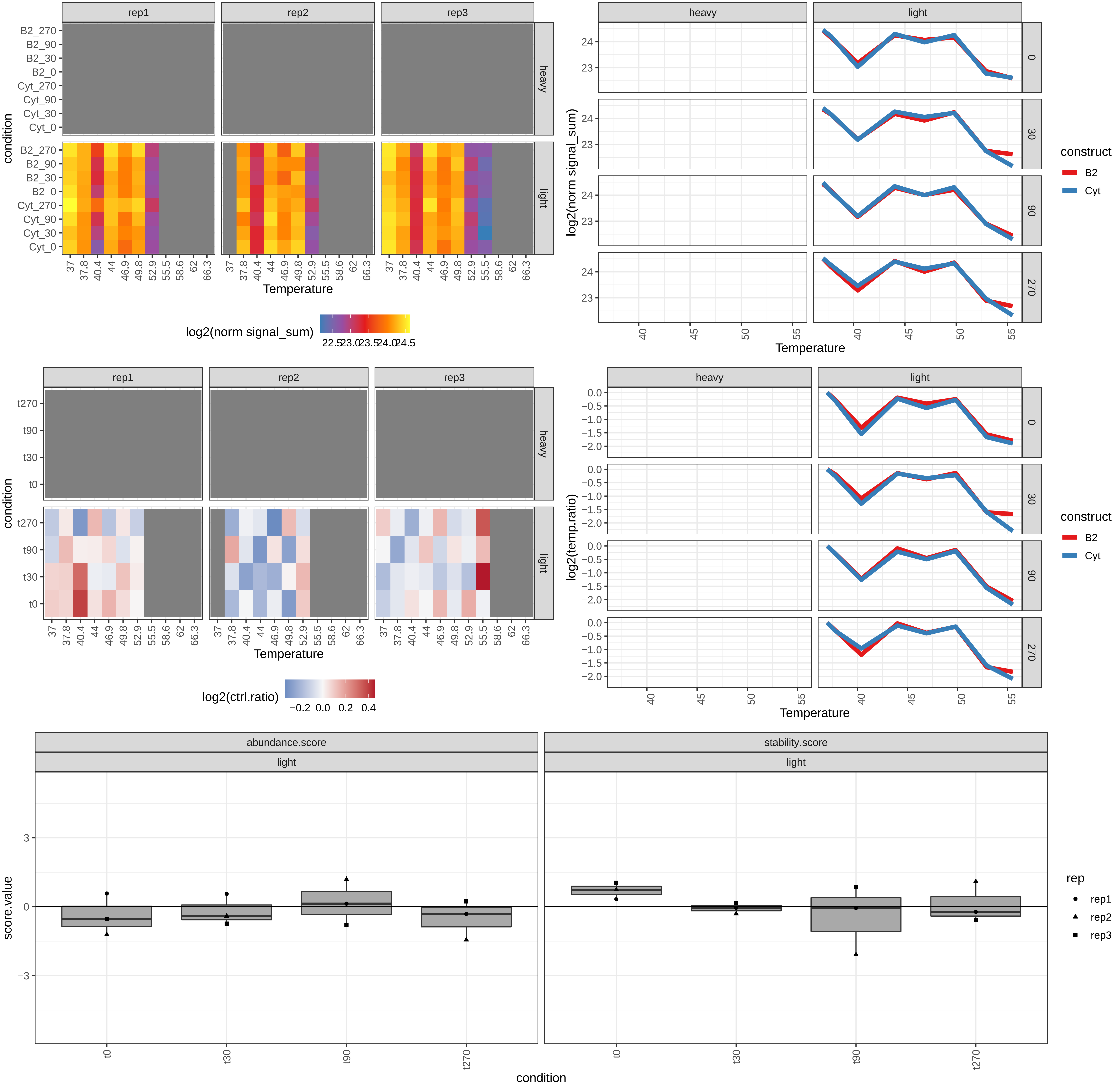

P32337 − Importin subunit beta−3 OS=Saccharomyces cerevisiae (strain ATCC 204508 / S288c) GN=PSE1 PE=1 SV=2

PSK1 − average.top3: 6.72893352941176 − max.qupm: 5

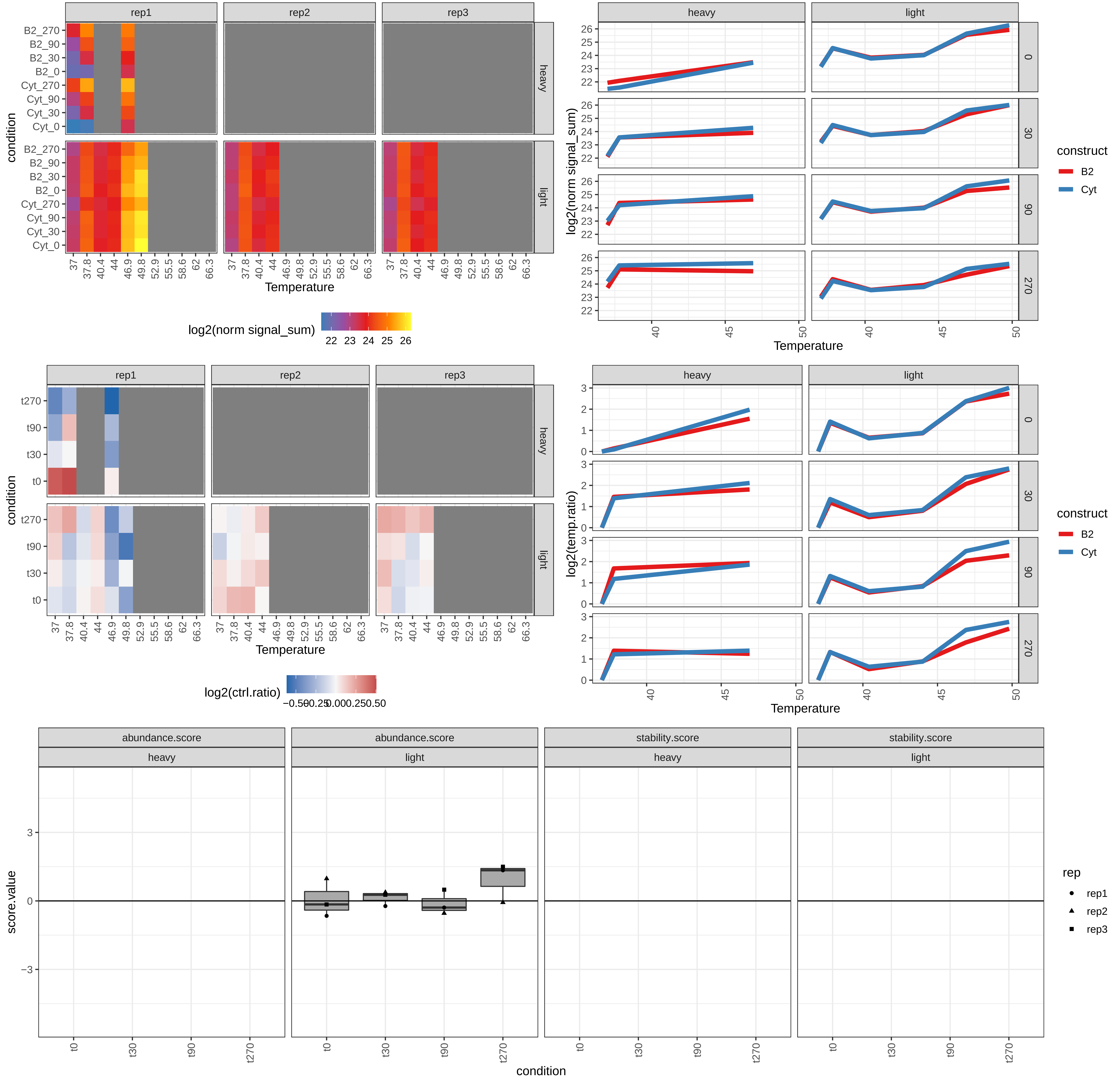

P31374 − Serine/threonine−protein kinase PSK1 OS=Saccharomyces cerevisiae (strain ATCC 204508 / S288c) GN=PSK1 PE=1 SV=2

PSP2 − average.top3: 6.70293689655172 − max.qupm: 5

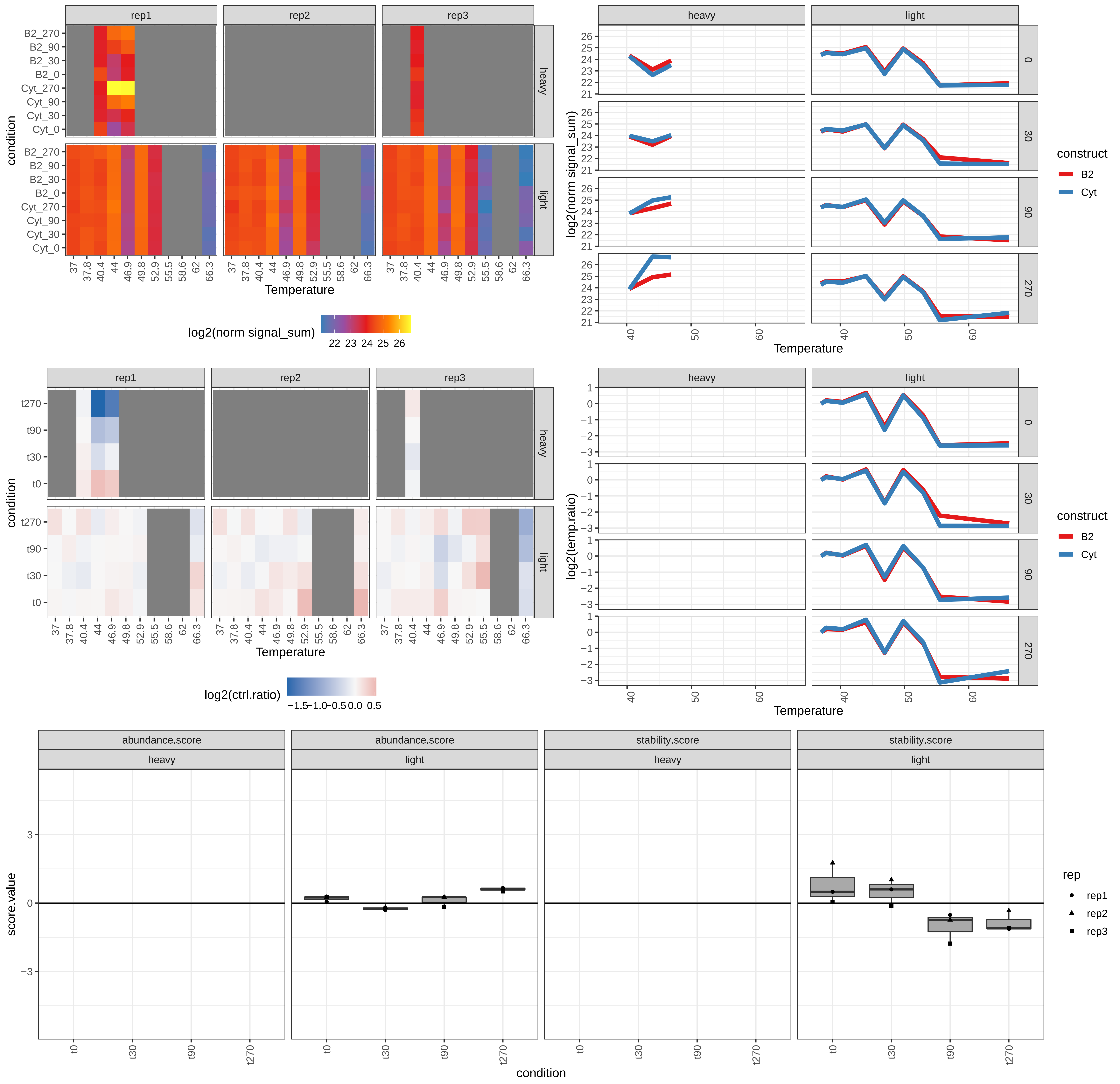

P50109 − Protein PSP2 OS=Saccharomyces cerevisiae (strain ATCC 204508 / S288c) GN=PSP2 PE=1 SV=2

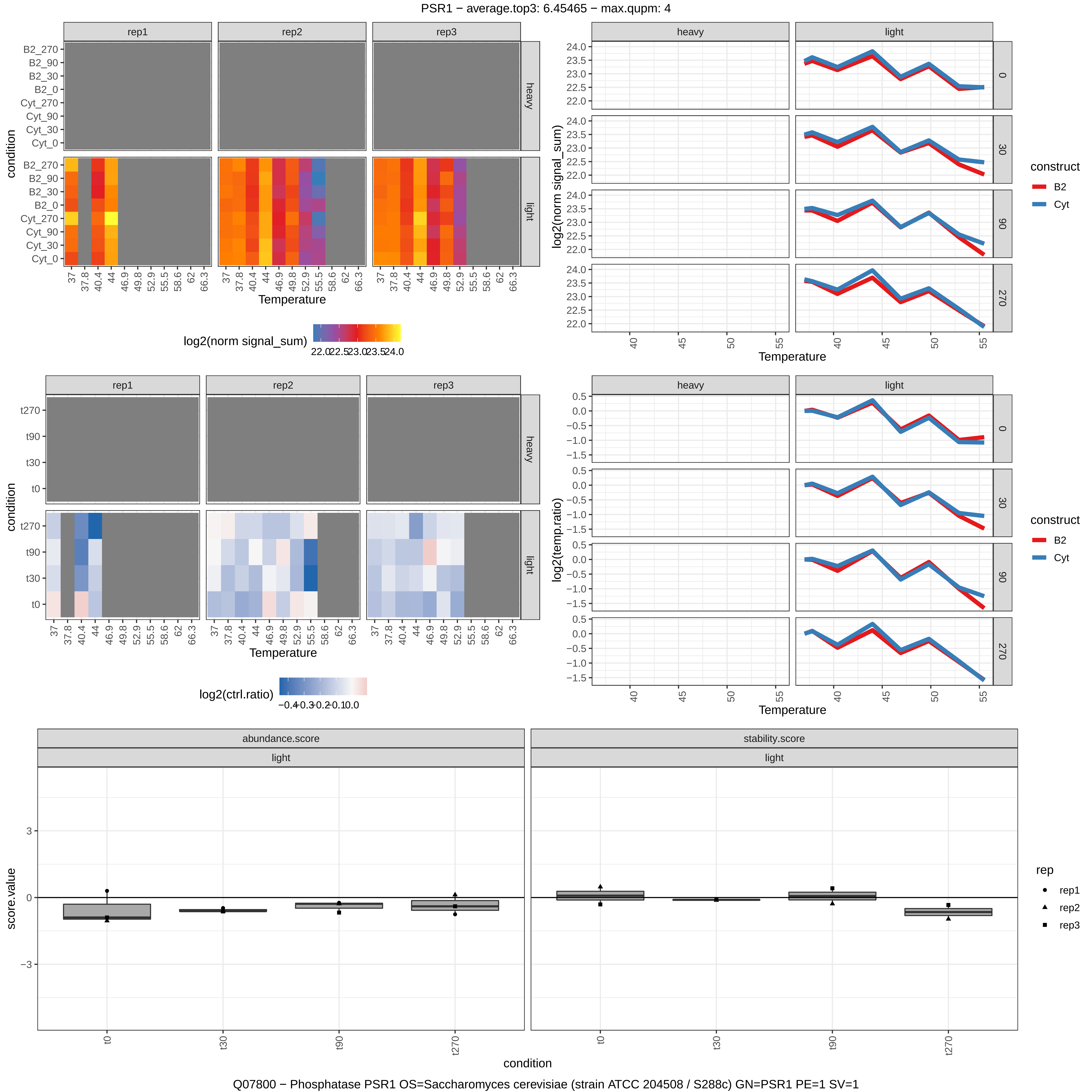
PSR1 − average.top3: 6.45465 − max.qupm: 4
rep1
rep2
rep3
heavy
light
condition
B2_270
B2_90
B2_30
B2_0
Cyt_270
Cyt_90
Cyt_30
Cyt_0
Temperature
log2(norm signal_sum)
log2(norm signal_sum)
construct
B2
Cyt
t270
t90
t30
t0
log2(ctrl.ratio)
log2(temp.ratio)
abundance.score
stability.score
score.value
rep
rep1
rep2
rep3
condition
Q07800 − Phosphatase PSR1 OS=Saccharomyces cerevisiae (strain ATCC 204508 / S288c) GN=PSR1 PE=1 SV=1

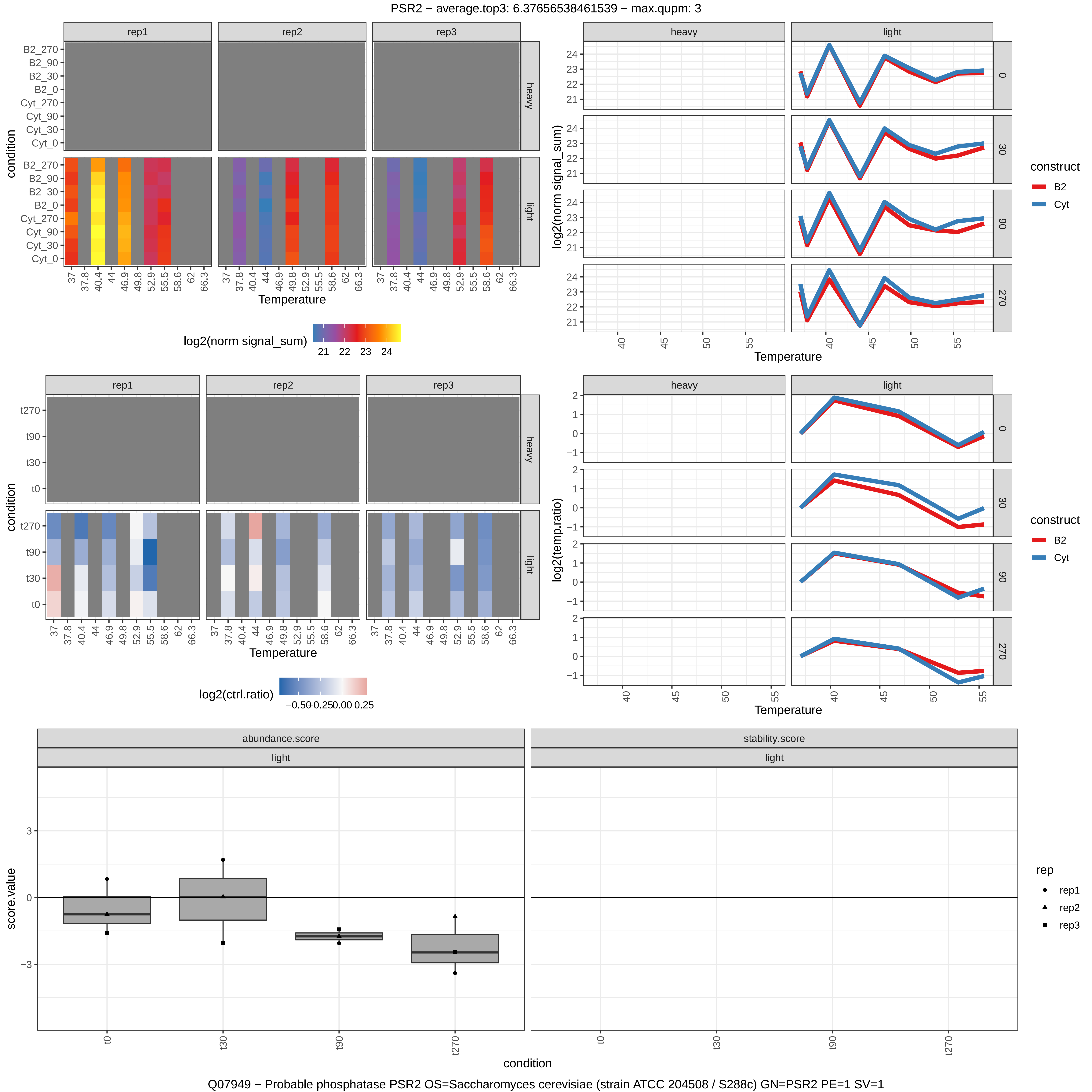
PSR2 − average.top3: 6.37656538461539 − max.qupm: 3
rep1
rep2
rep3
heavy
light
condition
Temperature
log2(norm signal_sum)
log2(norm signal_sum)
construct
B2
Cyt
log2(ctrl.ratio)
log2(temp.ratio)
abundance.score
stability.score
score.value
rep
rep1
rep2
rep3
Q07949 − Probable phosphatase PSR2 OS=Saccharomyces cerevisiae (strain ATCC 204508 / S288c) GN=PSR2 PE=1 SV=1

PST2 − average.top3: 7.07012419354839 − max.qupm: 5

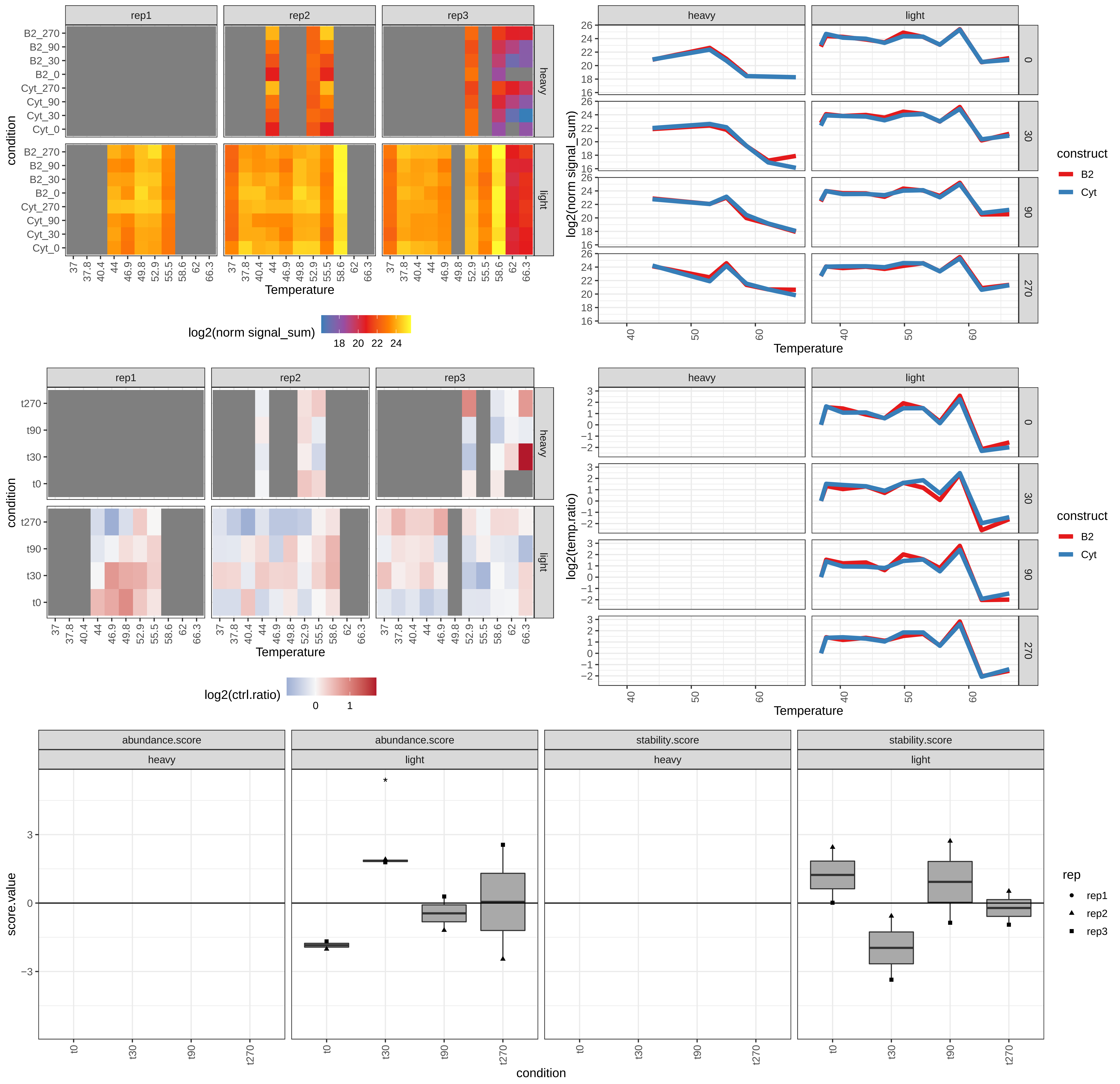

Q12335 − Protoplast secreted protein 2 OS=Saccharomyces cerevisiae (strain ATCC 204508 / S288c) GN=PST2 PE=1 SV=1

PSY2 − average.top3: 6.26891818181818 − max.qupm: 4

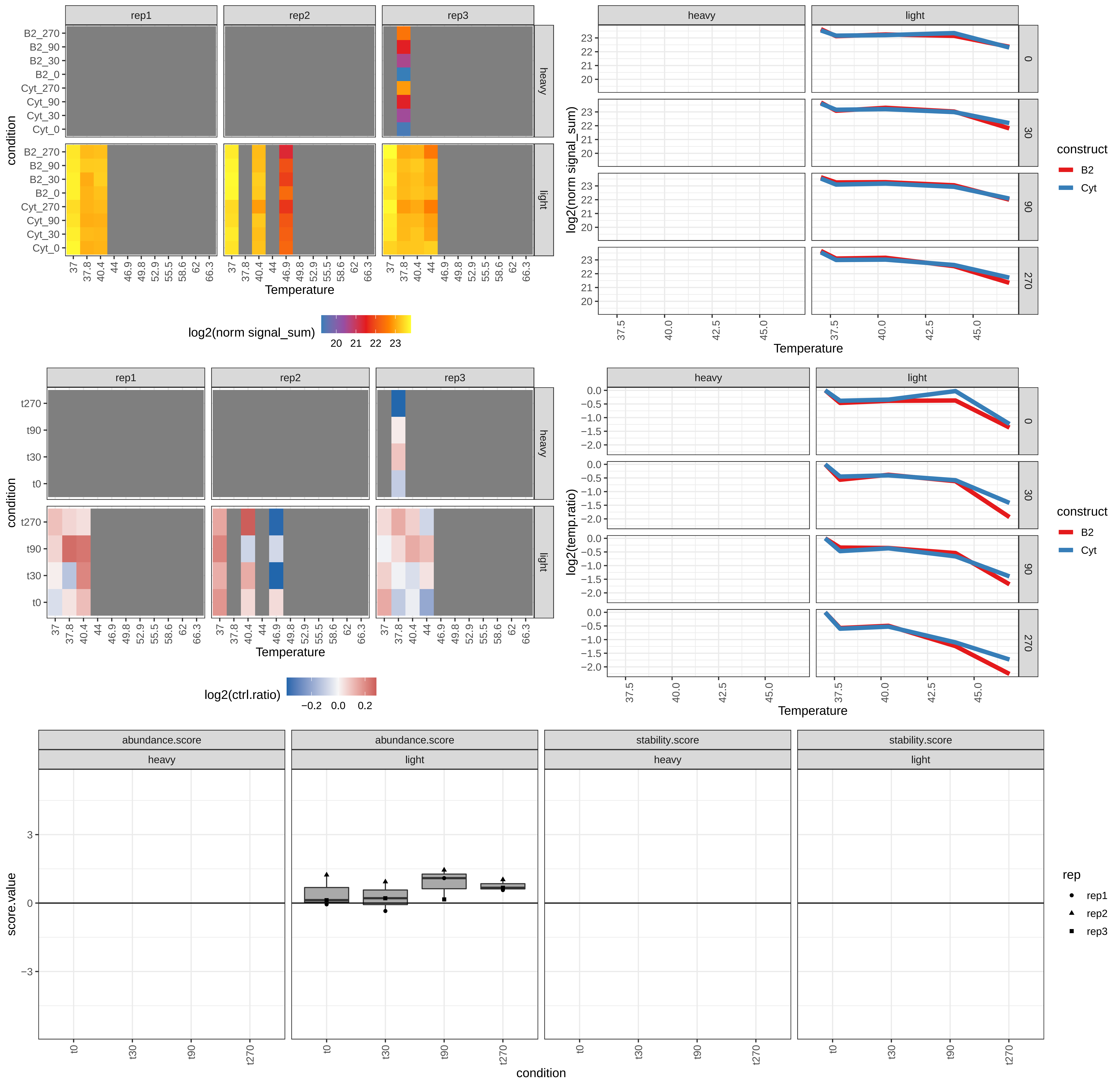

P40164 − Serine/threonine−protein phosphatase 4 regulatory subunit 3 OS=Saccharomyces cerevisiae (strain ATCC 204508 / S288c) GN=PSY2 PE=1 SV=1

PSY4 − average.top3: 6.58661444444444 − max.qupm: 2

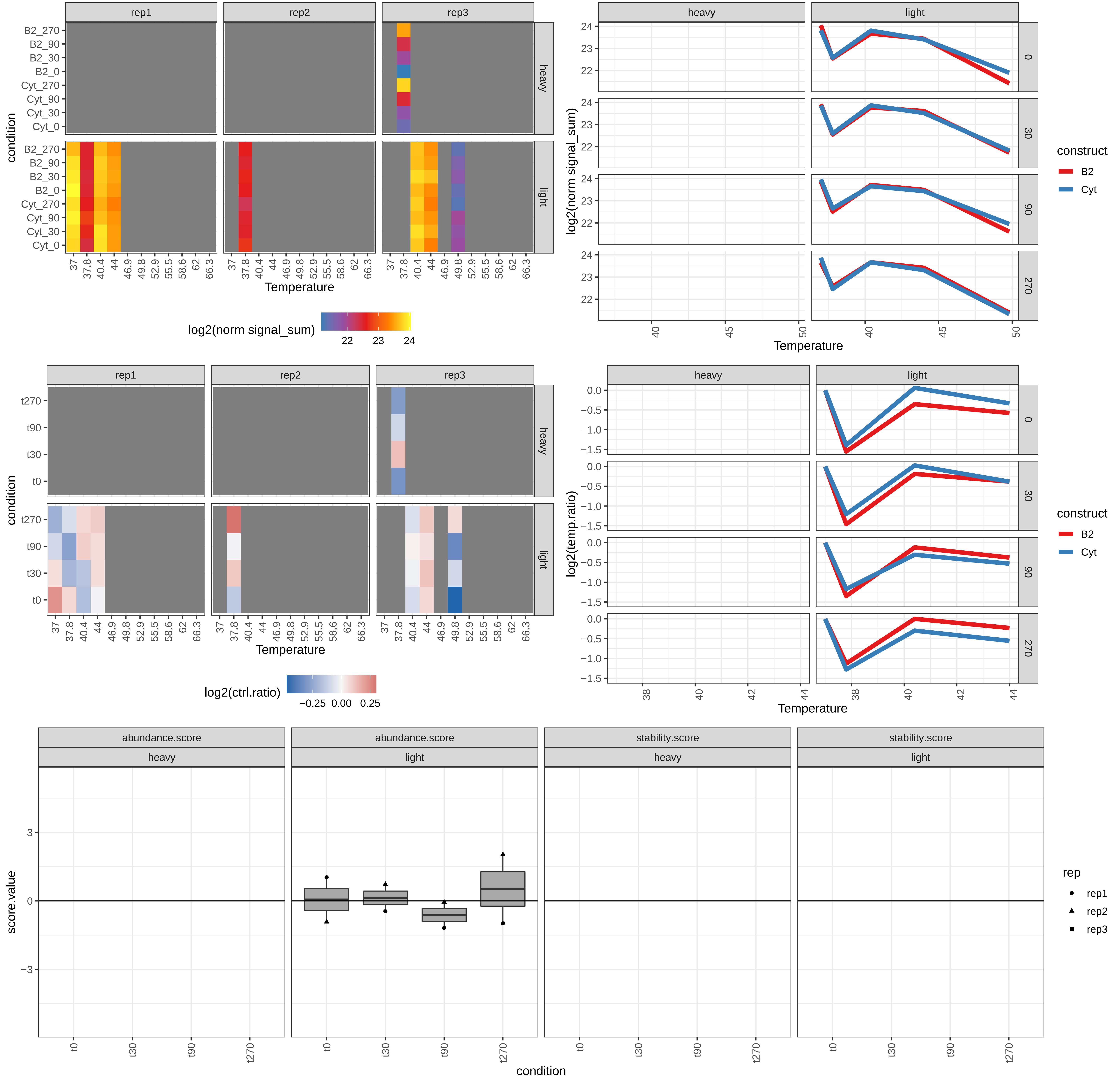

P38193 − Serine/threonine−protein phosphatase 4 regulatory subunit 2 OS=Saccharomyces cerevisiae (strain ATCC 204508 / S288c) GN=PSY4 PE=1 SV=1

PTA1 − average.top3: 6.66832533333333 − max.qupm: 6

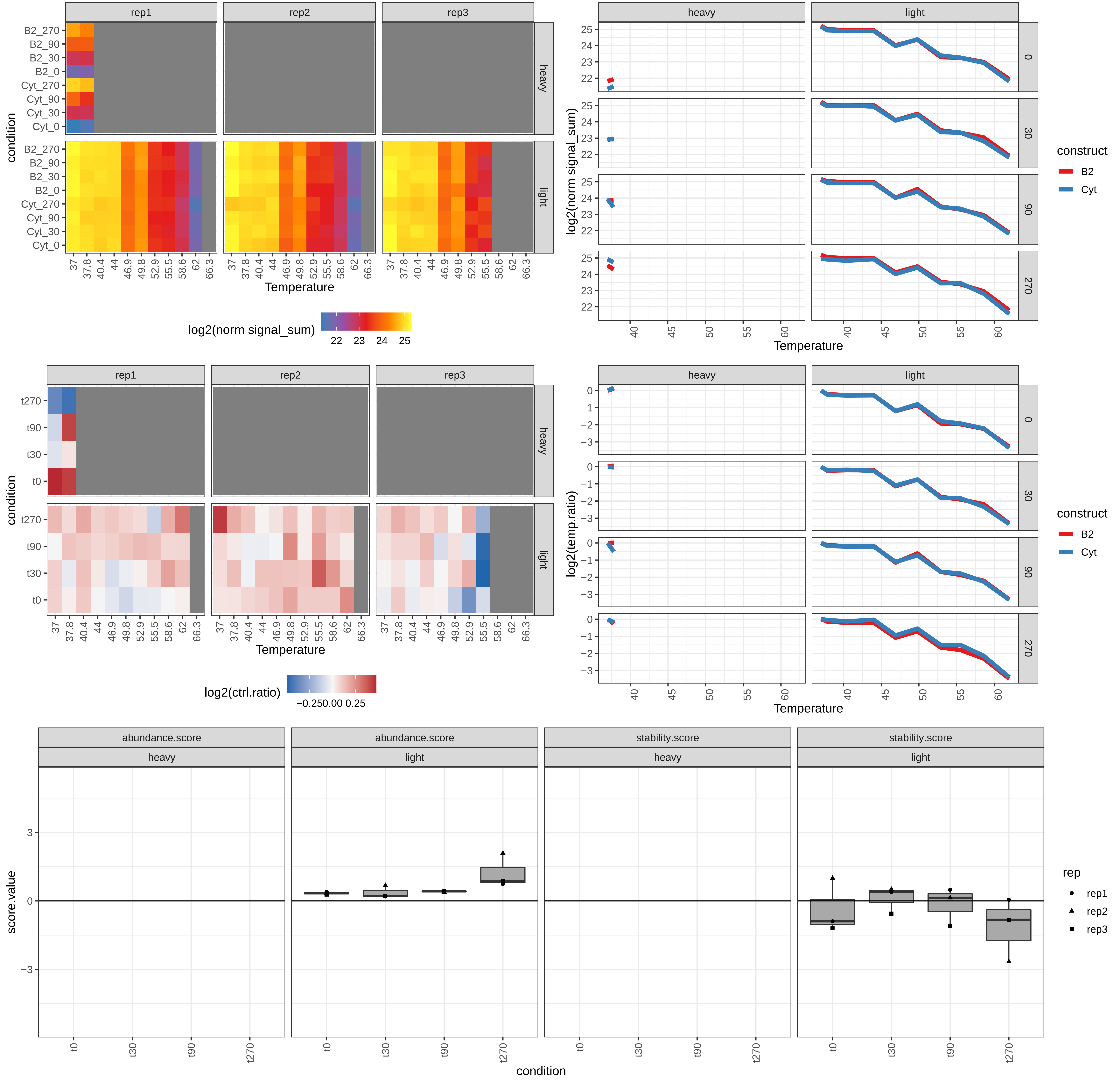

Q01329 − Pre−tRNA−processing protein PTA1 OS=Saccharomyces cerevisiae (strain ATCC 204508 / S288c) GN=PTA1 PE=1 SV=1

PTC1 − average.top3: 6.627858 − max.qupm: 3

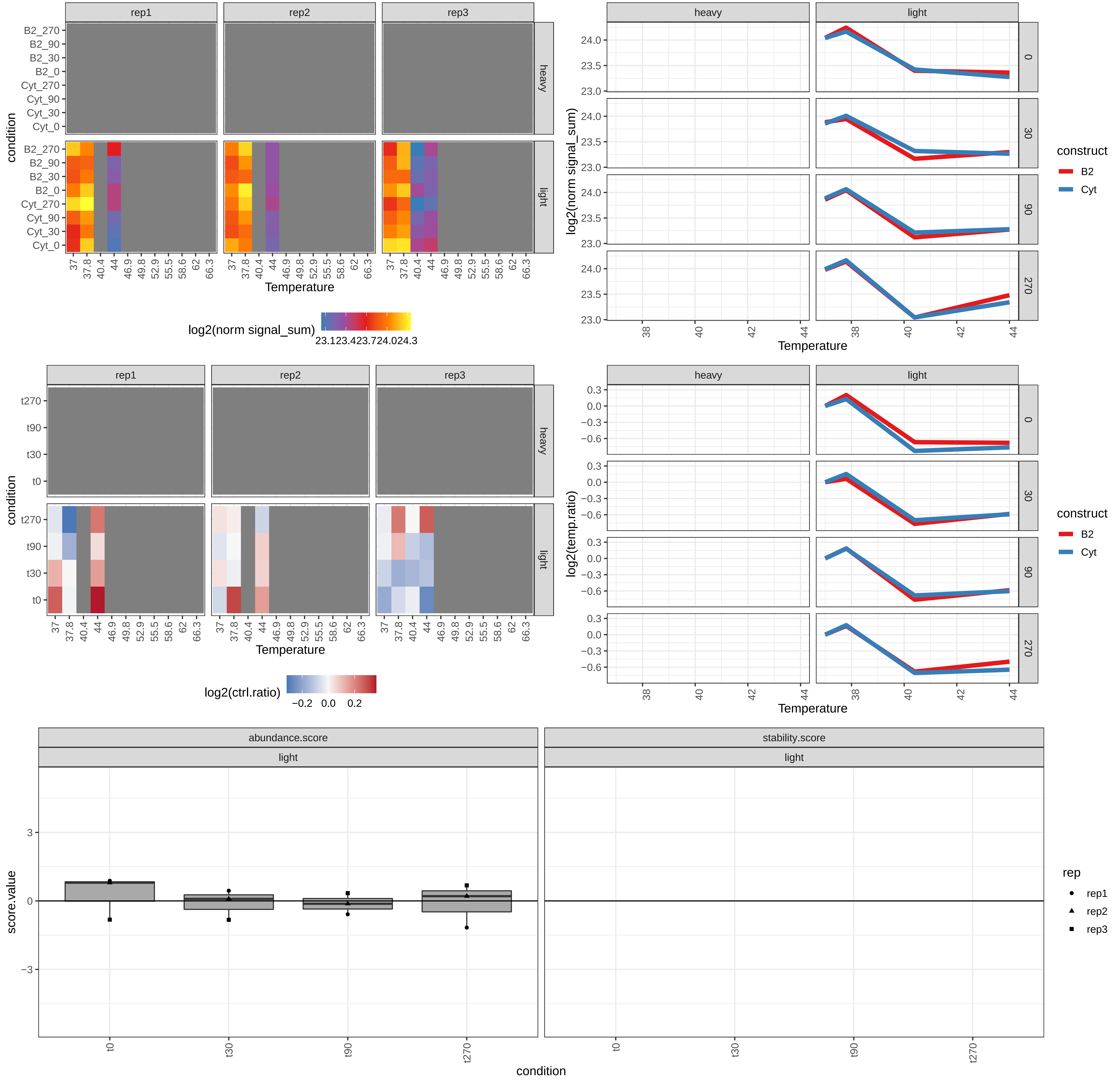

P35182 − Protein phosphatase 2C homolog 1 OS=Saccharomyces cerevisiae (strain ATCC 204508 / S288c) GN=PTC1 PE=1 SV=1

PTC2 − average.top3: 7.00551442307692 − max.qupm: 9

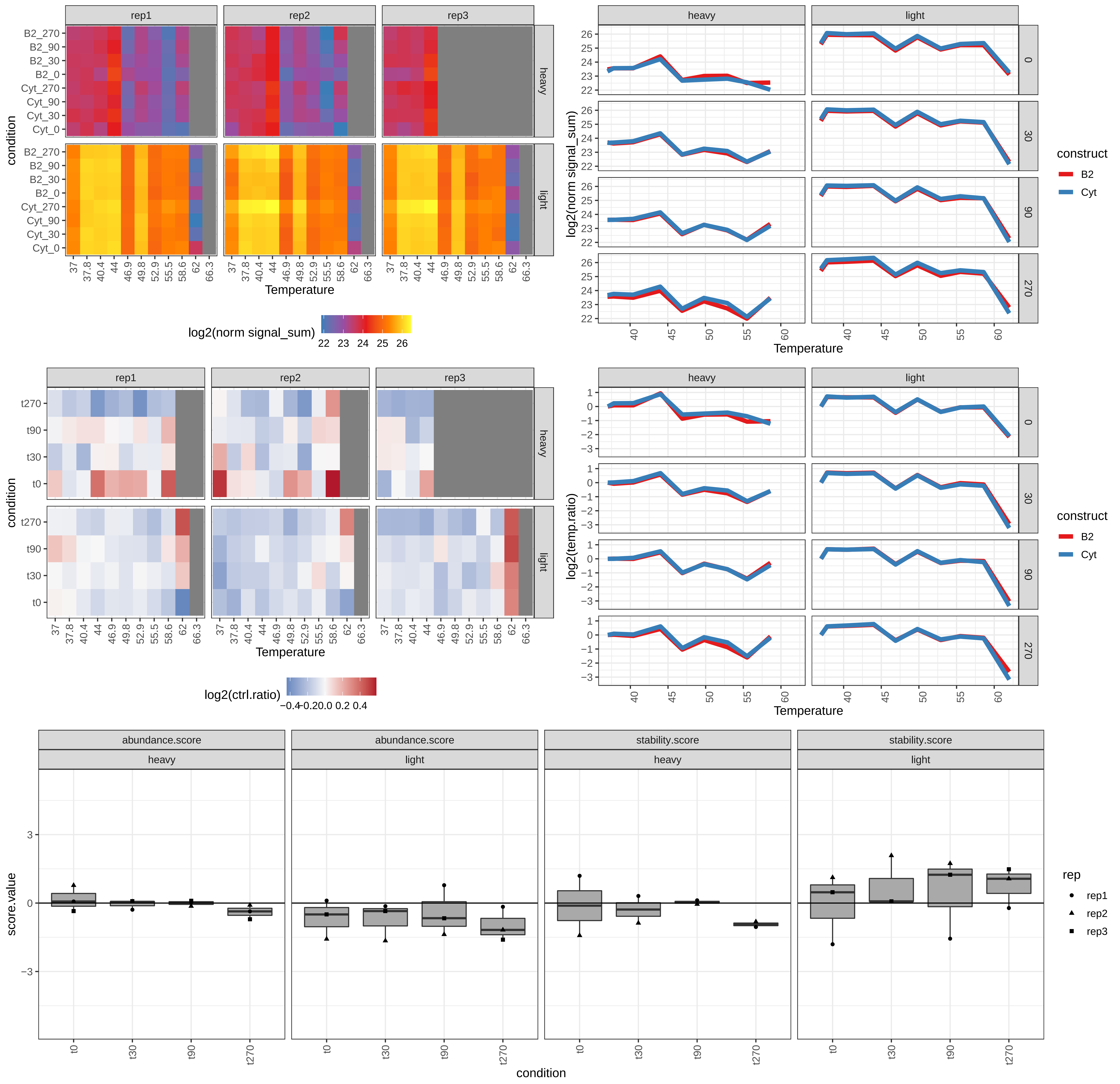

P39966 − Protein phosphatase 2C homolog 2 OS=Saccharomyces cerevisiae (strain ATCC 204508 / S288c) GN=PTC2 PE=1 SV=1

PTC3 − average.top3: 7.0918221875 − max.qupm: 5

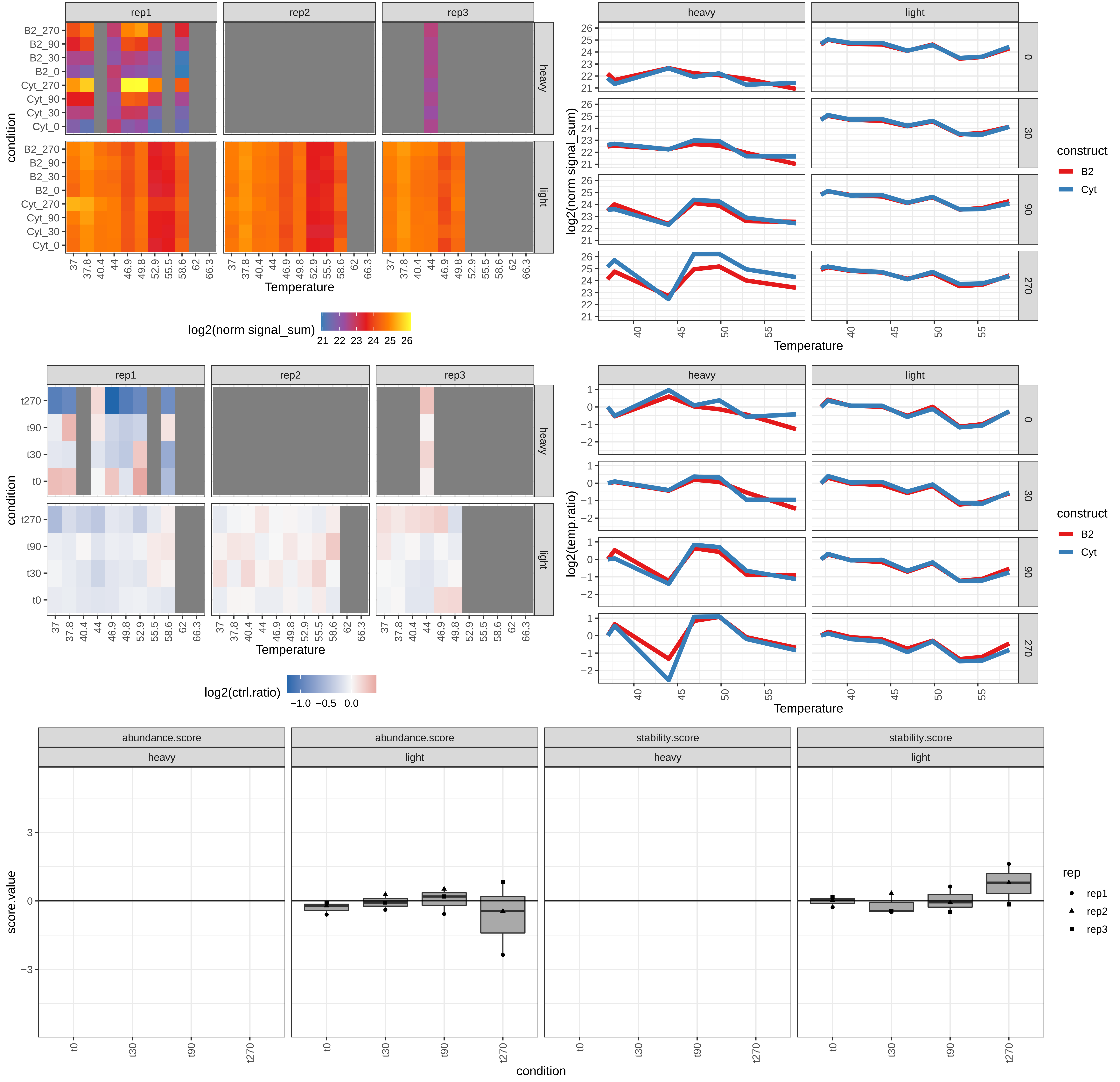

P34221 − Protein phosphatase 2C homolog 3 OS=Saccharomyces cerevisiae (strain ATCC 204508 / S288c) GN=PTC3 PE=1 SV=4

PTC4 − average.top3: 6.99051838709677 − max.qupm: 10

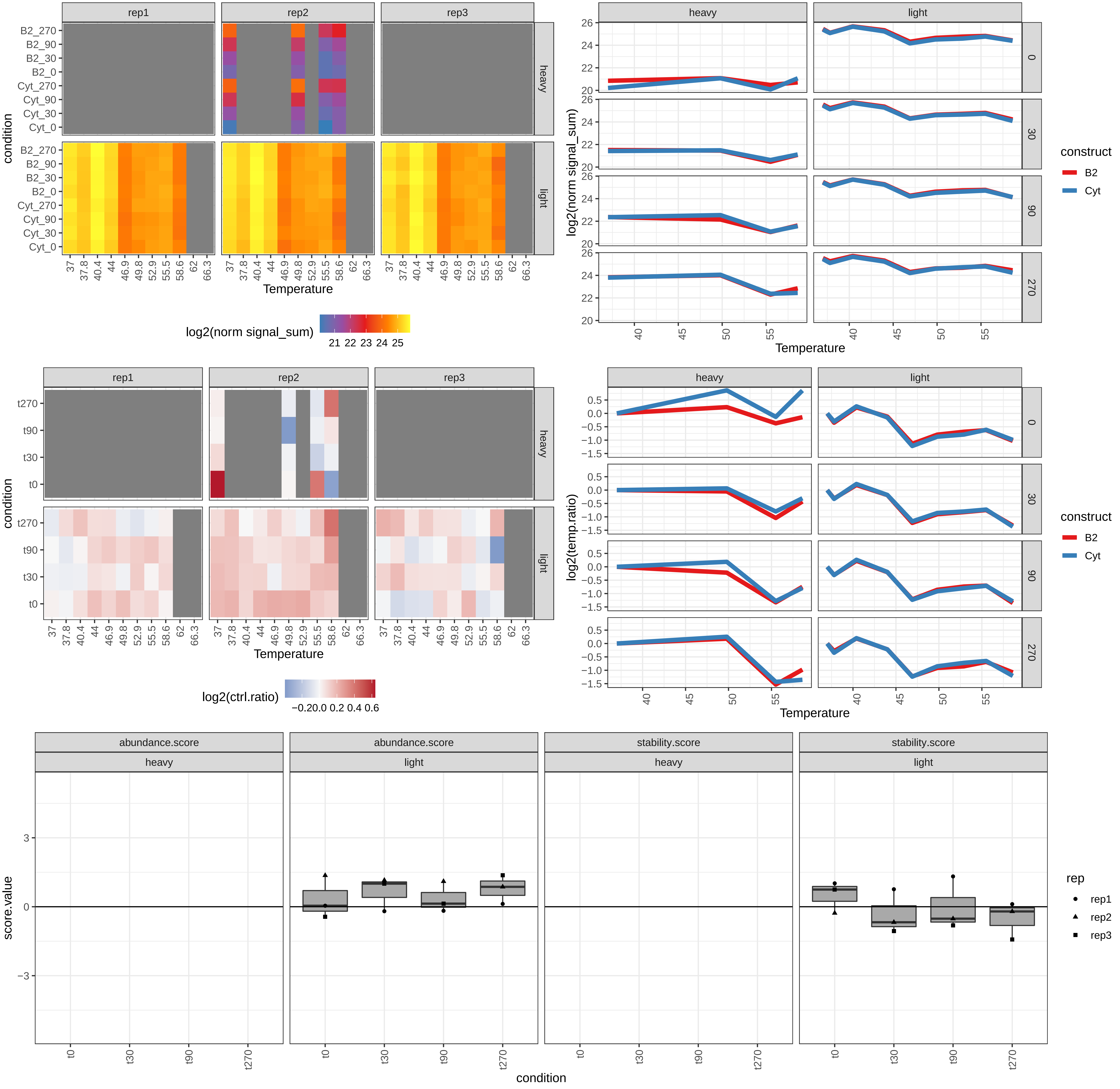

P38089 − Protein phosphatase 2C homolog 4 OS=Saccharomyces cerevisiae (strain ATCC 204508 / S288c) GN=PTC4 PE=1 SV=1

PTC5 − average.top3: 6.464535625 − max.qupm: 6

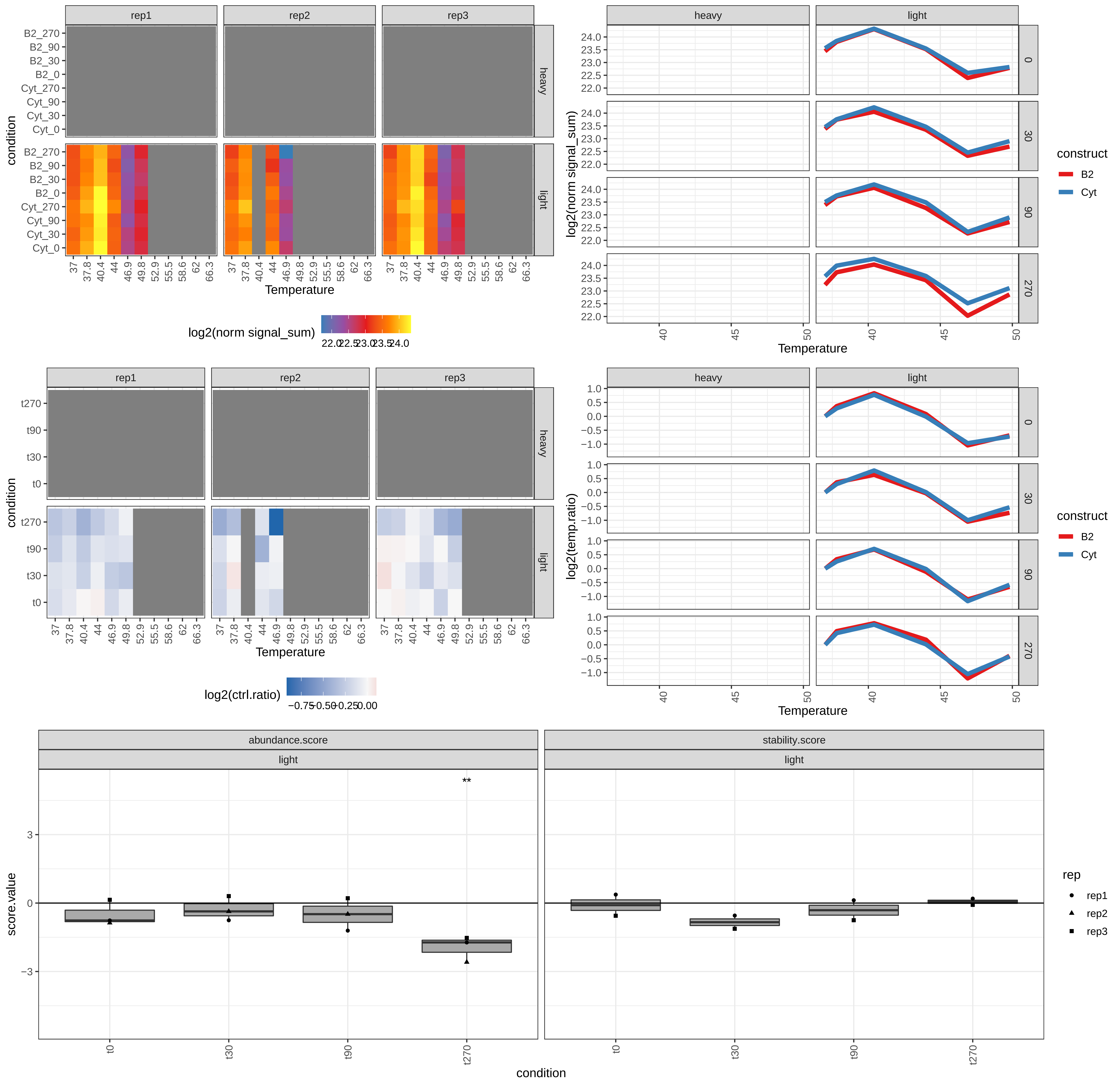

Q12511 − [Pyruvate dehydrogenase [acetyl−transferring]]−phosphatase 1, mitochondrial OS=Saccharomyces cerevisiae (strain ATCC 204508 / S288c) GN=PTC5 PE=1 SV=1

PTC6 − average.top3: 6.52636166666667 − max.qupm: 5

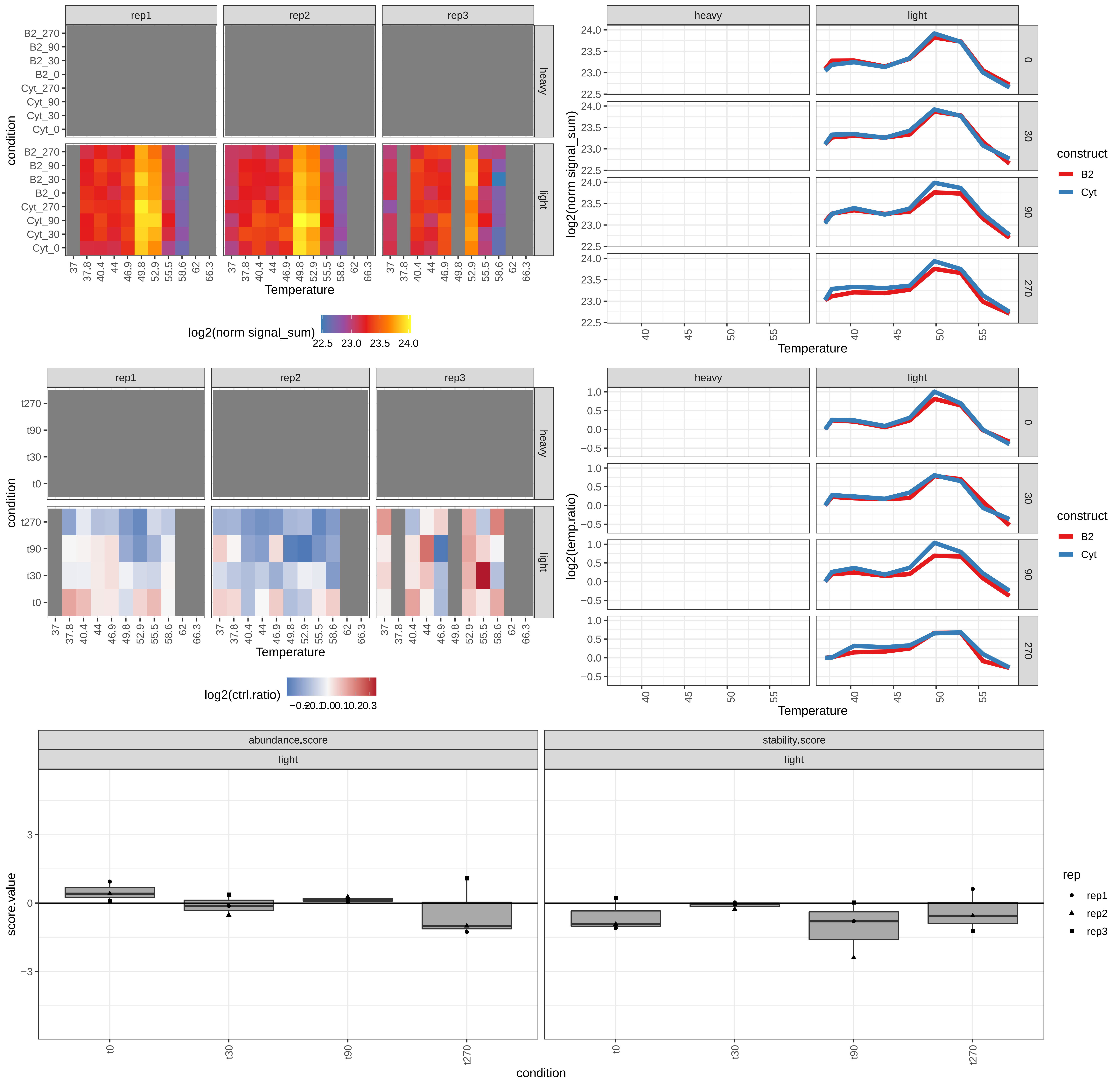

P25646 − [Pyruvate dehydrogenase [acetyl−transferring]]−phosphatase 2, mitochondrial OS=Saccharomyces cerevisiae (strain ATCC 204508 / S288c) GN=PTC6 PE=1 SV=2

PTC7 − average.top3: 7.225959 − max.qupm: 7

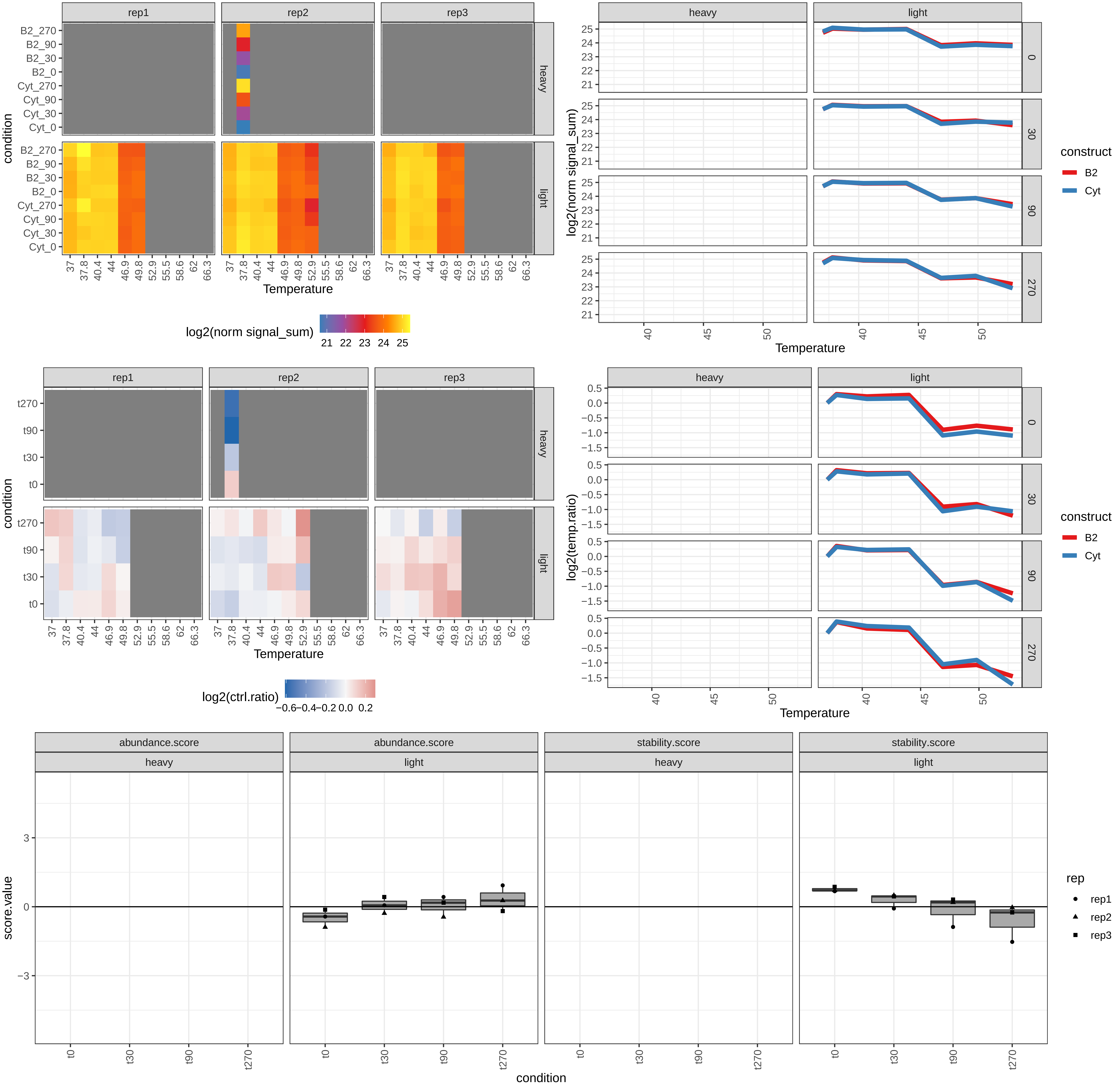

P38797 − Protein phosphatase 2C homolog 7, mitochondrial OS=Saccharomyces cerevisiae (strain ATCC 204508 / S288c) GN=PTC7 PE=1 SV=2

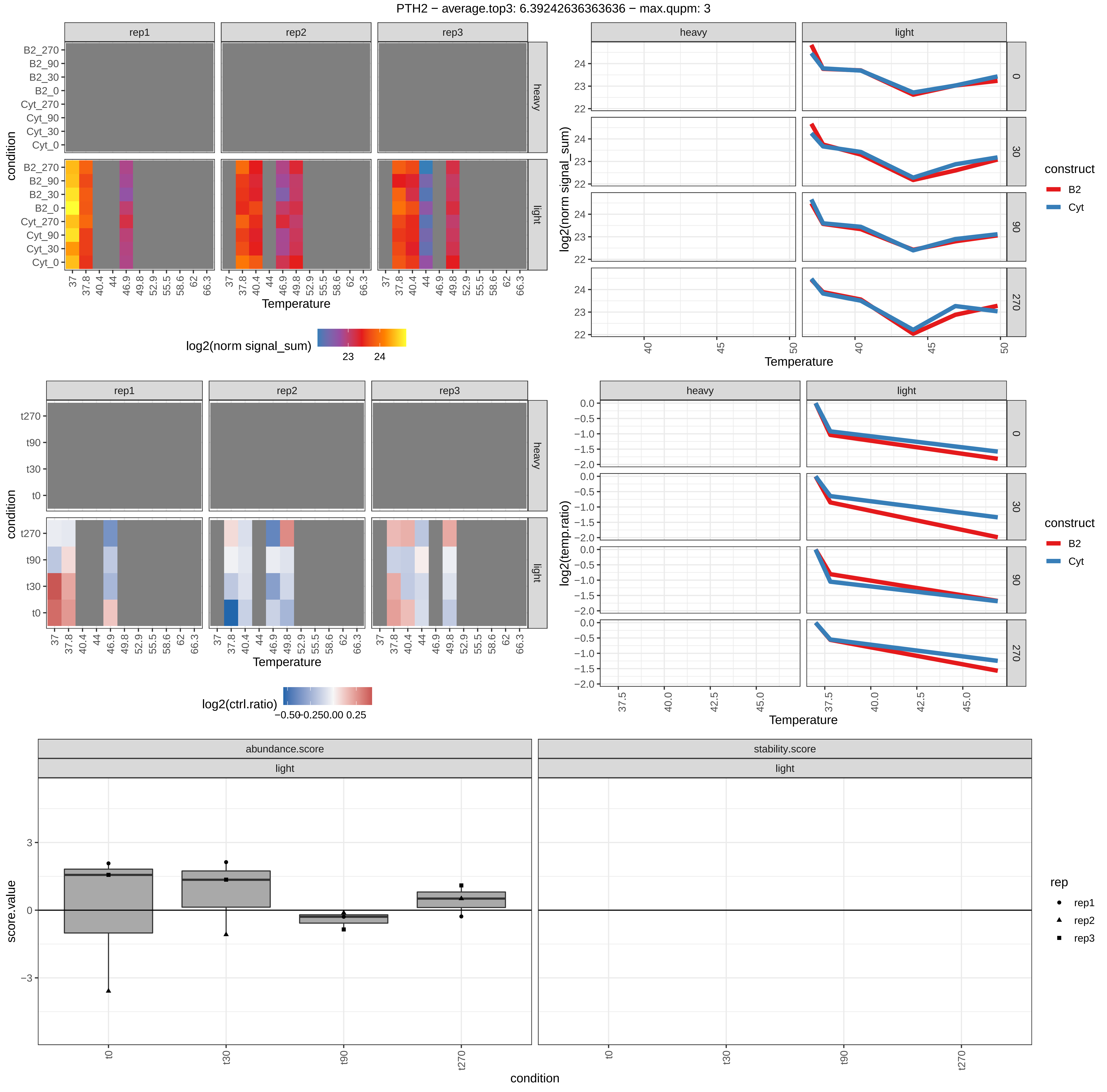

P34222 − Peptidyl−tRNA hydrolase 2 OS=Saccharomyces cerevisiae (strain ATCC 204508 / S288c) GN=PTH2 PE=1 SV=2

PTK2 − average.top3: 7.254155 − max.qupm: 9

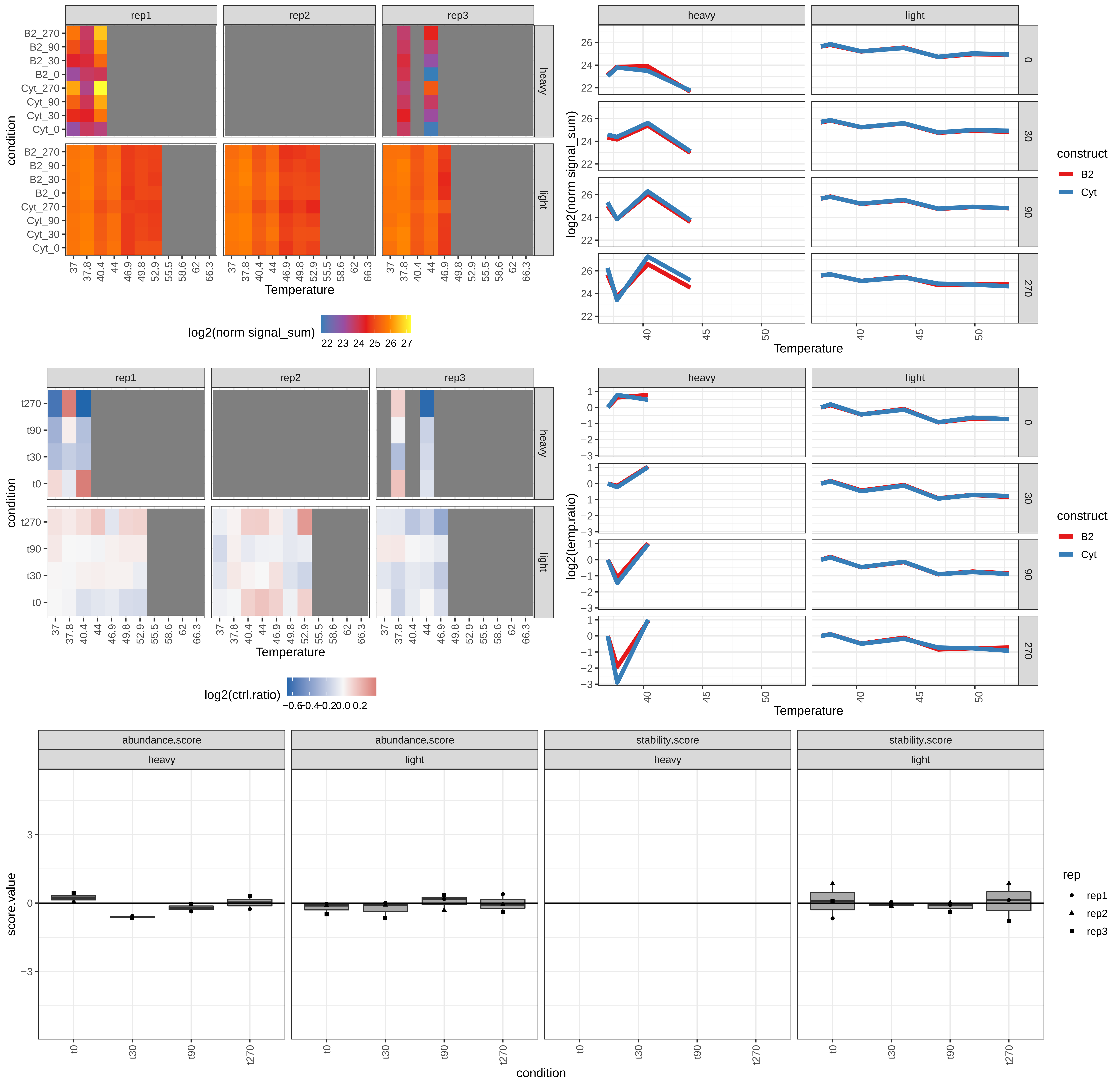

P47116 − Serine/threonine−protein kinase PTK2/STK2 OS=Saccharomyces cerevisiae (strain ATCC 204508 / S288c) GN=PTK2 PE=1 SV=1

PTM1 − average.top3: 7.34223076923077 − max.qupm: 4

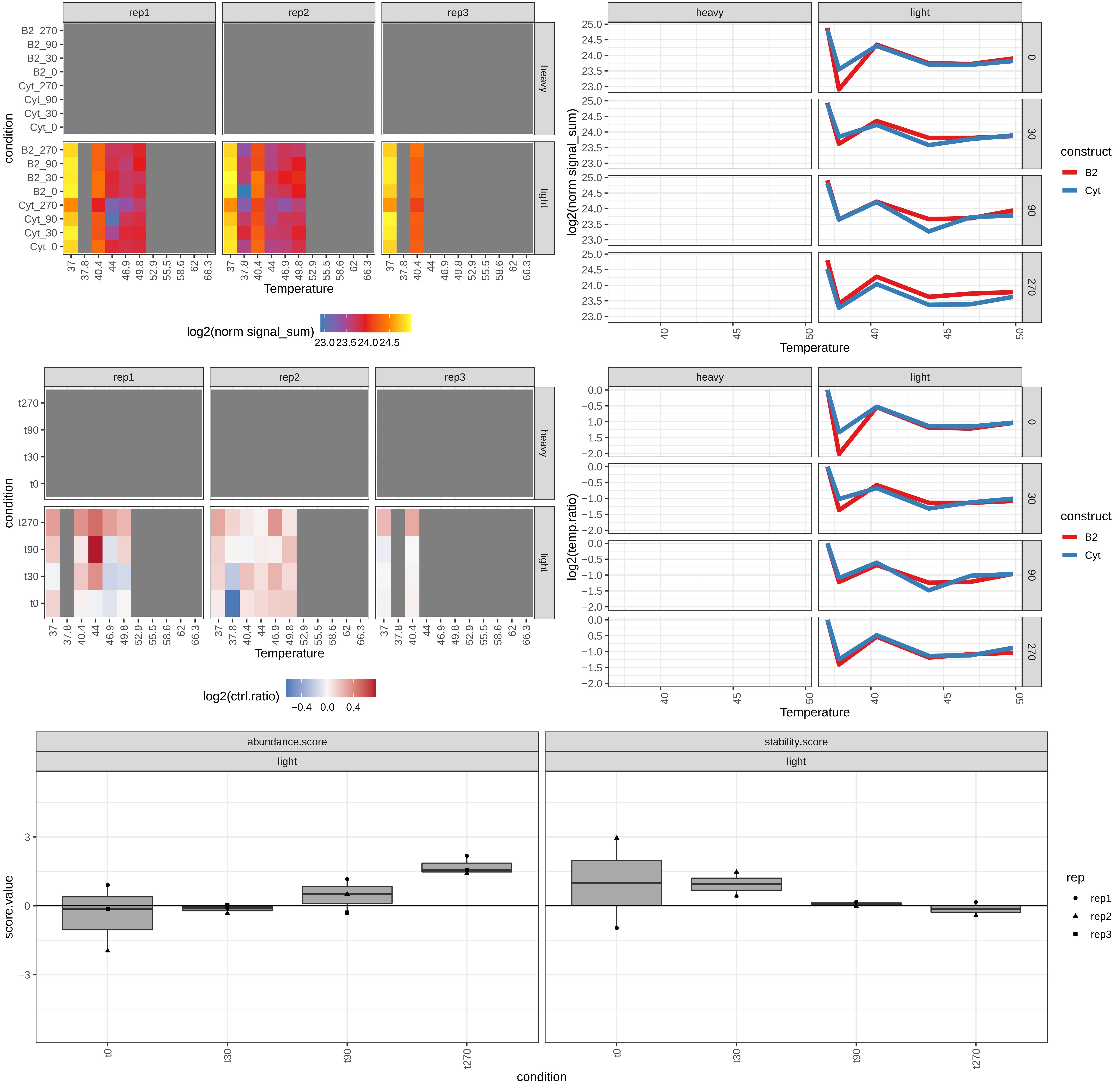

P32857 − Membrane protein PTM1 OS=Saccharomyces cerevisiae (strain ATCC 204508 / S288c) GN=PTM1 PE=1 SV=2

PTP1 − average.top3: 6.75081875 − max.qupm: 7

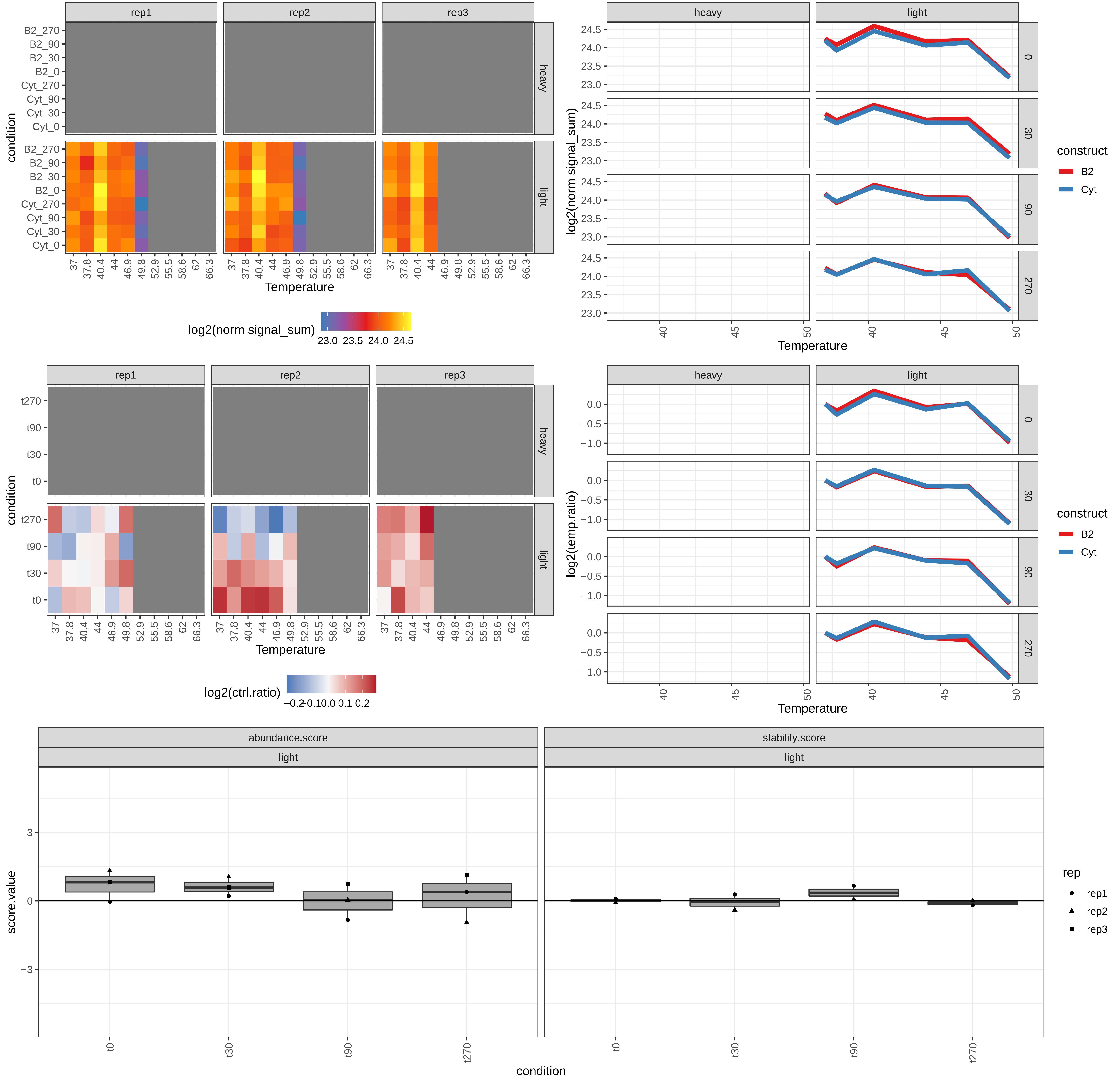

P25044 − Tyrosine−protein phosphatase 1 OS=Saccharomyces cerevisiae (strain ATCC 204508 / S288c) GN=PTP1 PE=1 SV=1

PUB1 − average.top3: 7.15897553191489 − max.qupm: 7

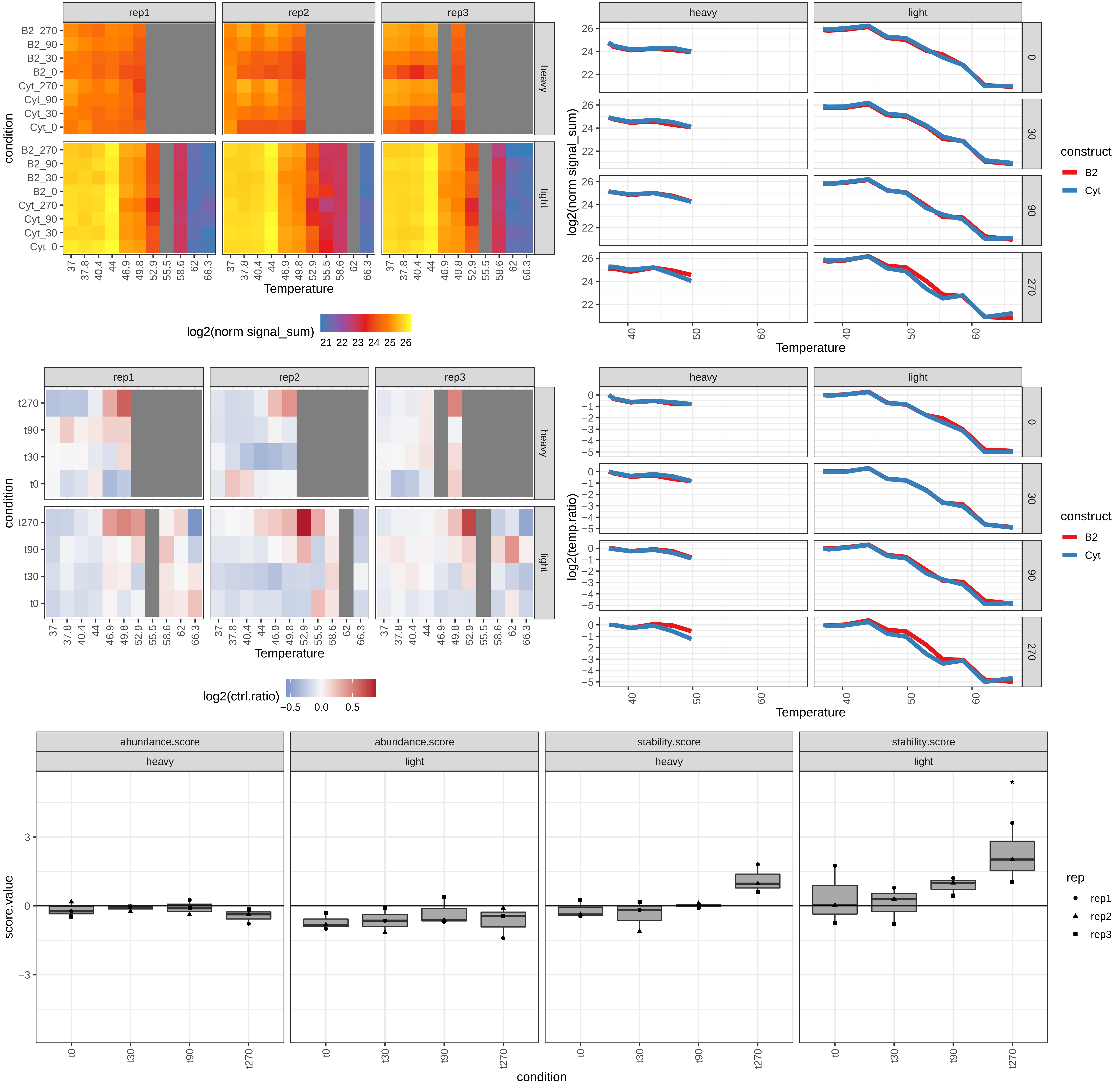

P32588 − Nuclear and cytoplasmic polyadenylated RNA−binding protein PUB1 OS=Saccharomyces cerevisiae (strain ATCC 204508 / S288c) GN=PUB1 PE=1 SV=4

PUF2 − average.top3: 6.81224428571429 − max.qupm: 3

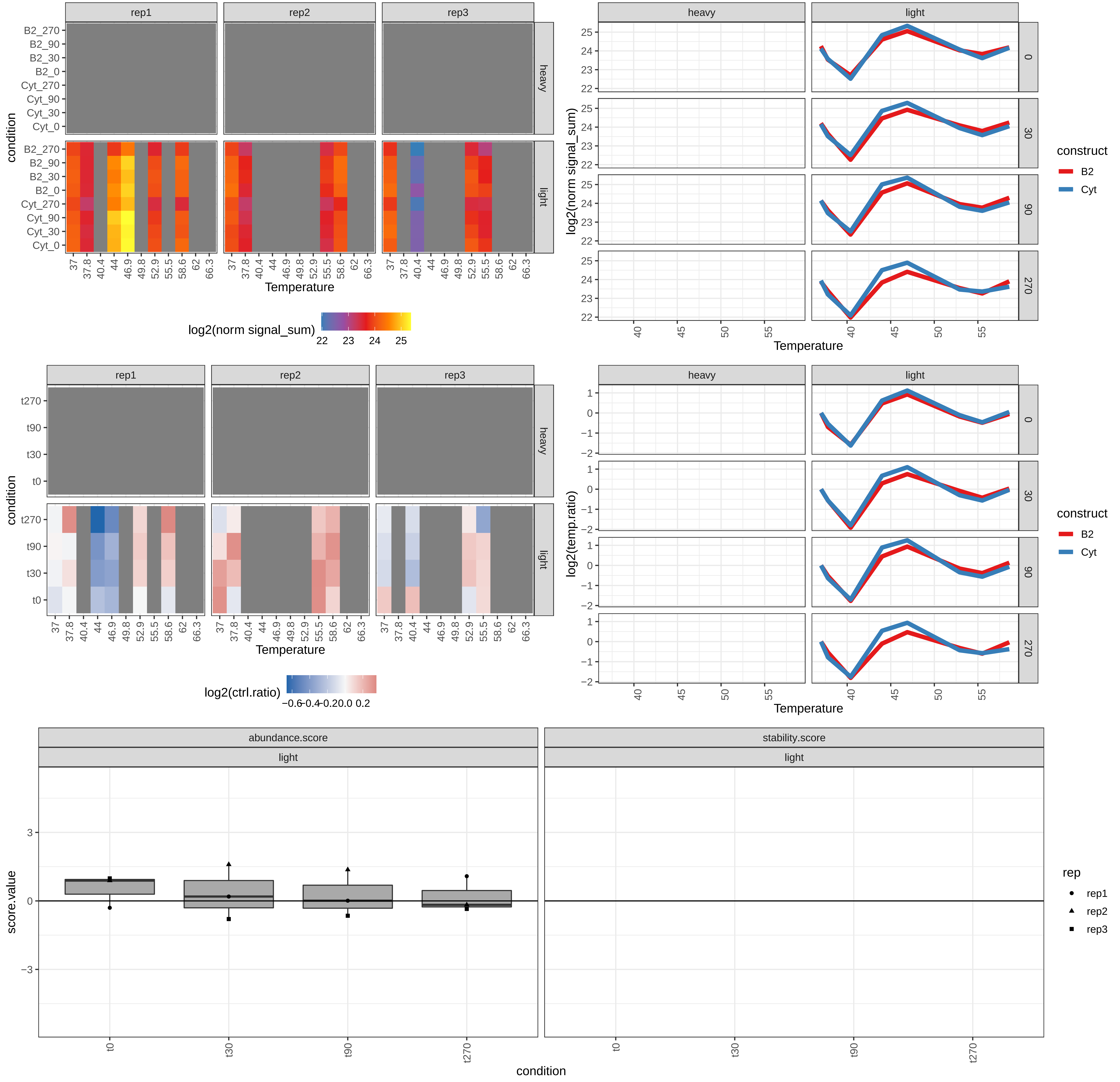

Q12221 − mRNA−binding protein PUF2 OS=Saccharomyces cerevisiae (strain ATCC 204508 / S288c) GN=PUF2 PE=1 SV=1

PUF3 − average.top3: 7.03650685714286 − max.qupm: 16

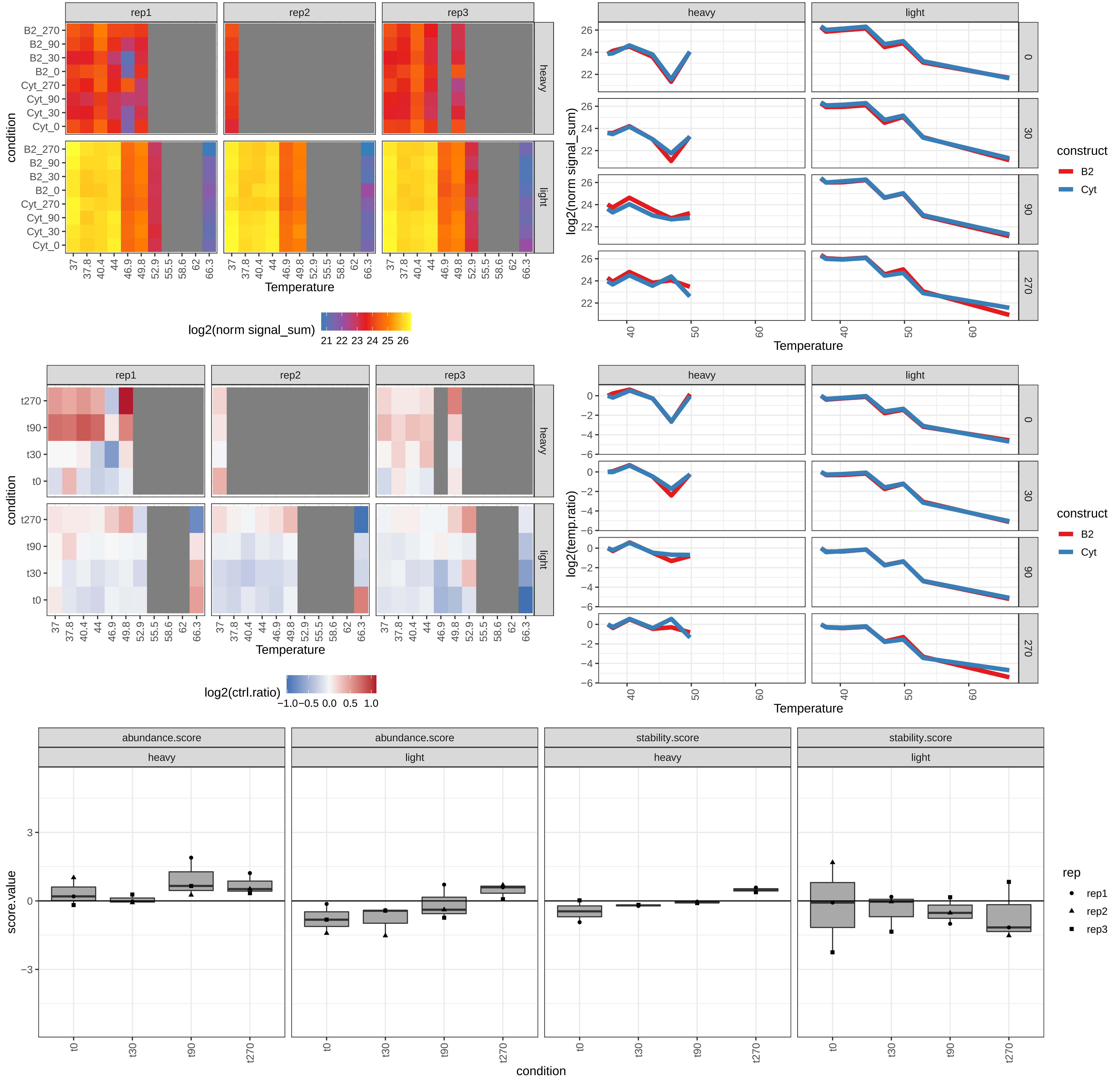

Q07807 − mRNA−binding protein PUF3 OS=Saccharomyces cerevisiae (strain ATCC 204508 / S288c) GN=PUF3 PE=1 SV=1

PUF4 − average.top3: 6.632946 − max.qupm: 5

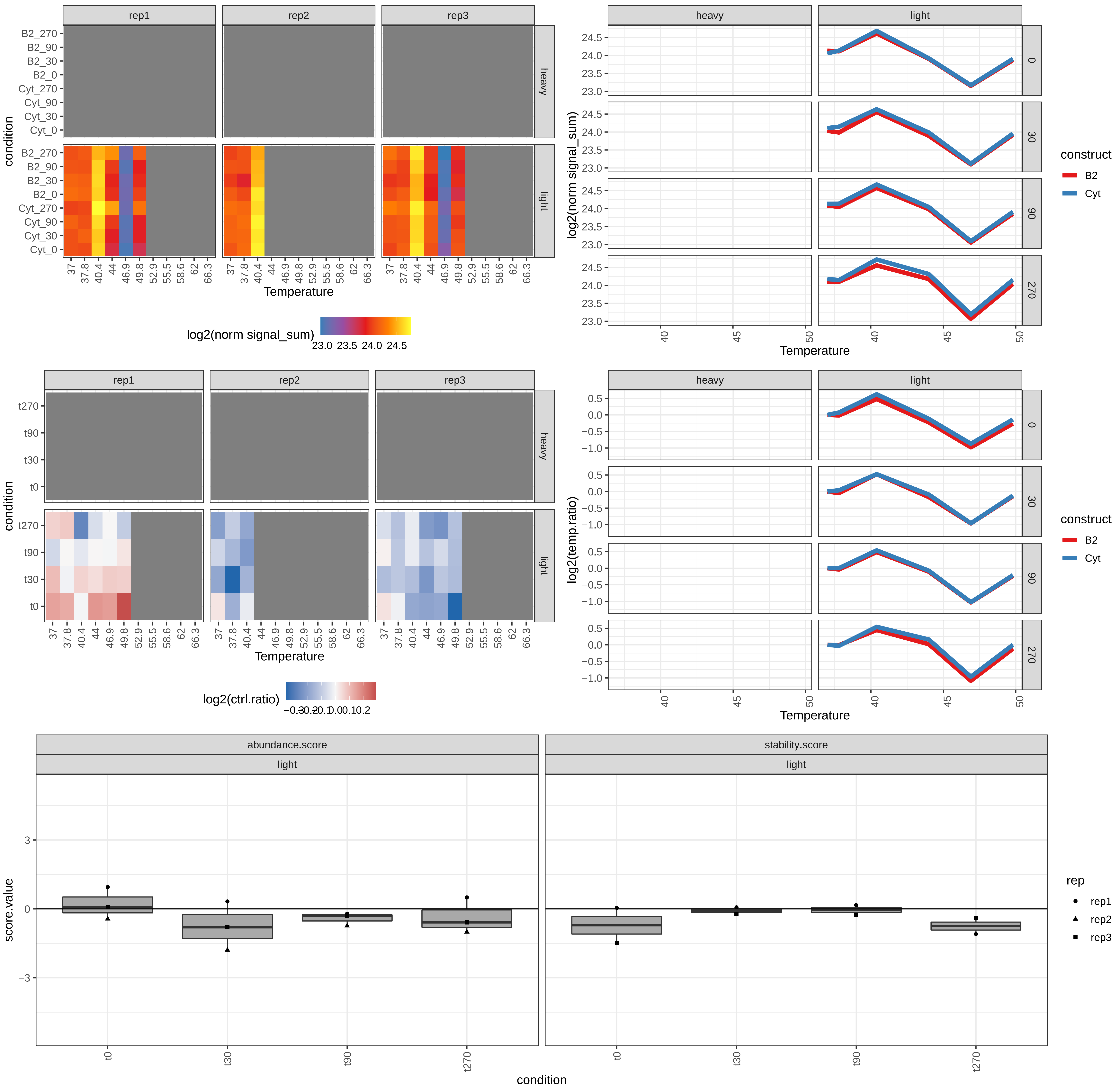

P25339 − Pumilio homology domain family member 4 OS=Saccharomyces cerevisiae (strain ATCC 204508 / S288c) GN=PUF4 PE=1 SV=2

PUF6 − average.top3: 6.95934428571429 − max.qupm: 14

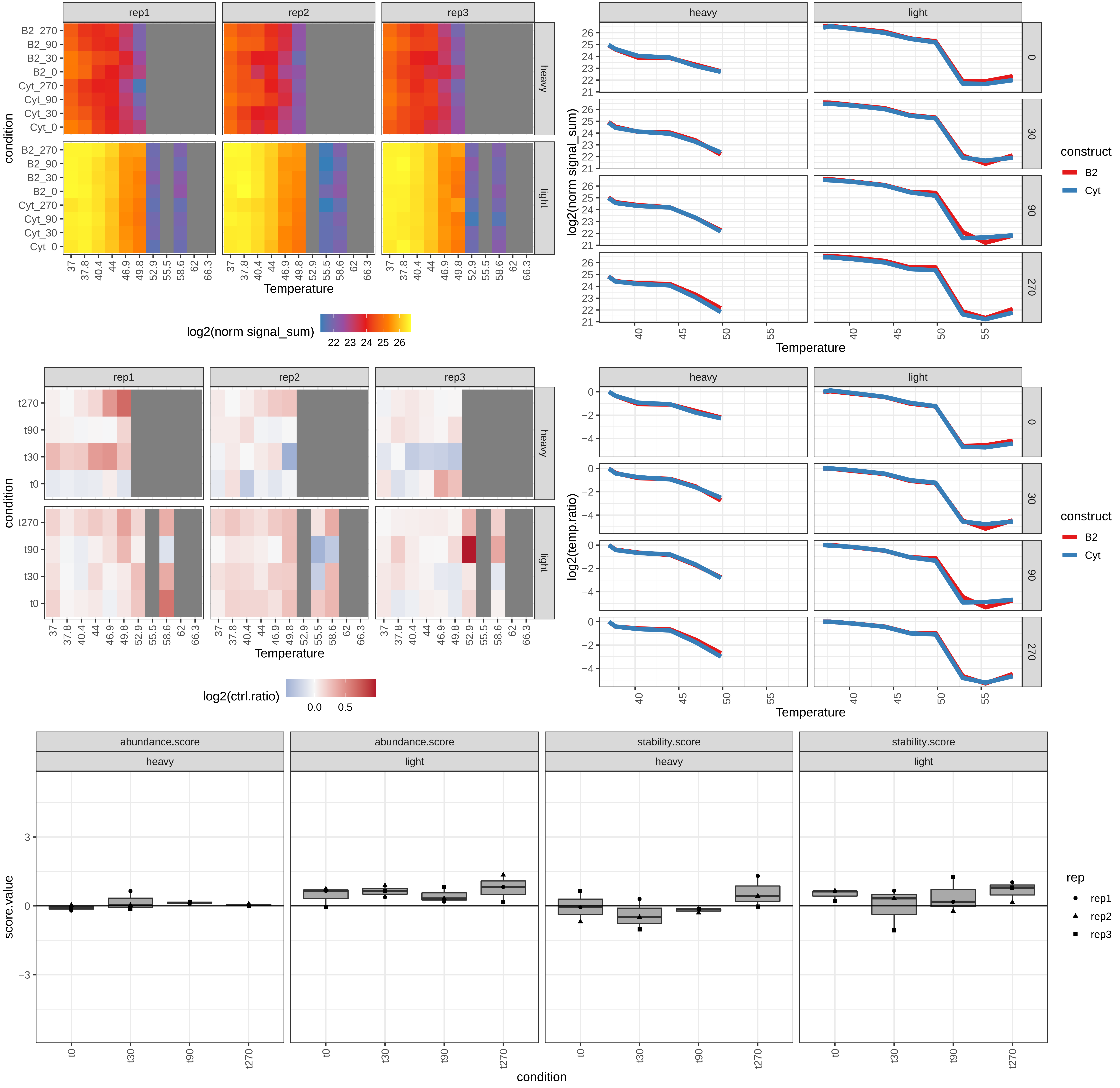

Q04373 − Pumilio homology domain family member 6 OS=Saccharomyces cerevisiae (strain ATCC 204508 / S288c) GN=PUF6 PE=1 SV=1

PUN1 − average.top3: 6.76413666666667 − max.qupm: 3

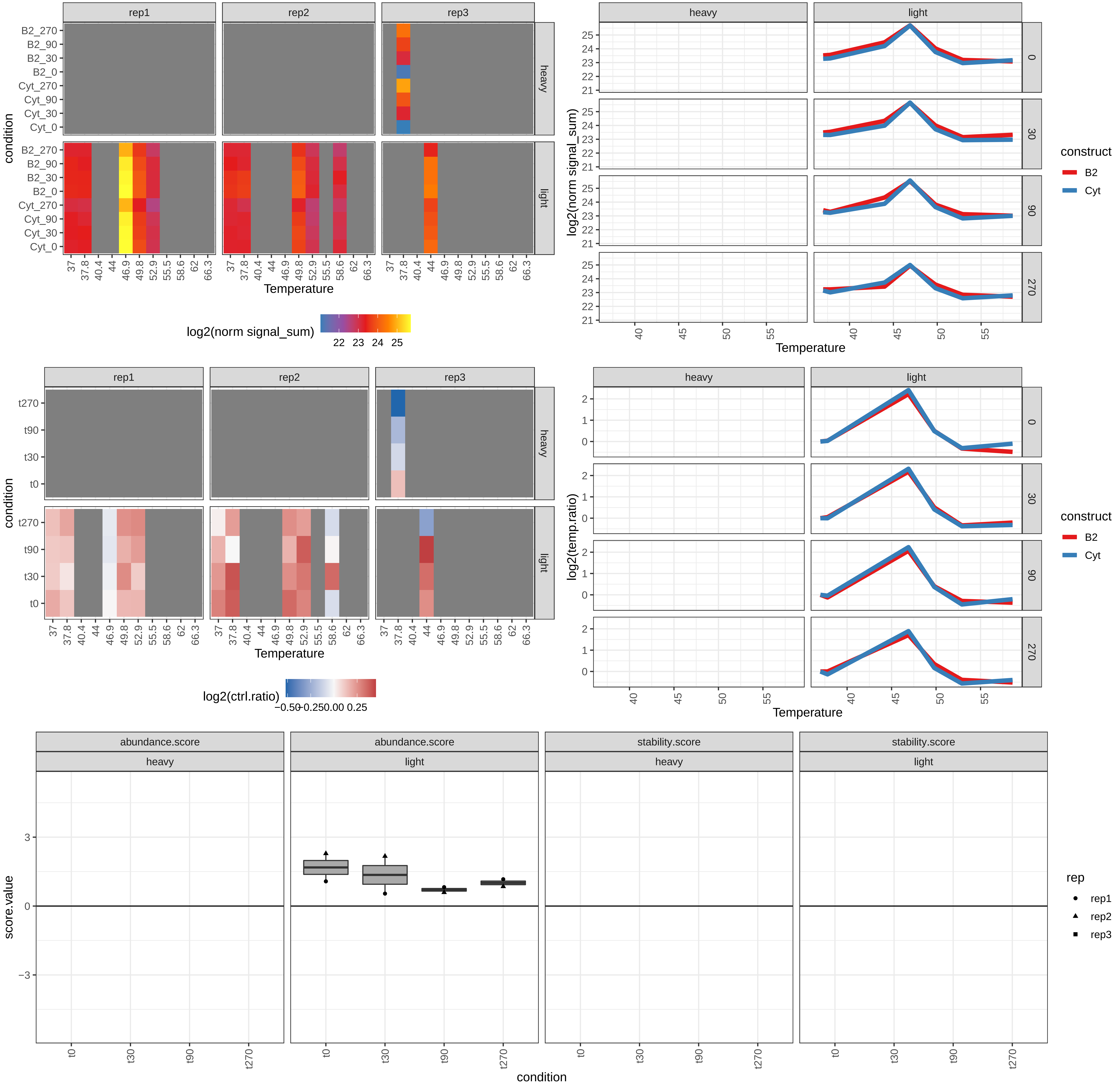

Q06991 − Protein PUN1 OS=Saccharomyces cerevisiae (strain ATCC 204508 / S288c) GN=PUN1 PE=1 SV=1

PUP1 − average.top3: 7.34693744186047 − max.qupm: 5

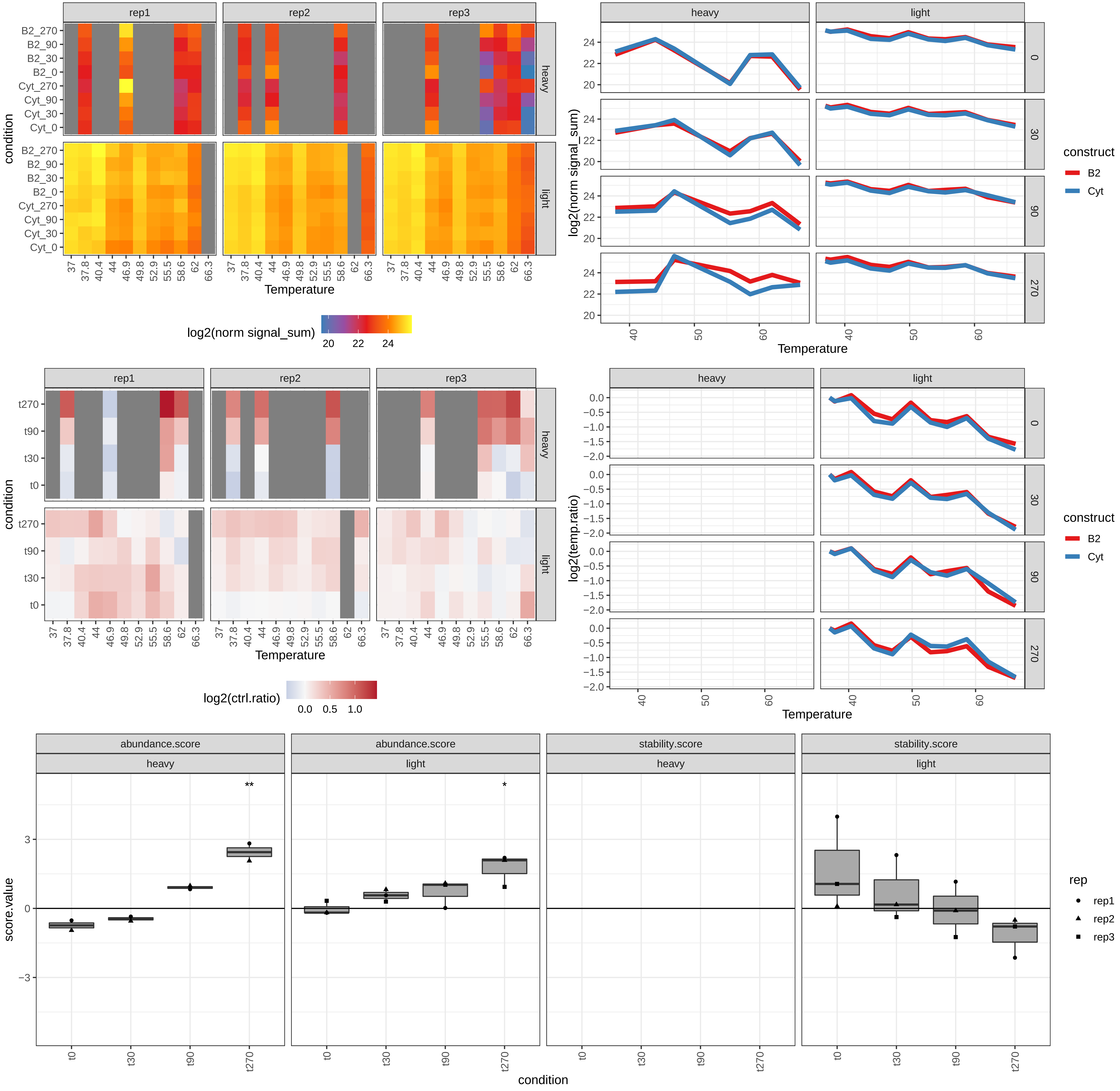

P25043 − Proteasome subunit beta type−2 OS=Saccharomyces cerevisiae (strain ATCC 204508 / S288c) GN=PUP1 PE=1 SV=1

PUP2 − average.top3: 7.32607151515152 − max.qupm: 11

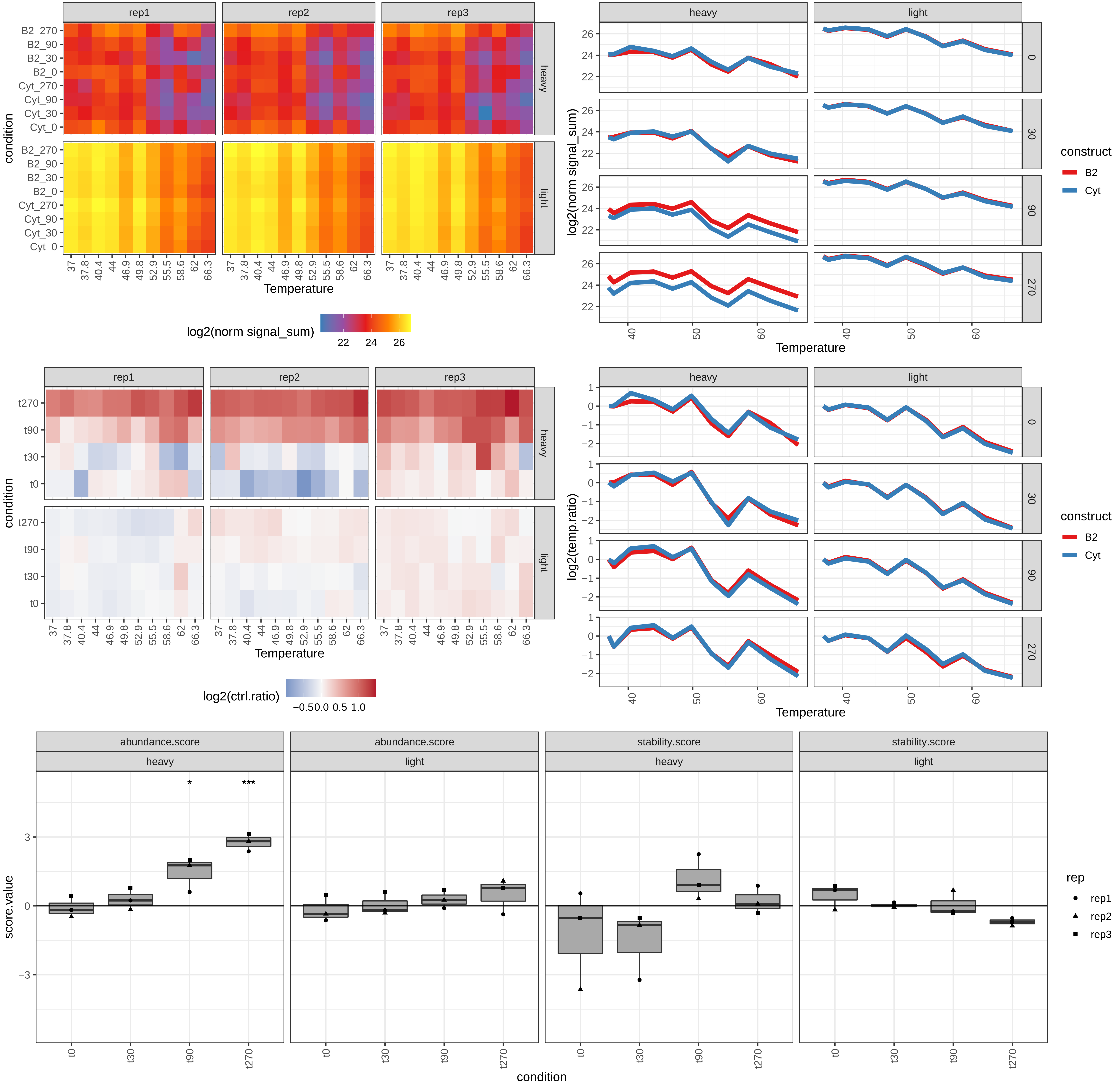

P32379 − Proteasome subunit alpha type−5 OS=Saccharomyces cerevisiae (strain ATCC 204508 / S288c) GN=PUP2 PE=1 SV=2

PUP3 − average.top3: 6.92742769230769 − max.qupm: 3

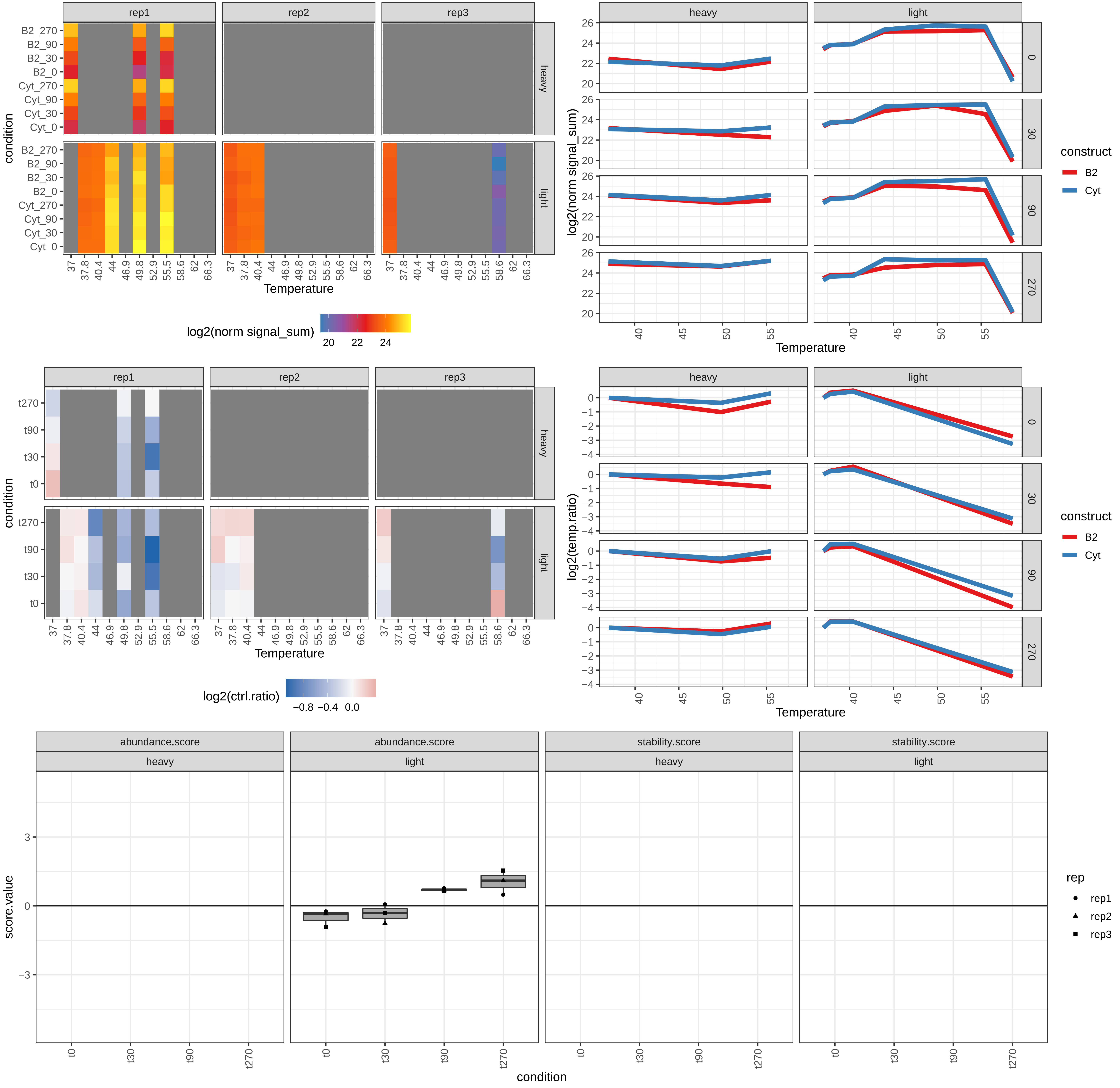

P25451 − Proteasome subunit beta type−3 OS=Saccharomyces cerevisiae (strain ATCC 204508 / S288c) GN=PUP3 PE=1 SV=1

PUS1 − average.top3: 6.99756825 − max.qupm: 16

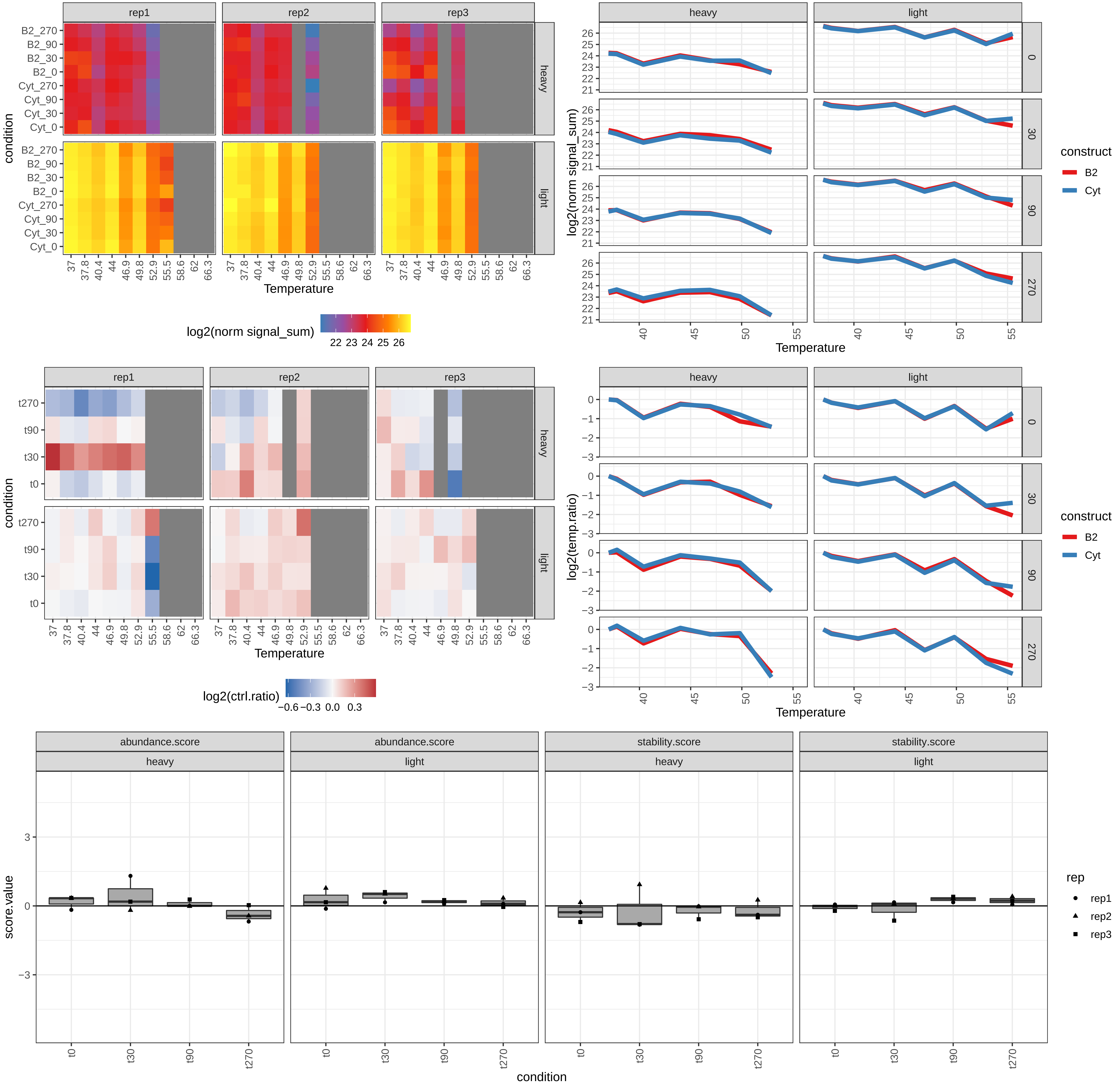

Q12211 − tRNA pseudouridine synthase 1 OS=Saccharomyces cerevisiae (strain ATCC 204508 / S288c) GN=PUS1 PE=1 SV=1

PUS4 − average.top3: 7.02003125 − max.qupm: 6

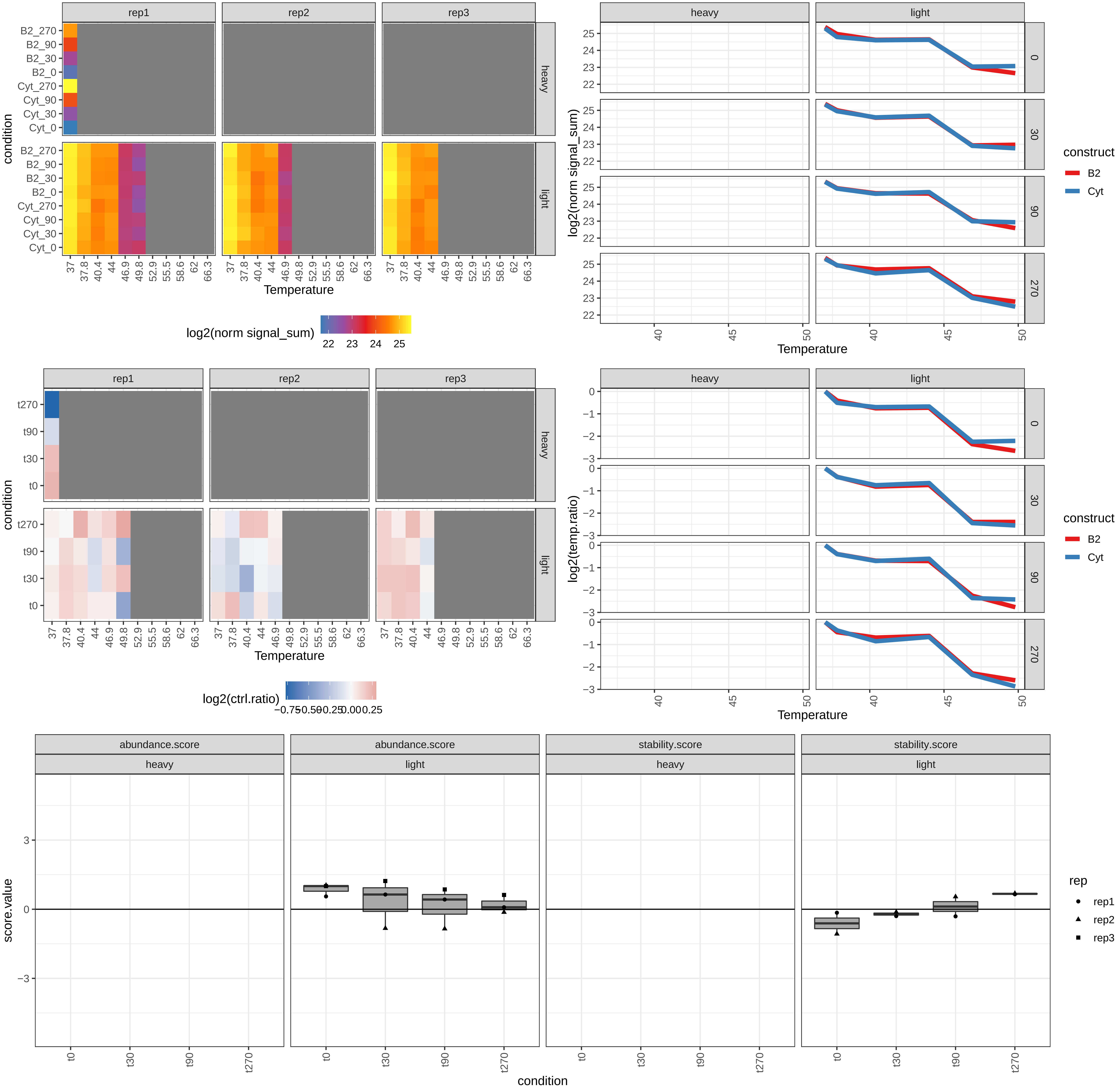

P48567 − tRNA pseudouridine synthase 4 OS=Saccharomyces cerevisiae (strain ATCC 204508 / S288c) GN=PUS4 PE=1 SV=1

PUS7 − average.top3: 6.8125562962963 − max.qupm: 12

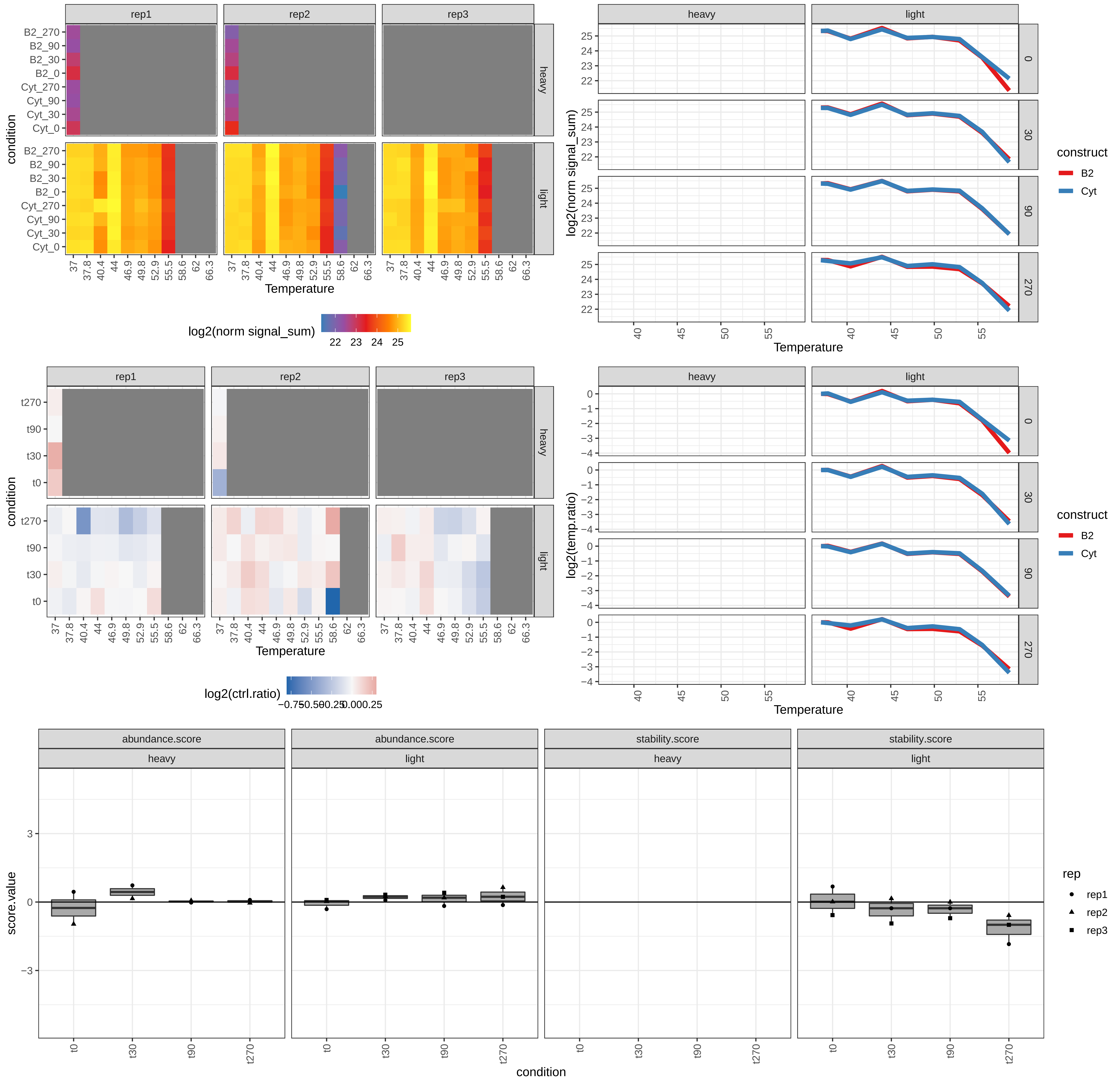

Q08647 − Multisubstrate pseudouridine synthase 7 OS=Saccharomyces cerevisiae (strain ATCC 204508 / S288c) GN=PUS7 PE=1 SV=1

PUT2 − average.top3: 7.38876344827586 − max.qupm: 16

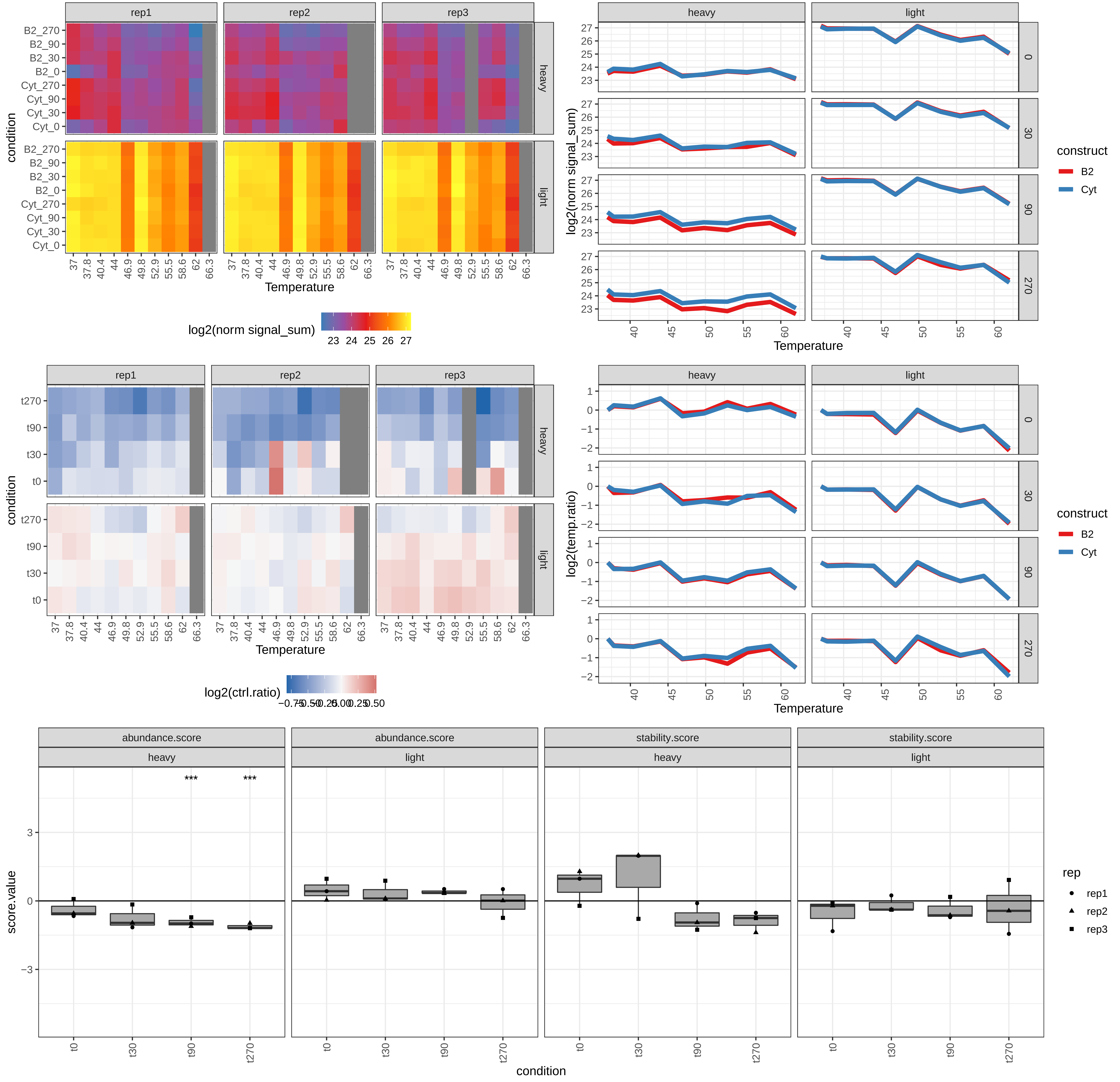

P07275 − Delta−1−pyrroline−5−carboxylate dehydrogenase, mitochondrial OS=Saccharomyces cerevisiae (strain ATCC 204508 / S288c) GN=PUT2 PE=1 SV=2

PWP1 − average.top3: 6.88362034482759 − max.qupm: 9

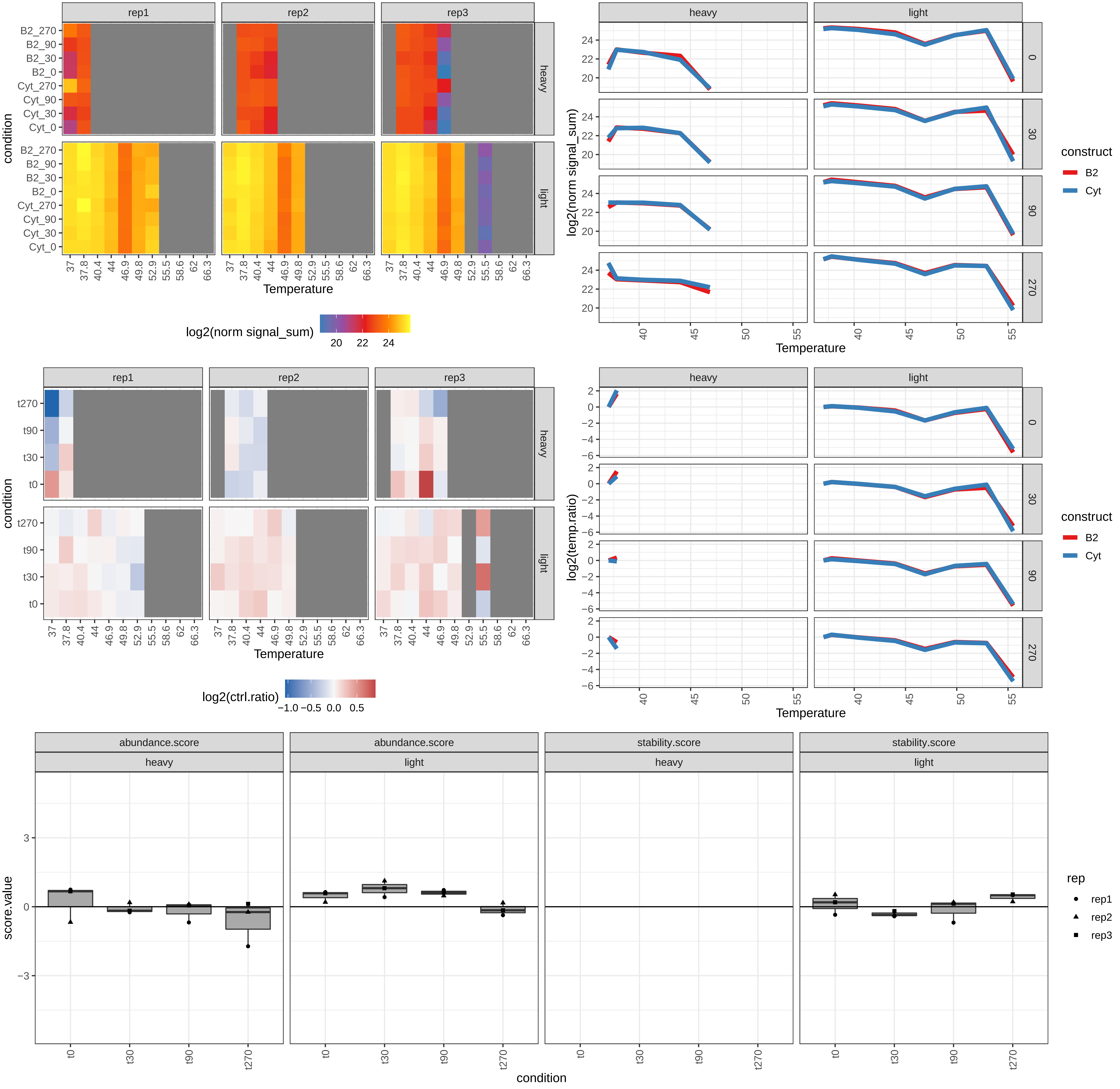

P21304 − Periodic tryptophan protein 1 OS=Saccharomyces cerevisiae (strain ATCC 204508 / S288c) GN=PWP1 PE=1 SV=1

PWP2 − average.top3: 6.9423848 − max.qupm: 11

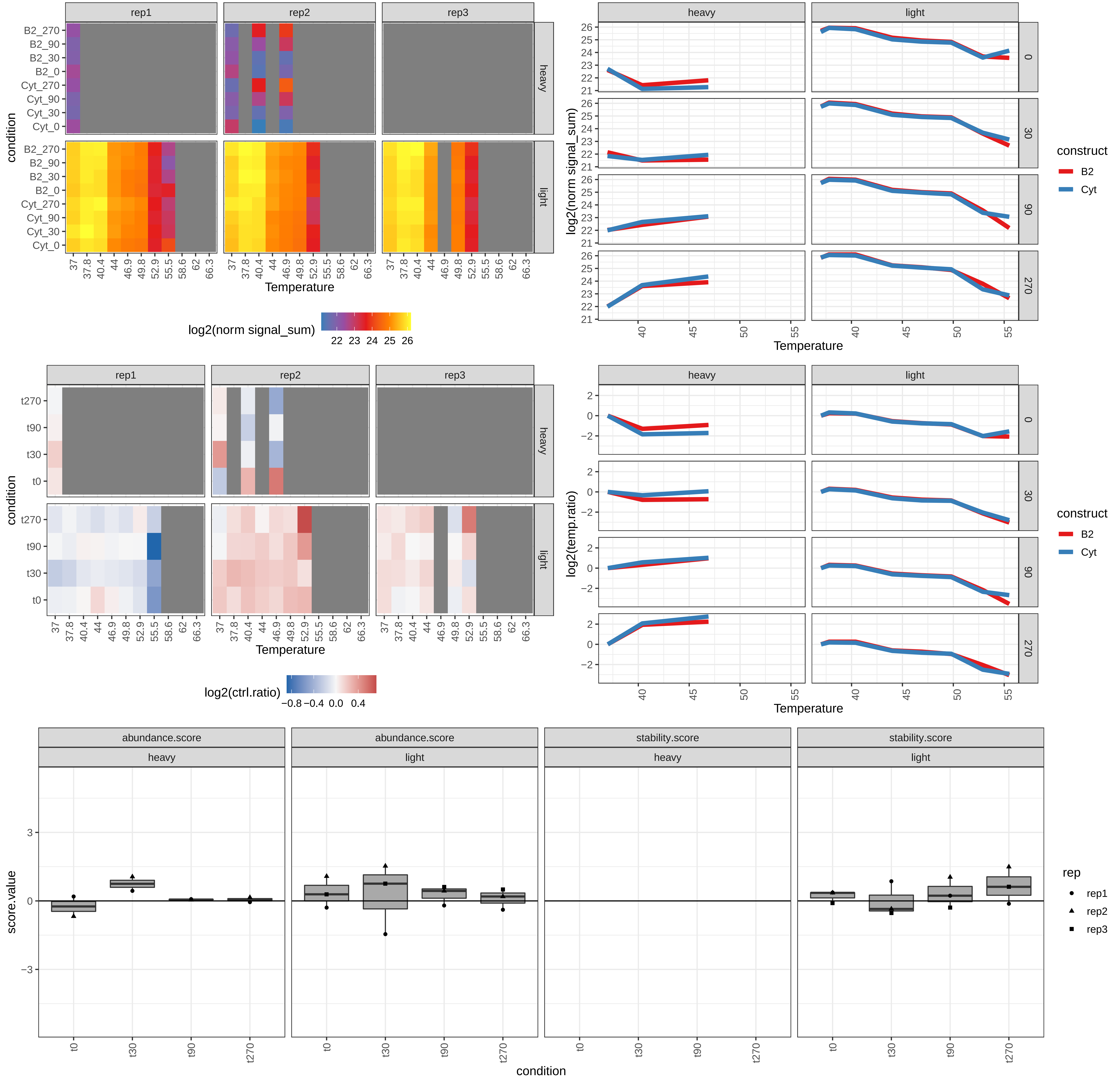

P25635 − Periodic tryptophan protein 2 OS=Saccharomyces cerevisiae (strain ATCC 204508 / S288c) GN=PWP2 PE=1 SV=2

PXA1 − average.top3: 6.47949454545455 − max.qupm: 4

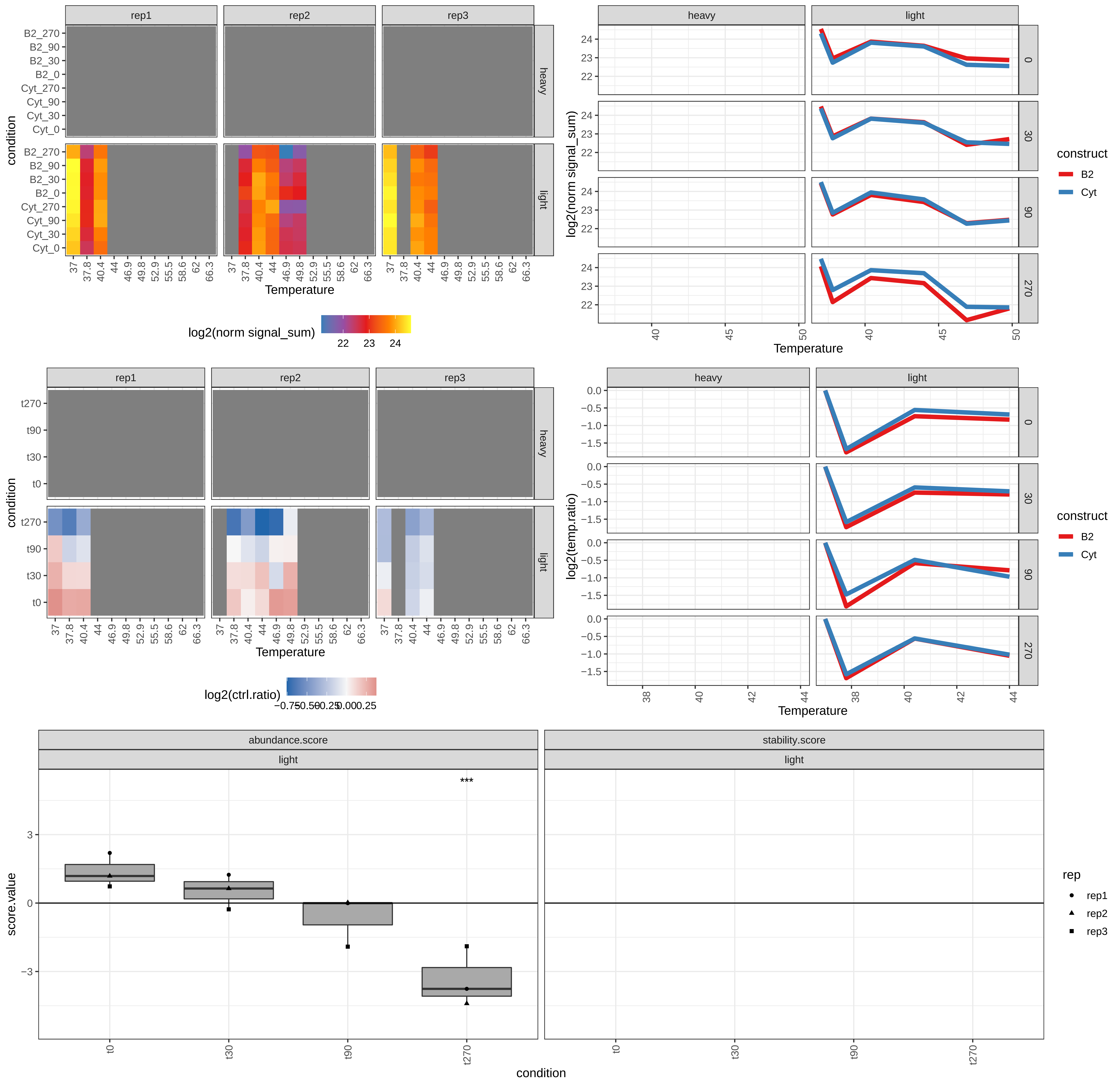

P41909 − Peroxisomal long−chain fatty acid import protein 2 OS=Saccharomyces cerevisiae (strain ATCC 204508 / S288c) GN=PXA1 PE=1 SV=2

PXA2 − average.top3: 6.22772555555556 − max.qupm: 6

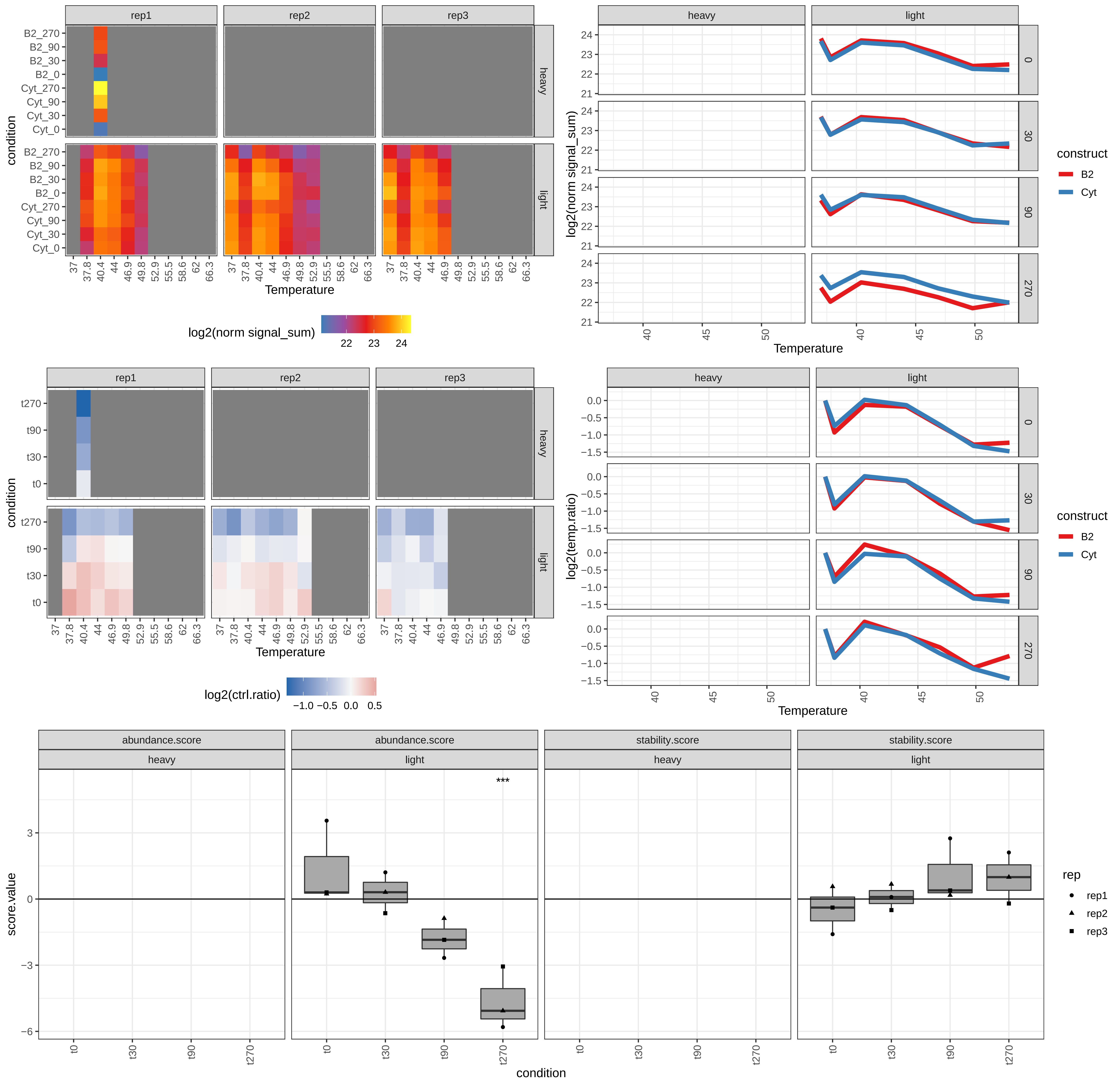

P34230 − Peroxisomal long−chain fatty acid import protein 1 OS=Saccharomyces cerevisiae (strain ATCC 204508 / S288c) GN=PXA2 PE=1 SV=1

PYC1 − average.top3: 7.75479213114754 − max.qupm: 12

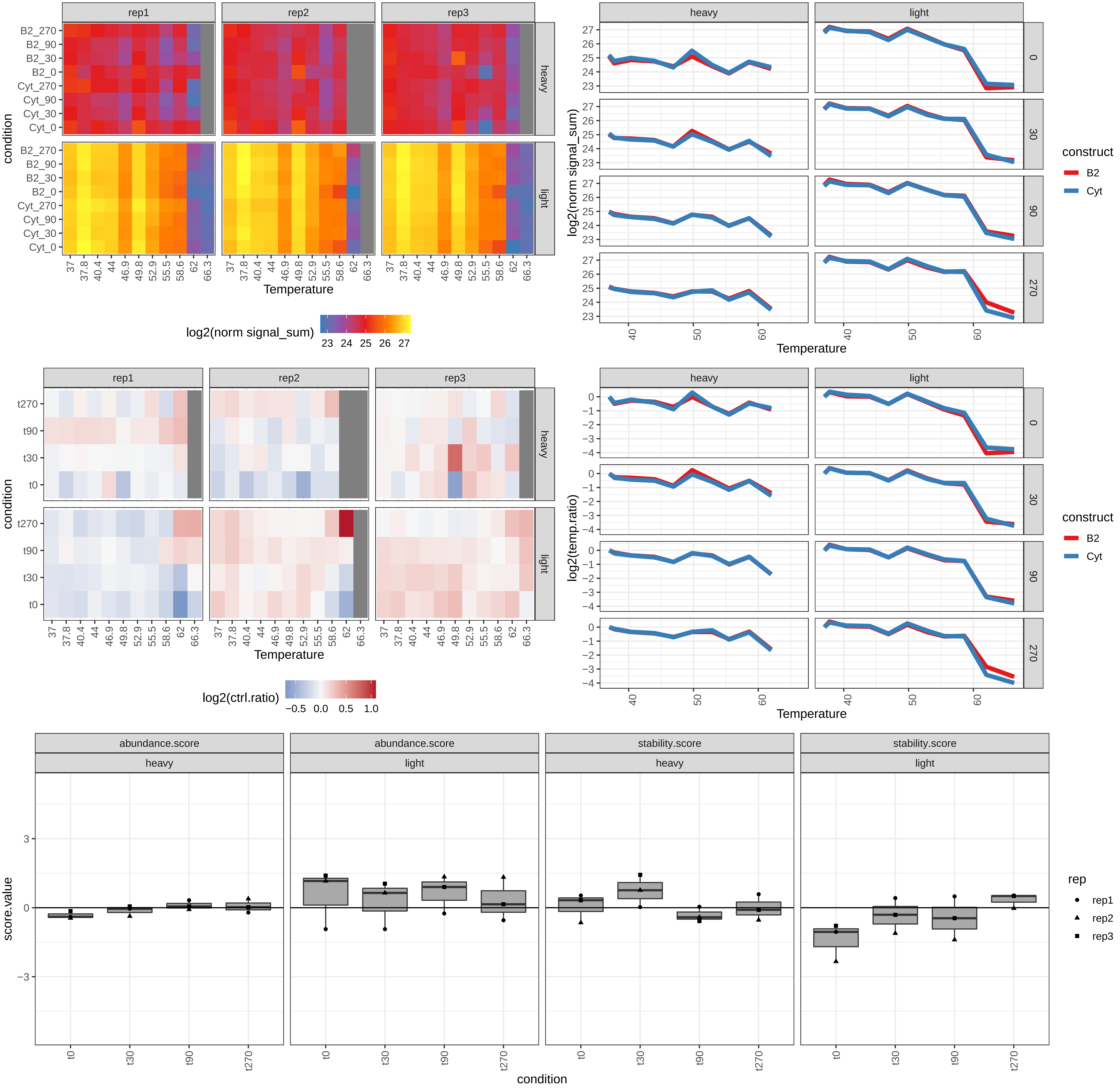

P11154 − Pyruvate carboxylase 1 OS=Saccharomyces cerevisiae (strain ATCC 204508 / S288c) GN=PYC1 PE=1 SV=2

PYC2 − average.top3: 7.46150214285714 − max.qupm: 12

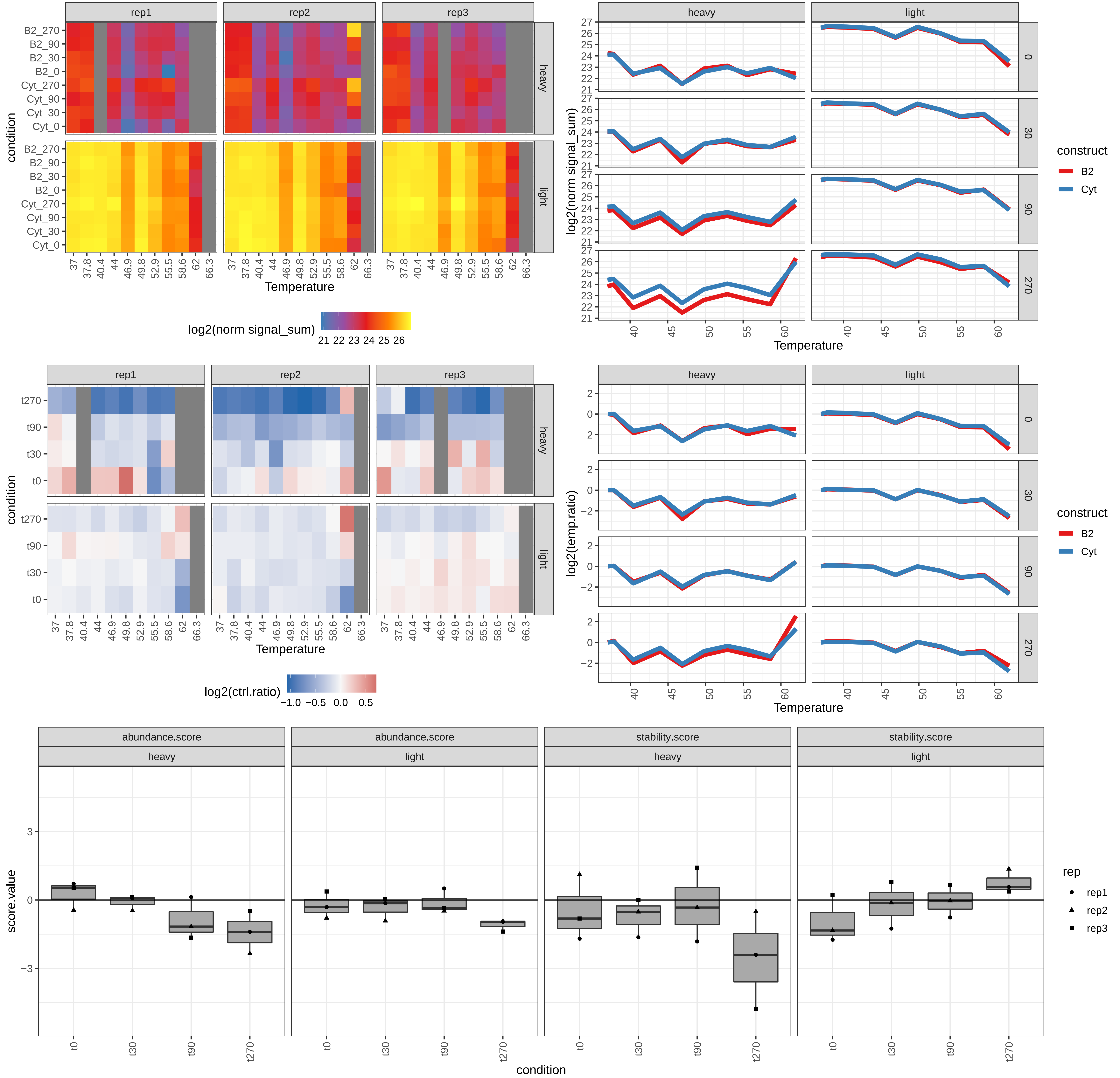

P32327 − Pyruvate carboxylase 2 OS=Saccharomyces cerevisiae (strain ATCC 204508 / S288c) GN=PYC2 PE=1 SV=2

PYK2 − average.top3: 7.06198297297297 − max.qupm: 9

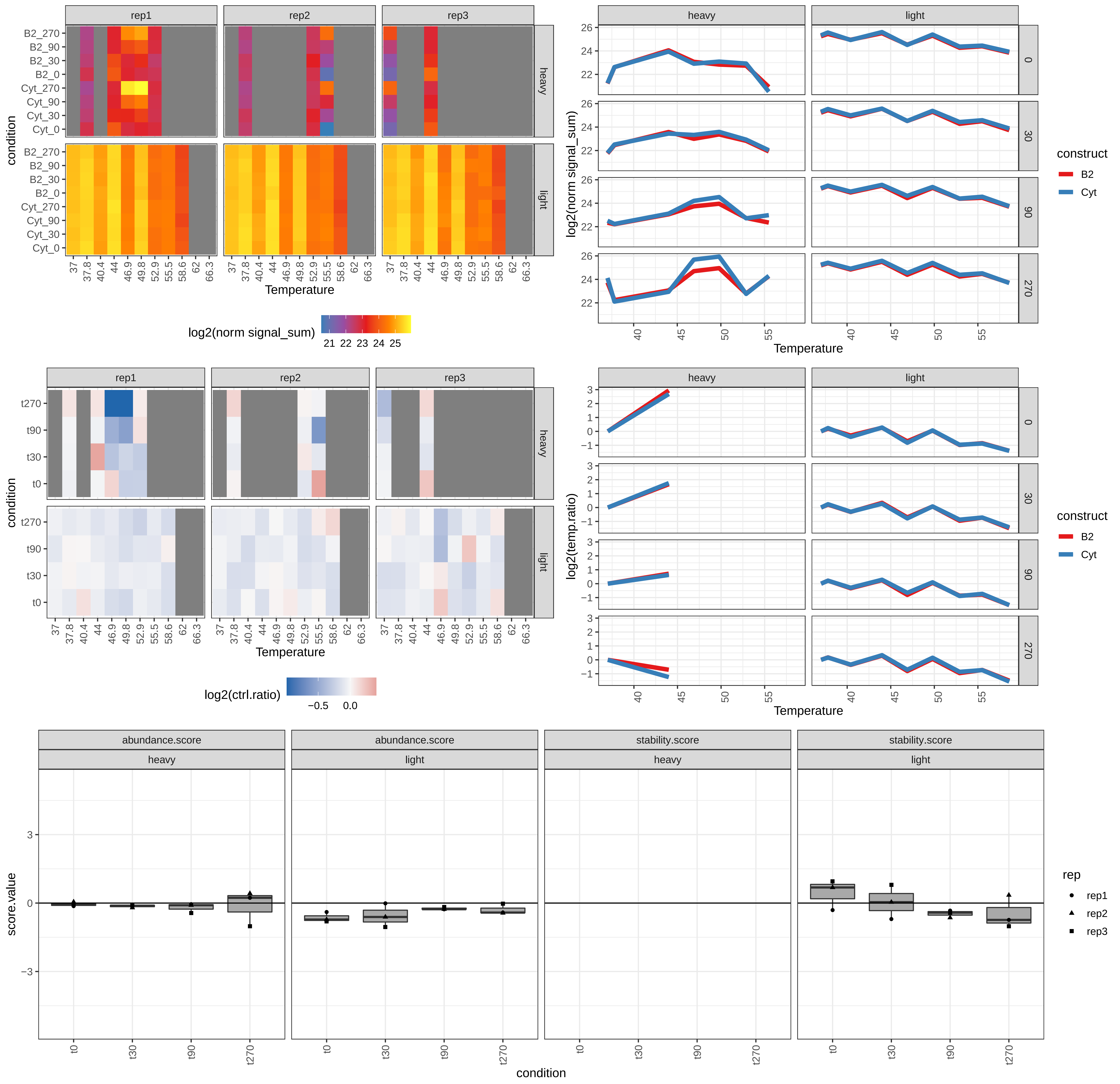

P52489 − Pyruvate kinase 2 OS=Saccharomyces cerevisiae (strain ATCC 204508 / S288c) GN=PYK2 PE=1 SV=1

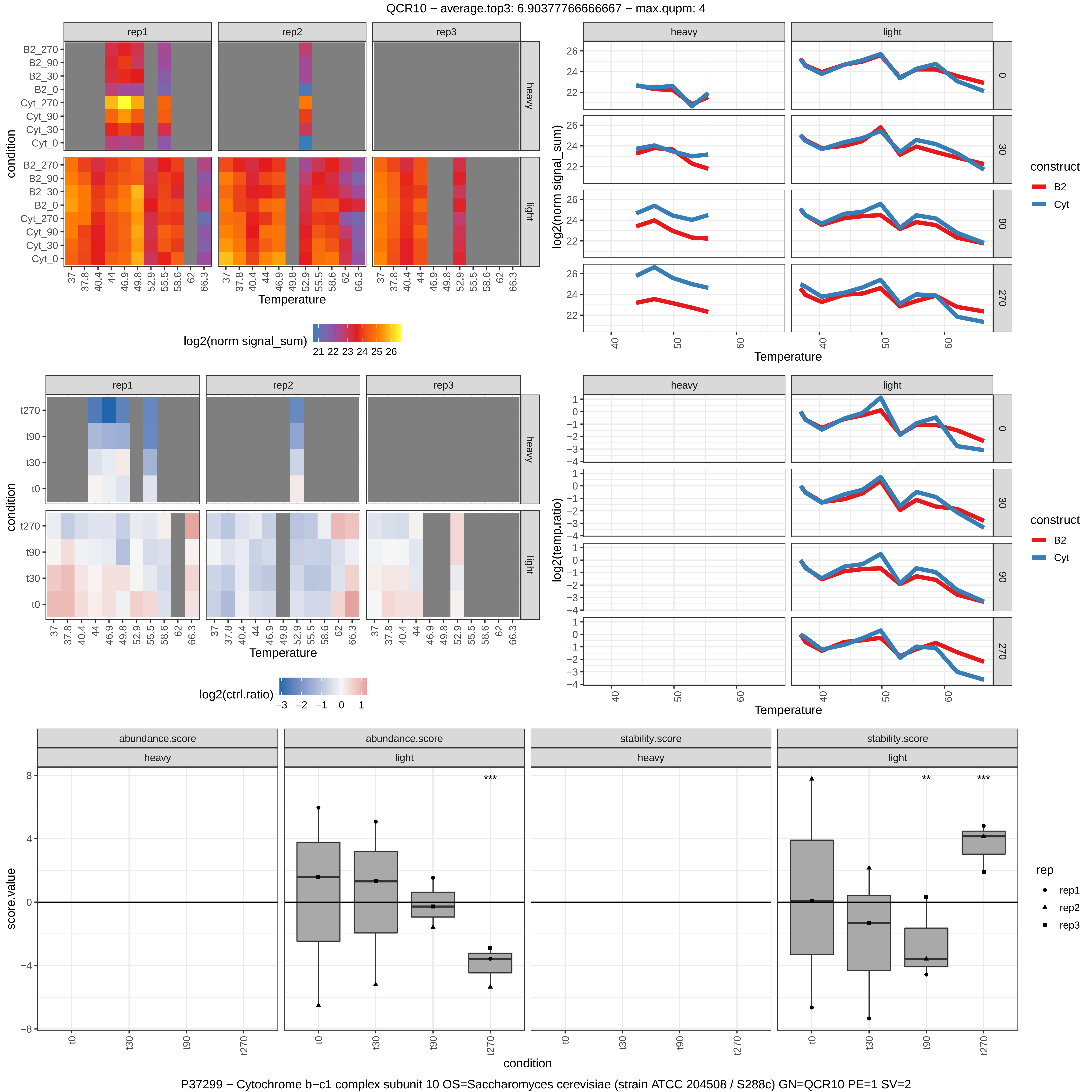
QCR10 − average.top3: 6.90377766666667 − max.qupm: 4
rep1
rep2
rep3
heavy
light
condition
Temperature
log2(norm signal_sum)
21 22 23 24 25 26
log2(norm signal_sum)
construct
B2
Cyt
log2(ctrl.ratio)
−3 −2 −1 0 1
log2(temp.ratio)
abundance.score
stability.score
score.value
rep
rep1
rep2
rep3
***
**
***
condition
P37299 − Cytochrome b−c1 complex subunit 10 OS=Saccharomyces cerevisiae (strain ATCC 204508 / S288c) GN=QCR10 PE=1 SV=2

QCR2 − average.top3: 7.99959050847458 − max.qupm: 22

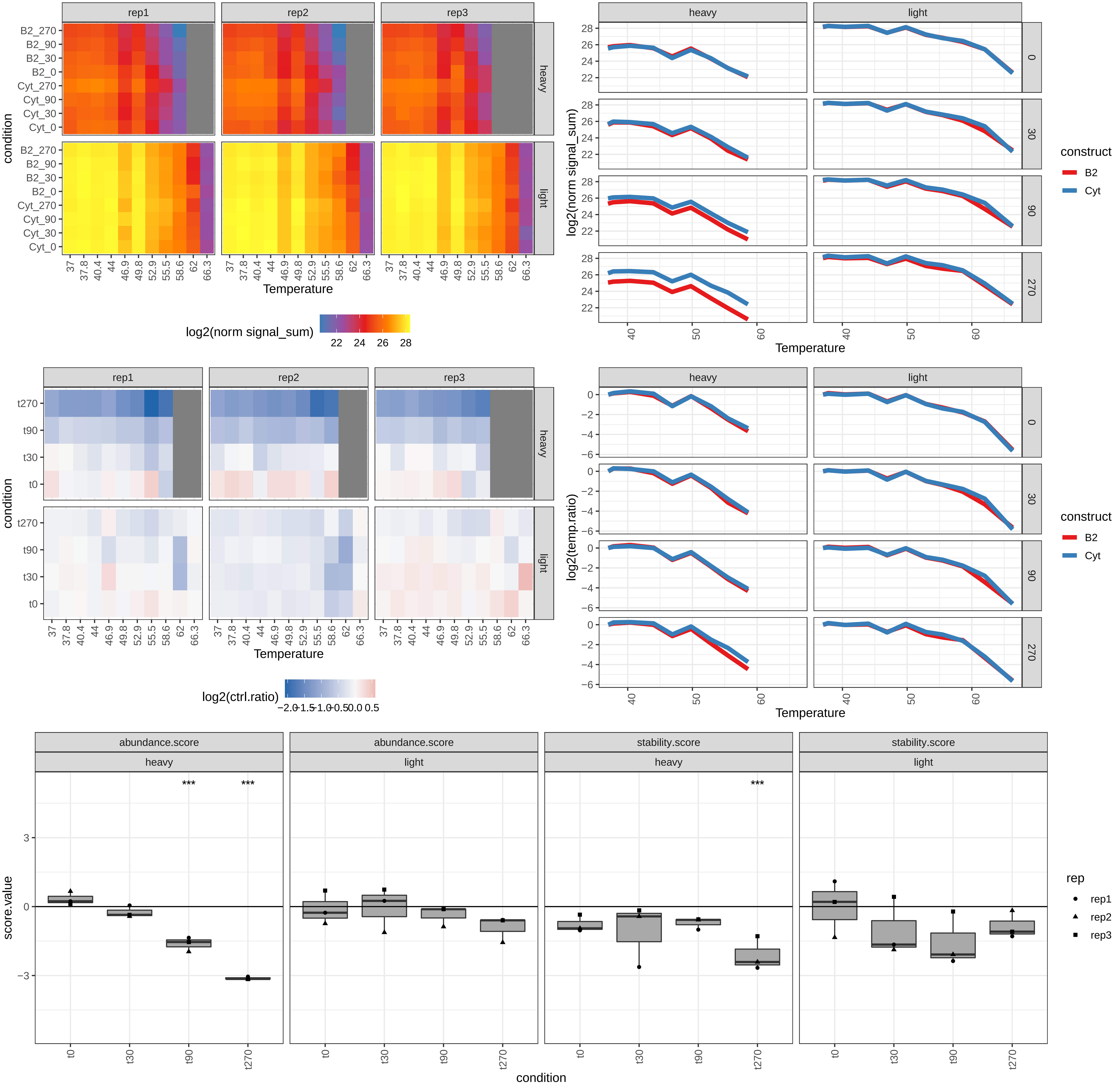

P07257 − Cytochrome b−c1 complex subunit 2, mitochondrial OS=Saccharomyces cerevisiae (strain ATCC 204508 / S288c) GN=QCR2 PE=1 SV=1

QCR6 − average.top3: 7.06486277777778 − max.qupm: 3

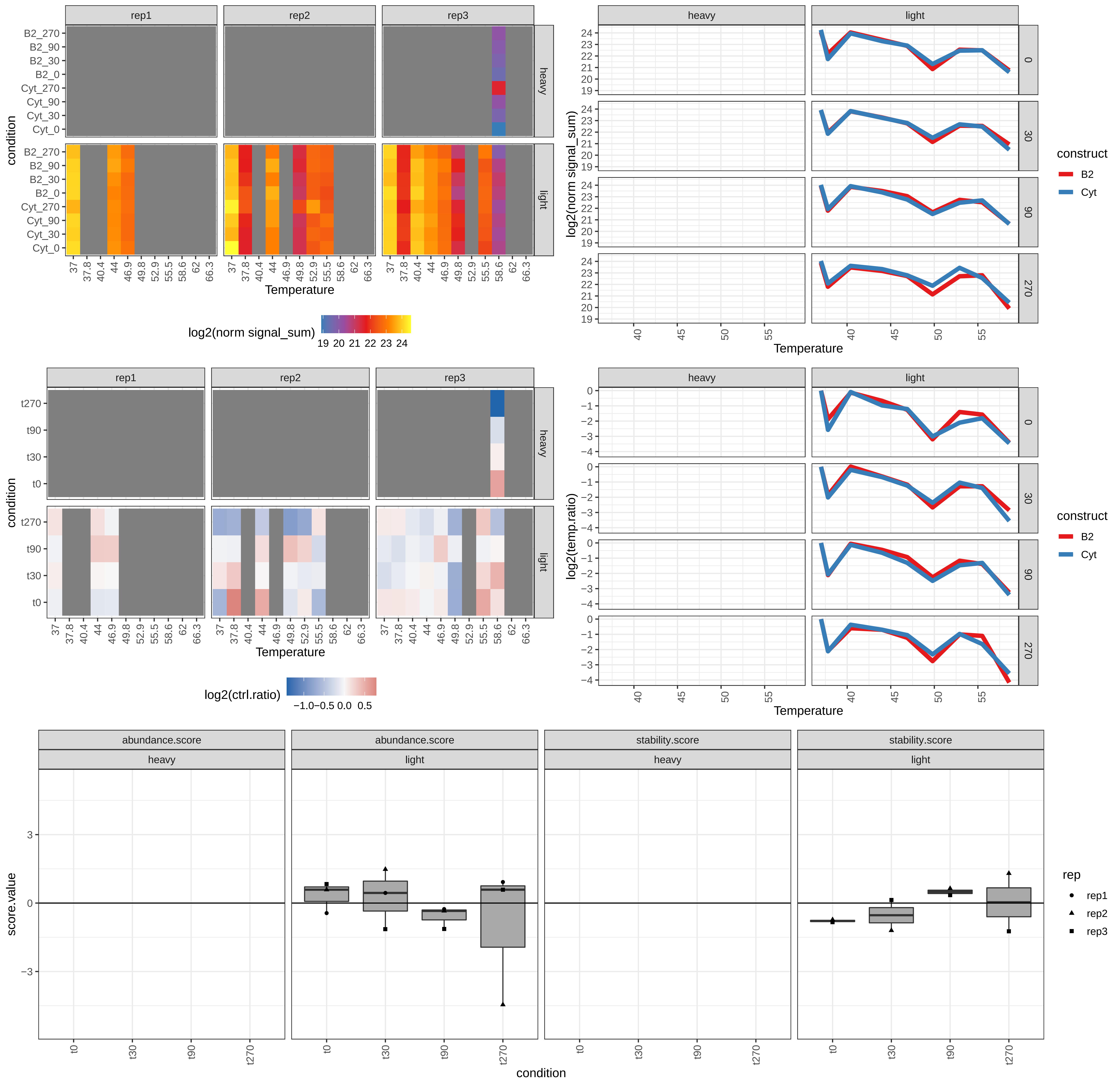

P00127 − Cytochrome b−c1 complex subunit 6 OS=Saccharomyces cerevisiae (strain ATCC 204508 / S288c) GN=QCR6 PE=1 SV=2

QCR7 − average.top3: 7.26847851851852 − max.qupm: 7

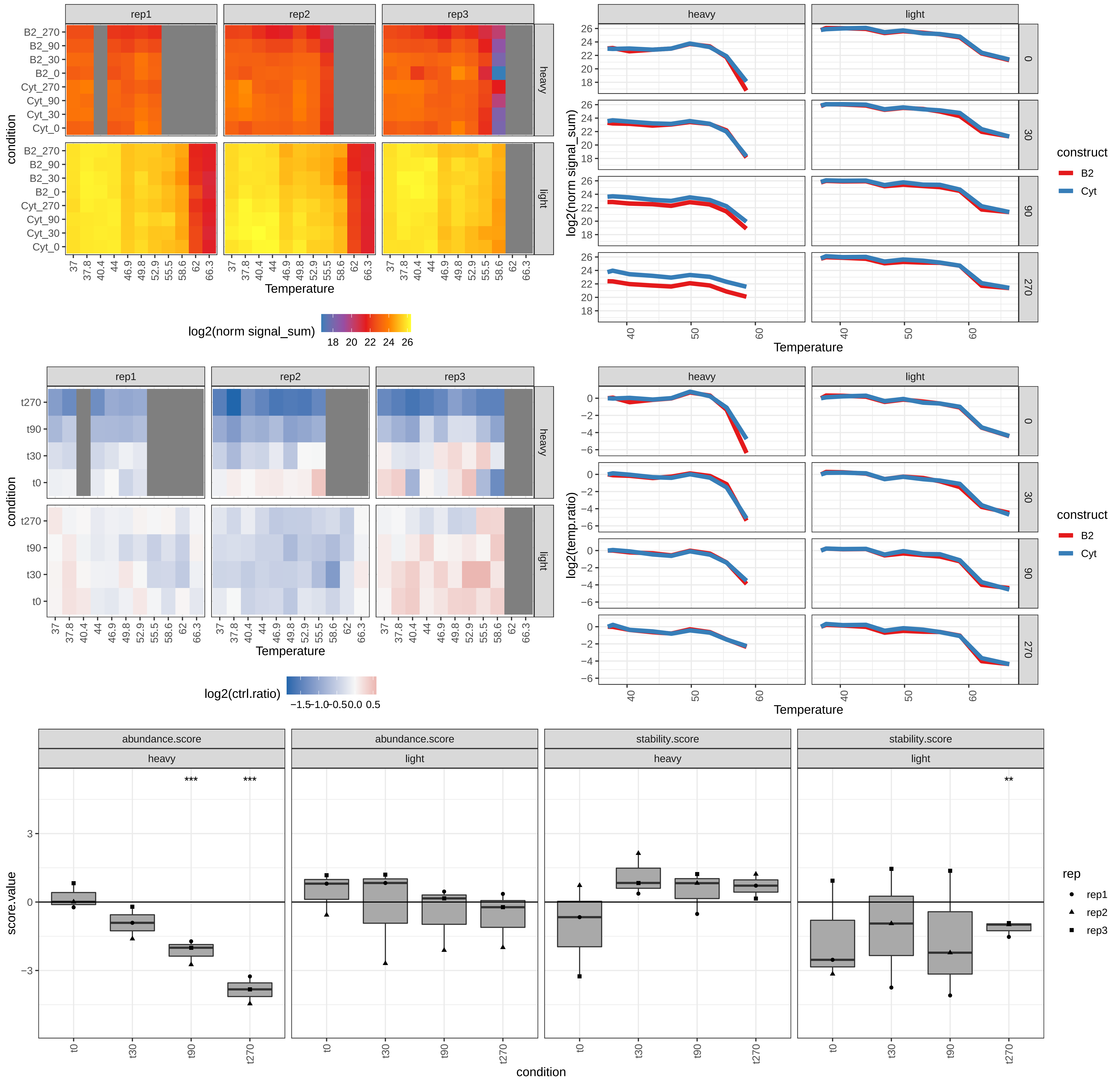

P00128 − Cytochrome b−c1 complex subunit 7 OS=Saccharomyces cerevisiae (strain ATCC 204508 / S288c) GN=QCR7 PE=1 SV=2

QCR8 − average.top3: 7.38471538461538 − max.qupm: 4

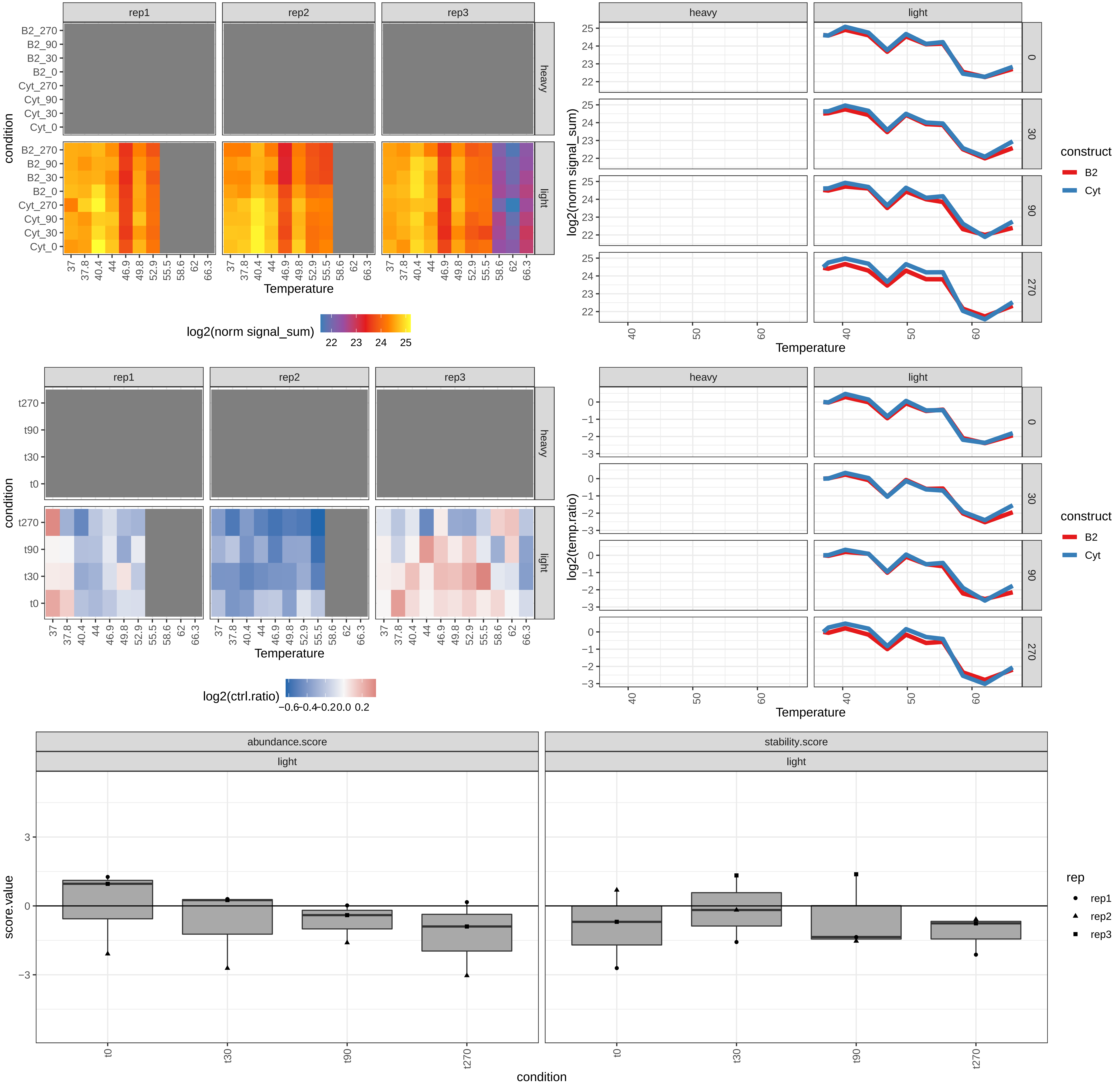

P08525 − Cytochrome b−c1 complex subunit 8 OS=Saccharomyces cerevisiae (strain ATCC 204508 / S288c) GN=QCR8 PE=1 SV=2

QCR9 − average.top3: 7.5713908 − max.qupm: 2

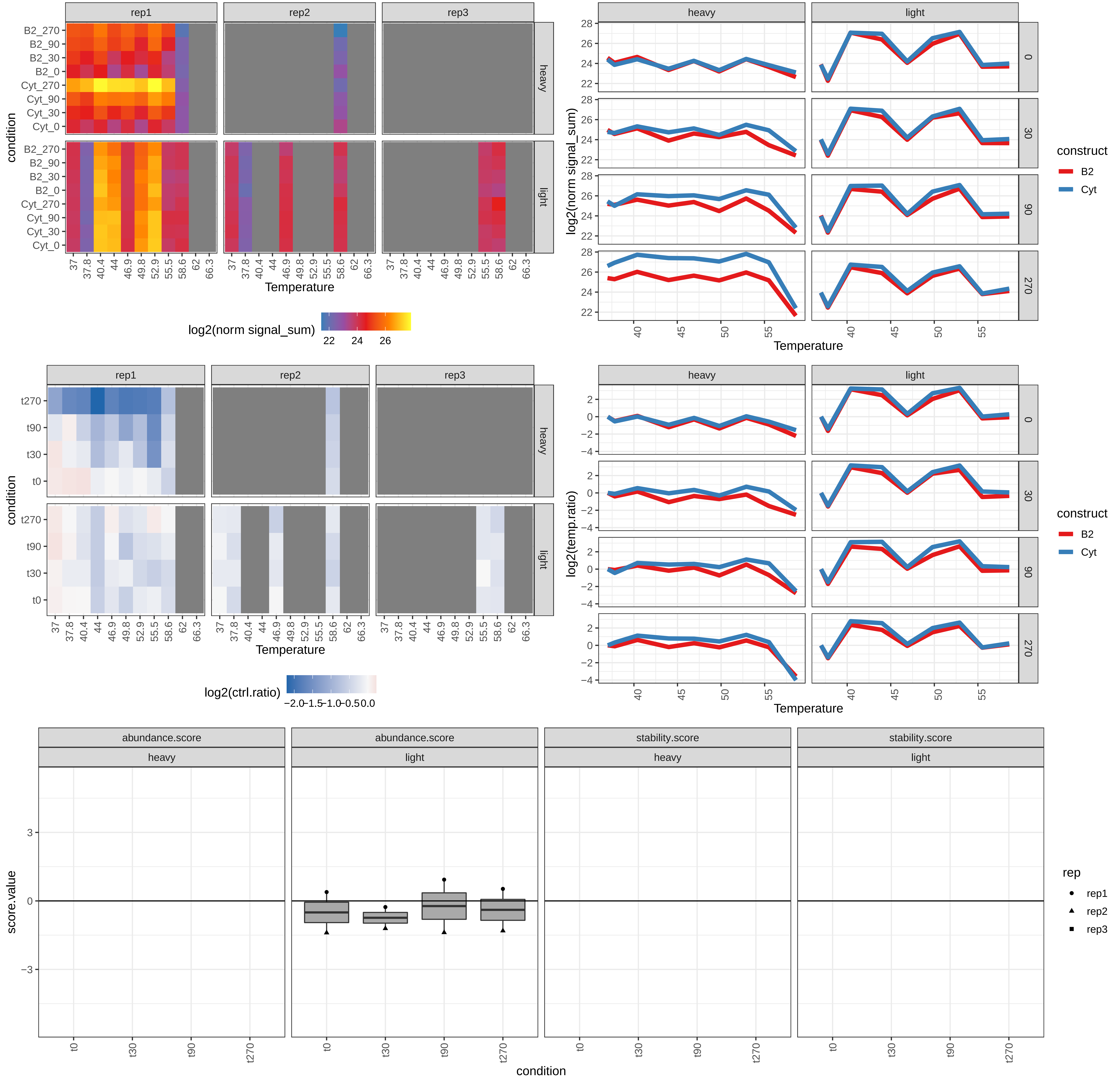

P22289 − Cytochrome b−c1 complex subunit 9 OS=Saccharomyces cerevisiae (strain ATCC 204508 / S288c) GN=QCR9 PE=1 SV=2

QNS1 − average.top3: 6.92018921568627 − max.qupm: 14

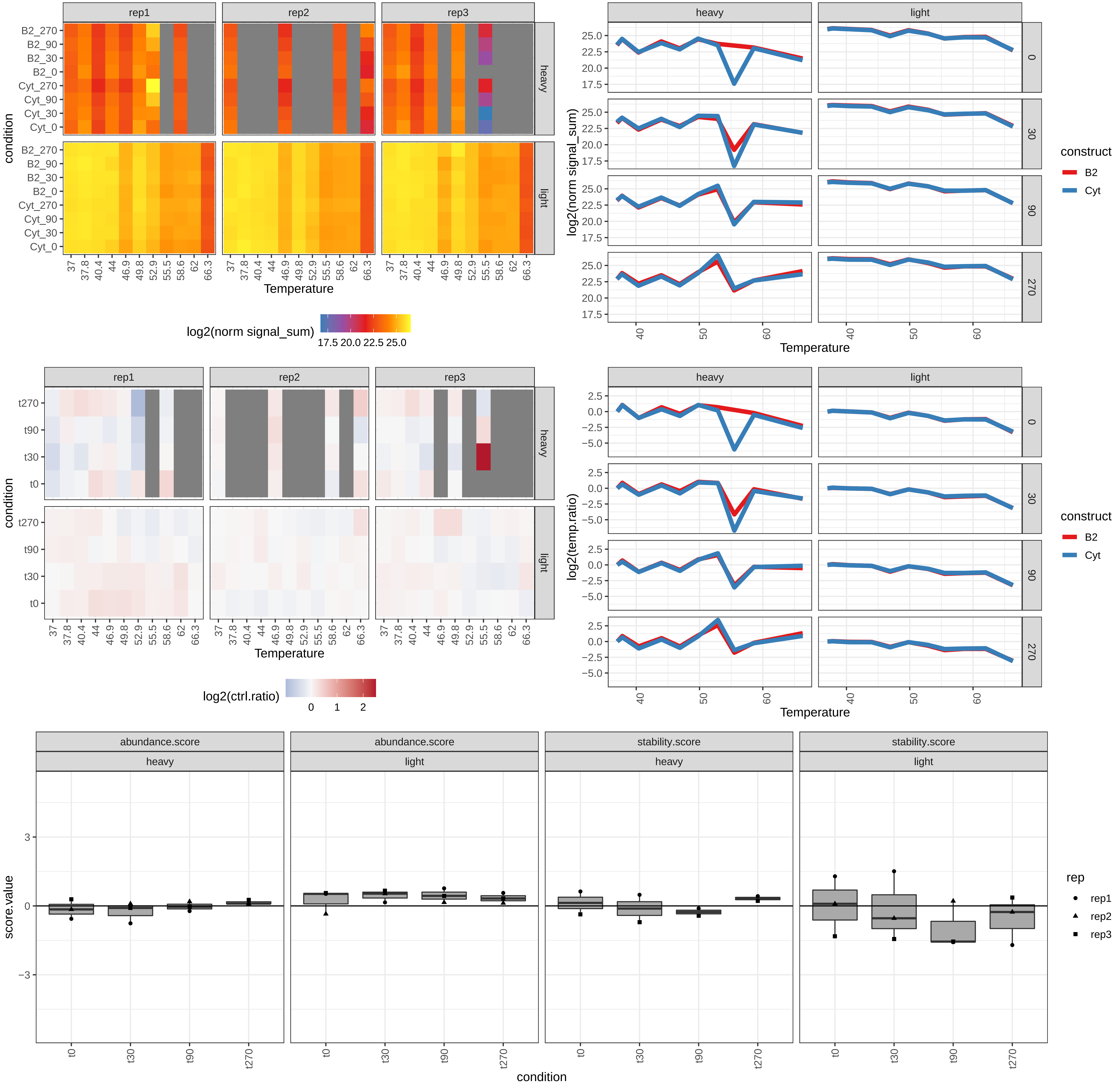

P38795 − Glutamine−dependent NAD(+) synthetase OS=Saccharomyces cerevisiae (strain ATCC 204508 / S288c) GN=QNS1 PE=1 SV=1

QRI1 − average.top3: 7.12750540540541 − max.qupm: 19

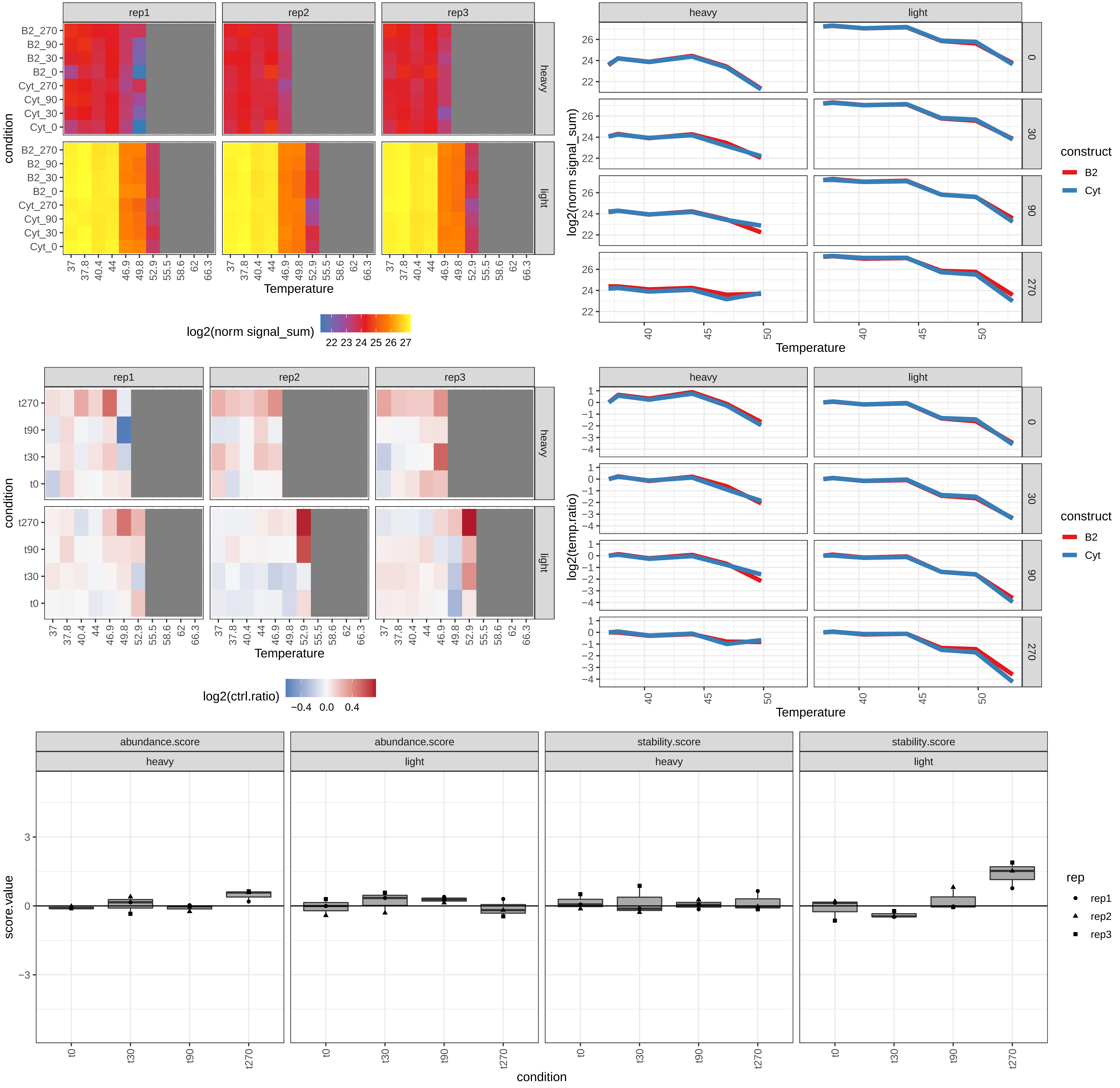

P43123 − UDP−N−acetylglucosamine pyrophosphorylase OS=Saccharomyces cerevisiae (strain ATCC 204508 / S288c) GN=QRI1 PE=1 SV=1

QRI7 − average.top3: 6.60526333333333 − max.qupm: 7

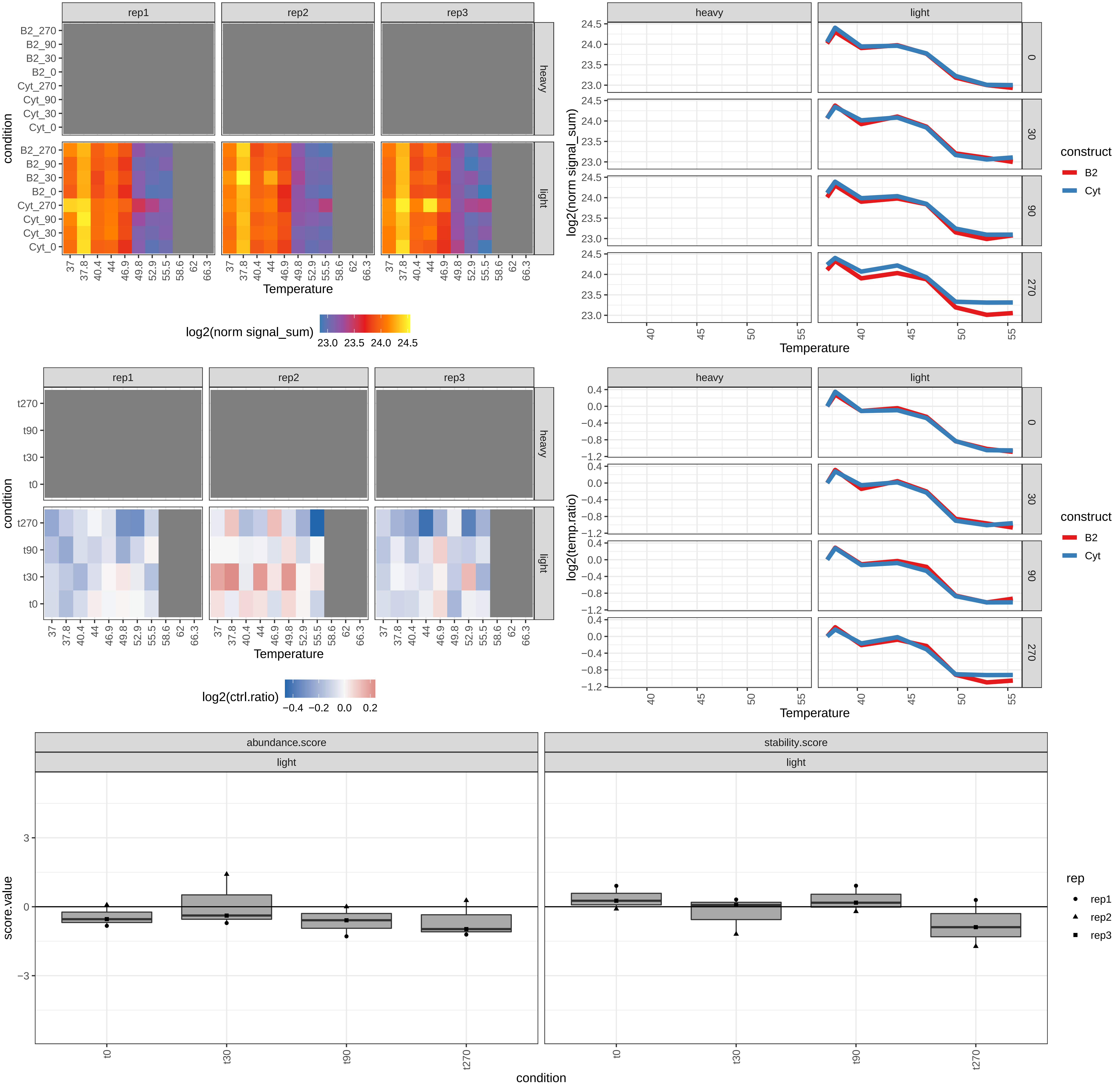

P43122 − tRNA N6−adenosine threonylcarbamoyltransferase, mitochondrial OS=Saccharomyces cerevisiae (strain ATCC 204508 / S288c) GN=QRI7 PE=1 SV=1

RAD17 − average.top3: 6.07153818181818 − max.qupm: 3

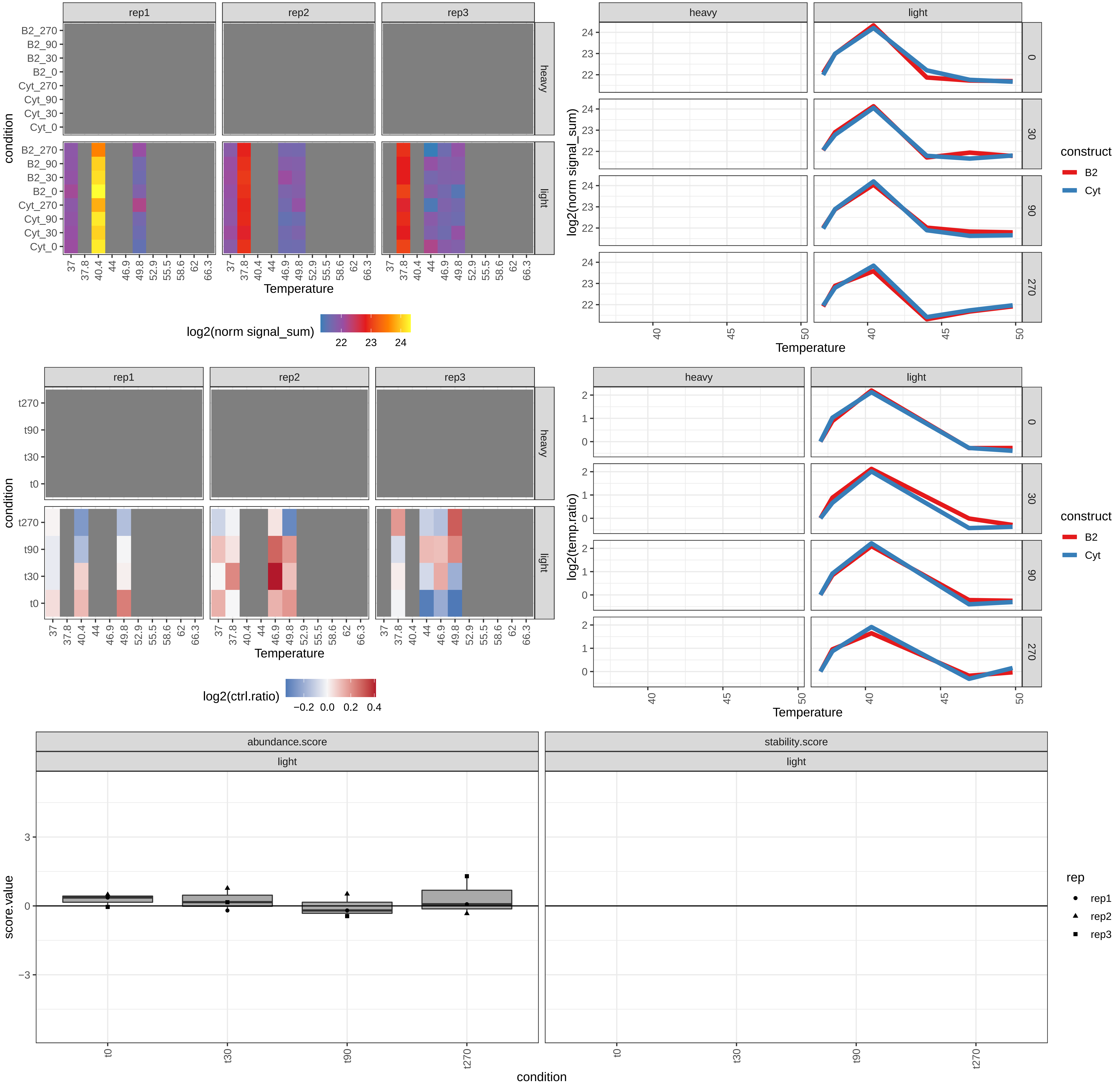

P48581 − DNA damage checkpoint control protein RAD17 OS=Saccharomyces cerevisiae (strain ATCC 204508 / S288c) GN=RAD17 PE=1 SV=1

RAD23 − average.top3: 6.82364509803922 − max.qupm: 8

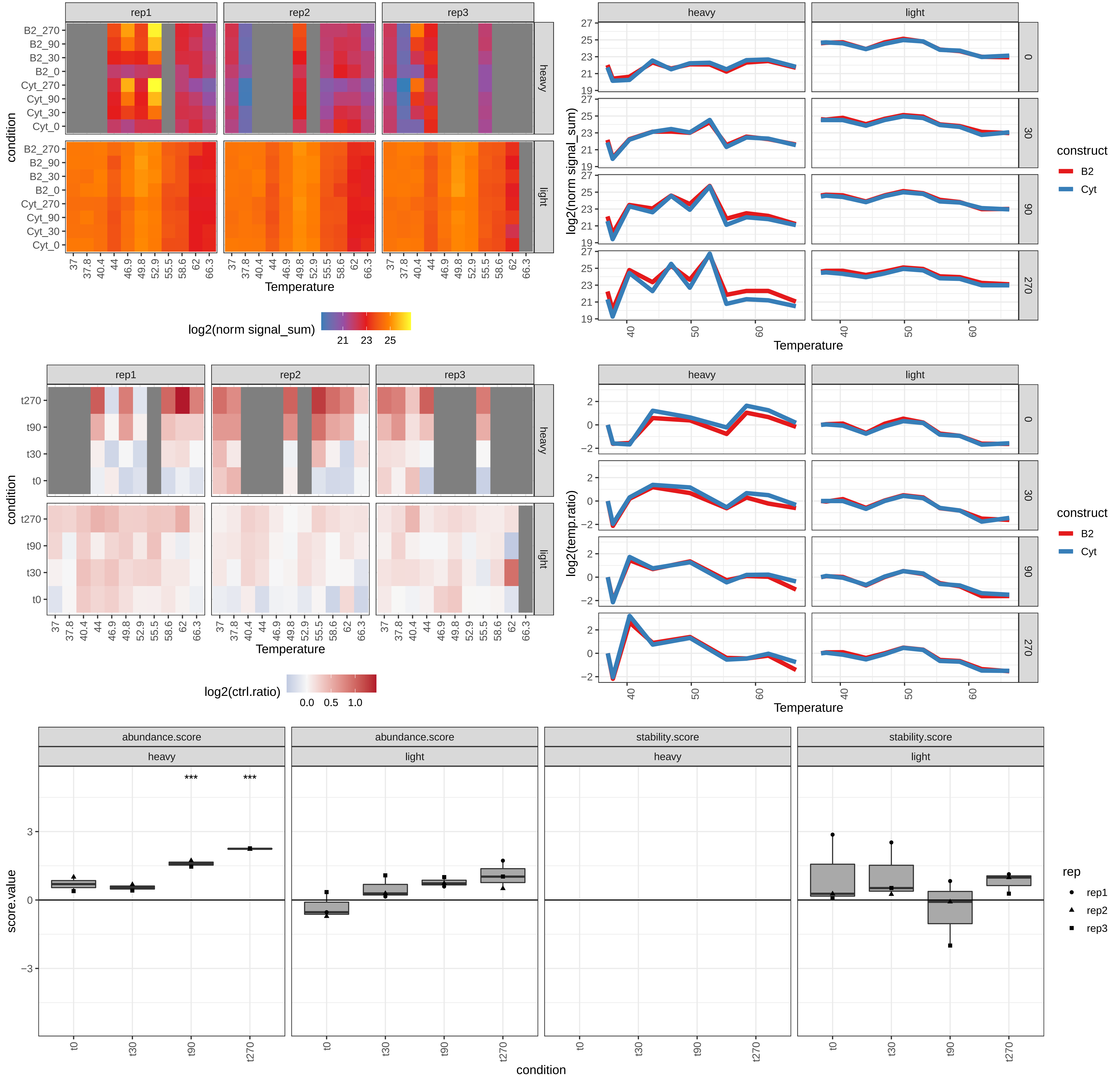

P32628 − UV excision repair protein RAD23 OS=Saccharomyces cerevisiae (strain ATCC 204508 / S288c) GN=RAD23 PE=1 SV=1

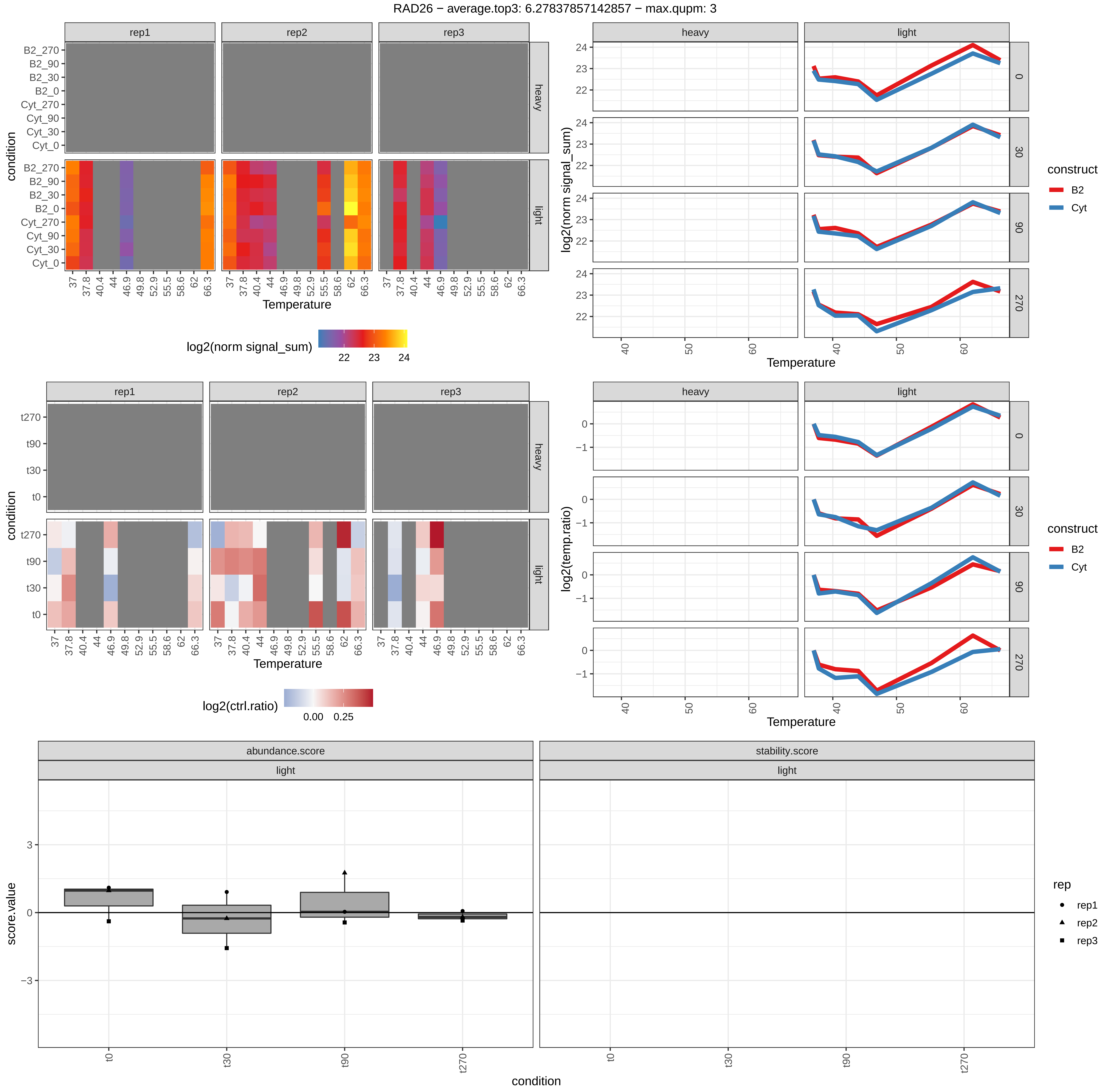

P40352 − DNA repair and recombination protein RAD26 OS=Saccharomyces cerevisiae (strain ATCC 204508 / S288c) GN=RAD26 PE=1 SV=1

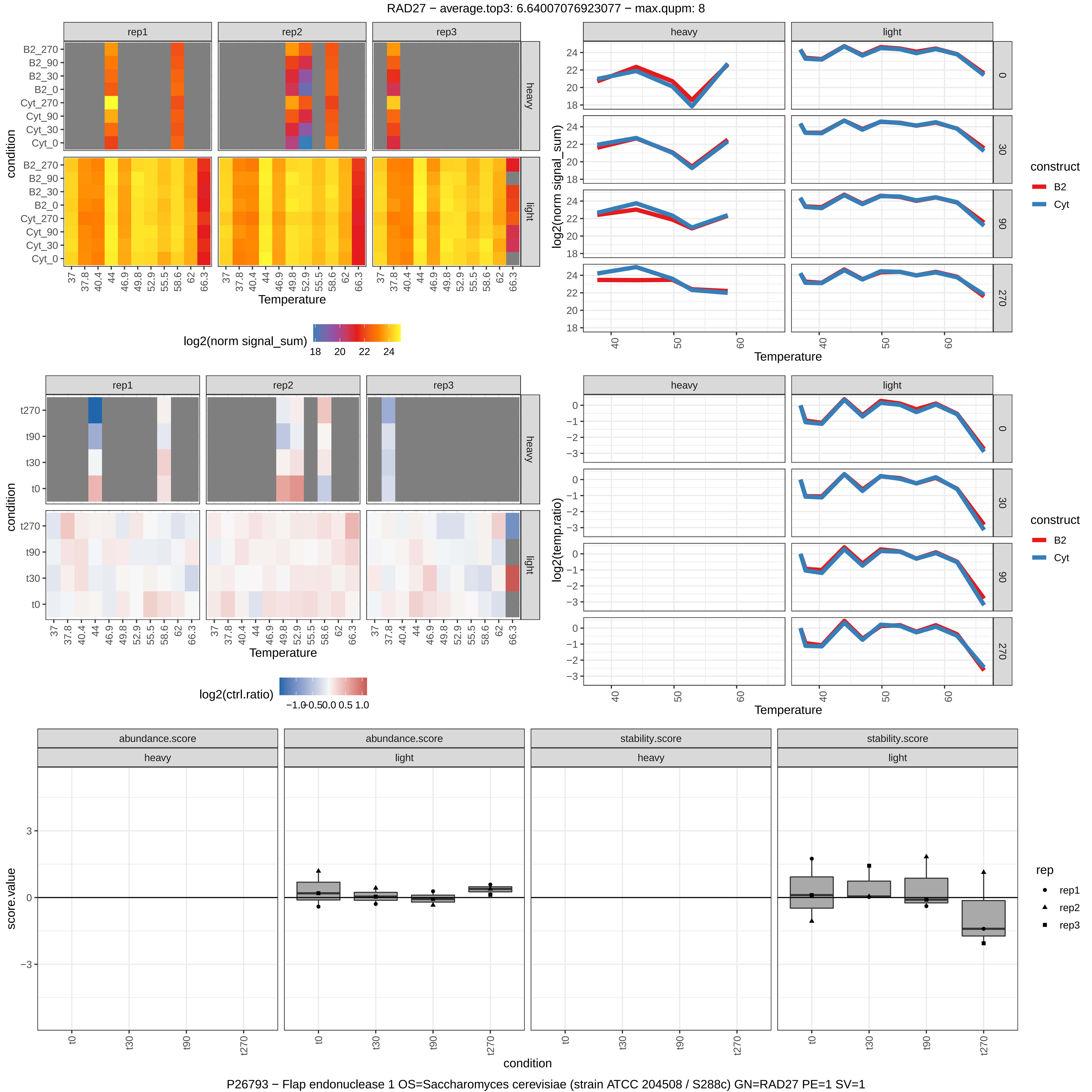

RAD27 − average.top3: 6.64007076923077 − max.qupm: 8
rep1
rep2
rep3
heavy
light
condition
Temperature
log2(norm signal_sum)
construct
B2
Cyt
log2(temp.ratio)
log2(ctrl.ratio)
abundance.score
stability.score
score.value
rep
P26793 − Flap endonuclease 1 OS=Saccharomyces cerevisiae (strain ATCC 204508 / S288c) GN=RAD27 PE=1 SV=1

RAD3 − average.top3: 6.447742 − max.qupm: 7

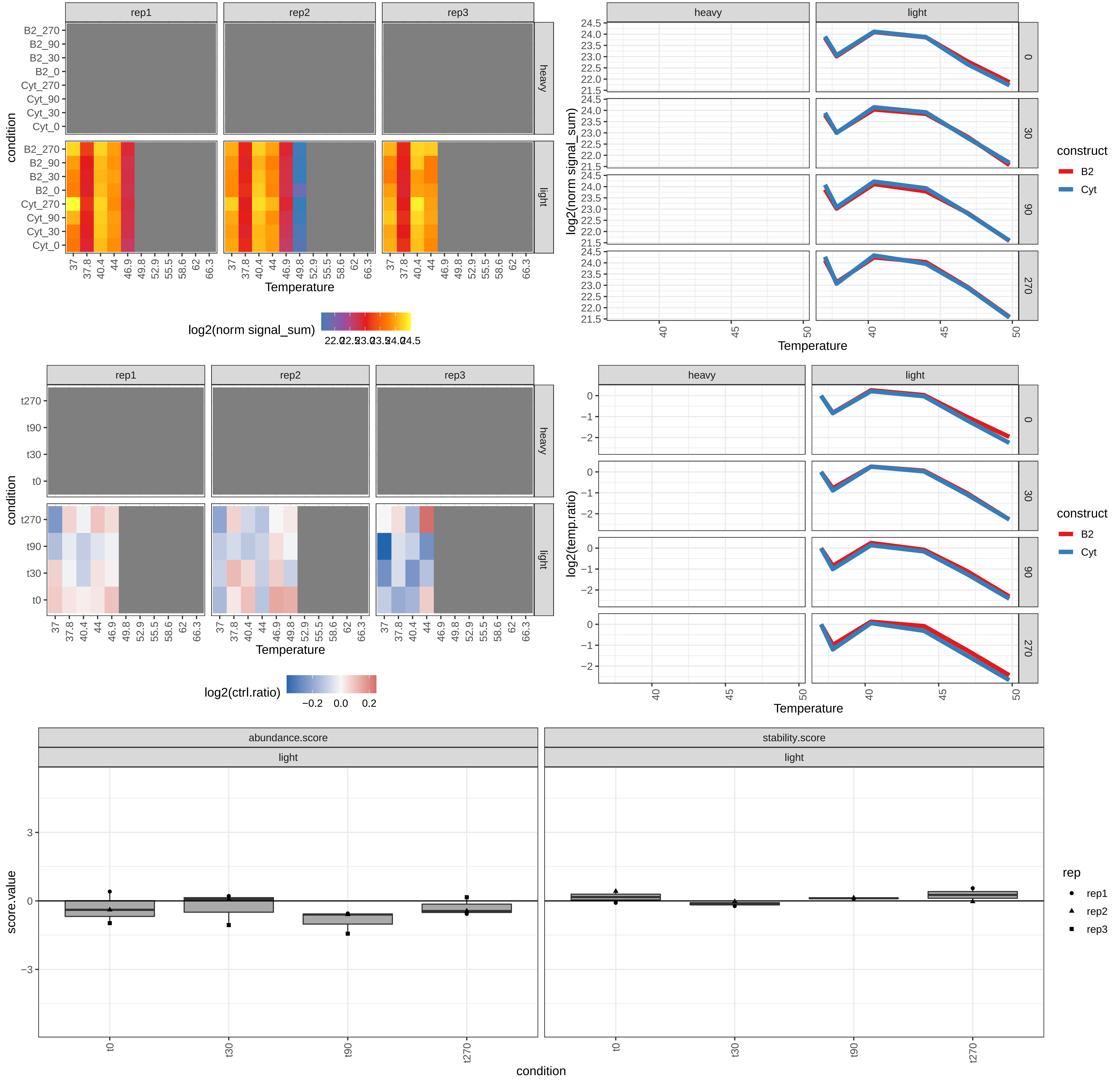

P06839 − DNA repair helicase RAD3 OS=Saccharomyces cerevisiae (strain ATCC 204508 / S288c) GN=RAD3 PE=1 SV=1

RAD50 − average.top3: 6.61792333333333 − max.qupm: 12

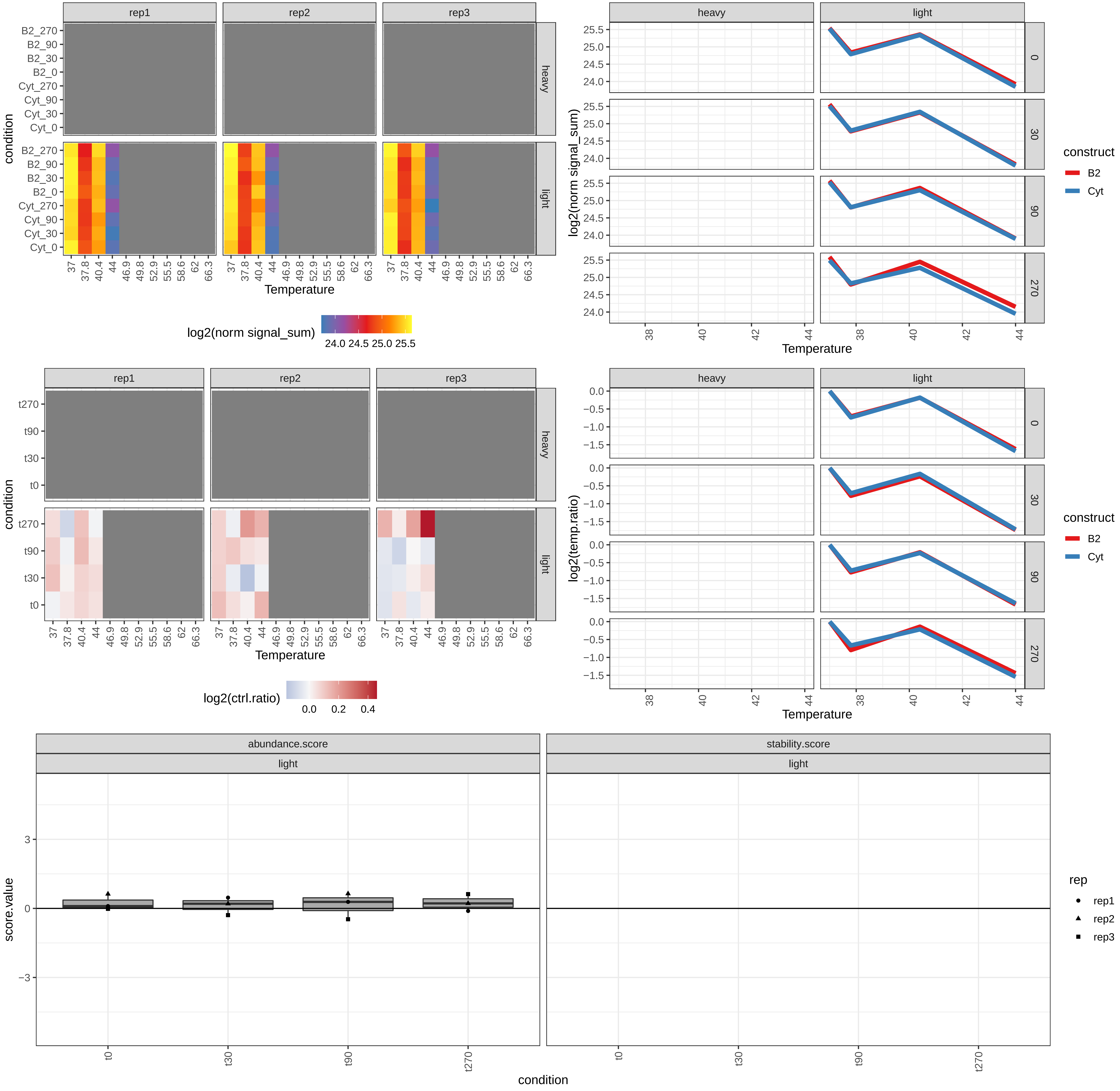

P12753 − DNA repair protein RAD50 OS=Saccharomyces cerevisiae (strain ATCC 204508 / S288c) GN=RAD50 PE=1 SV=1

RAD52 − average.top3: 6.47626454545455 − max.qupm: 3

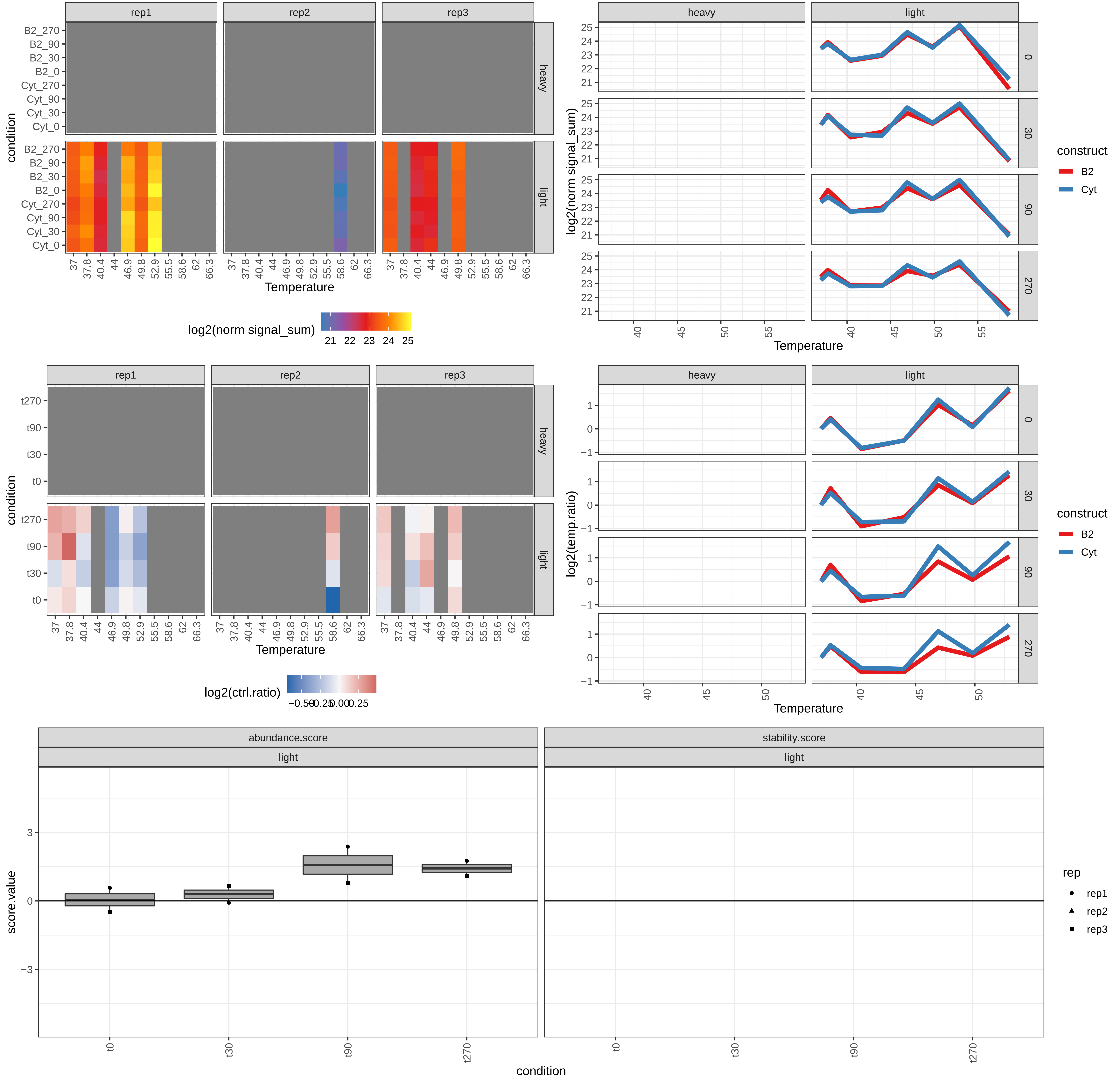

in ATCC 204508 / S288c) GN=RAD52 PE=1 SV=2|Isoform 2 of DNA repair and recombination protein RAD52 OS=Saccharomyces cerevisiae (strain ATCC 204508 / S288c) GN=RAD52|Isoform 3 of DNA r

RAD53 − average.top3: 6.44508071428571 − max.qupm: 5

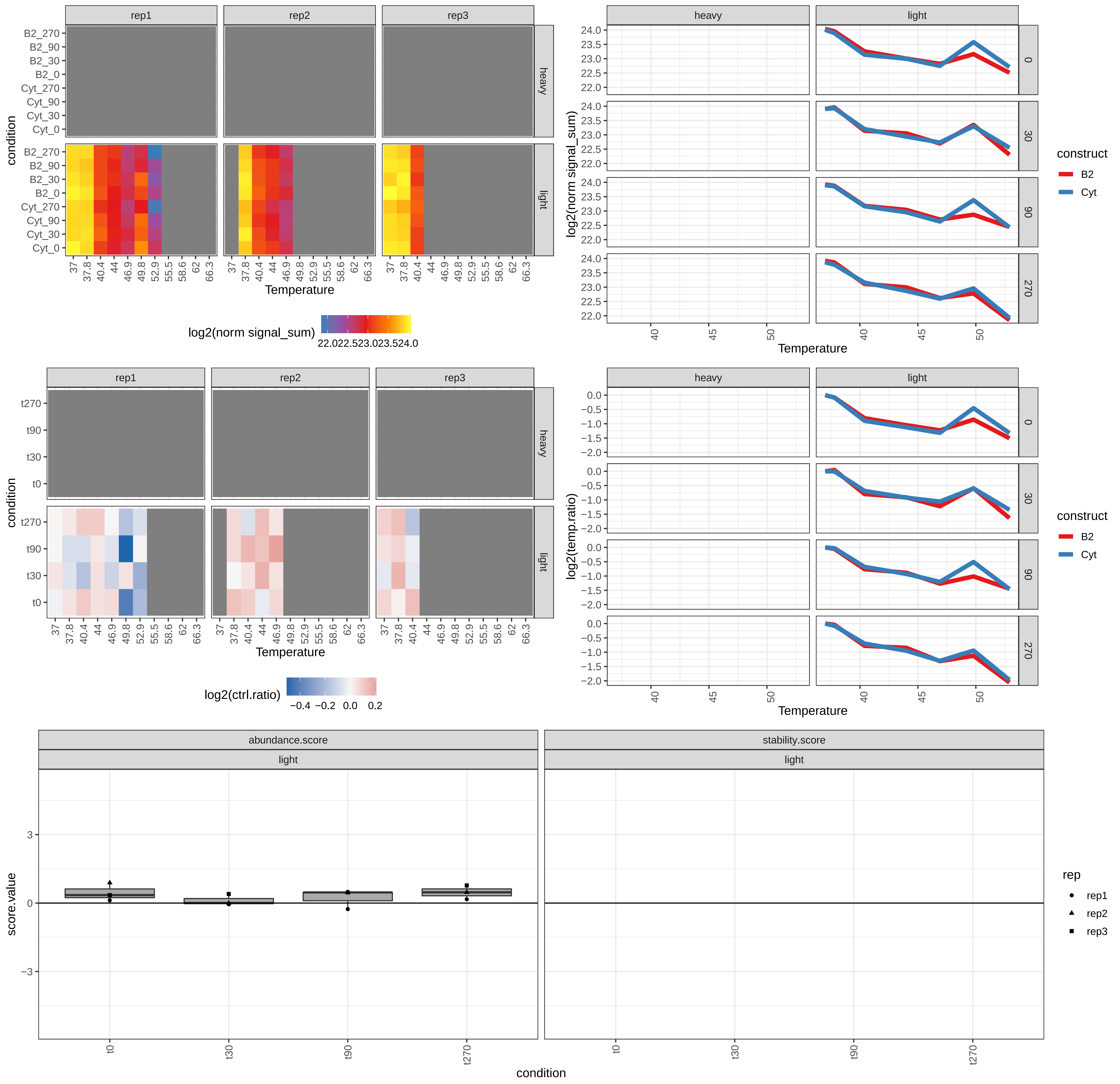

P22216 − Serine/threonine−protein kinase RAD53 OS=Saccharomyces cerevisiae (strain ATCC 204508 / S288c) GN=RAD53 PE=1 SV=1

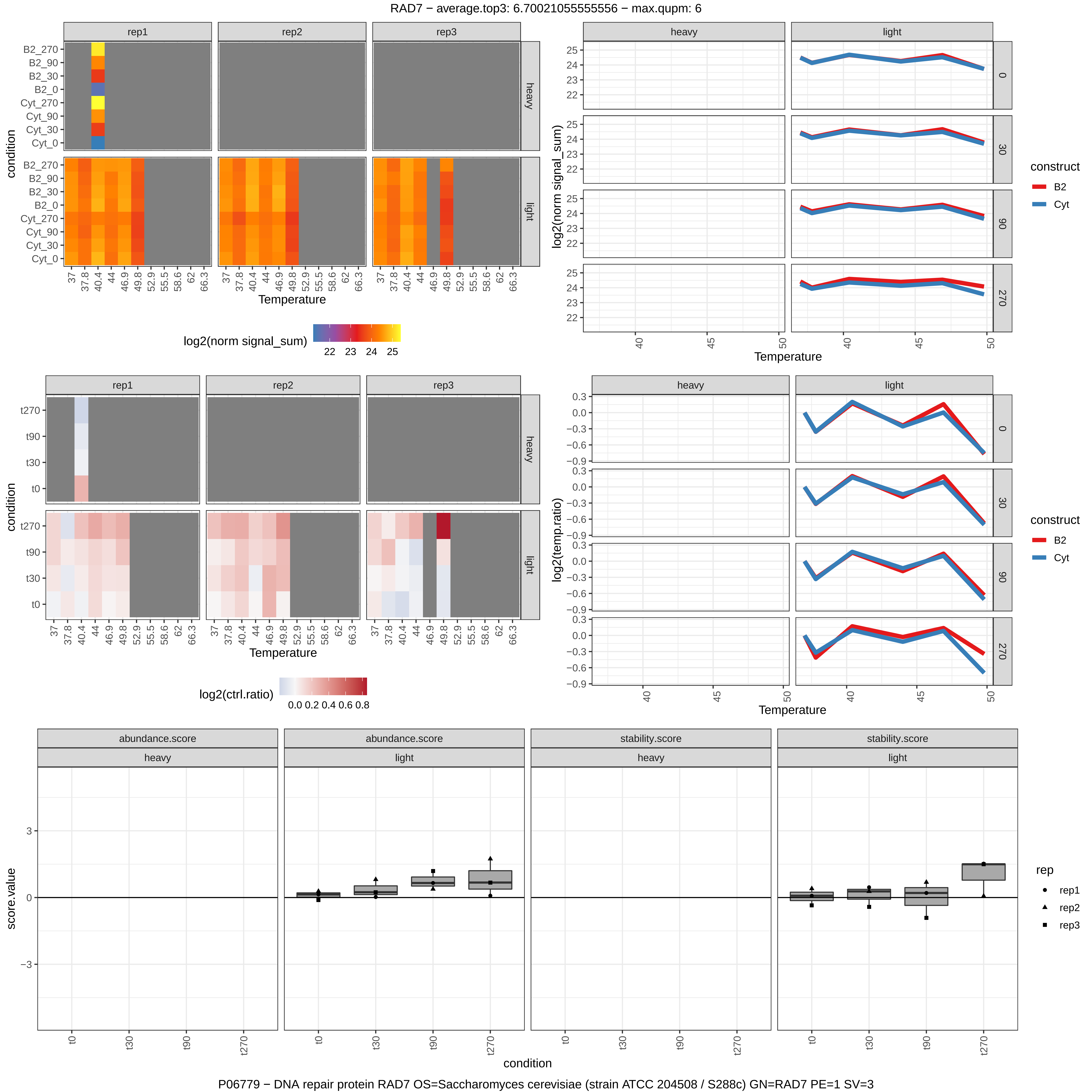
RAD7 − average.top3: 6.70021055555556 − max.qupm: 6
rep1
rep2
rep3
heavy
light
condition
Temperature
log2(norm signal_sum)
log2(ctrl.ratio)
log2(temp.ratio)
construct
B2
Cyt
abundance.score
stability.score
score.value
rep
P06779 − DNA repair protein RAD7 OS=Saccharomyces cerevisiae (strain ATCC 204508 / S288c) GN=RAD7 PE=1 SV=3

RAF1 − average.top3: 6.930557 − max.qupm: 5

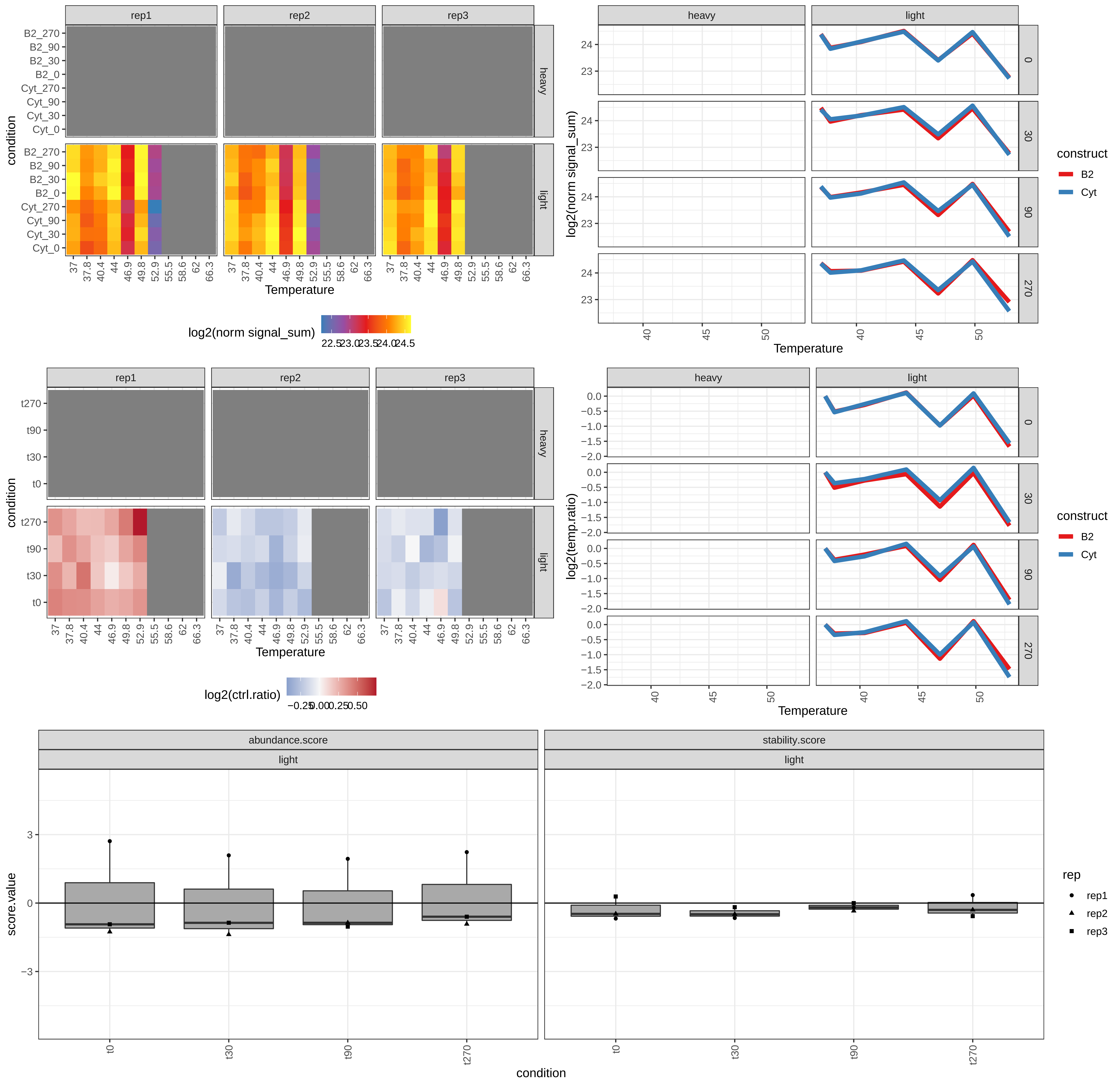

Q06891 − Trans−acting factor D OS=Saccharomyces cerevisiae (strain ATCC 204508 / S288c) GN=RAF1 PE=2 SV=1

RAI1 − average.top3: 7.02621111111111 − max.qupm: 11

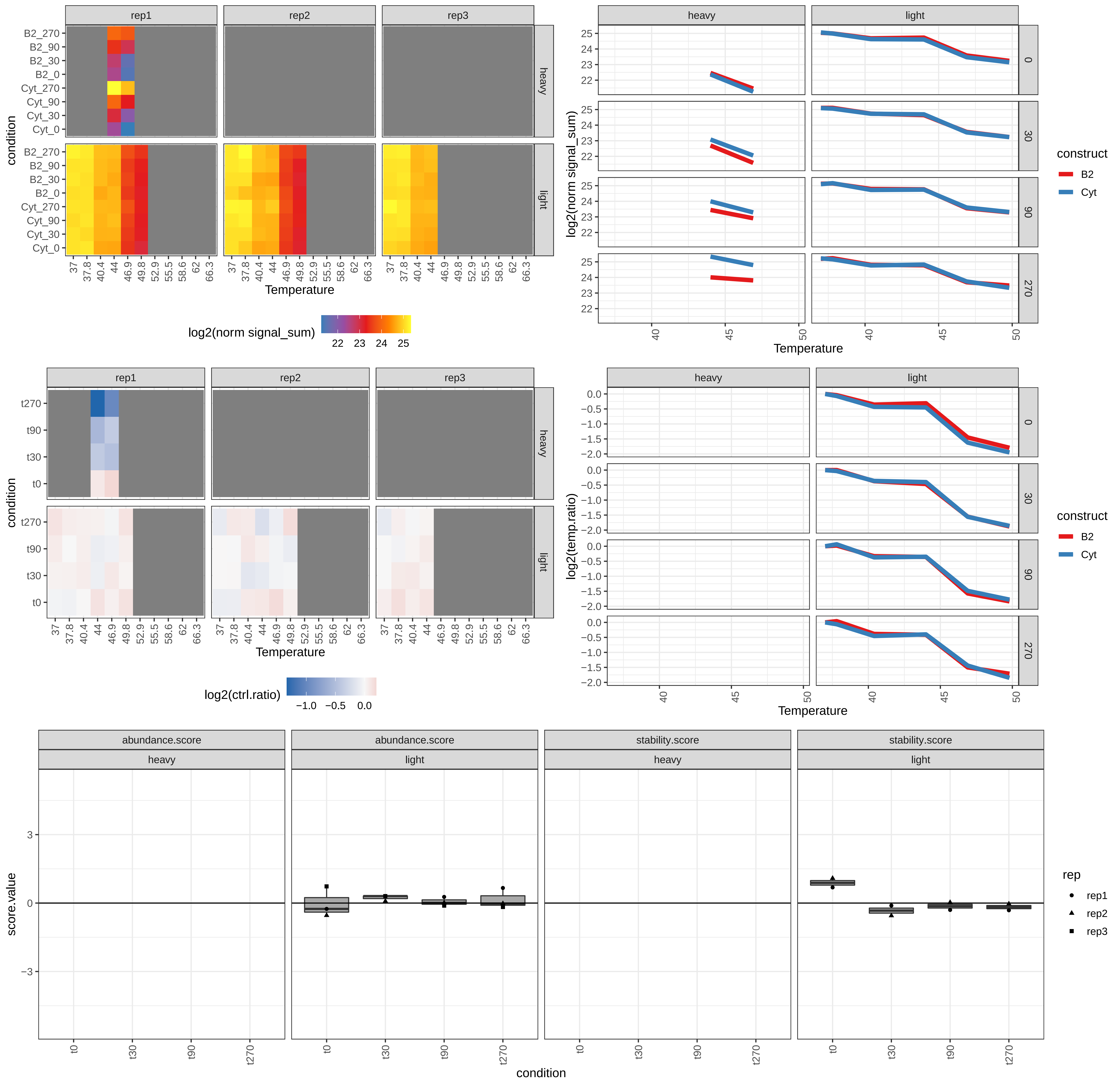

P53063 − Decapping nuclease RAI1 OS=Saccharomyces cerevisiae (strain ATCC 204508 / S288c) GN=RAI1 PE=1 SV=3

RAM2 − average.top3: 6.92655411764706 − max.qupm: 4

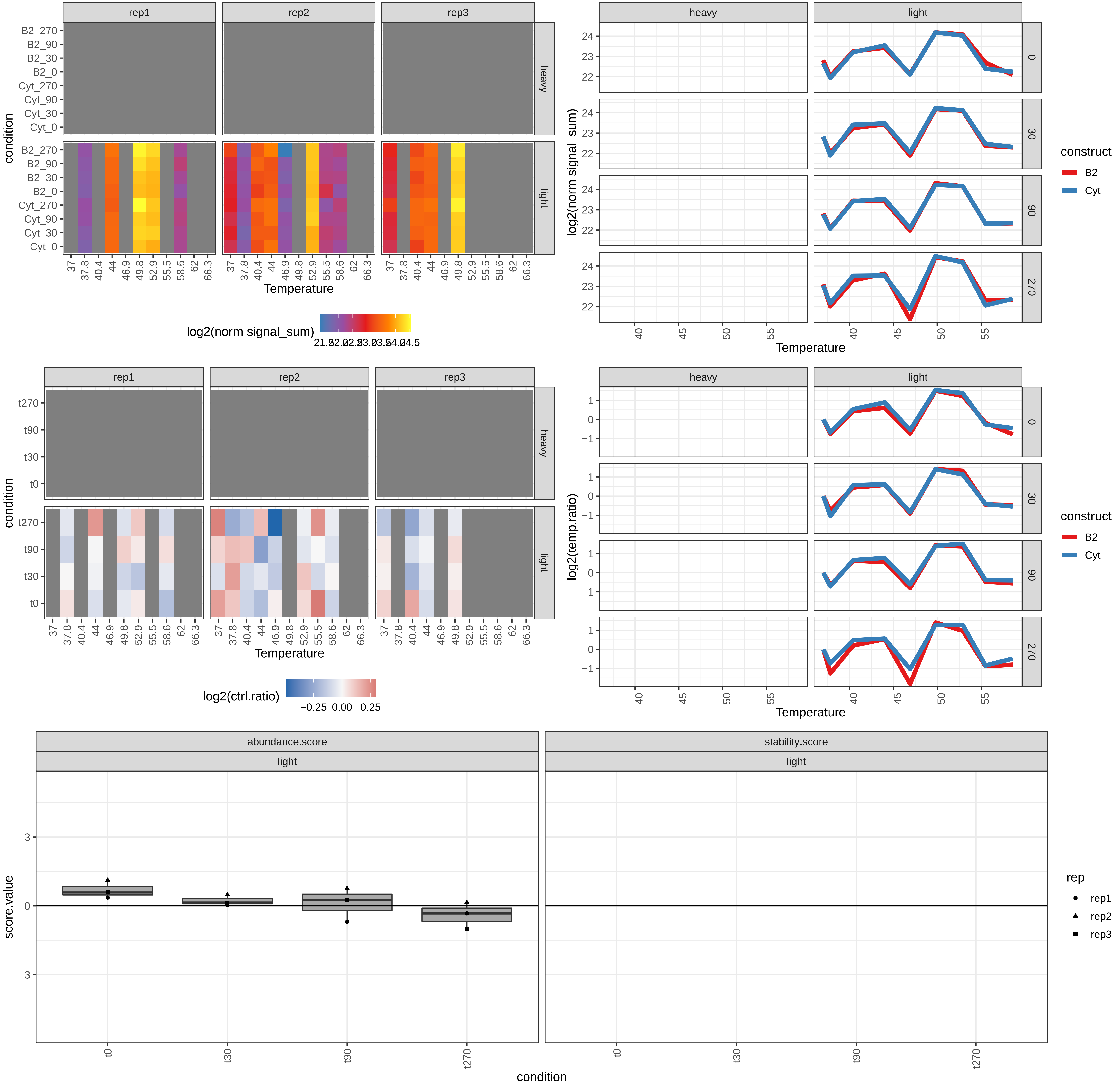

P29703 − Protein farnesyltransferase/geranylgeranyltransferase type−1 subunit alpha OS=Saccharomyces cerevisiae (strain ATCC 204508 / S288c) GN=RAM2 PE=1 SV=1

RAP1 − average.top3: 6.69052684210526 − max.qupm: 7

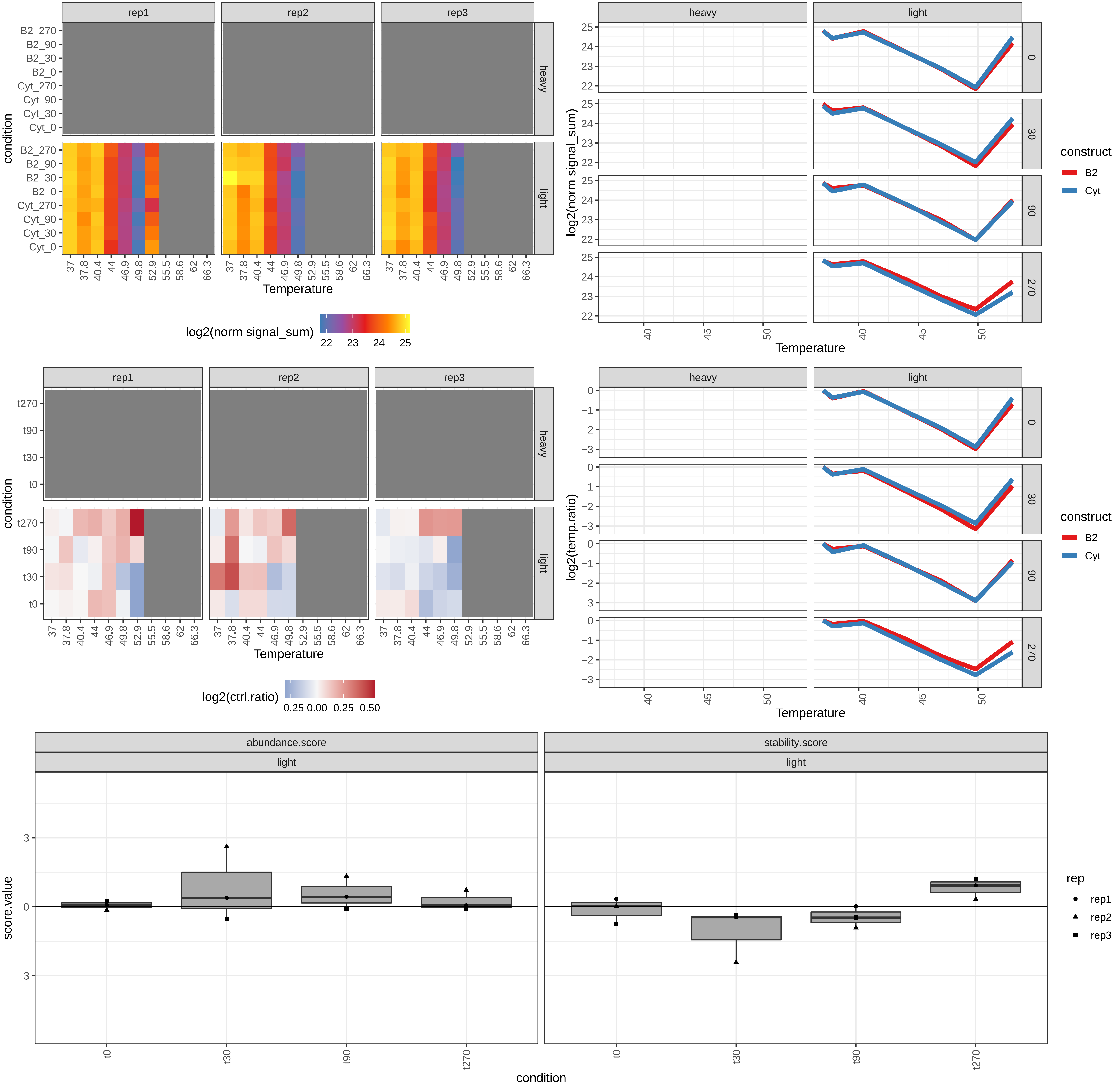

P11938 − DNA−binding protein RAP1 OS=Saccharomyces cerevisiae (strain ATCC 204508 / S288c) GN=RAP1 PE=1 SV=2

RAS1 − average.top3: 6.62575590909091 − max.qupm: 5

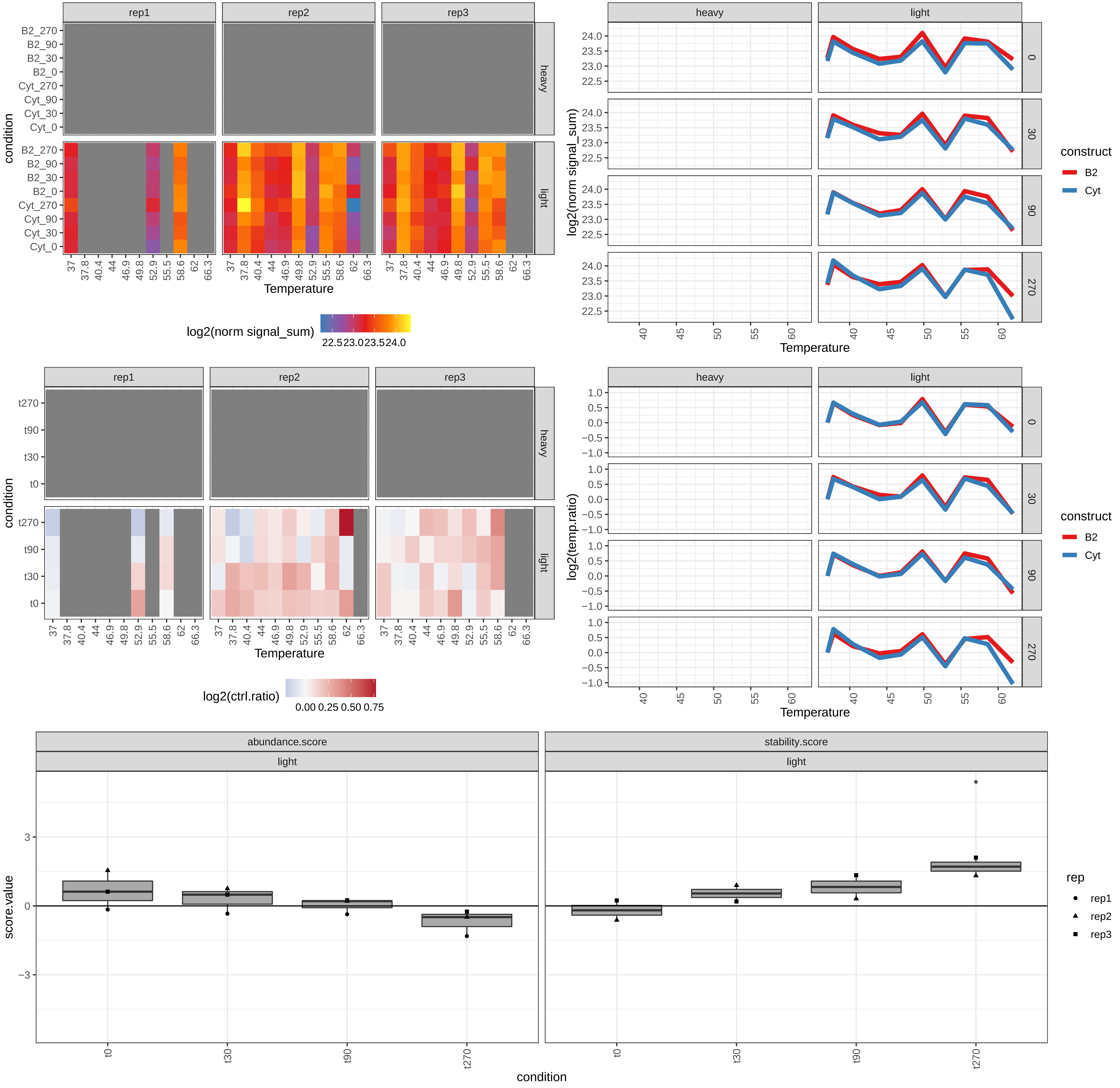

P01119 − Ras−like protein 1 OS=Saccharomyces cerevisiae (strain ATCC 204508 / S288c) GN=RAS1 PE=1 SV=2

RAS2 − average.top3: 7.14925269230769 − max.qupm: 10

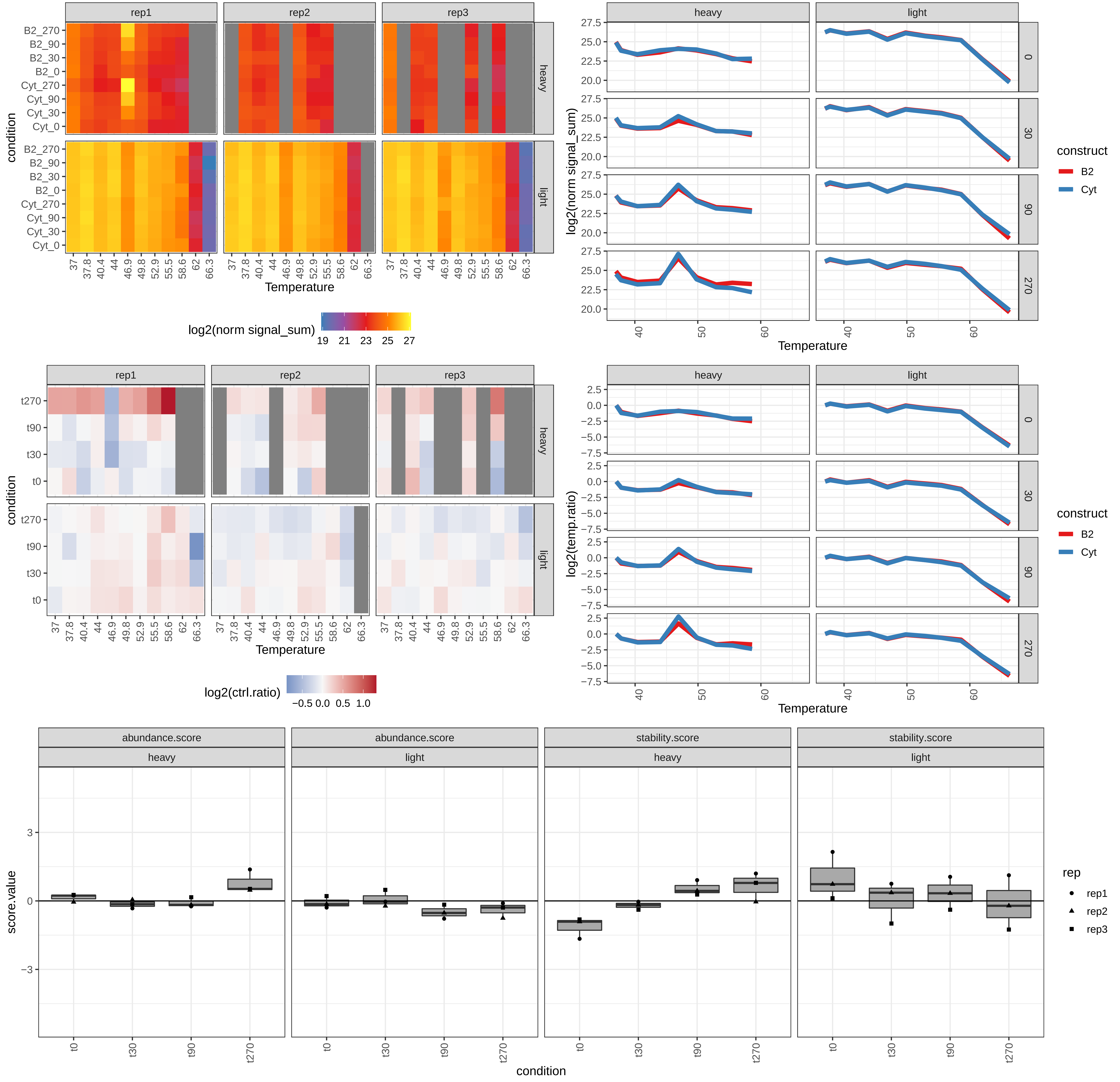

P01120 − Ras−like protein 2 OS=Saccharomyces cerevisiae (strain ATCC 204508 / S288c) GN=RAS2 PE=1 SV=4

RAT1 − average.top3: 6.85553111111111 − max.qupm: 13

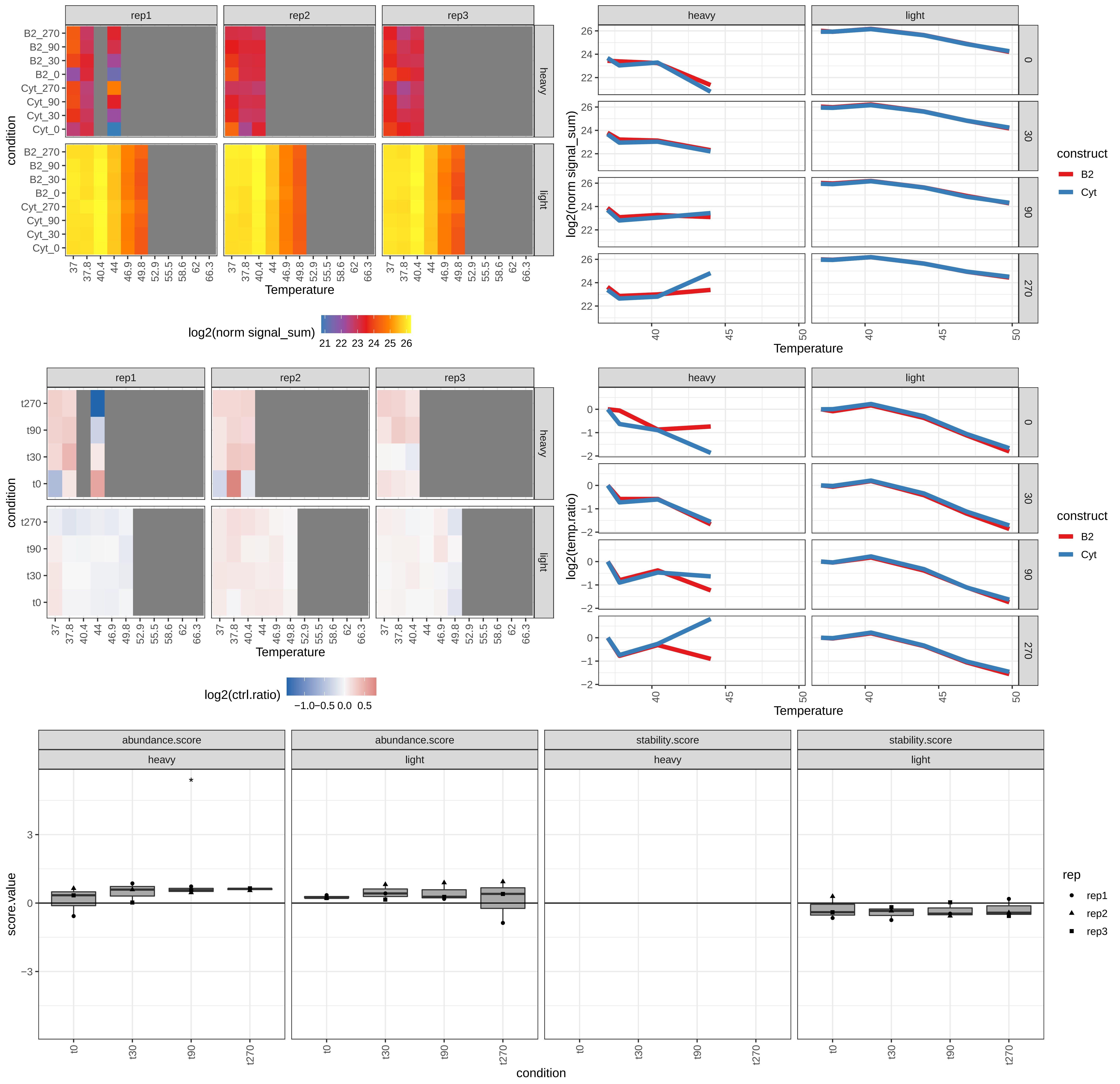

Q02792 − 5'−3' exoribonuclease 2 OS=Saccharomyces cerevisiae (strain ATCC 204508 / S288c) GN=RAT1 PE=1 SV=3

RAV1 − average.top3: 6.89154884615385 − max.qupm: 12

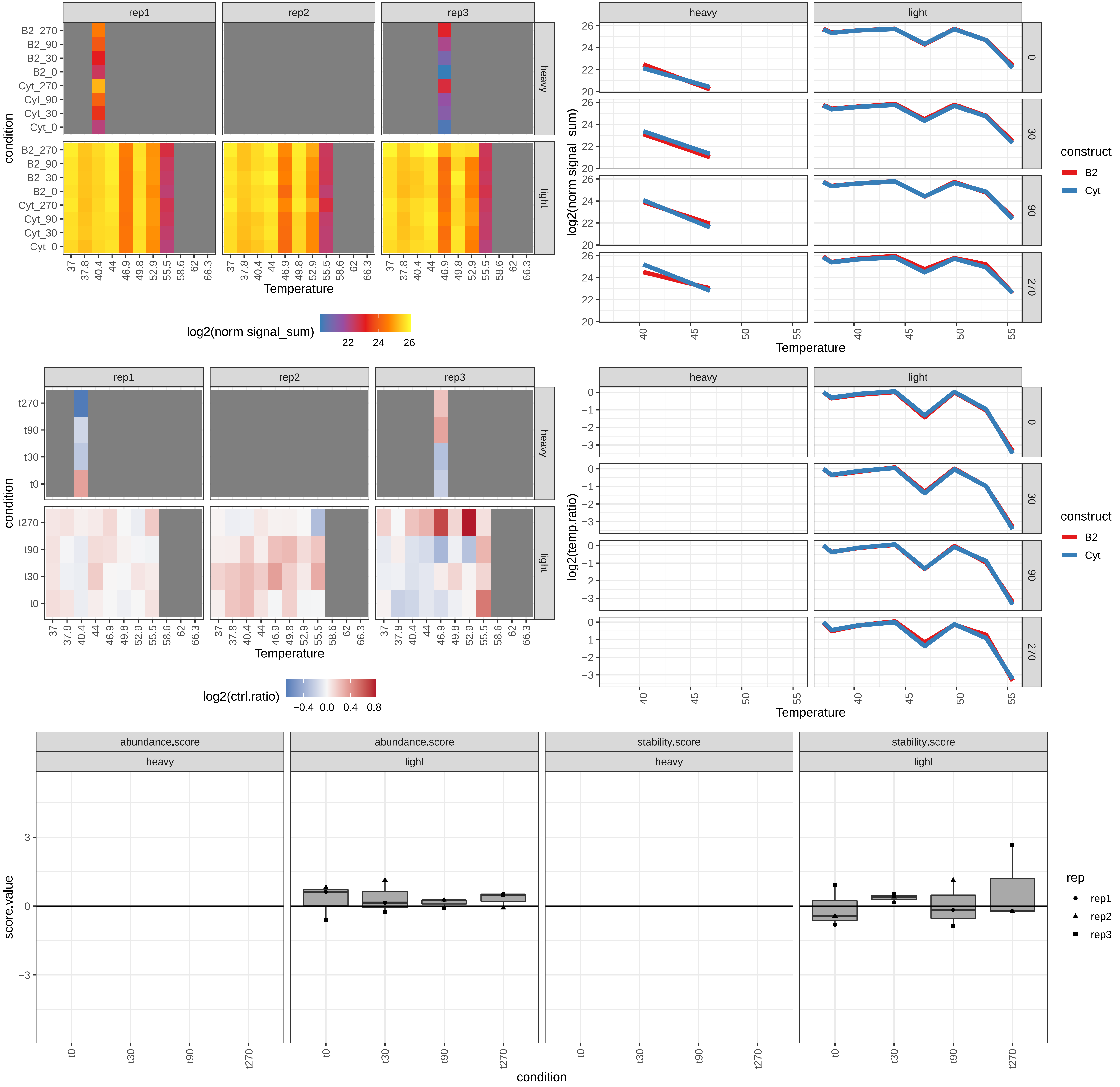

P47104 − Regulator of V−ATPase in vacuolar membrane protein 1 OS=Saccharomyces cerevisiae (strain ATCC 204508 / S288c) GN=RAV1 PE=1 SV=1

RAX2 − average.top3: 6.67040388888889 − max.qupm: 4

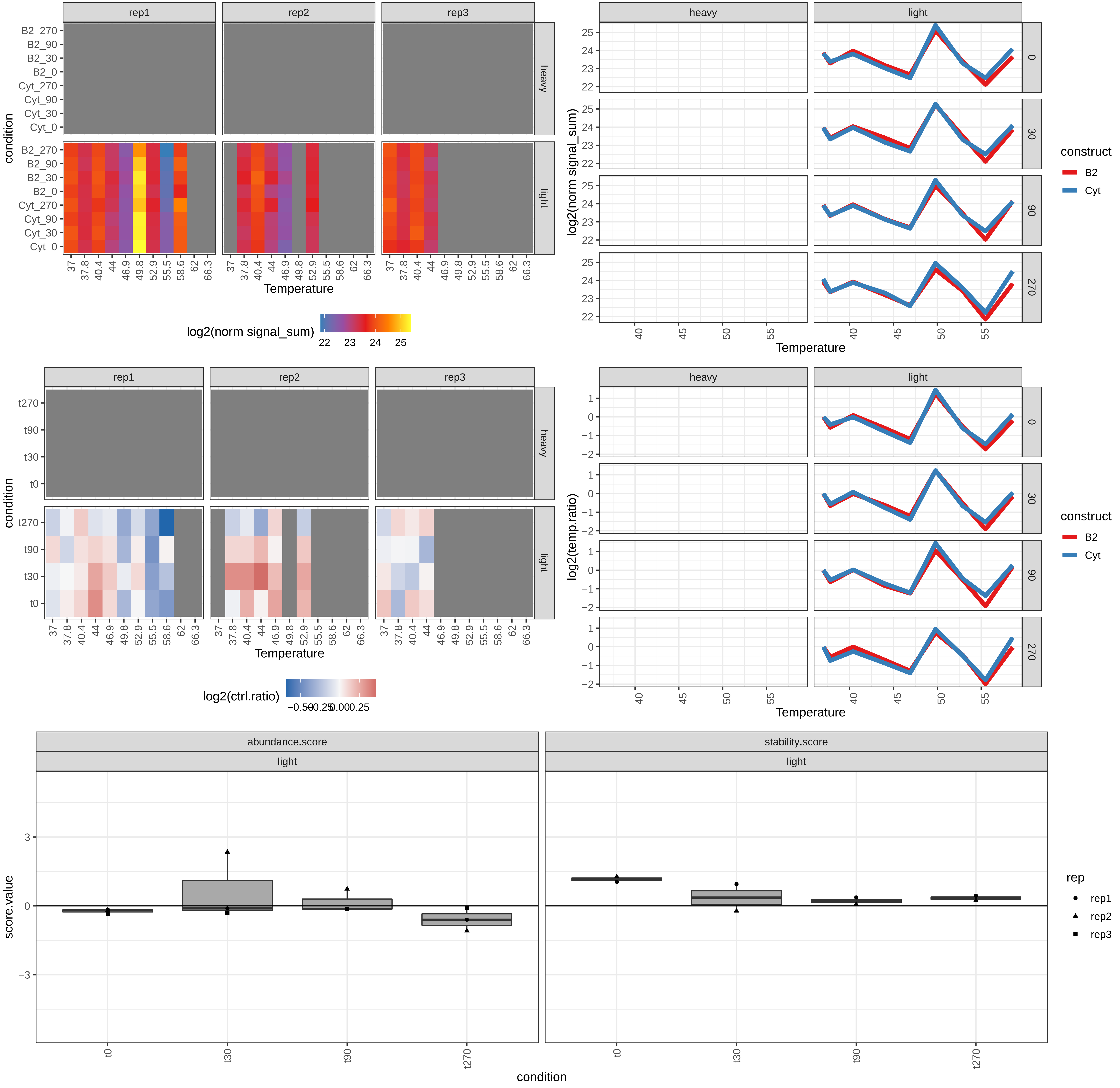

Q12465 − Bud site selection protein RAX2 OS=Saccharomyces cerevisiae (strain ATCC 204508 / S288c) GN=RAX2 PE=1 SV=1

RBD2 − average.top3: 7.36372333333333 − max.qupm: 4

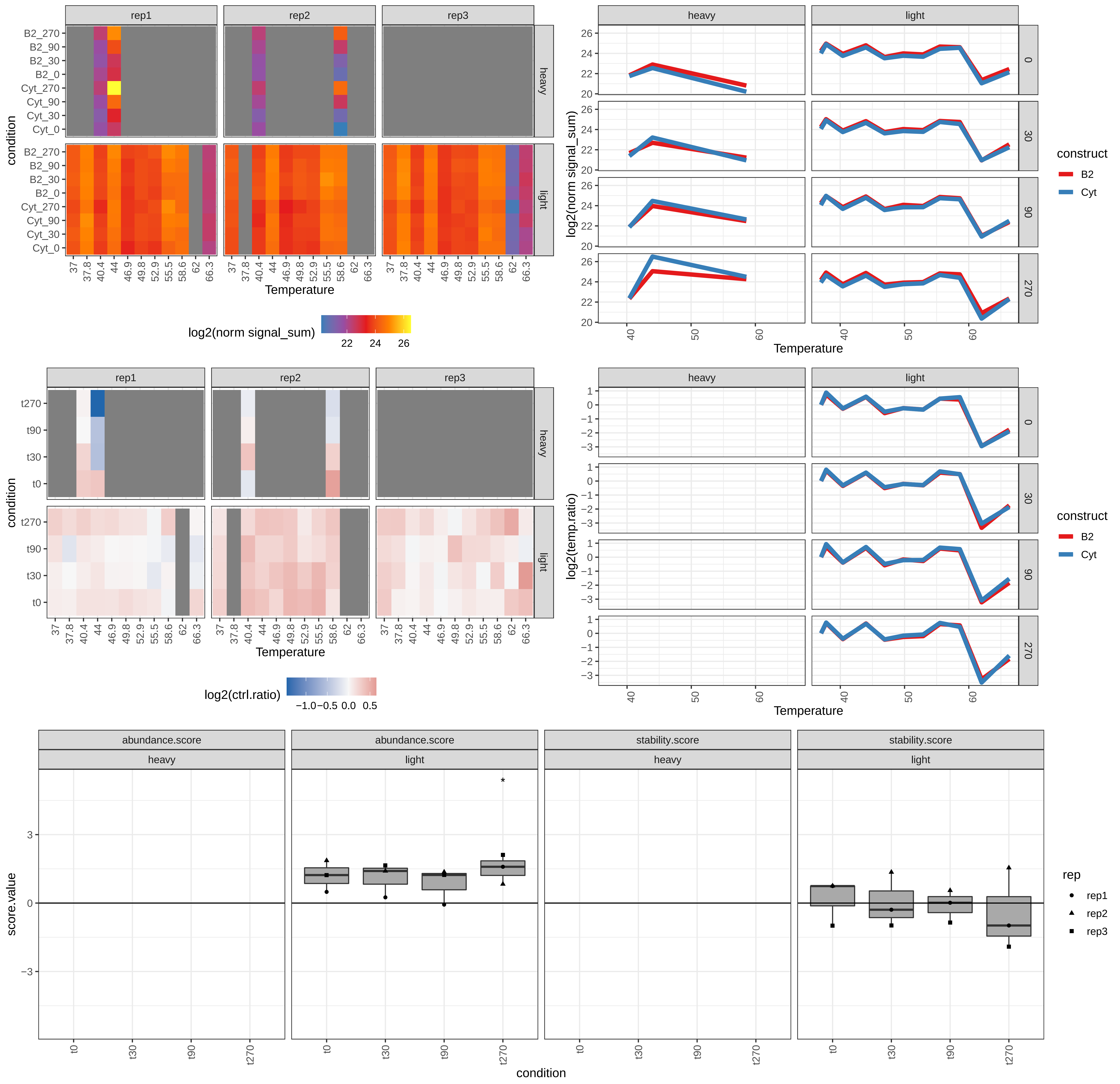

Q12270 − Rhomboid protein 2 OS=Saccharomyces cerevisiae (strain ATCC 204508 / S288c) GN=RBD2 PE=1 SV=1

RBG1 − average.top3: 7.1620665625 − max.qupm: 12

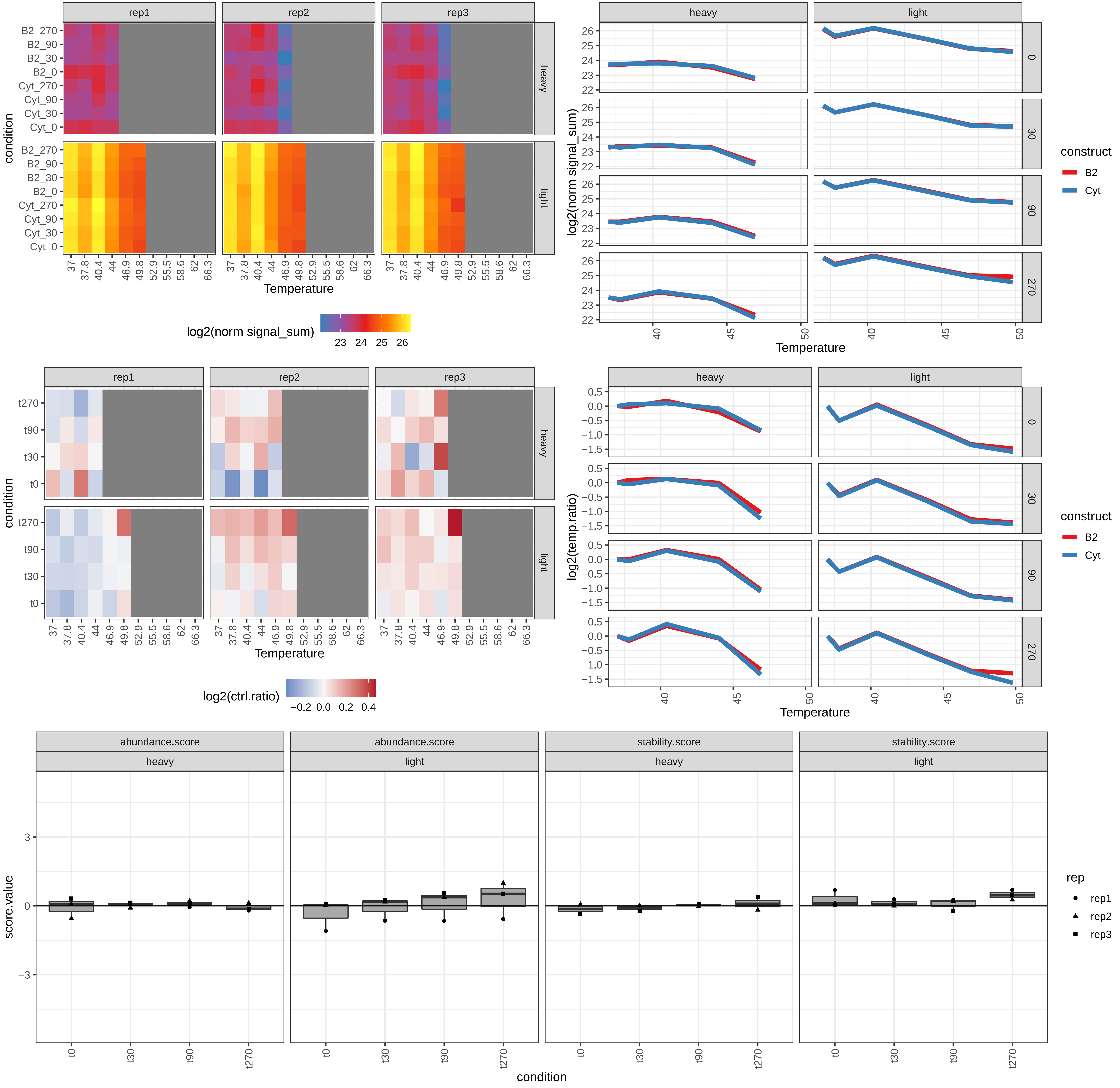

P39729 − Ribosome−interacting GTPase 1 OS=Saccharomyces cerevisiae (strain ATCC 204508 / S288c) GN=RBG1 PE=1 SV=1

RBG2 − average.top3: 7.13042391304348 − max.qupm: 10

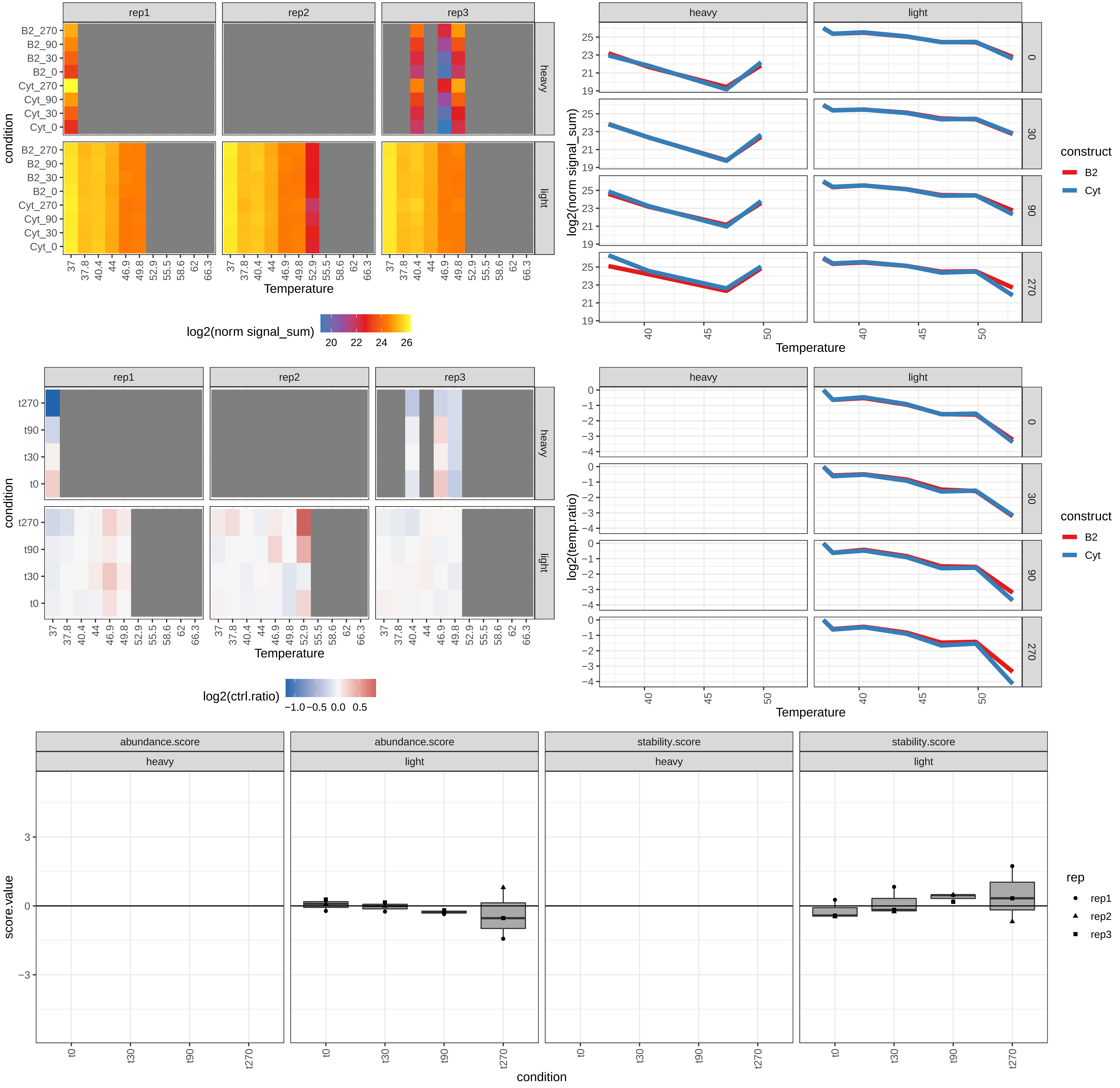

P53295 − Ribosome−interacting GTPase 2 OS=Saccharomyces cerevisiae (strain ATCC 204508 / S288c) GN=RBG2 PE=1 SV=1

RBK1 − average.top3: 6.9451408 − max.qupm: 10

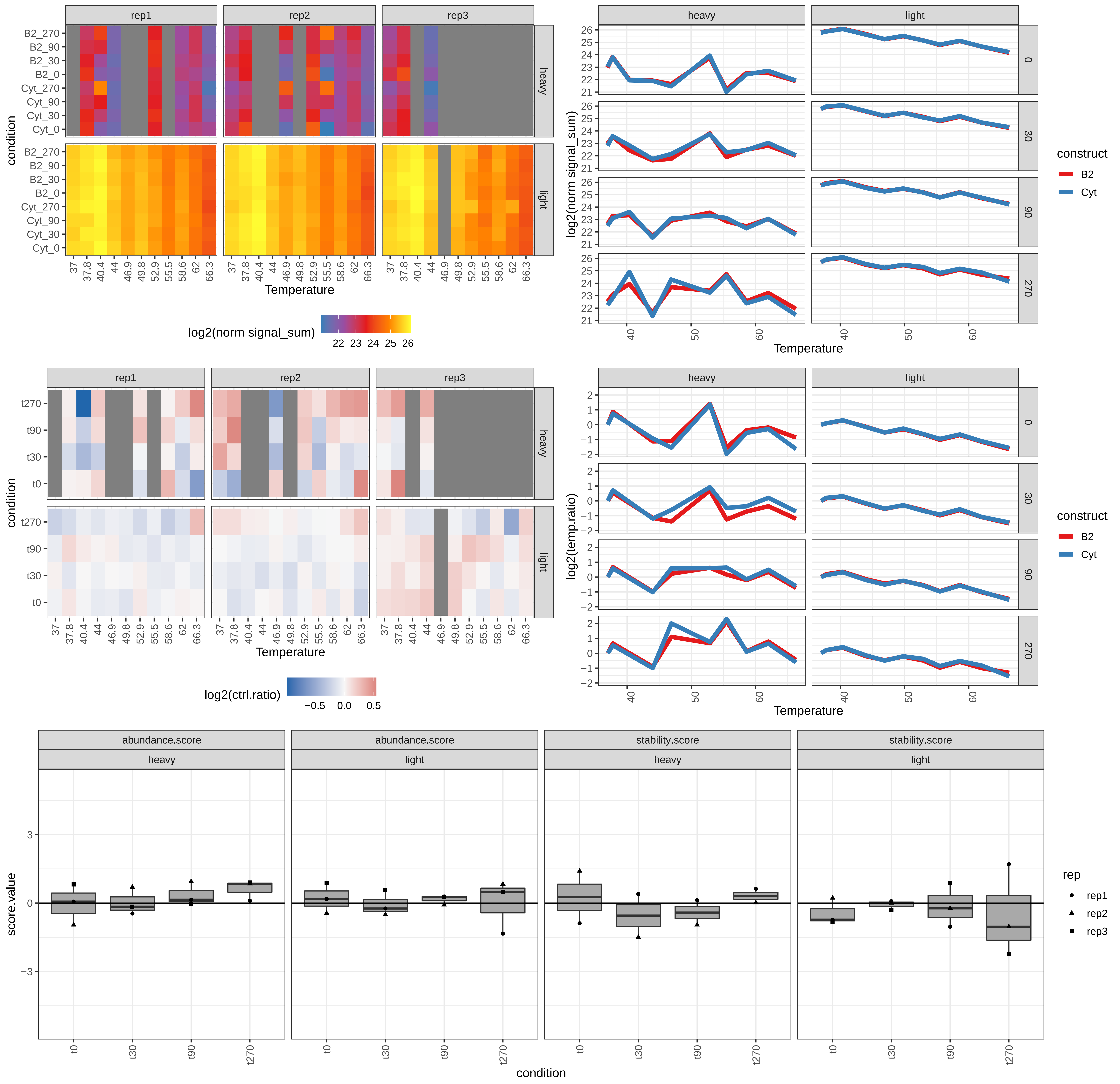

P25332 − Ribokinase OS=Saccharomyces cerevisiae (strain ATCC 204508 / S288c) GN=RBK1 PE=1 SV=2

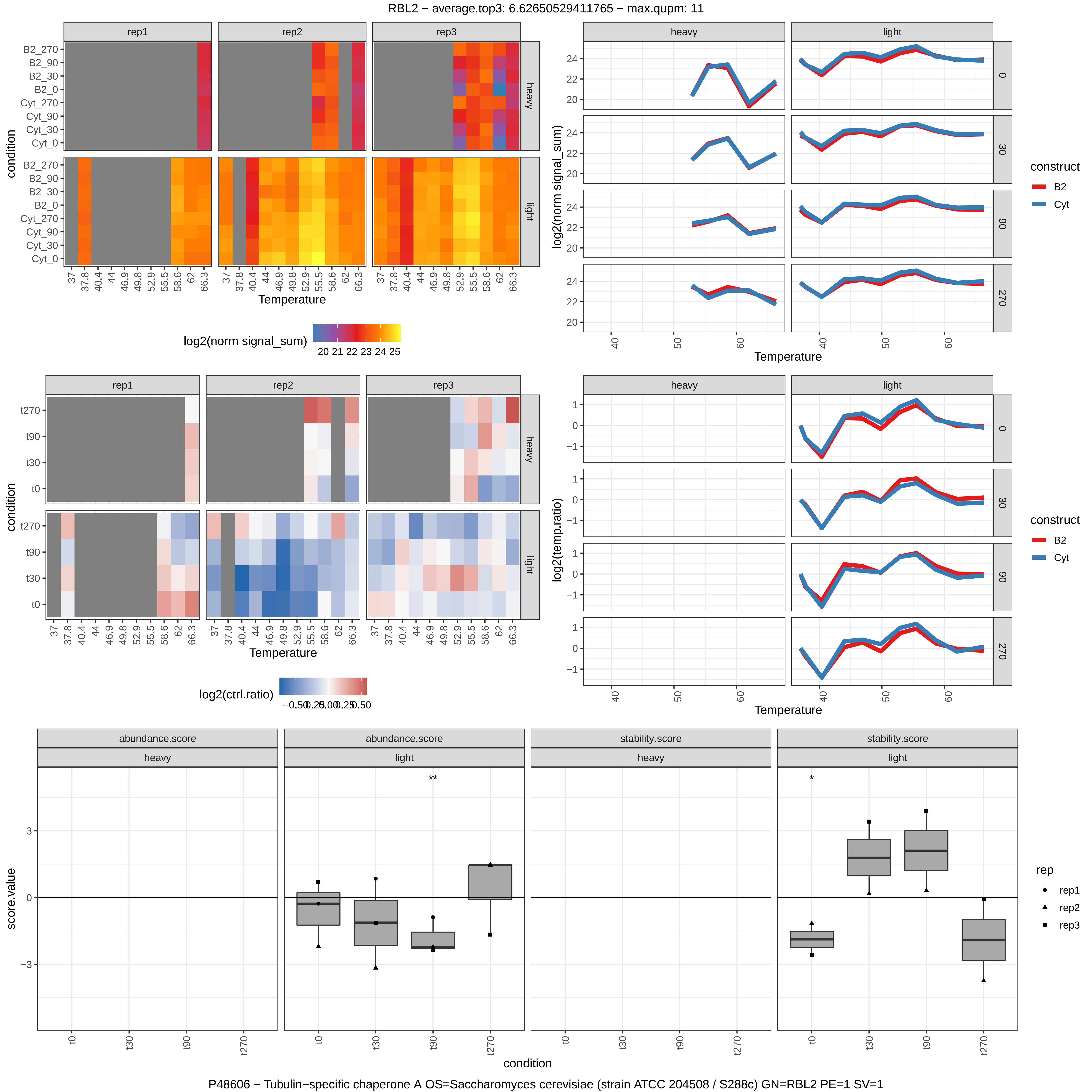
RBL2 − average.top3: 6.62650529411765 − max.qupm: 11
rep1
rep2
rep3
heavy
light
condition
Temperature
log2(norm signal_sum)
construct
B2
Cyt
log2(temp.ratio)
log2(ctrl.ratio)
abundance.score
stability.score
score.value
rep
rep1
rep2
rep3
P48606 − Tubulin−specific chaperone A OS=Saccharomyces cerevisiae (strain ATCC 204508 / S288c) GN=RBL2 PE=1 SV=1

RCF1 − average.top3: 7.096563125 − max.qupm: 6

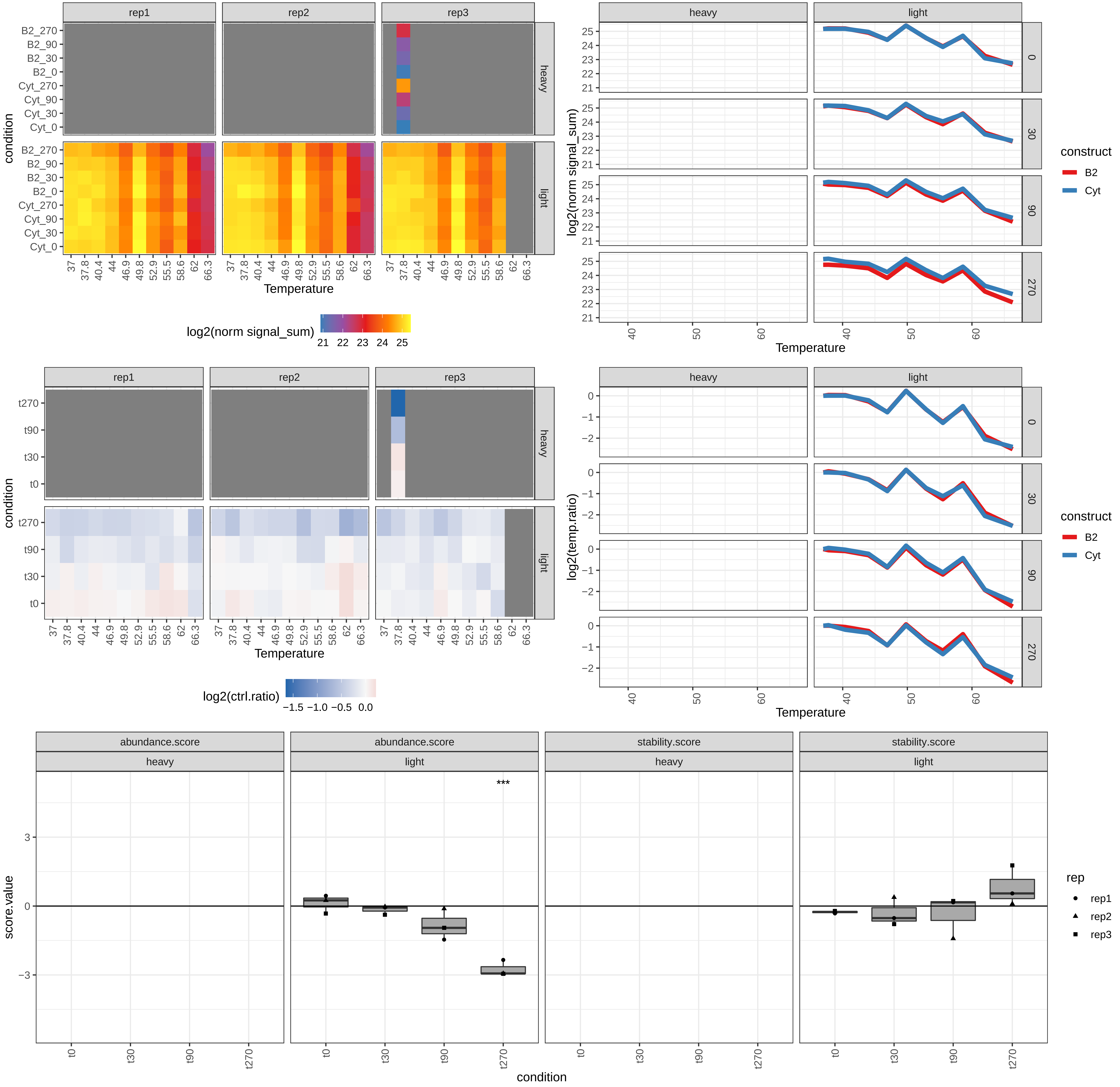

Q03713 − Respiratory supercomplex factor 1, mitochondrial OS=Saccharomyces cerevisiae (strain ATCC 204508 / S288c) GN=RCF1 PE=1 SV=1

RCF2 − average.top3: 6.97487535714286 − max.qupm: 5

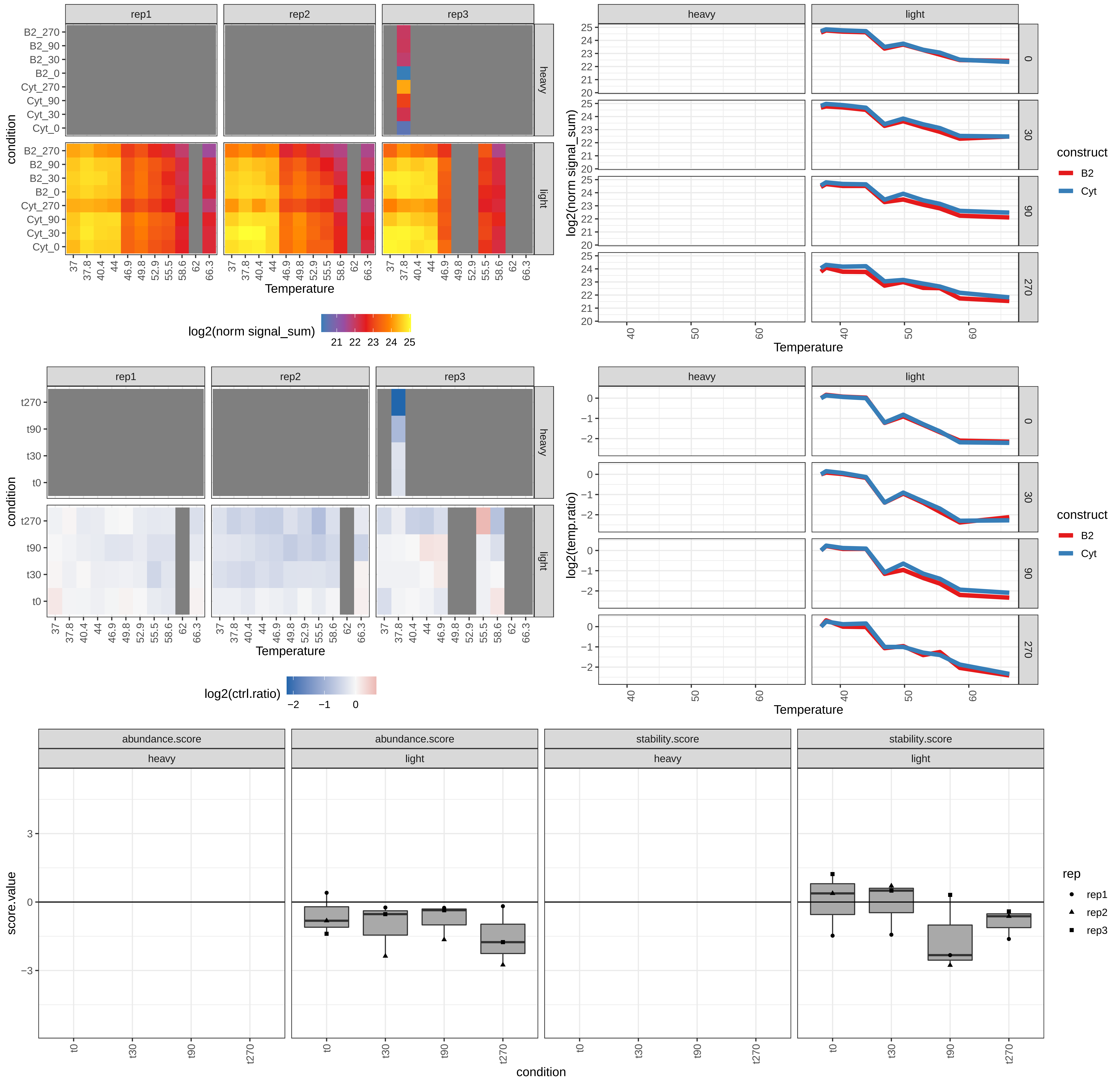

P53721 − Respiratory supercomplex factor 2, mitochondrial OS=Saccharomyces cerevisiae (strain ATCC 204508 / S288c) GN=RCF2 PE=1 SV=1

RCK2 − average.top3: 7.1684762962963 − max.qupm: 11

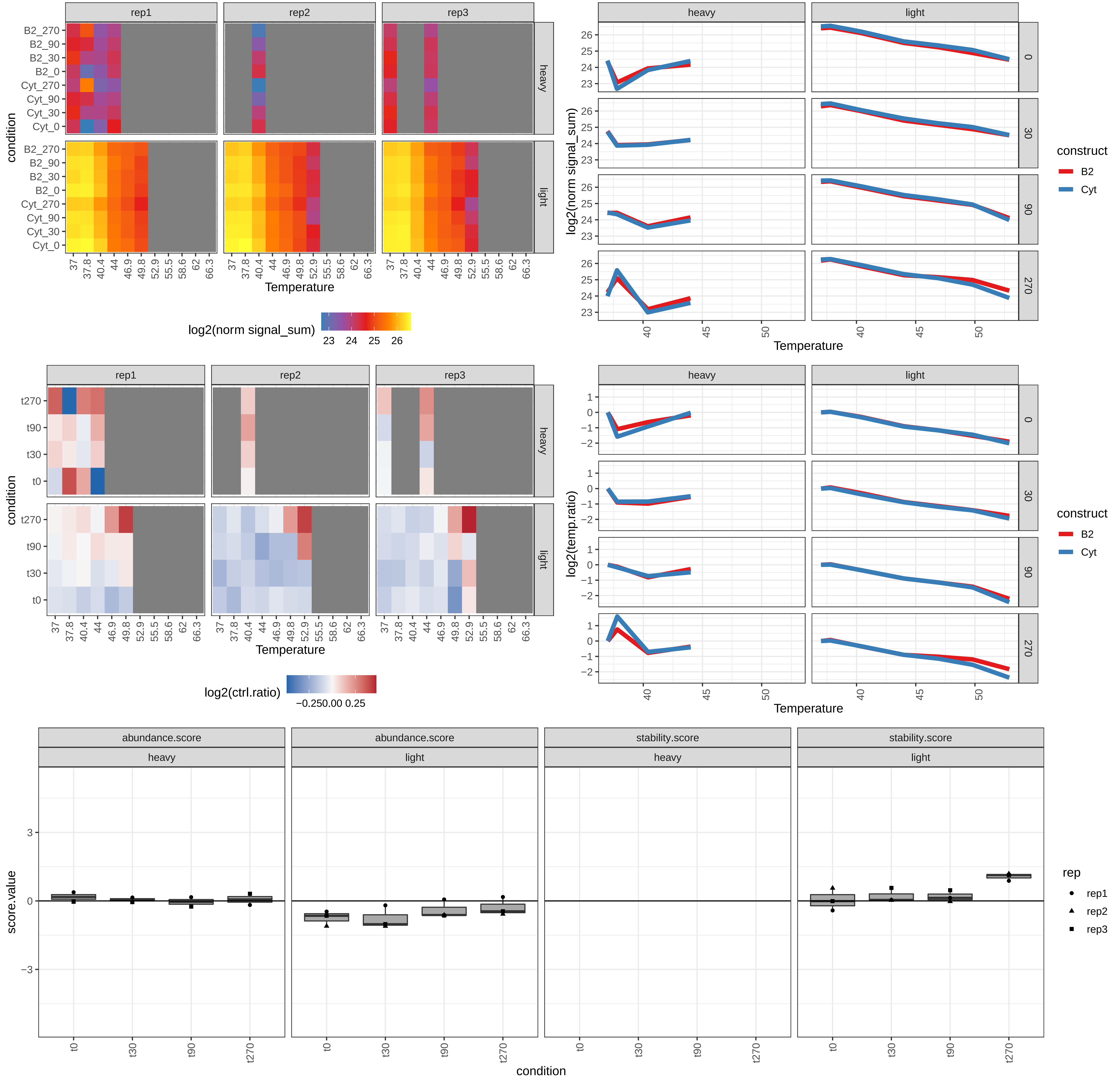

P38623 − Serine/threonine−protein kinase RCK2 OS=Saccharomyces cerevisiae (strain ATCC 204508 / S288c) GN=RCK2 PE=1 SV=3

RCL1 − average.top3: 7.06802933333333 − max.qupm: 8

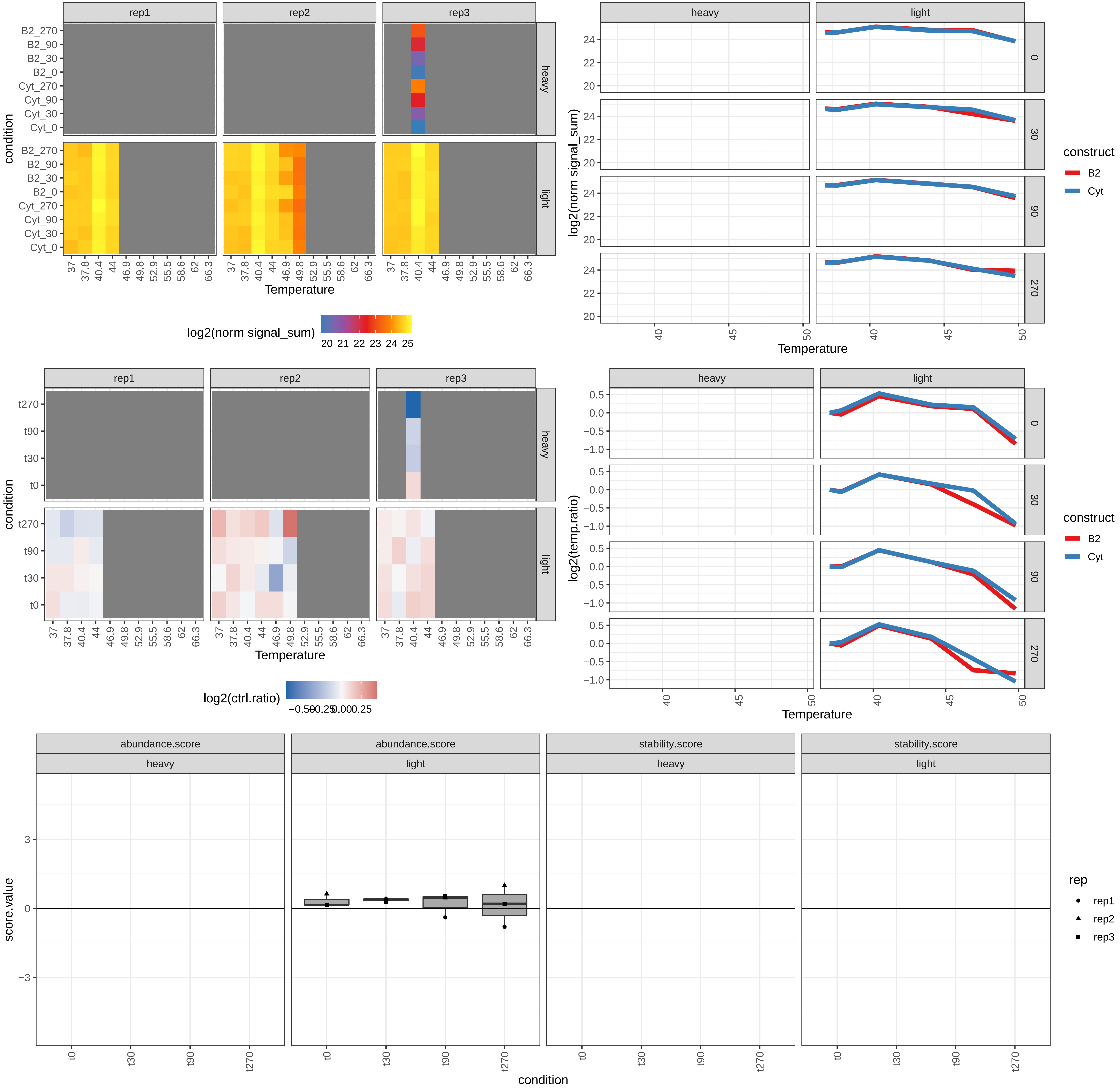

Q08096 − RNA 3'−terminal phosphate cyclase−like protein OS=Saccharomyces cerevisiae (strain ATCC 204508 / S288c) GN=RCL1 PE=1 SV=1

RCM1 − average.top3: 6.31135666666667 − max.qupm: 3

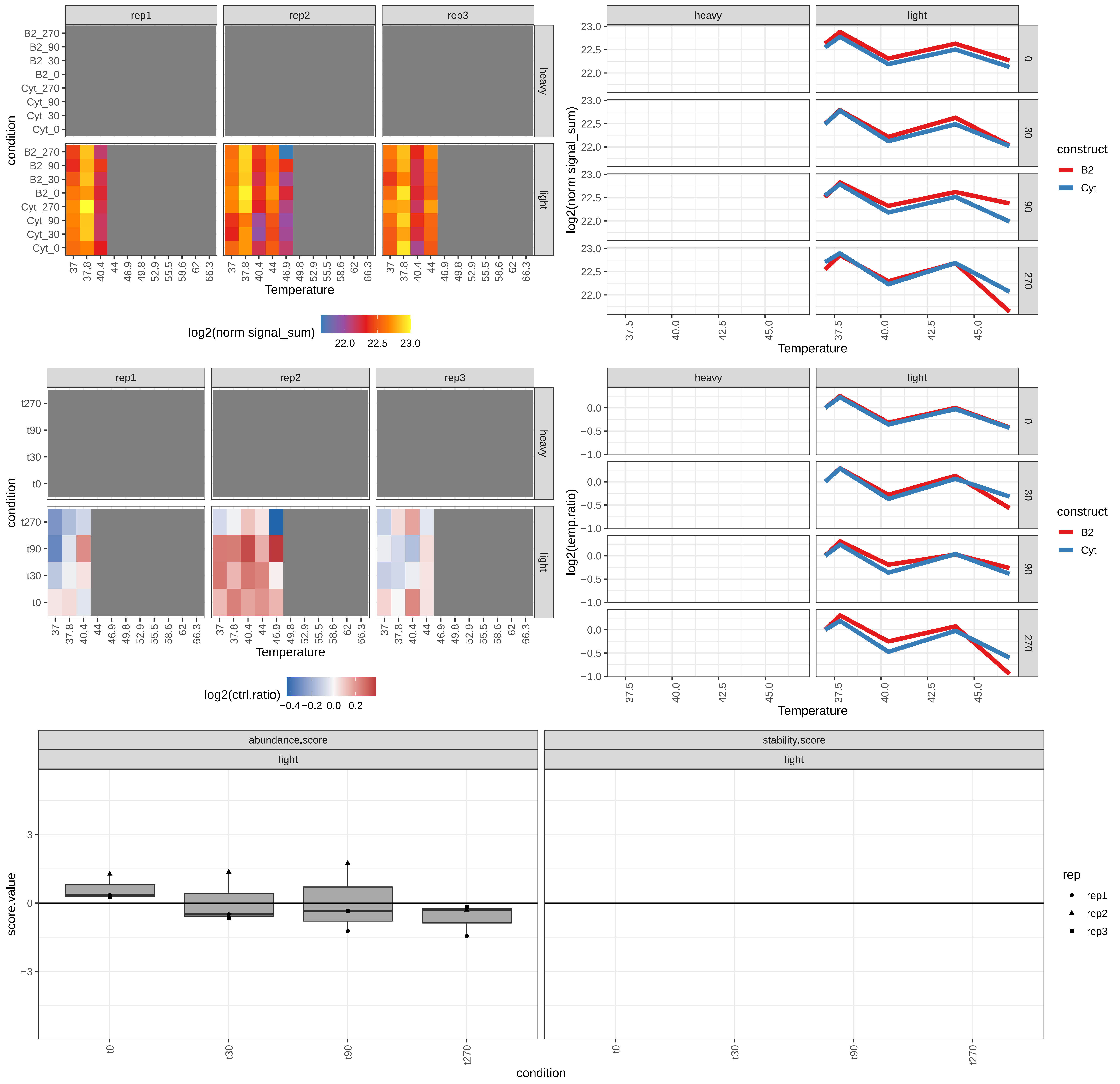

P53972 − 25S rRNA (cytosine(2278)−C(5))−methyltransferase OS=Saccharomyces cerevisiae (strain ATCC 204508 / S288c) GN=RCM1 PE=1 SV=1

RCN2 − average.top3: 6.72939266666667 − max.qupm: 3

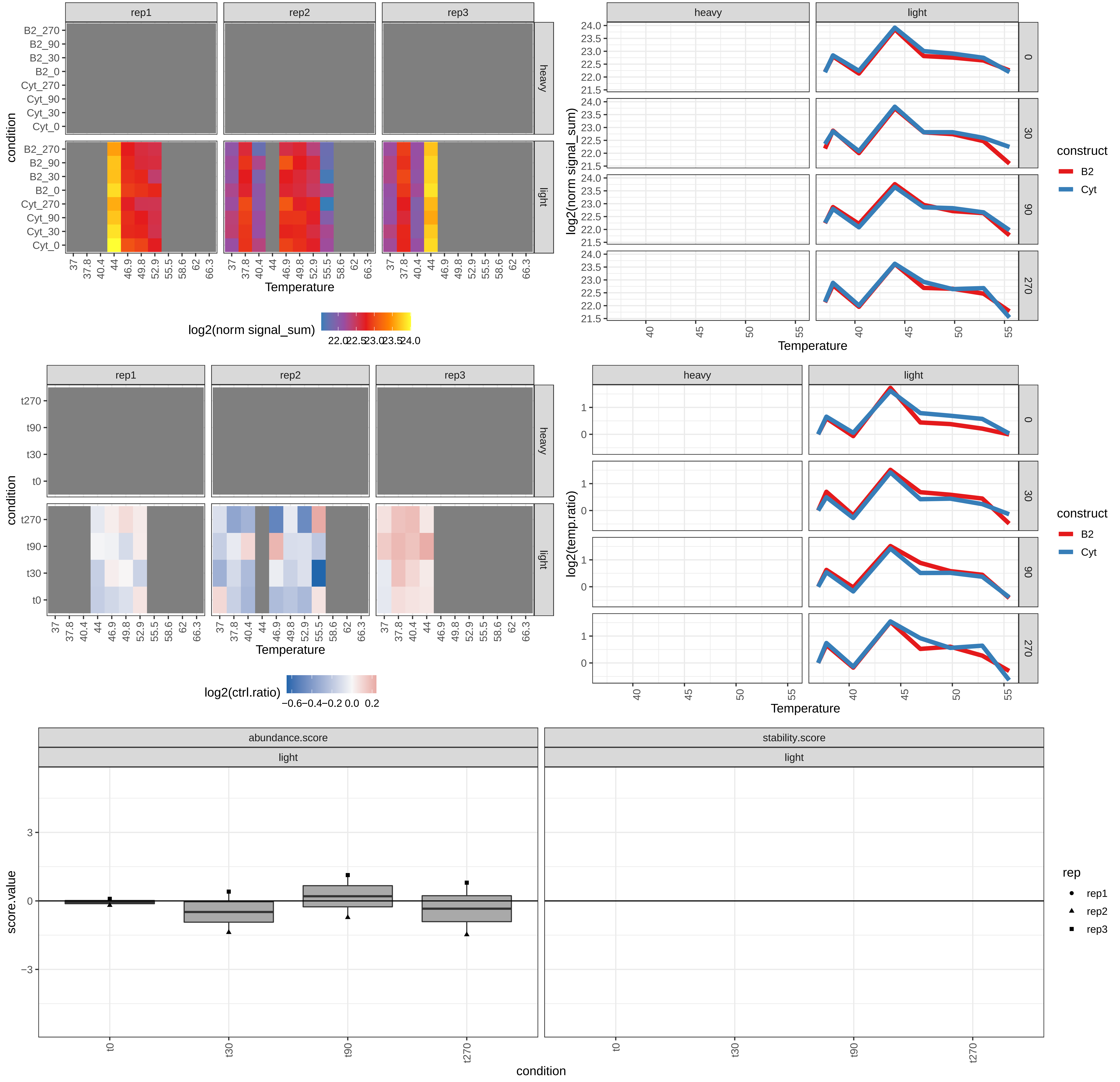

Q12044 − Regulator of calcineurin 2 OS=Saccharomyces cerevisiae (strain ATCC 204508 / S288c) GN=RCN2 PE=1 SV=1

RCO1 − average.top3: 6.461913 − max.qupm: 4

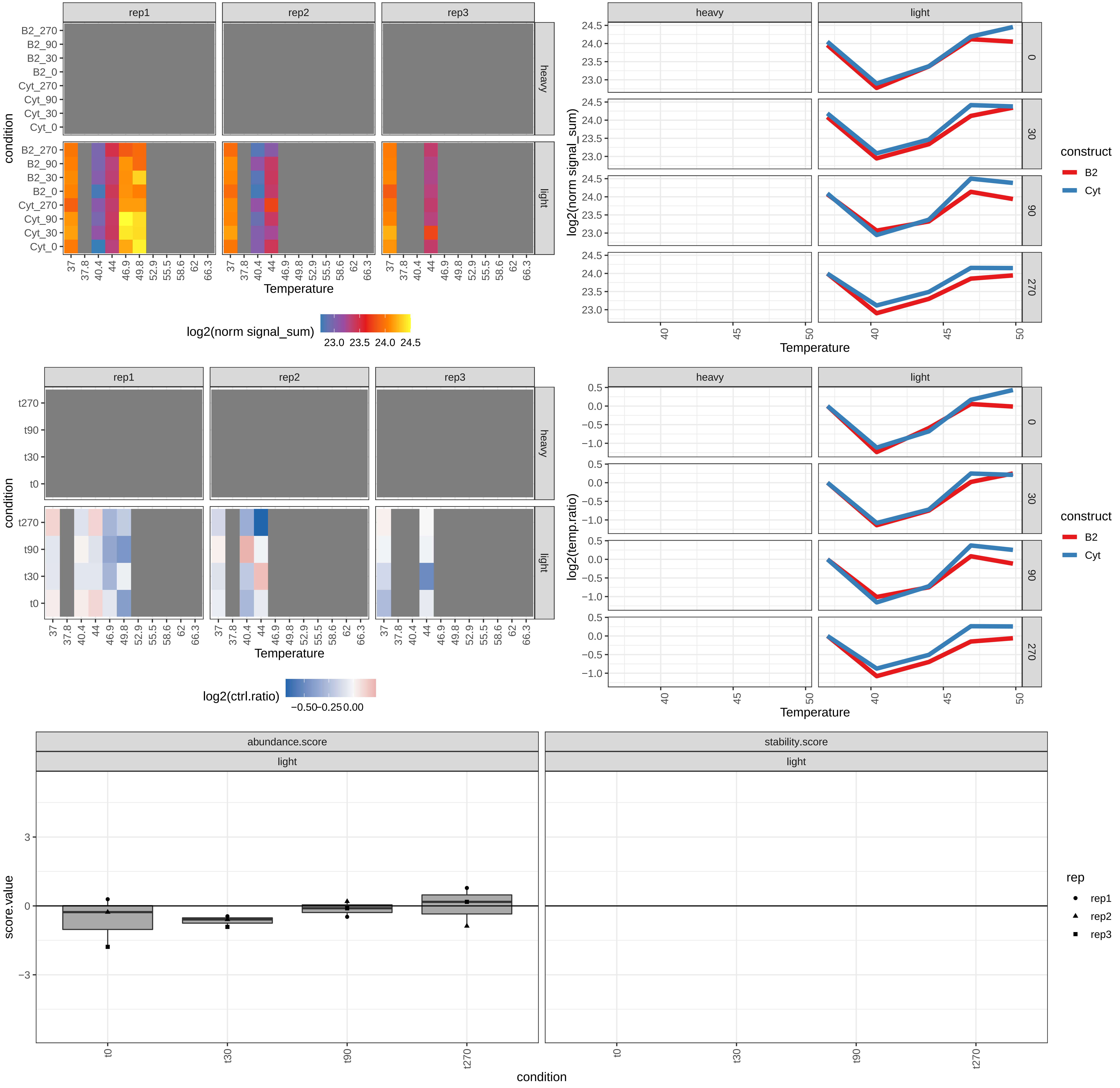

Q04779 − Transcriptional regulatory protein RCO1 OS=Saccharomyces cerevisiae (strain ATCC 204508 / S288c) GN=RCO1 PE=1 SV=1

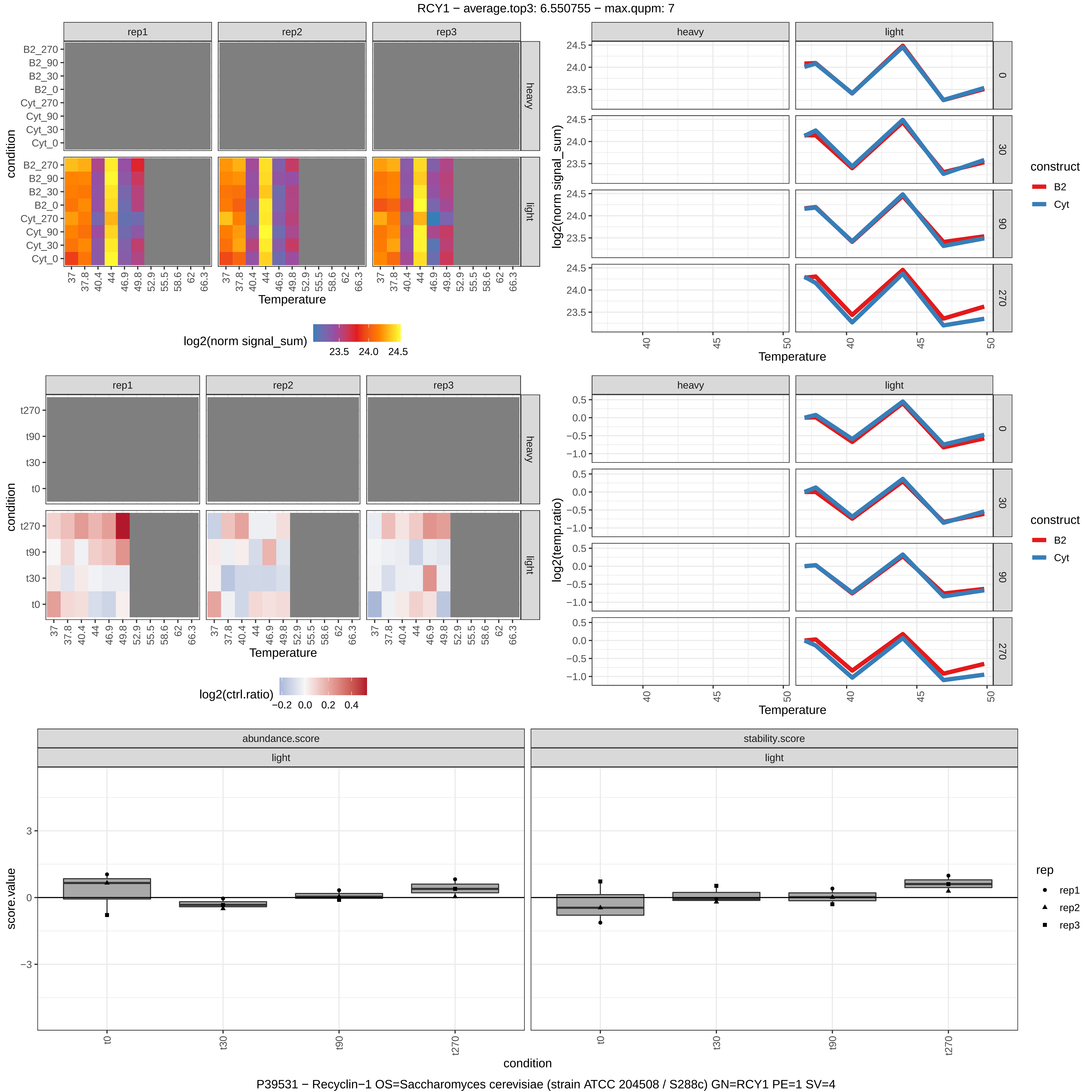
RCY1 − average.top3: 6.550755 − max.qupm: 7
rep1
rep2
rep3
heavy
light
condition
Temperature
log2(norm signal_sum)
log2(ctrl.ratio)
log2(temp.ratio)
construct
B2
Cyt
abundance.score
stability.score
score.value
rep
rep1
rep2
rep3
condition
P39531 − Recyclin−1 OS=Saccharomyces cerevisiae (strain ATCC 204508 / S288c) GN=RCY1 PE=1 SV=4

RDI1 − average.top3: 7.3008996875 − max.qupm: 10

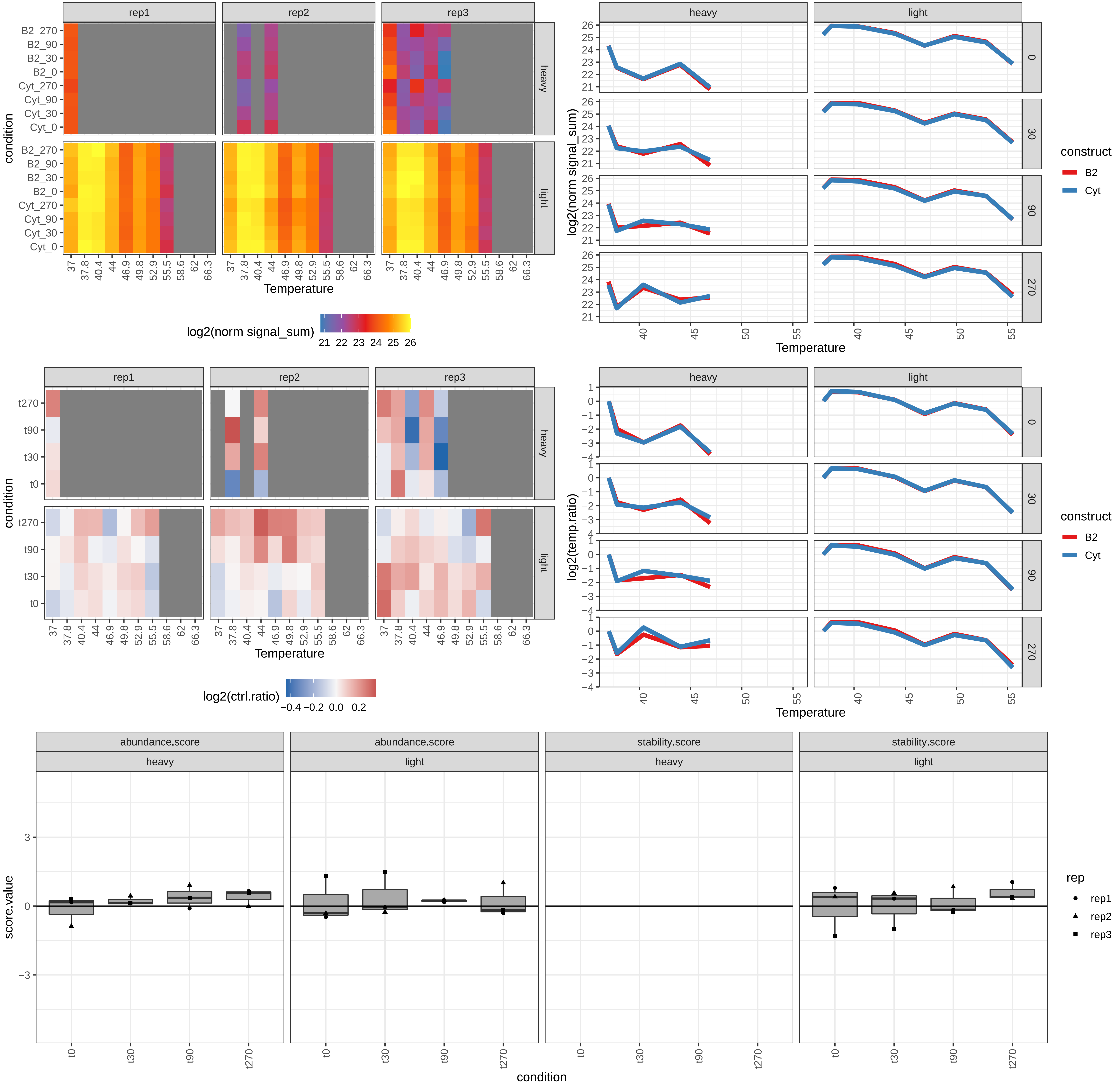

Q12434 − Rho GDP−dissociation inhibitor OS=Saccharomyces cerevisiae (strain ATCC 204508 / S288c) GN=RDI1 PE=1 SV=1

RDL1 − average.top3: 6.70605642857143 − max.qupm: 4

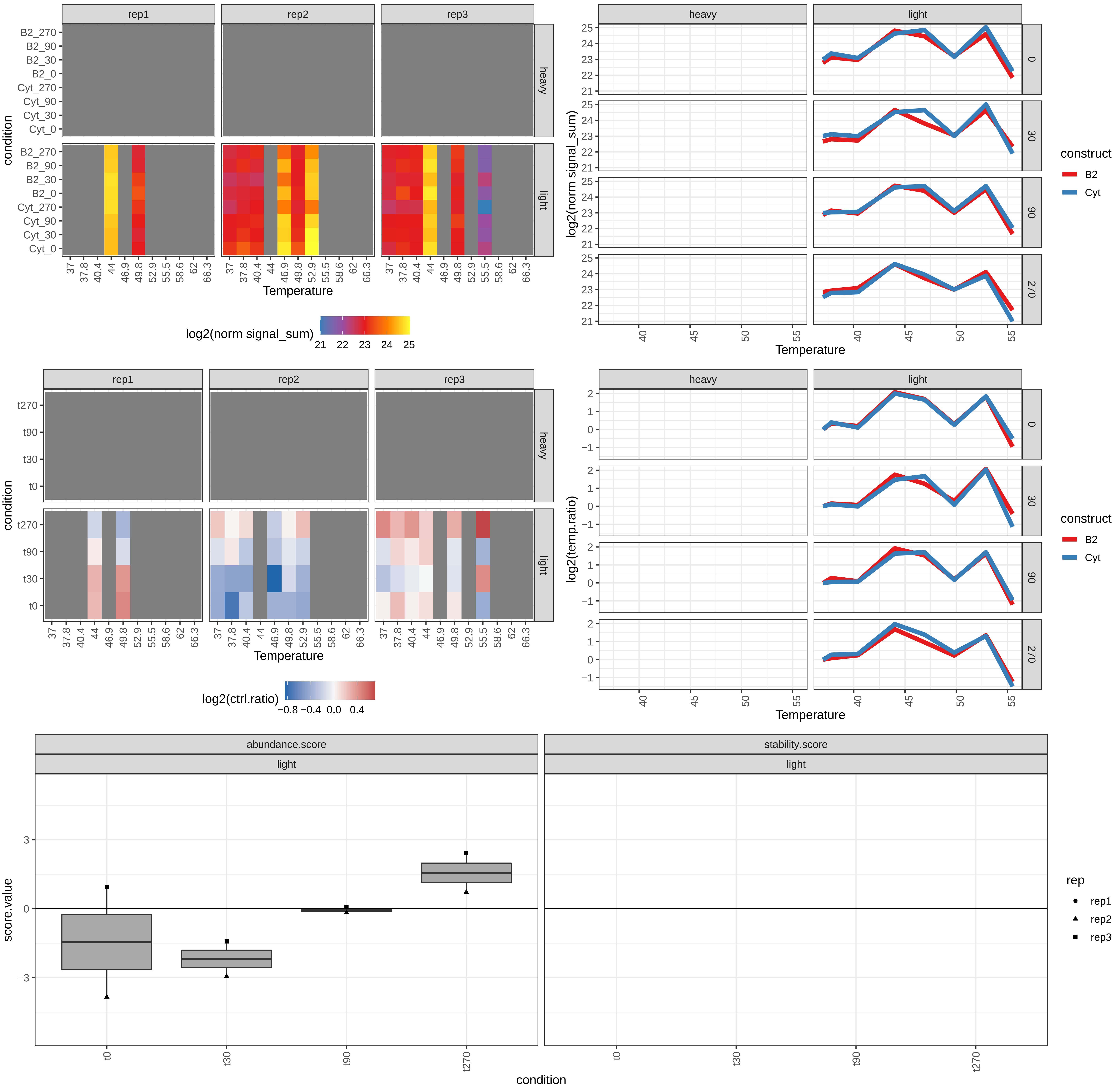

Q12305 − Thiosulfate sulfurtransferase RDL1, mitochondrial OS=Saccharomyces cerevisiae (strain ATCC 204508 / S288c) GN=RDL1 PE=1 SV=1

REB1 − average.top3: 6.48755193548387 − max.qupm: 8

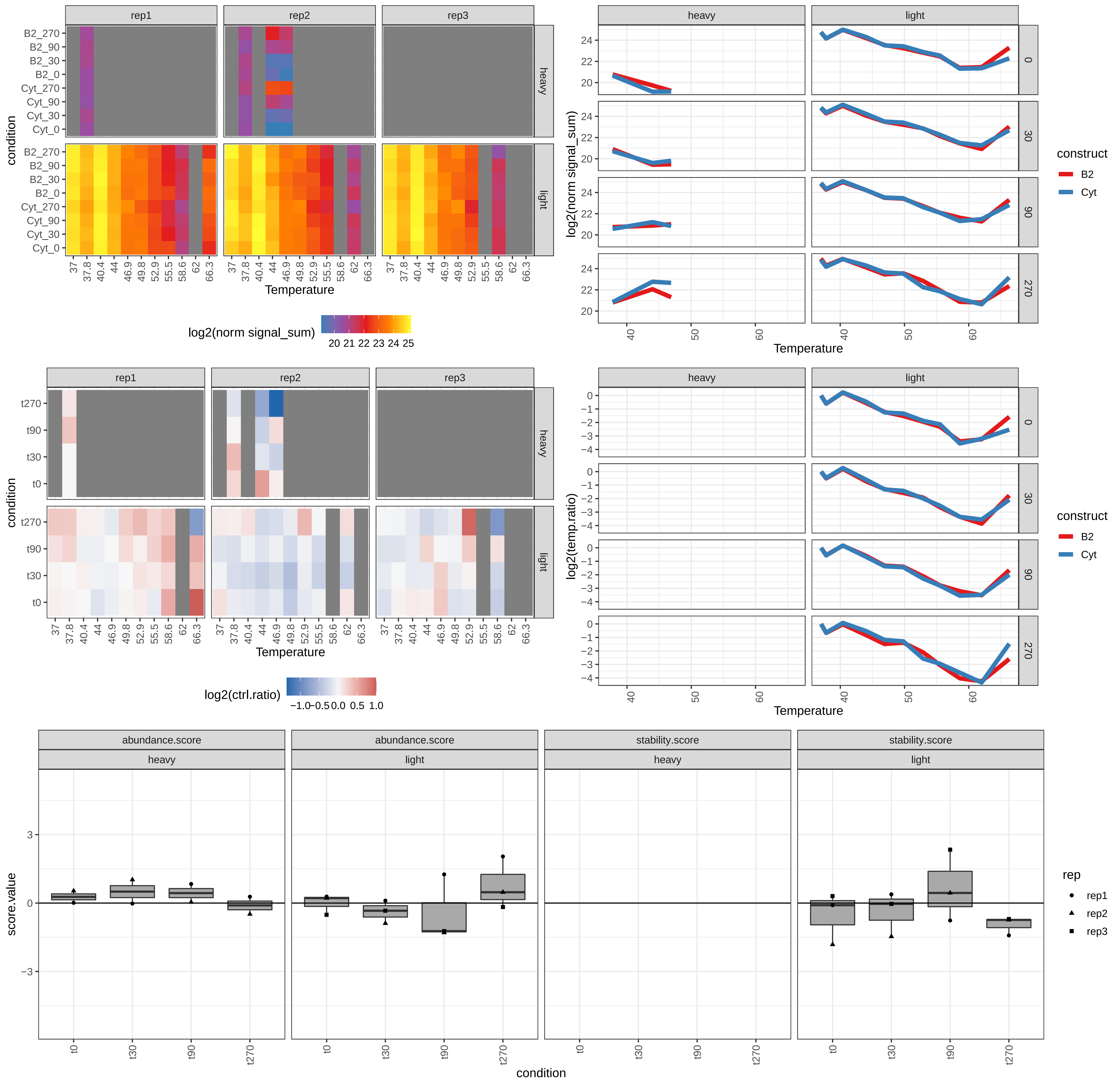

P21538 − DNA−binding protein REB1 OS=Saccharomyces cerevisiae (strain ATCC 204508 / S288c) GN=REB1 PE=1 SV=2

REE1 − average.top3: 7.76145176470588 − max.qupm: 10

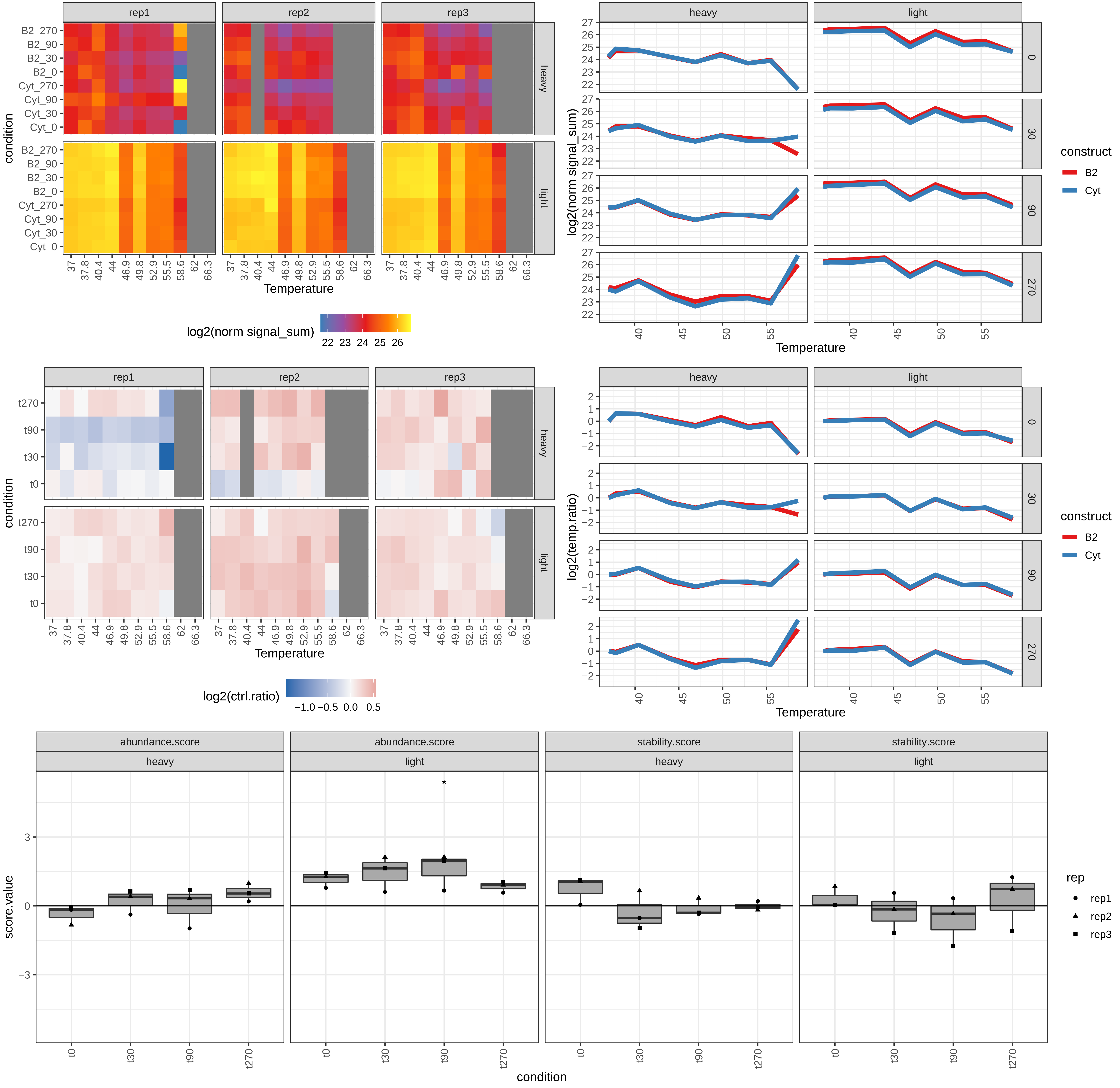

P40893 − Regulation of enolase protein 1 OS=Saccharomyces cerevisiae (strain ATCC 204508 / S288c) GN=REE1 PE=1 SV=1

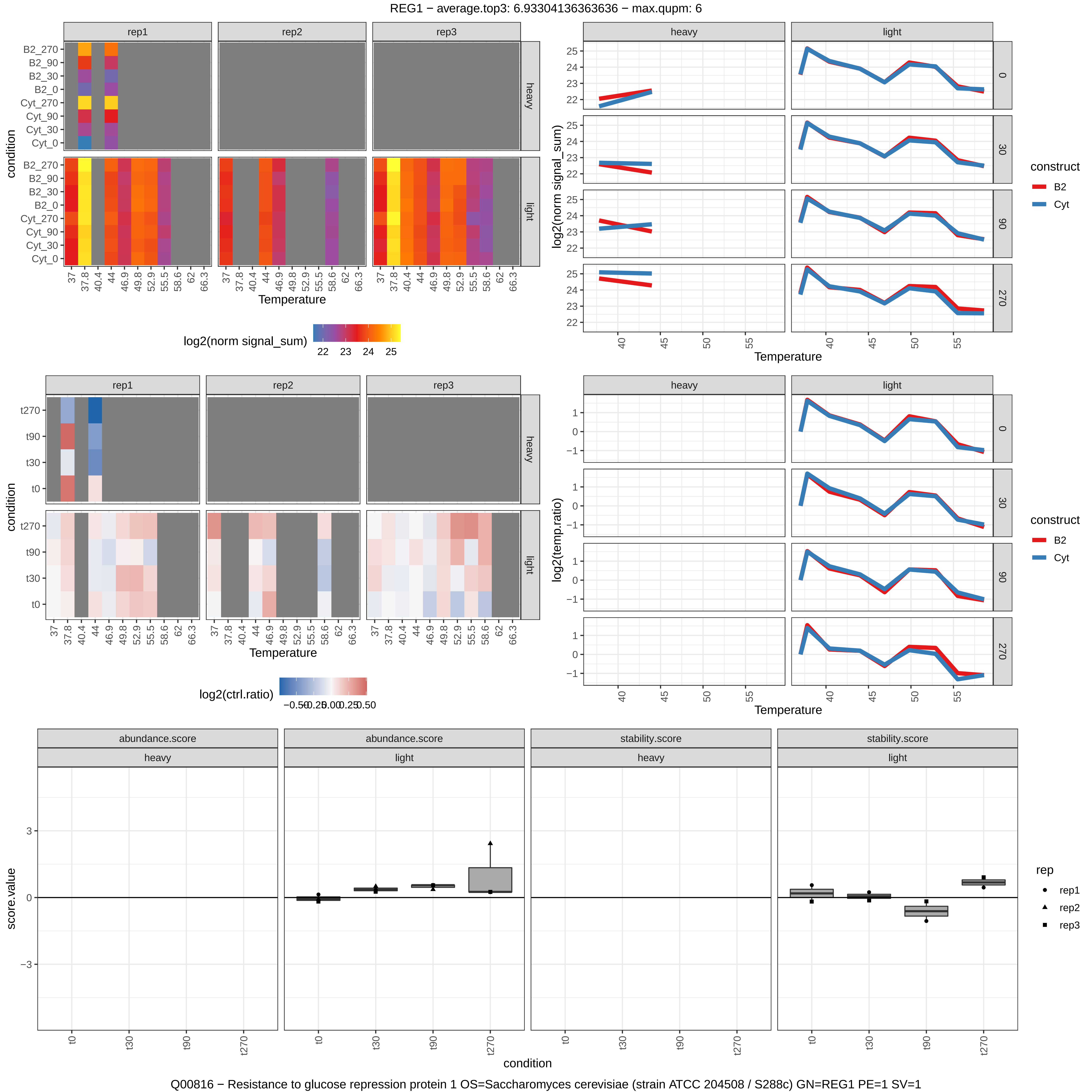
REG1 − average.top3: 6.93304136363636 − max.qupm: 6
rep1
rep2
rep3
heavy
light
condition
Temperature
log2(norm signal_sum)
construct
B2
Cyt
log2(temp.ratio)
log2(ctrl.ratio)
abundance.score
stability.score
score.value
rep
rep1
rep2
rep3
Q00816 − Resistance to glucose repression protein 1 OS=Saccharomyces cerevisiae (strain ATCC 204508 / S288c) GN=REG1 PE=1 SV=1

REH1 − average.top3: 6.60495708333333 − max.qupm: 6

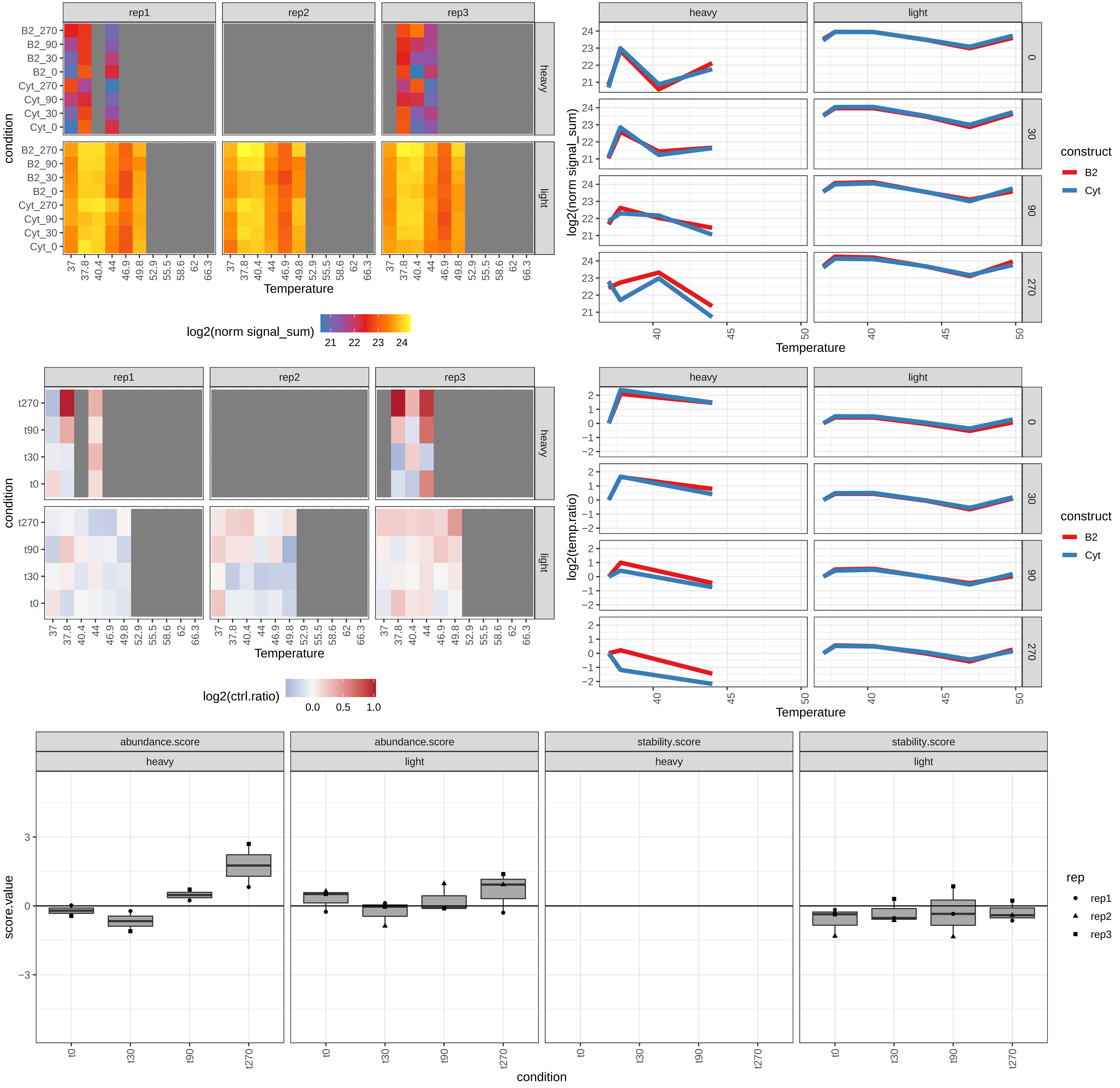

Q06709 − Cytoplasmic 60S subunit biogenesis factor REH1 OS=Saccharomyces cerevisiae (strain ATCC 204508 / S288c) GN=REH1 PE=1 SV=1

REI1 − average.top3: 6.40360142857143 − max.qupm: 5

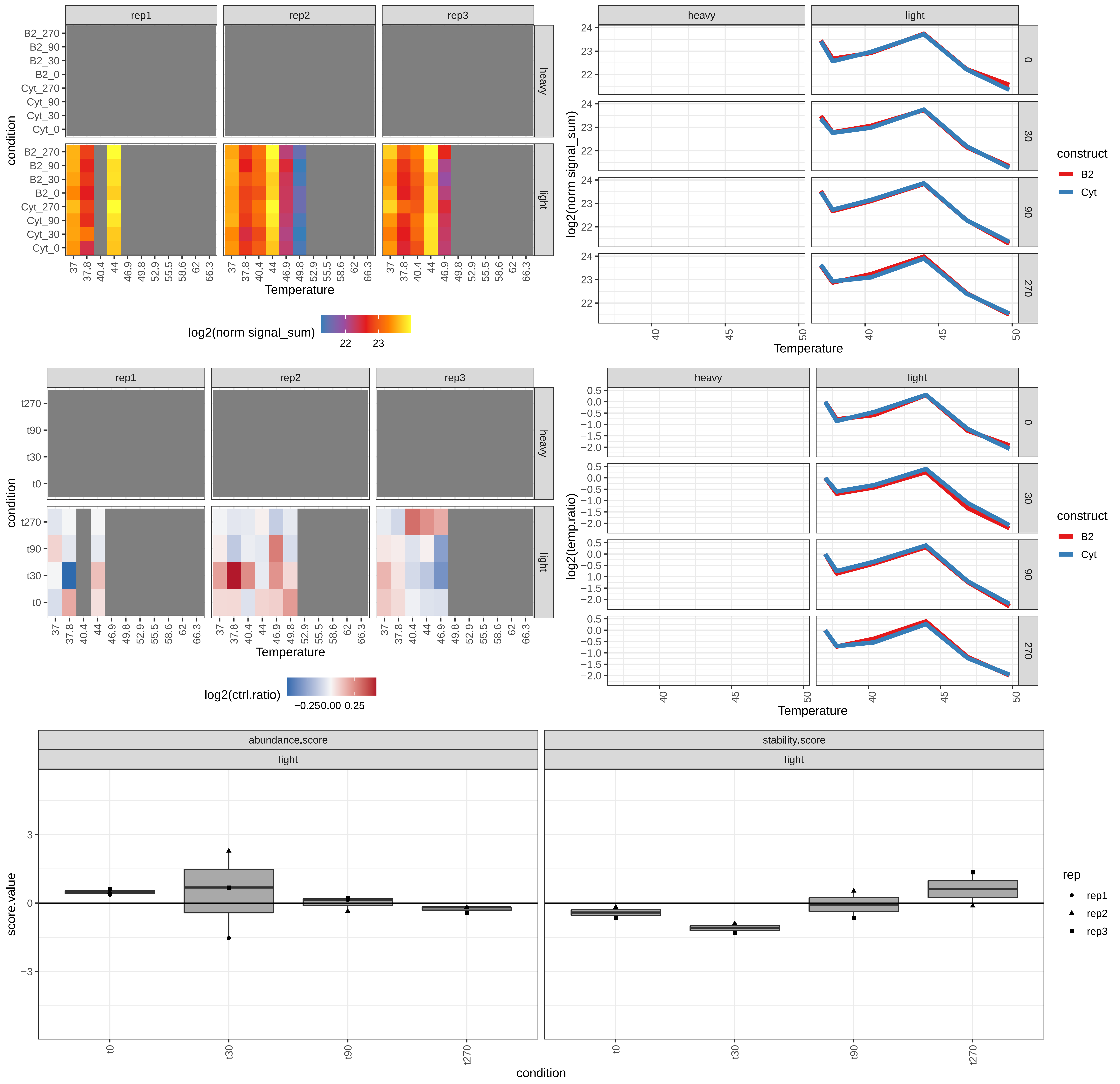

P38344 − Cytoplasmic 60S subunit biogenesis factor REI1 OS=Saccharomyces cerevisiae (strain ATCC 204508 / S288c) GN=REI1 PE=1 SV=3

REP1 − average.top3: 7.3687224 − max.qupm: 10

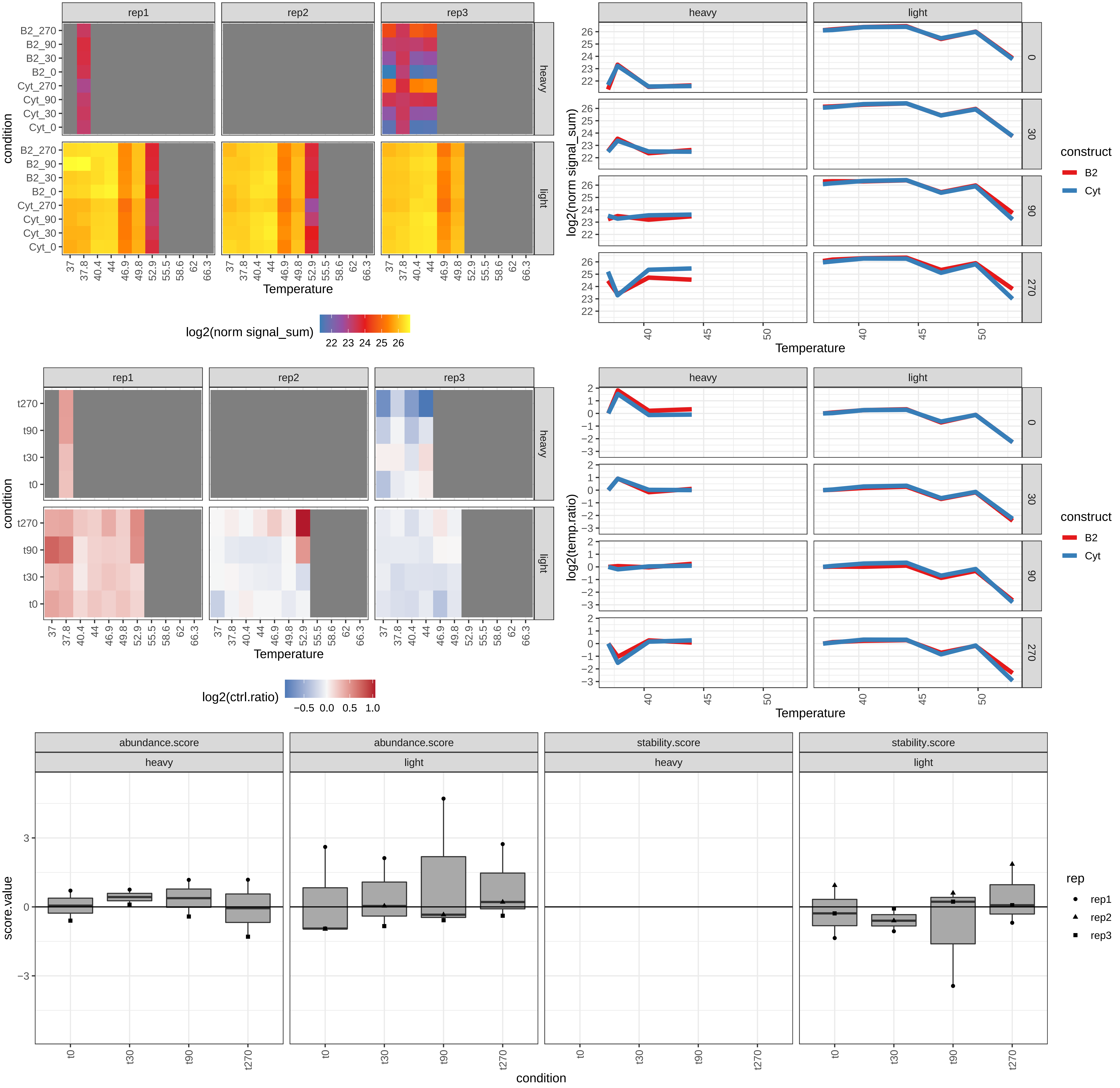

P03871 − Partitioning protein REP1 OS=Saccharomyces cerevisiae (strain ATCC 204508 / S288c) GN=REP1 PE=1 SV=1

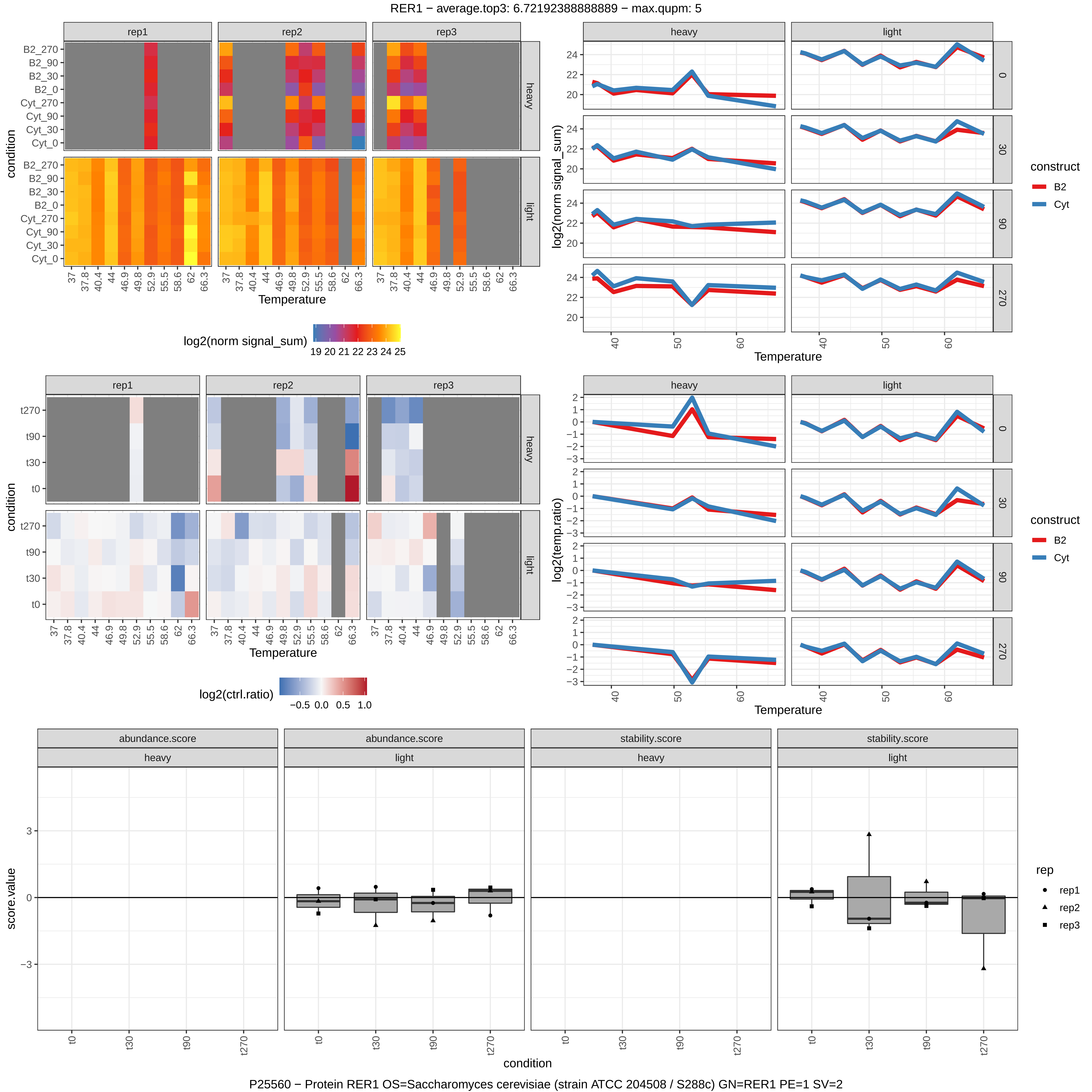

RER1 − average.top3: 6.72192388888889 − max.qupm: 5
rep1
rep2
rep3
heavy
light
condition
Temperature
log2(norm signal_sum)
construct
B2
Cyt
log2(temp.ratio)
log2(ctrl.ratio)
abundance.score
stability.score
score.value
rep
rep1
rep2
rep3
P25560 − Protein RER1 OS=Saccharomyces cerevisiae (strain ATCC 204508 / S288c) GN=RER1 PE=1 SV=2

RER2 − average.top3: 6.40742882352941 − max.qupm: 5

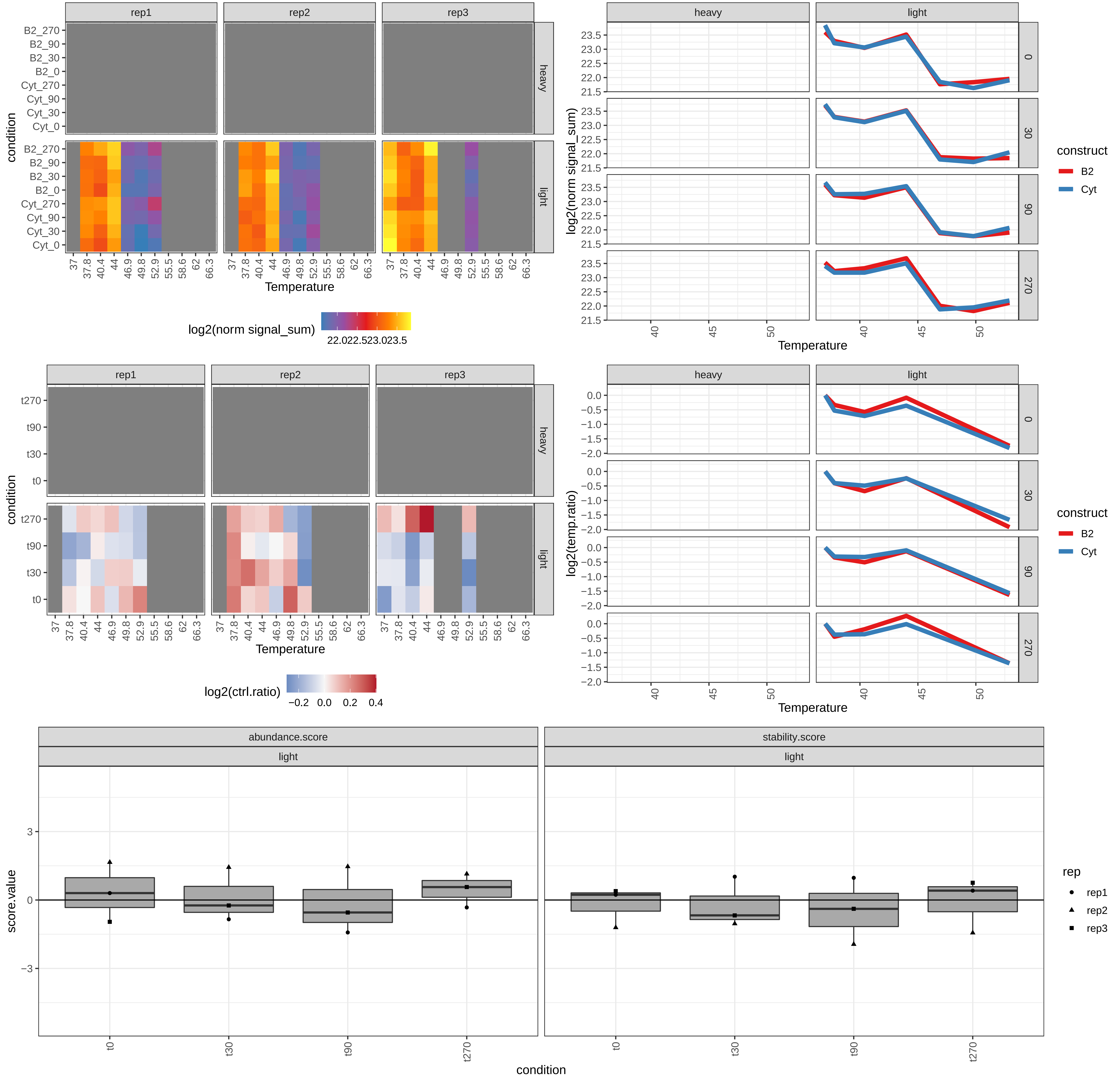

P35196 − Dehydrodolichyl diphosphate synthase complex subunit RER2 OS=Saccharomyces cerevisiae (strain ATCC 204508 / S288c) GN=RER2 PE=1 SV=1

RET1 − average.top3: 6.94587083333333 − max.qupm: 18

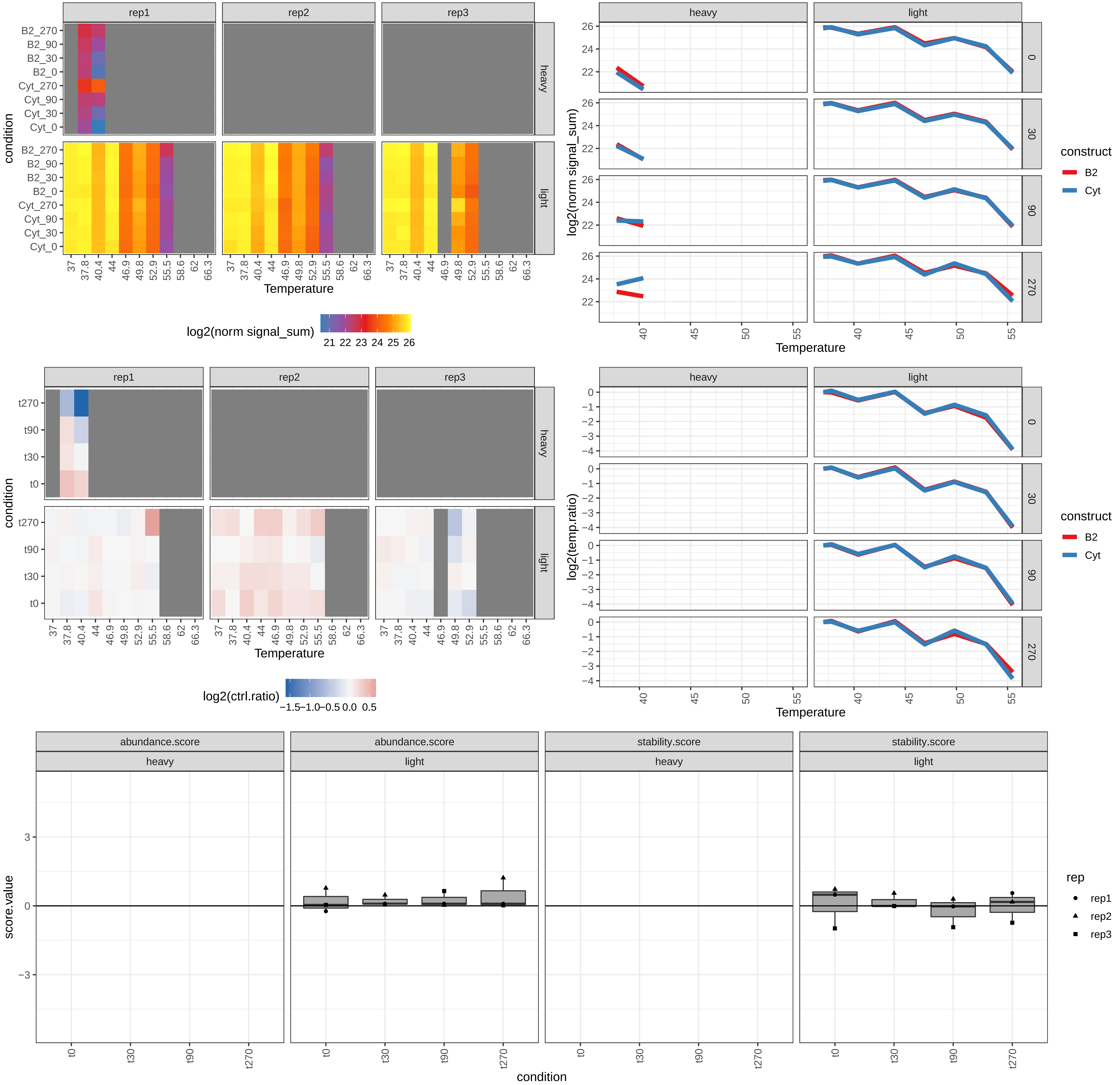

P22276 − DNA−directed RNA polymerase III subunit RPC2 OS=Saccharomyces cerevisiae (strain ATCC 204508 / S288c) GN=RET1 PE=1 SV=2

RET2 − average.top3: 7.46078254545455 − max.qupm: 14

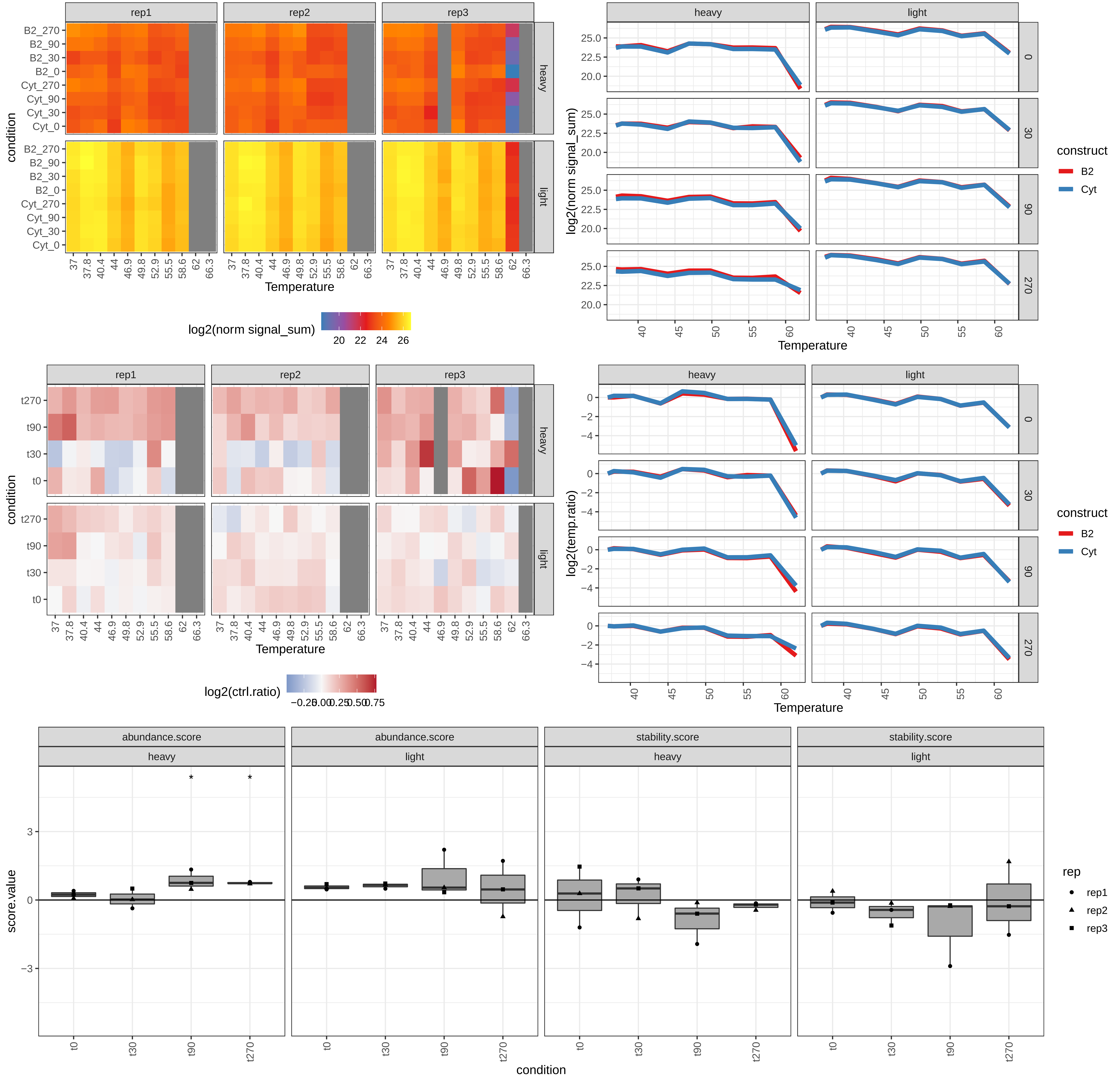

P43621 − Coatomer subunit delta OS=Saccharomyces cerevisiae (strain ATCC 204508 / S288c) GN=RET2 PE=1 SV=3

RET3 − average.top3: 6.64668740740741 − max.qupm: 2

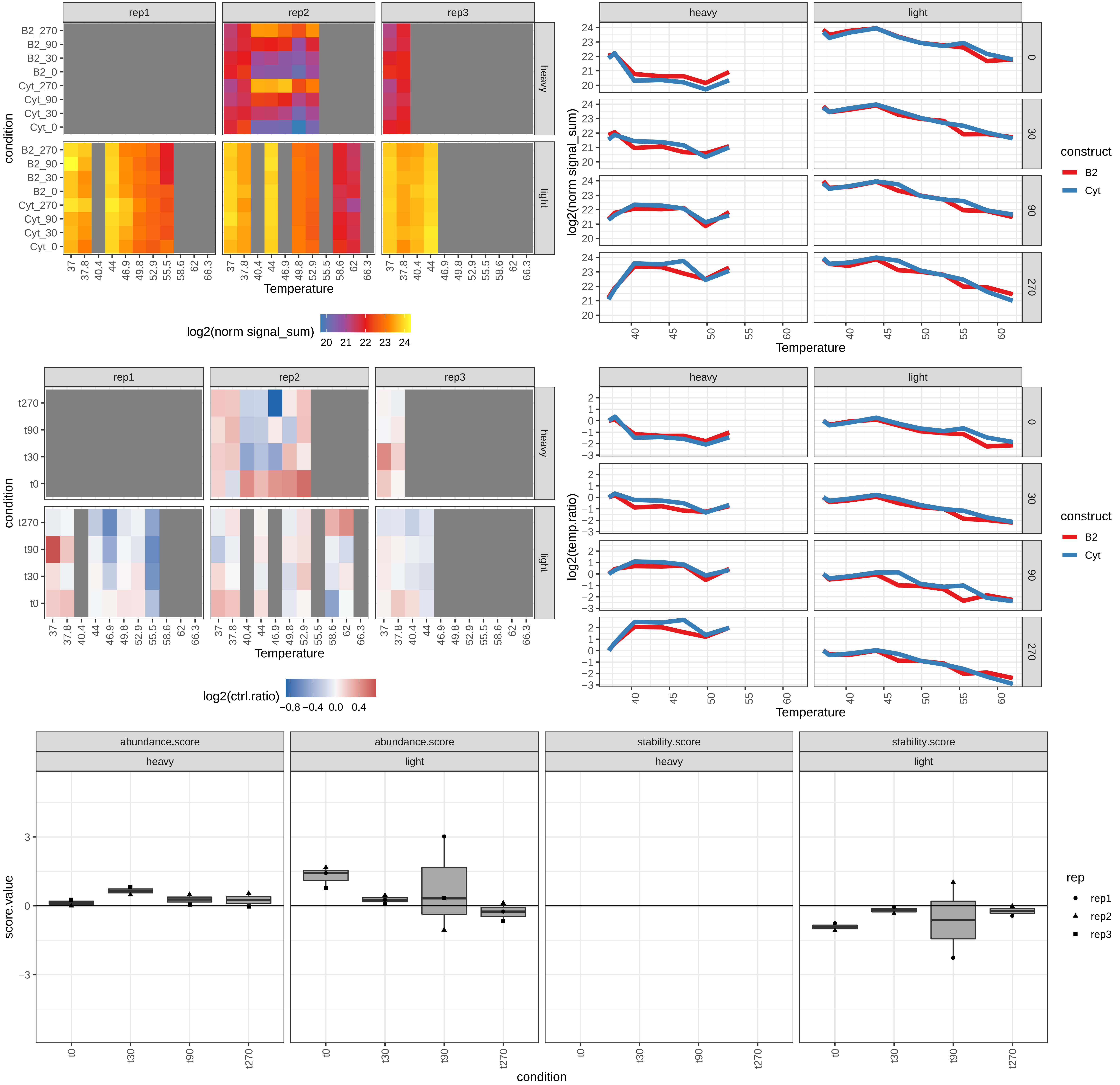

P53600 − Coatomer subunit zeta OS=Saccharomyces cerevisiae (strain ATCC 204508 / S288c) GN=RET3 PE=1 SV=1

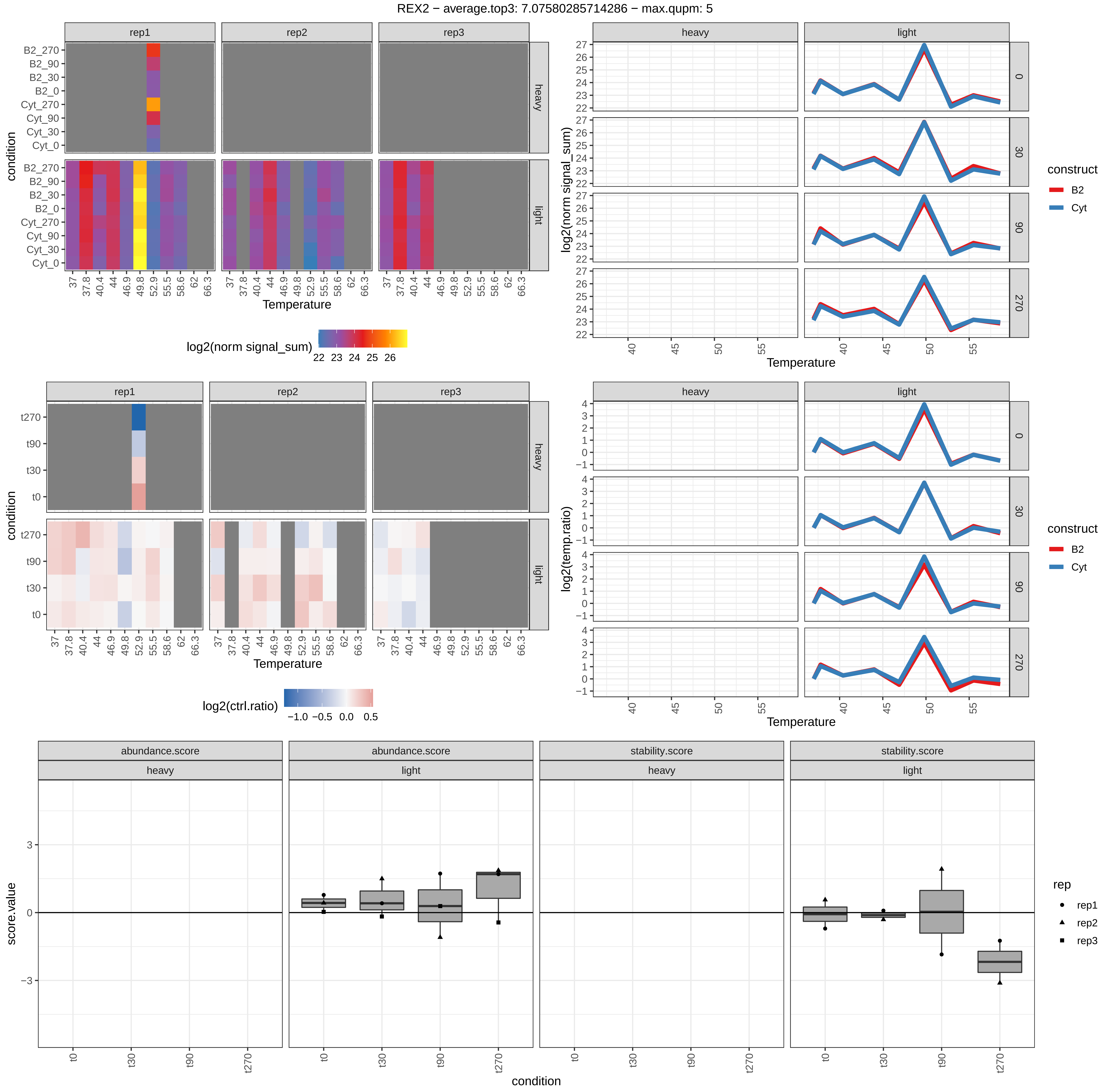

P54964 − Oligoribonuclease, mitochondrial OS=Saccharomyces cerevisiae (strain ATCC 204508 / S288c) GN=REX2 PE=1 SV=1

RFA1 − average.top3: 7.11136509803922 − max.qupm: 18

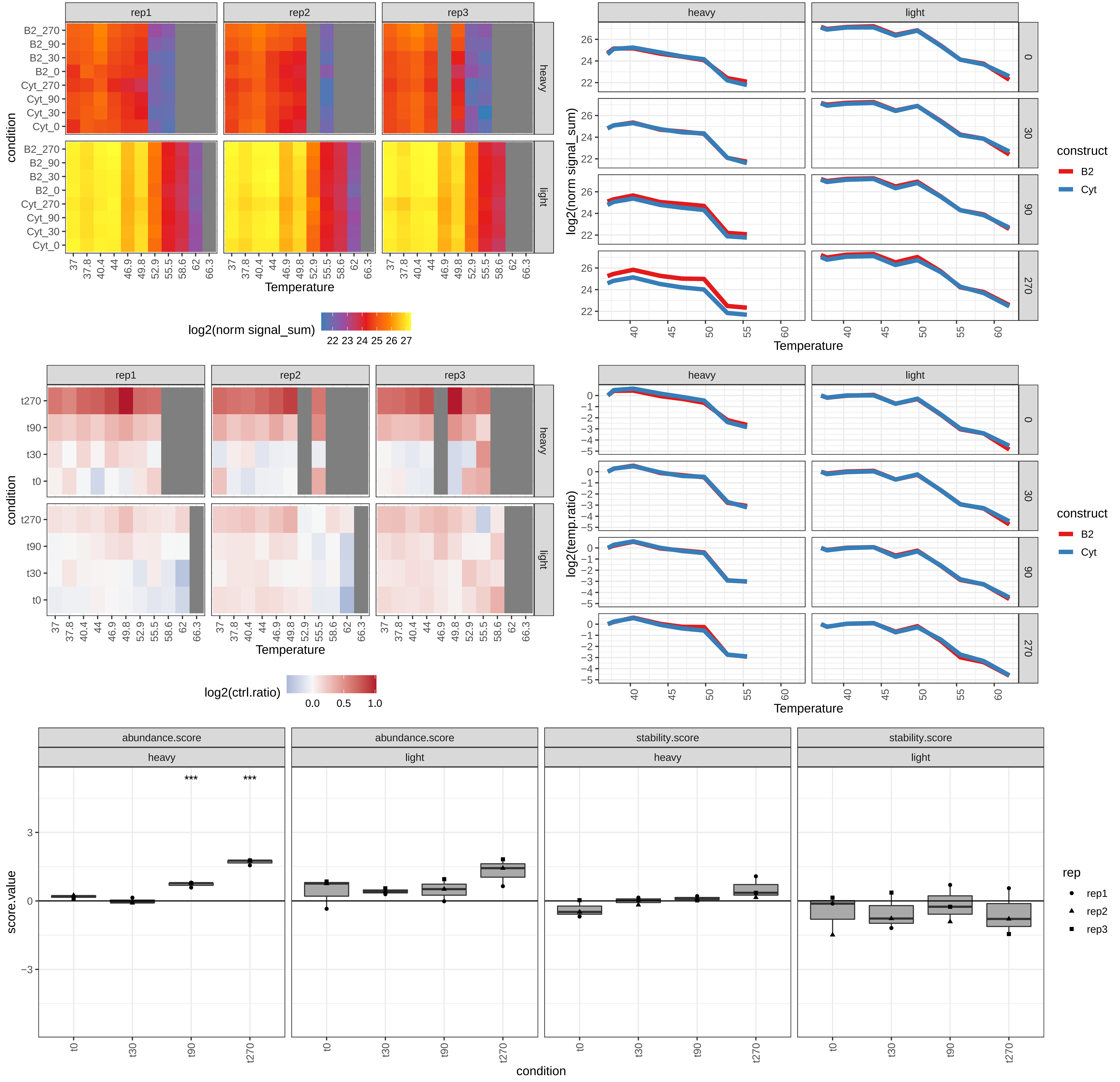

P22336 − Replication factor A protein 1 OS=Saccharomyces cerevisiae (strain ATCC 204508 / S288c) GN=RFA1 PE=1 SV=1

RFA2 − average.top3: 6.66889533333333 − max.qupm: 5

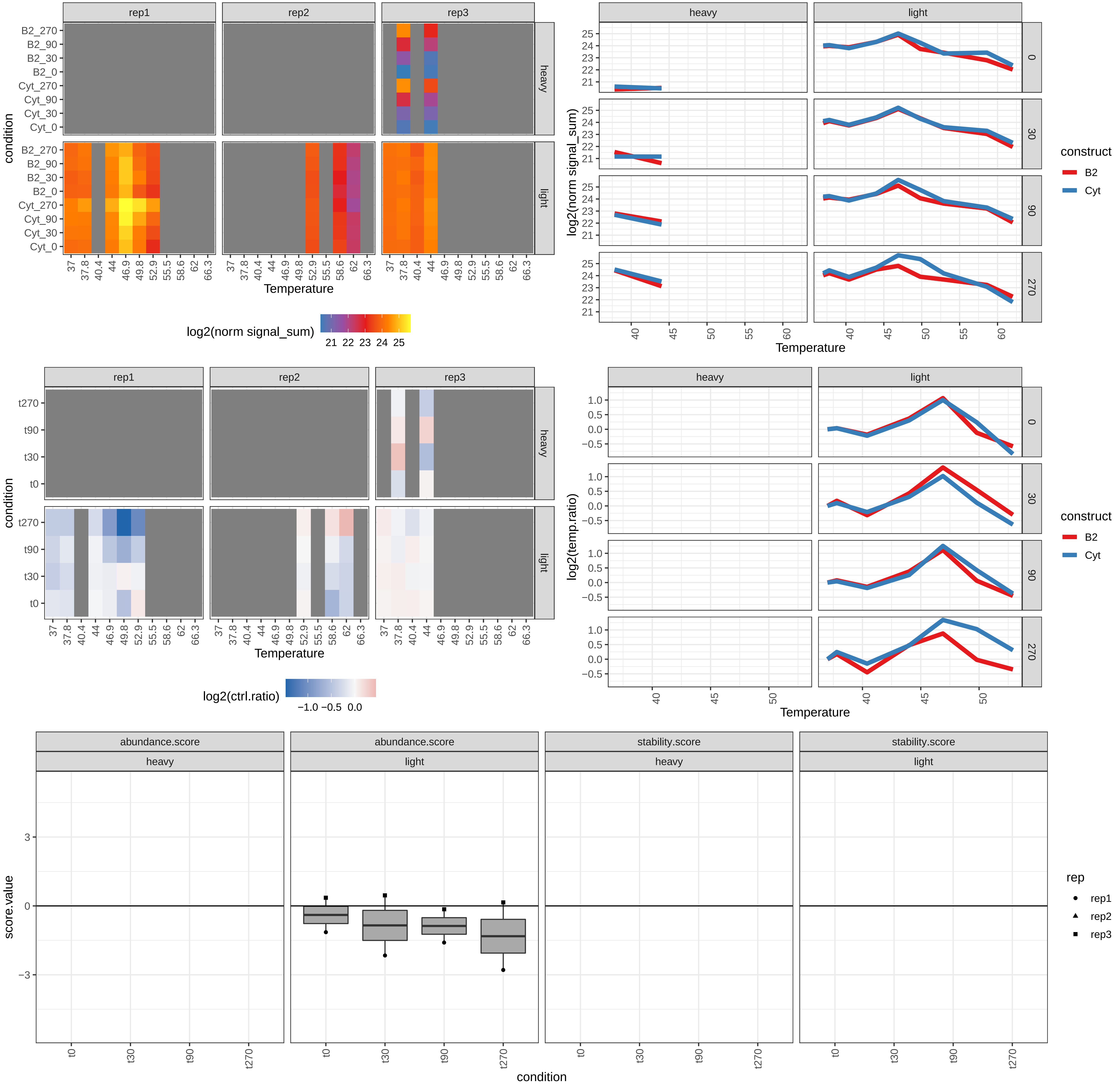

P26754 − Replication factor A protein 2 OS=Saccharomyces cerevisiae (strain ATCC 204508 / S288c) GN=RFA2 PE=1 SV=1

RFC1 − average.top3: 6.93153882352941 − max.qupm: 9

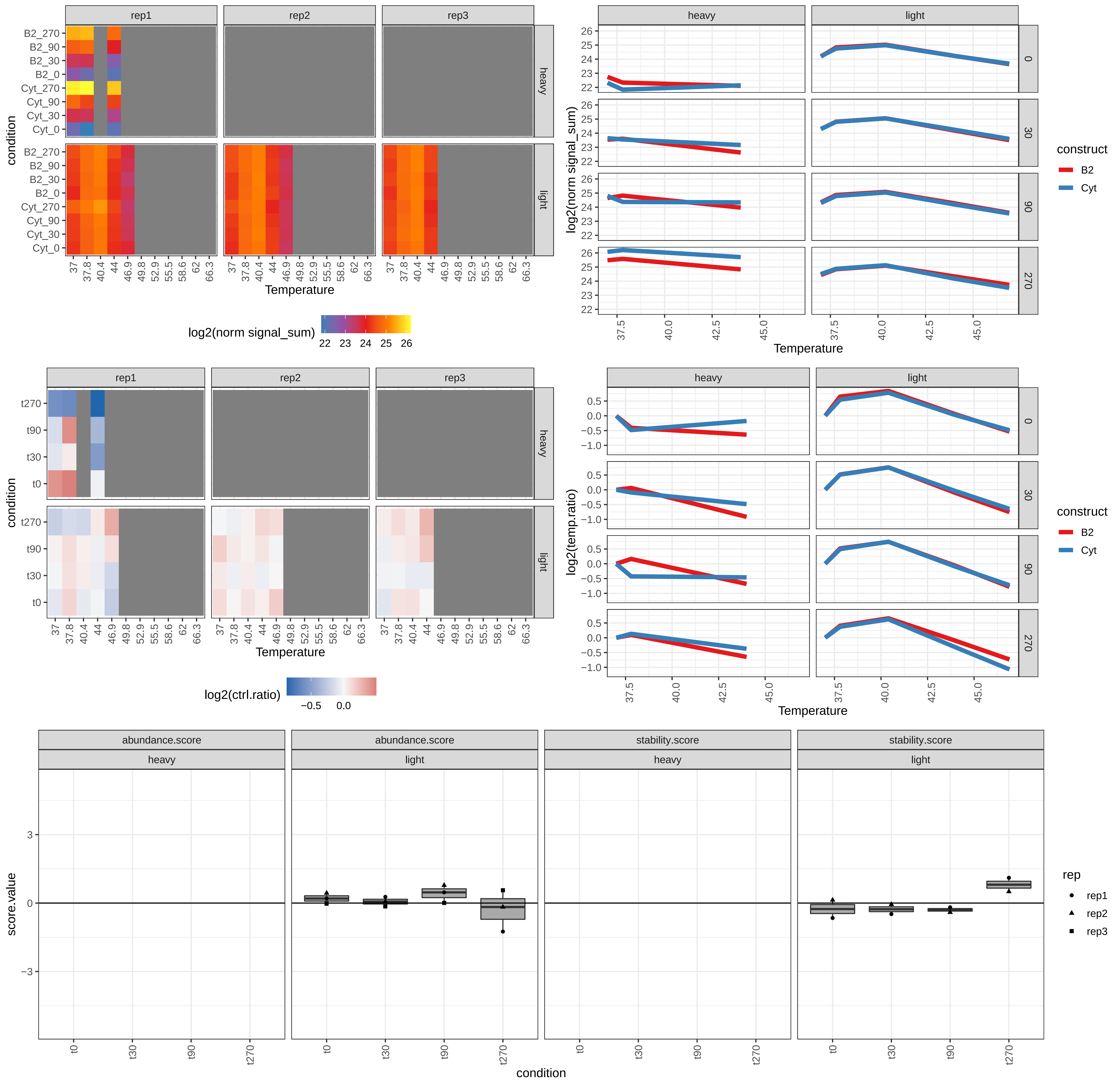

P38630 − Replication factor C subunit 1 OS=Saccharomyces cerevisiae (strain ATCC 204508 / S288c) GN=RFC1 PE=1 SV=1

RFC2 − average.top3: 6.9540948 − max.qupm: 12

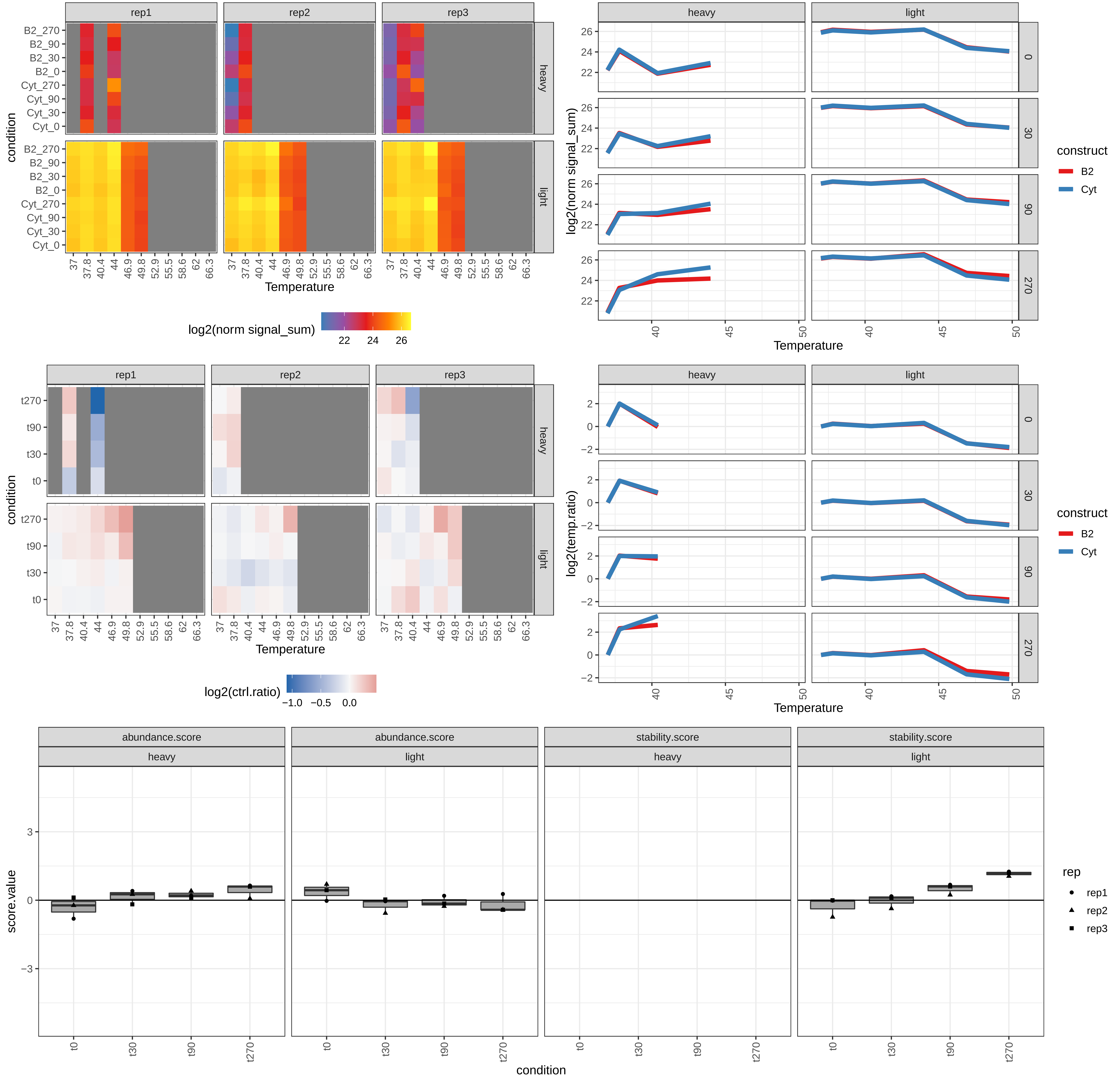

P40348 − Replication factor C subunit 2 OS=Saccharomyces cerevisiae (strain ATCC 204508 / S288c) GN=RFC2 PE=1 SV=1

RFC3 − average.top3: 6.86435608695652 − max.qupm: 10

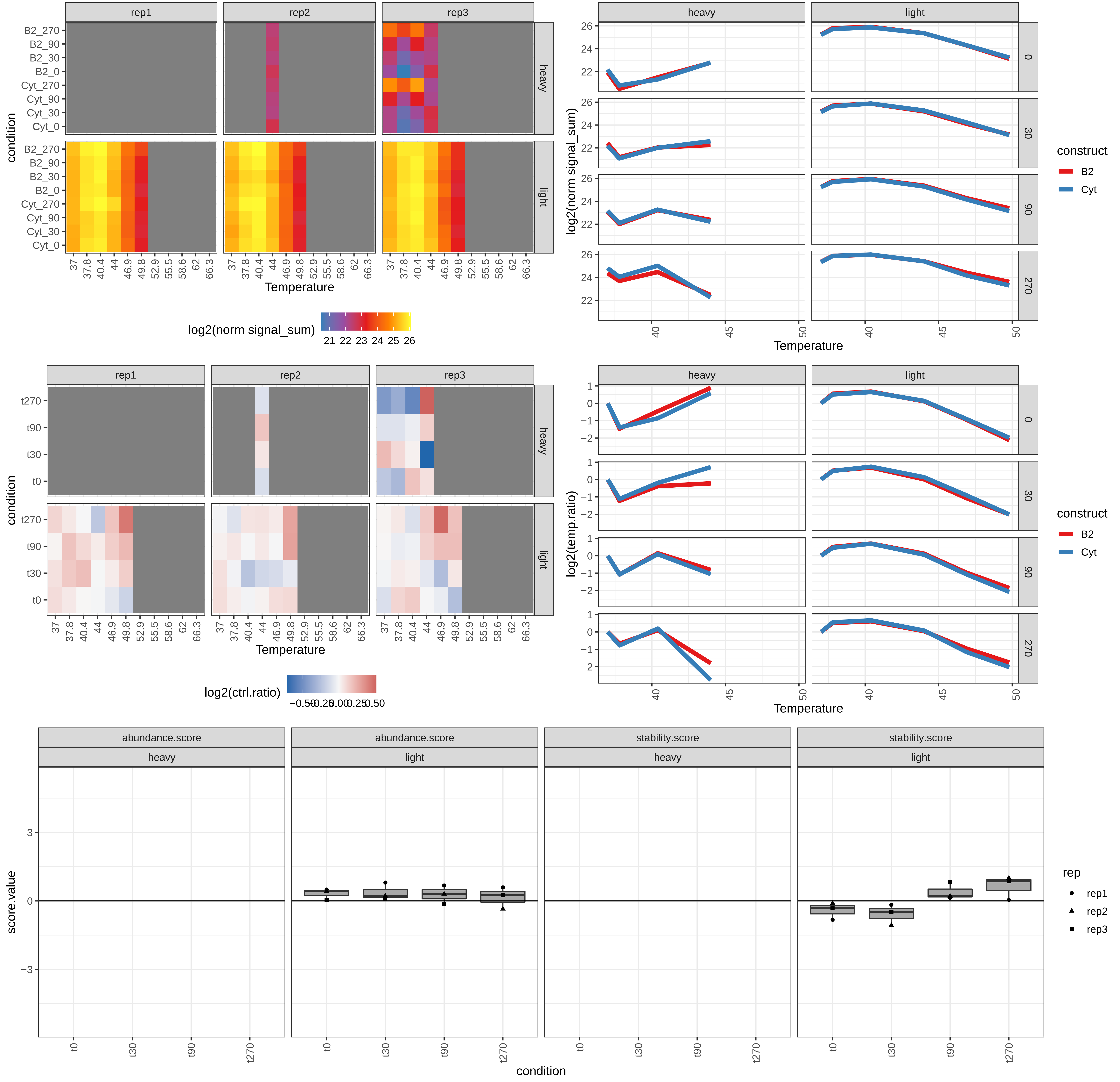

P38629 − Replication factor C subunit 3 OS=Saccharomyces cerevisiae (strain ATCC 204508 / S288c) GN=RFC3 PE=1 SV=1

RFC4 − average.top3: 6.78430571428571 − max.qupm: 9

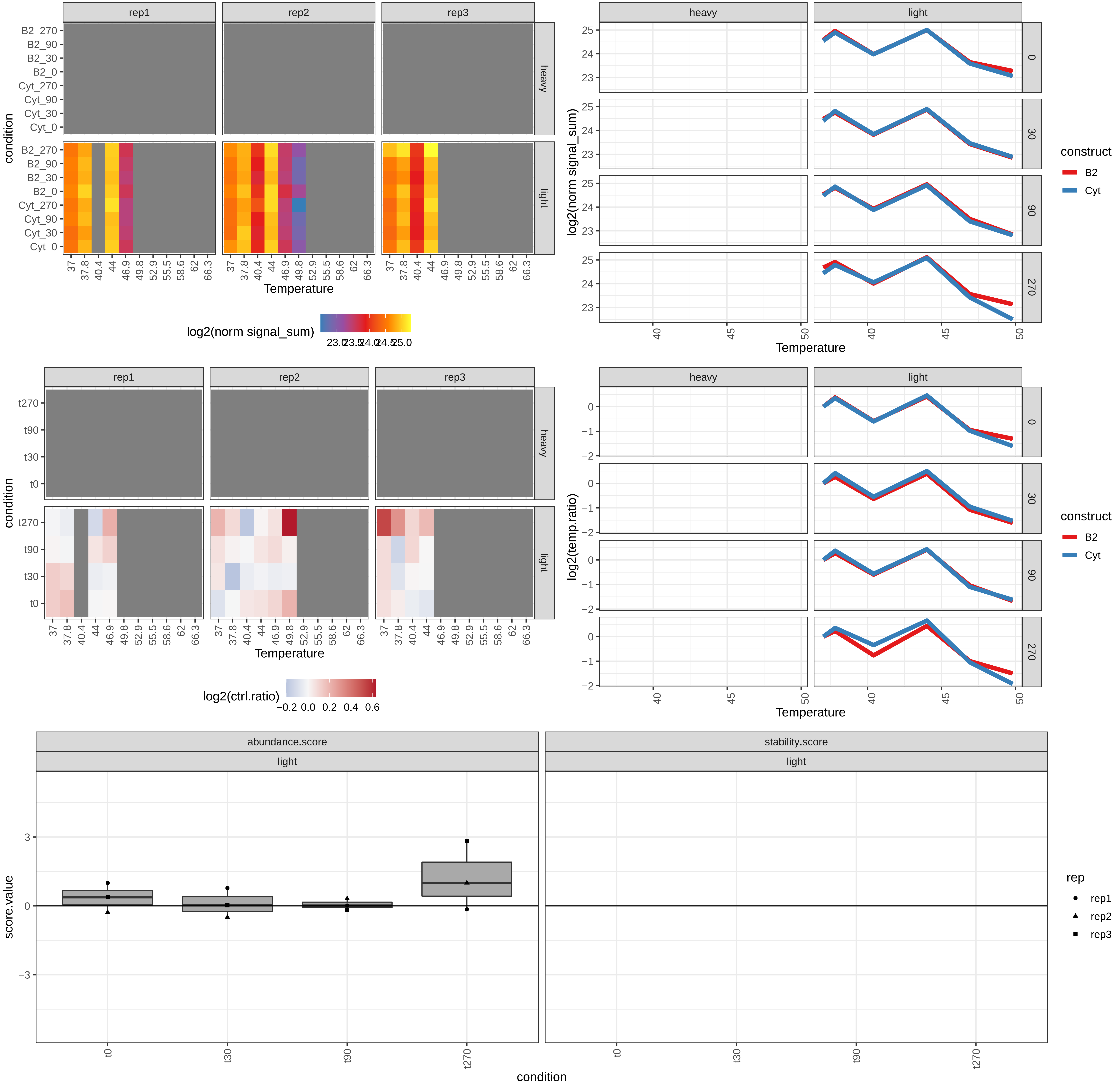

P40339 − Replication factor C subunit 4 OS=Saccharomyces cerevisiae (strain ATCC 204508 / S288c) GN=RFC4 PE=1 SV=1

RFC5 − average.top3: 6.88432 − max.qupm: 9

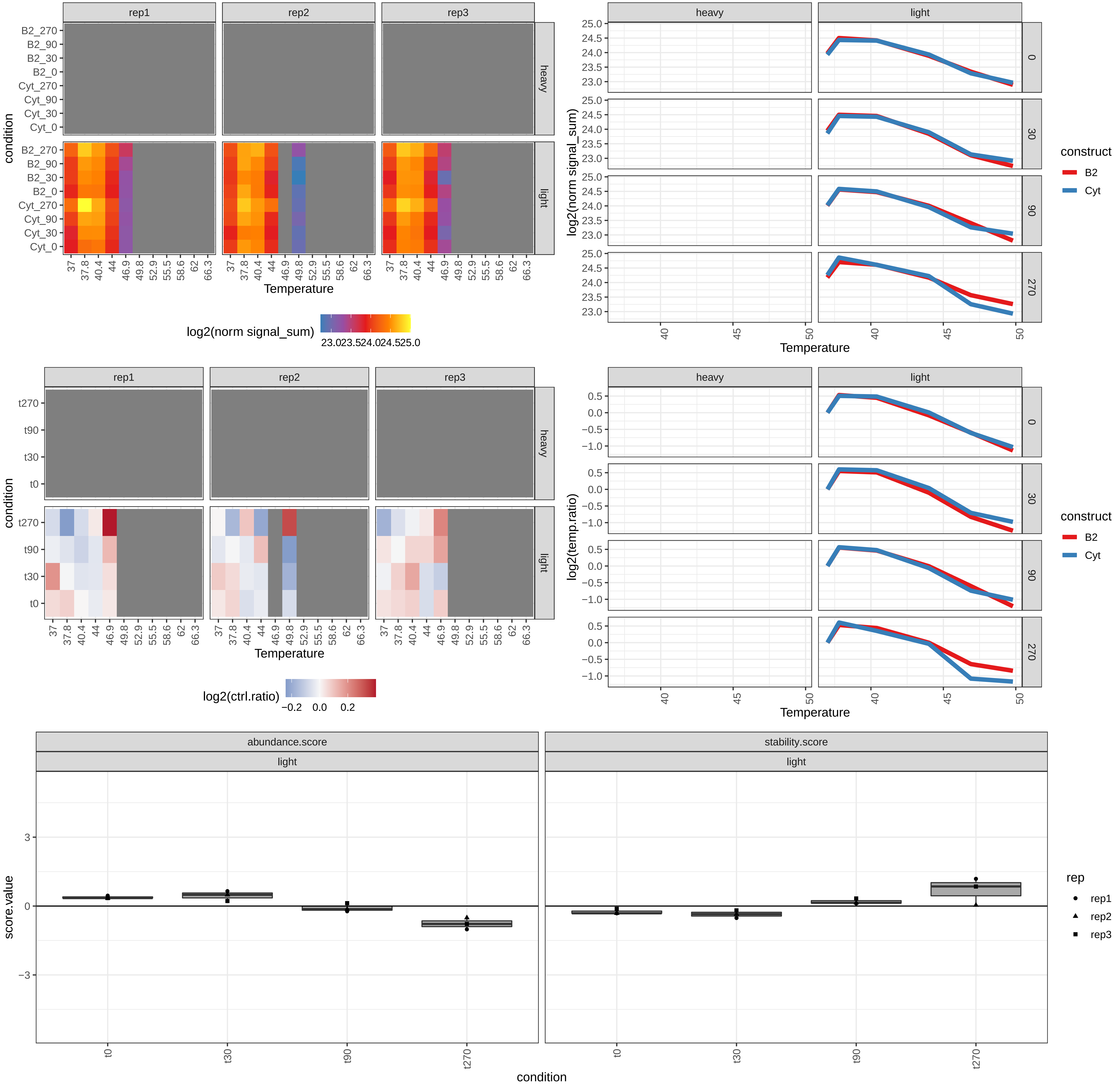

P38251 − Replication factor C subunit 5 OS=Saccharomyces cerevisiae (strain ATCC 204508 / S288c) GN=RFC5 PE=1 SV=1

RFS1 − average.top3: 6.78291227272727 − max.qupm: 4

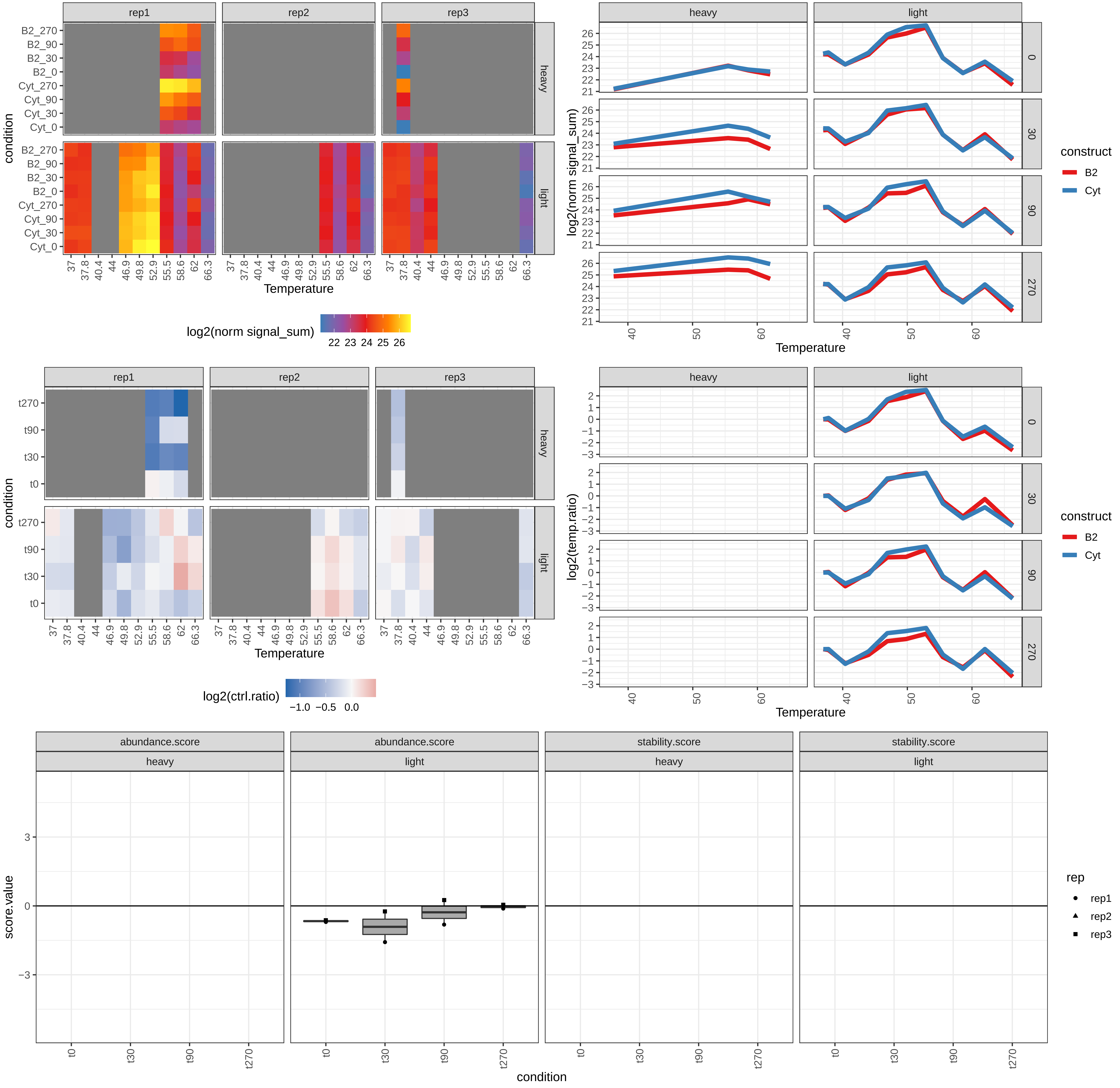

P38234 − Protein RFS1 OS=Saccharomyces cerevisiae (strain ATCC 204508 / S288c) GN=RFS1 PE=1 SV=1

RFT1 − average.top3: 6.51064222222222 − max.qupm: 5

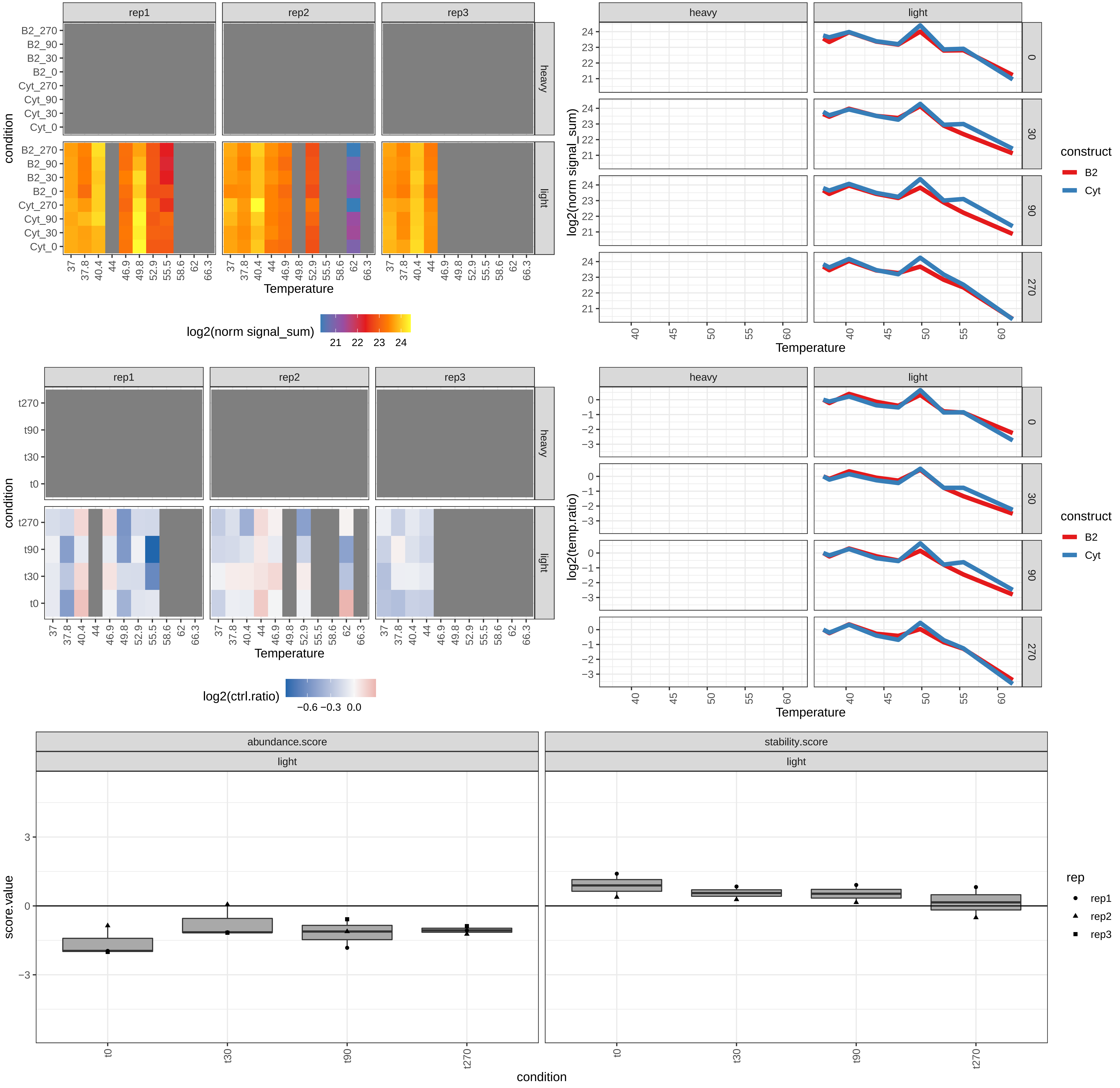

P38206 − Oligosaccharide translocation protein RFT1 OS=Saccharomyces cerevisiae (strain ATCC 204508 / S288c) GN=RFT1 PE=1 SV=1

RGA2 − average.top3: 6.23257066666667 − max.qupm: 3

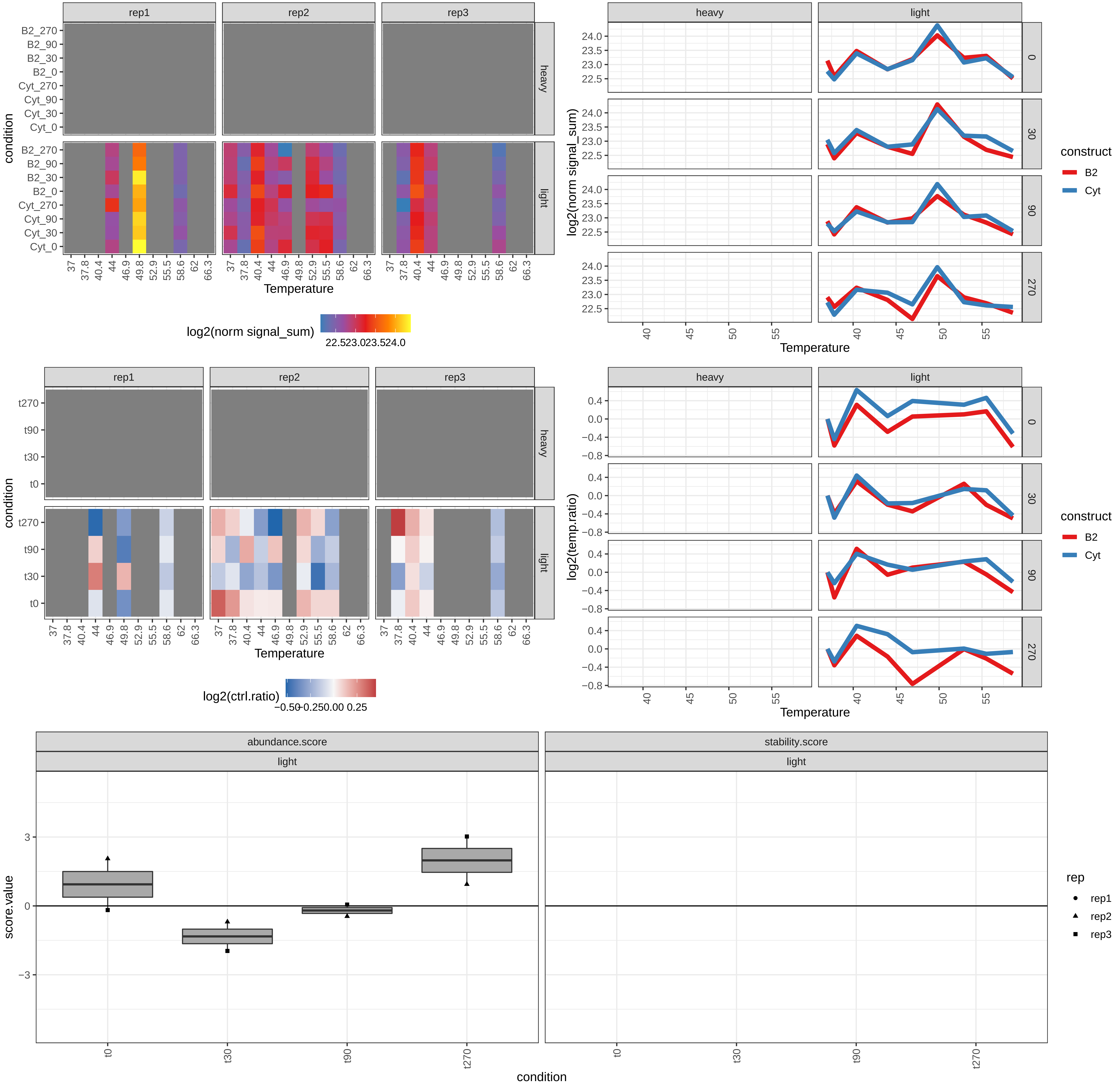

Q06407 − Rho−type GTPase−activating protein 2 OS=Saccharomyces cerevisiae (strain ATCC 204508 / S288c) GN=RGA2 PE=1 SV=1

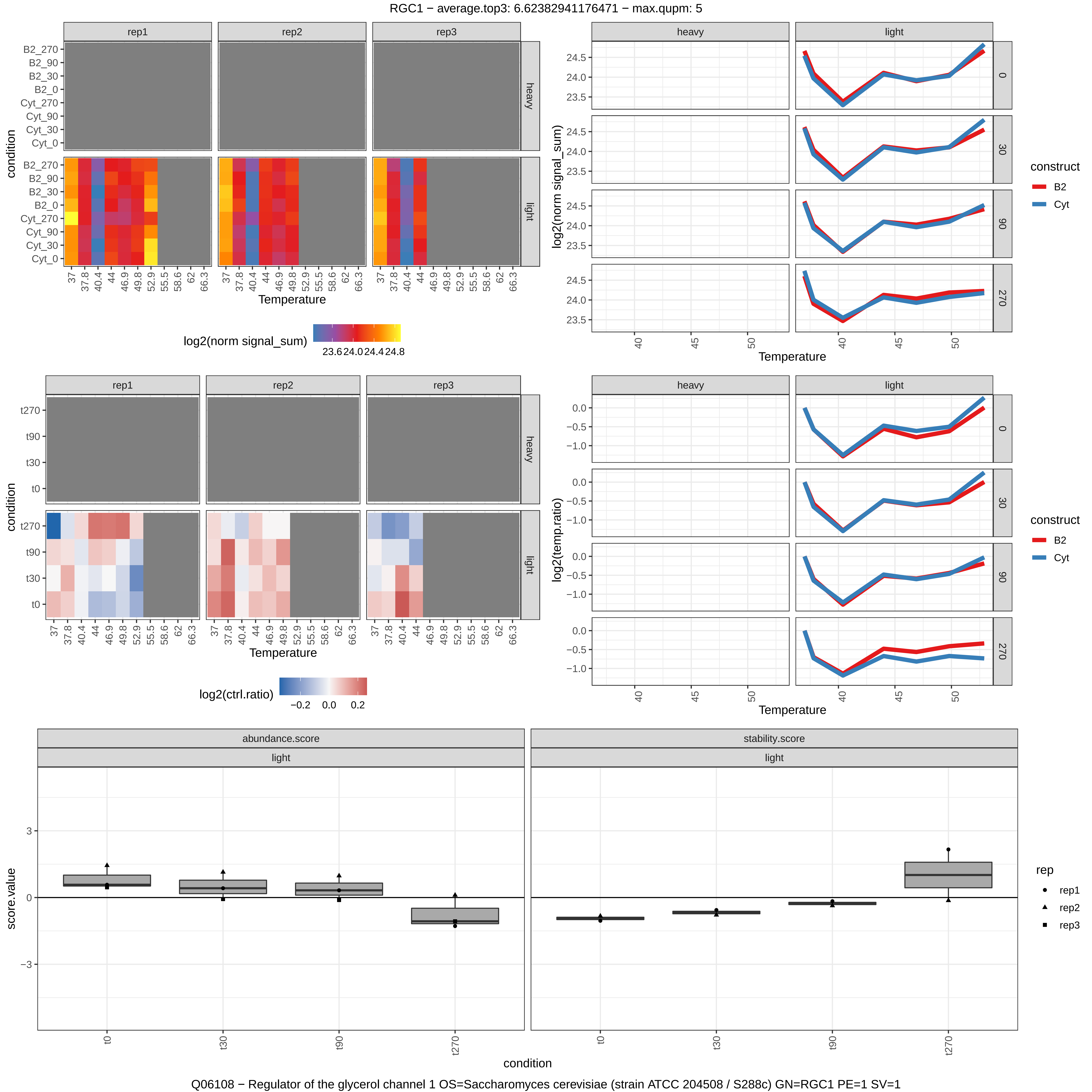
RGC1 − average.top3: 6.62382941176471 − max.qupm: 5
rep1
rep2
rep3
heavy
light
condition
Temperature
log2(norm signal_sum)
23.6 24.0 24.4 24.8
log2(norm signal_sum)
construct
B2
Cyt
log2(ctrl.ratio)
−0.2 0.0 0.2
log2(temp.ratio)
abundance.score
stability.score
score.value
rep
rep1
rep2
rep3
Q06108 − Regulator of the glycerol channel 1 OS=Saccharomyces cerevisiae (strain ATCC 204508 / S288c) GN=RGC1 PE=1 SV=1

RGD1 − average.top3: 6.90525444444444 − max.qupm: 7

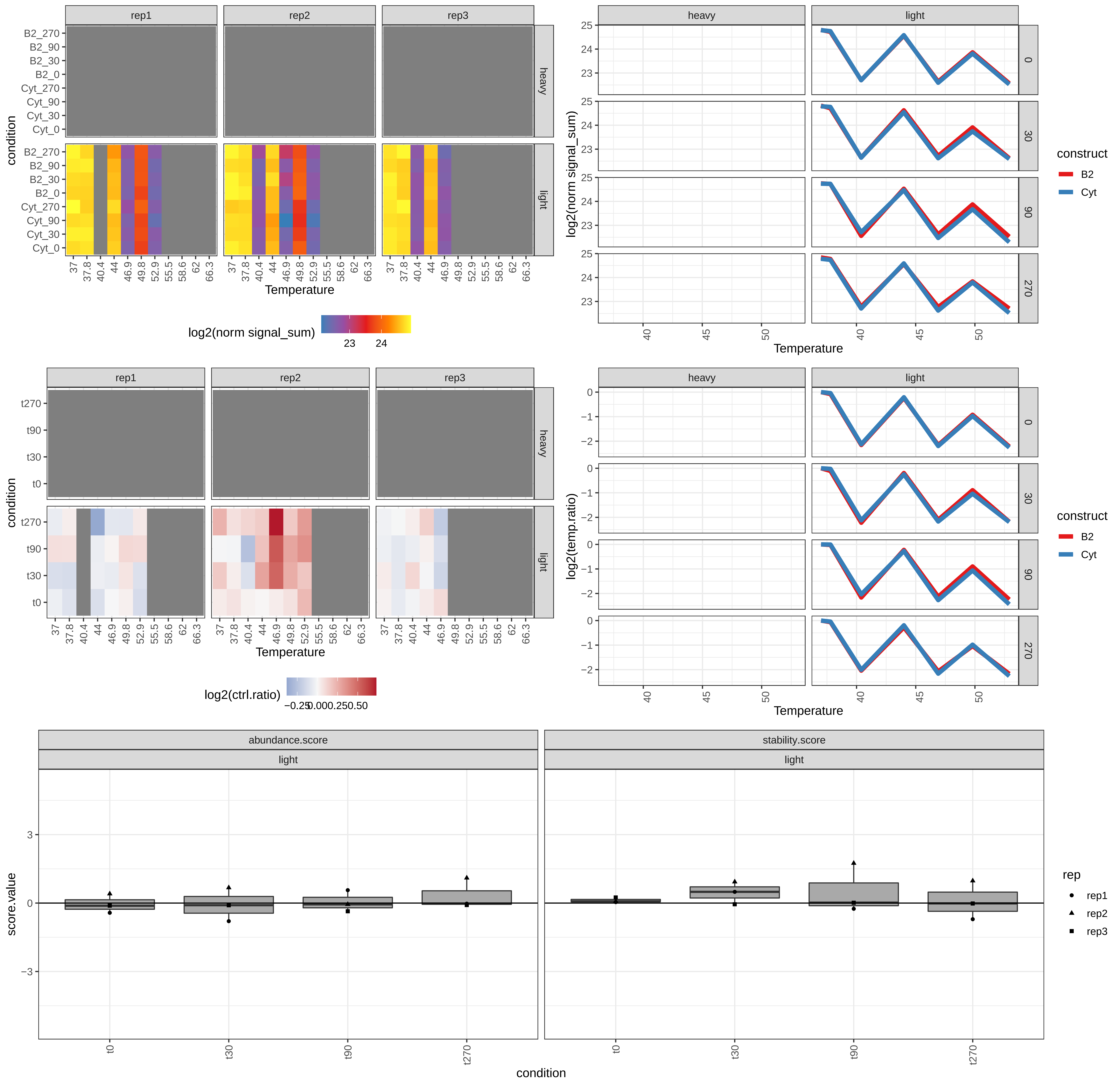

P38339 − RHO GTPase−activating protein RGD1 OS=Saccharomyces cerevisiae (strain ATCC 204508 / S288c) GN=RGD1 PE=1 SV=1

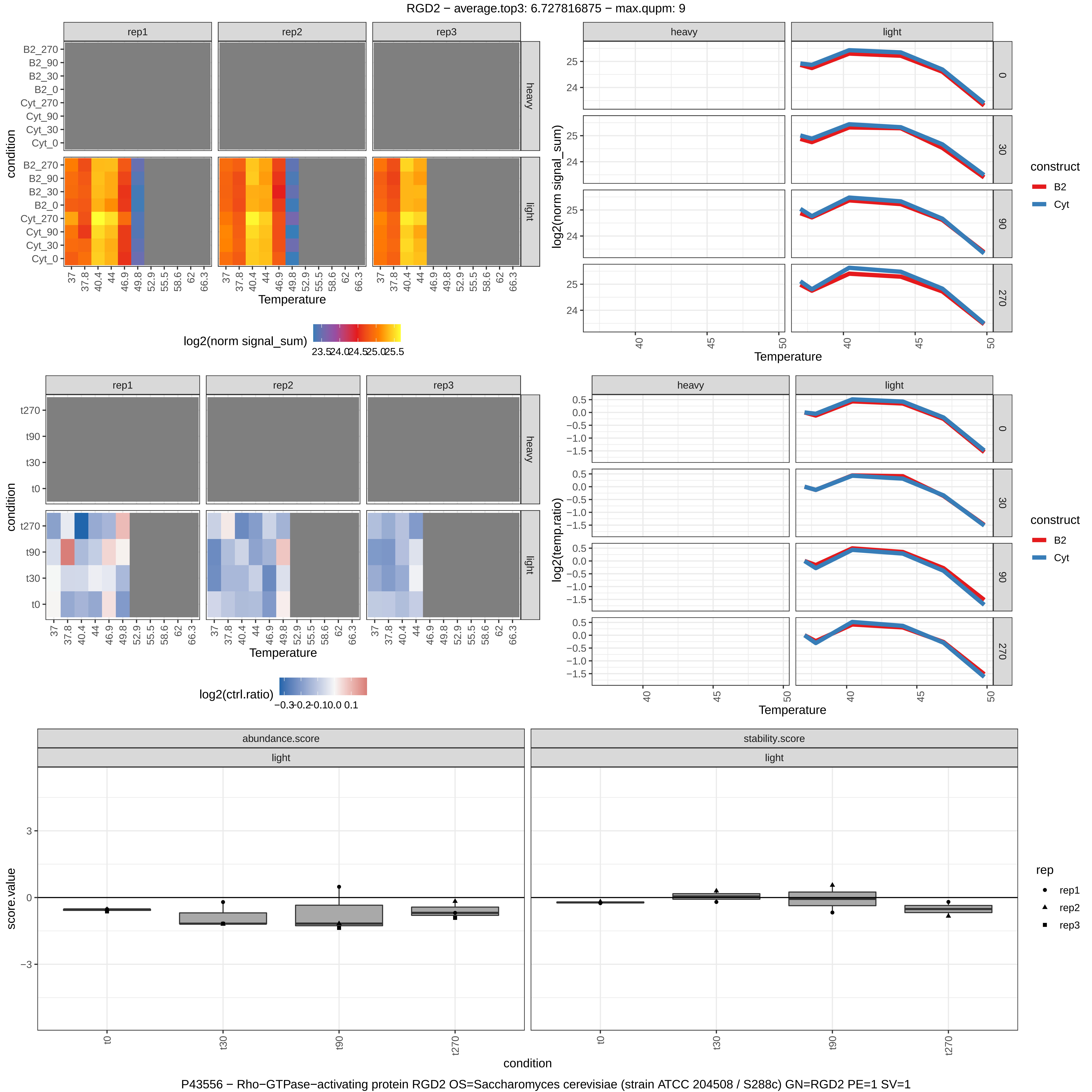

RGD2 − average.top3: 6.727816875 − max.qupm: 9
rep1
rep2
rep3
heavy
light
condition
B2_270
B2_90
B2_30
B2_0
Cyt_270
Cyt_90
Cyt_30
Cyt_0
Temperature
log2(norm signal_sum)
23.5 24.0 24.5 25.0 25.5
construct
B2
Cyt
t270
t90
t30
t0
log2(temp.ratio)
log2(ctrl.ratio)
−0.3 −0.2 −0.1 0.0 0.1
abundance.score
stability.score
score.value
rep
rep1
rep2
rep3
P43556 − Rho−GTPase−activating protein RGD2 OS=Saccharomyces cerevisiae (strain ATCC 204508 / S288c) GN=RGD2 PE=1 SV=1

RGI1 − average.top3: 7.07854214285714 − max.qupm: 5

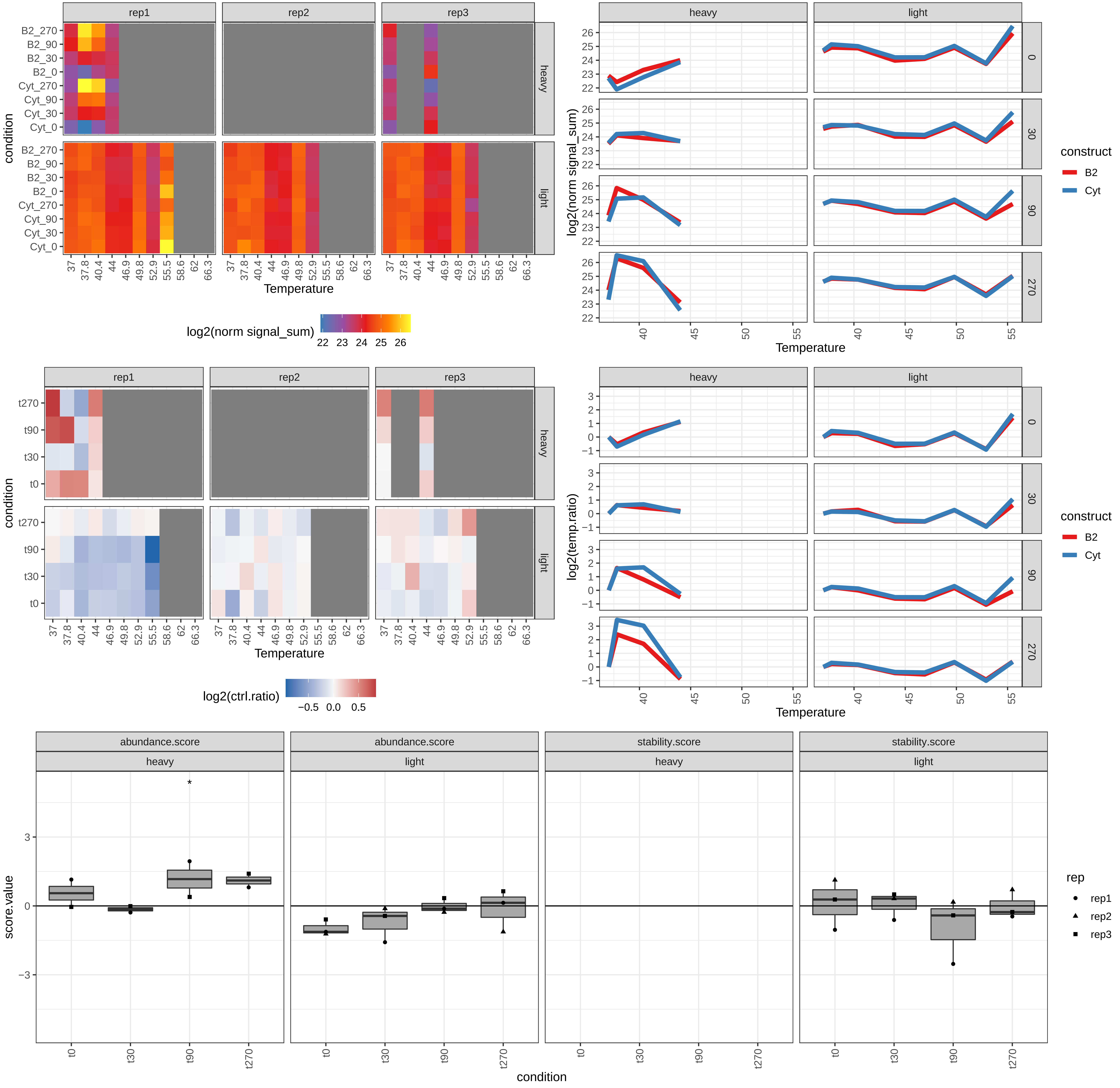

P40043 − Respiratory growth induced protein 1 OS=Saccharomyces cerevisiae (strain ATCC 204508 / S288c) GN=RGI1 PE=1 SV=1

RGI2 − average.top3: 6.73415210526316 − max.qupm: 5

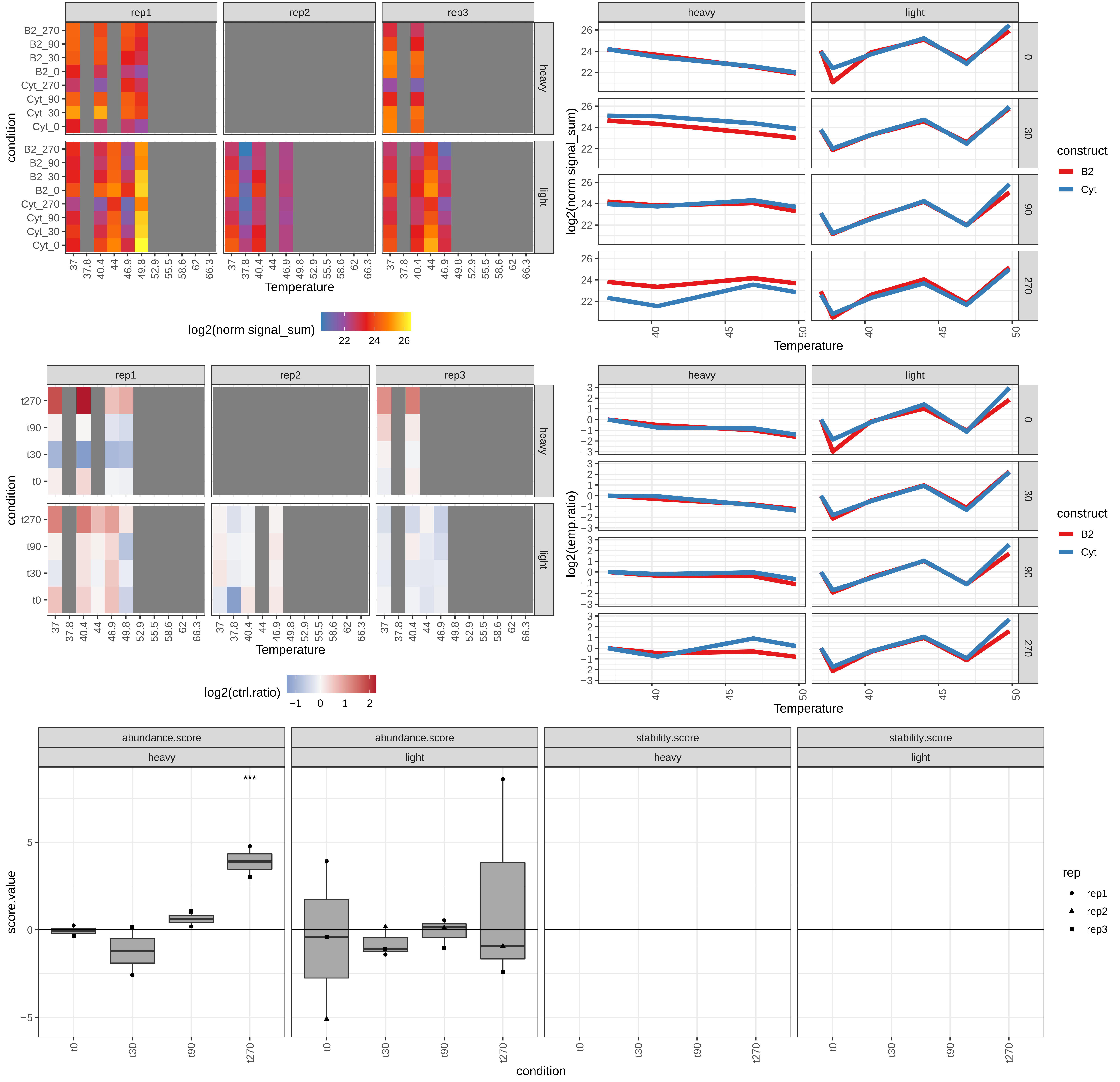

P40188 − Respiratory growth induced protein 2 OS=Saccharomyces cerevisiae (strain ATCC 204508 / S288c) GN=RGI2 PE=1 SV=1

RGR1 − average.top3: 6.83276866666667 − max.qupm: 6

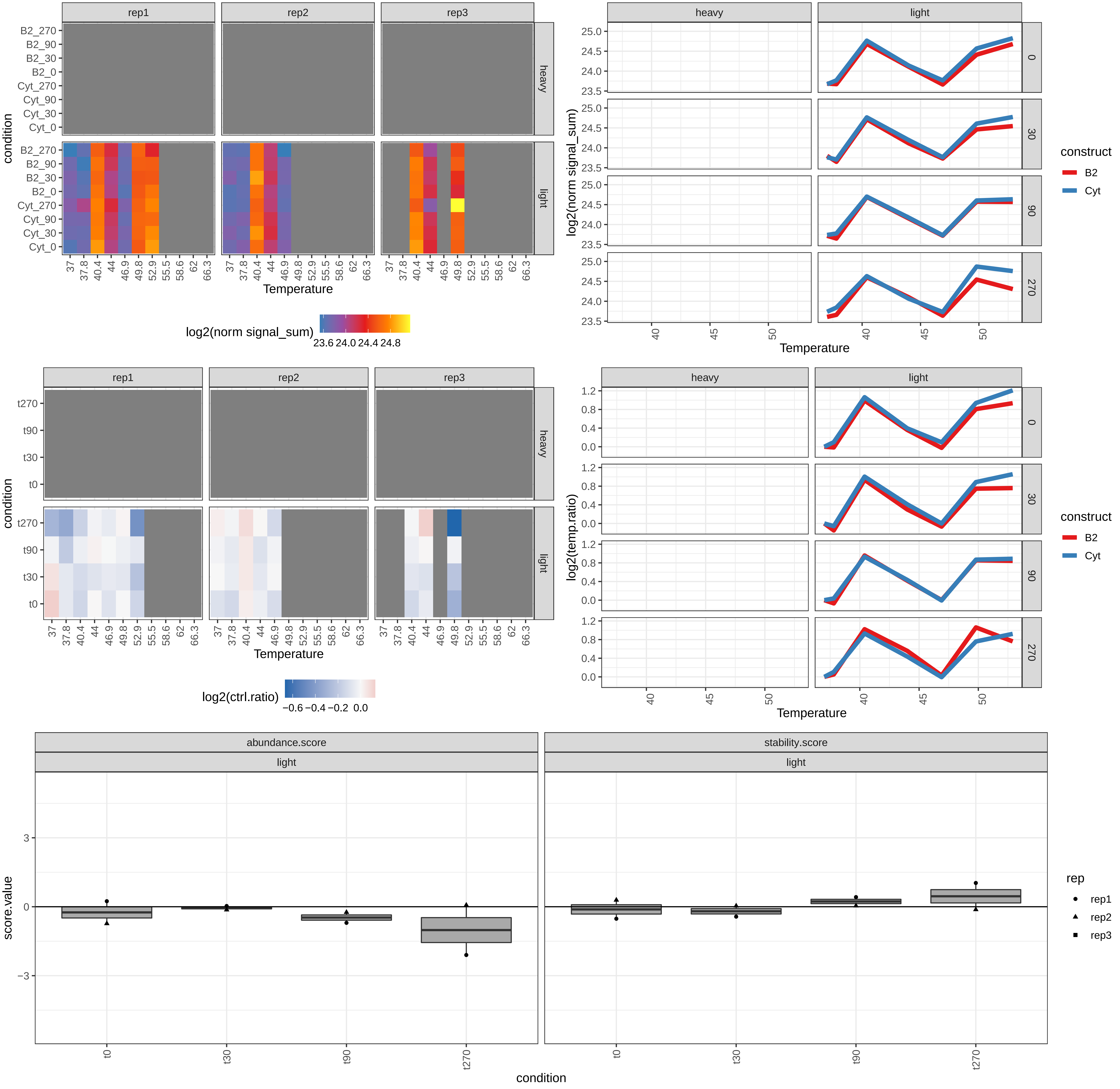

P19263 − Mediator of RNA polymerase II transcription subunit 14 OS=Saccharomyces cerevisiae (strain ATCC 204508 / S288c) GN=RGR1 PE=1 SV=2

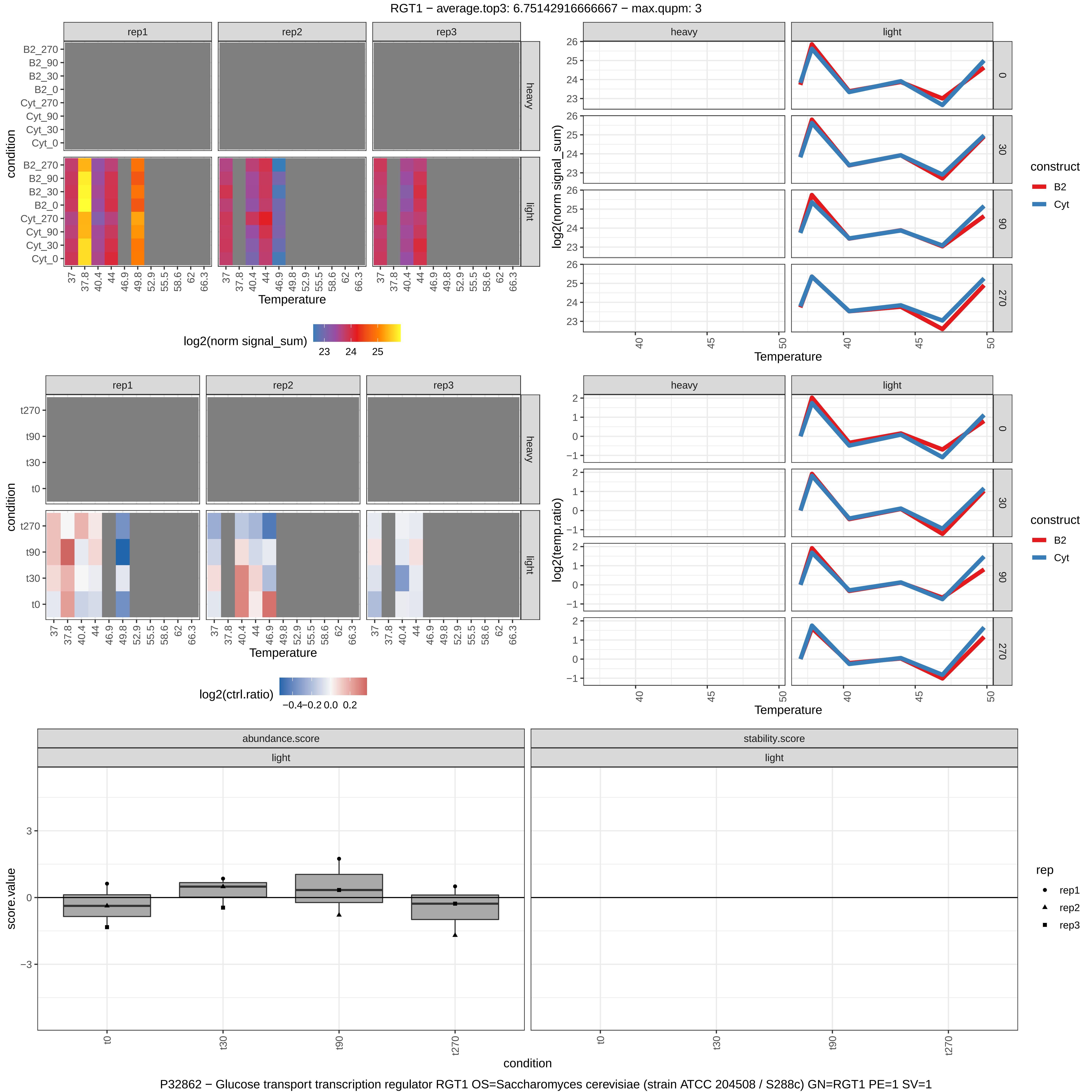
RGT1 − average.top3: 6.75142916666667 − max.qupm: 3
rep1
rep2
rep3
heavy
light
condition
Temperature
log2(norm signal_sum)
log2(ctrl.ratio)
log2(temp.ratio)
construct
B2
Cyt
abundance.score
stability.score
score.value
rep
P32862 − Glucose transport transcription regulator RGT1 OS=Saccharomyces cerevisiae (strain ATCC 204508 / S288c) GN=RGT1 PE=1 SV=1

RHO1 − average.top3: 7.47388072727273 − max.qupm: 6

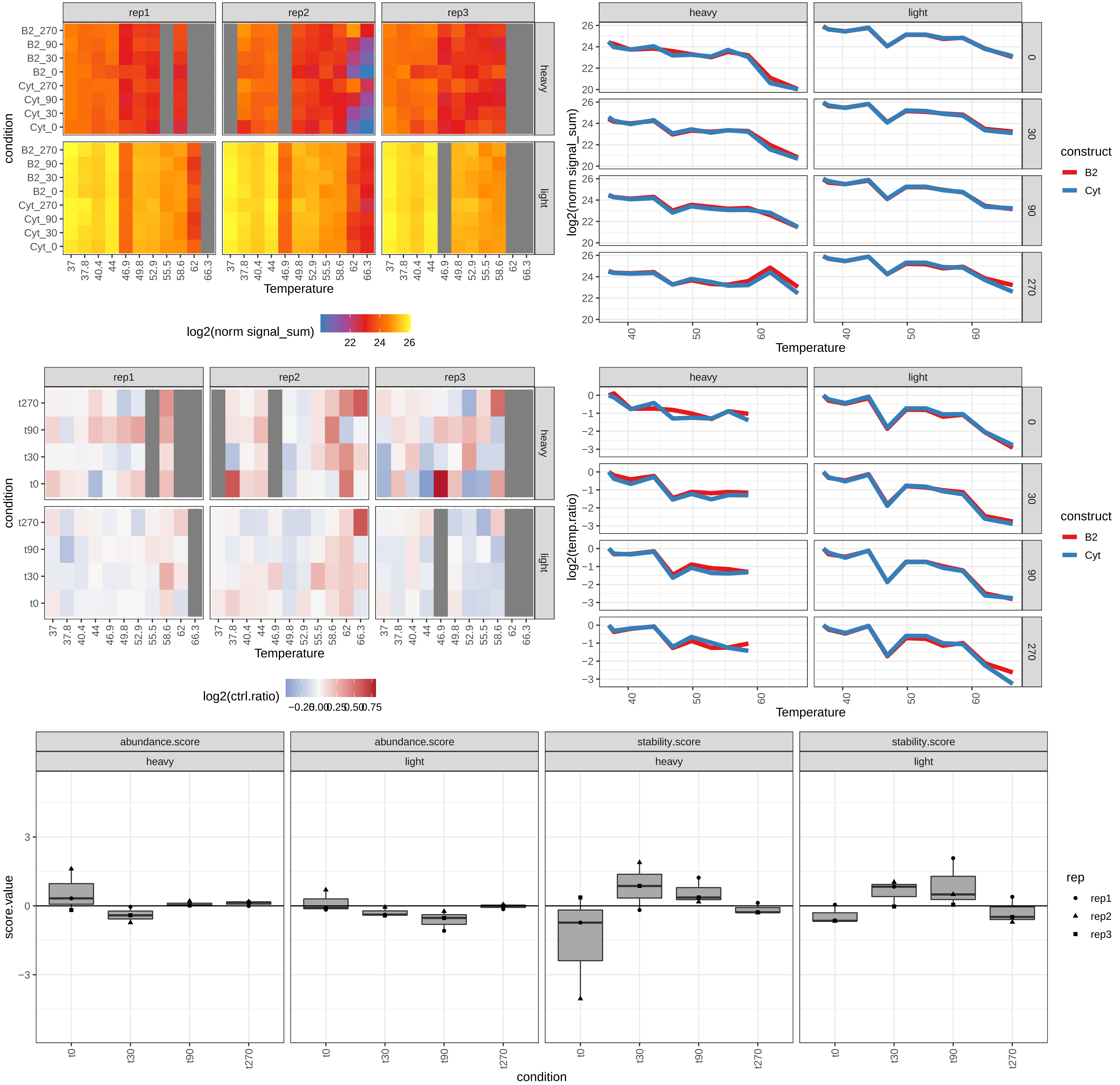

P06780 − GTP−binding protein RHO1 OS=Saccharomyces cerevisiae (strain ATCC 204508 / S288c) GN=RHO1 PE=1 SV=3

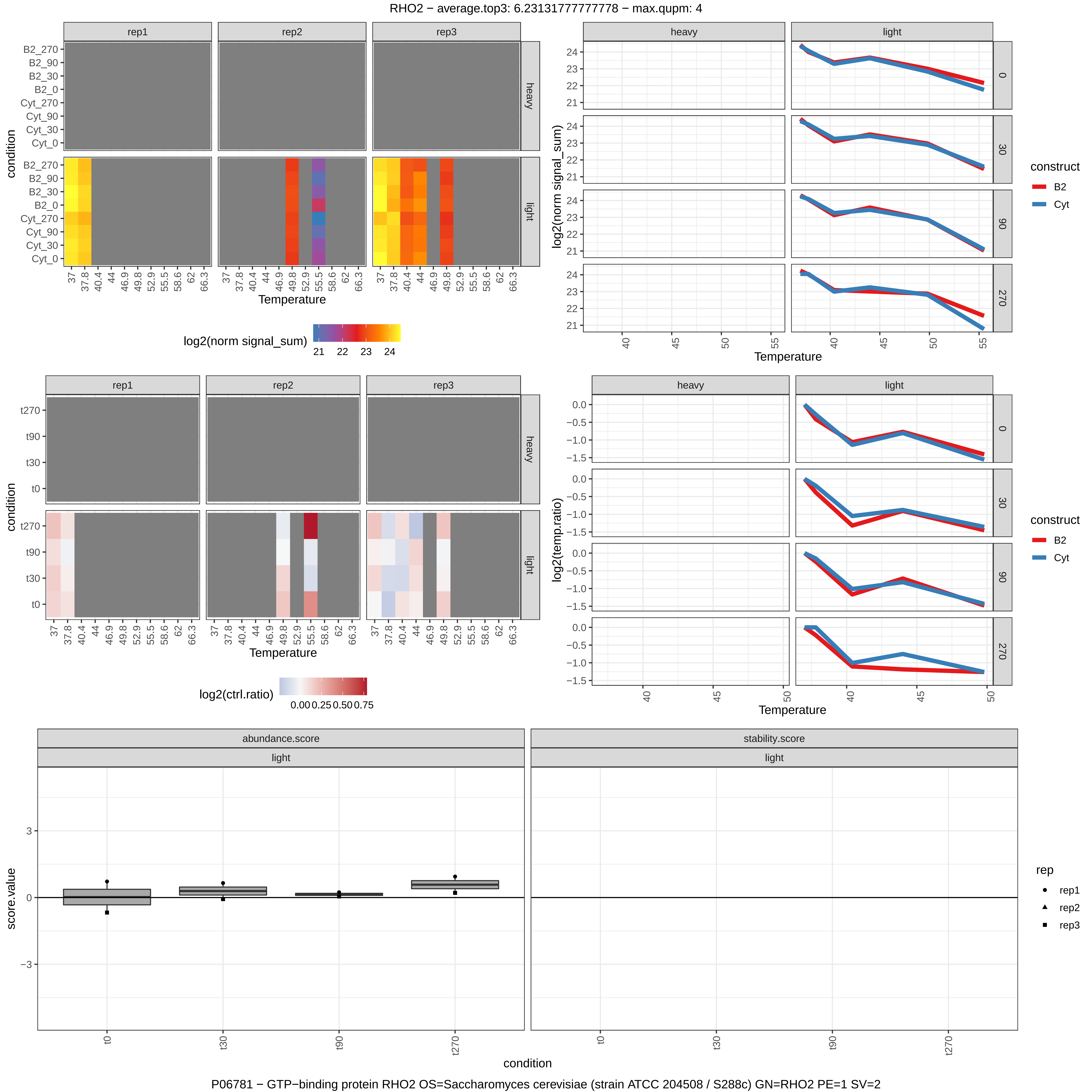

RHO2 − average.top3: 6.23131777777778 − max.qupm: 4
rep1
rep2
rep3
heavy
light
condition
Temperature
log2(norm signal_sum)
construct
B2
Cyt
log2(ctrl.ratio)
log2(temp.ratio)
abundance.score
stability.score
score.value
rep
rep1
rep2
rep3
P06781 − GTP−binding protein RHO2 OS=Saccharomyces cerevisiae (strain ATCC 204508 / S288c) GN=RHO2 PE=1 SV=2

RHO3 − average.top3: 6.84179272727273 − max.qupm: 3

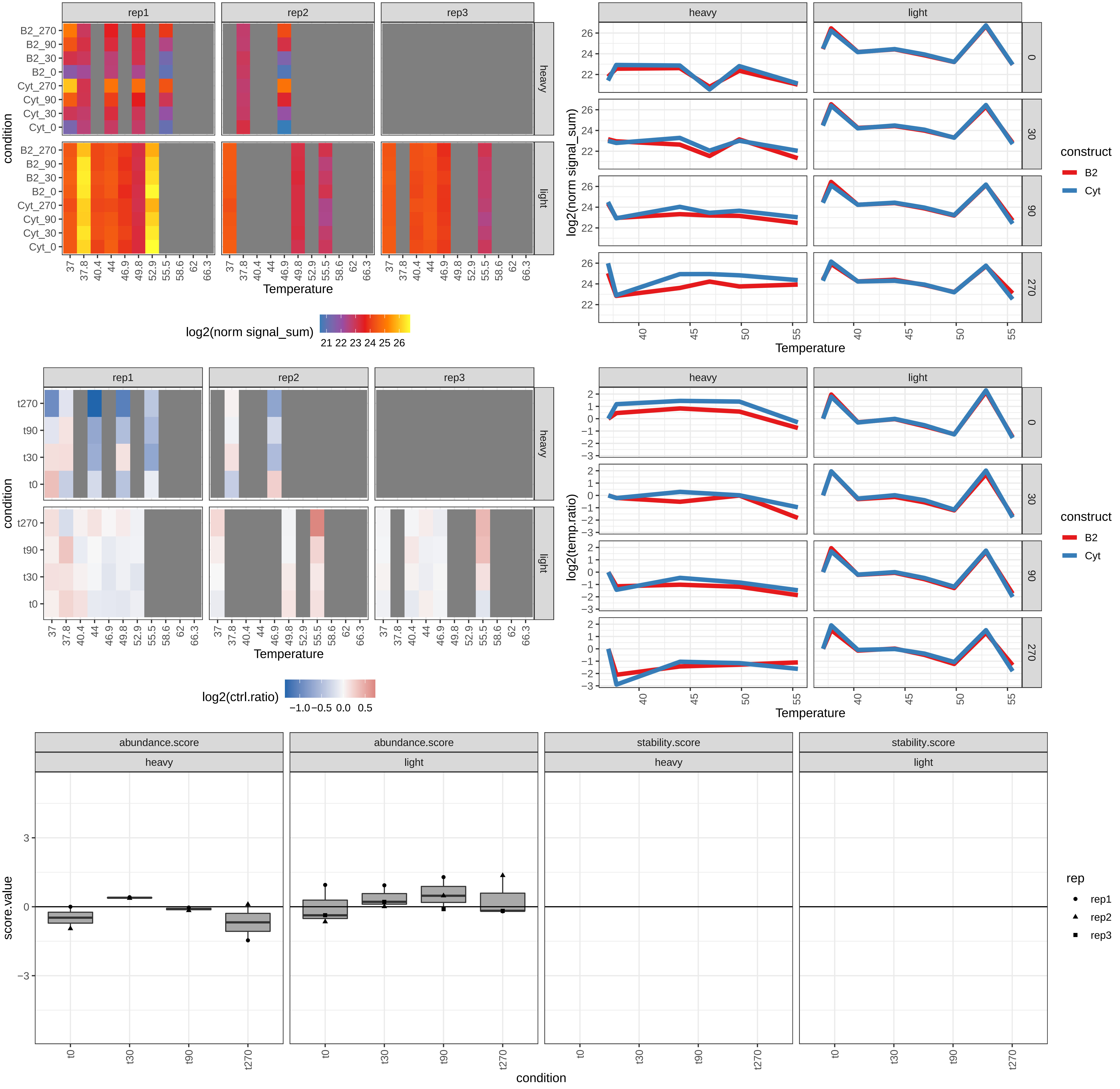

Q00245 − GTP−binding protein RHO3 OS=Saccharomyces cerevisiae (strain ATCC 204508 / S288c) GN=RHO3 PE=1 SV=2

RHO4 − average.top3: 6.7021325 − max.qupm: 3

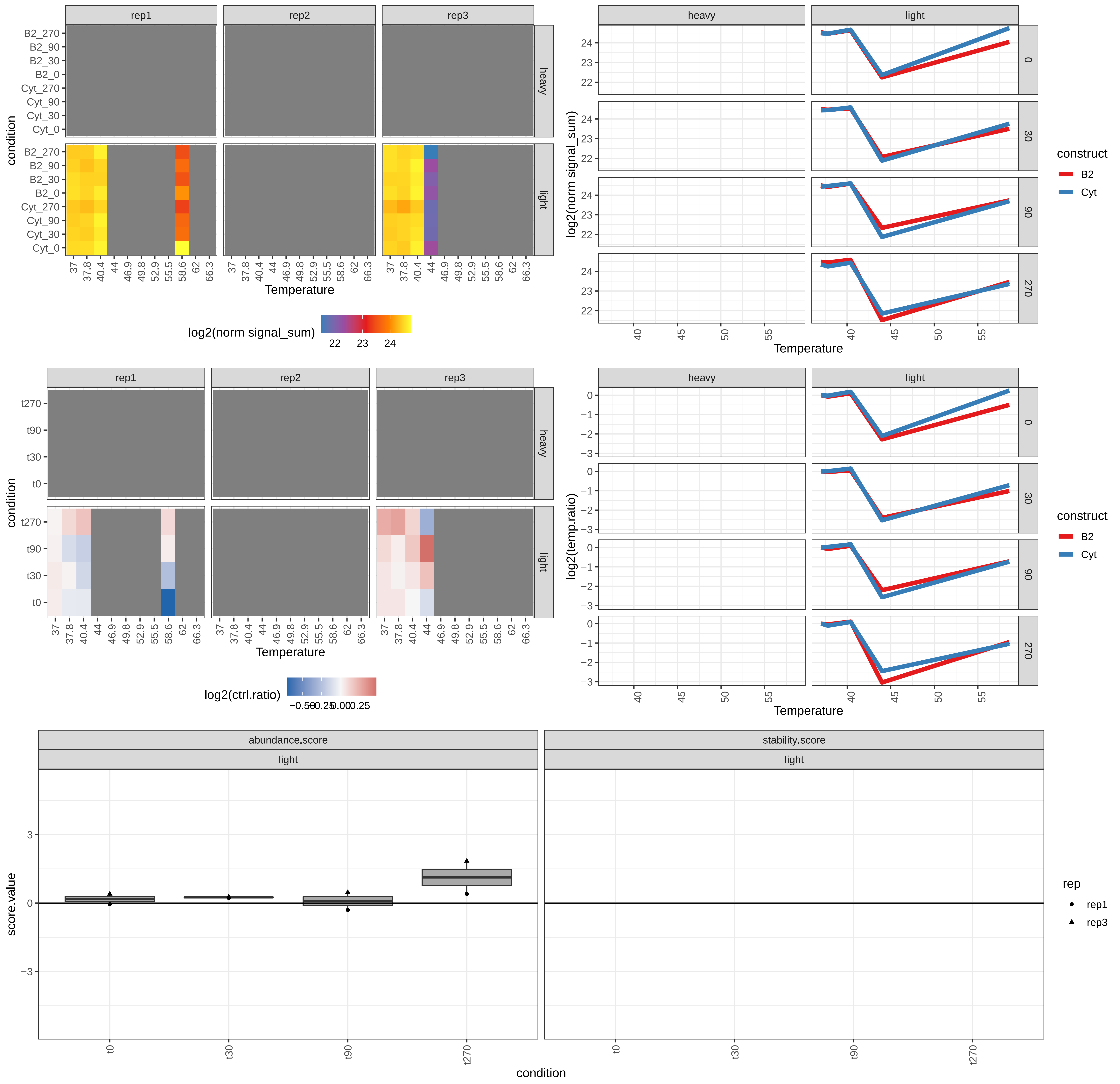

Q00246 − GTP−binding protein RHO4 OS=Saccharomyces cerevisiae (strain ATCC 204508 / S288c) GN=RHO4 PE=1 SV=2

RHO5 − average.top3: 6.80055952380952 − max.qupm: 7

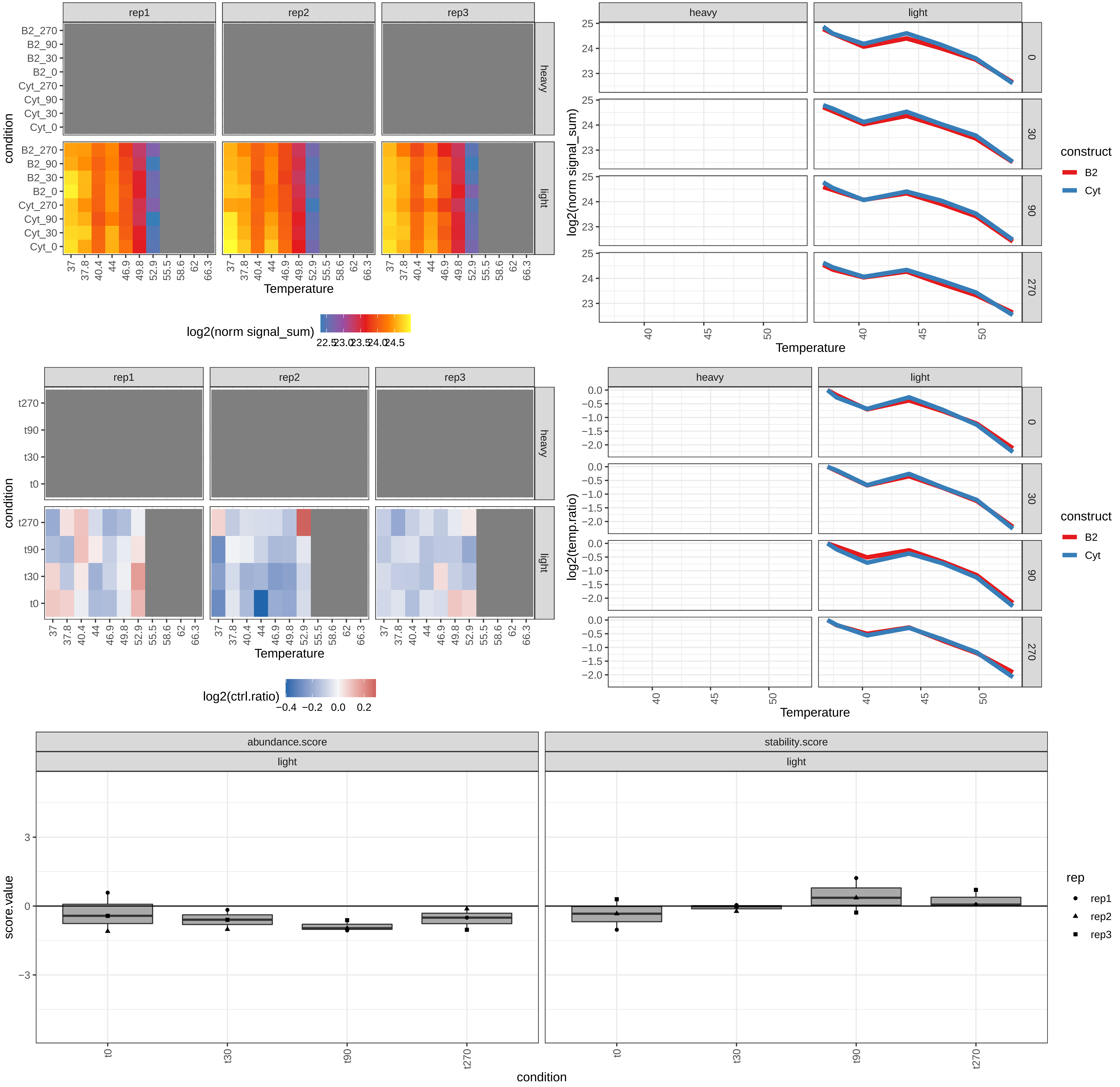

P53879 − GTP−binding protein RHO5 OS=Saccharomyces cerevisiae (strain ATCC 204508 / S288c) GN=RHO5 PE=1 SV=2

RIA1 − average.top3: 6.72698846153846 − max.qupm: 10

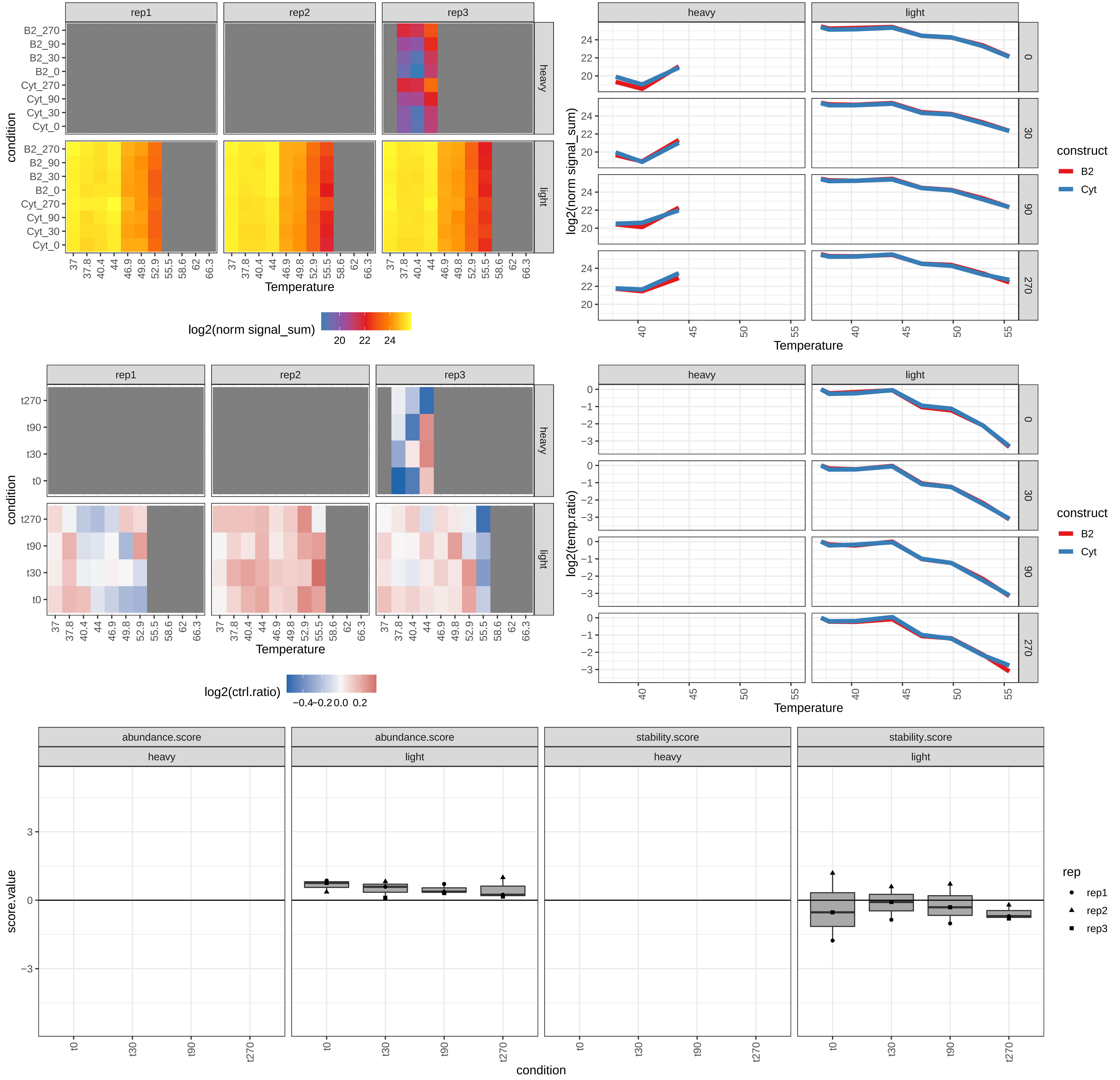

P53893 − Ribosome assembly protein 1 OS=Saccharomyces cerevisiae (strain ATCC 204508 / S288c) GN=RIA1 PE=1 SV=1

RIB1 − average.top3: 6.99153952380952 − max.qupm: 9

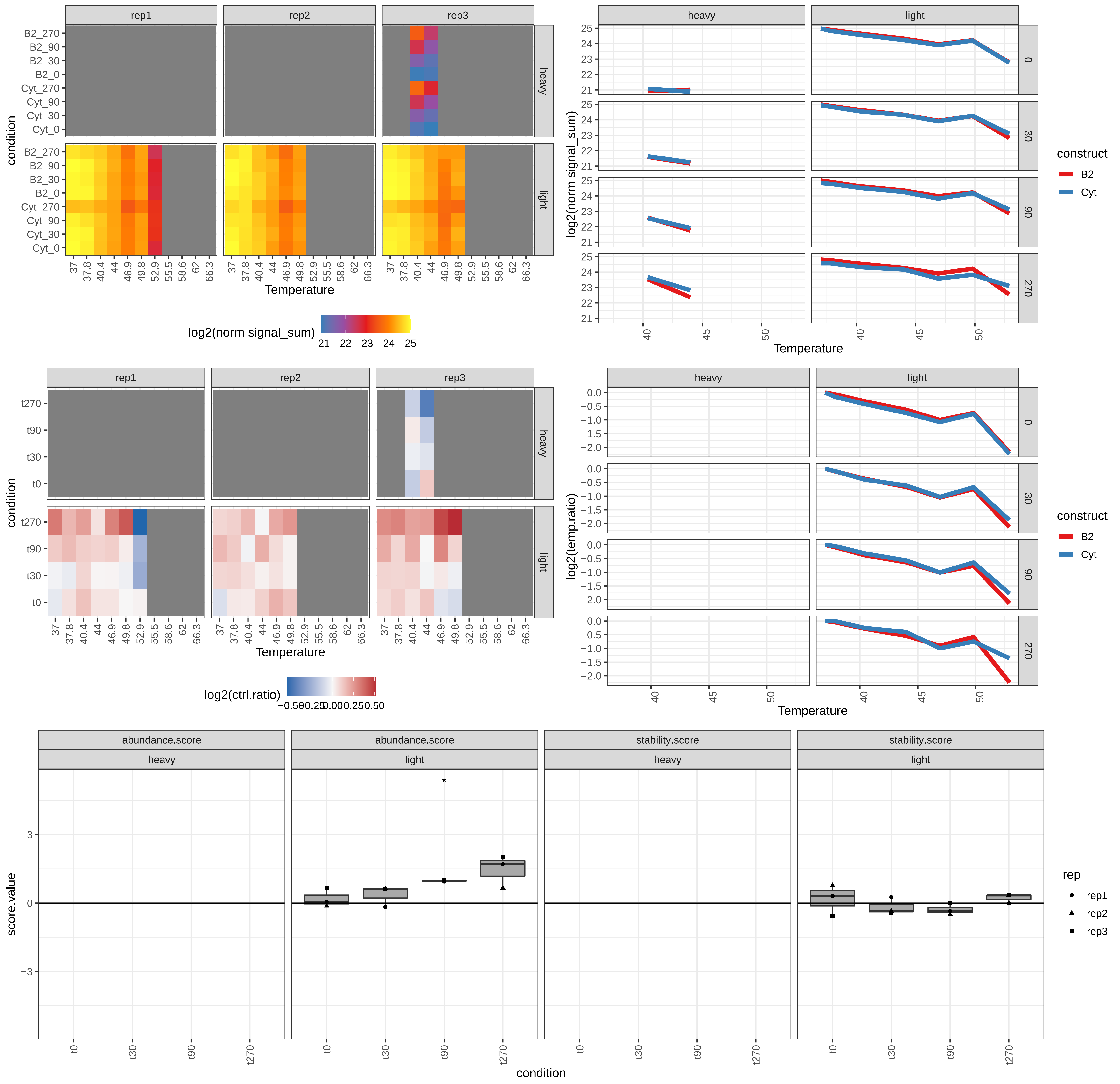

P38066 − GTP cyclohydrolase−2 OS=Saccharomyces cerevisiae (strain ATCC 204508 / S288c) GN=RIB1 PE=1 SV=2

RIB2 − average.top3: 6.42887785714286 − max.qupm: 5

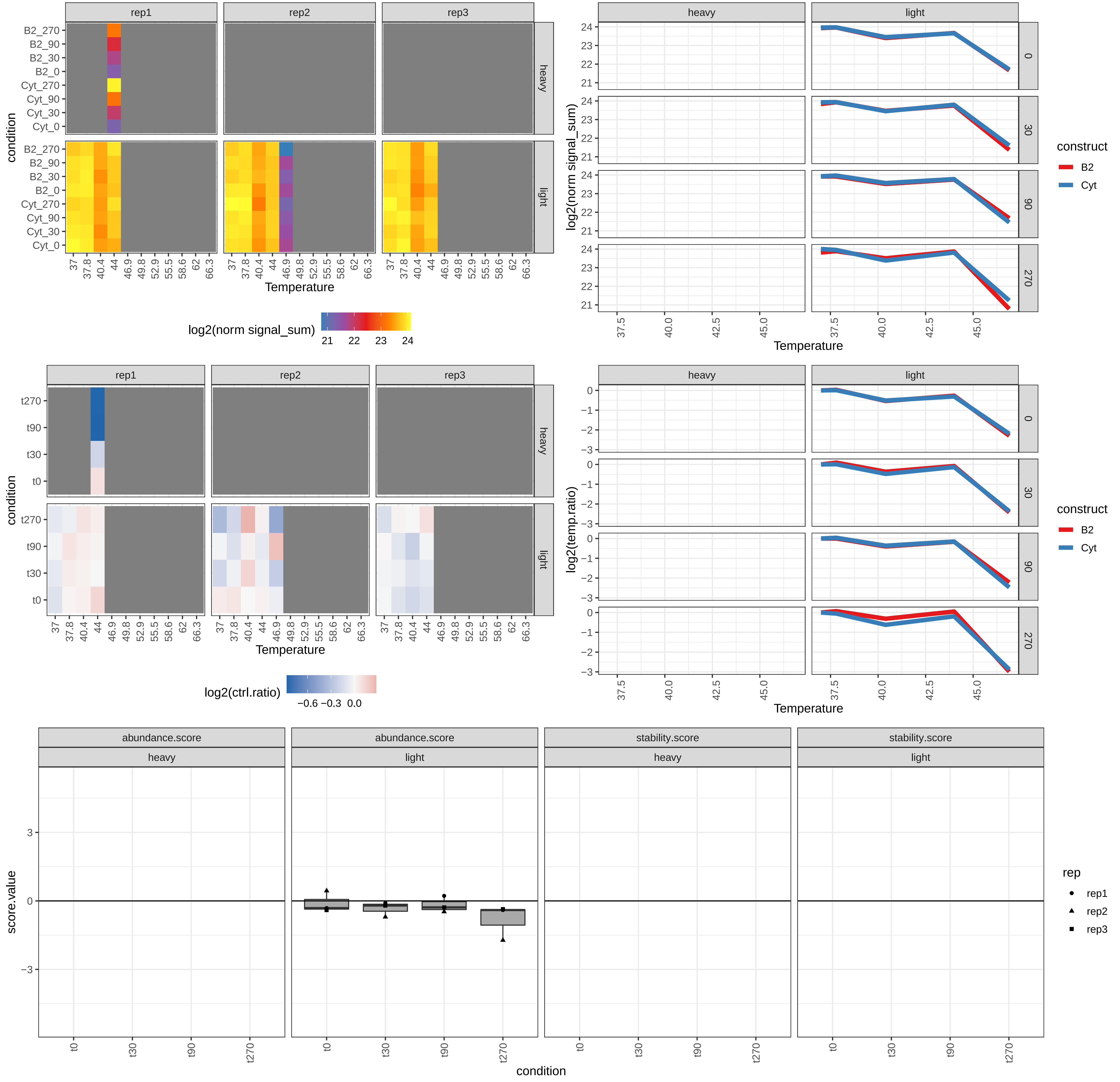

Q12362 − Bifunctional protein RIB2 OS=Saccharomyces cerevisiae (strain ATCC 204508 / S288c) GN=RIB2 PE=1 SV=1

RIB3 − average.top3: 7.36250307692308 − max.qupm: 8

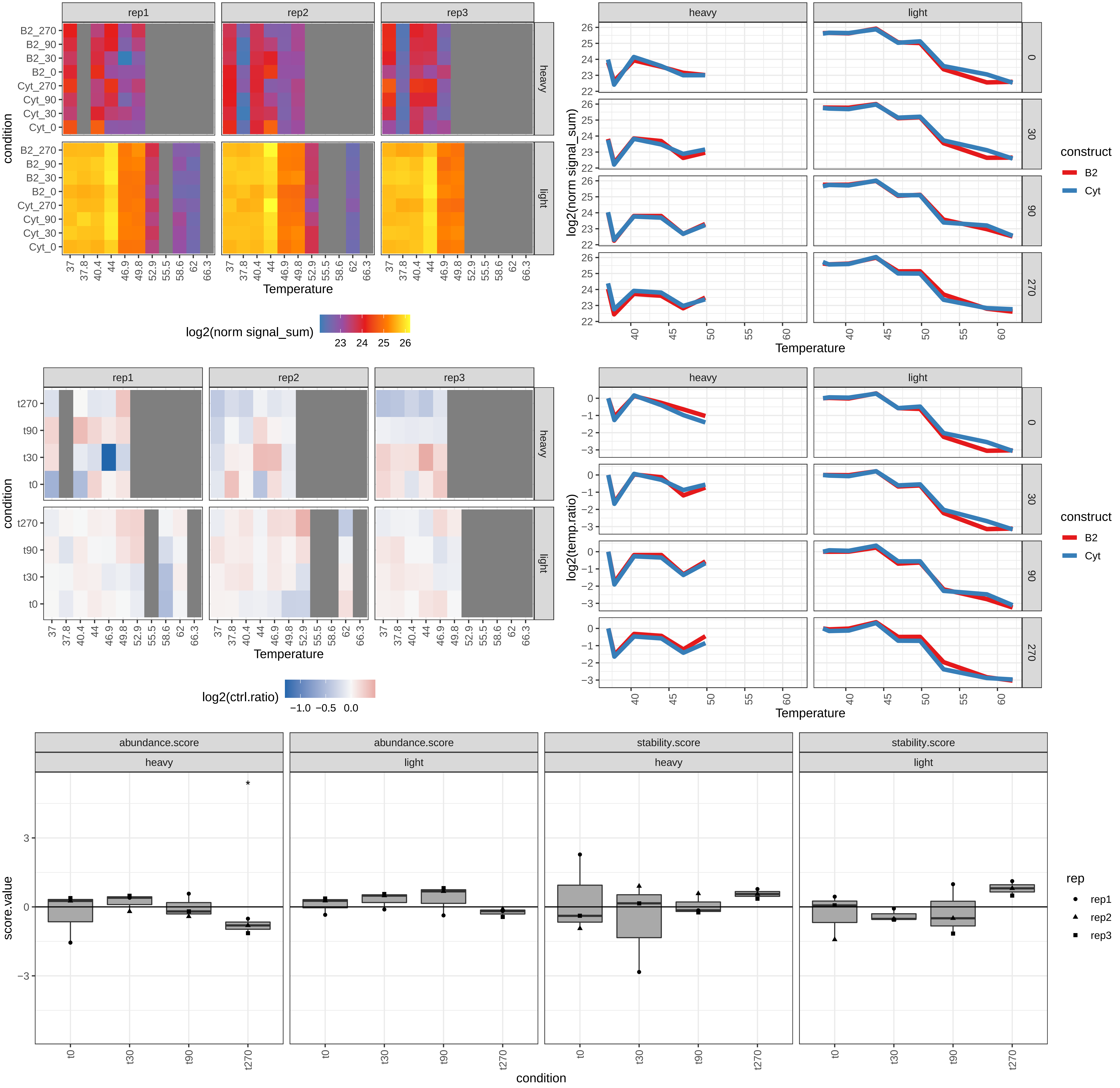

Q99258 − 3,4−dihydroxy−2−butanone 4−phosphate synthase OS=Saccharomyces cerevisiae (strain ATCC 204508 / S288c) GN=RIB3 PE=1 SV=1

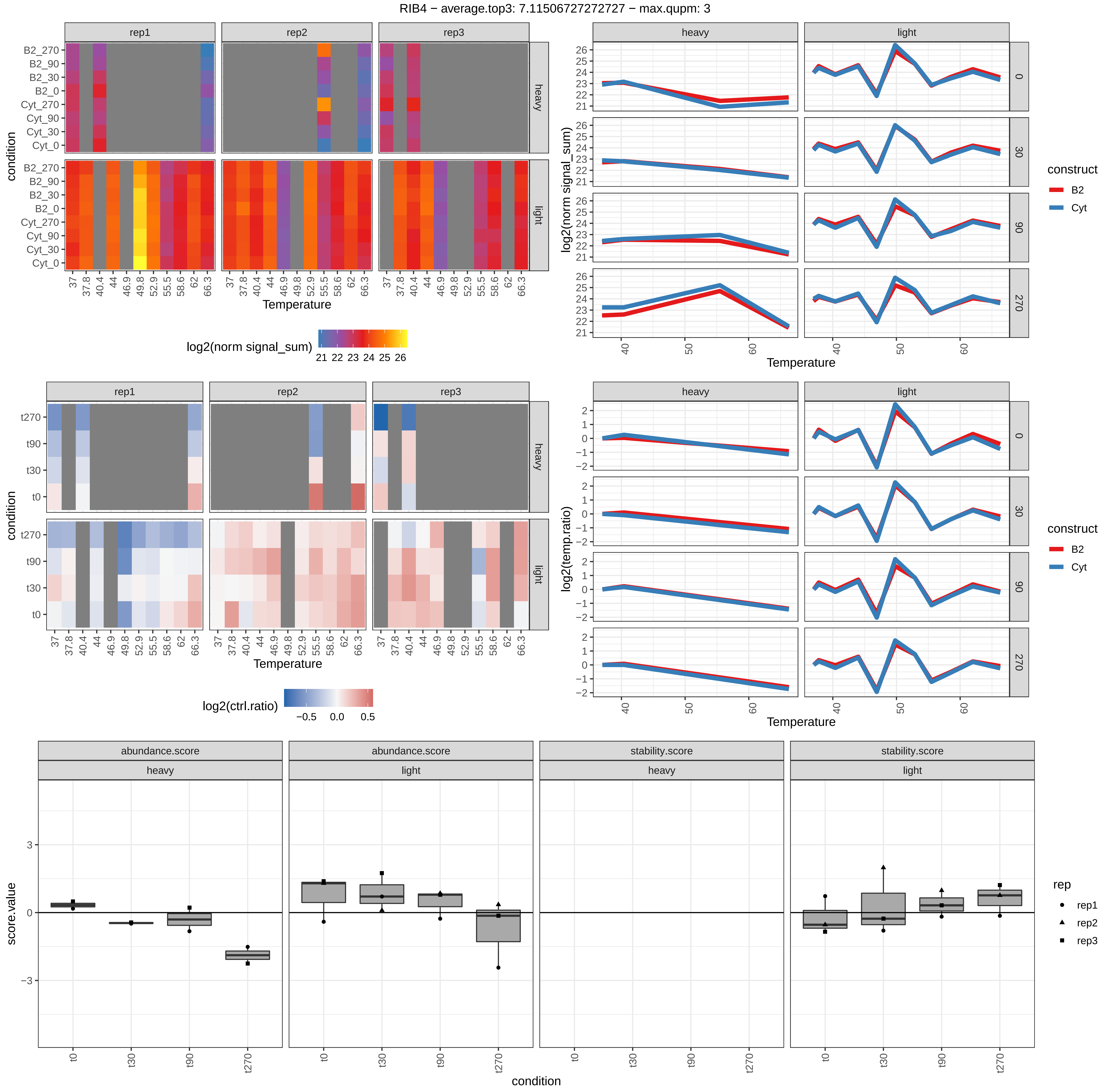

P50861 − 6,7−dimethyl−8−ribityllumazine synthase OS=Saccharomyces cerevisiae (strain ATCC 204508 / S288c) GN=RIB4 PE=1 SV=2

RIB5 − average.top3: 7.14971583333333 − max.qupm: 5

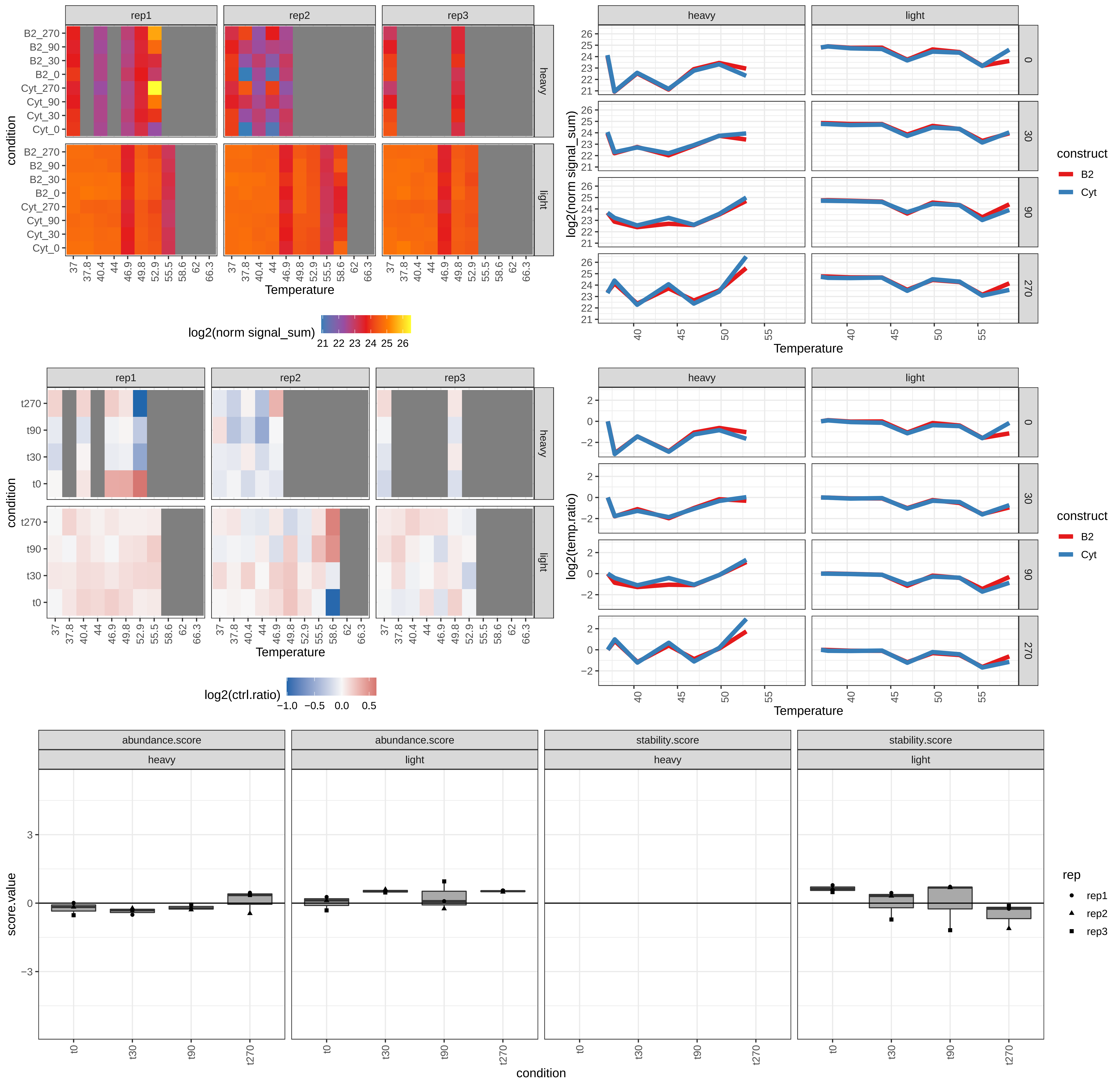

P38145 − Riboflavin synthase OS=Saccharomyces cerevisiae (strain ATCC 204508 / S288c) GN=RIB5 PE=1 SV=1

RIC1 − average.top3: 6.66035136363636 − max.qupm: 8

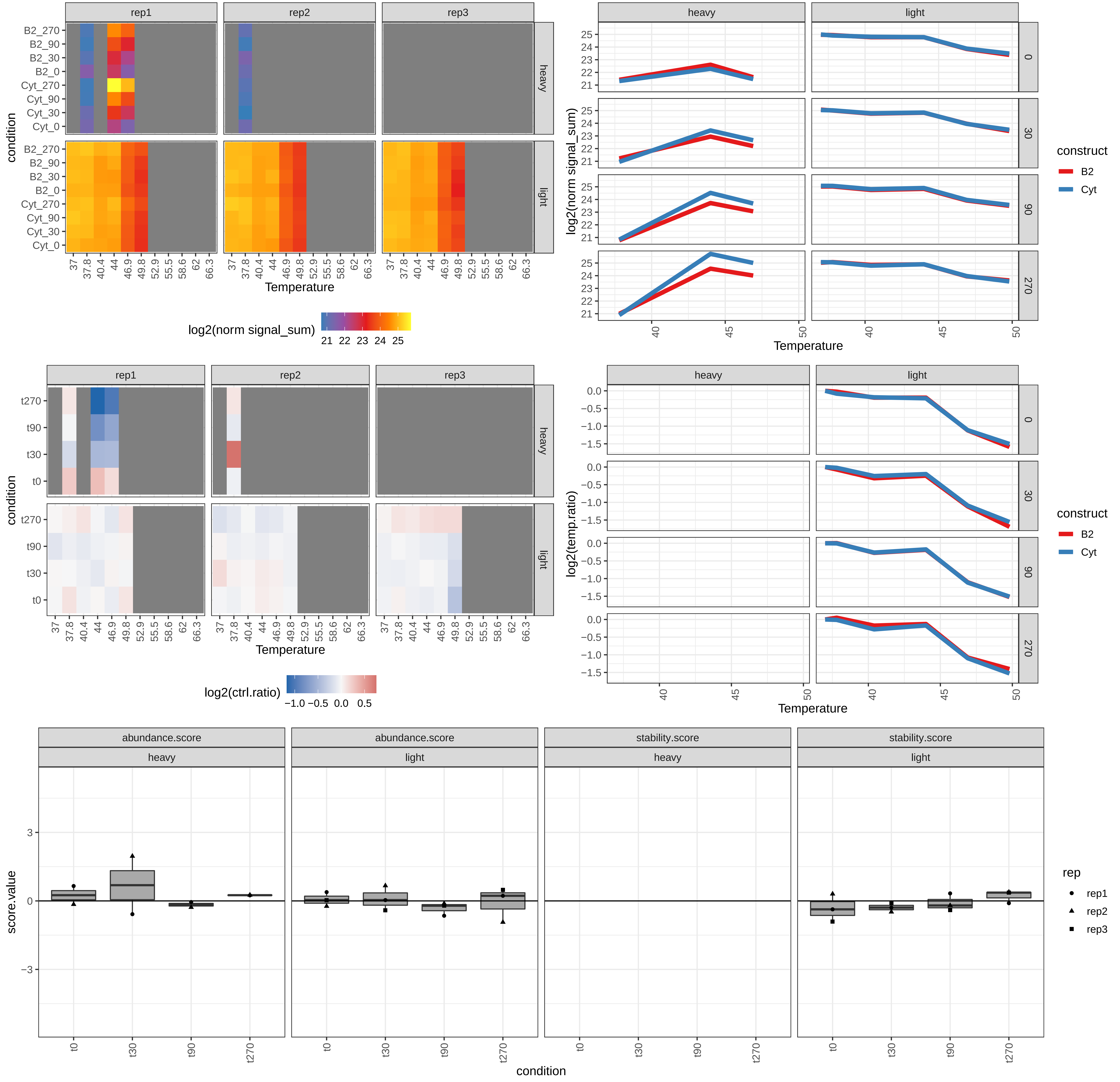

P40395 − Guanine nucleotide exchange factor subunit RIC1 OS=Saccharomyces cerevisiae (strain ATCC 204508 / S288c) GN=RIC1 PE=1 SV=1

RIM1 − average.top3: 7.4385580952381 − max.qupm: 6

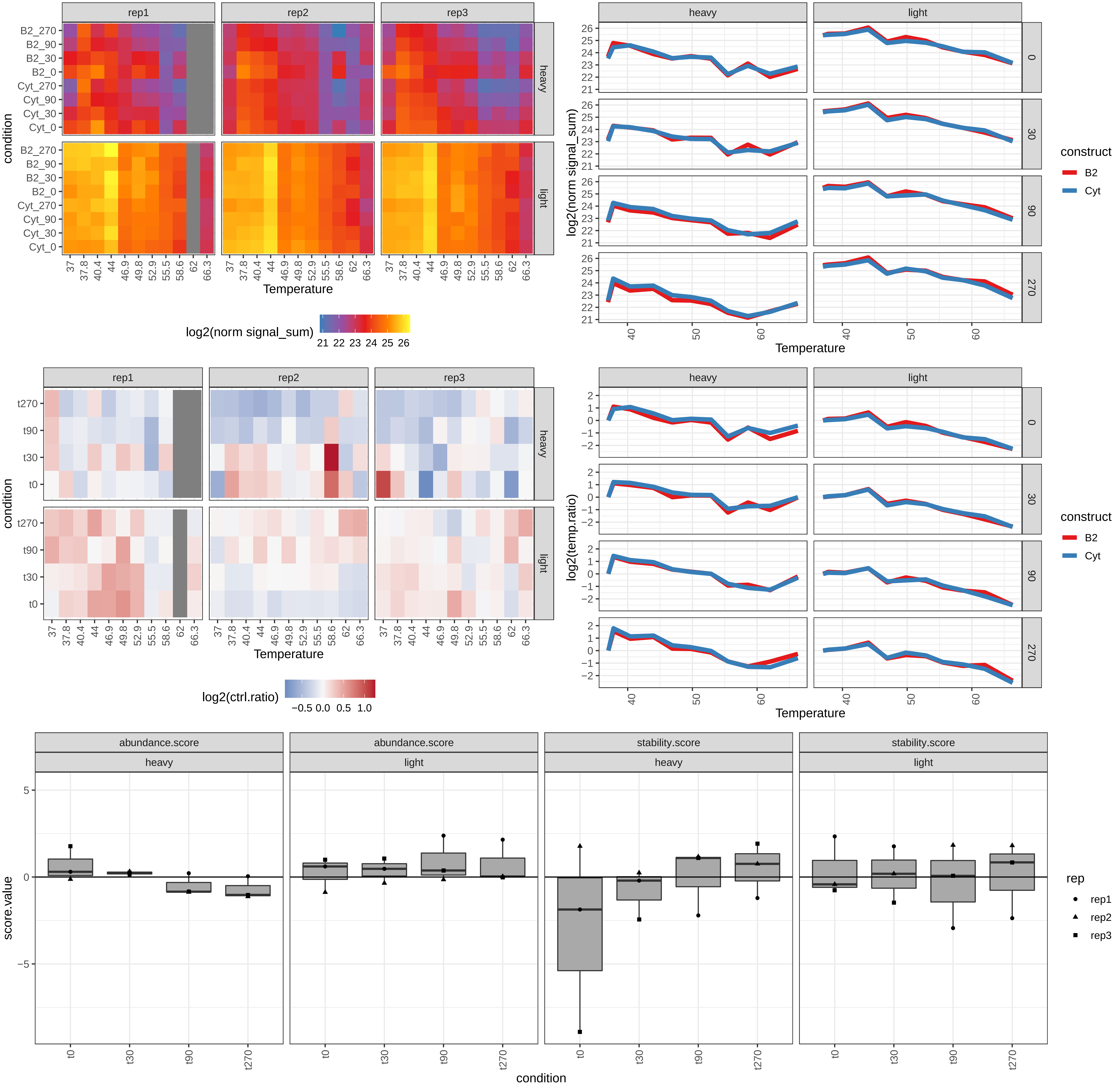

P32445 − Single−stranded DNA−binding protein RIM1, mitochondrial OS=Saccharomyces cerevisiae (strain ATCC 204508 / S288c) GN=RIM1 PE=1 SV=1

RIM11 − average.top3: 6.61926307692308 − max.qupm: 4

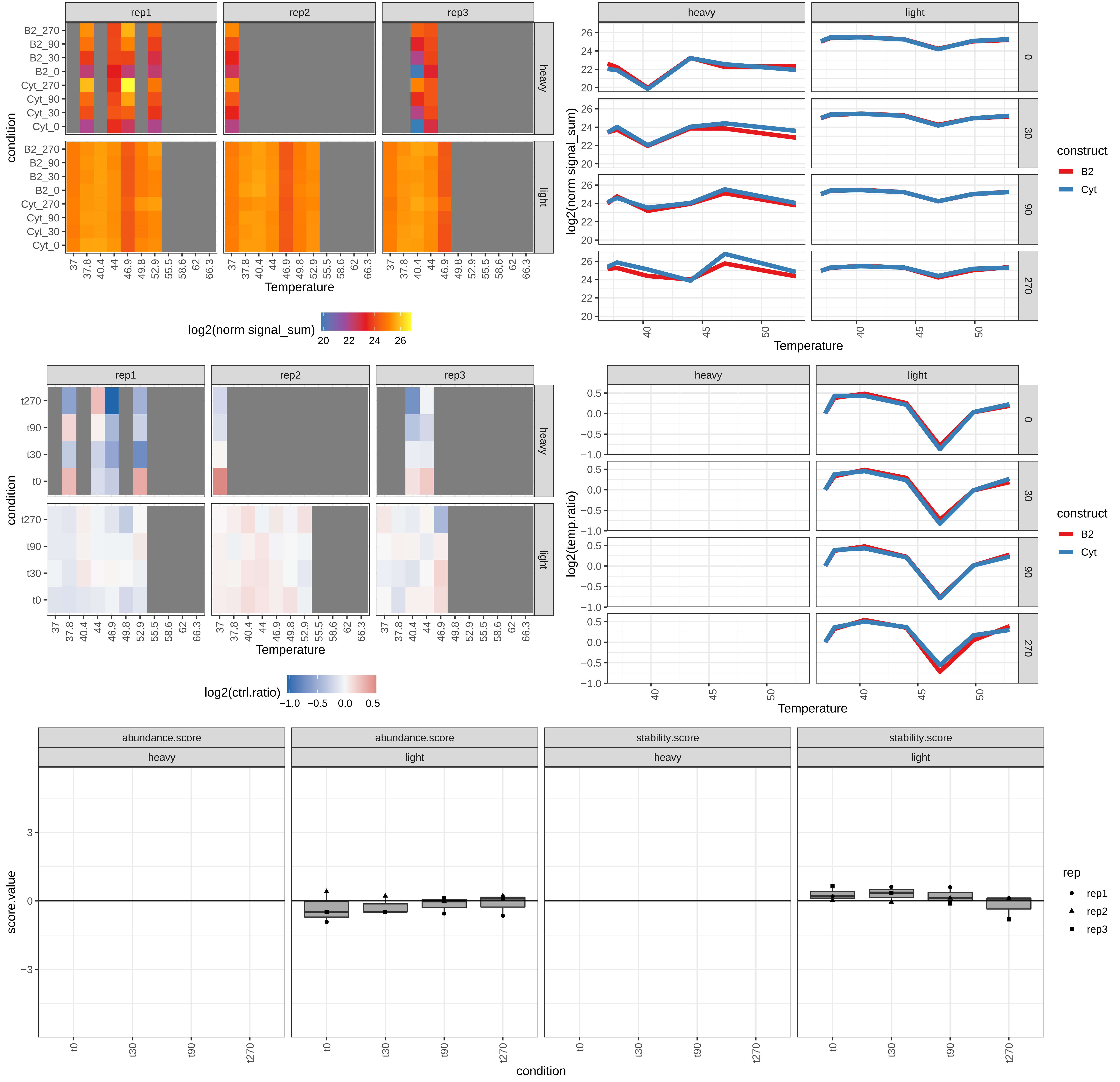

P38615 − Serine/threonine−protein kinase RIM11/MSD1 OS=Saccharomyces cerevisiae (strain ATCC 204508 / S288c) GN=RIM11 PE=1 SV=1

RIM2 − average.top3: 6.88719833333333 − max.qupm: 6

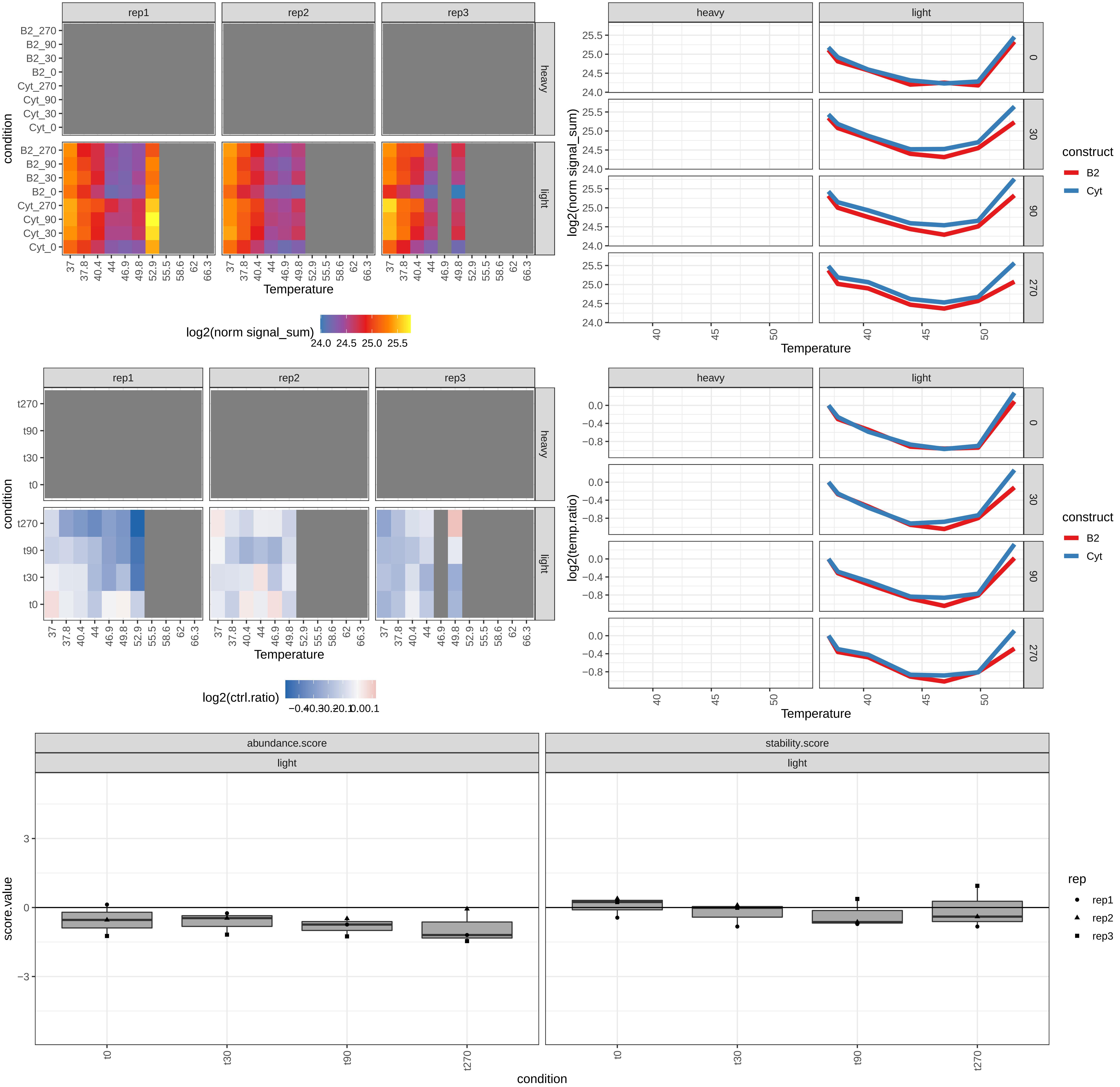

P38127 − Mitochondrial carrier protein RIM2 OS=Saccharomyces cerevisiae (strain ATCC 204508 / S288c) GN=RIM2 PE=3 SV=1

RIM20 − average.top3: 6.62334411764706 − max.qupm: 11

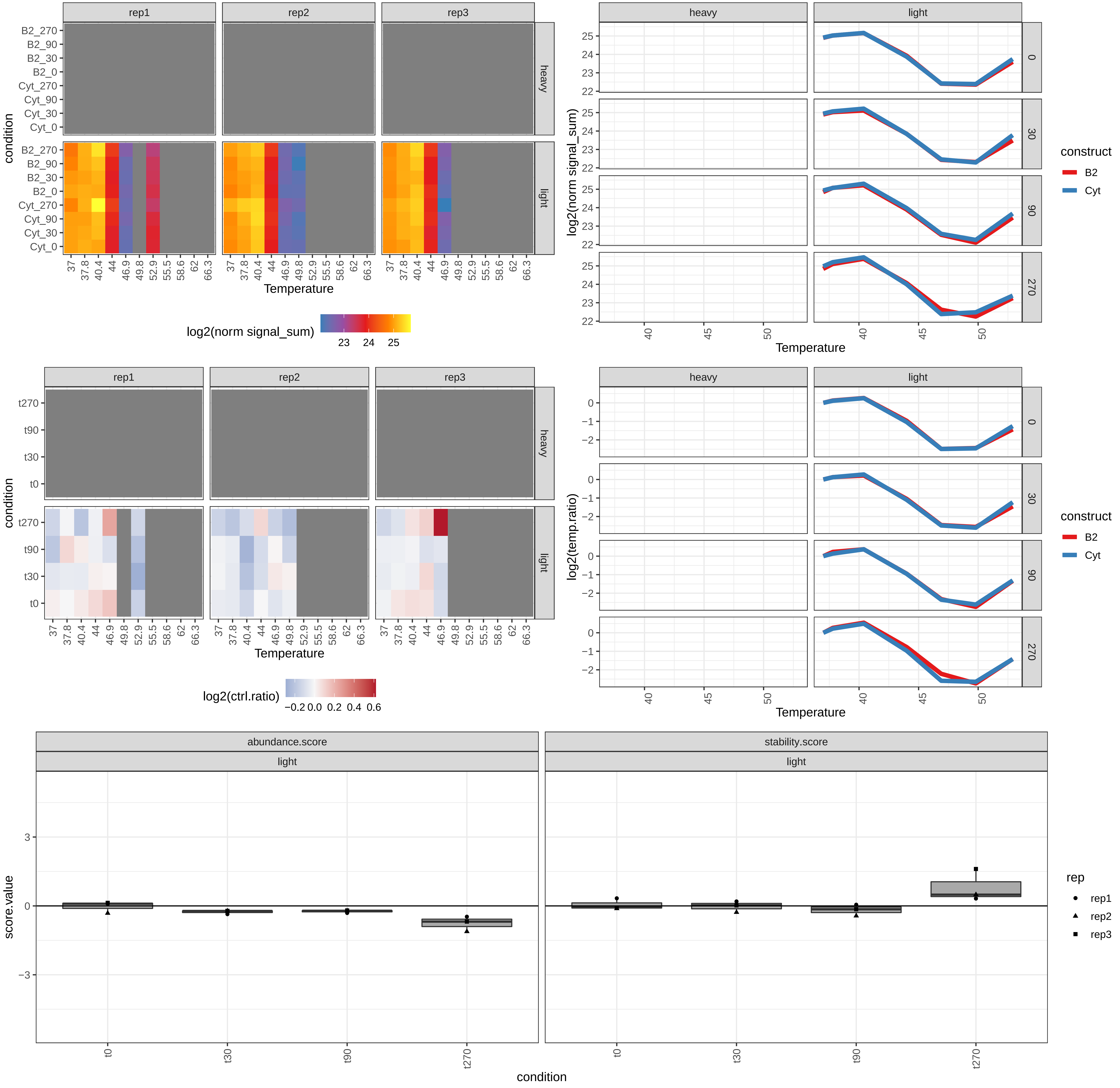

Q12033 − pH−response regulator protein palA/RIM20 OS=Saccharomyces cerevisiae (strain ATCC 204508 / S288c) GN=RIM20 PE=1 SV=1

RIM4 − average.top3: 6.49242117647059 − max.qupm: 3

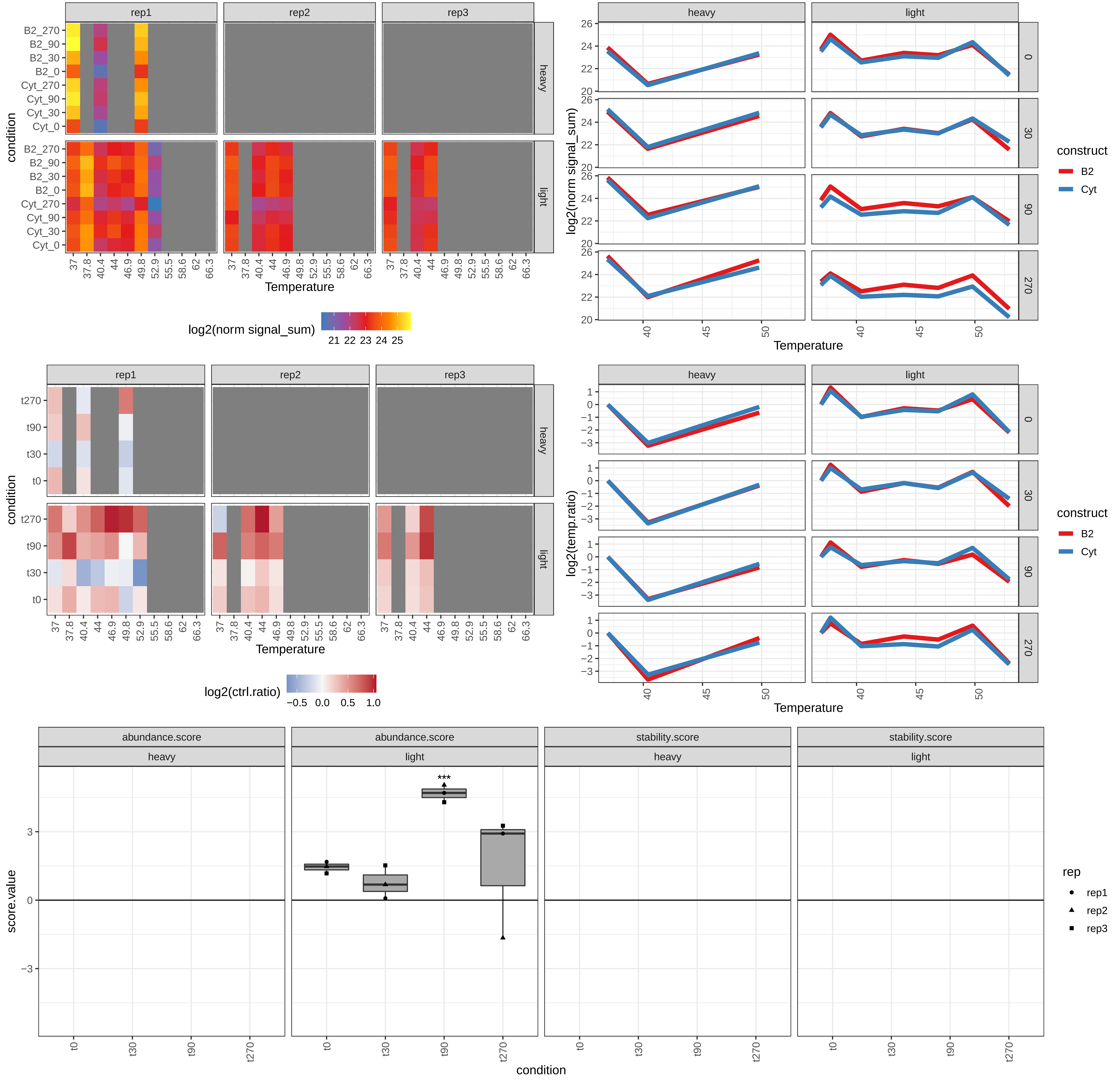

P38741 − Meiotic activator RIM4 OS=Saccharomyces cerevisiae (strain ATCC 204508 / S288c) GN=RIM4 PE=1 SV=1

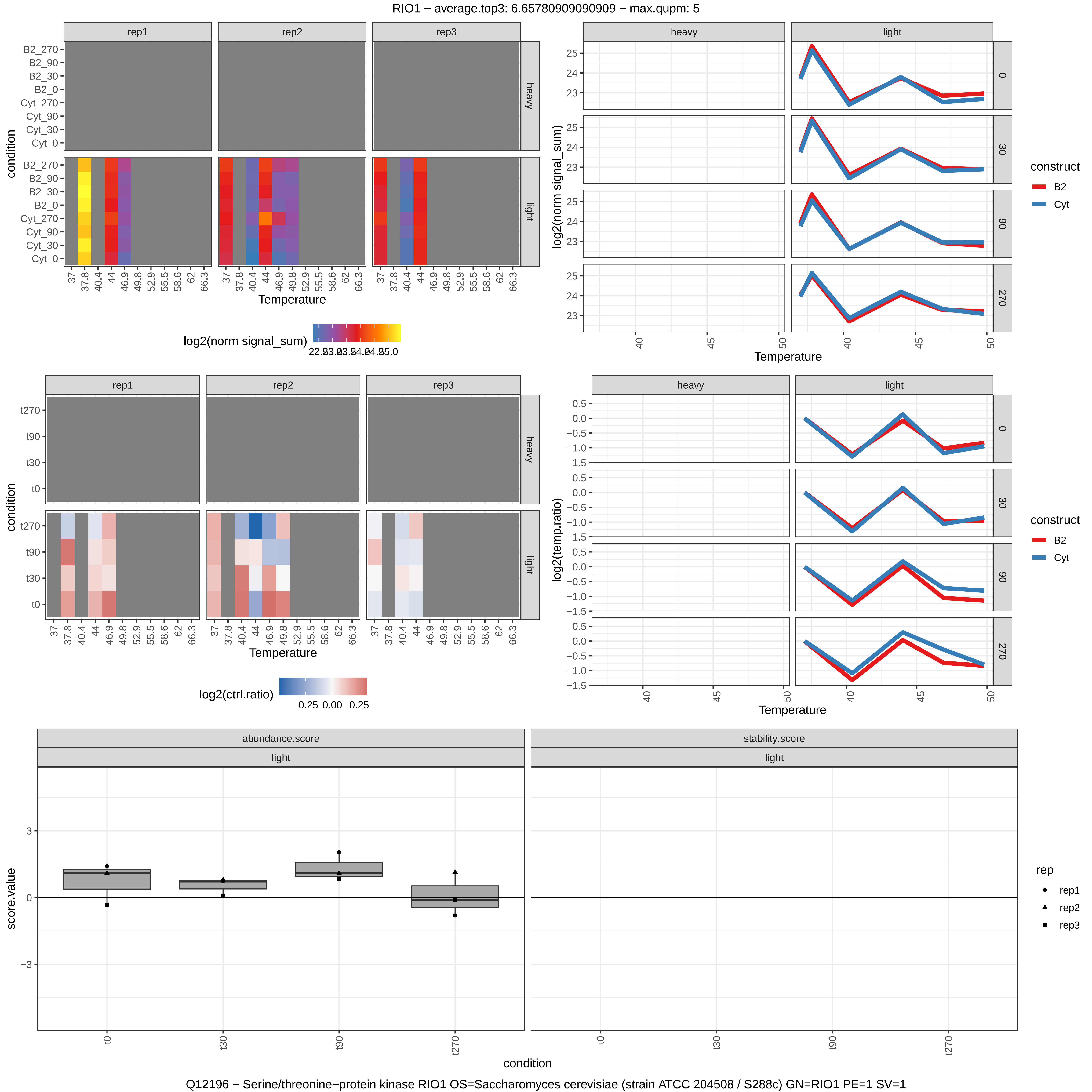

RIO1 − average.top3: 6.65780909090909 − max.qupm: 5
rep1
rep2
rep3
heavy
light
condition
Temperature
log2(norm signal_sum)
log2(ctrl.ratio)
log2(temp.ratio)
construct
B2
Cyt
abundance.score
stability.score
score.value
rep
rep1
rep2
rep3
Q12196 − Serine/threonine−protein kinase RIO1 OS=Saccharomyces cerevisiae (strain ATCC 204508 / S288c) GN=RIO1 PE=1 SV=1

RIO2 − average.top3: 7.10343133333333 − max.qupm: 8

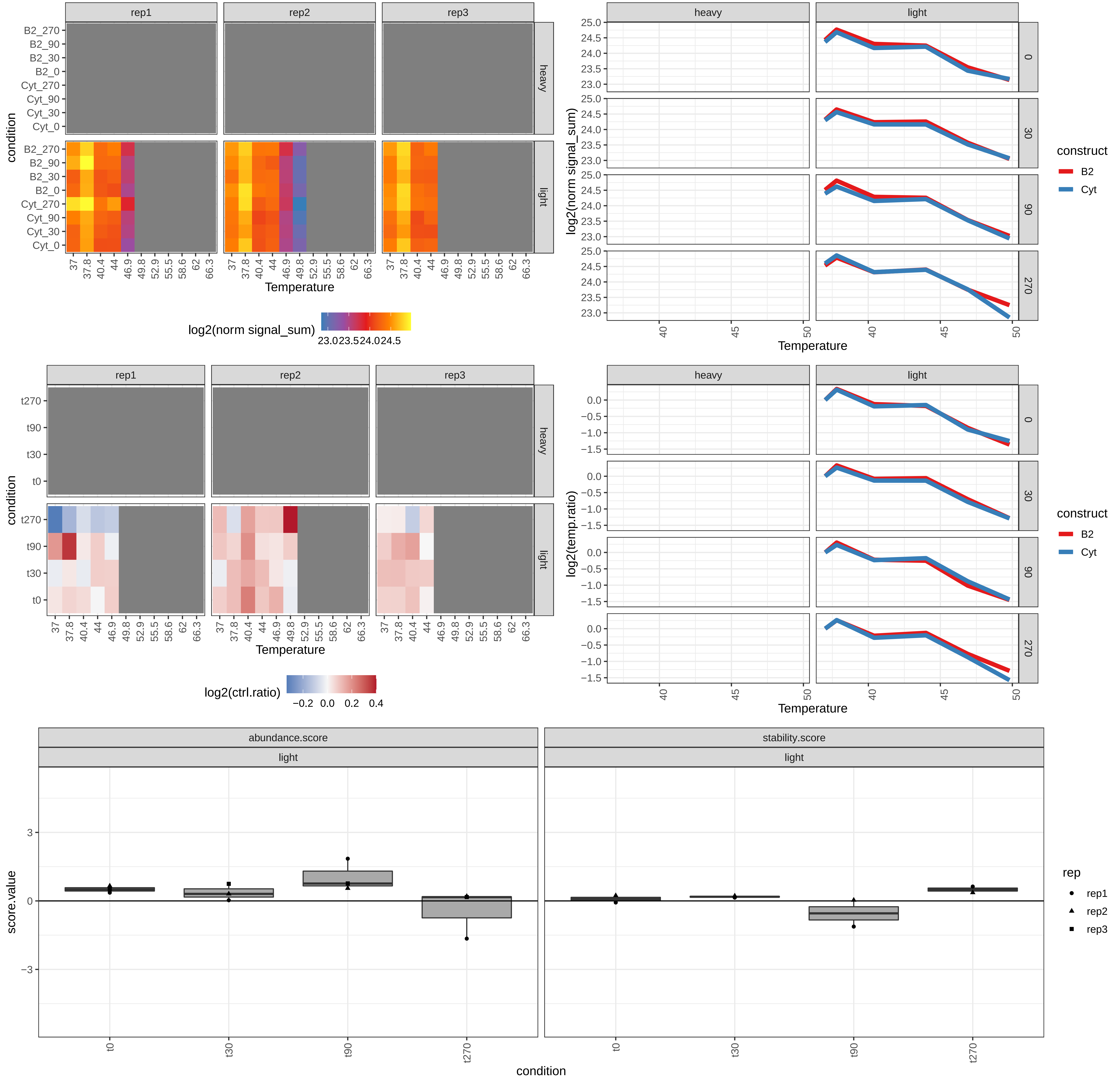

P40160 − Serine/threonine−protein kinase RIO2 OS=Saccharomyces cerevisiae (strain ATCC 204508 / S288c) GN=RIO2 PE=1 SV=1

RIP1 − average.top3: 7.209105 − max.qupm: 7

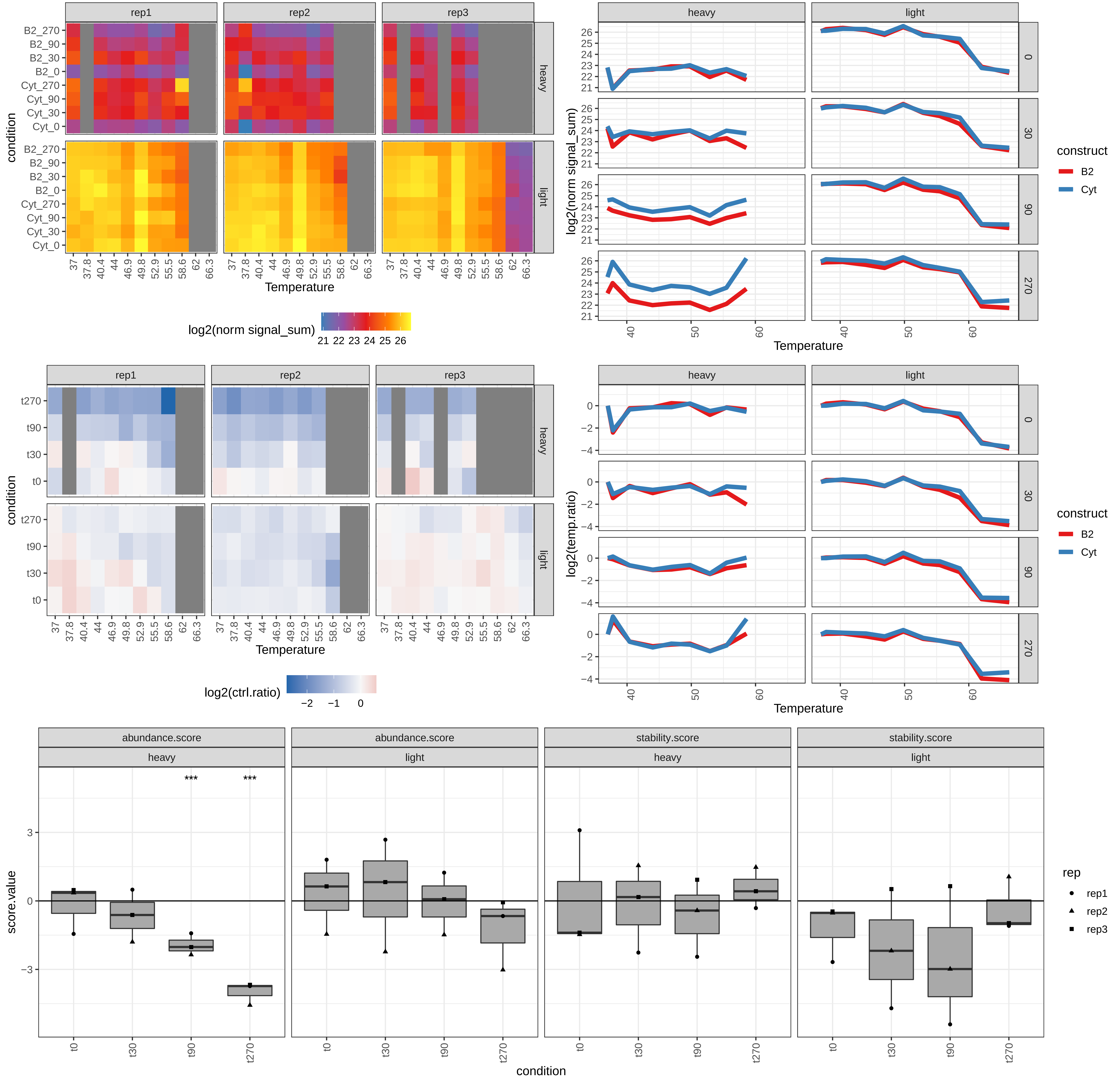

P08067 − Cytochrome b−c1 complex subunit Rieske, mitochondrial OS=Saccharomyces cerevisiae (strain ATCC 204508 / S288c) GN=RIP1 PE=1 SV=1

RIX1 − average.top3: 6.66492066666667 − max.qupm: 6

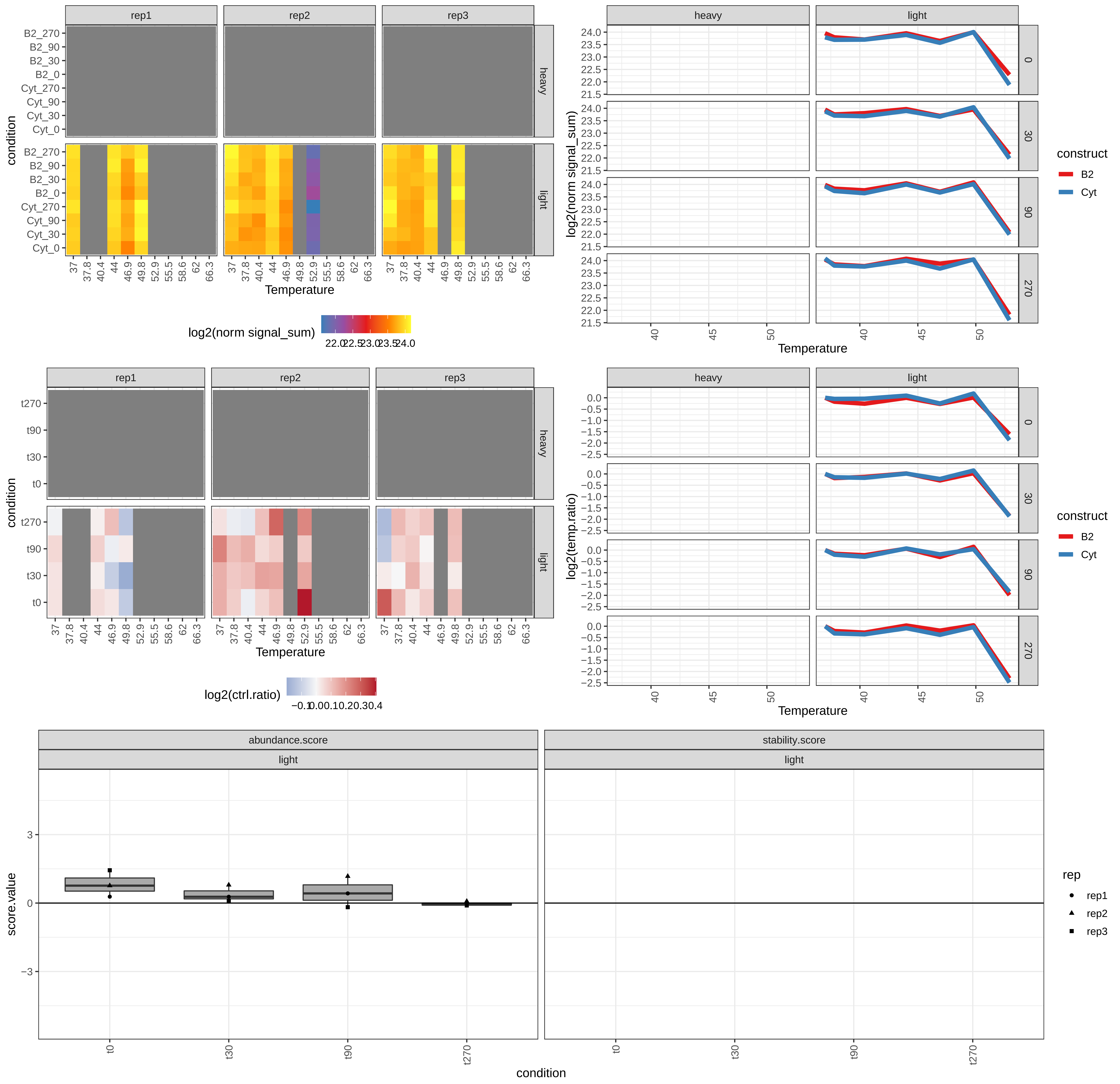

P38883 − Pre−rRNA−processing protein RIX1 OS=Saccharomyces cerevisiae (strain ATCC 204508 / S288c) GN=RIX1 PE=1 SV=2

RIX7 − average.top3: 6.57174222222222 − max.qupm: 5

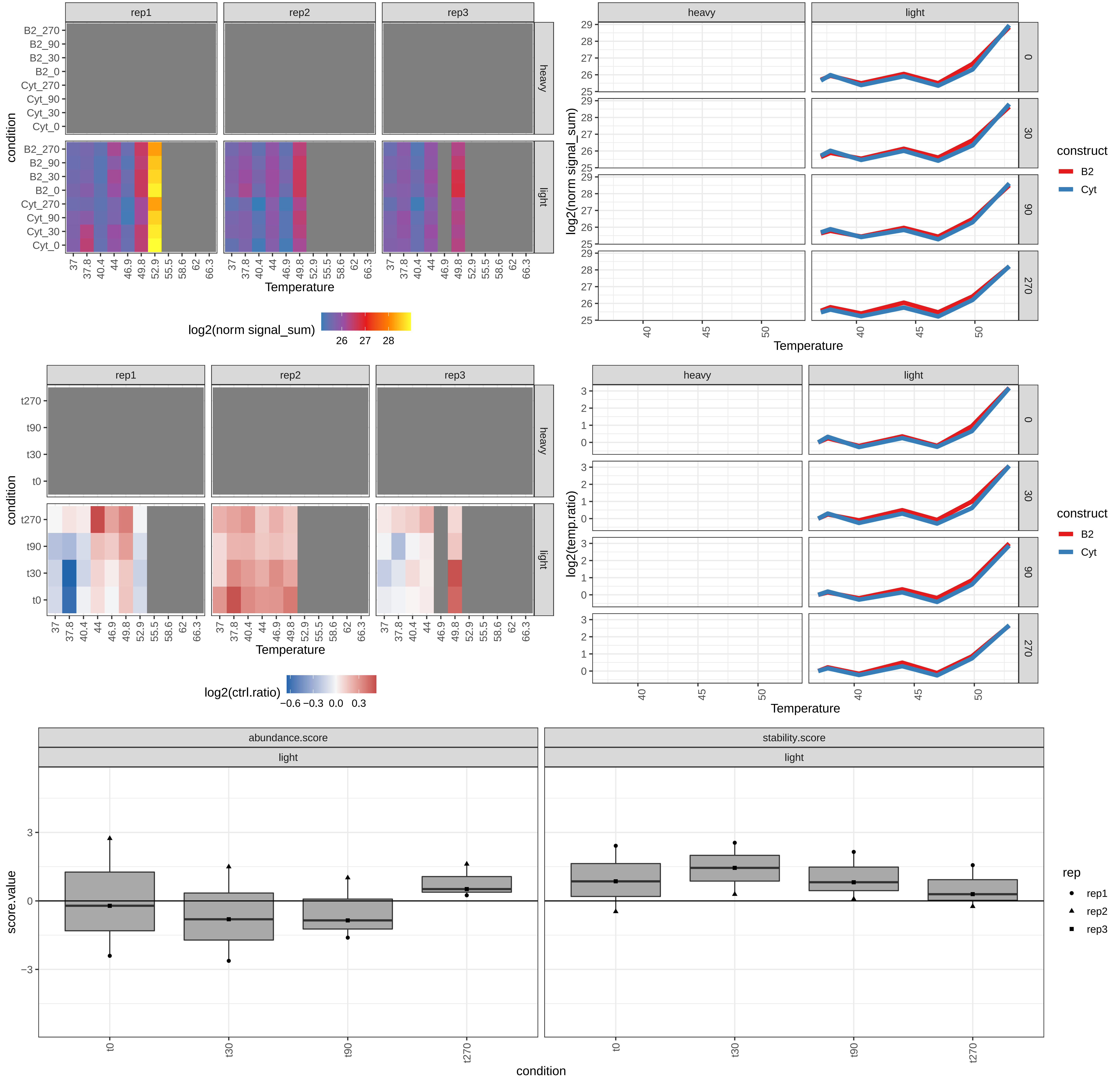

Q07844 − Ribosome biogenesis ATPase RIX7 OS=Saccharomyces cerevisiae (strain ATCC 204508 / S288c) GN=RIX7 PE=1 SV=1

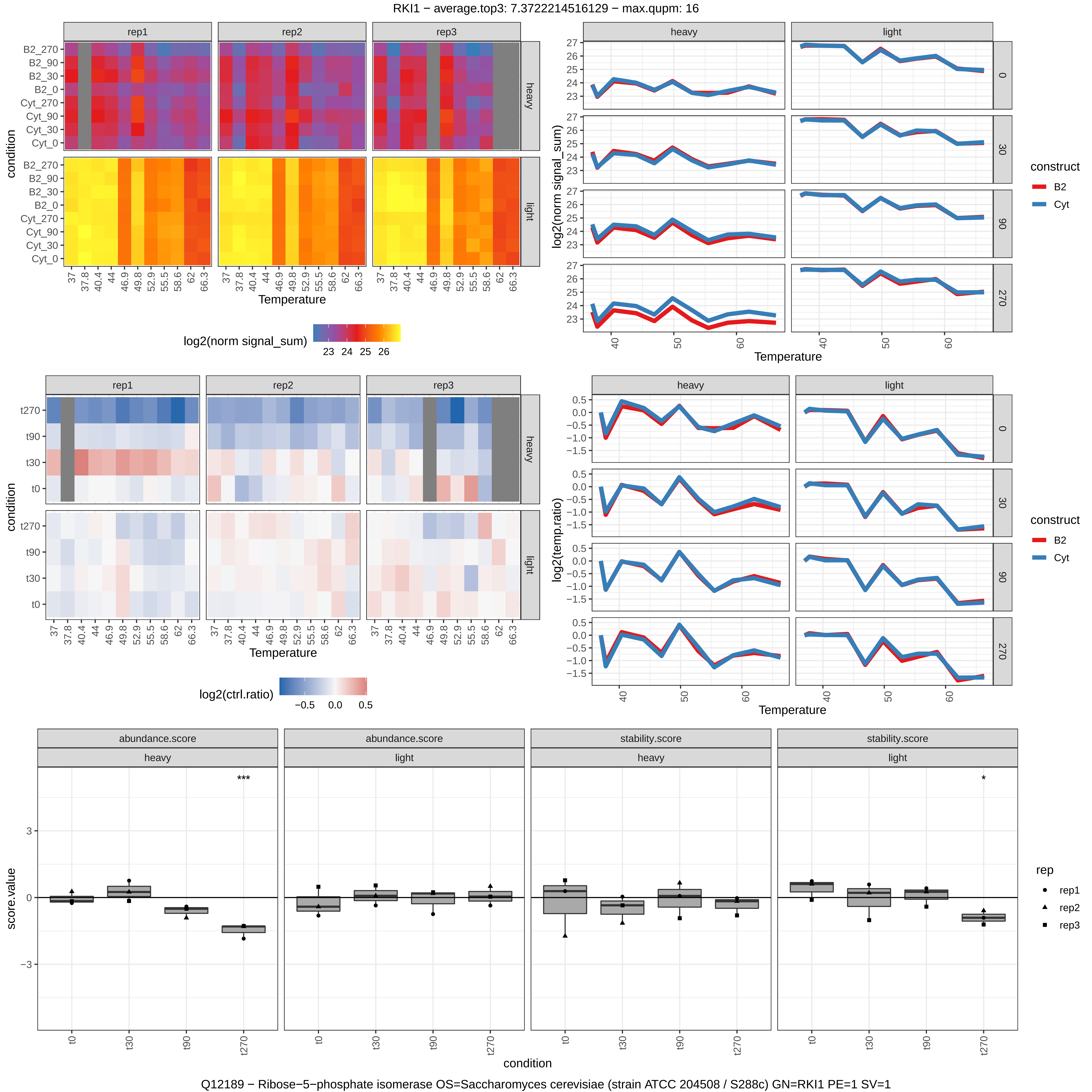
RKI1 − average.top3: 7.3722214516129 − max.qupm: 16
rep1
rep2
rep3
heavy
light
condition
Temperature
log2(norm signal_sum)
construct
B2
Cyt
log2(ctrl.ratio)
log2(temp.ratio)
abundance.score
stability.score
score.value
rep
rep1
rep2
rep3
***
*
Q12189 − Ribose−5−phosphate isomerase OS=Saccharomyces cerevisiae (strain ATCC 204508 / S288c) GN=RKI1 PE=1 SV=1

RKM1 − average.top3: 6.644575 − max.qupm: 6

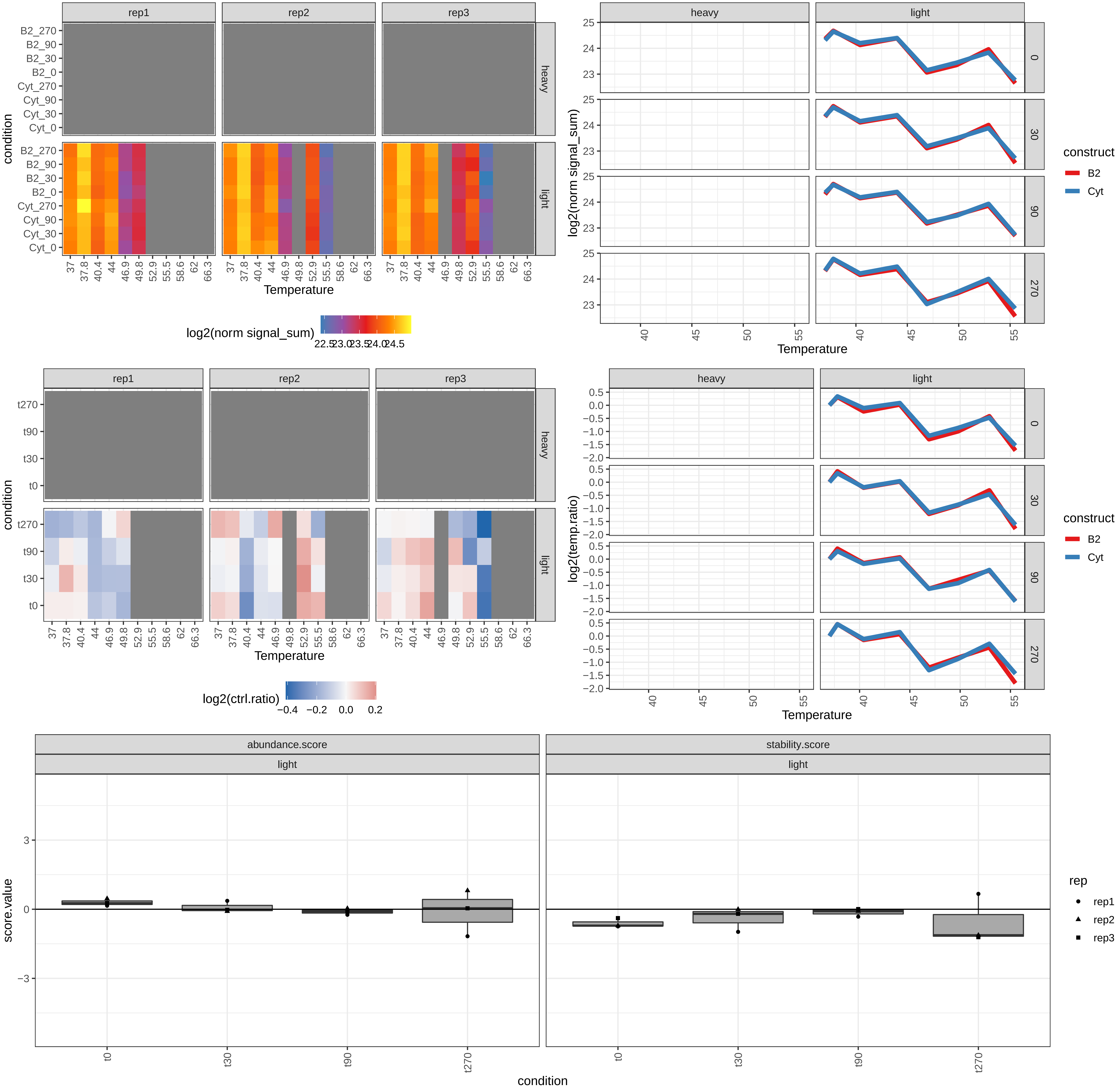

Q08961 − Ribosomal lysine N−methyltransferase 1 OS=Saccharomyces cerevisiae (strain ATCC 204508 / S288c) GN=RKM1 PE=1 SV=1

RKM2 − average.top3: 6.59612692307692 − max.qupm: 4

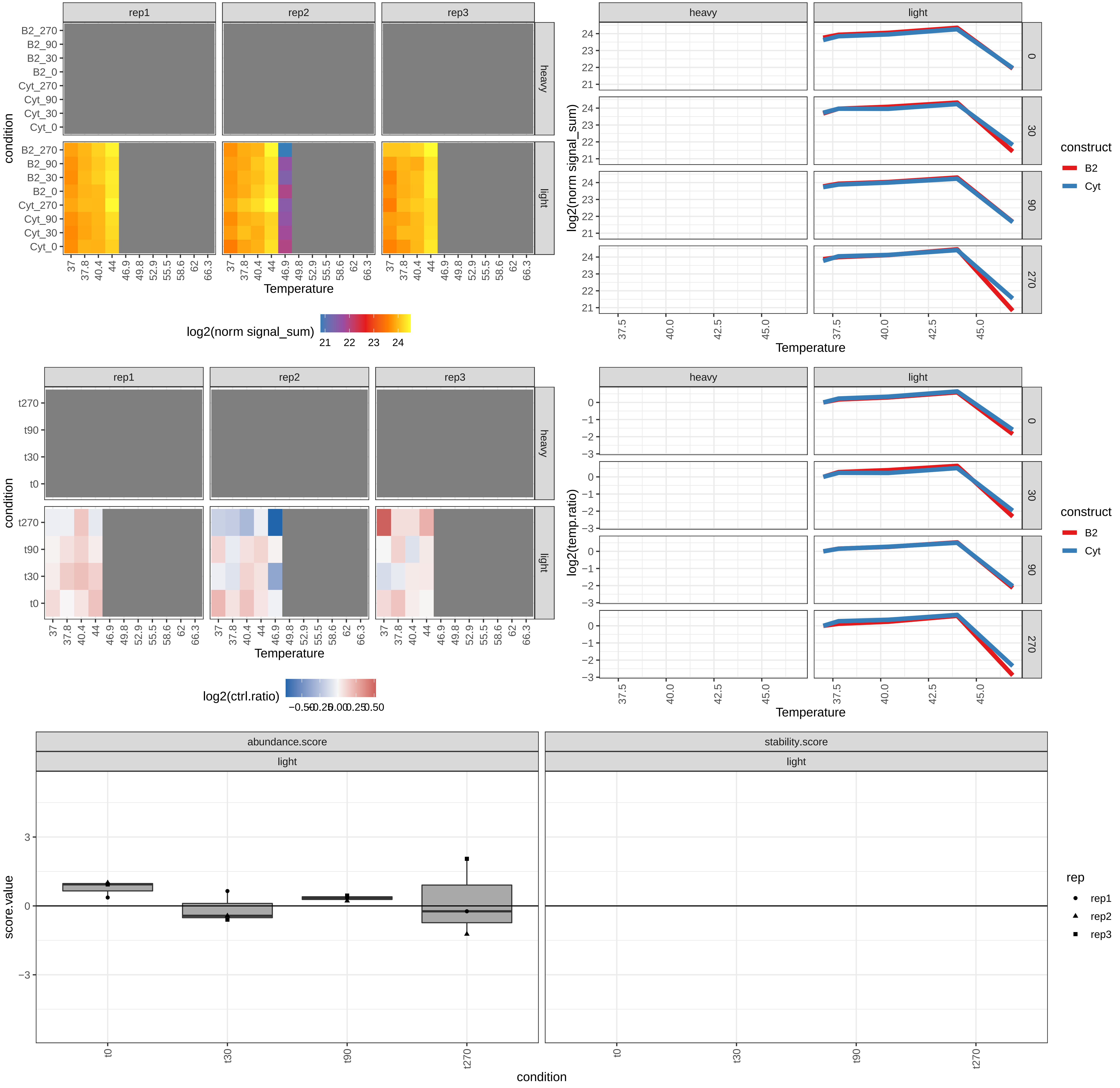

Q03942 − Ribosomal lysine N−methyltransferase 2 OS=Saccharomyces cerevisiae (strain ATCC 204508 / S288c) GN=RKM2 PE=1 SV=1

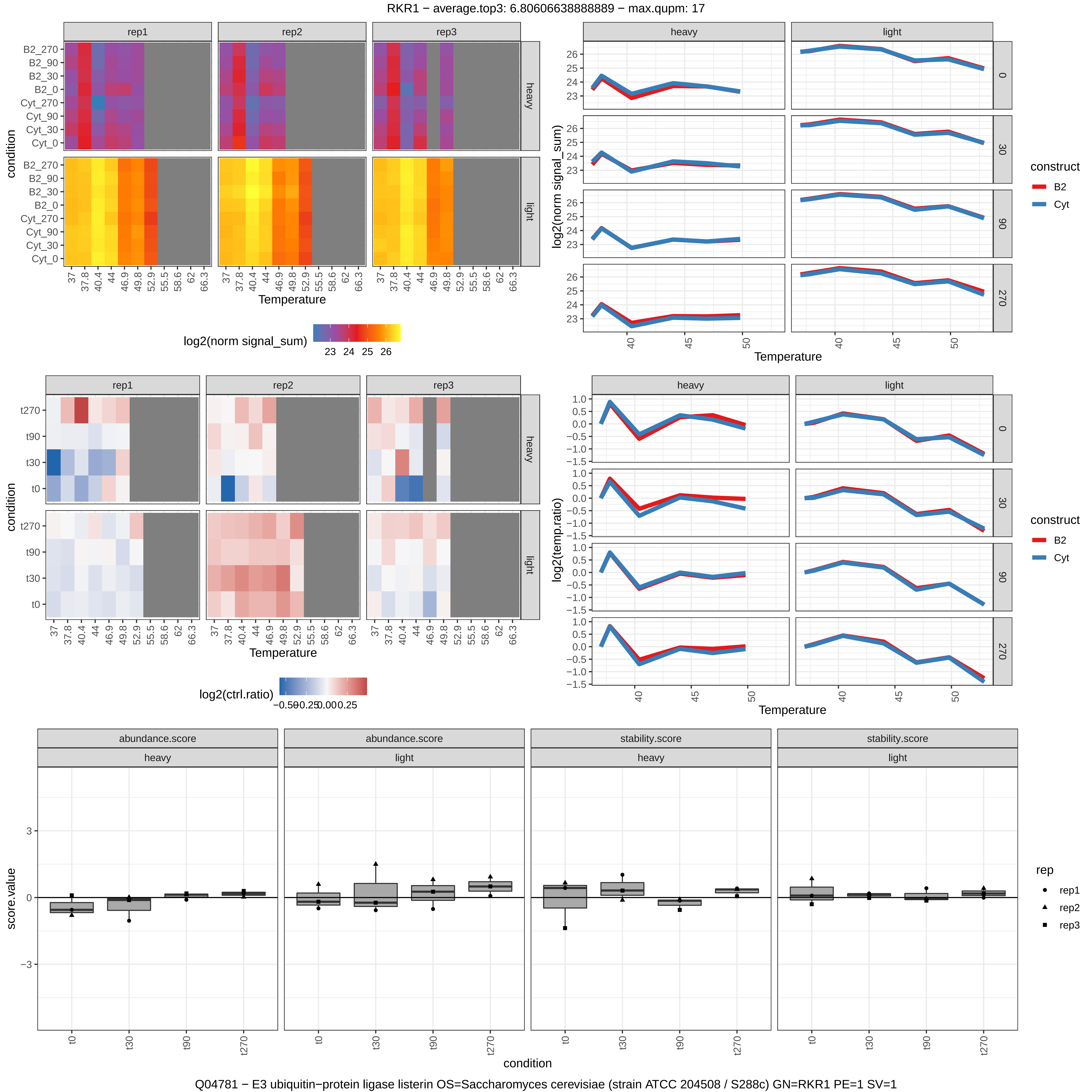
RKR1 − average.top3: 6.80606638888889 − max.qupm: 17
rep1
rep2
rep3
heavy
light
B2_270
B2_90
B2_30
B2_0
Cyt_270
Cyt_90
Cyt_30
Cyt_0
condition
Temperature
log2(norm signal_sum)
23 24 25 26
log2(norm signal_sum)
construct
B2
Cyt
t270
t90
t30
t0
log2(ctrl.ratio)
log2(temp.ratio)
abundance.score
stability.score
score.value
rep
rep1
rep2
rep3
Q04781 − E3 ubiquitin−protein ligase listerin OS=Saccharomyces cerevisiae (strain ATCC 204508 / S288c) GN=RKR1 PE=1 SV=1

RLI1 − average.top3: 7.25488966666667 − max.qupm: 24

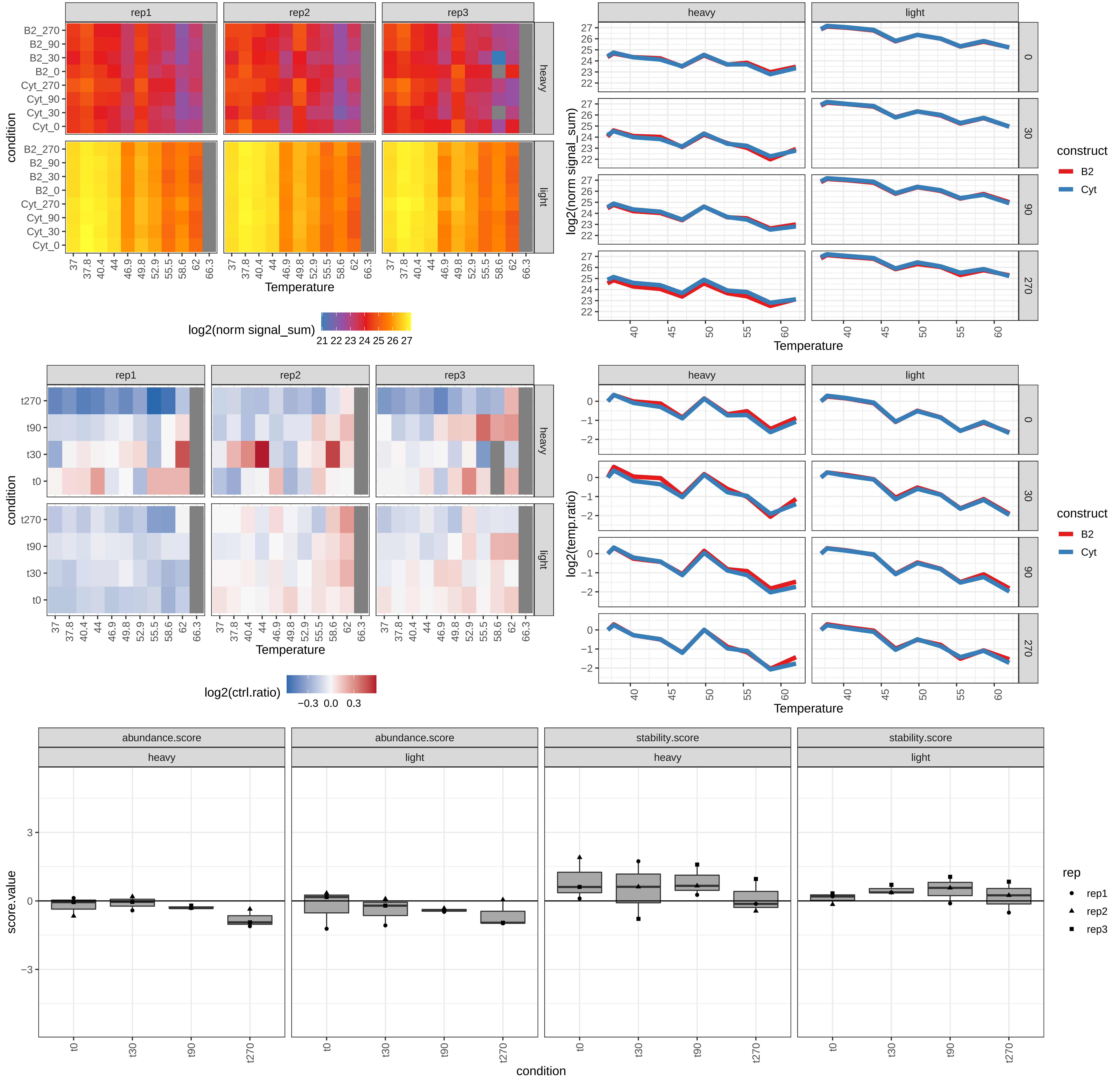

Q03195 − Translation initiation factor RLI1 OS=Saccharomyces cerevisiae (strain ATCC 204508 / S288c) GN=RLI1 PE=1 SV=1

RLP7 − average.top3: 6.73418703703704 − max.qupm: 6

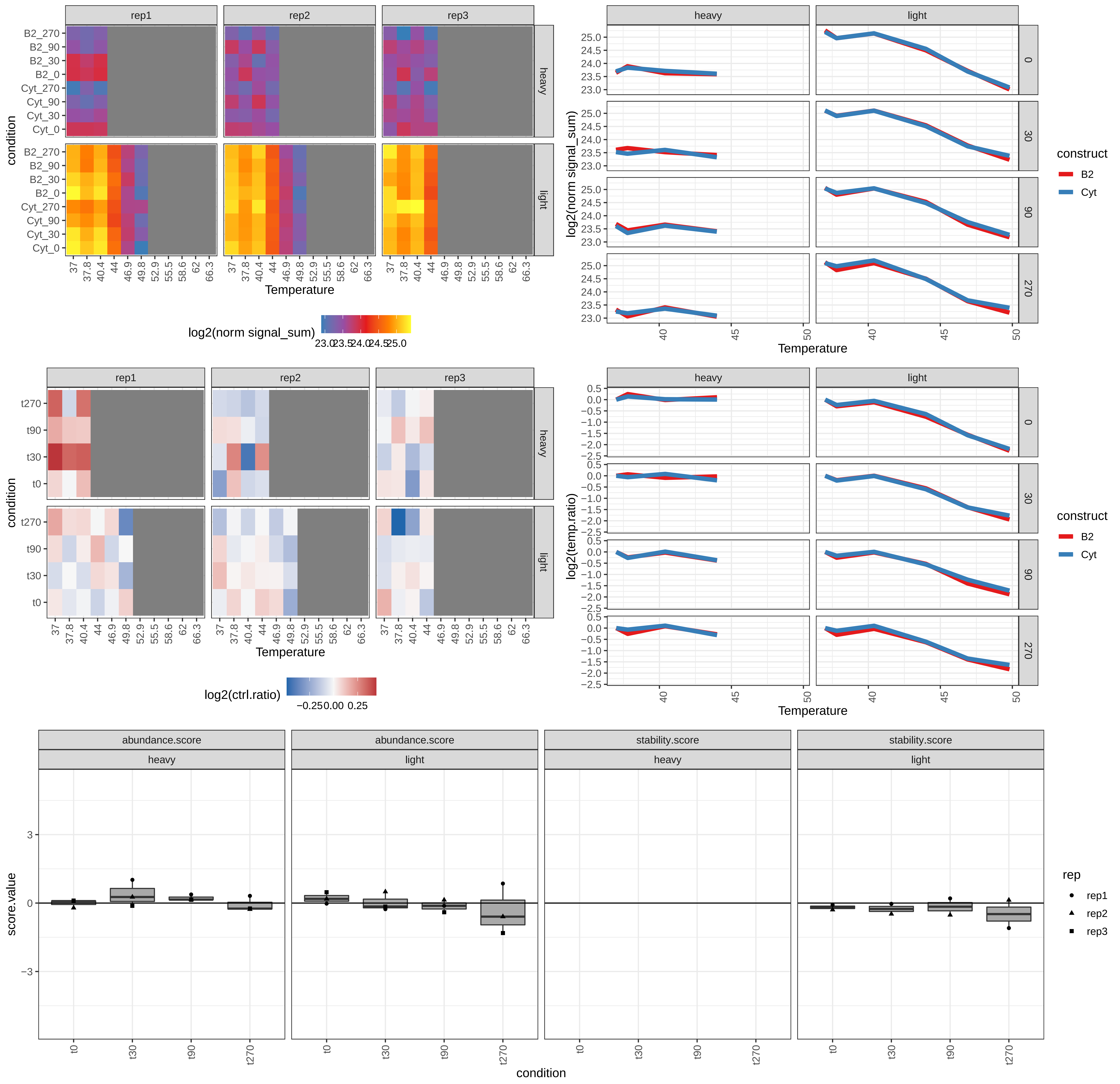

P40693 − Ribosome biogenesis protein RLP7 OS=Saccharomyces cerevisiae (strain ATCC 204508 / S288c) GN=RLP7 PE=1 SV=1

RMD1 − average.top3: 6.25703666666667 − max.qupm: 4

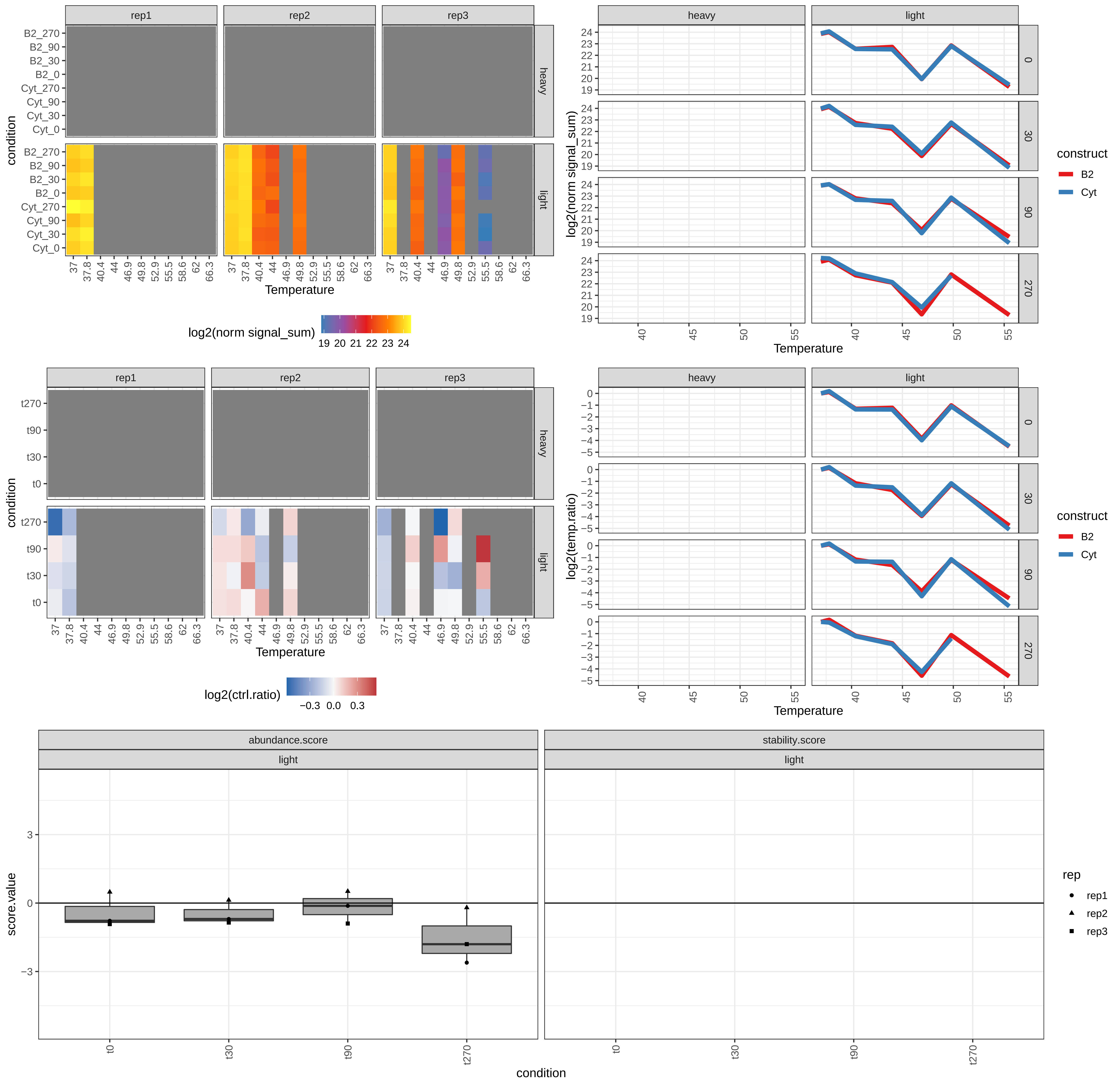

Q03441 − Sporulation protein RMD1 OS=Saccharomyces cerevisiae (strain ATCC 204508 / S288c) GN=RMD1 PE=1 SV=1

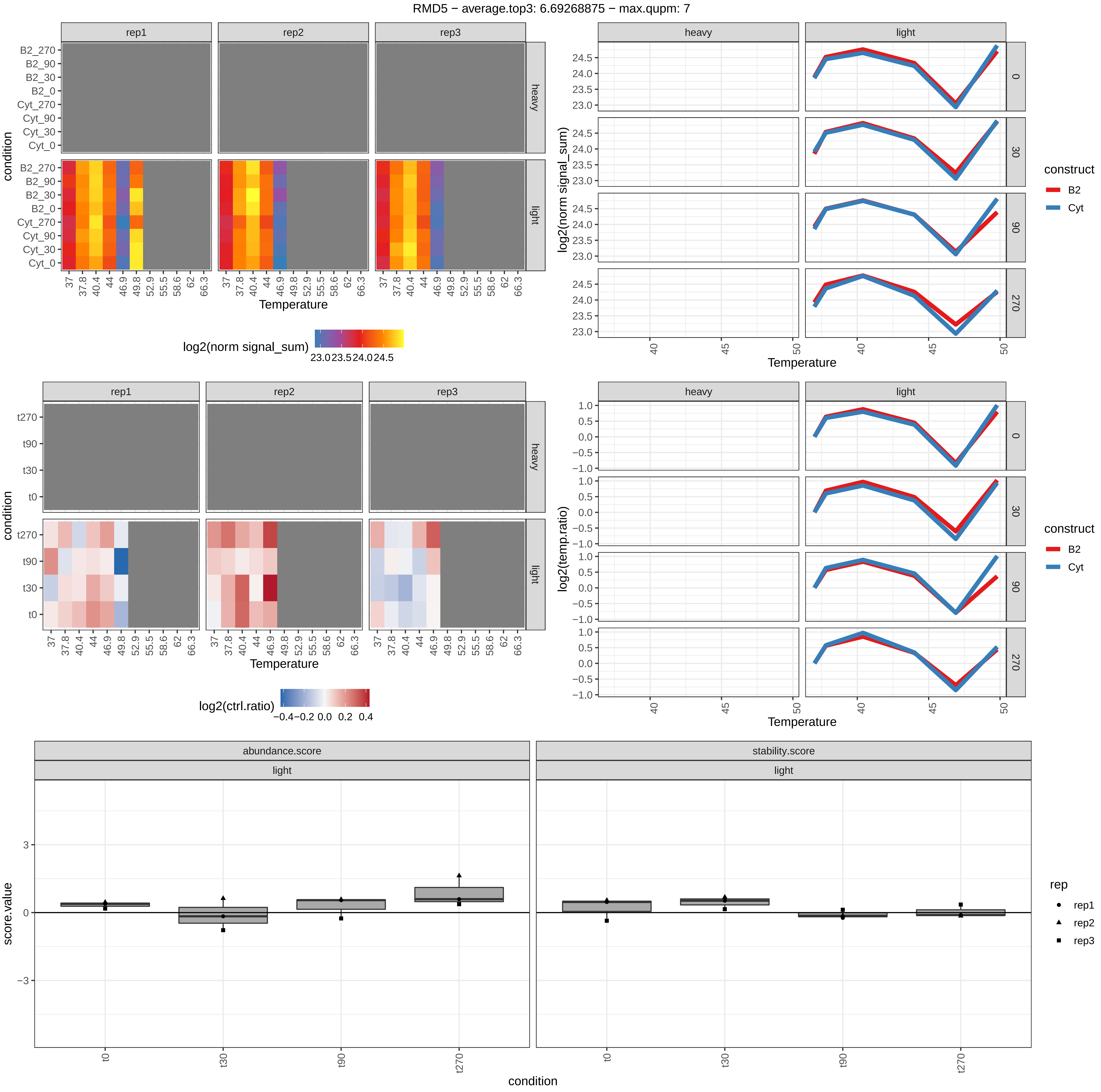

Q12508 − Sporulation protein RMD5 OS=Saccharomyces cerevisiae (strain ATCC 204508 / S288c) GN=RMD5 PE=1 SV=1

RMD9 − average.top3: 6.75850470588235 − max.qupm: 17

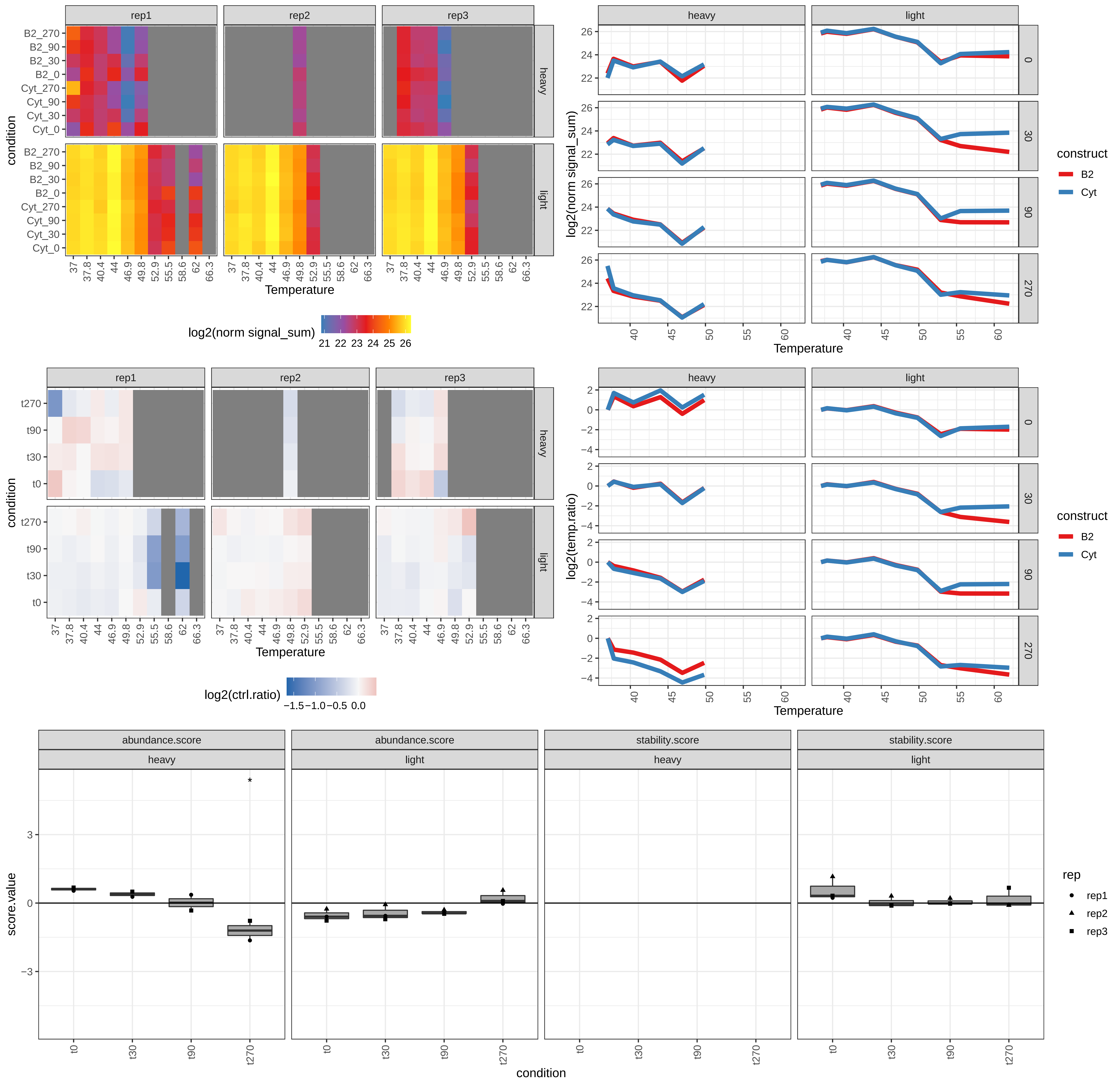

P53140 − Protein RMD9, mitochondrial OS=Saccharomyces cerevisiae (strain ATCC 204508 / S288c) GN=RMD9 PE=1 SV=1

RML2 − average.top3: 6.69725416666667 − max.qupm: 5

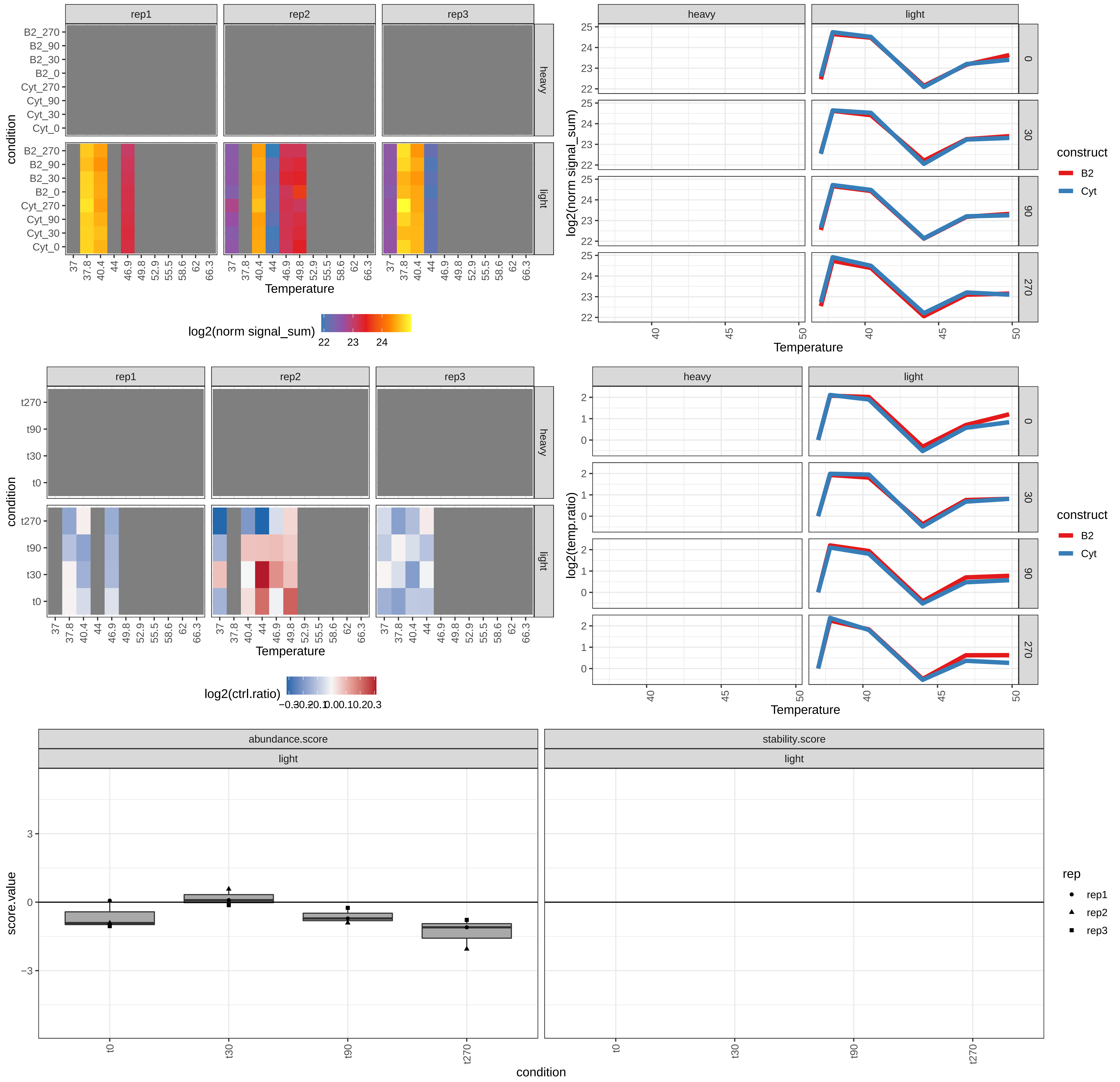

P32611 − 54S ribosomal protein RML2, mitochondrial OS=Saccharomyces cerevisiae (strain ATCC 204508 / S288c) GN=RML2 PE=1 SV=1

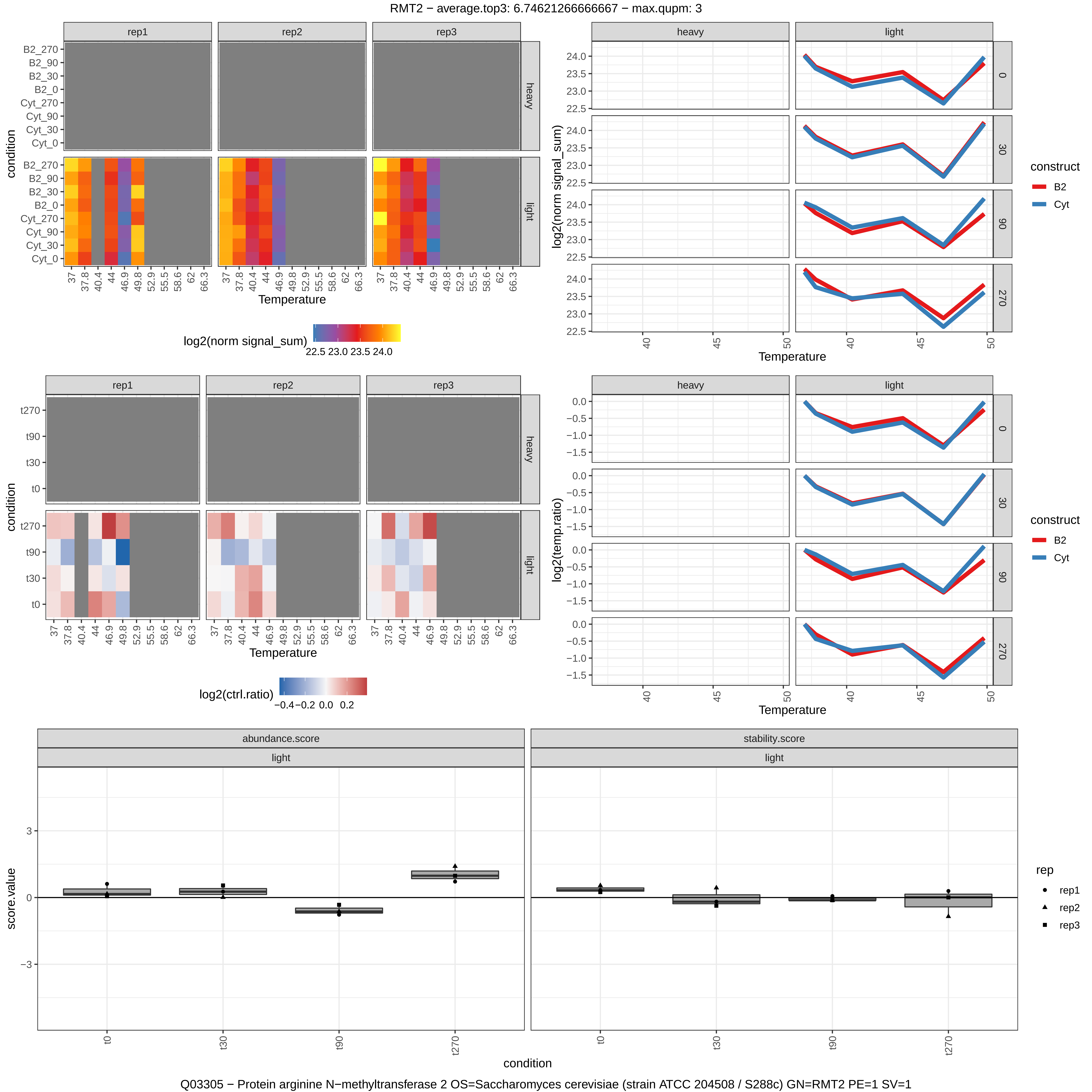

RMT2 − average.top3: 6.74621266666667 − max.qupm: 3
rep1
rep2
rep3
heavy
light
condition
Temperature
log2(norm signal_sum)
22.5 23.0 23.5 24.0
log2(norm signal_sum)
construct
B2
Cyt
log2(ctrl.ratio)
−0.4 −0.2 0.0 0.2
log2(temp.ratio)
abundance.score
stability.score
score.value
rep
rep1
rep2
rep3
Q03305 − Protein arginine N−methyltransferase 2 OS=Saccharomyces cerevisiae (strain ATCC 204508 / S288c) GN=RMT2 PE=1 SV=1

RNA1 − average.top3: 6.85171542857143 − max.qupm: 12

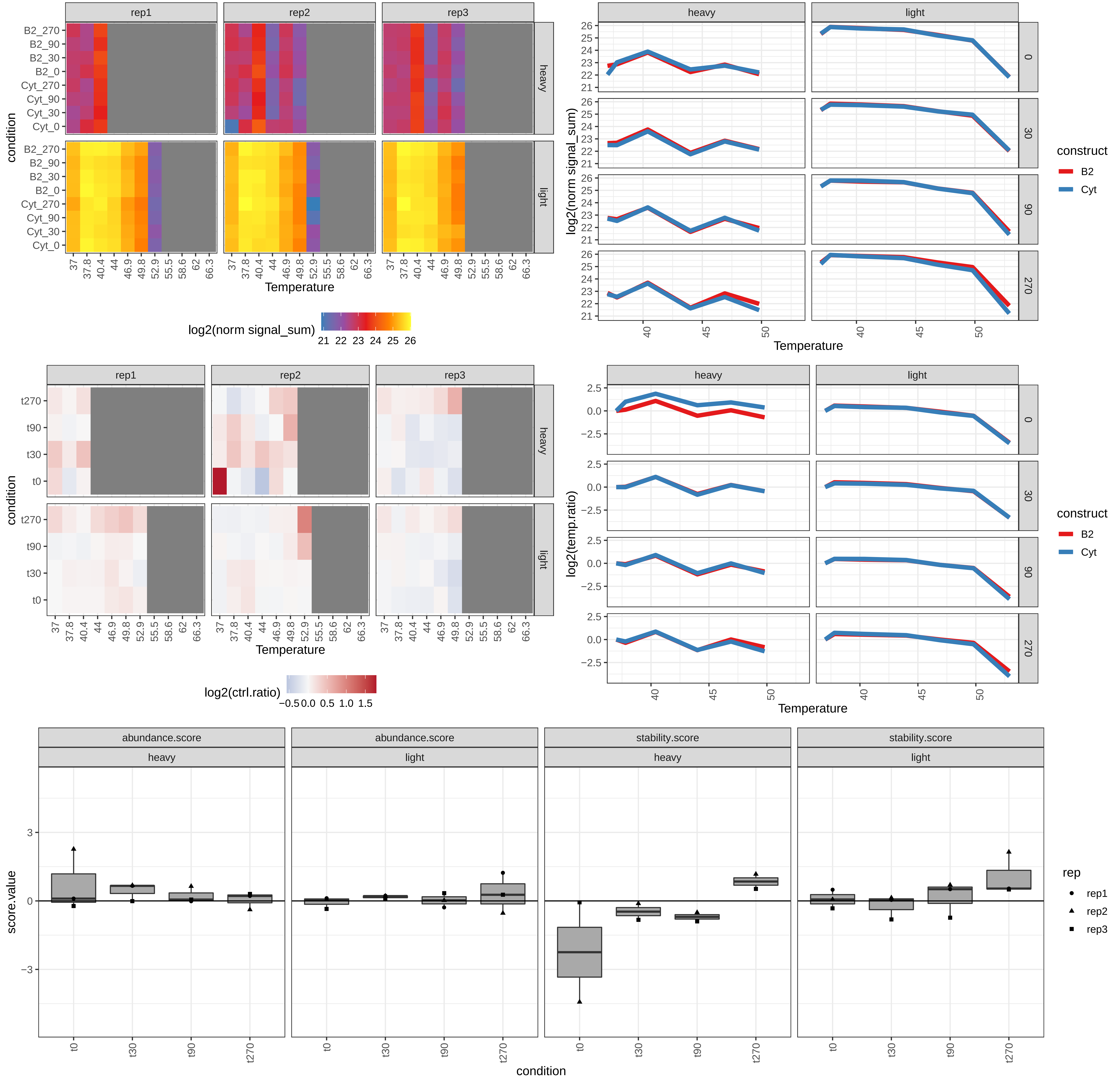

P11745 − Ran GTPase−activating protein 1 OS=Saccharomyces cerevisiae (strain ATCC 204508 / S288c) GN=RNA1 PE=1 SV=2

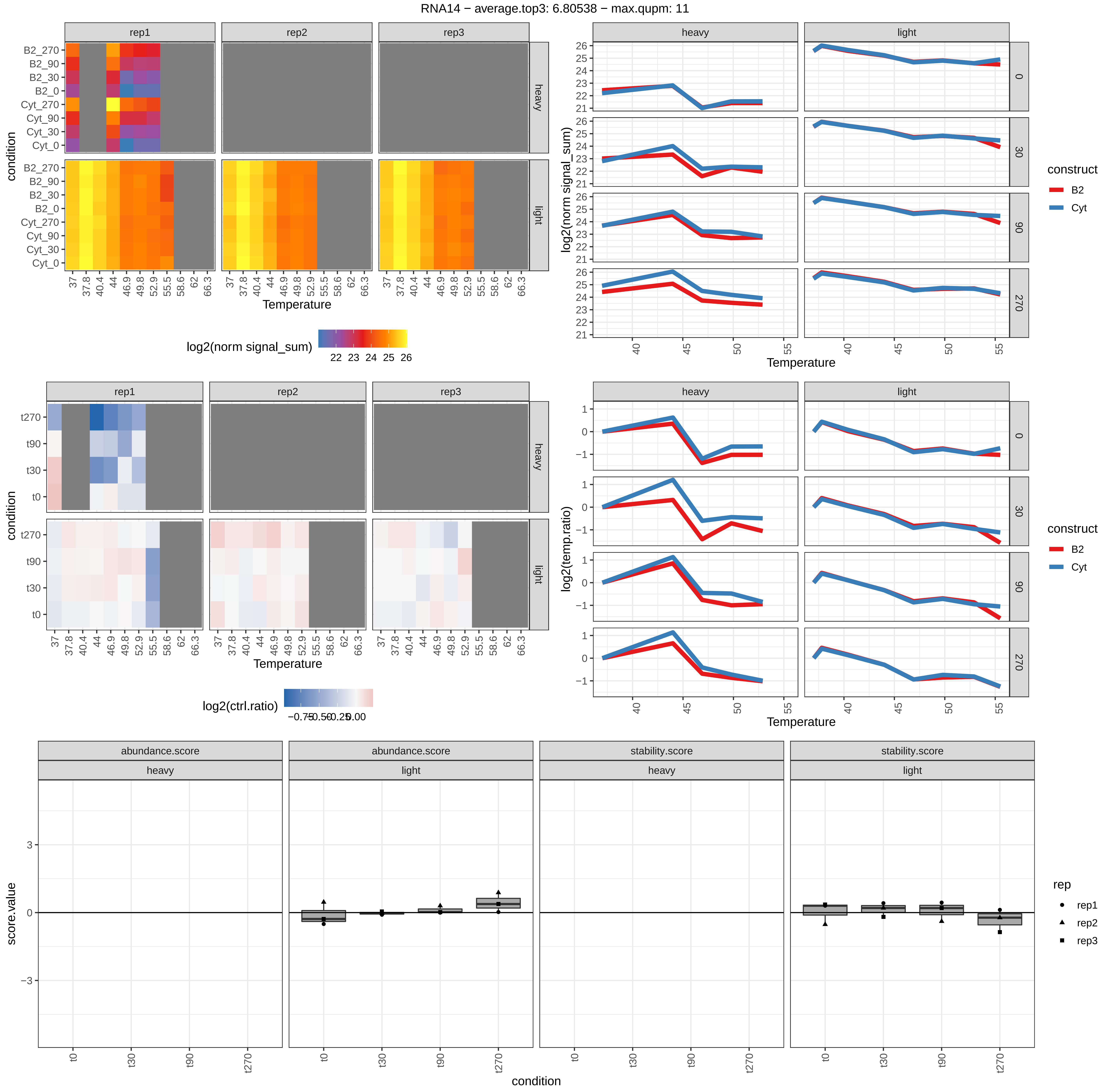

P25298 − mRNA 3'−end−processing protein RNA14 OS=Saccharomyces cerevisiae (strain ATCC 204508 / S288c) GN=RNA14 PE=1 SV=2

RNH70 − average.top3: 6.417758526315789 − max.qupm: 7

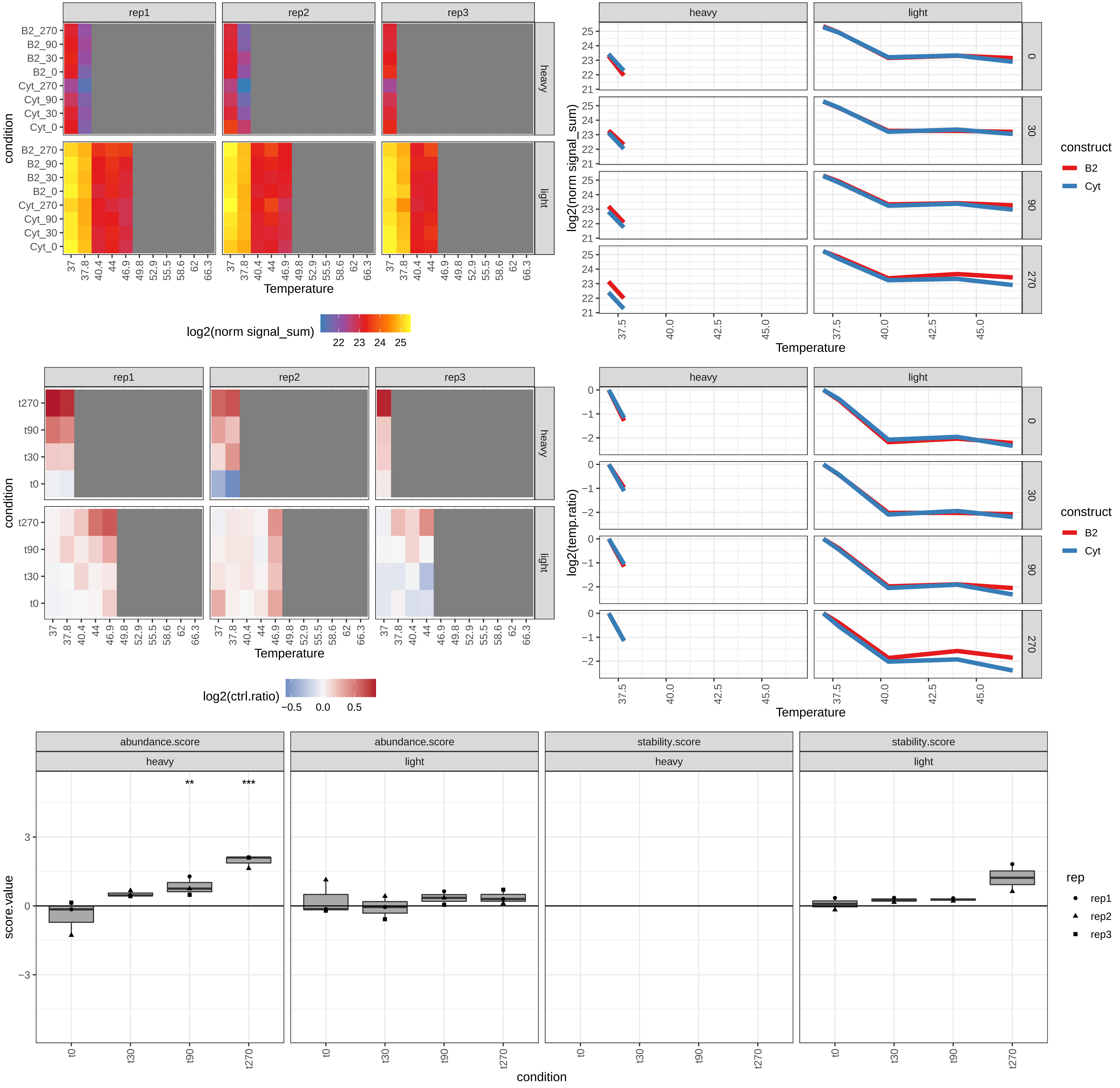

P53331 − RNA exonuclease 1 OS=Saccharomyces cerevisiae (strain ATCC 204508 / S288c) GN=RNH70 PE=1 SV=1

RNR1 − average.top3: 7.16227285714286 − max.qupm: 15

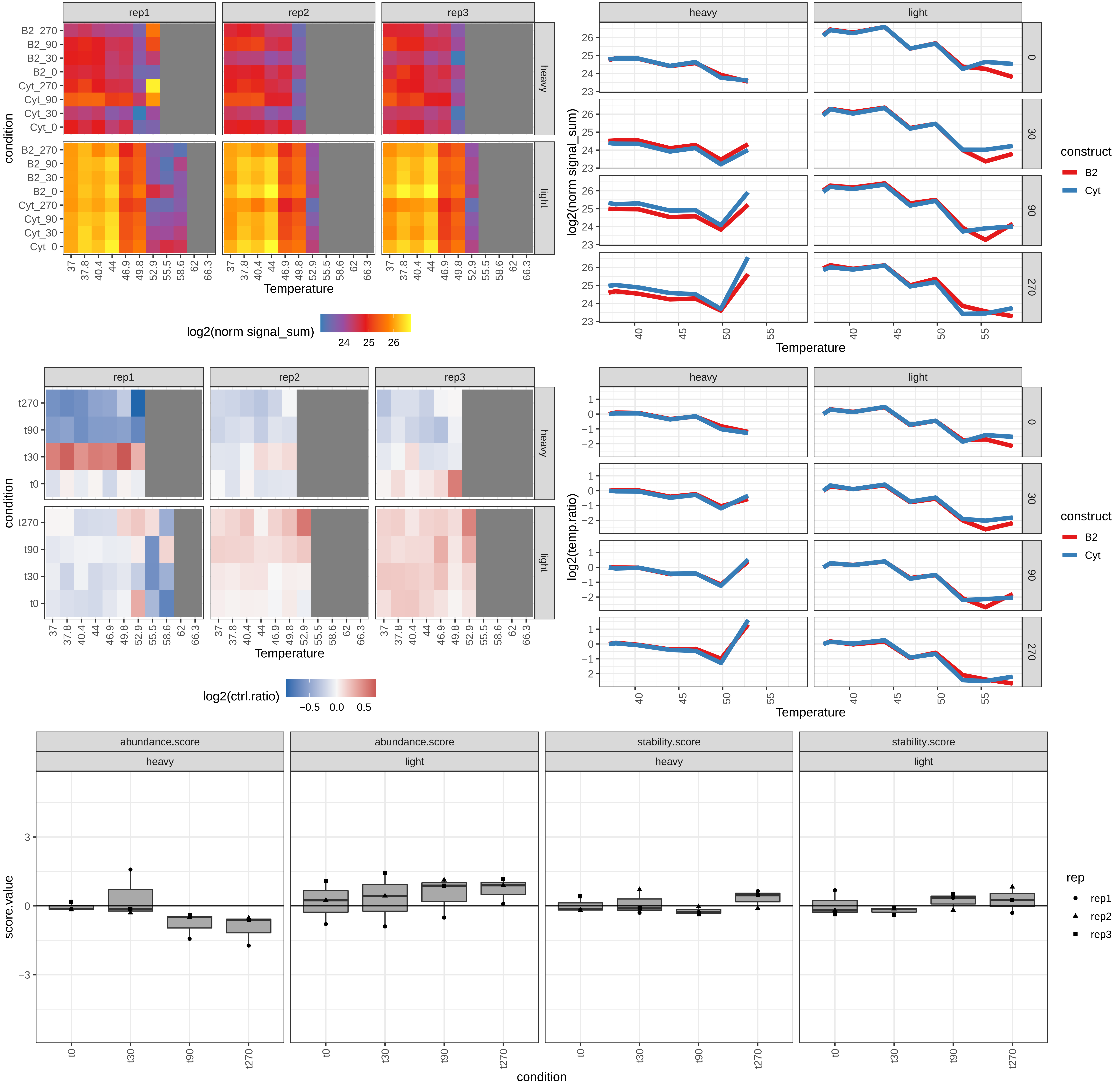

P21524 − Ribonucleoside−diphosphate reductase large chain 1 OS=Saccharomyces cerevisiae (strain ATCC 204508 / S288c) GN=RNR1 PE=1 SV=2

RNR2 − average.top3: 7.68834828125 − max.qupm: 18

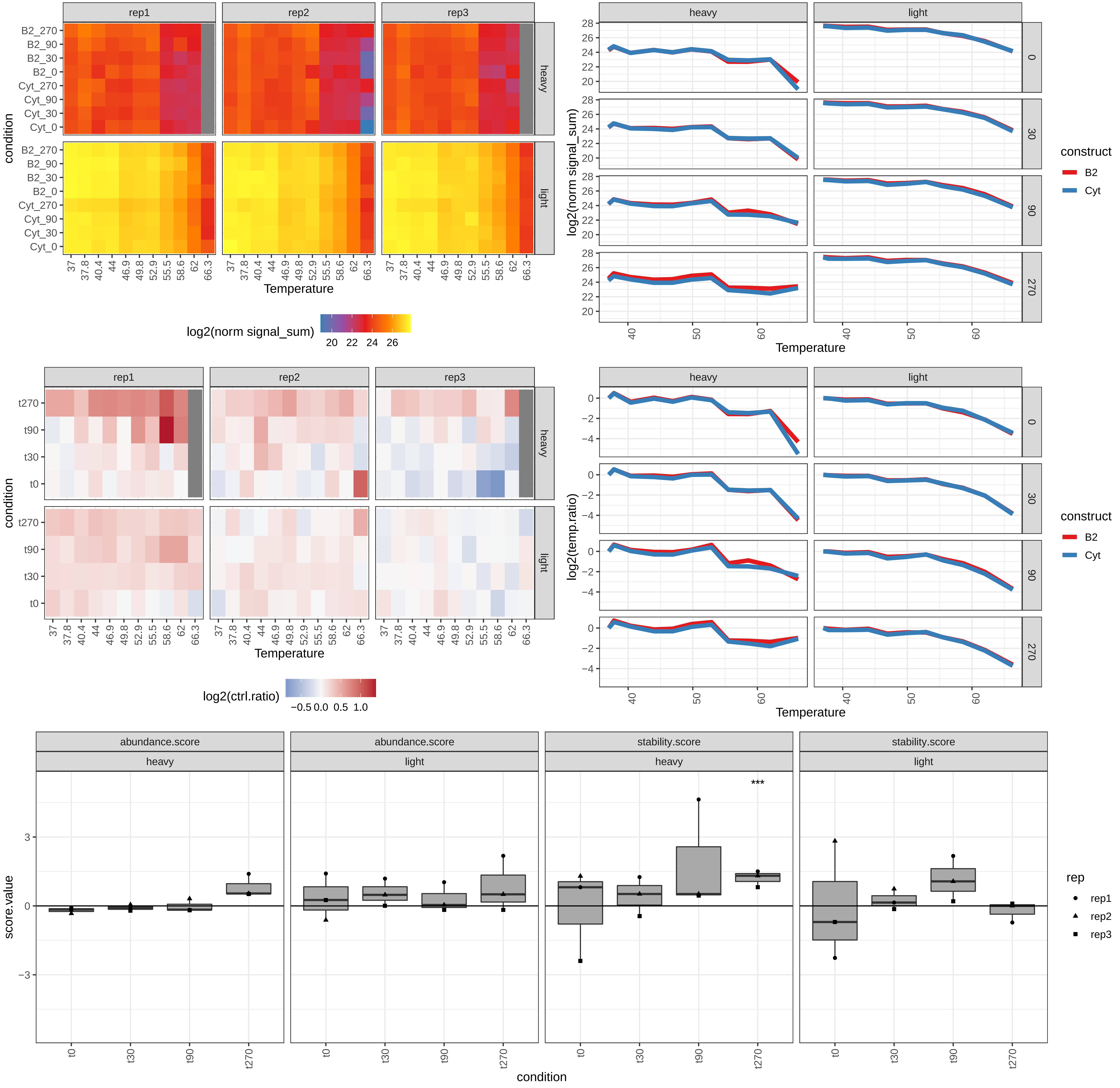

P09938 − Ribonucleoside−diphosphate reductase small chain 1 OS=Saccharomyces cerevisiae (strain ATCC 204508 / S288c) GN=RNR2 PE=1 SV=2

RNR4 − average.top3: 7.80804646153846 − max.qupm: 16

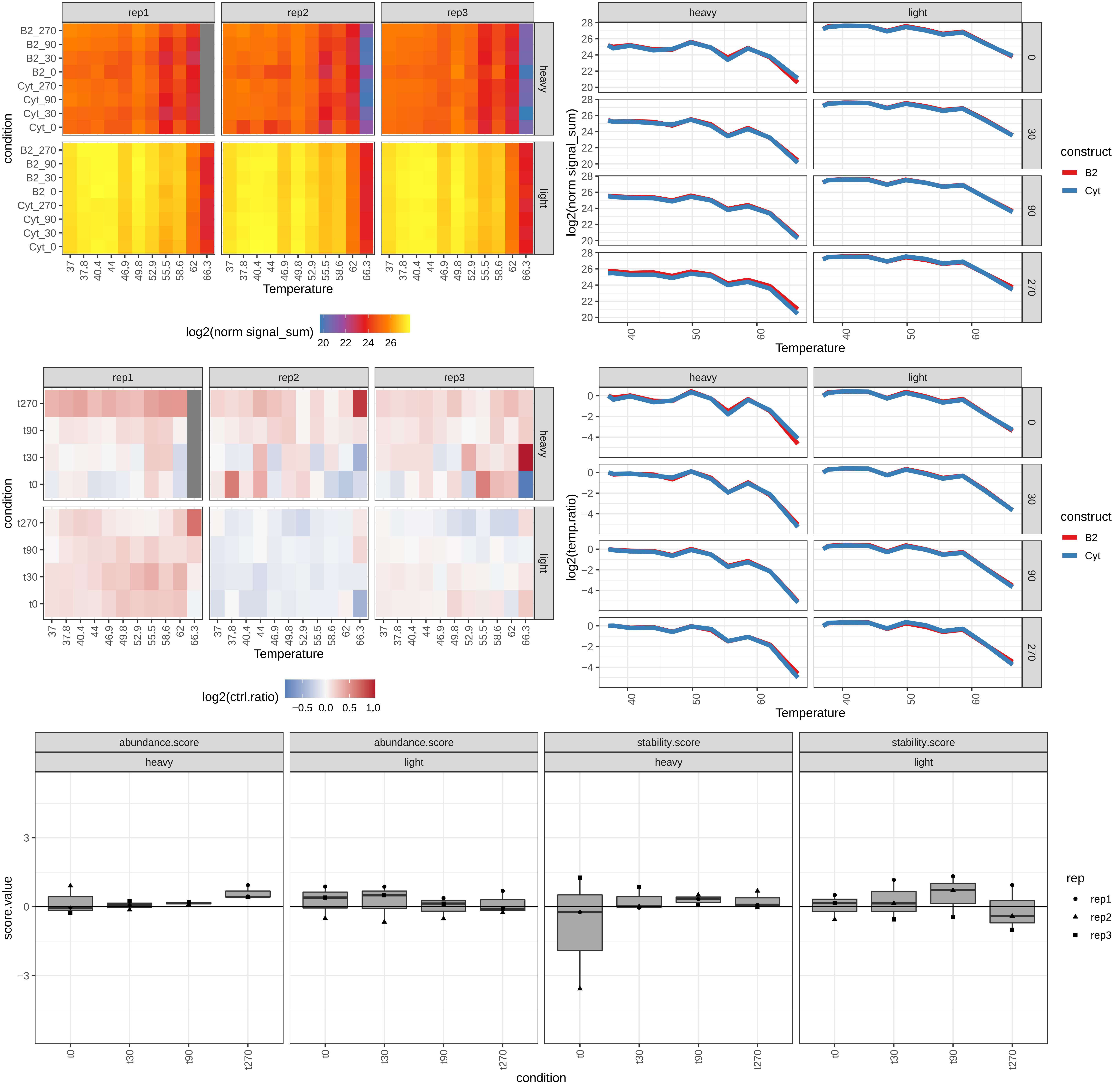

P49723 − Ribonucleoside−diphosphate reductase small chain 2 OS=Saccharomyces cerevisiae (strain ATCC 204508 / S288c) GN=RNR4 PE=1 SV=1

RNT1 − average.top3: 6.35631333333333 − max.qupm: 4

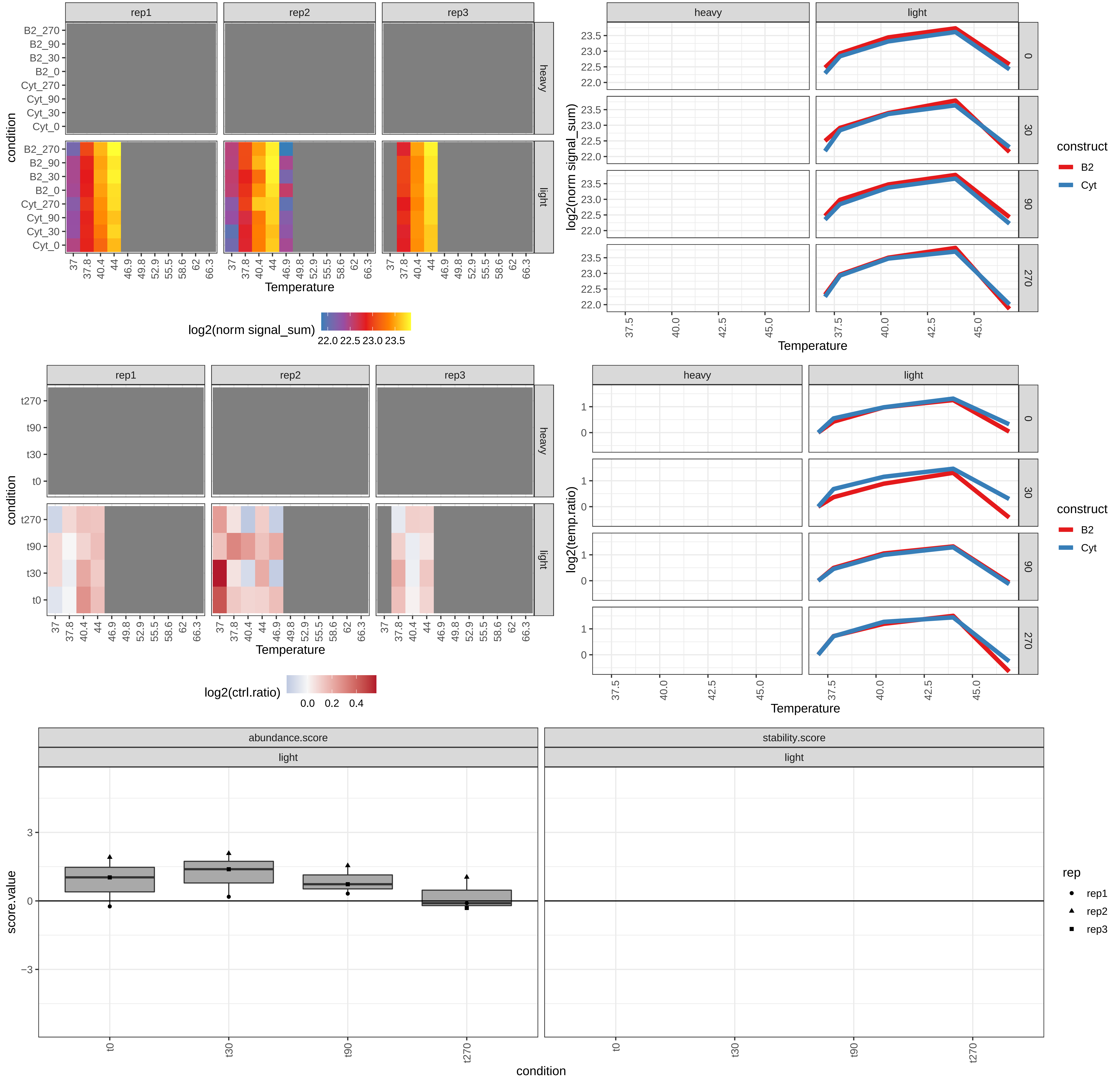

Q02555 − Ribonuclease 3 OS=Saccharomyces cerevisiae (strain ATCC 204508 / S288c) GN=RNT1 PE=1 SV=1

RNY1 − average.top3: 7.02092913043478 − max.qupm: 9

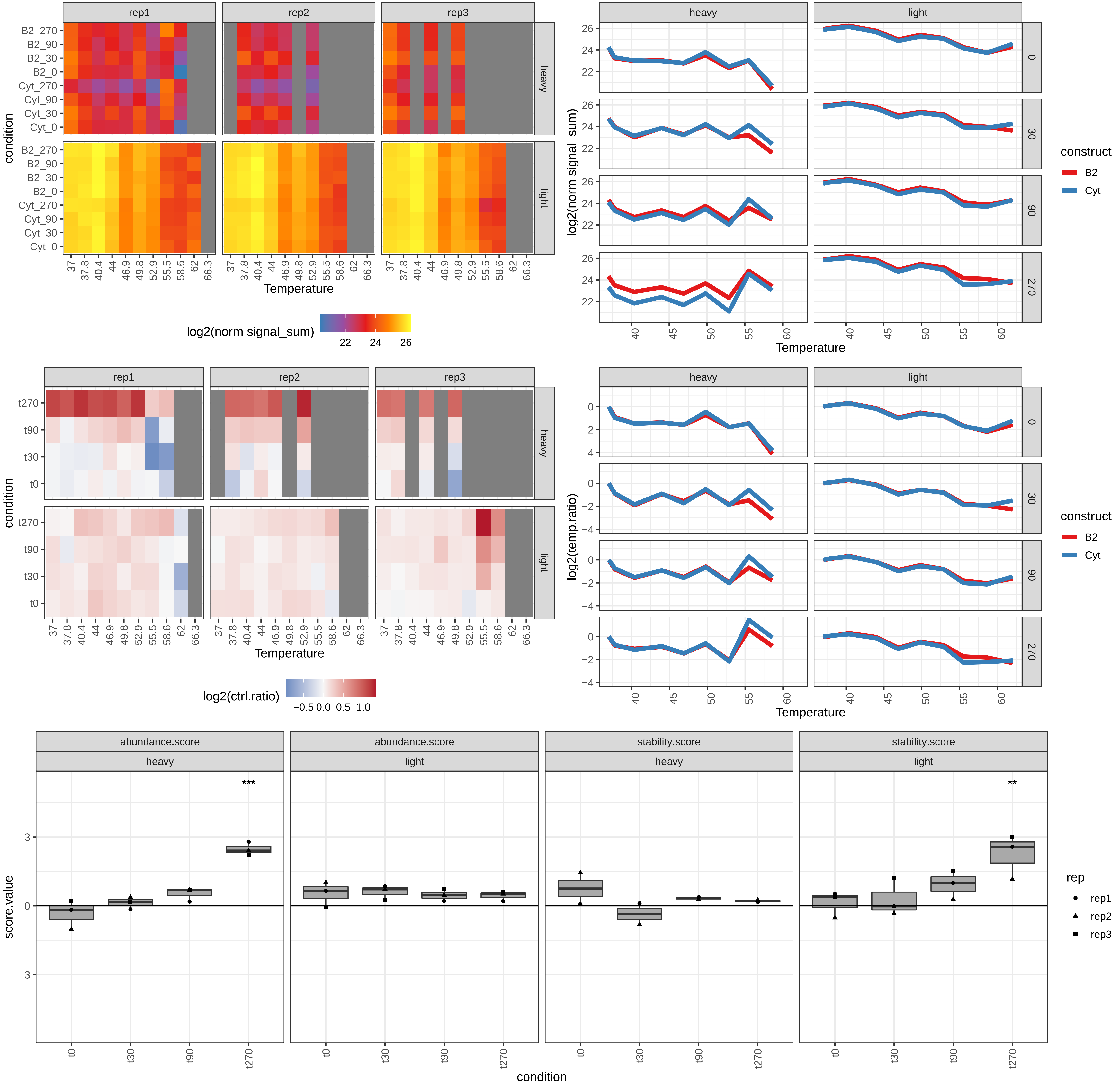

Q02933 − Ribonuclease T2−like OS=Saccharomyces cerevisiae (strain ATCC 204508 / S288c) GN=RNY1 PE=1 SV=1

ROK1 − average.top3: 6.66361476190476 − max.qupm: 4

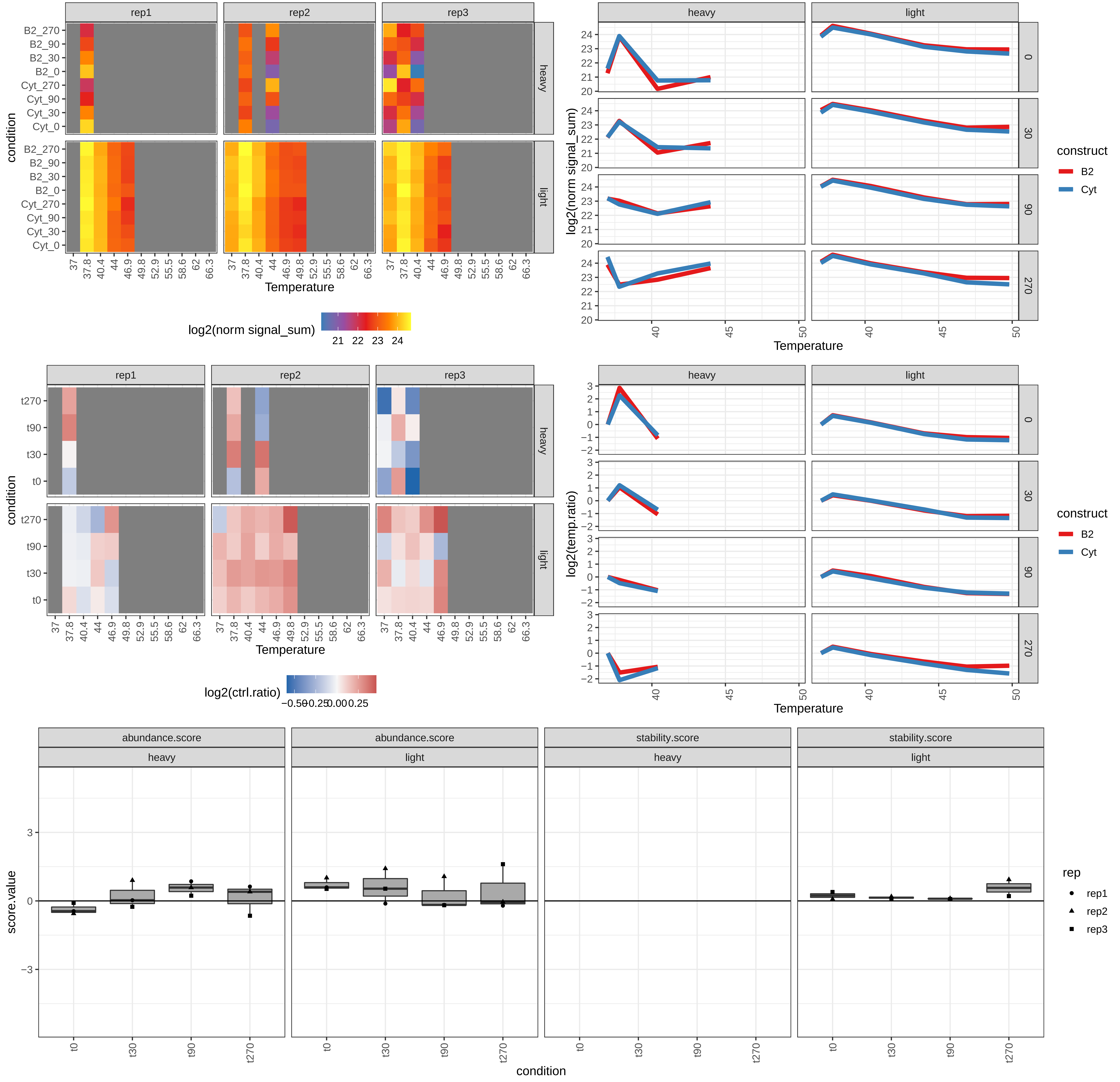

P45818 − ATP−dependent RNA helicase ROK1 OS=Saccharomyces cerevisiae (strain ATCC 204508 / S288c) GN=ROK1 PE=1 SV=1

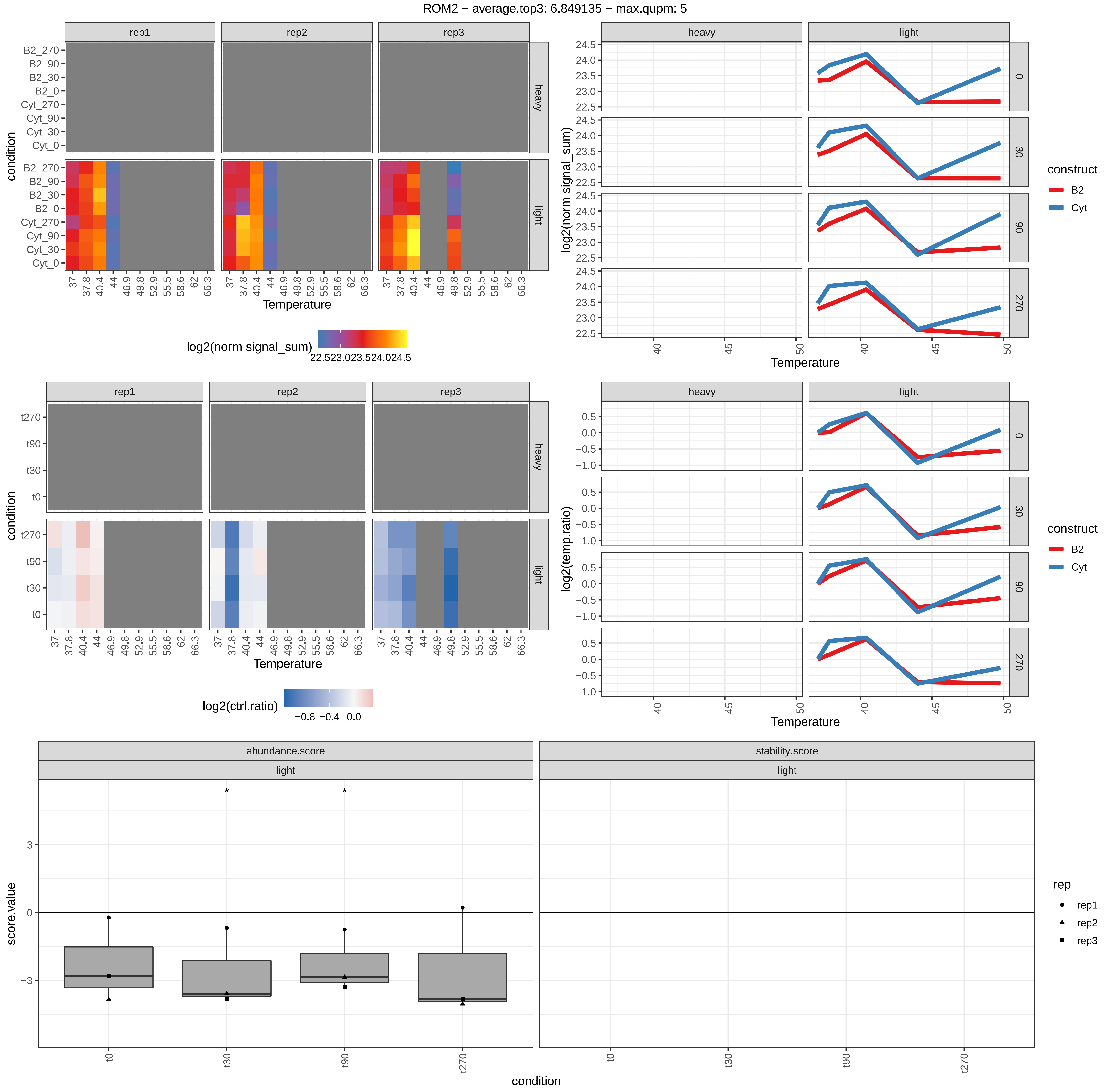

P51862 − RHO1 GDP−GTP exchange protein 2 OS=Saccharomyces cerevisiae (strain ATCC 204508 / S288c) GN=ROM2 PE=1 SV=1

ROT2 − average.top3: 7.09190689655172 − max.qupm: 13

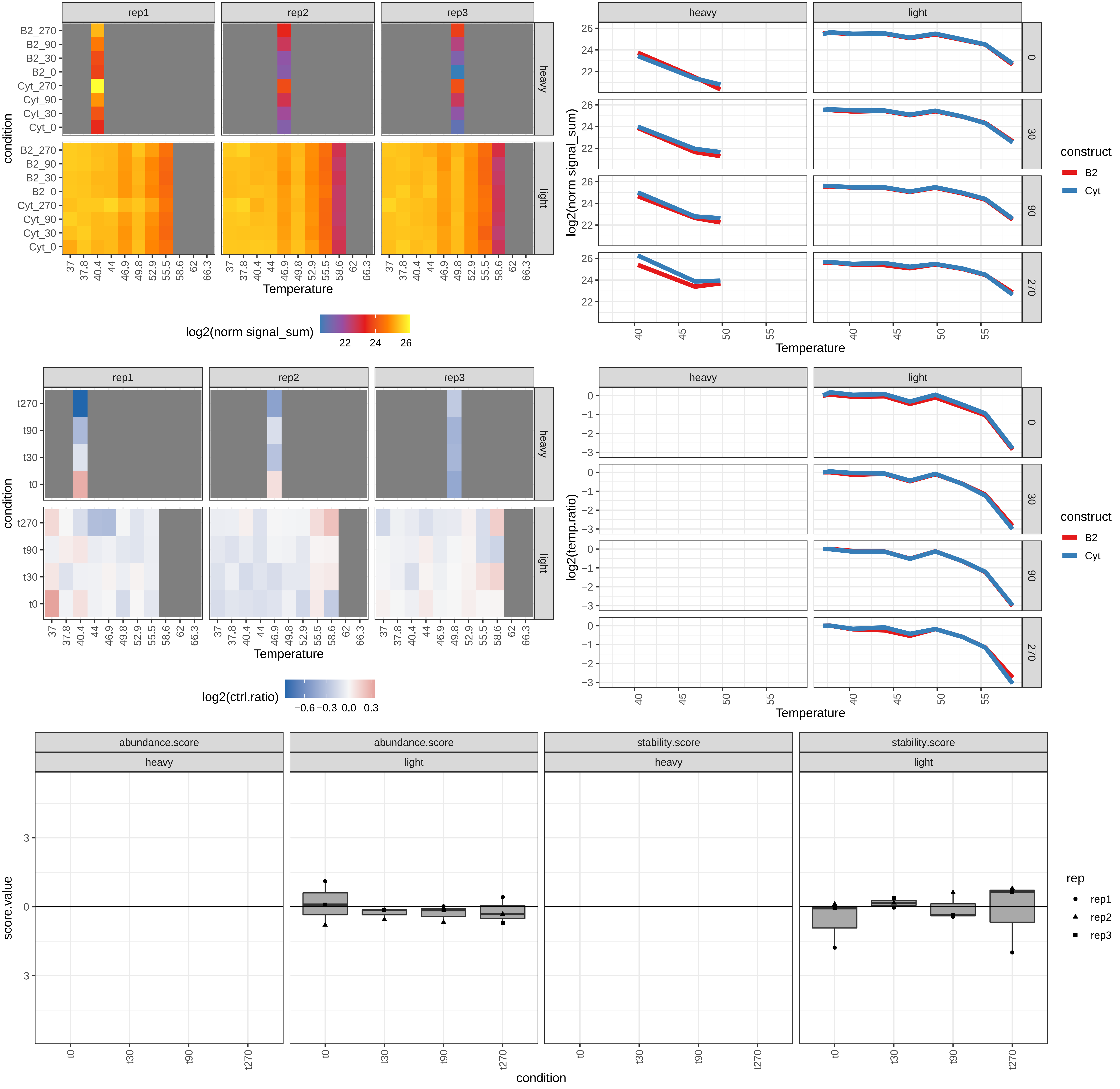

P38138 − Glucosidase 2 subunit alpha OS=Saccharomyces cerevisiae (strain ATCC 204508 / S288c) GN=ROT2 PE=1 SV=1

ROY1 − average.top3: 6.70614033333333 − max.qupm: 9

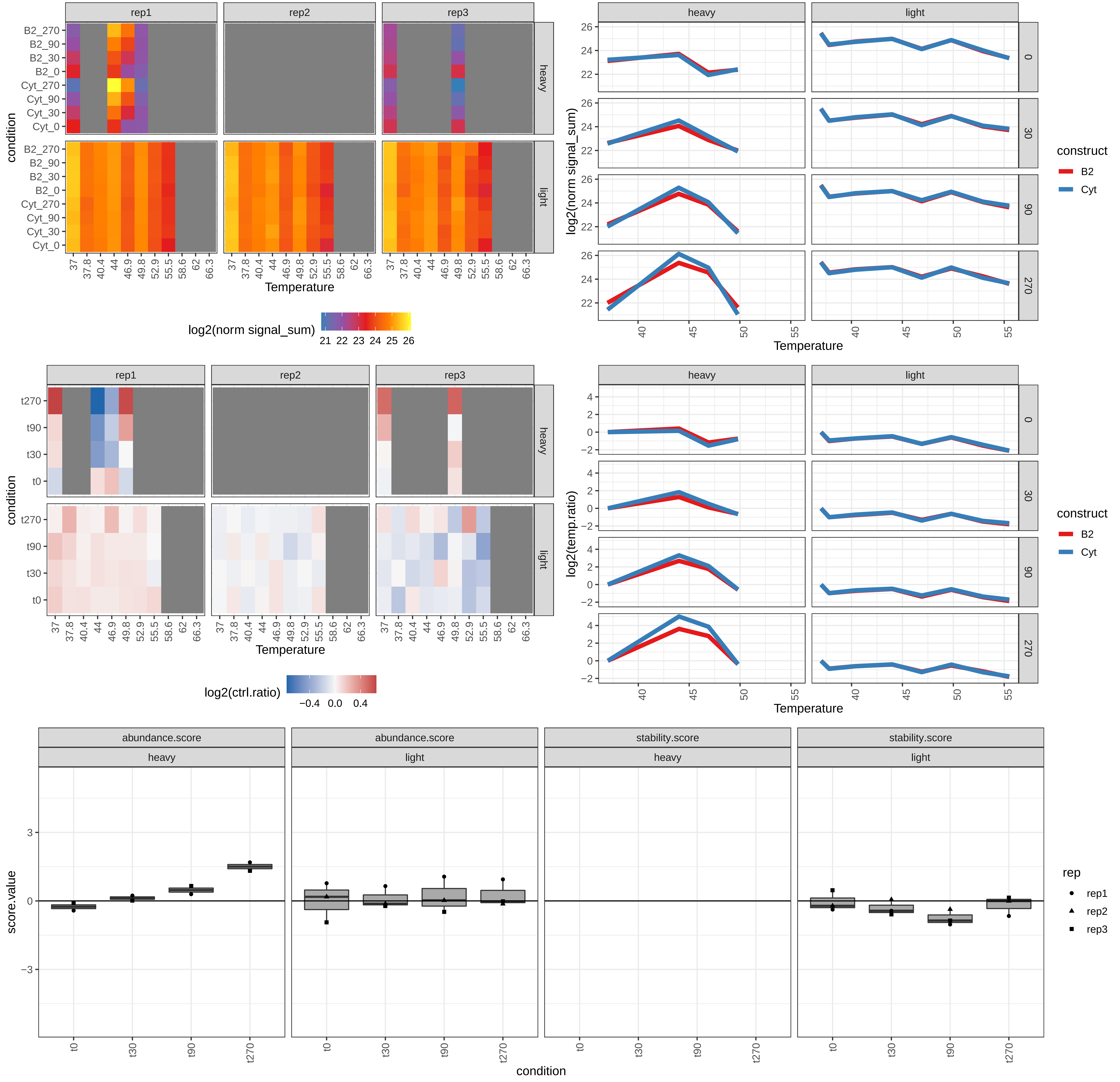

Q04847 − Non−SCF−type F−box protein ROY1 OS=Saccharomyces cerevisiae (strain ATCC 204508 / S288c) GN=ROY1 PE=1 SV=1

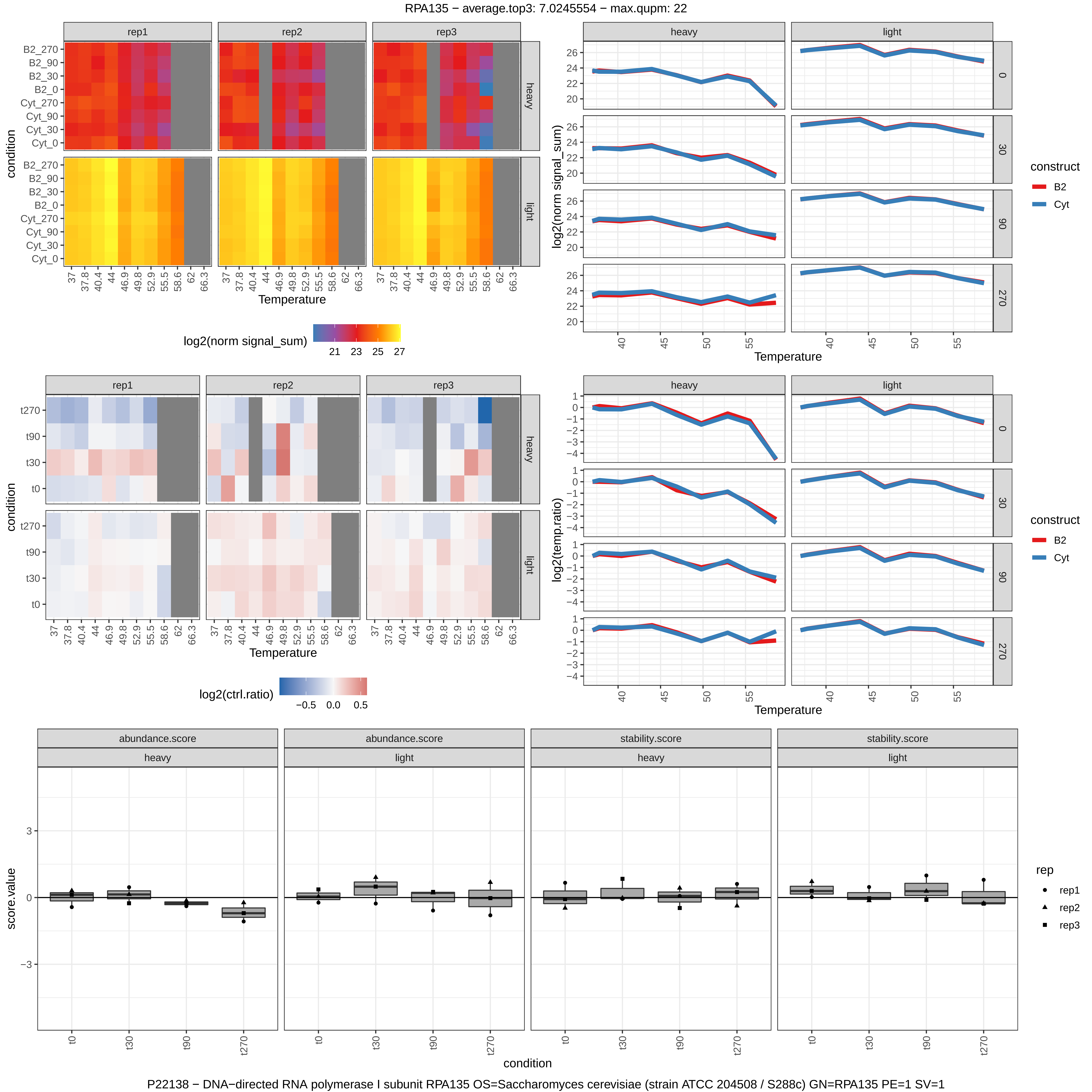
RPA135 − average.top3: 7.0245554 − max.qupm: 22
rep1
rep2
rep3
heavy
light
condition
Temperature
log2(norm signal_sum)
21 23 25 27
log2(norm signal_sum)
construct
B2
Cyt
log2(ctrl.ratio)
−0.5 0.0 0.5
log2(temp.ratio)
abundance.score
stability.score
score.value
rep
condition
P22138 − DNA−directed RNA polymerase I subunit RPA135 OS=Saccharomyces cerevisiae (strain ATCC 204508 / S288c) GN=RPA135 PE=1 SV=1

RPA190 − average.top3: 7.10493365384615 − max.qupm: 36

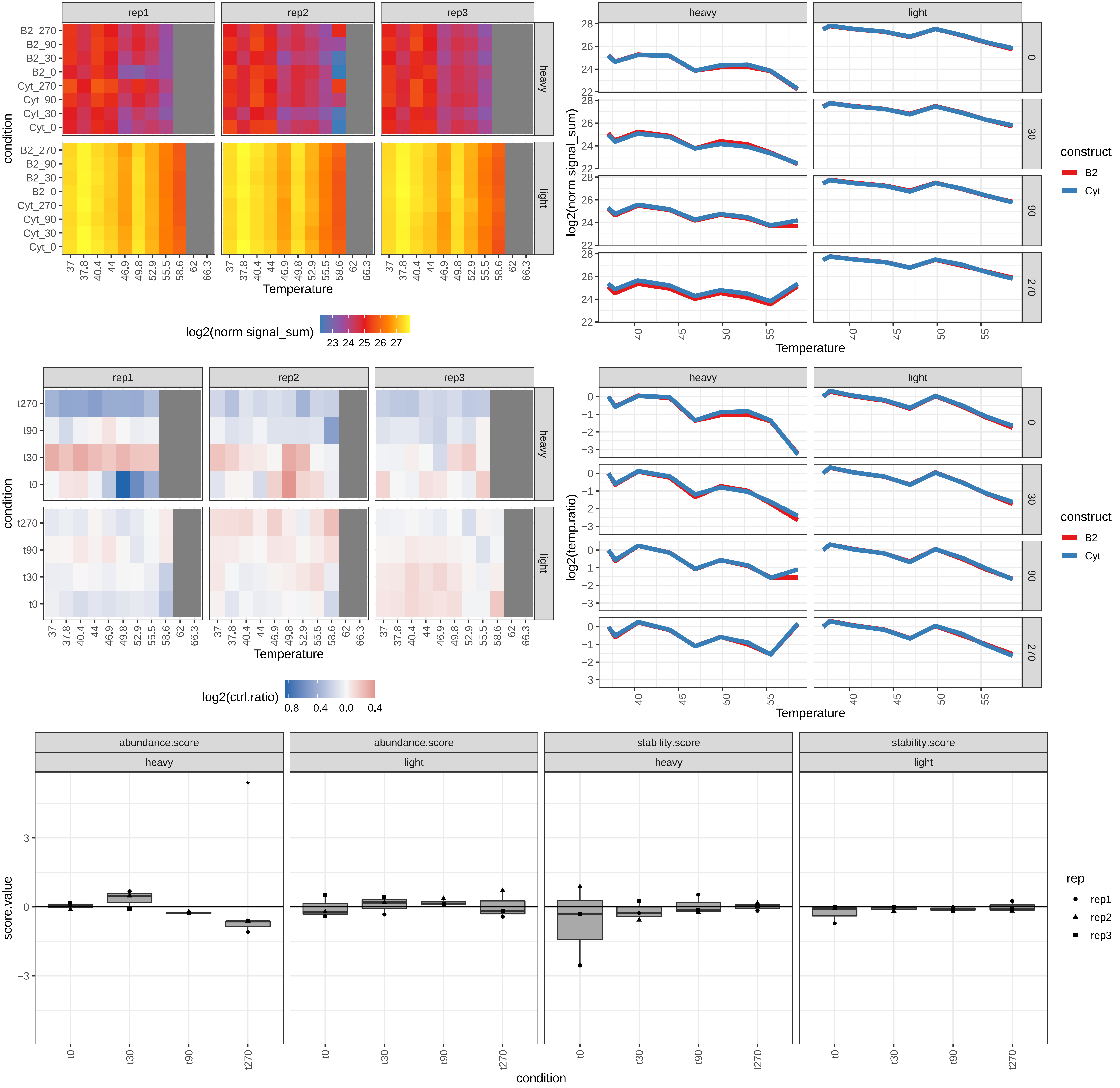

P10964 − DNA−directed RNA polymerase I subunit RPA190 OS=Saccharomyces cerevisiae (strain ATCC 204508 / S288c) GN=RPA190 PE=1 SV=2

RPA34 − average.top3: 6.68218 − max.qupm: 4

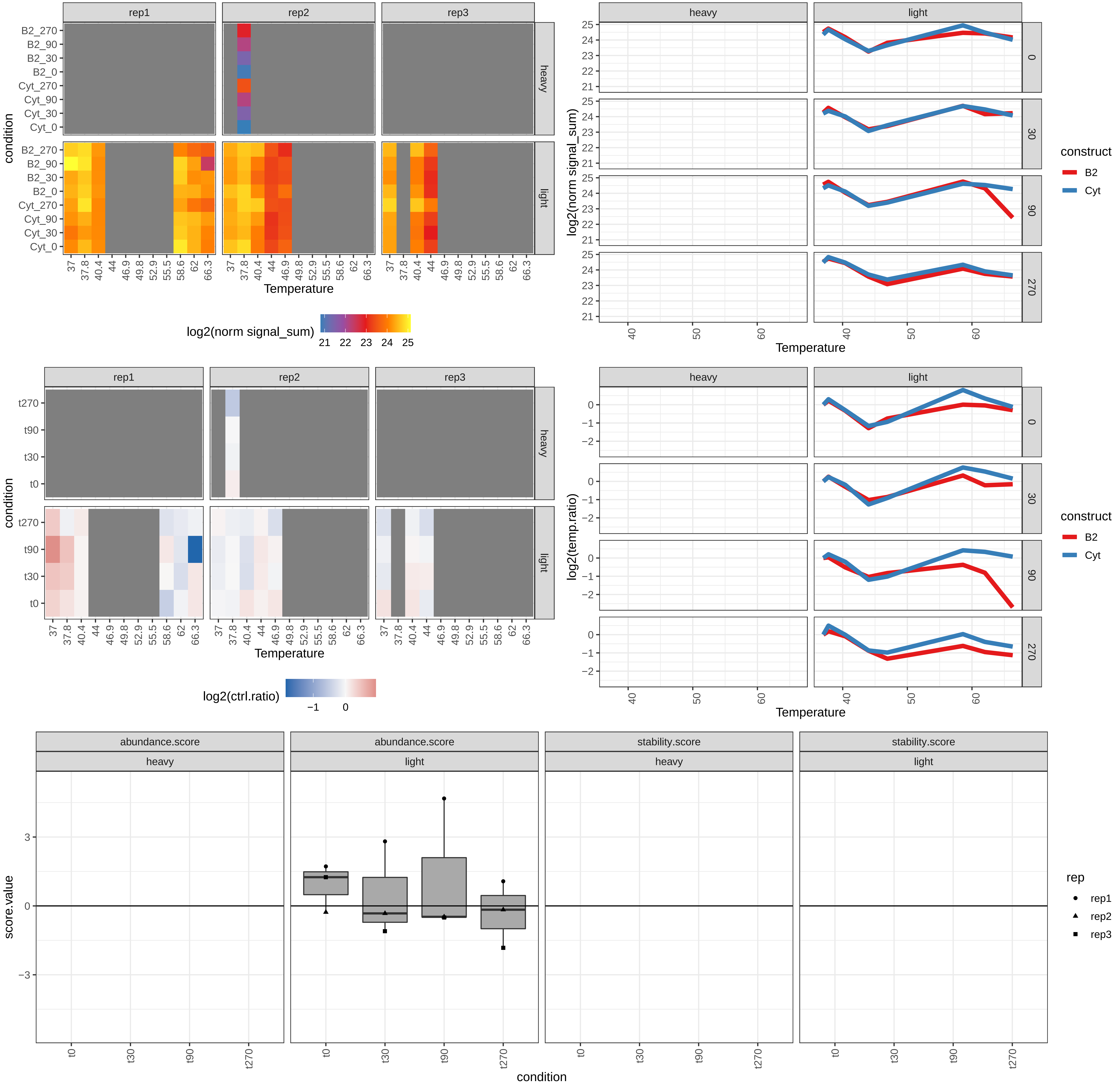

P47006 − DNA−directed RNA polymerase I subunit RPA34 OS=Saccharomyces cerevisiae (strain ATCC 204508 / S288c) GN=RPA34 PE=1 SV=1

RPA43 − average.top3: 6.76315148148148 − max.qupm: 6

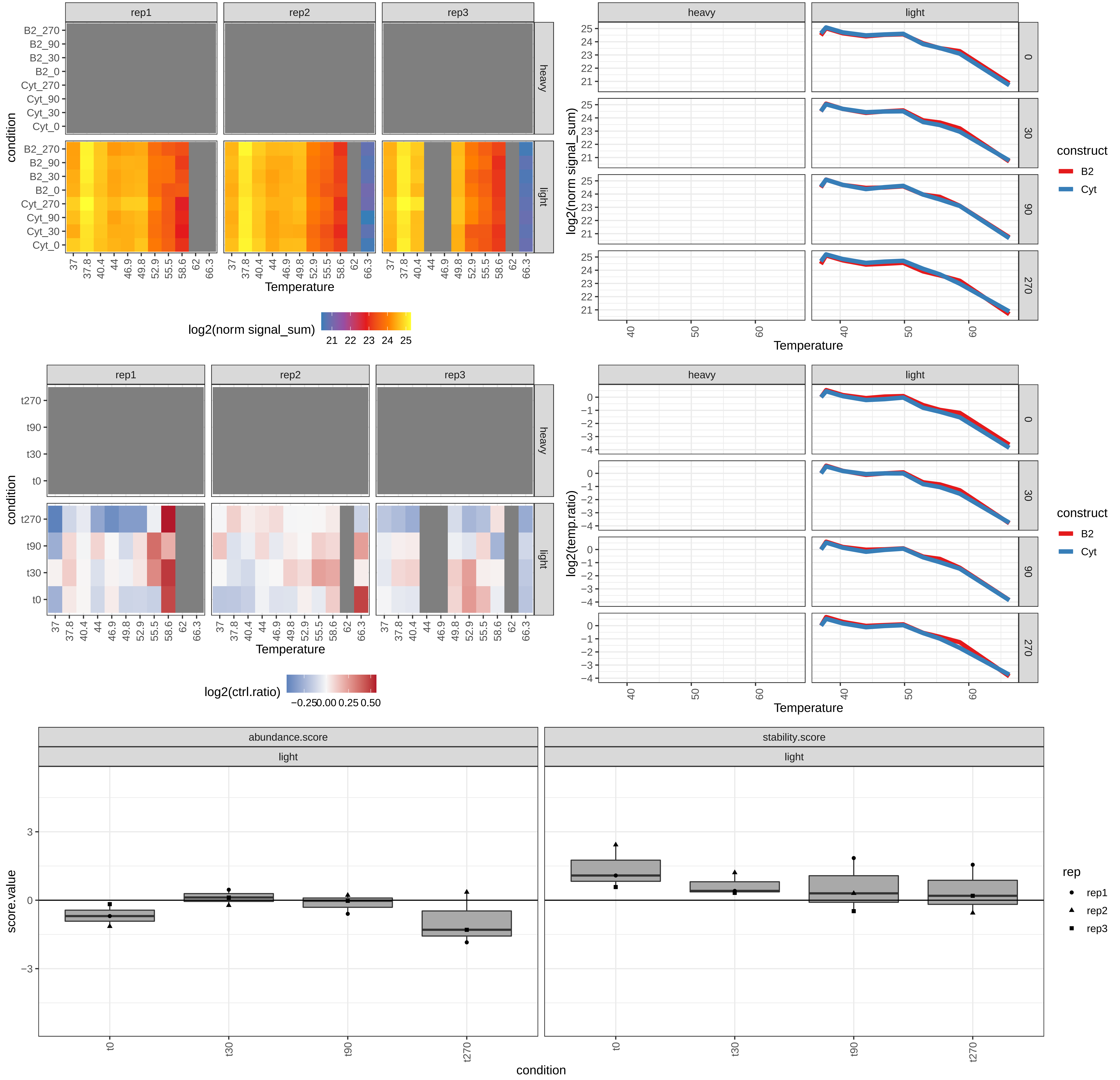

P46669 − DNA−directed RNA polymerase I subunit RPA43 OS=Saccharomyces cerevisiae (strain ATCC 204508 / S288c) GN=RPA43 PE=1 SV=2

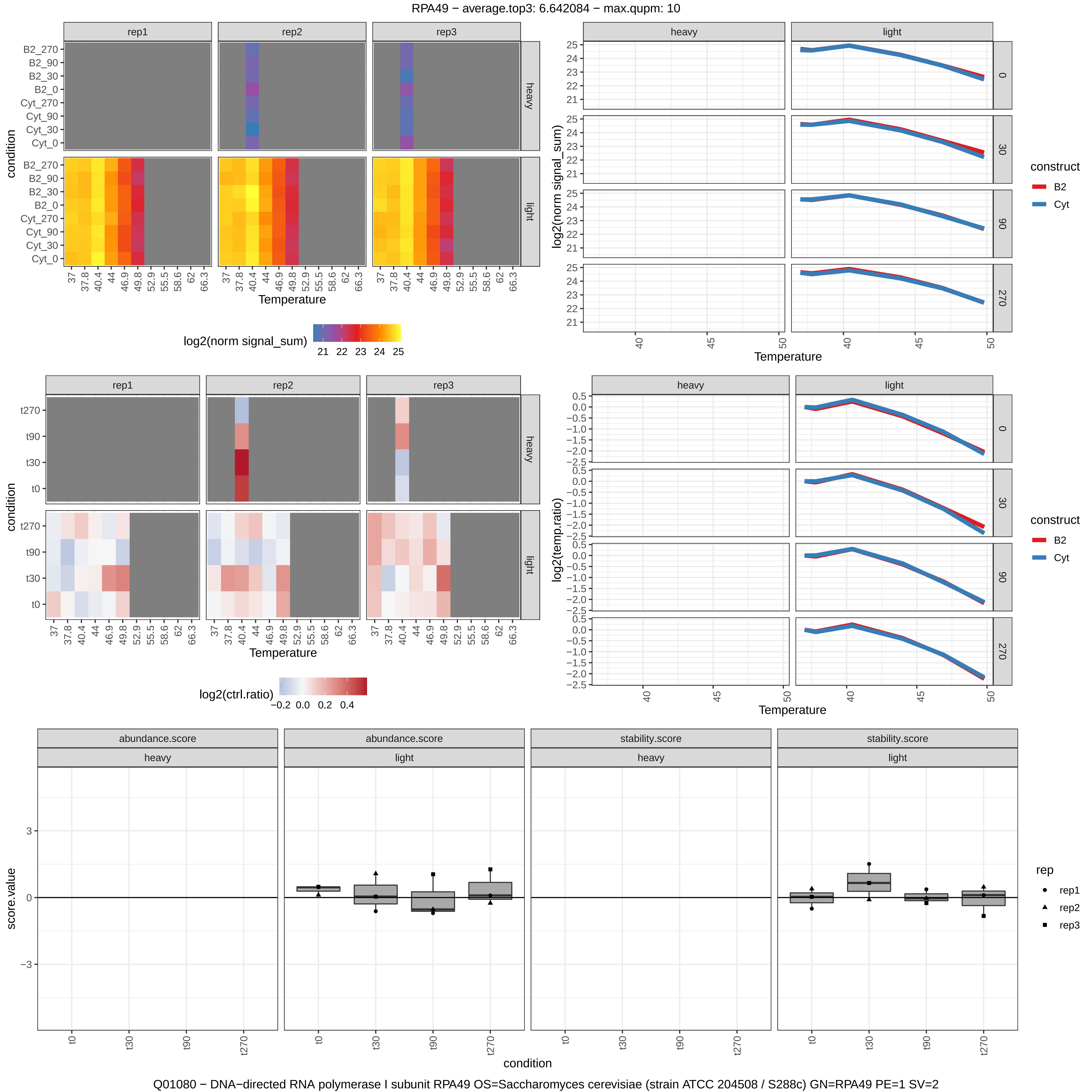

RPA49 − average.top3: 6.642084 − max.qupm: 10
rep1
rep2
rep3
heavy
light
condition
Temperature
log2(norm signal_sum)
log2(norm signal_sum)
construct
B2
Cyt
log2(ctrl.ratio)
log2(temp.ratio)
abundance.score
stability.score
score.value
rep
rep1
rep2
rep3
Q01080 − DNA−directed RNA polymerase I subunit RPA49 OS=Saccharomyces cerevisiae (strain ATCC 204508 / S288c) GN=RPA49 PE=1 SV=2

RPB11 − average.top3: 7.393783 − max.qupm: 5

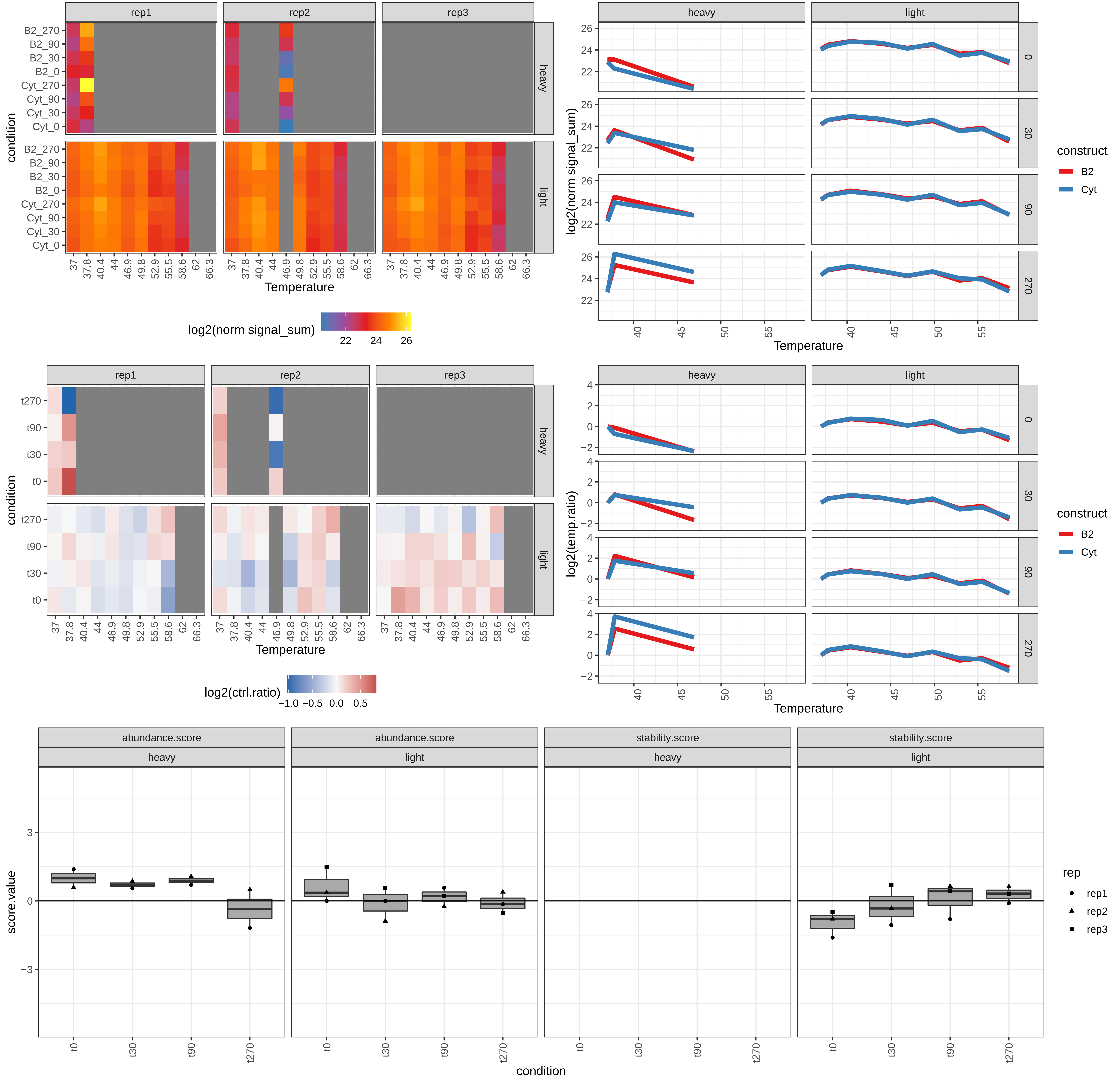

P38902 − DNA−directed RNA polymerase II subunit RPB11 OS=Saccharomyces cerevisiae (strain ATCC 204508 / S288c) GN=RPB11 PE=1 SV=1

RPB2 − average.top3: 7.39917192307692 − max.qupm: 34

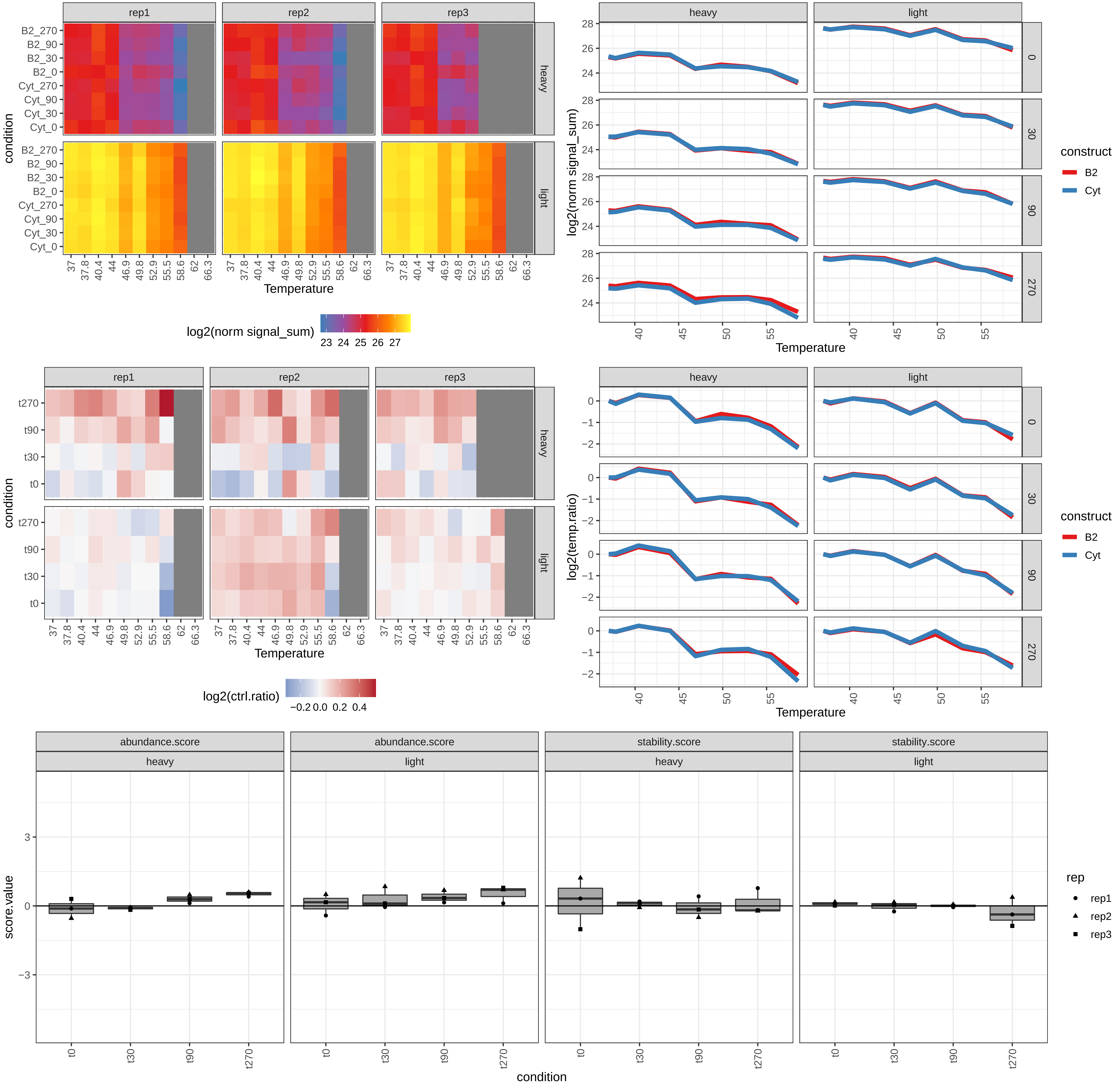

P08518 − DNA−directed RNA polymerase II subunit RPB2 OS=Saccharomyces cerevisiae (strain ATCC 204508 / S288c) GN=RPB2 PE=1 SV=2

RPB3 − average.top3: 7.04886 − max.qupm: 4

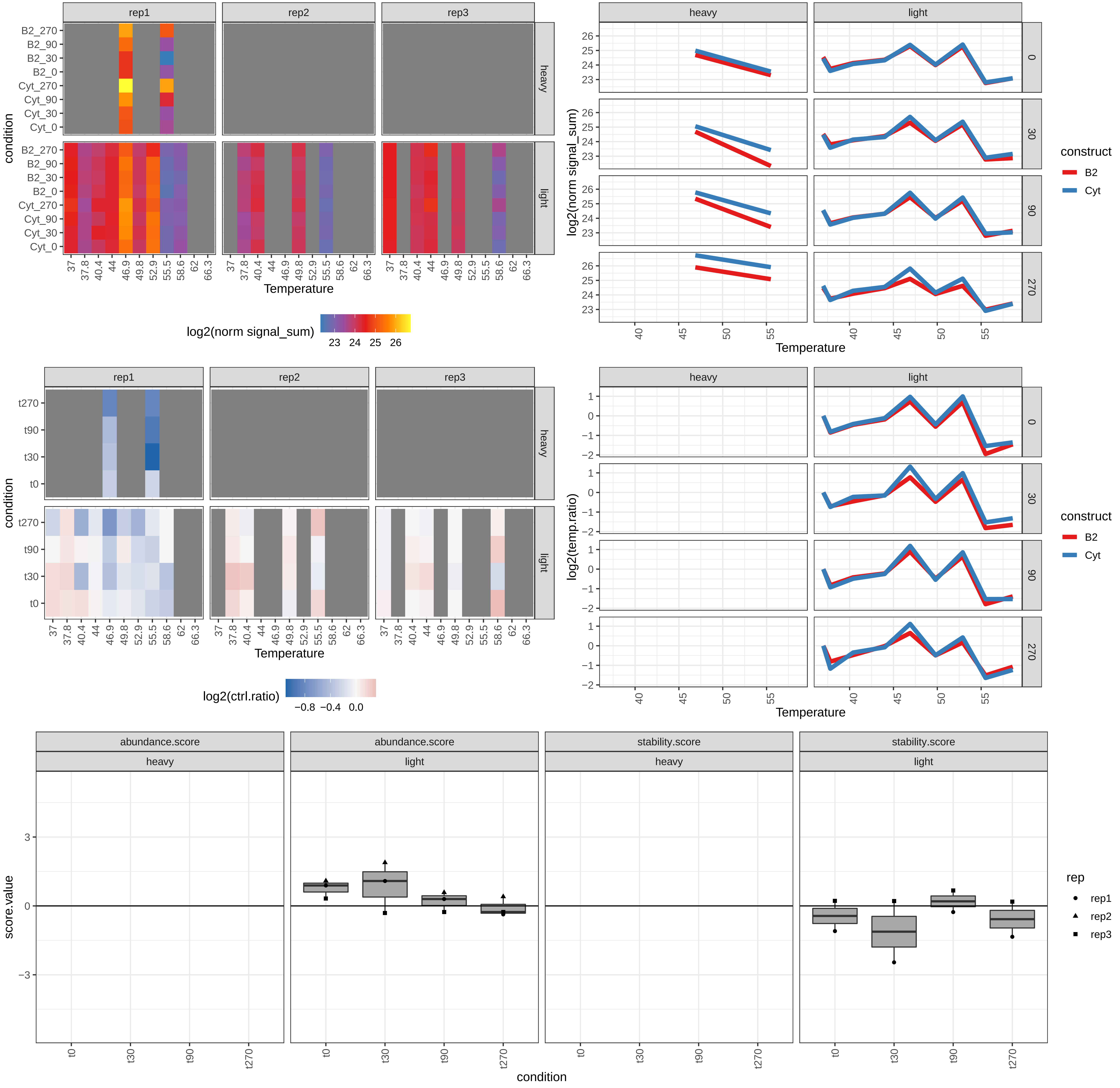

P16370 − DNA−directed RNA polymerase II subunit RPB3 OS=Saccharomyces cerevisiae (strain ATCC 204508 / S288c) GN=RPB3 PE=1 SV=2

RPB4 − average.top3: 6.688415 − max.qupm: 3

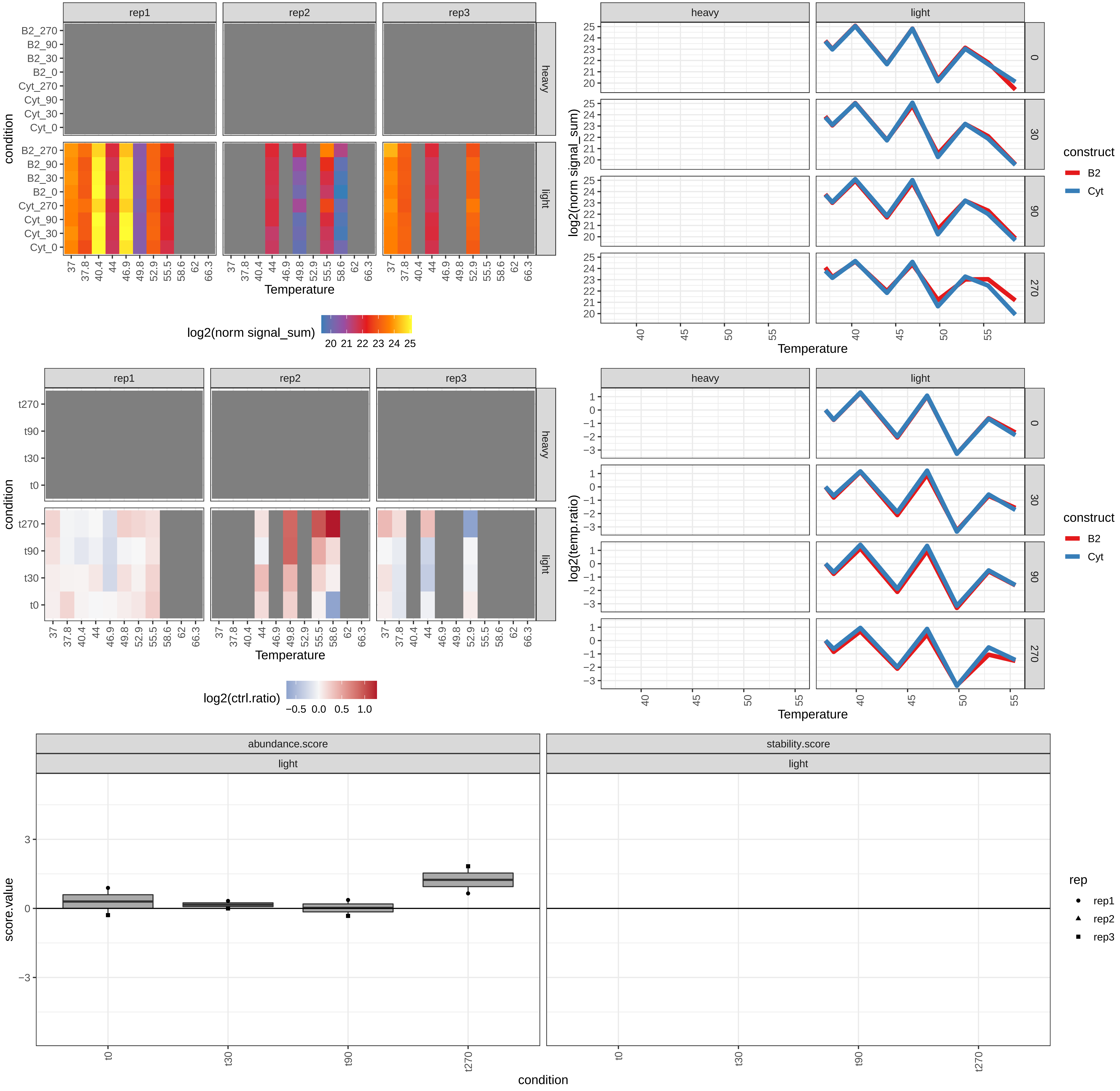

P20433 − DNA−directed RNA polymerase II subunit RPB4 OS=Saccharomyces cerevisiae (strain ATCC 204508 / S288c) GN=RPB4 PE=1 SV=1

RPB5 − average.top3: 7.316285 − max.qupm: 5

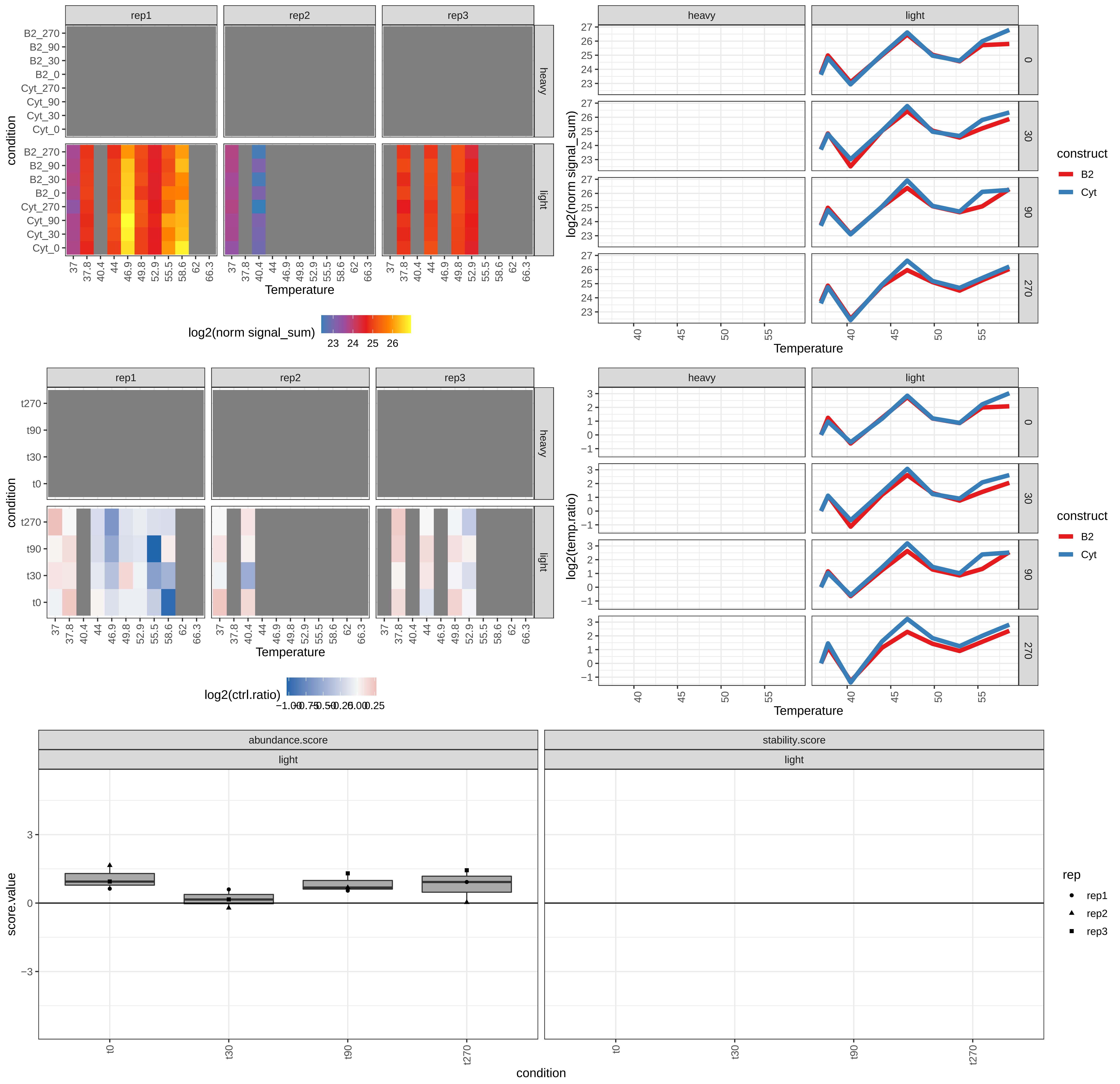

P20434 − DNA−directed RNA polymerases I, II, and III subunit RPABC1 OS=Saccharomyces cerevisiae (strain ATCC 204508 / S288c) GN=RPB5 PE=1 SV=1

RPB7 − average.top3: 7.03595366666667 − max.qupm: 3

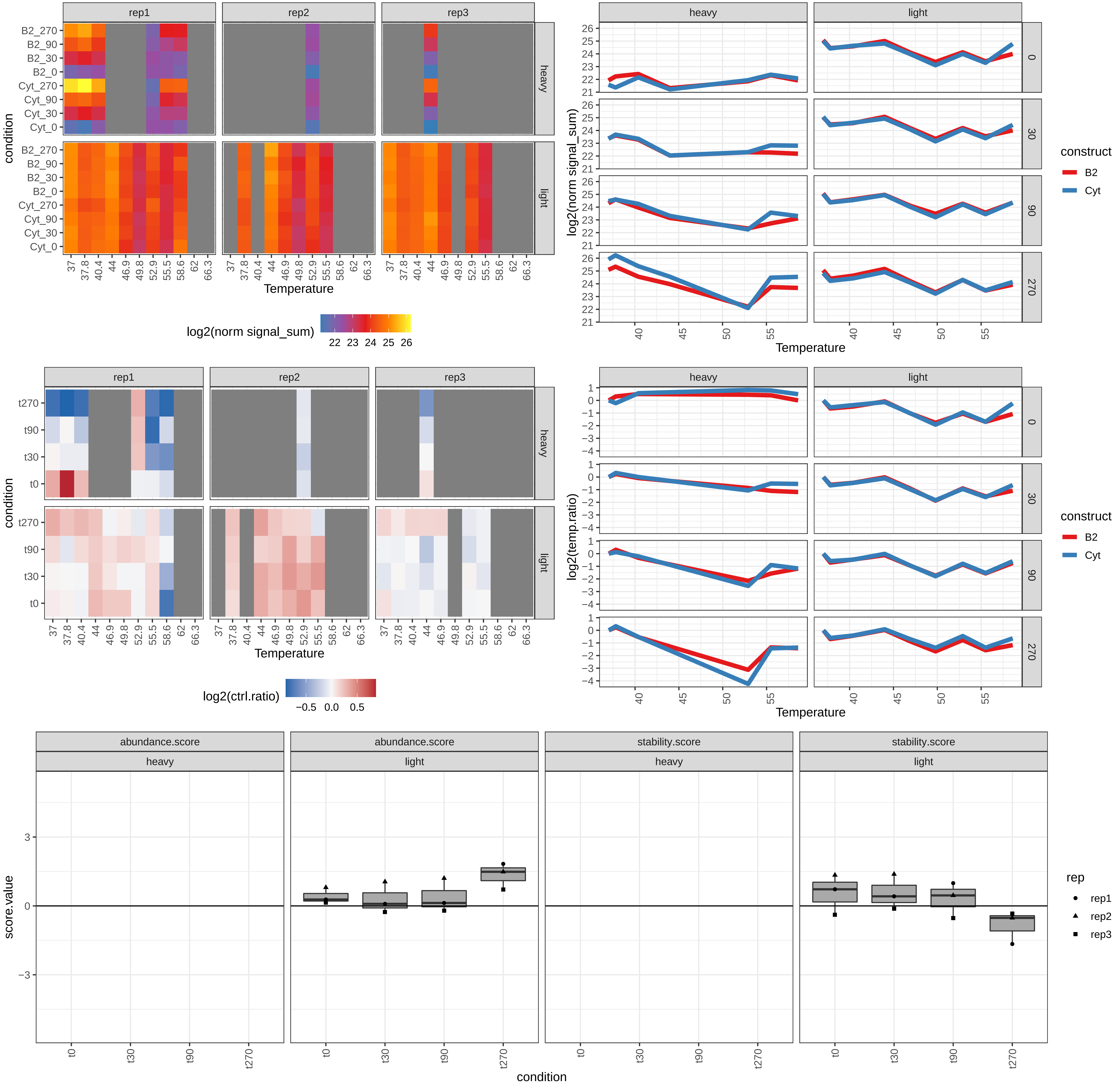

P34087 − DNA−directed RNA polymerase II subunit RPB7 OS=Saccharomyces cerevisiae (strain ATCC 204508 / S288c) GN=RPB7 PE=1 SV=1

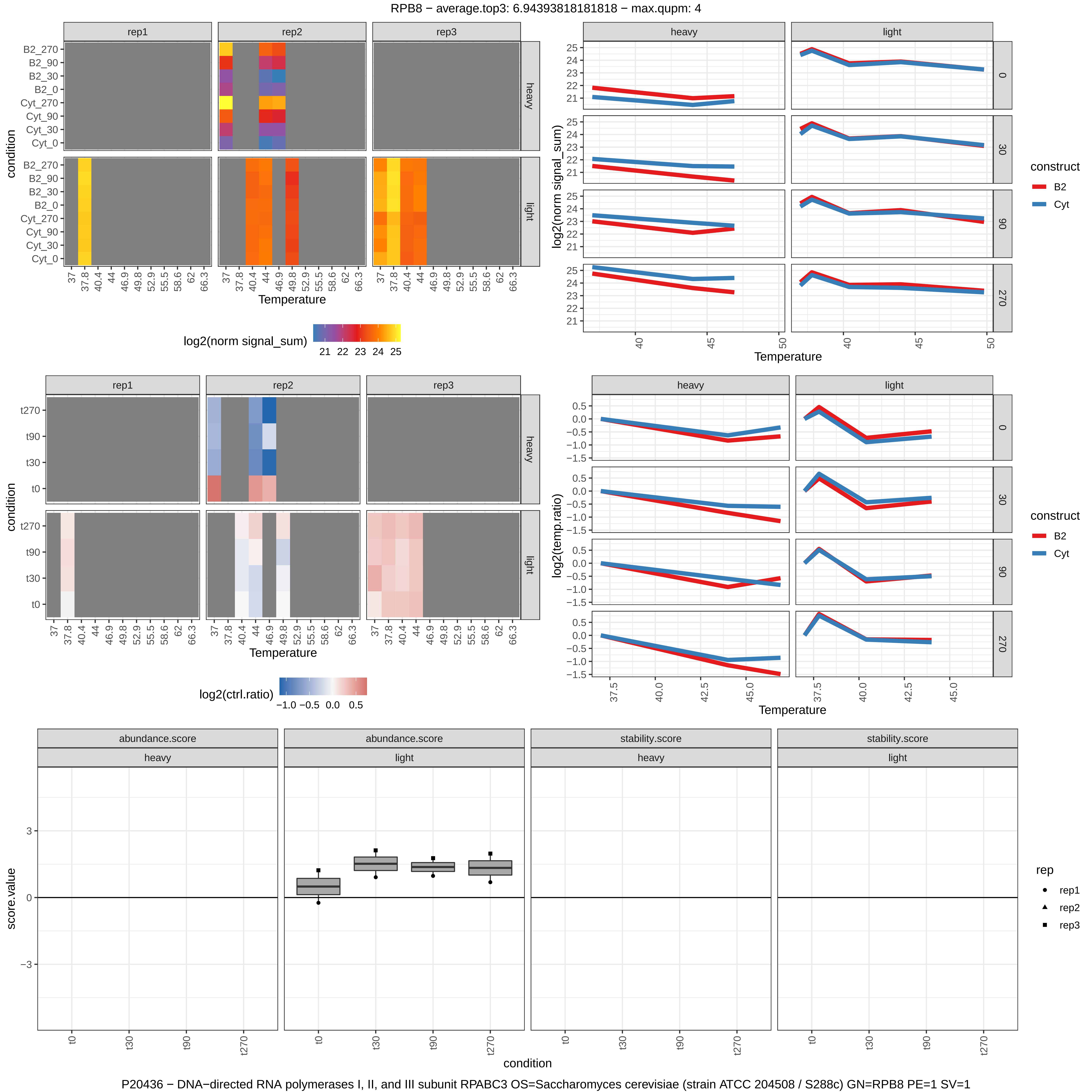

RPB8 − average.top3: 6.94393818181818 − max.qupm: 4
rep1
rep2
rep3
heavy
light
condition
Temperature
log2(norm signal_sum)
log2(temp.ratio)
log2(ctrl.ratio)
construct
B2
Cyt
abundance.score
stability.score
score.value
rep
P20436 − DNA−directed RNA polymerases I, II, and III subunit RPABC3 OS=Saccharomyces cerevisiae (strain ATCC 204508 / S288c) GN=RPB8 PE=1 SV=1

RPB9 − average.top3: 6.95756551724138 − max.qupm: 4

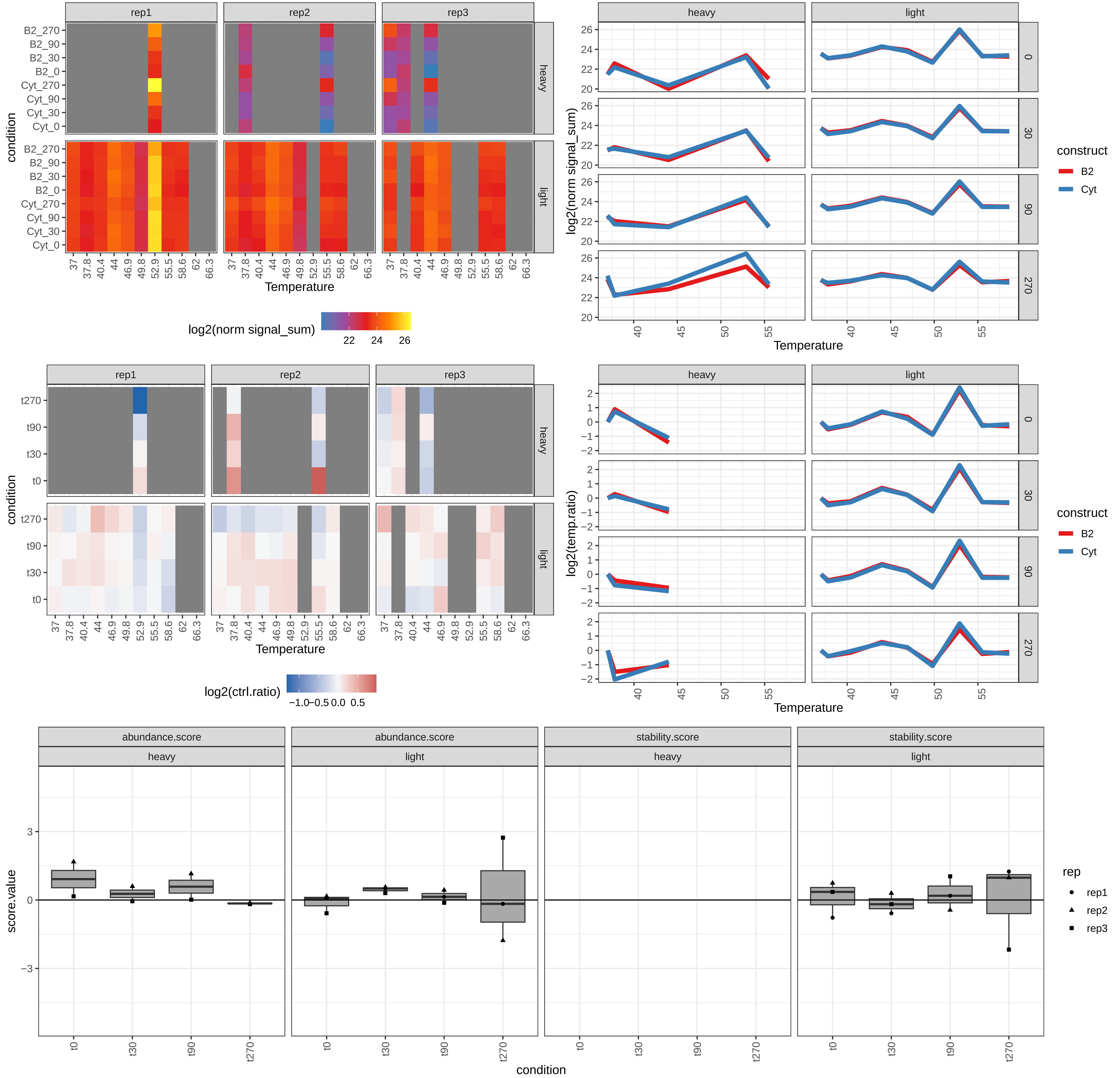

P27999 − DNA−directed RNA polymerase II subunit RPB9 OS=Saccharomyces cerevisiae (strain ATCC 204508 / S288c) GN=RPB9 PE=1 SV=1

RPC10 − average.top3: 7.150975 − max.qupm: 2

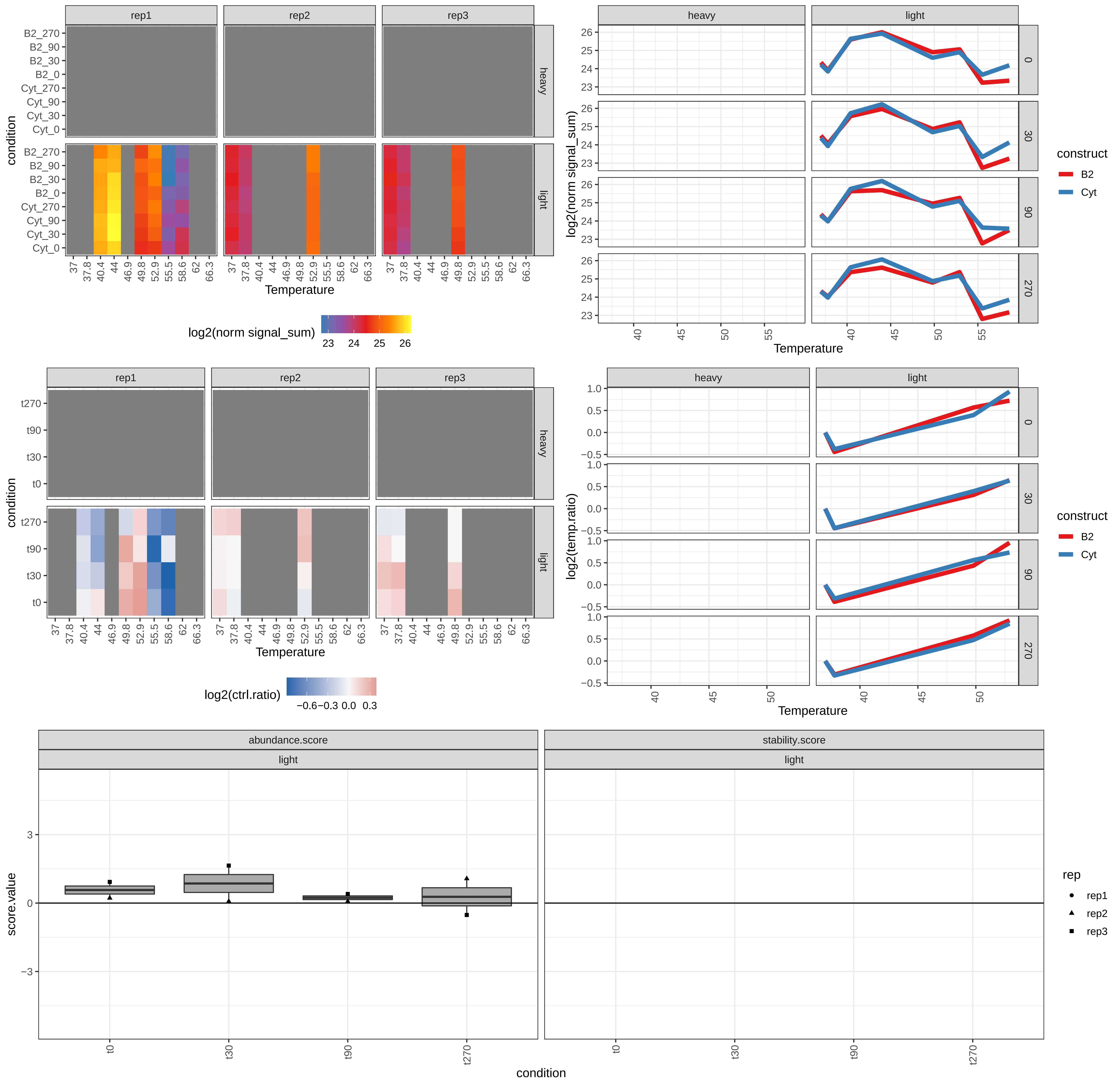

P40422 − DNA−directed RNA polymerases I, II, and III subunit RPABC4 OS=Saccharomyces cerevisiae (strain ATCC 204508 / S288c) GN=RPC10 PE=1 SV=1

RPC11 − average.top3: 6.30439384615385 − max.qupm: 3

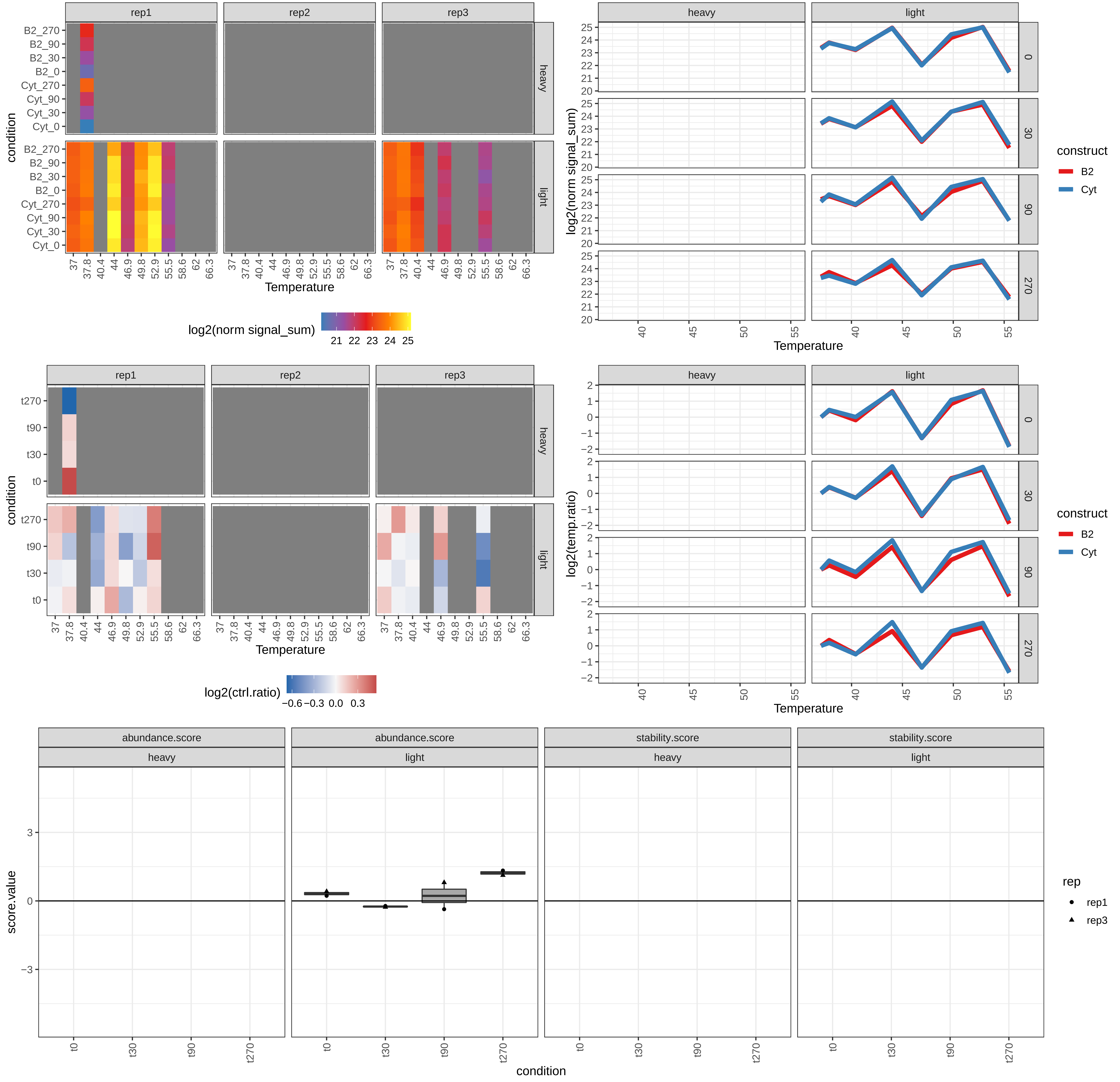

Q04307 − DNA−directed RNA polymerase III subunit RPC10 OS=Saccharomyces cerevisiae (strain ATCC 204508 / S288c) GN=RPC11 PE=1 SV=1

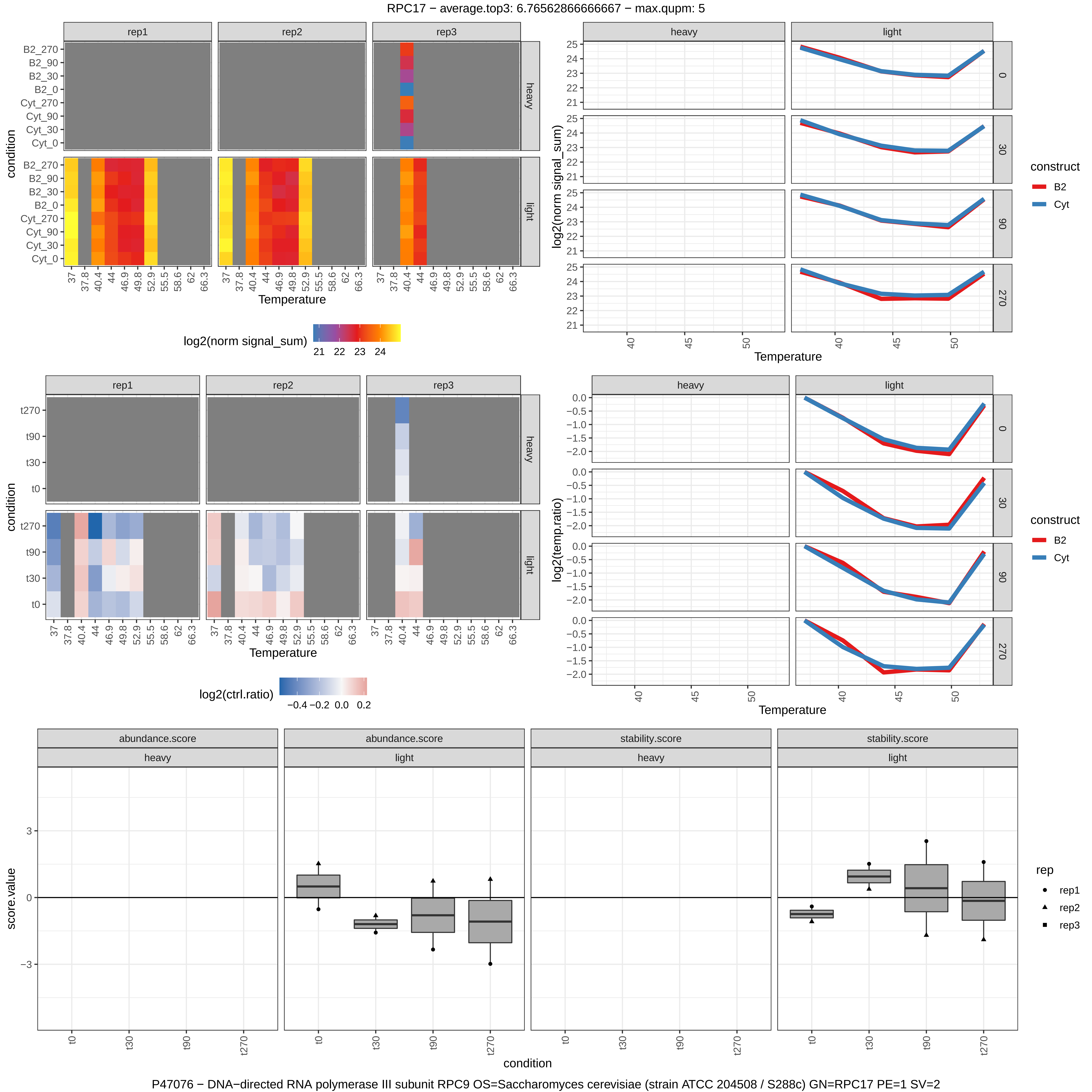

RPC17 − average.top3: 6.76562866666667 − max.qupm: 5
rep1
rep2
rep3
heavy
light
condition
Temperature
log2(norm signal_sum)
log2(ctrl.ratio)
log2(temp.ratio)
construct
B2
Cyt
abundance.score
stability.score
score.value
rep
rep1
rep2
rep3
P47076 − DNA−directed RNA polymerase III subunit RPC9 OS=Saccharomyces cerevisiae (strain ATCC 204508 / S288c) GN=RPC17 PE=1 SV=2

RPC25 − average.top3: 6.779218 − max.qupm: 4

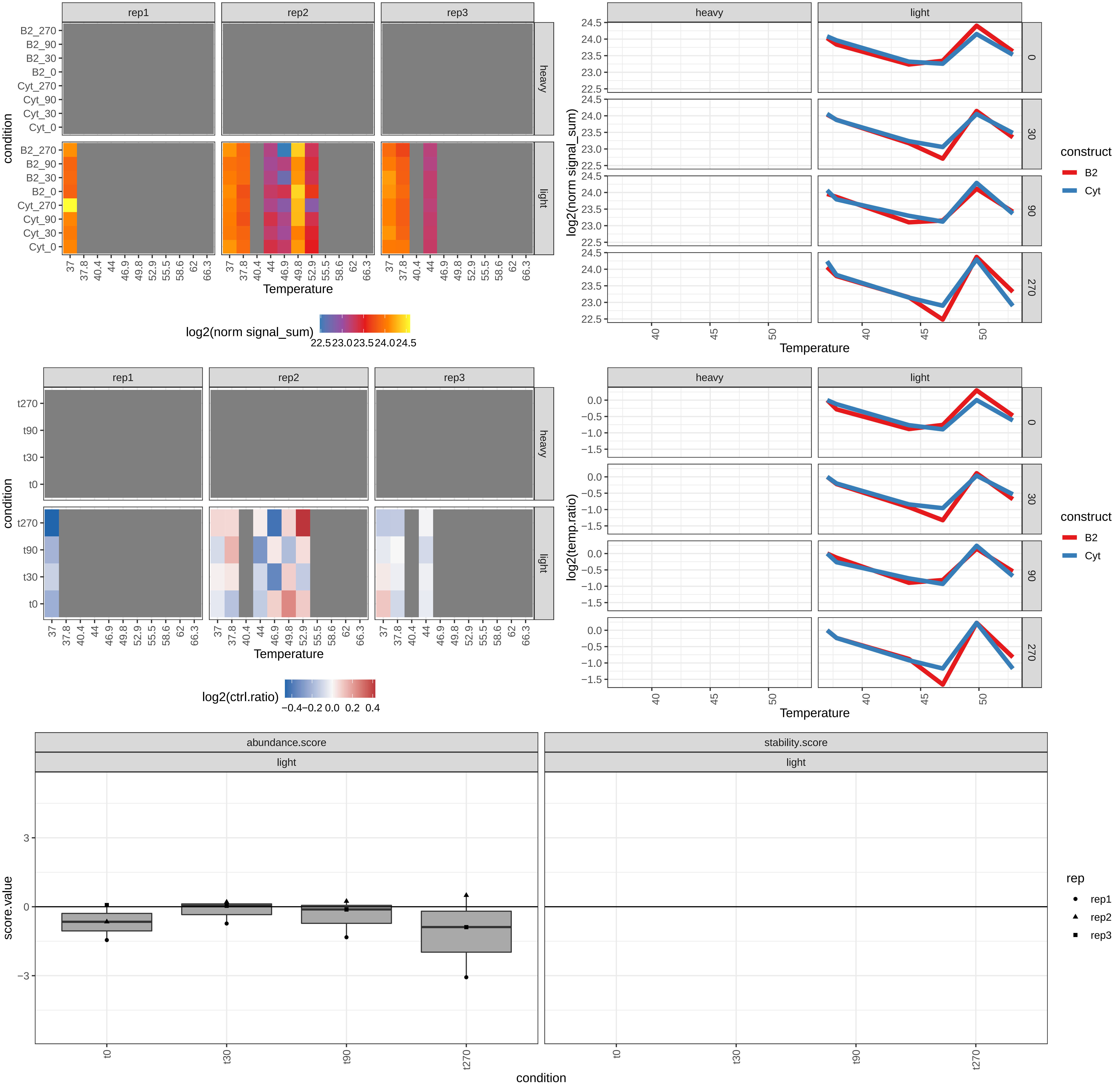

P35718 − DNA−directed RNA polymerase III subunit RPC8 OS=Saccharomyces cerevisiae (strain ATCC 204508 / S288c) GN=RPC25 PE=1 SV=1

RPC37 − average.top3: 6.69852166666667 − max.qupm: 4

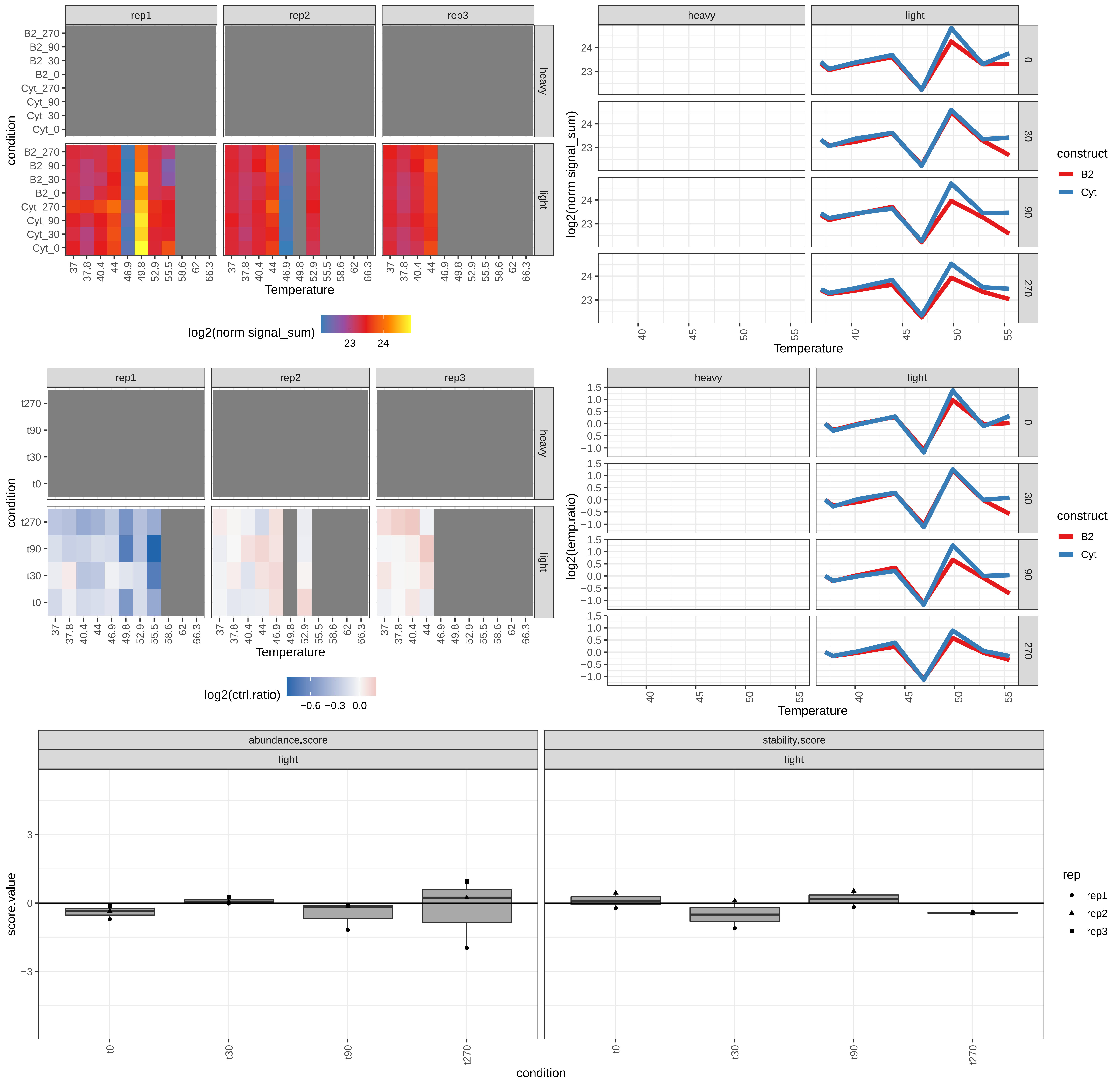

P36121 − DNA−directed RNA polymerase III subunit RPC5 OS=Saccharomyces cerevisiae (strain ATCC 204508 / S288c) GN=RPC37 PE=1 SV=1

RPC40 − average.top3: 7.11399233333333 − max.qupm: 7

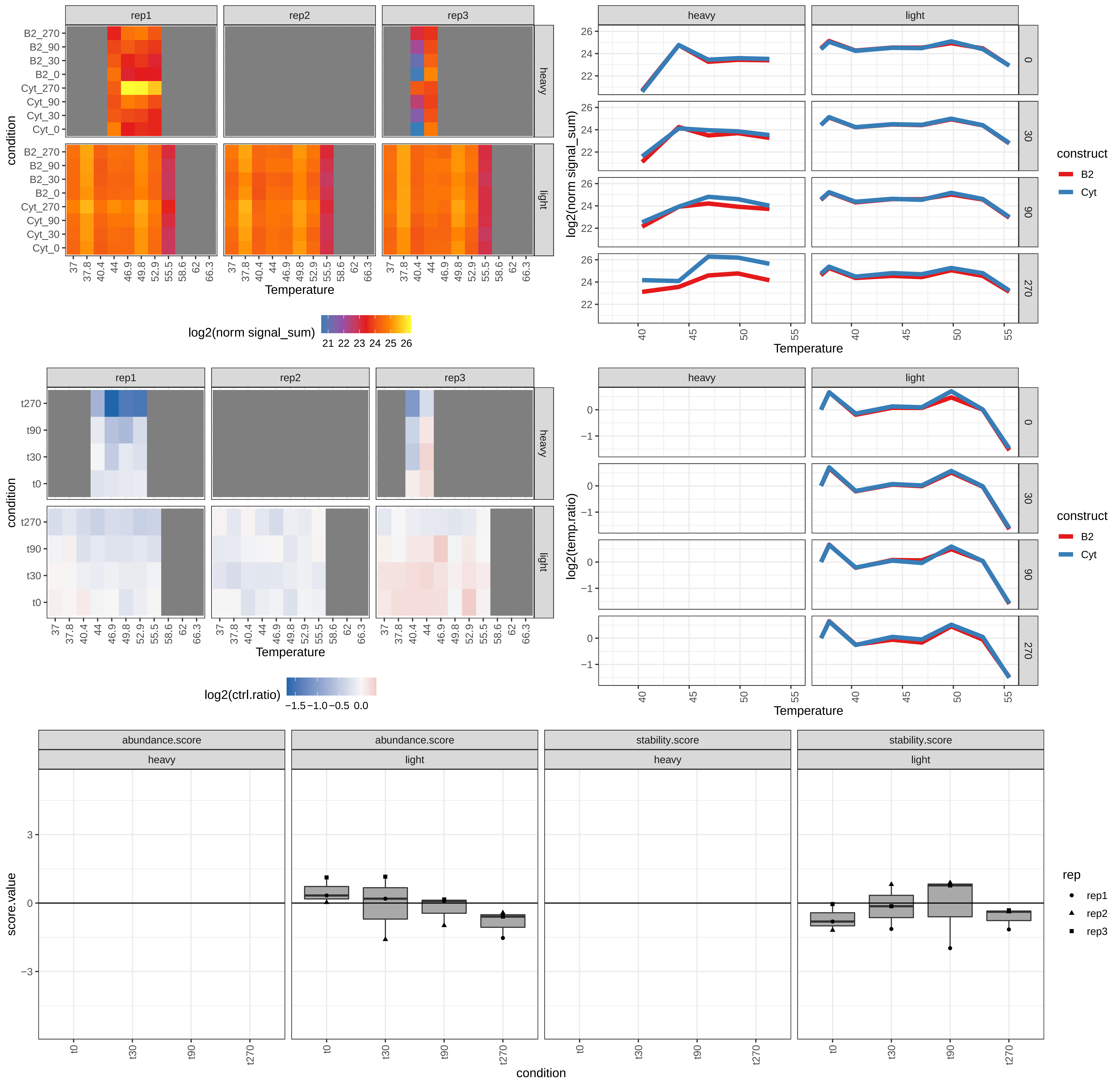

P07703 − DNA−directed RNA polymerases I and III subunit RPAC1 OS=Saccharomyces cerevisiae (strain ATCC 204508 / S288c) GN=RPC40 PE=1 SV=1

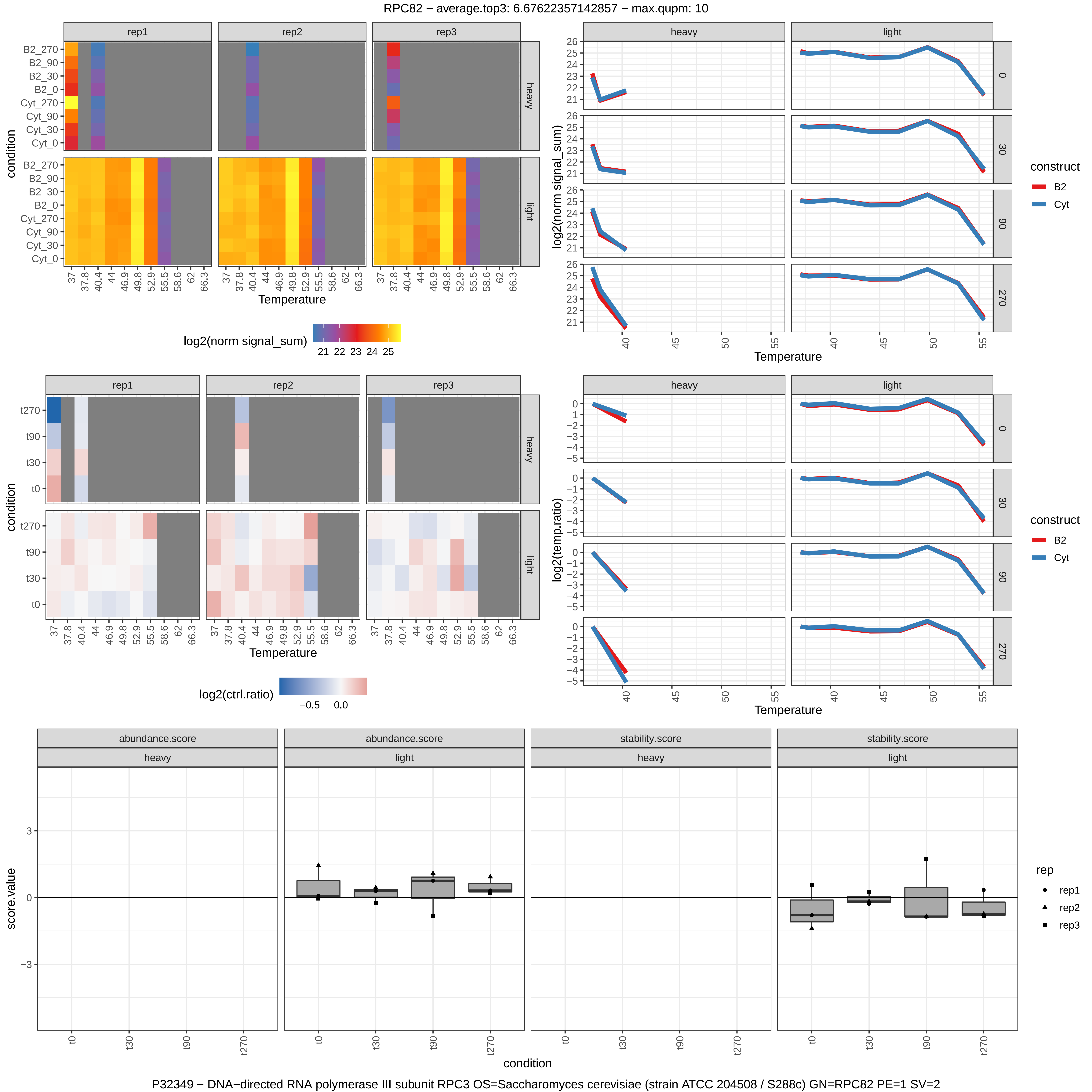

RPC82 − average.top3: 6.67622357142857 − max.qupm: 10
rep1
rep2
rep3
heavy
light
condition
Temperature
log2(norm signal_sum)
log2(temp.ratio)
construct
B2
Cyt
log2(ctrl.ratio)
abundance.score
stability.score
score.value
rep
rep1
rep2
rep3
P32349 − DNA−directed RNA polymerase III subunit RPC3 OS=Saccharomyces cerevisiae (strain ATCC 204508 / S288c) GN=RPC82 PE=1 SV=2

RPD3 − average.top3: 6.77168058823529 − max.qupm: 7

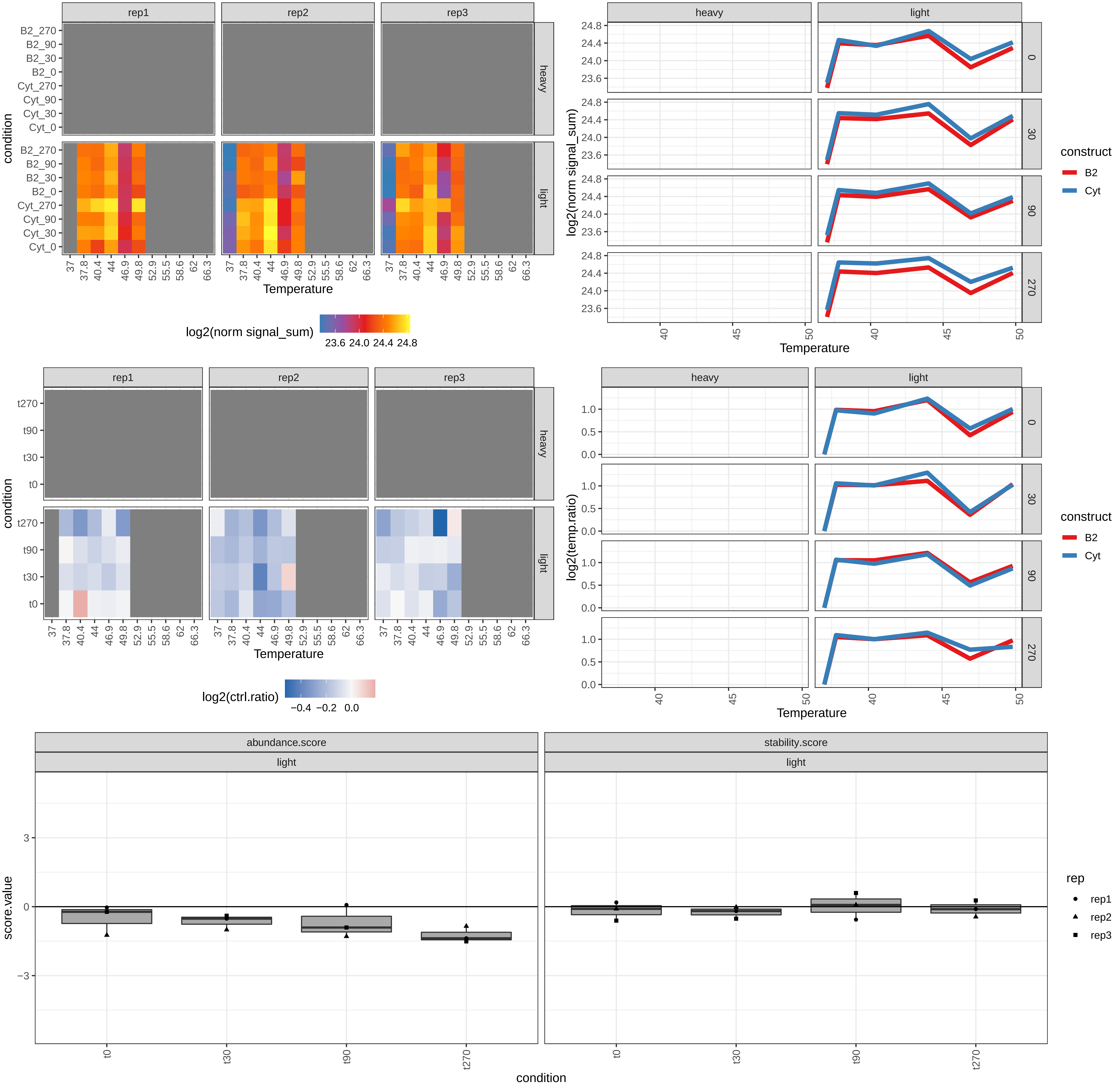

P32561 − Histone deacetylase RPD3 OS=Saccharomyces cerevisiae (strain ATCC 204508 / S288c) GN=RPD3 PE=1 SV=1

RPE1 − average.top3: 7.14414222222222 − max.qupm: 4

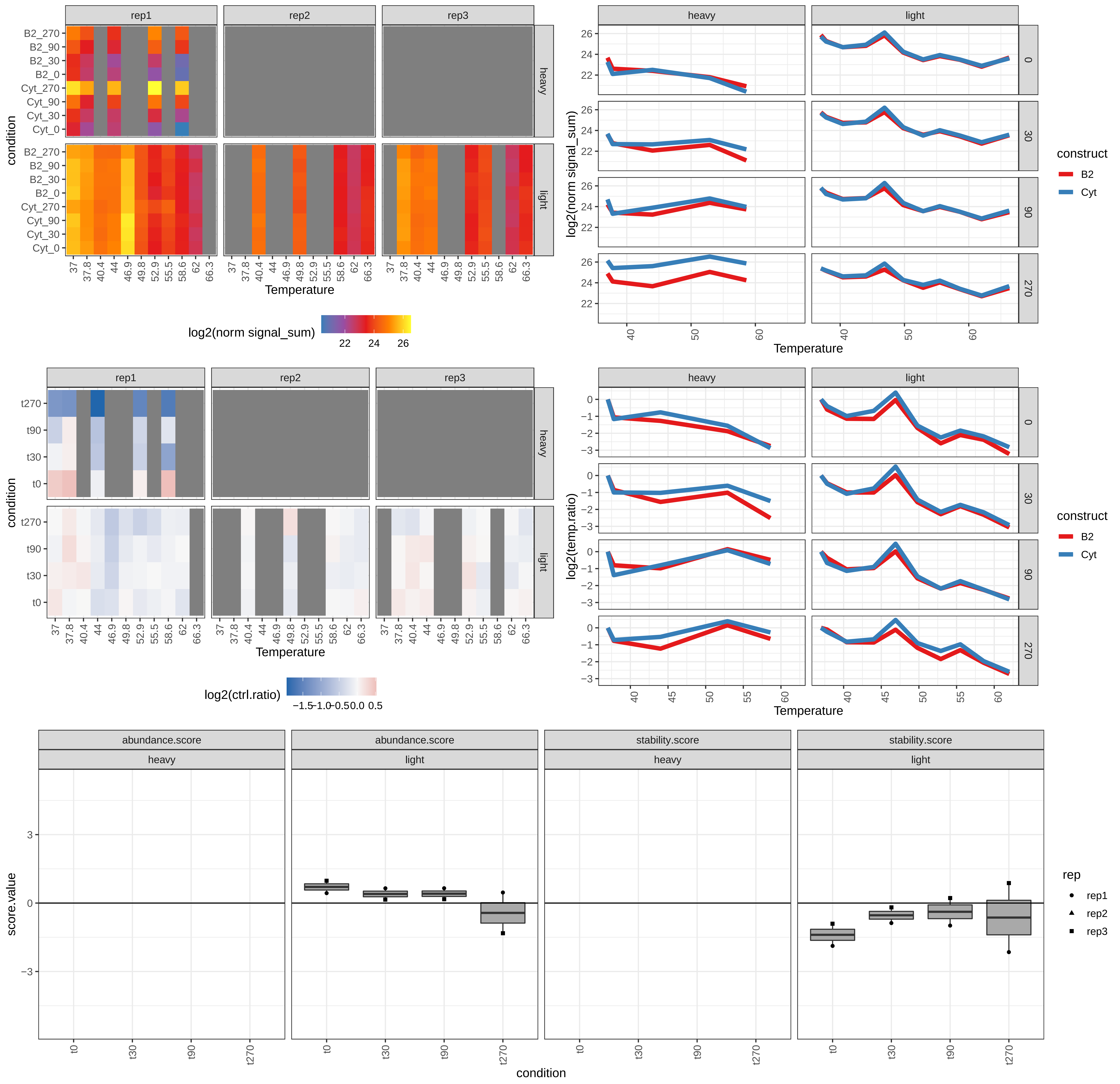

P46969 − Ribulose−phosphate 3−epimerase OS=Saccharomyces cerevisiae (strain ATCC 204508 / S288c) GN=RPE1 PE=1 SV=1

RPF1 − average.top3: 6.41185047619048 − max.qupm: 6

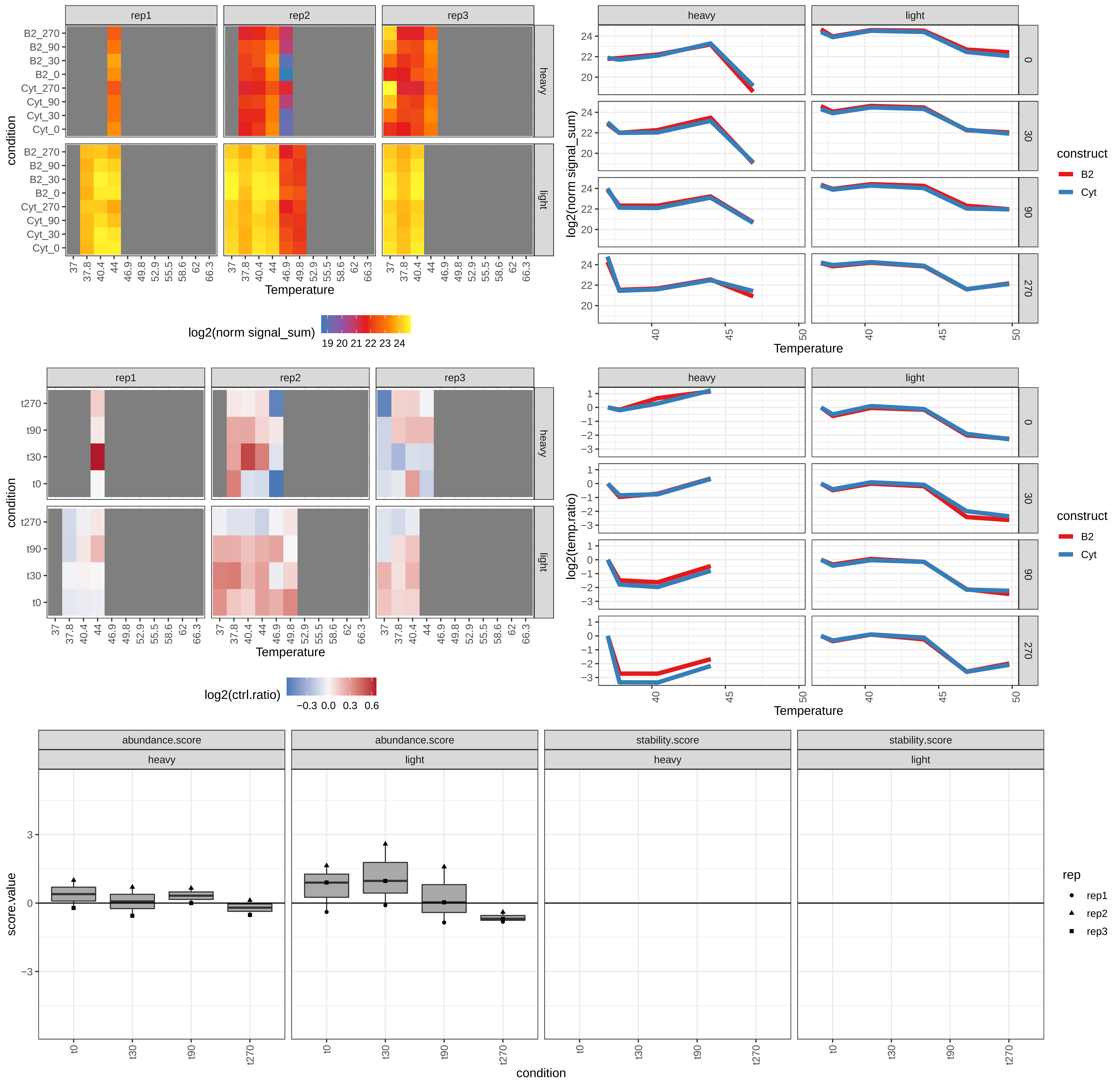

P38805 − Ribosome production factor 1 OS=Saccharomyces cerevisiae (strain ATCC 204508 / S288c) GN=RPF1 PE=1 SV=1

RPF2 − average.top3: 7.07278346153846 − max.qupm: 7

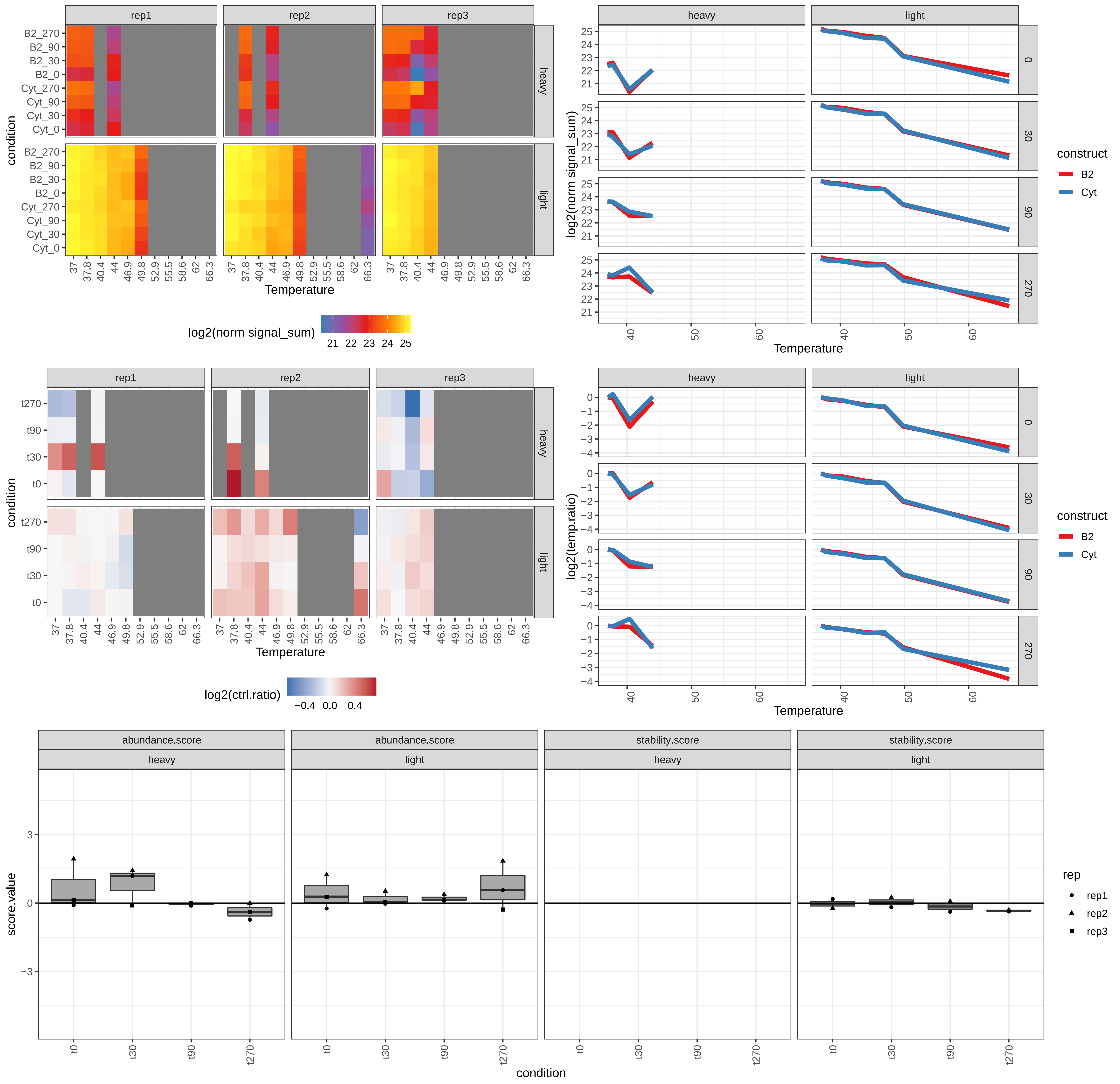

P36160 − Ribosome biogenesis protein RPF2 OS=Saccharomyces cerevisiae (strain ATCC 204508 / S288c) GN=RPF2 PE=1 SV=1

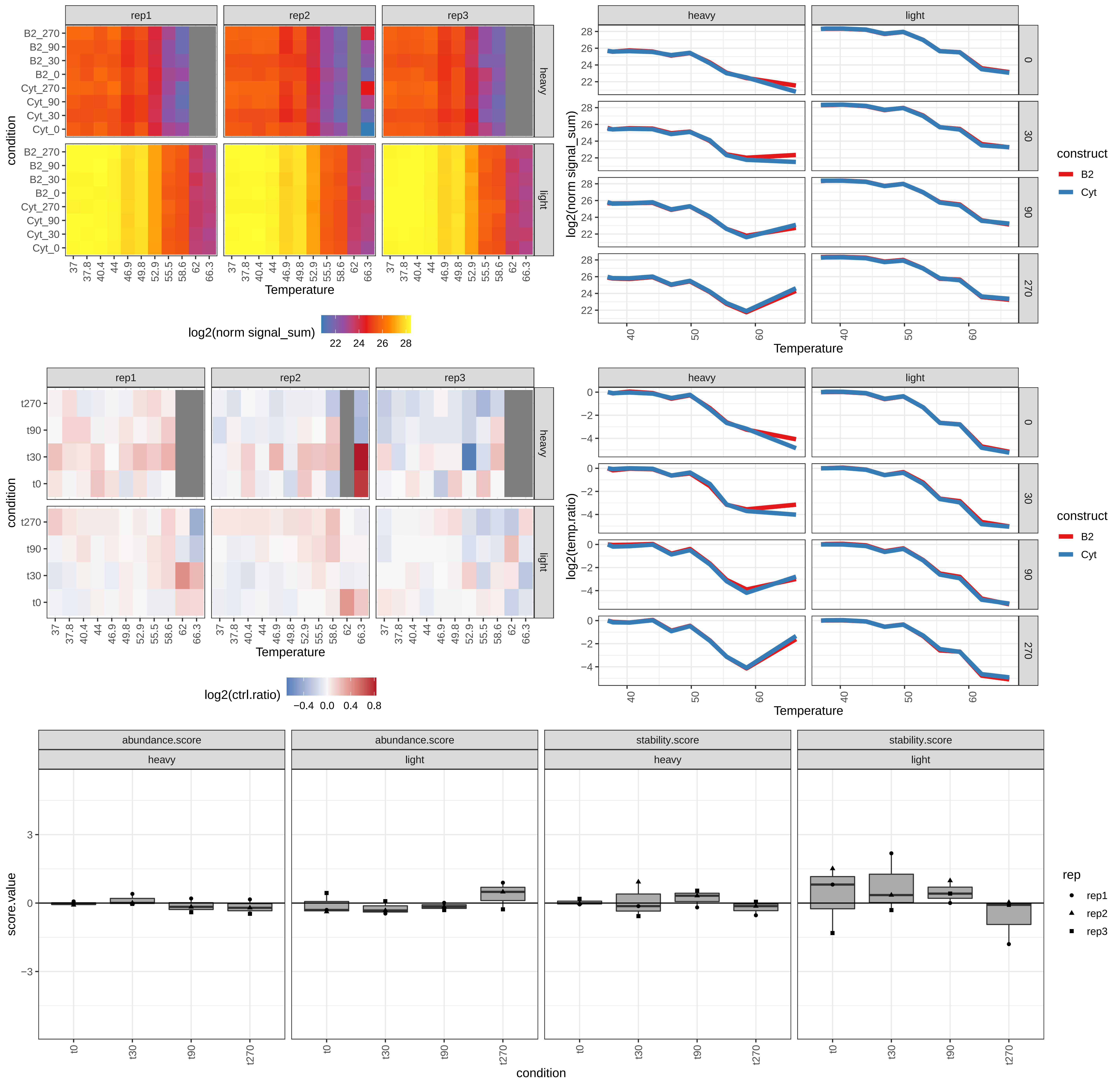

P38249 − Eukaryotic translation initiation factor 3 subunit A OS=Saccharomyces cerevisiae (strain ATCC 204508 / S288c) GN=RPG1 PE=1 SV=1

RPL10 − average.top3: 8.07949945454545 − max.qupm: 20

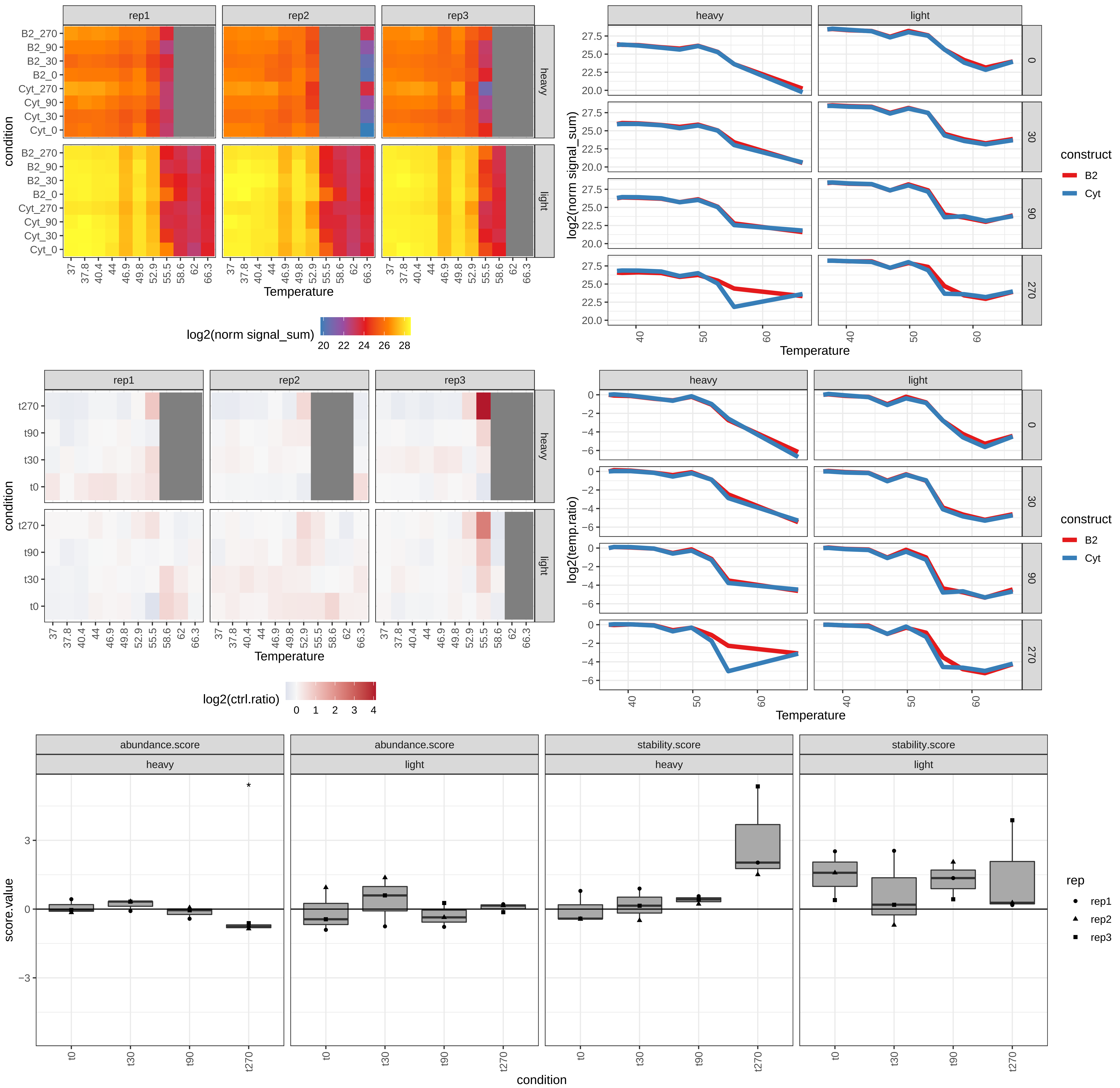

P41805 − 60S ribosomal protein L10 OS=Saccharomyces cerevisiae (strain ATCC 204508 / S288c) GN=RPL10 PE=1 SV=1

RPL11A|RPL11B − average.top3: 8.1343268 − max.qupm: 13

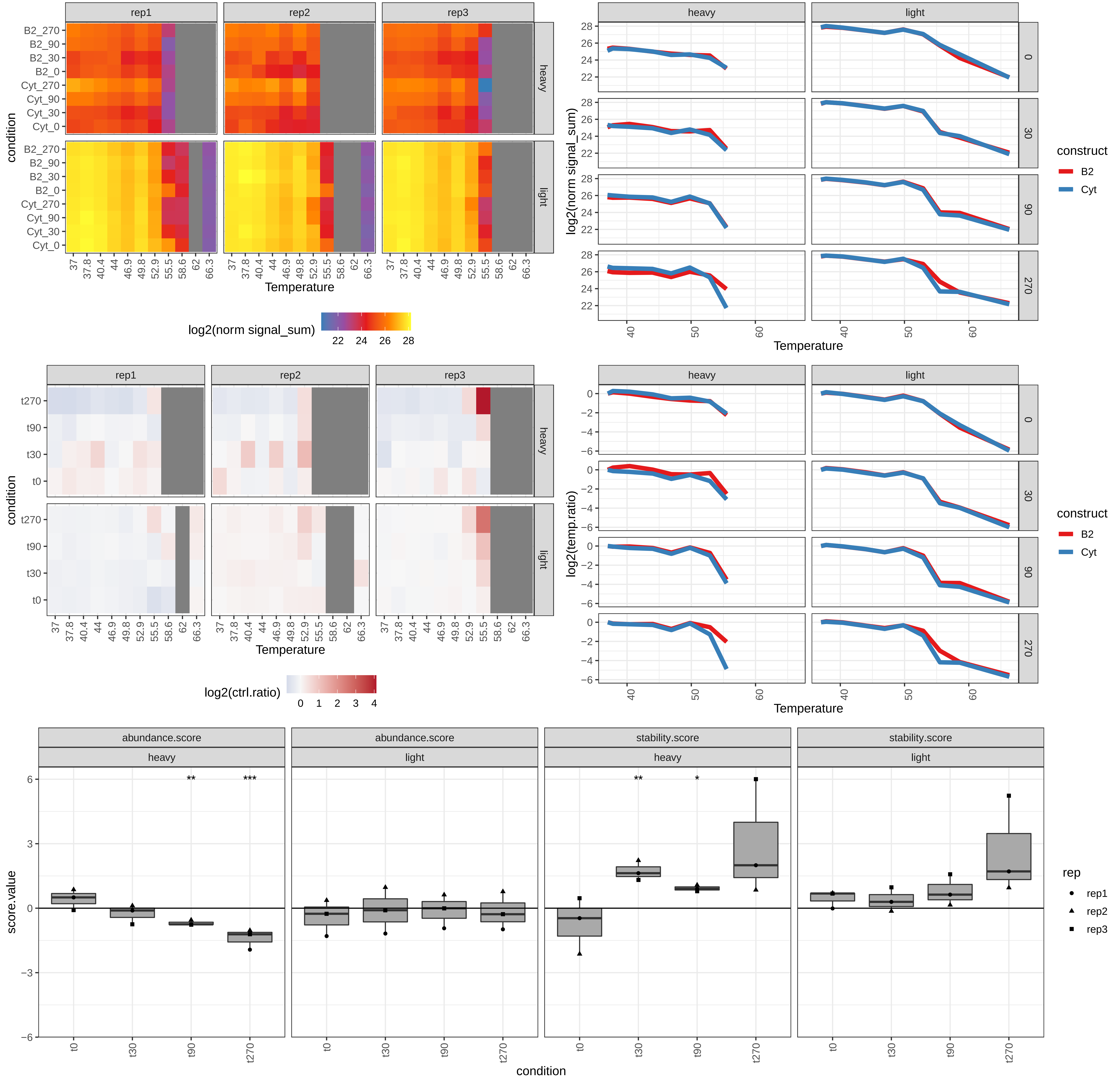

osomal protein L11−A OS=Saccharomyces cerevisiae (strain ATCC 204508 / S288c) GN=RPL11A PE=1 SV=2|60S ribosomal protein L11−B OS=Saccharomyces cerevisiae (strain ATCC 204508 / S288c

RPL12A|RPL12B − average.top3: 7.95079365384615 − max.qupm: 10

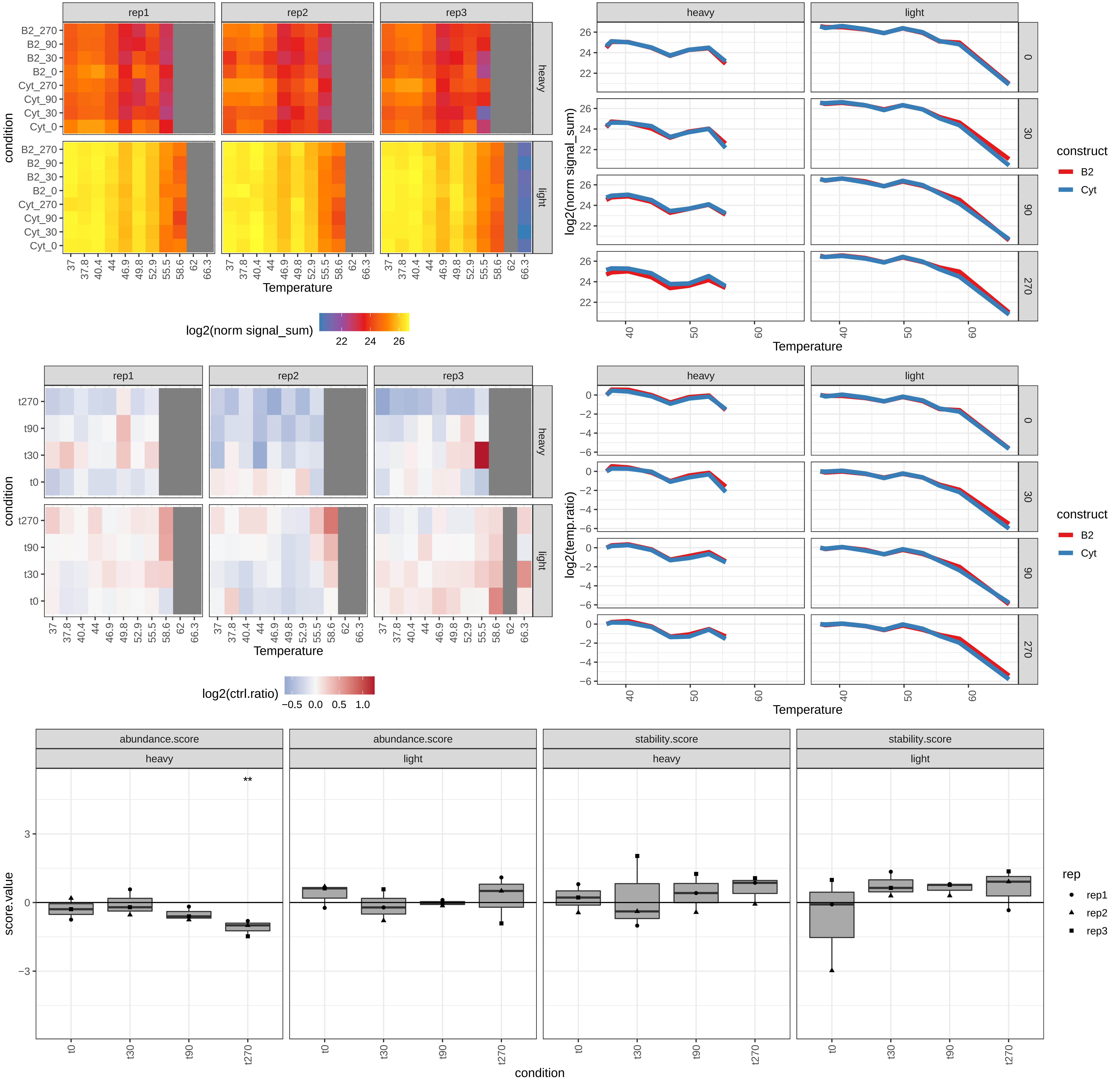

osomal protein L12−A OS=Saccharomyces cerevisiae (strain ATCC 204508 / S288c) GN=RPL12A PE=1 SV=1|60S ribosomal protein L12−B OS=Saccharomyces cerevisiae (strain ATCC 204508 / S288c)

RPL13B|RPL13A − average.top3: 8.0609840625 − max.qupm: 14

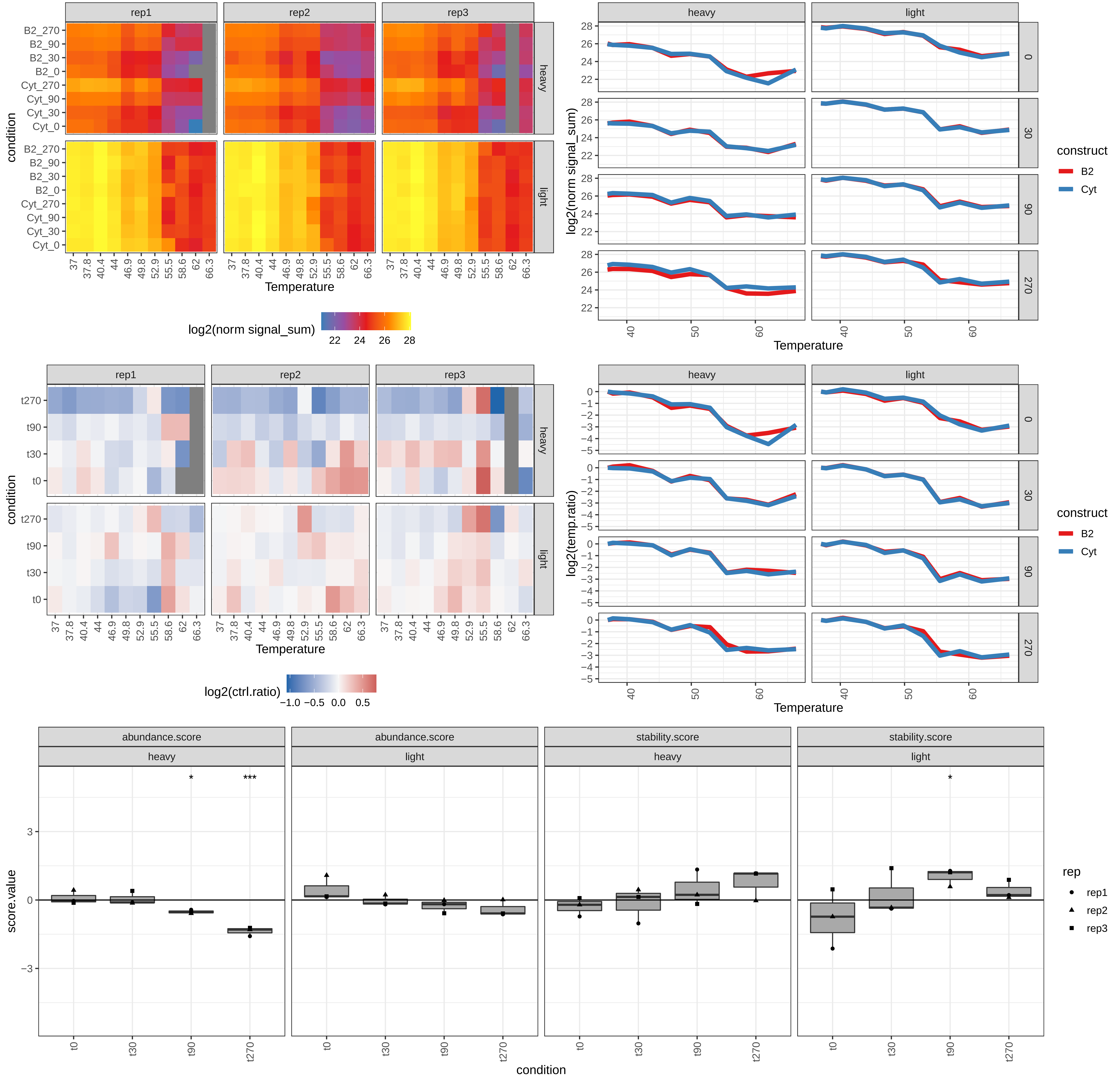

osomal protein L13−B OS=Saccharomyces cerevisiae (strain ATCC 204508 / S288c) GN=RPL13B PE=1 SV=1|60S ribosomal protein L13−A OS=Saccharomyces cerevisiae (strain ATCC 204508 / S288c)

RPL15A − average.top3: 7.91086058823529 − max.qupm: 8

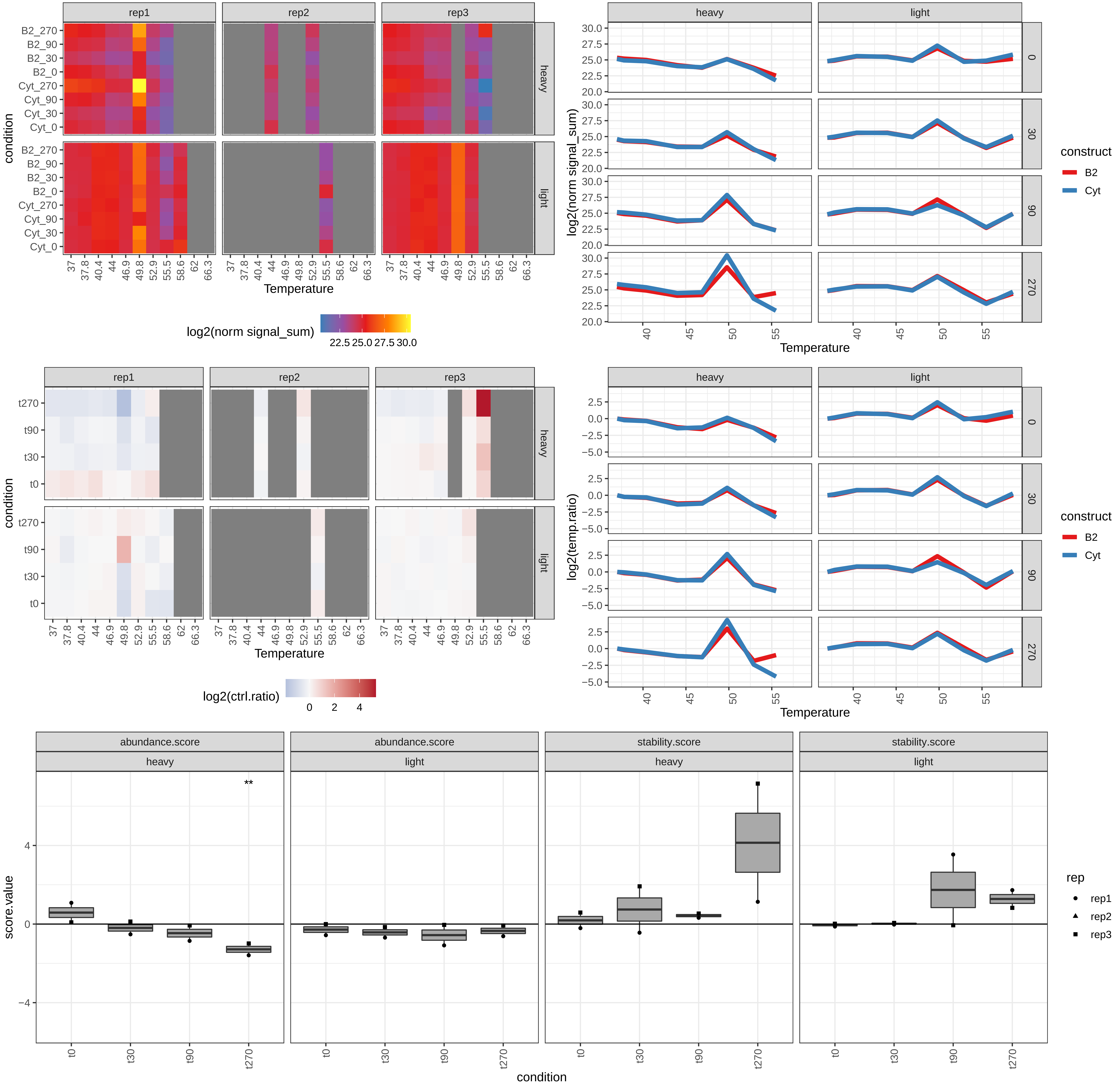

P05748 − 60S ribosomal protein L15−A OS=Saccharomyces cerevisiae (strain ATCC 204508 / S288c) GN=RPL15A PE=1 SV=3

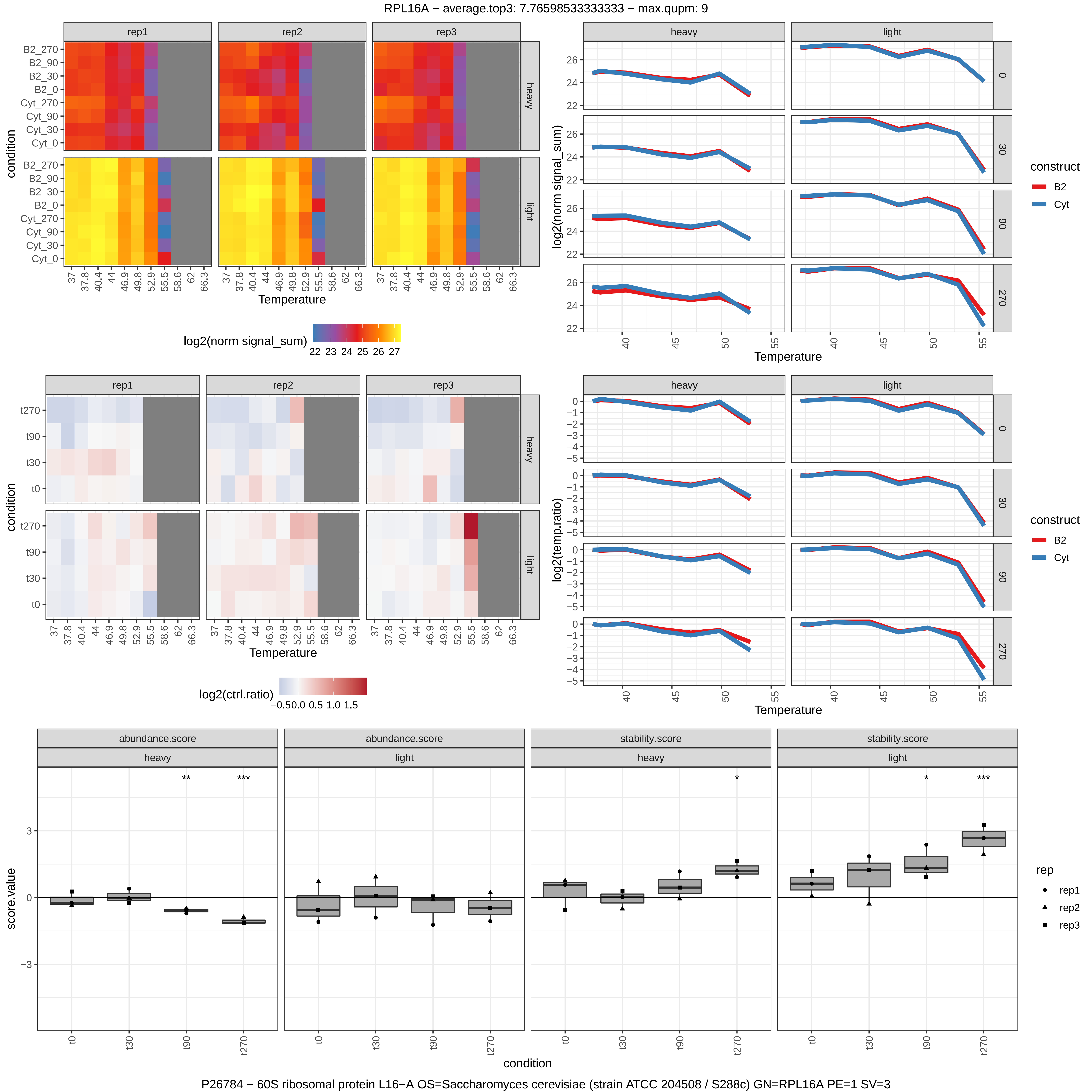

RPL16A − average.top3: 7.76598533333333 − max.qupm: 9
rep1
rep2
rep3
heavy
light
condition
Temperature
log2(norm signal_sum)
construct
B2
Cyt
log2(temp.ratio)
log2(ctrl.ratio)
abundance.score
stability.score
score.value
rep
rep1
rep2
rep3
P26784 − 60S ribosomal protein L16−A OS=Saccharomyces cerevisiae (strain ATCC 204508 / S288c) GN=RPL16A PE=1 SV=3

RPL16B − average.top3: 7.90700791666667 − max.qupm: 9

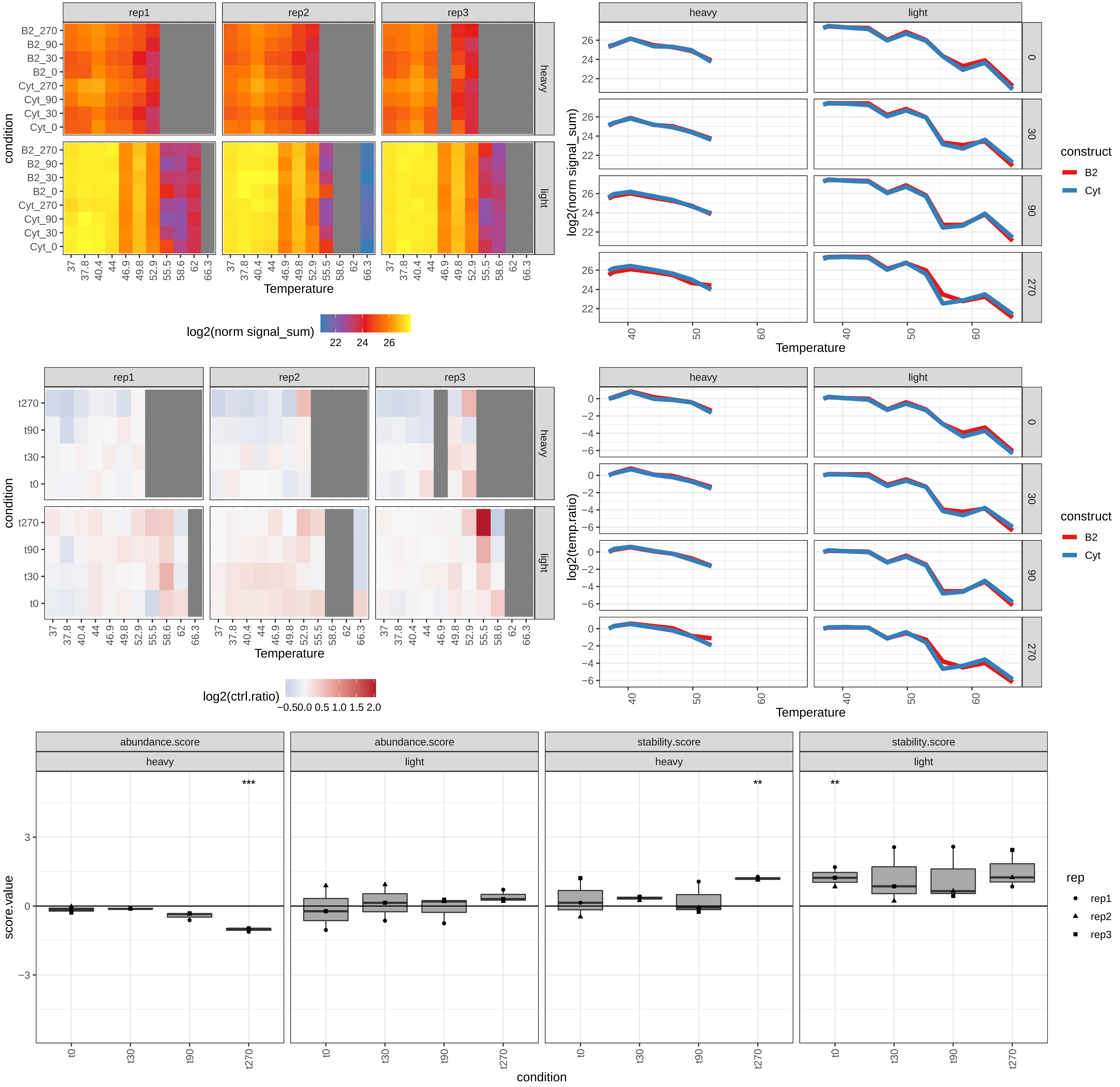

P26785 − 60S ribosomal protein L16−B OS=Saccharomyces cerevisiae (strain ATCC 204508 / S288c) GN=RPL16B PE=1 SV=3

RPL18A|RPL18B − average.top3: 8.33100274509804 − max.qupm: 14

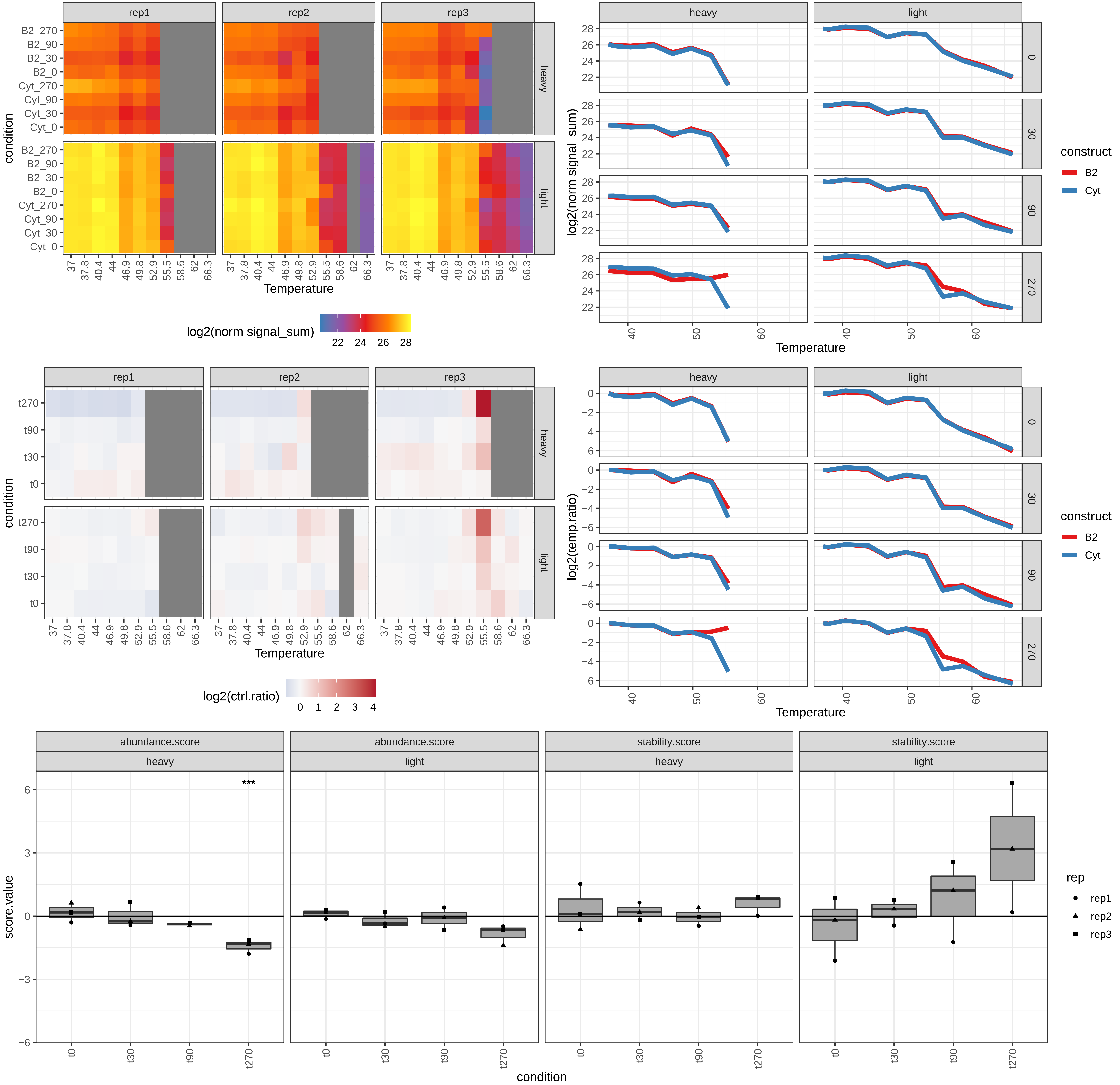

osomal protein L18−A OS=Saccharomyces cerevisiae (strain ATCC 204508 / S288c) GN=RPL18A PE=1 SV=1|60S ribosomal protein L18−B OS=Saccharomyces cerevisiae (strain ATCC 204508 / S288c)

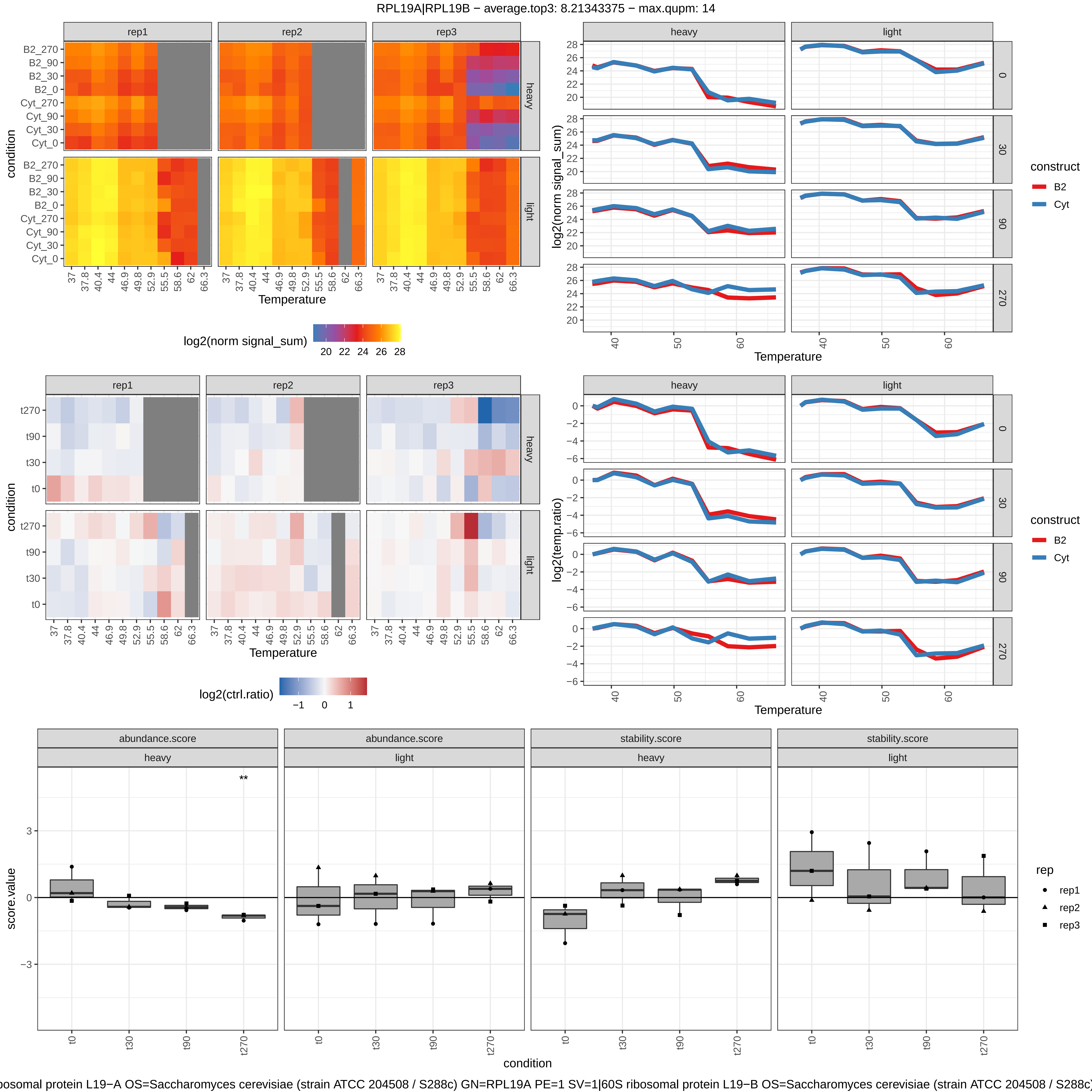
RPL19A|RPL19B − average.top3: 8.21343375 − max.qupm: 14
rep1
rep2
rep3
heavy
light
condition
Temperature
log2(norm signal_sum)
log2(temp.ratio)
log2(ctrl.ratio)
construct
B2
Cyt
abundance.score
stability.score
score.value
rep
rep1
rep2
rep3
**
osomal protein L19−A OS=Saccharomyces cerevisiae (strain ATCC 204508 / S288c) GN=RPL19A PE=1 SV=1|60S ribosomal protein L19−B OS=Saccharomyces cerevisiae (strain ATCC 204508 / S288c)

RPL1A|RPL1B − average.top3: 8.063035 − max.qupm: 13

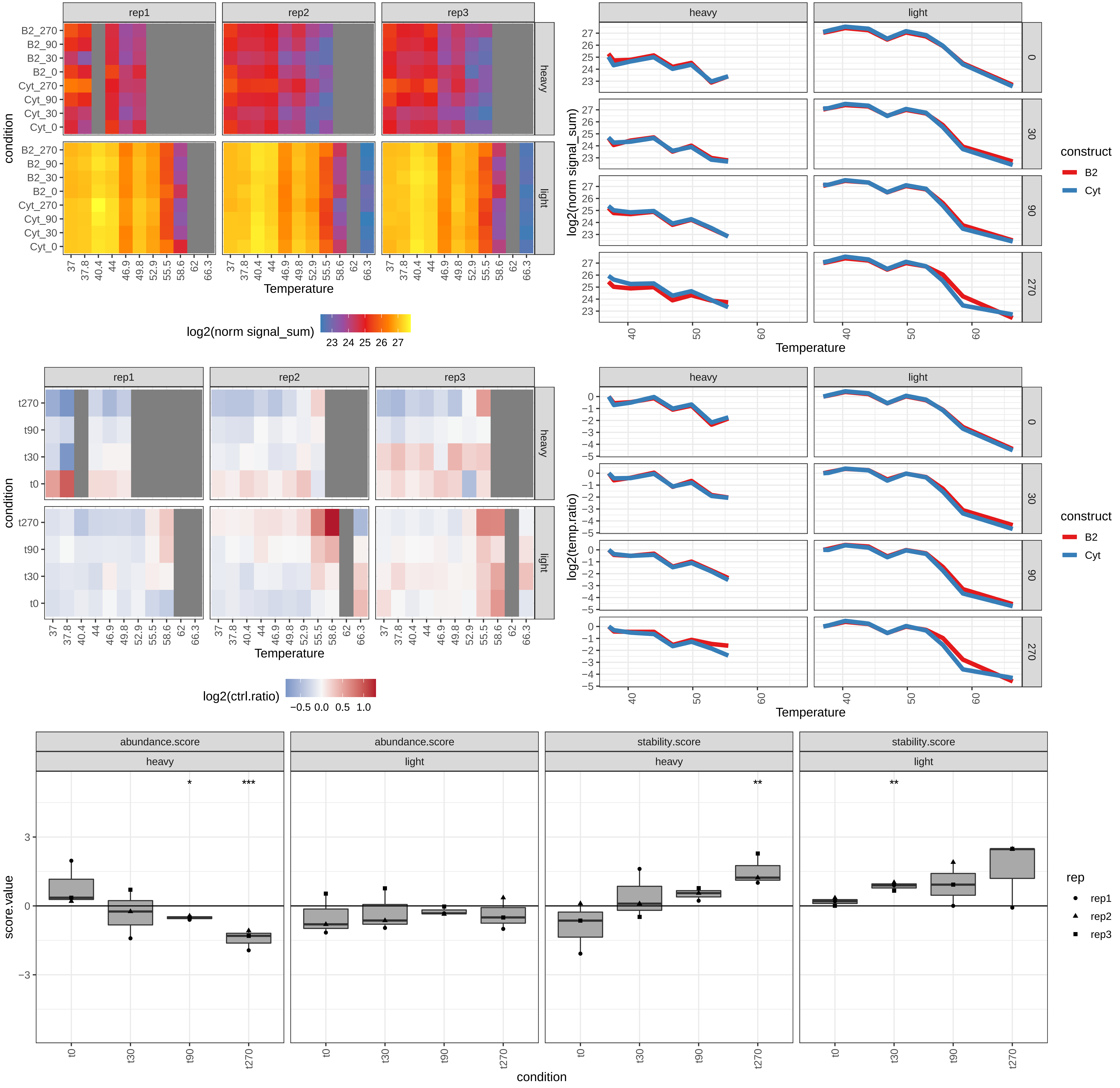

ribosomal protein L1−A OS=Saccharomyces cerevisiae (strain ATCC 204508 / S288c) GN=RPL1A PE=1 SV=1|60S ribosomal protein L1−B OS=Saccharomyces cerevisiae (strain ATCC 204508 / S288c)

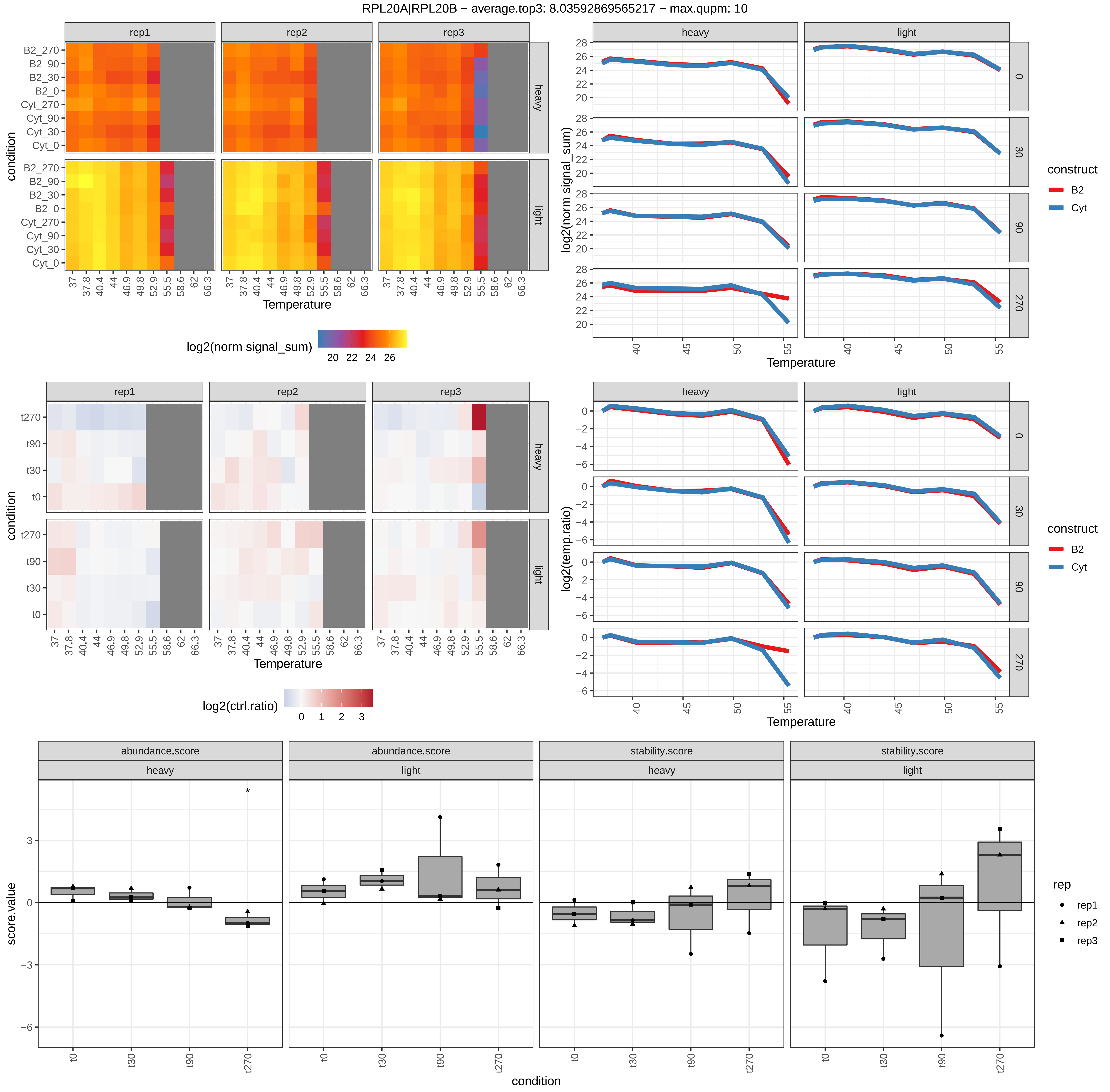

osomal protein L20−A OS=Saccharomyces cerevisiae (strain ATCC 204508 / S288c) GN=RPL20A PE=1 SV=1|60S ribosomal protein L20−B OS=Saccharomyces cerevisiae (strain ATCC 204508 / S288c)

RPL21A|RPL21B − average.top3: 8.21071325 − max.qupm: 7

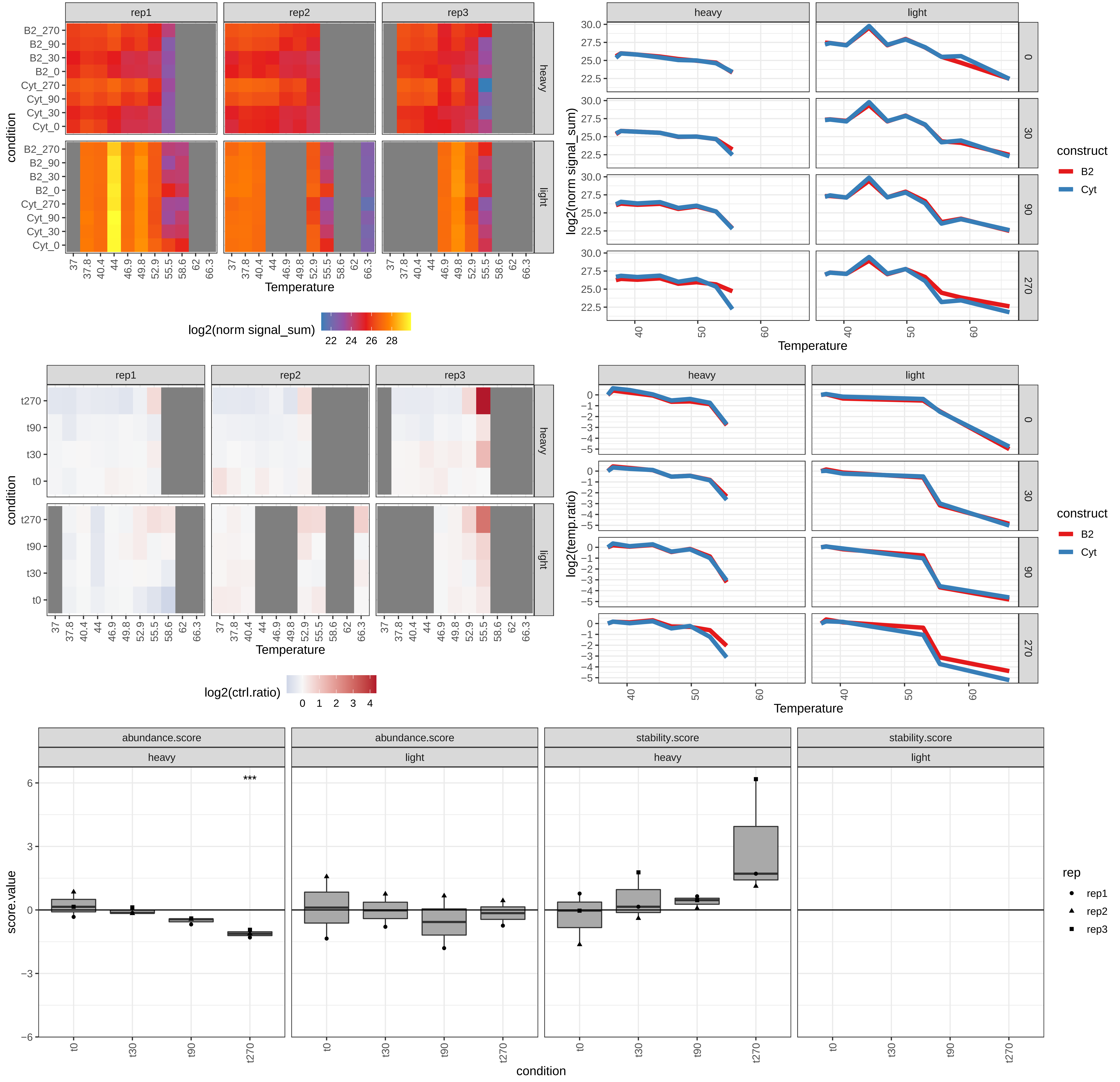

osomal protein L21−A OS=Saccharomyces cerevisiae (strain ATCC 204508 / S288c) GN=RPL21A PE=1 SV=1|60S ribosomal protein L21−B OS=Saccharomyces cerevisiae (strain ATCC 204508 / S288c)

RPL22A − average.top3: 6.77078555555556 − max.qupm: 3

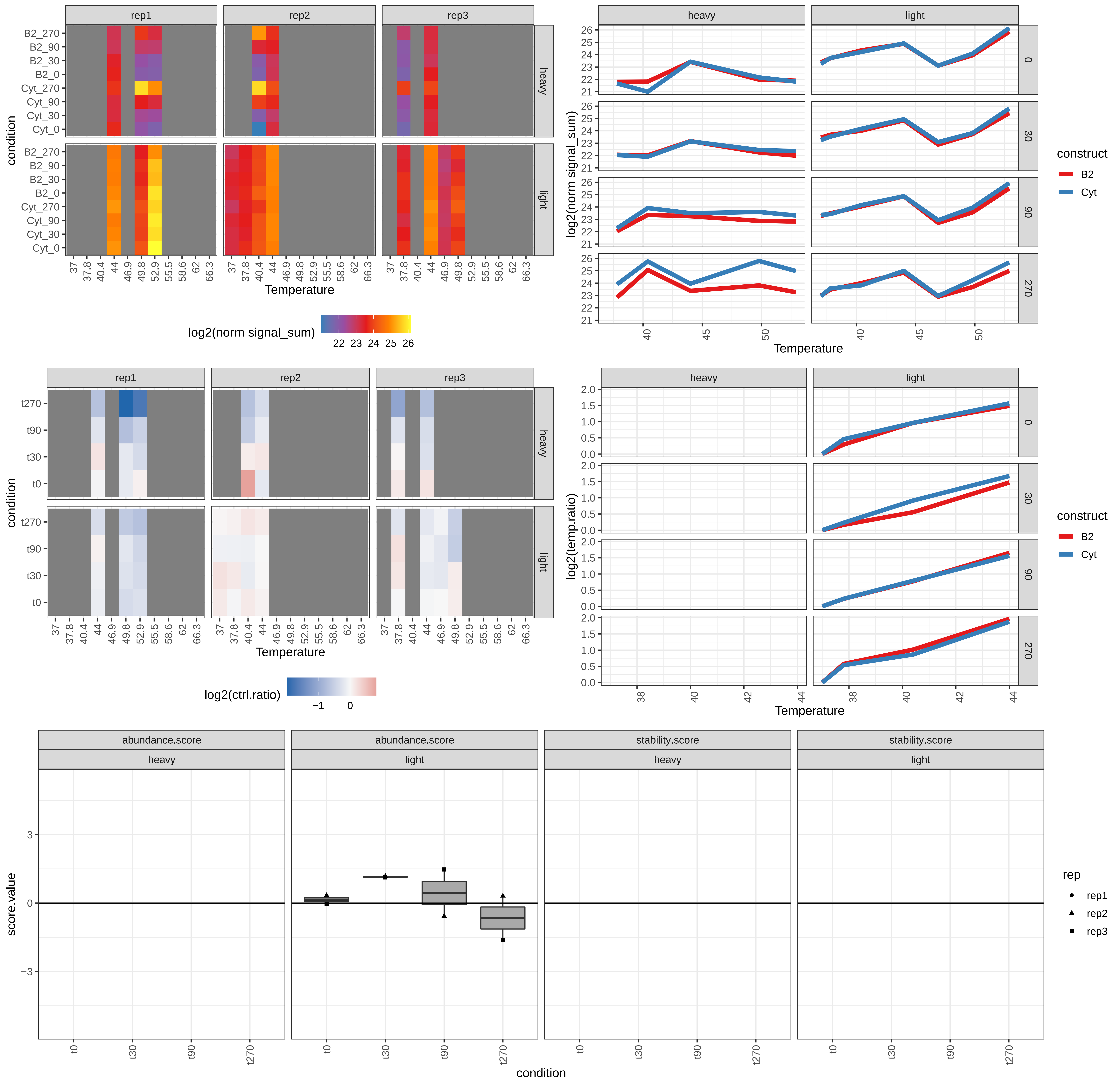

P05749 − 60S ribosomal protein L22−A OS=Saccharomyces cerevisiae (strain ATCC 204508 / S288c) GN=RPL22A PE=1 SV=3

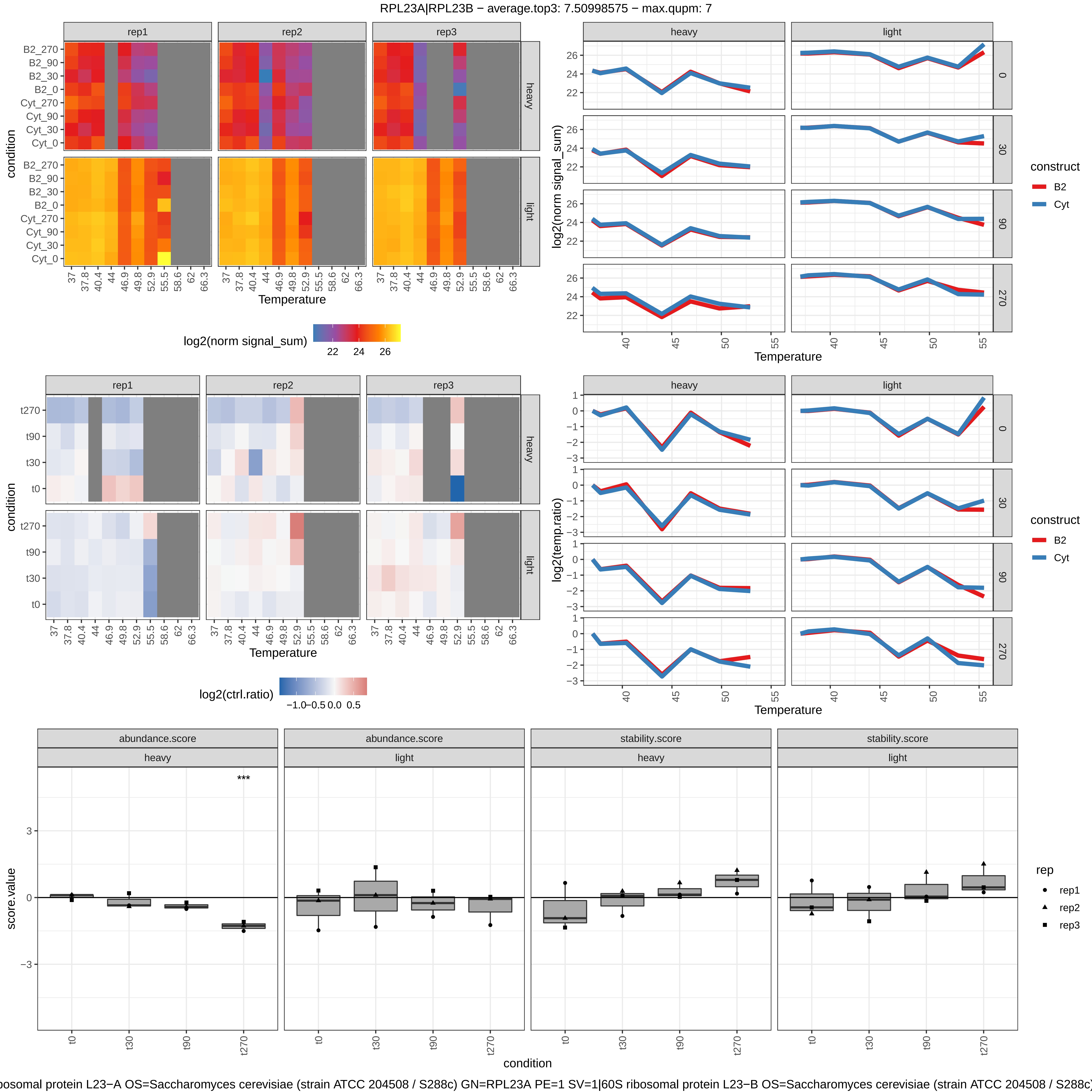

RPL23A|RPL23B − average.top3: 7.50998575 − max.qupm: 7
rep1
rep2
rep3
heavy
light
condition
B2_270
B2_90
B2_30
B2_0
Cyt_270
Cyt_90
Cyt_30
Cyt_0
Temperature
37
37.8
40.4
44
46.9
49.8
52.9
55.5
58.6
62
66.3
log2(norm signal_sum)
22
24
26
0
30
90
270
40
45
50
55
construct
B2
Cyt
t270
t90
t30
t0
log2(ctrl.ratio)
−1.0
−0.5
0.0
0.5
log2(temp.ratio)
1
−1
−2
−3
abundance.score
stability.score
score.value
3
−3
***
rep
rep1
rep2
rep3
osomal protein L23−A OS=Saccharomyces cerevisiae (strain ATCC 204508 / S288c) GN=RPL23A PE=1 SV=1|60S ribosomal protein L23−B OS=Saccharomyces cerevisiae (strain ATCC 204508 / S288c)

RPL24A − average.top3: 7.4082335 − max.qupm: 8

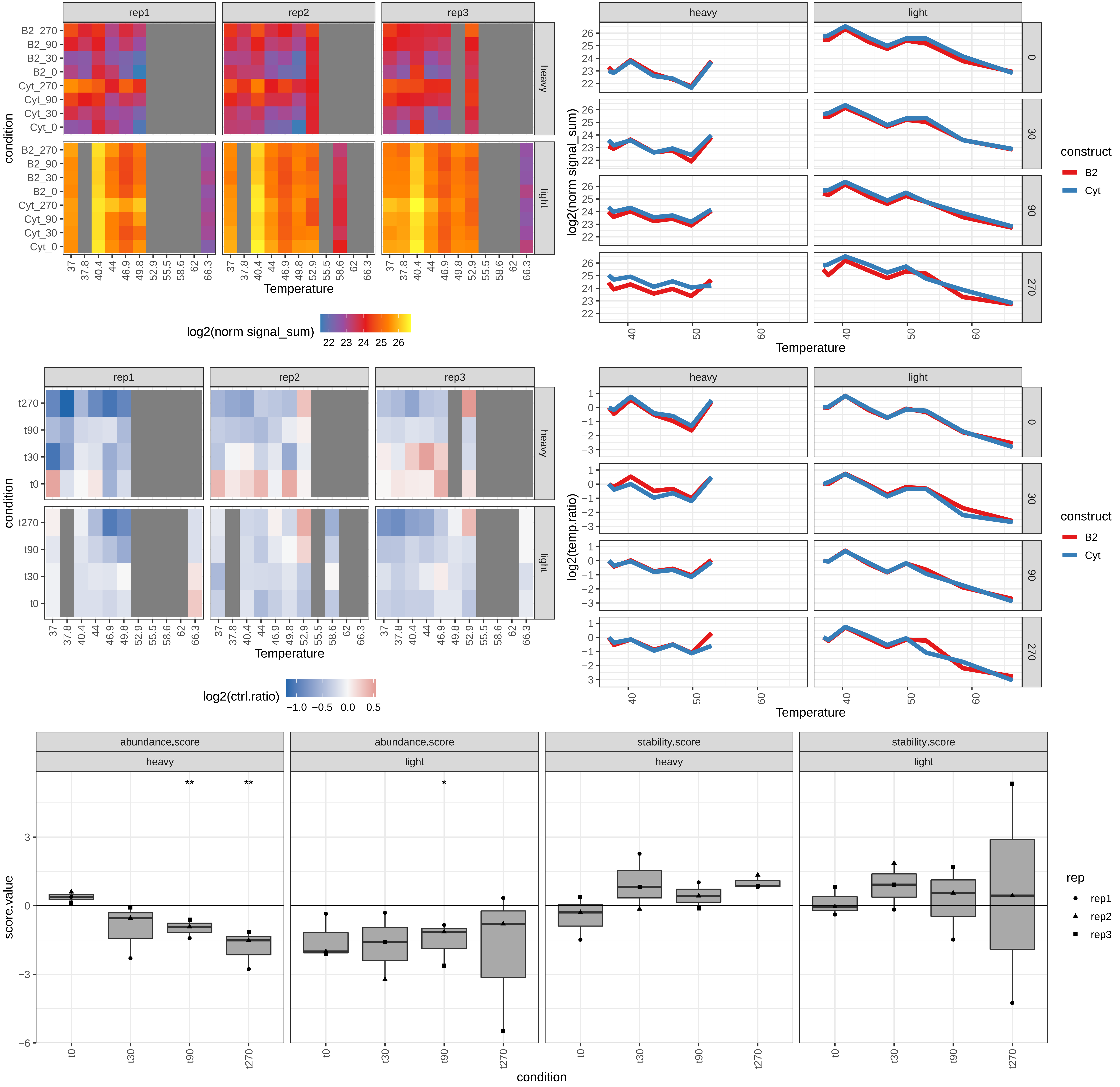

P04449 − 60S ribosomal protein L24−A OS=Saccharomyces cerevisiae (strain ATCC 204508 / S288c) GN=RPL24A PE=1 SV=1

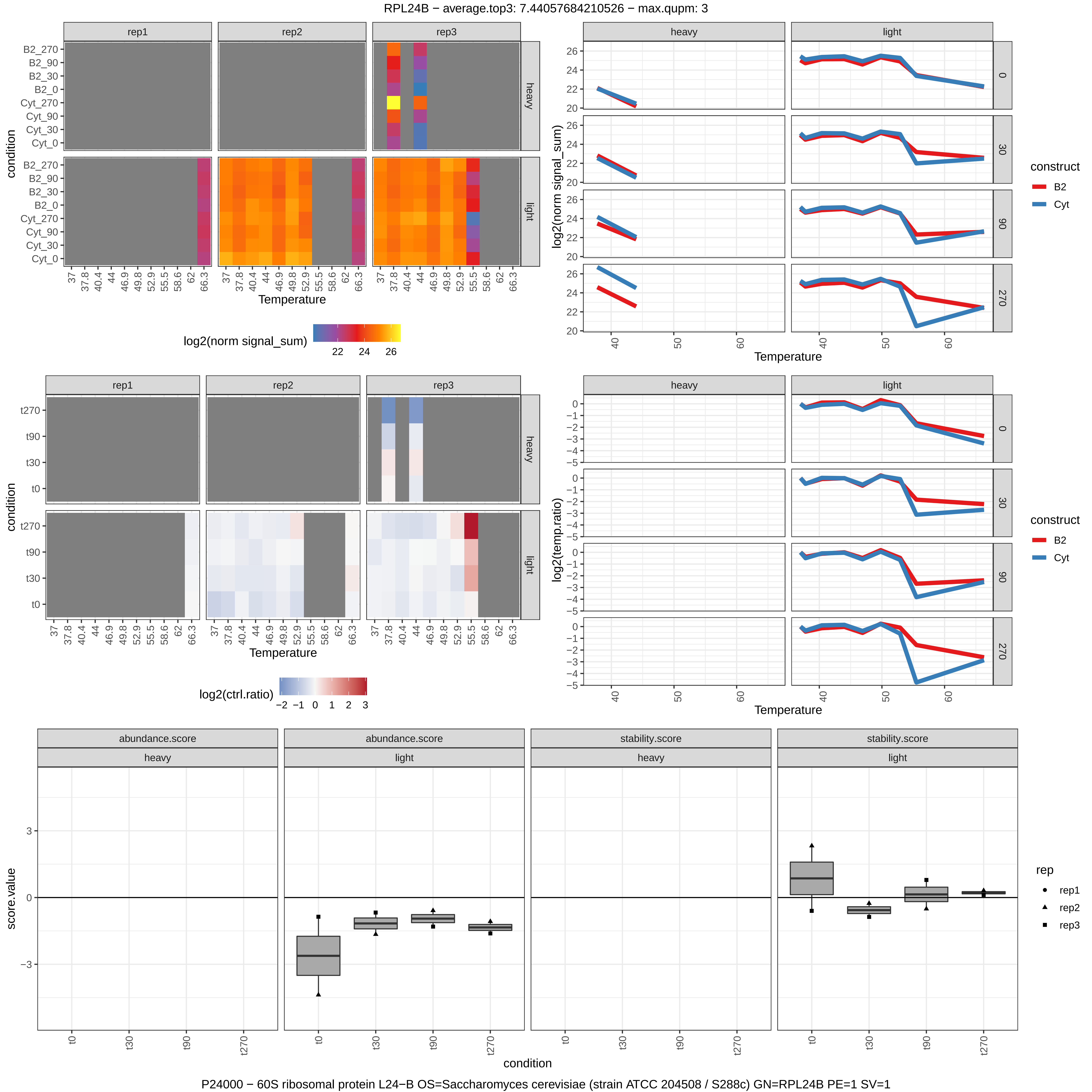

RPL24B − average.top3: 7.44057684210526 − max.qupm: 3
rep1
rep2
rep3
heavy
light
condition
Temperature
log2(norm signal_sum)
log2(ctrl.ratio)
log2(temp.ratio)
construct
B2
Cyt
abundance.score
stability.score
score.value
rep
rep1
rep2
rep3
P24000 − 60S ribosomal protein L24−B OS=Saccharomyces cerevisiae (strain ATCC 204508 / S288c) GN=RPL24B PE=1 SV=1

RPL25 − average.top3: 7.73014591836735 − max.qupm: 7

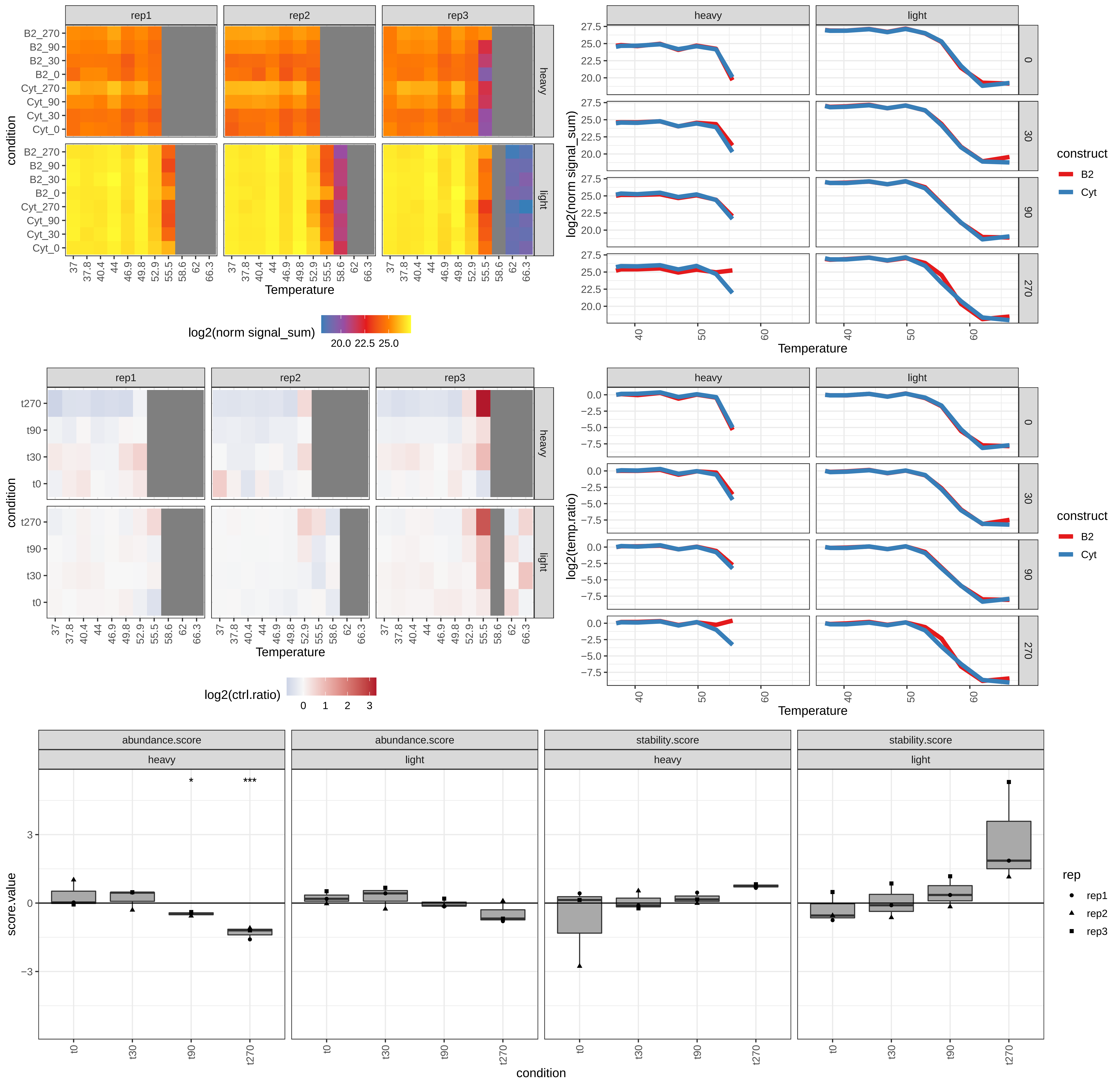

P04456 − 60S ribosomal protein L25 OS=Saccharomyces cerevisiae (strain ATCC 204508 / S288c) GN=RPL25 PE=1 SV=4

RPL26B − average.top3: 8.27344458333333 − max.qupm: 13

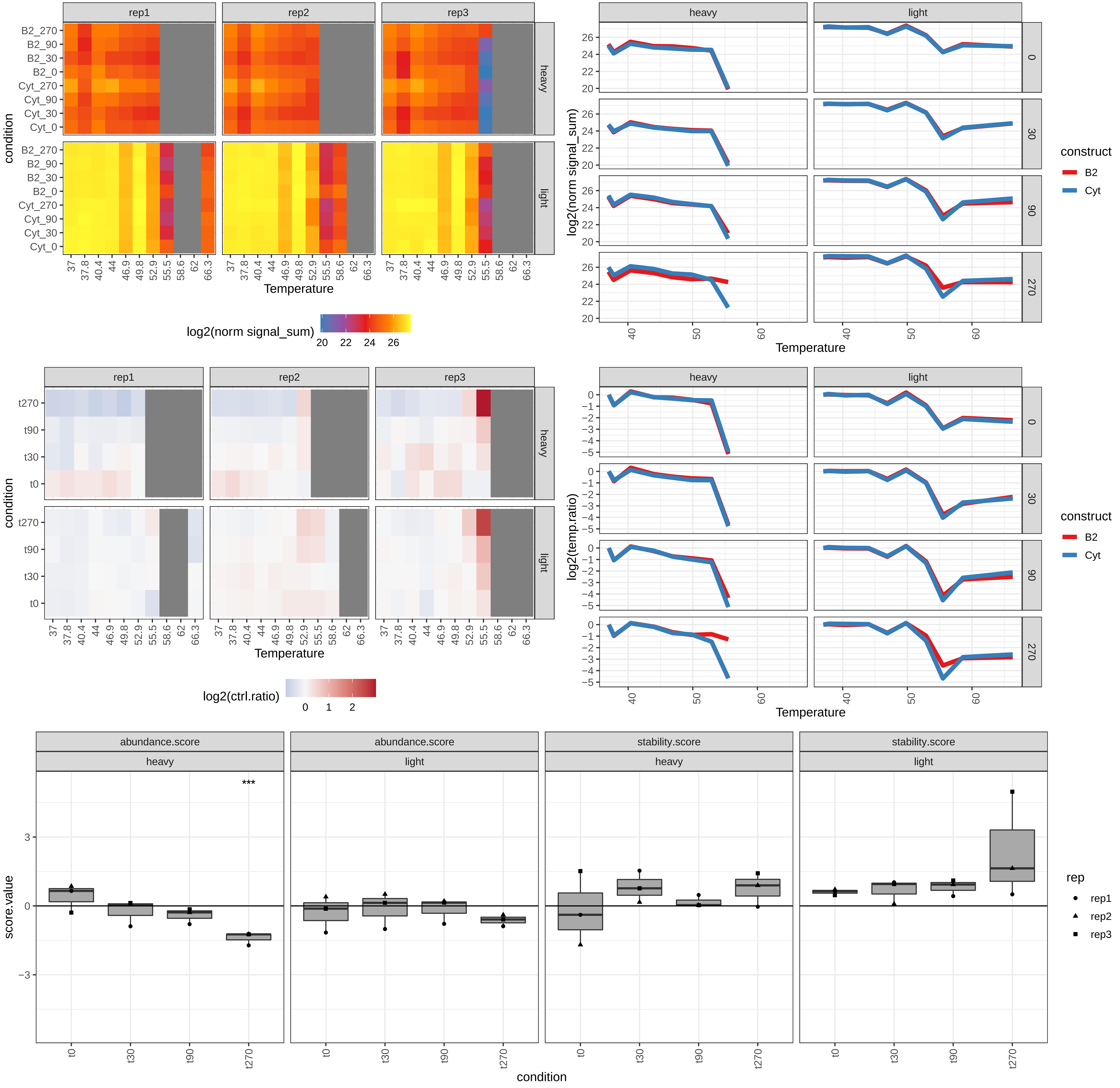

P53221 − 60S ribosomal protein L26−B OS=Saccharomyces cerevisiae (strain ATCC 204508 / S288c) GN=RPL26B PE=1 SV=2

RPL27A|RPL27B − average.top3: 7.99987744680851 − max.qupm: 7

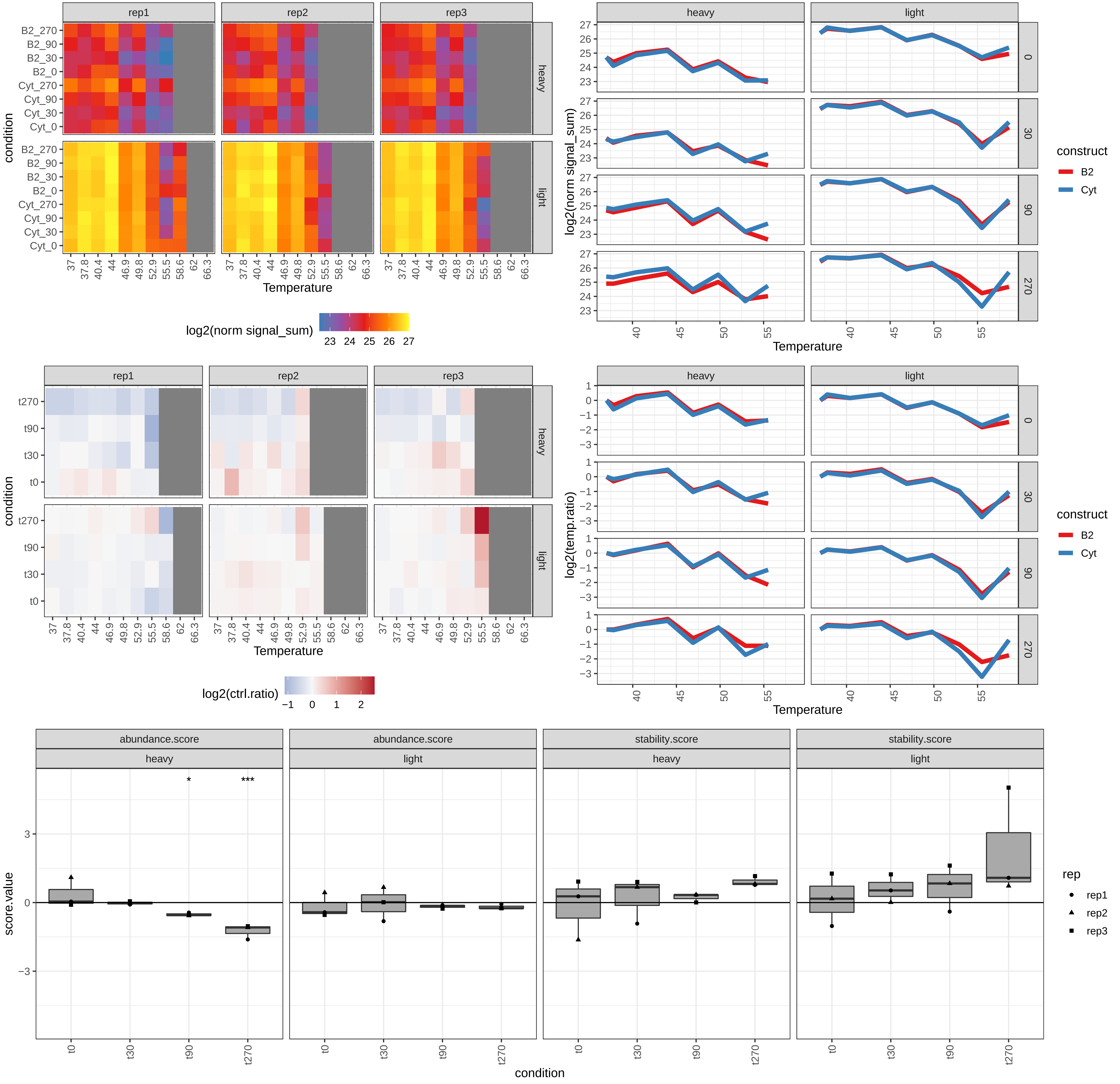

osomal protein L27−A OS=Saccharomyces cerevisiae (strain ATCC 204508 / S288c) GN=RPL27A PE=1 SV=1|60S ribosomal protein L27−B OS=Saccharomyces cerevisiae (strain ATCC 204508 / S288c

RPL28 − average.top3: 7.70223 − max.qupm: 11

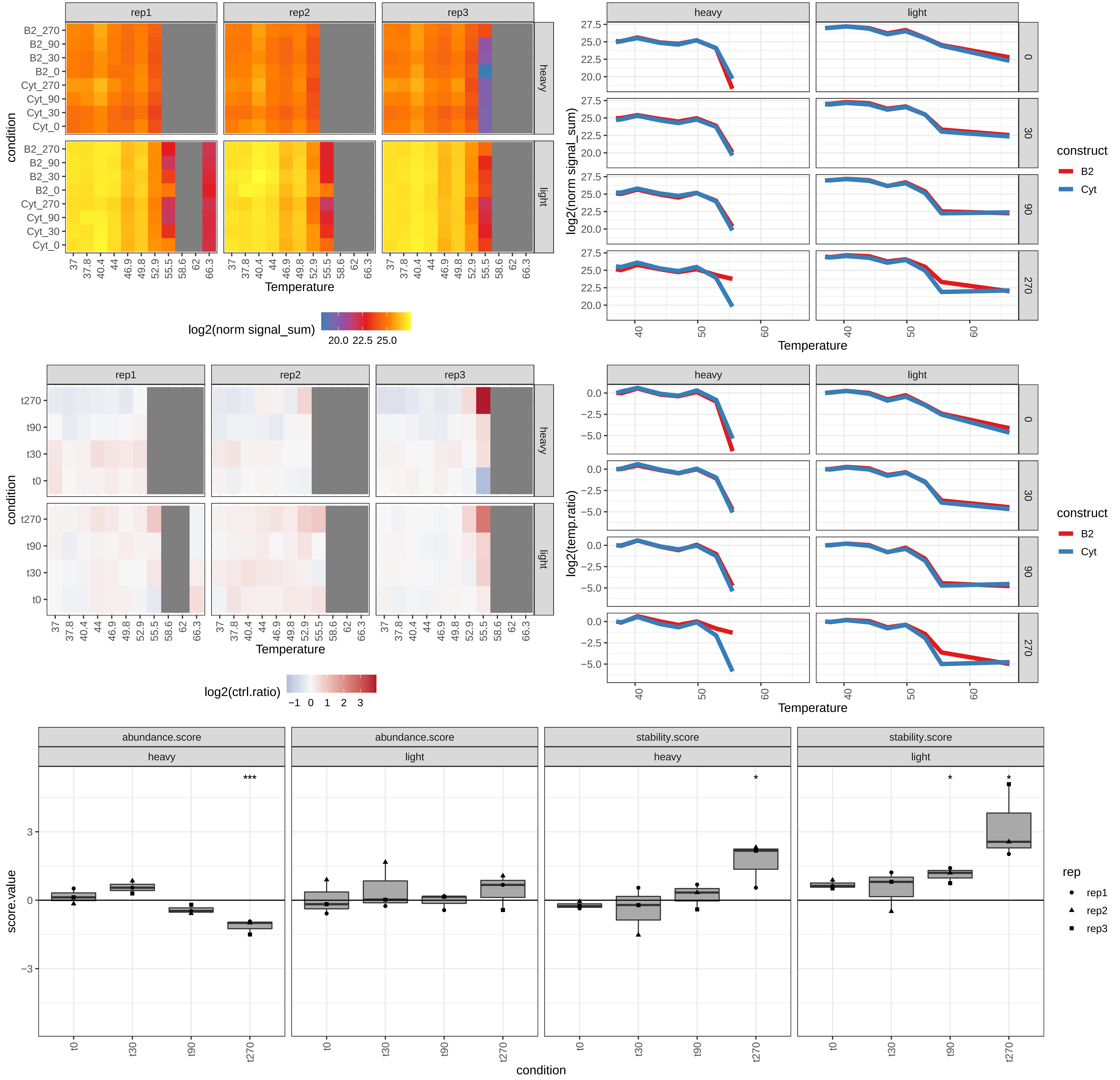

P02406 − 60S ribosomal protein L28 OS=Saccharomyces cerevisiae (strain ATCC 204508 / S288c) GN=RPL28 PE=1 SV=3

RPL29 − average.top3: 7.30545757575758 − max.qupm: 5

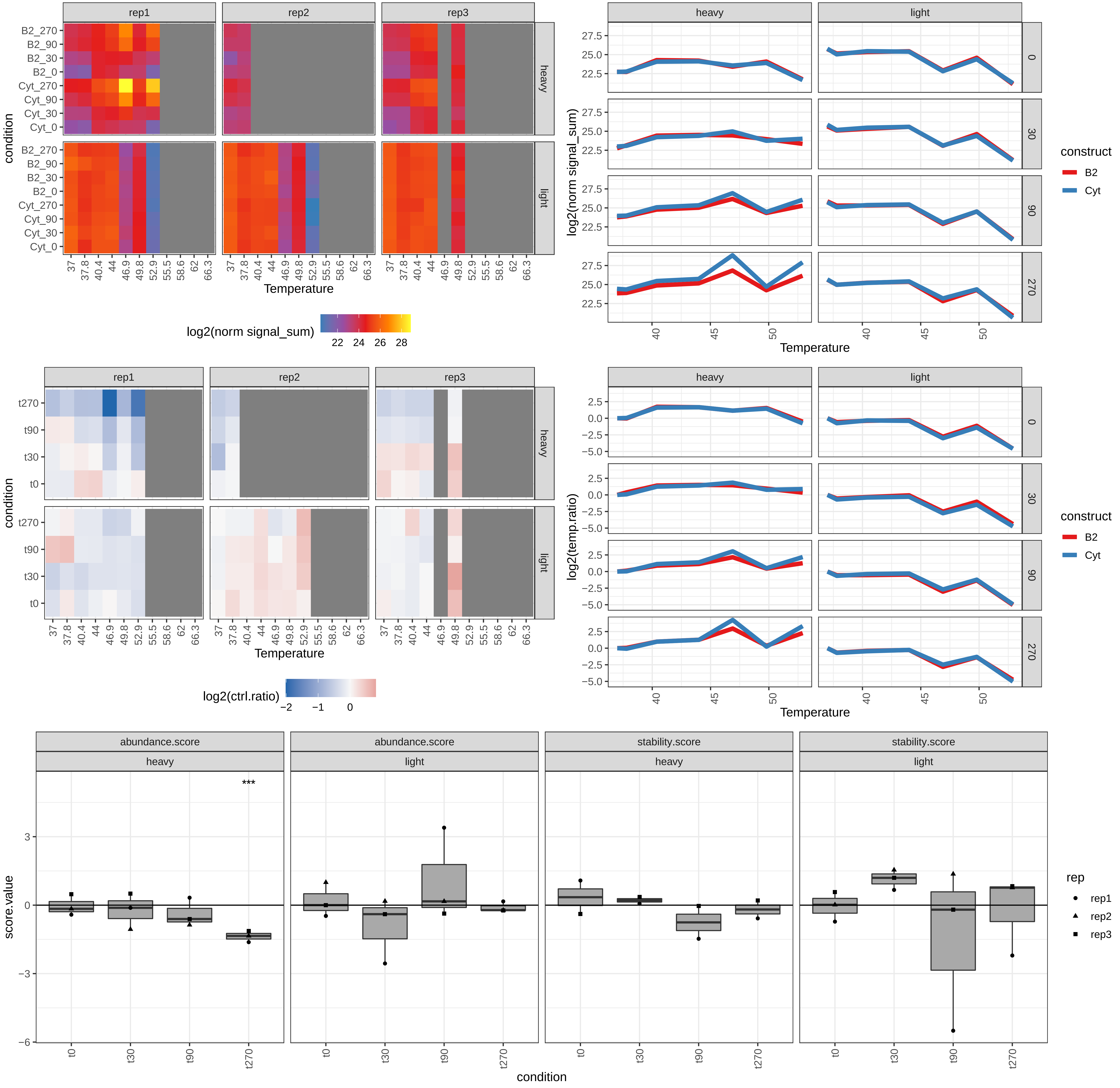

P05747 − 60S ribosomal protein L29 OS=Saccharomyces cerevisiae (strain ATCC 204508 / S288c) GN=RPL29 PE=1 SV=3

RPL2A|RPL2B − average.top3: 8.34396653846154 − max.qupm: 17

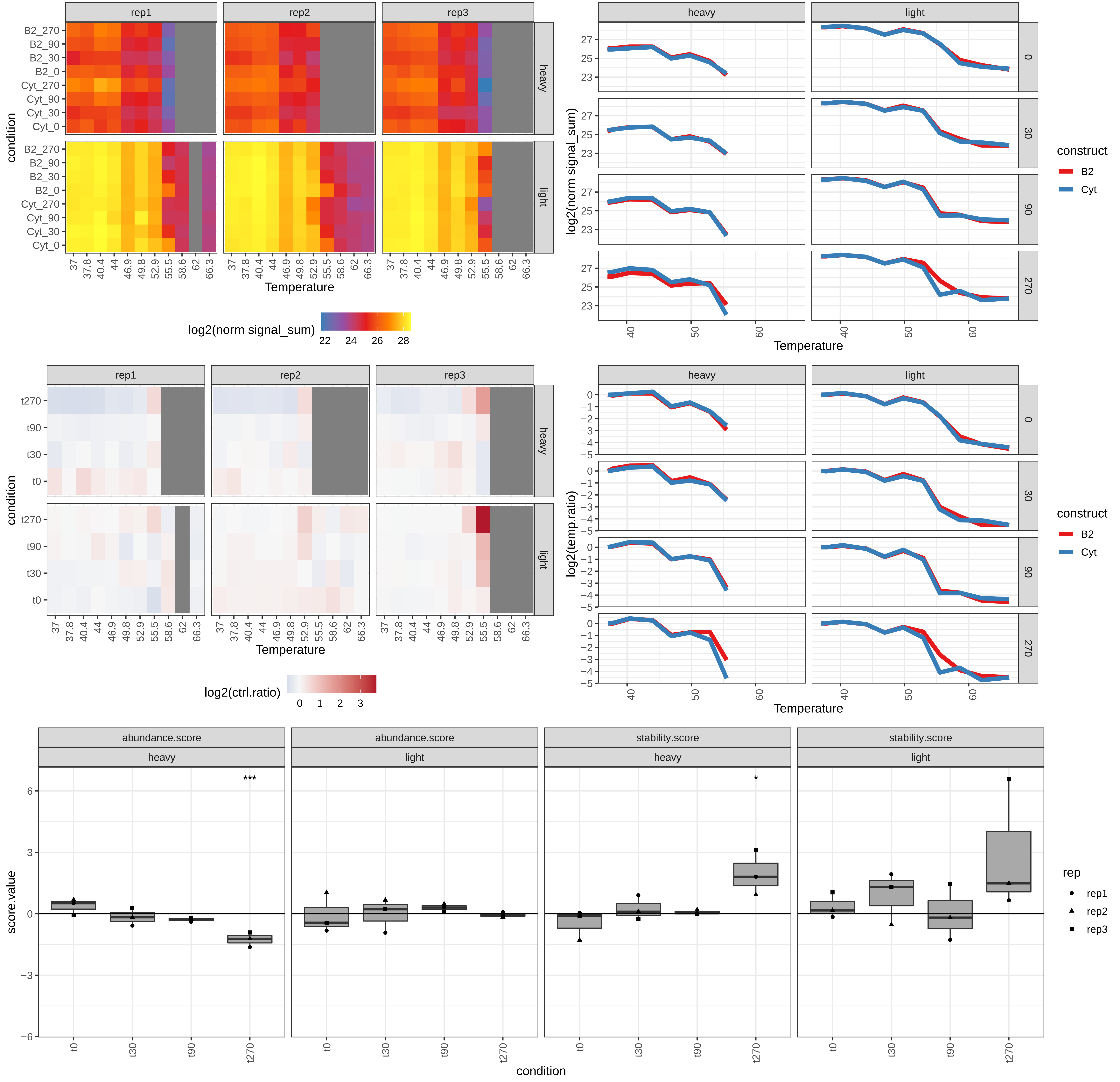

ribosomal protein L2−A OS=Saccharomyces cerevisiae (strain ATCC 204508 / S288c) GN=RPL2A PE=1 SV=1|60S ribosomal protein L2−B OS=Saccharomyces cerevisiae (strain ATCC 204508 / S288c)

RPL3 − average.top3: 8.33515039215686 − max.qupm: 26

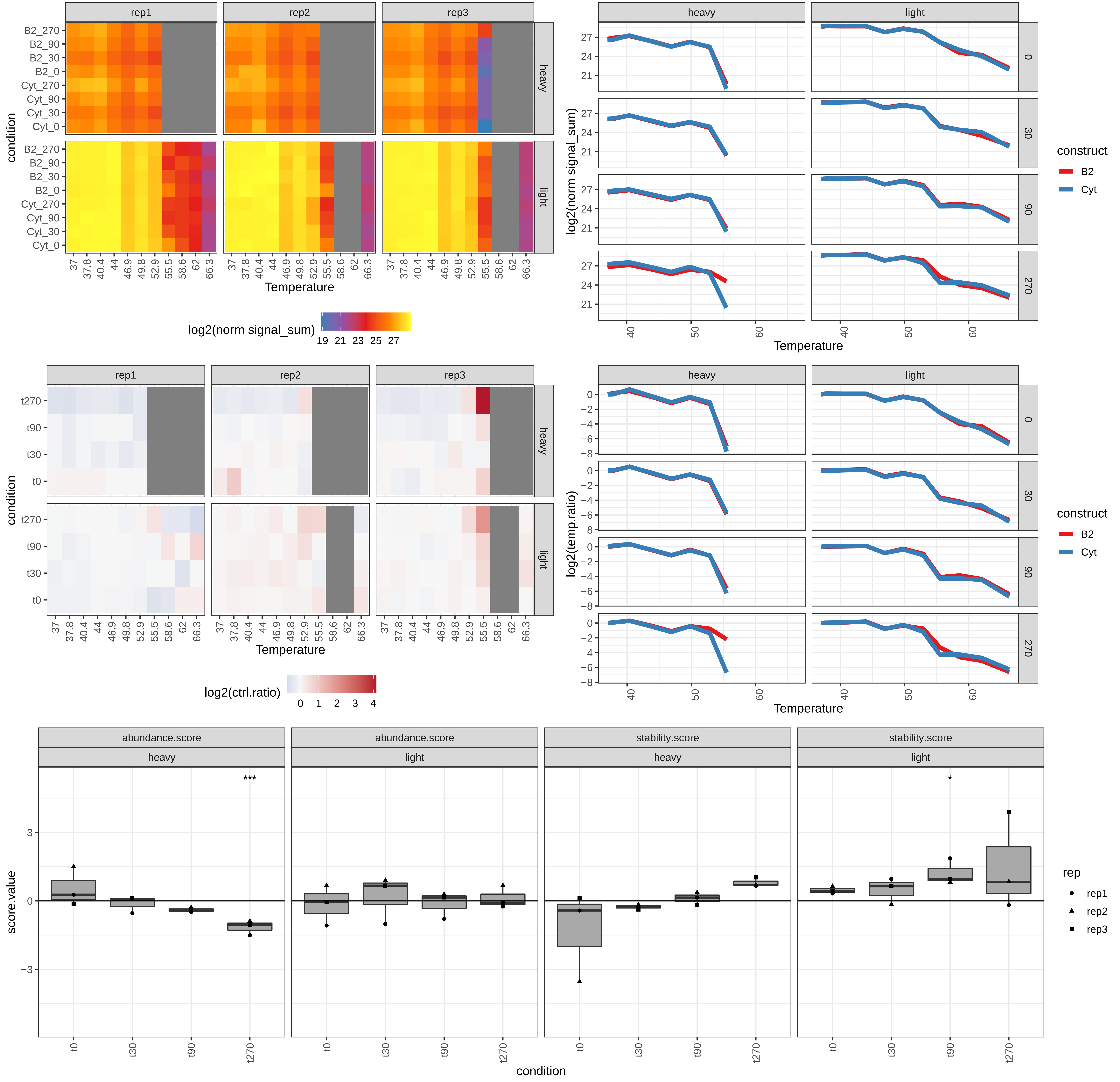

P14126 − 60S ribosomal protein L3 OS=Saccharomyces cerevisiae (strain ATCC 204508 / S288c) GN=RPL3 PE=1 SV=4

RPL30 − average.top3: 7.71353708333333 − max.qupm: 6

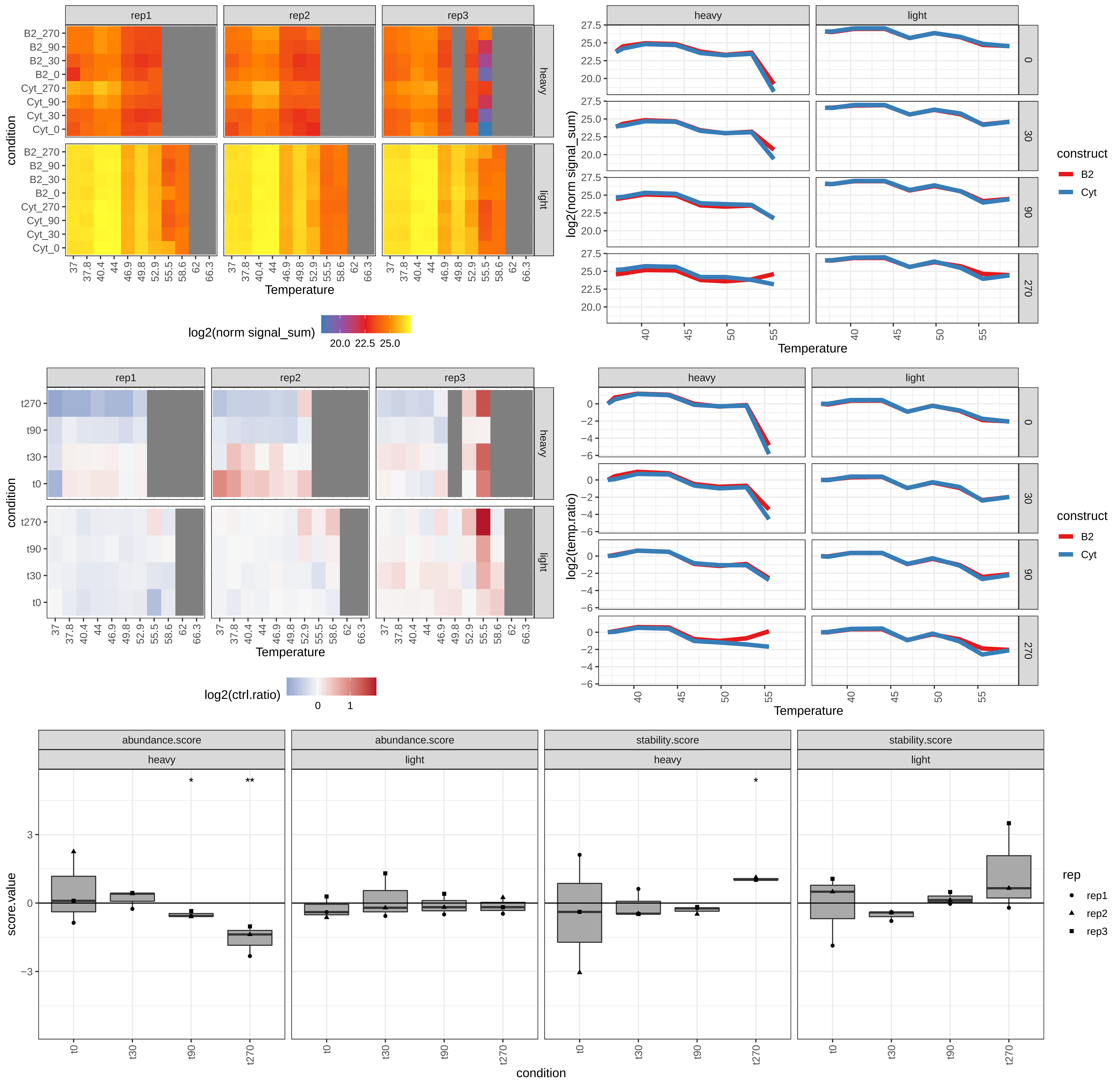

P14120 − 60S ribosomal protein L30 OS=Saccharomyces cerevisiae (strain ATCC 204508 / S288c) GN=RPL30 PE=1 SV=3

RPL32 − average.top3: 8.33892177777778 − max.qupm: 9

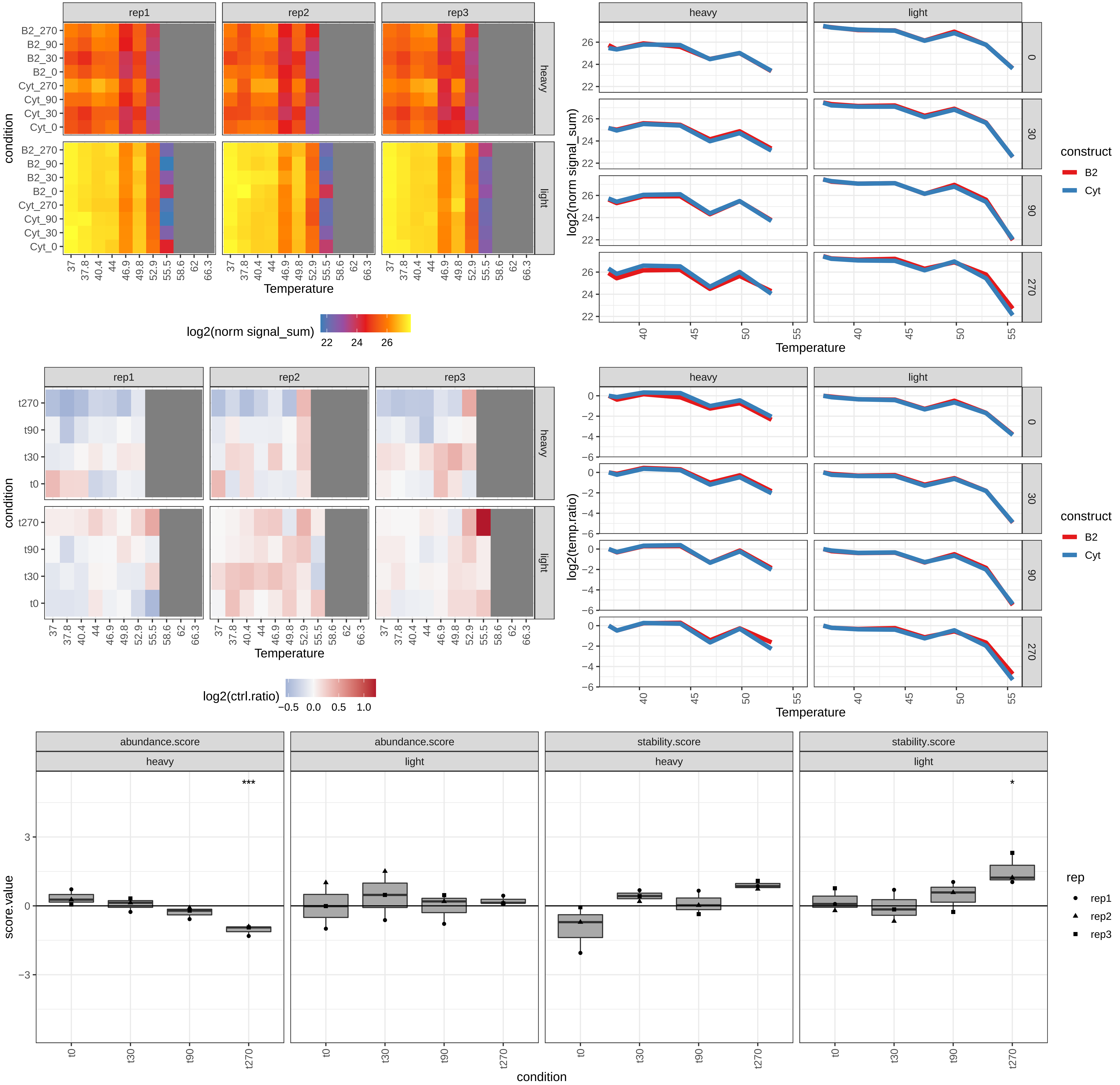

P38061 − 60S ribosomal protein L32 OS=Saccharomyces cerevisiae (strain ATCC 204508 / S288c) GN=RPL32 PE=1 SV=1

RPL34B|RPL34A − average.top3: 7.93044625 − max.qupm: 6

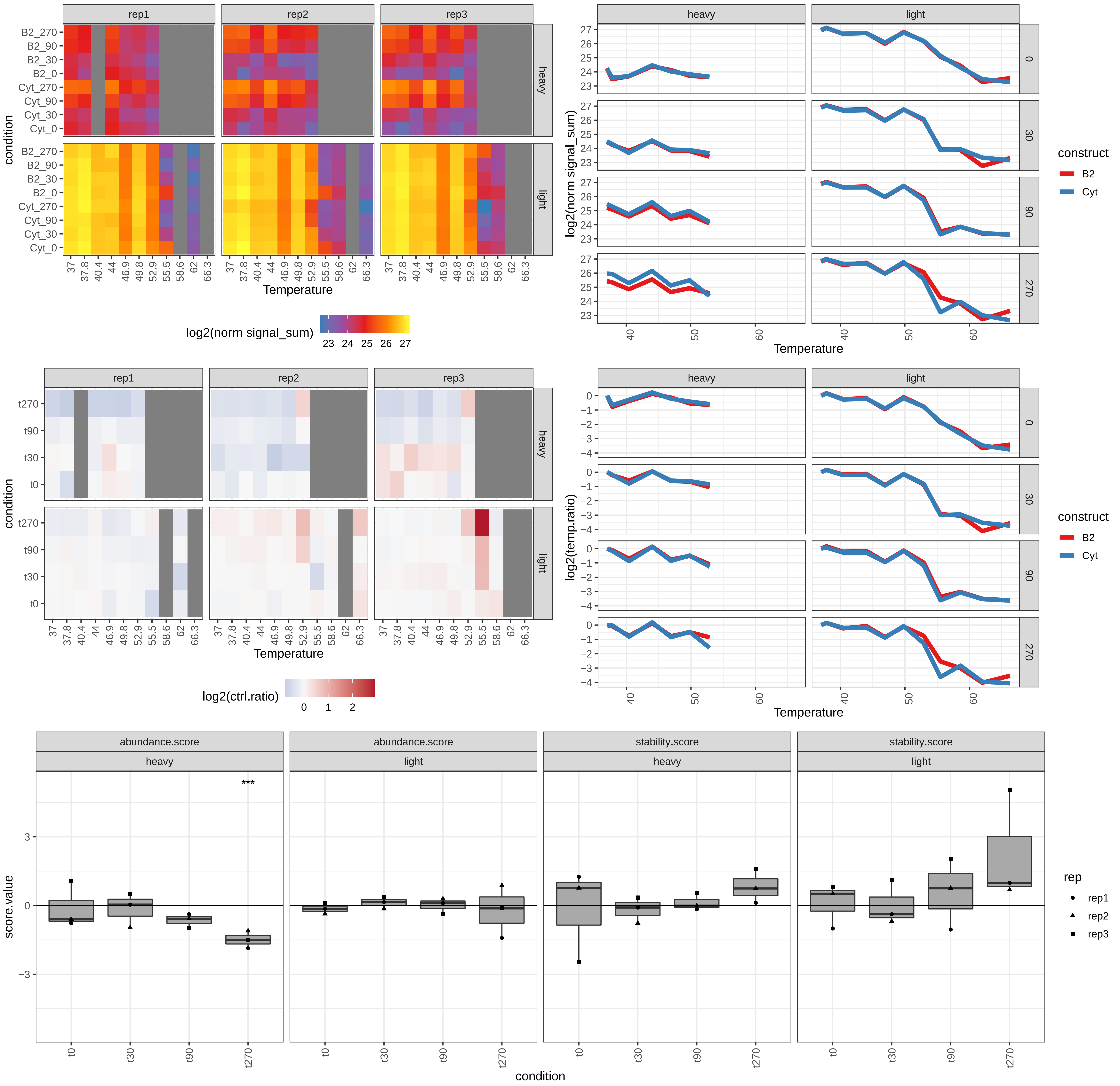

osomal protein L34−B OS=Saccharomyces cerevisiae (strain ATCC 204508 / S288c) GN=RPL34B PE=1 SV=1|60S ribosomal protein L34−A OS=Saccharomyces cerevisiae (strain ATCC 204508 / S288c)

RPL35A|RPL35B − average.top3: 8.12178567567568 − max.qupm: 5

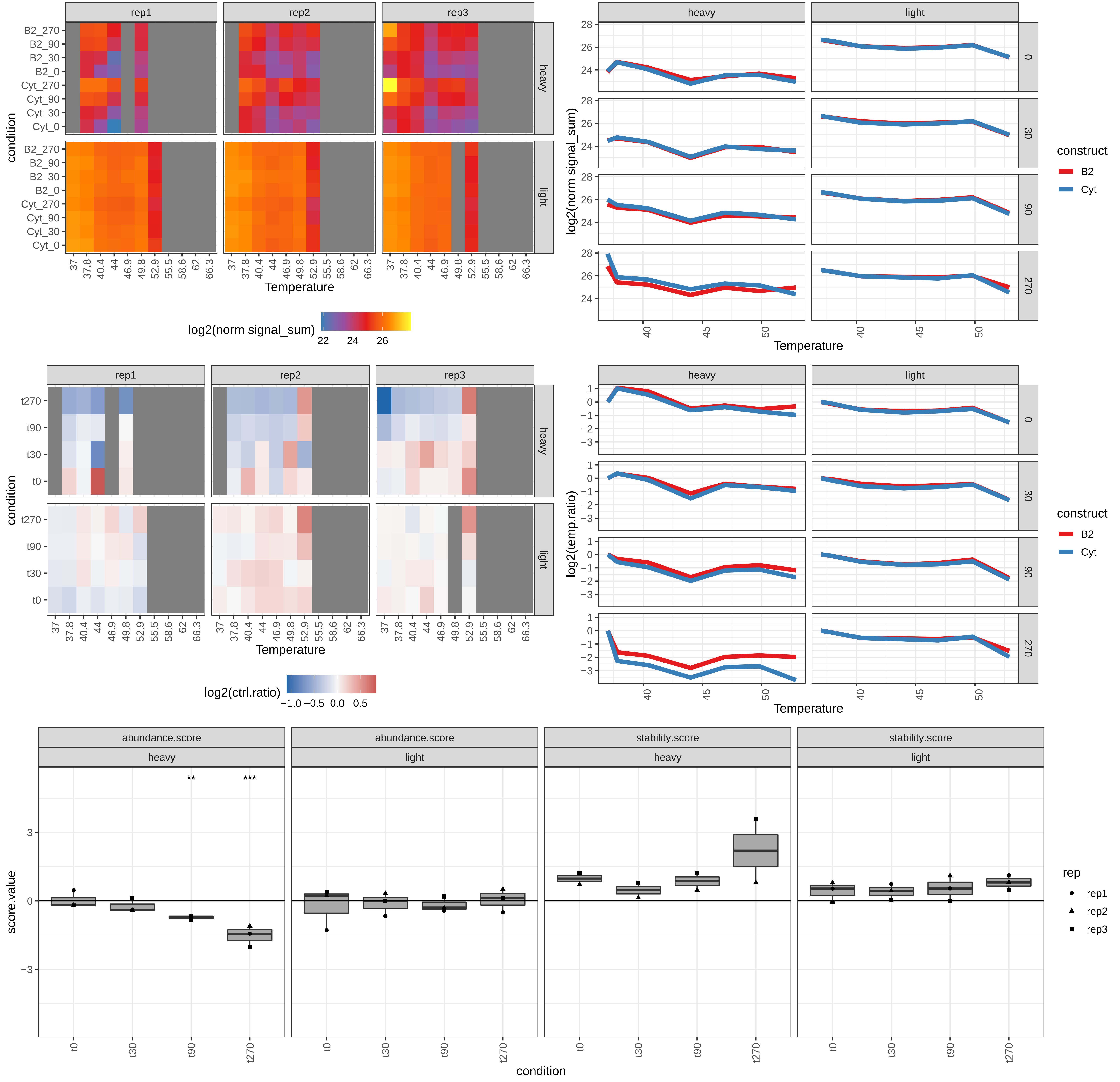

osomal protein L35−A OS=Saccharomyces cerevisiae (strain ATCC 204508 / S288c) GN=RPL35A PE=1 SV=1|60S ribosomal protein L35−B OS=Saccharomyces cerevisiae (strain ATCC 204508 / S288c)

RPL36A − average.top3: 7.2848275 − max.qupm: 2

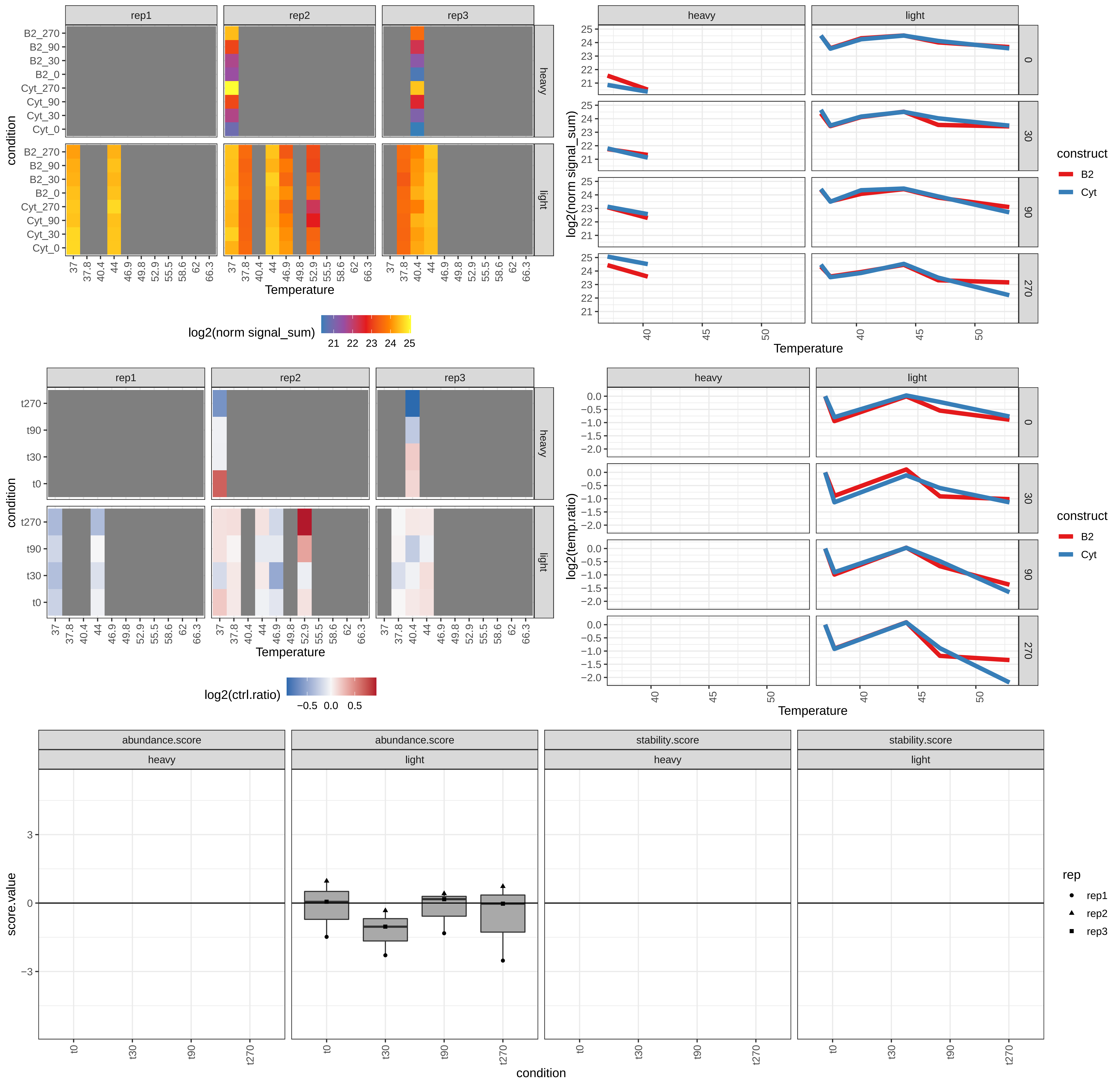

P05745 − 60S ribosomal protein L36−A OS=Saccharomyces cerevisiae (strain ATCC 204508 / S288c) GN=RPL36A PE=1 SV=3

RPL36B − average.top3: 7.24336285714286 − max.qupm: 3

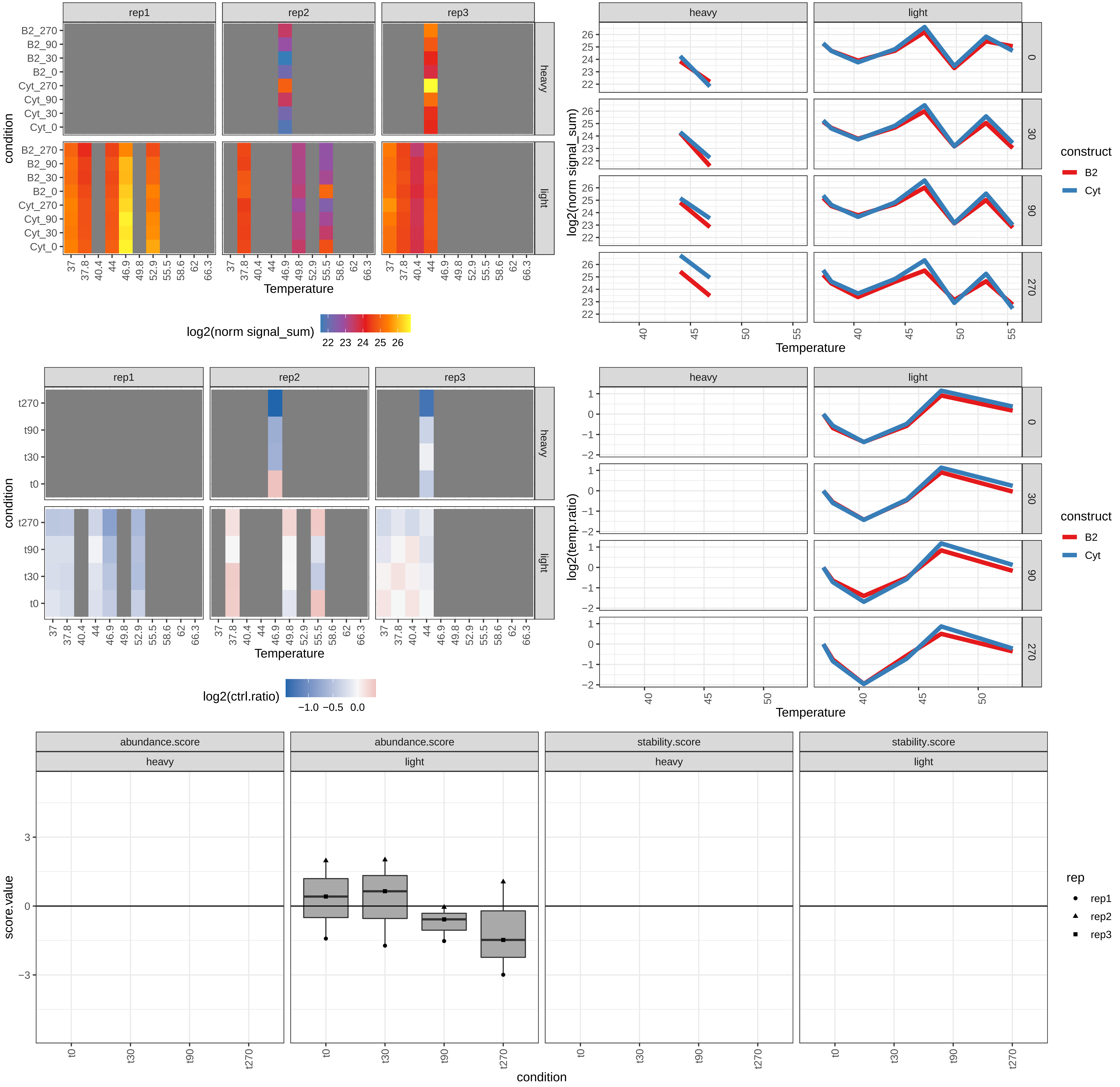

O14455 − 60S ribosomal protein L36−B OS=Saccharomyces cerevisiae (strain ATCC 204508 / S288c) GN=RPL36B PE=1 SV=3

RPL37A − average.top3: 7.80543720930233 − max.qupm: 4

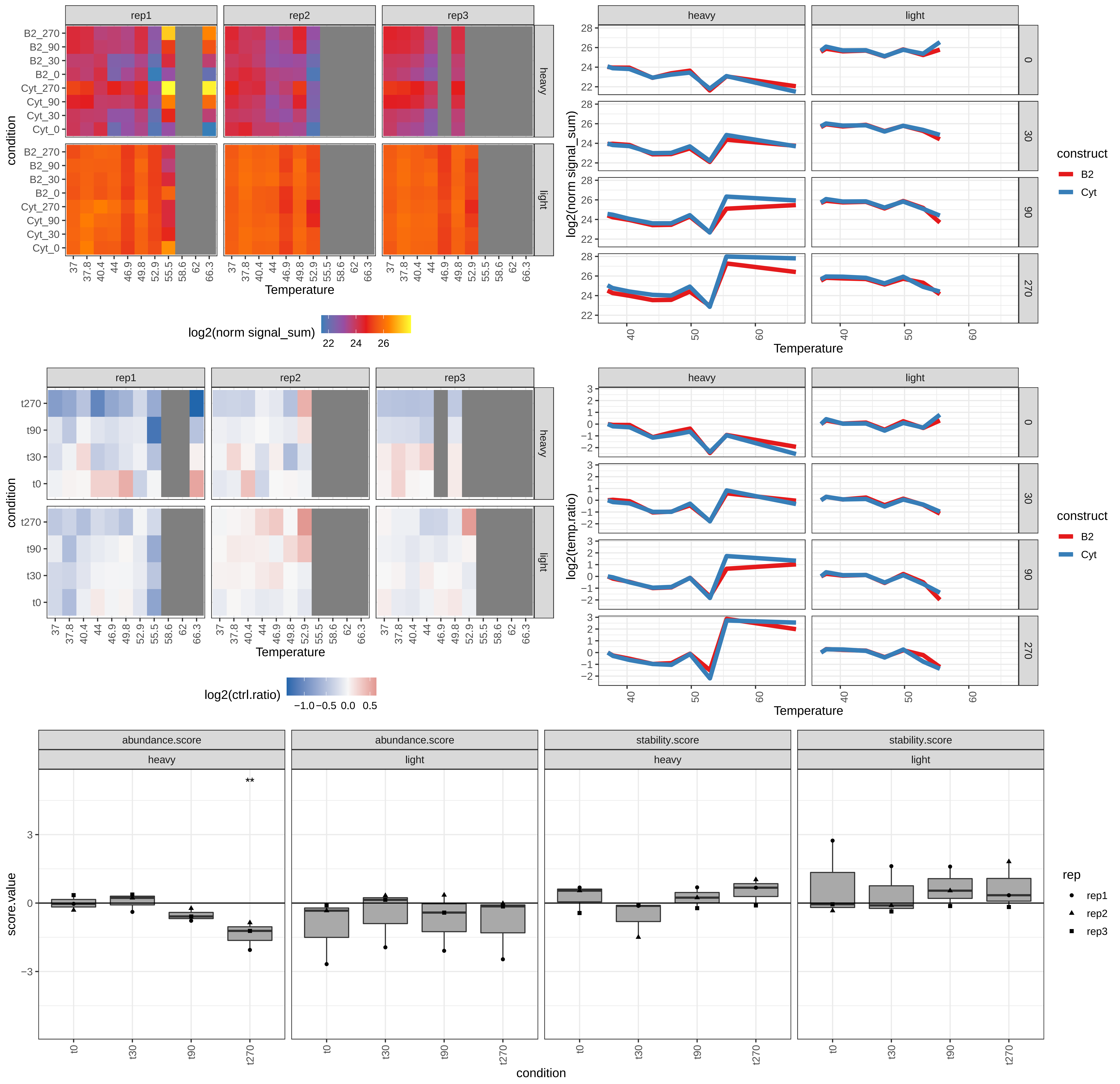

P49166 − 60S ribosomal protein L37−A OS=Saccharomyces cerevisiae (strain ATCC 204508 / S288c) GN=RPL37A PE=1 SV=2

RPL37B − average.top3: 7.70842146341463 − max.qupm: 4

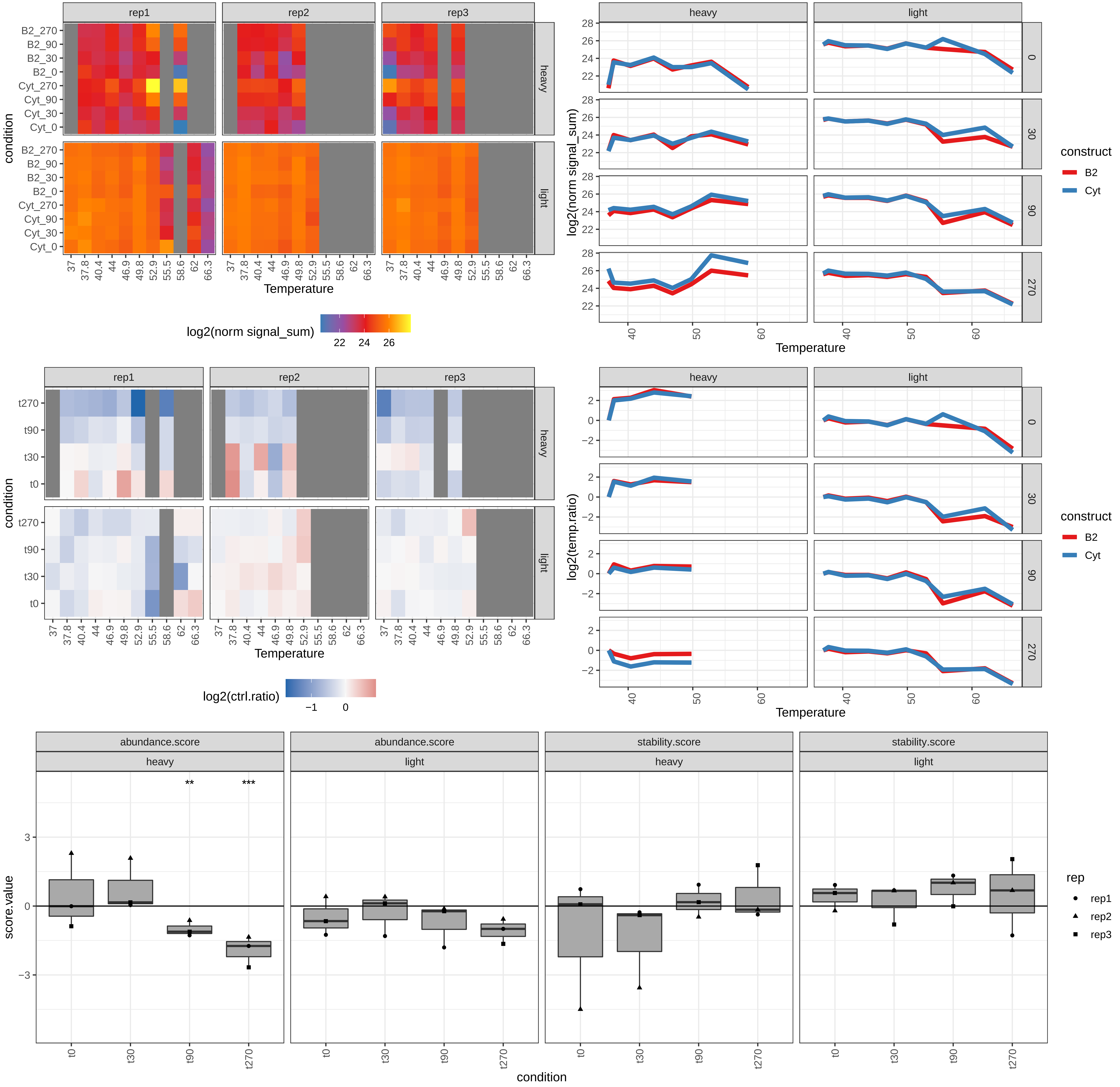

P51402 − 60S ribosomal protein L37−B OS=Saccharomyces cerevisiae (strain ATCC 204508 / S288c) GN=RPL37B PE=1 SV=2

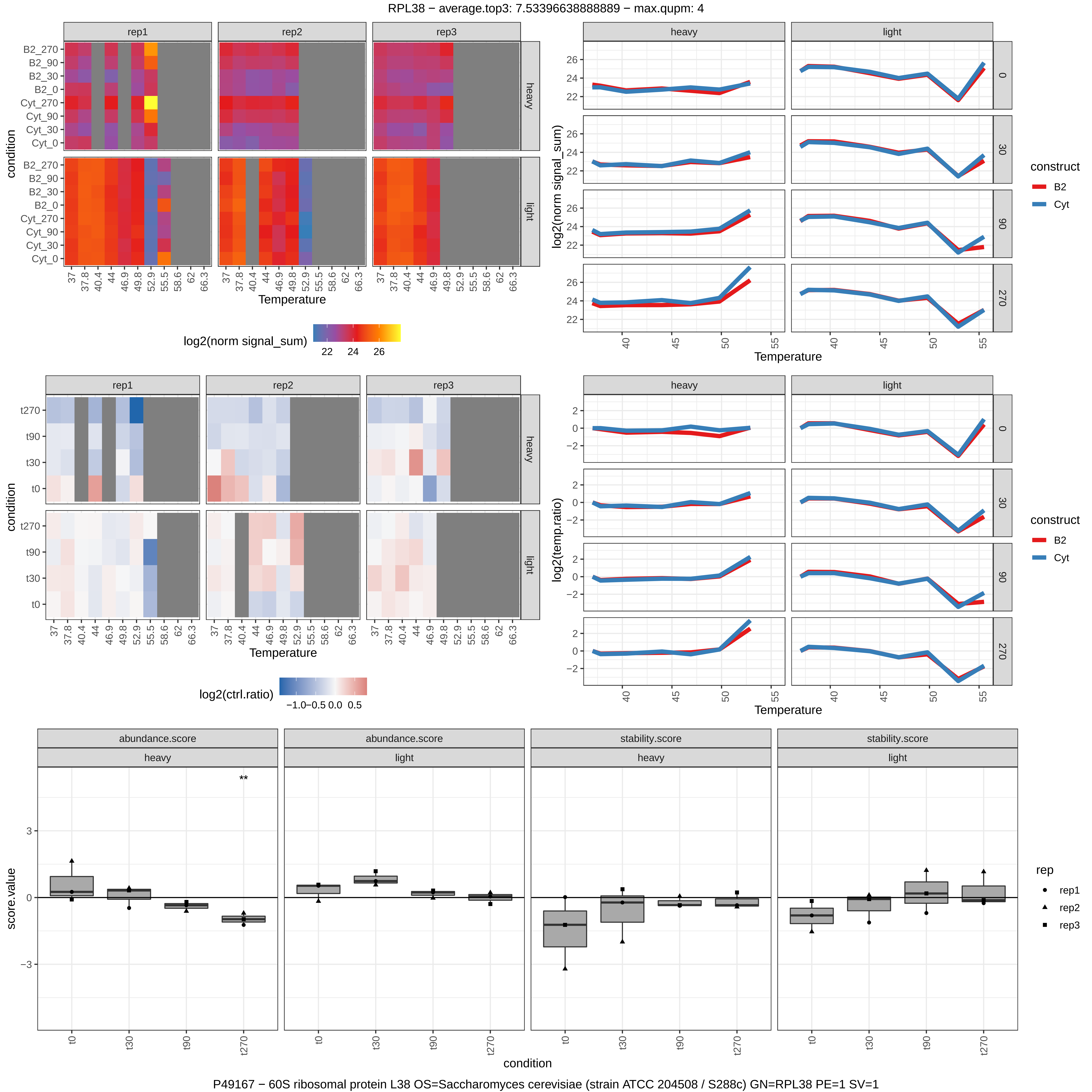

RPL38 − average.top3: 7.53396638888889 − max.qupm: 4
rep1
rep2
rep3
heavy
light
condition
Temperature
log2(norm signal_sum)
construct
B2
Cyt
log2(ctrl.ratio)
log2(temp.ratio)
abundance.score
stability.score
score.value
rep
rep1
rep2
rep3
P49167 − 60S ribosomal protein L38 OS=Saccharomyces cerevisiae (strain ATCC 204508 / S288c) GN=RPL38 PE=1 SV=1

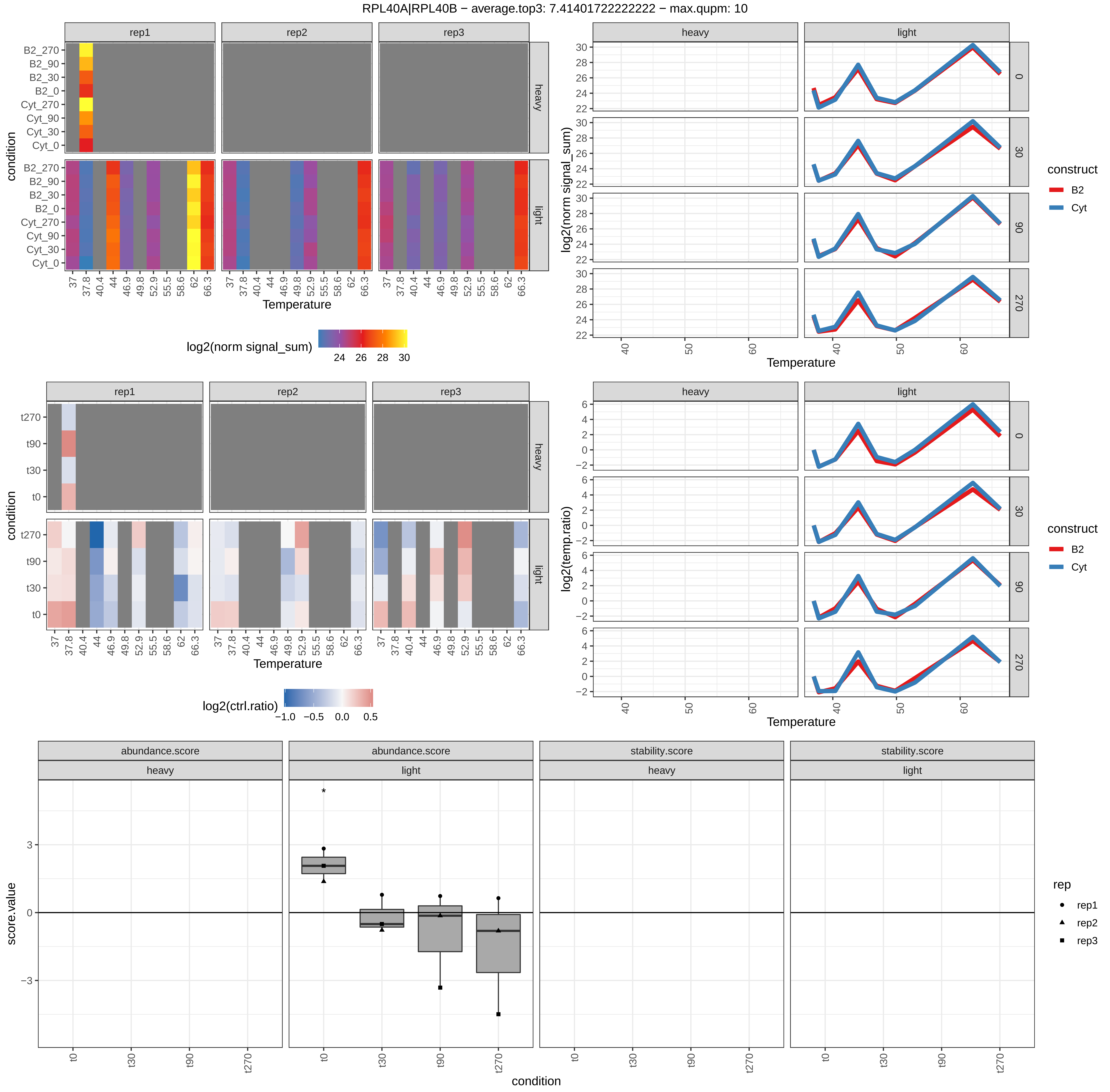

S ribosomal protein L40 OS=Saccharomyces cerevisiae (strain ATCC 204508 / S288c) GN=RPL40A PE=1 SV=1|Ubiquitin−60S ribosomal protein L40 OS=Saccharomyces cerevisiae (strain ATCC 204508 /

RPL42A|RPL42B − average.top3: 7.96091976744186 − max.qupm: 7

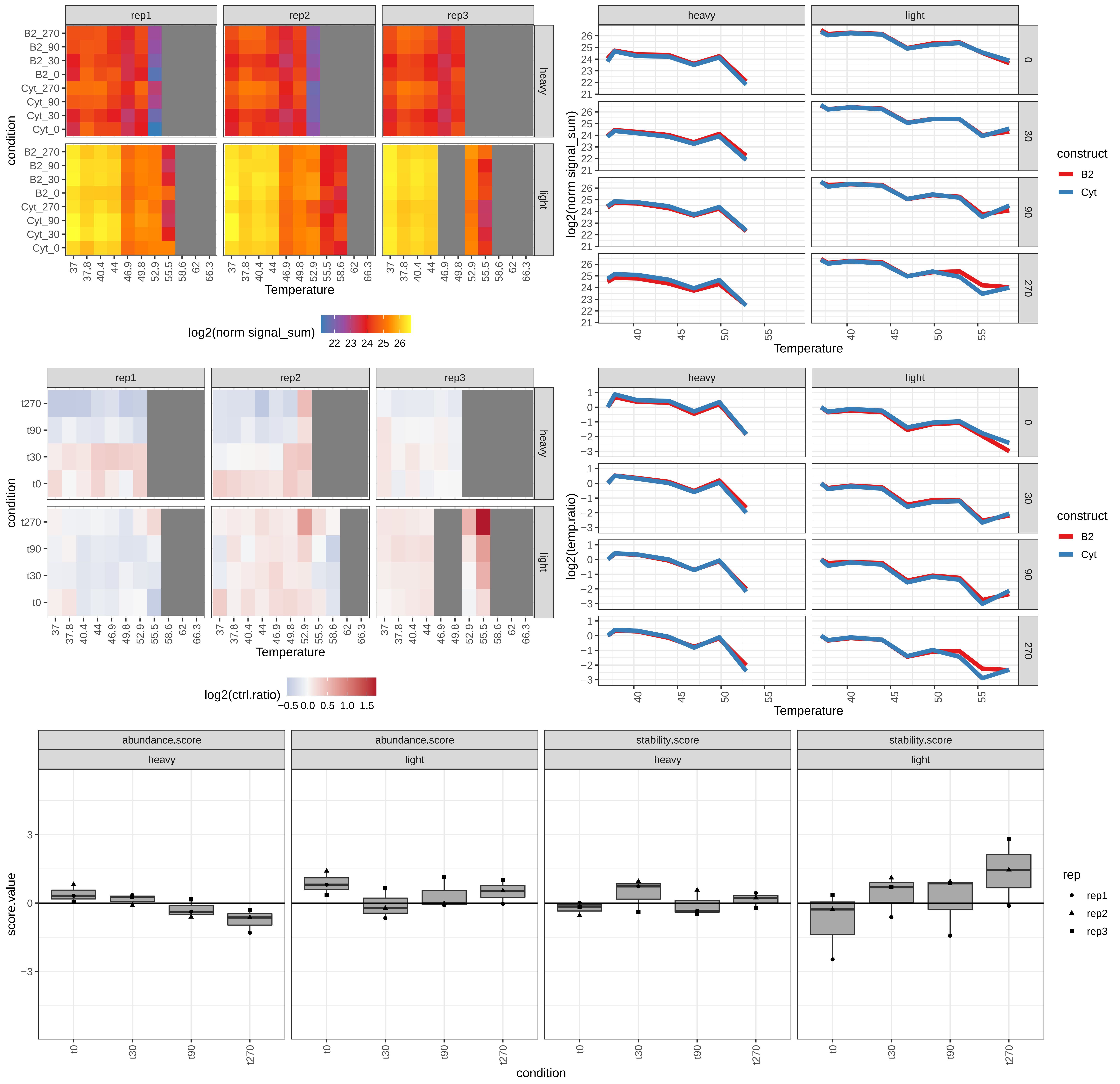

bosomal protein L42−A OS=Saccharomyces cerevisiae (strain ATCC 204508 / S288c) GN=RPL42A PE=1 SV=1|60S ribosomal protein L42−B OS=Saccharomyces cerevisiae (strain ATCC 204508 / S288c)

RPL43A|RPL43B − average.top3: 8.0197625 − max.qupm: 7

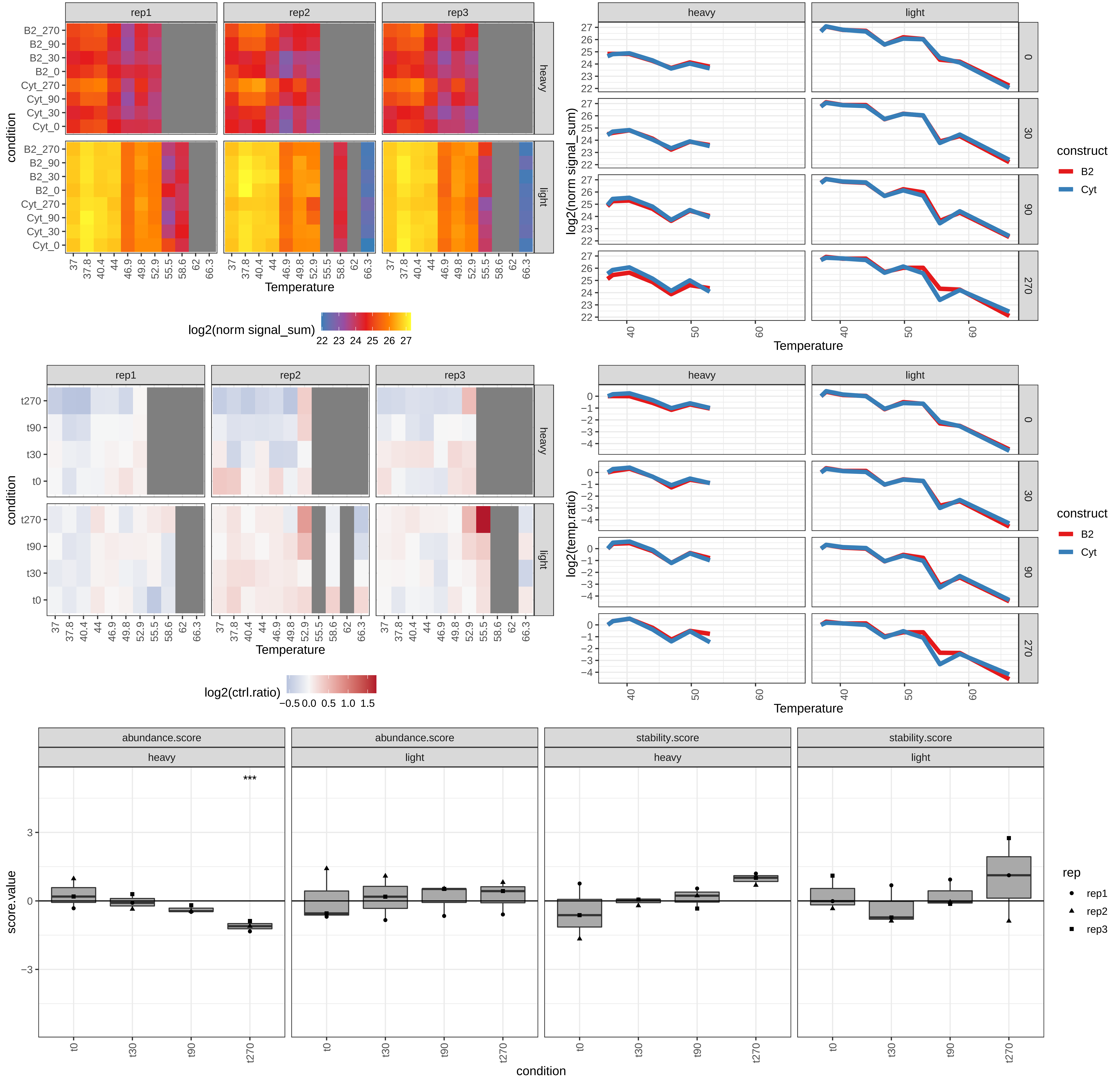

osomal protein L43−A OS=Saccharomyces cerevisiae (strain ATCC 204508 / S288c) GN=RPL43A PE=1 SV=1|60S ribosomal protein L43−B OS=Saccharomyces cerevisiae (strain ATCC 204508 / S288c

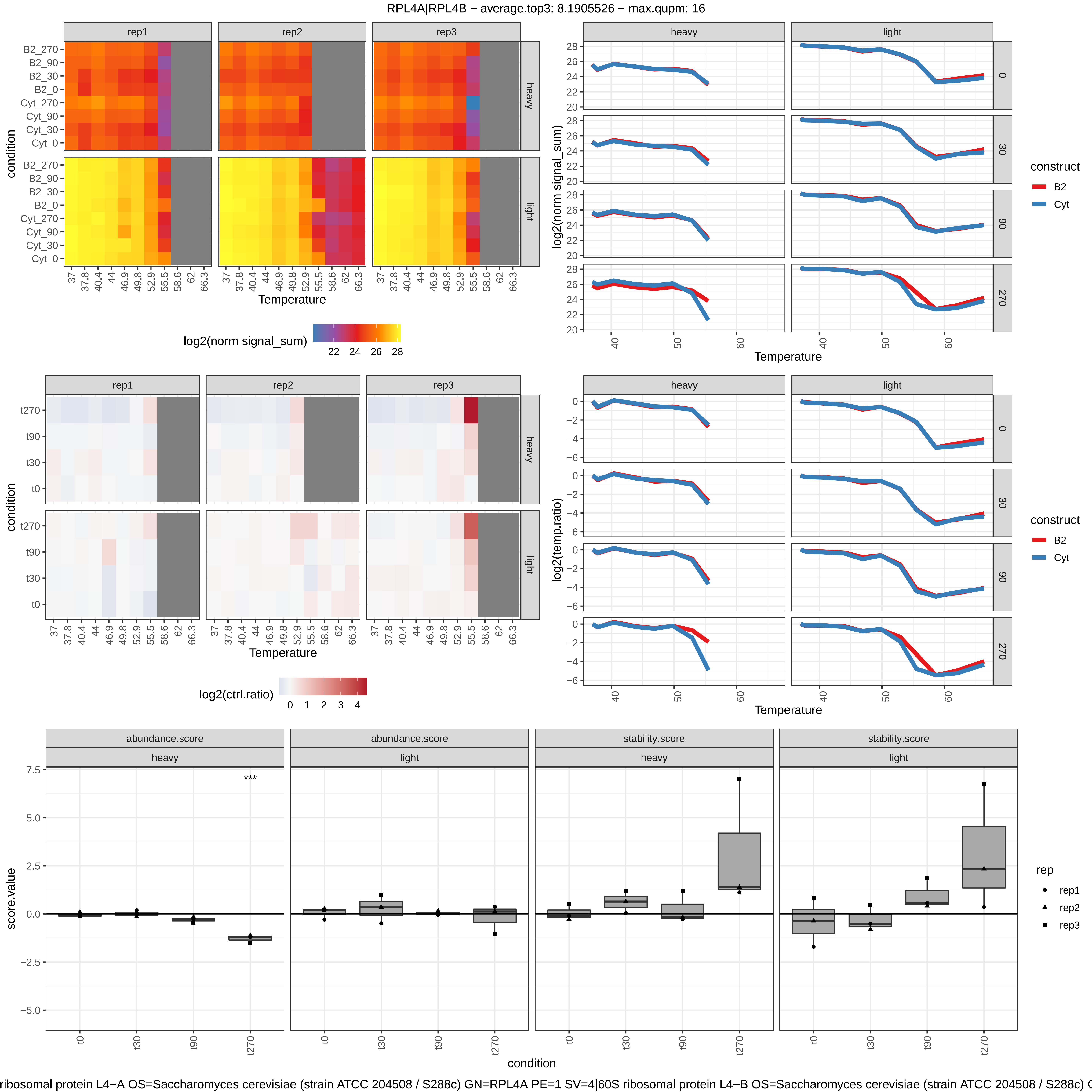

RPL4A|RPL4B − average.top3: 8.1905526 − max.qupm: 16
rep1
rep2
rep3
heavy
light
B2_270
B2_90
B2_30
B2_0
Cyt_270
Cyt_90
Cyt_30
Cyt_0
condition
37
37.8
40.4
44
46.9
49.8
52.9
55.5
58.6
62
66.3
Temperature
log2(norm signal_sum)
22
24
26
28
0
30
90
270
construct
B2
Cyt
t270
t90
t30
t0
log2(ctrl.ratio)
0
1
2
3
4
log2(temp.ratio)
abundance.score
stability.score
score.value
7.5
5.0
2.5
0.0
−2.5
−5.0
***
rep
rep1
rep2
rep3
ribosomal protein L4−A OS=Saccharomyces cerevisiae (strain ATCC 204508 / S288c) GN=RPL4A PE=1 SV=4|60S ribosomal protein L4−B OS=Saccharomyces cerevisiae (strain ATCC 204508 / S288c) G

RPL5 − average.top3: 7.9843625 − max.qupm: 11

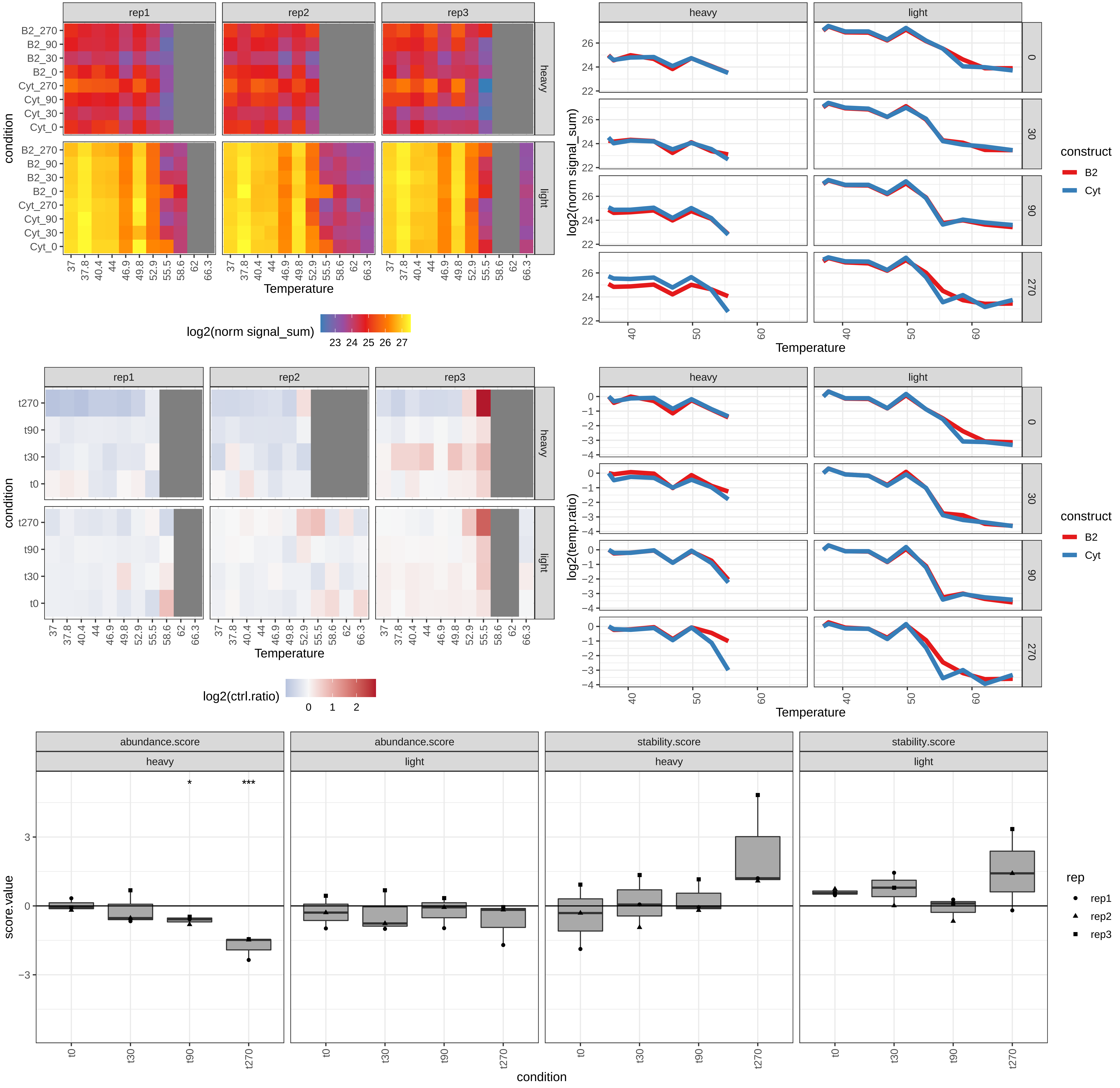

P26321 − 60S ribosomal protein L5 OS=Saccharomyces cerevisiae (strain ATCC 204508 / S288c) GN=RPL5 PE=1 SV=4

RPL6A − average.top3: 7.60609244444444 − max.qupm: 7

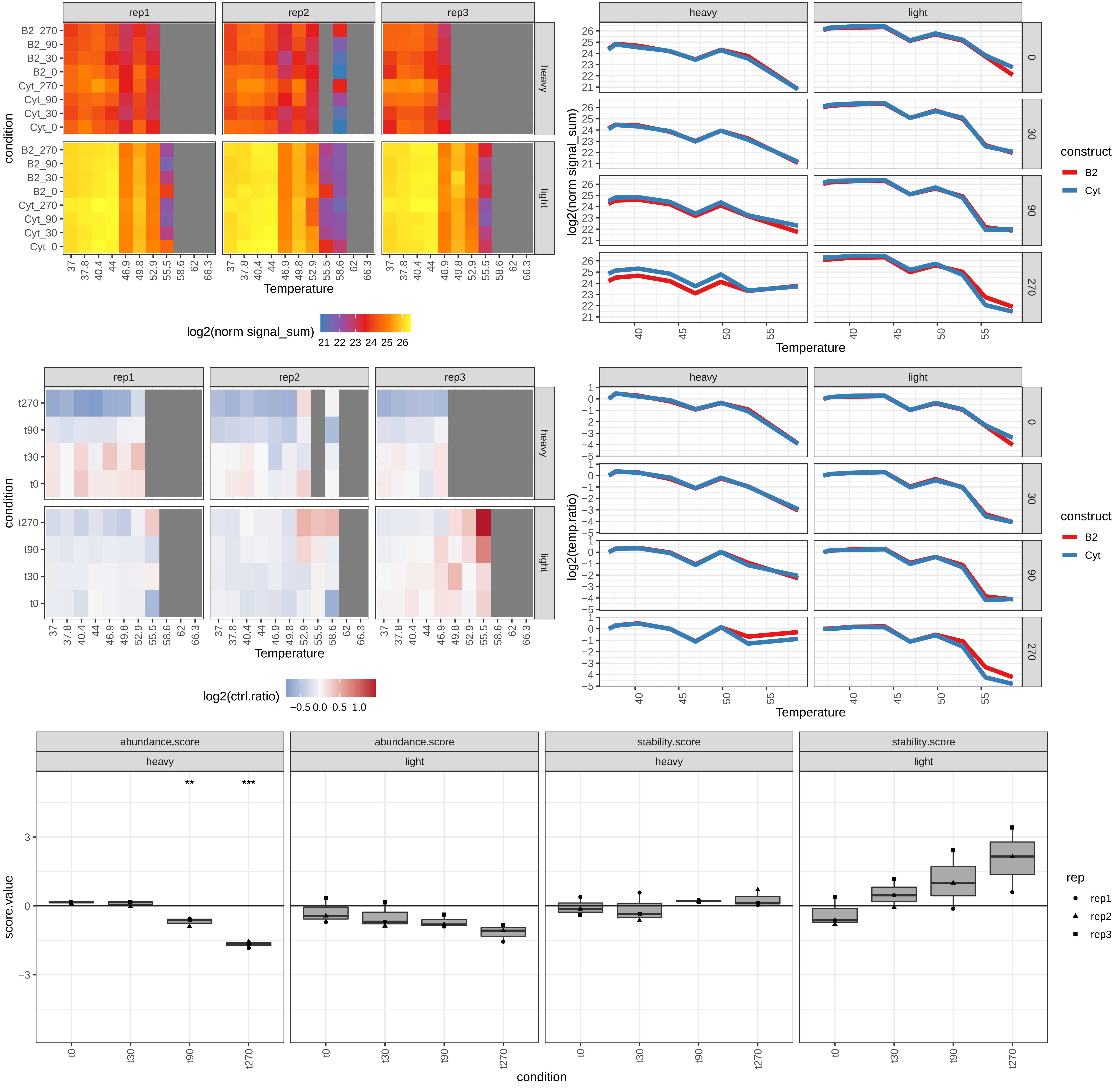

Q02326 − 60S ribosomal protein L6−A OS=Saccharomyces cerevisiae (strain ATCC 204508 / S288c) GN=RPL6A PE=1 SV=2

RPL6B − average.top3: 7.41635888888889 − max.qupm: 8

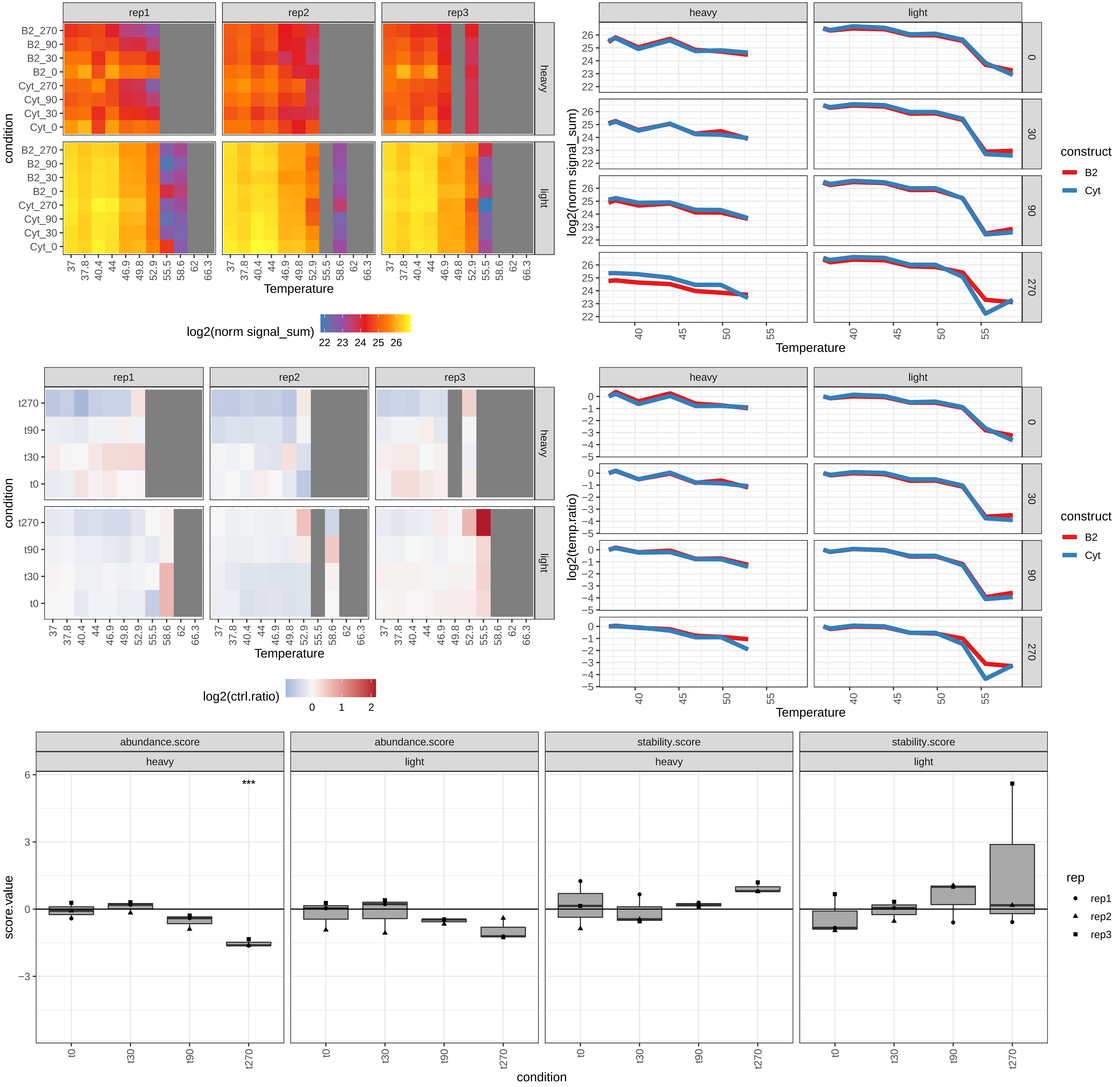

P05739 − 60S ribosomal protein L6−B OS=Saccharomyces cerevisiae (strain ATCC 204508 / S288c) GN=RPL6B PE=1 SV=4

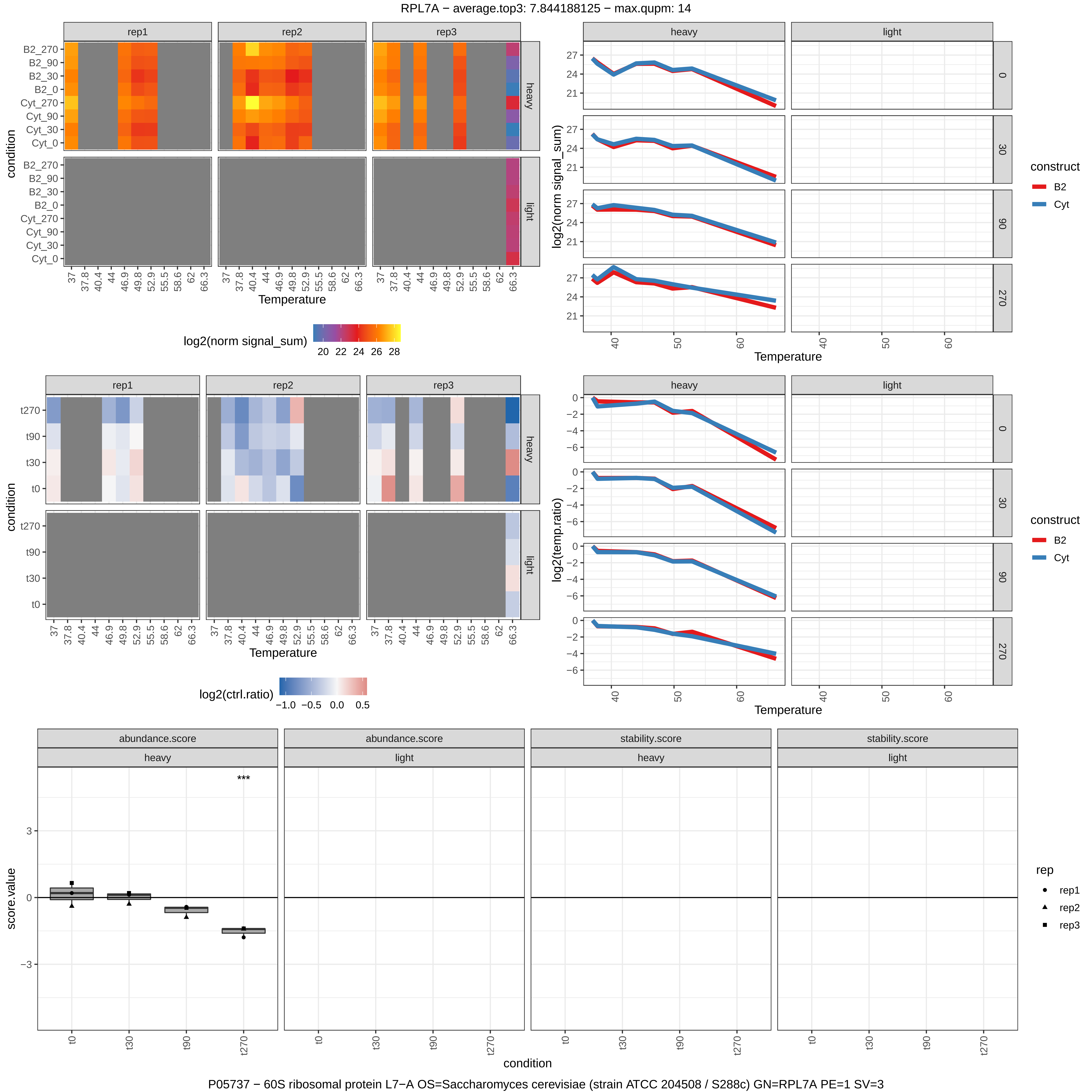

RPL7A − average.top3: 7.844188125 − max.qupm: 14
rep1
rep2
rep3
heavy
light
condition
Temperature
log2(norm signal_sum)
log2(ctrl.ratio)
log2(temp.ratio)
construct
B2
Cyt
abundance.score
stability.score
score.value
rep
rep1
rep2
rep3
***
P05737 − 60S ribosomal protein L7−A OS=Saccharomyces cerevisiae (strain ATCC 204508 / S288c) GN=RPL7A PE=1 SV=3

RPL8A − average.top3: 7.51320777777778 − max.qupm: 3

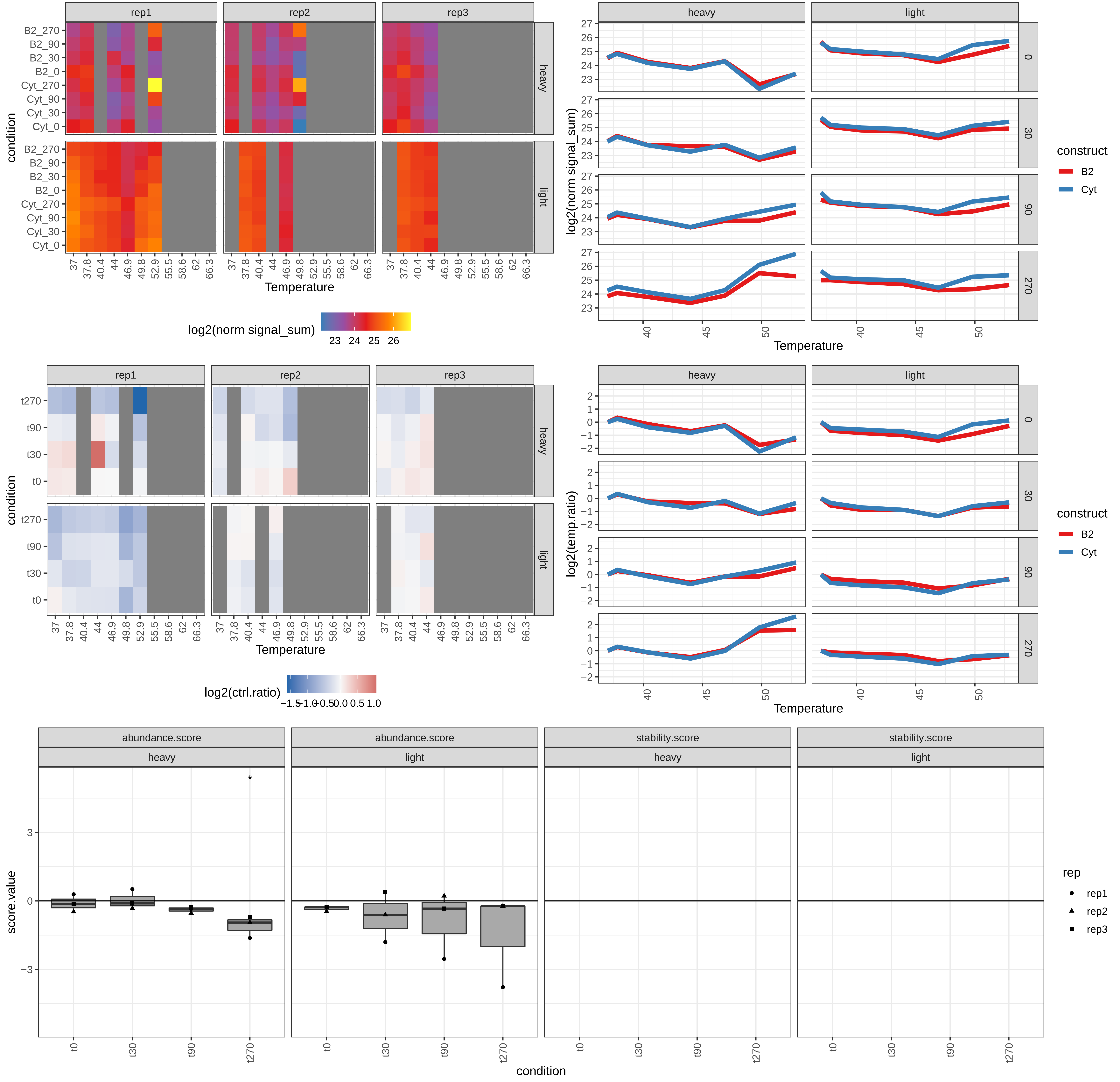

P17076 − 60S ribosomal protein L8−A OS=Saccharomyces cerevisiae (strain ATCC 204508 / S288c) GN=RPL8A PE=1 SV=4

RPL8B − average.top3: 7.29423964285714 − max.qupm: 4

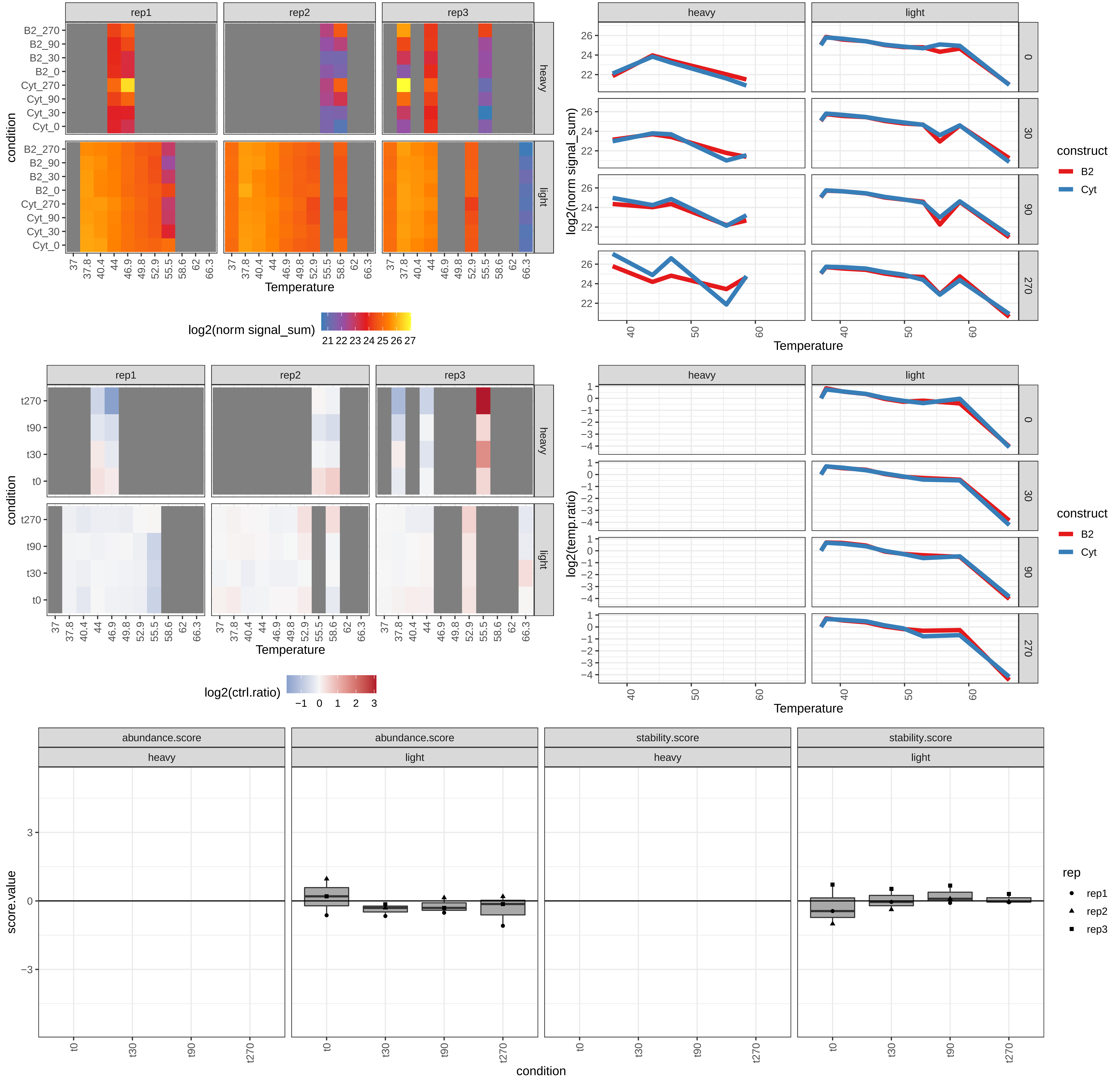

P29453 − 60S ribosomal protein L8−B OS=Saccharomyces cerevisiae (strain ATCC 204508 / S288c) GN=RPL8B PE=1 SV=3

RPL9B − average.top3: 7.47052 − max.qupm: 8

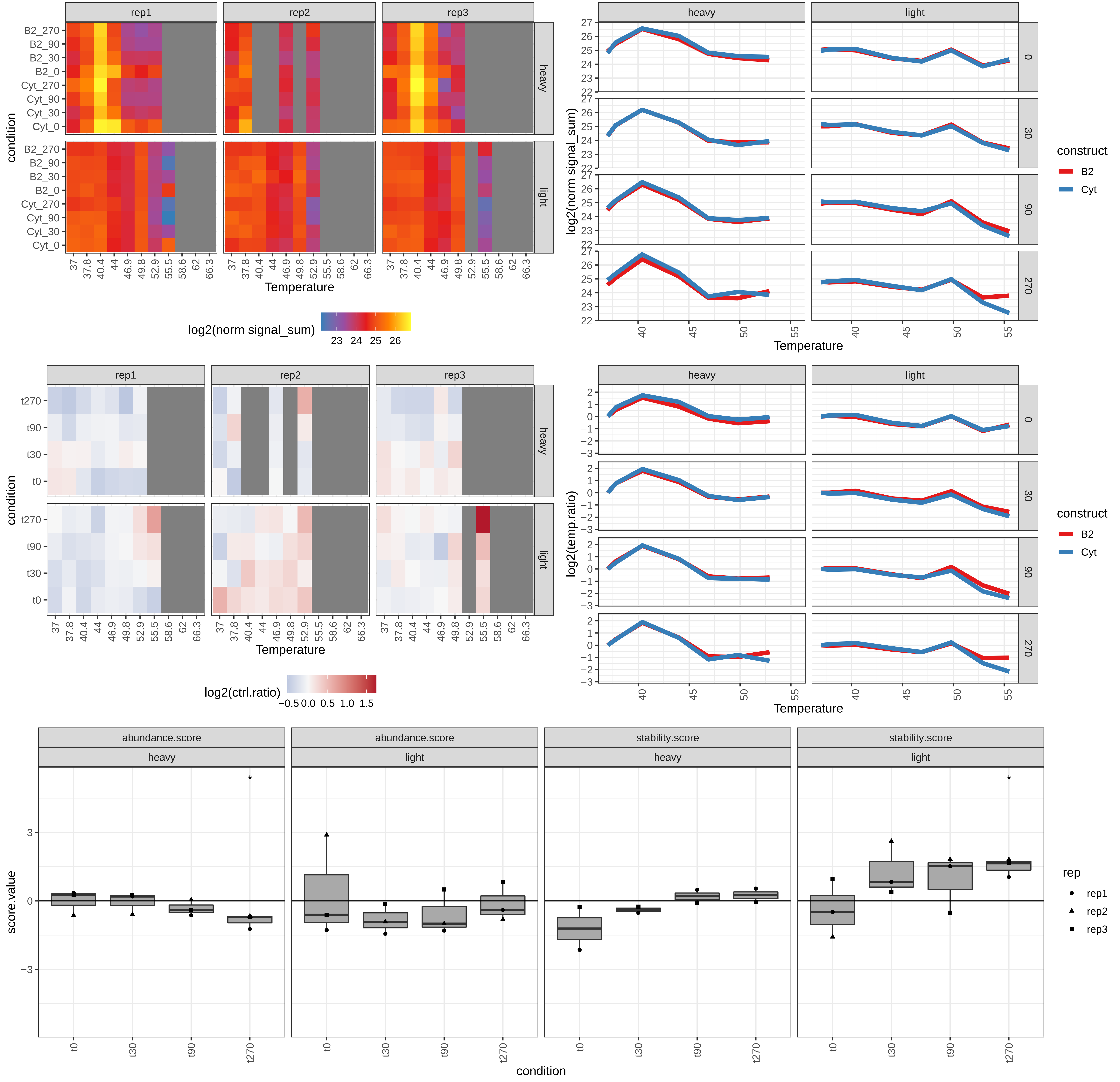

P51401 − 60S ribosomal protein L9−B OS=Saccharomyces cerevisiae (strain ATCC 204508 / S288c) GN=RPL9B PE=1 SV=1

RPM2 − average.top3: 6.57466391304348 − max.qupm: 8

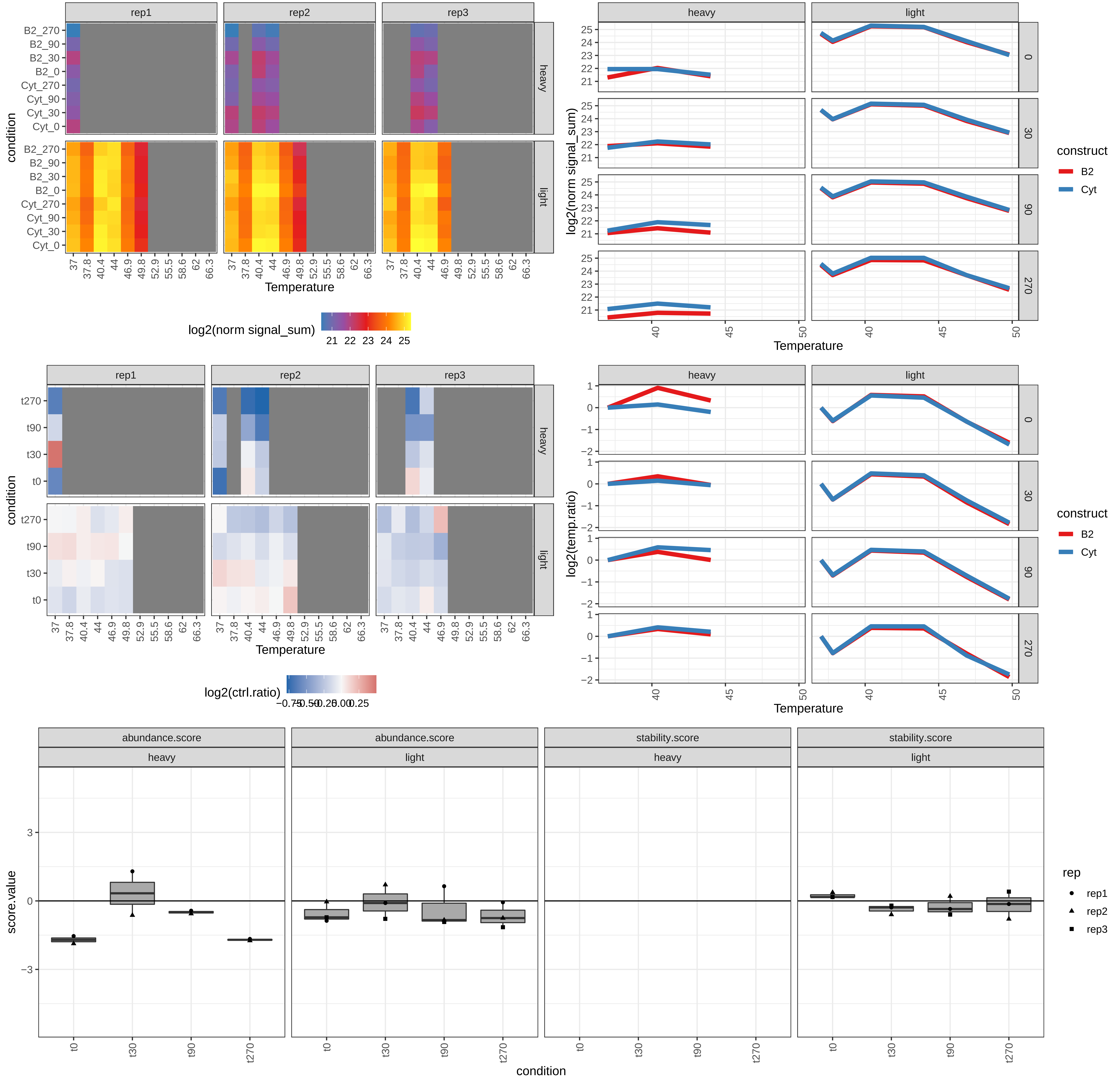

Q02773 − Ribonuclease P protein component, mitochondrial OS=Saccharomyces cerevisiae (strain ATCC 204508 / S288c) GN=RPM2 PE=1 SV=1

RPN1 − average.top3: 7.31472229166667 − max.qupm: 25

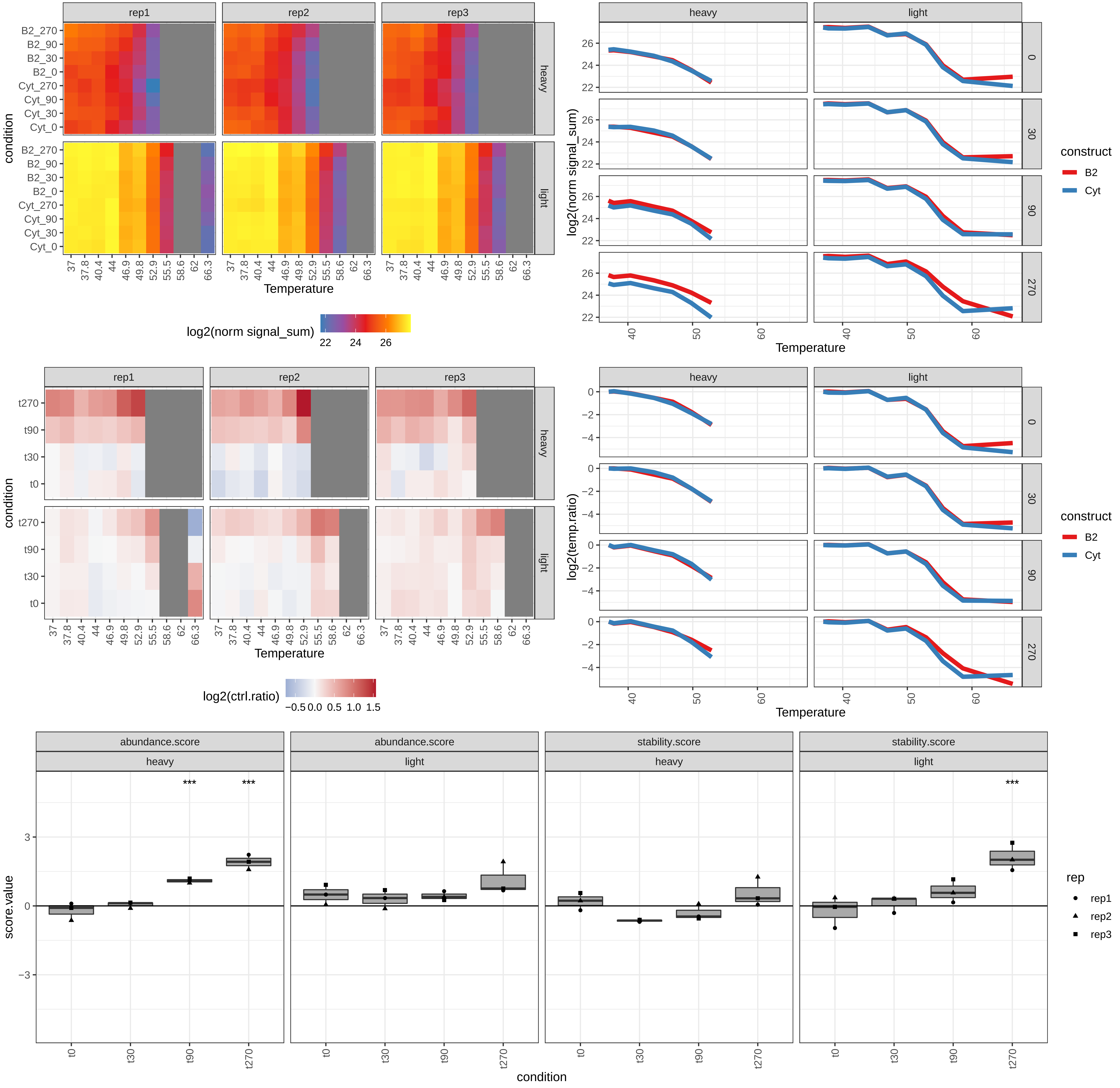

P38764 − 26S proteasome regulatory subunit RPN1 OS=Saccharomyces cerevisiae (strain ATCC 204508 / S288c) GN=RPN1 PE=1 SV=3

RPN10 − average.top3: 6.6348835 − max.qupm: 4

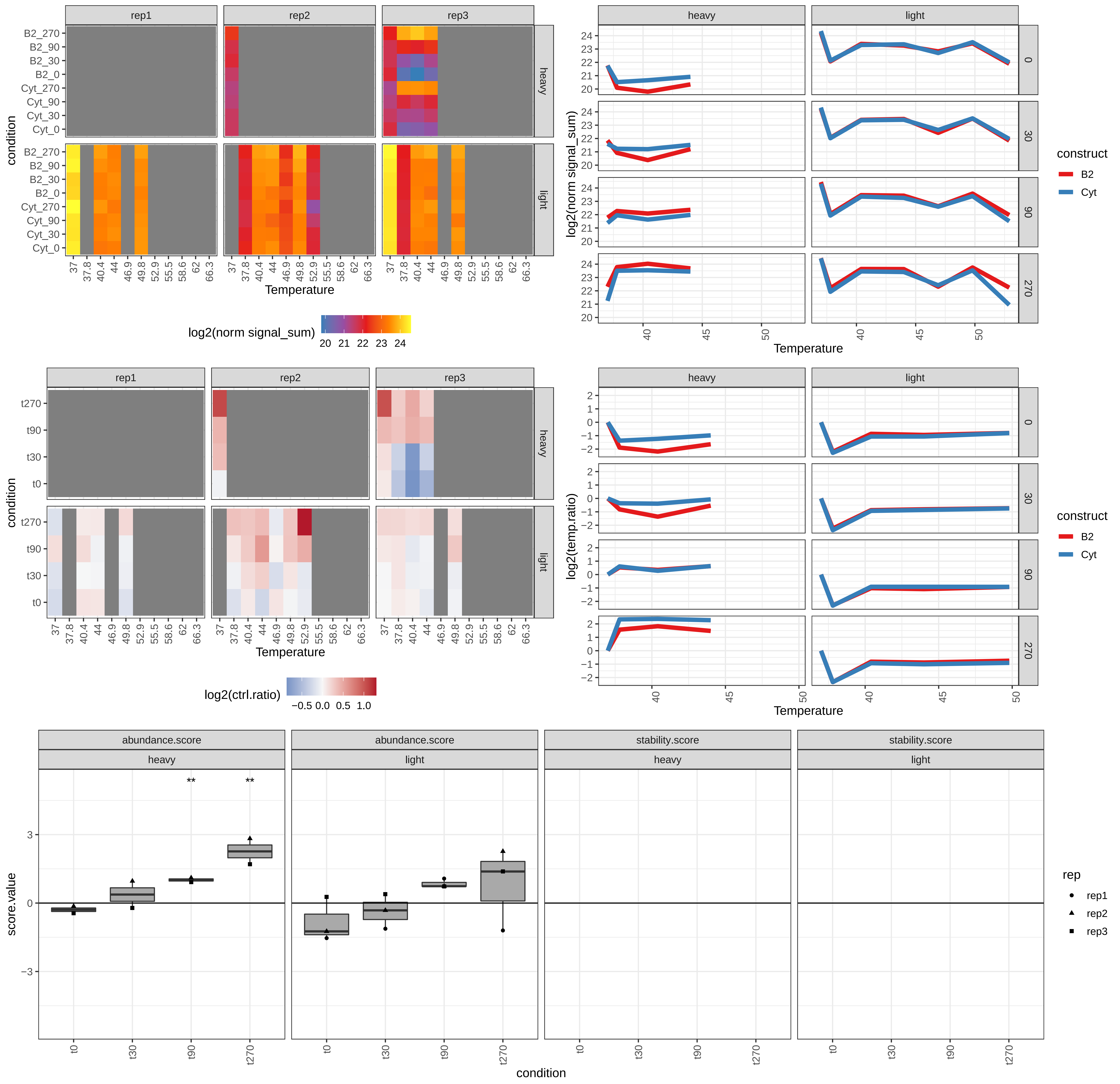

P38886 − 26S proteasome regulatory subunit RPN10 OS=Saccharomyces cerevisiae (strain ATCC 204508 / S288c) GN=RPN10 PE=1 SV=3

RPN11 − average.top3: 7.25369585365854 − max.qupm: 12

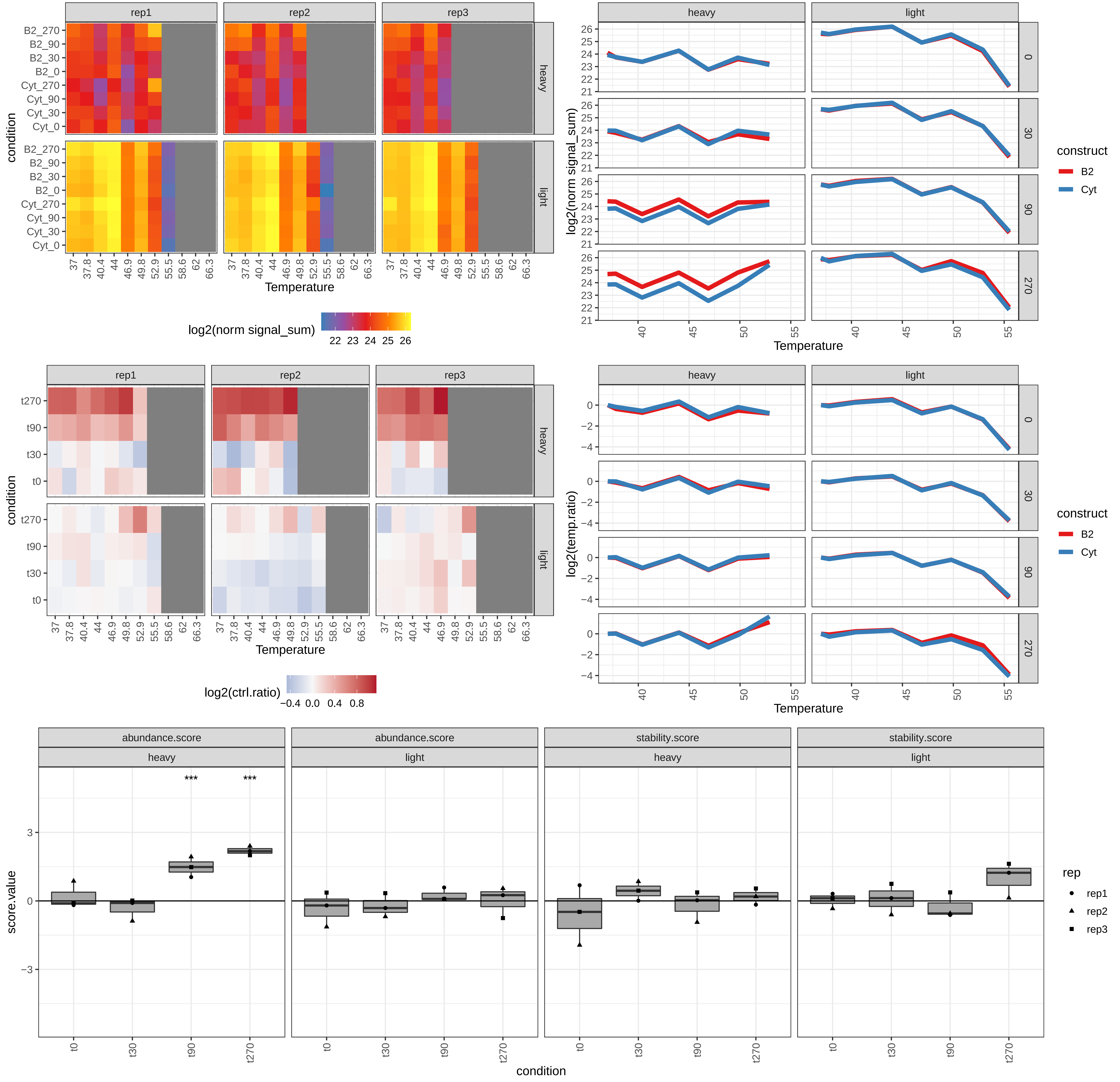

P43588 − Ubiquitin carboxyl−terminal hydrolase RPN11 OS=Saccharomyces cerevisiae (strain ATCC 204508 / S288c) GN=RPN11 PE=1 SV=1

RPN12 − average.top3: 7.04700692307692 − max.qupm: 10

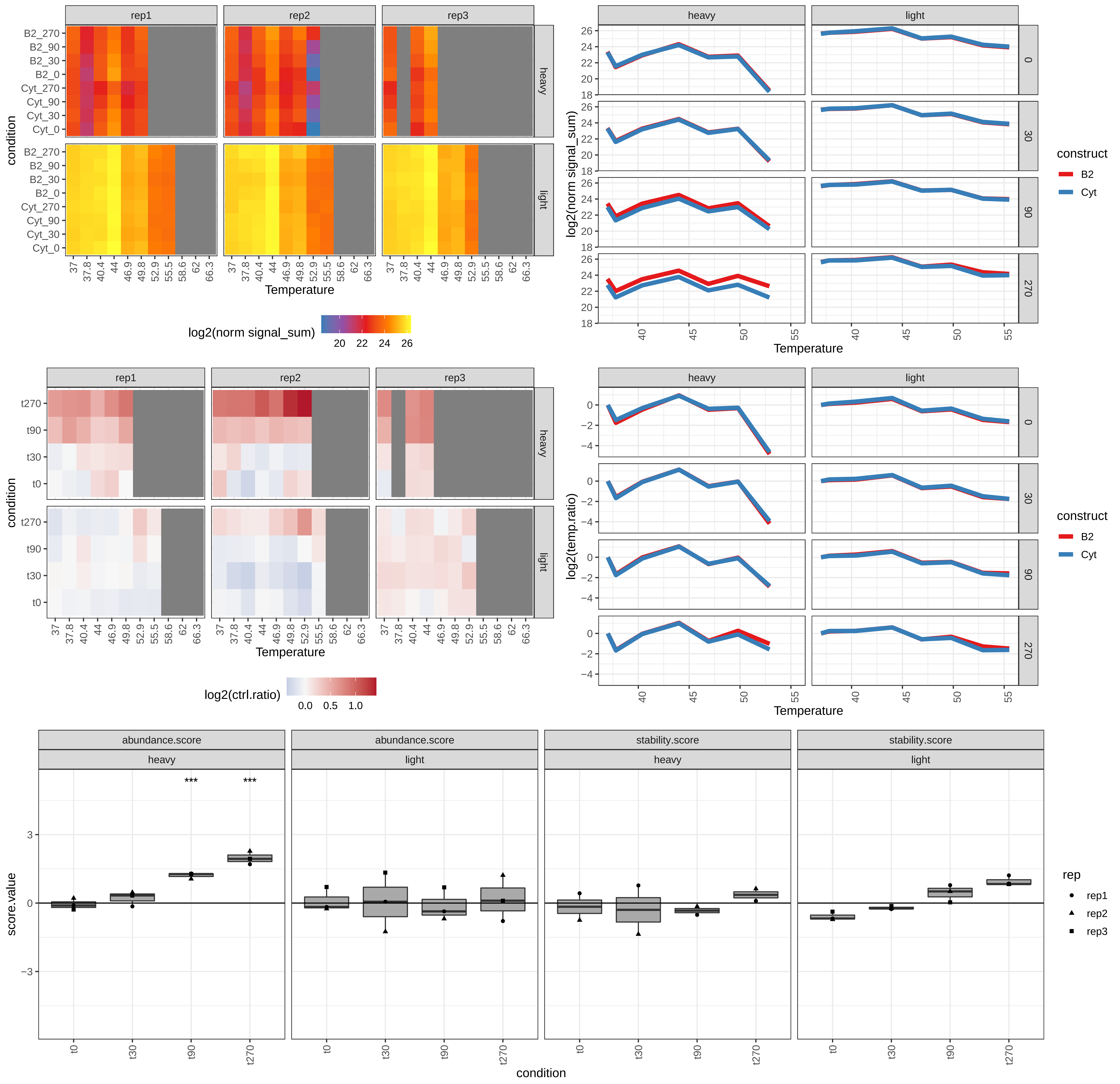

P32496 − 26S proteasome regulatory subunit RPN12 OS=Saccharomyces cerevisiae (strain ATCC 204508 / S288c) GN=RPN12 PE=1 SV=3

RPN13 − average.top3: 7.16040083333333 − max.qupm: 5

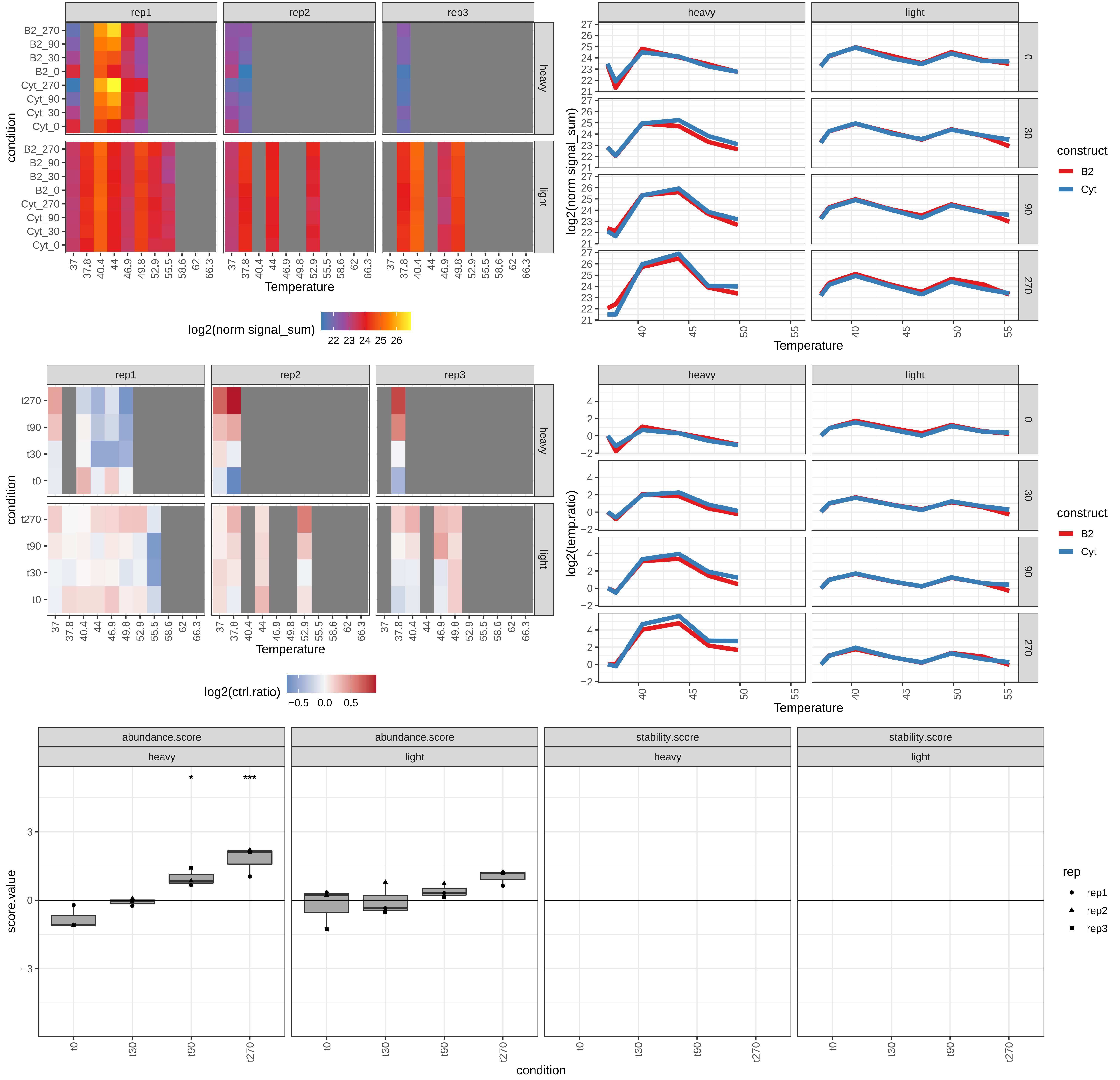

egulatory subunit RPN13 OS=Saccharomyces cerevisiae (strain ATCC 204508 / S288c) GN=RPN13 PE=1 SV=1|Isoform 2 of 26S proteasome regulatory subunit RPN13 OS=Saccharomyces cerevisiae (st

RPN14 − average.top3: 7.10366083333333 − max.qupm: 11

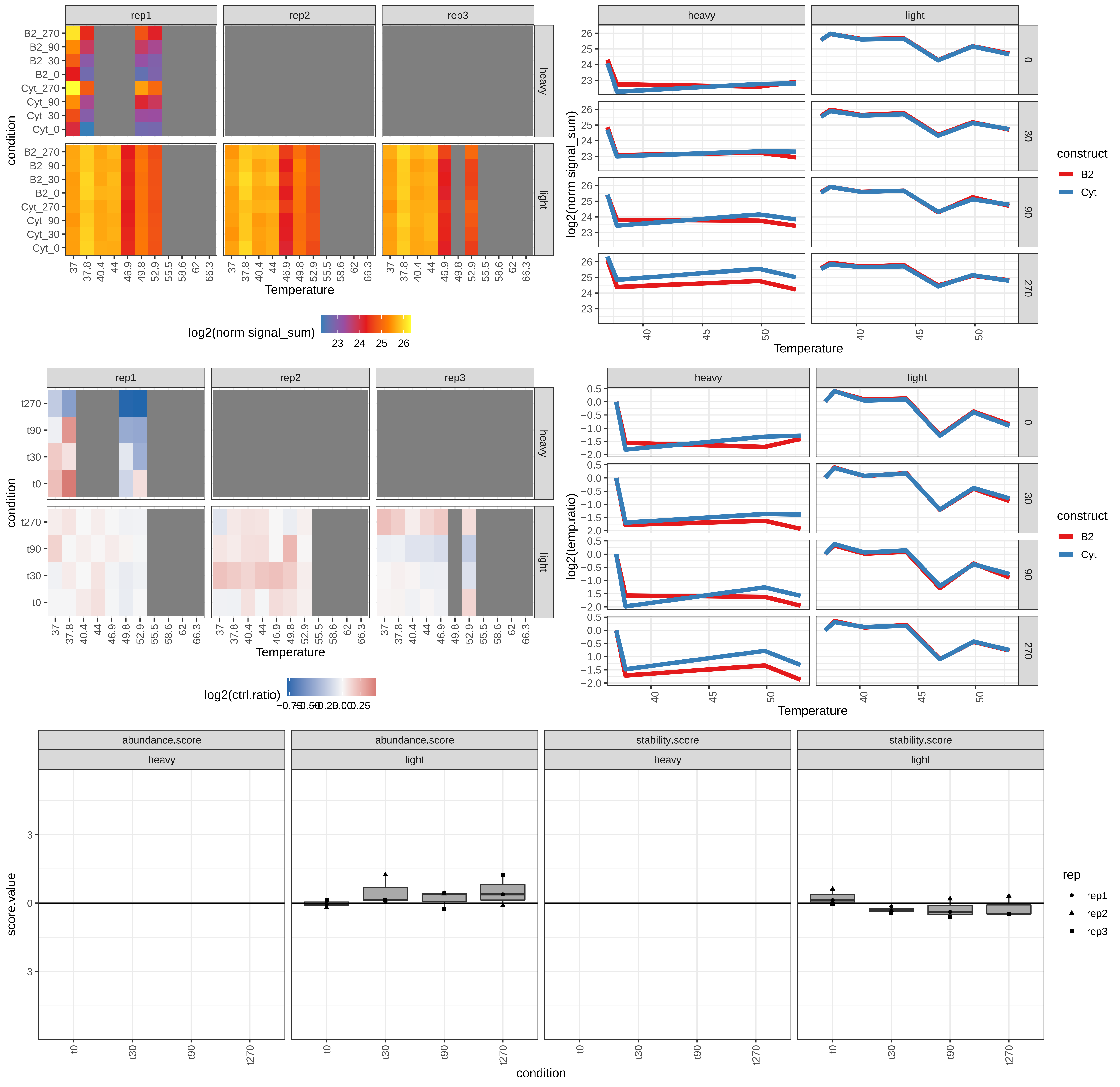

P53196 − 26S proteasome regulatory subunit RPN14 OS=Saccharomyces cerevisiae (strain ATCC 204508 / S288c) GN=RPN14 PE=1 SV=1

RPN2 − average.top3: 7.39329288461538 − max.qupm: 31

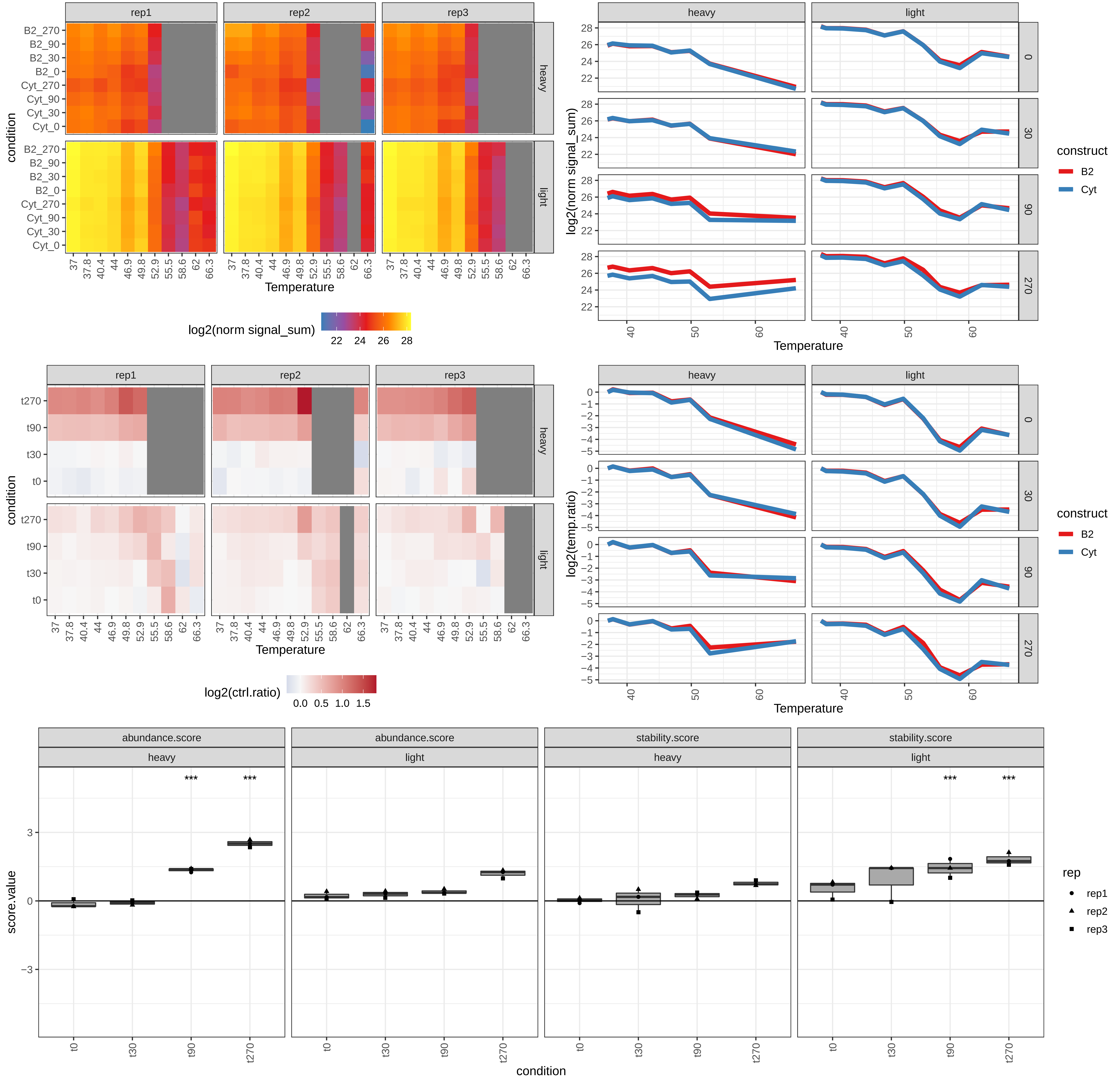

P32565 − 26S proteasome regulatory subunit RPN2 OS=Saccharomyces cerevisiae (strain ATCC 204508 / S288c) GN=RPN2 PE=1 SV=4

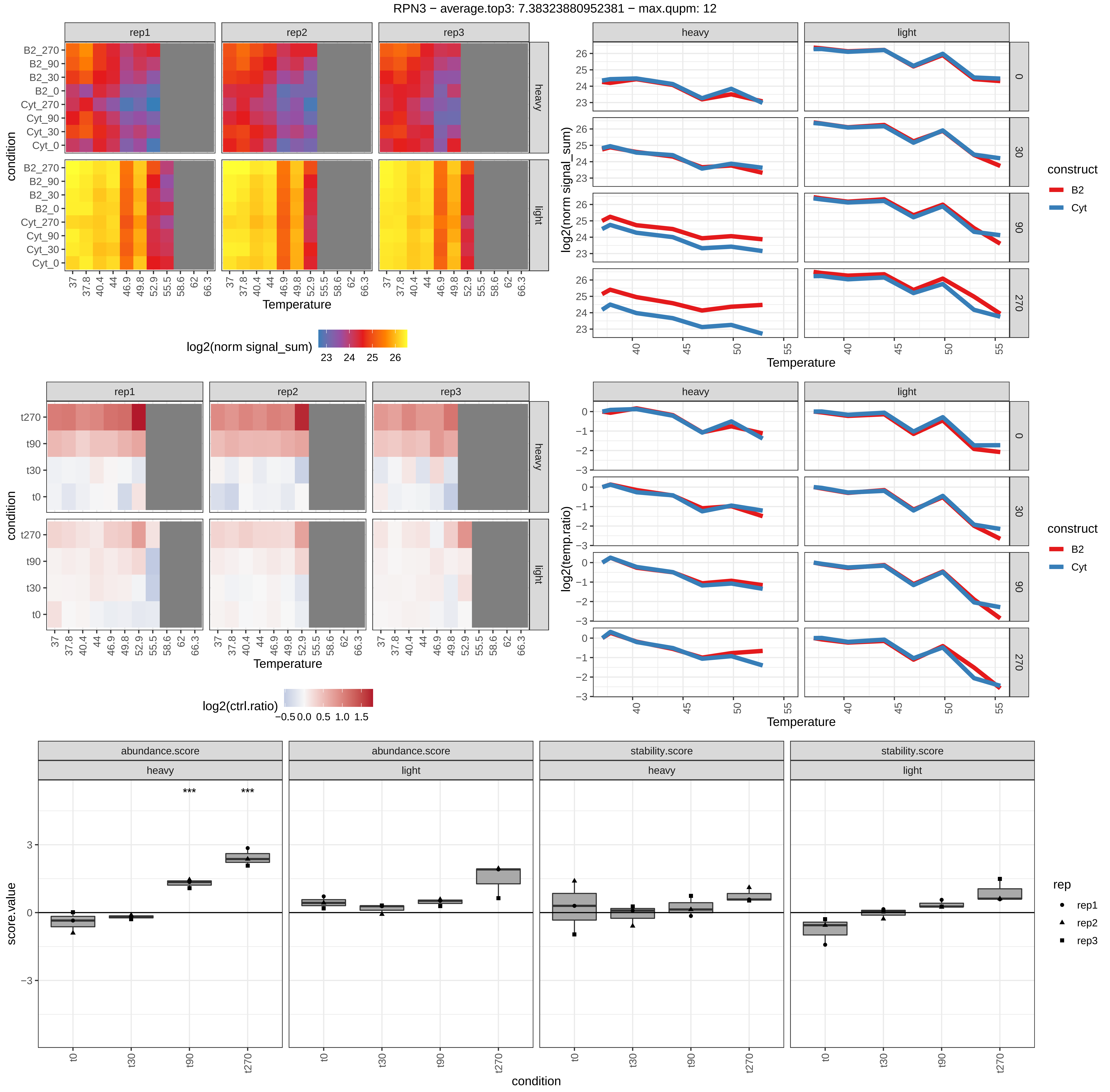

P40016 − 26S proteasome regulatory subunit RPN3 OS=Saccharomyces cerevisiae (strain ATCC 204508 / S288c) GN=RPN3 PE=1 SV=5

RPN5 − average.top3: 7.424139 − max.qupm: 16

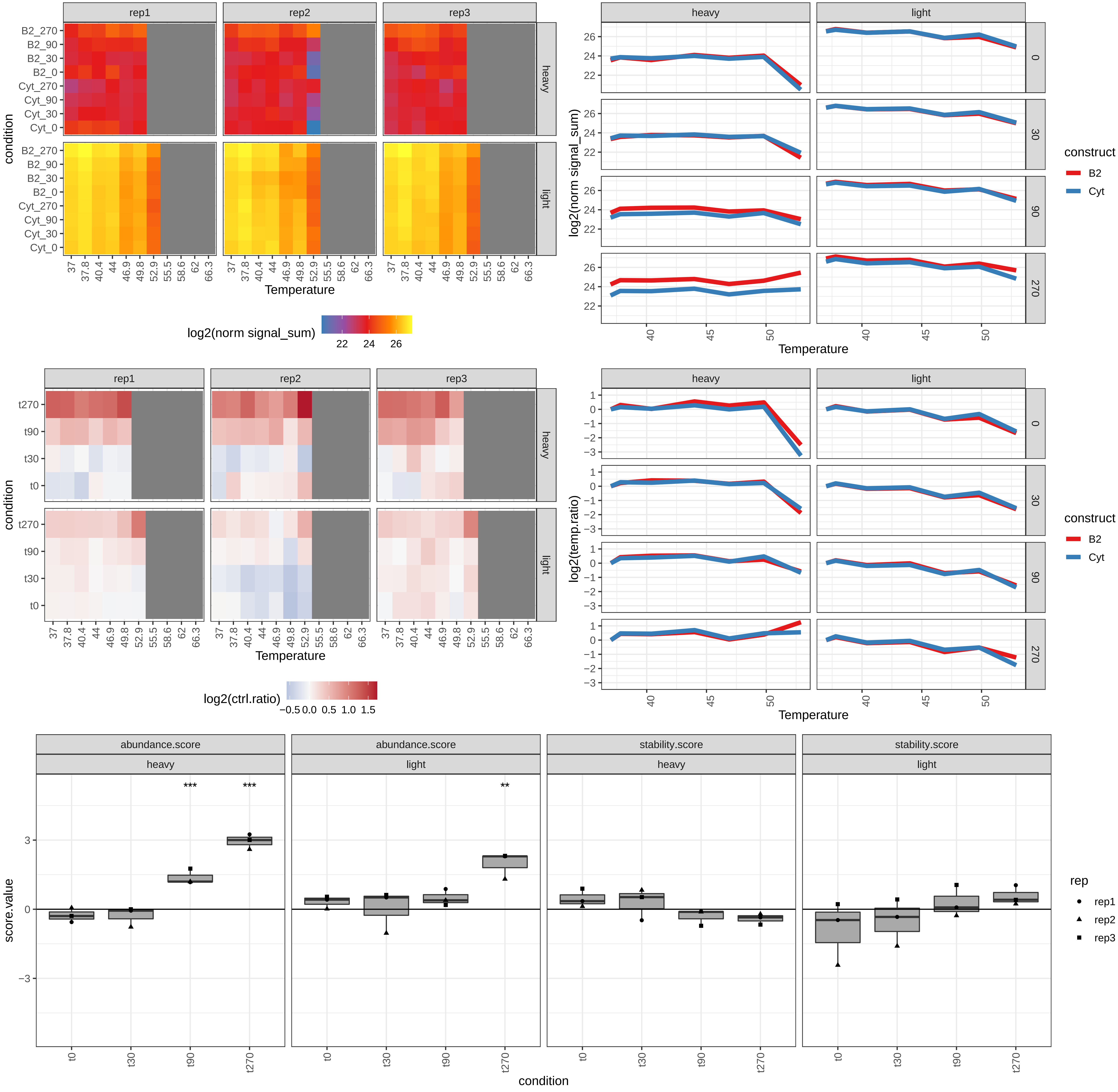

Q12250 − 26S proteasome regulatory subunit RPN5 OS=Saccharomyces cerevisiae (strain ATCC 204508 / S288c) GN=RPN5 PE=1 SV=3

RPN6 − average.top3: 7.35842025 − max.qupm: 14

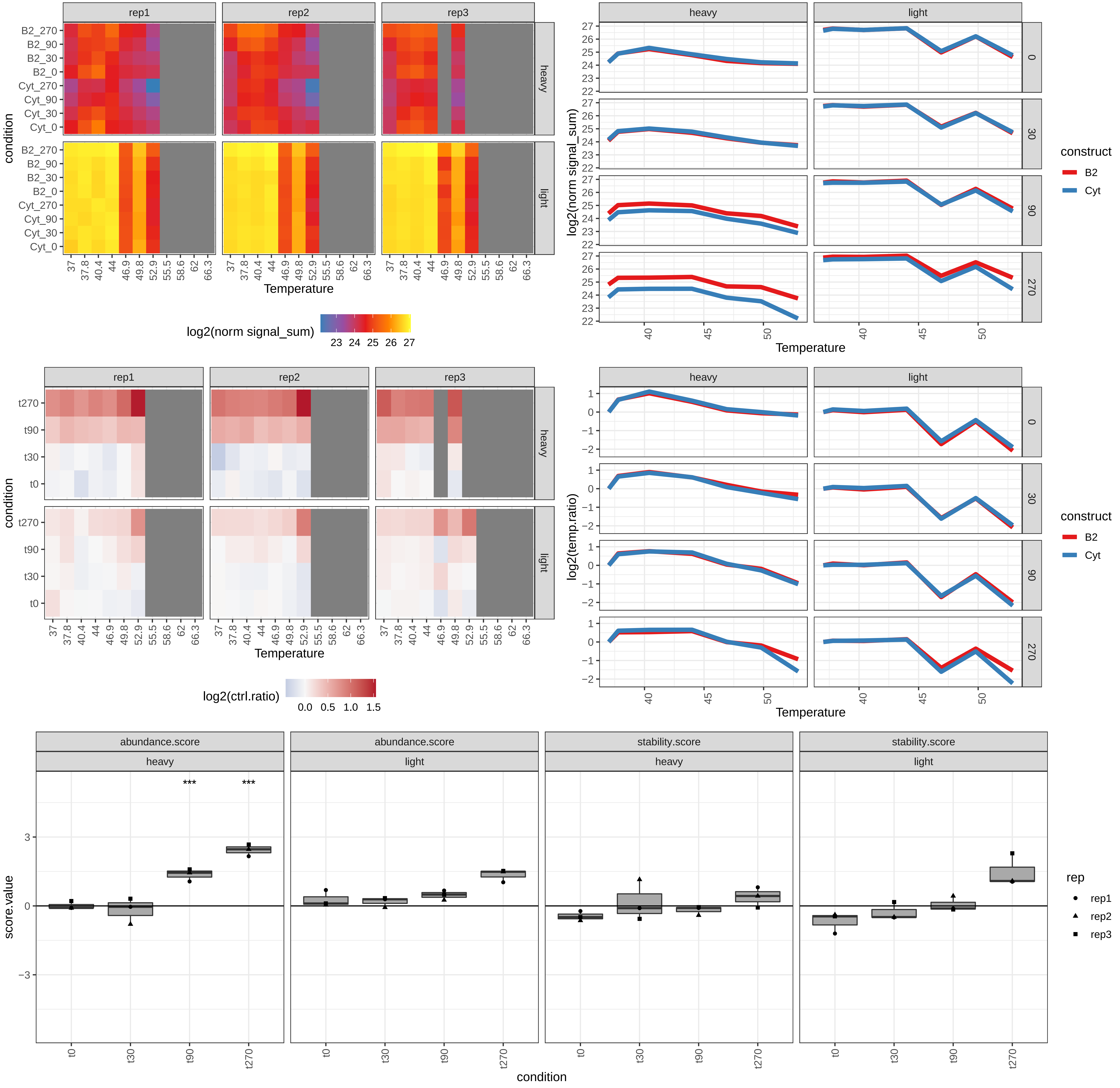

Q12377 − 26S proteasome regulatory subunit RPN6 OS=Saccharomyces cerevisiae (strain ATCC 204508 / S288c) GN=RPN6 PE=1 SV=3

RPN7 − average.top3: 7.32036219512195 − max.qupm: 17

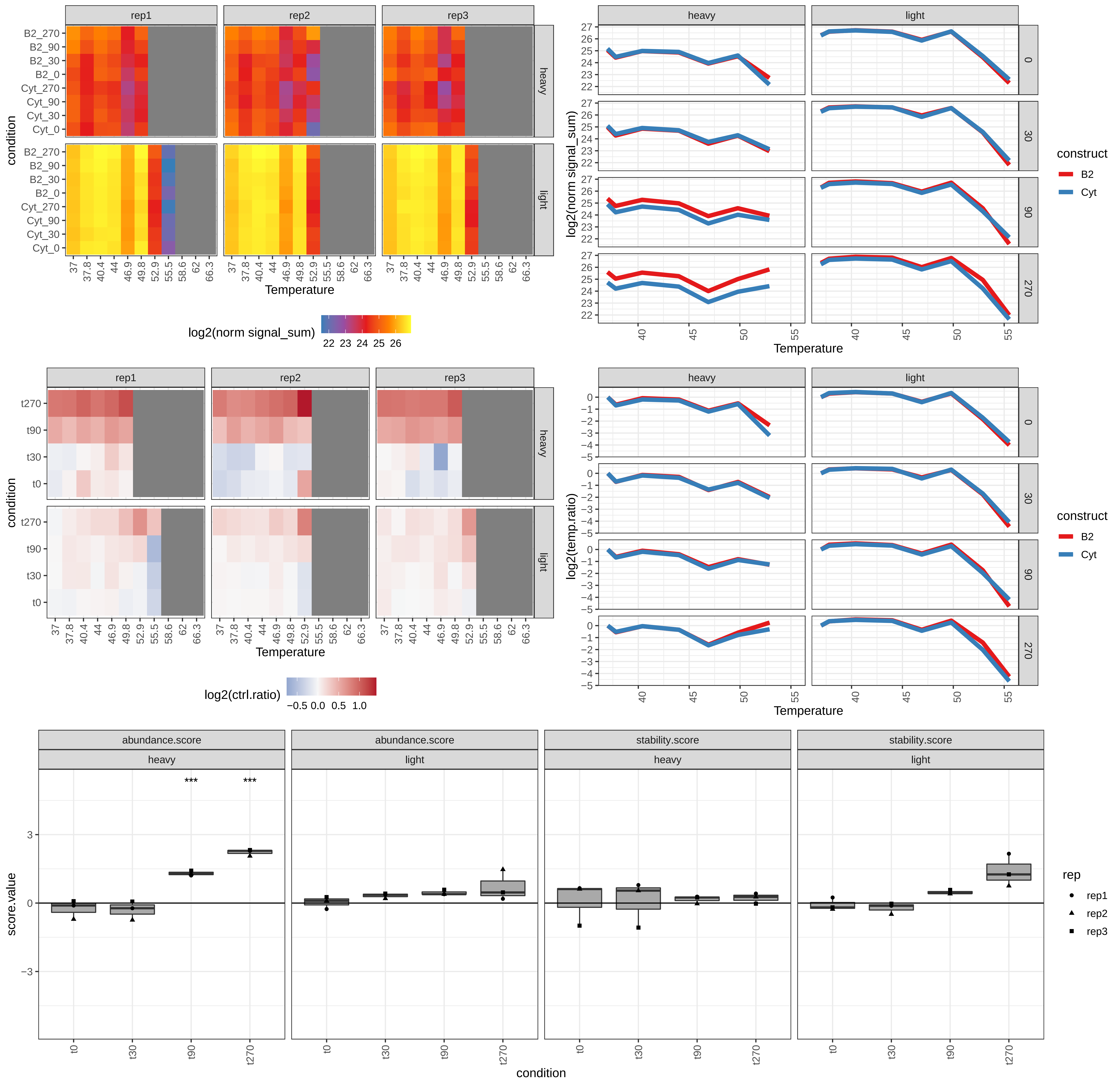

Q06103 − 26S proteasome regulatory subunit RPN7 OS=Saccharomyces cerevisiae (strain ATCC 204508 / S288c) GN=RPN7 PE=1 SV=3

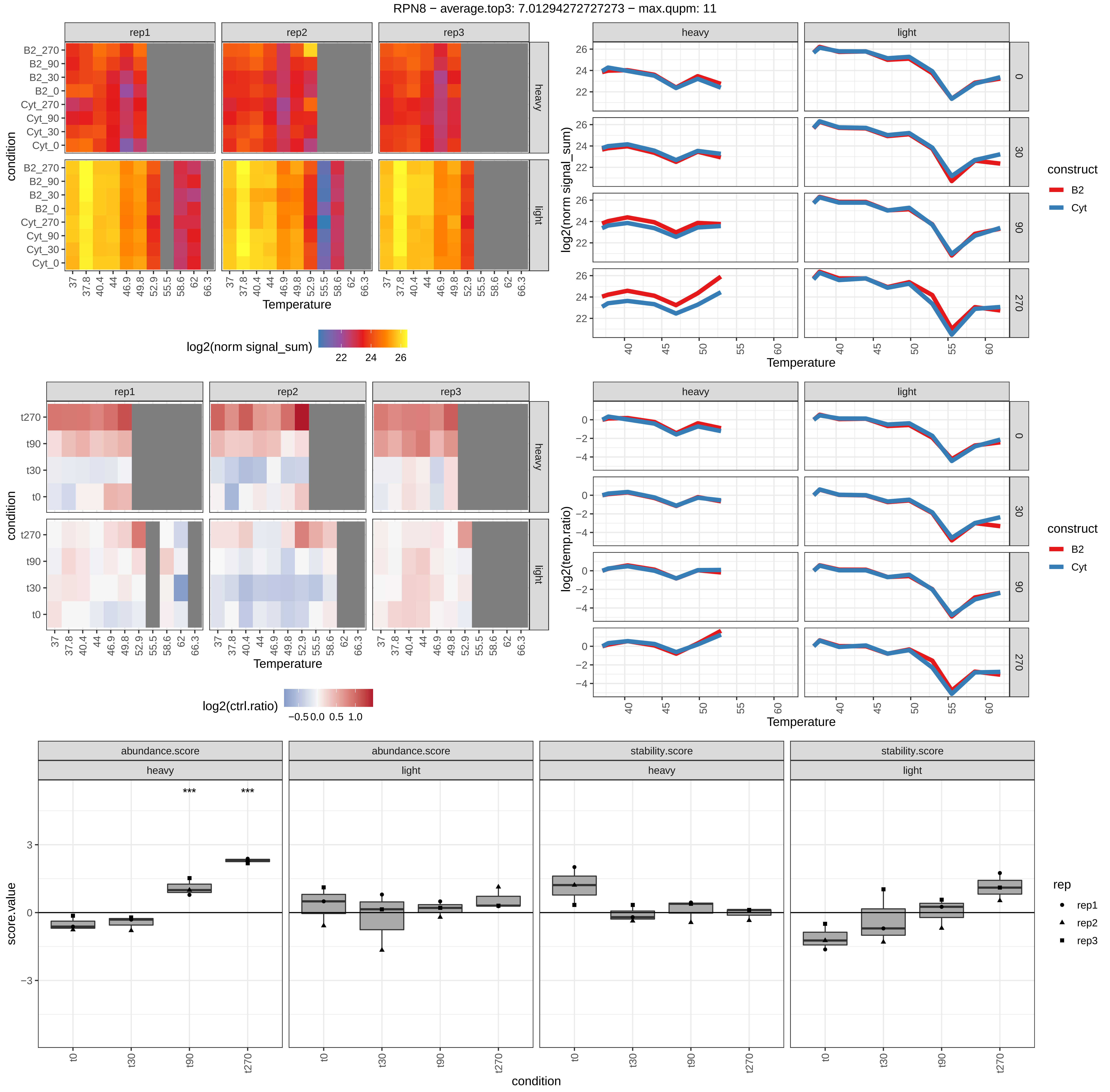

Q08723 − 26S proteasome regulatory subunit RPN8 OS=Saccharomyces cerevisiae (strain ATCC 204508 / S288c) GN=RPN8 PE=1 SV=3

RPN9 − average.top3: 7.41009088888889 − max.qupm: 15

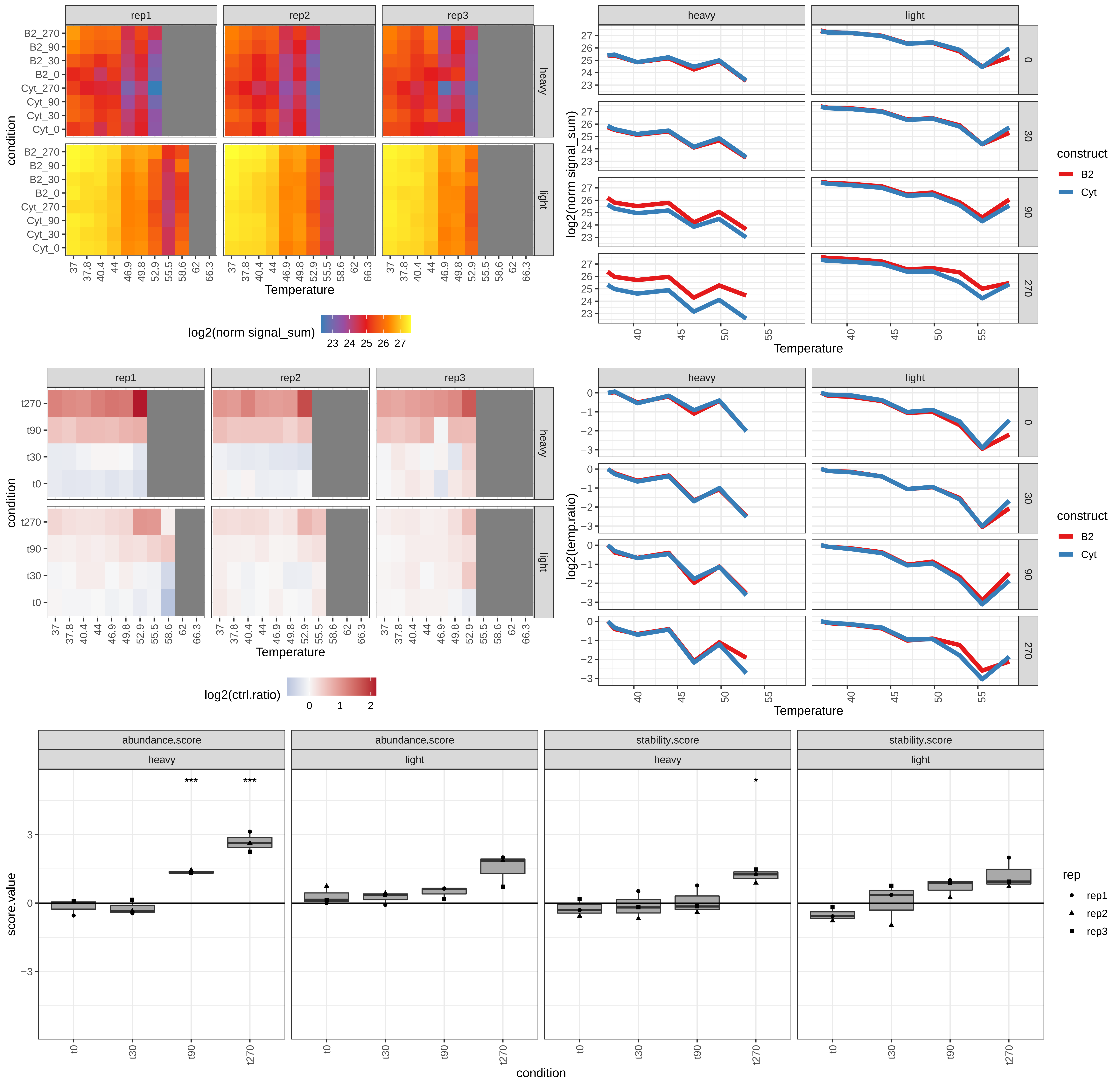

Q04062 − 26S proteasome regulatory subunit RPN9 OS=Saccharomyces cerevisiae (strain ATCC 204508 / S288c) GN=RPN9 PE=1 SV=1

RPO21 − average.top3: 7.44688592592593 − max.qupm: 38

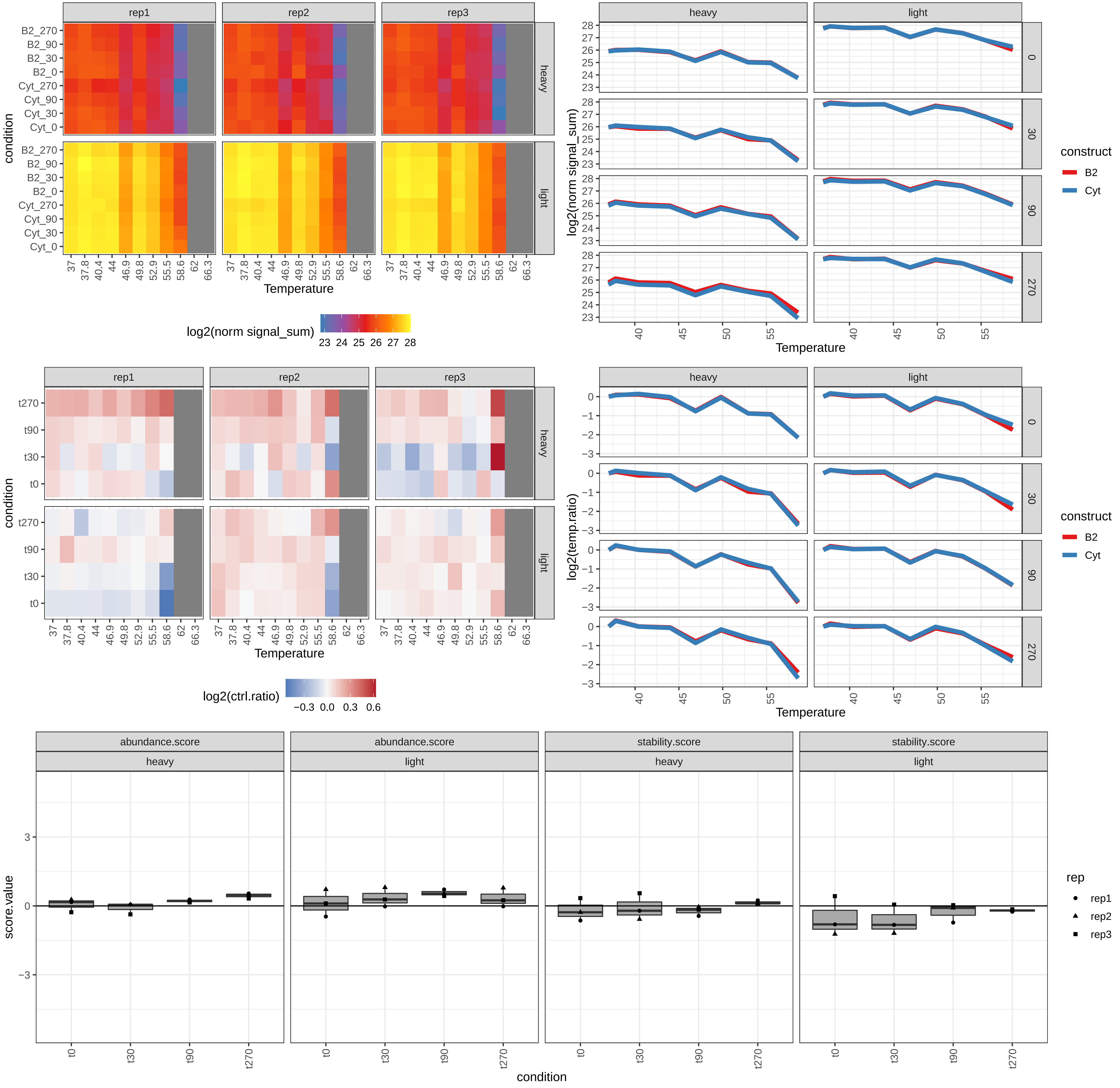

P04050 − DNA−directed RNA polymerase II subunit RPB1 OS=Saccharomyces cerevisiae (strain ATCC 204508 / S288c) GN=RPO21 PE=1 SV=2

RPO26 − average.top3: 6.94389909090909 − max.qupm: 4

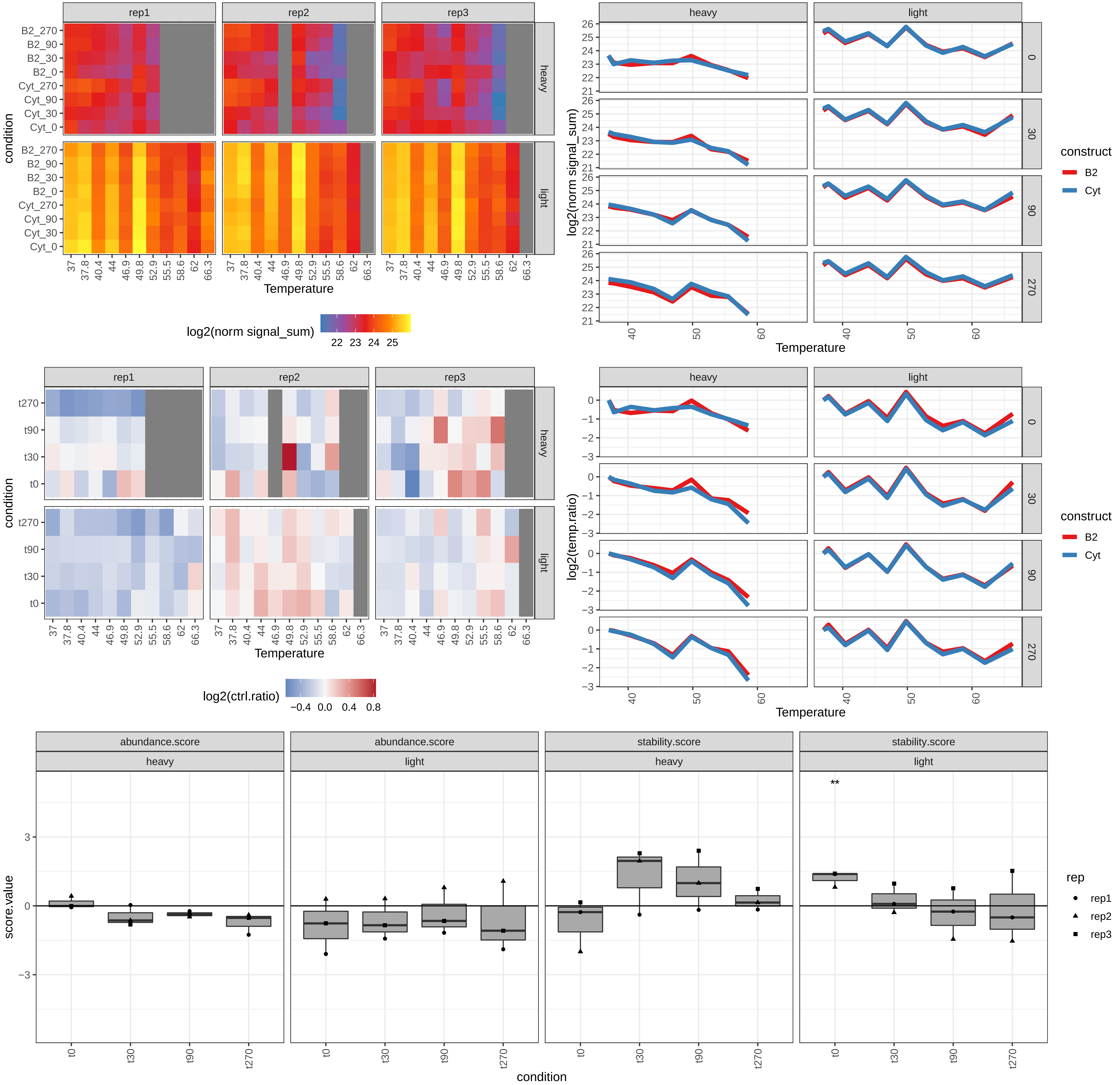

P20435 − DNA−directed RNA polymerases I, II, and III subunit RPABC2 OS=Saccharomyces cerevisiae (strain ATCC 204508 / S288c) GN=RPO26 PE=1 SV=1

RPO31 − average.top3: 6.858367 − max.qupm: 26

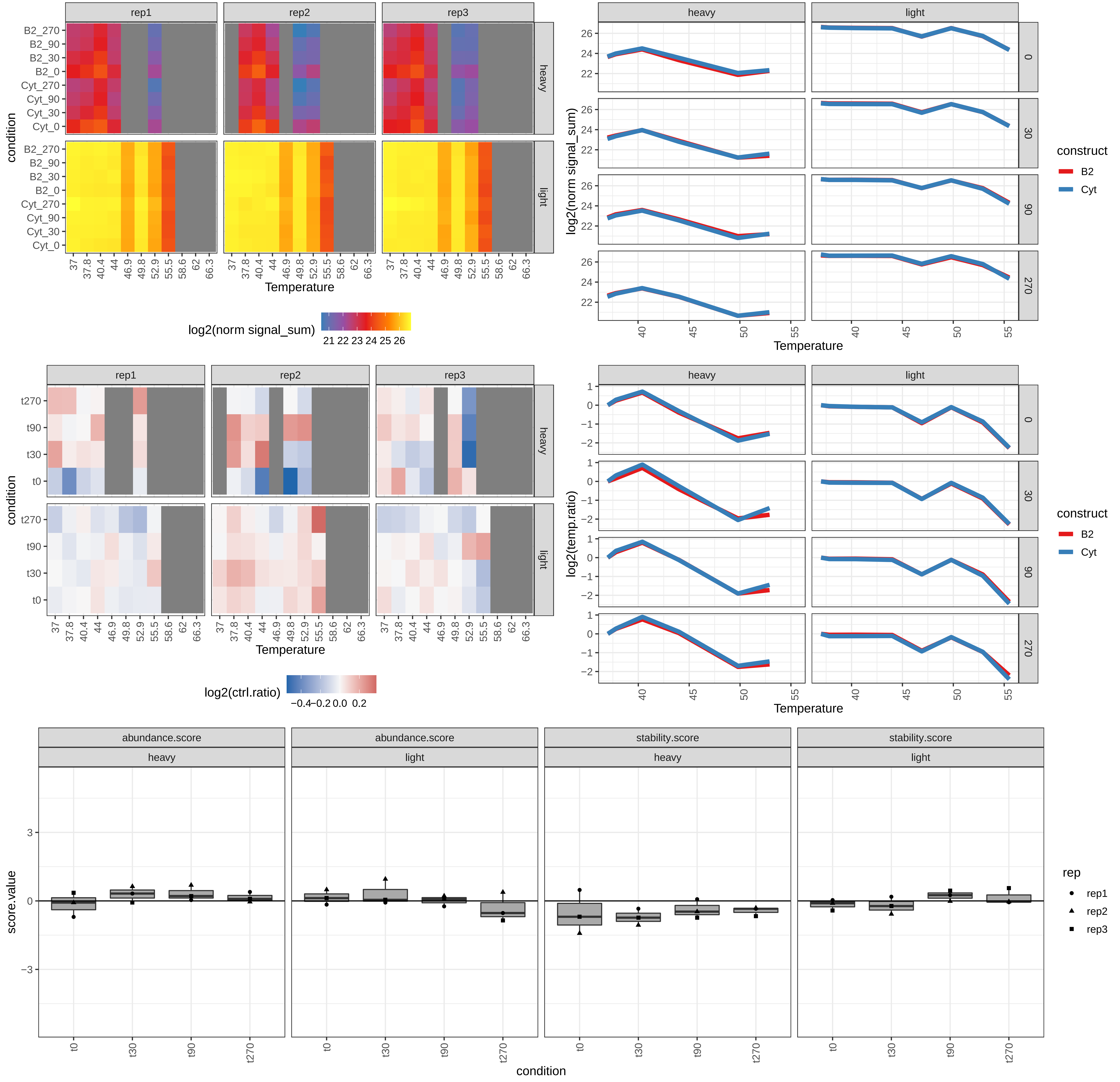

P04051 − DNA−directed RNA polymerase III subunit RPC1 OS=Saccharomyces cerevisiae (strain ATCC 204508 / S288c) GN=RPO31 PE=1 SV=1

RPO41 − average.top3: 6.41966625 − max.qupm: 5

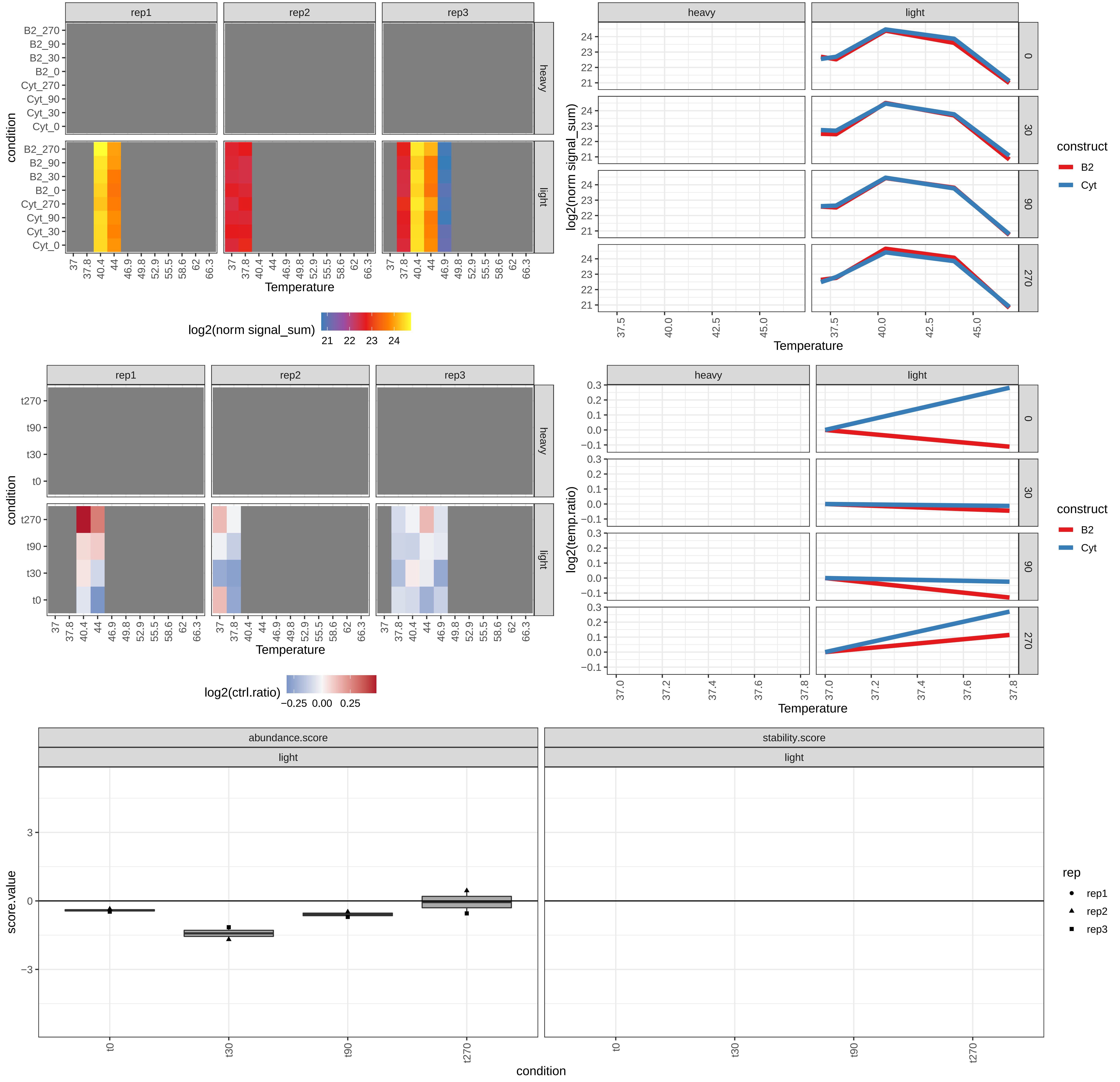

P13433 − DNA−directed RNA polymerase, mitochondrial OS=Saccharomyces cerevisiae (strain ATCC 204508 / S288c) GN=RPO41 PE=3 SV=2

RPP0 − average.top3: 8.20913322033898 − max.qupm: 15

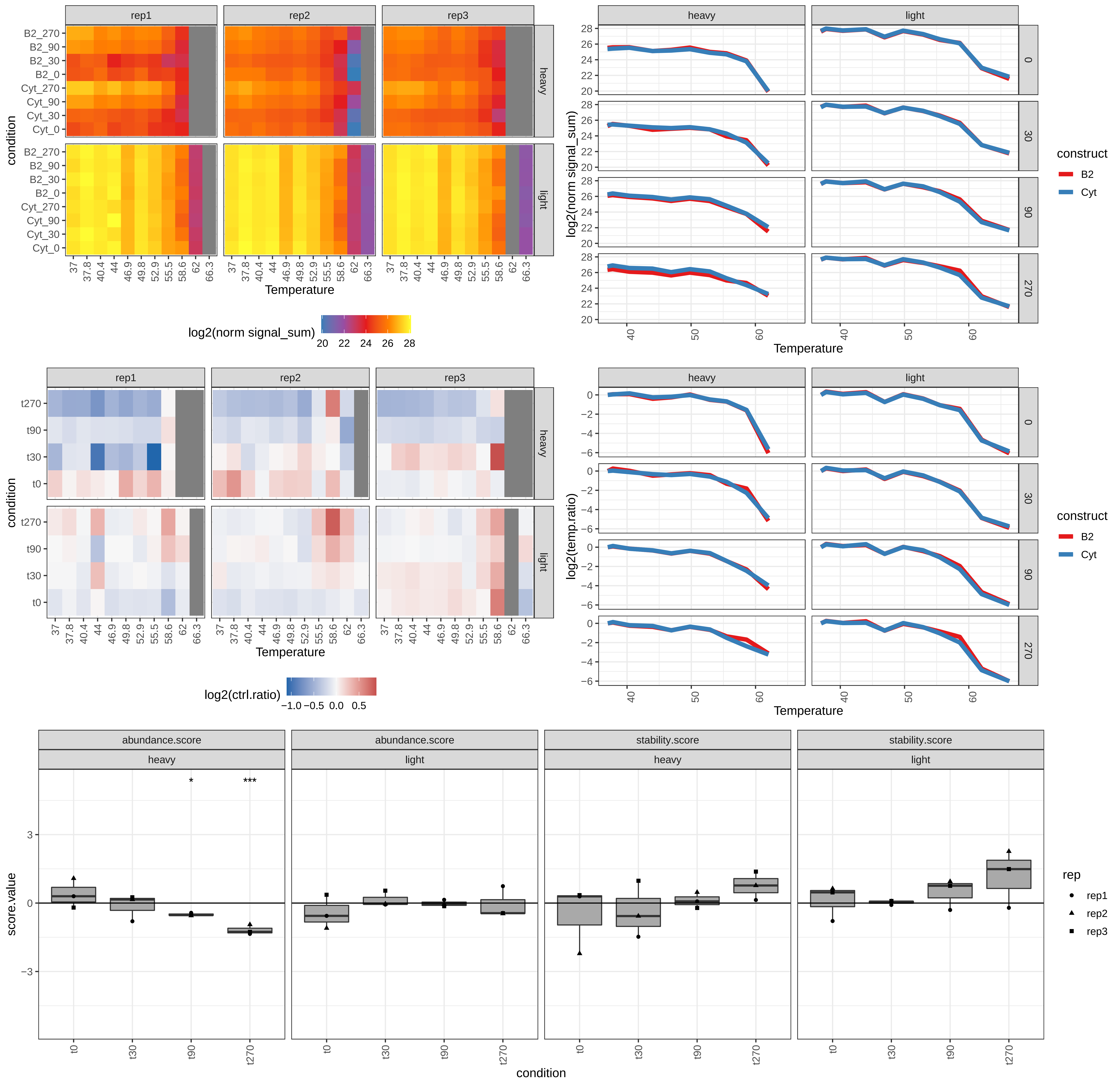

P05317 − 60S acidic ribosomal protein P0 OS=Saccharomyces cerevisiae (strain ATCC 204508 / S288c) GN=RPP0 PE=1 SV=2

RPP1 − average.top3: 7.03558421052632 − max.qupm: 7

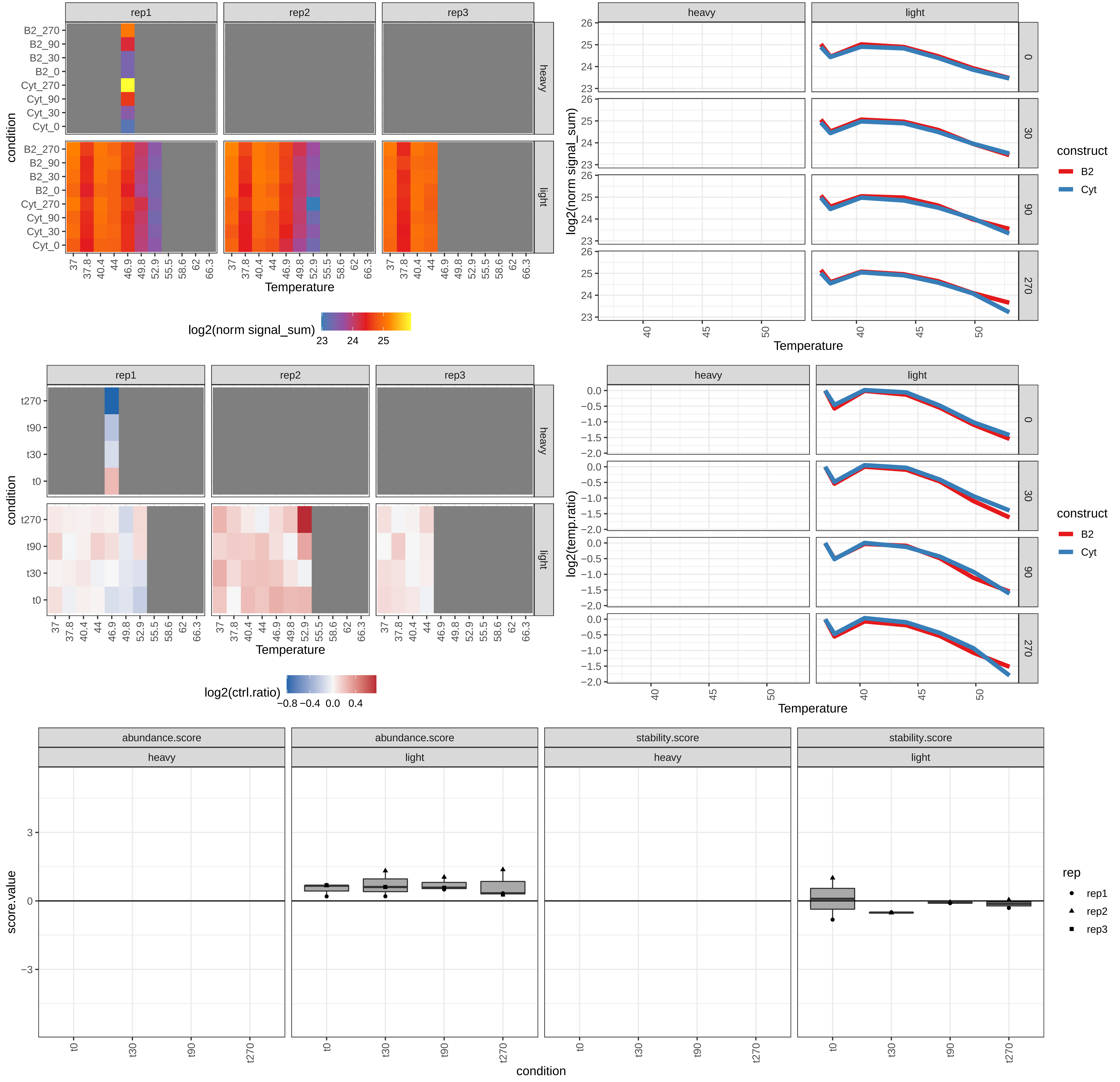

P38786 − Ribonuclease P/MRP protein subunit RPP1 OS=Saccharomyces cerevisiae (strain ATCC 204508 / S288c) GN=RPP1 PE=1 SV=1

RPP2A − average.top3: 6.56578086956522 − max.qupm: 2

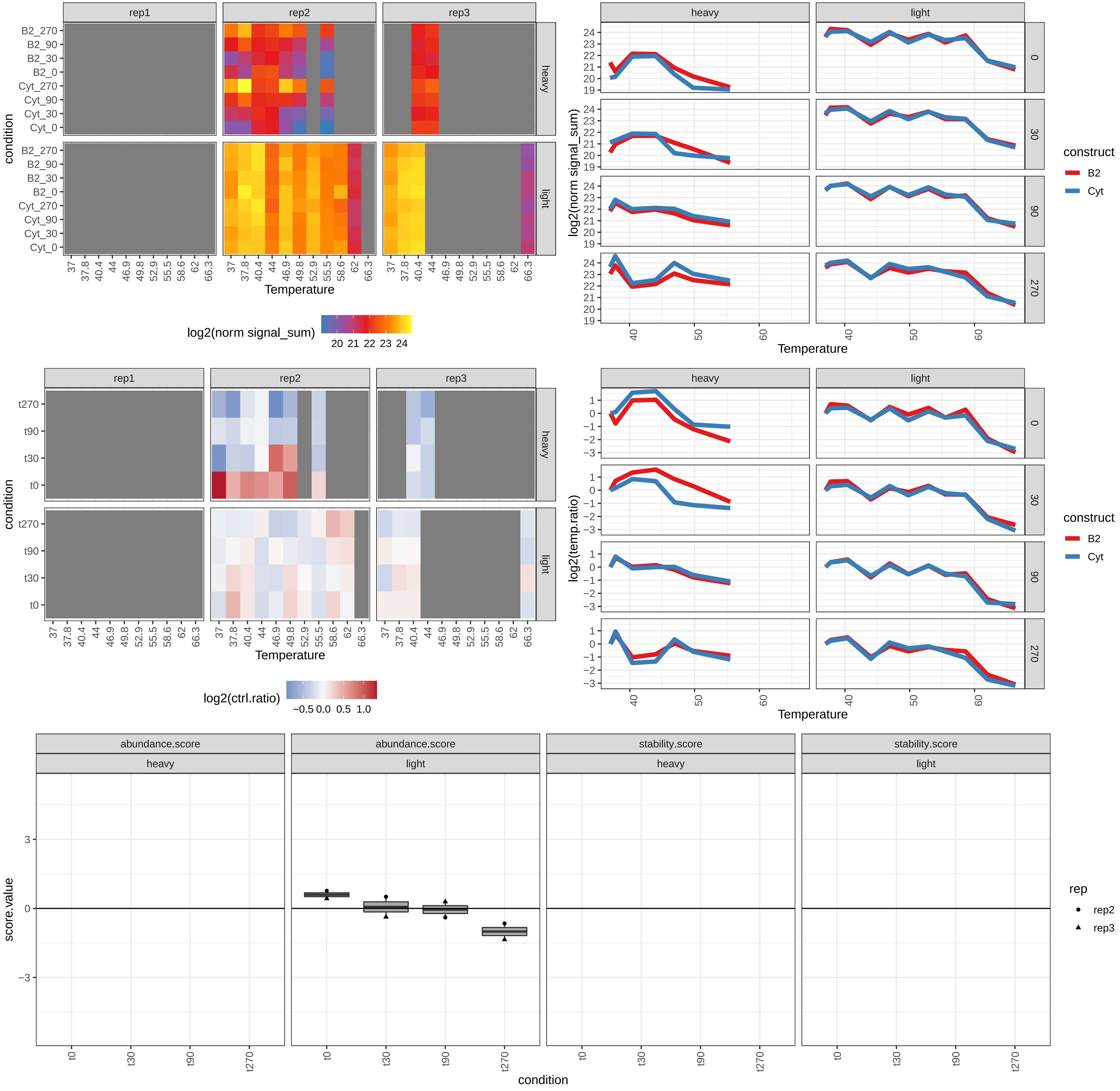

P05319 − 60S acidic ribosomal protein P2−alpha OS=Saccharomyces cerevisiae (strain ATCC 204508 / S288c) GN=RPP2A PE=1 SV=1

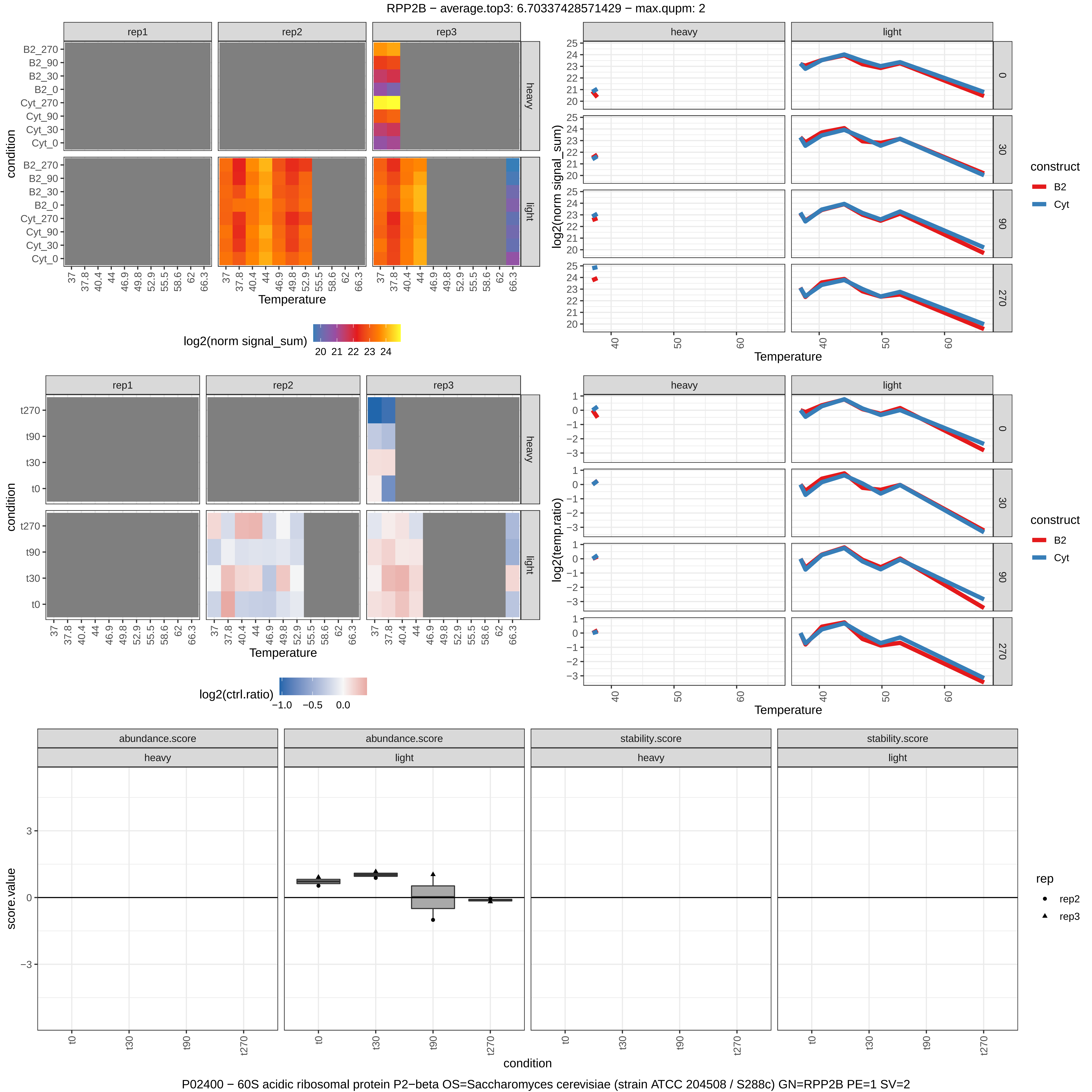

RPP2B − average.top3: 6.70337428571429 − max.qupm: 2
rep1
rep2
rep3
heavy
light
condition
Temperature
log2(norm signal_sum)
log2(ctrl.ratio)
log2(temp.ratio)
construct
B2
Cyt
abundance.score
stability.score
score.value
rep
rep2
rep3
P02400 − 60S acidic ribosomal protein P2−beta OS=Saccharomyces cerevisiae (strain ATCC 204508 / S288c) GN=RPP2B PE=1 SV=2

RPS0A − average.top3: 7.965608 − max.qupm: 9

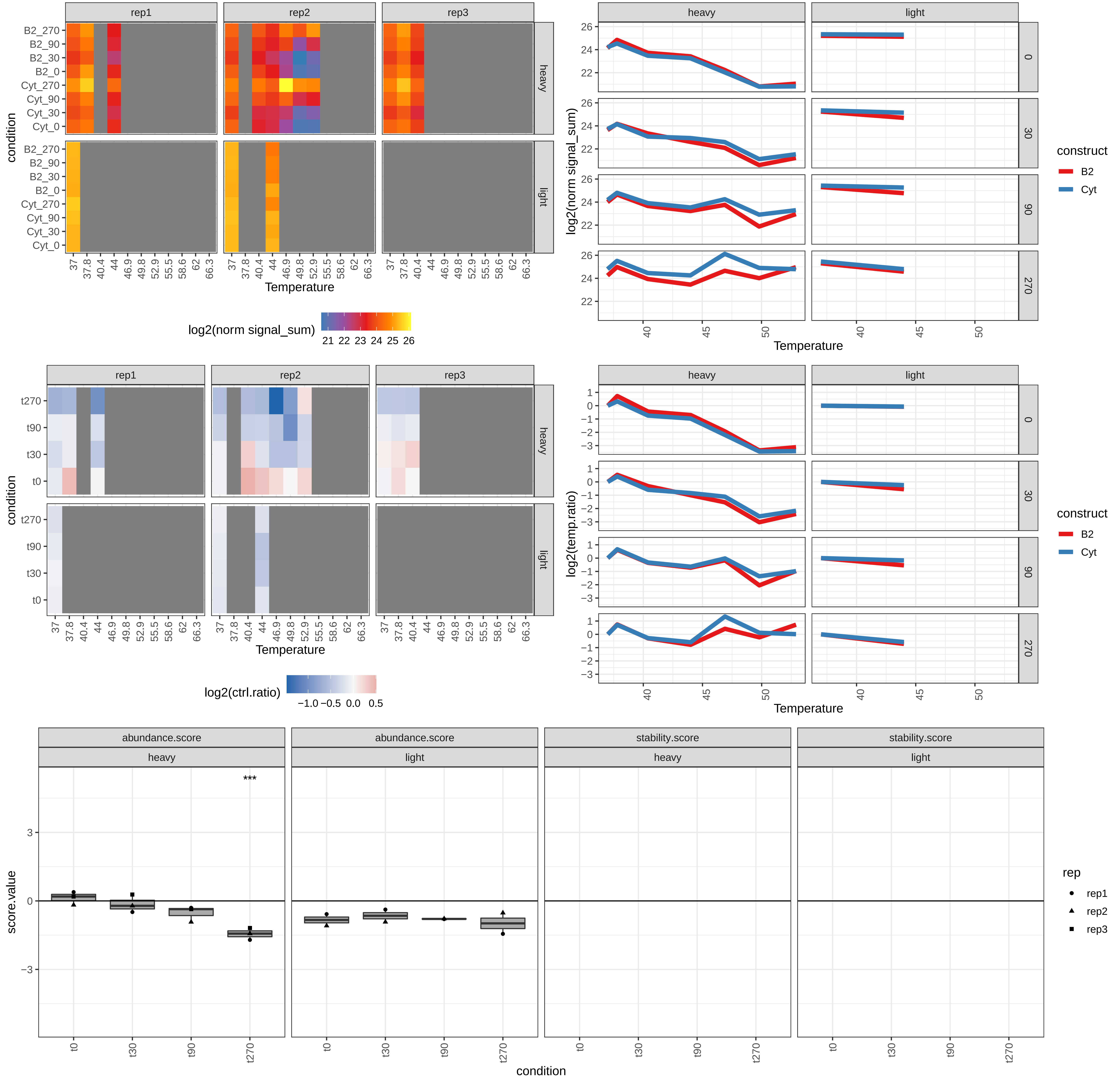

P32905 − 40S ribosomal protein S0−A OS=Saccharomyces cerevisiae (strain ATCC 204508 / S288c) GN=RPS0A PE=1 SV=3

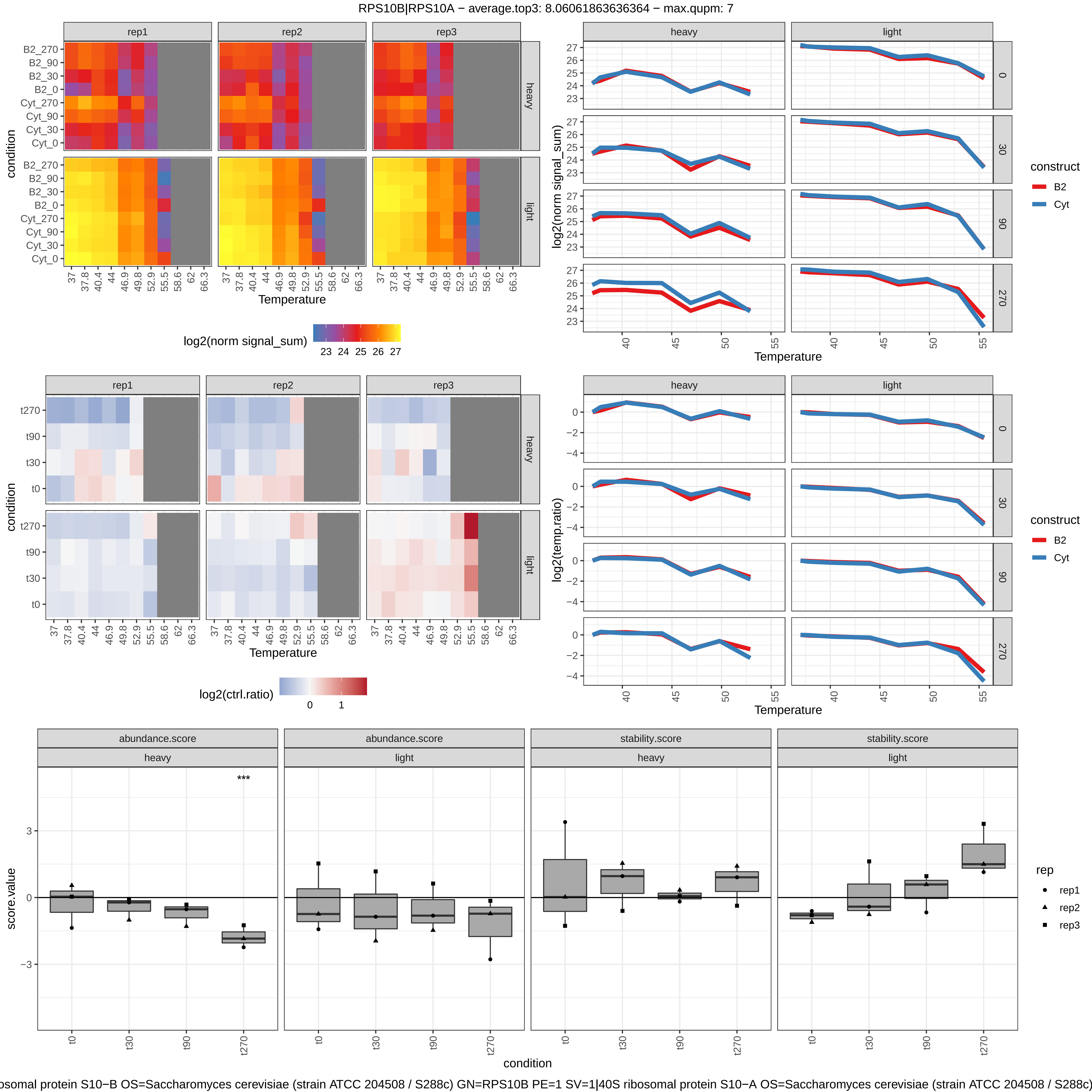

RPS10B|RPS10A − average.top3: 8.06061863636364 − max.qupm: 7
rep1
rep2
rep3
heavy
light
condition
B2_270
B2_90
B2_30
B2_0
Cyt_270
Cyt_90
Cyt_30
Cyt_0
37
37.8
40.4
44
46.9
49.8
52.9
55.5
58.6
62
66.3
Temperature
log2(norm signal_sum)
23 24 25 26 27
log2(norm signal_sum)
construct
B2
Cyt
0
30
90
270
t270
t90
t30
t0
log2(ctrl.ratio)
0 1
log2(temp.ratio)
abundance.score
stability.score
score.value
rep
rep1
rep2
rep3
***
condition
osomal protein S10−B OS=Saccharomyces cerevisiae (strain ATCC 204508 / S288c) GN=RPS10B PE=1 SV=1|40S ribosomal protein S10−A OS=Saccharomyces cerevisiae (strain ATCC 204508 / S288c)

RPS11A|RPS11B − average.top3: 8.04474041666667 − max.qupm: 11

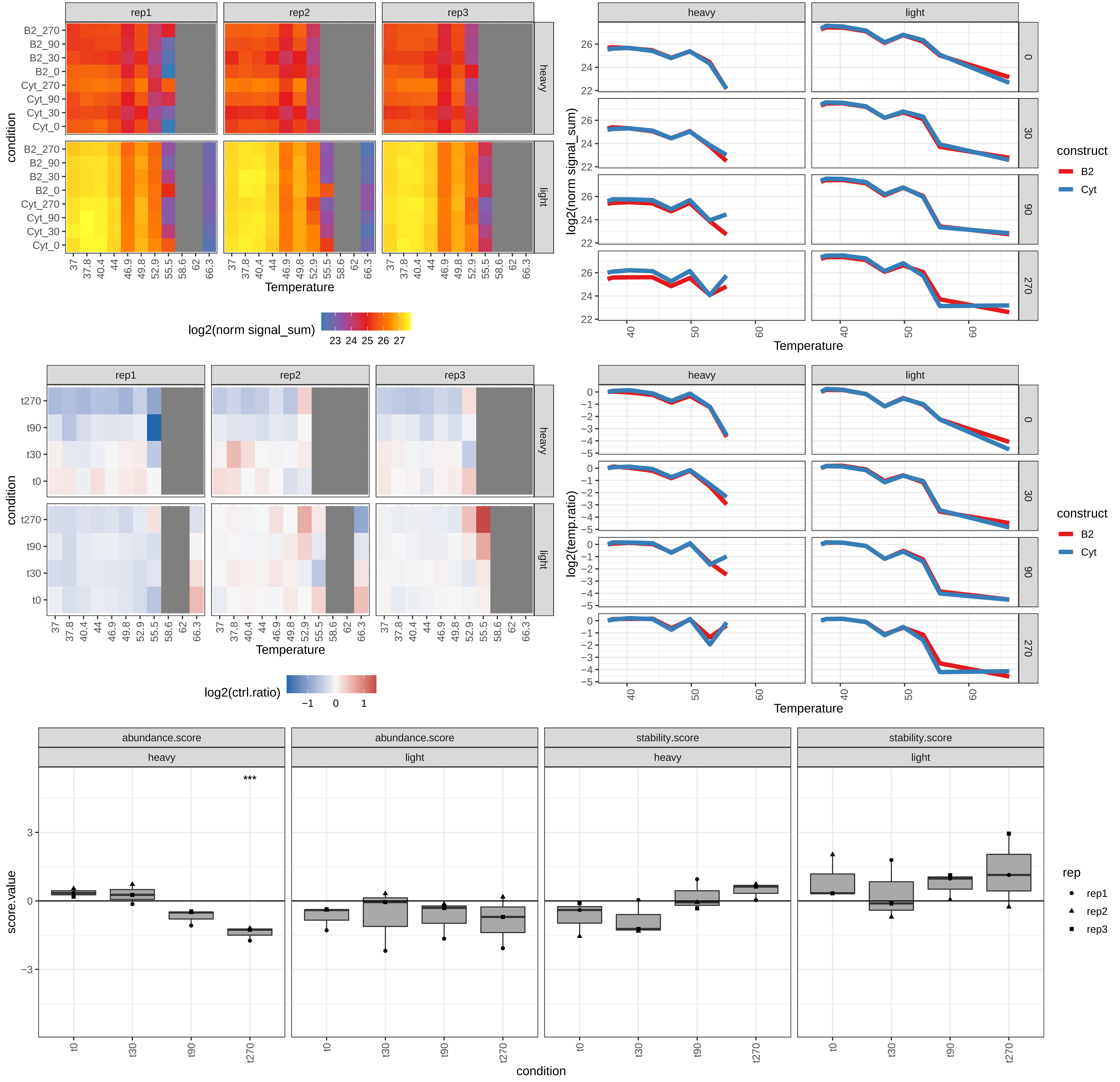

osomal protein S11−A OS=Saccharomyces cerevisiae (strain ATCC 204508 / S288c) GN=RPS11A PE=1 SV=1|40S ribosomal protein S11−B OS=Saccharomyces cerevisiae (strain ATCC 204508 / S288c

RPS12 − average.top3: 7.8468024 − max.qupm: 5

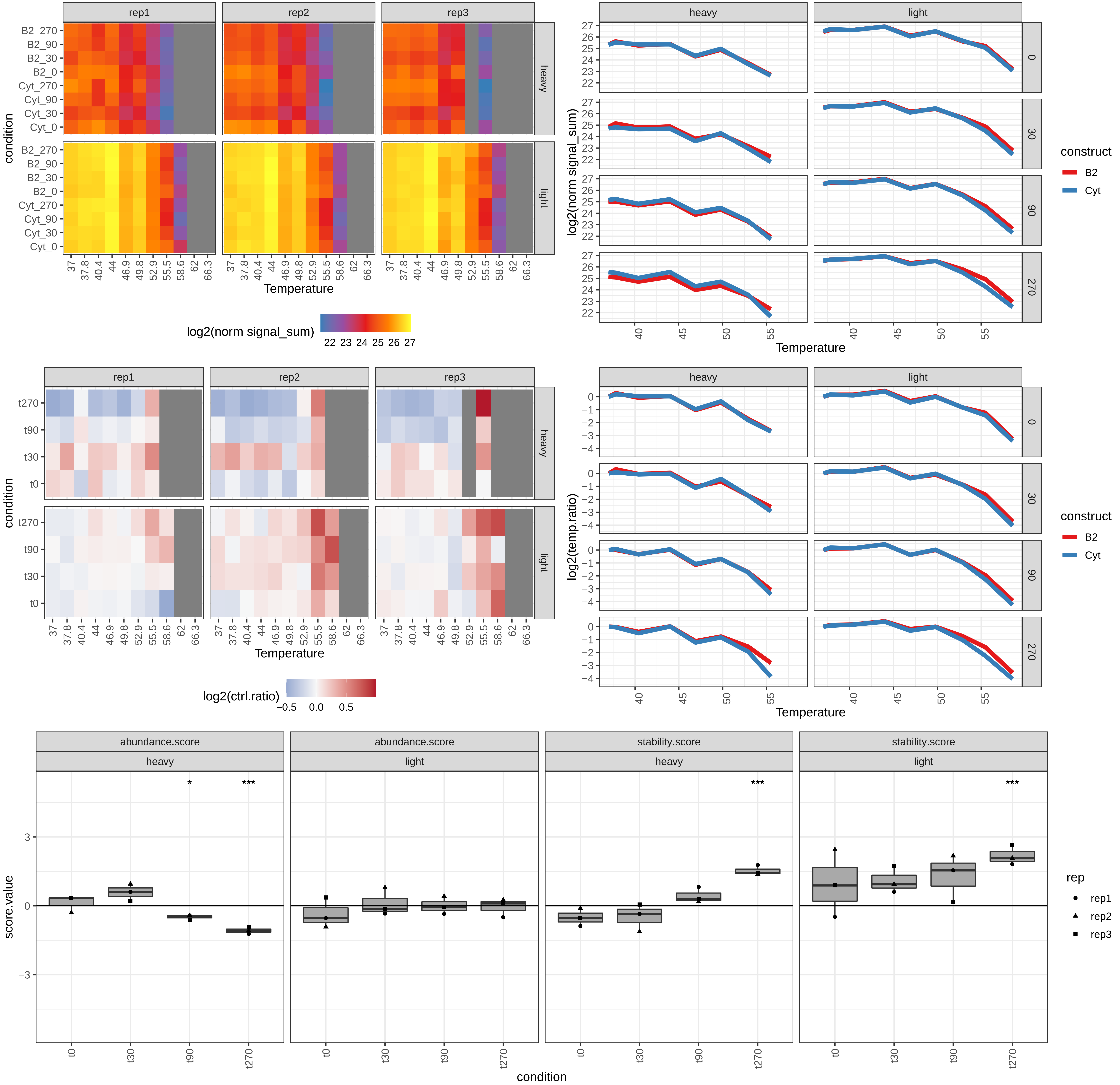

P48589 − 40S ribosomal protein S12 OS=Saccharomyces cerevisiae (strain ATCC 204508 / S288c) GN=RPS12 PE=1 SV=1

RPS13 − average.top3: 7.85934914893617 − max.qupm: 11

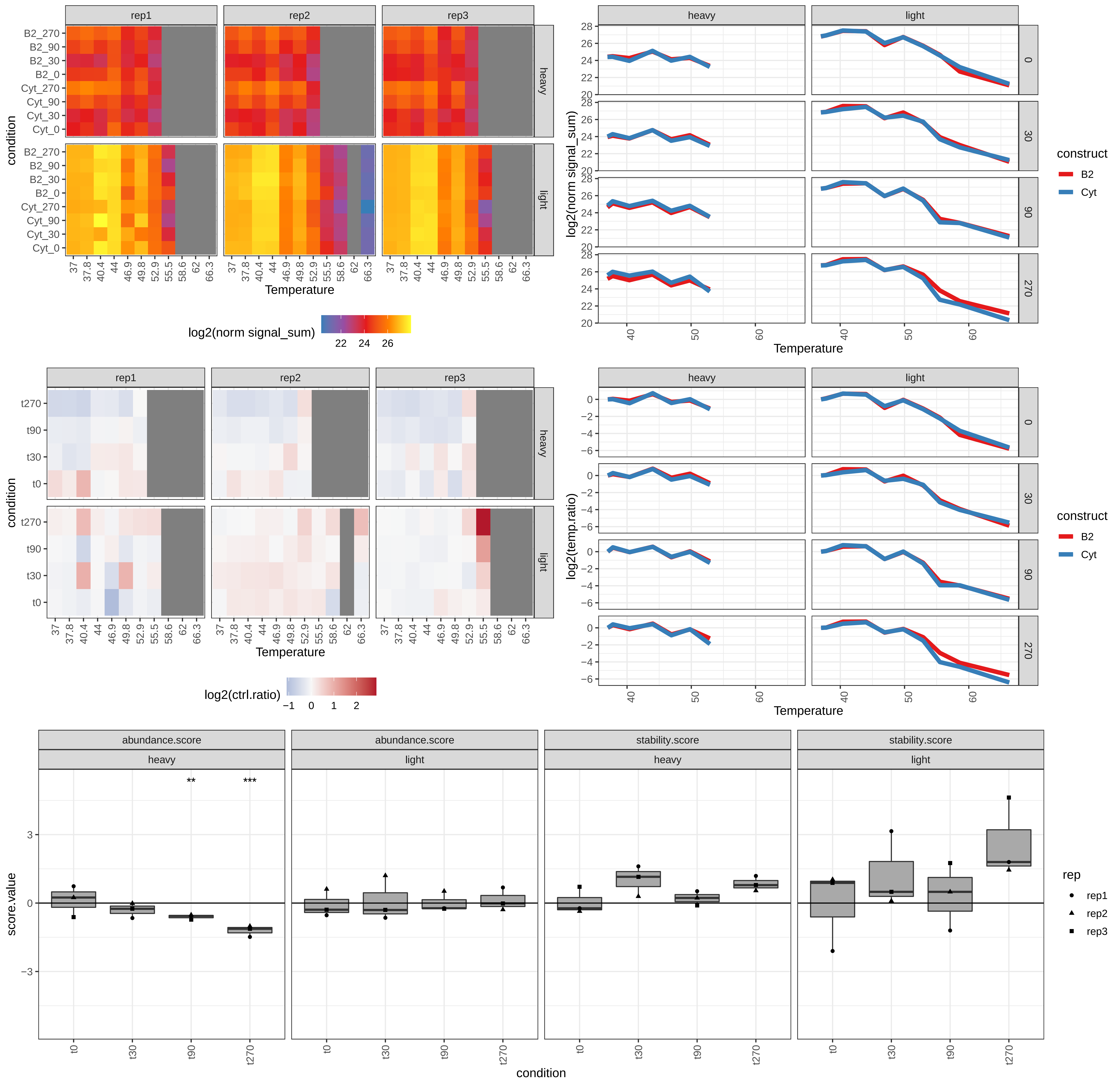

P05756 − 40S ribosomal protein S13 OS=Saccharomyces cerevisiae (strain ATCC 204508 / S288c) GN=RPS13 PE=1 SV=3

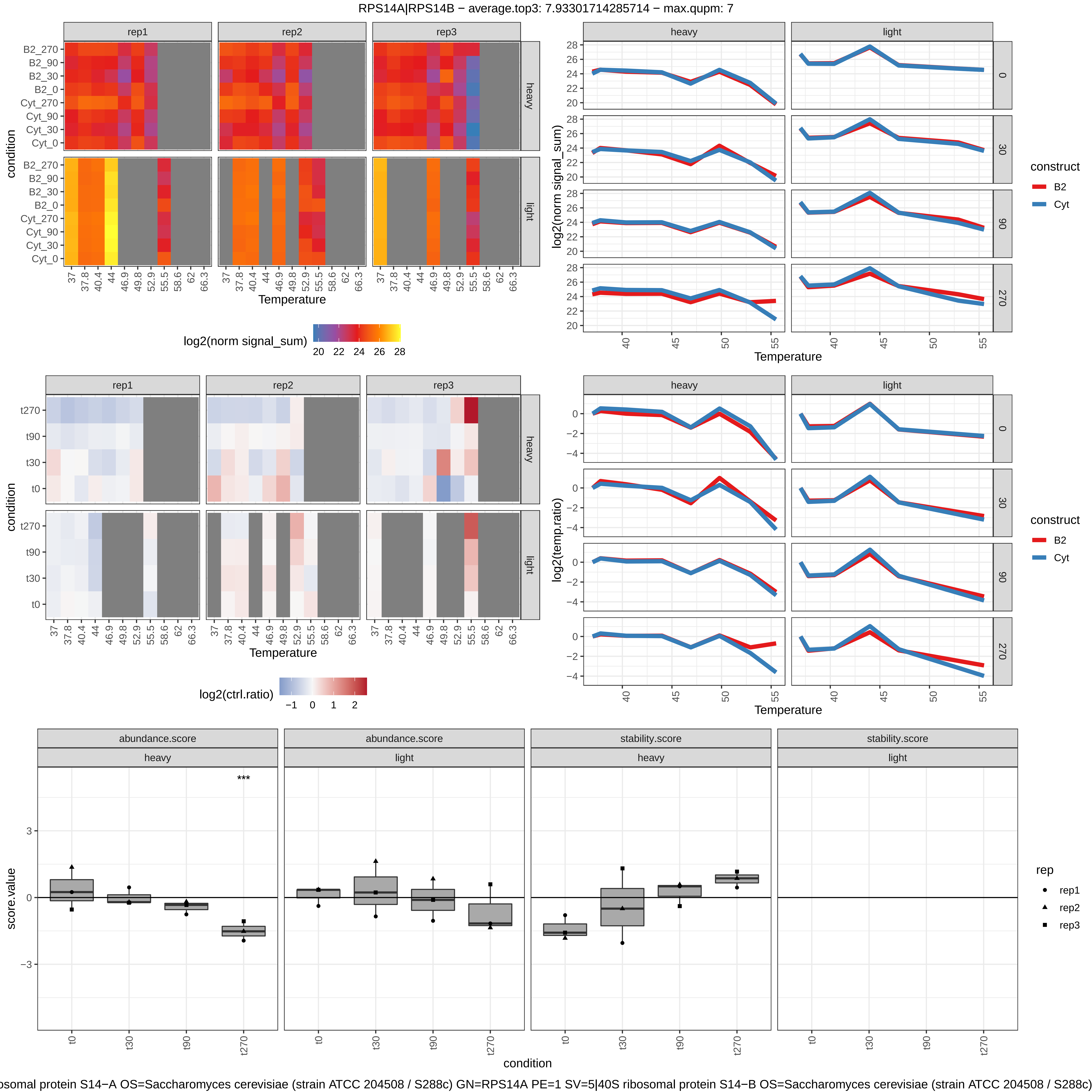
RPS14A|RPS14B − average.top3: 7.93301714285714 − max.qupm: 7
rep1
rep2
rep3
heavy
light
condition
B2_270
B2_90
B2_30
B2_0
Cyt_270
Cyt_90
Cyt_30
Cyt_0
Temperature
log2(norm signal_sum)
20 22 24 26 28
construct
B2
Cyt
0
30
90
270
t270
t90
t30
t0
log2(temp.ratio)
log2(ctrl.ratio)
−1 0 1 2
abundance.score
stability.score
score.value
rep
rep1
rep2
rep3
***
osomal protein S14−A OS=Saccharomyces cerevisiae (strain ATCC 204508 / S288c) GN=RPS14A PE=1 SV=5|40S ribosomal protein S14−B OS=Saccharomyces cerevisiae (strain ATCC 204508 / S288c)

RPS15 − average.top3: 7.89405204081633 − max.qupm: 8

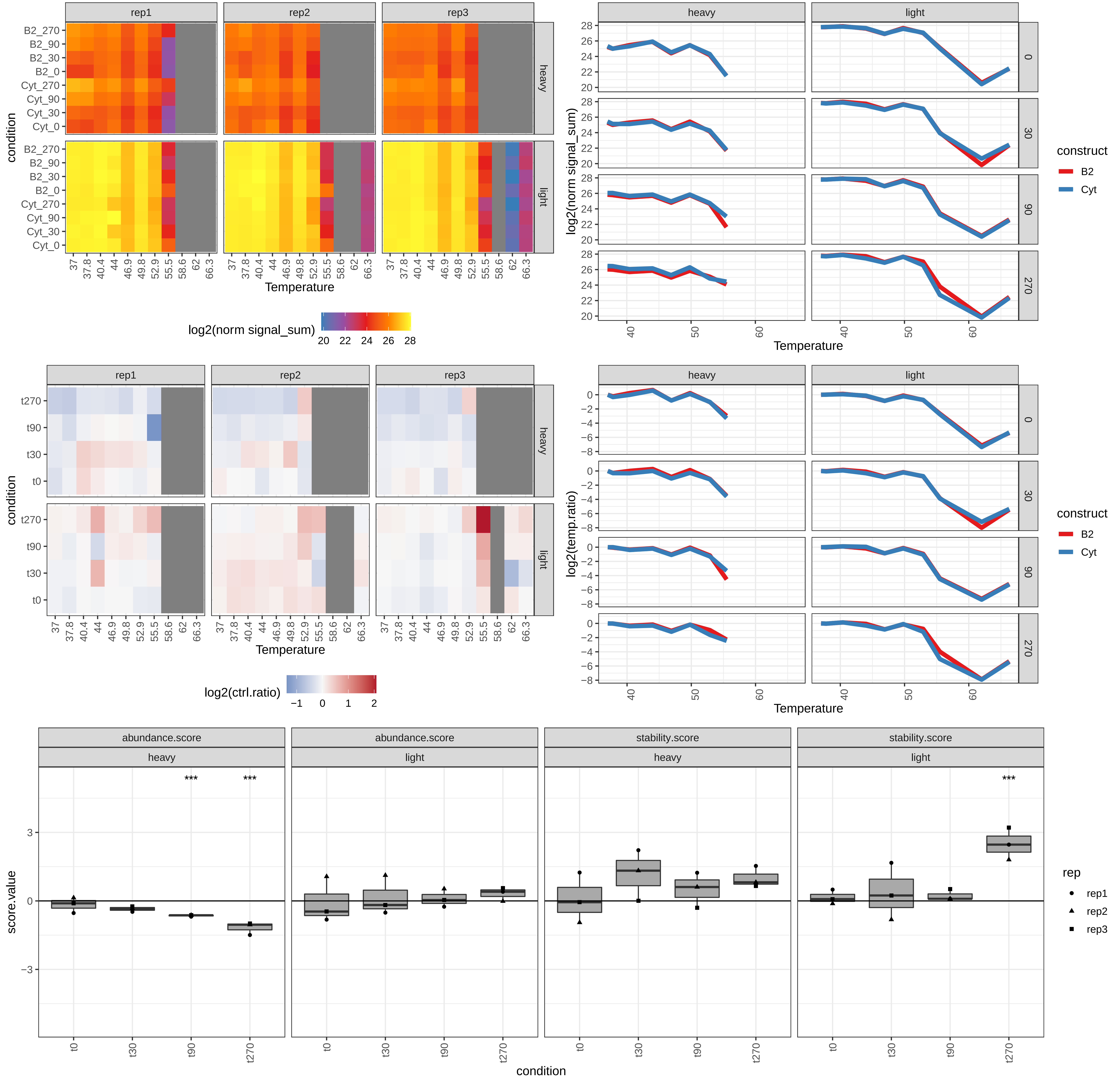

Q01855 − 40S ribosomal protein S15 OS=Saccharomyces cerevisiae (strain ATCC 204508 / S288c) GN=RPS15 PE=1 SV=1

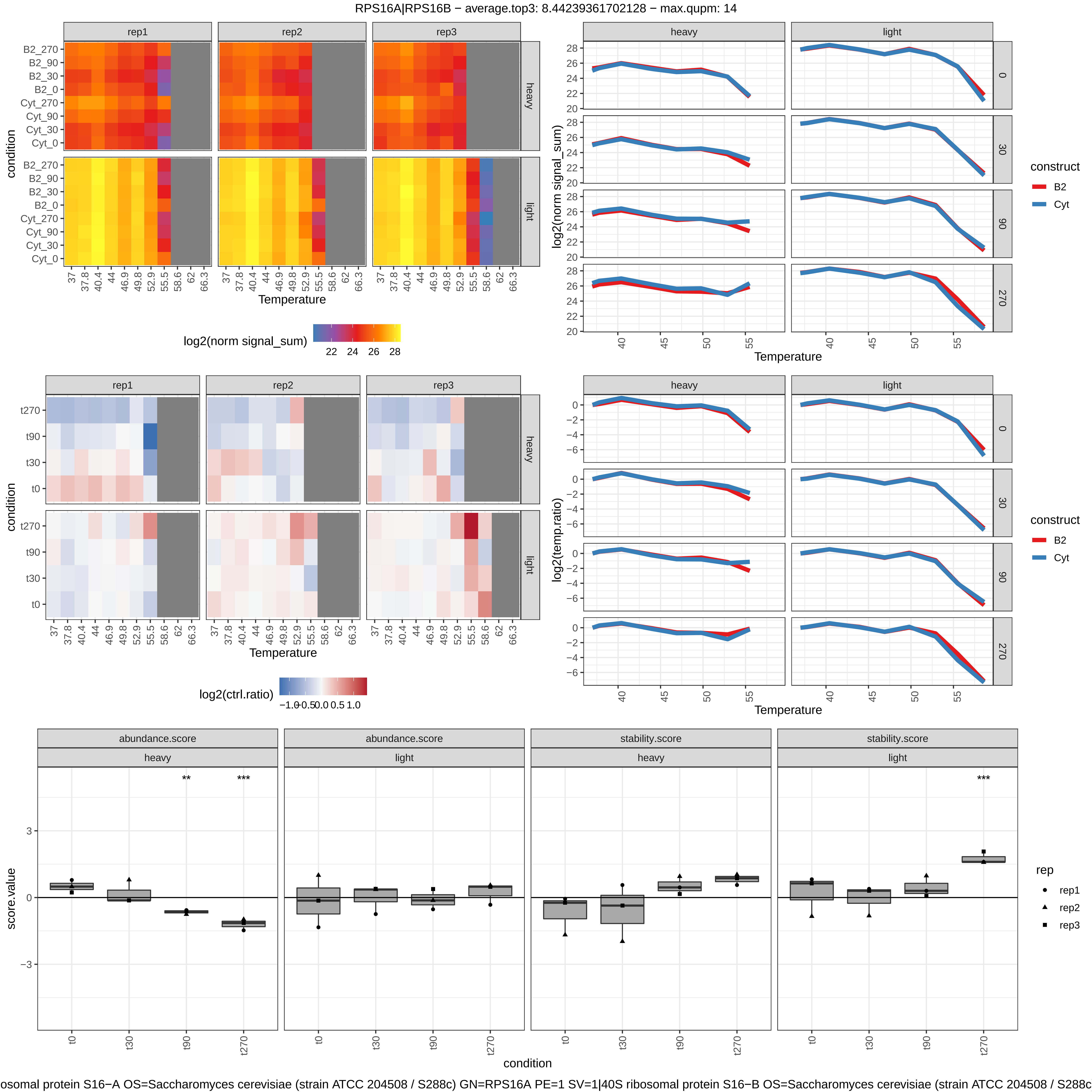

RPS16A|RPS16B − average.top3: 8.44239361702128 − max.qupm: 14
rep1
rep2
rep3
heavy
light
B2_270
B2_90
B2_30
B2_0
Cyt_270
Cyt_90
Cyt_30
Cyt_0
condition
Temperature
log2(norm signal_sum)
construct
B2
Cyt
0
30
90
270
log2(ctrl.ratio)
t270
t90
t30
t0
log2(temp.ratio)
abundance.score
stability.score
score.value
rep
**
***
osomal protein S16−A OS=Saccharomyces cerevisiae (strain ATCC 204508 / S288c) GN=RPS16A PE=1 SV=1|40S ribosomal protein S16−B OS=Saccharomyces cerevisiae (strain ATCC 204508 / S288c

RPS17A|RPS17B − average.top3: 7.86062733333333 − max.qupm: 9

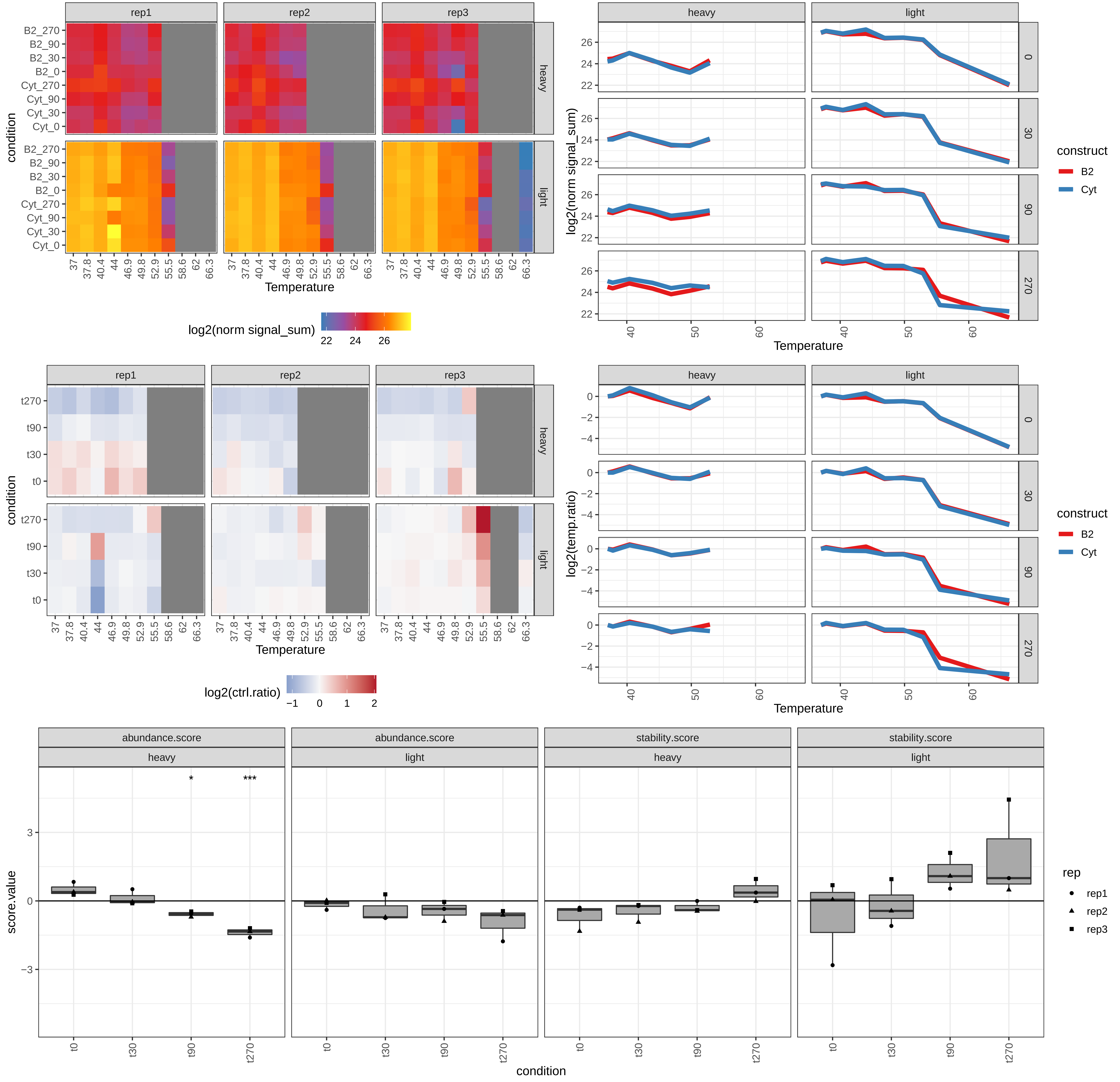

osomal protein S17−A OS=Saccharomyces cerevisiae (strain ATCC 204508 / S288c) GN=RPS17A PE=1 SV=1|40S ribosomal protein S17−B OS=Saccharomyces cerevisiae (strain ATCC 204508 / S288c)

RPS18A|RPS18B − average.top3: 8.14481981132075 − max.qupm: 13

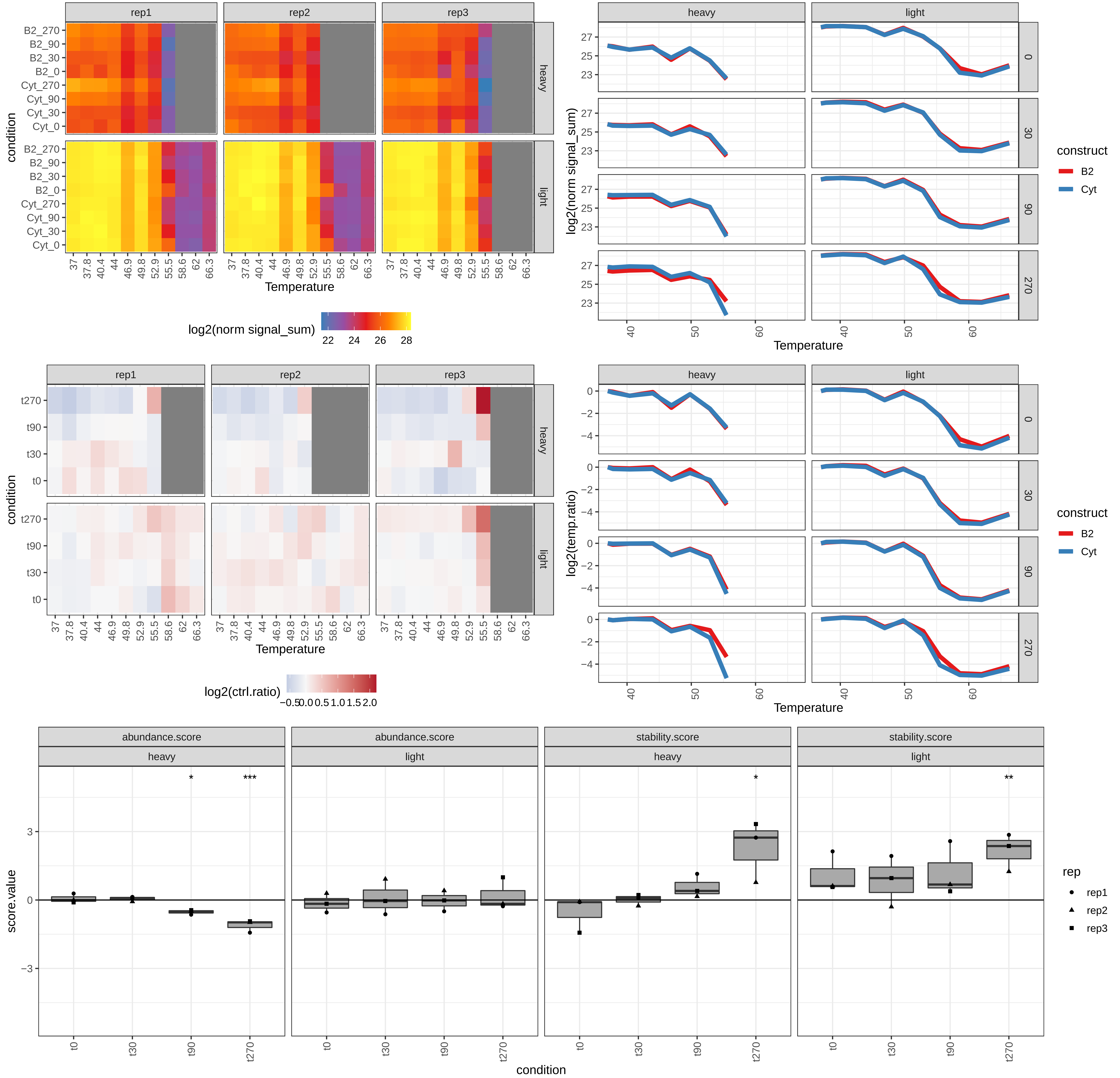

osomal protein S18−A OS=Saccharomyces cerevisiae (strain ATCC 204508 / S288c) GN=RPS18A PE=1 SV=1|40S ribosomal protein S18−B OS=Saccharomyces cerevisiae (strain ATCC 204508 / S288c

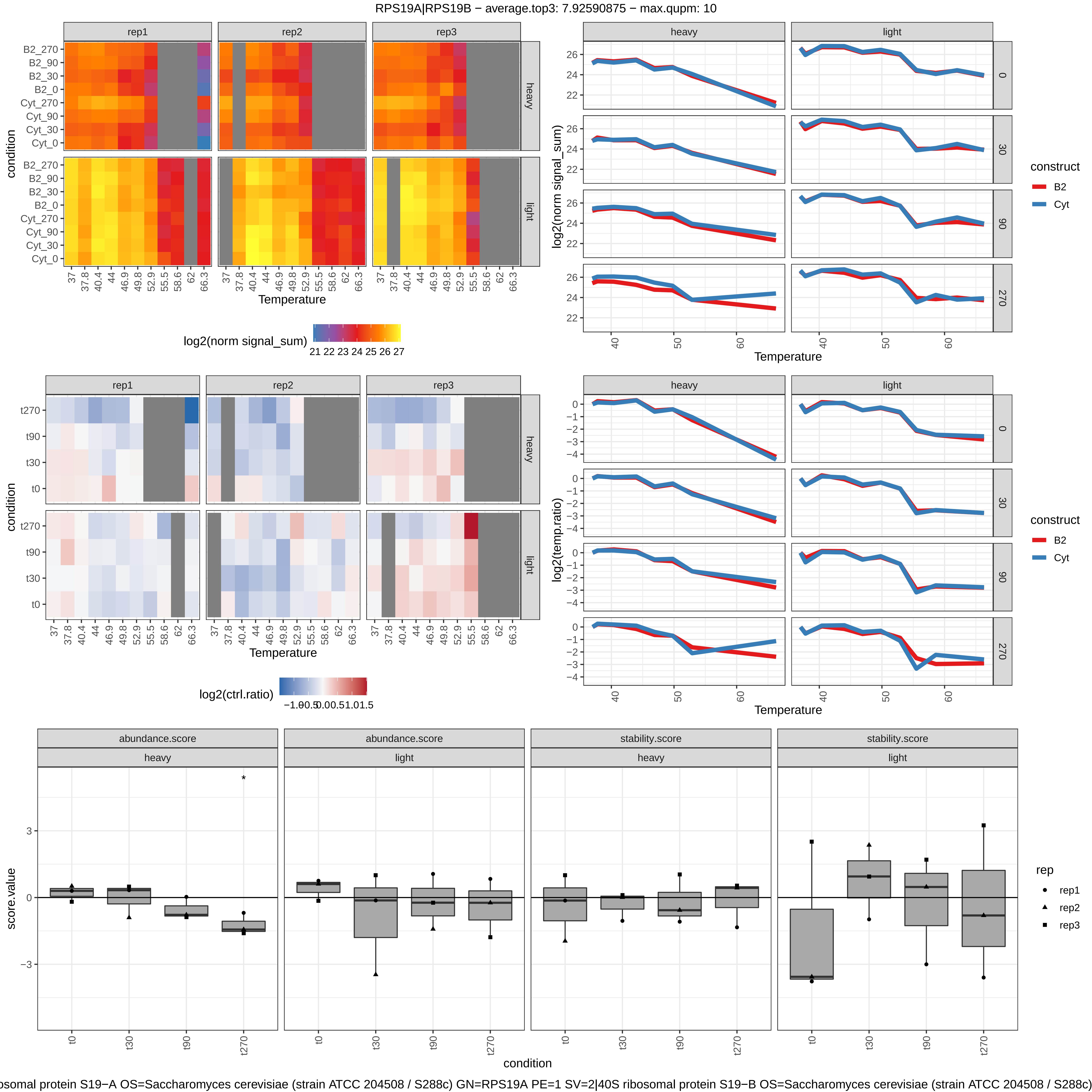
RPS19A|RPS19B − average.top3: 7.92590875 − max.qupm: 10
rep1
rep2
rep3
heavy
light
B2_270
B2_90
B2_30
B2_0
Cyt_270
Cyt_90
Cyt_30
Cyt_0
condition
37 37.8 40.4 44 46.9 49.8 52.9 55.5 58.6 62 66.3
Temperature
log2(norm signal_sum)
21 22 23 24 25 26 27
log2(norm signal_sum)
0
30
90
270
construct
B2
Cyt
t270
t90
t30
t0
log2(ctrl.ratio)
−1.0 −0.5 0.0 0.5 1.0 1.5
log2(temp.ratio)
abundance.score
stability.score
score.value
rep
rep1
rep2
rep3
condition
osomal protein S19−A OS=Saccharomyces cerevisiae (strain ATCC 204508 / S288c) GN=RPS19A PE=1 SV=2|40S ribosomal protein S19−B OS=Saccharomyces cerevisiae (strain ATCC 204508 / S288c)

RPS1A − average.top3: 7.67498222222222 − max.qupm: 5

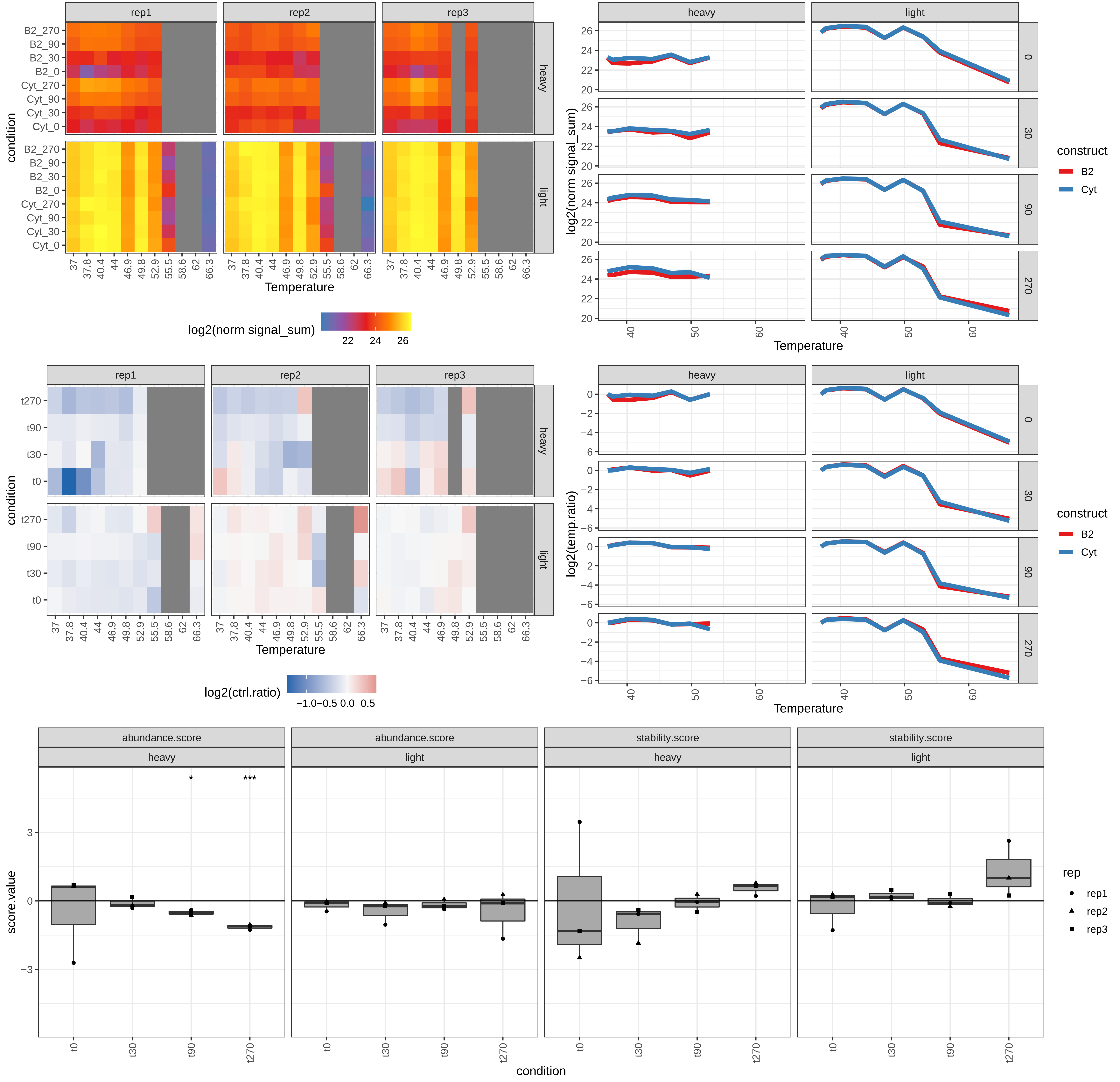

P33442 − 40S ribosomal protein S1−A OS=Saccharomyces cerevisiae (strain ATCC 204508 / S288c) GN=RPS1A PE=1 SV=2

RPS1B − average.top3: 7.74474761904762 − max.qupm: 10

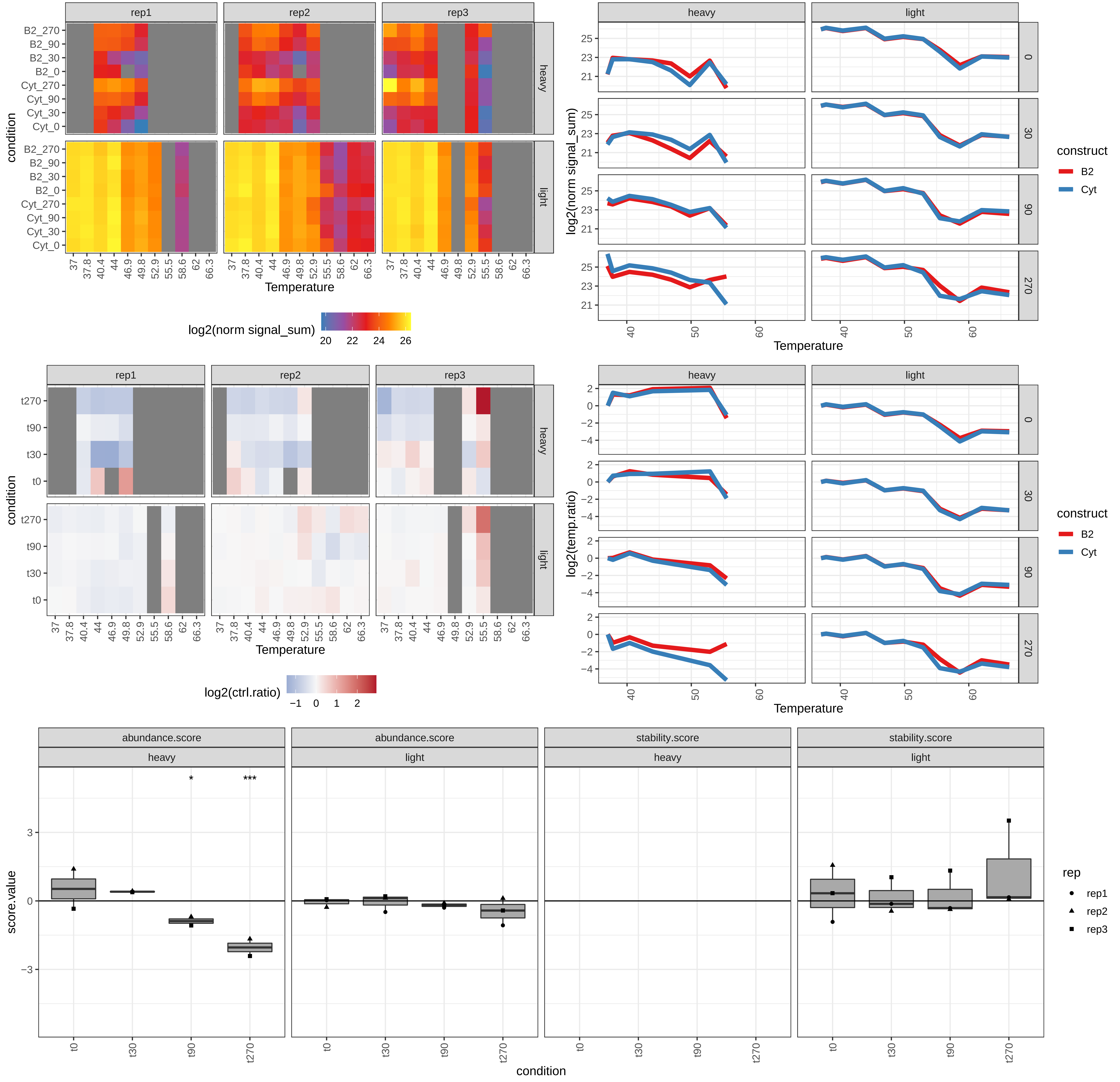

P23248 − 40S ribosomal protein S1−B OS=Saccharomyces cerevisiae (strain ATCC 204508 / S288c) GN=RPS1B PE=1 SV=3

RPS2 − average.top3: 8.12109583333333 − max.qupm: 12

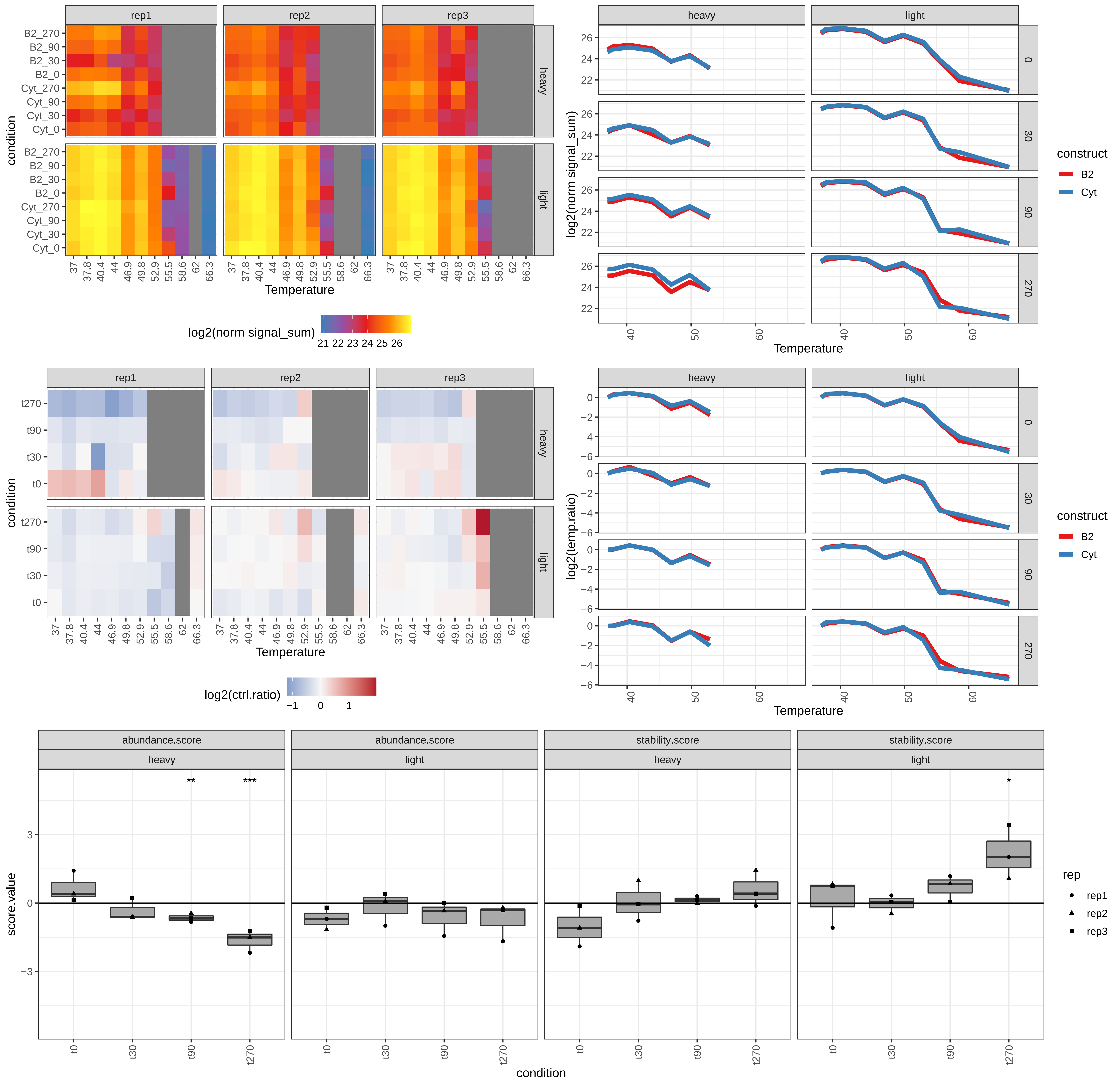

P25443 − 40S ribosomal protein S2 OS=Saccharomyces cerevisiae (strain ATCC 204508 / S288c) GN=RPS2 PE=1 SV=3

RPS20 − average.top3: 8.11393595744681 − max.qupm: 10

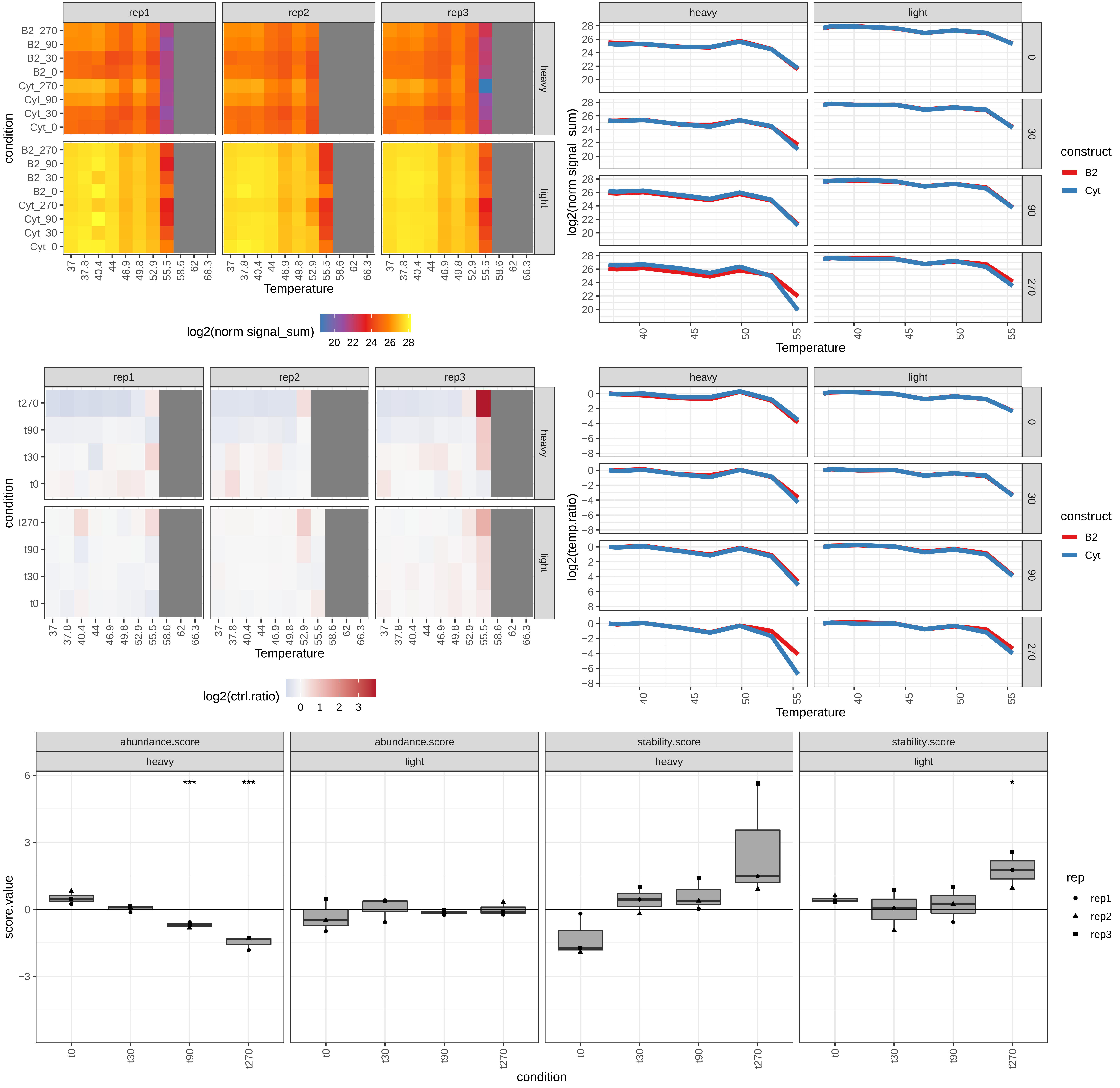

P38701 − 40S ribosomal protein S20 OS=Saccharomyces cerevisiae (strain ATCC 204508 / S288c) GN=RPS20 PE=1 SV=3

RPS21A|RPS21B − average.top3: 7.802683 − max.qupm: 7

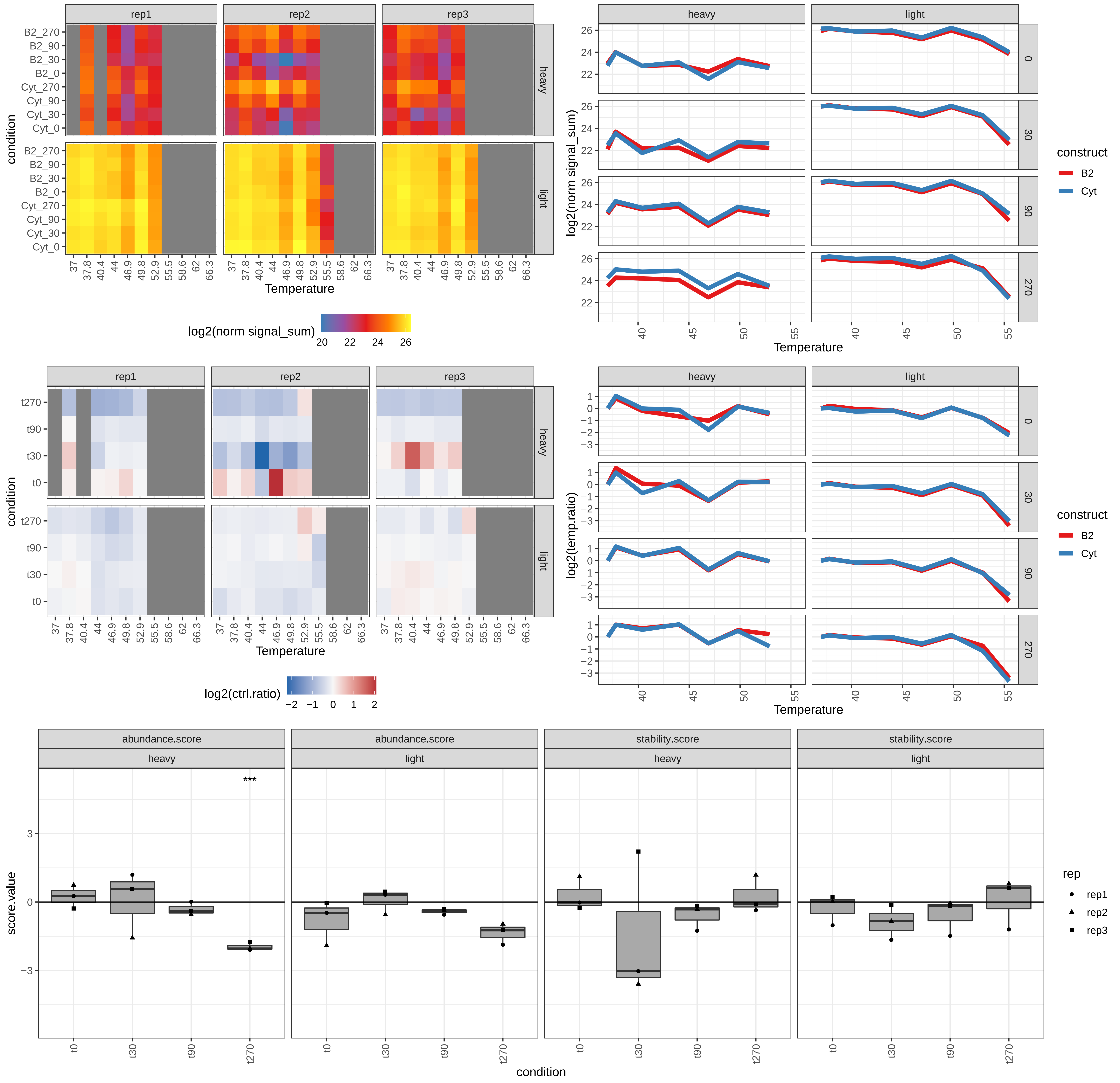

osomal protein S21−A OS=Saccharomyces cerevisiae (strain ATCC 204508 / S288c) GN=RPS21A PE=1 SV=1|40S ribosomal protein S21−B OS=Saccharomyces cerevisiae (strain ATCC 204508 / S288c

RPS23A|RPS23B − average.top3: 8.16265066666667 − max.qupm: 8

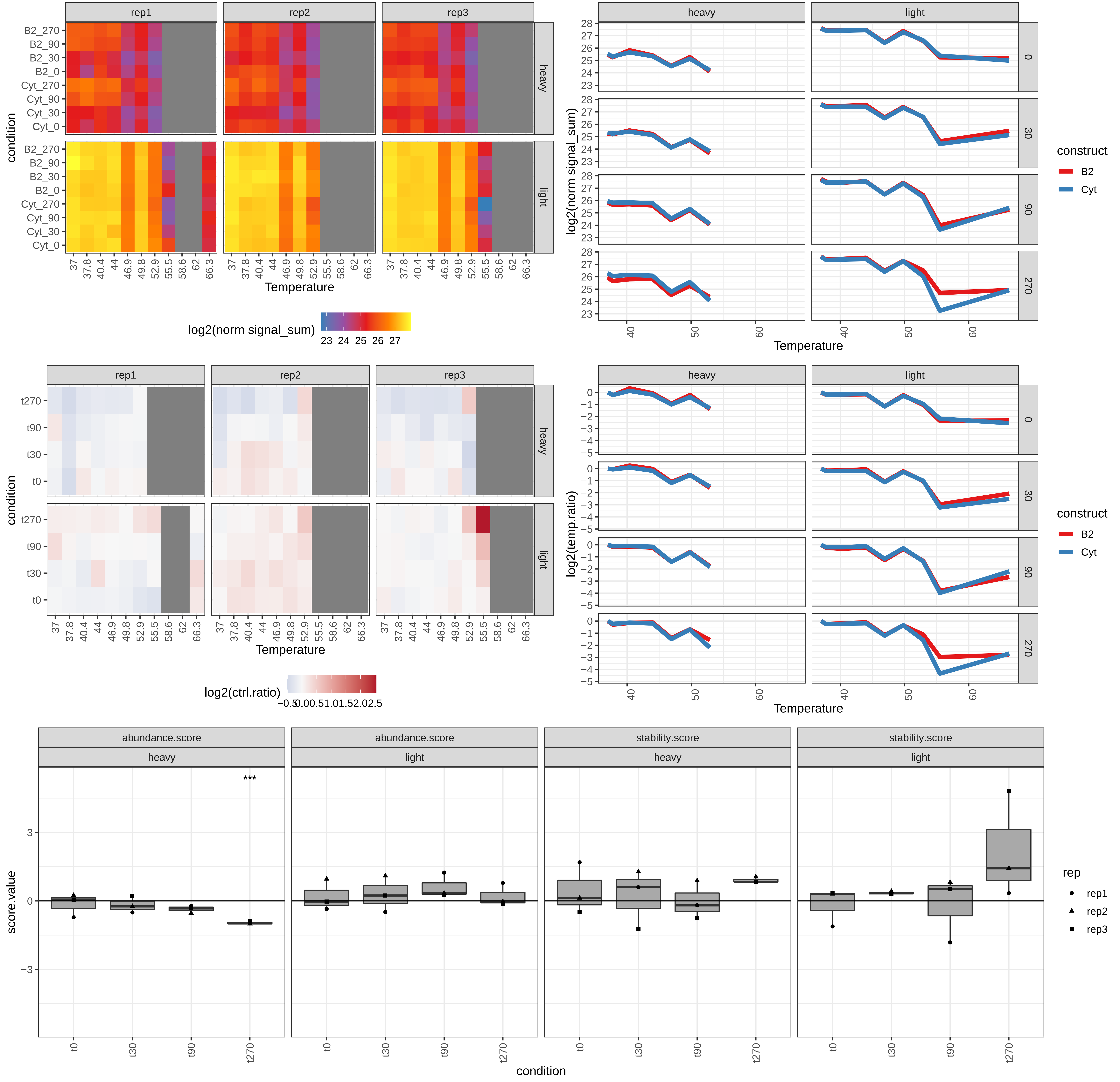

osomal protein S23−A OS=Saccharomyces cerevisiae (strain ATCC 204508 / S288c) GN=RPS23A PE=1 SV=1|40S ribosomal protein S23−B OS=Saccharomyces cerevisiae (strain ATCC 204508 / S288c

RPS24A|RPS24B − average.top3: 8.27426063829787 − max.qupm: 12

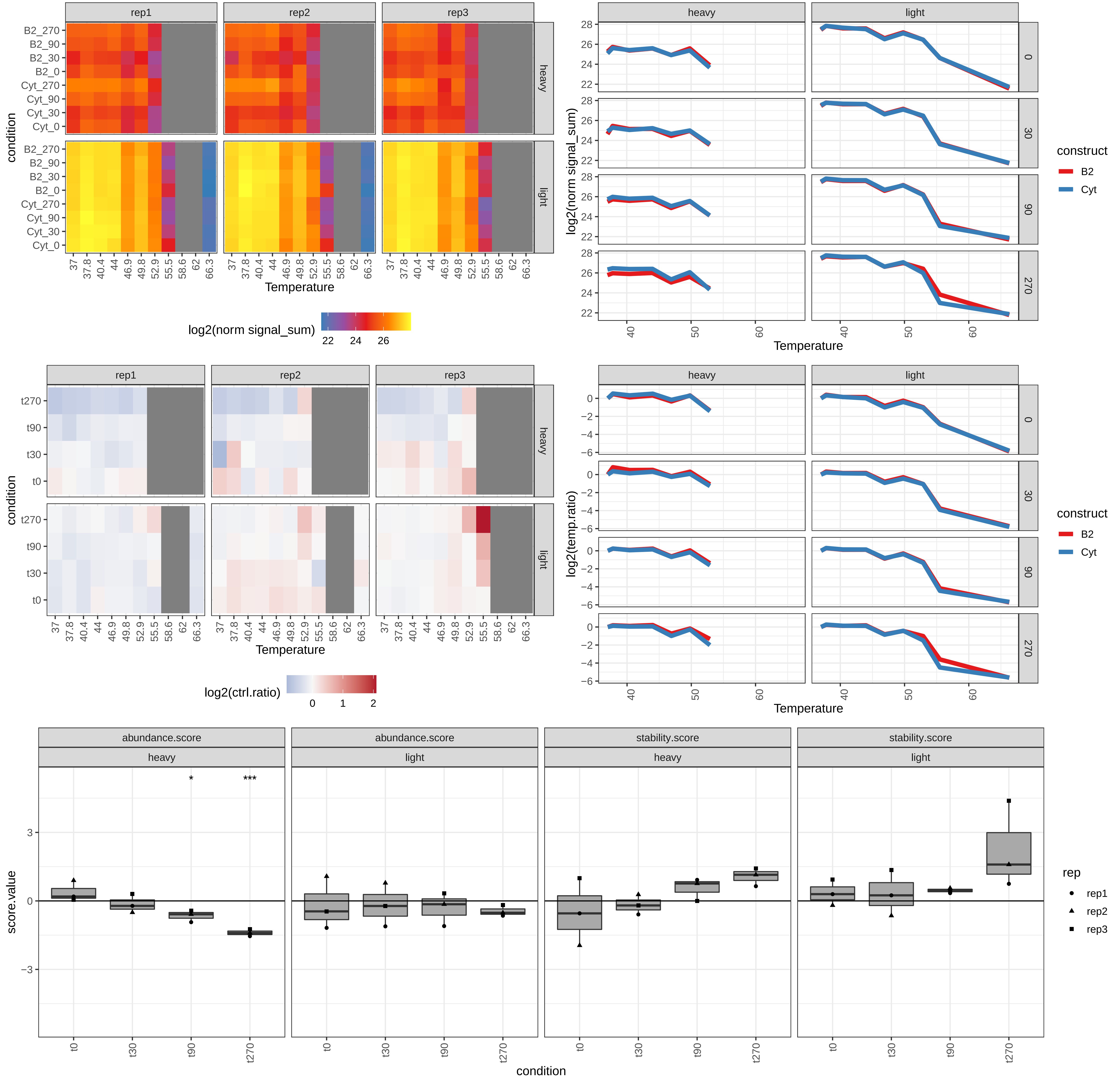

osomal protein S24−A OS=Saccharomyces cerevisiae (strain ATCC 204508 / S288c) GN=RPS24A PE=1 SV=1|40S ribosomal protein S24−B OS=Saccharomyces cerevisiae (strain ATCC 204508 / S288c

RPS25B|RPS25A − average.top3: 8.30268733333333 − max.qupm: 7

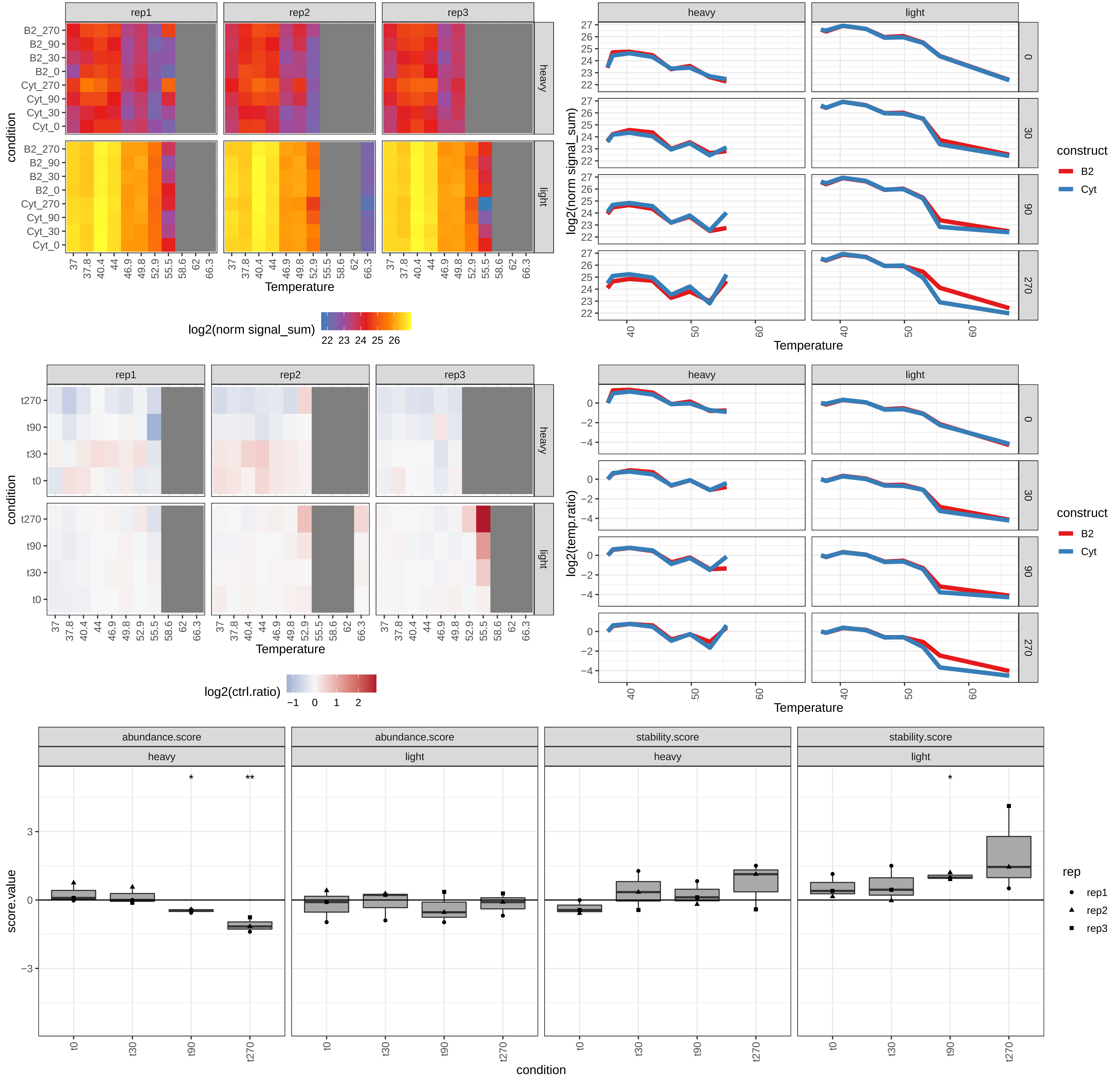

osomal protein S25−B OS=Saccharomyces cerevisiae (strain ATCC 204508 / S288c) GN=RPS25B PE=1 SV=1|40S ribosomal protein S25−A OS=Saccharomyces cerevisiae (strain ATCC 204508 / S288c)

RPS26A − average.top3: 7.86689590909091 − max.qupm: 5

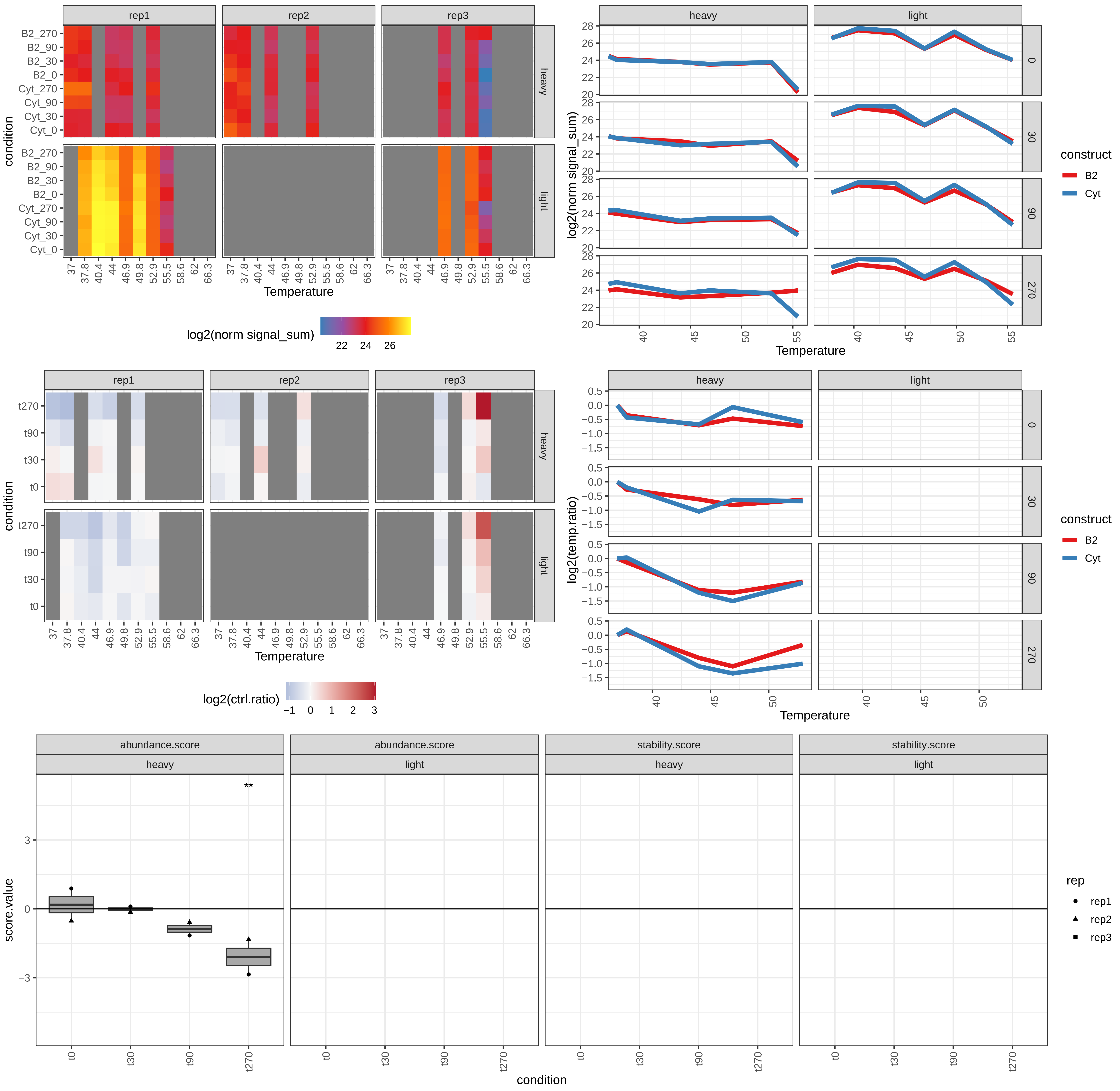

P39938 − 40S ribosomal protein S26−A OS=Saccharomyces cerevisiae (strain ATCC 204508 / S288c) GN=RPS26A PE=1 SV=1

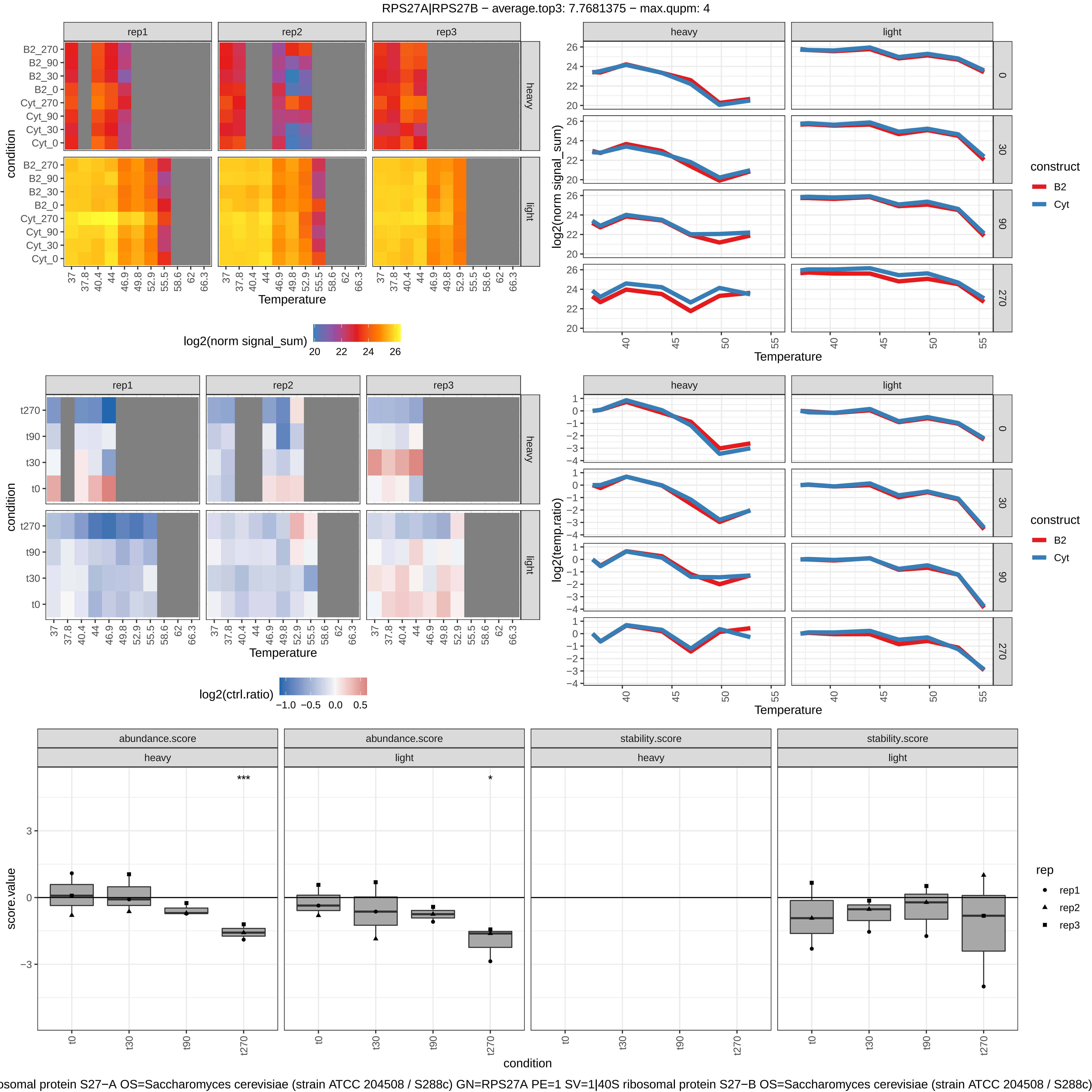

RPS27A|RPS27B − average.top3: 7.7681375 − max.qupm: 4
rep1
rep2
rep3
heavy
light
B2_270
B2_90
B2_30
B2_0
Cyt_270
Cyt_90
Cyt_30
Cyt_0
condition
37
37.8
40.4
44
46.9
49.8
52.9
55.5
58.6
62
66.3
Temperature
log2(norm signal_sum)
20
22
24
26
log2(norm signal_sum)
0
30
90
270
40
45
50
55
construct
B2
Cyt
t270
t90
t30
t0
log2(ctrl.ratio)
−1.0
−0.5
0.0
0.5
log2(temp.ratio)
abundance.score
stability.score
***
*
score.value
3
0
−3
rep
rep1
rep2
rep3
condition
osomal protein S27−A OS=Saccharomyces cerevisiae (strain ATCC 204508 / S288c) GN=RPS27A PE=1 SV=1|40S ribosomal protein S27−B OS=Saccharomyces cerevisiae (strain ATCC 204508 / S288c)

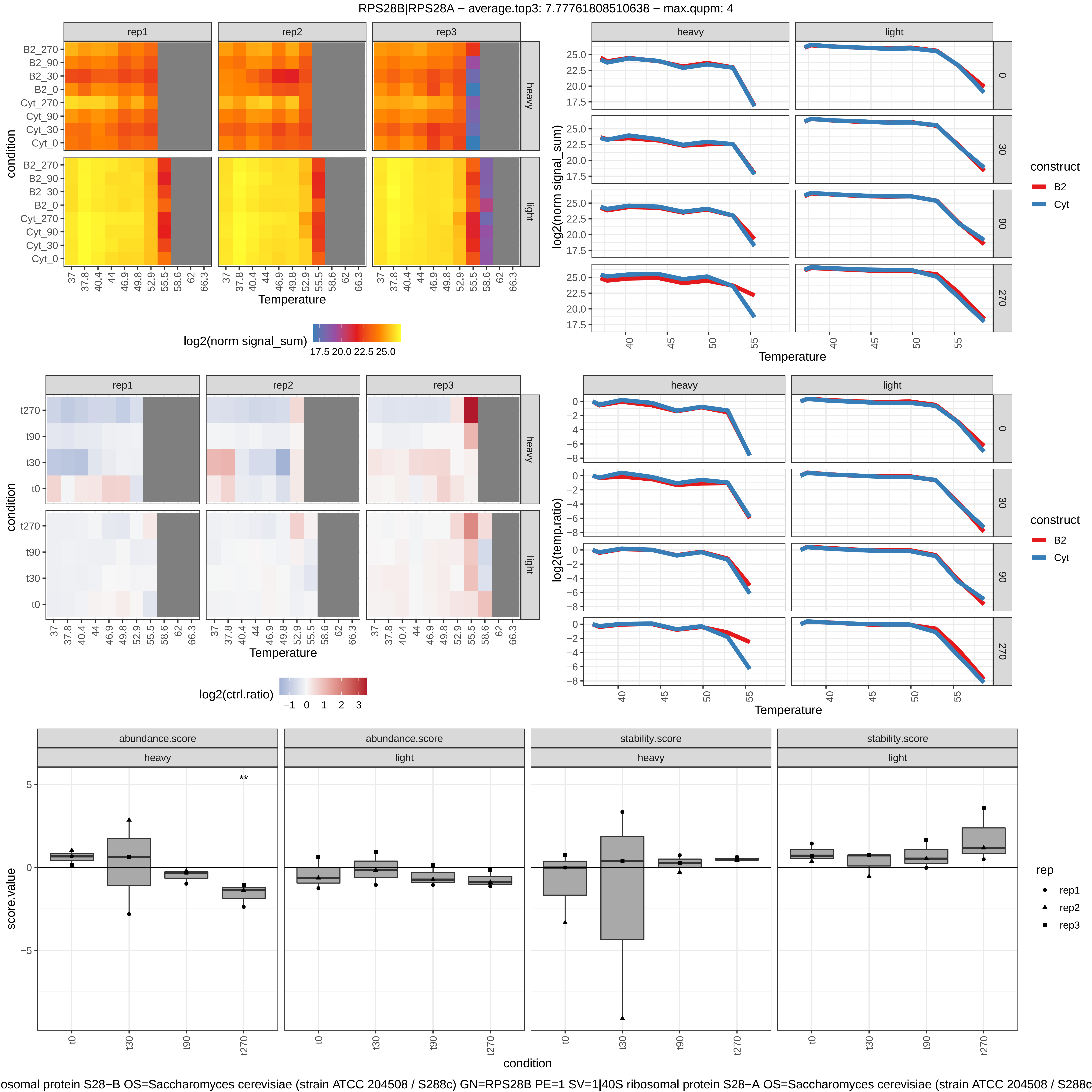
RPS28B|RPS28A − average.top3: 7.77761808510638 − max.qupm: 4
rep1
rep2
rep3
heavy
light
condition
B2_270
B2_90
B2_30
B2_0
Cyt_270
Cyt_90
Cyt_30
Cyt_0
Temperature
log2(norm signal_sum)
17.5 20.0 22.5 25.0
log2(norm signal_sum)
construct
B2
Cyt
t270
t90
t30
t0
log2(ctrl.ratio)
−1 0 1 2 3
log2(temp.ratio)
abundance.score
stability.score
score.value
**
rep
rep1
rep2
rep3
condition
osomal protein S28−B OS=Saccharomyces cerevisiae (strain ATCC 204508 / S288c) GN=RPS28B PE=1 SV=1|40S ribosomal protein S28−A OS=Saccharomyces cerevisiae (strain ATCC 204508 / S288c

RPS29A − average.top3: 7.72290095238095 − max.qupm: 5

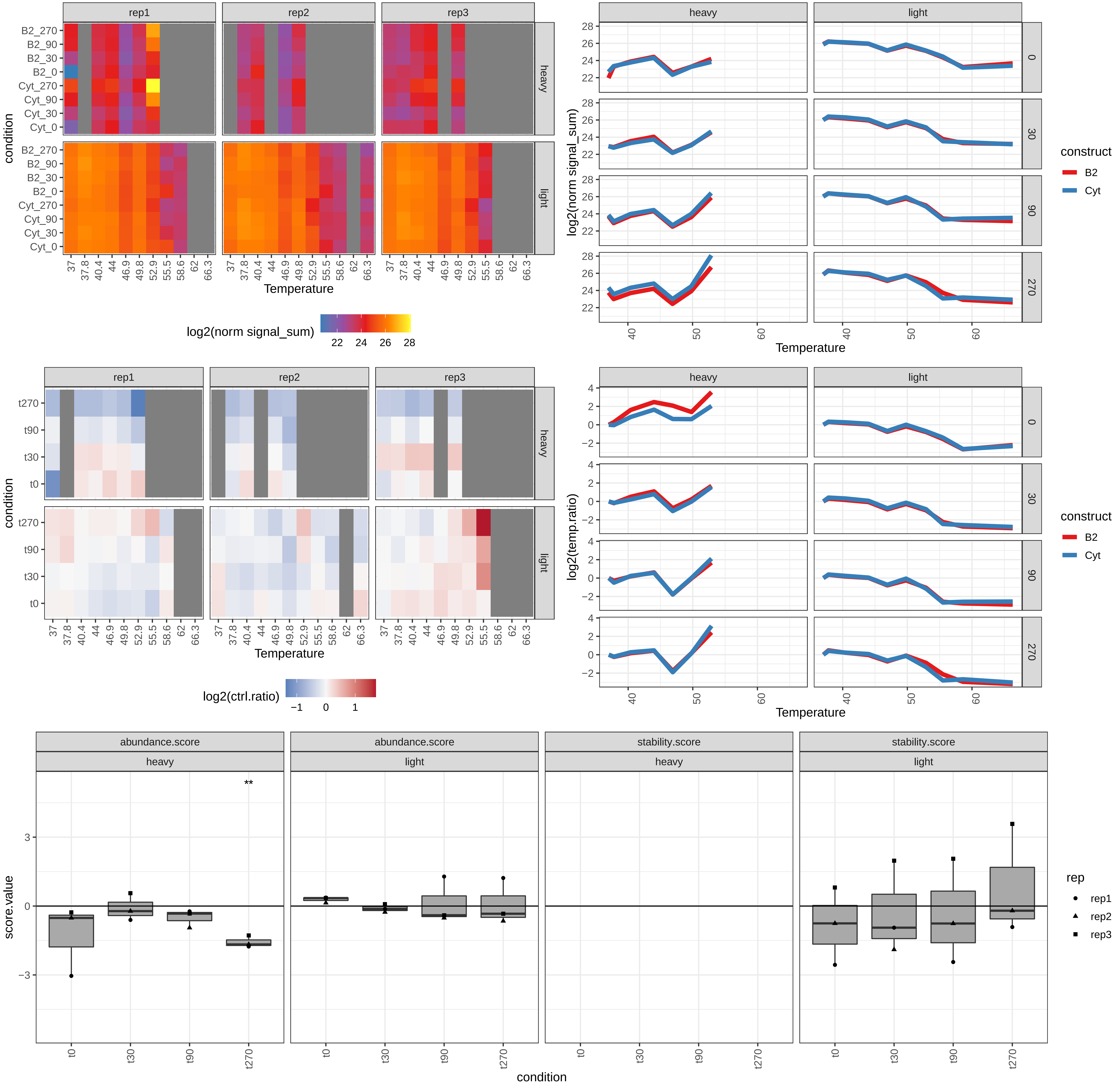

P41057 − 40S ribosomal protein S29−A OS=Saccharomyces cerevisiae (strain ATCC 204508 / S288c) GN=RPS29A PE=1 SV=3

RPS29B − average.top3: 7.68711973684211 − max.qupm: 5

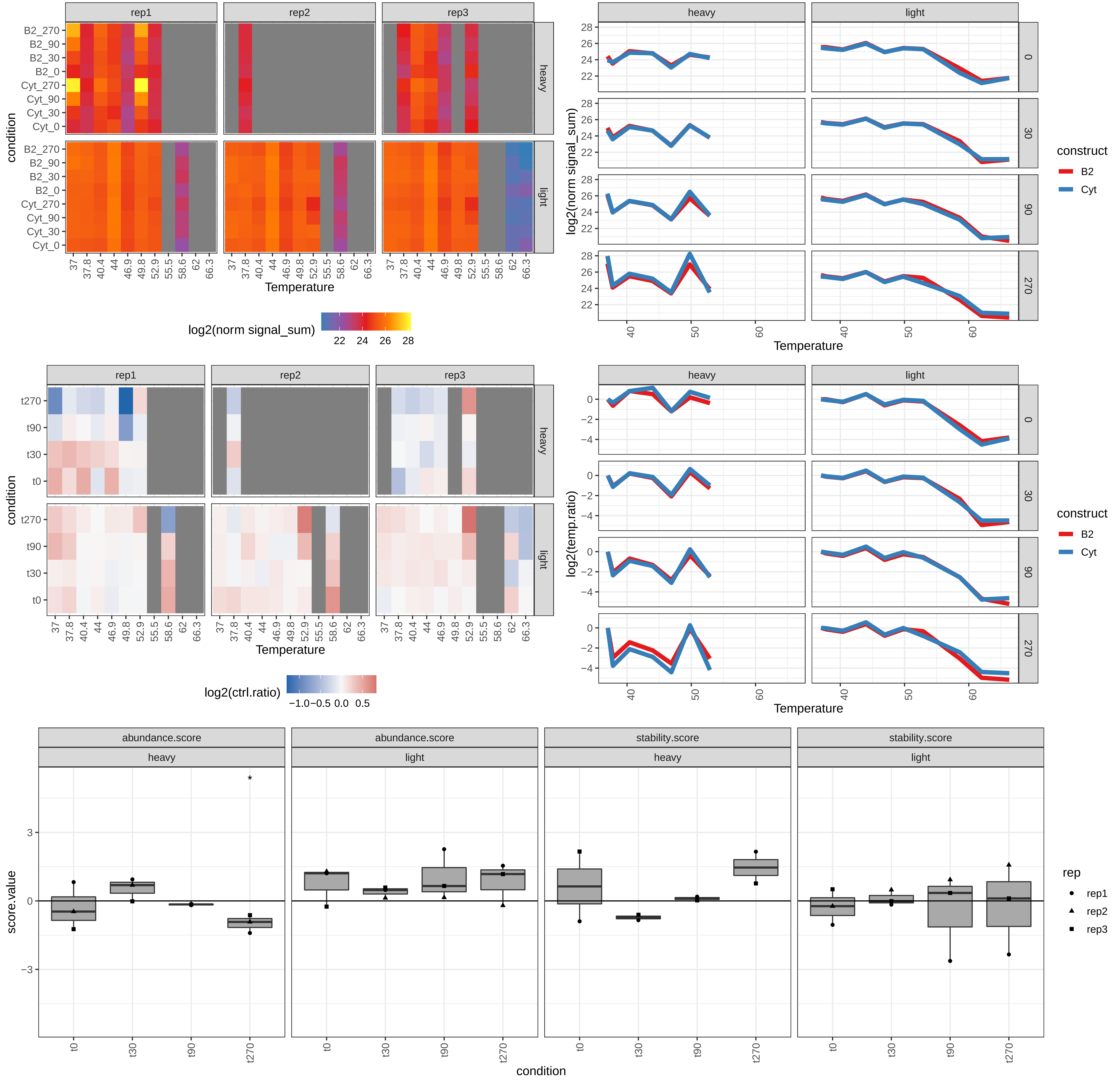

P41058 − 40S ribosomal protein S29−B OS=Saccharomyces cerevisiae (strain ATCC 204508 / S288c) GN=RPS29B PE=1 SV=3

RPS3 − average.top3: 8.13059518518519 − max.qupm: 19

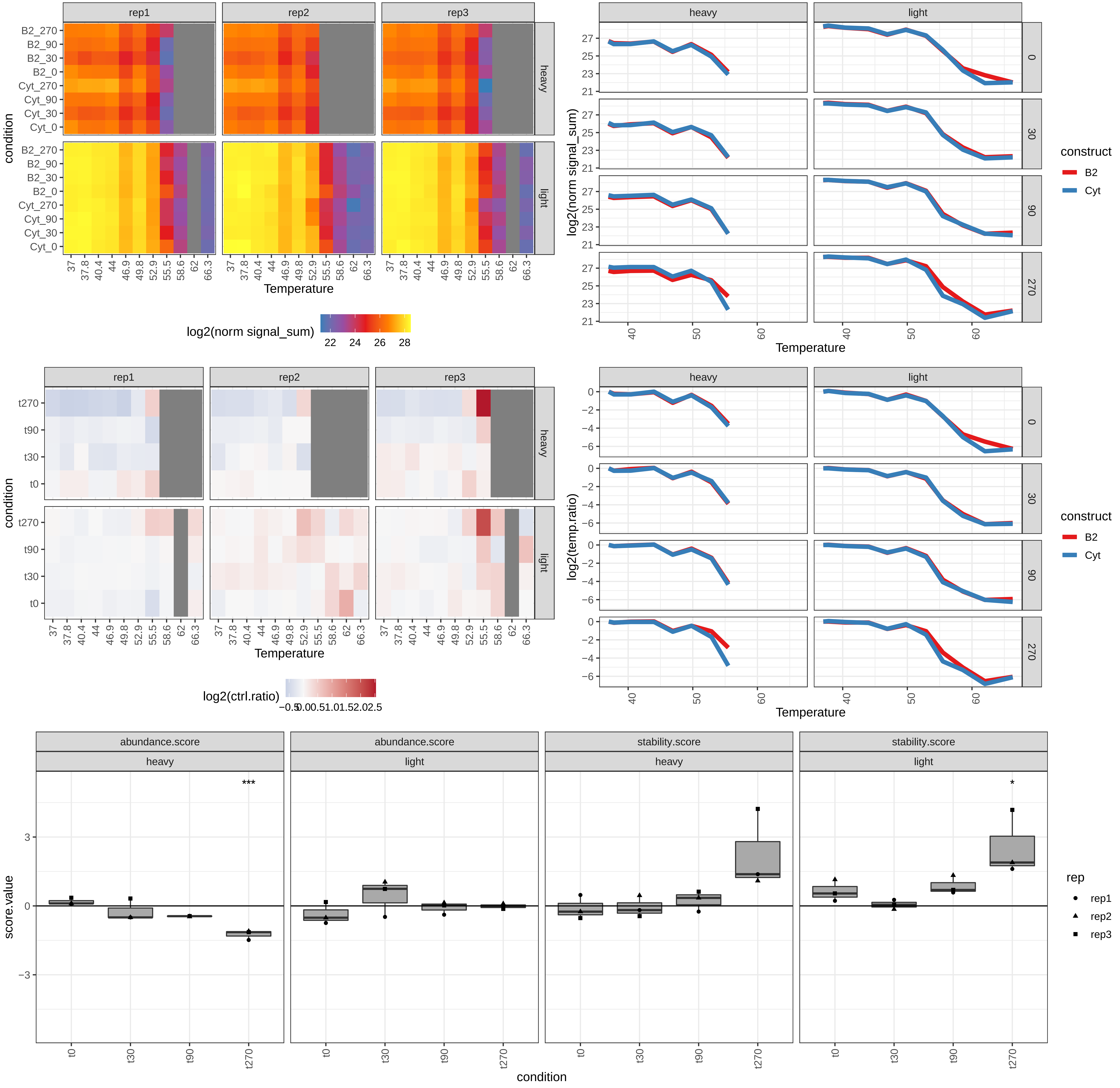

P05750 − 40S ribosomal protein S3 OS=Saccharomyces cerevisiae (strain ATCC 204508 / S288c) GN=RPS3 PE=1 SV=5

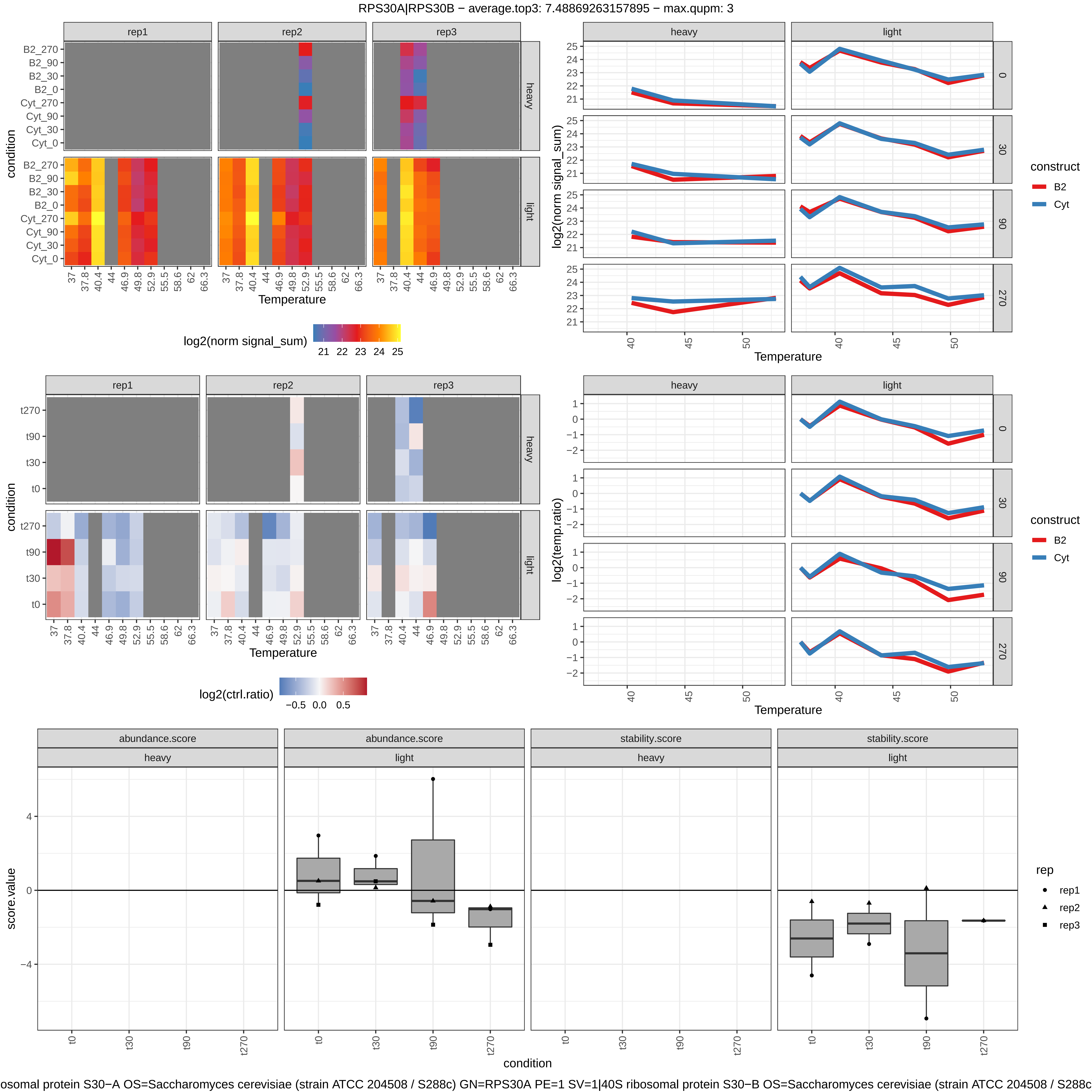
RPS30A|RPS30B − average.top3: 7.48869263157895 − max.qupm: 3
rep1
rep2
rep3
heavy
light
B2_270
B2_90
B2_30
B2_0
Cyt_270
Cyt_90
Cyt_30
Cyt_0
condition
37
37.8
40.4
44
46.9
49.8
52.9
55.5
58.6
62
66.3
Temperature
log2(norm signal_sum)
21 22 23 24 25
log2(norm signal_sum)
0
30
90
270
40 45 50
construct
B2
Cyt
t270
t90
t30
t0
log2(ctrl.ratio)
−0.5 0.0 0.5
log2(temp.ratio)
abundance.score
stability.score
score.value
4
0
−4
condition
rep
rep1
rep2
rep3
osomal protein S30−A OS=Saccharomyces cerevisiae (strain ATCC 204508 / S288c) GN=RPS30A PE=1 SV=1|40S ribosomal protein S30−B OS=Saccharomyces cerevisiae (strain ATCC 204508 / S288c

RPS31 − average.top3: 7.90635159090909 − max.qupm: 13

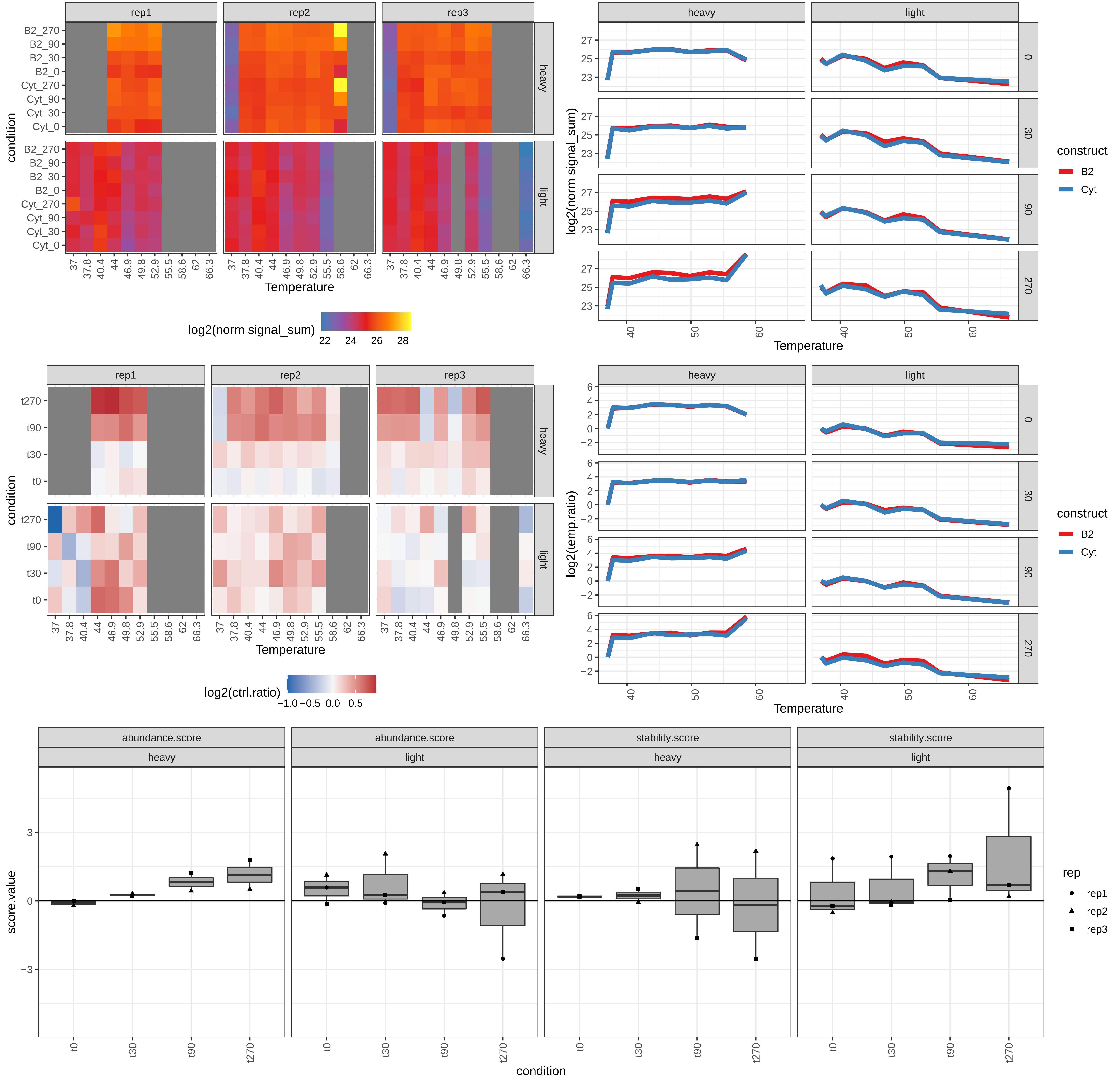

P05759 − Ubiquitin−40S ribosomal protein S31 OS=Saccharomyces cerevisiae (strain ATCC 204508 / S288c) GN=RPS31 PE=1 SV=3

RPS4A|RPS4B − average.top3: 8.22483745454545 − max.qupm: 23

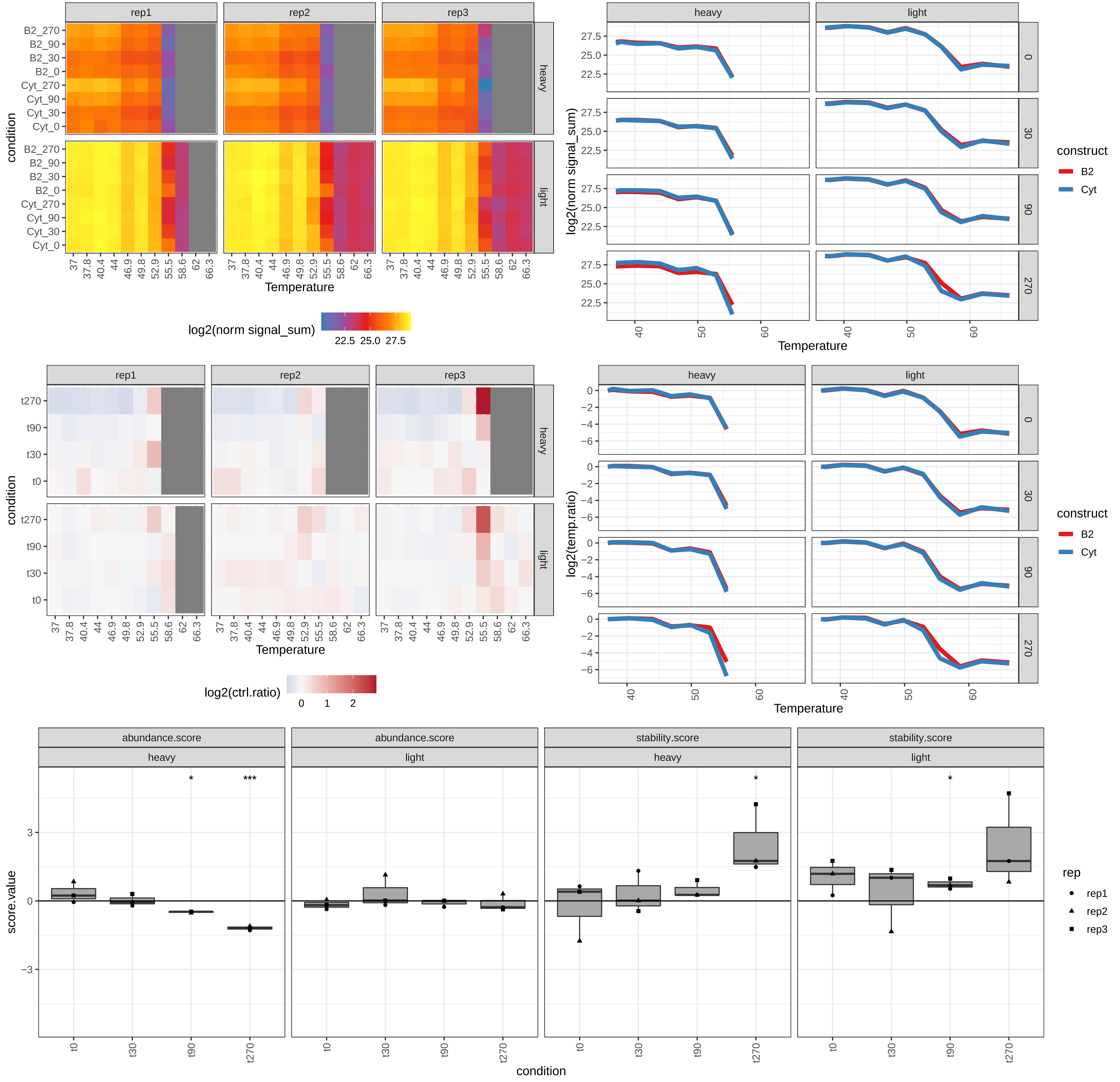

ribosomal protein S4−A OS=Saccharomyces cerevisiae (strain ATCC 204508 / S288c) GN=RPS4A PE=1 SV=1|40S ribosomal protein S4−B OS=Saccharomyces cerevisiae (strain ATCC 204508 / S288c)

RPS5 − average.top3: 8.14318098039216 − max.qupm: 12

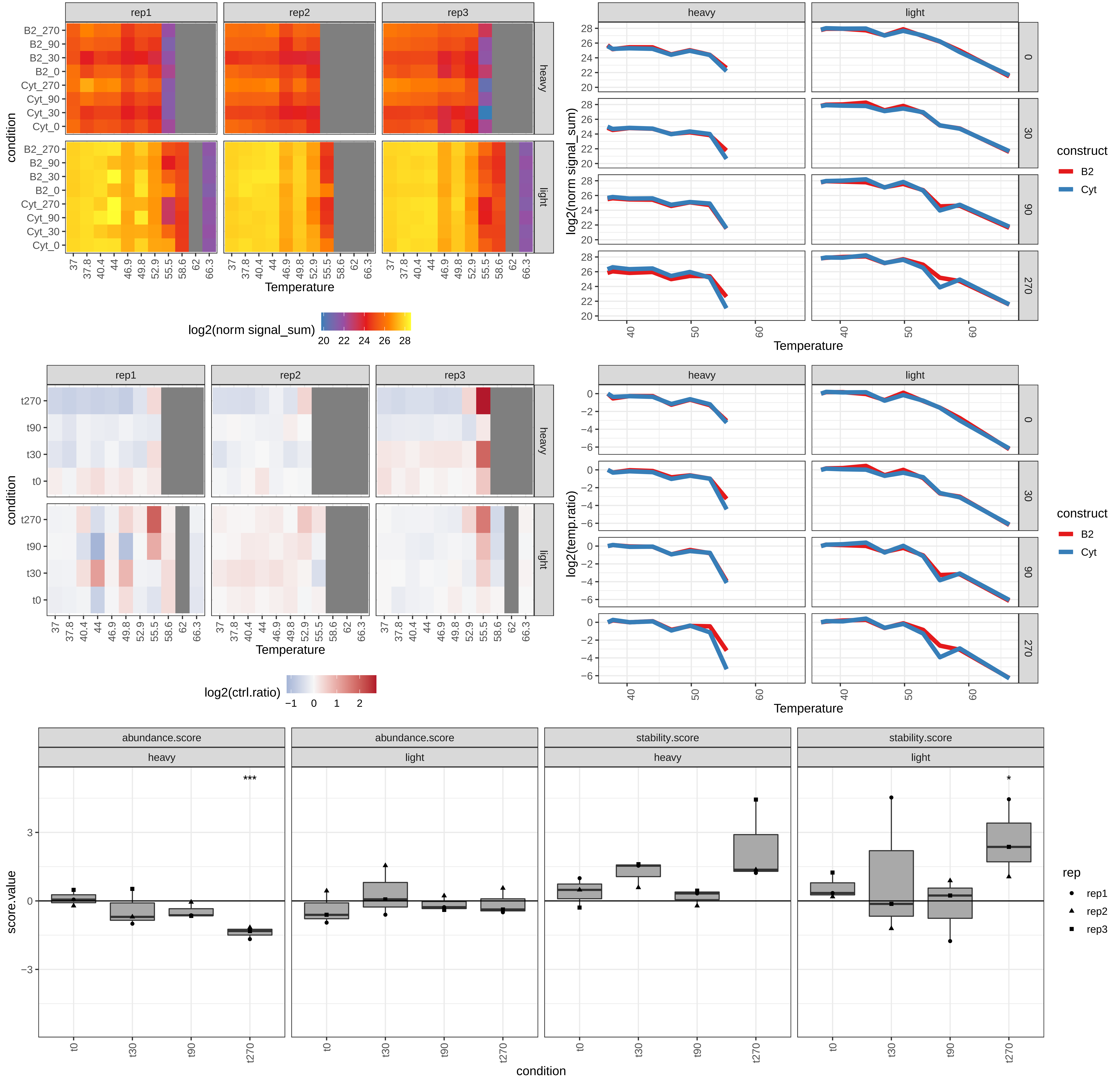

P26783 − 40S ribosomal protein S5 OS=Saccharomyces cerevisiae (strain ATCC 204508 / S288c) GN=RPS5 PE=1 SV=3

RPS6A|RPS6B − average.top3: 8.05033384615385 − max.qupm: 13

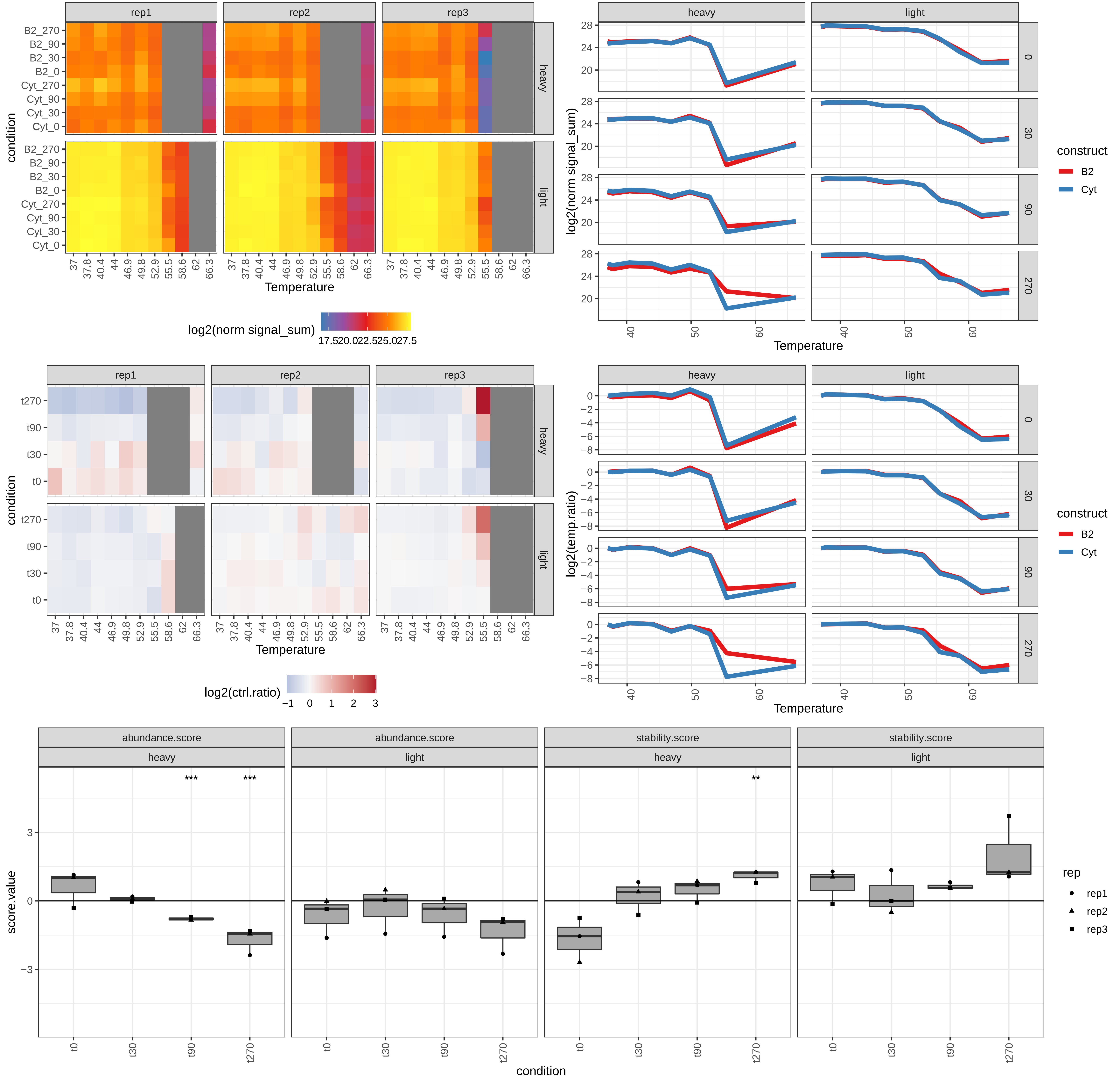

ribosomal protein S6−A OS=Saccharomyces cerevisiae (strain ATCC 204508 / S288c) GN=RPS6A PE=1 SV=1|40S ribosomal protein S6−B OS=Saccharomyces cerevisiae (strain ATCC 204508 / S288c)

RPS7A − average.top3: 7.71303978723404 − max.qupm: 5

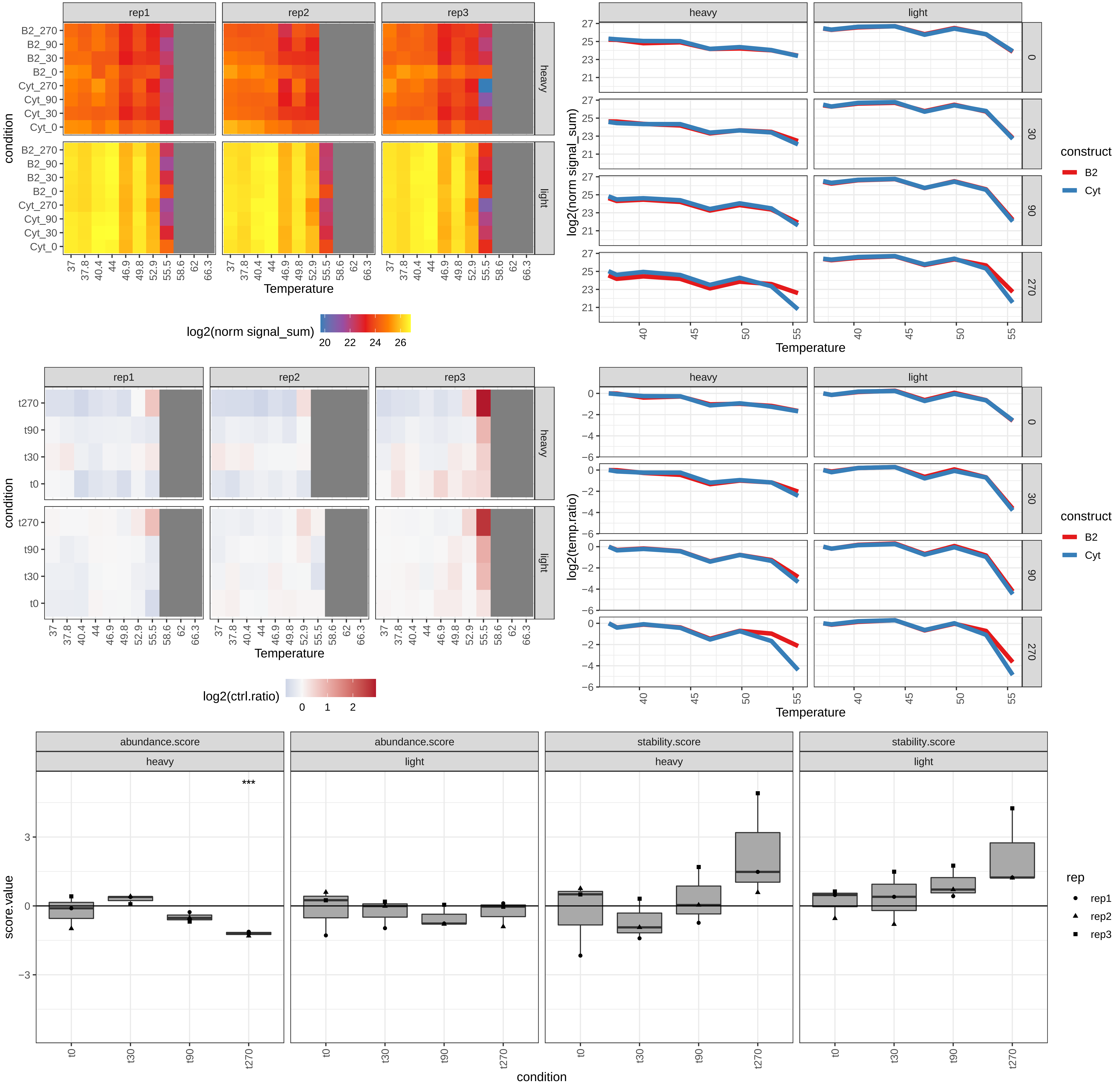

P26786 − 40S ribosomal protein S7−A OS=Saccharomyces cerevisiae (strain ATCC 204508 / S288c) GN=RPS7A PE=1 SV=4

RPS7B − average.top3: 7.60602355555556 − max.qupm: 7

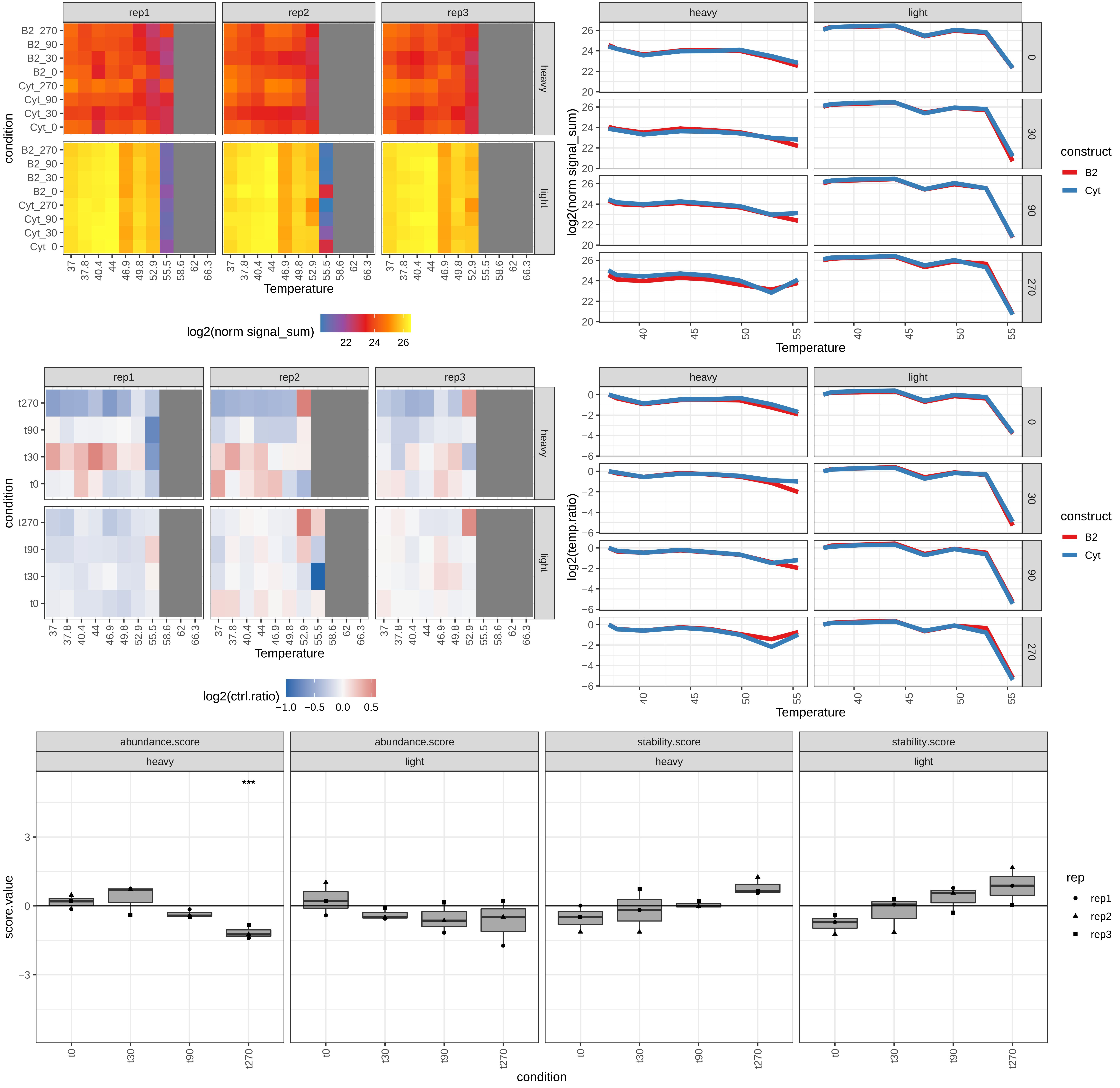

P48164 − 40S ribosomal protein S7−B OS=Saccharomyces cerevisiae (strain ATCC 204508 / S288c) GN=RPS7B PE=1 SV=1

RPS8A|RPS8B − average.top3: 7.95725875 − max.qupm: 9

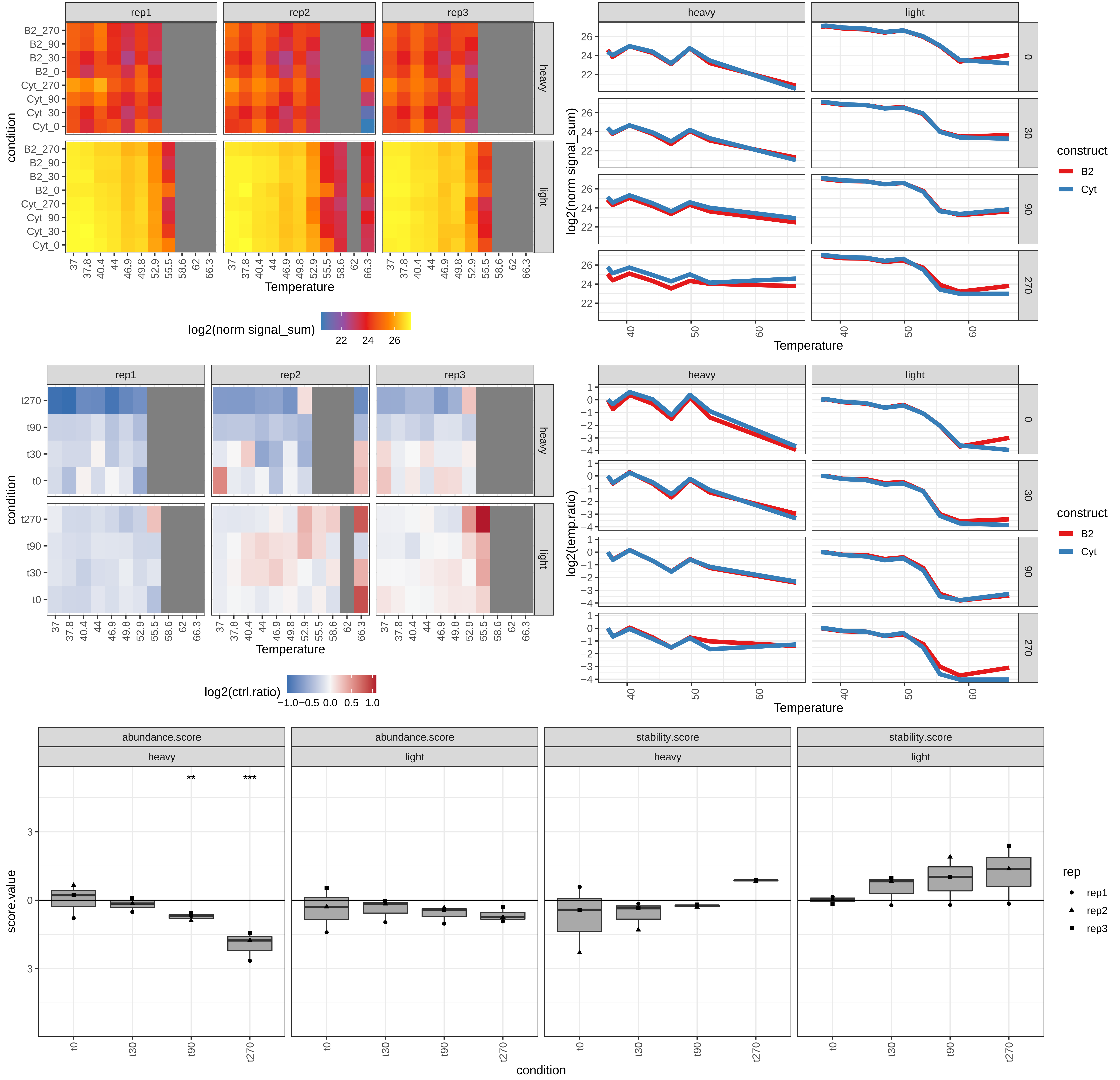

ribosomal protein S8−A OS=Saccharomyces cerevisiae (strain ATCC 204508 / S288c) GN=RPS8A PE=1 SV=1|40S ribosomal protein S8−B OS=Saccharomyces cerevisiae (strain ATCC 204508 / S288c)

RPT1 − average.top3: 7.07277166666667 − max.qupm: 17

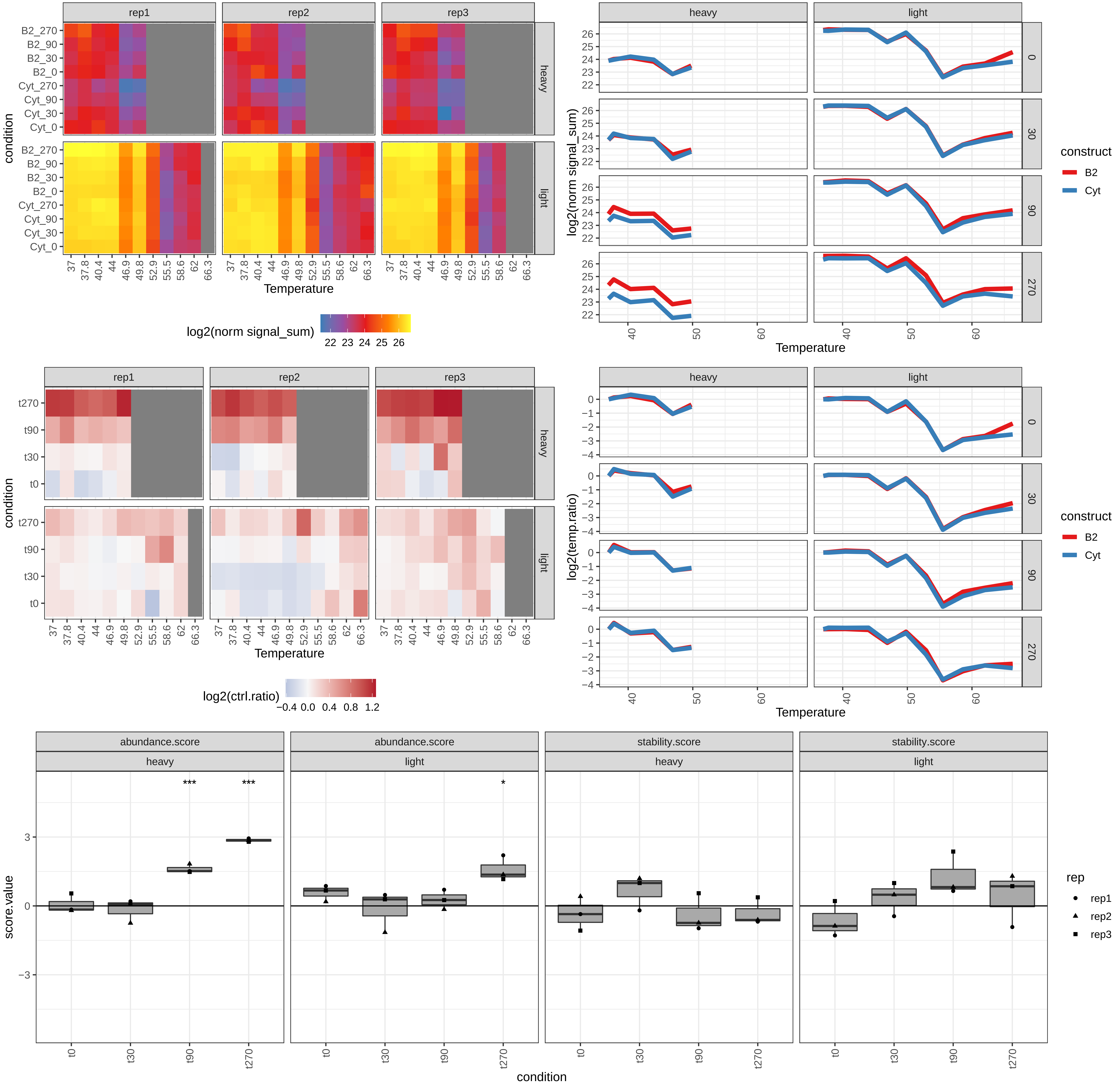

P33299 − 26S protease regulatory subunit 7 homolog OS=Saccharomyces cerevisiae (strain ATCC 204508 / S288c) GN=RPT1 PE=1 SV=1

RPT2 − average.top3: 7.02855282051282 − max.qupm: 12

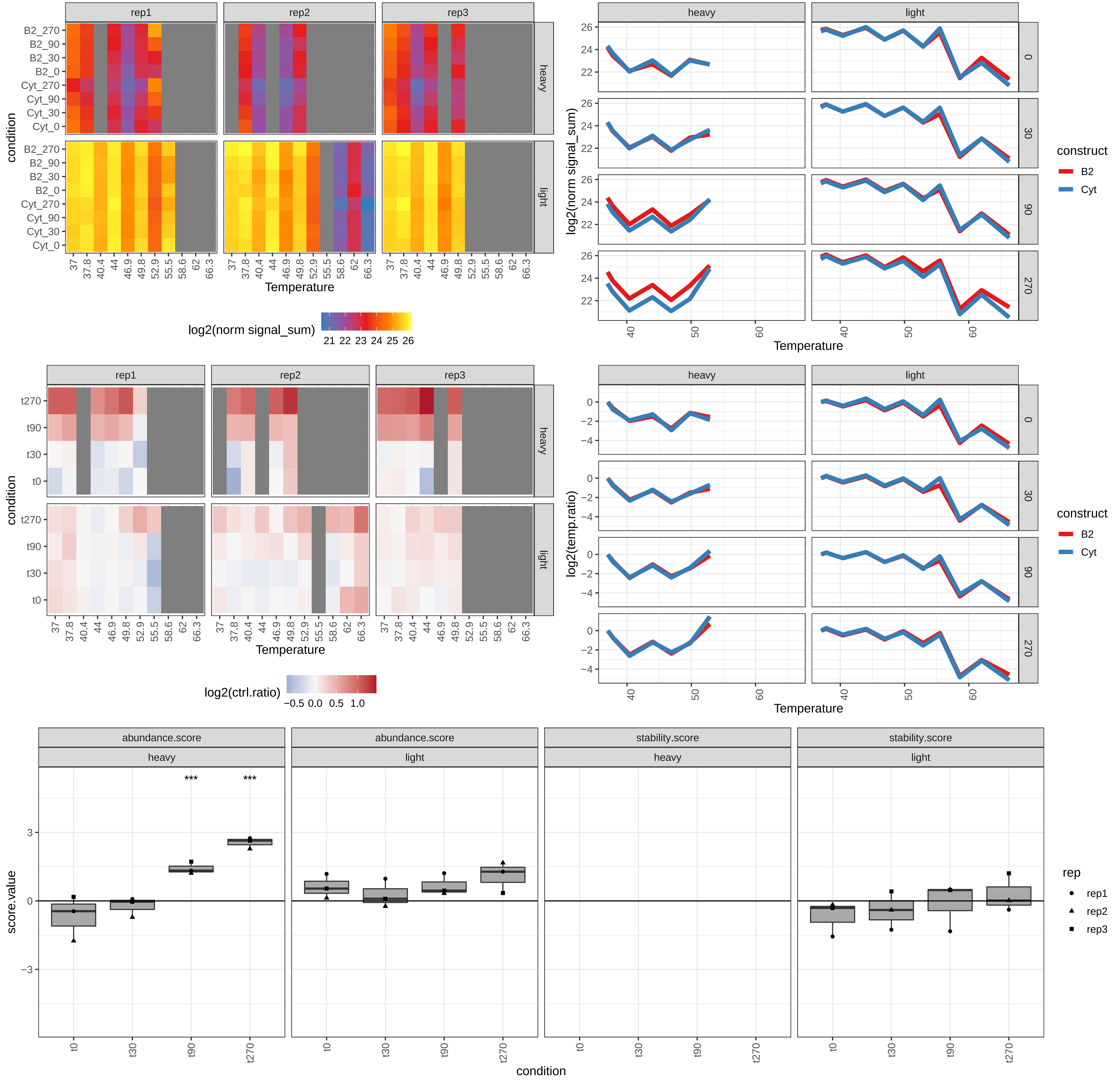

P40327 − 26S protease regulatory subunit 4 homolog OS=Saccharomyces cerevisiae (strain ATCC 204508 / S288c) GN=RPT2 PE=1 SV=3

RPT3 − average.top3: 7.10054277777778 − max.qupm: 12

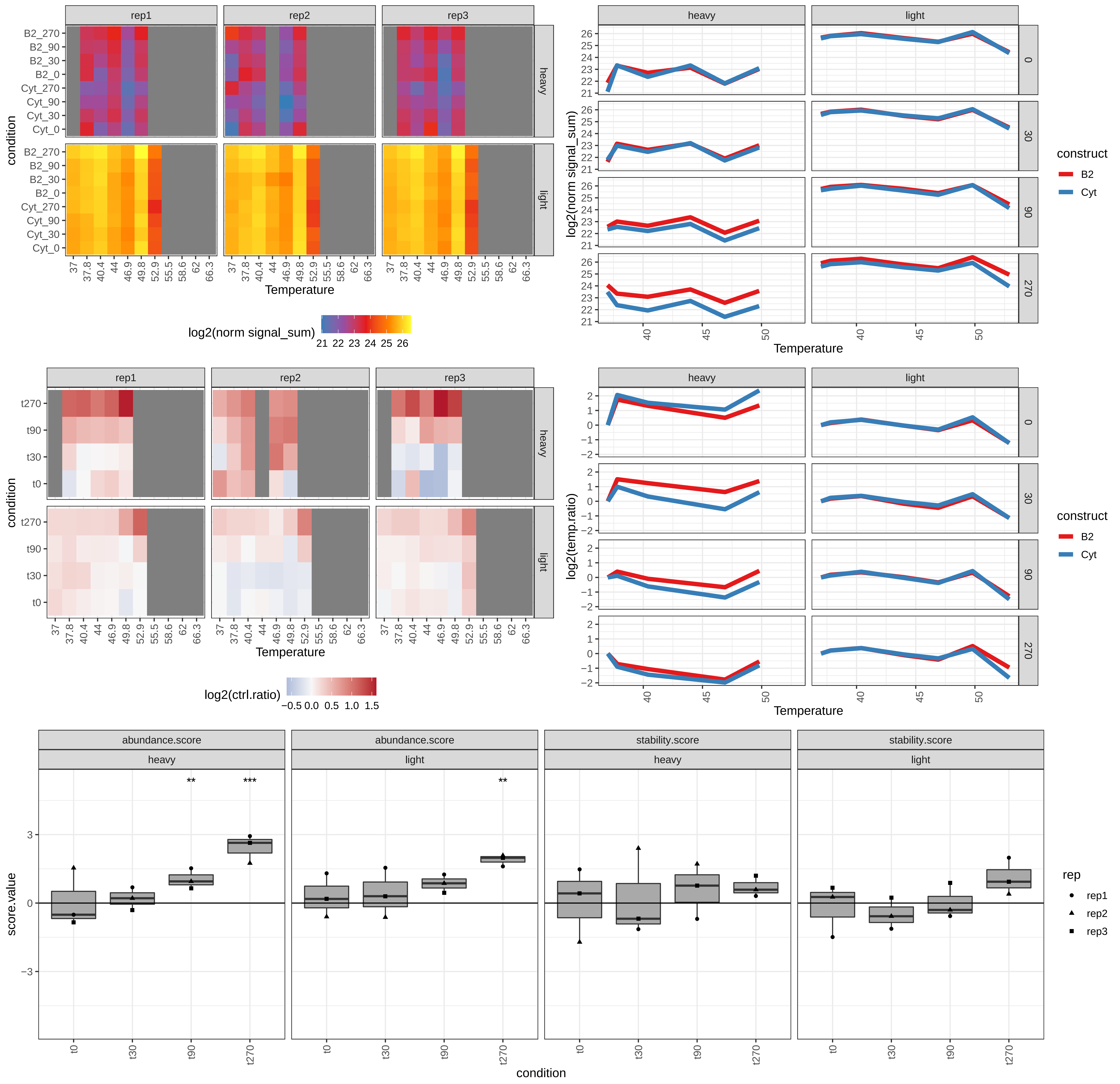

P33298 − 26S protease regulatory subunit 6B homolog OS=Saccharomyces cerevisiae (strain ATCC 204508 / S288c) GN=RPT3 PE=1 SV=2

RPT4 − average.top3: 7.17831133333333 − max.qupm: 13

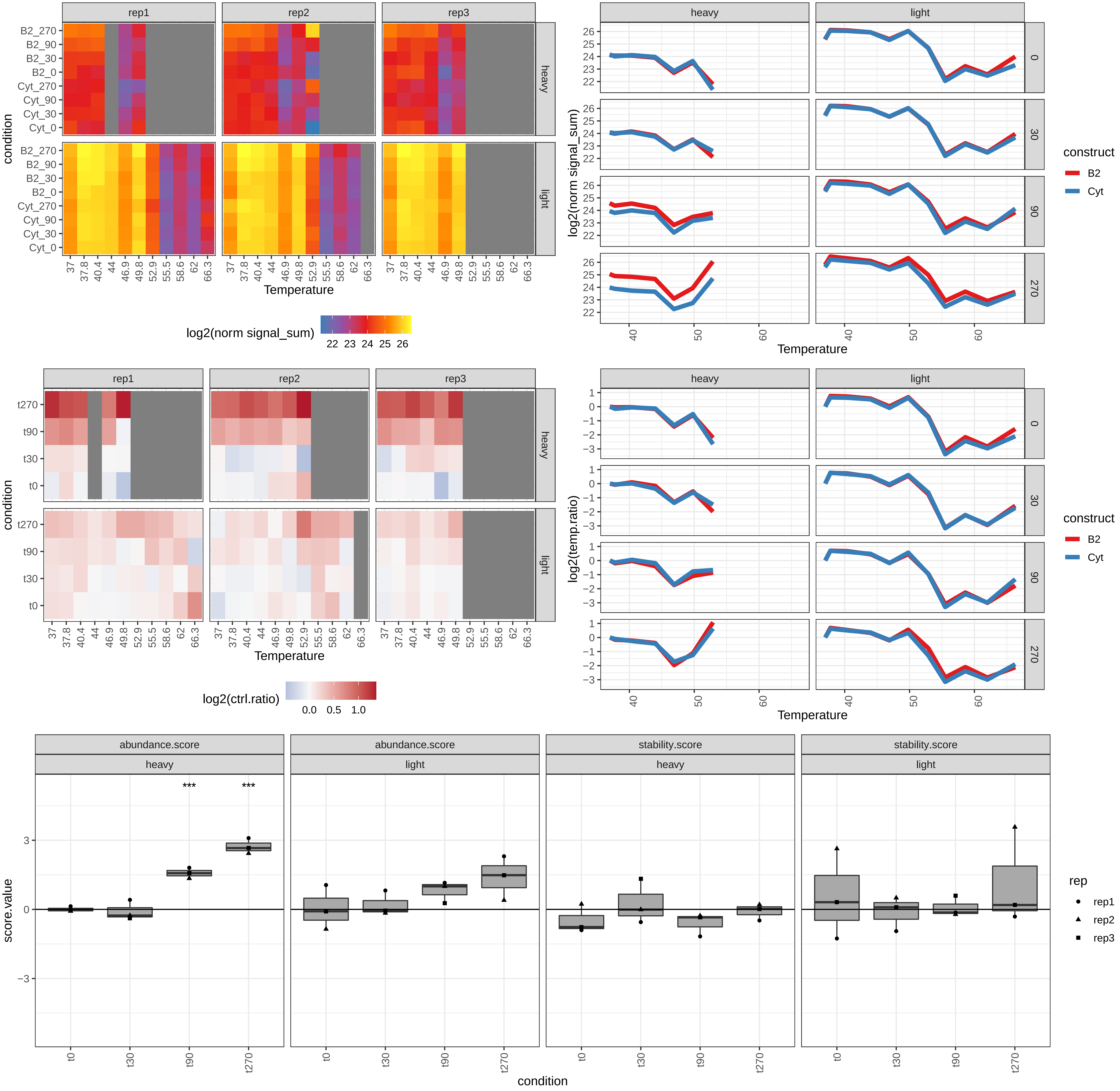

P53549 − 26S protease subunit RPT4 OS=Saccharomyces cerevisiae (strain ATCC 204508 / S288c) GN=RPT4 PE=1 SV=4

RPT5 − average.top3: 6.91516475 − max.qupm: 11

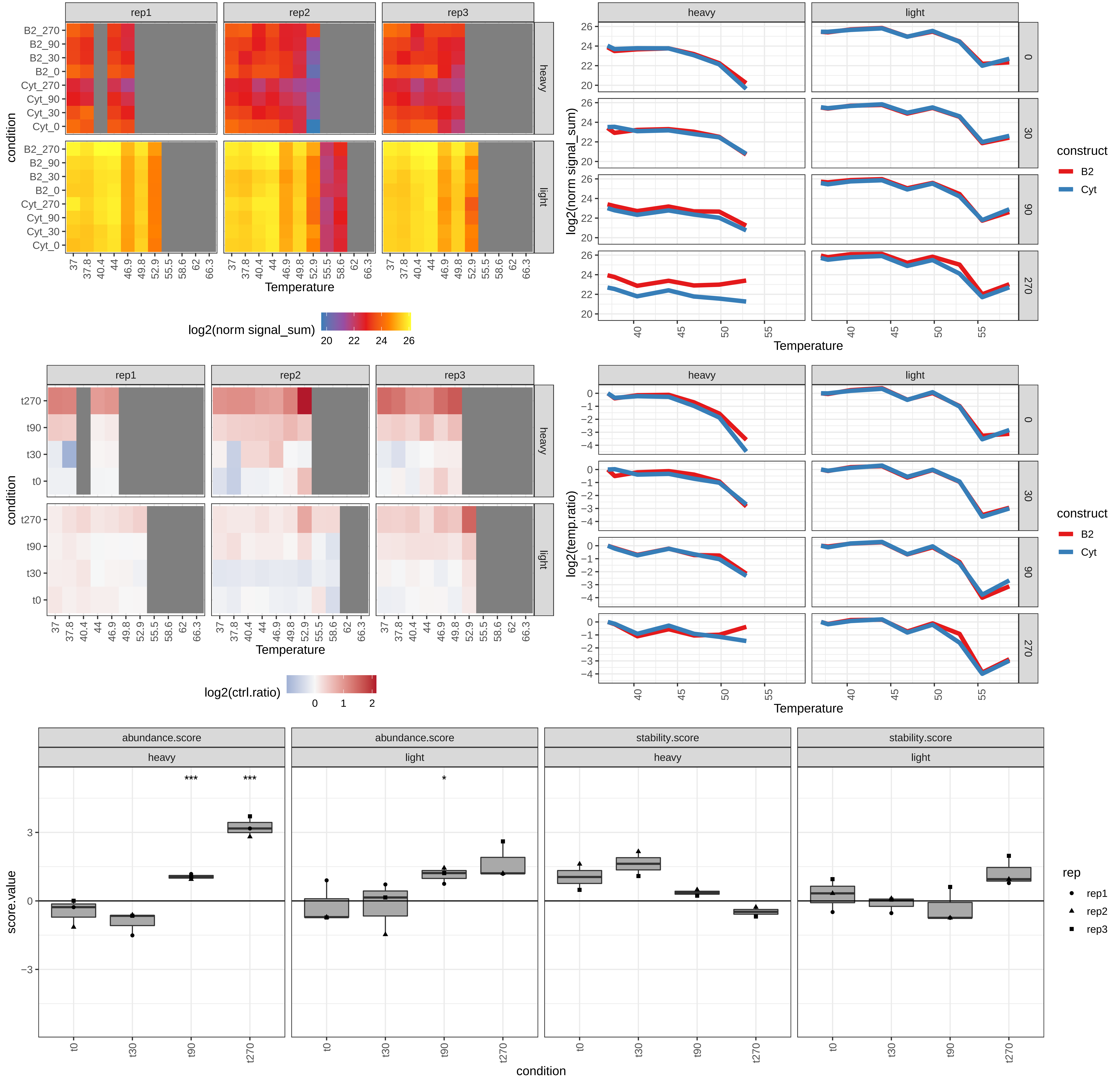

P33297 − 26S protease regulatory subunit 6A OS=Saccharomyces cerevisiae (strain ATCC 204508 / S288c) GN=RPT5 PE=1 SV=3

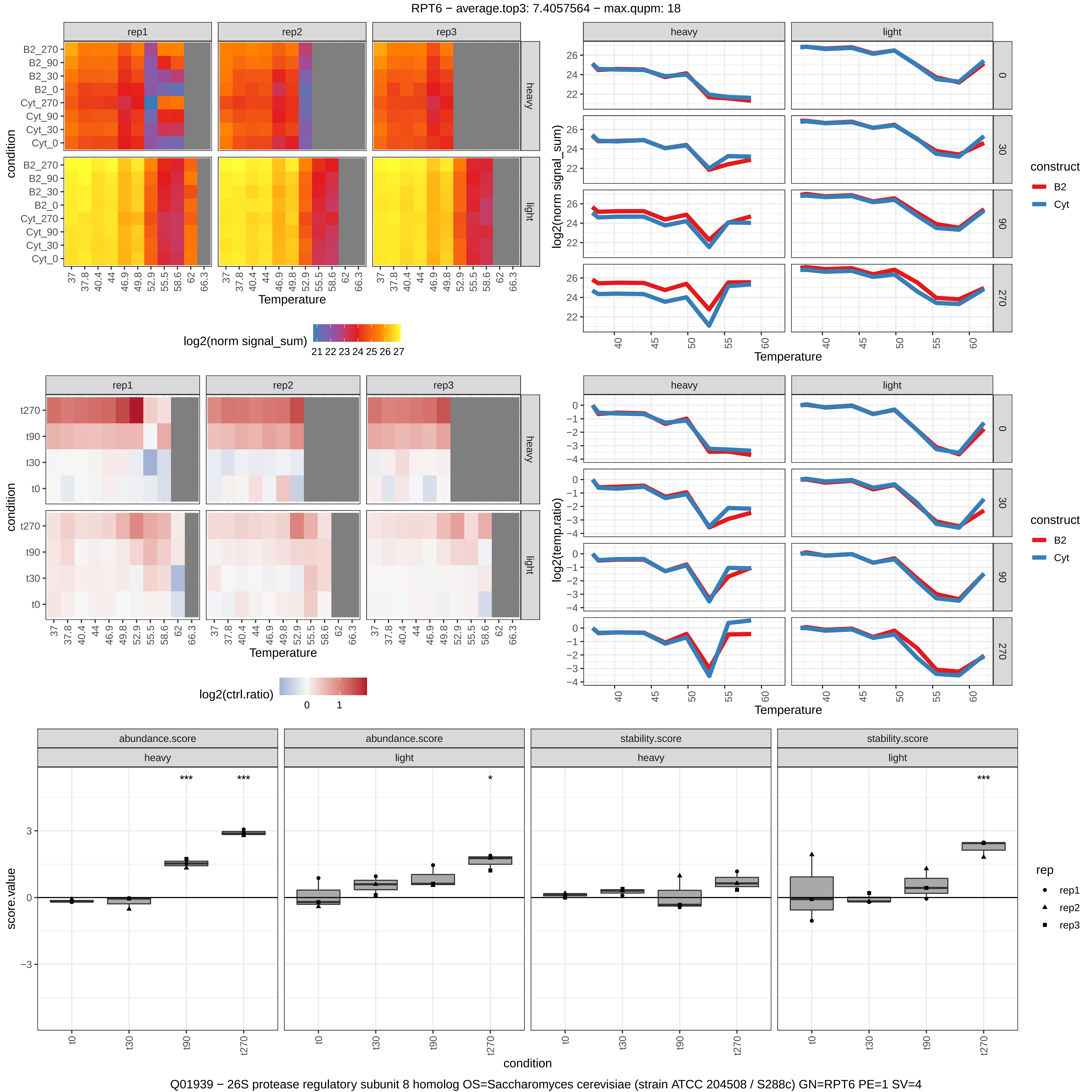

RPT6 − average.top3: 7.4057564 − max.qupm: 18
rep1
rep2
rep3
heavy
light
condition
B2_270
B2_90
B2_30
B2_0
Cyt_270
Cyt_90
Cyt_30
Cyt_0
Temperature
log2(norm signal_sum)
21 22 23 24 25 26 27
construct
B2
Cyt
t270
t90
t30
t0
log2(ctrl.ratio)
log2(temp.ratio)
abundance.score
stability.score
score.value
rep
rep1
rep2
rep3
Q01939 − 26S protease regulatory subunit 8 homolog OS=Saccharomyces cerevisiae (strain ATCC 204508 / S288c) GN=RPT6 PE=1 SV=4

RQC1 − average.top3: 6.82874111111111 − max.qupm: 7

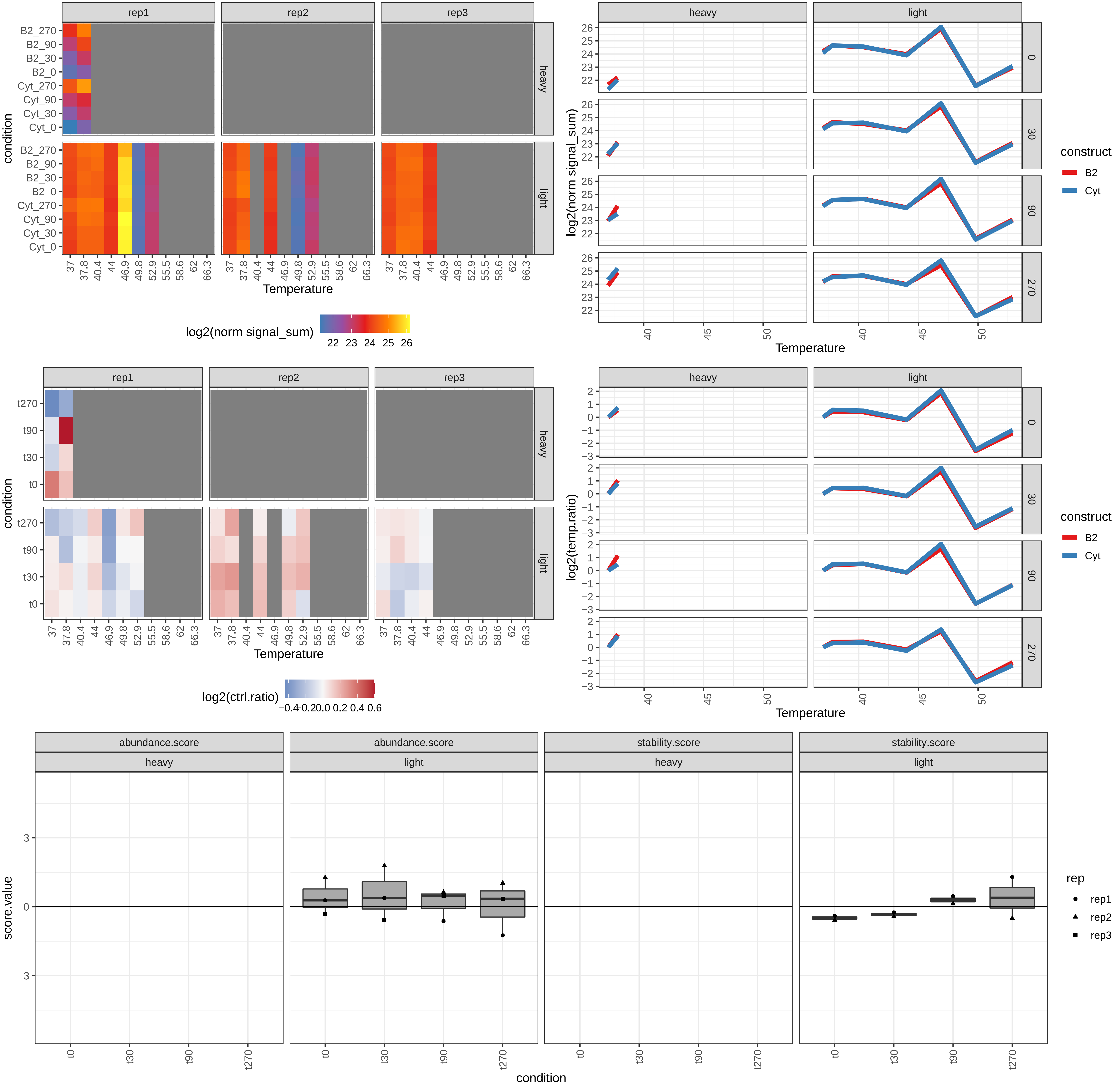

Q05468 − Ribosome quality control complex subunit 1 OS=Saccharomyces cerevisiae (strain ATCC 204508 / S288c) GN=RQC1 PE=1 SV=1

RQC2 − average.top3: 6.90393590909091 − max.qupm: 18

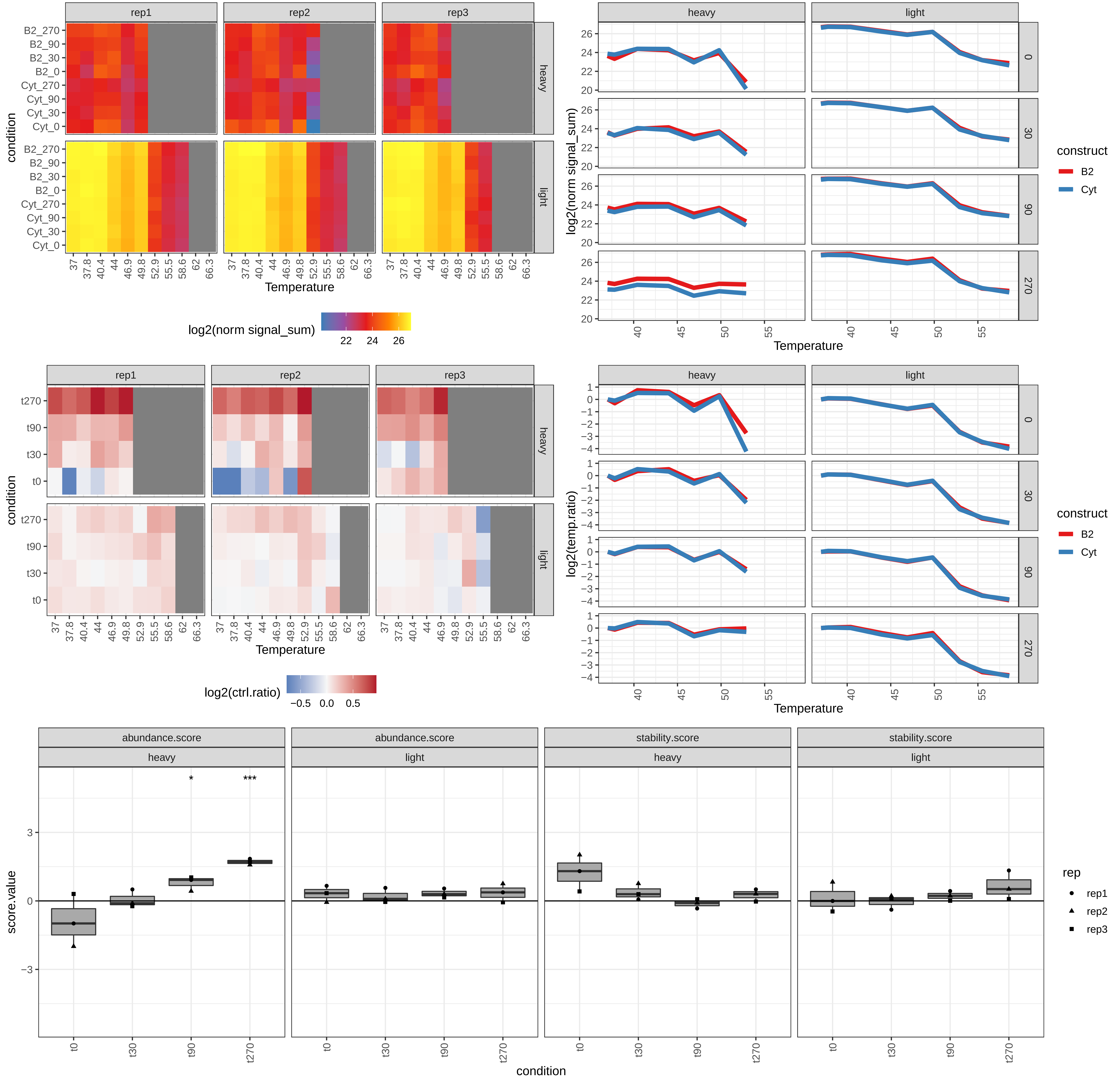

Q12532 − Ribosome quality control complex subunit 2 OS=Saccharomyces cerevisiae (strain ATCC 204508 / S288c) GN=RQC2 PE=1 SV=1

RRB1 − average.top3: 6.74688538461538 − max.qupm: 5

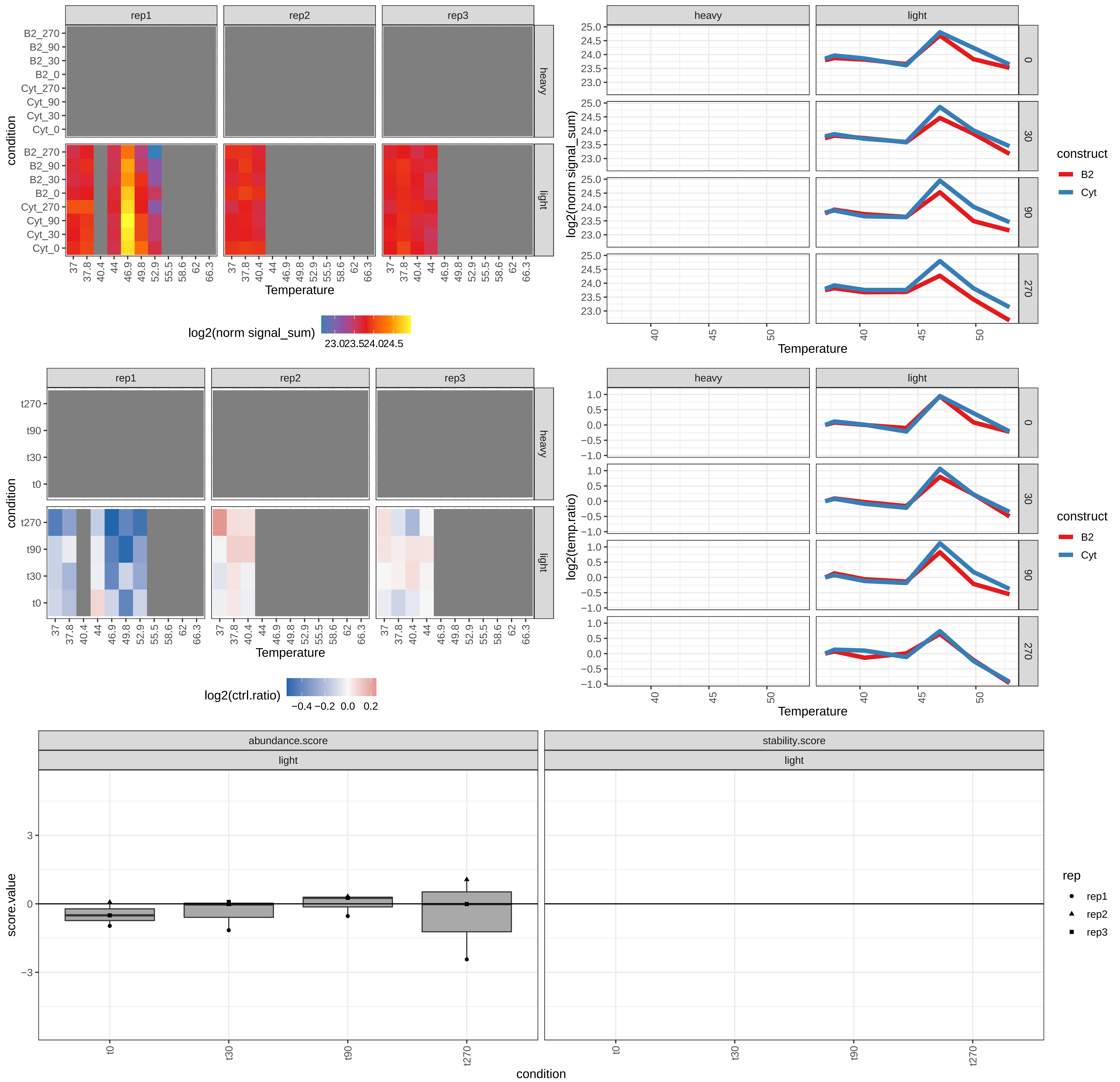

Q04225 − Ribosome assembly protein RRB1 OS=Saccharomyces cerevisiae (strain ATCC 204508 / S288c) GN=RRB1 PE=1 SV=1

RRF1 − average.top3: 6.76380210526316 − max.qupm: 7

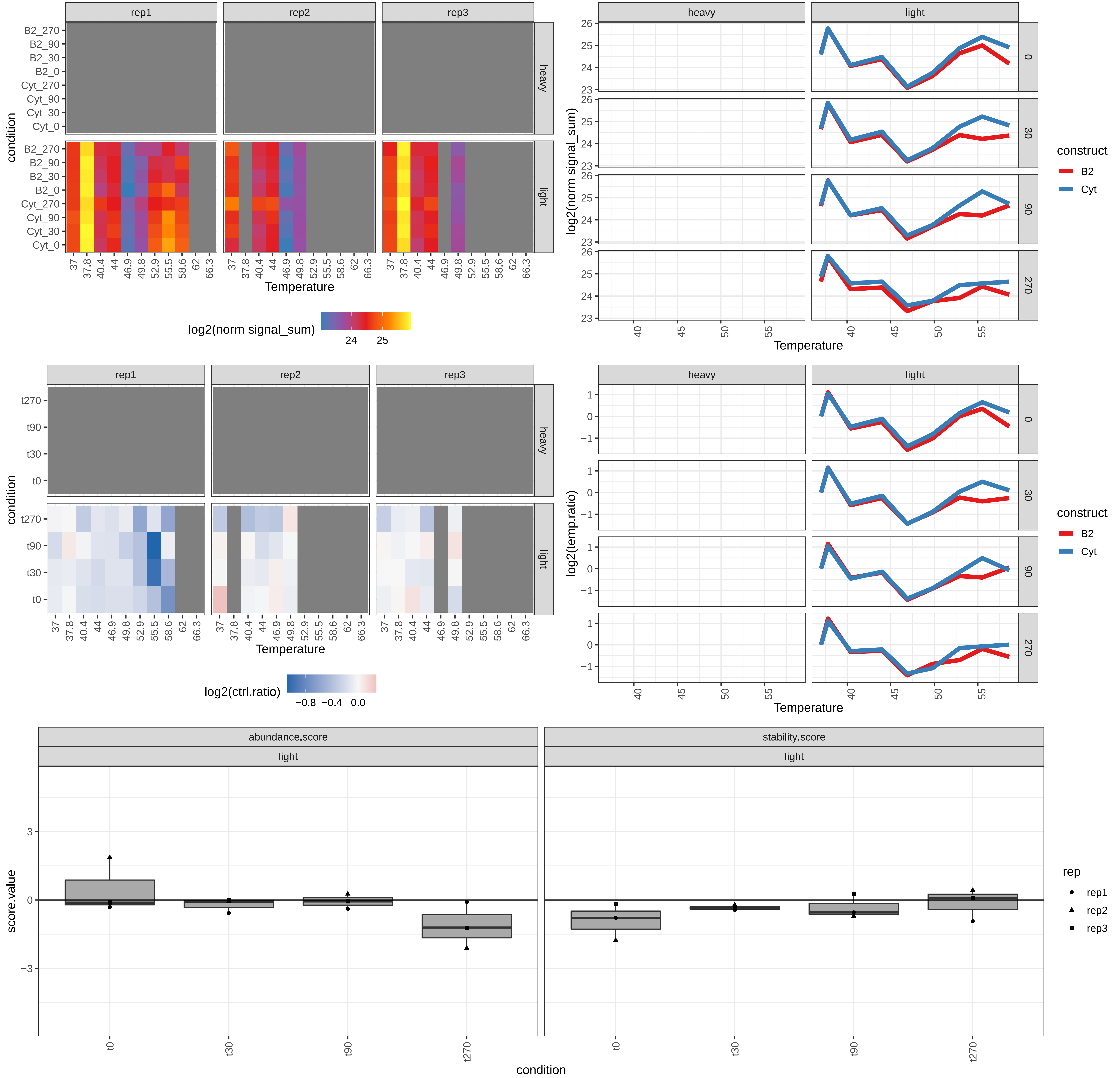

P38771 − Ribosome−recycling factor, mitochondrial OS=Saccharomyces cerevisiae (strain ATCC 204508 / S288c) GN=RRF1 PE=1 SV=1

RRP1 − average.top3: 6.96772954545455 − max.qupm: 8

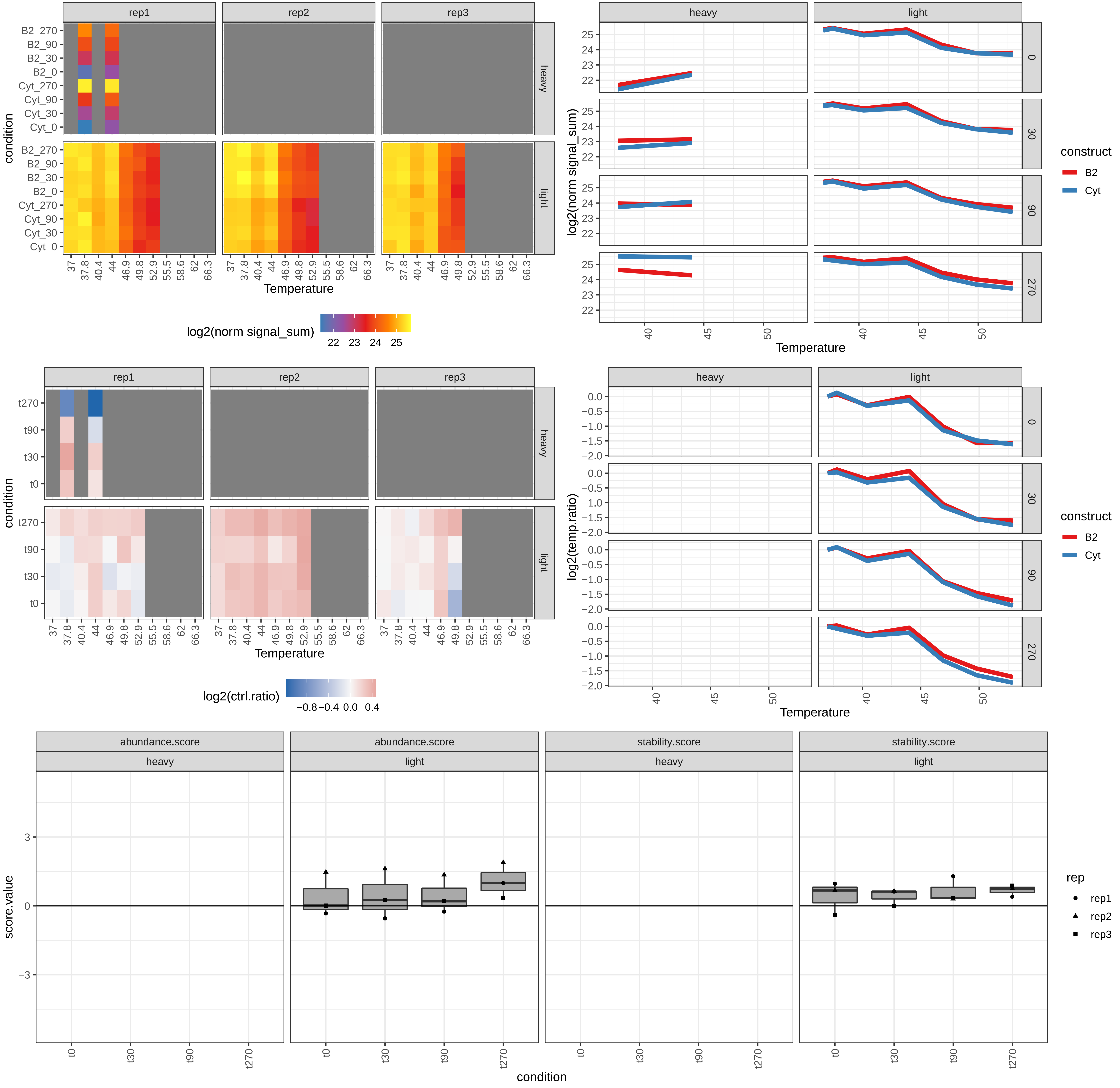

P35178 − Ribosomal RNA−processing protein 1 OS=Saccharomyces cerevisiae (strain ATCC 204508 / S288c) GN=RRP1 PE=1 SV=2

RRP12 − average.top3: 6.71509583333333 − max.qupm: 12

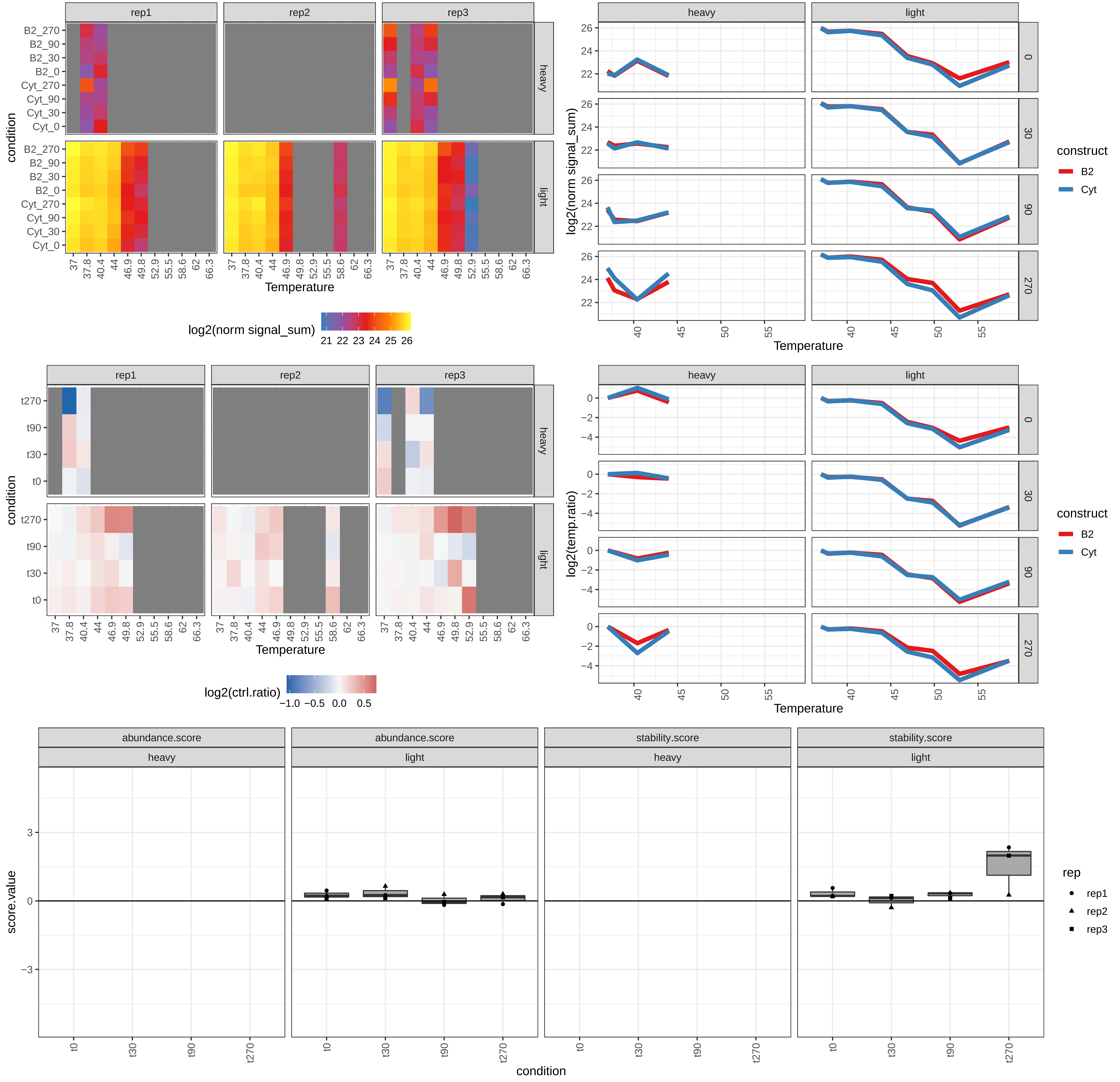

Q12754 − Ribosomal RNA−processing protein 12 OS=Saccharomyces cerevisiae (strain ATCC 204508 / S288c) GN=RRP12 PE=1 SV=1

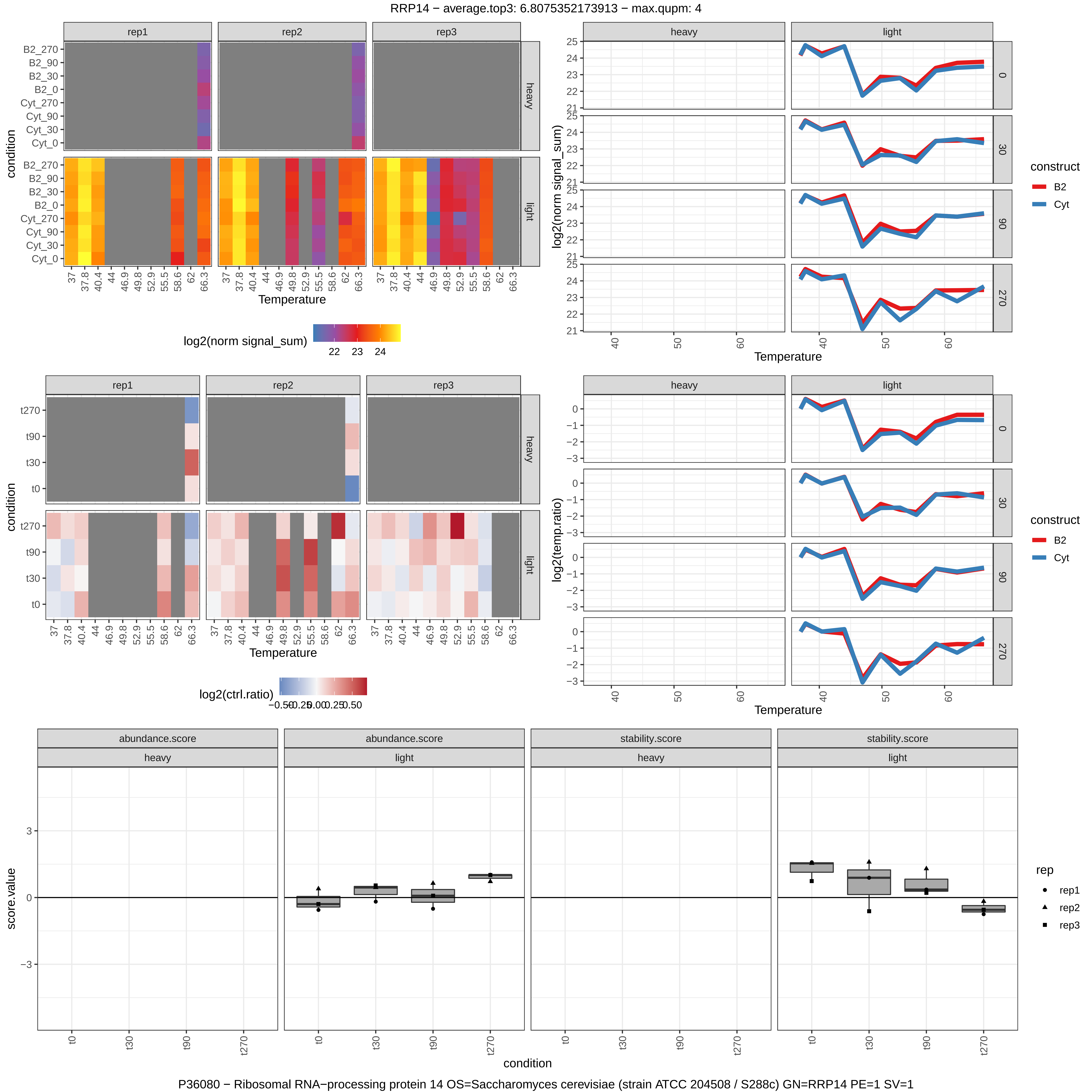

RRP14 − average.top3: 6.8075352173913 − max.qupm: 4
rep1
rep2
rep3
heavy
light
condition
Temperature
log2(norm signal_sum)
log2(temp.ratio)
log2(ctrl.ratio)
construct
B2
Cyt
abundance.score
stability.score
score.value
rep
rep1
rep2
rep3
P36080 − Ribosomal RNA−processing protein 14 OS=Saccharomyces cerevisiae (strain ATCC 204508 / S288c) GN=RRP14 PE=1 SV=1

RRP3 − average.top3: 6.6159385 − max.qupm: 7

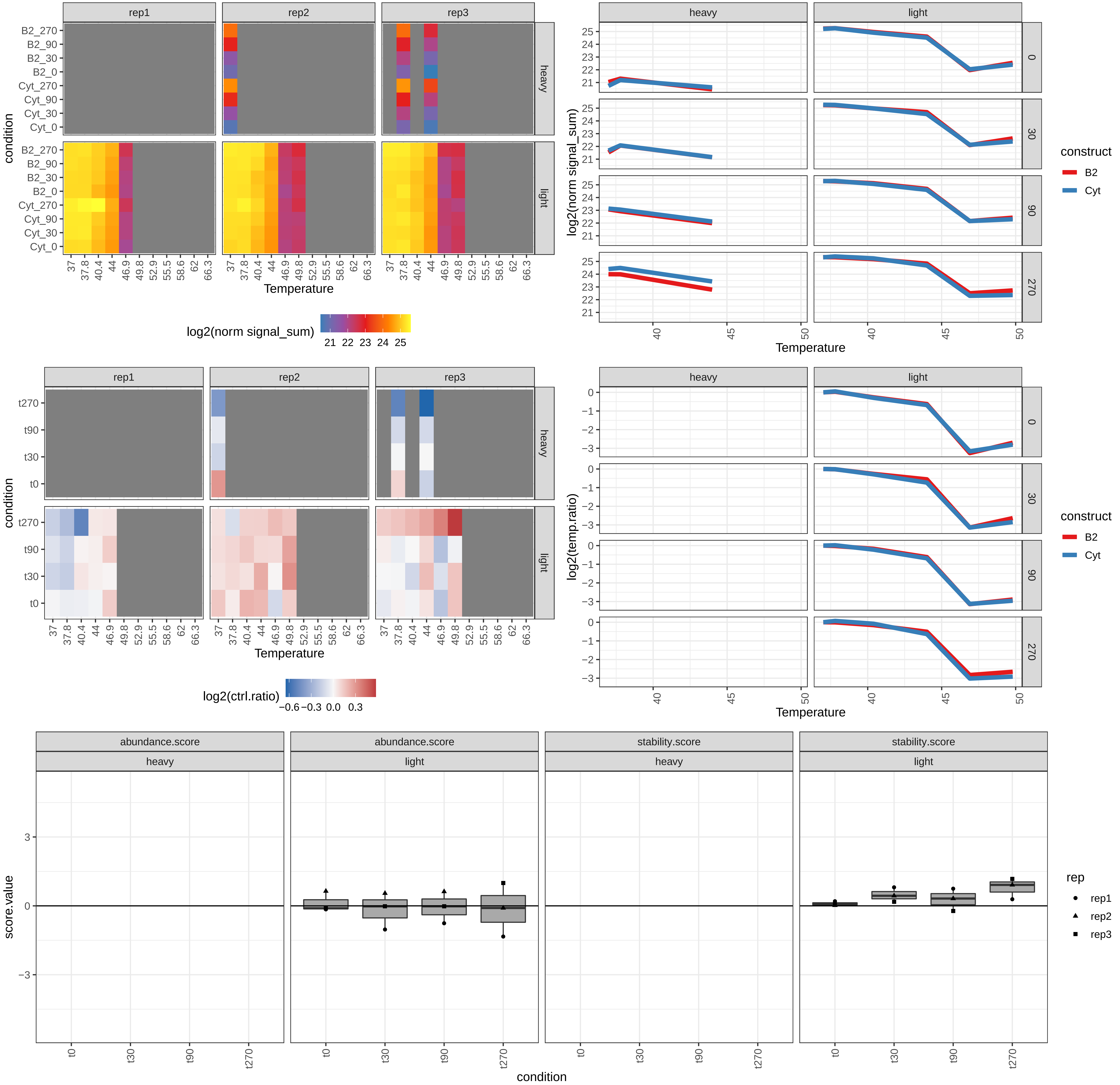

P38712 − ATP−dependent rRNA helicase RRP3 OS=Saccharomyces cerevisiae (strain ATCC 204508 / S288c) GN=RRP3 PE=1 SV=2

RRP36 − average.top3: 6.52982111111111 − max.qupm: 5

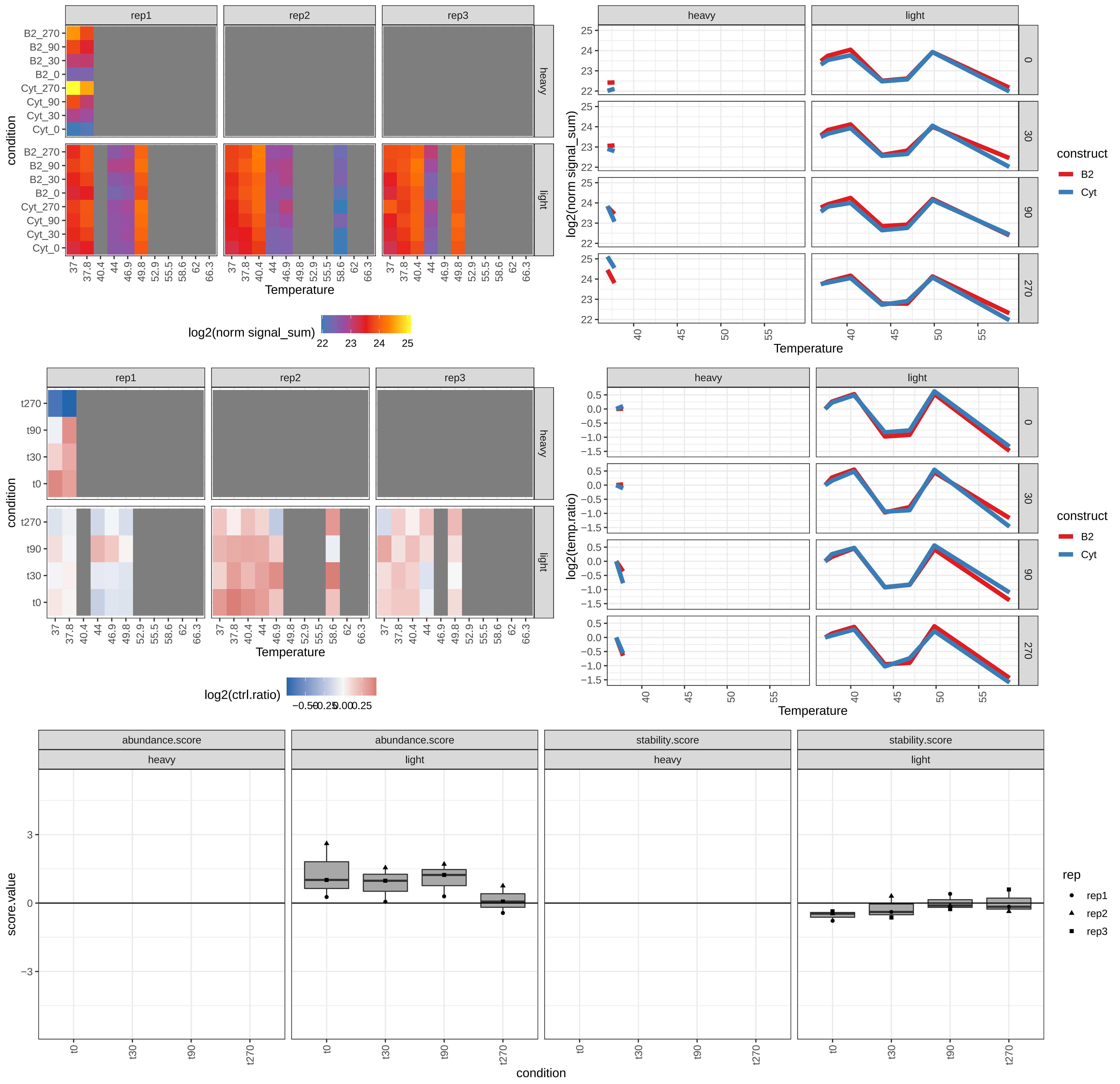

Q12481 − rRNA biogenesis protein RRP36 OS=Saccharomyces cerevisiae (strain ATCC 204508 / S288c) GN=RRP36 PE=1 SV=1

RRP4 − average.top3: 6.8791175 − max.qupm: 6

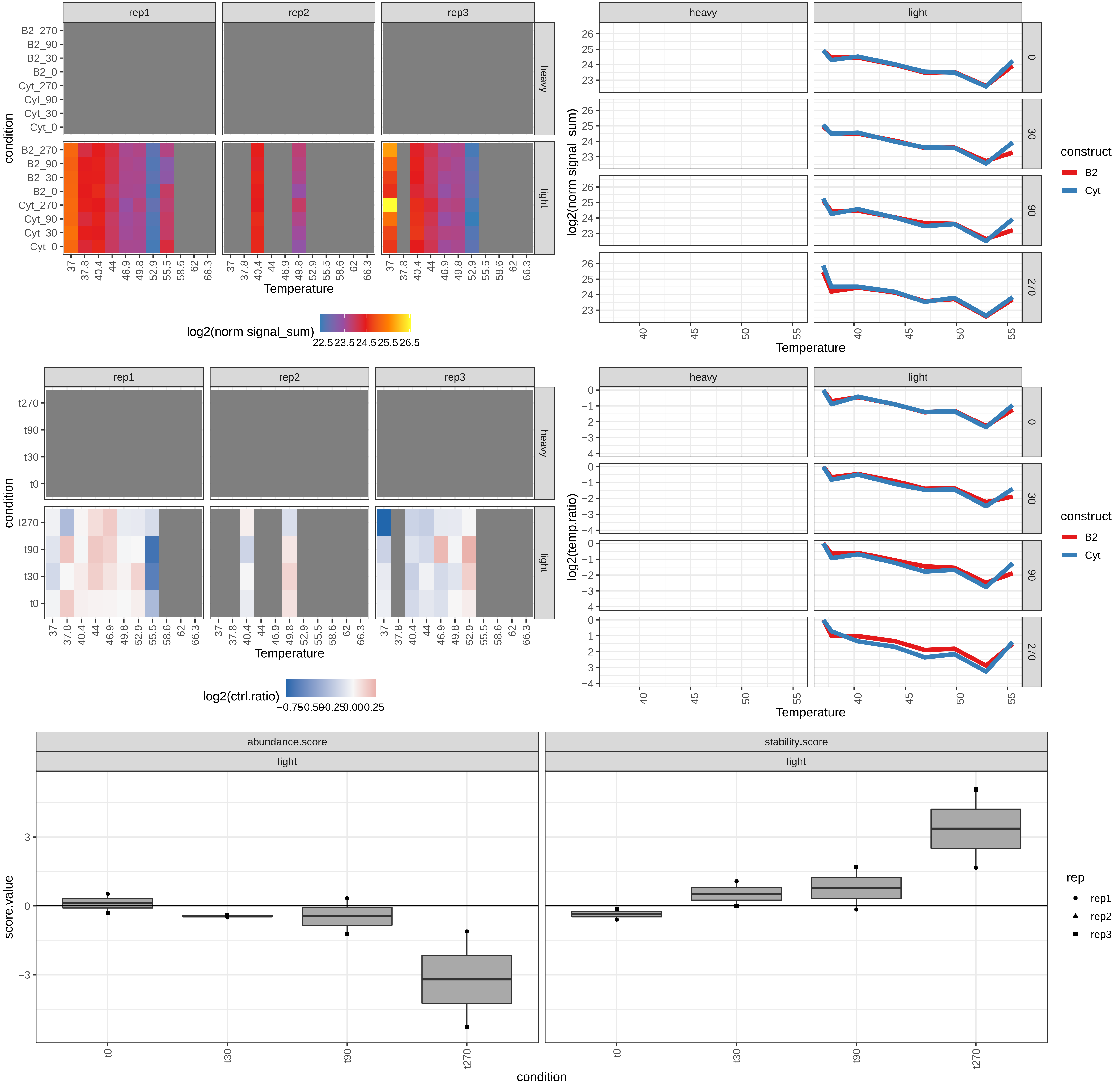

P38792 − Exosome complex component RRP4 OS=Saccharomyces cerevisiae (strain ATCC 204508 / S288c) GN=RRP4 PE=1 SV=1

RRP40 − average.top3: 7.04716263157895 − max.qupm: 5

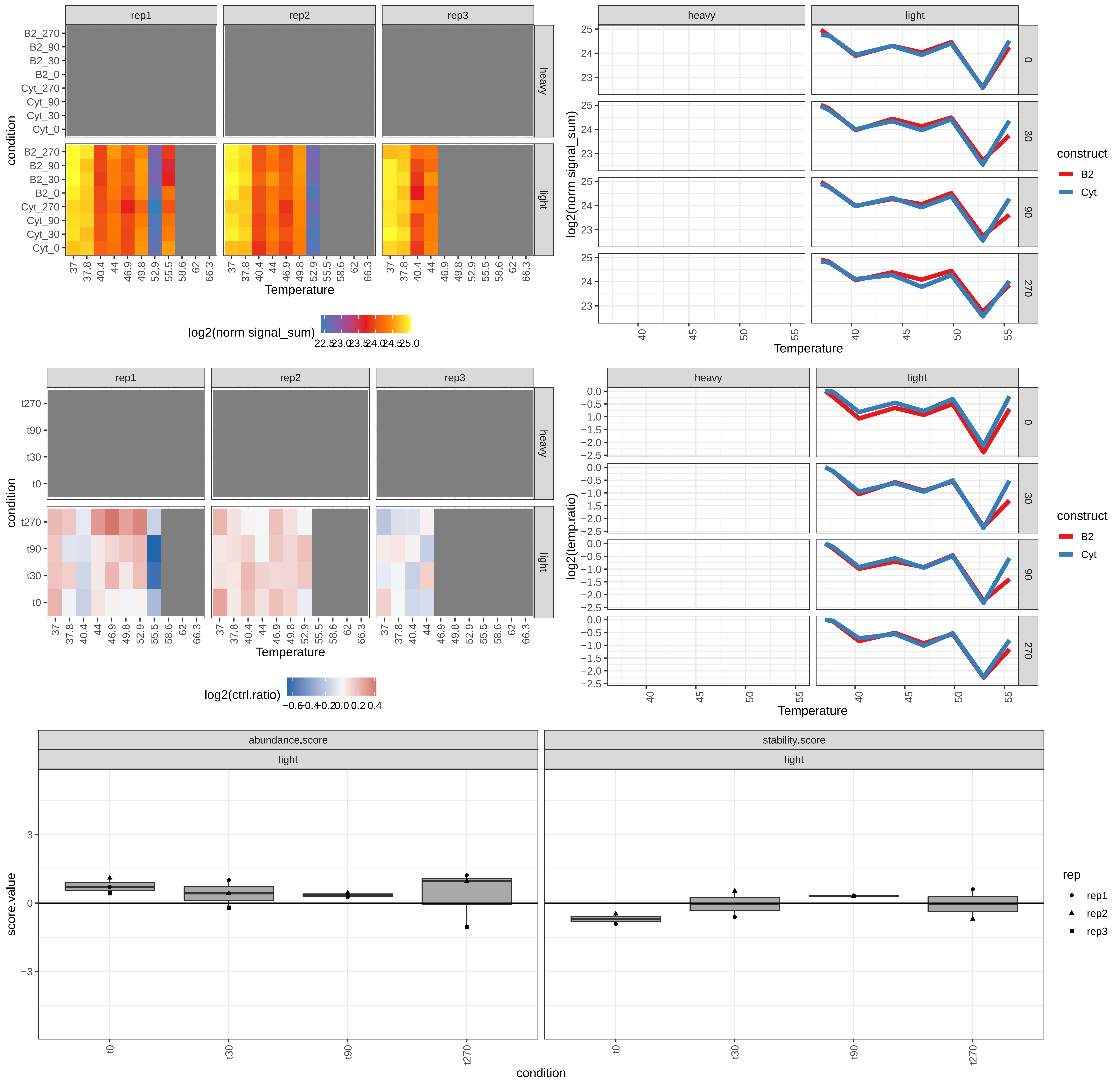

Q08285 − Exosome complex component RRP40 OS=Saccharomyces cerevisiae (strain ATCC 204508 / S288c) GN=RRP40 PE=1 SV=2

RRP42 − average.top3: 6.85441285714286 − max.qupm: 3

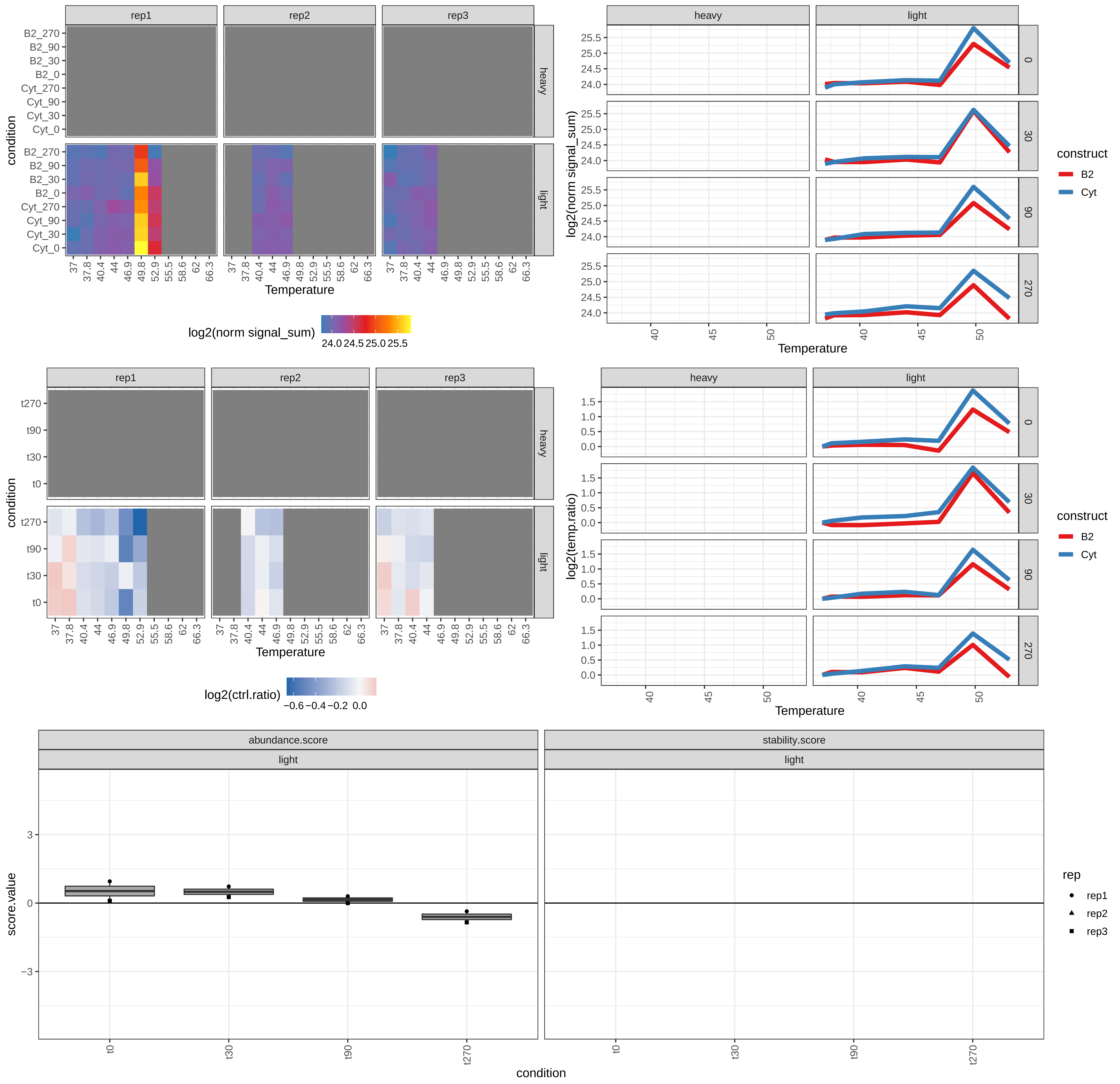

Q12277 − Exosome complex component RRP42 OS=Saccharomyces cerevisiae (strain ATCC 204508 / S288c) GN=RRP42 PE=1 SV=1

RRP43 − average.top3: 6.83445148148148 − max.qupm: 7

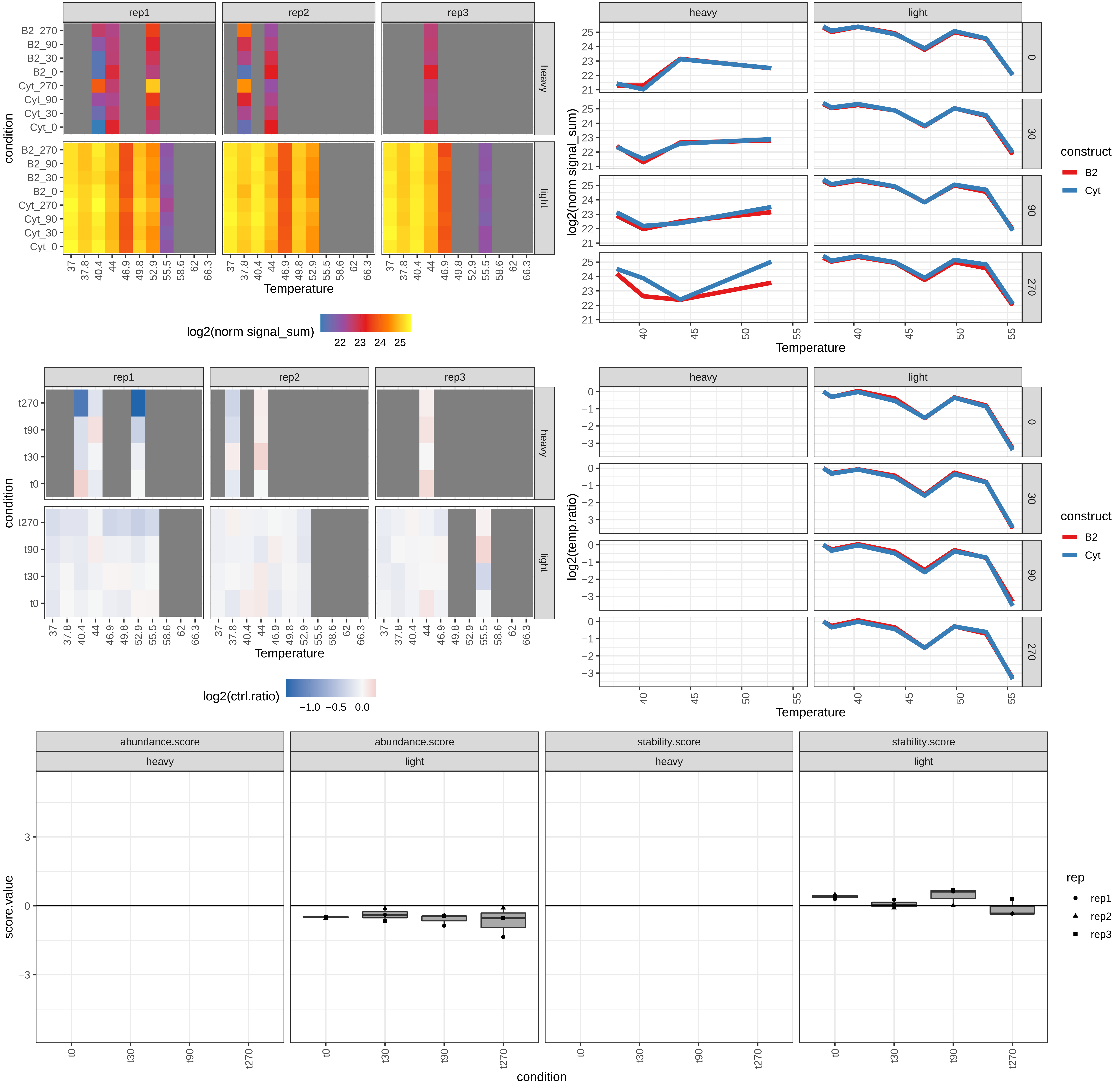

P25359 − Exosome complex component RRP43 OS=Saccharomyces cerevisiae (strain ATCC 204508 / S288c) GN=RRP43 PE=1 SV=2

RRP45 − average.top3: 6.74354454545455 − max.qupm: 3

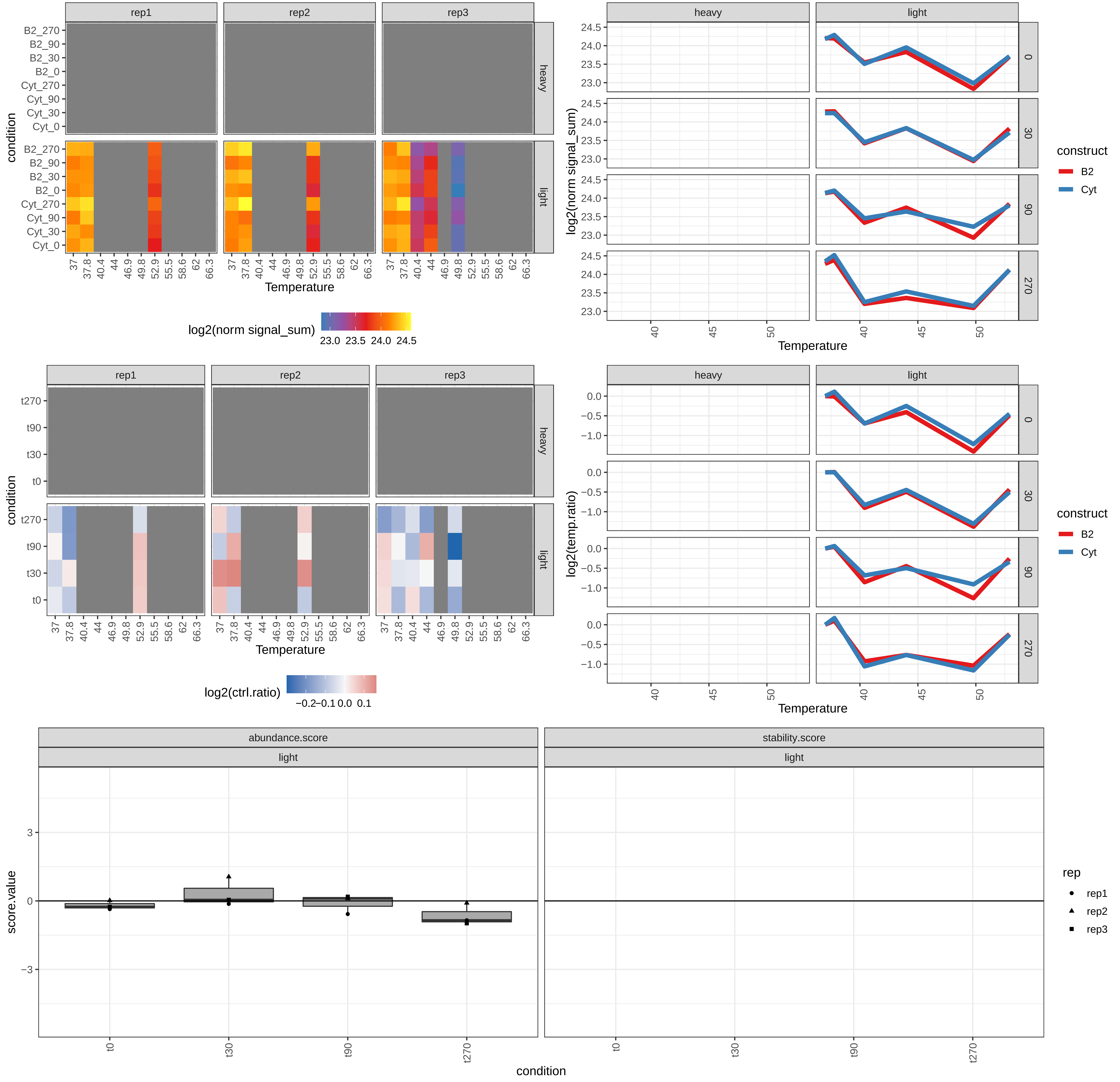

Q05636 − Exosome complex component RRP45 OS=Saccharomyces cerevisiae (strain ATCC 204508 / S288c) GN=RRP45 PE=1 SV=1

RRP46 − average.top3: 6.516491 − max.qupm: 3

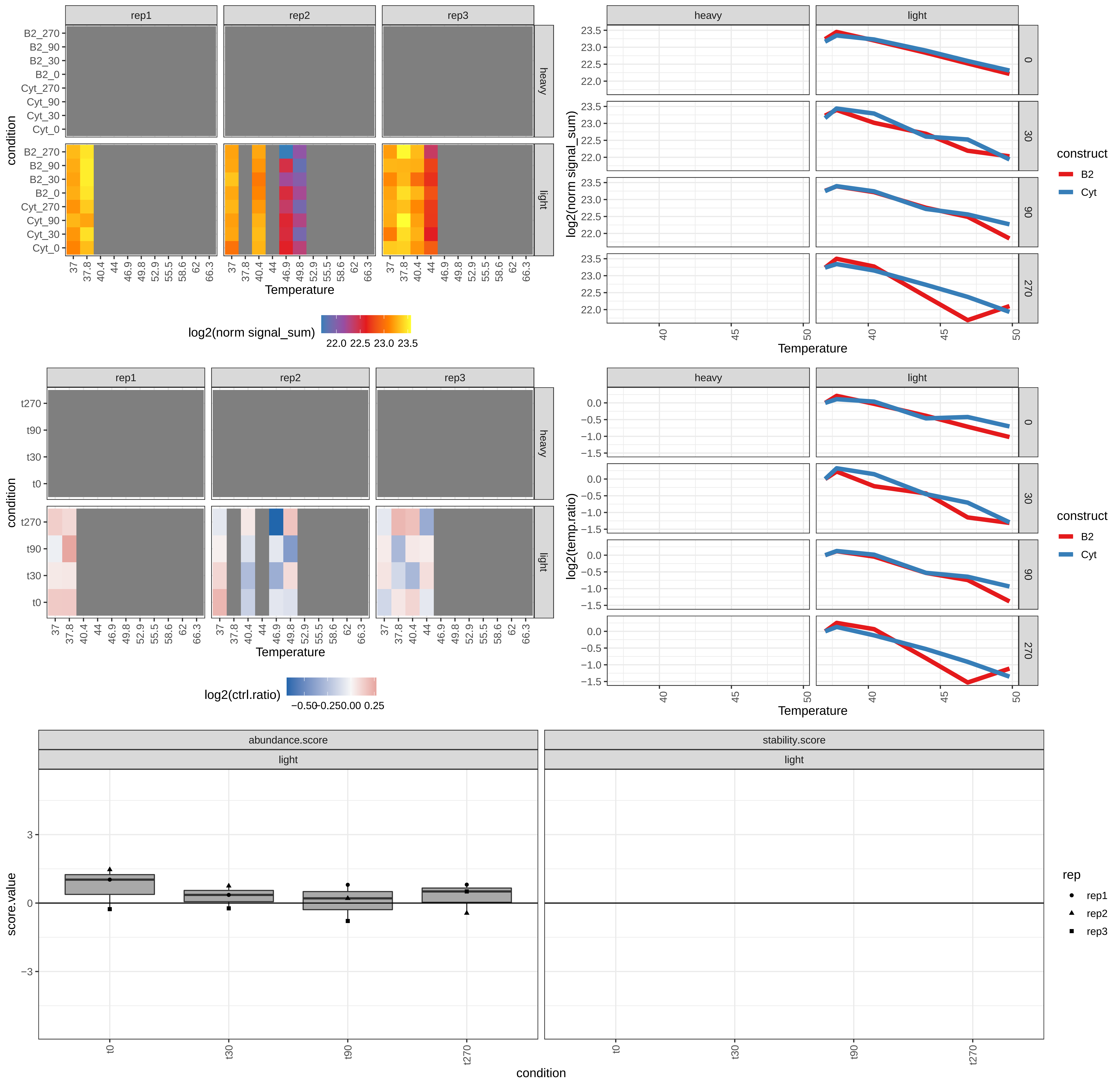

P53256 − Exosome complex component RRP46 OS=Saccharomyces cerevisiae (strain ATCC 204508 / S288c) GN=RRP46 PE=1 SV=2

RRP5 − average.top3: 7.23810205882353 − max.qupm: 49

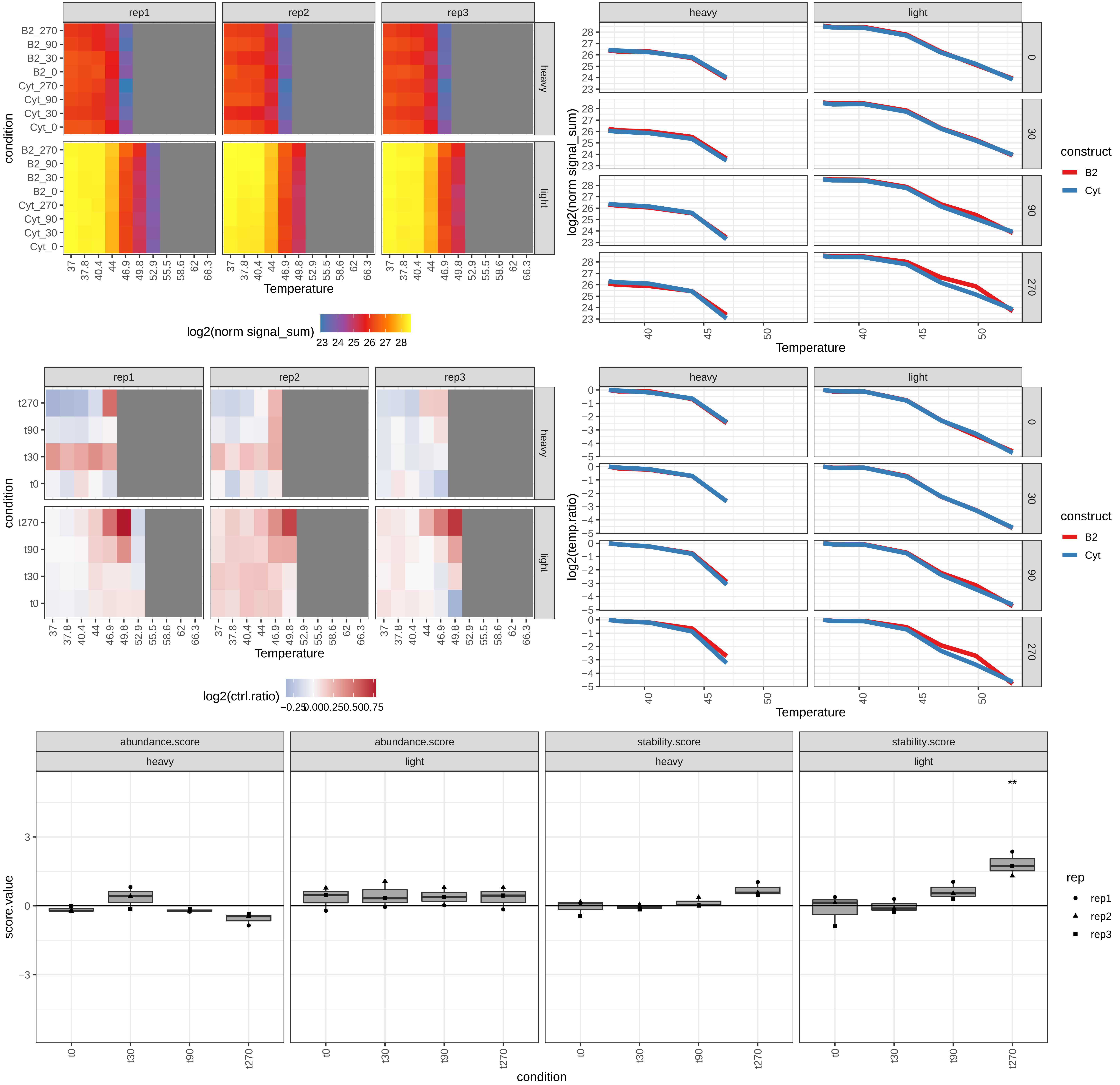

Q05022 − rRNA biogenesis protein RRP5 OS=Saccharomyces cerevisiae (strain ATCC 204508 / S288c) GN=RRP5 PE=1 SV=1

RRP6 − average.top3: 6.49537166666667 − max.qupm: 6

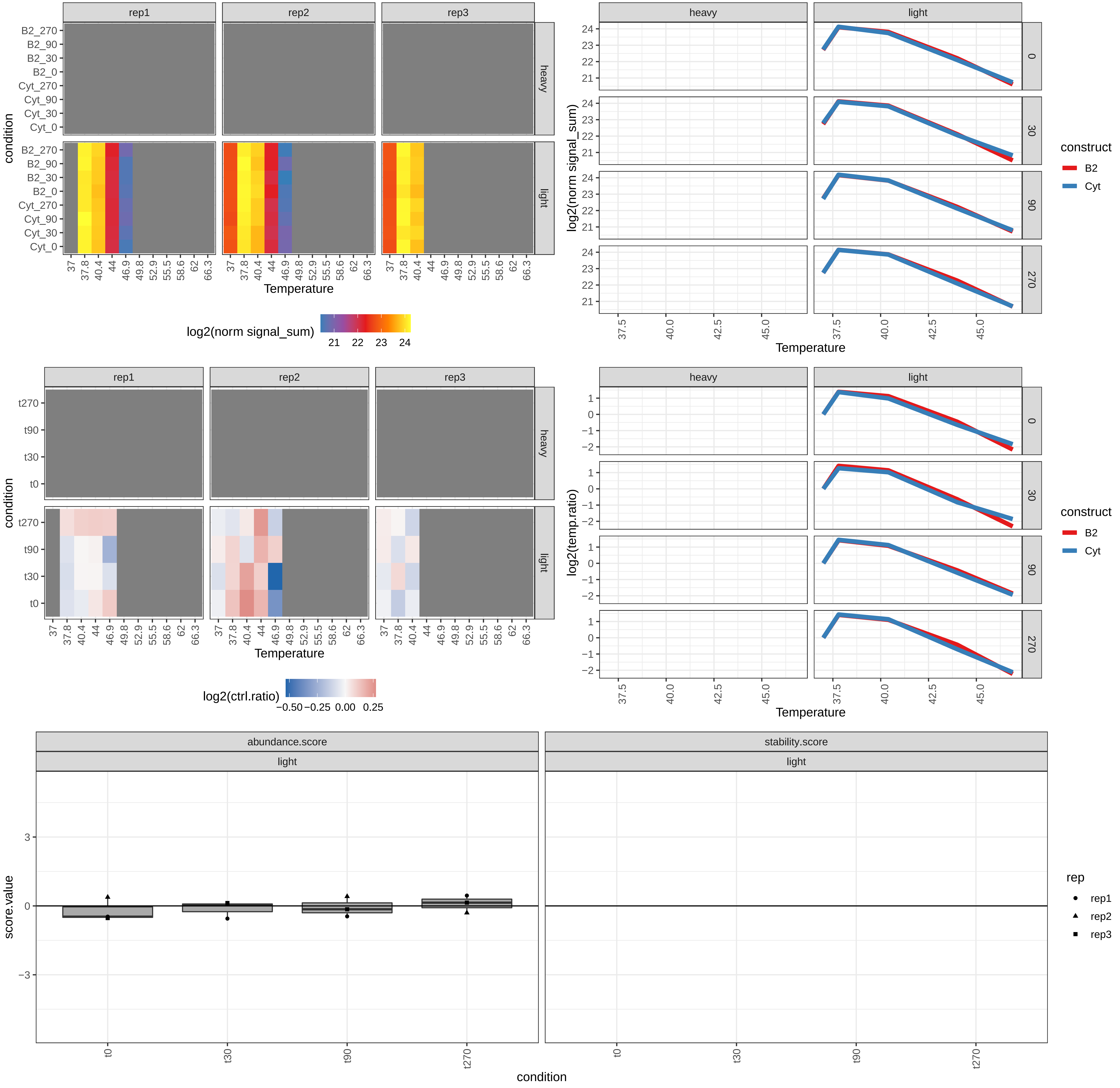

Q12149 − Exosome complex exonuclease RRP6 OS=Saccharomyces cerevisiae (strain ATCC 204508 / S288c) GN=RRP6 PE=1 SV=1

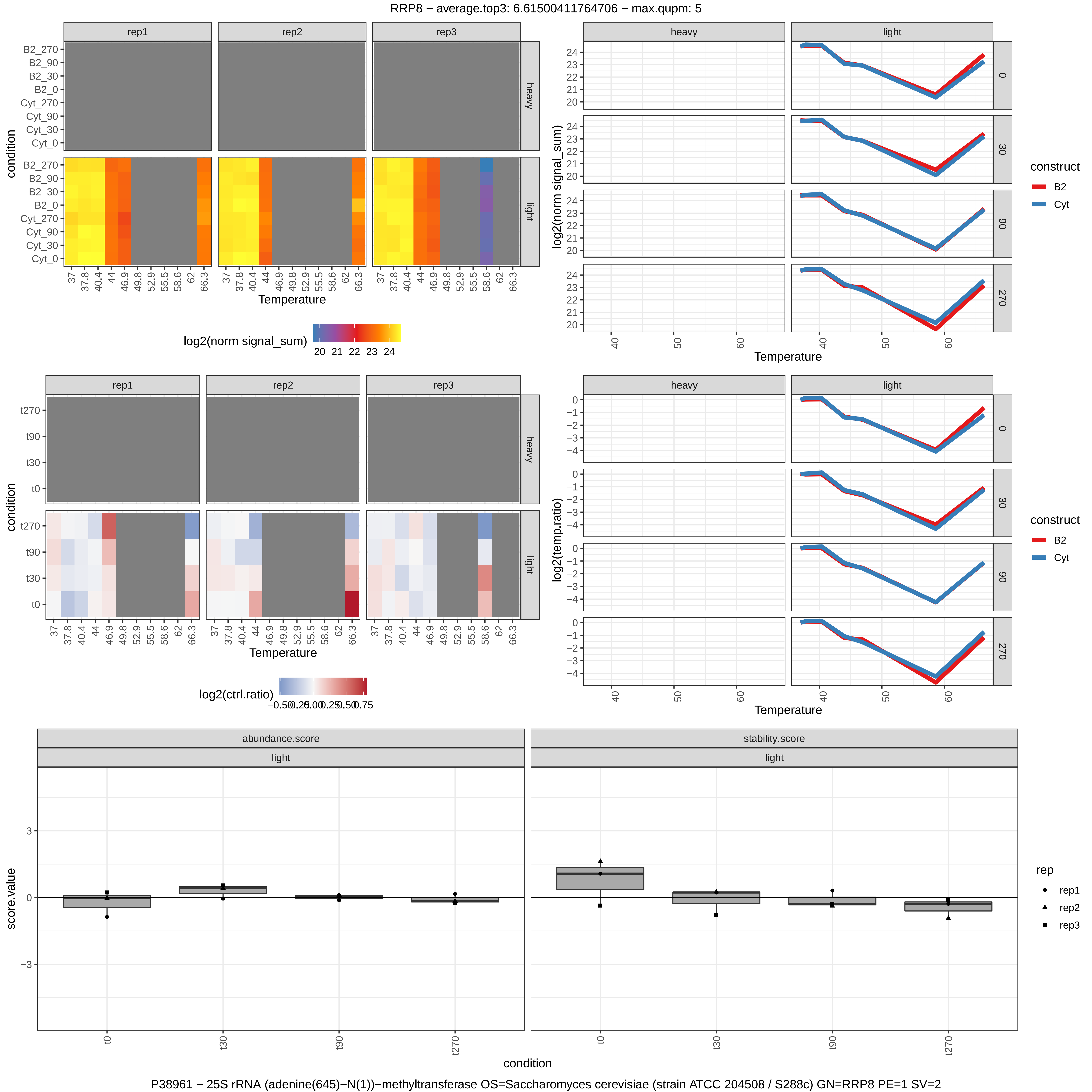

RRP8 − average.top3: 6.61500411764706 − max.qupm: 5
rep1
rep2
rep3
heavy
light
condition
Temperature
log2(norm signal_sum)
log2(ctrl.ratio)
log2(temp.ratio)
construct
B2
Cyt
abundance.score
stability.score
score.value
rep
rep1
rep2
rep3
P38961 − 25S rRNA (adenine(645)−N(1))−methyltransferase OS=Saccharomyces cerevisiae (strain ATCC 204508 / S288c) GN=RRP8 PE=1 SV=2

RRP9 − average.top3: 6.68279470588235 − max.qupm: 6

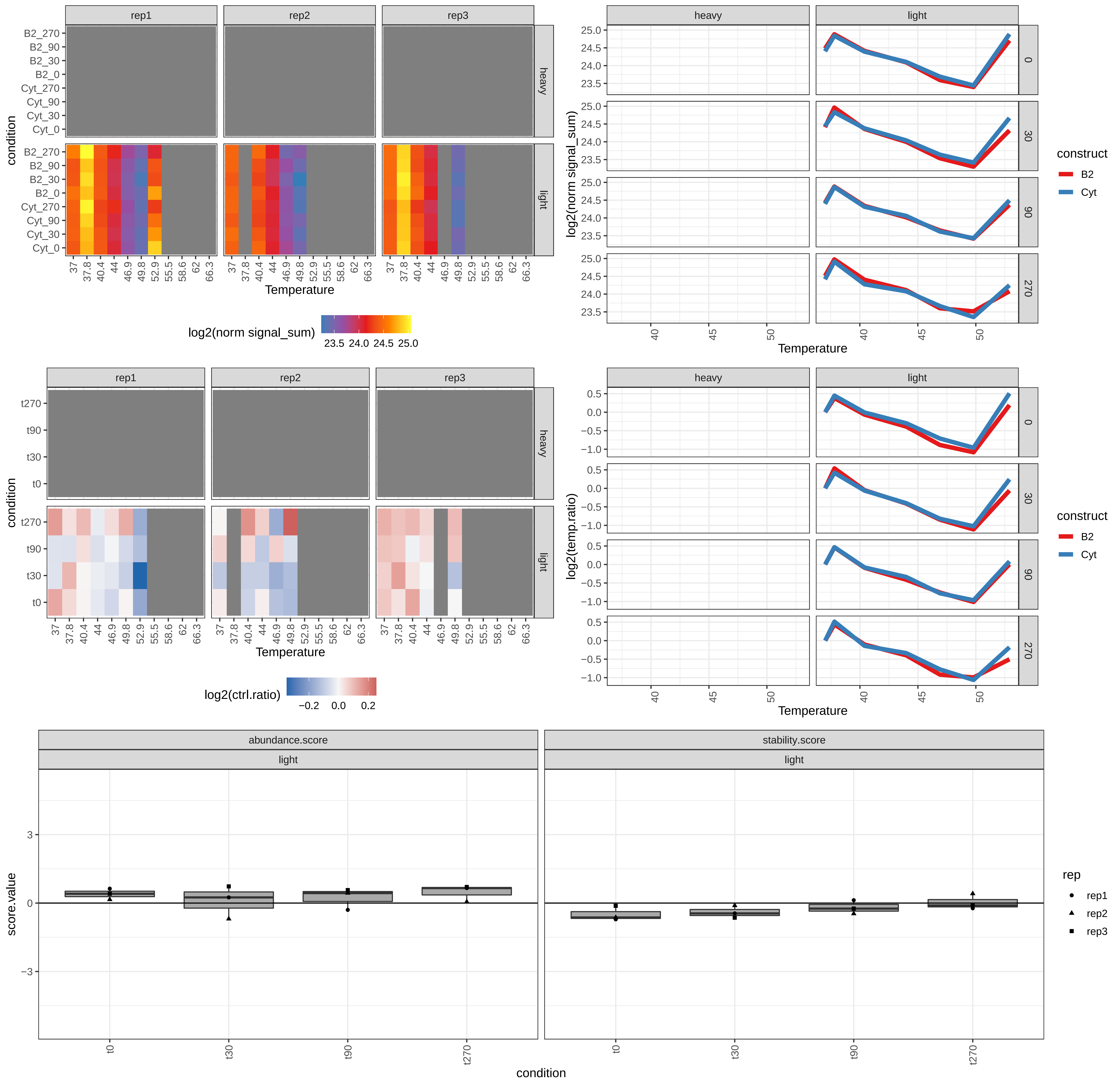

Q06506 − Ribosomal RNA−processing protein 9 OS=Saccharomyces cerevisiae (strain ATCC 204508 / S288c) GN=RRP9 PE=1 SV=1

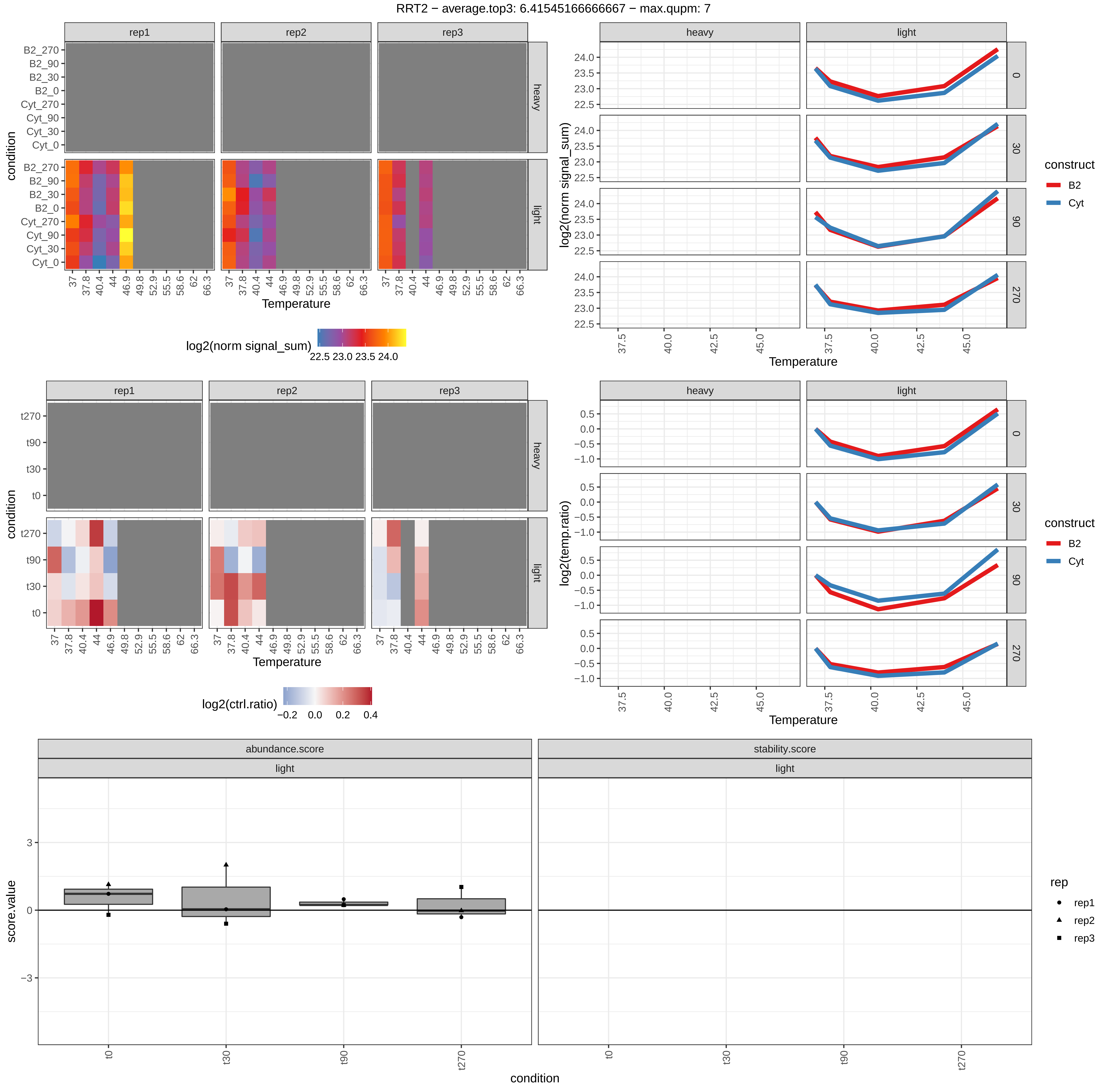

P38332 − Diphthine methyltransferase OS=Saccharomyces cerevisiae (strain ATCC 204508 / S288c) GN=RRT2 PE=1 SV=1

RSA3 − average.top3: 6.79356384615385 − max.qupm: 4

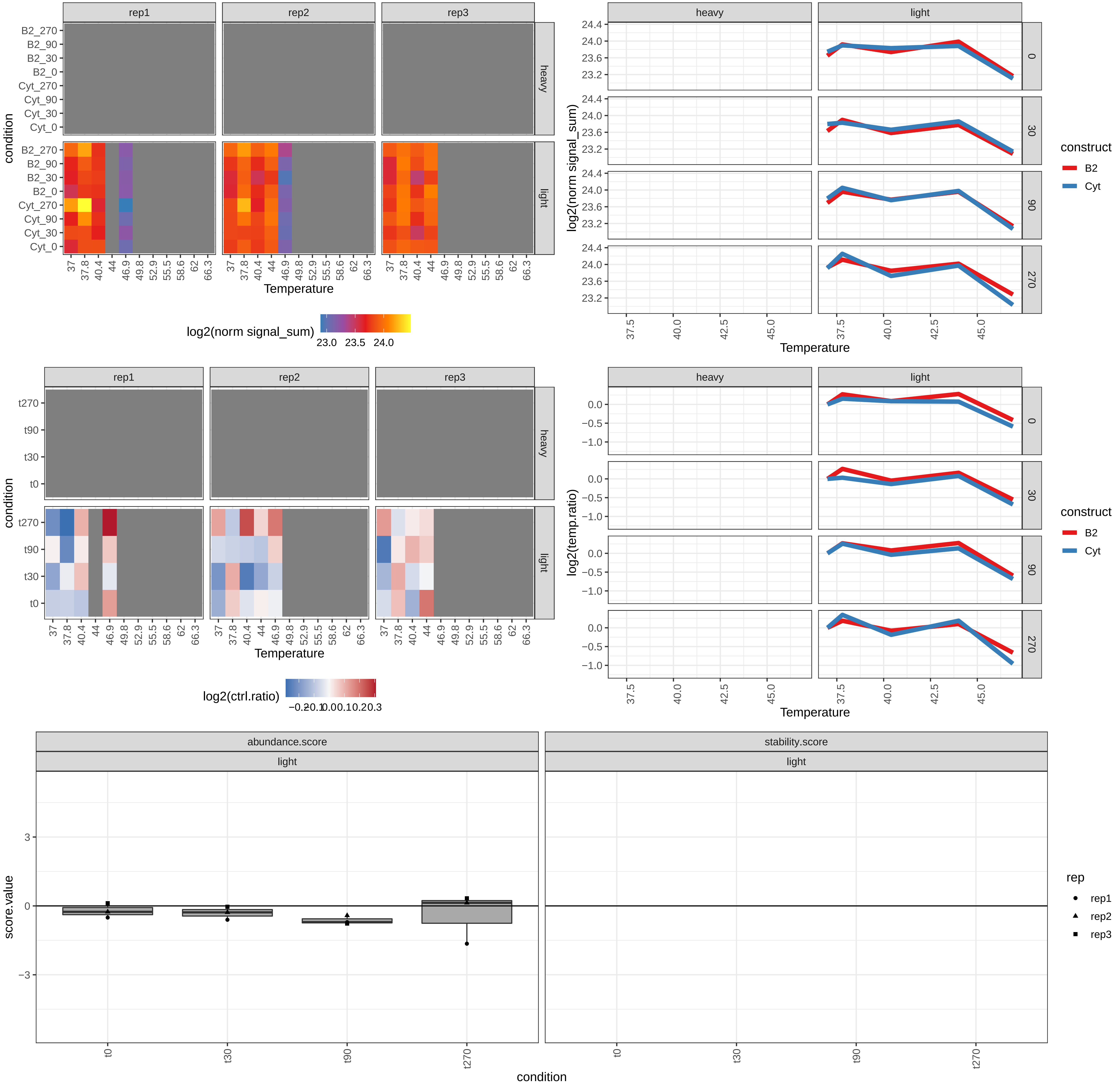

Q05942 − Ribosome assembly protein 3 OS=Saccharomyces cerevisiae (strain ATCC 204508 / S288c) GN=RSA3 PE=1 SV=1

RSA4 − average.top3: 7.09125090909091 − max.qupm: 10

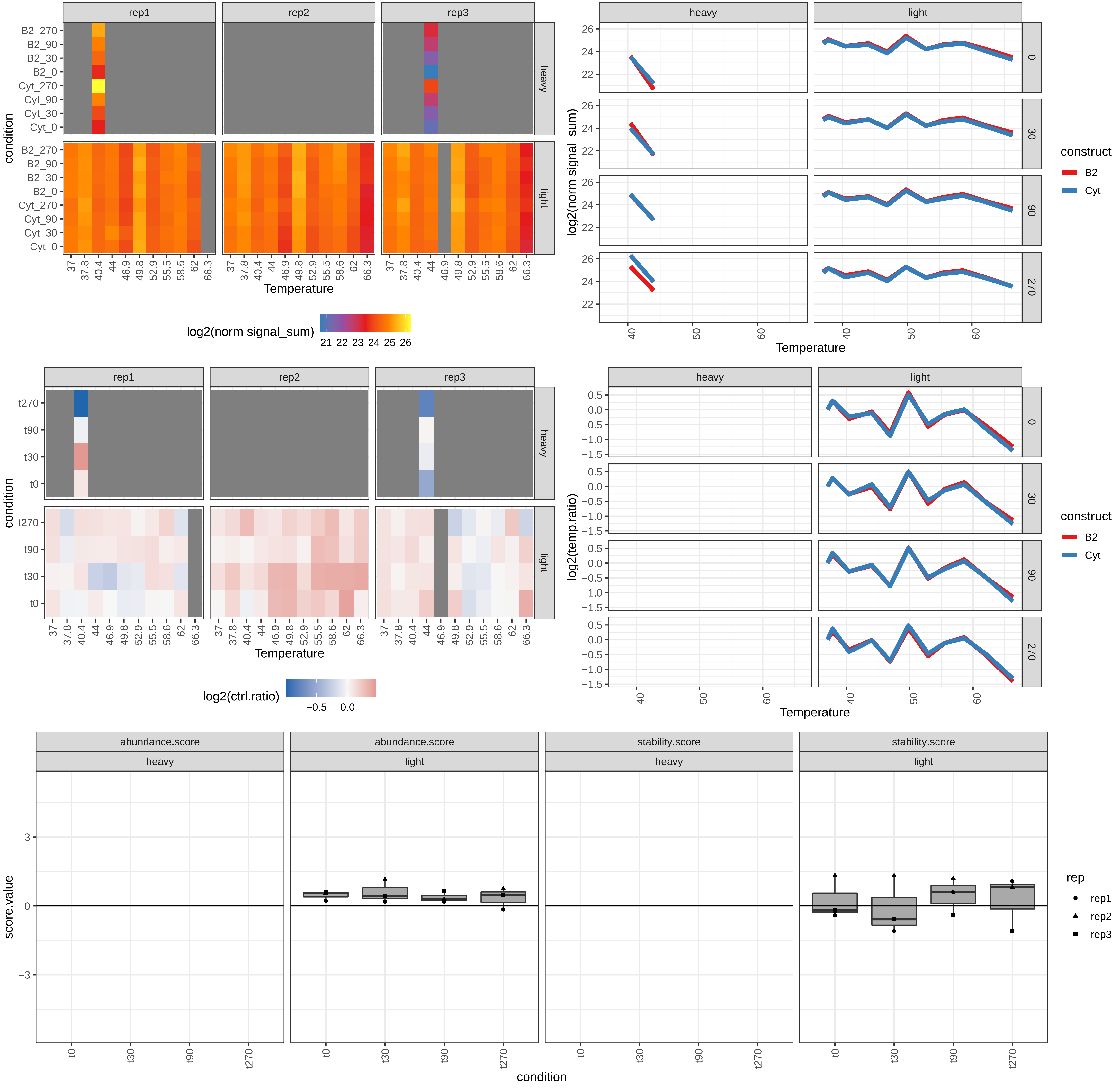

P25382 − Ribosome assembly protein 4 OS=Saccharomyces cerevisiae (strain ATCC 204508 / S288c) GN=RSA4 PE=1 SV=3

RSC1 − average.top3: 6.54738875 − max.qupm: 8

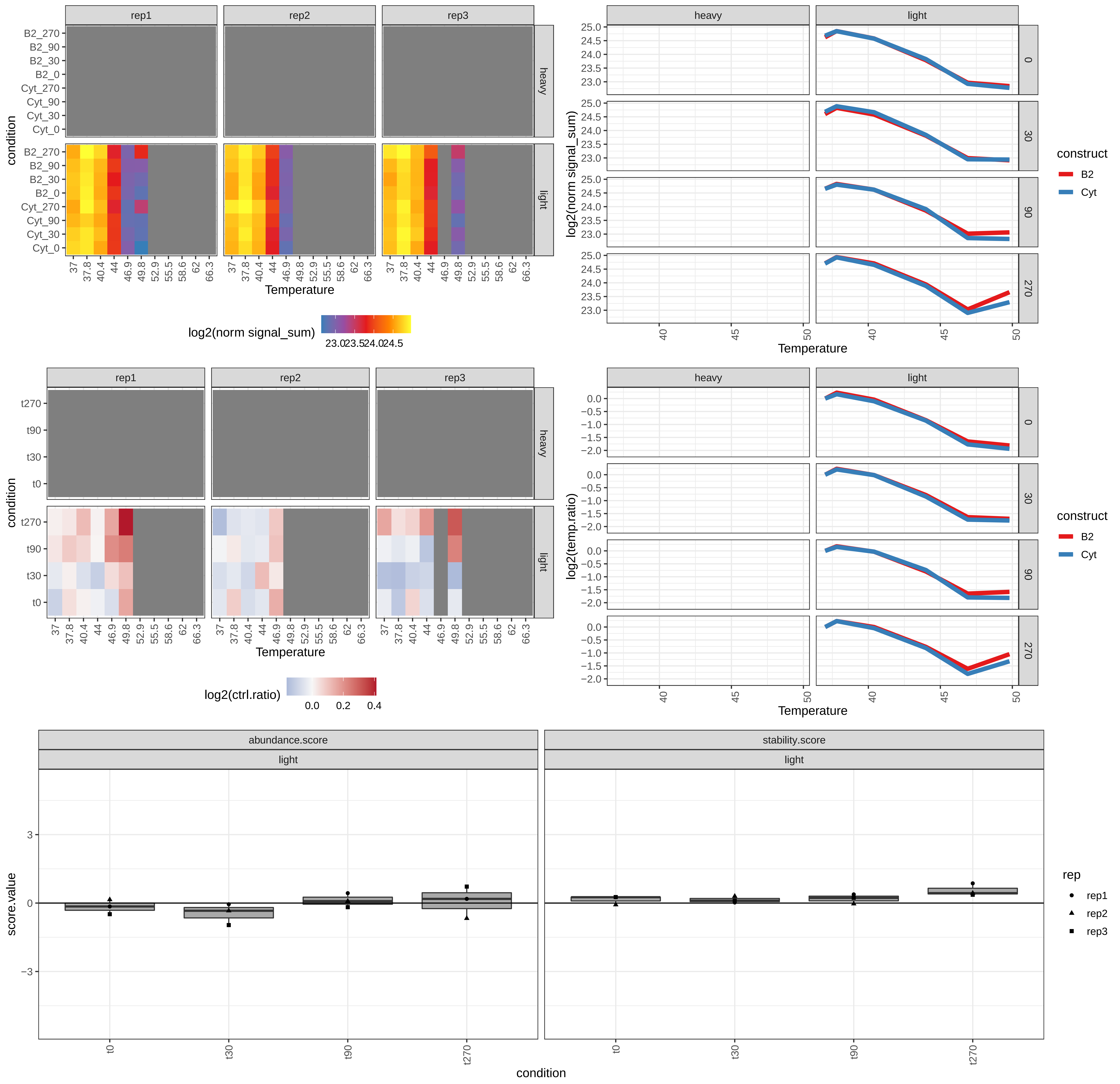

P53236 − Chromatin structure−remodeling complex subunit RSC1 OS=Saccharomyces cerevisiae (strain ATCC 204508 / S288c) GN=RSC1 PE=1 SV=1

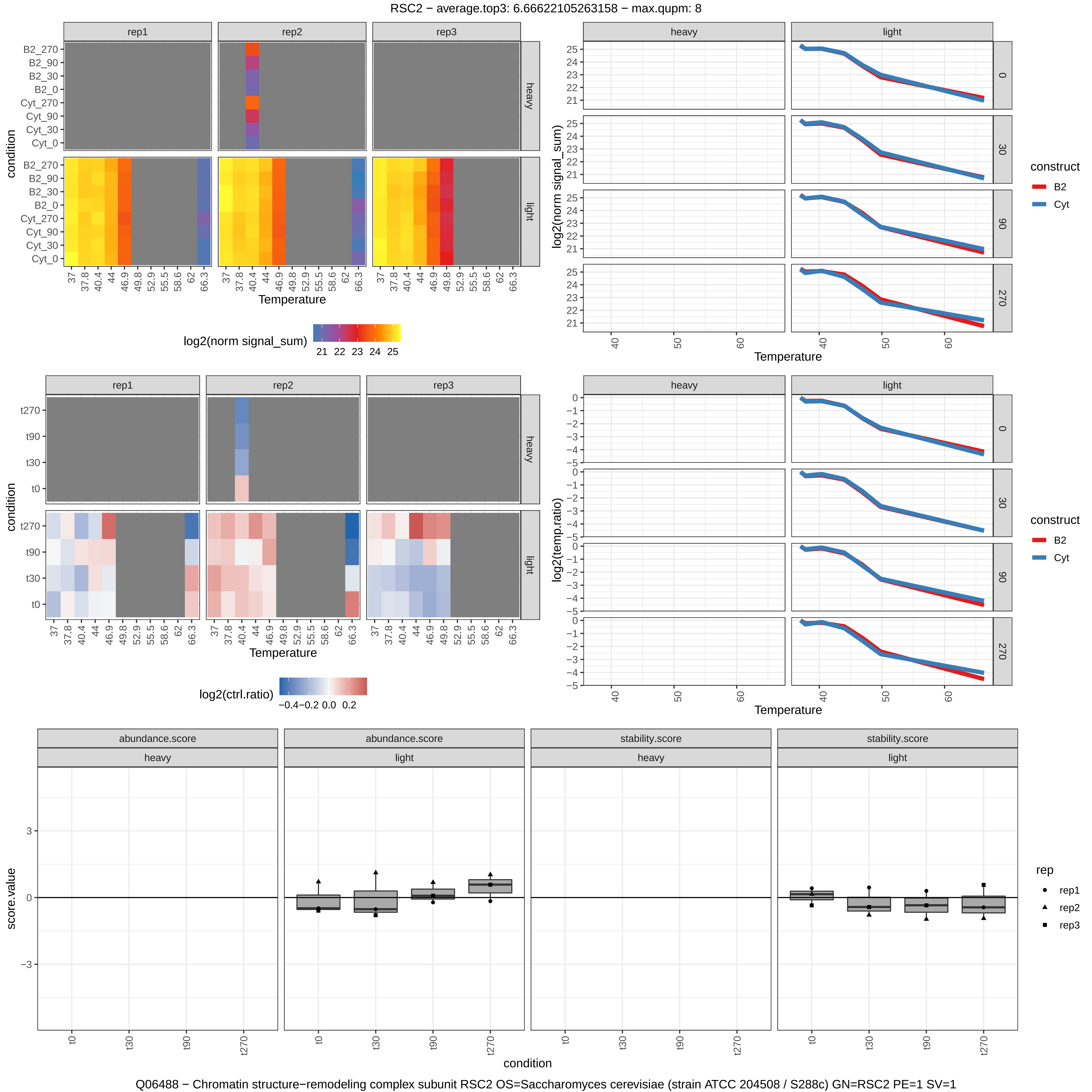
RSC2 − average.top3: 6.66622105263158 − max.qupm: 8
rep1
rep2
rep3
heavy
light
B2_270
B2_90
B2_30
B2_0
Cyt_270
Cyt_90
Cyt_30
Cyt_0
condition
Temperature
log2(norm signal_sum)
21 22 23 24 25
construct
B2
Cyt
log2(ctrl.ratio)
−0.4 −0.2 0.0 0.2
t270
t90
t30
t0
log2(temp.ratio)
abundance.score
stability.score
score.value
rep
rep1
rep2
rep3
condition
Q06488 − Chromatin structure−remodeling complex subunit RSC2 OS=Saccharomyces cerevisiae (strain ATCC 204508 / S288c) GN=RSC2 PE=1 SV=1

RSC3 − average.top3: 6.64116 − max.qupm: 6

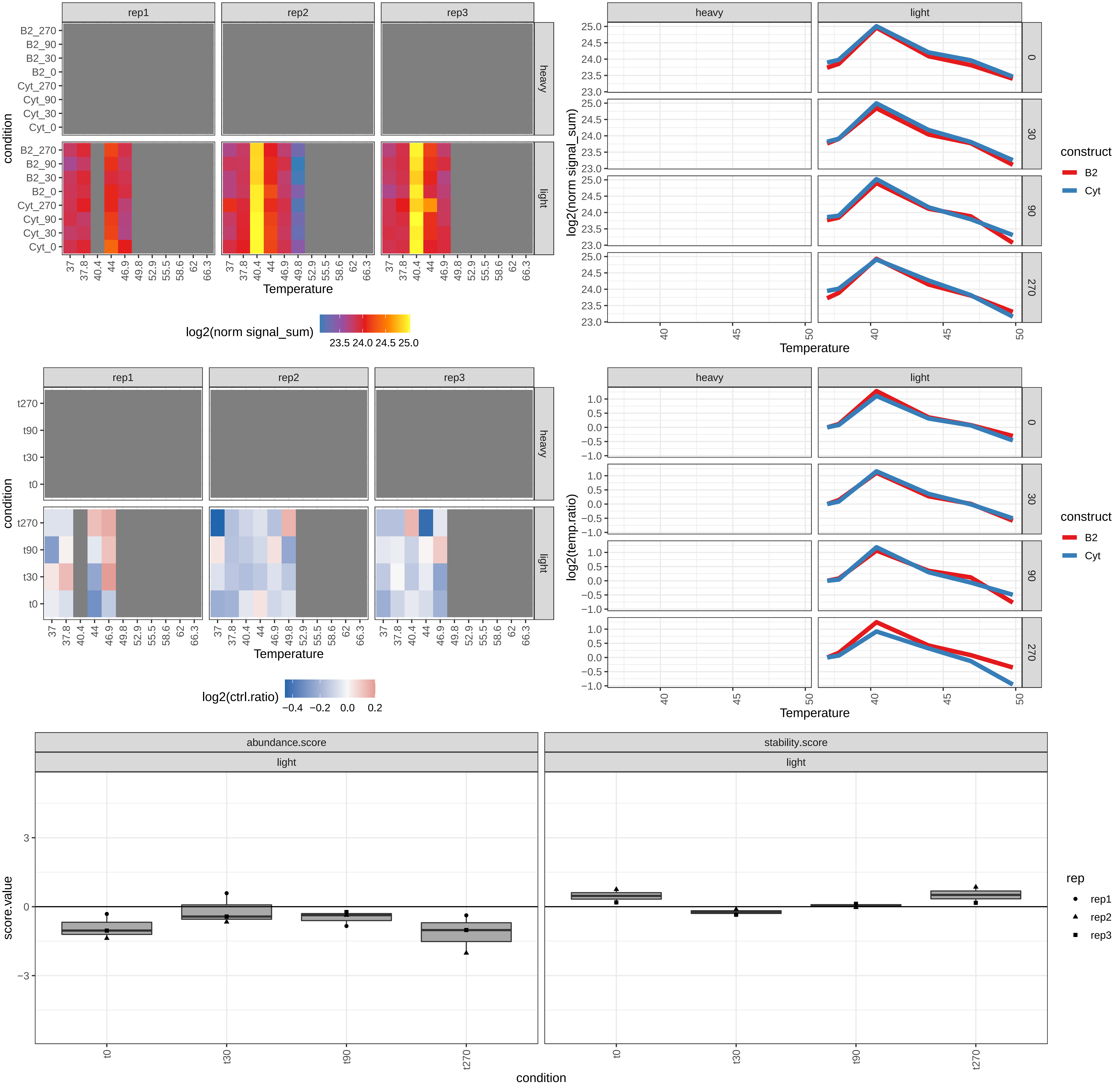

Q06639 − Chromatin structure−remodeling complex protein RSC3 OS=Saccharomyces cerevisiae (strain ATCC 204508 / S288c) GN=RSC3 PE=1 SV=1

RSC30 − average.top3: 6.49363090909091 − max.qupm: 5

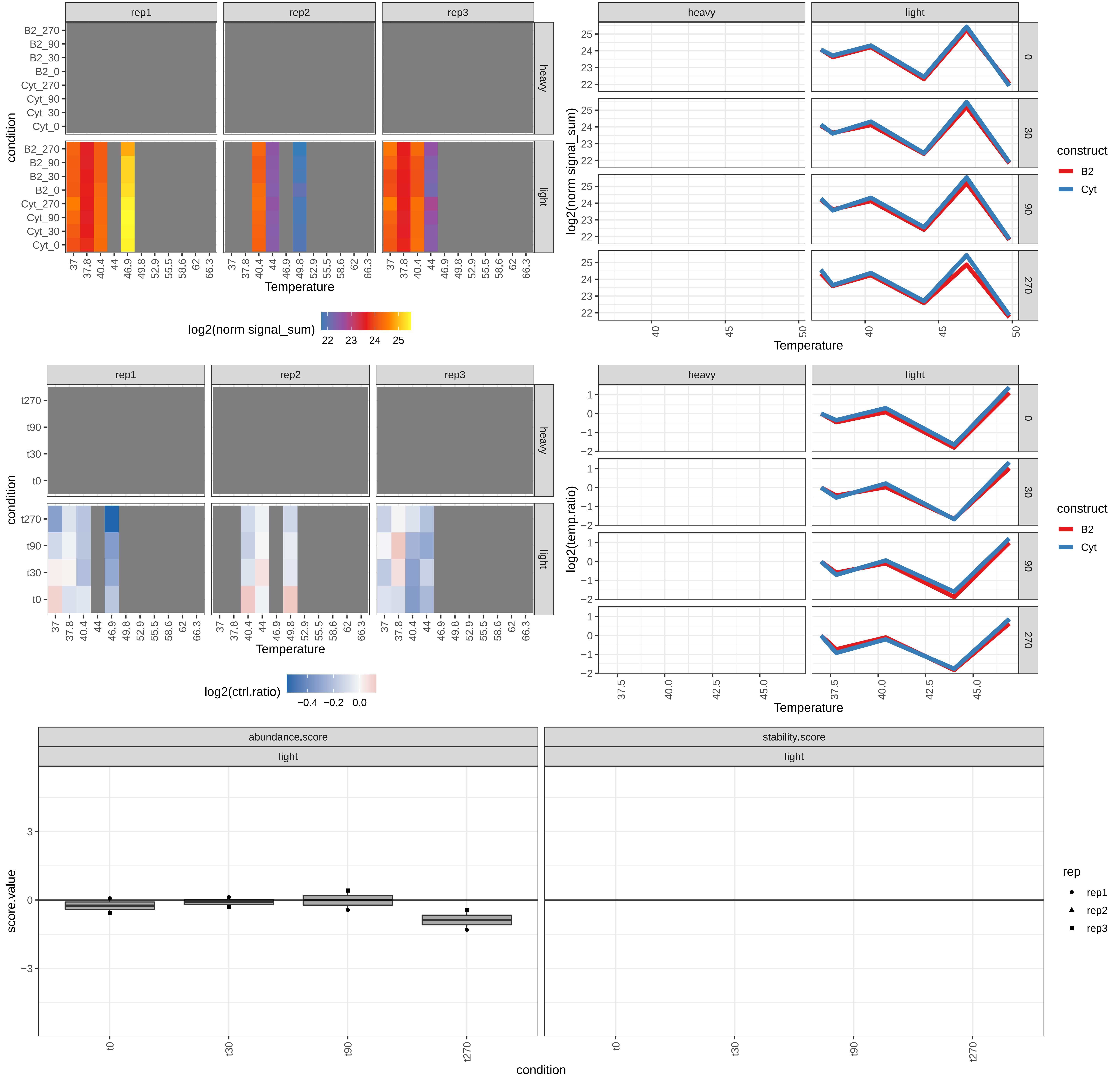

P38781 − Chromatin structure−remodeling complex protein RSC30 OS=Saccharomyces cerevisiae (strain ATCC 204508 / S288c) GN=RSC30 PE=1 SV=2

RSC4 − average.top3: 6.69975846153846 − max.qupm: 6

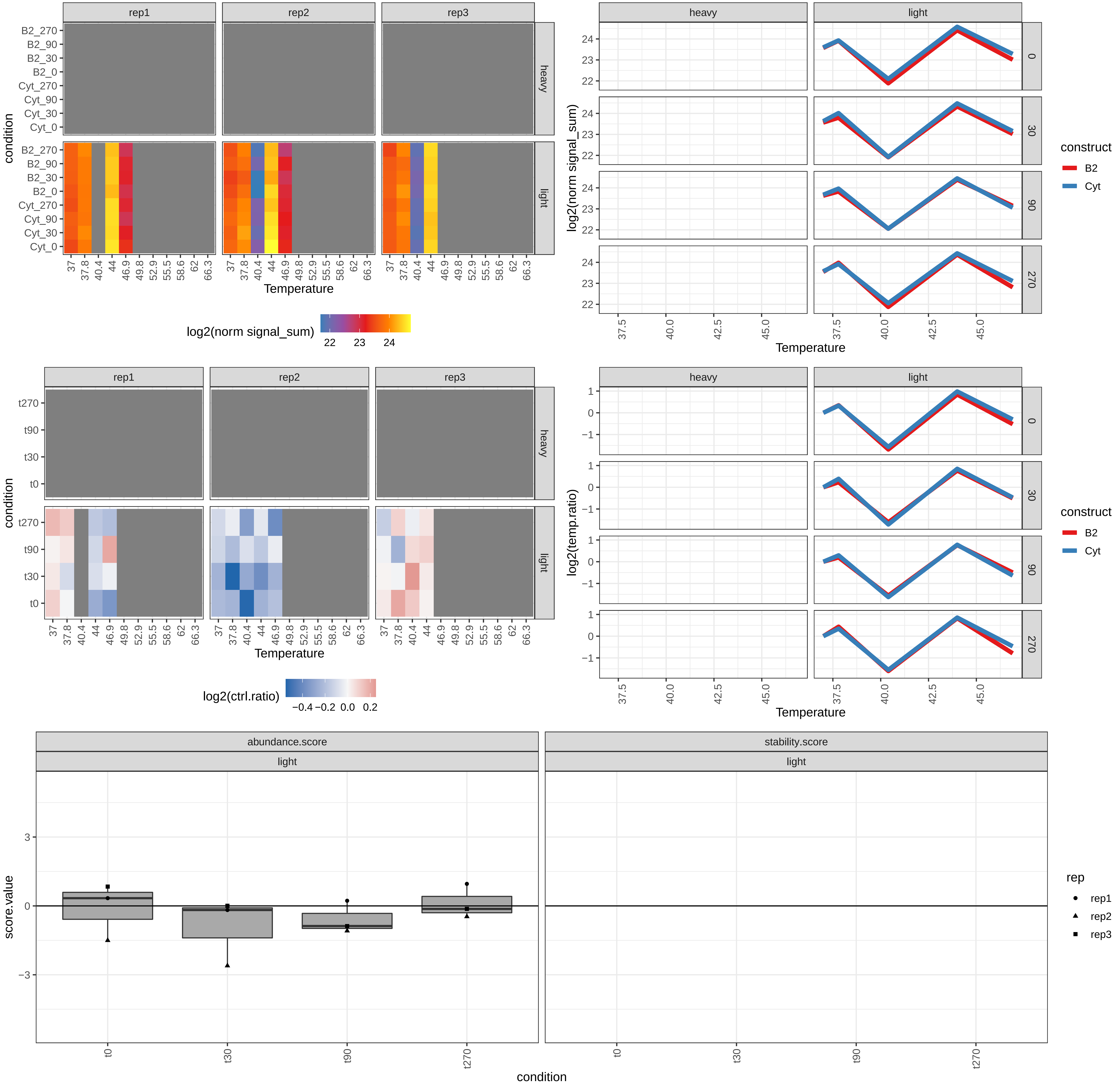

Q02206 − Chromatin structure−remodeling complex subunit RSC4 OS=Saccharomyces cerevisiae (strain ATCC 204508 / S288c) GN=RSC4 PE=1 SV=2

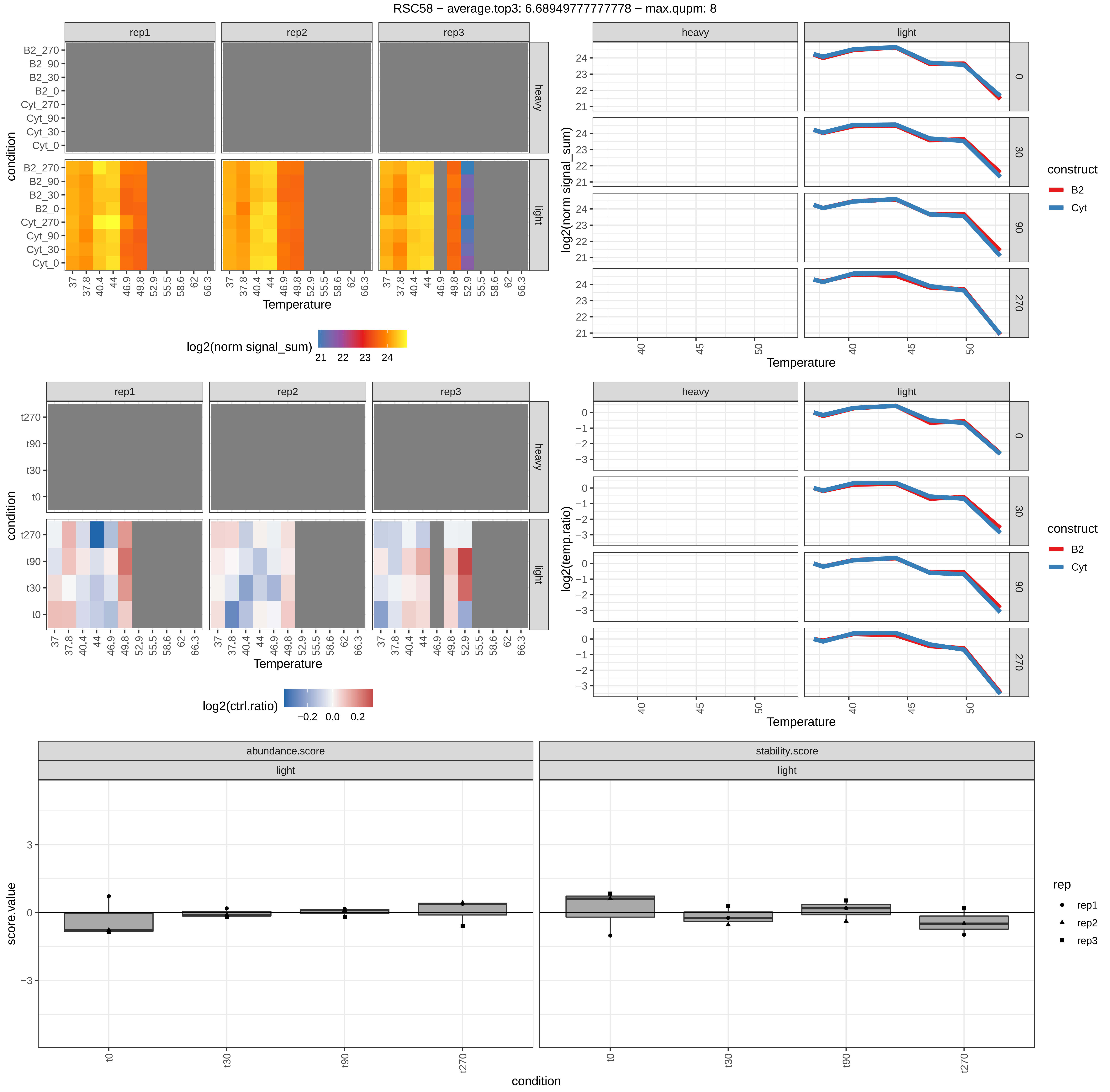

Q07979 − Chromatin structure−remodeling complex protein RSC58 OS=Saccharomyces cerevisiae (strain ATCC 204508 / S288c) GN=RSC58 PE=1 SV=1

RSC6 − average.top3: 6.63065 − max.qupm: 5

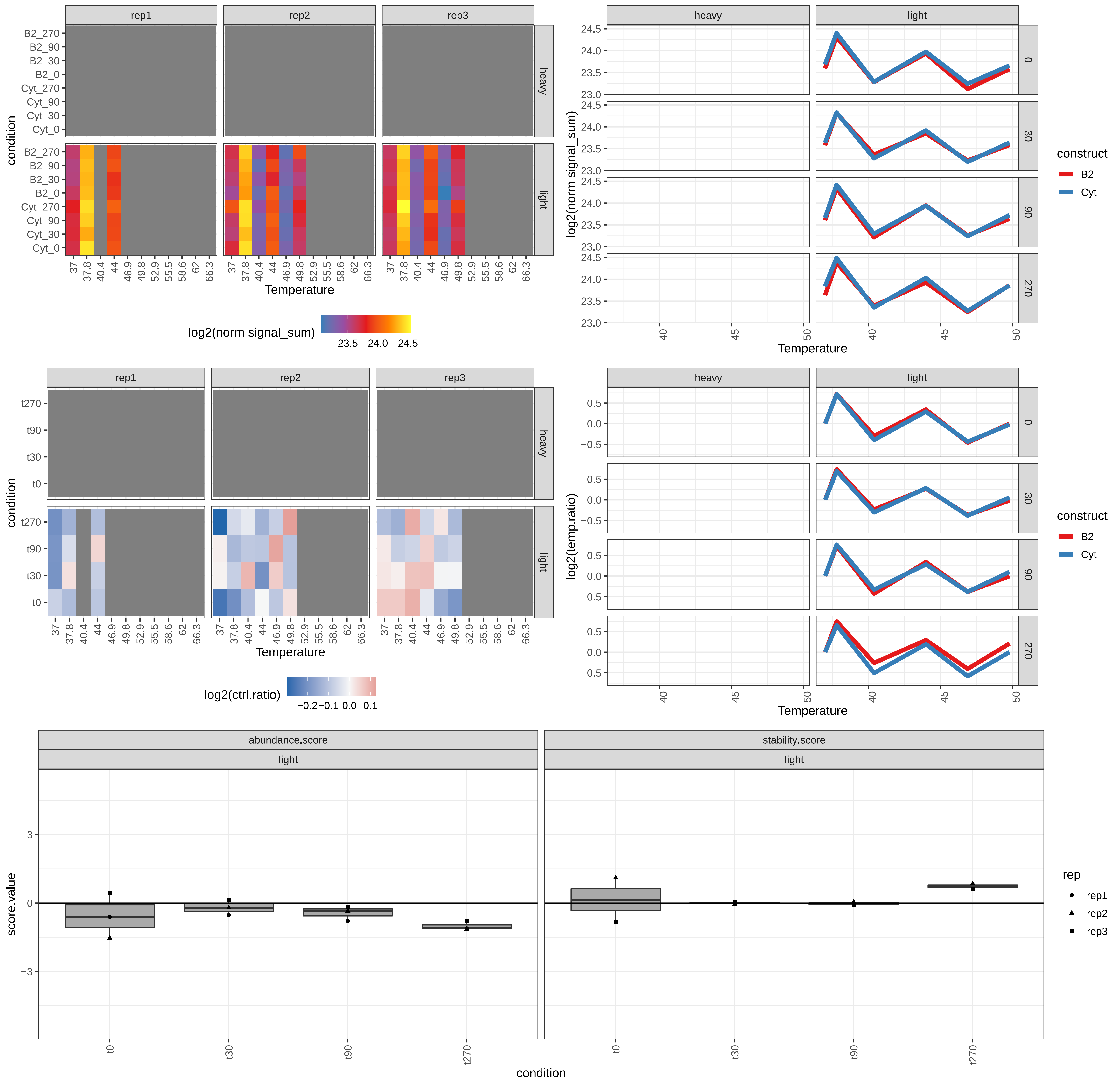

P25632 − Chromatin structure−remodeling complex protein RSC6 OS=Saccharomyces cerevisiae (strain ATCC 204508 / S288c) GN=RSC6 PE=1 SV=1

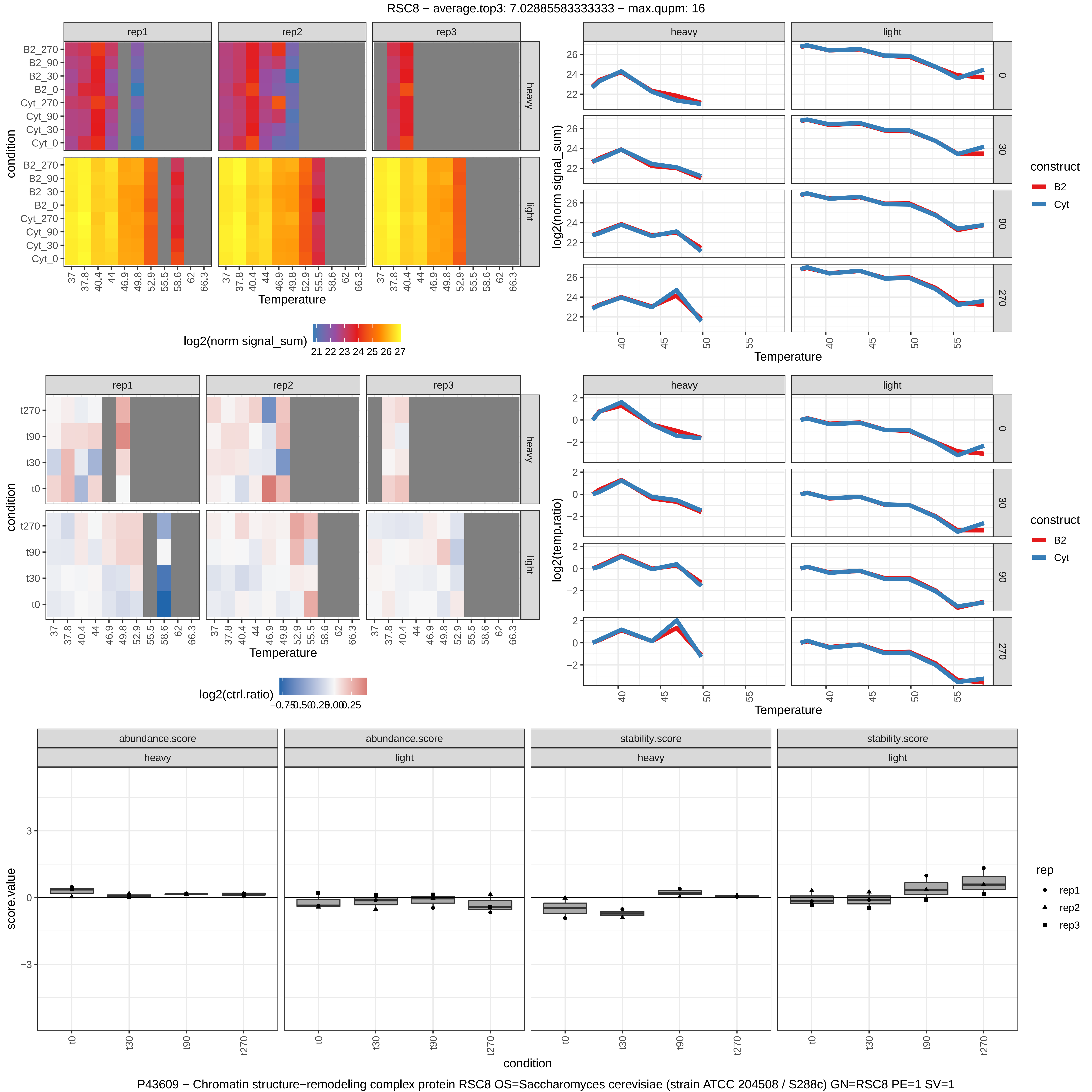

RSC8 − average.top3: 7.02885583333333 − max.qupm: 16
rep1
rep2
rep3
heavy
light
condition
Temperature
log2(norm signal_sum)
construct
B2
Cyt
log2(ctrl.ratio)
log2(temp.ratio)
abundance.score
stability.score
score.value
rep
P43609 − Chromatin structure−remodeling complex protein RSC8 OS=Saccharomyces cerevisiae (strain ATCC 204508 / S288c) GN=RSC8 PE=1 SV=1

RSC9 − average.top3: 6.82622619047619 − max.qupm: 10

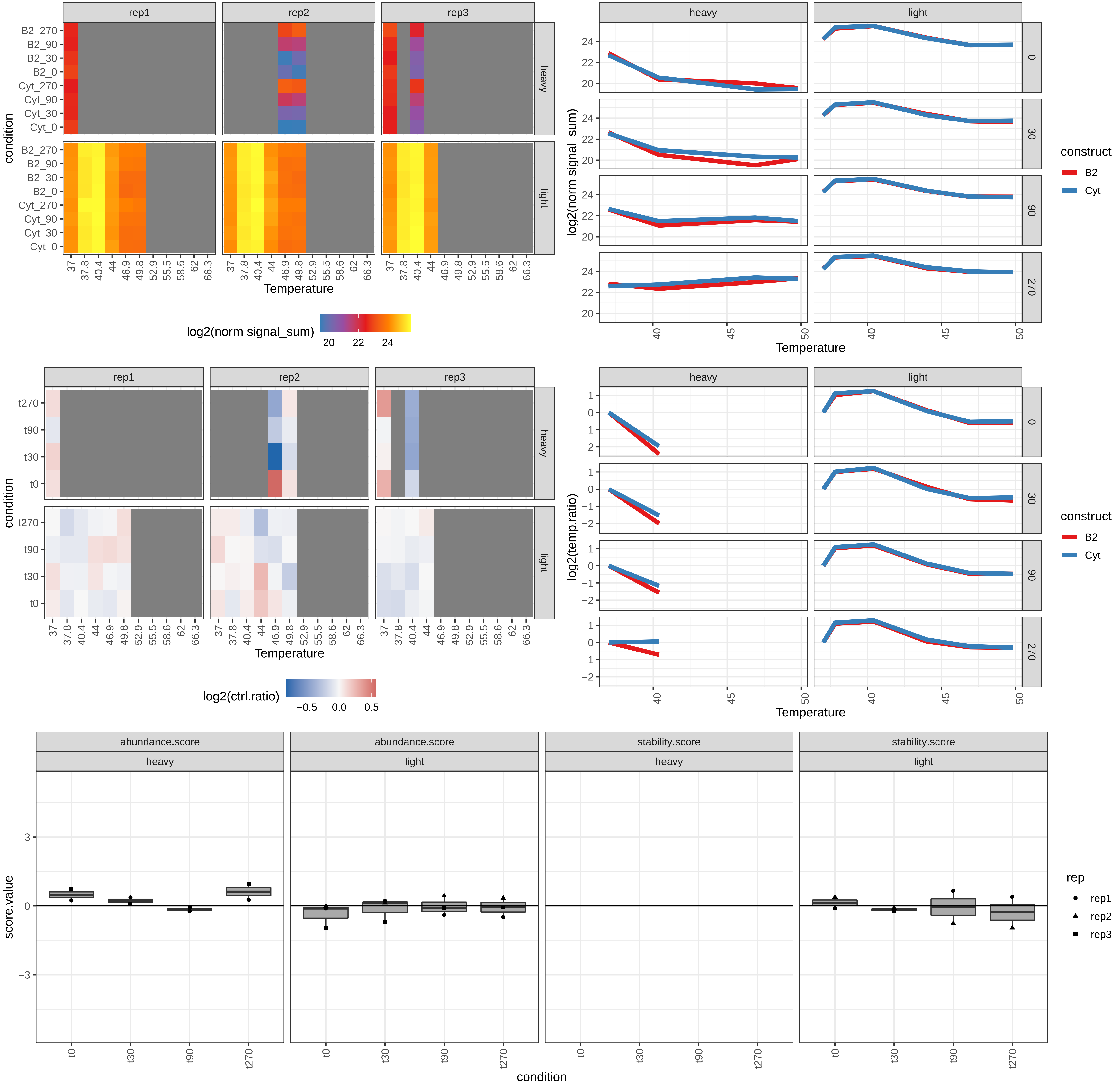

Q03124 − Chromatin structure−remodeling complex subunit RSC9 OS=Saccharomyces cerevisiae (strain ATCC 204508 / S288c) GN=RSC9 PE=1 SV=1

RSE1 − average.top3: 6.48320136363636 − max.qupm: 8

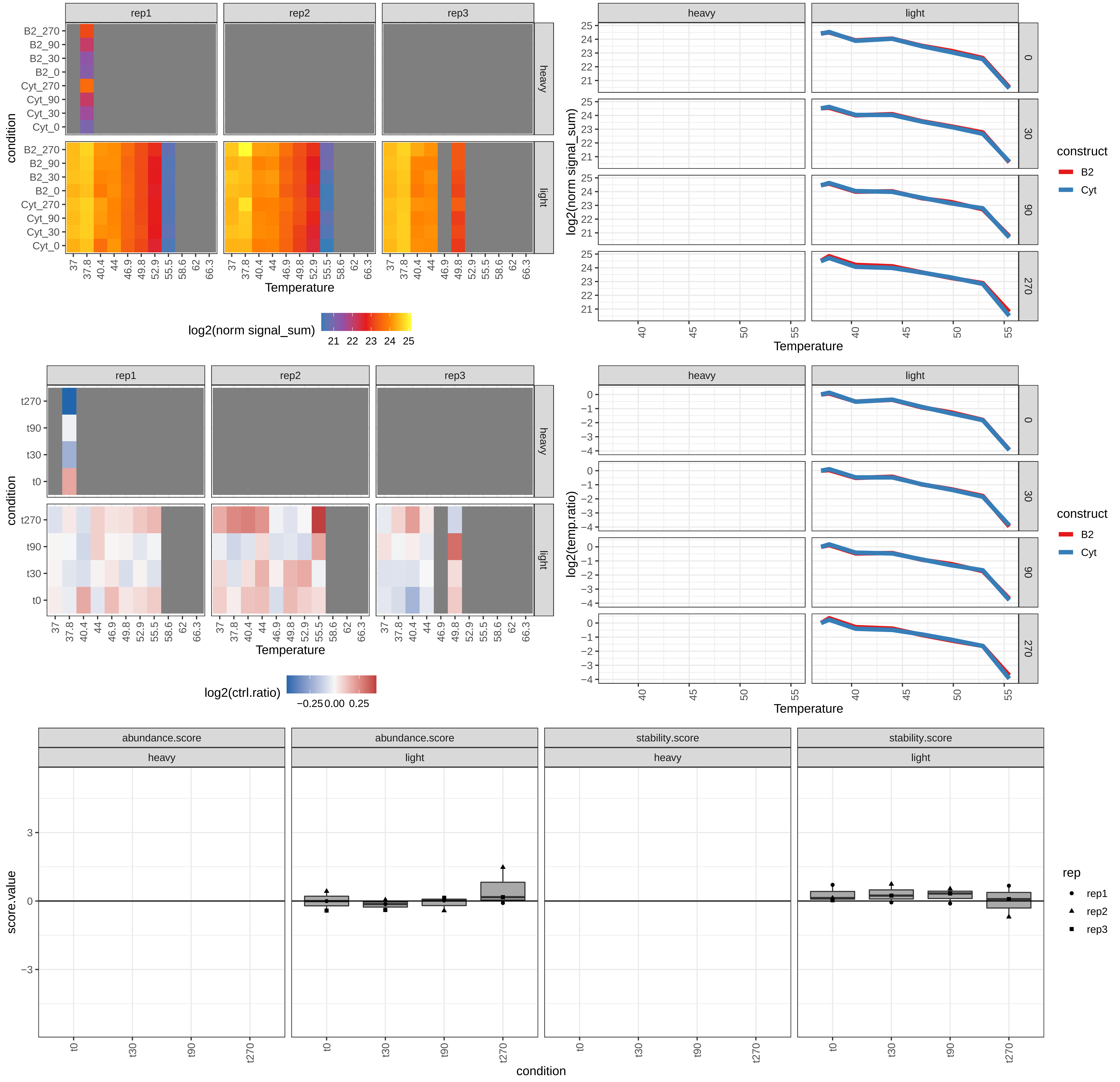

Q04693 − Pre−mRNA−splicing factor RSE1 OS=Saccharomyces cerevisiae (strain ATCC 204508 / S288c) GN=RSE1 PE=1 SV=1

RSM10 − average.top3: 6.45839666666667 − max.qupm: 7

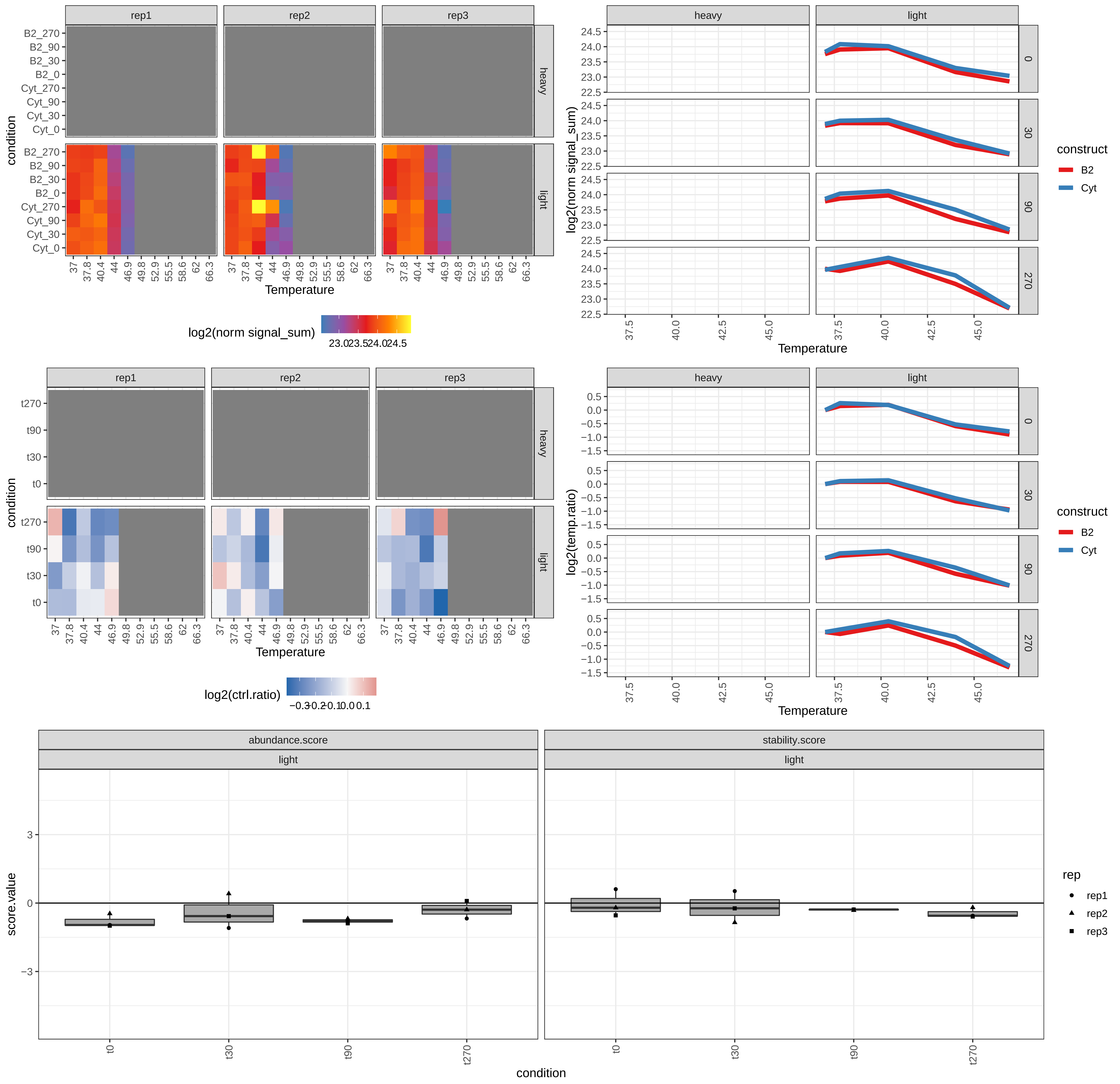

Q03201 − 37S ribosomal protein S10, mitochondrial OS=Saccharomyces cerevisiae (strain ATCC 204508 / S288c) GN=RSM10 PE=1 SV=1

RSM18 − average.top3: 6.89371666666667 − max.qupm: 5

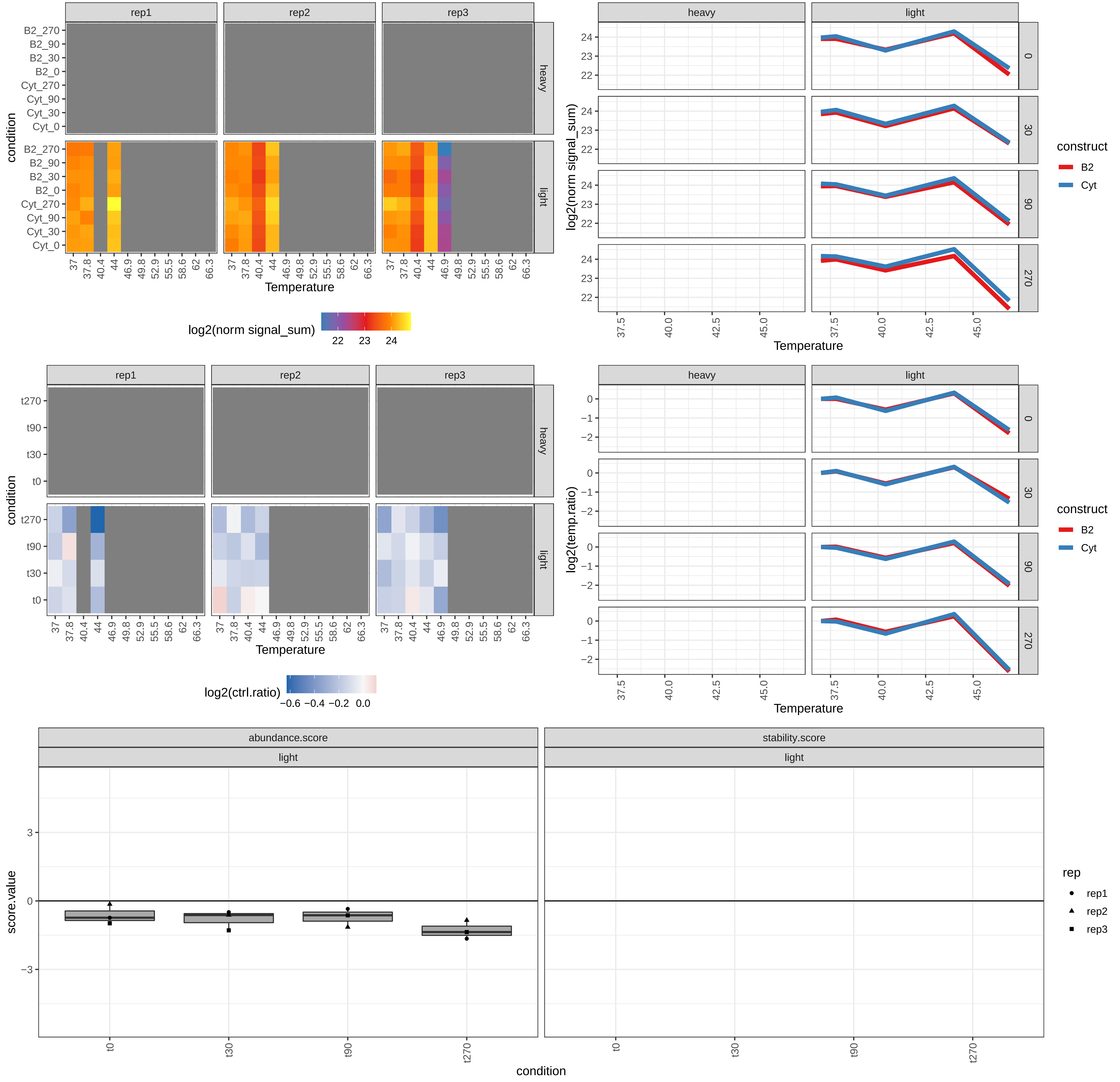

P40033 − 37S ribosomal protein RSM18, mitochondrial OS=Saccharomyces cerevisiae (strain ATCC 204508 / S288c) GN=RSM18 PE=1 SV=2

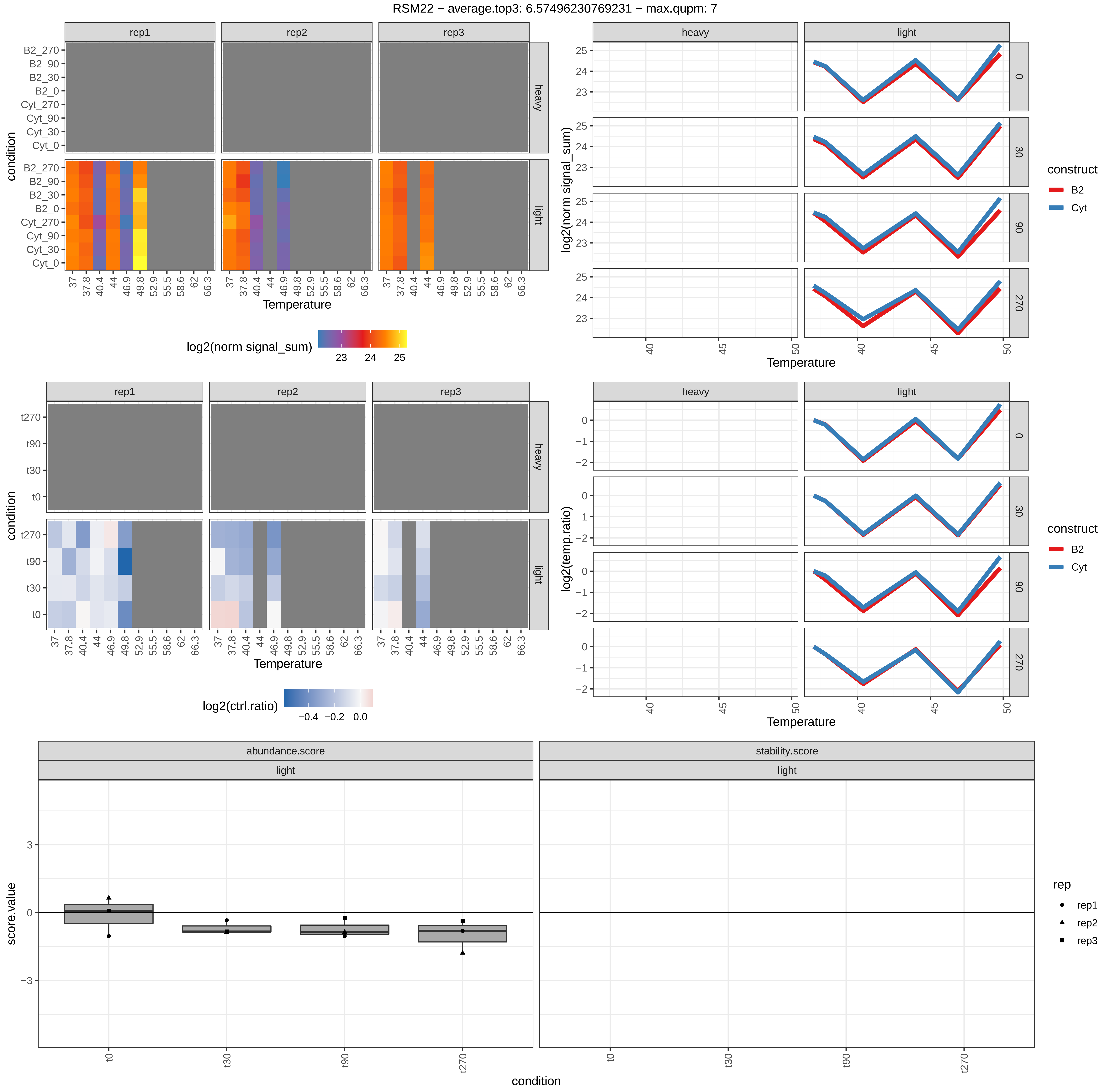

P36056 − 37S ribosomal protein S22, mitochondrial OS=Saccharomyces cerevisiae (strain ATCC 204508 / S288c) GN=RSM22 PE=1 SV=1

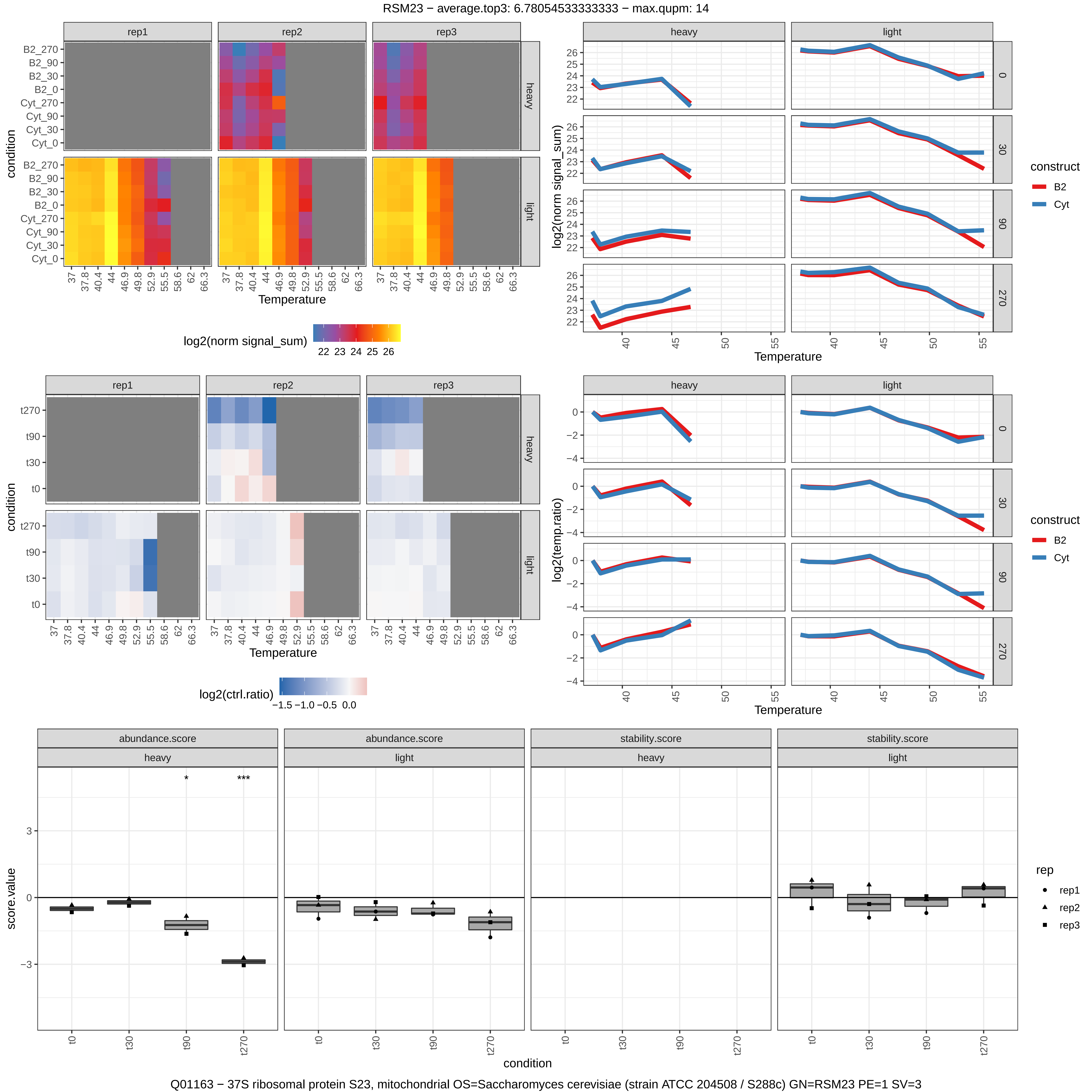

RSM23 − average.top3: 6.78054533333333 − max.qupm: 14
rep1
rep2
rep3
heavy
light
condition
Temperature
log2(norm signal_sum)
log2(ctrl.ratio)
construct
B2
Cyt
log2(temp.ratio)
abundance.score
stability.score
score.value
rep
rep1
rep2
rep3
Q01163 − 37S ribosomal protein S23, mitochondrial OS=Saccharomyces cerevisiae (strain ATCC 204508 / S288c) GN=RSM23 PE=1 SV=3

RSM24 − average.top3: 6.92624055555556 − max.qupm: 6

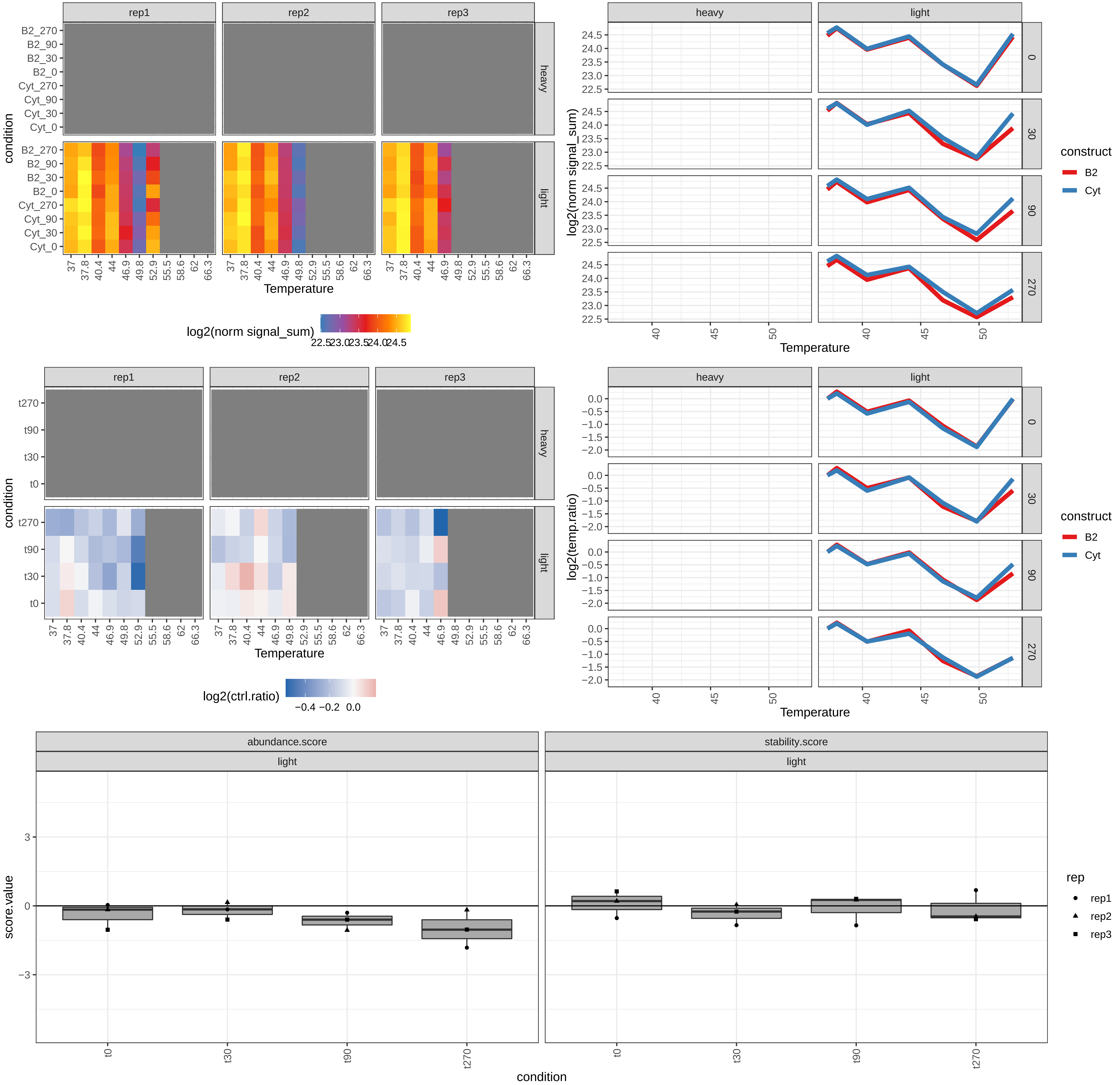

Q03976 − 37S ribosomal protein S24, mitochondrial OS=Saccharomyces cerevisiae (strain ATCC 204508 / S288c) GN=RSM24 PE=1 SV=1

RSM25 − average.top3: 6.871814 − max.qupm: 6

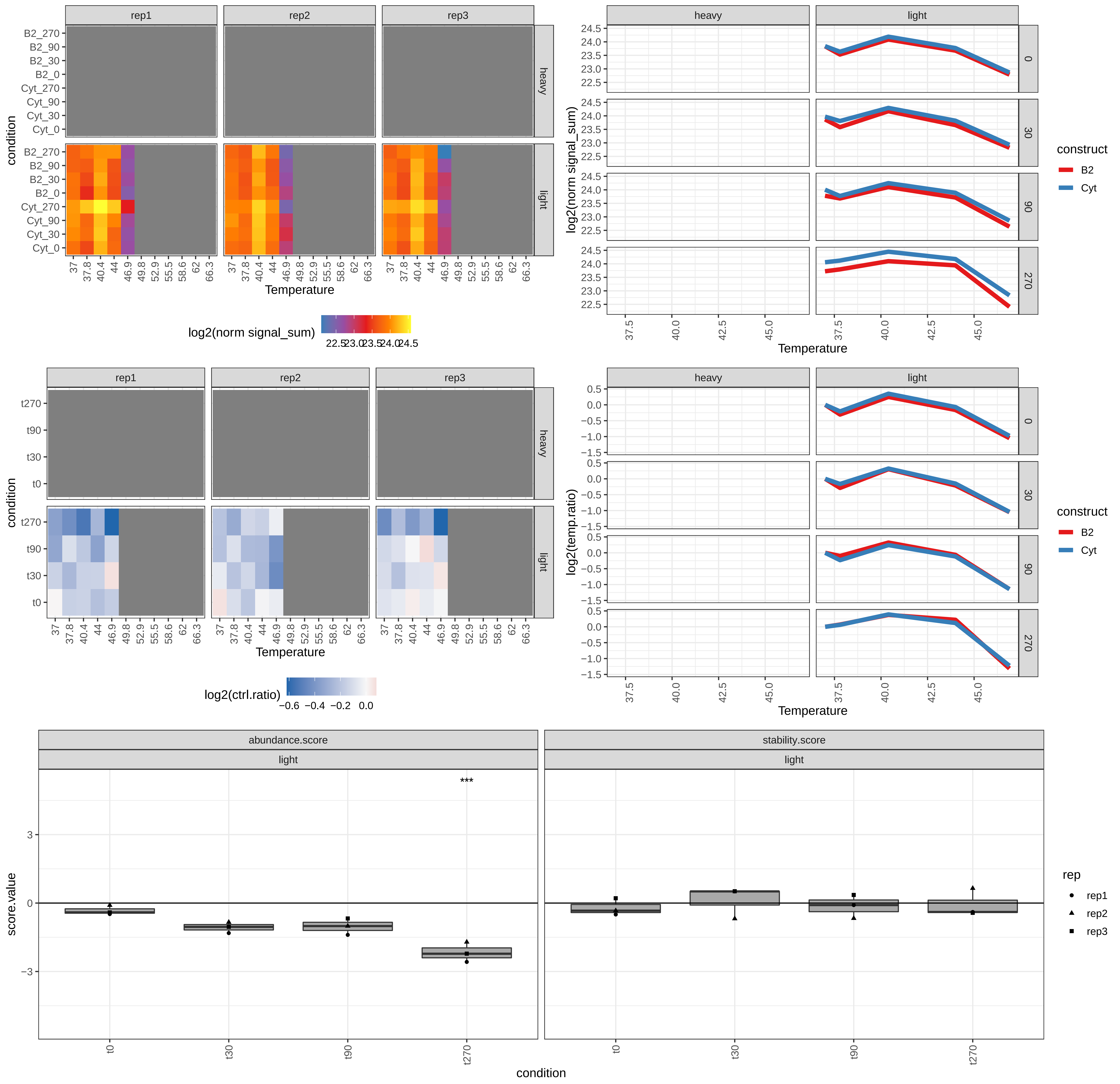

P40496 − 37S ribosomal protein S25, mitochondrial OS=Saccharomyces cerevisiae (strain ATCC 204508 / S288c) GN=RSM25 PE=1 SV=1

RSM7 − average.top3: 6.69972642857143 − max.qupm: 6

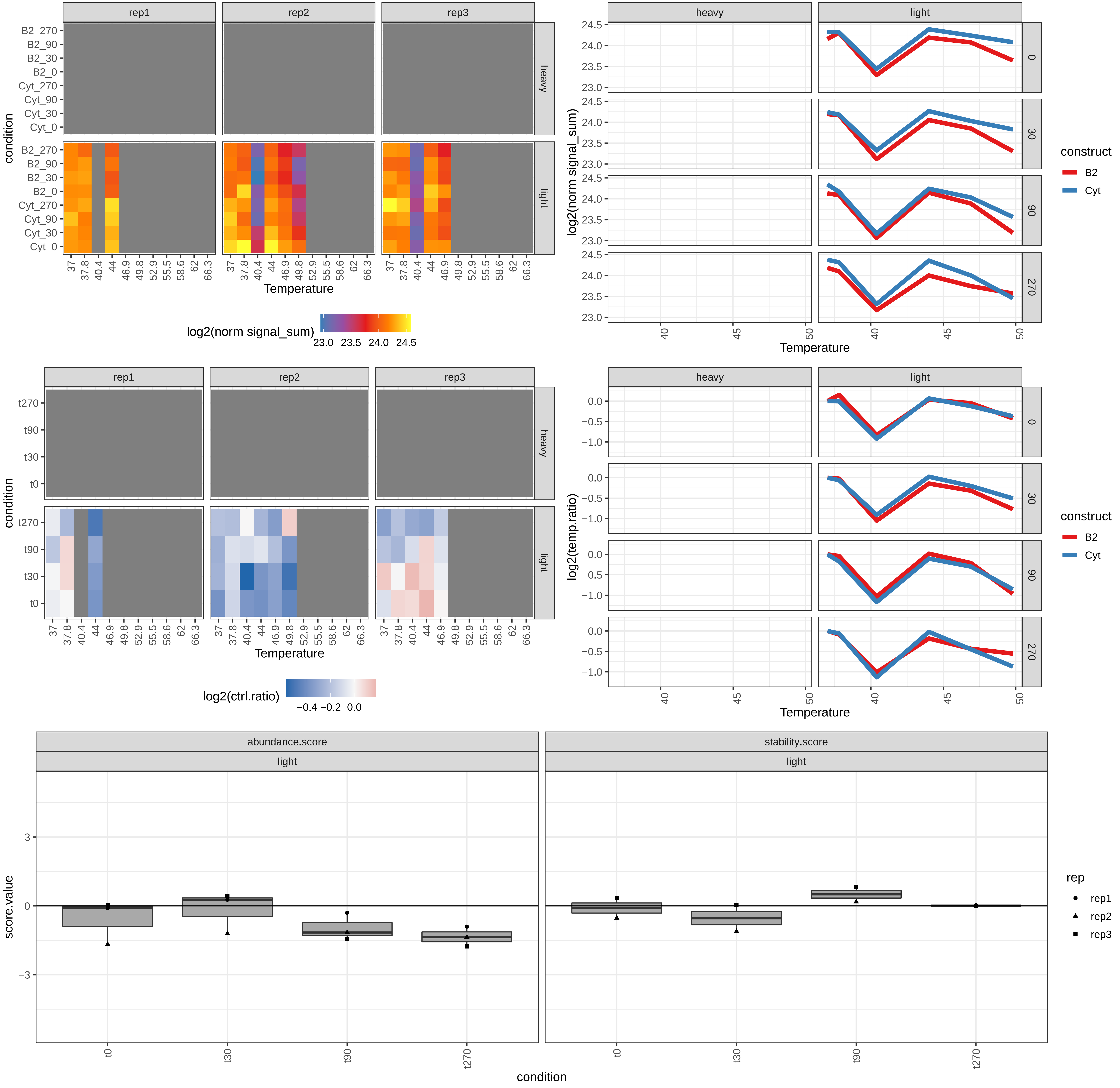

P47150 − 37S ribosomal protein S7, mitochondrial OS=Saccharomyces cerevisiae (strain ATCC 204508 / S288c) GN=RSM7 PE=1 SV=1

RSN1 − average.top3: 6.58778 − max.qupm: 4

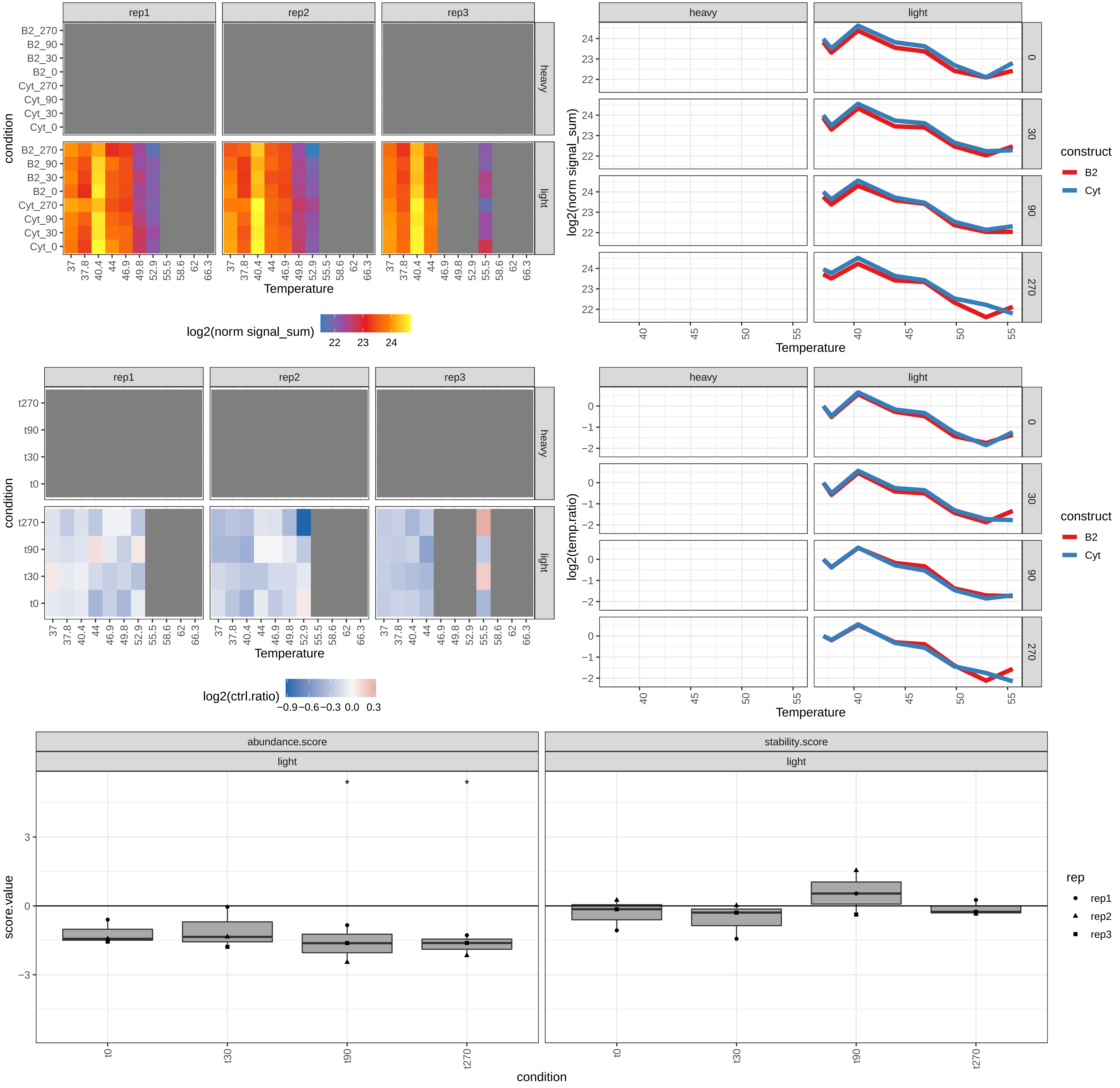

Q03516 − Uncharacterized protein RSN1 OS=Saccharomyces cerevisiae (strain ATCC 204508 / S288c) GN=RSN1 PE=1 SV=1

RSP5 − average.top3: 6.94951903846154 − max.qupm: 21

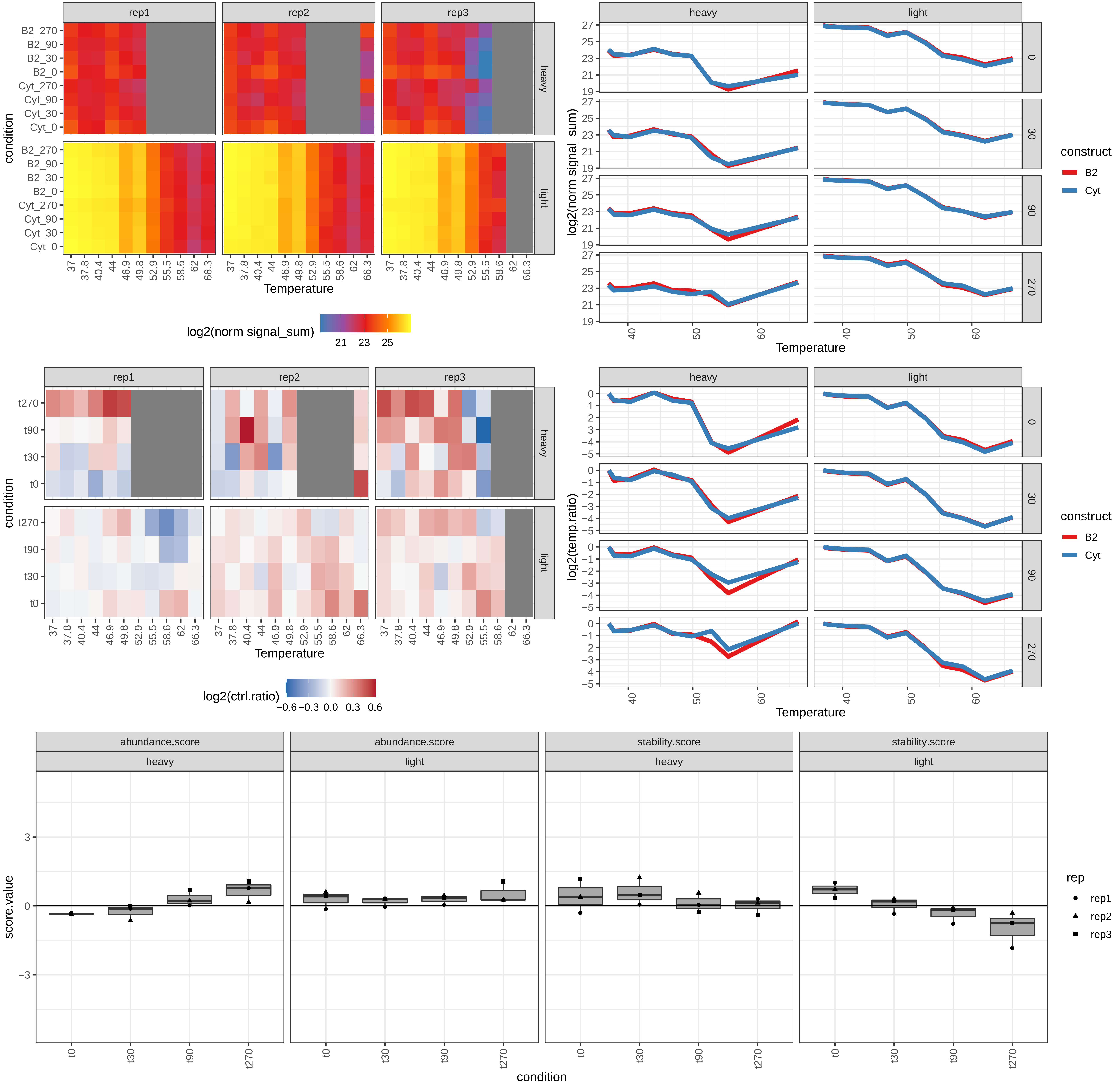

P39940 − E3 ubiquitin−protein ligase RSP5 OS=Saccharomyces cerevisiae (strain ATCC 204508 / S288c) GN=RSP5 PE=1 SV=1

RSR1 − average.top3: 6.70652958333333 − max.qupm: 4

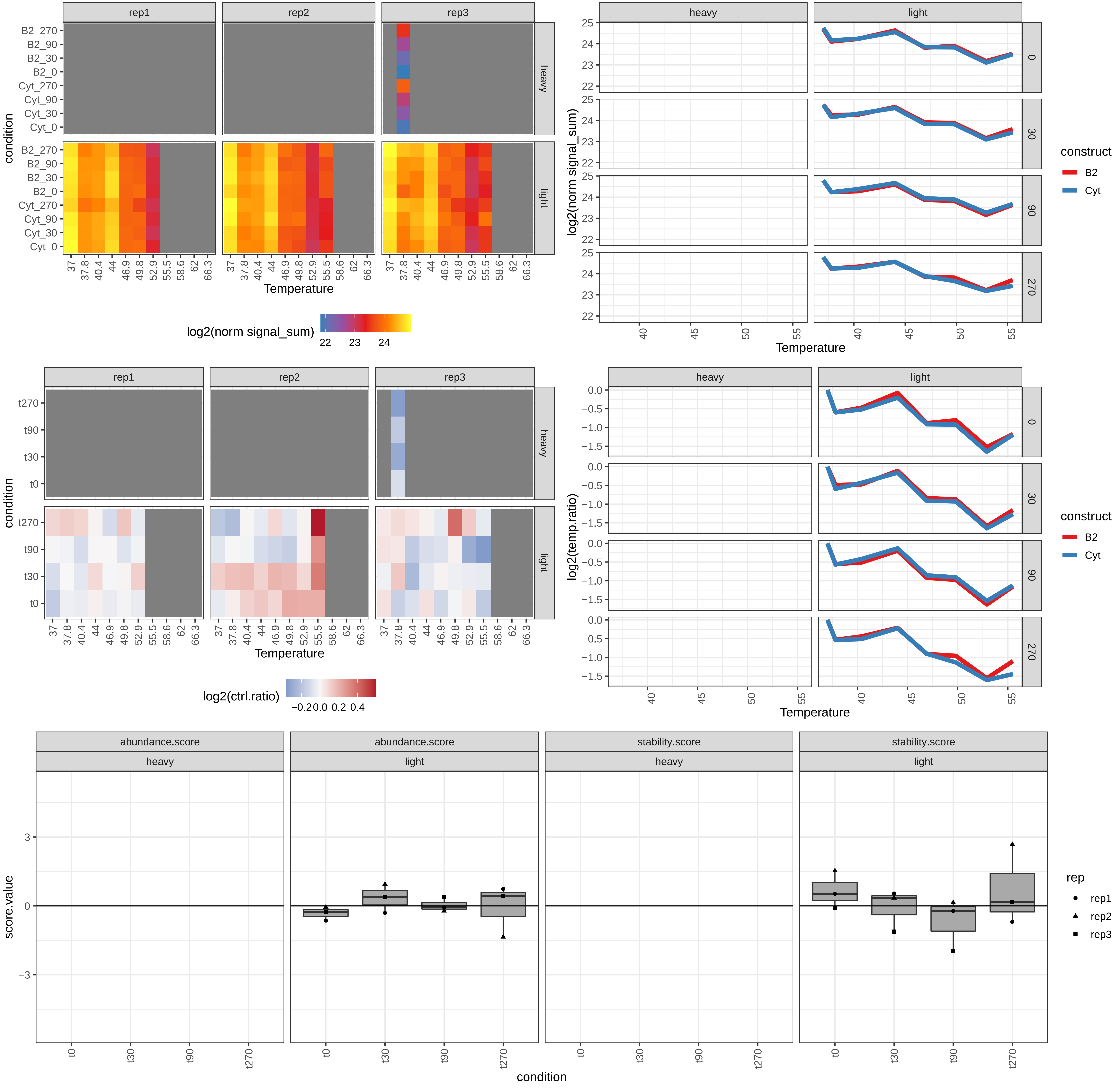

P13856 − Ras−related protein RSR1 OS=Saccharomyces cerevisiae (strain ATCC 204508 / S288c) GN=RSR1 PE=3 SV=1

RTC5 − average.top3: 6.52890894736842 − max.qupm: 11

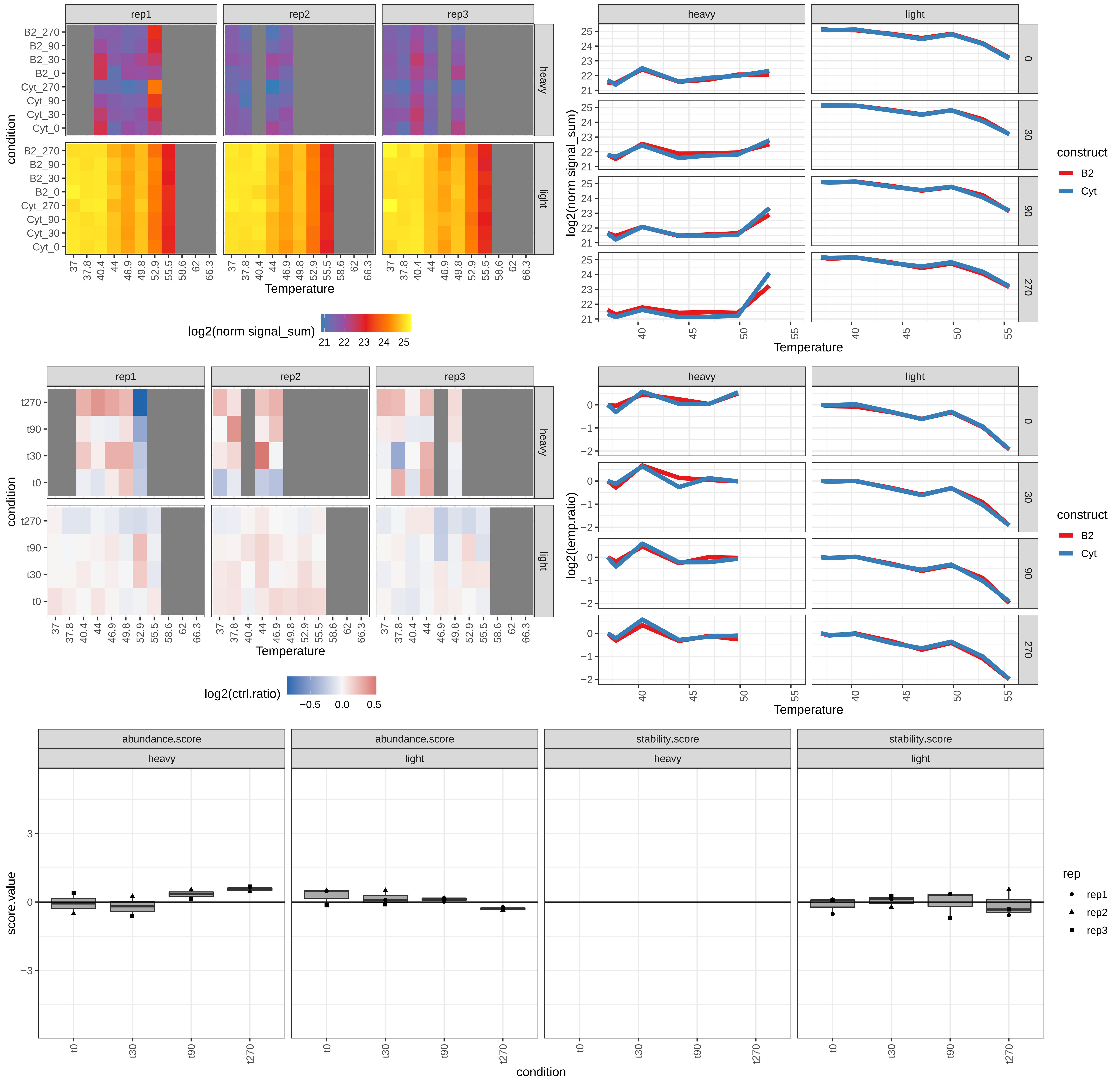

Q12108 − Restriction of telomere capping protein 5 OS=Saccharomyces cerevisiae (strain ATCC 204508 / S288c) GN=RTC5 PE=1 SV=1

RTF1 − average.top3: 6.77880464285714 − max.qupm: 9

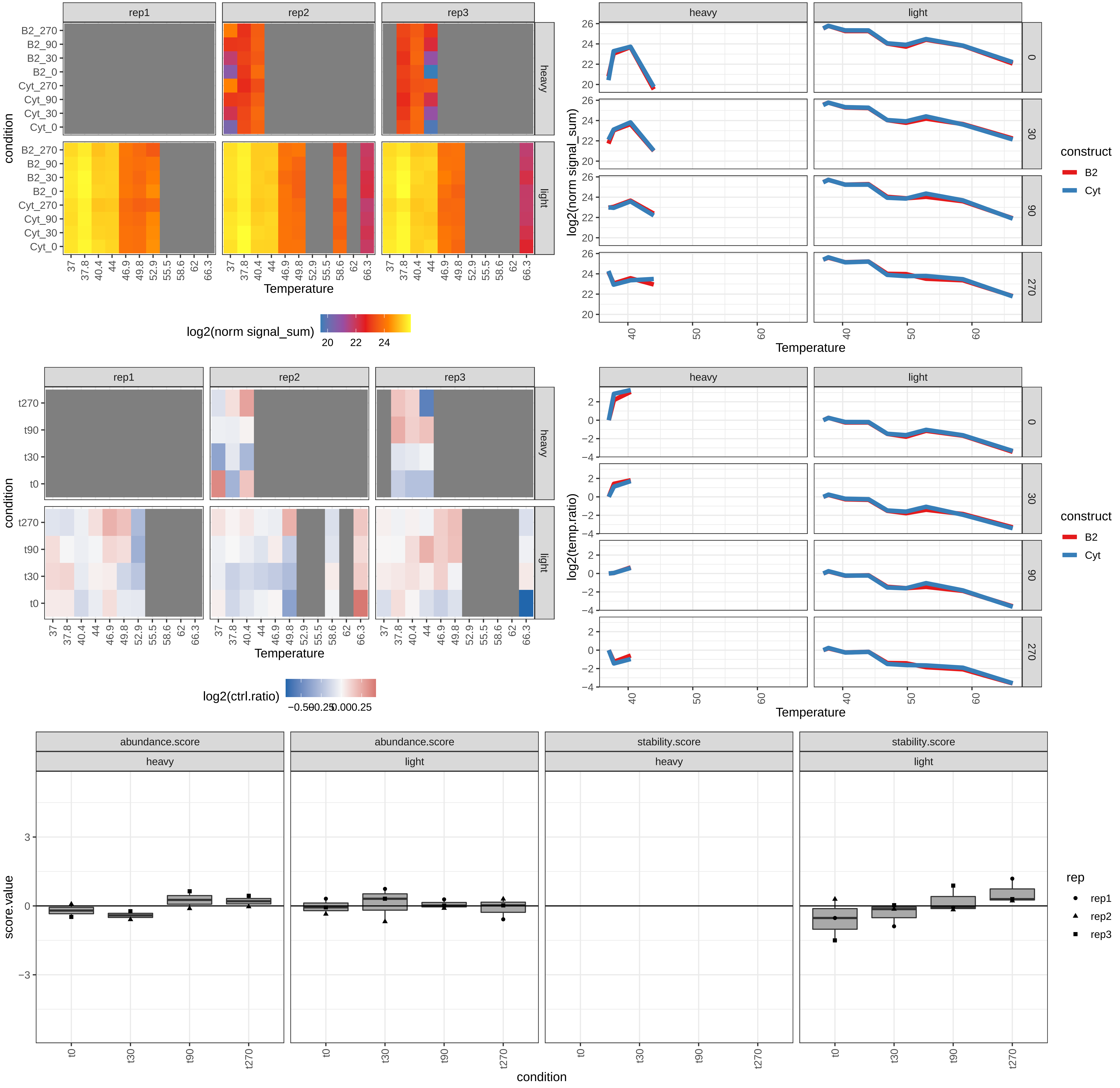

P53064 − RNA polymerase−associated protein RTF1 OS=Saccharomyces cerevisiae (strain ATCC 204508 / S288c) GN=RTF1 PE=1 SV=2

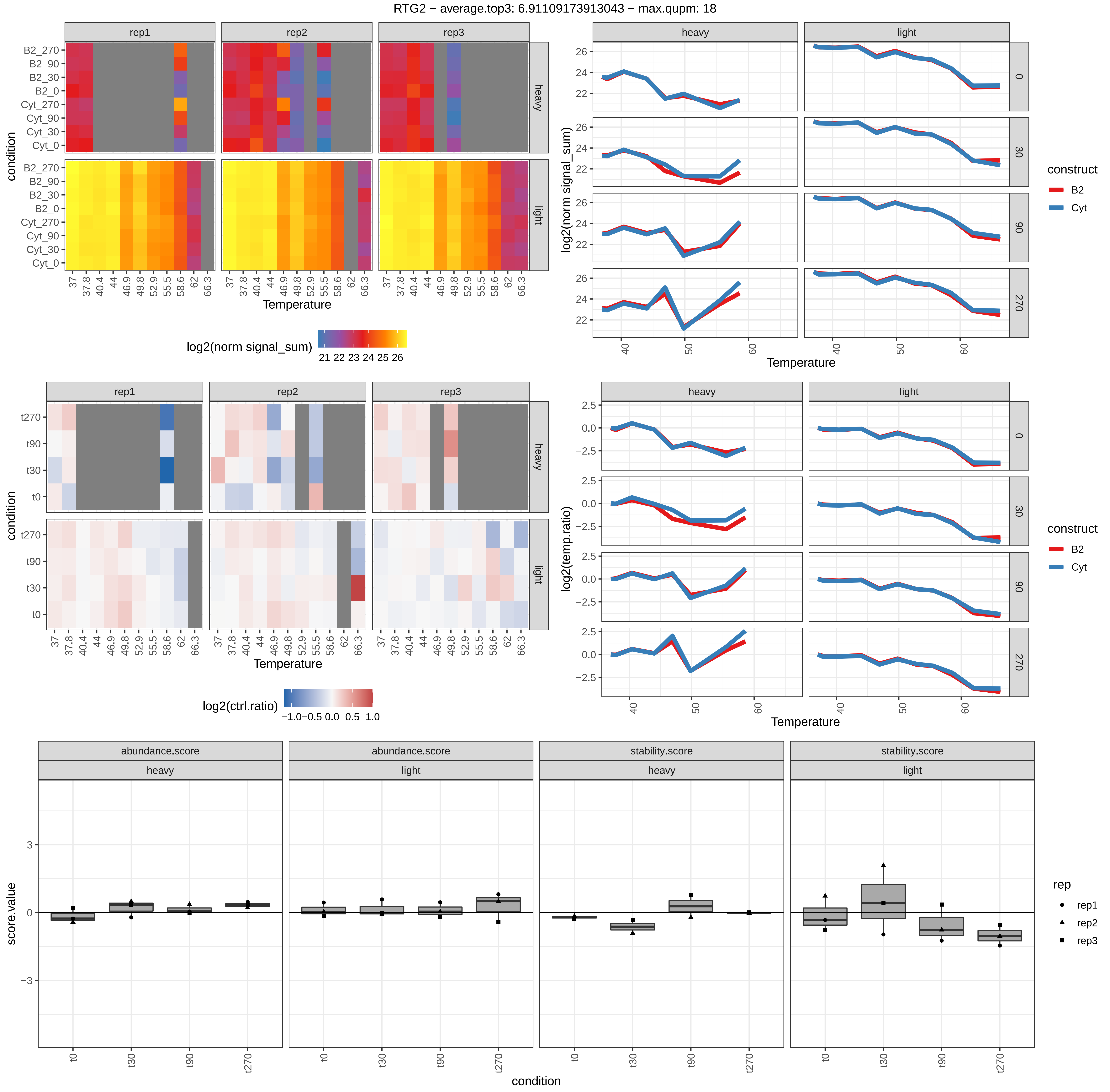

P32608 − Retrograde regulation protein 2 OS=Saccharomyces cerevisiae (strain ATCC 204508 / S288c) GN=RTG2 PE=1 SV=2

RTK1 − average.top3: 6.41283 − max.qupm: 4

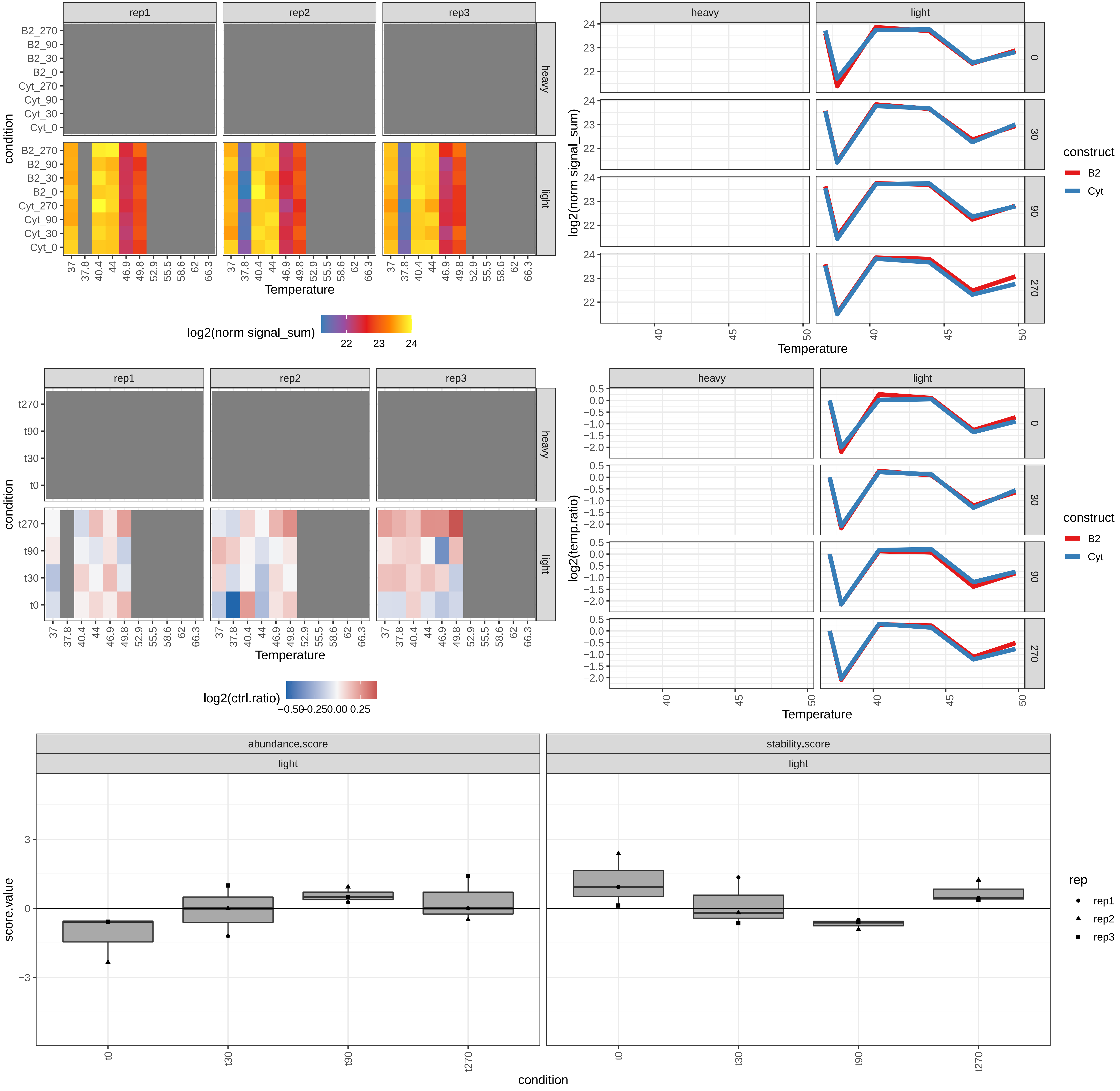

Q12100 − Probable serine/threonine−protein kinase RTK1 OS=Saccharomyces cerevisiae (strain ATCC 204508 / S288c) GN=RTK1 PE=1 SV=1

RTN1 − average.top3: 7.39229171875 − max.qupm: 13

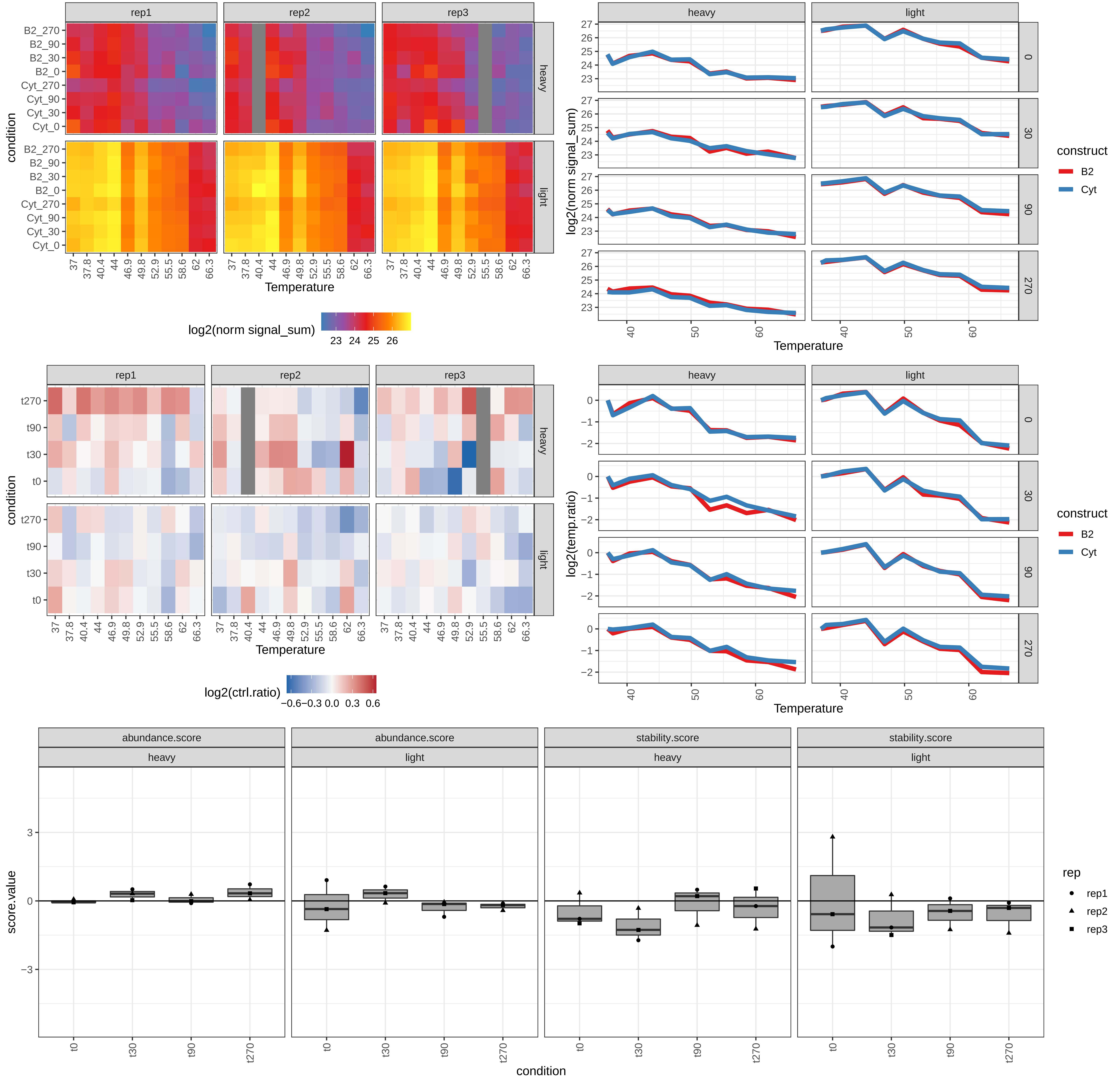

Q04947 − Reticulon−like protein 1 OS=Saccharomyces cerevisiae (strain ATCC 204508 / S288c) GN=RTN1 PE=1 SV=1

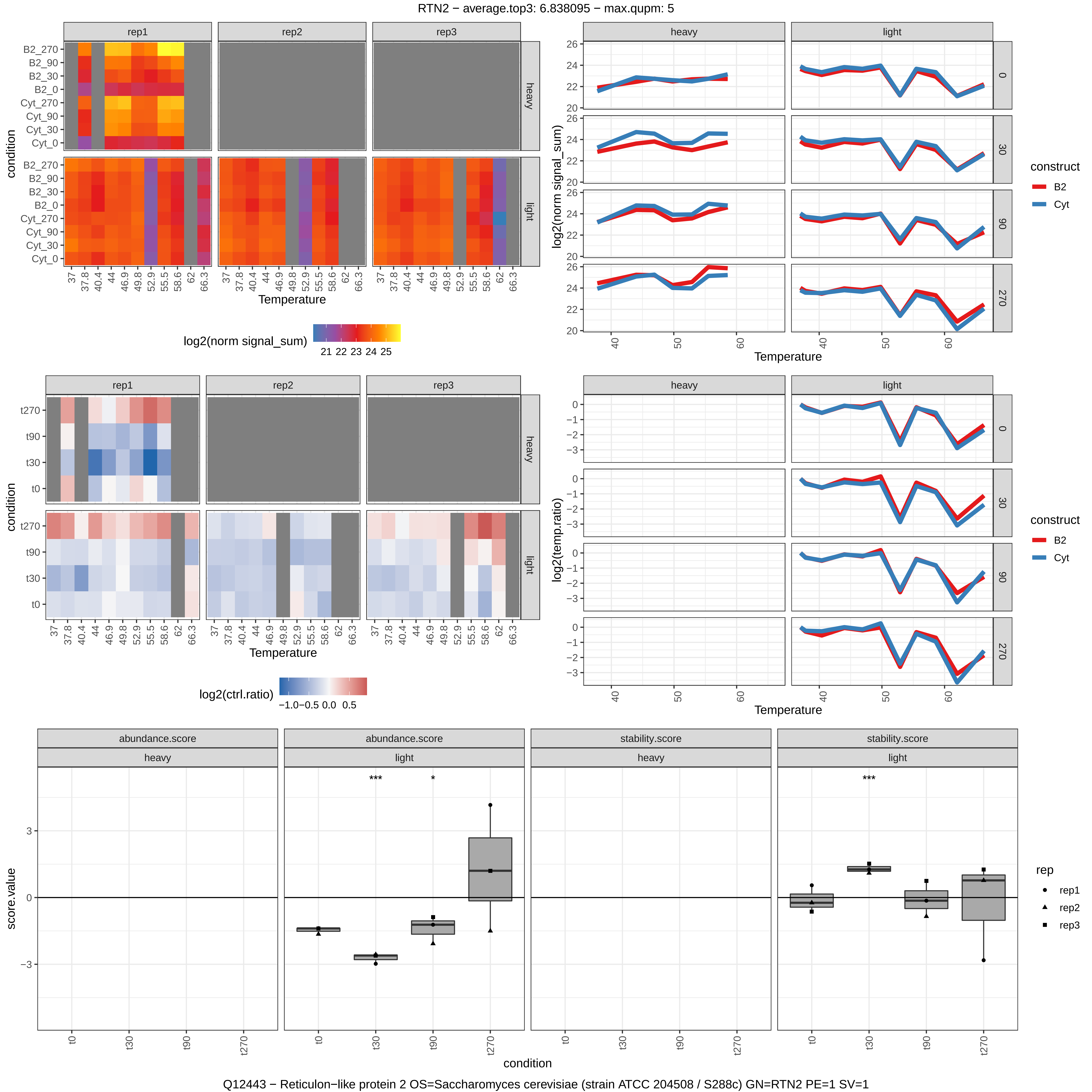

RTN2 − average.top3: 6.838095 − max.qupm: 5
rep1
rep2
rep3
heavy
light
condition
Temperature
log2(norm signal_sum)
construct
B2
Cyt
log2(ctrl.ratio)
log2(temp.ratio)
abundance.score
stability.score
score.value
rep
rep1
rep2
rep3
Q12443 − Reticulon−like protein 2 OS=Saccharomyces cerevisiae (strain ATCC 204508 / S288c) GN=RTN2 PE=1 SV=1

RTS1 − average.top3: 6.68112709677419 − max.qupm: 6

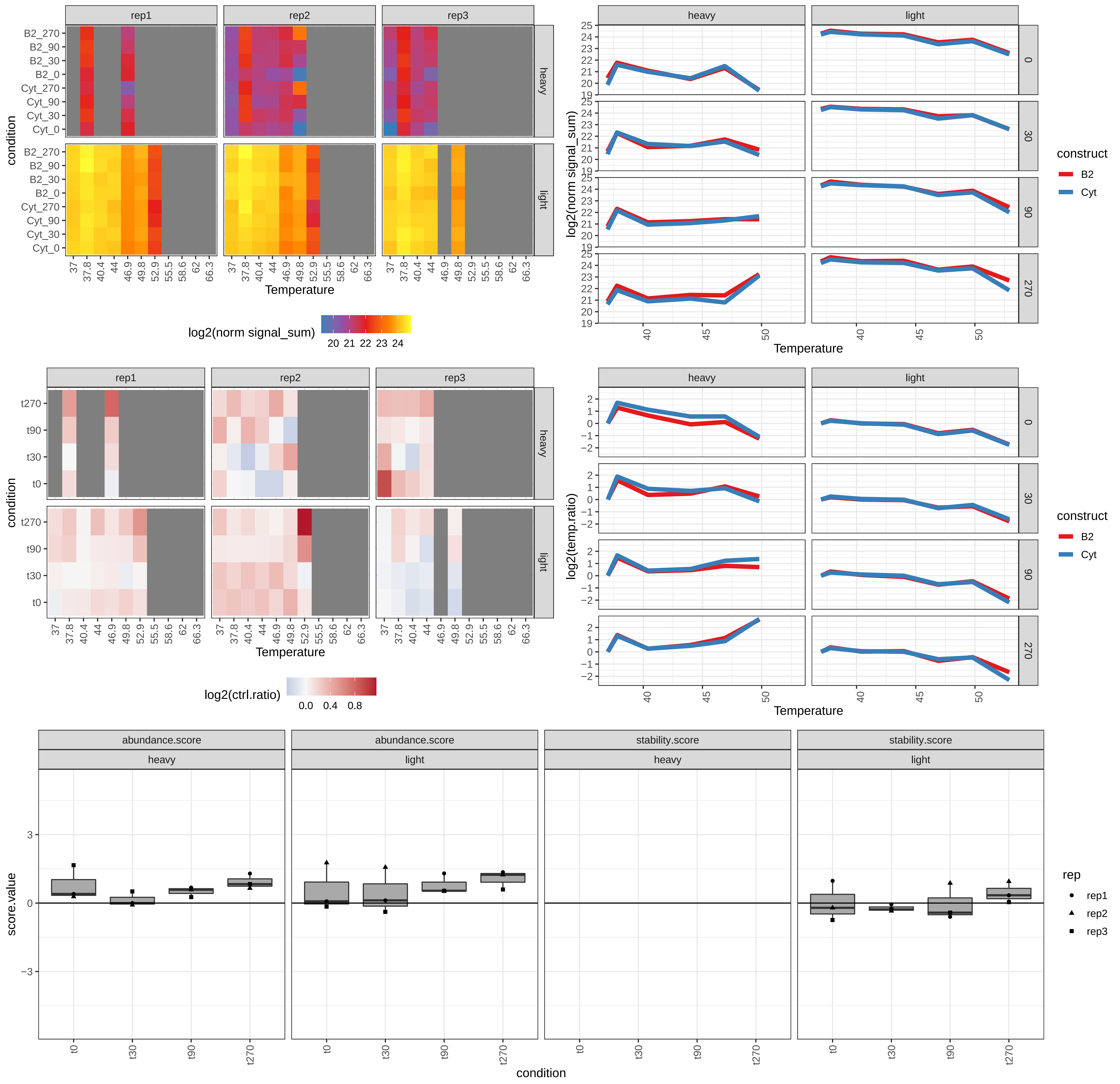

P38903 − Serine/threonine−protein phosphatase 2A 56 kDa regulatory subunit delta isoform OS=Saccharomyces cerevisiae (strain ATCC 204508 / S288c) GN=RTS1 PE=1 SV=2

RTT10 − average.top3: 6.94149121212121 − max.qupm: 15

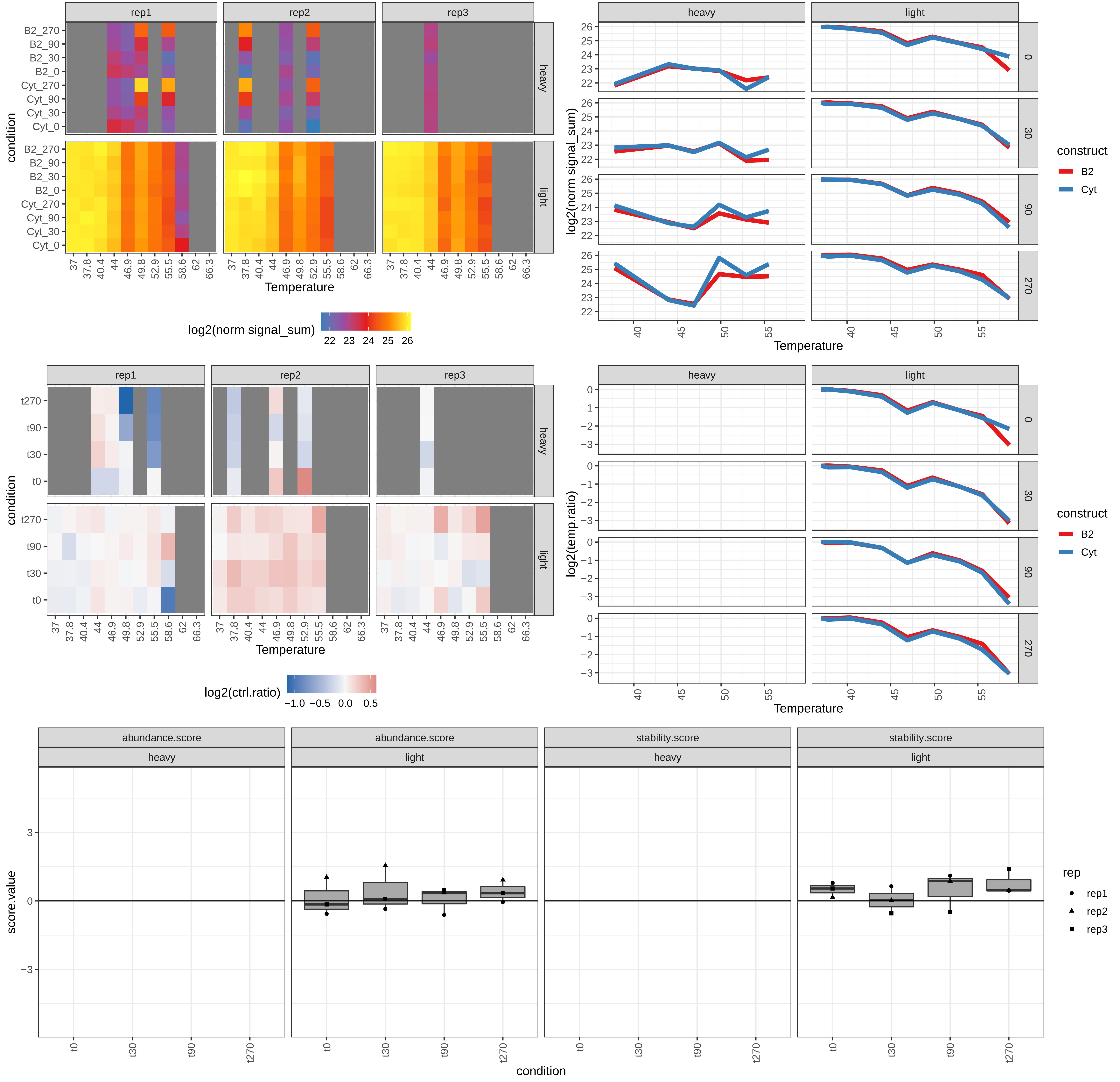

Q08924 − Regulator of Ty1 transposition protein 10 OS=Saccharomyces cerevisiae (strain ATCC 204508 / S288c) GN=RTT10 PE=1 SV=1

RTT101 − average.top3: 6.36789375 − max.qupm: 5

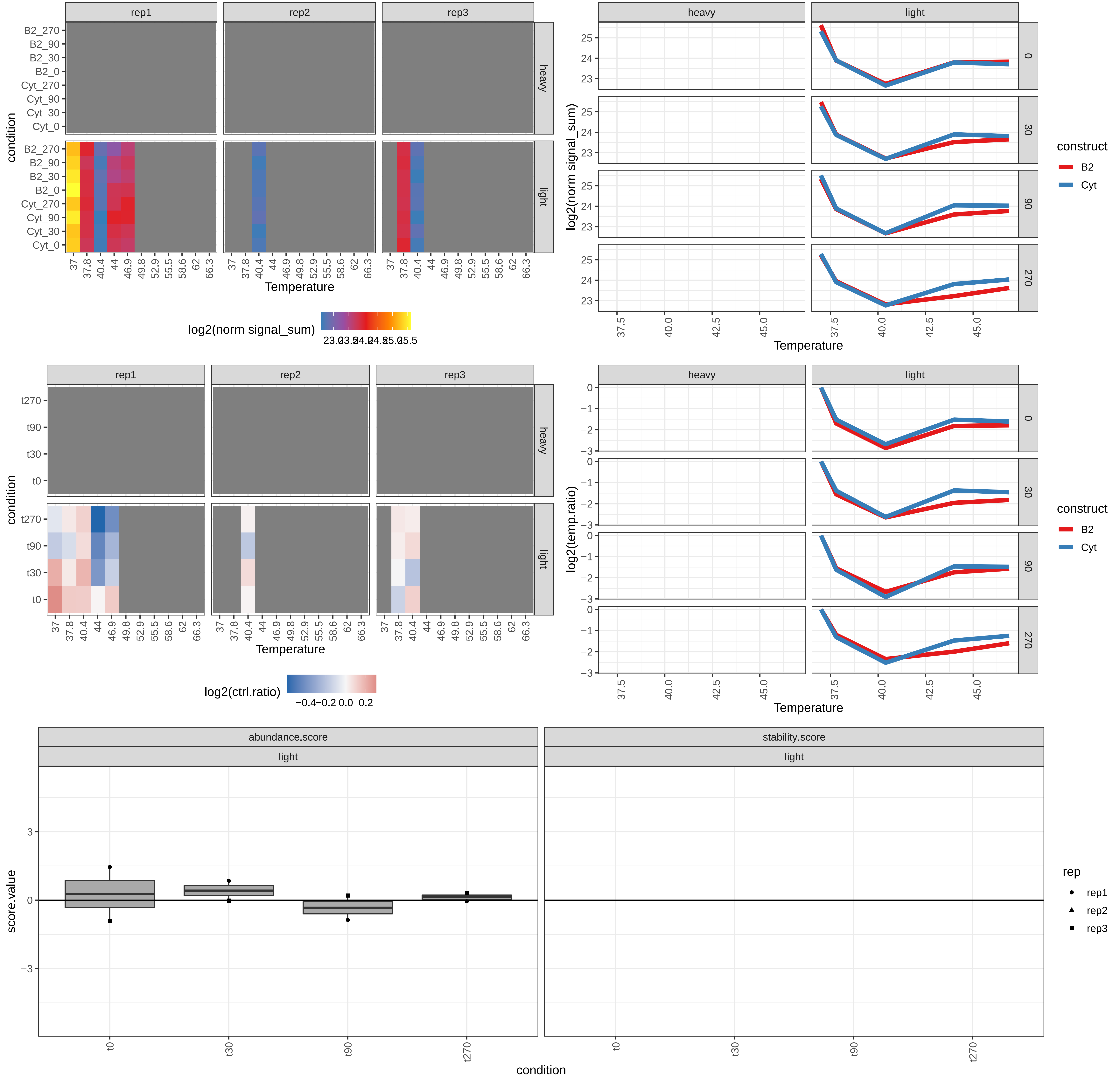

P47050 − Cullin−8 OS=Saccharomyces cerevisiae (strain ATCC 204508 / S288c) GN=RTT101 PE=1 SV=1

RTT103 − average.top3: 6.4196325 − max.qupm: 4

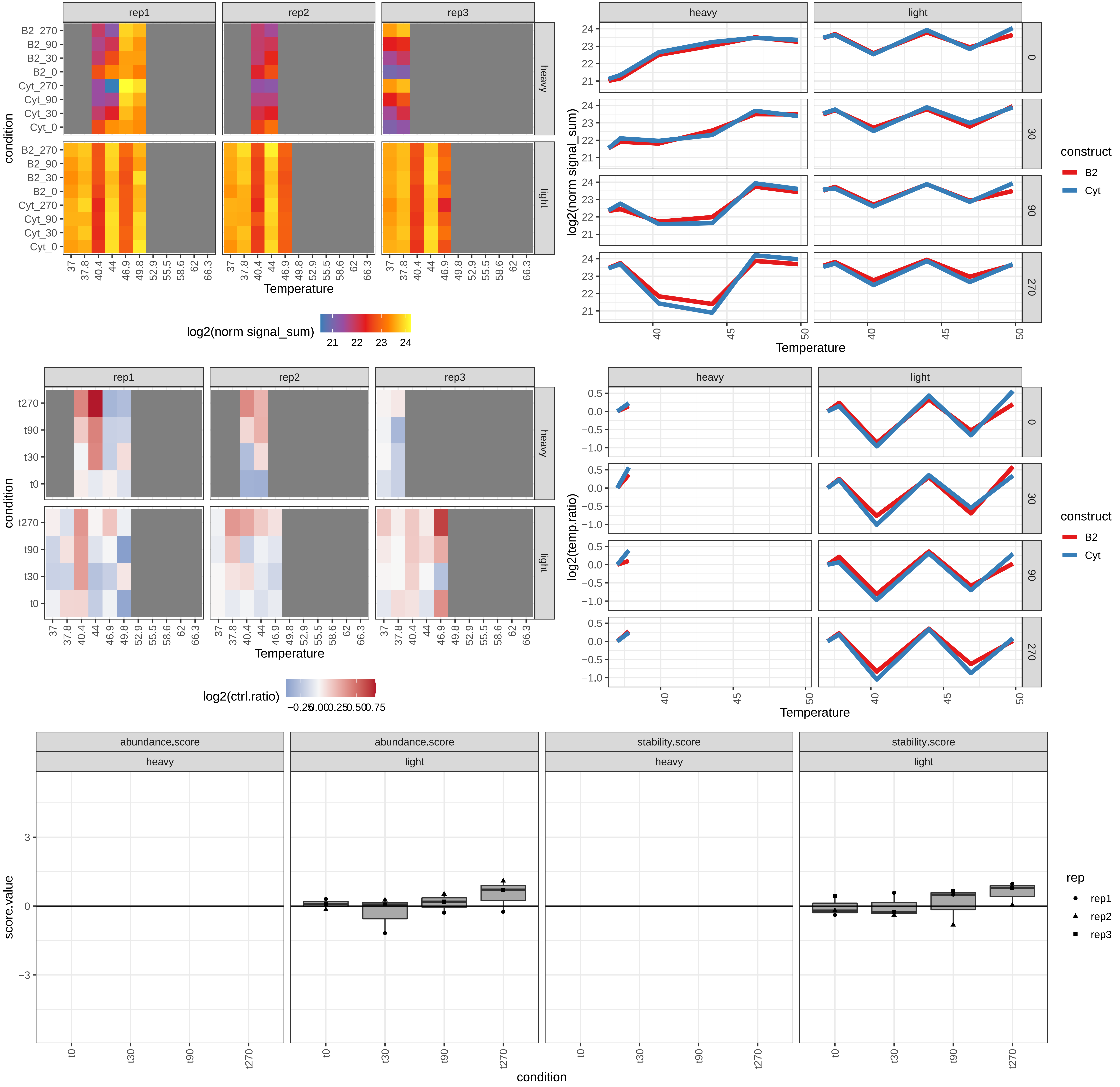

Q05543 − Regulator of Ty1 transposition protein 103 OS=Saccharomyces cerevisiae (strain ATCC 204508 / S288c) GN=RTT103 PE=1 SV=1

RTT106 − average.top3: 6.38375666666667 − max.qupm: 4

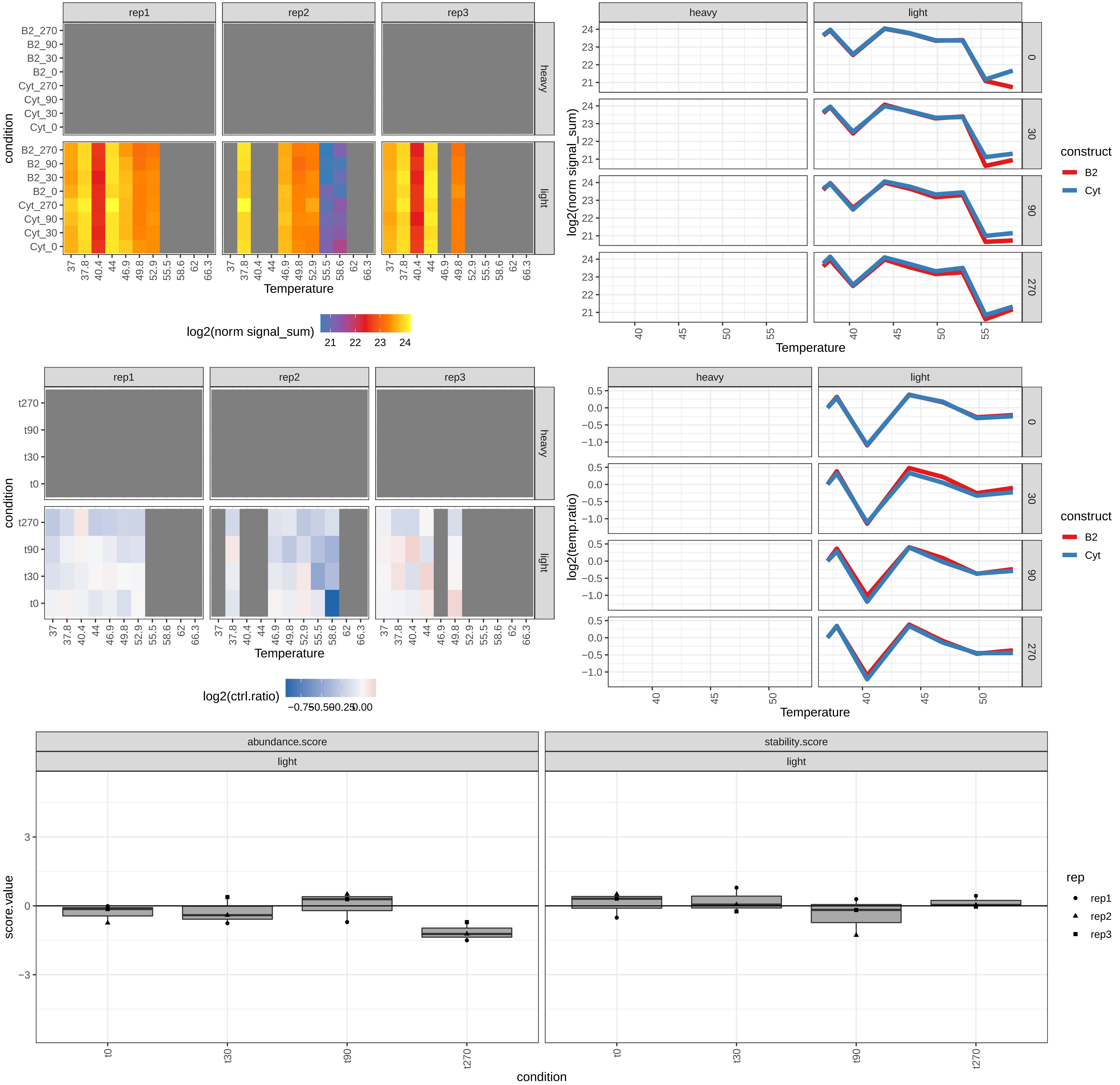

P40161 − Histone chaperone RTT106 OS=Saccharomyces cerevisiae (strain ATCC 204508 / S288c) GN=RTT106 PE=1 SV=1

RTT107 − average.top3: 6.44119227272727 − max.qupm: 6

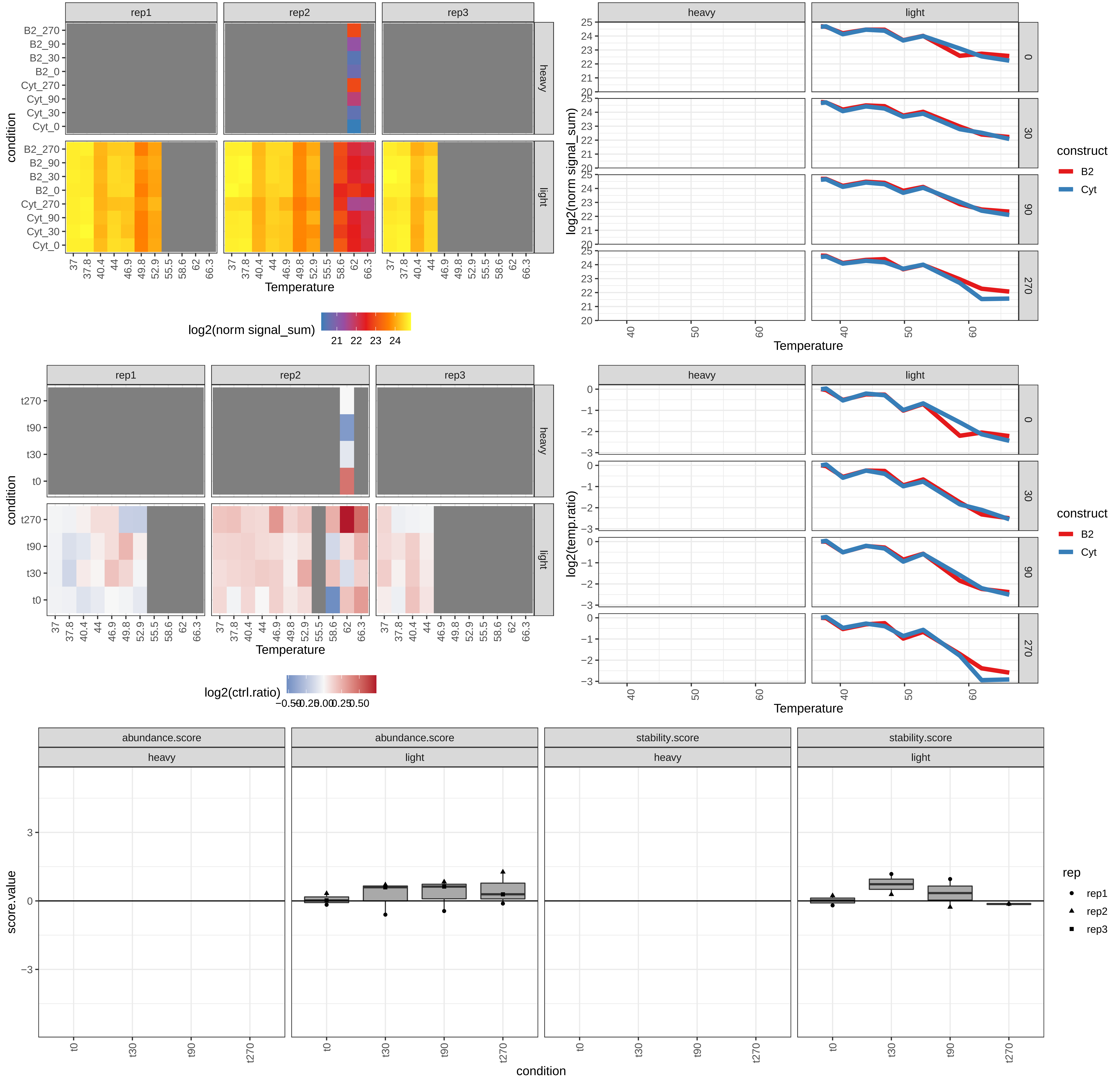

P38850 − Regulator of Ty1 transposition protein 107 OS=Saccharomyces cerevisiae (strain ATCC 204508 / S288c) GN=RTT107 PE=1 SV=1

RUB1 − average.top3: 7.35924038461538 − max.qupm: 5

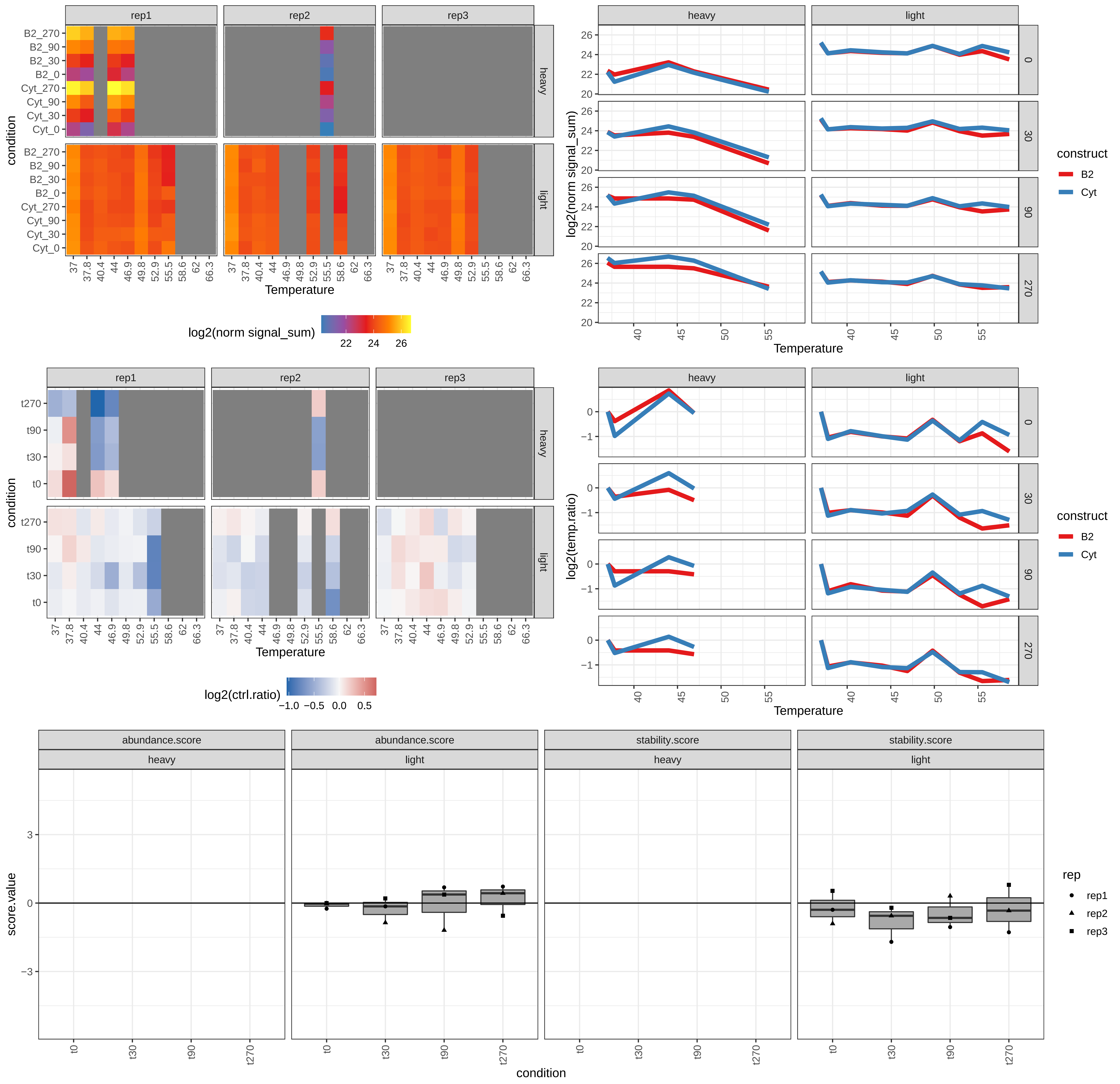

Q03919 − NEDD8−like protein RUB1 OS=Saccharomyces cerevisiae (strain ATCC 204508 / S288c) GN=RUB1 PE=1 SV=1

RUD3 − average.top3: 6.59623636363636 − max.qupm: 5

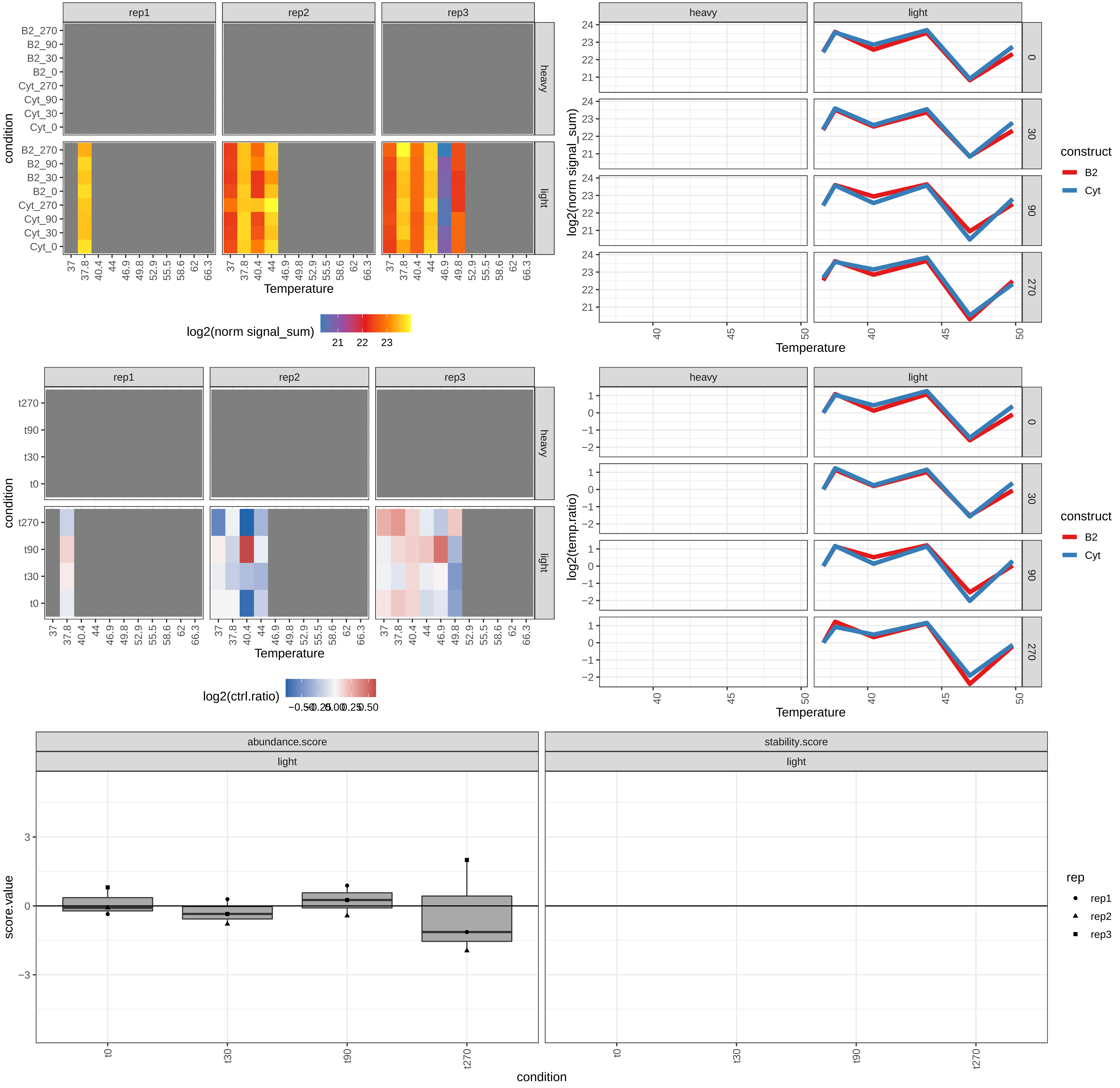

Q12234 − GRIP domain−containing protein RUD3 OS=Saccharomyces cerevisiae (strain ATCC 204508 / S288c) GN=RUD3 PE=1 SV=1

RUP1 − average.top3: 7.11352 − max.qupm: 10

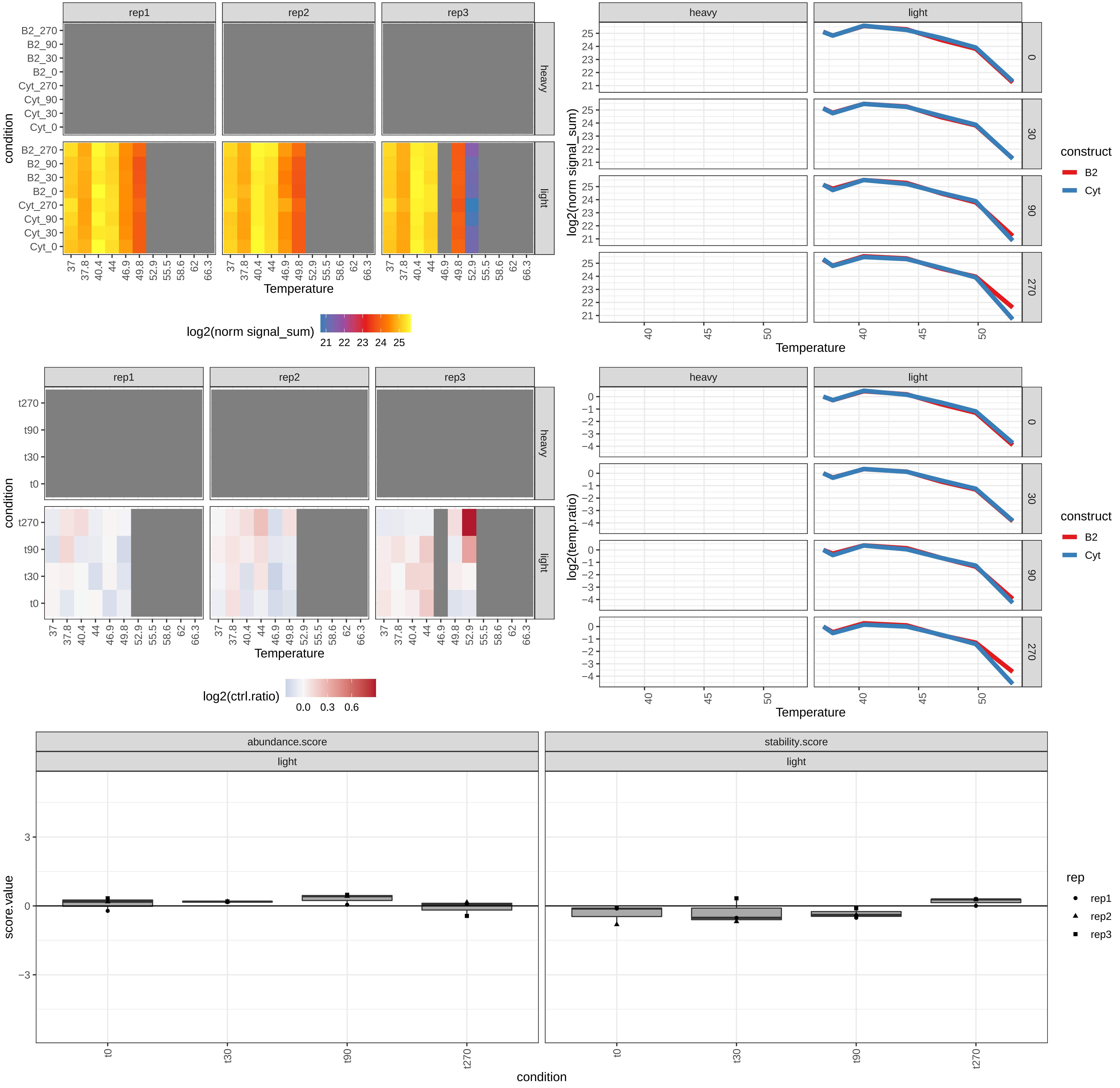

Q12242 − UBA domain−containing protein RUP1 OS=Saccharomyces cerevisiae (strain ATCC 204508 / S288c) GN=RUP1 PE=1 SV=1

RVB1 − average.top3: 7.09825162162162 − max.qupm: 11

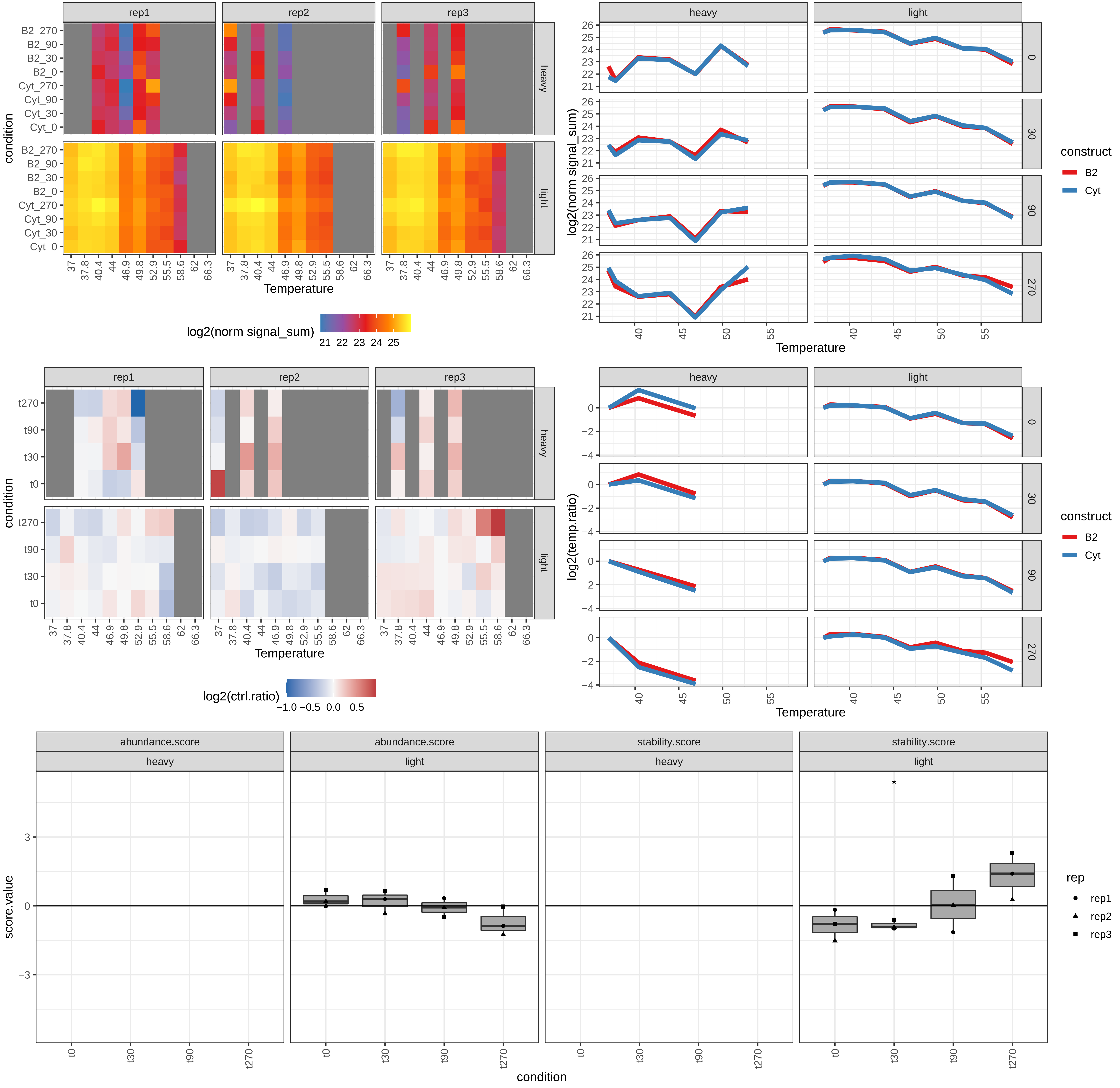

Q03940 − RuvB−like protein 1 OS=Saccharomyces cerevisiae (strain ATCC 204508 / S288c) GN=RVB1 PE=1 SV=1

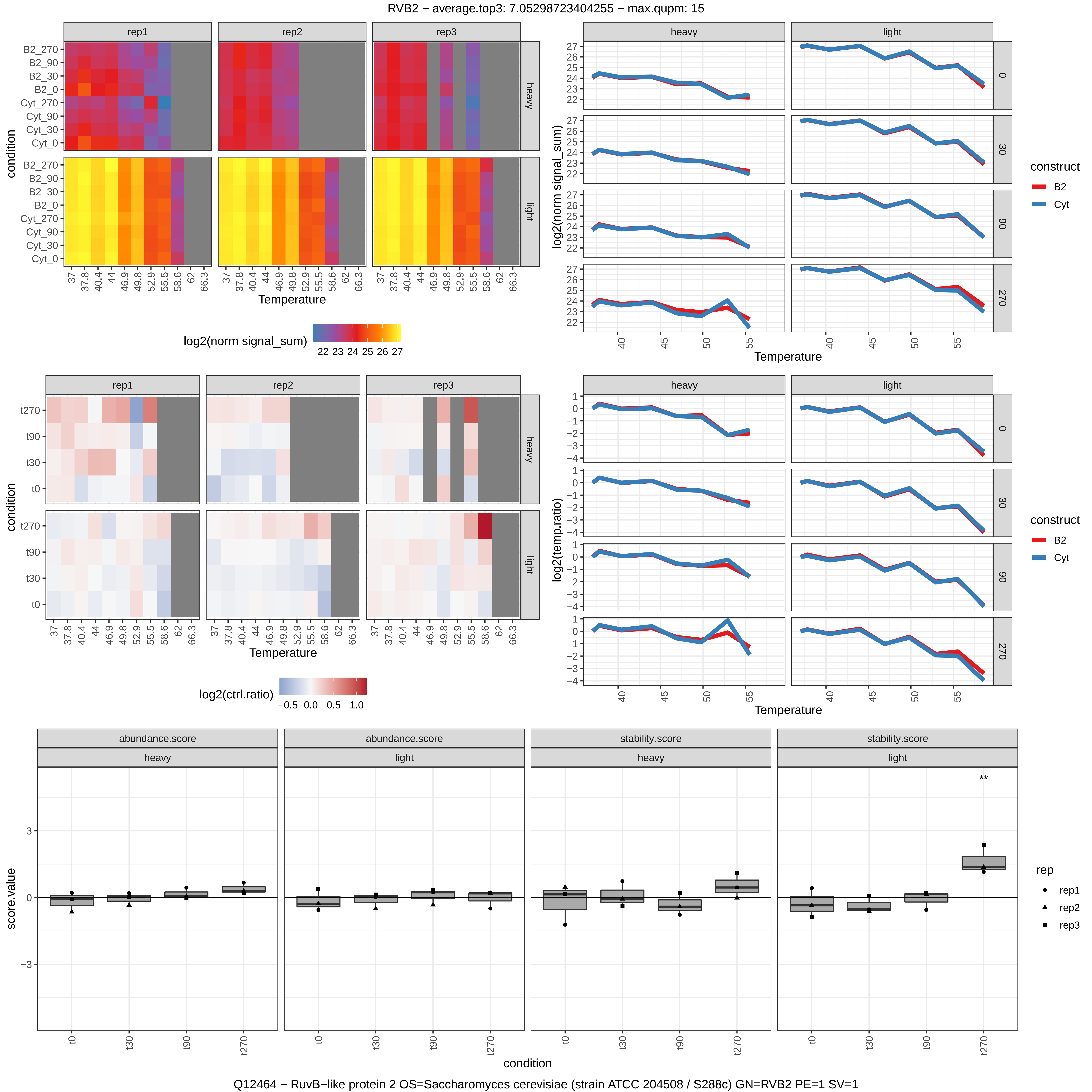

RVB2 − average.top3: 7.05298723404255 − max.qupm: 15
rep1
rep2
rep3
heavy
light
condition
Temperature
log2(norm signal_sum)
construct
B2
Cyt
log2(temp.ratio)
log2(ctrl.ratio)
abundance.score
stability.score
score.value
rep
rep1
rep2
rep3
**
Q12464 − RuvB−like protein 2 OS=Saccharomyces cerevisiae (strain ATCC 204508 / S288c) GN=RVB2 PE=1 SV=1

RVS161 − average.top3: 7.505045 − max.qupm: 15

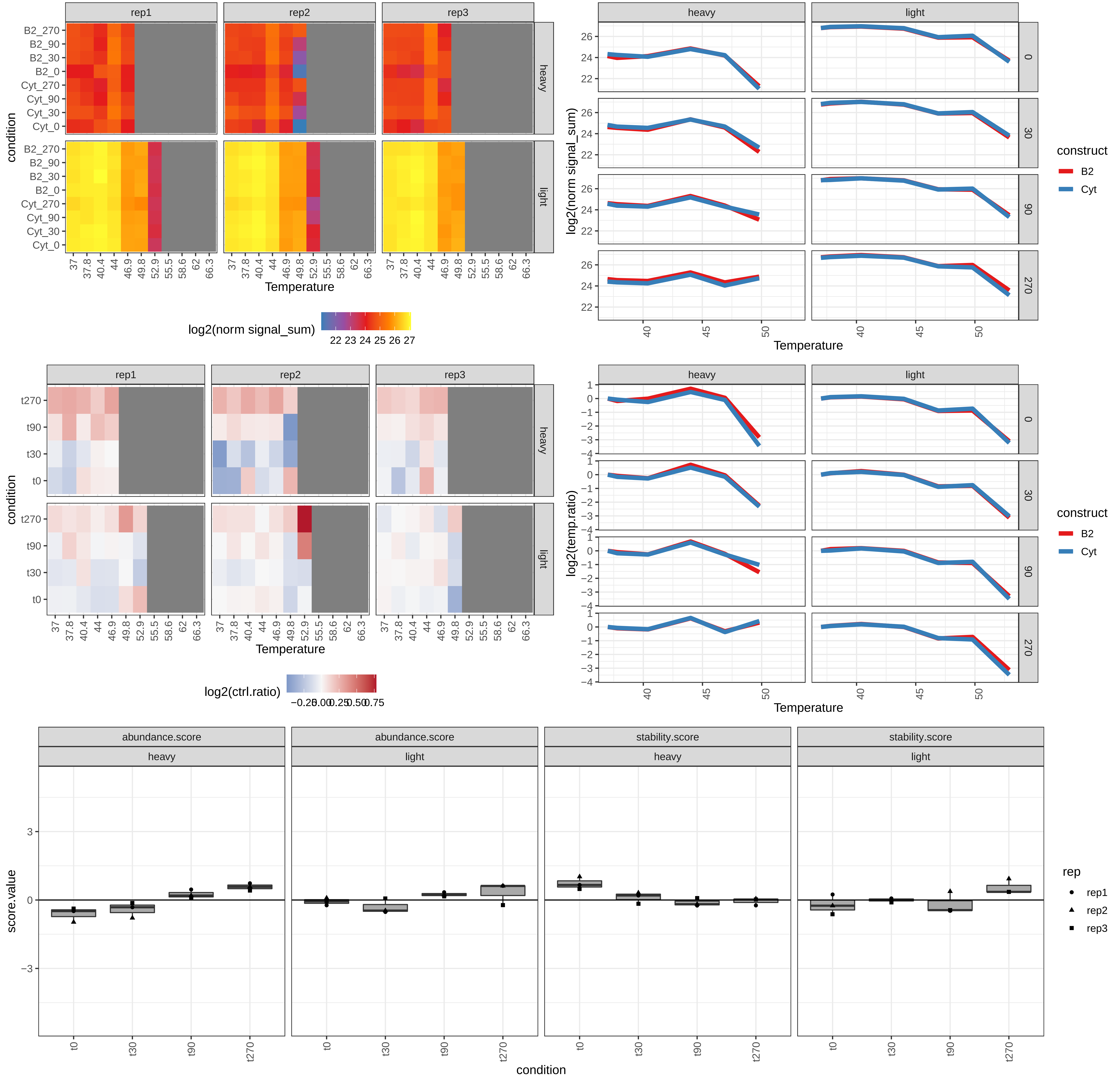

P25343 − Reduced viability upon starvation protein 161 OS=Saccharomyces cerevisiae (strain ATCC 204508 / S288c) GN=RVS161 PE=1 SV=1

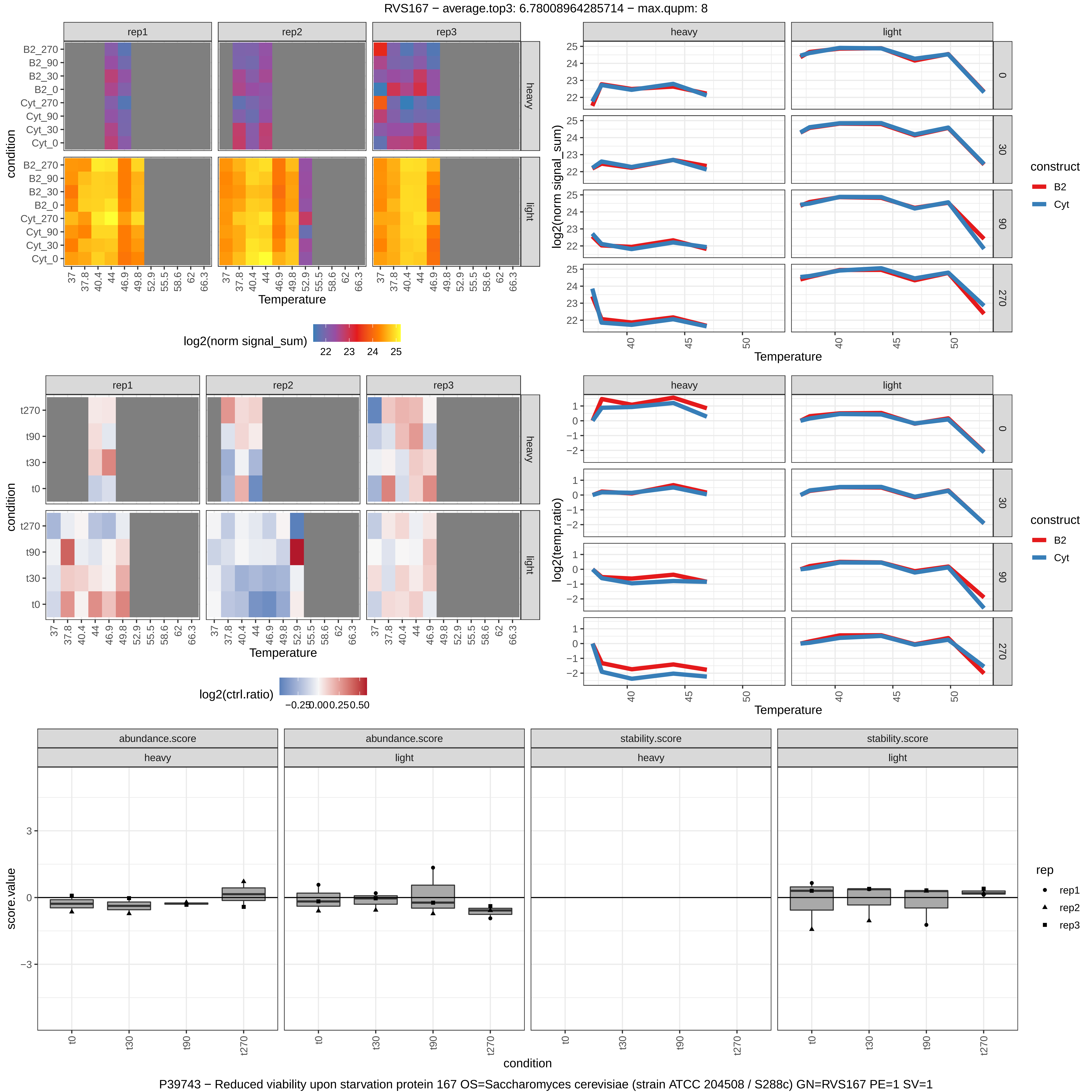

RVS167 − average.top3: 6.78008964285714 − max.qupm: 8
rep1
rep2
rep3
heavy
light
condition
Temperature
log2(norm signal_sum)
construct
B2
Cyt
log2(ctrl.ratio)
log2(temp.ratio)
abundance.score
stability.score
score.value
rep
rep1
rep2
rep3
P39743 − Reduced viability upon starvation protein 167 OS=Saccharomyces cerevisiae (strain ATCC 204508 / S288c) GN=RVS167 PE=1 SV=1

RXT2 − average.top3: 6.47970333333333 − max.qupm: 4

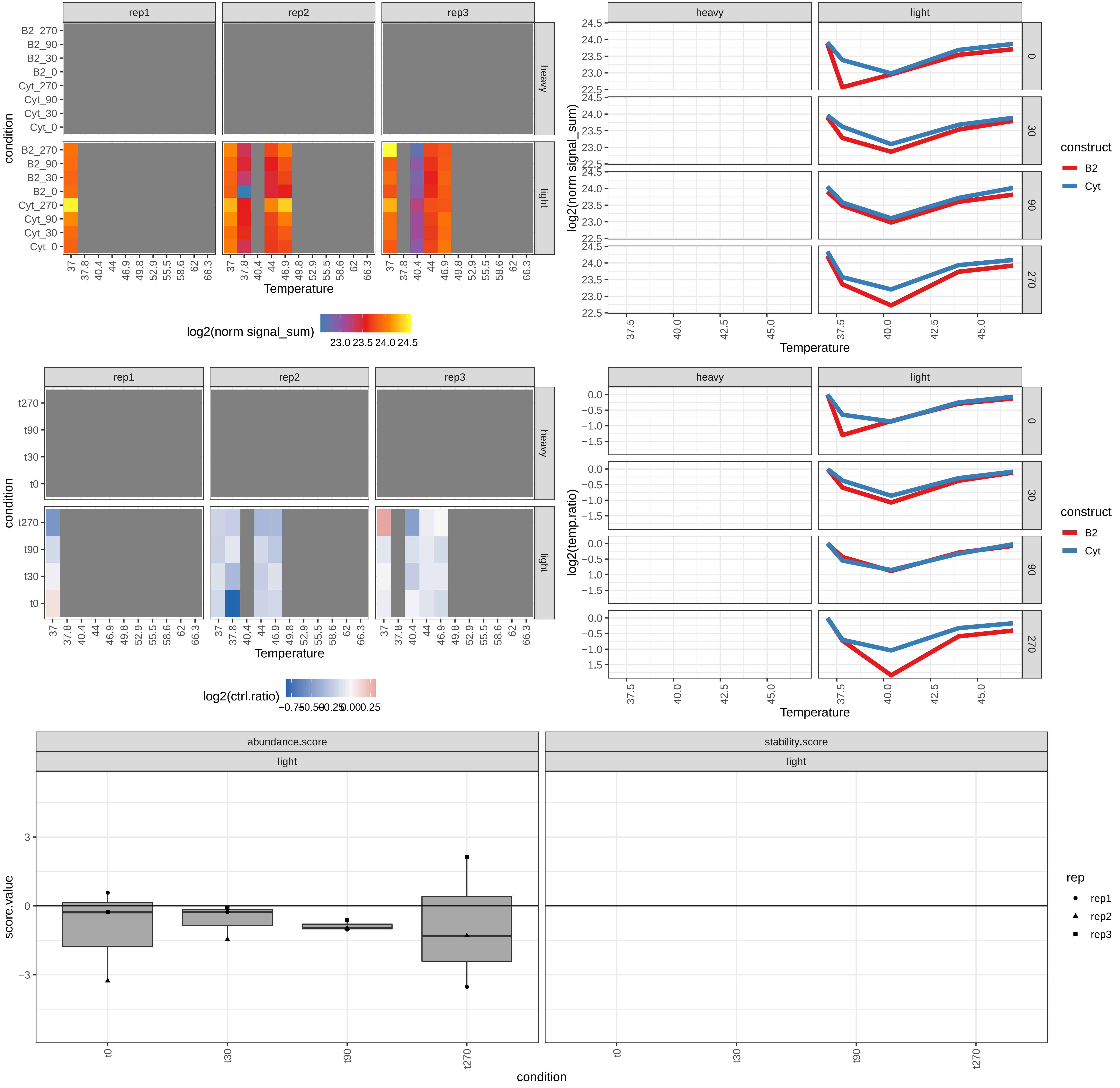

P38255 − Transcriptional regulatory protein RXT2 OS=Saccharomyces cerevisiae (strain ATCC 204508 / S288c) GN=RXT2 PE=1 SV=2

SAC1 − average.top3: 7.21857385964912 − max.qupm: 18

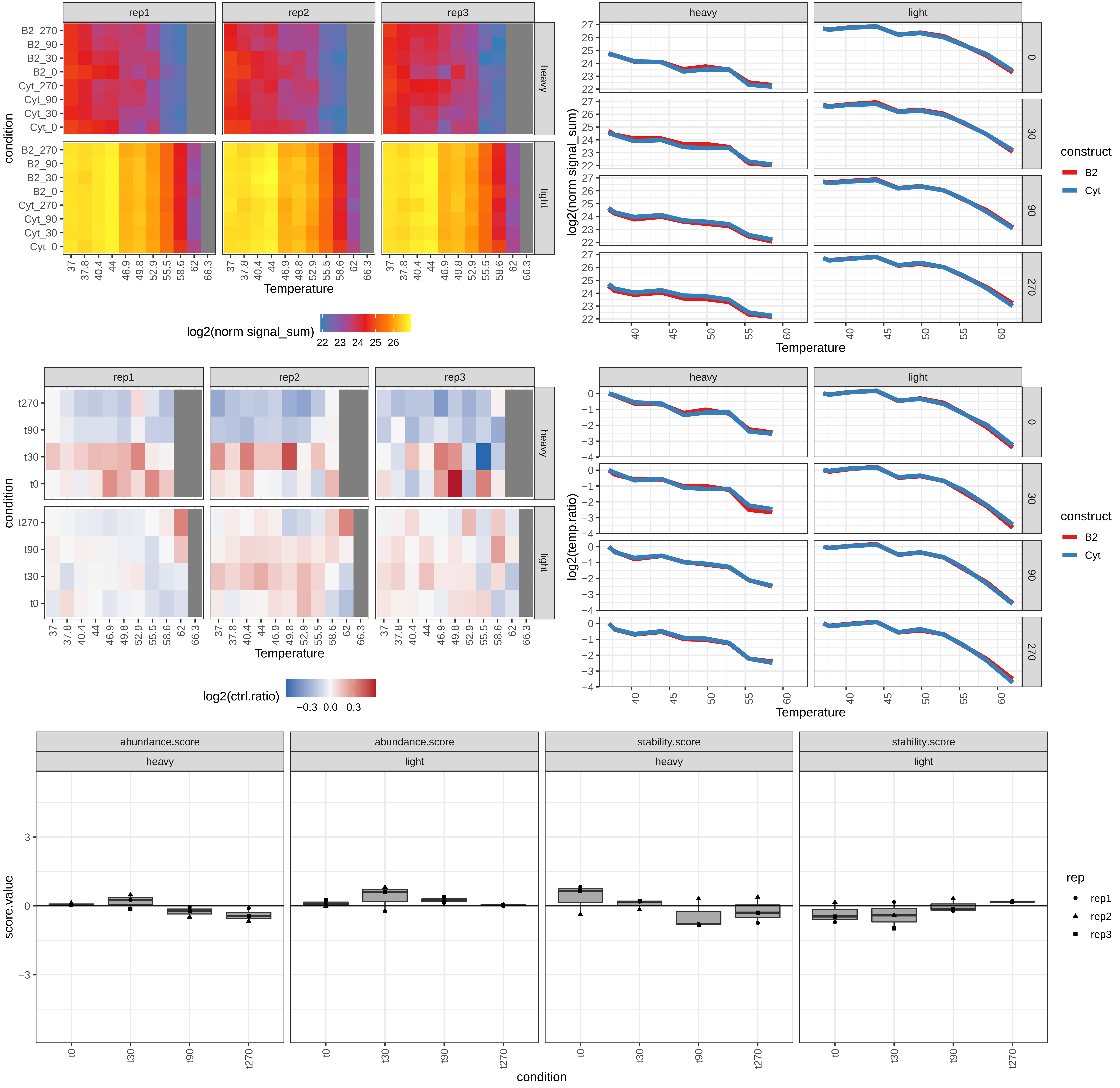

P32368 − Phosphoinositide phosphatase SAC1 OS=Saccharomyces cerevisiae (strain ATCC 204508 / S288c) GN=SAC1 PE=1 SV=1

SAC3 − average.top3: 6.49265055555556 − max.qupm: 5

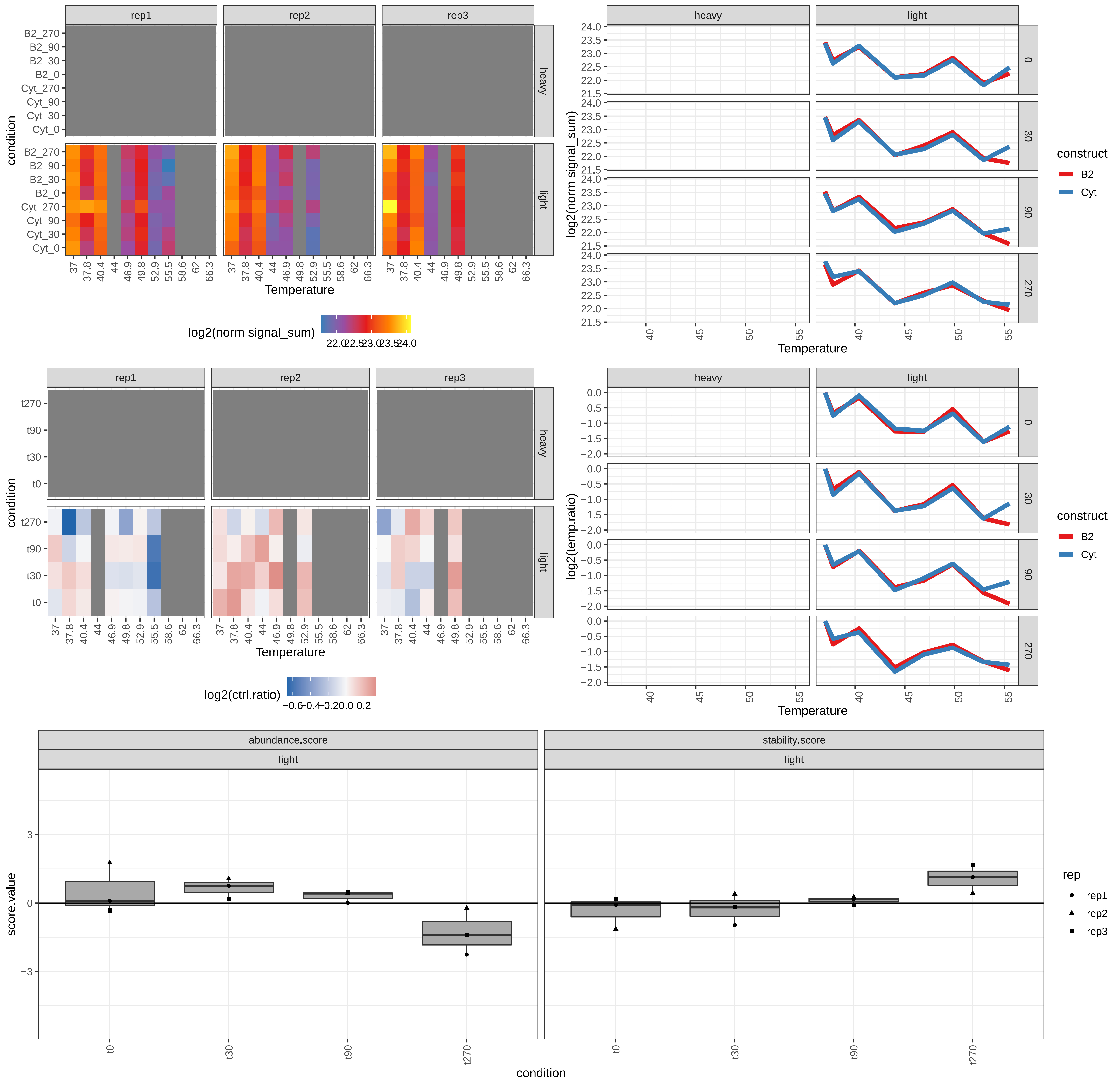

P46674 − Nuclear mRNA export protein SAC3 OS=Saccharomyces cerevisiae (strain ATCC 204508 / S288c) GN=SAC3 PE=1 SV=2

SAC6 − average.top3: 7.44300057692308 − max.qupm: 24

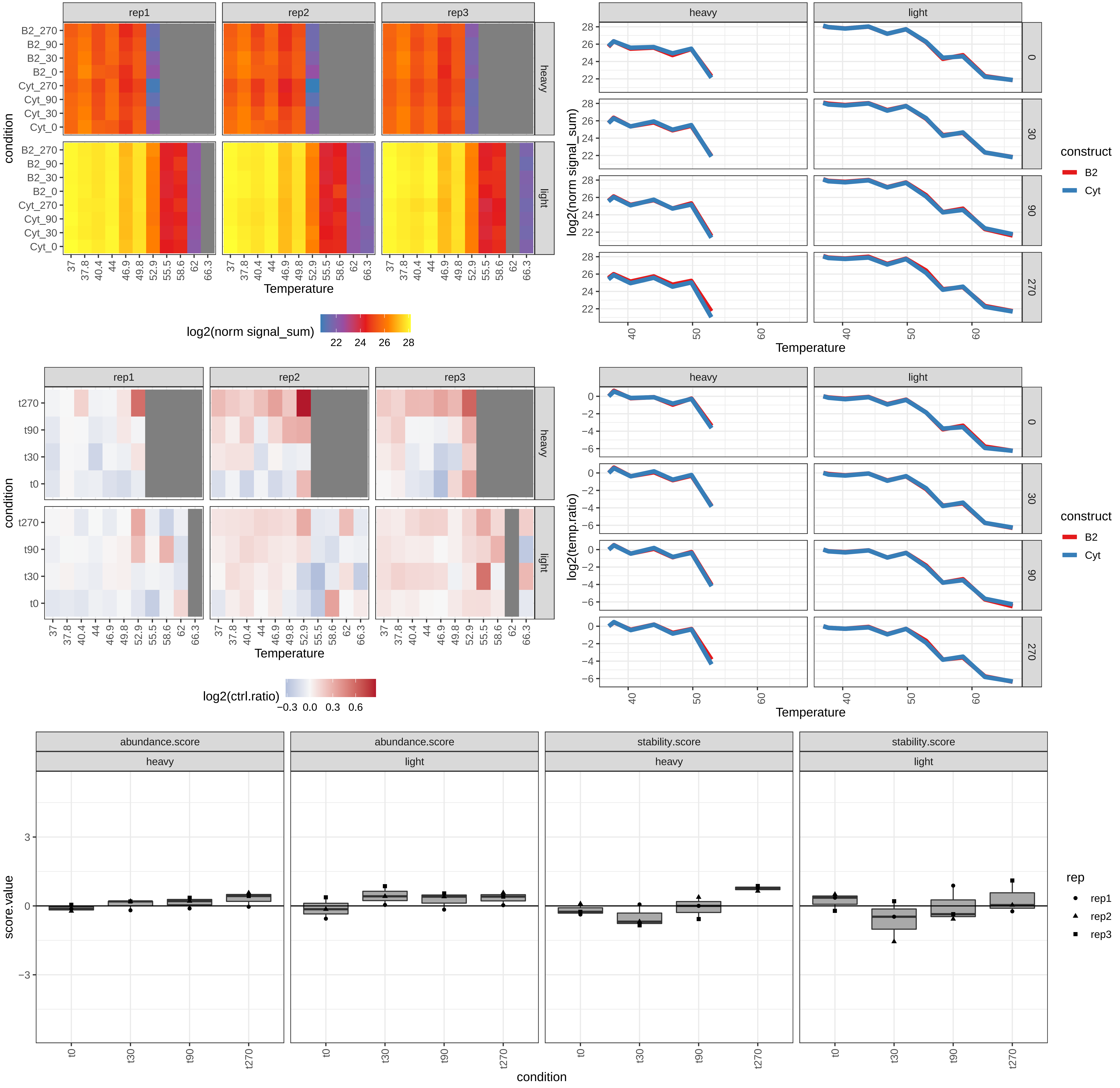

P32599 − Fimbrin OS=Saccharomyces cerevisiae (strain ATCC 204508 / S288c) GN=SAC6 PE=1 SV=1

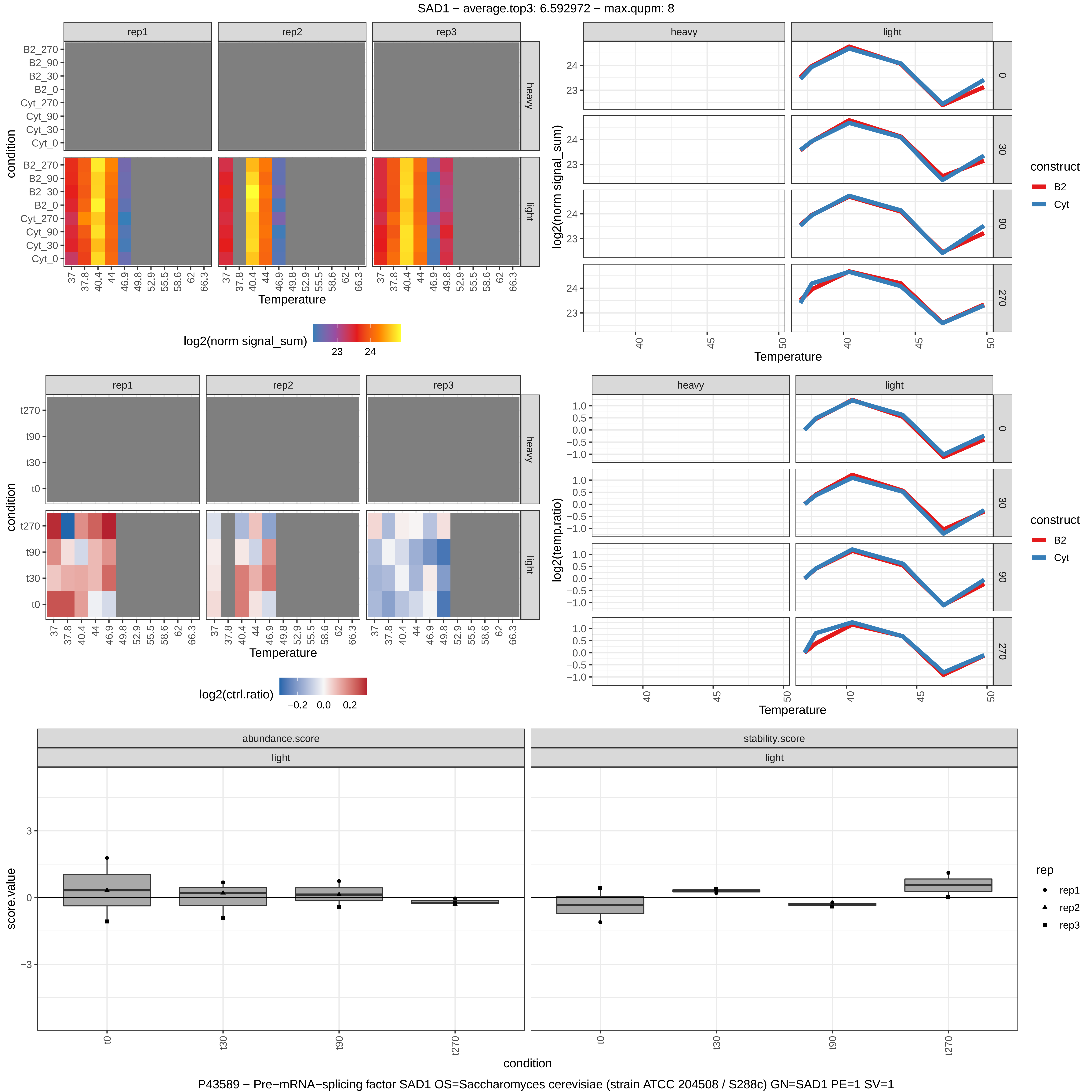
SAD1 − average.top3: 6.592972 − max.qupm: 8
rep1
rep2
rep3
heavy
light
condition
Temperature
log2(norm signal_sum)
log2(temp.ratio)
log2(ctrl.ratio)
construct
B2
Cyt
abundance.score
stability.score
score.value
rep
rep1
rep2
rep3
P43589 − Pre−mRNA−splicing factor SAD1 OS=Saccharomyces cerevisiae (strain ATCC 204508 / S288c) GN=SAD1 PE=1 SV=1

SAF1 − average.top3: 6.77655307692308 − max.qupm: 5

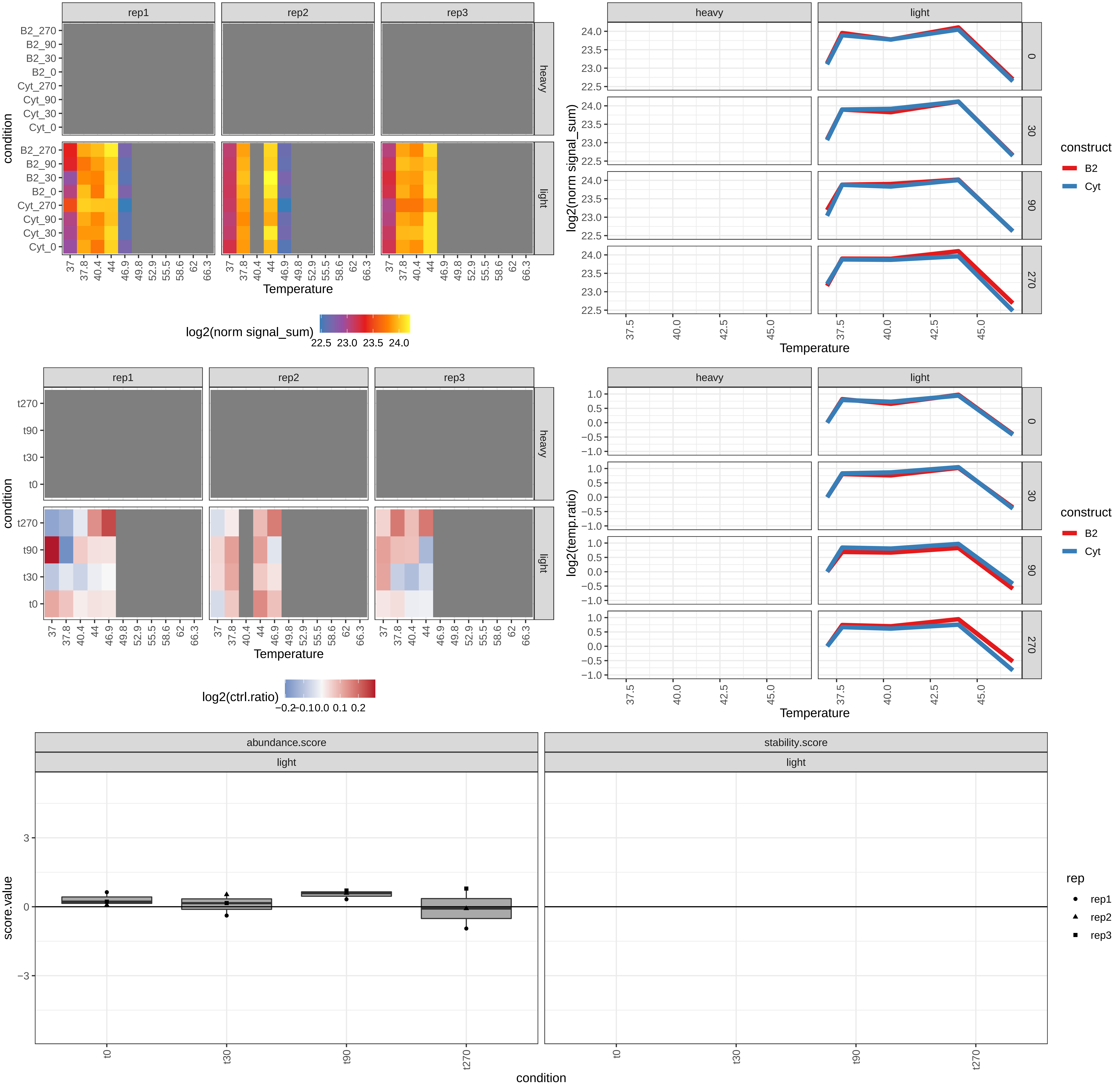

P38352 − SCF−associated factor 1 OS=Saccharomyces cerevisiae (strain ATCC 204508 / S288c) GN=SAF1 PE=1 SV=2

SAH1 − average.top3: 8.6490646969697 − max.qupm: 25

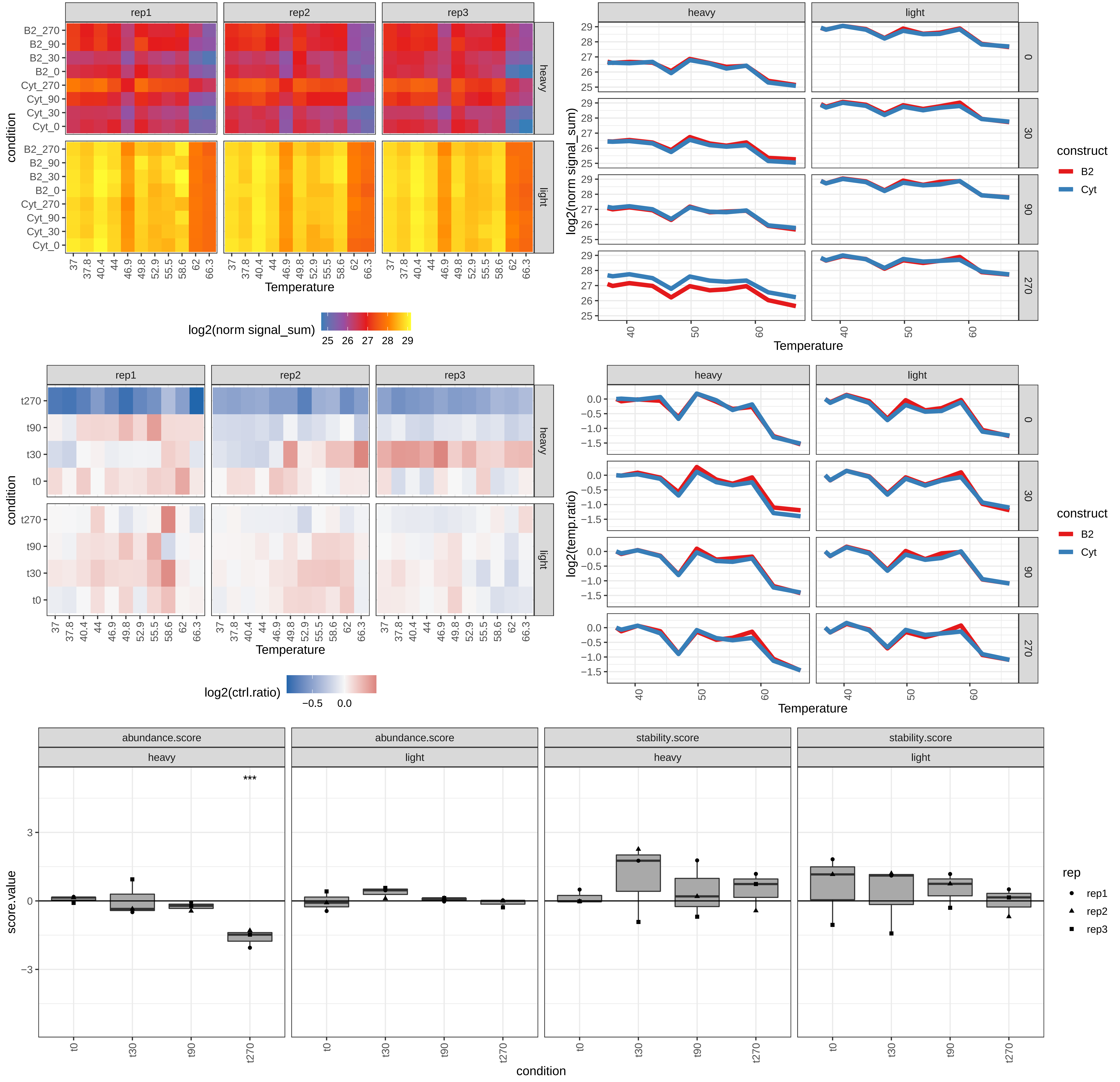

P39954 − Adenosylhomocysteinase OS=Saccharomyces cerevisiae (strain ATCC 204508 / S288c) GN=SAH1 PE=1 SV=1

SAM1 − average.top3: 7.10921375 − max.qupm: 7

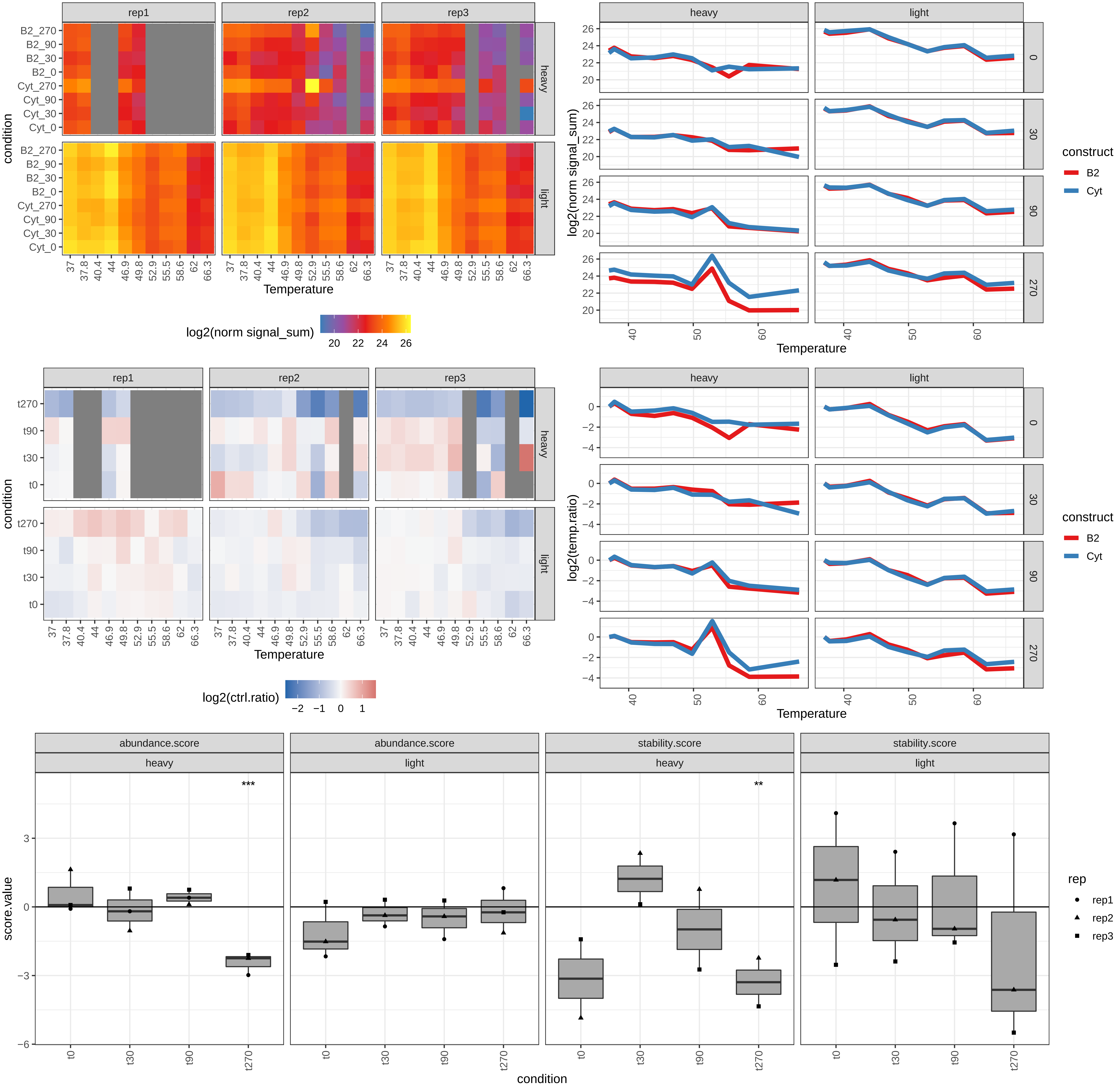

P10659 − S−adenosylmethionine synthase 1 OS=Saccharomyces cerevisiae (strain ATCC 204508 / S288c) GN=SAM1 PE=1 SV=2

SAM2 − average.top3: 7.31562901639344 − max.qupm: 8

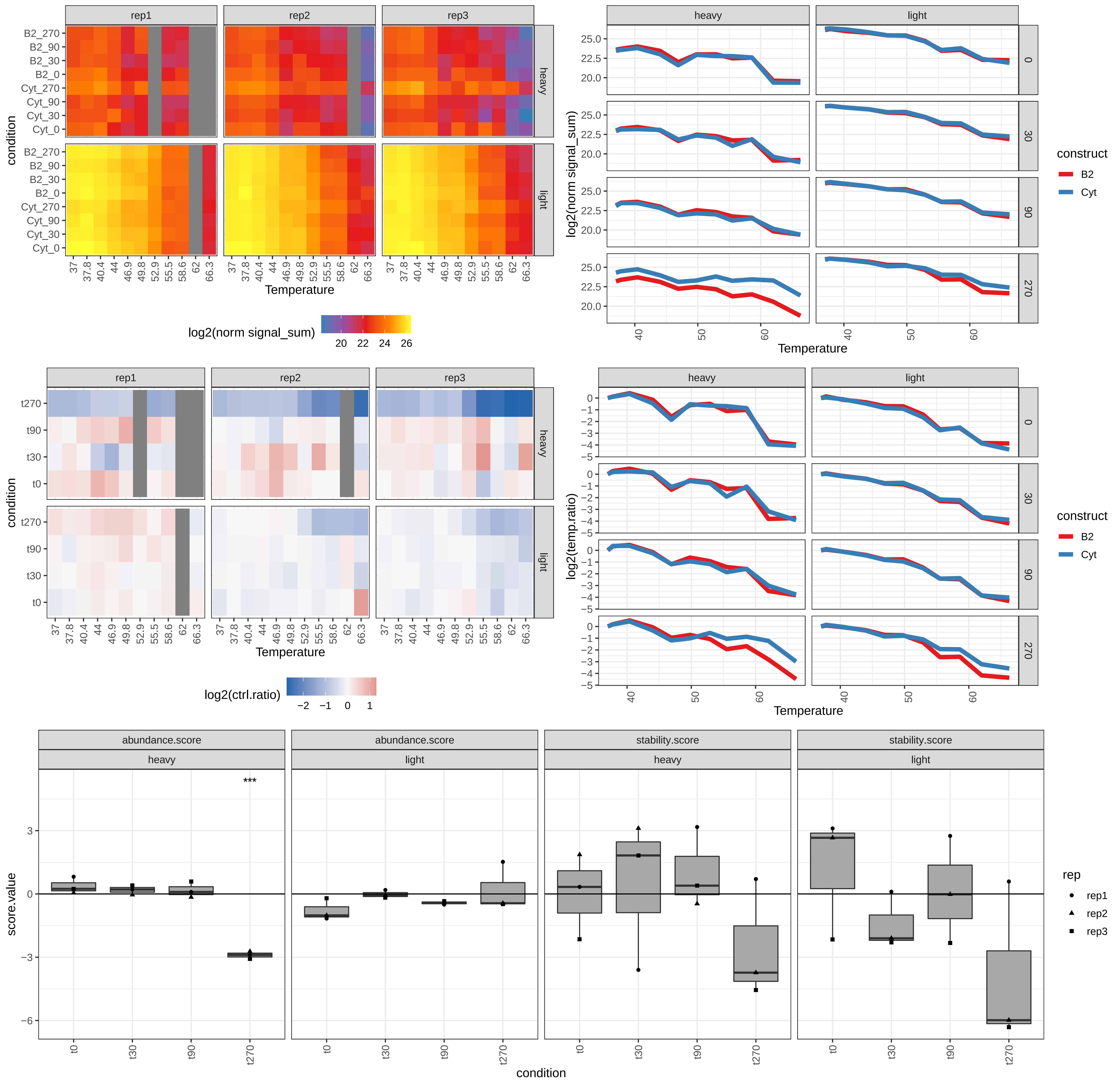

P19358 − S−adenosylmethionine synthase 2 OS=Saccharomyces cerevisiae (strain ATCC 204508 / S288c) GN=SAM2 PE=1 SV=3

SAM35 − average.top3: 6.71632863636364 − max.qupm: 7

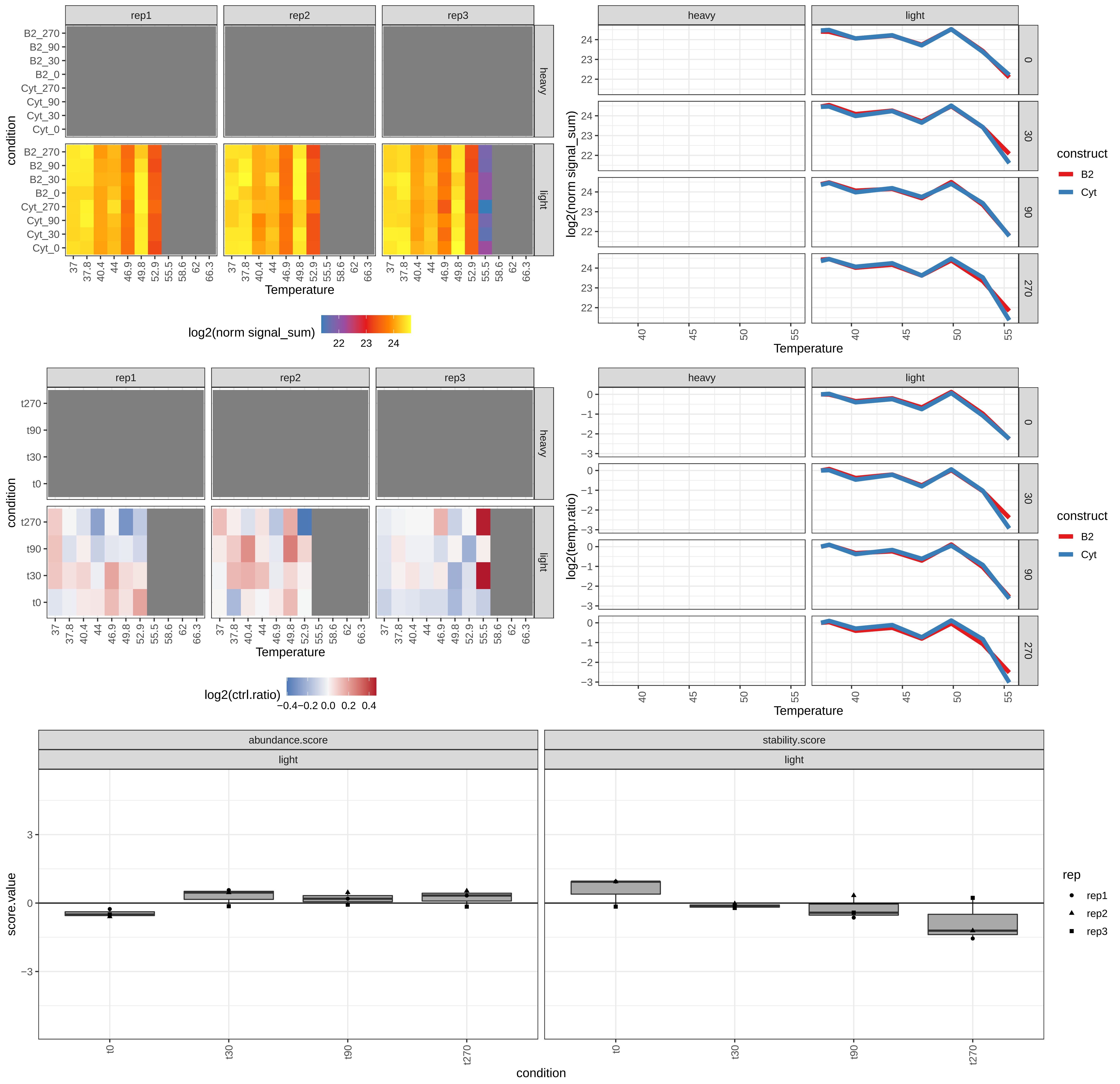

P14693 − Sorting assembly machinery 35 kDa subunit OS=Saccharomyces cerevisiae (strain ATCC 204508 / S288c) GN=SAM35 PE=1 SV=2

SAM37 − average.top3: 6.60383904761905 − max.qupm: 4

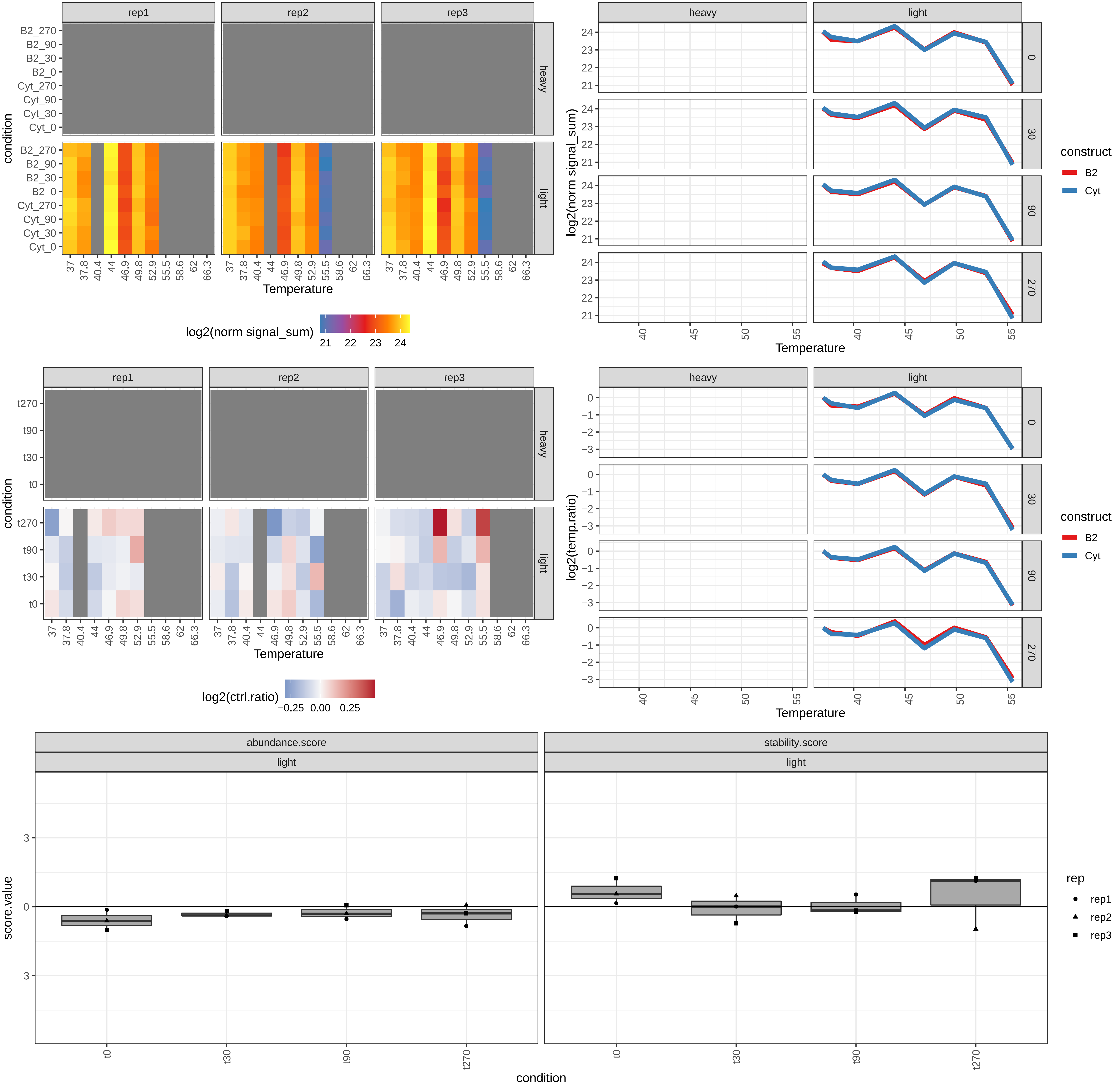

P50110 − Sorting assembly machinery 37 kDa subunit OS=Saccharomyces cerevisiae (strain ATCC 204508 / S288c) GN=SAM37 PE=1 SV=1

SAM4 − average.top3: 6.72994407407407 − max.qupm: 9

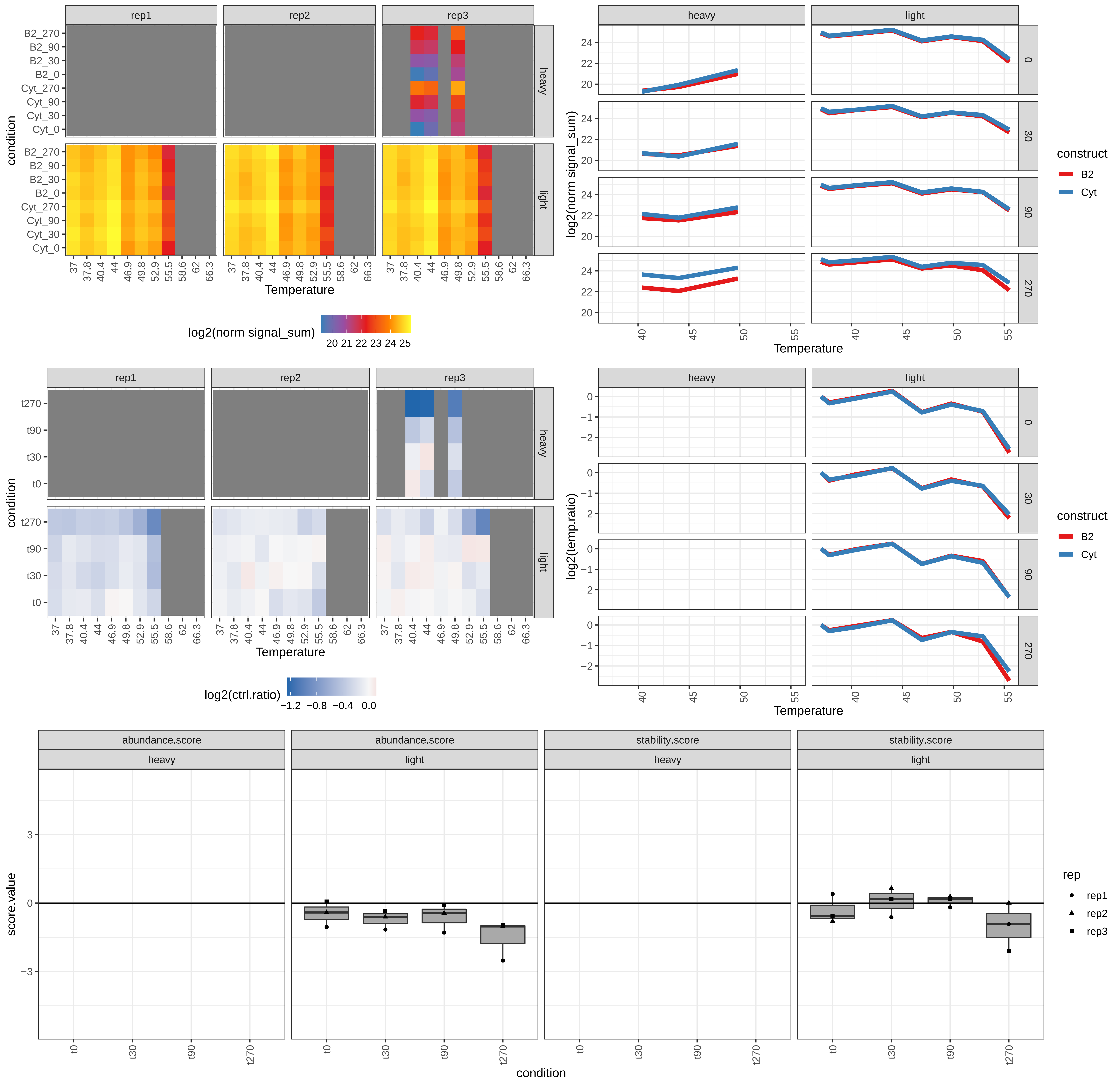

Q08985 − Homocysteine S−methyltransferase 2 OS=Saccharomyces cerevisiae (strain ATCC 204508 / S288c) GN=SAM4 PE=1 SV=1

SAM50 − average.top3: 6.927424 − max.qupm: 7

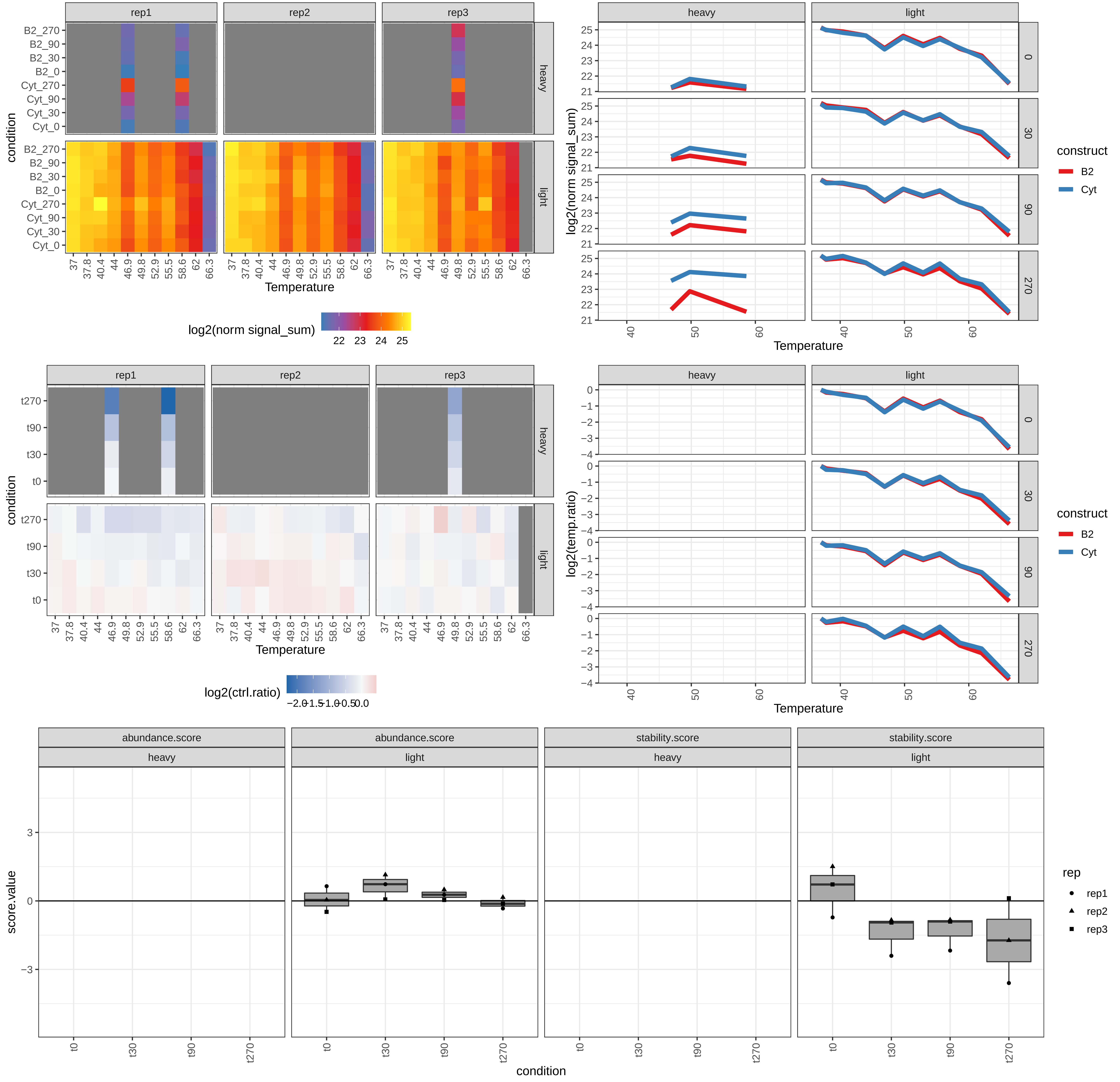

P53969 − Sorting assembly machinery 50 kDa subunit OS=Saccharomyces cerevisiae (strain ATCC 204508 / S288c) GN=SAM50 PE=1 SV=1

SAP1 − average.top3: 6.116669 − max.qupm: 4

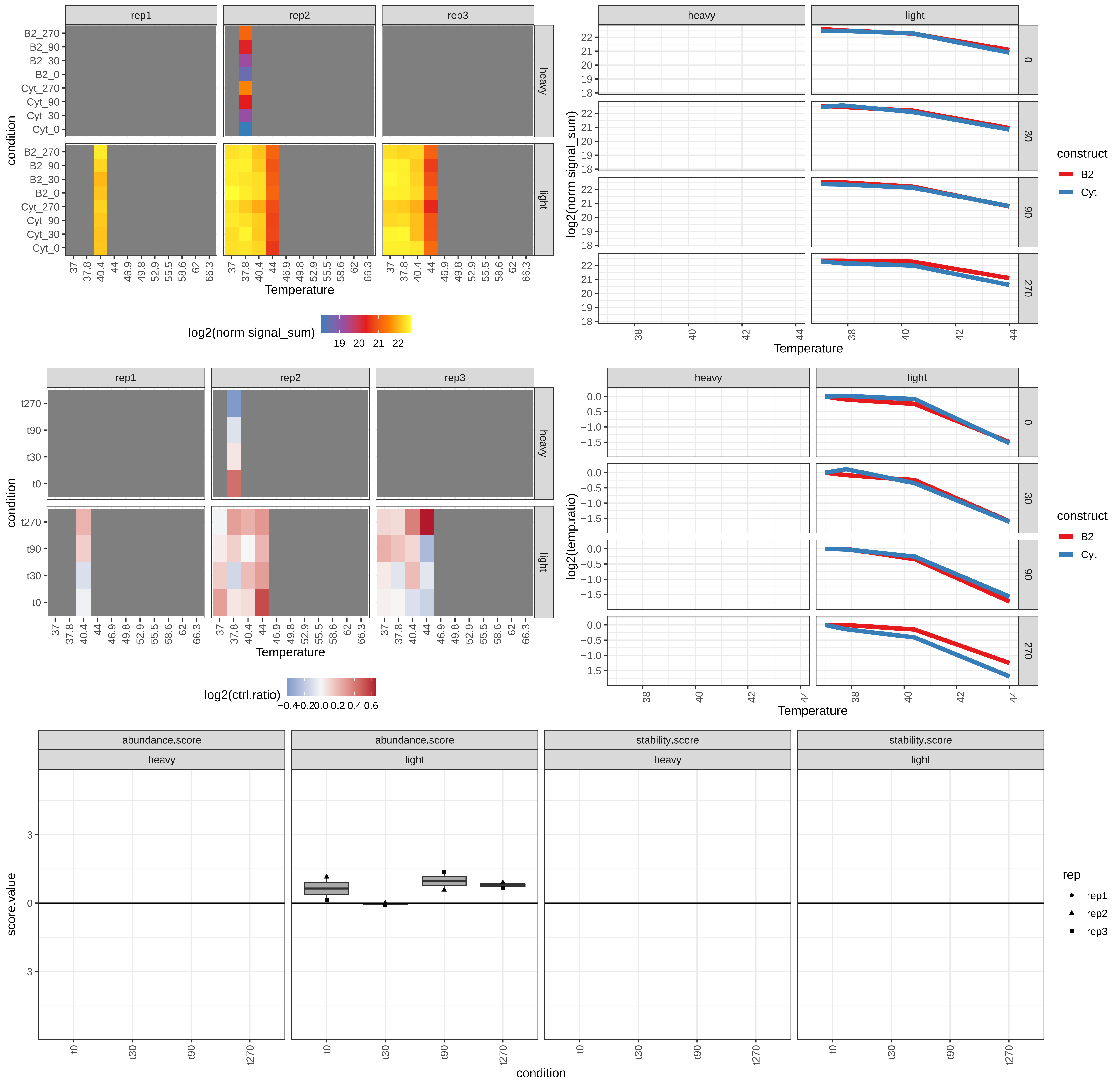

P39955 − Protein SAP1 OS=Saccharomyces cerevisiae (strain ATCC 204508 / S288c) GN=SAP1 PE=1 SV=1

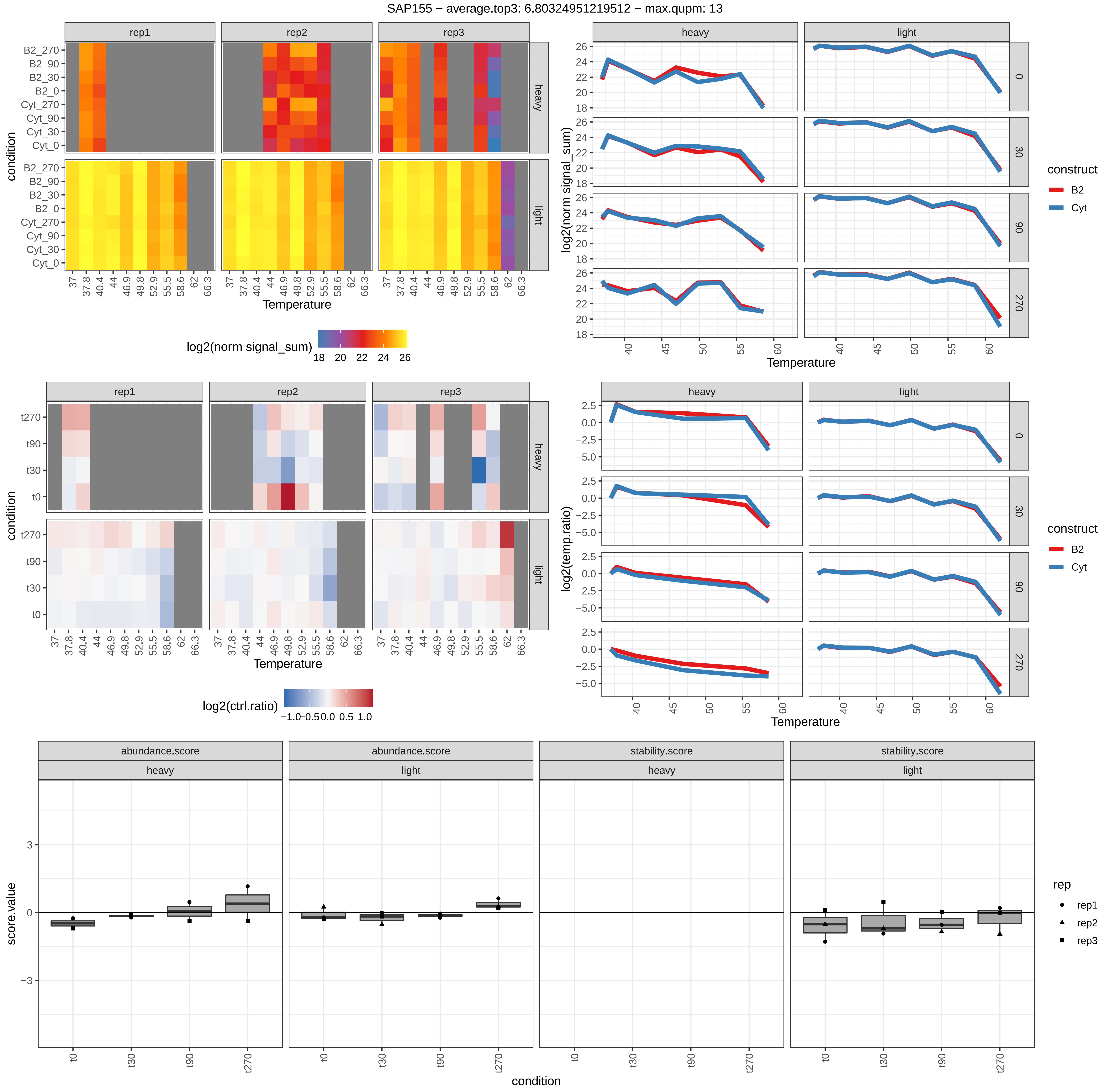

P43612 − SIT4−associating protein SAP155 OS=Saccharomyces cerevisiae (strain ATCC 204508 / S288c) GN=SAP155 PE=1 SV=4

SAP190 − average.top3: 6.563595 − max.qupm: 10

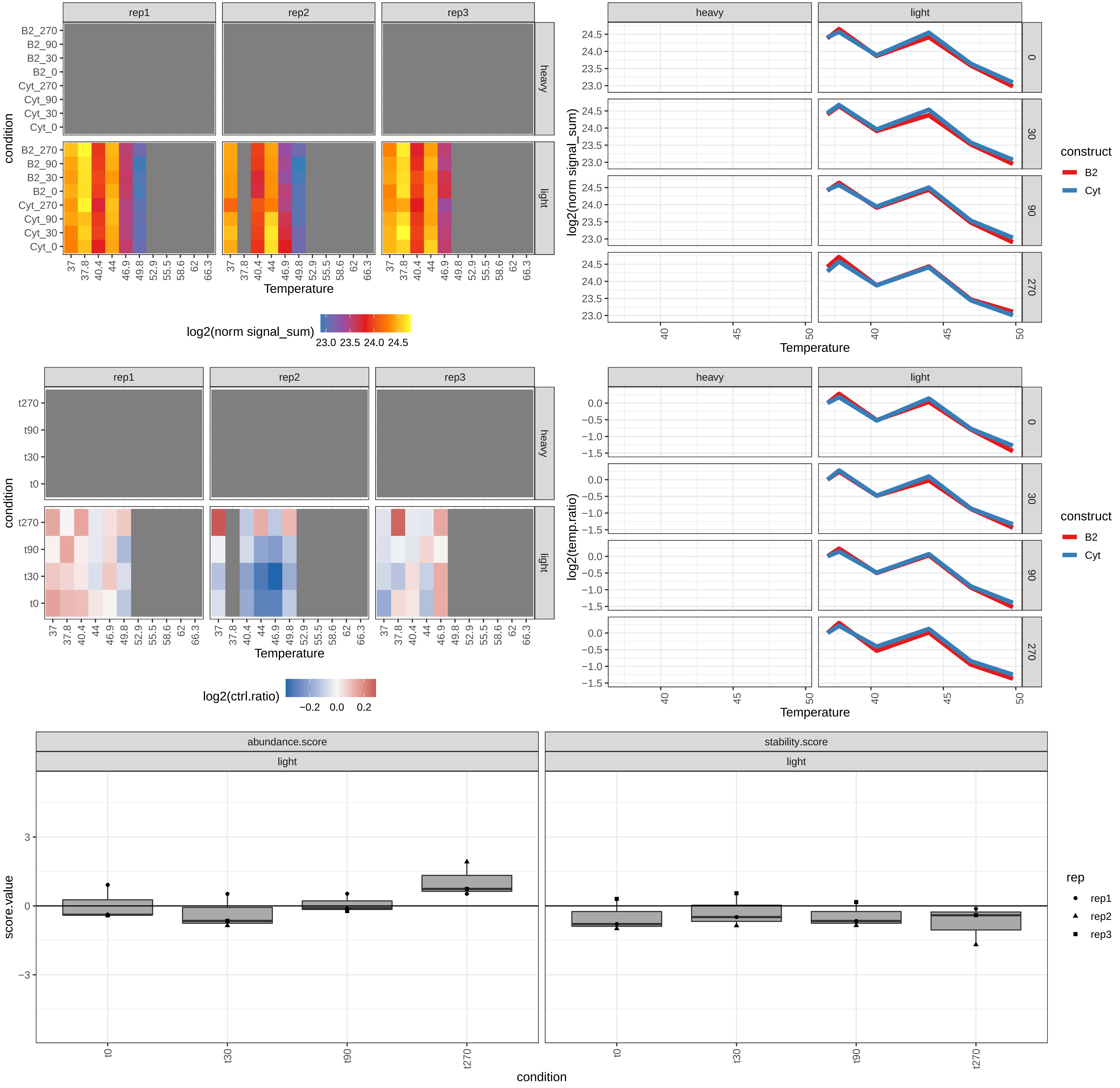

P36123 − SIT4−associating protein SAP190 OS=Saccharomyces cerevisiae (strain ATCC 204508 / S288c) GN=SAP190 PE=1 SV=2

SAP30 − average.top3: 6.52858076923077 − max.qupm: 6

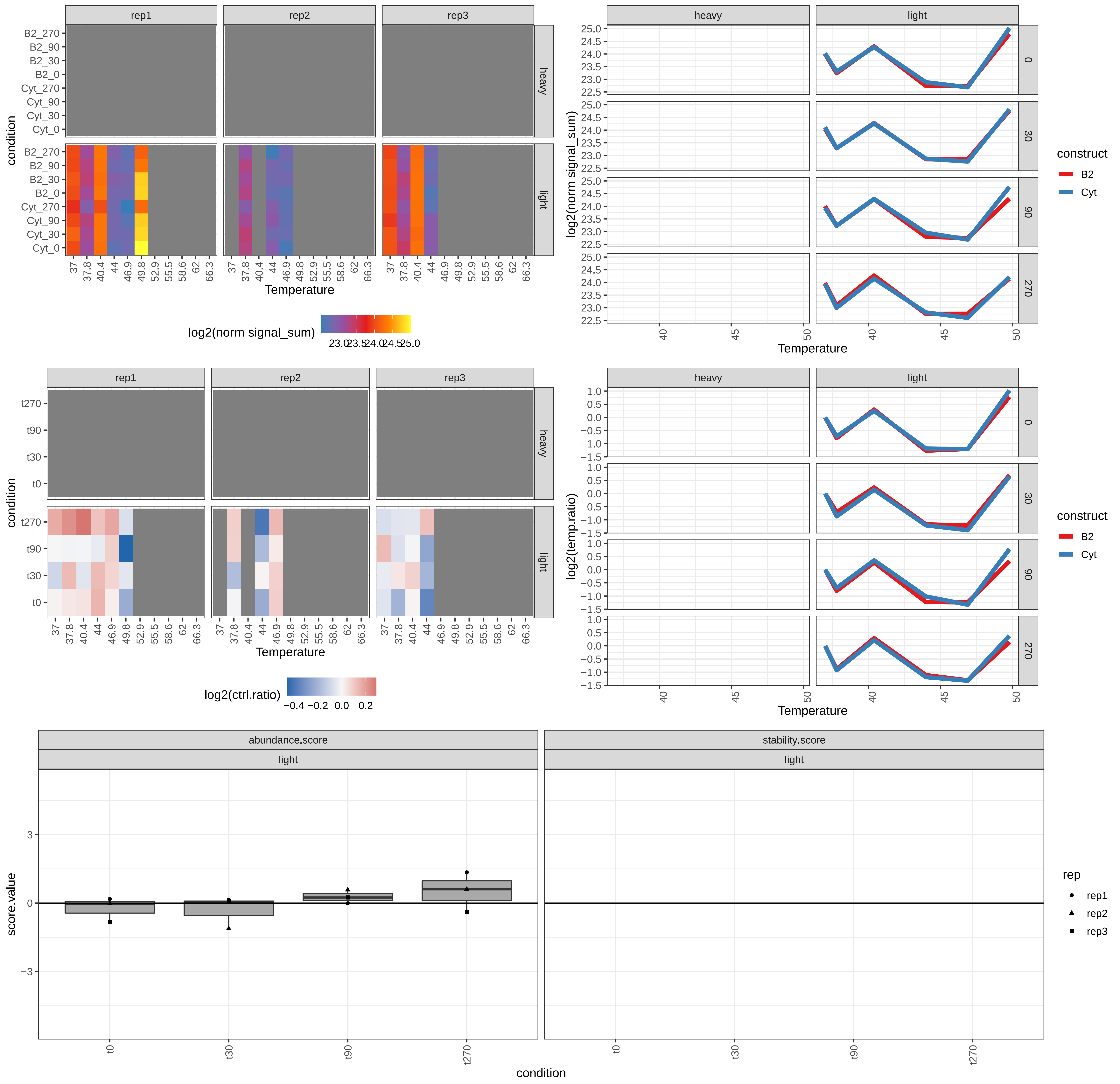

P38429 − Transcriptional regulatory protein SAP30 OS=Saccharomyces cerevisiae (strain ATCC 204508 / S288c) GN=SAP30 PE=1 SV=2

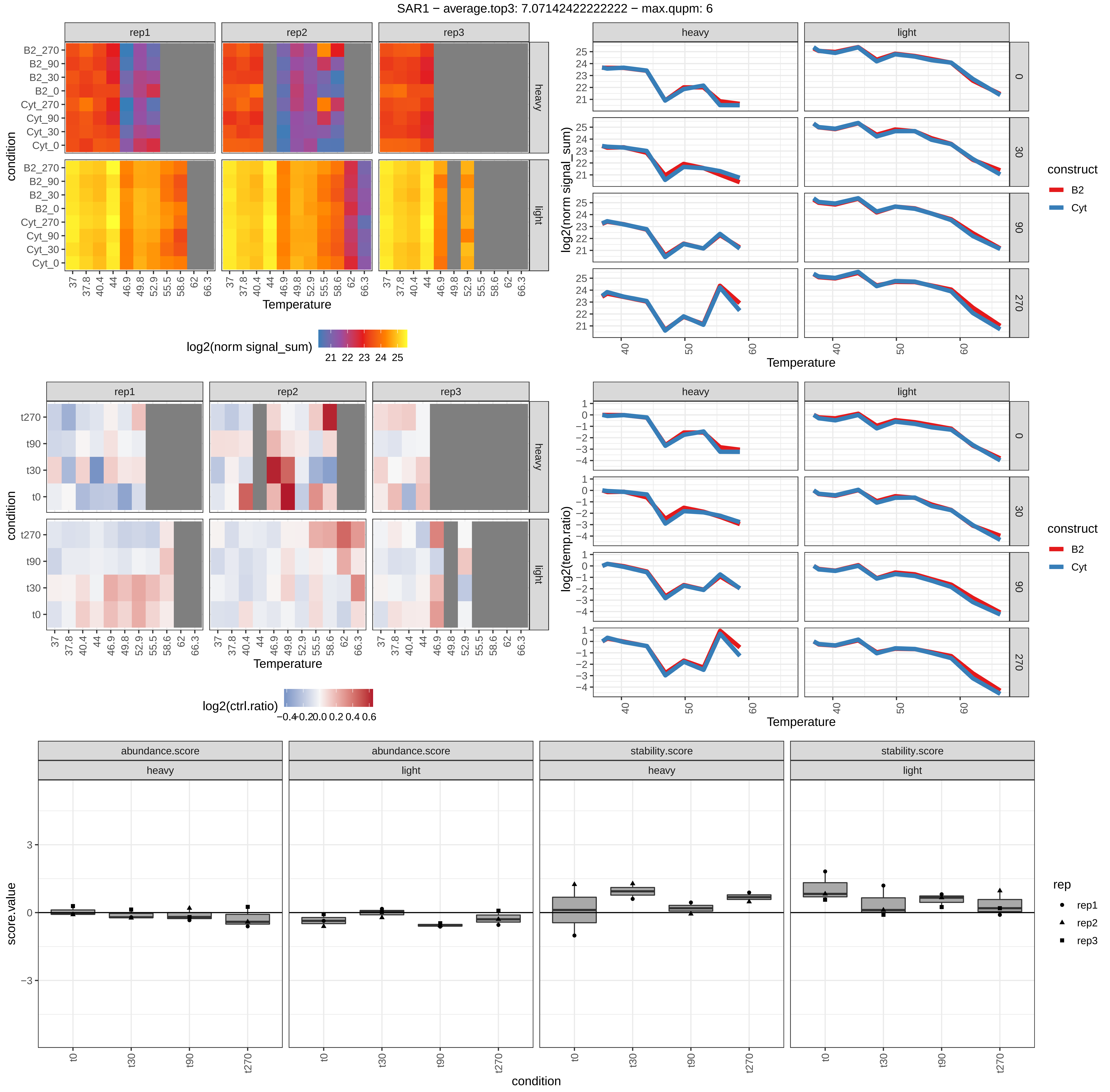

P20606 − Small COPII coat GTPase SAR1 OS=Saccharomyces cerevisiae (strain ATCC 204508 / S288c) GN=SAR1 PE=1 SV=1

SAS10 − average.top3: 6.6461625 − max.qupm: 4

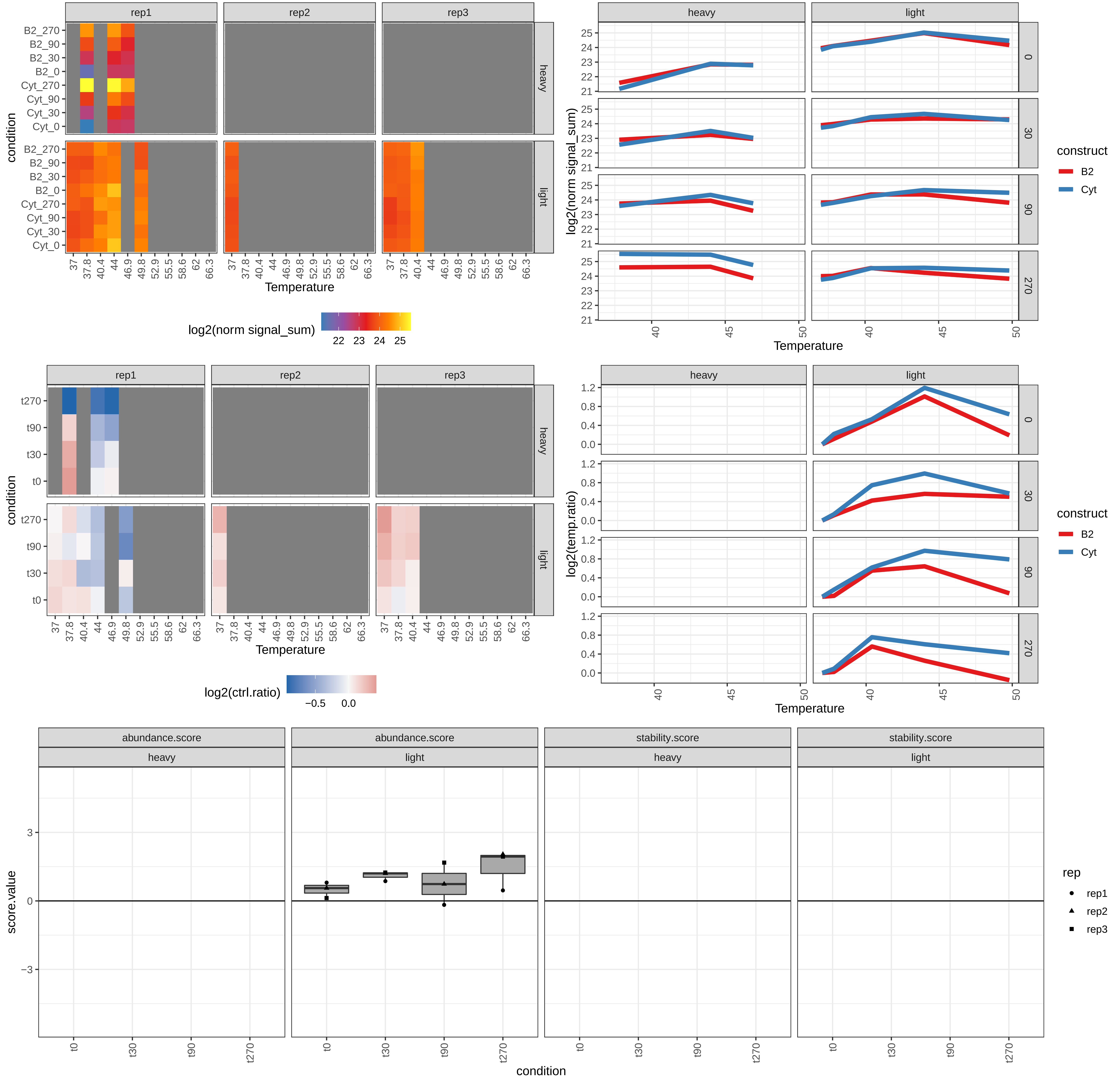

Q12136 − Something about silencing protein 10 OS=Saccharomyces cerevisiae (strain ATCC 204508 / S288c) GN=SAS10 PE=1 SV=1

SBA1 − average.top3: 7.39349272727273 − max.qupm: 5

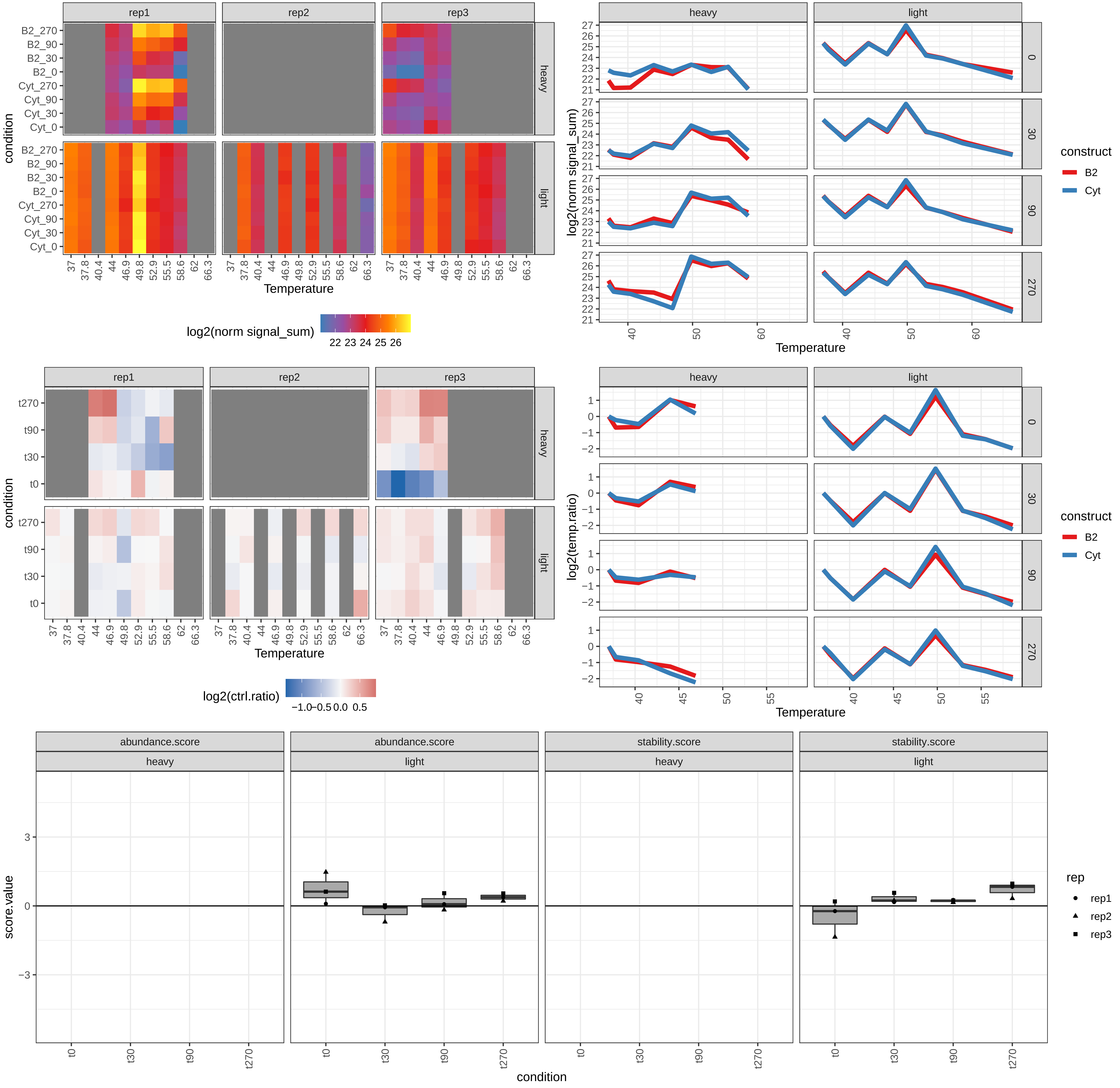

P28707 − Co−chaperone protein SBA1 OS=Saccharomyces cerevisiae (strain ATCC 204508 / S288c) GN=SBA1 PE=1 SV=3

SBE22 − average.top3: 6.5448775 − max.qupm: 4

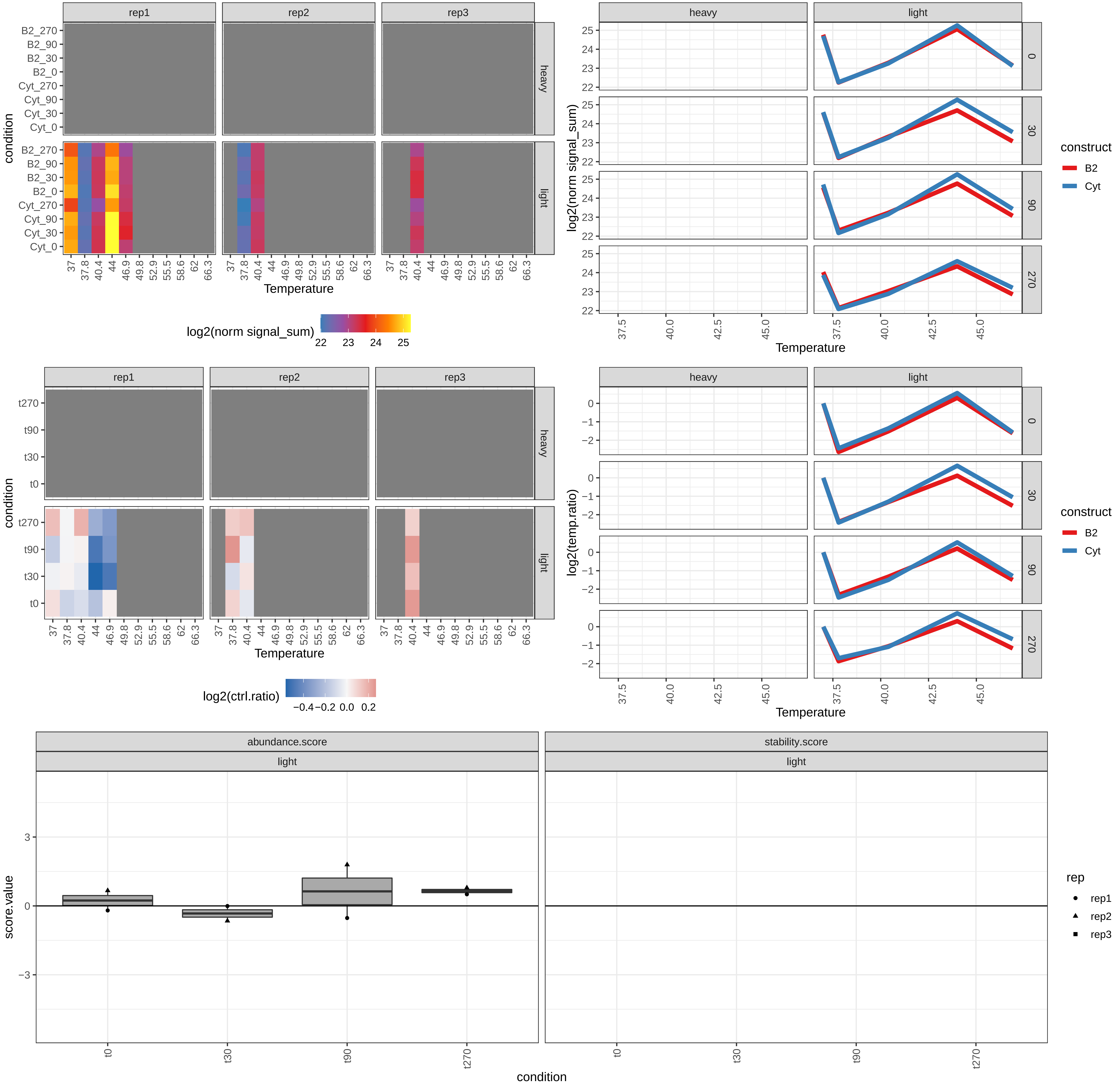

P38814 − Protein SBE22 OS=Saccharomyces cerevisiae (strain ATCC 204508 / S288c) GN=SBE22 PE=1 SV=1

SBH2 − average.top3: 7.29342318181818 − max.qupm: 4

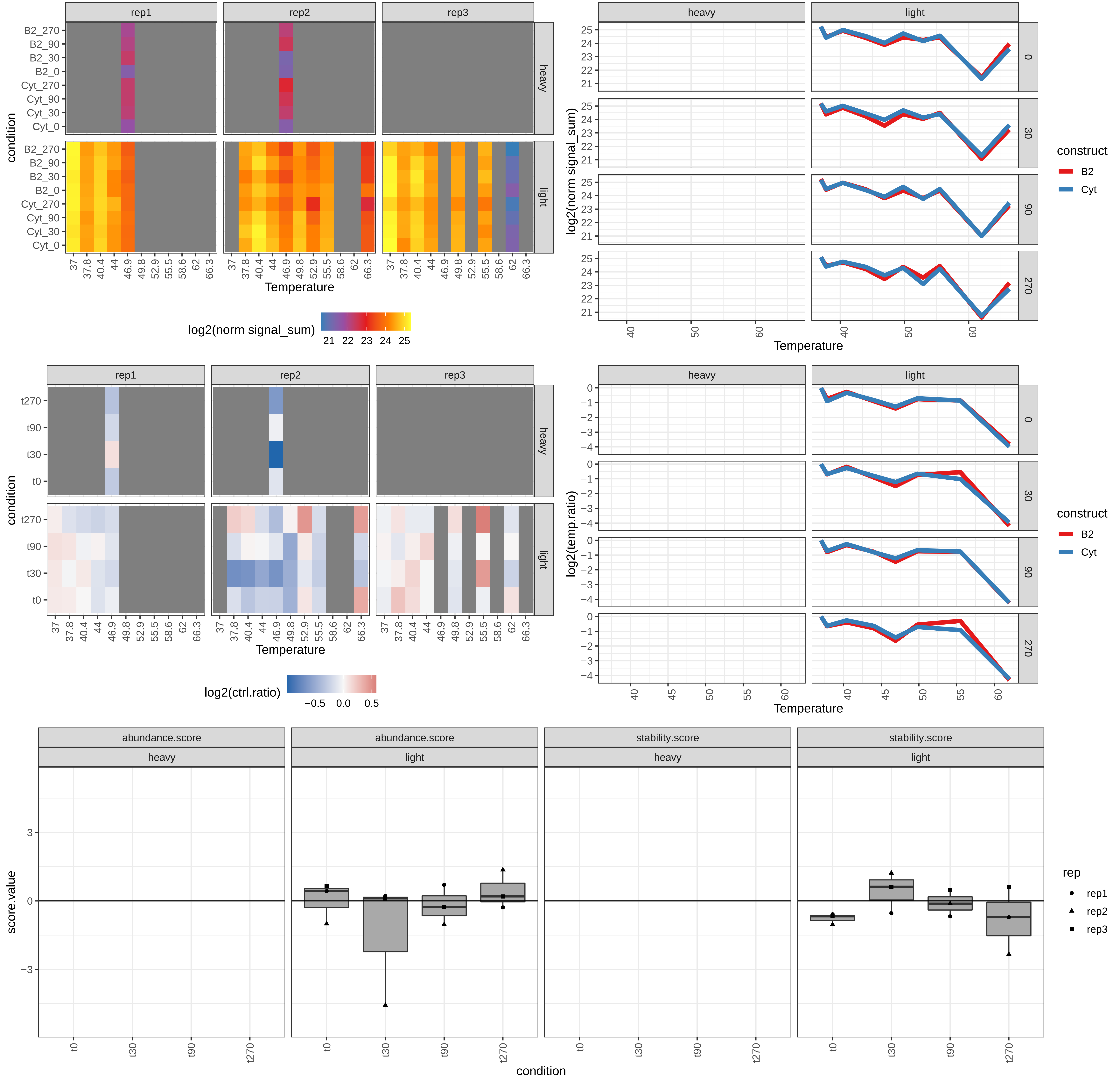

P52871 − Protein transport protein SBH2 OS=Saccharomyces cerevisiae (strain ATCC 204508 / S288c) GN=SBH2 PE=1 SV=1

SBP1 − average.top3: 7.55459507692308 − max.qupm: 11

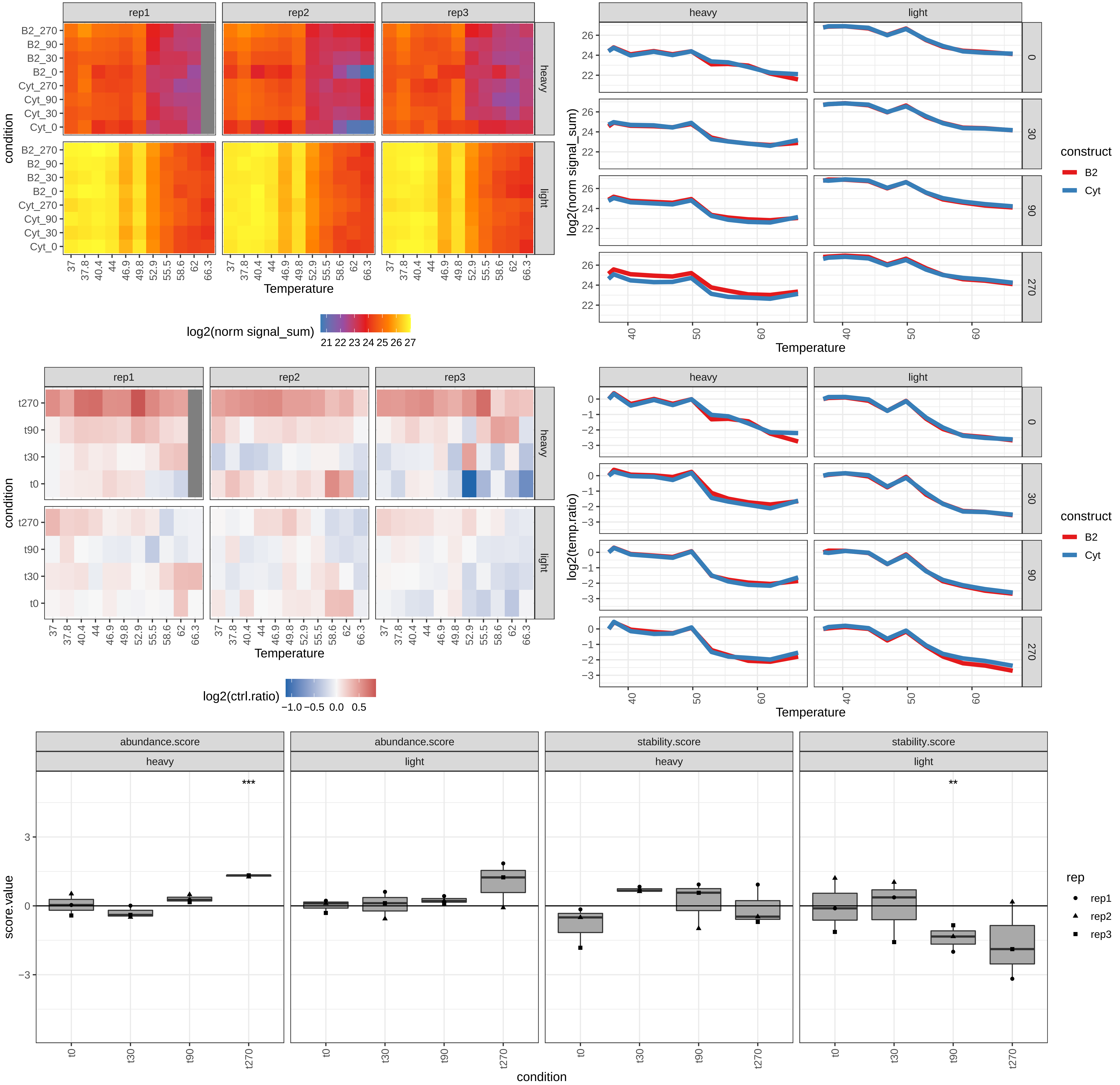

P10080 − Single−stranded nucleic acid−binding protein OS=Saccharomyces cerevisiae (strain ATCC 204508 / S288c) GN=SBP1 PE=1 SV=2

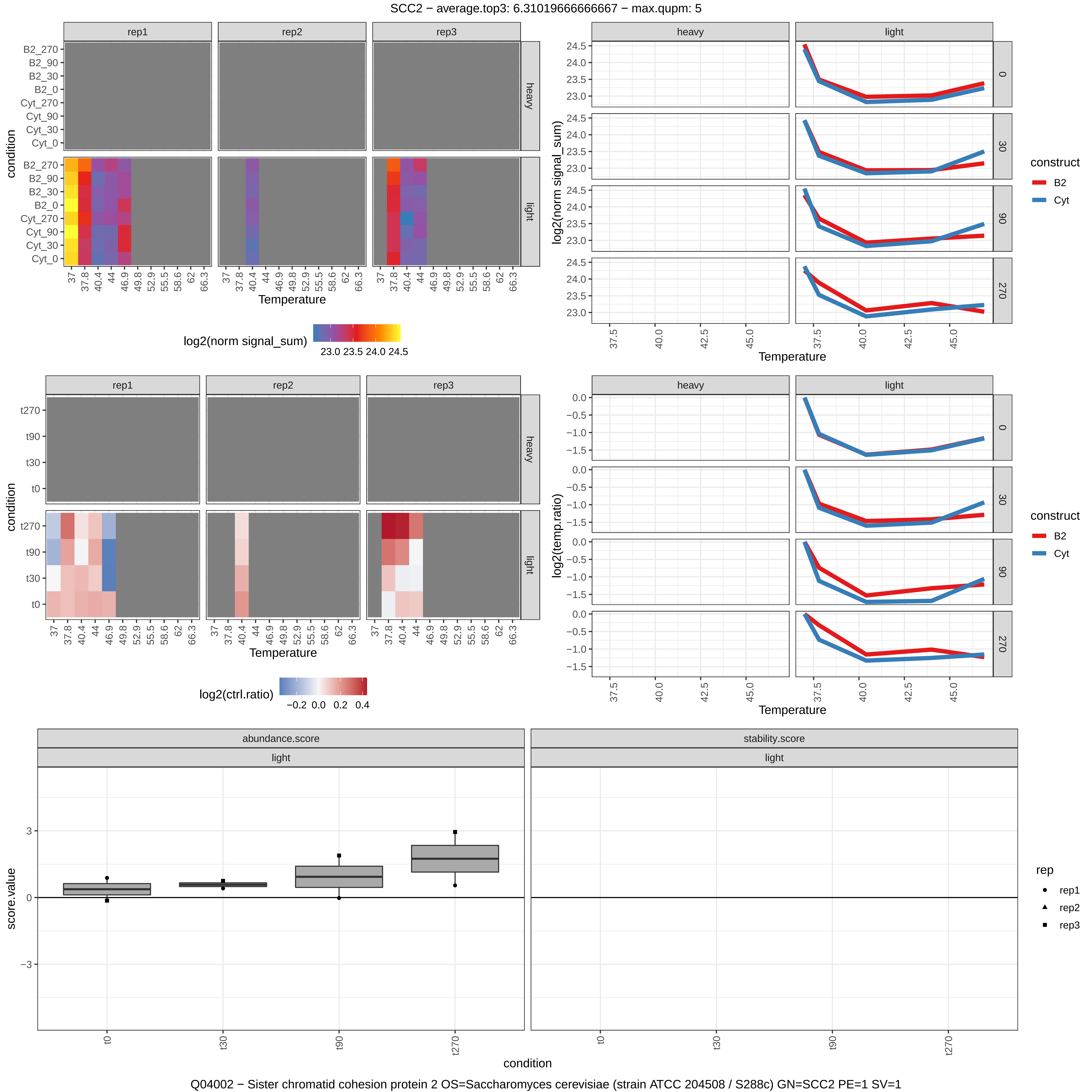
SCC2 − average.top3: 6.31019666666667 − max.qupm: 5
rep1
rep2
rep3
heavy
light
condition
Temperature
log2(norm signal_sum)
construct
B2
Cyt
log2(ctrl.ratio)
log2(temp.ratio)
abundance.score
stability.score
score.value
rep
rep1
rep2
rep3
t0
t30
t90
t270
Q04002 − Sister chromatid cohesion protein 2 OS=Saccharomyces cerevisiae (strain ATCC 204508 / S288c) GN=SCC2 PE=1 SV=1

SCC4 − average.top3: 6.31399625 − max.qupm: 6

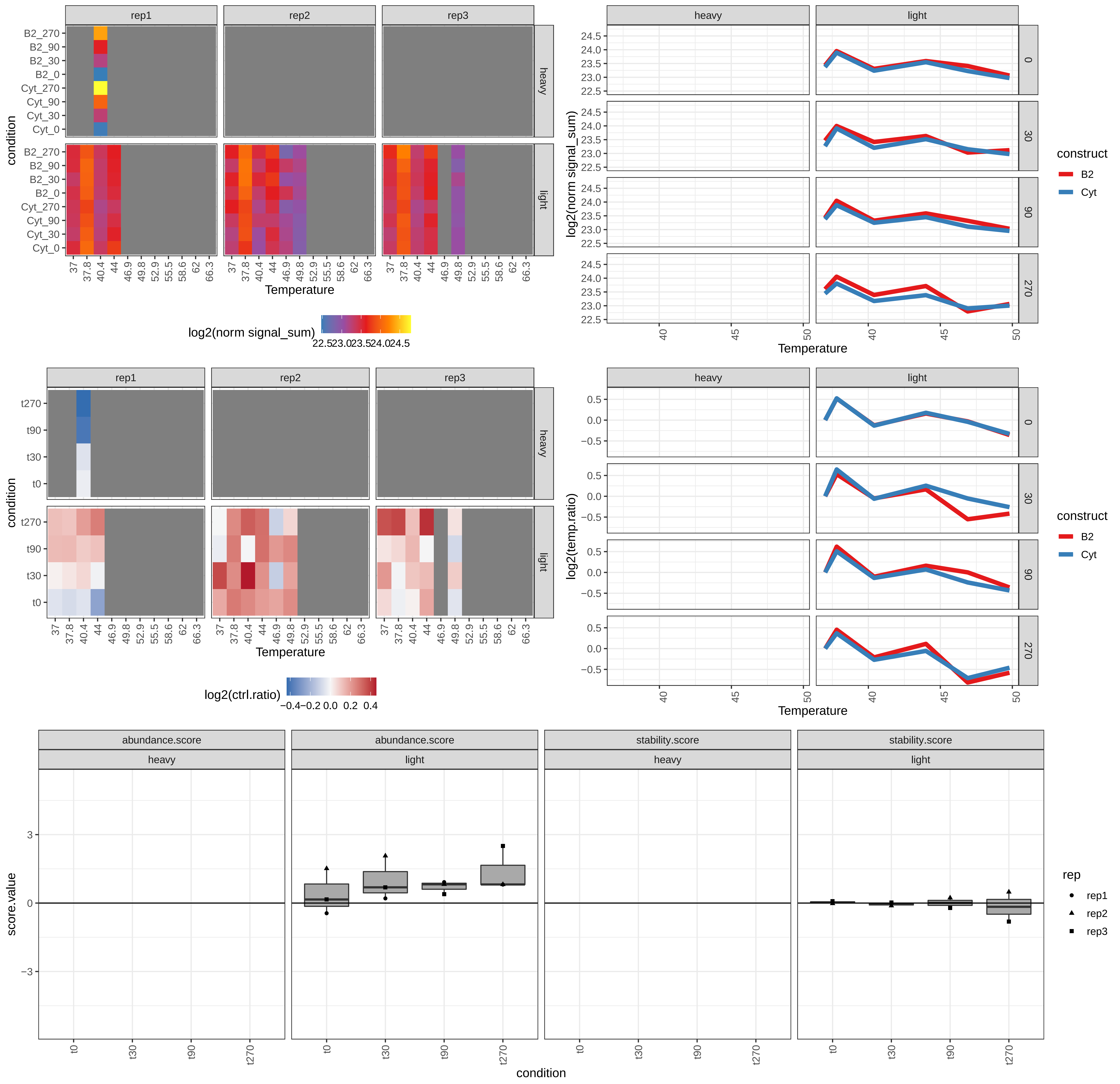

P40090 − MAU2 chromatid cohesion factor homolog OS=Saccharomyces cerevisiae (strain ATCC 204508 / S288c) GN=SCC4 PE=1 SV=1

SCD6 − average.top3: 6.87295 − max.qupm: 6

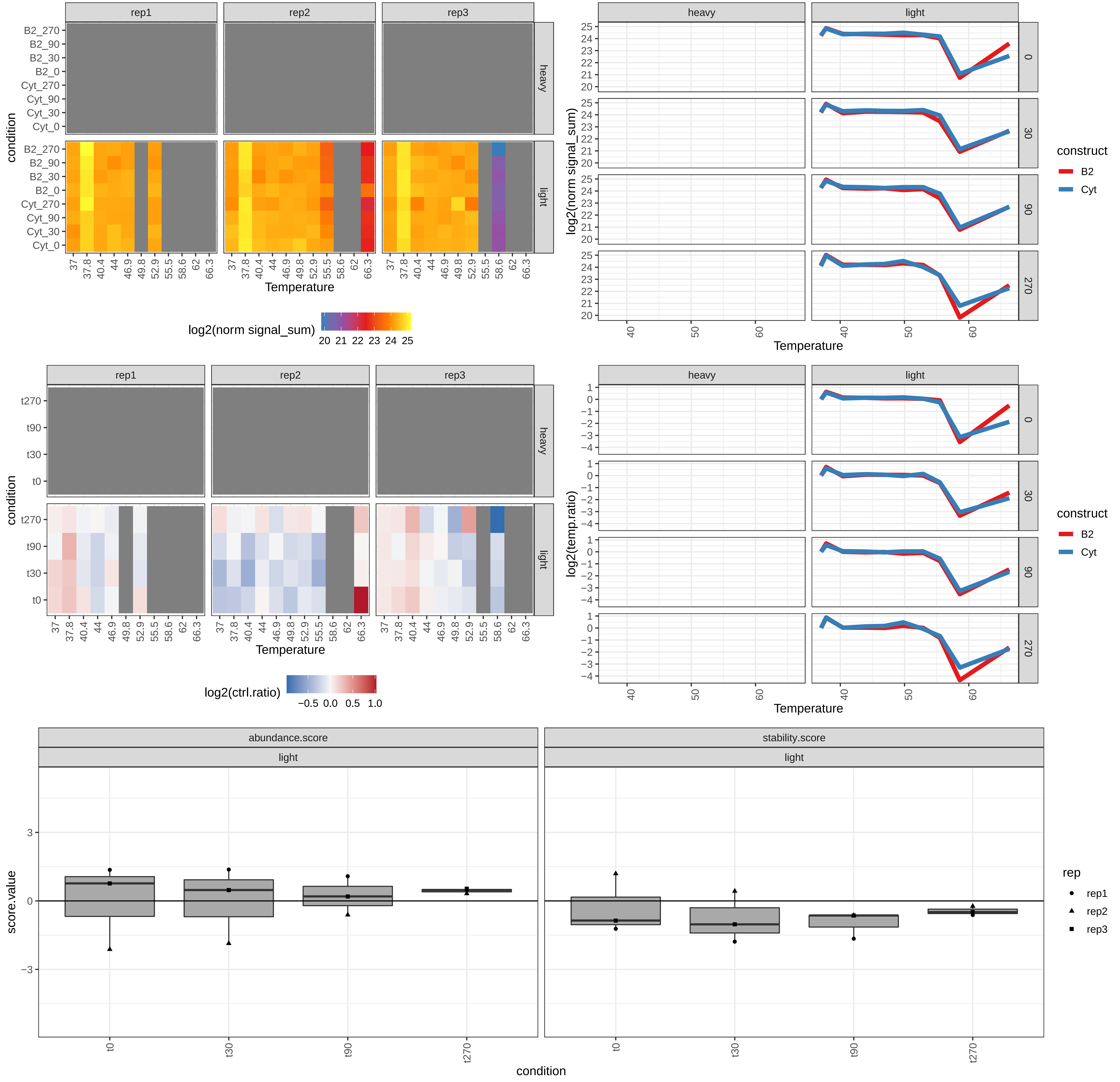

P45978 − Protein SCD6 OS=Saccharomyces cerevisiae (strain ATCC 204508 / S288c) GN=SCD6 PE=1 SV=1

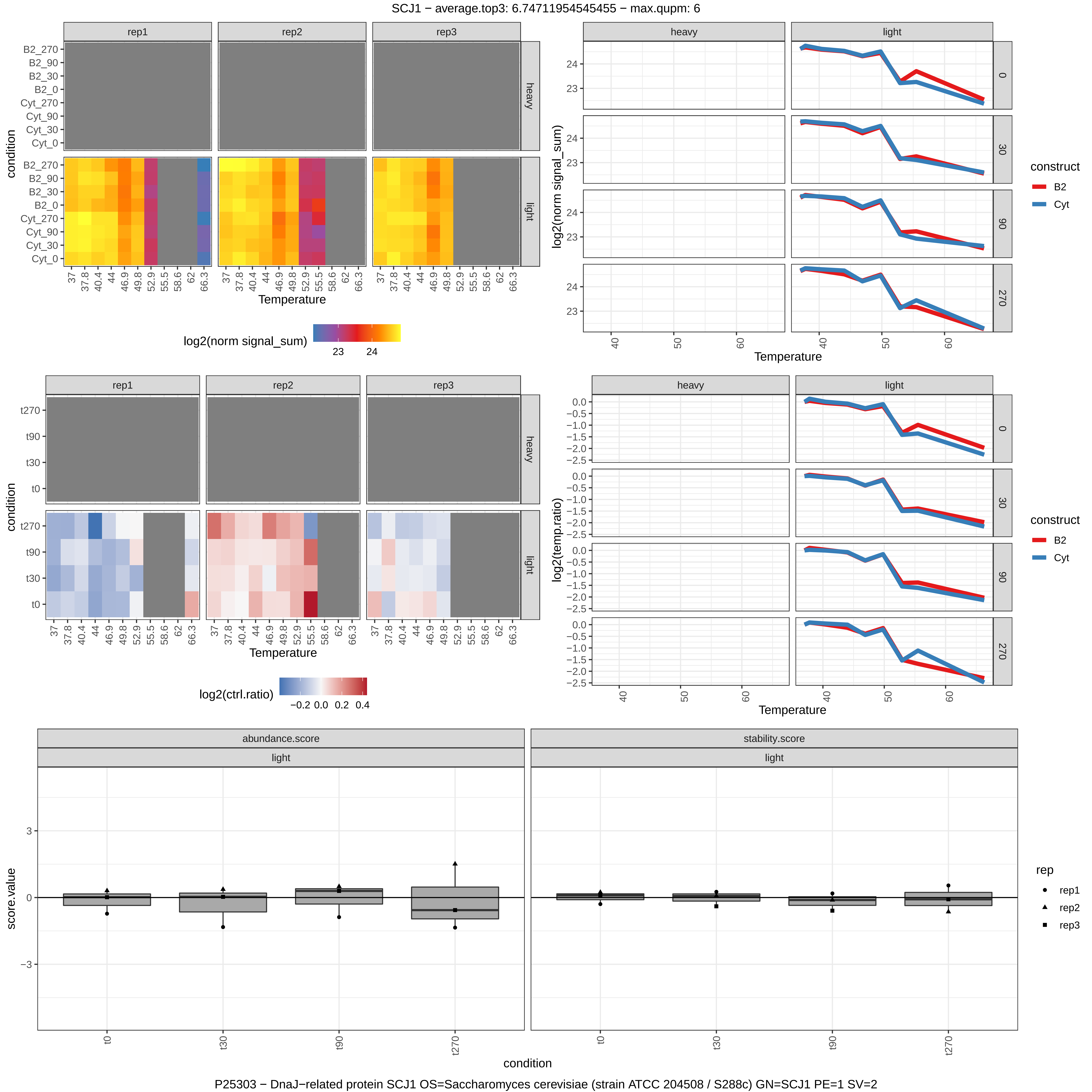
SCJ1 − average.top3: 6.74711954545455 − max.qupm: 6
rep1
rep2
rep3
heavy
light
condition
B2_270
B2_90
B2_30
B2_0
Cyt_270
Cyt_90
Cyt_30
Cyt_0
Temperature
37
37.8
40.4
44
46.9
49.8
52.9
55.5
58.6
62
66.3
log2(norm signal_sum)
23
24
log2(norm signal_sum)
0
30
90
270
construct
B2
Cyt
40
50
60
t270
t90
t30
t0
log2(ctrl.ratio)
−0.2
0.0
0.2
0.4
log2(temp.ratio)
0.0
−0.5
−1.0
−1.5
−2.0
−2.5
abundance.score
stability.score
score.value
3
0
−3
condition
rep
rep1
rep2
rep3
P25303 − DnaJ−related protein SCJ1 OS=Saccharomyces cerevisiae (strain ATCC 204508 / S288c) GN=SCJ1 PE=1 SV=2

SCL1 − average.top3: 7.19312015873016 − max.qupm: 15

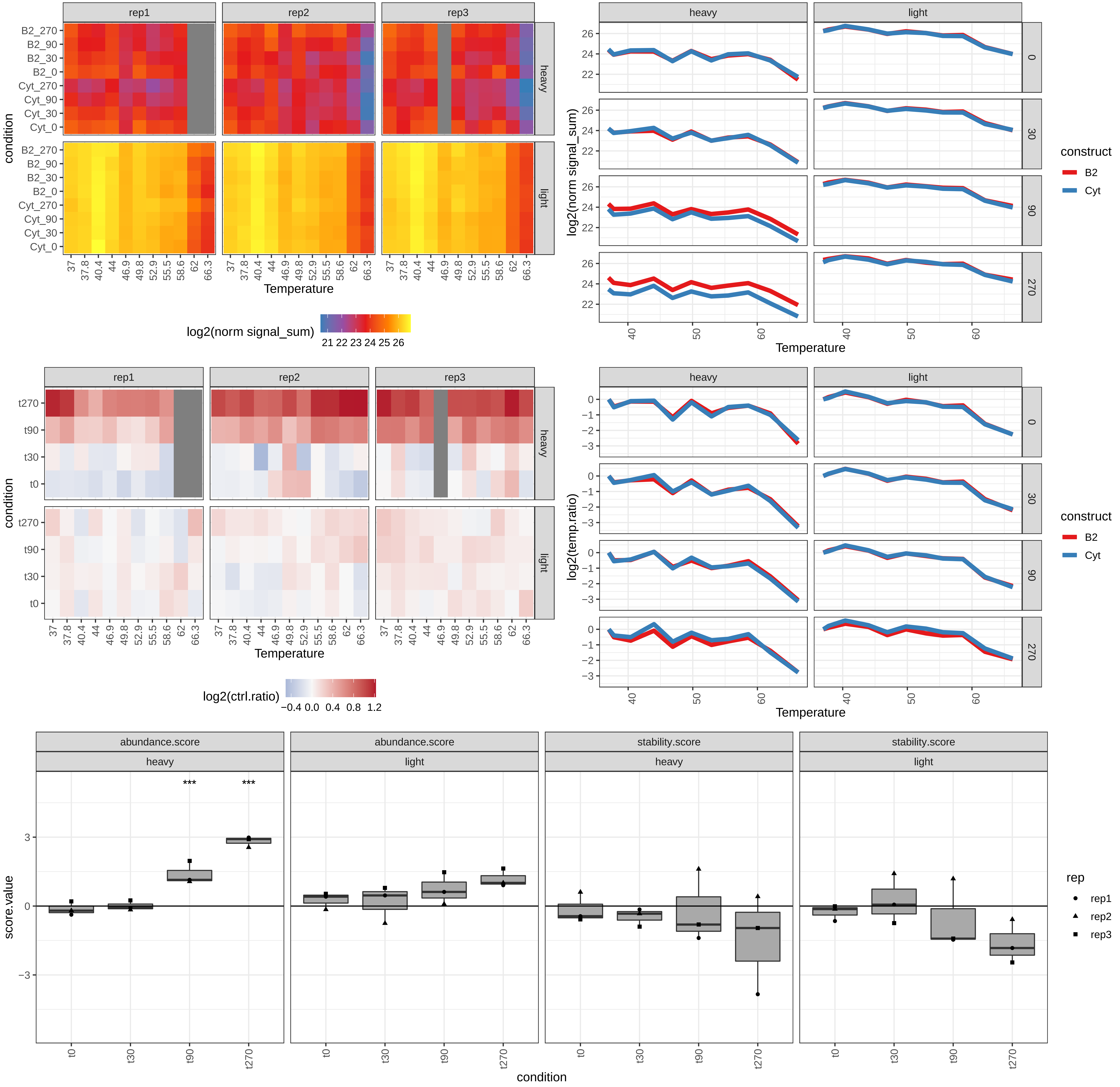

P21243 − Proteasome subunit alpha type−1 OS=Saccharomyces cerevisiae (strain ATCC 204508 / S288c) GN=SCL1 PE=1 SV=1

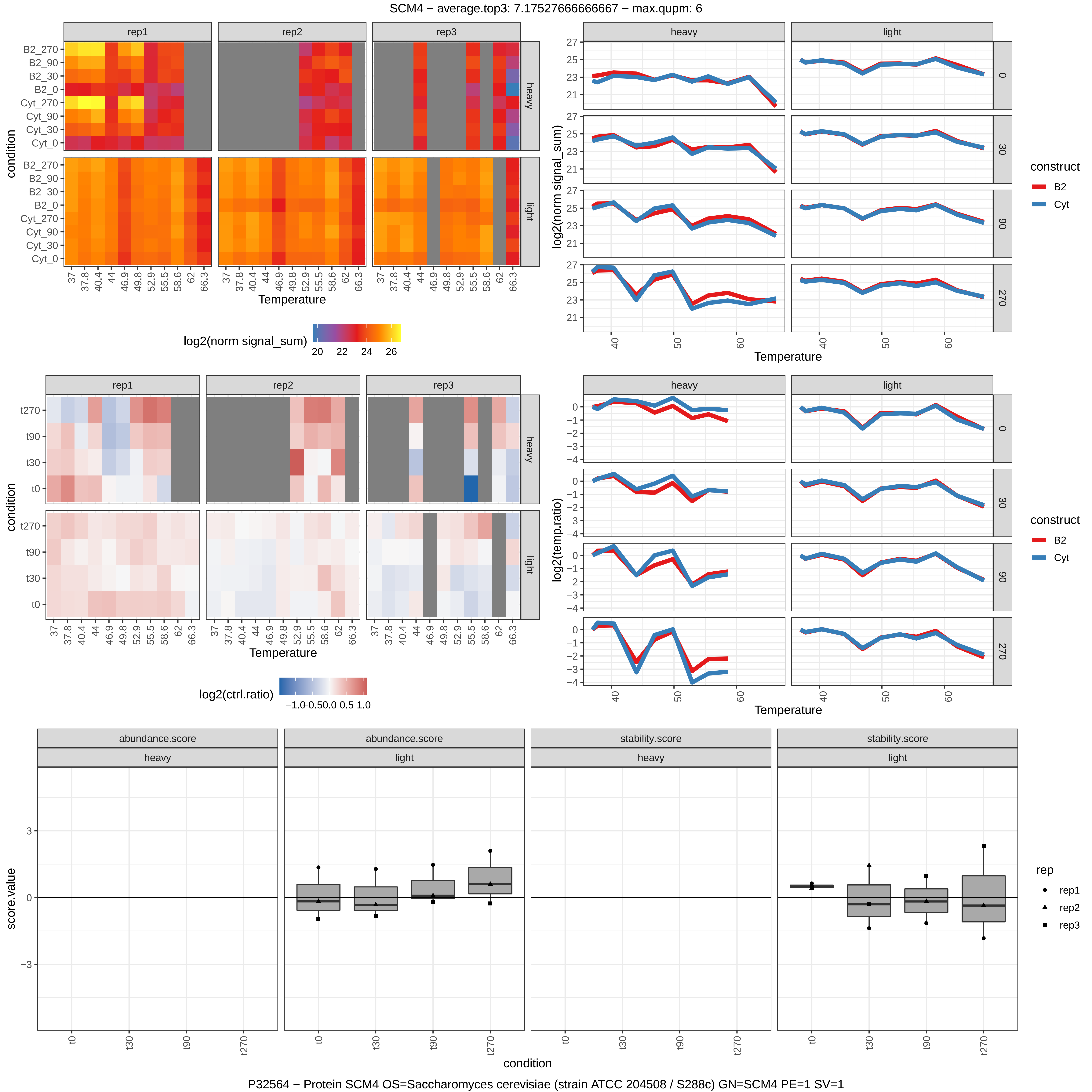
SCM4 − average.top3: 7.17527666666667 − max.qupm: 6
rep1
rep2
rep3
heavy
light
condition
Temperature
log2(norm signal_sum)
log2(ctrl.ratio)
log2(temp.ratio)
construct
B2
Cyt
score.value
abundance.score
stability.score
rep
rep1
rep2
rep3
P32564 − Protein SCM4 OS=Saccharomyces cerevisiae (strain ATCC 204508 / S288c) GN=SCM4 PE=1 SV=1

SCO1 − average.top3: 6.958204 − max.qupm: 6

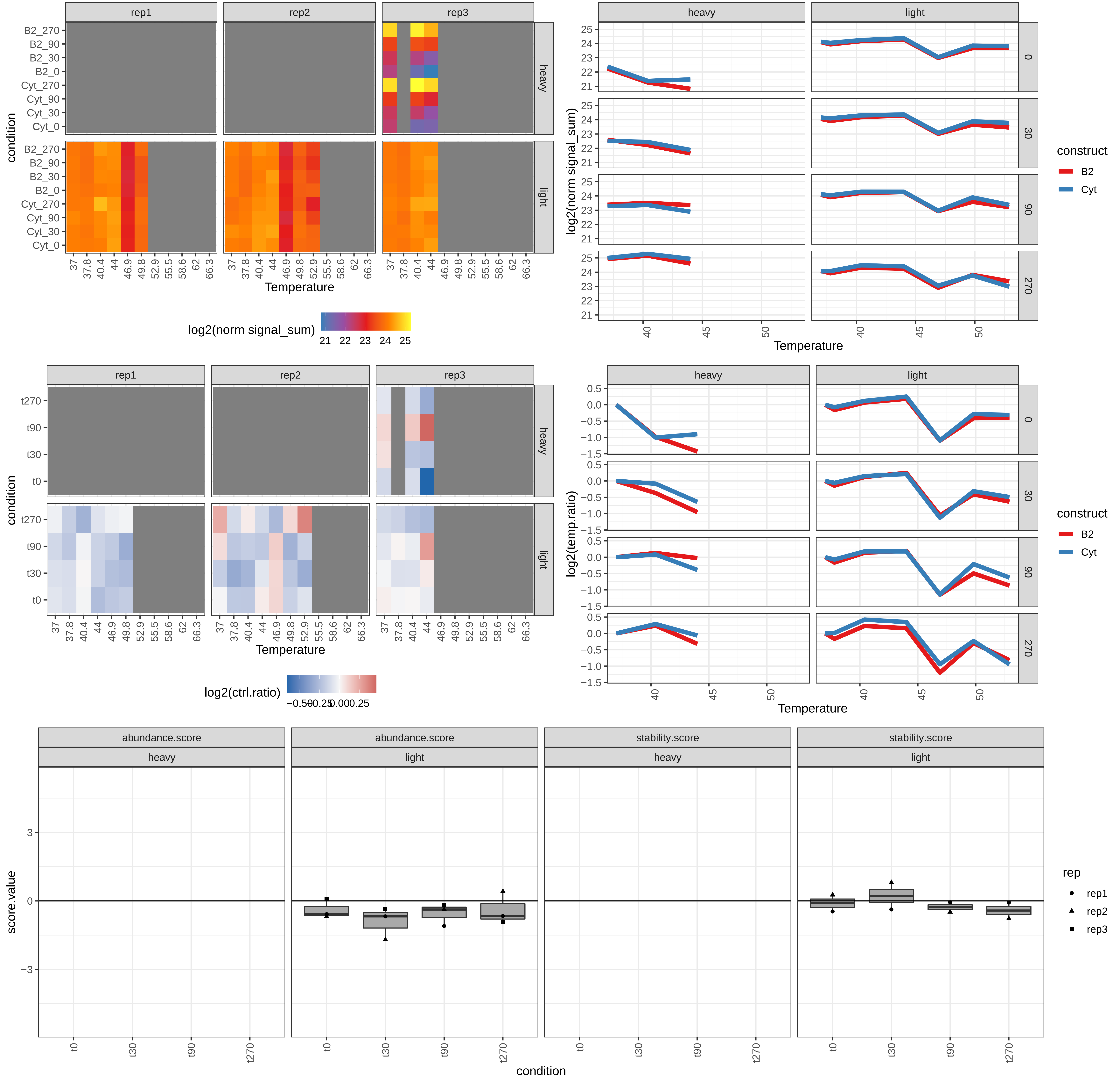

P23833 − Protein SCO1, mitochondrial OS=Saccharomyces cerevisiae (strain ATCC 204508 / S288c) GN=SCO1 PE=1 SV=1

SCO2 − average.top3: 7.01683282051282 − max.qupm: 6

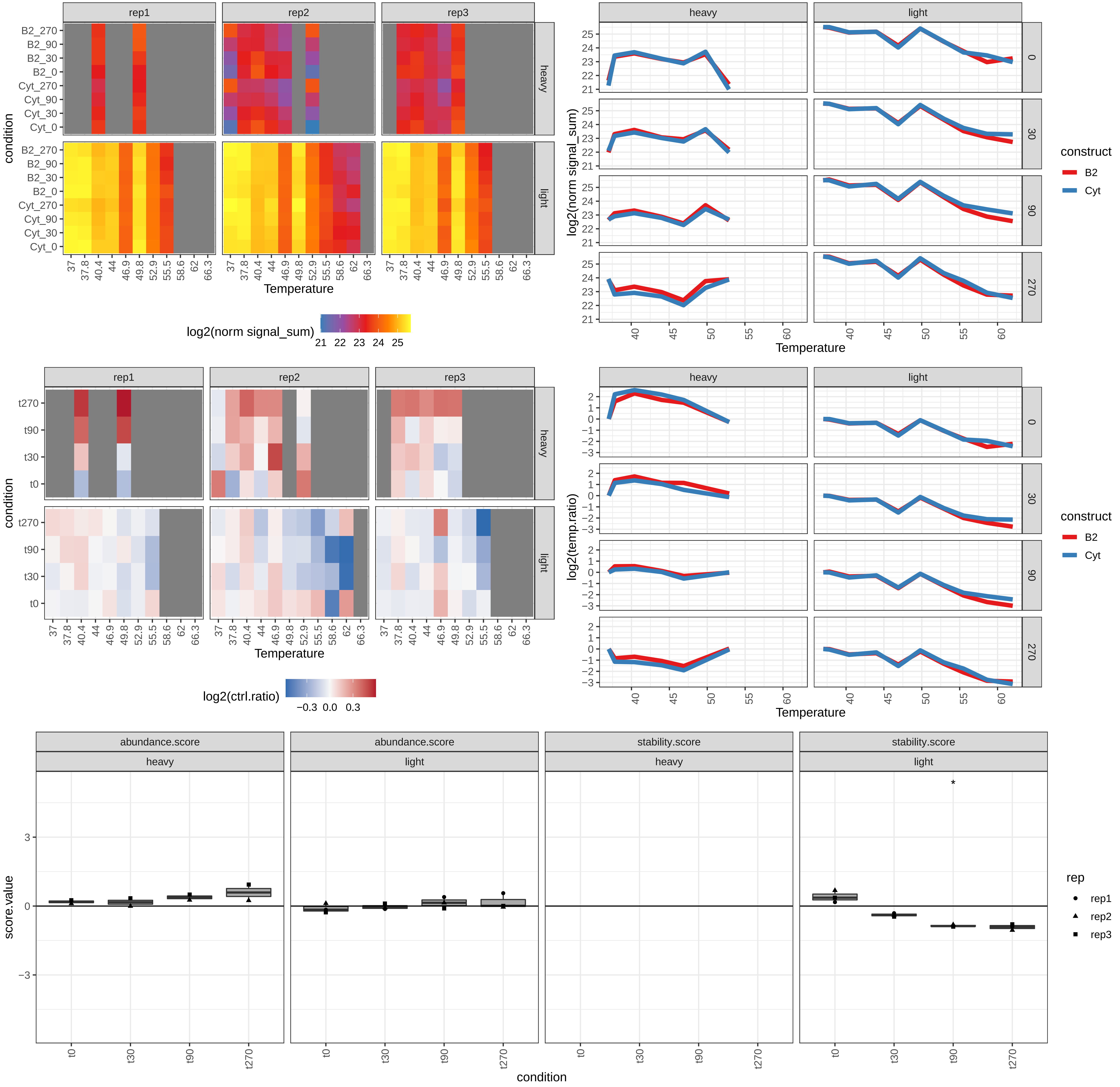

P38072 − Protein SCO2, mitochondrial OS=Saccharomyces cerevisiae (strain ATCC 204508 / S288c) GN=SCO2 PE=1 SV=1

SCP160 − average.top3: 7.34867553191489 − max.qupm: 42

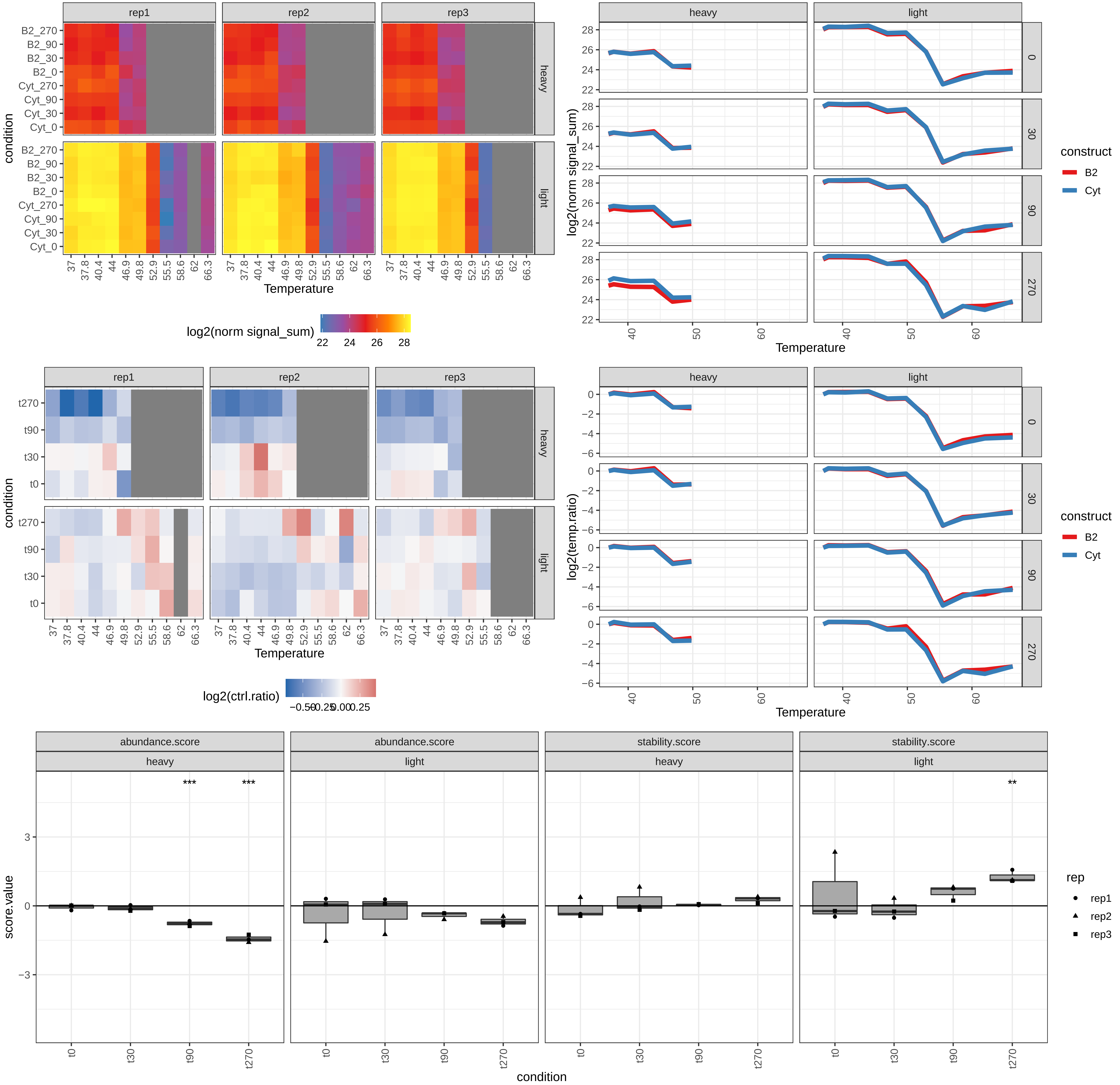

P06105 − Protein SCP160 OS=Saccharomyces cerevisiae (strain ATCC 204508 / S288c) GN=SCP160 PE=1 SV=3

SCS3 − average.top3: 6.48362071428571 − max.qupm: 3

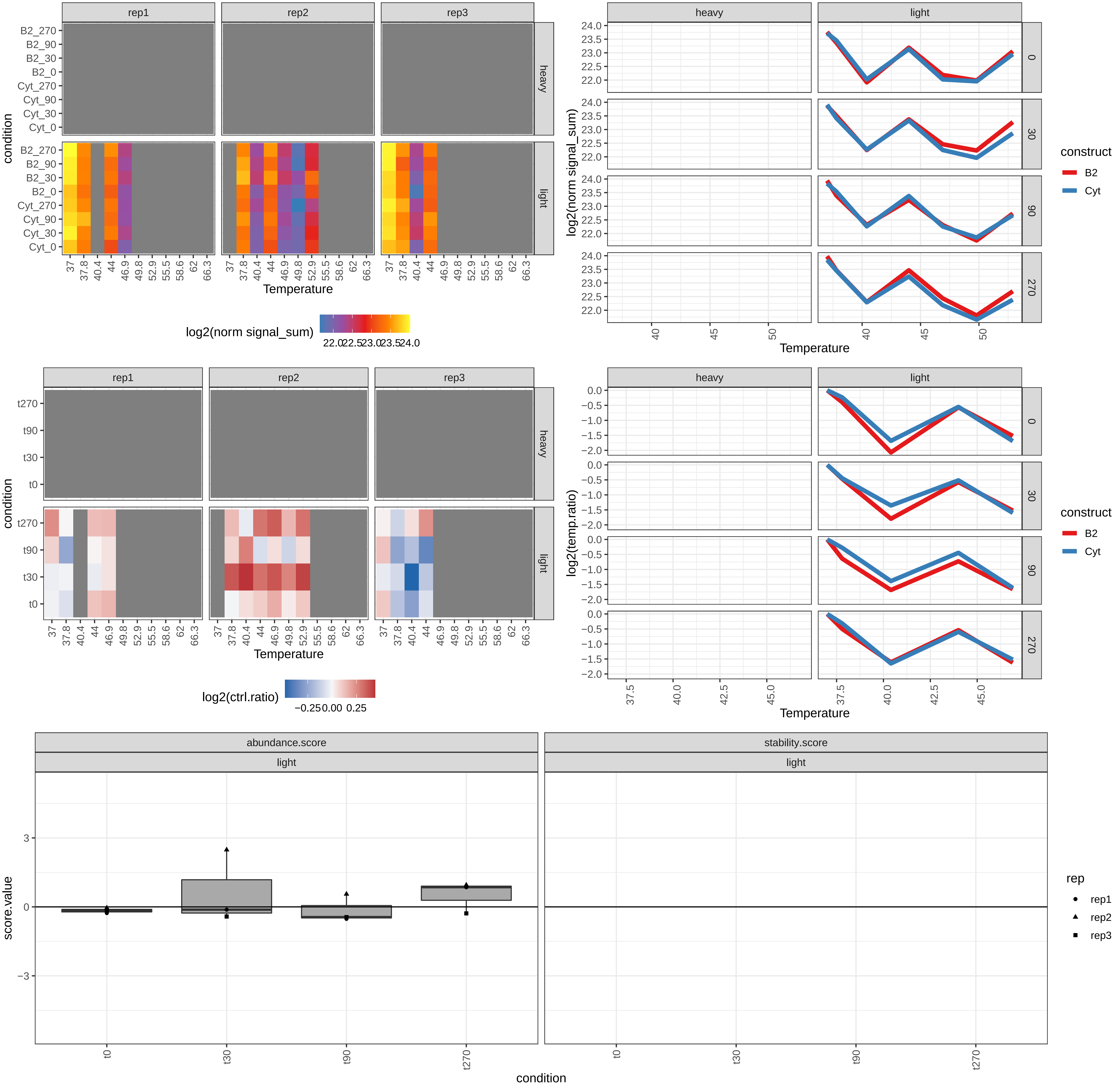

P53012 − FIT family protein SCS3 OS=Saccharomyces cerevisiae (strain ATCC 204508 / S288c) GN=SCS3 PE=1 SV=1

SCS7 − average.top3: 6.37990705882353 − max.qupm: 4

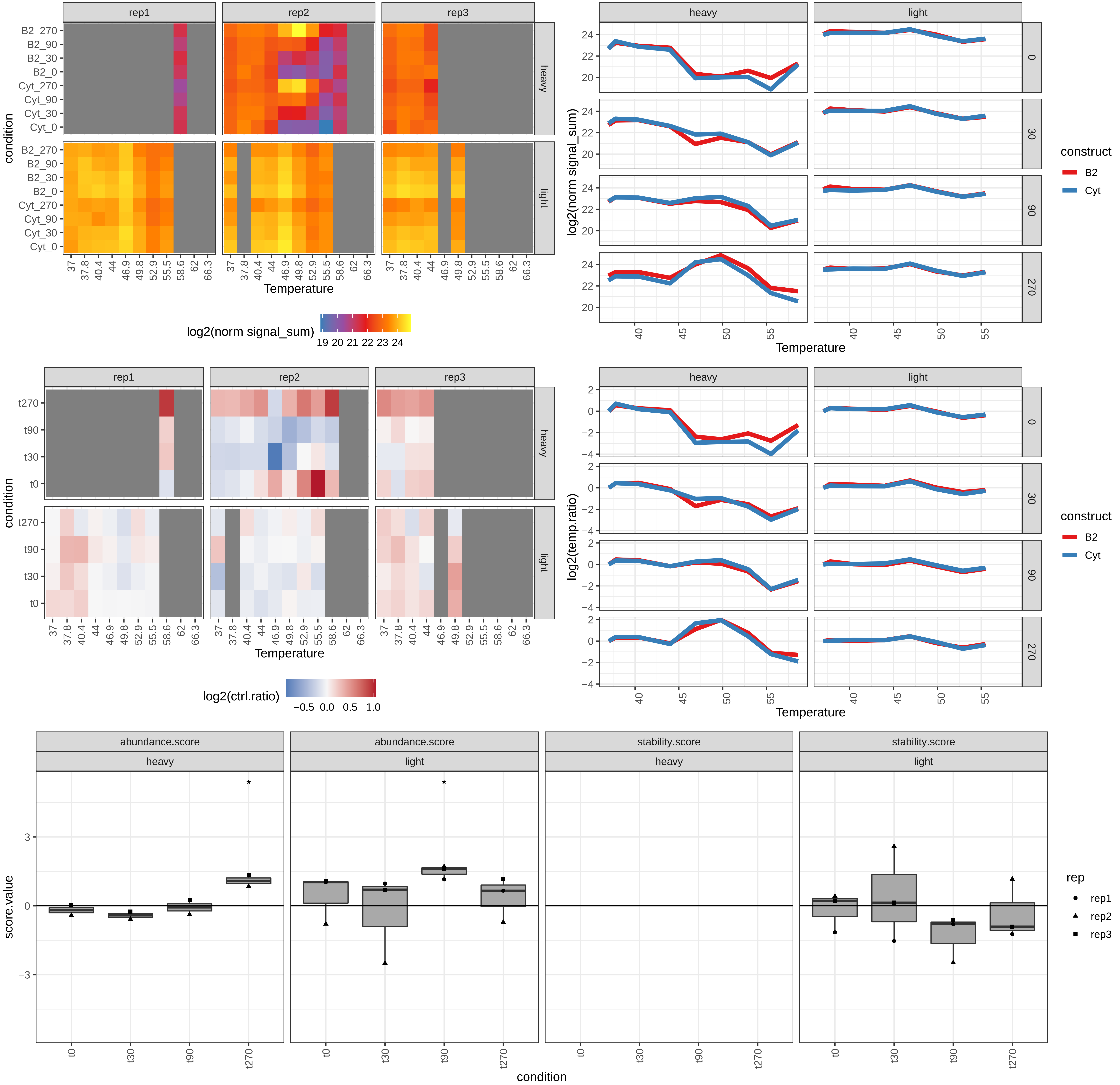

Q03529 − Ceramide very long chain fatty acid hydroxylase SCS7 OS=Saccharomyces cerevisiae (strain ATCC 204508 / S288c) GN=SCS7 PE=1 SV=1

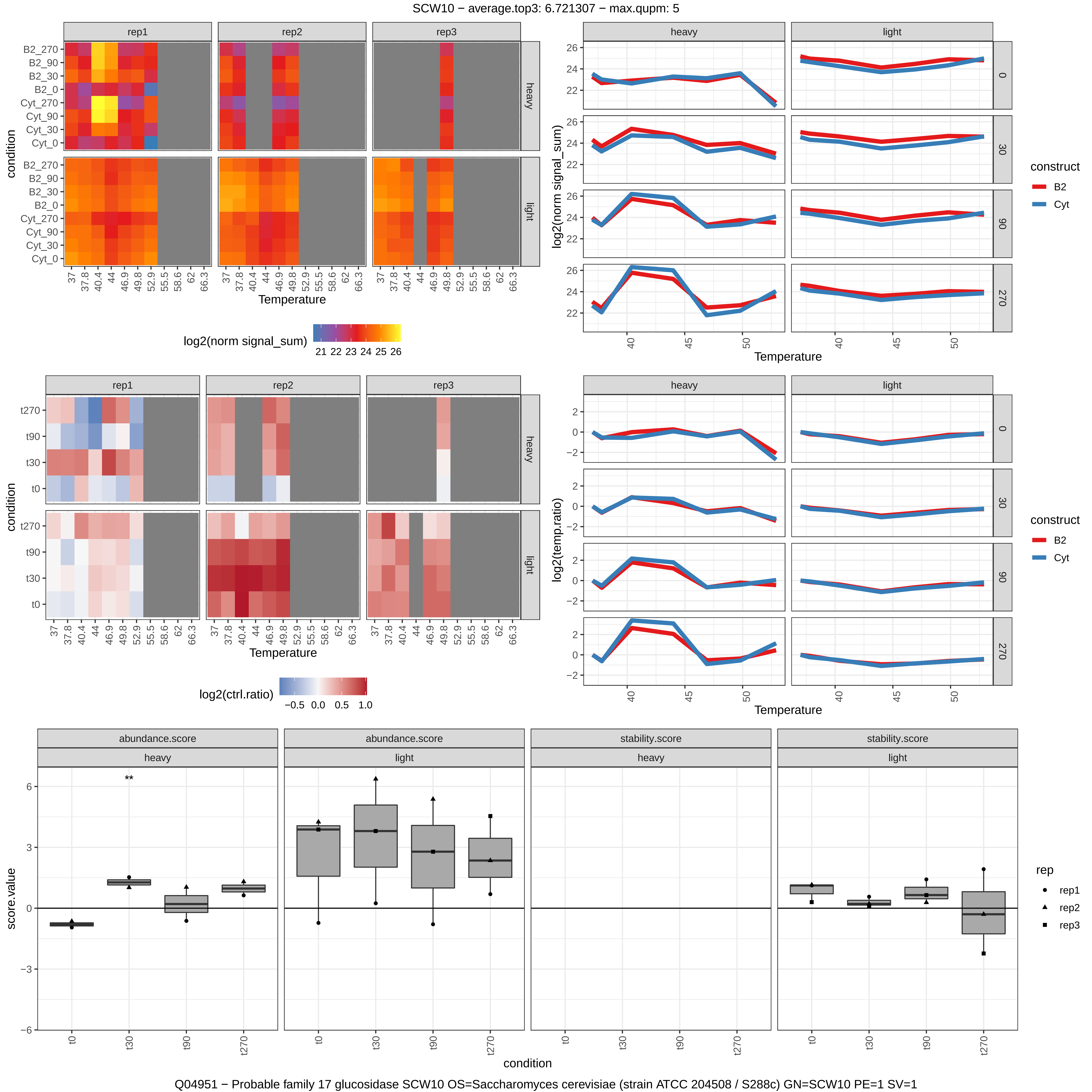

SCW10 − average.top3: 6.721307 − max.qupm: 5
rep1
rep2
rep3
heavy
light
condition
B2_270
B2_90
B2_30
B2_0
Cyt_270
Cyt_90
Cyt_30
Cyt_0
Temperature
log2(norm signal_sum)
21 22 23 24 25 26
log2(norm signal_sum)
construct
B2
Cyt
t270
t90
t30
t0
log2(ctrl.ratio)
−0.5 0.0 0.5 1.0
log2(temp.ratio)
abundance.score
stability.score
score.value
condition
rep
rep1
rep2
rep3
Q04951 − Probable family 17 glucosidase SCW10 OS=Saccharomyces cerevisiae (strain ATCC 204508 / S288c) GN=SCW10 PE=1 SV=1

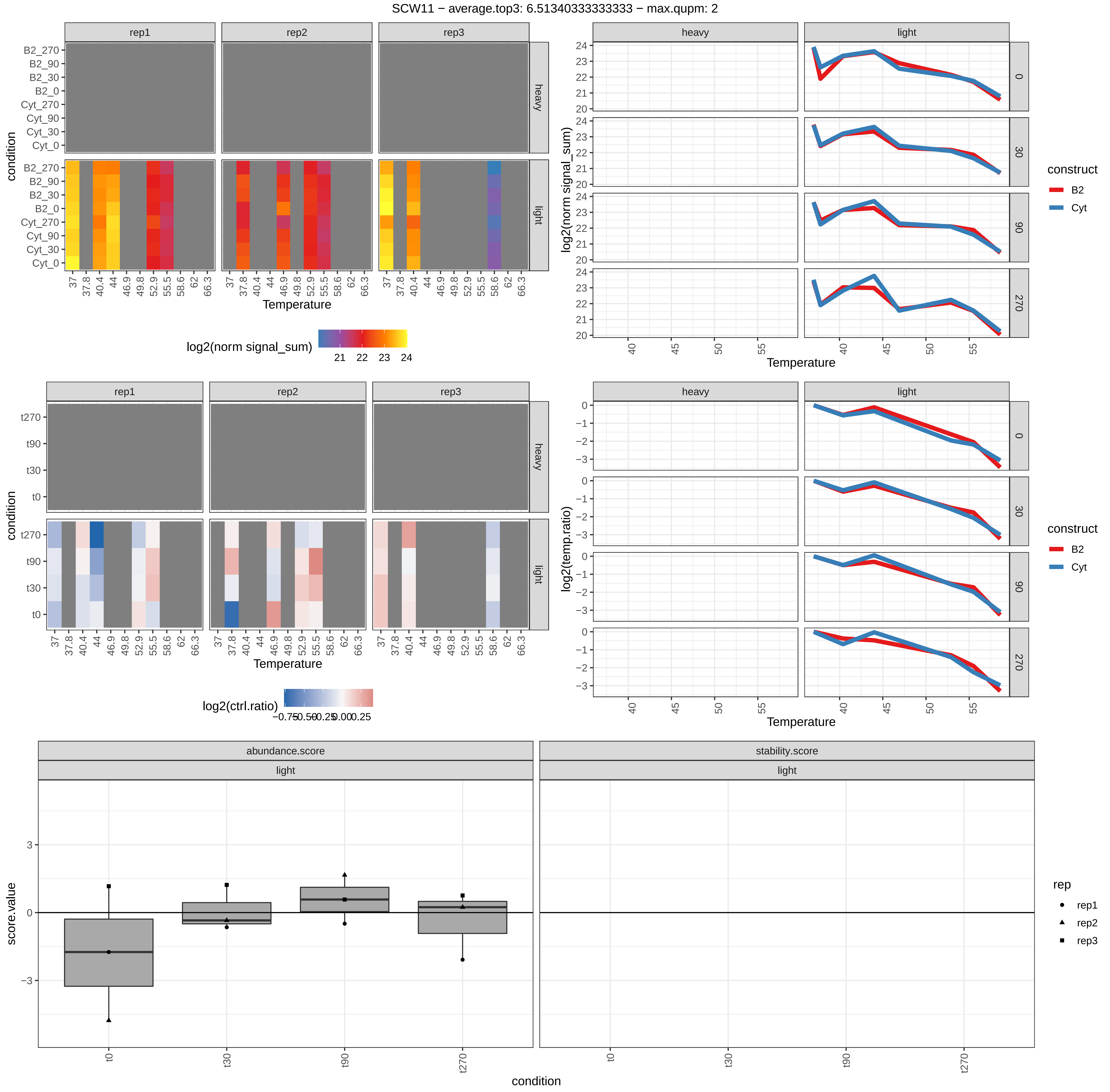

P53189 − Probable family 17 glucosidase SCW11 OS=Saccharomyces cerevisiae (strain ATCC 204508 / S288c) GN=SCW11 PE=1 SV=1

SCW4 − average.top3: 7.07223389830509 − max.qupm: 7

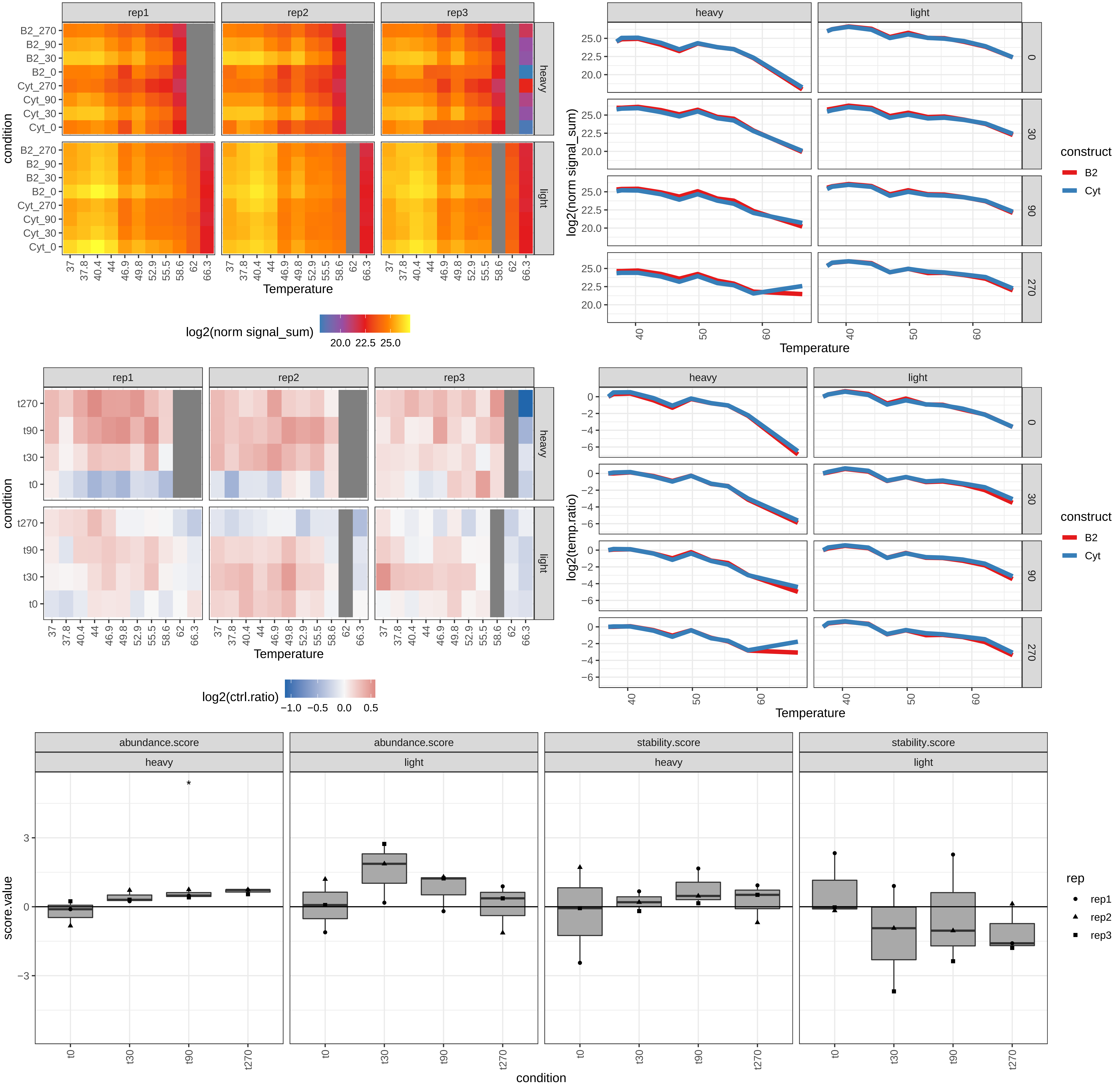

P53334 − Probable family 17 glucosidase SCW4 OS=Saccharomyces cerevisiae (strain ATCC 204508 / S288c) GN=SCW4 PE=1 SV=1

SCY1 − average.top3: 6.67888375 − max.qupm: 7

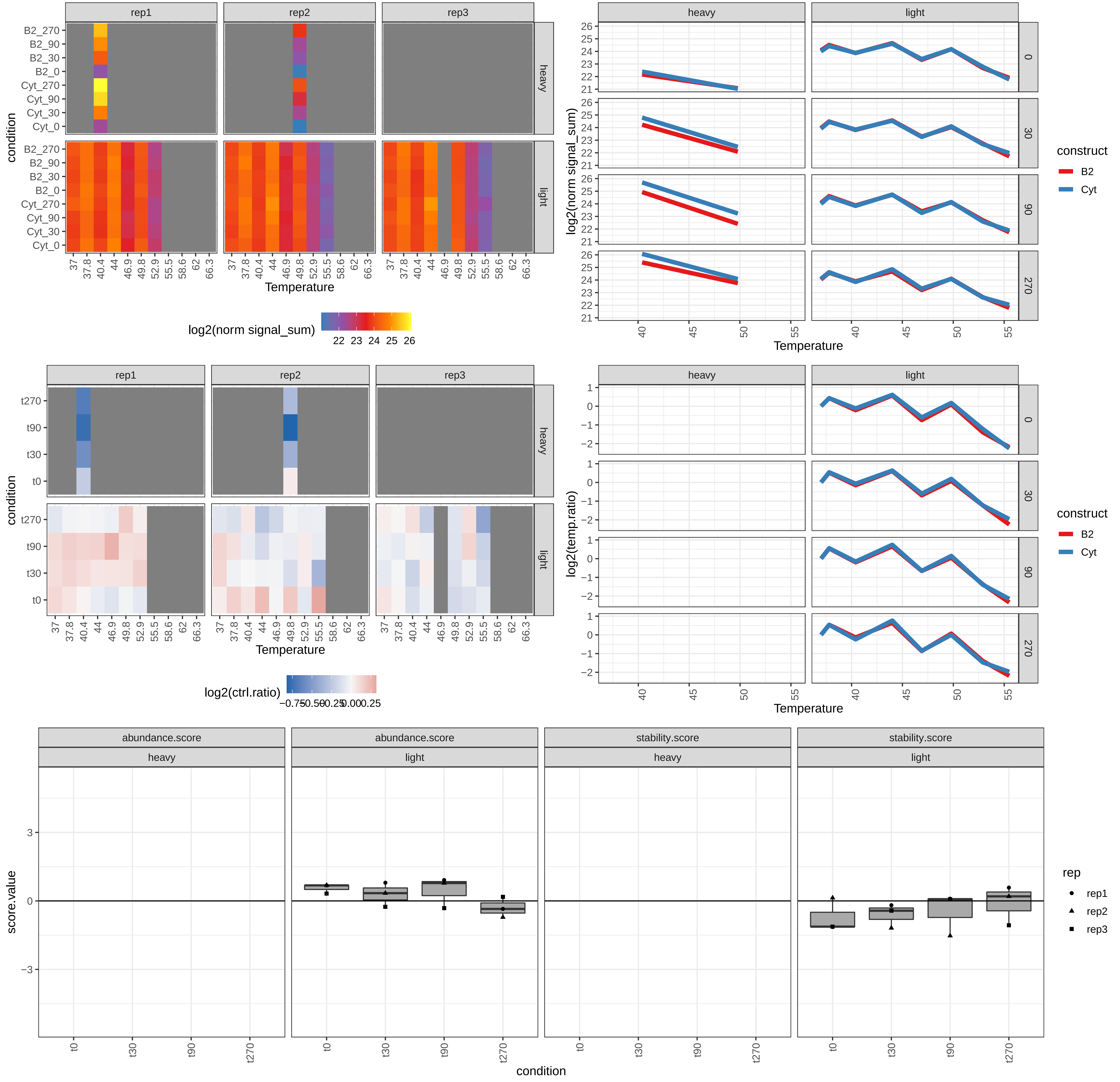

P53009 − Protein kinase−like protein SCY1 OS=Saccharomyces cerevisiae (strain ATCC 204508 / S288c) GN=SCY1 PE=1 SV=1

SDH1 − average.top3: 7.96068412698413 − max.qupm: 24

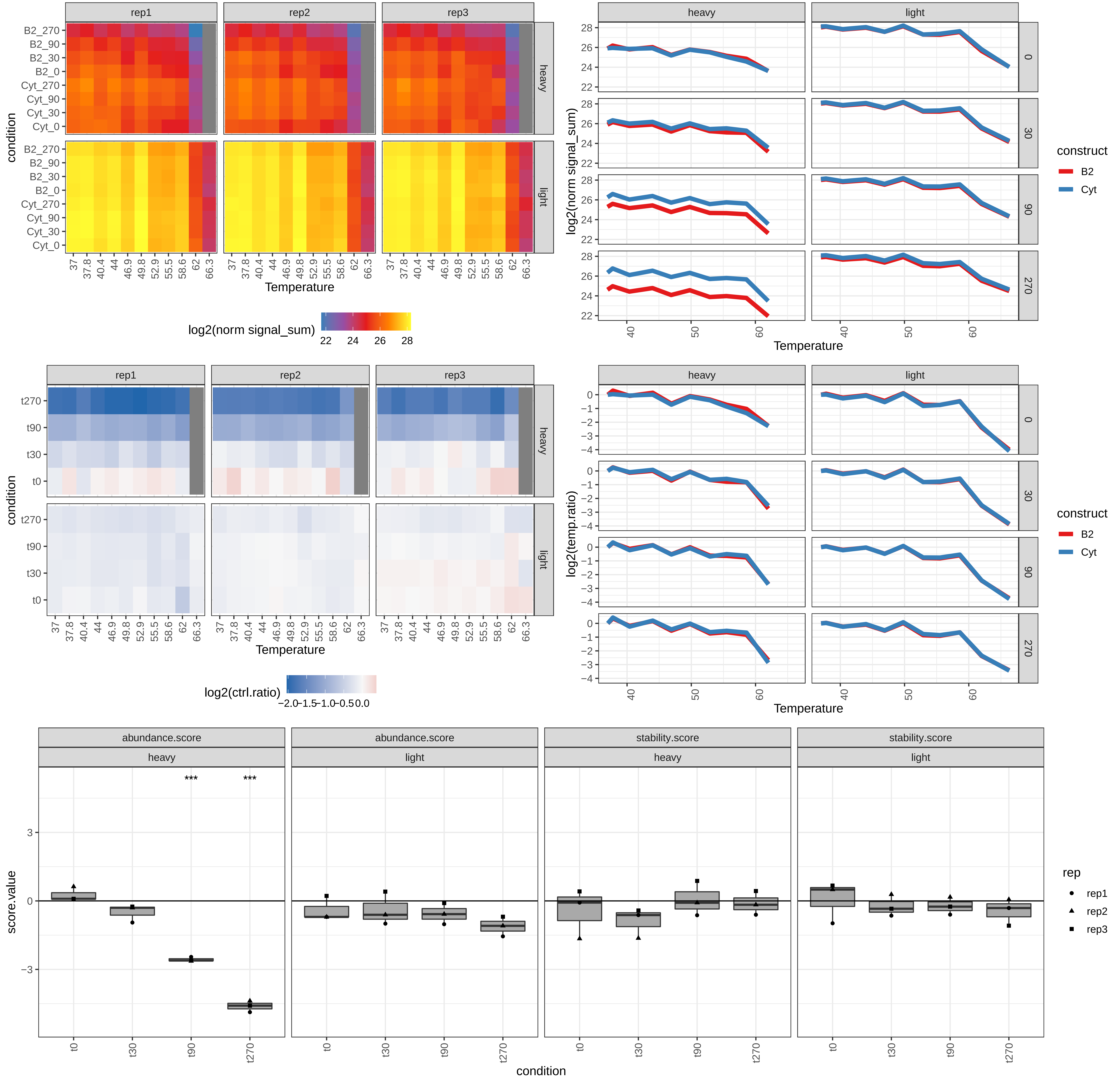

Q00711 − Succinate dehydrogenase [ubiquinone] flavoprotein subunit, mitochondrial OS=Saccharomyces cerevisiae (strain ATCC 204508 / S288c) GN=SDH1 PE=1 SV=1

SDH2 − average.top3: 7.85590949152542 − max.qupm: 14

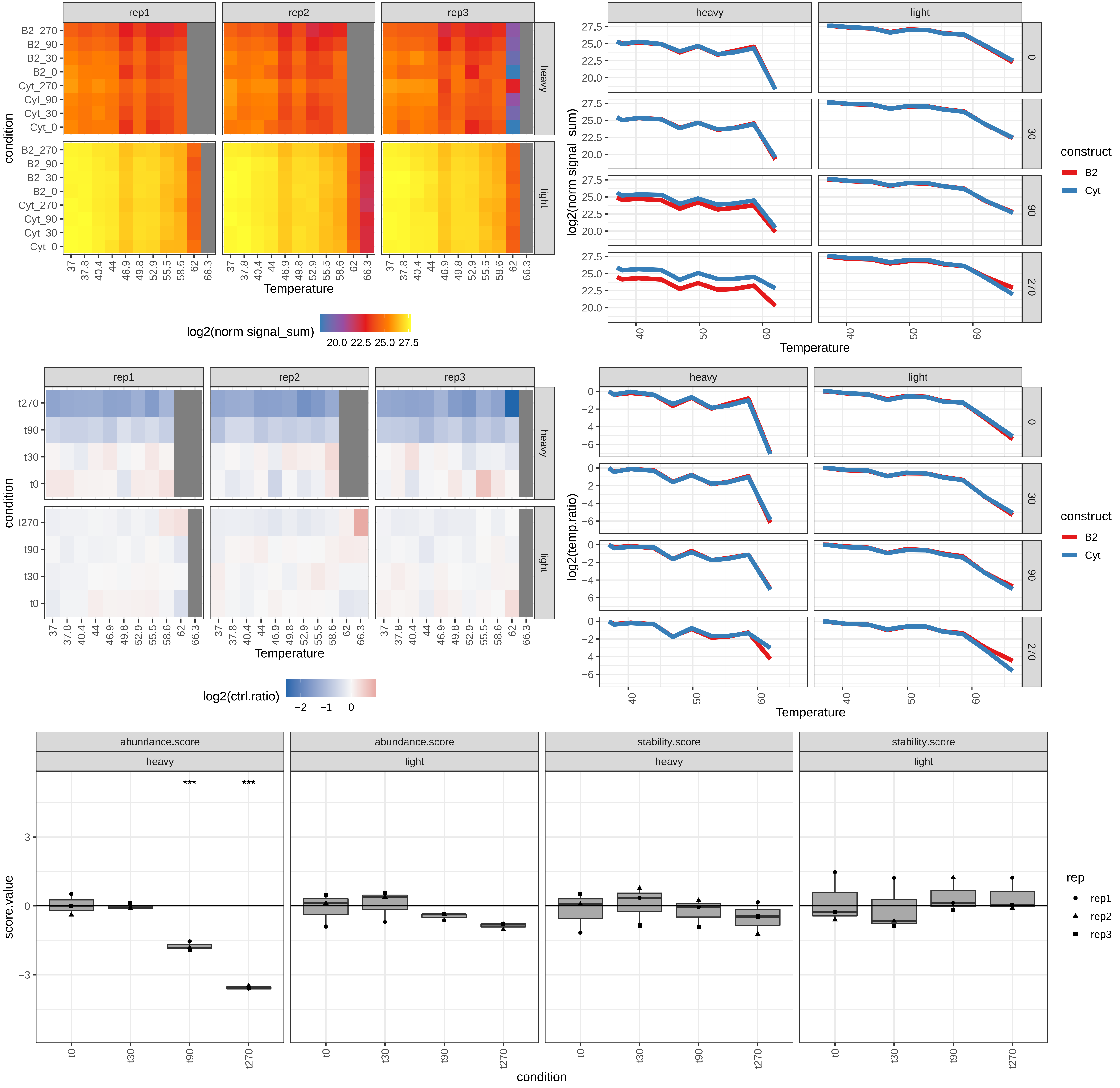

P21801 − Succinate dehydrogenase [ubiquinone] iron−sulfur subunit, mitochondrial OS=Saccharomyces cerevisiae (strain ATCC 204508 / S288c) GN=SDH2 PE=1 SV=1

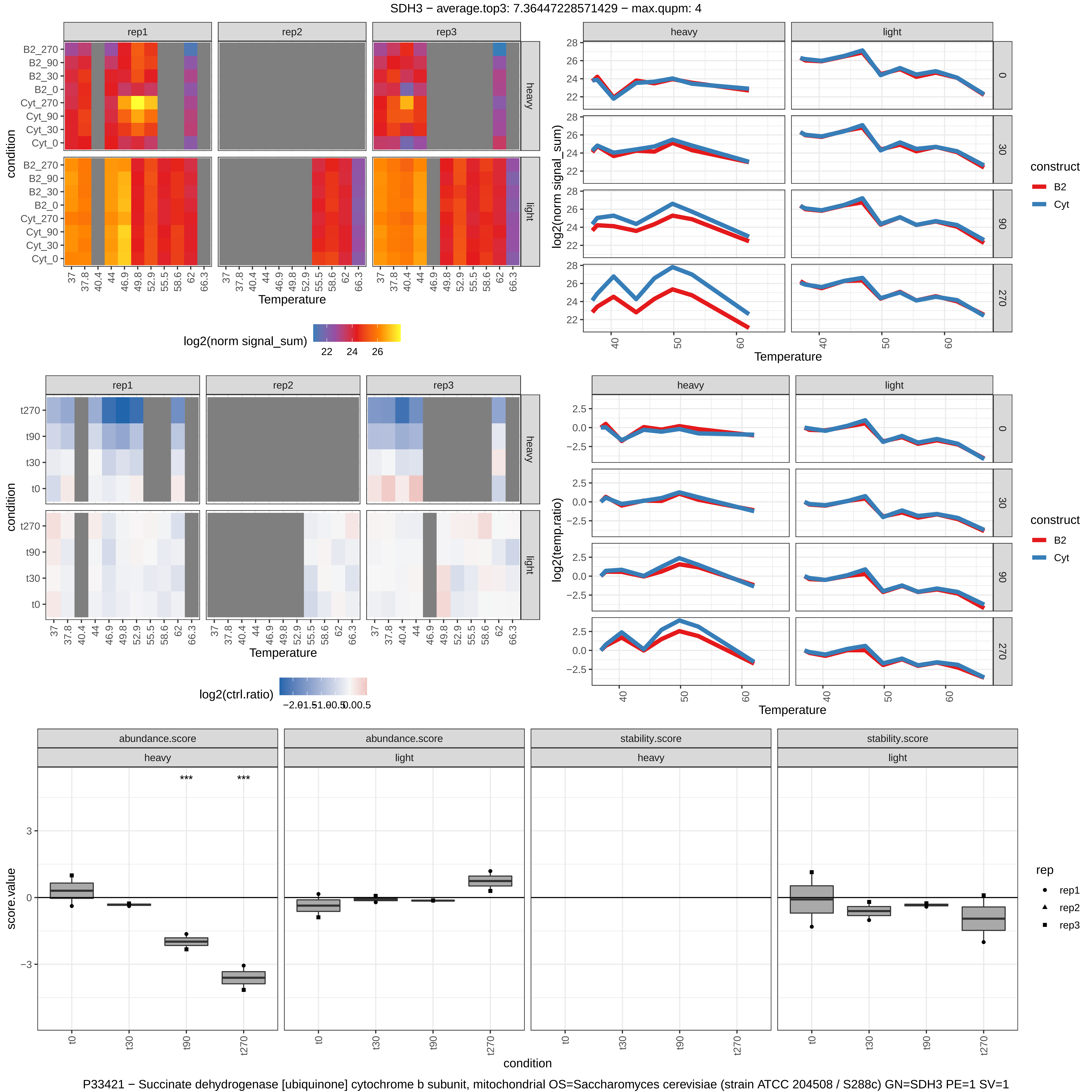
SDH3 − average.top3: 7.36447228571429 − max.qupm: 4
rep1
rep2
rep3
heavy
light
condition
Temperature
log2(norm signal_sum)
construct
B2
Cyt
log2(ctrl.ratio)
log2(temp.ratio)
abundance.score
stability.score
score.value
rep
rep1
rep2
rep3
P33421 − Succinate dehydrogenase [ubiquinone] cytochrome b subunit, mitochondrial OS=Saccharomyces cerevisiae (strain ATCC 204508 / S288c) GN=SDH3 PE=1 SV=1

SDH4 − average.top3: 7.19652789473684 − max.qupm: 5

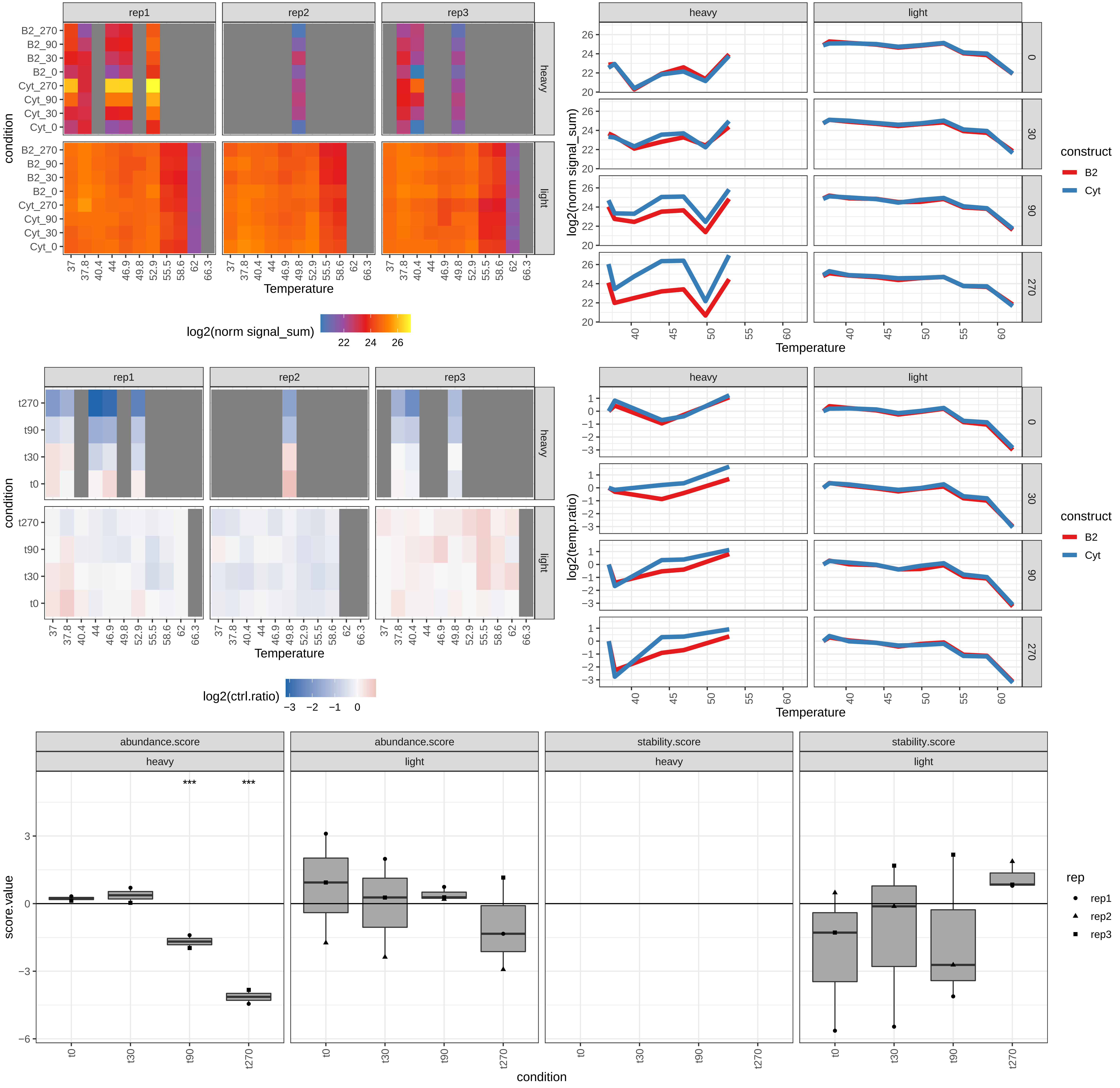

P37298 − Succinate dehydrogenase [ubiquinone] cytochrome b small subunit, mitochondrial OS=Saccharomyces cerevisiae (strain ATCC 204508 / S288c) GN=SDH4 PE=1 SV=1

SDH5 − average.top3: 6.91682173913043 − max.qupm: 4

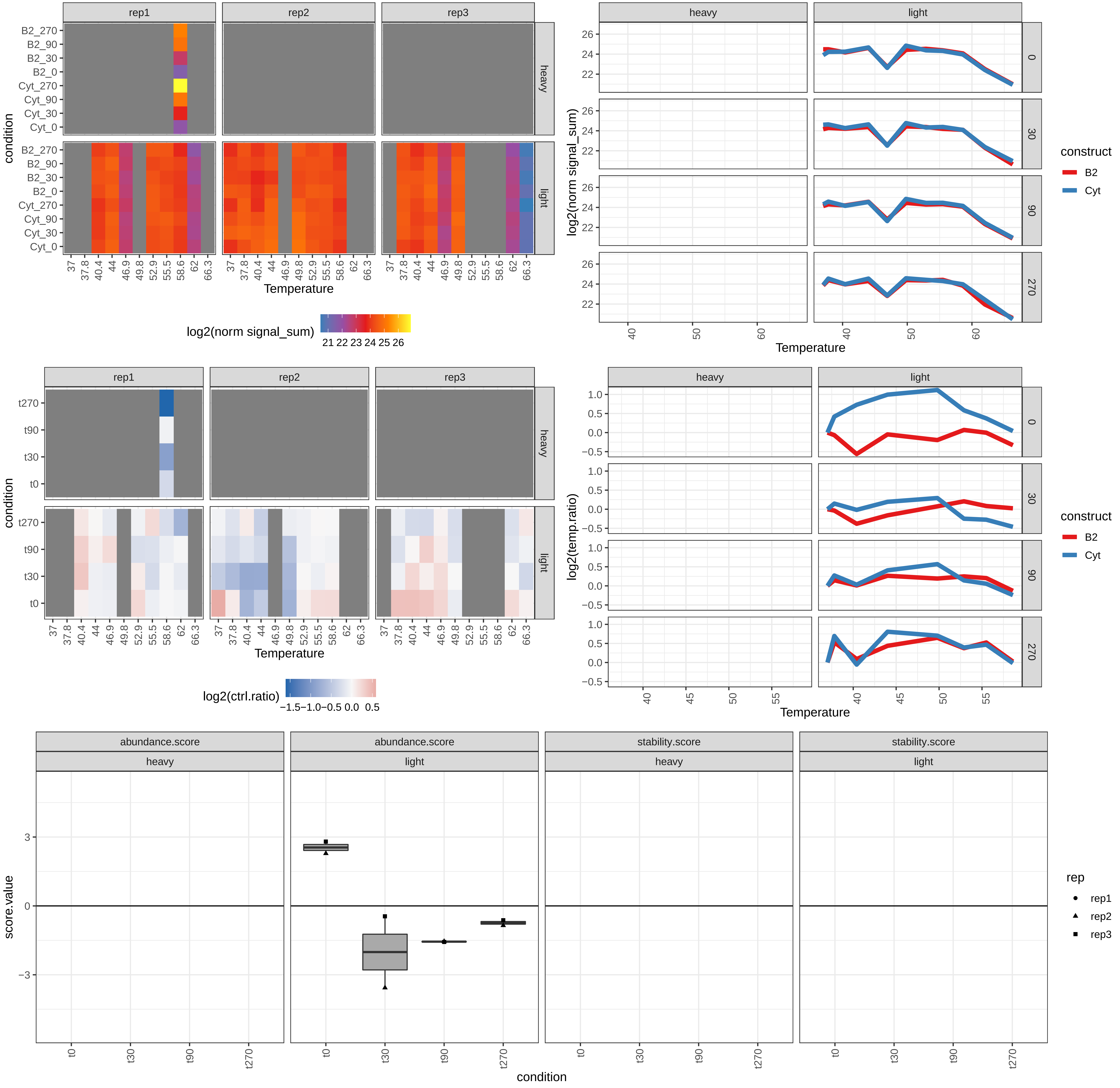

Q08230 − Succinate dehydrogenase assembly factor 2, mitochondrial OS=Saccharomyces cerevisiae (strain ATCC 204508 / S288c) GN=SDH5 PE=1 SV=1

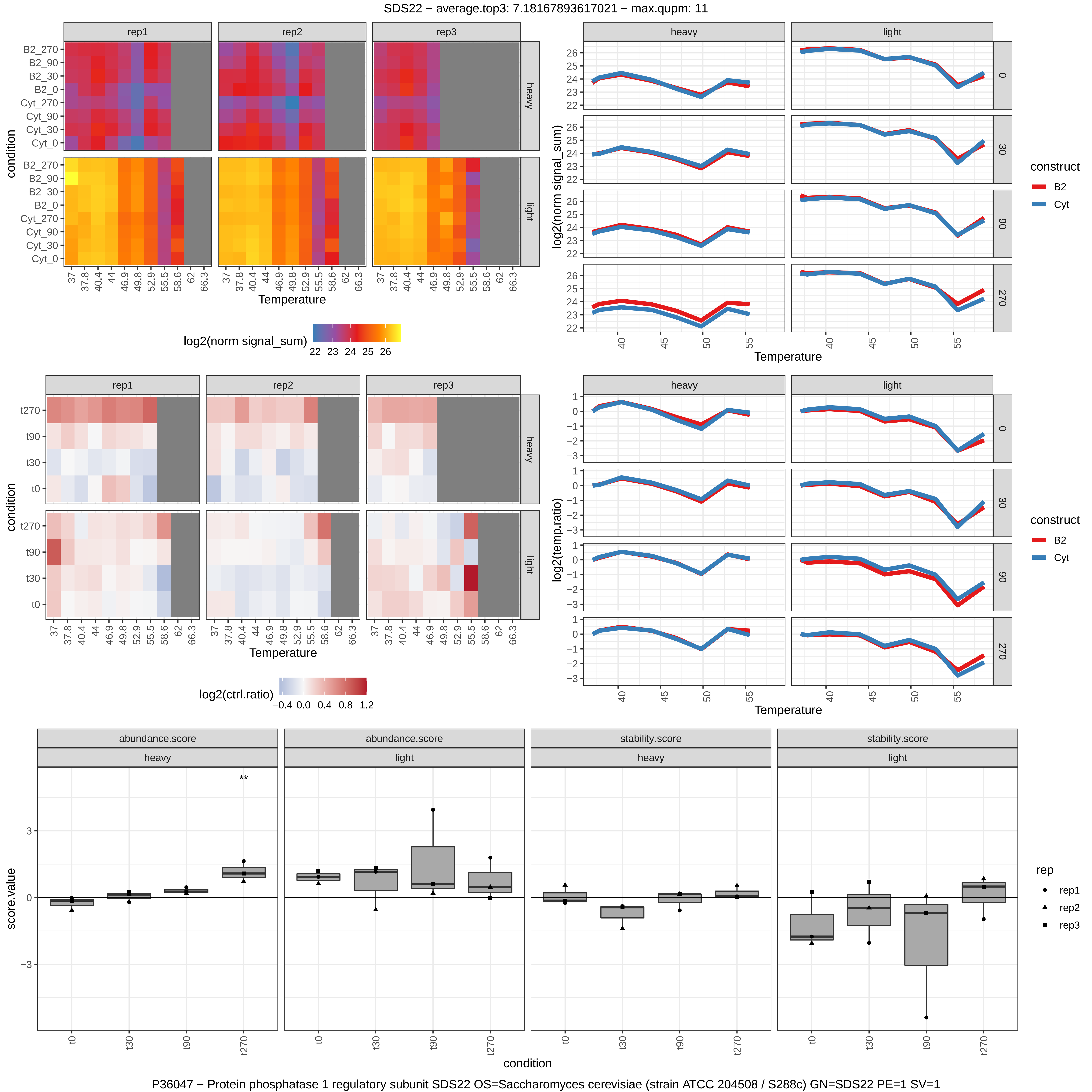

SDS22 − average.top3: 7.18167893617021 − max.qupm: 11
rep1
rep2
rep3
heavy
light
condition
Temperature
log2(norm signal_sum)
log2(norm signal_sum)
construct
B2
Cyt
log2(ctrl.ratio)
log2(temp.ratio)
abundance.score
stability.score
score.value
rep
rep1
rep2
rep3
P36047 − Protein phosphatase 1 regulatory subunit SDS22 OS=Saccharomyces cerevisiae (strain ATCC 204508 / S288c) GN=SDS22 PE=1 SV=1

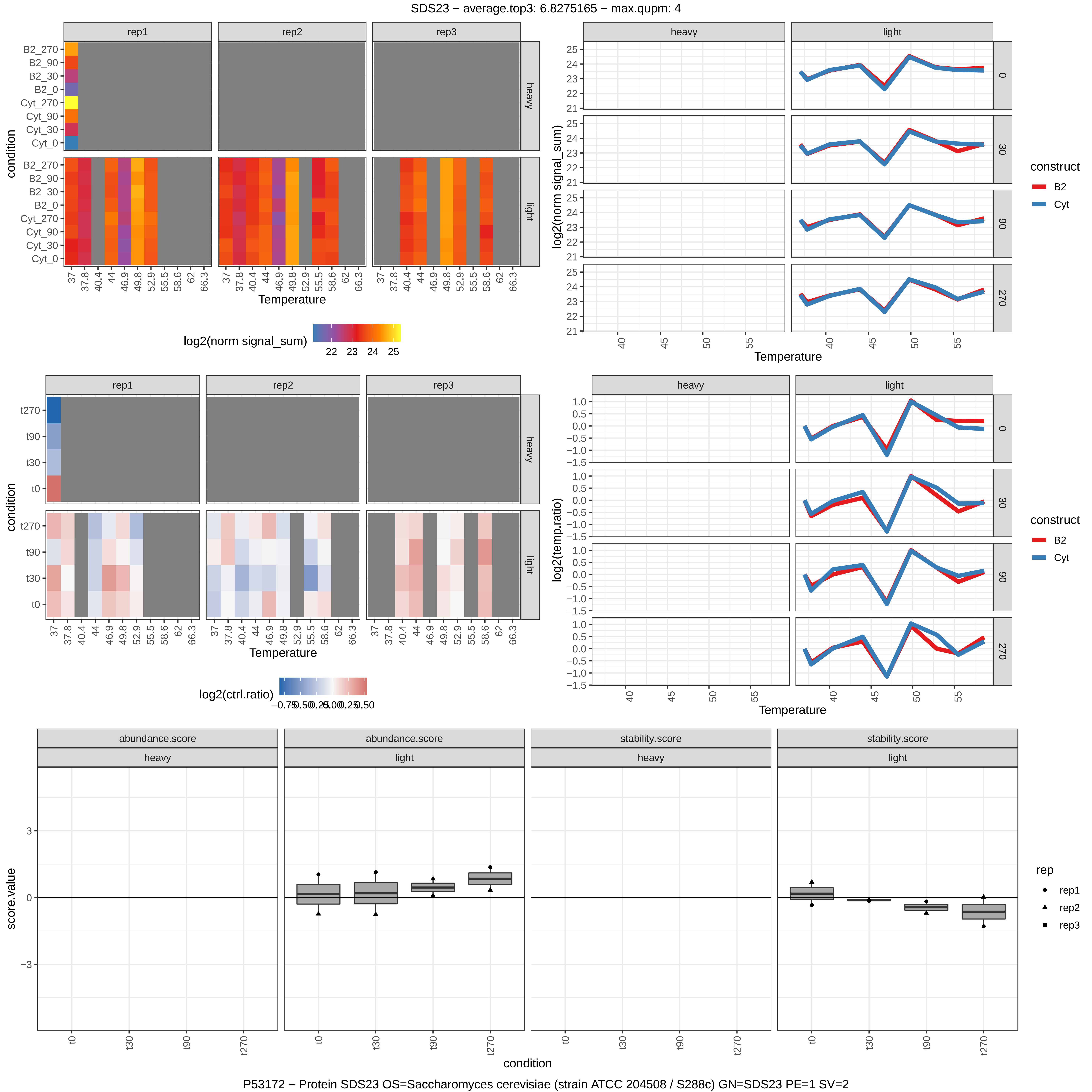

SDS23 − average.top3: 6.8275165 − max.qupm: 4
rep1
rep2
rep3
heavy
light
condition
Temperature
log2(norm signal_sum)
log2(ctrl.ratio)
log2(temp.ratio)
construct
B2
Cyt
abundance.score
stability.score
score.value
rep
rep1
rep2
rep3
P53172 − Protein SDS23 OS=Saccharomyces cerevisiae (strain ATCC 204508 / S288c) GN=SDS23 PE=1 SV=2

SDS24 − average.top3: 6.9799258974359 − max.qupm: 6

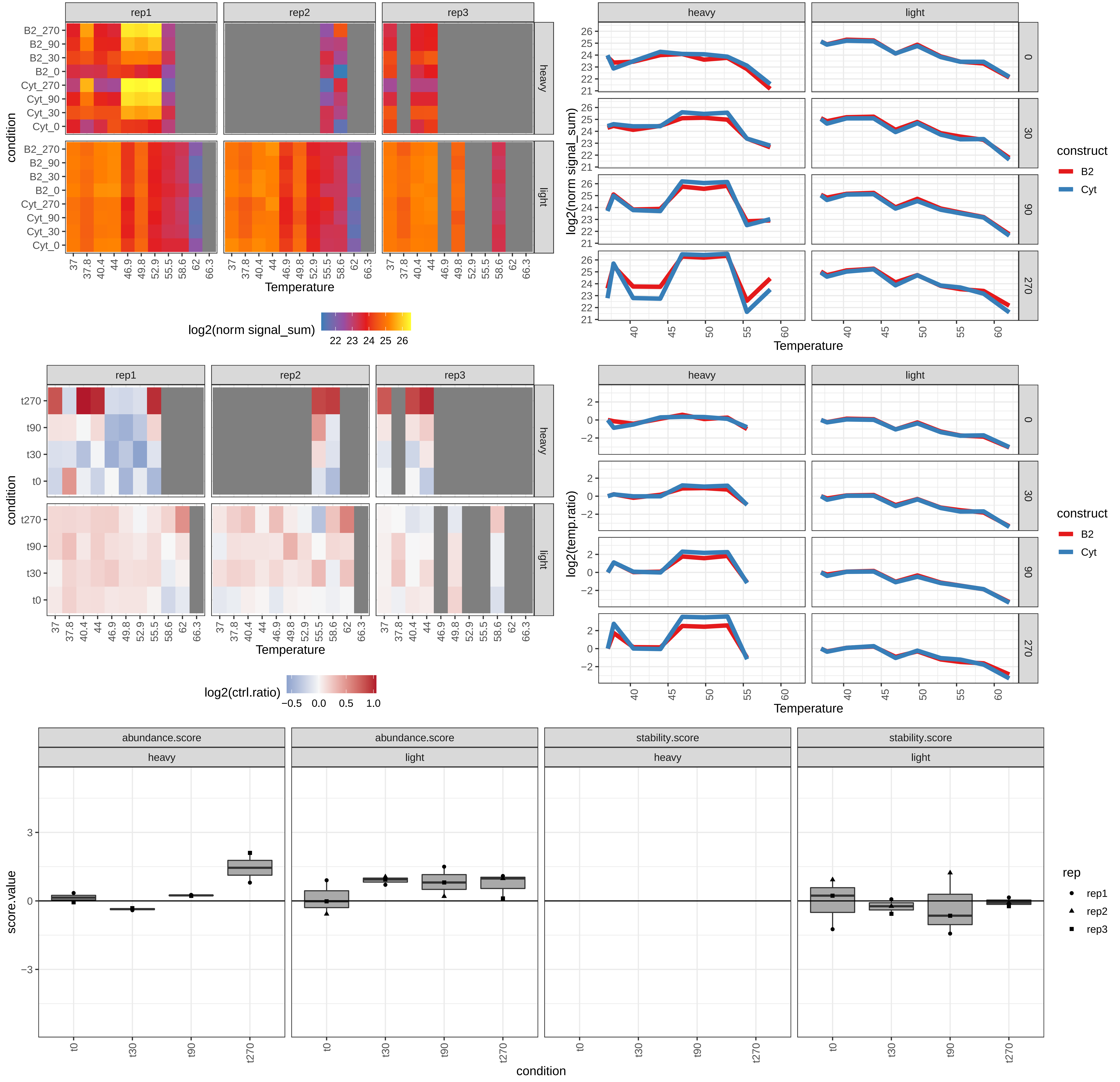

P38314 − Protein SDS24 OS=Saccharomyces cerevisiae (strain ATCC 204508 / S288c) GN=SDS24 PE=1 SV=1

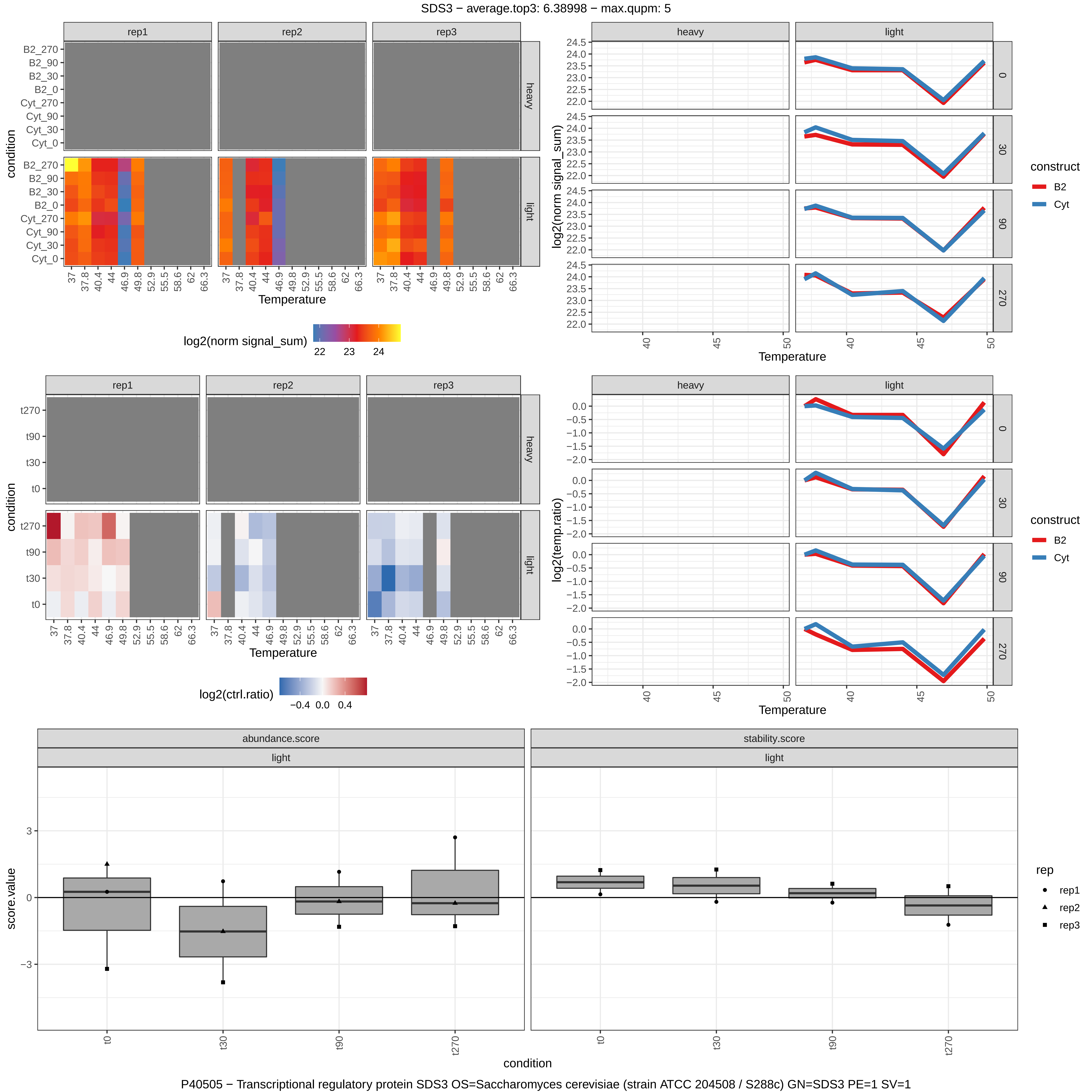
SDS3 − average.top3: 6.38998 − max.qupm: 5
rep1
rep2
rep3
heavy
light
condition
B2_270
B2_90
B2_30
B2_0
Cyt_270
Cyt_90
Cyt_30
Cyt_0
Temperature
log2(norm signal_sum)
log2(norm signal_sum)
construct
B2
Cyt
t270
t90
t30
t0
log2(ctrl.ratio)
log2(temp.ratio)
abundance.score
stability.score
score.value
rep
condition
P40505 − Transcriptional regulatory protein SDS3 OS=Saccharomyces cerevisiae (strain ATCC 204508 / S288c) GN=SDS3 PE=1 SV=1

SEA4 − average.top3: 6.75193722222222 − max.qupm: 9

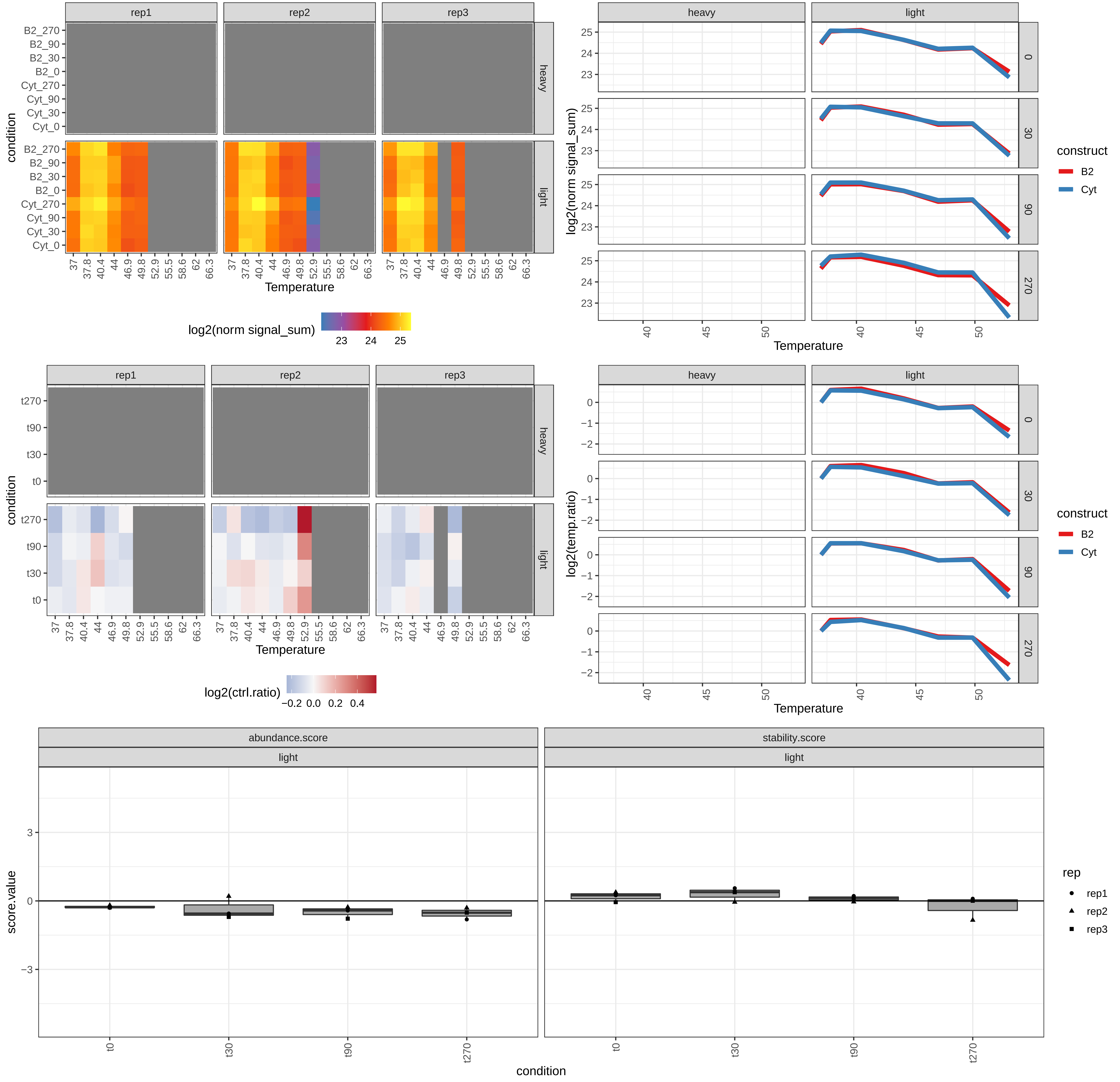

P38164 − SEH−associated protein 4 OS=Saccharomyces cerevisiae (strain ATCC 204508 / S288c) GN=SEA4 PE=1 SV=2

SEC1 − average.top3: 6.90871238095238 − max.qupm: 8

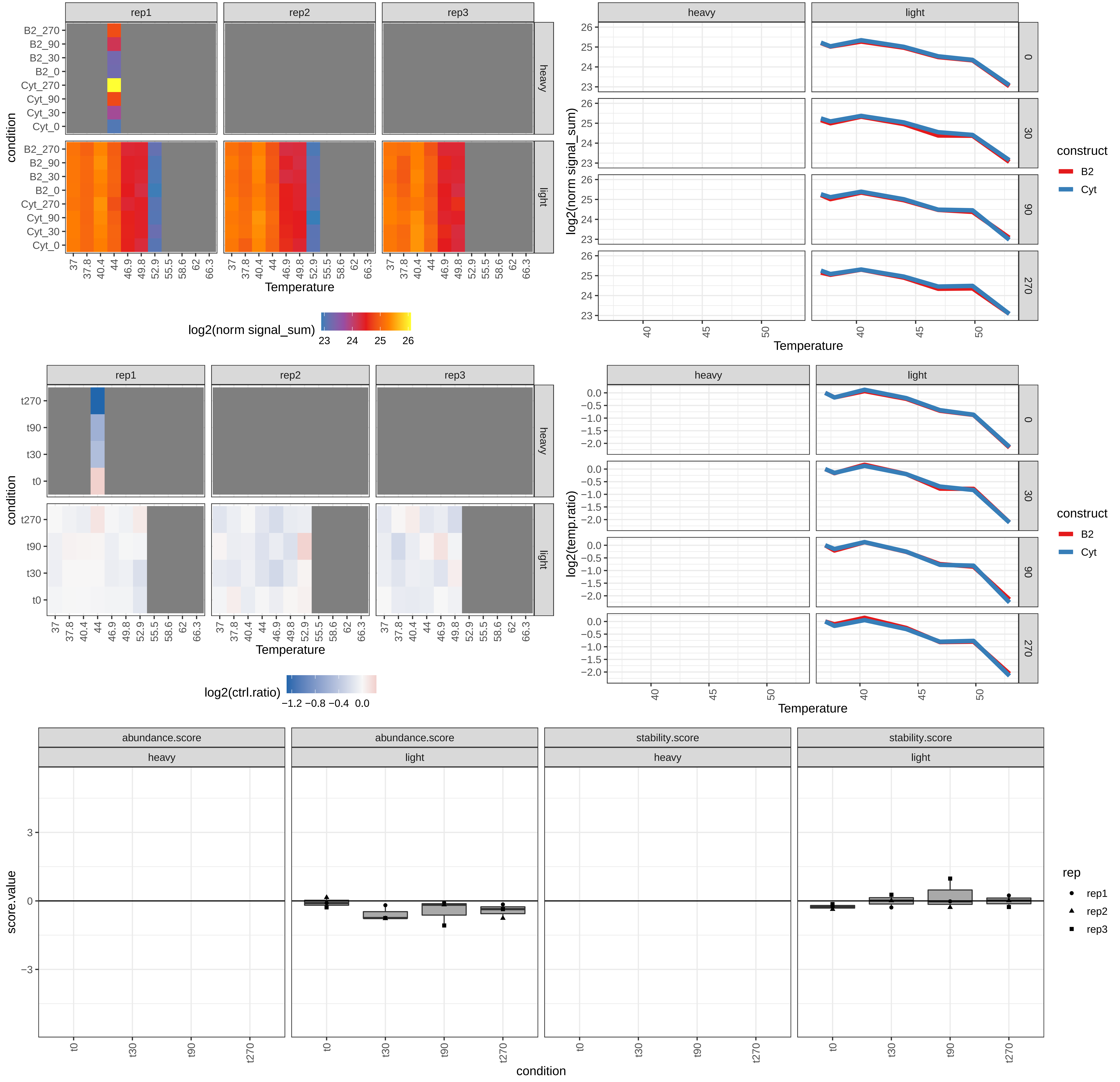

P30619 − Protein transport protein SEC1 OS=Saccharomyces cerevisiae (strain ATCC 204508 / S288c) GN=SEC1 PE=1 SV=1

SEC10 − average.top3: 6.79122588235294 − max.qupm: 6

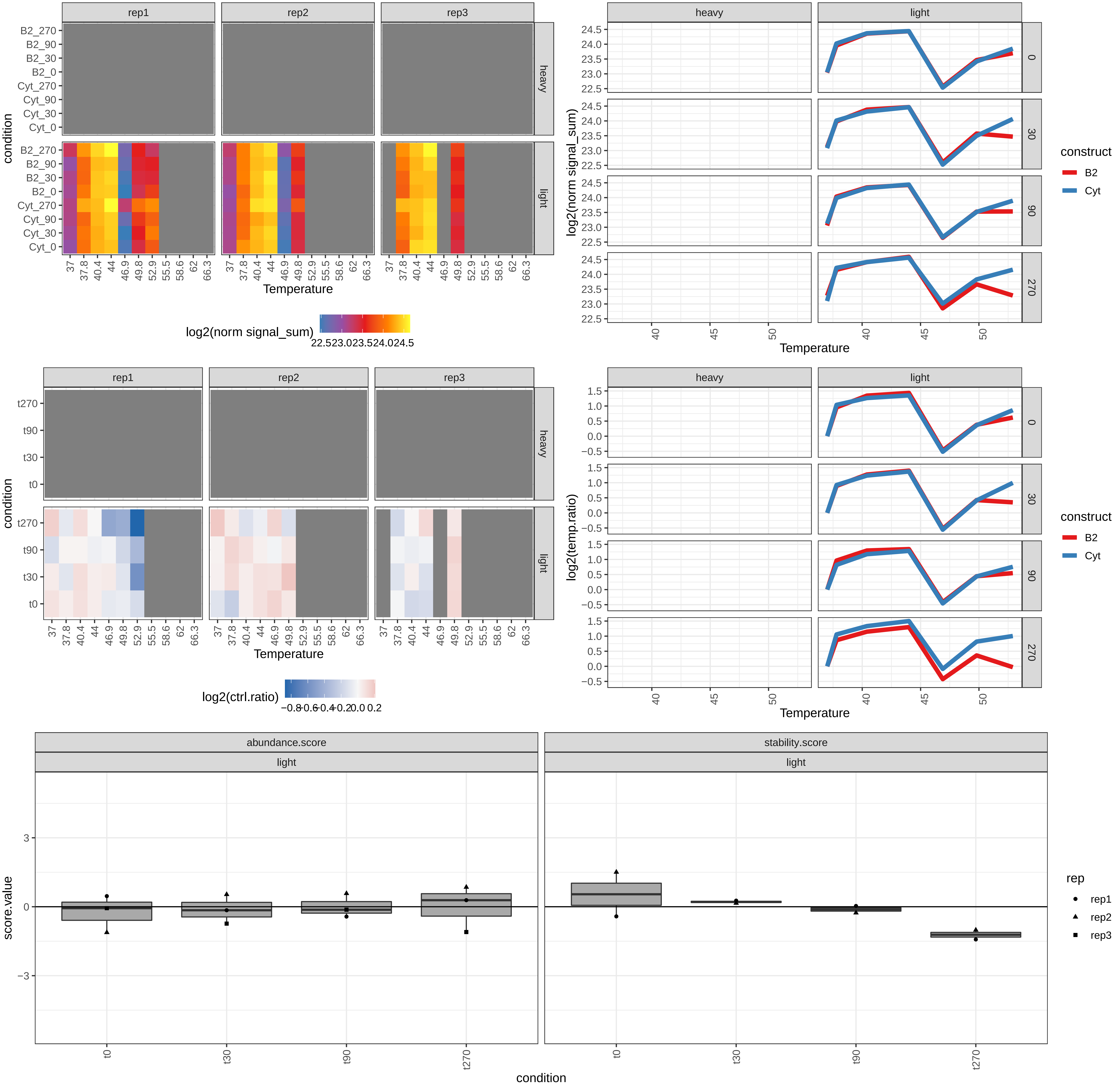

Q06245 − Exocyst complex component SEC10 OS=Saccharomyces cerevisiae (strain ATCC 204508 / S288c) GN=SEC10 PE=1 SV=1

SEC11 − average.top3: 6.88296142857143 − max.qupm: 3

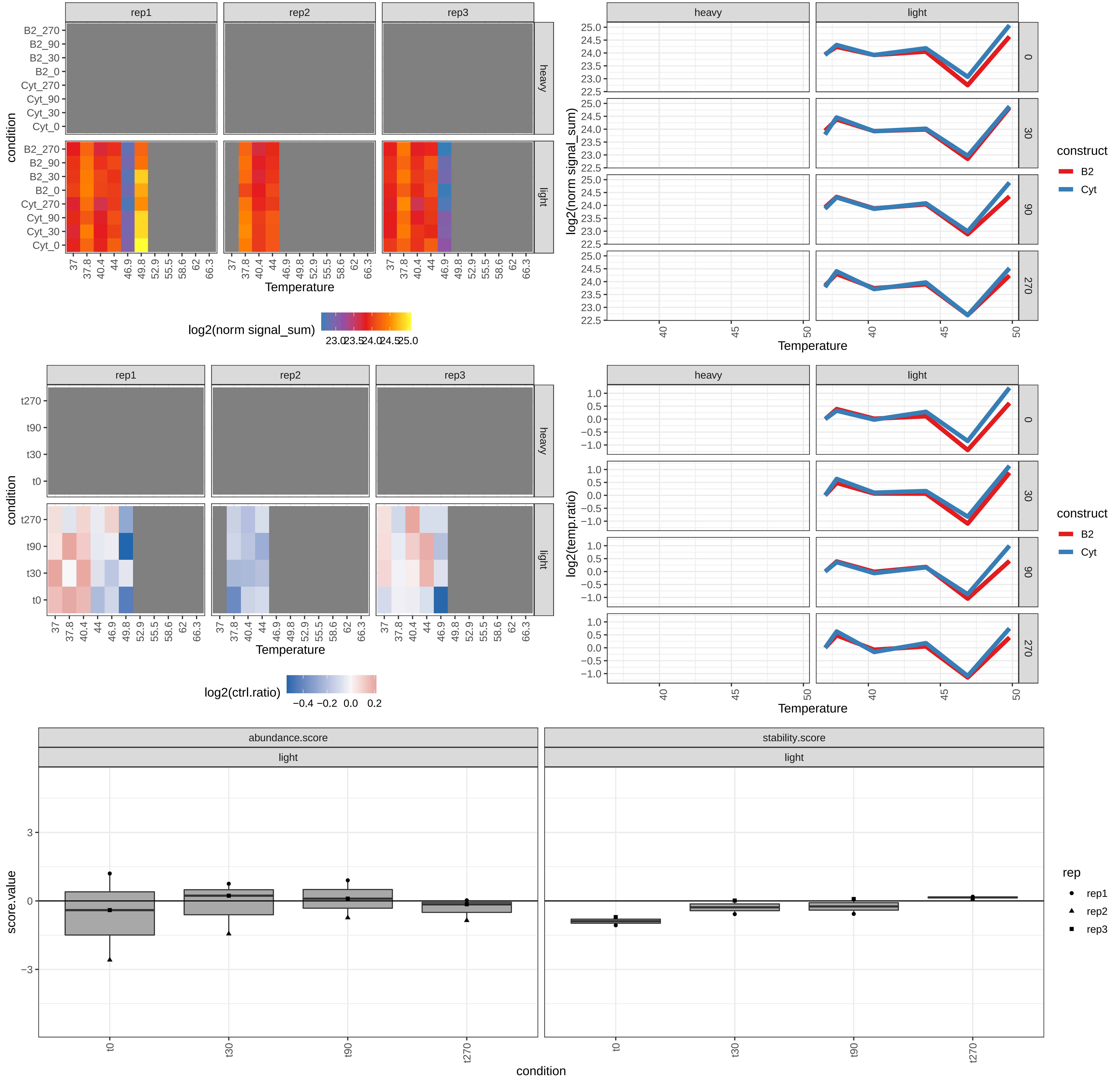

P15367 − Signal peptidase complex catalytic subunit SEC11 OS=Saccharomyces cerevisiae (strain ATCC 204508 / S288c) GN=SEC11 PE=1 SV=2

SEC12 − average.top3: 6.92627516129032 − max.qupm: 5

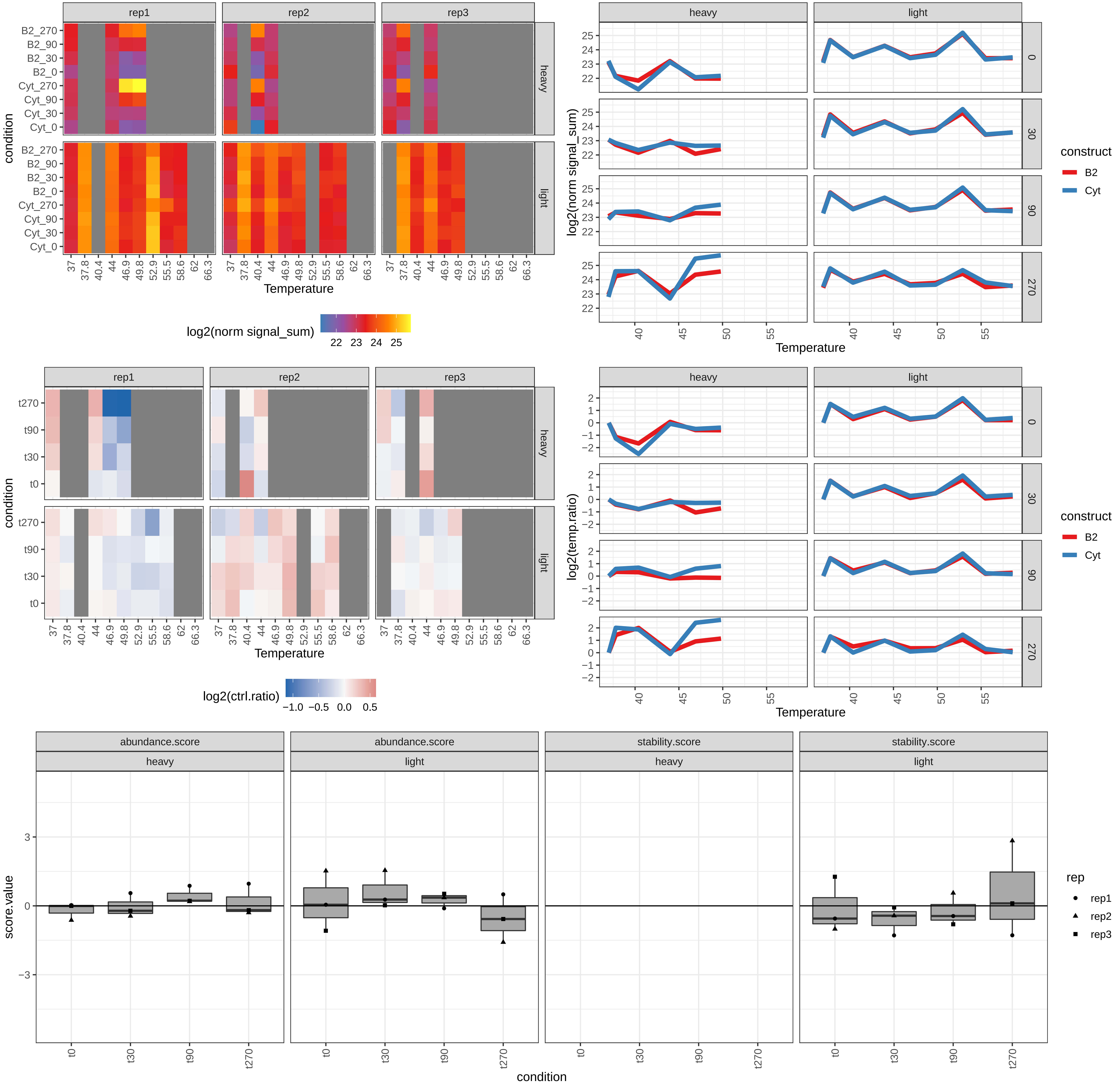

P11655 − Guanine nucleotide−exchange factor SEC12 OS=Saccharomyces cerevisiae (strain ATCC 204508 / S288c) GN=SEC12 PE=1 SV=1

SEC13 − average.top3: 7.26846425 − max.qupm: 10

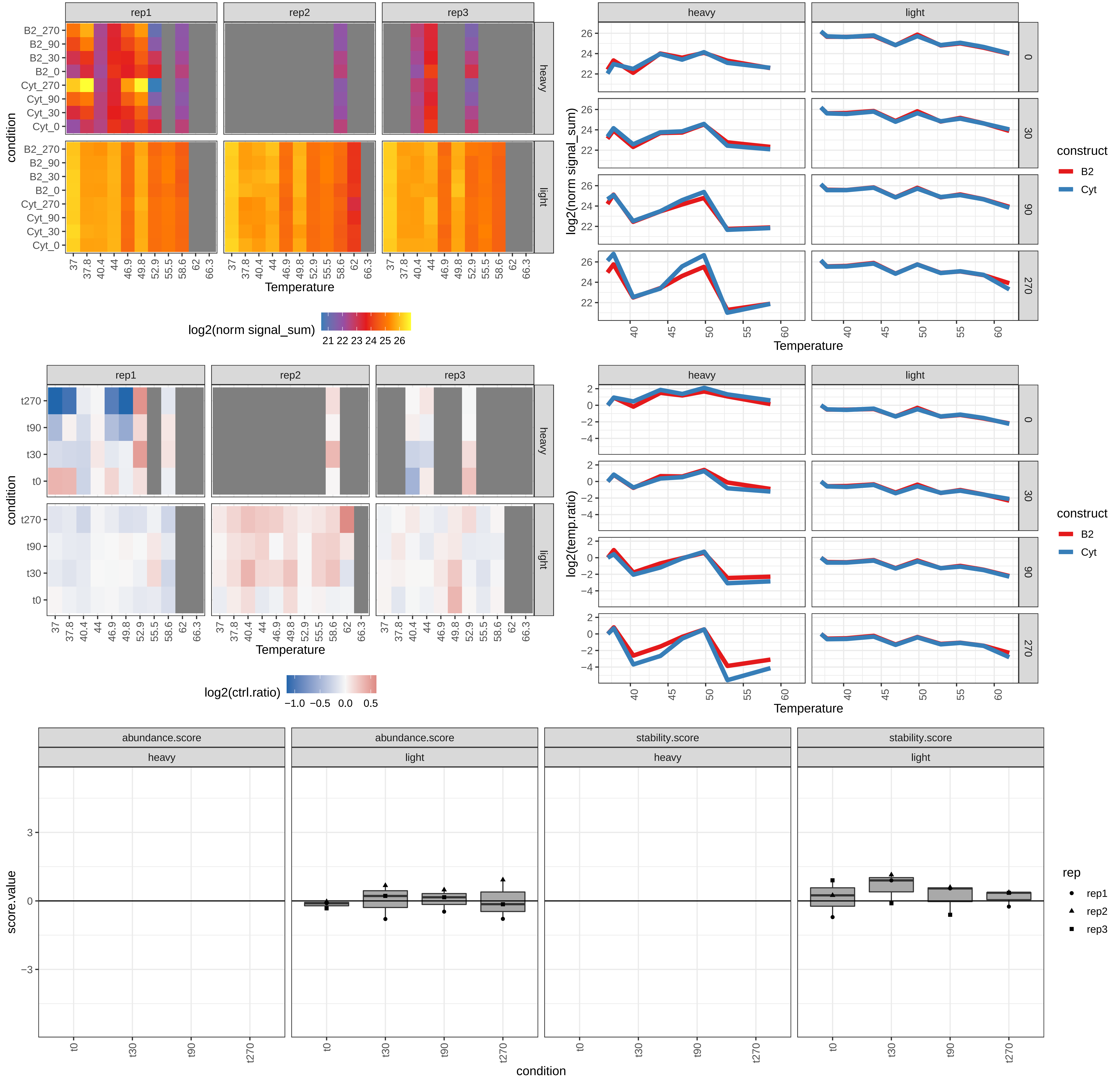

Q04491 − Protein transport protein SEC13 OS=Saccharomyces cerevisiae (strain ATCC 204508 / S288c) GN=SEC13 PE=1 SV=1

SEC14 − average.top3: 7.37820234042553 − max.qupm: 16

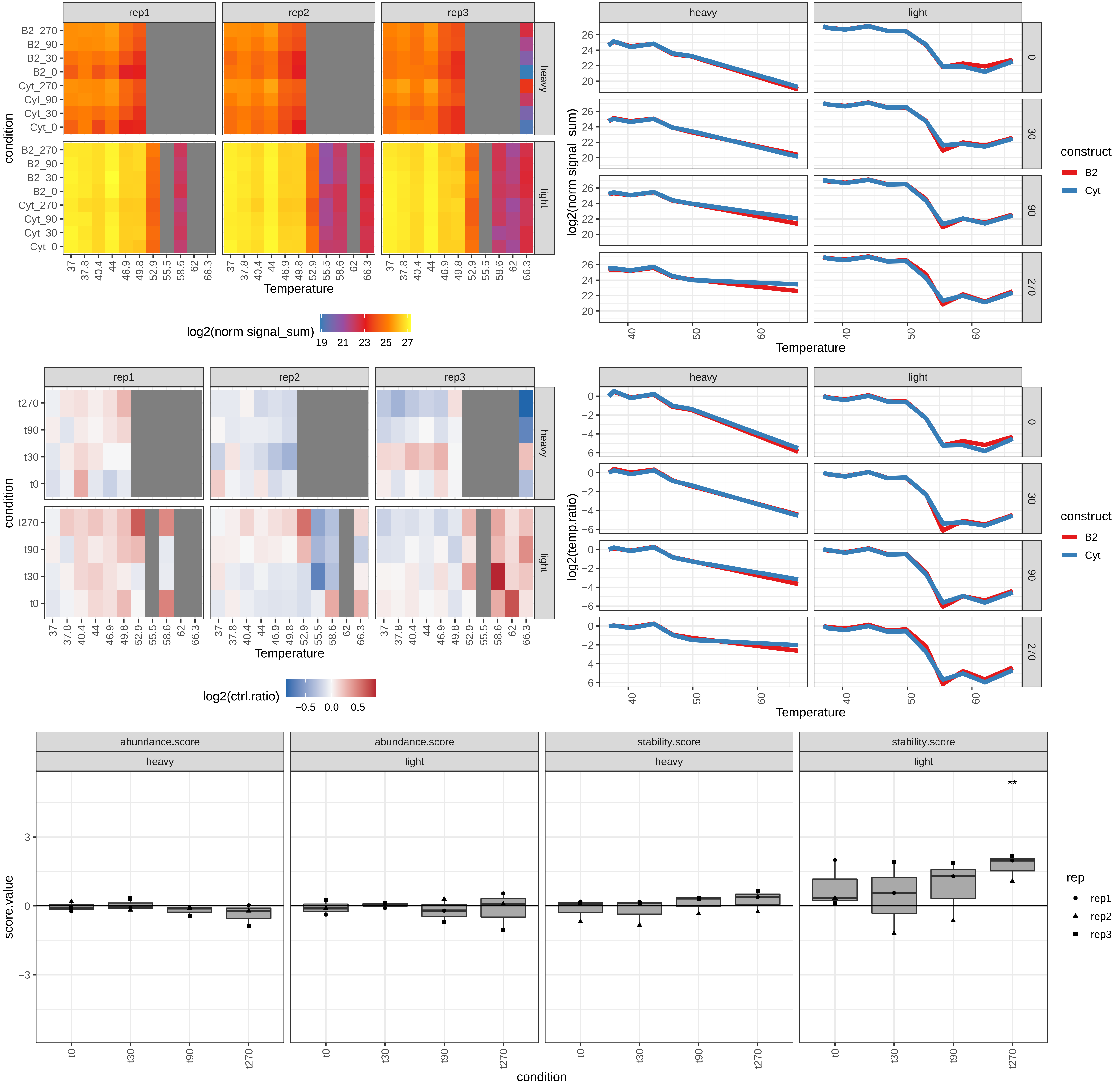

P24280 − SEC14 cytosolic factor OS=Saccharomyces cerevisiae (strain ATCC 204508 / S288c) GN=SEC14 PE=1 SV=3

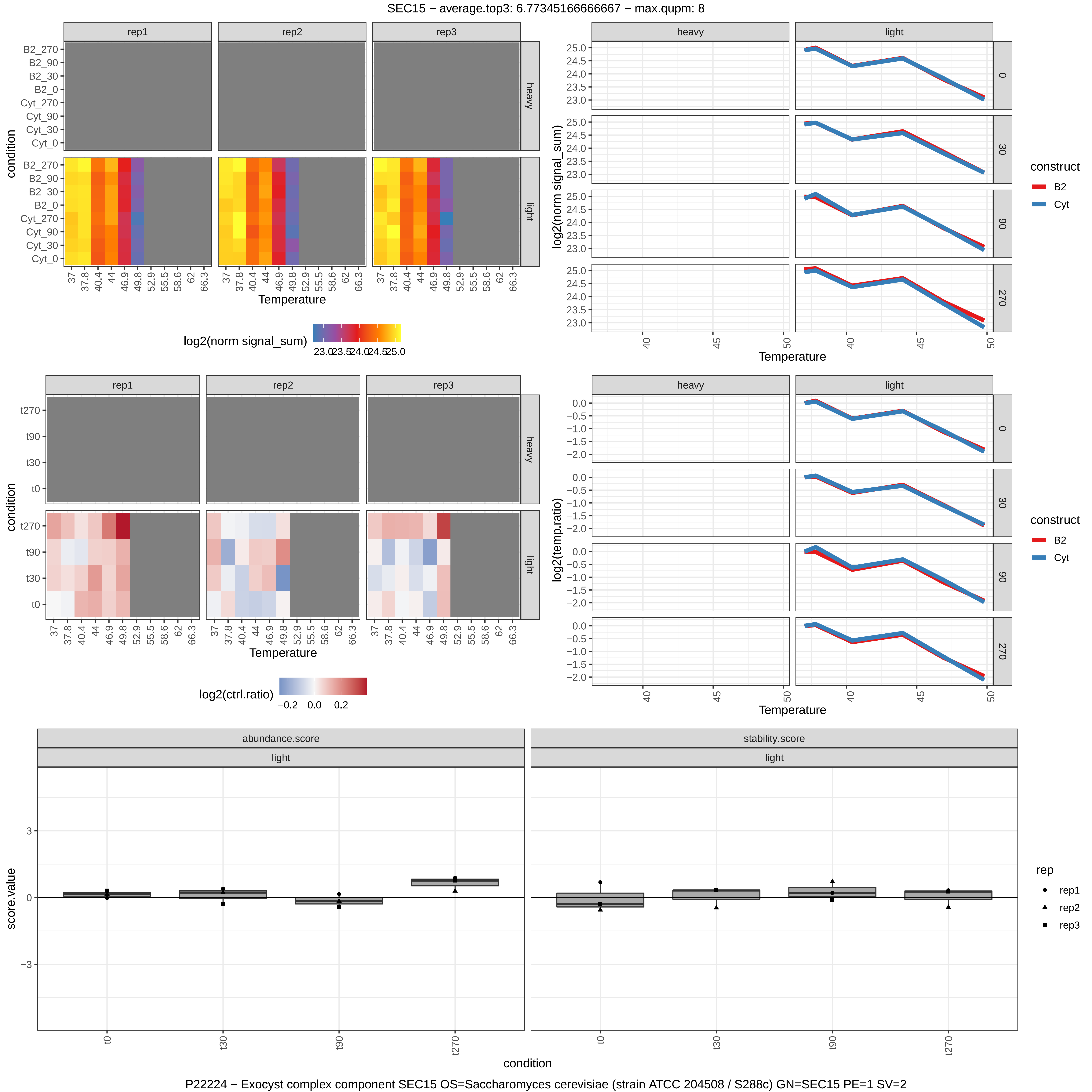

SEC15 − average.top3: 6.77345166666667 − max.qupm: 8
rep1
rep2
rep3
heavy
light
condition
Temperature
log2(norm signal_sum)
log2(temp.ratio)
log2(ctrl.ratio)
construct
B2
Cyt
abundance.score
stability.score
score.value
rep
rep1
rep2
rep3
P22224 − Exocyst complex component SEC15 OS=Saccharomyces cerevisiae (strain ATCC 204508 / S288c) GN=SEC15 PE=1 SV=2

SEC16 − average.top3: 6.87214206896552 − max.qupm: 9

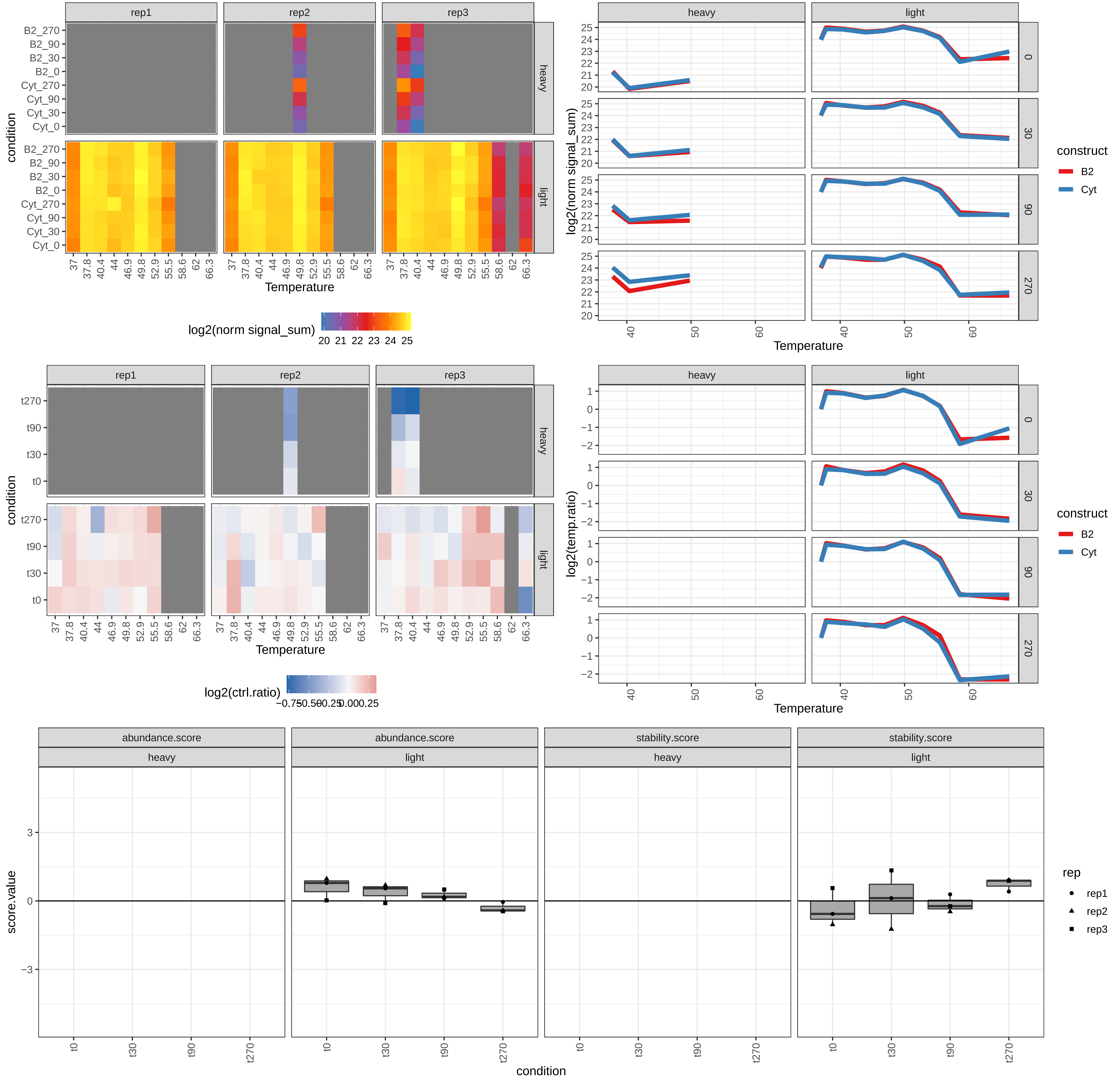

P48415 − COPII coat assembly protein SEC16 OS=Saccharomyces cerevisiae (strain ATCC 204508 / S288c) GN=SEC16 PE=1 SV=2

SEC17 − average.top3: 6.67989 − max.qupm: 8

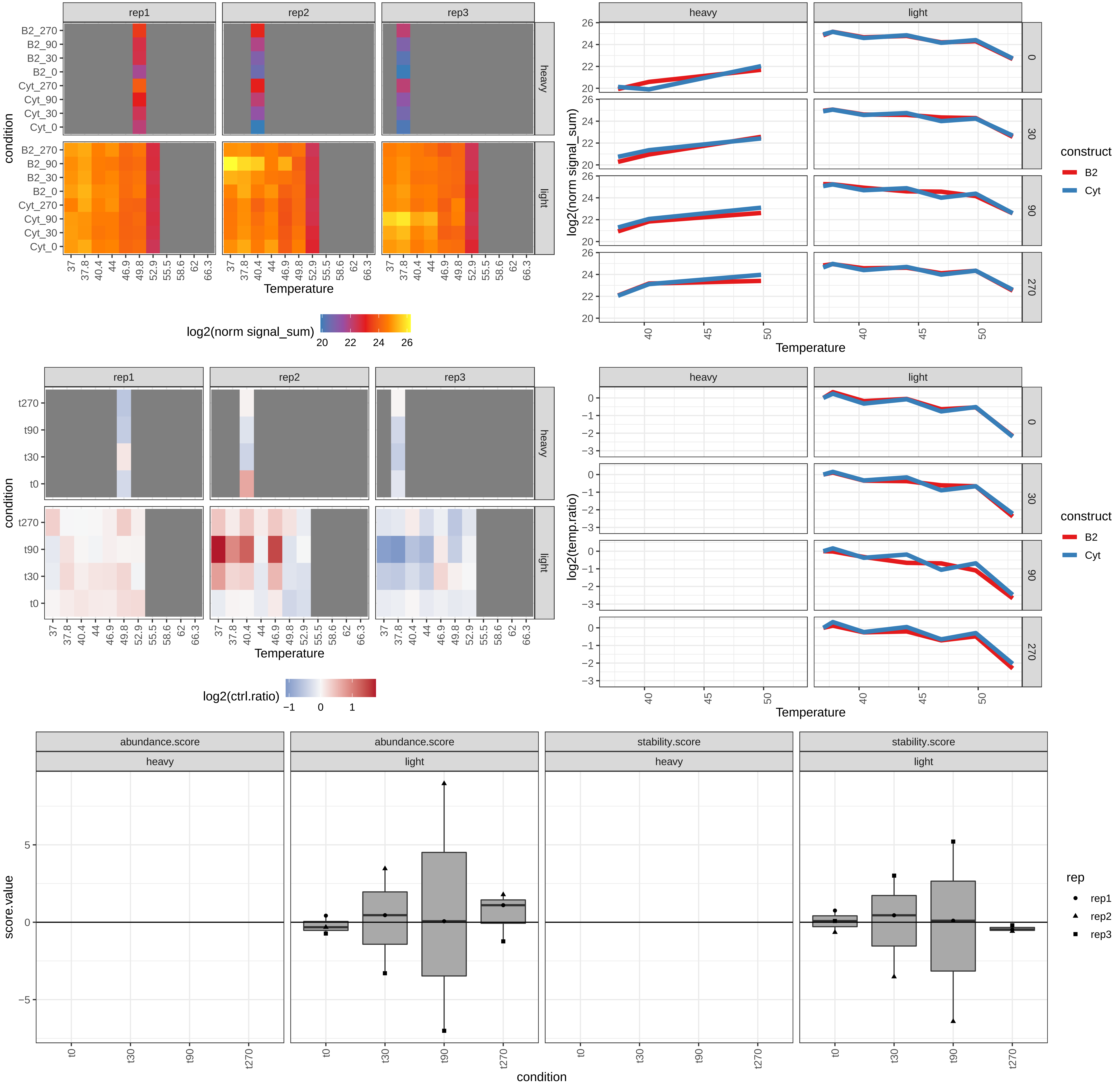

P32602 − Alpha−soluble NSF attachment protein OS=Saccharomyces cerevisiae (strain ATCC 204508 / S288c) GN=SEC17 PE=1 SV=4

SEC18 − average.top3: 7.19231916666667 − max.qupm: 17

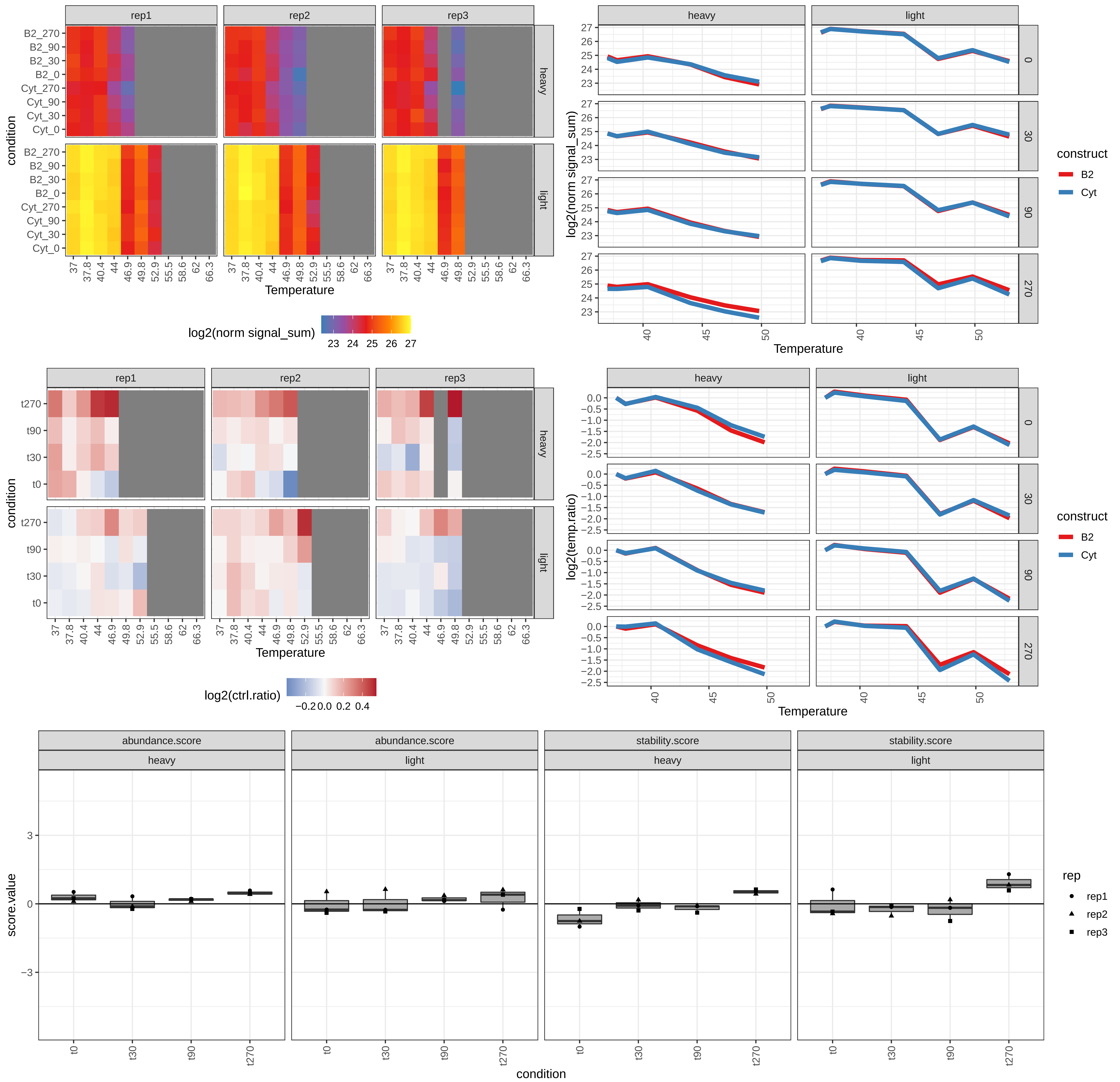

P18759 − Vesicular−fusion protein SEC18 OS=Saccharomyces cerevisiae (strain ATCC 204508 / S288c) GN=SEC18 PE=1 SV=2

SEC2 − average.top3: 6.47497875 − max.qupm: 5

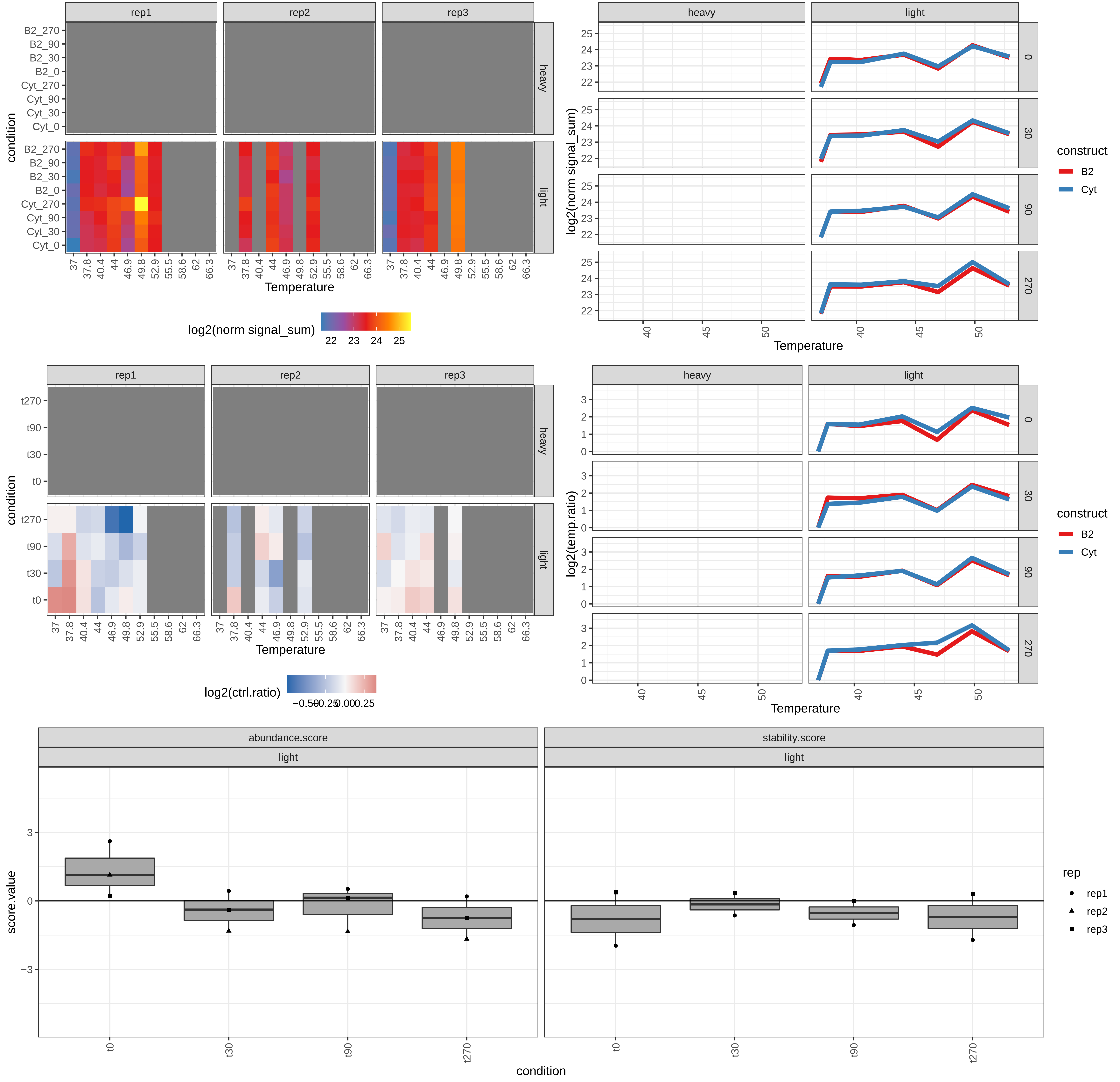

P17065 − Rab guanine nucleotide exchange factor SEC2 OS=Saccharomyces cerevisiae (strain ATCC 204508 / S288c) GN=SEC2 PE=1 SV=1

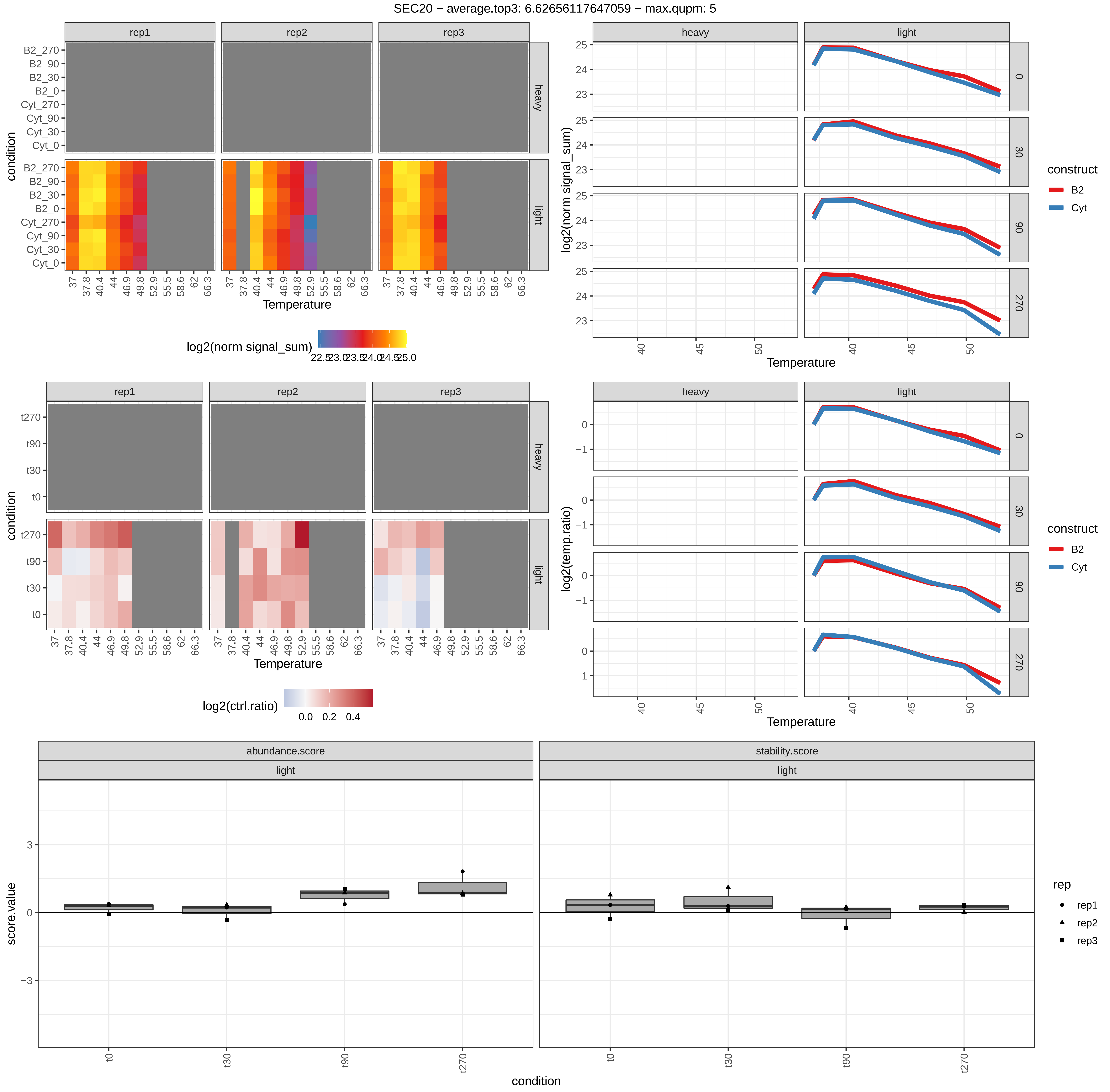

P28791 − Protein transport protein SEC20 OS=Saccharomyces cerevisiae (strain ATCC 204508 / S288c) GN=SEC20 PE=1 SV=1

SEC21 − average.top3: 7.27543730769231 − max.qupm: 24

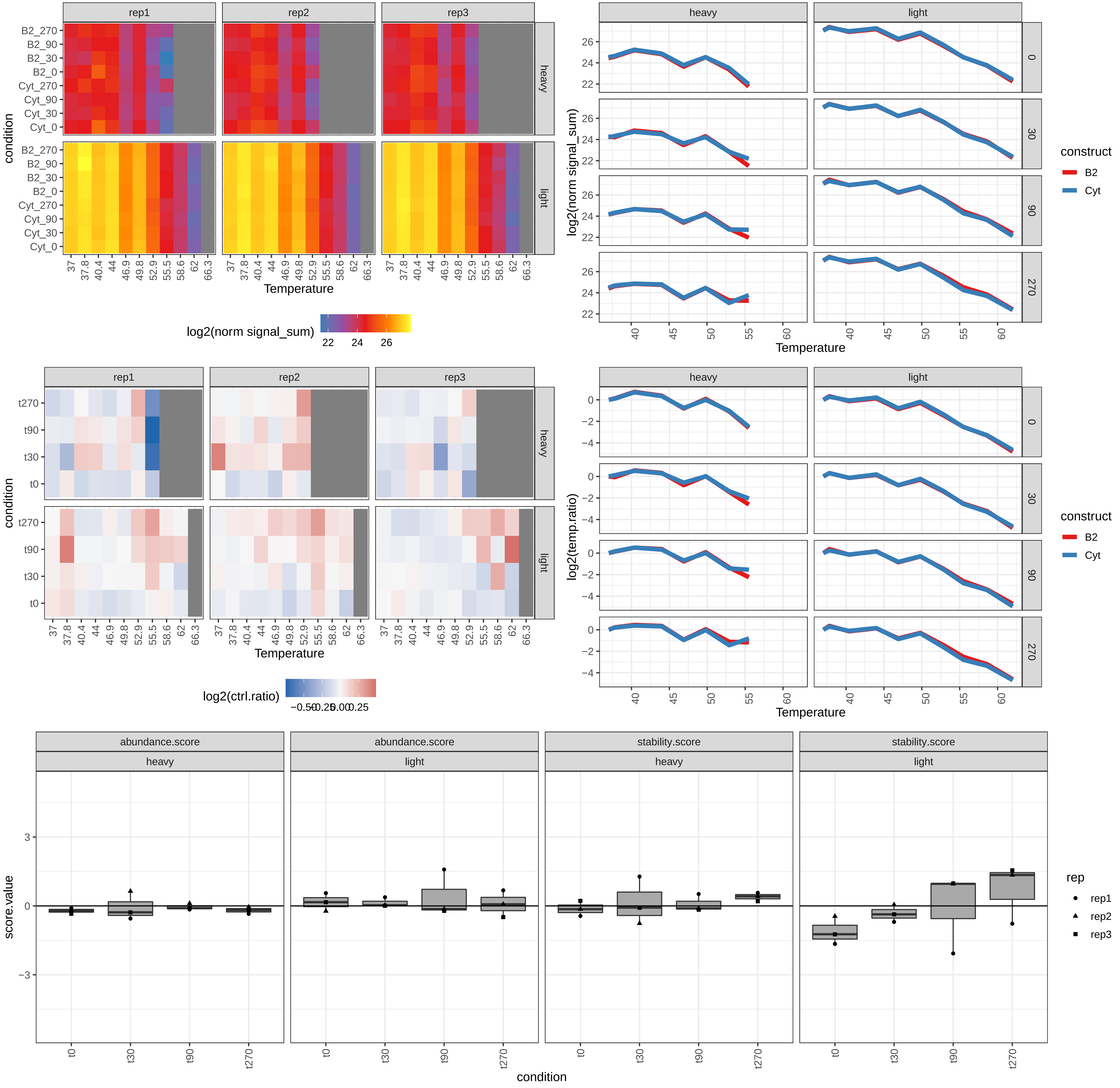

P32074 − Coatomer subunit gamma OS=Saccharomyces cerevisiae (strain ATCC 204508 / S288c) GN=SEC21 PE=1 SV=2

SEC22 − average.top3: 6.77865363636364 − max.qupm: 4

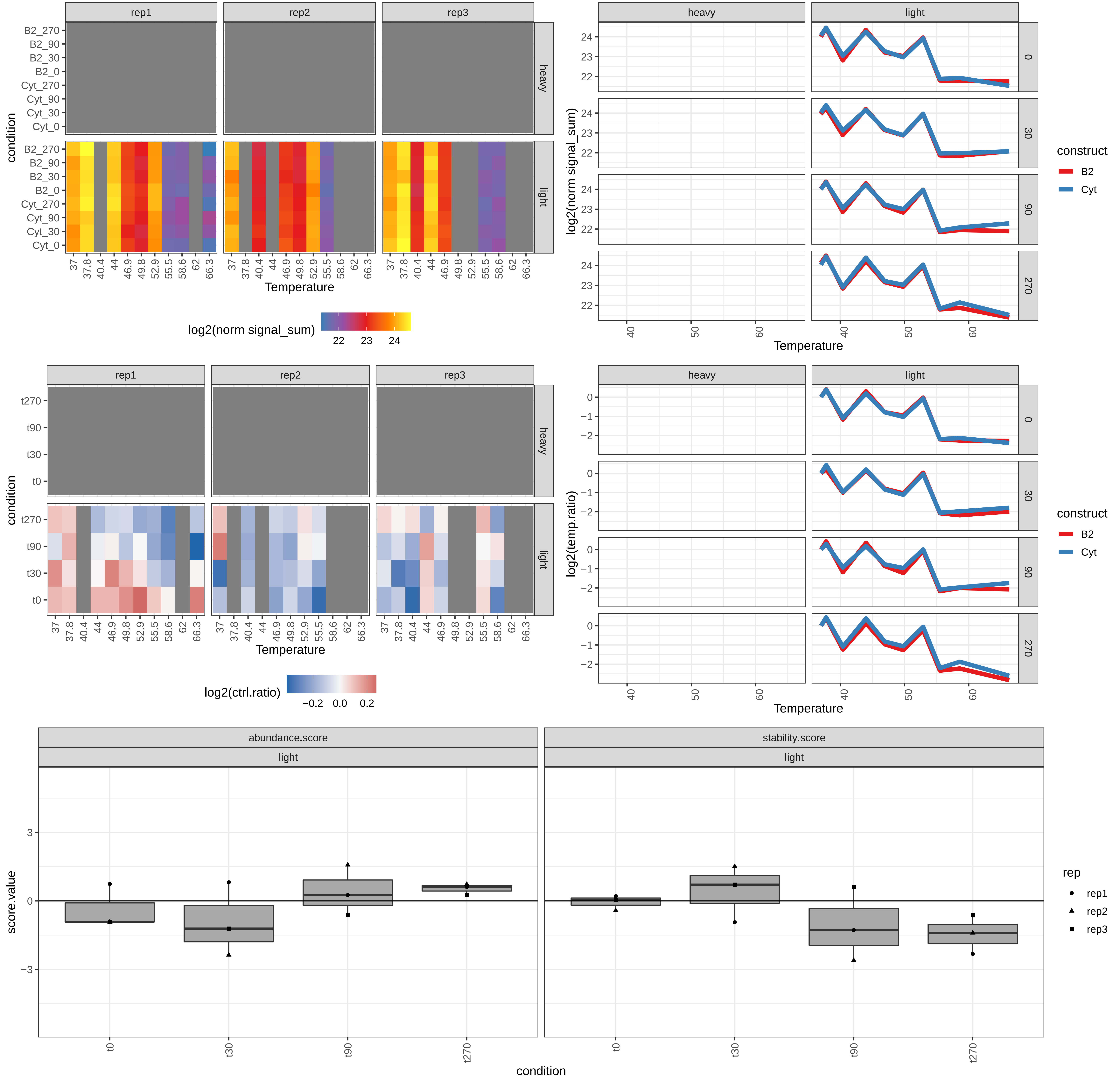

P22214 − Protein transport protein SEC22 OS=Saccharomyces cerevisiae (strain ATCC 204508 / S288c) GN=SEC22 PE=1 SV=1

SEC23 − average.top3: 7.4014382051282 − max.qupm: 16

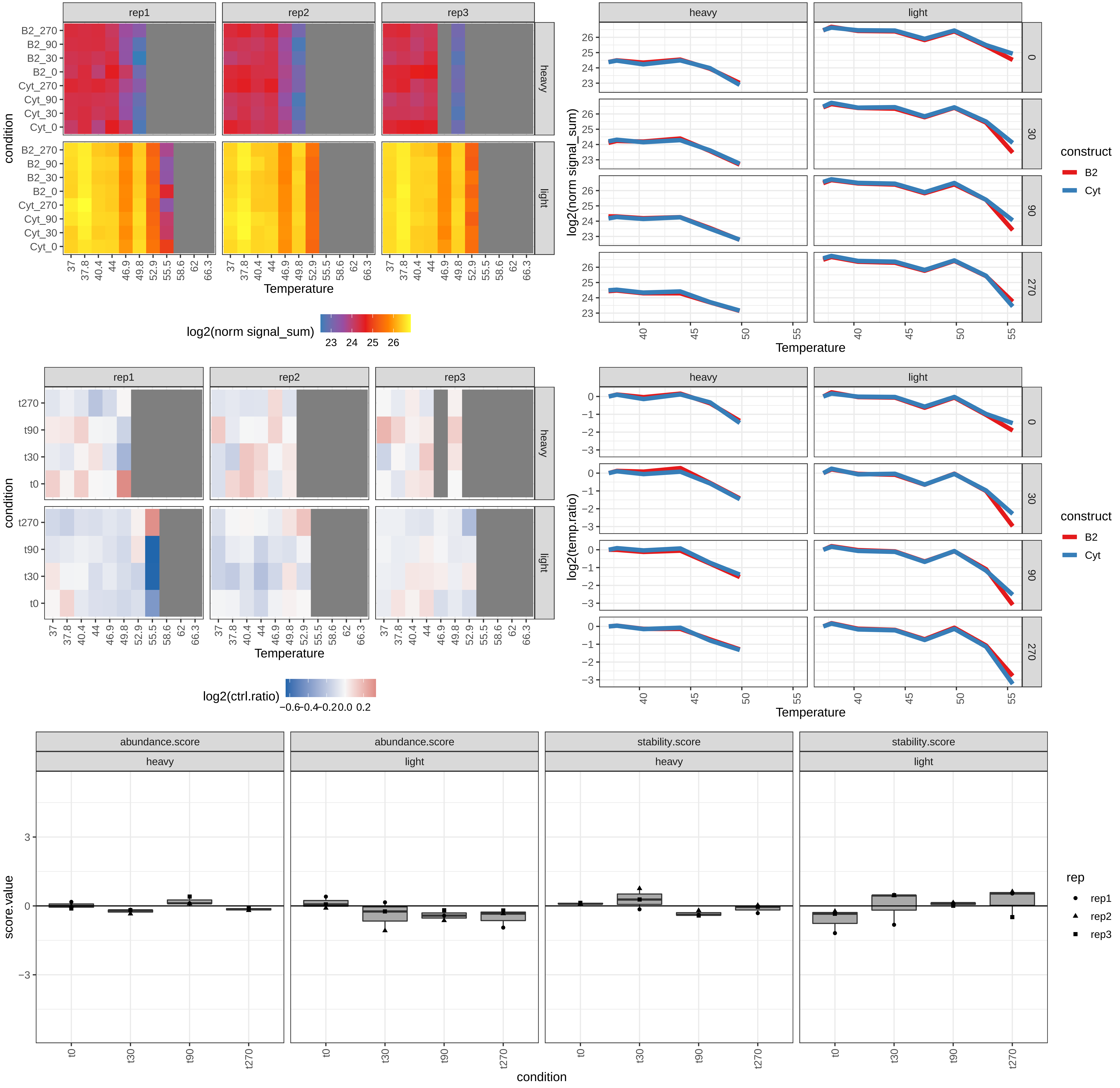

P15303 − Protein transport protein SEC23 OS=Saccharomyces cerevisiae (strain ATCC 204508 / S288c) GN=SEC23 PE=1 SV=1

SEC24 − average.top3: 7.34442733333333 − max.qupm: 18

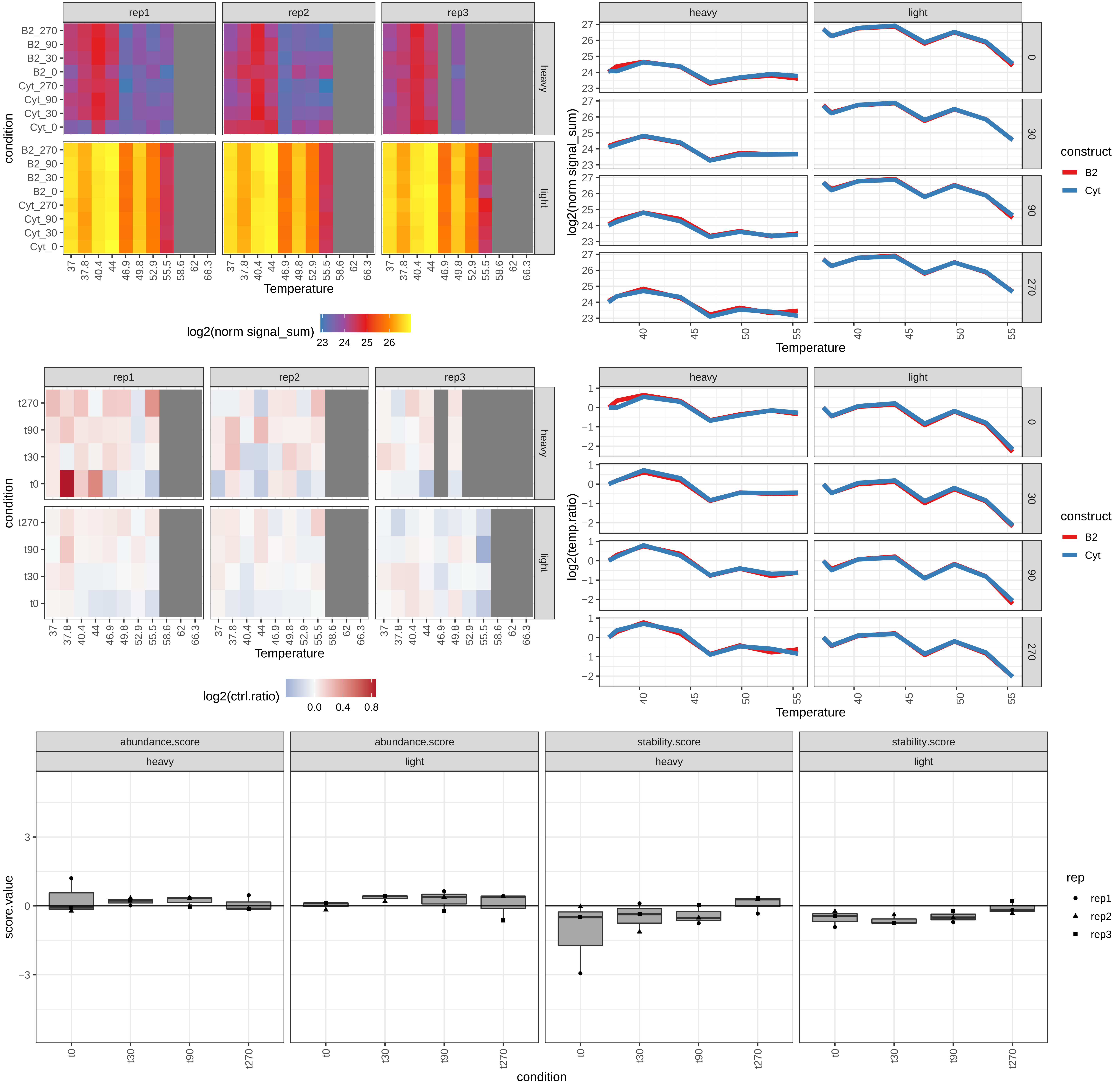

P40482 − Protein transport protein SEC24 OS=Saccharomyces cerevisiae (strain ATCC 204508 / S288c) GN=SEC24 PE=1 SV=1

SEC26 − average.top3: 7.36726954545455 − max.qupm: 30

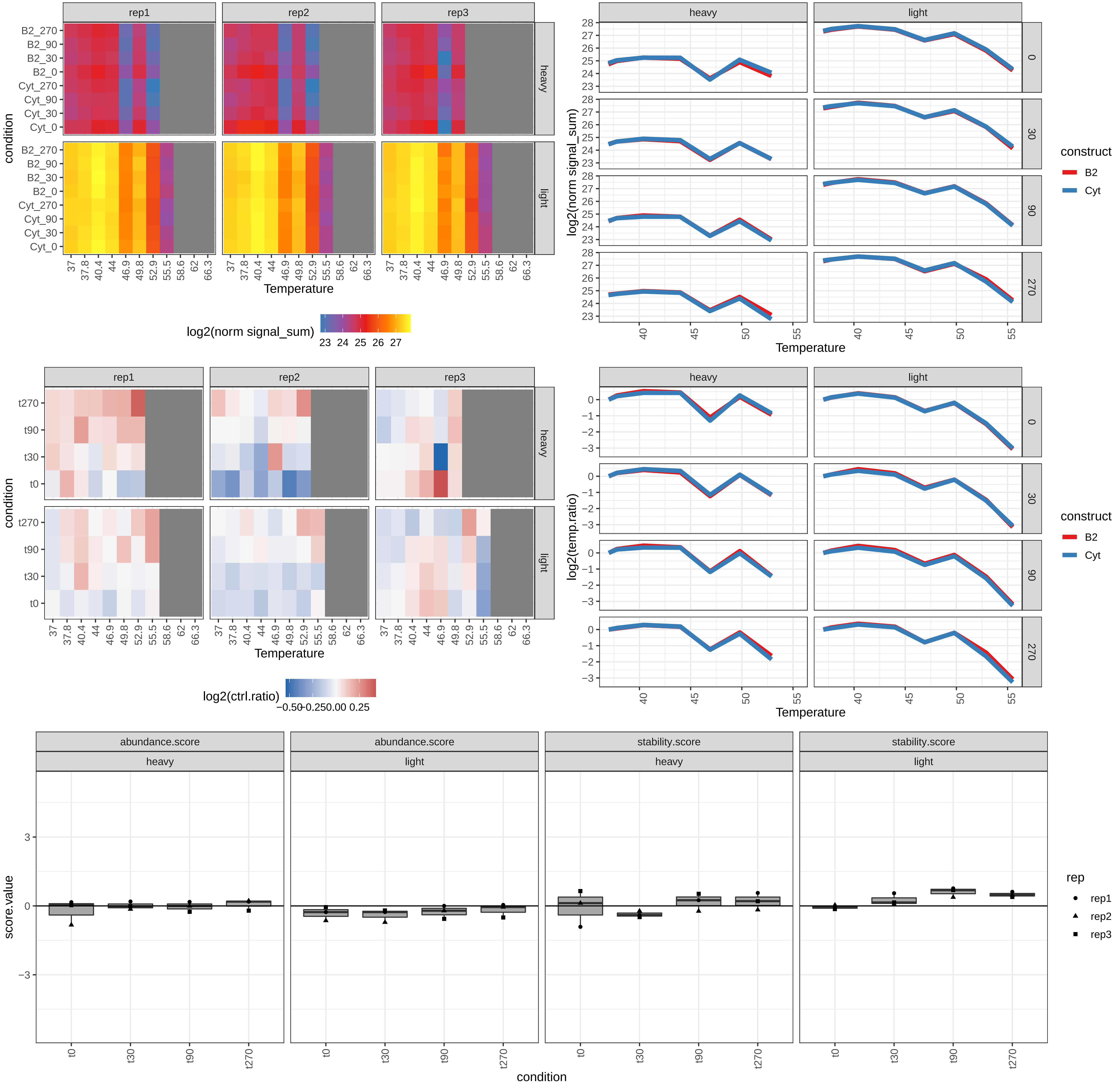

P41810 − Coatomer subunit beta OS=Saccharomyces cerevisiae (strain ATCC 204508 / S288c) GN=SEC26 PE=1 SV=2

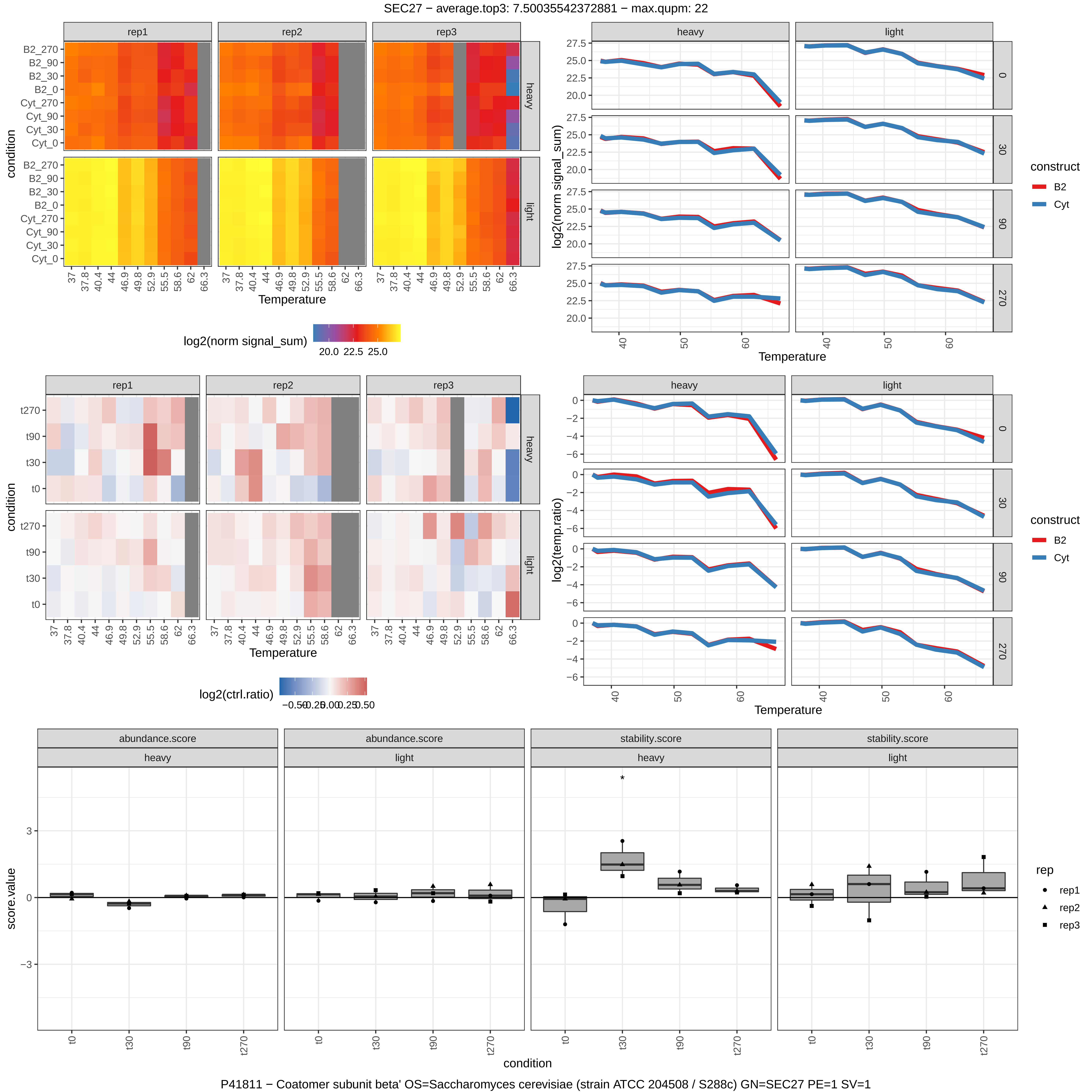
SEC27 − average.top3: 7.50035542372881 − max.qupm: 22
rep1
rep2
rep3
heavy
light
condition
Temperature
log2(norm signal_sum)
log2(ctrl.ratio)
log2(temp.ratio)
construct
B2
Cyt
abundance.score
stability.score
score.value
rep
P41811 − Coatomer subunit beta' OS=Saccharomyces cerevisiae (strain ATCC 204508 / S288c) GN=SEC27 PE=1 SV=1

SEC28 − average.top3: 6.33043615384615 − max.qupm: 6

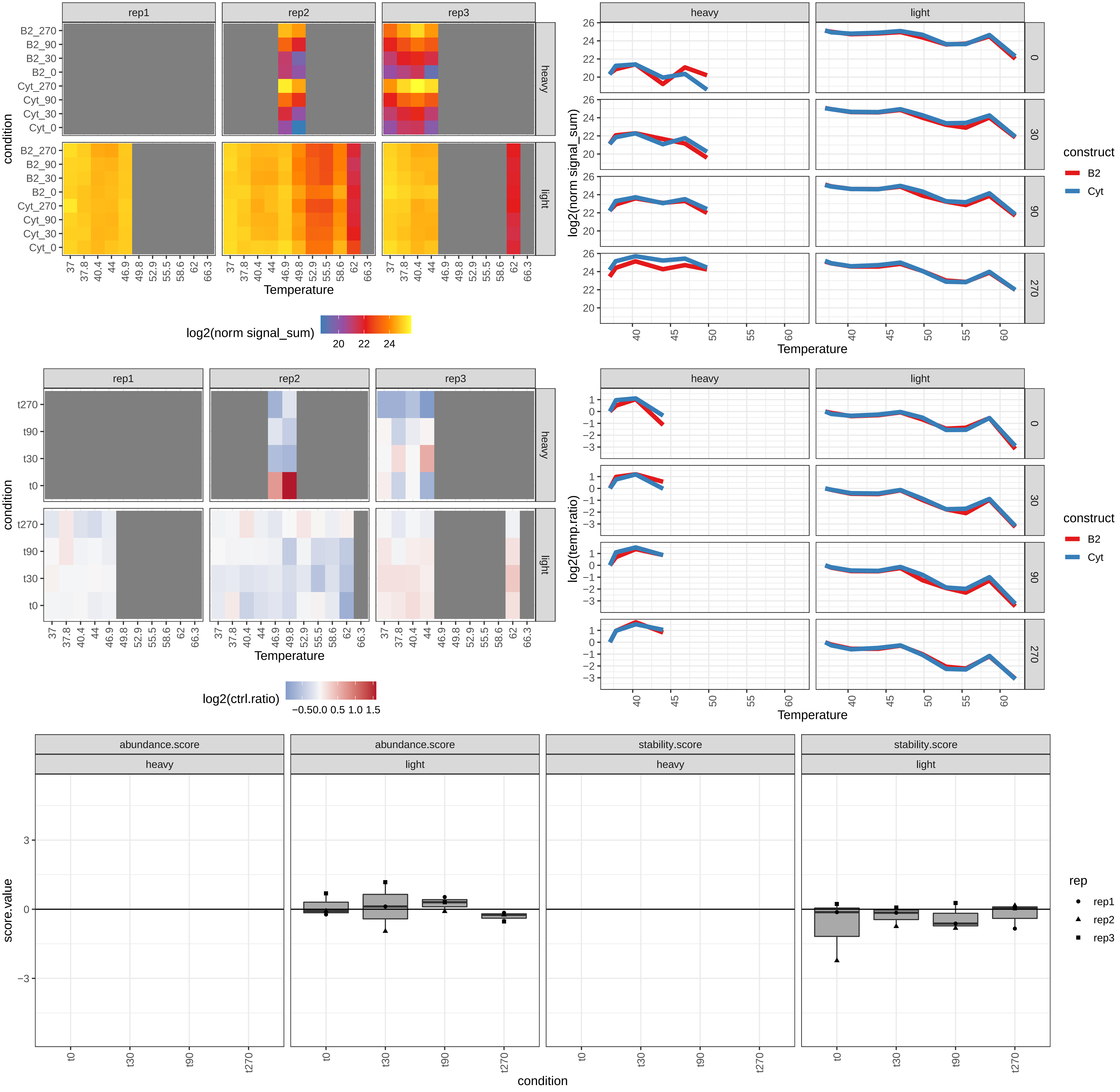

P40509 − Coatomer subunit epsilon OS=Saccharomyces cerevisiae (strain ATCC 204508 / S288c) GN=SEC28 PE=1 SV=2

SEC3 − average.top3: 6.77419636363636 − max.qupm: 11

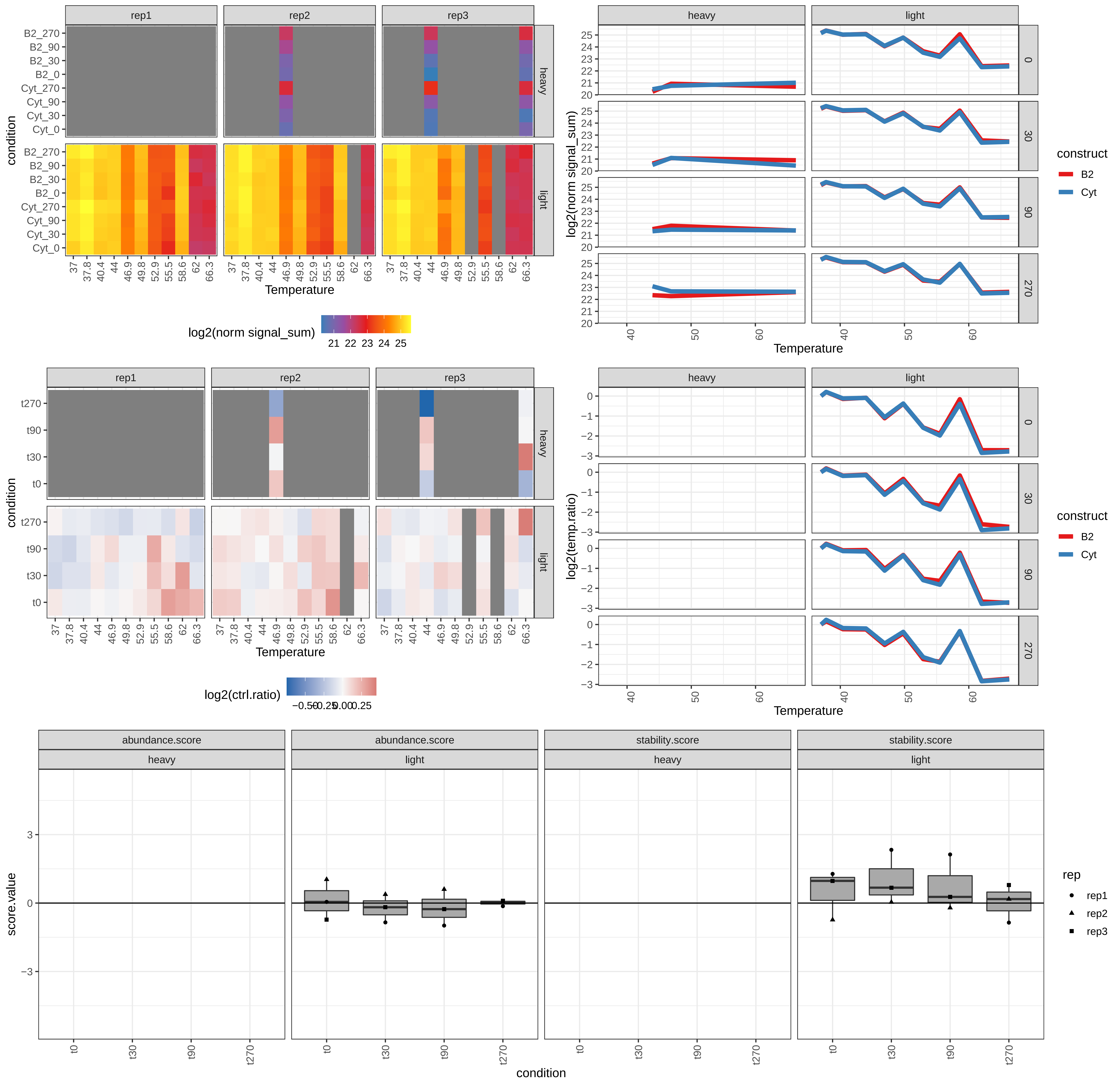

P33332 − Exocyst complex component SEC3 OS=Saccharomyces cerevisiae (strain ATCC 204508 / S288c) GN=SEC3 PE=1 SV=1

SEC31 − average.top3: 7.28931362068966 − max.qupm: 19

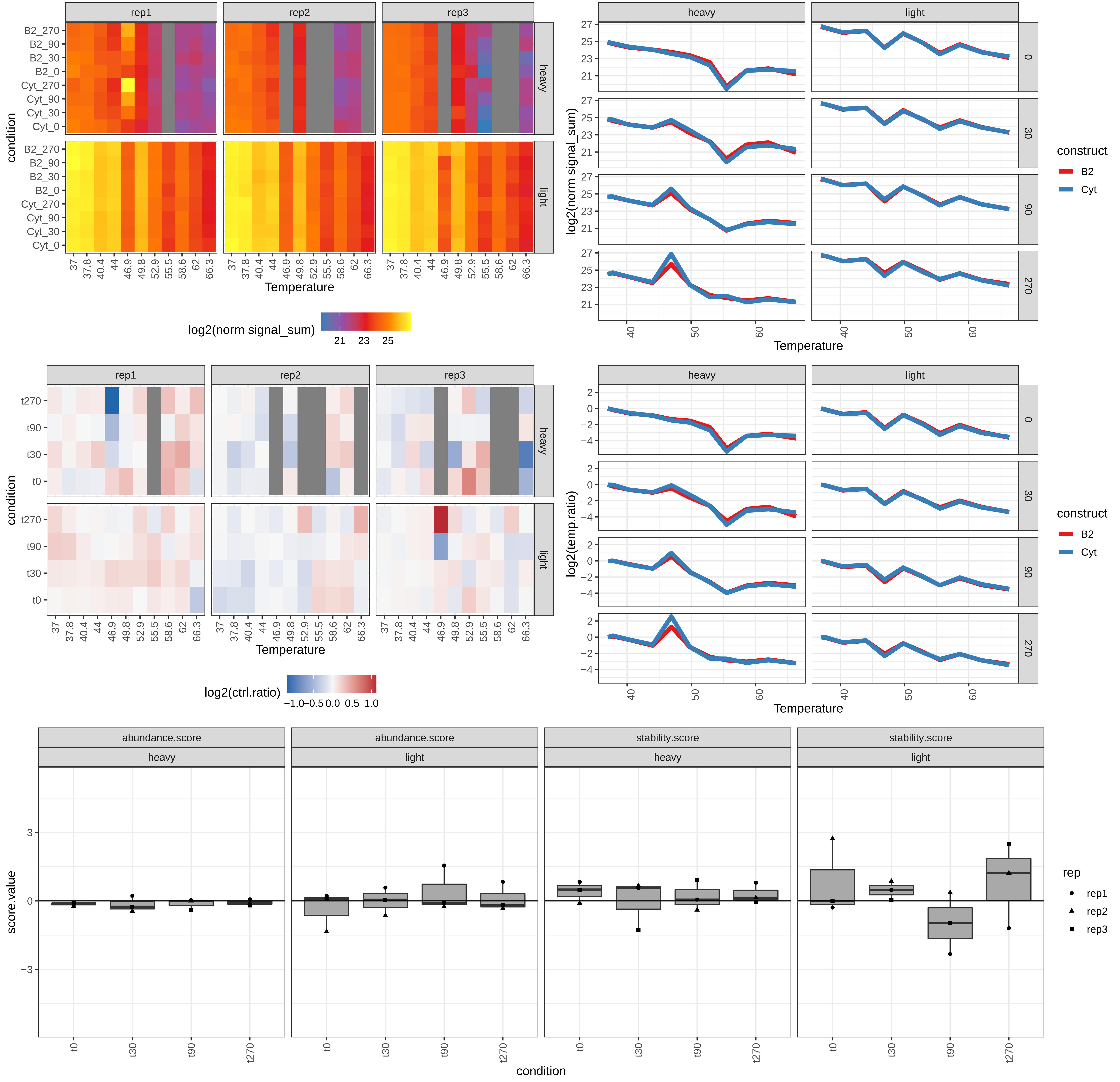

P38968 − Protein transport protein SEC31 OS=Saccharomyces cerevisiae (strain ATCC 204508 / S288c) GN=SEC31 PE=1 SV=3

SEC39 − average.top3: 6.46242714285714 − max.qupm: 5

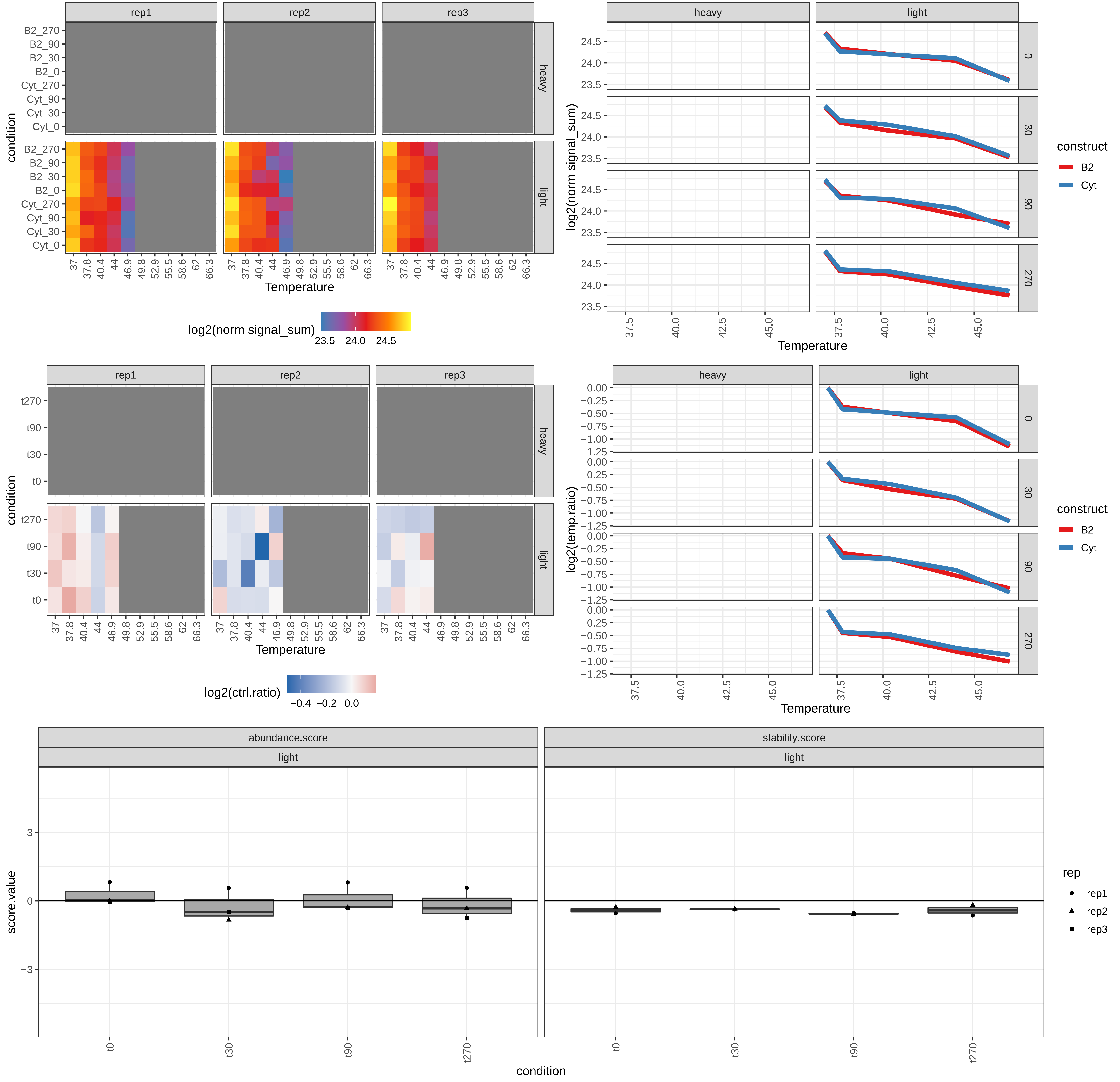

Q12745 − Protein transport protein SEC39 OS=Saccharomyces cerevisiae (strain ATCC 204508 / S288c) GN=SEC39 PE=1 SV=1

SEC4 − average.top3: 7.37981459016393 − max.qupm: 12

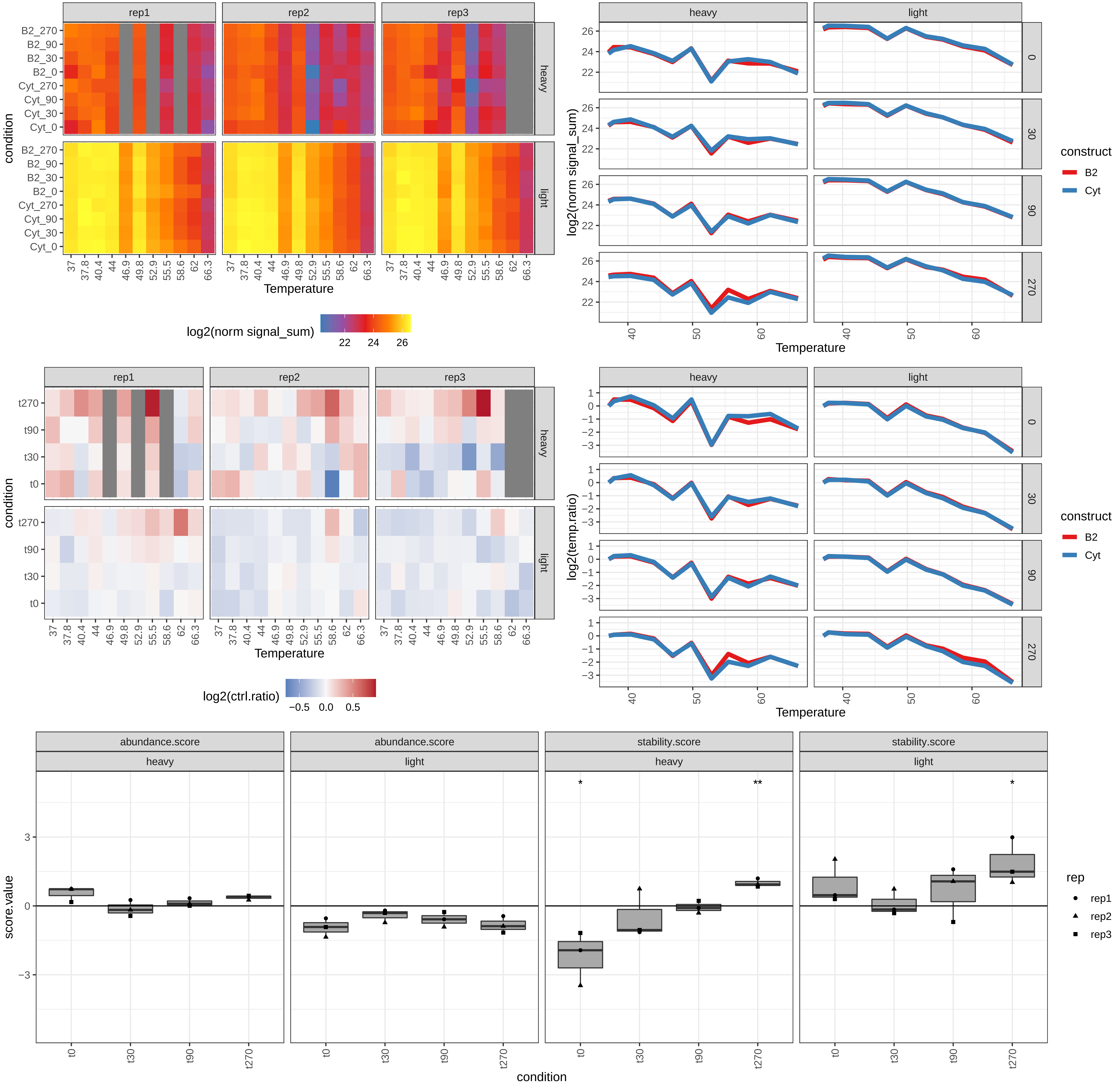

P07560 − Ras−related protein SEC4 OS=Saccharomyces cerevisiae (strain ATCC 204508 / S288c) GN=SEC4 PE=1 SV=1

SEC5 − average.top3: 6.57287066666667 − max.qupm: 7

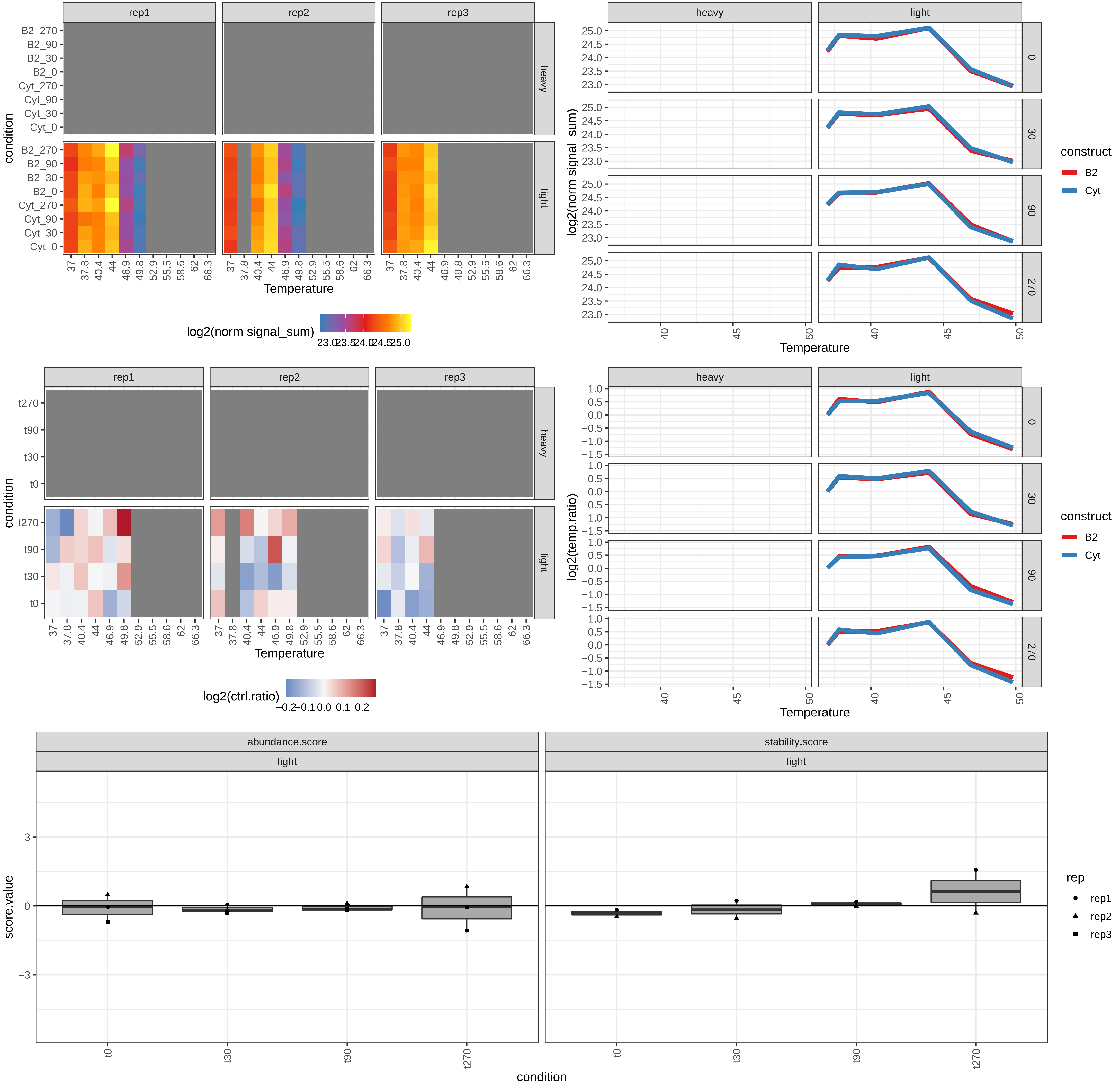

P89102 − Exocyst complex component SEC5 OS=Saccharomyces cerevisiae (strain ATCC 204508 / S288c) GN=SEC5 PE=1 SV=1

SEC53 − average.top3: 7.56670490196078 − max.qupm: 18

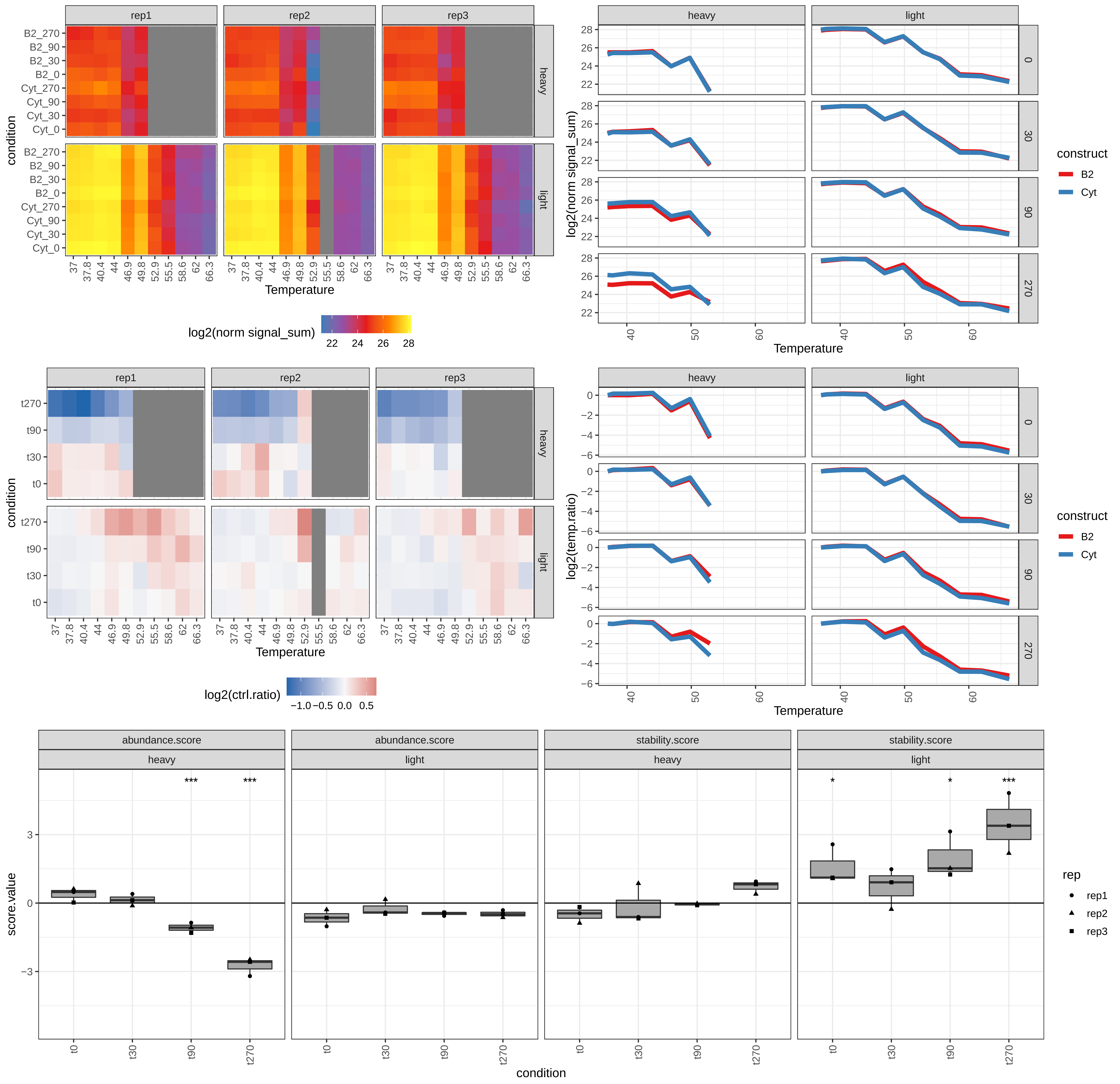

P07283 − Phosphomannomutase OS=Saccharomyces cerevisiae (strain ATCC 204508 / S288c) GN=SEC53 PE=1 SV=1

SEC6 − average.top3: 6.61129647058824 − max.qupm: 8

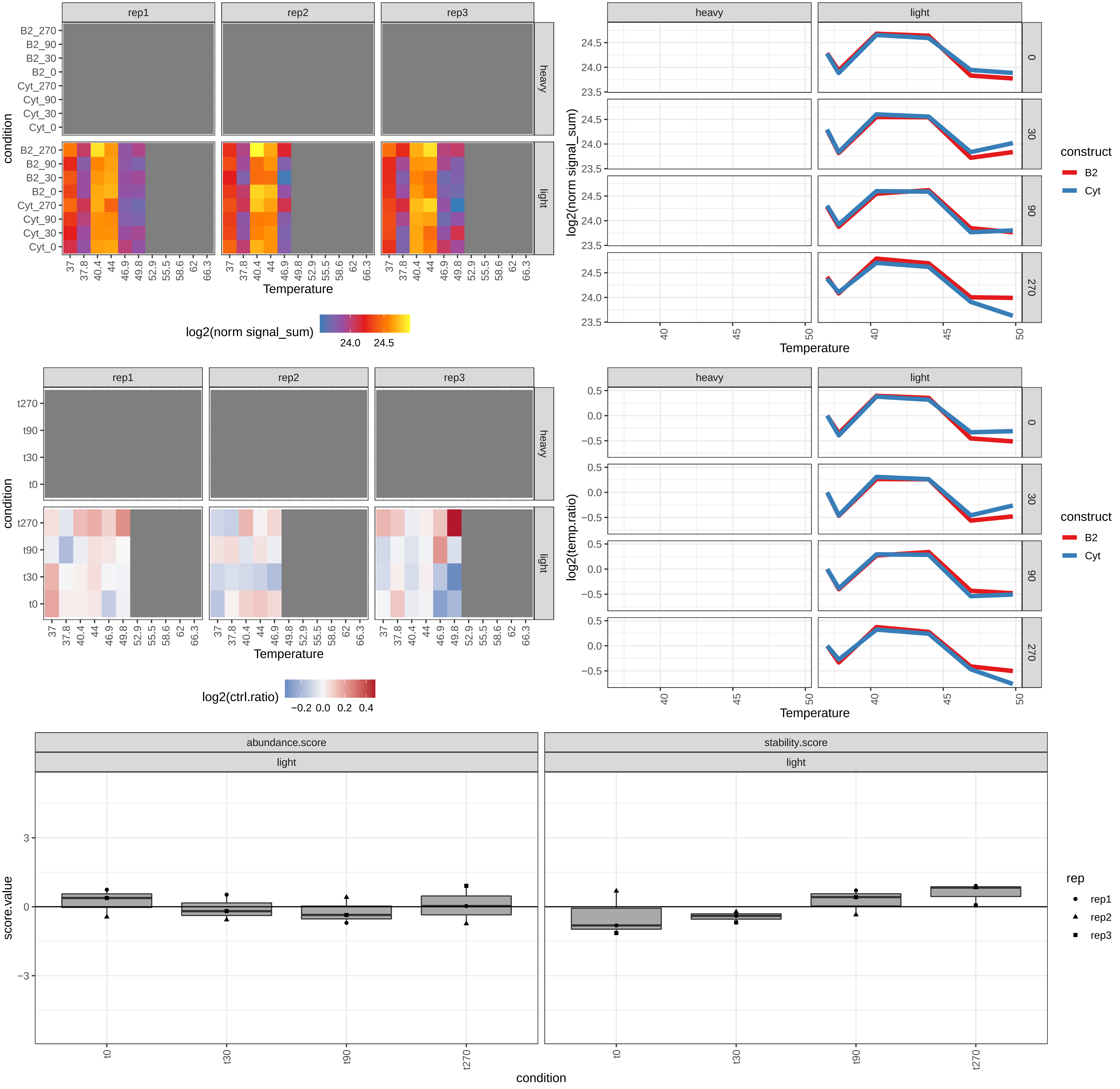

P32844 − Exocyst complex component SEC6 OS=Saccharomyces cerevisiae (strain ATCC 204508 / S288c) GN=SEC6 PE=1 SV=2

SEC61 − average.top3: 7.23924781818182 − max.qupm: 9

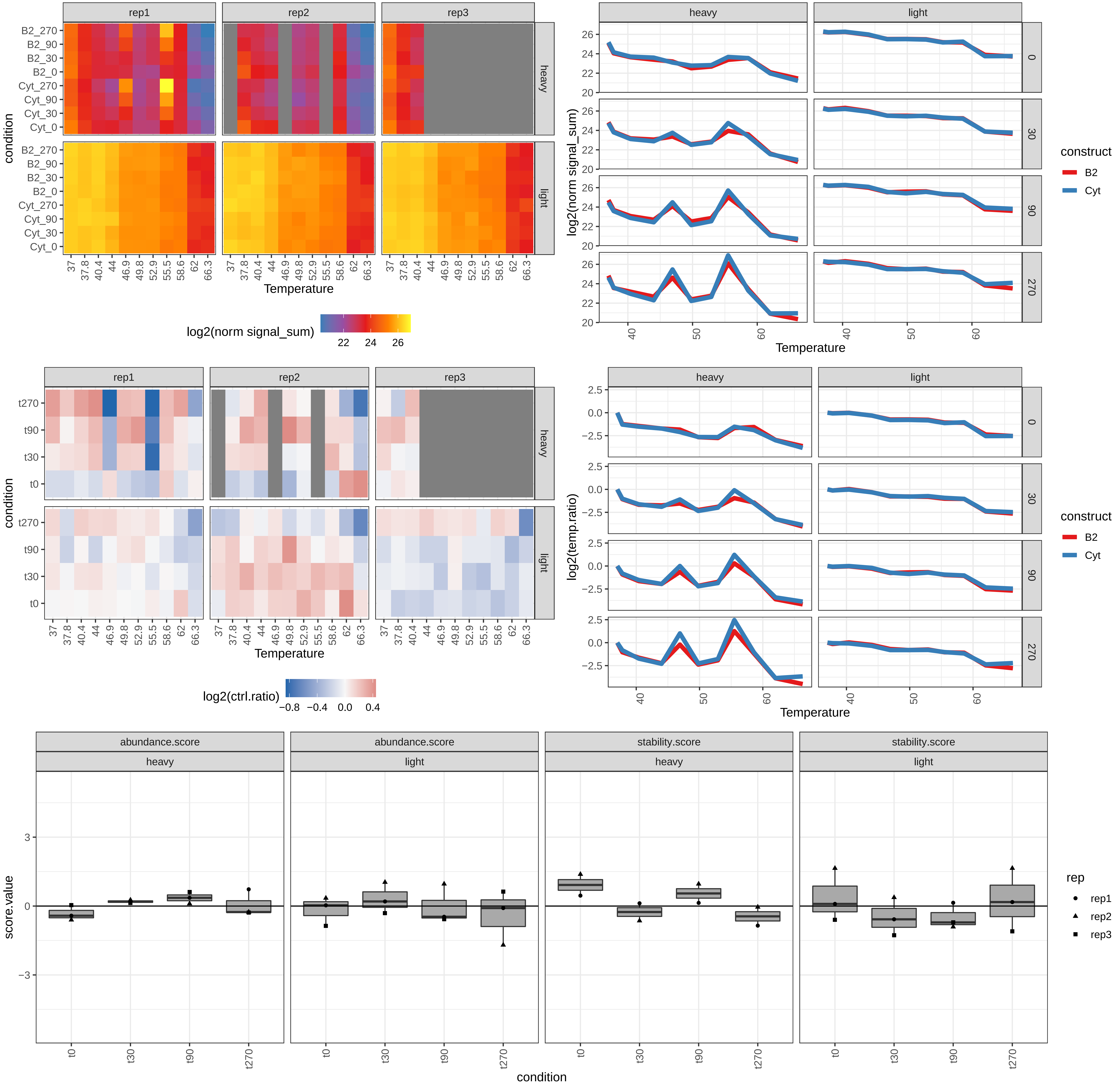

P32915 − Protein transport protein SEC61 OS=Saccharomyces cerevisiae (strain ATCC 204508 / S288c) GN=SEC61 PE=1 SV=1

SEC62 − average.top3: 6.80036933333333 − max.qupm: 5

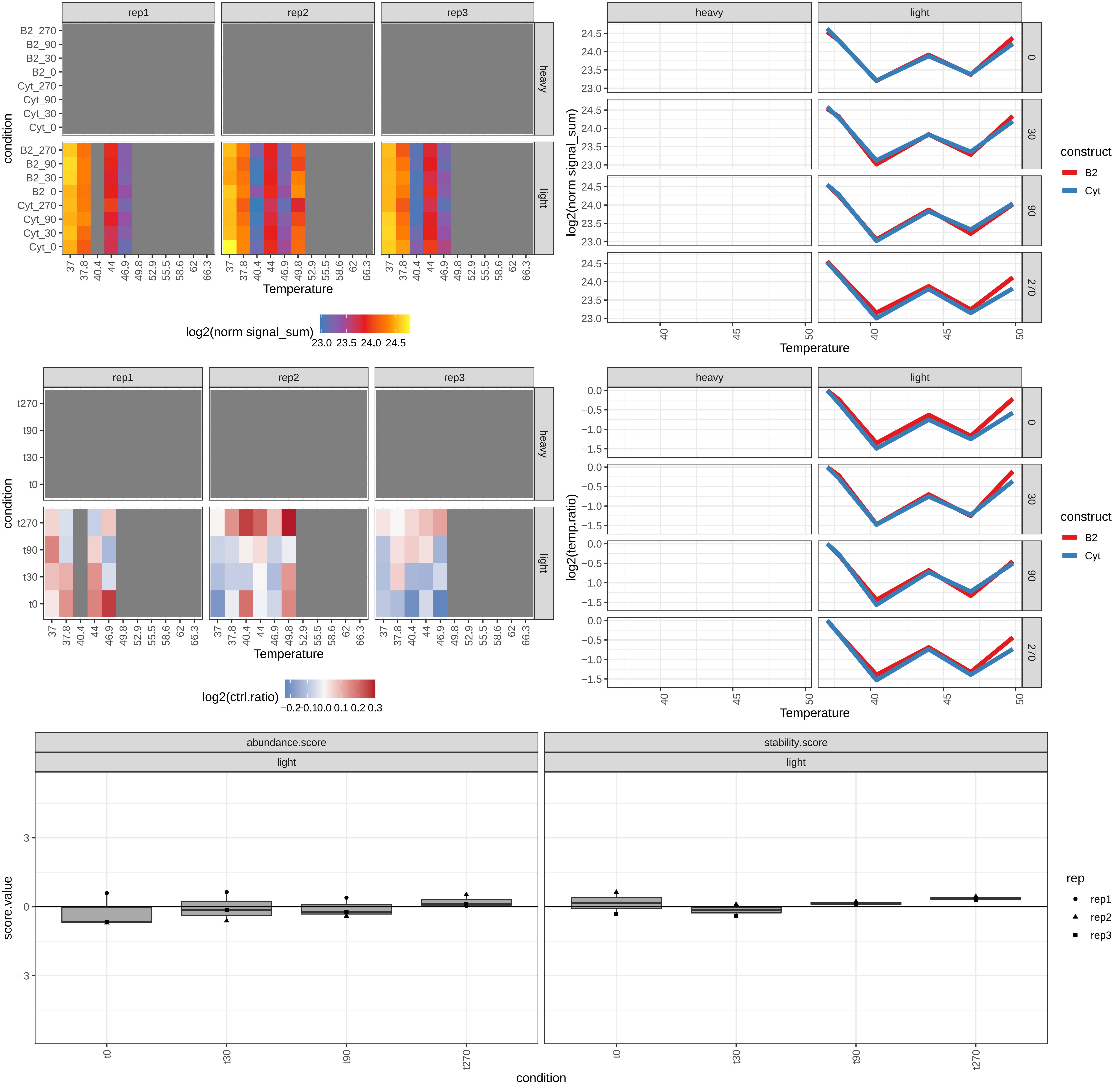

P21825 − Translocation protein SEC62 OS=Saccharomyces cerevisiae (strain ATCC 204508 / S288c) GN=SEC62 PE=1 SV=2

SEC63 − average.top3: 6.96218240740741 − max.qupm: 16

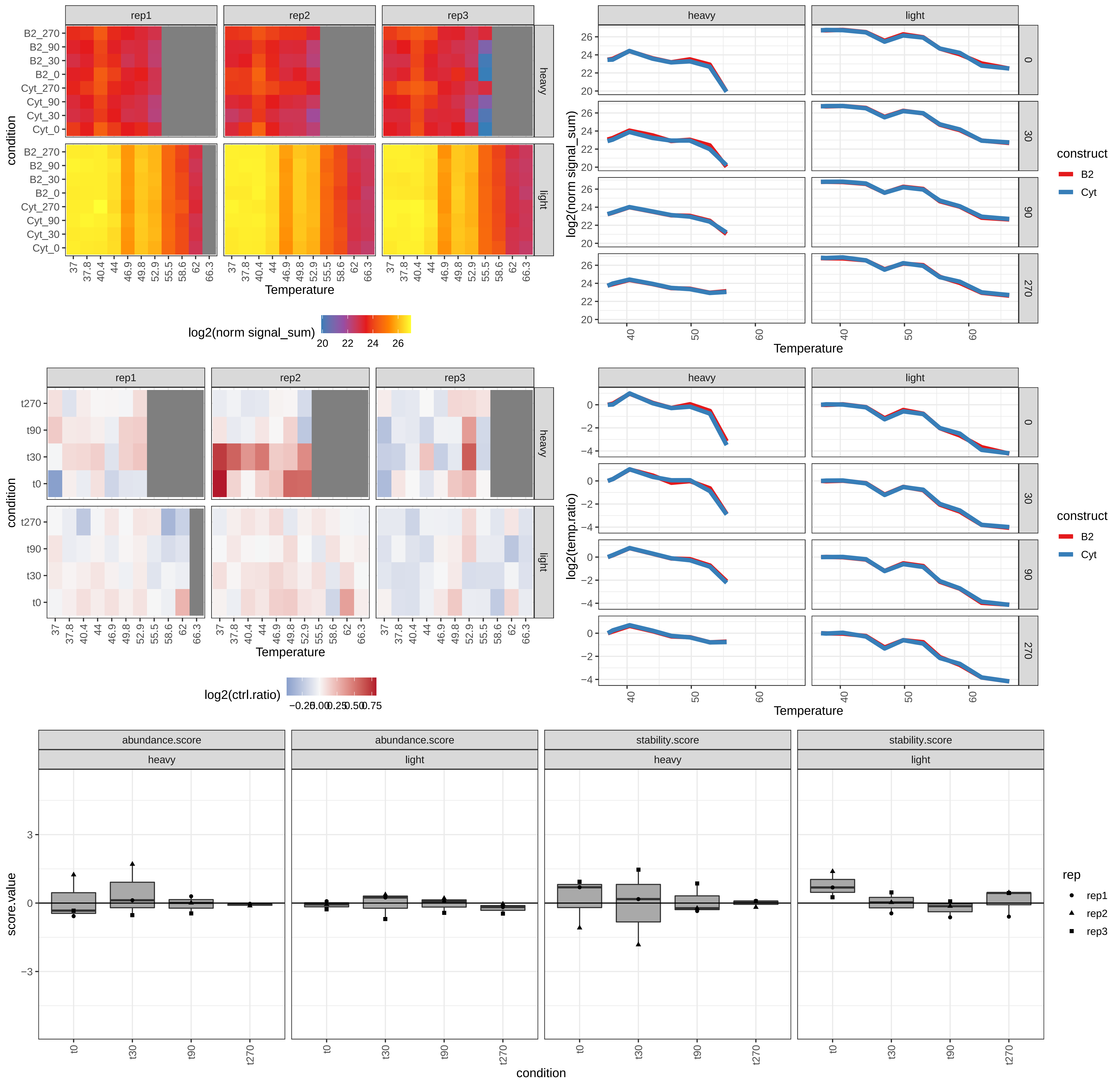

P14906 − Protein translocation protein SEC63 OS=Saccharomyces cerevisiae (strain ATCC 204508 / S288c) GN=SEC63 PE=1 SV=2

SEC65 − average.top3: 6.9500519047619 − max.qupm: 6

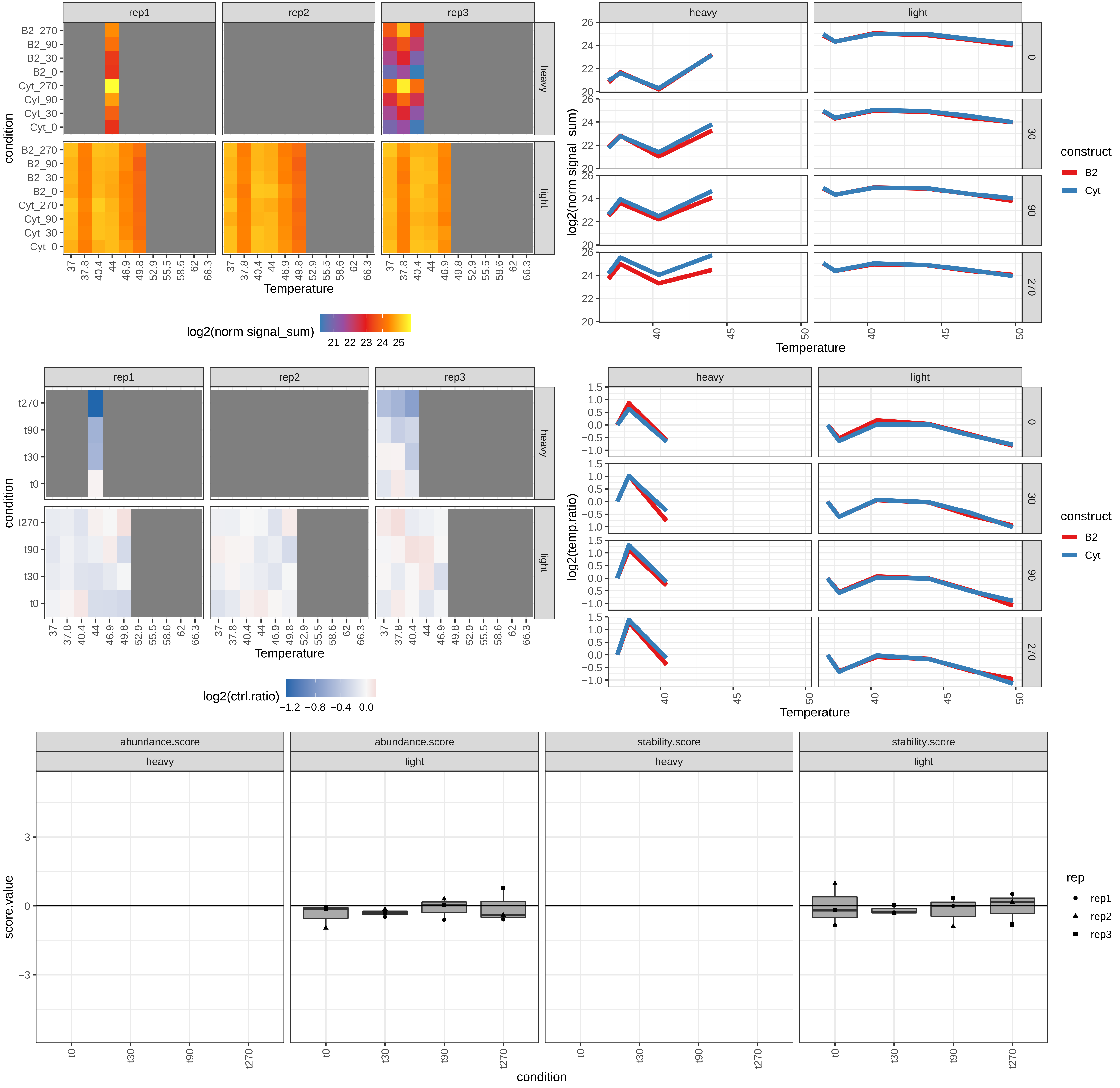

P29478 − Signal recognition particle subunit SEC65 OS=Saccharomyces cerevisiae (strain ATCC 204508 / S288c) GN=SEC65 PE=1 SV=1

SEC66 − average.top3: 7.23059185185185 − max.qupm: 7

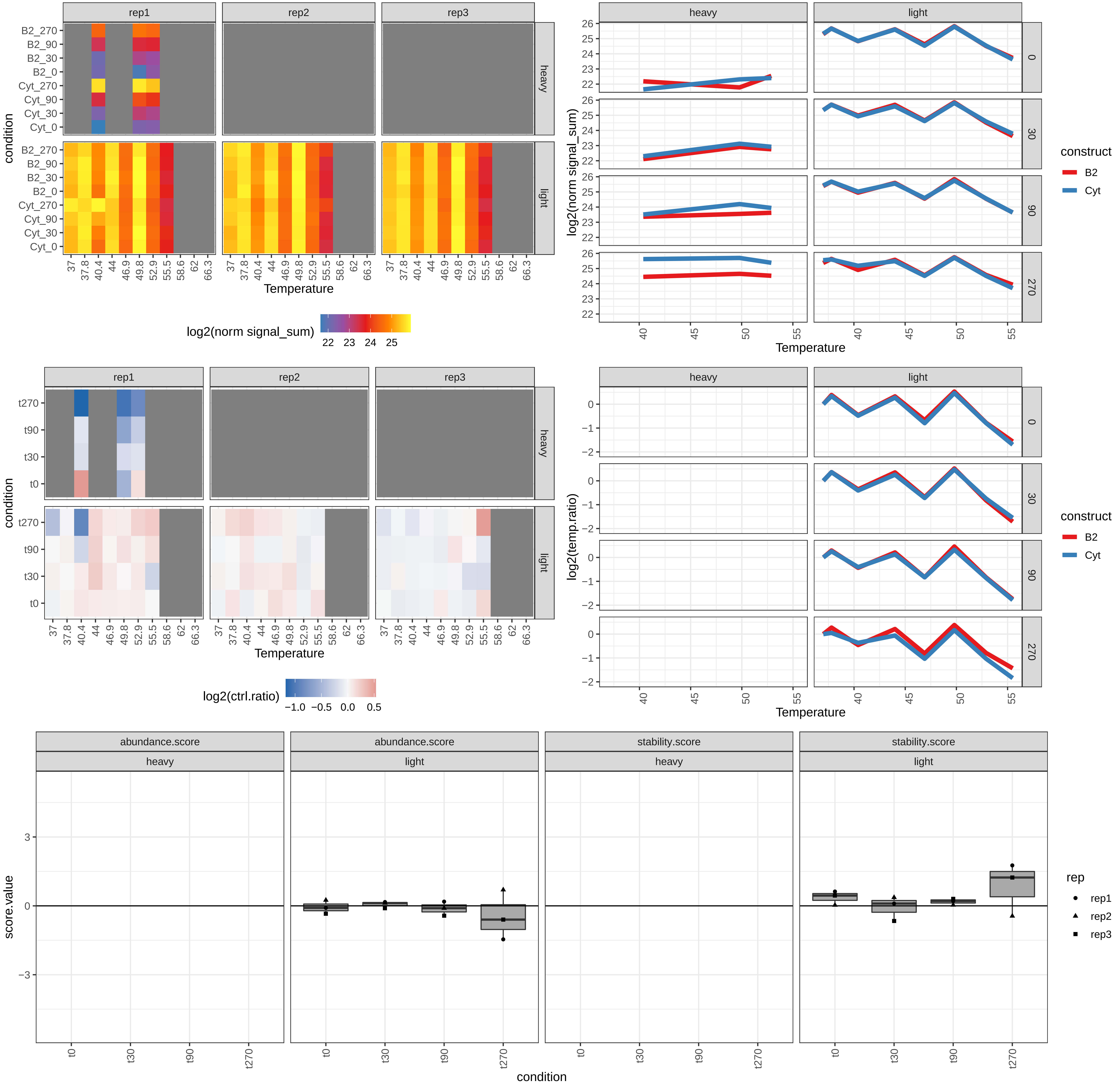

P33754 − Translocation protein SEC66 OS=Saccharomyces cerevisiae (strain ATCC 204508 / S288c) GN=SEC66 PE=1 SV=1

SEC7 − average.top3: 7.06369838709677 − max.qupm: 12

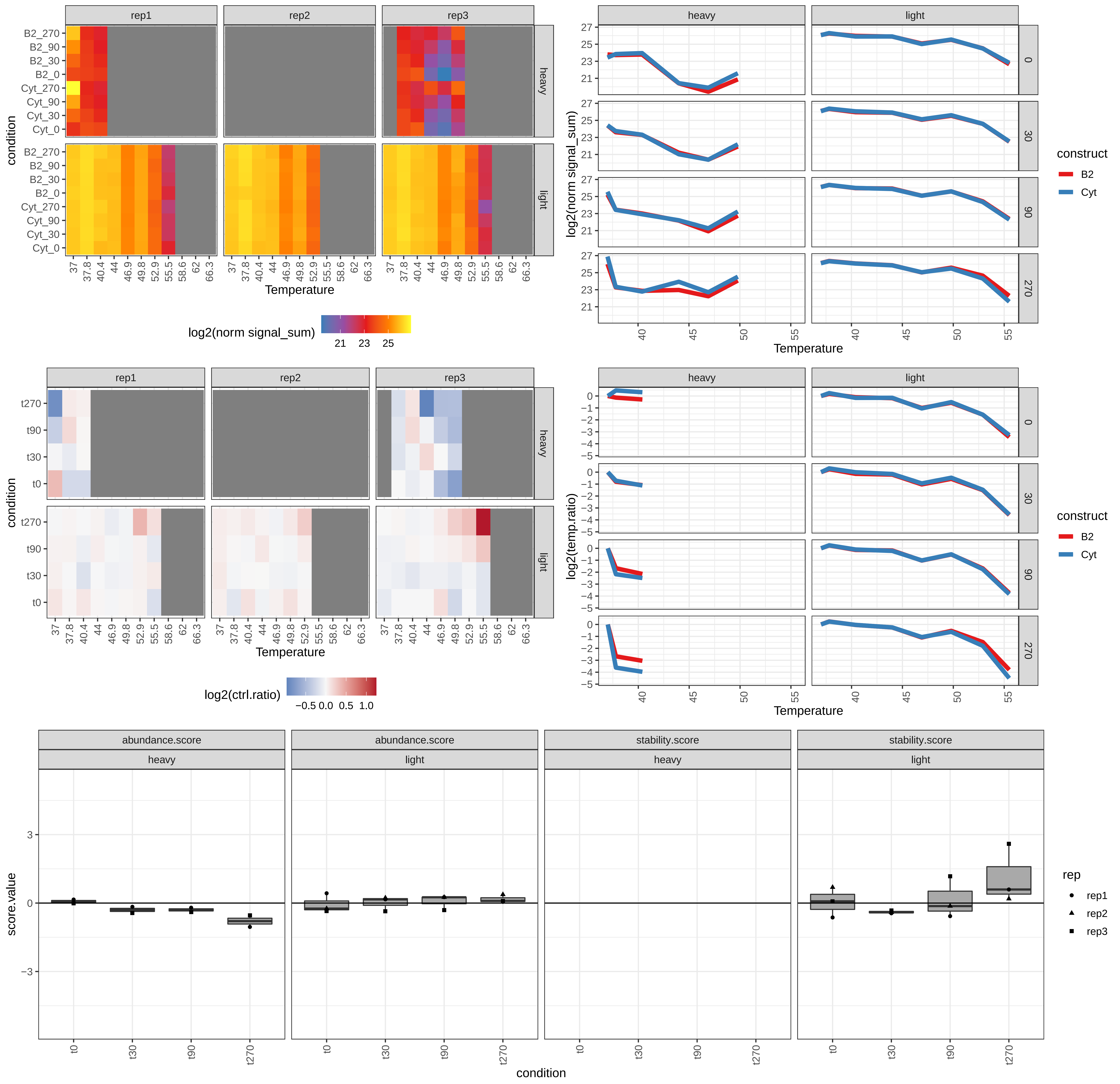

P11075 − Protein transport protein SEC7 OS=Saccharomyces cerevisiae (strain ATCC 204508 / S288c) GN=SEC7 PE=1 SV=2

SEC72 − average.top3: 6.94198333333333 − max.qupm: 6

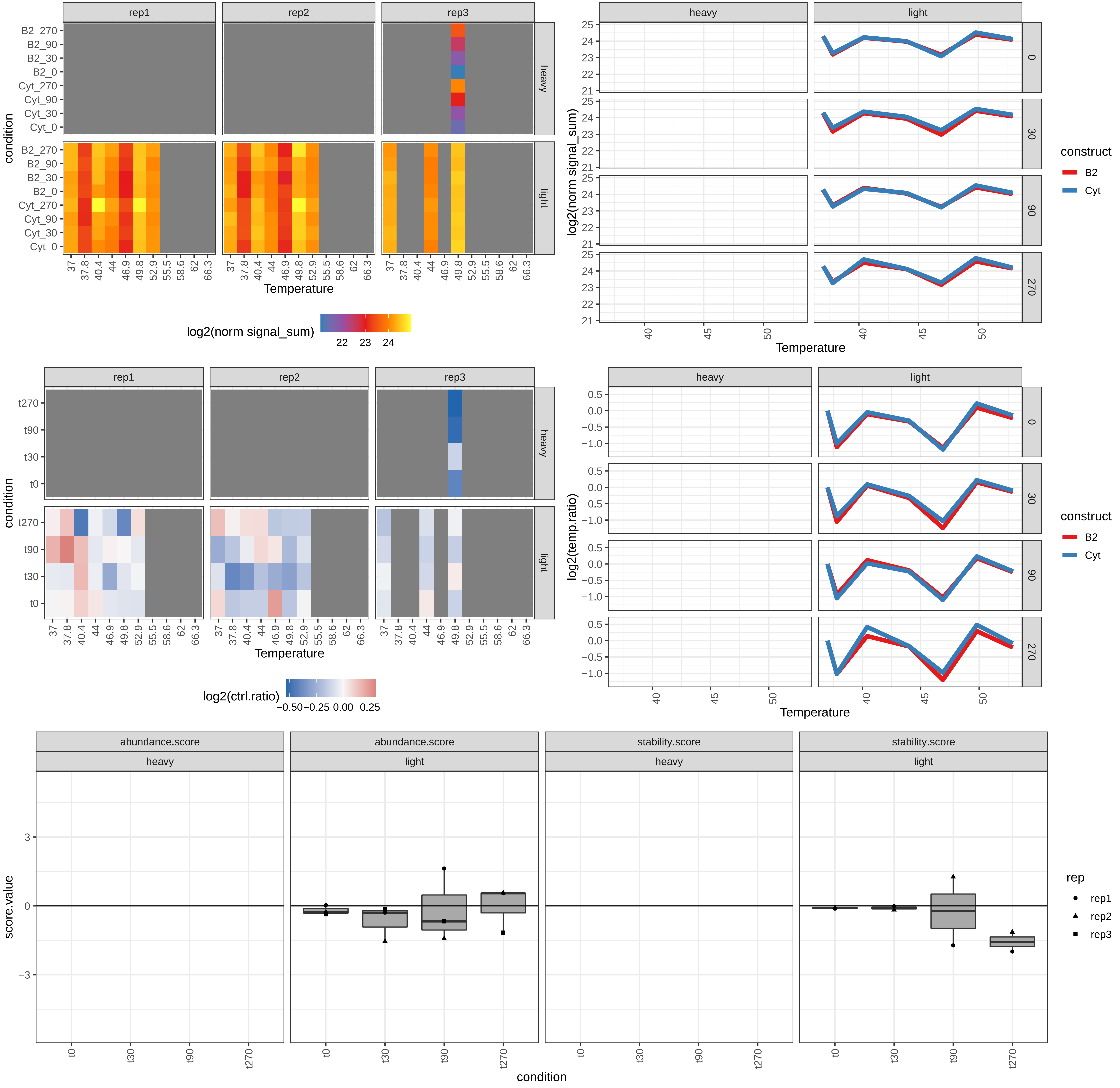

P39742 − Translocation protein SEC72 OS=Saccharomyces cerevisiae (strain ATCC 204508 / S288c) GN=SEC72 PE=1 SV=3

SEC8 − average.top3: 6.78986 − max.qupm: 6

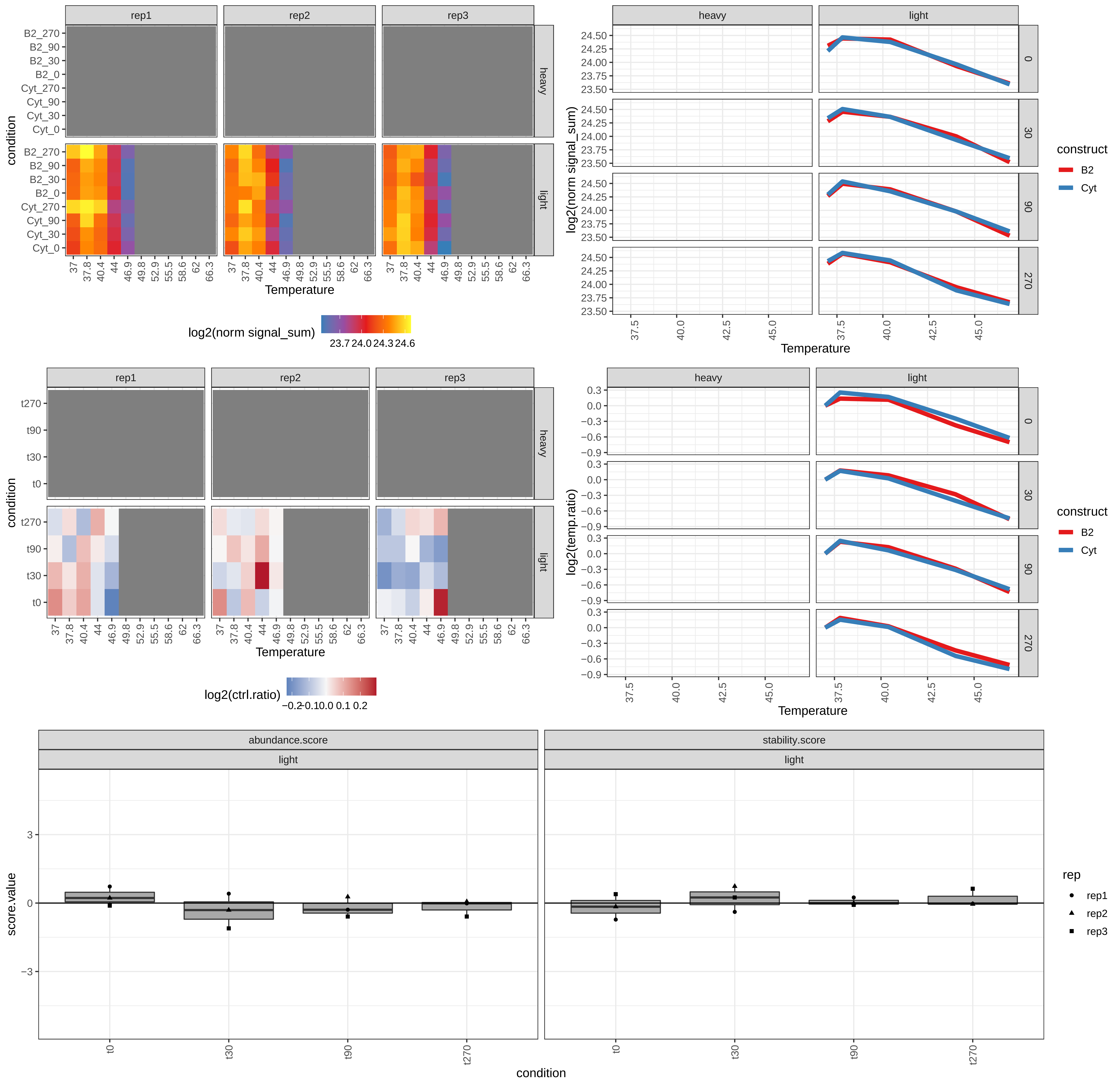

P32855 − Exocyst complex component SEC8 OS=Saccharomyces cerevisiae (strain ATCC 204508 / S288c) GN=SEC8 PE=1 SV=1

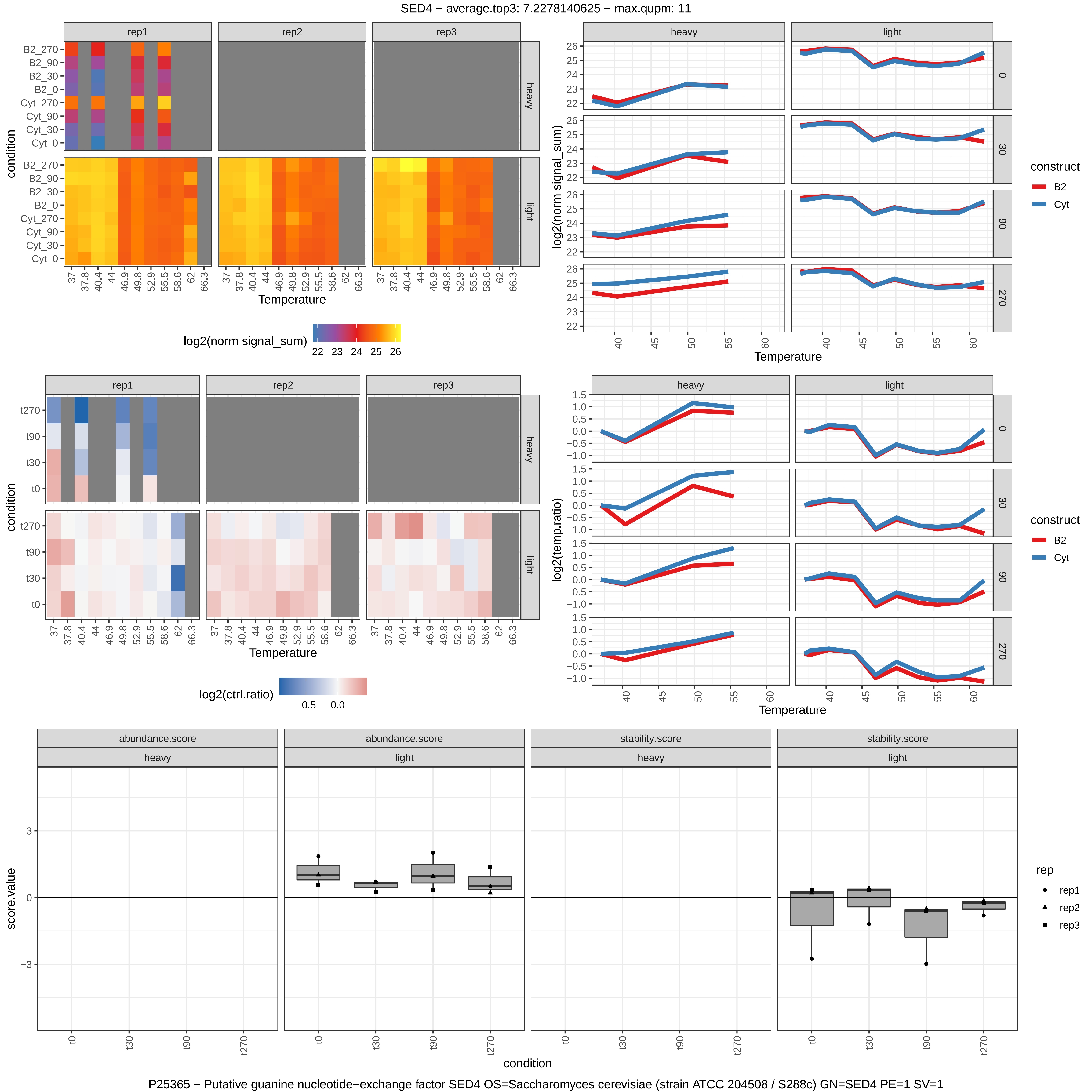

SED4 − average.top3: 7.2278140625 − max.qupm: 11
rep1
rep2
rep3
heavy
light
condition
Temperature
log2(norm signal_sum)
construct
B2
Cyt
log2(ctrl.ratio)
log2(temp.ratio)
abundance.score
stability.score
score.value
rep
rep1
rep2
rep3
P25365 − Putative guanine nucleotide−exchange factor SED4 OS=Saccharomyces cerevisiae (strain ATCC 204508 / S288c) GN=SED4 PE=1 SV=1

SEF1 − average.top3: 6.95714774193548 − max.qupm: 9

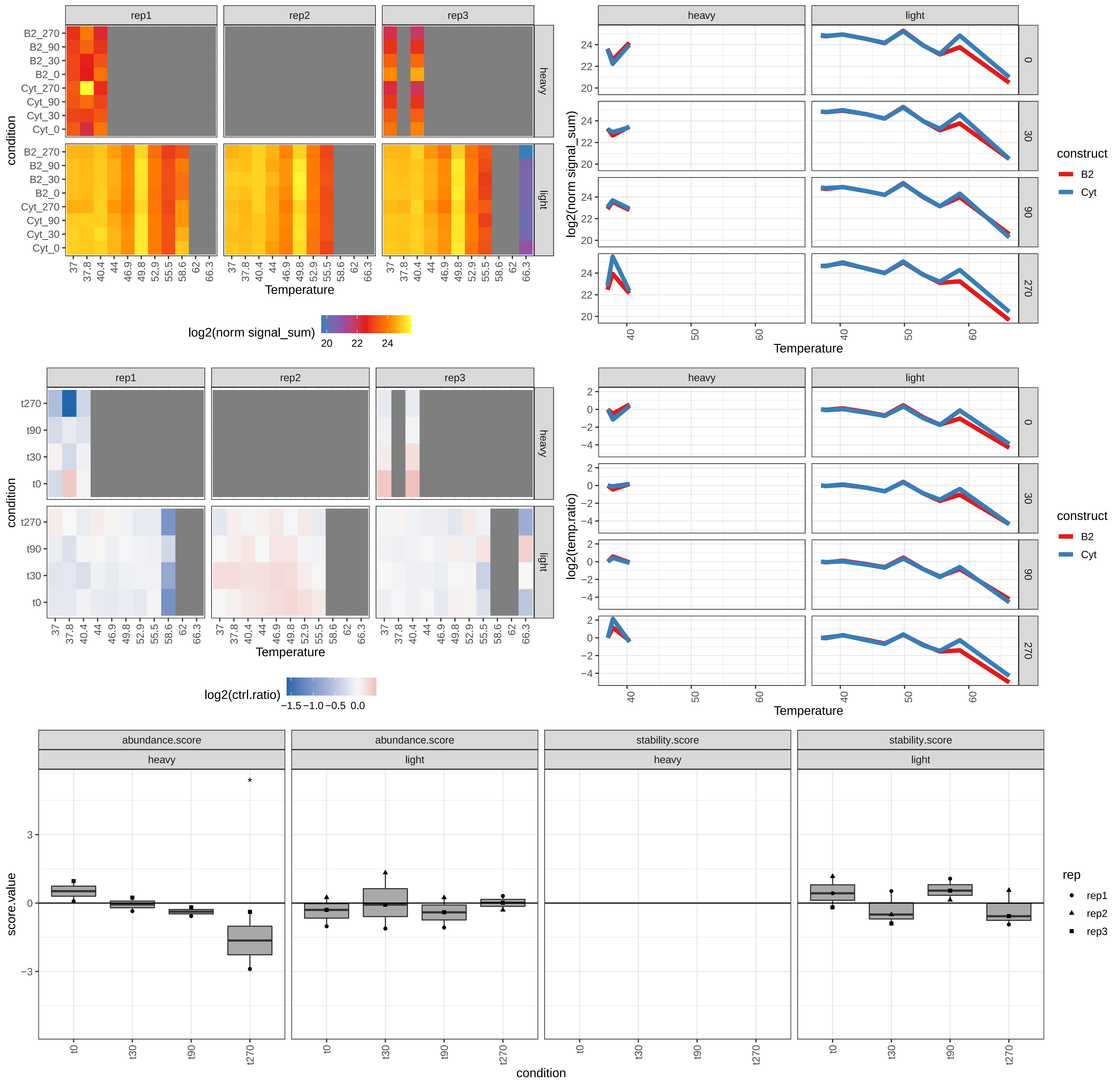

P34228 − Putative transcription factor SEF1 OS=Saccharomyces cerevisiae (strain ATCC 204508 / S288c) GN=SEF1 PE=1 SV=4

SEH1 − average.top3: 7.1587316 − max.qupm: 12

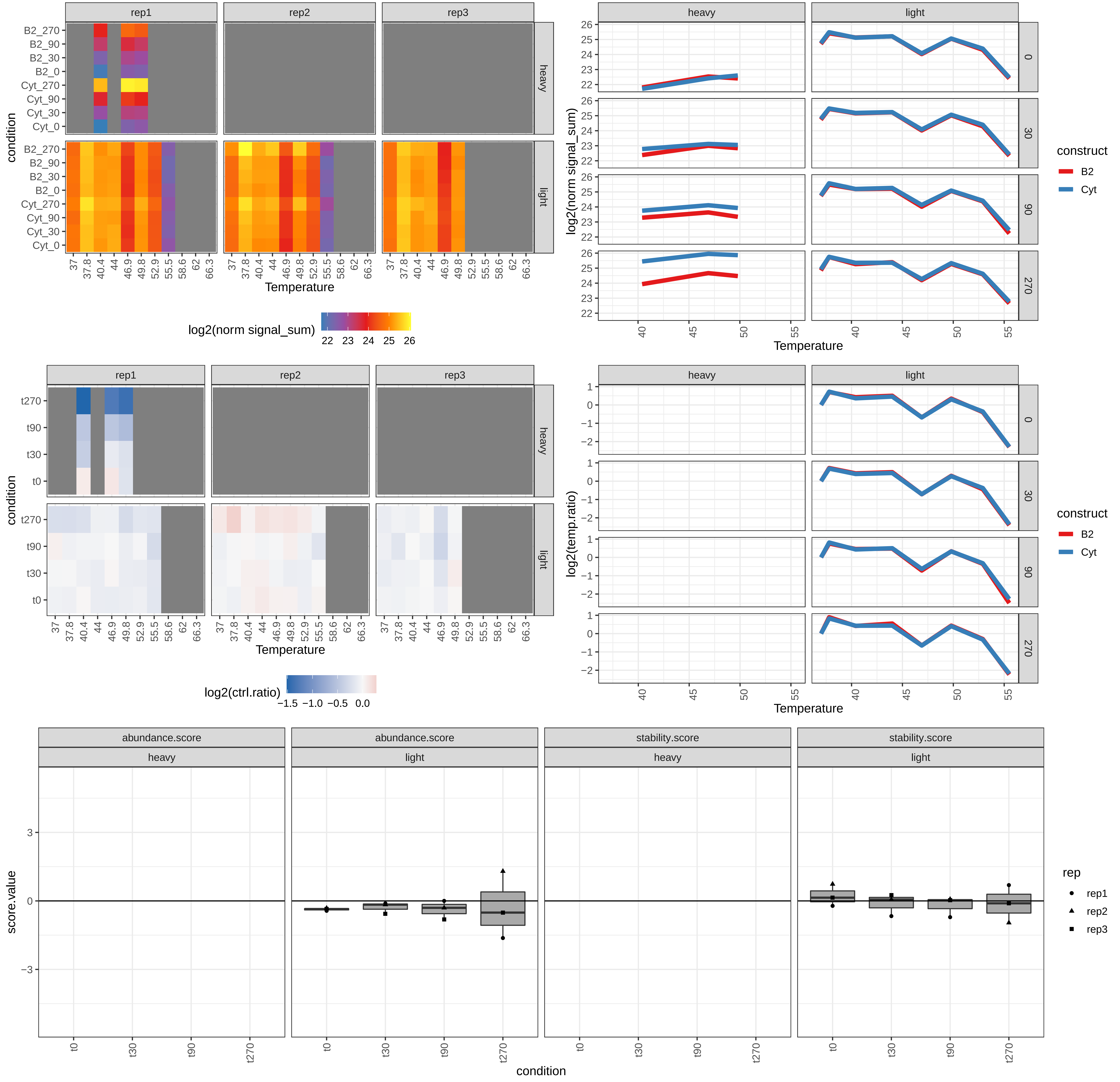

P53011 − Nucleoporin SEH1 OS=Saccharomyces cerevisiae (strain ATCC 204508 / S288c) GN=SEH1 PE=1 SV=1

SEN1 − average.top3: 6.39021695652174 − max.qupm: 10

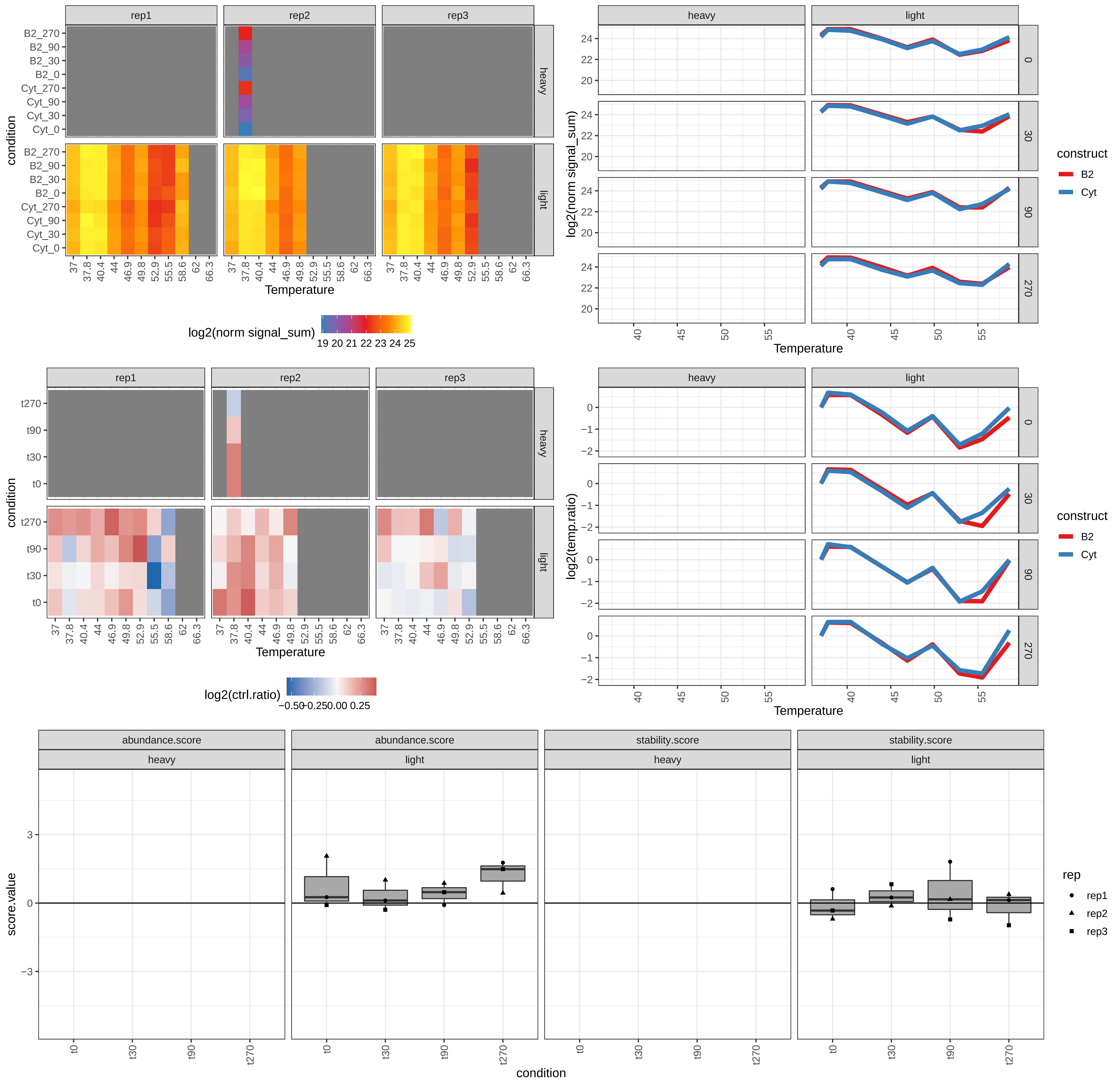

Q00416 − Helicase SEN1 OS=Saccharomyces cerevisiae (strain ATCC 204508 / S288c) GN=SEN1 PE=1 SV=2

SEN2 − average.top3: 6.10797666666667 − max.qupm: 3

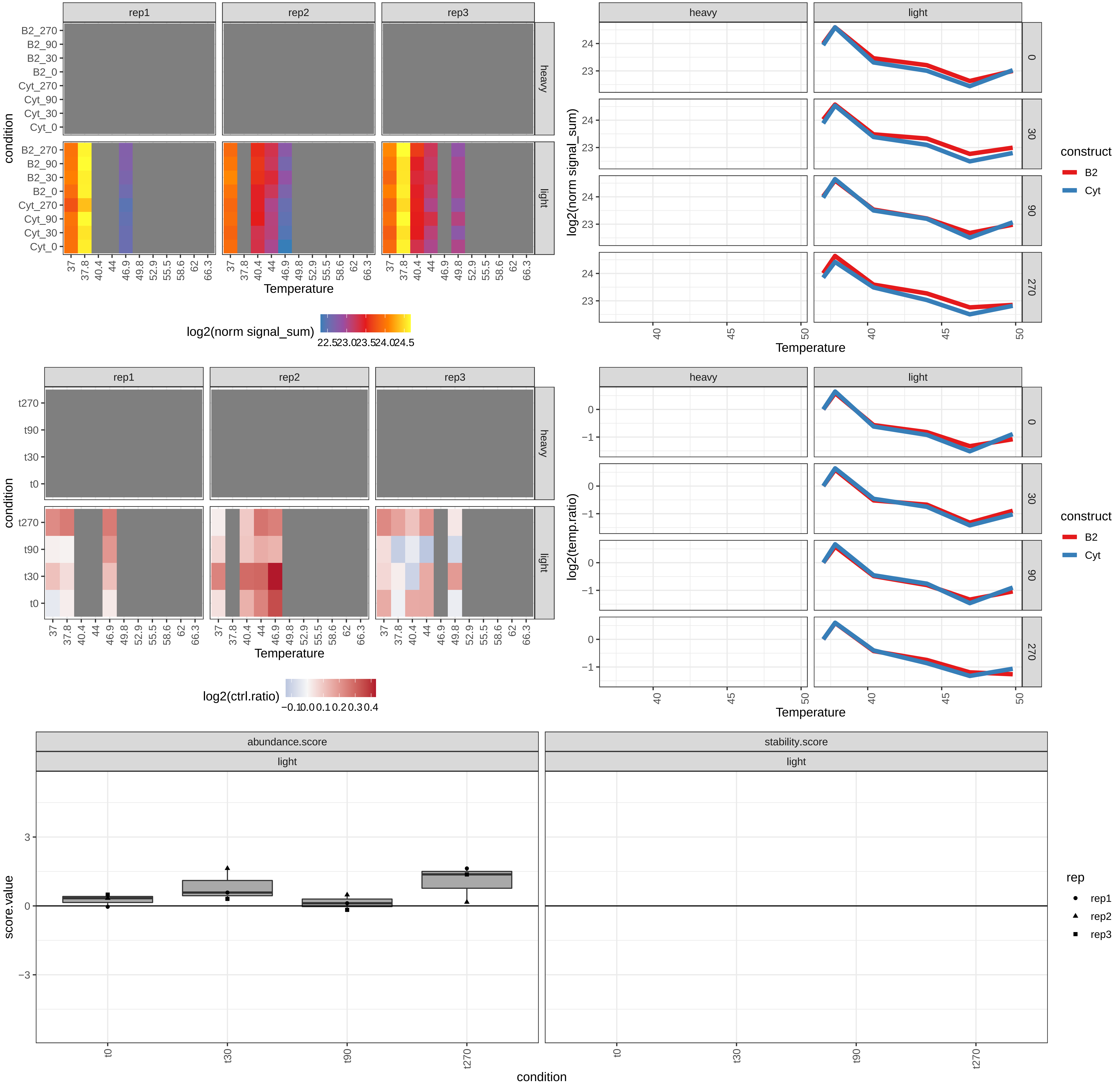

P16658 − tRNA−splicing endonuclease subunit SEN2 OS=Saccharomyces cerevisiae (strain ATCC 204508 / S288c) GN=SEN2 PE=1 SV=1

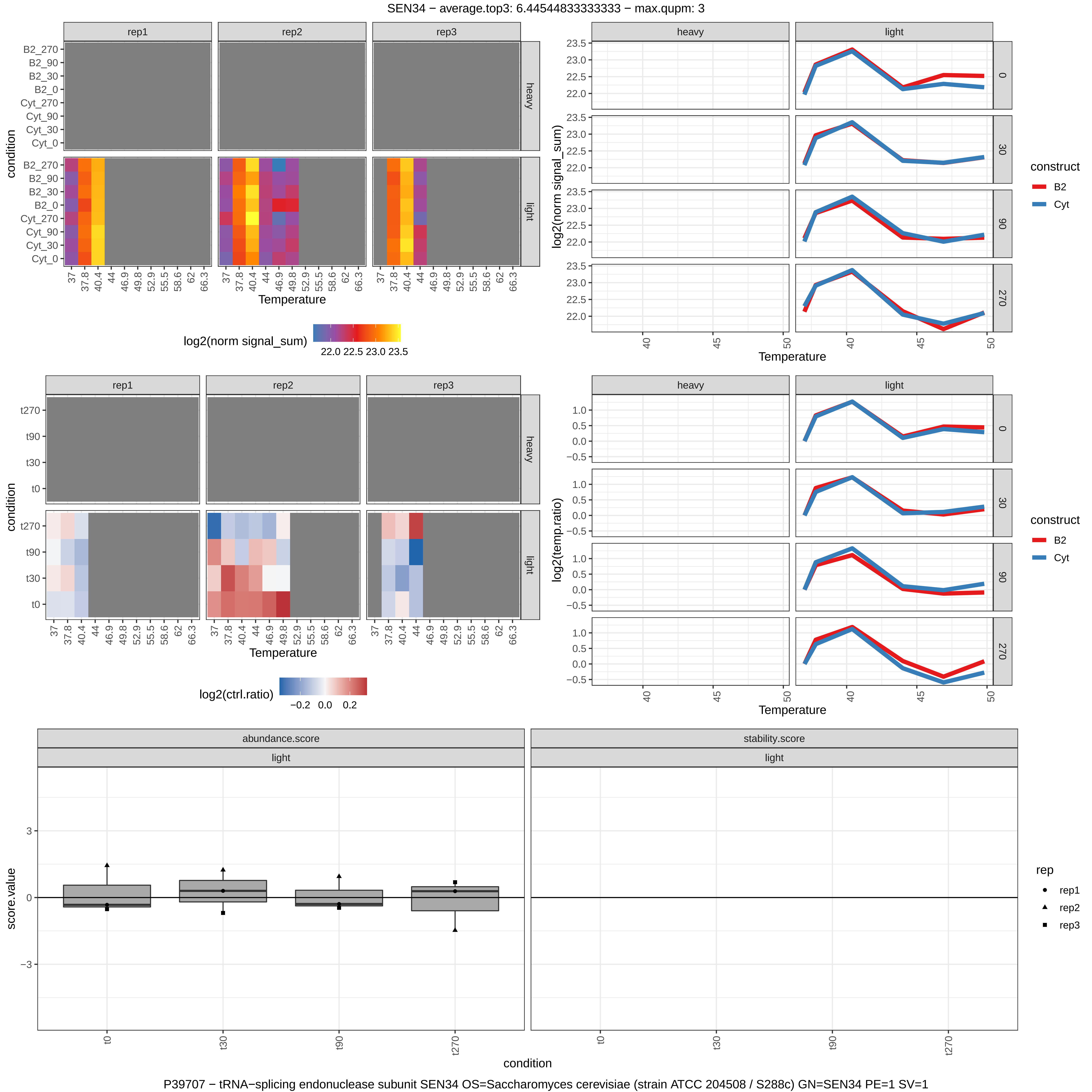
SEN34 − average.top3: 6.445448333333333 − max.qupm: 3
rep1
rep2
rep3
heavy
light
condition
Temperature
log2(norm signal_sum)
construct
B2
Cyt
log2(ctrl.ratio)
log2(temp.ratio)
abundance.score
stability.score
score.value
rep
rep1
rep2
rep3
P39707 − tRNA−splicing endonuclease subunit SEN34 OS=Saccharomyces cerevisiae (strain ATCC 204508 / S288c) GN=SEN34 PE=1 SV=1

SER1 − average.top3: 7.15305871794872 − max.qupm: 14

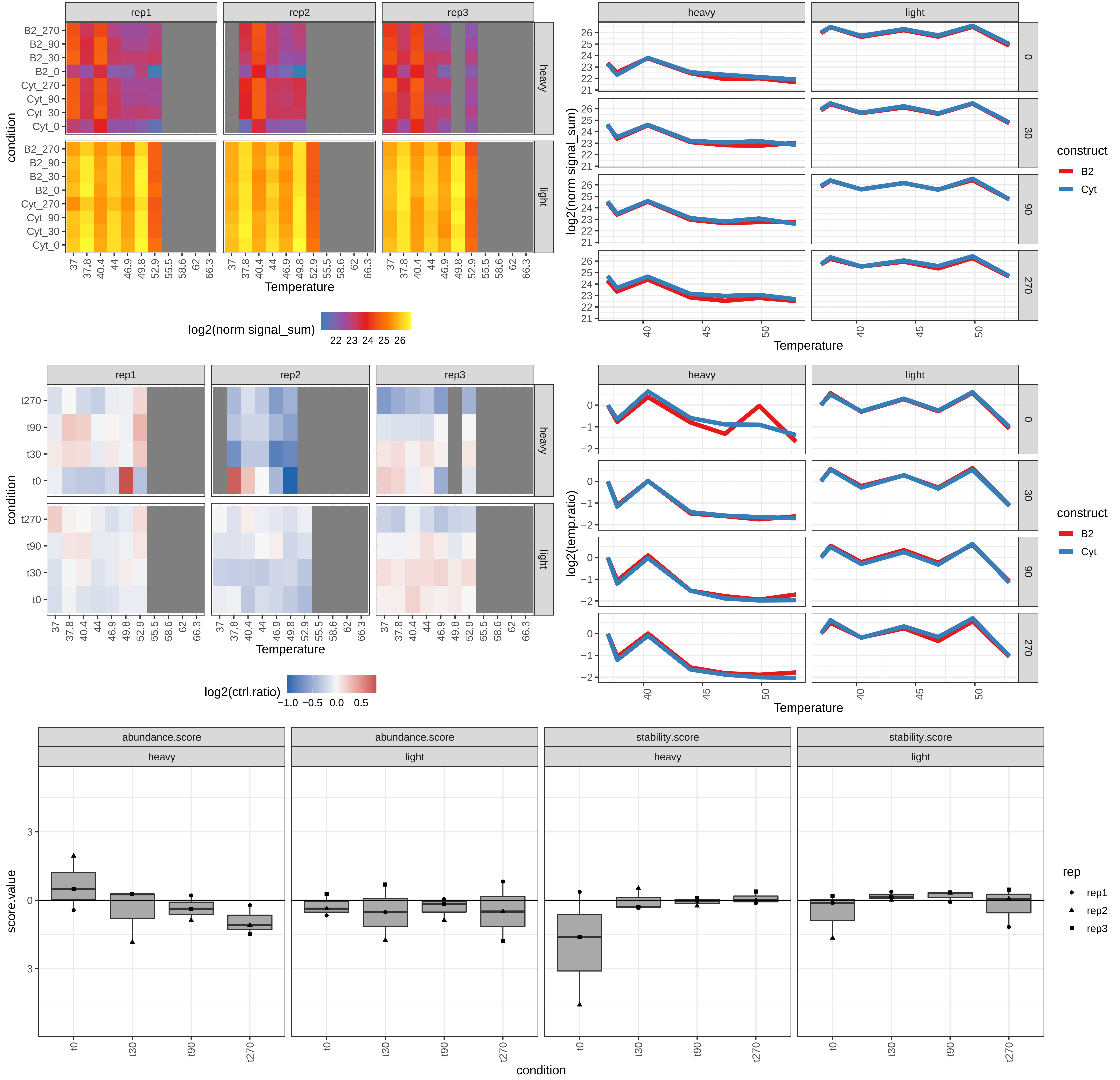

P33330 − Phosphoserine aminotransferase OS=Saccharomyces cerevisiae (strain ATCC 204508 / S288c) GN=SER1 PE=1 SV=1

SER2 − average.top3: 6.86297625 − max.qupm: 7

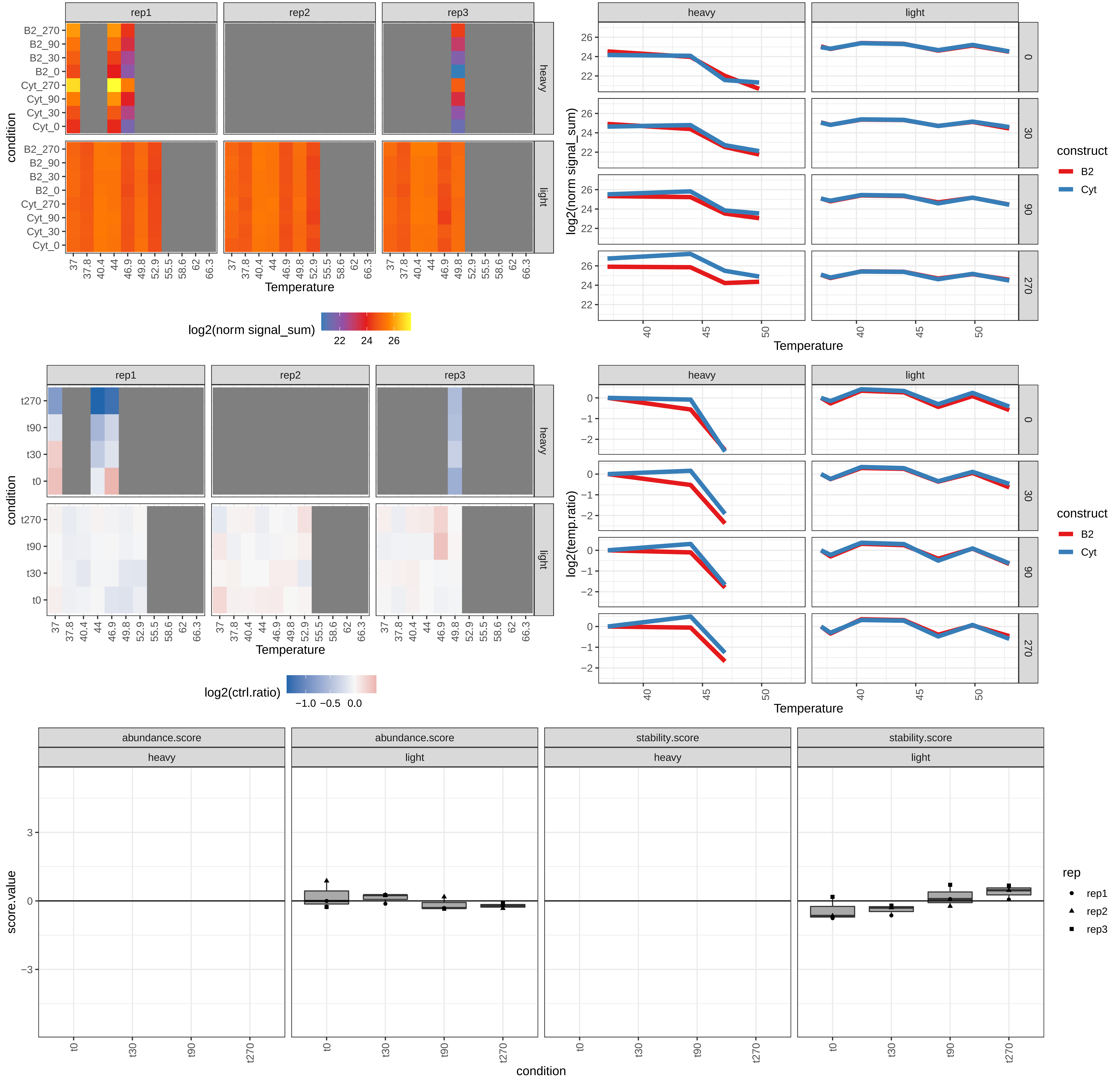

P42941 − Phosphoserine phosphatase OS=Saccharomyces cerevisiae (strain ATCC 204508 / S288c) GN=SER2 PE=1 SV=1

SER33 − average.top3: 7.08957227272727 − max.qupm: 7

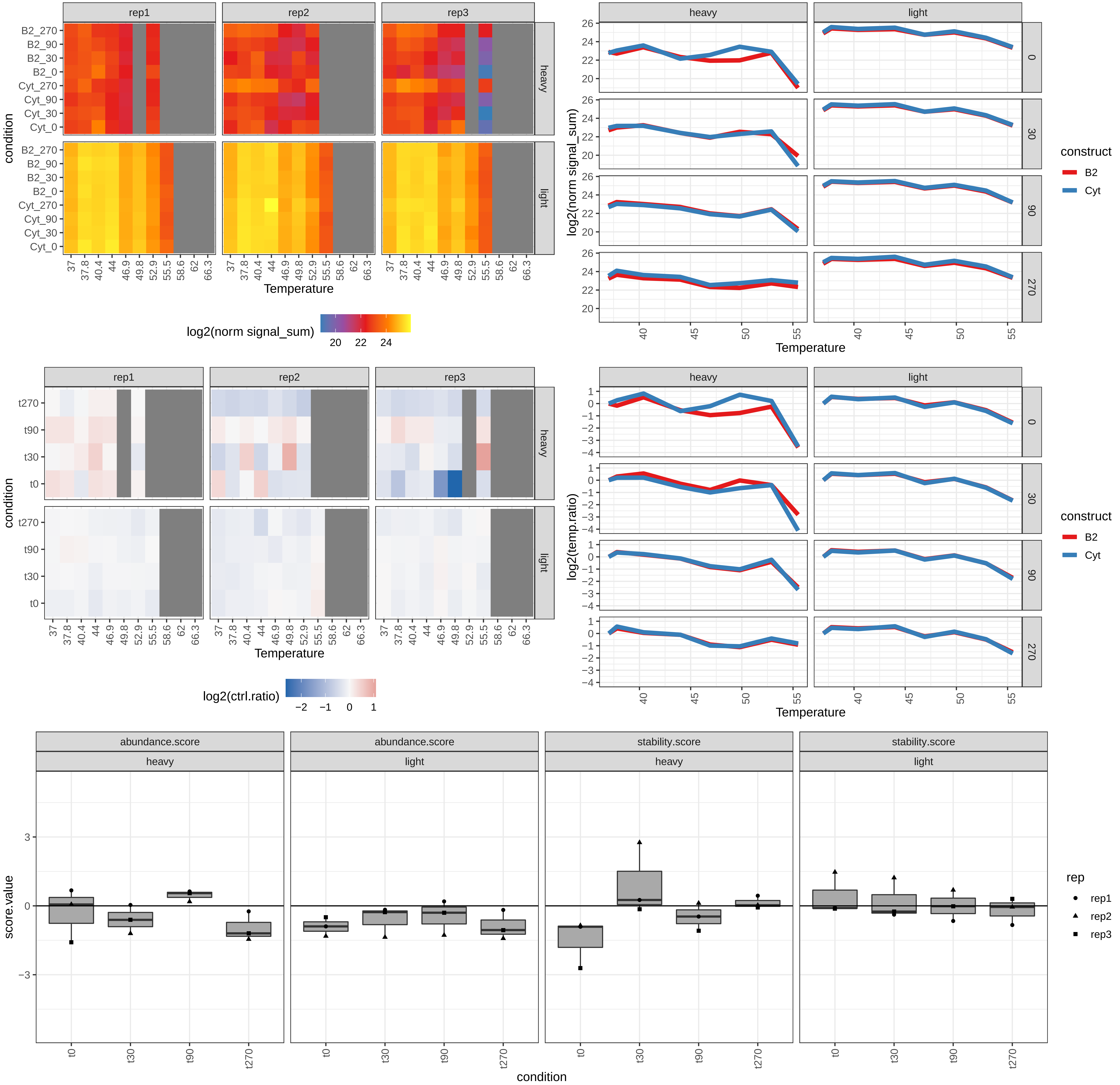

P40510 − D−3−phosphoglycerate dehydrogenase 2 OS=Saccharomyces cerevisiae (strain ATCC 204508 / S288c) GN=SER33 PE=1 SV=1

SES1 − average.top3: 7.5878053125 − max.qupm: 21

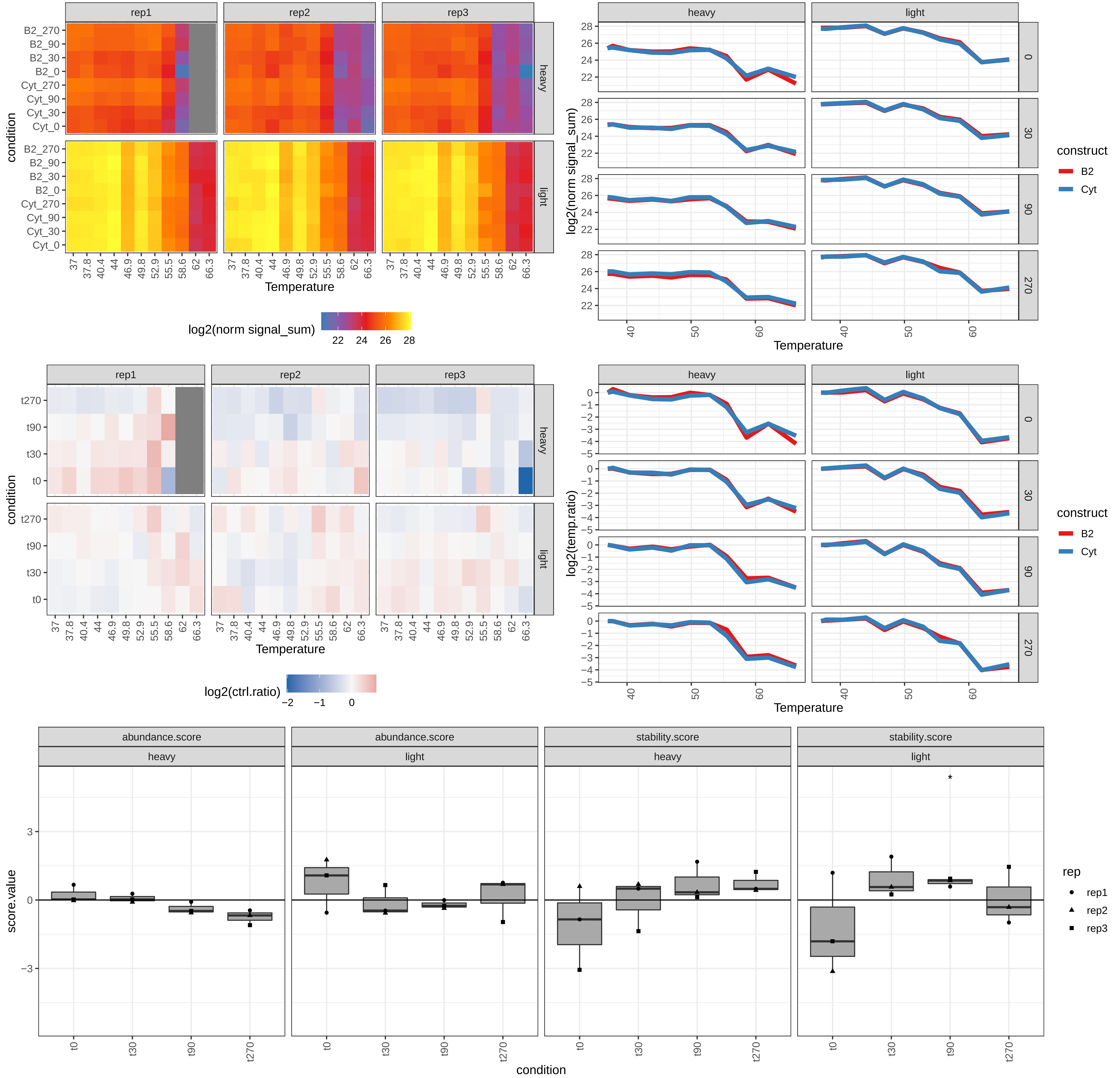

P07284 − Serine−−tRNA ligase, cytoplasmic OS=Saccharomyces cerevisiae (strain ATCC 204508 / S288c) GN=SES1 PE=1 SV=2

SET1 − average.top3: 6.693082 − max.qupm: 6

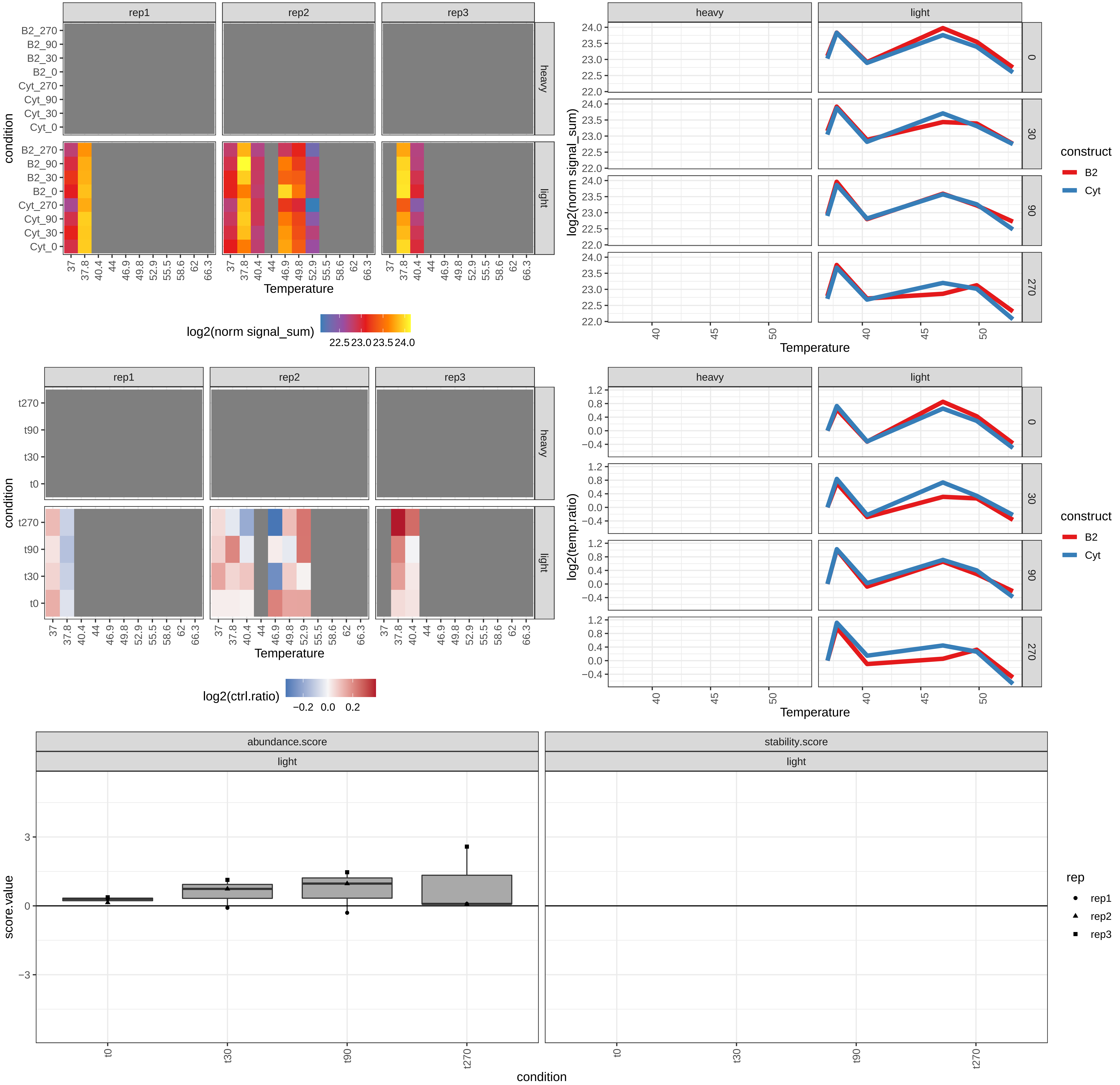

P38827 − Histone−lysine N−methyltransferase, H3 lysine−4 specific OS=Saccharomyces cerevisiae (strain ATCC 204508 / S288c) GN=SET1 PE=1 SV=1

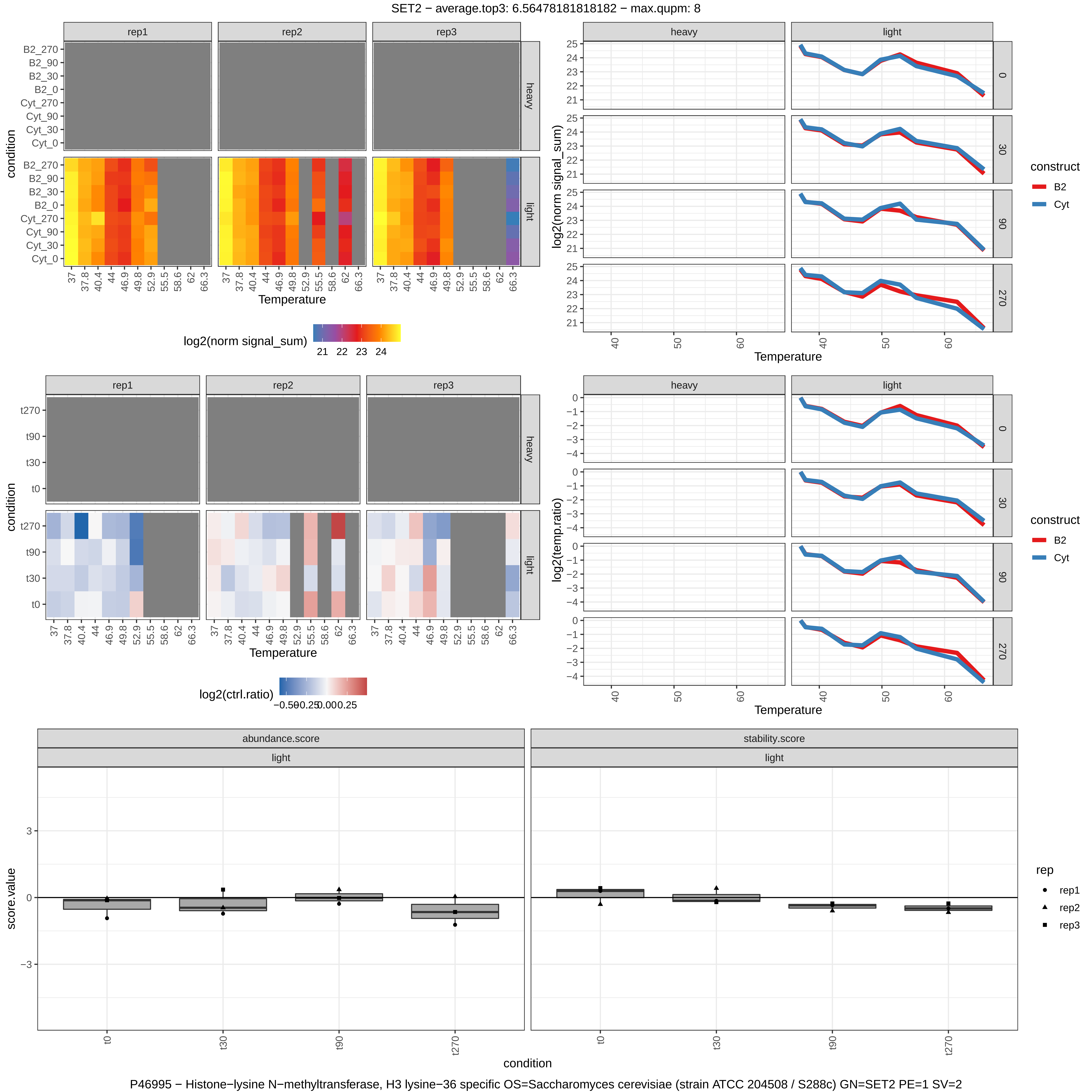
SET2 − average.top3: 6.56478181818182 − max.qupm: 8
rep1
rep2
rep3
heavy
light
condition
Temperature
log2(norm signal_sum)
log2(ctrl.ratio)
log2(temp.ratio)
construct
B2
Cyt
abundance.score
stability.score
score.value
rep
rep1
rep2
rep3
P46995 − Histone−lysine N−methyltransferase, H3 lysine−36 specific OS=Saccharomyces cerevisiae (strain ATCC 204508 / S288c) GN=SET2 PE=1 SV=2

SET3 − average.top3: 6.60519428571429 − max.qupm: 6

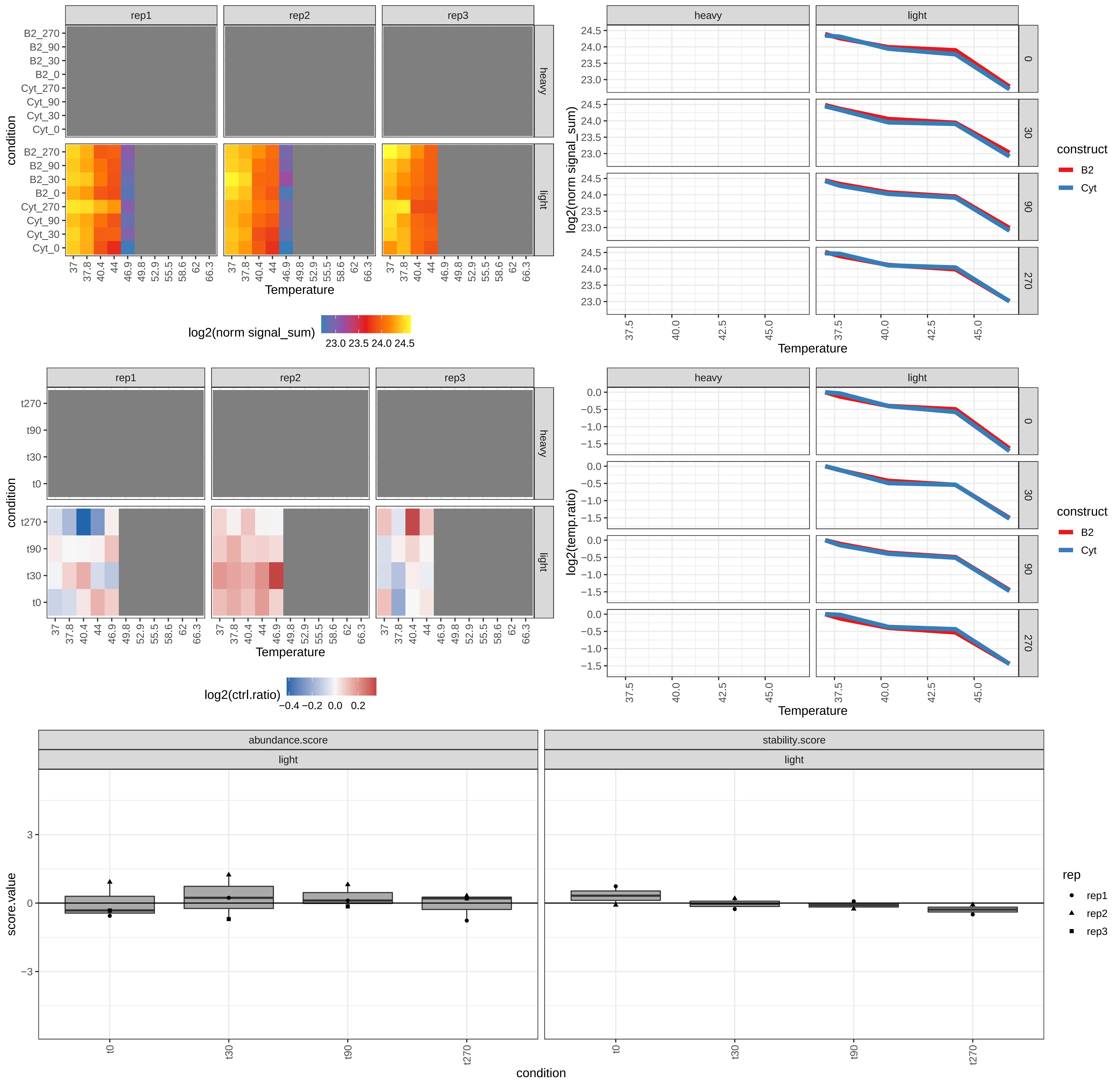

P36124 − SET domain−containing protein 3 OS=Saccharomyces cerevisiae (strain ATCC 204508 / S288c) GN=SET3 PE=1 SV=1

SET5 − average.top3: 6.7817930952381 − max.qupm: 9

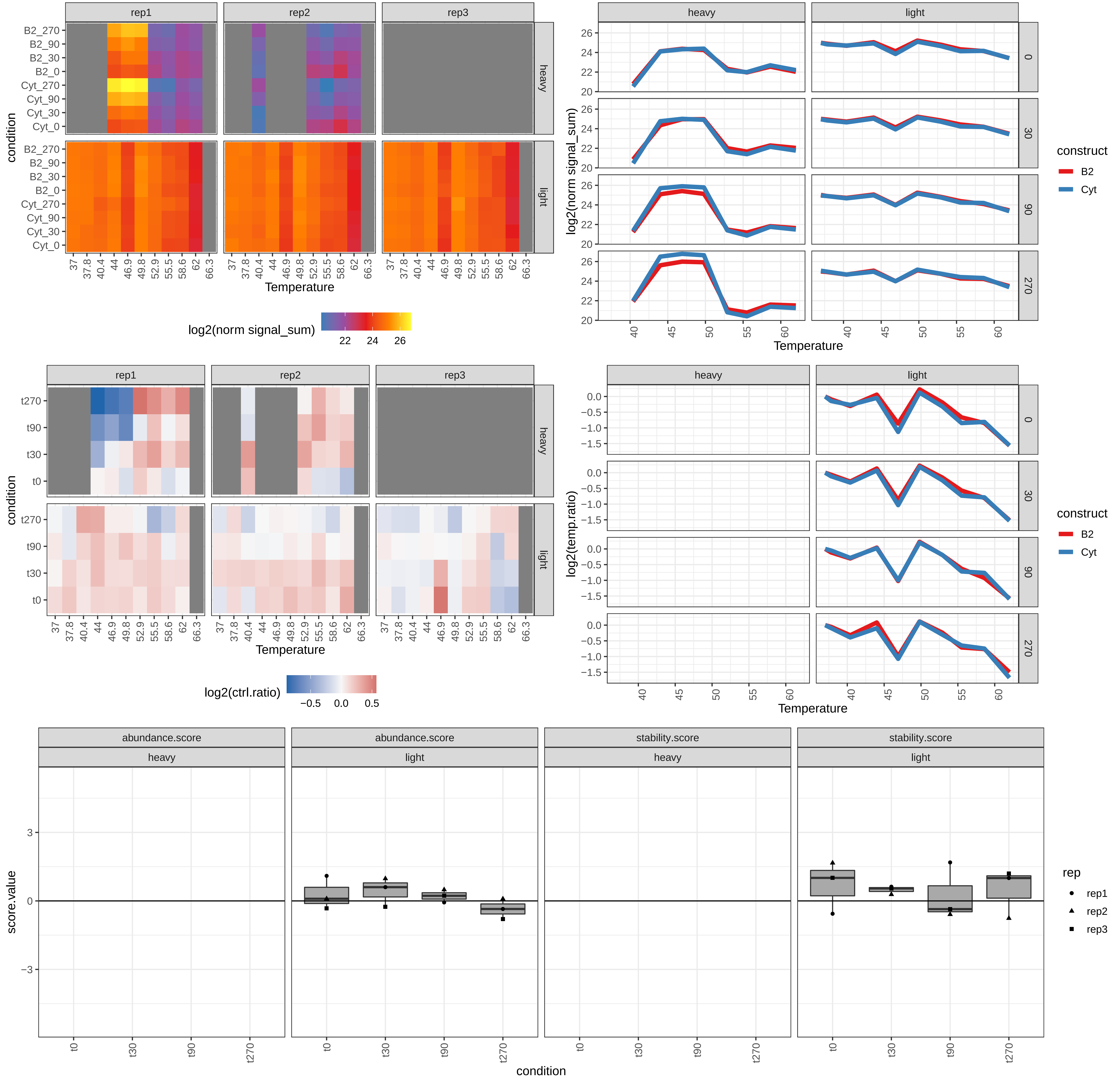

P38890 − Putative protein lysine methyltransferase SET5 OS=Saccharomyces cerevisiae (strain ATCC 204508 / S288c) GN=SET5 PE=1 SV=1

SEY1 − average.top3: 7.12792743589744 − max.qupm: 25

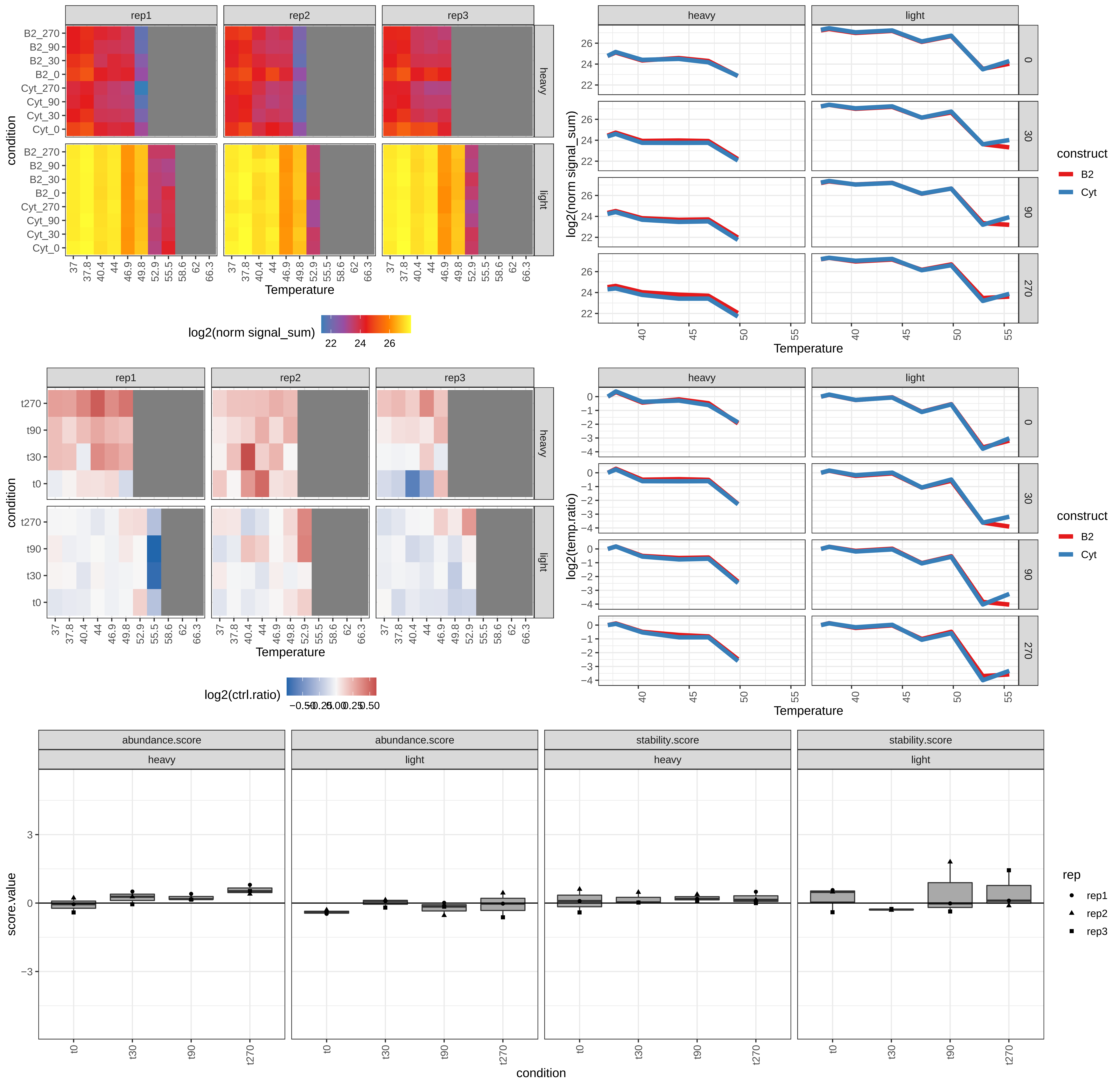

Q99287 − Protein SEY1 OS=Saccharomyces cerevisiae (strain ATCC 204508 / S288c) GN=SEY1 PE=1 SV=1

SFA1 − average.top3: 7.61971654545455 − max.qupm: 13

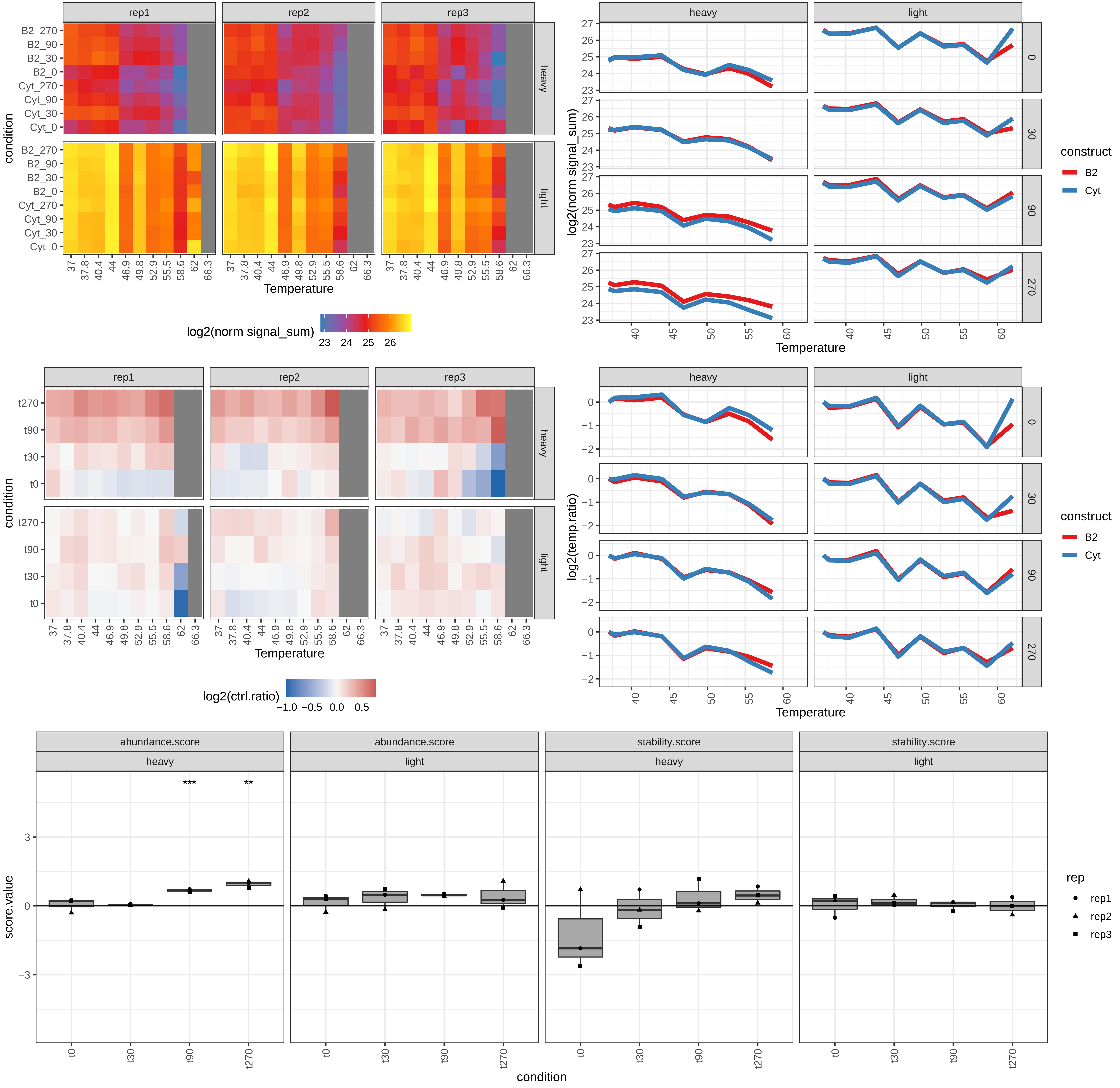

P32771 − S−(hydroxymethyl)glutathione dehydrogenase OS=Saccharomyces cerevisiae (strain ATCC 204508 / S288c) GN=SFA1 PE=1 SV=1

SFB2 − average.top3: 6.20458076923077 − max.qupm: 6

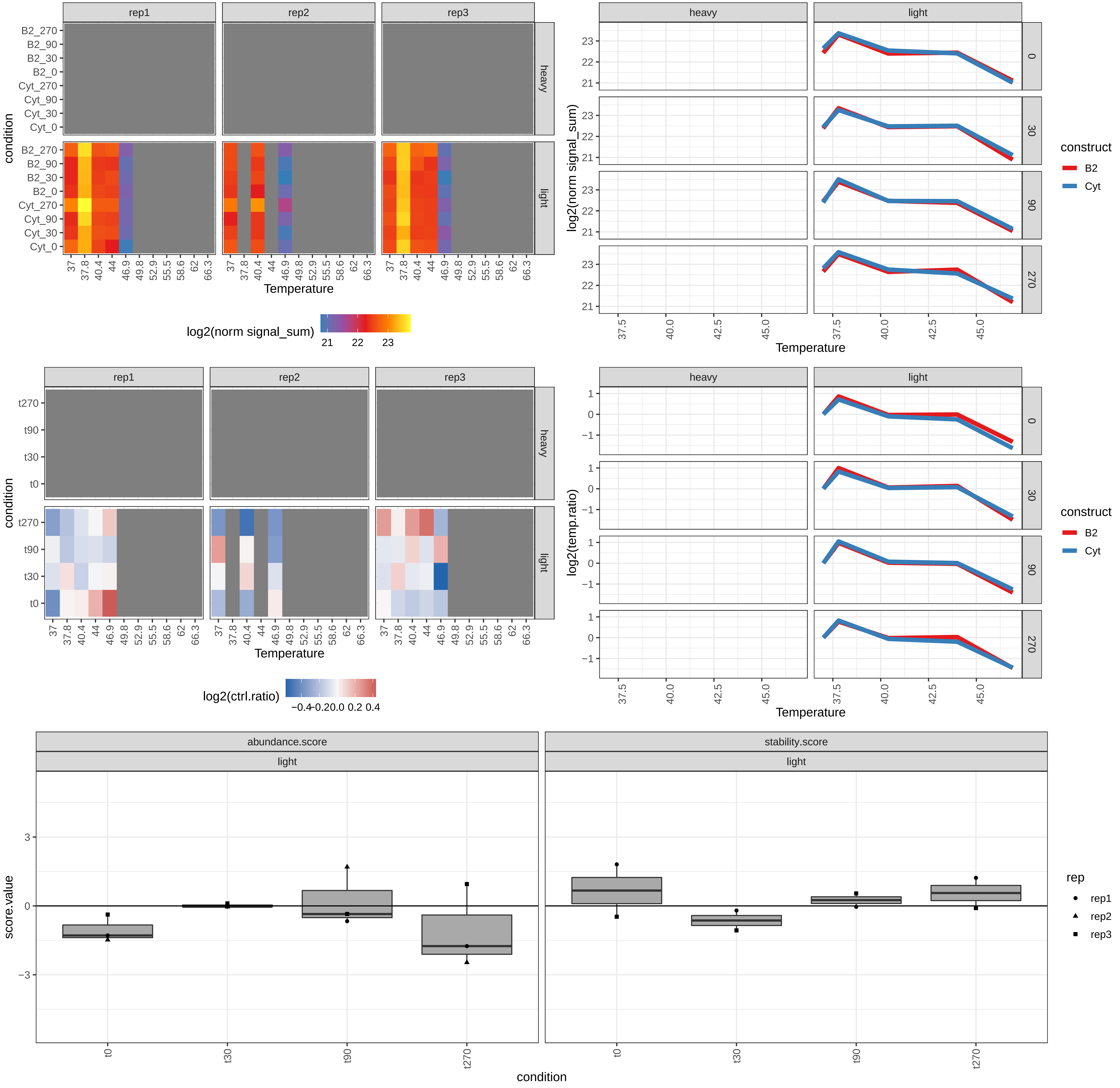

P53953 − SED5−binding protein 2 OS=Saccharomyces cerevisiae (strain ATCC 204508 / S288c) GN=SFB2 PE=1 SV=1

SFB3 − average.top3: 6.9453725 − max.qupm: 11

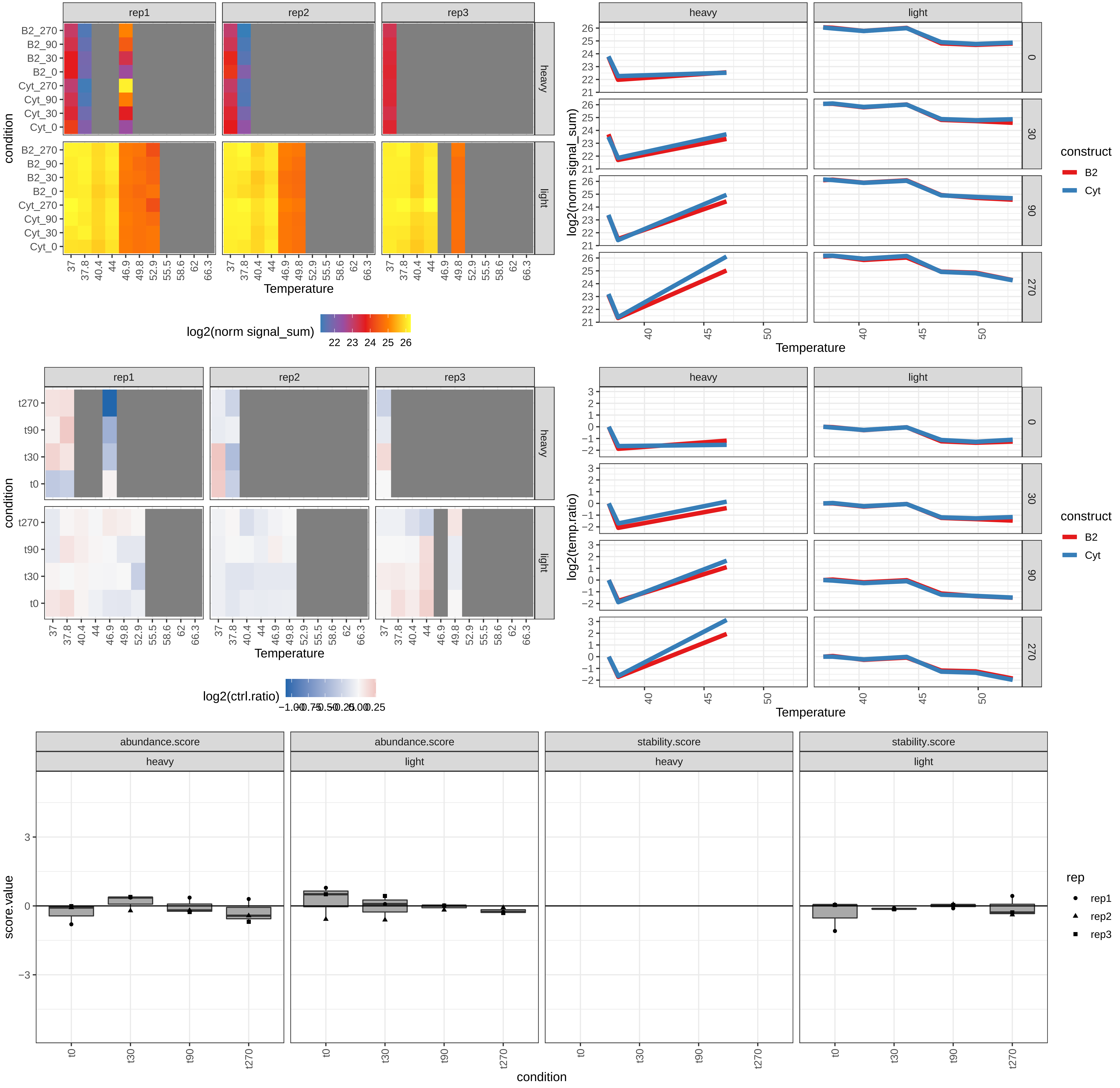

P38810 − SED5−binding protein 3 OS=Saccharomyces cerevisiae (strain ATCC 204508 / S288c) GN=SFB3 PE=1 SV=1

SFC1 − average.top3: 7.18987558139535 − max.qupm: 7

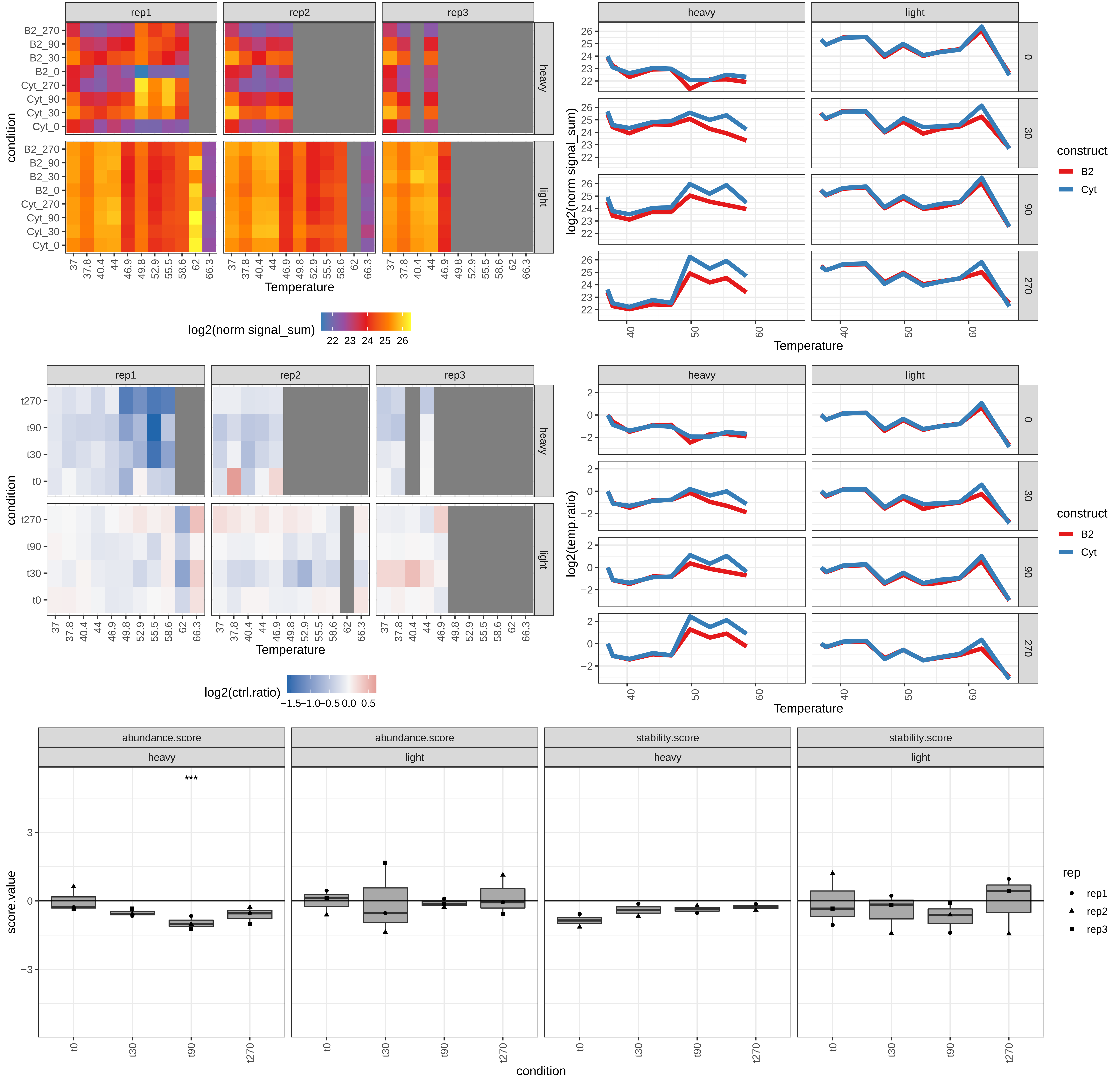

P33303 − Succinate/fumarate mitochondrial transporter OS=Saccharomyces cerevisiae (strain ATCC 204508 / S288c) GN=SFC1 PE=1 SV=2

SFH5 − average.top3: 7.1170686 − max.qupm: 13

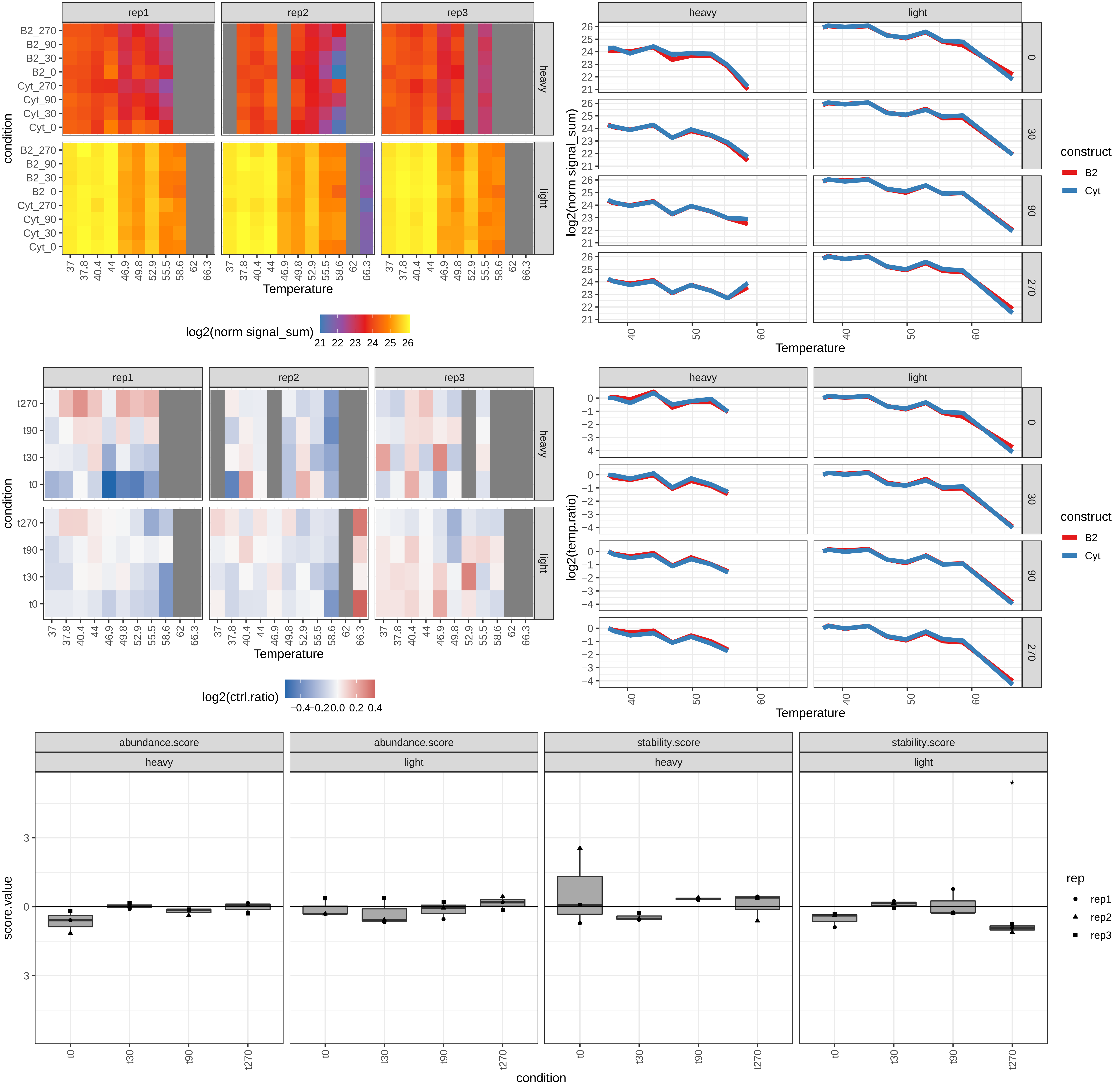

P47008 − Phosphatidylinositol transfer protein SFH5 OS=Saccharomyces cerevisiae (strain ATCC 204508 / S288c) GN=SFH5 PE=1 SV=1

SFM1 − average.top3: 7.30884673913044 − max.qupm: 14

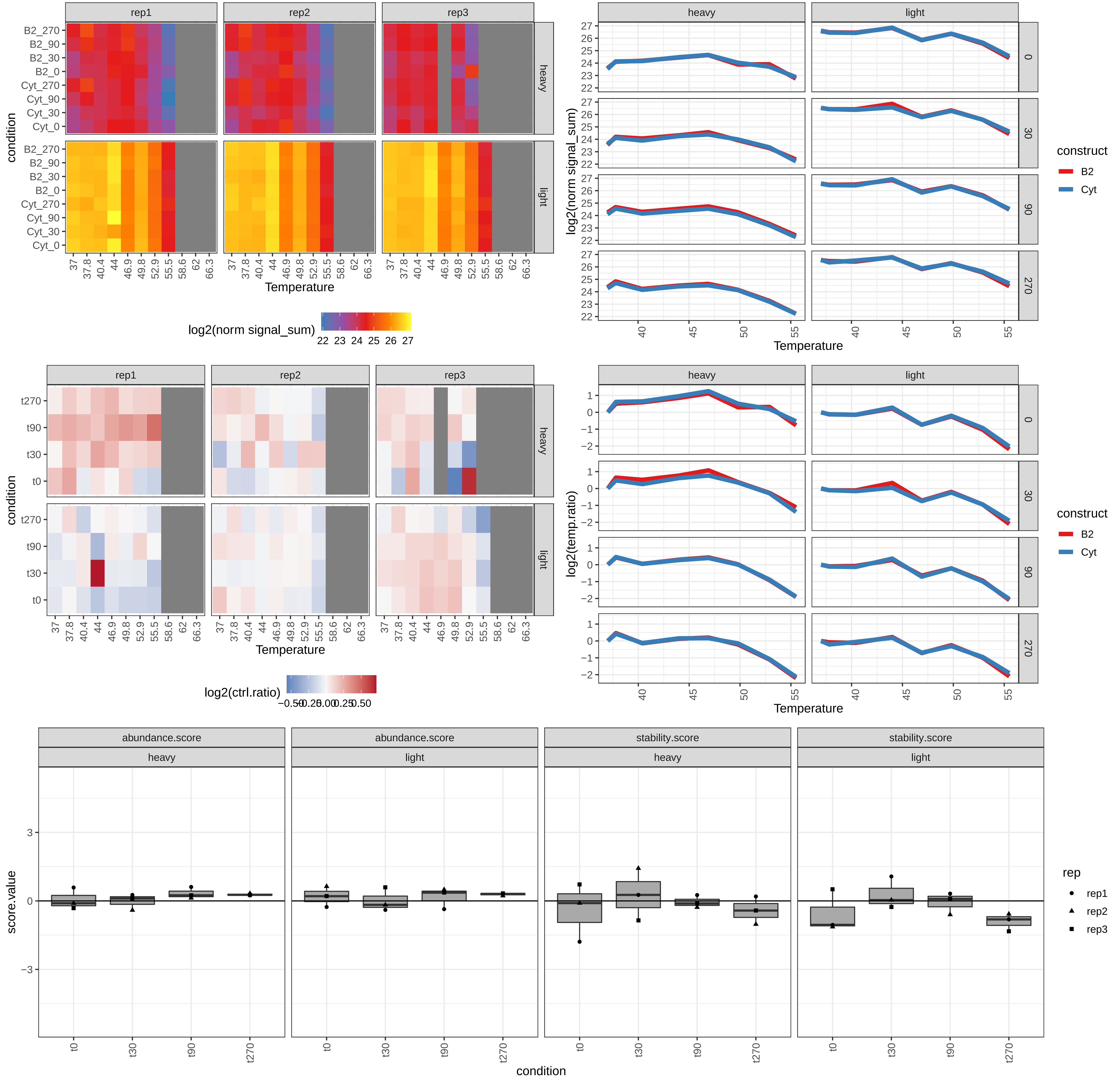

Q12314 − Protein arginine N−methyltransferase SFM1 OS=Saccharomyces cerevisiae (strain ATCC 204508 / S288c) GN=SFM1 PE=1 SV=1

SFT2 − average.top3: 6.61344260869565 − max.qupm: 3

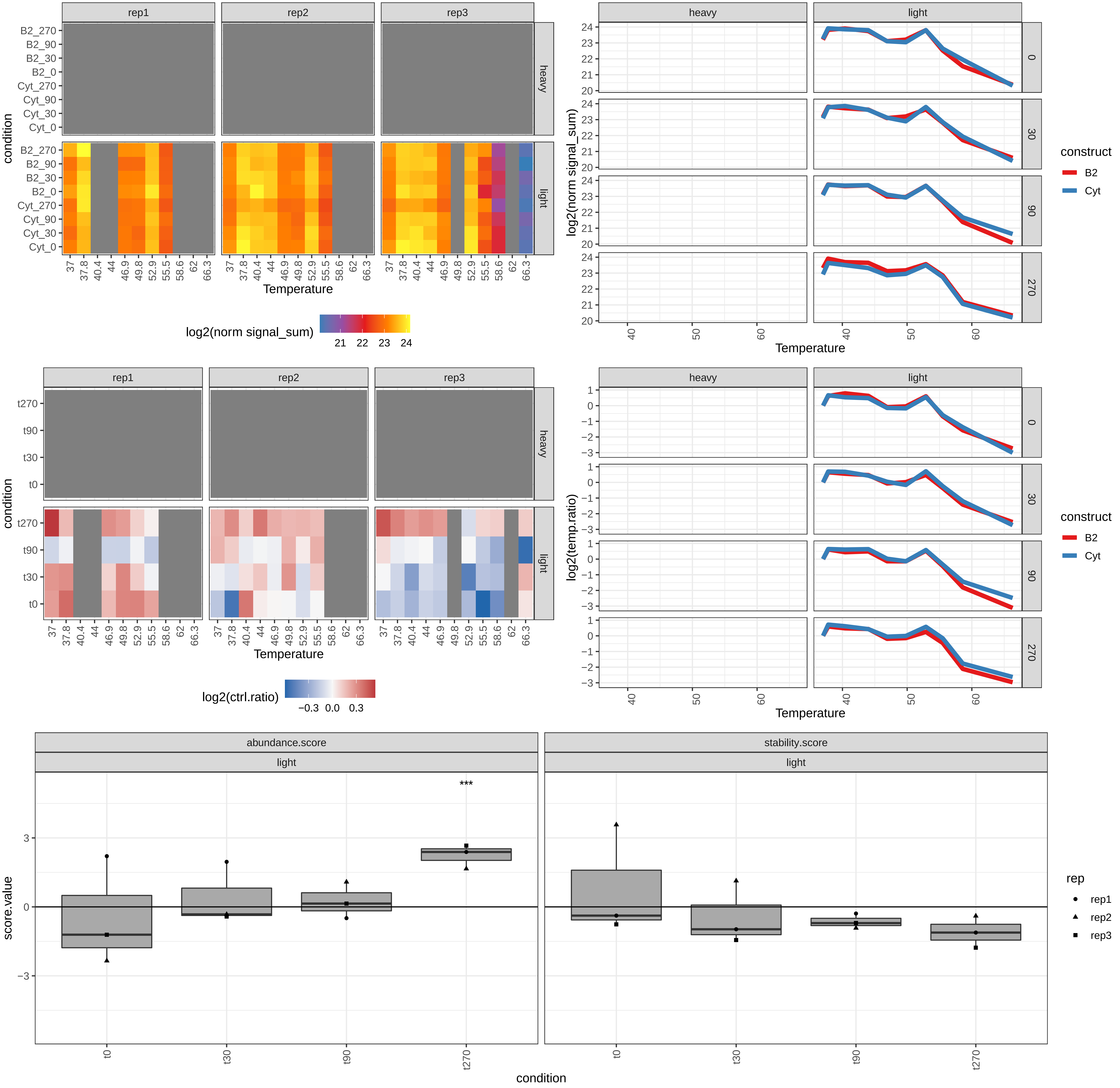

P38166 − Protein transport protein SFT2 OS=Saccharomyces cerevisiae (strain ATCC 204508 / S288c) GN=SFT2 PE=1 SV=1

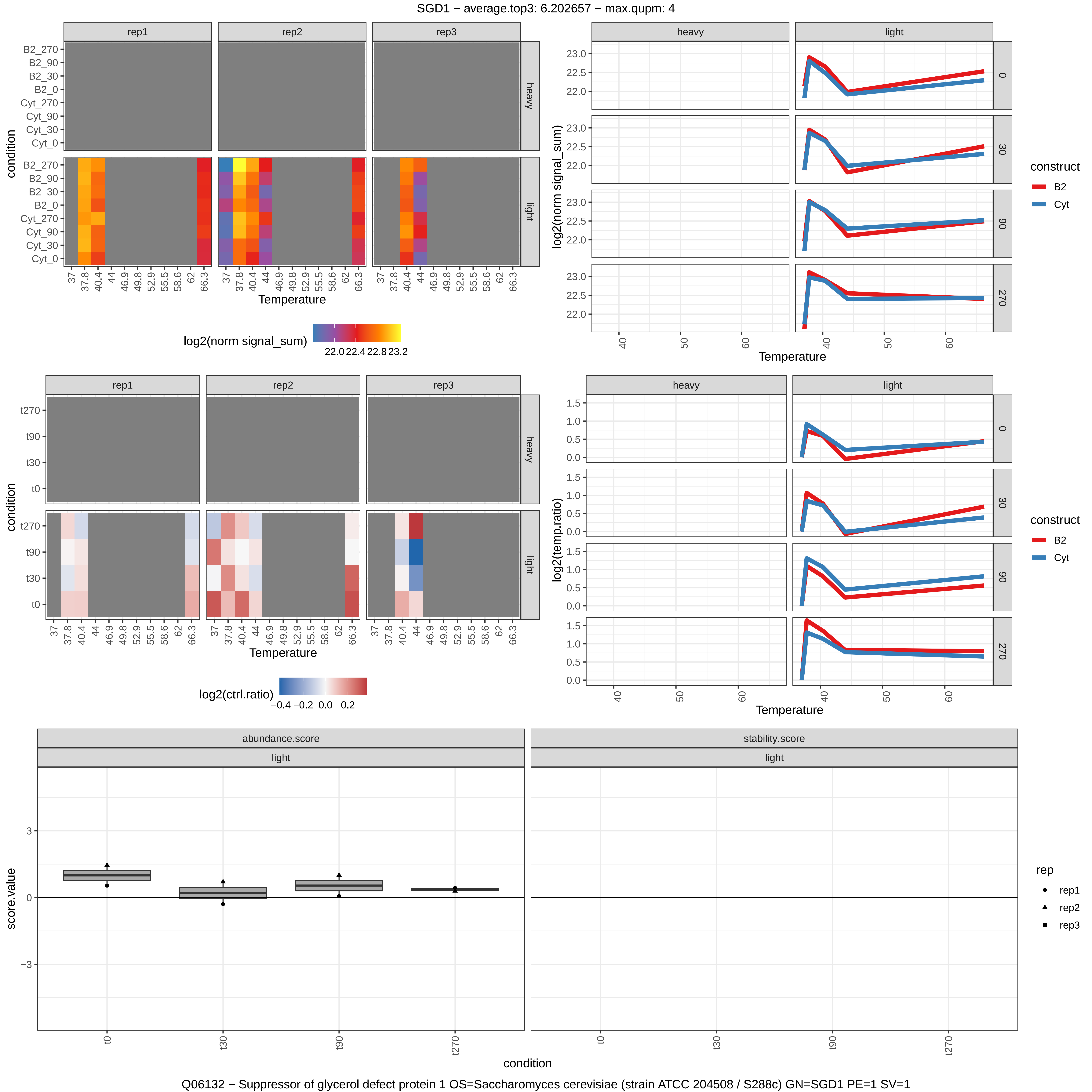
SGD1 − average.top3: 6.202657 − max.qupm: 4
rep1
rep2
rep3
heavy
light
condition
Temperature
log2(norm signal_sum)
construct
B2
Cyt
log2(ctrl.ratio)
log2(temp.ratio)
abundance.score
stability.score
score.value
rep
Q06132 − Suppressor of glycerol defect protein 1 OS=Saccharomyces cerevisiae (strain ATCC 204508 / S288c) GN=SGD1 PE=1 SV=1

SGF73 − average.top3: 6.36970357142857 − max.qupm: 4

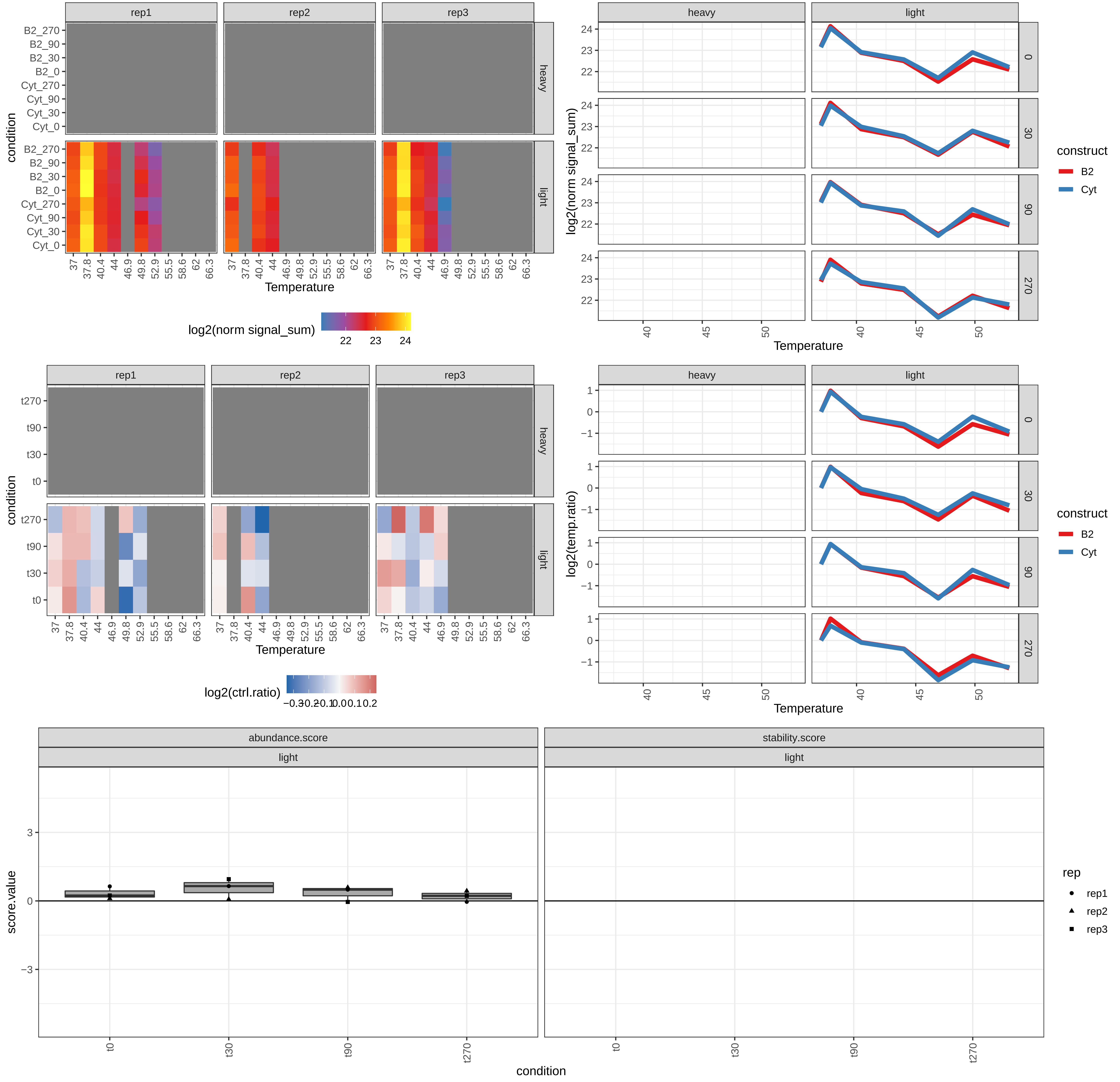

P53165 − SAGA−associated factor 73 OS=Saccharomyces cerevisiae (strain ATCC 204508 / S288c) GN=SGF73 PE=1 SV=1

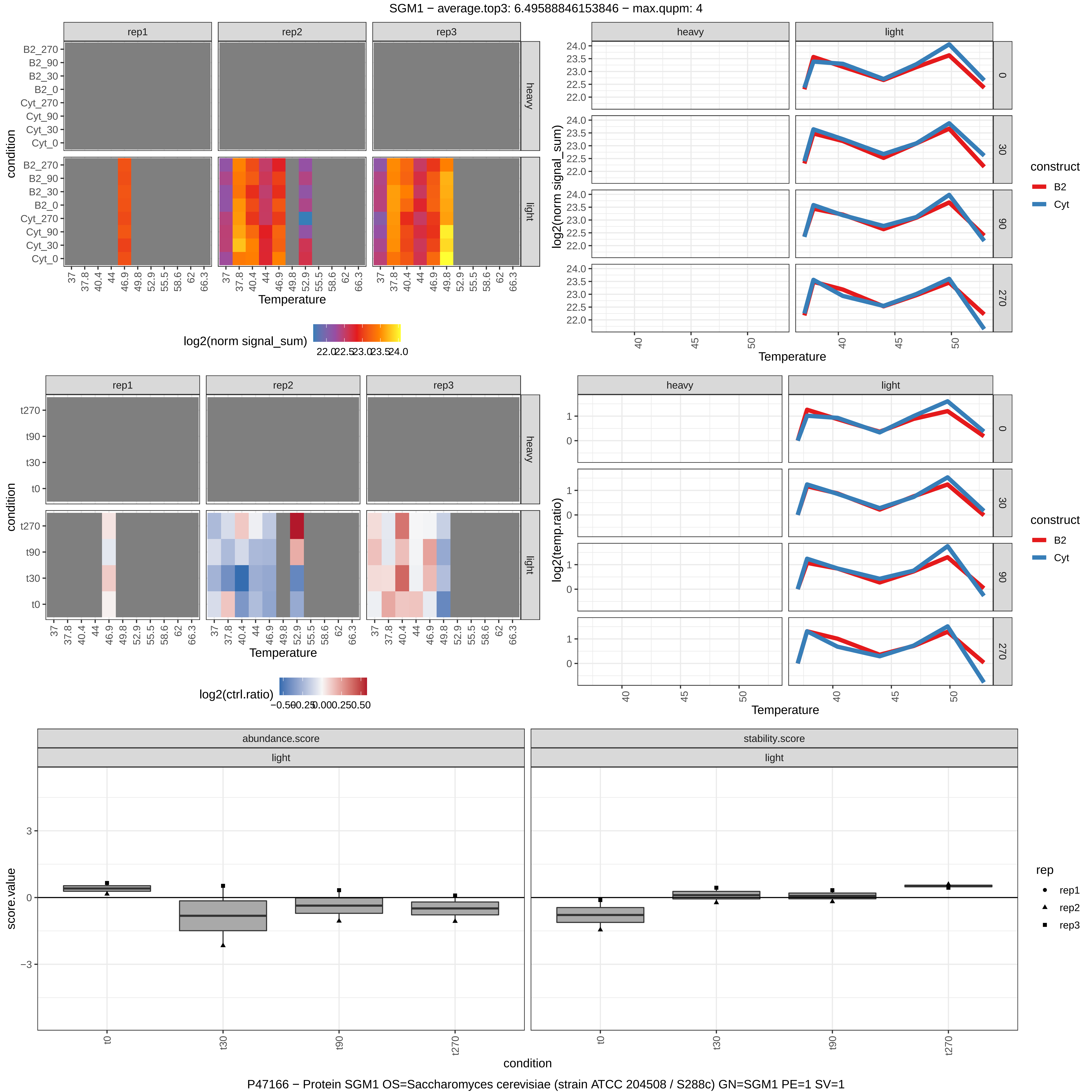
SGM1 − average.top3: 6.49588846153846 − max.qupm: 4
rep1
rep2
rep3
heavy
light
B2_270
B2_90
B2_30
B2_0
Cyt_270
Cyt_90
Cyt_30
Cyt_0
condition
37
37.8
40.4
44
46.9
49.8
52.9
55.5
58.6
62
66.3
Temperature
log2(norm signal_sum)
22.0
22.5
23.0
23.5
24.0
log2(norm signal_sum)
construct
B2
Cyt
0
30
90
270
40
45
50
t270
t90
t30
t0
log2(ctrl.ratio)
−0.50
−0.25
0.00
0.25
0.50
log2(temp.ratio)
abundance.score
stability.score
score.value
3
0
−3
rep
rep1
rep2
rep3
P47166 − Protein SGM1 OS=Saccharomyces cerevisiae (strain ATCC 204508 / S288c) GN=SGM1 PE=1 SV=1

SGN1 − average.top3: 6.586599 − max.qupm: 4

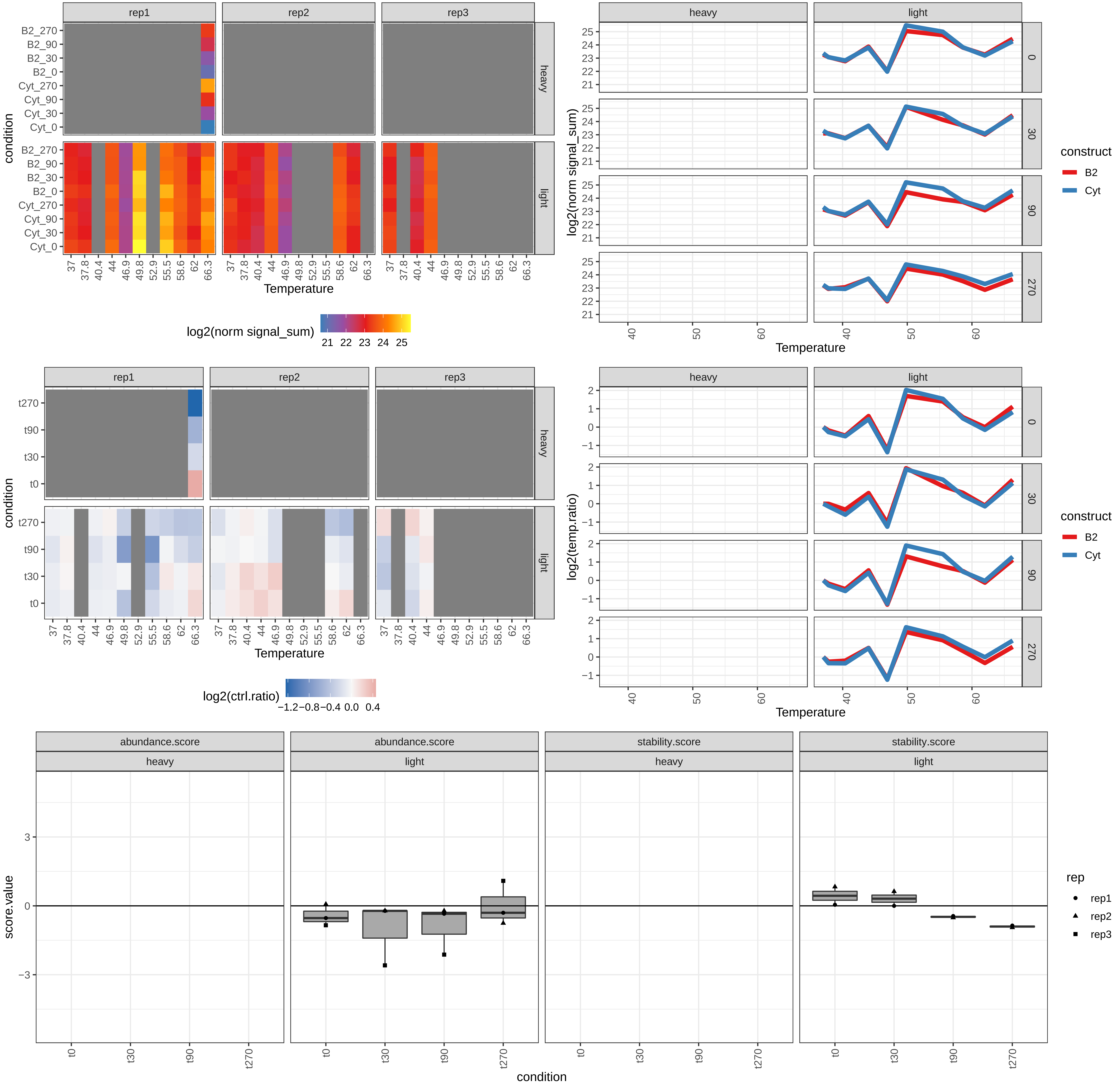

P40561 − RNA−binding protein SGN1 OS=Saccharomyces cerevisiae (strain ATCC 204508 / S288c) GN=SGN1 PE=1 SV=1

SGT1 − average.top3: 6.80607869565217 − max.qupm: 8

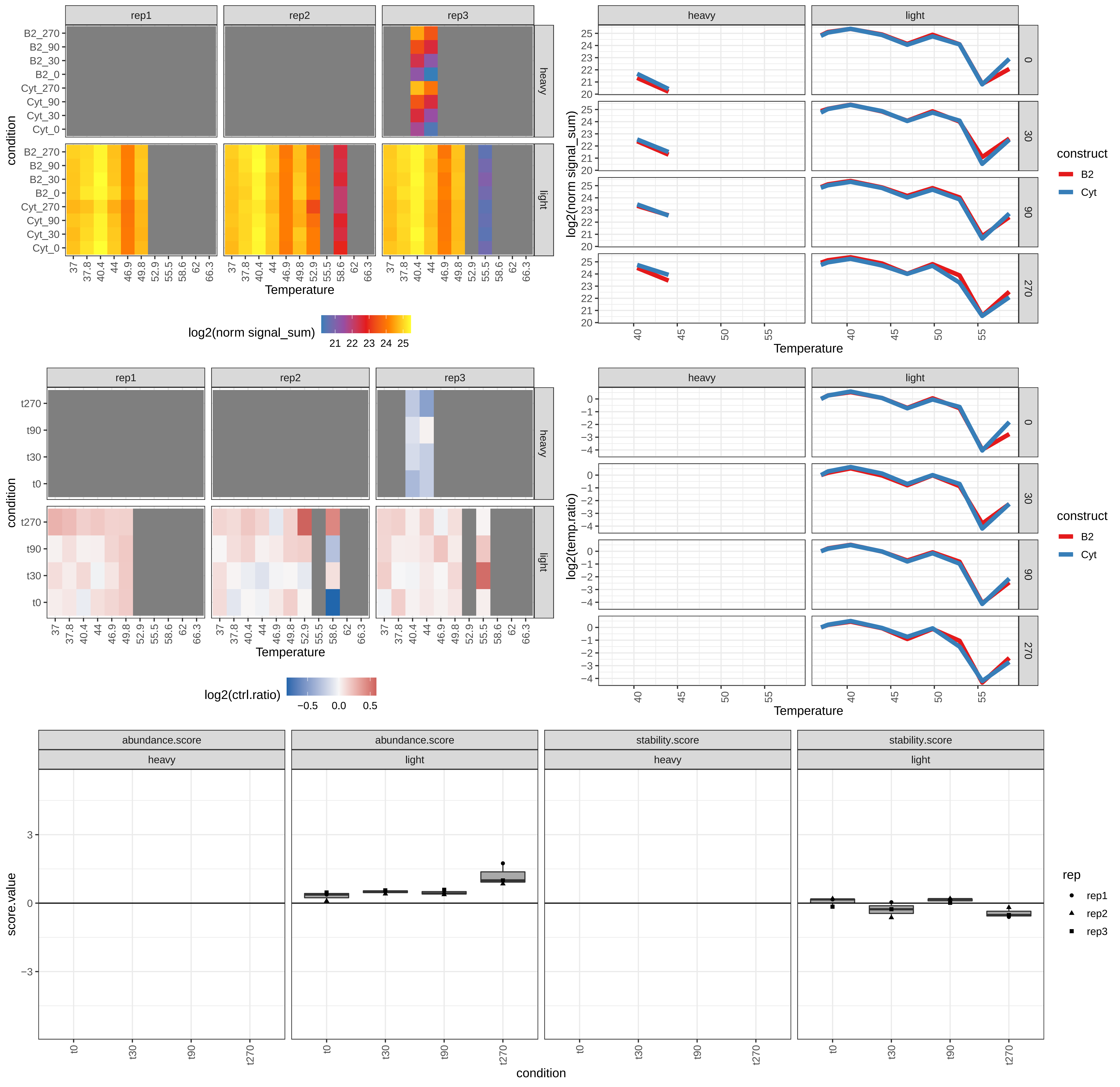

Q08446 − Protein SGT1 OS=Saccharomyces cerevisiae (strain ATCC 204508 / S288c) GN=SGT1 PE=1 SV=1

SGT2 − average.top3: 7.07674966101695 − max.qupm: 13

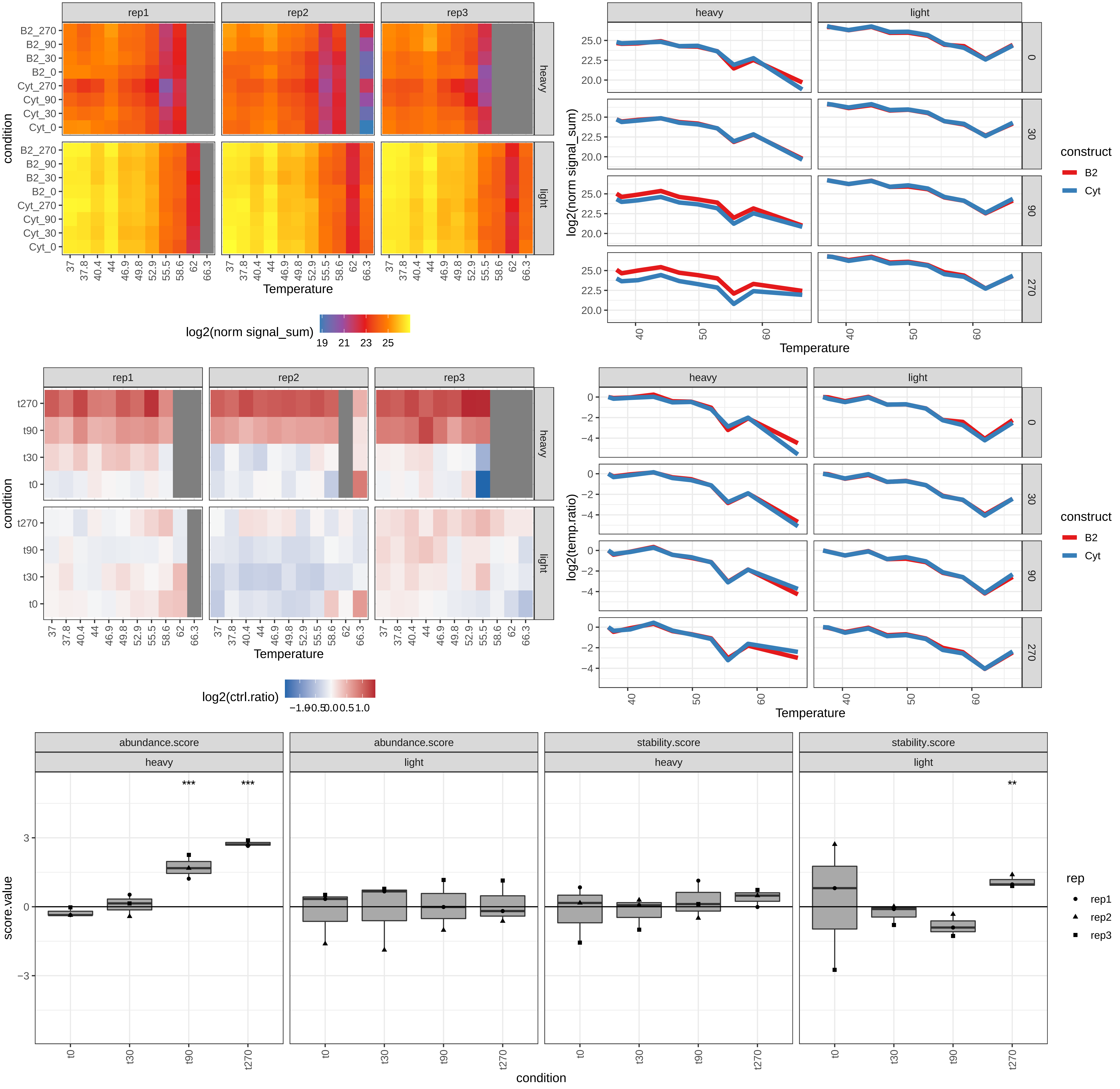

Q12118 − Small glutamine−rich tetratricopeptide repeat−containing protein 2 OS=Saccharomyces cerevisiae (strain ATCC 204508 / S288c) GN=SGT2 PE=1 SV=1

SGV1 − average.top3: 6.90618809523809 − max.qupm: 10

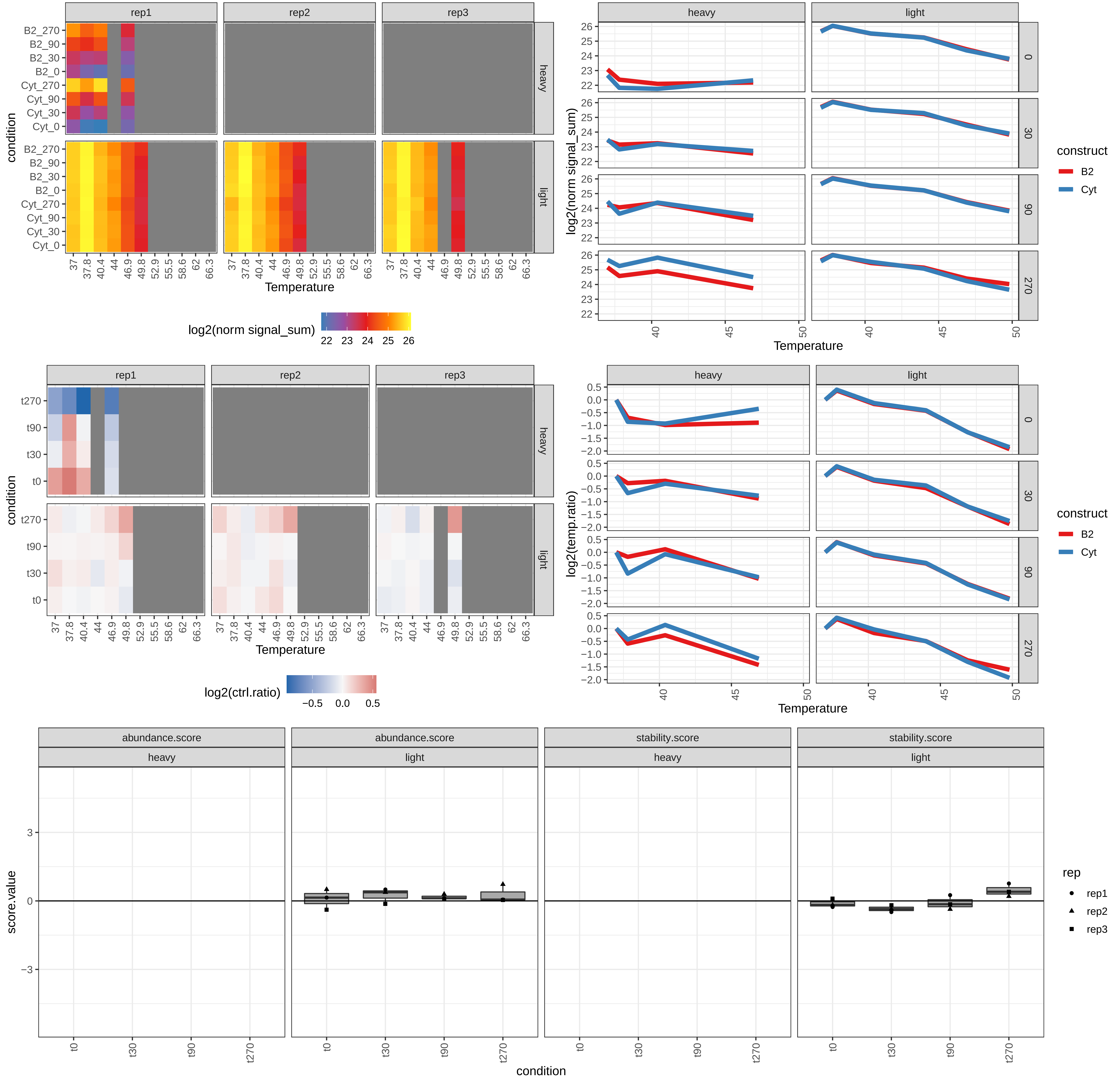

P23293 − Serine/threonine−protein kinase BUR1 OS=Saccharomyces cerevisiae (strain ATCC 204508 / S288c) GN=SGV1 PE=1 SV=1

SHB17 − average.top3: 7.2077937254902 − max.qupm: 10

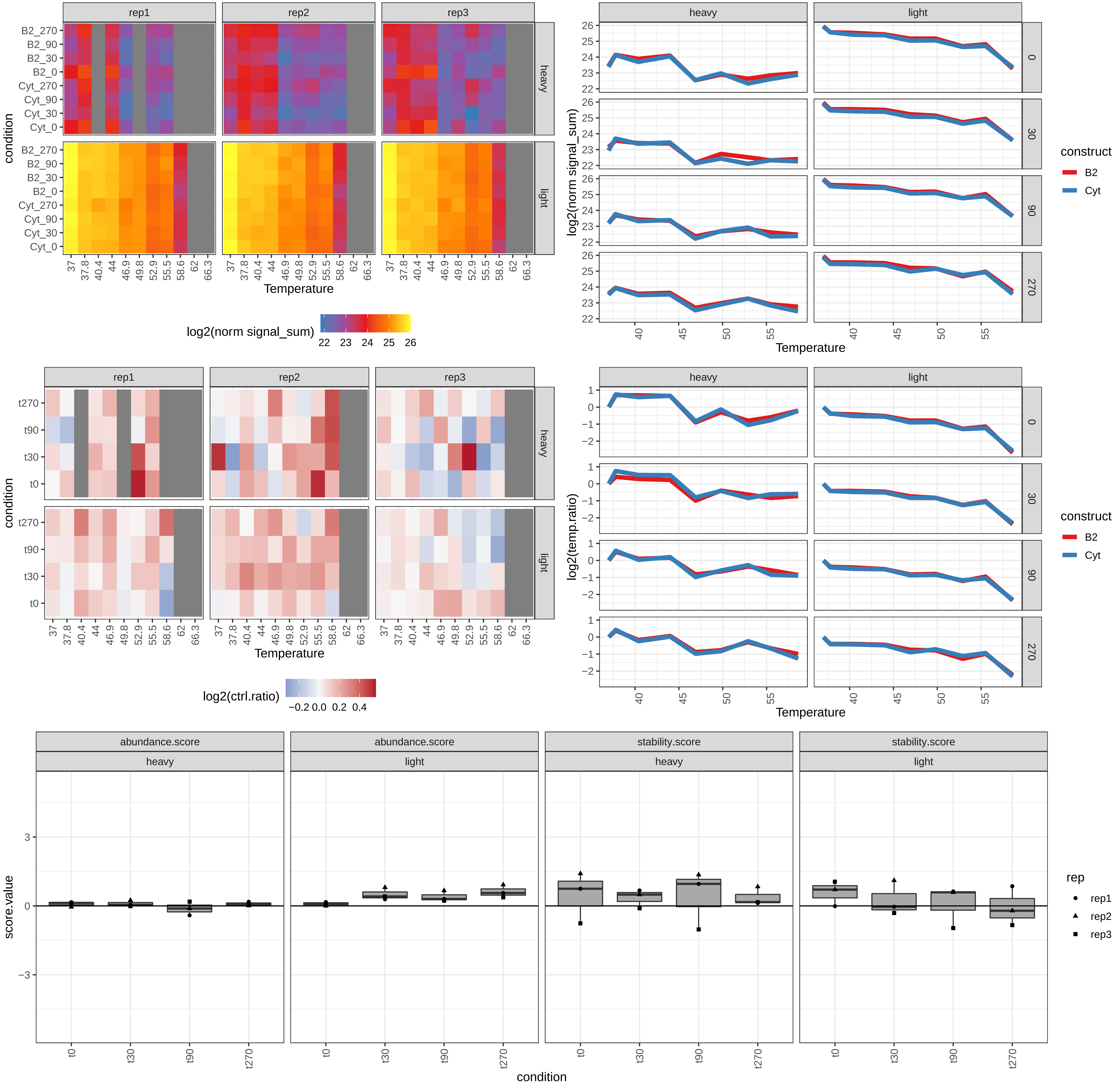

P36136 − Sedoheptulose 1,7−bisphosphatase OS=Saccharomyces cerevisiae (strain ATCC 204508 / S288c) GN=SHB17 PE=1 SV=1

SHE10 − average.top3: 7.12672056603774 − max.qupm: 15

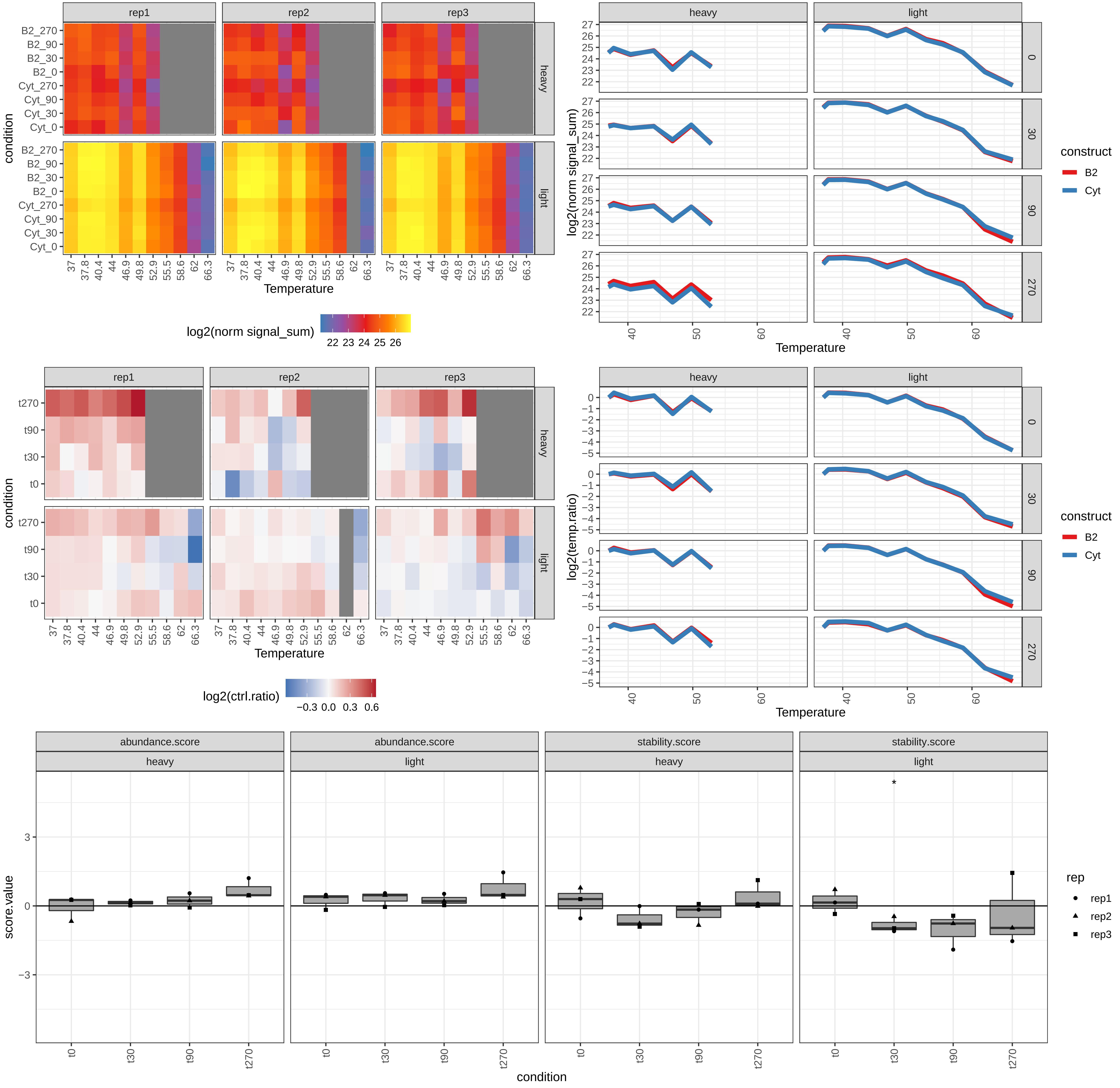

P53075 − Outer spore wall assembly protein SHE10 OS=Saccharomyces cerevisiae (strain ATCC 204508 / S288c) GN=SHE10 PE=1 SV=1

SHE4 − average.top3: 6.67212884615385 − max.qupm: 10

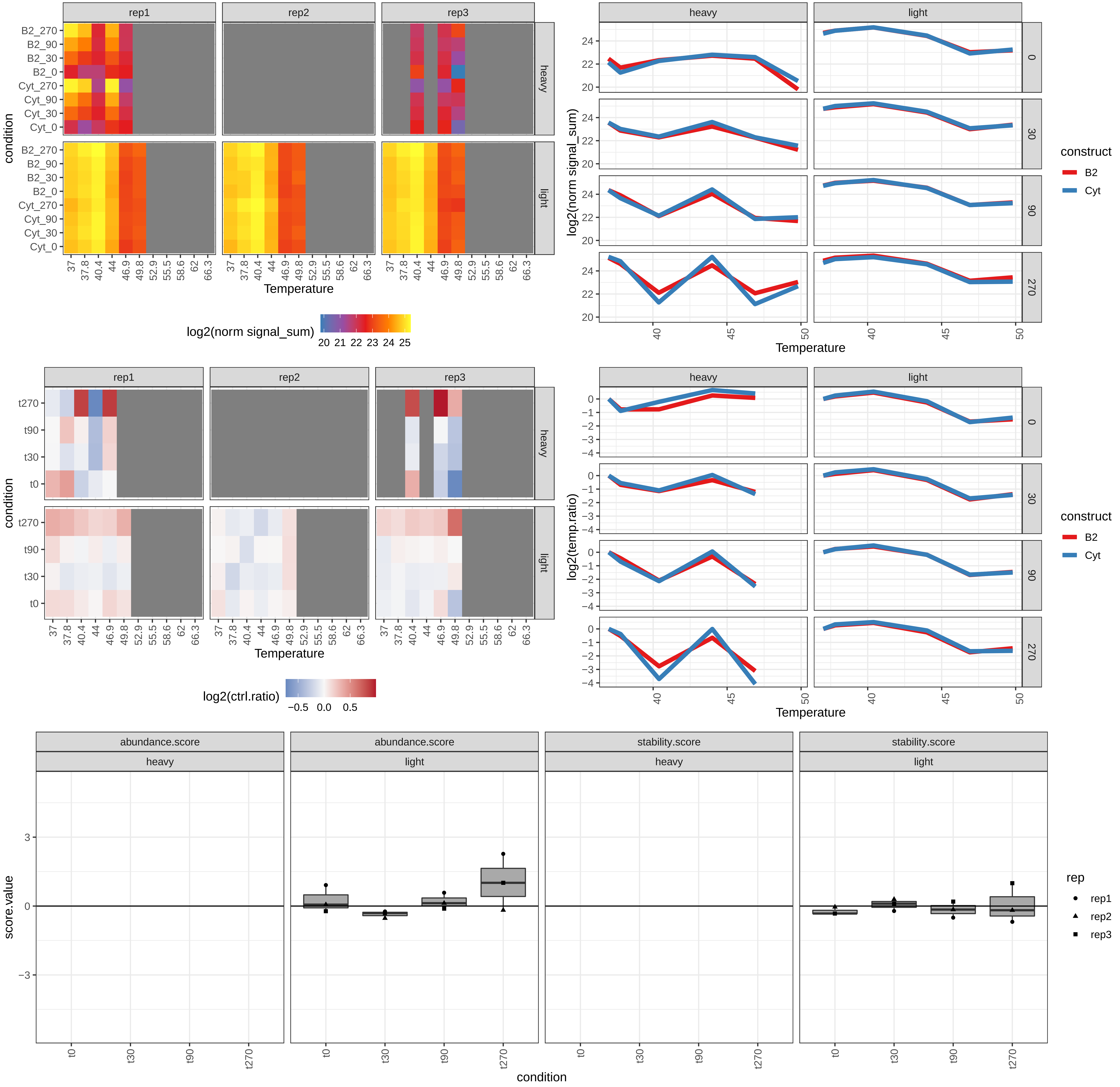

P51534 − SWI5−dependent HO expression protein 4 OS=Saccharomyces cerevisiae (strain ATCC 204508 / S288c) GN=SHE4 PE=1 SV=2

SHE9 − average.top3: 6.64403733333333 − max.qupm: 5

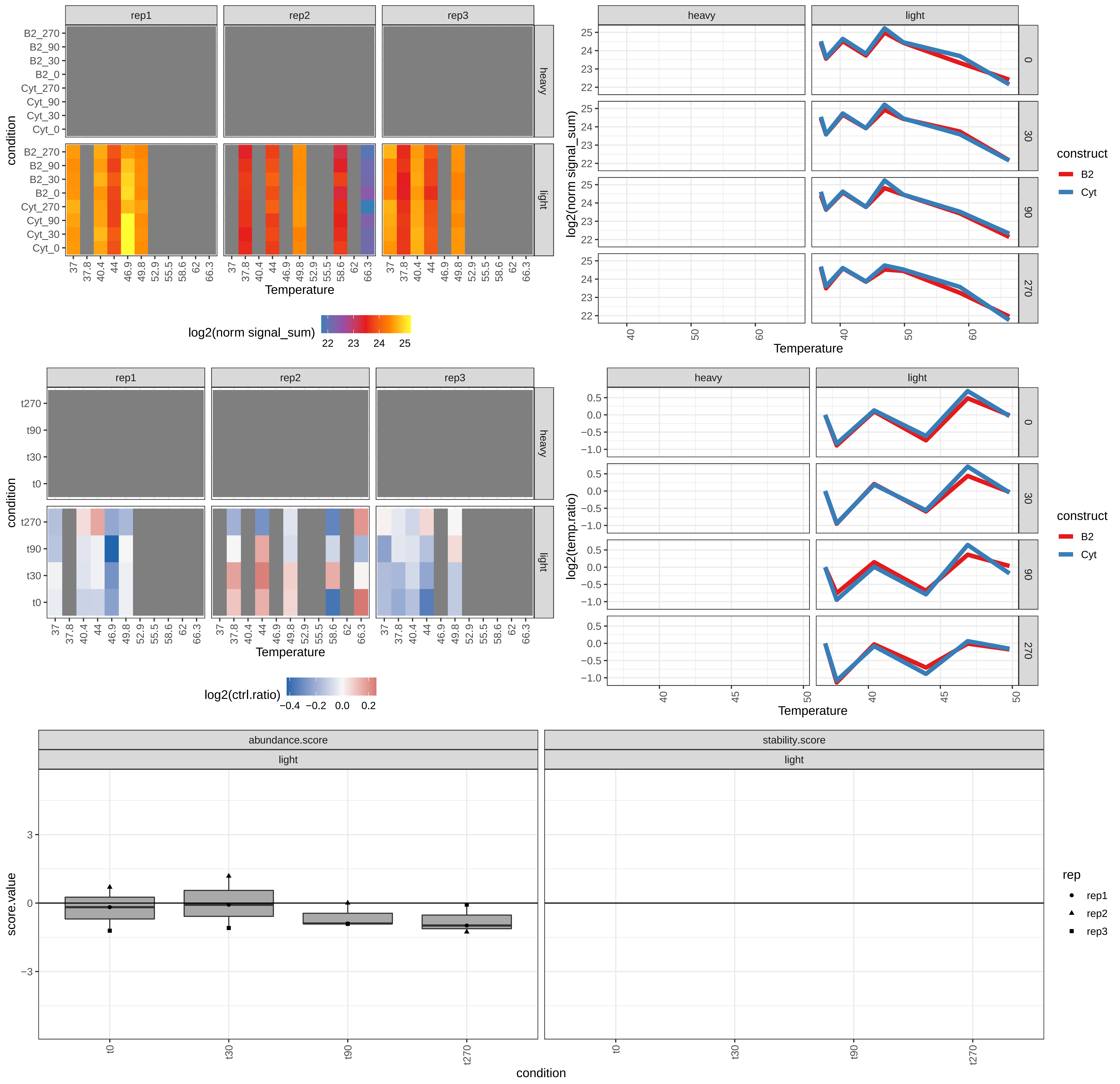

Q04172 − Sensitive to high expression protein 9, mitochondrial OS=Saccharomyces cerevisiae (strain ATCC 204508 / S288c) GN=SHE9 PE=1 SV=1

SHM1 − average.top3: 7.89785439393939 − max.qupm: 25

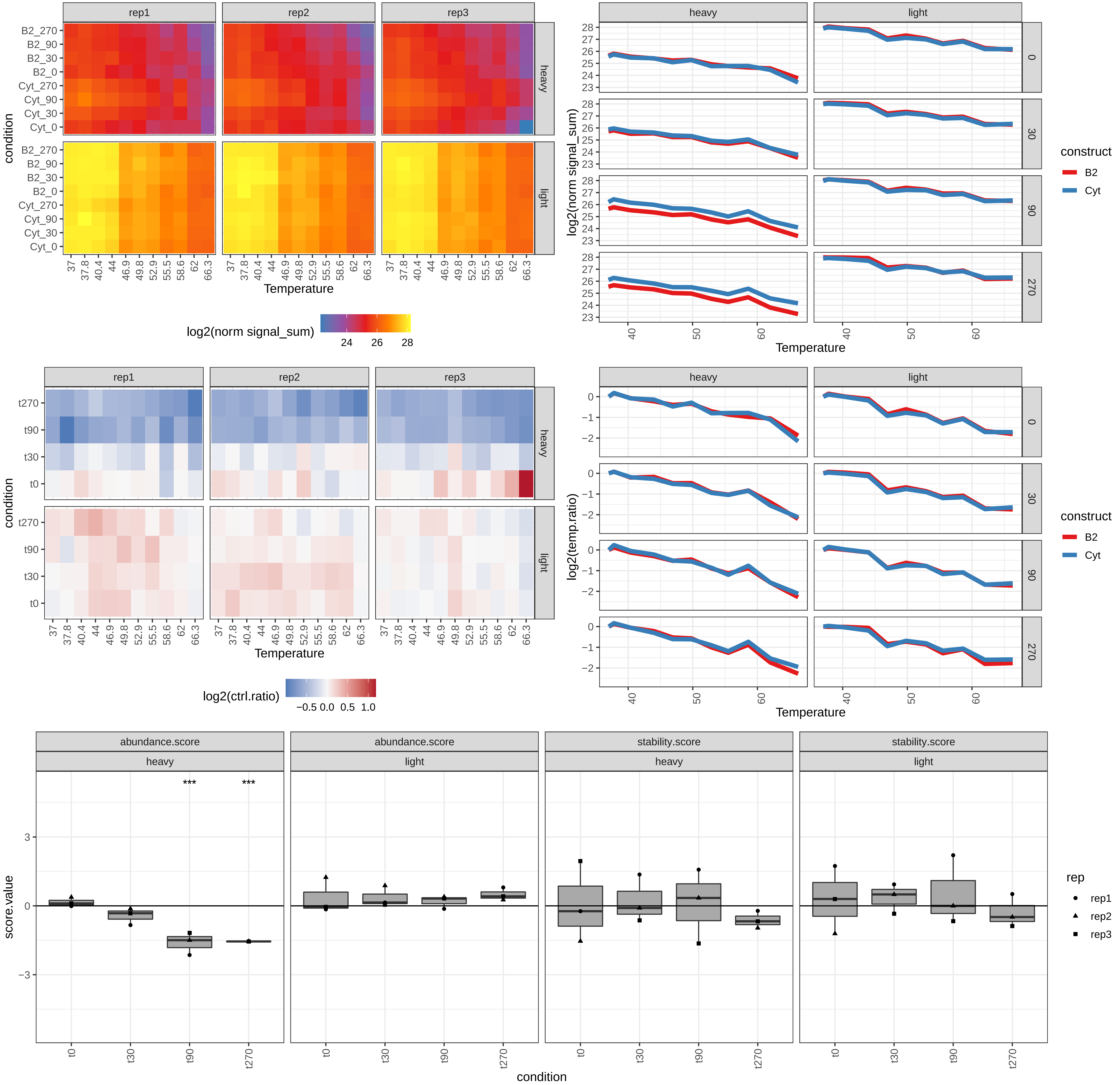

P37292 − Serine hydroxymethyltransferase, mitochondrial OS=Saccharomyces cerevisiae (strain ATCC 204508 / S288c) GN=SHM1 PE=1 SV=2

SHM2 − average.top3: 7.57000426229508 − max.qupm: 21

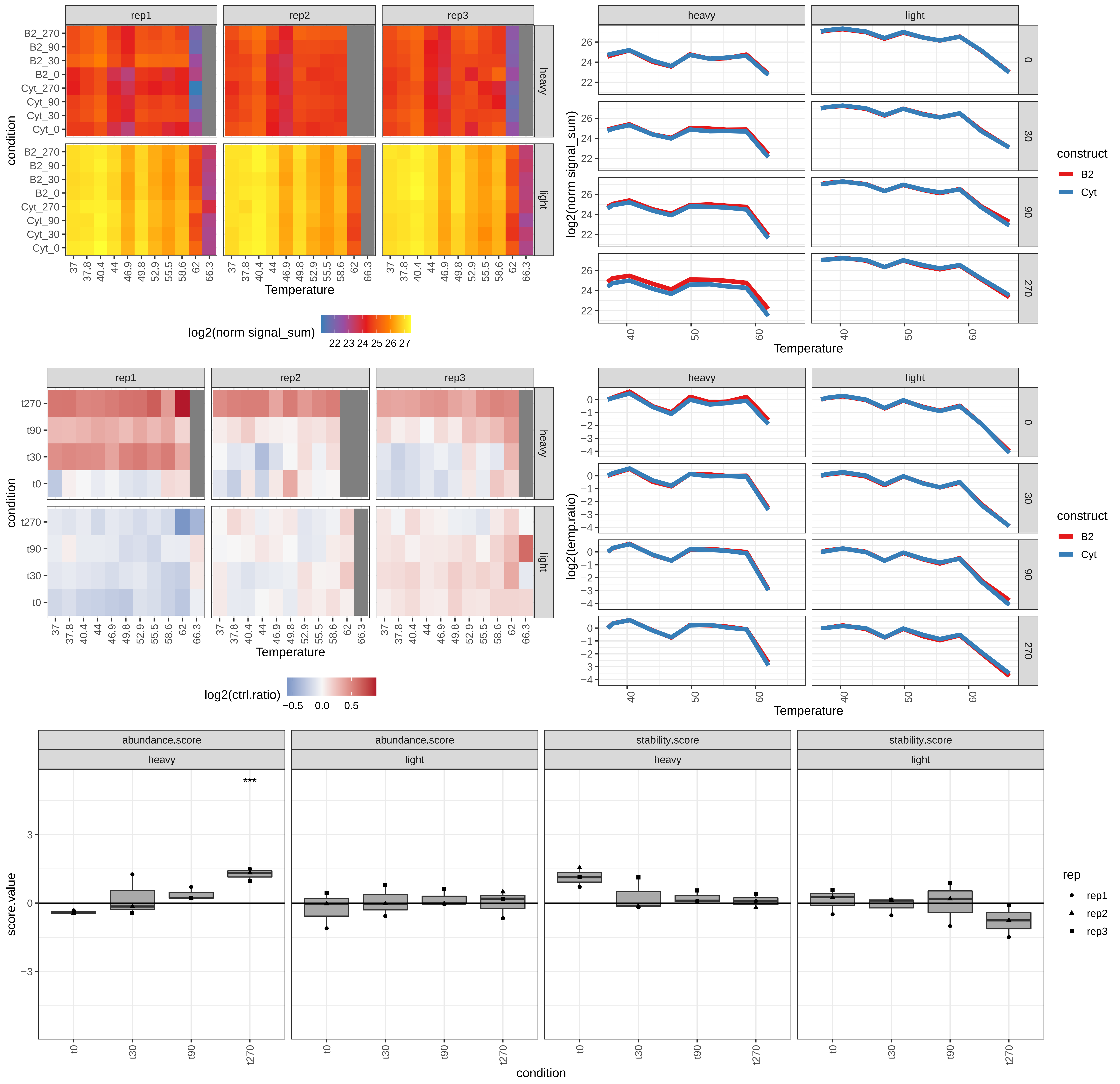

P37291 − Serine hydroxymethyltransferase, cytosolic OS=Saccharomyces cerevisiae (strain ATCC 204508 / S288c) GN=SHM2 PE=1 SV=2

SHP1 − average.top3: 6.70240697674419 − max.qupm: 7

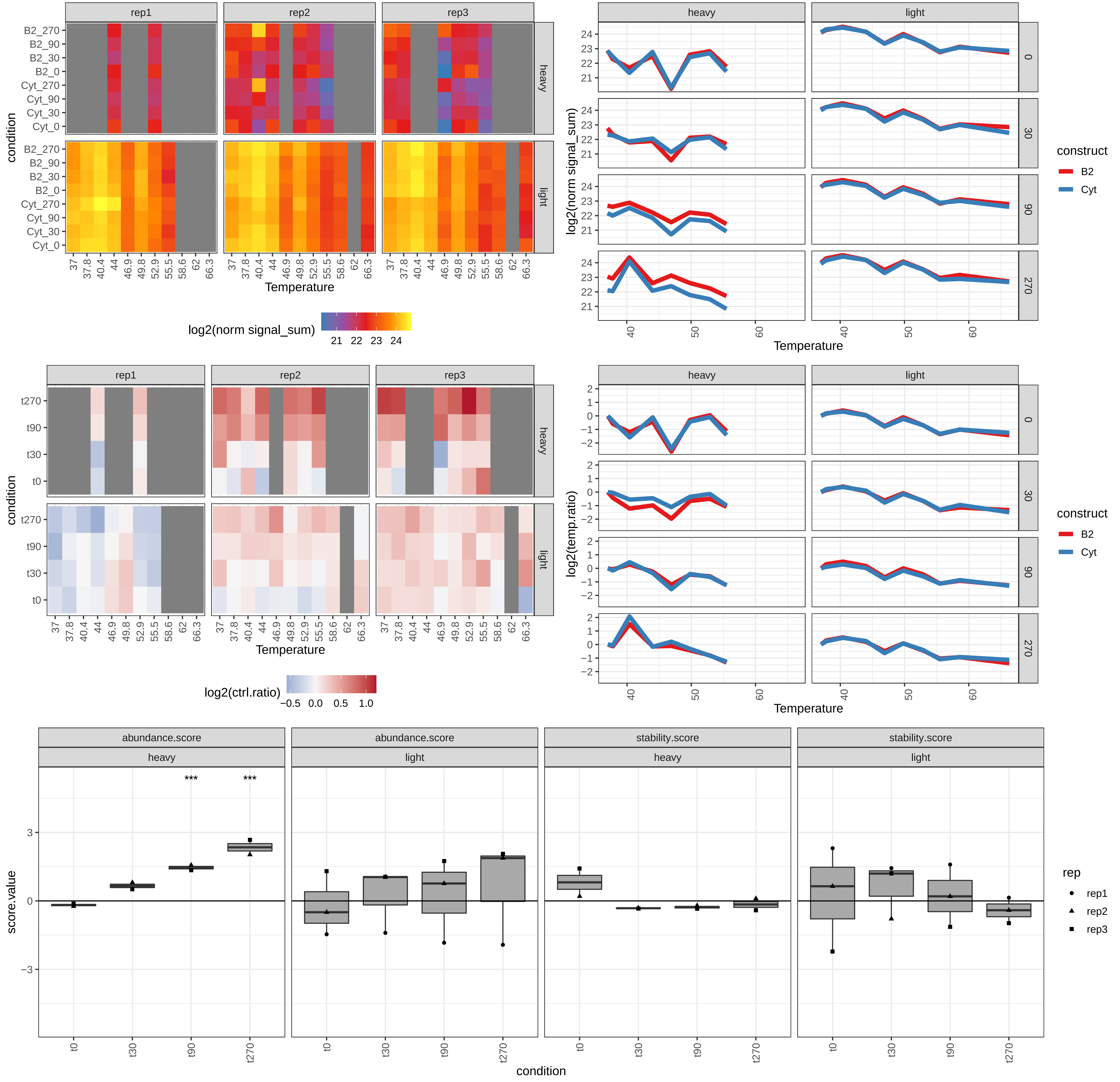

P34223 − UBX domain−containing protein 1 OS=Saccharomyces cerevisiae (strain ATCC 204508 / S288c) GN=SHP1 PE=1 SV=1

SHQ1 − average.top3: 6.53154823529412 − max.qupm: 7

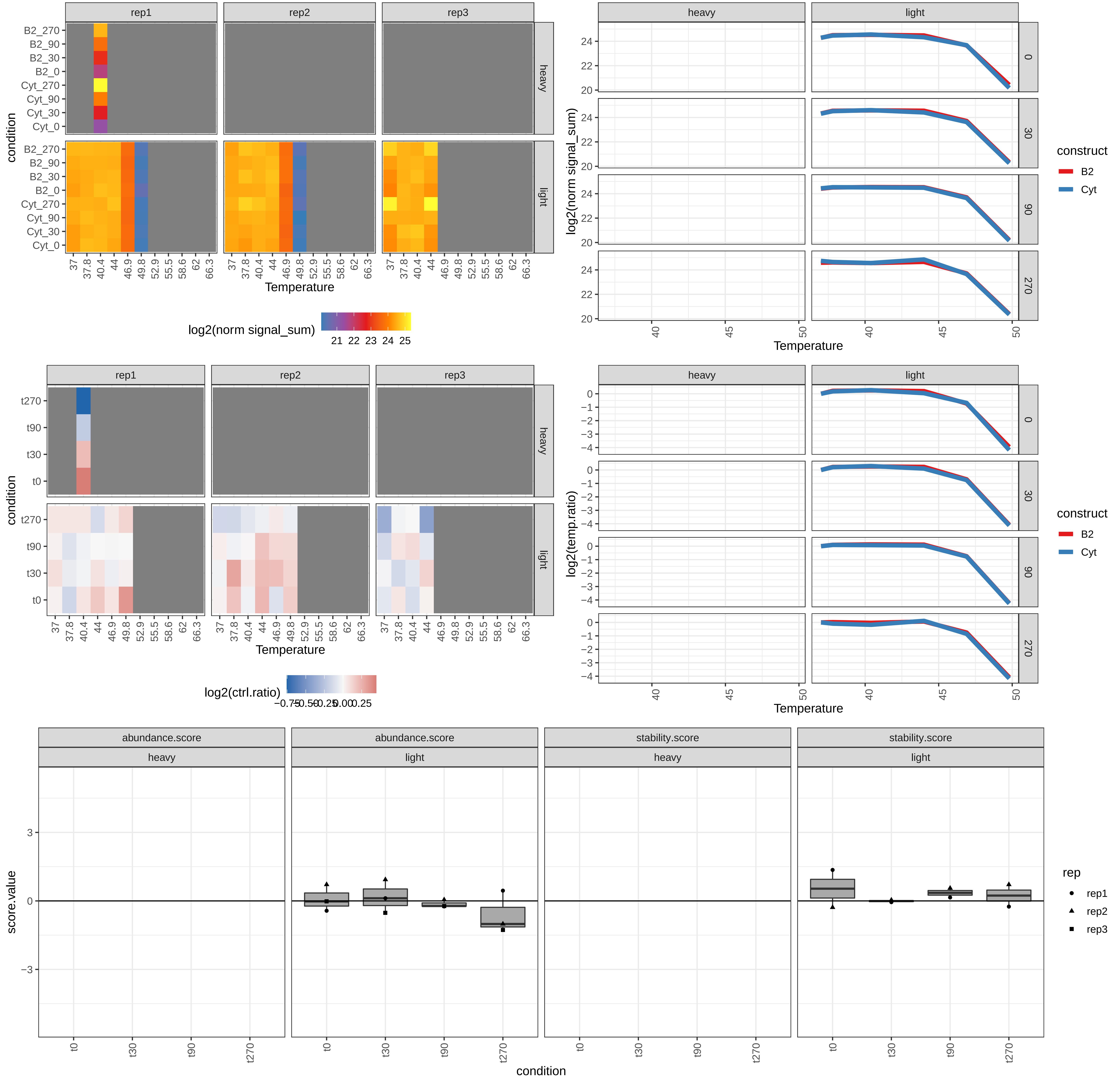

P40486 − Protein SHQ1 OS=Saccharomyces cerevisiae (strain ATCC 204508 / S288c) GN=SHQ1 PE=1 SV=1

SHR3 − average.top3: 7.50994588235294 − max.qupm: 3

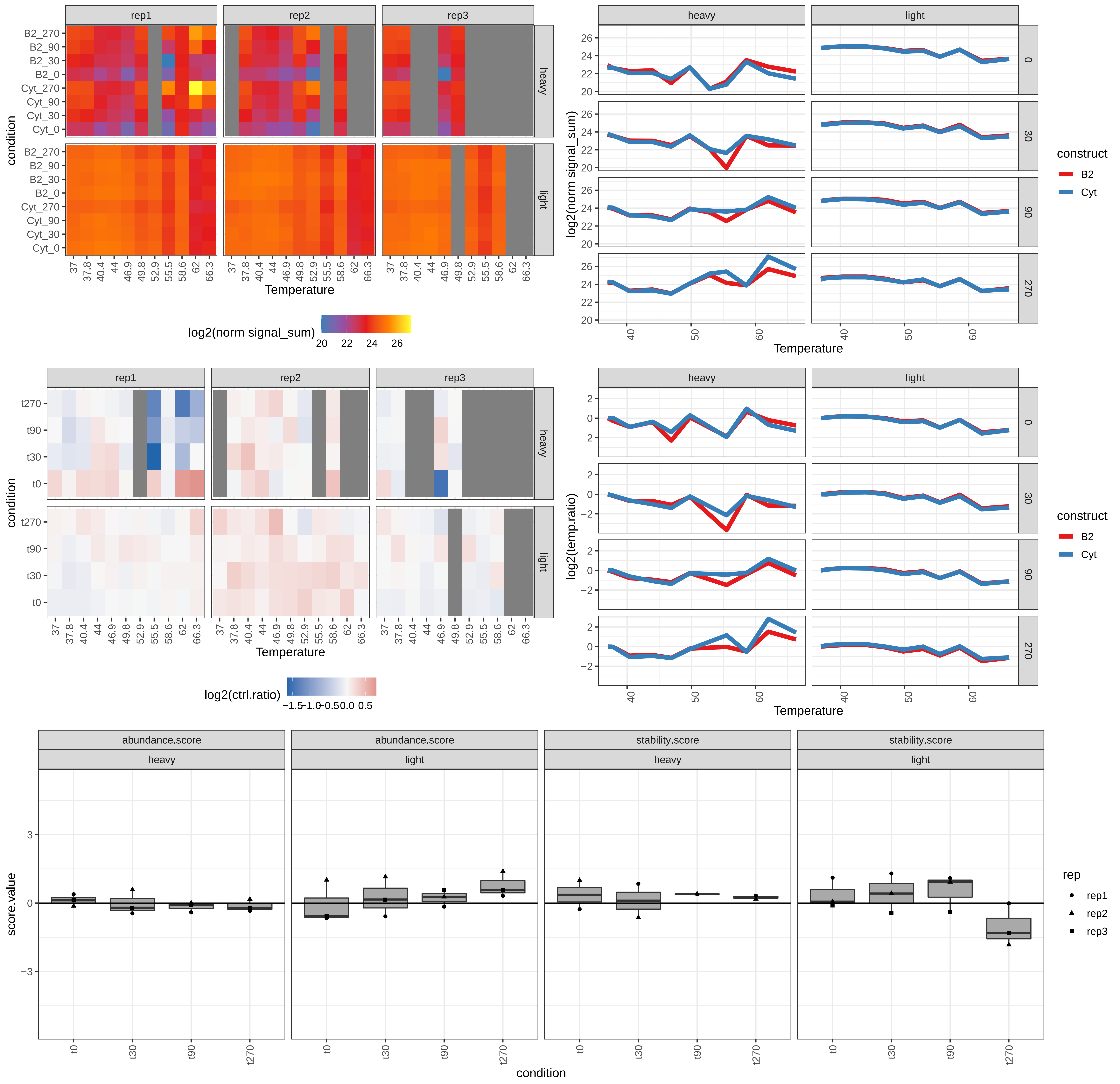

Q02774 − Secretory component protein SHR3 OS=Saccharomyces cerevisiae (strain ATCC 204508 / S288c) GN=SHR3 PE=1 SV=2

SHS1 − average.top3: 6.84128975 − max.qupm: 9

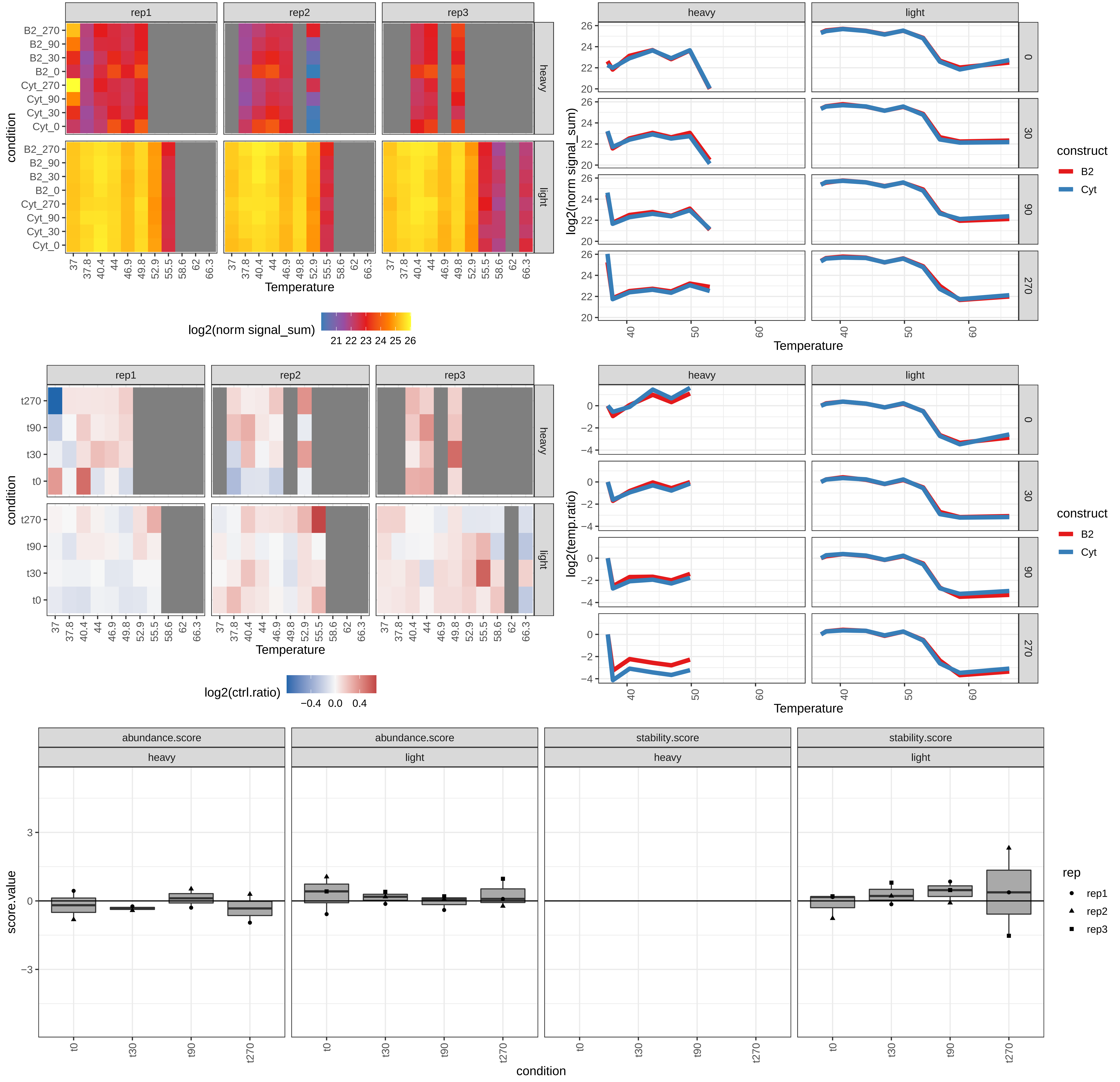

Q07657 − Seventh homolog of septin 1 OS=Saccharomyces cerevisiae (strain ATCC 204508 / S288c) GN=SHS1 PE=1 SV=1

SIF2 − average.top3: 6.82269545454546 − max.qupm: 6

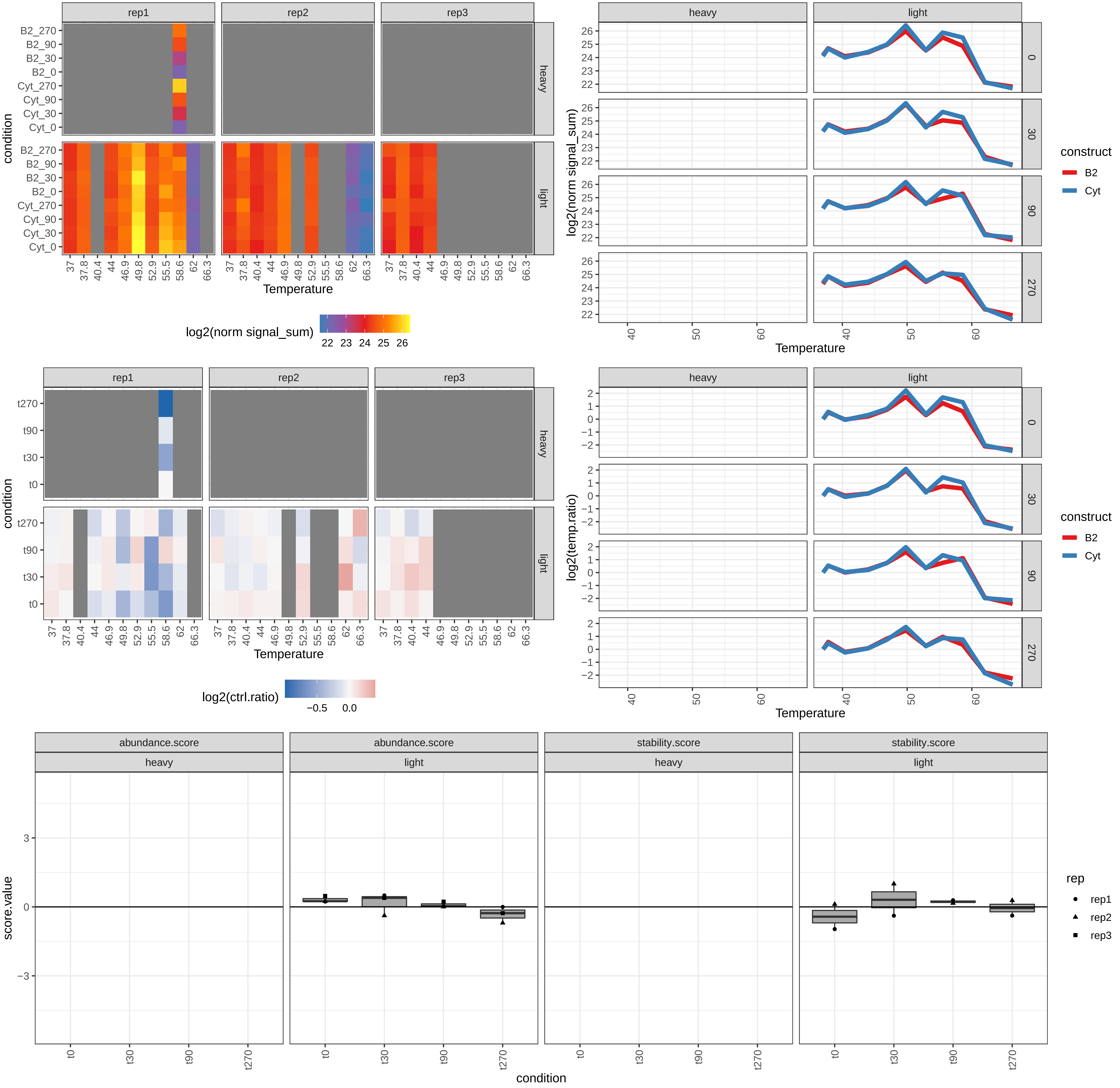

P38262 − SIR4−interacting protein SIF2 OS=Saccharomyces cerevisiae (strain ATCC 204508 / S288c) GN=SIF2 PE=1 SV=2

SIL1 − average.top3: 6.47786954545455 − max.qupm: 5

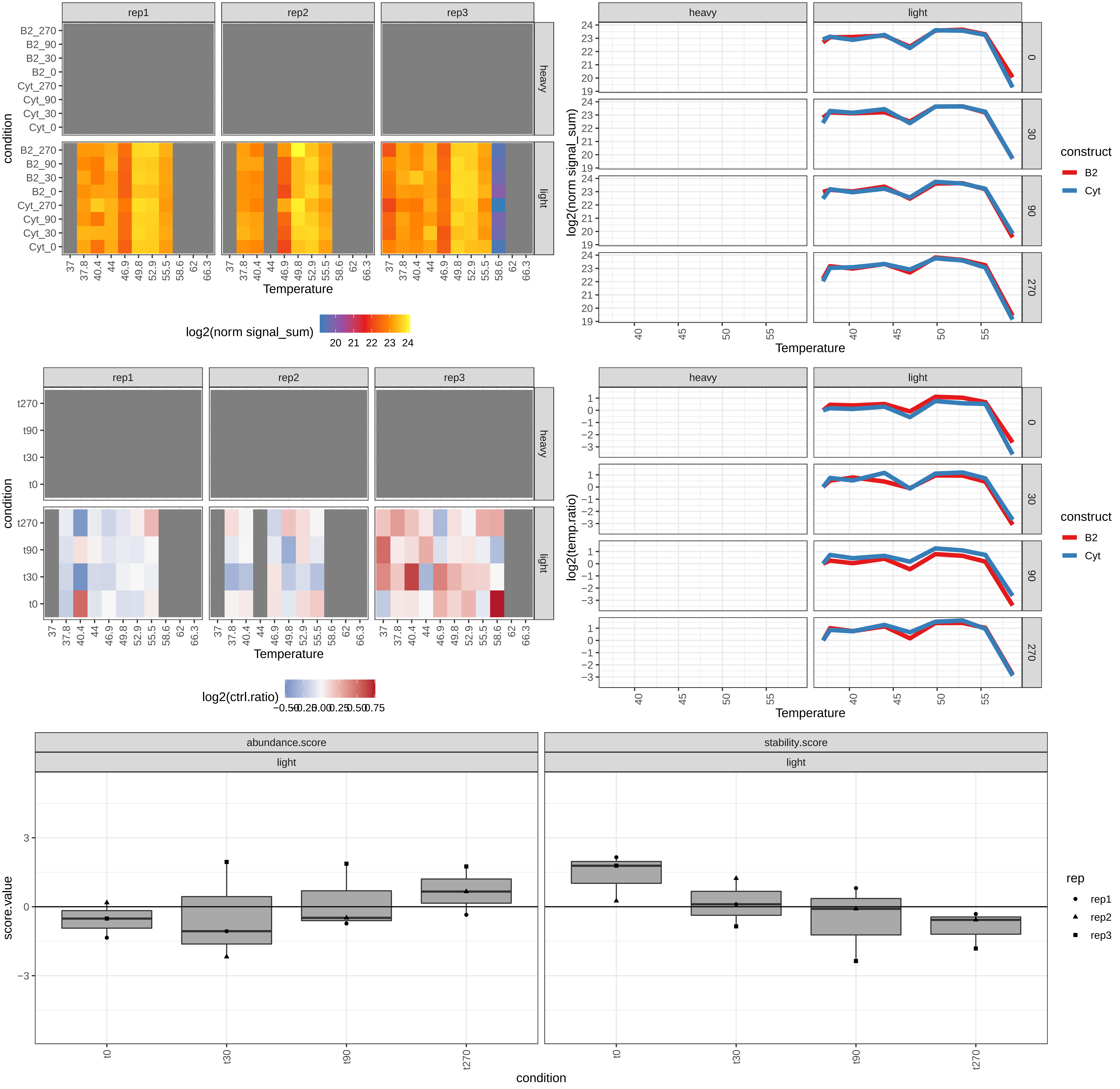

Q08199 − Nucleotide exchange factor SIL1 OS=Saccharomyces cerevisiae (strain ATCC 204508 / S288c) GN=SIL1 PE=1 SV=1

SIM1 − average.top3: 6.82167956521739 − max.qupm: 4

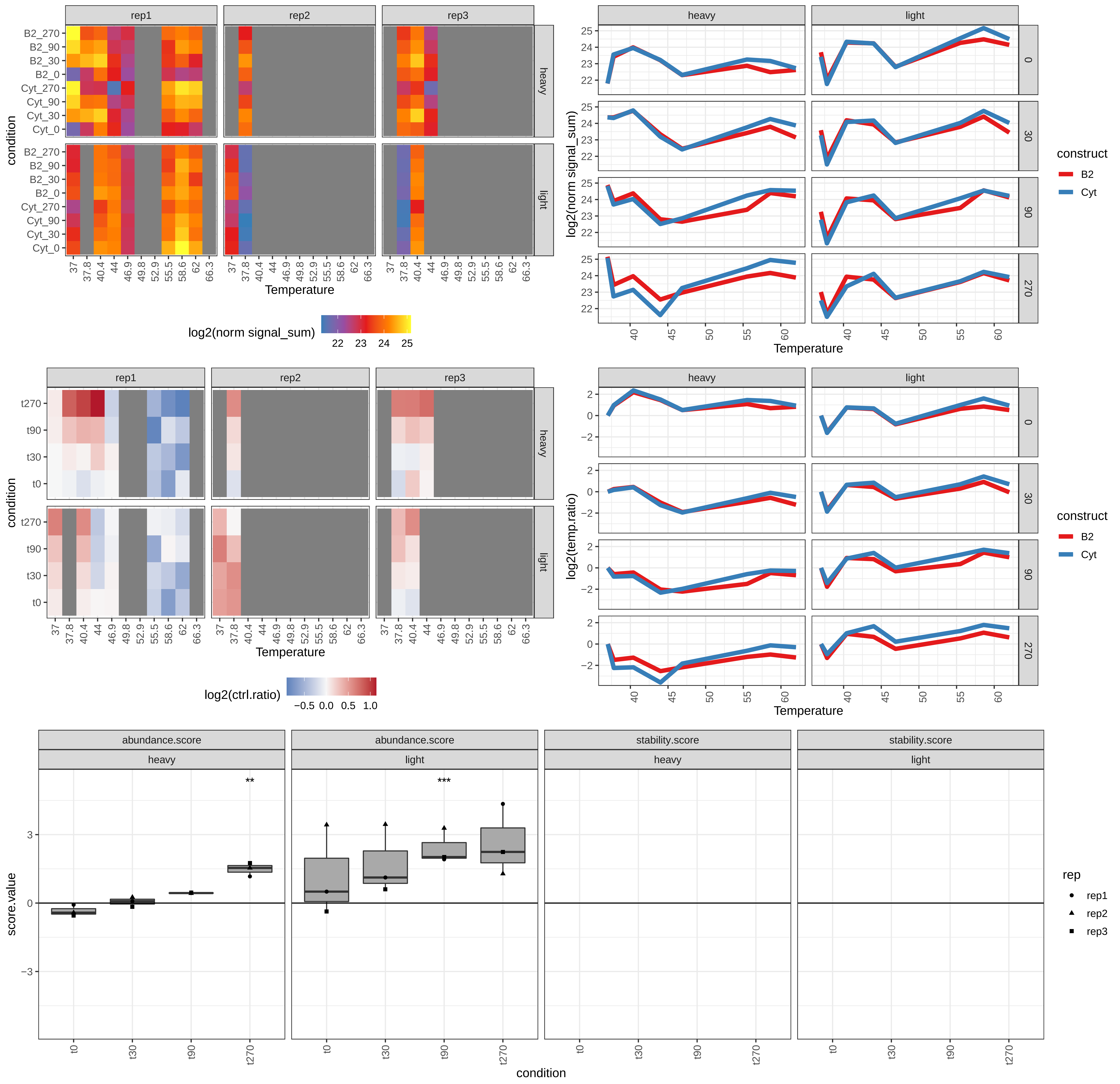

P40472 − Probable secreted beta−glucosidase SIM1 OS=Saccharomyces cerevisiae (strain ATCC 204508 / S288c) GN=SIM1 PE=1 SV=2

SIN3 − average.top3: 7.00775666666667 − max.qupm: 11

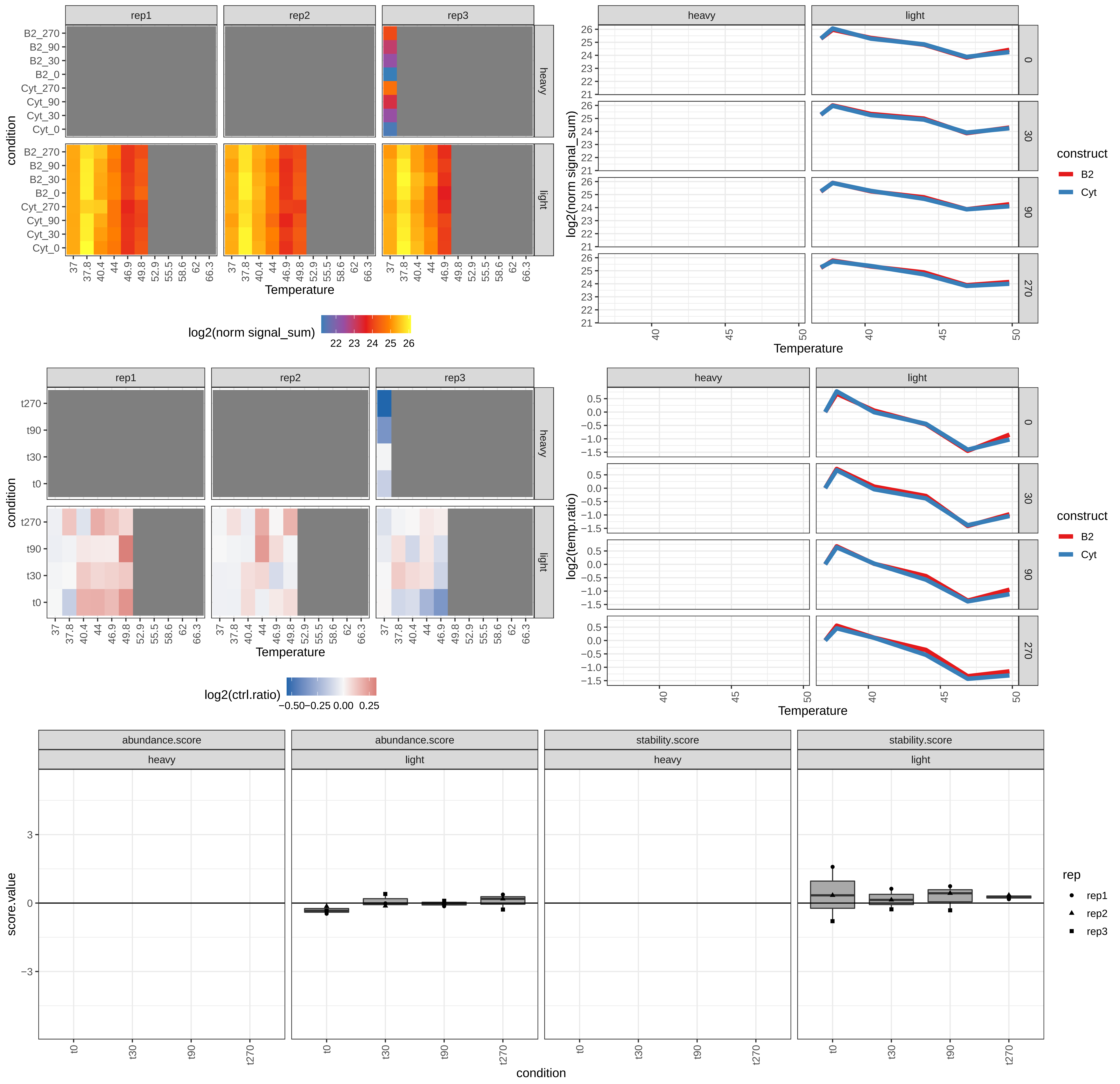

P22579 − Transcriptional regulatory protein SIN3 OS=Saccharomyces cerevisiae (strain ATCC 204508 / S288c) GN=SIN3 PE=1 SV=2

SIN4 − average.top3: 6.6052755 − max.qupm: 9

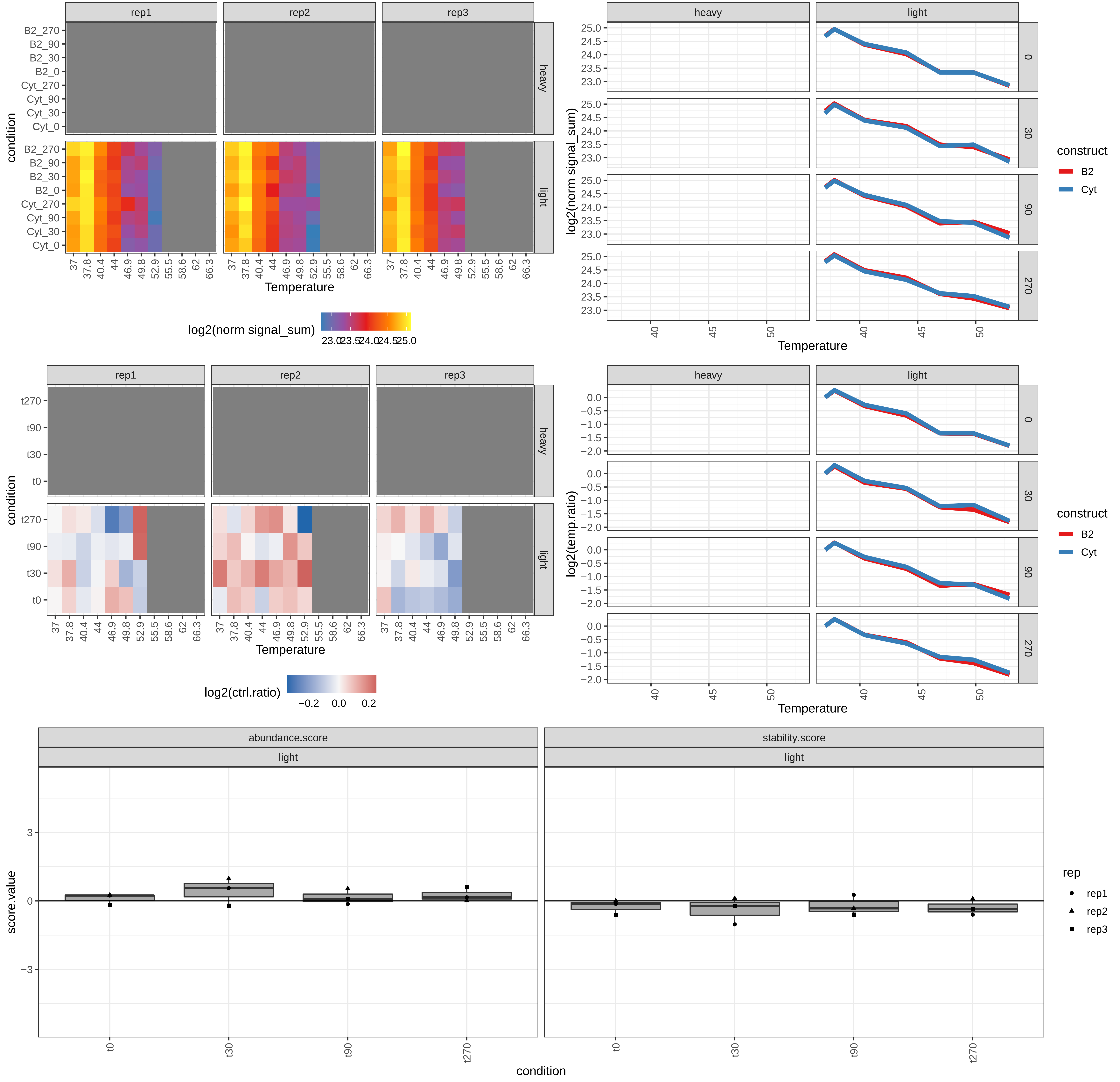

P32259 − Mediator of RNA polymerase II transcription subunit 16 OS=Saccharomyces cerevisiae (strain ATCC 204508 / S288c) GN=SIN4 PE=1 SV=1

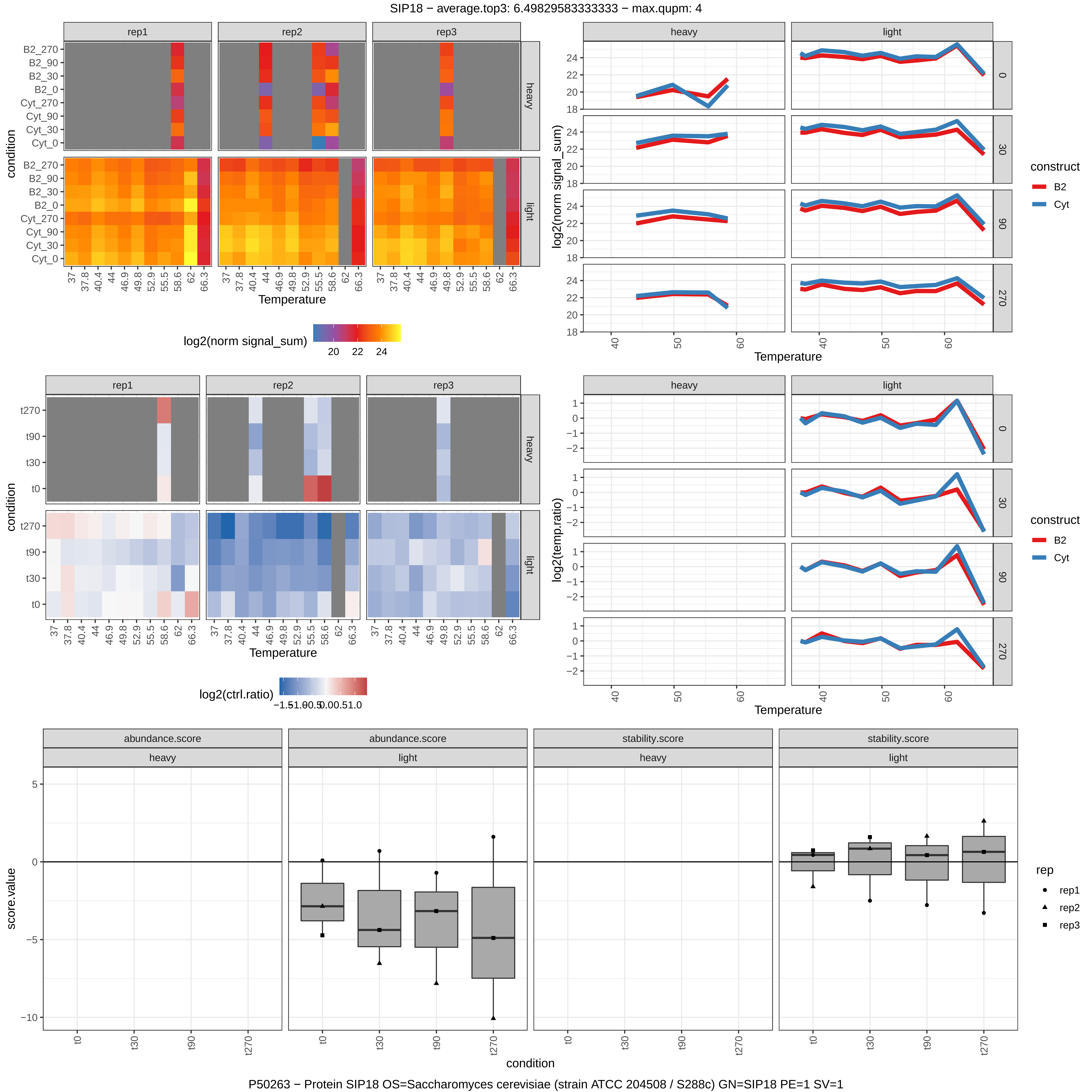
SIP18 − average.top3: 6.49829583333333 − max.qupm: 4
rep1
rep2
rep3
heavy
light
condition
Temperature
log2(norm signal_sum)
construct
B2
Cyt
log2(ctrl.ratio)
log2(temp.ratio)
abundance.score
stability.score
score.value
rep
condition
P50263 − Protein SIP18 OS=Saccharomyces cerevisiae (strain ATCC 204508 / S288c) GN=SIP18 PE=1 SV=1

SIP2 − average.top3: 6.8506675 − max.qupm: 4

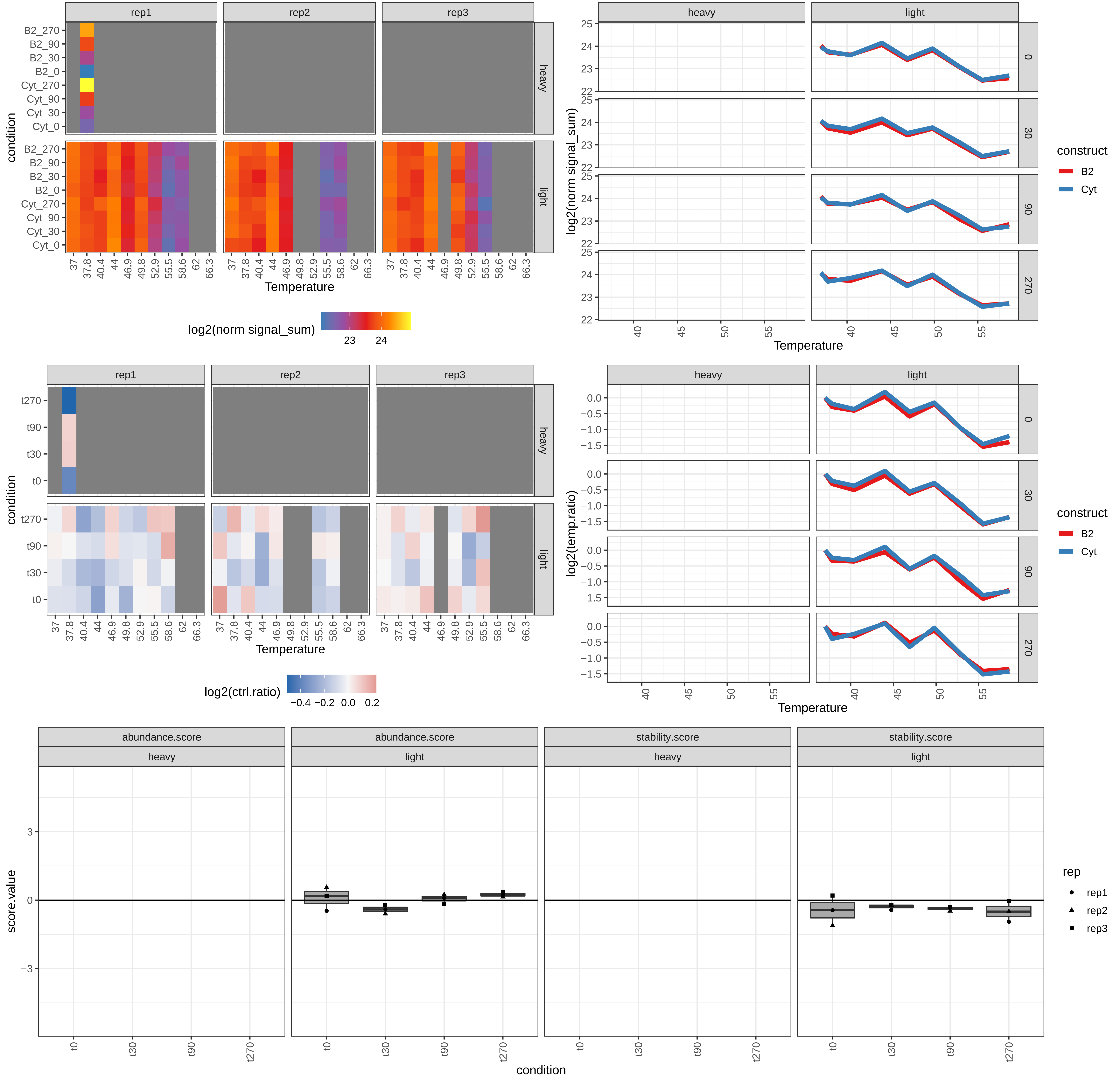

P34164 − SNF1 protein kinase subunit beta−2 OS=Saccharomyces cerevisiae (strain ATCC 204508 / S288c) GN=SIP2 PE=1 SV=3

SIP3 − average.top3: 6.20714 − max.qupm: 6

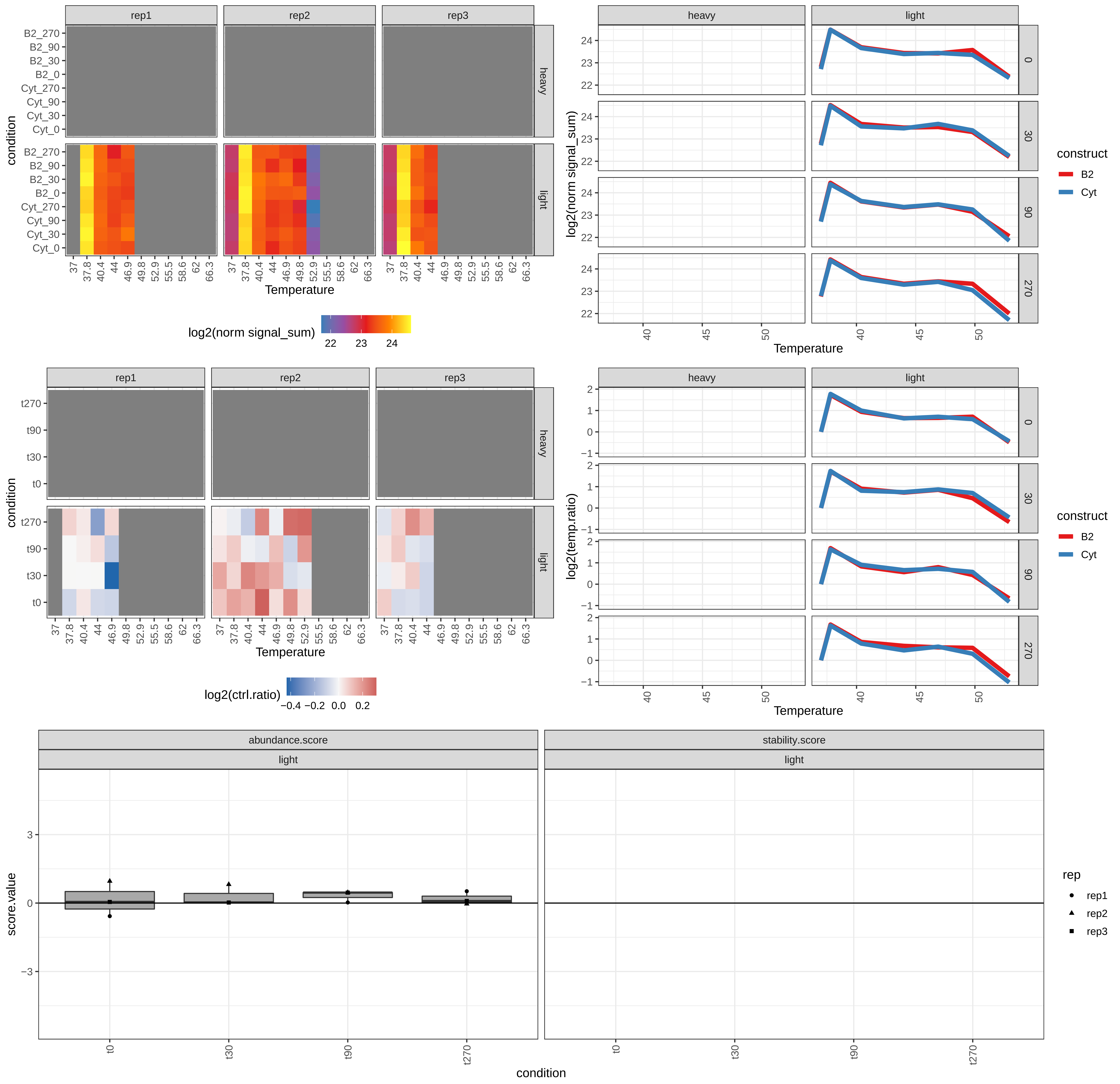

P38717 − Membrane−anchored lipid−binding protein SIP3 OS=Saccharomyces cerevisiae (strain ATCC 204508 / S288c) GN=SIP3 PE=1 SV=1

SIP5 − average.top3: 6.71137058823529 − max.qupm: 6

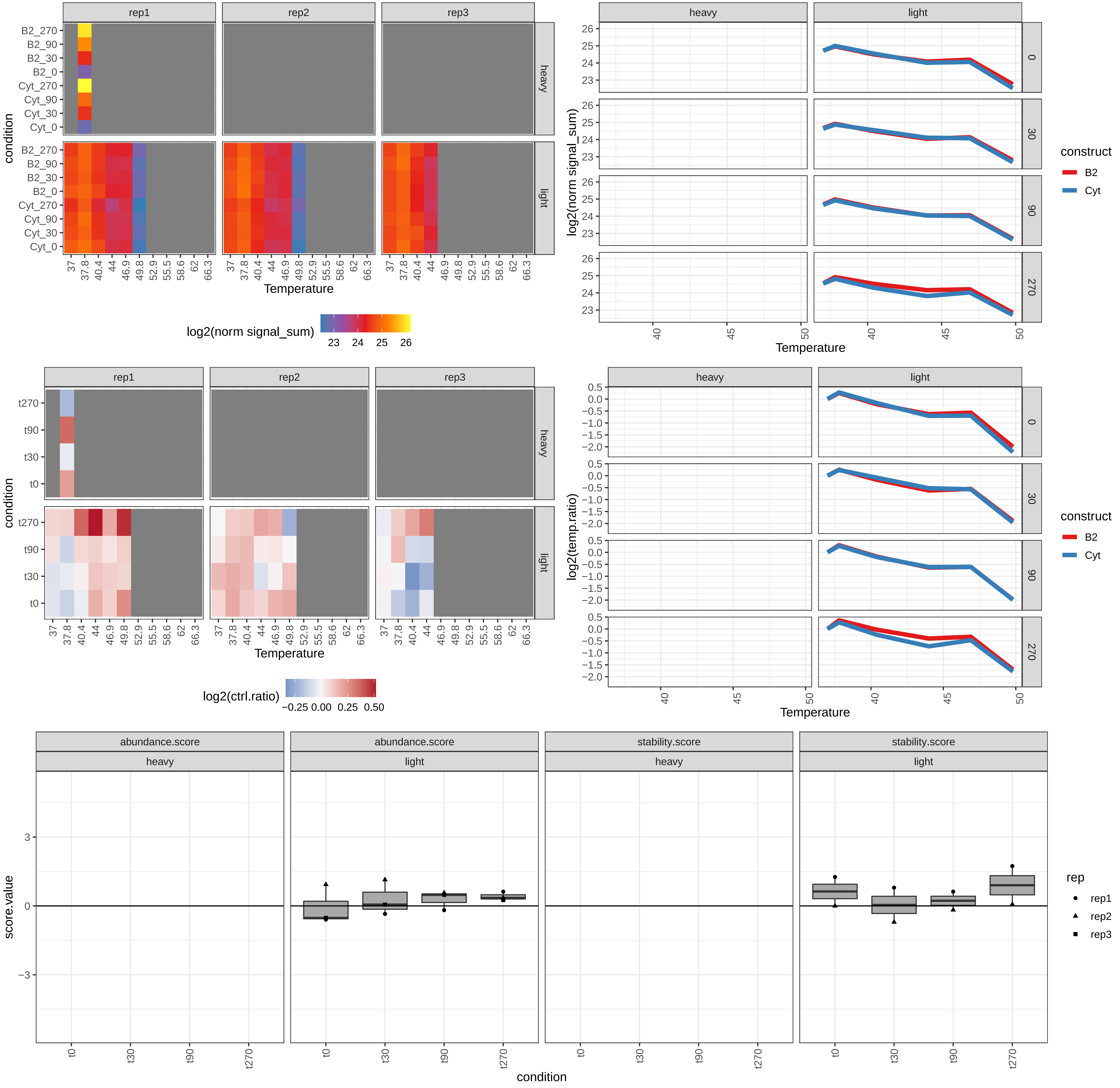

P40210 − Protein SIP5 OS=Saccharomyces cerevisiae (strain ATCC 204508 / S288c) GN=SIP5 PE=1 SV=1

SIR3 − average.top3: 6.502836875 − max.qupm: 6

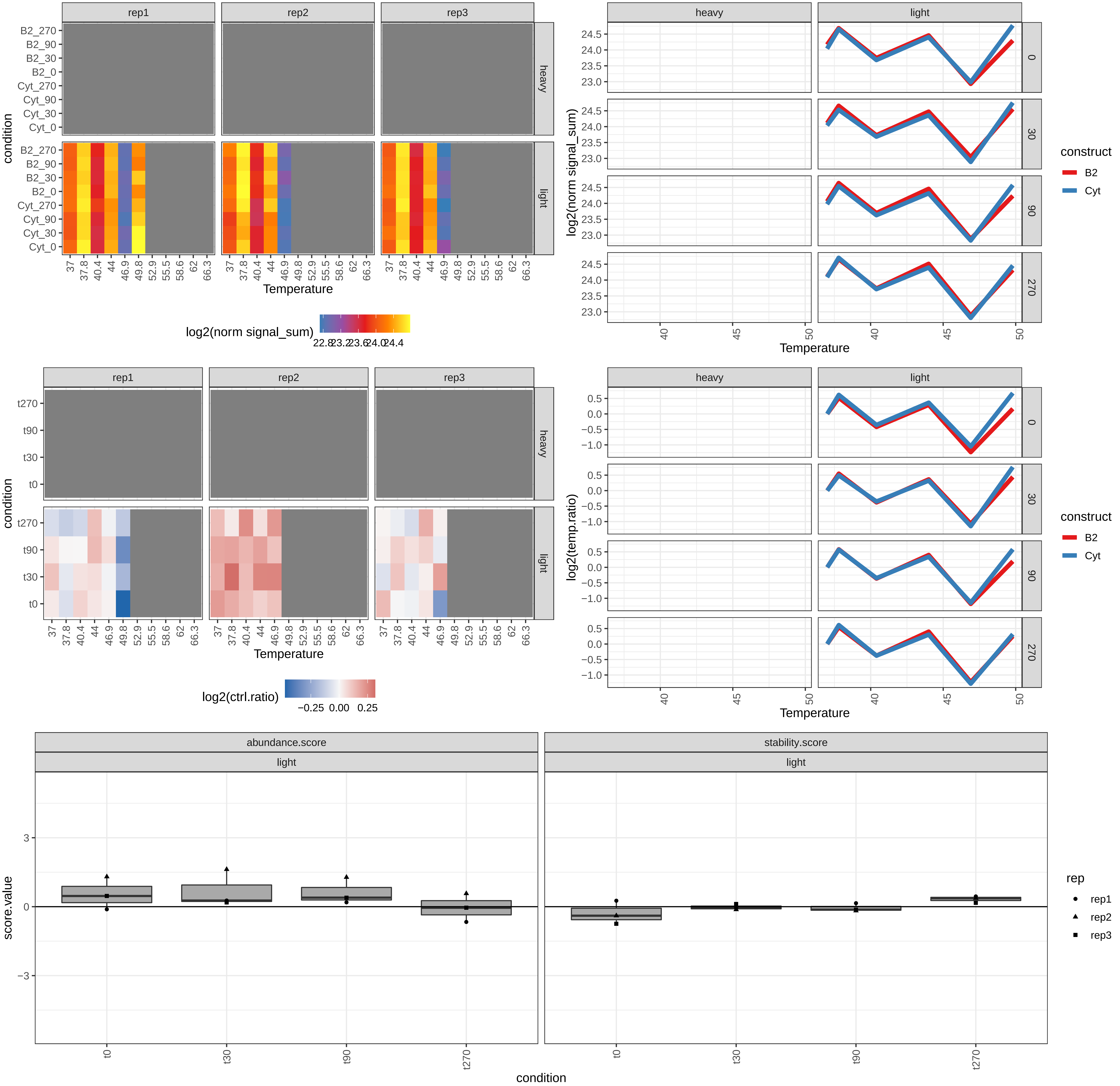

P06701 − Regulatory protein SIR3 OS=Saccharomyces cerevisiae (strain ATCC 204508 / S288c) GN=SIR3 PE=1 SV=6

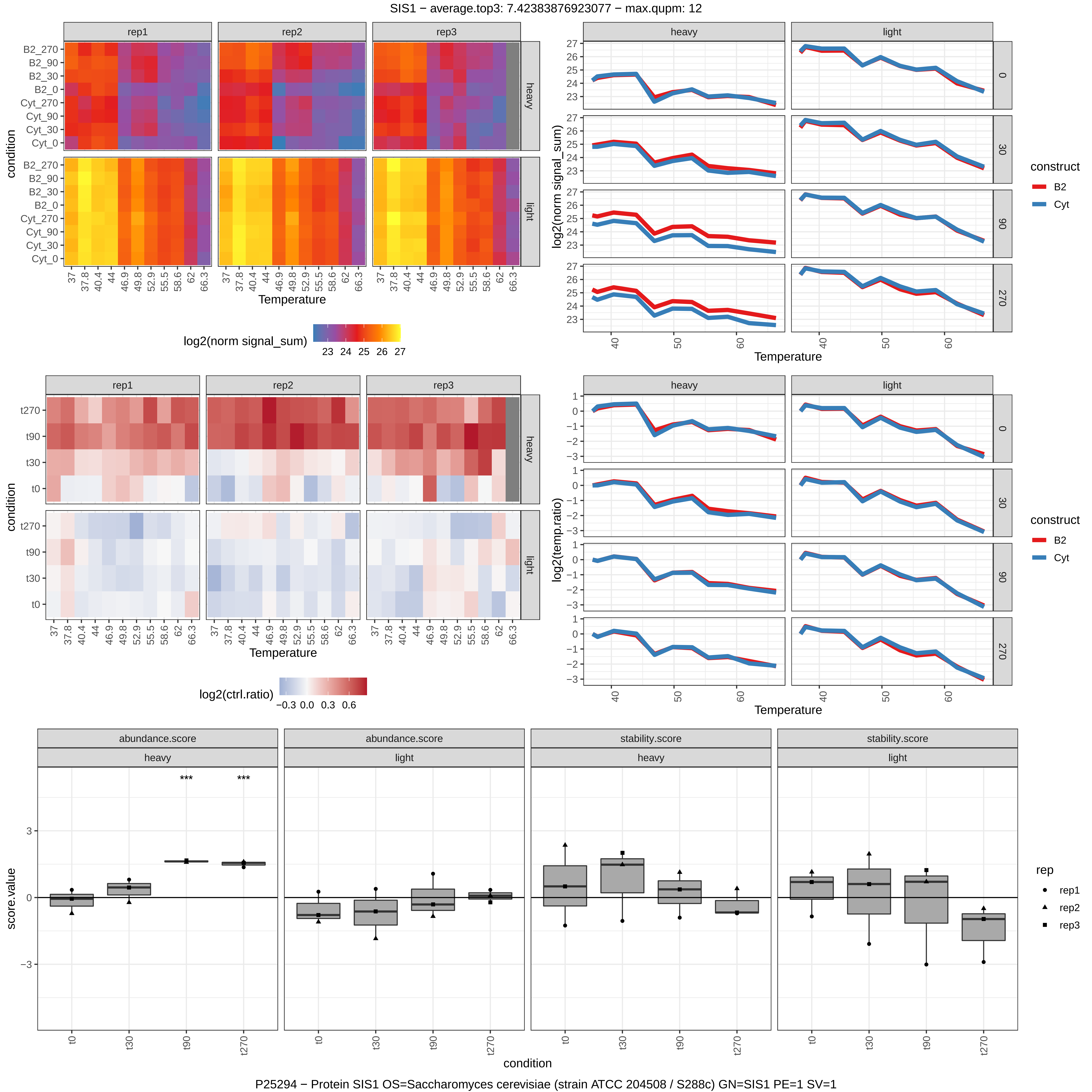
SIS1 − average.top3: 7.42383876923077 − max.qupm: 12
rep1
rep2
rep3
heavy
light
B2_270
B2_90
B2_30
B2_0
Cyt_270
Cyt_90
Cyt_30
Cyt_0
condition
Temperature
37
37.8
40.4
44
46.9
49.8
52.9
55.5
58.6
62
66.3
log2(norm signal_sum)
23
24
25
26
27
0
30
90
270
40
50
60
construct
B2
Cyt
t270
t90
t30
t0
log2(ctrl.ratio)
−0.3
0.0
0.3
0.6
log2(temp.ratio)
1
0
−1
−2
−3
abundance.score
stability.score
***
3
0
−3
score.value
rep
rep1
rep2
rep3
condition
P25294 − Protein SIS1 OS=Saccharomyces cerevisiae (strain ATCC 204508 / S288c) GN=SIS1 PE=1 SV=1

SIT4 − average.top3: 6.8037268 − max.qupm: 8

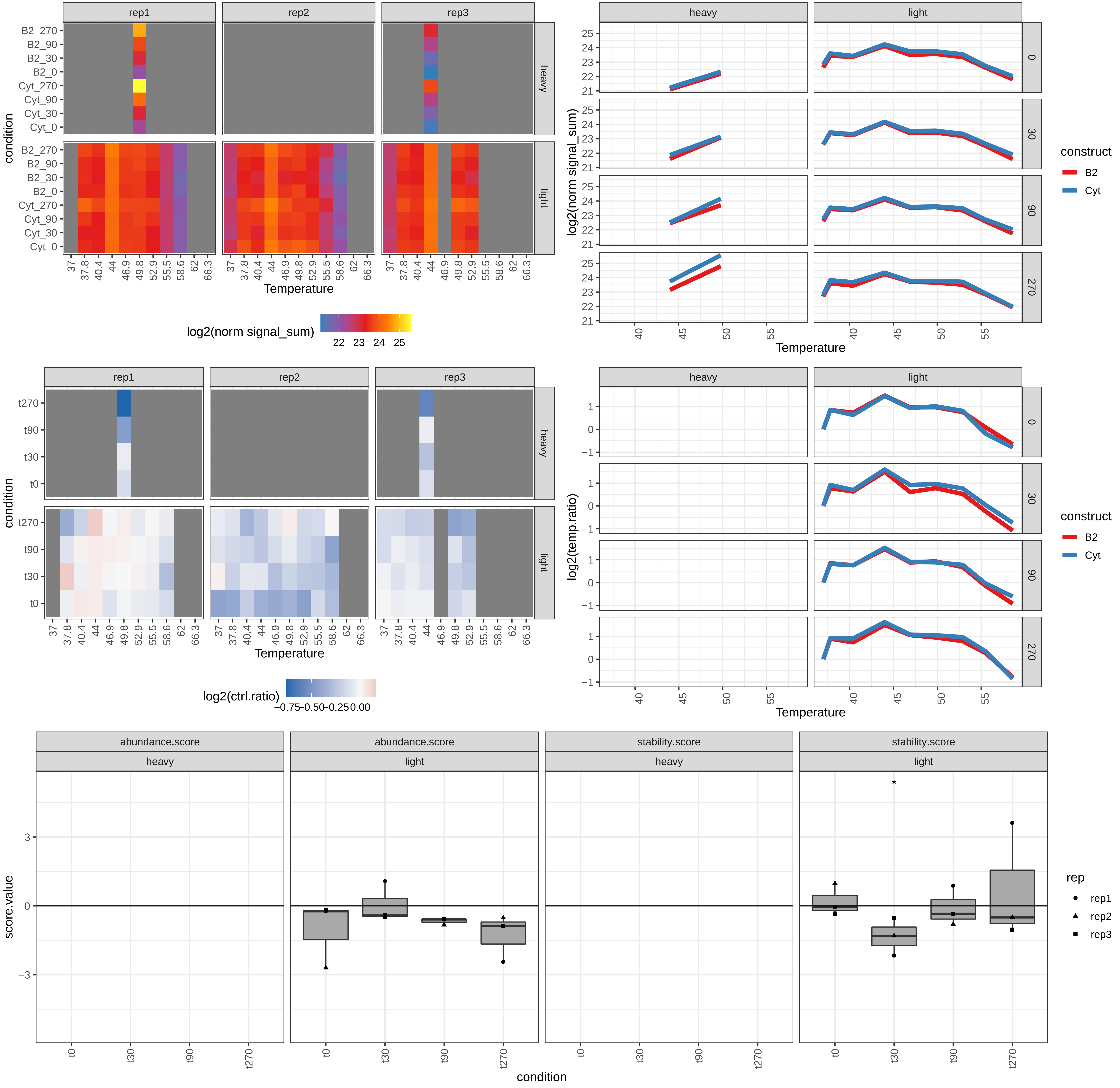

P20604 − Serine/threonine−protein phosphatase PP1−1 OS=Saccharomyces cerevisiae (strain ATCC 204508 / S288c) GN=SIT4 PE=1 SV=1

SKI2 − average.top3: 6.88195151515152 − max.qupm: 21

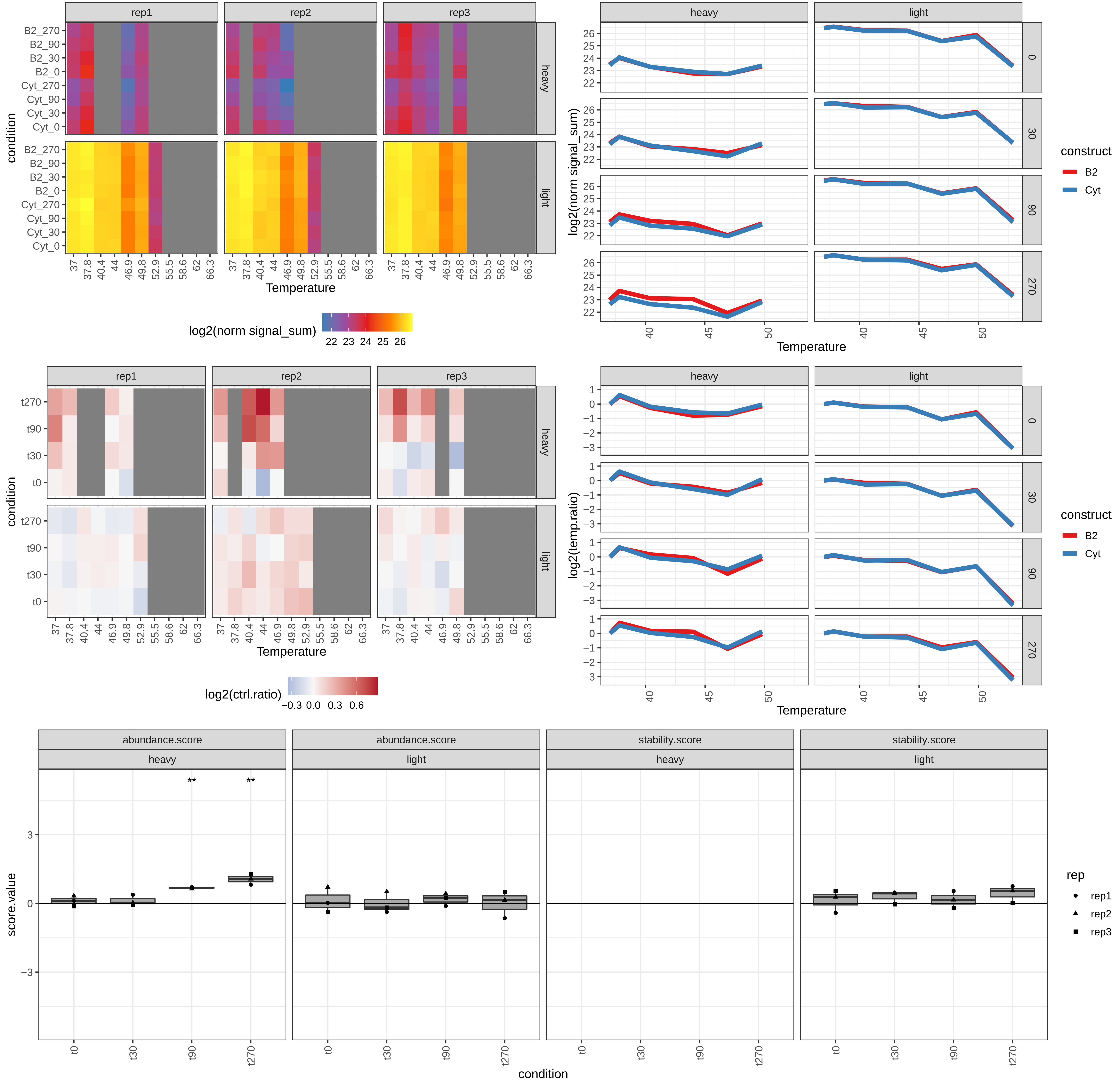

P35207 − Antiviral helicase SKI2 OS=Saccharomyces cerevisiae (strain ATCC 204508 / S288c) GN=SKI2 PE=1 SV=2

SKI3 − average.top3: 6.6813603030303 − max.qupm: 17

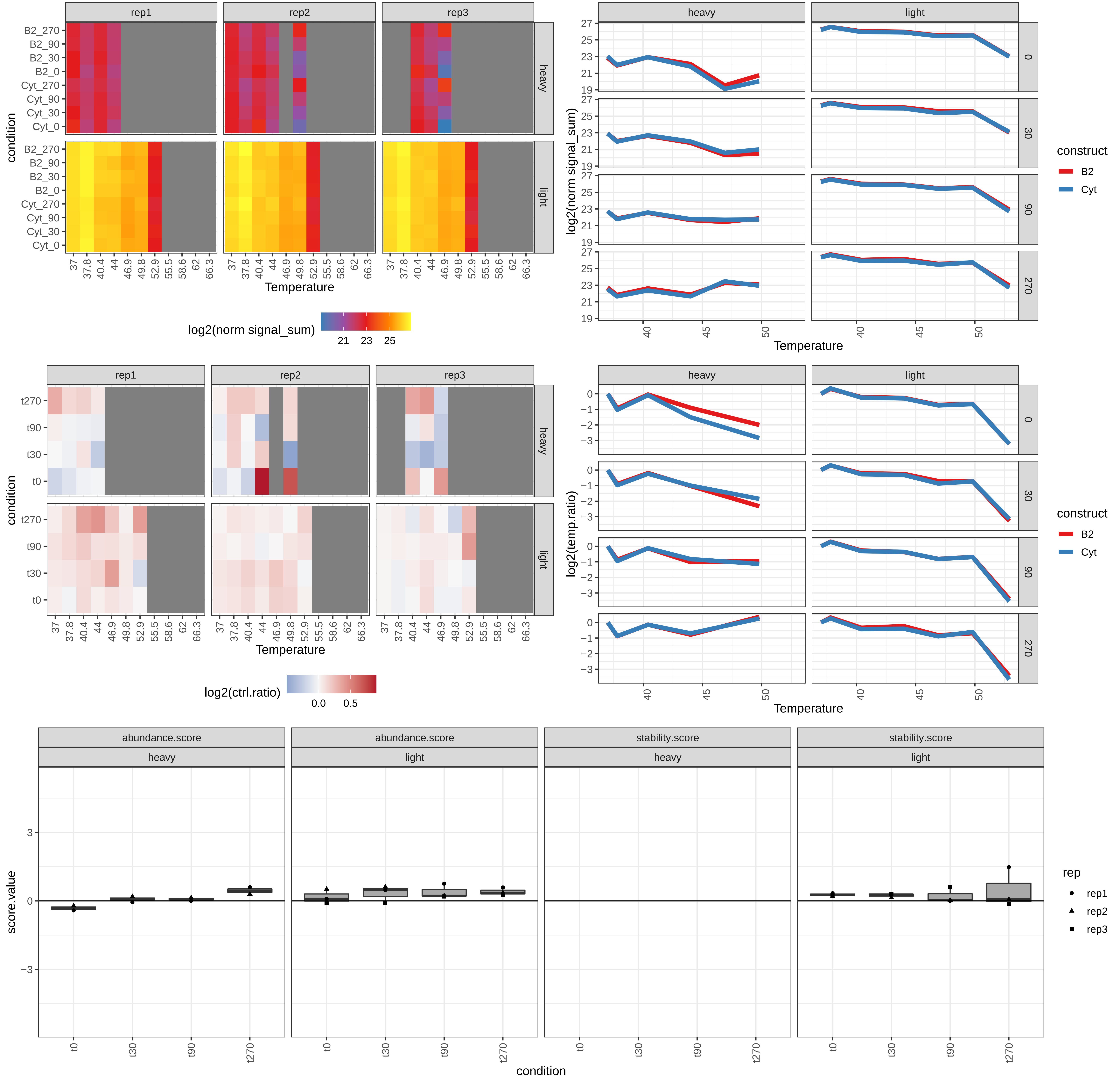

P17883 − Superkiller protein 3 OS=Saccharomyces cerevisiae (strain ATCC 204508 / S288c) GN=SKI3 PE=1 SV=2

SKI8 − average.top3: 7.17177590909091 − max.qupm: 6

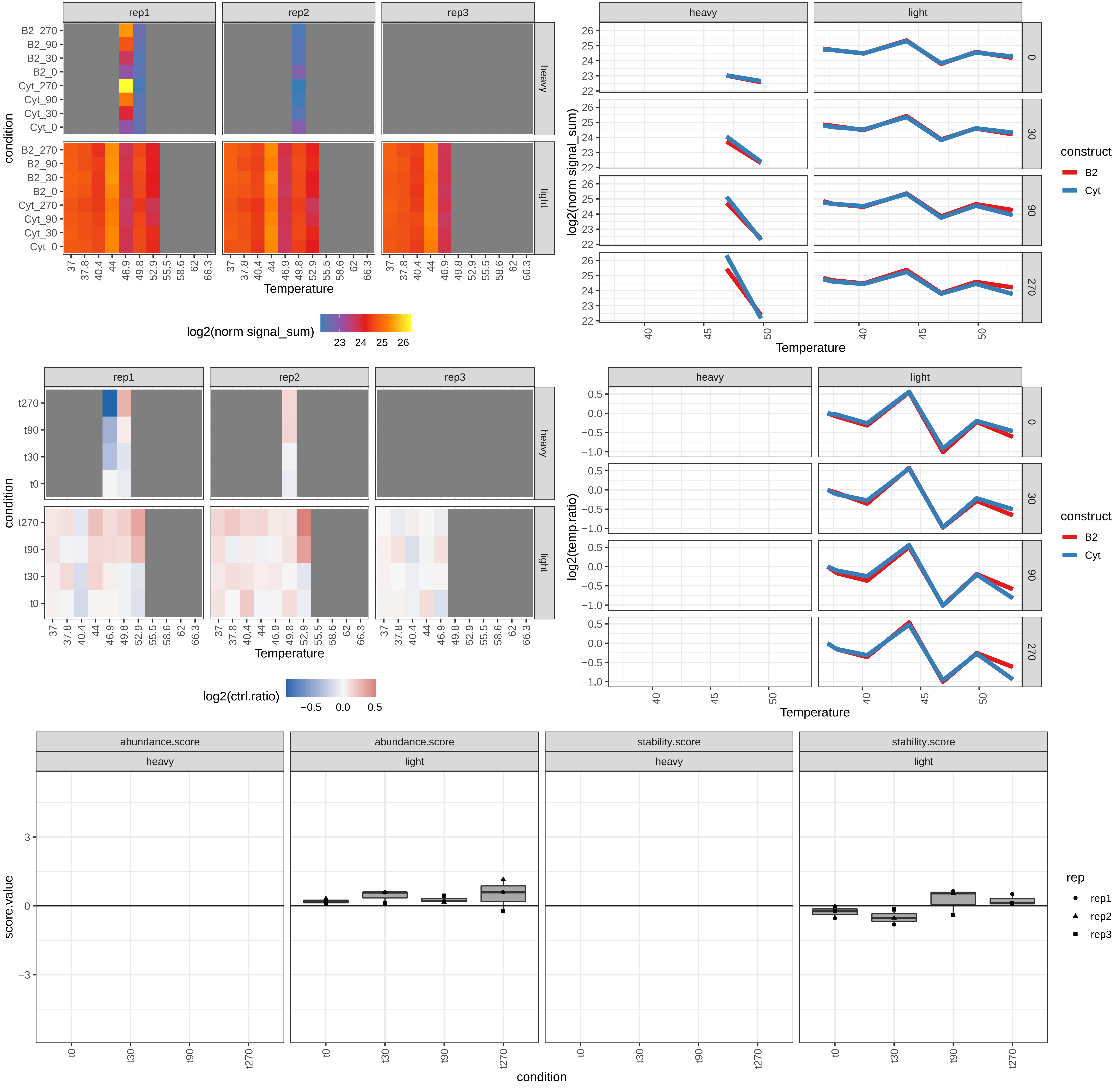

Q02793 − Antiviral protein SKI8 OS=Saccharomyces cerevisiae (strain ATCC 204508 / S288c) GN=SKI8 PE=1 SV=1

SKP1 − average.top3: 7.10221742857143 − max.qupm: 11

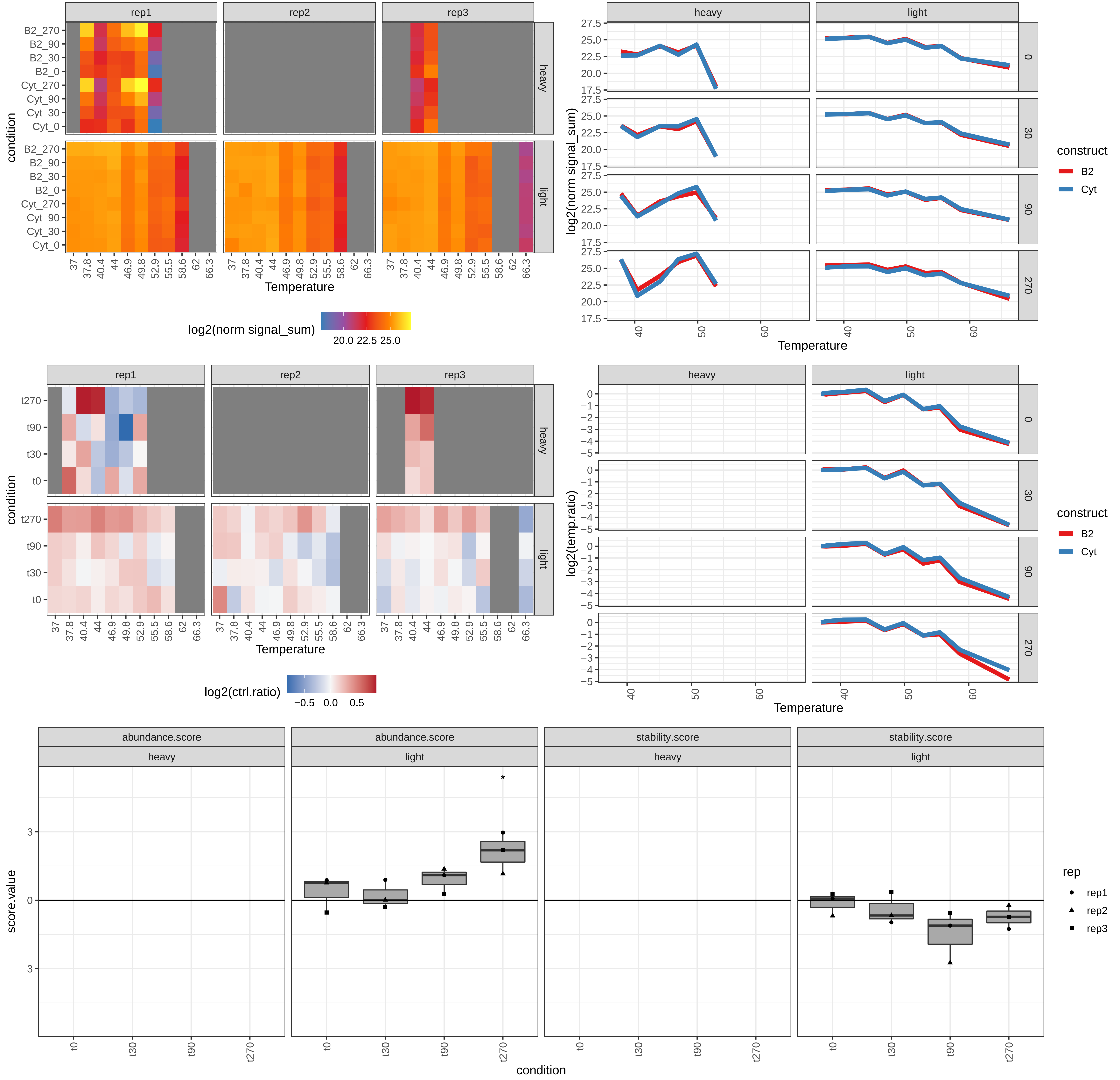

P52286 − Suppressor of kinetochore protein 1 OS=Saccharomyces cerevisiae (strain ATCC 204508 / S288c) GN=SKP1 PE=1 SV=2

SKY1 − average.top3: 6.522633 − max.qupm: 5

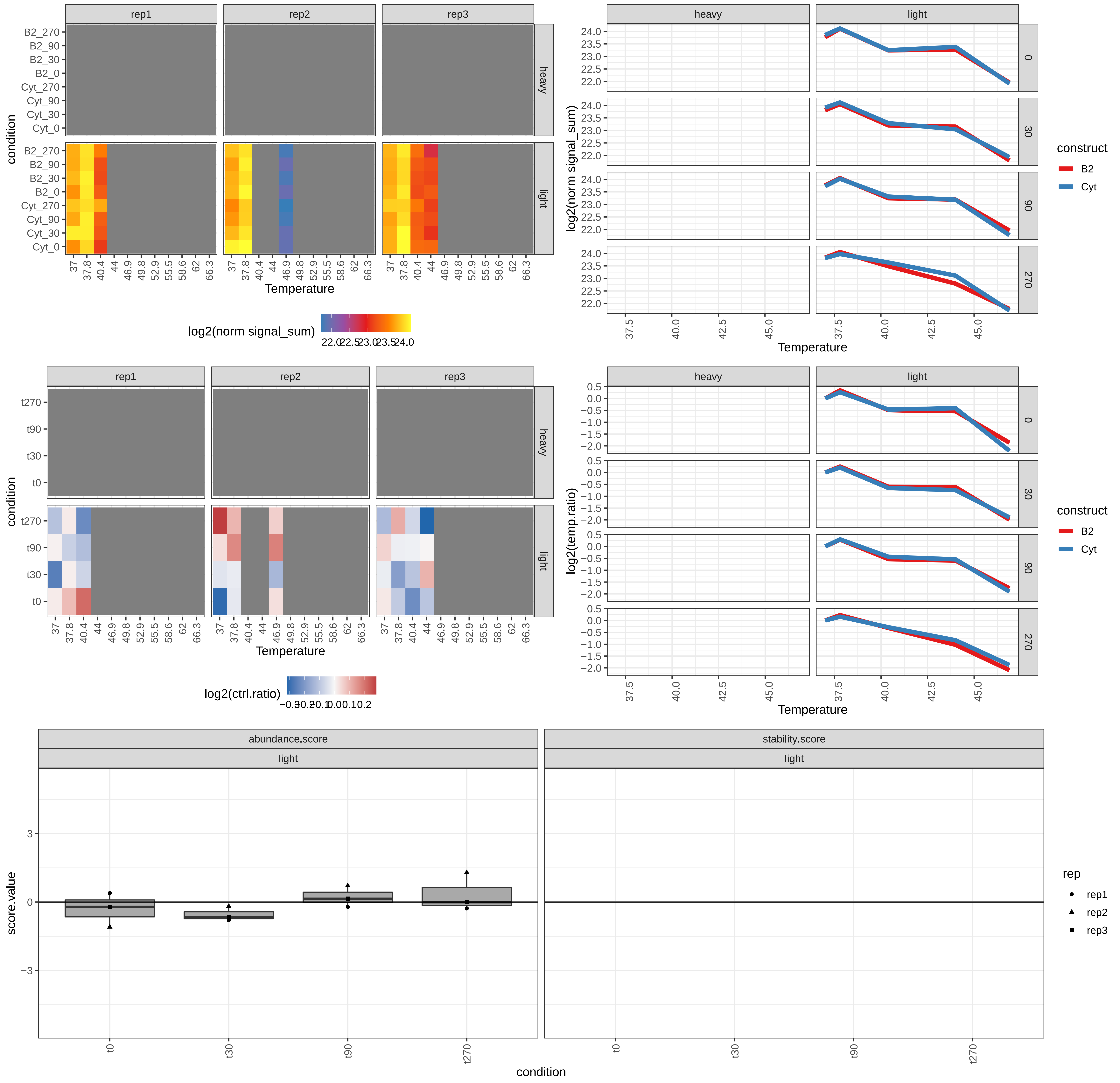

Q03656 − Serine/threonine−protein kinase SKY1 OS=Saccharomyces cerevisiae (strain ATCC 204508 / S288c) GN=SKY1 PE=1 SV=1

SLA1 − average.top3: 6.94945795918367 − max.qupm: 14

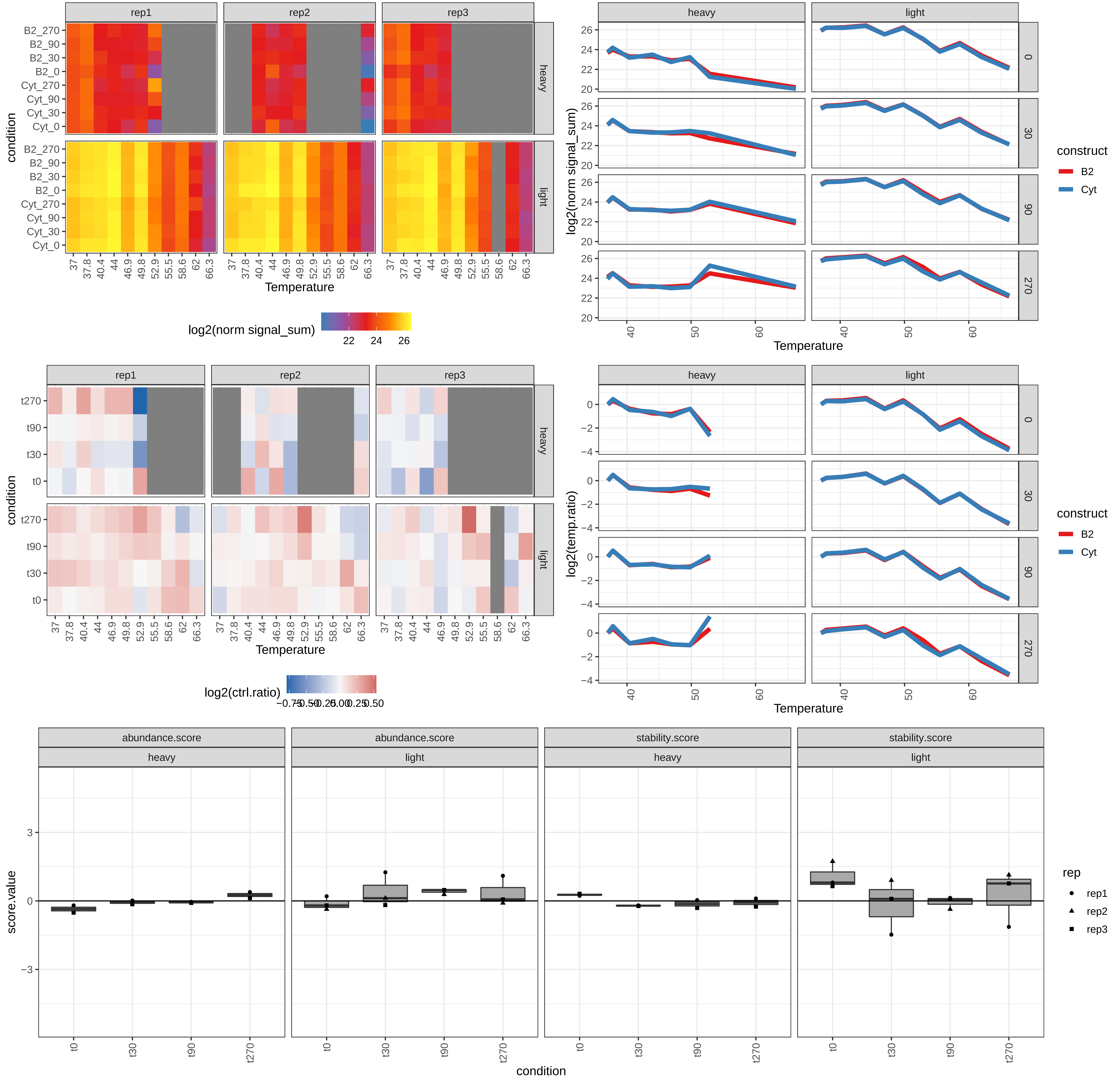

P32790 − Actin cytoskeleton−regulatory complex protein SLA1 OS=Saccharomyces cerevisiae (strain ATCC 204508 / S288c) GN=SLA1 PE=1 SV=1

SLA2 − average.top3: 6.92307722222222 − max.qupm: 11

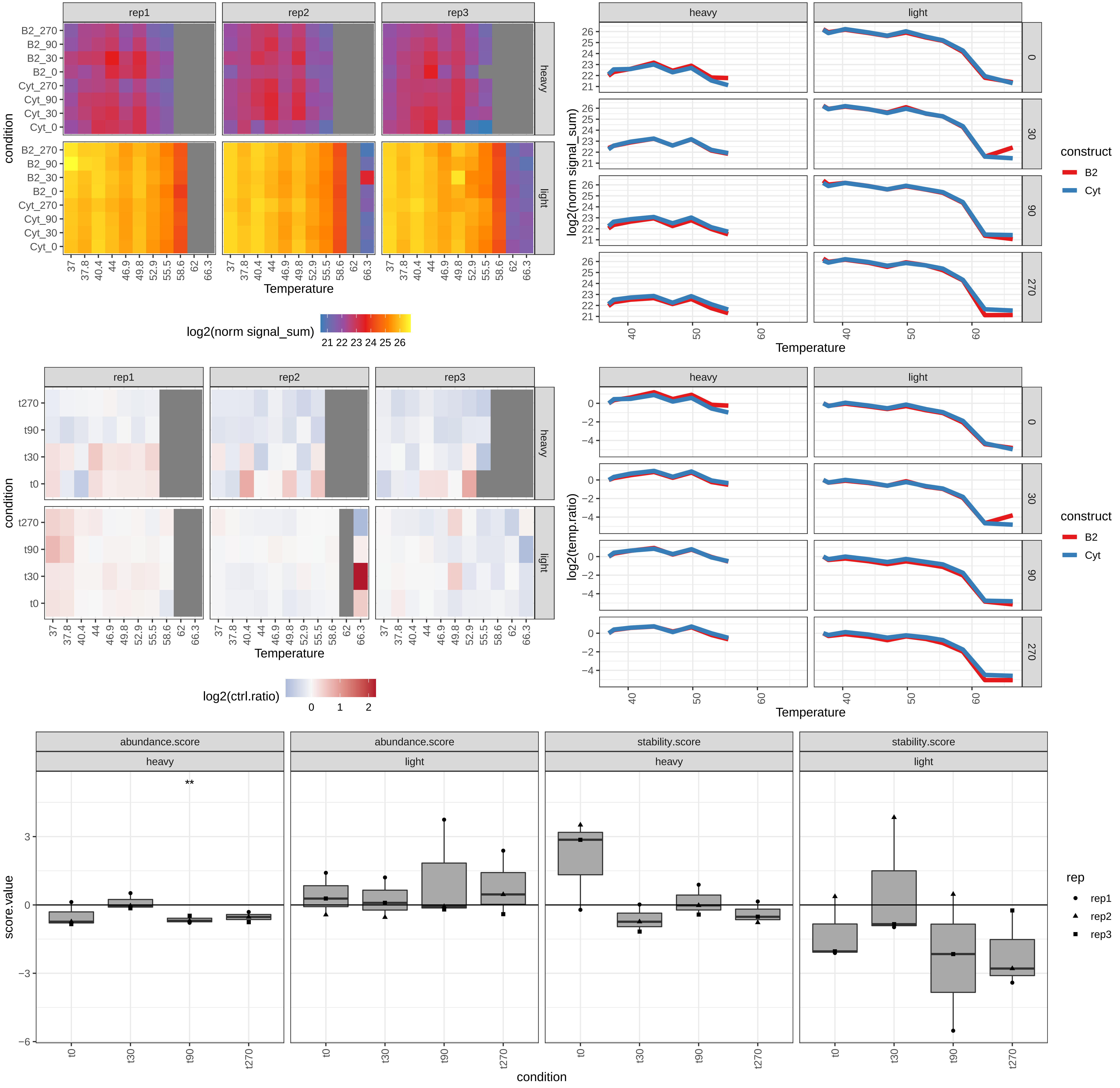

P33338 − Protein SLA2 OS=Saccharomyces cerevisiae (strain ATCC 204508 / S288c) GN=SLA2 PE=1 SV=5

SLC1 − average.top3: 6.84893888888889 − max.qupm: 4

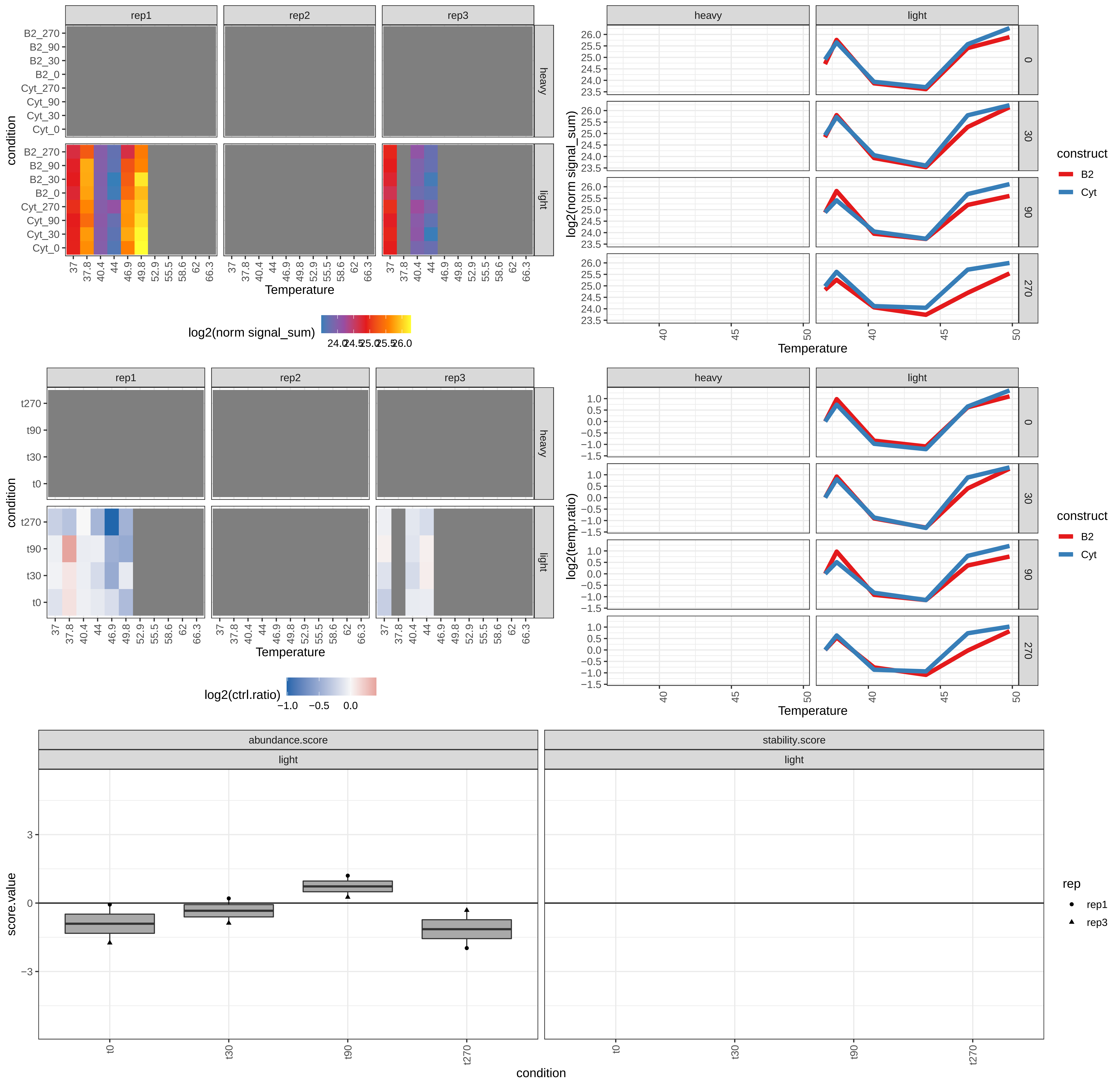

P33333 − Probable 1−acyl−sn−glycerol−3−phosphate acyltransferase OS=Saccharomyces cerevisiae (strain ATCC 204508 / S288c) GN=SLC1 PE=1 SV=1

SLK19 − average.top3: 6.78582846153846 − max.qupm: 5

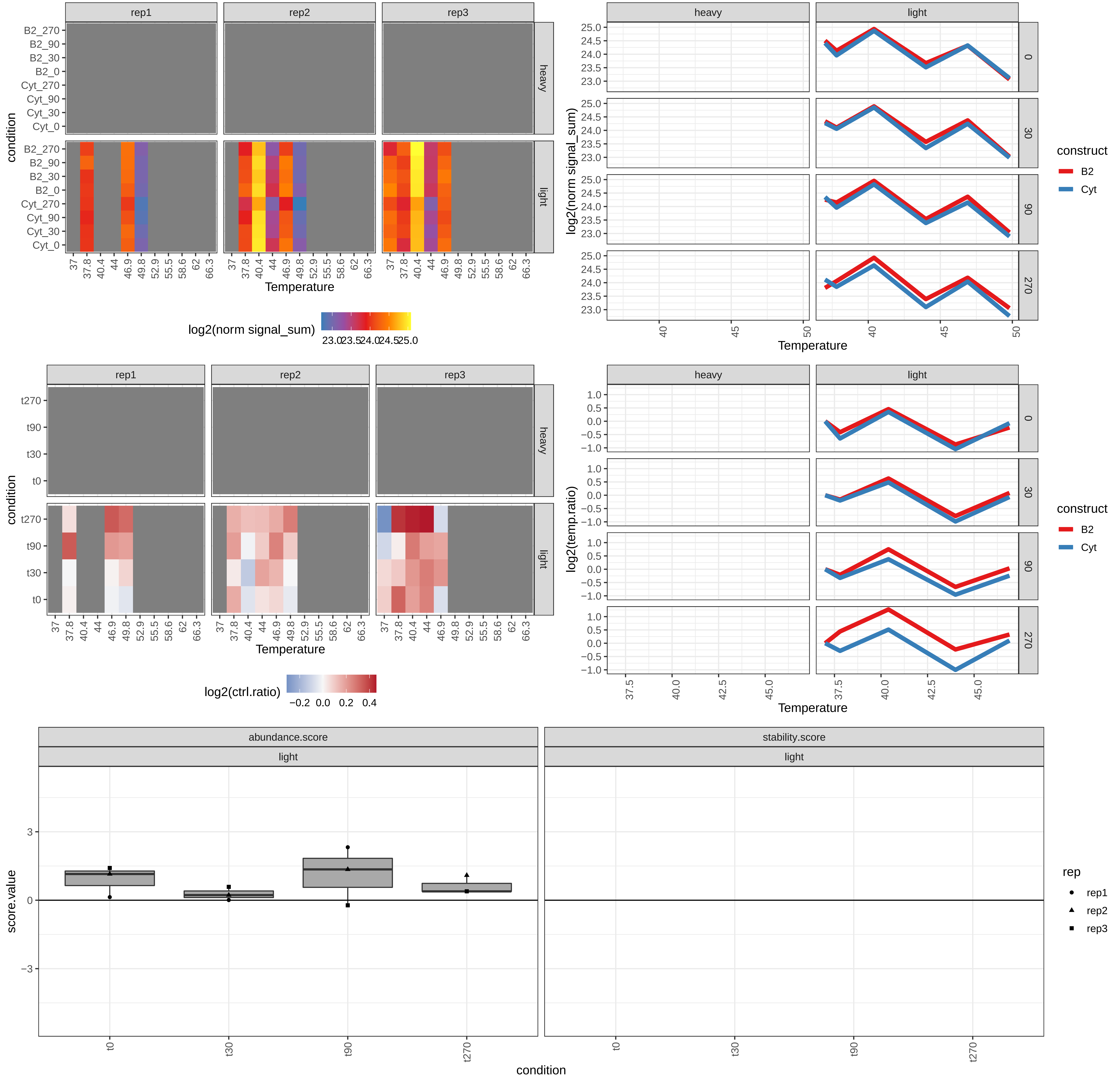

Q08581 − Kinetochore protein SLK19 OS=Saccharomyces cerevisiae (strain ATCC 204508 / S288c) GN=SLK19 PE=1 SV=1

SLM1 − average.top3: 6.9893895 − max.qupm: 10

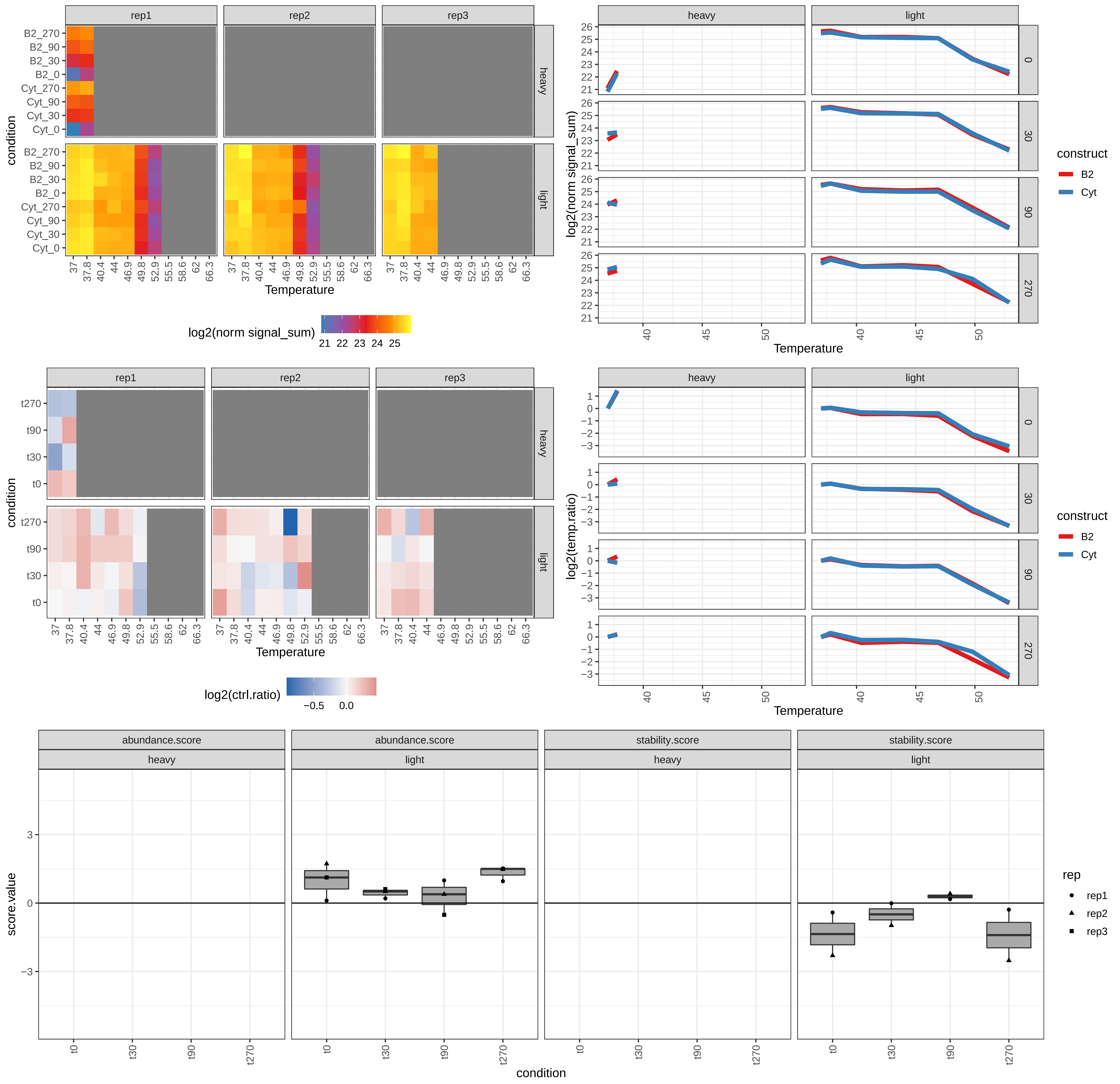

P40485 − Phosphatidylinositol 4,5−bisphosphate−binding protein SLM1 OS=Saccharomyces cerevisiae (strain ATCC 204508 / S288c) GN=SLM1 PE=1 SV=1

SLM5 − average.top3: 6.38827153846154 − max.qupm: 6

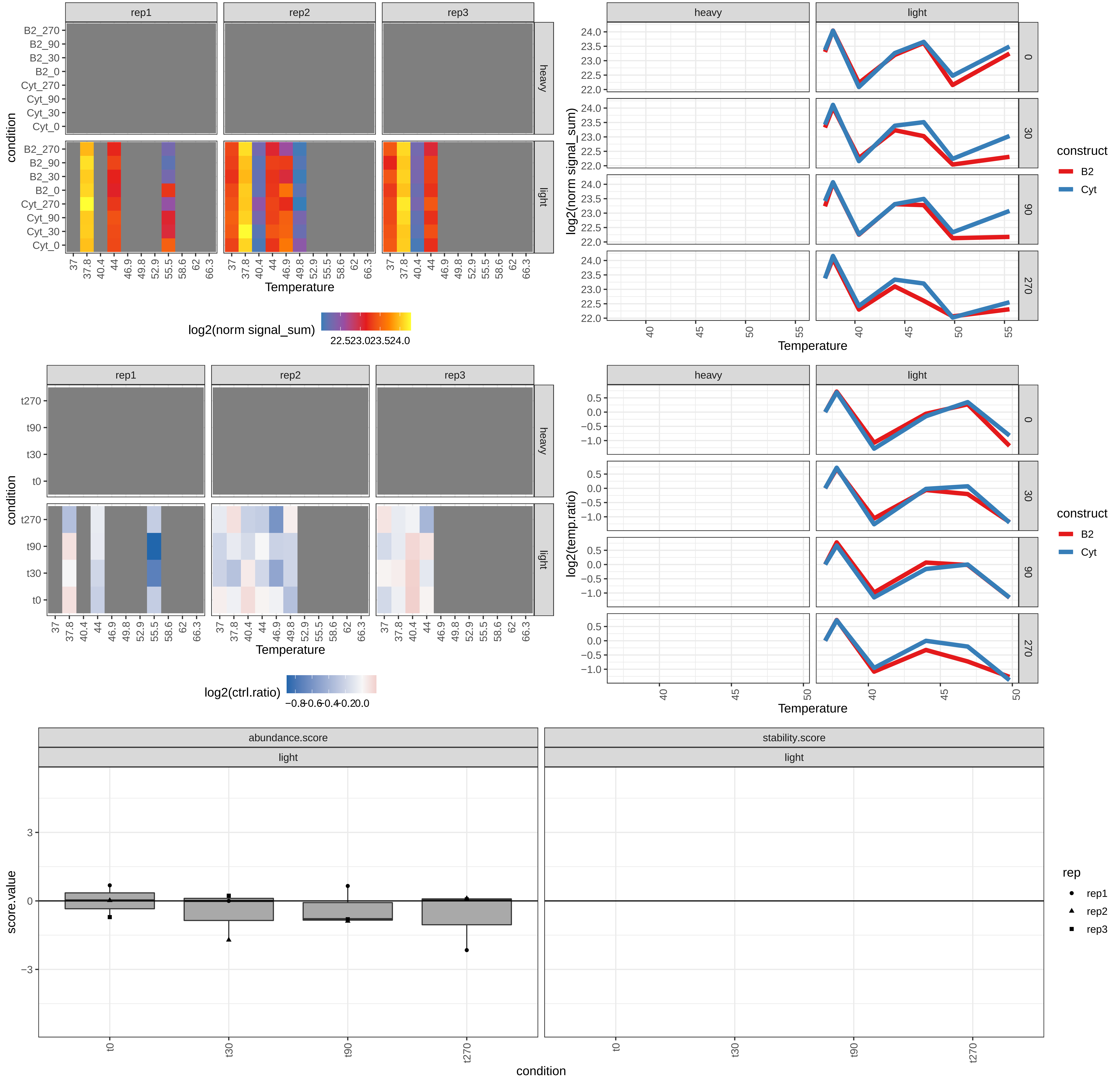

P25345 − Asparagine−−tRNA ligase, mitochondrial OS=Saccharomyces cerevisiae (strain ATCC 204508 / S288c) GN=SLM5 PE=1 SV=1

SLT2 − average.top3: 6.87107764705882 − max.qupm: 5

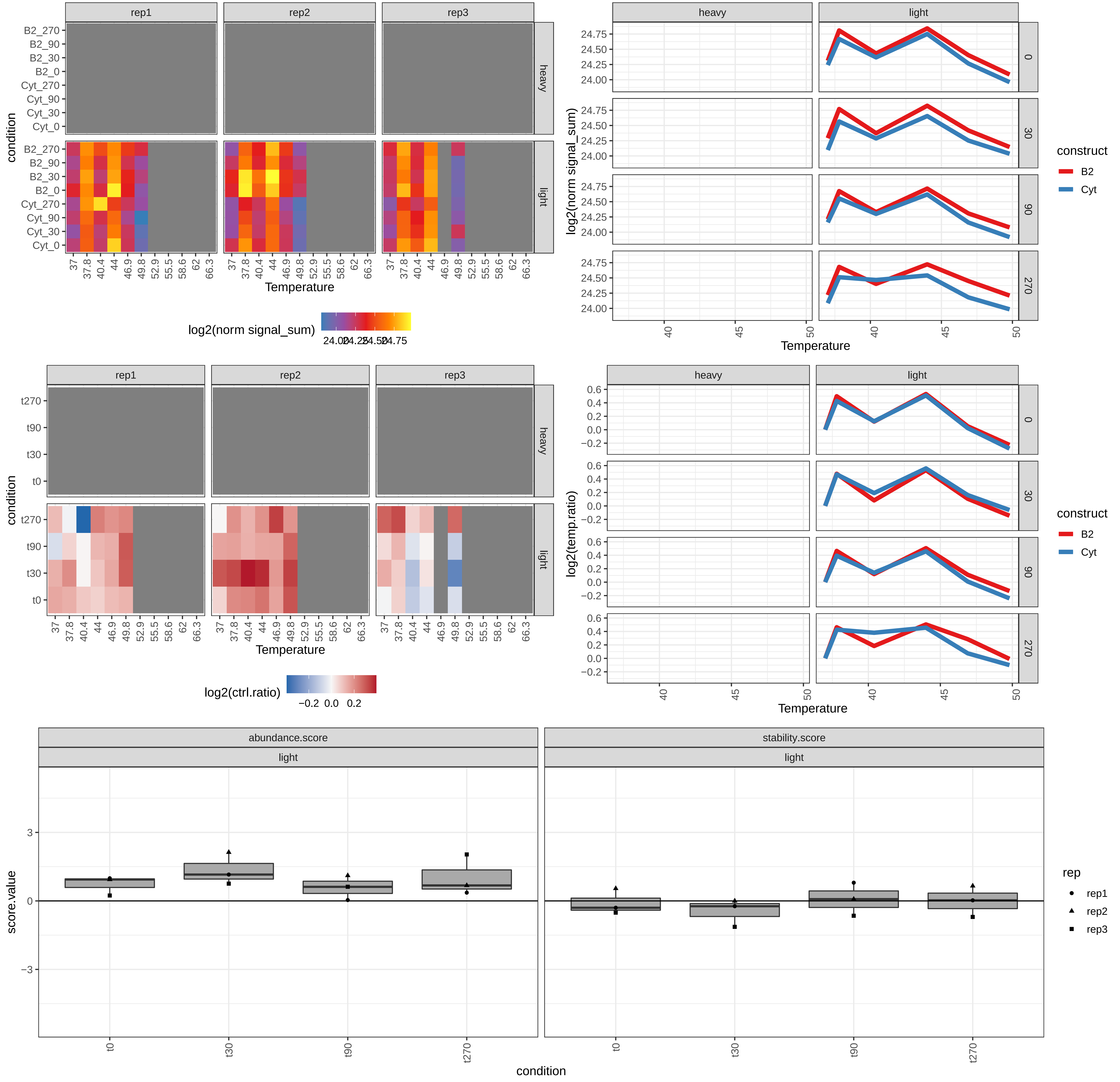

Q00772 − Mitogen−activated protein kinase SLT2/MPK1 OS=Saccharomyces cerevisiae (strain ATCC 204508 / S288c) GN=SLT2 PE=1 SV=2

SLY1 − average.top3: 6.53087125 − max.qupm: 9

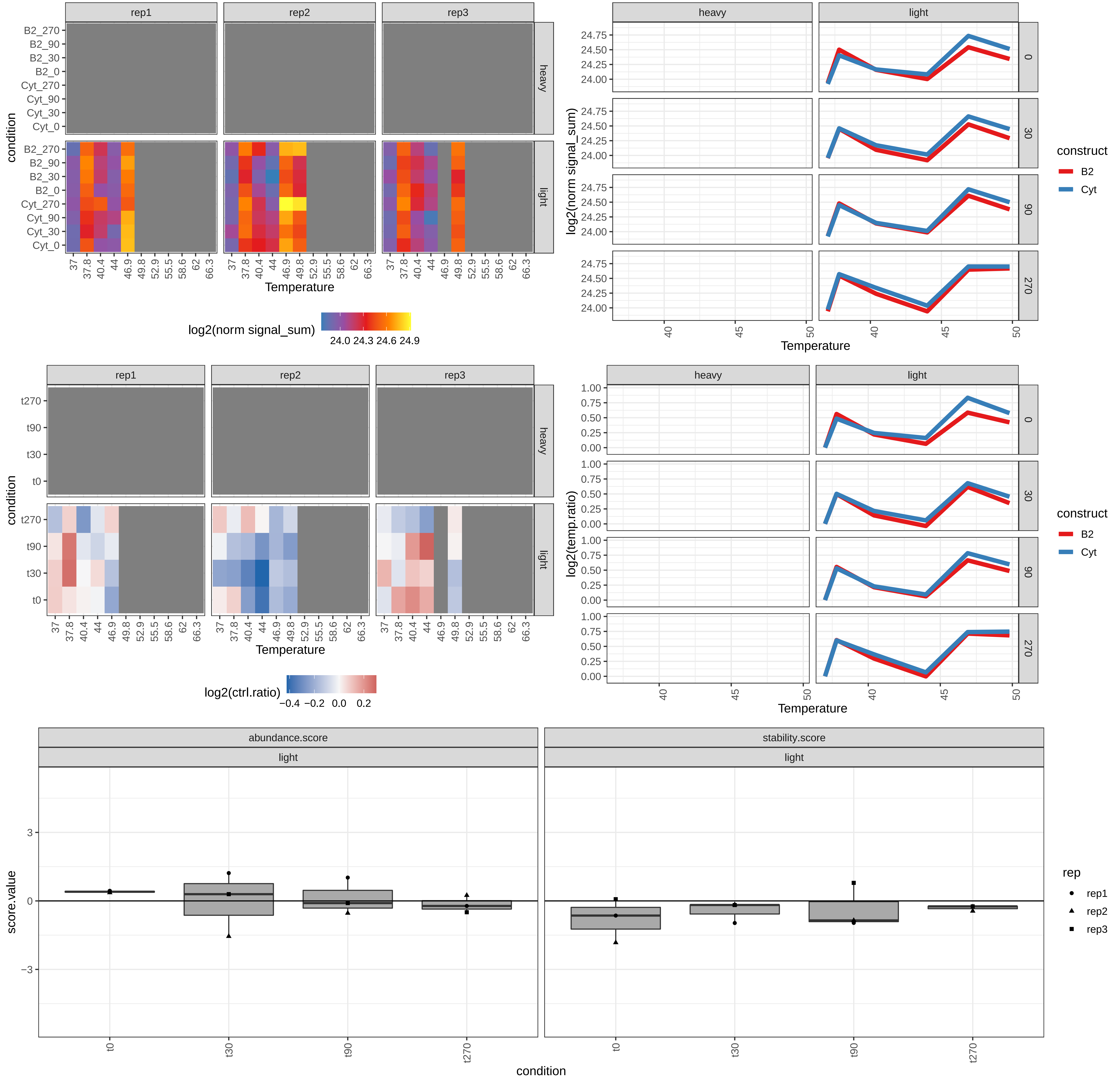

P22213 − Protein SLY1 OS=Saccharomyces cerevisiae (strain ATCC 204508 / S288c) GN=SLY1 PE=1 SV=1

SMB1 − average.top3: 6.85922416666667 − max.qupm: 5

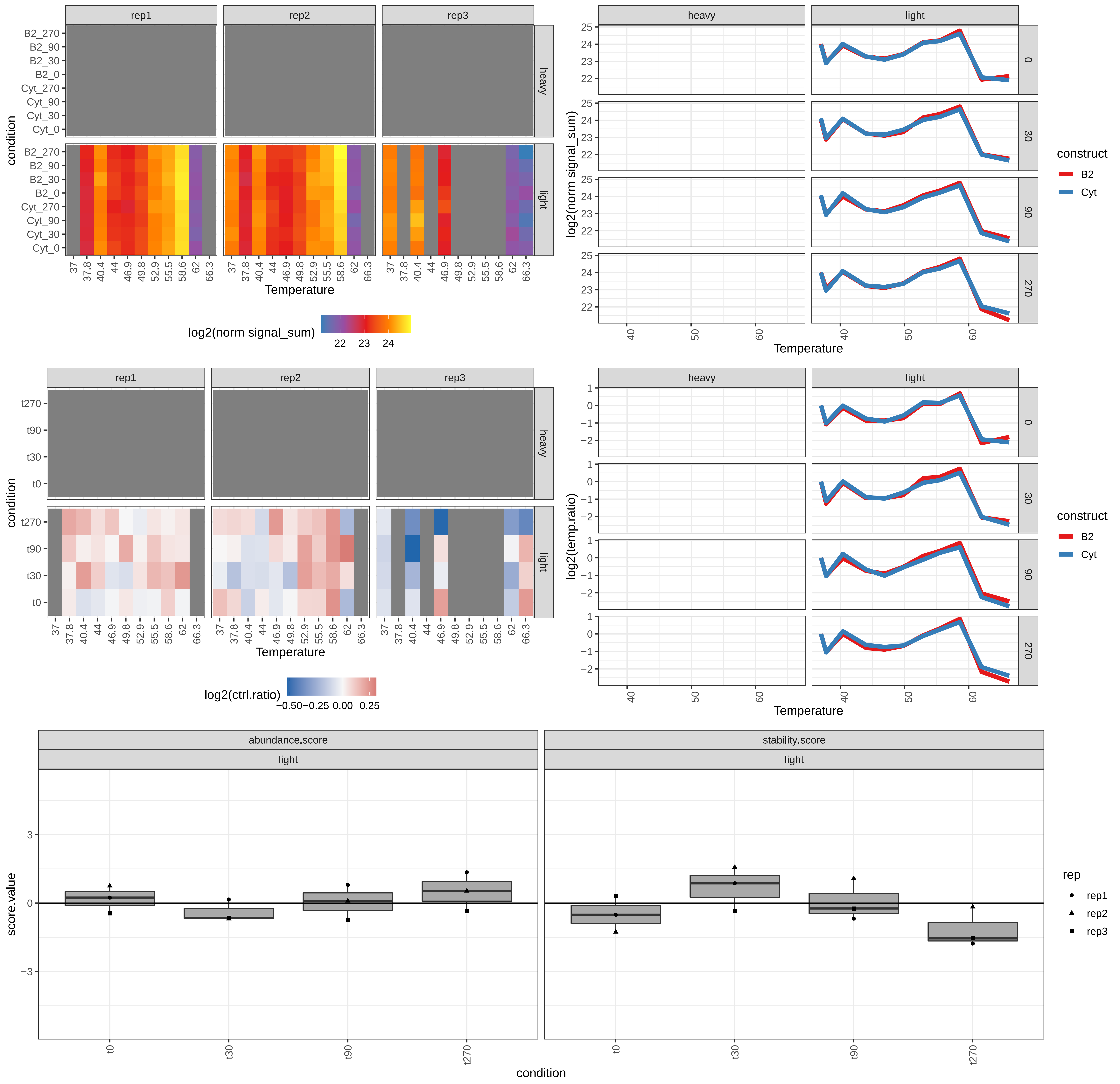

P40018 − Small nuclear ribonucleoprotein−associated protein B OS=Saccharomyces cerevisiae (strain ATCC 204508 / S288c) GN=SMB1 PE=1 SV=1

SMC1 − average.top3: 6.80198 − max.qupm: 19

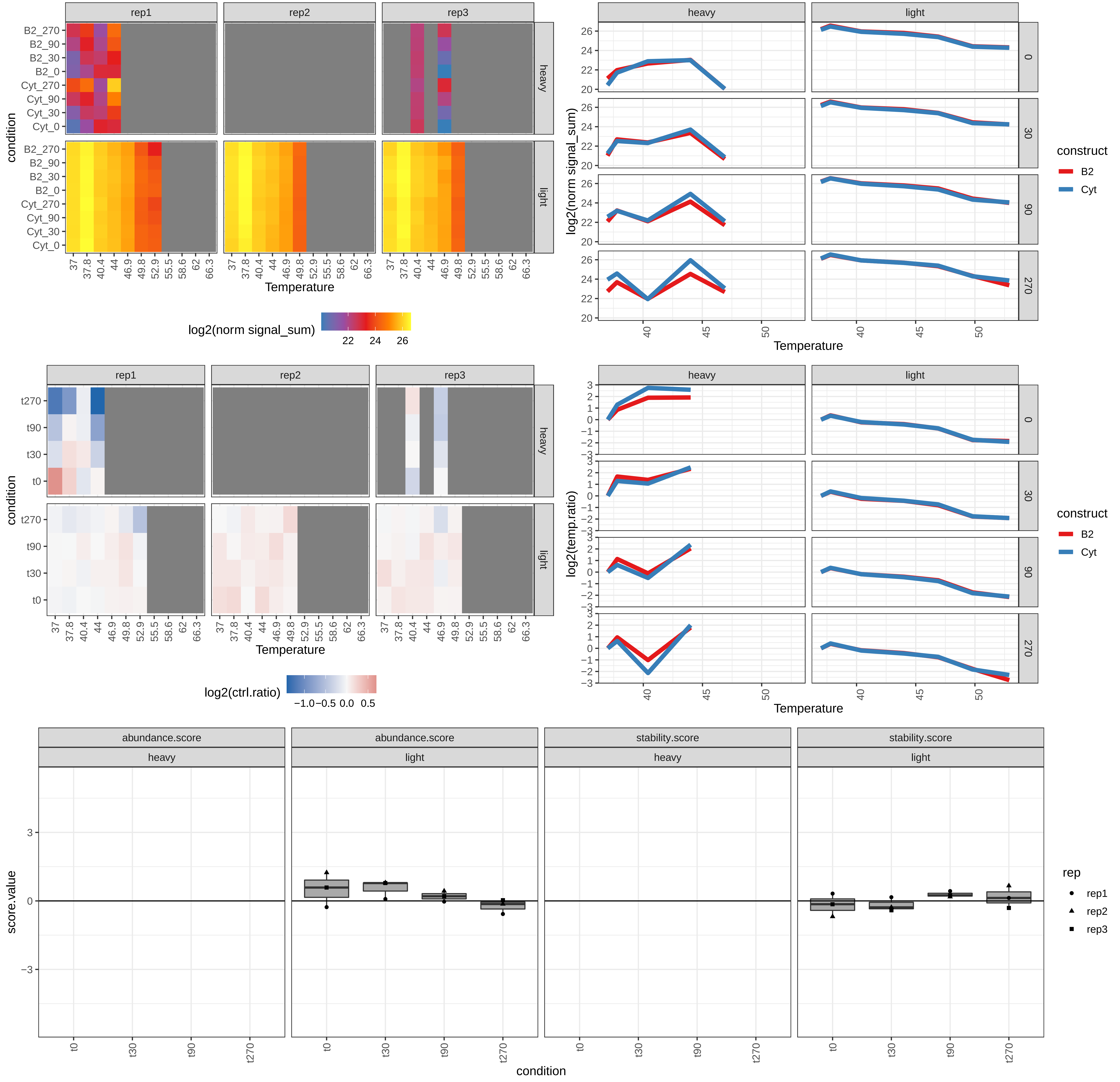

P32908 − Structural maintenance of chromosomes protein 1 OS=Saccharomyces cerevisiae (strain ATCC 204508 / S288c) GN=SMC1 PE=1 SV=1

SMC2 − average.top3: 6.46602416666667 − max.qupm: 7

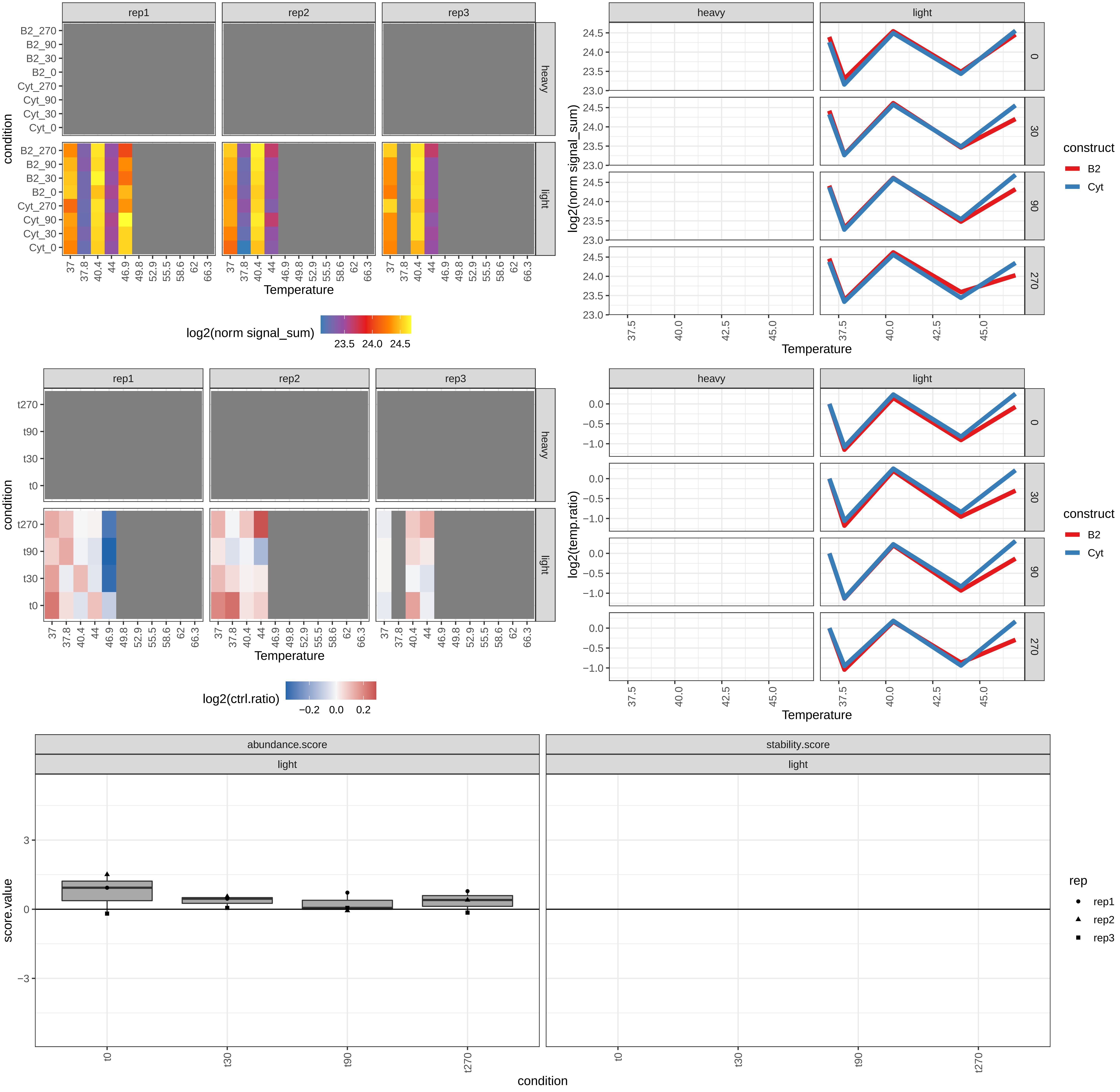

P38989 − Structural maintenance of chromosomes protein 2 OS=Saccharomyces cerevisiae (strain ATCC 204508 / S288c) GN=SMC2 PE=1 SV=1

SMC3 − average.top3: 6.771119 − max.qupm: 25

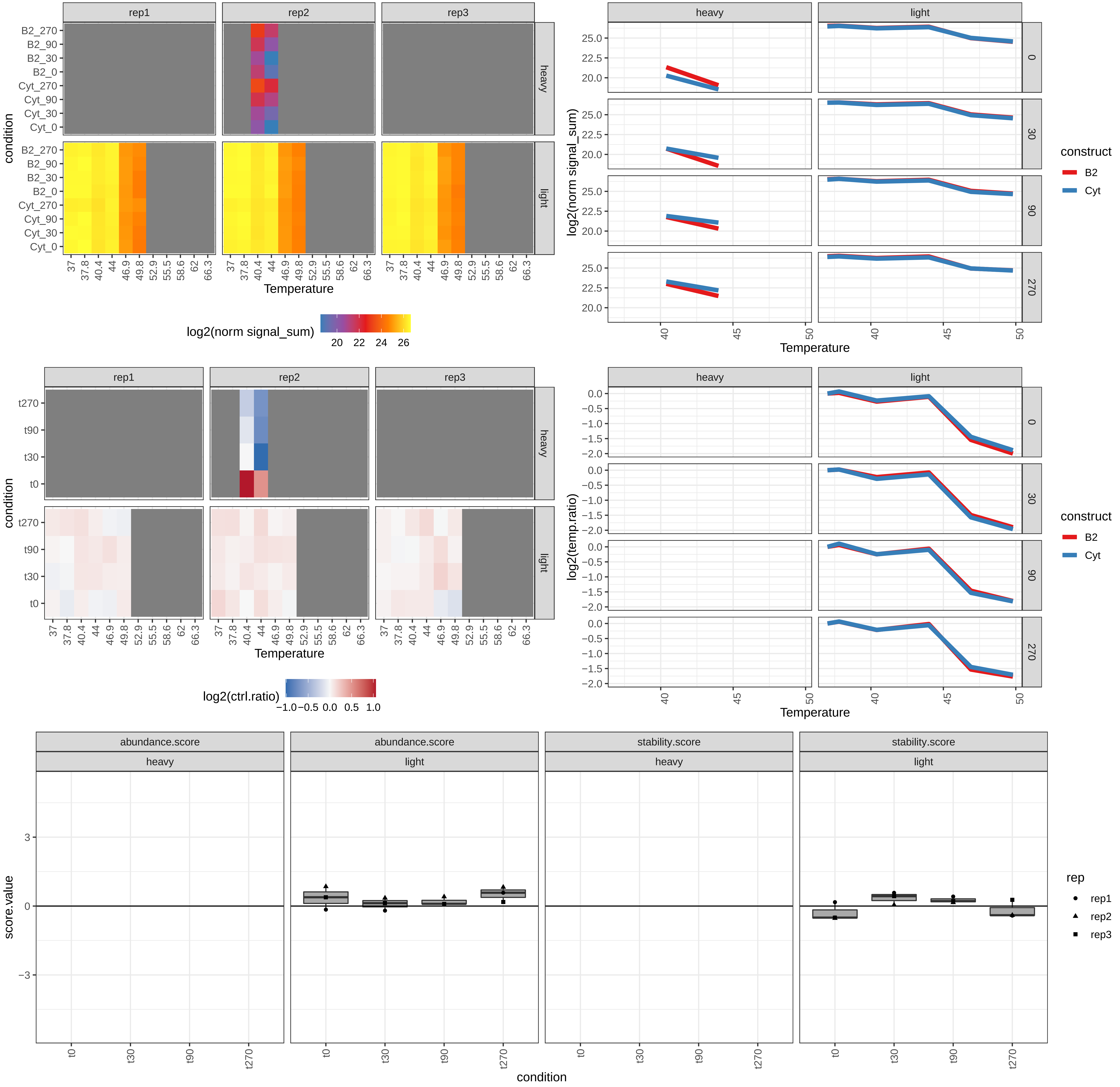

P47037 − Structural maintenance of chromosomes protein 3 OS=Saccharomyces cerevisiae (strain ATCC 204508 / S288c) GN=SMC3 PE=1 SV=1

SMC4 − average.top3: 6.42590928571429 − max.qupm: 9

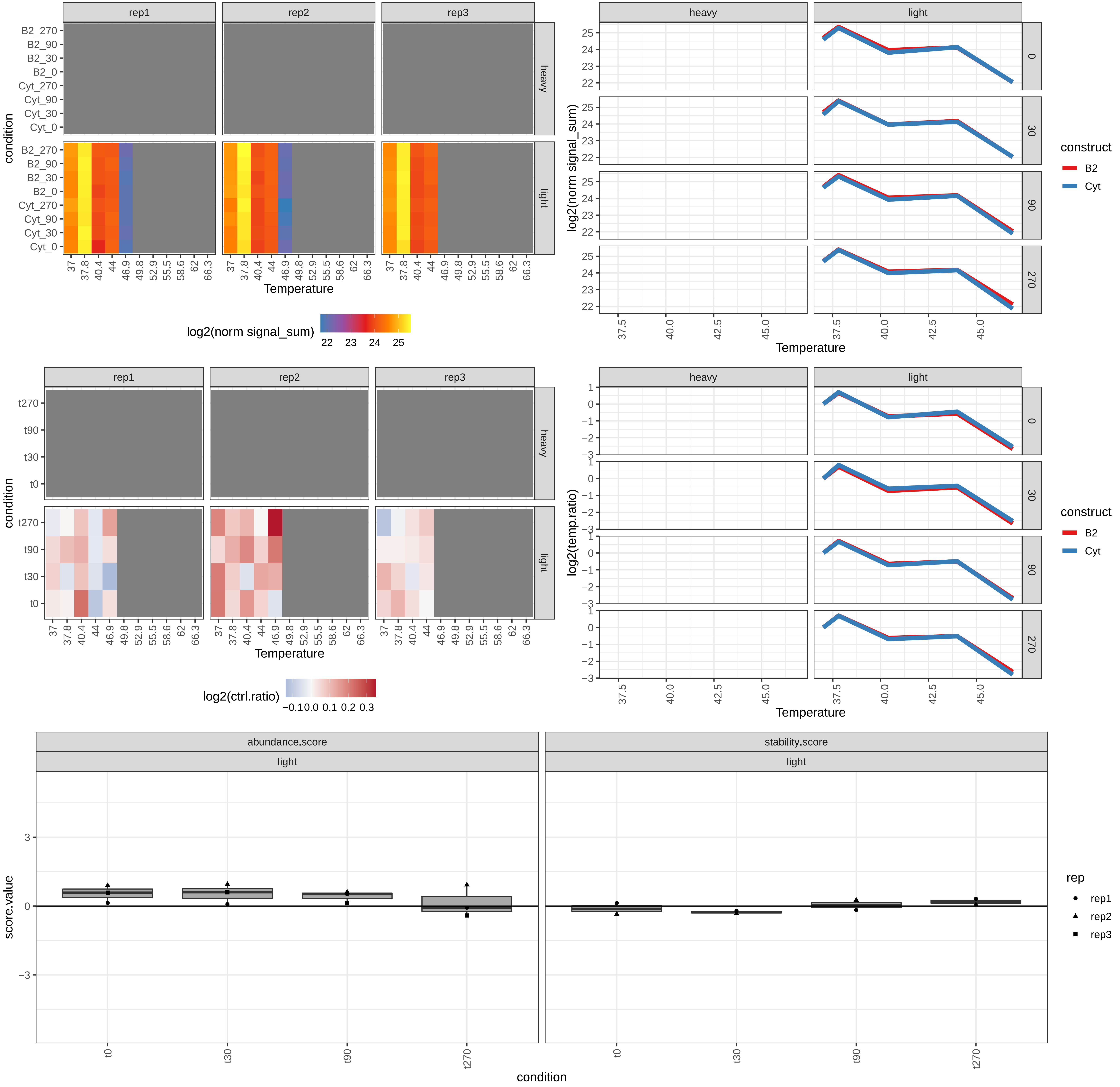

Q12267 − Structural maintenance of chromosomes protein 4 OS=Saccharomyces cerevisiae (strain ATCC 204508 / S288c) GN=SMC4 PE=1 SV=1

SMC5 − average.top3: 6.26581333333333 − max.qupm: 5

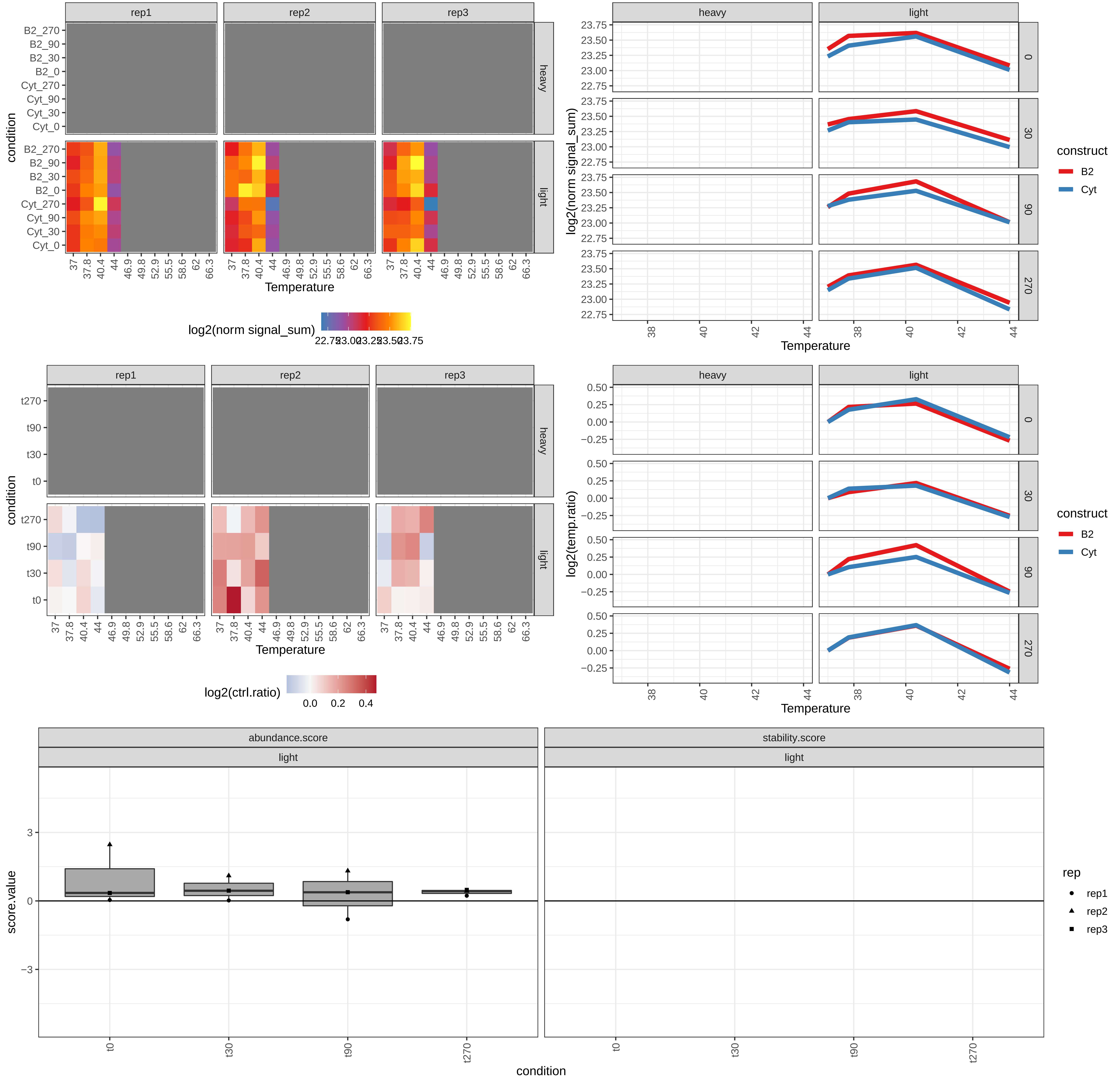

Q08204 − Structural maintenance of chromosomes protein 5 OS=Saccharomyces cerevisiae (strain ATCC 204508 / S288c) GN=SMC5 PE=1 SV=1

SMD1 − average.top3: 6.586252 − max.qupm: 3

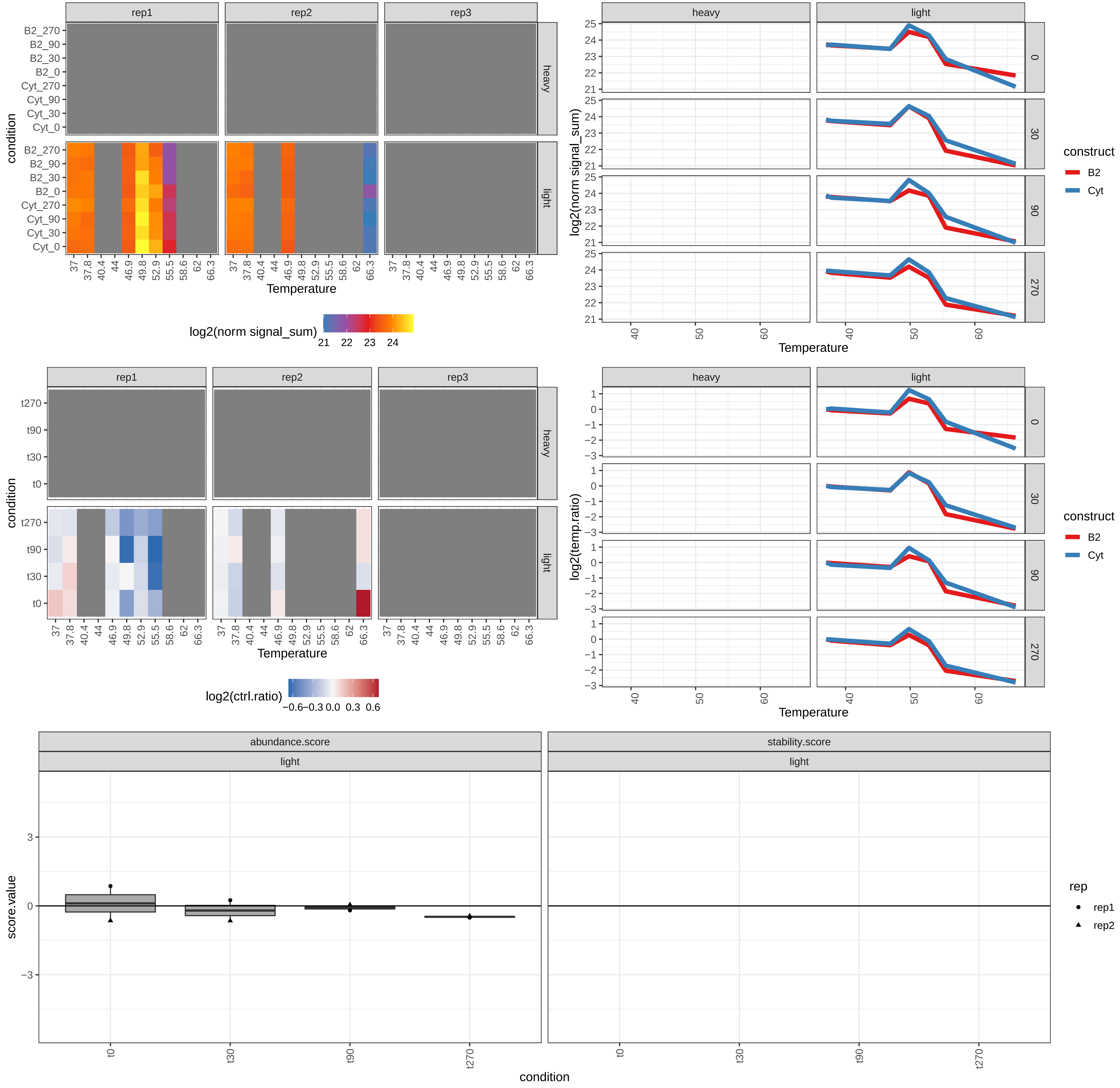

Q02260 − Small nuclear ribonucleoprotein Sm D1 OS=Saccharomyces cerevisiae (strain ATCC 204508 / S288c) GN=SMD1 PE=1 SV=1

SMD2 − average.top3: 6.68609666666667 − max.qupm: 4

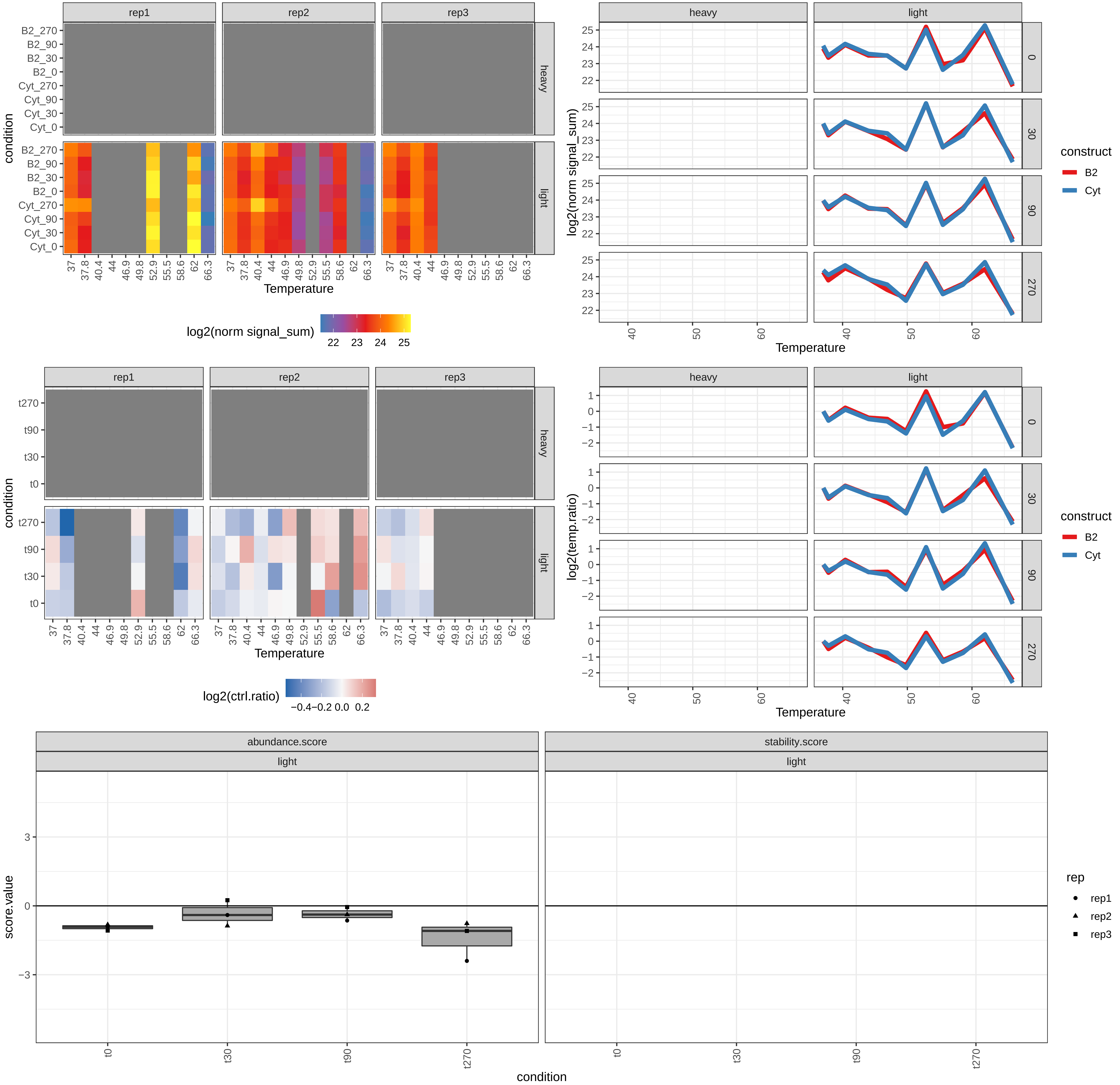

Q06217 − Small nuclear ribonucleoprotein Sm D2 OS=Saccharomyces cerevisiae (strain ATCC 204508 / S288c) GN=SMD2 PE=1 SV=2

SMD3 − average.top3: 6.30827625 − max.qupm: 4

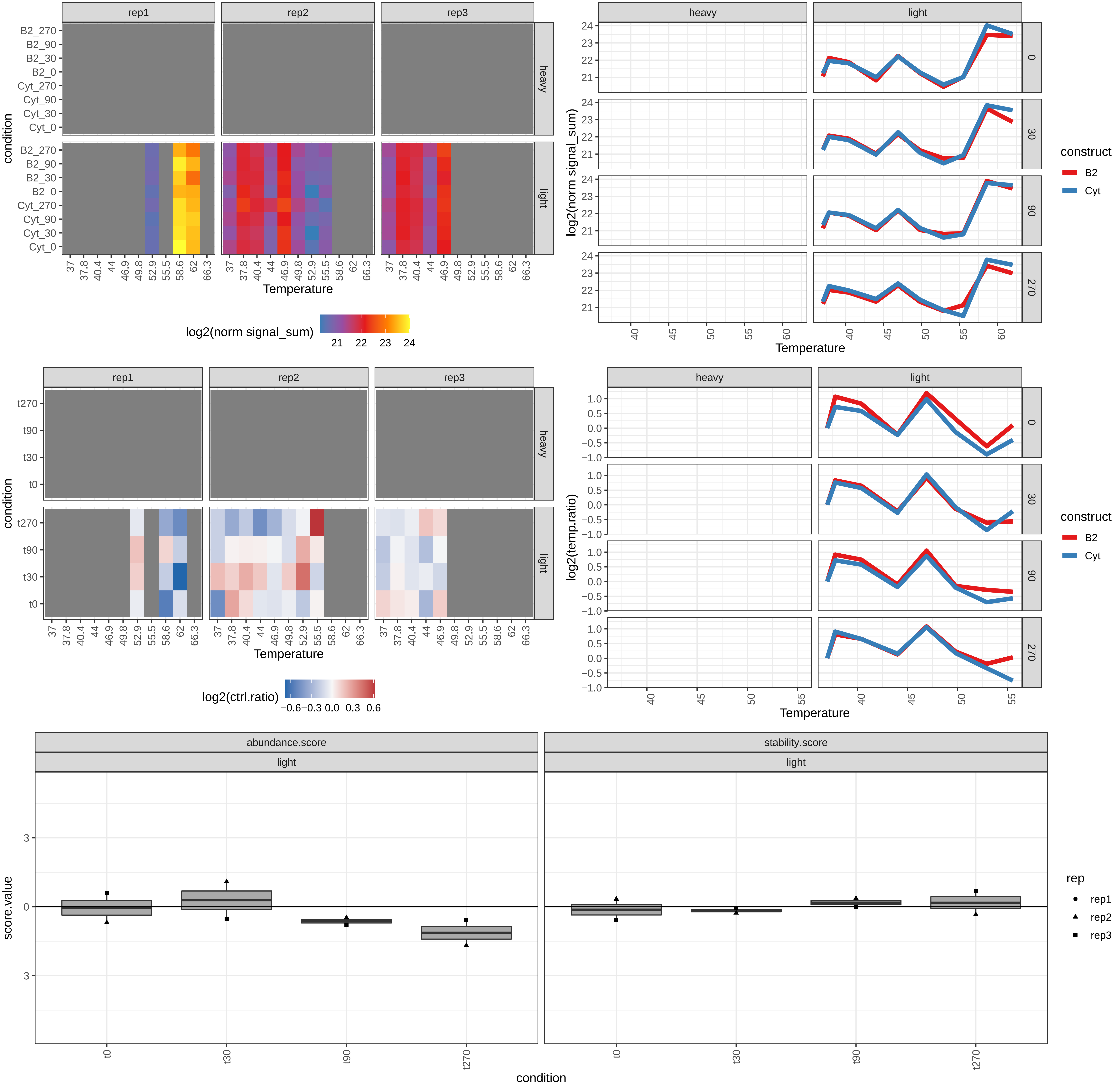

P43321 − Small nuclear ribonucleoprotein Sm D3 OS=Saccharomyces cerevisiae (strain ATCC 204508 / S288c) GN=SMD3 PE=1 SV=1

SMI1 − average.top3: 6.9325505 − max.qupm: 4

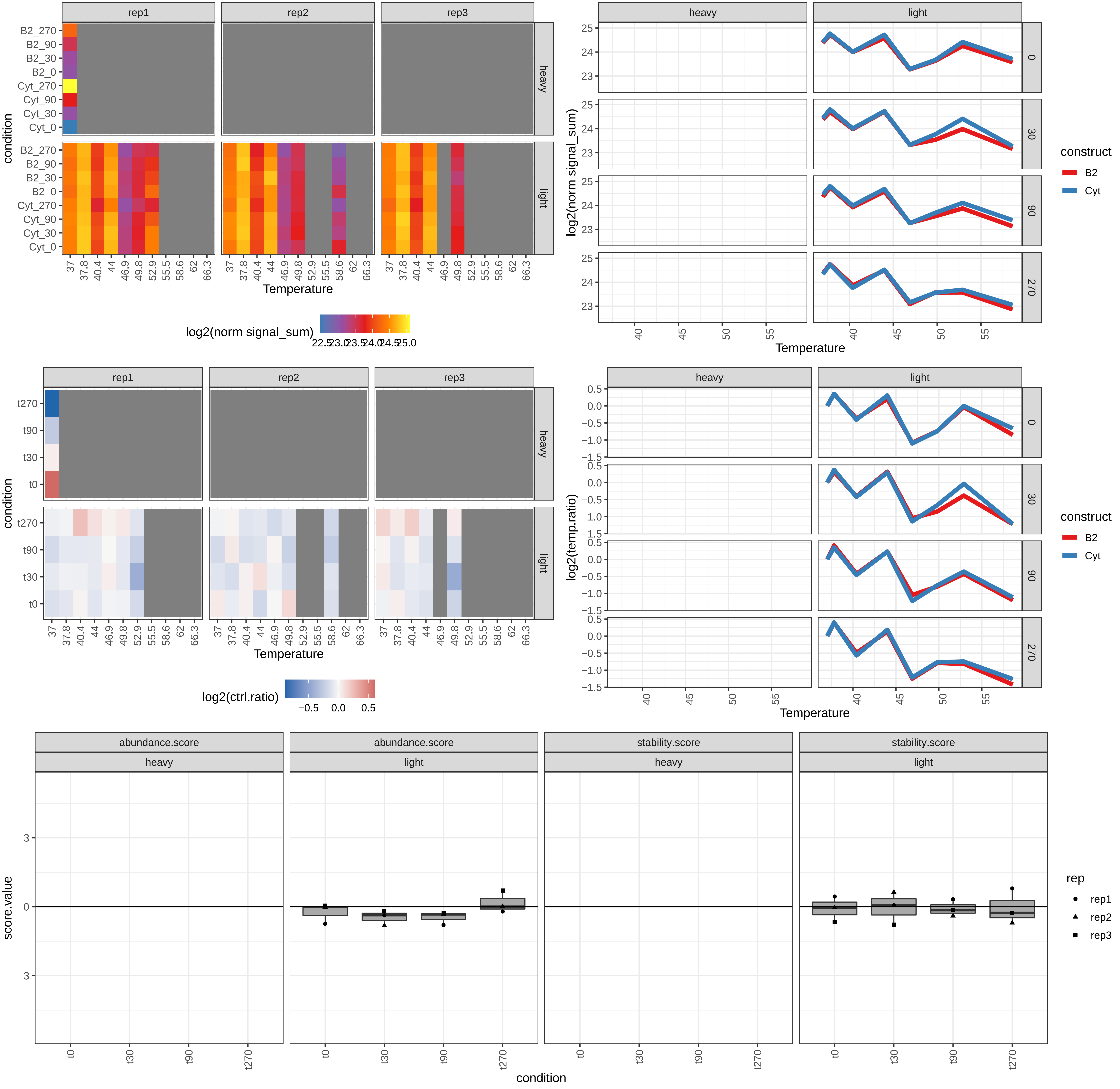

P32566 − Cell wall assembly regulator SMI1 OS=Saccharomyces cerevisiae (strain ATCC 204508 / S288c) GN=SMI1 PE=1 SV=1

SMM1 − average.top3: 6.63456058823529 − max.qupm: 4

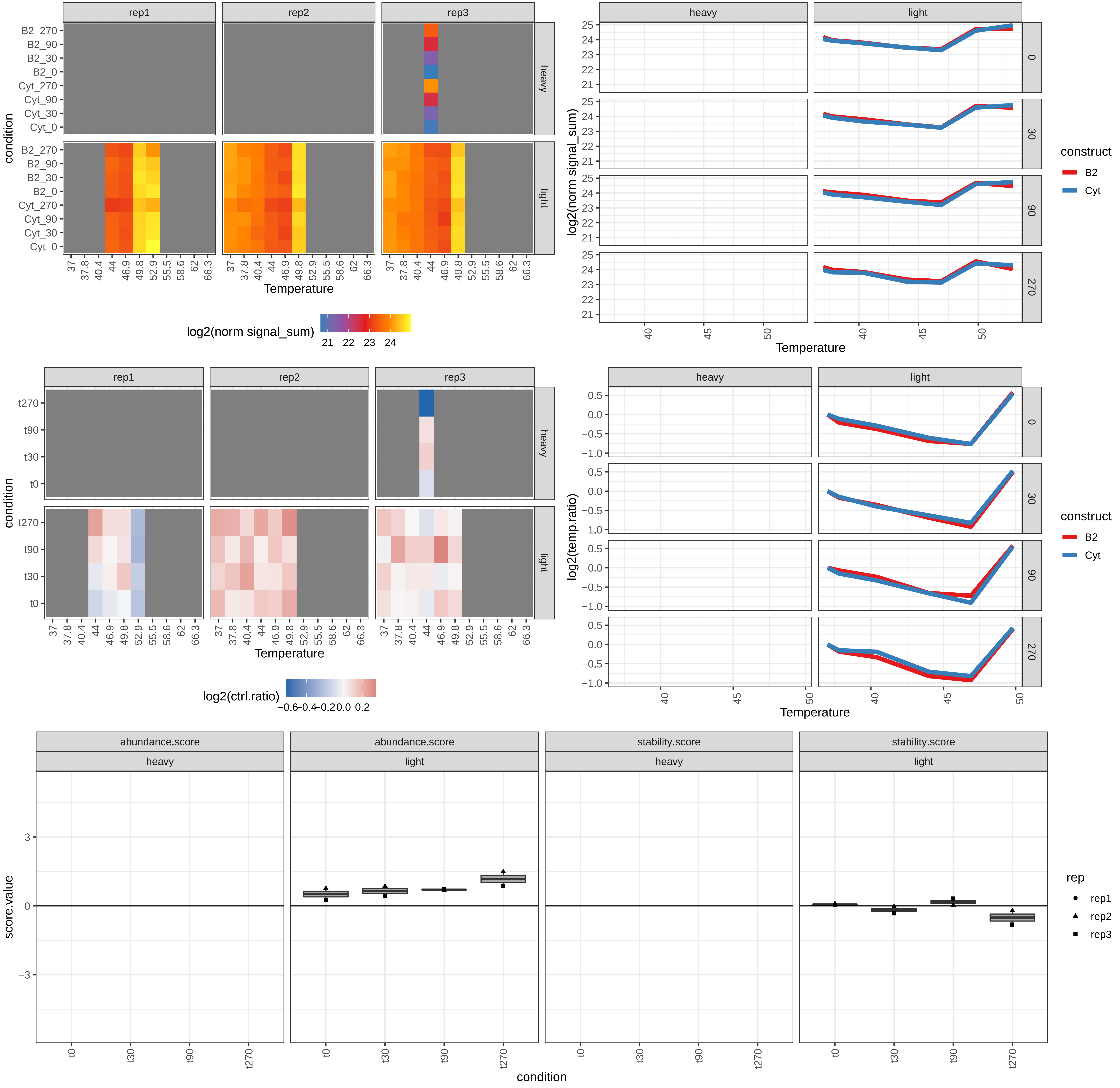

P53720 − tRNA−dihydrouridine(20) synthase [NAD(P)+] OS=Saccharomyces cerevisiae (strain ATCC 204508 / S288c) GN=SMM1 PE=1 SV=1

SMT3 − average.top3: 7.54327409836066 − max.qupm: 5

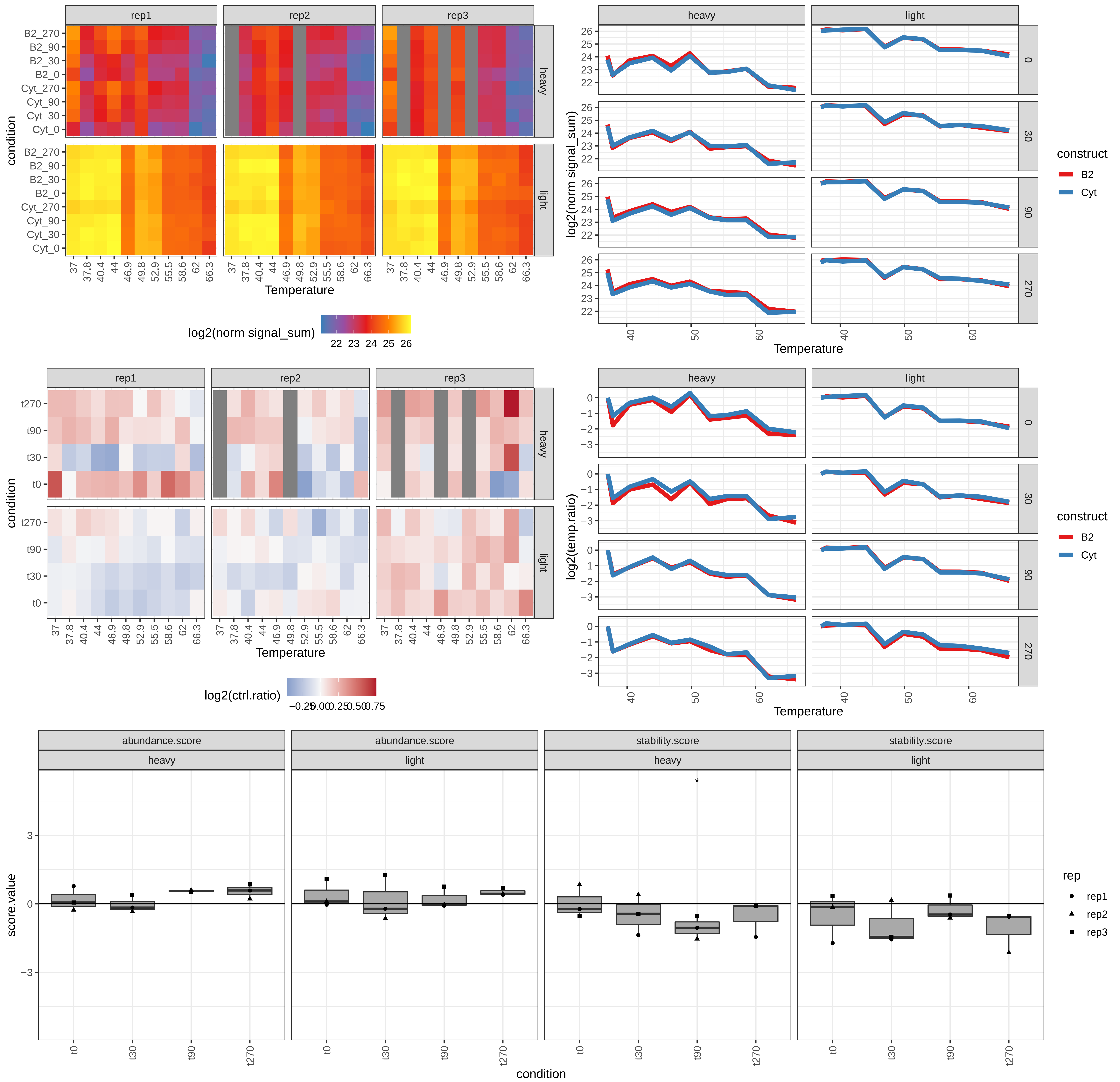

Q12306 − Ubiquitin−like protein SMT3 OS=Saccharomyces cerevisiae (strain ATCC 204508 / S288c) GN=SMT3 PE=1 SV=1

SMY1 − average.top3: 6.4142425 − max.qupm: 7

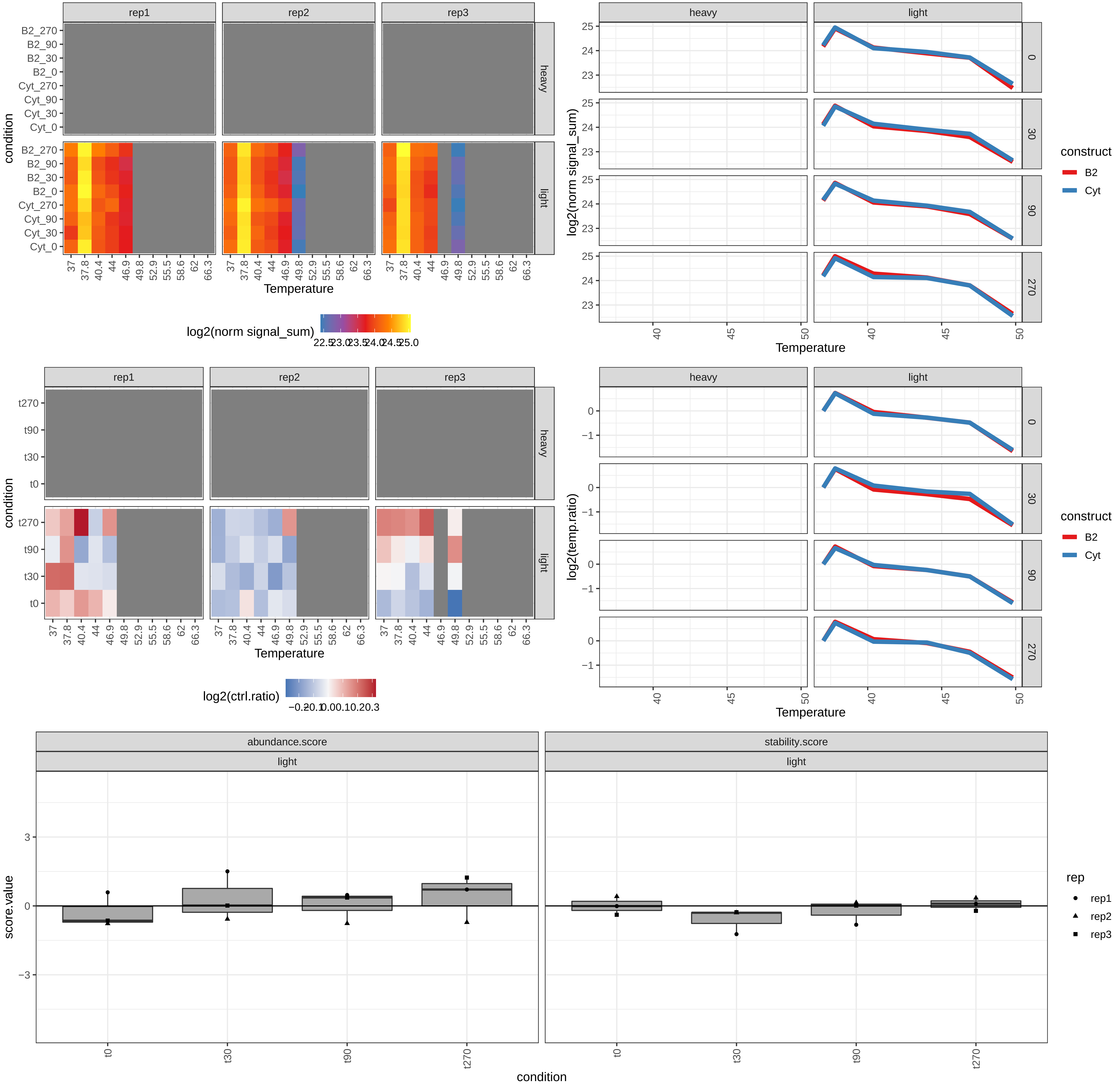

P32364 − Kinesin−related protein SMY1 OS=Saccharomyces cerevisiae (strain ATCC 204508 / S288c) GN=SMY1 PE=1 SV=1

SMY2 − average.top3: 6.8586356 − max.qupm: 5

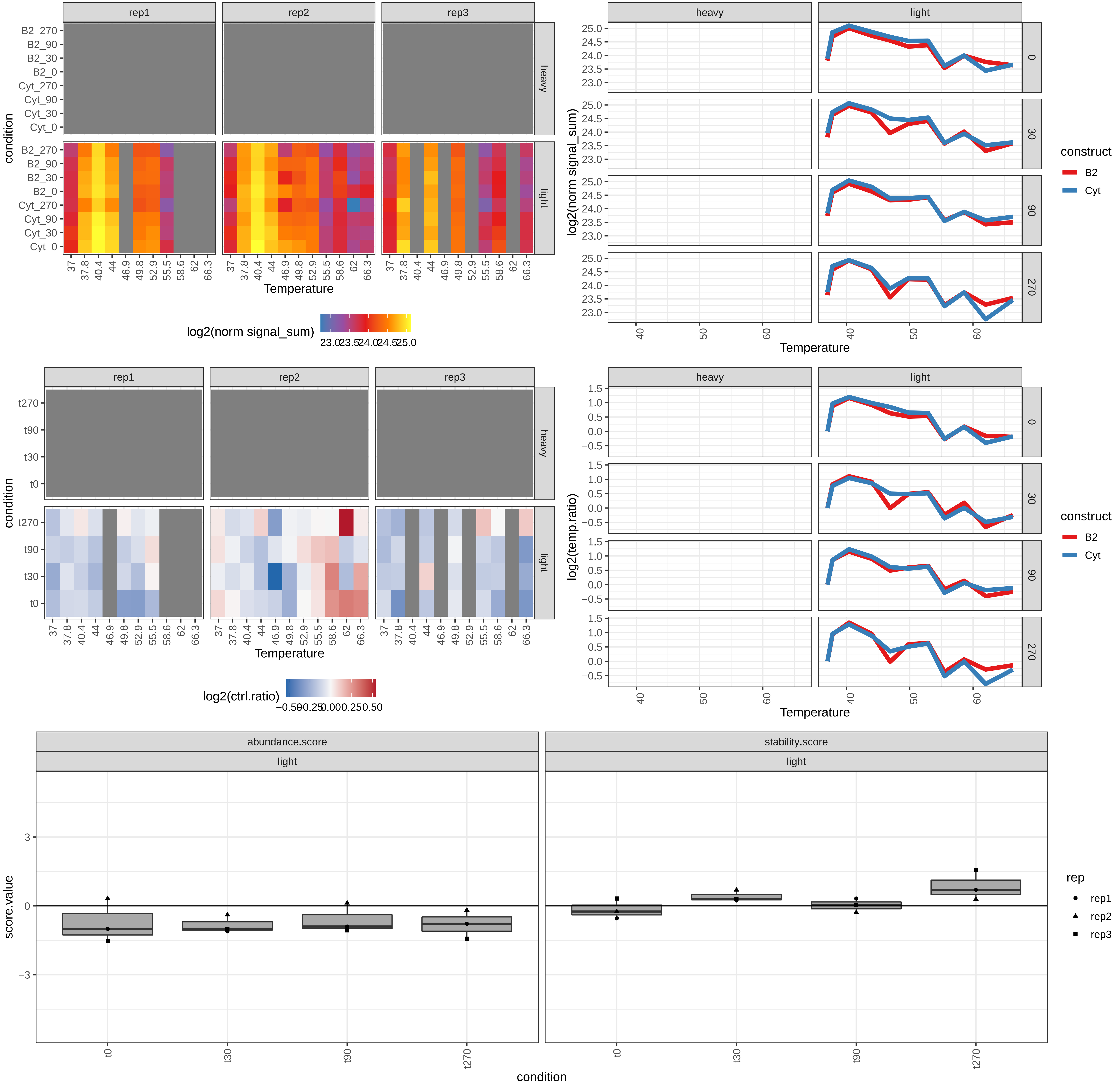

P32909 − Protein SMY2 OS=Saccharomyces cerevisiae (strain ATCC 204508 / S288c) GN=SMY2 PE=1 SV=2

SNF1 − average.top3: 6.98782341463415 − max.qupm: 12

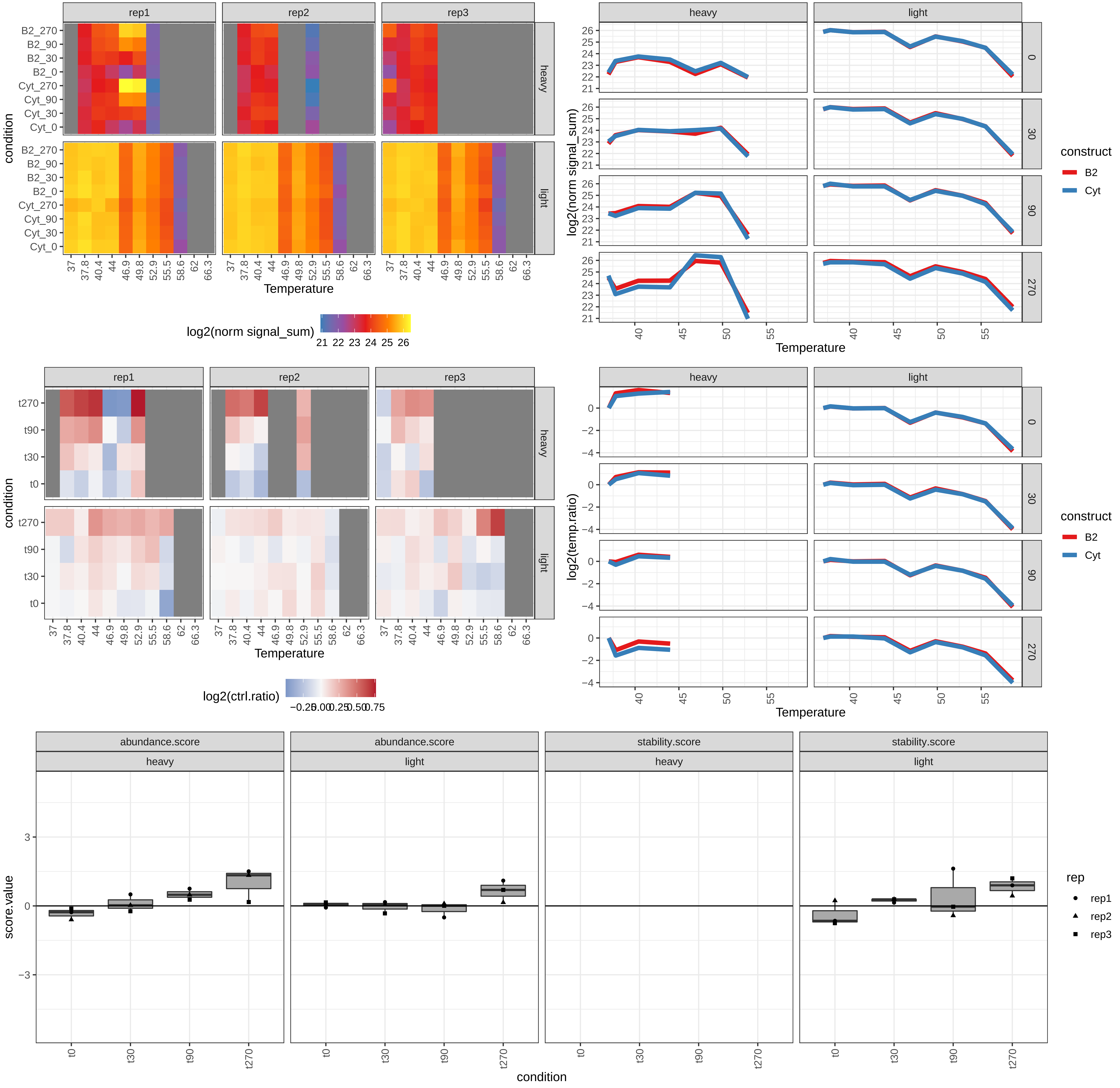

P06782 − Carbon catabolite−derepressing protein kinase OS=Saccharomyces cerevisiae (strain ATCC 204508 / S288c) GN=SNF1 PE=1 SV=1

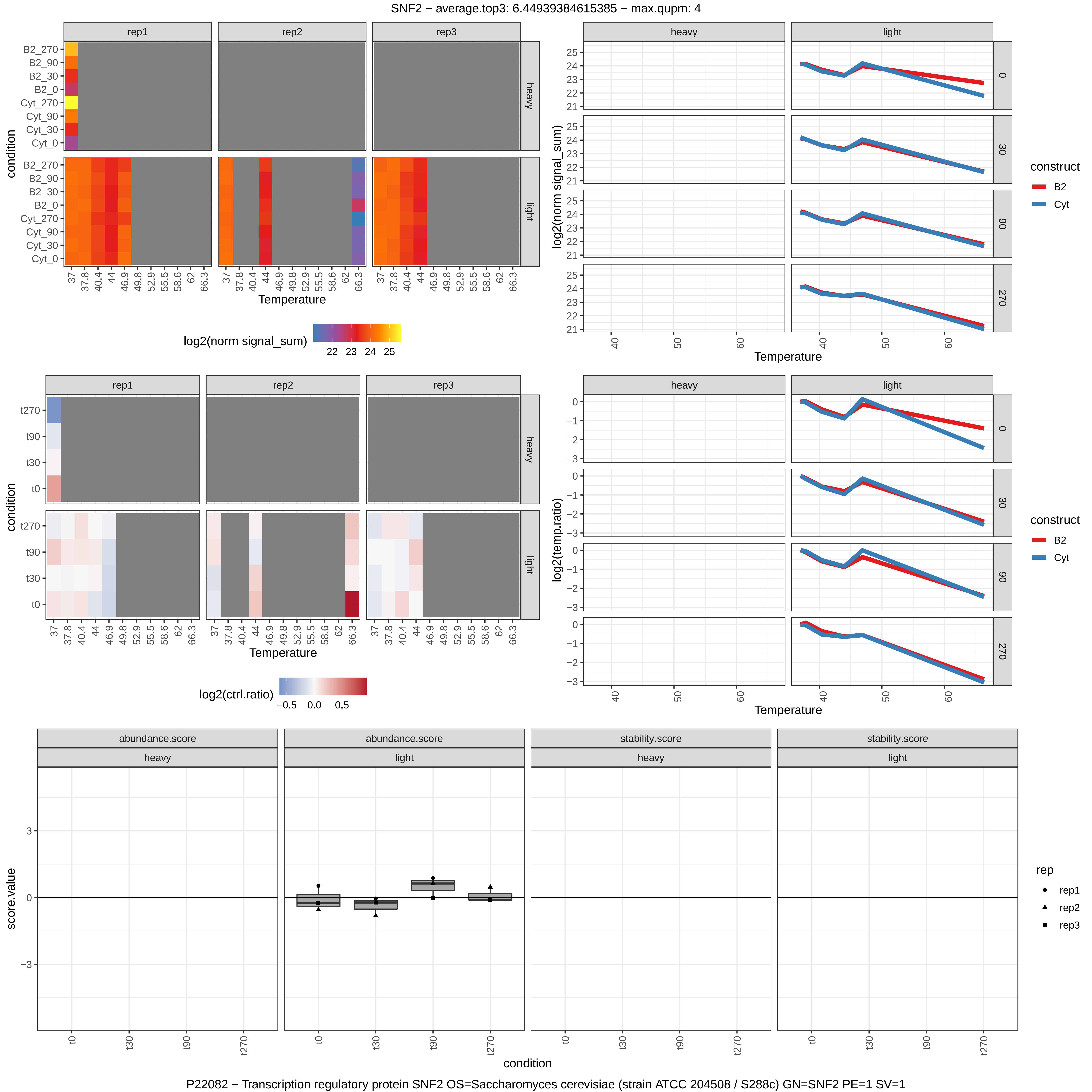

SNF2 − average.top3: 6.44939384615385 − max.qupm: 4
rep1
rep2
rep3
heavy
light
condition
Temperature
log2(norm signal_sum)
construct
B2
Cyt
log2(ctrl.ratio)
log2(temp.ratio)
abundance.score
stability.score
score.value
rep
P22082 − Transcription regulatory protein SNF2 OS=Saccharomyces cerevisiae (strain ATCC 204508 / S288c) GN=SNF2 PE=1 SV=1

SNF4 − average.top3: 6.988145625 − max.qupm: 10

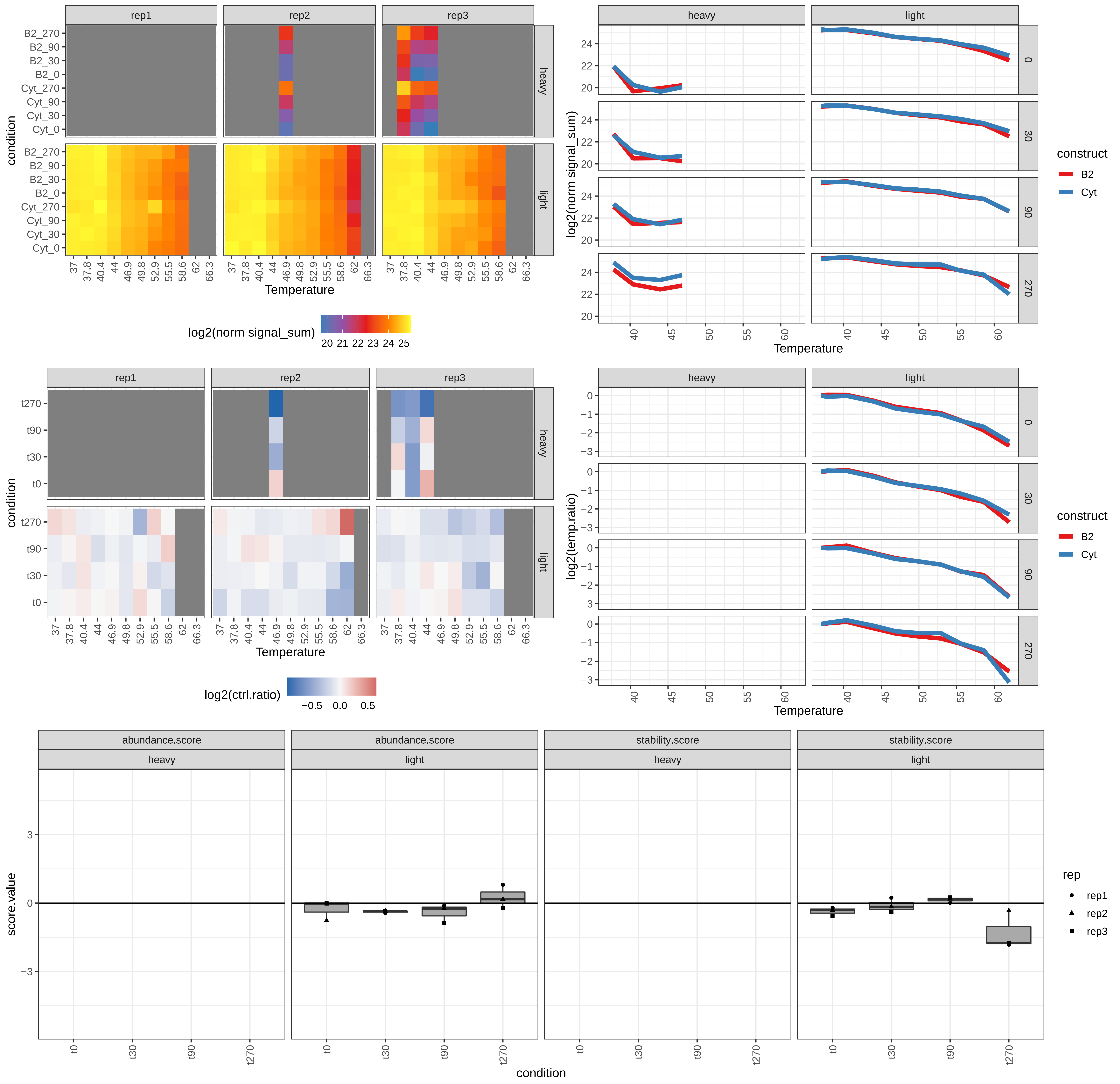

P12904 − 5'−AMP−activated protein kinase subunit gamma OS=Saccharomyces cerevisiae (strain ATCC 204508 / S288c) GN=SNF4 PE=1 SV=1

SNG1 − average.top3: 6.46175727272727 − max.qupm: 3

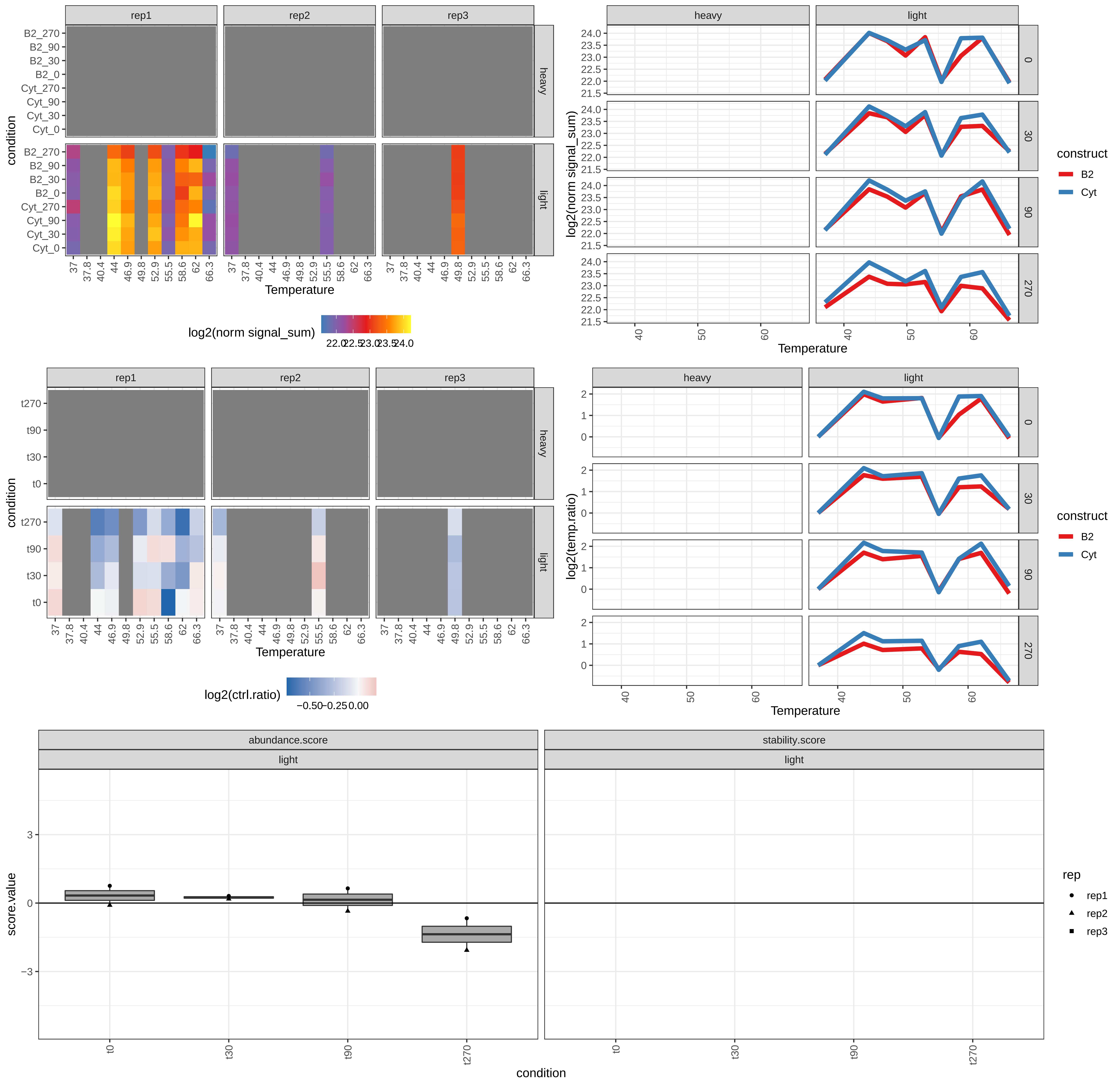

P46950 − Nitrosoguanidine resistance protein SNG1 OS=Saccharomyces cerevisiae (strain ATCC 204508 / S288c) GN=SNG1 PE=1 SV=1

SNL1 − average.top3: 6.89015 − max.qupm: 4

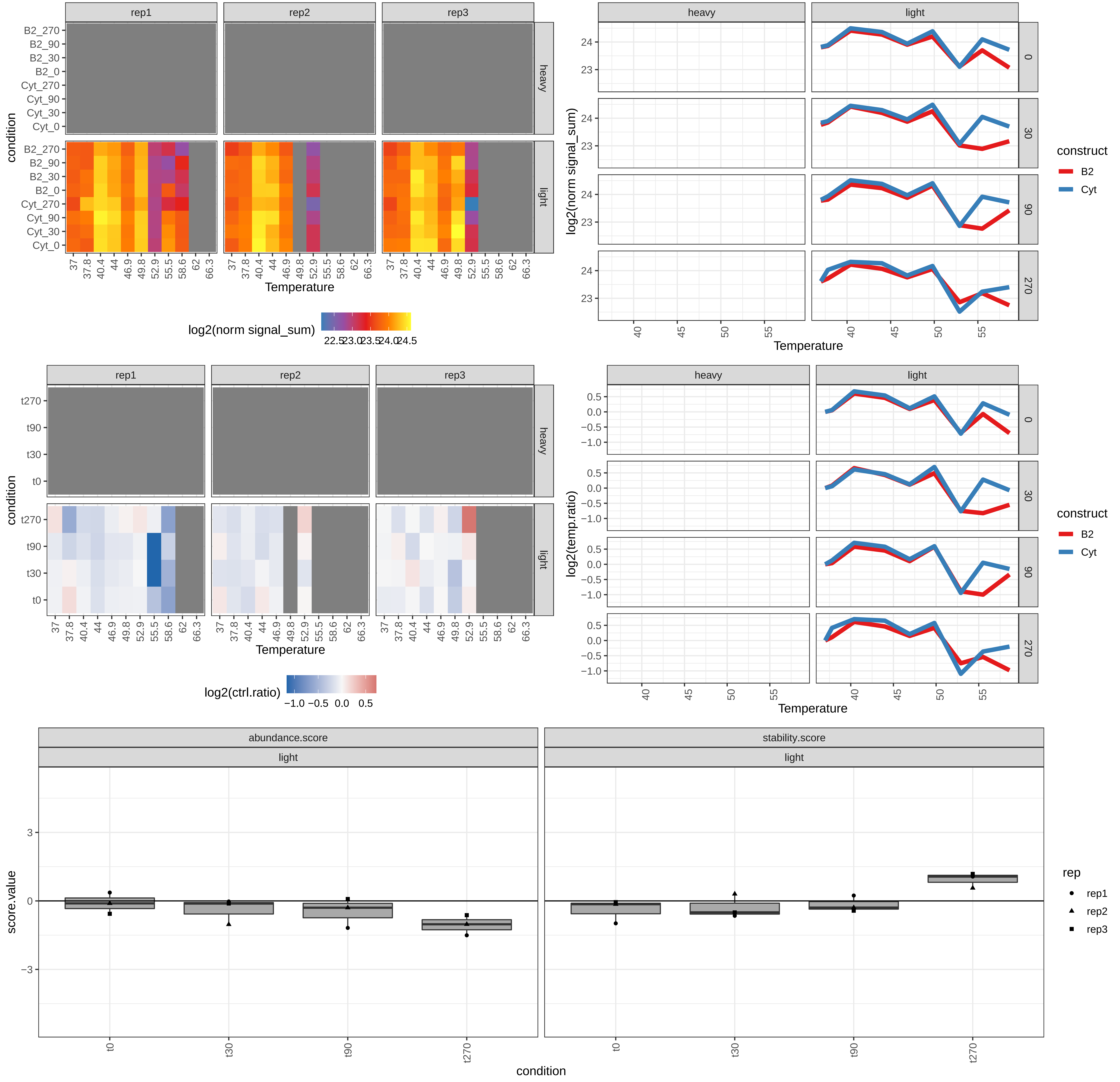

P40548 − HSP70 co−chaperone SNL1 OS=Saccharomyces cerevisiae (strain ATCC 204508 / S288c) GN=SNL1 PE=1 SV=1

SNO4|HSP33|HSP32 − average.top3: 6.885135 − max.qupm: 5

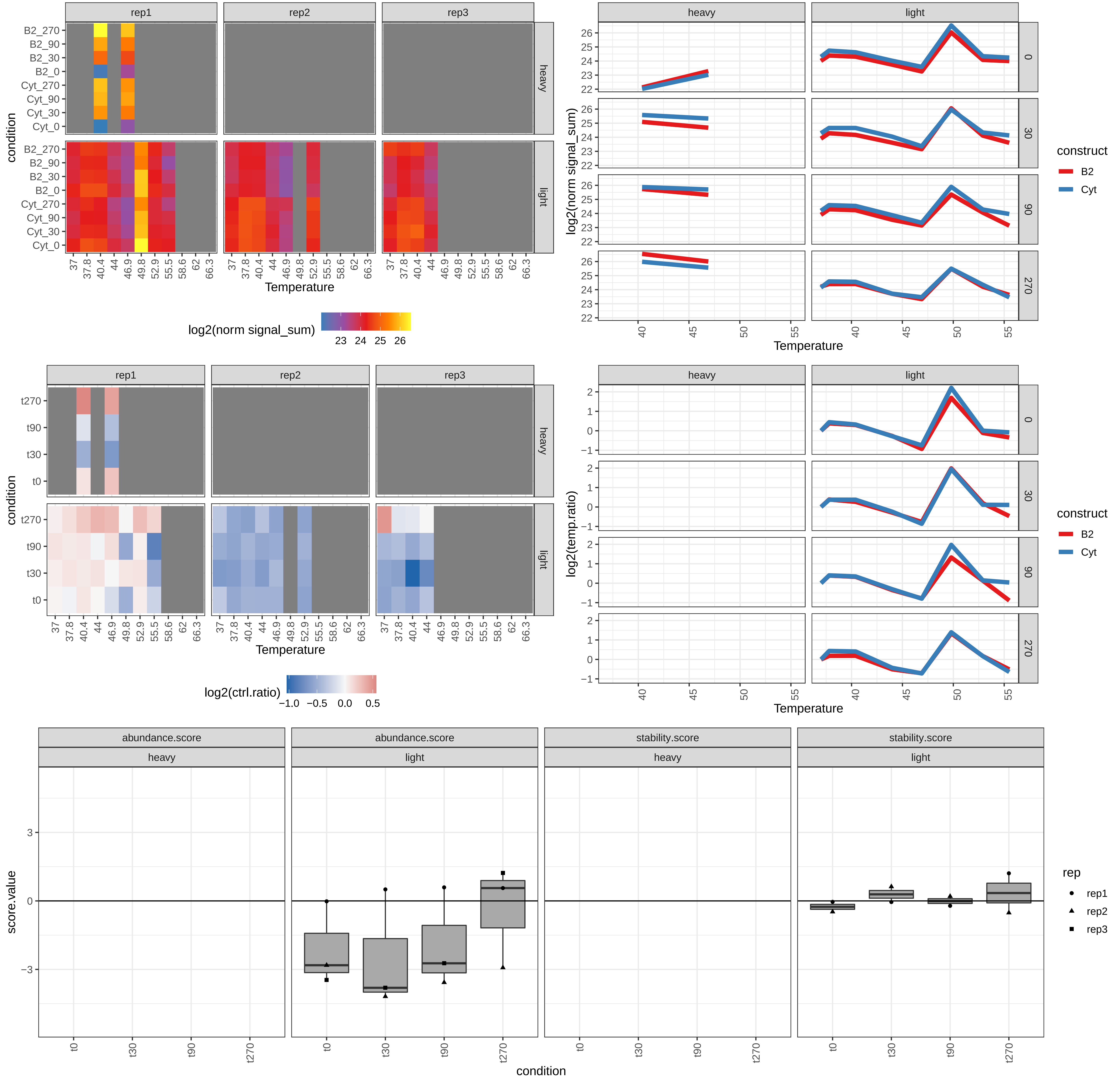

CC 204508 / S288c) GN=SNO4 PE=3 SV=1|Probable glutathione−independent glyoxalase HSP33 OS=Saccharomyces cerevisiae (strain ATCC 204508 / S288c) GN=HSP33 PE=1 SV=1|Probable glutathic

SNP1 − average.top3: 6.27813888888889 − max.qupm: 2

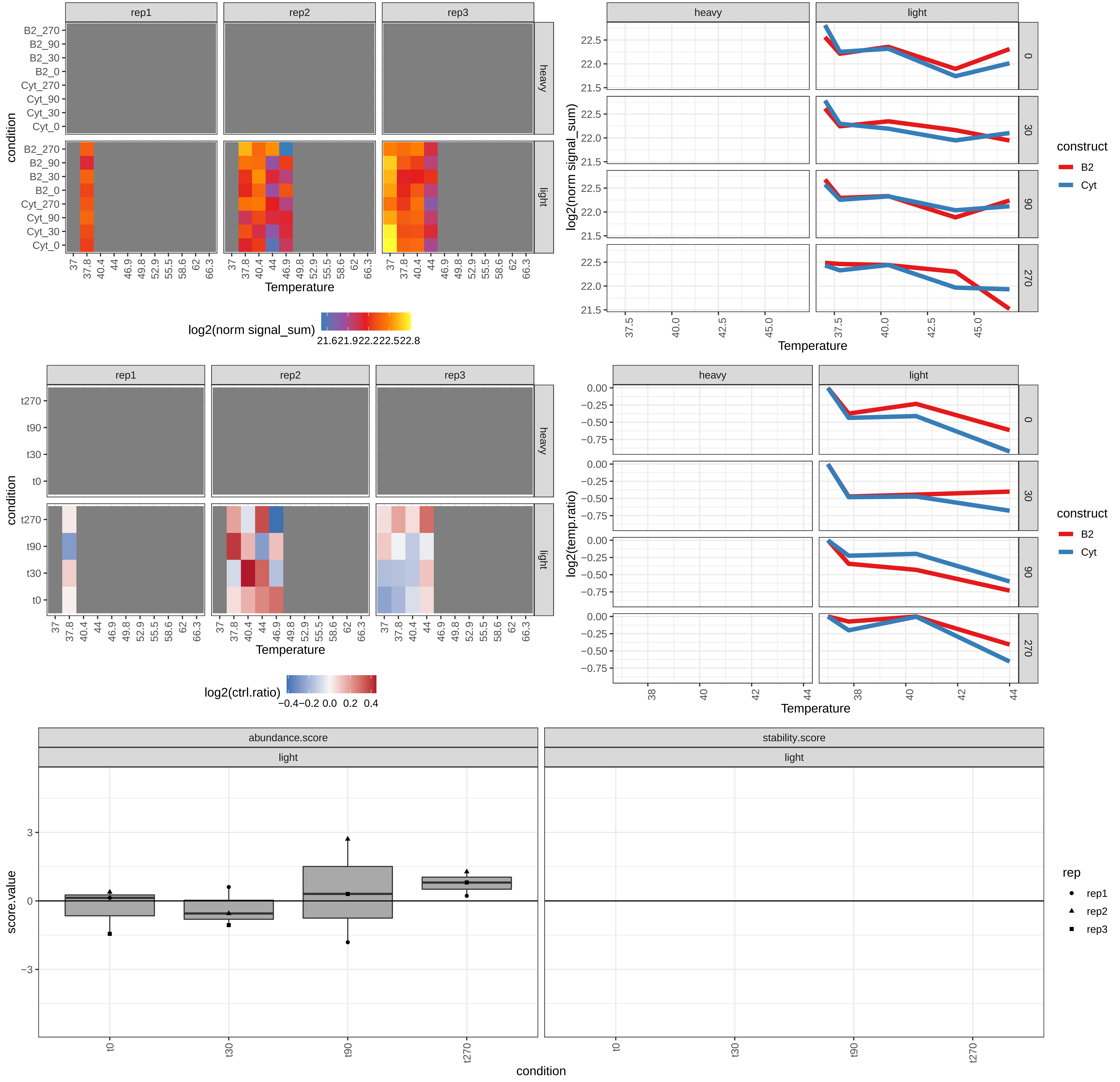

Q00916 − U1 small nuclear ribonucleoprotein 70 kDa homolog OS=Saccharomyces cerevisiae (strain ATCC 204508 / S288c) GN=SNP1 PE=1 SV=1

SNQ2 − average.top3: 6.52093027027027 − max.qupm: 10

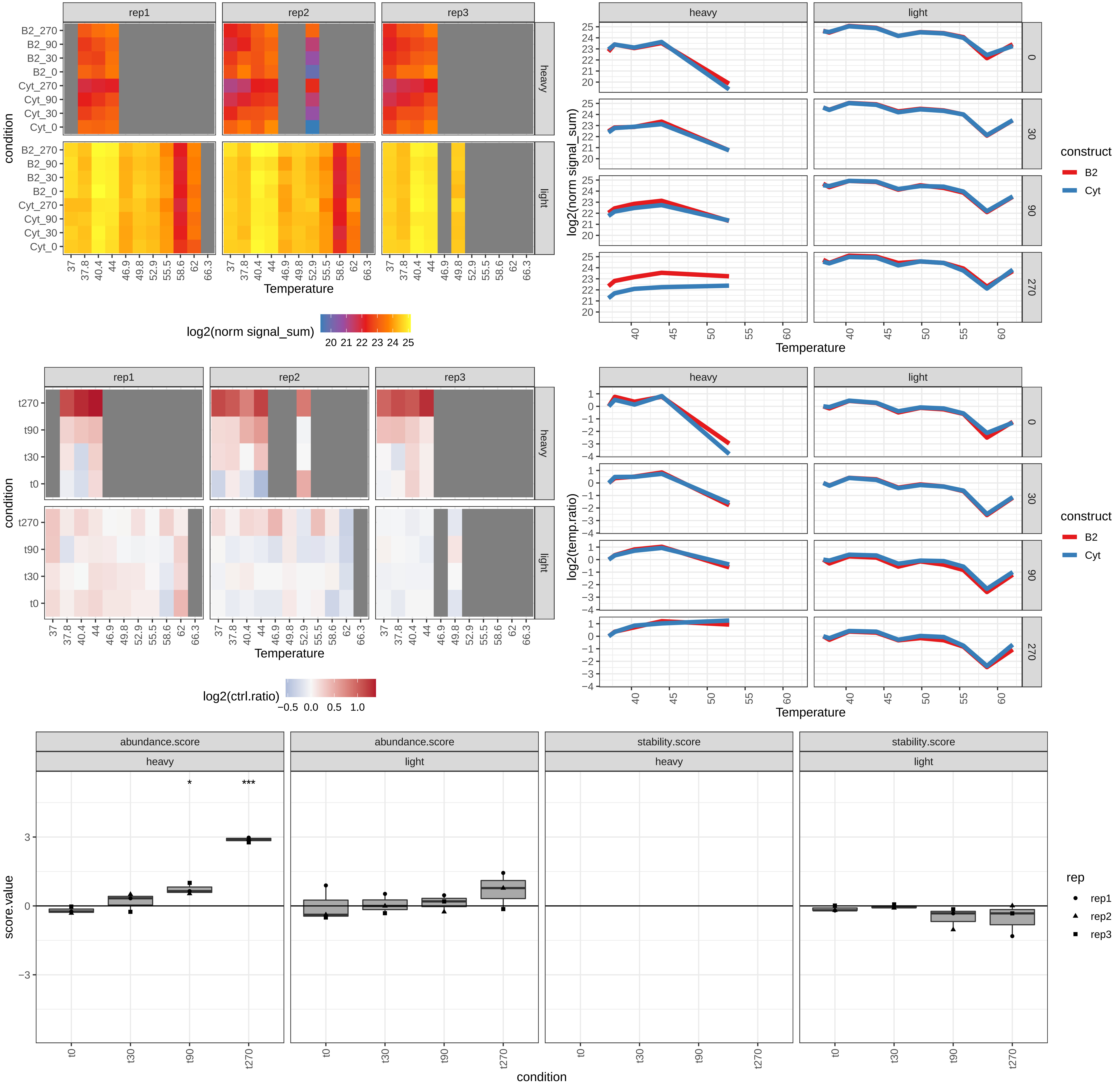

P32568 − Protein SNQ2 OS=Saccharomyces cerevisiae (strain ATCC 204508 / S288c) GN=SNQ2 PE=1 SV=2

SNT1 − average.top3: 6.54949538461538 − max.qupm: 7

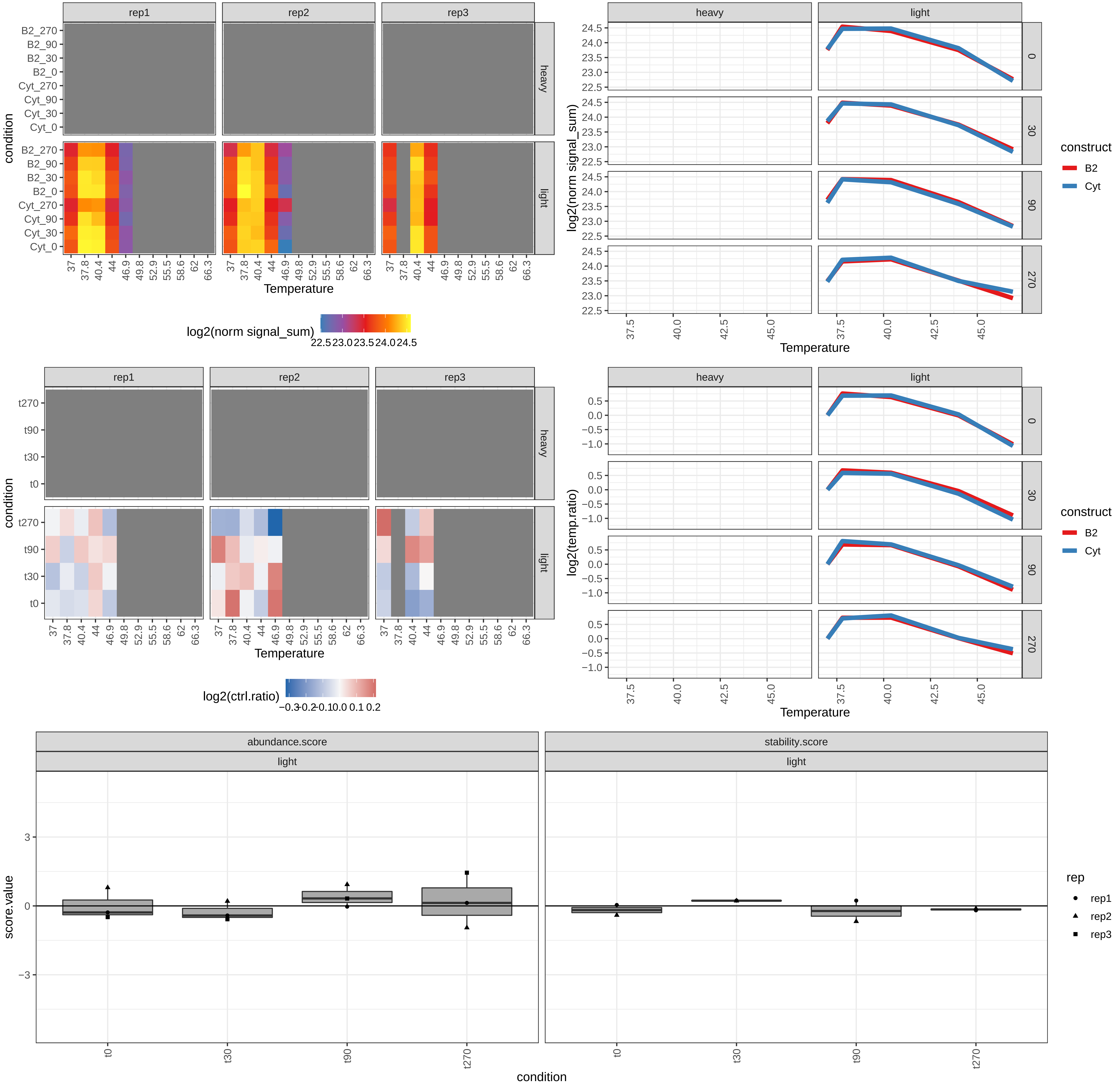

P25357 − Probable DNA−binding protein SNT1 OS=Saccharomyces cerevisiae (strain ATCC 204508 / S288c) GN=SNT1 PE=1 SV=2

SNU114 − average.top3: 6.97917647058824 − max.qupm: 10

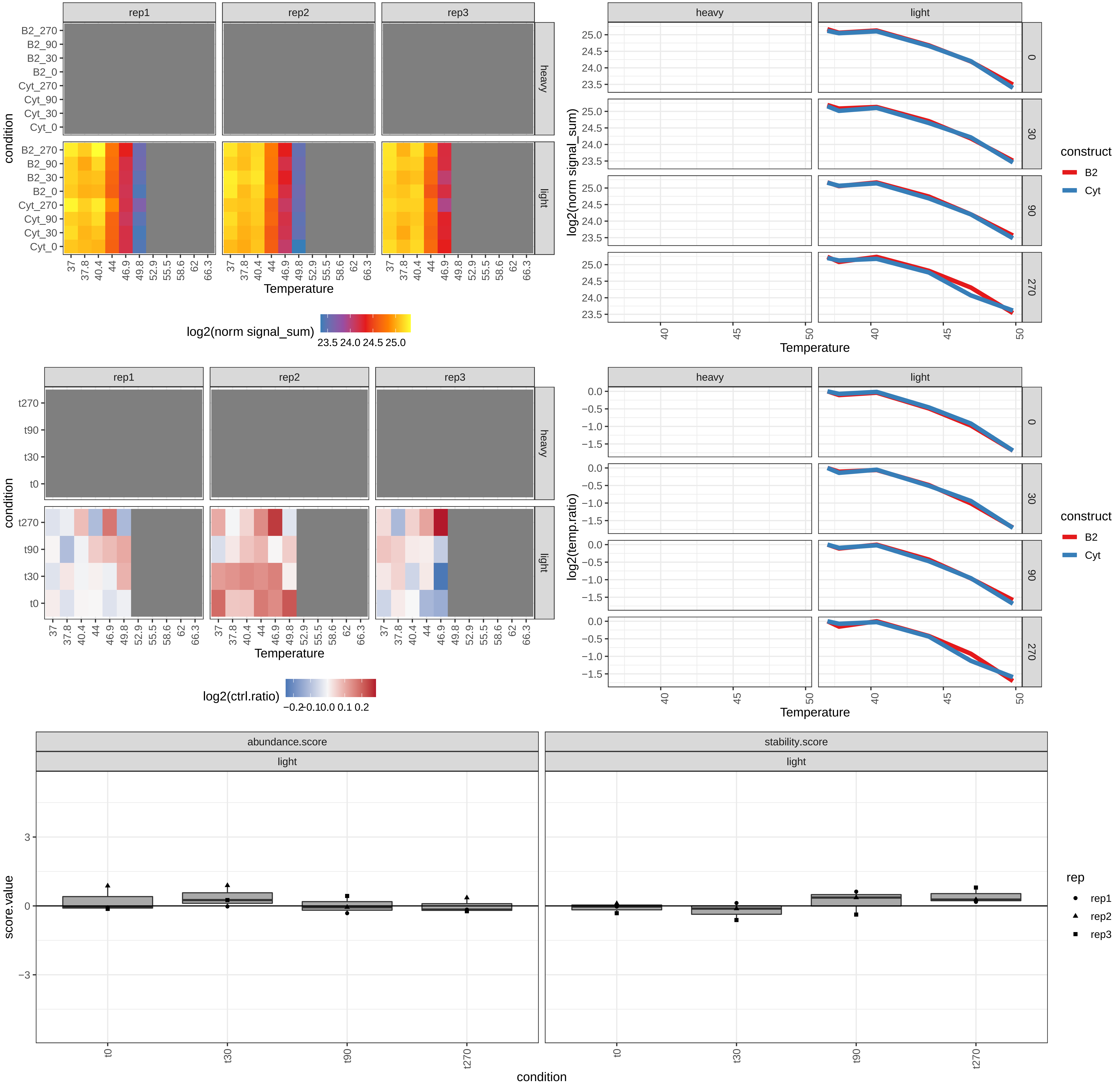

P36048 − Pre−mRNA−splicing factor SNU114 OS=Saccharomyces cerevisiae (strain ATCC 204508 / S288c) GN=SNU114 PE=1 SV=1

SNU13 − average.top3: 6.56205884615385 − max.qupm: 3

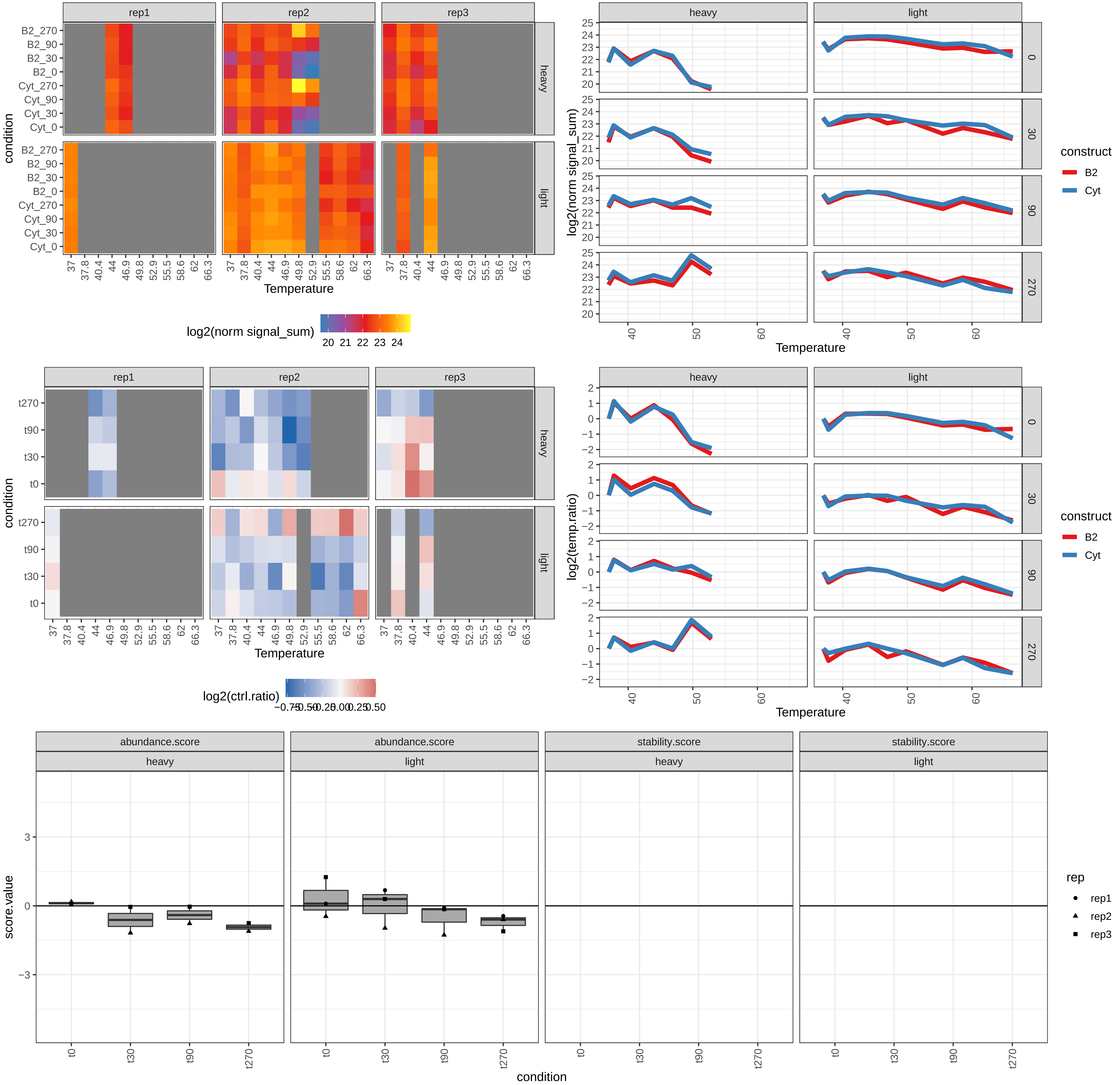

P39990 − 13 kDa ribonucleoprotein−associated protein OS=Saccharomyces cerevisiae (strain ATCC 204508 / S288c) GN=SNU13 PE=1 SV=1

SNU71 − average.top3: 6.416911875 − max.qupm: 7

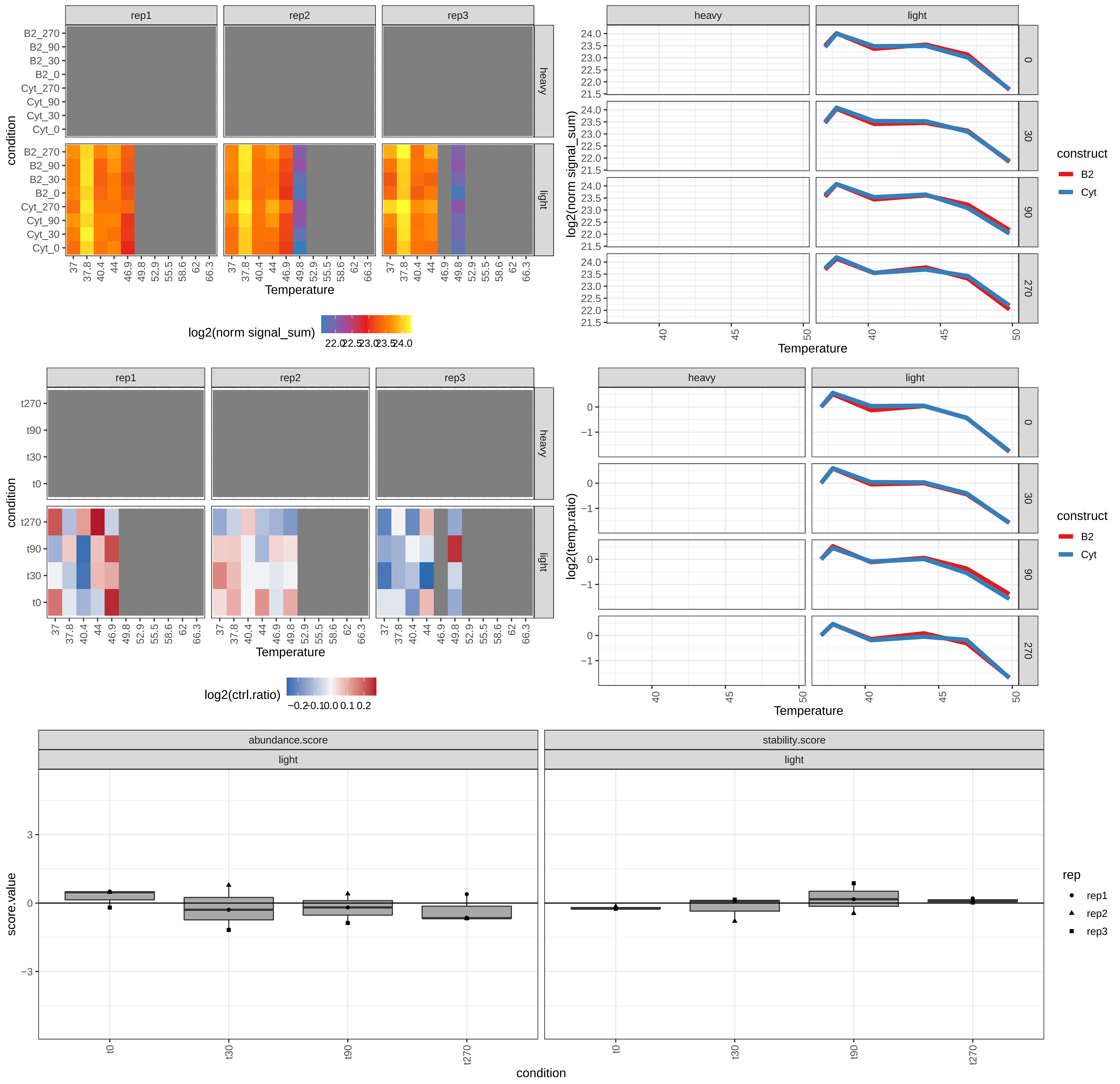

P53207 − U1 small nuclear ribonucleoprotein component SNU71 OS=Saccharomyces cerevisiae (strain ATCC 204508 / S288c) GN=SNU71 PE=1 SV=1

SNX3 − average.top3: 7.157814 − max.qupm: 7

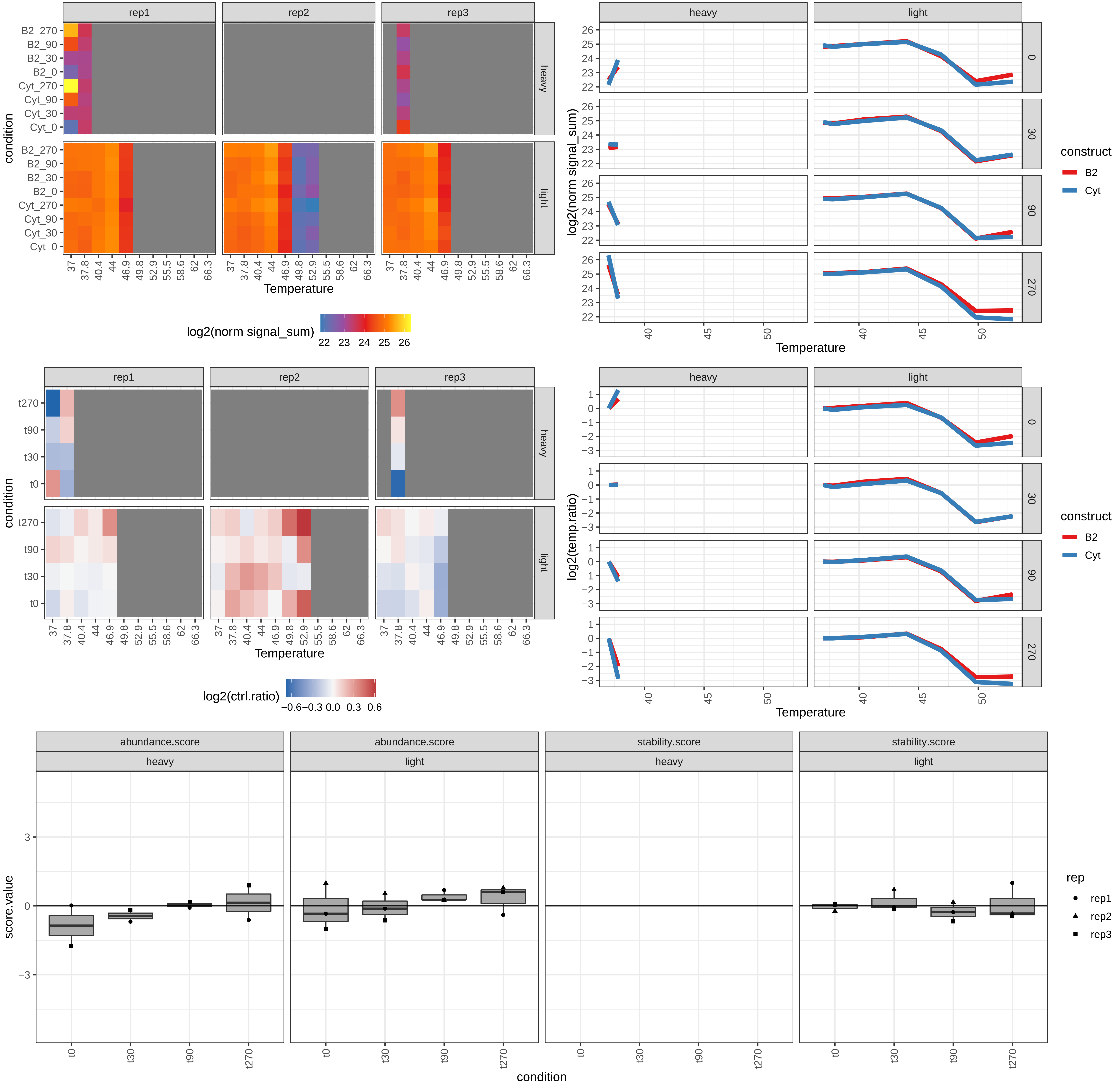

Q08826 − Sorting nexin−3 OS=Saccharomyces cerevisiae (strain ATCC 204508 / S288c) GN=SNX3 PE=1 SV=1

SNX4 − average.top3: 6.97431352941176 − max.qupm: 7

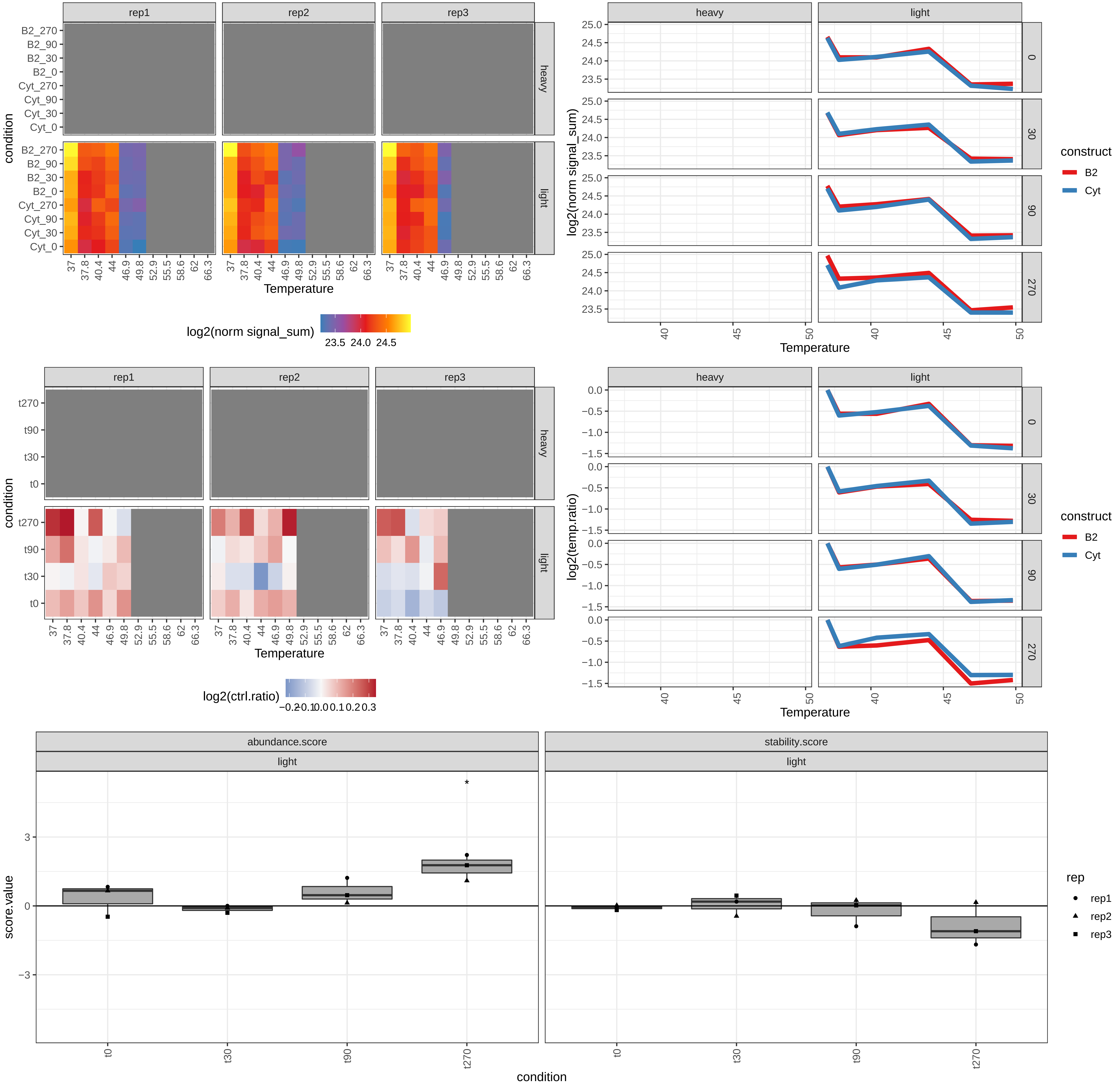

P47057 − Sorting nexin−4 OS=Saccharomyces cerevisiae (strain ATCC 204508 / S288c) GN=SNX4 PE=1 SV=1

SNX41 − average.top3: 6.628274545454555 − max.qupm: 4

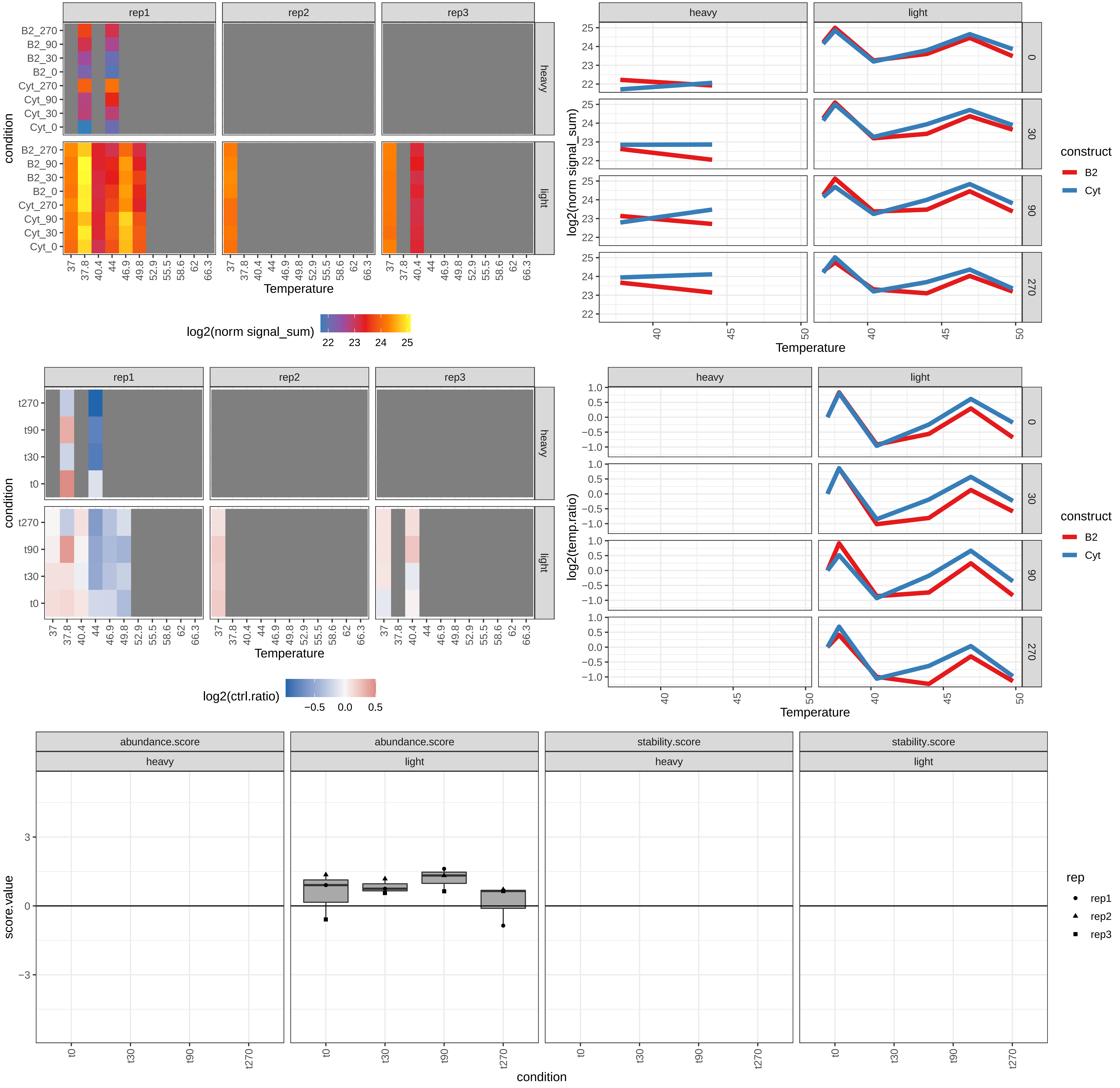

Q04053 − Sorting nexin−41 OS=Saccharomyces cerevisiae (strain ATCC 204508 / S288c) GN=SNX41 PE=1 SV=1

SNZ3|SNZ2 − average.top3: 7.17612436363636 − max.qupm: 13

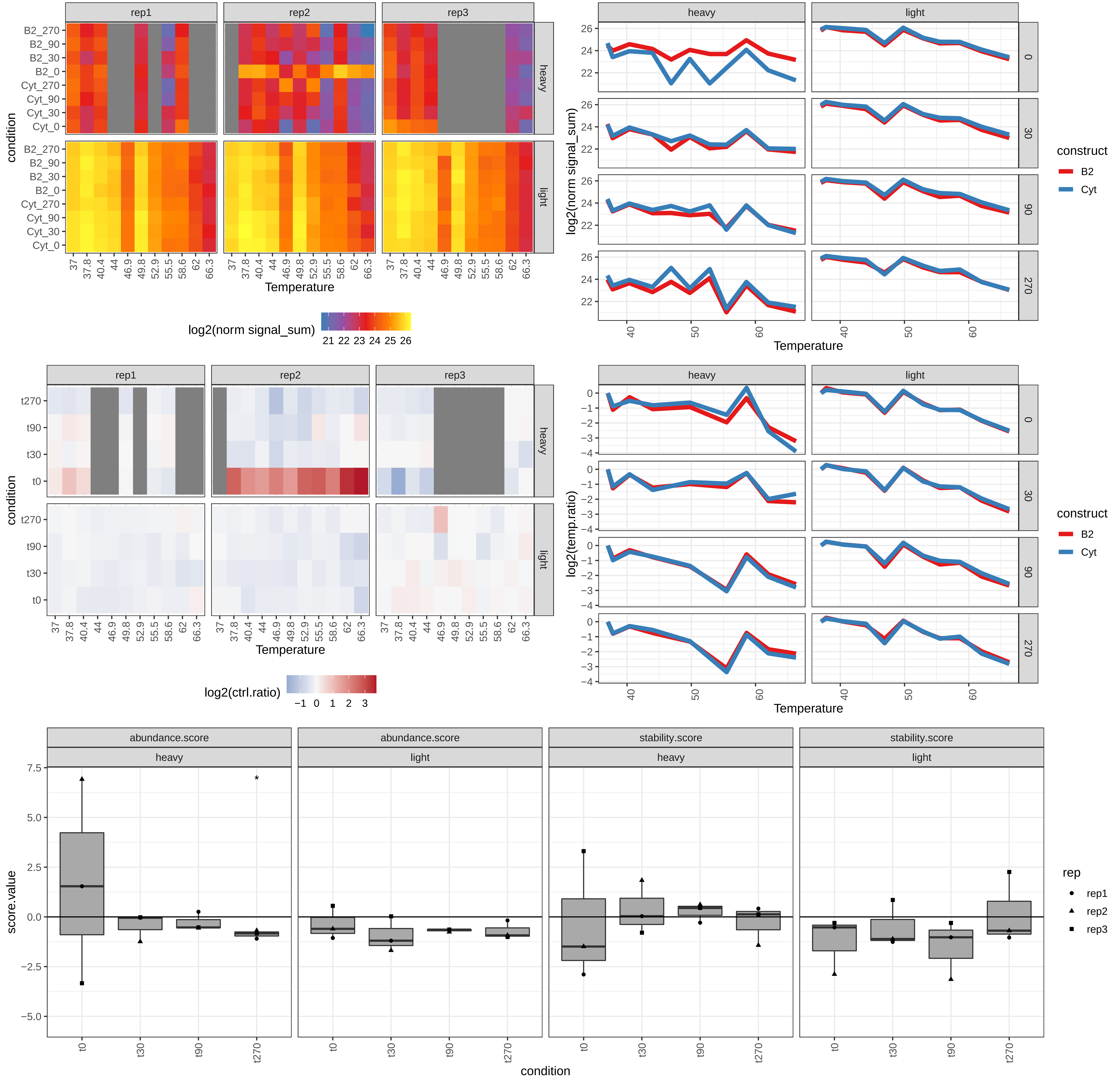

 synthase subunit SNZ3 OS=Saccharomyces cerevisiae (strain ATCC 204508 / S288c) GN=SNZ3 PE=2 SV=1|Probable pyridoxal 5'−phosphate synthase subunit SNZ2 OS=Saccharomyces cerevisiae (st

SOD1 − average.top3: 8.54499287878788 − max.qupm: 12

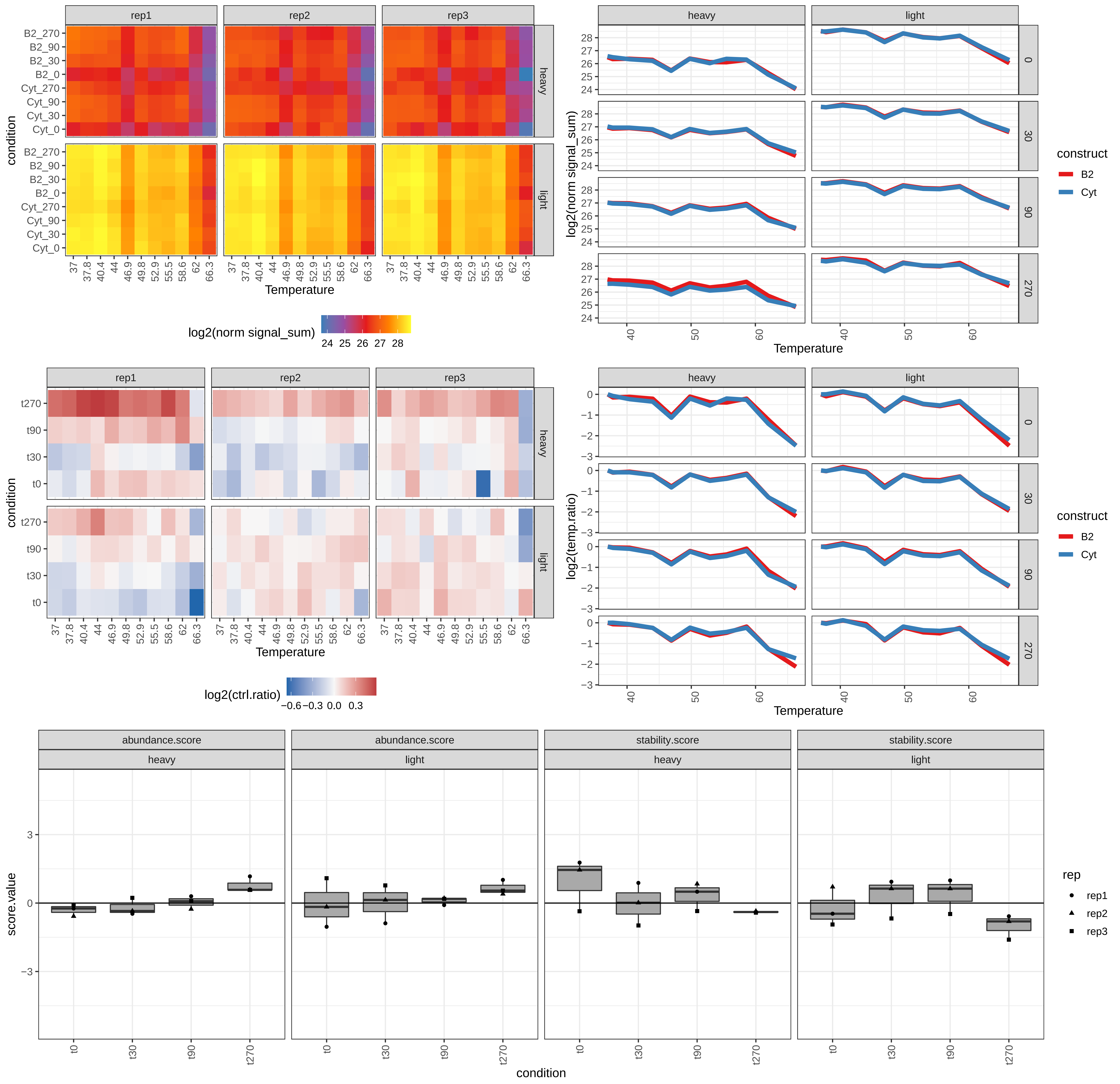

P00445 − Superoxide dismutase [Cu−Zn] OS=Saccharomyces cerevisiae (strain ATCC 204508 / S288c) GN=SOD1 PE=1 SV=2

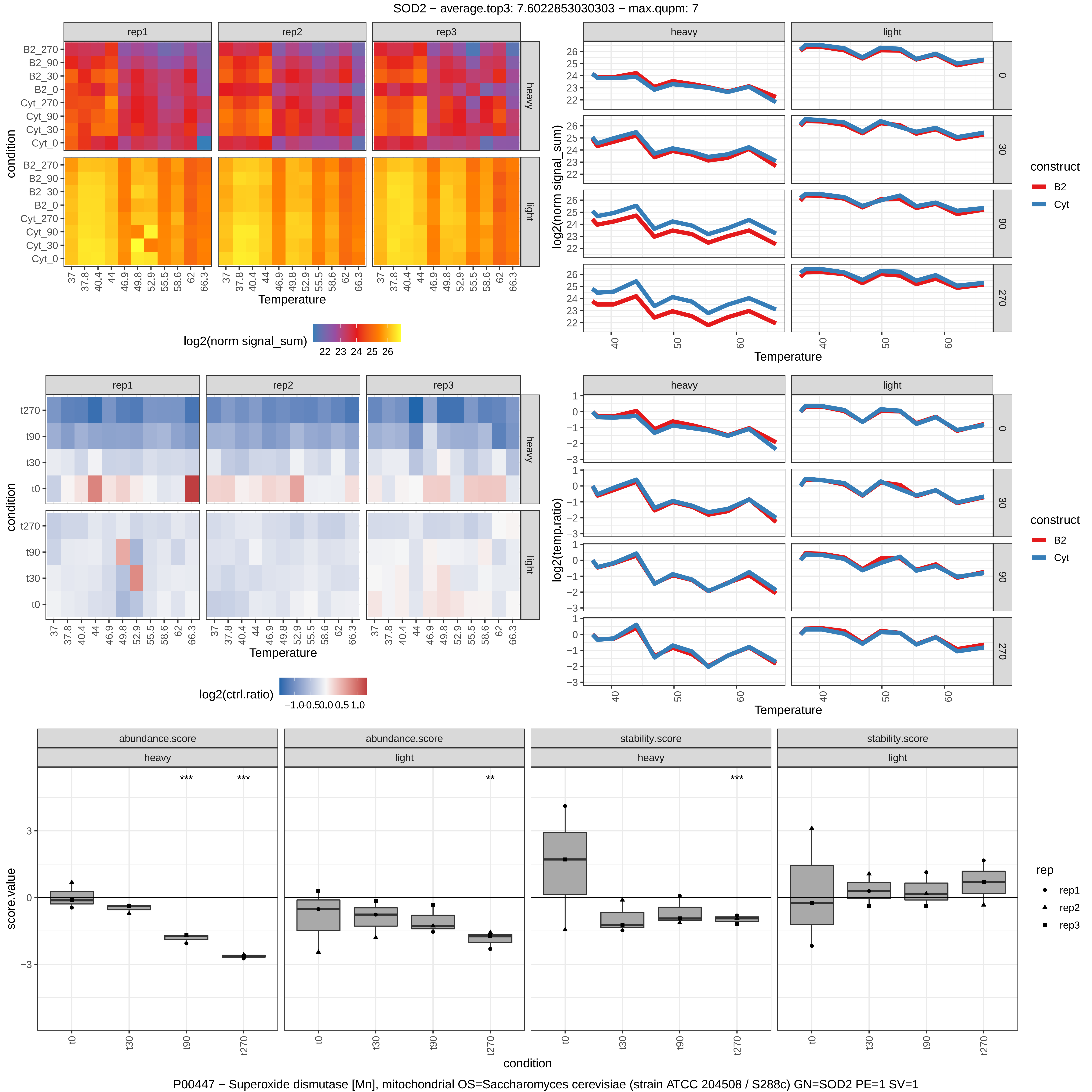
SOD2 − average.top3: 7.6022853030303 − max.qupm: 7
rep1
rep2
rep3
heavy
light
B2_270
B2_90
B2_30
B2_0
Cyt_270
Cyt_90
Cyt_30
Cyt_0
condition
37
37.8
40.4
44
46.9
49.8
52.9
55.5
58.6
62
66.3
Temperature
log2(norm signal_sum)
22 23 24 25 26
log2(norm signal_sum)
0
30
90
270
construct
B2
Cyt
t270
t90
t30
t0
log2(ctrl.ratio)
−1.0 −0.5 0.0 0.5 1.0
log2(temp.ratio)
abundance.score
stability.score
score.value
***
**
rep
rep1
rep2
rep3
condition
P00447 − Superoxide dismutase [Mn], mitochondrial OS=Saccharomyces cerevisiae (strain ATCC 204508 / S288c) GN=SOD2 PE=1 SV=1

SOF1 − average.top3: 6.92877826086957 − max.qupm: 7

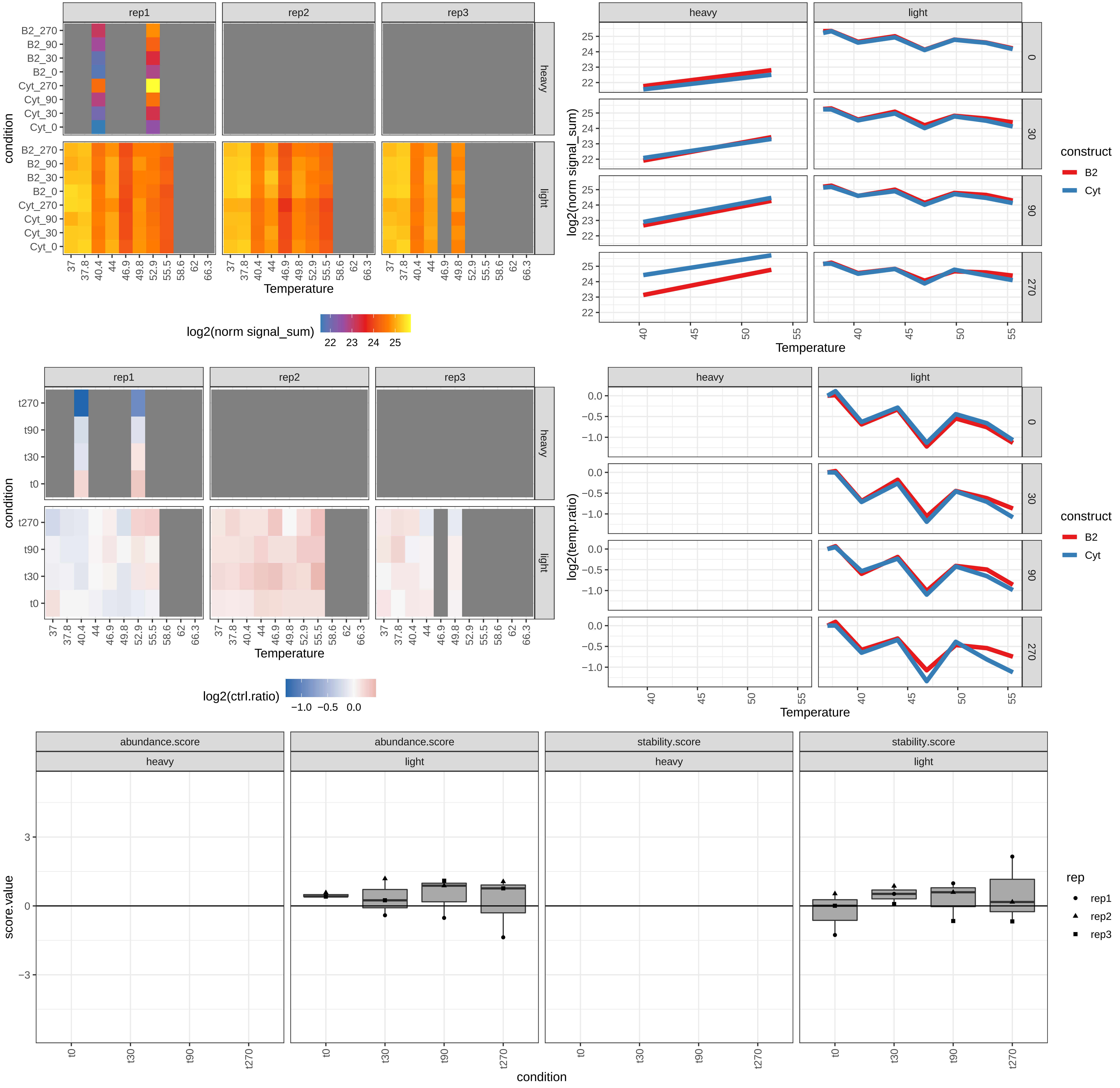

P33750 − Protein SOF1 OS=Saccharomyces cerevisiae (strain ATCC 204508 / S288c) GN=SOF1 PE=1 SV=1

SOG2 − average.top3: 7.03739388888889 − max.qupm: 5

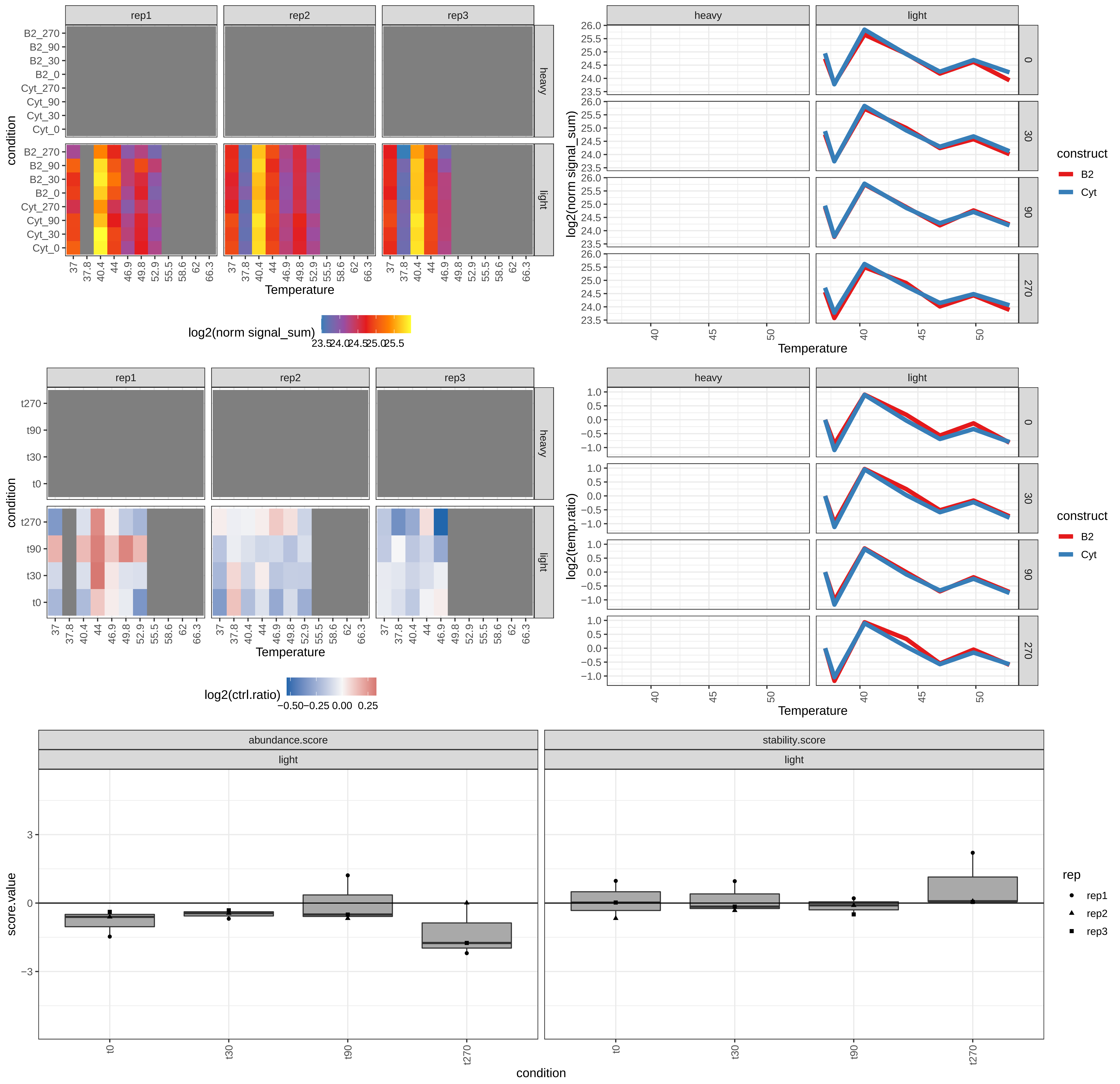

Q08817 − Leucine−rich repeat−containing protein SOG2 OS=Saccharomyces cerevisiae (strain ATCC 204508 / S288c) GN=SOG2 PE=1 SV=1

SOL1 − average.top3: 6.81762705882353 − max.qupm: 8

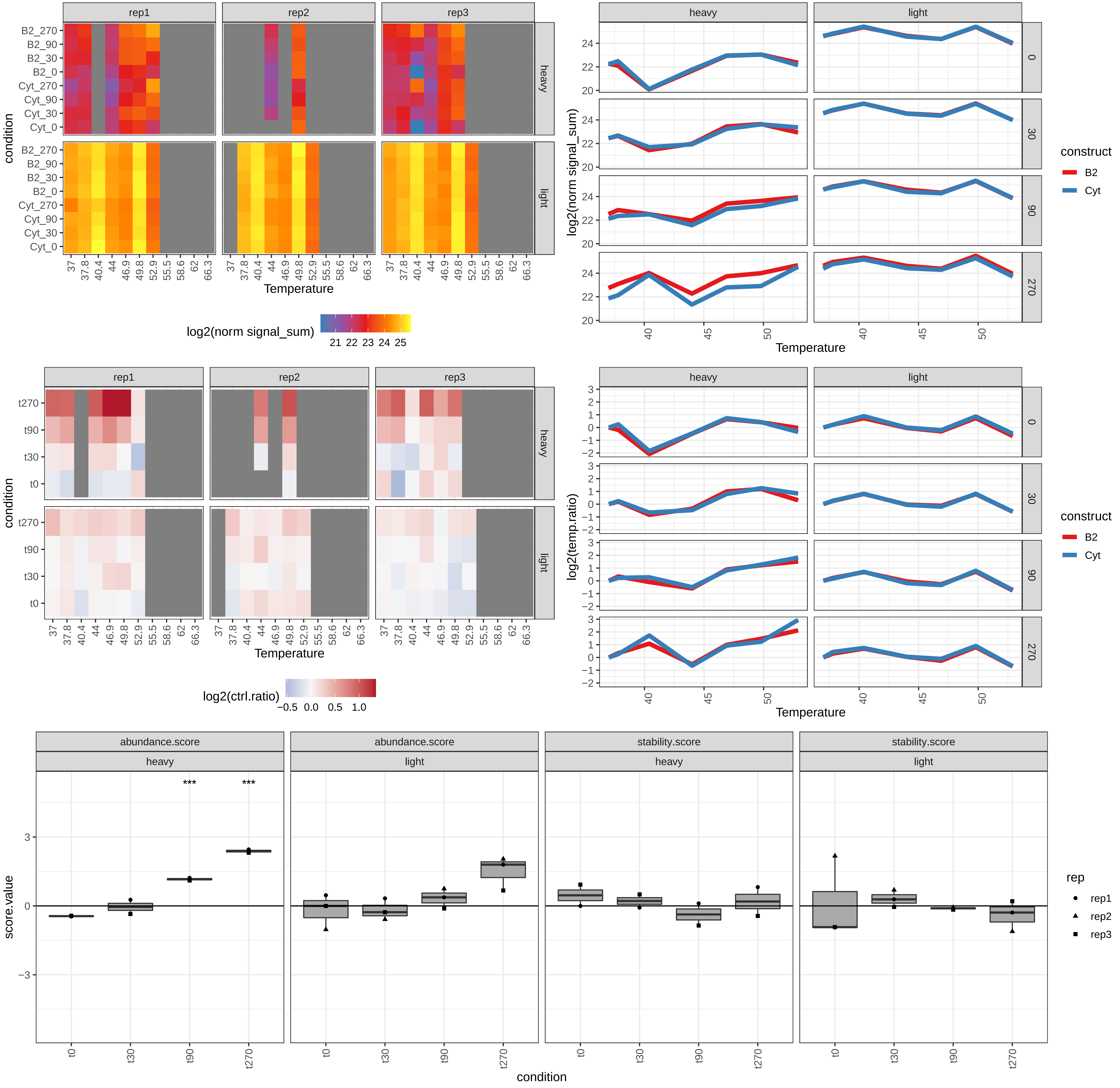

P50278 − 6−phosphogluconolactonase−like protein 1 OS=Saccharomyces cerevisiae (strain ATCC 204508 / S288c) GN=SOL1 PE=1 SV=1

SOL2 − average.top3: 6.82709032258064 − max.qupm: 6

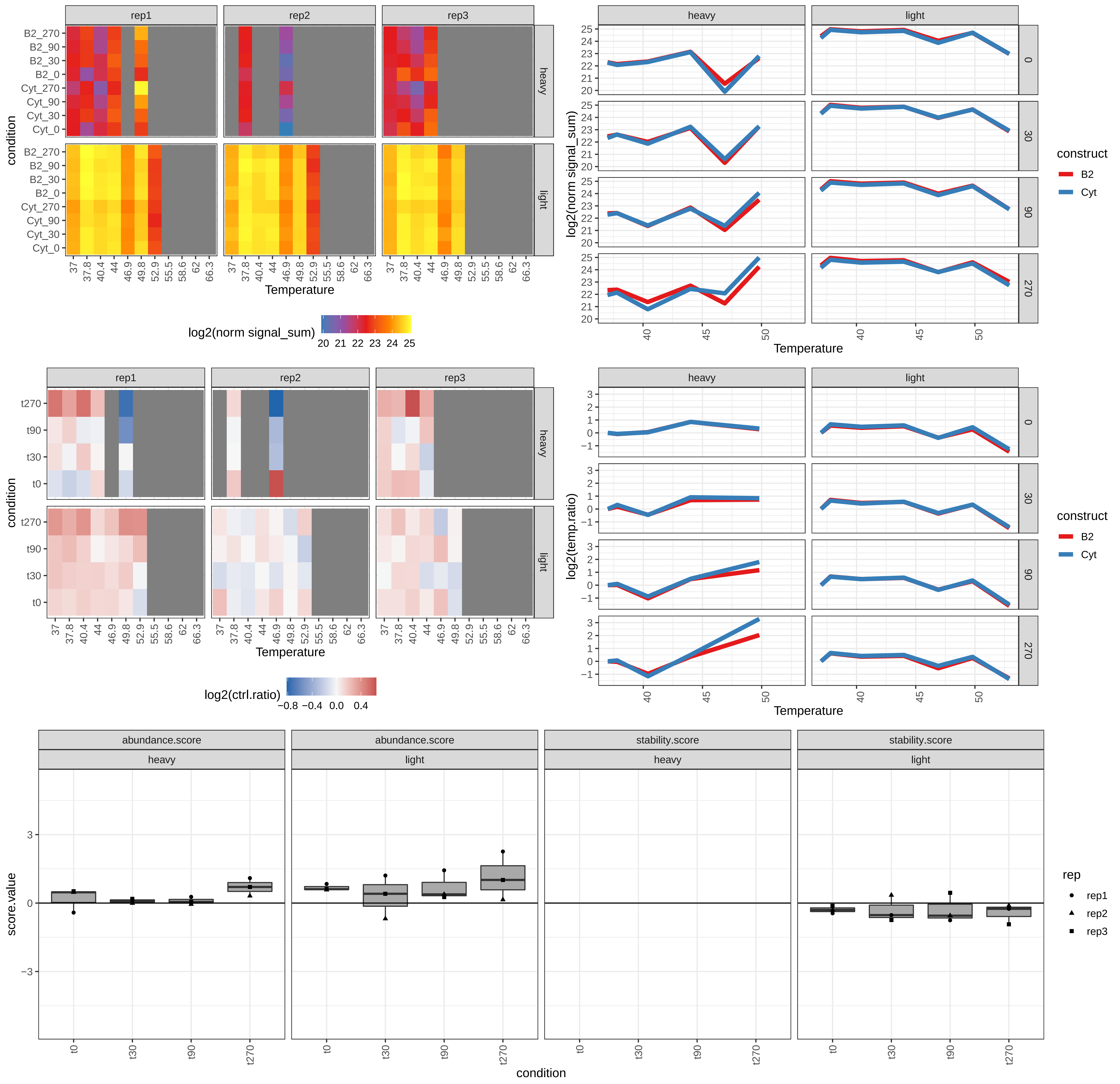

P37262 − 6−phosphogluconolactonase−like protein 2 OS=Saccharomyces cerevisiae (strain ATCC 204508 / S288c) GN=SOL2 PE=1 SV=2

SOL3 − average.top3: 7.12466416666667 − max.qupm: 11

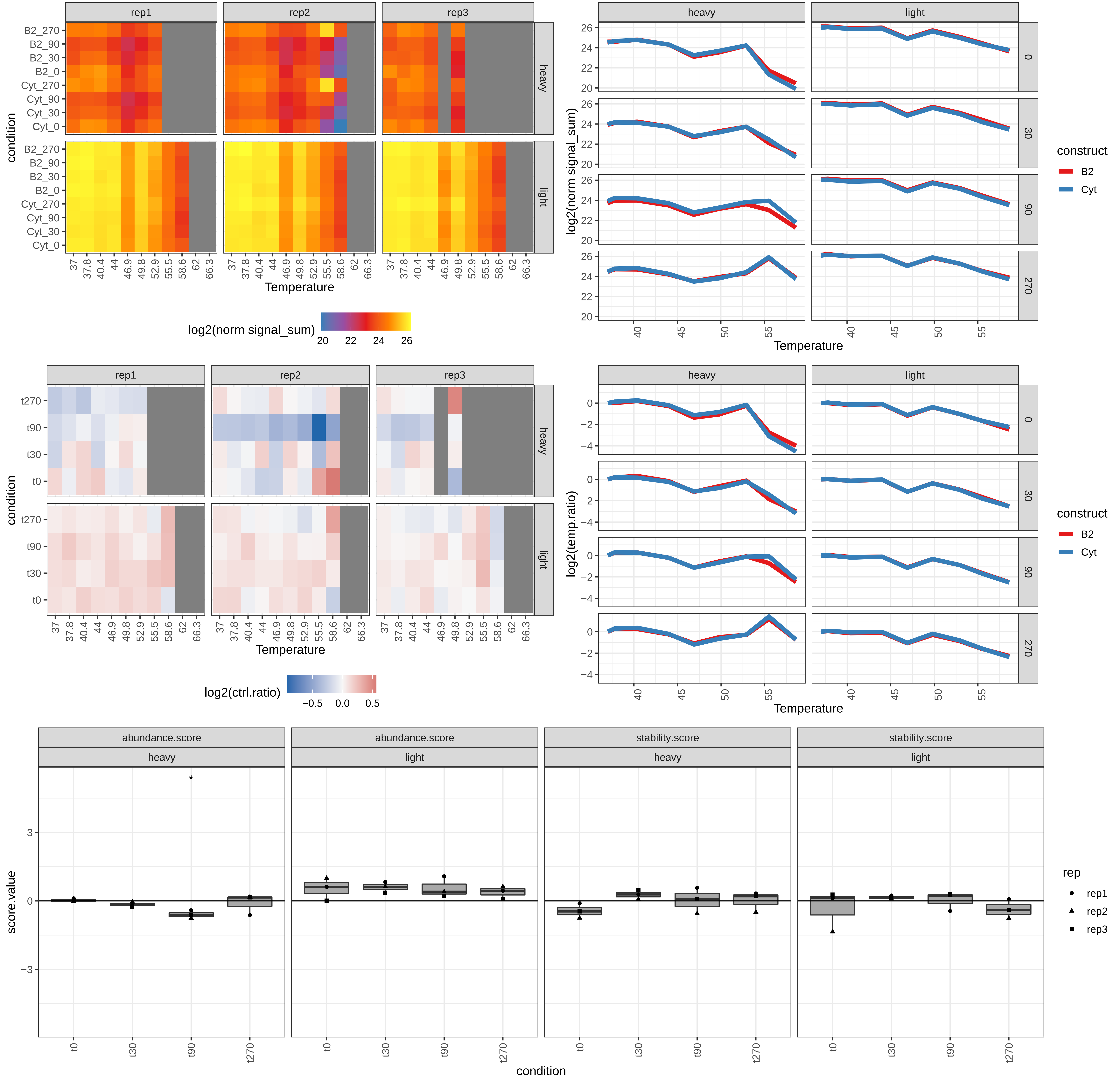

P38858 − 6−phosphogluconolactonase 3 OS=Saccharomyces cerevisiae (strain ATCC 204508 / S288c) GN=SOL3 PE=1 SV=2

SOL4 − average.top3: 7.00672166666667 − max.qupm: 5

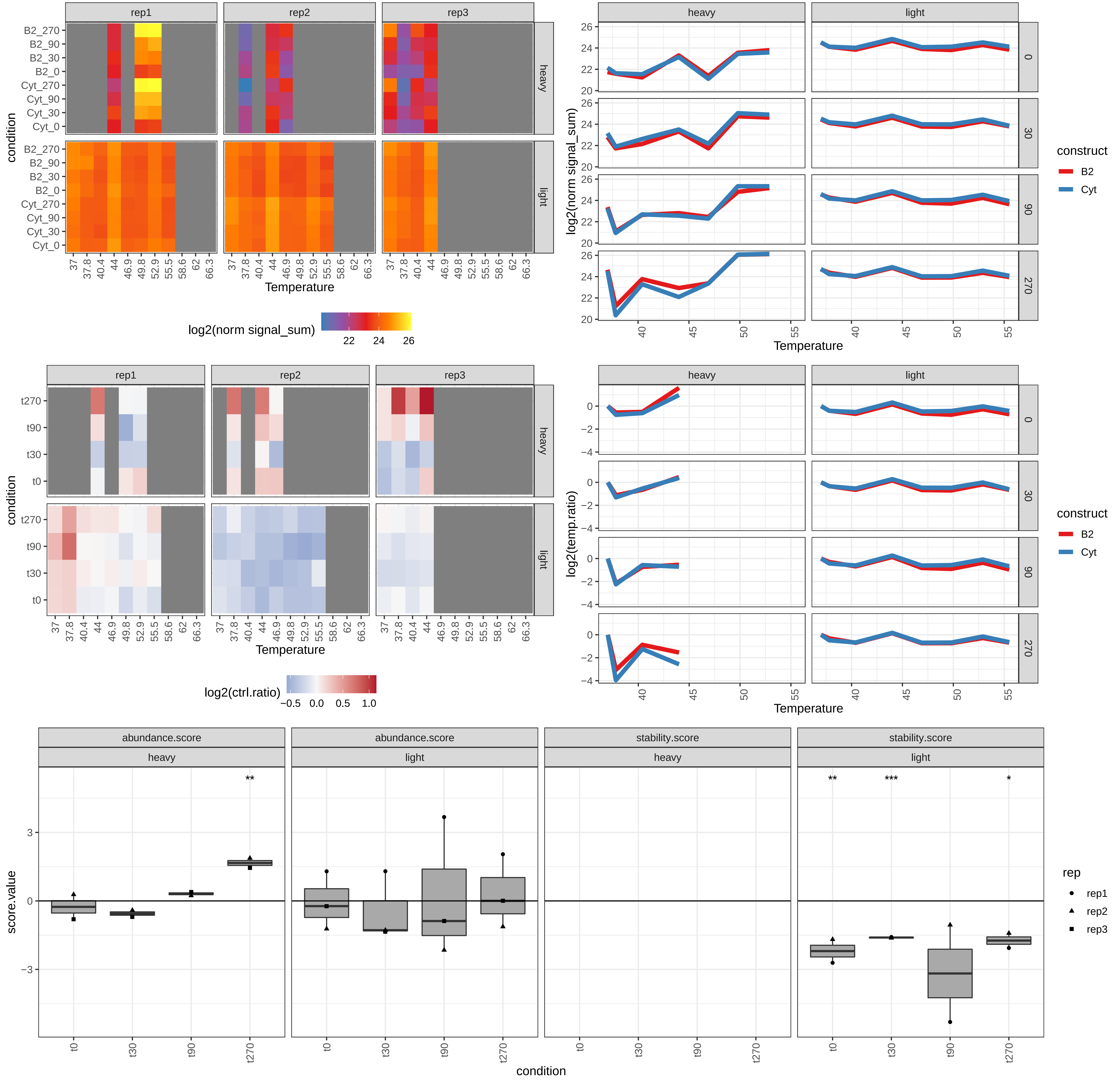

P53315 − 6−phosphogluconolactonase 4 OS=Saccharomyces cerevisiae (strain ATCC 204508 / S288c) GN=SOL4 PE=1 SV=1

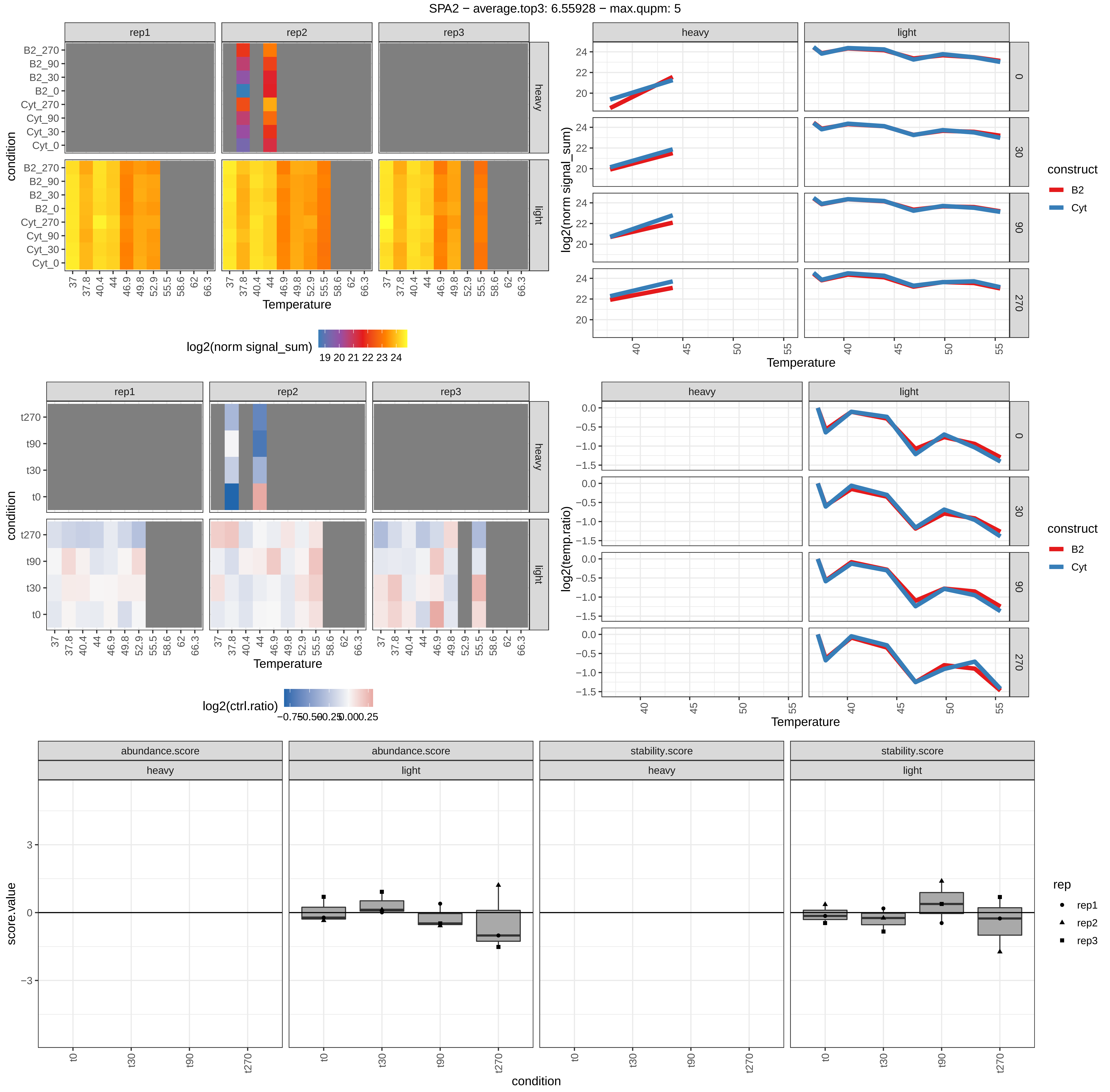

P23201 − Protein SPA2 OS=Saccharomyces cerevisiae (strain ATCC 204508 / S288c) GN=SPA2 PE=1 SV=1

SPB1 − average.top3: 6.76513620689655 − max.qupm: 9

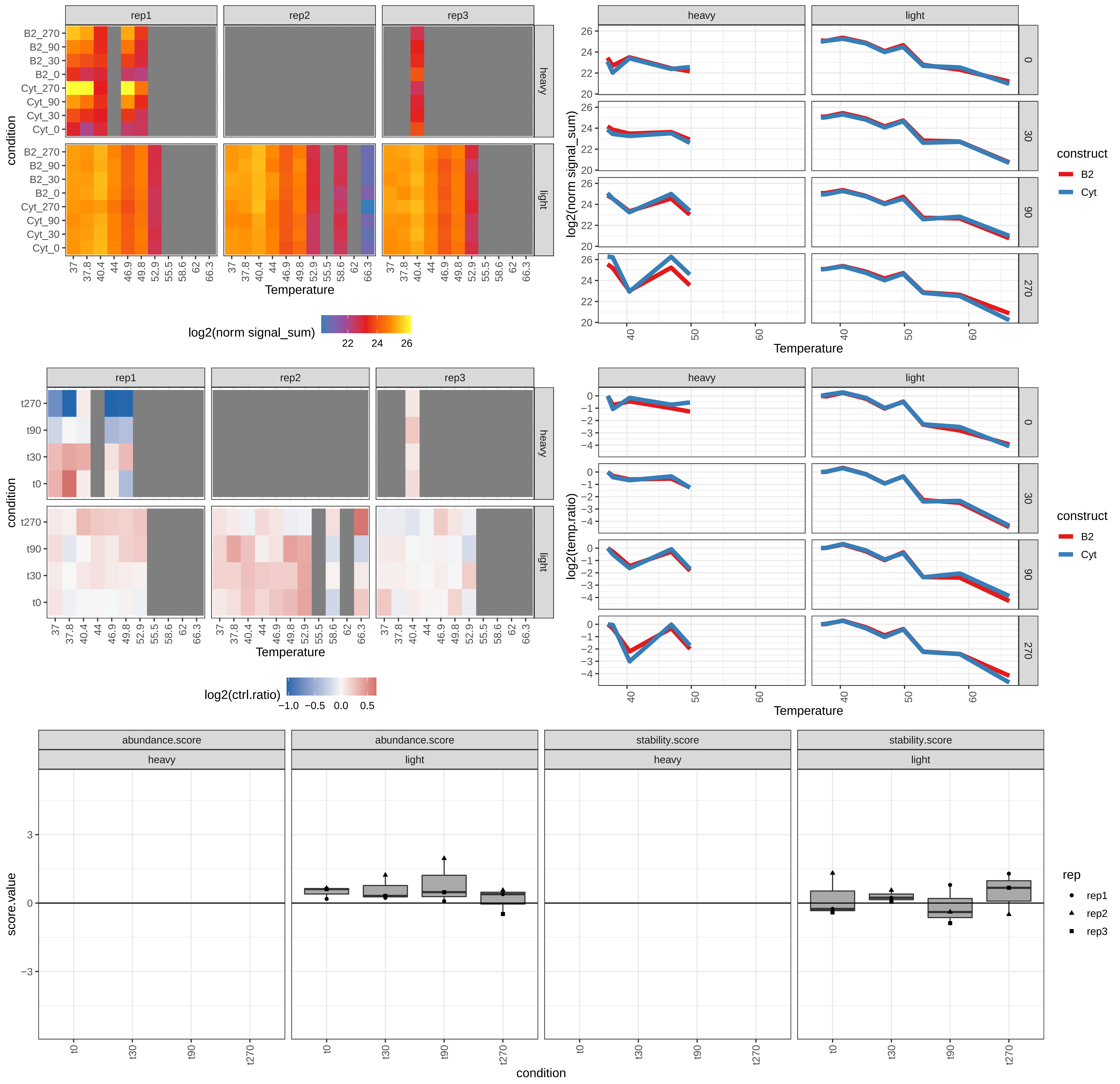

P25582 − 27S pre−rRNA (guanosine(2922)−2'−O)−methyltransferase OS=Saccharomyces cerevisiae (strain ATCC 204508 / S288c) GN=SPB1 PE=1 SV=2

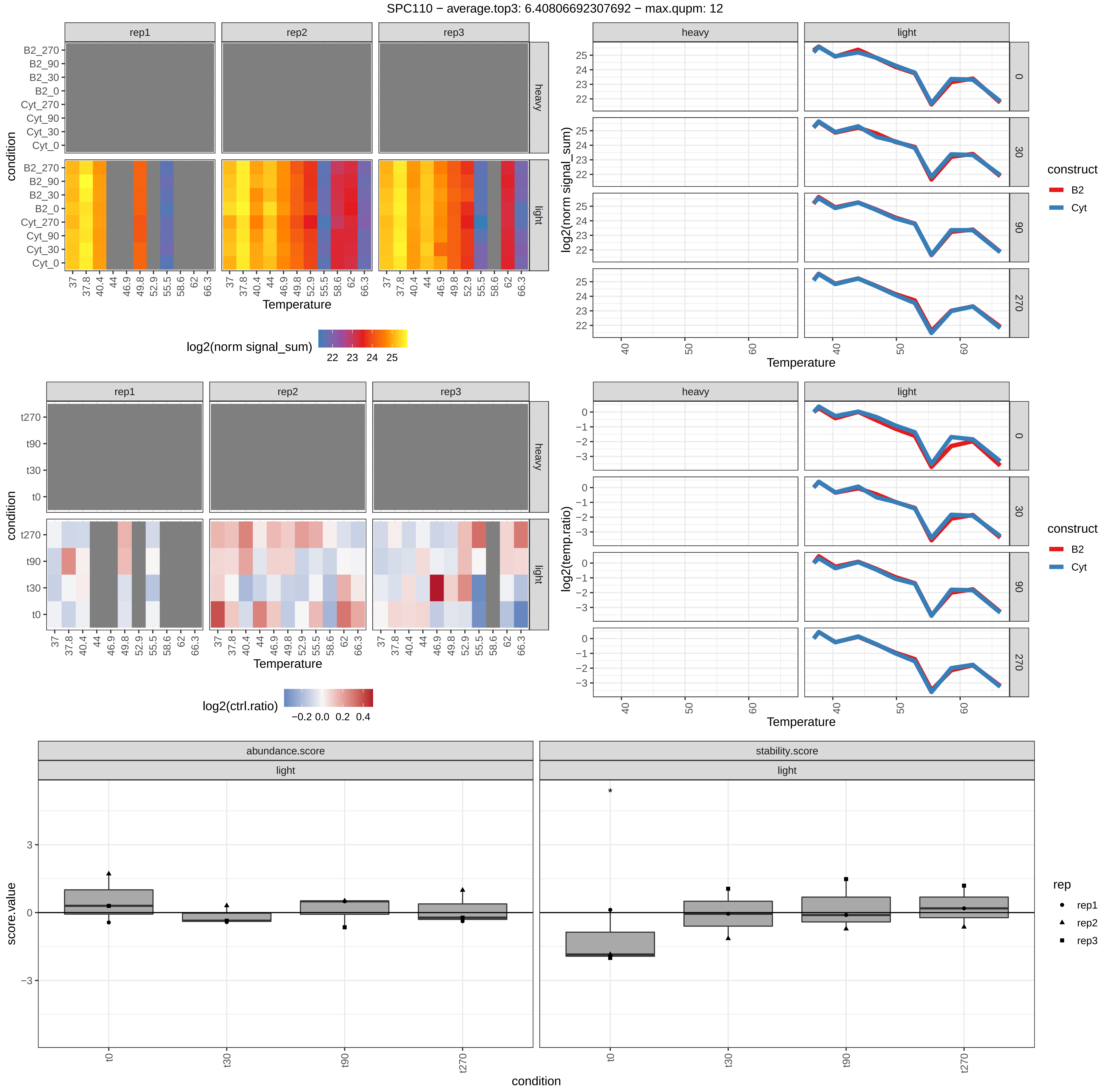

P32380 − Spindle pole body component 110 OS=Saccharomyces cerevisiae (strain ATCC 204508 / S288c) GN=SPC110 PE=1 SV=1

SPC2 − average.top3: 7.06597136363636 − max.qupm: 5

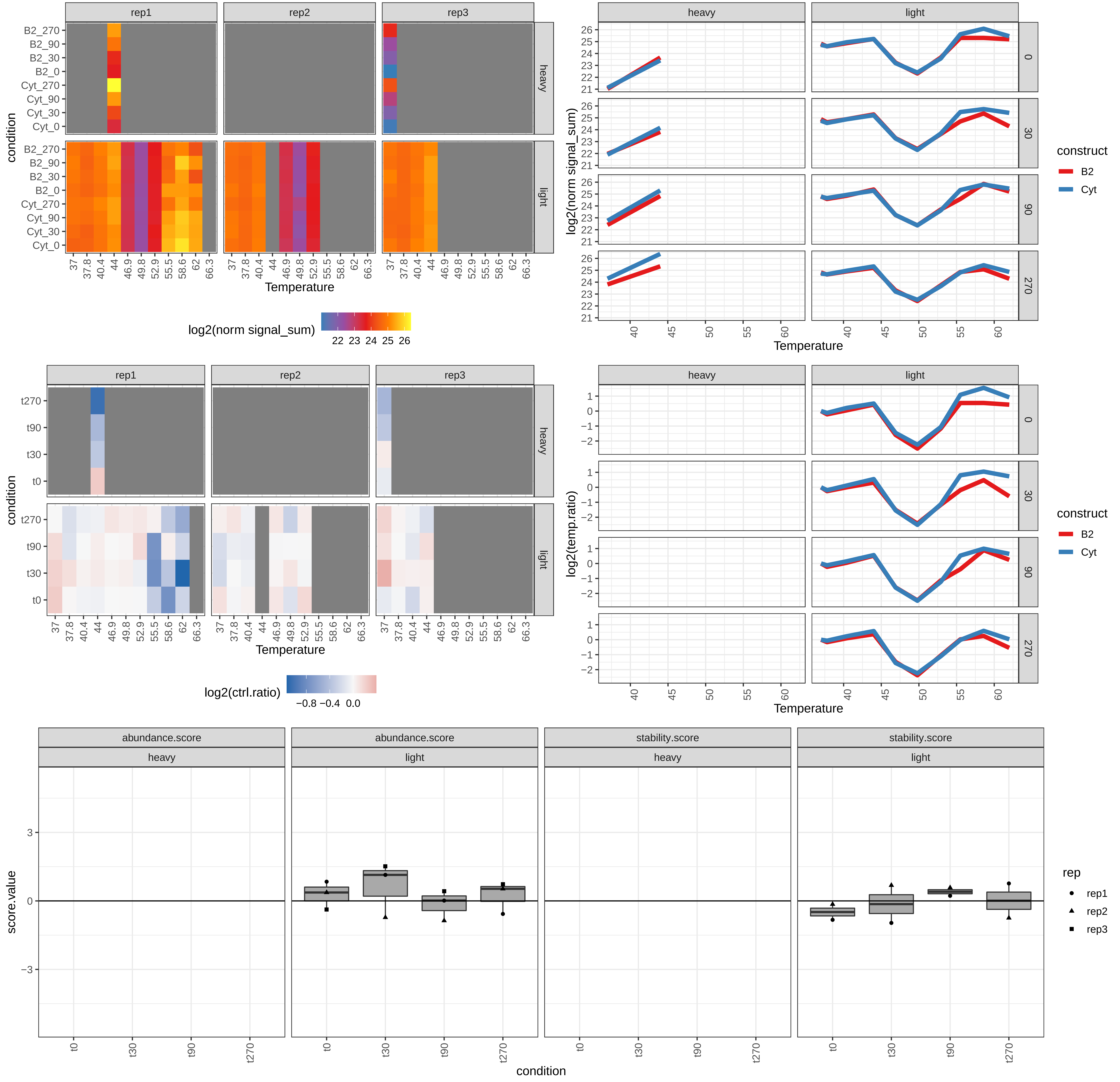

Q04969 − Signal peptidase complex subunit SPC2 OS=Saccharomyces cerevisiae (strain ATCC 204508 / S288c) GN=SPC2 PE=1 SV=1

SPC25 − average.top3: 6.64702363636364 − max.qupm: 4

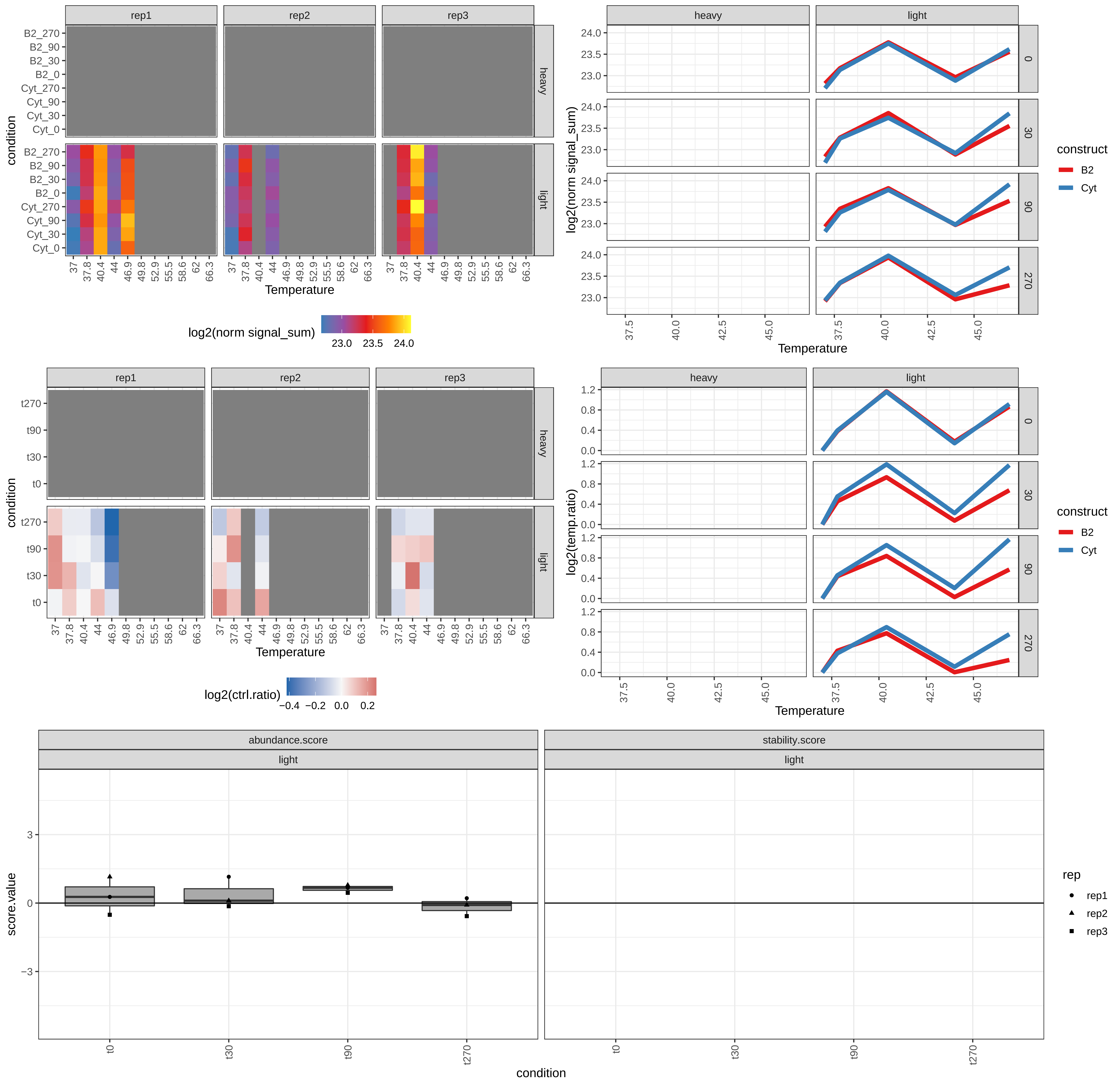

P40014 − Kinetochore protein SPC25 OS=Saccharomyces cerevisiae (strain ATCC 204508 / S288c) GN=SPC25 PE=1 SV=1

SPC3 − average.top3: 7.01545411764706 − max.qupm: 5

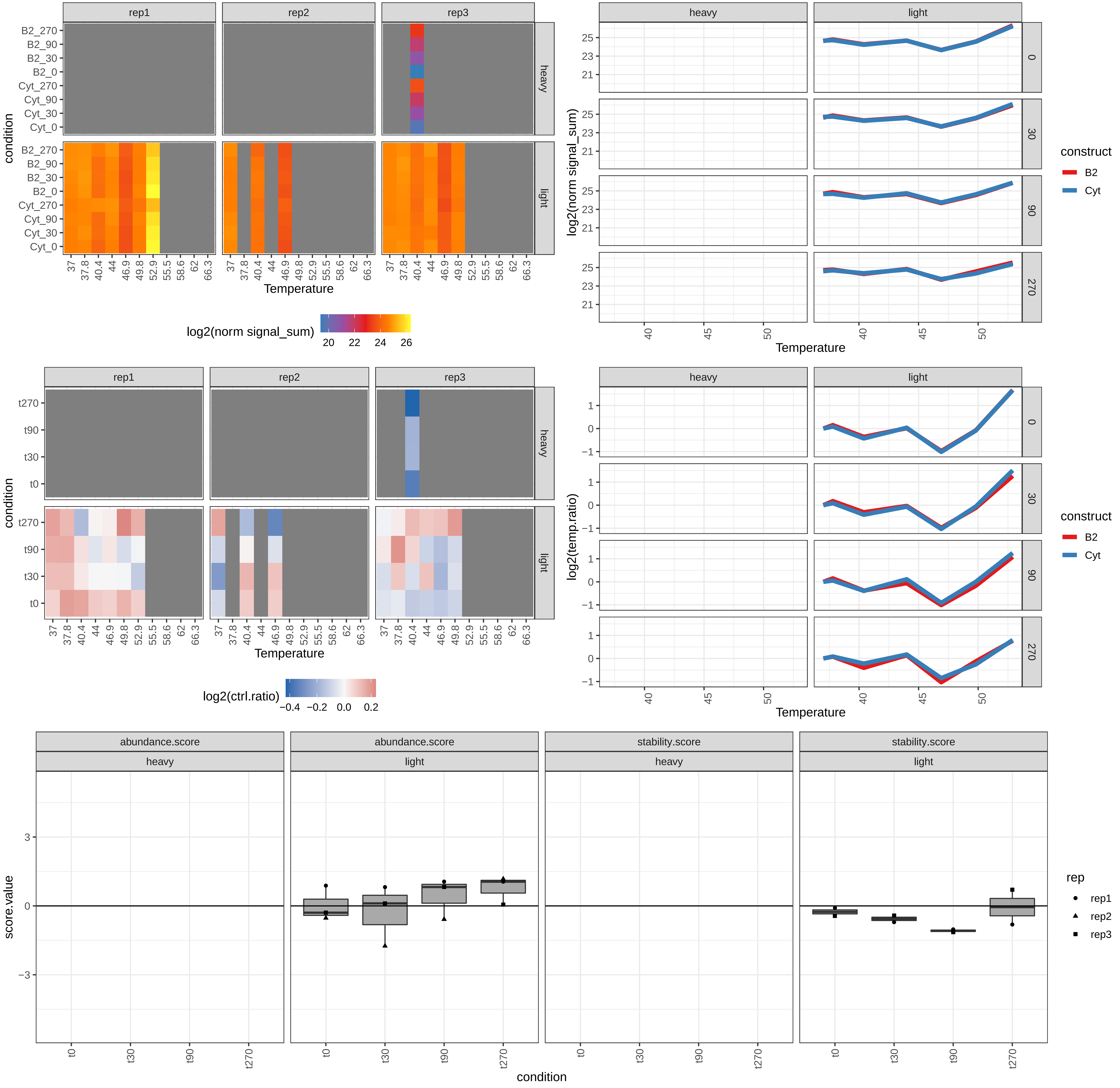

Q12133 − Signal peptidase complex subunit SPC3 OS=Saccharomyces cerevisiae (strain ATCC 204508 / S288c) GN=SPC3 PE=1 SV=1

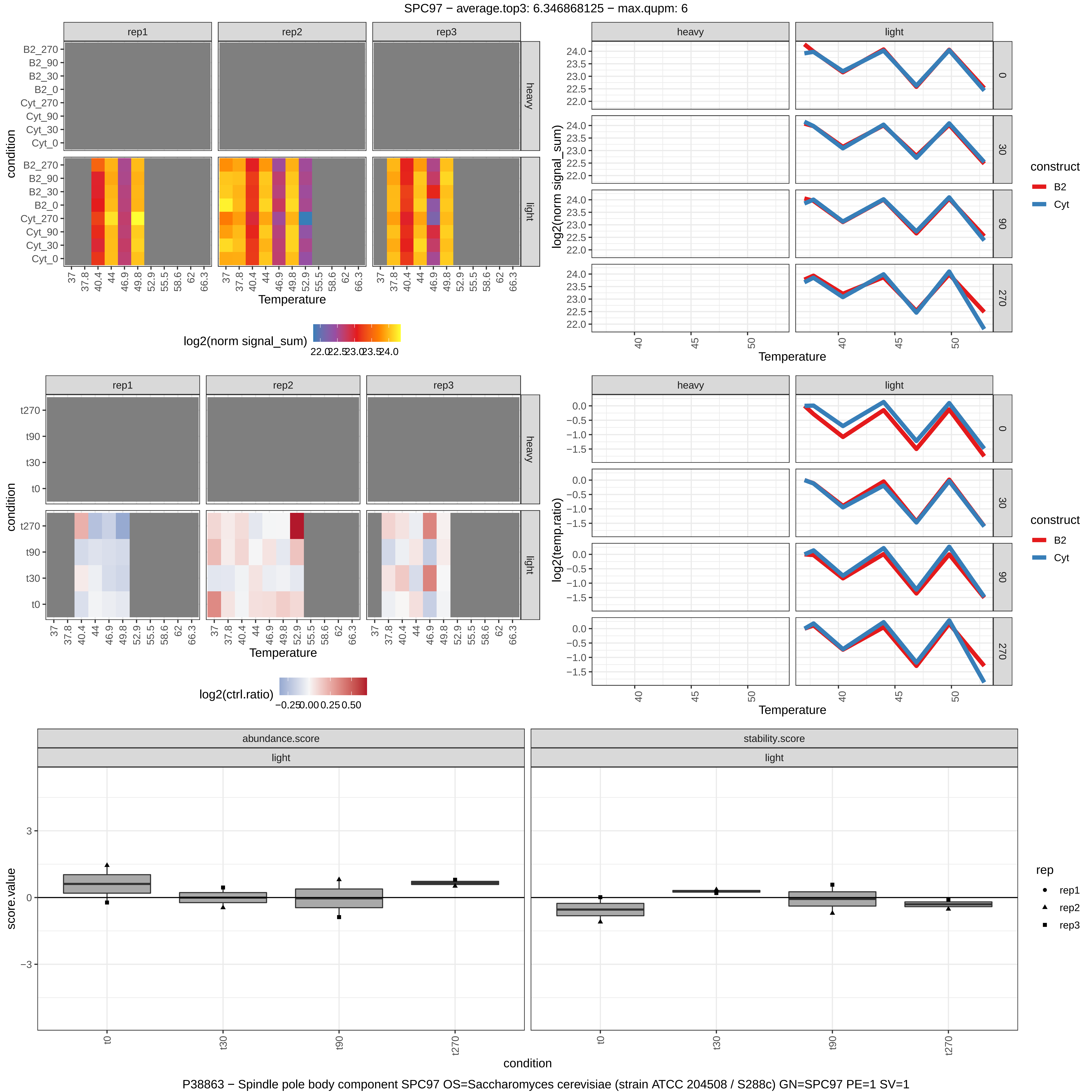

SPC97 − average.top3: 6.346868125 − max.qupm: 6
rep1
rep2
rep3
heavy
light
condition
Temperature
log2(norm signal_sum)
log2(ctrl.ratio)
log2(temp.ratio)
construct
B2
Cyt
abundance.score
stability.score
score.value
rep
rep1
rep2
rep3
P38863 − Spindle pole body component SPC97 OS=Saccharomyces cerevisiae (strain ATCC 204508 / S288c) GN=SPC97 PE=1 SV=1

SPC98 − average.top3: 6.33111166666667 − max.qupm: 6

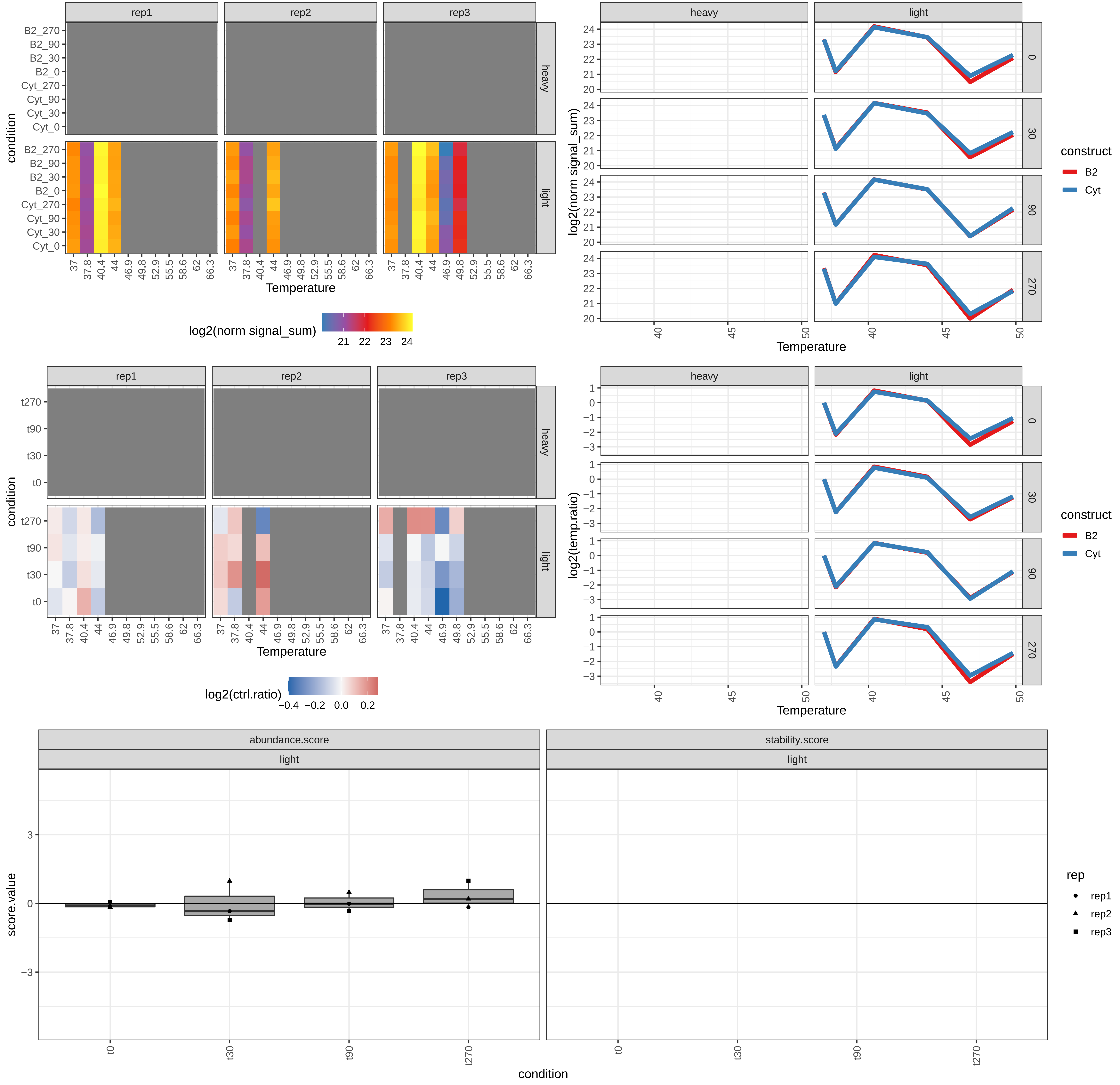

P53540 − Spindle pole body component SPC98 OS=Saccharomyces cerevisiae (strain ATCC 204508 / S288c) GN=SPC98 PE=1 SV=1

SPE2 − average.top3: 6.99991111111111 − max.qupm: 7

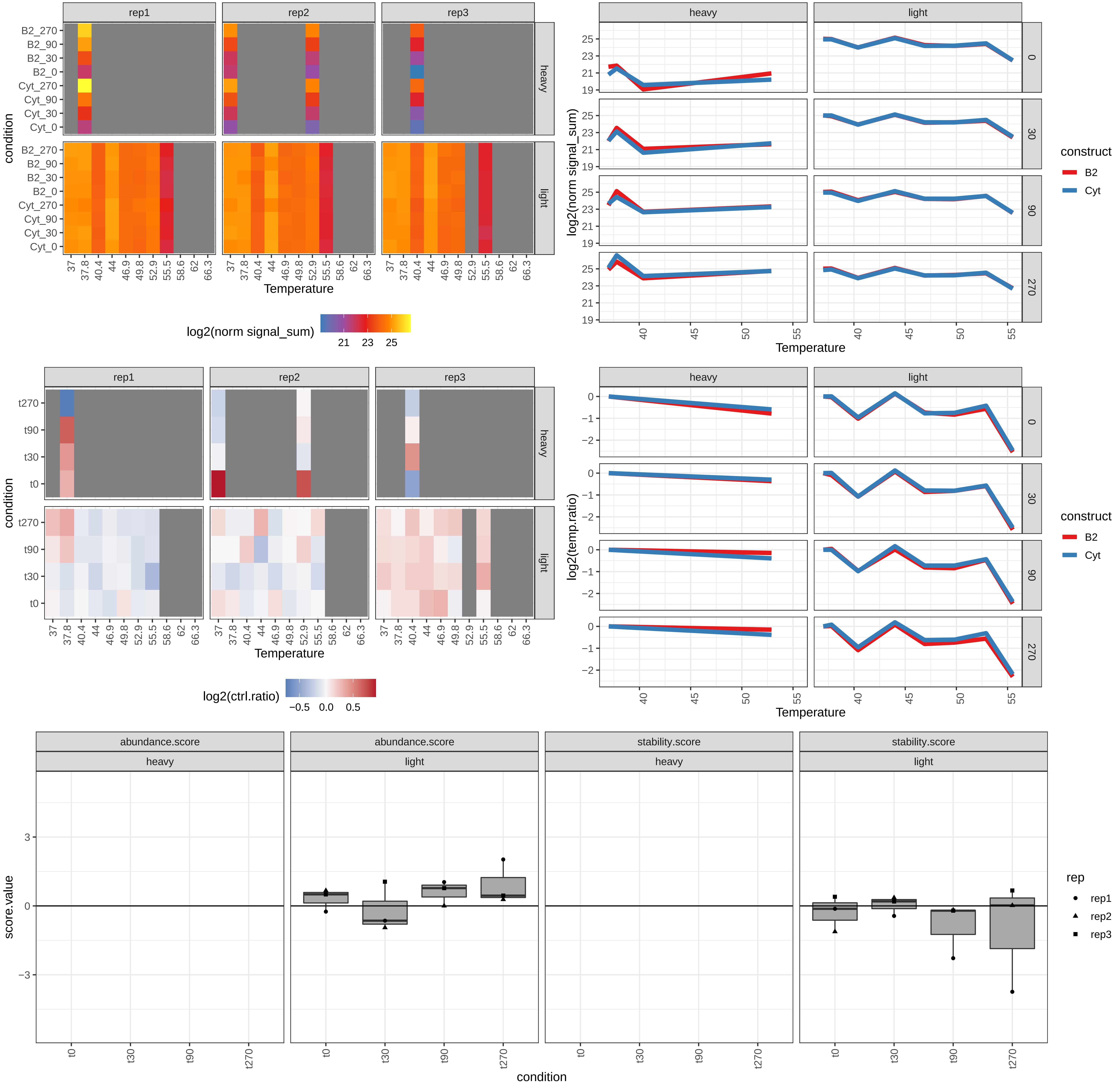

P21182 − S−adenosylmethionine decarboxylase proenzyme OS=Saccharomyces cerevisiae (strain ATCC 204508 / S288c) GN=SPE2 PE=1 SV=1

SPE3 − average.top3: 7.3955895 − max.qupm: 8

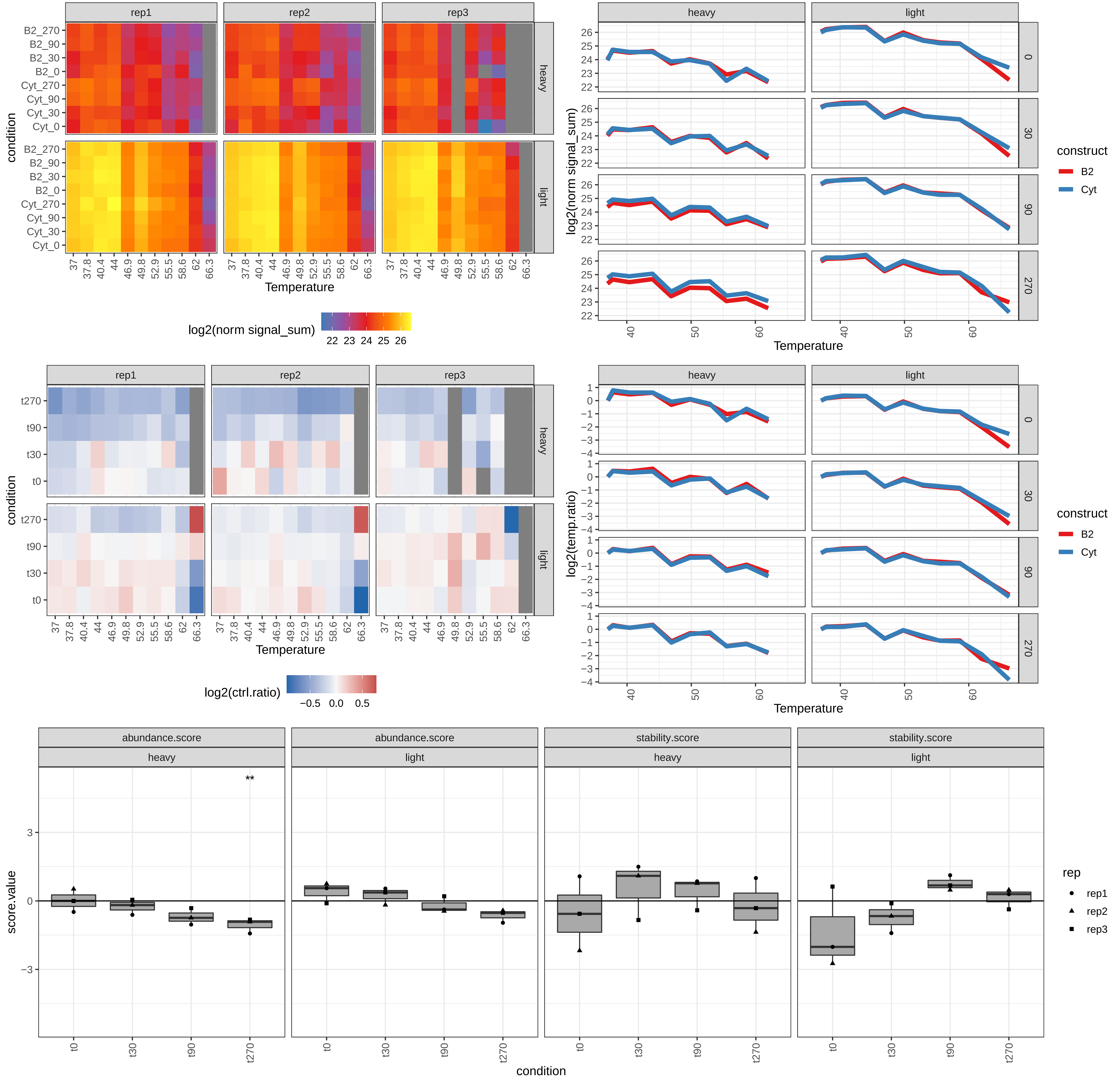

Q12074 − Spermidine synthase OS=Saccharomyces cerevisiae (strain ATCC 204508 / S288c) GN=SPE3 PE=1 SV=1

SPE4 − average.top3: 6.7714255 − max.qupm: 4

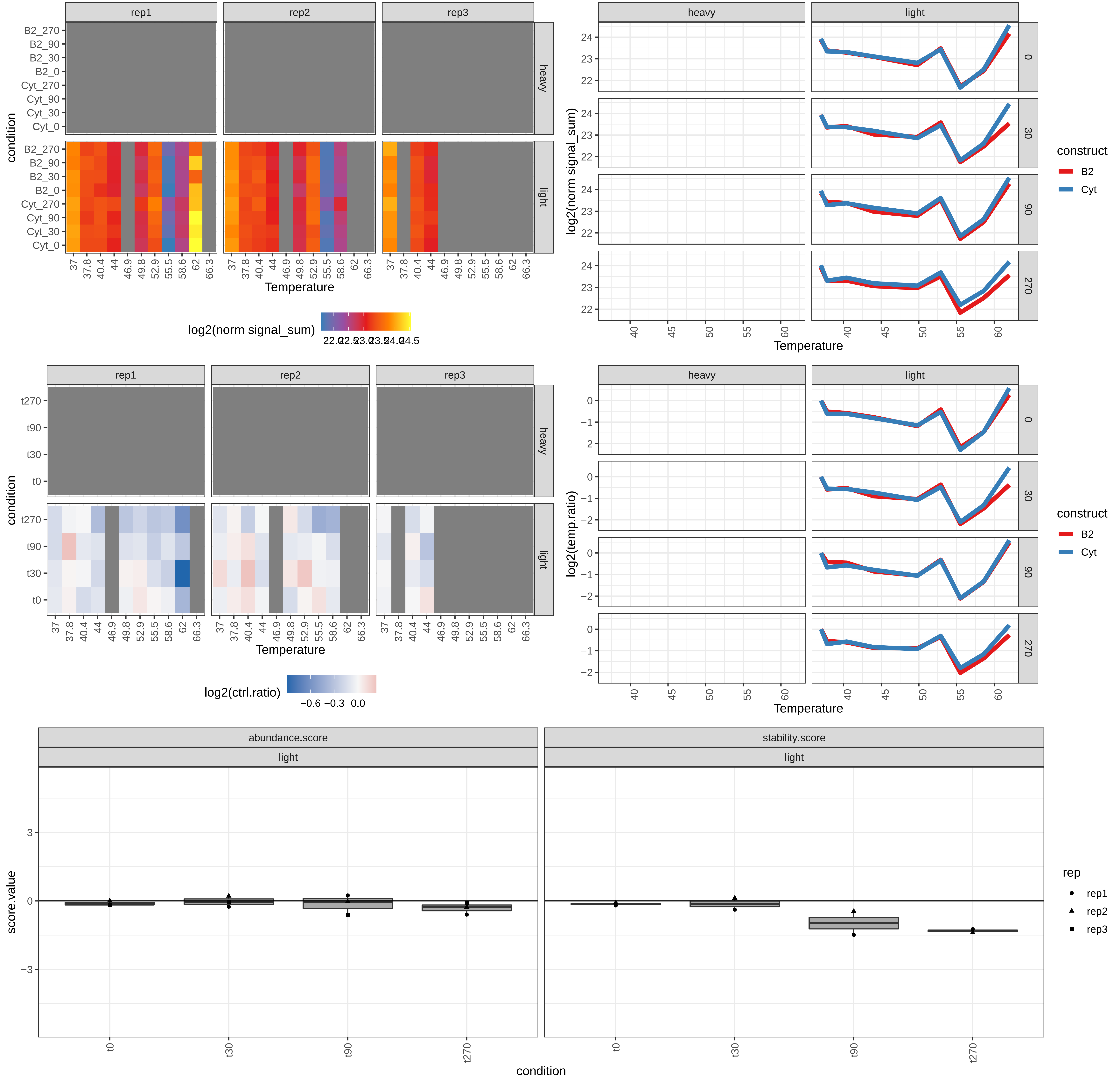

Q12455 − Spermine synthase OS=Saccharomyces cerevisiae (strain ATCC 204508 / S288c) GN=SPE4 PE=1 SV=1

SPF1 − average.top3: 7.18834872340426 − max.qupm: 23

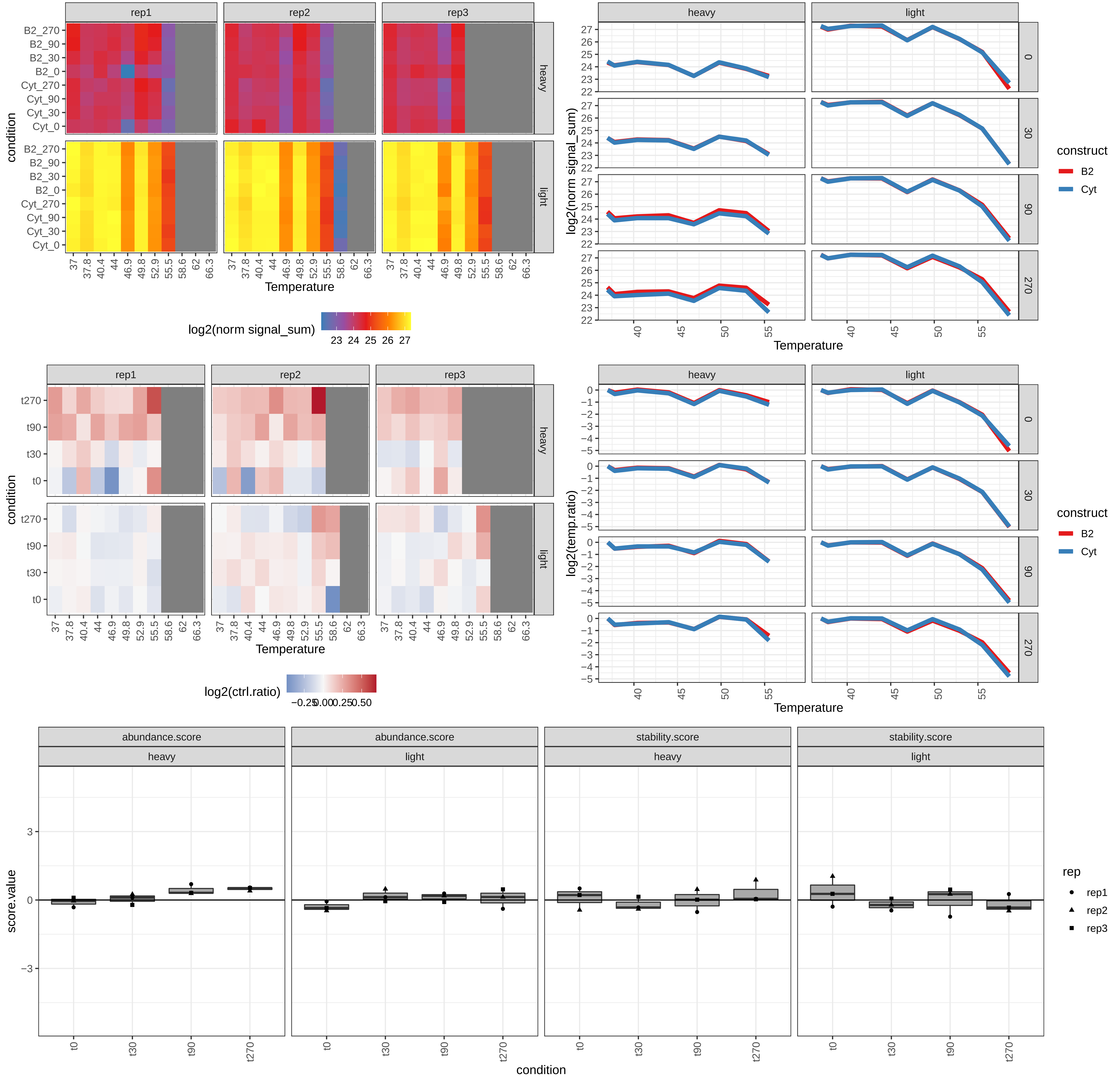

P39986 − Manganese−transporting ATPase 1 OS=Saccharomyces cerevisiae (strain ATCC 204508 / S288c) GN=SPF1 PE=1 SV=1

SPN1 − average.top3: 6.26739090909091 − max.qupm: 4

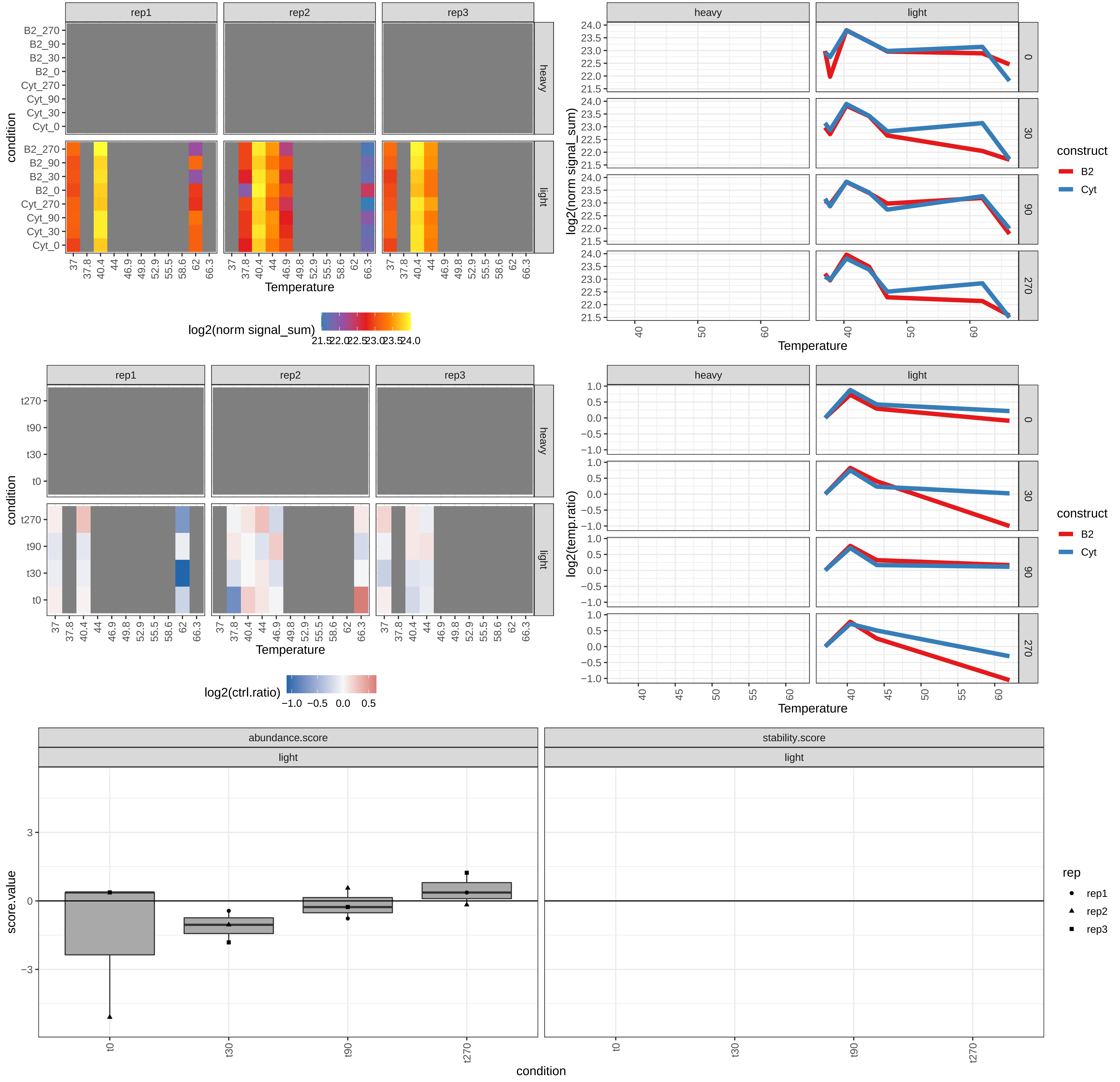

Q06505 − Transcription factor SPN1 OS=Saccharomyces cerevisiae (strain ATCC 204508 / S288c) GN=SPN1 PE=1 SV=1

SPO14 − average.top3: 6.42615722222222 − max.qupm: 6

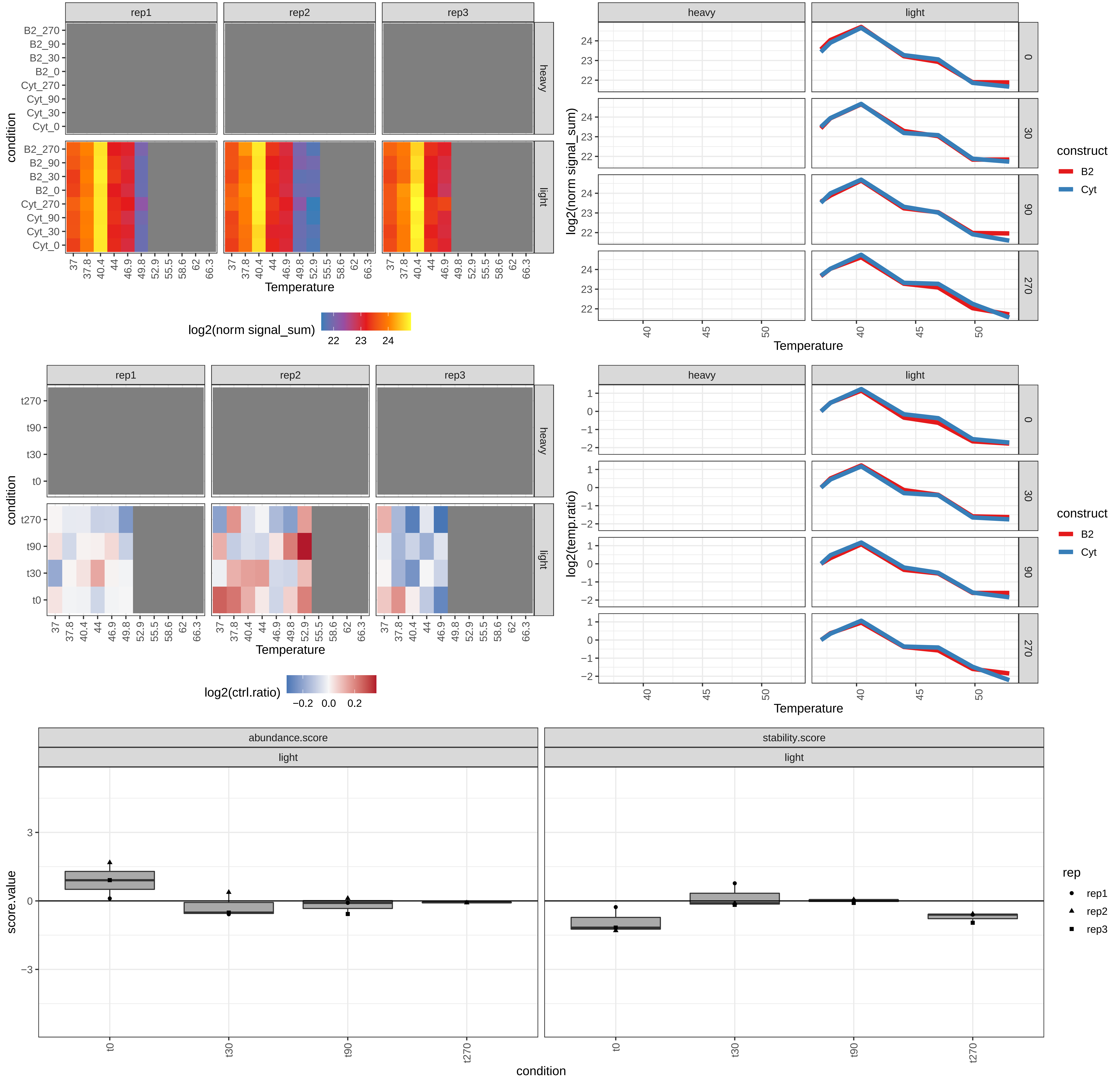

P36126 − Phospholipase D1 OS=Saccharomyces cerevisiae (strain ATCC 204508 / S288c) GN=SPO14 PE=1 SV=3

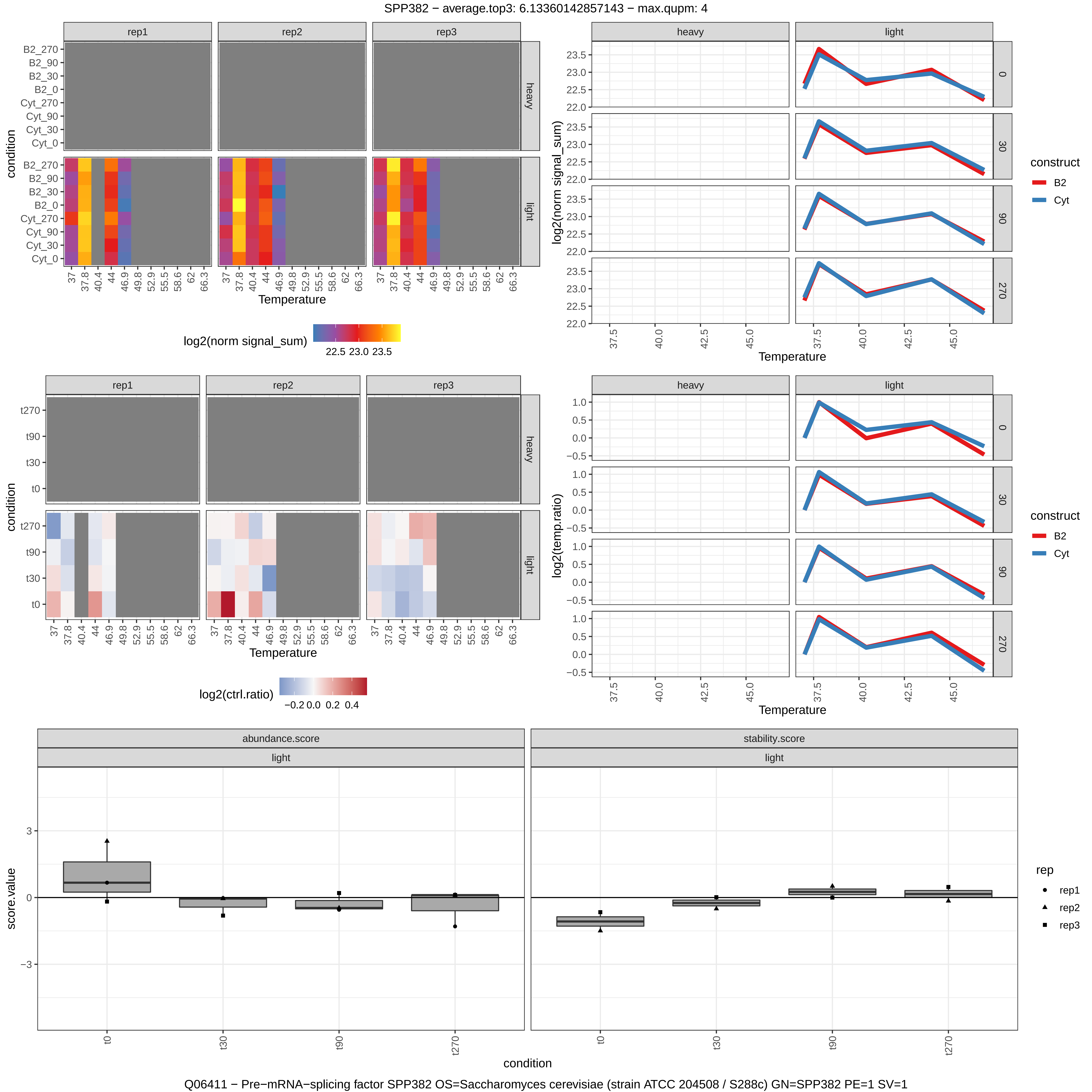

SPP382 − average.top3: 6.13360142857143 − max.qupm: 4
rep1
rep2
rep3
heavy
light
condition
Temperature
log2(norm signal_sum)
log2(ctrl.ratio)
log2(temp.ratio)
construct
B2
Cyt
abundance.score
stability.score
score.value
rep
Q06411 − Pre−mRNA−splicing factor SPP382 OS=Saccharomyces cerevisiae (strain ATCC 204508 / S288c) GN=SPP382 PE=1 SV=1

SPS19 − average.top3: 7.56433931034483 − max.qupm: 11

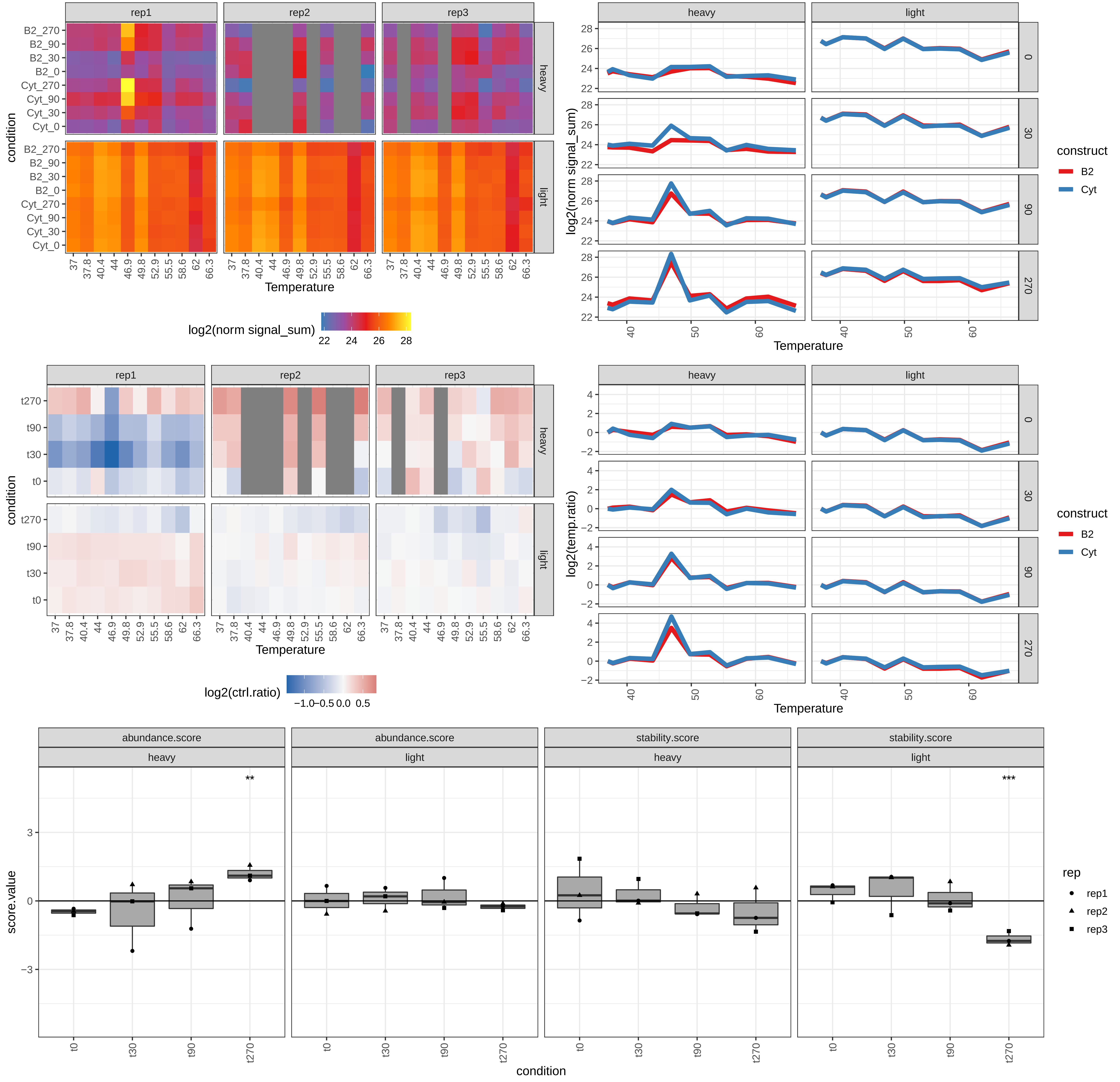

P32573 − Peroxisomal 2,4−dienoyl−CoA reductase SPS19 OS=Saccharomyces cerevisiae (strain ATCC 204508 / S288c) GN=SPS19 PE=1 SV=4

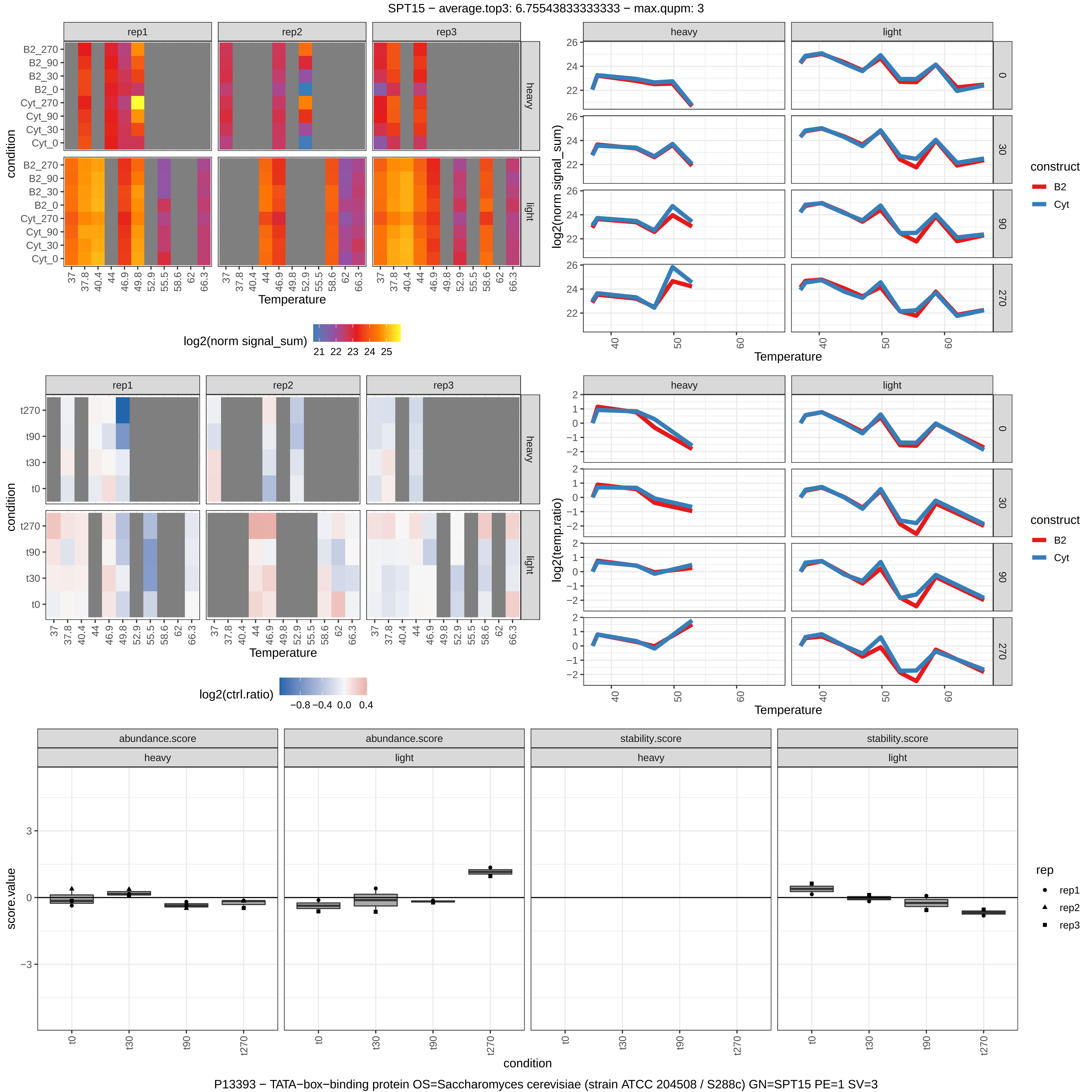
SPT15 − average.top3: 6.75543833333333 − max.qupm: 3
rep1
rep2
rep3
heavy
light
condition
Temperature
log2(norm signal_sum)
log2(ctrl.ratio)
log2(temp.ratio)
construct
B2
Cyt
abundance.score
stability.score
score.value
rep
rep1
rep2
rep3
P13393 − TATA−box−binding protein OS=Saccharomyces cerevisiae (strain ATCC 204508 / S288c) GN=SPT15 PE=1 SV=3

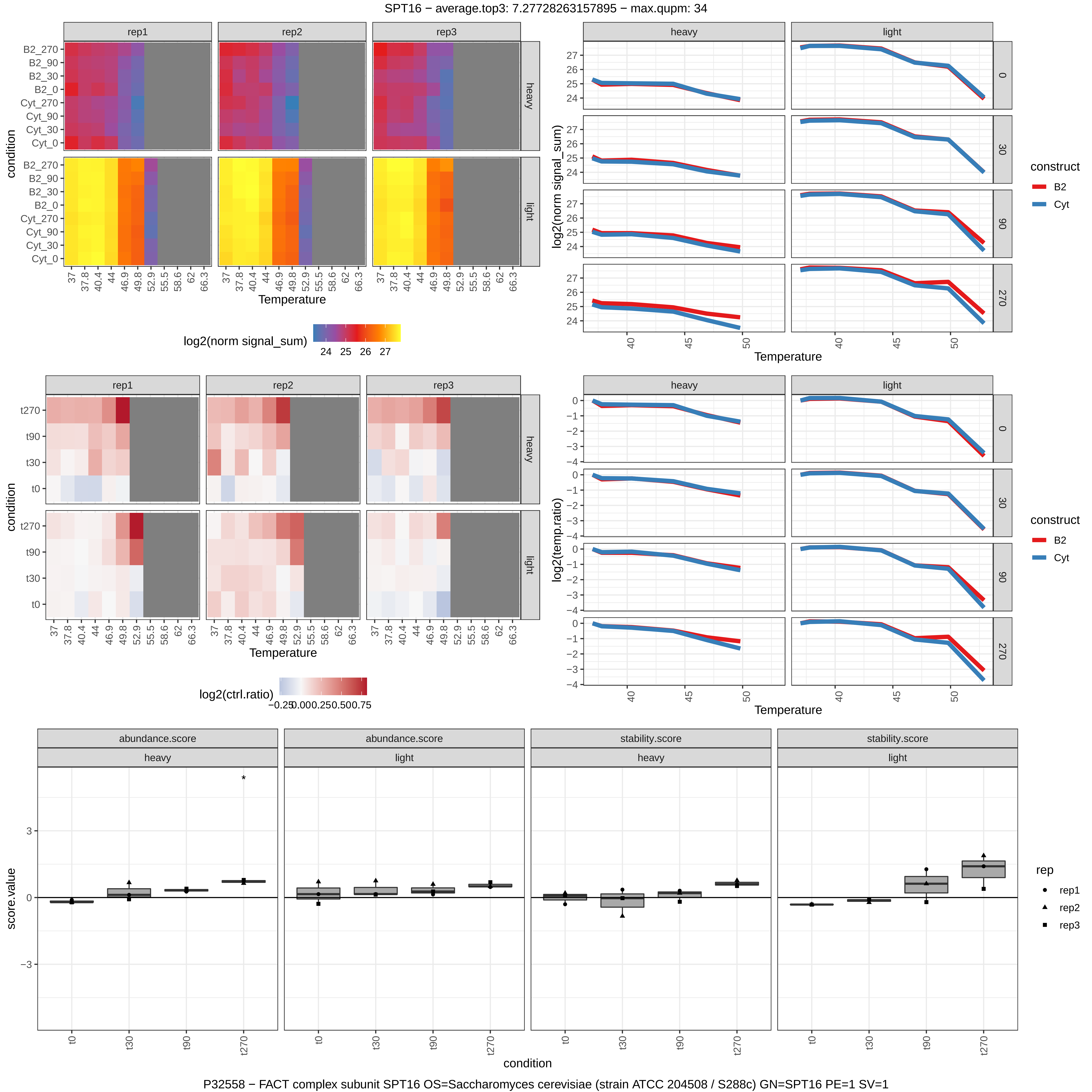
SPT16 − average.top3: 7.27728263157895 − max.qupm: 34
rep1
rep2
rep3
heavy
light
B2_270
B2_90
B2_30
B2_0
Cyt_270
Cyt_90
Cyt_30
Cyt_0
condition
37
37.8
40.4
44
46.9
49.8
52.9
55.5
58.6
62
66.3
Temperature
log2(norm signal_sum)
24
25
26
27
log2(norm signal_sum)
0
30
90
270
40
45
50
construct
B2
Cyt
t270
t90
t30
t0
log2(ctrl.ratio)
−0.25
0.00
0.25
0.50
0.75
log2(temp.ratio)
abundance.score
stability.score
score.value
3
0
−3
rep
rep1
rep2
rep3
*
P32558 − FACT complex subunit SPT16 OS=Saccharomyces cerevisiae (strain ATCC 204508 / S288c) GN=SPT16 PE=1 SV=1

SPT5 − average.top3: 7.00383232142857 − max.qupm: 22

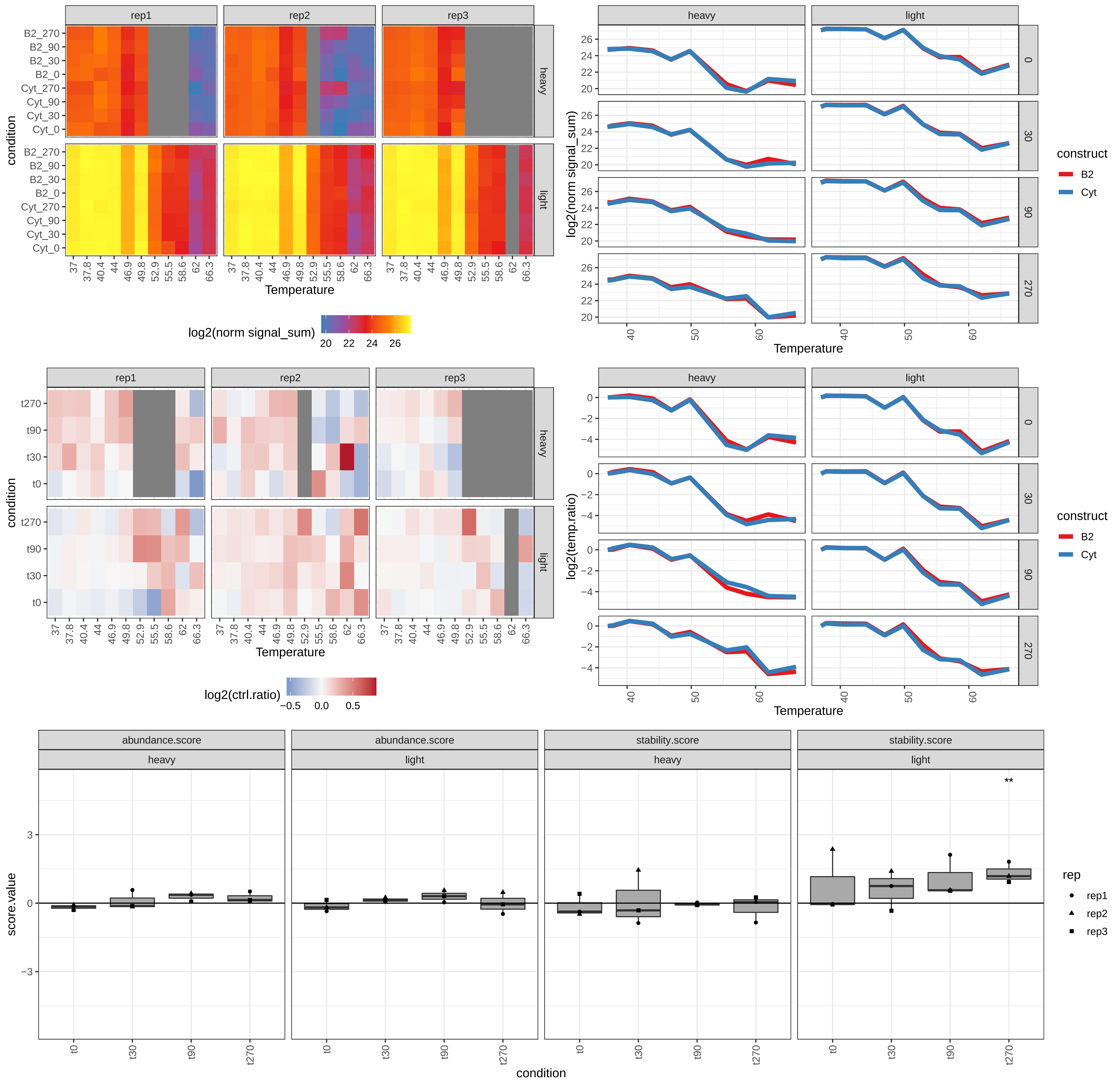

P27692 − Transcription elongation factor SPT5 OS=Saccharomyces cerevisiae (strain ATCC 204508 / S288c) GN=SPT5 PE=1 SV=1

SPT6 − average.top3: 7.19145628571429 − max.qupm: 36

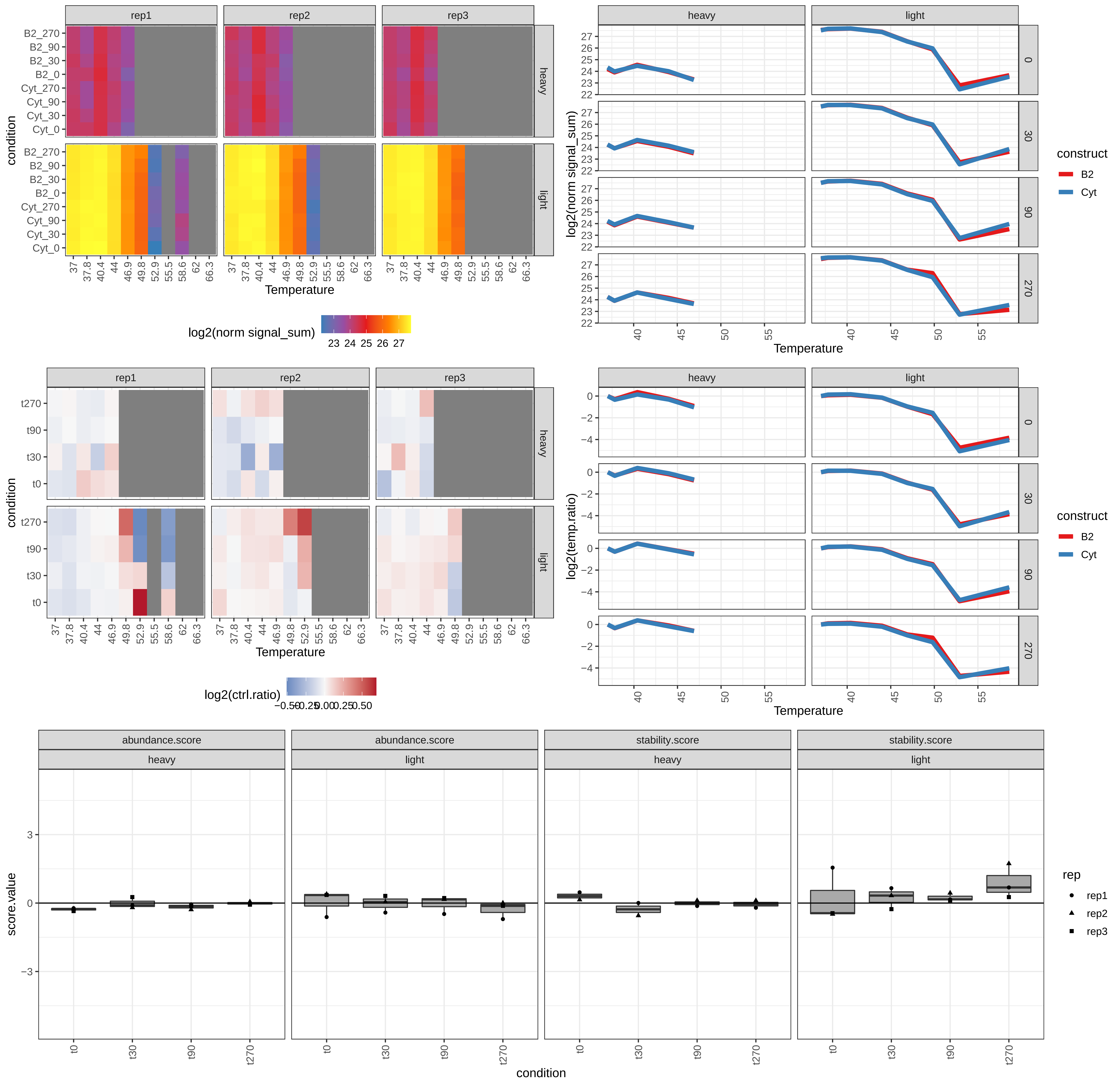

P23615 − Transcription elongation factor SPT6 OS=Saccharomyces cerevisiae (strain ATCC 204508 / S288c) GN=SPT6 PE=1 SV=1

SPT7 − average.top3: 6.48826615384615 − max.qupm: 5

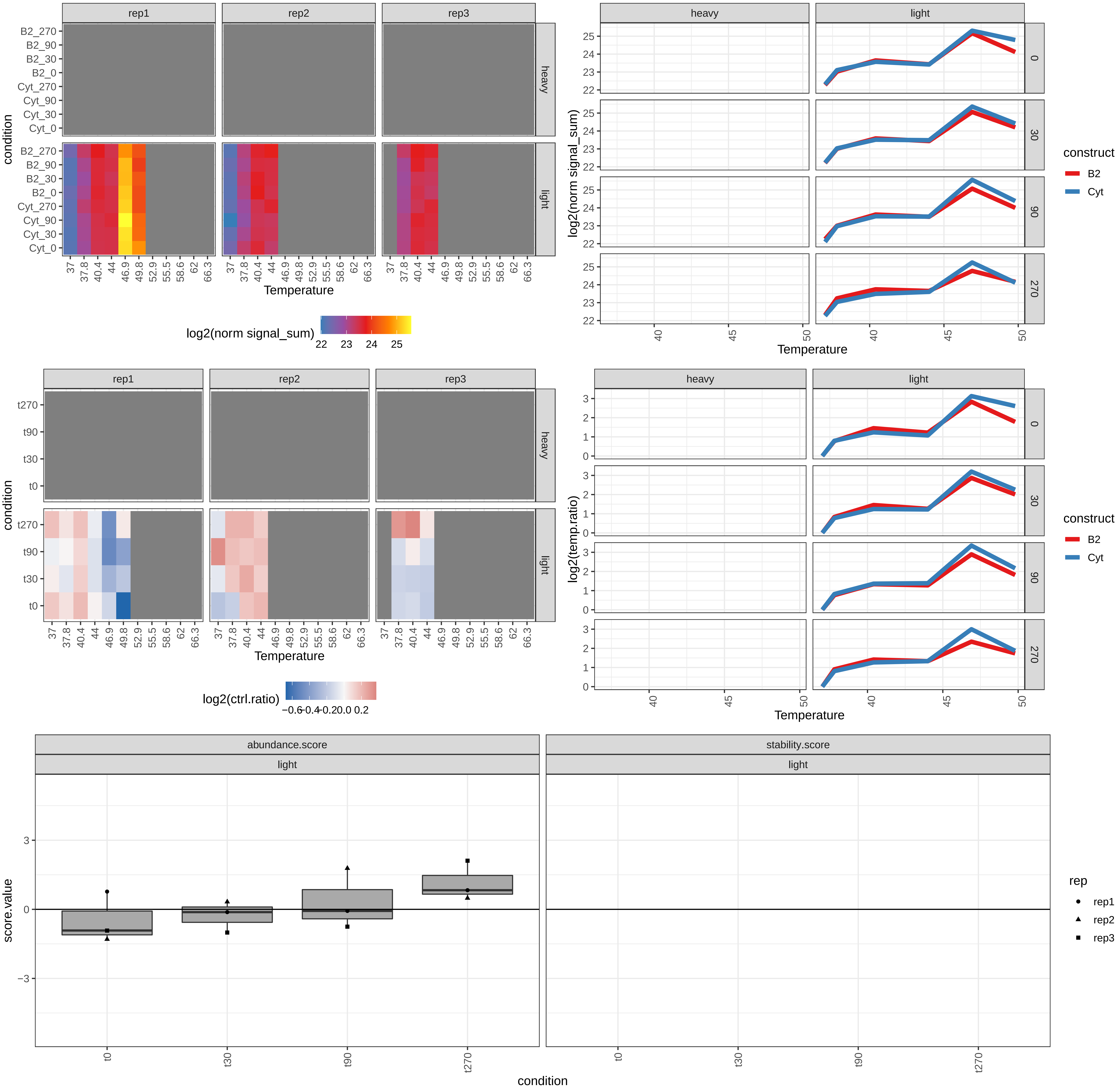

P35177 − Transcriptional activator SPT7 OS=Saccharomyces cerevisiae (strain ATCC 204508 / S288c) GN=SPT7 PE=1 SV=1

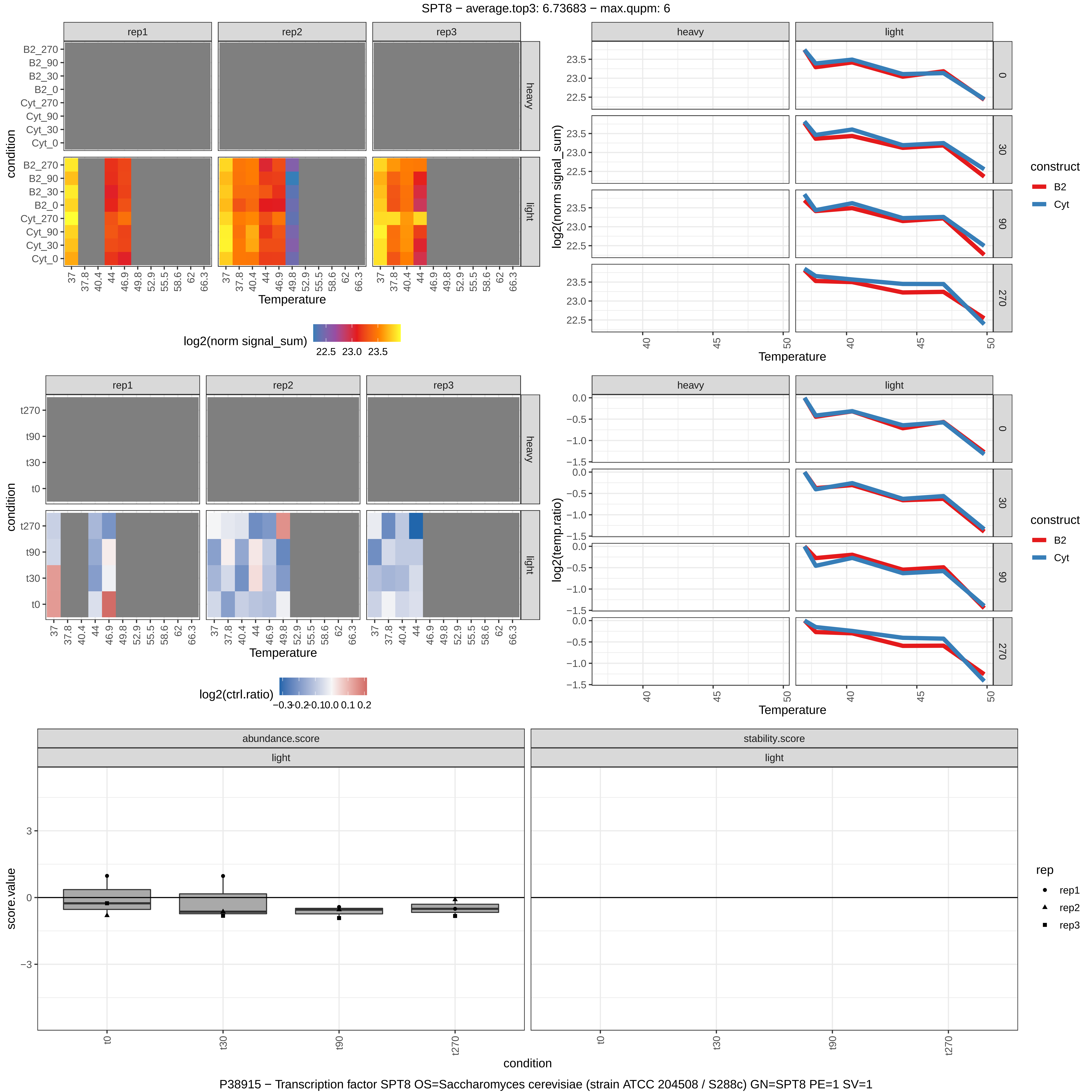
SPT8 − average.top3: 6.73683 − max.qupm: 6
rep1
rep2
rep3
heavy
light
condition
Temperature
log2(norm signal_sum)
log2(ctrl.ratio)
log2(temp.ratio)
construct
B2
Cyt
abundance.score
stability.score
score.value
rep
rep1
rep2
rep3
P38915 − Transcription factor SPT8 OS=Saccharomyces cerevisiae (strain ATCC 204508 / S288c) GN=SPT8 PE=1 SV=1

SQT1 − average.top3: 7.07853833333333 − max.qupm: 5

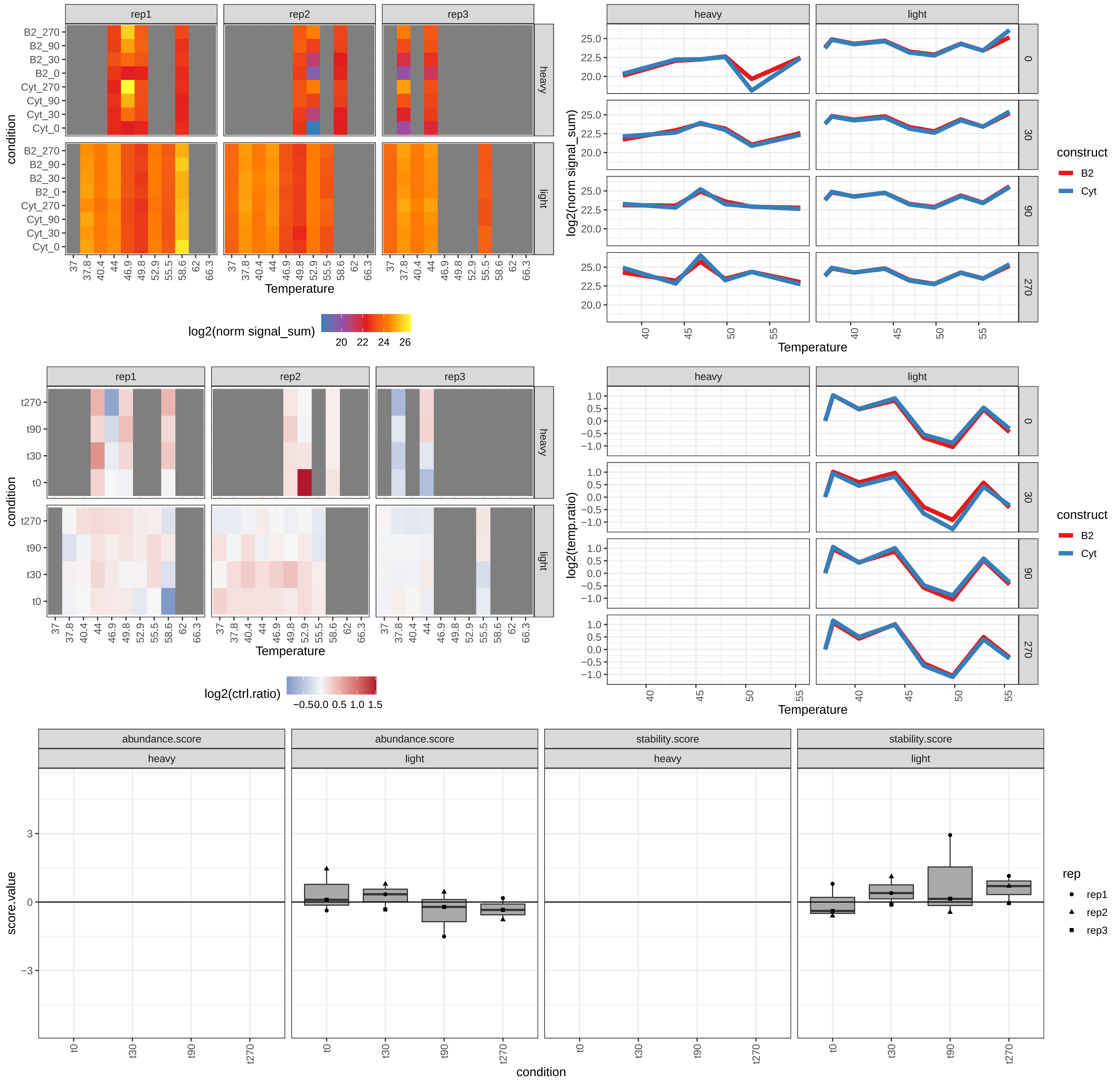

P35184 − Ribosome assembly protein SQT1 OS=Saccharomyces cerevisiae (strain ATCC 204508 / S288c) GN=SQT1 PE=1 SV=2

SRB2 − average.top3: 6.364816 − max.qupm: 5

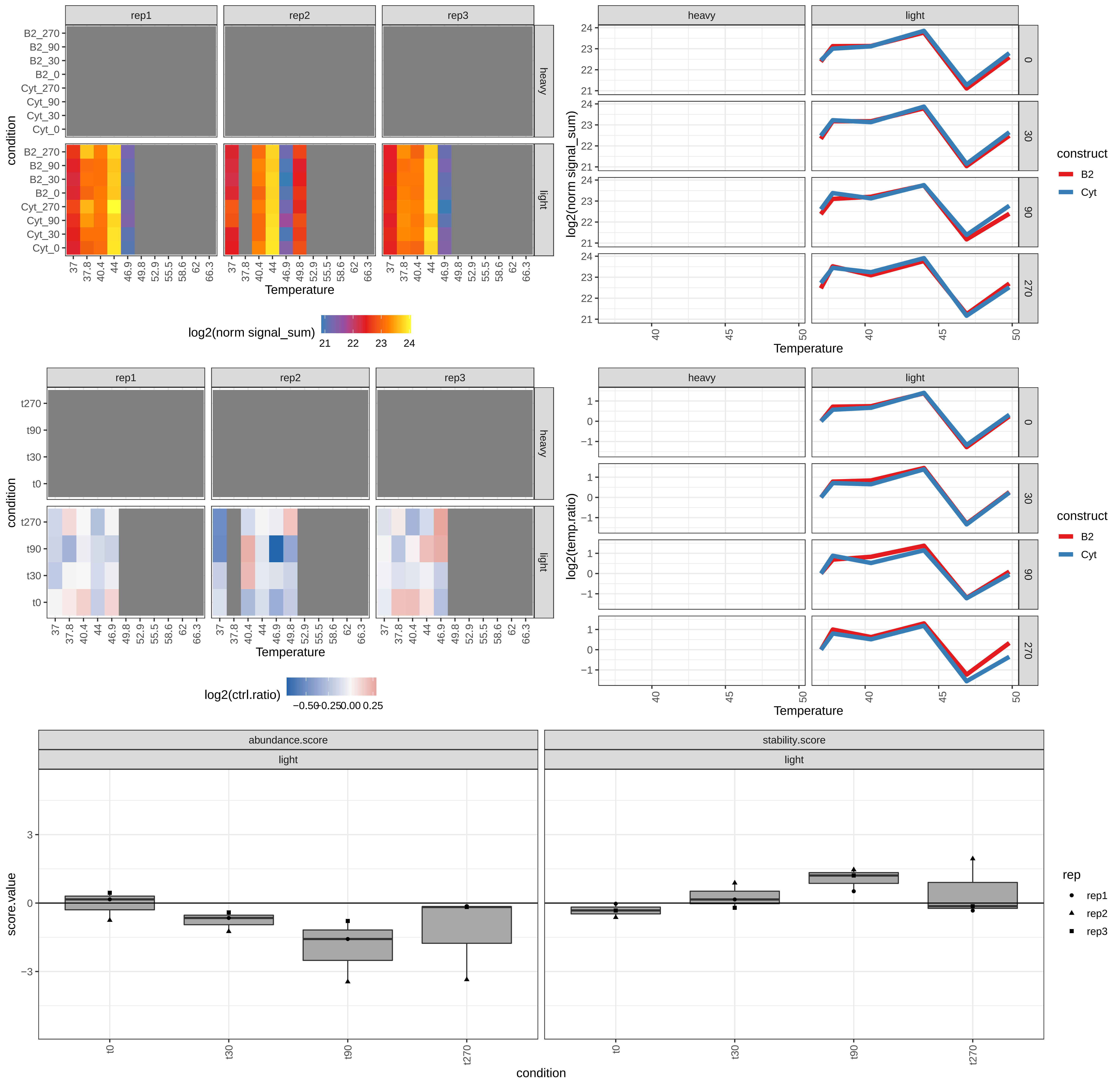

P34162 − Mediator of RNA polymerase II transcription subunit 20 OS=Saccharomyces cerevisiae (strain ATCC 204508 / S288c) GN=SRB2 PE=1 SV=1

SRB4 − average.top3: 6.8185252173913 − max.qupm: 9

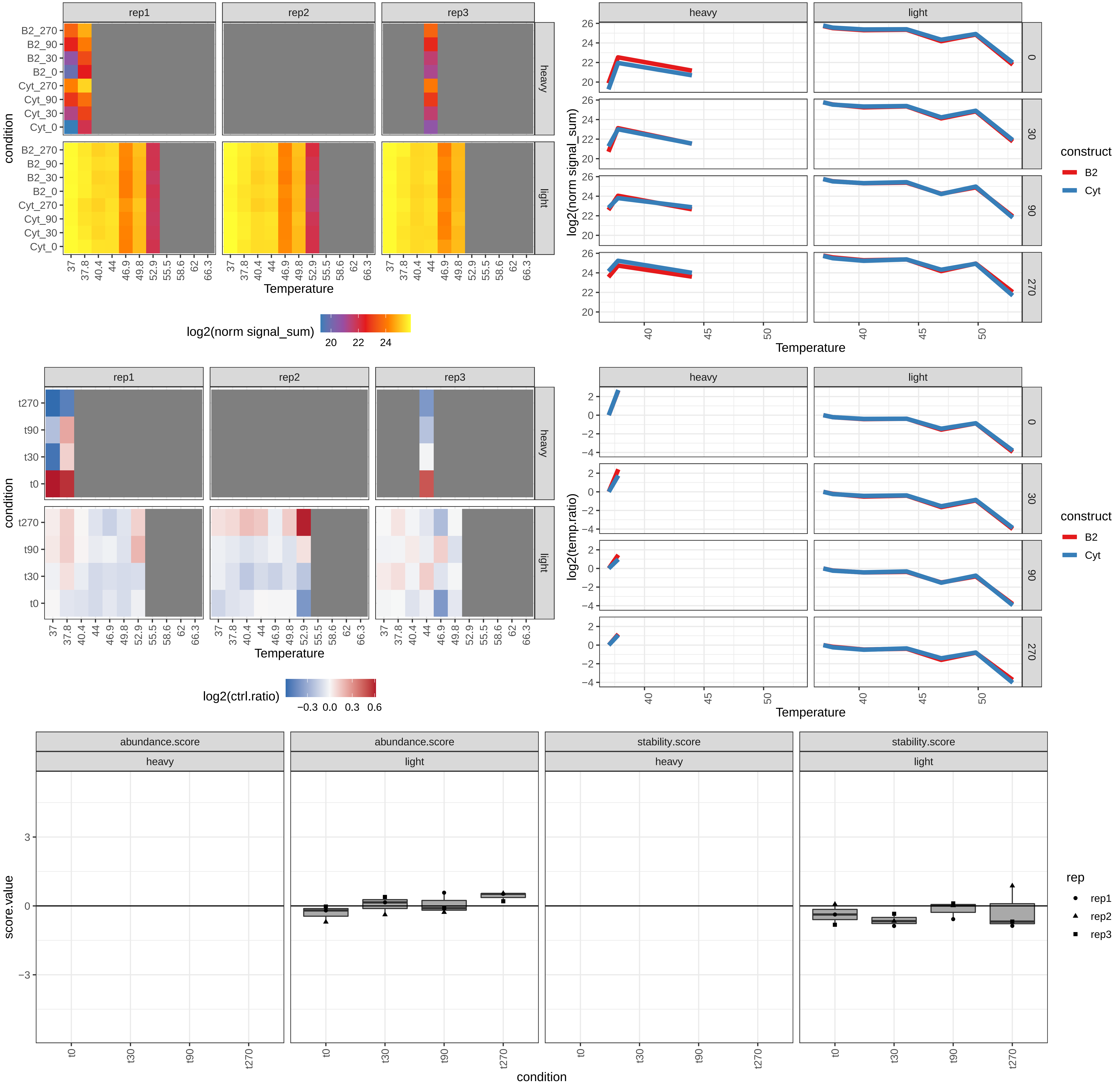

P32569 − Mediator of RNA polymerase II transcription subunit 17 OS=Saccharomyces cerevisiae (strain ATCC 204508 / S288c) GN=SRB4 PE=1 SV=1

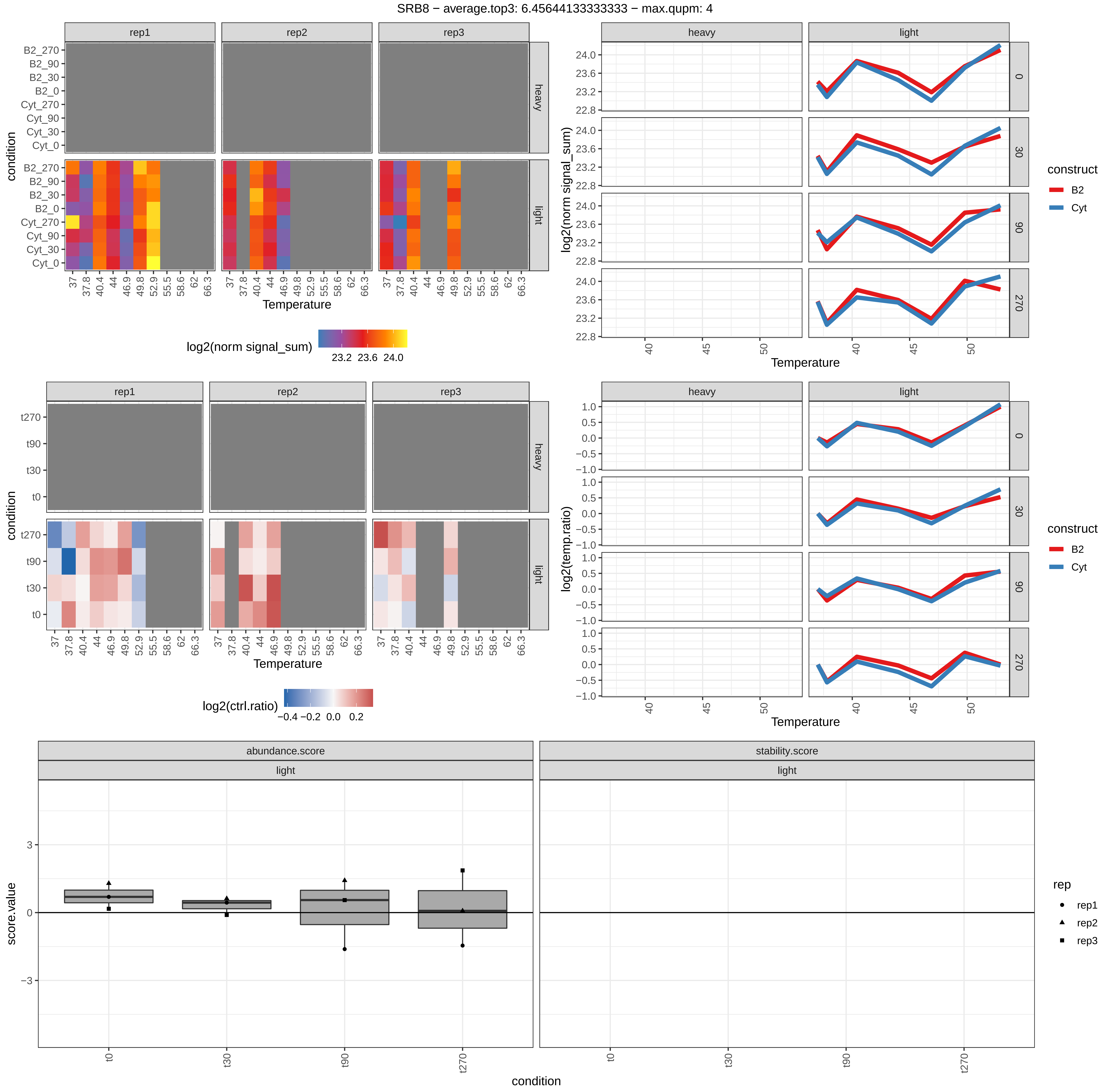

P25648 − Mediator of RNA polymerase II transcription subunit 12 OS=Saccharomyces cerevisiae (strain ATCC 204508 / S288c) GN=SRB8 PE=1 SV=2

SRC1 − average.top3: 6.49684941176471 − max.qupm: 6

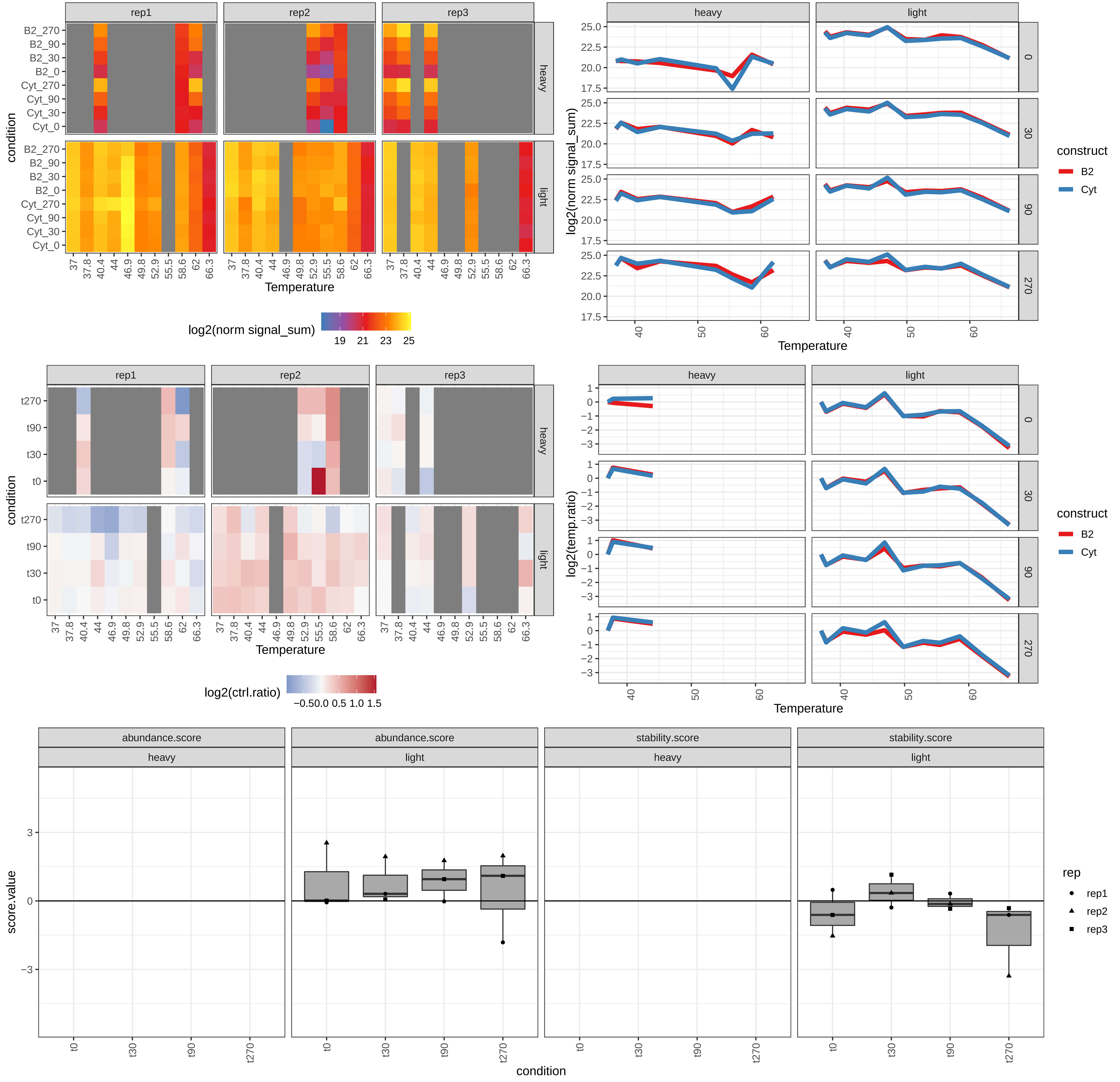

Q03707 − Inner nuclear membrane protein SRC1 OS=Saccharomyces cerevisiae (strain ATCC 204508 / S288c) GN=SRC1 PE=1 SV=2

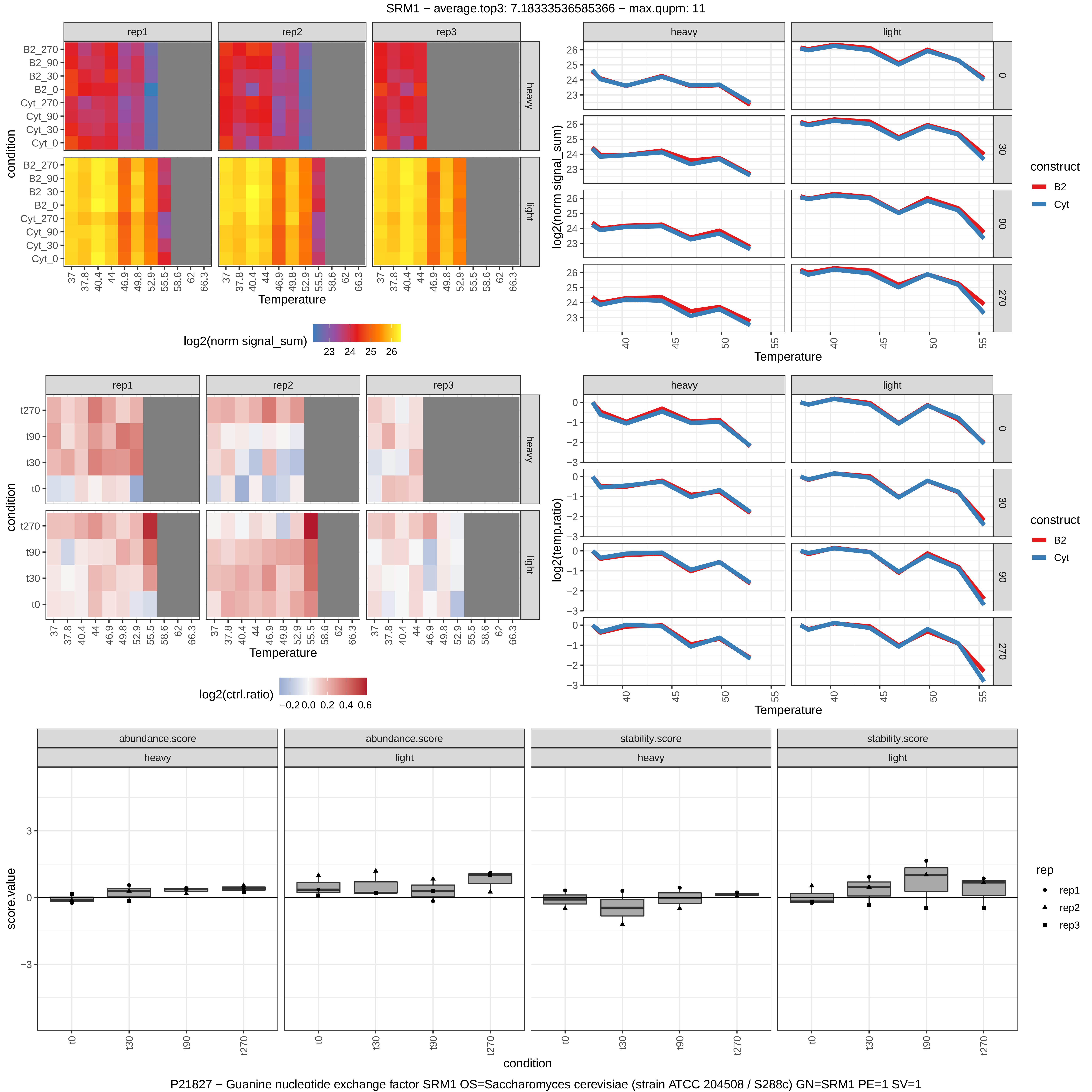
SRM1 − average.top3: 7.18333536585366 − max.qupm: 11
rep1
rep2
rep3
heavy
light
condition
Temperature
log2(norm signal_sum)
log2(temp.ratio)
log2(ctrl.ratio)
construct
B2
Cyt
abundance.score
stability.score
score.value
rep
rep1
rep2
rep3
P21827 − Guanine nucleotide exchange factor SRM1 OS=Saccharomyces cerevisiae (strain ATCC 204508 / S288c) GN=SRM1 PE=1 SV=1

SRN2 − average.top3: 6.3970975 − max.qupm: 6

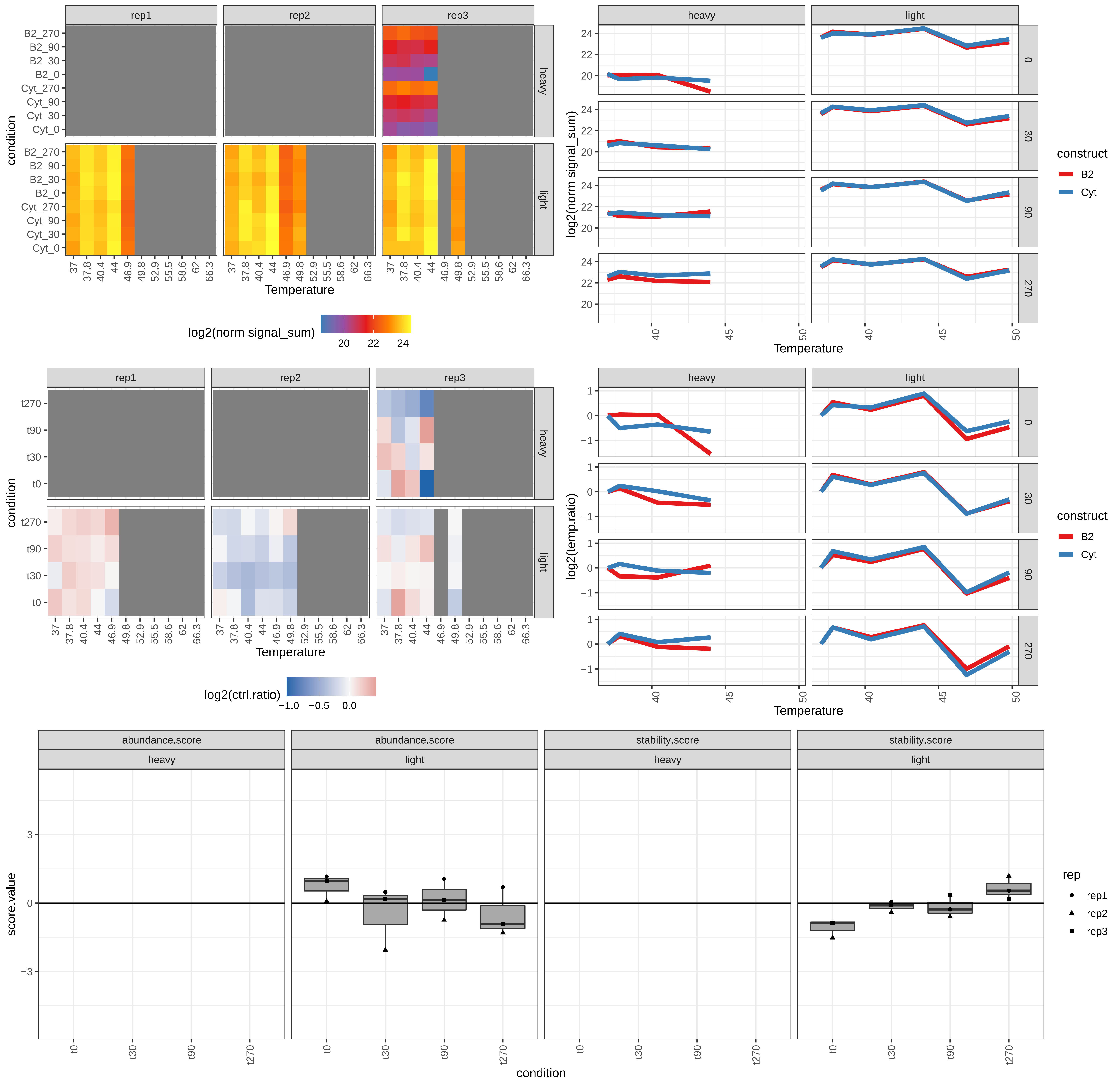

Q99176 − Protein SRN2 OS=Saccharomyces cerevisiae (strain ATCC 204508 / S288c) GN=SRN2 PE=1 SV=1

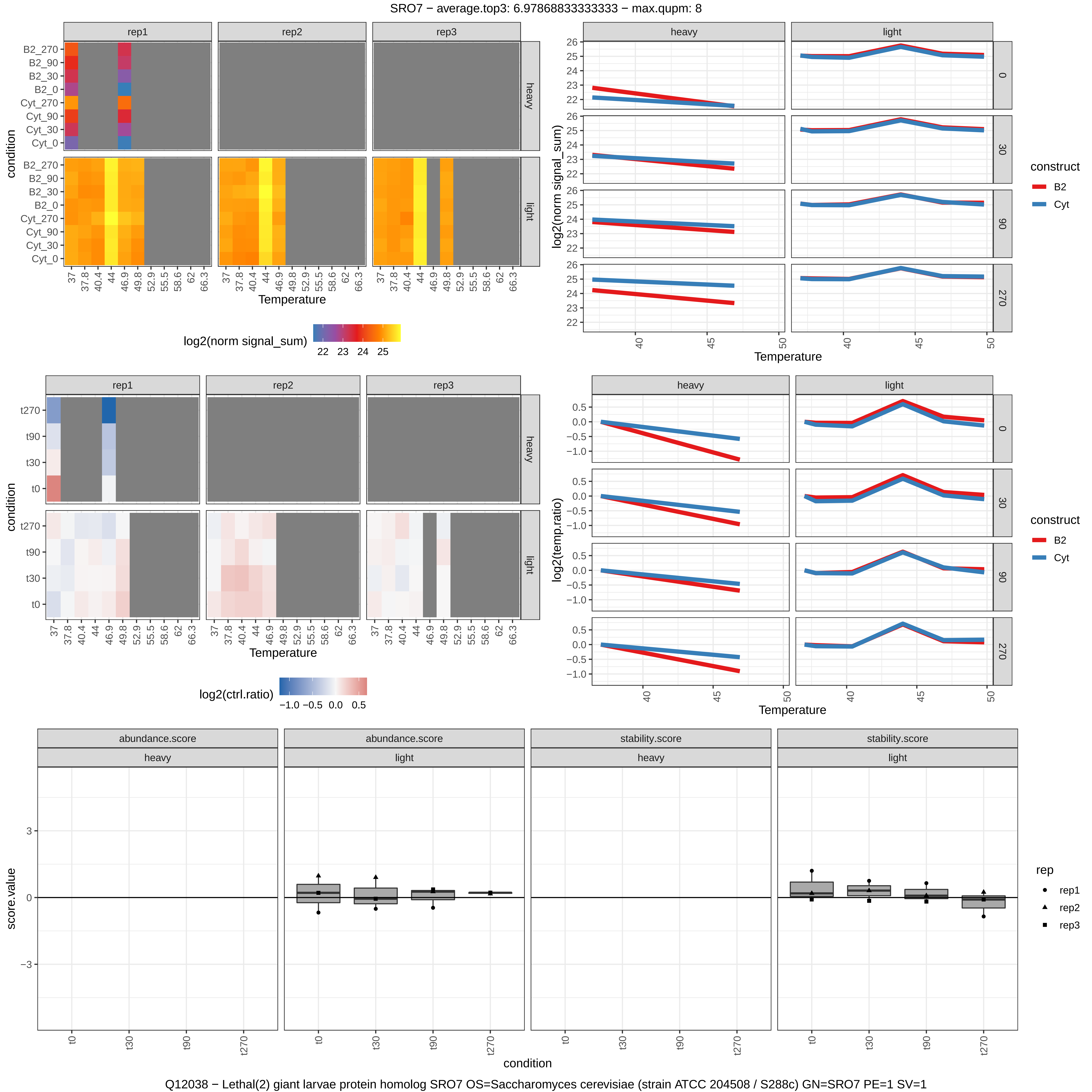

SRO7 − average.top3: 6.97868833333333 − max.qupm: 8
rep1
rep2
rep3
heavy
light
condition
B2_270
B2_90
B2_30
B2_0
Cyt_270
Cyt_90
Cyt_30
Cyt_0
37
37.8
40.4
44
46.9
49.8
52.9
55.5
58.6
62
66.3
Temperature
log2(norm signal_sum)
22
23
24
25
log2(norm signal_sum)
0
30
90
270
construct
B2
Cyt
t270
t90
t30
t0
log2(ctrl.ratio)
−1.0
−0.5
0.0
0.5
log2(temp.ratio)
abundance.score
stability.score
score.value
3
0
−3
rep
rep1
rep2
rep3
condition
Q12038 − Lethal(2) giant larvae protein homolog SRO7 OS=Saccharomyces cerevisiae (strain ATCC 204508 / S288c) GN=SRO7 PE=1 SV=1

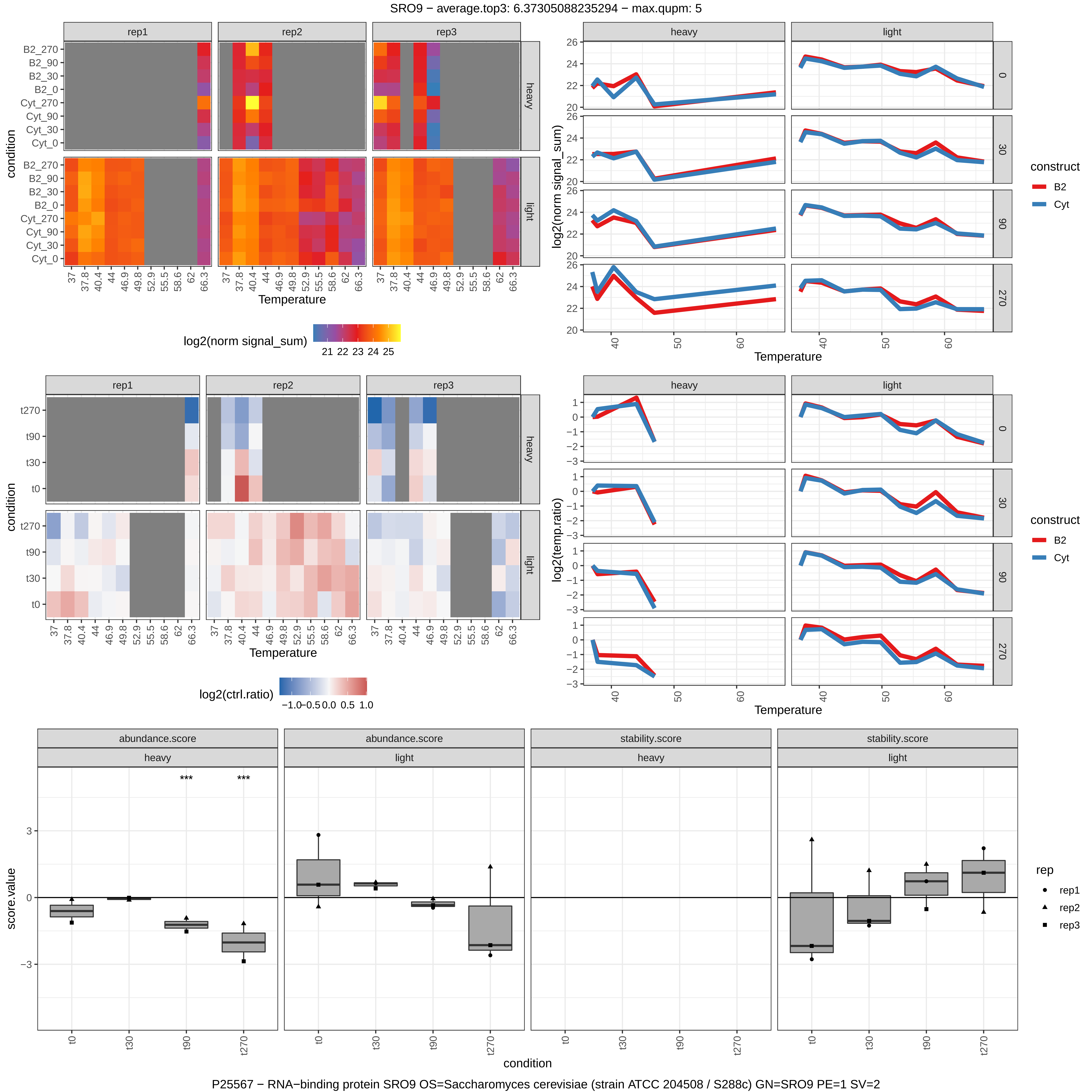

SRO9 − average.top3: 6.37305088235294 − max.qupm: 5
rep1
rep2
rep3
heavy
light
condition
Temperature
log2(norm signal_sum)
log2(norm signal_sum)
construct
B2
Cyt
log2(ctrl.ratio)
log2(temp.ratio)
abundance.score
stability.score
score.value
rep
rep1
rep2
rep3
***
P25567 − RNA−binding protein SRO9 OS=Saccharomyces cerevisiae (strain ATCC 204508 / S288c) GN=SRO9 PE=1 SV=2

SRP1 − average.top3: 7.09432142857143 − max.qupm: 9

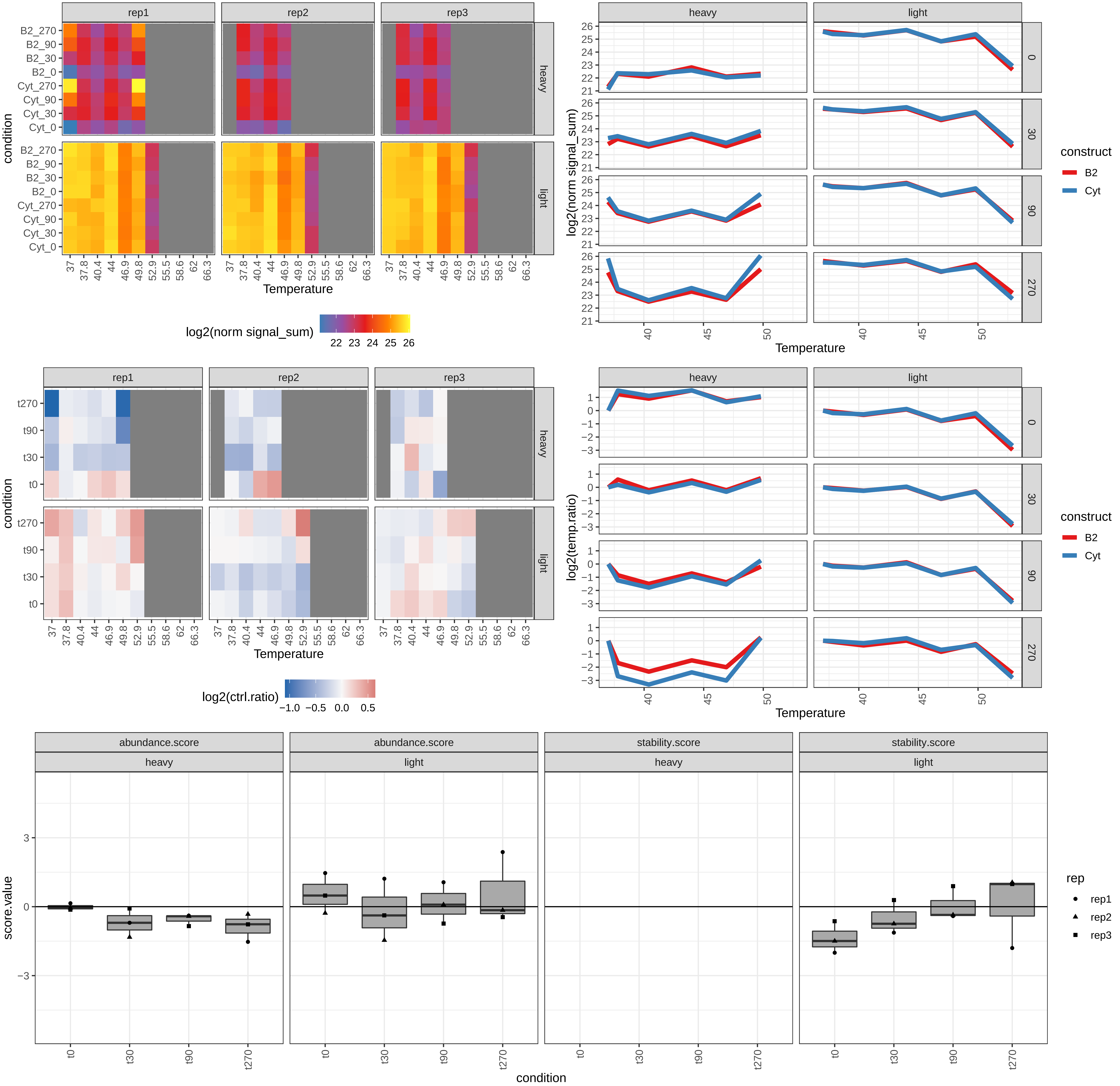

Q02821 − Importin subunit alpha OS=Saccharomyces cerevisiae (strain ATCC 204508 / S288c) GN=SRP1 PE=1 SV=1

SRP101 − average.top3: 6.87565166666667 − max.qupm: 5

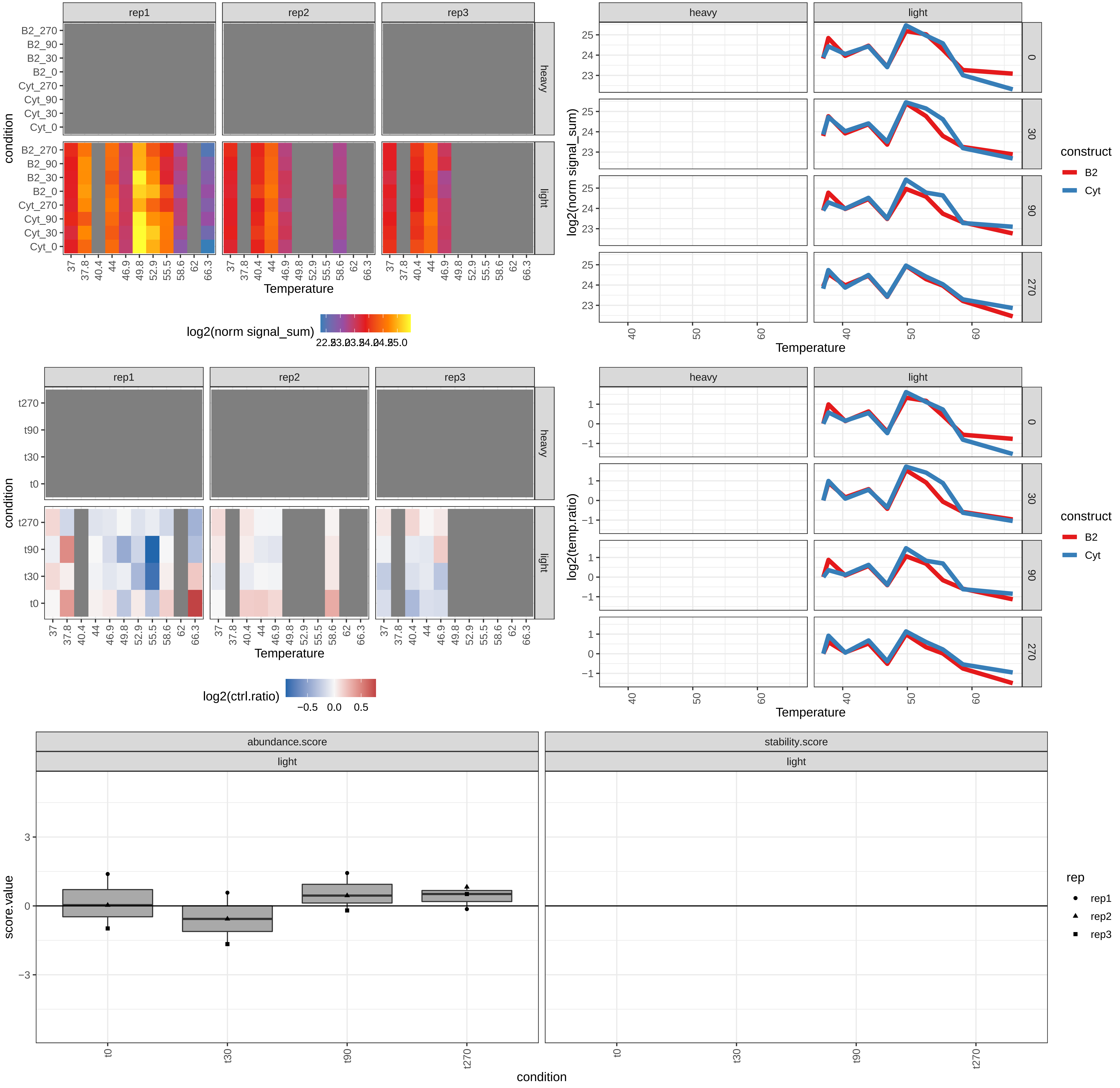

P32916 − Signal recognition particle receptor subunit alpha homolog OS=Saccharomyces cerevisiae (strain ATCC 204508 / S288c) GN=SRP101 PE=1 SV=2

SRP102 − average.top3: 7.0018580952381 − max.qupm: 5

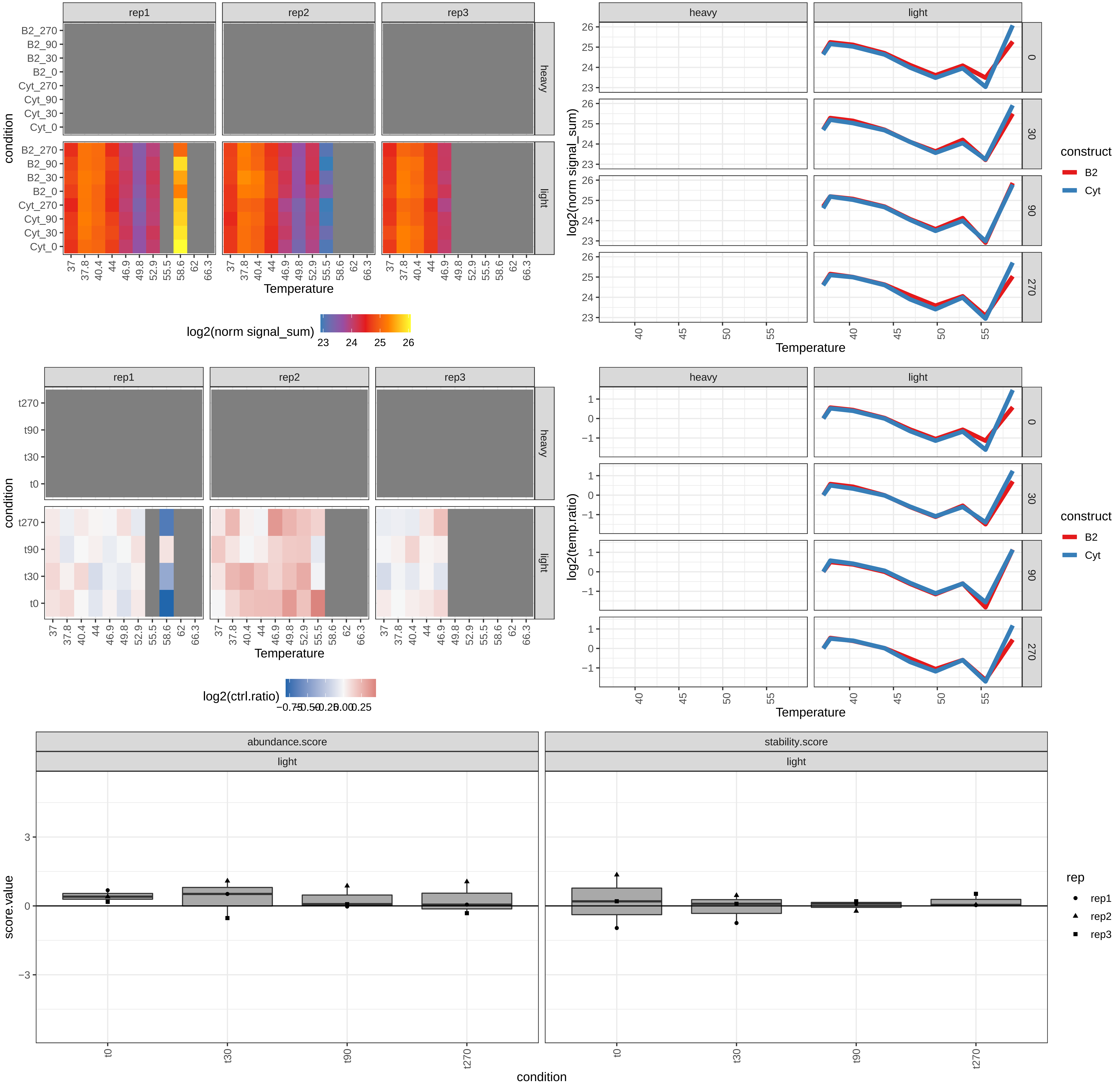

P36057 − Signal recognition particle receptor subunit beta OS=Saccharomyces cerevisiae (strain ATCC 204508 / S288c) GN=SRP102 PE=1 SV=1

SRP40 − average.top3: 6.64745090909091 − max.qupm: 3

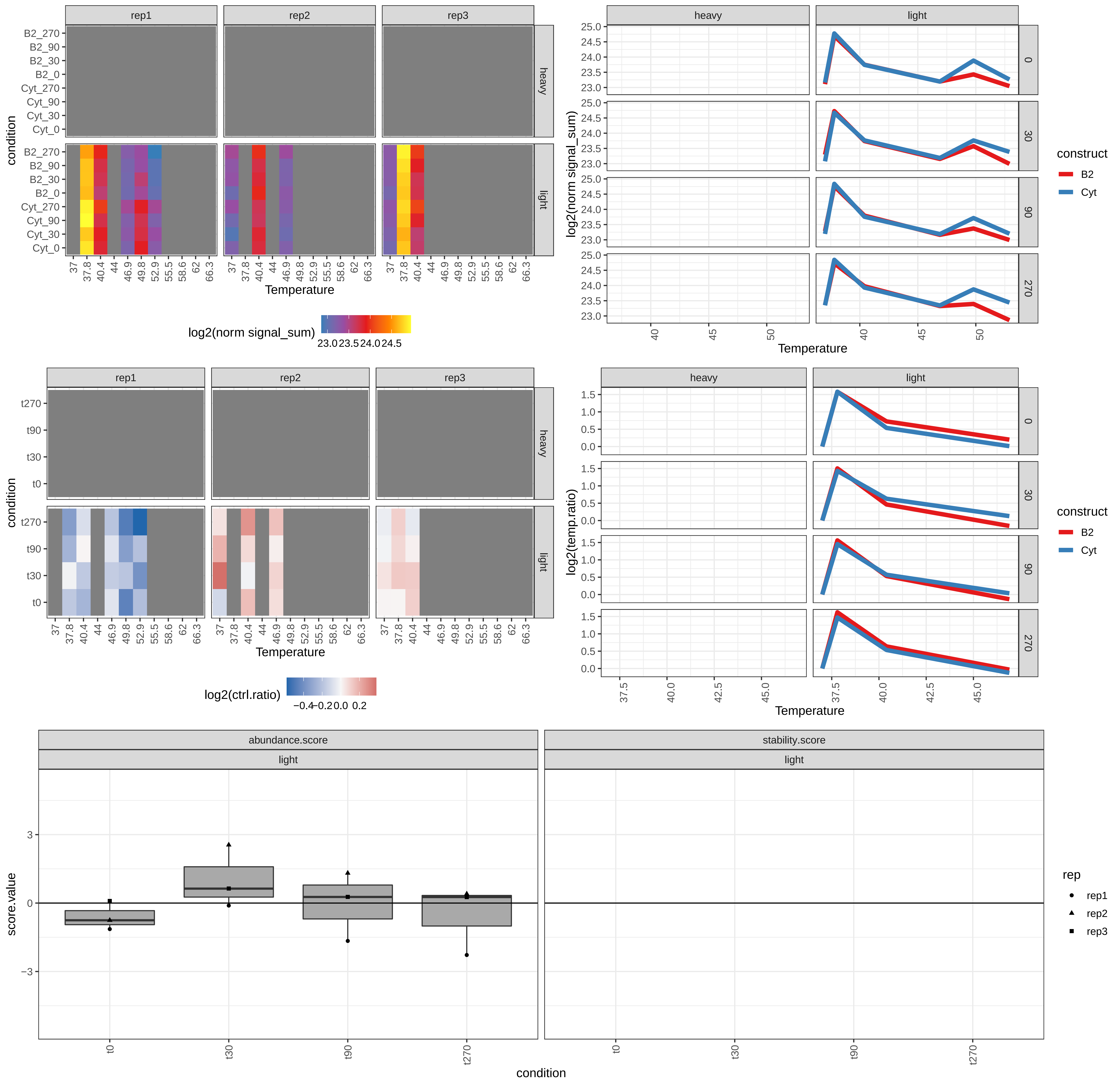

P32583 − Suppressor protein SRP40 OS=Saccharomyces cerevisiae (strain ATCC 204508 / S288c) GN=SRP40 PE=1 SV=2

SRP54 − average.top3: 6.84071869565217 − max.qupm: 9

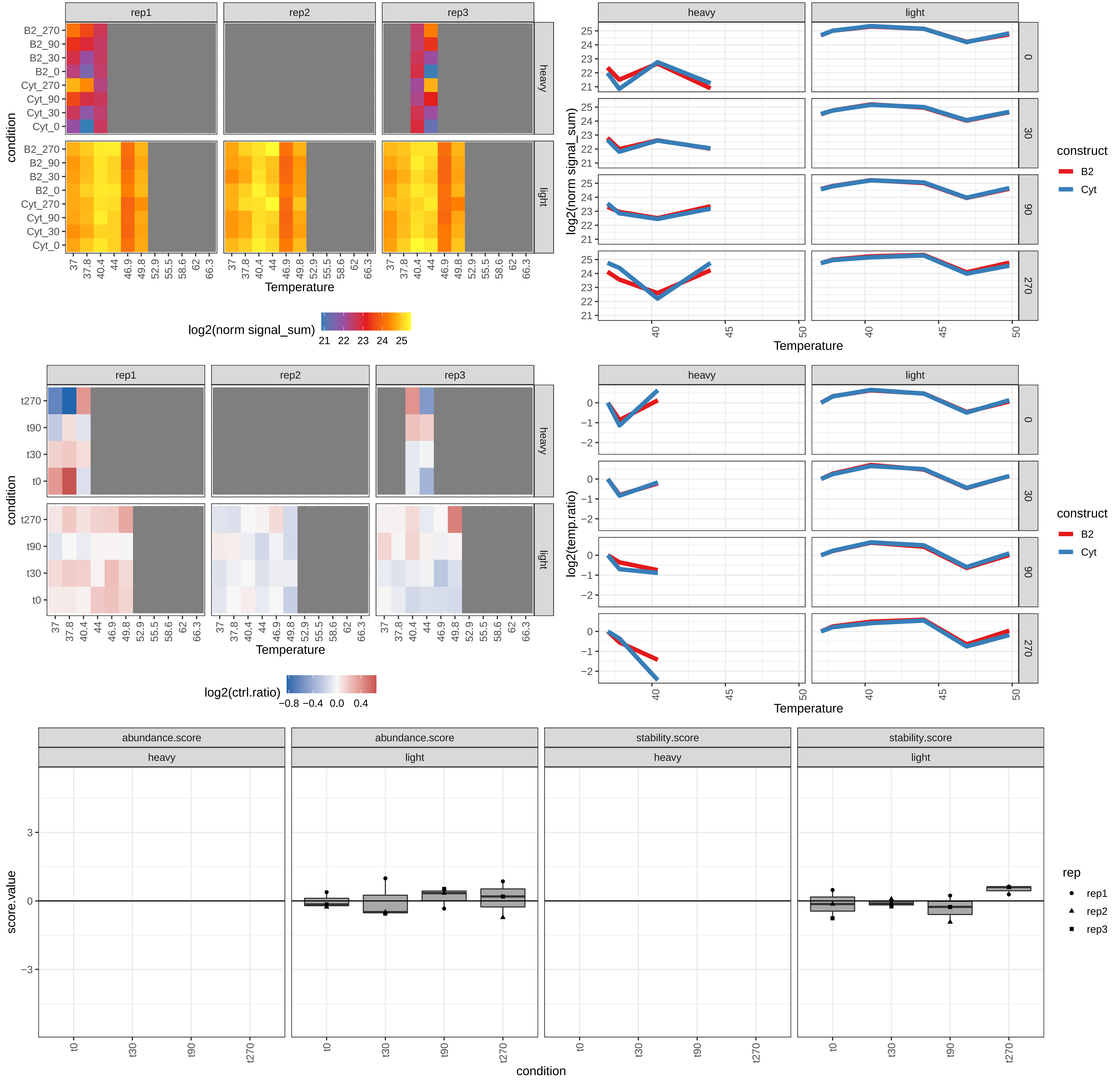

P20424 − Signal recognition particle subunit SRP54 OS=Saccharomyces cerevisiae (strain ATCC 204508 / S288c) GN=SRP54 PE=1 SV=2

SRP68 − average.top3: 6.84233965517241 − max.qupm: 13

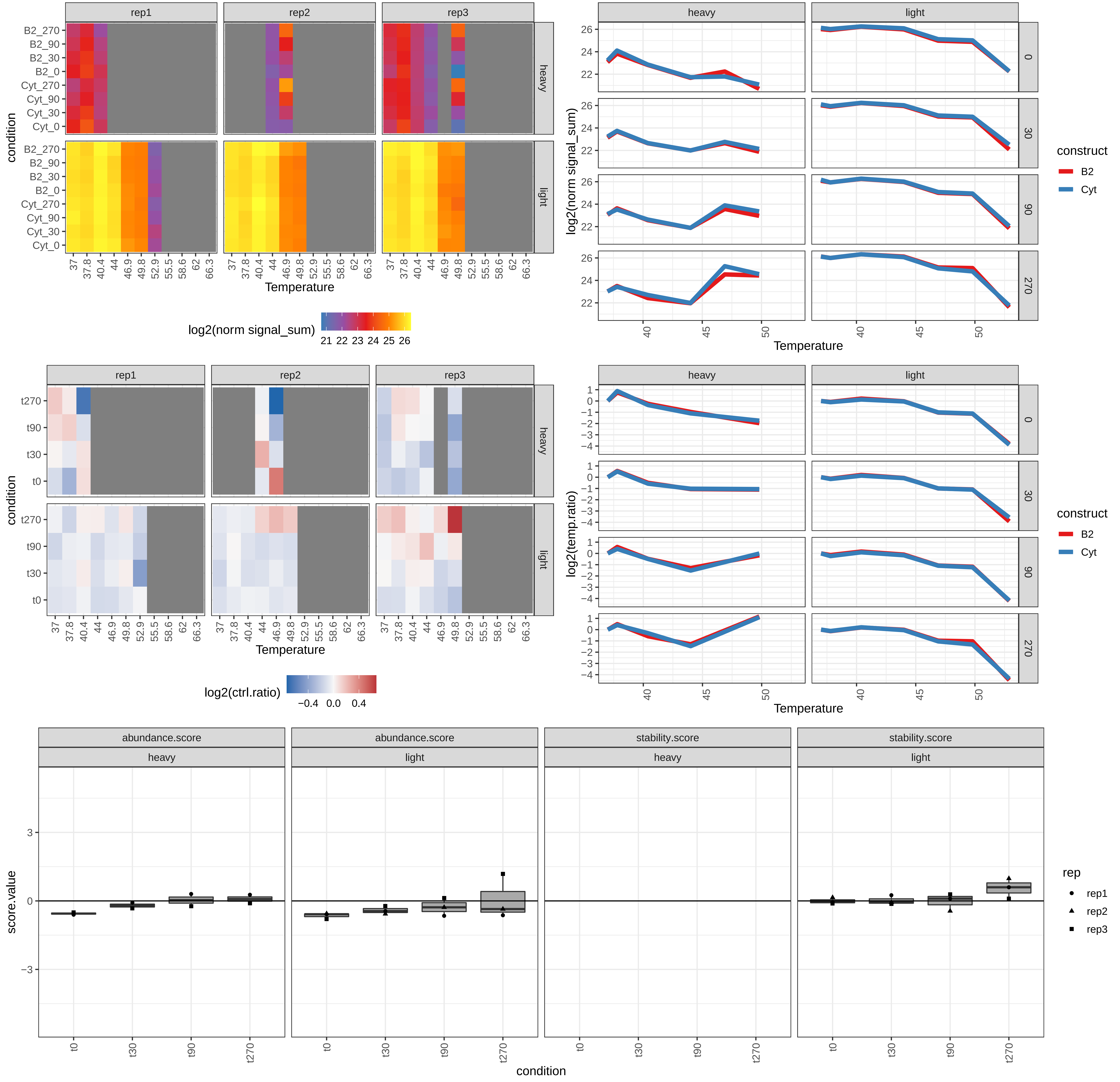

P38687 − Signal recognition particle subunit SRP68 OS=Saccharomyces cerevisiae (strain ATCC 204508 / S288c) GN=SRP68 PE=1 SV=1

SRP72 − average.top3: 7.04713242424242 − max.qupm: 17

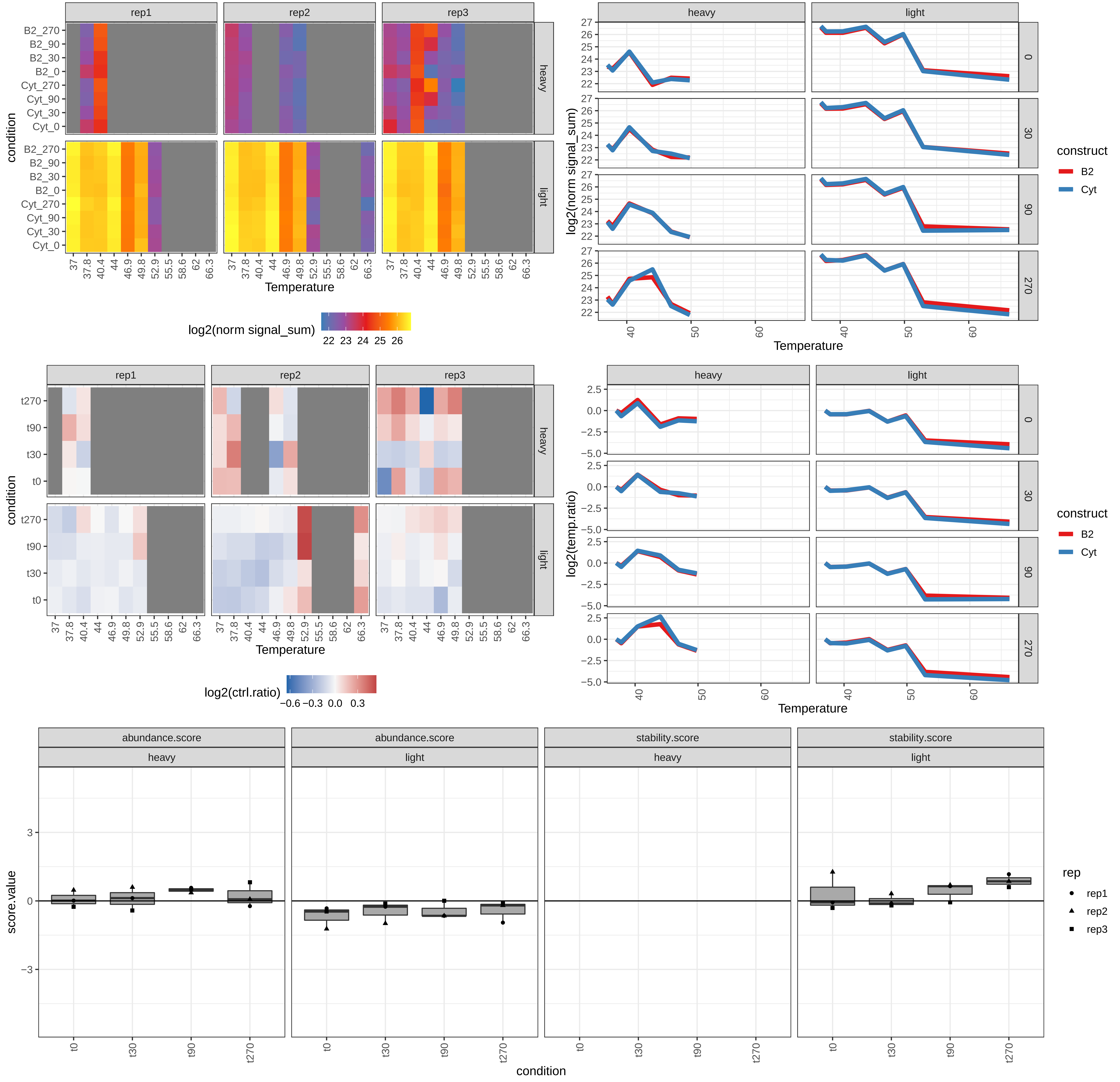

P38688 − Signal recognition particle subunit SRP72 OS=Saccharomyces cerevisiae (strain ATCC 204508 / S288c) GN=SRP72 PE=1 SV=2

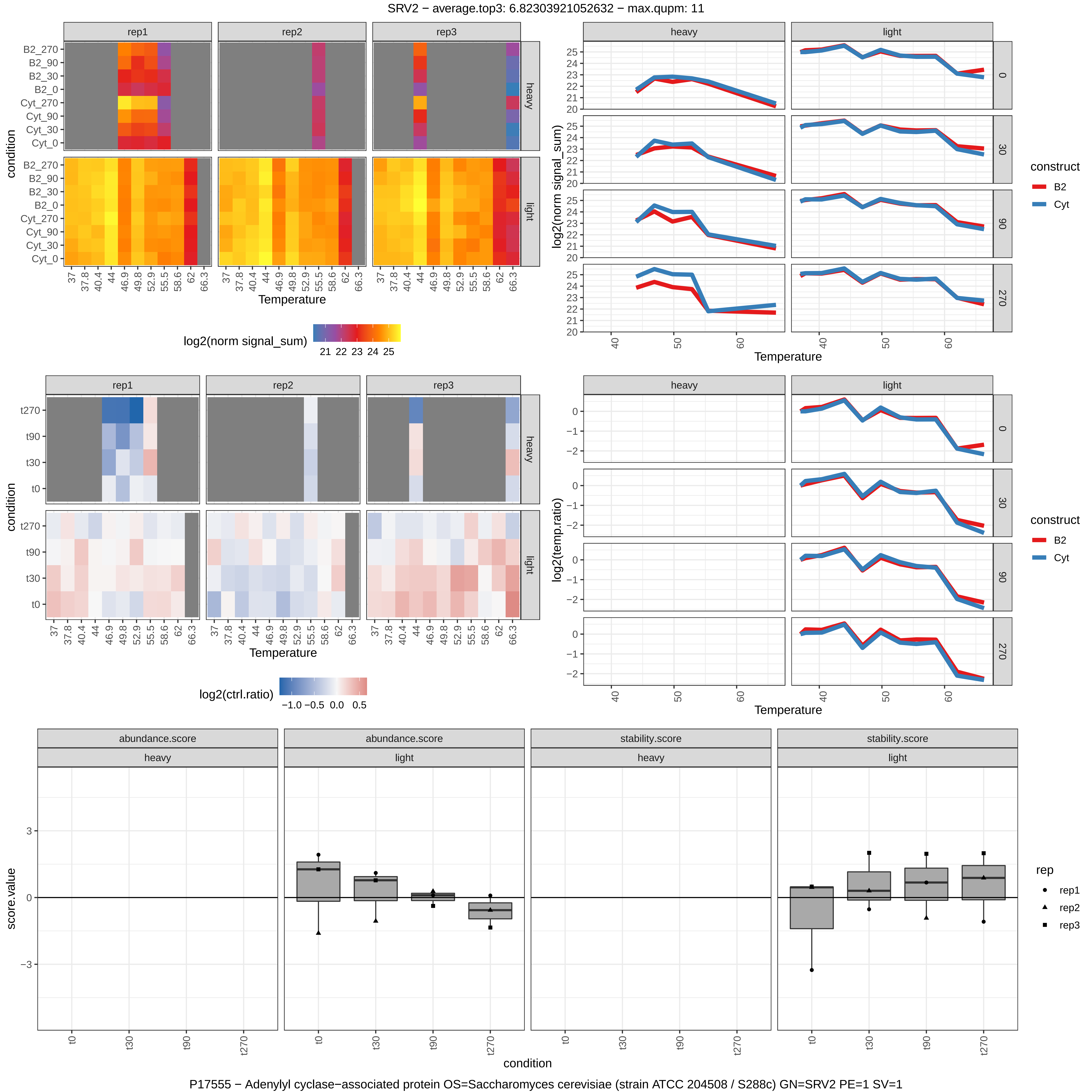
SRV2 − average.top3: 6.82303921052632 − max.qupm: 11
rep1
rep2
rep3
heavy
light
condition
Temperature
log2(norm signal_sum)
construct
B2
Cyt
log2(ctrl.ratio)
log2(temp.ratio)
abundance.score
stability.score
score.value
rep
rep1
rep2
rep3
P17555 − Adenylyl cyclase−associated protein OS=Saccharomyces cerevisiae (strain ATCC 204508 / S288c) GN=SRV2 PE=1 SV=1

SSA1 − average.top3: 7.70313676923077 − max.qupm: 9

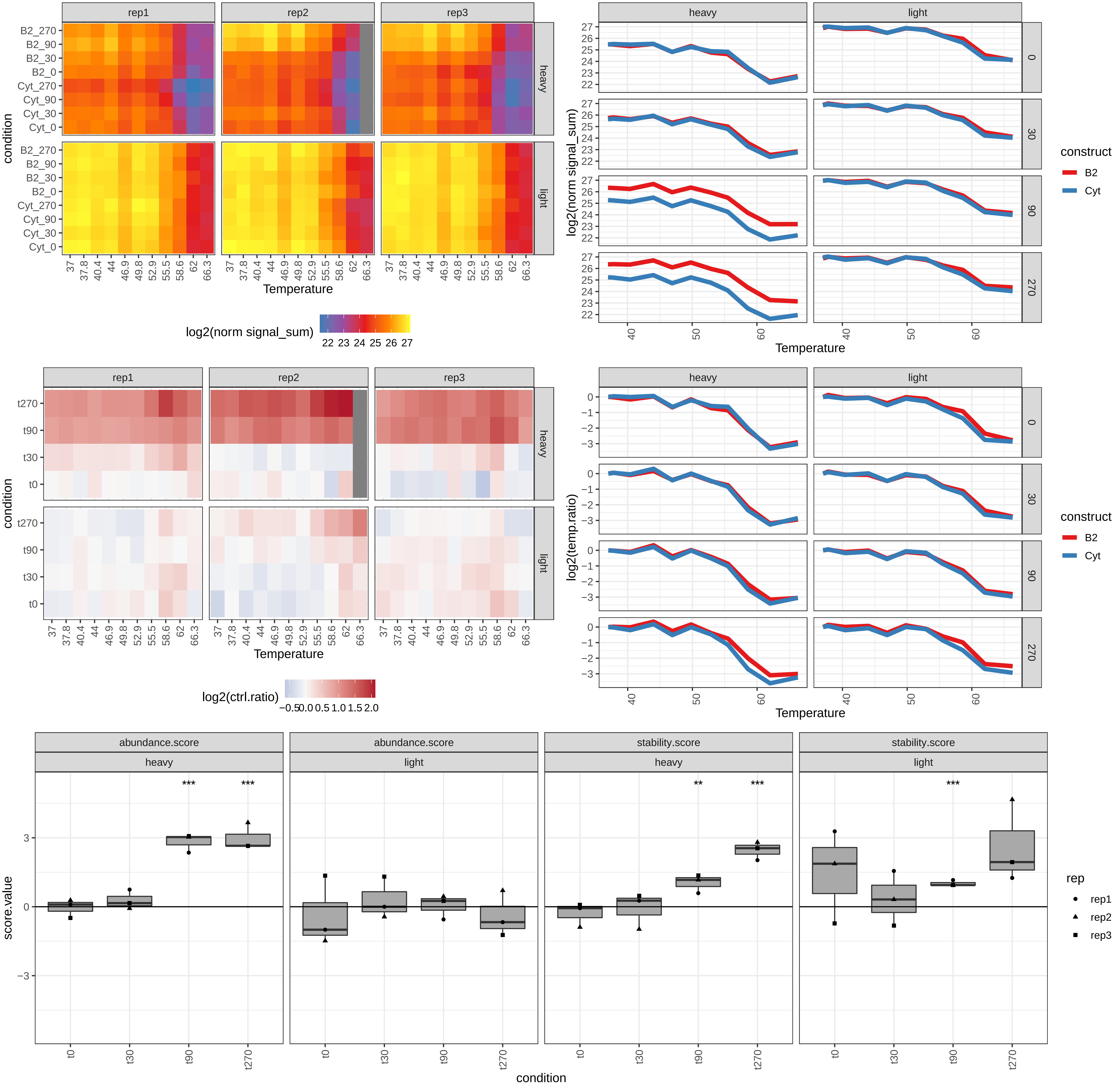

P10591 − Heat shock protein SSA1 OS=Saccharomyces cerevisiae (strain ATCC 204508 / S288c) GN=SSA1 PE=1 SV=4

SSA2 − average.top3: 7.04150392857143 − max.qupm: 8

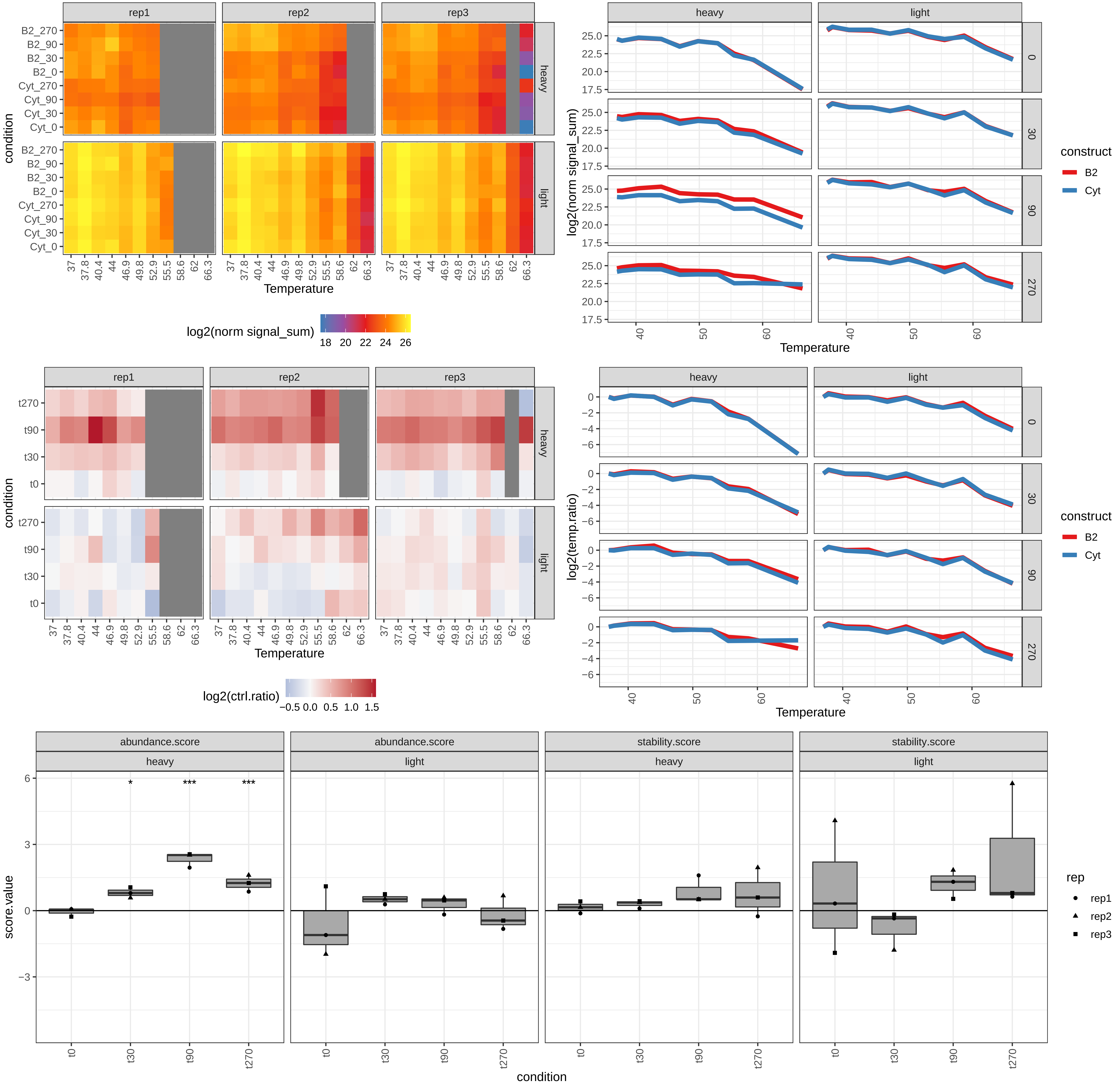

P10592 − Heat shock protein SSA2 OS=Saccharomyces cerevisiae (strain ATCC 204508 / S288c) GN=SSA2 PE=1 SV=3

SSA3 − average.top3: 6.81748238095238 − max.qupm: 4

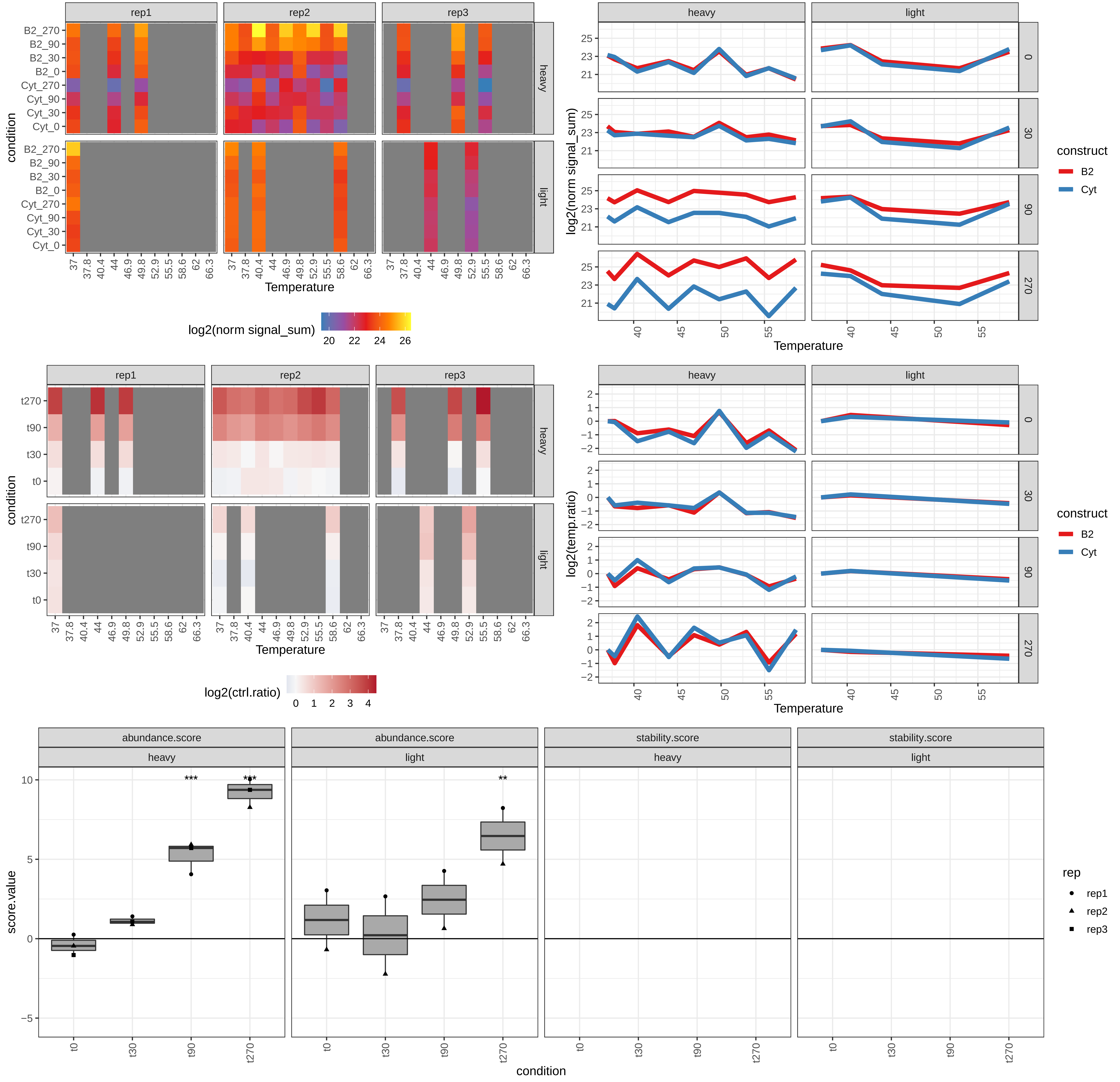

P09435 − Heat shock protein SSA3 OS=Saccharomyces cerevisiae (strain ATCC 204508 / S288c) GN=SSA3 PE=1 SV=3

SSA4 − average.top3: 7.07595524590164 − max.qupm: 14

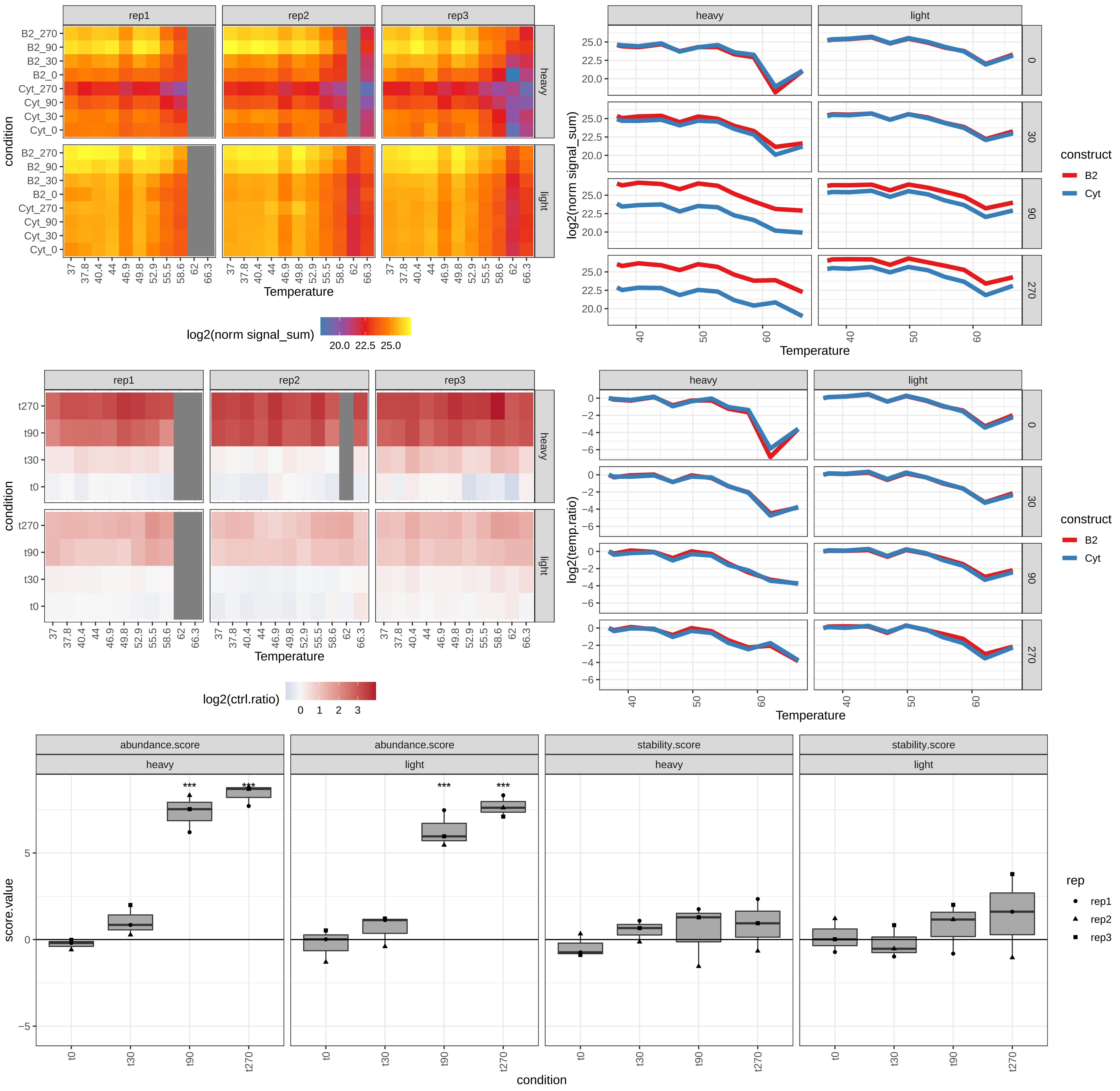

P22202 − Heat shock protein SSA4 OS=Saccharomyces cerevisiae (strain ATCC 204508 / S288c) GN=SSA4 PE=1 SV=3

SSC1 − average.top3: 7.59706846153846 − max.qupm: 21

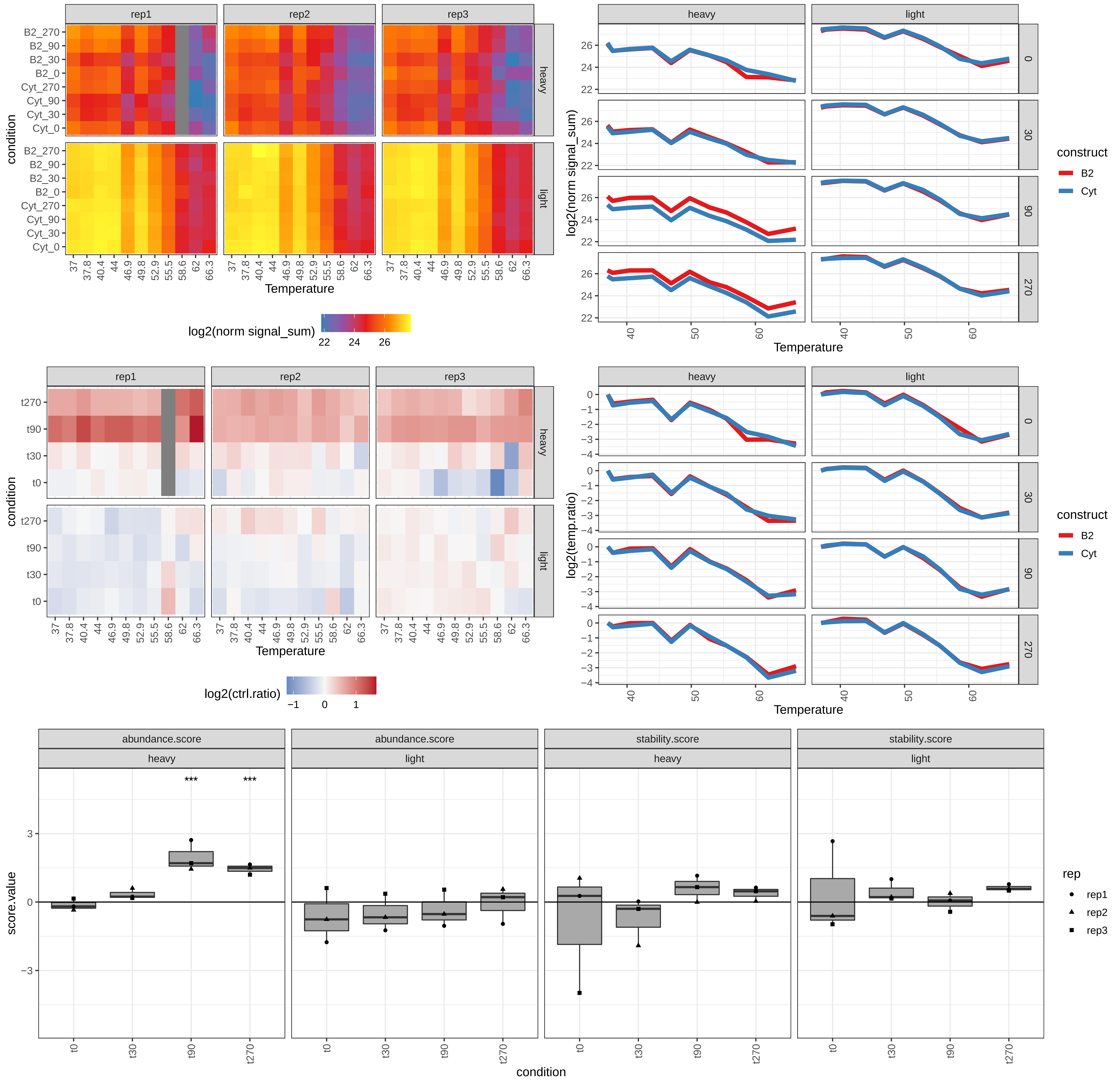

P0CS90 − Heat shock protein SSC1, mitochondrial OS=Saccharomyces cerevisiae (strain ATCC 204508 / S288c) GN=SSC1 PE=1 SV=1

SSE1 − average.top3: 7.56957018867925 − max.qupm: 27

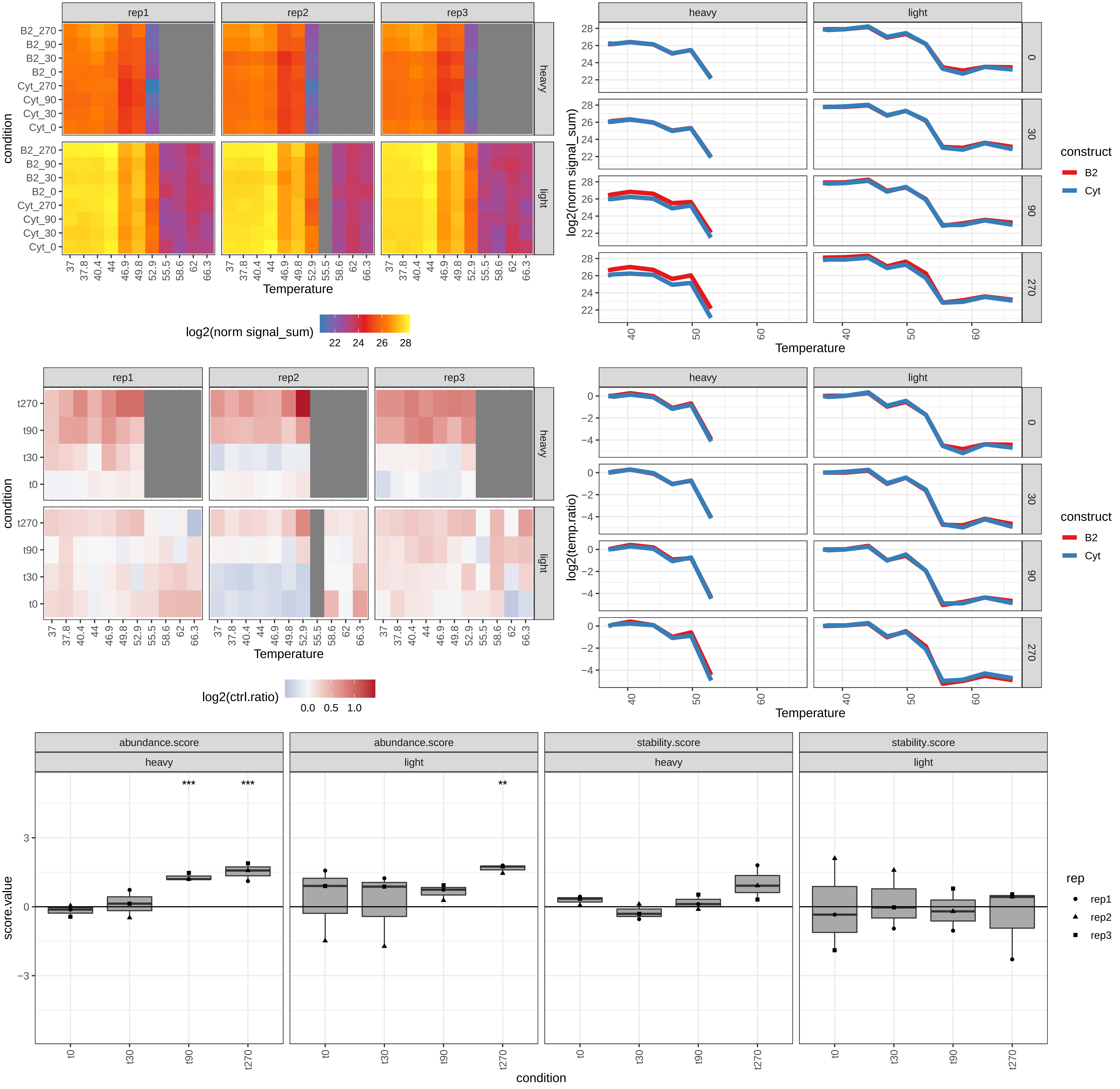

P32589 − Heat shock protein homolog SSE1 OS=Saccharomyces cerevisiae (strain ATCC 204508 / S288c) GN=SSE1 PE=1 SV=4

SSE2 − average.top3: 7.28452660377358 − max.qupm: 21

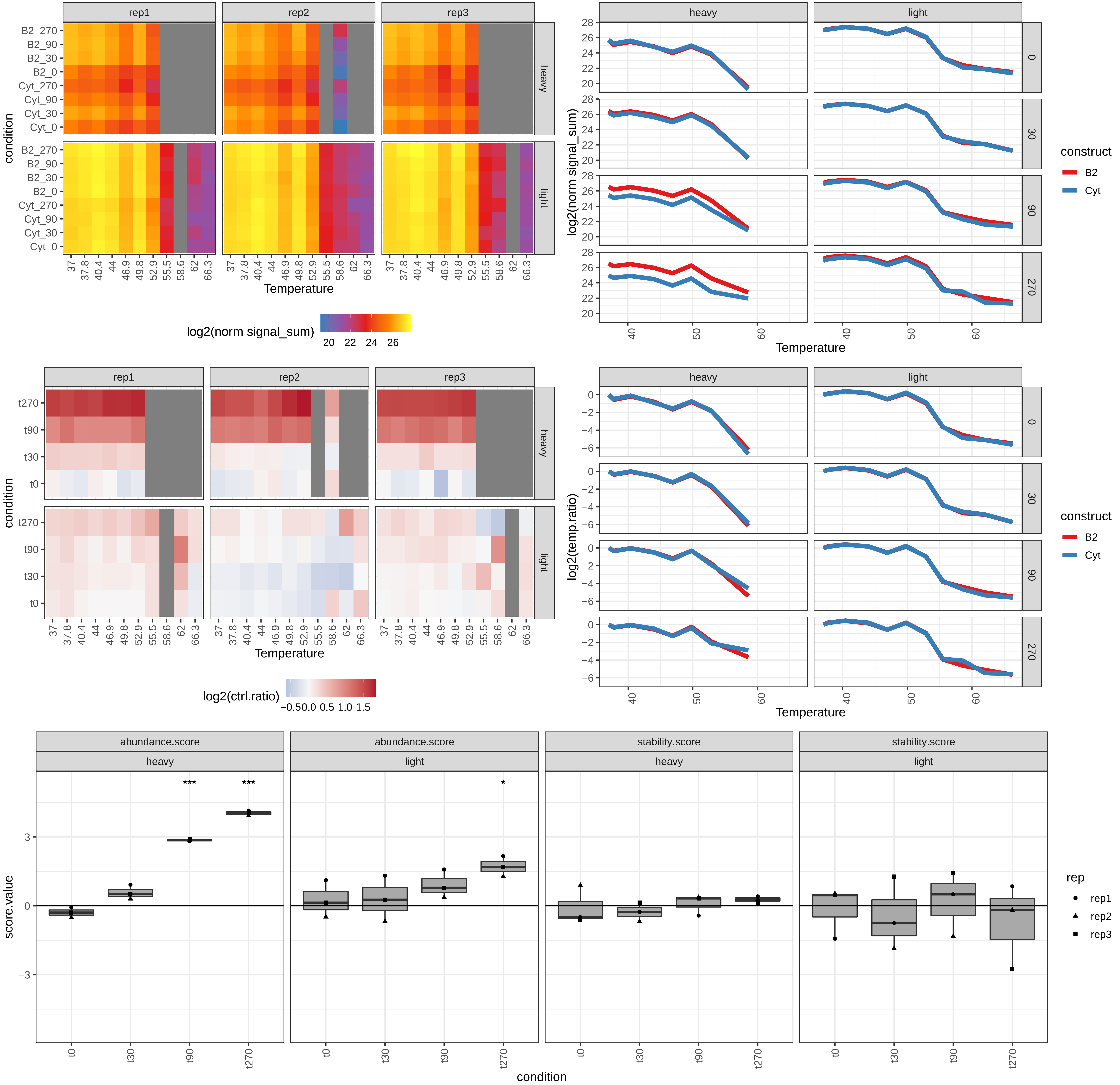

P32590 − Heat shock protein homolog SSE2 OS=Saccharomyces cerevisiae (strain ATCC 204508 / S288c) GN=SSE2 PE=1 SV=3

SSF1 − average.top3: 6.7440135 − max.qupm: 6

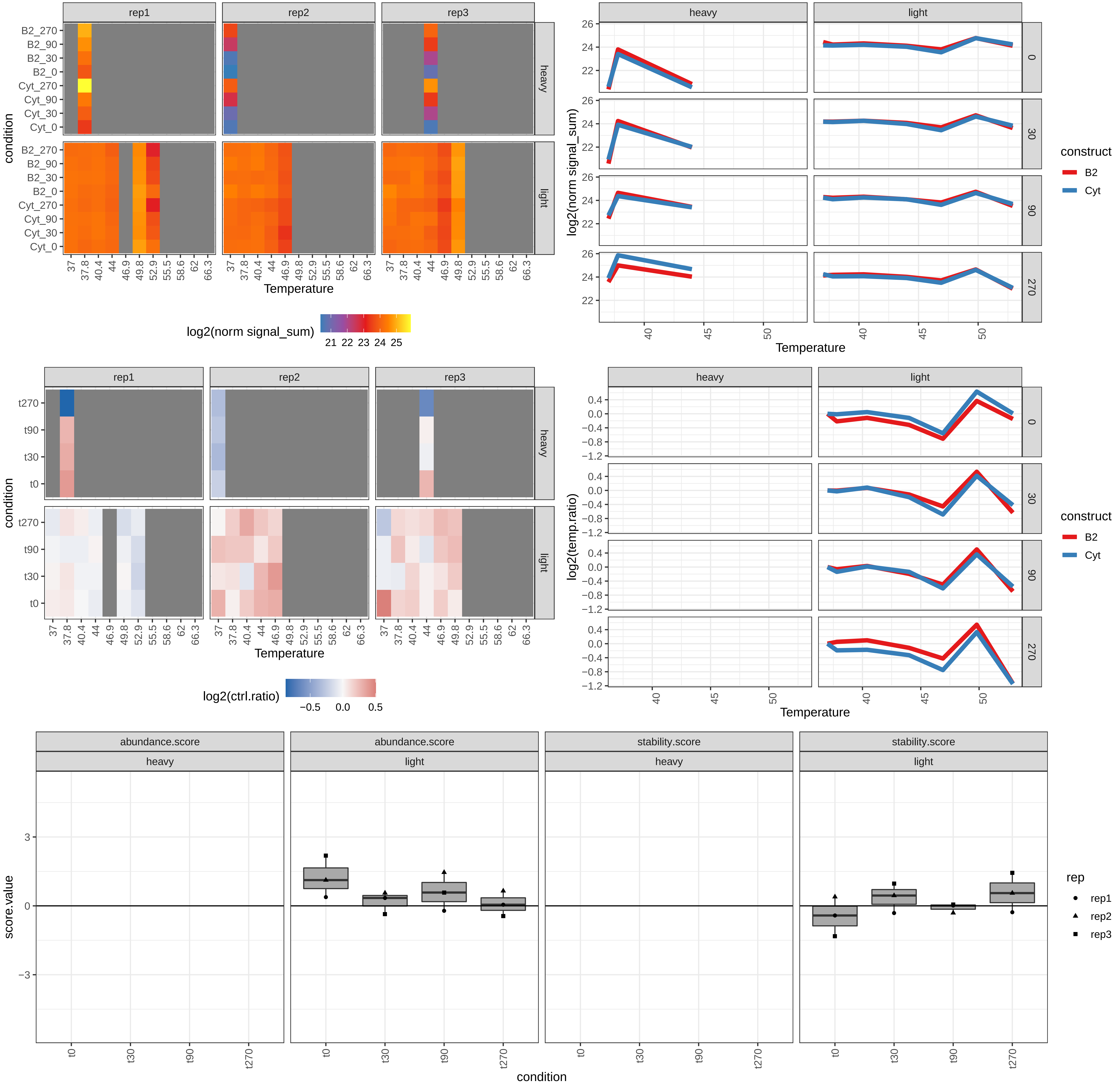

P38789 − Ribosome biogenesis protein SSF1 OS=Saccharomyces cerevisiae (strain ATCC 204508 / S288c) GN=SSF1 PE=1 SV=1

SSF2 − average.top3: 6.68976 − max.qupm: 3

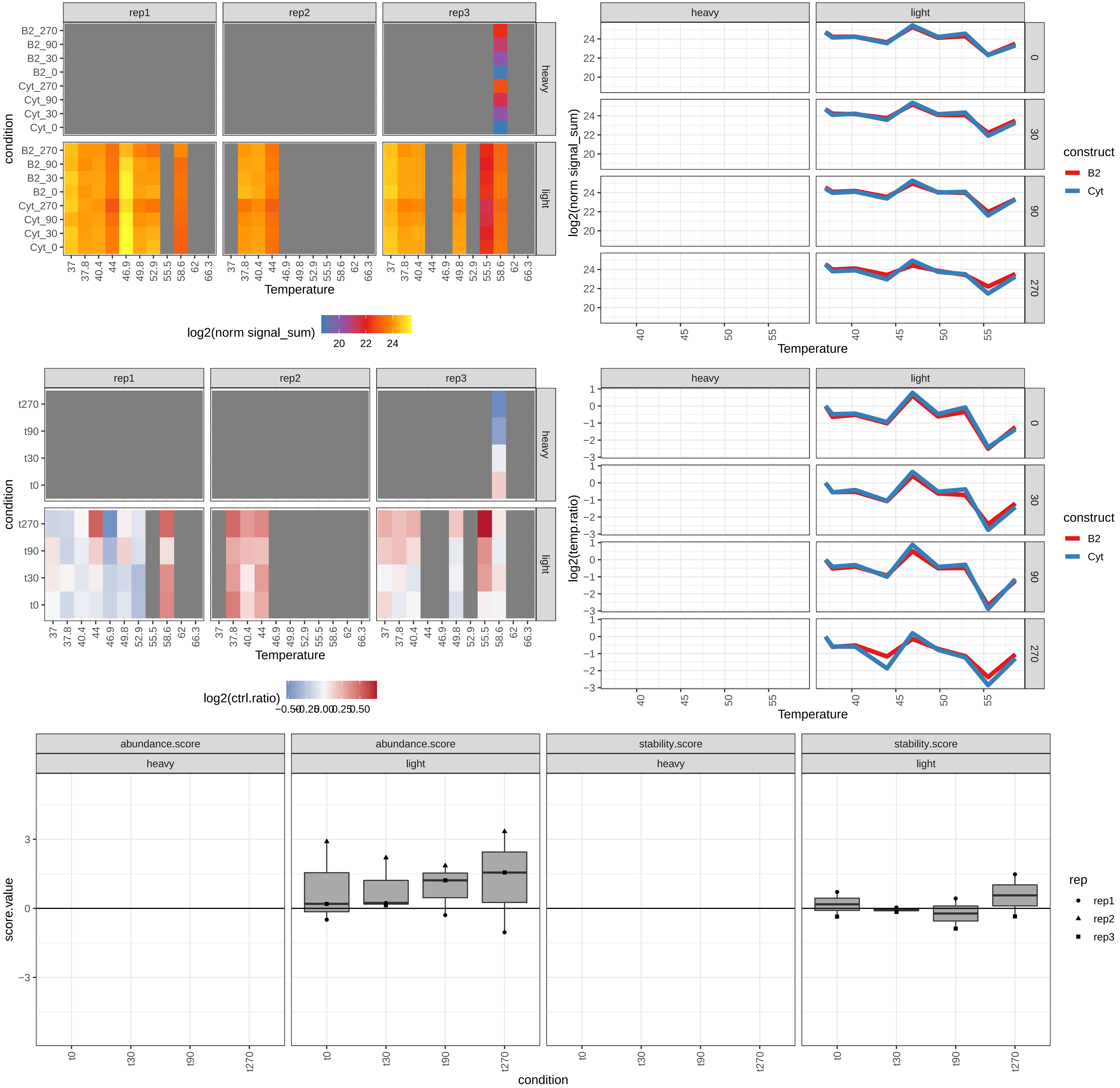

Q12153 − Ribosome biogenesis protein SSF2 OS=Saccharomyces cerevisiae (strain ATCC 204508 / S288c) GN=SSF2 PE=1 SV=1

SSH1 − average.top3: 7.20528639344262 − max.qupm: 10

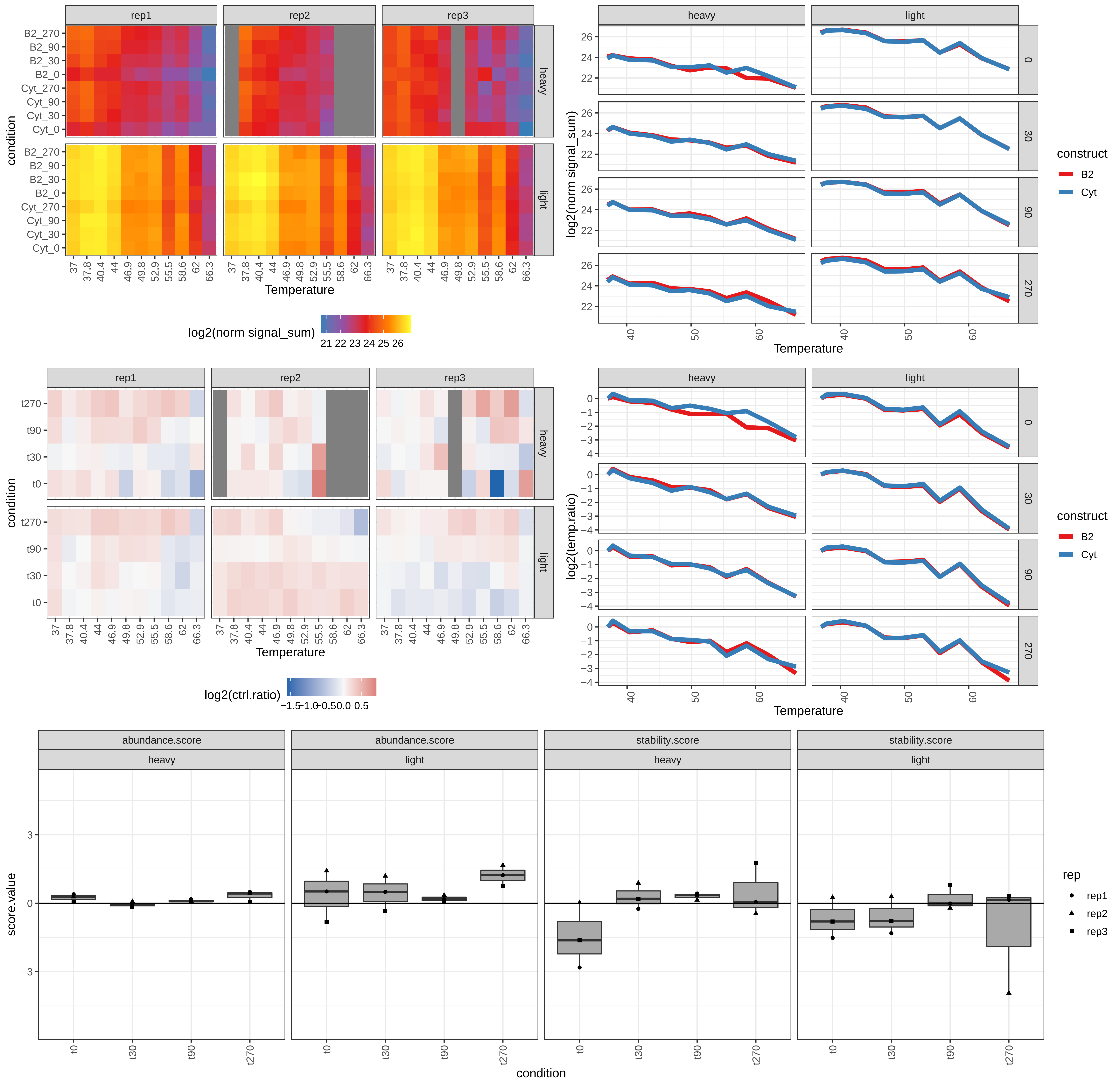

P38353 − Sec sixty−one protein homolog OS=Saccharomyces cerevisiae (strain ATCC 204508 / S288c) GN=SSH1 PE=1 SV=1

SSL1 − average.top3: 6.70813894736842 − max.qupm: 7

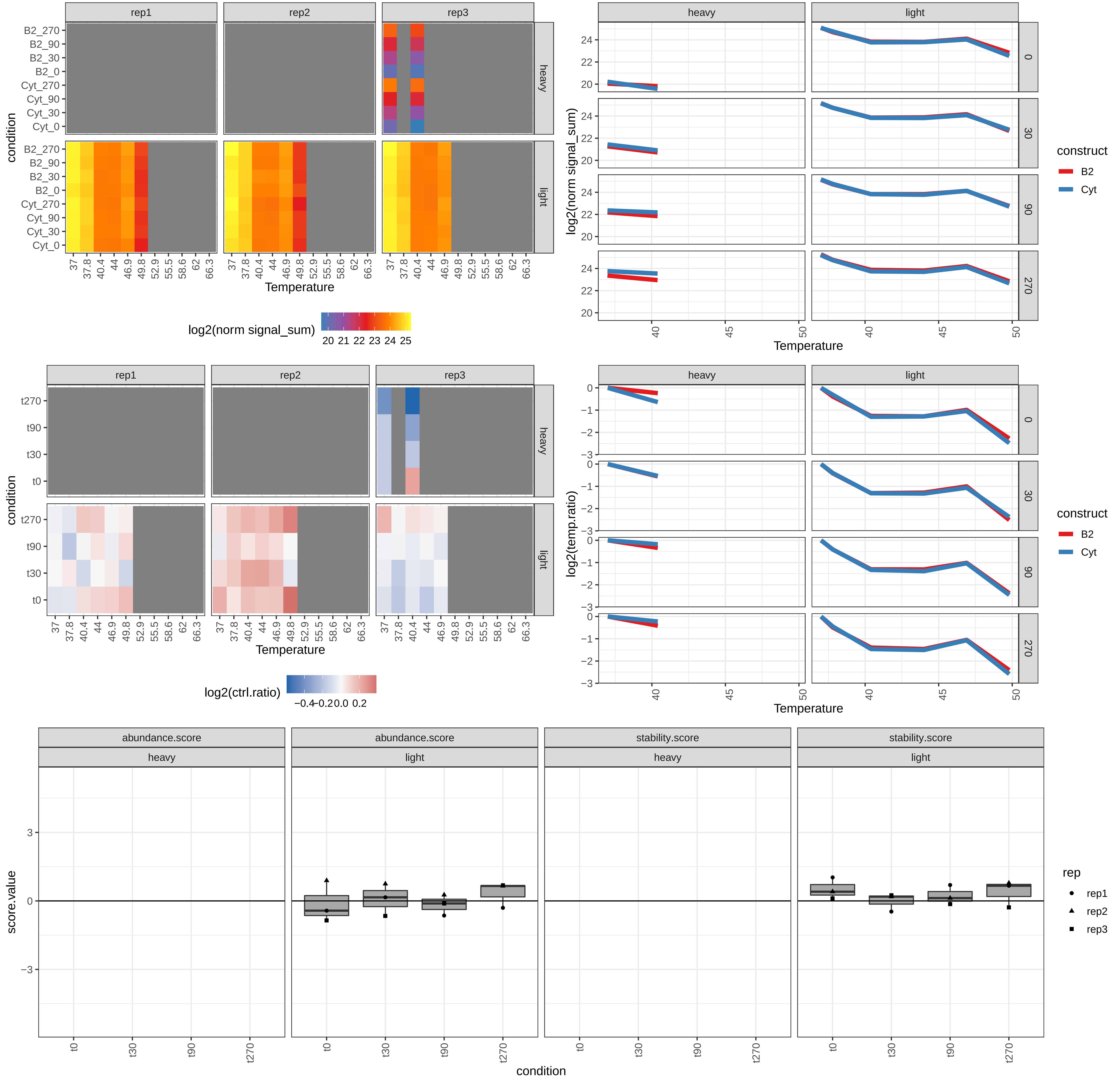

Q04673 − Suppressor of stem−loop protein 1 OS=Saccharomyces cerevisiae (strain ATCC 204508 / S288c) GN=SSL1 PE=1 SV=1

SSL2 − average.top3: 6.223075 − max.qupm: 4

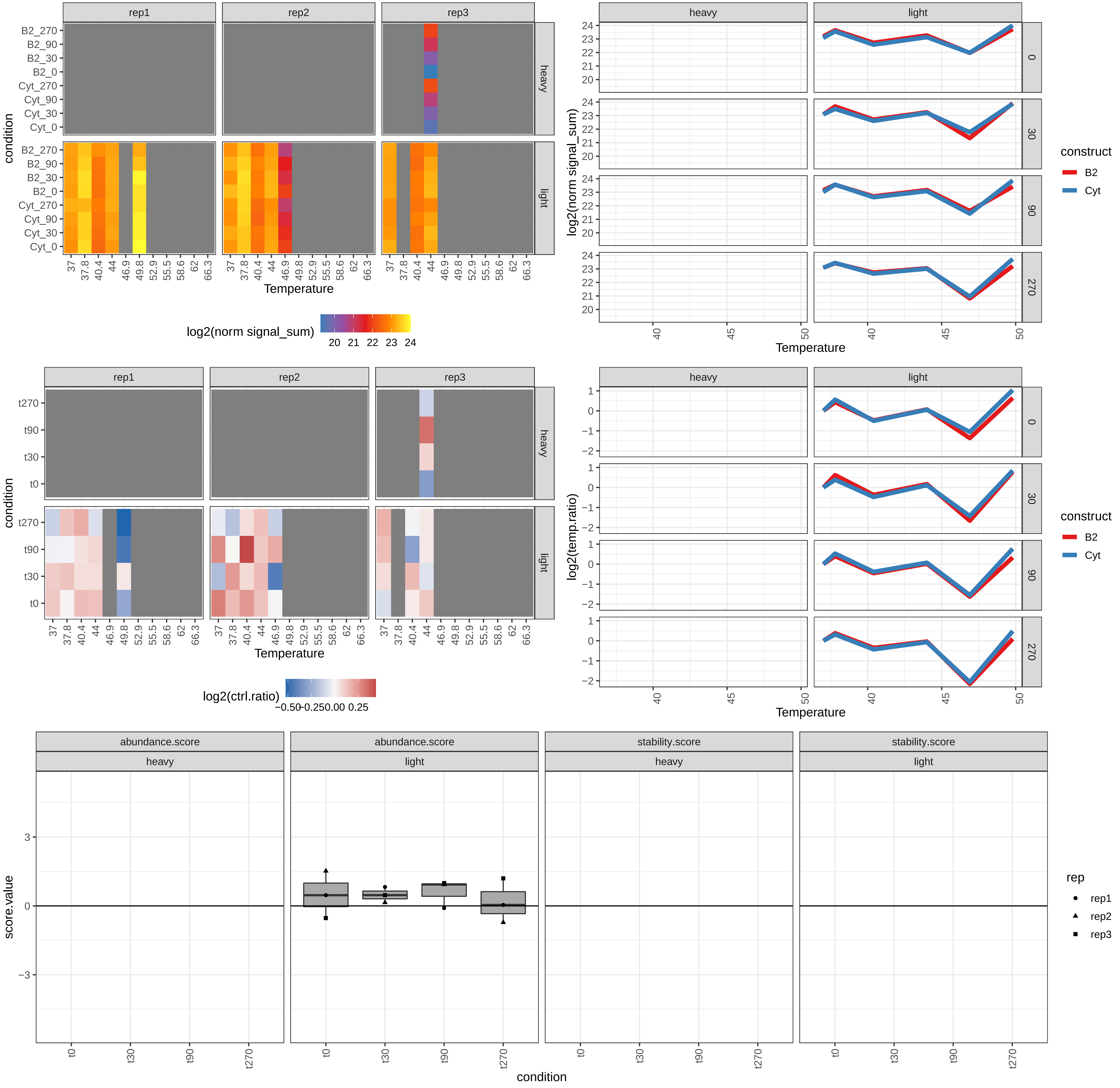

Q00578 − DNA repair helicase RAD25 OS=Saccharomyces cerevisiae (strain ATCC 204508 / S288c) GN=SSL2 PE=1 SV=1

SSM4 − average.top3: 6.70952391304348 − max.qupm: 7

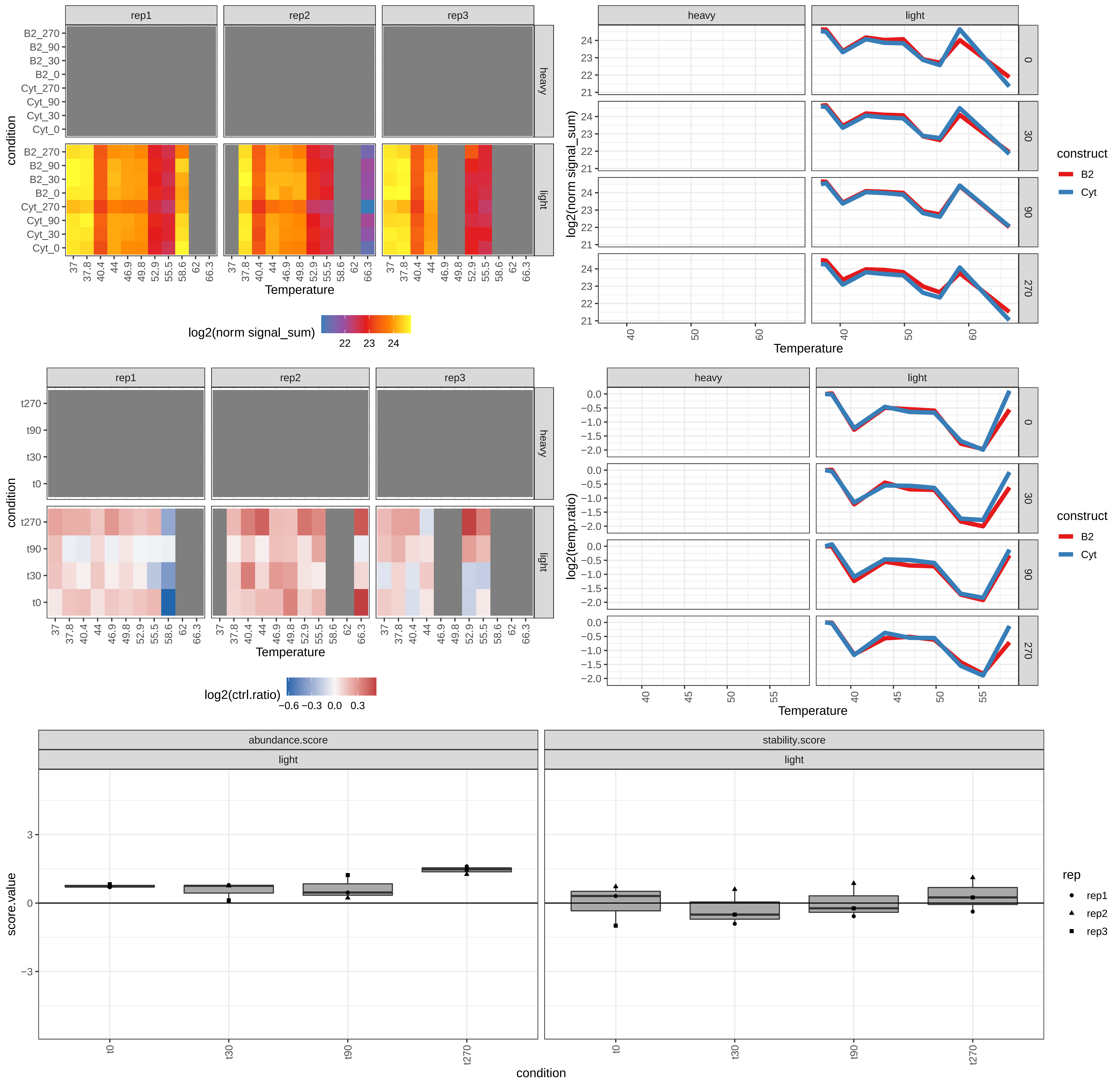

P40318 − ERAD−associated E3 ubiquitin−protein ligase DOA10 OS=Saccharomyces cerevisiae (strain ATCC 204508 / S288c) GN=SSM4 PE=1 SV=1

SSO1 − average.top3: 7.10295894736842 − max.qupm: 9

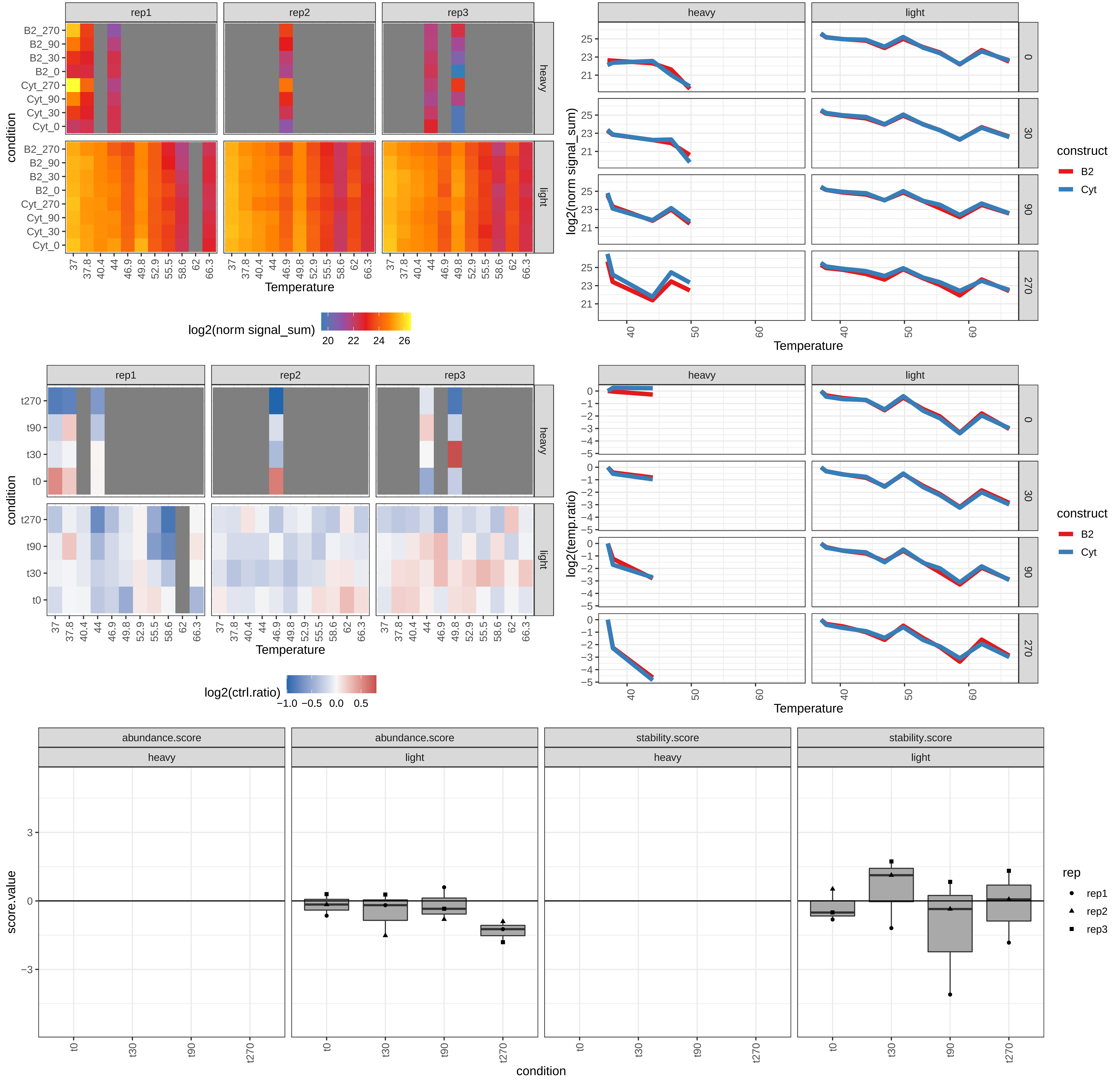

P32867 − Protein SSO1 OS=Saccharomyces cerevisiae (strain ATCC 204508 / S288c) GN=SSO1 PE=1 SV=2

SSO2 − average.top3: 6.82917465116279 − max.qupm: 7

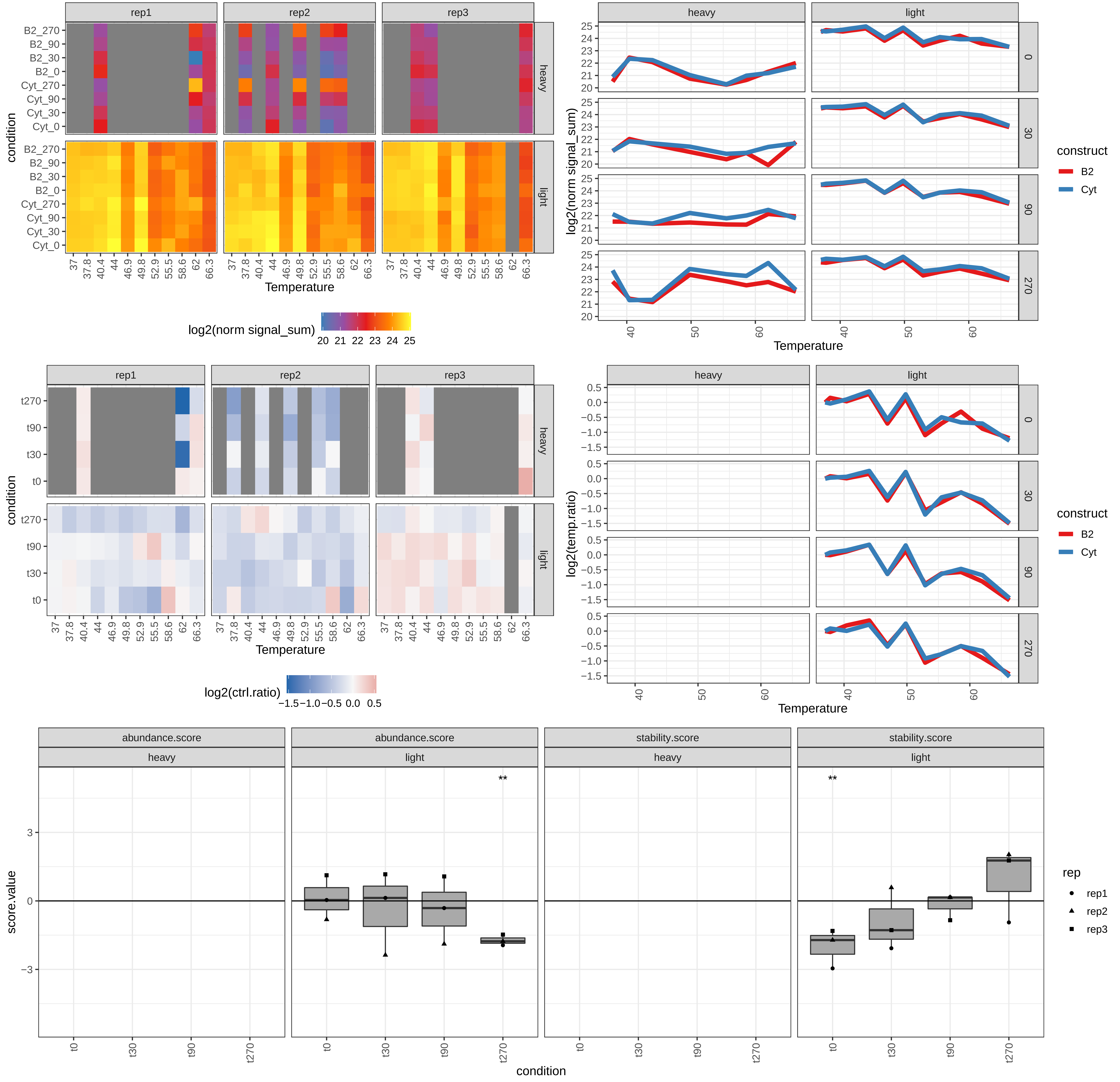

P39926 − Protein SSO2 OS=Saccharomyces cerevisiae (strain ATCC 204508 / S288c) GN=SSO2 PE=1 SV=2

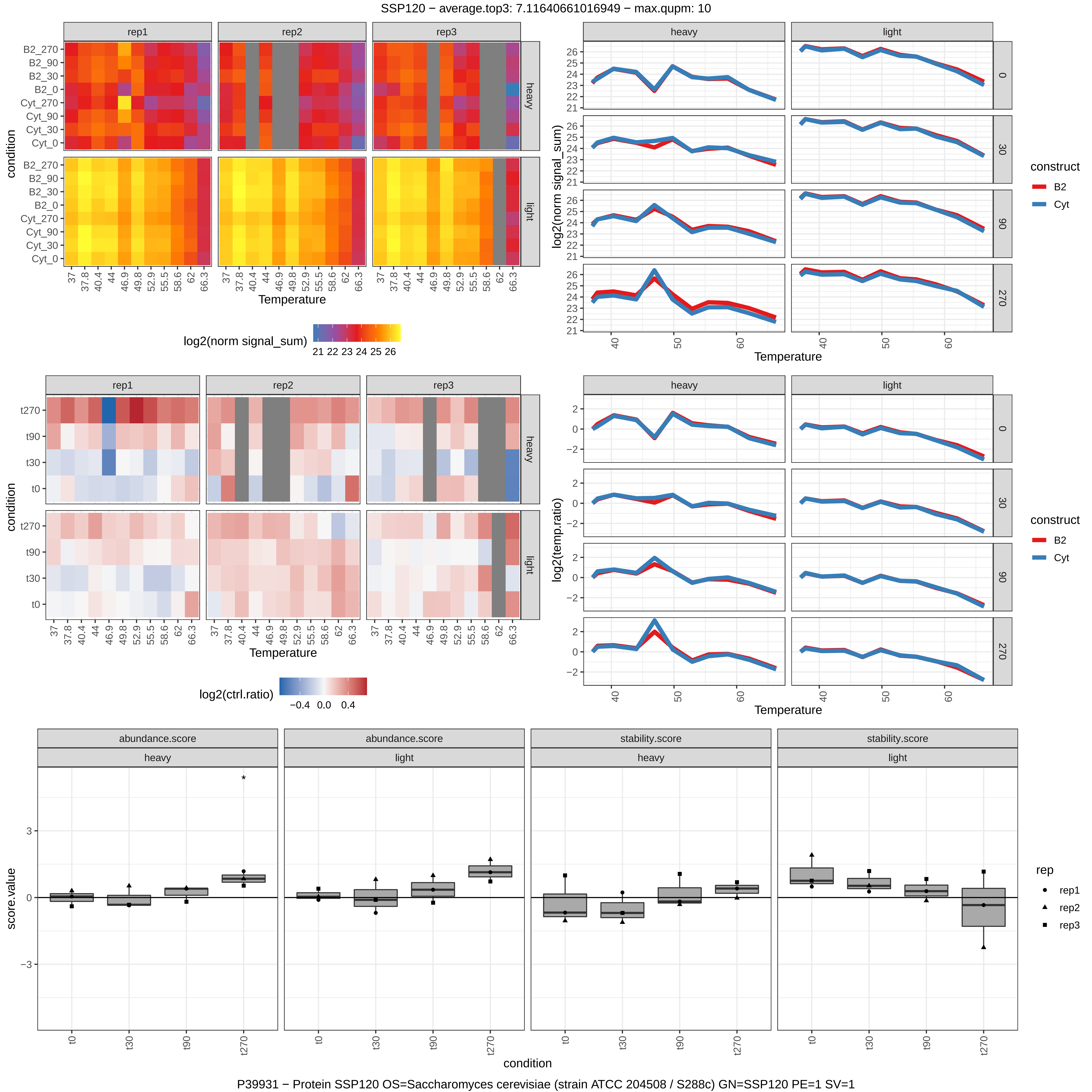
SSP120 − average.top3: 7.11640661016949 − max.qupm: 10
rep1
rep2
rep3
heavy
light
condition
Temperature
log2(norm signal_sum)
21 22 23 24 25 26
log2(norm signal_sum)
construct
B2
Cyt
log2(ctrl.ratio)
−0.4 0.0 0.4
log2(temp.ratio)
abundance.score
stability.score
score.value
rep
rep1
rep2
rep3
P39931 − Protein SSP120 OS=Saccharomyces cerevisiae (strain ATCC 204508 / S288c) GN=SSP120 PE=1 SV=1

SSQ1 − average.top3: 6.5821696 − max.qupm: 7

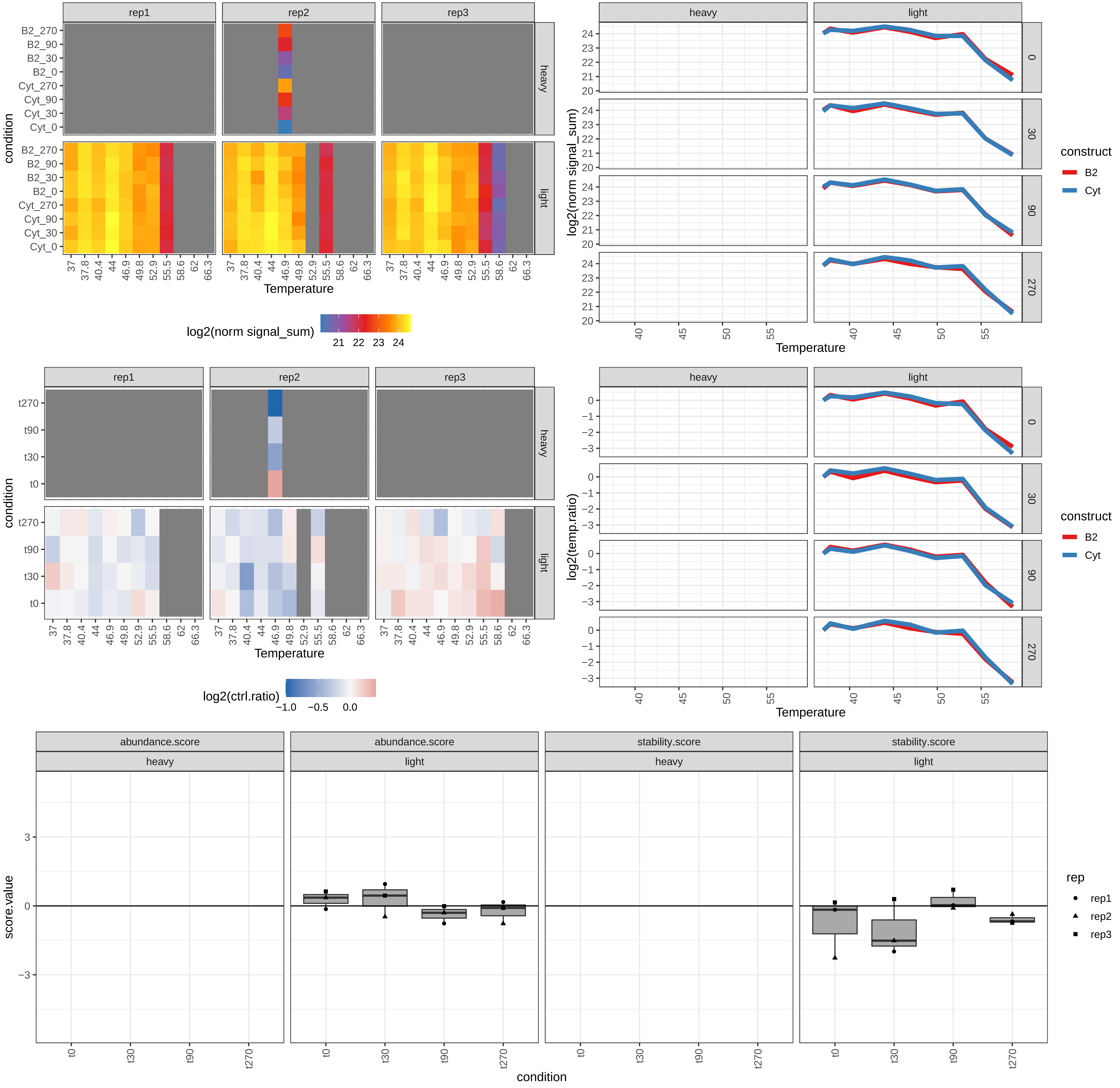

Q05931 − Heat shock protein SSQ1, mitochondrial OS=Saccharomyces cerevisiae (strain ATCC 204508 / S288c) GN=SSQ1 PE=1 SV=1

SSS1 − average.top3: 7.33973178571429 − max.qupm: 5

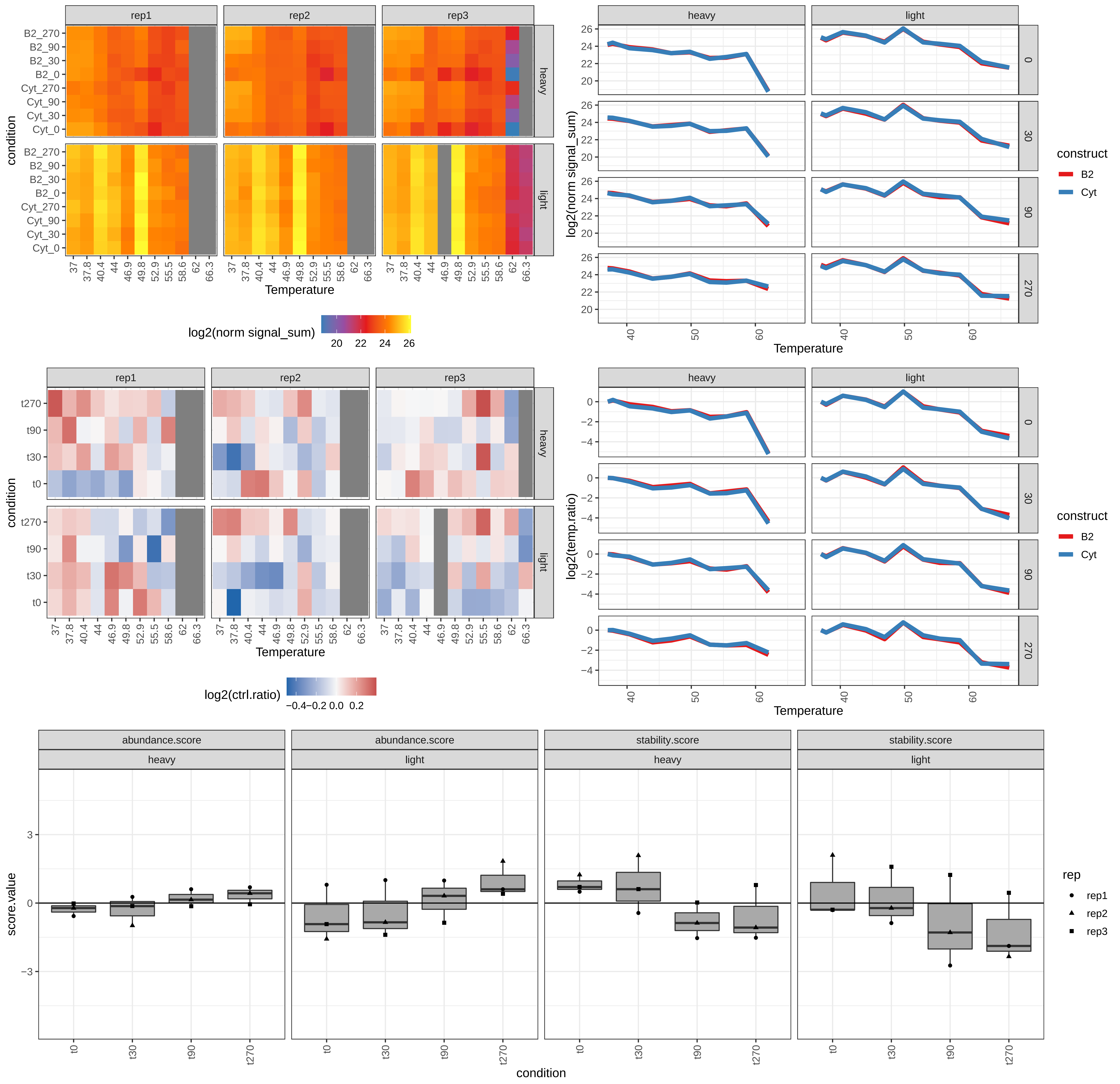

P35179 − Protein transport protein SSS1 OS=Saccharomyces cerevisiae (strain ATCC 204508 / S288c) GN=SSS1 PE=1 SV=2

SSZ1 − average.top3: 7.49610574468085 − max.qupm: 15

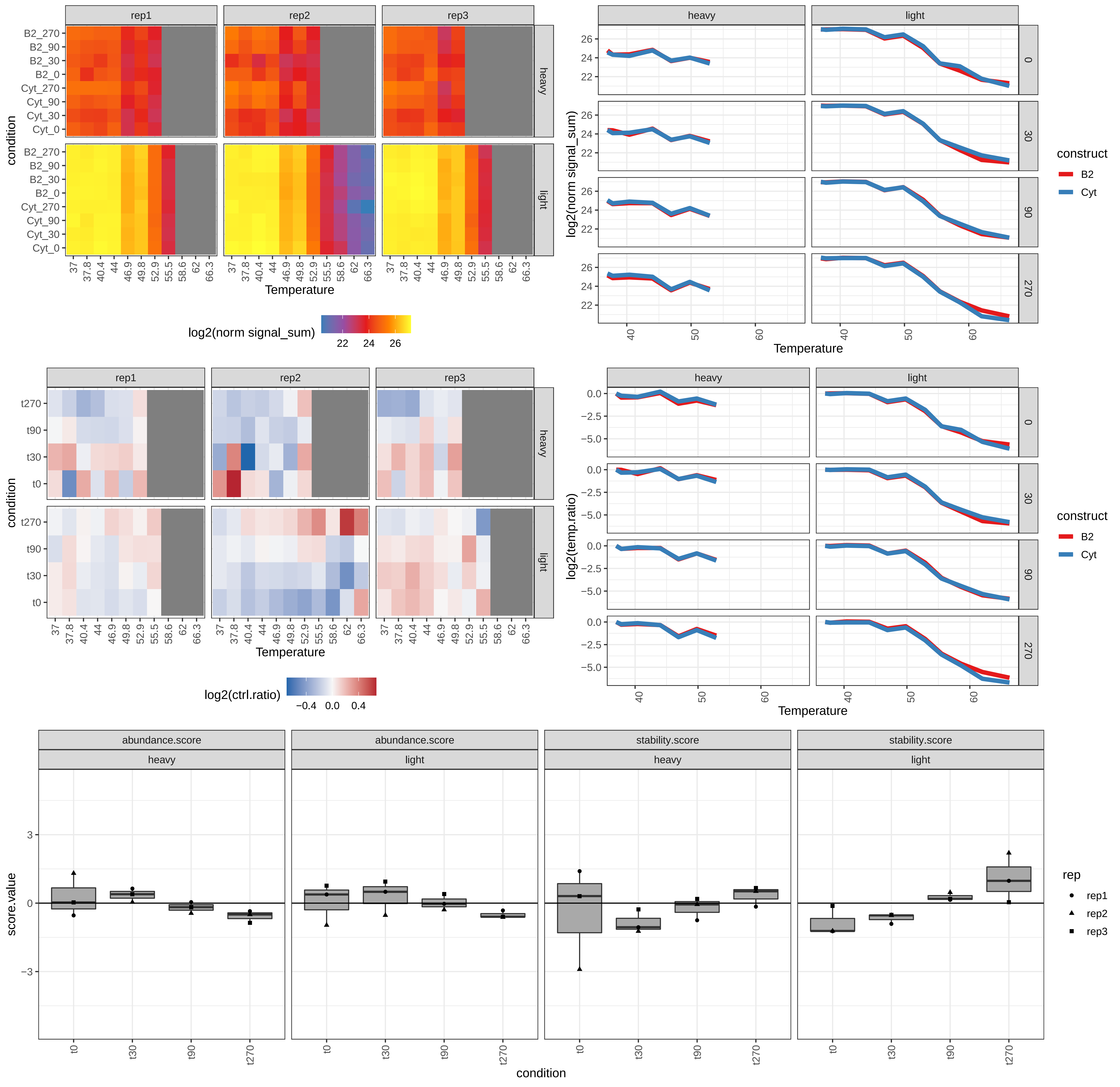

P38788 − Ribosome−associated complex subunit SSZ1 OS=Saccharomyces cerevisiae (strain ATCC 204508 / S288c) GN=SSZ1 PE=1 SV=2

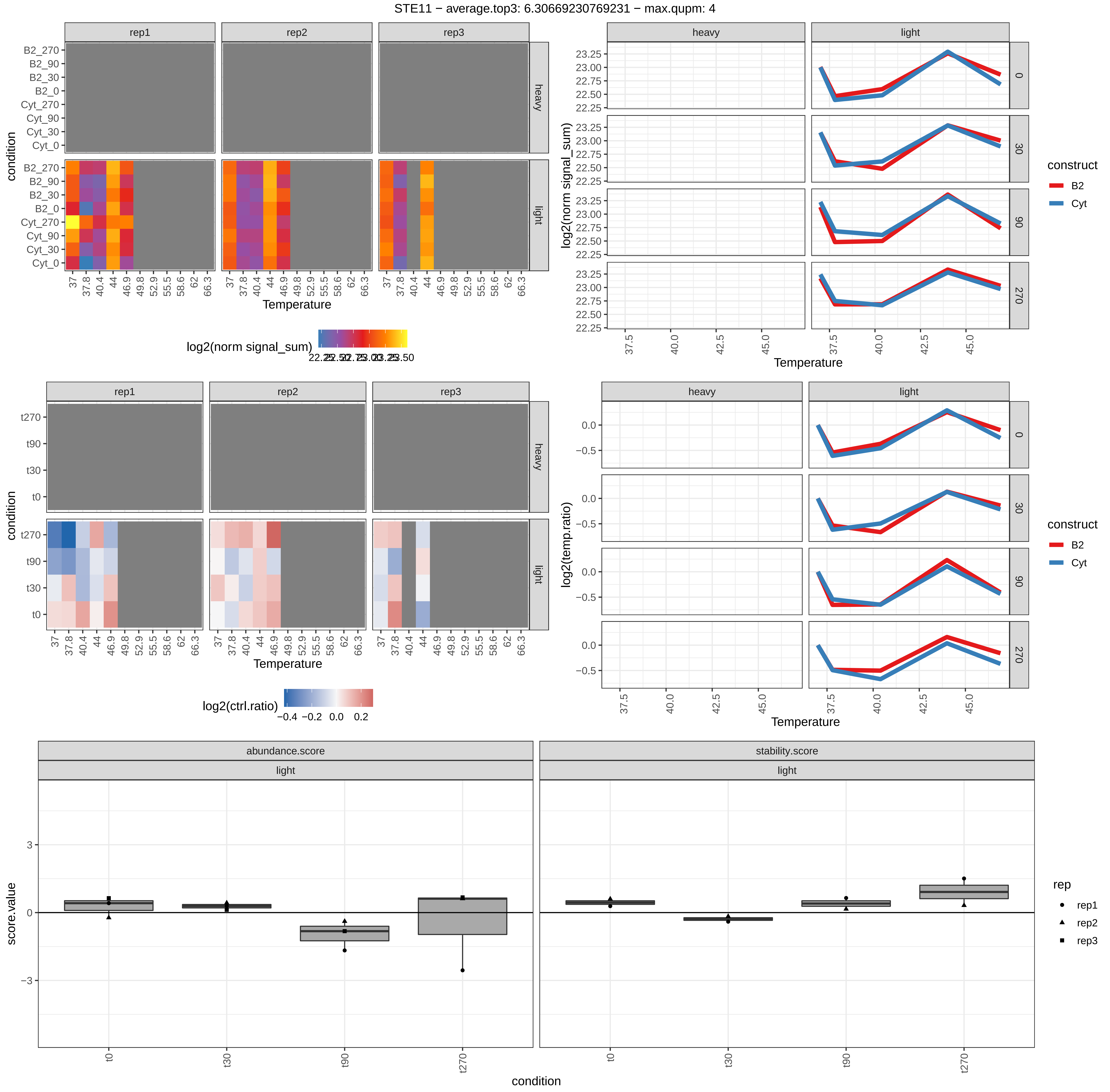

P23561 − Serine/threonine−protein kinase STE11 OS=Saccharomyces cerevisiae (strain ATCC 204508 / S288c) GN=STE11 PE=1 SV=3

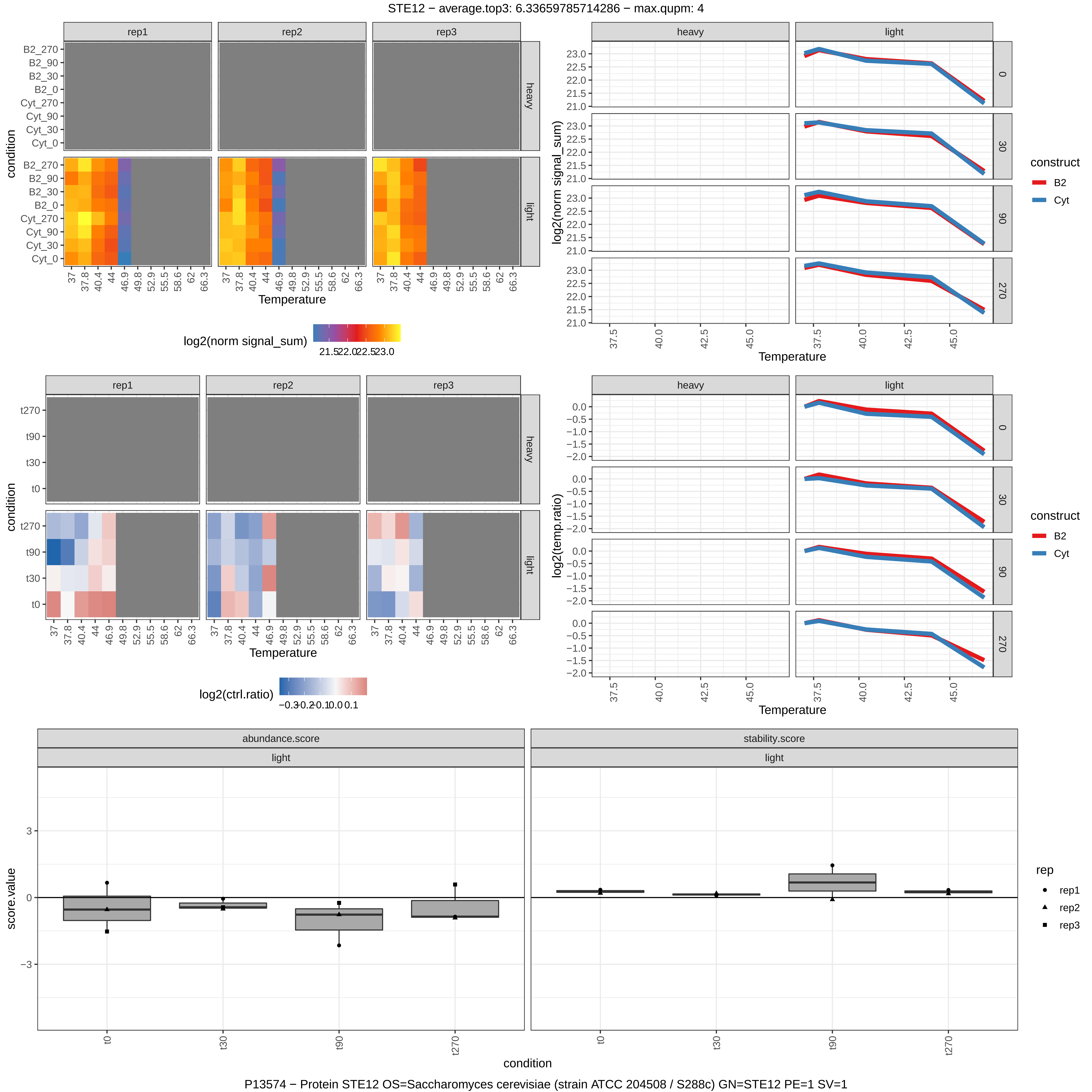

STE12 − average.top3: 6.33659785714286 − max.qupm: 4
rep1
rep2
rep3
heavy
light
condition
Temperature
log2(norm signal_sum)
construct
B2
Cyt
log2(ctrl.ratio)
log2(temp.ratio)
abundance.score
stability.score
score.value
rep
rep1
rep2
rep3
P13574 − Protein STE12 OS=Saccharomyces cerevisiae (strain ATCC 204508 / S288c) GN=STE12 PE=1 SV=1

STE13 − average.top3: 6.42227909090909 − max.qupm: 10

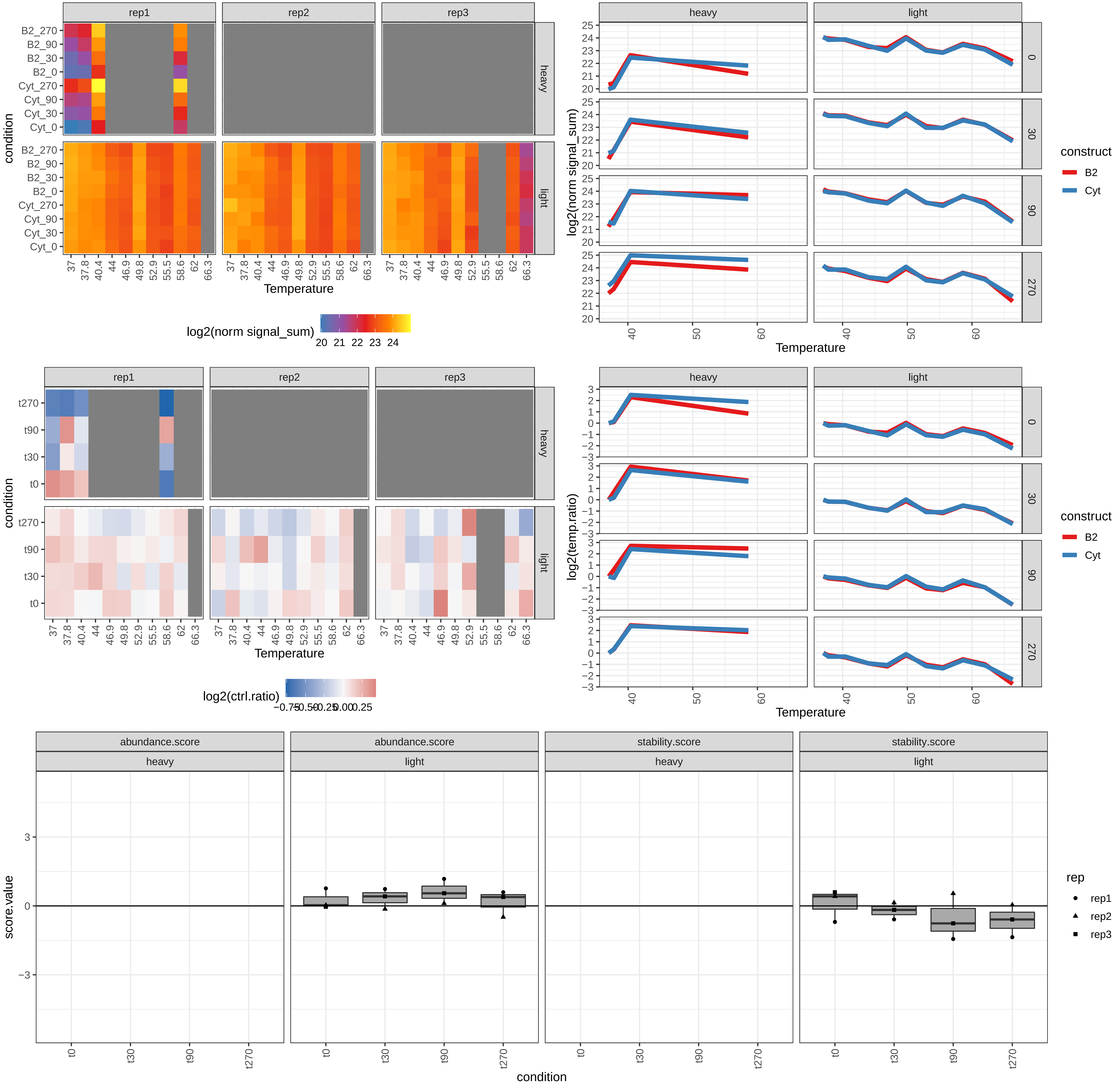

P33894 − Dipeptidyl aminopeptidase A OS=Saccharomyces cerevisiae (strain ATCC 204508 / S288c) GN=STE13 PE=1 SV=1

STE23 − average.top3: 6.79686431818182 − max.qupm: 25

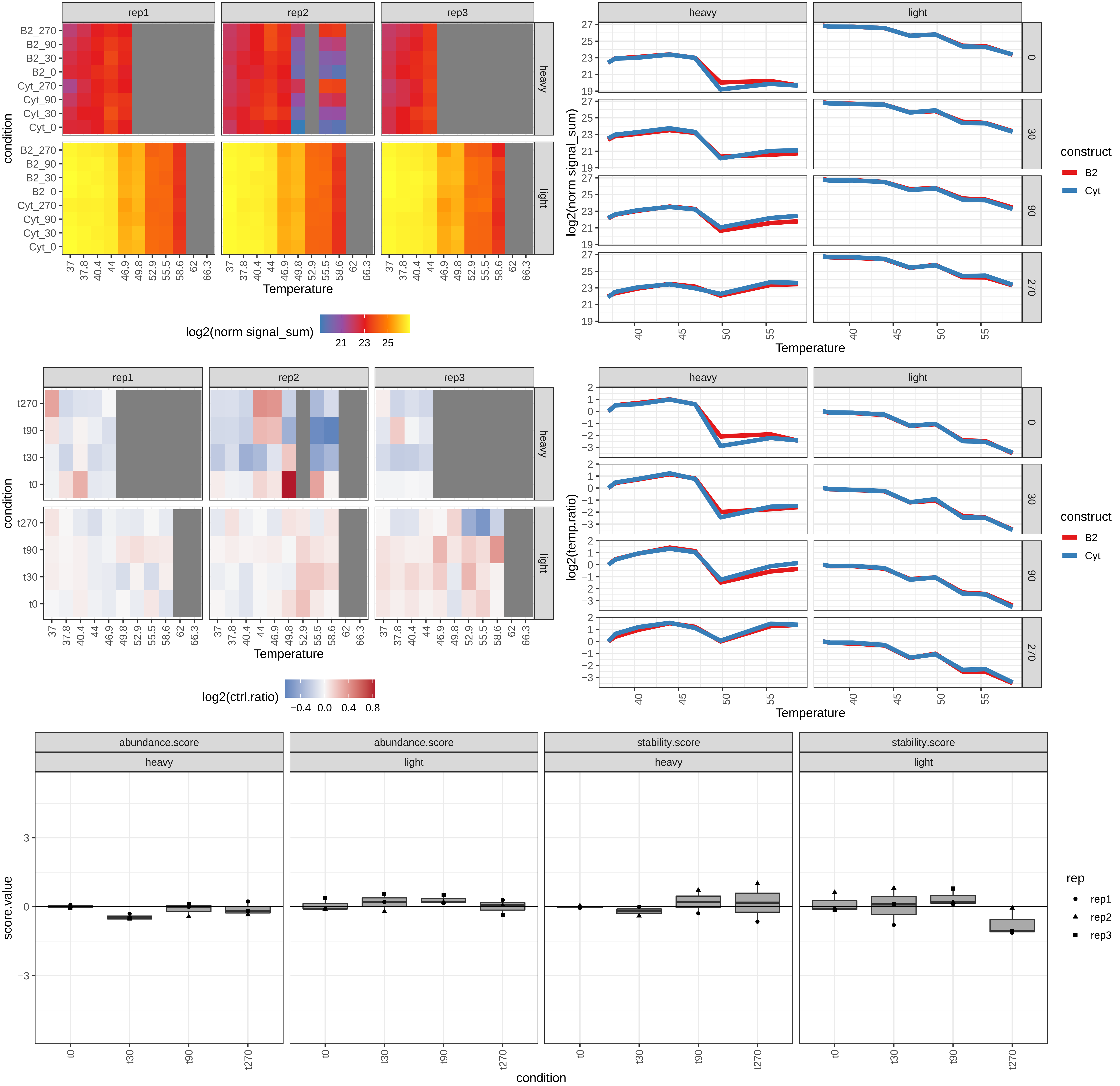

Q06010 − A−factor−processing enzyme OS=Saccharomyces cerevisiae (strain ATCC 204508 / S288c) GN=STE23 PE=1 SV=2

STE24 − average.top3: 7.17658911111111 − max.qupm: 12

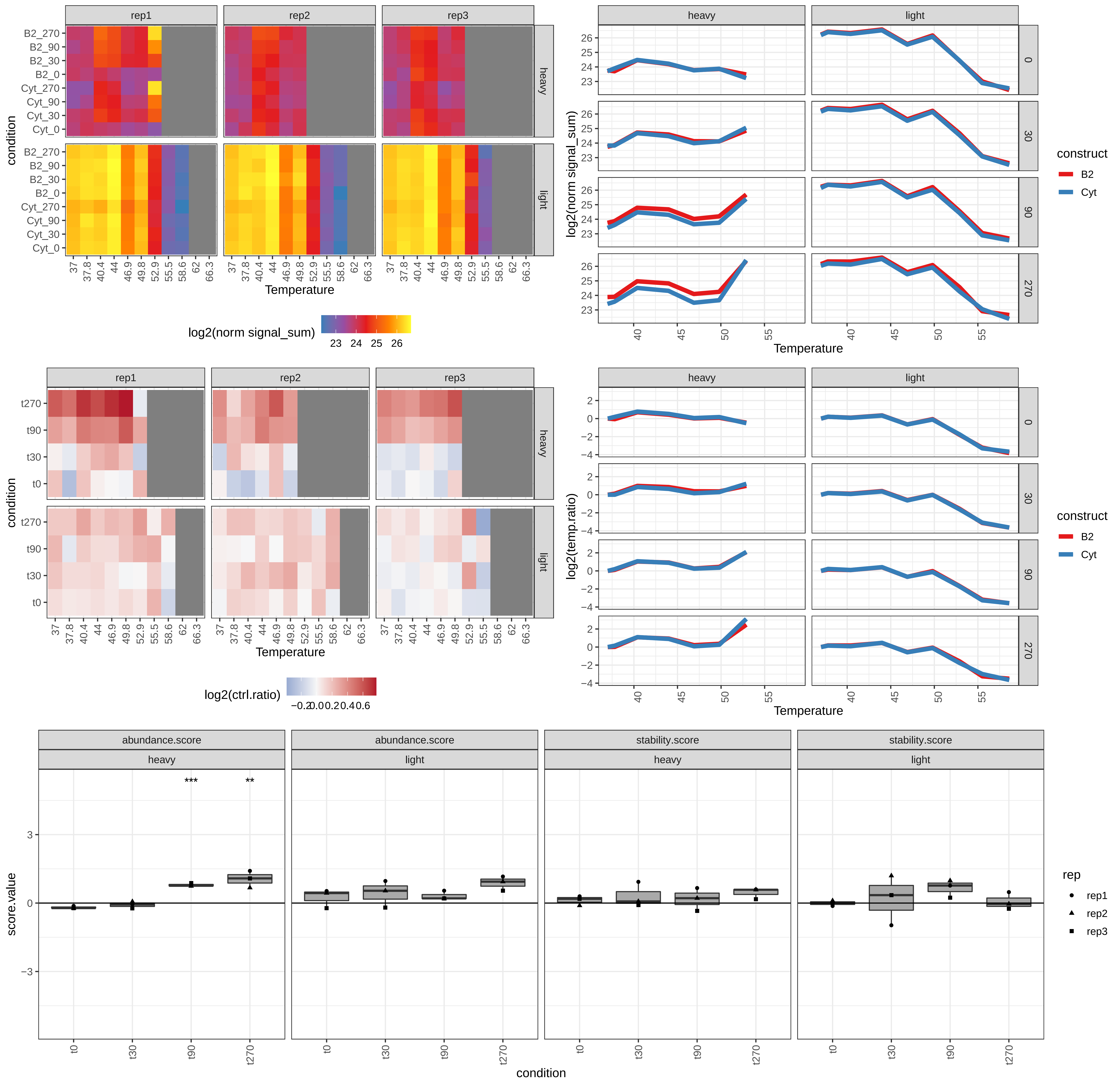

P47154 − CAAX prenyl protease 1 OS=Saccharomyces cerevisiae (strain ATCC 204508 / S288c) GN=STE24 PE=1 SV=1

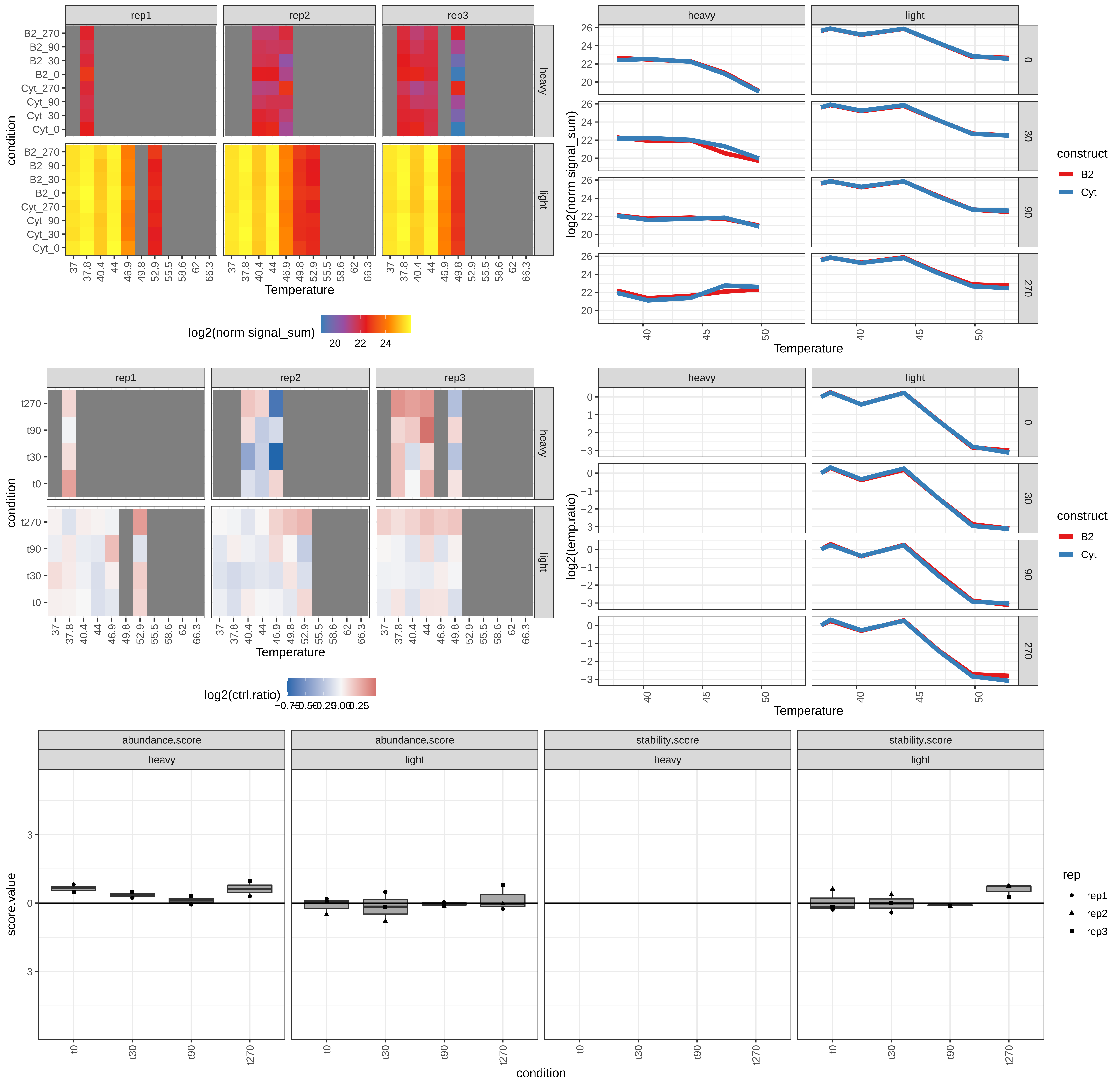

P32597 − Nuclear protein STH1/NPS1 OS=Saccharomyces cerevisiae (strain ATCC 204508 / S288c) GN=STH1 PE=1 SV=1

STI1 − average.top3: 7.55902621212121 − max.qupm: 35

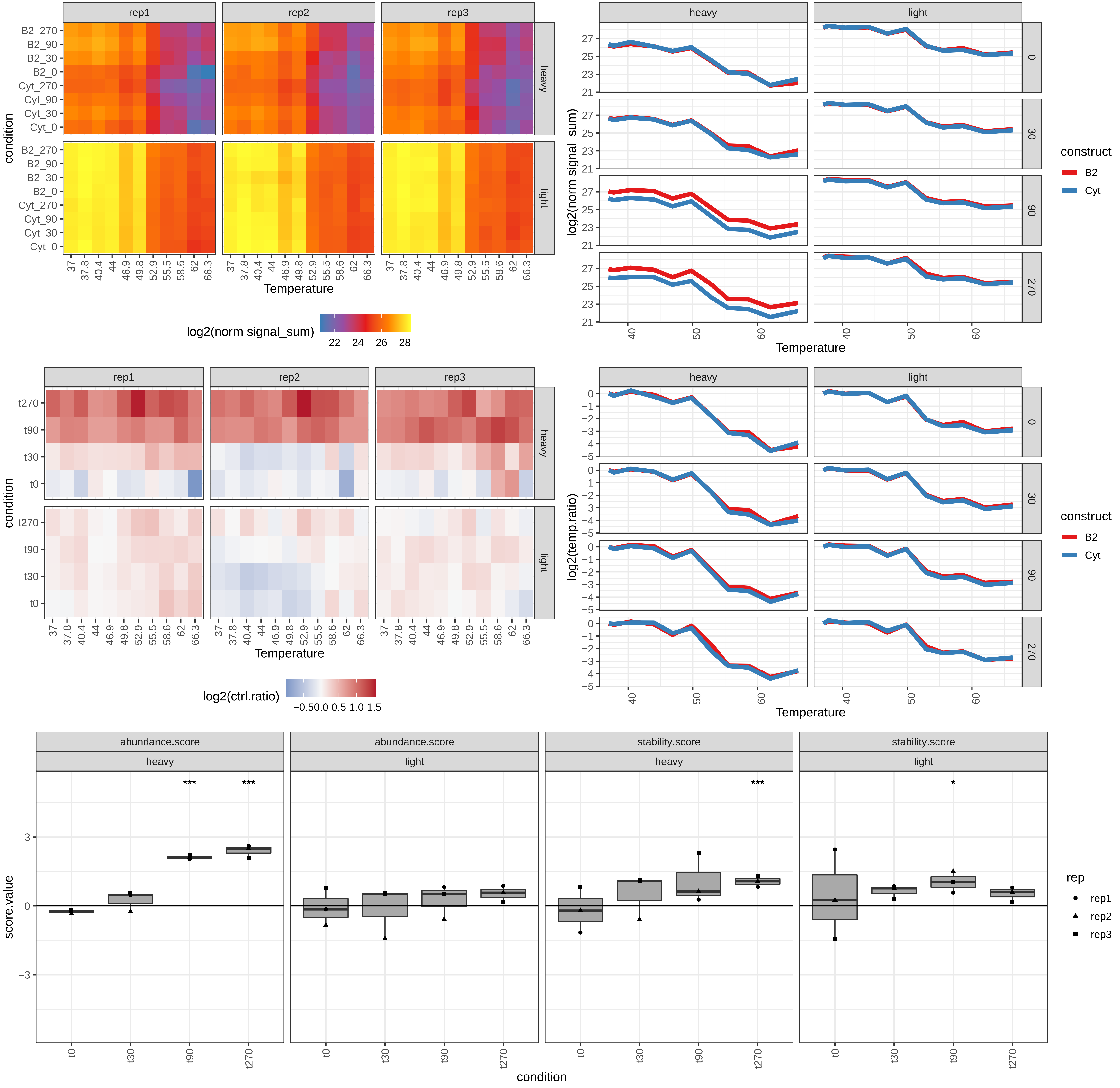

P15705 − Heat shock protein STI1 OS=Saccharomyces cerevisiae (strain ATCC 204508 / S288c) GN=STI1 PE=1 SV=1

STM1 − average.top3: 7.43282283333333 − max.qupm: 10

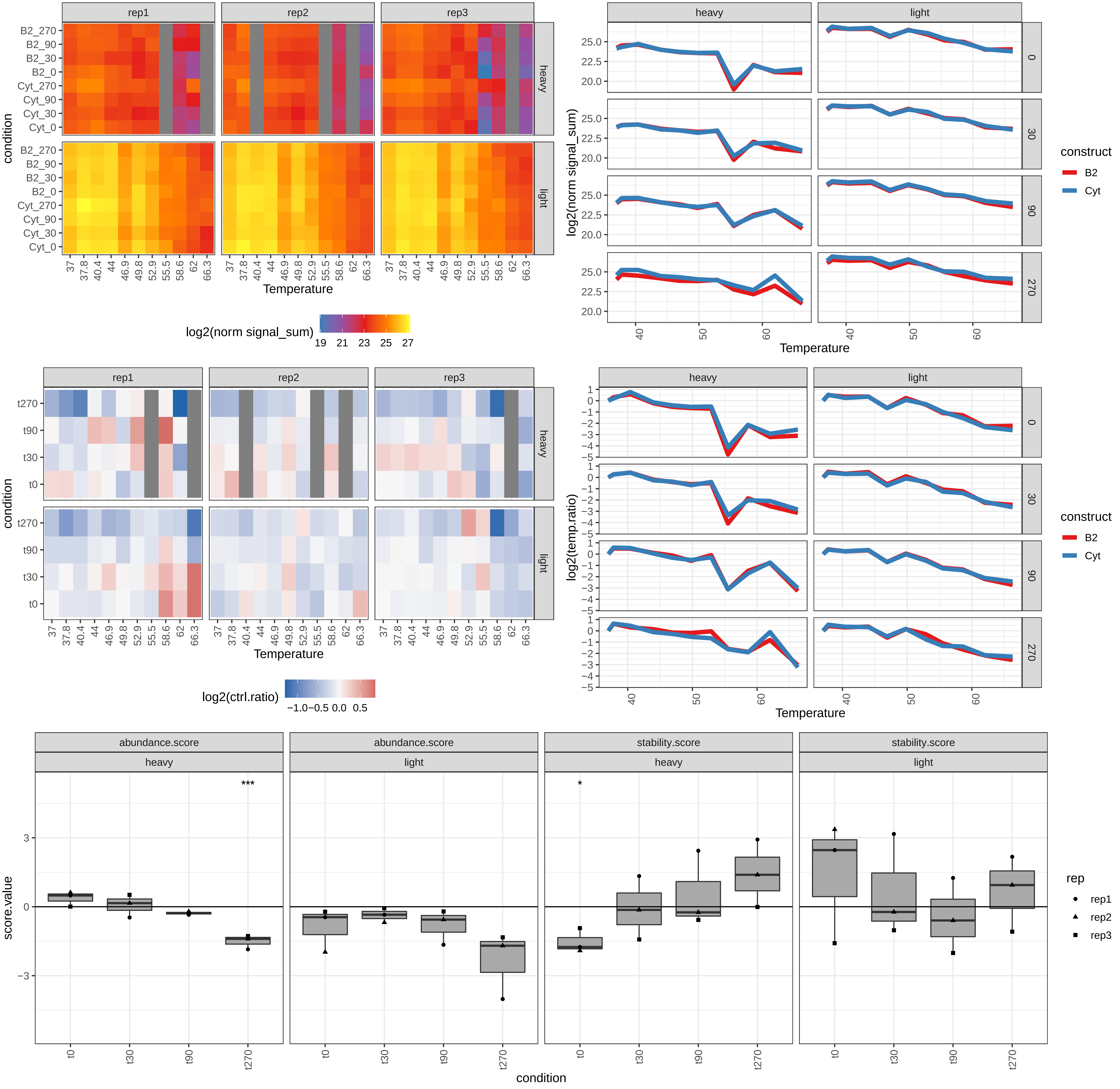

P39015 − Suppressor protein STM1 OS=Saccharomyces cerevisiae (strain ATCC 204508 / S288c) GN=STM1 PE=1 SV=3

STO1 − average.top3: 7.0535485106383 − max.qupm: 17

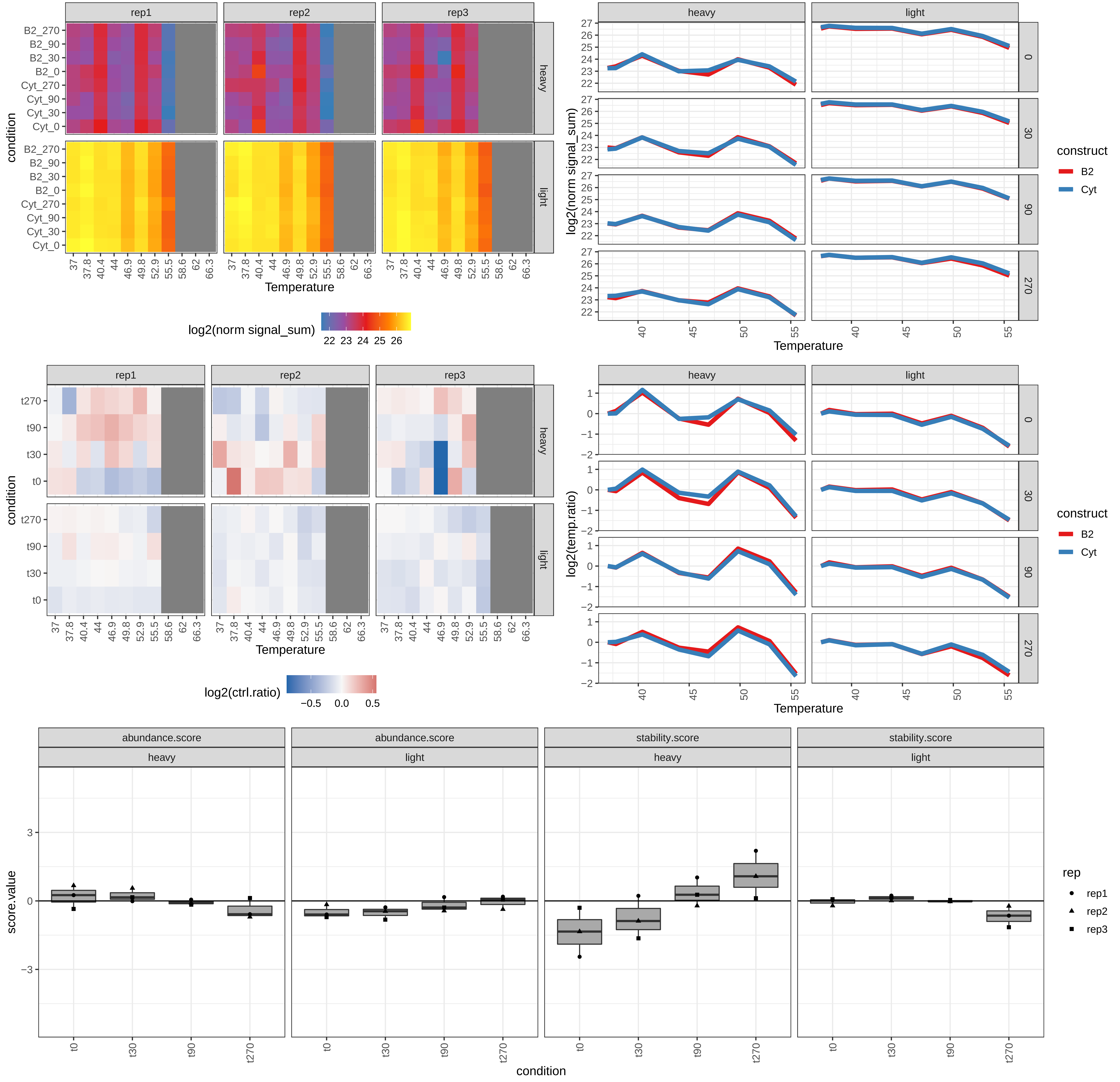

P34160 − Nuclear cap−binding protein complex subunit 1 OS=Saccharomyces cerevisiae (strain ATCC 204508 / S288c) GN=STO1 PE=1 SV=2

STP22 − average.top3: 6.33118111111111 − max.qupm: 3

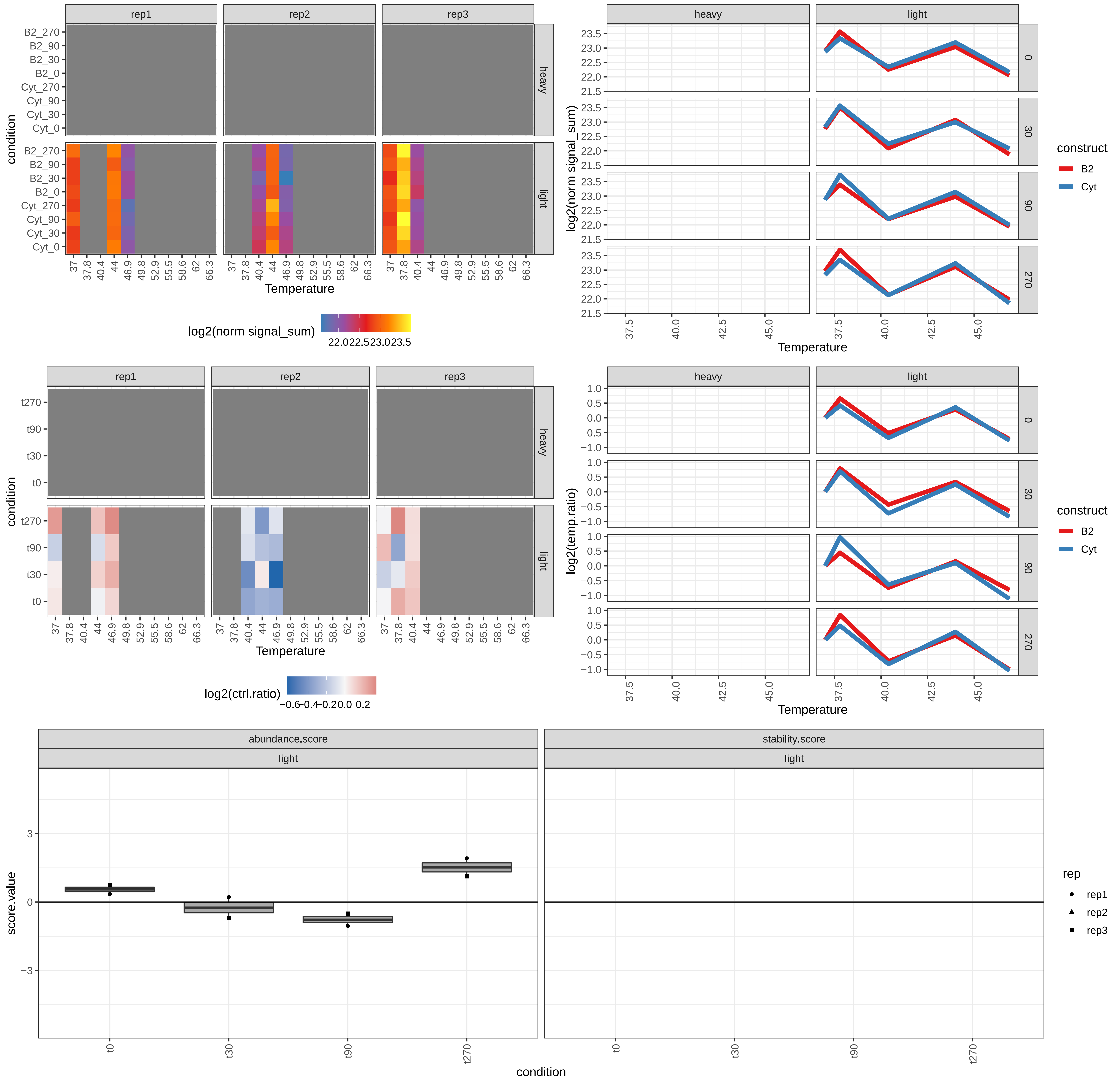

P25604 − Suppressor protein STP22 of temperature−sensitive alpha−factor receptor and arginine permease OS=Saccharomyces cerevisiae (strain ATCC 204508 / S288c) GN=STP22 PE=1 SV=3

STR2 − average.top3: 6.70433323529412 − max.qupm: 9

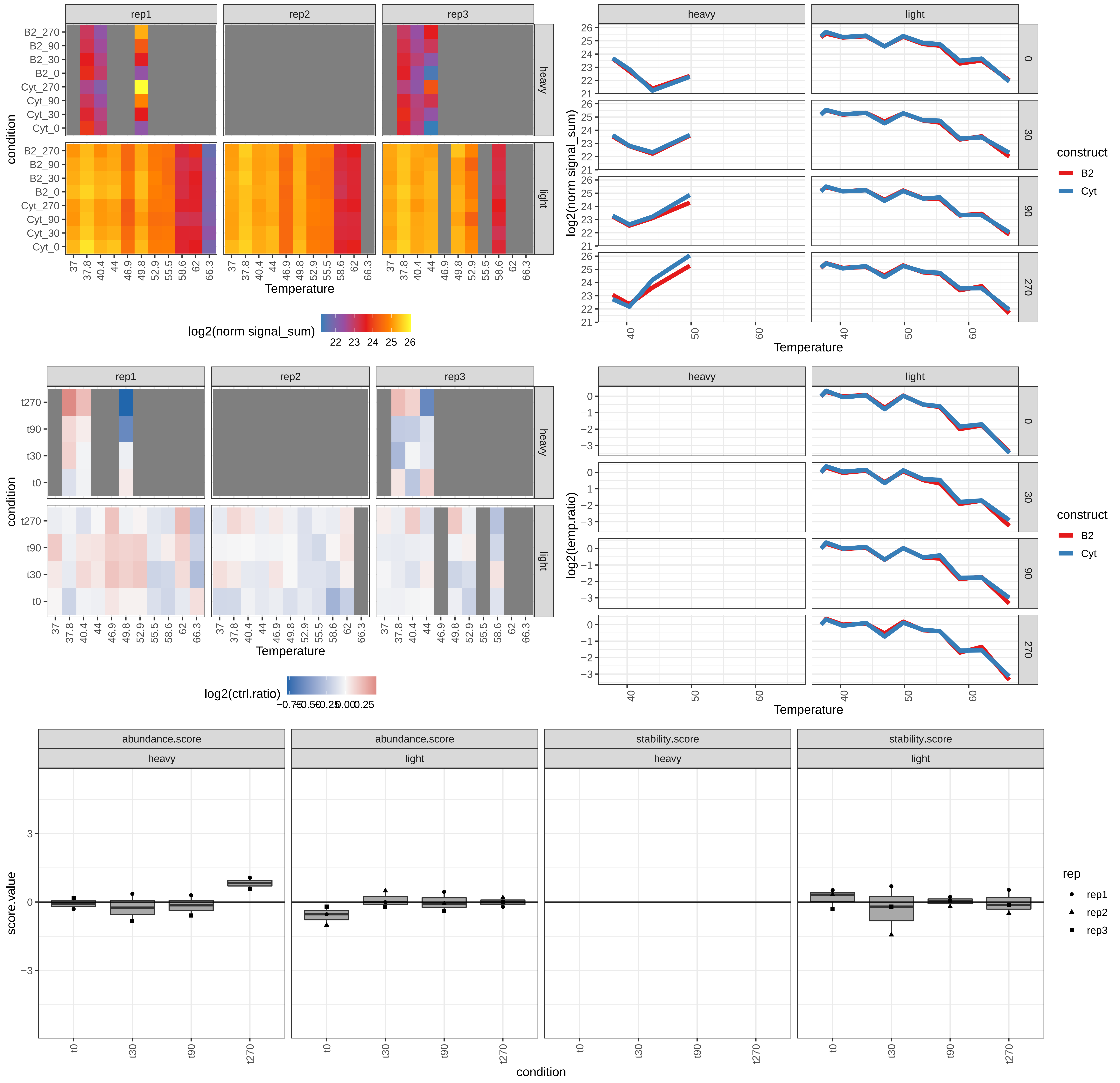

P47164 − Cystathionine gamma−synthase OS=Saccharomyces cerevisiae (strain ATCC 204508 / S288c) GN=STR2 PE=1 SV=1

STR3 − average.top3: 6.313042 − max.qupm: 3

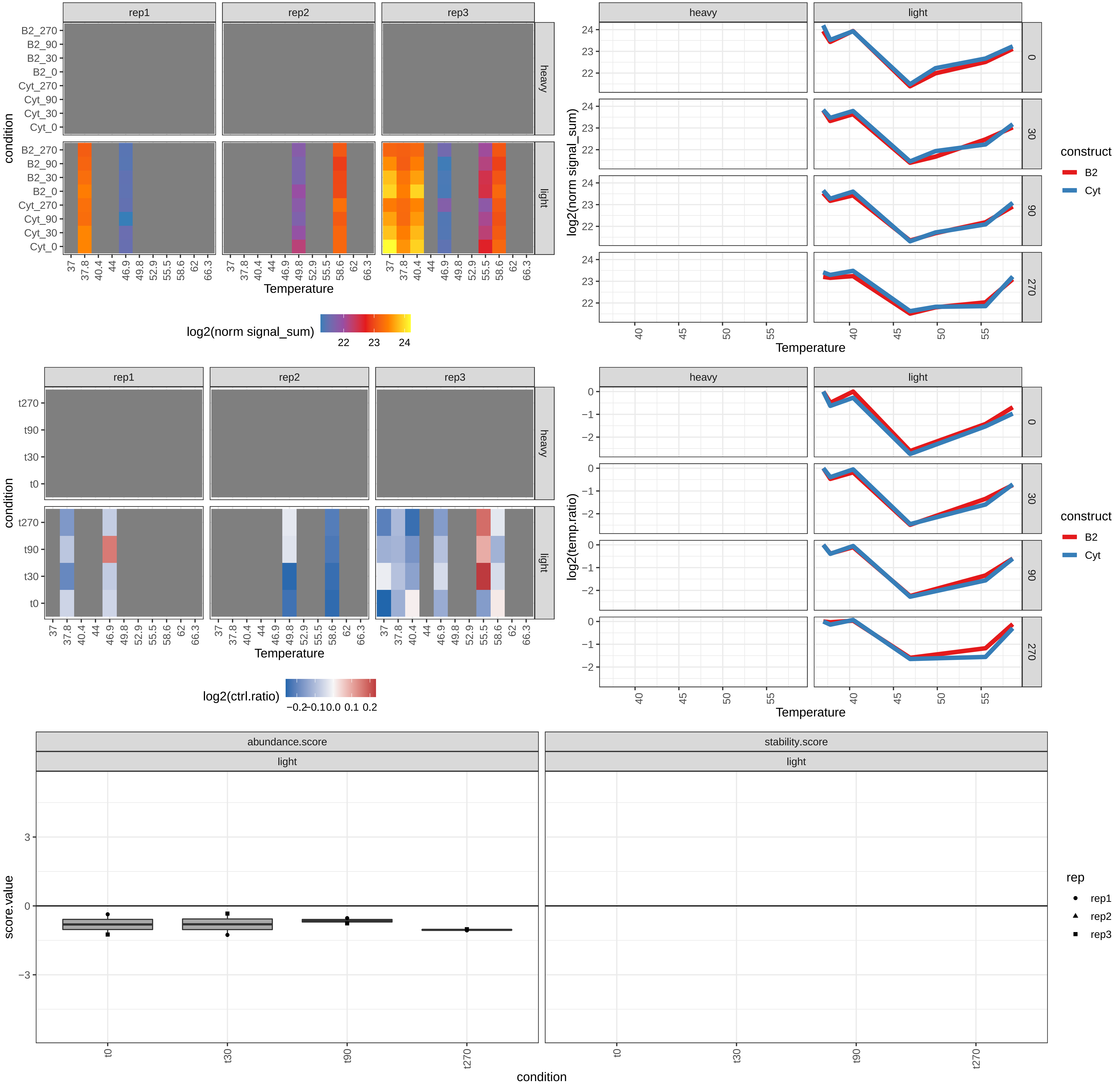

P53101 − Cystathionine beta−lyase OS=Saccharomyces cerevisiae (strain ATCC 204508 / S288c) GN=STR3 PE=1 SV=1

STT3 − average.top3: 7.05144035714286 − max.qupm: 13

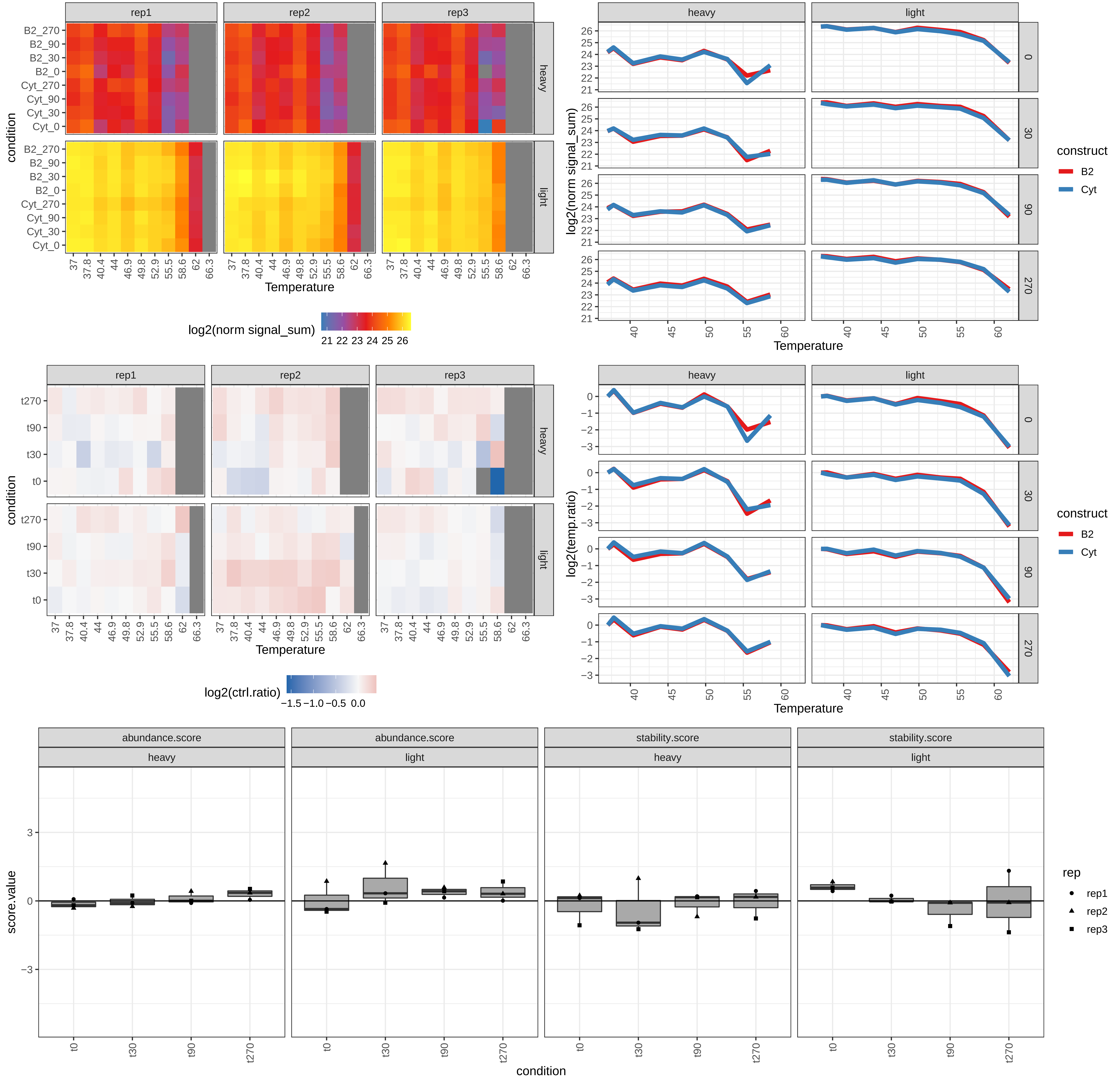

P39007 − Dolichyl−diphosphooligosaccharide−−protein glycosyltransferase subunit STT3 OS=Saccharomyces cerevisiae (strain ATCC 204508 / S288c) GN=STT3 PE=1 SV=2

STT4 − average.top3: 6.78684652173913 − max.qupm: 14

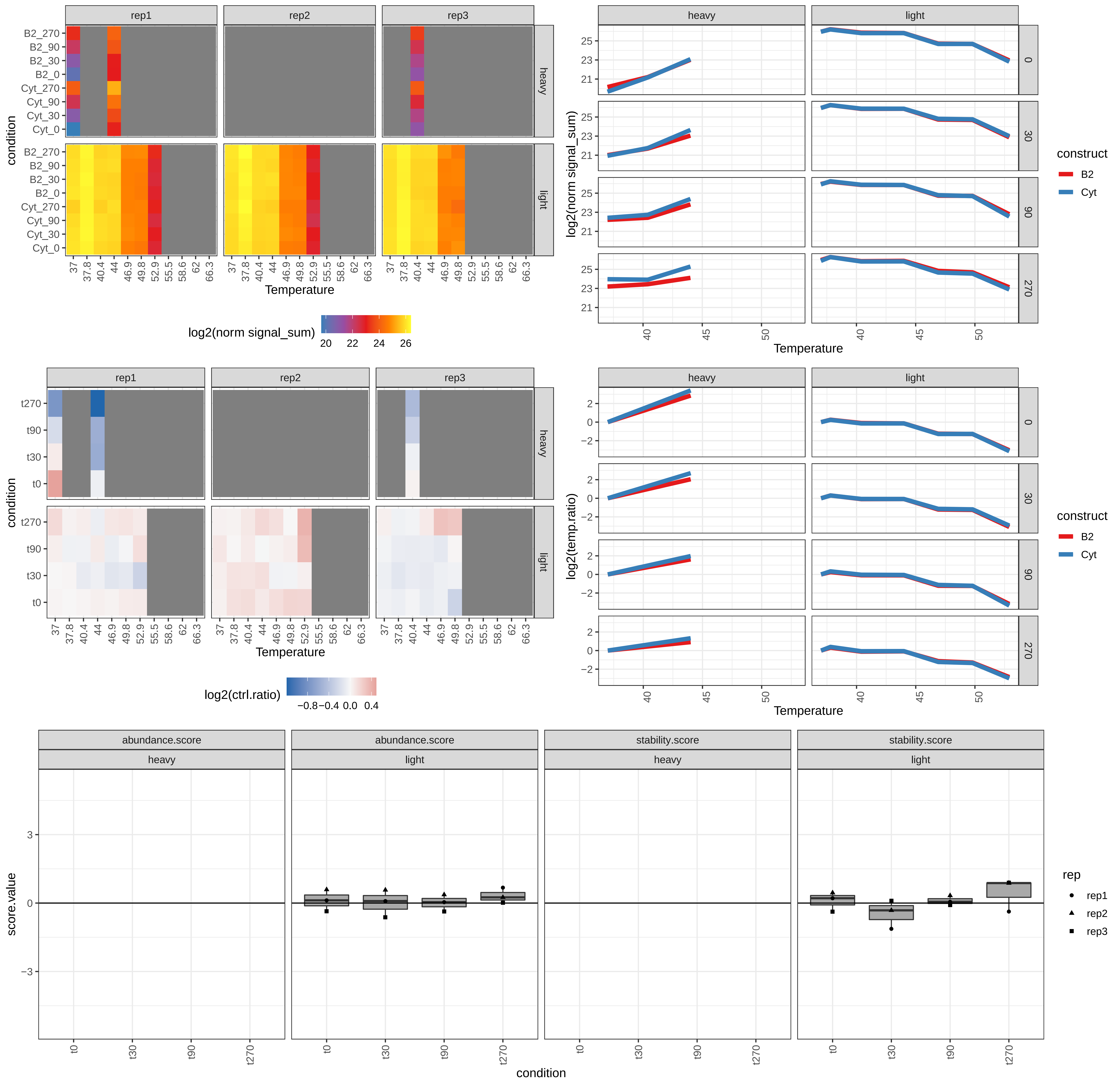

P37297 − Phosphatidylinositol 4−kinase STT4 OS=Saccharomyces cerevisiae (strain ATCC 204508 / S288c) GN=STT4 PE=1 SV=1

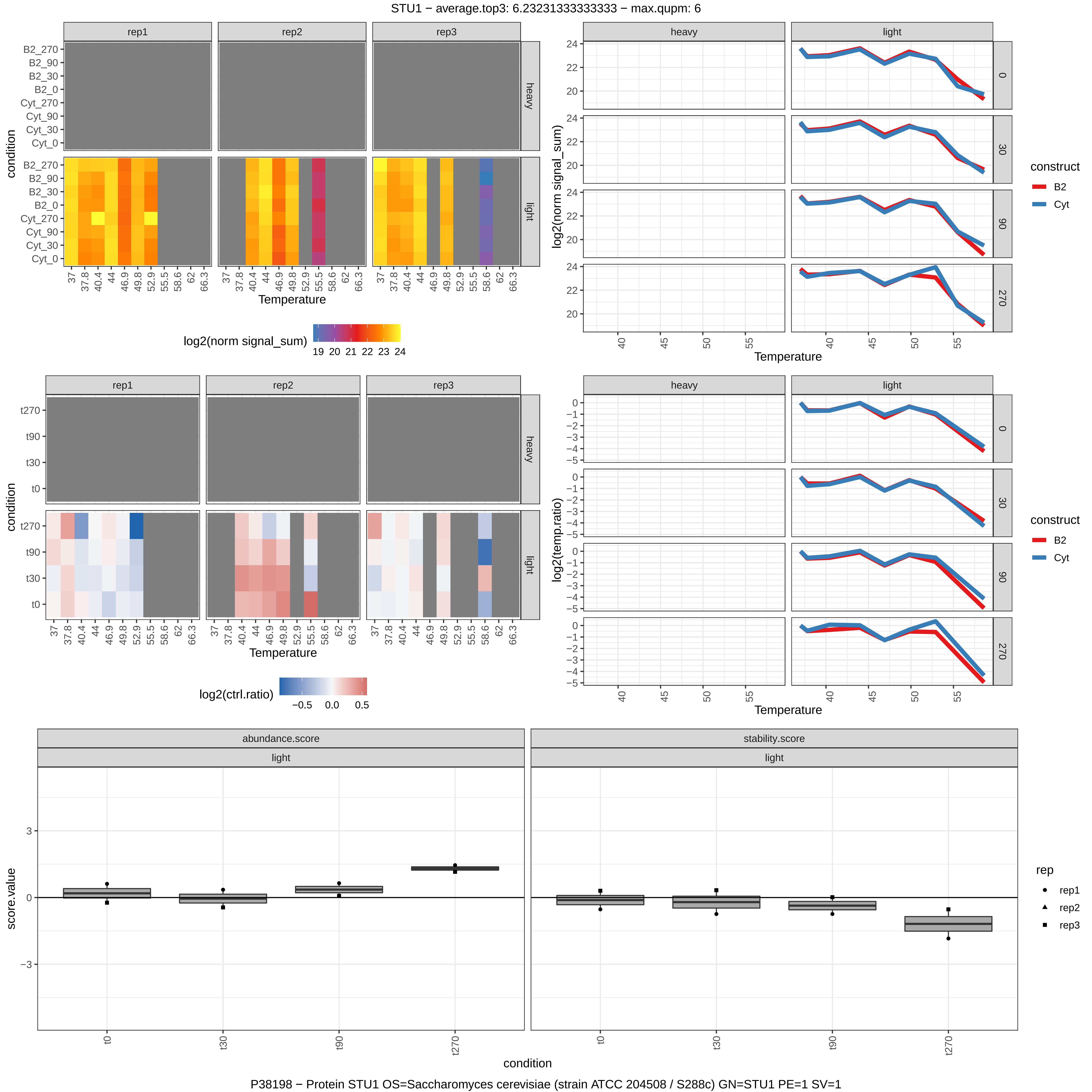

STU1 − average.top3: 6.23231333333333 − max.qupm: 6
rep1
rep2
rep3
heavy
light
condition
Temperature
log2(norm signal_sum)
log2(ctrl.ratio)
log2(temp.ratio)
construct
B2
Cyt
abundance.score
stability.score
score.value
rep
rep1
rep2
rep3
P38198 − Protein STU1 OS=Saccharomyces cerevisiae (strain ATCC 204508 / S288c) GN=STU1 PE=1 SV=1

STU2 − average.top3: 6.52332777777778 − max.qupm: 4

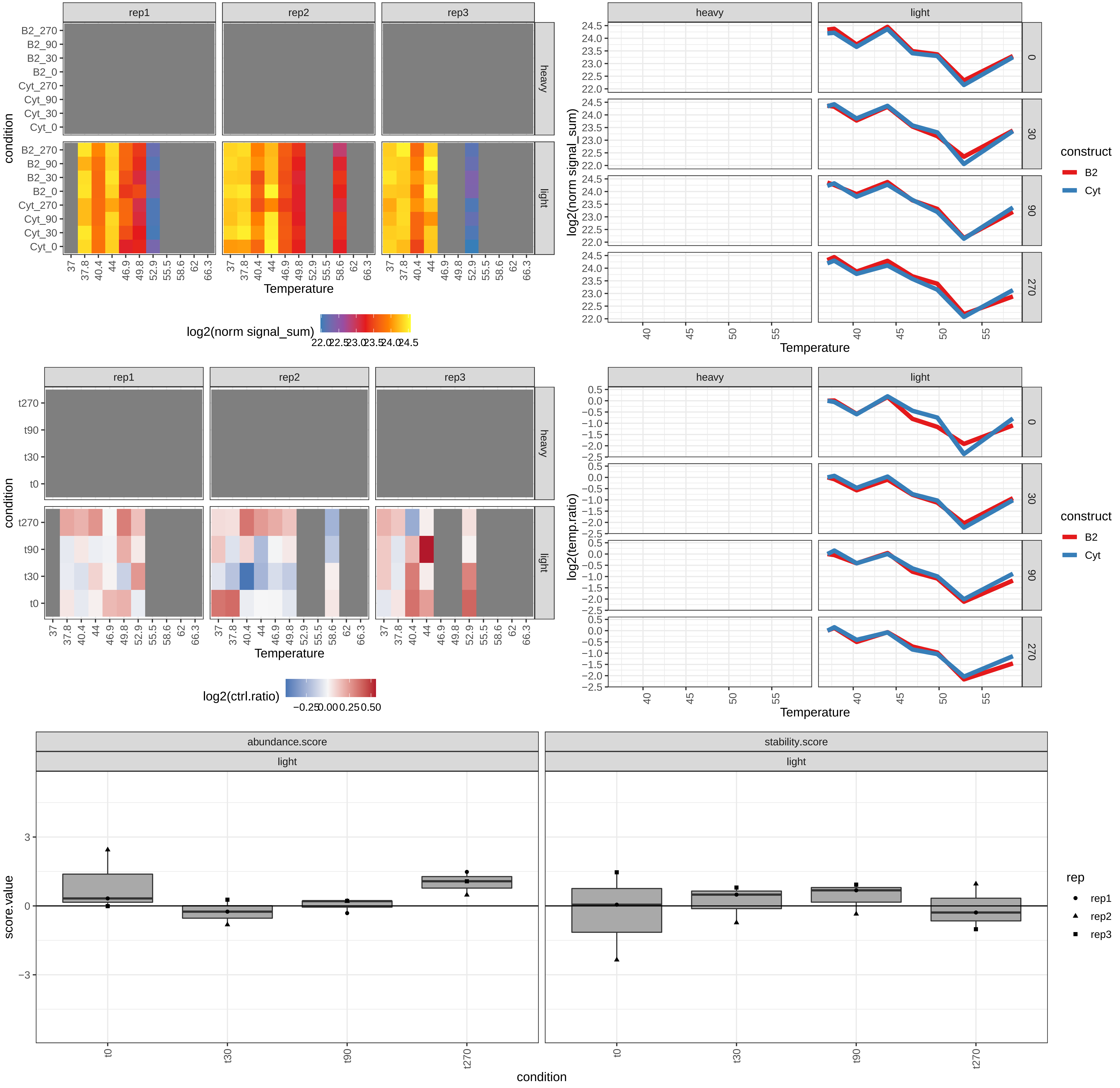

P46675 − Protein STU2 OS=Saccharomyces cerevisiae (strain ATCC 204508 / S288c) GN=STU2 PE=1 SV=1

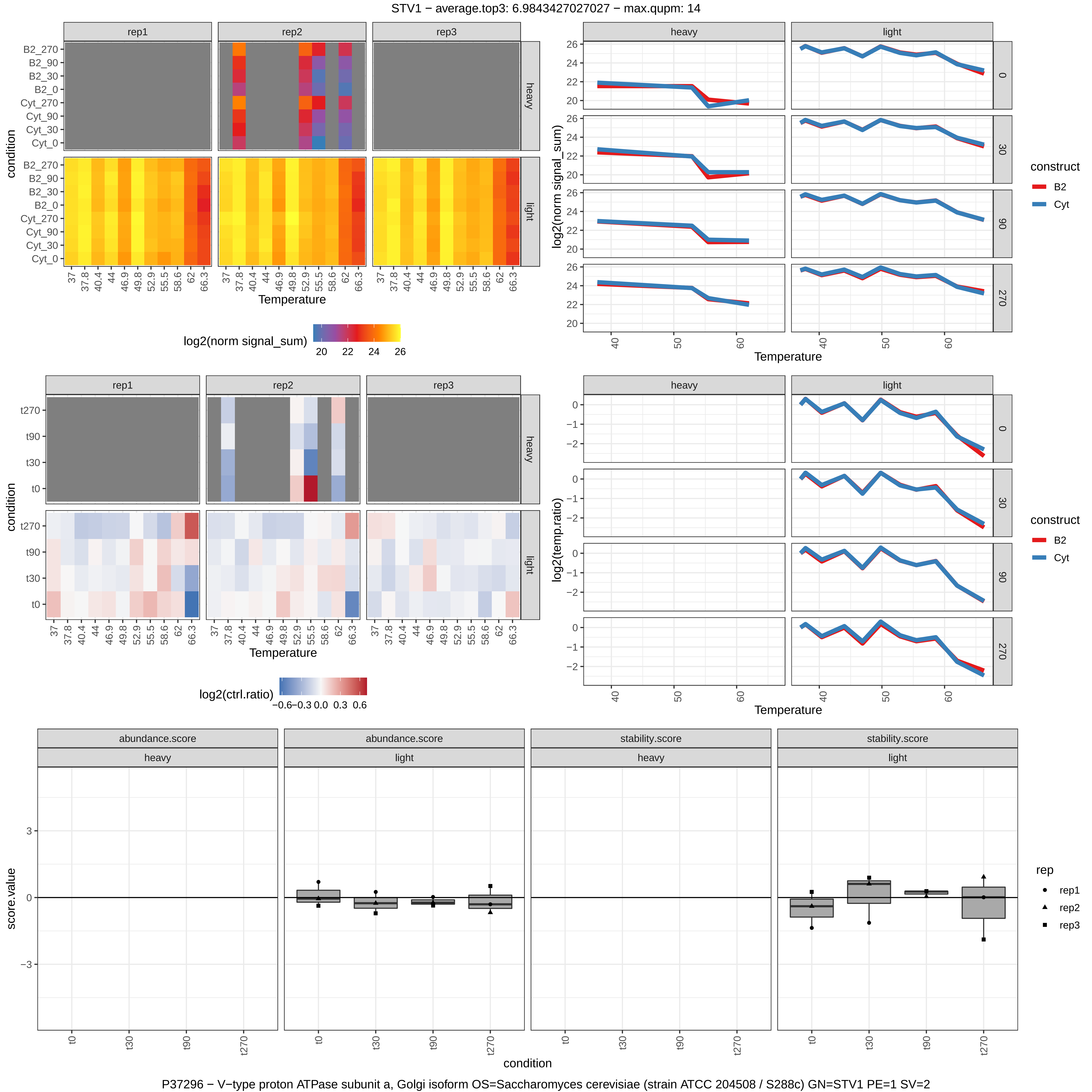
STV1 − average.top3: 6.9843427027027 − max.qupm: 14
rep1
rep2
rep3
heavy
light
condition
Temperature
log2(norm signal_sum)
construct
B2
Cyt
log2(ctrl.ratio)
log2(temp.ratio)
abundance.score
stability.score
score.value
rep
rep1
rep2
rep3
P37296 − V−type proton ATPase subunit a, Golgi isoform OS=Saccharomyces cerevisiae (strain ATCC 204508 / S288c) GN=STV1 PE=1 SV=2

SUA5 − average.top3: 6.93408388888889 − max.qupm: 5

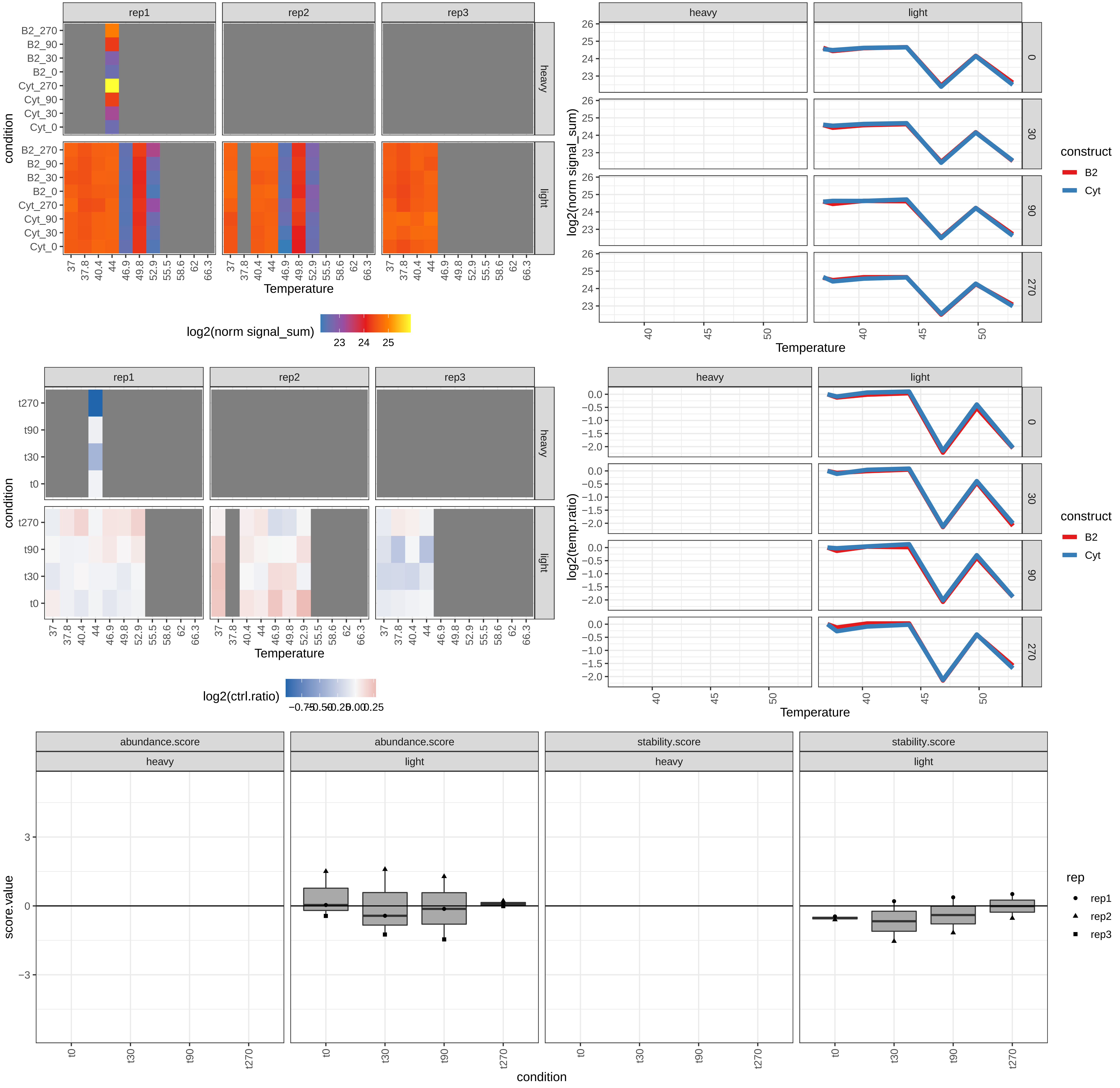

P32579 − Threonylcarbamoyl−AMP synthase OS=Saccharomyces cerevisiae (strain ATCC 204508 / S288c) GN=SUA5 PE=1 SV=1

SUB1 − average.top3: 7.09488541666667 − max.qupm: 5

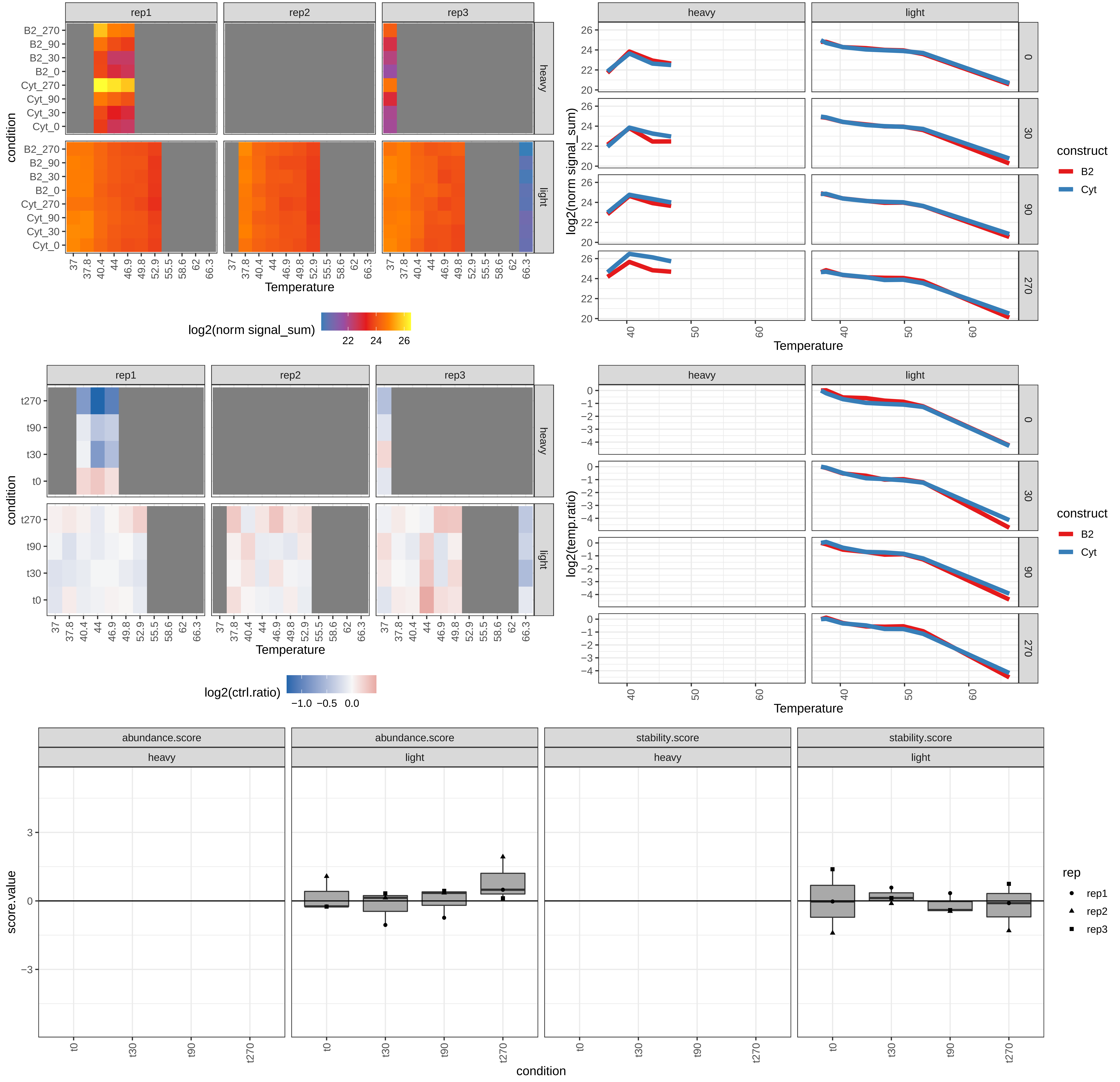

P54000 − RNA polymerase II transcriptional coactivator SUB1 OS=Saccharomyces cerevisiae (strain ATCC 204508 / S288c) GN=SUB1 PE=1 SV=1

SUB2 − average.top3: 7.60519888888889 − max.qupm: 17

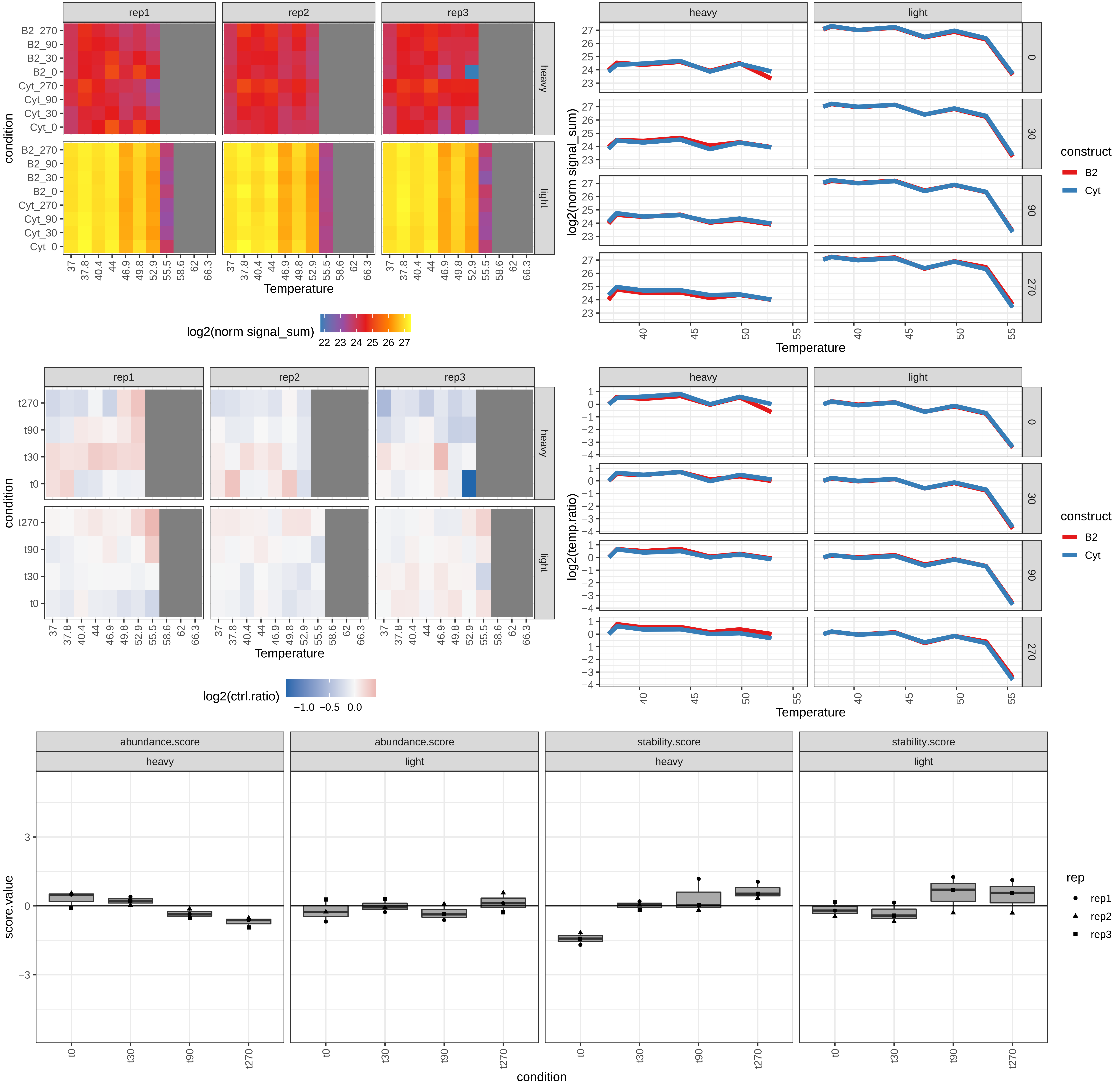

Q07478 − ATP−dependent RNA helicase SUB2 OS=Saccharomyces cerevisiae (strain ATCC 204508 / S288c) GN=SUB2 PE=1 SV=1

SUC2 − average.top3: 7.73269727272727 − max.qupm: 13

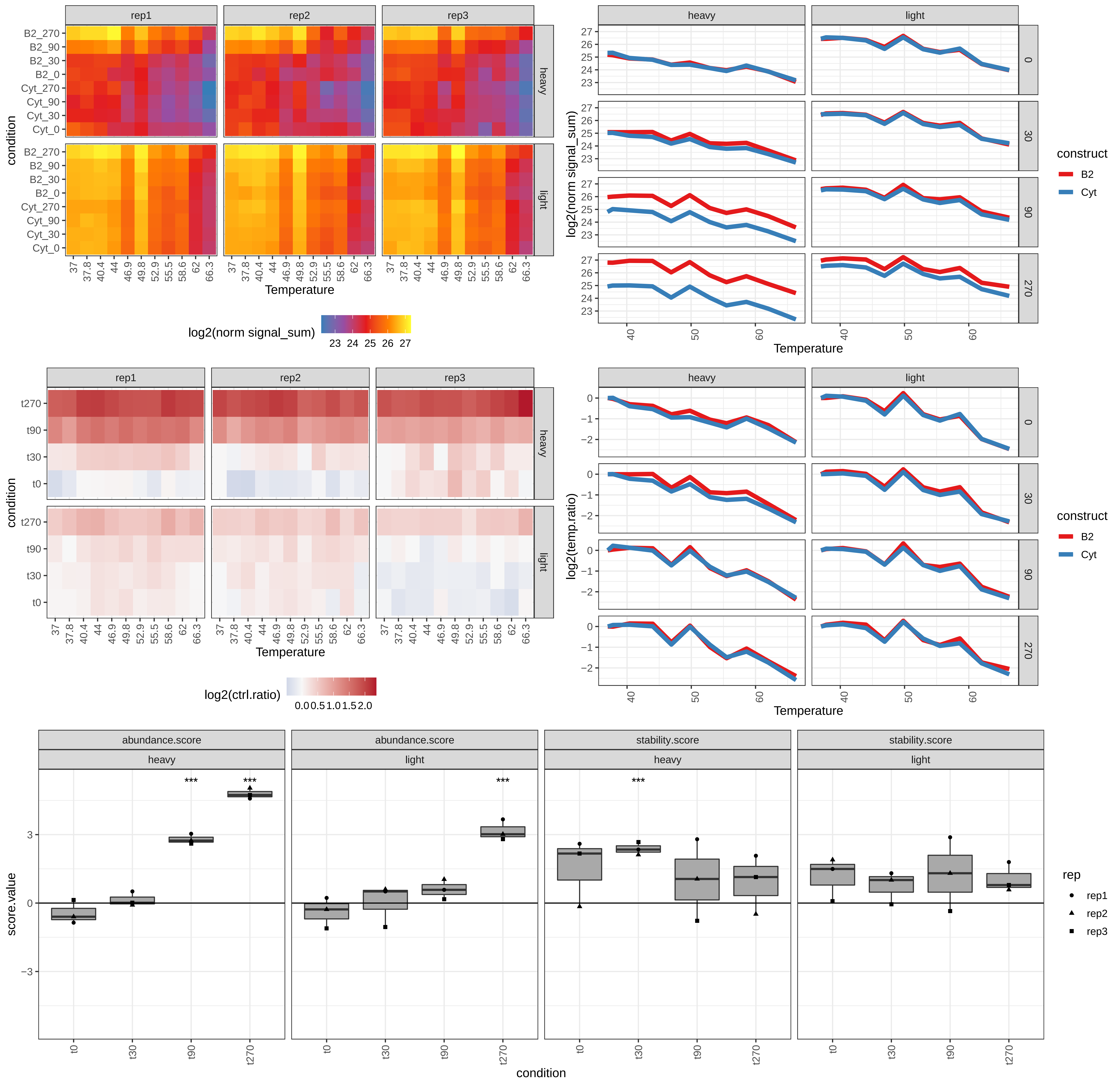

P36724−2 − Invertase 2 OS=Saccharomyces cerevisiae (strain ATCC 204508 / S288c) GN=SUC2 PE=1 SV=1|Isoform Intracellular of Invertase 2 OS=Saccharomyces cerevisiae (strain ATCC 204508 / S288c

SUI1 − average.top3: 6.5699143902439 − max.qupm: 6

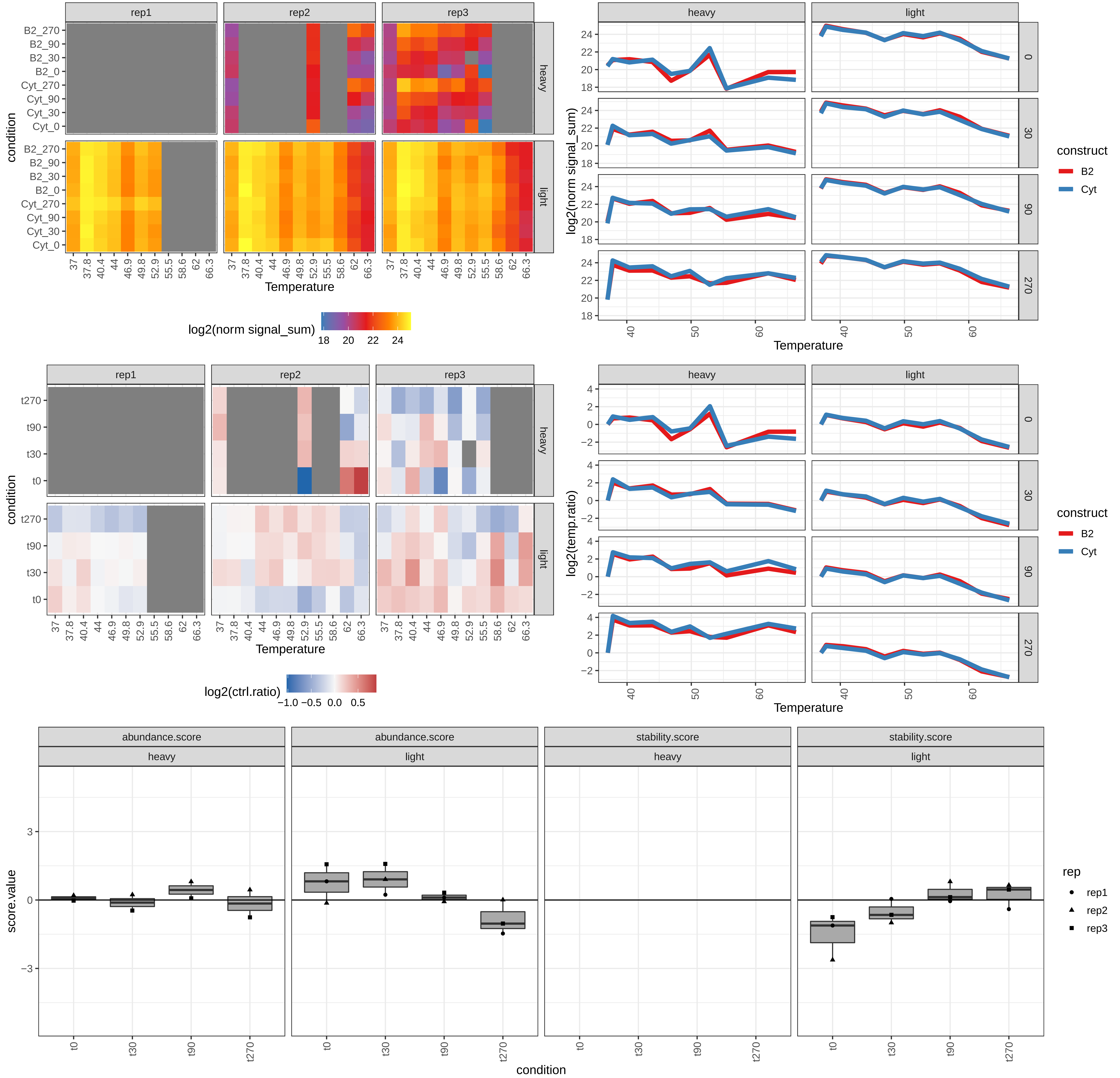

P32911 − Eukaryotic translation initiation factor eIF−1 OS=Saccharomyces cerevisiae (strain ATCC 204508 / S288c) GN=SUI1 PE=1 SV=1

SUI2 − average.top3: 7.42508303571429 − max.qupm: 13

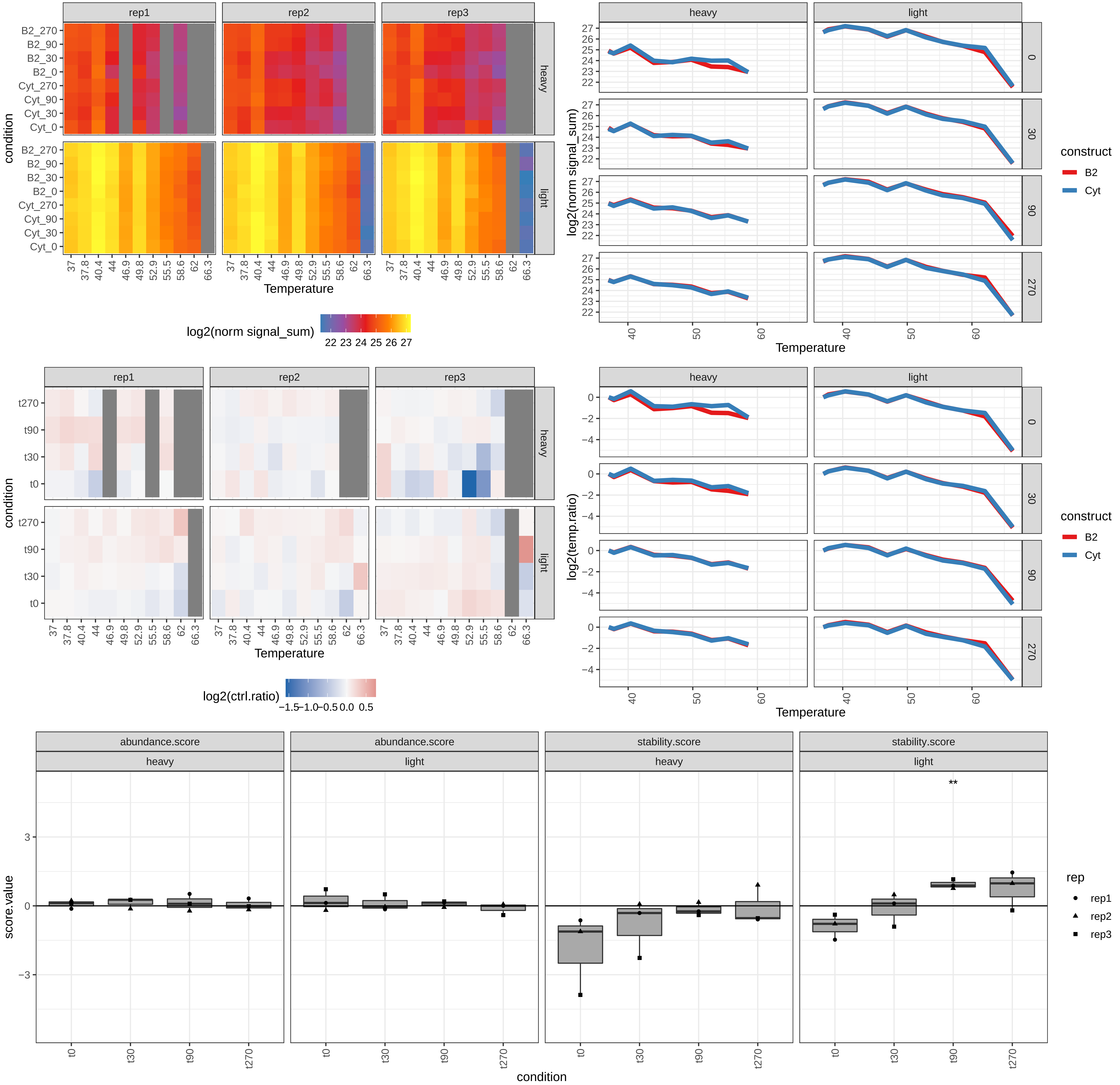

P20459 − Eukaryotic translation initiation factor 2 subunit alpha OS=Saccharomyces cerevisiae (strain ATCC 204508 / S288c) GN=SUI2 PE=1 SV=1

SUI3 − average.top3: 7.38185107692308 − max.qupm: 13

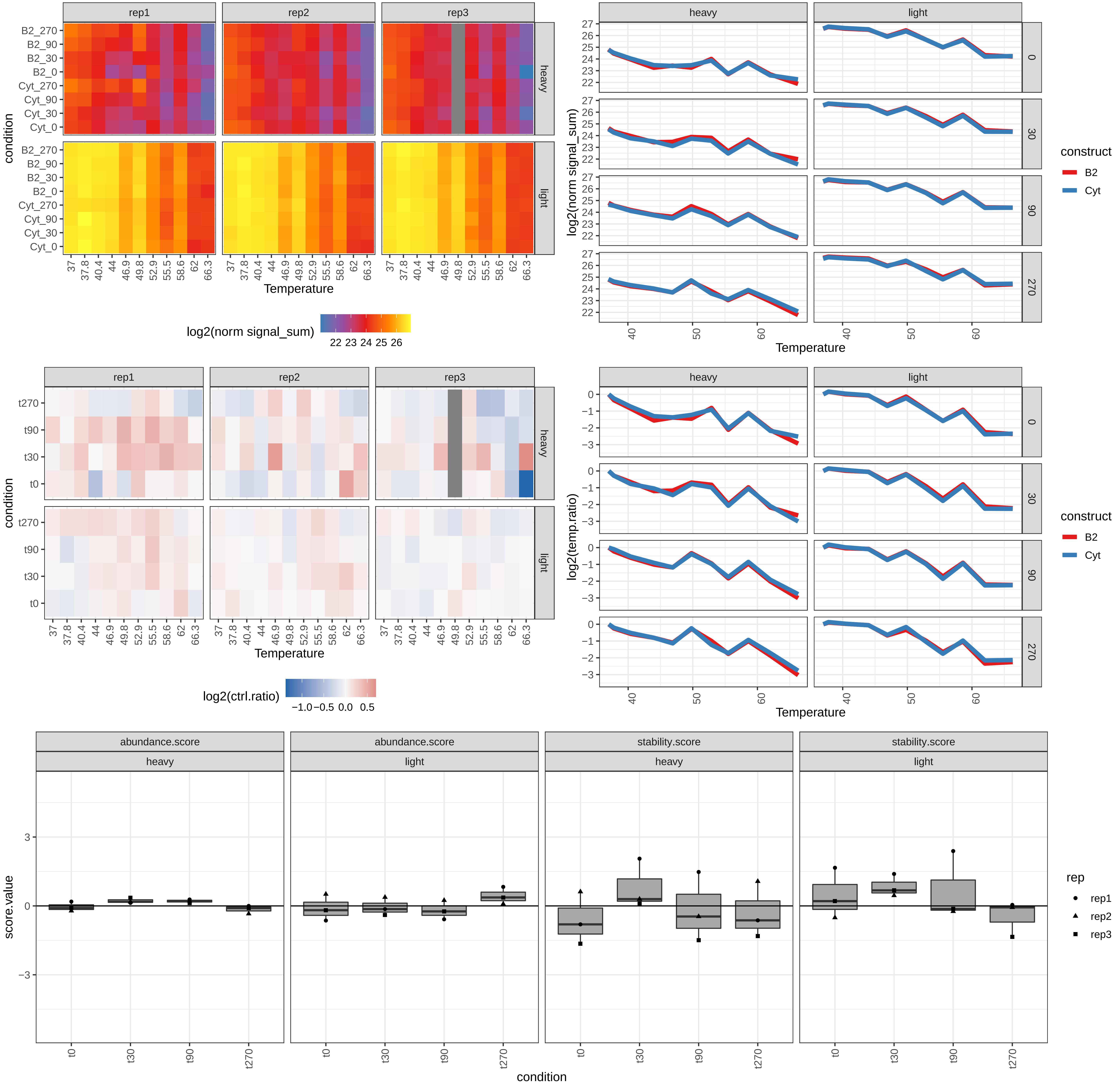

P09064 − Eukaryotic translation initiation factor 2 subunit beta OS=Saccharomyces cerevisiae (strain ATCC 204508 / S288c) GN=SUI3 PE=1 SV=2

SUP35 − average.top3: 7.46682513513514 − max.qupm: 17

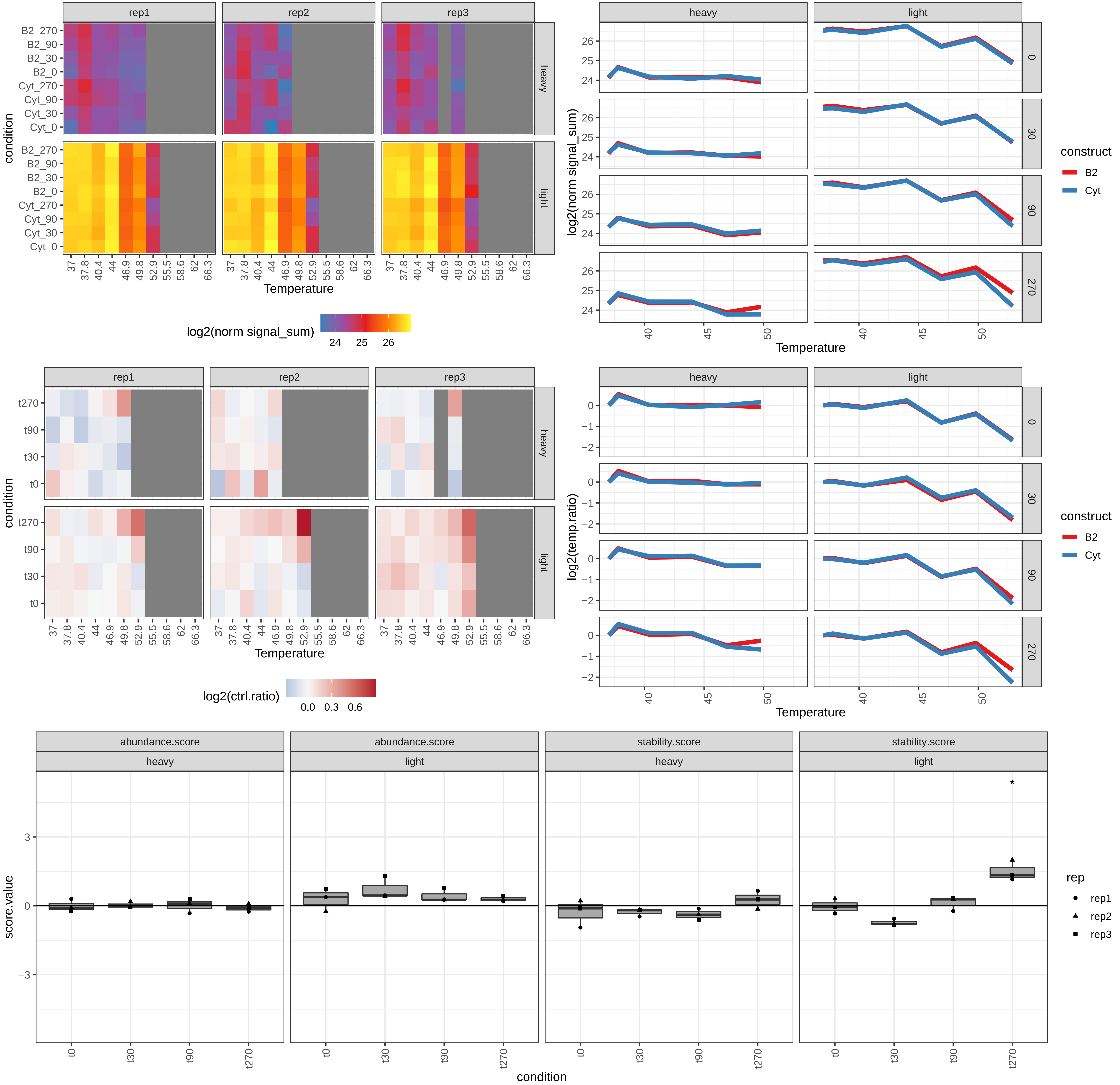

P05453 − Eukaryotic peptide chain release factor GTP−binding subunit OS=Saccharomyces cerevisiae (strain ATCC 204508 / S288c) GN=SUP35 PE=1 SV=1

SUP45 − average.top3: 7.26970157894737 − max.qupm: 14

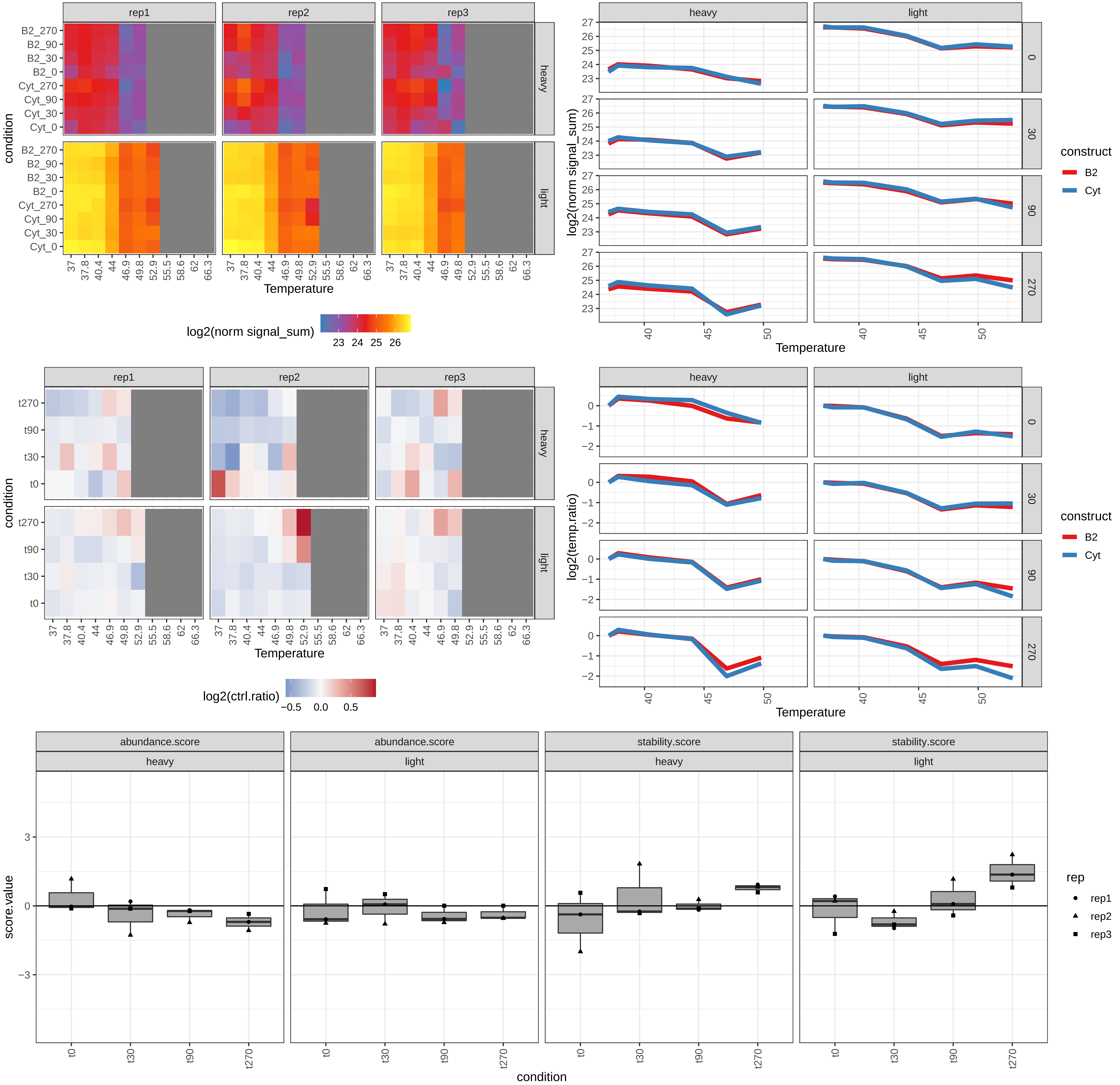

P12385 − Eukaryotic peptide chain release factor subunit 1 OS=Saccharomyces cerevisiae (strain ATCC 204508 / S288c) GN=SUP45 PE=1 SV=2

SUR2 − average.top3: 6.84746379310345 − max.qupm: 5

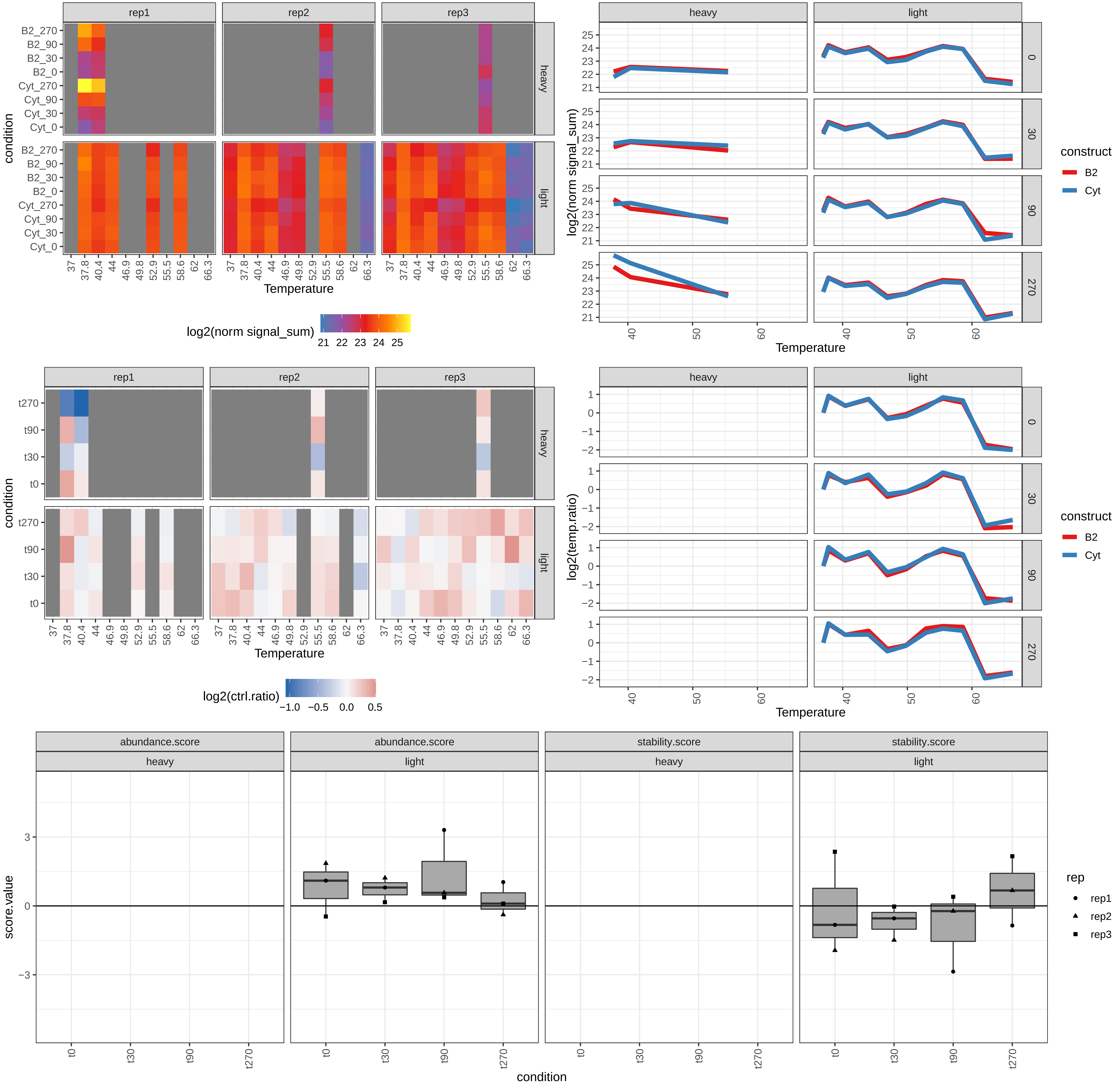

P38992 − Sphingolipid C4−hydroxylase SUR2 OS=Saccharomyces cerevisiae (strain ATCC 204508 / S288c) GN=SUR2 PE=1 SV=1

SUR7 − average.top3: 6.92956375 − max.qupm: 4

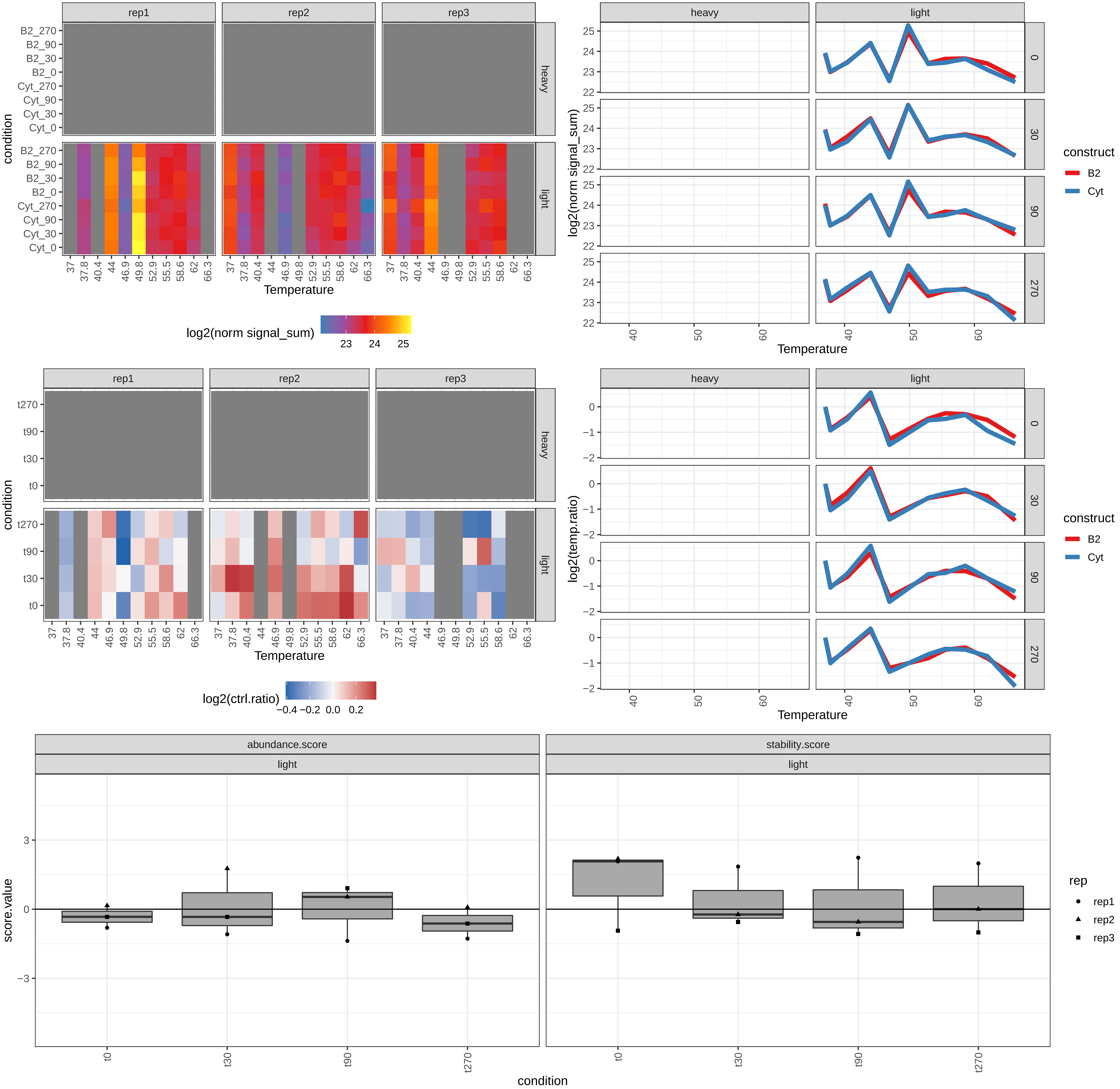

P54003 − Protein SUR7 OS=Saccharomyces cerevisiae (strain ATCC 204508 / S288c) GN=SUR7 PE=1 SV=1

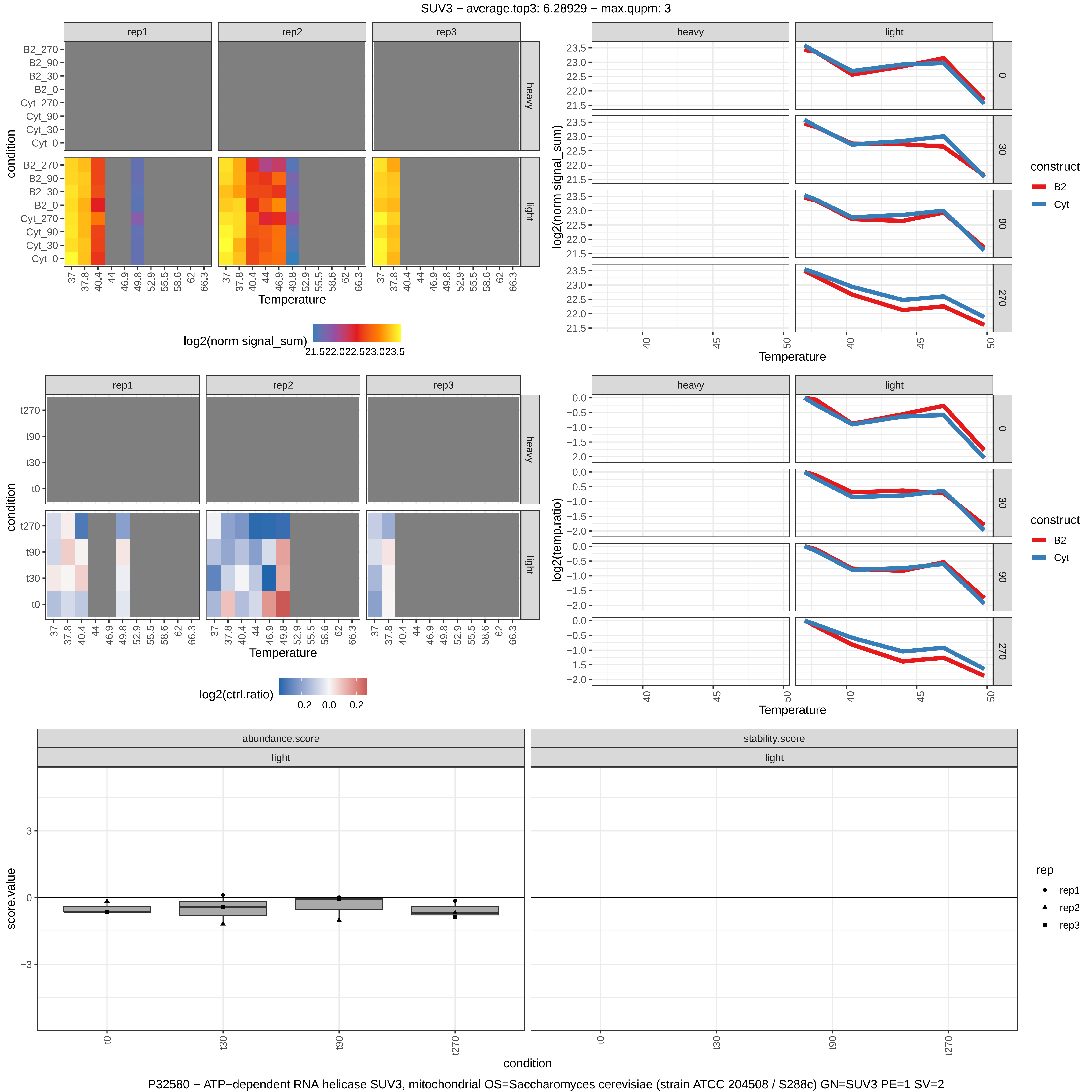
SUV3 − average.top3: 6.28929 − max.qupm: 3
rep1
rep2
rep3
heavy
light
condition
B2_270
B2_90
B2_30
B2_0
Cyt_270
Cyt_90
Cyt_30
Cyt_0
37 37.8 40.4 44 46.9 49.8 52.9 55.5 58.6 62 66.3
Temperature
log2(norm signal_sum)
21.522.022.523.023.5
log2(norm signal_sum)
23.5 23.0 22.5 22.0 21.5
0
30
90
270
40 45 50
construct
B2
Cyt
t270
t90
t30
t0
log2(ctrl.ratio)
−0.2 0.0 0.2
log2(temp.ratio)
0.0 −0.5 −1.0 −1.5 −2.0
abundance.score
stability.score
score.value
3
0
−3
rep
rep1
rep2
rep3
P32580 − ATP−dependent RNA helicase SUV3, mitochondrial OS=Saccharomyces cerevisiae (strain ATCC 204508 / S288c) GN=SUV3 PE=1 SV=2

SVF1 − average.top3: 6.82862258064516 − max.qupm: 7

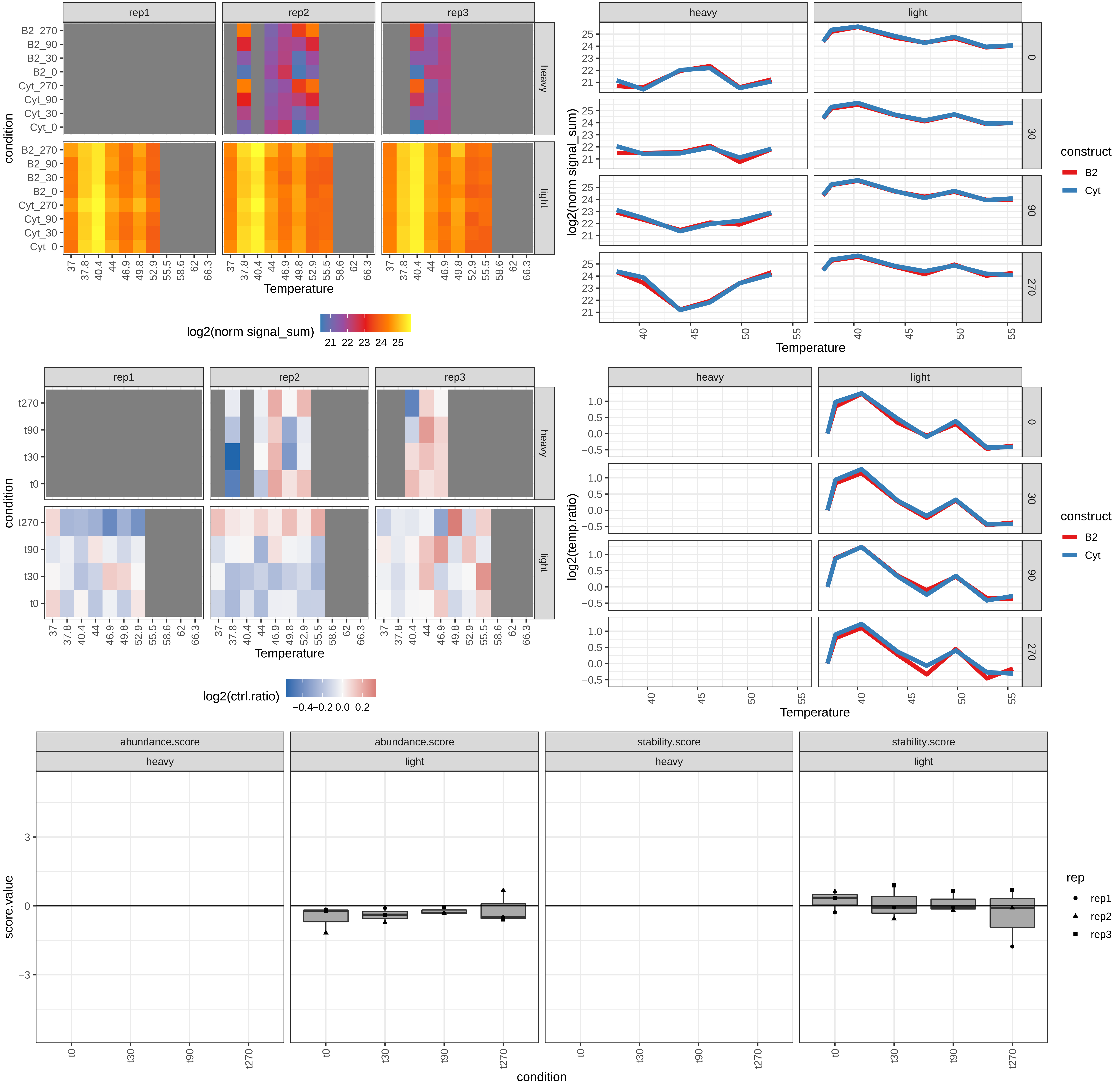

Q05515 − Survival factor 1 OS=Saccharomyces cerevisiae (strain ATCC 204508 / S288c) GN=SVF1 PE=1 SV=1

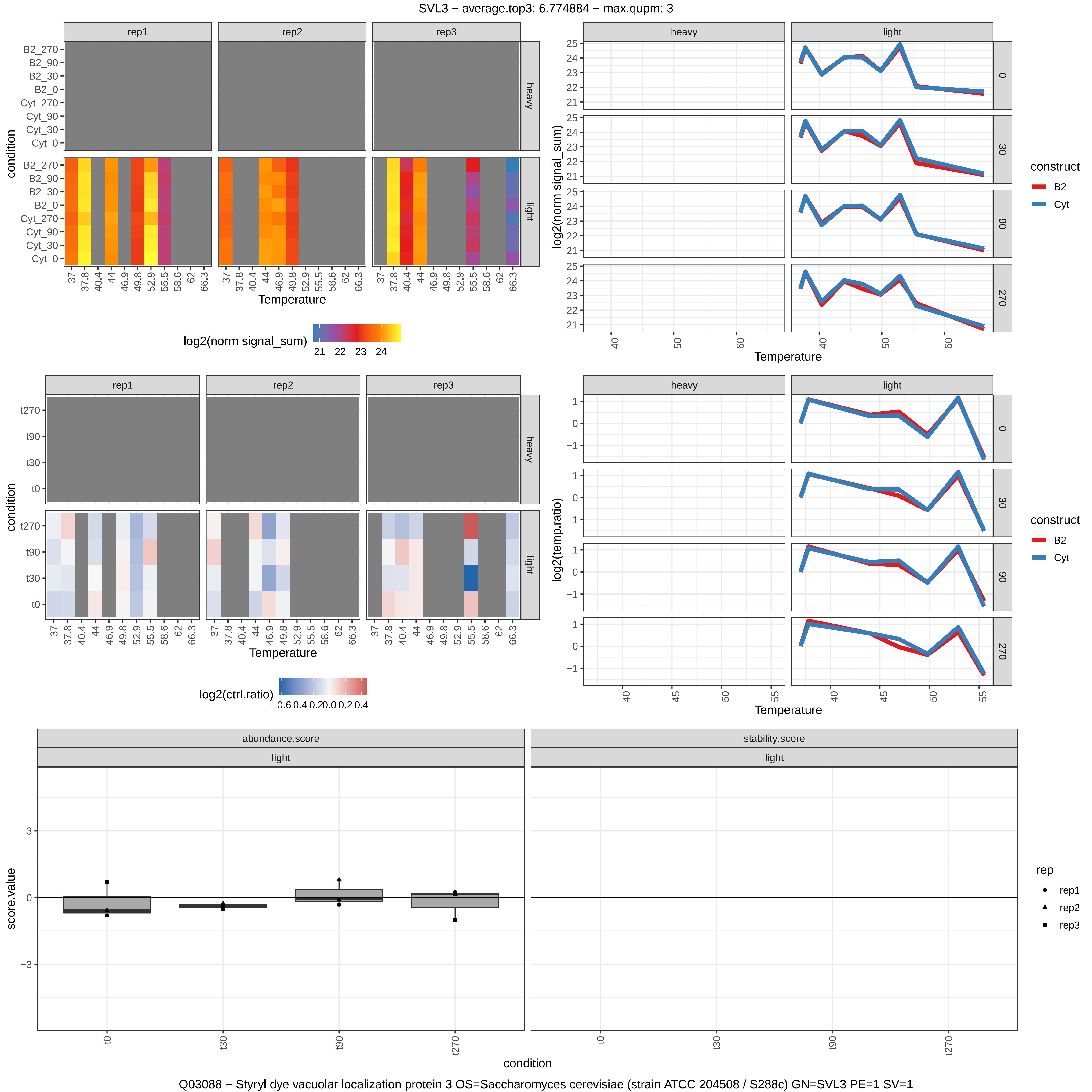

SVL3 − average.top3: 6.774884 − max.qupm: 3
rep1
rep2
rep3
heavy
light
condition
Temperature
log2(norm signal_sum)
log2(ctrl.ratio)
log2(temp.ratio)
construct
B2
Cyt
abundance.score
stability.score
score.value
rep
rep1
rep2
rep3
Q03088 − Styryl dye vacuolar localization protein 3 OS=Saccharomyces cerevisiae (strain ATCC 204508 / S288c) GN=SVL3 PE=1 SV=1

SVP26 − average.top3: 6.93006736842105 − max.qupm: 4

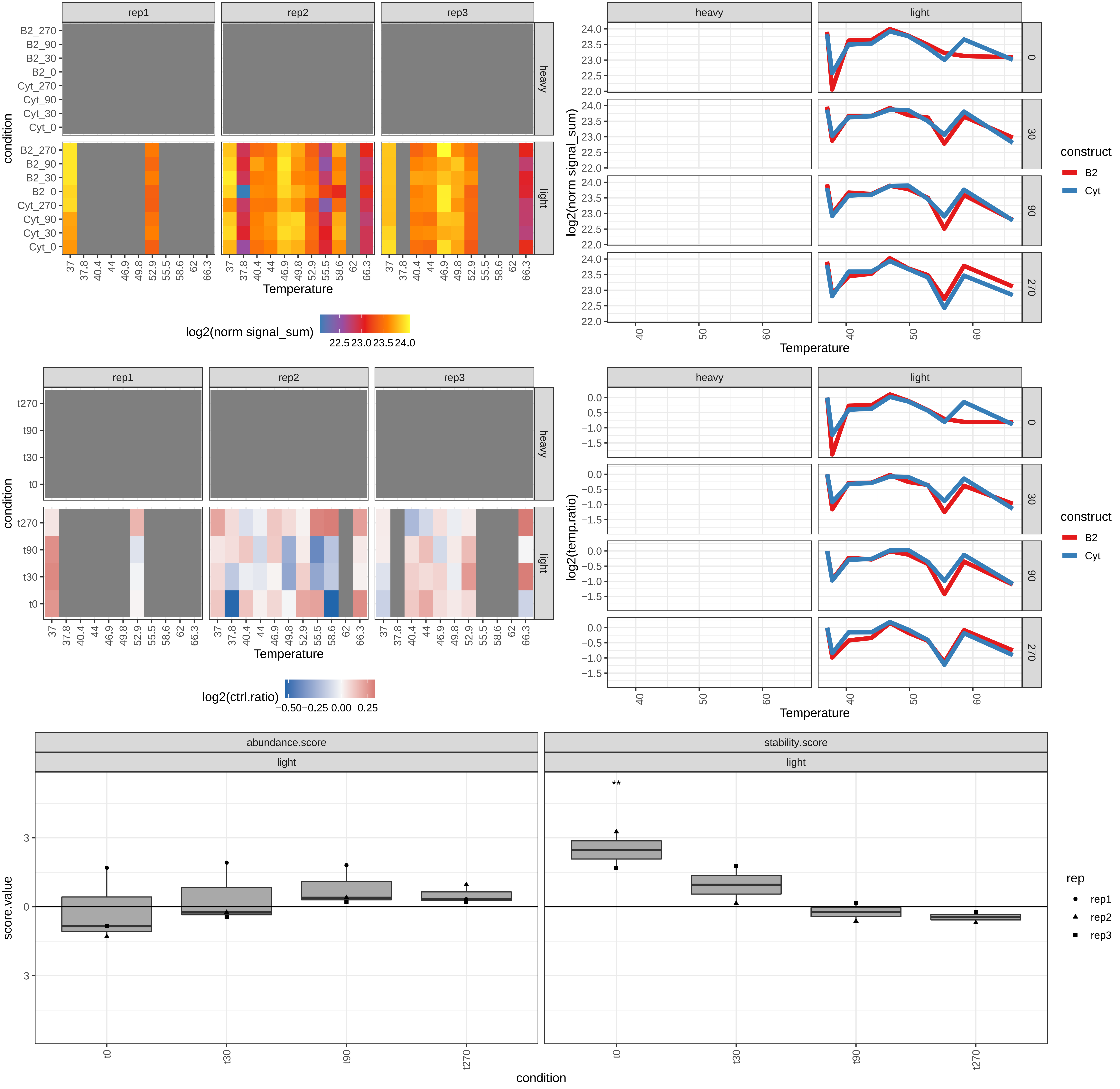

P38869 − Protein SVP26 OS=Saccharomyces cerevisiae (strain ATCC 204508 / S288c) GN=SVP26 PE=1 SV=1

SWA2 − average.top3: 6.62897375 − max.qupm: 3

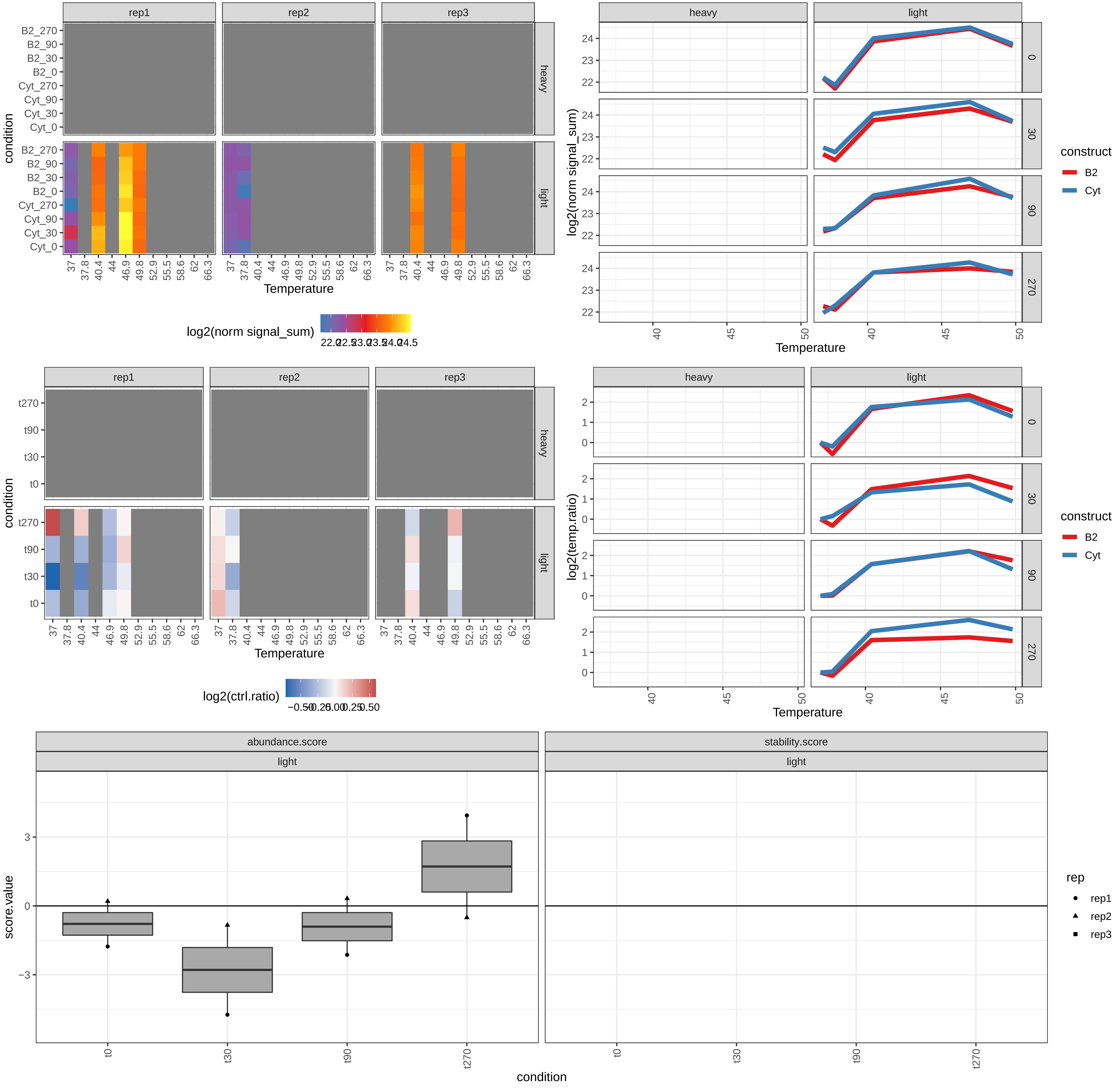

Q06677 − Auxilin−like clathrin uncoating factor SWA2 OS=Saccharomyces cerevisiae (strain ATCC 204508 / S288c) GN=SWA2 PE=1 SV=1

SWD2 − average.top3: 6.762858 − max.qupm: 5

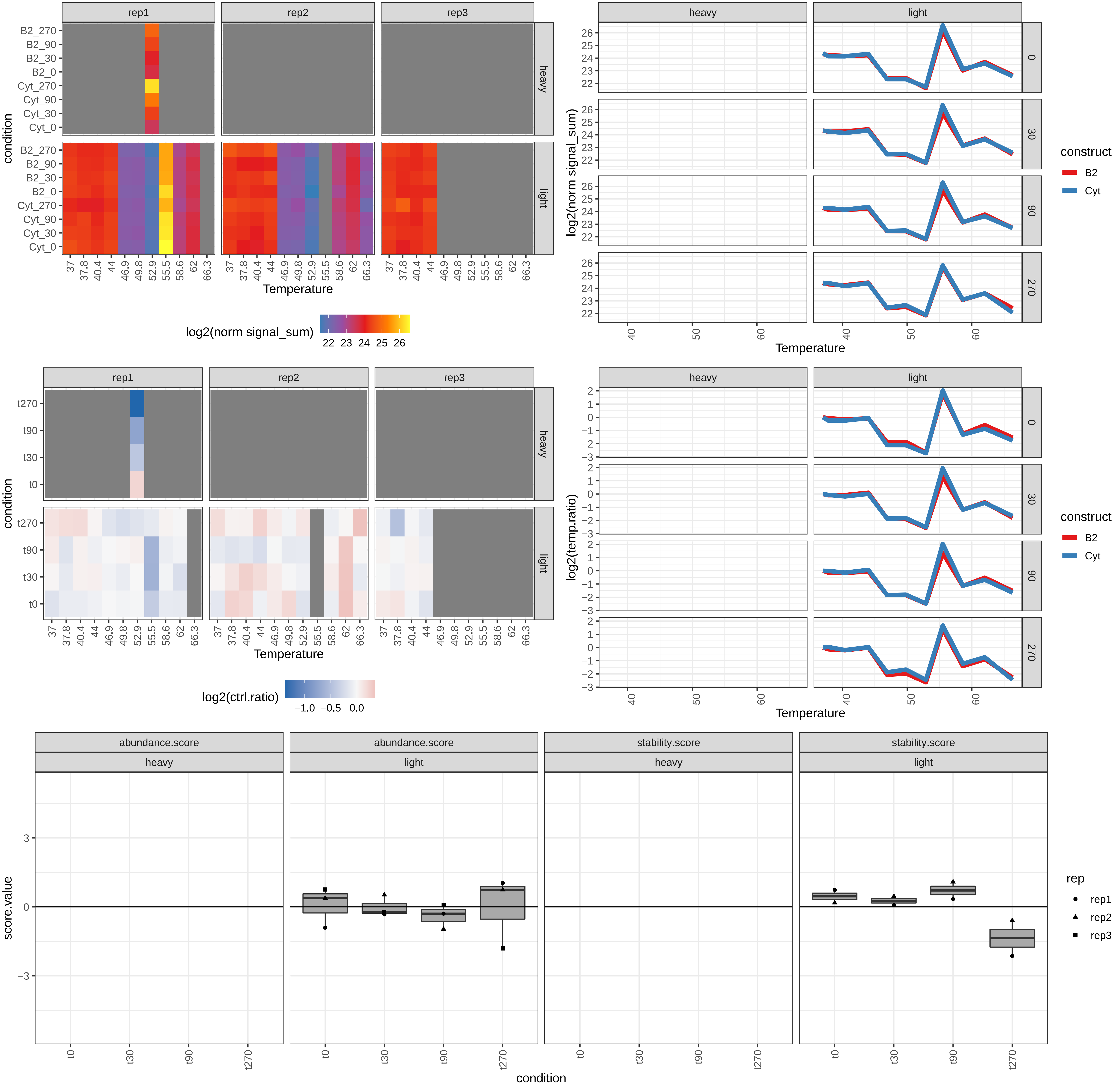

P36104 − COMPASS component SWD2 OS=Saccharomyces cerevisiae (strain ATCC 204508 / S288c) GN=SWD2 PE=1 SV=1

SWH1 − average.top3: 7.02733217391304 − max.qupm: 15

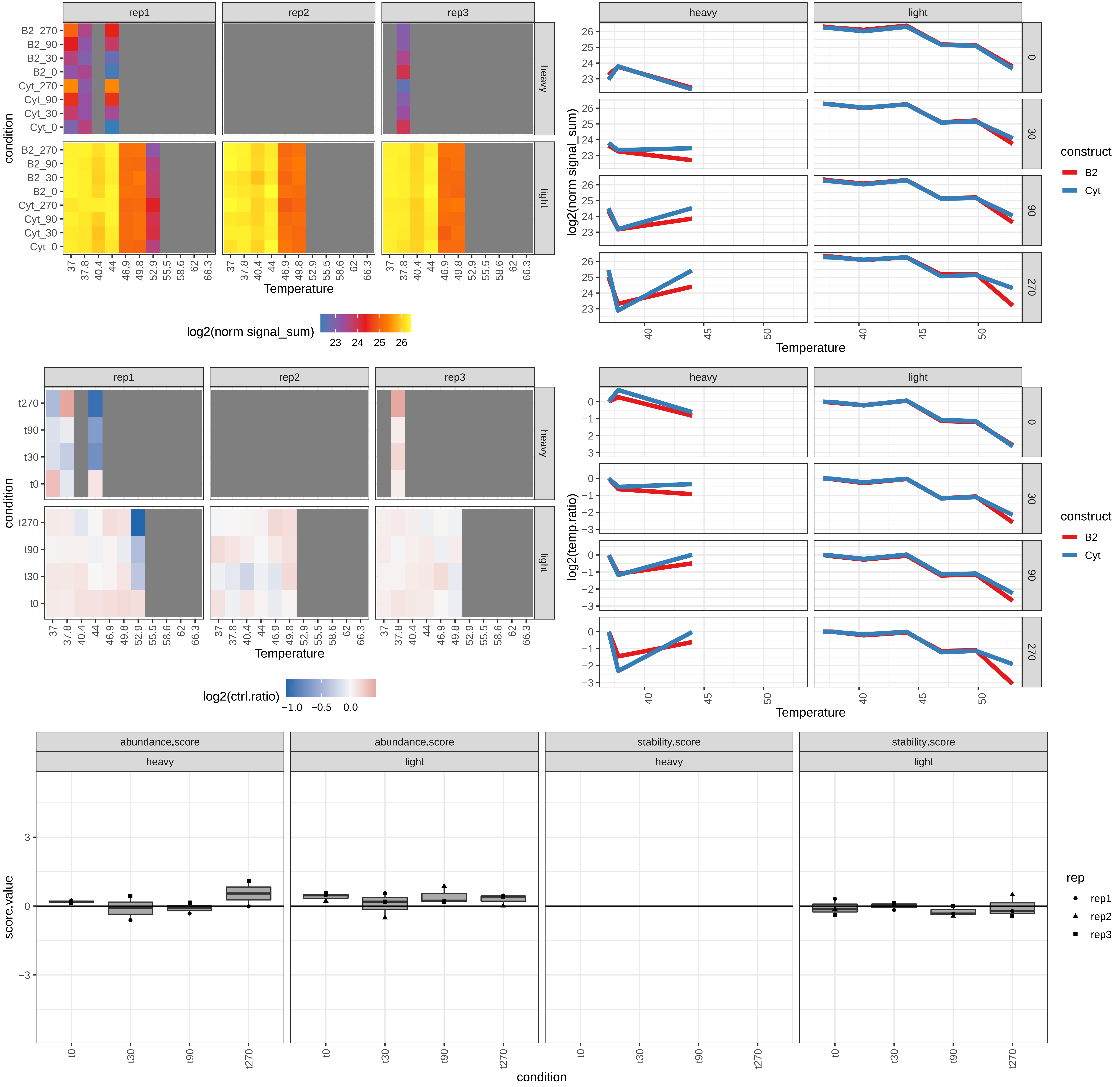

P35845 − Oxysterol−binding protein homolog 1 OS=Saccharomyces cerevisiae (strain ATCC 204508 / S288c) GN=SWH1 PE=1 SV=4

SWI1 − average.top3: 6.81448071428571 − max.qupm: 8

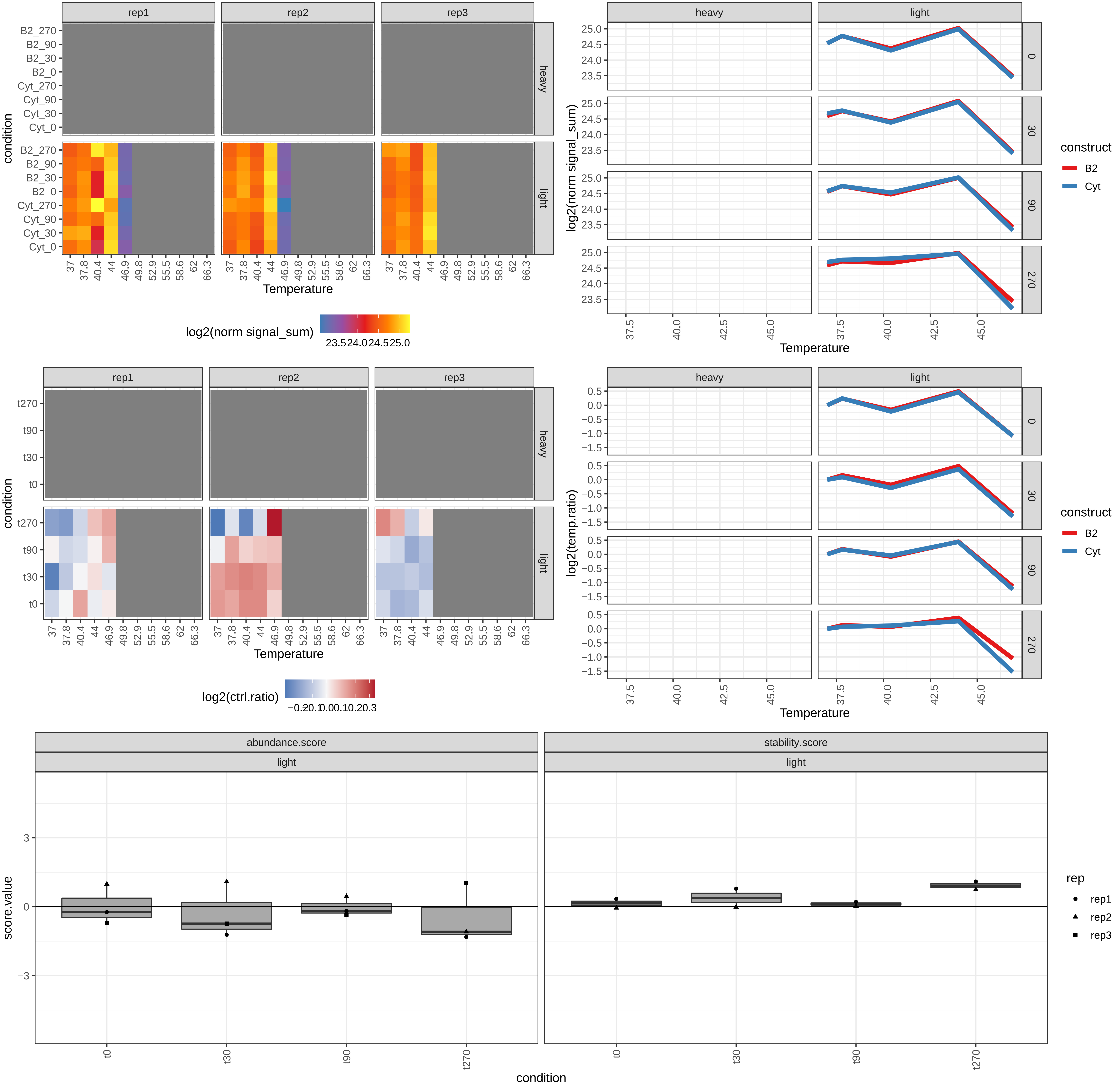

P09547 − SWI/SNF chromatin−remodeling complex subunit SWI1 OS=Saccharomyces cerevisiae (strain ATCC 204508 / S288c) GN=SWI1 PE=1 SV=1

SWI3 − average.top3: 6.62490875 − max.qupm: 9

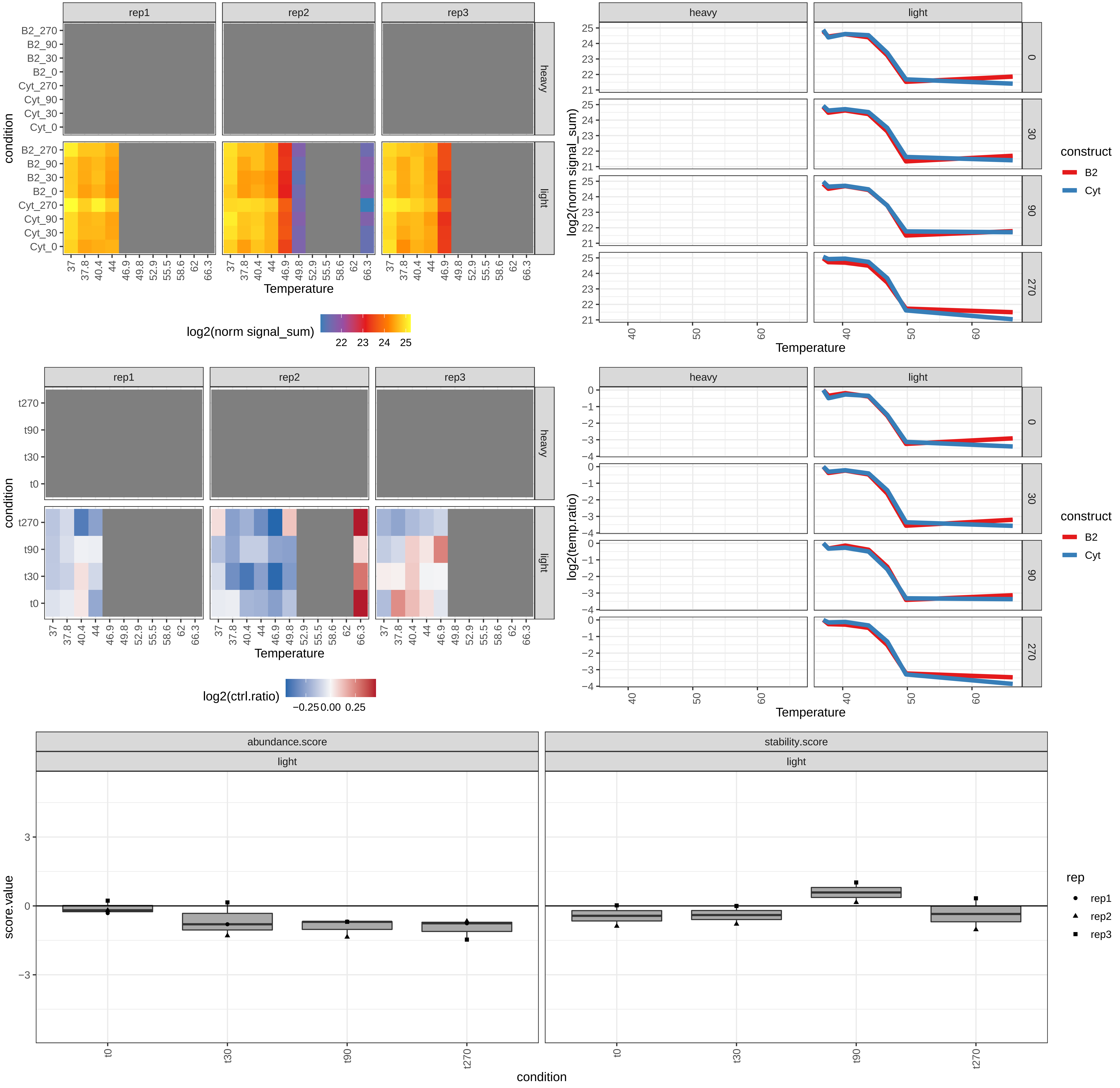

P32591 − SWI/SNF complex subunit SWI3 OS=Saccharomyces cerevisiae (strain ATCC 204508 / S288c) GN=SWI3 PE=1 SV=1

SWI6 − average.top3: 6.22905142857143 − max.qupm: 4

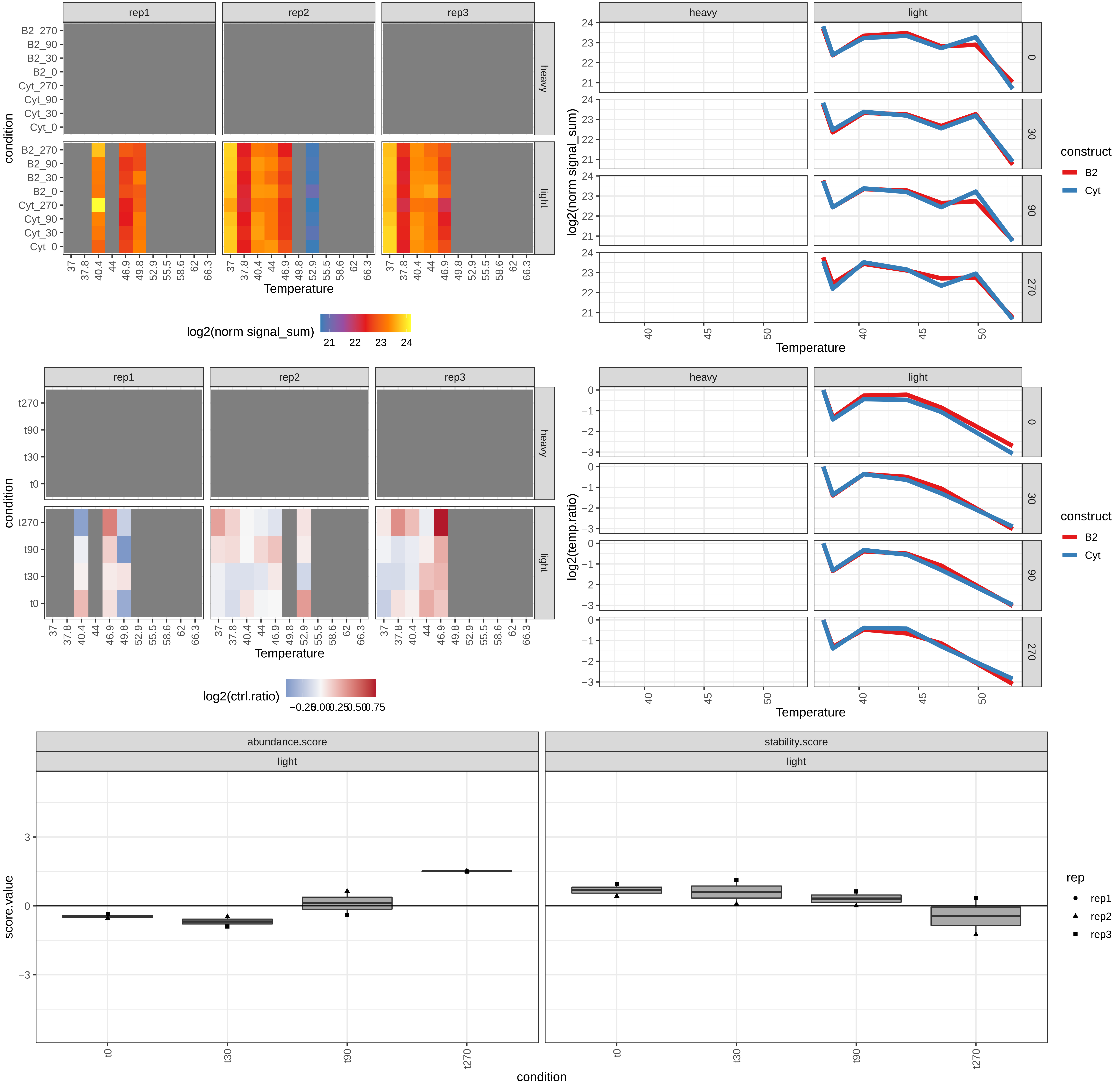

P09959 − Regulatory protein SWI6 OS=Saccharomyces cerevisiae (strain ATCC 204508 / S288c) GN=SWI6 PE=1 SV=1

SWP1 − average.top3: 6.87812578947368 − max.qupm: 3

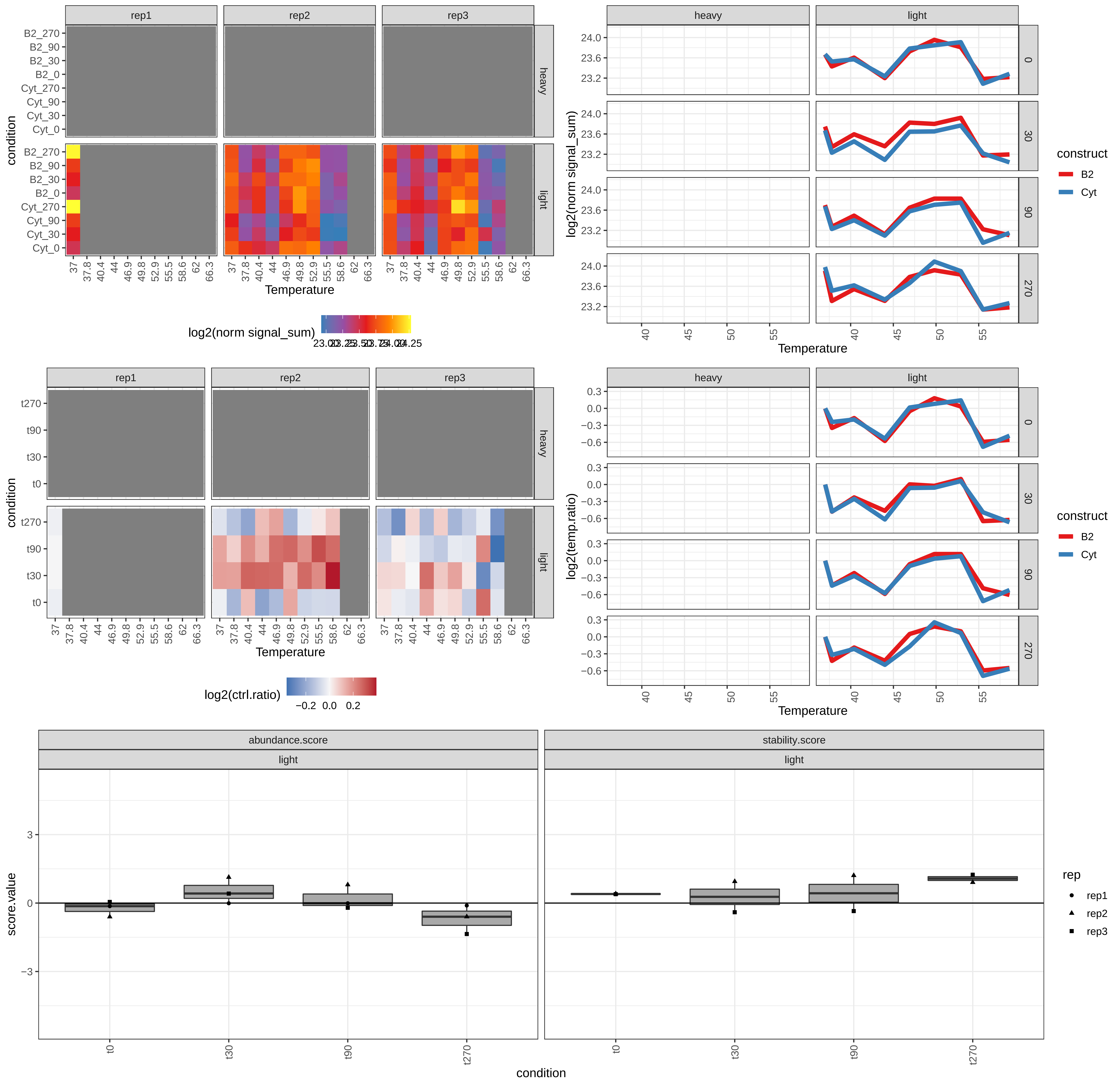

Q02795 − Dolichyl−diphosphooligosaccharide−−protein glycosyltransferase subunit SWP1 OS=Saccharomyces cerevisiae (strain ATCC 204508 / S288c) GN=SWP1 PE=1 SV=1

SXM1 − average.top3: 6.65397859649123 − max.qupm: 11

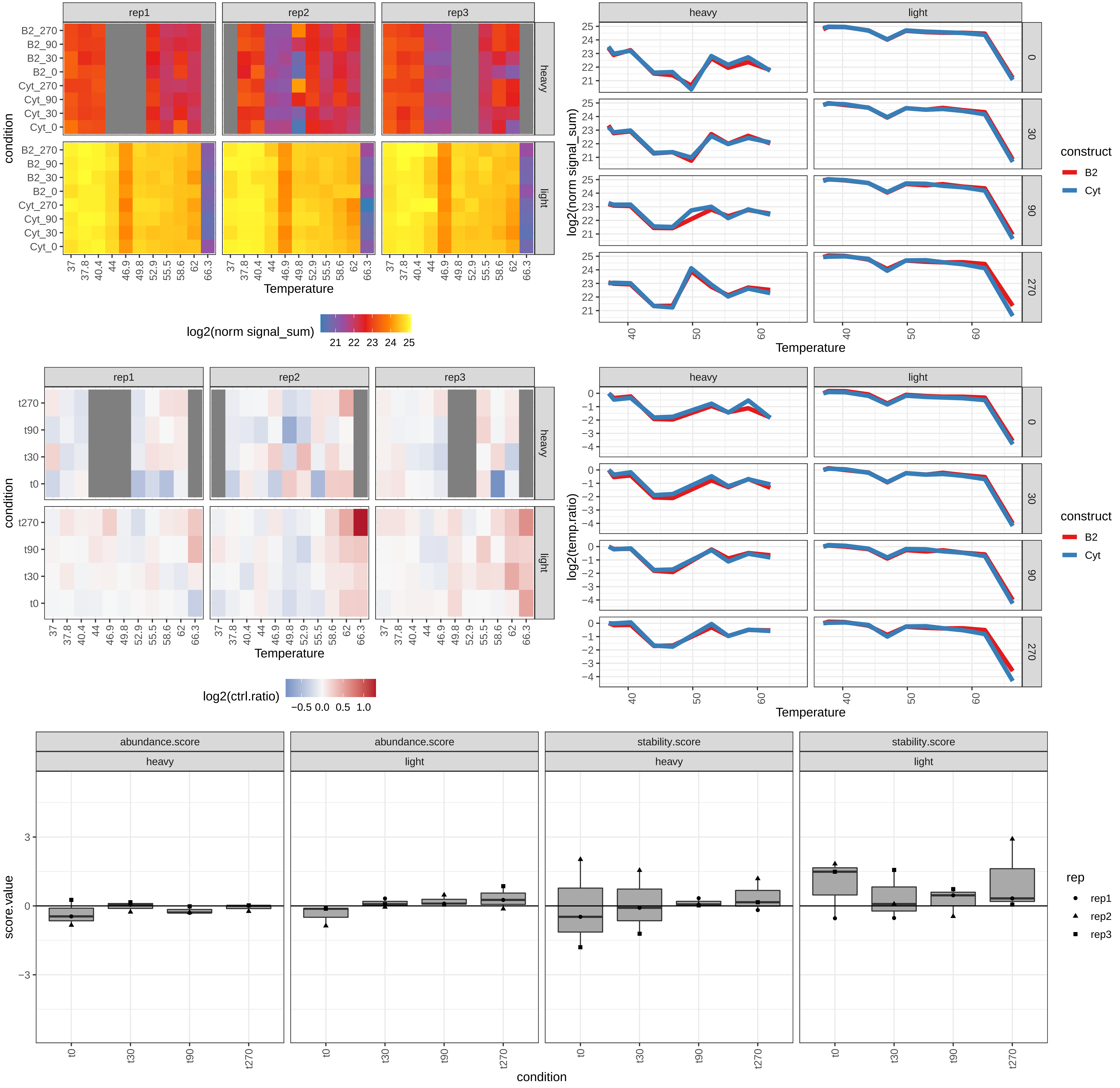

Q04175 − Importin beta SMX1 OS=Saccharomyces cerevisiae (strain ATCC 204508 / S288c) GN=SXM1 PE=1 SV=1

SYC1 − average.top3: 6.53114352941176 − max.qupm: 4

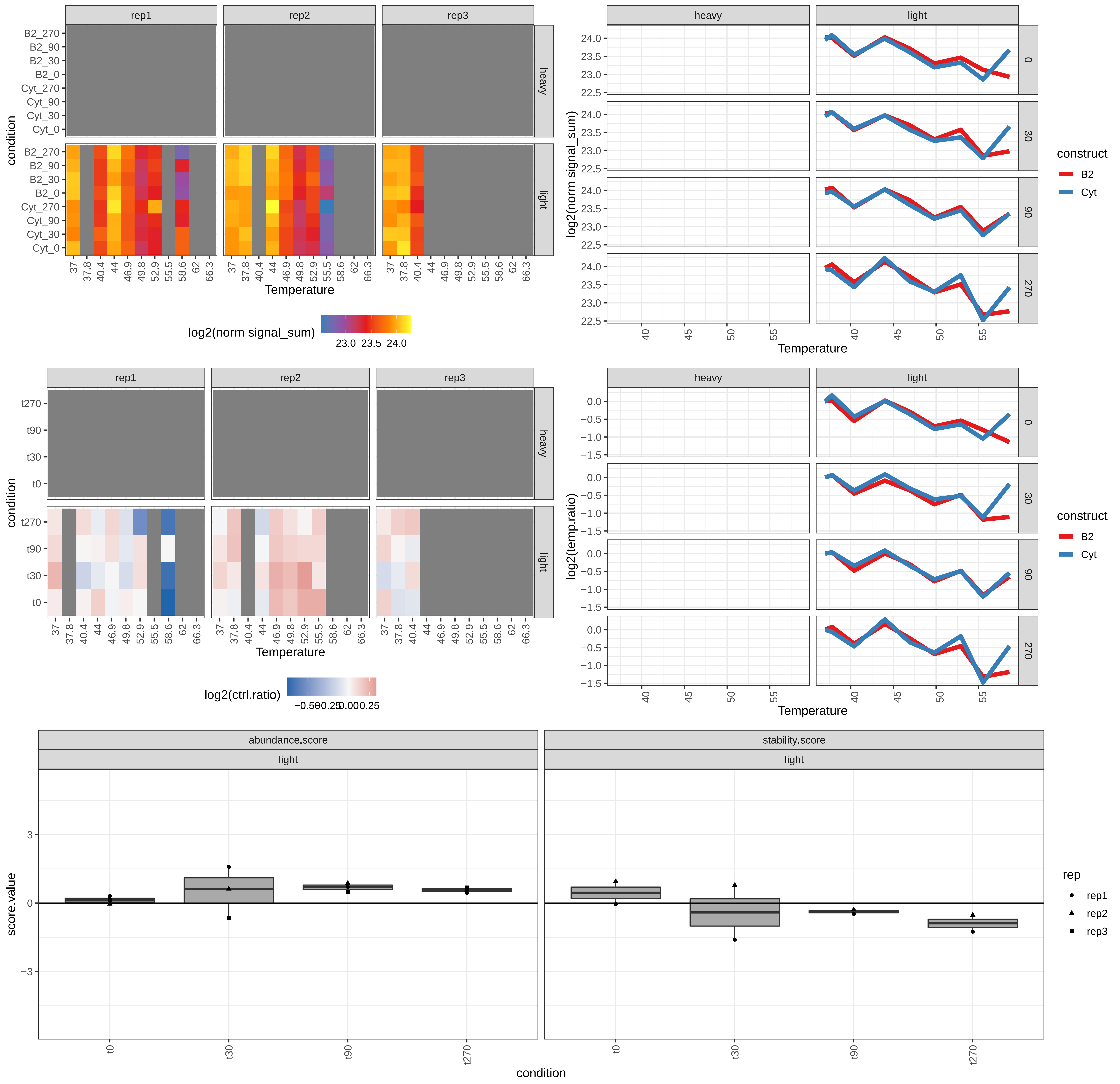

Q08553 − Protein SYC1 OS=Saccharomyces cerevisiae (strain ATCC 204508 / S288c) GN=SYC1 PE=1 SV=1

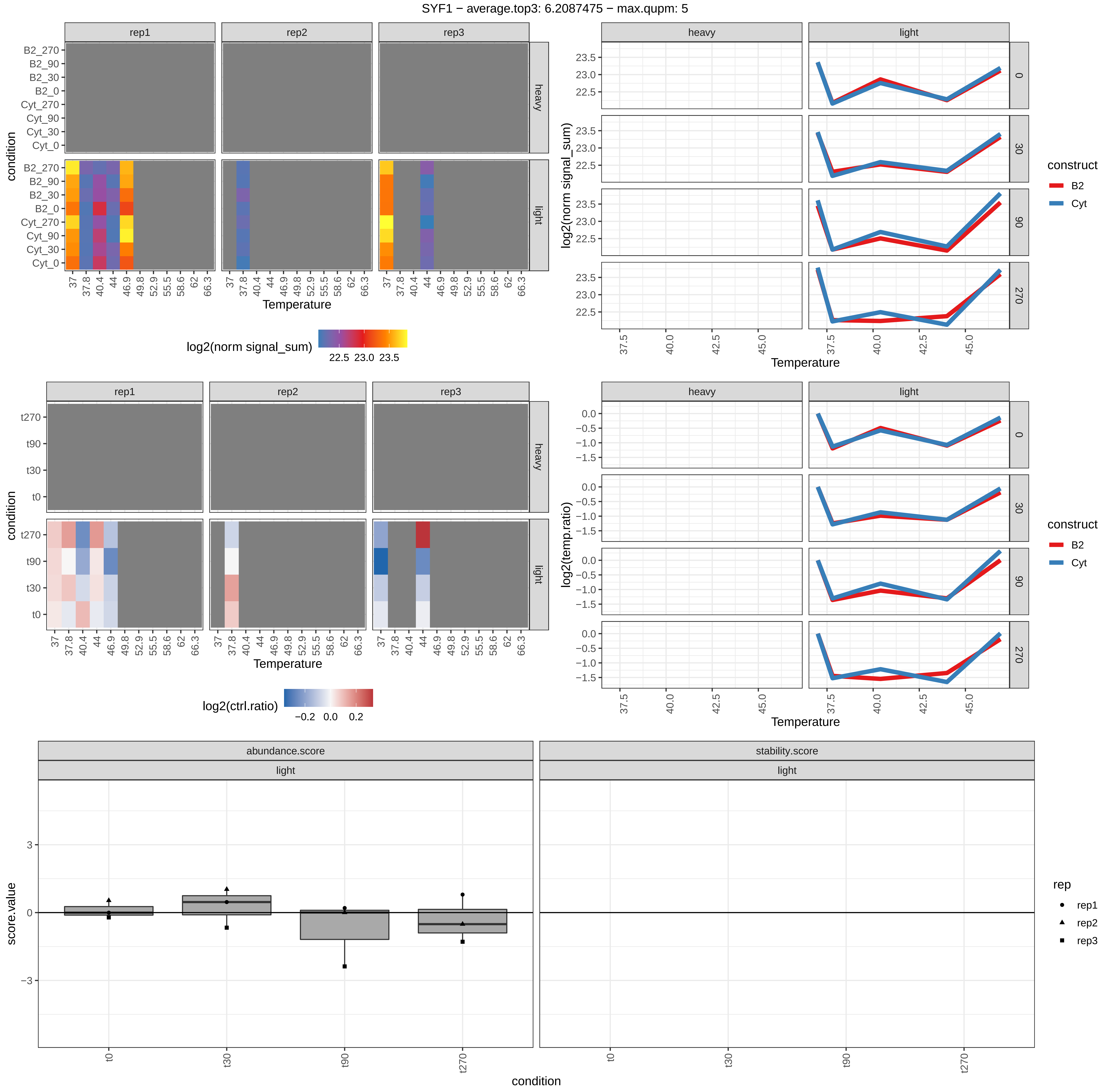

Q04048 − Pre−mRNA−splicing factor SYF1 OS=Saccharomyces cerevisiae (strain ATCC 204508 / S288c) GN=SYF1 PE=1 SV=1

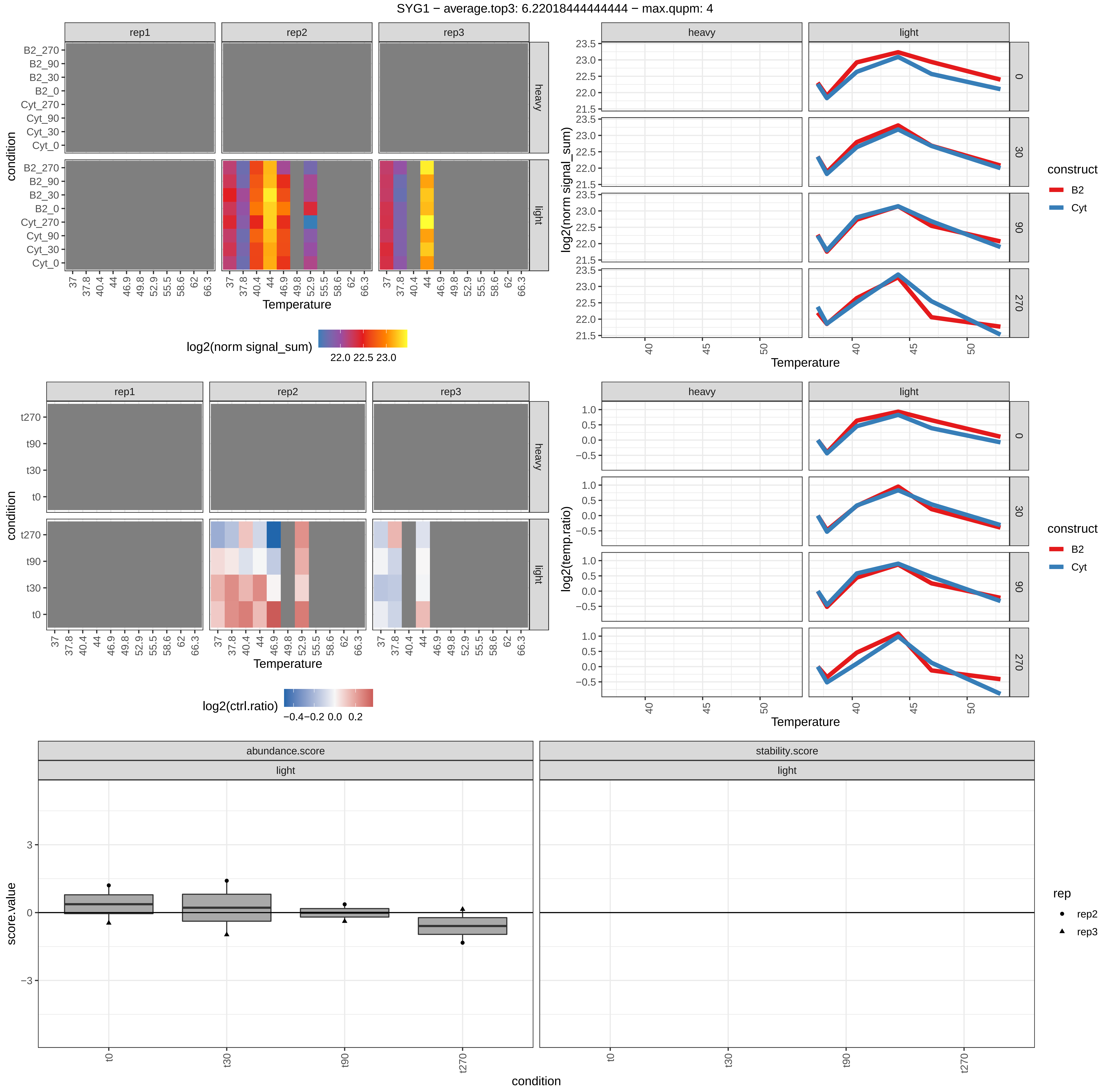

P40528 − Protein SYG1 OS=Saccharomyces cerevisiae (strain ATCC 204508 / S288c) GN=SYG1 PE=1 SV=1

SYH1 − average.top3: 6.72435333333333 − max.qupm: 2

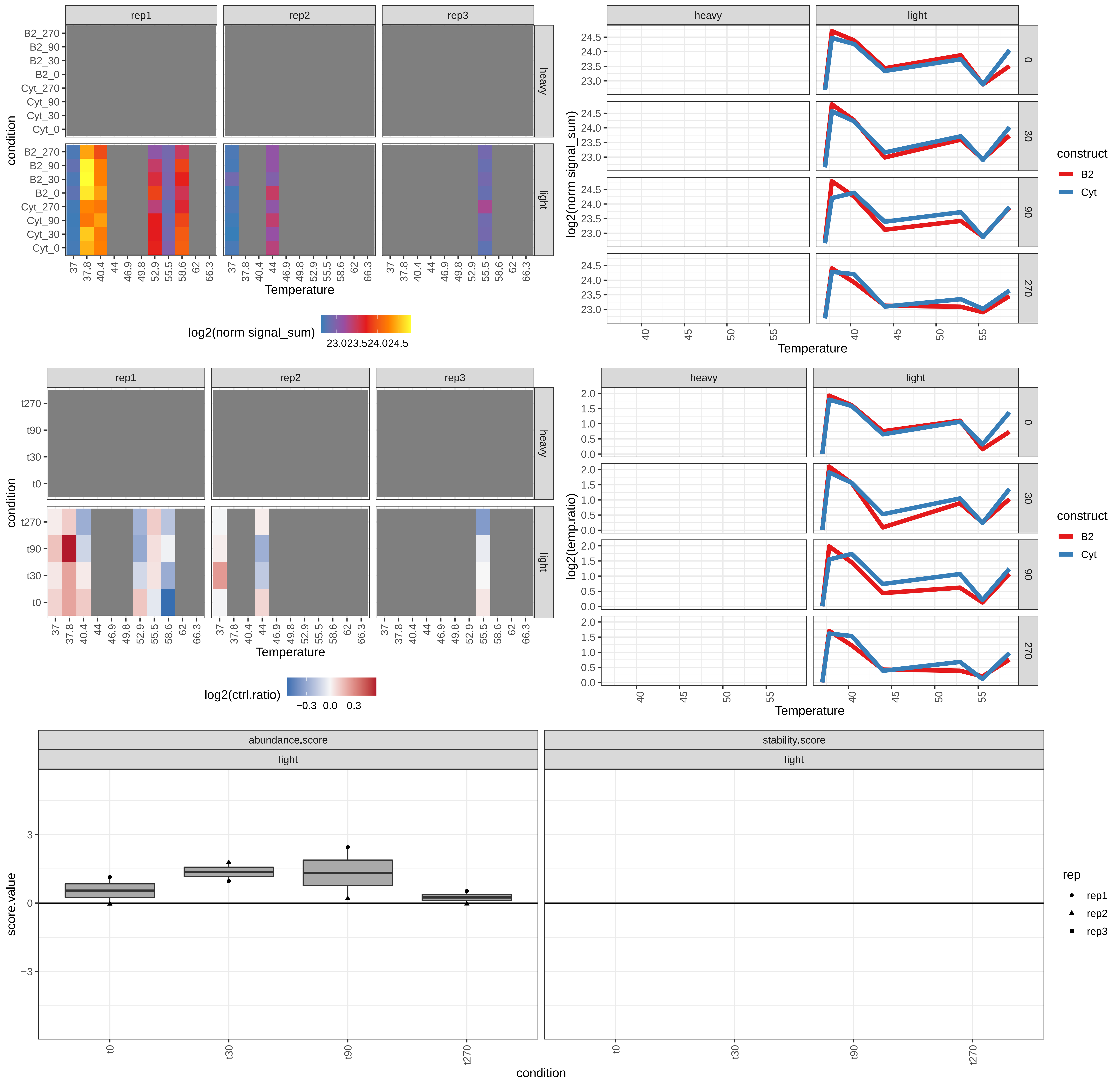

Q02875 − SMY2 homolog 2 OS=Saccharomyces cerevisiae (strain ATCC 204508 / S288c) GN=SYH1 PE=1 SV=1

SYP1 − average.top3: 6.98254966666667 − max.qupm: 10

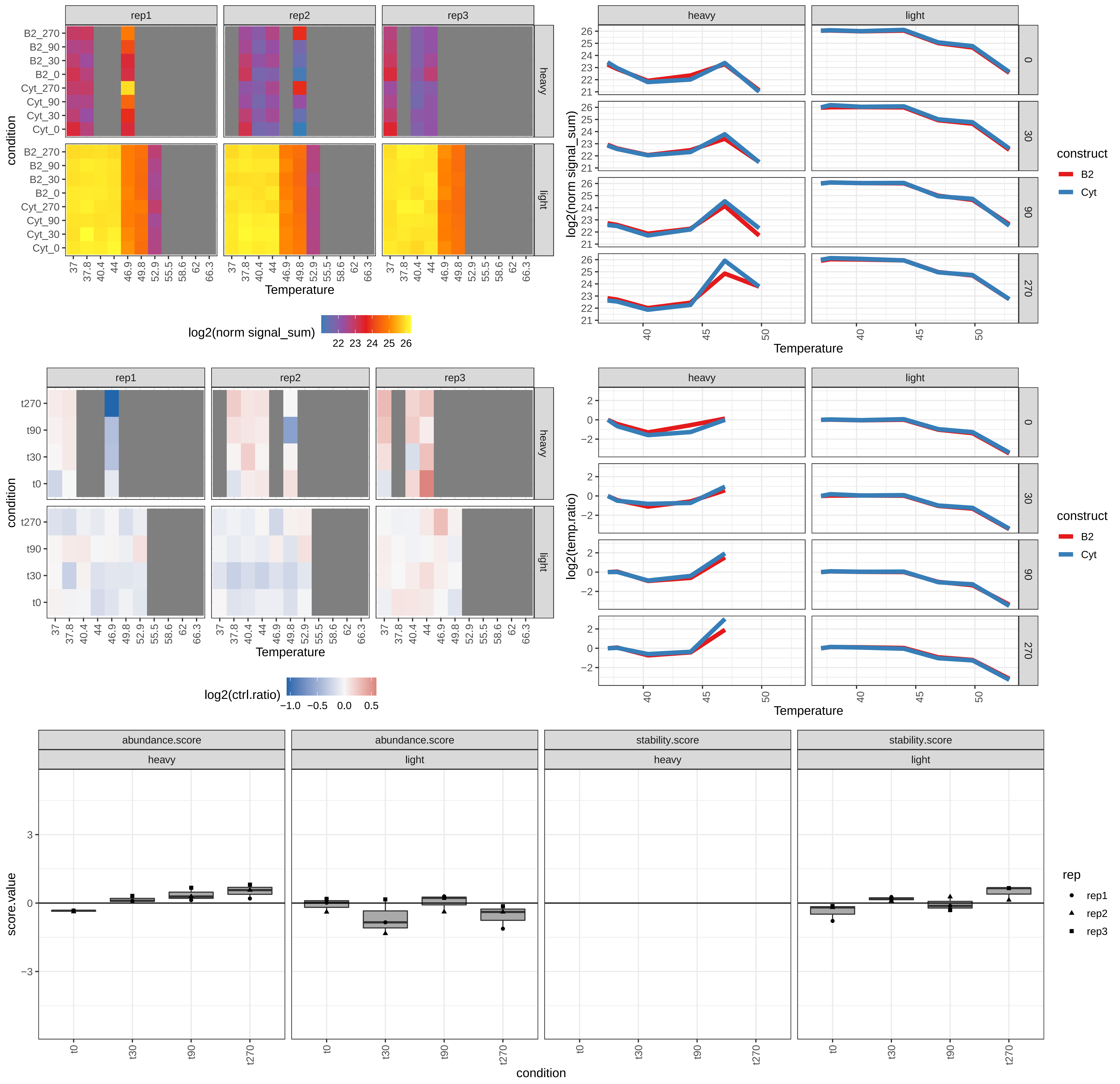

P25623 − Suppressor of yeast profilin deletion OS=Saccharomyces cerevisiae (strain ATCC 204508 / S288c) GN=SYP1 PE=1 SV=3

TAF1 − average.top3: 6.60218153846154 − max.qupm: 5

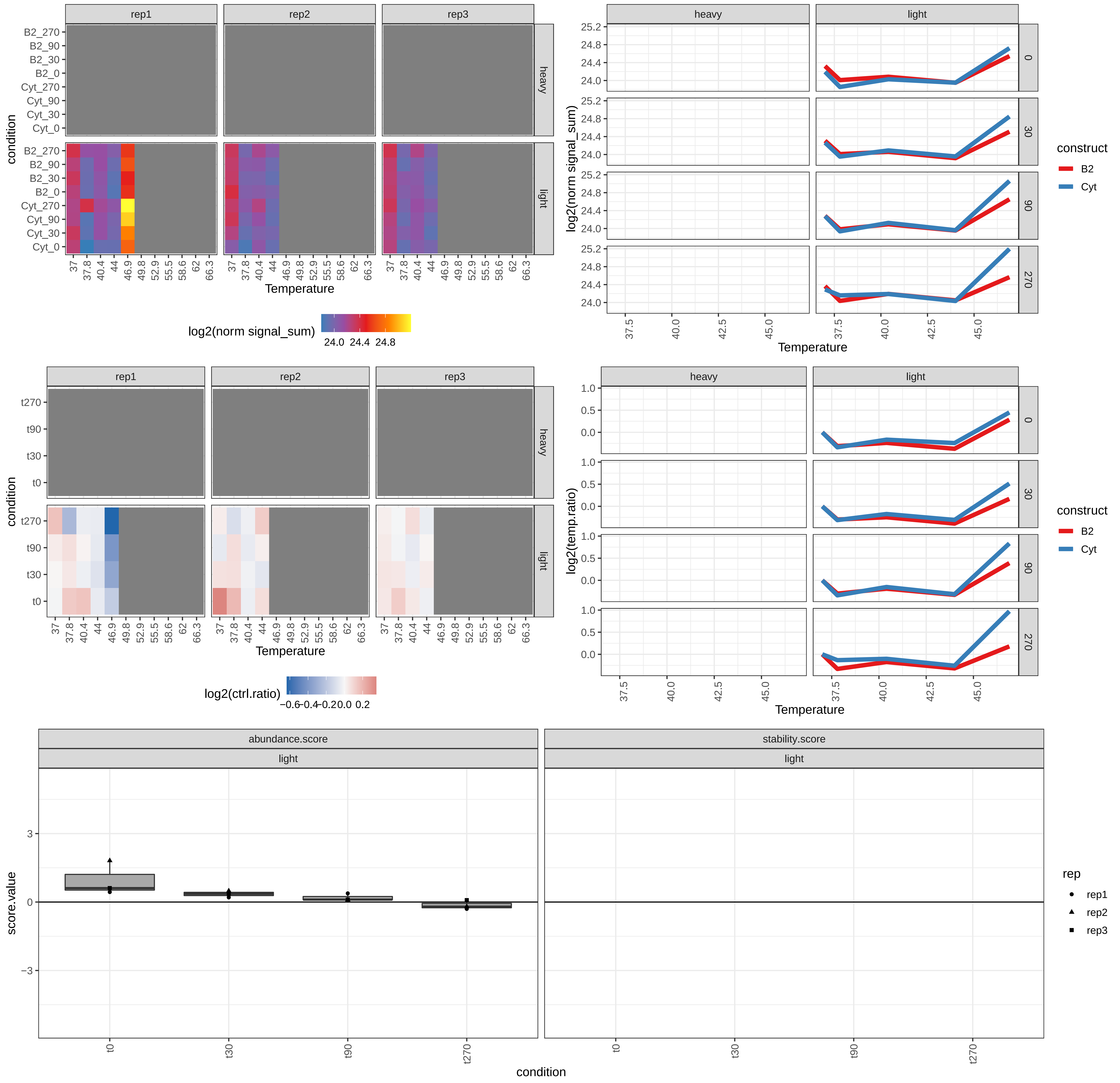

P46677 − Transcription initiation factor TFIID subunit 1 OS=Saccharomyces cerevisiae (strain ATCC 204508 / S288c) GN=TAF1 PE=1 SV=1

TAF11 − average.top3: 6.54998444444444 − max.qupm: 3

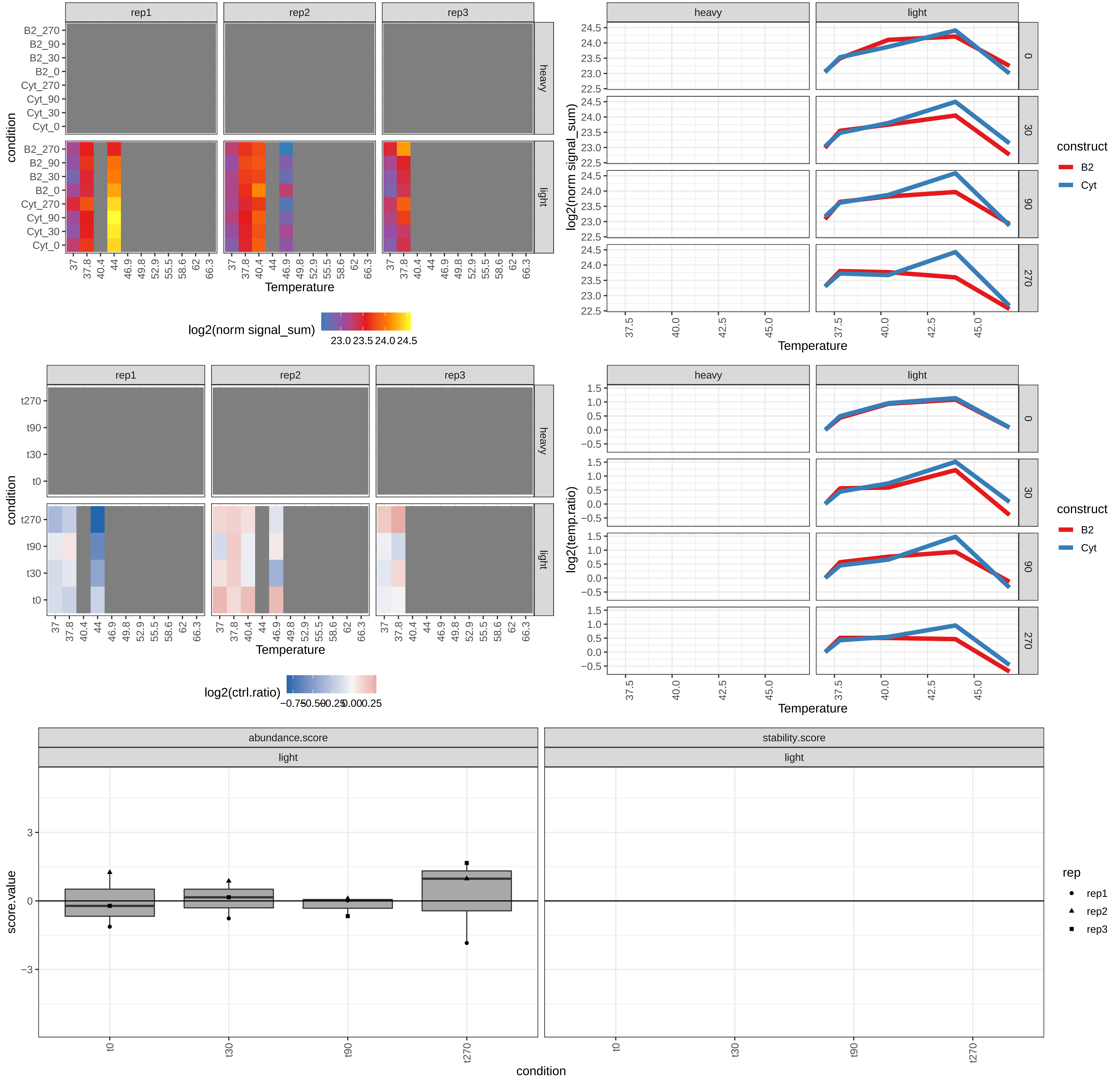

Q04226 − Transcription initiation factor TFIID subunit 11 OS=Saccharomyces cerevisiae (strain ATCC 204508 / S288c) GN=TAF11 PE=1 SV=1

TAF12 − average.top3: 6.790175 − max.qupm: 5

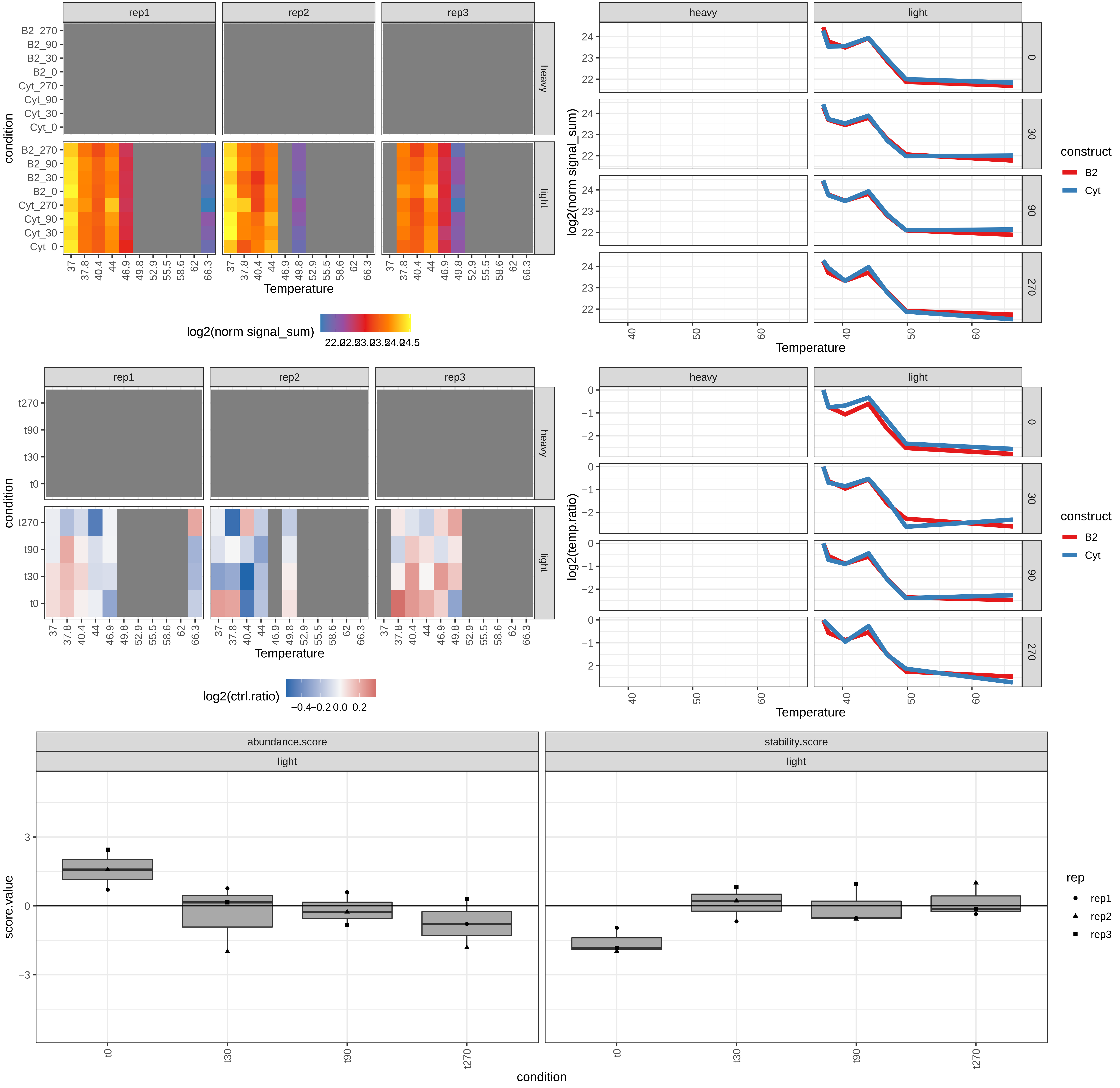

Q03761 − Transcription initiation factor TFIID subunit 12 OS=Saccharomyces cerevisiae (strain ATCC 204508 / S288c) GN=TAF12 PE=1 SV=1

TAF14 − average.top3: 6.72495357142857 − max.qupm: 4

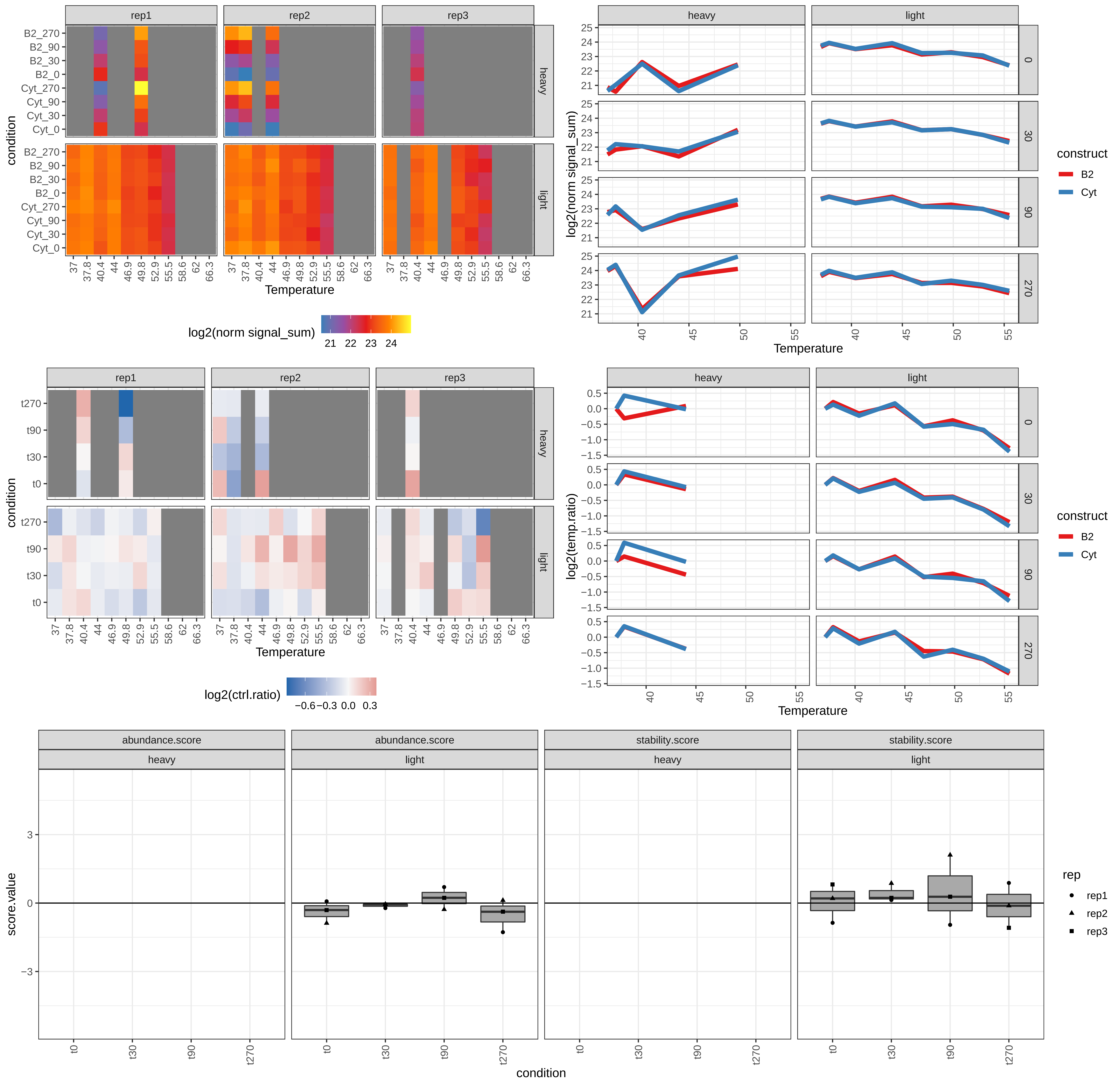

P35189 − Transcription initiation factor TFIID subunit 14 OS=Saccharomyces cerevisiae (strain ATCC 204508 / S288c) GN=TAF14 PE=1 SV=1

TAF2 − average.top3: 6.61448192307692 − max.qupm: 15

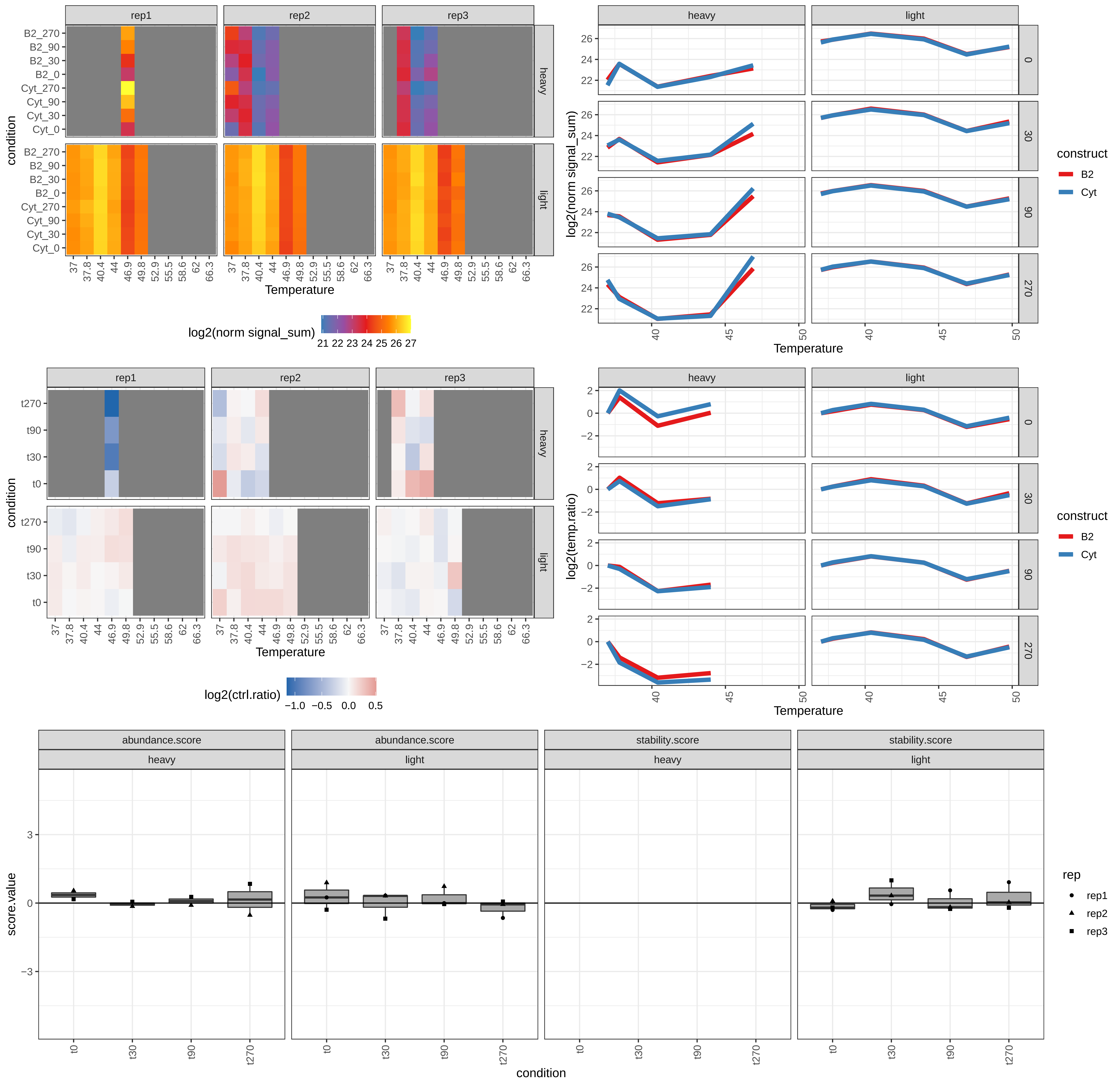

P23255 − Transcription initiation factor TFIID subunit 2 OS=Saccharomyces cerevisiae (strain ATCC 204508 / S288c) GN=TAF2 PE=1 SV=3

TAF4 − average.top3: 6.52345636363636 − max.qupm: 4

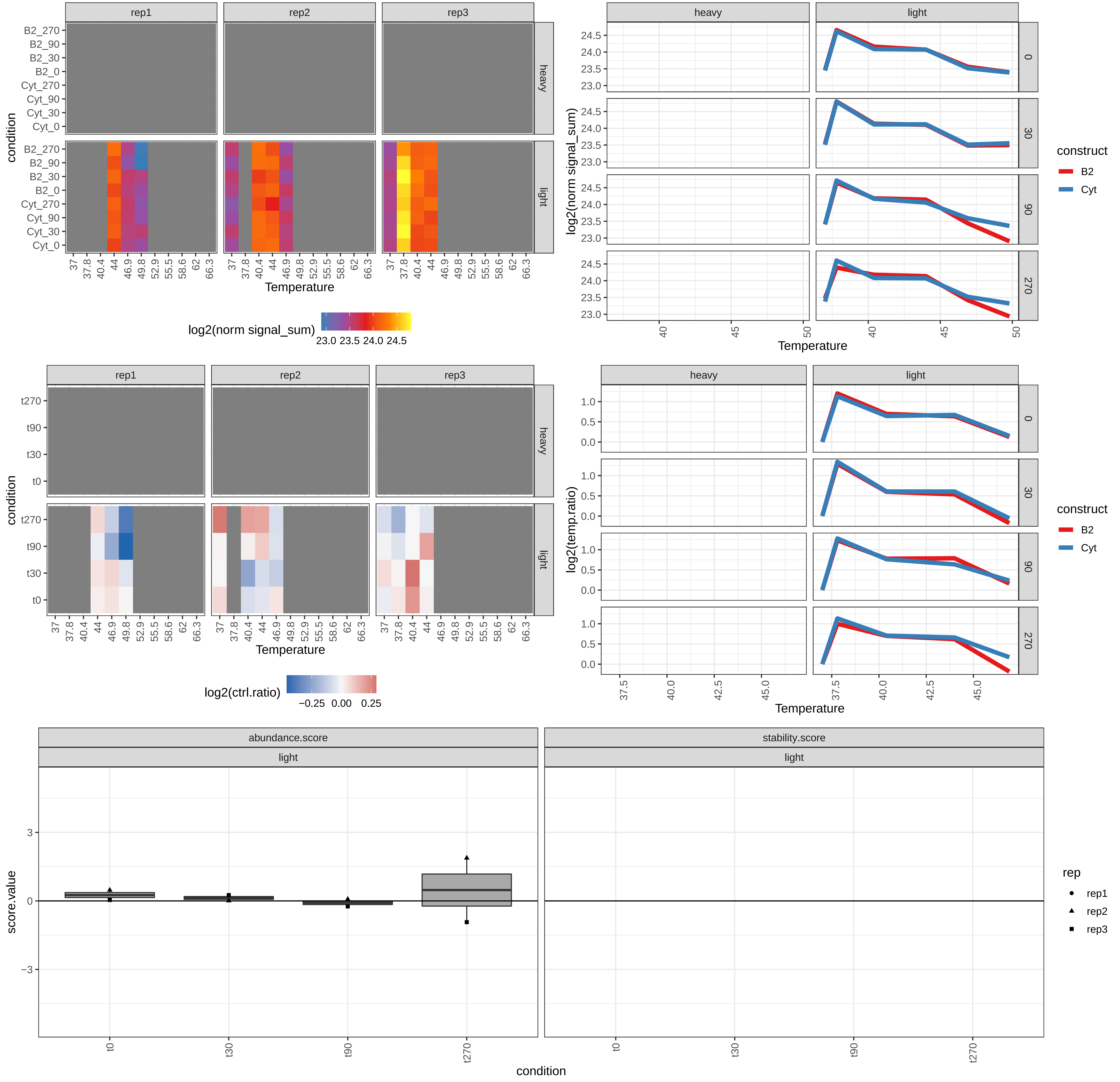

P50105 − Transcription initiation factor TFIID subunit 4 OS=Saccharomyces cerevisiae (strain ATCC 204508 / S288c) GN=TAF4 PE=1 SV=1

TAF5 − average.top3: 6.82197411764706 − max.qupm: 9

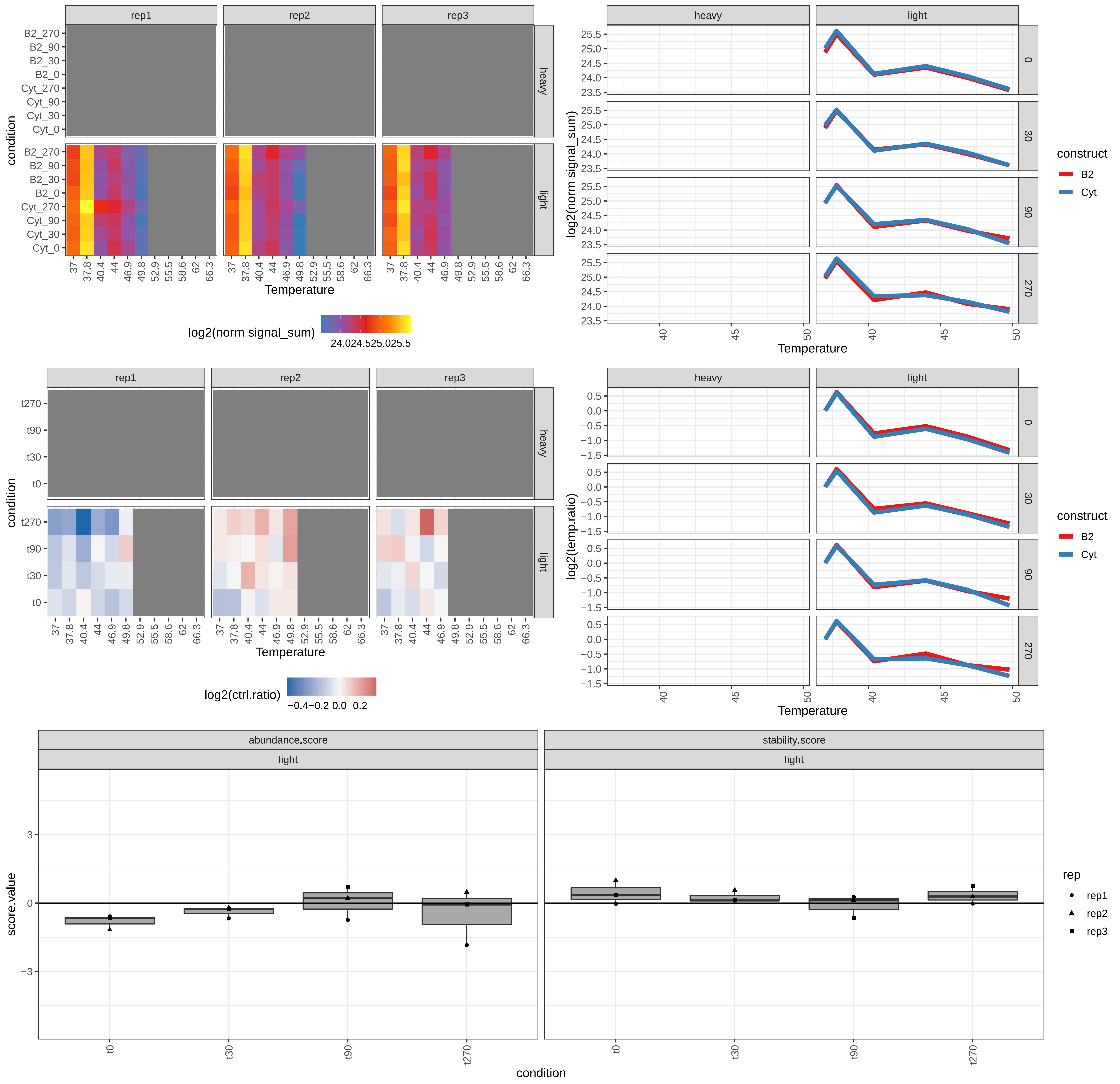

P38129 − Transcription initiation factor TFIID subunit 5 OS=Saccharomyces cerevisiae (strain ATCC 204508 / S288c) GN=TAF5 PE=1 SV=1

TAF6 − average.top3: 6.941375625 − max.qupm: 6

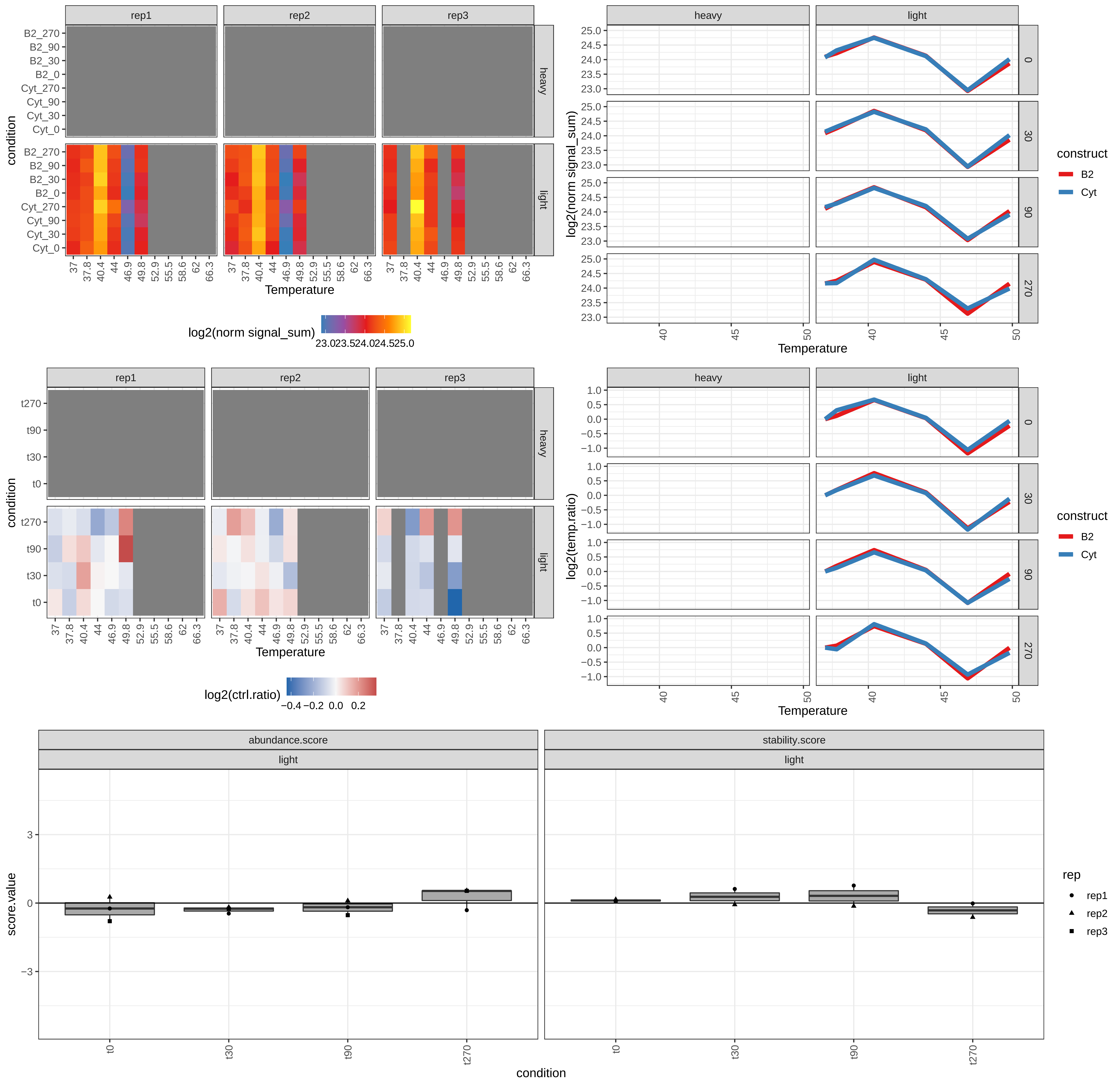

P53040 − Transcription initiation factor TFIID subunit 6 OS=Saccharomyces cerevisiae (strain ATCC 204508 / S288c) GN=TAF6 PE=1 SV=1

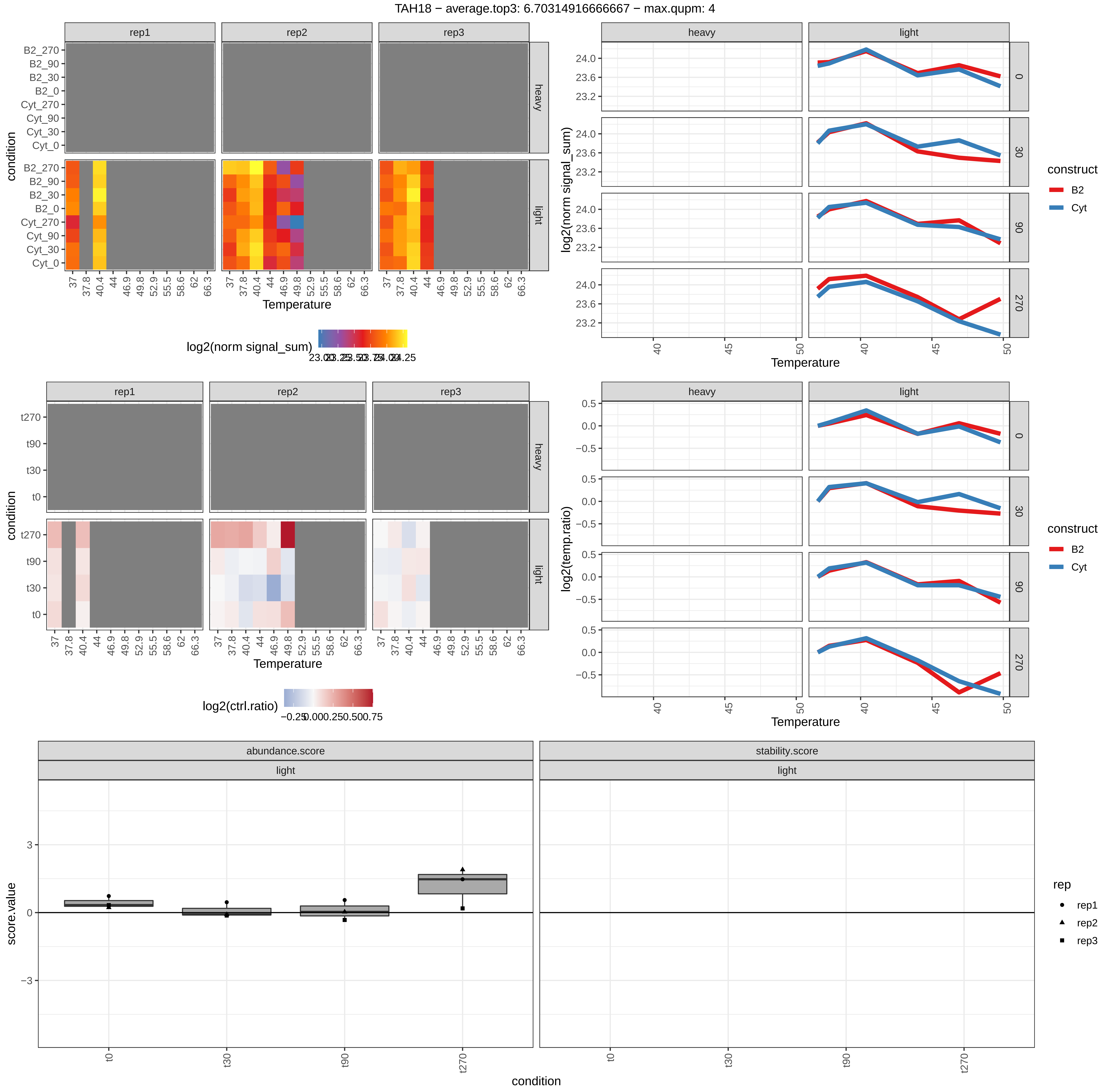

Q12181 − NADPH−dependent diflavin oxidoreductase 1 OS=Saccharomyces cerevisiae (strain ATCC 204508 / S288c) GN=TAH18 PE=1 SV=1

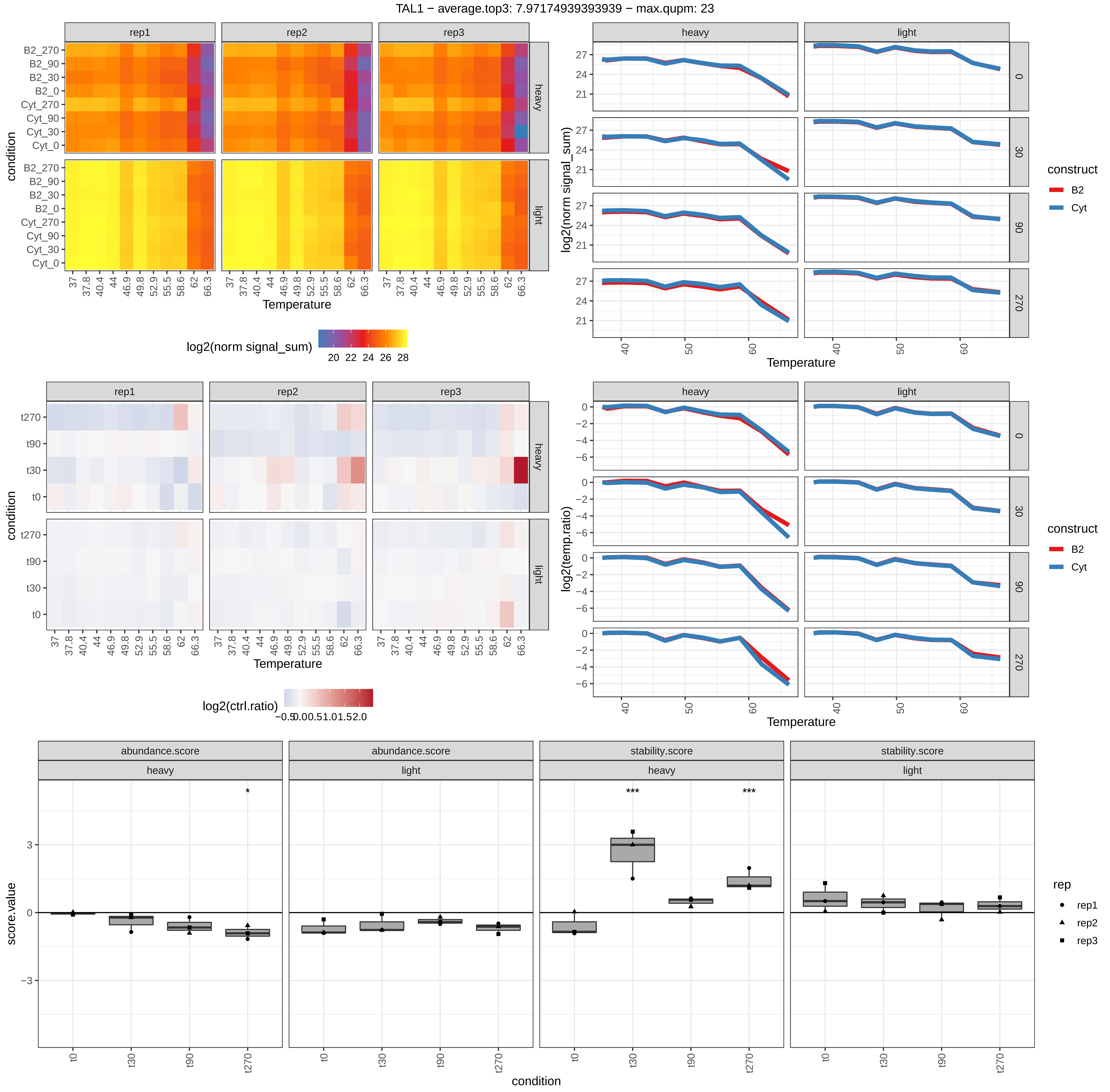

P15019 − Transaldolase OS=Saccharomyces cerevisiae (strain ATCC 204508 / S288c) GN=TAL1 PE=1 SV=4

TAN1 − average.top3: 6.89180447368421 − max.qupm: 13

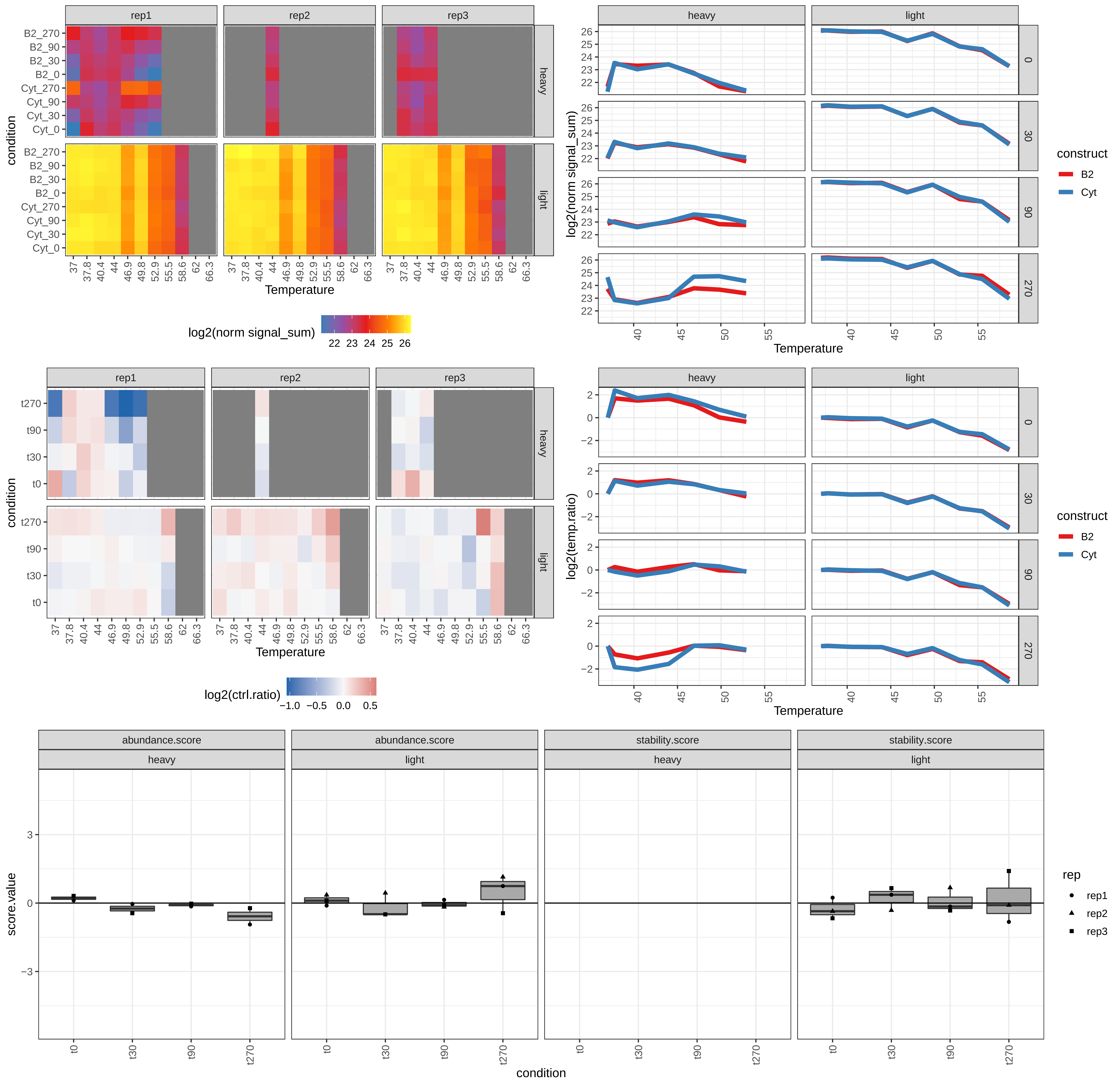

P53072 − tRNA acetyltransferase TAN1 OS=Saccharomyces cerevisiae (strain ATCC 204508 / S288c) GN=TAN1 PE=1 SV=1

TAO3 − average.top3: 6.651924375 − max.qupm: 8

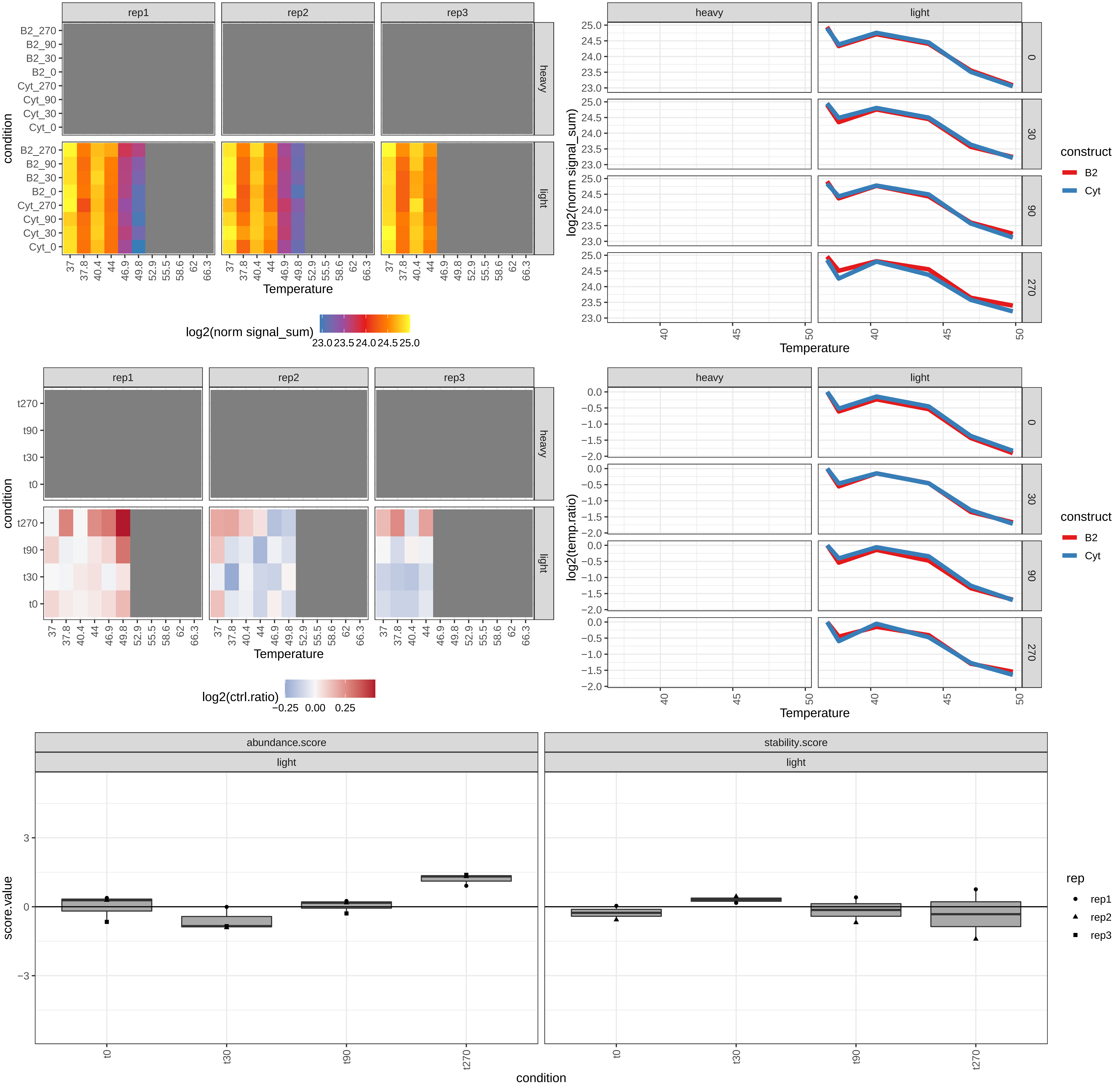

P40468 − Cell morphogenesis protein PAG1 OS=Saccharomyces cerevisiae (strain ATCC 204508 / S288c) GN=TAO3 PE=1 SV=1

TAT1 − average.top3: 6.99509976190476 − max.qupm: 5

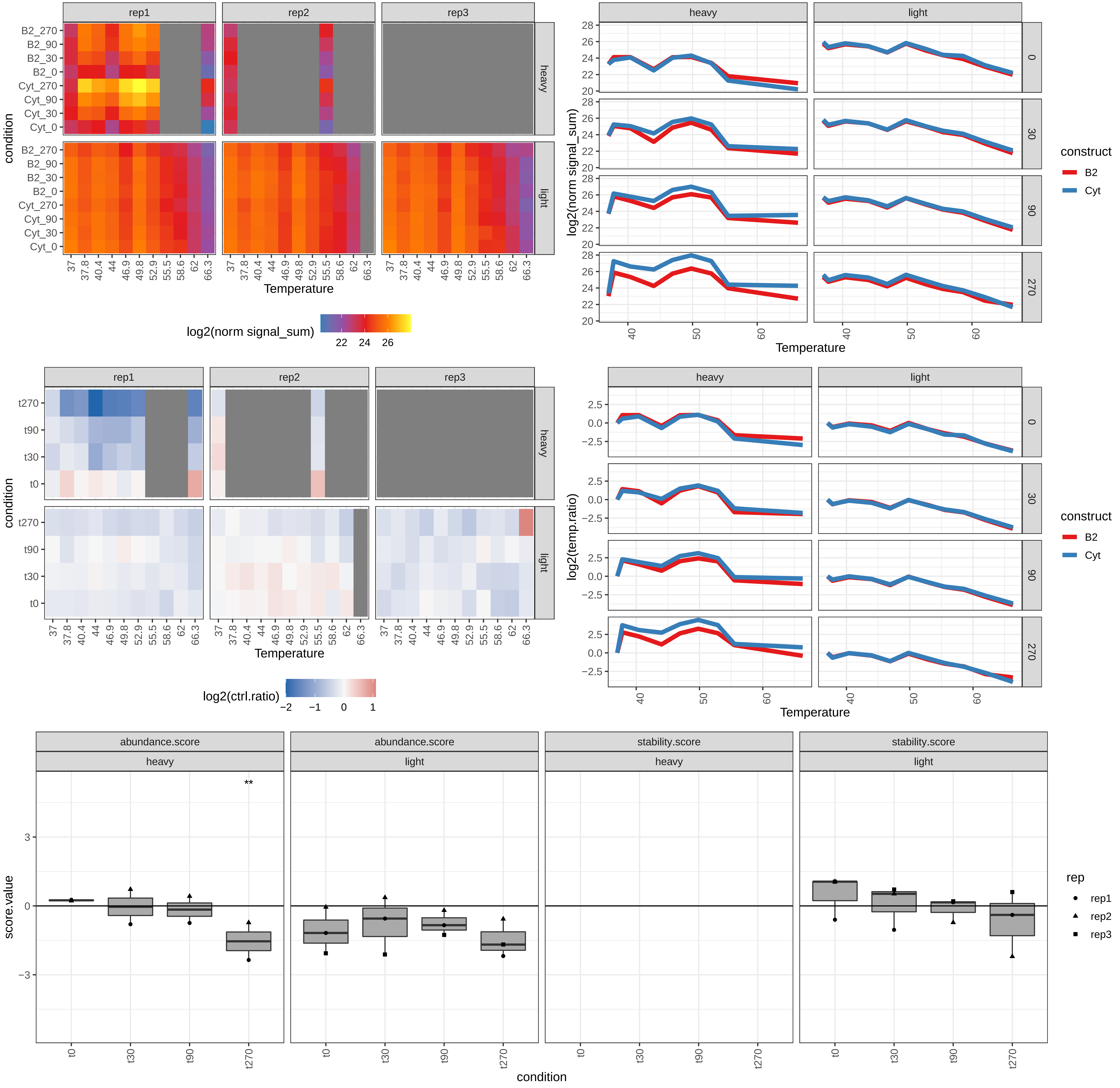

P38085 − Valine/tyrosine/tryptophan amino−acid permease 1 OS=Saccharomyces cerevisiae (strain ATCC 204508 / S288c) GN=TAT1 PE=1 SV=1

TAT2 − average.top3: 6.74690076923077 − max.qupm: 3

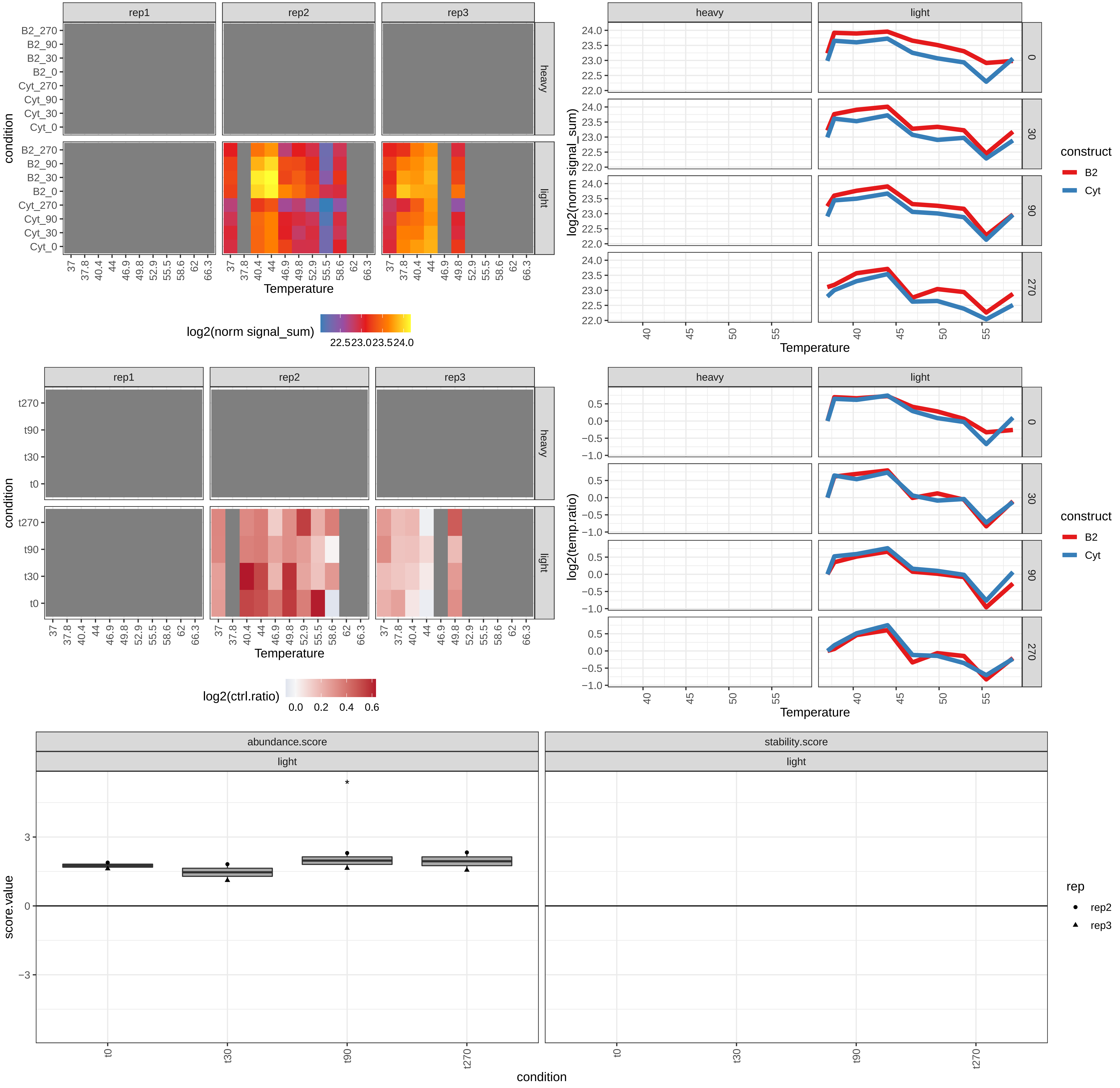

P38967 − Tryptophan permease OS=Saccharomyces cerevisiae (strain ATCC 204508 / S288c) GN=TAT2 PE=1 SV=1

TAZ1 − average.top3: 6.79850125 − max.qupm: 7

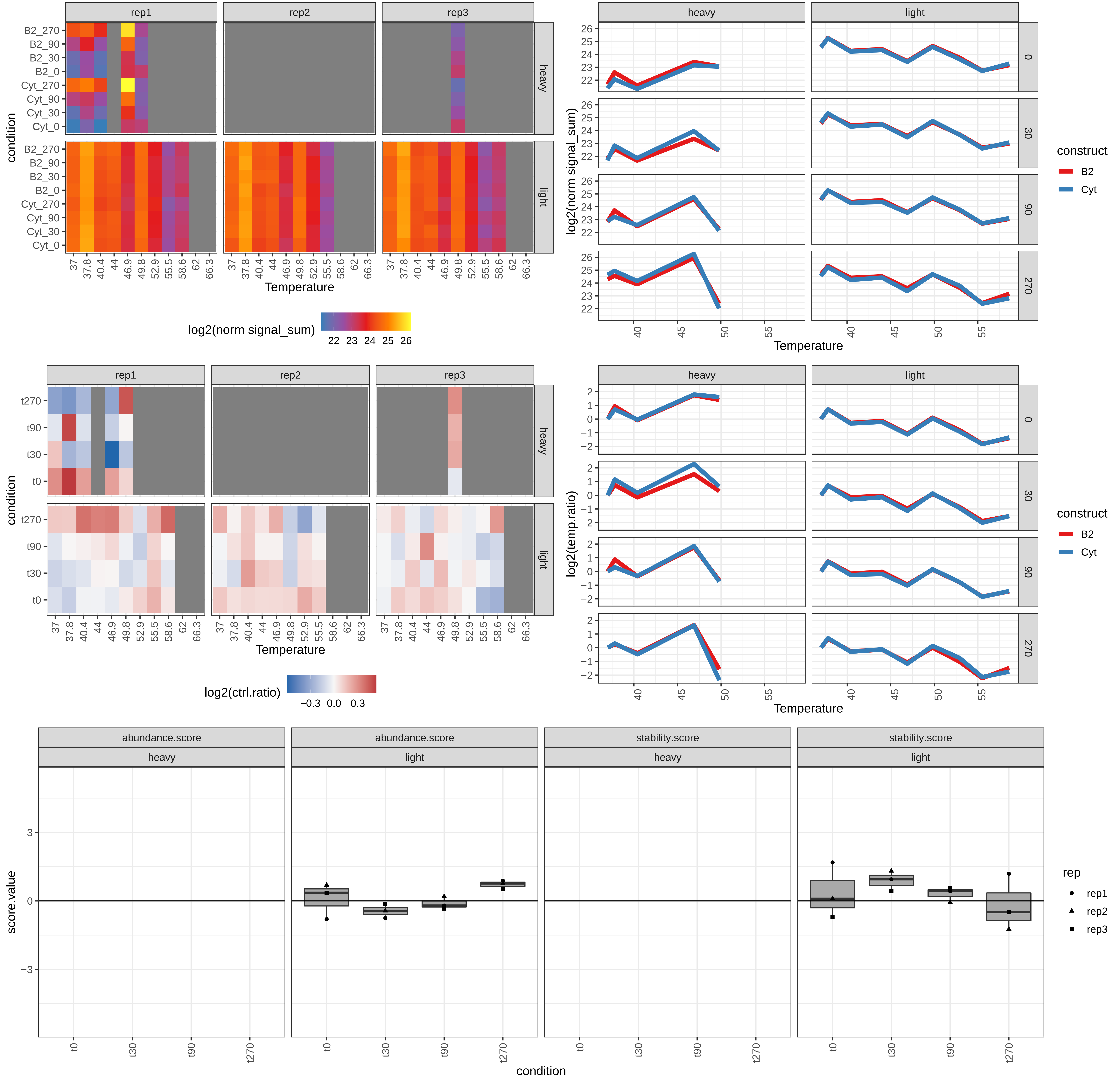

Q06510 − Lysophosphatidylcholine acyltransferase OS=Saccharomyces cerevisiae (strain ATCC 204508 / S288c) GN=TAZ1 PE=1 SV=1

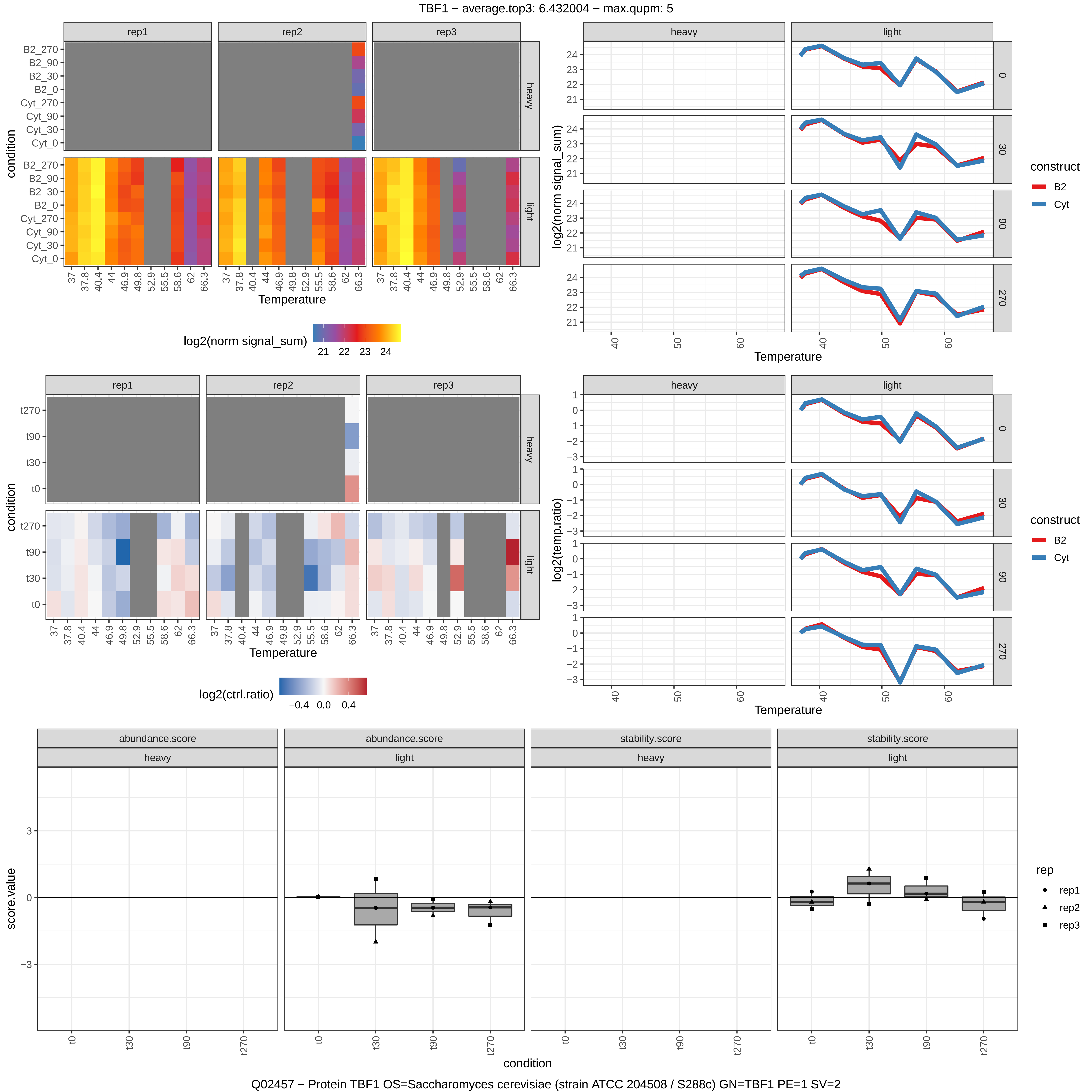
TBF1 − average.top3: 6.432004 − max.qupm: 5
rep1
rep2
rep3
heavy
light
B2_270
B2_90
B2_30
B2_0
Cyt_270
Cyt_90
Cyt_30
Cyt_0
condition
37 37.8 40.4 44 46.9 49.8 52.9 55.5 58.6 62 66.3
Temperature
log2(norm signal_sum)
21 22 23 24
log2(norm signal_sum)
0
30
90
270
construct
B2
Cyt
t270
t90
t30
t0
log2(ctrl.ratio)
−0.4 0.0 0.4
log2(temp.ratio)
abundance.score
stability.score
score.value
condition
rep
Q02457 − Protein TBF1 OS=Saccharomyces cerevisiae (strain ATCC 204508 / S288c) GN=TBF1 PE=1 SV=2

TBS1 − average.top3: 6.59962777777778 − max.qupm: 4

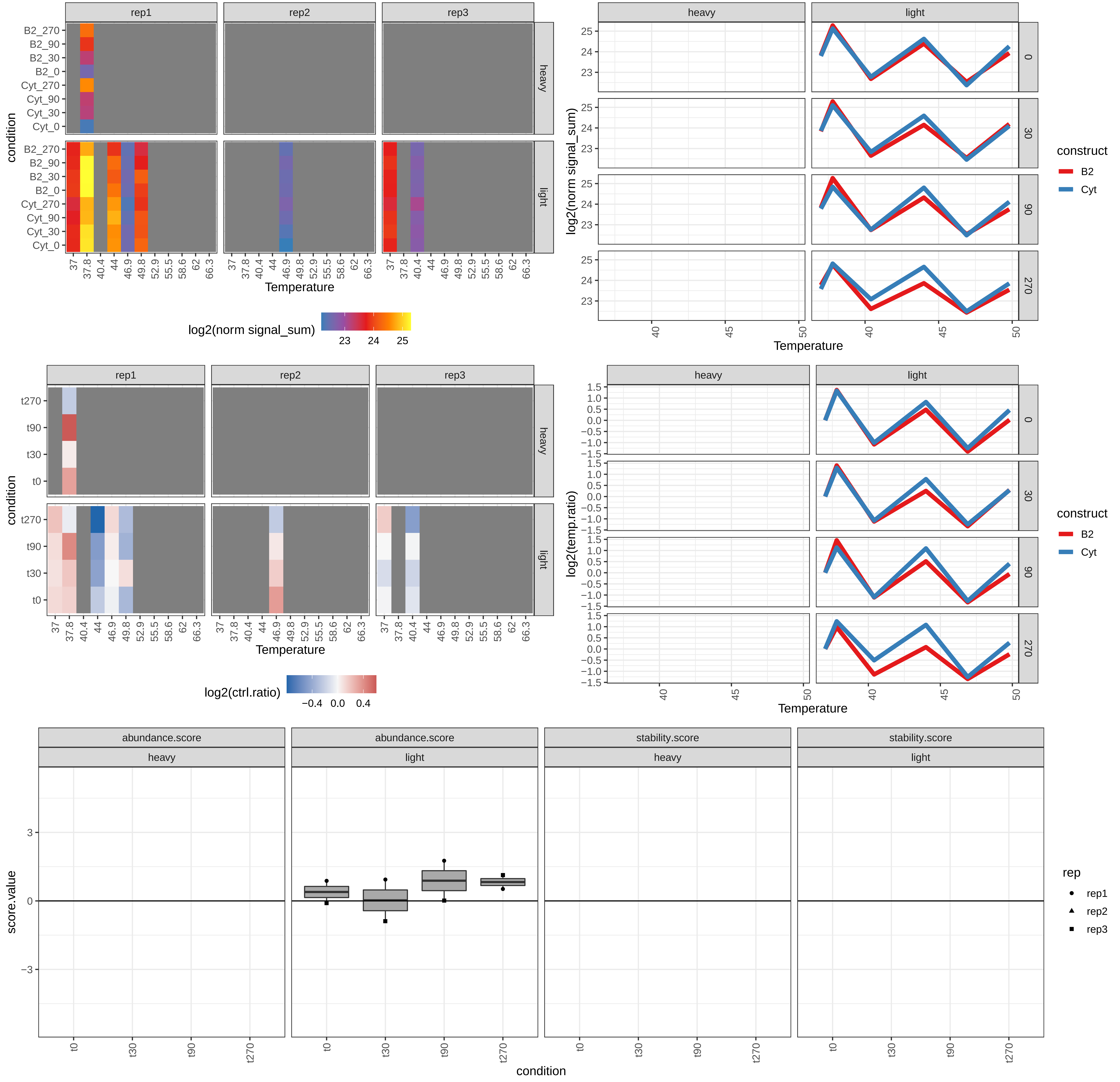

P38114 − Uncharacterized transcriptional regulatory protein TBS1 OS=Saccharomyces cerevisiae (strain ATCC 204508 / S288c) GN=TBS1 PE=1 SV=1

TCB1 − average.top3: 7.03567676470588 − max.qupm: 24

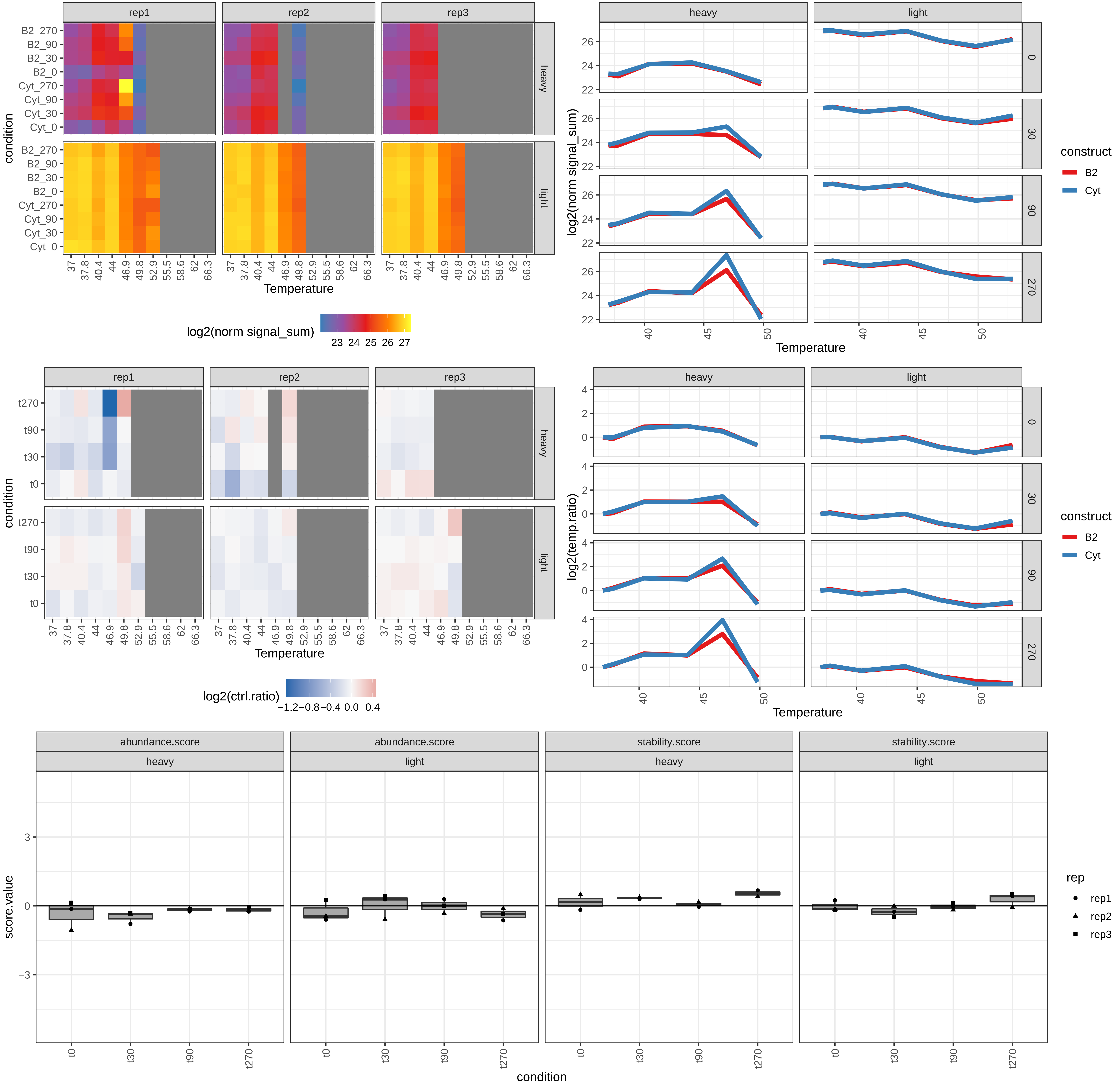

Q12466 − Tricalbin−1 OS=Saccharomyces cerevisiae (strain ATCC 204508 / S288c) GN=TCB1 PE=1 SV=1

TCB2 − average.top3: 6.84927307692308 − max.qupm: 13

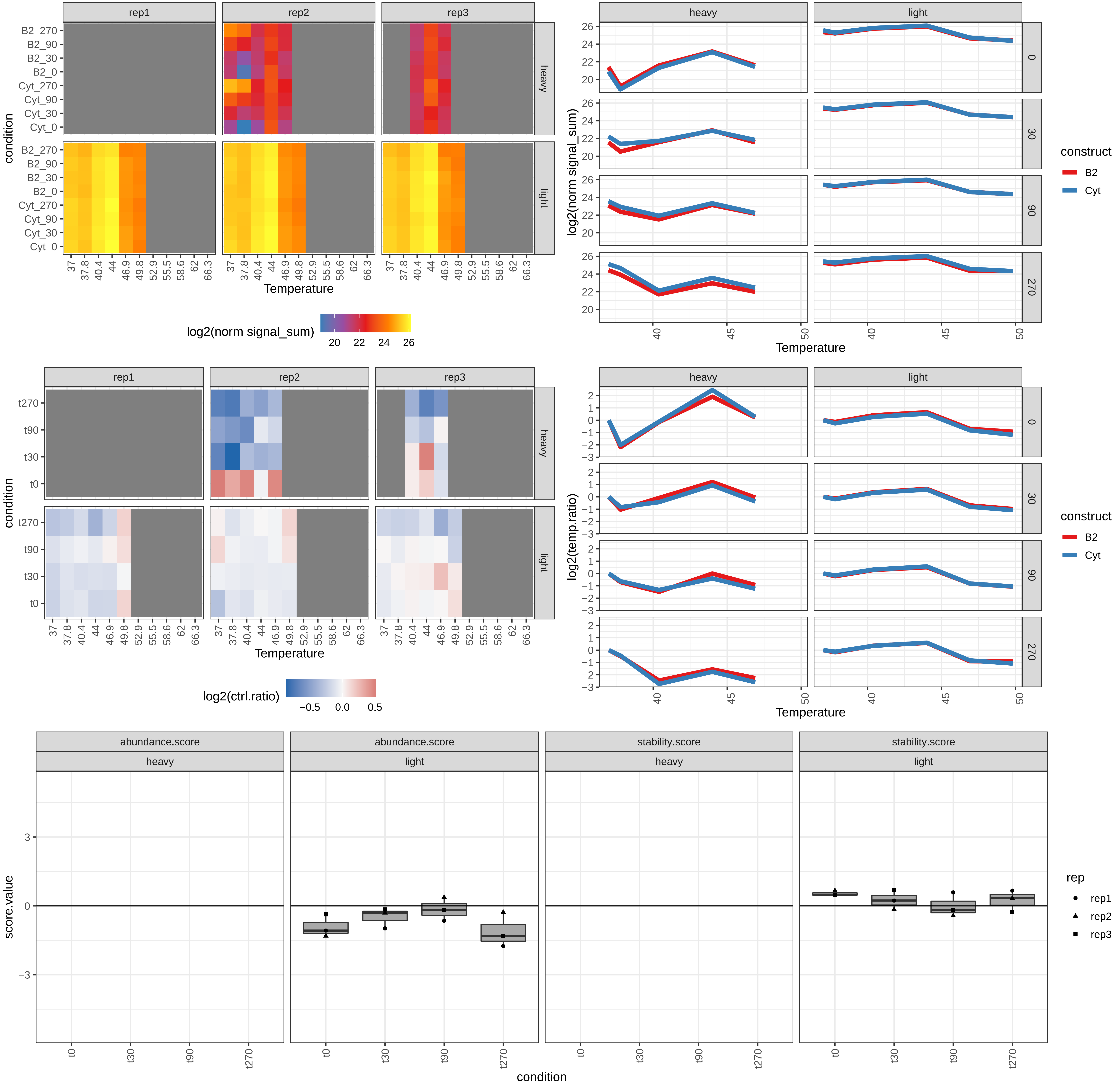

P48231 − Tricalbin−2 OS=Saccharomyces cerevisiae (strain ATCC 204508 / S288c) GN=TCB2 PE=1 SV=1

TCB3 − average.top3: 6.847154375 − max.qupm: 17

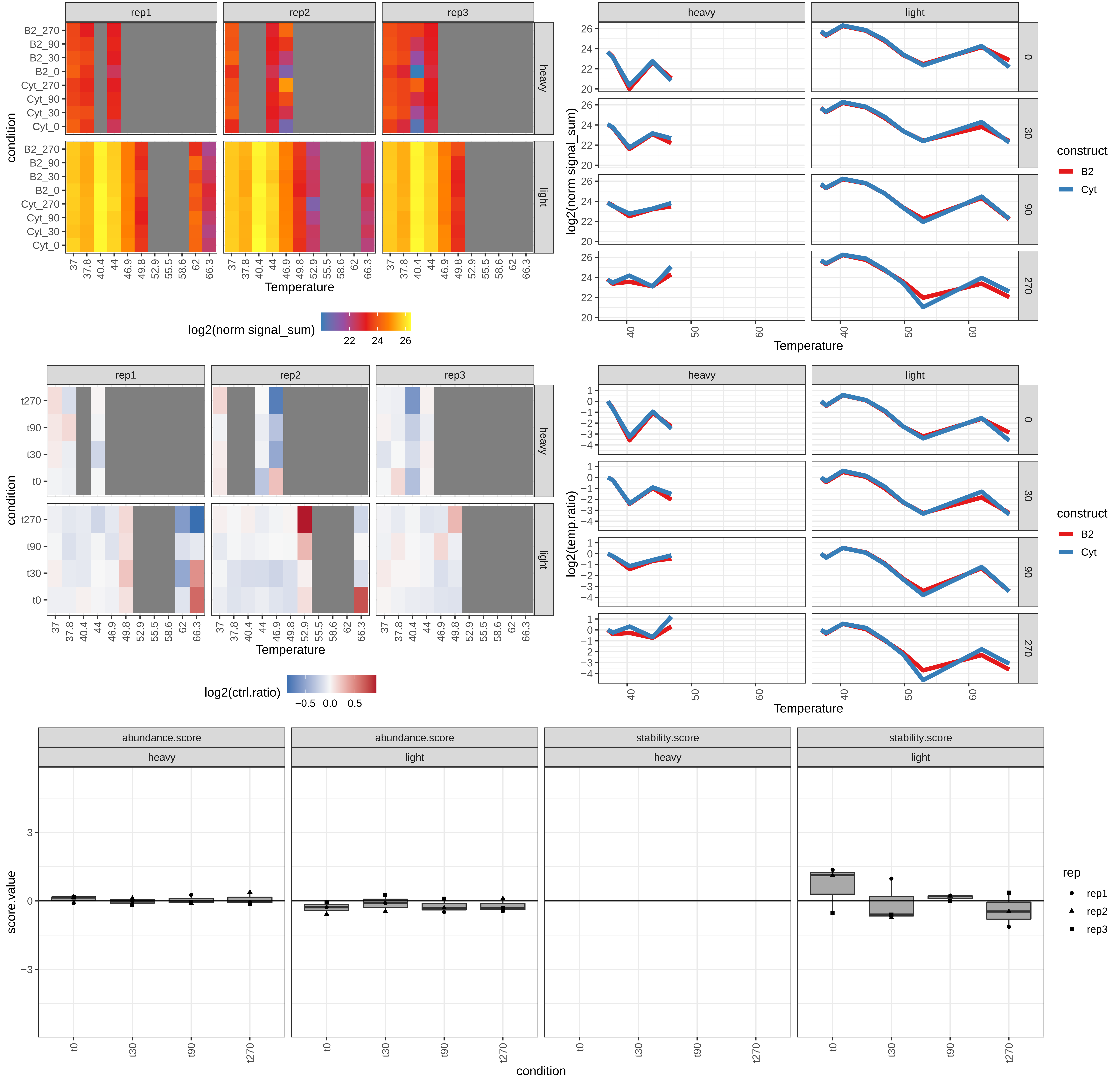

Q03640 − Tricalbin−3 OS=Saccharomyces cerevisiae (strain ATCC 204508 / S288c) GN=TCB3 PE=1 SV=1

TCD1 − average.top3: 7.00452208333333 − max.qupm: 7

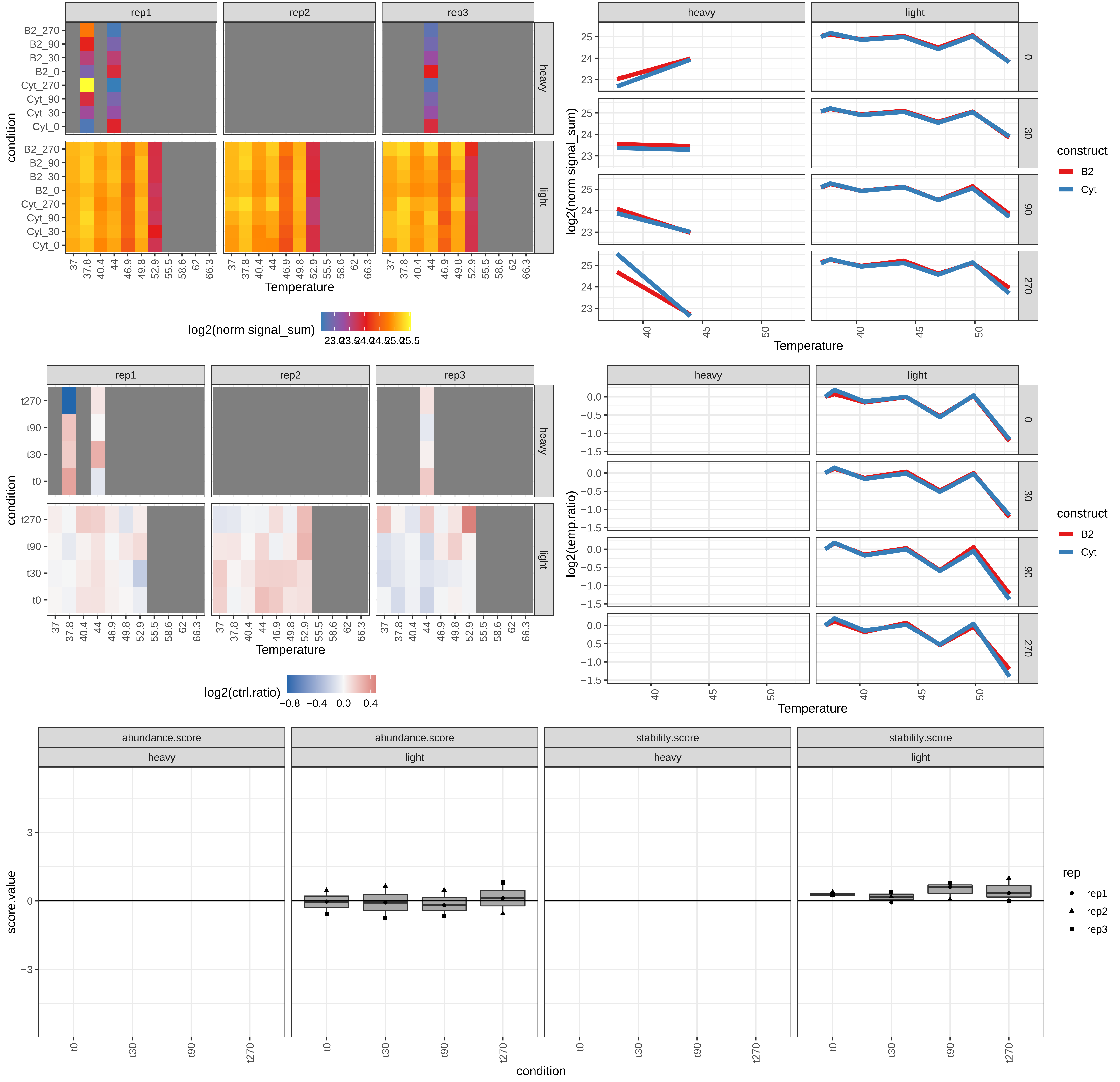

P38756 − tRNA threonylcarbamoyladenosine dehydratase 1 OS=Saccharomyces cerevisiae (strain ATCC 204508 / S288c) GN=TCD1 PE=1 SV=1

TCD2 − average.top3: 6.6647715625 − max.qupm: 10

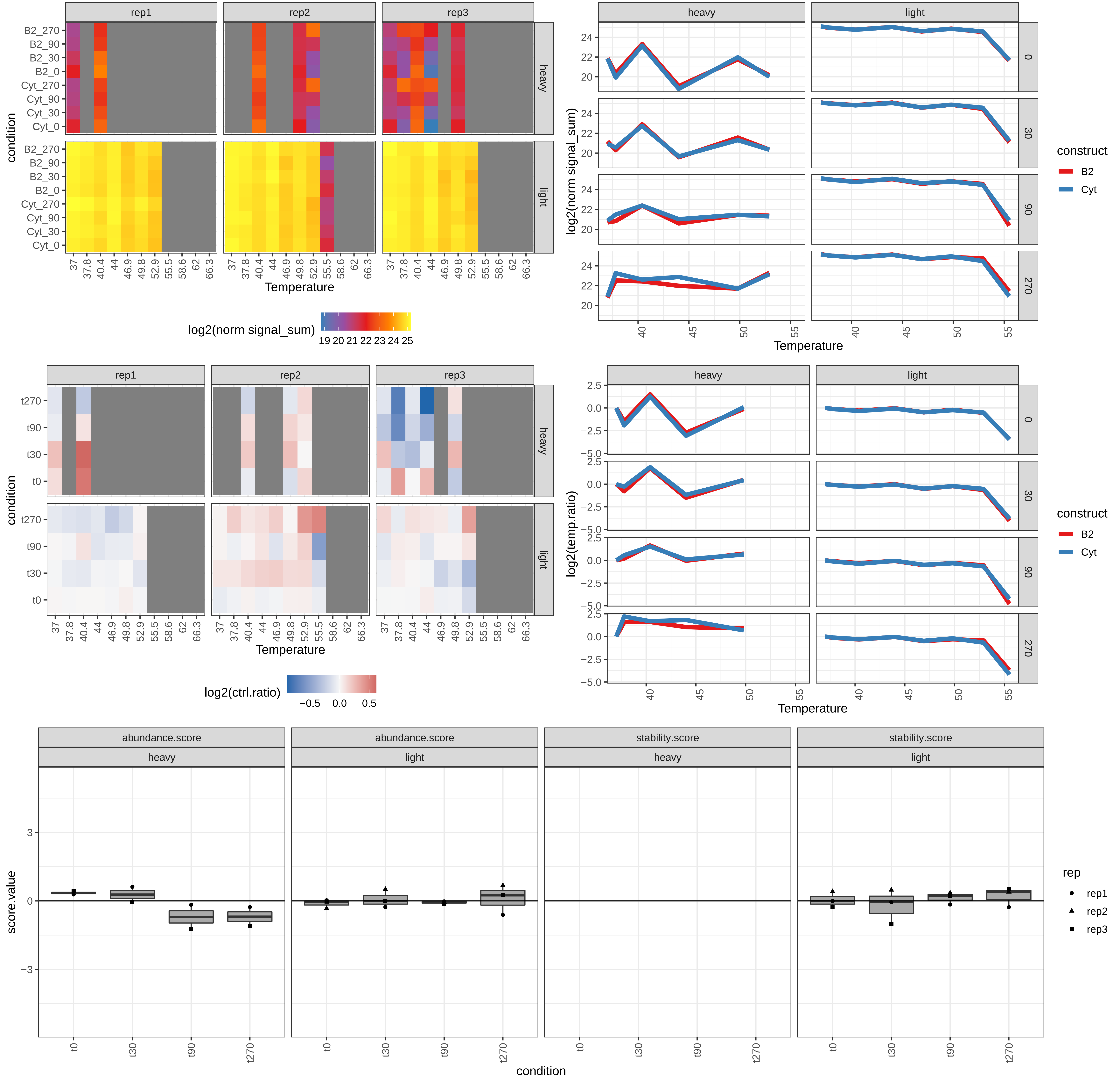

P36101 − tRNA threonylcarbamoyladenosine dehydratase 2 OS=Saccharomyces cerevisiae (strain ATCC 204508 / S288c) GN=TCD2 PE=1 SV=1

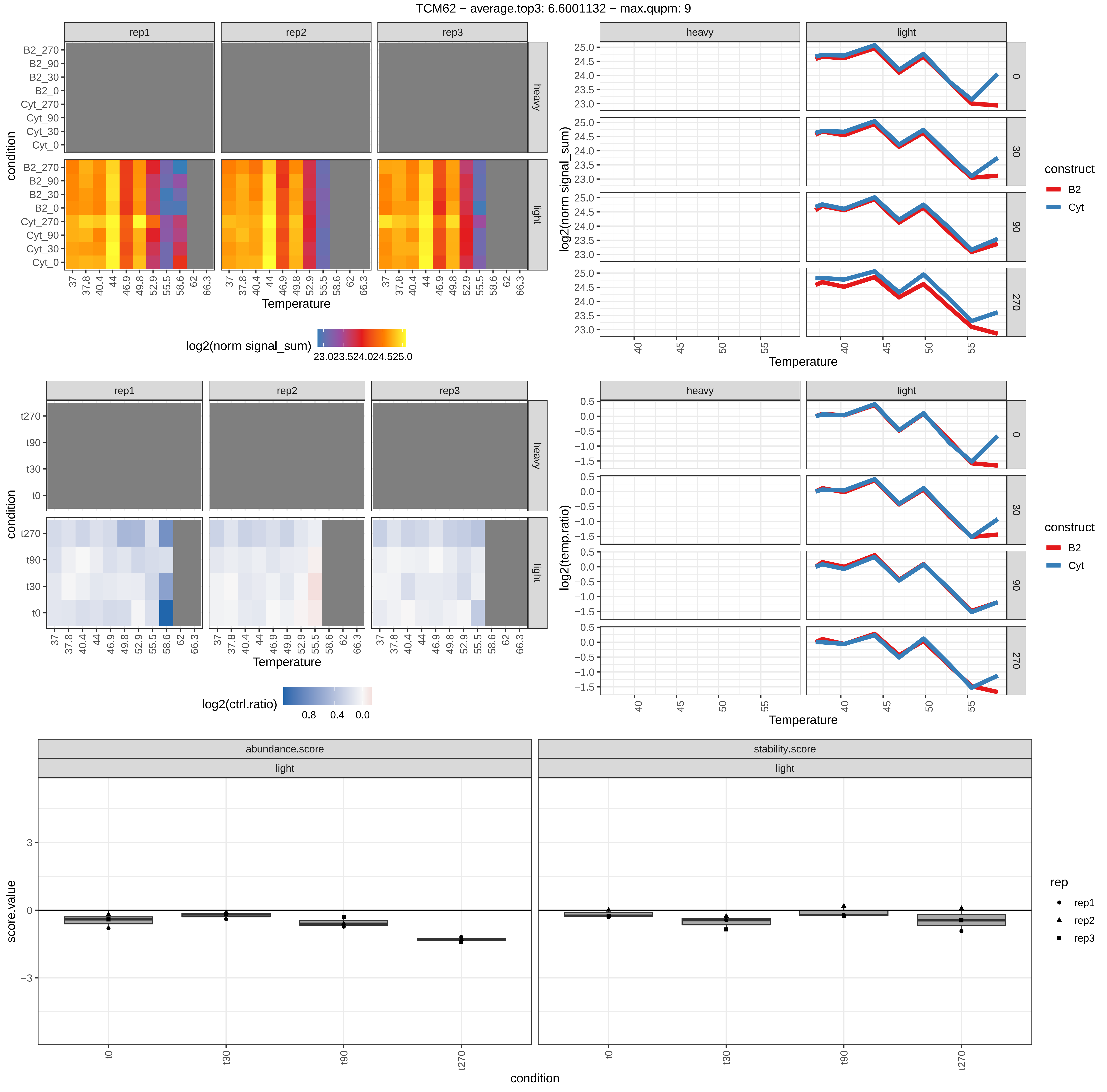

P38228 − Mitochondrial chaperone TCM62 OS=Saccharomyces cerevisiae (strain ATCC 204508 / S288c) GN=TCM62 PE=1 SV=2

TCP1 − average.top3: 7.29417980769231 − max.qupm: 17

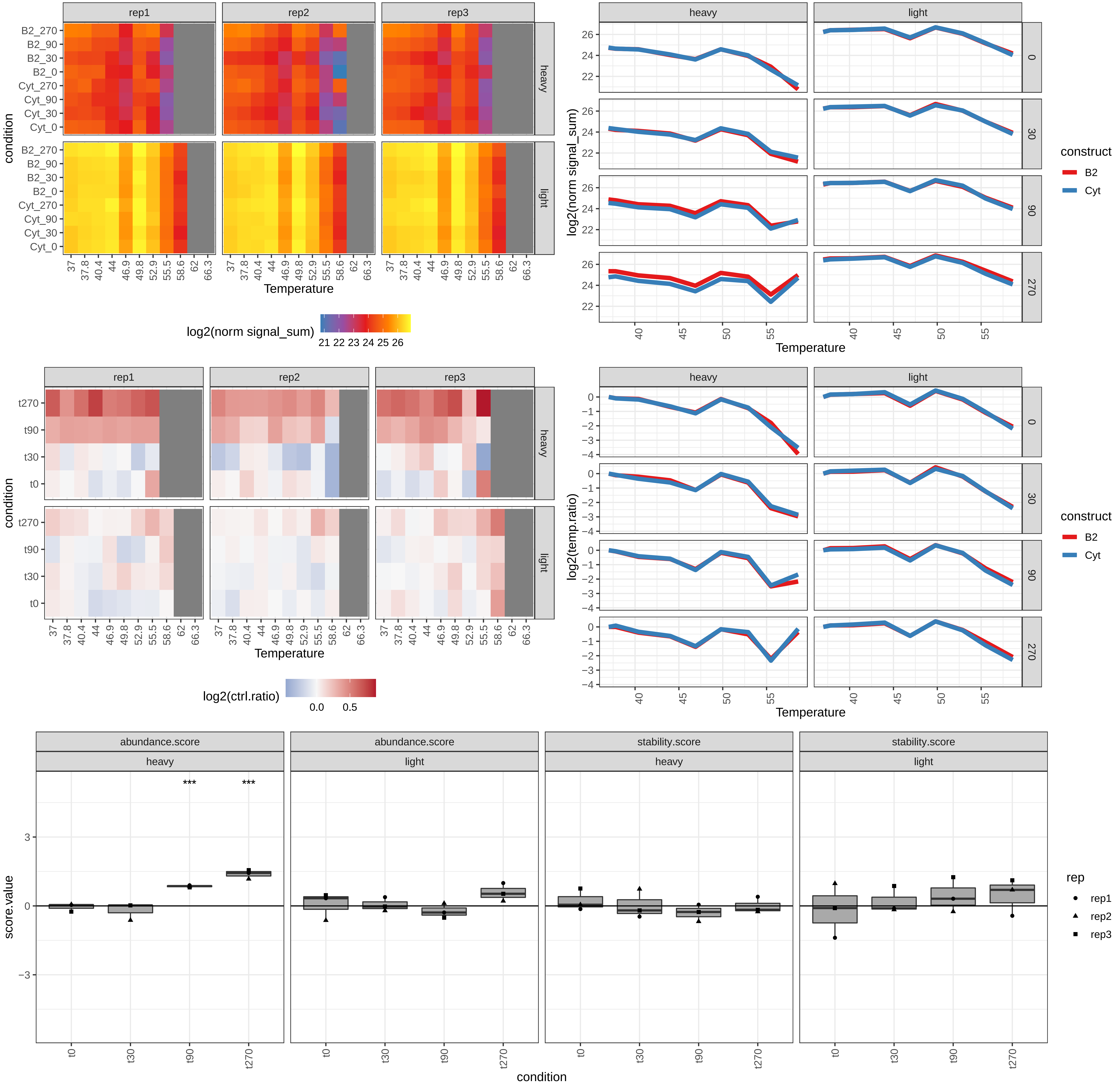

P12612 − T−complex protein 1 subunit alpha OS=Saccharomyces cerevisiae (strain ATCC 204508 / S288c) GN=TCP1 PE=1 SV=2

TDA1 − average.top3: 6.86977380952381 − max.qupm: 5

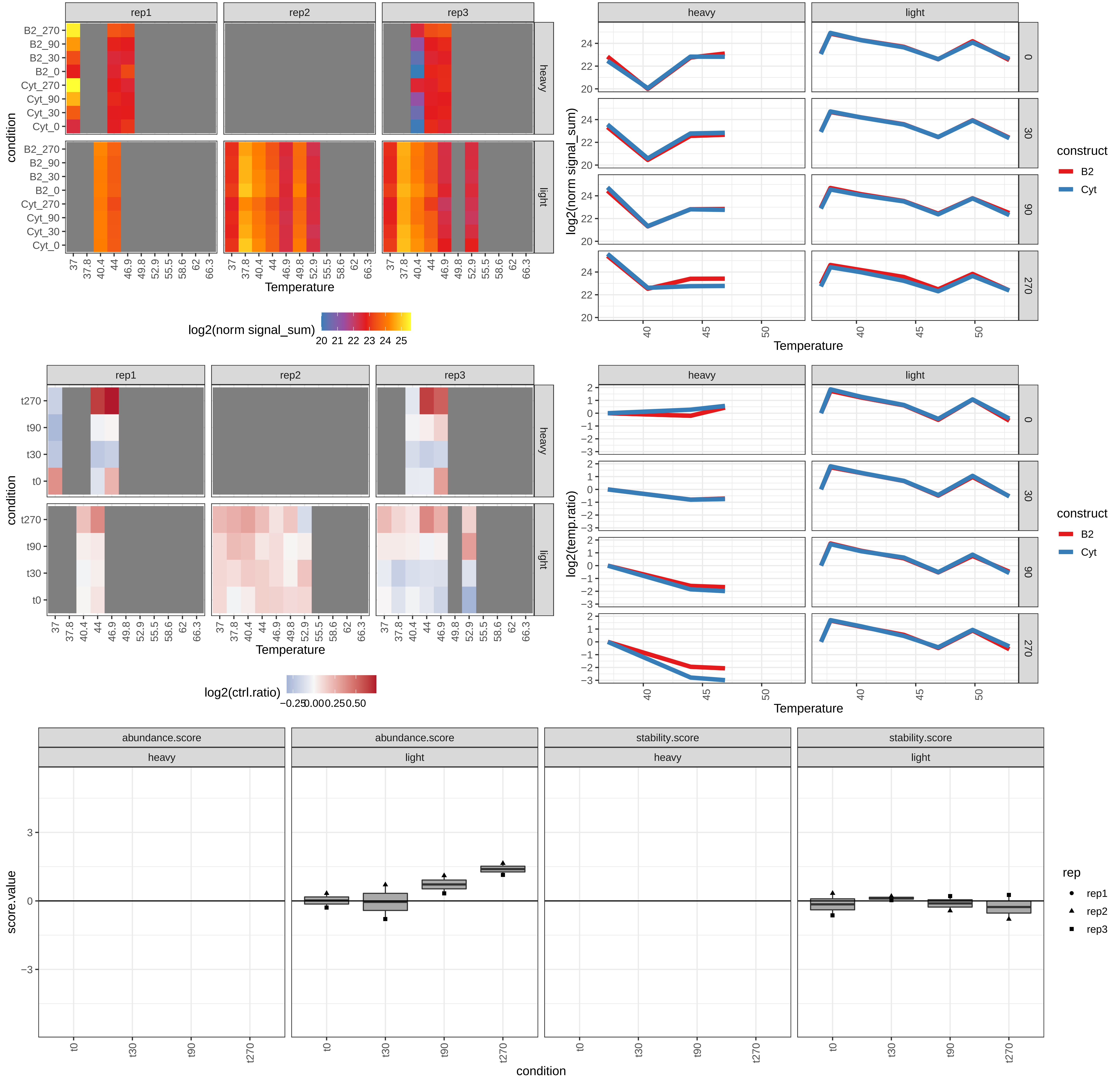

Q03533 − Serine/threonine−protein kinase TDA1 OS=Saccharomyces cerevisiae (strain ATCC 204508 / S288c) GN=TDA1 PE=1 SV=1

TDA10 − average.top3: 7.302817 − max.qupm: 8

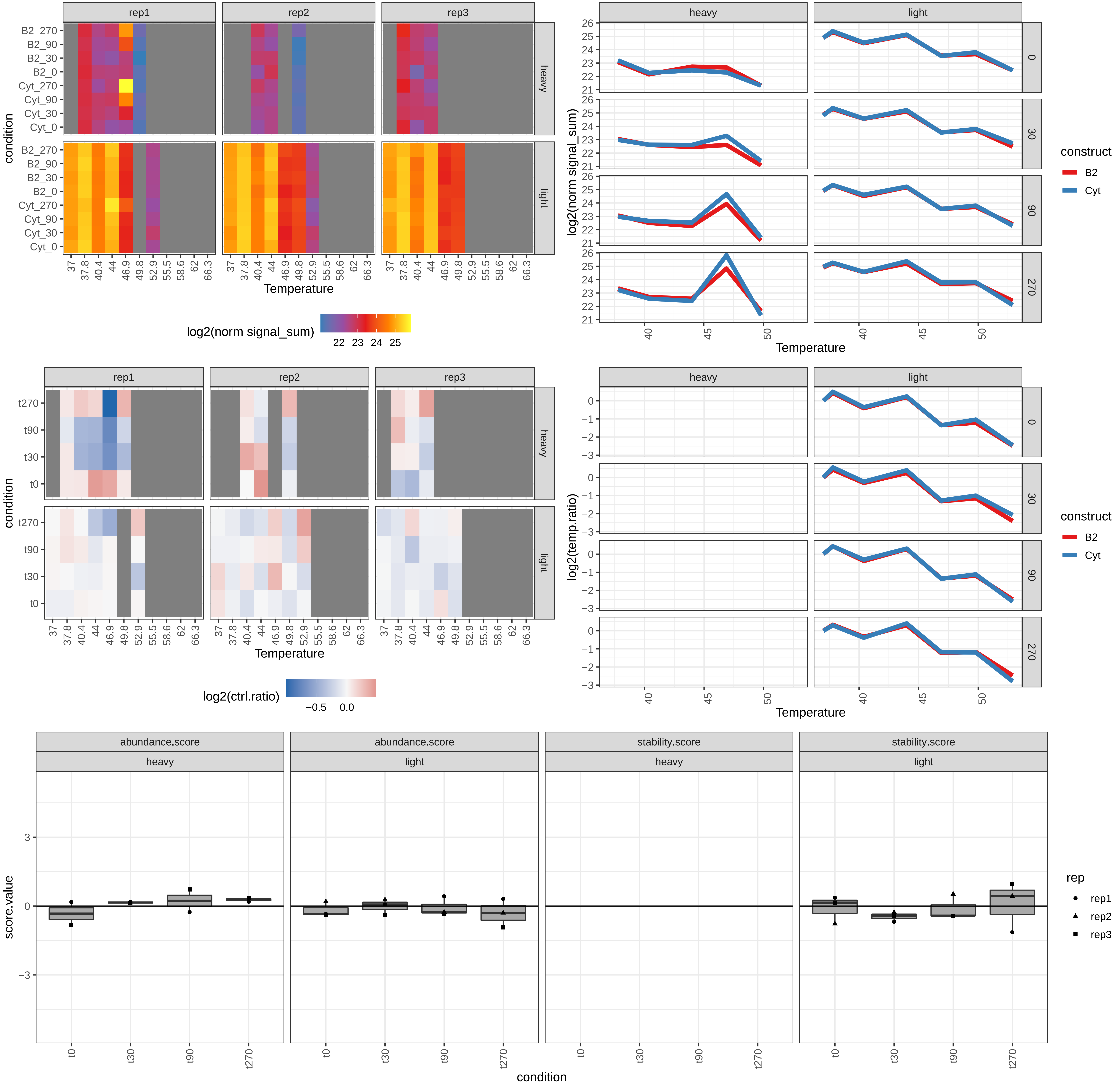

P42938 − Probable ATP−dependent kinase TDA10 OS=Saccharomyces cerevisiae (strain ATCC 204508 / S288c) GN=TDA10 PE=1 SV=1

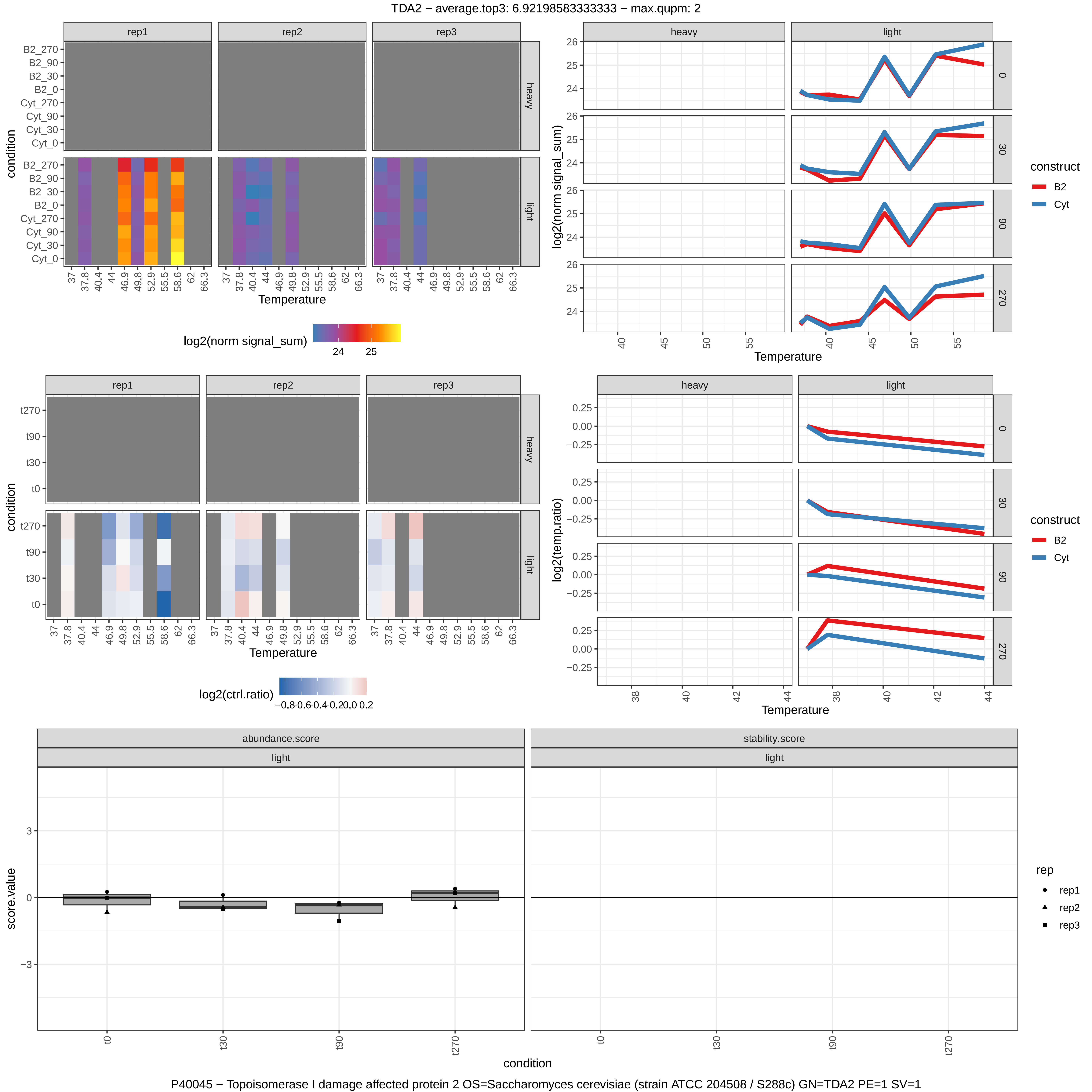

TDA2 − average.top3: 6.92198583333333 − max.qupm: 2
rep1
rep2
rep3
heavy
light
B2_270
B2_90
B2_30
B2_0
Cyt_270
Cyt_90
Cyt_30
Cyt_0
condition
Temperature
37 37.8 40.4 44 46.9 49.8 52.9 55.5 58.6 62 66.3
log2(norm signal_sum)
24 25
log2(norm signal_sum)
26 25 24
0 30 90 270
40 45 50 55
construct
B2
Cyt
t270
t90
t30
t0
log2(ctrl.ratio)
−0.8 −0.6 −0.4 −0.2 0.0 0.2
log2(temp.ratio)
0.25 0.00 −0.25
38 40 42 44
abundance.score
stability.score
score.value
3 0 −3
condition
rep
rep1
rep2
rep3
P40045 − Topoisomerase I damage affected protein 2 OS=Saccharomyces cerevisiae (strain ATCC 204508 / S288c) GN=TDA2 PE=1 SV=1

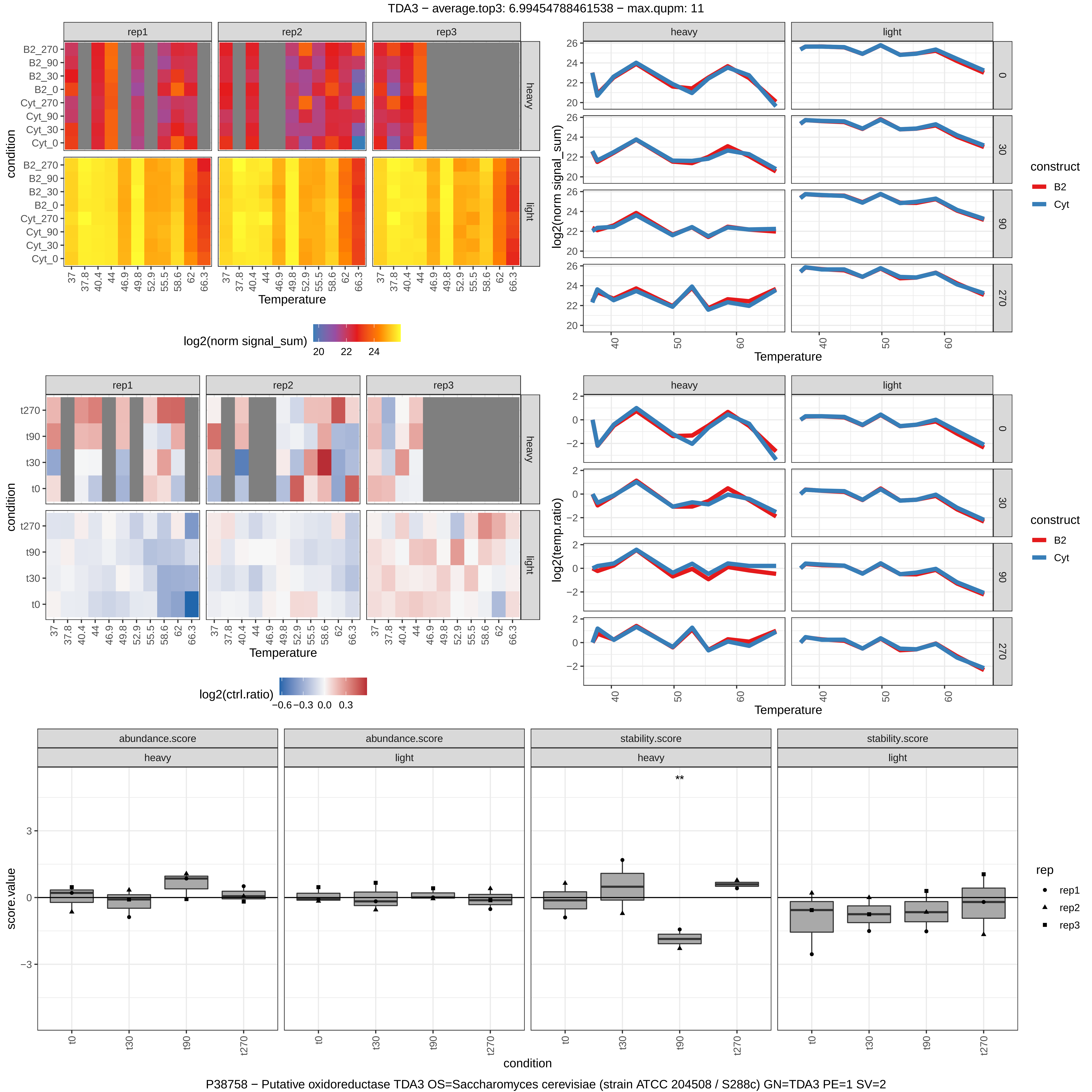
TDA3 − average.top3: 6.99454788461538 − max.qupm: 11
rep1
rep2
rep3
heavy
light
condition
B2_270
B2_90
B2_30
B2_0
Cyt_270
Cyt_90
Cyt_30
Cyt_0
Temperature
37
37.8
40.4
44
46.9
49.8
52.9
55.5
58.6
62
66.3
log2(norm signal_sum)
20
22
24
log2(norm signal_sum)
construct
B2
Cyt
0
30
90
270
40
50
60
t270
t90
t30
t0
log2(ctrl.ratio)
−0.6
−0.3
0.0
0.3
log2(temp.ratio)
abundance.score
stability.score
score.value
rep
rep1
rep2
rep3
**
P38758 − Putative oxidoreductase TDA3 OS=Saccharomyces cerevisiae (strain ATCC 204508 / S288c) GN=TDA3 PE=1 SV=2

TDH1 − average.top3: 7.88205257575758 − max.qupm: 15

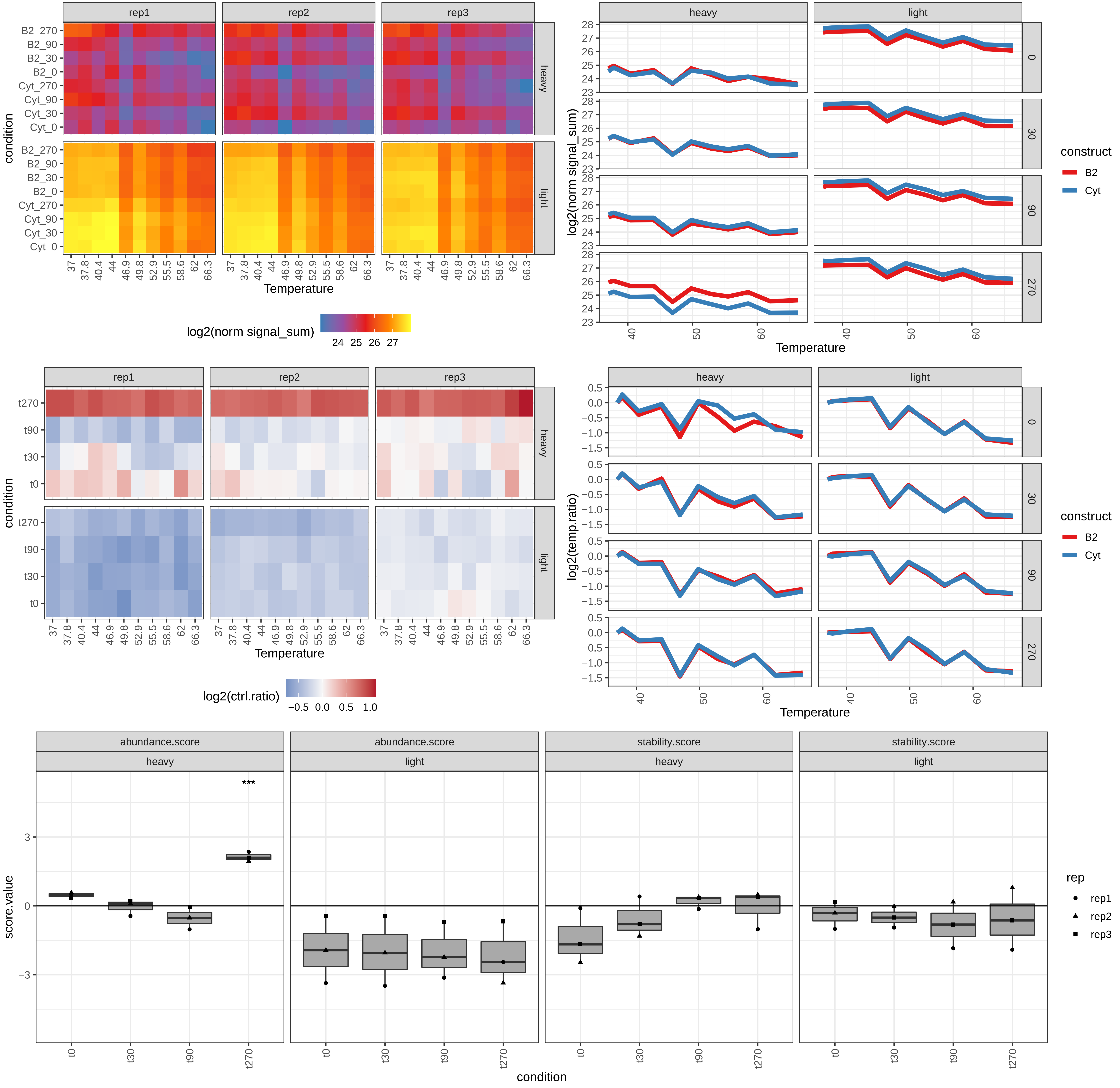

P00360 − Glyceraldehyde−3−phosphate dehydrogenase 1 OS=Saccharomyces cerevisiae (strain ATCC 204508 / S288c) GN=TDH1 PE=1 SV=3

TDH2 − average.top3: 7.78897169491525 − max.qupm: 3

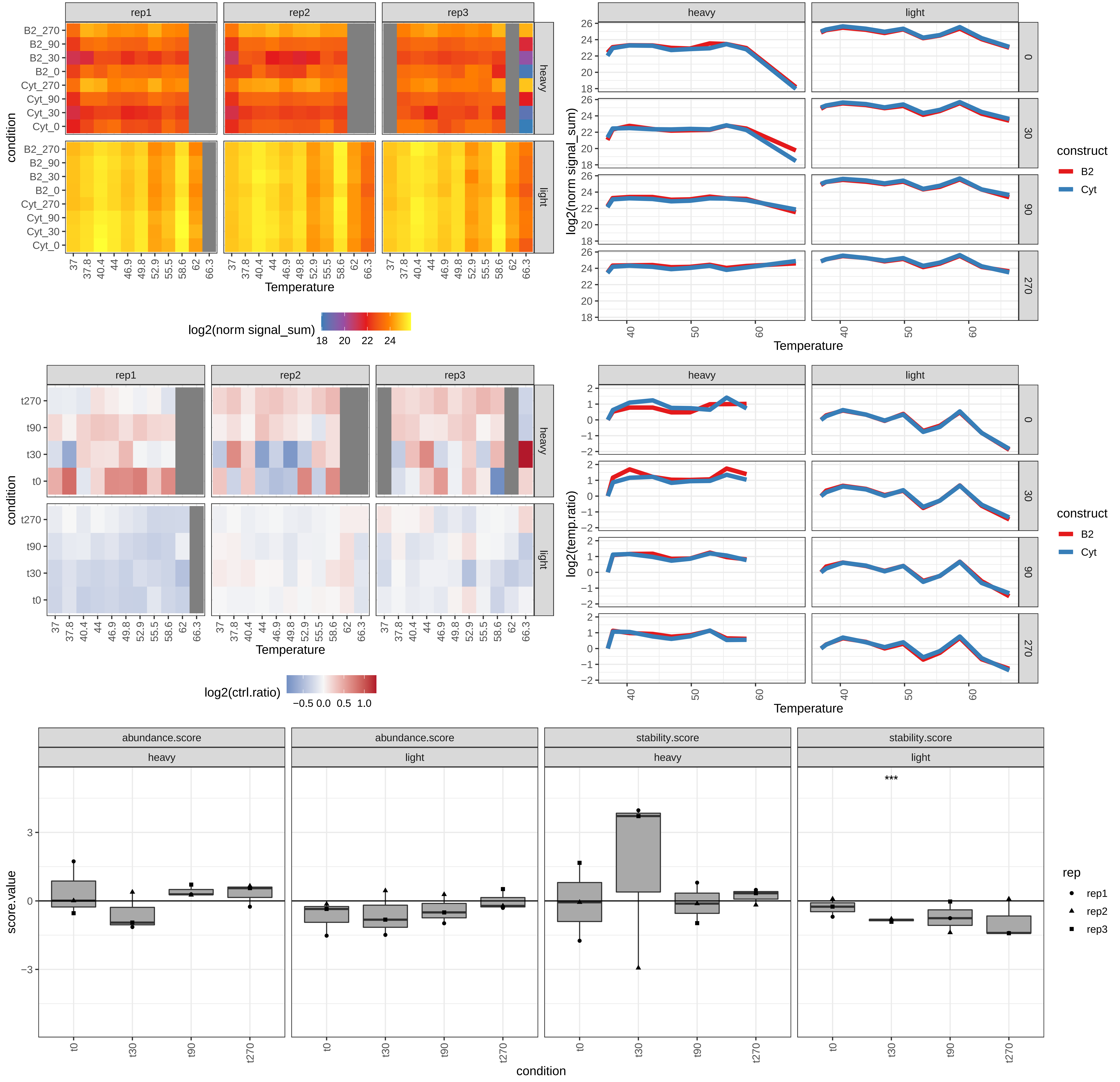

P00358 − Glyceraldehyde−3−phosphate dehydrogenase 2 OS=Saccharomyces cerevisiae (strain ATCC 204508 / S288c) GN=TDH2 PE=1 SV=3

TDH3 − average.top3: 7.84351369230769 − max.qupm: 5

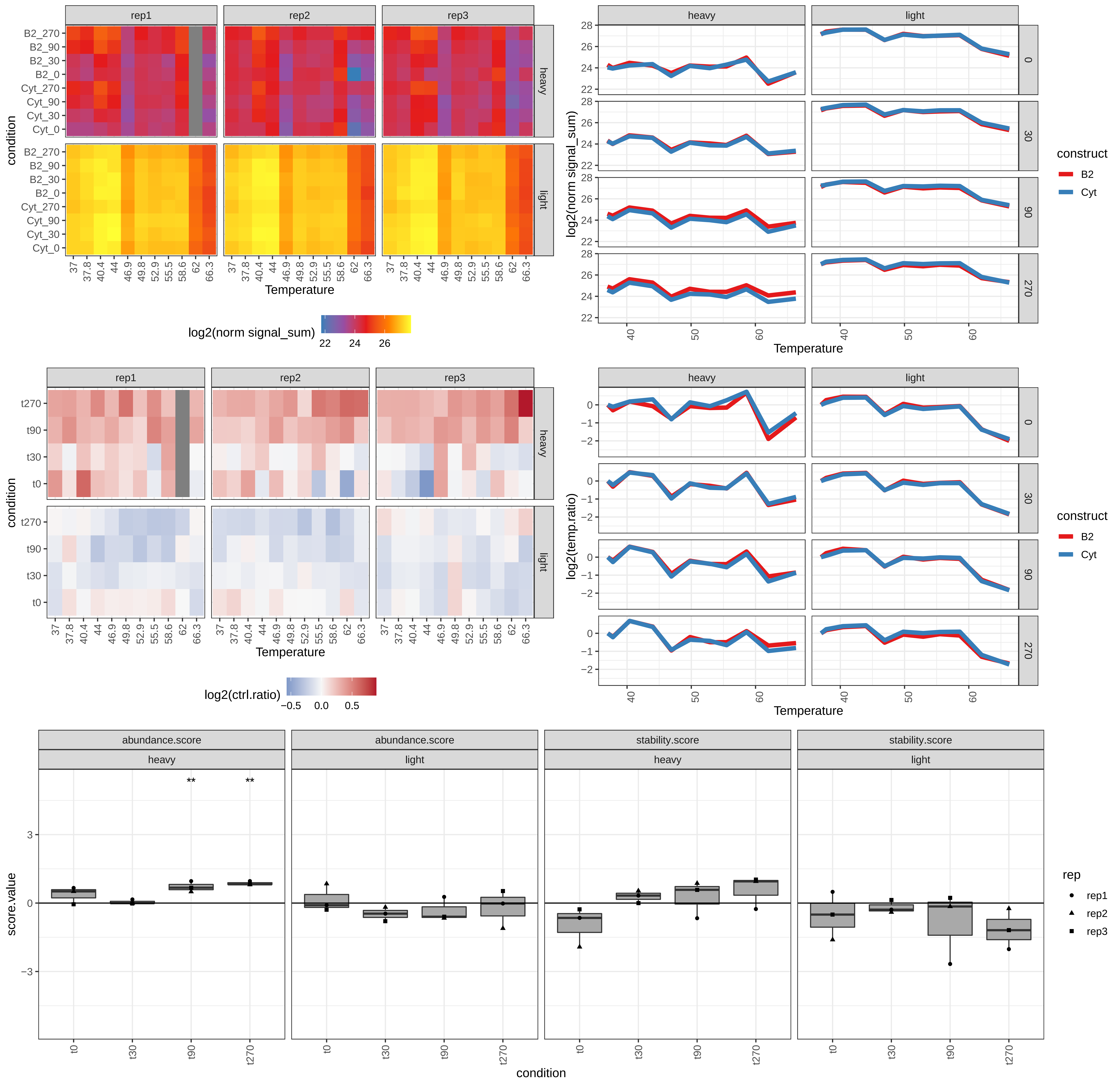

P00359 − Glyceraldehyde−3−phosphate dehydrogenase 3 OS=Saccharomyces cerevisiae (strain ATCC 204508 / S288c) GN=TDH3 PE=1 SV=3

TED1 − average.top3: 7.05561866666667 − max.qupm: 11

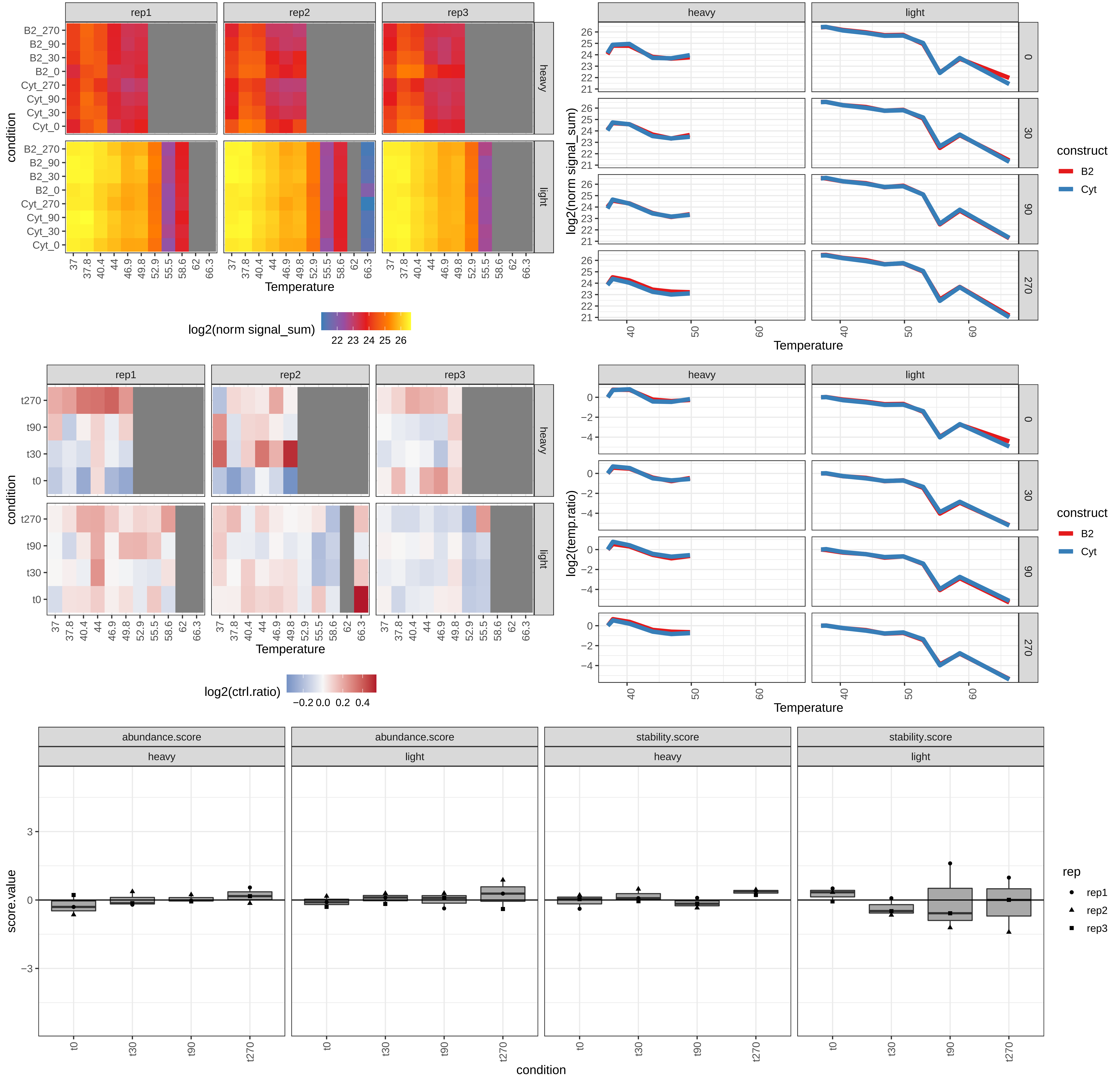

P40533 − Protein TED1 OS=Saccharomyces cerevisiae (strain ATCC 204508 / S288c) GN=TED1 PE=1 SV=1

TEF1 − average.top3: 8.42543681818182 − max.qupm: 25

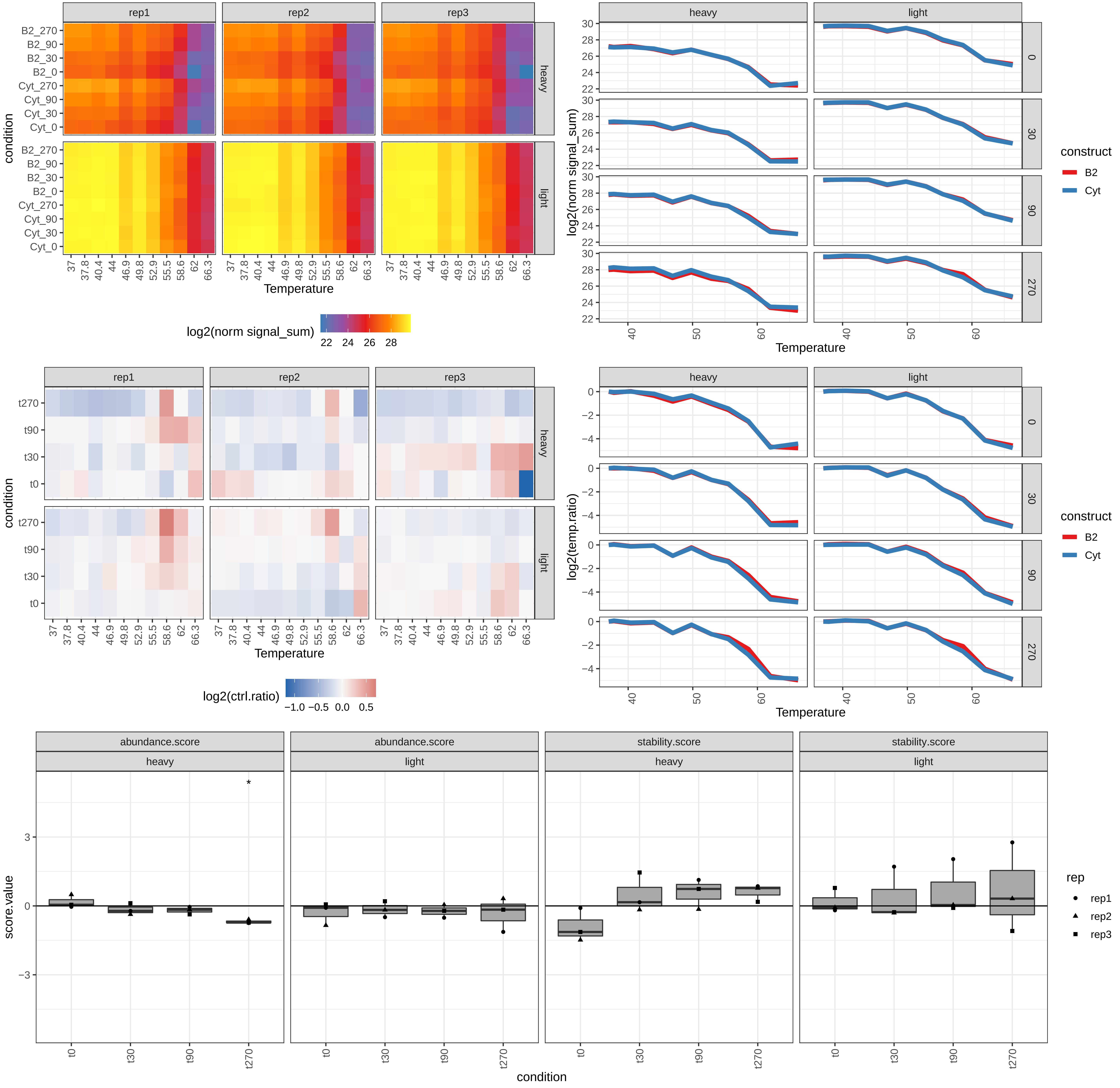

P02994 − Elongation factor 1−alpha OS=Saccharomyces cerevisiae (strain ATCC 204508 / S288c) GN=TEF1 PE=1 SV=1

TEF4 − average.top3: 7.5931655 − max.qupm: 9

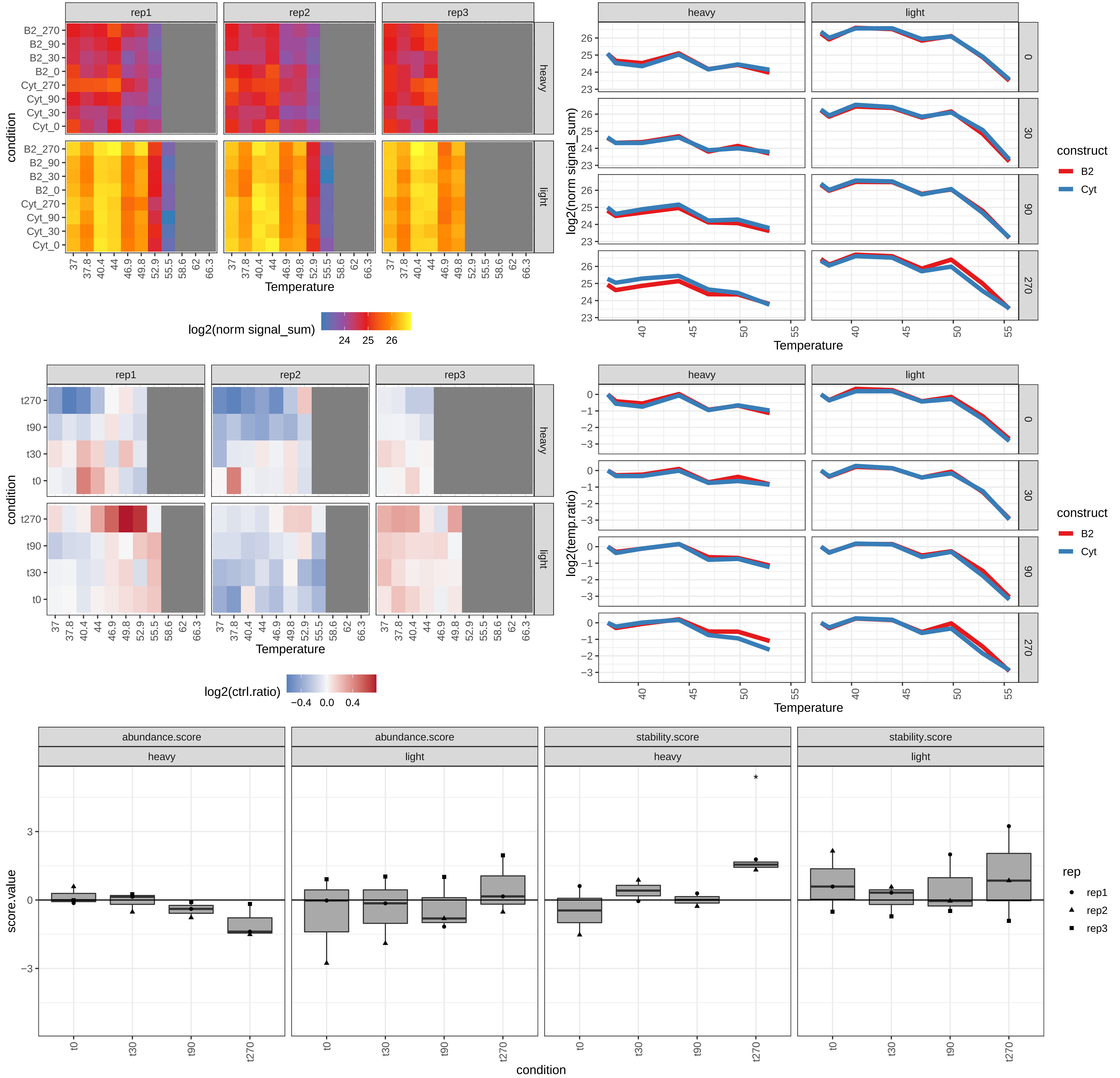

P36008 − Elongation factor 1−gamma 2 OS=Saccharomyces cerevisiae (strain ATCC 204508 / S288c) GN=TEF4 PE=1 SV=1

TES1 − average.top3: 7.52365217391304 − max.qupm: 14

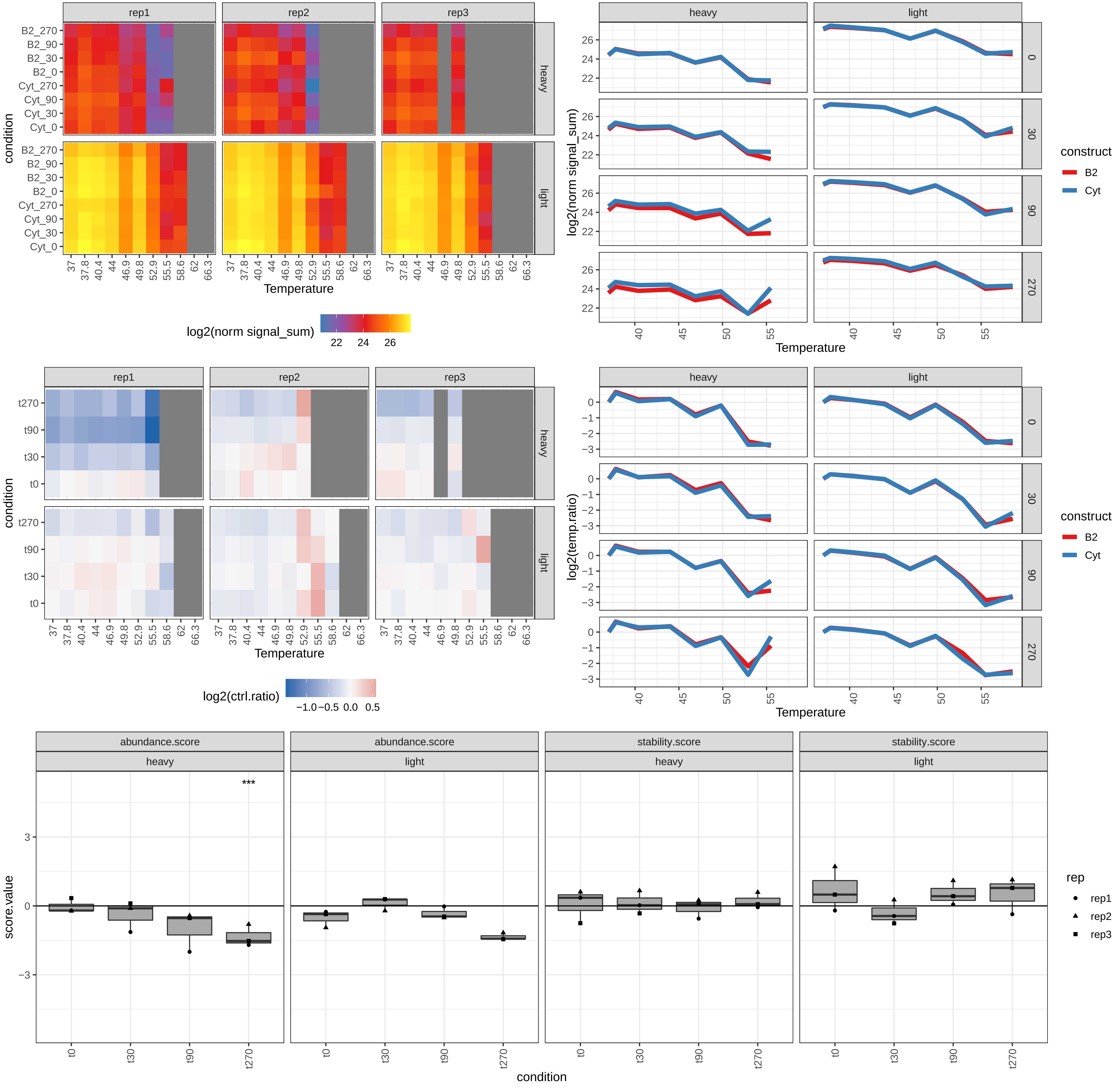

P41903 − Peroxisomal acyl−coenzyme A thioester hydrolase 1 OS=Saccharomyces cerevisiae (strain ATCC 204508 / S288c) GN=TES1 PE=1 SV=1

TEX1 − average.top3: 6.487491 − max.qupm: 7

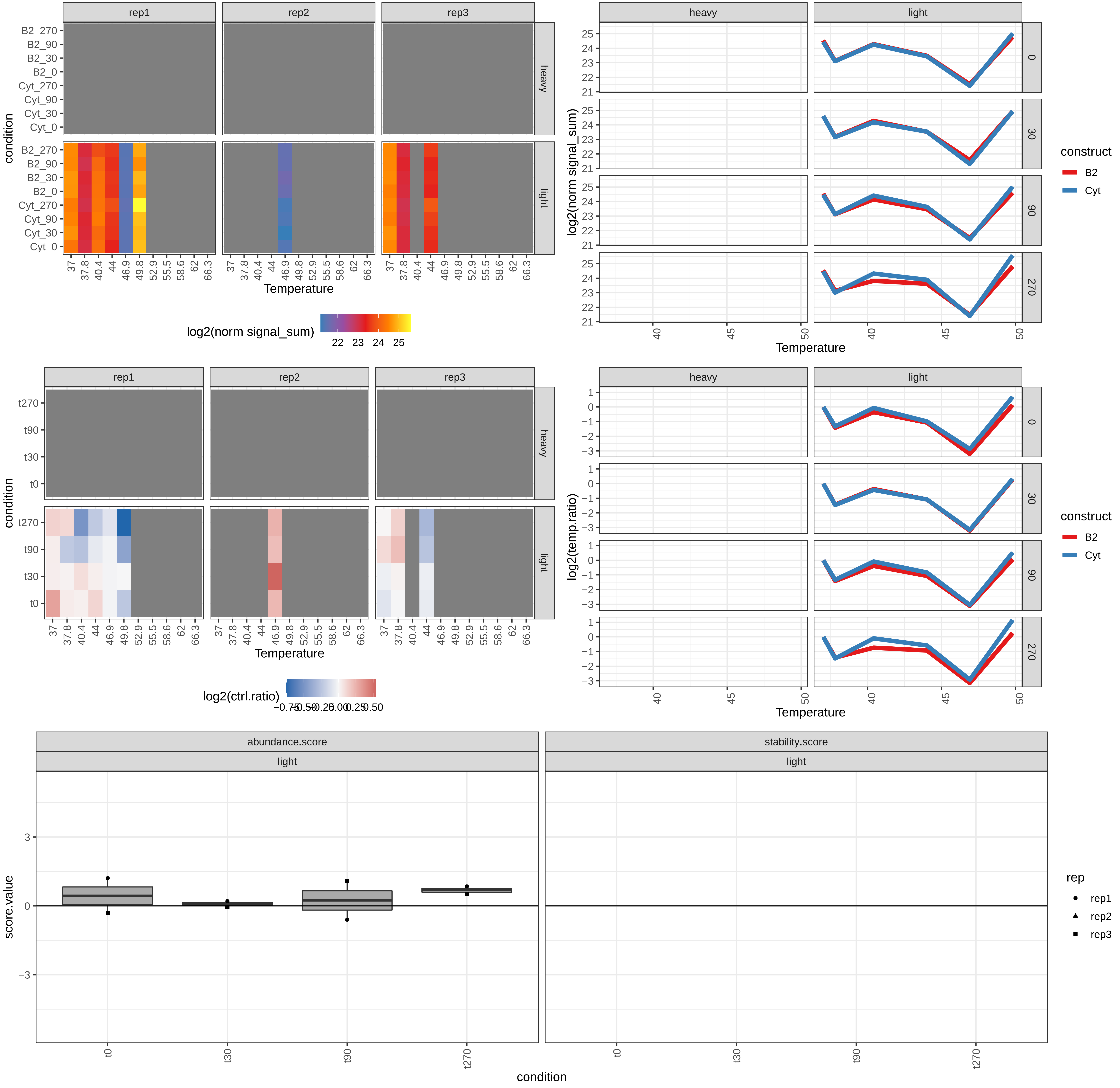

P53851 − Protein TEX1 OS=Saccharomyces cerevisiae (strain ATCC 204508 / S288c) GN=TEX1 PE=1 SV=1

TFA1 − average.top3: 6.69716111111111 − max.qupm: 5

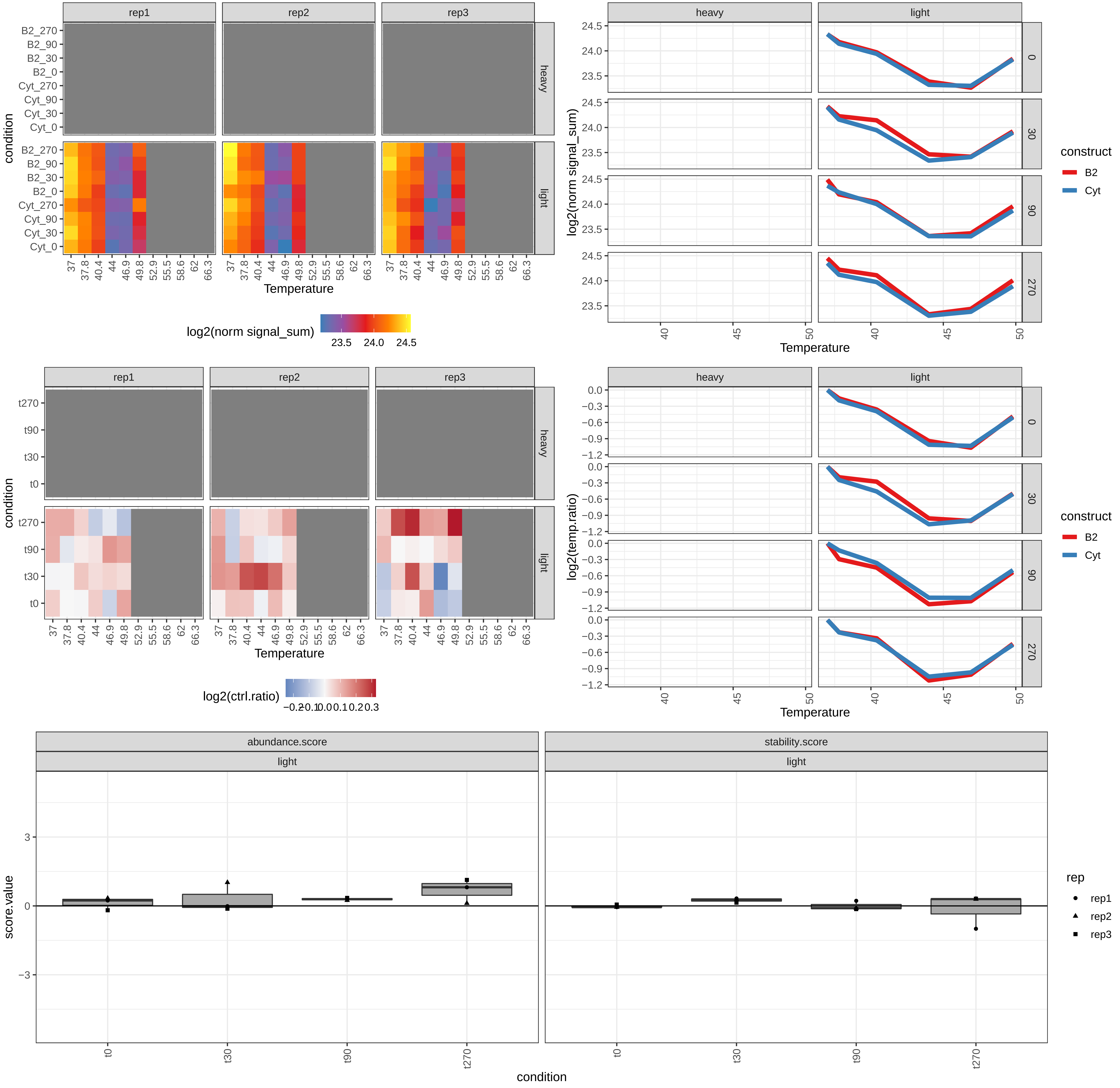

P36100 − Transcription initiation factor IIE subunit alpha OS=Saccharomyces cerevisiae (strain ATCC 204508 / S288c) GN=TFA1 PE=1 SV=1

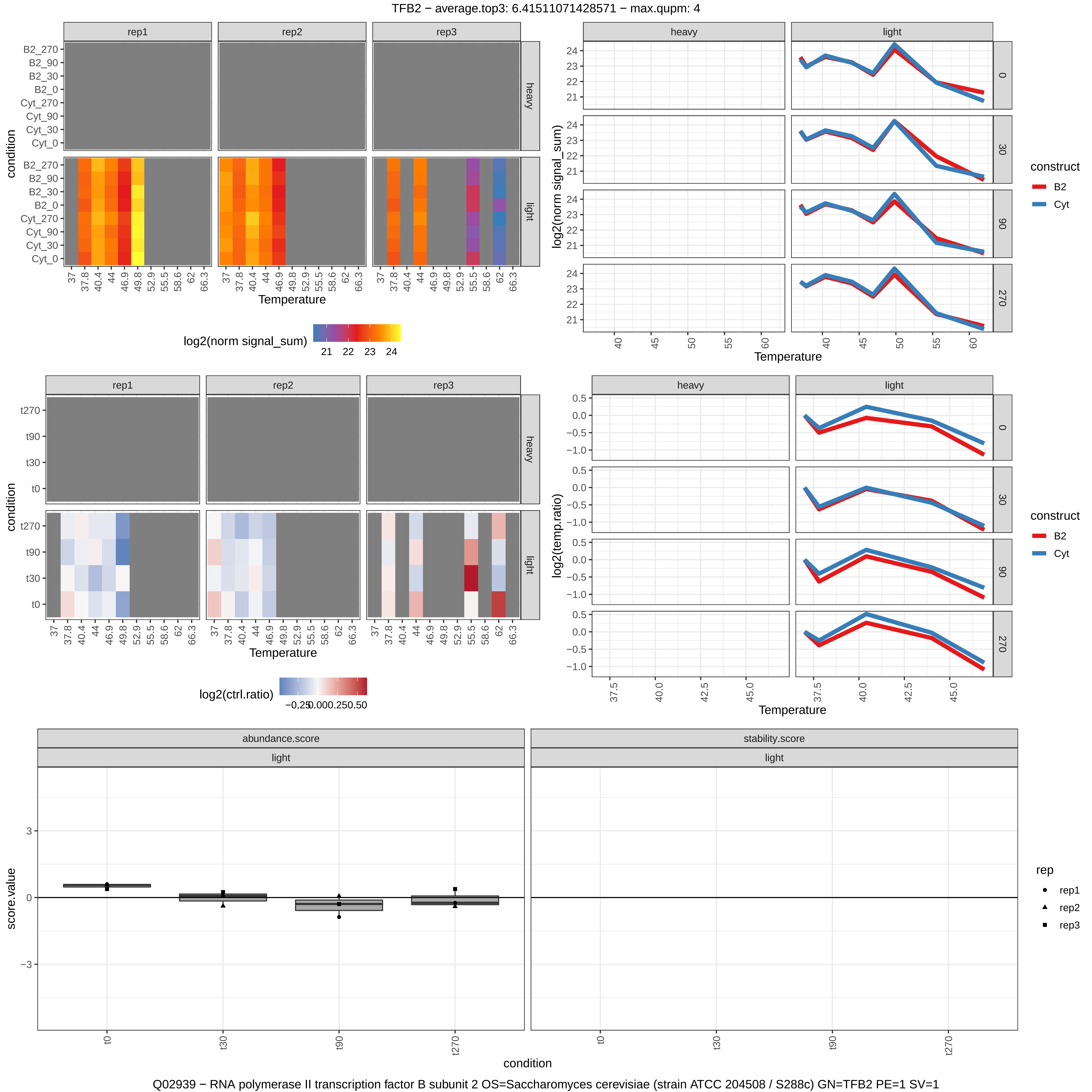
TFB2 − average.top3: 6.41511071428571 − max.qupm: 4
rep1
rep2
rep3
heavy
light
condition
B2_270
B2_90
B2_30
B2_0
Cyt_270
Cyt_90
Cyt_30
Cyt_0
Temperature
log2(norm signal_sum)
construct
B2
Cyt
0
30
90
270
t270
t90
t30
t0
log2(ctrl.ratio)
log2(temp.ratio)
abundance.score
stability.score
score.value
rep
Q02939 − RNA polymerase II transcription factor B subunit 2 OS=Saccharomyces cerevisiae (strain ATCC 204508 / S288c) GN=TFB2 PE=1 SV=1

TFB3 − average.top3: 6.534405 − max.qupm: 4

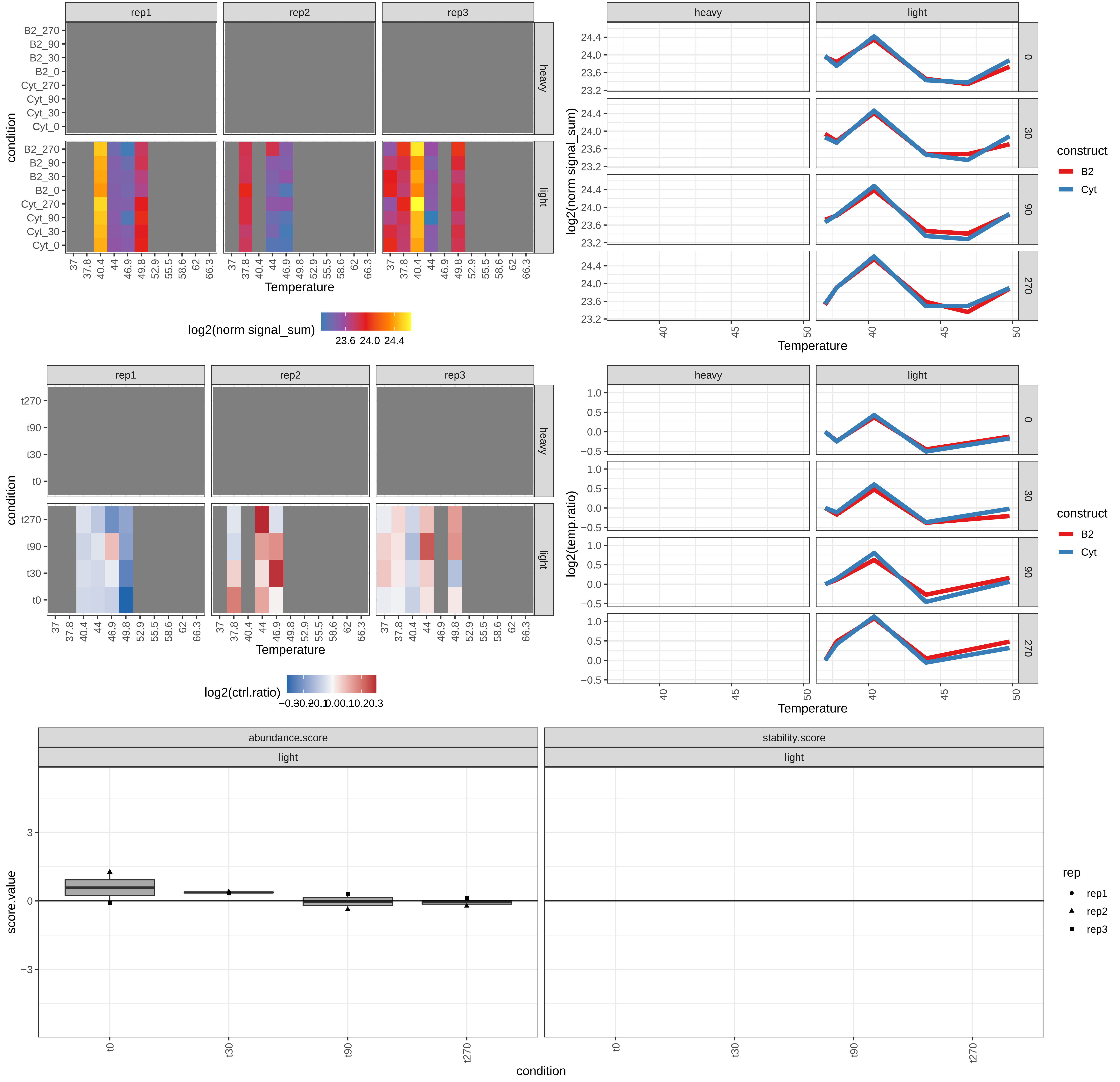

Q03290 − RNA polymerase II transcription factor B subunit 3 OS=Saccharomyces cerevisiae (strain ATCC 204508 / S288c) GN=TFB3 PE=1 SV=1

TFB4 − average.top3: 6.38499714285714 − max.qupm: 5

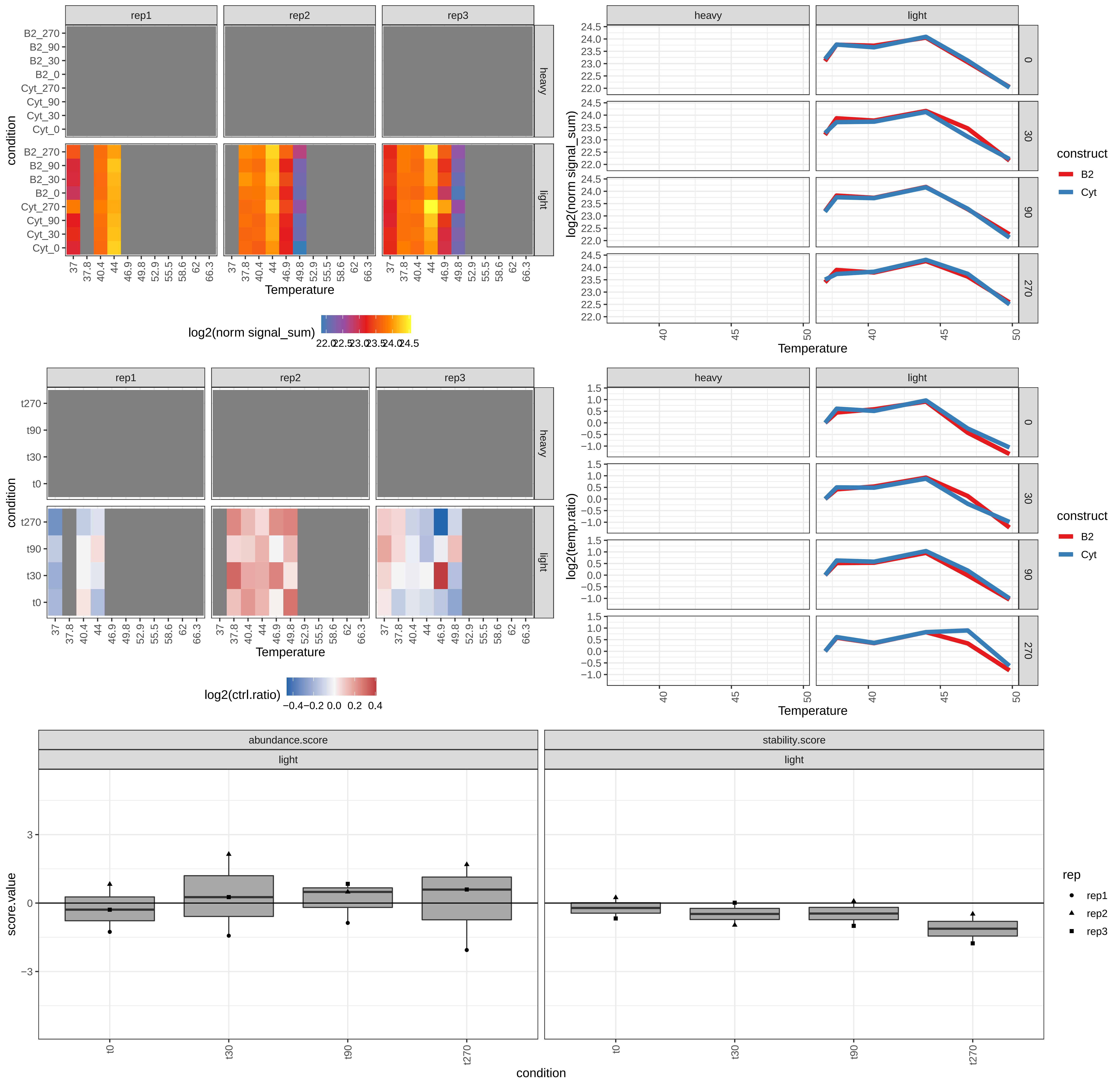

Q12004 − RNA polymerase II transcription factor B subunit 4 OS=Saccharomyces cerevisiae (strain ATCC 204508 / S288c) GN=TFB4 PE=1 SV=1

TFC3 − average.top3: 6.27467333333333 − max.qupm: 6

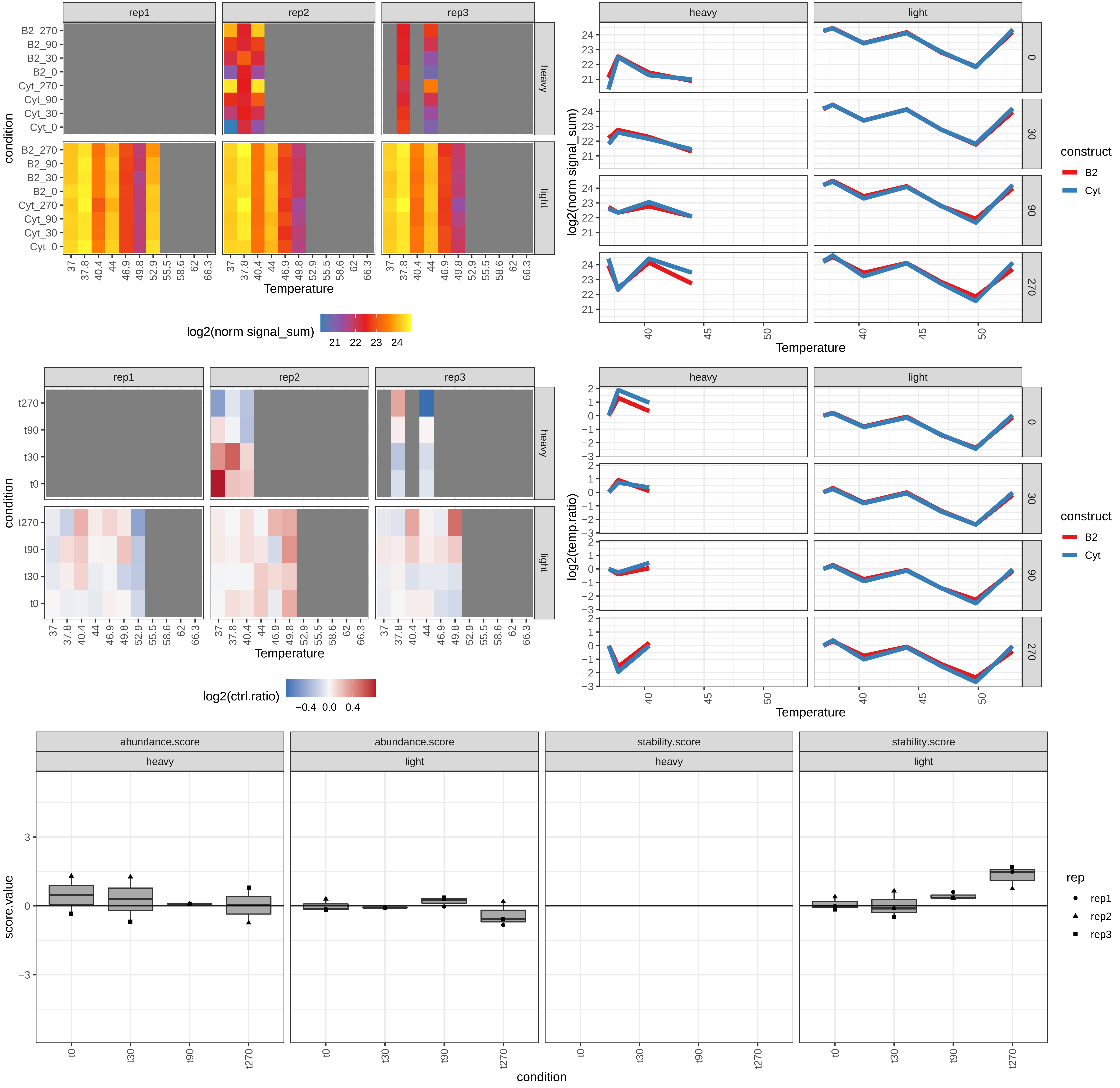

P34111 − Transcription factor tau 138 kDa subunit OS=Saccharomyces cerevisiae (strain ATCC 204508 / S288c) GN=TFC3 PE=1 SV=1

TFC4 − average.top3: 6.80657117647059 − max.qupm: 7

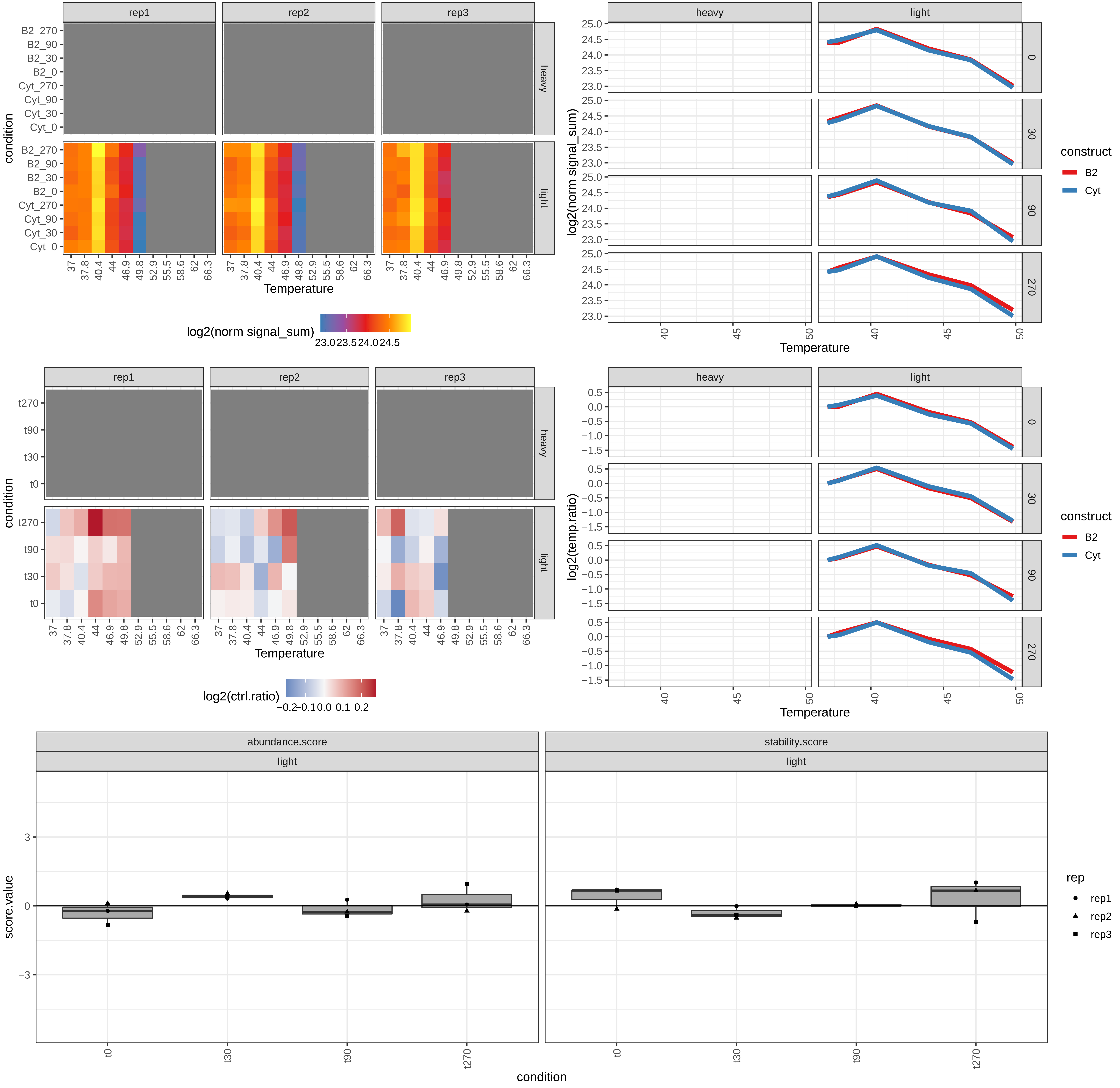

P33339 − Transcription factor tau 131 kDa subunit OS=Saccharomyces cerevisiae (strain ATCC 204508 / S288c) GN=TFC4 PE=1 SV=1

TFC8 − average.top3: 6.63043058823529 − max.qupm: 5

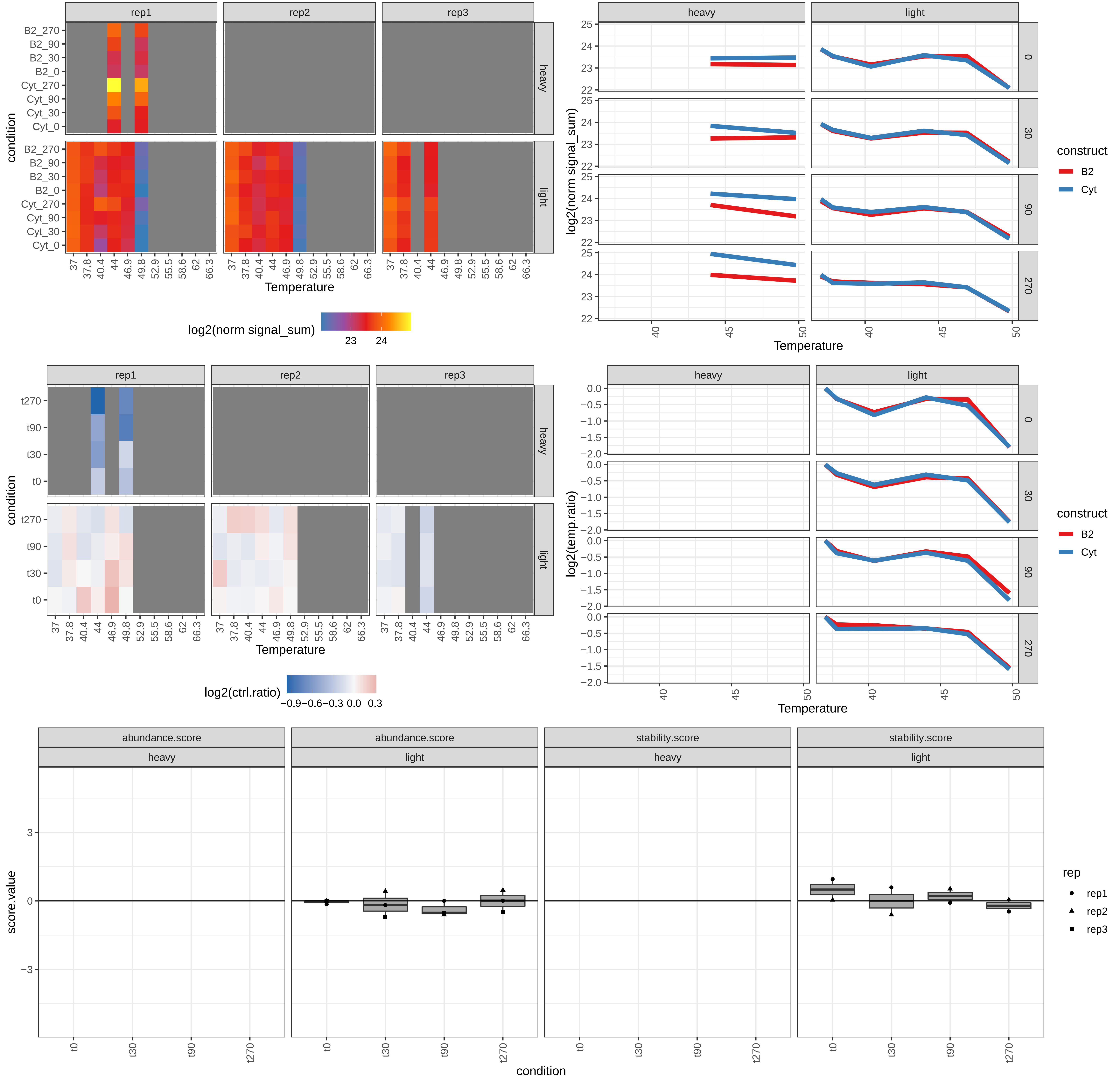

Q12308 − Transcription factor tau 60 kDa subunit OS=Saccharomyces cerevisiae (strain ATCC 204508 / S288c) GN=TFC8 PE=1 SV=1

TFG1 − average.top3: 6.53140705882353 − max.qupm: 5

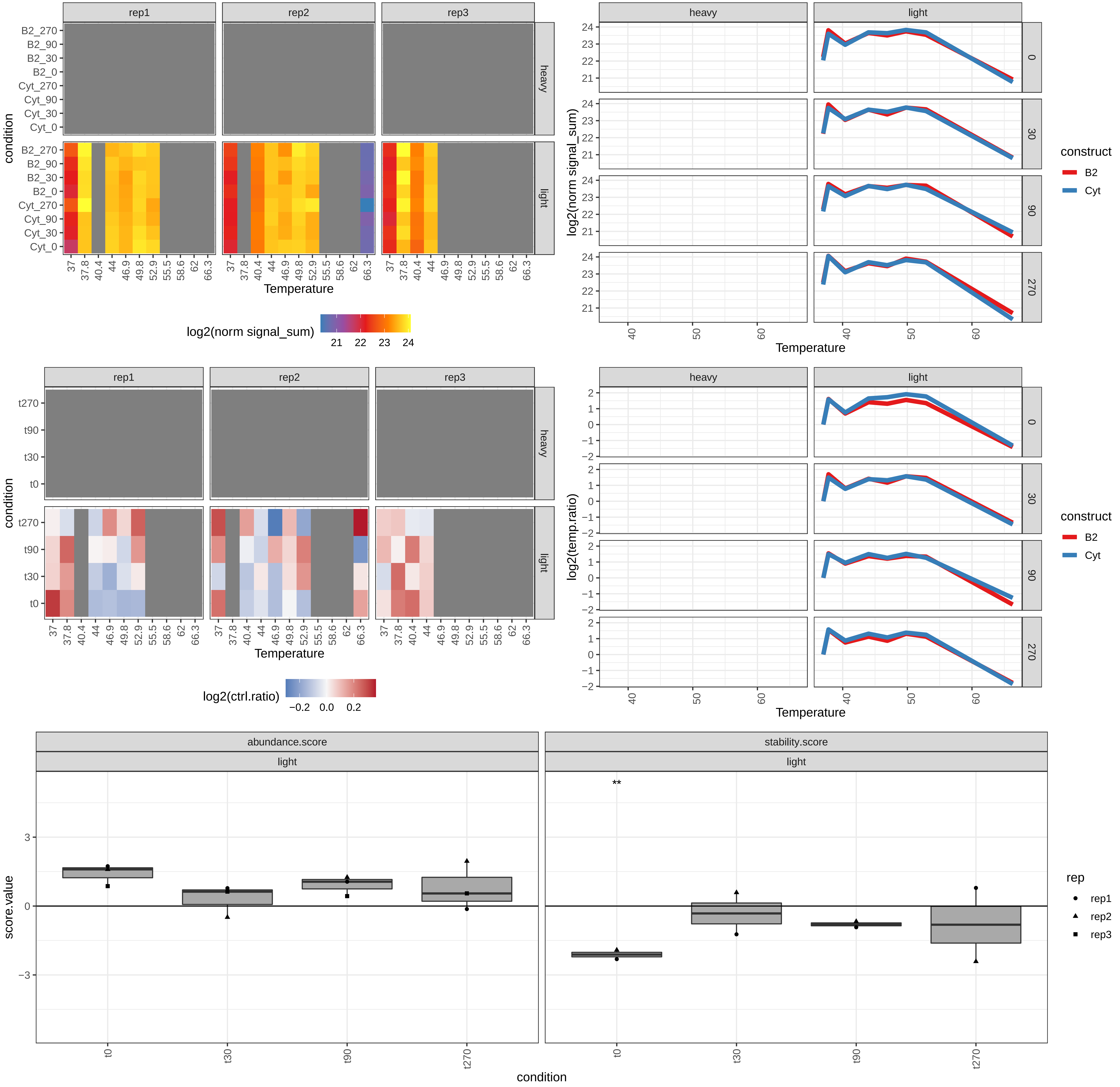

P41895 − Transcription initiation factor IIF subunit alpha OS=Saccharomyces cerevisiae (strain ATCC 204508 / S288c) GN=TFG1 PE=1 SV=2

TFG2 − average.top3: 6.71151913043478 − max.qupm: 4

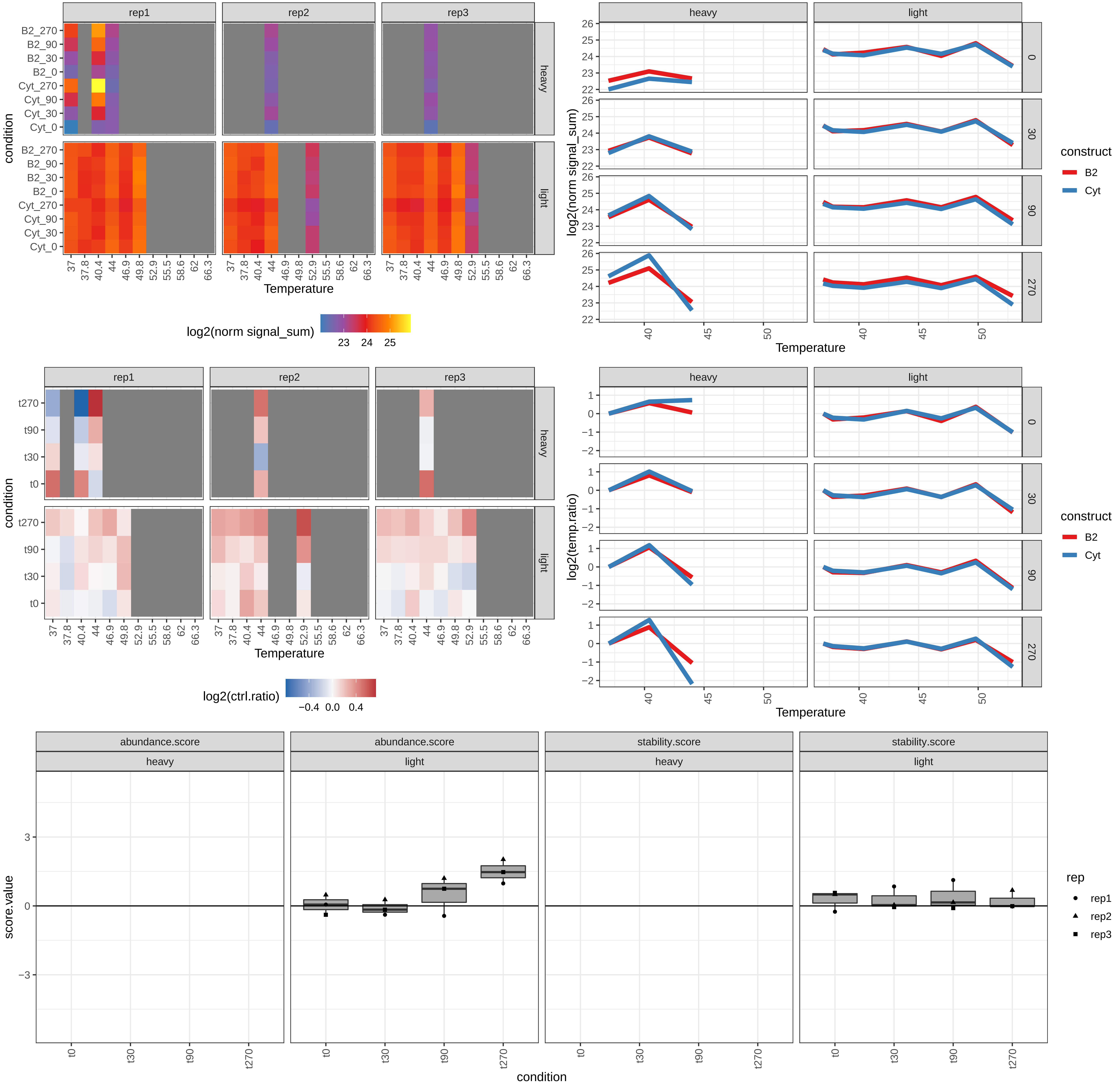

P41896 − Transcription initiation factor IIF subunit beta OS=Saccharomyces cerevisiae (strain ATCC 204508 / S288c) GN=TFG2 PE=1 SV=2

TFS1 − average.top3: 6.87845323529412 − max.qupm: 4

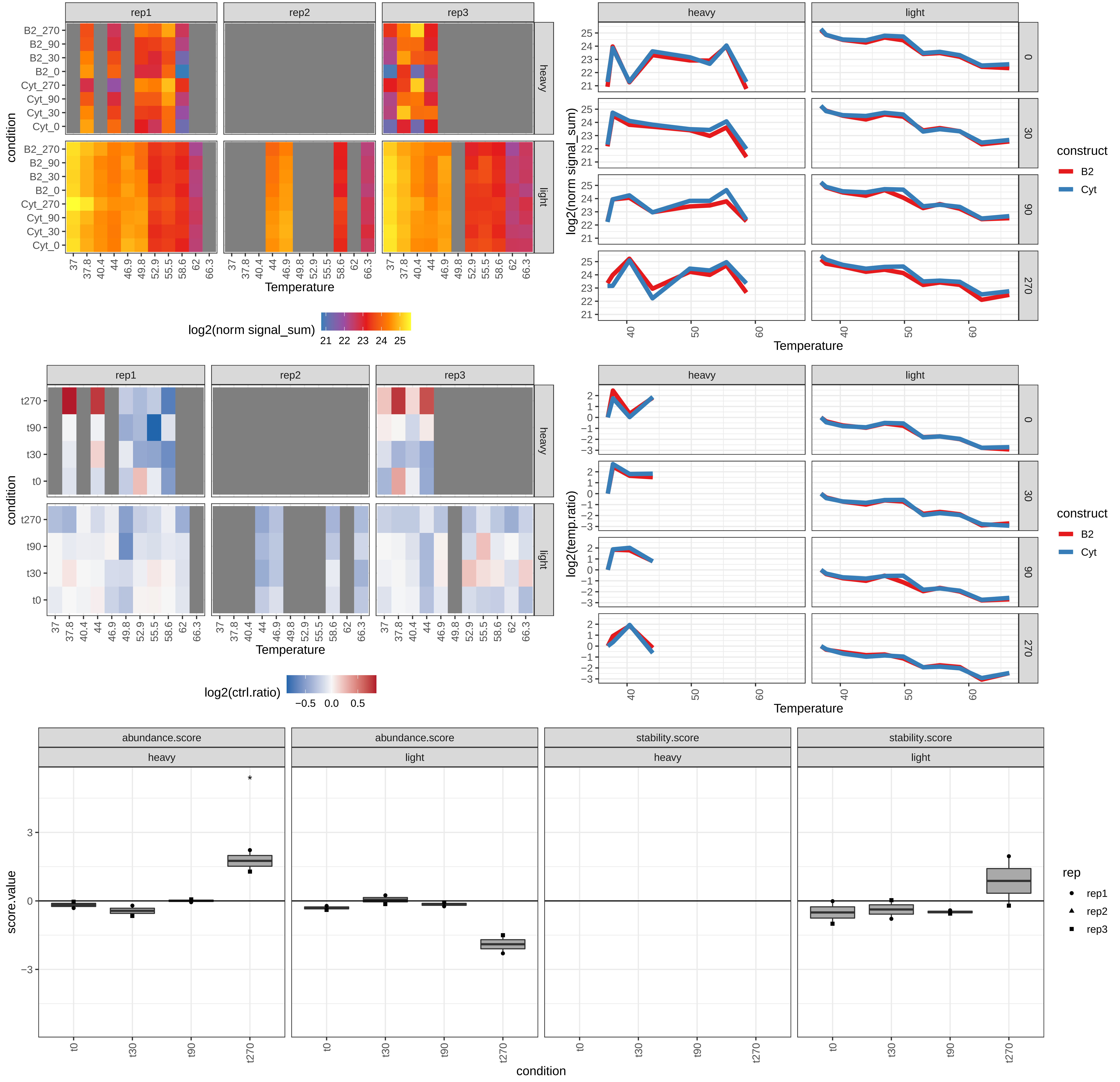

P14306 − Carboxypeptidase Y inhibitor OS=Saccharomyces cerevisiae (strain ATCC 204508 / S288c) GN=TFS1 PE=1 SV=2

TGL1 − average.top3: 6.90335428571429 − max.qupm: 5

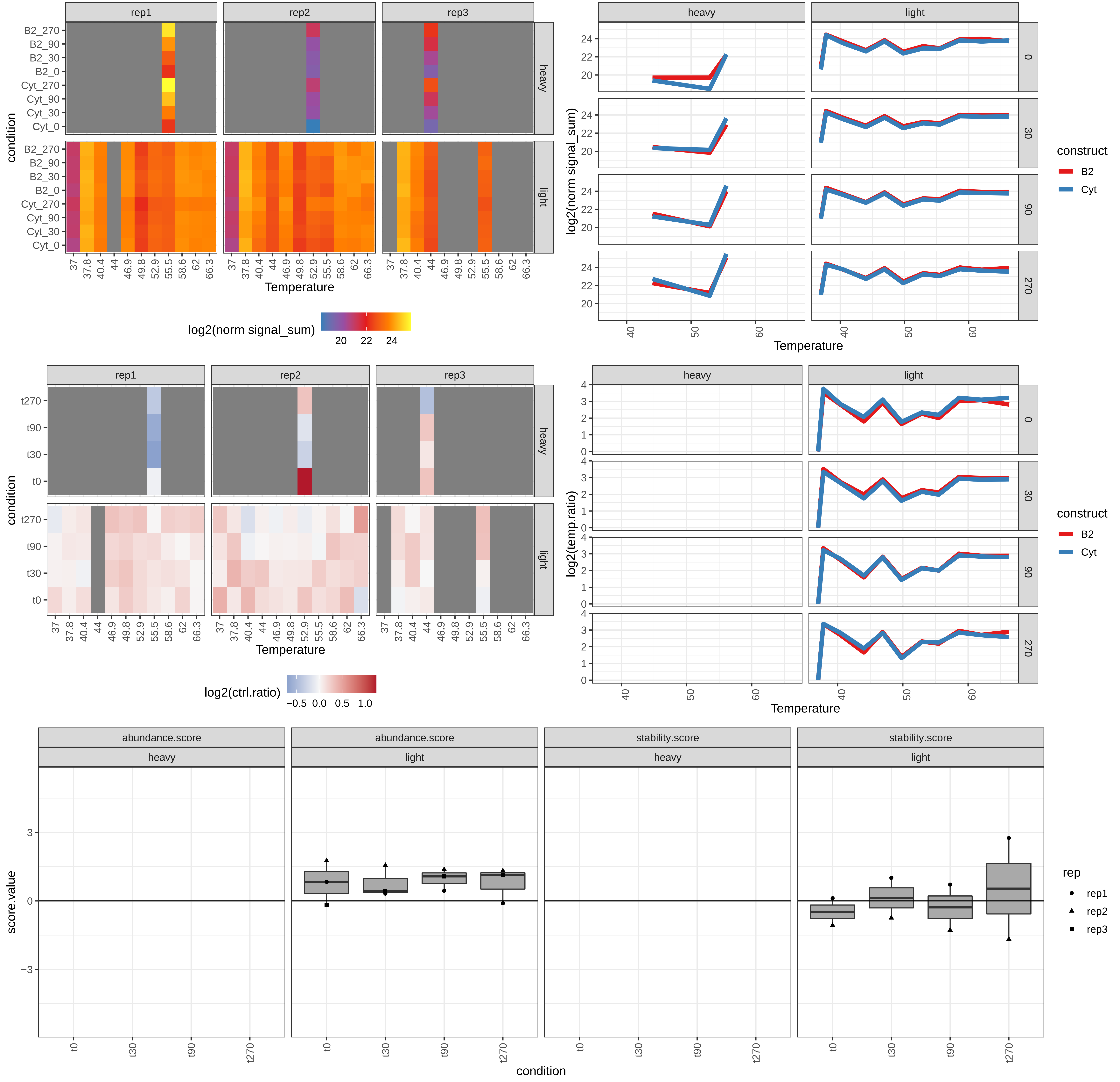

P34163 − Sterol esterase TGL1 OS=Saccharomyces cerevisiae (strain ATCC 204508 / S288c) GN=TGL1 PE=1 SV=1

TGL3 − average.top3: 6.36672 − max.qupm: 6

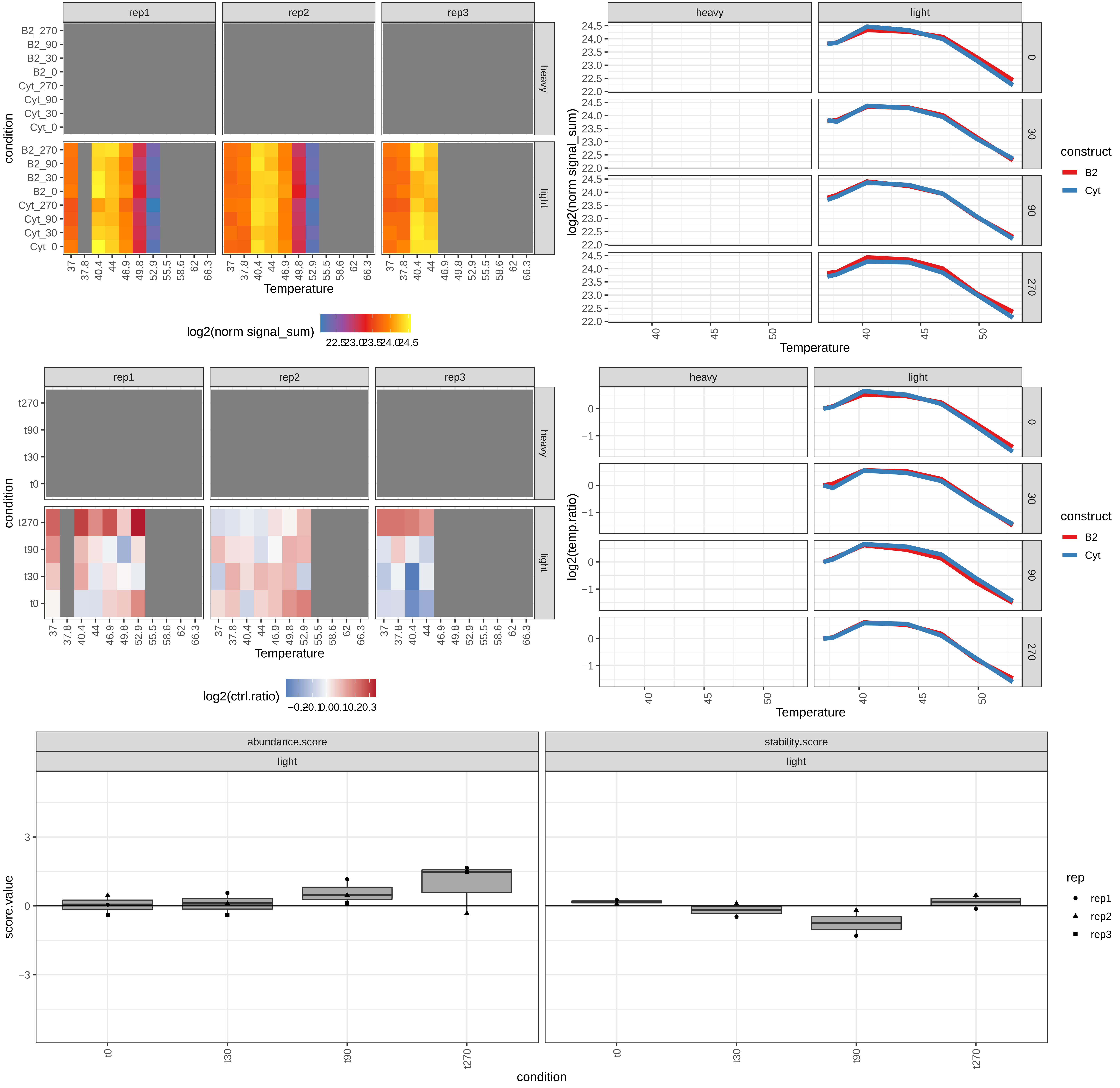

P40308 − Lipase 3 OS=Saccharomyces cerevisiae (strain ATCC 204508 / S288c) GN=TGL3 PE=1 SV=2

TGL4 − average.top3: 6.70342 − max.qupm: 6

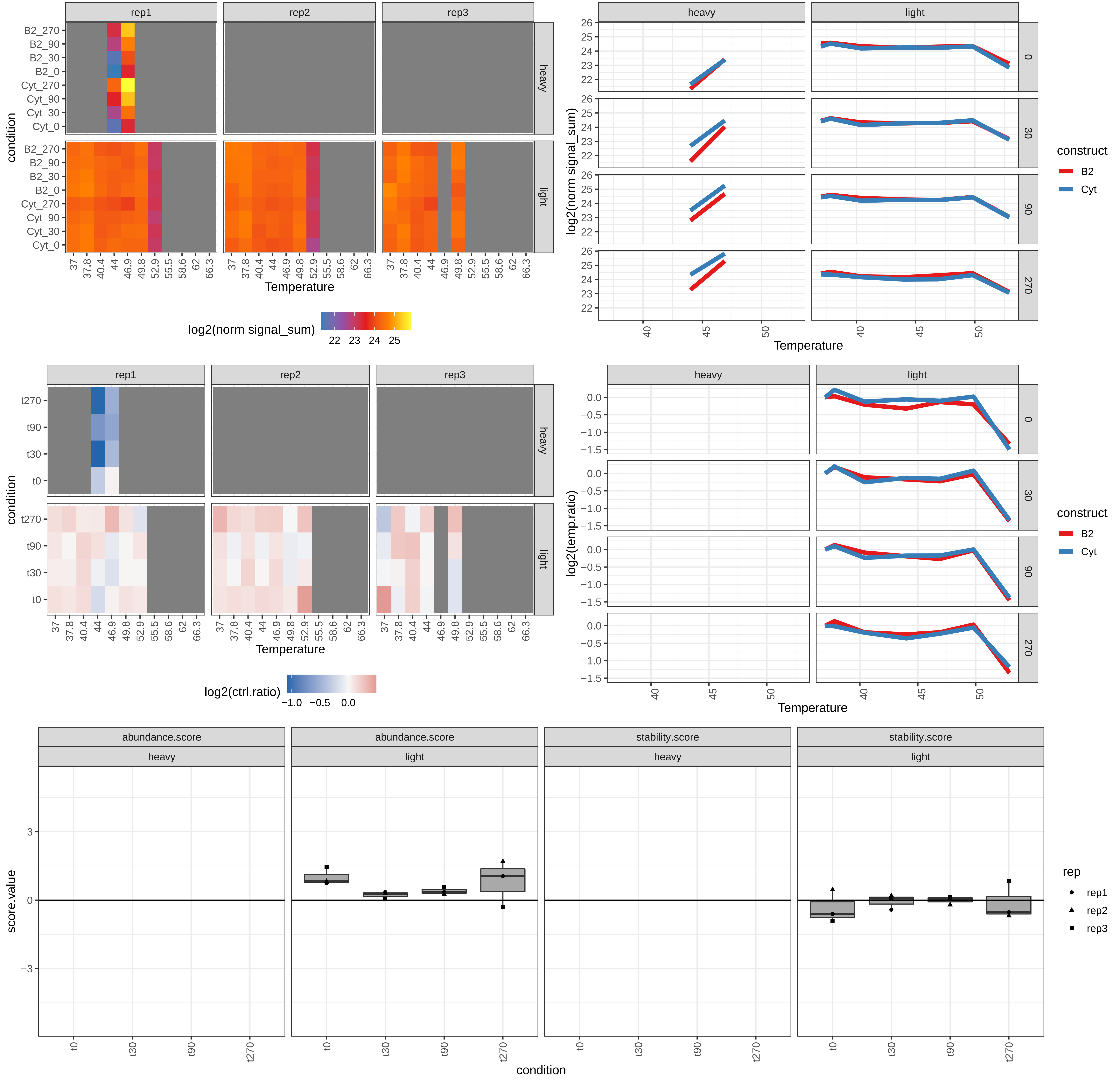

P36165 − Lipase 4 OS=Saccharomyces cerevisiae (strain ATCC 204508 / S288c) GN=TGL4 PE=1 SV=1

THI20 − average.top3: 6.61938166666667 − max.qupm: 4

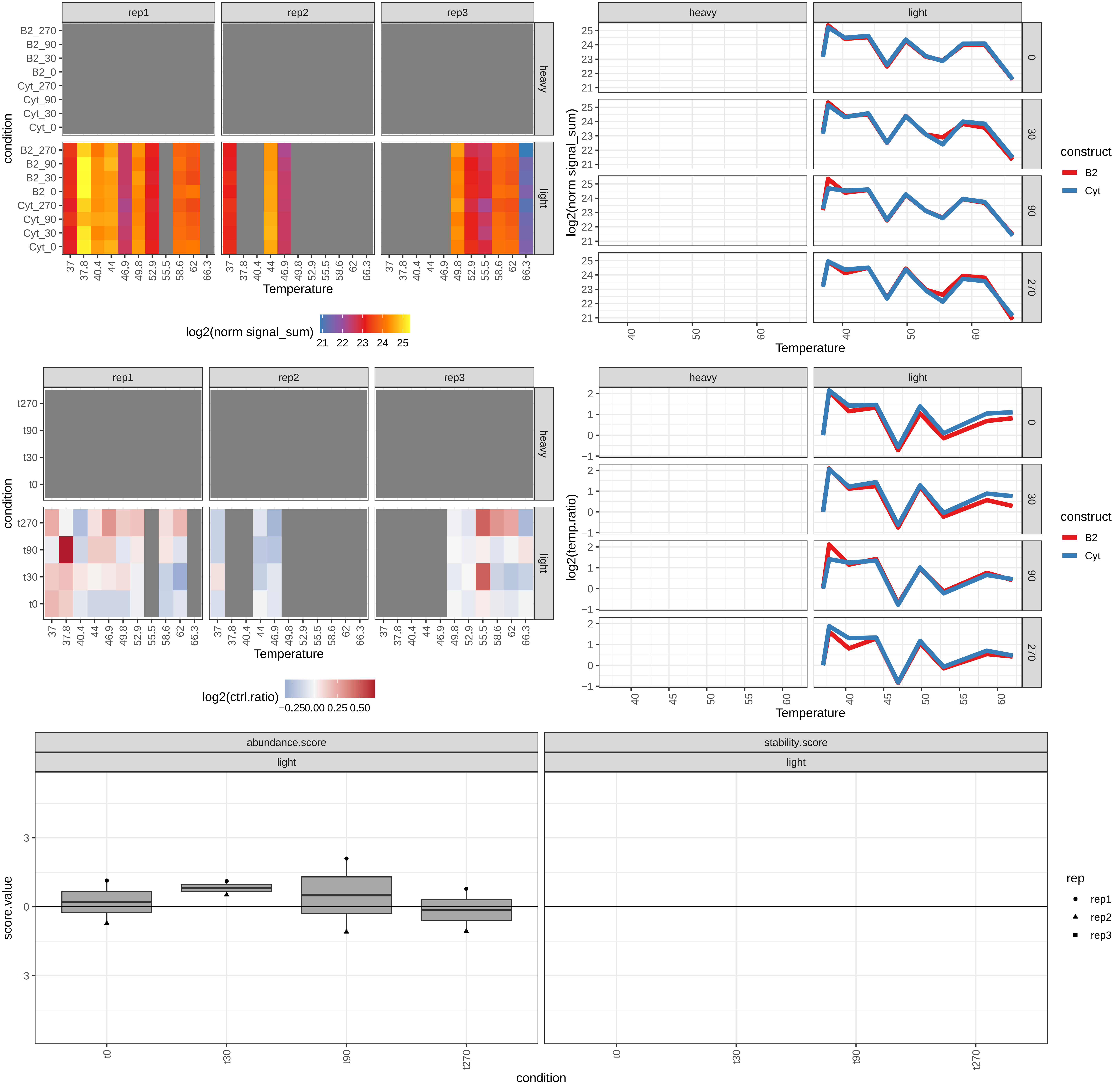

Q08224 − Hydroxymethylpyrimidine/phosphomethylpyrimidine kinase THI20 OS=Saccharomyces cerevisiae (strain ATCC 204508 / S288c) GN=THI20 PE=1 SV=1

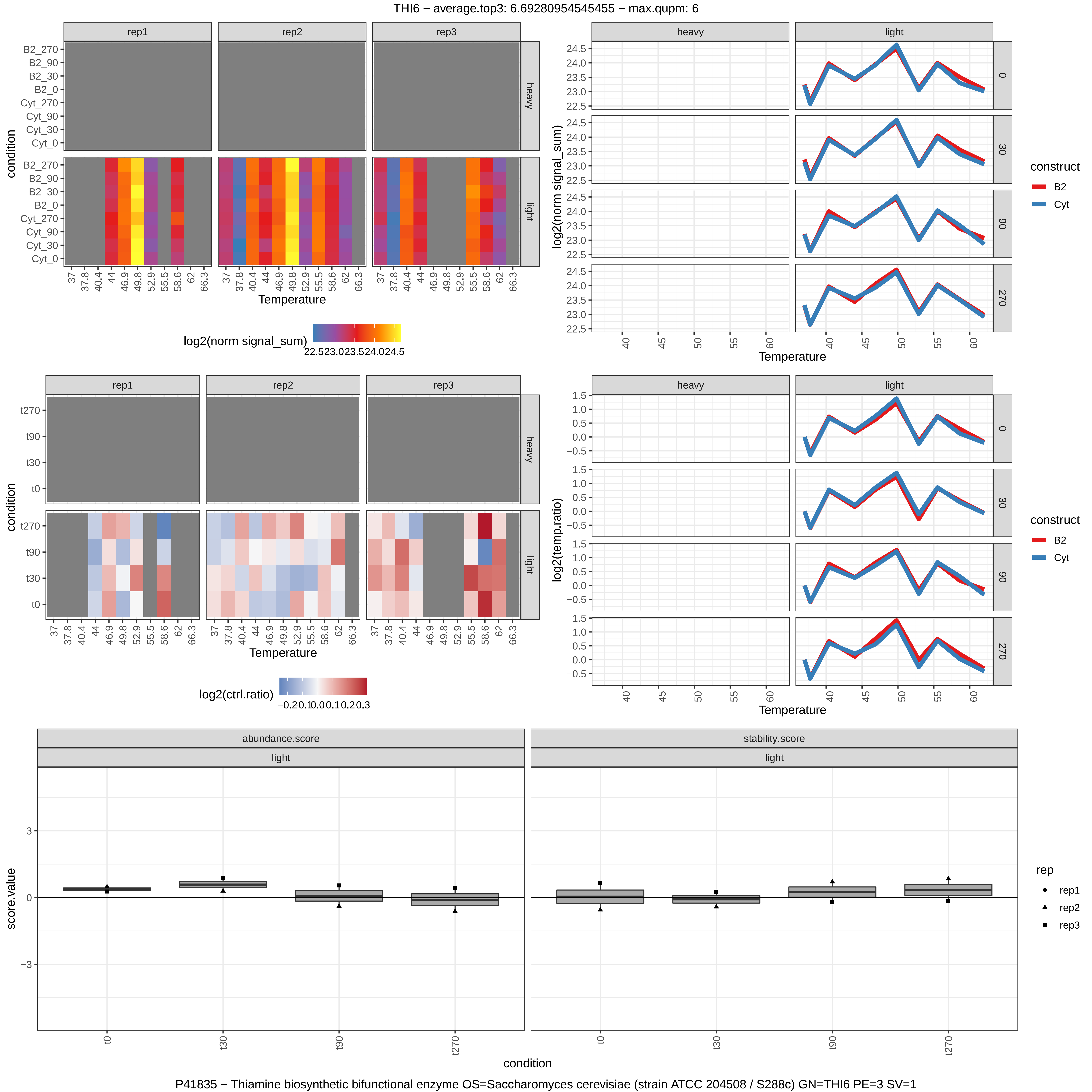
THI6 − average.top3: 6.69280954545455 − max.qupm: 6
rep1
rep2
rep3
heavy
light
condition
Temperature
log2(norm signal_sum)
construct
B2
Cyt
log2(ctrl.ratio)
log2(temp.ratio)
abundance.score
stability.score
score.value
rep
rep1
rep2
rep3
t0
t30
t90
t270
P41835 − Thiamine biosynthetic bifunctional enzyme OS=Saccharomyces cerevisiae (strain ATCC 204508 / S288c) GN=THI6 PE=3 SV=1

THI7 − average.top3: 6.68331727272727 − max.qupm: 5

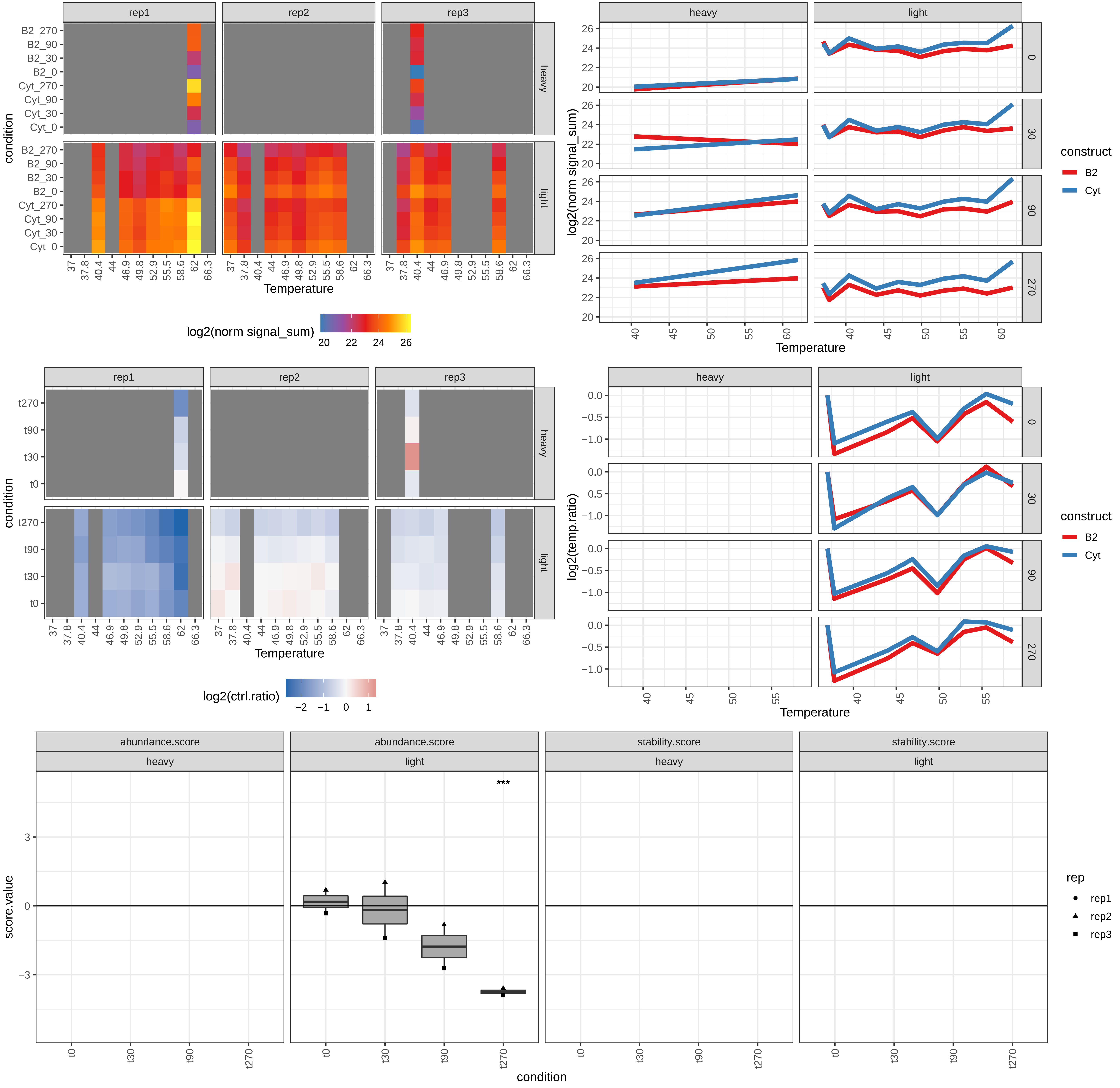

Q05998 − Thiamine transporter OS=Saccharomyces cerevisiae (strain ATCC 204508 / S288c) GN=THI7 PE=1 SV=1

THI80 − average.top3: 6.64973709677419 − max.qupm: 8

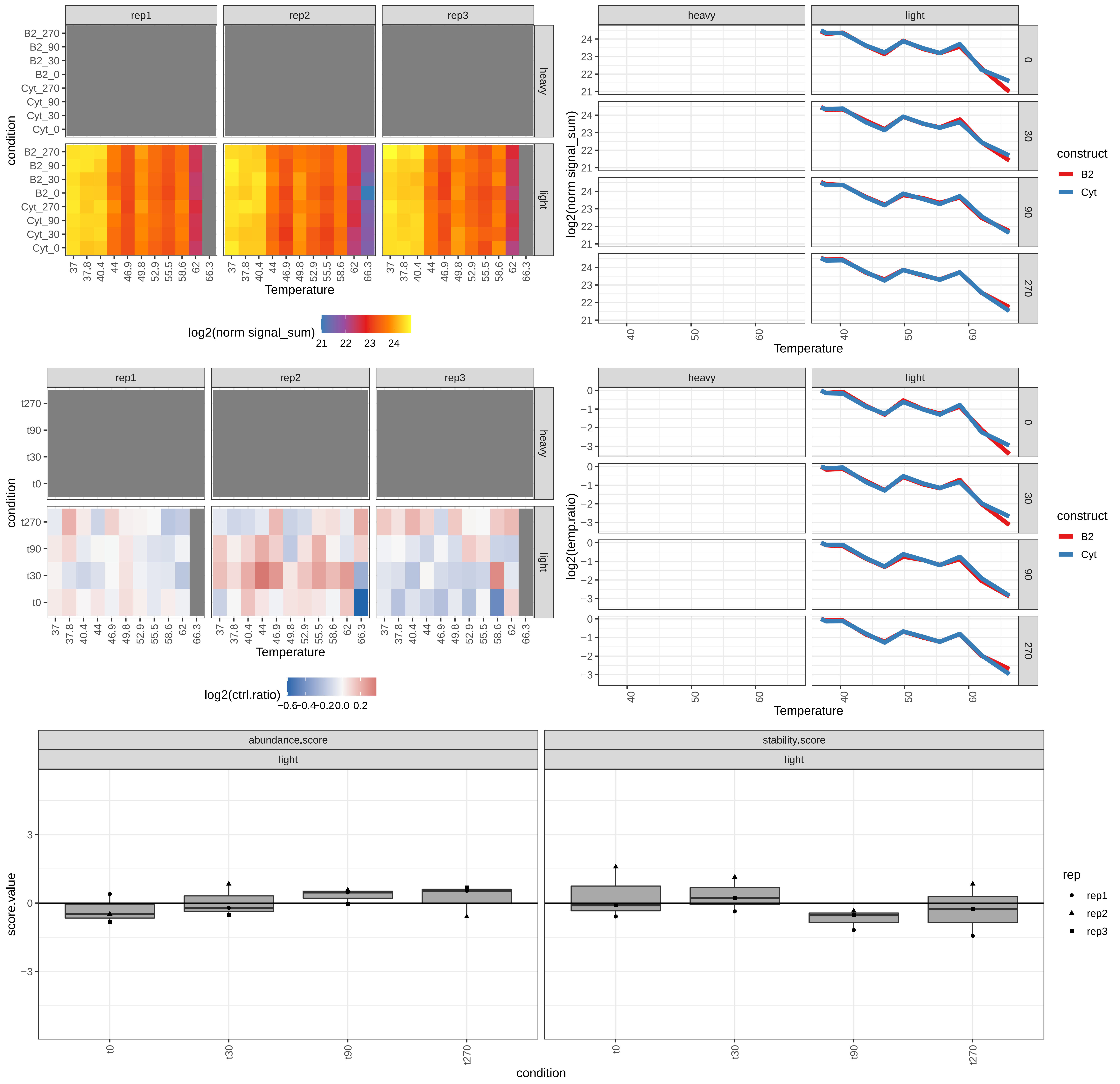

P35202 − Thiamine pyrophosphokinase OS=Saccharomyces cerevisiae (strain ATCC 204508 / S288c) GN=THI80 PE=1 SV=1

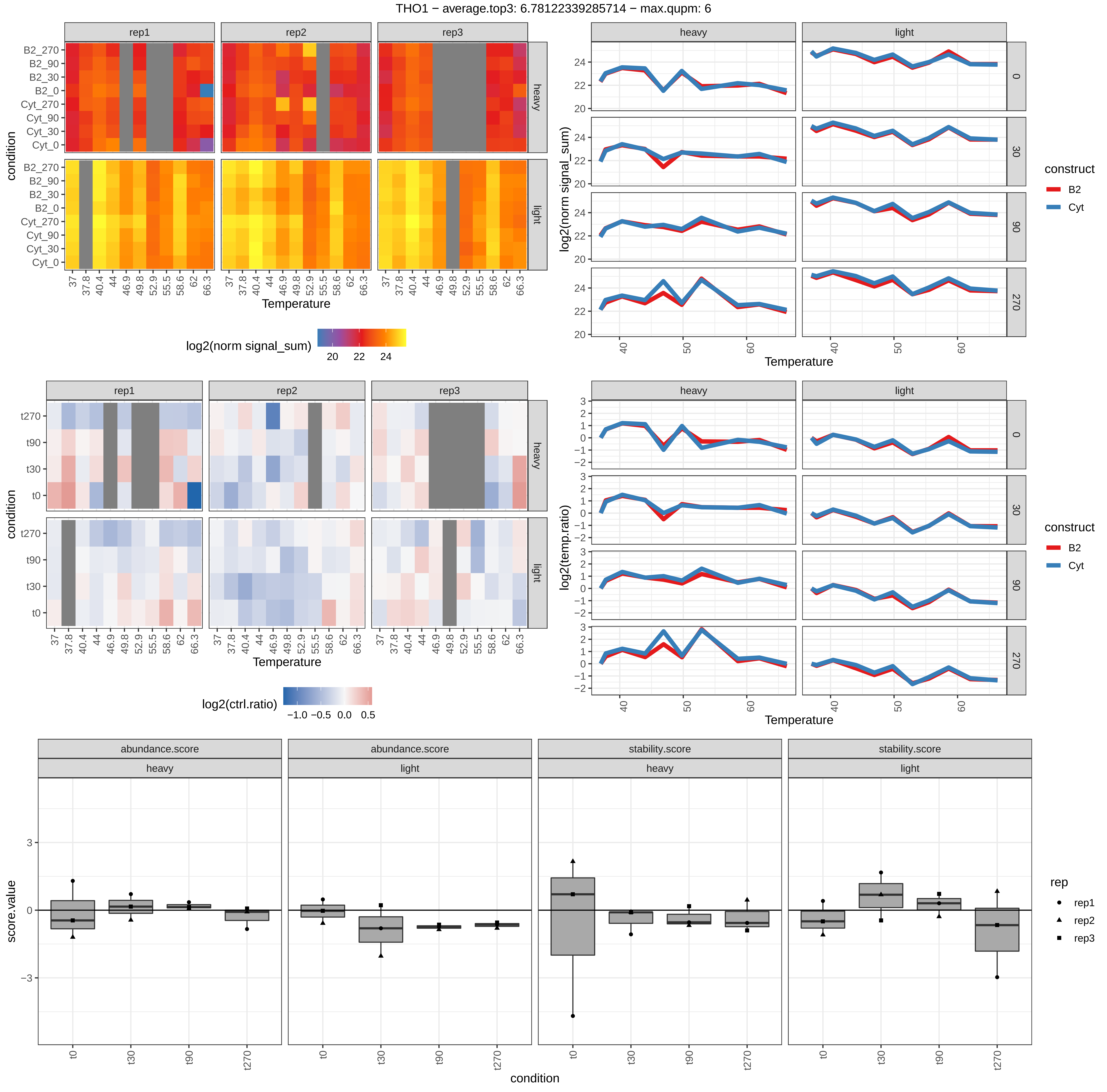

P40040 − Protein THO1 OS=Saccharomyces cerevisiae (strain ATCC 204508 / S288c) GN=THO1 PE=1 SV=1

THO2 − average.top3: 6.78031807692308 − max.qupm: 14

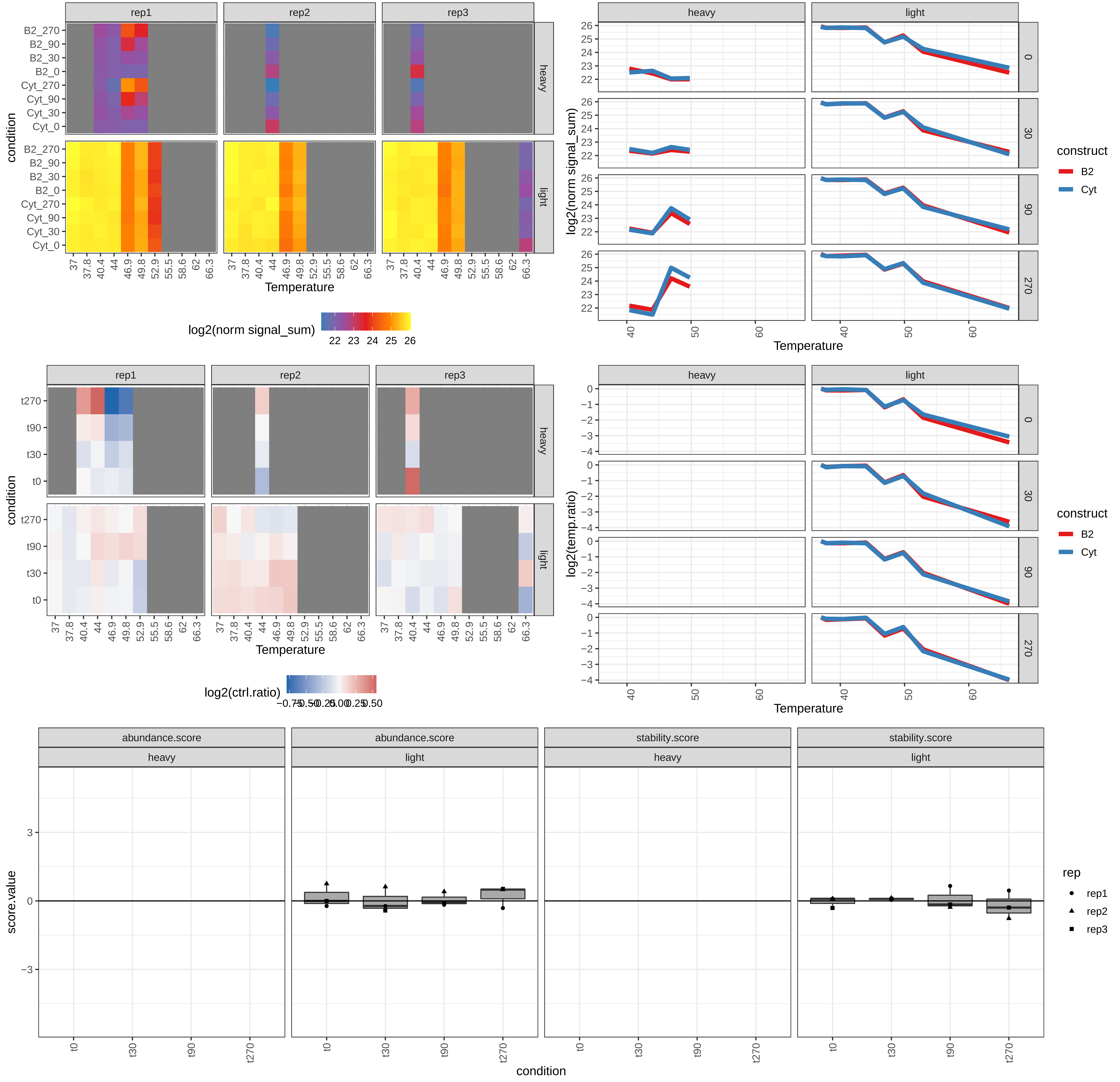

P53552 − THO complex subunit 2 OS=Saccharomyces cerevisiae (strain ATCC 204508 / S288c) GN=THO2 PE=1 SV=1

THP3 − average.top3: 6.38924052631579 − max.qupm: 4

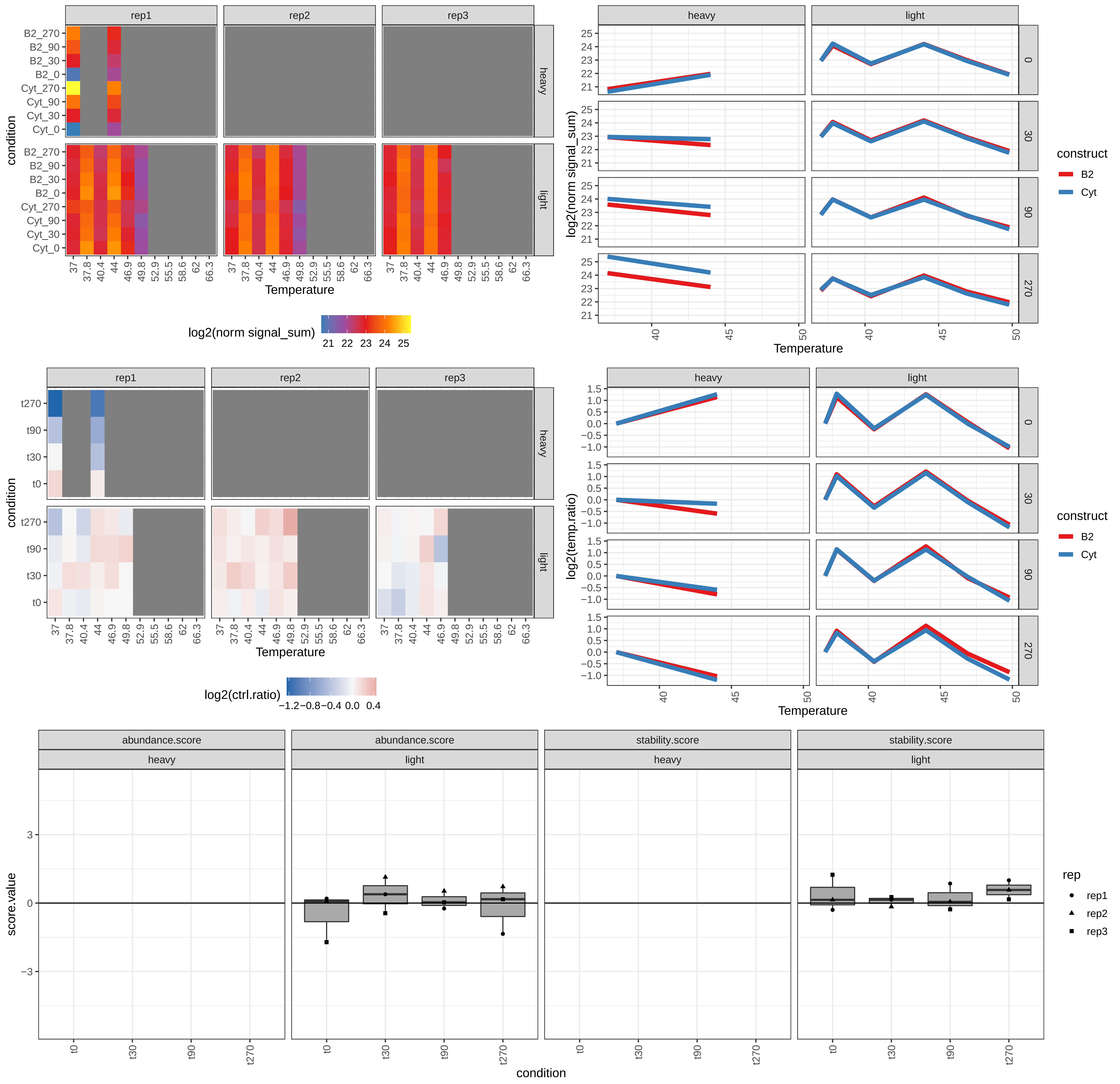

Q12049 − Protein THP3 OS=Saccharomyces cerevisiae (strain ATCC 204508 / S288c) GN=THP3 PE=1 SV=1

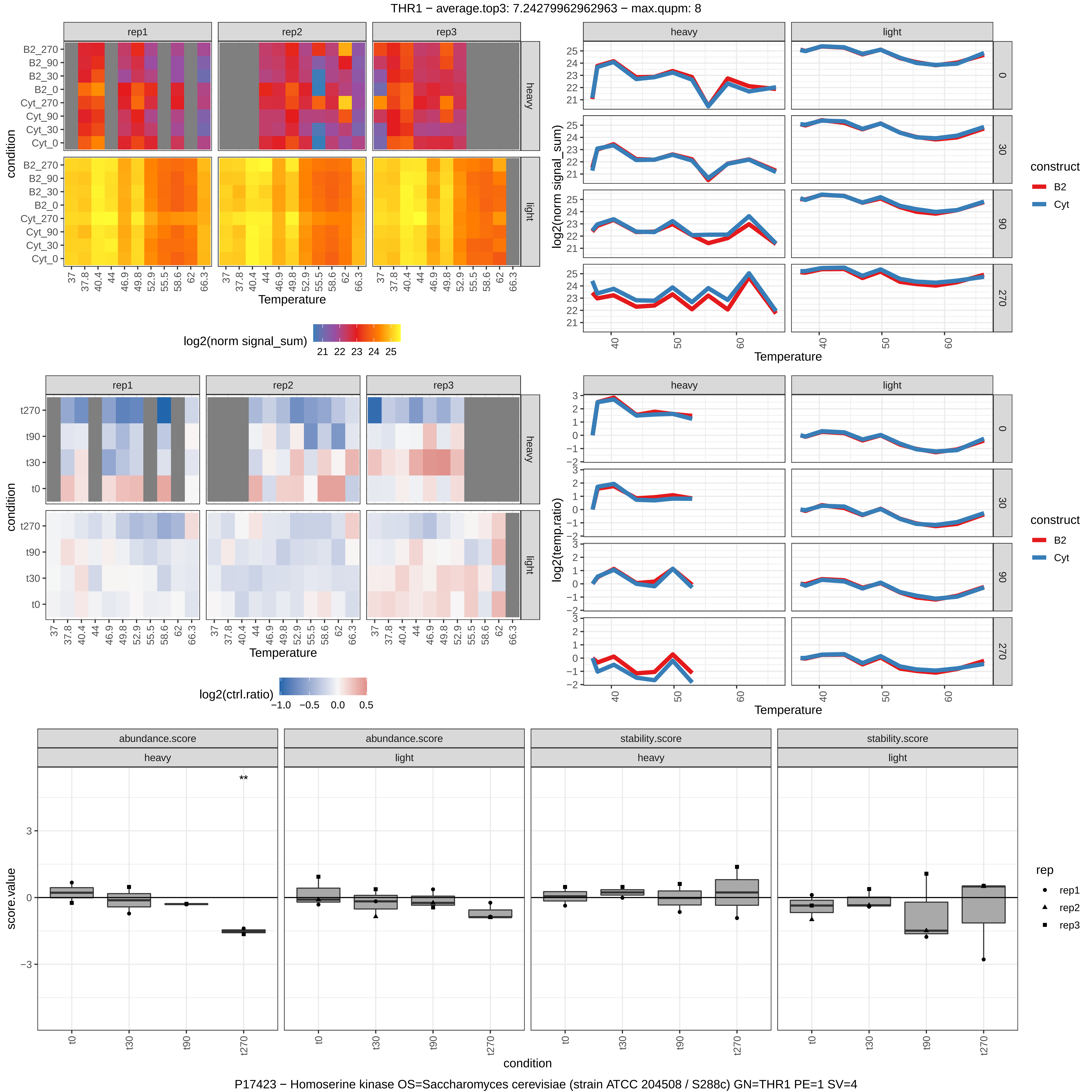
THR1 − average.top3: 7.24279962962963 − max.qupm: 8
rep1
rep2
rep3
heavy
light
B2_270
B2_90
B2_30
B2_0
Cyt_270
Cyt_90
Cyt_30
Cyt_0
condition
Temperature
37 37.8 40.4 44 46.9 49.8 52.9 55.5 58.6 62 66.3
log2(norm signal_sum)
21 22 23 24 25
log2(norm signal_sum)
0
30
90
270
40 50 60
construct
B2
Cyt
t270
t90
t30
t0
log2(ctrl.ratio)
−1.0 −0.5 0.0 0.5
log2(temp.ratio)
abundance.score
stability.score
score.value
condition
**
rep
rep1
rep2
rep3
P17423 − Homoserine kinase OS=Saccharomyces cerevisiae (strain ATCC 204508 / S288c) GN=THR1 PE=1 SV=4

THR4 − average.top3: 7.59402838709677 − max.qupm: 22

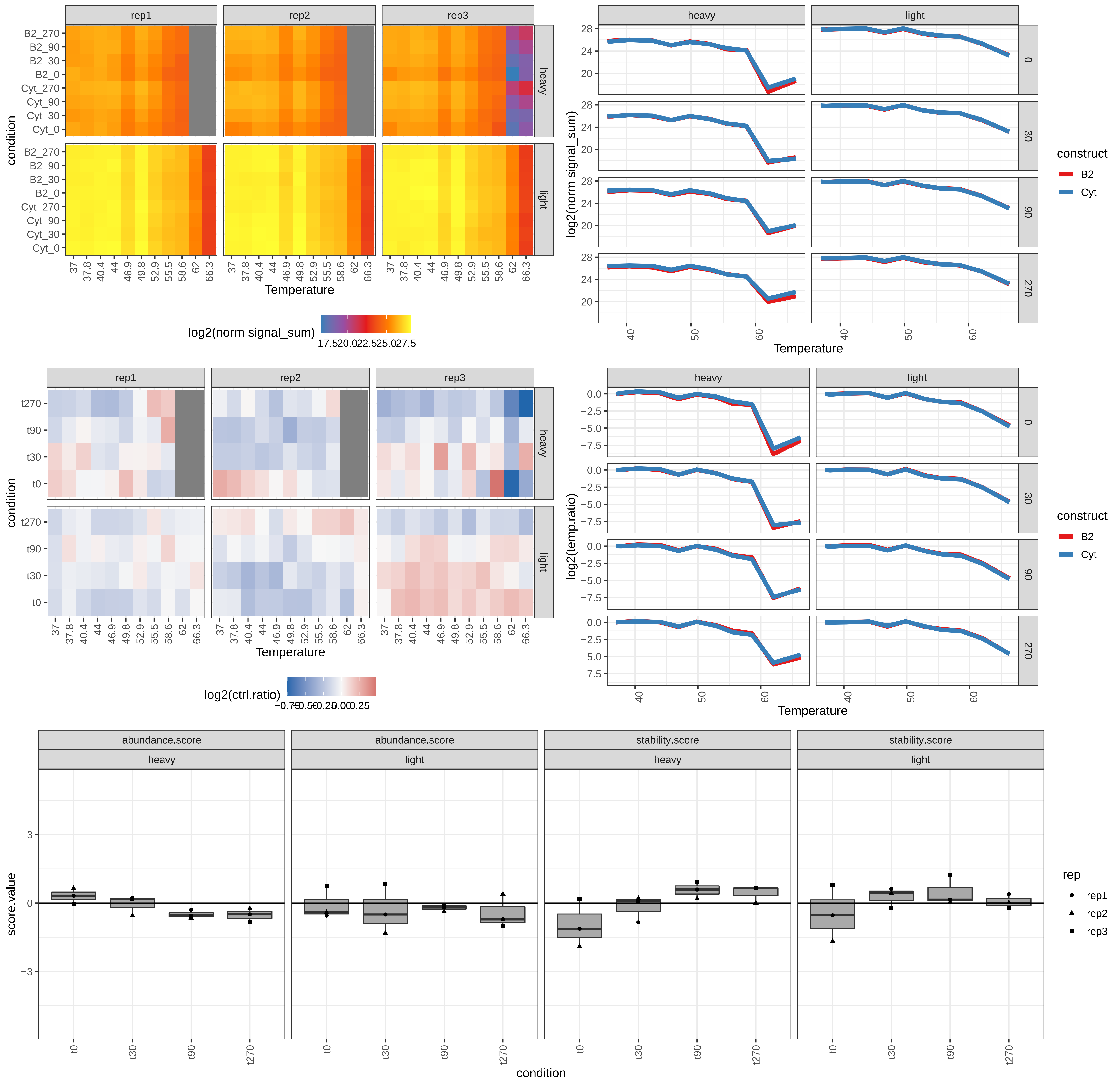

P16120 − Threonine synthase OS=Saccharomyces cerevisiae (strain ATCC 204508 / S288c) GN=THR4 PE=1 SV=1

THS1 − average.top3: 7.59268333333333 − max.qupm: 32

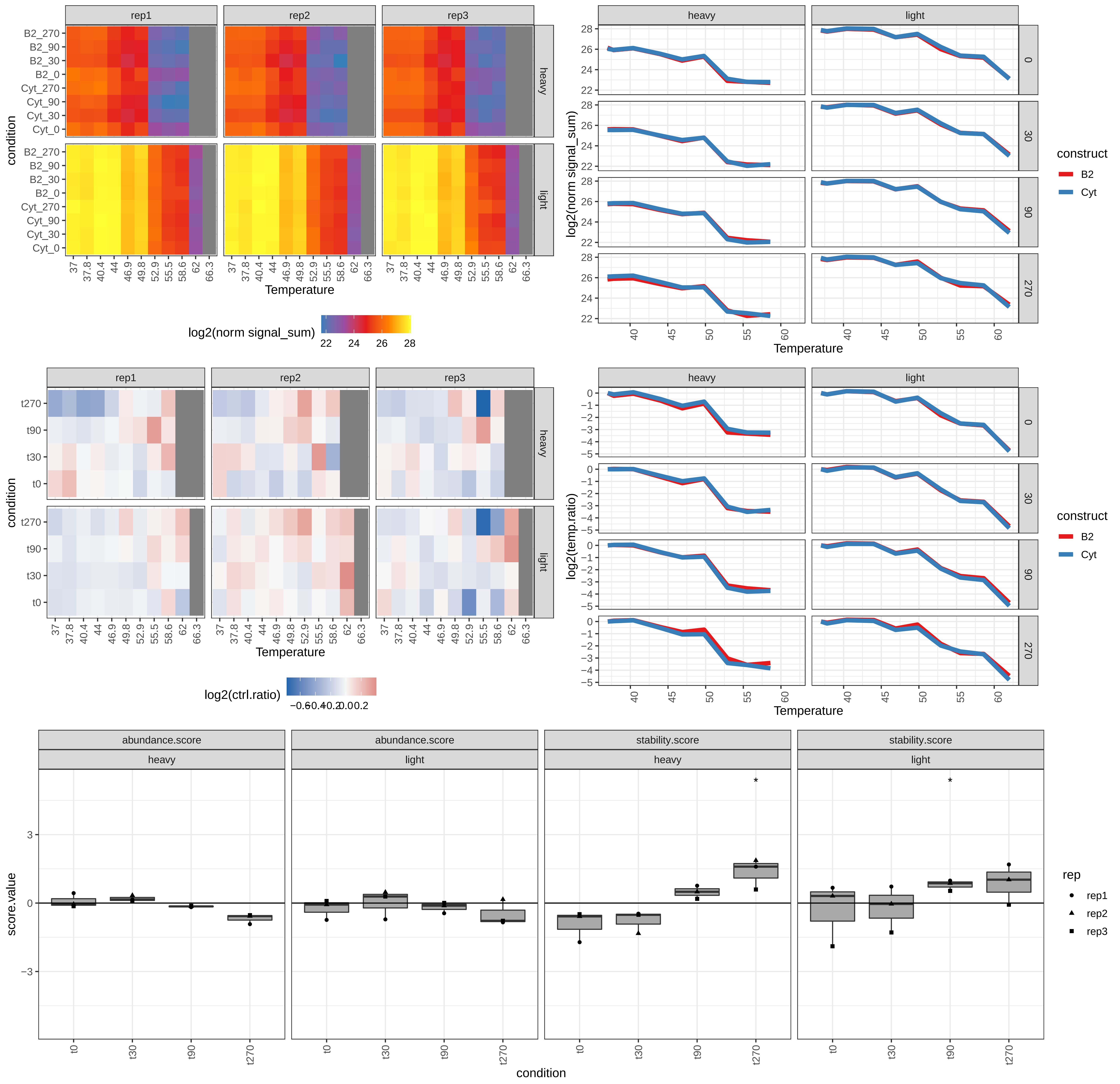

P04801 − Threonine−−tRNA ligase, cytoplasmic OS=Saccharomyces cerevisiae (strain ATCC 204508 / S288c) GN=THS1 PE=1 SV=2

TIF1 − average.top3: 7.76190220338983 − max.qupm: 22

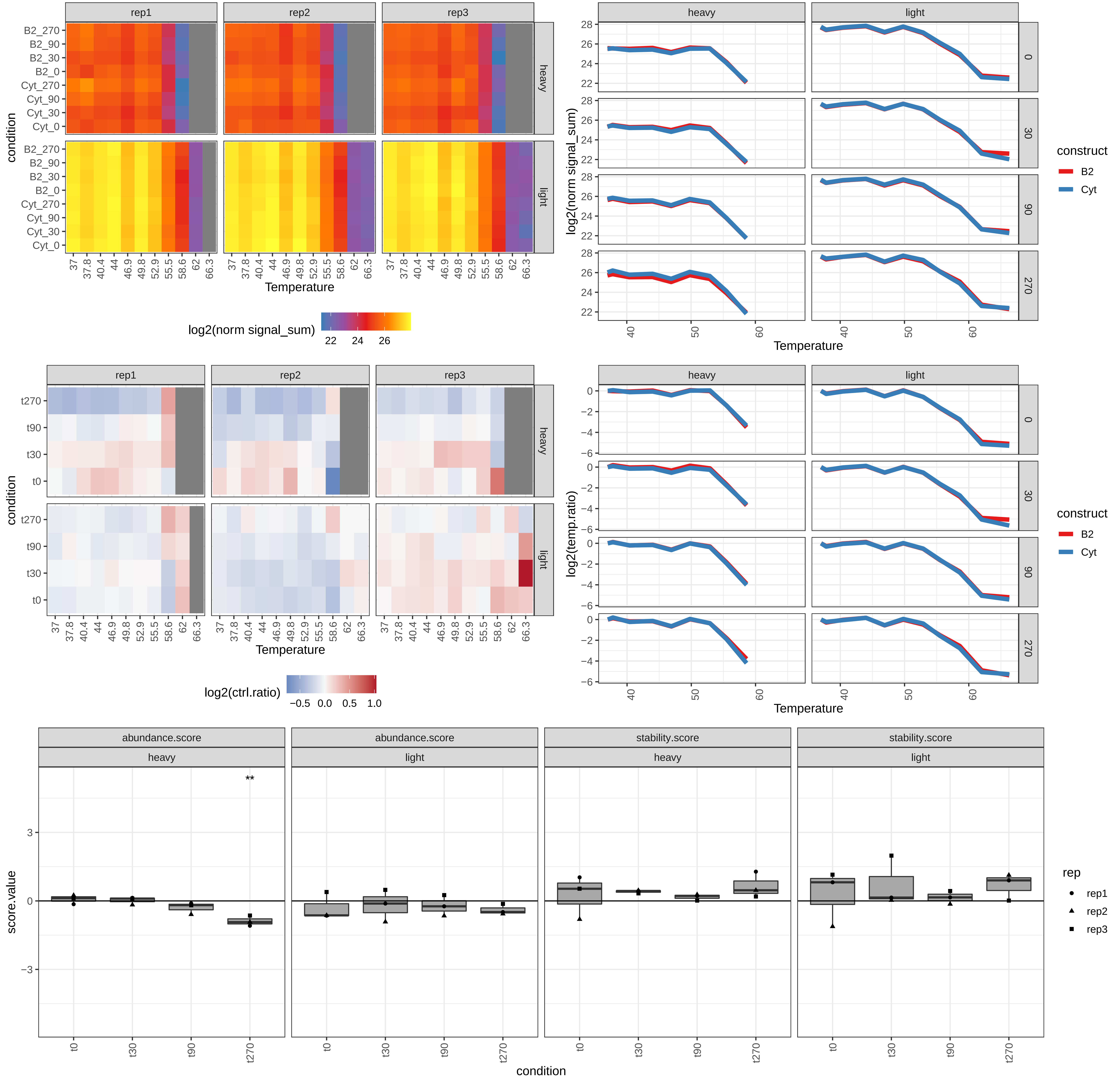

P10081 − ATP−dependent RNA helicase eIF4A OS=Saccharomyces cerevisiae (strain ATCC 204508 / S288c) GN=TIF1 PE=1 SV=3

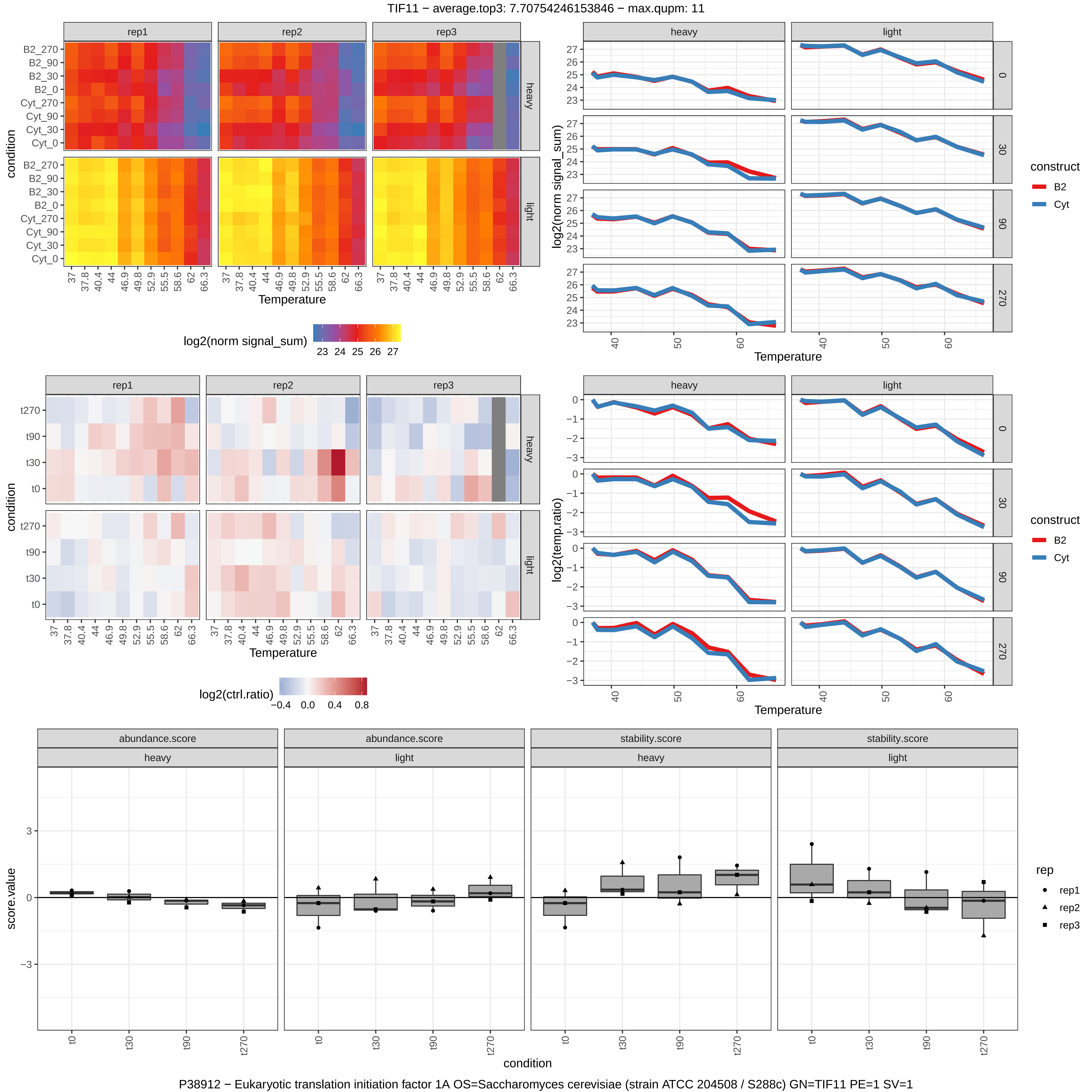
TIF11 − average.top3: 7.70754246153846 − max.qupm: 11
rep1
rep2
rep3
heavy
light
condition
Temperature
log2(norm signal_sum)
log2(ctrl.ratio)
log2(temp.ratio)
construct
B2
Cyt
abundance.score
stability.score
score.value
rep
rep1
rep2
rep3
P38912 − Eukaryotic translation initiation factor 1A OS=Saccharomyces cerevisiae (strain ATCC 204508 / S288c) GN=TIF11 PE=1 SV=1

TIF3 − average.top3: 7.29182905660377 − max.qupm: 16

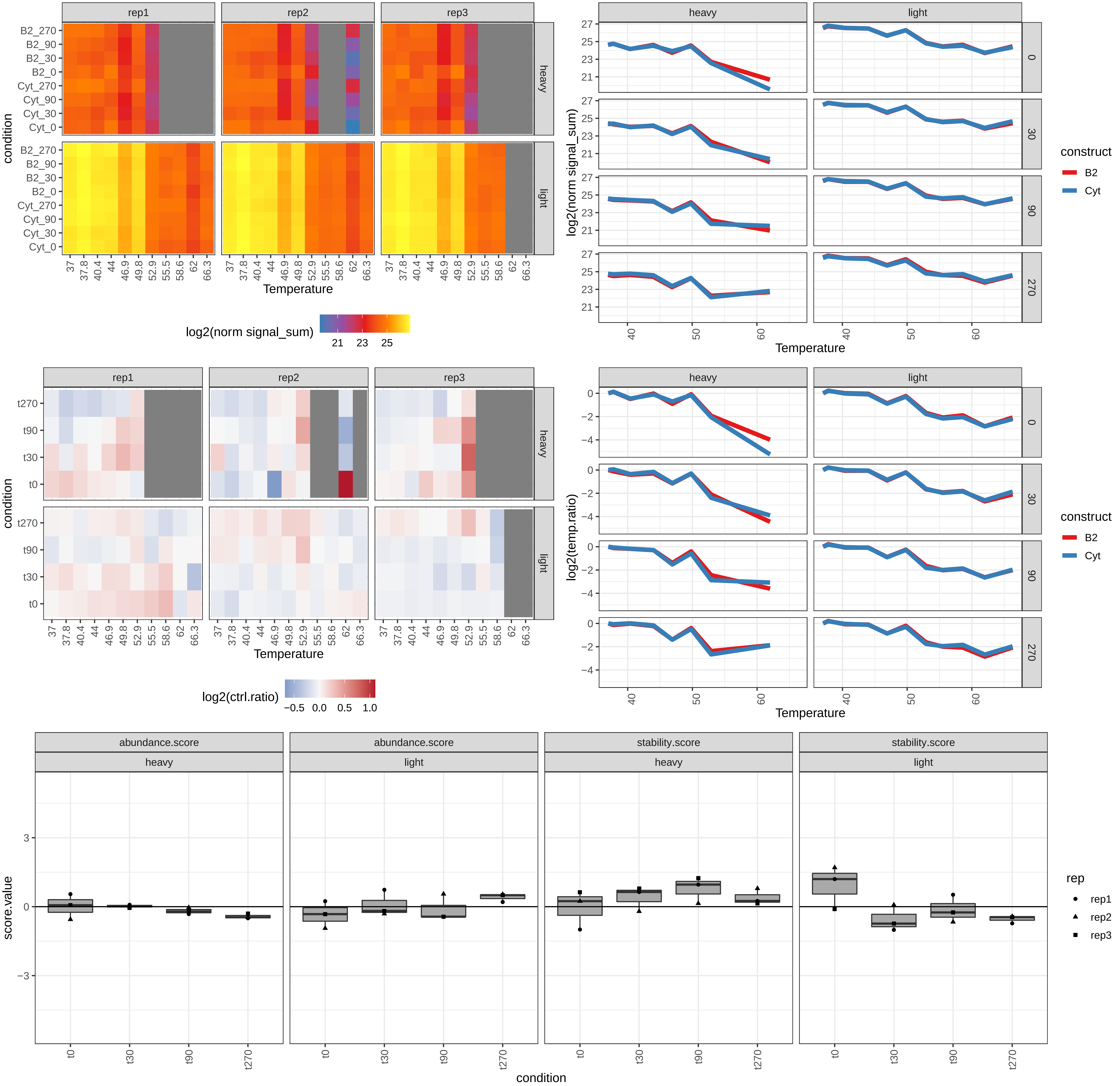

P34167 − Eukaryotic translation initiation factor 4B OS=Saccharomyces cerevisiae (strain ATCC 204508 / S288c) GN=TIF3 PE=1 SV=1

TIF34 − average.top3: 7.11093 − max.qupm: 13

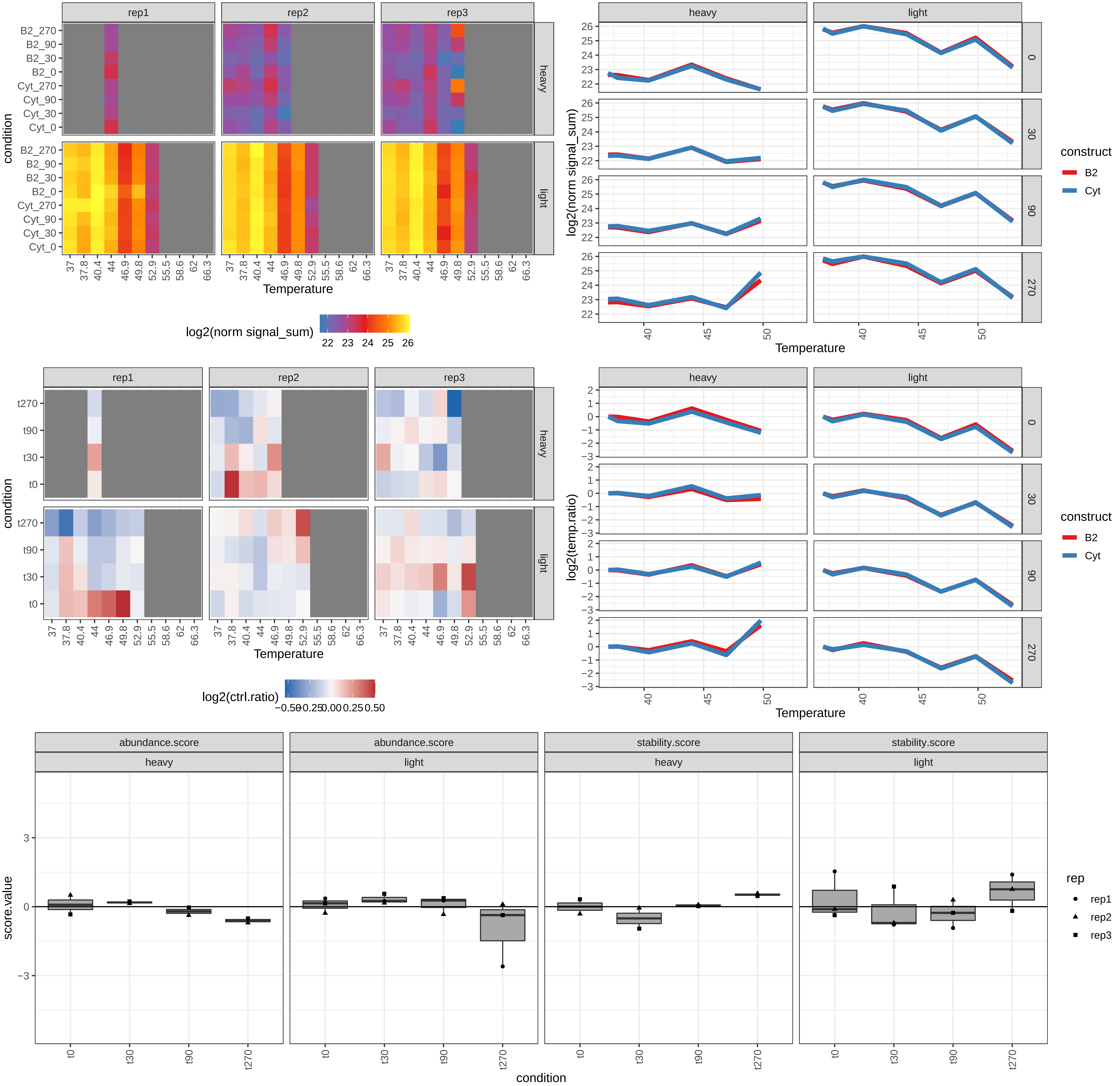

P40217 − Eukaryotic translation initiation factor 3 subunit I OS=Saccharomyces cerevisiae (strain ATCC 204508 / S288c) GN=TIF34 PE=1 SV=1

TIF35 − average.top3: 7.27376735294118 − max.qupm: 9

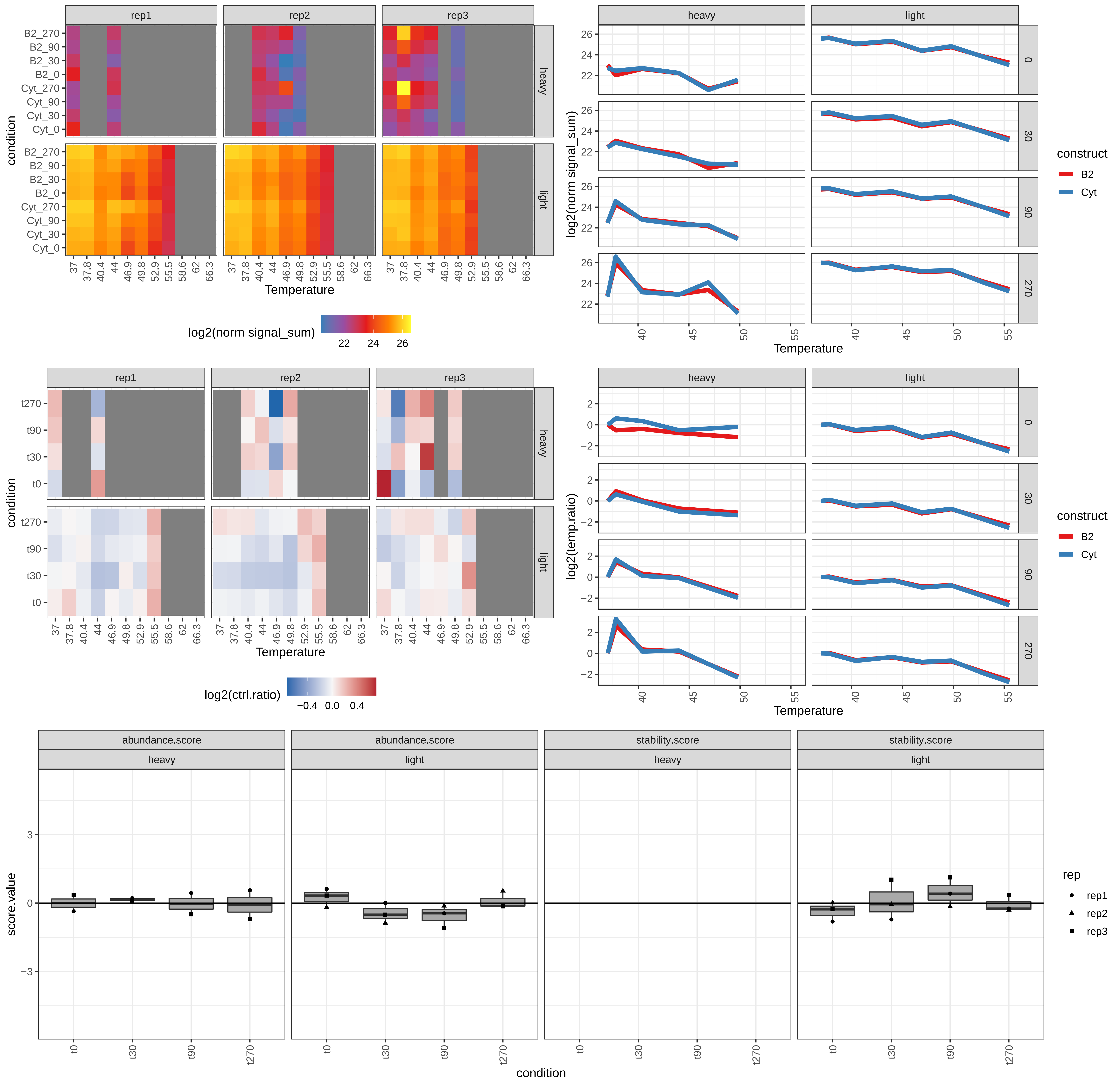

Q04067 − Eukaryotic translation initiation factor 3 subunit G OS=Saccharomyces cerevisiae (strain ATCC 204508 / S288c) GN=TIF35 PE=1 SV=1

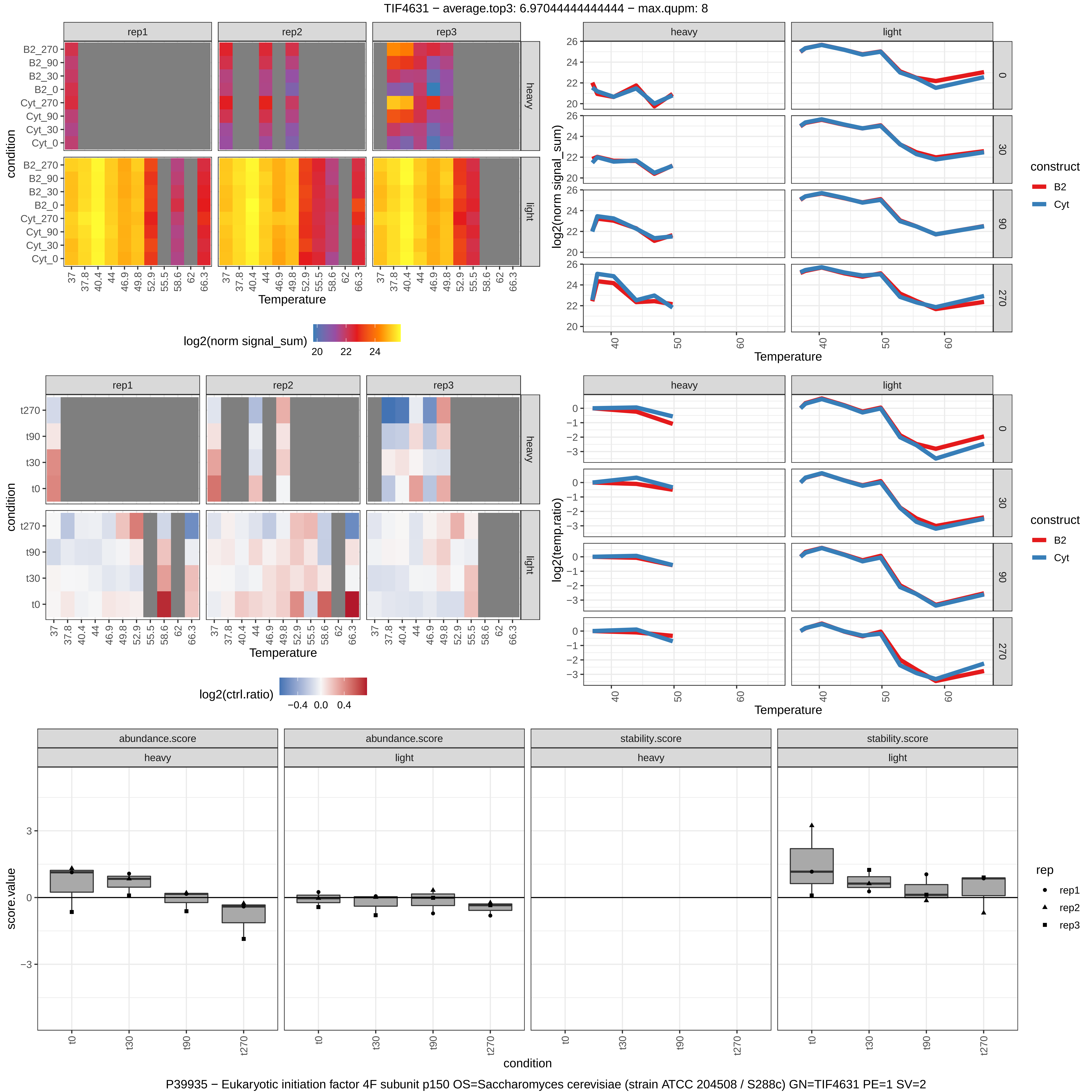

TIF4631 − average.top3: 6.97044444444444 − max.qupm: 8
rep1
rep2
rep3
heavy
light
condition
Temperature
log2(norm signal_sum)
log2(temp.ratio)
log2(ctrl.ratio)
construct
B2
Cyt
abundance.score
stability.score
score.value
rep
rep1
rep2
rep3
P39935 − Eukaryotic initiation factor 4F subunit p150 OS=Saccharomyces cerevisiae (strain ATCC 204508 / S288c) GN=TIF4631 PE=1 SV=2

TIF4632 − average.top3: 6.78184083333333 − max.qupm: 4

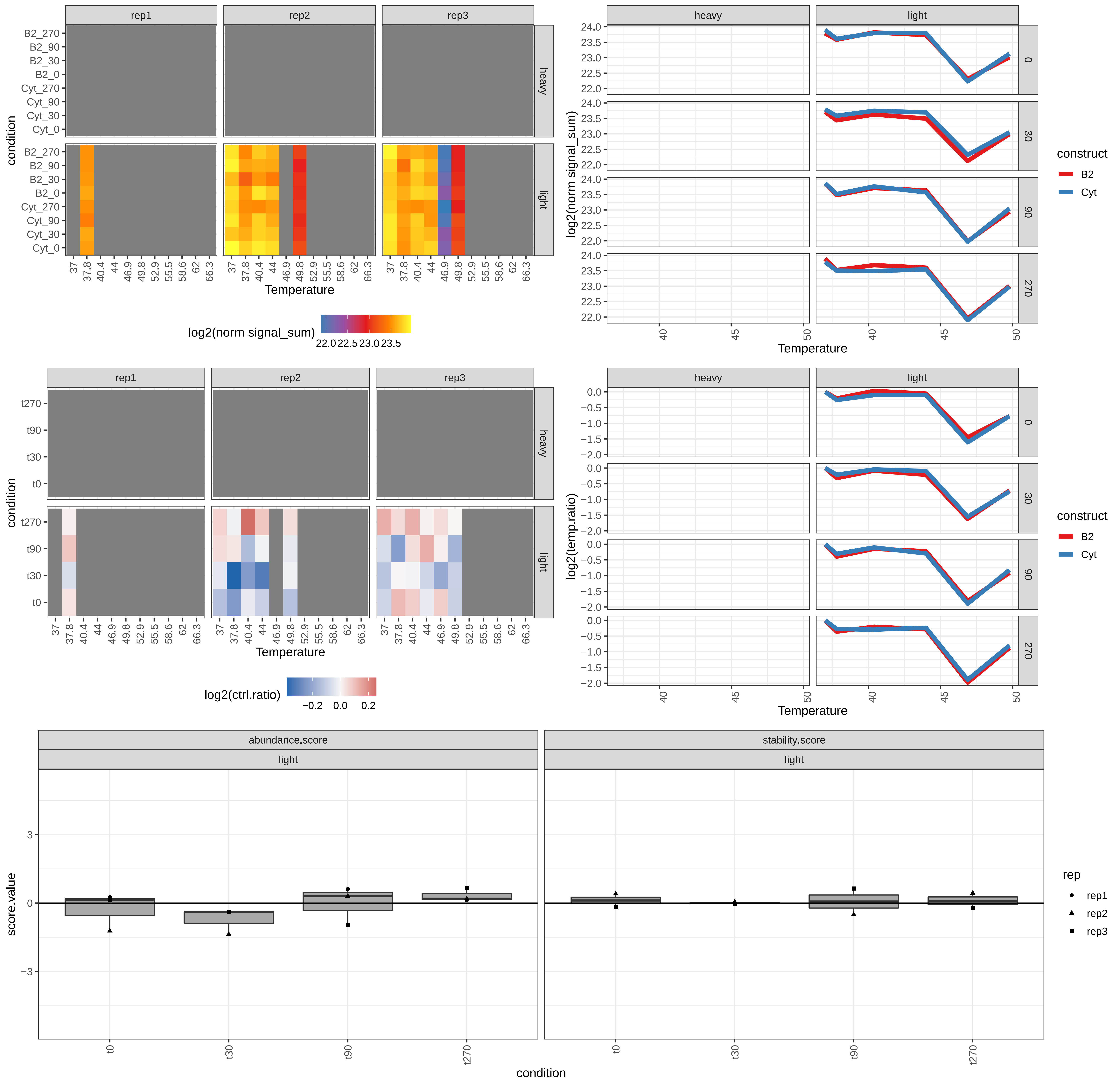

P39936 − Eukaryotic initiation factor 4F subunit p130 OS=Saccharomyces cerevisiae (strain ATCC 204508 / S288c) GN=TIF4632 PE=1 SV=1

TIF5 − average.top3: 7.26636301886792 − max.qupm: 15

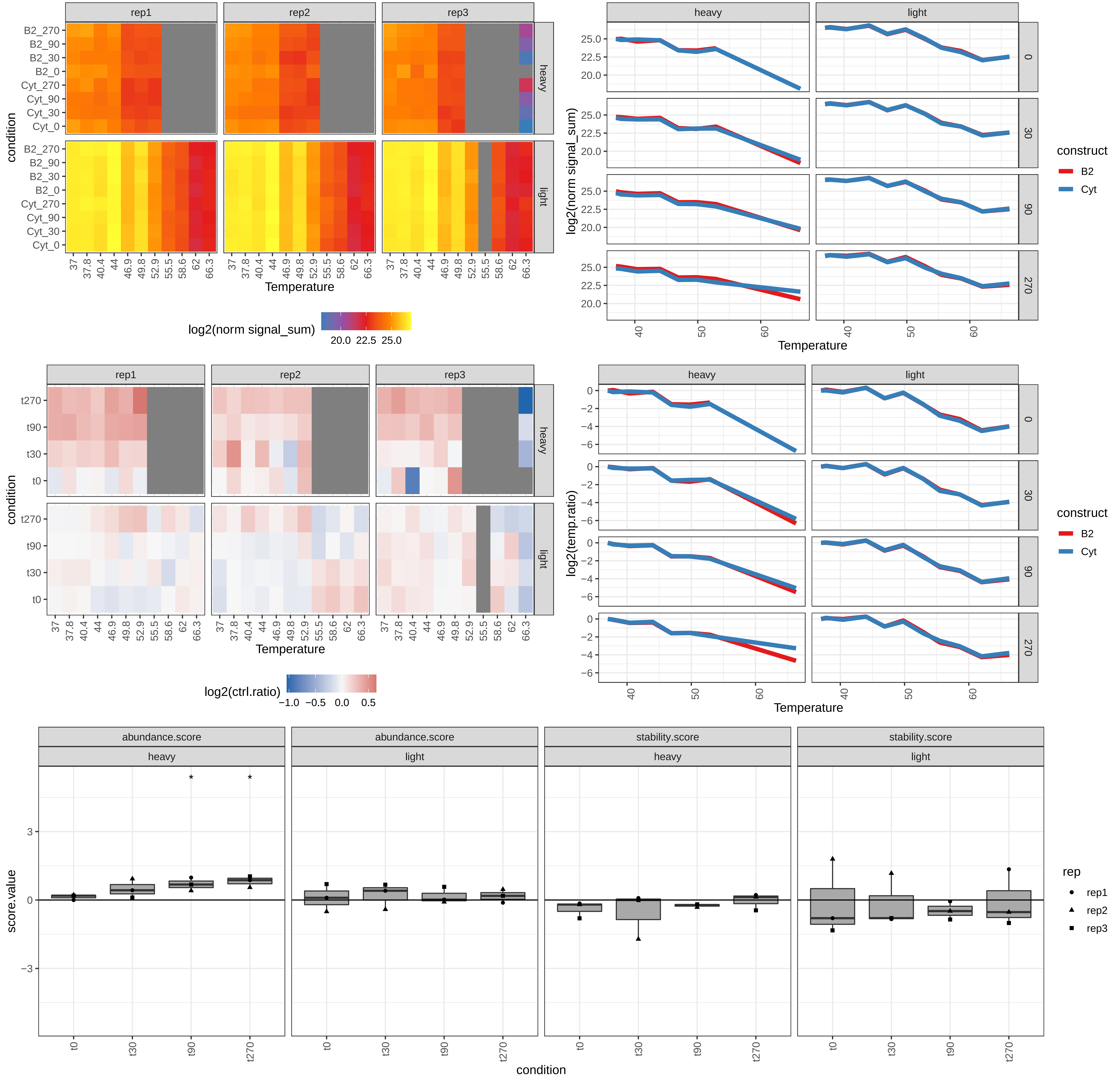

nslation initiation factor 5 OS=Saccharomyces cerevisiae (strain ATCC 204508 / S288c) GN=TIF5 PE=1 SV=1|Isoform Short of Eukaryotic translation initiation factor 5 OS=Saccharomyces cerevisiae (strai

TIF6 − average.top3: 7.0699075 − max.qupm: 4

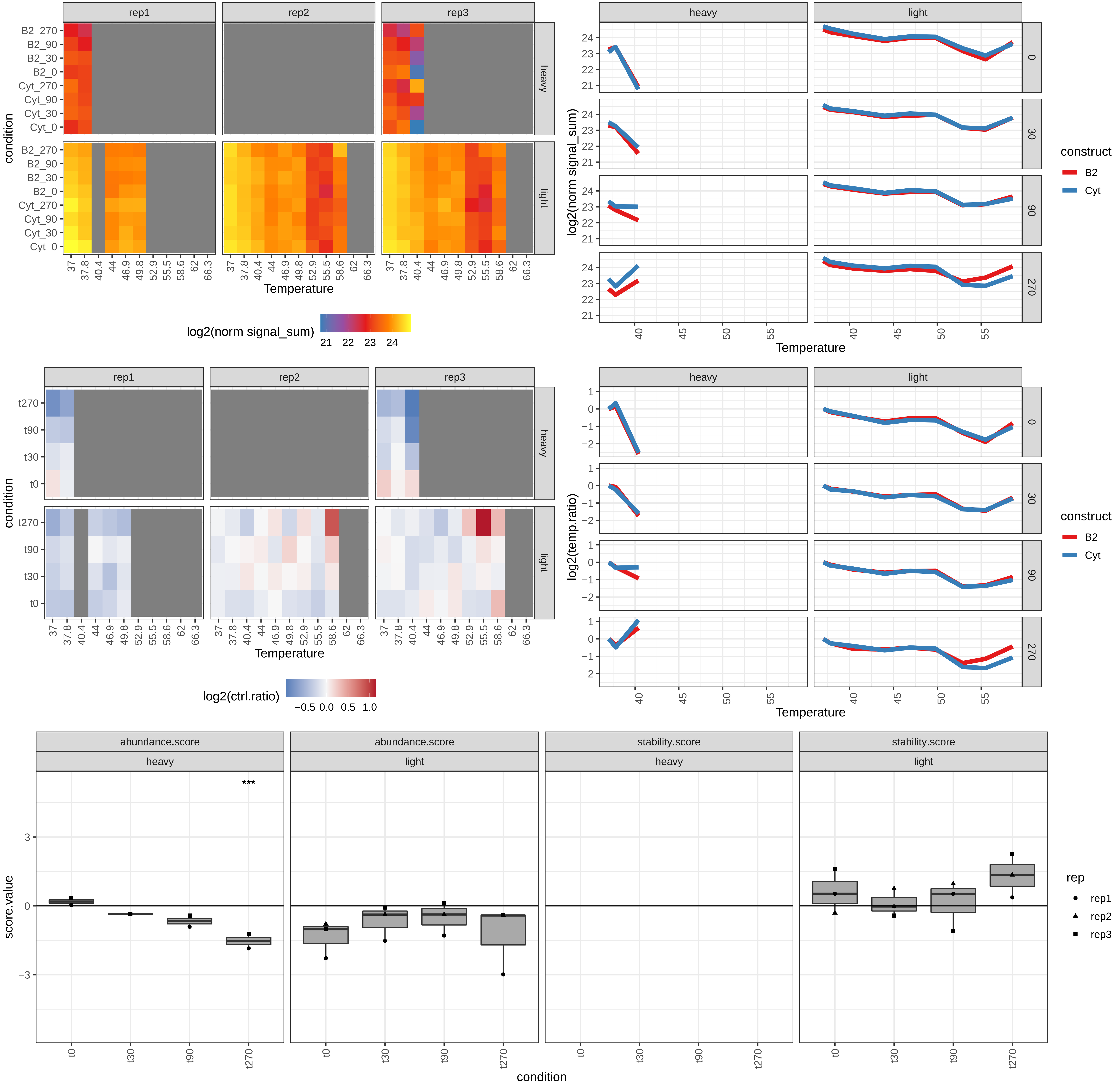

Q12522 − Eukaryotic translation initiation factor 6 OS=Saccharomyces cerevisiae (strain ATCC 204508 / S288c) GN=TIF6 PE=1 SV=1

TIM11 − average.top3: 7.16521444444444 − max.qupm: 5

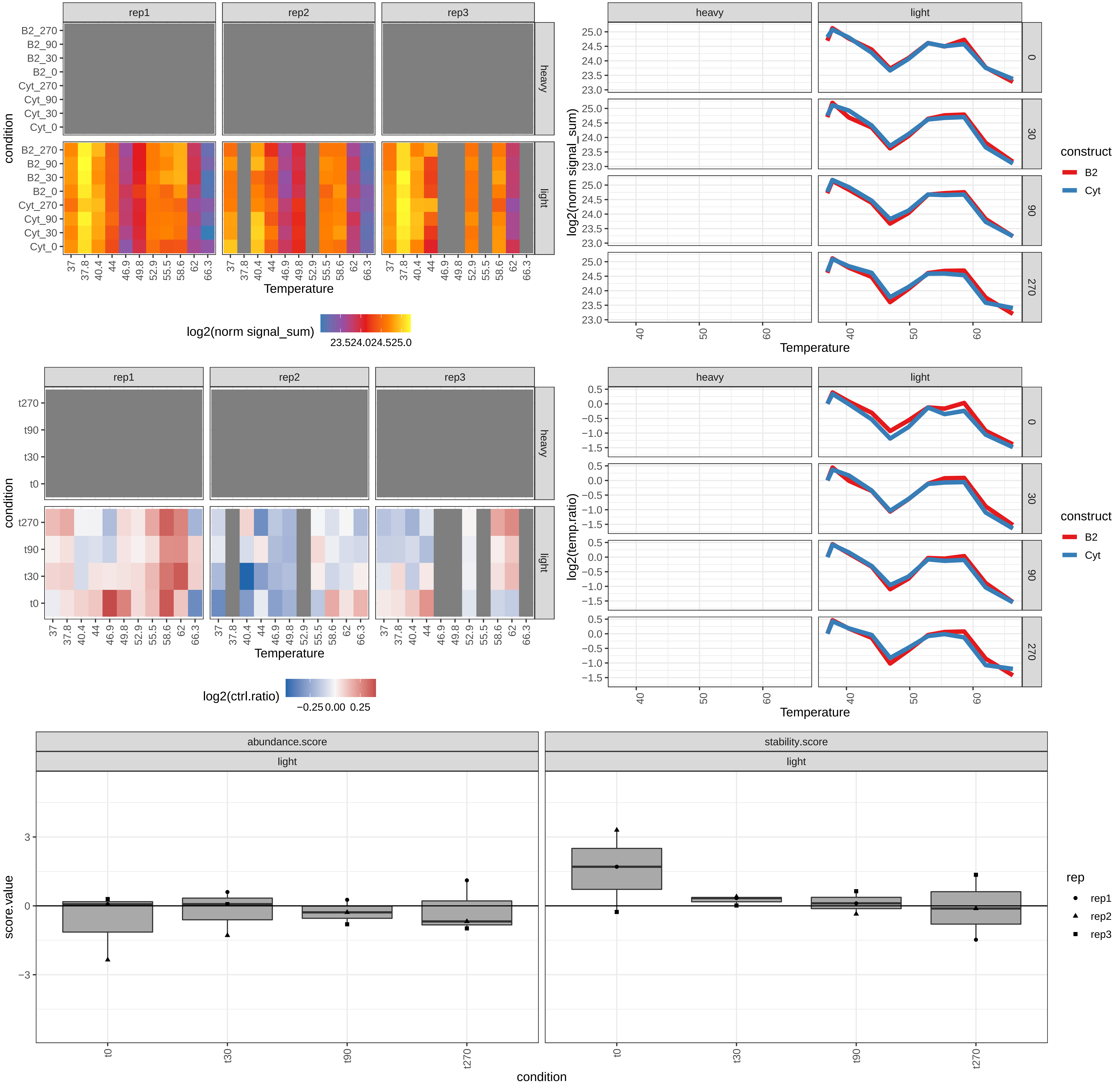

P81449 − ATP synthase subunit e, mitochondrial OS=Saccharomyces cerevisiae (strain ATCC 204508 / S288c) GN=TIM11 PE=1 SV=2

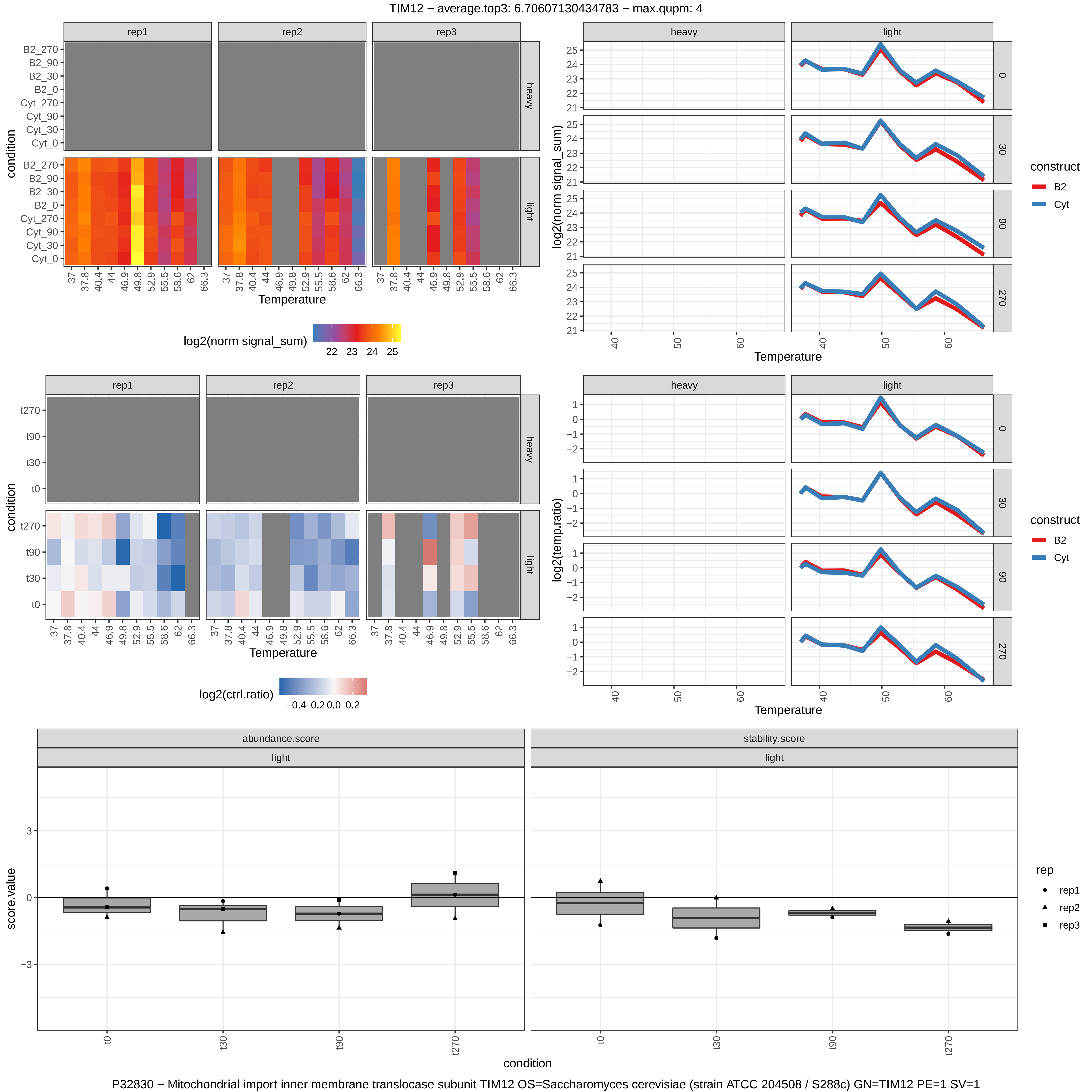

TIM12 − average.top3: 6.70607130434783 − max.qupm: 4
rep1
rep2
rep3
heavy
light
condition
Temperature
log2(norm signal_sum)
construct
B2
Cyt
log2(temp.ratio)
log2(ctrl.ratio)
abundance.score
stability.score
score.value
rep
rep1
rep2
rep3
P32830 − Mitochondrial import inner membrane translocase subunit TIM12 OS=Saccharomyces cerevisiae (strain ATCC 204508 / S288c) GN=TIM12 PE=1 SV=1

TIM17 − average.top3: 7.28873727272727 − max.qupm: 3

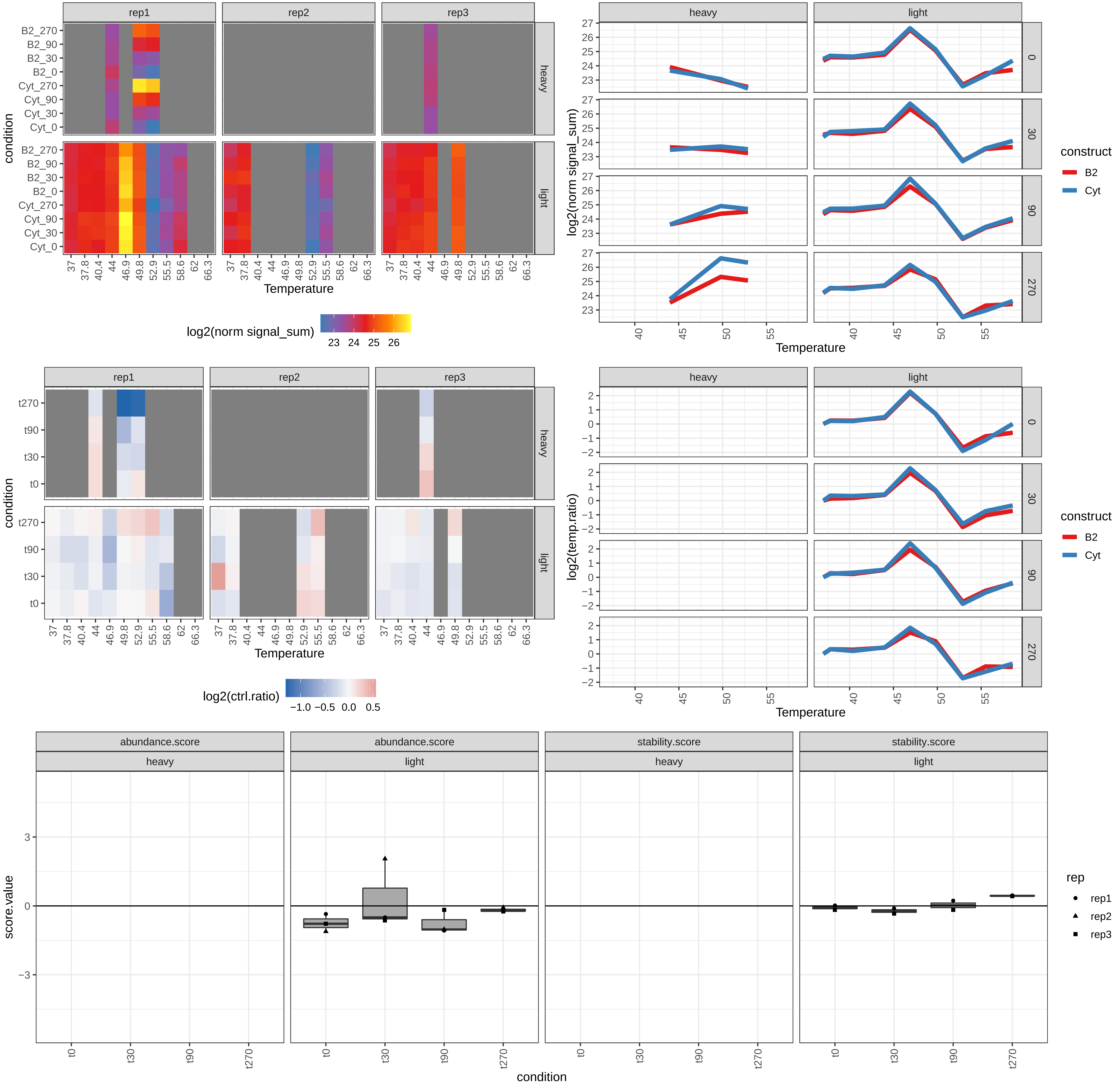

P39515 − Mitochondrial import inner membrane translocase subunit TIM17 OS=Saccharomyces cerevisiae (strain ATCC 204508 / S288c) GN=TIM17 PE=1 SV=1

TIM21 − average.top3: 7.11693947368421 − max.qupm: 4

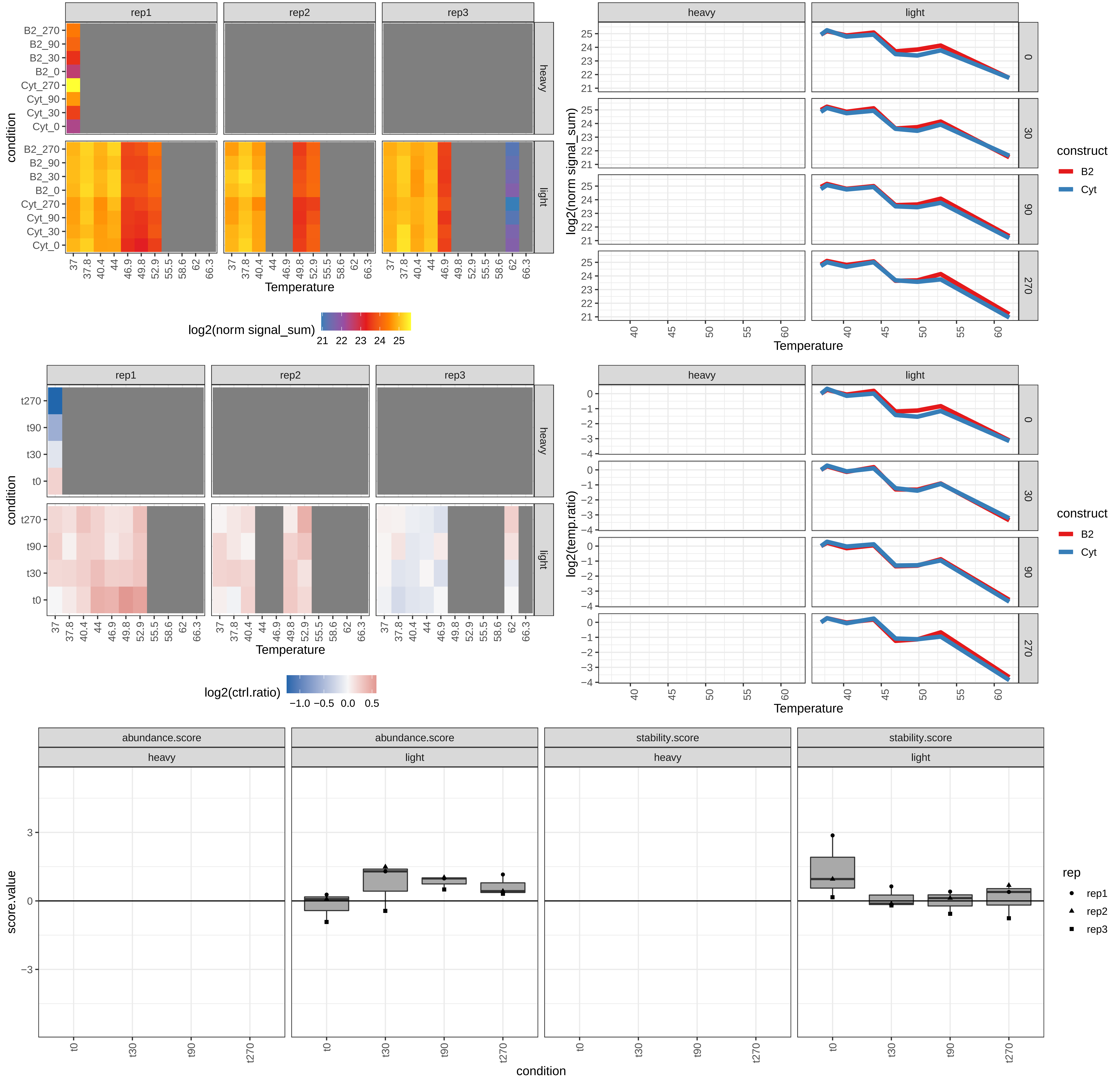

P53220 − Mitochondrial import inner membrane translocase subunit TIM21 OS=Saccharomyces cerevisiae (strain ATCC 204508 / S288c) GN=TIM21 PE=1 SV=1

TIM44 − average.top3: 7.1956272972973 − max.qupm: 10

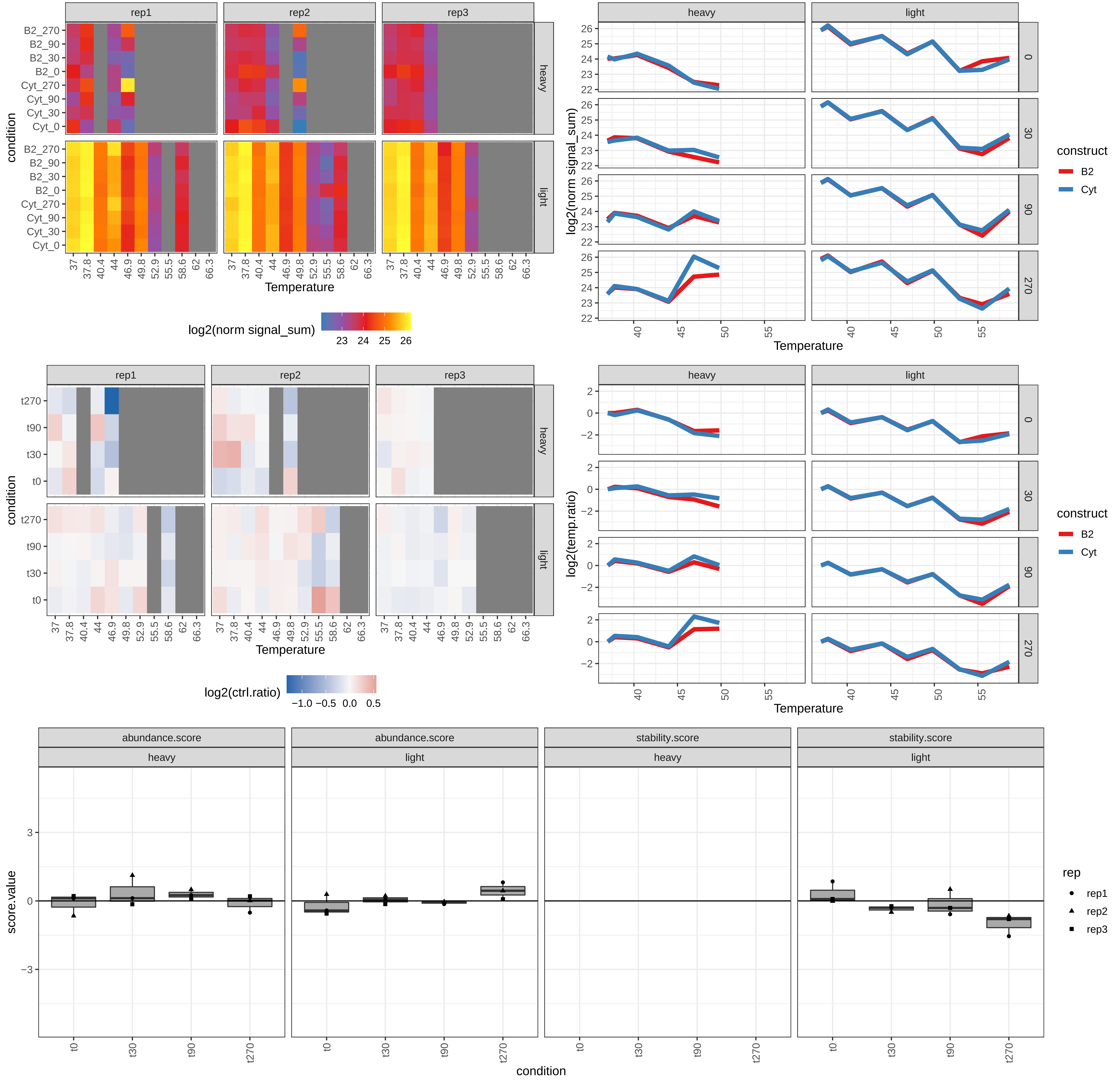

Q01852 − Mitochondrial import inner membrane translocase subunit TIM44 OS=Saccharomyces cerevisiae (strain ATCC 204508 / S288c) GN=TIM44 PE=1 SV=1

TIM50 − average.top3: 6.94532878787879 − max.qupm: 9

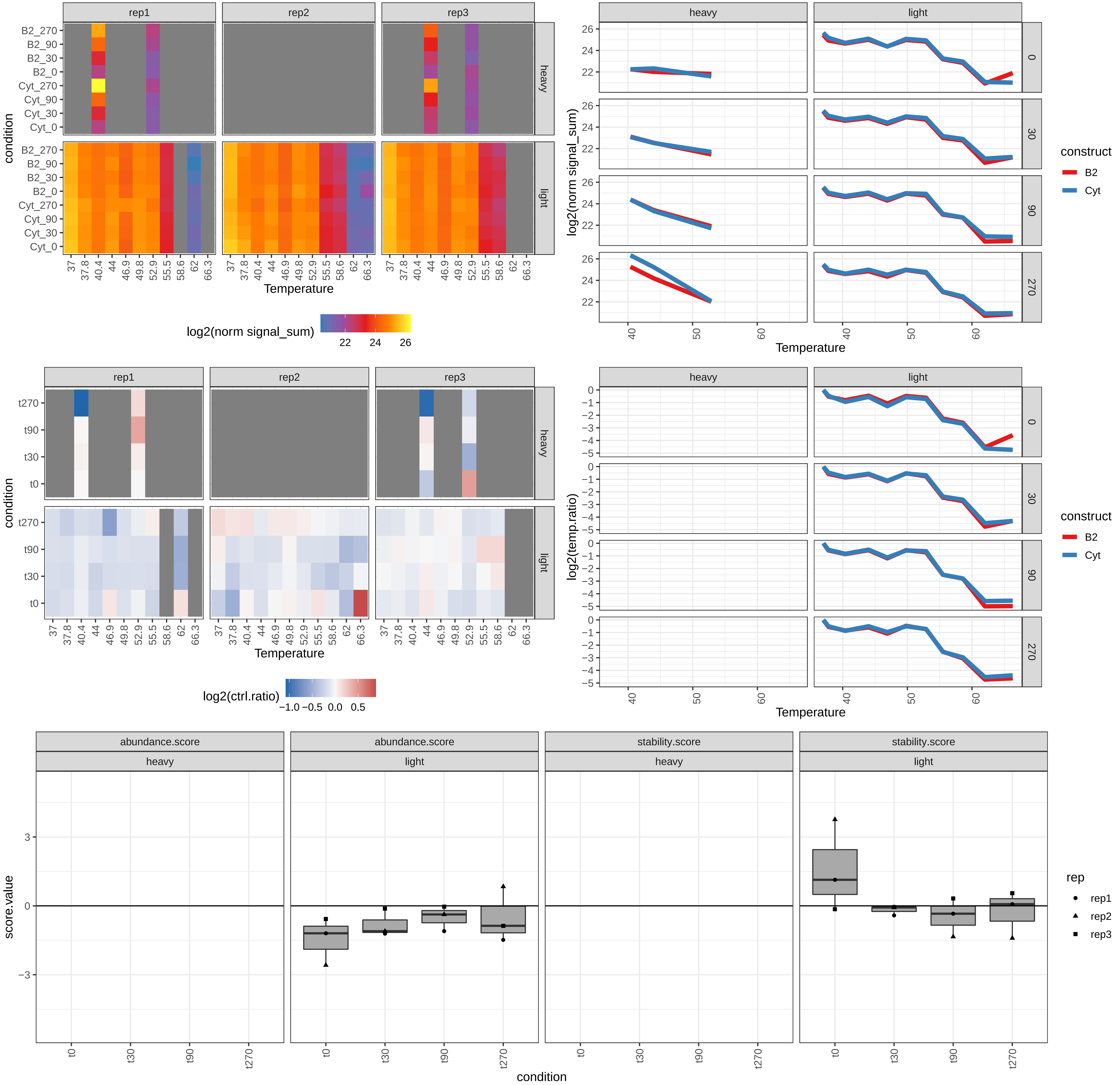

Q02776 − Mitochondrial import inner membrane translocase subunit TIM50 OS=Saccharomyces cerevisiae (strain ATCC 204508 / S288c) GN=TIM50 PE=1 SV=1

TIM9 − average.top3: 7.08011279069767 − max.qupm: 5

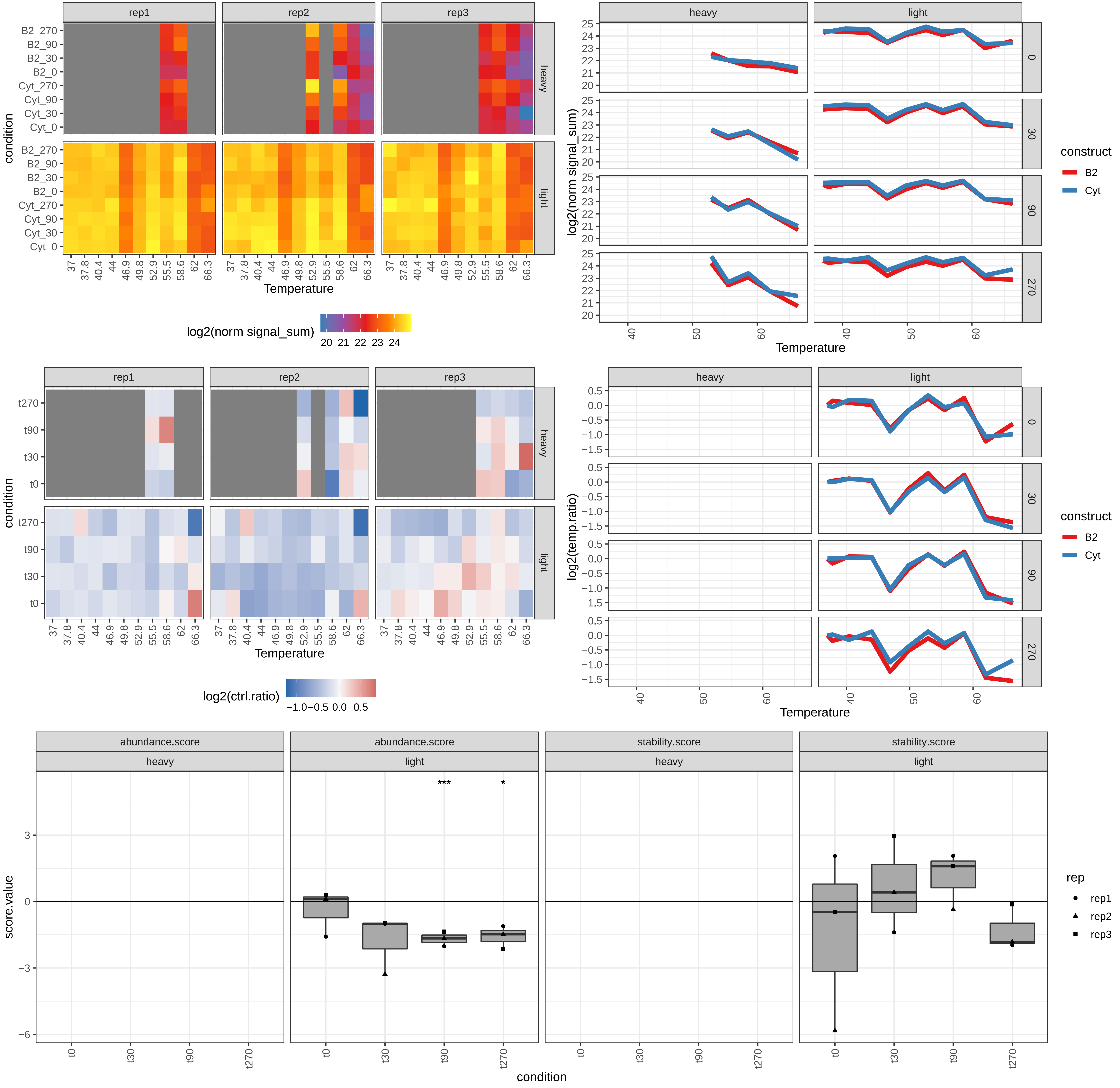

O74700 − Mitochondrial import inner membrane translocase subunit TIM9 OS=Saccharomyces cerevisiae (strain ATCC 204508 / S288c) GN=TIM9 PE=1 SV=1

TIP20 − average.top3: 6.65838588235294 − max.qupm: 8

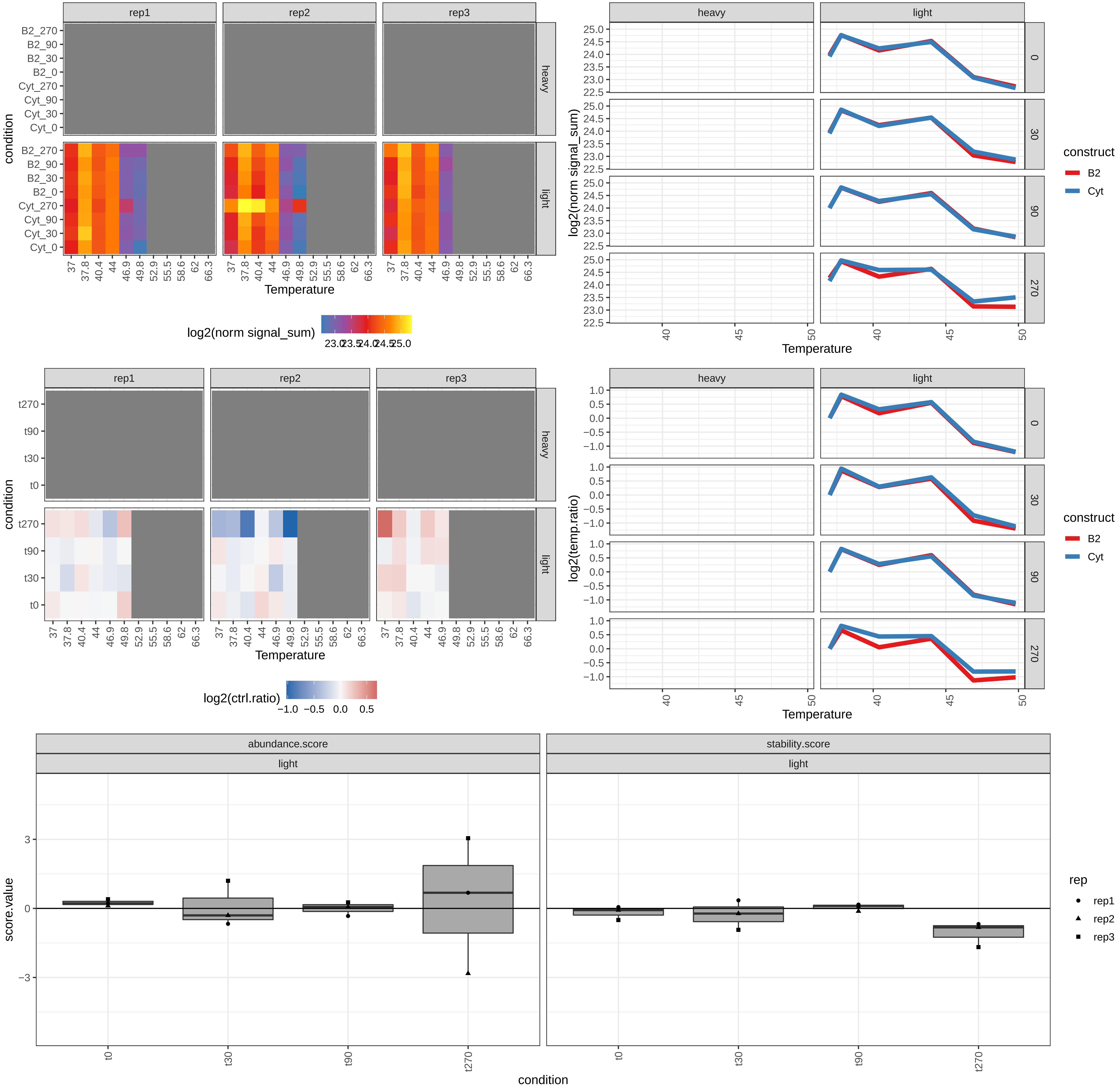

P33891 − Protein transport protein TIP20 OS=Saccharomyces cerevisiae (strain ATCC 204508 / S288c) GN=TIP20 PE=1 SV=1

TIP41 − average.top3: 6.86796769230769 − max.qupm: 7

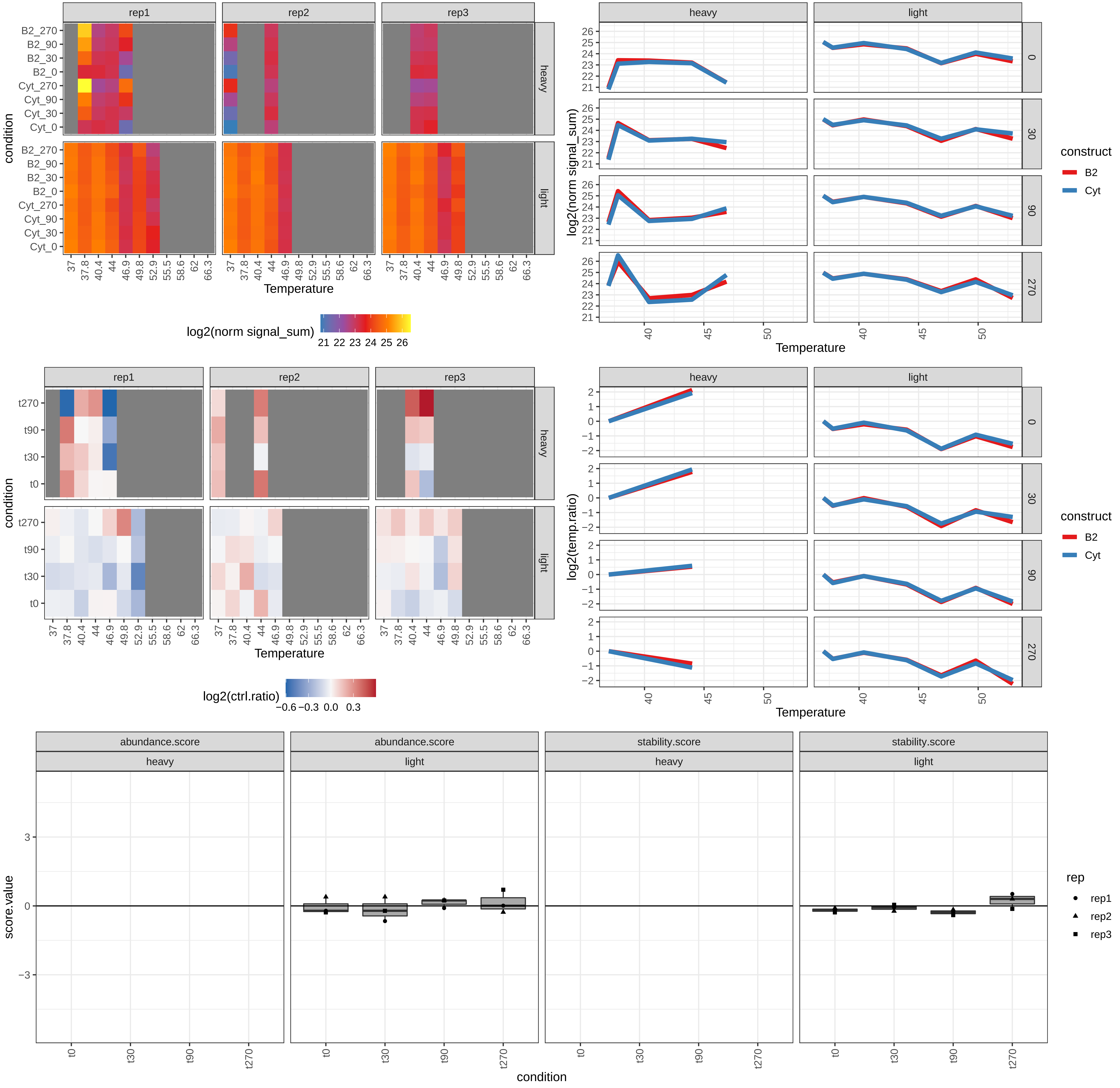

Q12199 − Type 2A phosphatase activator TIP41 OS=Saccharomyces cerevisiae (strain ATCC 204508 / S288c) GN=TIP41 PE=1 SV=1

TKL1 − average.top3: 8.1512546969697 − max.qupm: 28

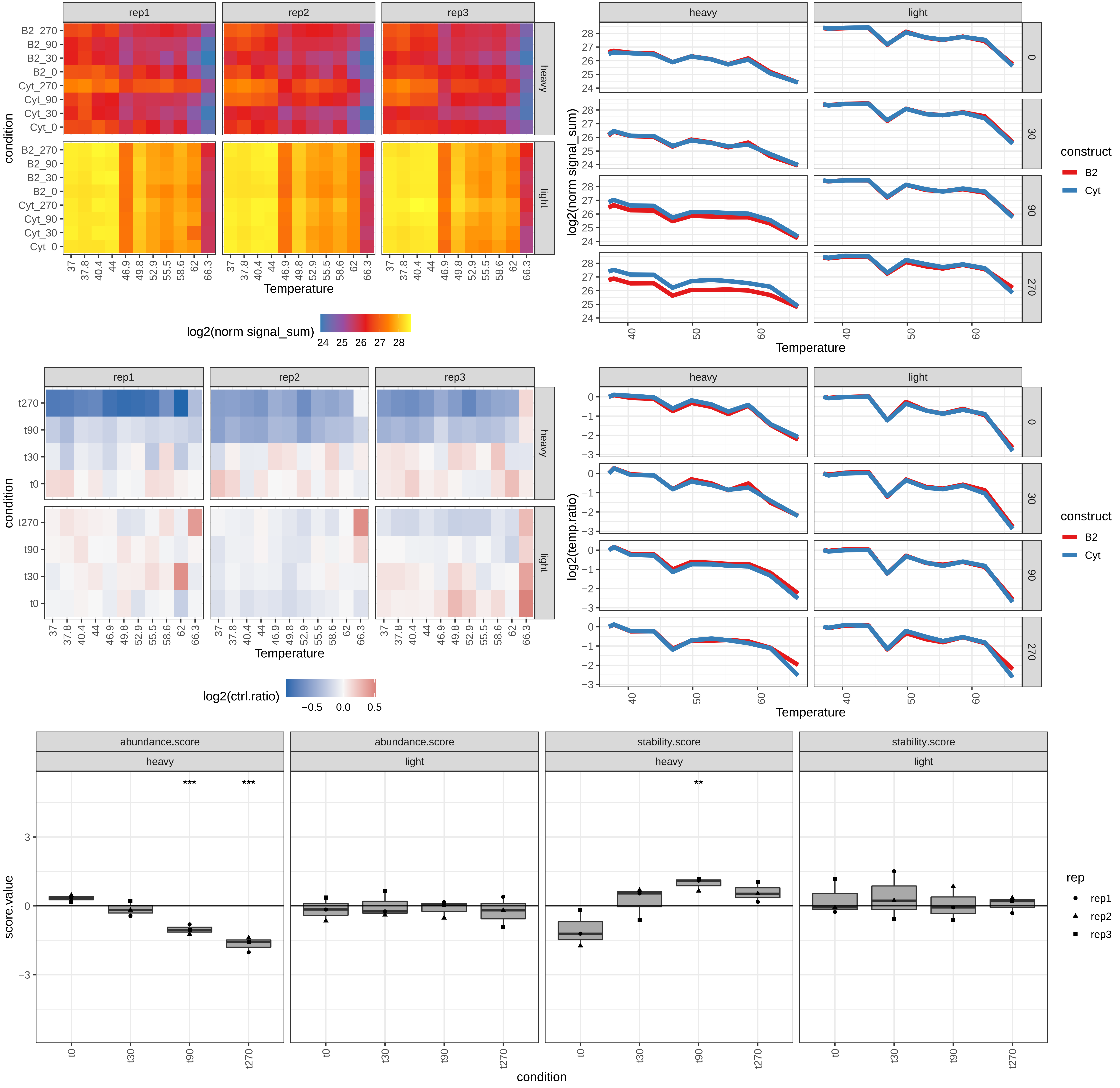

P23254 − Transketolase 1 OS=Saccharomyces cerevisiae (strain ATCC 204508 / S288c) GN=TKL1 PE=1 SV=4

TKL2 − average.top3: 6.707268125 − max.qupm: 11

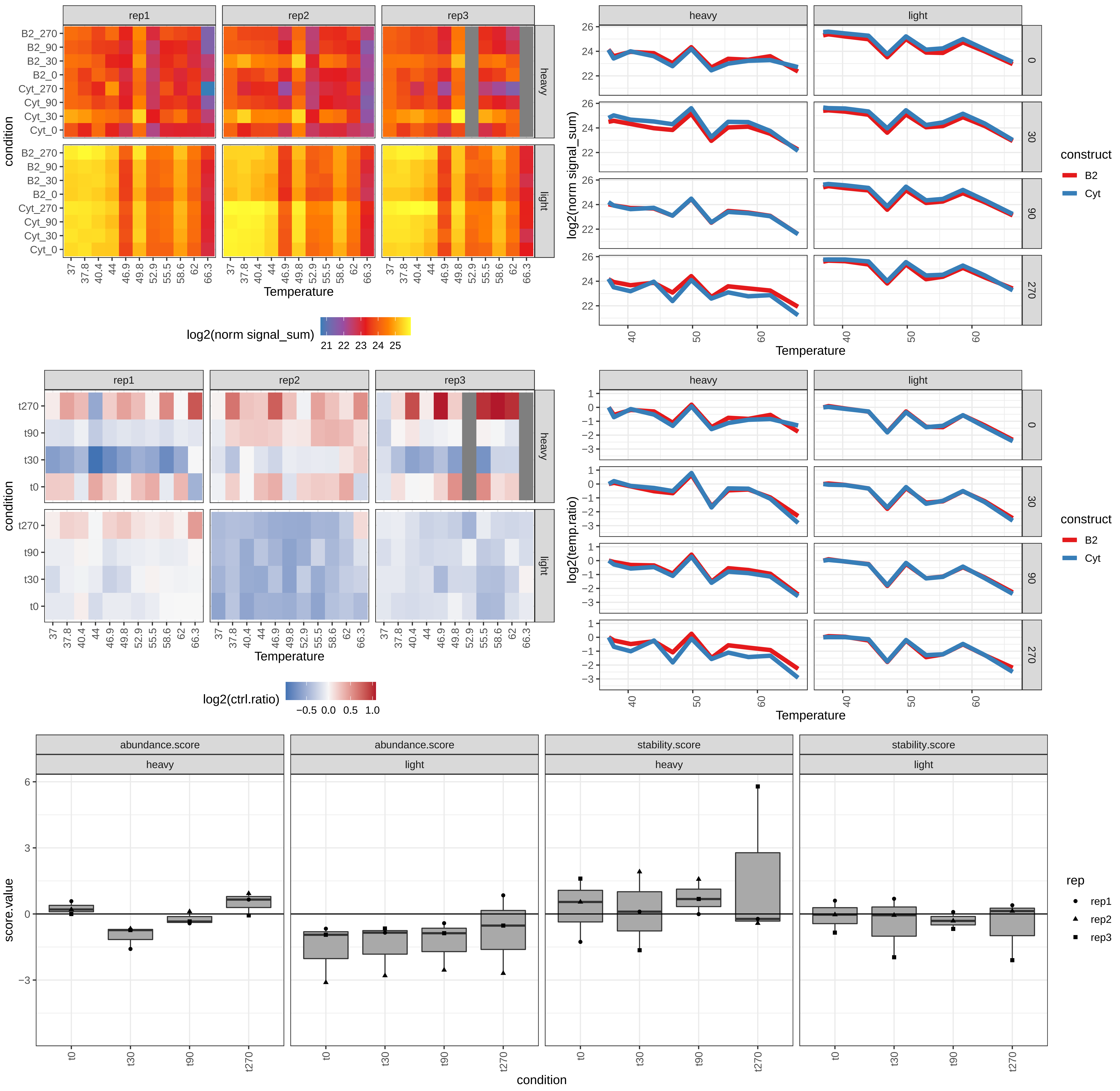

P33315 − Transketolase 2 OS=Saccharomyces cerevisiae (strain ATCC 204508 / S288c) GN=TKL2 PE=1 SV=1

TLG2 − average.top3: 6.502415 − max.qupm: 8

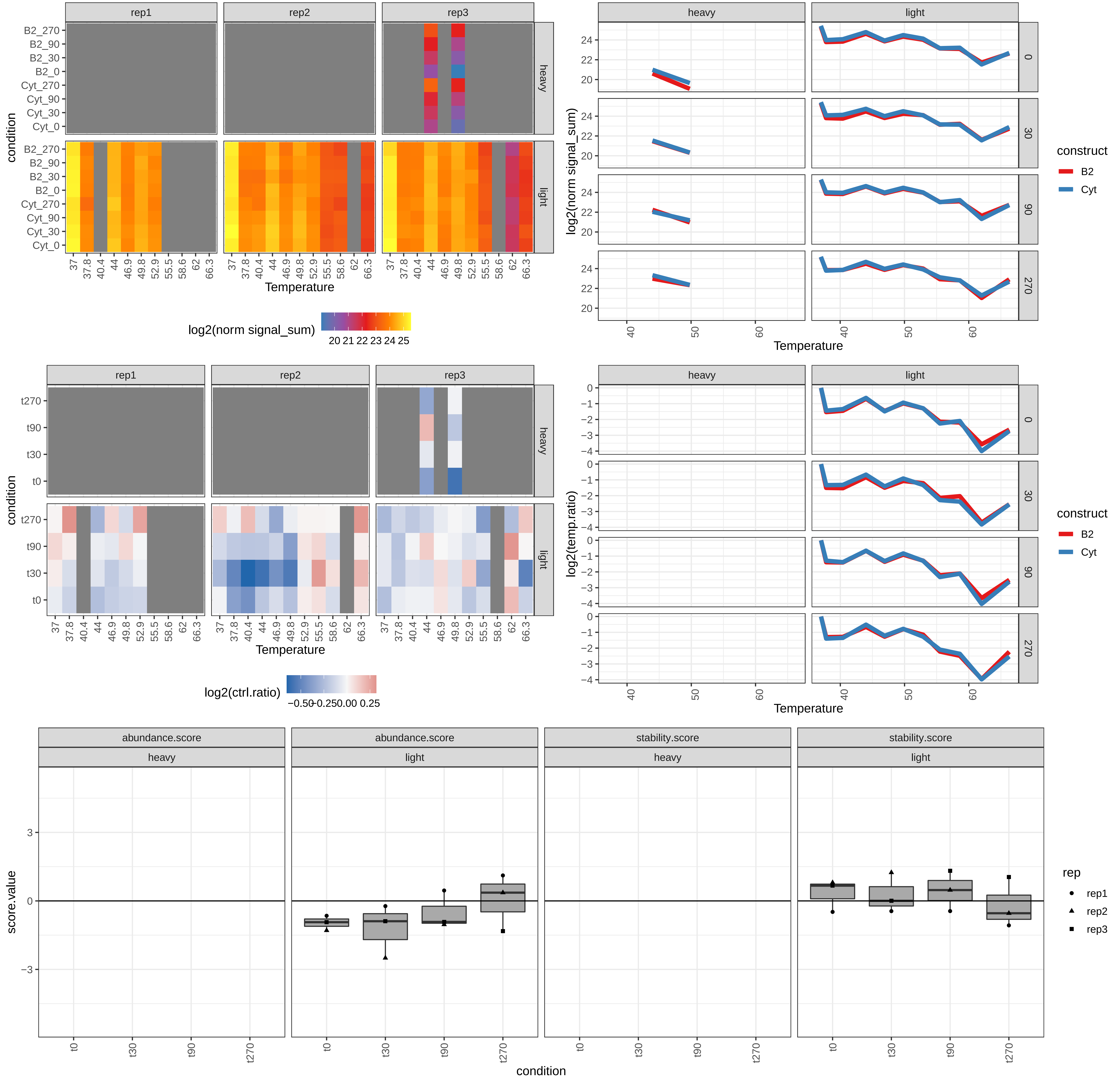

Q08144 − T−SNARE affecting a late Golgi compartment protein 2 OS=Saccharomyces cerevisiae (strain ATCC 204508 / S288c) GN=TLG2 PE=1 SV=1

TMA108 − average.top3: 6.9630965 − max.qupm: 15

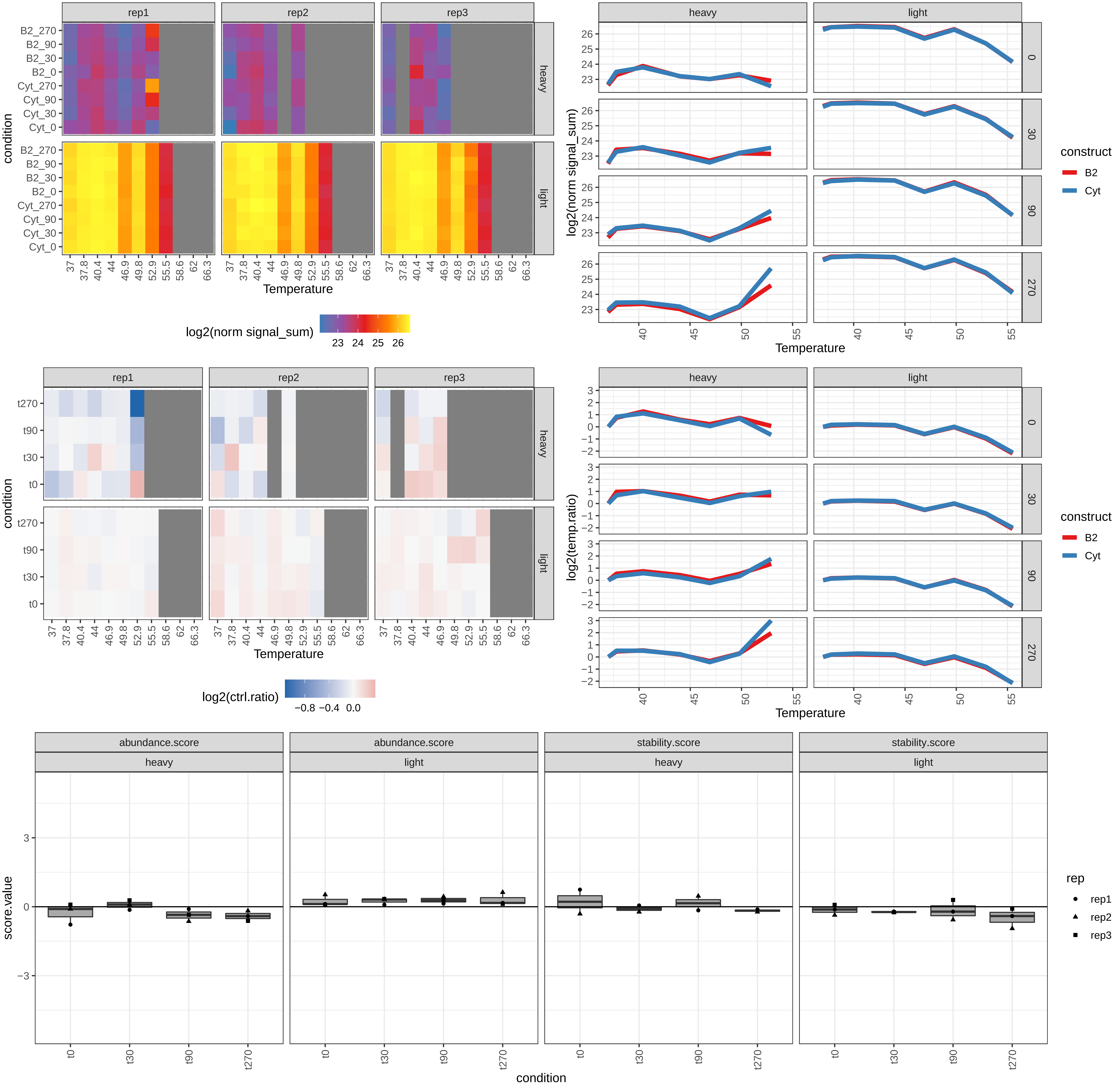

P40462 − Protein TMA108 OS=Saccharomyces cerevisiae (strain ATCC 204508 / S288c) GN=TMA108 PE=1 SV=1

TMA16 − average.top3: 6.4624315 − max.qupm: 6

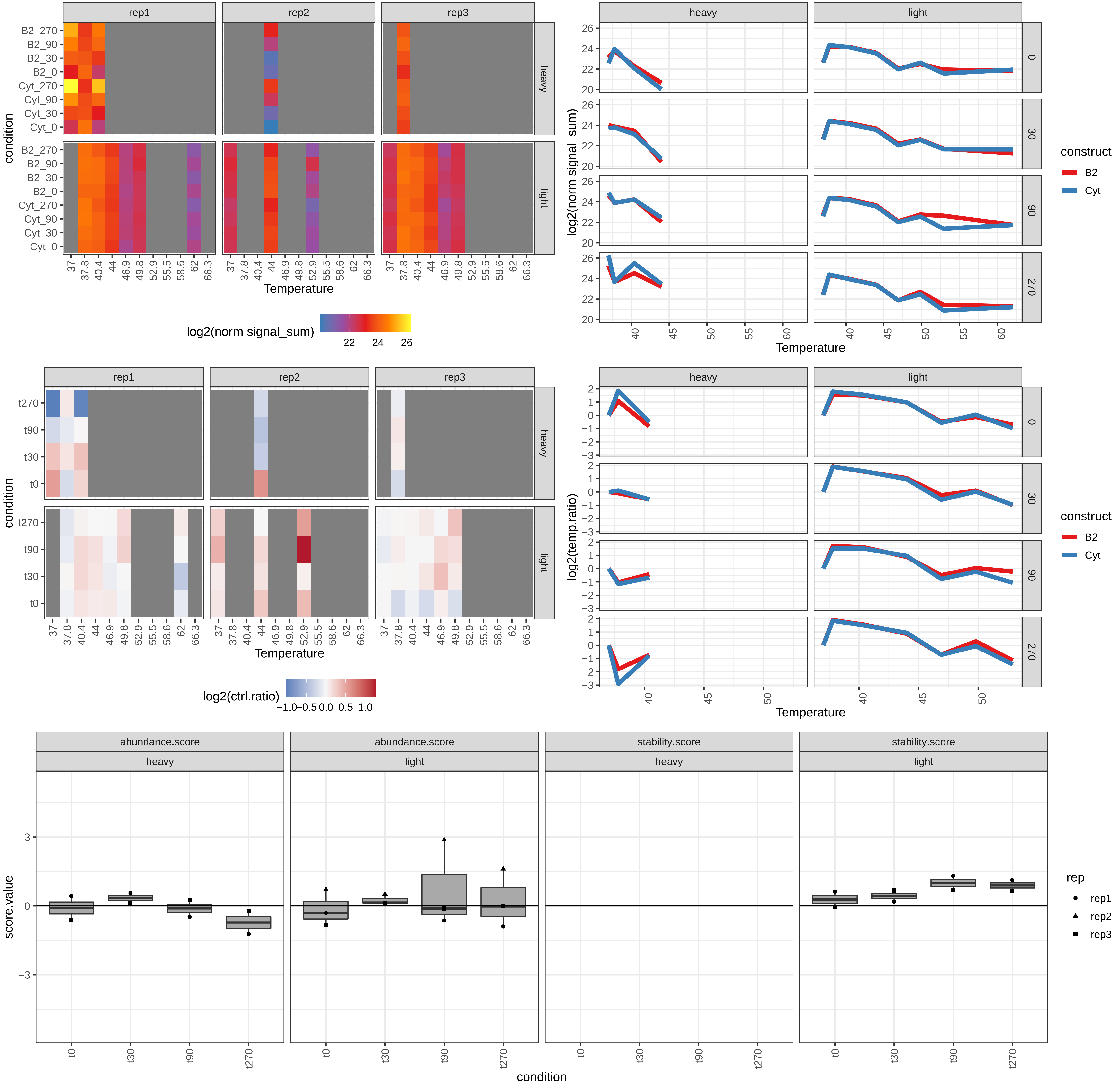

Q08687 − Translation machinery−associated protein 16 OS=Saccharomyces cerevisiae (strain ATCC 204508 / S288c) GN=TMA16 PE=1 SV=2

TMA17 − average.top3: 7.0457158 − max.qupm: 7

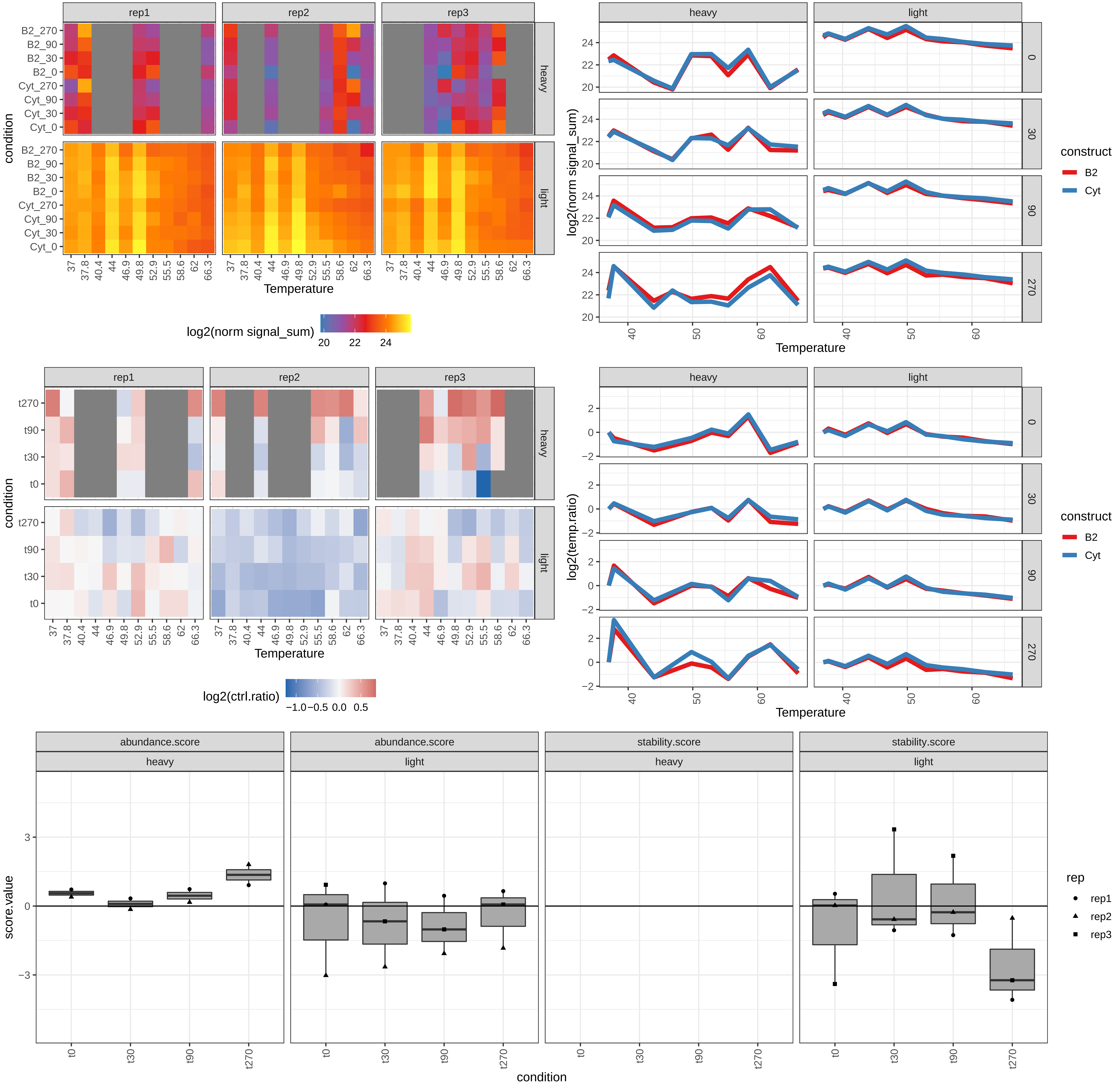

Q12513 − Translation machinery−associated protein 17 OS=Saccharomyces cerevisiae (strain ATCC 204508 / S288c) GN=TMA17 PE=1 SV=1

TMA19 − average.top3: 7.78446471698113 − max.qupm: 10

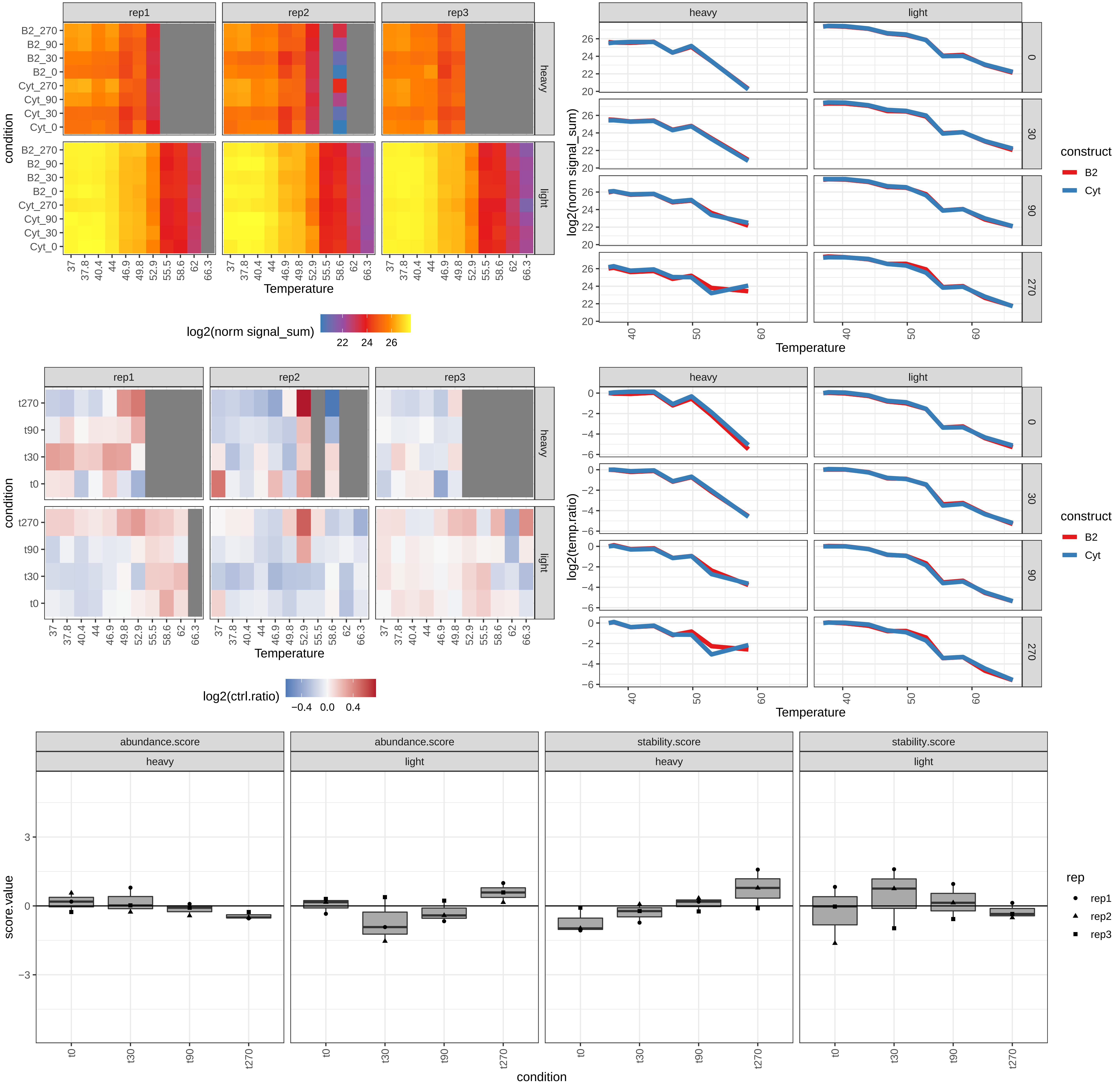

P35691 − Translationally−controlled tumor protein homolog OS=Saccharomyces cerevisiae (strain ATCC 204508 / S288c) GN=TMA19 PE=1 SV=1

TMA20 − average.top3: 6.99808846153846 − max.qupm: 7

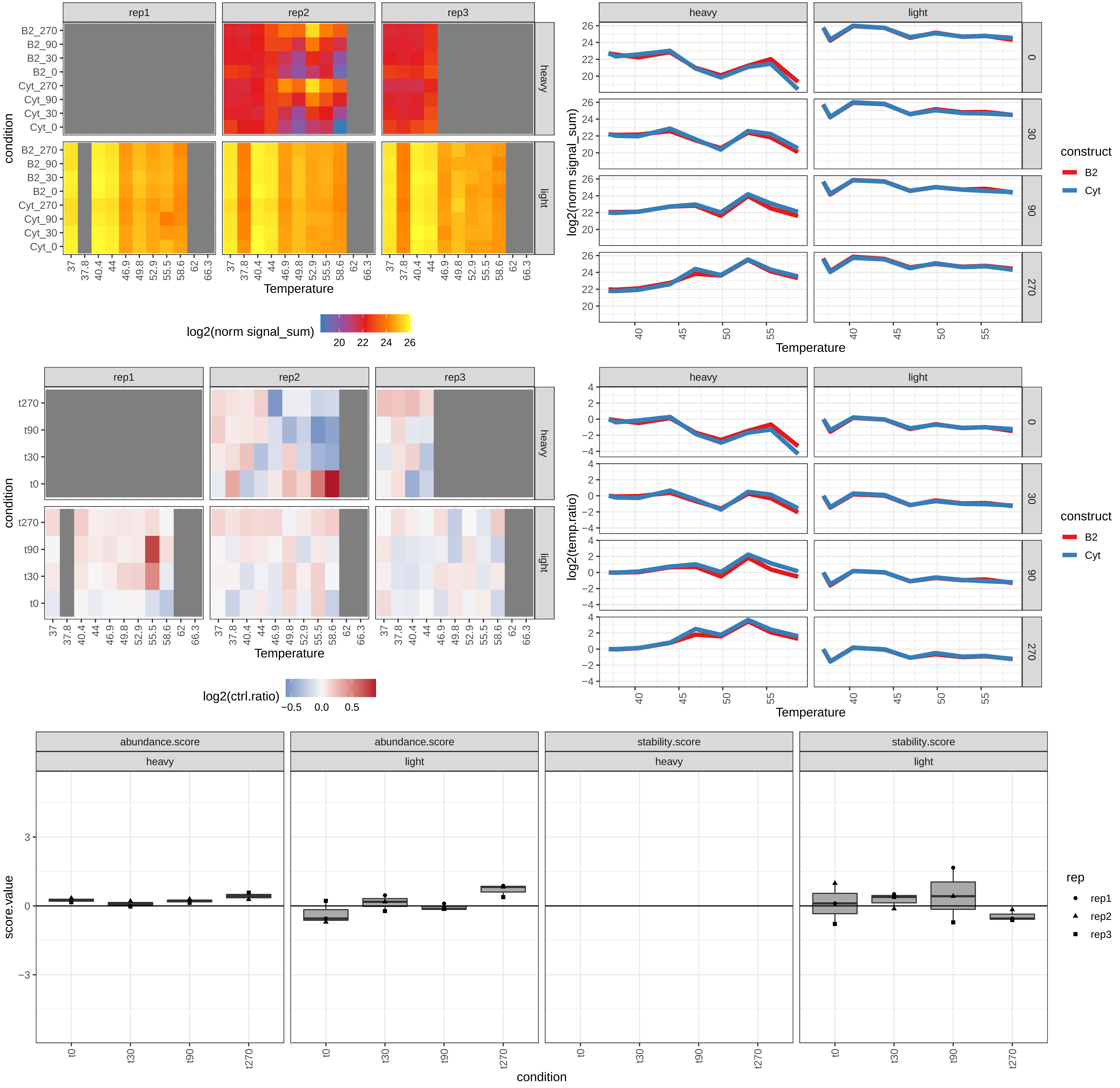

P89886 − Translation machinery−associated protein 20 OS=Saccharomyces cerevisiae (strain ATCC 204508 / S288c) GN=TMA20 PE=1 SV=1

TMA22 − average.top3: 6.9200532 − max.qupm: 6

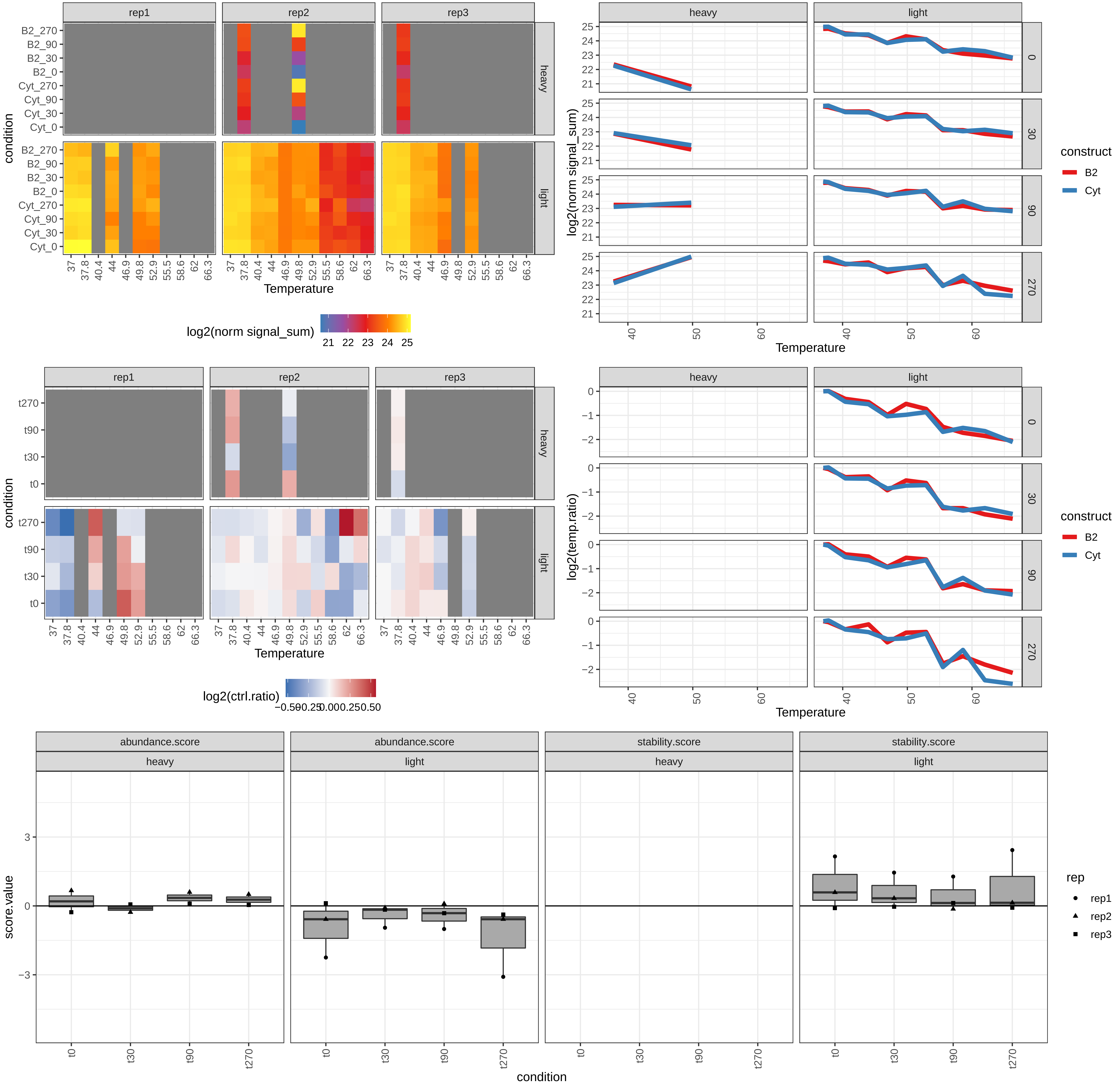

P47089 − Translation machinery−associated protein 22 OS=Saccharomyces cerevisiae (strain ATCC 204508 / S288c) GN=TMA22 PE=1 SV=1

TMA46 − average.top3: 6.77328145833333 − max.qupm: 9

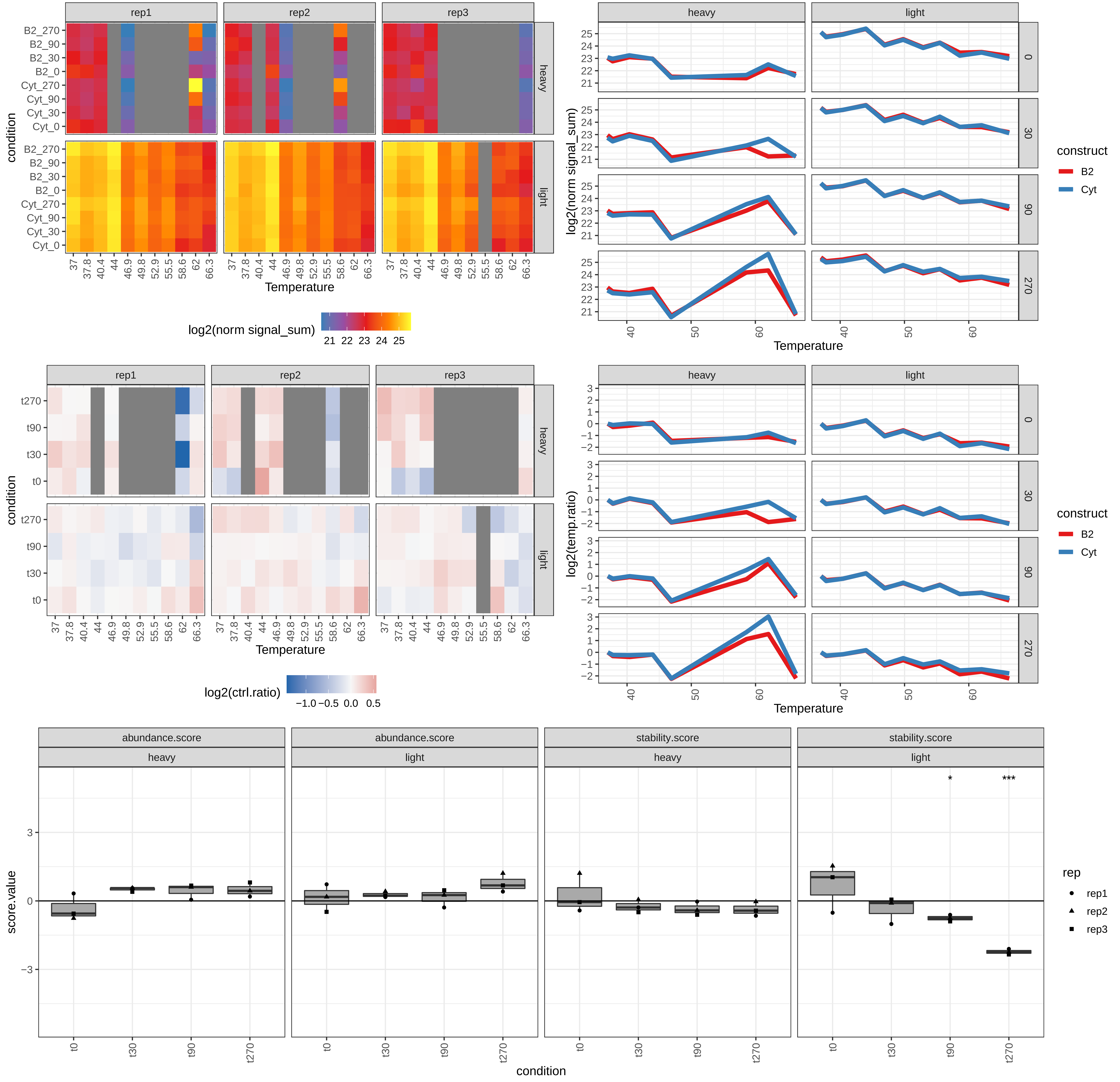

Q12000 − Translation machinery−associated protein 46 OS=Saccharomyces cerevisiae (strain ATCC 204508 / S288c) GN=TMA46 PE=1 SV=2

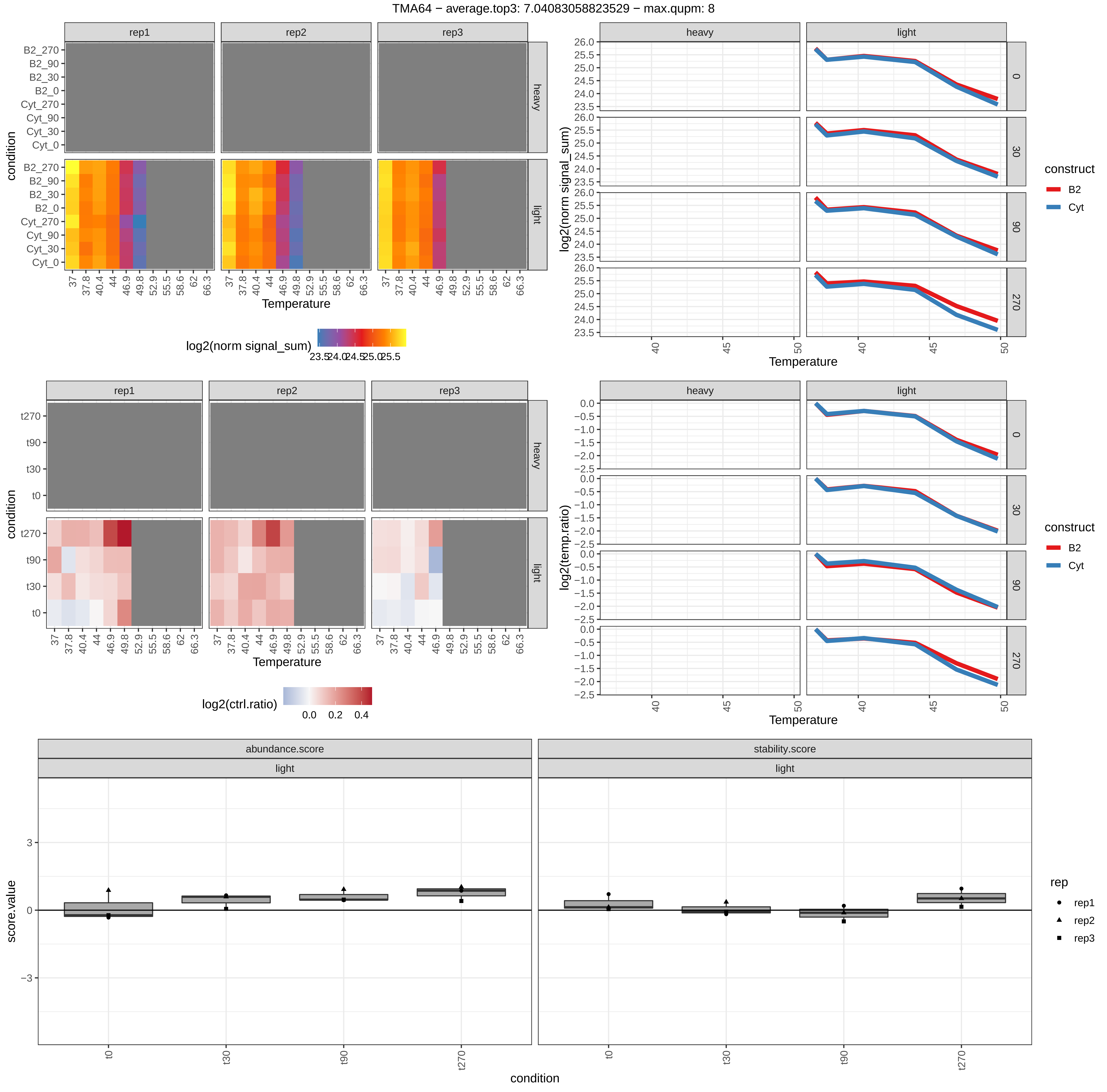

Q04600 − Translation machinery−associated protein 64 OS=Saccharomyces cerevisiae (strain ATCC 204508 / S288c) GN=TMA64 PE=1 SV=1

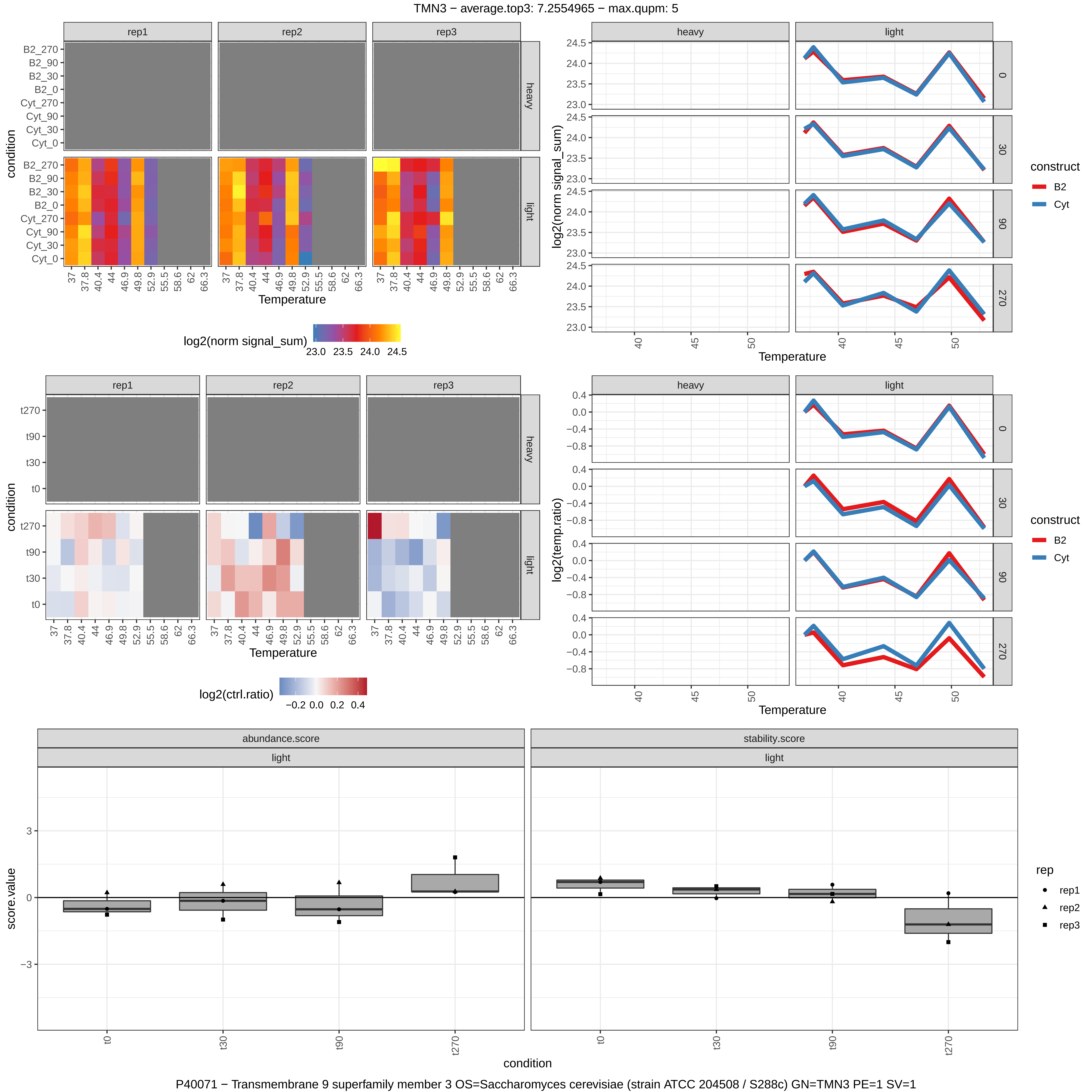
TMN3 − average.top3: 7.2554965 − max.qupm: 5
rep1
rep2
rep3
heavy
light
condition
Temperature
log2(norm signal_sum)
construct
B2
Cyt
log2(ctrl.ratio)
log2(temp.ratio)
abundance.score
stability.score
score.value
rep
rep1
rep2
rep3
P40071 − Transmembrane 9 superfamily member 3 OS=Saccharomyces cerevisiae (strain ATCC 204508 / S288c) GN=TMN3 PE=1 SV=1

TMT1 − average.top3: 7.20267209302326 − max.qupm: 11

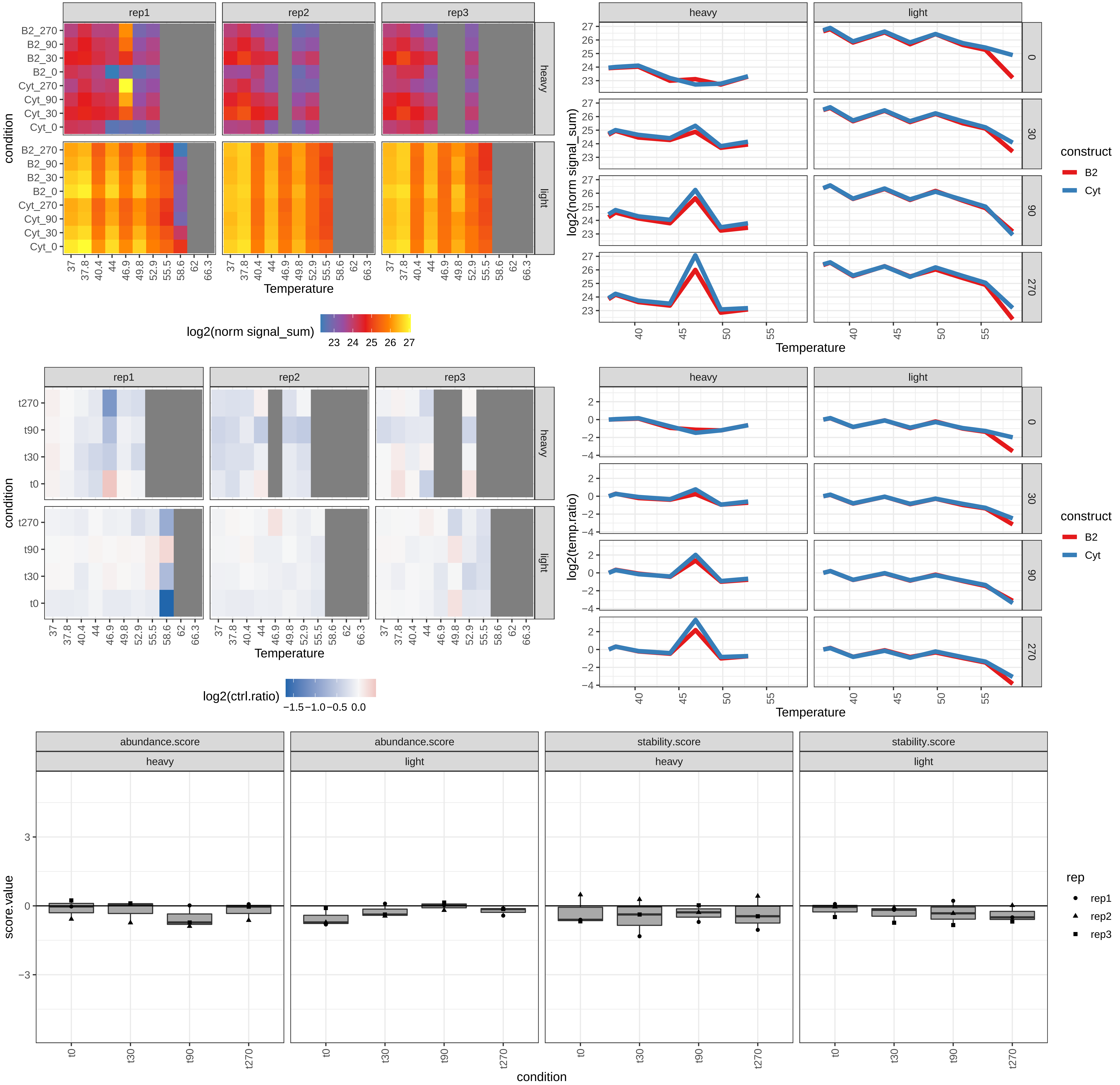

P32643 − Trans−aconitate 3−methyltransferase OS=Saccharomyces cerevisiae (strain ATCC 204508 / S288c) GN=TMT1 PE=1 SV=1

TNA1 − average.top3: 6.94802153846154 − max.qupm: 6

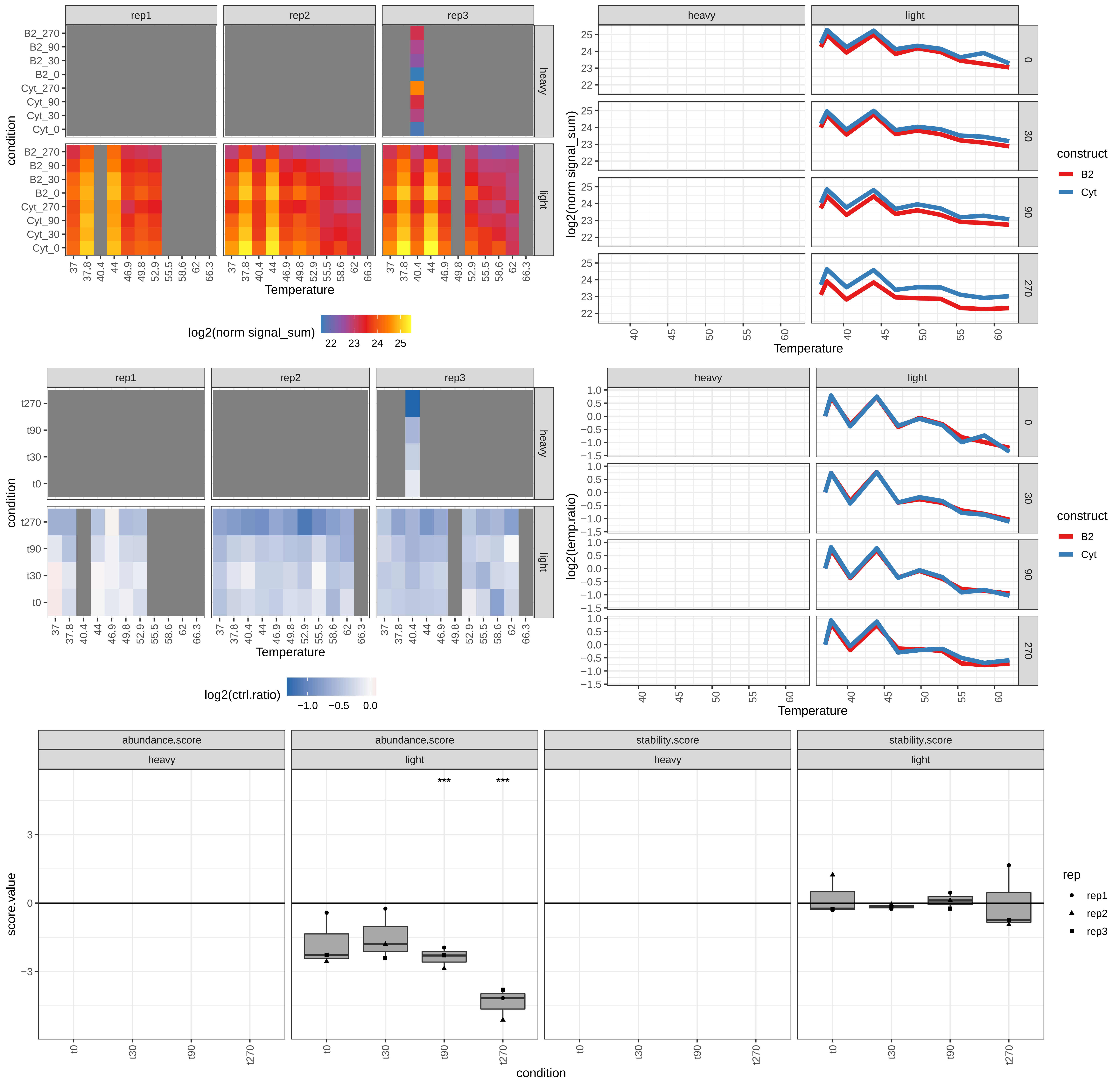

P53322 − High−affinity nicotinic acid transporter OS=Saccharomyces cerevisiae (strain ATCC 204508 / S288c) GN=TNA1 PE=1 SV=1

TOA2 − average.top3: 6.93944942857143 − max.qupm: 5

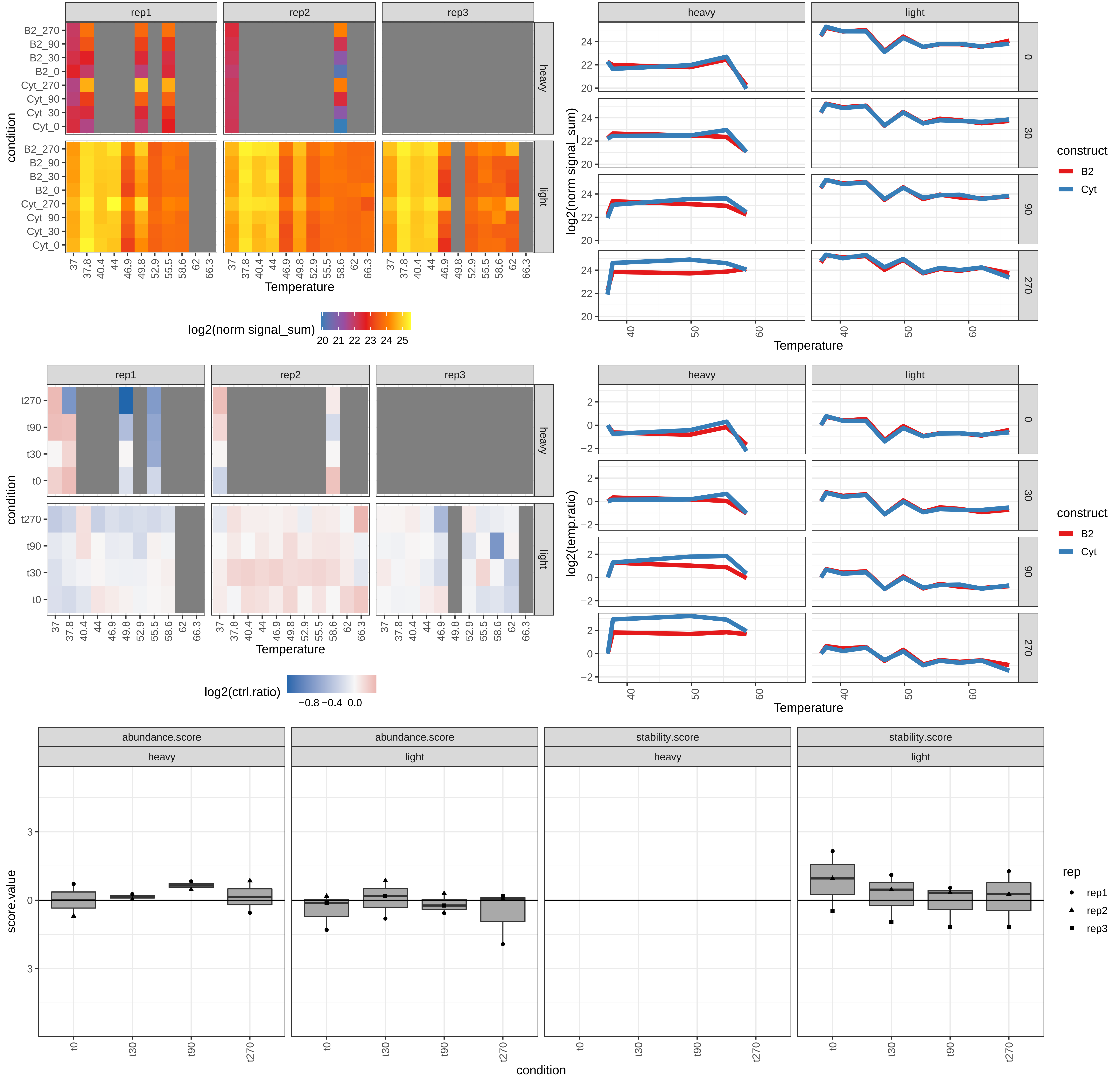

P32774 − Transcription initiation factor IIA subunit 2 OS=Saccharomyces cerevisiae (strain ATCC 204508 / S288c) GN=TOA2 PE=1 SV=1

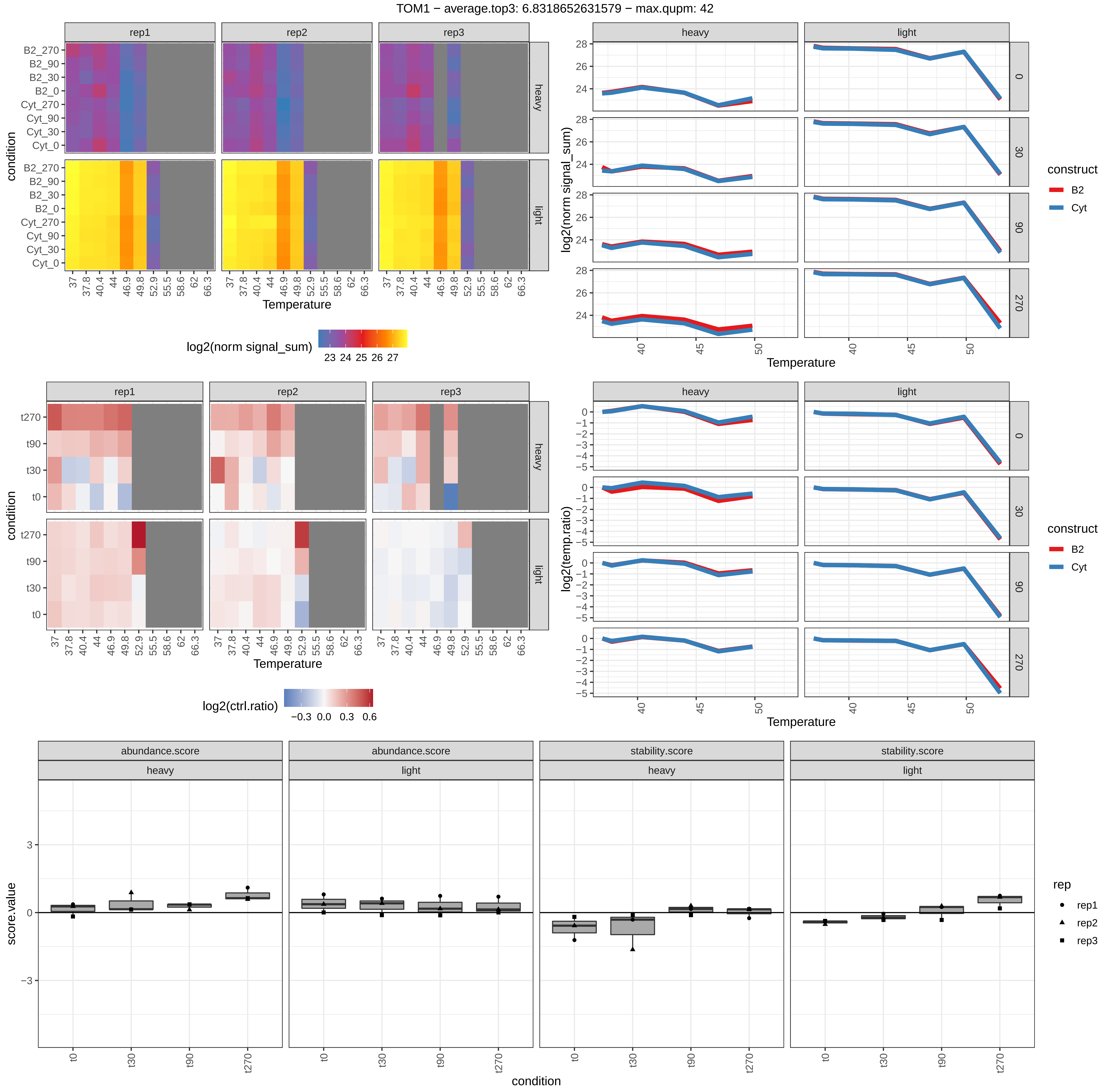

Q03280 − E3 ubiquitin−protein ligase TOM1 OS=Saccharomyces cerevisiae (strain ATCC 204508 / S288c) GN=TOM1 PE=1 SV=1

TOM40 − average.top3: 7.27031166666667 − max.qupm: 6

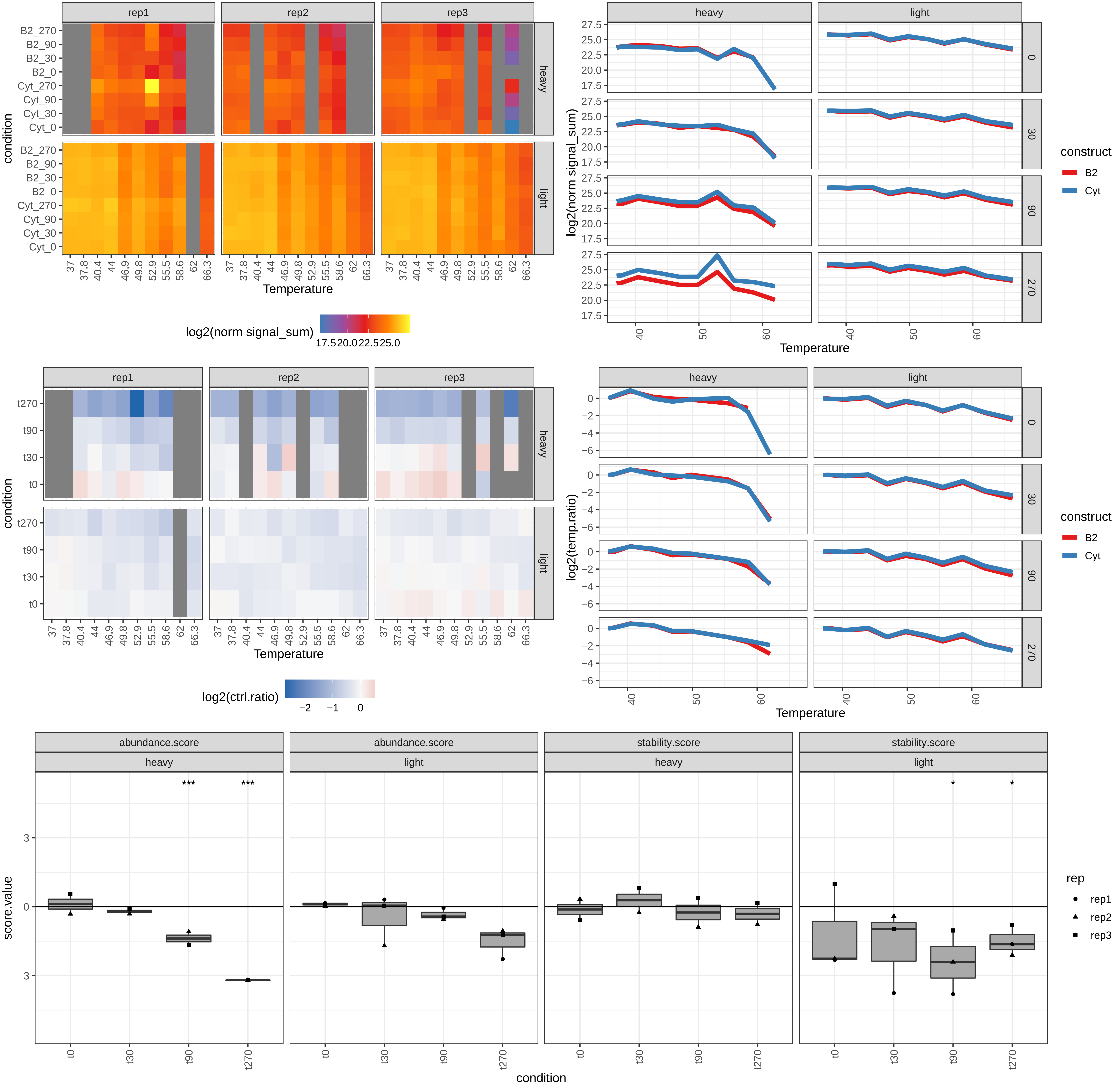

P23644 − Mitochondrial import receptor subunit TOM40 OS=Saccharomyces cerevisiae (strain ATCC 204508 / S288c) GN=TOM40 PE=1 SV=1

TOM5 − average.top3: 6.6293296969697 − max.qupm: 4

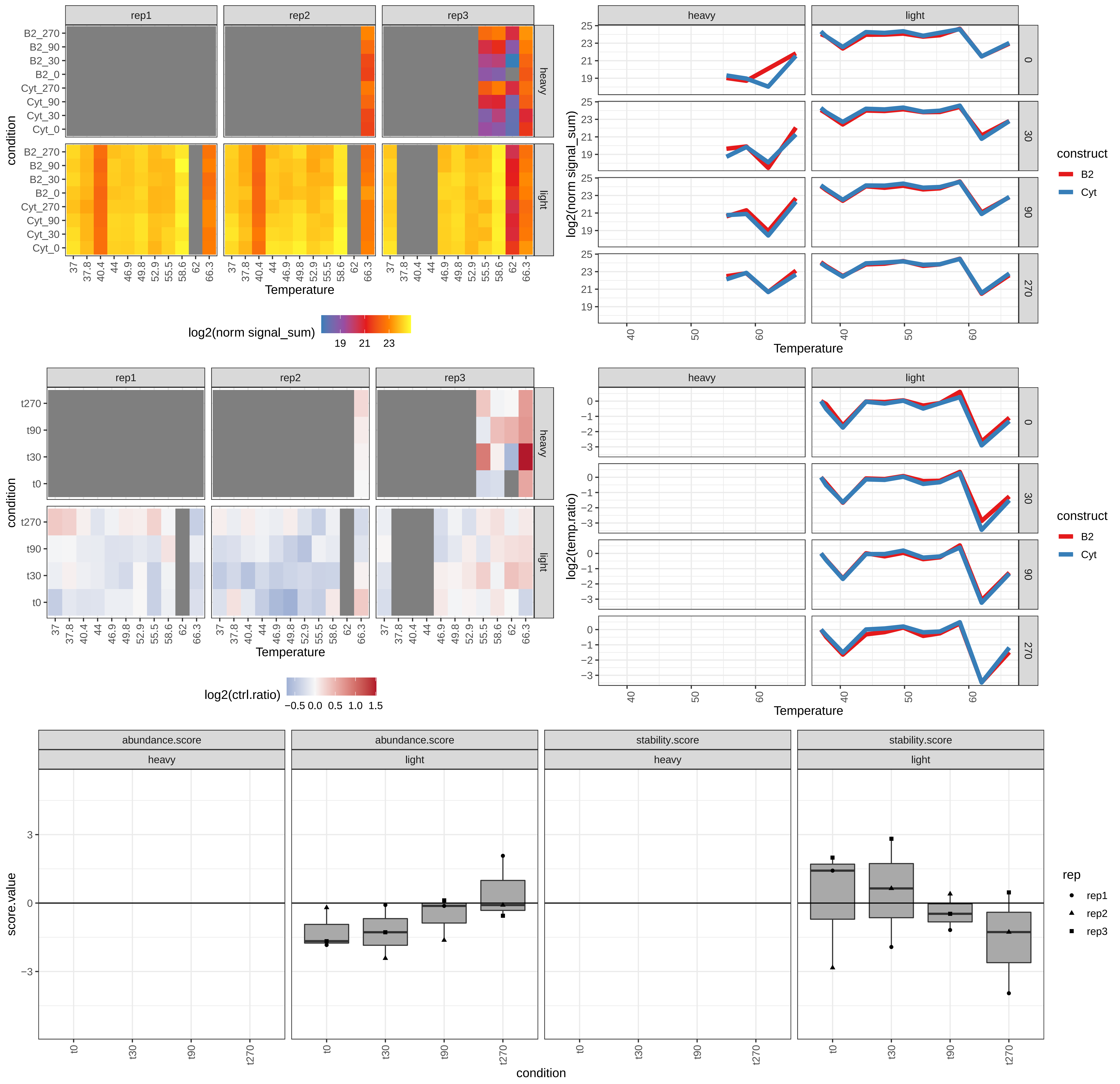

P80967 − Mitochondrial import receptor subunit TOM5 OS=Saccharomyces cerevisiae (strain ATCC 204508 / S288c) GN=TOM5 PE=1 SV=1

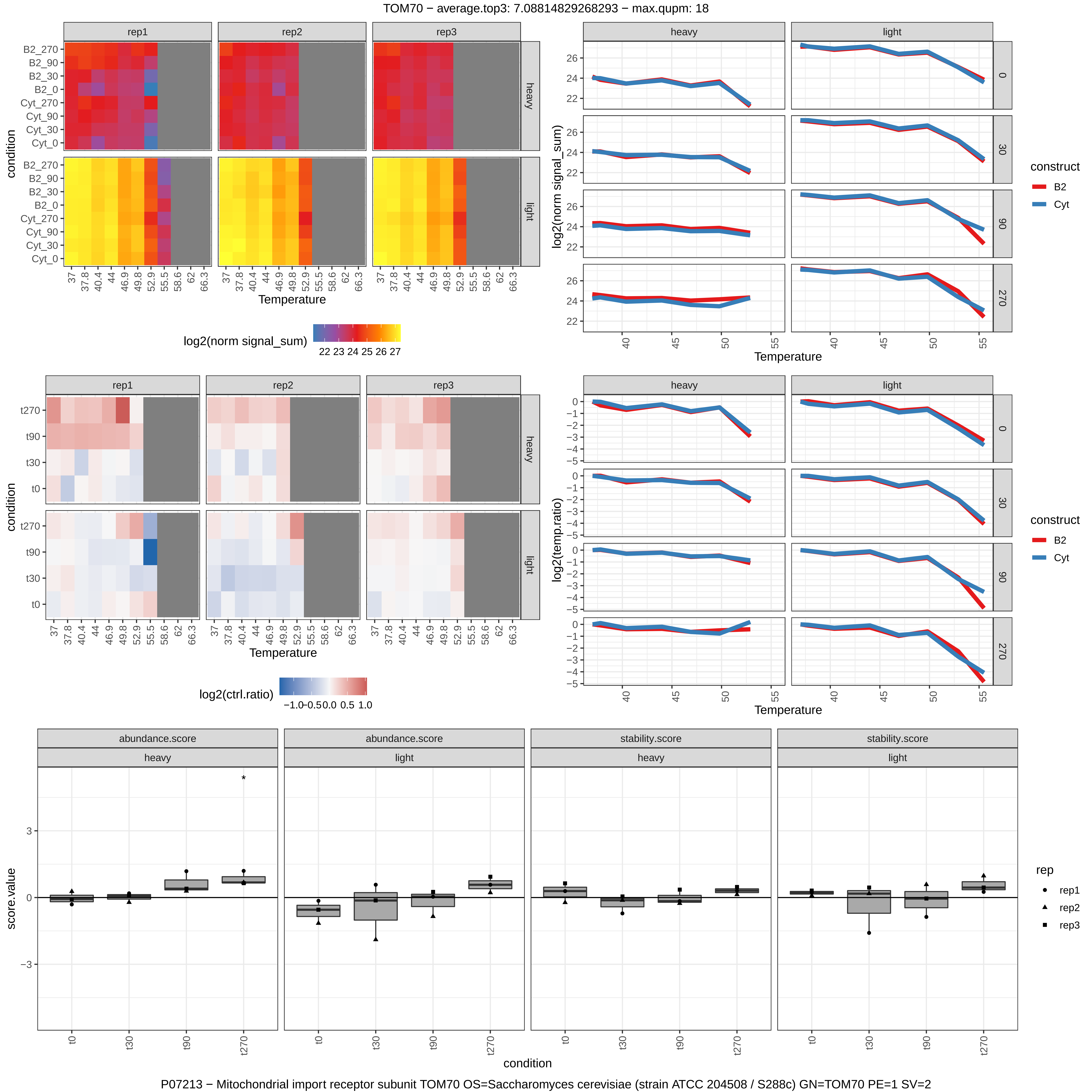

TOM70 − average.top3: 7.08814829268293 − max.qupm: 18
rep1
rep2
rep3
heavy
light
condition
Temperature
log2(norm signal_sum)
construct
B2
Cyt
log2(ctrl.ratio)
log2(temp.ratio)
abundance.score
stability.score
score.value
rep
rep1
rep2
rep3
P07213 − Mitochondrial import receptor subunit TOM70 OS=Saccharomyces cerevisiae (strain ATCC 204508 / S288c) GN=TOM70 PE=1 SV=2

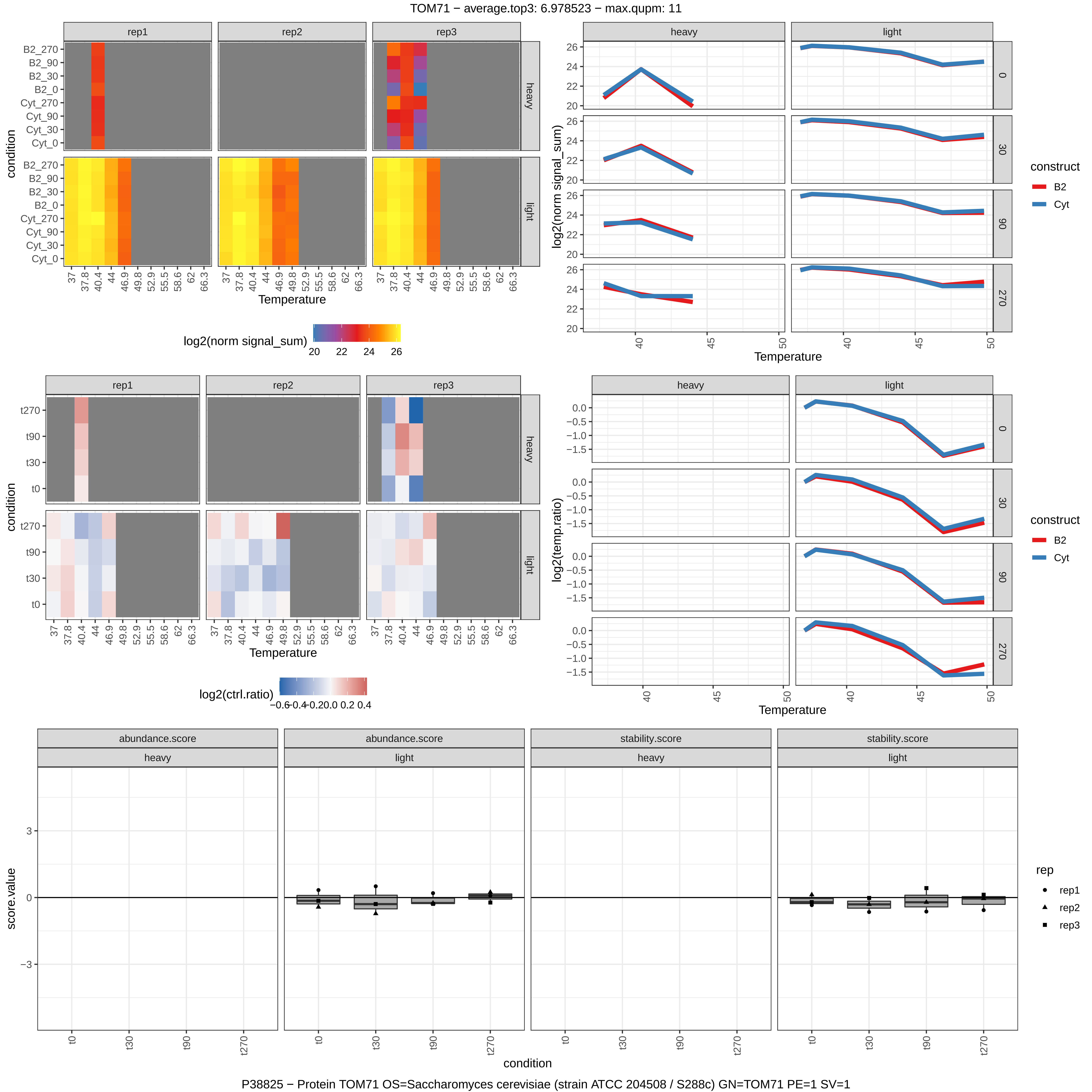

TOM71 − average.top3: 6.978523 − max.qupm: 11
rep1
rep2
rep3
heavy
light
B2_270
B2_90
B2_30
B2_0
Cyt_270
Cyt_90
Cyt_30
Cyt_0
condition
37
37.8
40.4
44
46.9
49.8
52.9
55.5
58.6
62
66.3
Temperature
log2(norm signal_sum)
20
22
24
26
0
30
90
270
log2(norm signal_sum)
construct
B2
Cyt
40
45
50
t270
t90
t30
t0
log2(ctrl.ratio)
−0.6
−0.4
−0.2
0.0
0.2
0.4
0.0
−0.5
−1.0
−1.5
log2(temp.ratio)
abundance.score
stability.score
score.value
3
0
−3
rep
rep1
rep2
rep3
condition
P38825 − Protein TOM71 OS=Saccharomyces cerevisiae (strain ATCC 204508 / S288c) GN=TOM71 PE=1 SV=1

TOP1 − average.top3: 6.85611363636364 − max.qupm: 18

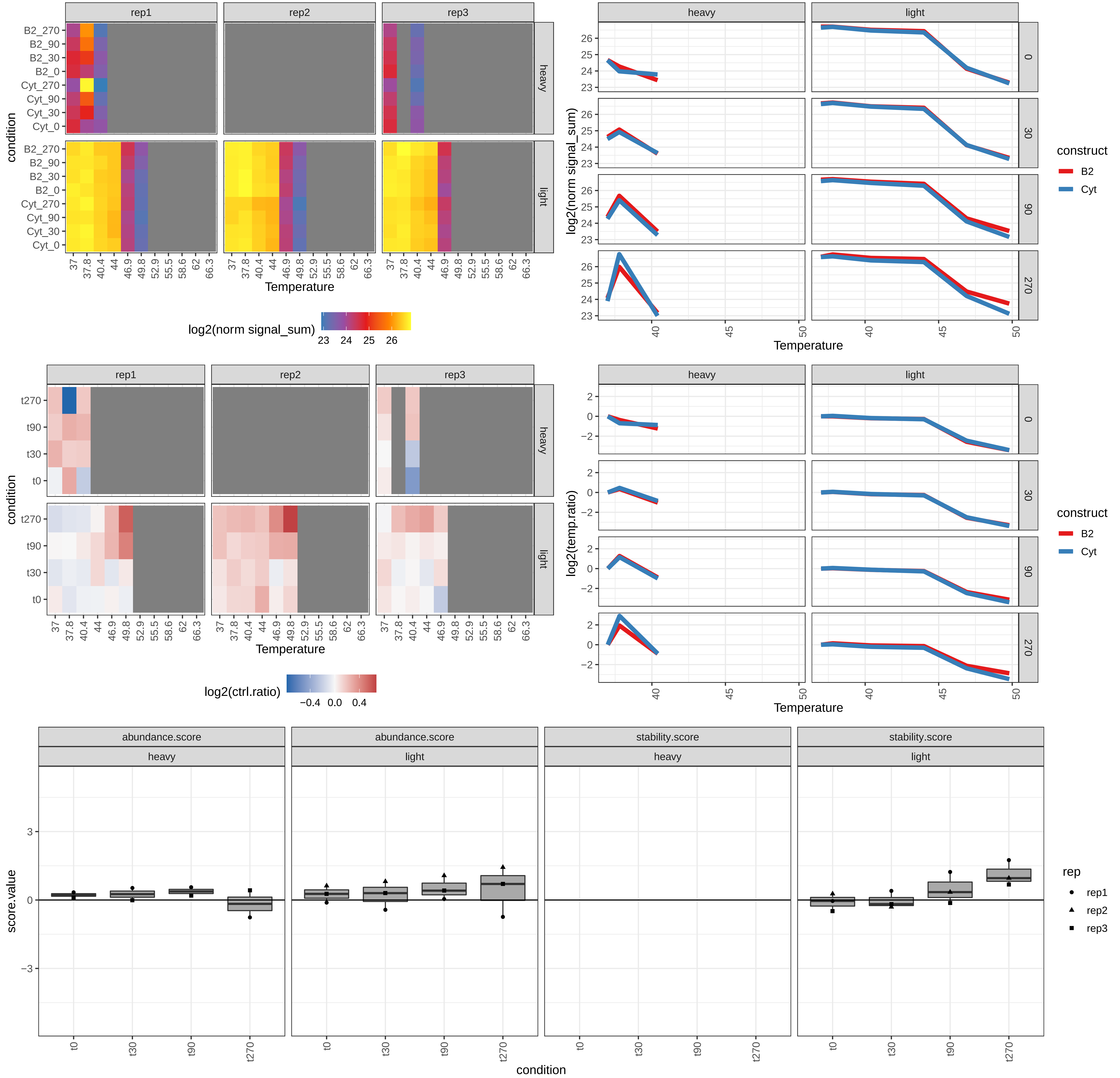

P04786 − DNA topoisomerase 1 OS=Saccharomyces cerevisiae (strain ATCC 204508 / S288c) GN=TOP1 PE=1 SV=2

TOP2 − average.top3: 6.98240823529412 − max.qupm: 34

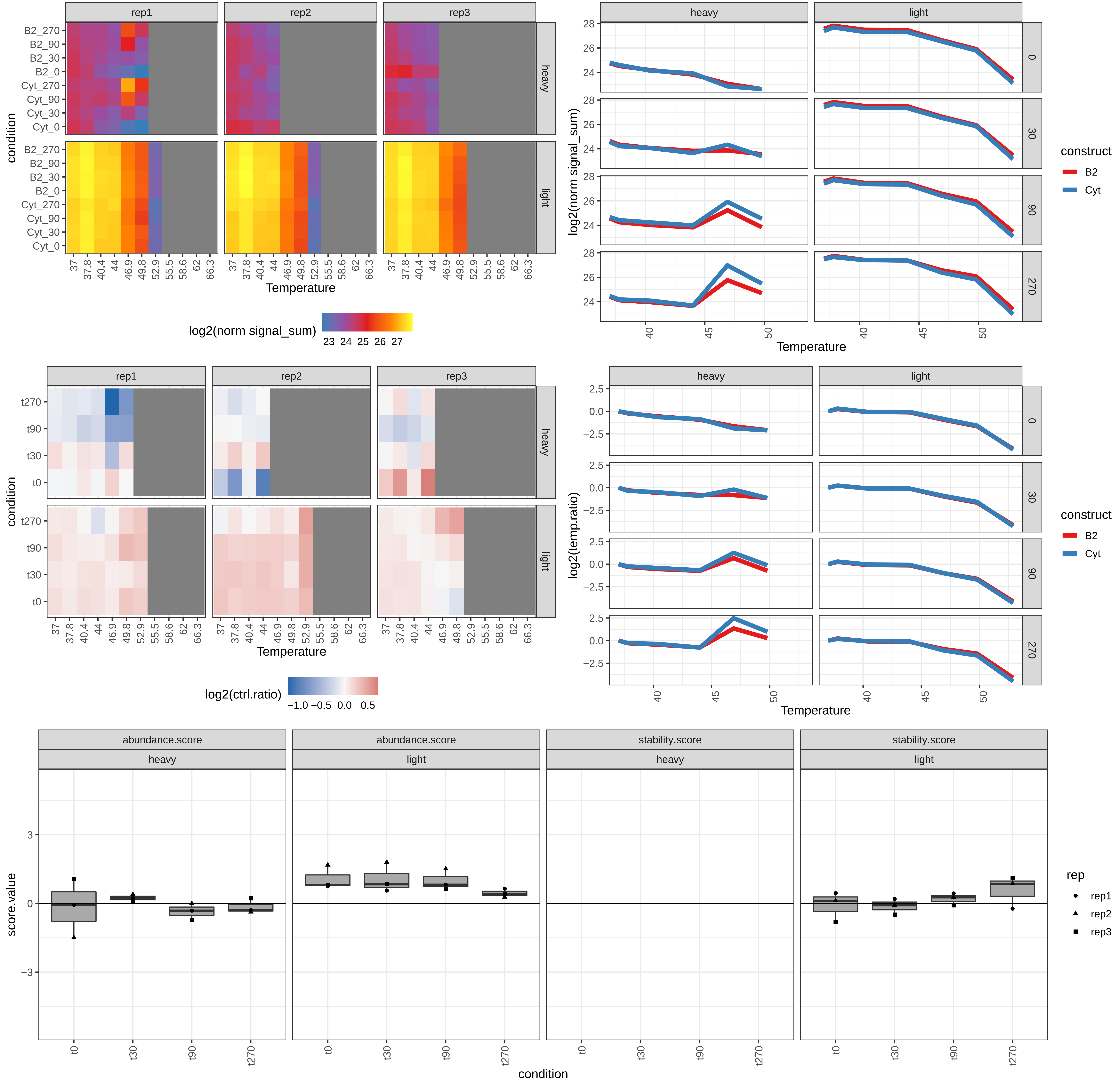

P06786 − DNA topoisomerase 2 OS=Saccharomyces cerevisiae (strain ATCC 204508 / S288c) GN=TOP2 PE=1 SV=2

TOP3 − average.top3: 7.24258909090909 − max.qupm: 5

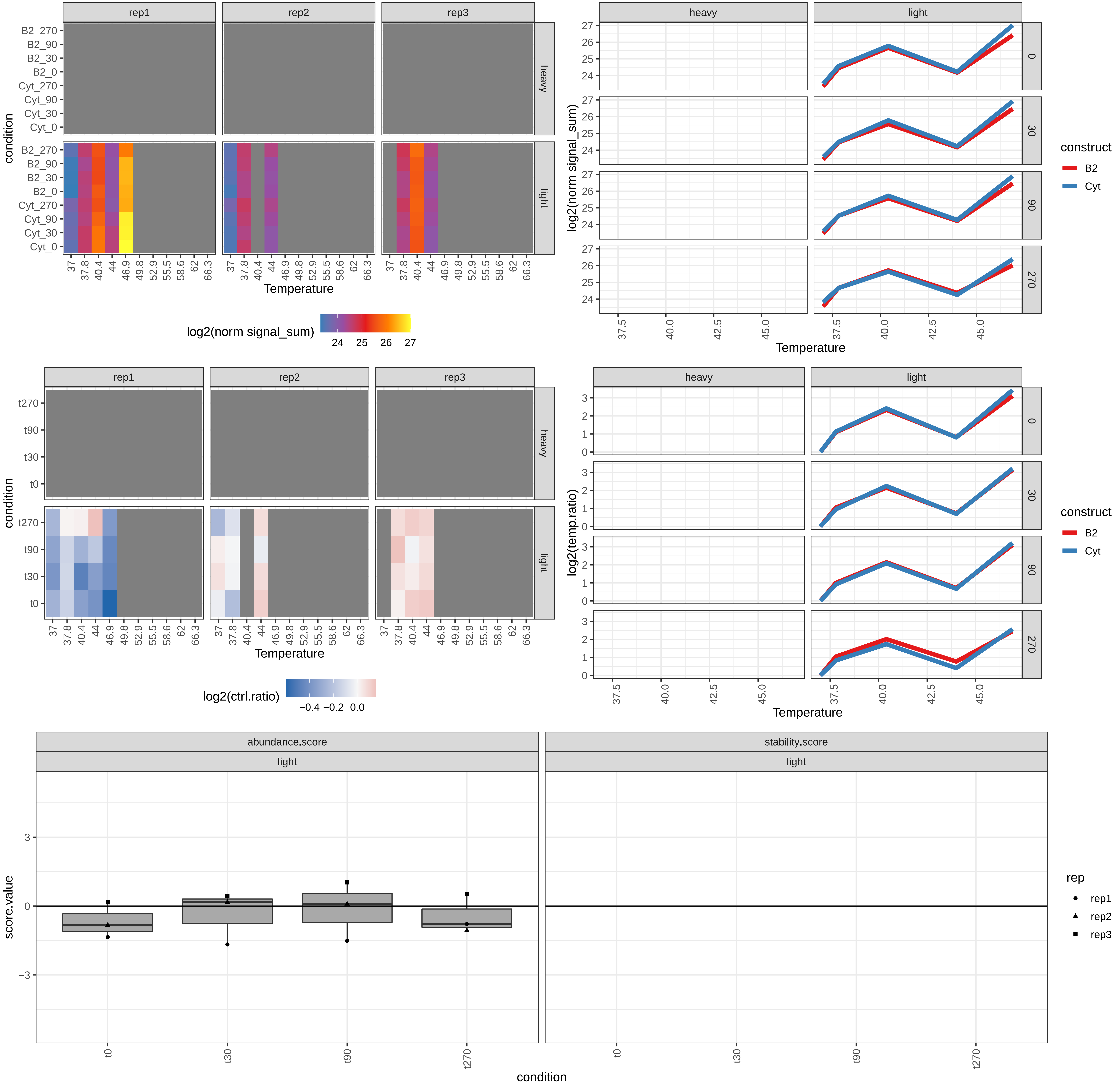

P13099 − DNA topoisomerase 3 OS=Saccharomyces cerevisiae (strain ATCC 204508 / S288c) GN=TOP3 PE=1 SV=1

TOR2 − average.top3: 6.16883928571429 − max.qupm: 7

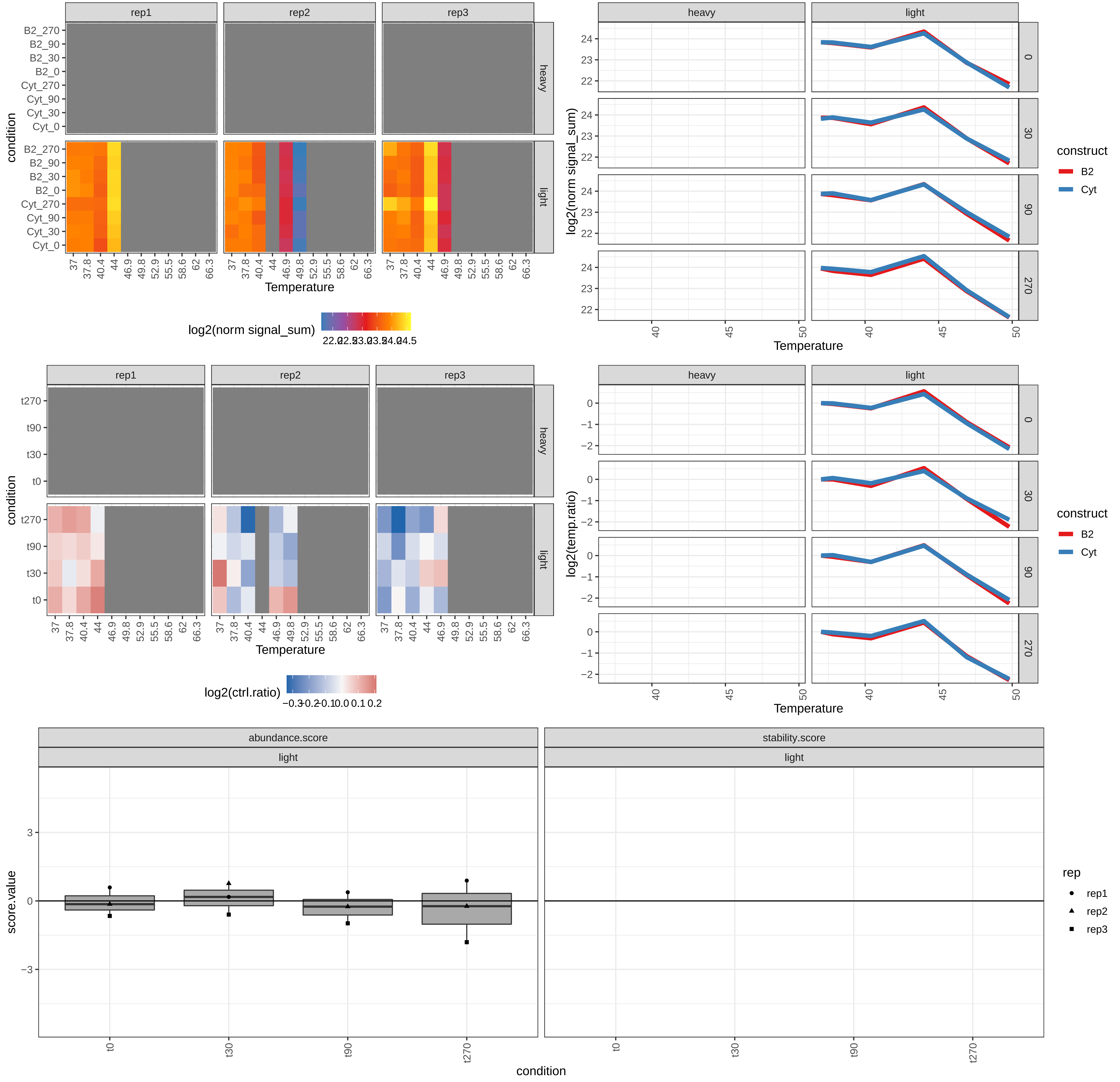

P32600 − Serine/threonine−protein kinase TOR2 OS=Saccharomyces cerevisiae (strain ATCC 204508 / S288c) GN=TOR2 PE=1 SV=3

TOS1 − average.top3: 6.96206378378378 − max.qupm: 6

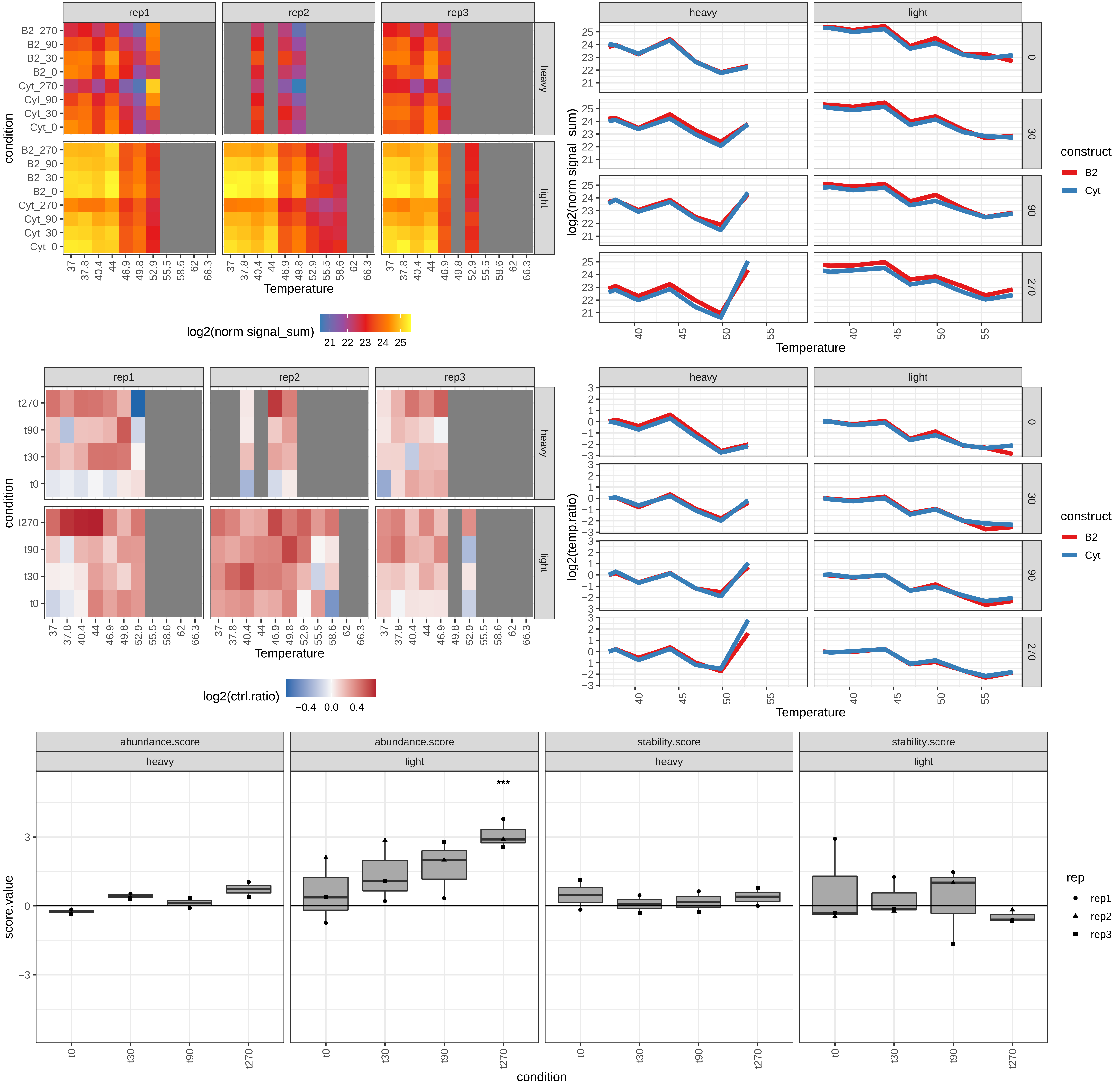

P38288 − Protein TOS1 OS=Saccharomyces cerevisiae (strain ATCC 204508 / S288c) GN=TOS1 PE=1 SV=1

TPA1 − average.top3: 6.75856 − max.qupm: 10

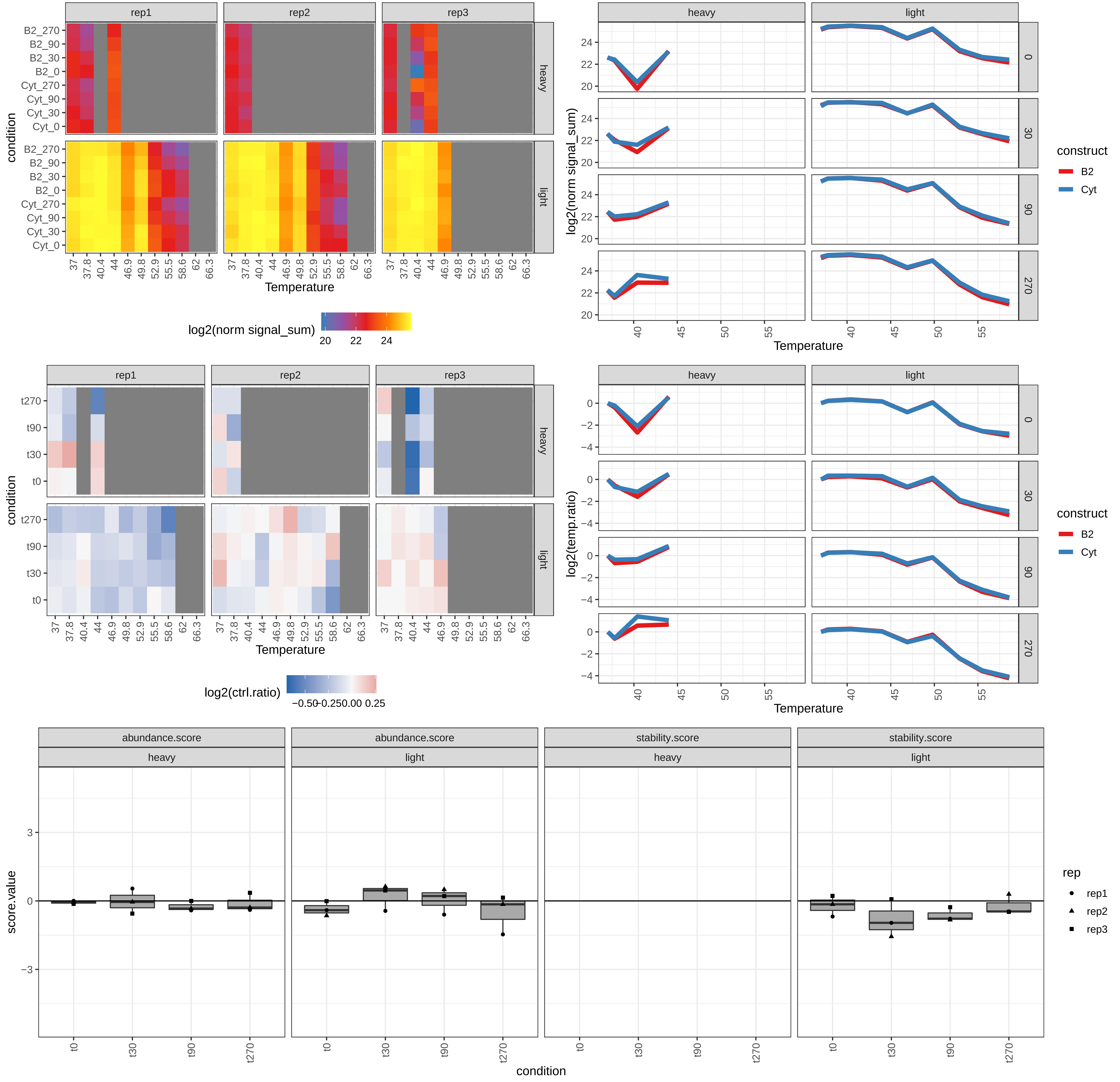

P40032 − Prolyl 3,4−dihydroxylase TPA1 OS=Saccharomyces cerevisiae (strain ATCC 204508 / S288c) GN=TPA1 PE=1 SV=1

TPD3 − average.top3: 7.38162054545455 − max.qupm: 17

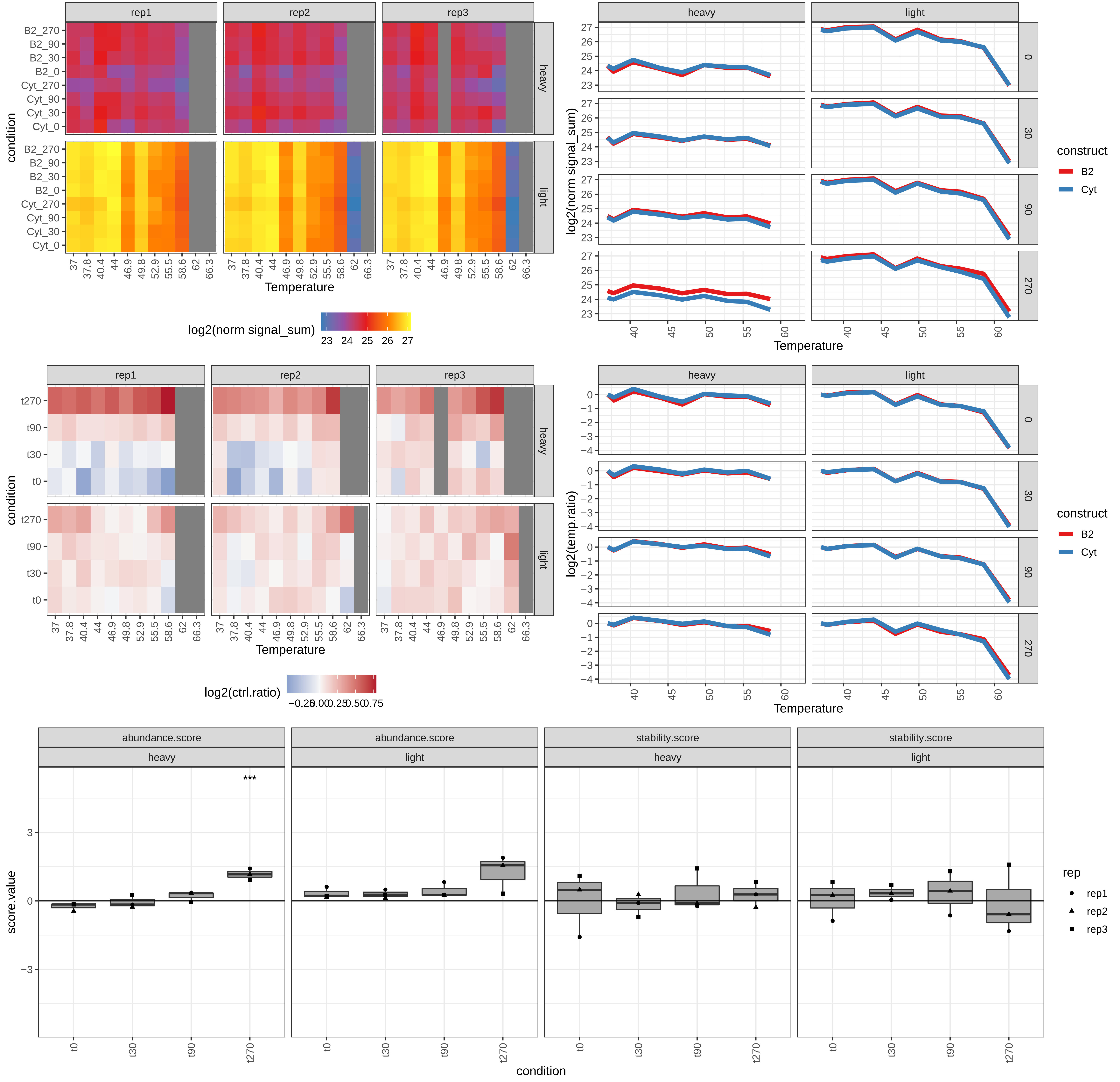

P31383 − Protein phosphatase PP2A regulatory subunit A OS=Saccharomyces cerevisiae (strain ATCC 204508 / S288c) GN=TPD3 PE=1 SV=3

TPI1 − average.top3: 8.2112226984127 − max.qupm: 14

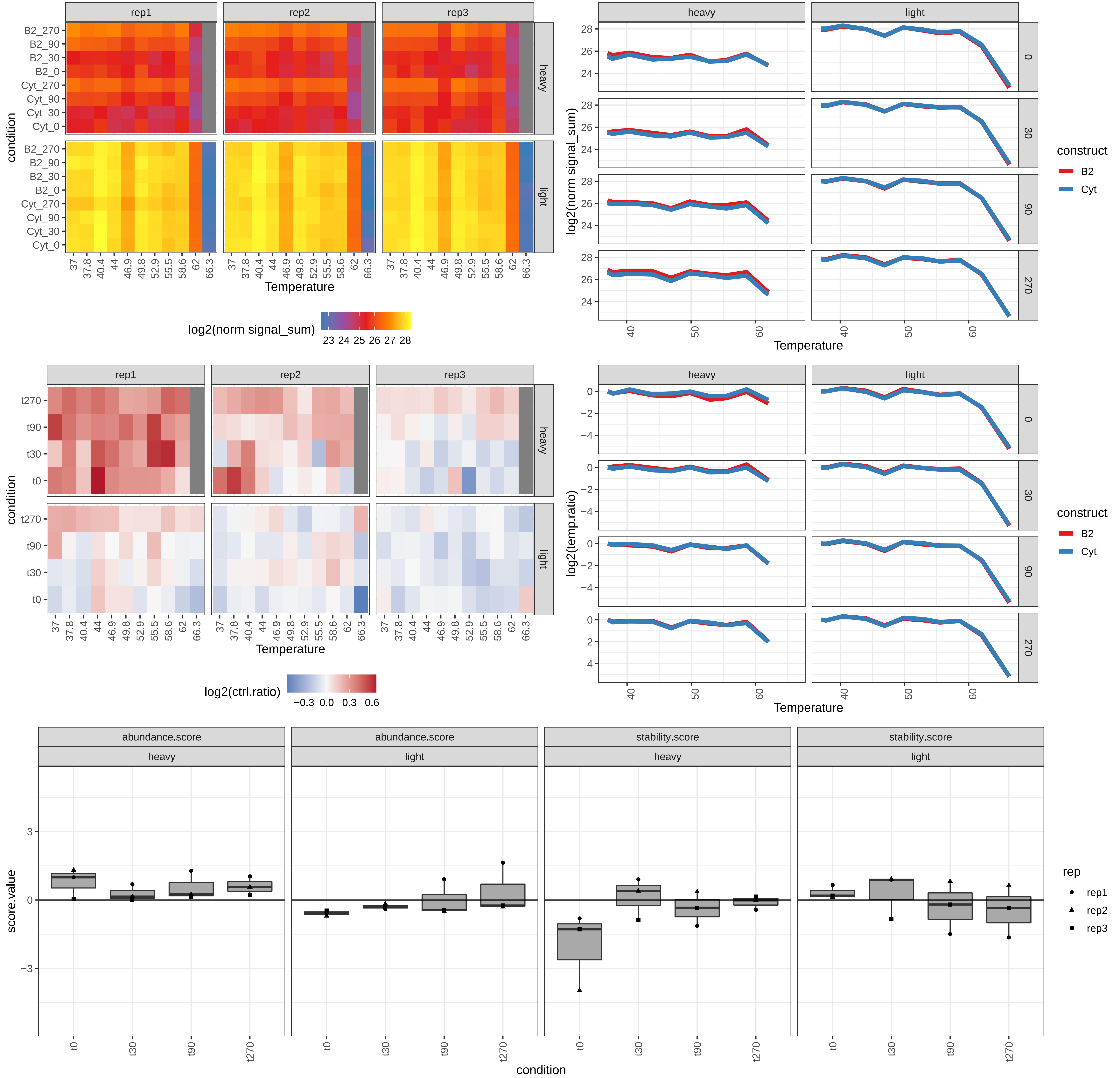

P00942 − Triosephosphate isomerase OS=Saccharomyces cerevisiae (strain ATCC 204508 / S288c) GN=TPI1 PE=1 SV=2

TPK1 − average.top3: 6.595547 − max.qupm: 6

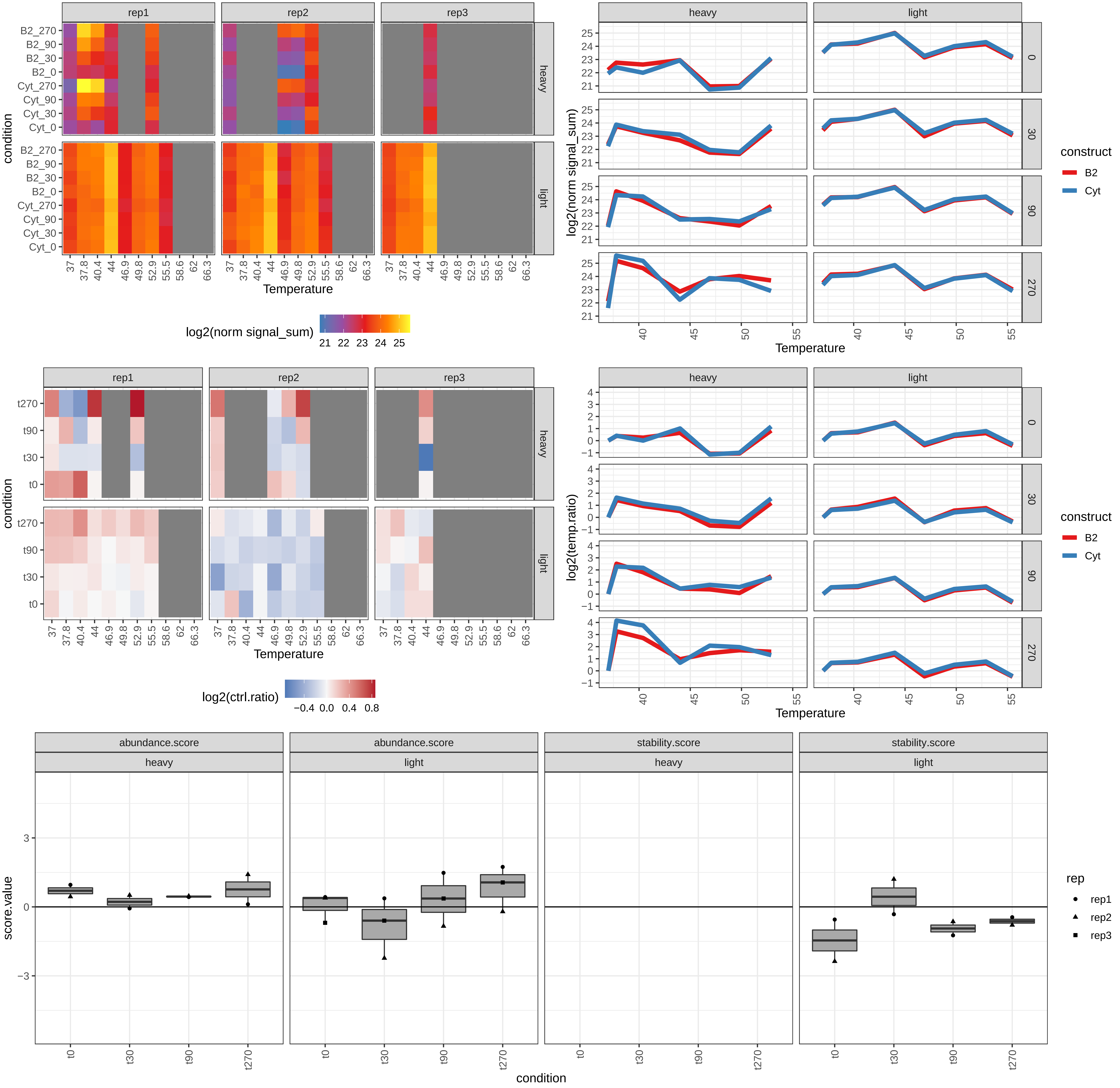

P06244 − cAMP−dependent protein kinase type 1 OS=Saccharomyces cerevisiae (strain ATCC 204508 / S288c) GN=TPK1 PE=1 SV=3

TPK2 − average.top3: 7.11311 − max.qupm: 6

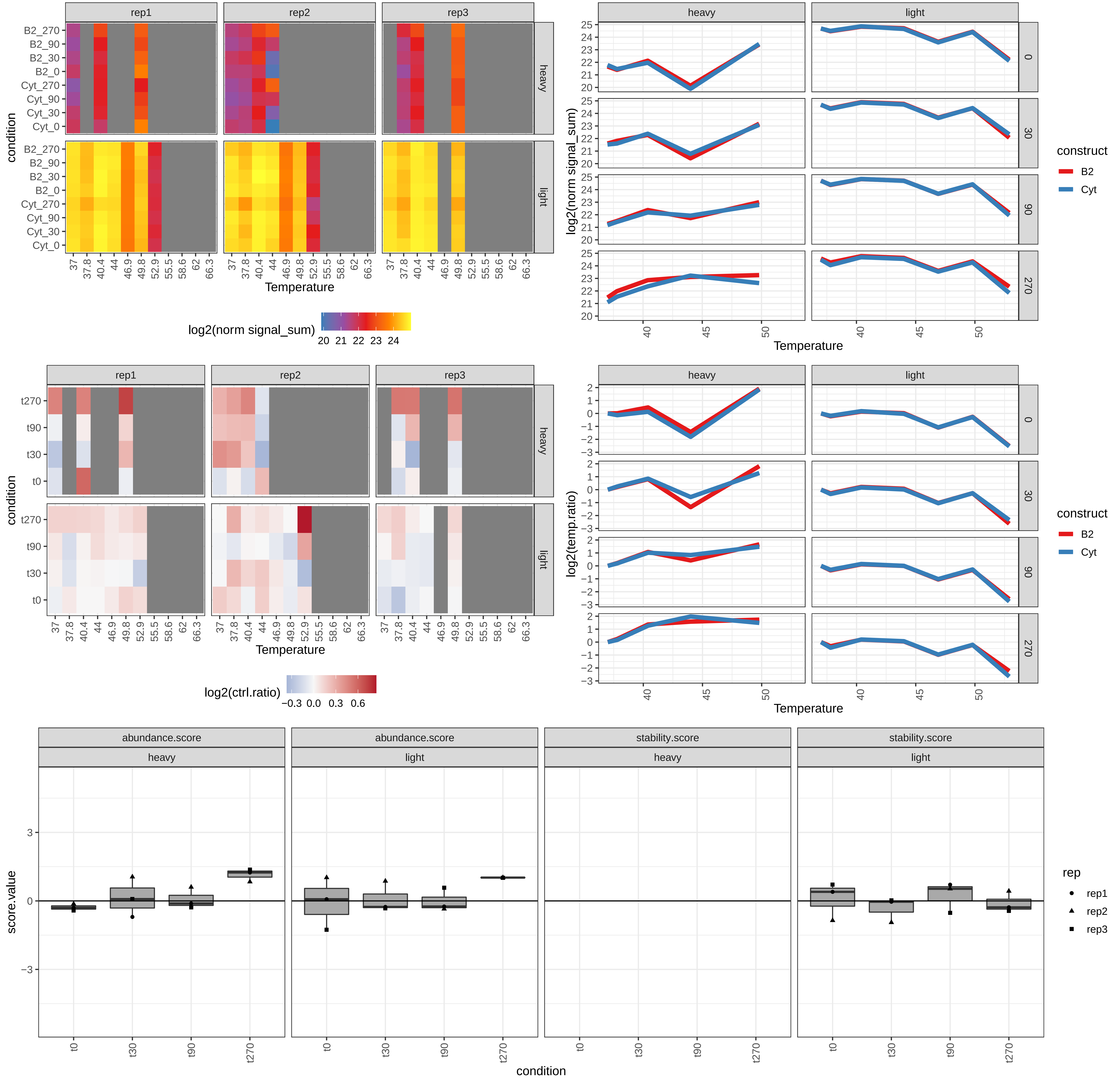

P06245 − cAMP−dependent protein kinase type 2 OS=Saccharomyces cerevisiae (strain ATCC 204508 / S288c) GN=TPK2 PE=1 SV=2

TPM1 − average.top3: 6.99517101694915 − max.qupm: 16

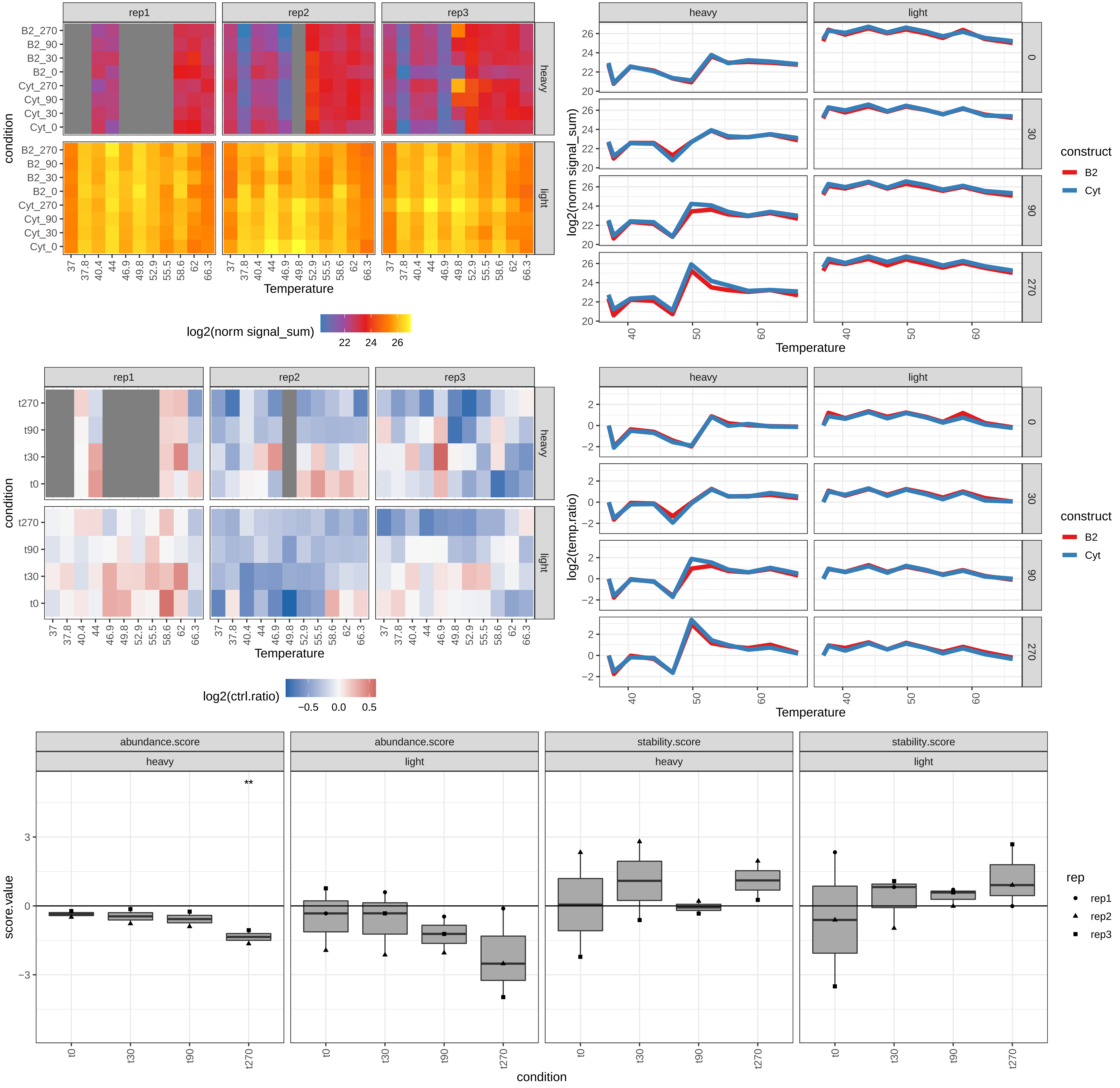

P17536 − Tropomyosin−1 OS=Saccharomyces cerevisiae (strain ATCC 204508 / S288c) GN=TPM1 PE=1 SV=1

TPM2 − average.top3: 6.54832810810811 − max.qupm: 9

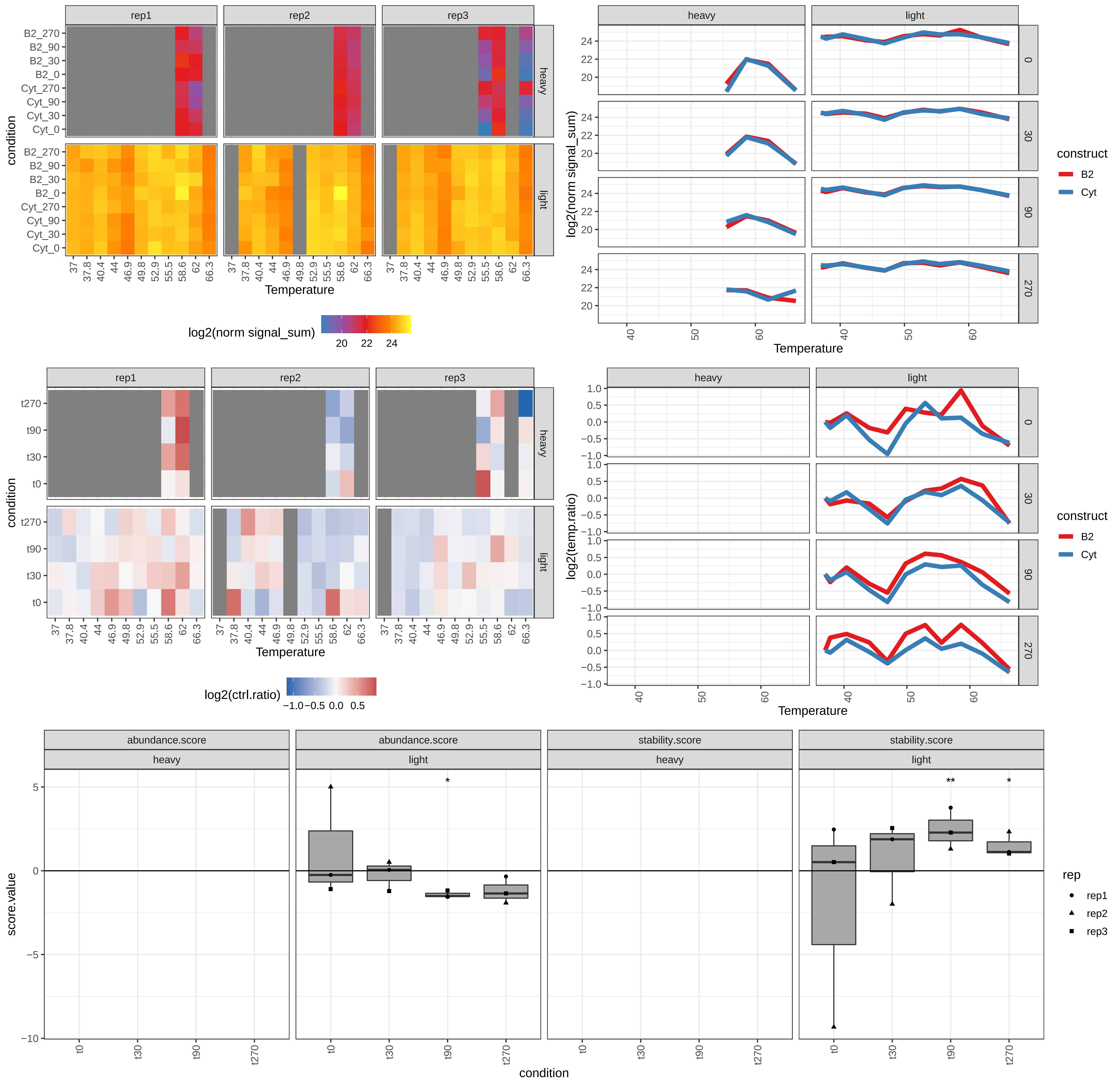

P40414 − Tropomyosin−2 OS=Saccharomyces cerevisiae (strain ATCC 204508 / S288c) GN=TPM2 PE=1 SV=1

TPO5 − average.top3: 6.5623125 − max.qupm: 2

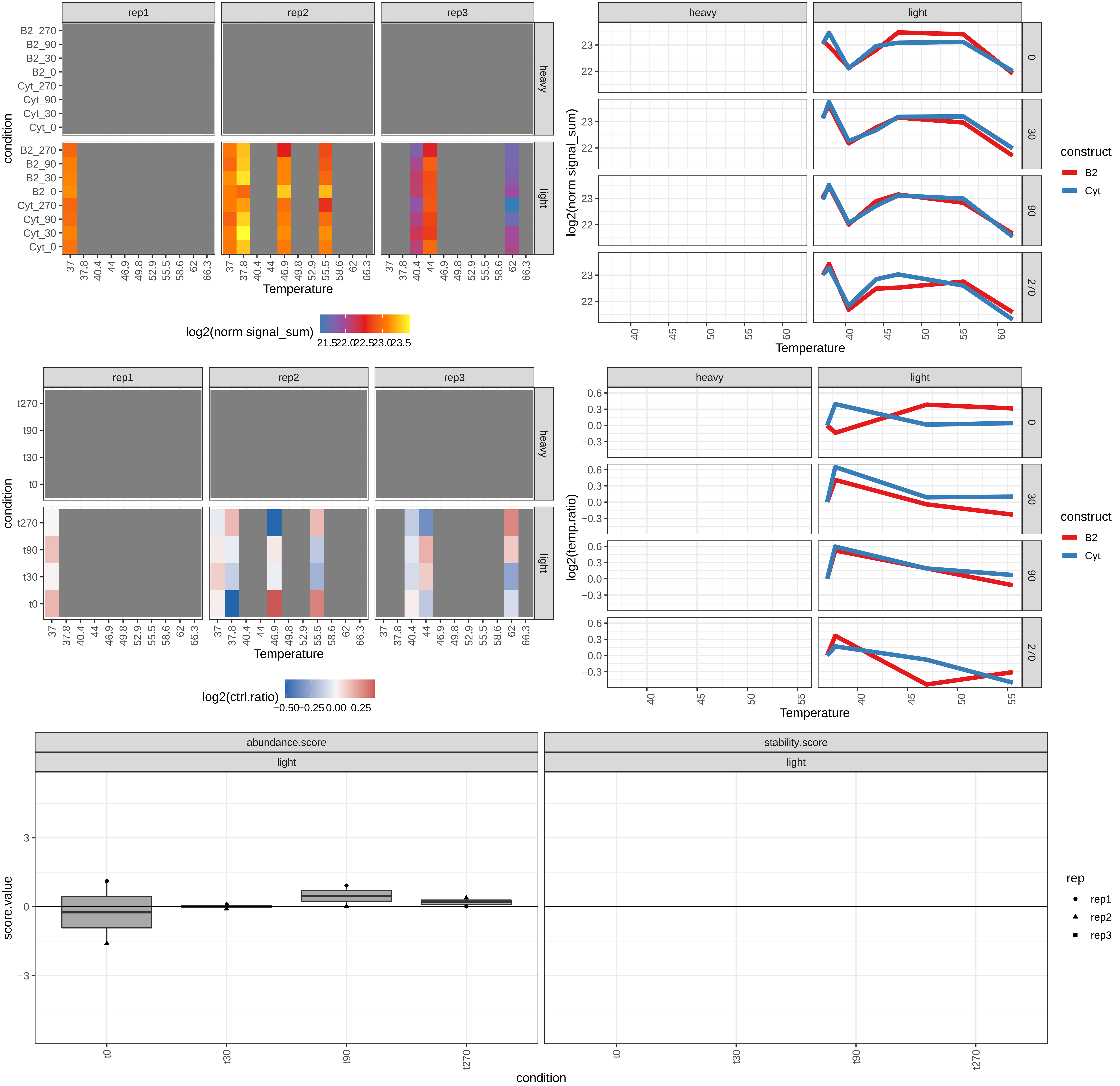

P36029 − Polyamine transporter TPO5 OS=Saccharomyces cerevisiae (strain ATCC 204508 / S288c) GN=TPO5 PE=1 SV=1

TPS1 − average.top3: 7.40037781818182 − max.qupm: 11

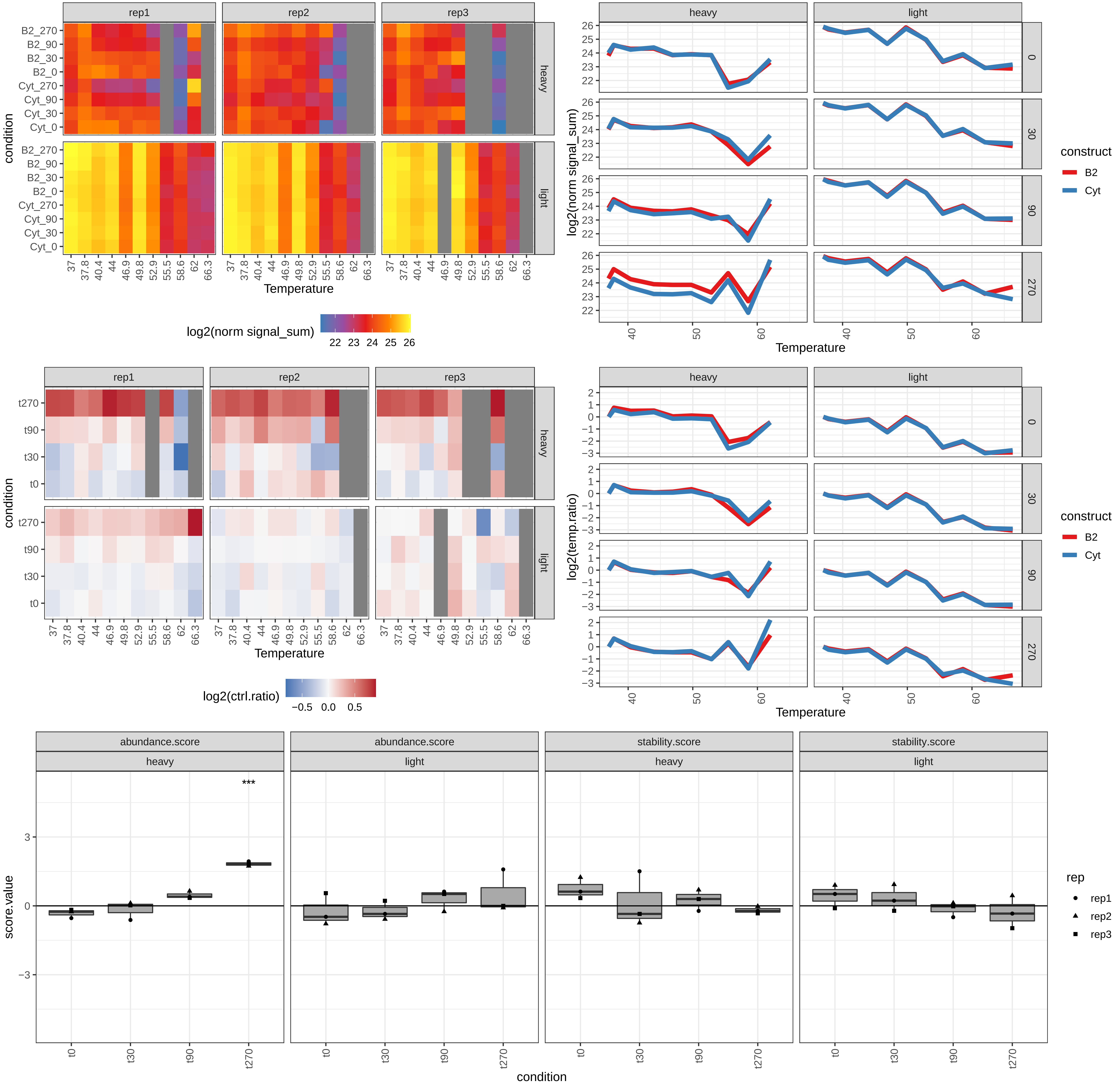

Q00764 − Alpha,alpha−trehalose−phosphate synthase [UDP−forming] 56 kDa subunit OS=Saccharomyces cerevisiae (strain ATCC 204508 / S288c) GN=TPS1 PE=1 SV=2

TPS2 − average.top3: 7.58778438596491 − max.qupm: 25

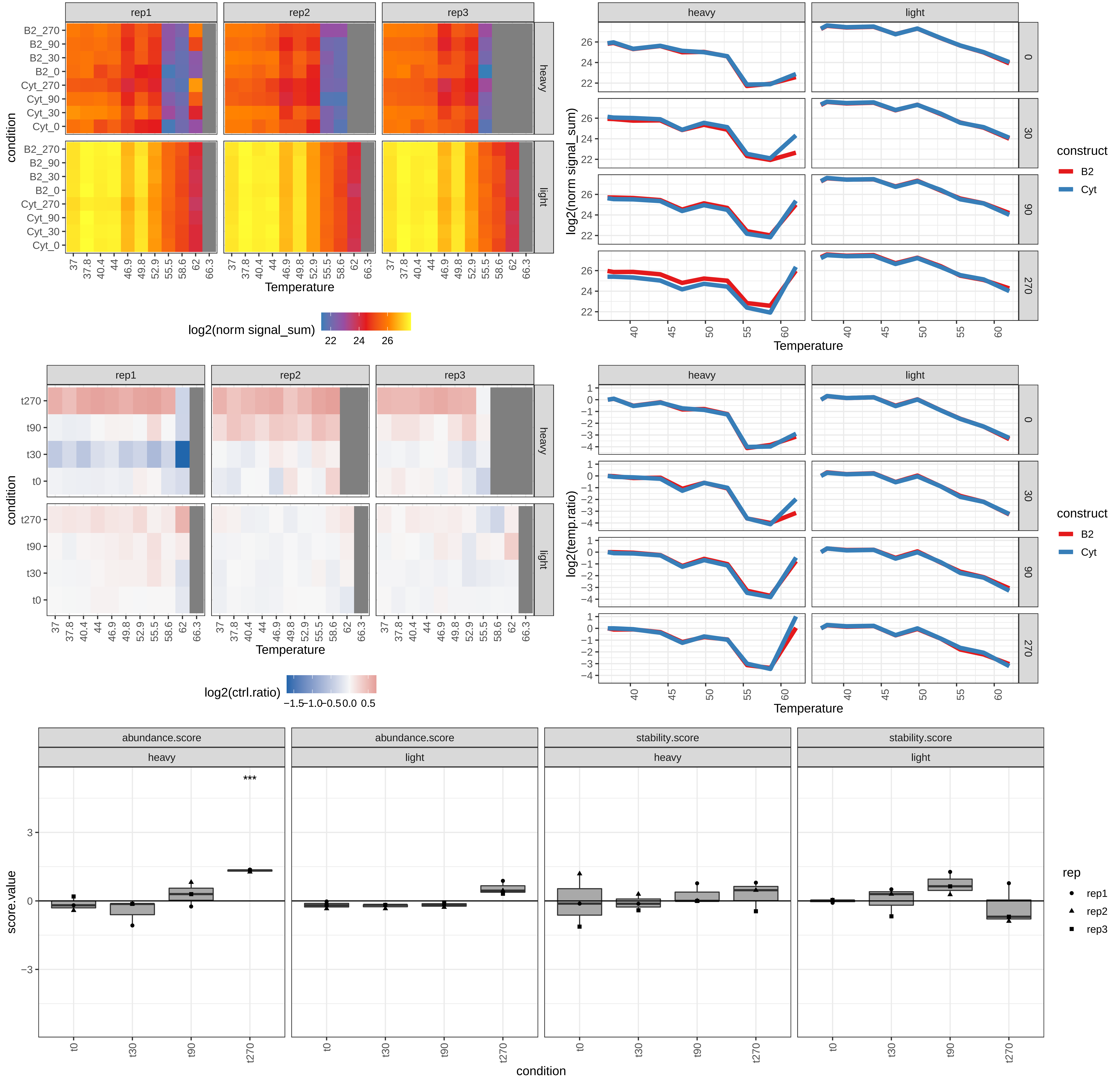

P31688 − Trehalose−phosphatase OS=Saccharomyces cerevisiae (strain ATCC 204508 / S288c) GN=TPS2 PE=1 SV=3

TPS3 − average.top3: 6.86747307692308 − max.qupm: 14

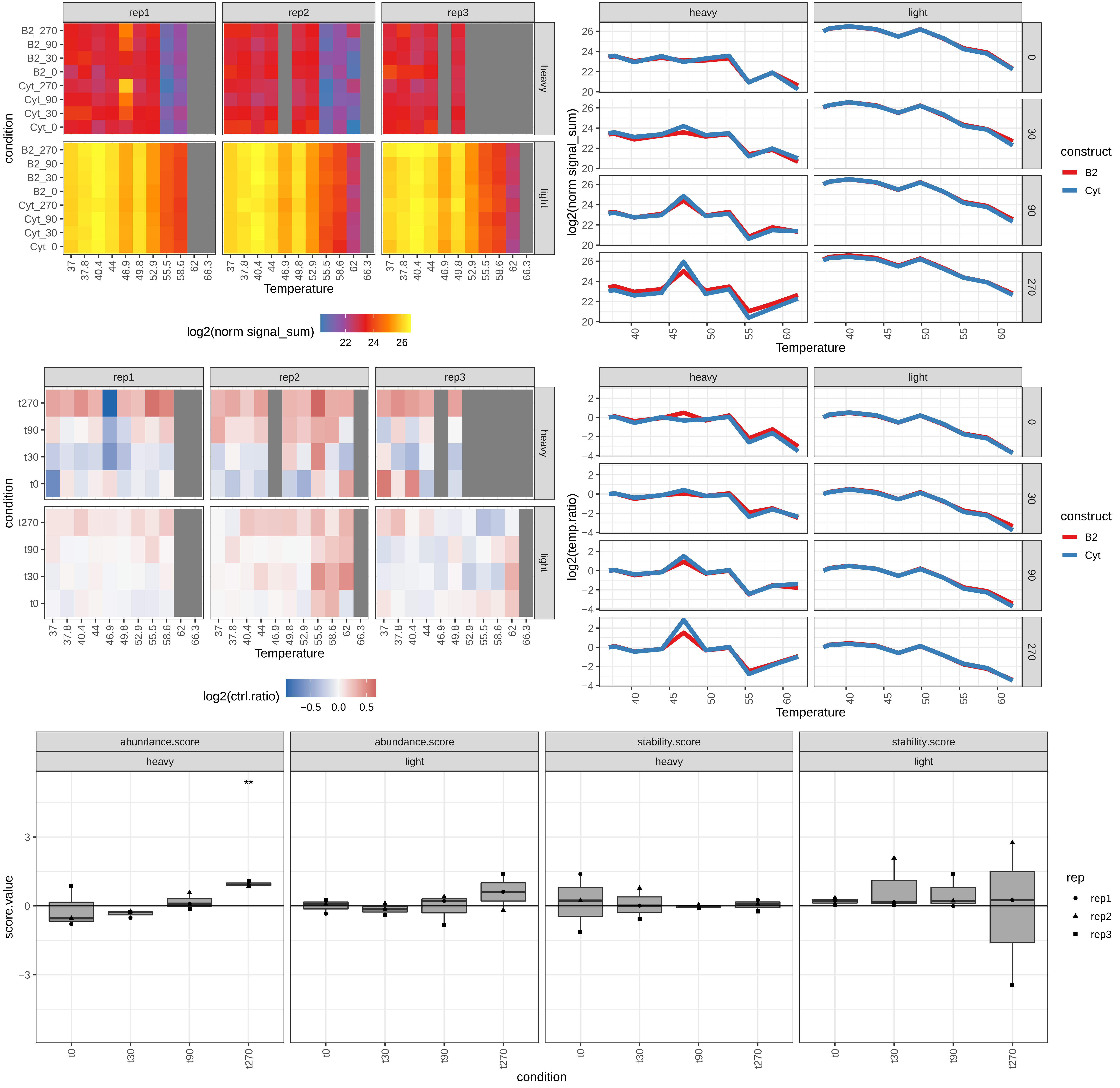

P38426 − Trehalose synthase complex regulatory subunit TPS3 OS=Saccharomyces cerevisiae (strain ATCC 204508 / S288c) GN=TPS3 PE=1 SV=3

TRA1 − average.top3: 6.80316074074074 − max.qupm: 34

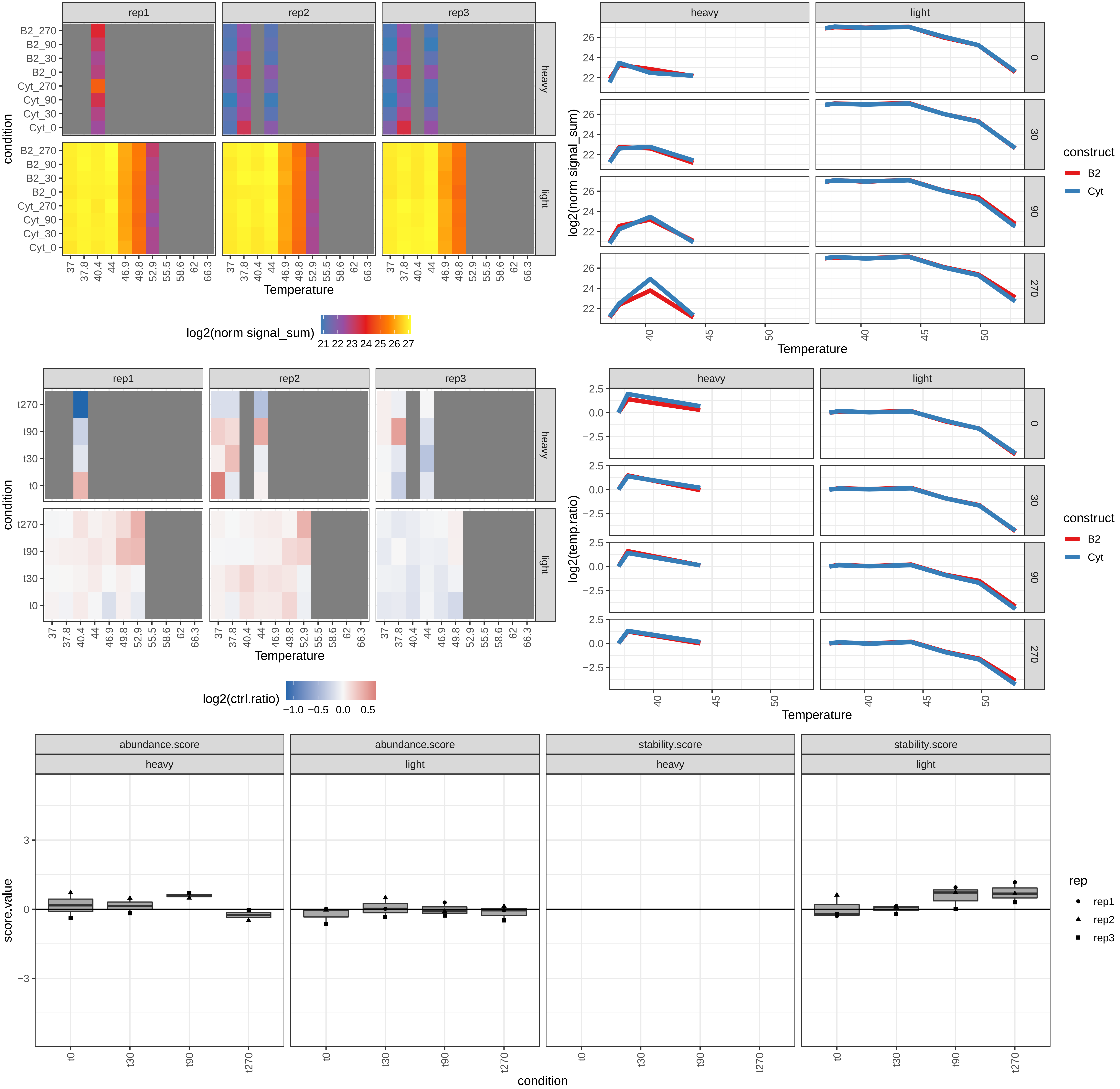

P38811 − Transcription−associated protein 1 OS=Saccharomyces cerevisiae (strain ATCC 204508 / S288c) GN=TRA1 PE=1 SV=1

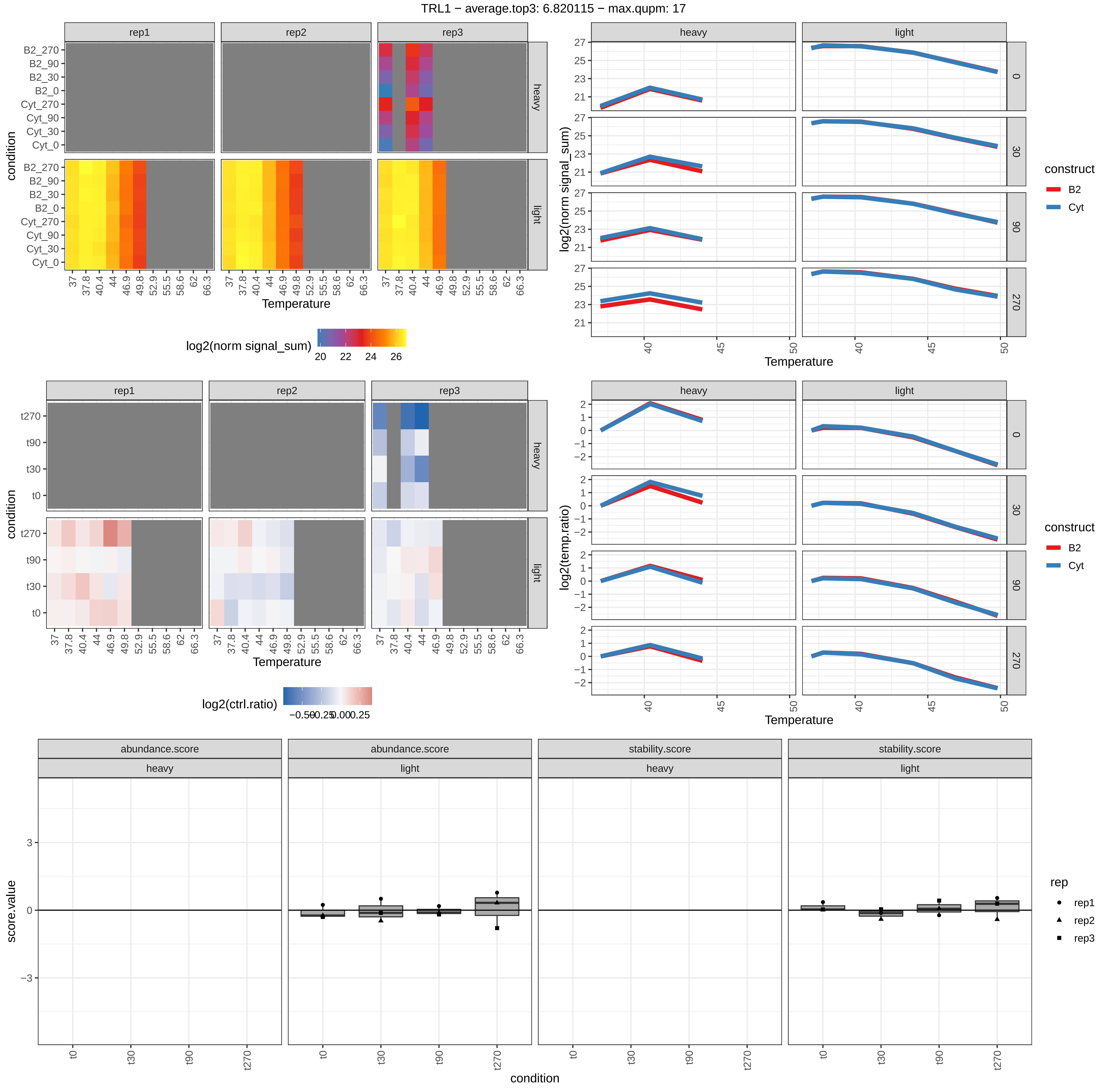

P09880 − tRNA ligase OS=Saccharomyces cerevisiae (strain ATCC 204508 / S288c) GN=TRL1 PE=1 SV=1

TRM1 − average.top3: 6.68033346938776 − max.qupm: 11

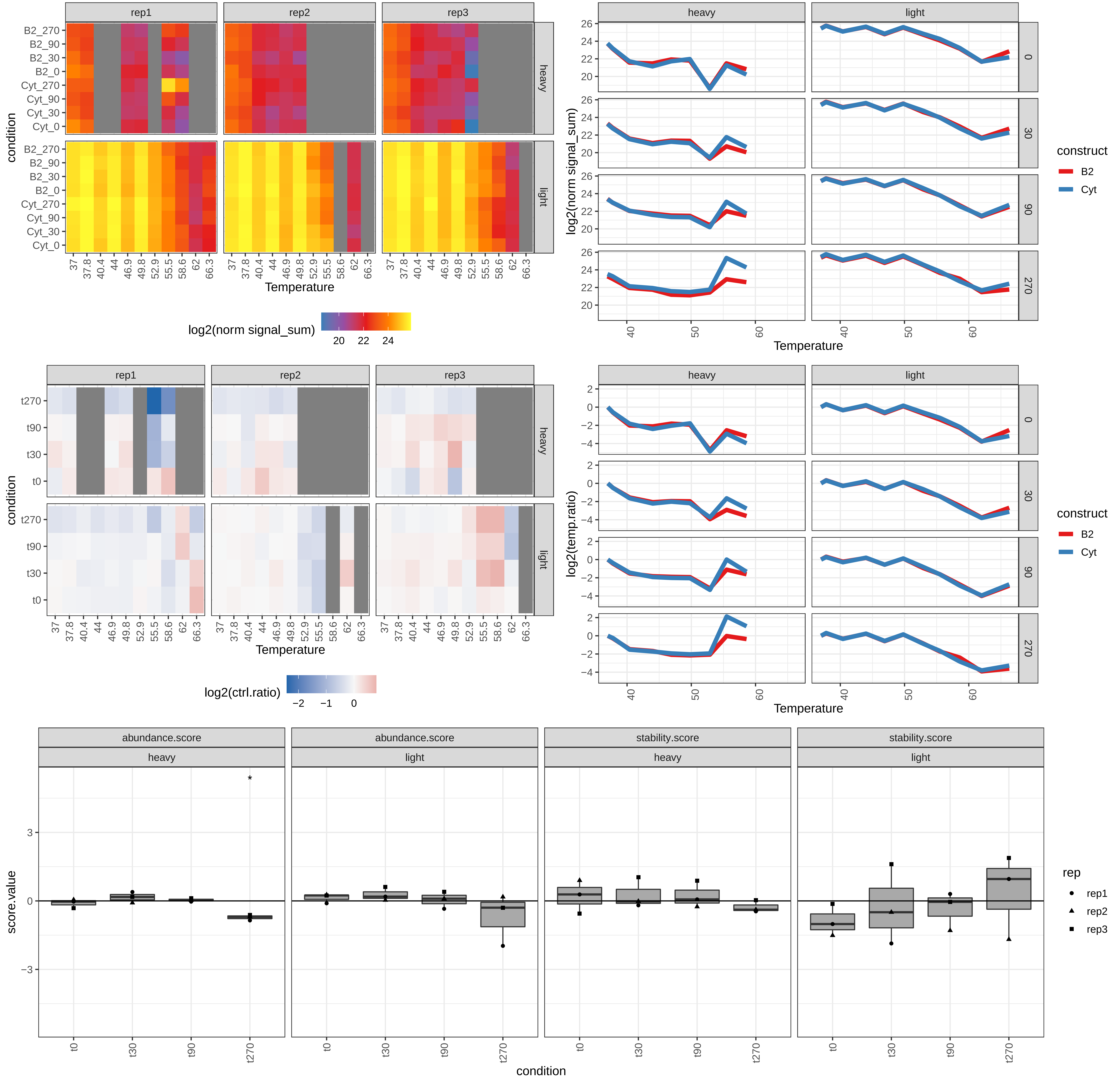

transferase, mitochondrial OS=Saccharomyces cerevisiae (strain ATCC 204508 / S288c) GN=TRM1 PE=1 SV=1|Isoform 2 of tRNA (guanine(26)−N(2))−dimethyltransferase, mitochondrial OS=Saccharomy

TRM10 − average.top3: 6.99081904761905 − max.qupm: 8

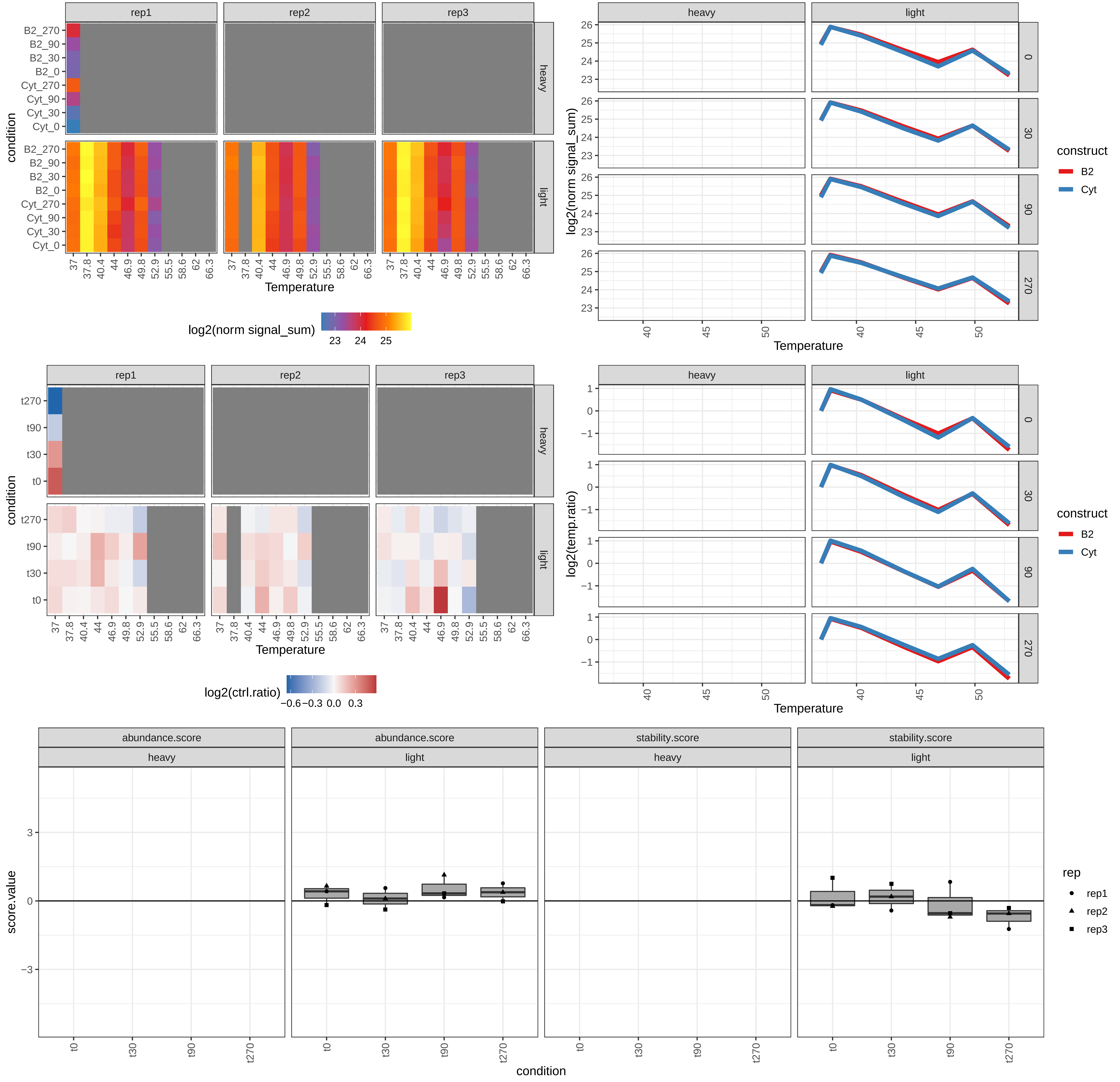

Q12400 − tRNA (guanine(9)−N1)−methyltransferase OS=Saccharomyces cerevisiae (strain ATCC 204508 / S288c) GN=TRM10 PE=1 SV=1

TRM11 − average.top3: 6.65232363636364 − max.qupm: 5

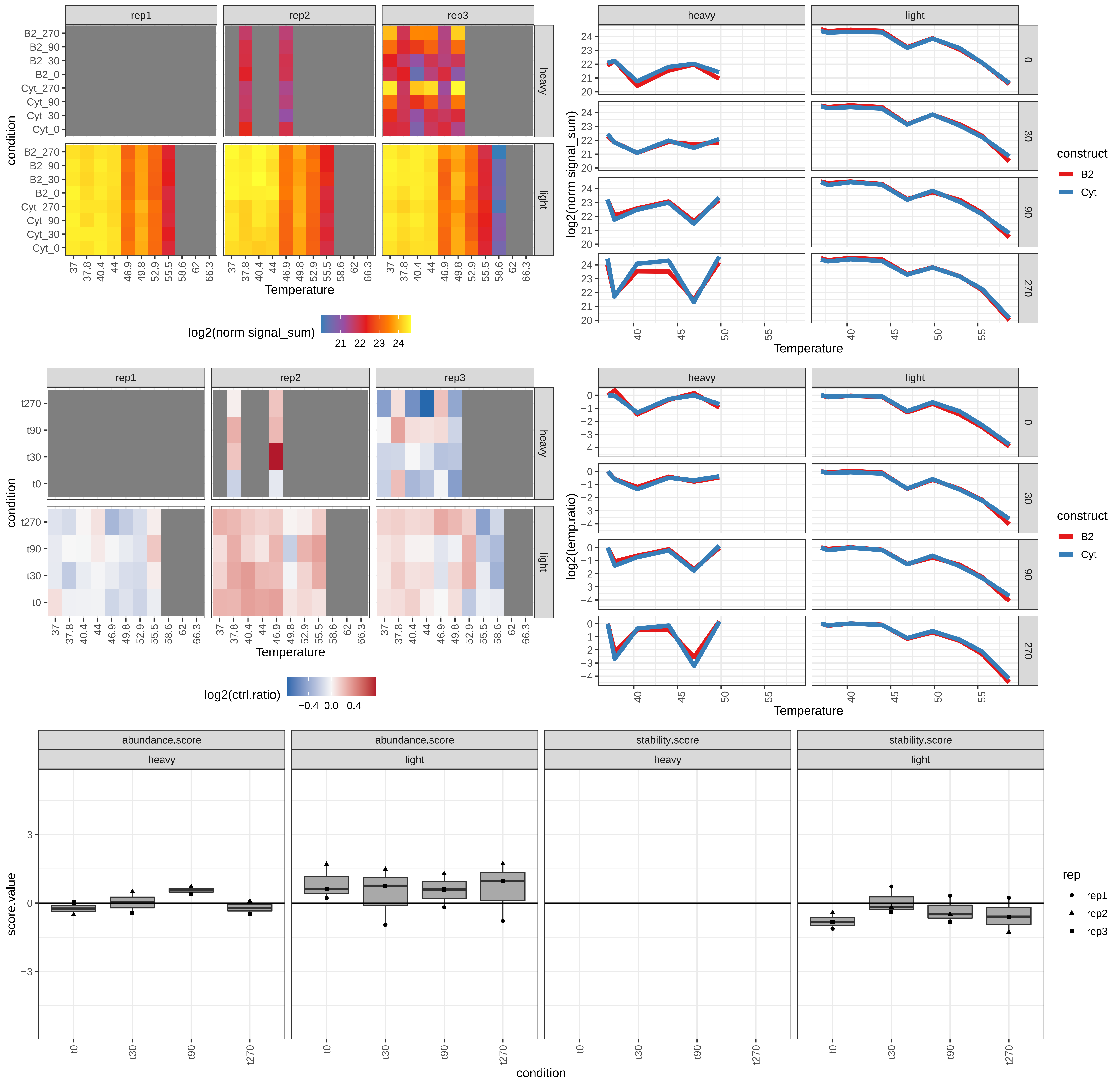

Q12463 − tRNA (guanine(10)−N2)−methyltransferase OS=Saccharomyces cerevisiae (strain ATCC 204508 / S288c) GN=TRM11 PE=1 SV=1

TRM112 − average.top3: 7.1160052173913 − max.qupm: 3

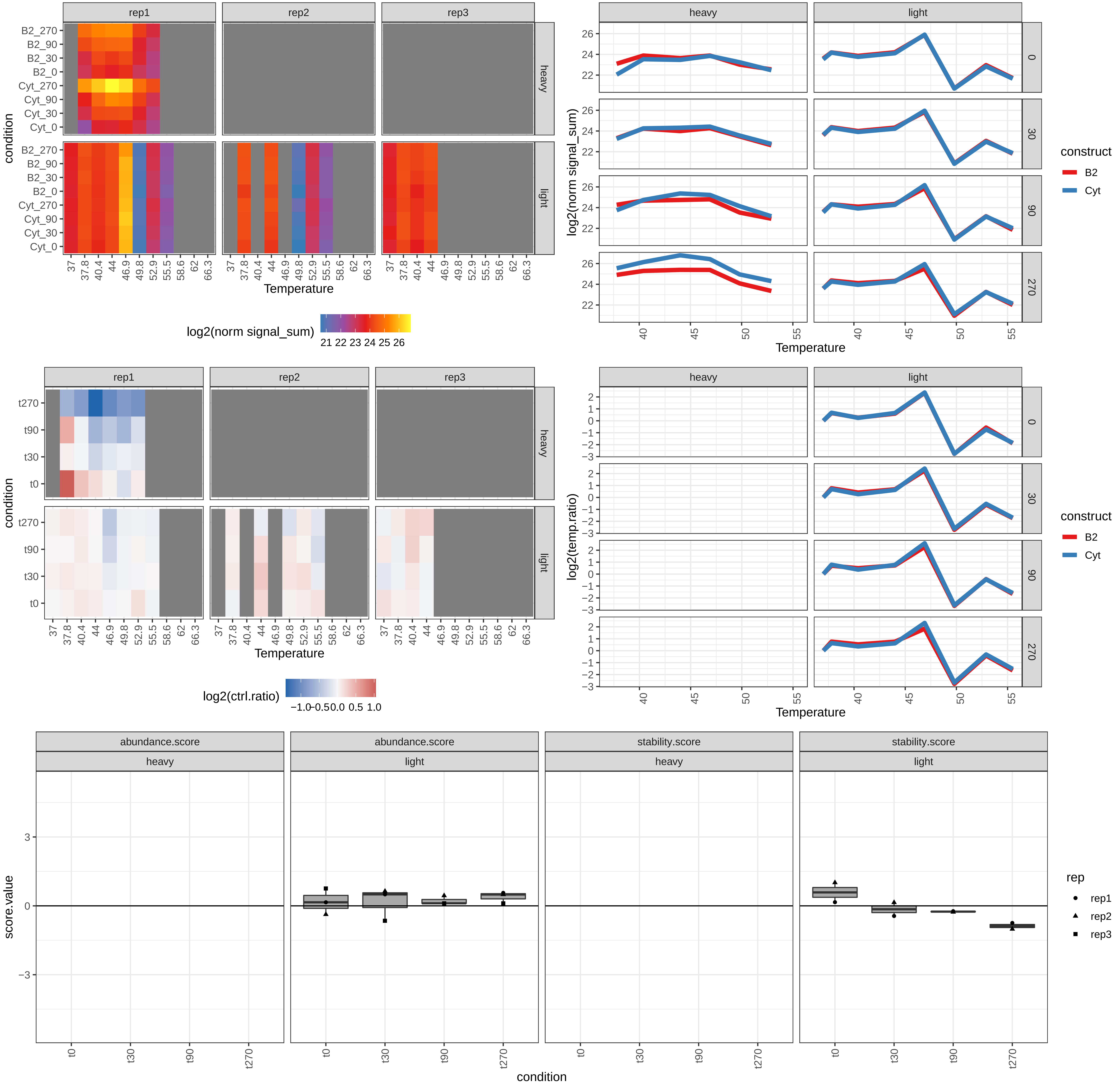

P53738 − Multifunctional methyltransferase subunit TRM112 OS=Saccharomyces cerevisiae (strain ATCC 204508 / S288c) GN=TRM112 PE=1 SV=1

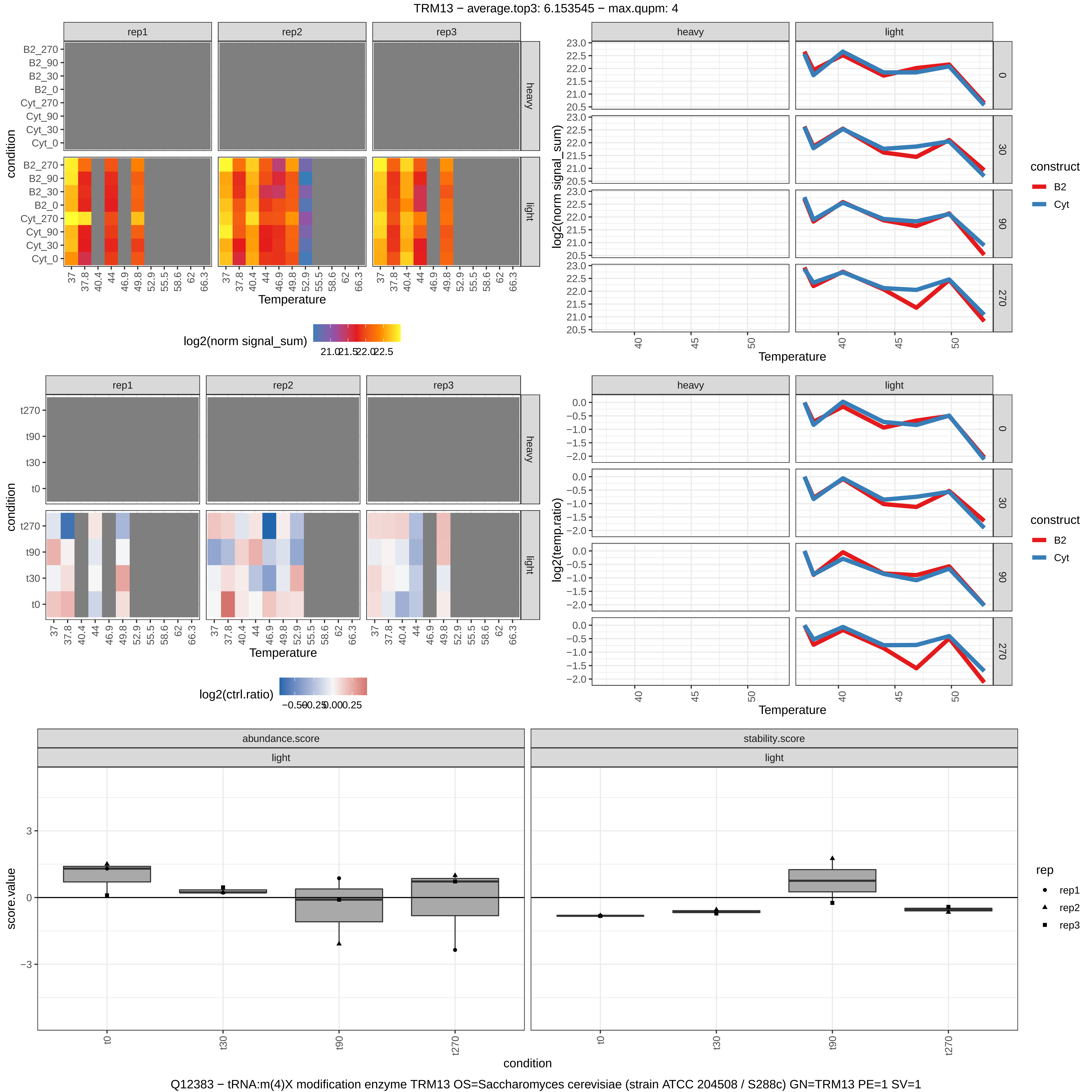
TRM13 − average.top3: 6.153545 − max.qupm: 4
rep1
rep2
rep3
heavy
light
condition
Temperature
log2(norm signal_sum)
log2(ctrl.ratio)
log2(temp.ratio)
construct
B2
Cyt
abundance.score
stability.score
score.value
rep
rep1
rep2
rep3
Q12383 − tRNA:m(4)X modification enzyme TRM13 OS=Saccharomyces cerevisiae (strain ATCC 204508 / S288c) GN=TRM13 PE=1 SV=1

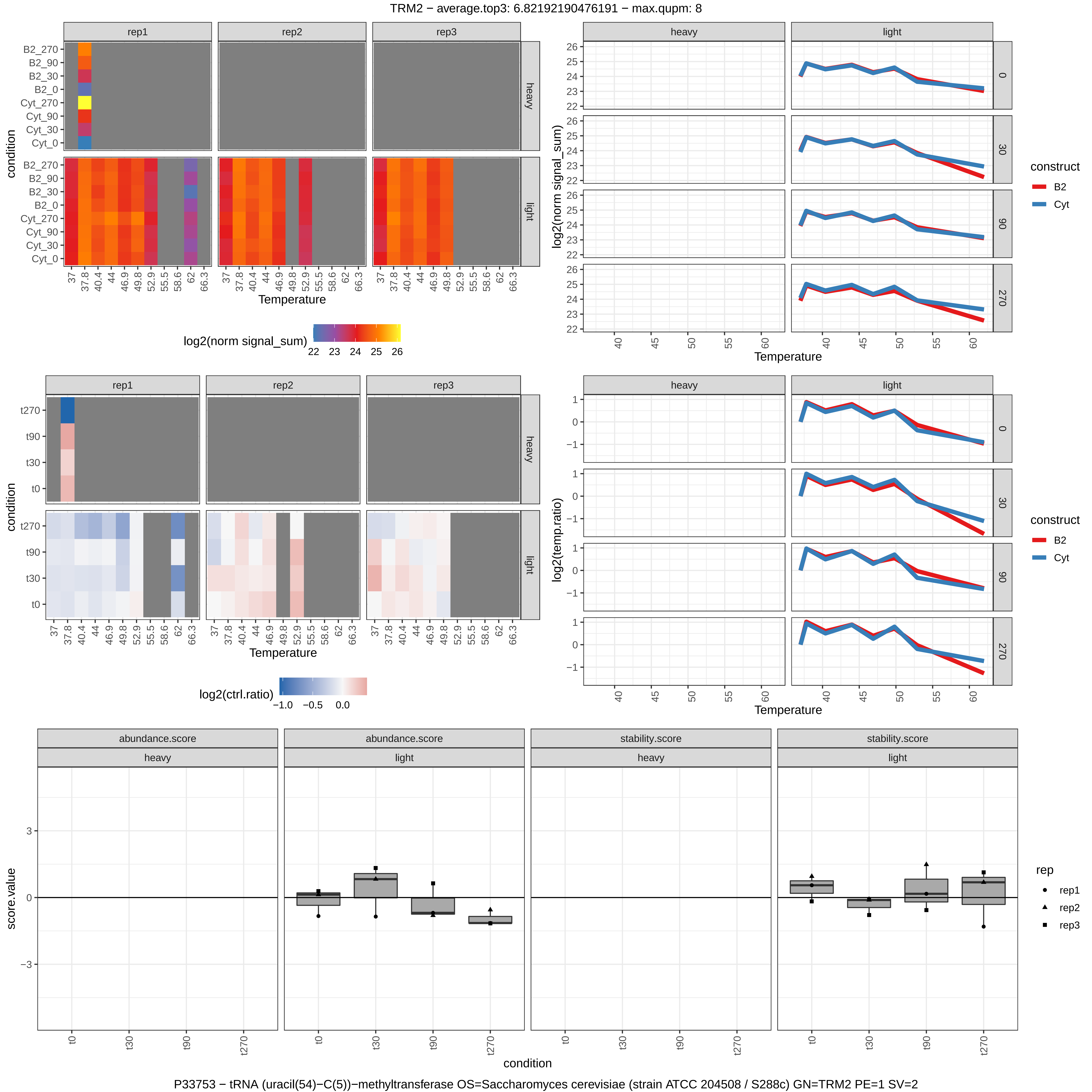

TRM2 − average.top3: 6.82192190476191 − max.qupm: 8
rep1
rep2
rep3
heavy
light
condition
Temperature
log2(norm signal_sum)
log2(ctrl.ratio)
log2(temp.ratio)
construct
B2
Cyt
abundance.score
stability.score
score.value
rep
rep1
rep2
rep3
P33753 − tRNA (uracil(54)−C(5))−methyltransferase OS=Saccharomyces cerevisiae (strain ATCC 204508 / S288c) GN=TRM2 PE=1 SV=2

TRM3 − average.top3: 6.9615848 − max.qupm: 18

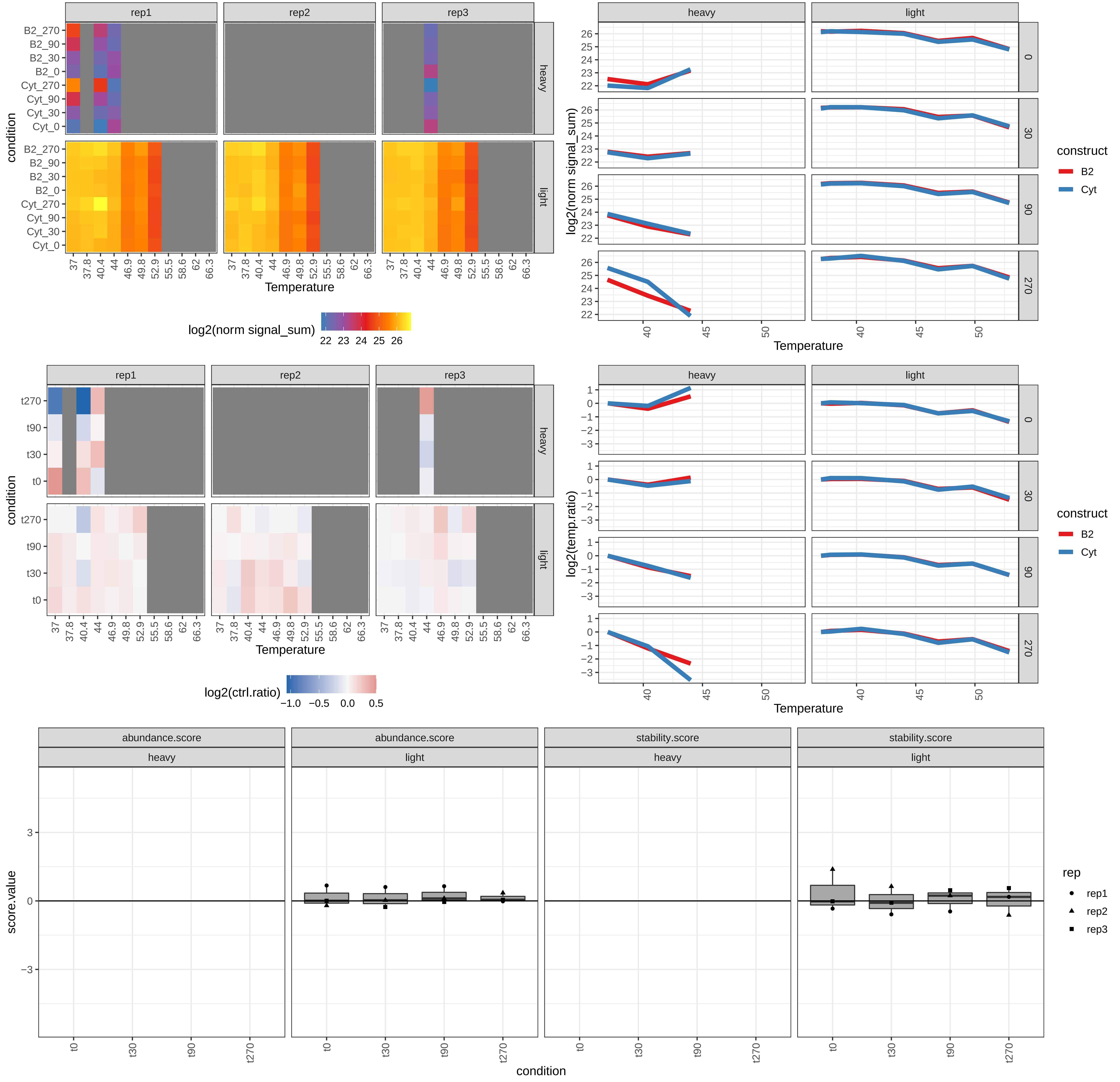

Q07527 − tRNA (guanosine(18)−2'−O)−methyltransferase OS=Saccharomyces cerevisiae (strain ATCC 204508 / S288c) GN=TRM3 PE=1 SV=1

TRM44 − average.top3: 6.86787578947368 − max.qupm: 7

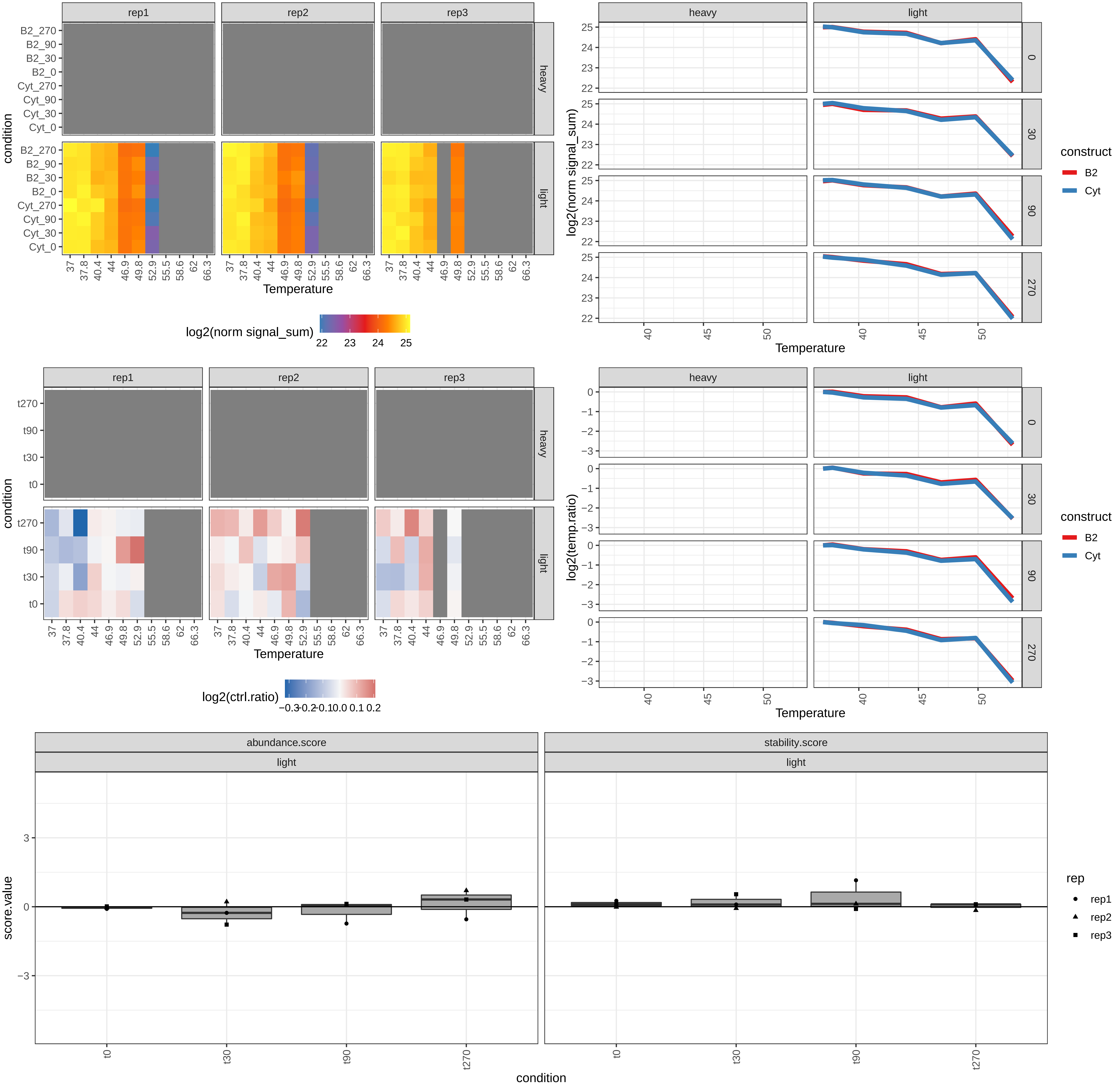

Q02648 − tRNA (uracil−O(2)−)−methyltransferase OS=Saccharomyces cerevisiae (strain ATCC 204508 / S288c) GN=TRM44 PE=1 SV=1

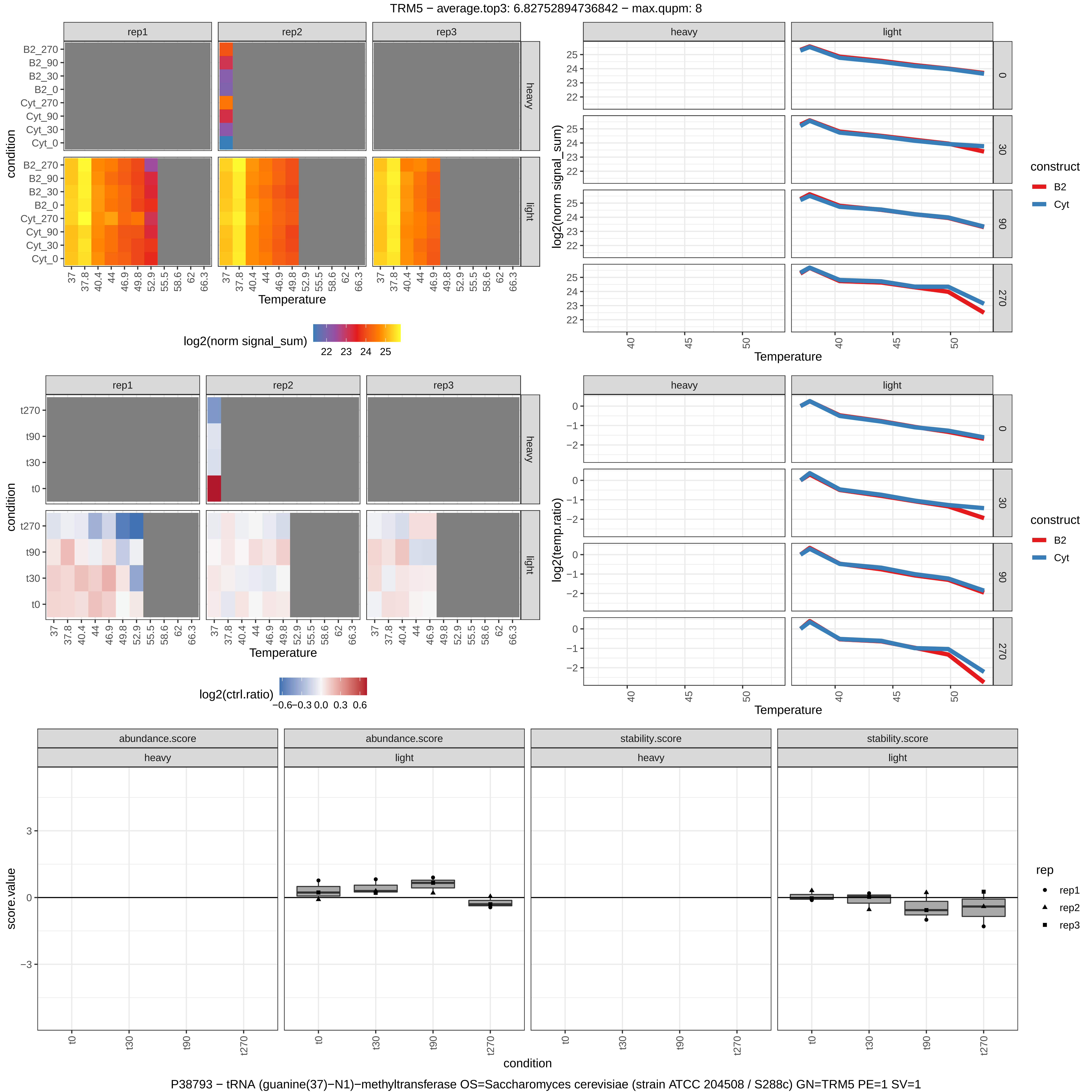
TRM5 − average.top3: 6.82752894736842 − max.qupm: 8
rep1
rep2
rep3
heavy
light
condition
Temperature
log2(norm signal_sum)
log2(ctrl.ratio)
log2(temp.ratio)
construct
B2
Cyt
abundance.score
stability.score
score.value
rep
rep1
rep2
rep3
P38793 − tRNA (guanine(37)−N1)−methyltransferase OS=Saccharomyces cerevisiae (strain ATCC 204508 / S288c) GN=TRM5 PE=1 SV=1

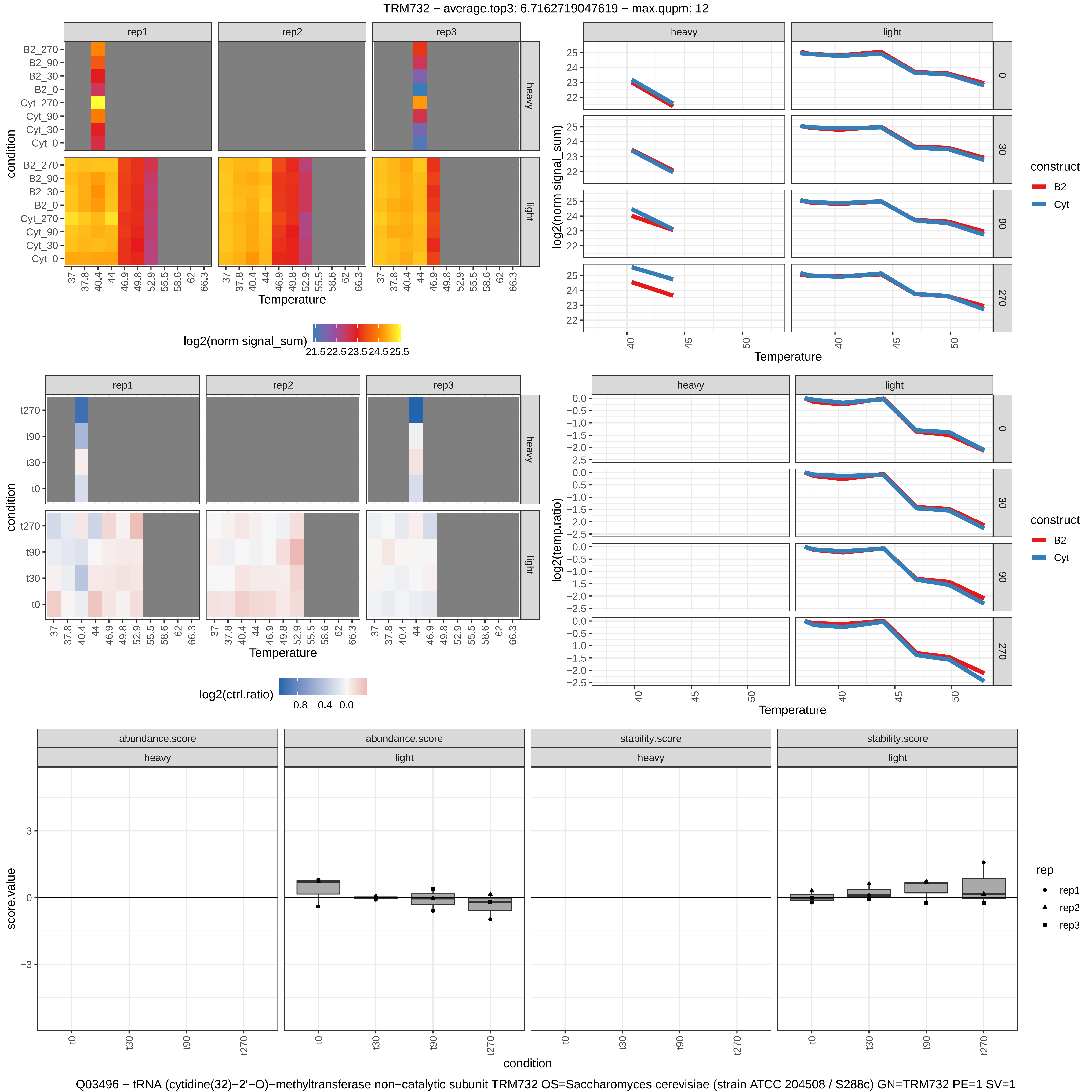
TRM732 − average.top3: 6.7162719047619 − max.qupm: 12
rep1
rep2
rep3
heavy
light
condition
Temperature
log2(norm signal_sum)
log2(temp.ratio)
log2(ctrl.ratio)
construct
B2
Cyt
abundance.score
stability.score
score.value
rep
rep1
rep2
rep3
Q03496 − tRNA (cytidine(32)−2'−O)−methyltransferase non−catalytic subunit TRM732 OS=Saccharomyces cerevisiae (strain ATCC 204508 / S288c) GN=TRM732 PE=1 SV=1

TRM8 − average.top3: 6.95630733333333 − max.qupm: 8

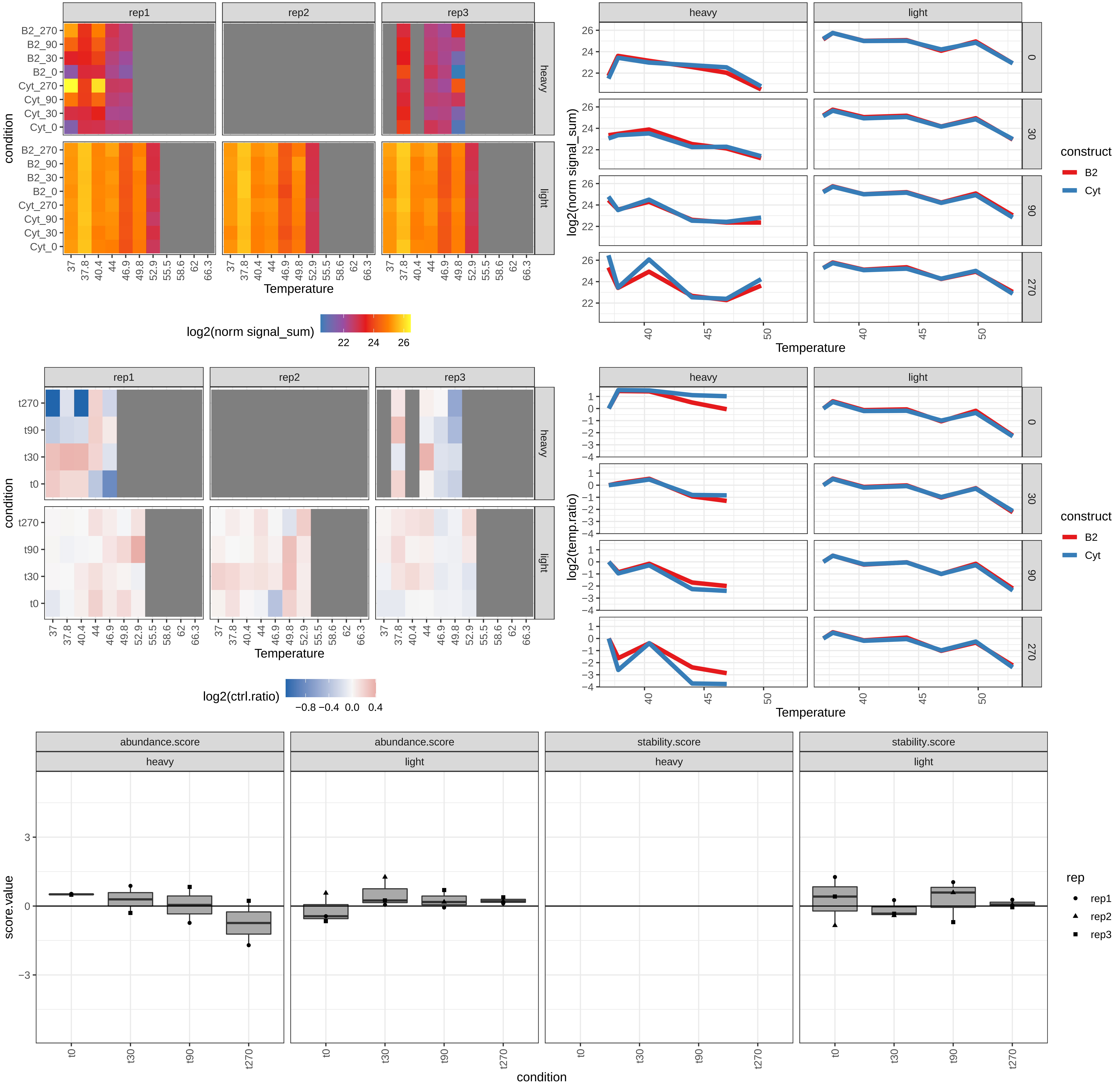

Q12009 − tRNA (guanine−N(7)−)−methyltransferase OS=Saccharomyces cerevisiae (strain ATCC 204508 / S288c) GN=TRM8 PE=1 SV=1

TRM82 − average.top3: 6.80678363636364 − max.qupm: 9

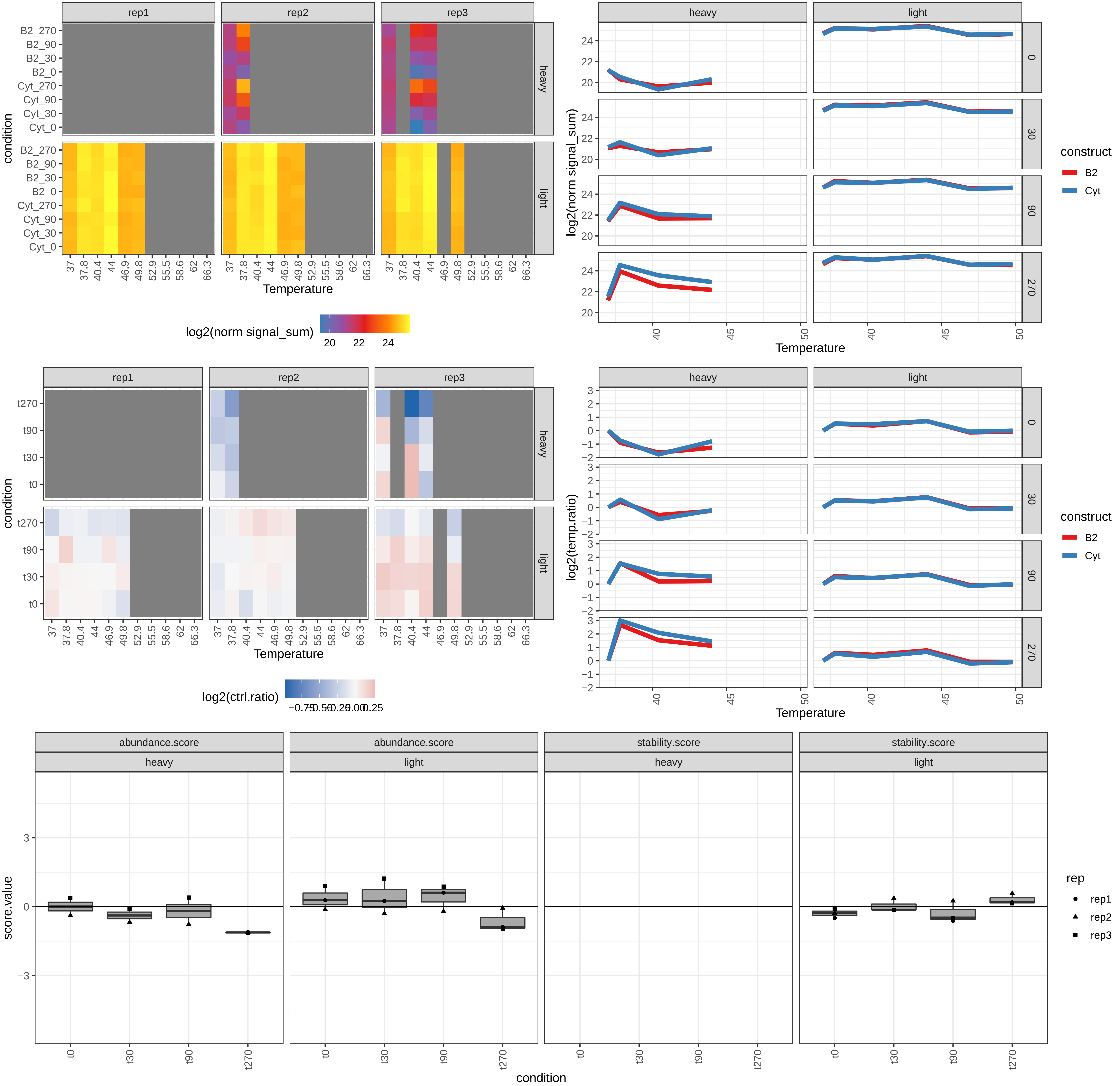

Q03774 − tRNA (guanine−N(7)−)−methyltransferase non−catalytic subunit TRM82 OS=Saccharomyces cerevisiae (strain ATCC 204508 / S288c) GN=TRM82 PE=1 SV=1

TRM9 − average.top3: 6.74634166666667 − max.qupm: 9

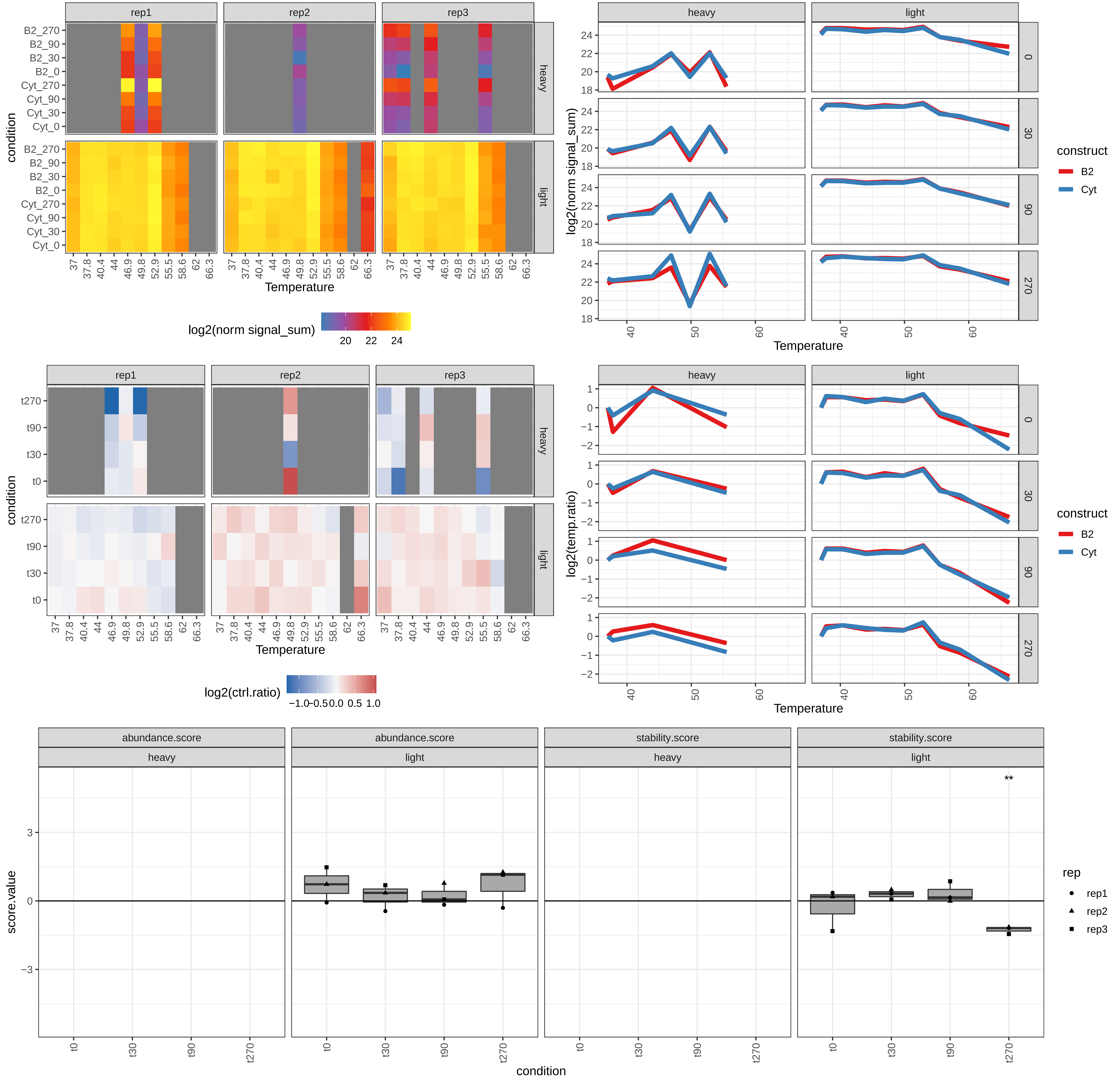

P49957 − tRNA (carboxymethyluridine(34)−5−O)−methyltransferase OS=Saccharomyces cerevisiae (strain ATCC 204508 / S288c) GN=TRM9 PE=1 SV=1

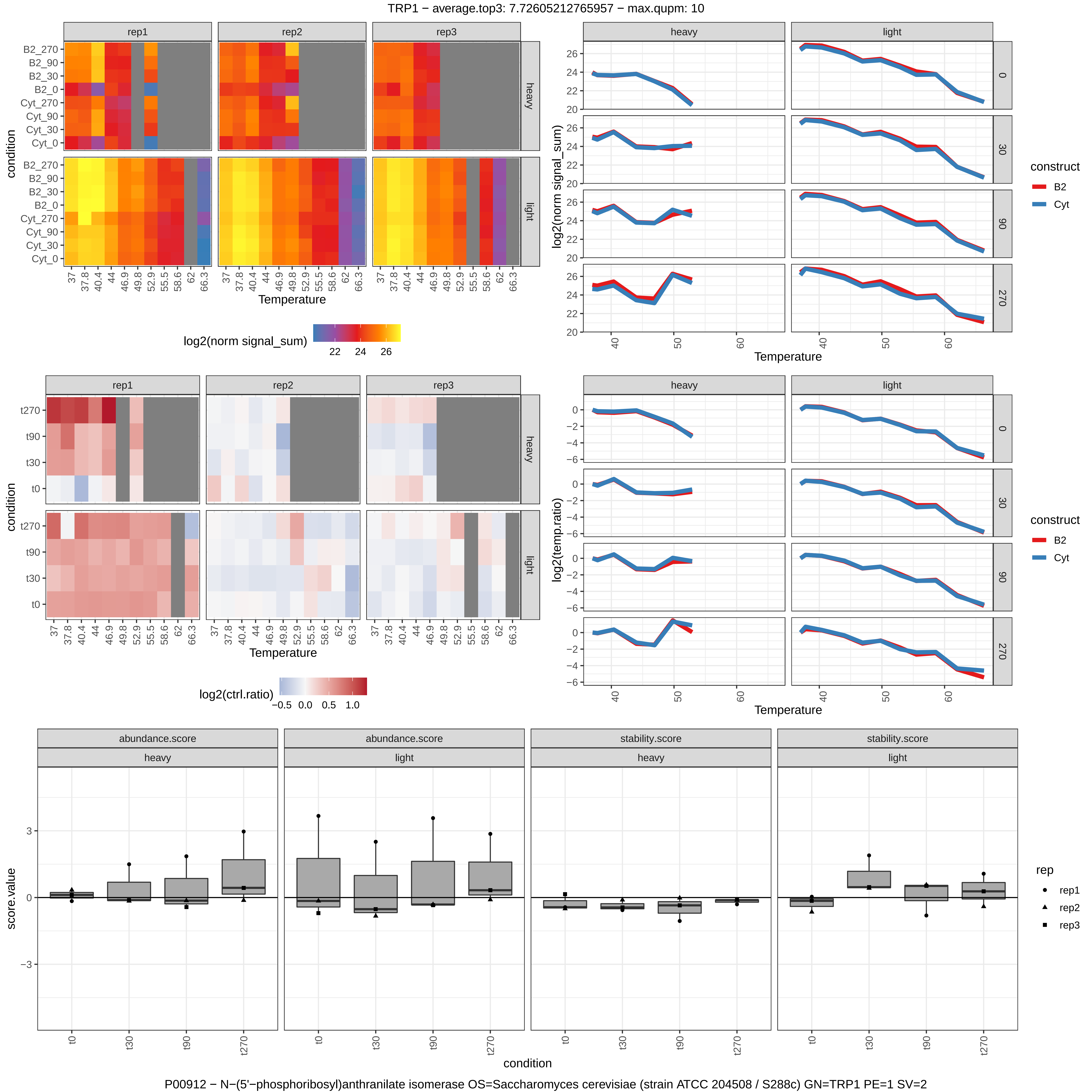
TRP1 − average.top3: 7.72605212765957 − max.qupm: 10
rep1
rep2
rep3
heavy
light
B2_270
B2_90
B2_30
B2_0
Cyt_270
Cyt_90
Cyt_30
Cyt_0
condition
Temperature
log2(norm signal_sum)
22
24
26
log2(norm signal_sum)
construct
B2
Cyt
0
30
90
270
t270
t90
t30
t0
log2(ctrl.ratio)
−0.5
0.0
0.5
1.0
log2(temp.ratio)
abundance.score
stability.score
score.value
condition
rep
P00912 − N−(5'−phosphoribosyl)anthranilate isomerase OS=Saccharomyces cerevisiae (strain ATCC 204508 / S288c) GN=TRP1 PE=1 SV=2

TRP2 − average.top3: 7.09278950819672 − max.qupm: 19

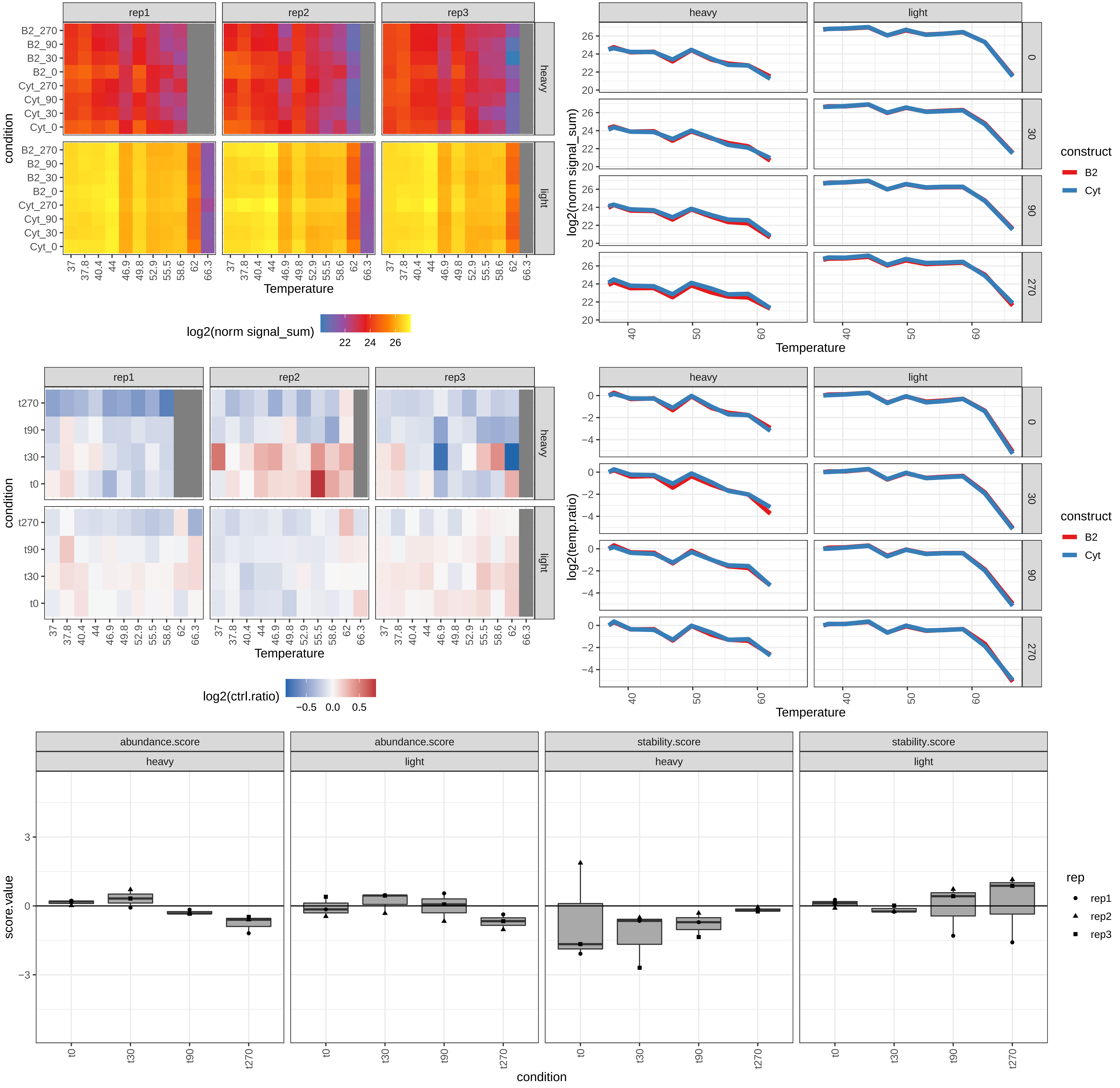

P00899 − Anthranilate synthase component 1 OS=Saccharomyces cerevisiae (strain ATCC 204508 / S288c) GN=TRP2 PE=1 SV=4

TRP3 − average.top3: 7.5006265 − max.qupm: 18

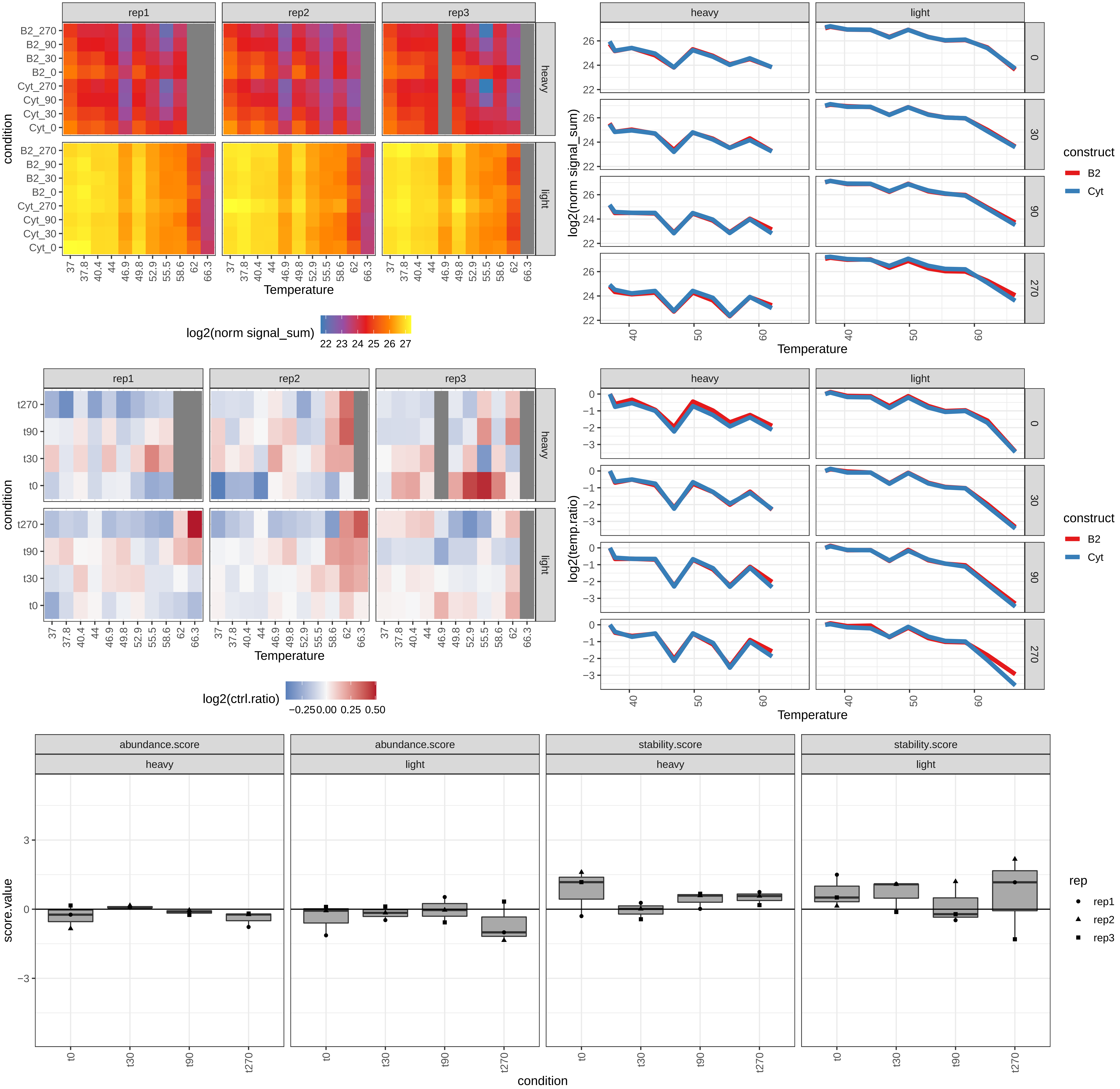

P00937 − Multifunctional tryptophan biosynthesis protein OS=Saccharomyces cerevisiae (strain ATCC 204508 / S288c) GN=TRP3 PE=1 SV=2

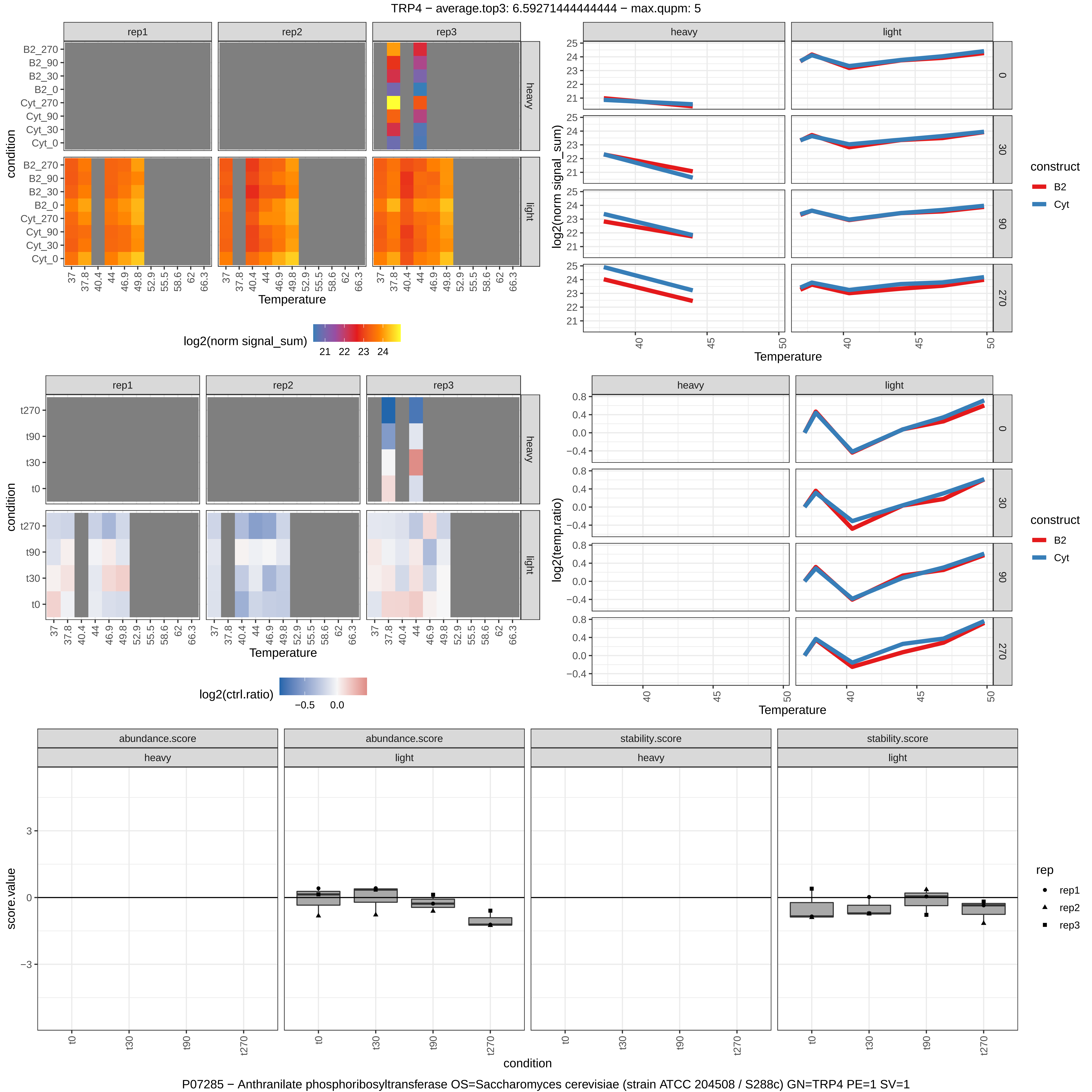
TRP4 − average.top3: 6.59271444444444 − max.qupm: 5
rep1
rep2
rep3
heavy
light
B2_270
B2_90
B2_30
B2_0
Cyt_270
Cyt_90
Cyt_30
Cyt_0
condition
37
37.8
40.4
44
46.9
49.8
52.9
55.5
58.6
62
66.3
Temperature
log2(norm signal_sum)
21
22
23
24
log2(norm signal_sum)
0
30
90
270
construct
B2
Cyt
t270
t90
t30
t0
log2(ctrl.ratio)
−0.5
0.0
log2(temp.ratio)
abundance.score
stability.score
score.value
condition
rep
rep1
rep2
rep3
P07285 − Anthranilate phosphoribosyltransferase OS=Saccharomyces cerevisiae (strain ATCC 204508 / S288c) GN=TRP4 PE=1 SV=1

TRP5 − average.top3: 7.73637484848485 − max.qupm: 27

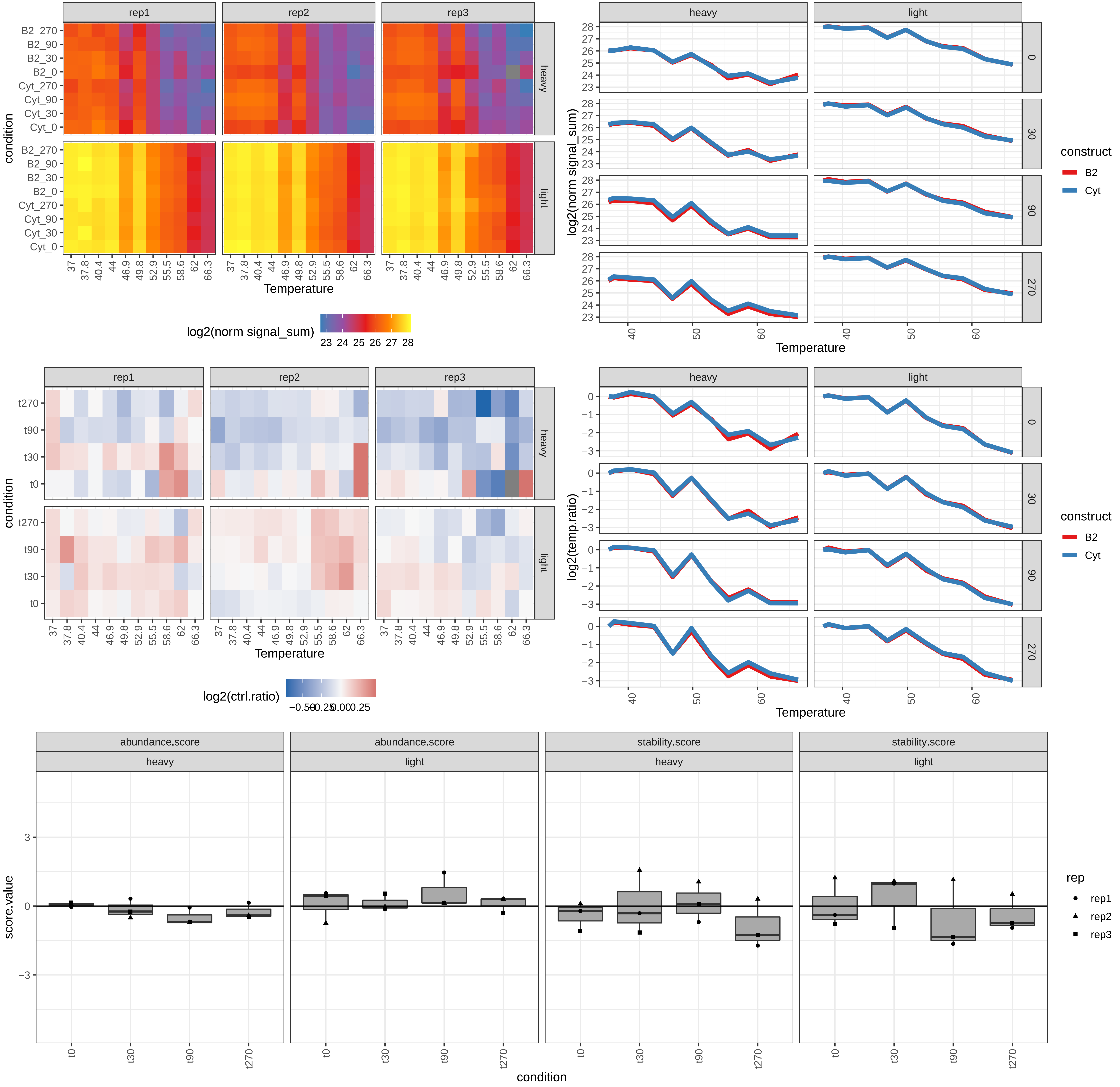

P00931 − Tryptophan synthase OS=Saccharomyces cerevisiae (strain ATCC 204508 / S288c) GN=TRP5 PE=1 SV=1

TRR1 − average.top3: 7.08254905660377 − max.qupm: 8

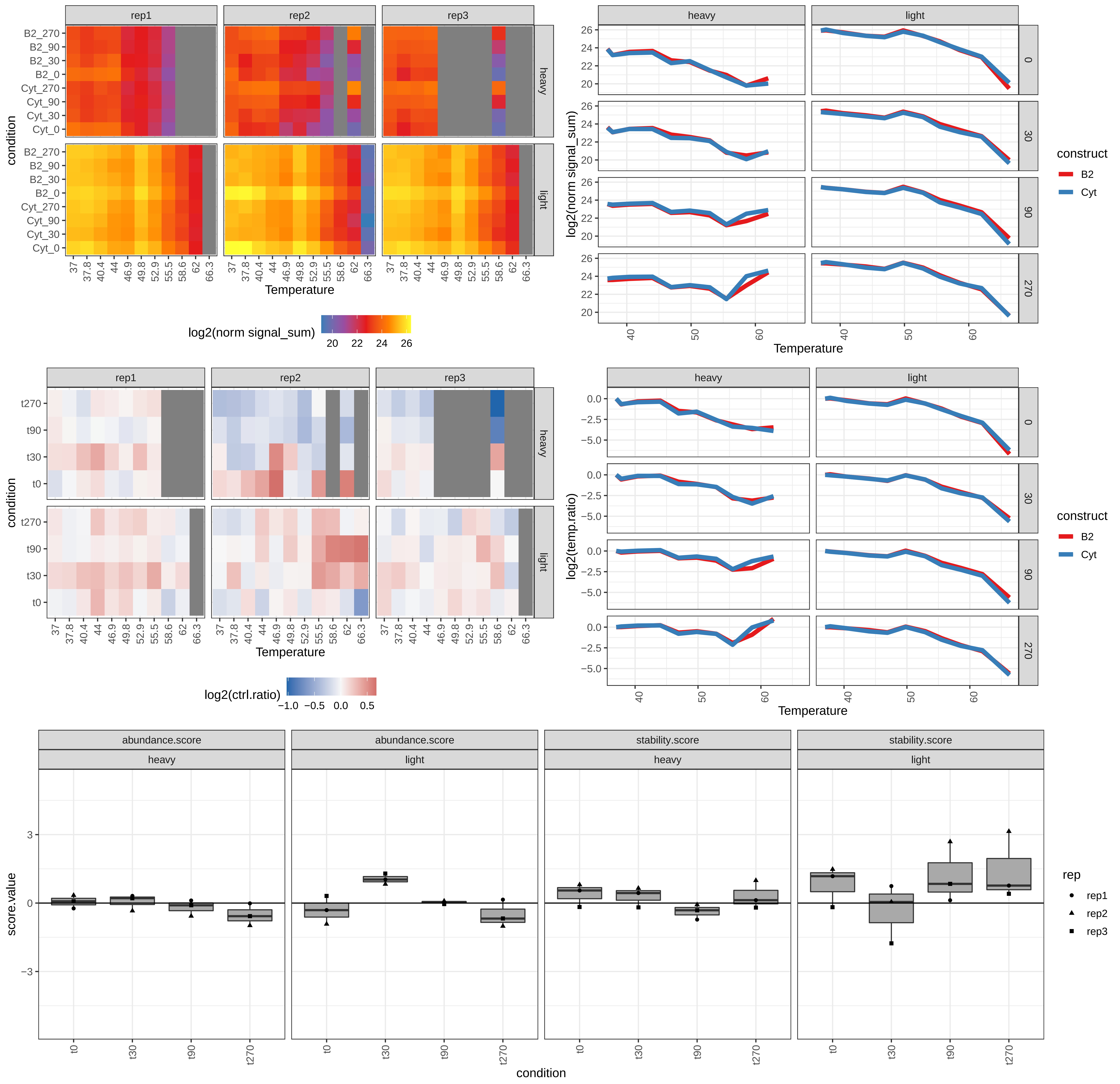

P29509 − Thioredoxin reductase 1 OS=Saccharomyces cerevisiae (strain ATCC 204508 / S288c) GN=TRR1 PE=1 SV=3

TRR2 − average.top3: 6.71463409090909 − max.qupm: 5

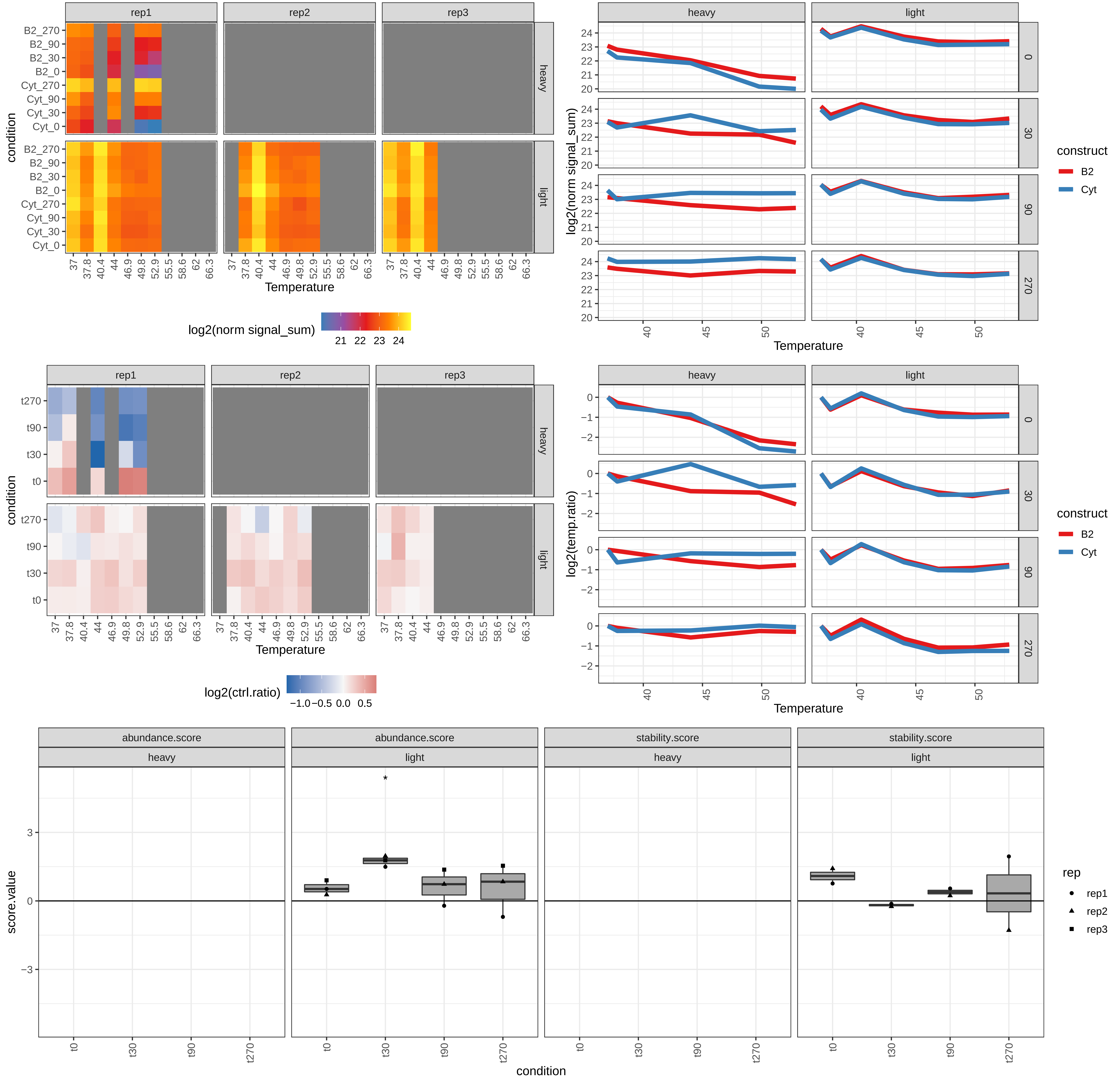

P38816 − Thioredoxin reductase 2, mitochondrial OS=Saccharomyces cerevisiae (strain ATCC 204508 / S288c) GN=TRR2 PE=1 SV=1

TRS120 − average.top3: 6.49580166666667 − max.qupm: 6

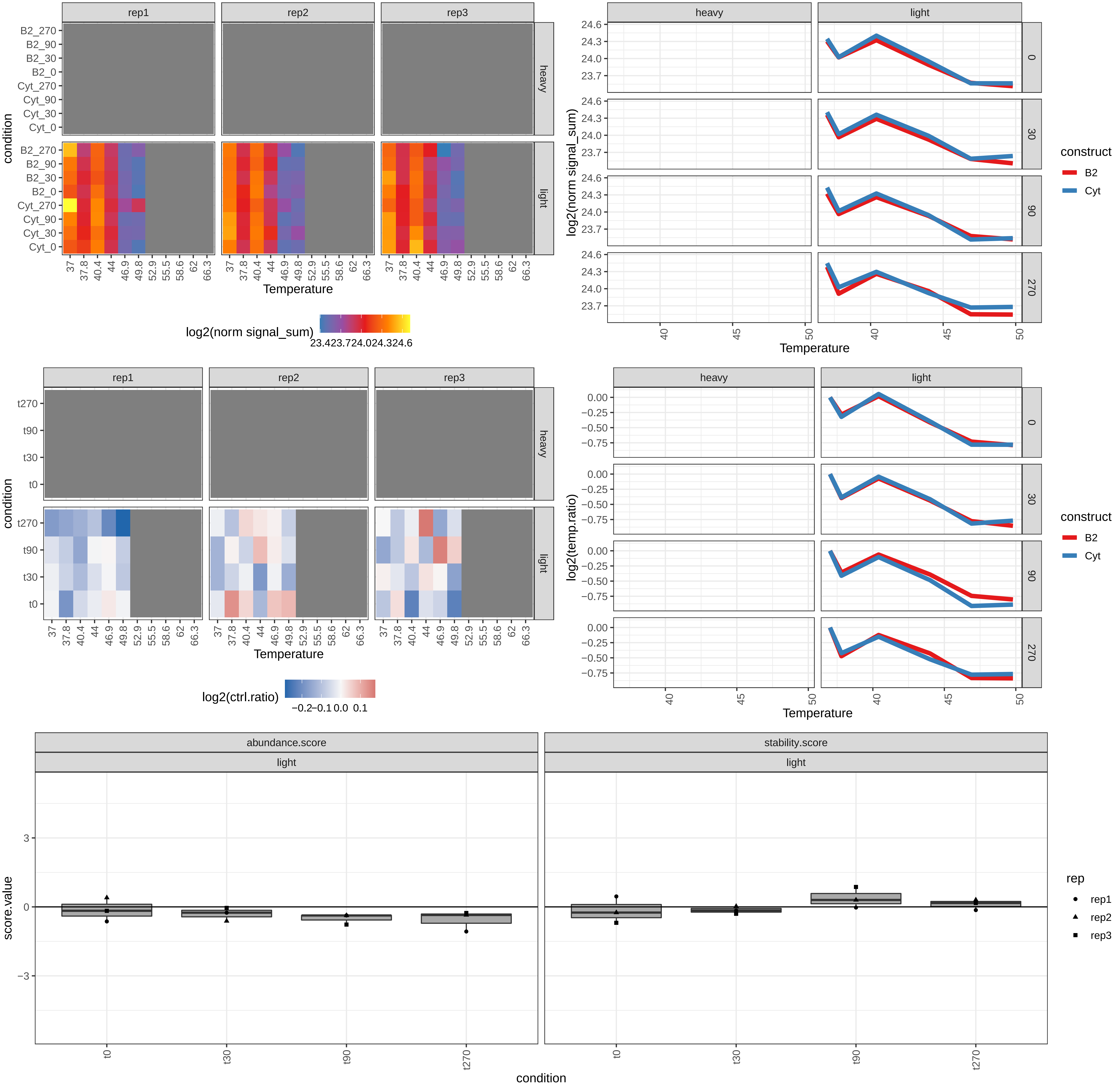

Q04183 − Trafficking protein particle complex II−specific subunit 120 OS=Saccharomyces cerevisiae (strain ATCC 204508 / S288c) GN=TRS120 PE=1 SV=1

TRS130 − average.top3: 6.68459857142857 − max.qupm: 5

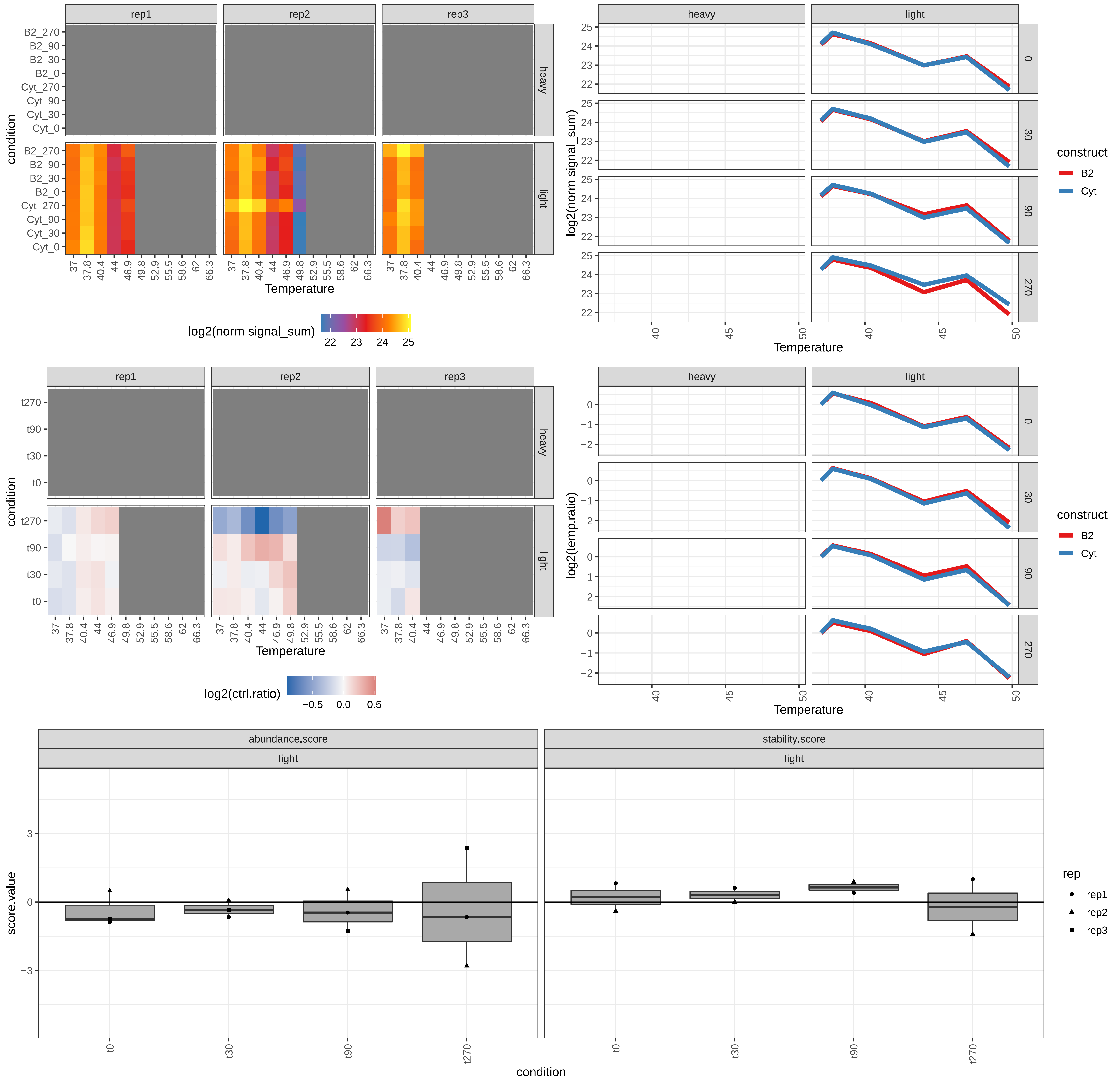

Q03660 − Trafficking protein particle complex II−specific subunit 130 OS=Saccharomyces cerevisiae (strain ATCC 204508 / S288c) GN=TRS130 PE=1 SV=1

TRS23 − average.top3: 6.78026181818182 − max.qupm: 4

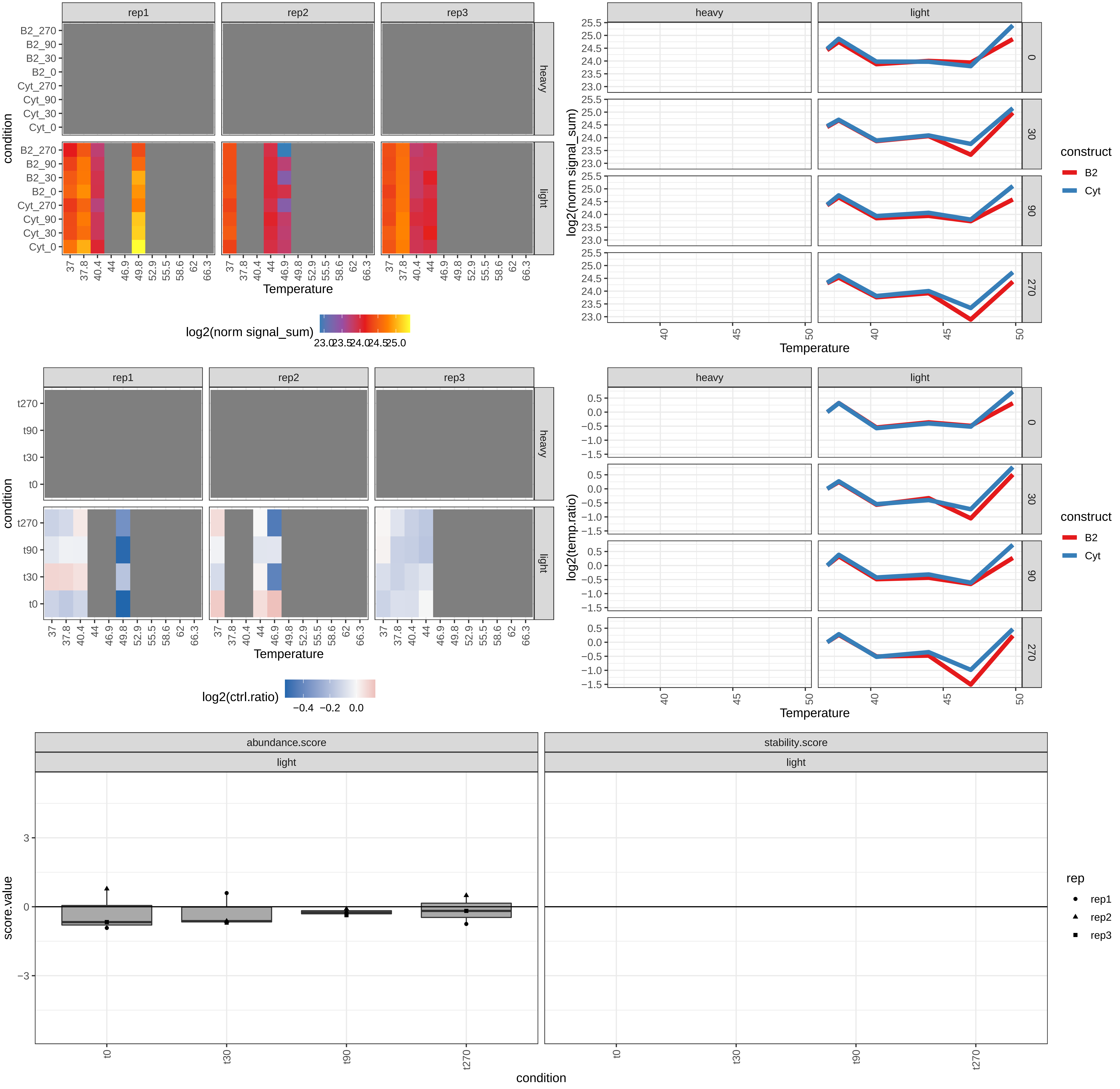

Q03784 − Trafficking protein particle complex subunit 23 OS=Saccharomyces cerevisiae (strain ATCC 204508 / S288c) GN=TRS23 PE=1 SV=1

TRS31 − average.top3: 6.2219825 − max.qupm: 3

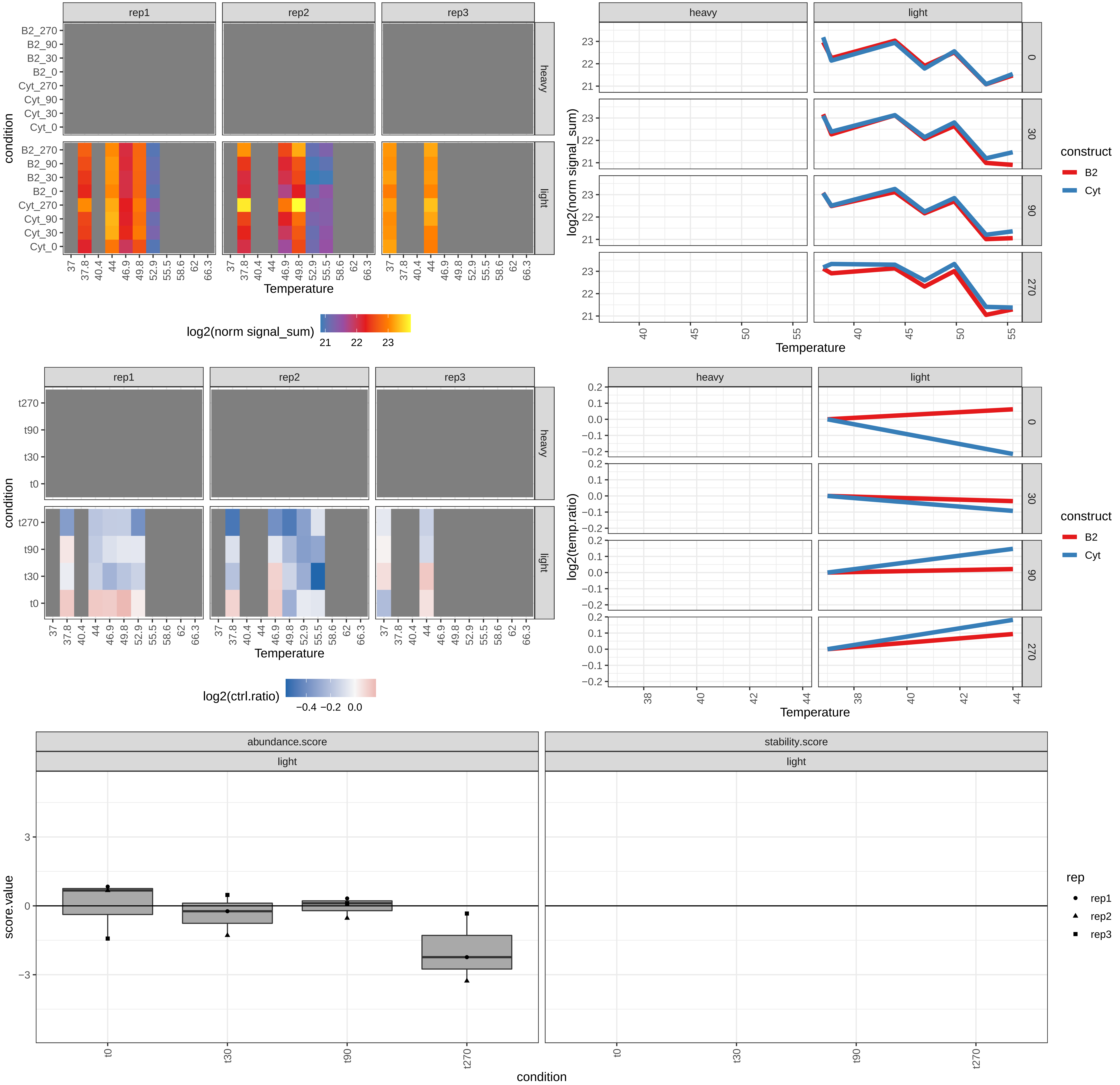

Q03337 − Trafficking protein particle complex subunit 31 OS=Saccharomyces cerevisiae (strain ATCC 204508 / S288c) GN=TRS31 PE=1 SV=1

TRS85 − average.top3: 6.65904619047619 − max.qupm: 6

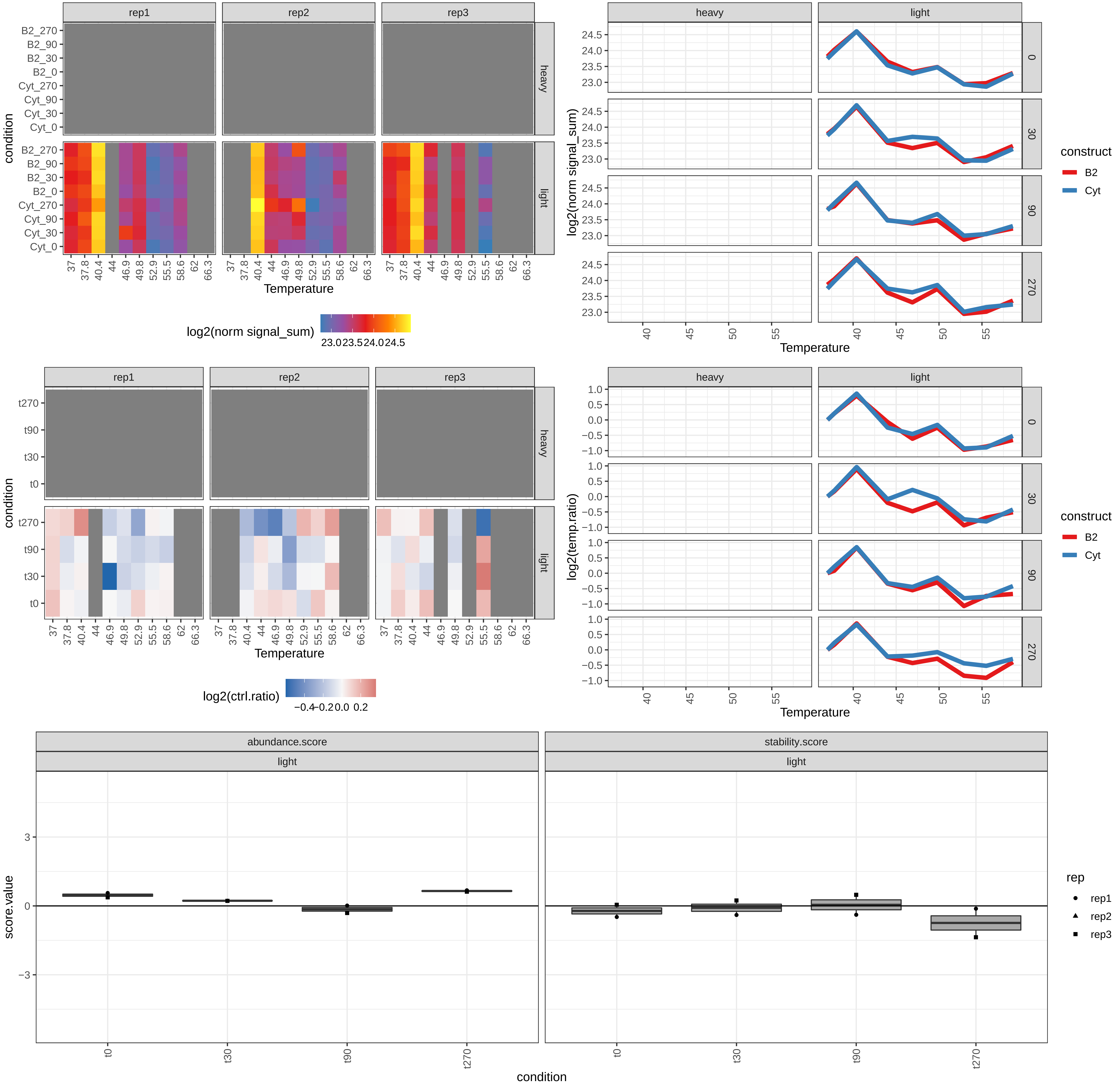

P46944 − Trafficking protein particle complex III−specific subunit 85 OS=Saccharomyces cerevisiae (strain ATCC 204508 / S288c) GN=TRS85 PE=1 SV=2

TRX1 − average.top3: 6.97892178571429 − max.qupm: 4

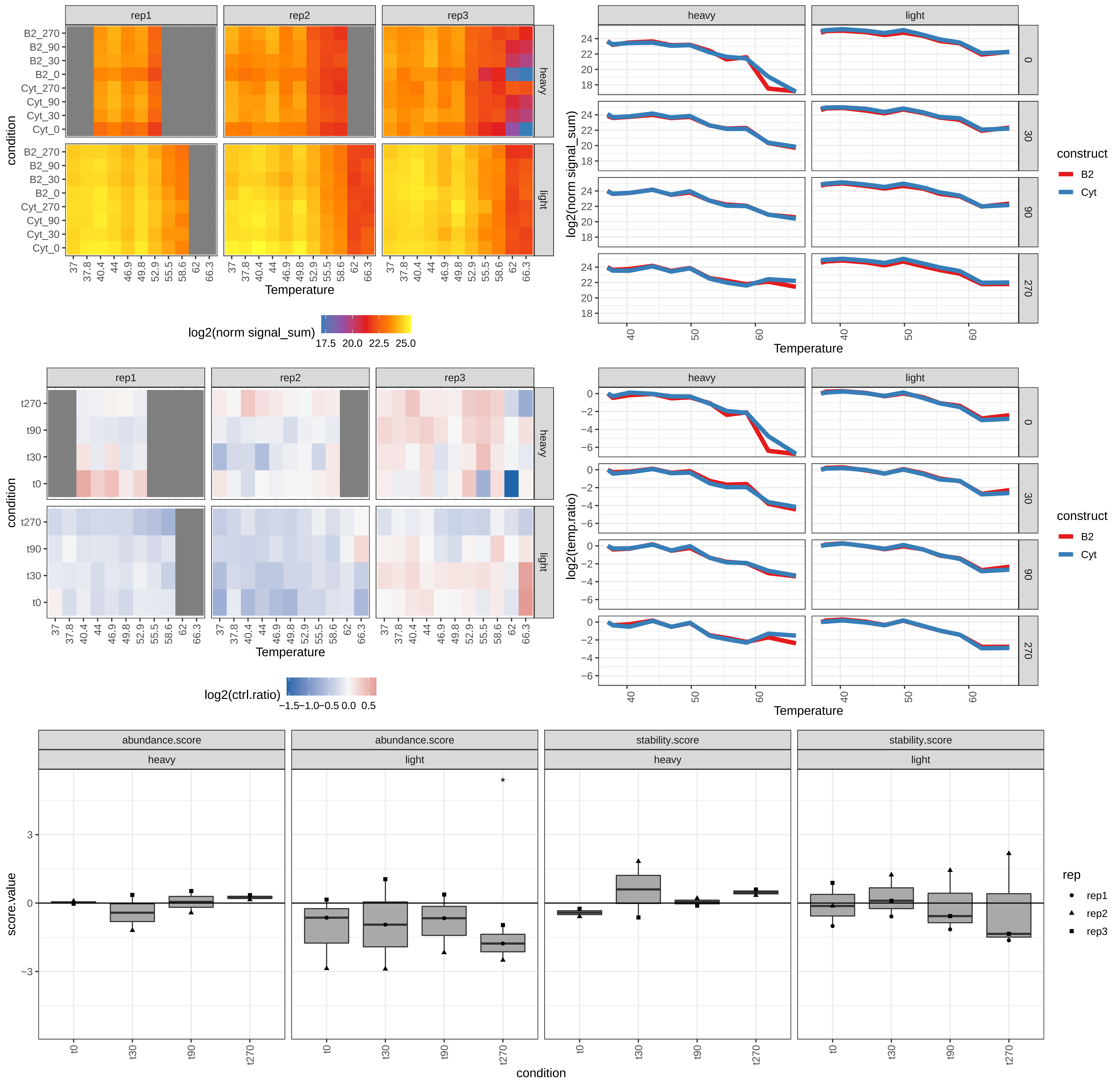

P22217 − Thioredoxin−1 OS=Saccharomyces cerevisiae (strain ATCC 204508 / S288c) GN=TRX1 PE=1 SV=3

TRX2 − average.top3: 7.09425117647059 − max.qupm: 3

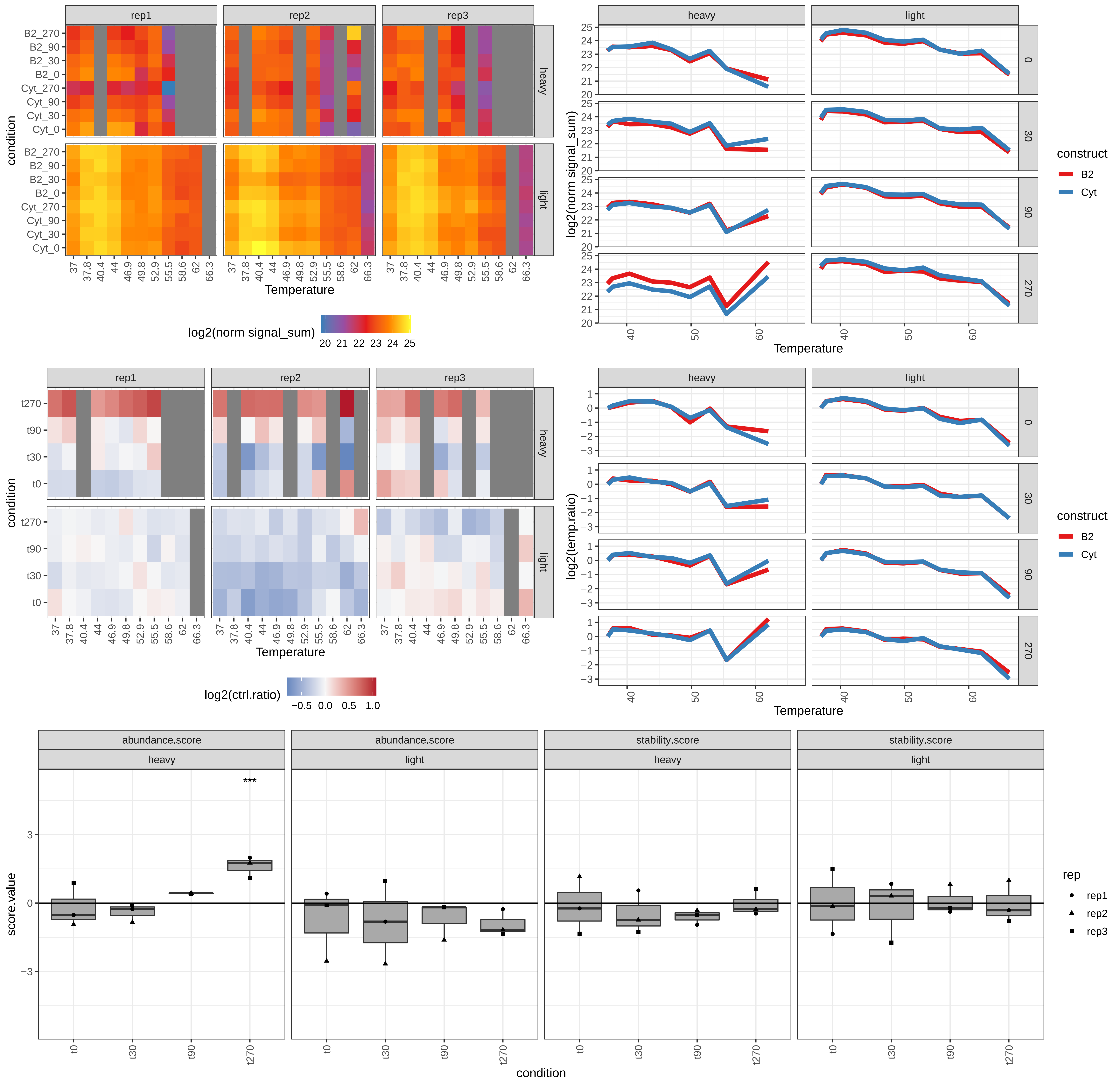

P22803 − Thioredoxin−2 OS=Saccharomyces cerevisiae (strain ATCC 204508 / S288c) GN=TRX2 PE=1 SV=3

TRX3 − average.top3: 7.19711428571429 − max.qupm: 4

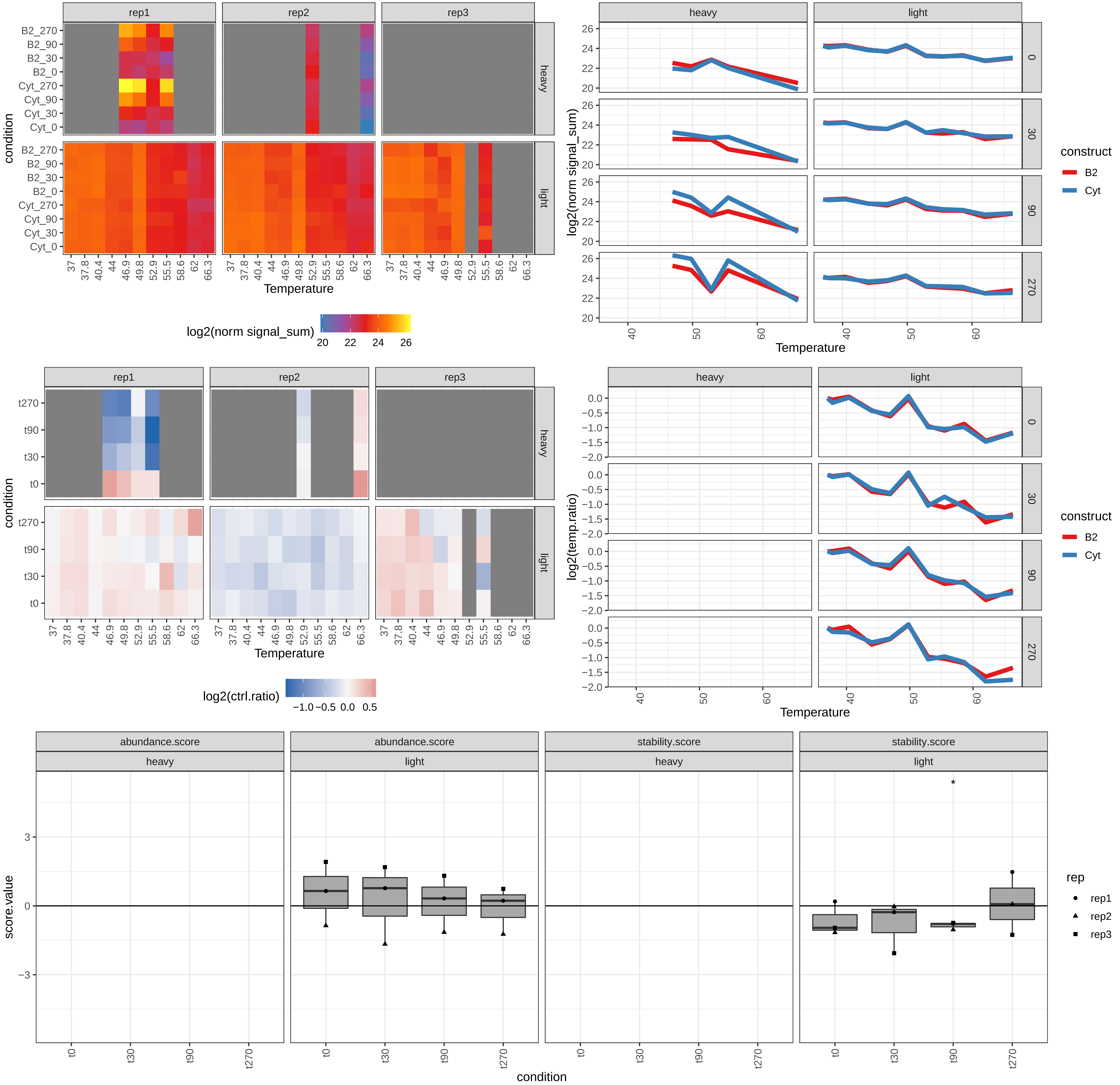

P25372 − Thioredoxin−3, mitochondrial OS=Saccharomyces cerevisiae (strain ATCC 204508 / S288c) GN=TRX3 PE=1 SV=1

TRZ1 − average.top3: 6.71424285714286 − max.qupm: 10

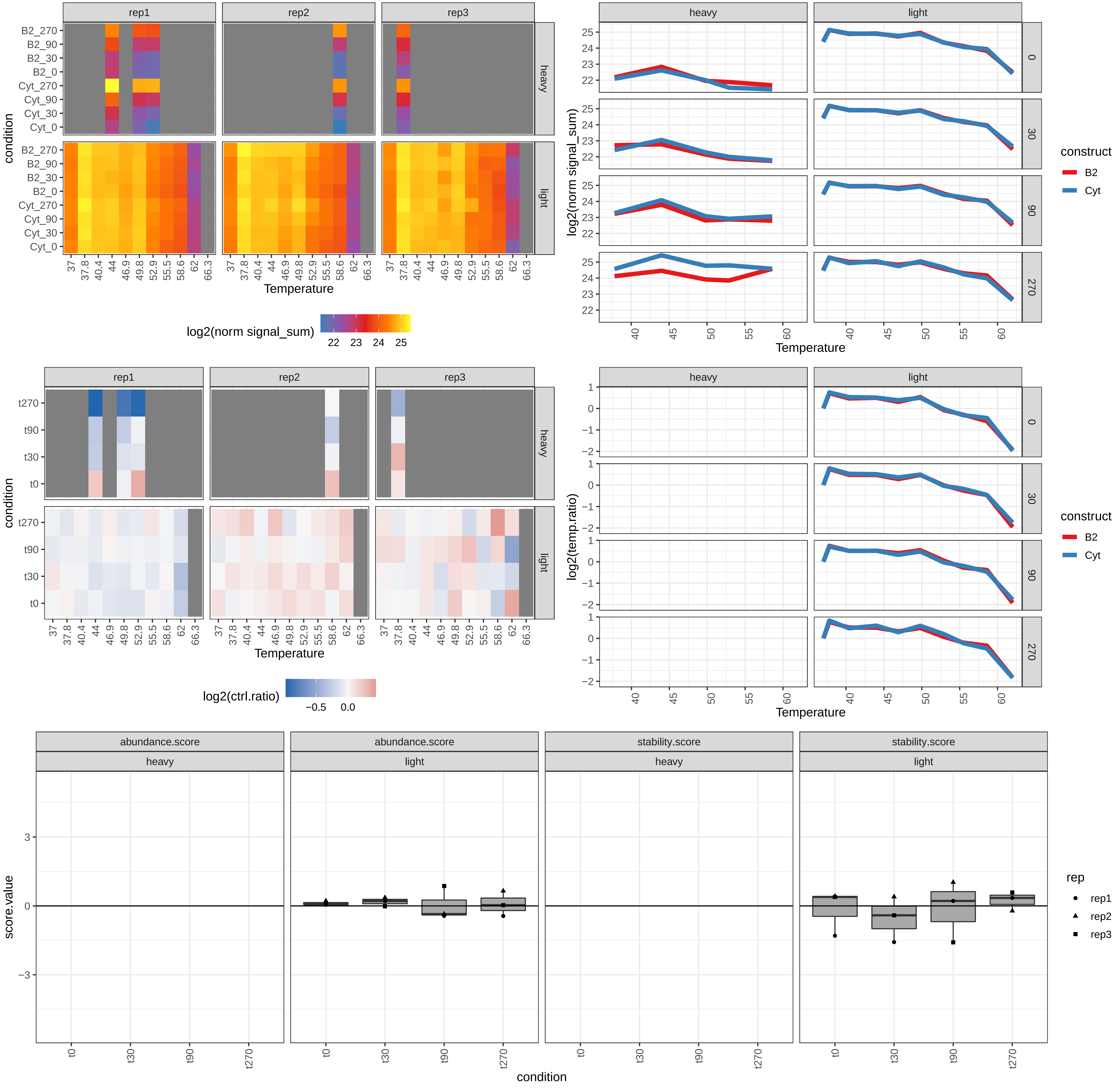

P36159 − Ribonuclease Z OS=Saccharomyces cerevisiae (strain ATCC 204508 / S288c) GN=TRZ1 PE=1 SV=1

TSA1 − average.top3: 7.87698621212121 − max.qupm: 12

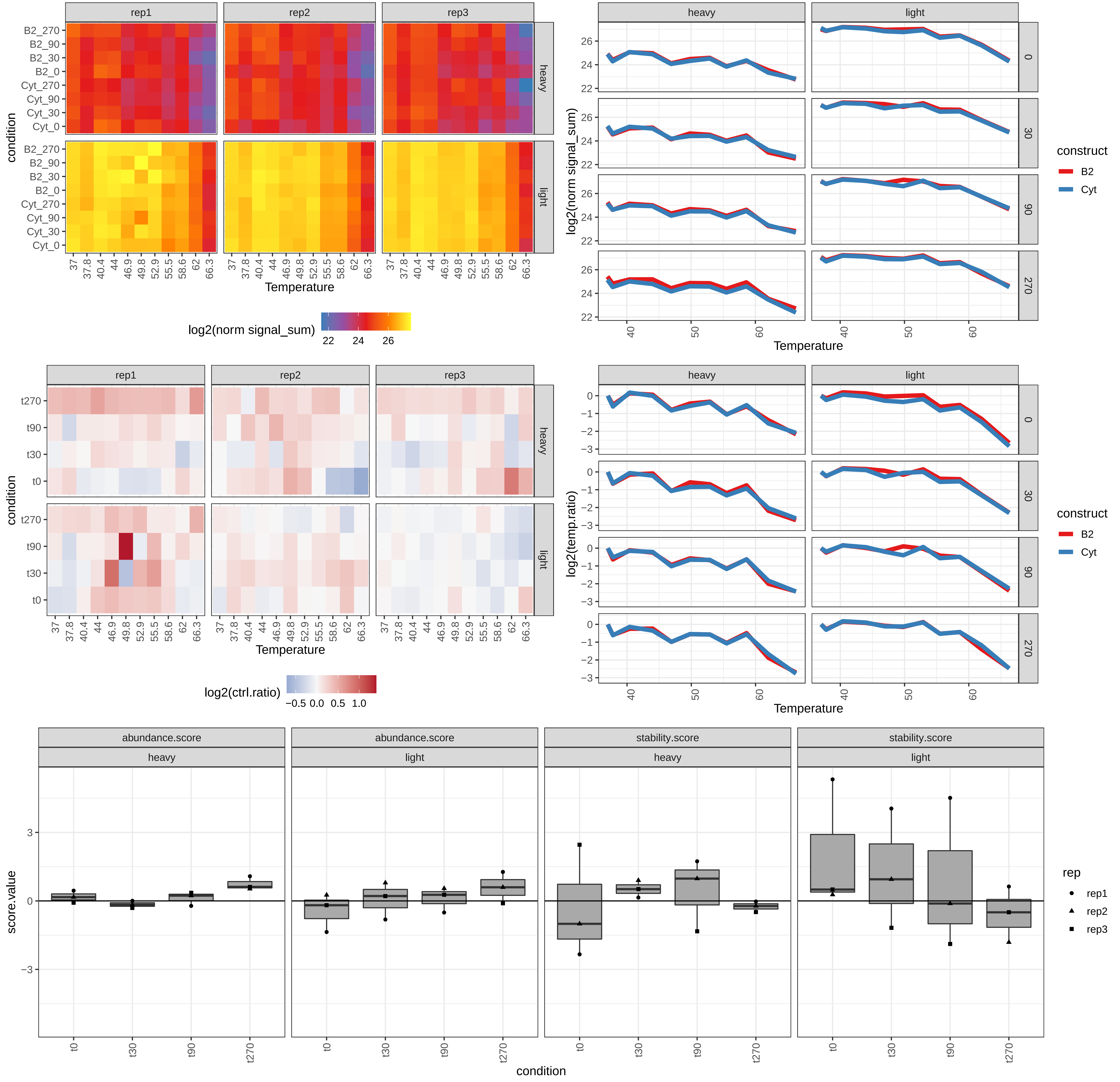

P34760 − Peroxiredoxin TSA1 OS=Saccharomyces cerevisiae (strain ATCC 204508 / S288c) GN=TSA1 PE=1 SV=3

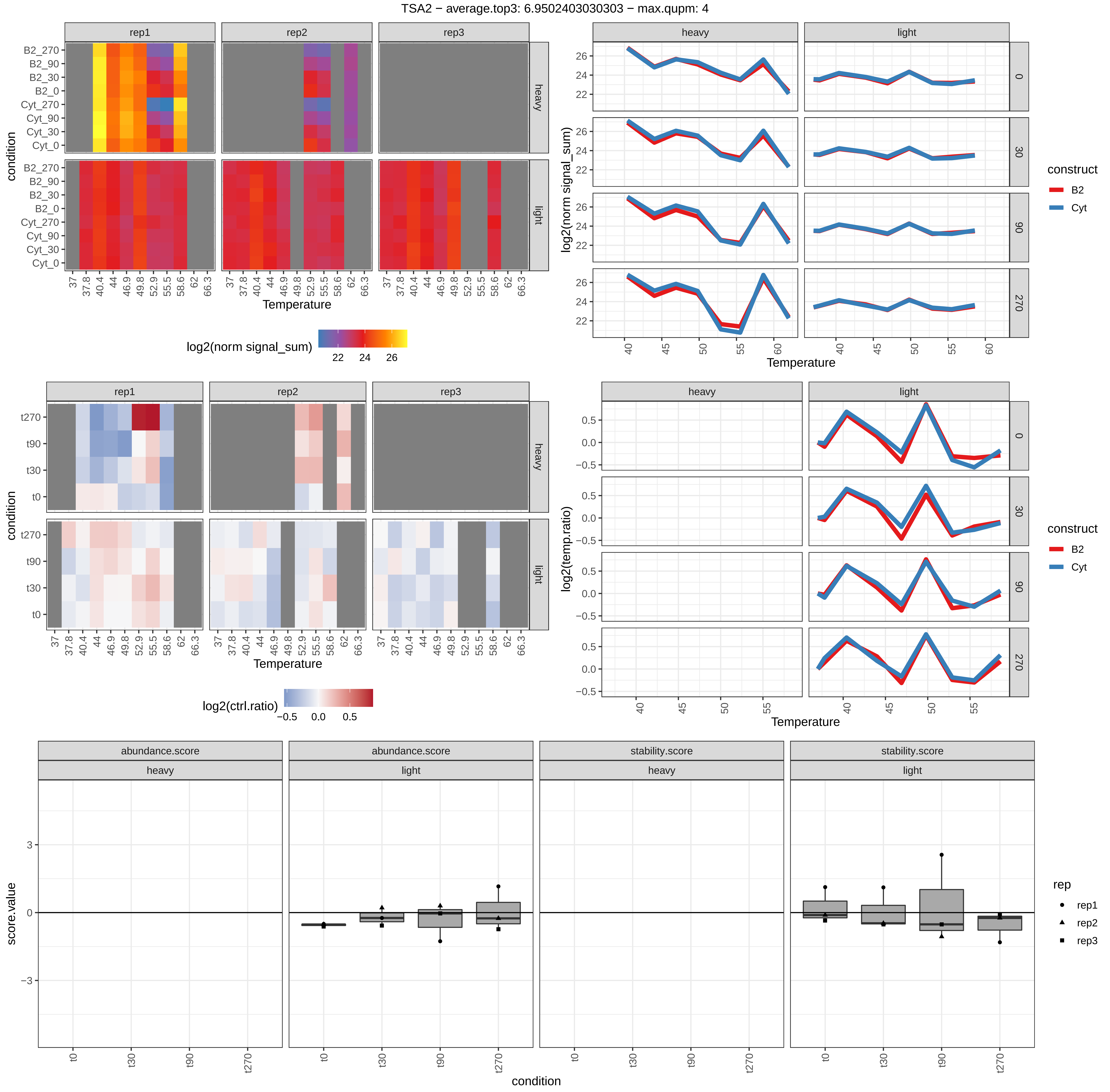

Q04120 − Peroxiredoxin TSA2 OS=Saccharomyces cerevisiae (strain ATCC 204508 / S288c) GN=TSA2 PE=1 SV=3

TSC10 − average.top3: 6.69017794117647 − max.qupm: 6

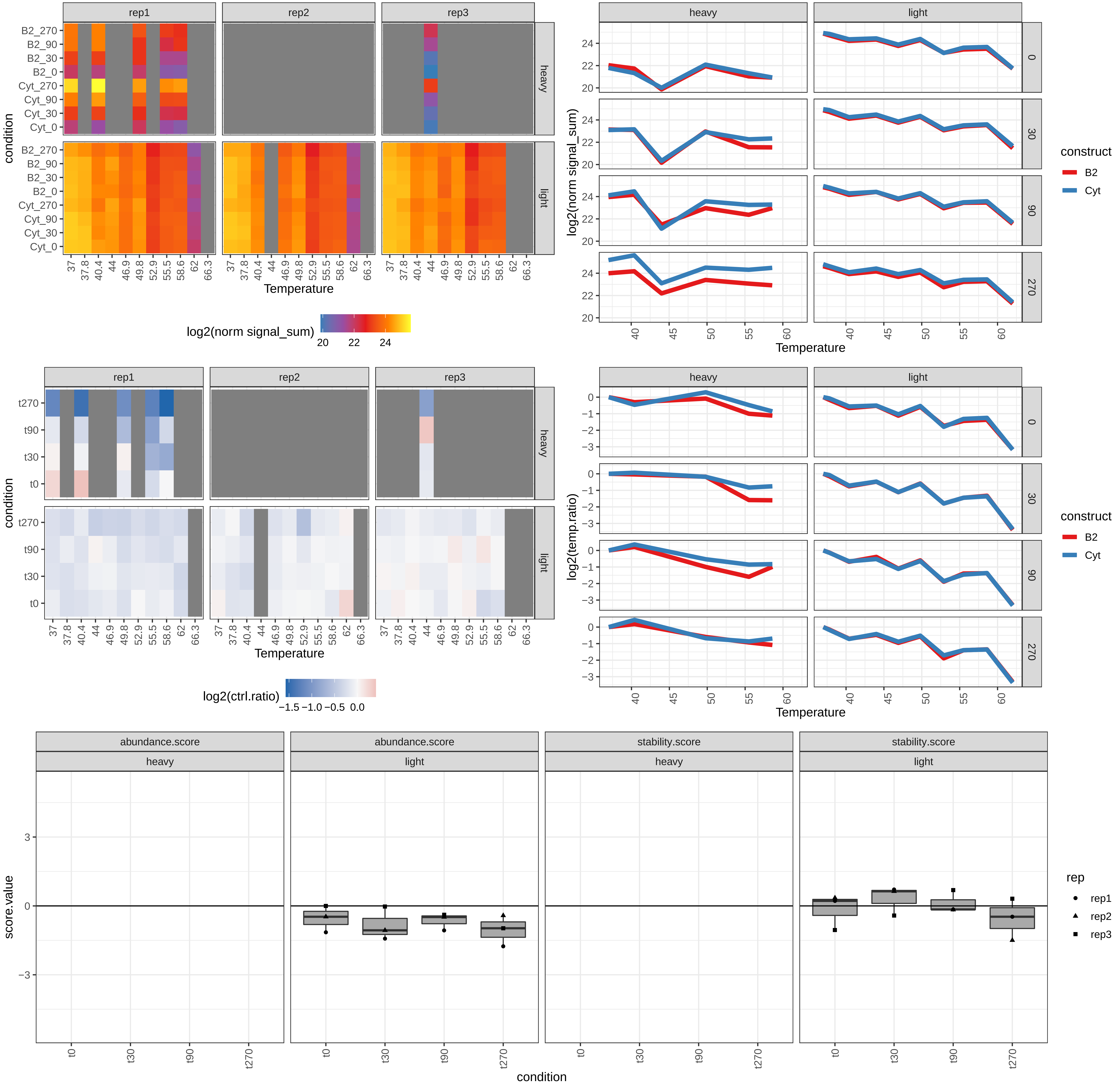

P38342 − 3−ketodihydrosphingosine reductase TSC10 OS=Saccharomyces cerevisiae (strain ATCC 204508 / S288c) GN=TSC10 PE=1 SV=2

TSC13 − average.top3: 7.16401888888889 − max.qupm: 6

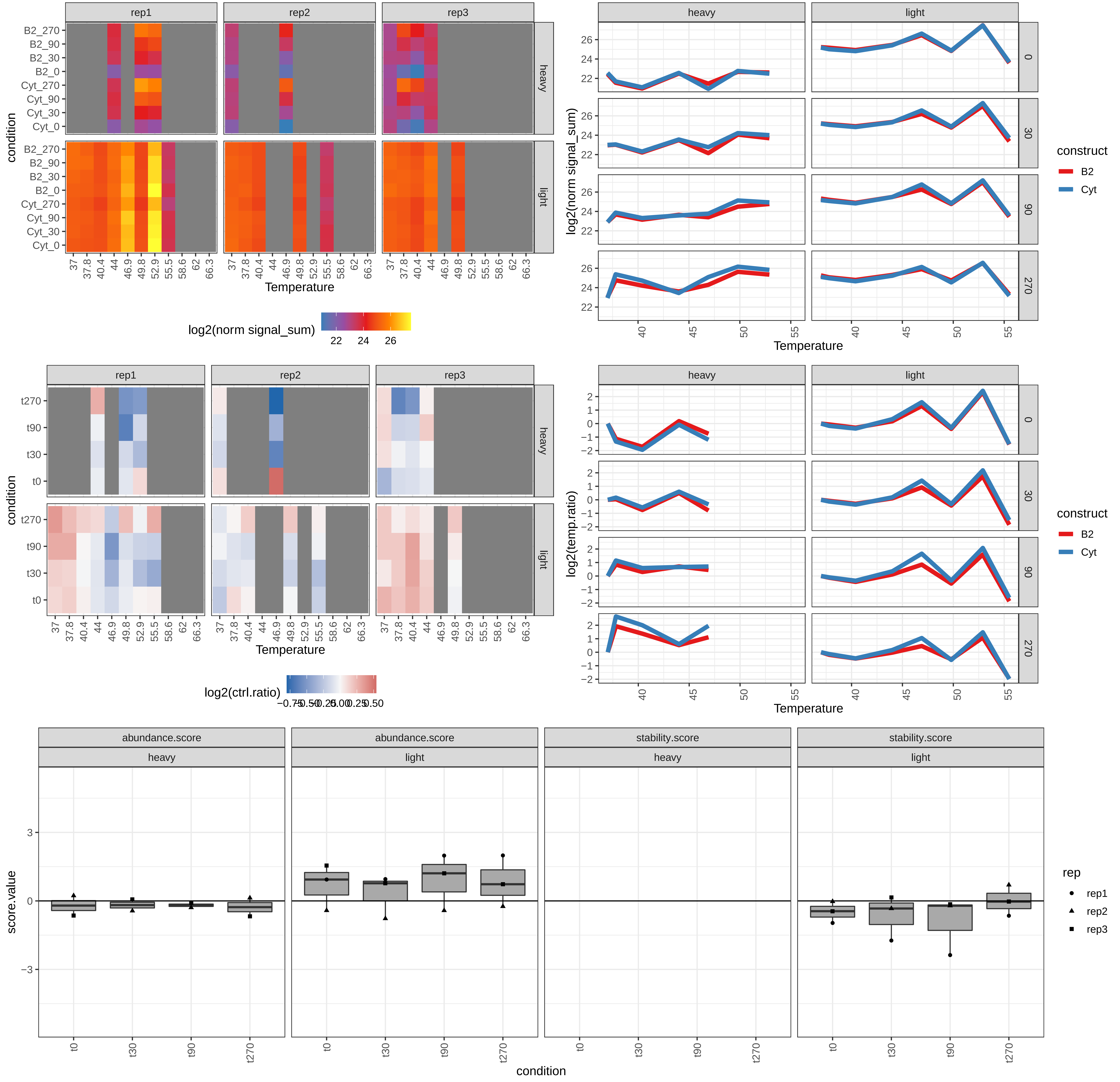

Q99190 − Very−long−chain enoyl−CoA reductase OS=Saccharomyces cerevisiae (strain ATCC 204508 / S288c) GN=TSC13 PE=1 SV=1

TSL1 − average.top3: 7.26109929824561 − max.qupm: 23

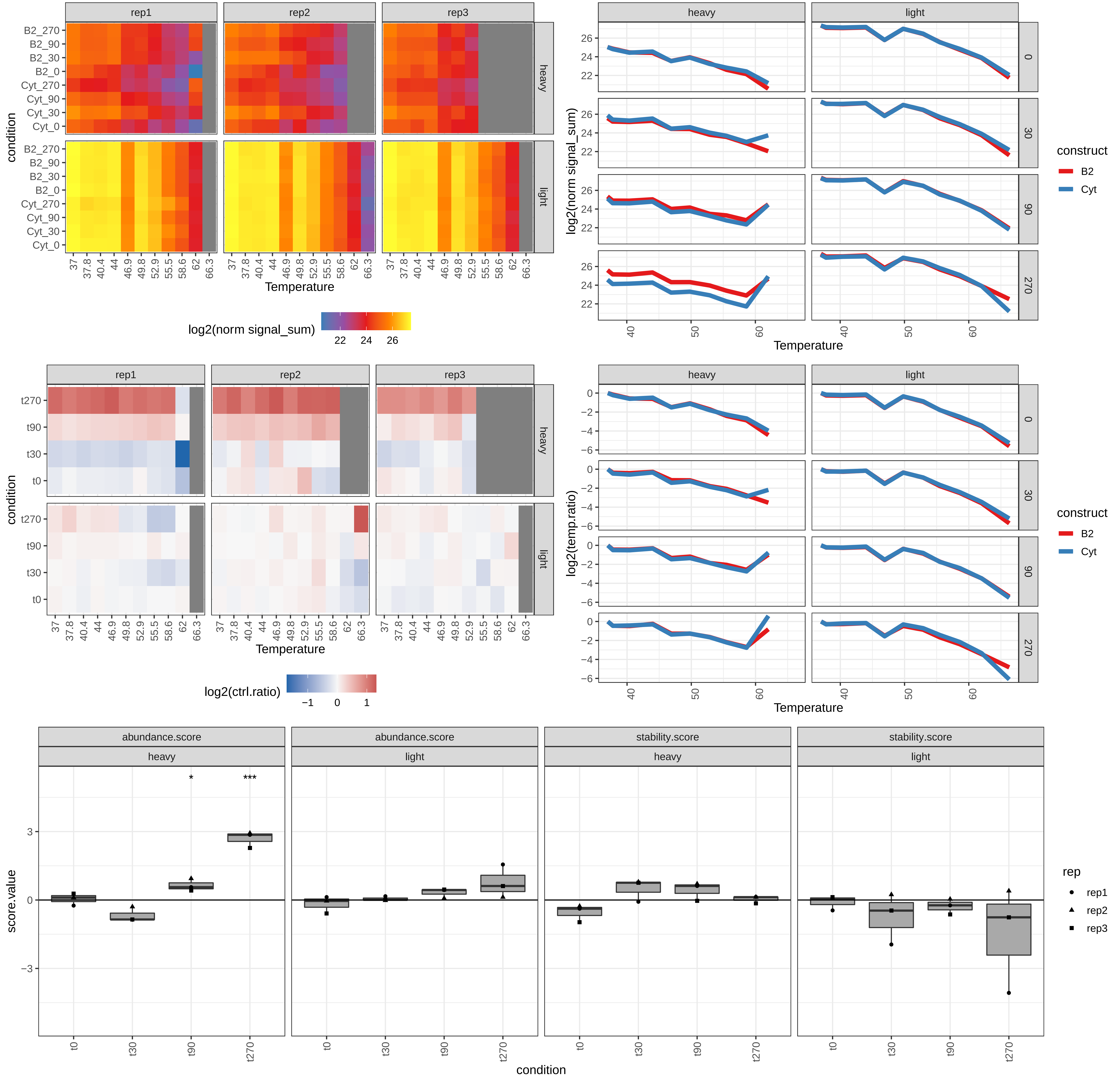

P38427 − Trehalose synthase complex regulatory subunit TSL1 OS=Saccharomyces cerevisiae (strain ATCC 204508 / S288c) GN=TSL1 PE=1 SV=1

TSR1 − average.top3: 7.10951115384615 − max.qupm: 9

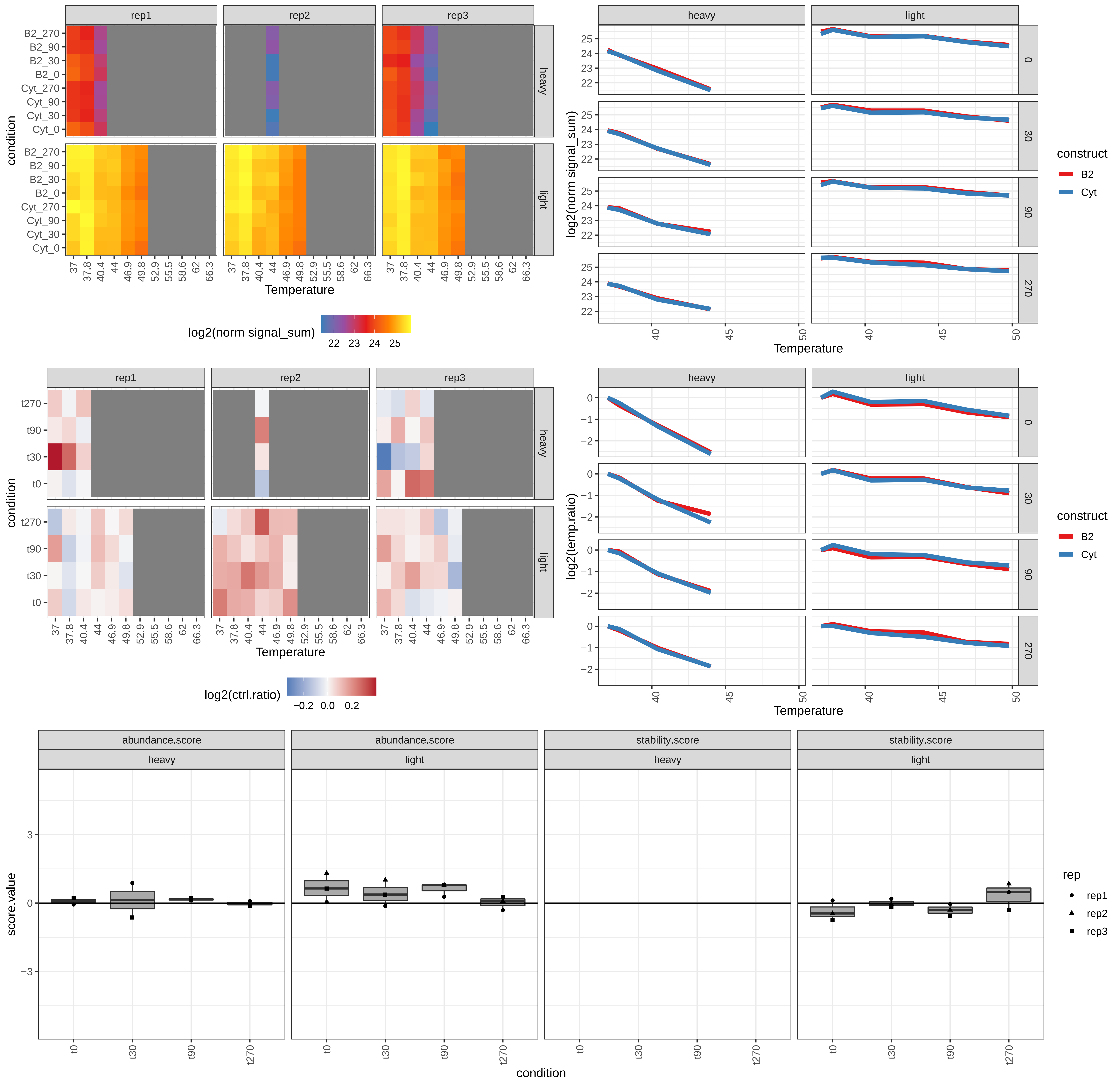

Q07381 − Ribosome biogenesis protein TSR1 OS=Saccharomyces cerevisiae (strain ATCC 204508 / S288c) GN=TSR1 PE=1 SV=1

TSR3 − average.top3: 6.63440380952381 − max.qupm: 4

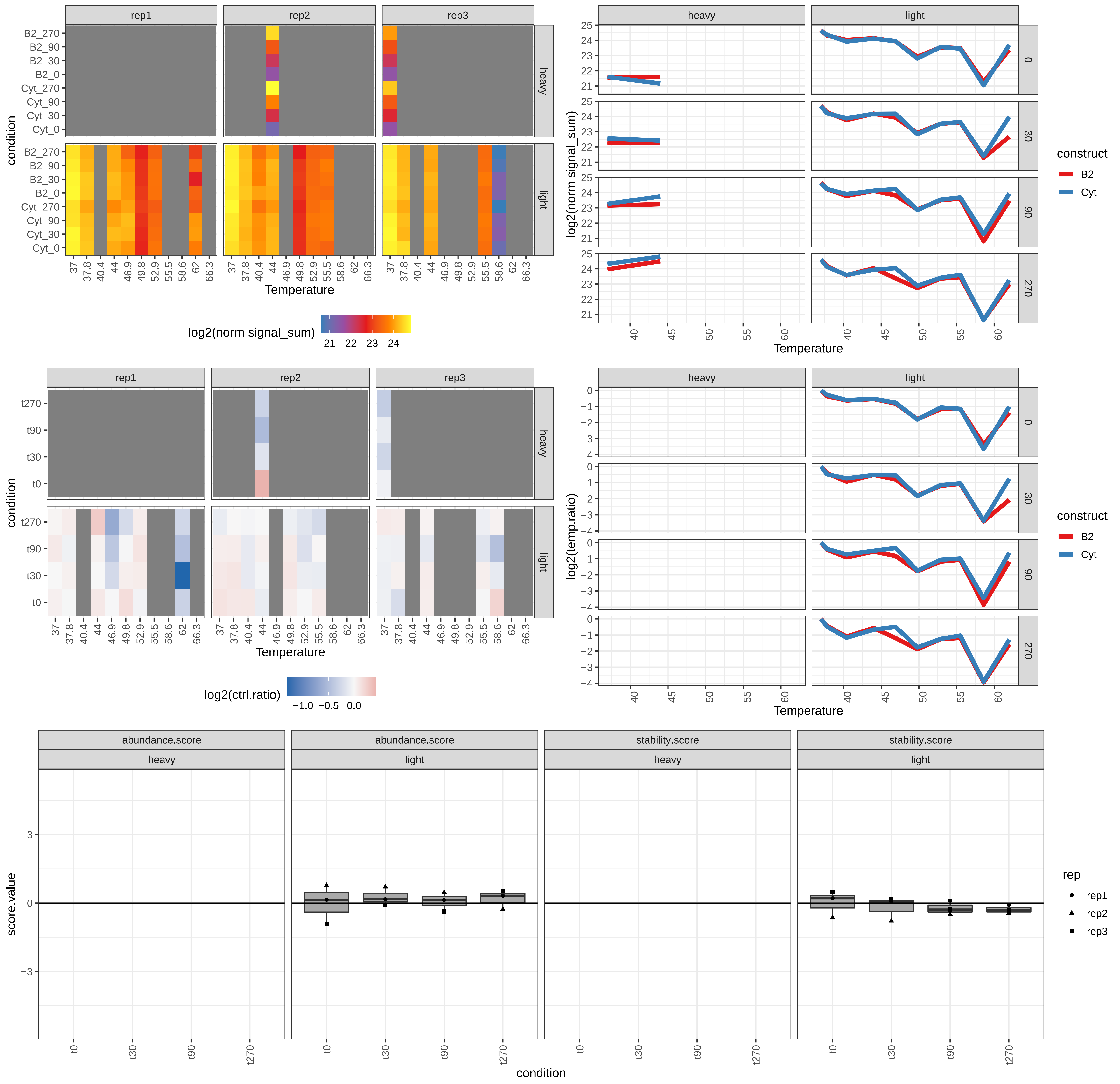

Q12094 − Ribosome biogenesis protein TSR3 OS=Saccharomyces cerevisiae (strain ATCC 204508 / S288c) GN=TSR3 PE=1 SV=1

TSR4 − average.top3: 6.339981 − max.qupm: 5

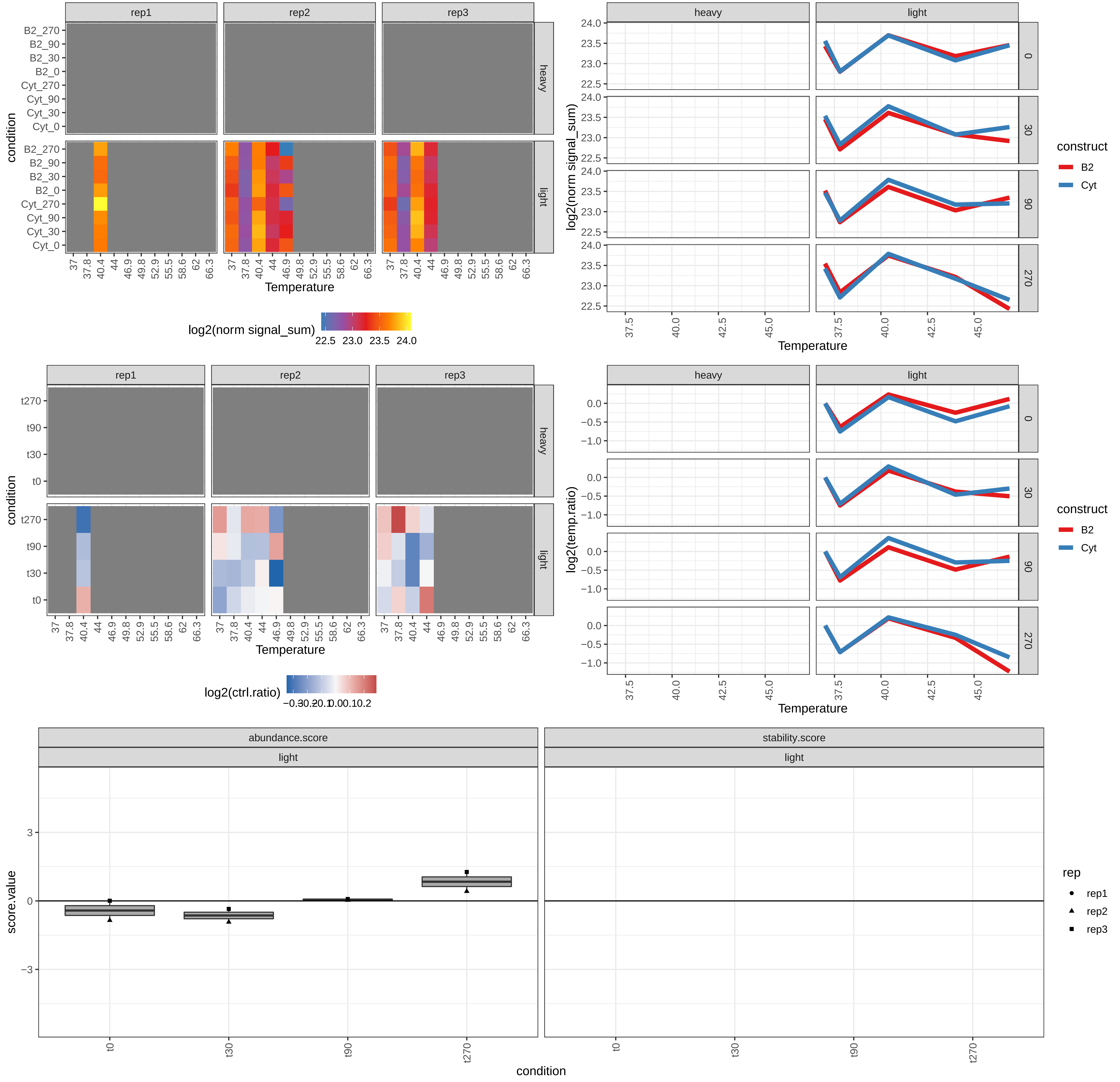

P25040 − 20S rRNA accumulation protein 4 OS=Saccharomyces cerevisiae (strain ATCC 204508 / S288c) GN=TSR4 PE=1 SV=2

TUB1 − average.top3: 7.01590292682927 − max.qupm: 4

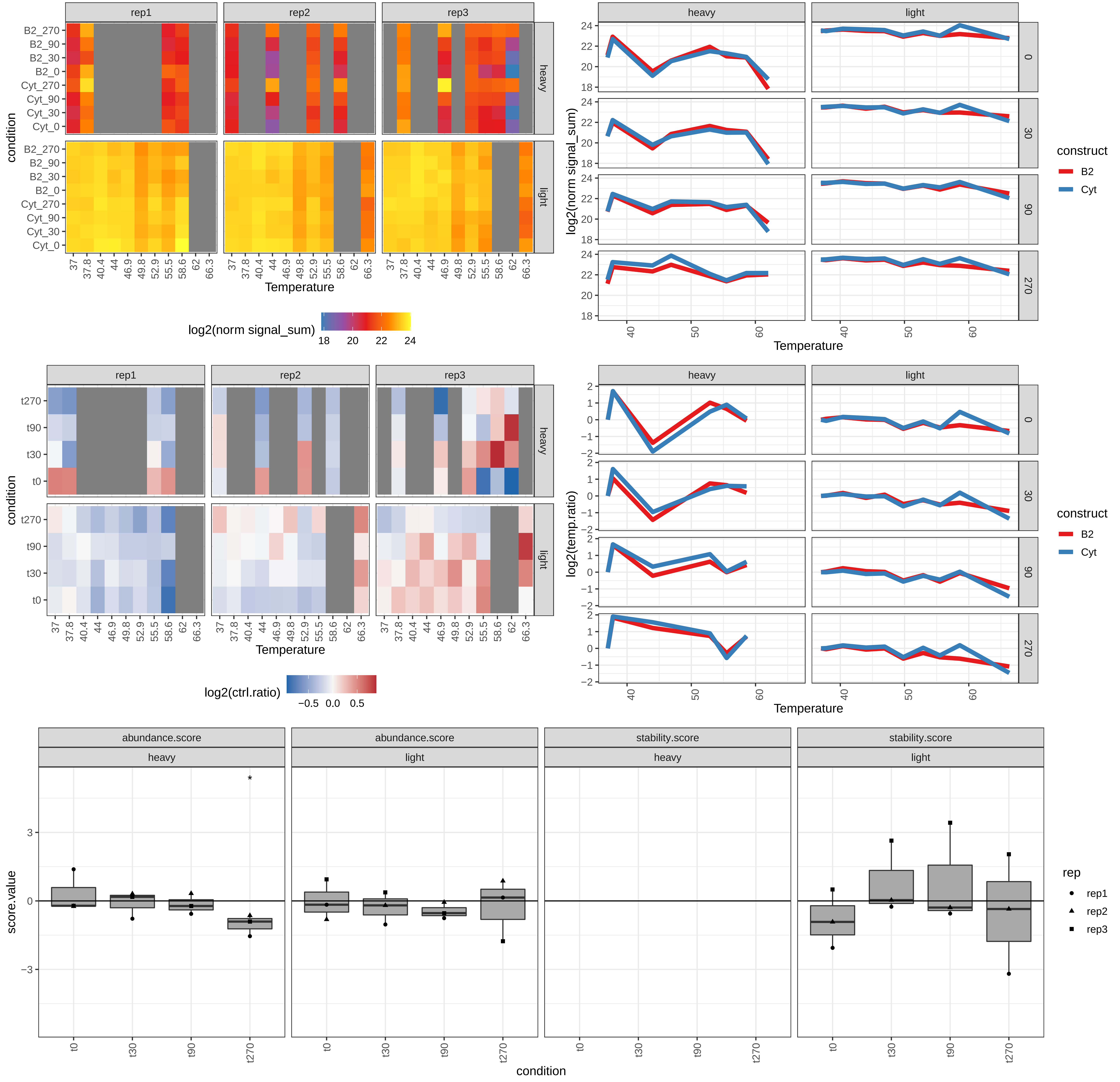

P09733 − Tubulin alpha−1 chain OS=Saccharomyces cerevisiae (strain ATCC 204508 / S288c) GN=TUB1 PE=1 SV=2

TUB2 − average.top3: 7.29001333333333 − max.qupm: 9

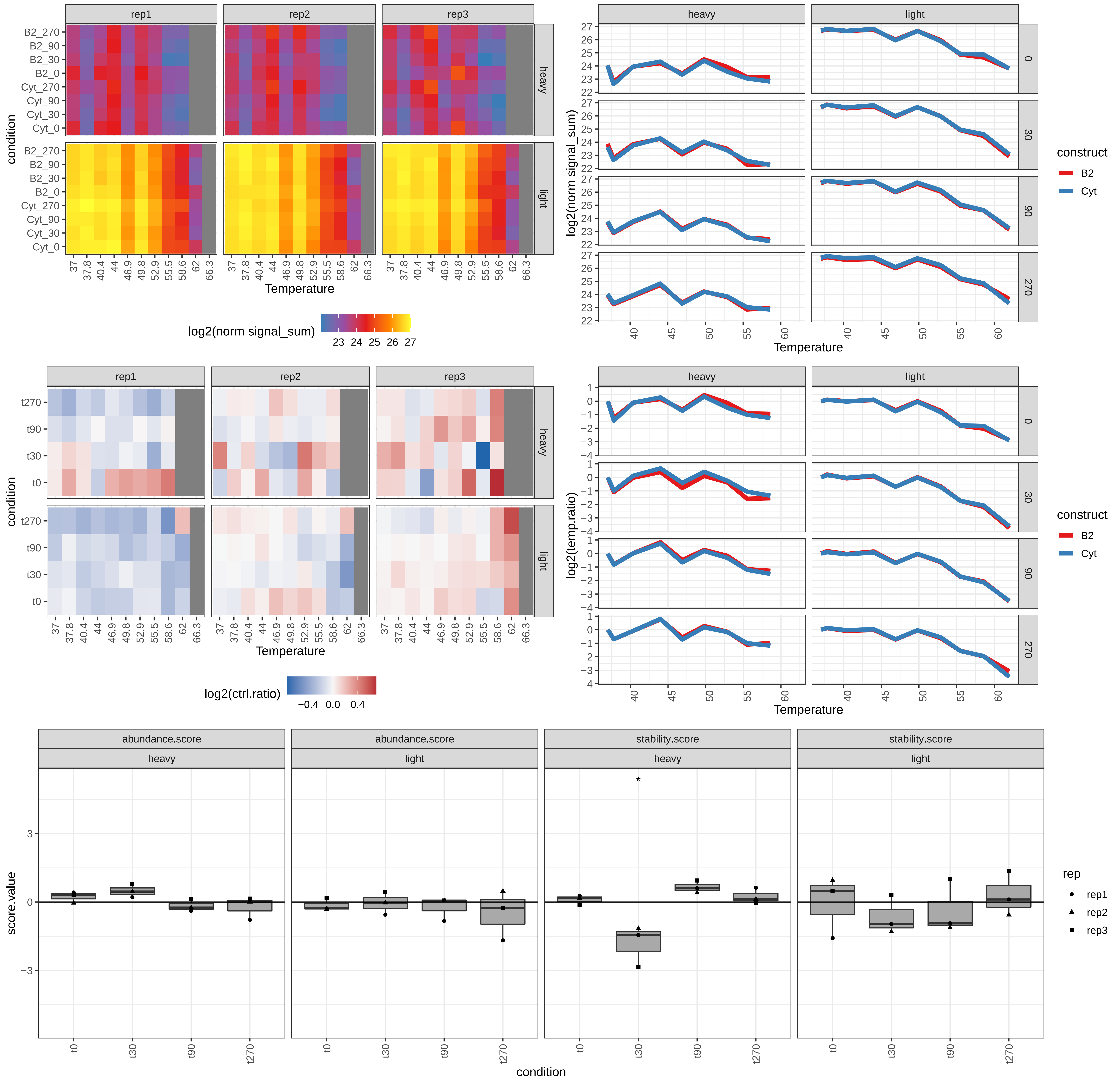

P02557 − Tubulin beta chain OS=Saccharomyces cerevisiae (strain ATCC 204508 / S288c) GN=TUB2 PE=1 SV=2

TUB3 − average.top3: 7.105910625 − max.qupm: 6

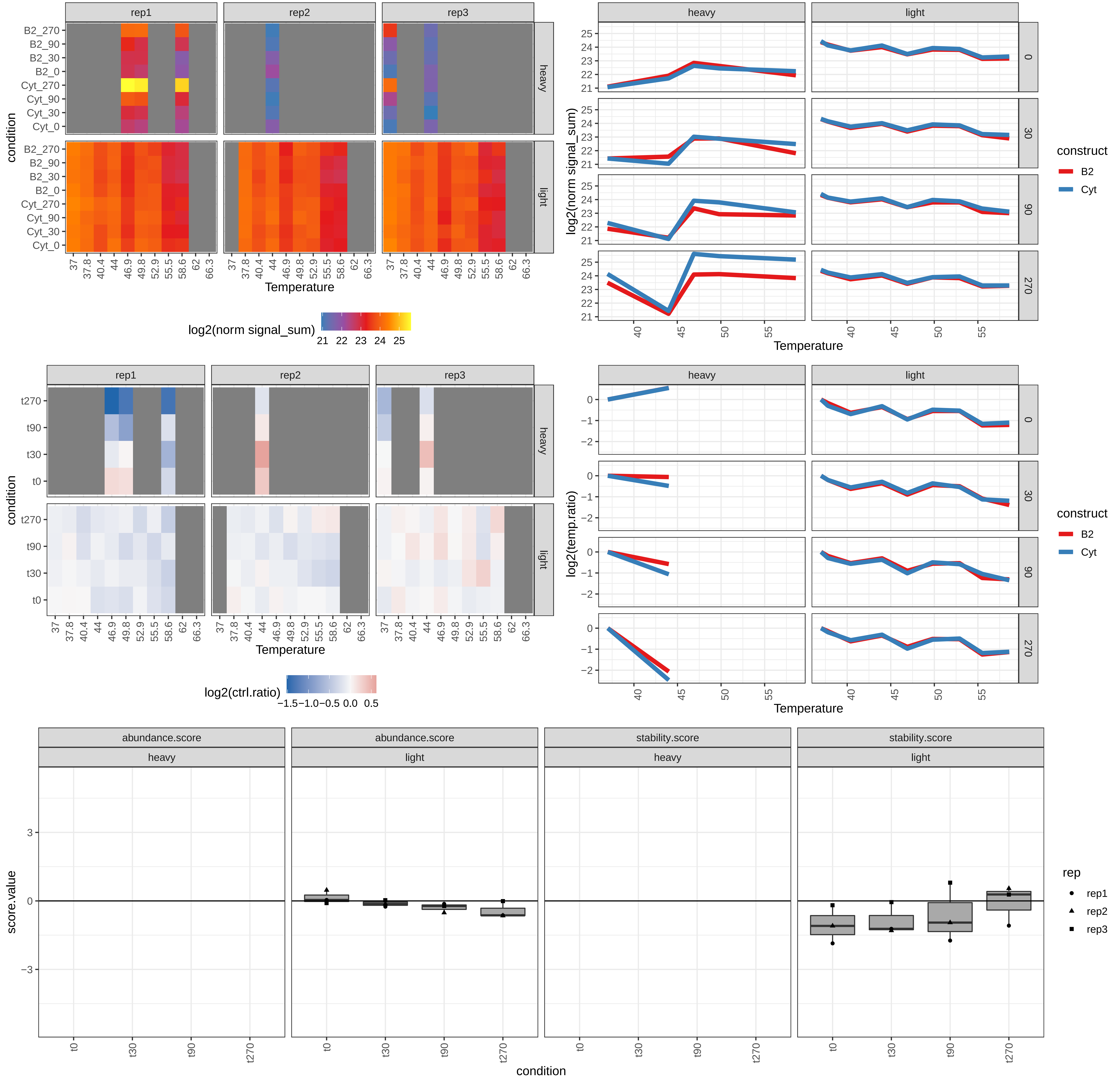

P09734 − Tubulin alpha−3 chain OS=Saccharomyces cerevisiae (strain ATCC 204508 / S288c) GN=TUB3 PE=1 SV=1

TUF1 − average.top3: 7.41719681818182 − max.qupm: 16

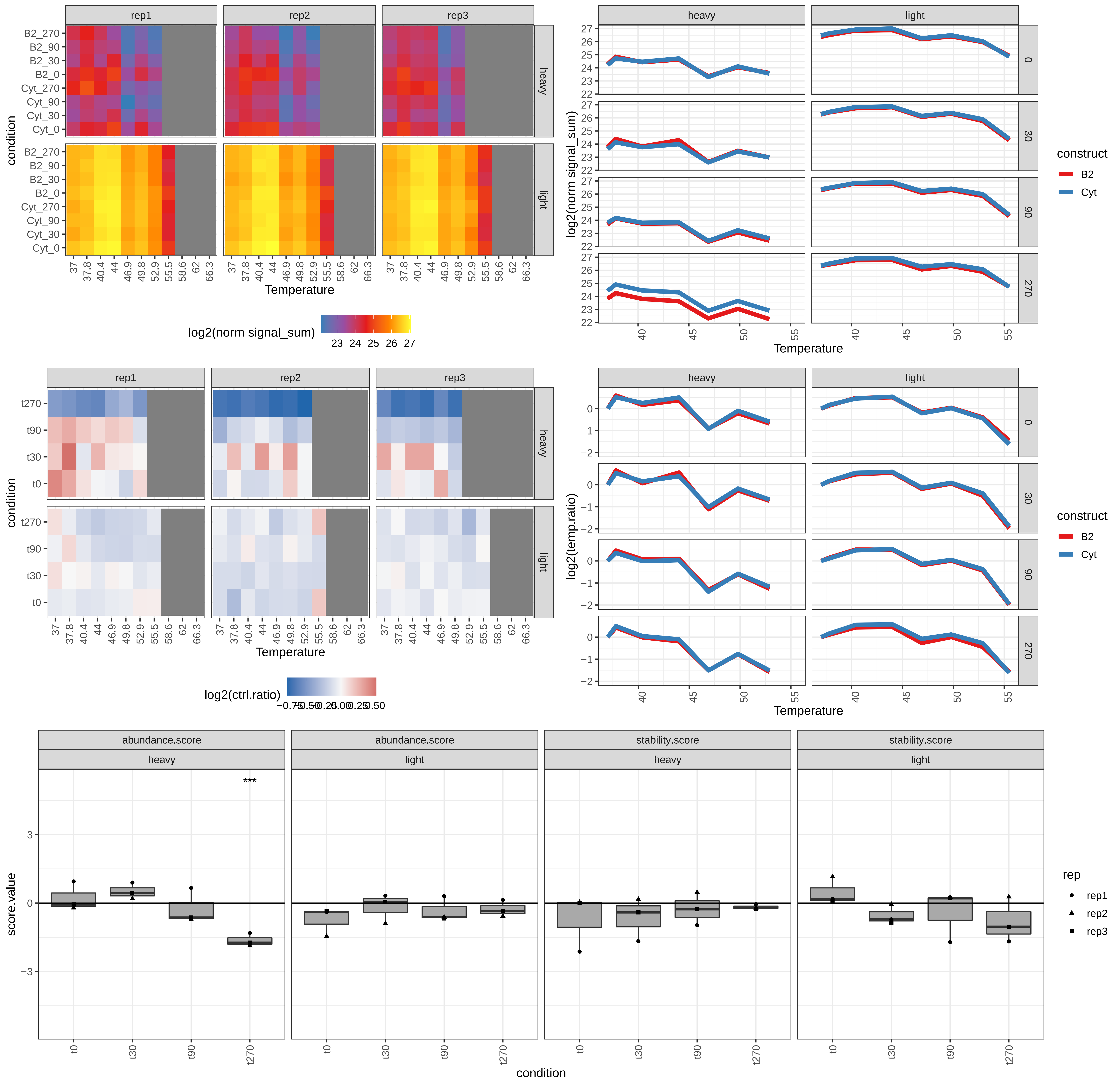

P02992 − Elongation factor Tu, mitochondrial OS=Saccharomyces cerevisiae (strain ATCC 204508 / S288c) GN=TUF1 PE=1 SV=1

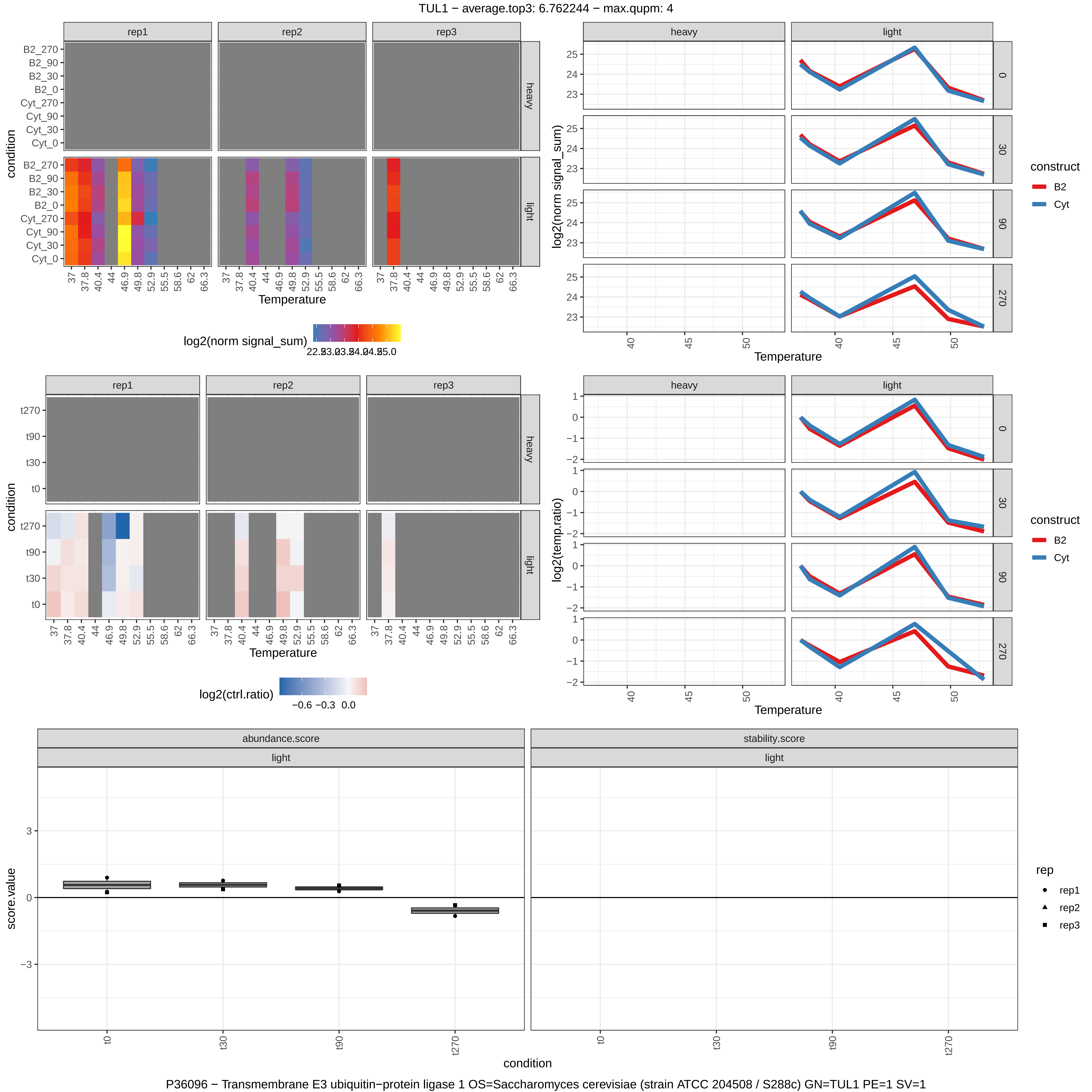
TUL1 − average.top3: 6.762244 − max.qupm: 4
rep1
rep2
rep3
heavy
light
condition
B2_270
B2_90
B2_30
B2_0
Cyt_270
Cyt_90
Cyt_30
Cyt_0
Temperature
log2(norm signal_sum)
log2(norm signal_sum)
construct
B2
Cyt
t270
t90
t30
t0
log2(ctrl.ratio)
−0.6 −0.3 0.0
log2(temp.ratio)
abundance.score
stability.score
score.value
rep
rep1
rep2
rep3
condition
P36096 − Transmembrane E3 ubiquitin−protein ligase 1 OS=Saccharomyces cerevisiae (strain ATCC 204508 / S288c) GN=TUL1 PE=1 SV=1

TUM1 − average.top3: 6.975176 − max.qupm: 8

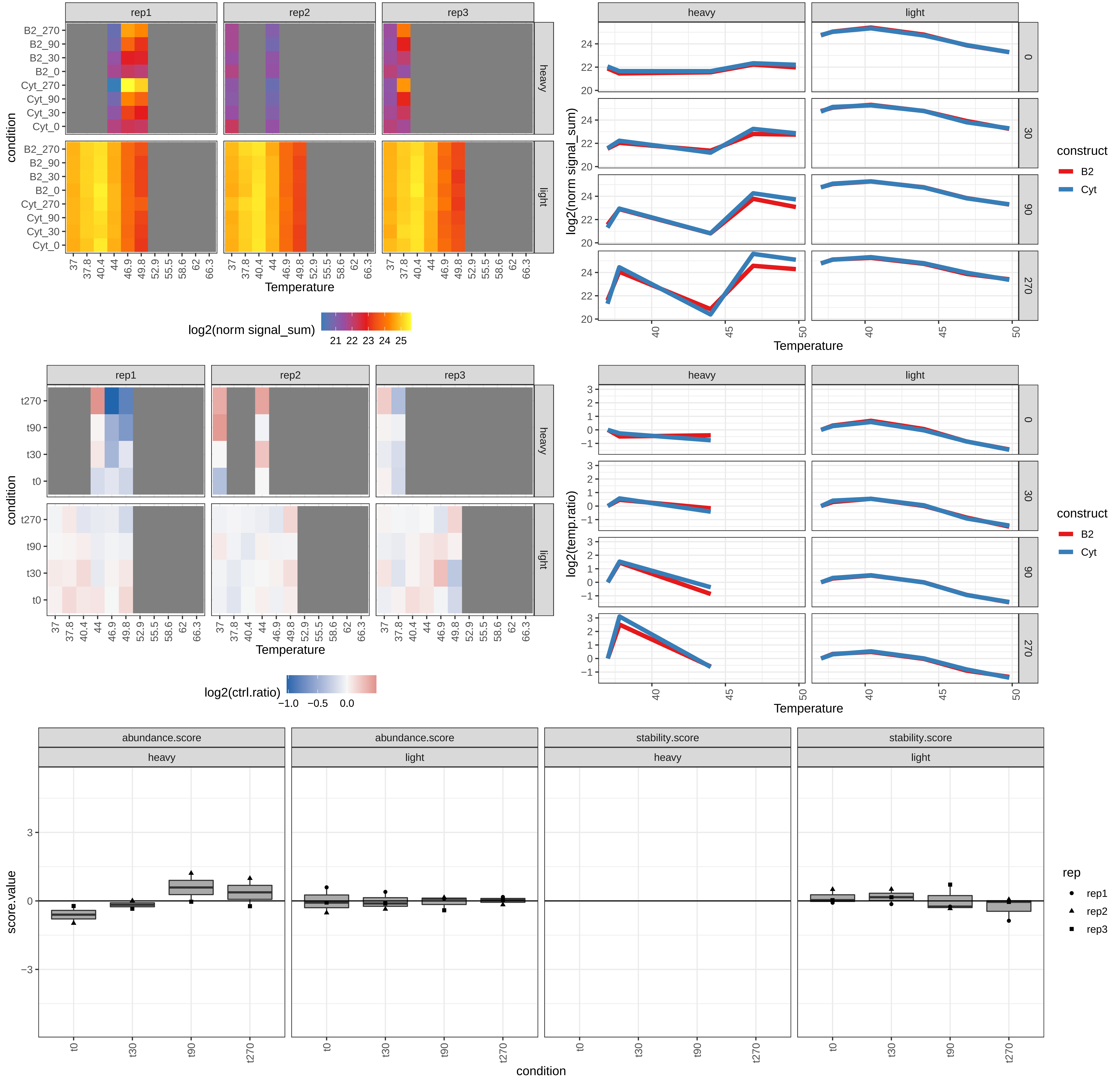

Q08686 − Thiosulfate sulfurtransferase TUM1 OS=Saccharomyces cerevisiae (strain ATCC 204508 / S288c) GN=TUM1 PE=1 SV=1

TUP1 − average.top3: 7.20168444444444 − max.qupm: 16

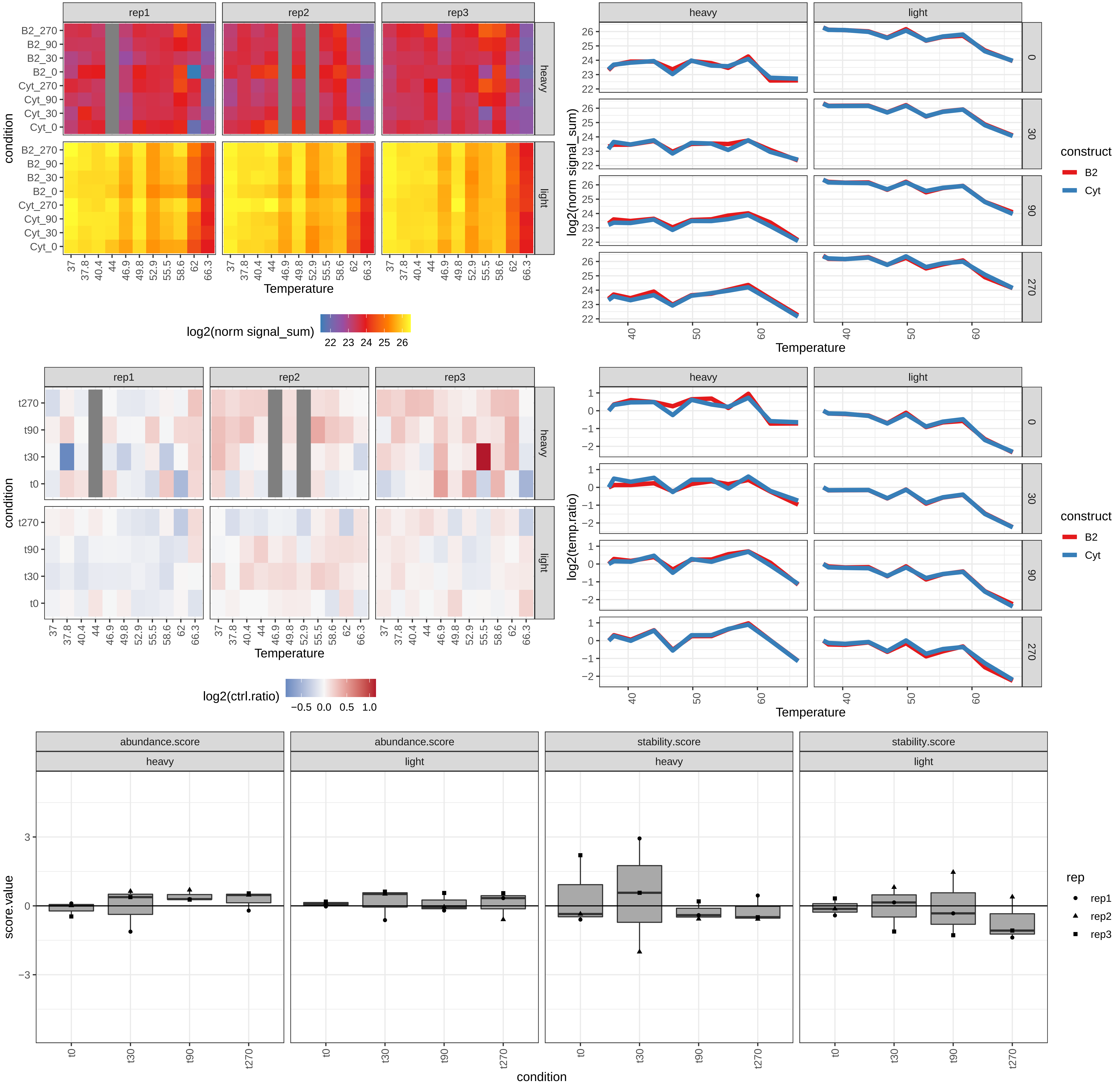

P16649 − General transcriptional corepressor TUP1 OS=Saccharomyces cerevisiae (strain ATCC 204508 / S288c) GN=TUP1 PE=1 SV=2

TVP23 − average.top3: 6.82650416666667 − max.qupm: 3

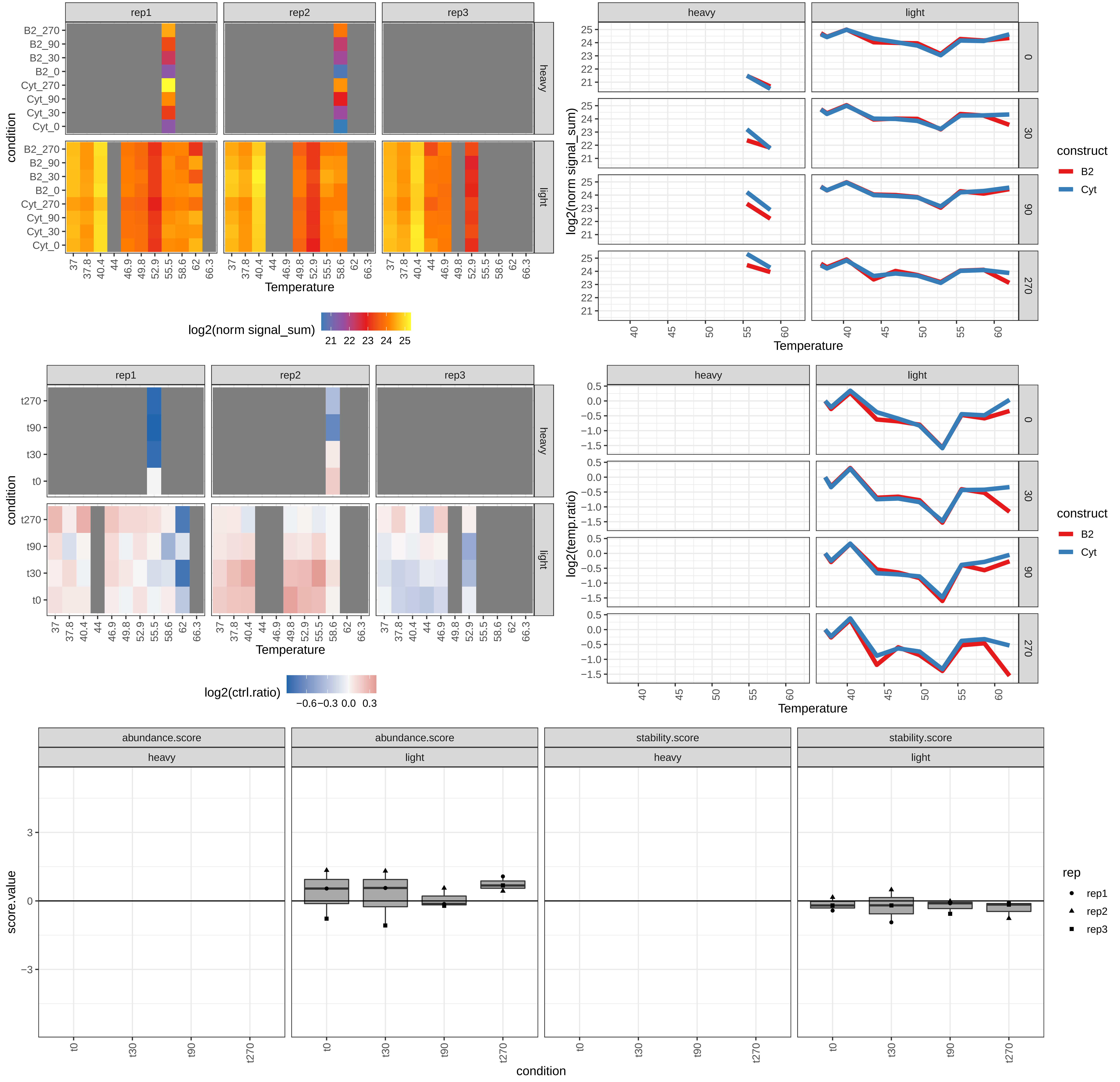

P38962 − Golgi apparatus membrane protein TVP23 OS=Saccharomyces cerevisiae (strain ATCC 204508 / S288c) GN=TVP23 PE=1 SV=1

TWF1 − average.top3: 6.53358346153846 − max.qupm: 5

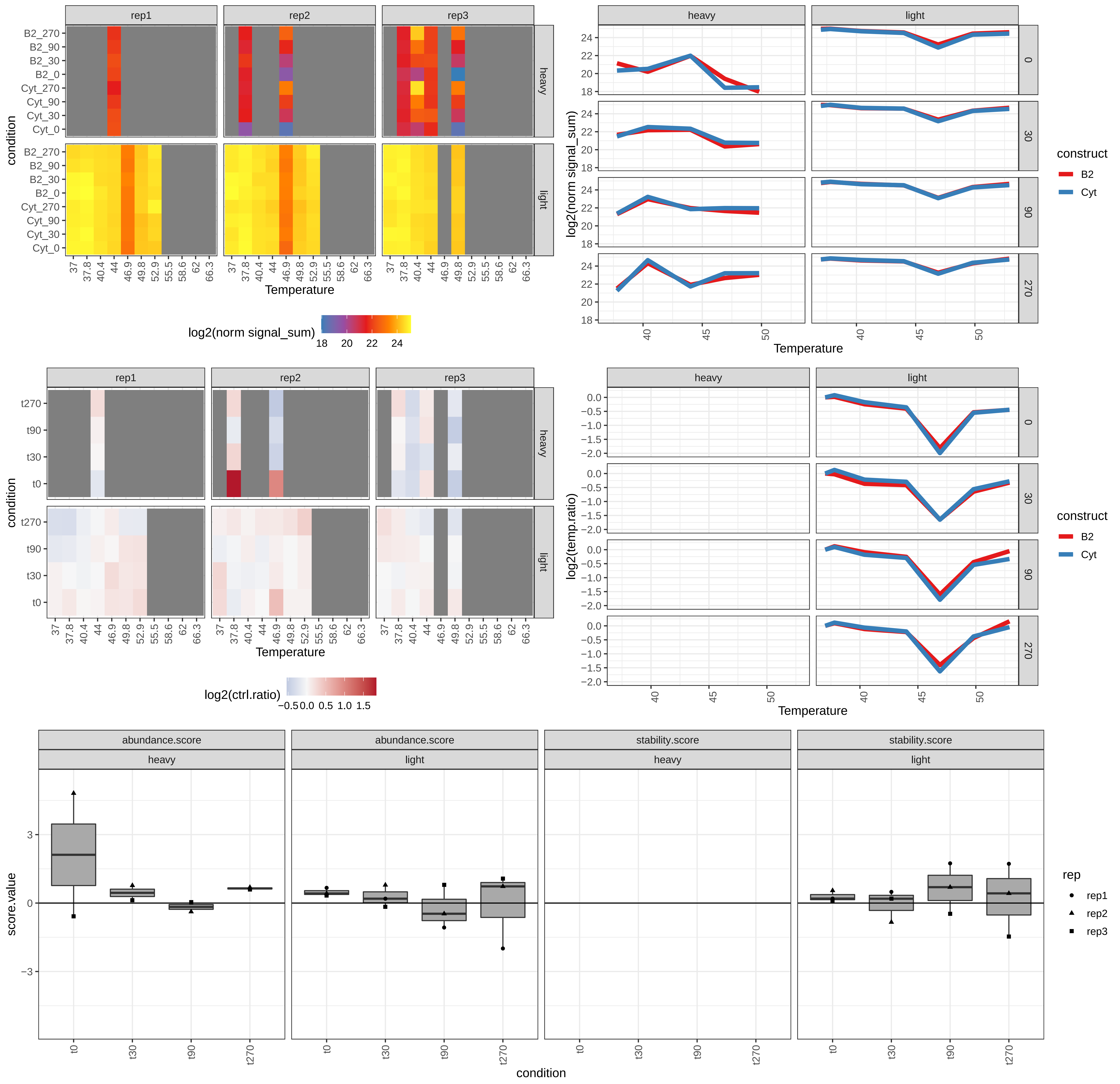

P53250 − Twinfilin−1 OS=Saccharomyces cerevisiae (strain ATCC 204508 / S288c) GN=TWF1 PE=1 SV=1

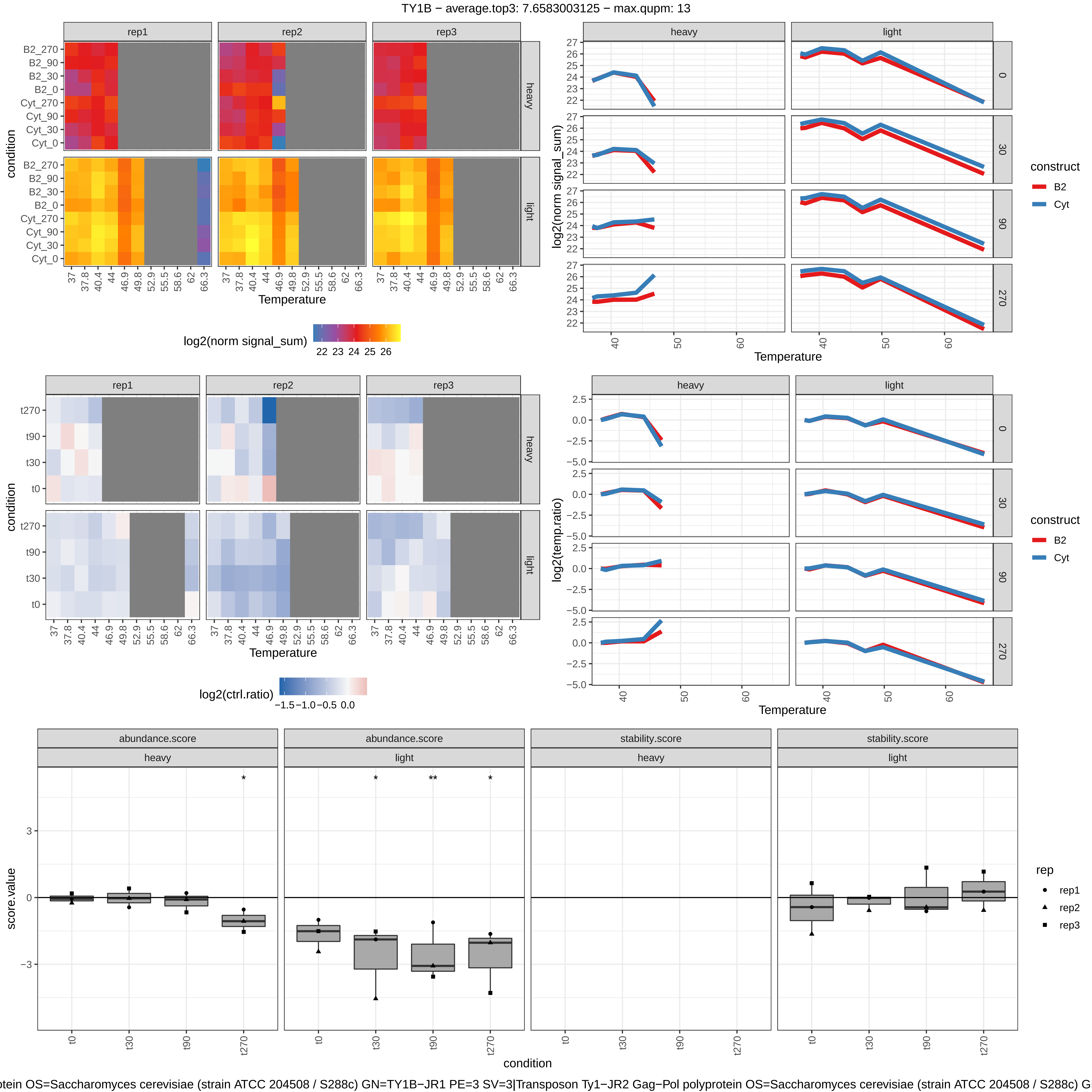
TY1B − average.top3: 7.6583003125 − max.qupm: 13
rep1
rep2
rep3
heavy
light
B2_270
B2_90
B2_30
B2_0
Cyt_270
Cyt_90
Cyt_30
Cyt_0
condition
37 37.8 40.4 44 46.9 49.8 52.9 55.5 58.6 62 66.3
Temperature
log2(norm signal_sum)
22 23 24 25 26
log2(norm signal_sum)
0
30
90
270
construct
B2
Cyt
t270
t90
t30
t0
log2(ctrl.ratio)
−1.5 −1.0 −0.5 0.0
log2(temp.ratio)
abundance.score
stability.score
score.value
rep
rep1
rep2
rep3
condition
tein OS=Saccharomyces cerevisiae (strain ATCC 204508 / S288c) GN=TY1B−JR1 PE=3 SV=3|Transposon Ty1−JR2 Gag−Pol polyprotein OS=Saccharomyces cerevisiae (strain ATCC 204508 / S288c) G

TYR1 − average.top3: 6.680955 − max.qupm: 8

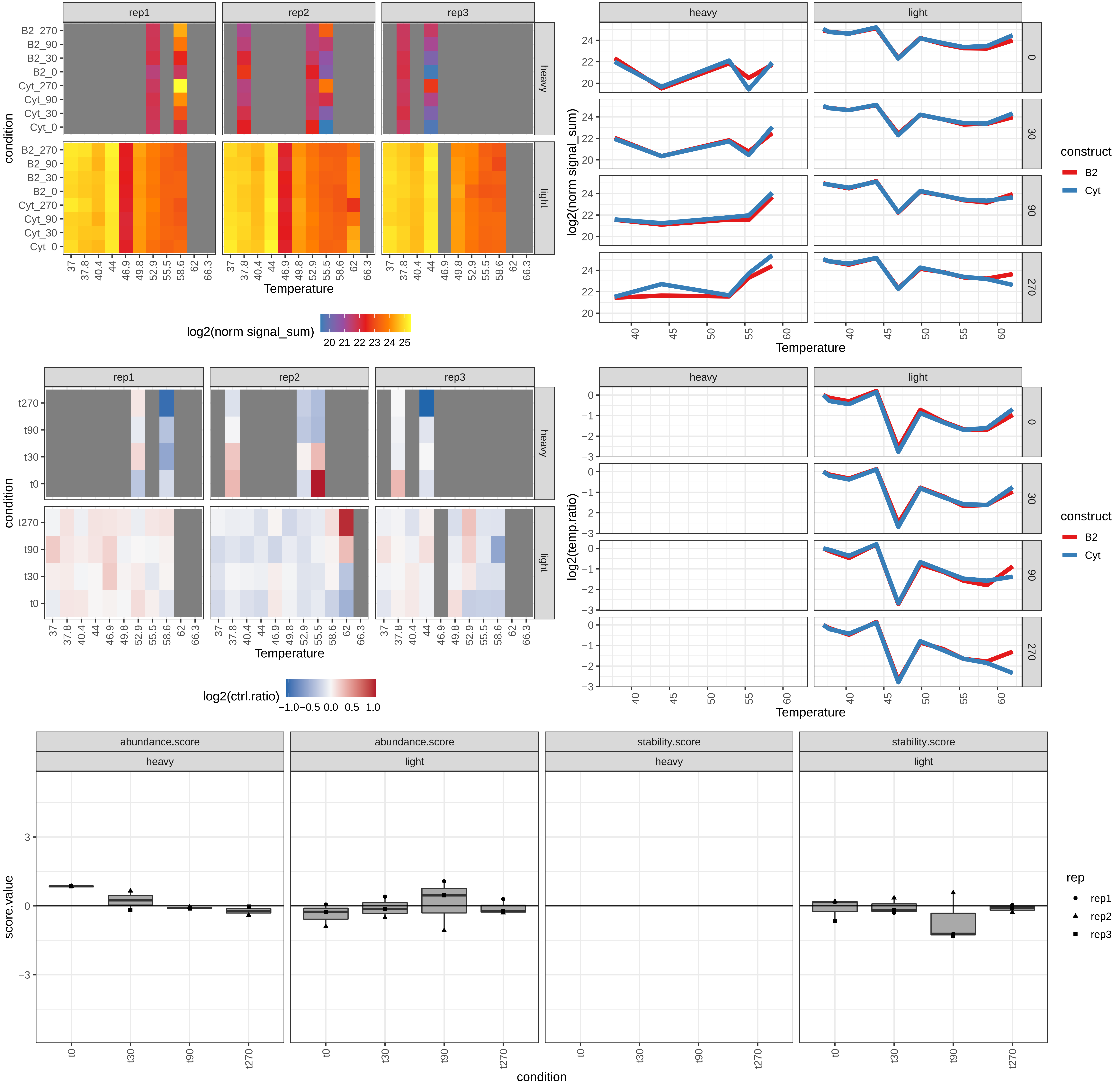

P20049 − Prephenate dehydrogenase [NADP(+)] OS=Saccharomyces cerevisiae (strain ATCC 204508 / S288c) GN=TYR1 PE=1 SV=2

TYS1 − average.top3: 7.57875650793651 − max.qupm: 18

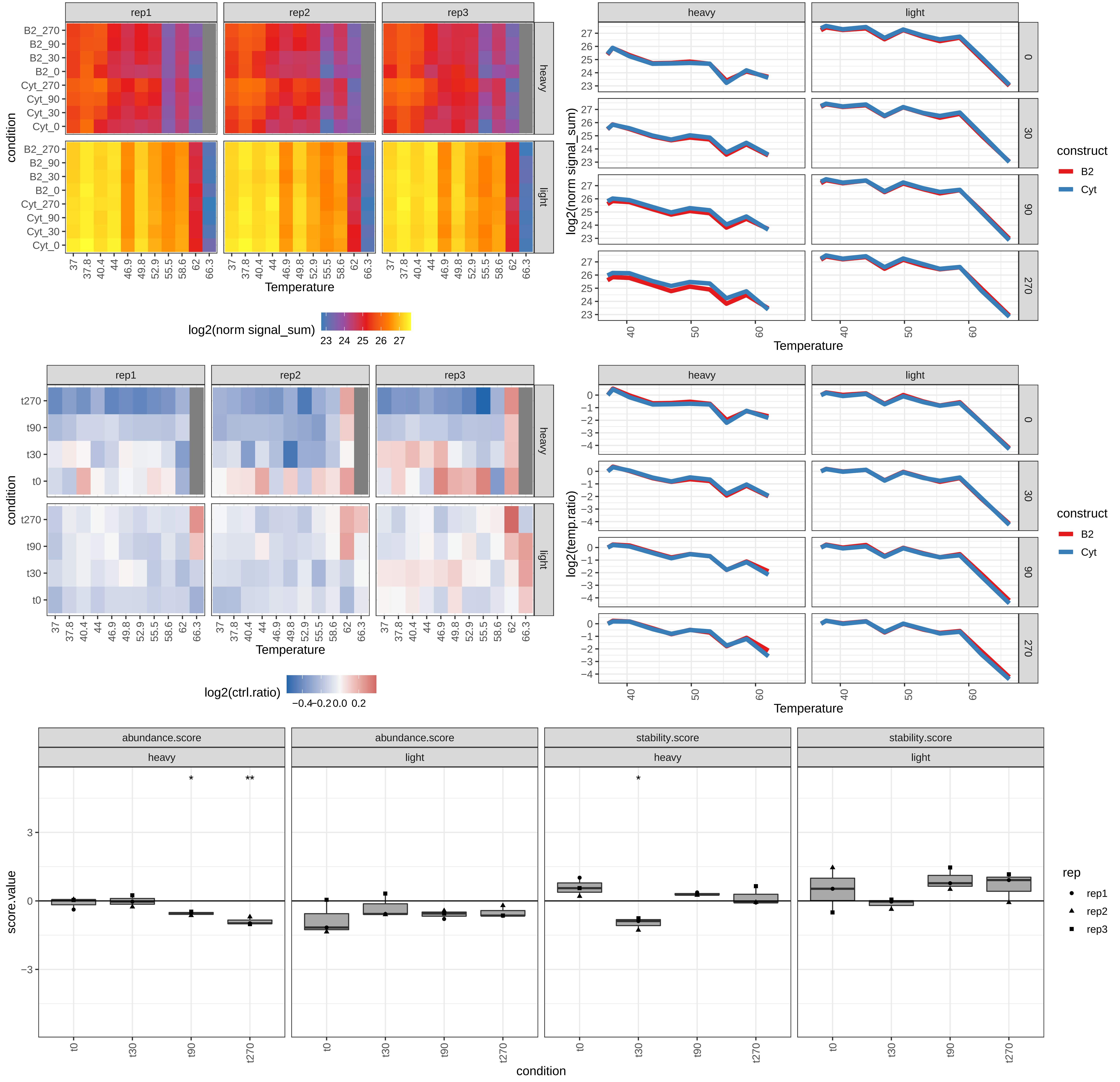

P36421 − Tyrosine−−tRNA ligase, cytoplasmic OS=Saccharomyces cerevisiae (strain ATCC 204508 / S288c) GN=TYS1 PE=1 SV=3

TYW1 − average.top3: 6.96398444444444 − max.qupm: 13

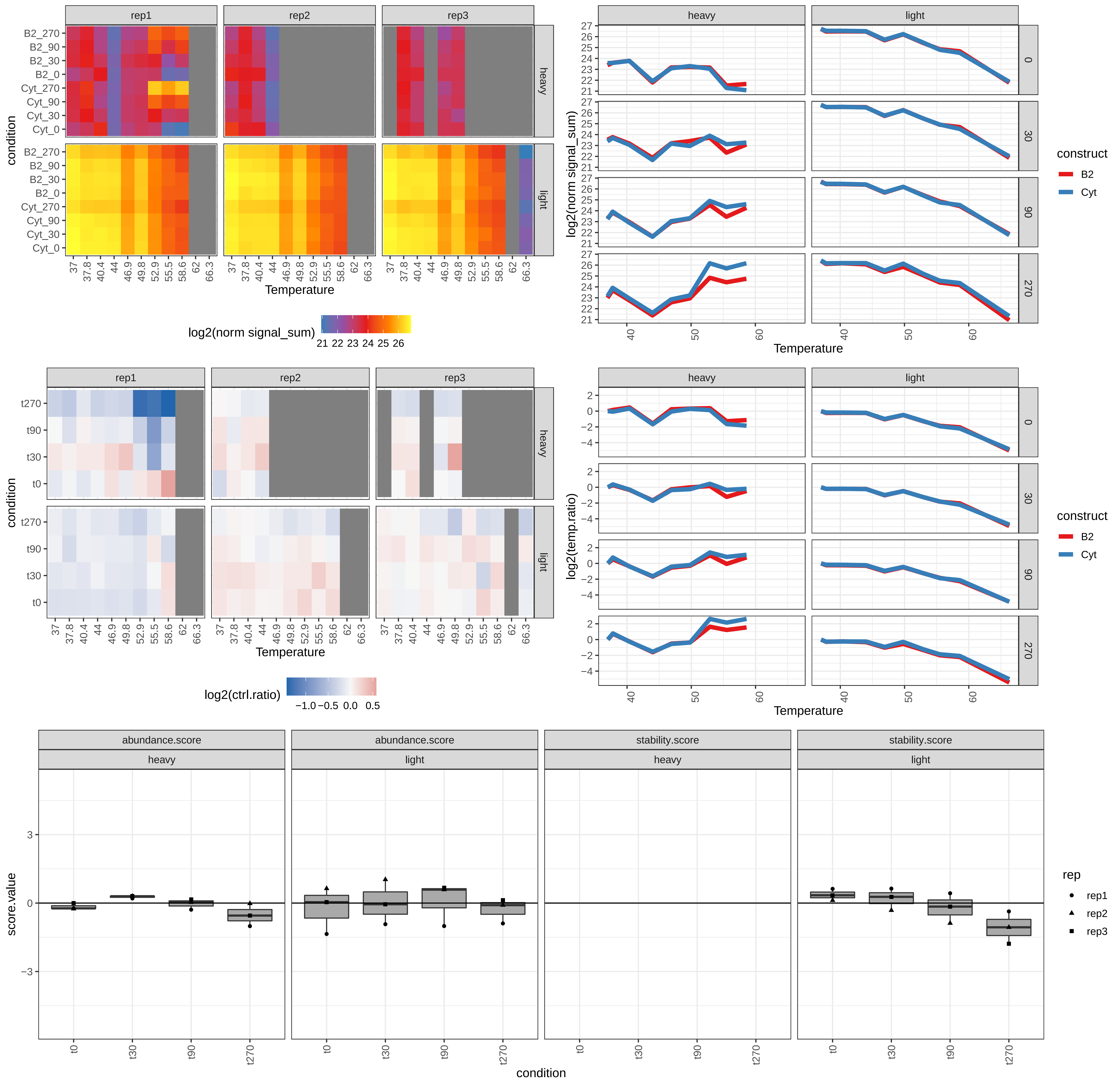

Q08960 − S−adenosyl−L−methionine−dependent tRNA 4−demethylwyosine synthase OS=Saccharomyces cerevisiae (strain ATCC 204508 / S288c) GN=TYW1 PE=1 SV=1

TYW3 − average.top3: 6.63947914285714 − max.qupm: 9

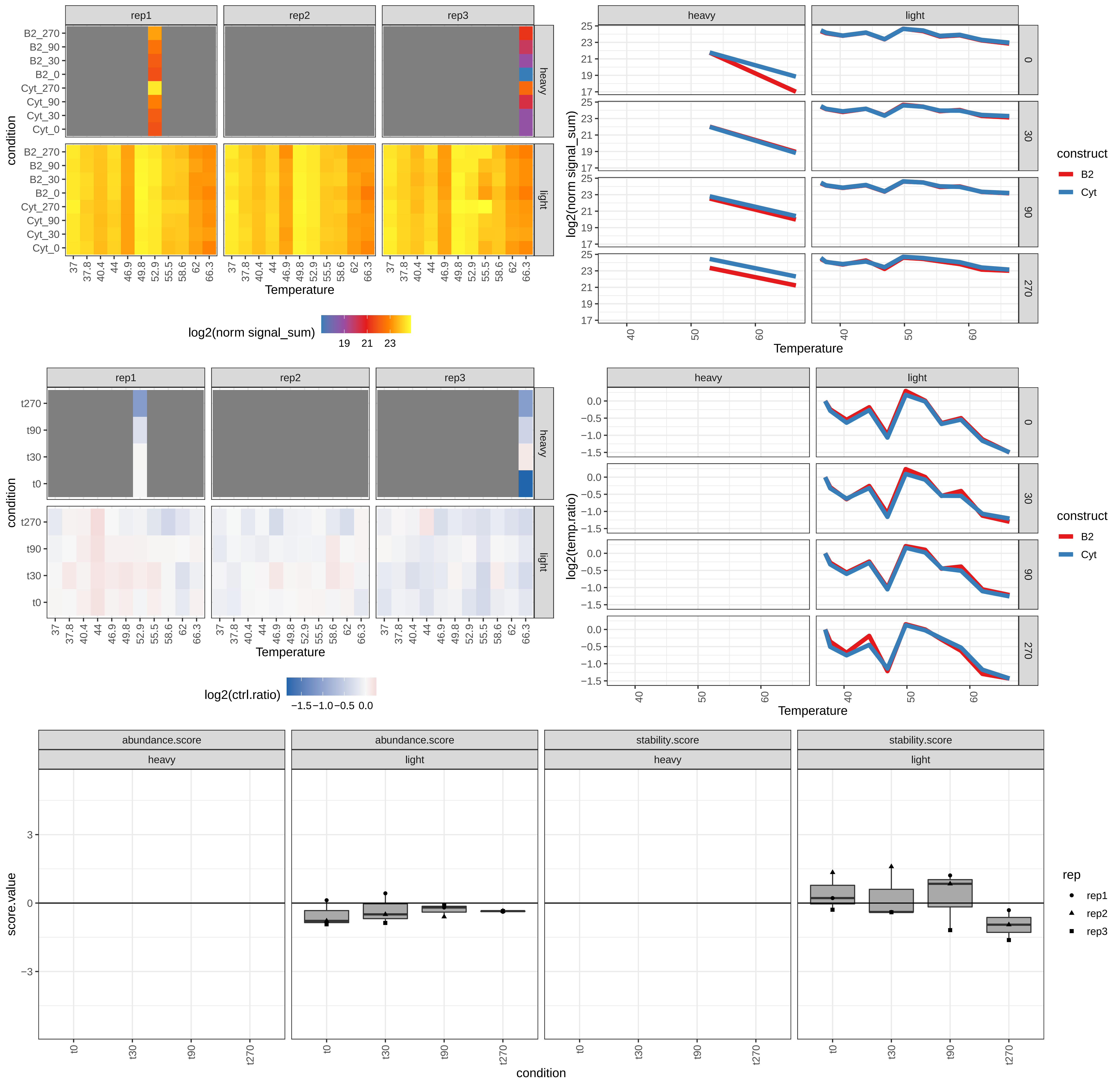

P53177 − tRNA wybutosine−synthesizing protein 3 OS=Saccharomyces cerevisiae (strain ATCC 204508 / S288c) GN=TYW3 PE=1 SV=1

UBA1 − average.top3: 7.767704 − max.qupm: 36

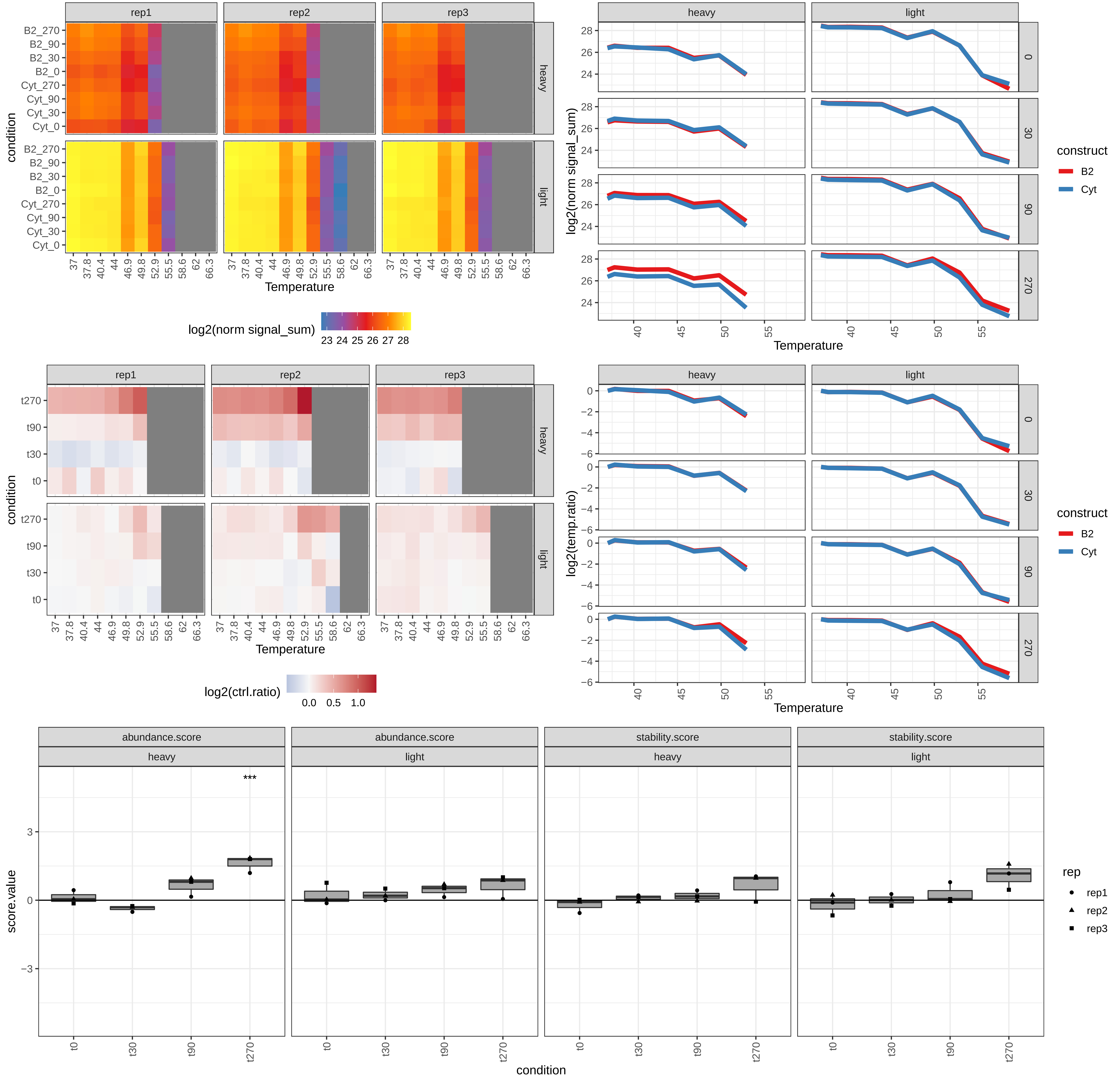

P22515 − Ubiquitin−activating enzyme E1 1 OS=Saccharomyces cerevisiae (strain ATCC 204508 / S288c) GN=UBA1 PE=1 SV=2

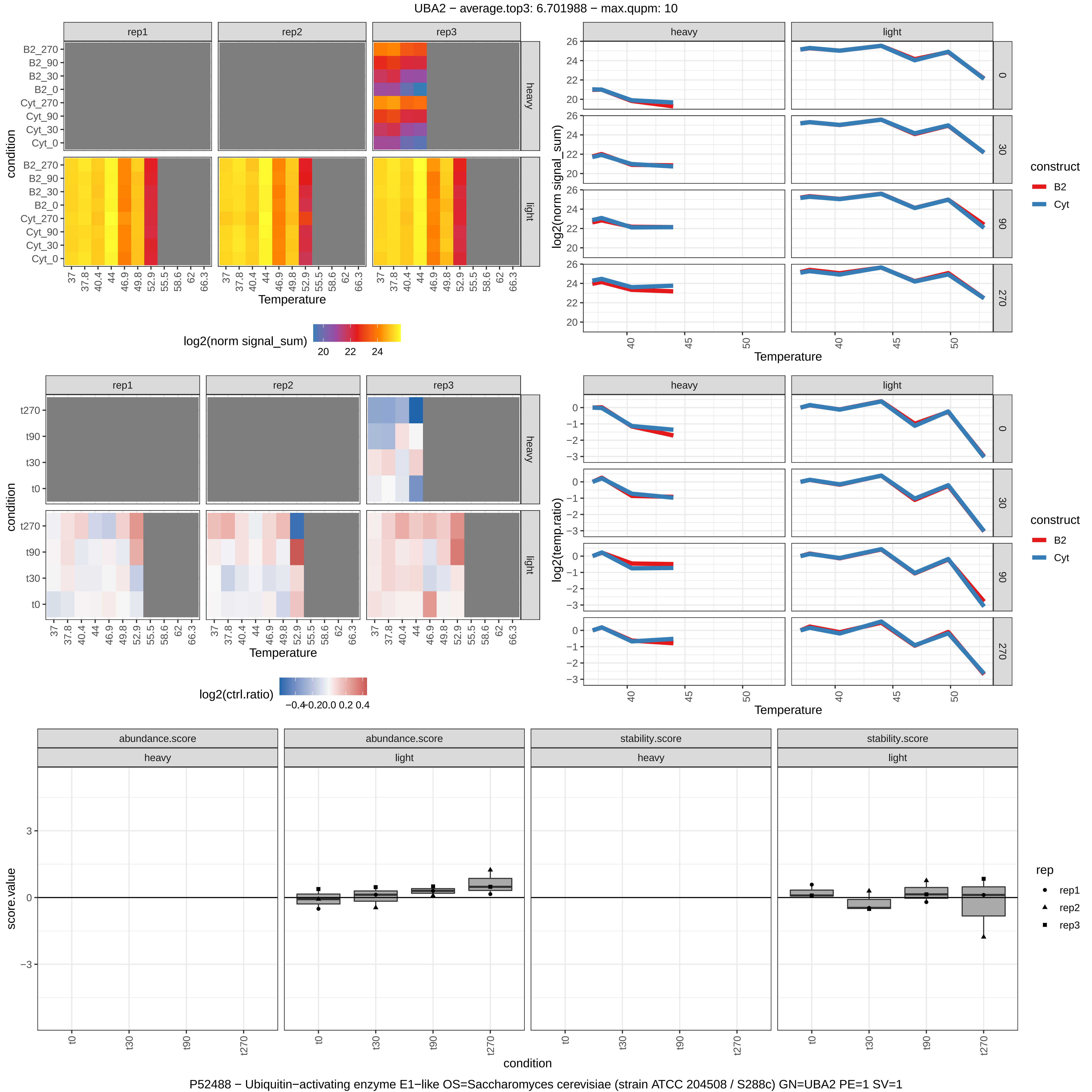

UBA2 − average.top3: 6.701988 − max.qupm: 10
rep1
rep2
rep3
heavy
light
condition
Temperature
log2(norm signal_sum)
construct
B2
Cyt
log2(ctrl.ratio)
log2(temp.ratio)
abundance.score
stability.score
score.value
rep
rep1
rep2
rep3
P52488 − Ubiquitin−activating enzyme E1−like OS=Saccharomyces cerevisiae (strain ATCC 204508 / S288c) GN=UBA2 PE=1 SV=1

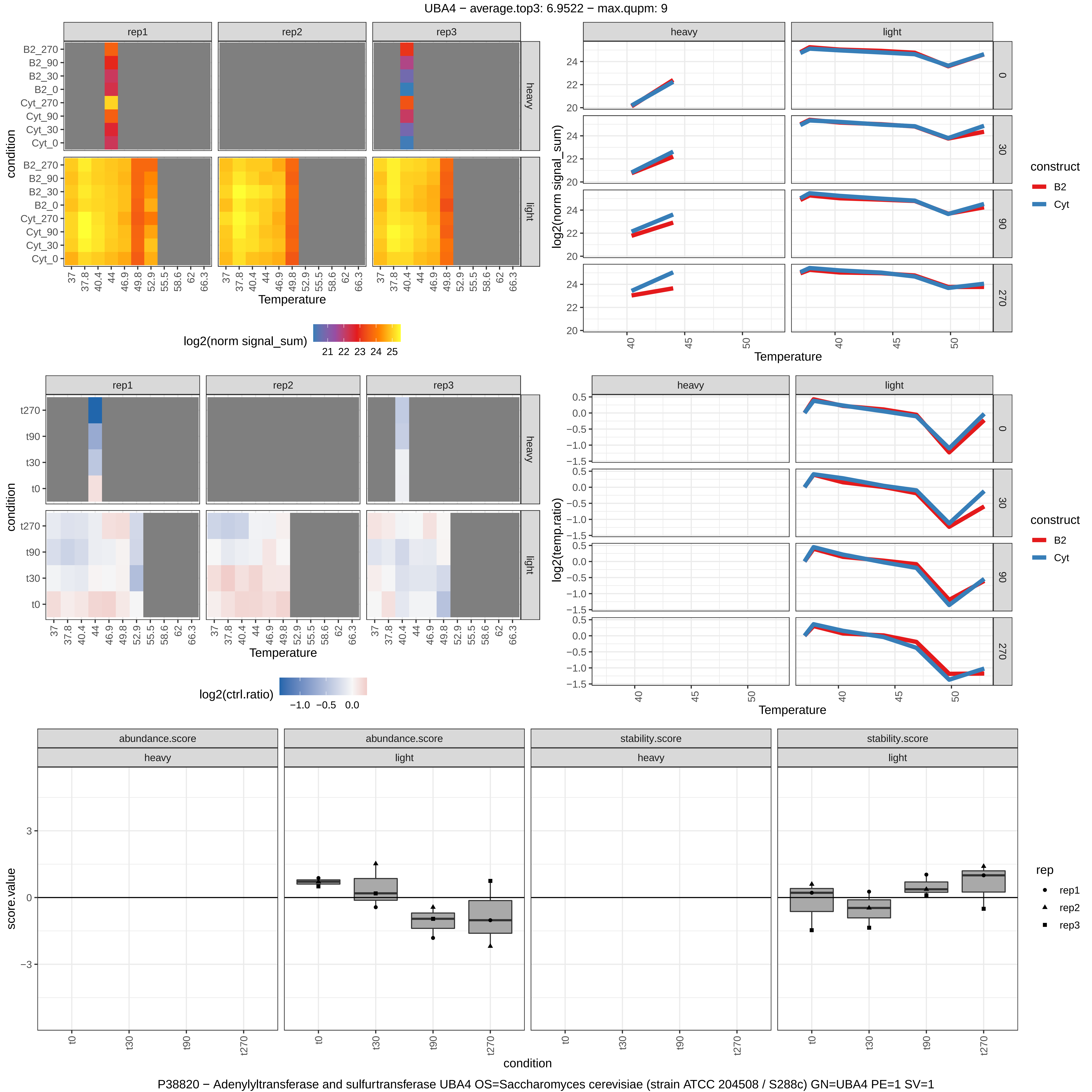

UBA4 − average.top3: 6.9522 − max.qupm: 9
rep1
rep2
rep3
heavy
light
condition
Temperature
log2(norm signal_sum)
construct
B2
Cyt
log2(ctrl.ratio)
log2(temp.ratio)
abundance.score
stability.score
score.value
rep
rep1
rep2
rep3
P38820 − Adenylyltransferase and sulfurtransferase UBA4 OS=Saccharomyces cerevisiae (strain ATCC 204508 / S288c) GN=UBA4 PE=1 SV=1

UBC1 − average.top3: 7.25642944444444 − max.qupm: 10

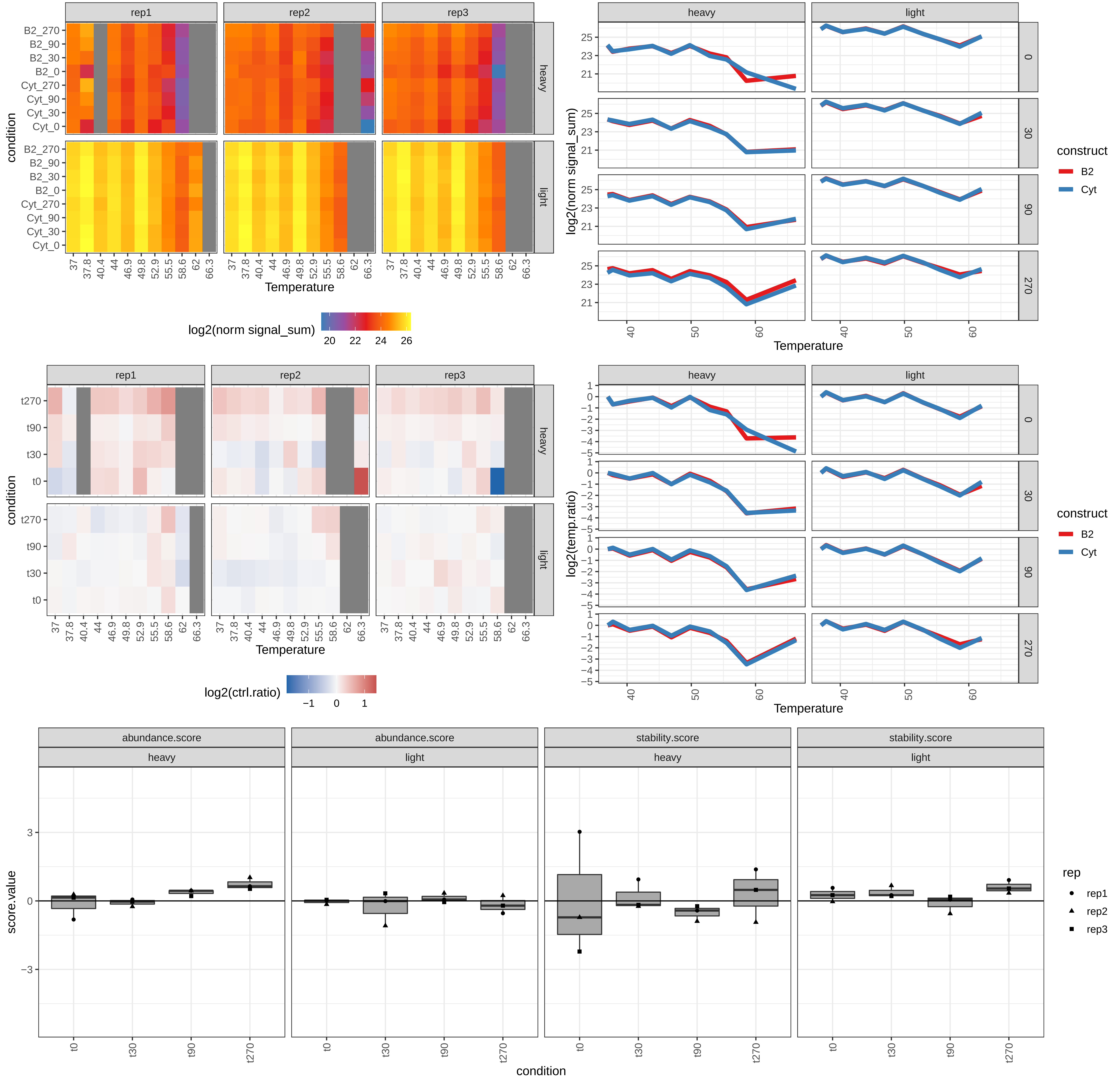

P21734 − Ubiquitin−conjugating enzyme E2 1 OS=Saccharomyces cerevisiae (strain ATCC 204508 / S288c) GN=UBC1 PE=1 SV=1

UBC8 − average.top3: 6.75700066666667 − max.qupm: 4

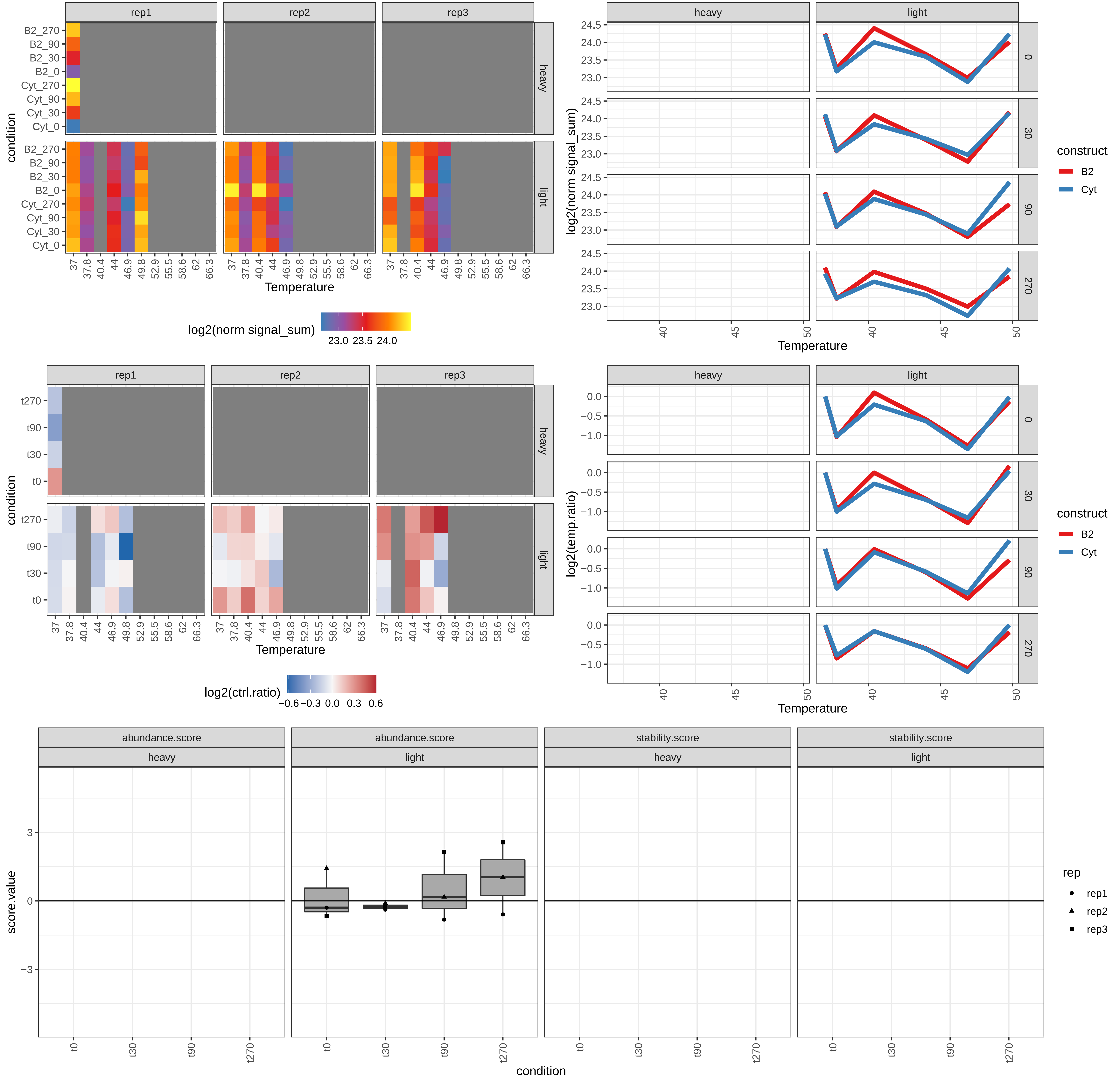

P28263 − Ubiquitin−conjugating enzyme E2−24 kDa OS=Saccharomyces cerevisiae (strain ATCC 204508 / S288c) GN=UBC8 PE=1 SV=2

UBP1 − average.top3: 6.87208888888889 − max.qupm: 9

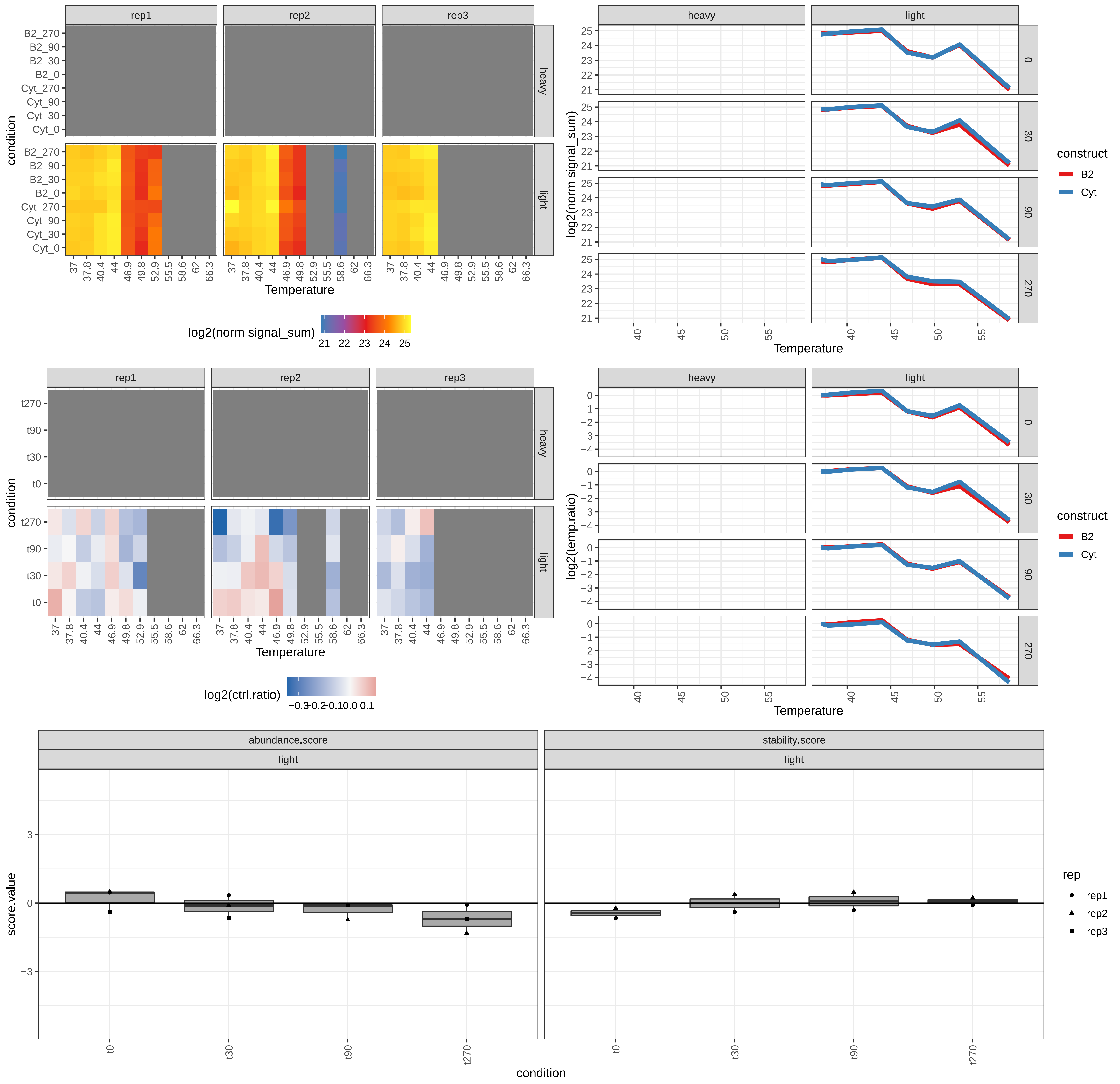

P25037 − Ubiquitin carboxyl−terminal hydrolase 1 OS=Saccharomyces cerevisiae (strain ATCC 204508 / S288c) GN=UBP1 PE=1 SV=2

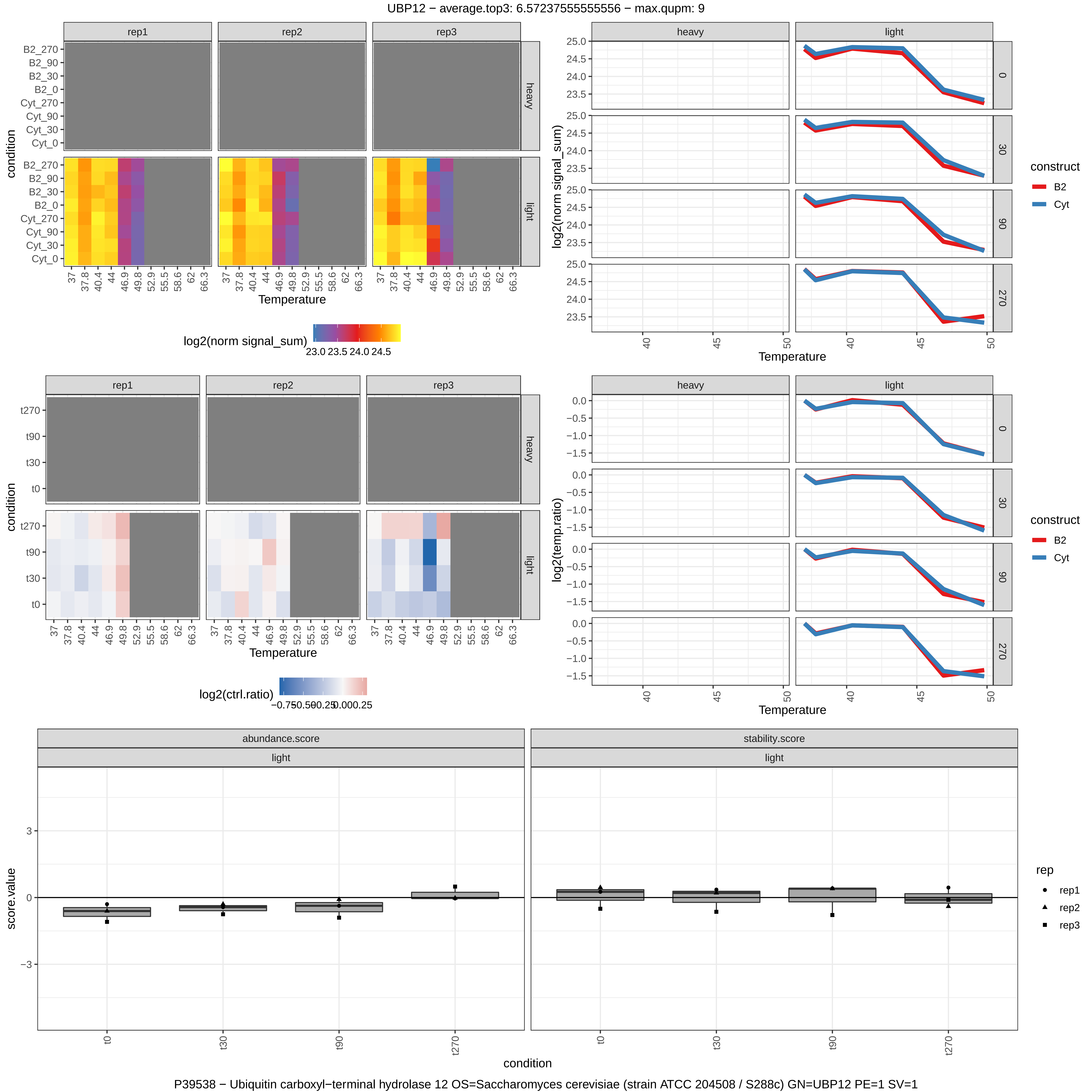

UBP12 − average.top3: 6.57237555555556 − max.qupm: 9
rep1
rep2
rep3
heavy
light
condition
Temperature
log2(norm signal_sum)
construct
B2
Cyt
log2(ctrl.ratio)
log2(temp.ratio)
abundance.score
stability.score
score.value
rep
rep1
rep2
rep3
P39538 − Ubiquitin carboxyl−terminal hydrolase 12 OS=Saccharomyces cerevisiae (strain ATCC 204508 / S288c) GN=UBP12 PE=1 SV=1

UBP14 − average.top3: 6.80562594594595 − max.qupm: 12

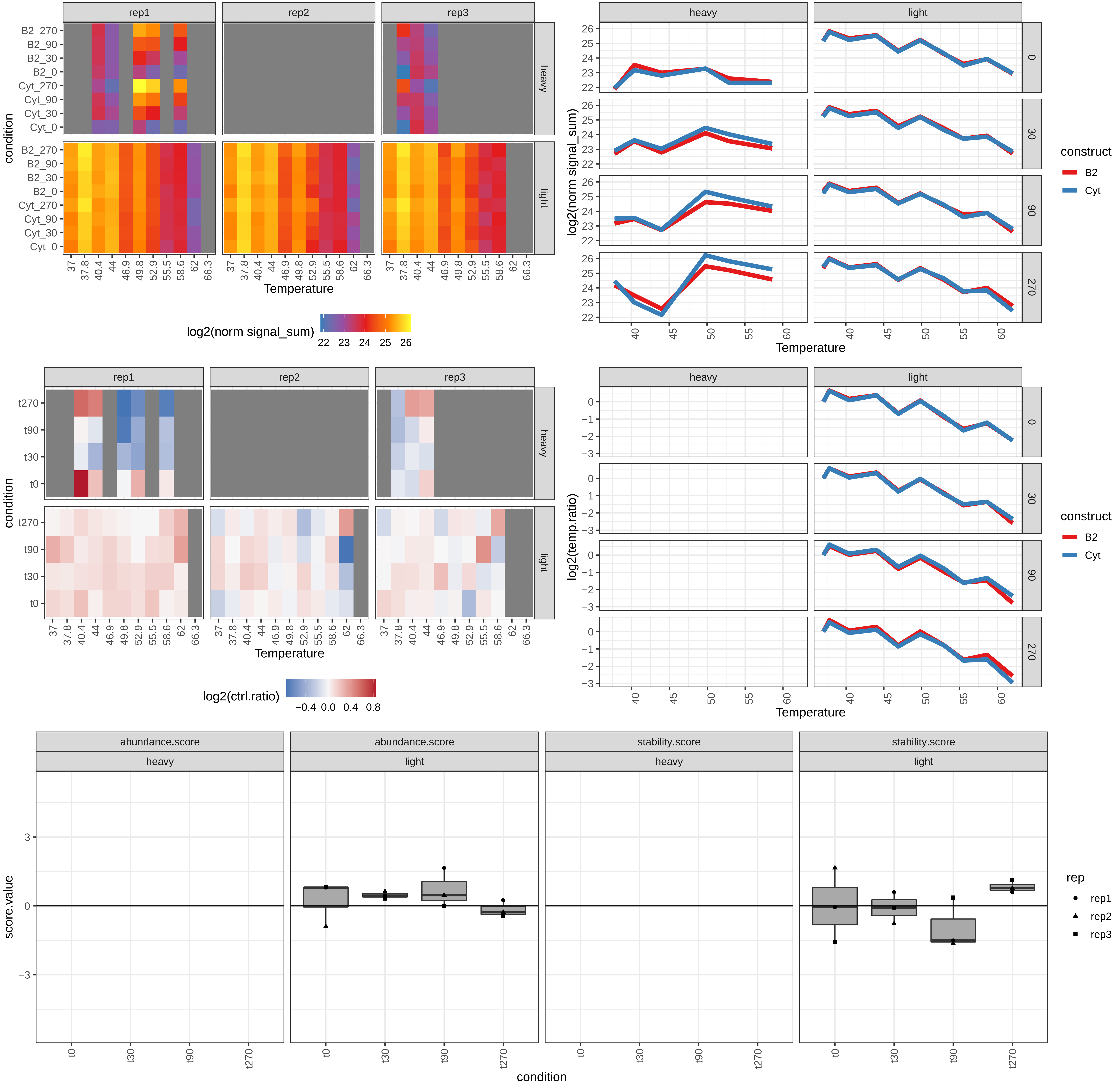

P38237 − Ubiquitin carboxyl−terminal hydrolase 14 OS=Saccharomyces cerevisiae (strain ATCC 204508 / S288c) GN=UBP14 PE=1 SV=2

UBP15 − average.top3: 6.819 − max.qupm: 19

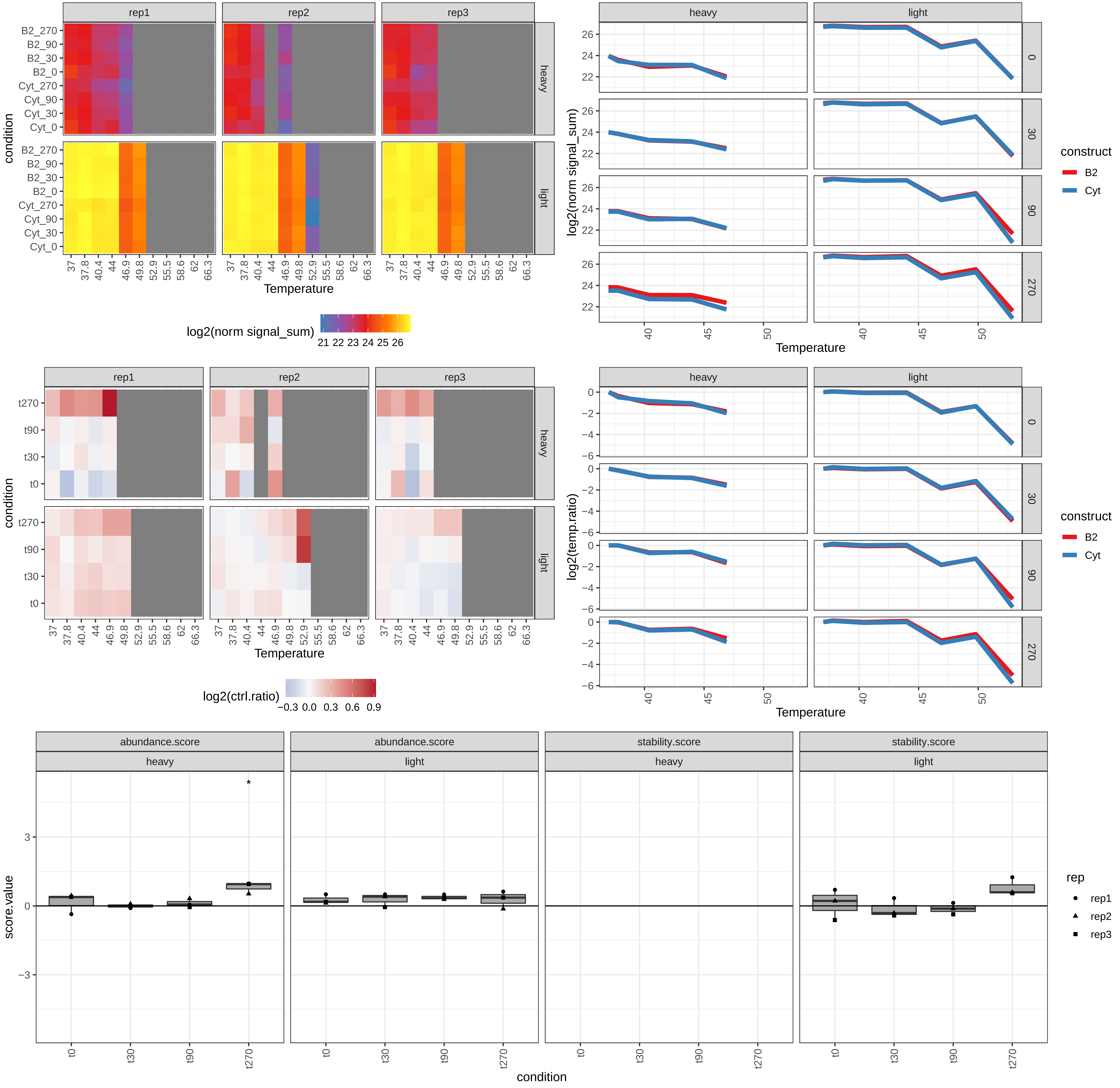

P50101 − Ubiquitin carboxyl−terminal hydrolase 15 OS=Saccharomyces cerevisiae (strain ATCC 204508 / S288c) GN=UBP15 PE=1 SV=1

UBP16 − average.top3: 6.48500666666667 − max.qupm: 5

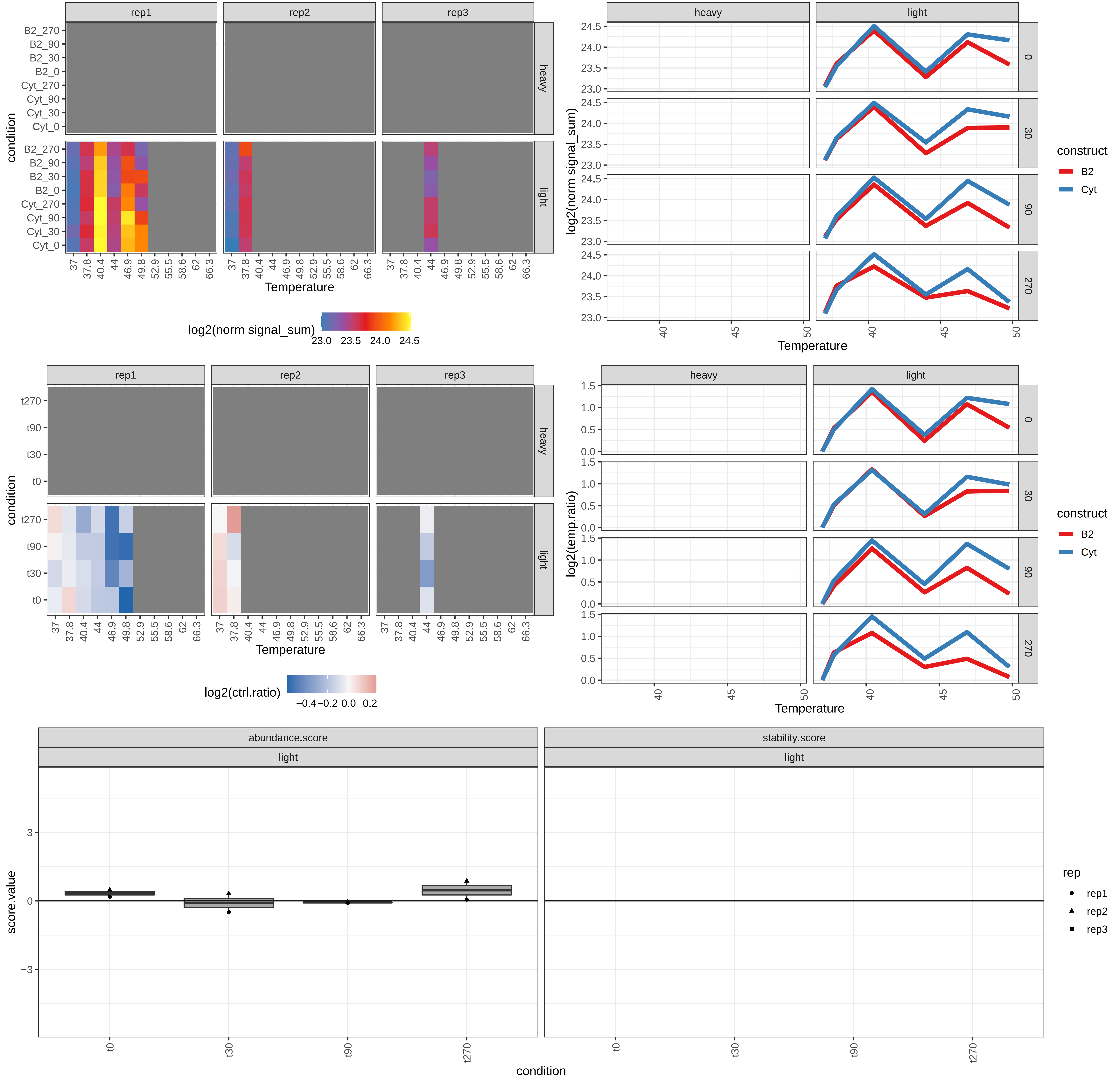

Q02863 − Ubiquitin carboxyl−terminal hydrolase 16 OS=Saccharomyces cerevisiae (strain ATCC 204508 / S288c) GN=UBP16 PE=1 SV=1

UBP2 − average.top3: 6.76145970588235 − max.qupm: 14

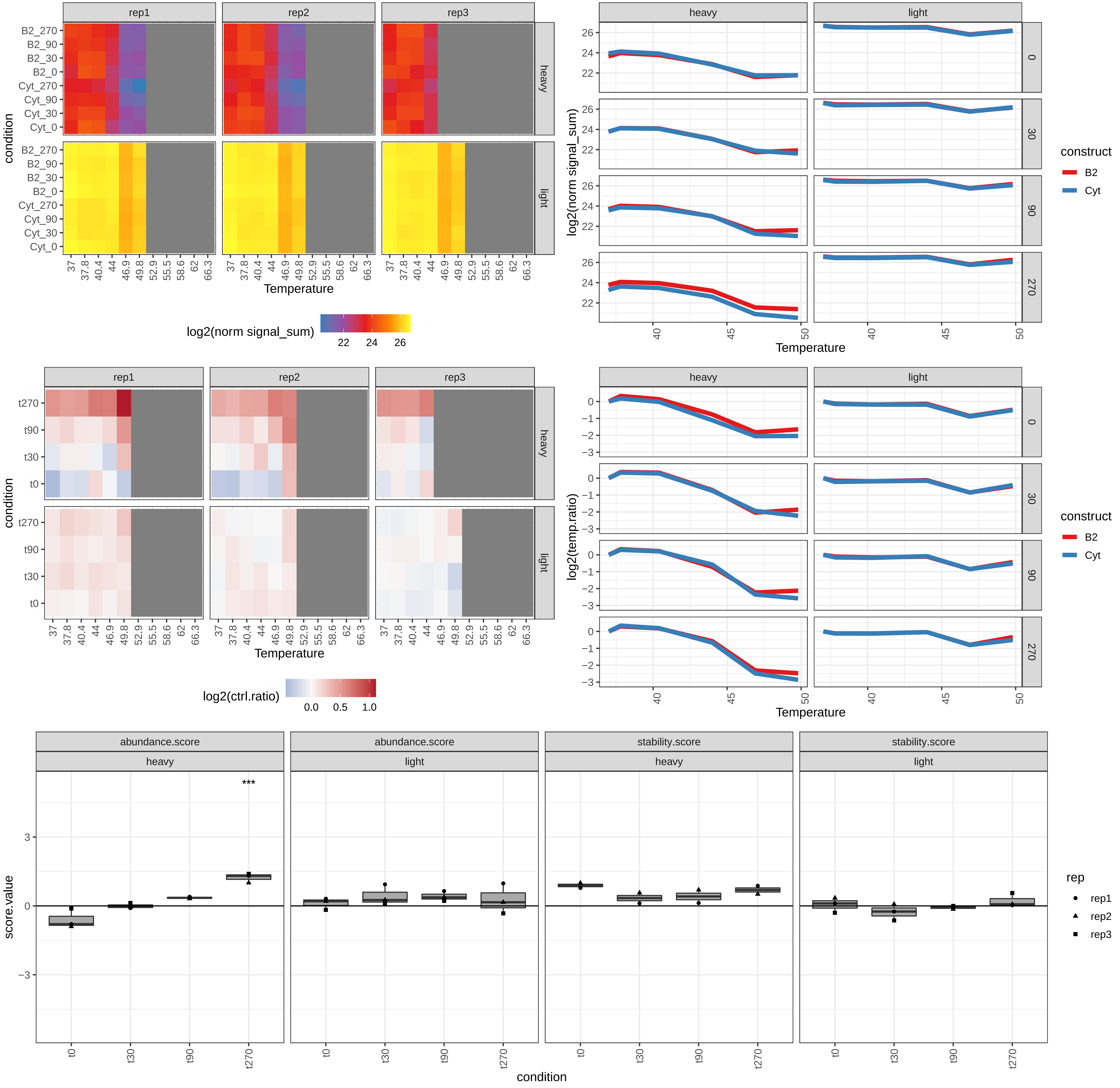

Q01476 − Ubiquitin carboxyl−terminal hydrolase 2 OS=Saccharomyces cerevisiae (strain ATCC 204508 / S288c) GN=UBP2 PE=1 SV=2

UBP3 − average.top3: 6.83167 − max.qupm: 10

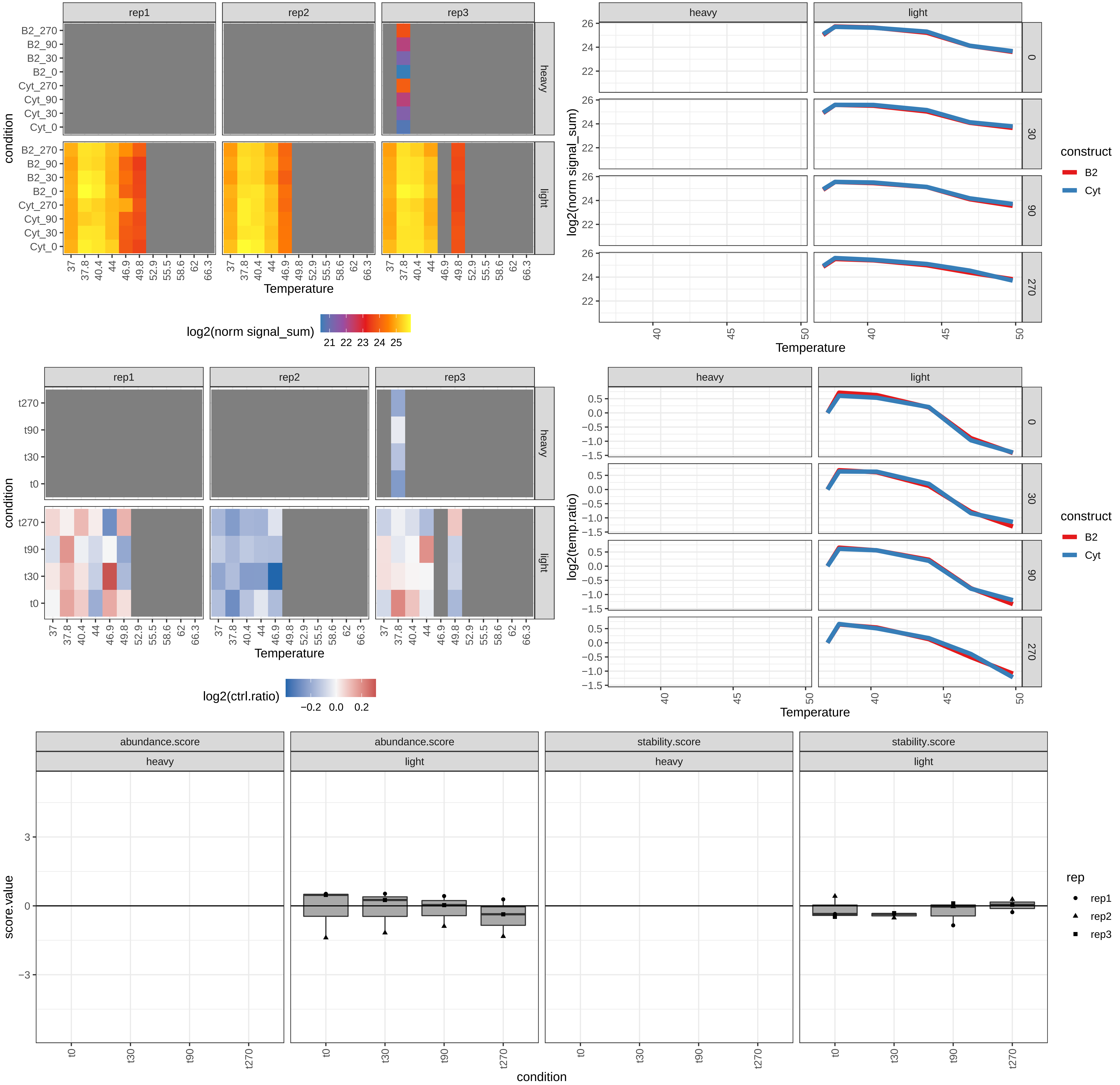

Q01477 − Ubiquitin carboxyl−terminal hydrolase 3 OS=Saccharomyces cerevisiae (strain ATCC 204508 / S288c) GN=UBP3 PE=1 SV=1

UBP5 − average.top3: 6.29574272727273 − max.qupm: 4

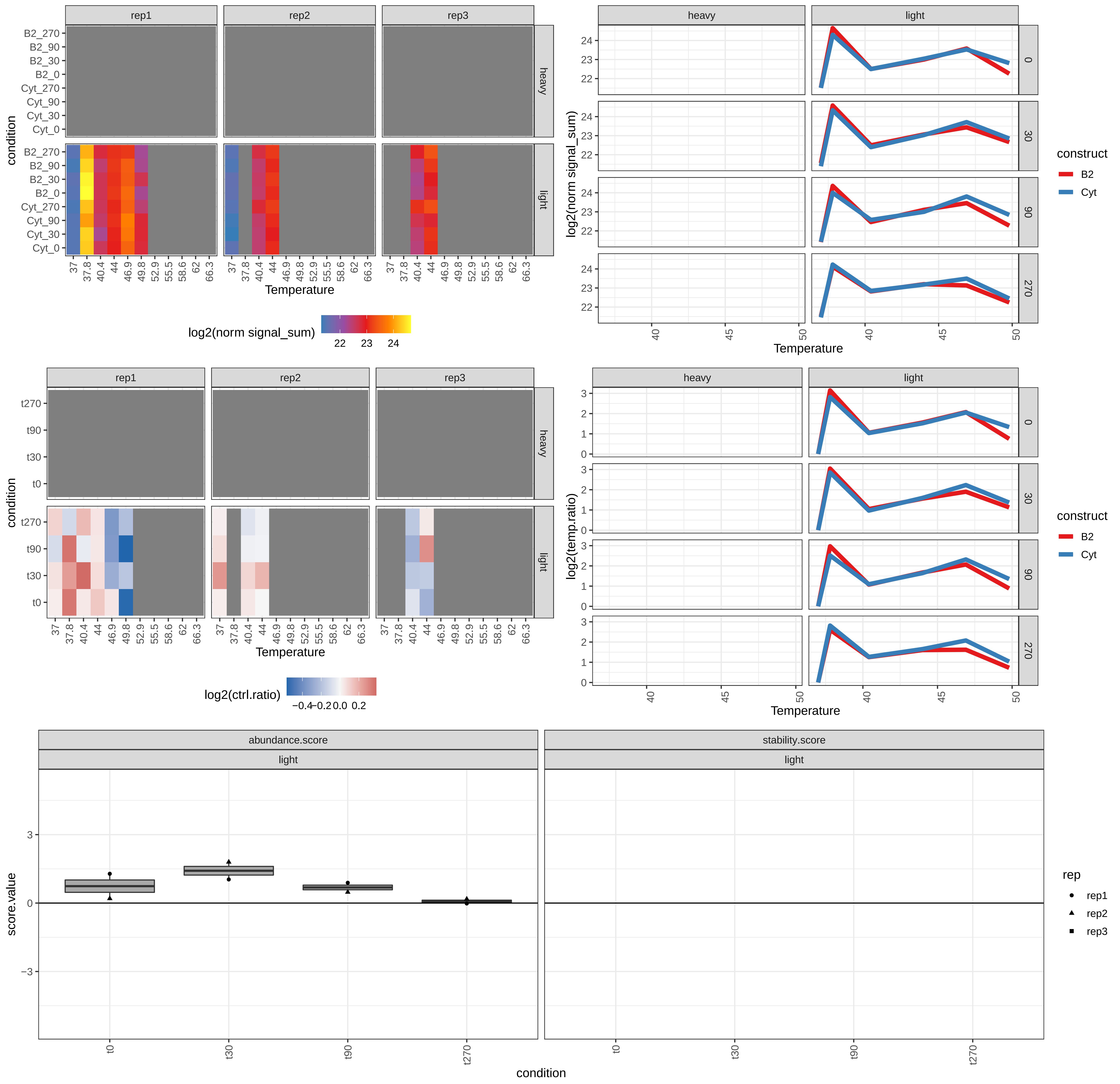

P39944 − Ubiquitin carboxyl−terminal hydrolase 5 OS=Saccharomyces cerevisiae (strain ATCC 204508 / S288c) GN=UBP5 PE=1 SV=1

UBP6 − average.top3: 7.17074111111111 − max.qupm: 13

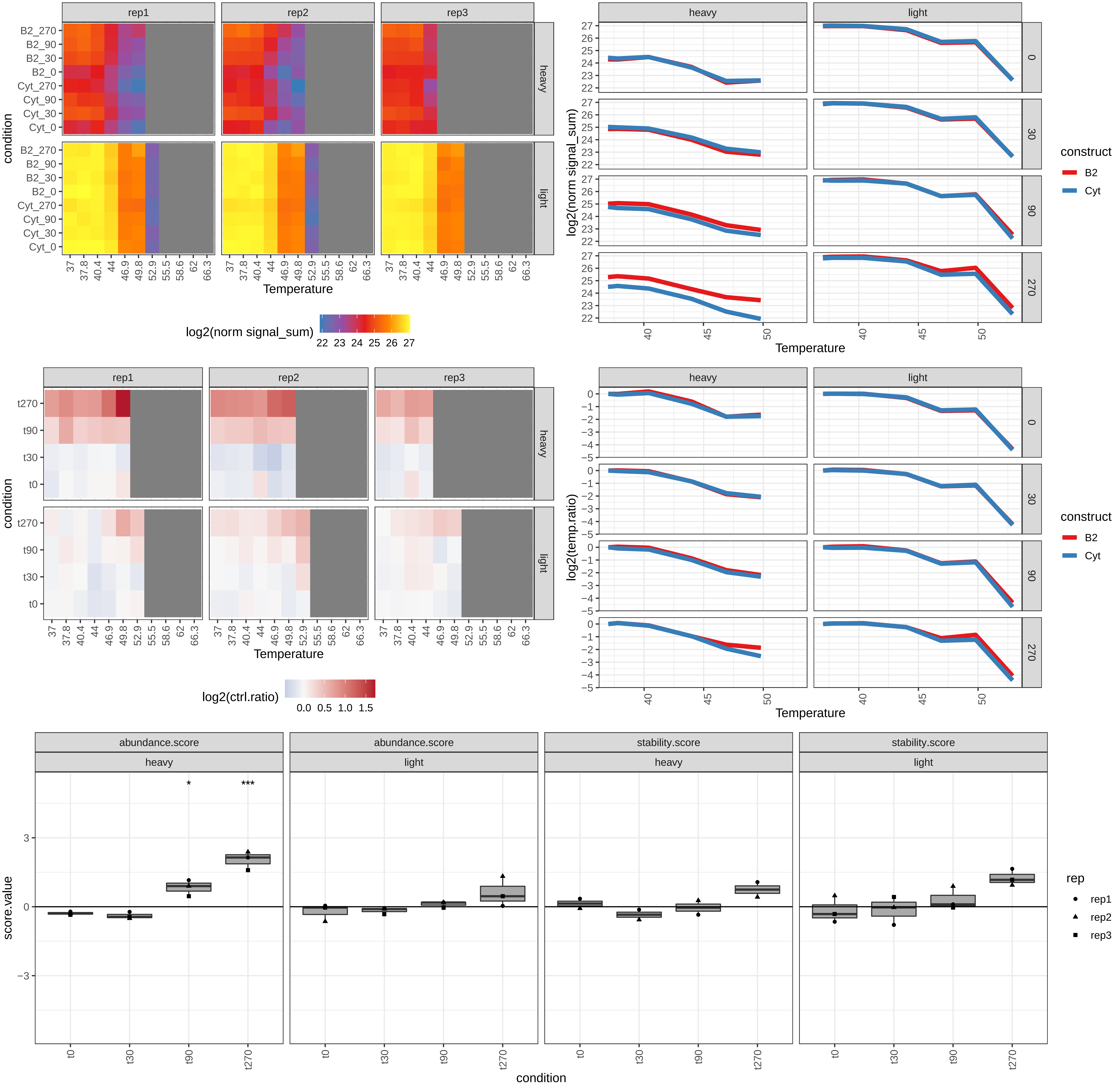

P43593 − Ubiquitin carboxyl−terminal hydrolase 6 OS=Saccharomyces cerevisiae (strain ATCC 204508 / S288c) GN=UBP6 PE=1 SV=1

UBR1 − average.top3: 6.66956279069767 − max.qupm: 19

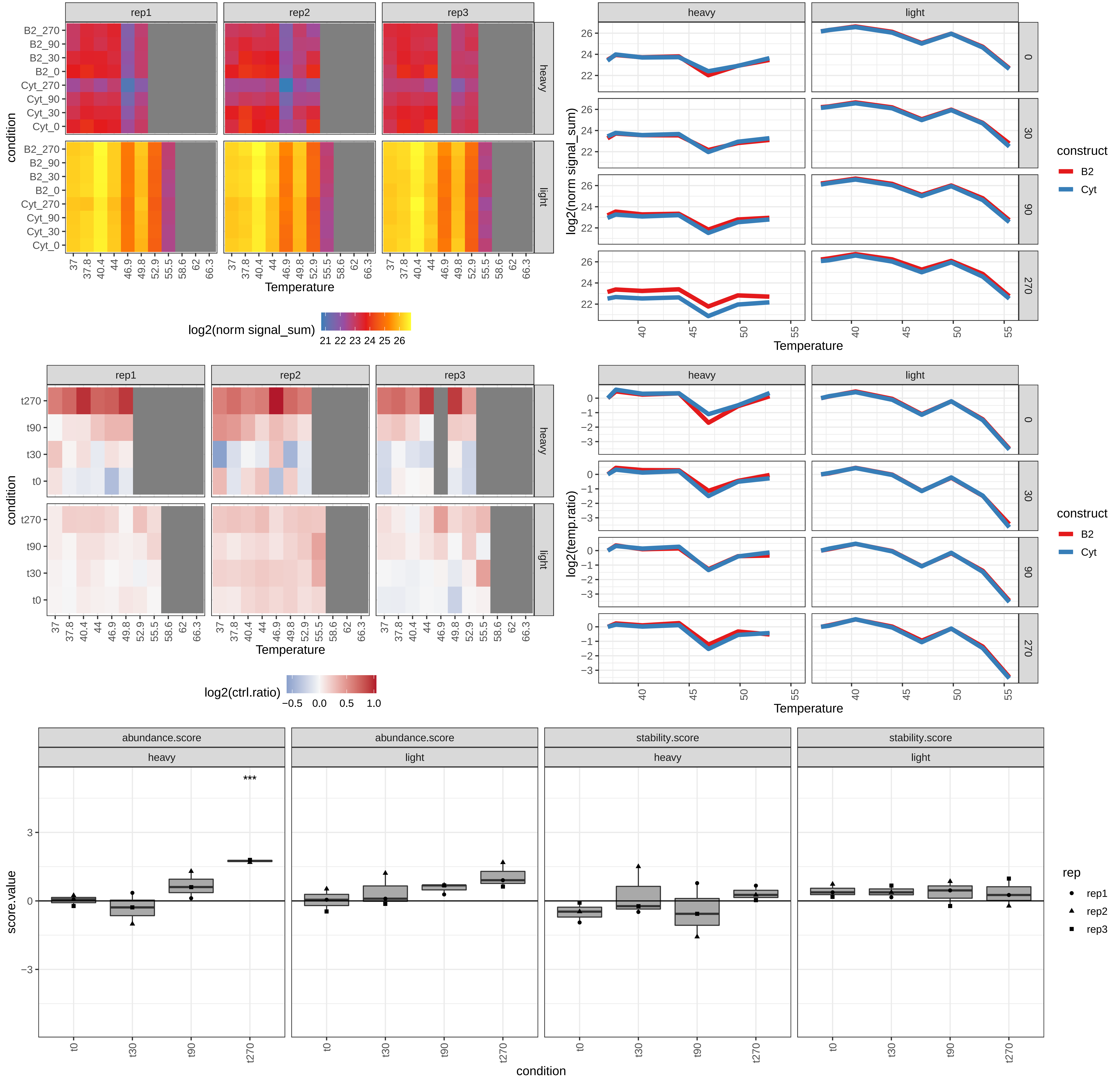

P19812 − E3 ubiquitin−protein ligase UBR1 OS=Saccharomyces cerevisiae (strain ATCC 204508 / S288c) GN=UBR1 PE=1 SV=1

UBX2 − average.top3: 6.54922692307692 − max.qupm: 4

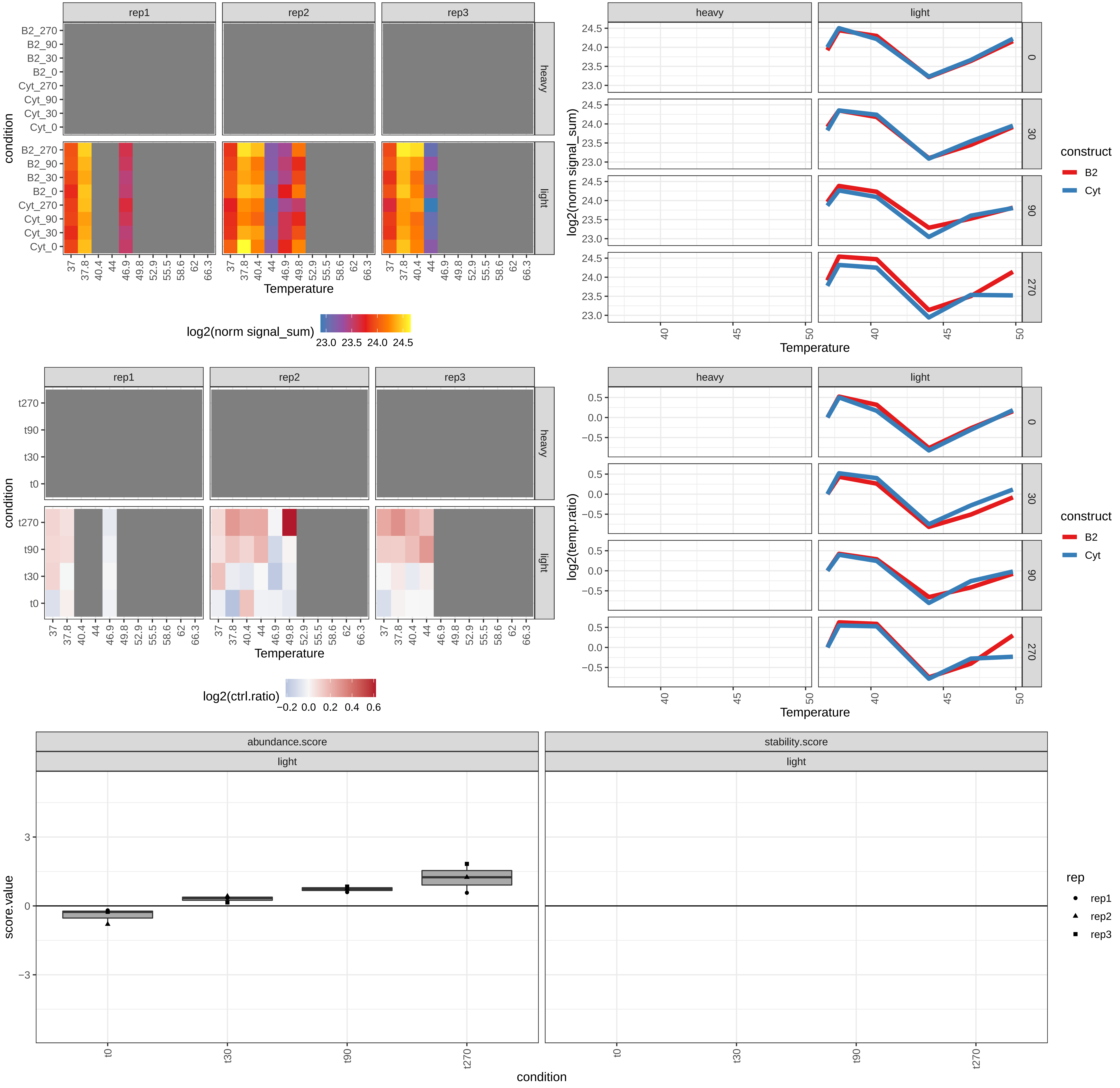

Q04228 − UBX domain−containing protein 2 OS=Saccharomyces cerevisiae (strain ATCC 204508 / S288c) GN=UBX2 PE=1 SV=1

UBX3 − average.top3: 6.55619928571429 − max.qupm: 6

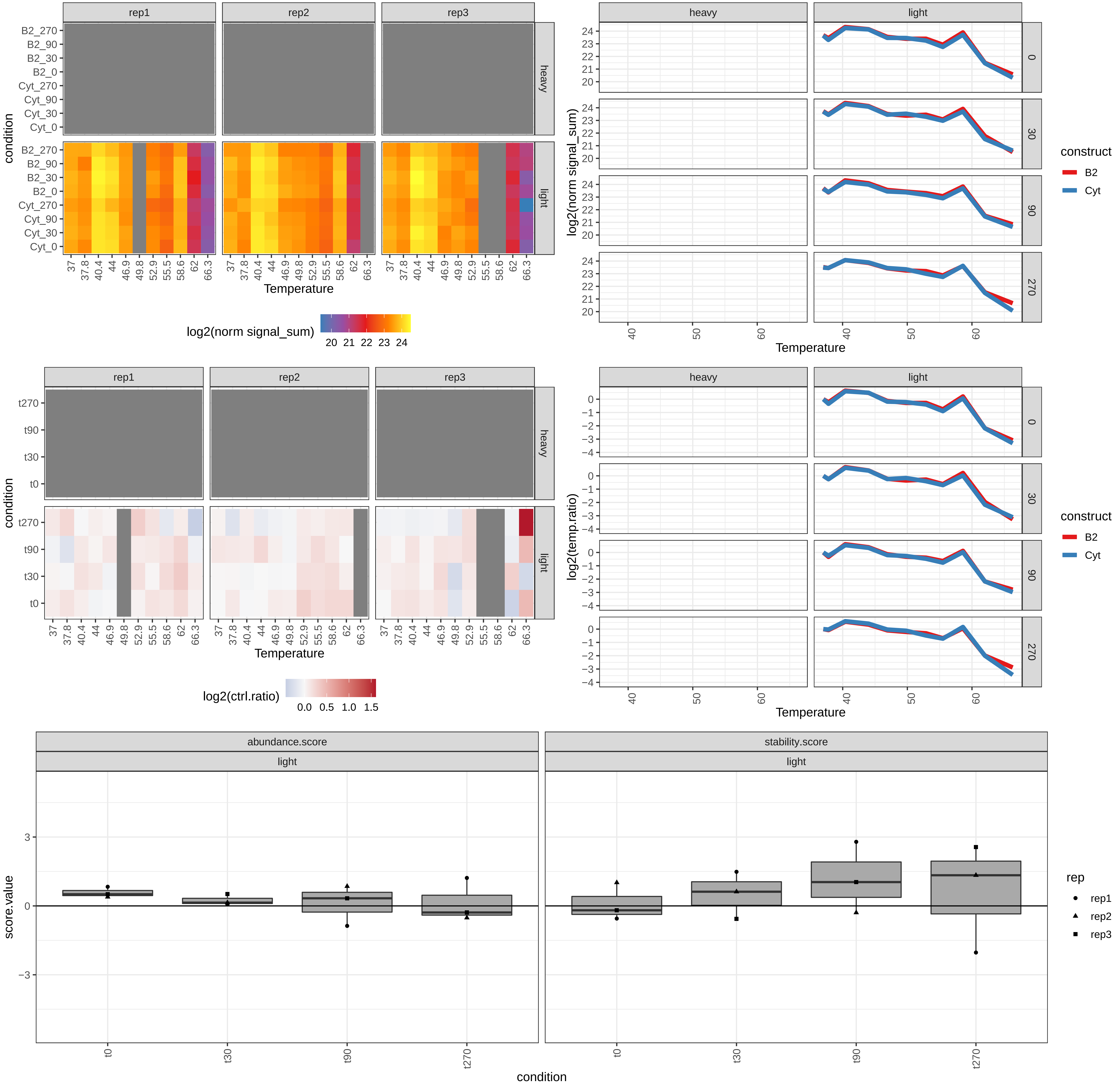

Q12229 − UBX domain−containing protein 3 OS=Saccharomyces cerevisiae (strain ATCC 204508 / S288c) GN=UBX3 PE=1 SV=1

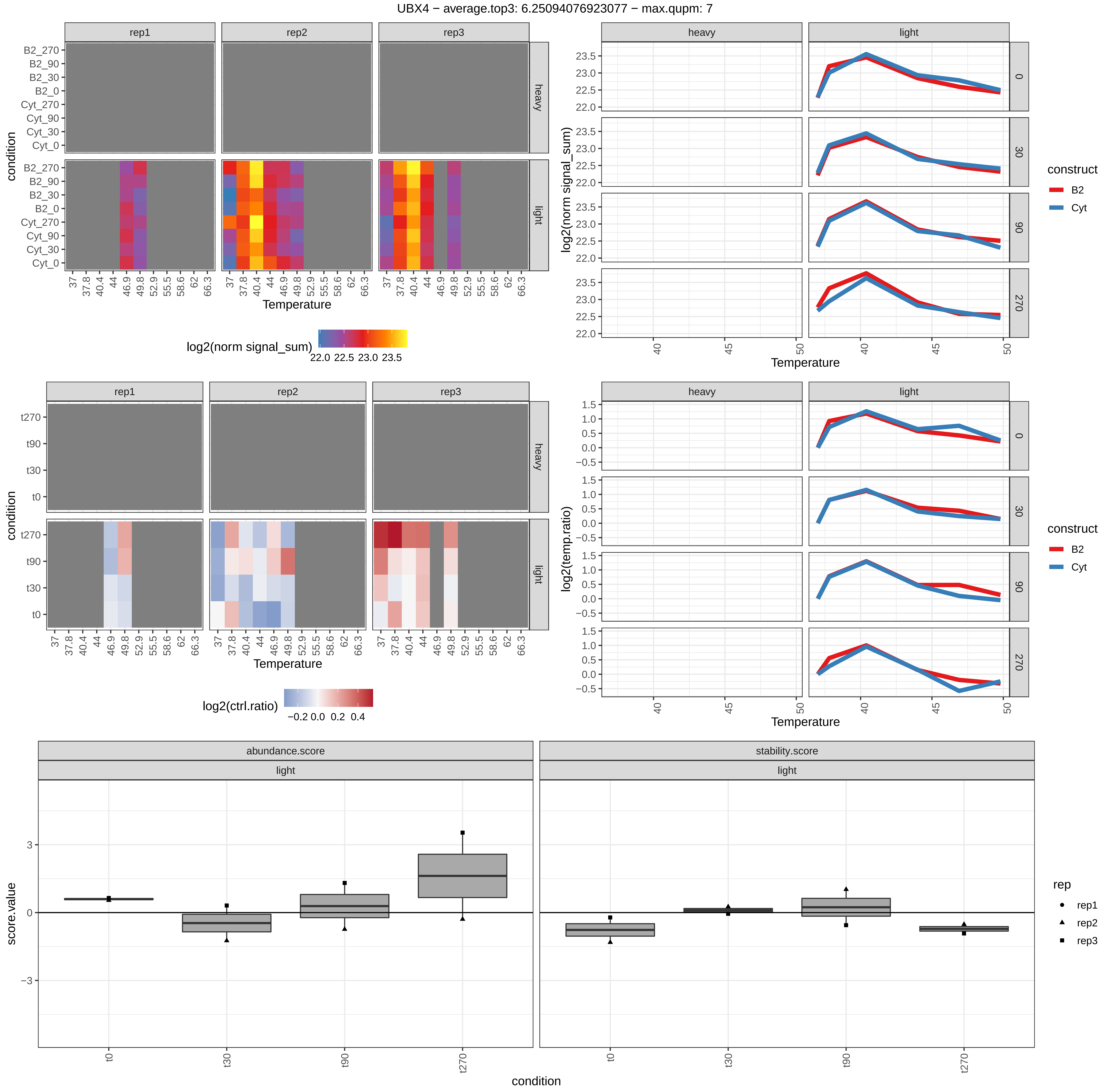

P54730 − UBX domain−containing protein 4 OS=Saccharomyces cerevisiae (strain ATCC 204508 / S288c) GN=UBX4 PE=1 SV=1

UBX5 − average.top3: 6.77452529411765 − max.qupm: 7

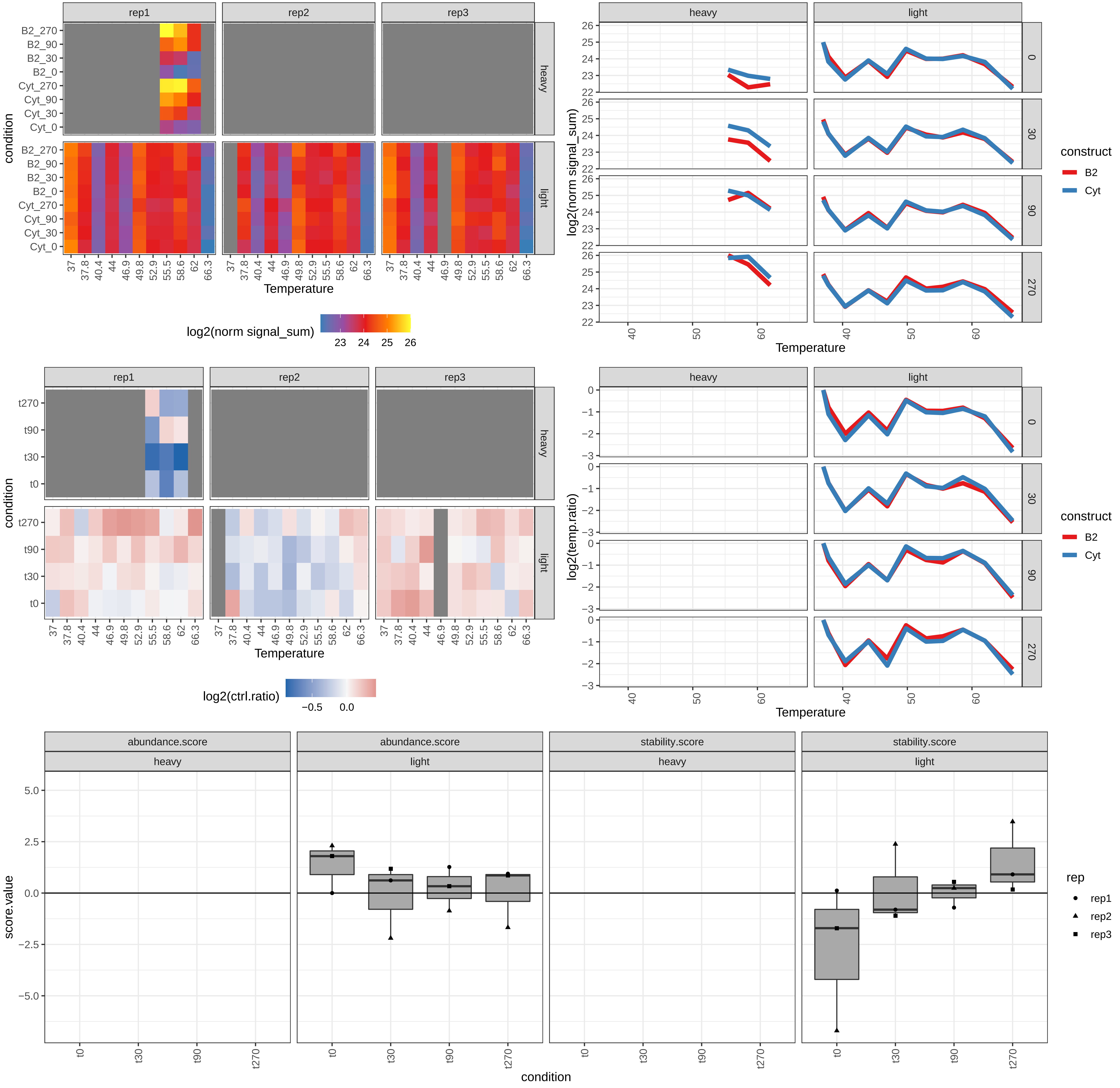

Q06682 − UBX domain−containing protein 5 OS=Saccharomyces cerevisiae (strain ATCC 204508 / S288c) GN=UBX5 PE=1 SV=1

UBX7 − average.top3: 6.51730421052632 − max.qupm: 6

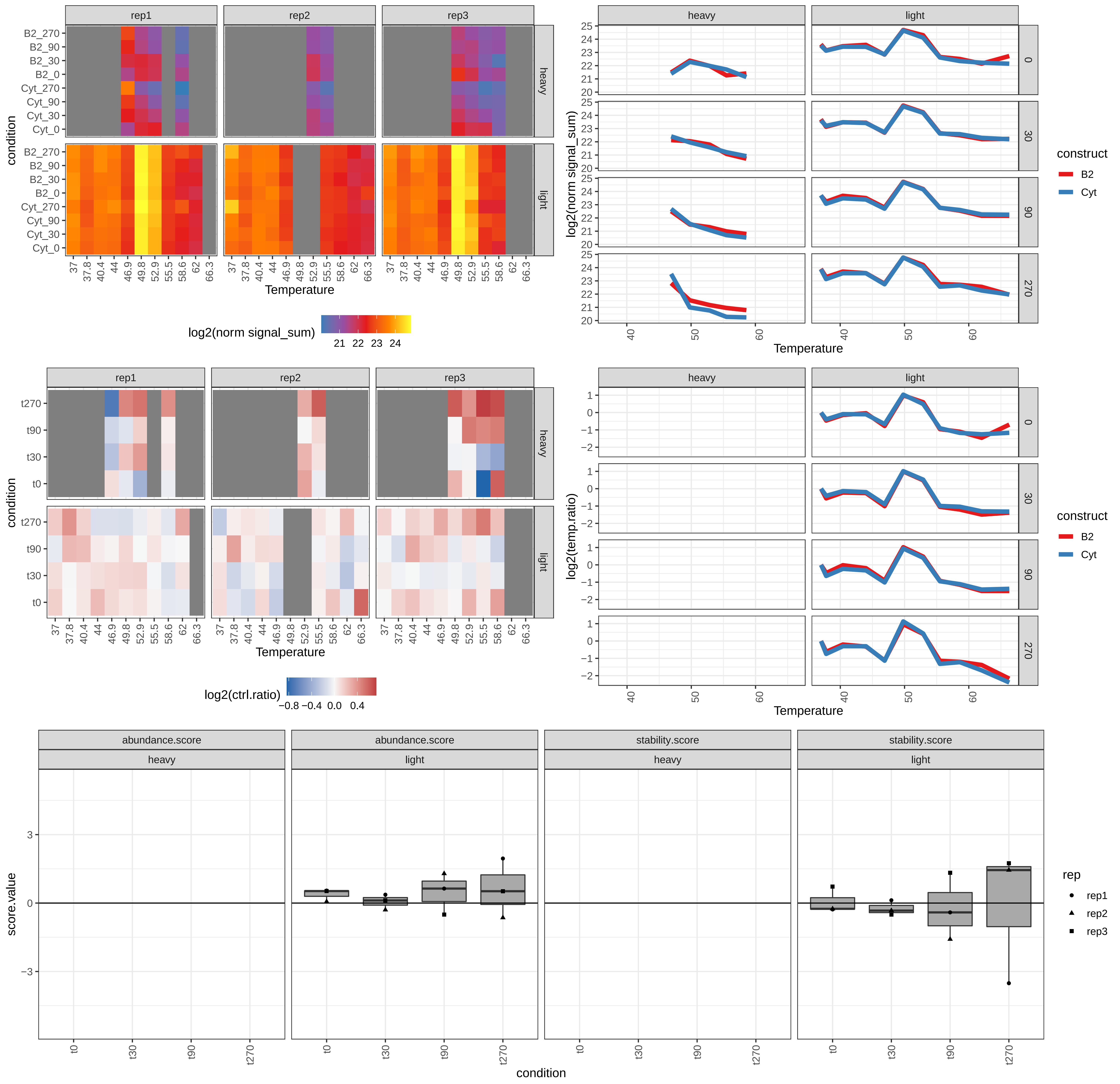

P38349 − UBX domain−containing protein 7 OS=Saccharomyces cerevisiae (strain ATCC 204508 / S288c) GN=UBX7 PE=1 SV=1

UFD1 − average.top3: 6.86700021276596 − max.qupm: 10

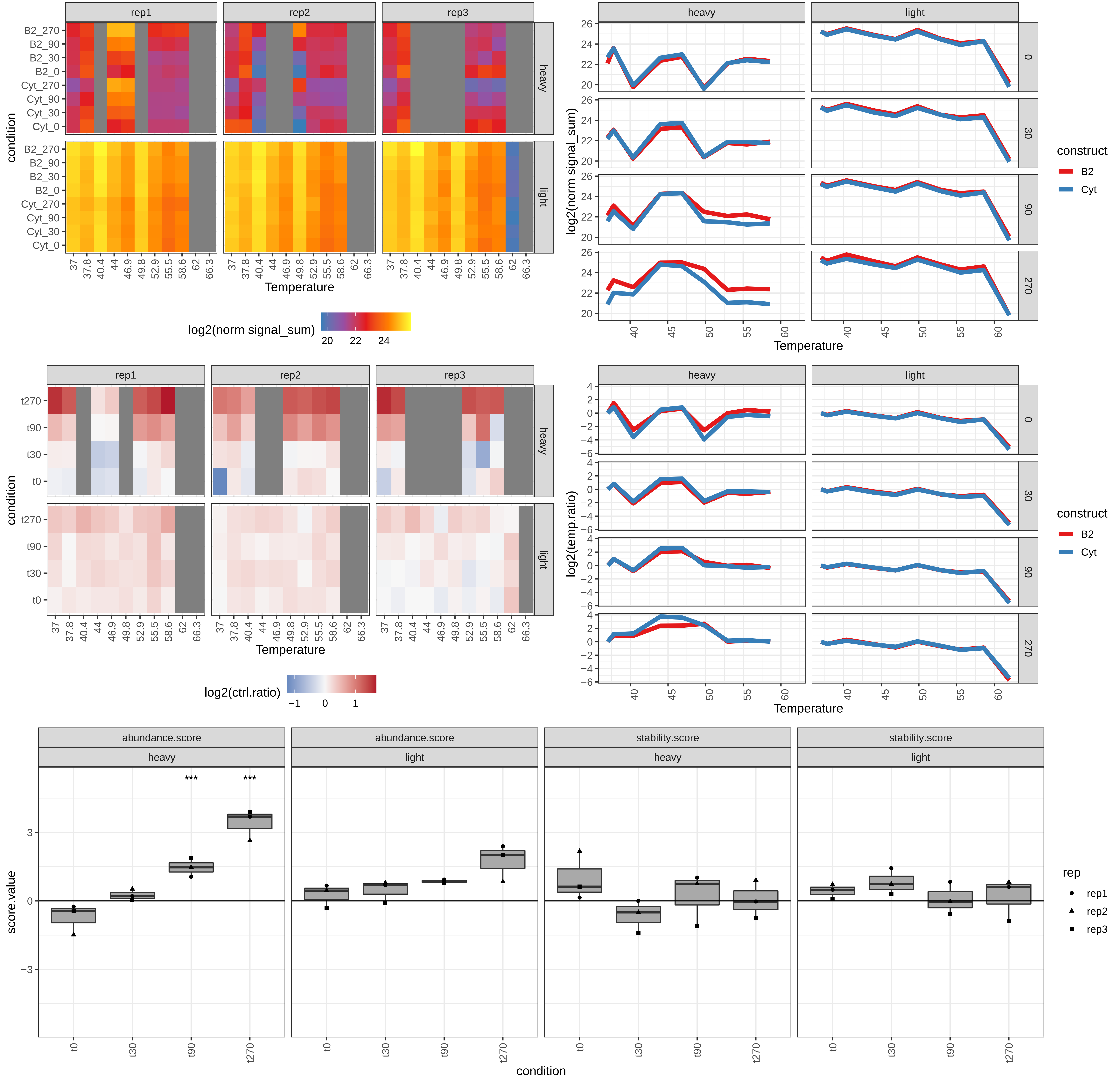

P53044 − Ubiquitin fusion degradation protein 1 OS=Saccharomyces cerevisiae (strain ATCC 204508 / S288c) GN=UFD1 PE=1 SV=1

UFD2 − average.top3: 7.17756666666667 − max.qupm: 13

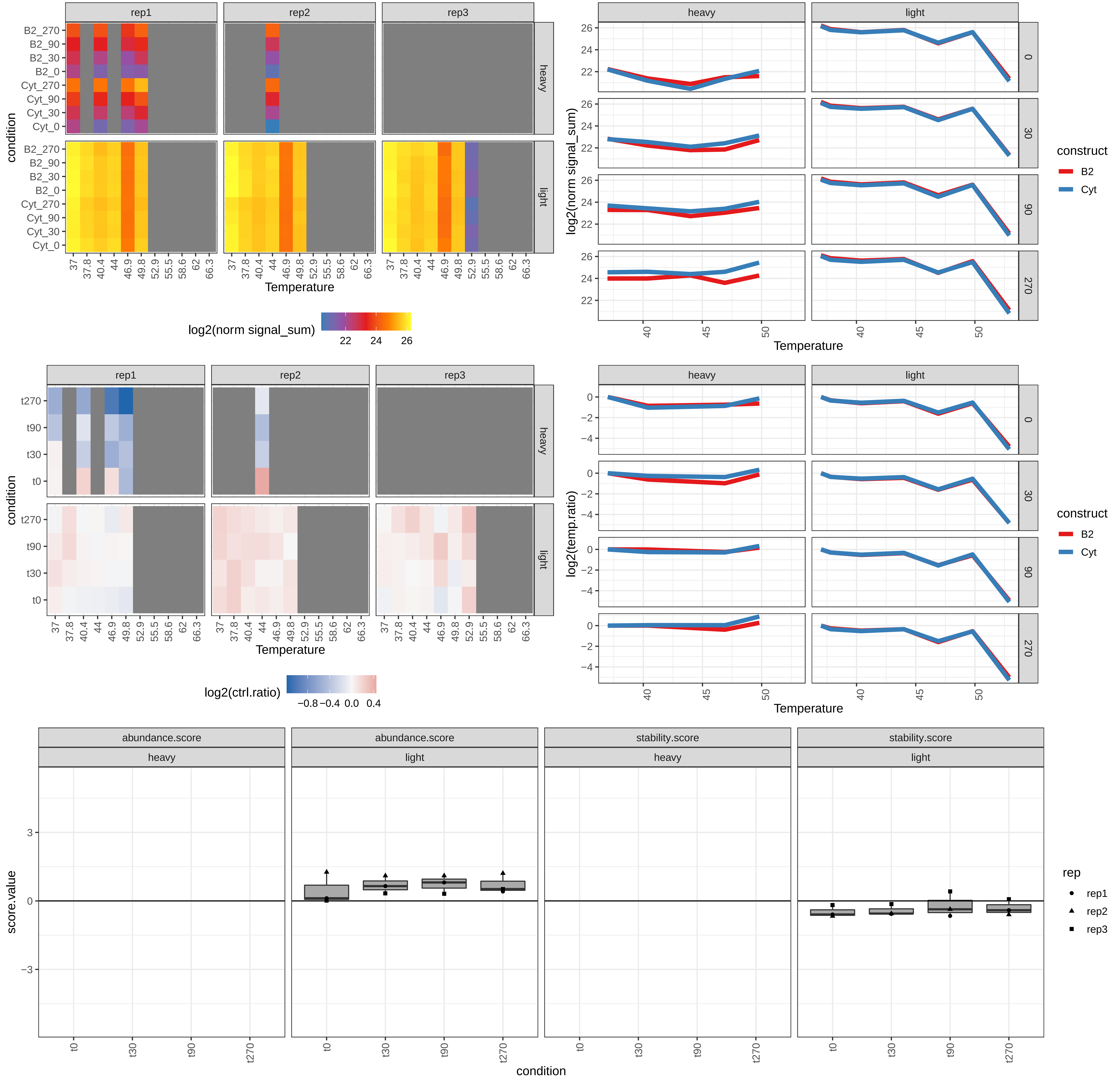

P54860 − E4 ubiquitin−protein ligase UFD2 OS=Saccharomyces cerevisiae (strain ATCC 204508 / S288c) GN=UFD2 PE=1 SV=3

UFD4 − average.top3: 6.93404588235294 − max.qupm: 12

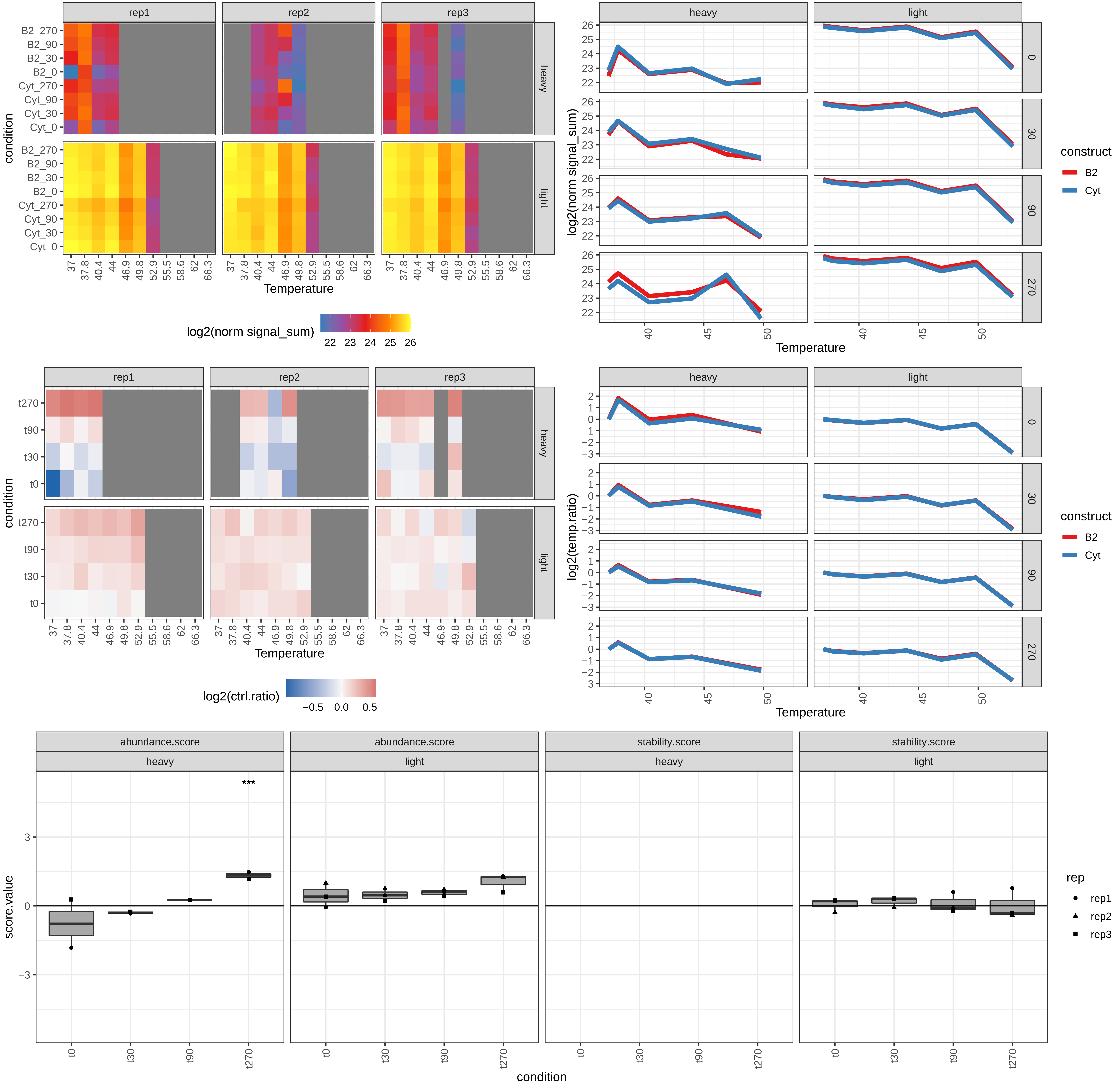

P33202 − Ubiquitin fusion degradation protein 4 OS=Saccharomyces cerevisiae (strain ATCC 204508 / S288c) GN=UFD4 PE=1 SV=1

UGA1 − average.top3: 7.18691075 − max.qupm: 18

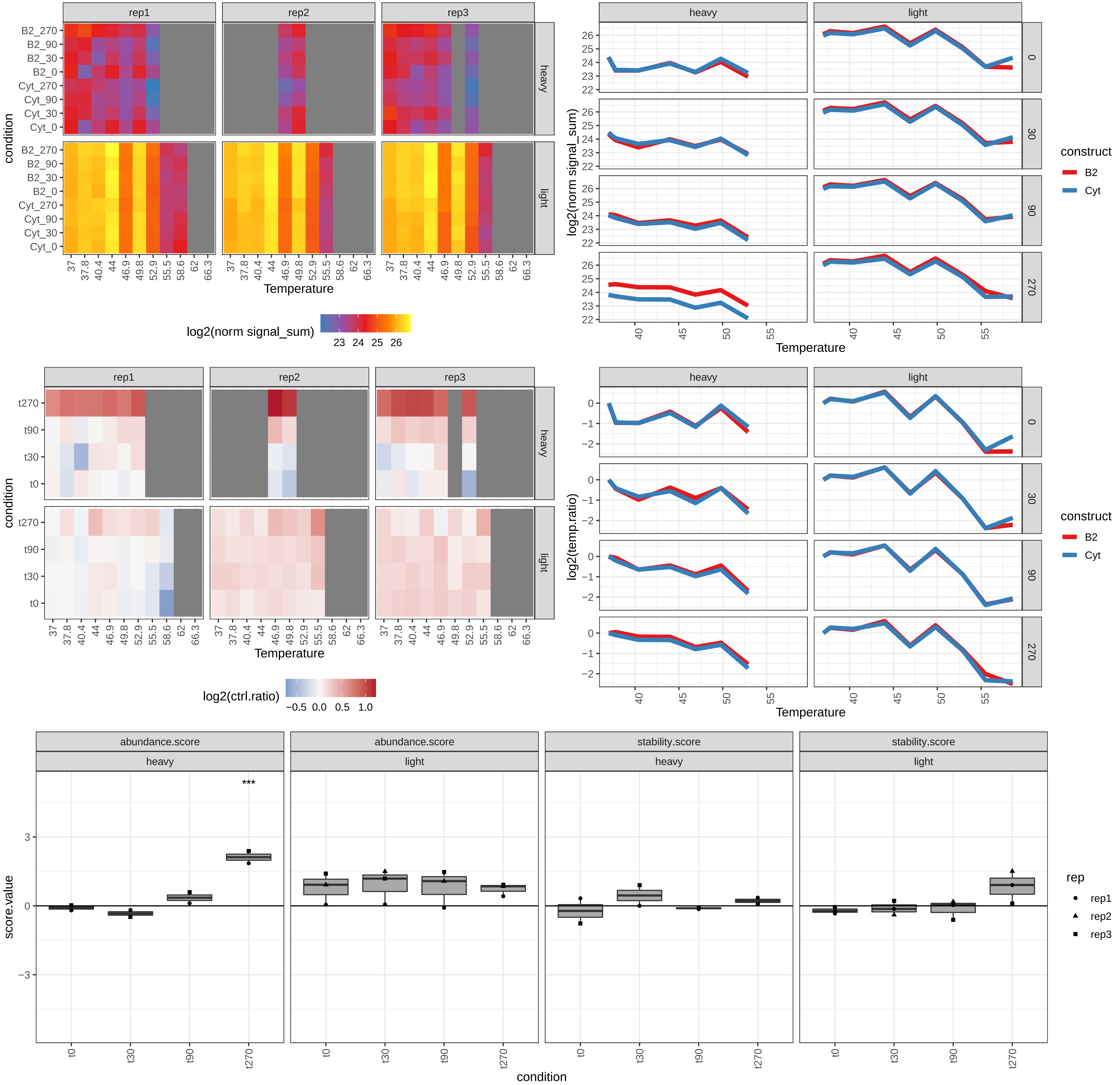

P17649 − 4−aminobutyrate aminotransferase OS=Saccharomyces cerevisiae (strain ATCC 204508 / S288c) GN=UGA1 PE=1 SV=2

UGA2 − average.top3: 7.04685745454545 − max.qupm: 13

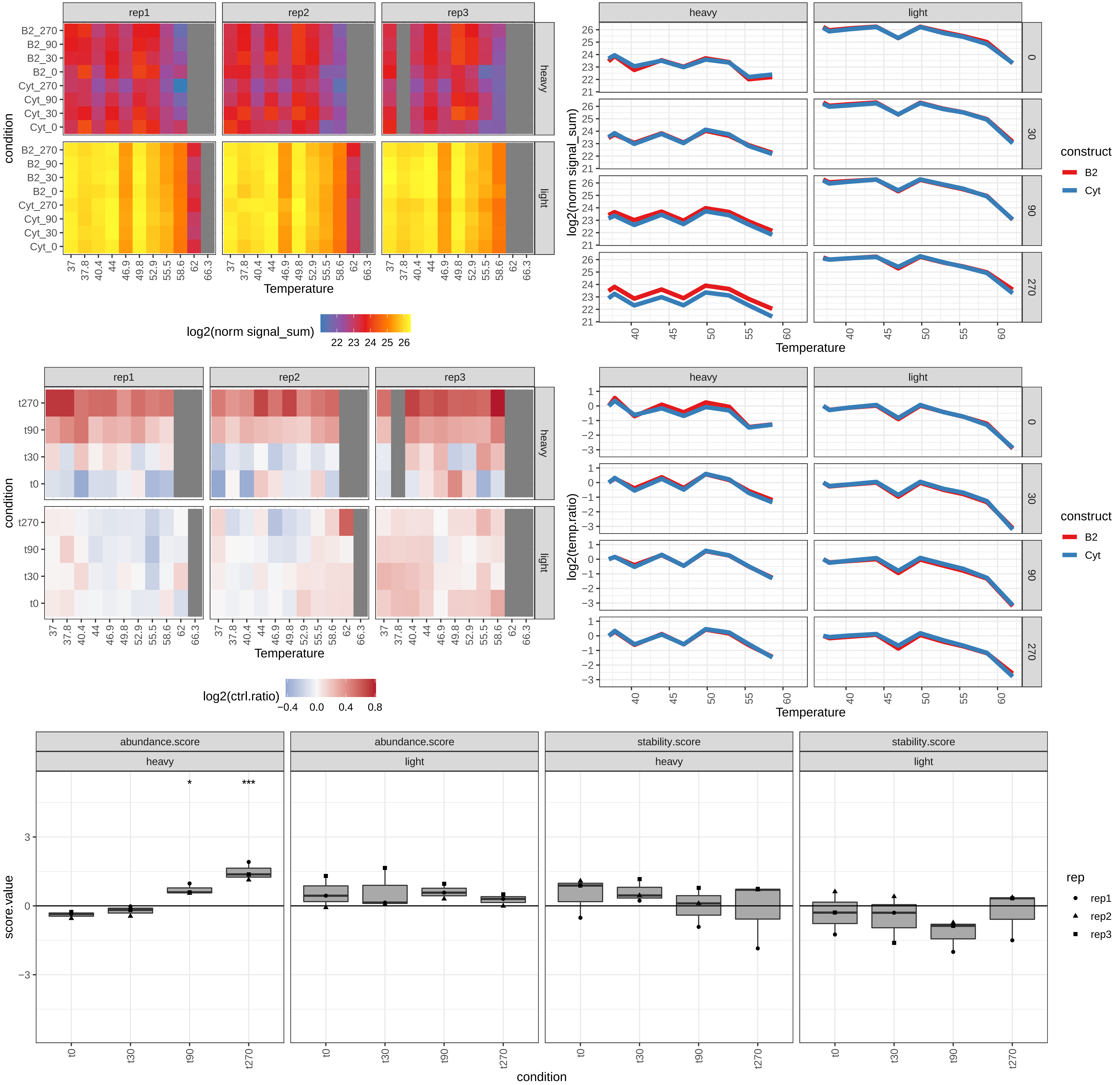

P38067 − Succinate−semialdehyde dehydrogenase [NADP(+)] OS=Saccharomyces cerevisiae (strain ATCC 204508 / S288c) GN=UGA2 PE=1 SV=2

UGP1 − average.top3: 7.86052901960784 − max.qupm: 20

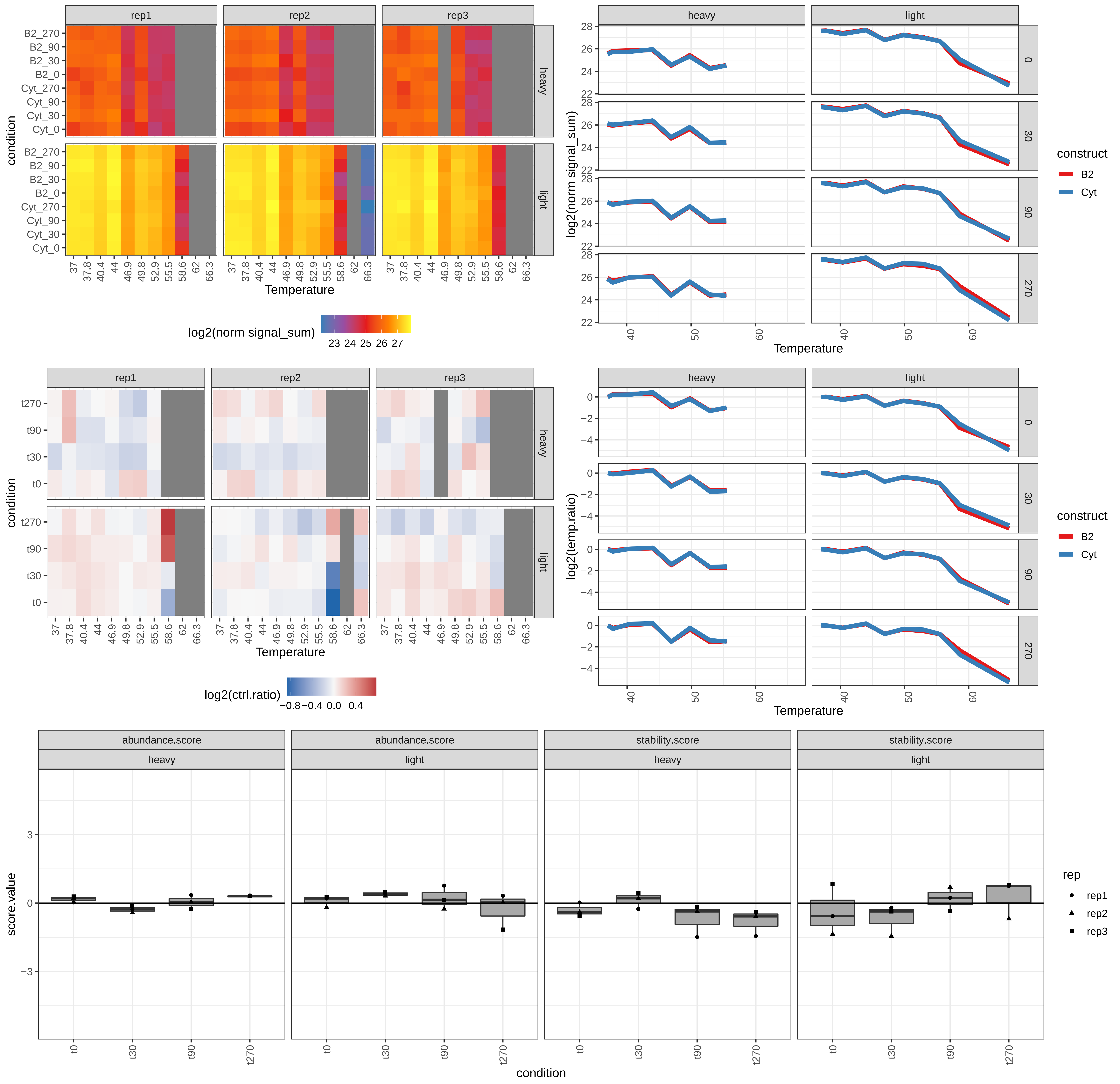

P32861 − UTP−−glucose−1−phosphate uridylyltransferase OS=Saccharomyces cerevisiae (strain ATCC 204508 / S288c) GN=UGP1 PE=1 SV=1

UIP4 − average.top3: 6.85152466666667 − max.qupm: 7

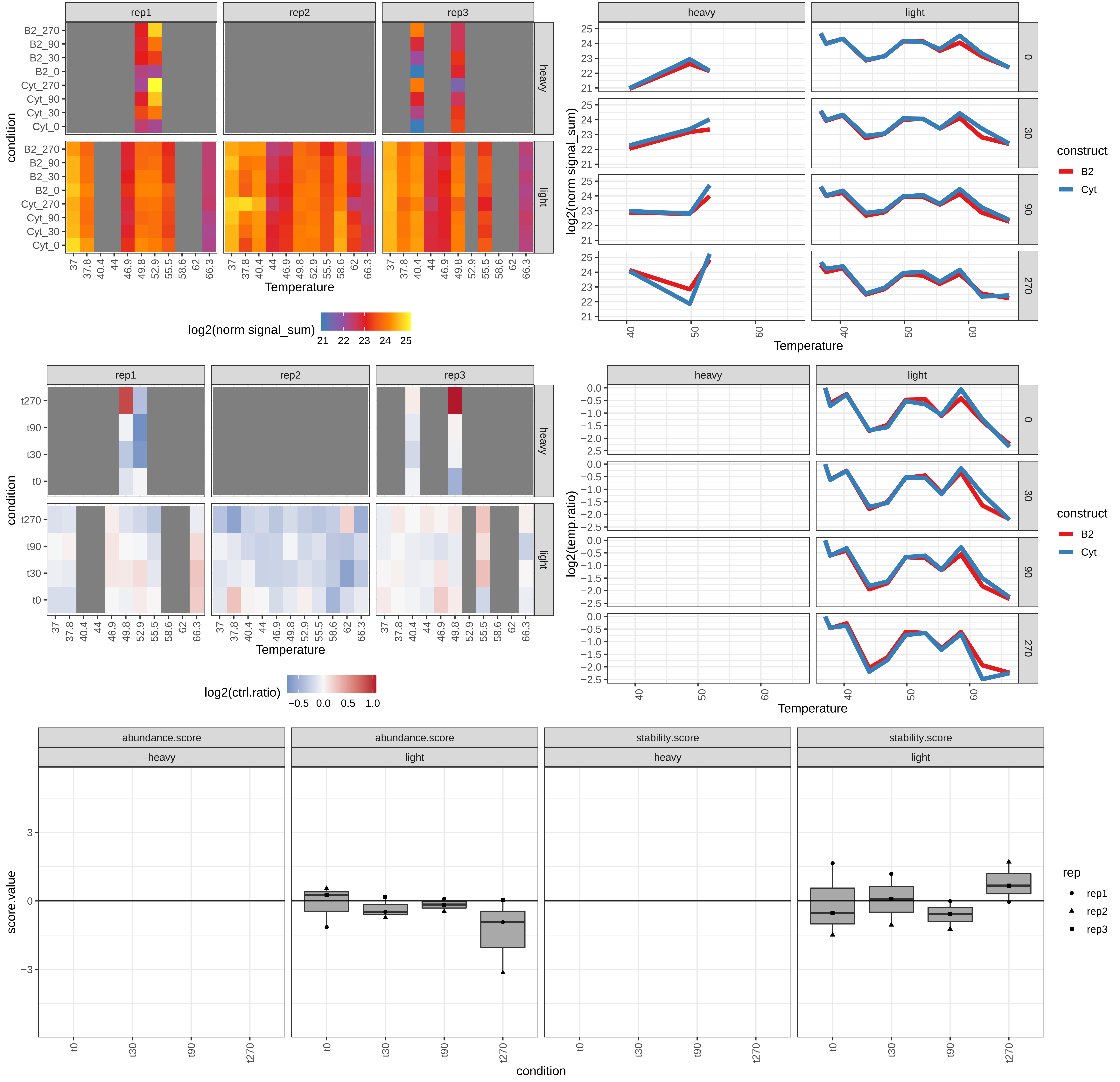

Q08926 − ULP1−interacting protein 4 OS=Saccharomyces cerevisiae (strain ATCC 204508 / S288c) GN=UIP4 PE=1 SV=1

ULA1 − average.top3: 6.35410111111111 − max.qupm: 3

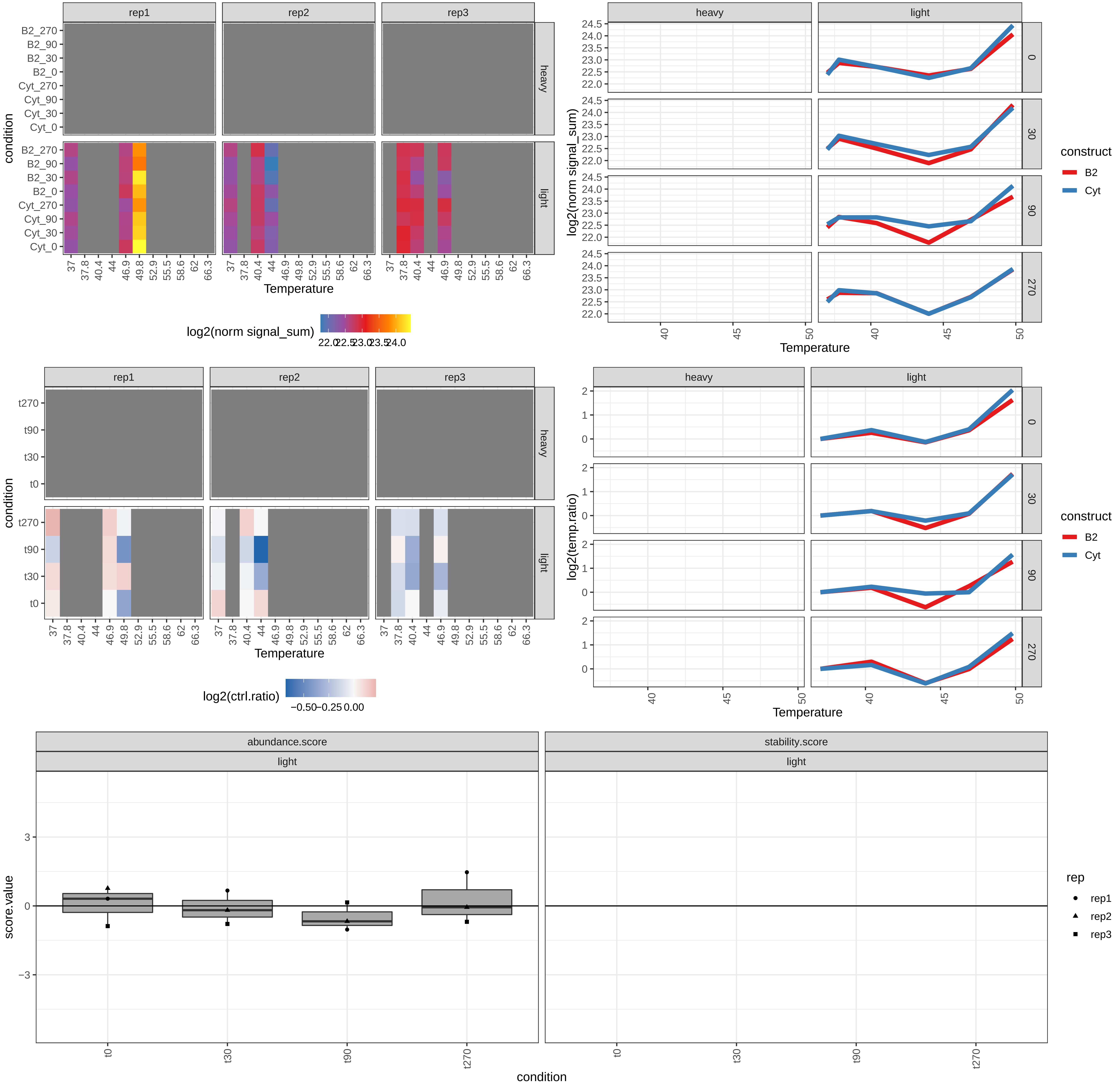

Q12059 − NEDD8−activating enzyme E1 regulatory subunit OS=Saccharomyces cerevisiae (strain ATCC 204508 / S288c) GN=ULA1 PE=1 SV=1

UME1 − average.top3: 7.08708588235294 − max.qupm: 7

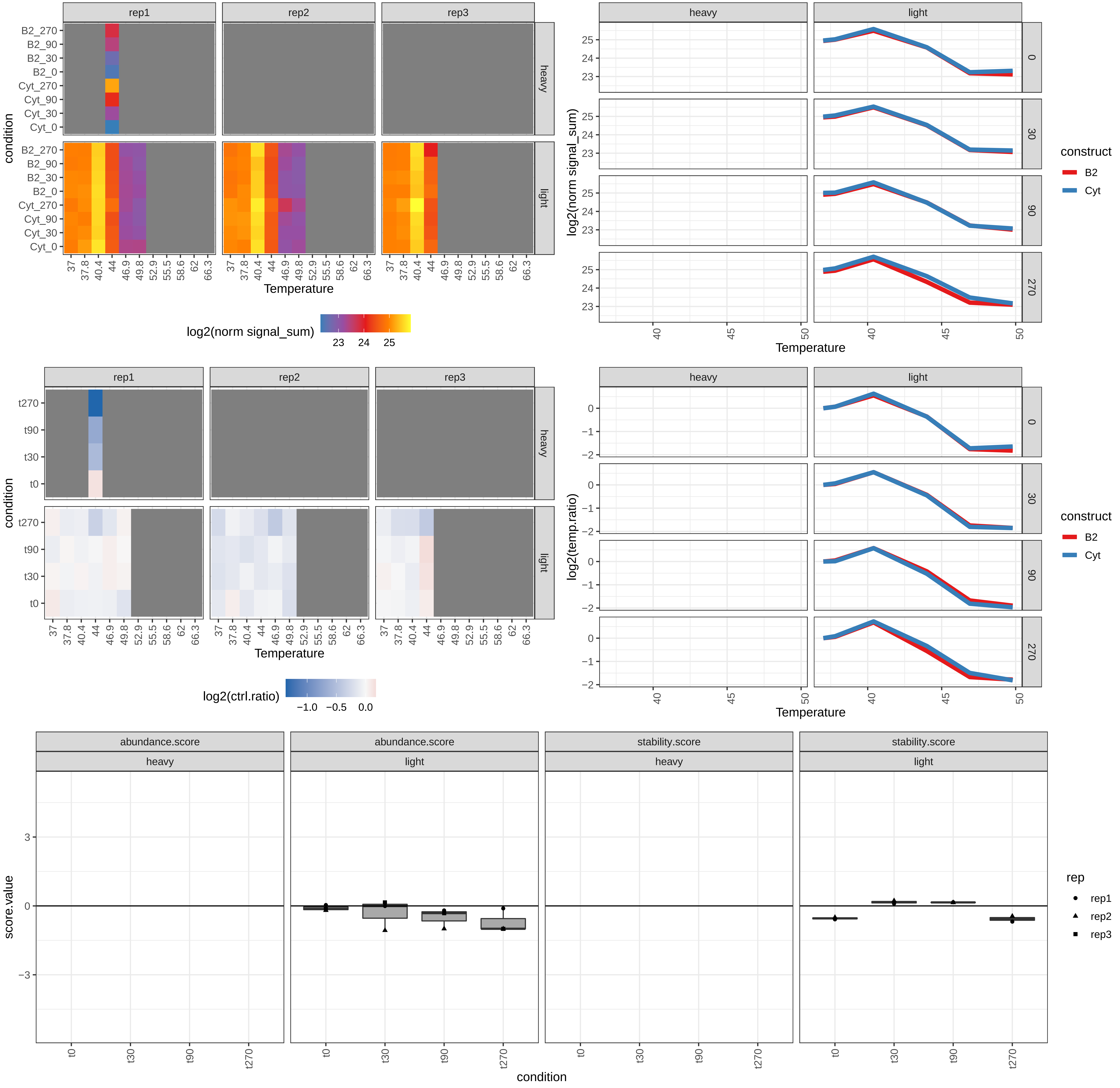

Q03010 − Transcriptional regulatory protein UME1 OS=Saccharomyces cerevisiae (strain ATCC 204508 / S288c) GN=UME1 PE=1 SV=1

UNG1 − average.top3: 6.22918 − max.qupm: 3

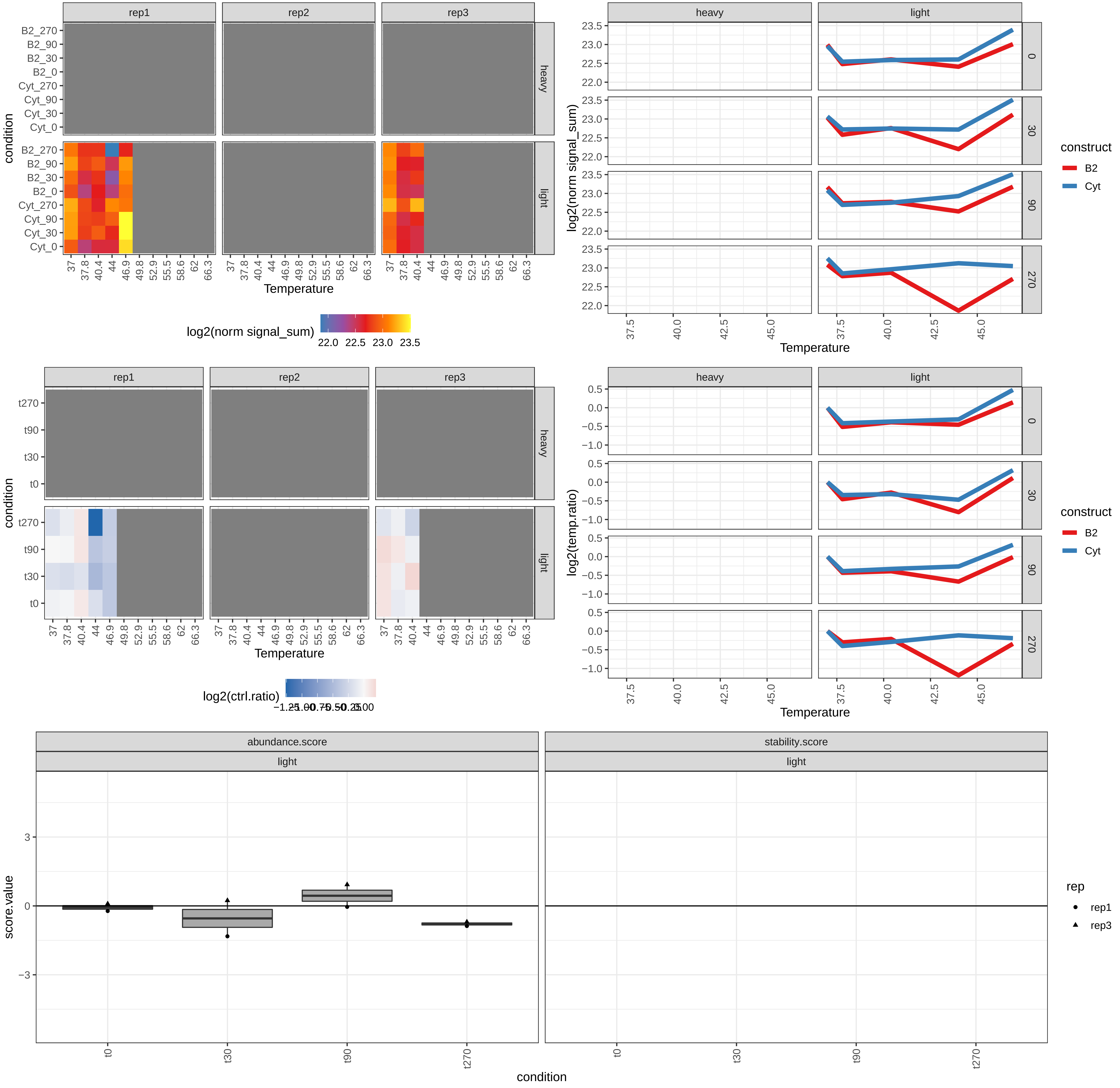

P12887 − Uracil−DNA glycosylase OS=Saccharomyces cerevisiae (strain ATCC 204508 / S288c) GN=UNG1 PE=1 SV=1

UPF3 − average.top3: 6.43650416666667 − max.qupm: 6

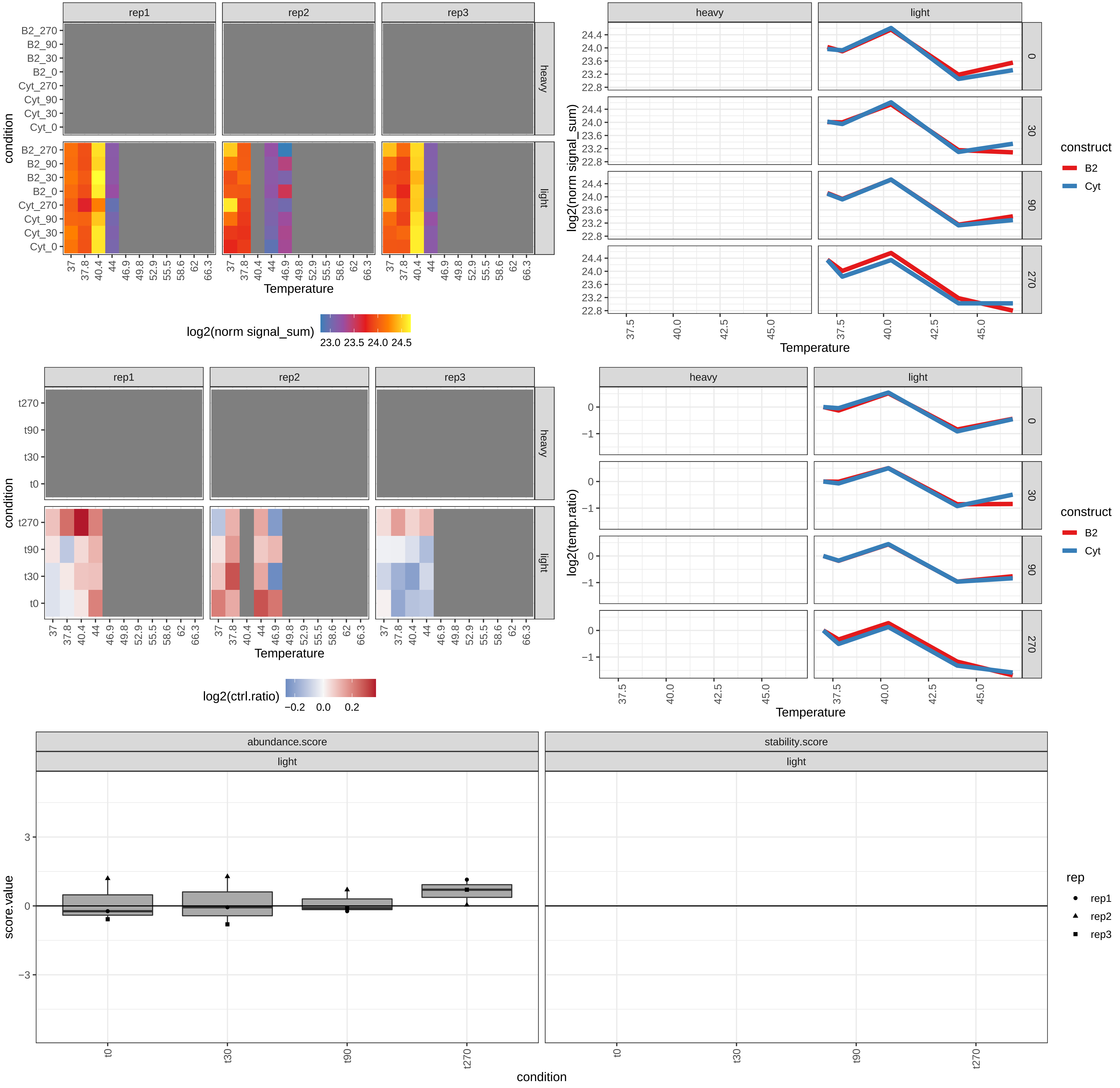

P48412 − Nonsense−mediated mRNA decay protein 3 OS=Saccharomyces cerevisiae (strain ATCC 204508 / S288c) GN=UPF3 PE=1 SV=1

URA1 − average.top3: 7.82307525423729 − max.qupm: 11

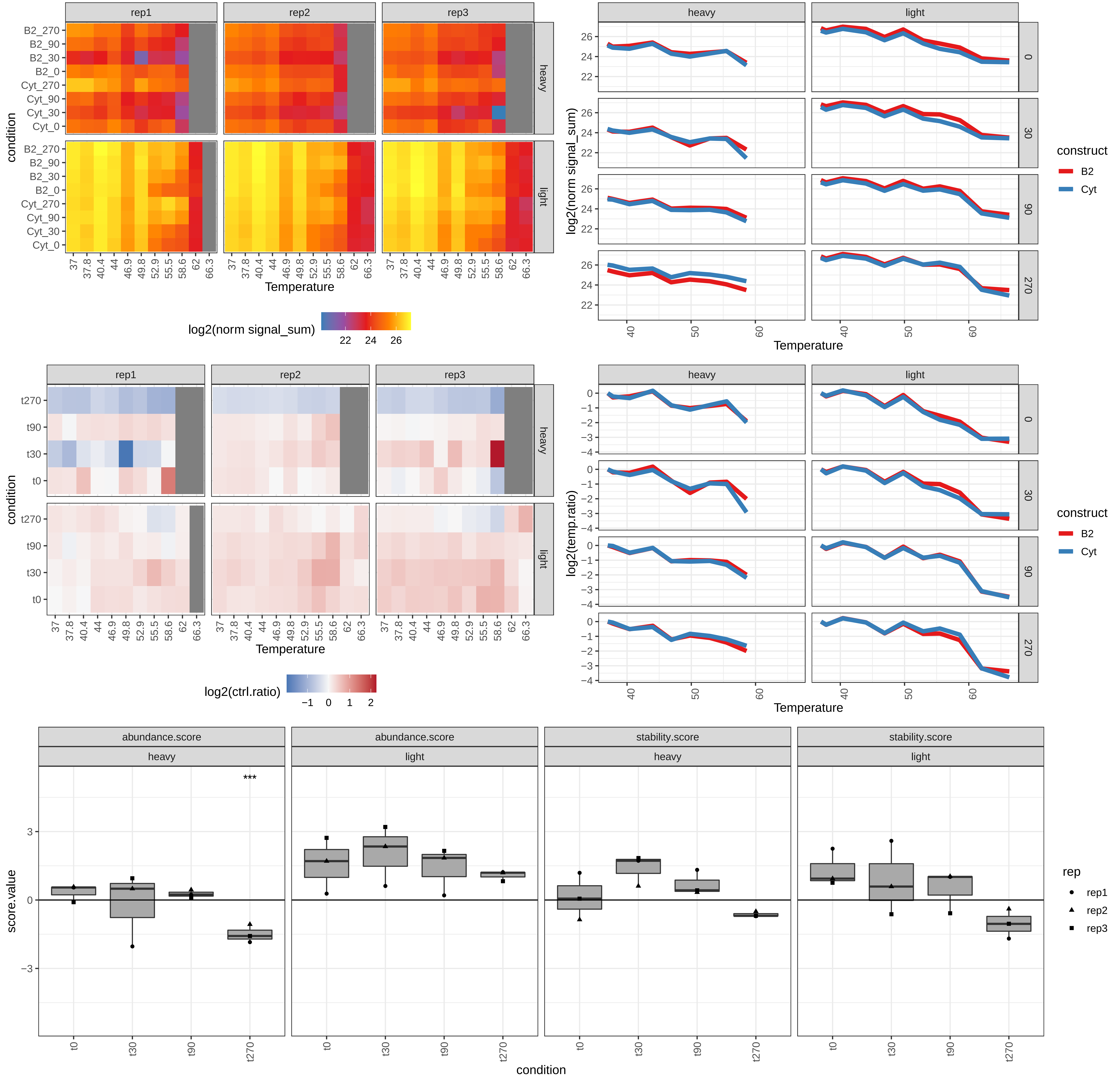

P28272 − Dihydroorotate dehydrogenase (fumarate) OS=Saccharomyces cerevisiae (strain ATCC 204508 / S288c) GN=URA1 PE=1 SV=1

URA10 − average.top3: 6.8834172 − max.qupm: 4

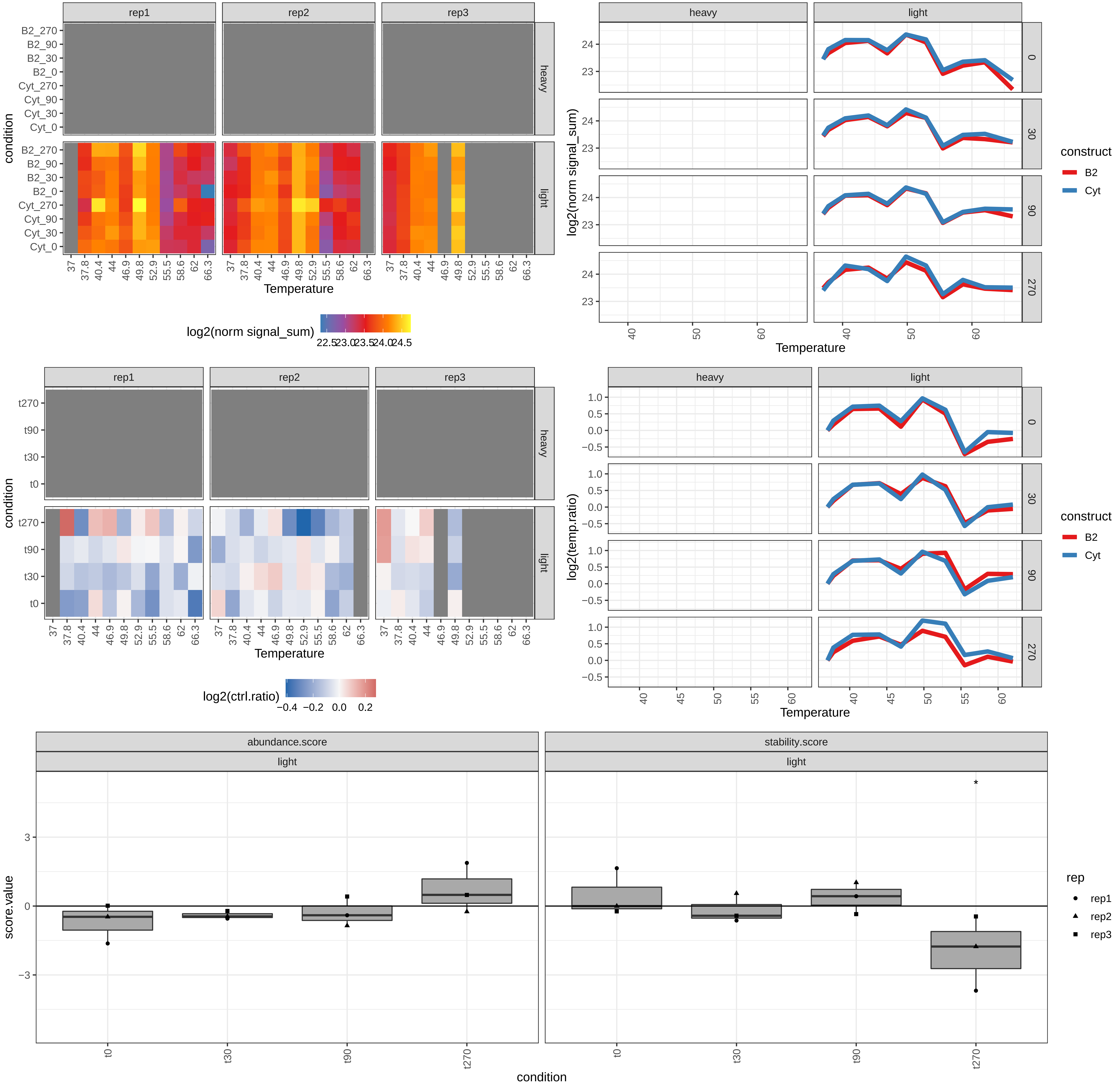

P30402 − Orotate phosphoribosyltransferase 2 OS=Saccharomyces cerevisiae (strain ATCC 204508 / S288c) GN=URA10 PE=1 SV=3

URA2 − average.top3: 7.54498890625 − max.qupm: 74

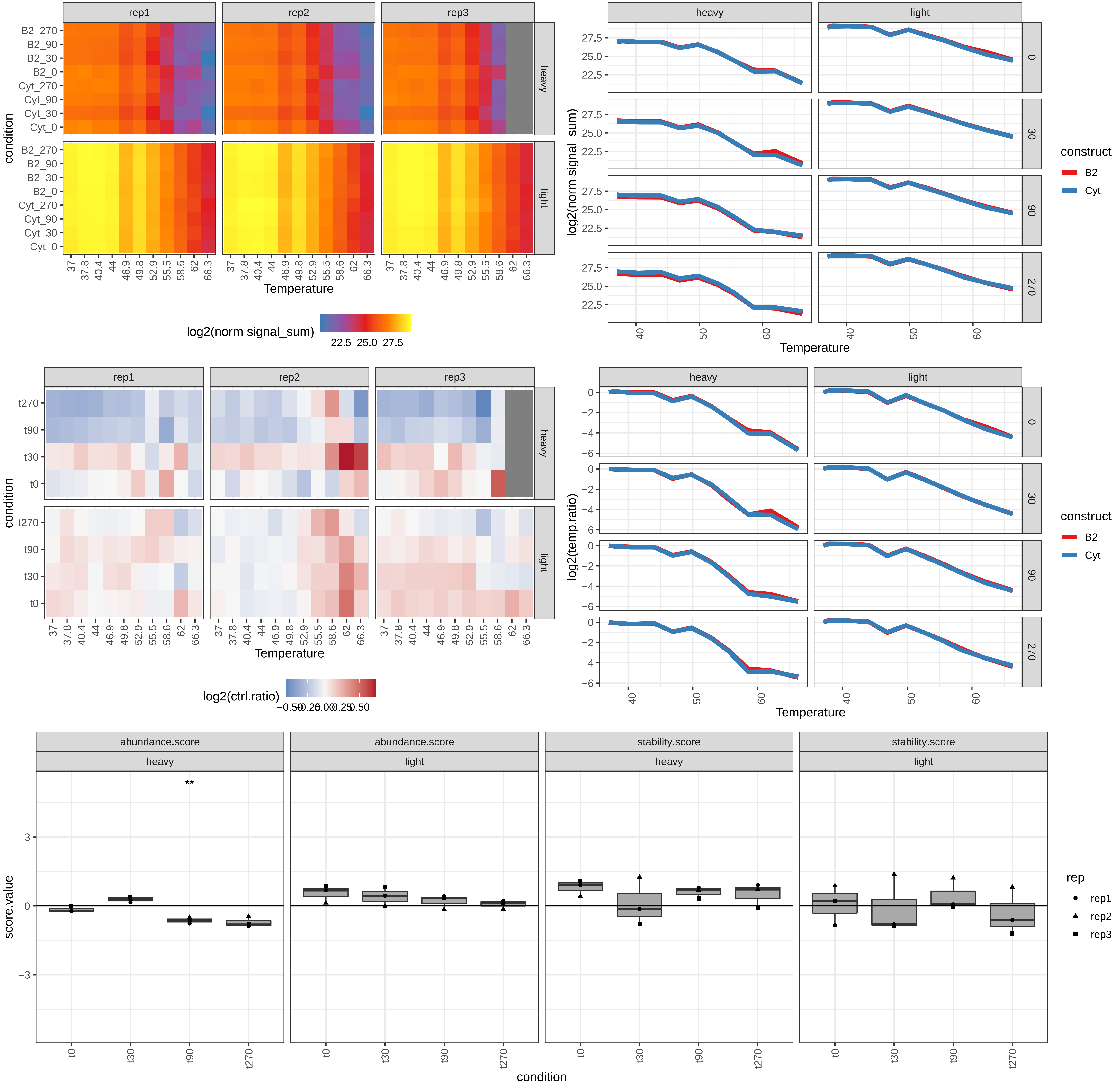

P07259 − Protein URA2 OS=Saccharomyces cerevisiae (strain ATCC 204508 / S288c) GN=URA2 PE=1 SV=5

URA4 − average.top3: 7.09350672131148 − max.qupm: 8

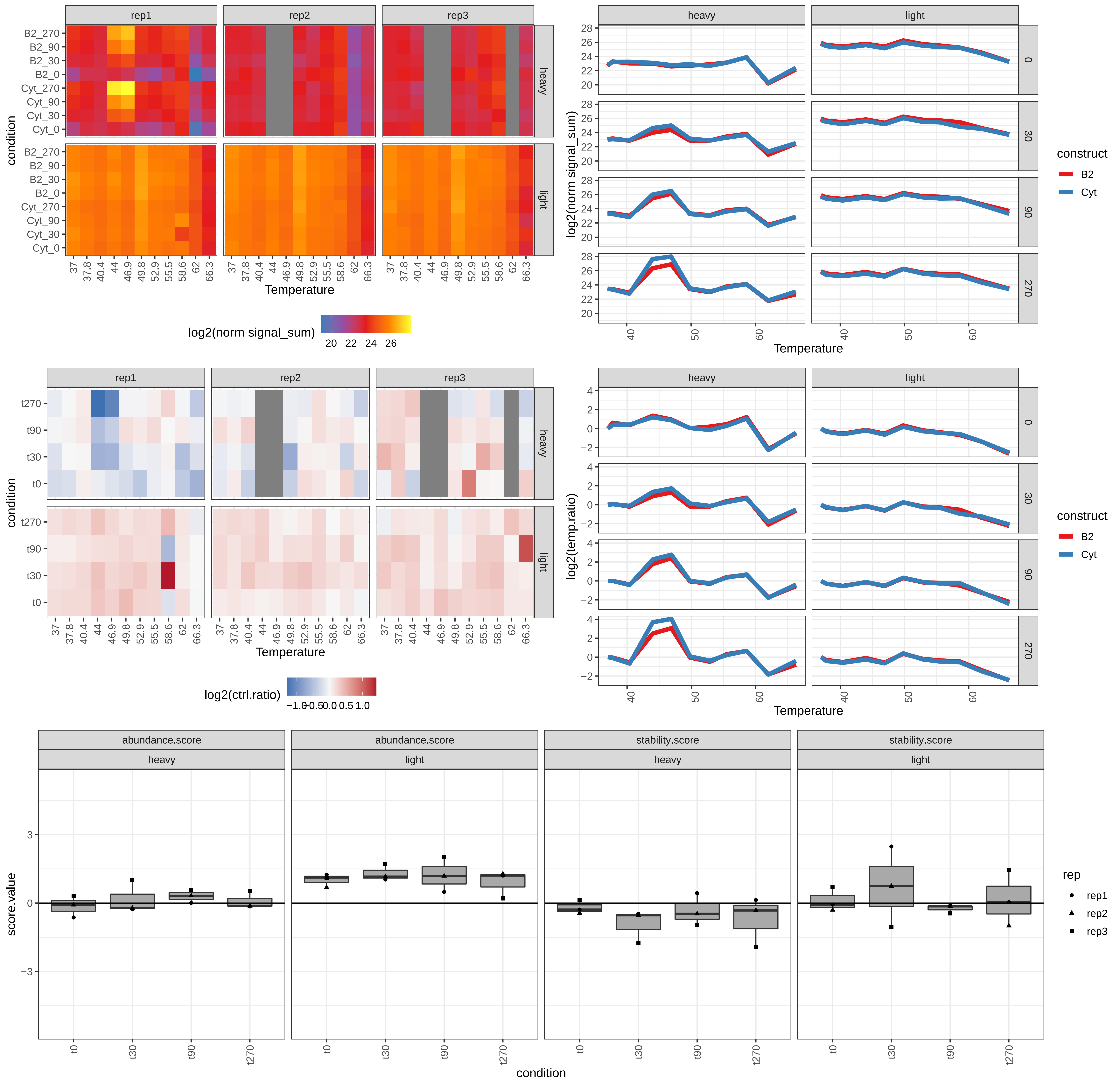

P20051 − Dihydroorotase OS=Saccharomyces cerevisiae (strain ATCC 204508 / S288c) GN=URA4 PE=1 SV=2

URA5 − average.top3: 7.34483328125 − max.qupm: 8

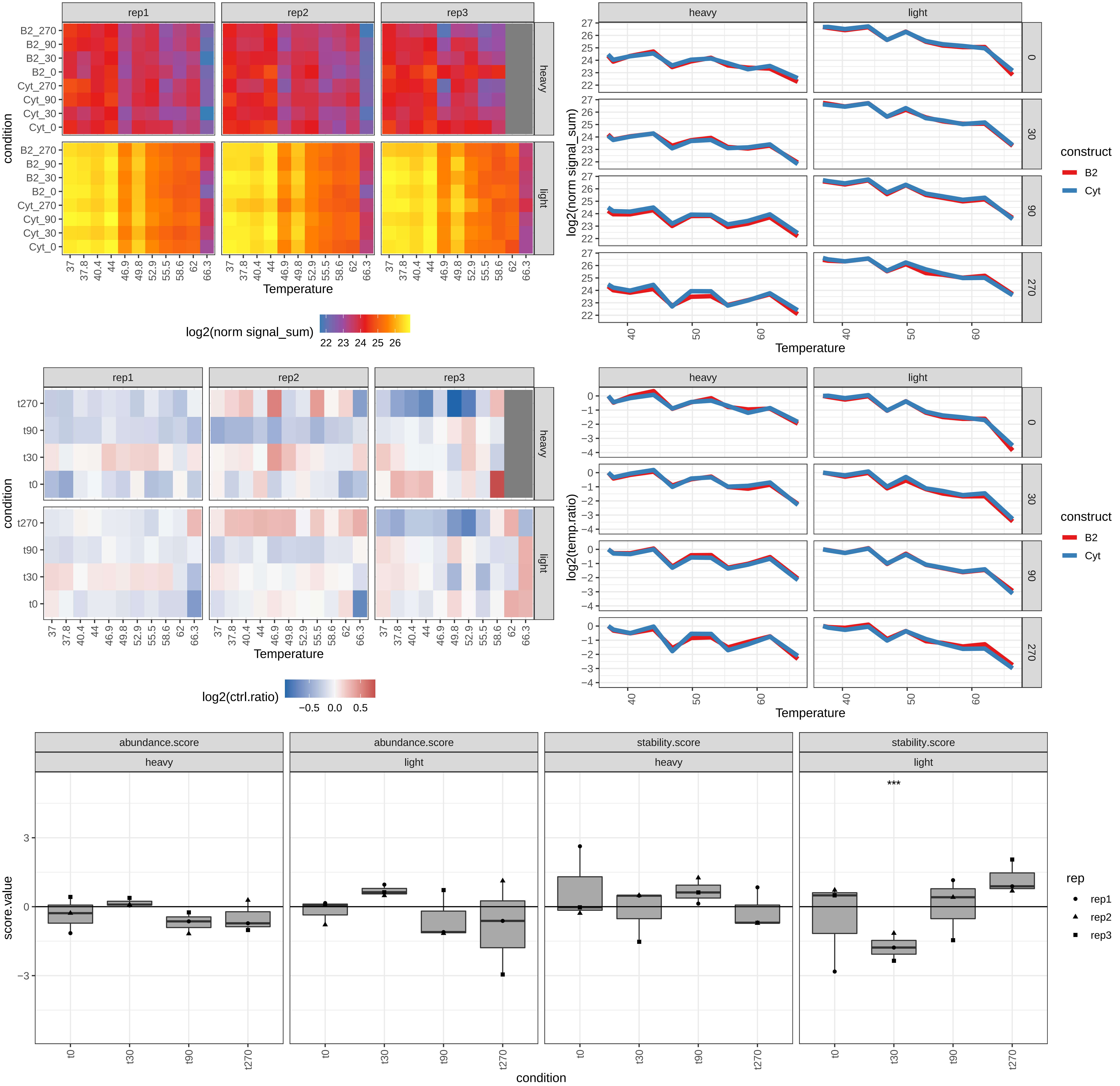

P13298 − Orotate phosphoribosyltransferase 1 OS=Saccharomyces cerevisiae (strain ATCC 204508 / S288c) GN=URA5 PE=1 SV=2

URA6 − average.top3: 7.38172536585366 − max.qupm: 13

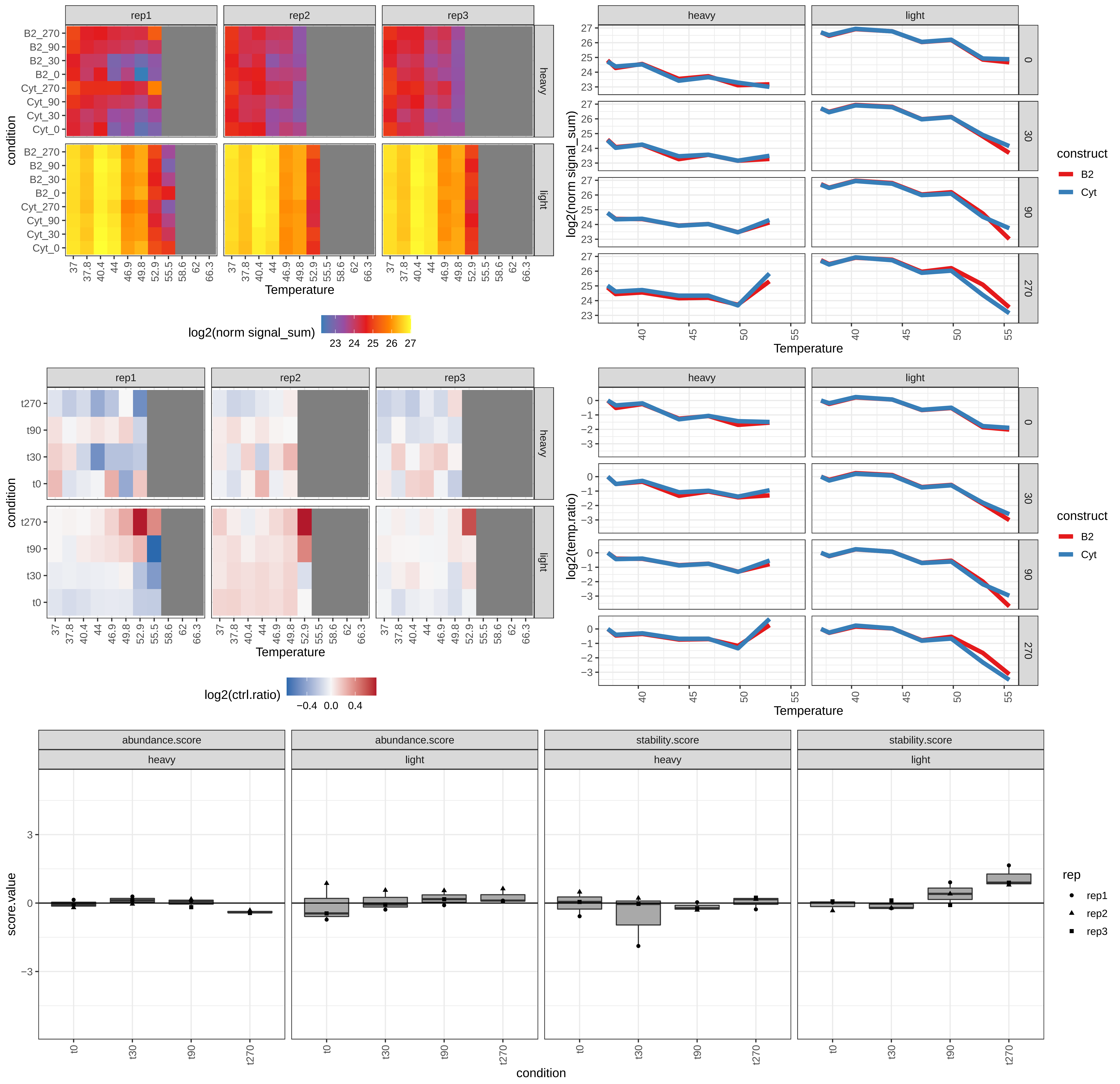

P15700 − Uridylate kinase OS=Saccharomyces cerevisiae (strain ATCC 204508 / S288c) GN=URA6 PE=1 SV=1

URA7 − average.top3: 7.03495108108108 − max.qupm: 15

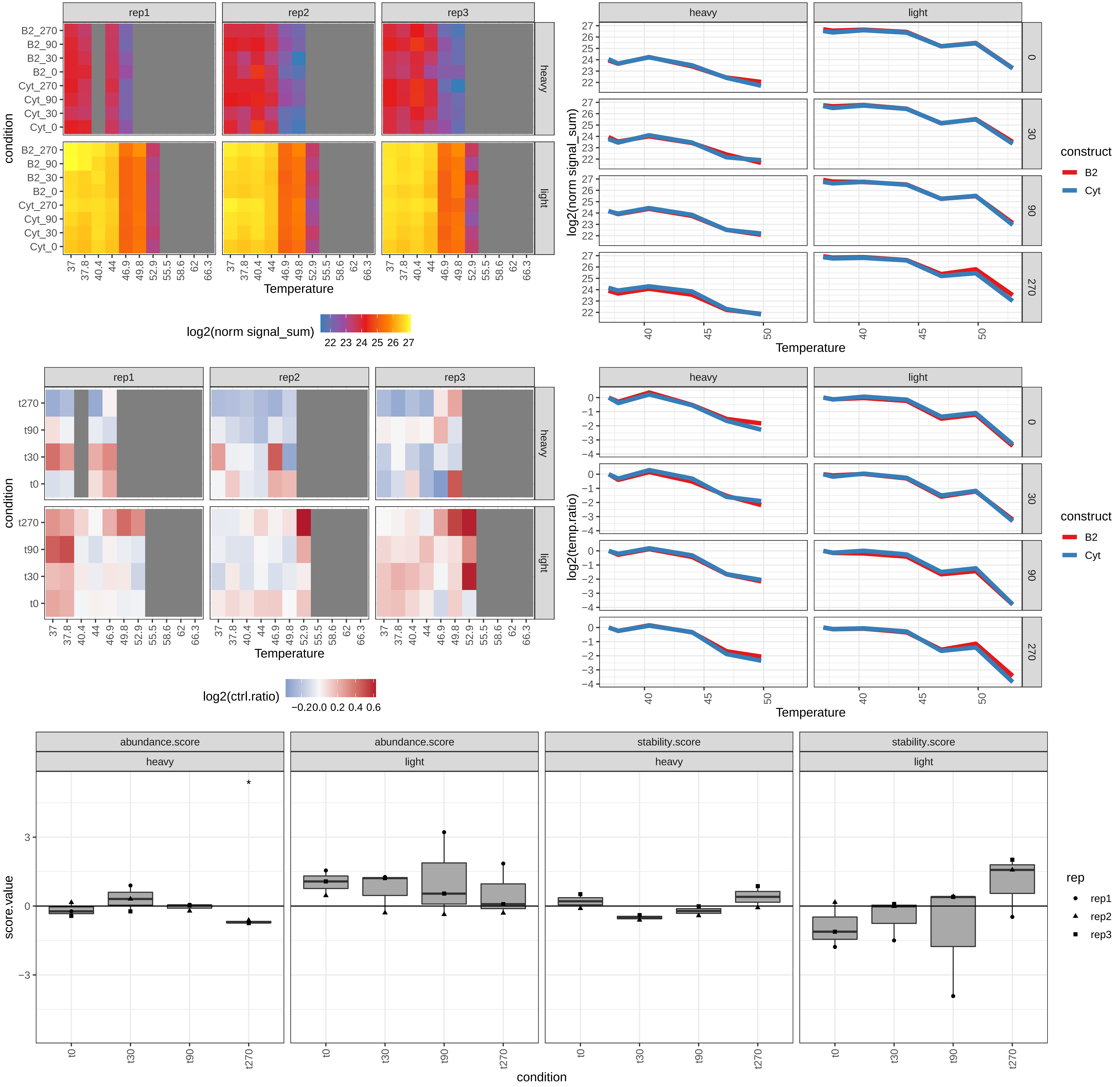

P28274 − CTP synthase 1 OS=Saccharomyces cerevisiae (strain ATCC 204508 / S288c) GN=URA7 PE=1 SV=2

URA8 − average.top3: 7.0464258974359 − max.qupm: 14

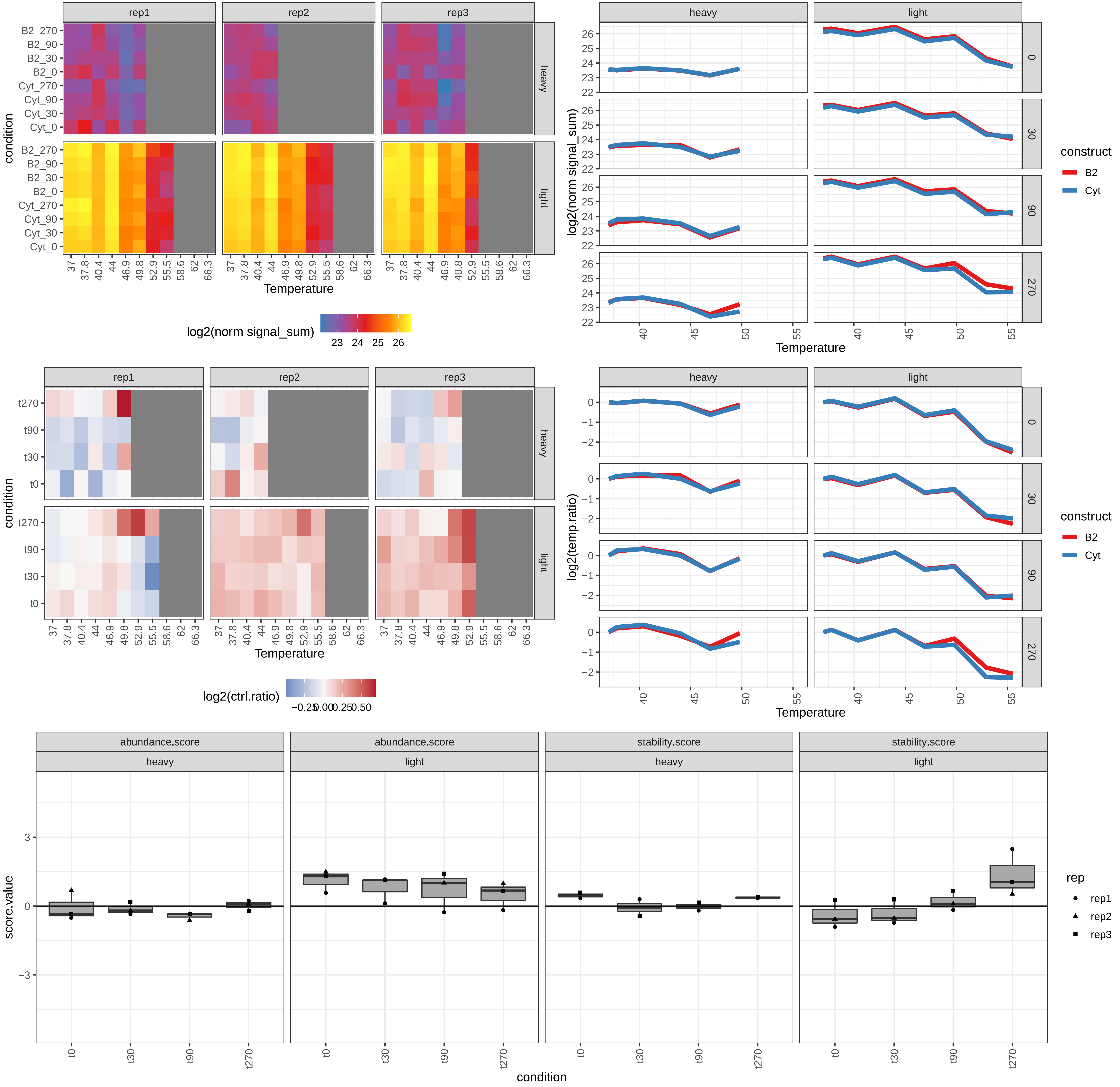

P38627 − CTP synthase 2 OS=Saccharomyces cerevisiae (strain ATCC 204508 / S288c) GN=URA8 PE=1 SV=4

URB1 − average.top3: 6.68435863636364 − max.qupm: 18

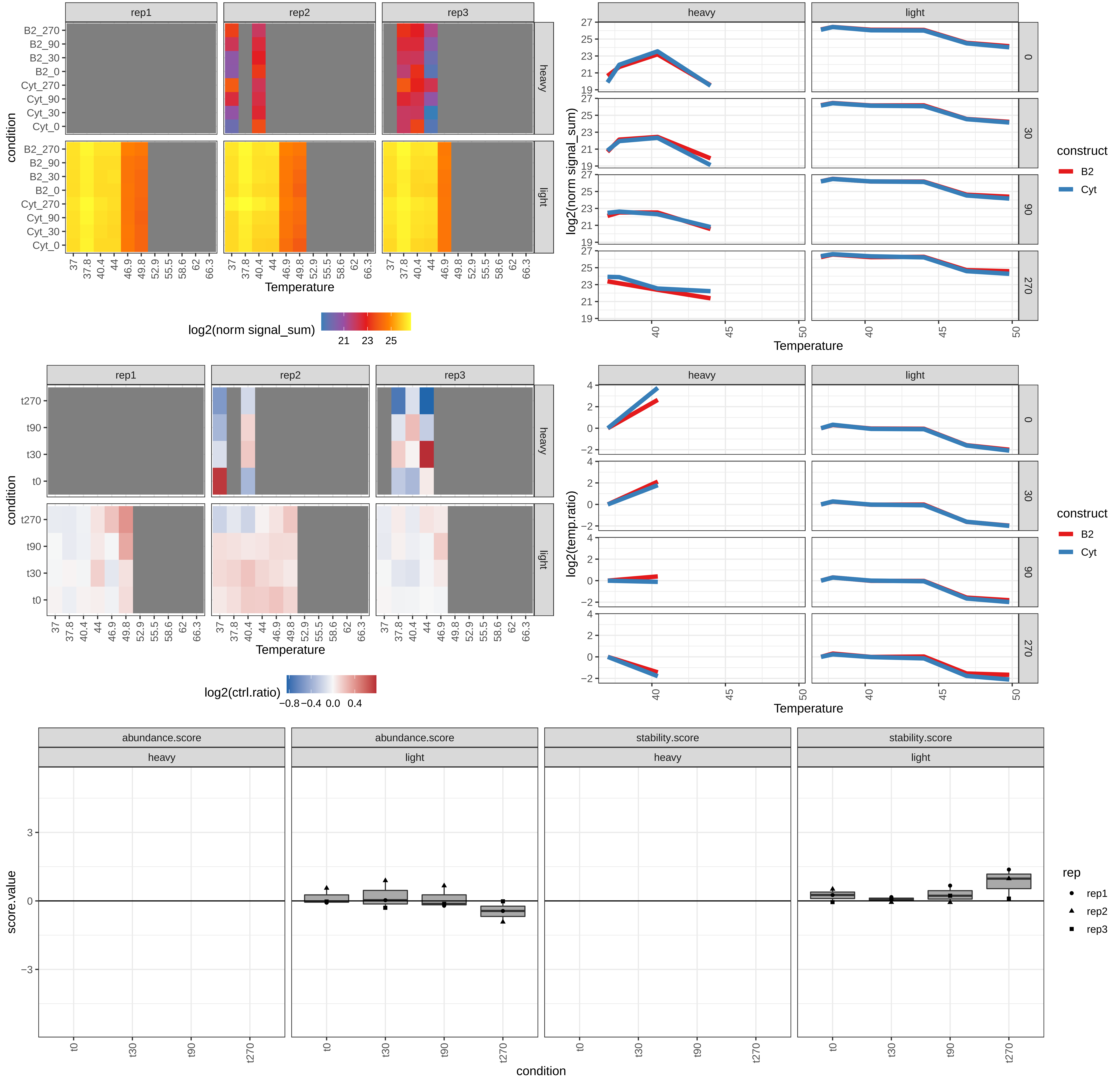

P34241 − Nucleolar pre−ribosomal−associated protein 1 OS=Saccharomyces cerevisiae (strain ATCC 204508 / S288c) GN=URB1 PE=1 SV=2

URB2 − average.top3: 6.69539090909091 − max.qupm: 9

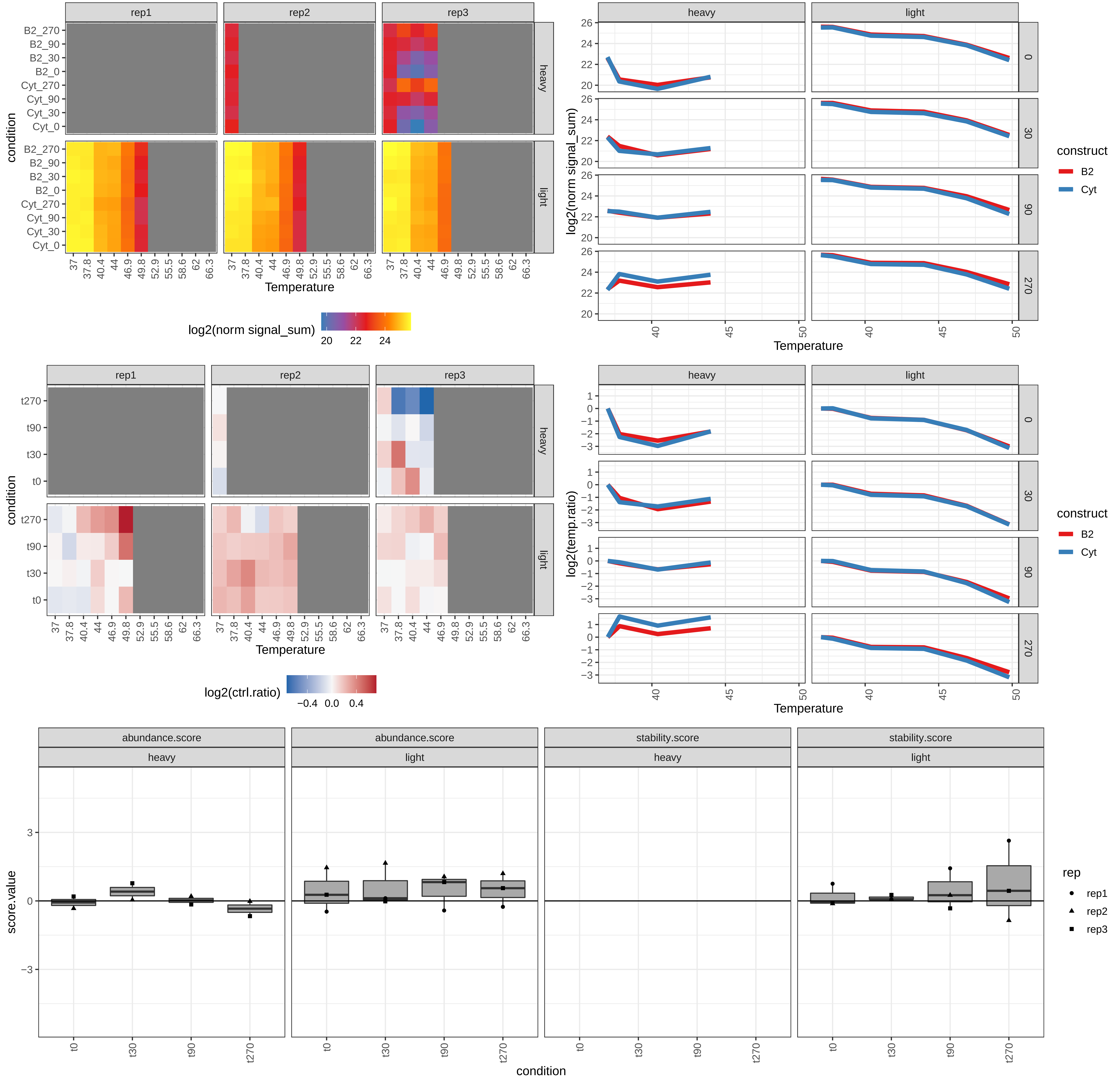

P47108 − Nucleolar pre−ribosomal−associated protein 2 OS=Saccharomyces cerevisiae (strain ATCC 204508 / S288c) GN=URB2 PE=1 SV=1

URE2 − average.top3: 6.95406057142857 − max.qupm: 6

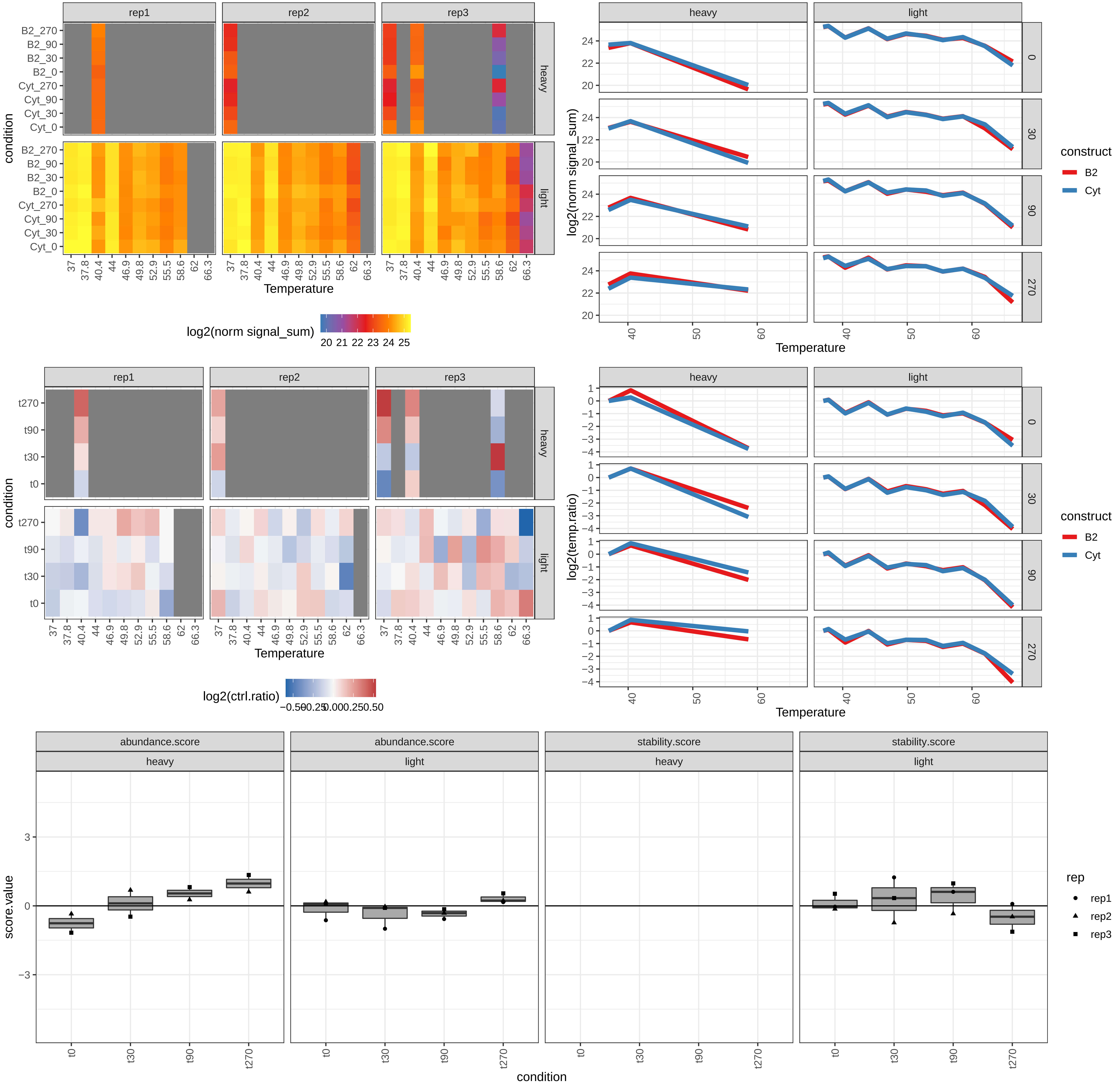

P23202 − Transcriptional regulator URE2 OS=Saccharomyces cerevisiae (strain ATCC 204508 / S288c) GN=URE2 PE=1 SV=1

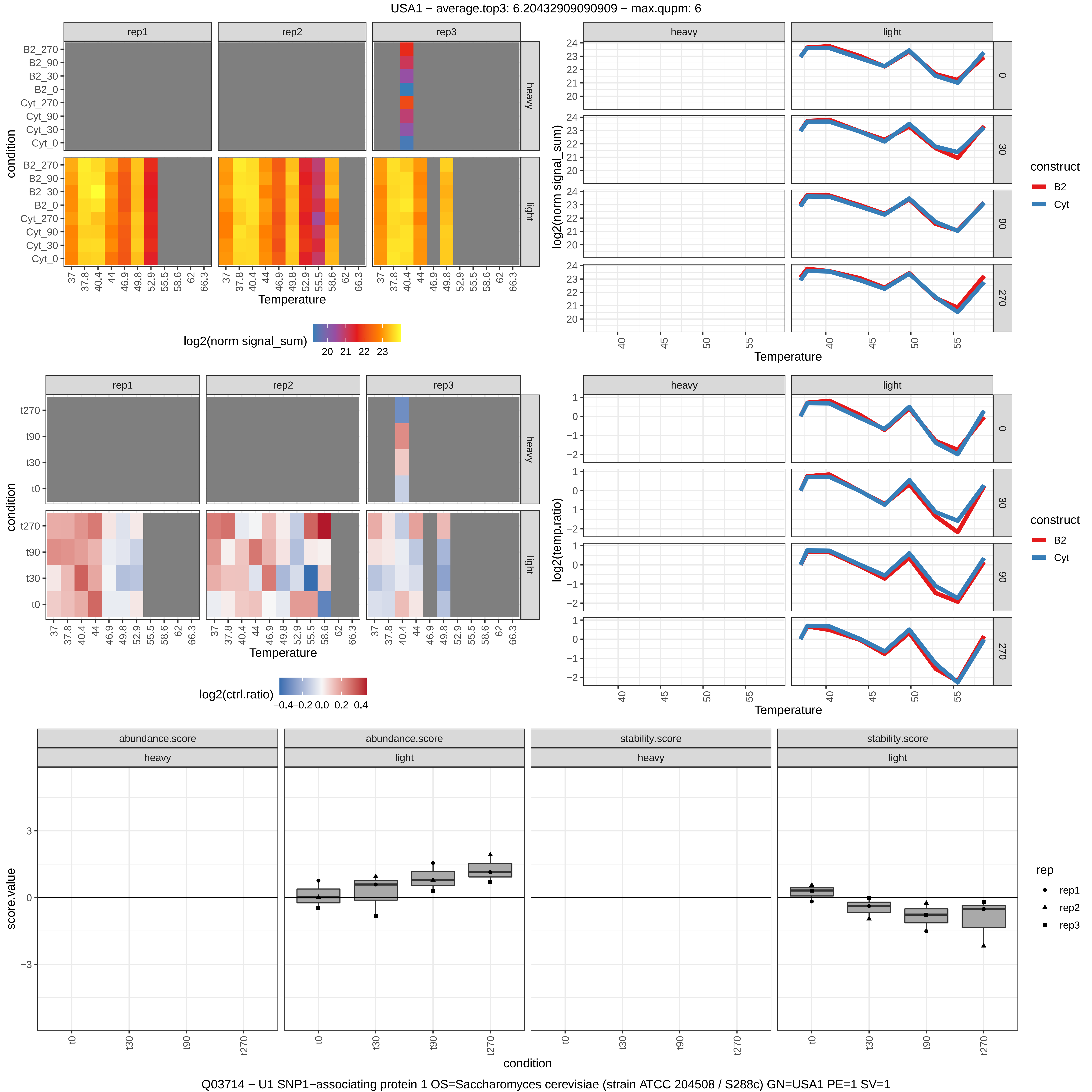
USA1 − average.top3: 6.20432909090909 − max.qupm: 6
rep1
rep2
rep3
heavy
light
condition
Temperature
log2(norm signal_sum)
construct
B2
Cyt
log2(ctrl.ratio)
log2(temp.ratio)
abundance.score
stability.score
score.value
rep
Q03714 − U1 SNP1−associating protein 1 OS=Saccharomyces cerevisiae (strain ATCC 204508 / S288c) GN=USA1 PE=1 SV=1

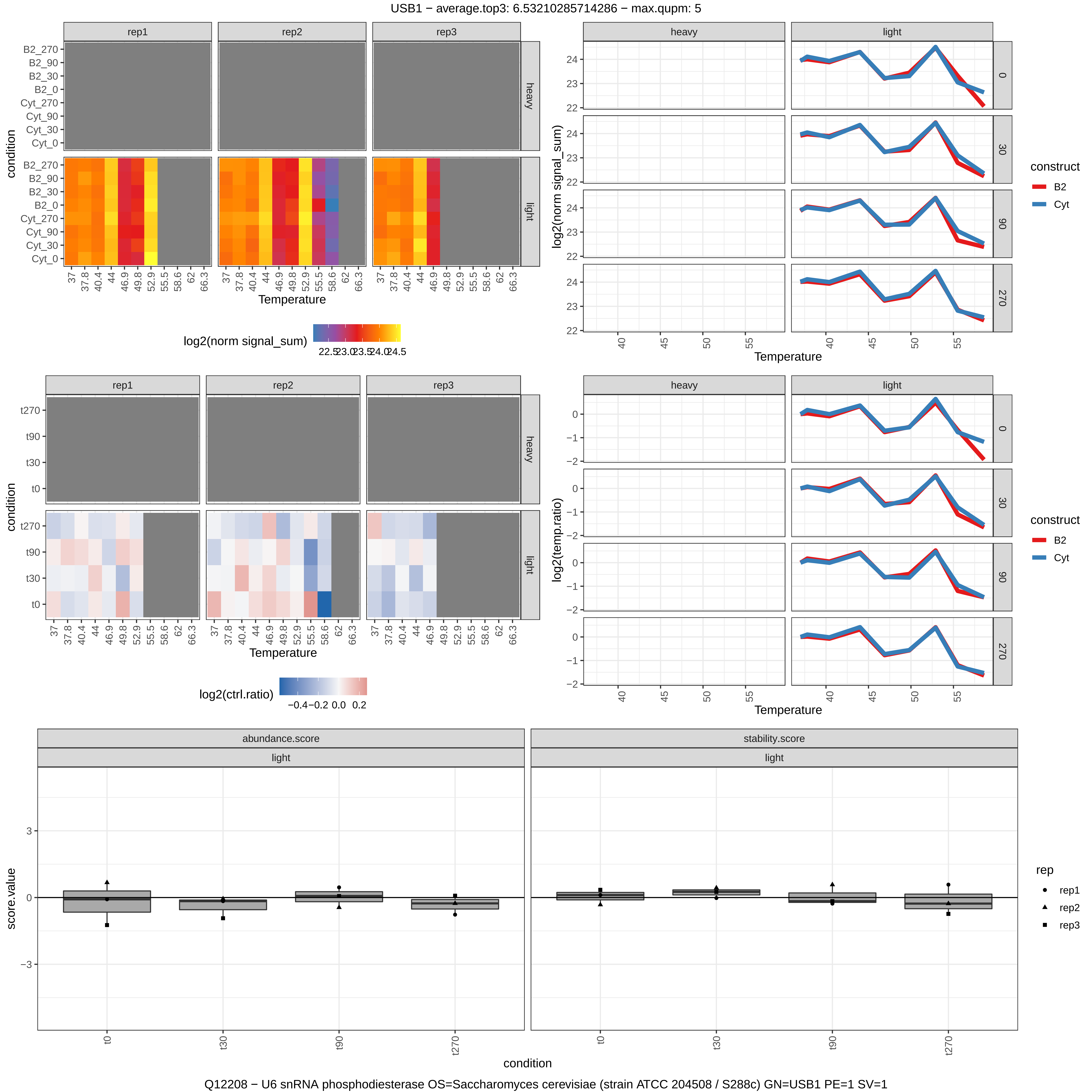

USB1 − average.top3: 6.53210285714286 − max.qupm: 5
rep1
rep2
rep3
heavy
light
condition
Temperature
log2(norm signal_sum)
log2(temp.ratio)
log2(ctrl.ratio)
construct
B2
Cyt
abundance.score
stability.score
score.value
rep
Q12208 − U6 snRNA phosphodiesterase OS=Saccharomyces cerevisiae (strain ATCC 204508 / S288c) GN=USB1 PE=1 SV=1

USO1 − average.top3: 6.56169078947368 − max.qupm: 25

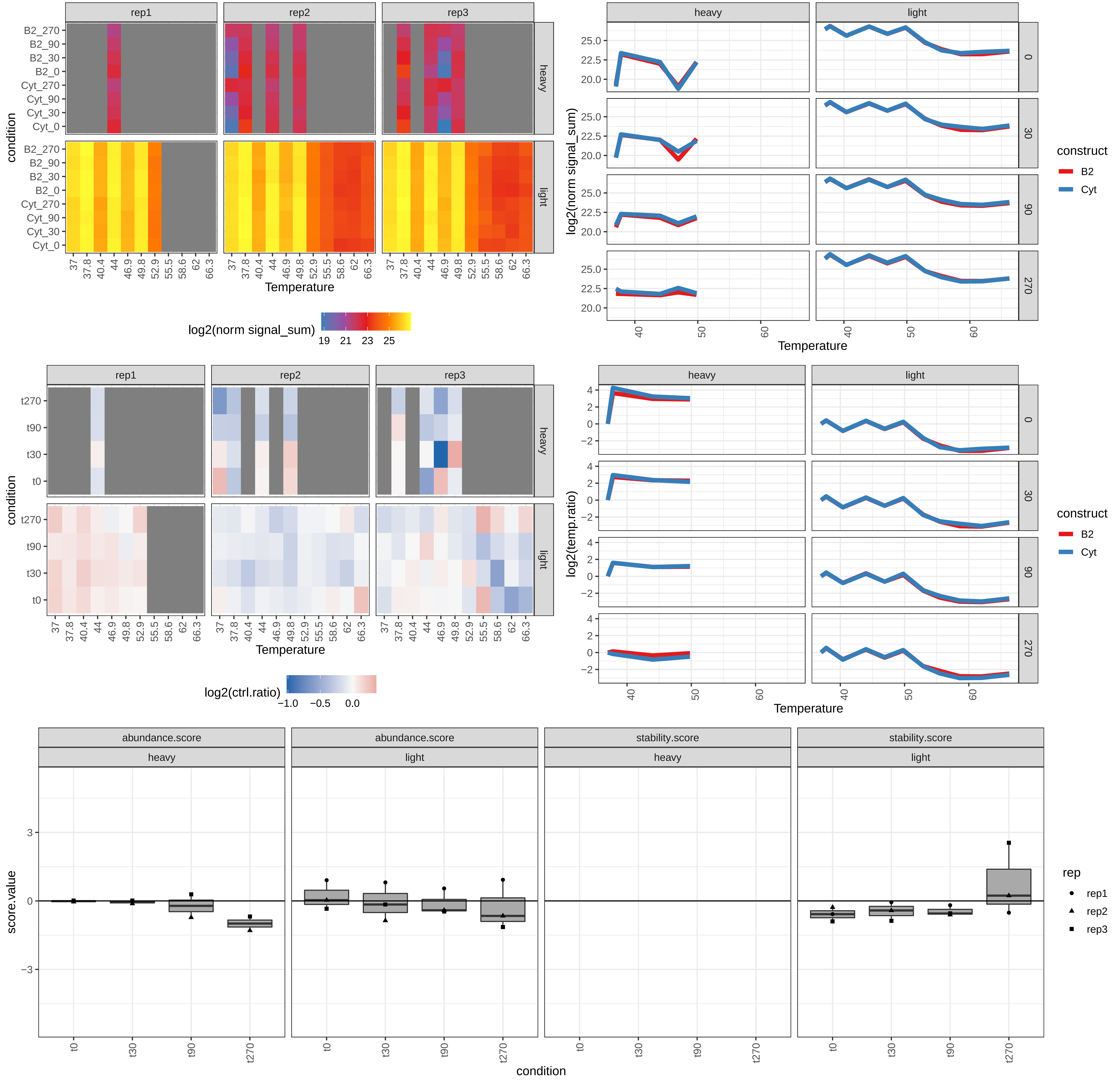

P25386 − Intracellular protein transport protein USO1 OS=Saccharomyces cerevisiae (strain ATCC 204508 / S288c) GN=USO1 PE=1 SV=2

UTH1 − average.top3: 7.28197510204082 − max.qupm: 5

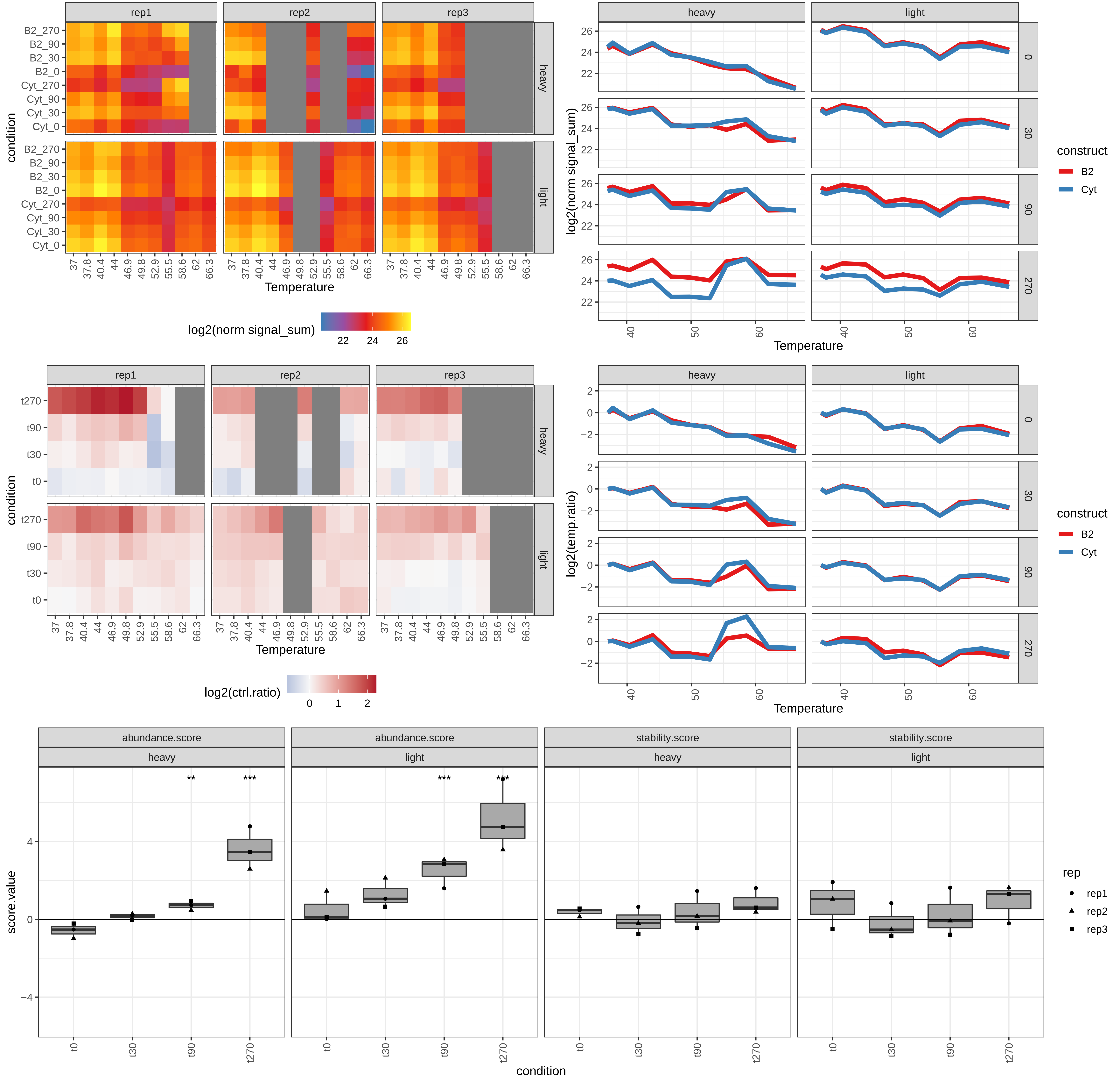

P36135 − Probable secreted beta−glucosidase UTH1 OS=Saccharomyces cerevisiae (strain ATCC 204508 / S288c) GN=UTH1 PE=1 SV=2

UTP10 − average.top3: 6.7685765625 − max.qupm: 23

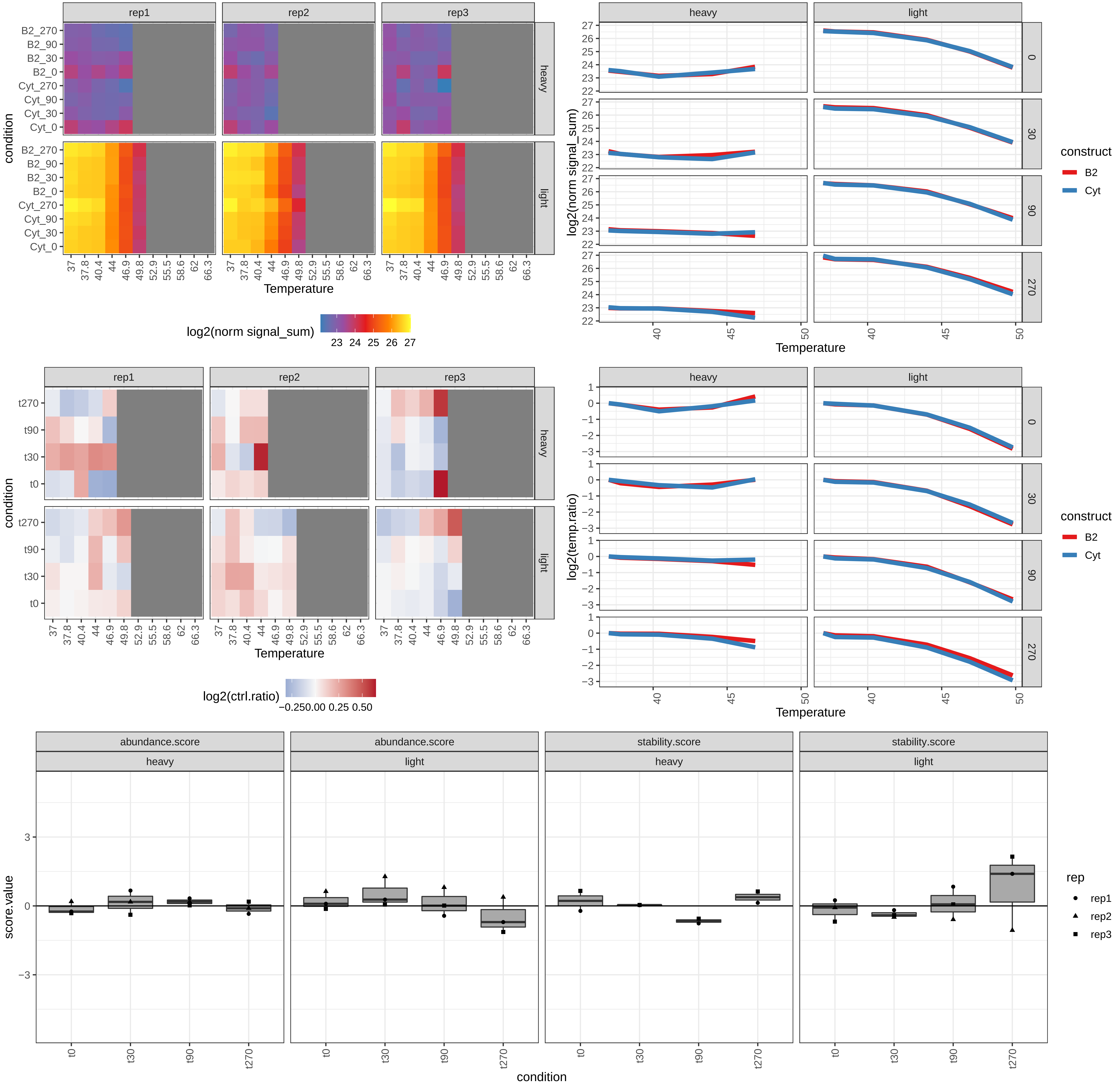

P42945 − U3 small nucleolar RNA−associated protein 10 OS=Saccharomyces cerevisiae (strain ATCC 204508 / S288c) GN=UTP10 PE=1 SV=1

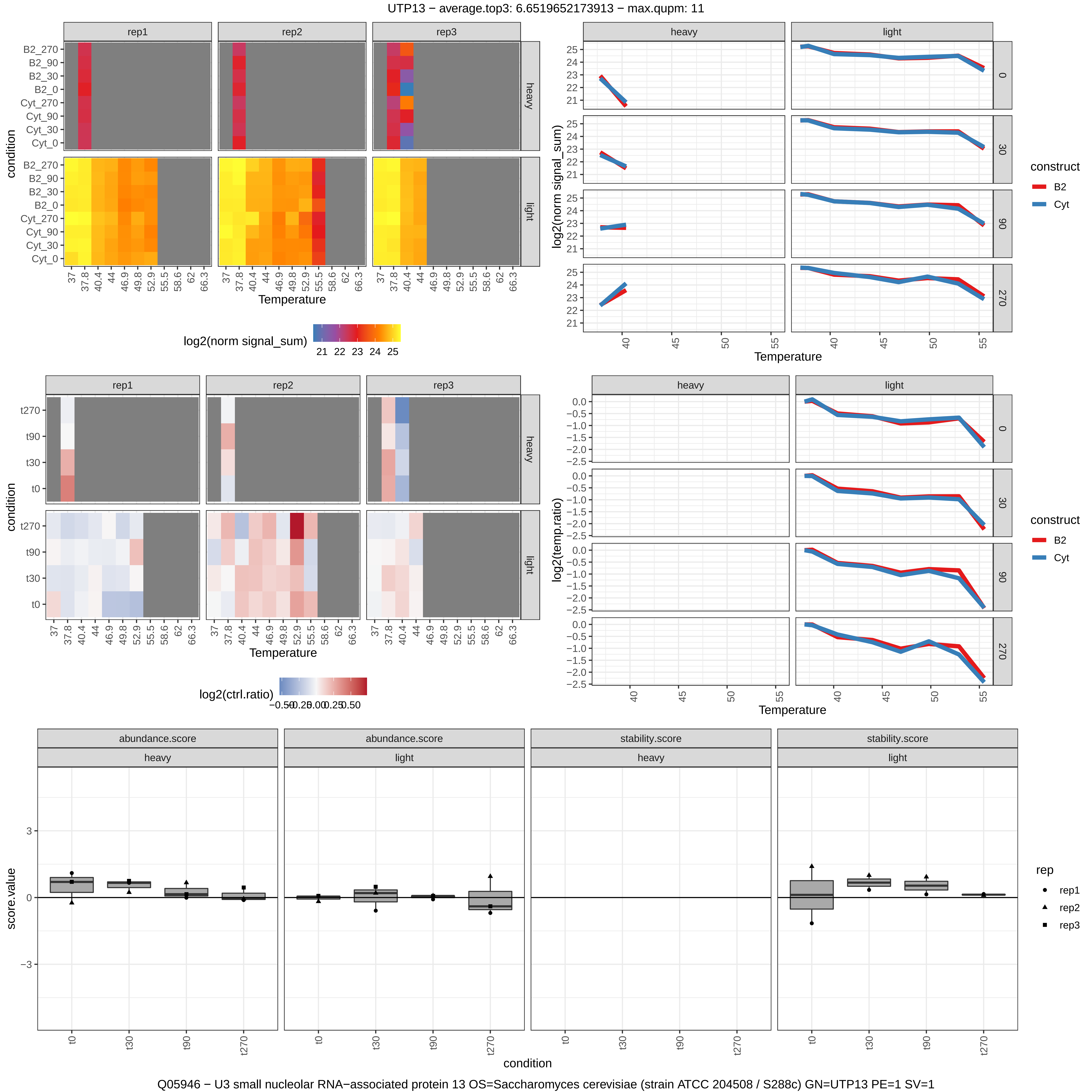

UTP13 − average.top3: 6.6519652173913 − max.qupm: 11
rep1
rep2
rep3
heavy
light
condition
Temperature
log2(norm signal_sum)
construct
B2
Cyt
log2(temp.ratio)
log2(ctrl.ratio)
abundance.score
stability.score
score.value
rep
rep1
rep2
rep3
Q05946 − U3 small nucleolar RNA−associated protein 13 OS=Saccharomyces cerevisiae (strain ATCC 204508 / S288c) GN=UTP13 PE=1 SV=1

UTP15 − average.top3: 6.68974714285714 − max.qupm: 6

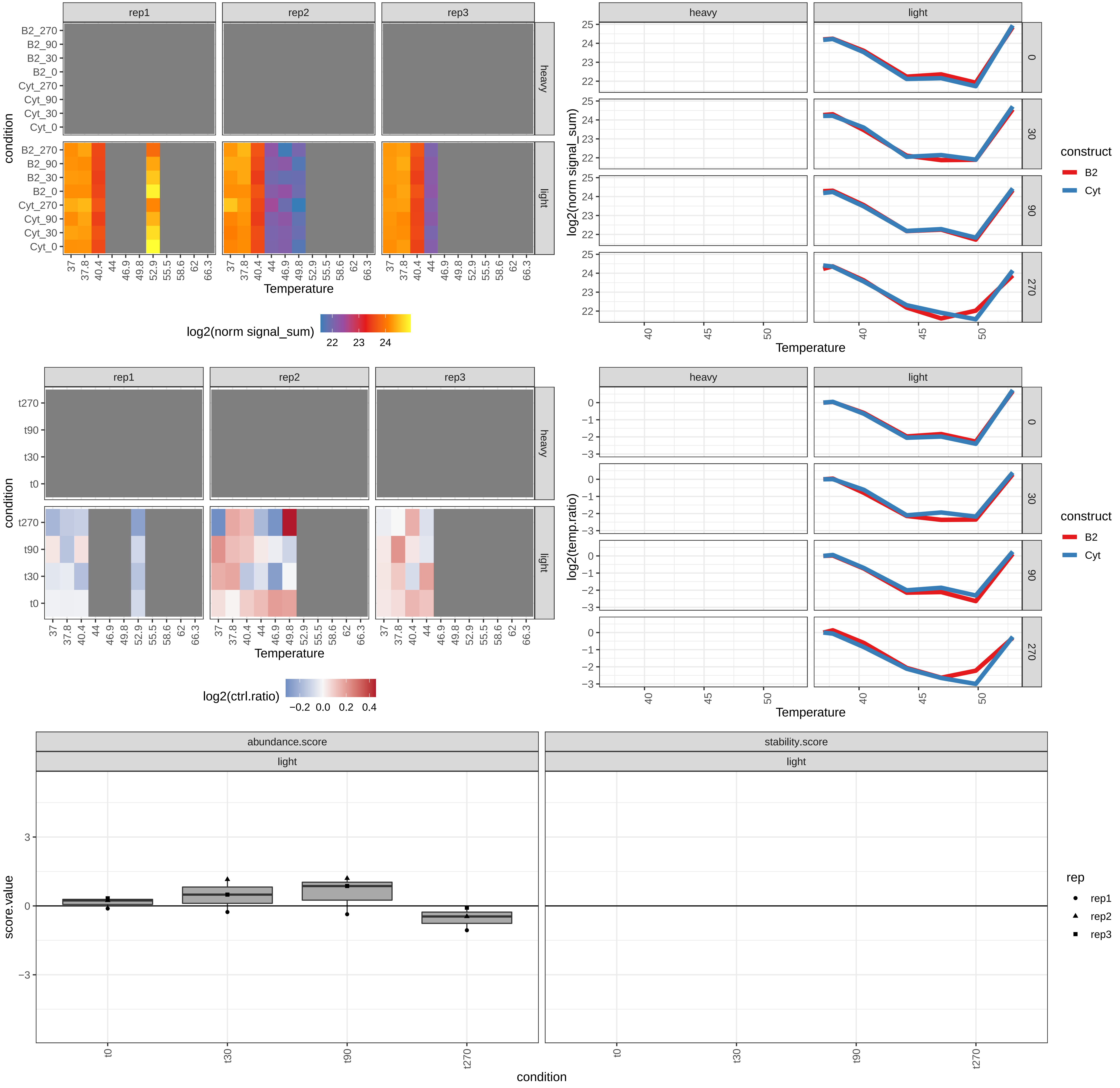

Q04305 − U3 small nucleolar RNA−associated protein 15 OS=Saccharomyces cerevisiae (strain ATCC 204508 / S288c) GN=UTP15 PE=1 SV=1

UTP20 − average.top3: 6.56171178571429 − max.qupm: 21

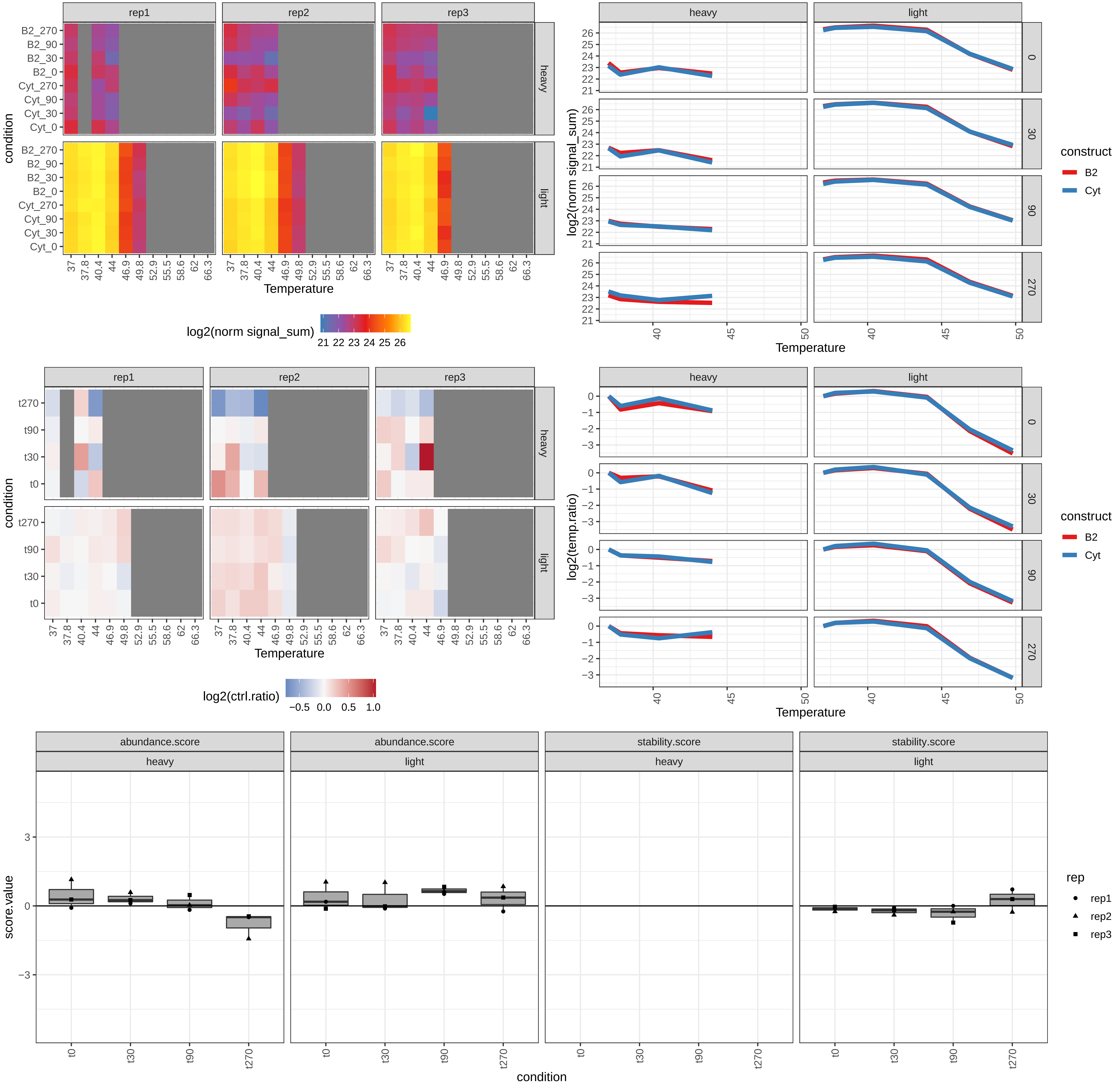

P35194 − U3 small nucleolar RNA−associated protein 20 OS=Saccharomyces cerevisiae (strain ATCC 204508 / S288c) GN=UTP20 PE=1 SV=3

UTP21 − average.top3: 6.75886785714286 − max.qupm: 10

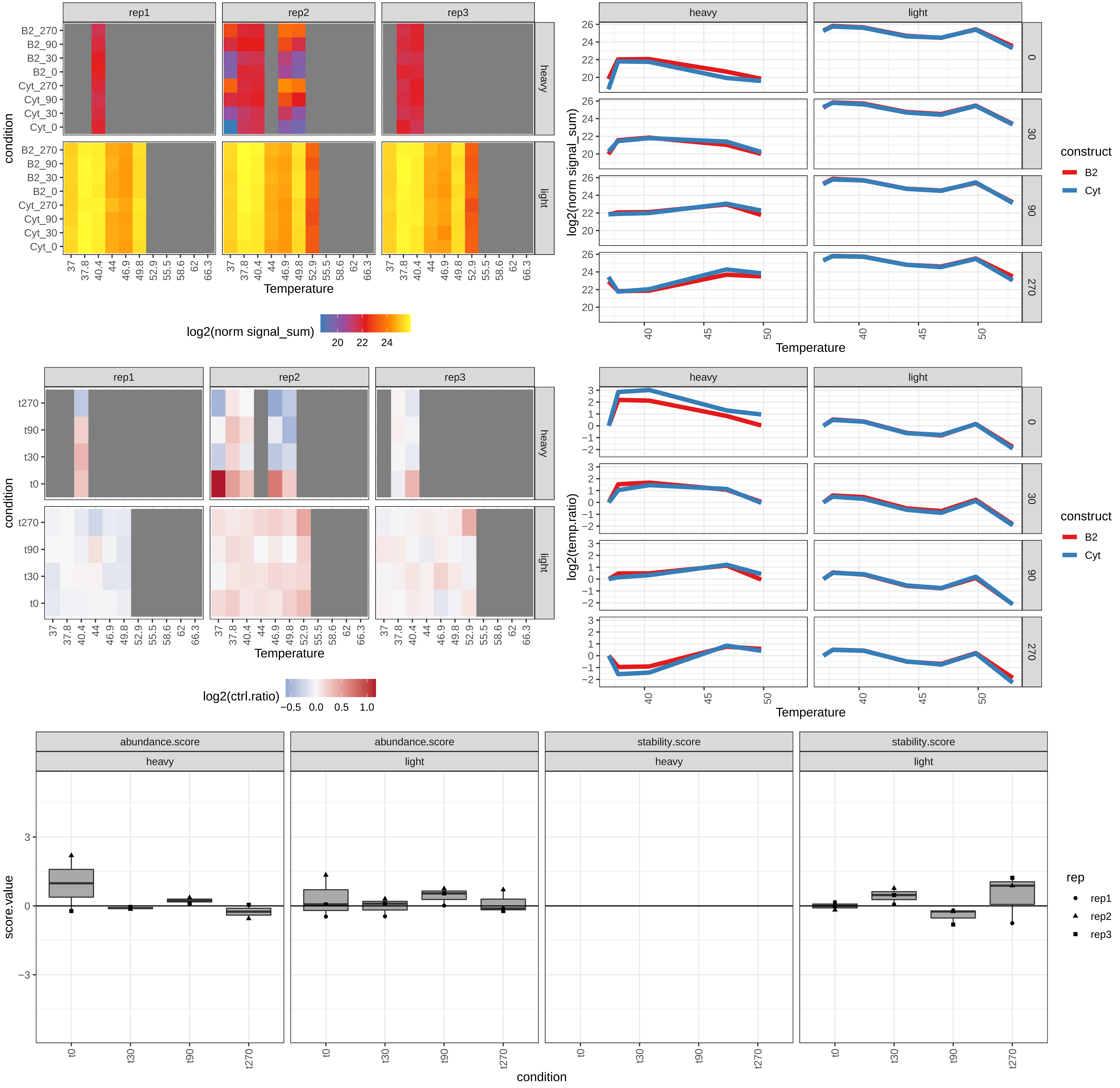

Q06078 − U3 small nucleolar RNA−associated protein 21 OS=Saccharomyces cerevisiae (strain ATCC 204508 / S288c) GN=UTP21 PE=1 SV=1

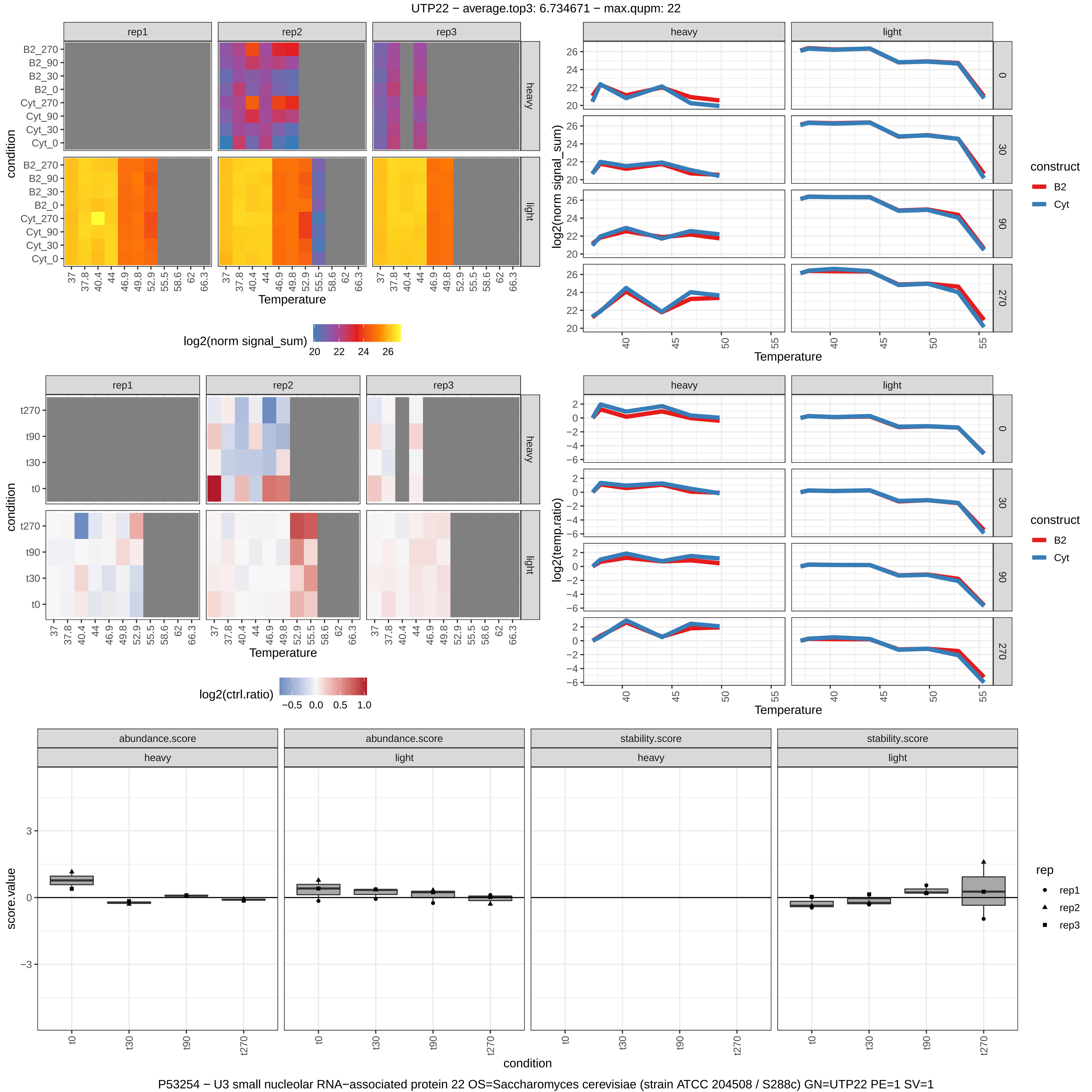
UTP22 − average.top3: 6.734671 − max.qupm: 22
rep1
rep2
rep3
heavy
light
condition
Temperature
log2(norm signal_sum)
log2(ctrl.ratio)
log2(temp.ratio)
construct
B2
Cyt
abundance.score
stability.score
score.value
rep
rep1
rep2
rep3
P53254 − U3 small nucleolar RNA−associated protein 22 OS=Saccharomyces cerevisiae (strain ATCC 204508 / S288c) GN=UTP22 PE=1 SV=1

UTP25 − average.top3: 6.8609695 − max.qupm: 16

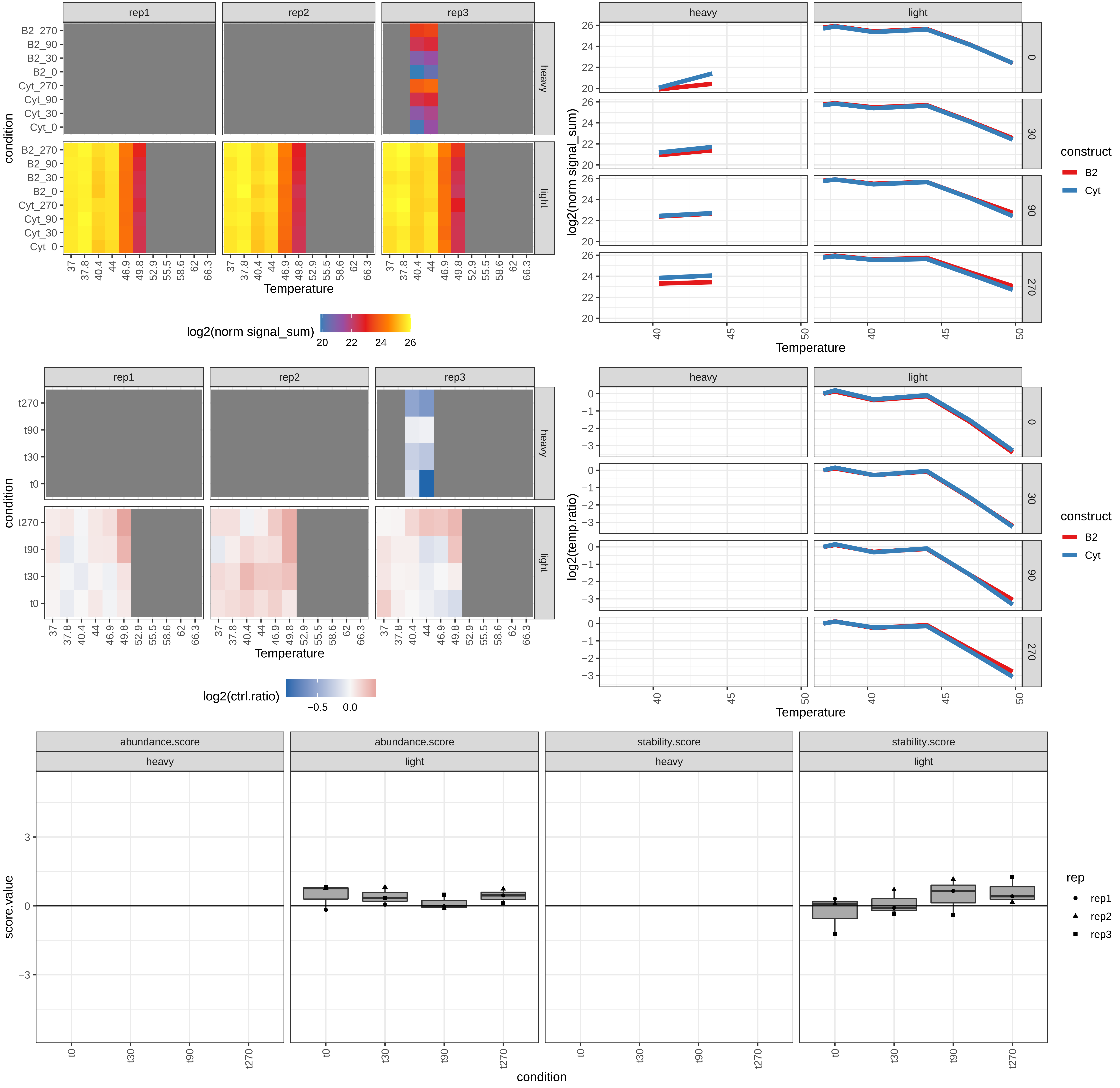

P40498 − U3 small nucleolar RNA−associated protein 25 OS=Saccharomyces cerevisiae (strain ATCC 204508 / S288c) GN=UTP25 PE=1 SV=1

UTP30 − average.top3: 6.37920666666667 − max.qupm: 4

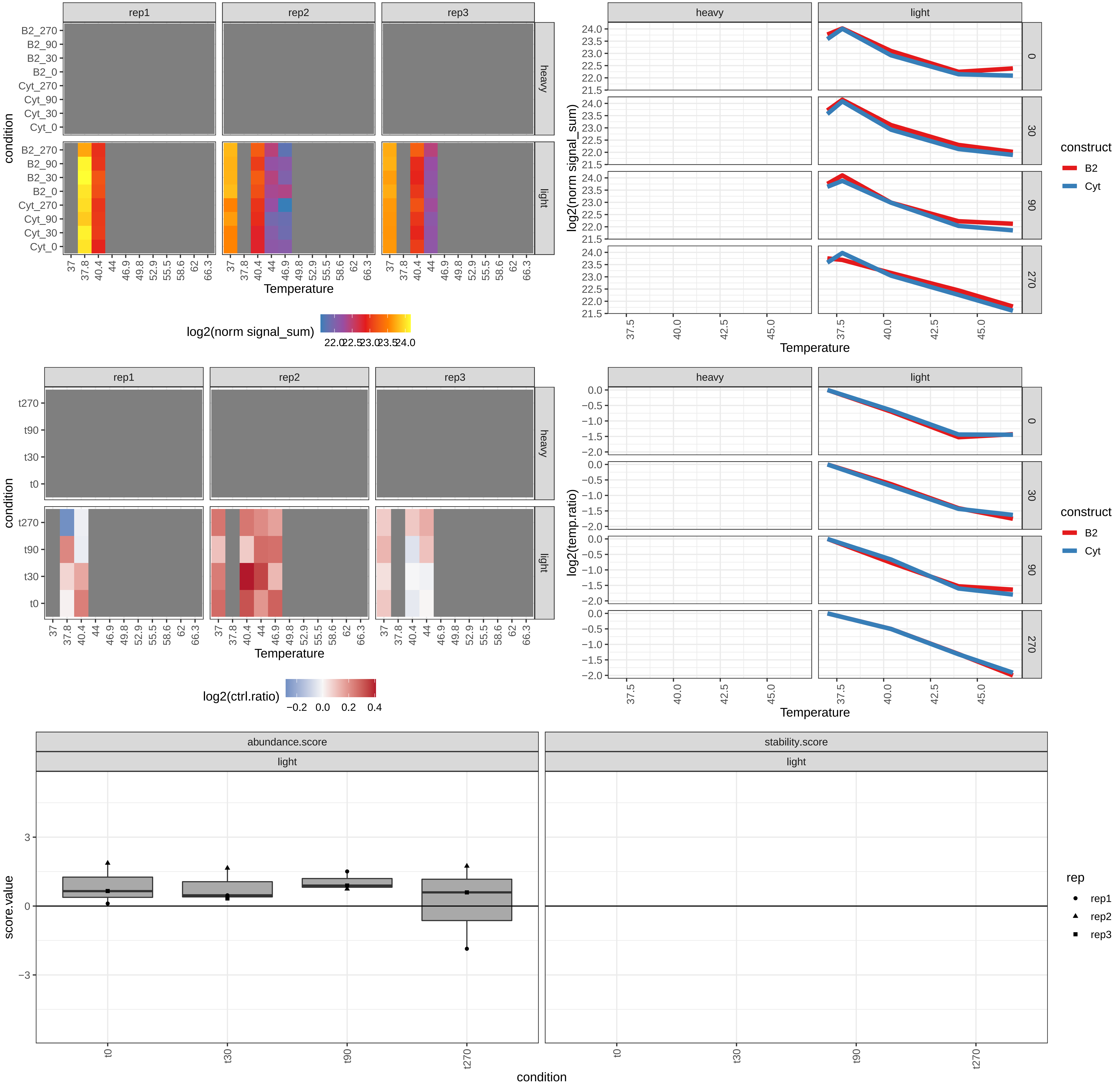

P36144 − Ribosome biogenesis protein UTP30 OS=Saccharomyces cerevisiae (strain ATCC 204508 / S288c) GN=UTP30 PE=1 SV=1

UTP4 − average.top3: 6.9040703125 − max.qupm: 12

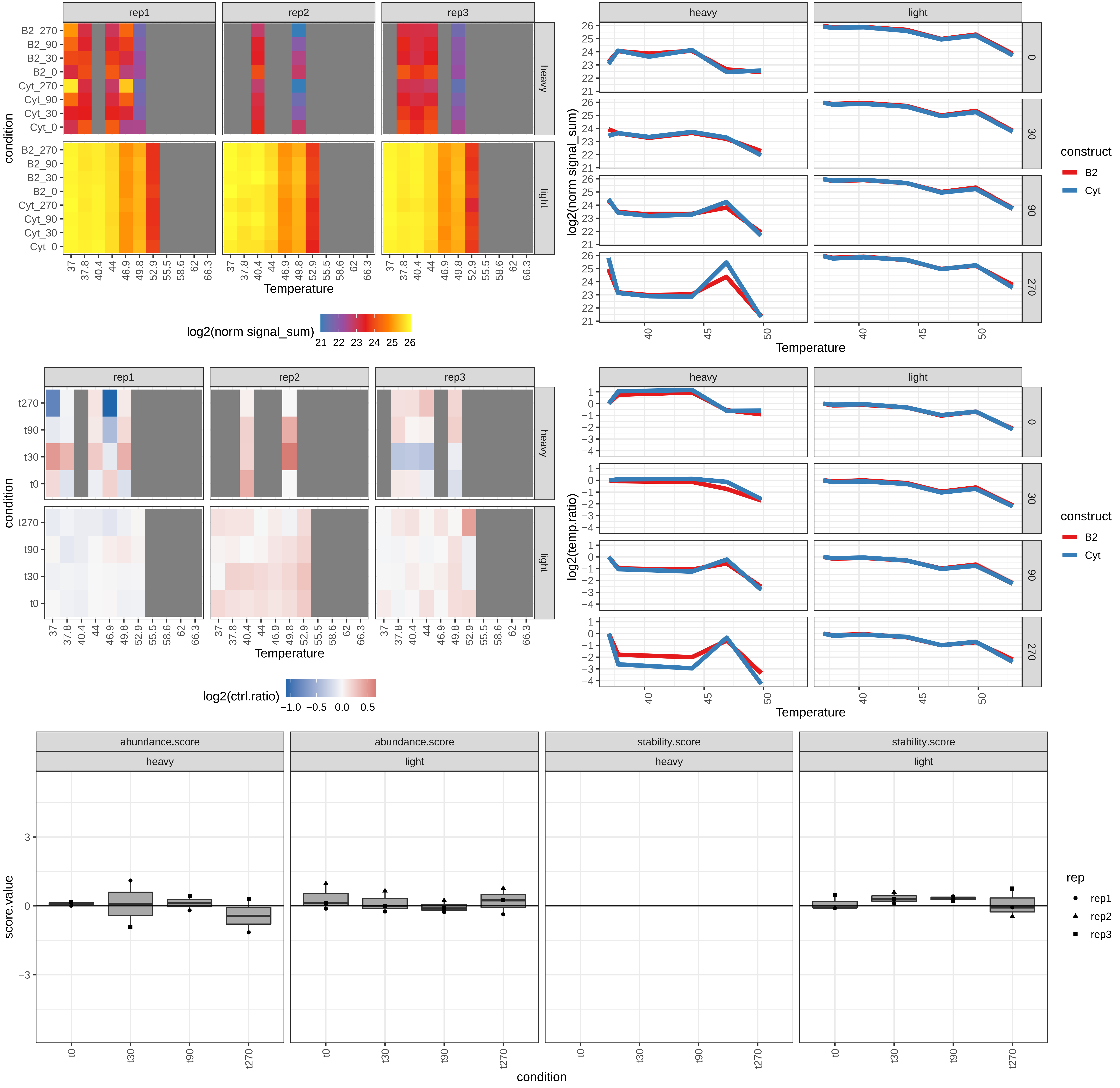

Q06679 − U3 small nucleolar RNA−associated protein 4 OS=Saccharomyces cerevisiae (strain ATCC 204508 / S288c) GN=UTP4 PE=1 SV=1

UTP5 − average.top3: 6.8661532 − max.qupm: 6

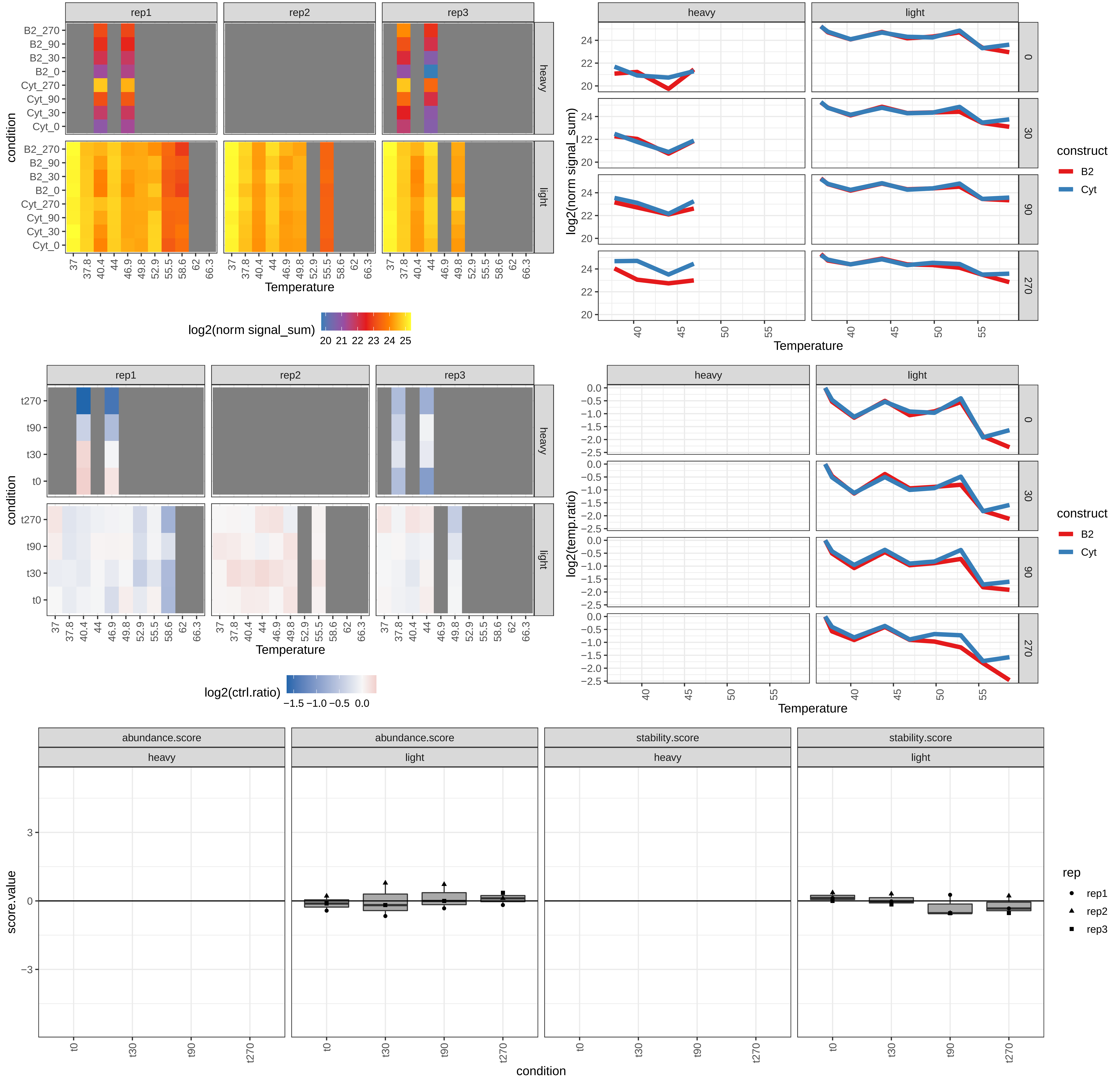

Q04177 − U3 small nucleolar RNA−associated protein 5 OS=Saccharomyces cerevisiae (strain ATCC 204508 / S288c) GN=UTP5 PE=1 SV=1

UTP6 − average.top3: 6.82246904761905 − max.qupm: 14

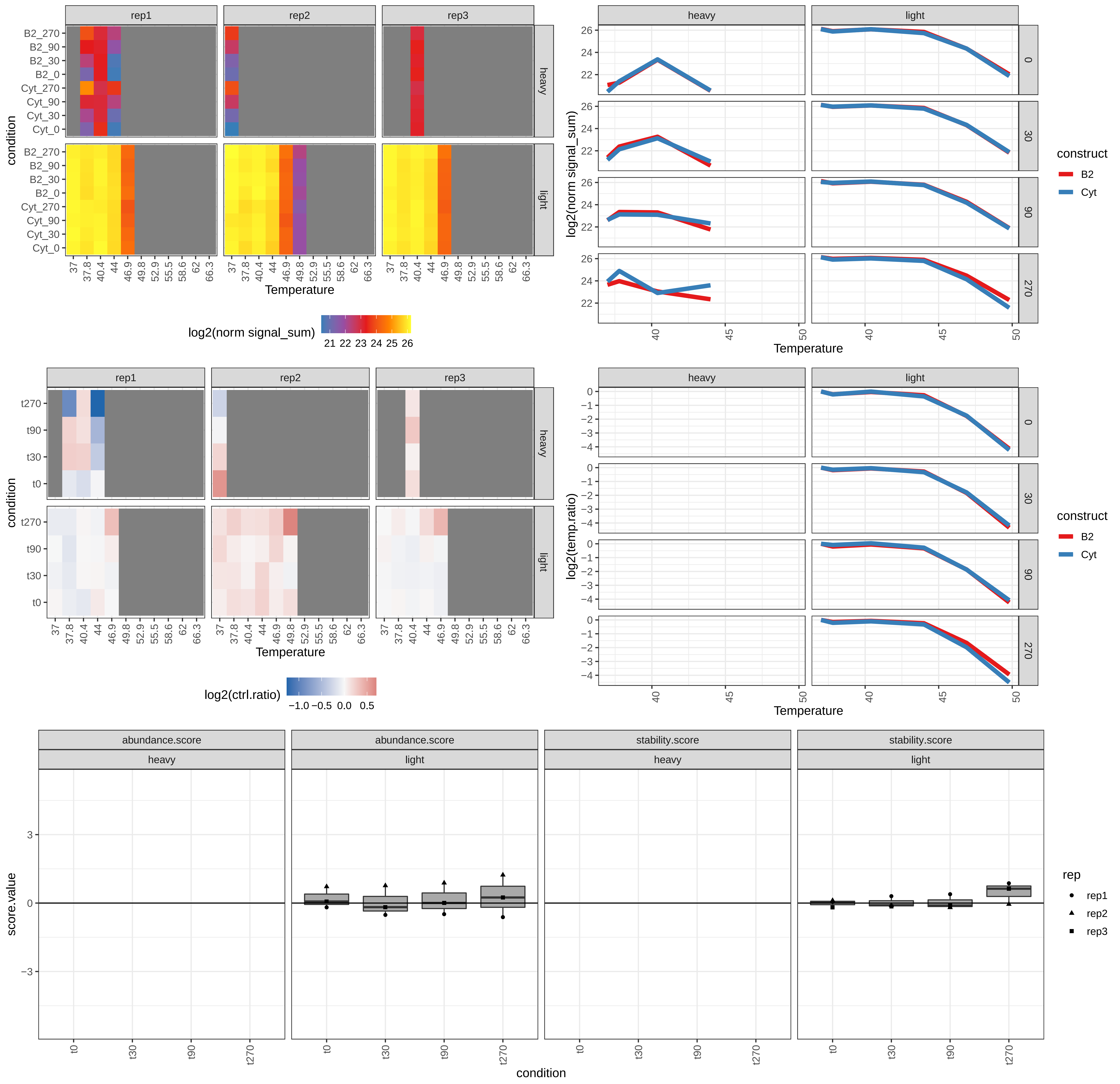

Q02354 − U3 small nucleolar RNA−associated protein 6 OS=Saccharomyces cerevisiae (strain ATCC 204508 / S288c) GN=UTP6 PE=1 SV=2

UTP7 − average.top3: 6.39556055555556 − max.qupm: 5

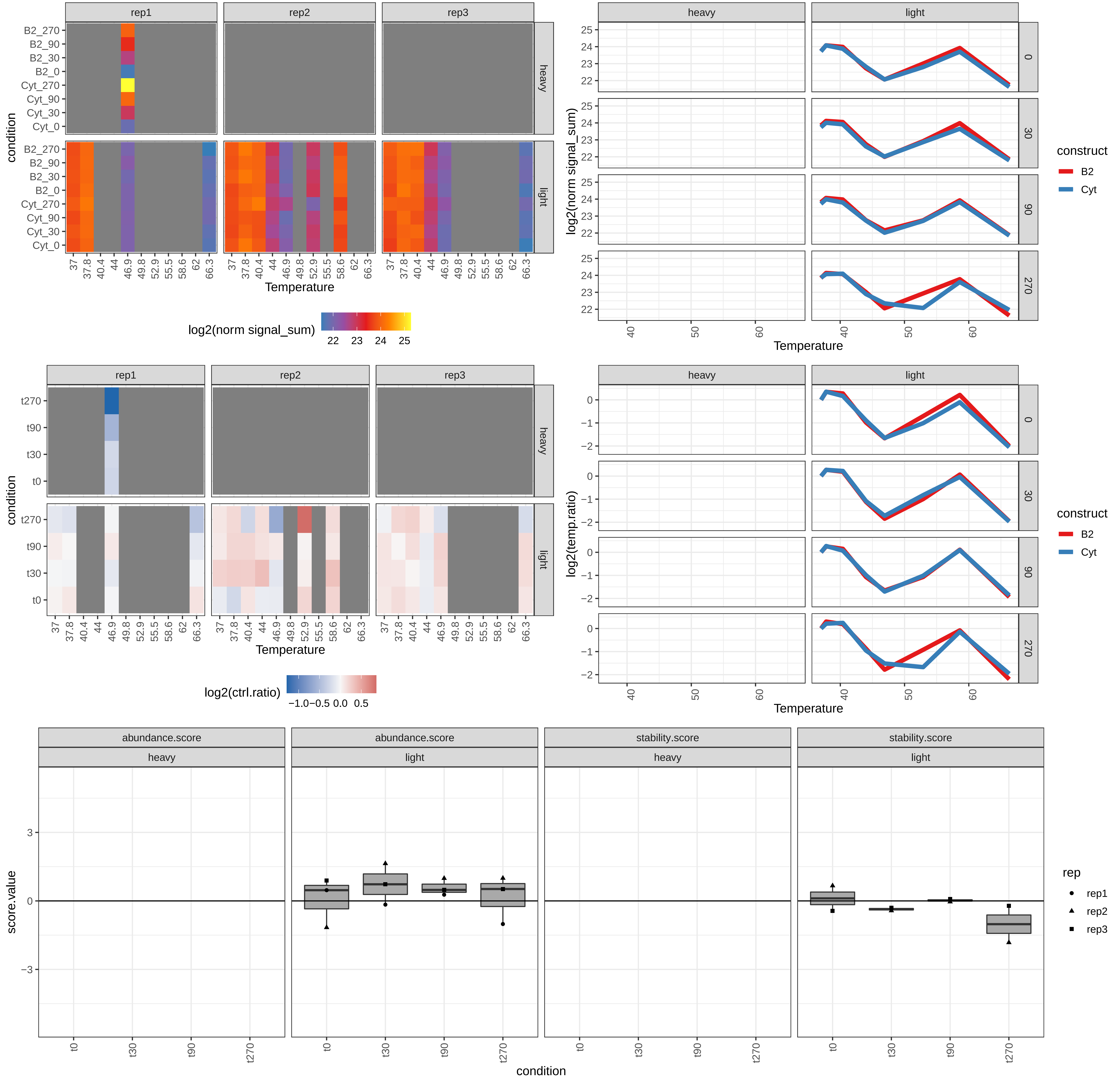

P40055 − U3 small nucleolar RNA−associated protein 7 OS=Saccharomyces cerevisiae (strain ATCC 204508 / S288c) GN=UTP7 PE=1 SV=1

UTP8 − average.top3: 6.810132 − max.qupm: 9

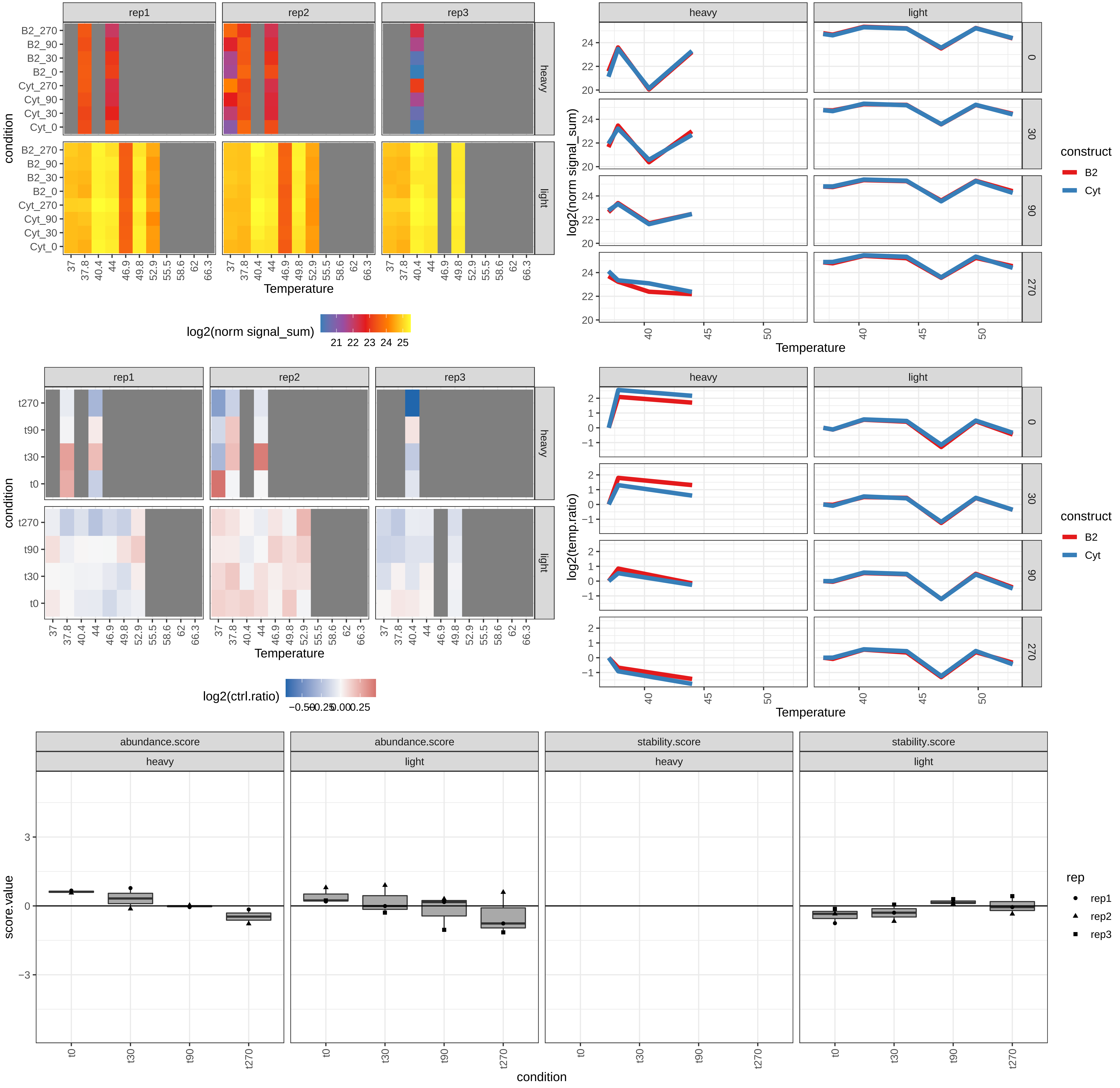

P53276 − U3 small nucleolar RNA−associated protein 8 OS=Saccharomyces cerevisiae (strain ATCC 204508 / S288c) GN=UTP8 PE=1 SV=1

UTP9 − average.top3: 7.00465931034483 − max.qupm: 11

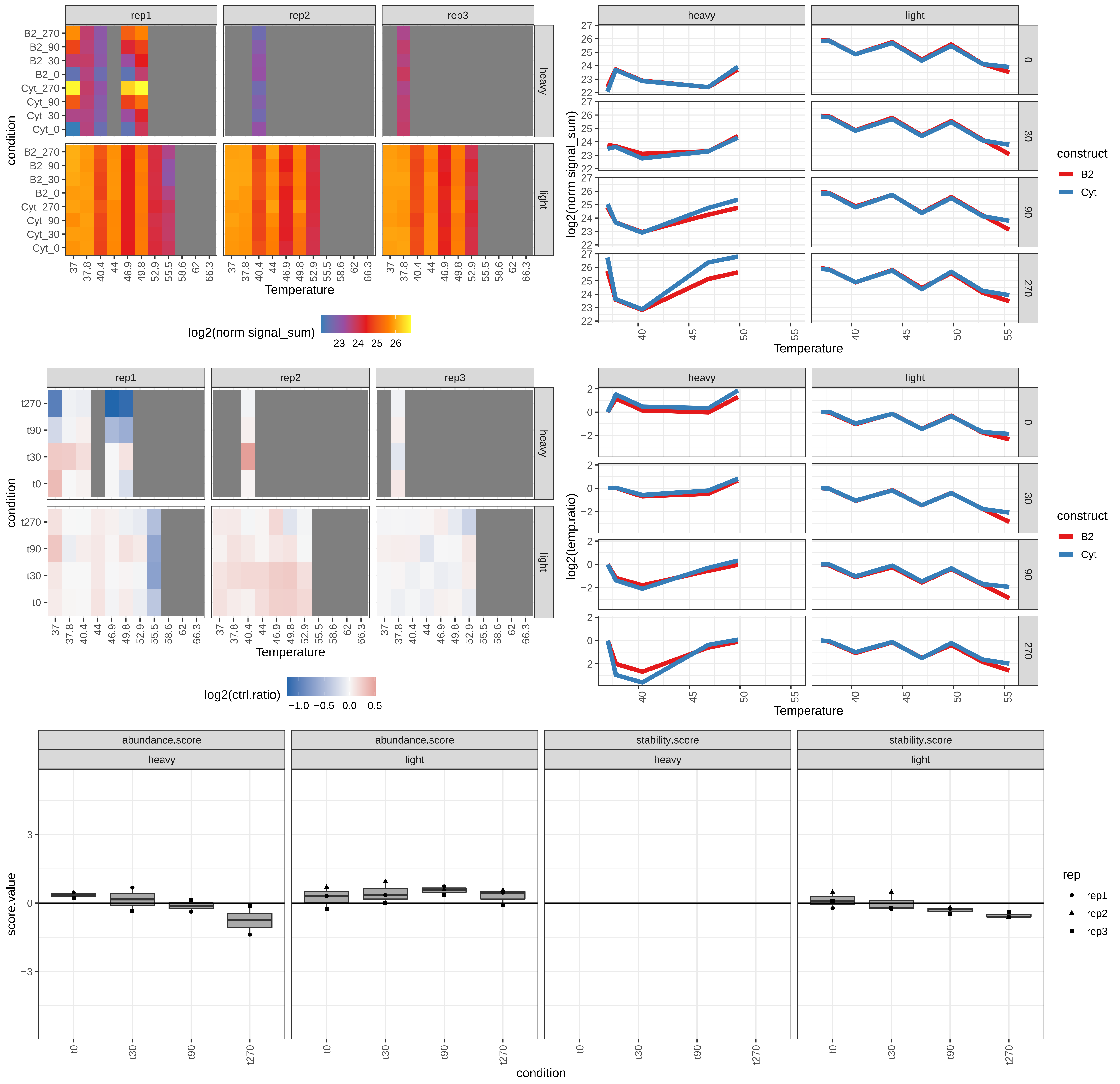

P38882 − U3 small nucleolar RNA−associated protein 9 OS=Saccharomyces cerevisiae (strain ATCC 204508 / S288c) GN=UTP9 PE=1 SV=1

UTR2 − average.top3: 7.08085375 − max.qupm: 3

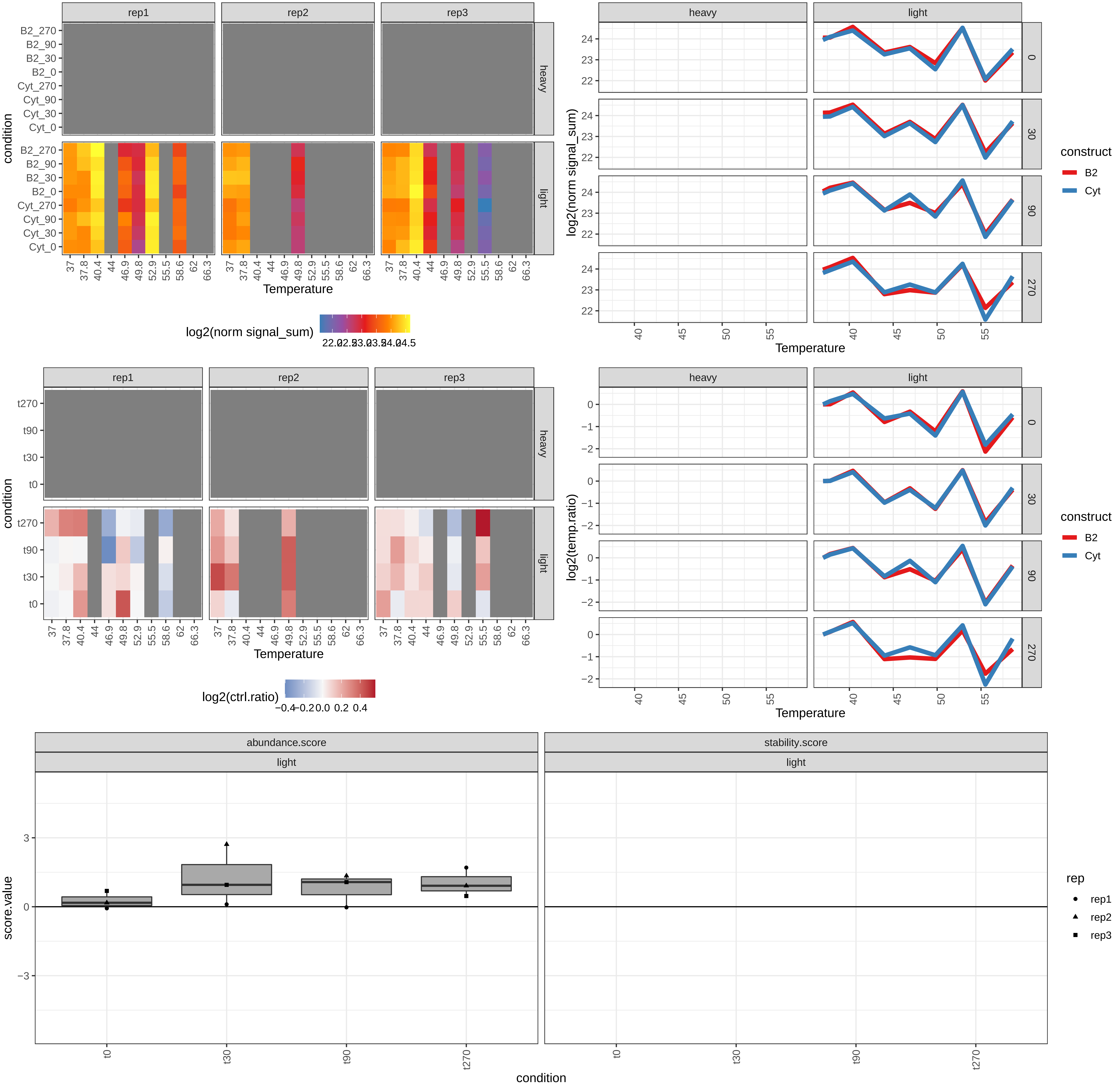

P32623 − Probable glycosidase CRH2 OS=Saccharomyces cerevisiae (strain ATCC 204508 / S288c) GN=UTR2 PE=1 SV=3

UTR4 − average.top3: 6.44178764705882 − max.qupm: 6

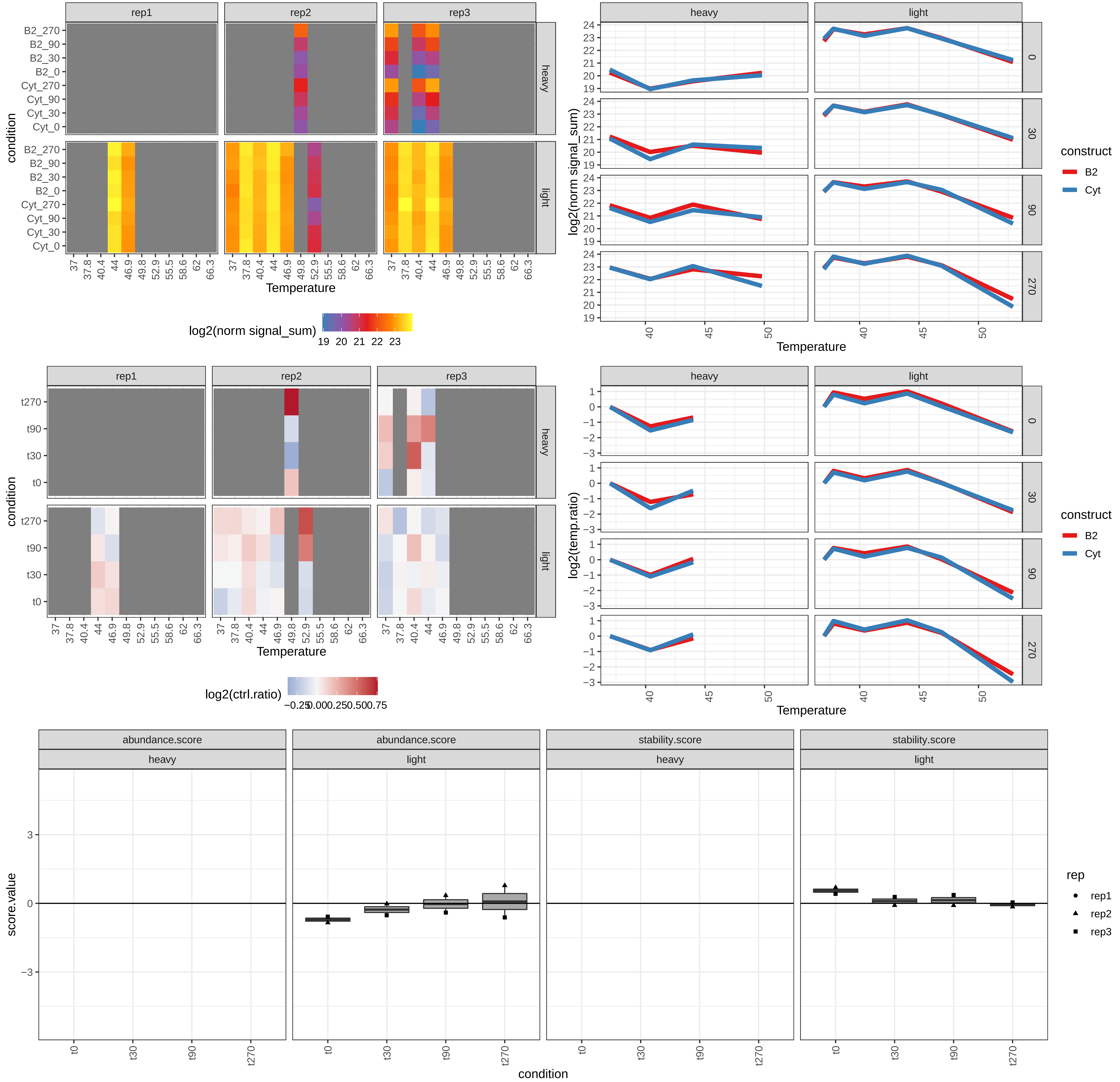

P32626 − Enolase−phosphatase E1 OS=Saccharomyces cerevisiae (strain ATCC 204508 / S288c) GN=UTR4 PE=1 SV=2

VAB2 − average.top3: 6.472425 − max.qupm: 4

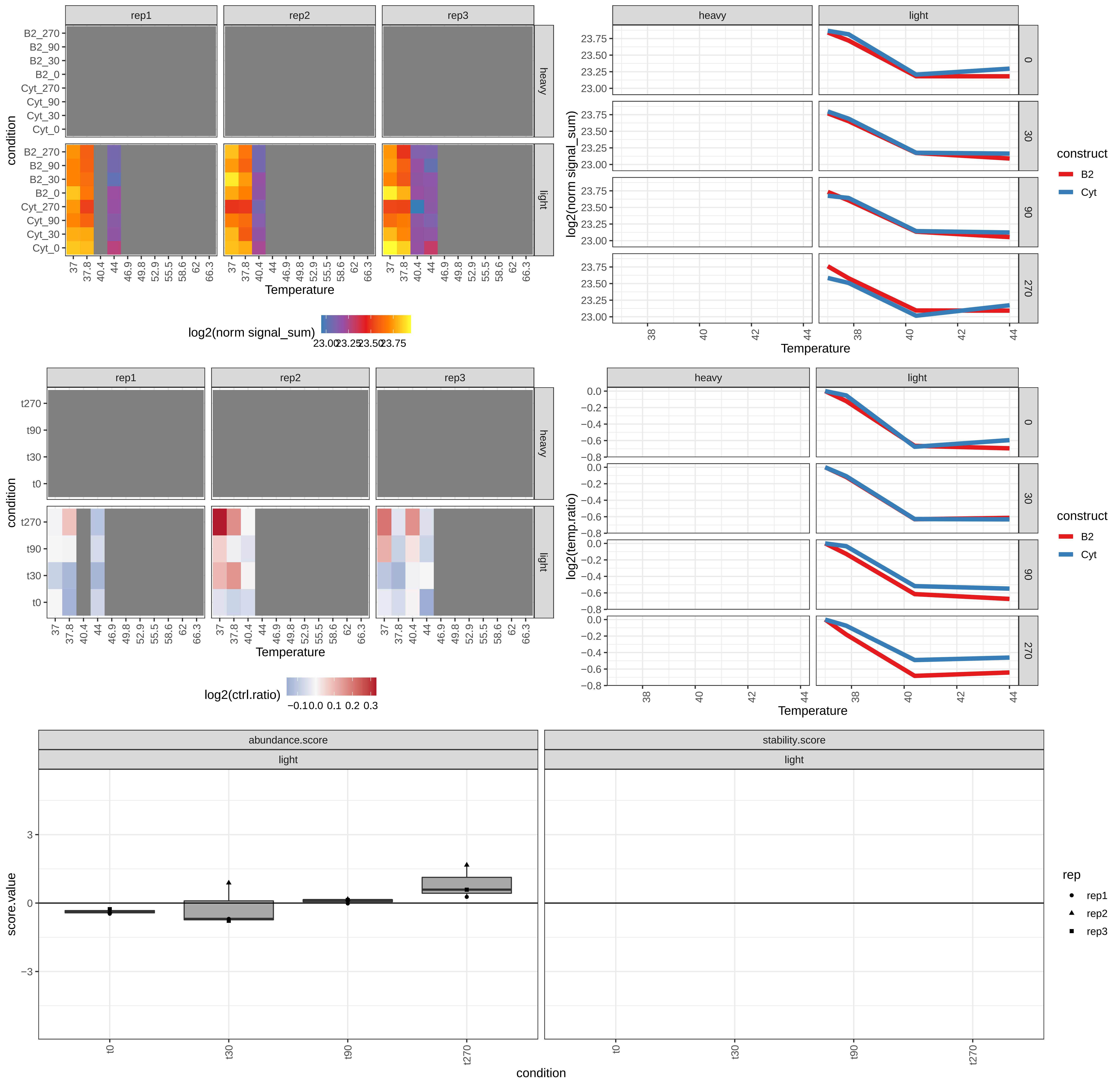

P40003 − Biogenesis of lysosome−related organelles complex 1 subunit VAB2 OS=Saccharomyces cerevisiae (strain ATCC 204508 / S288c) GN=VAB2 PE=1 SV=1

VAC14 − average.top3: 7.06587291666667 − max.qupm: 12

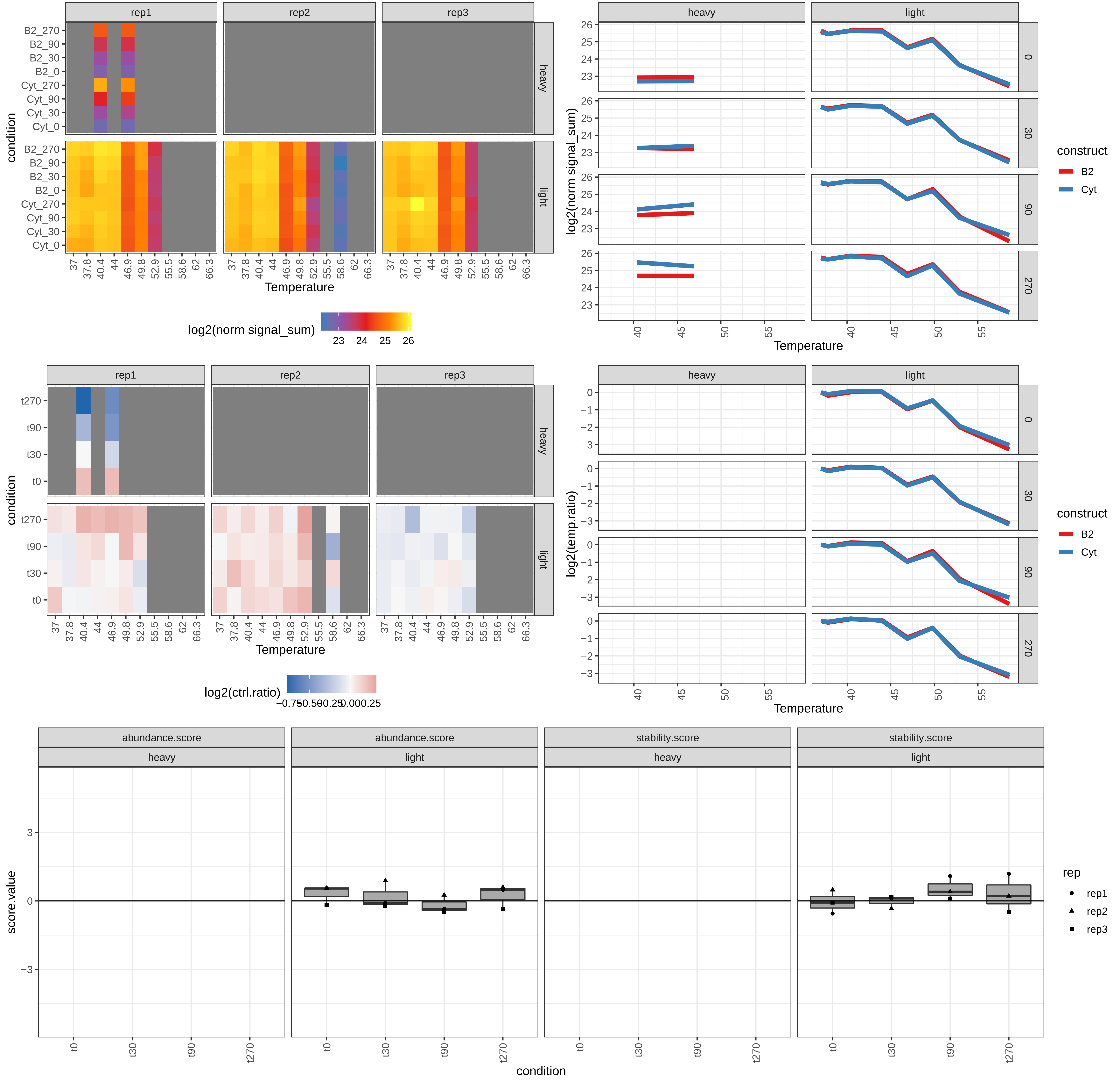

Q06708 − Vacuole morphology and inheritance protein 14 OS=Saccharomyces cerevisiae (strain ATCC 204508 / S288c) GN=VAC14 PE=1 SV=1

VAC8 − average.top3: 7.21124555555556 − max.qupm: 12

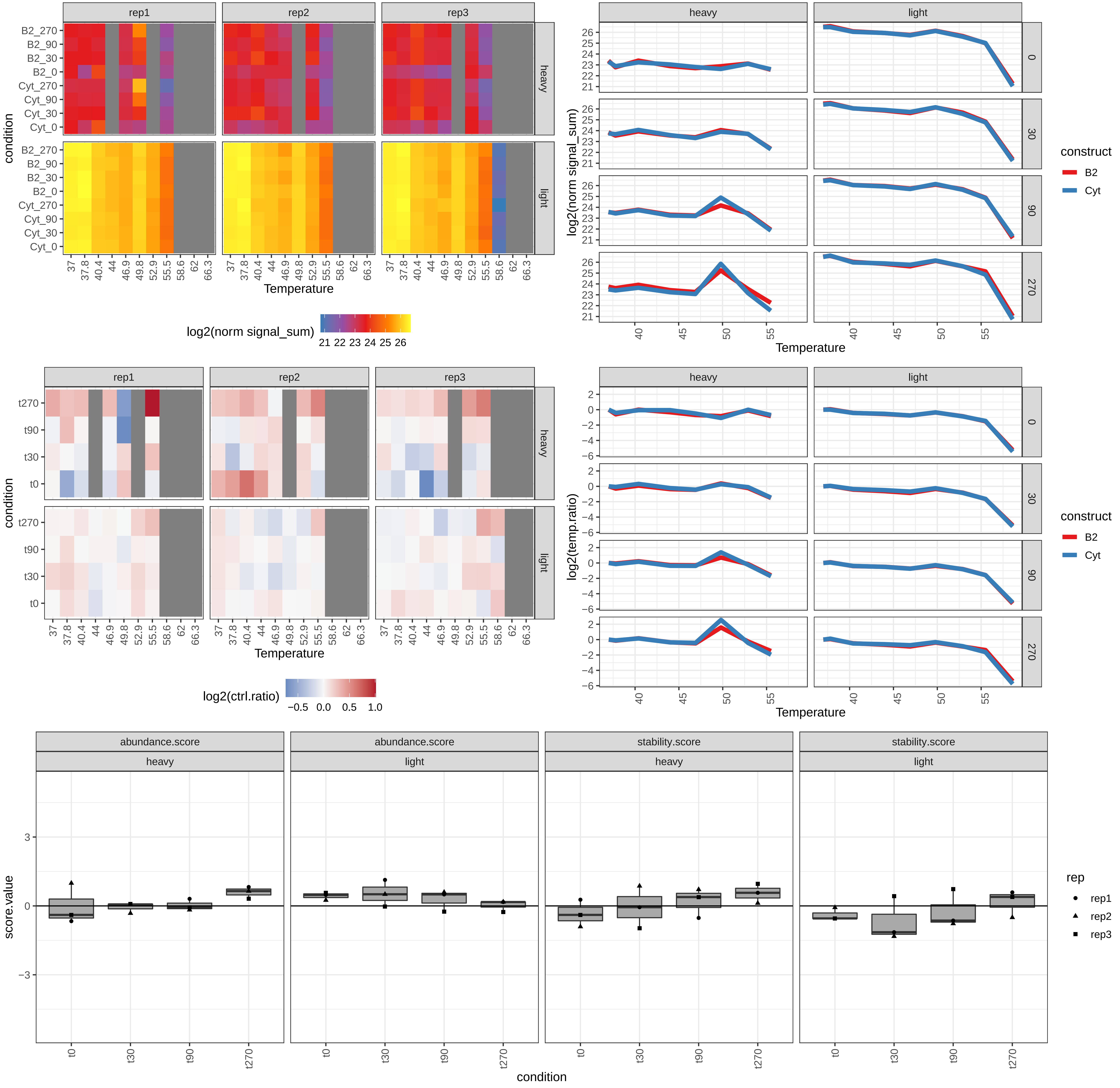

P39968 − Vacuolar protein 8 OS=Saccharomyces cerevisiae (strain ATCC 204508 / S288c) GN=VAC8 PE=1 SV=3

VAM6 − average.top3: 5.97350583333333 − max.qupm: 4

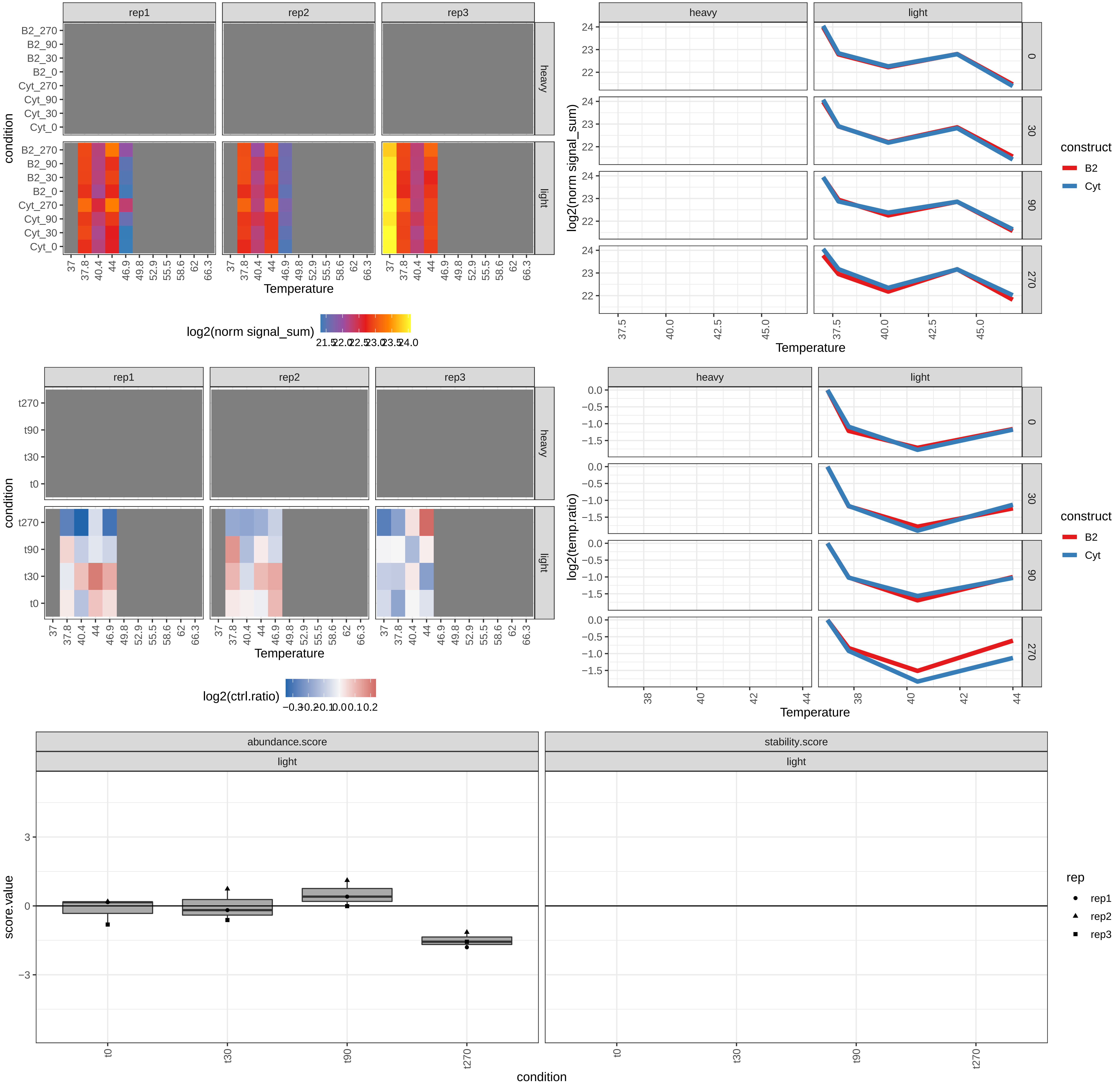

Q07468 − Vacuolar morphogenesis protein 6 OS=Saccharomyces cerevisiae (strain ATCC 204508 / S288c) GN=VAM6 PE=1 SV=1

VAN1 − average.top3: 6.79333466666667 − max.qupm: 5

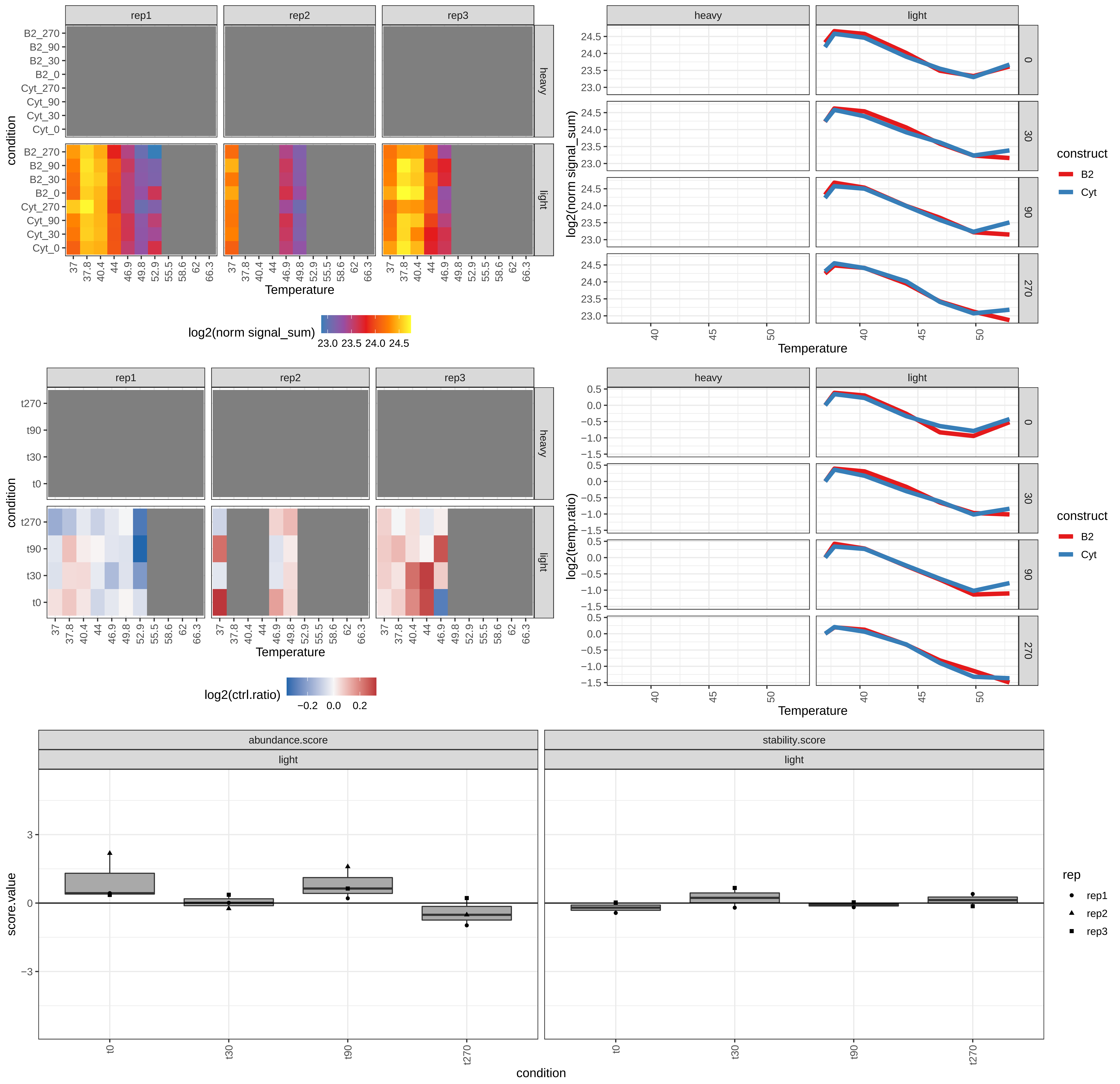

P23642 − Mannan polymerase I complex VAN1 subunit OS=Saccharomyces cerevisiae (strain ATCC 204508 / S288c) GN=VAN1 PE=1 SV=3

VAR1 − average.top3: 6.66101636363636 − max.qupm: 6

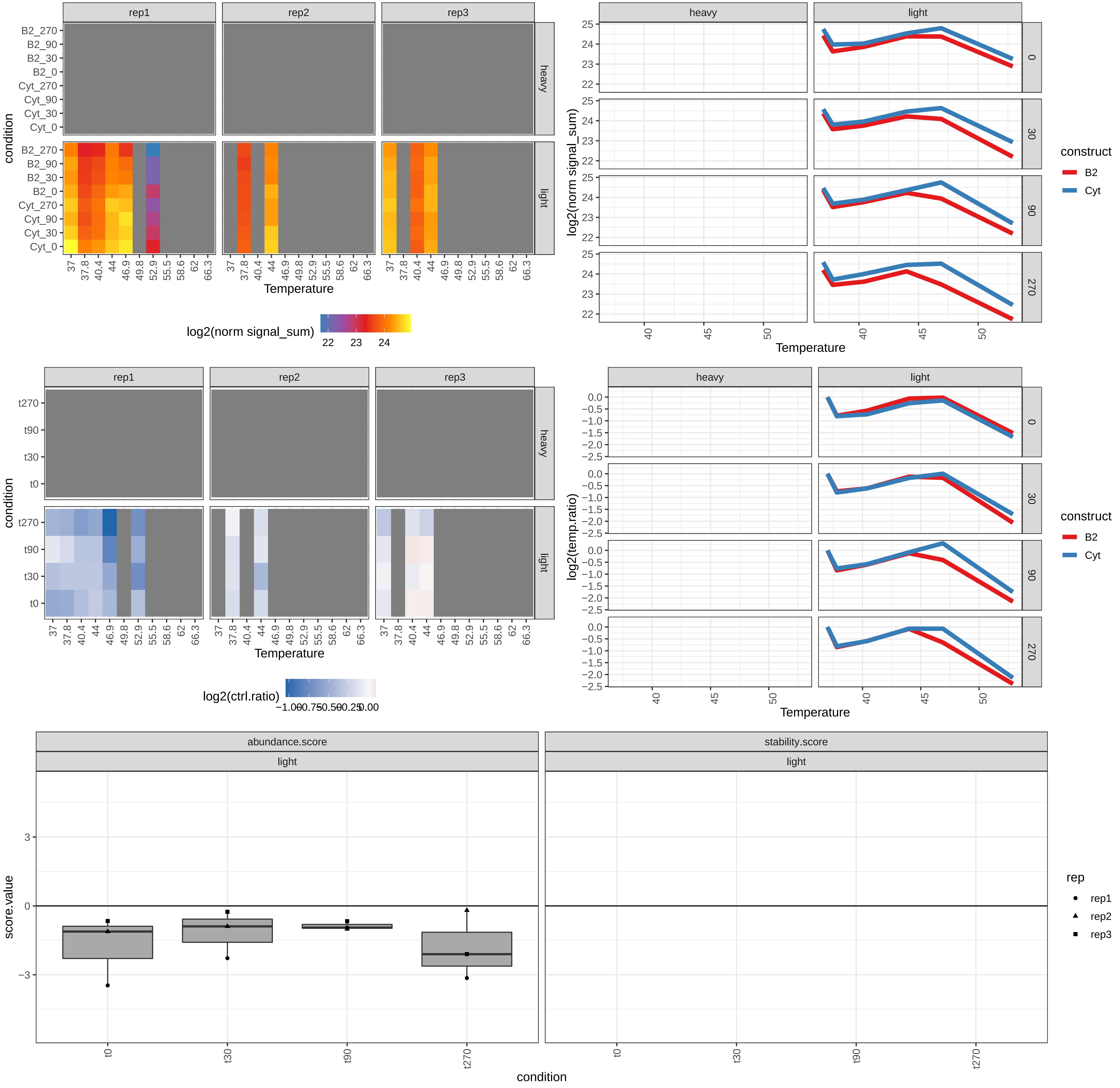

P02381 − Ribosomal protein VAR1, mitochondrial OS=Saccharomyces cerevisiae (strain ATCC 204508 / S288c) GN=VAR1 PE=1 SV=3

VAS1 − average.top3: 7.46080689655172 − max.qupm: 36

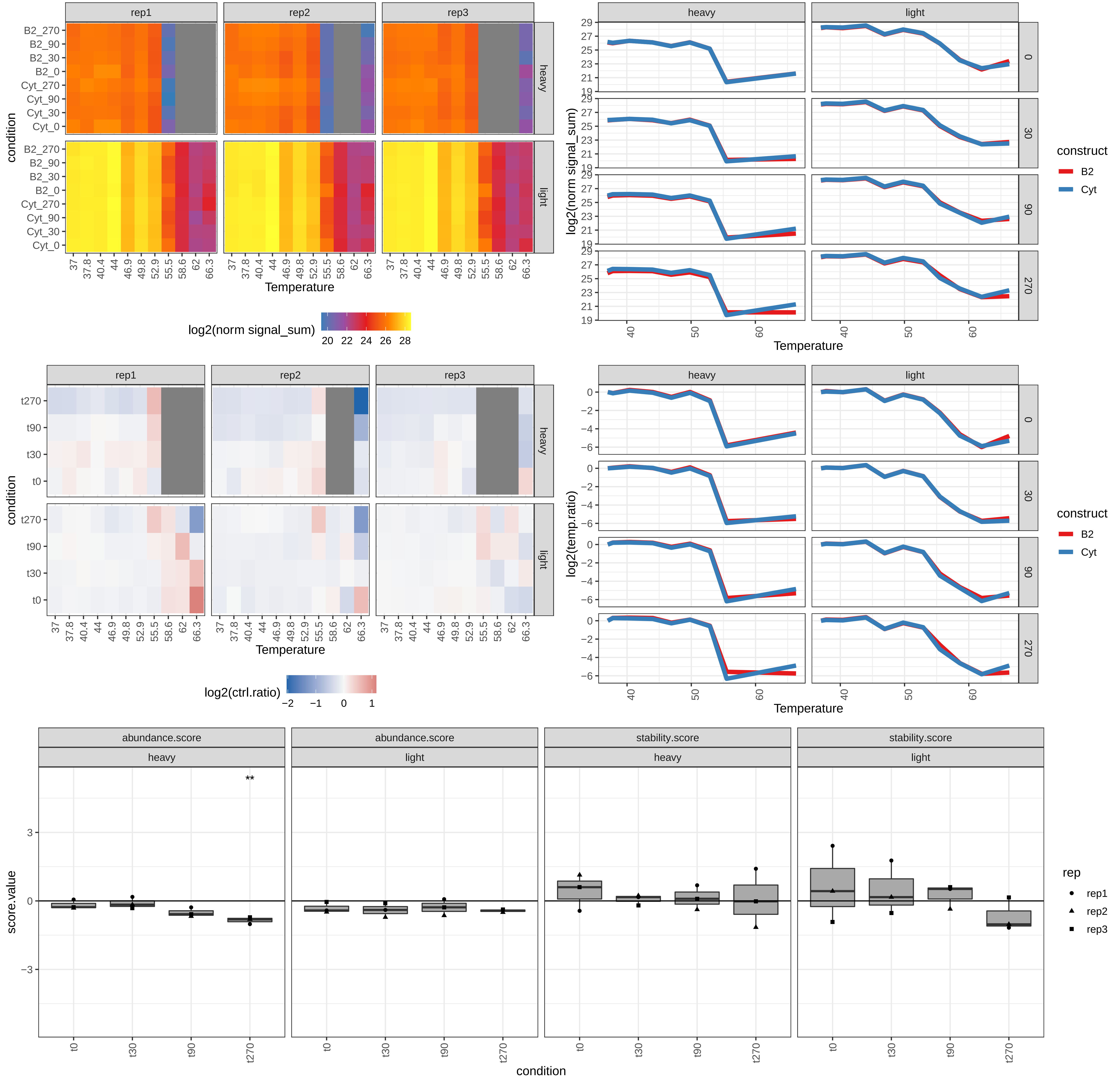

asmic of Valine−−tRNA ligase, mitochondrial OS=Saccharomyces cerevisiae (strain ATCC 204508 / S288c) GN=VAS1|Valine−−tRNA ligase, mitochondrial OS=Saccharomyces cerevisiae (strain ATCC 204

VBA1 − average.top3: 6.65956130434783 − max.qupm: 4

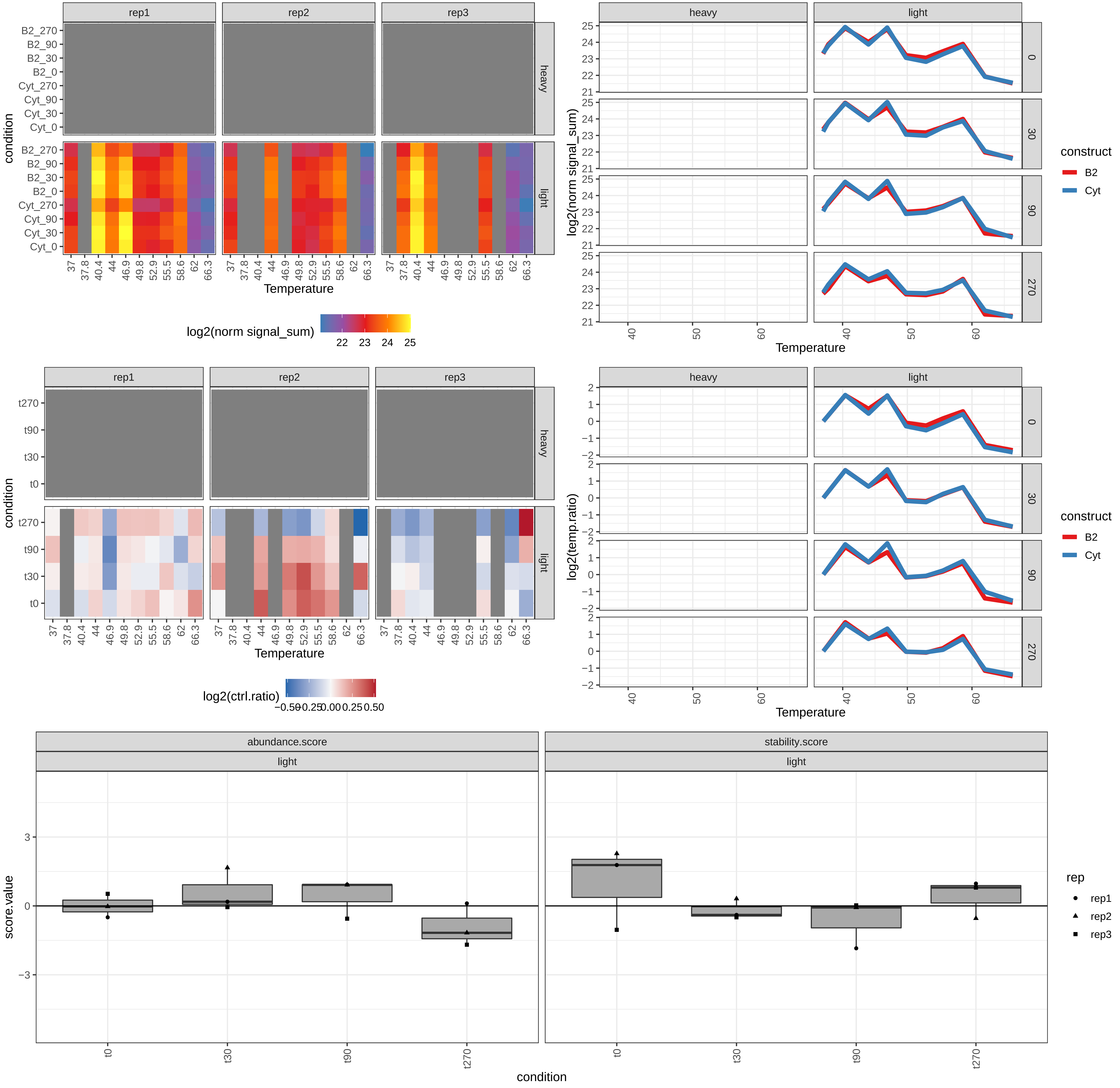

Q04301 − Vacuolar basic amino acid transporter 1 OS=Saccharomyces cerevisiae (strain ATCC 204508 / S288c) GN=VBA1 PE=1 SV=1

VCX1 − average.top3: 7.06008210526316 − max.qupm: 5

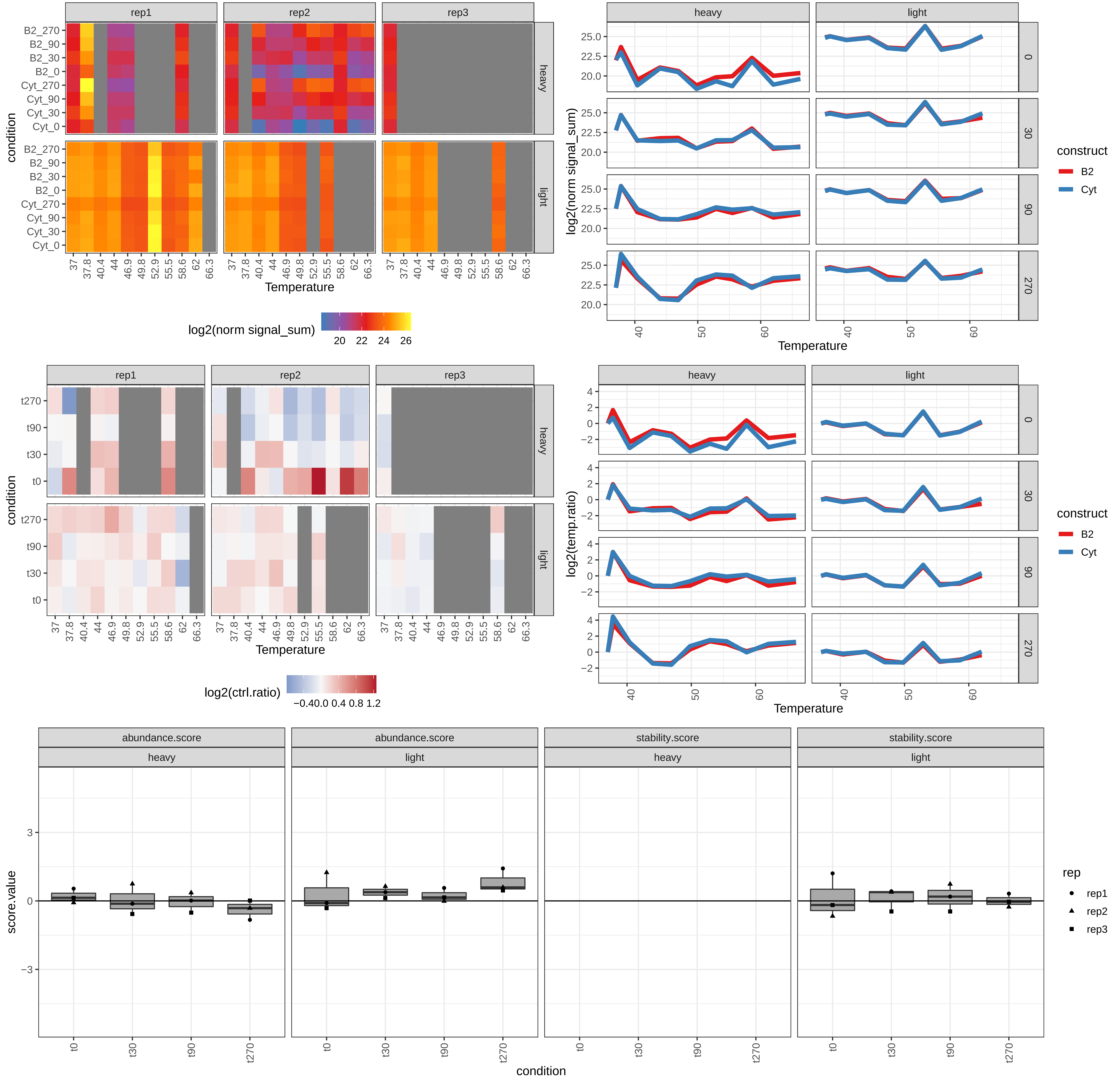

Q99385 − Vacuolar calcium ion transporter OS=Saccharomyces cerevisiae (strain ATCC 204508 / S288c) GN=VCX1 PE=1 SV=1

VHS3 − average.top3: 6.55895636363636 − max.qupm: 5

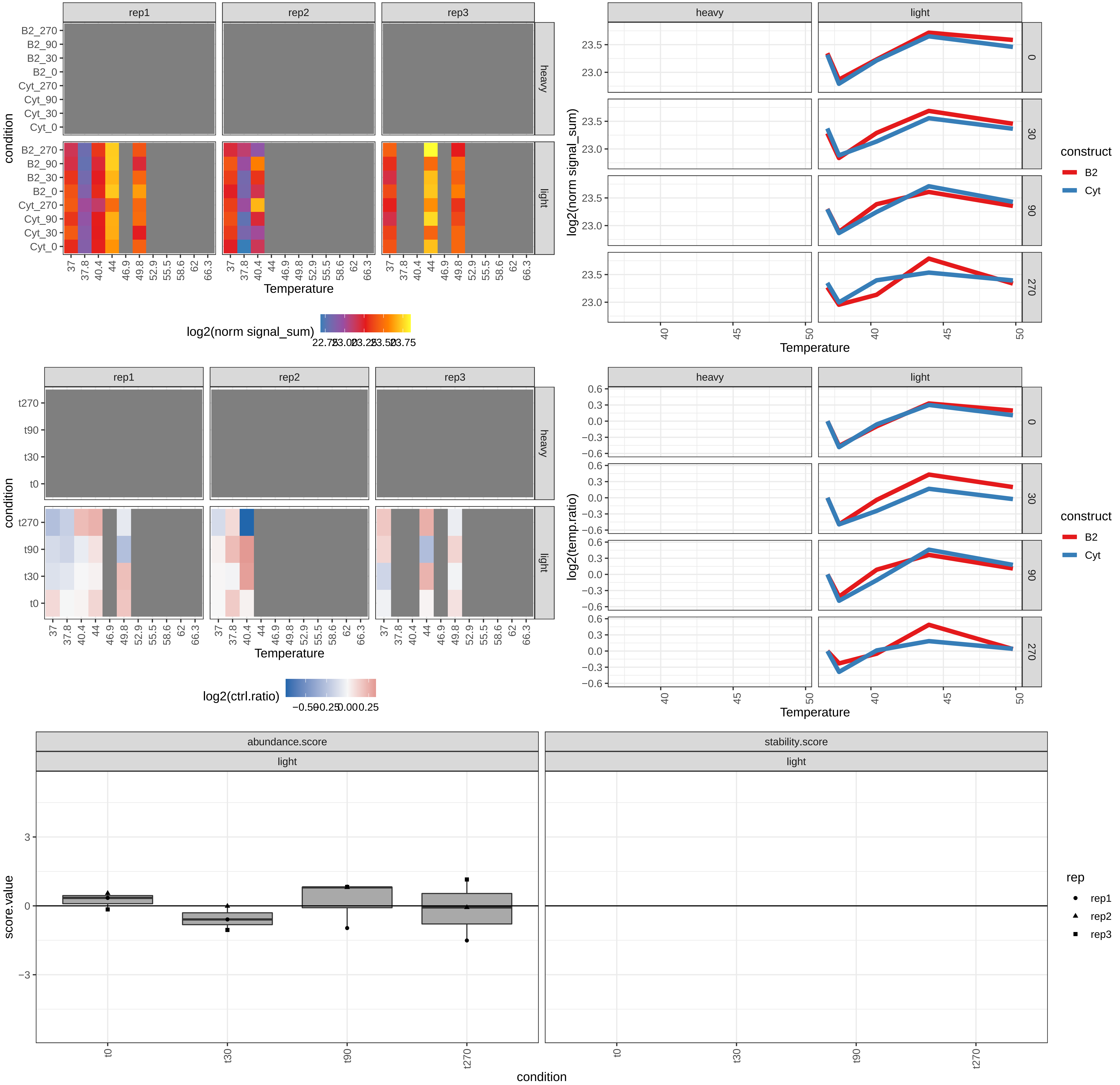

Q08438 − Phosphopantothenoylcysteine decarboxylase subunit VHS3 OS=Saccharomyces cerevisiae (strain ATCC 204508 / S288c) GN=VHS3 PE=1 SV=1

VHT1 − average.top3: 6.75639166666667 − max.qupm: 6

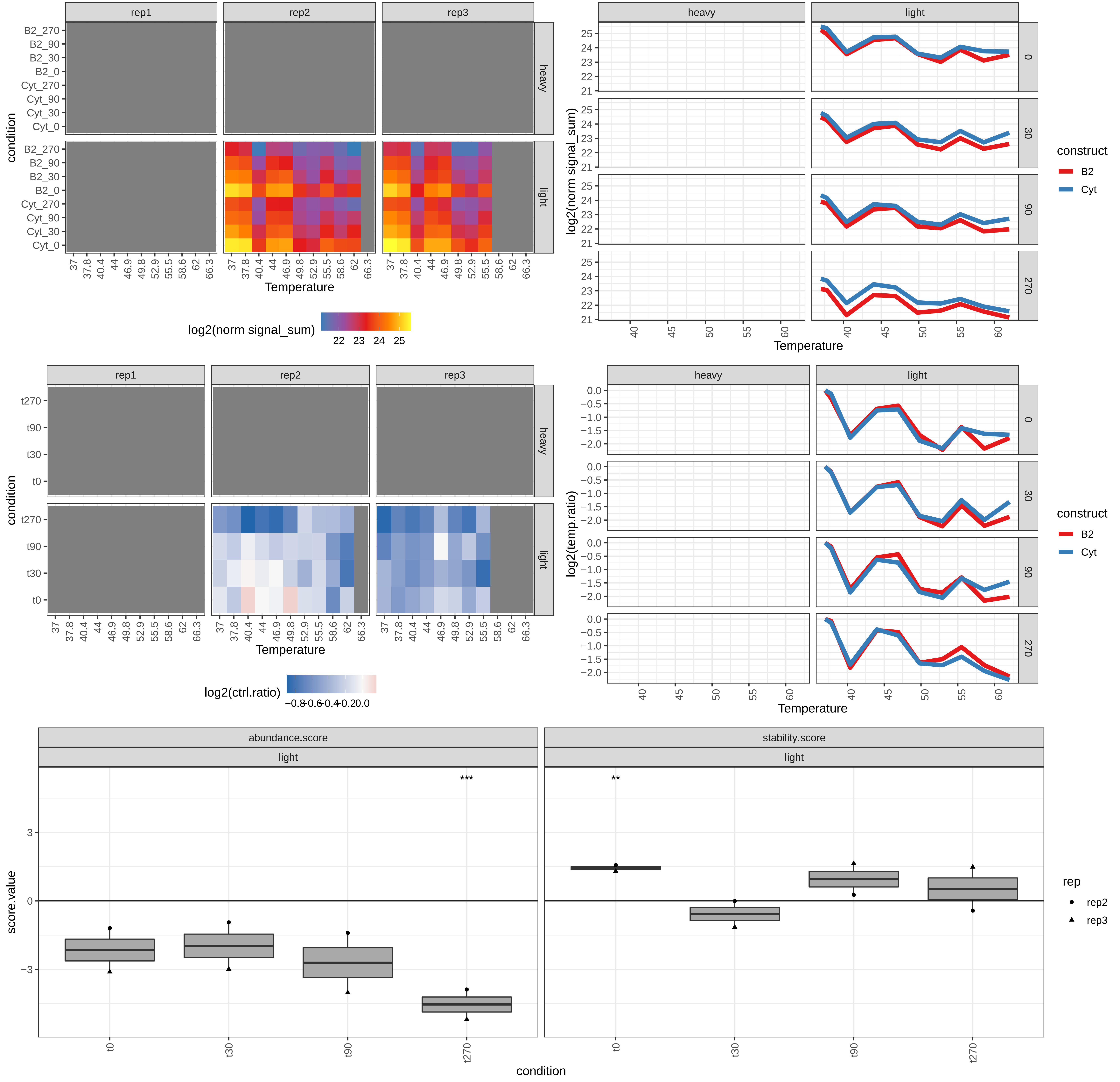

P53241 − Vitamin H transporter OS=Saccharomyces cerevisiae (strain ATCC 204508 / S288c) GN=VHT1 PE=1 SV=1

VID22 − average.top3: 6.52687142857143 − max.qupm: 5

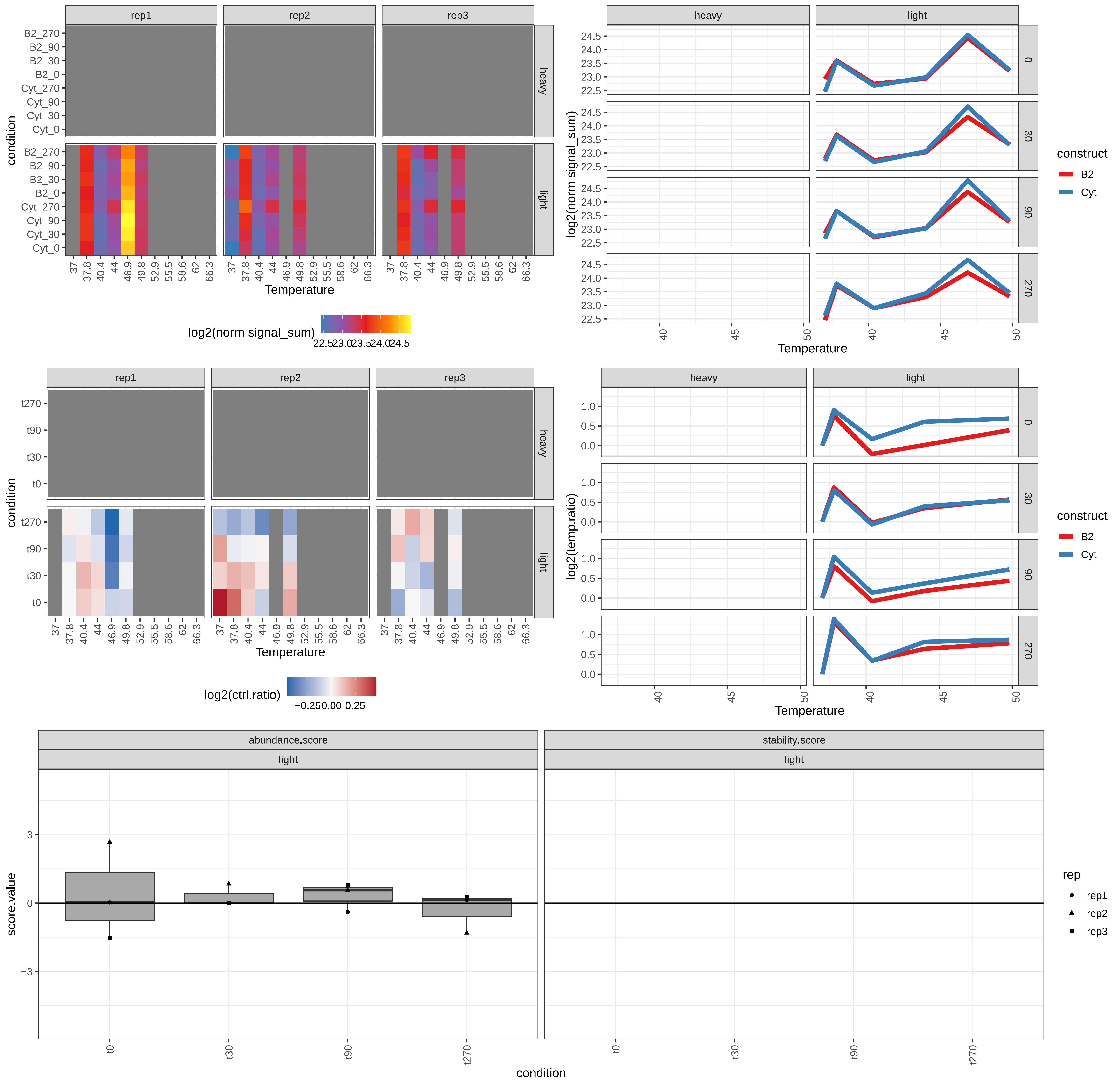

Q05934 − Vacuolar import and degradation protein 22 OS=Saccharomyces cerevisiae (strain ATCC 204508 / S288c) GN=VID22 PE=1 SV=1

VID27 − average.top3: 6.83201588235294 − max.qupm: 8

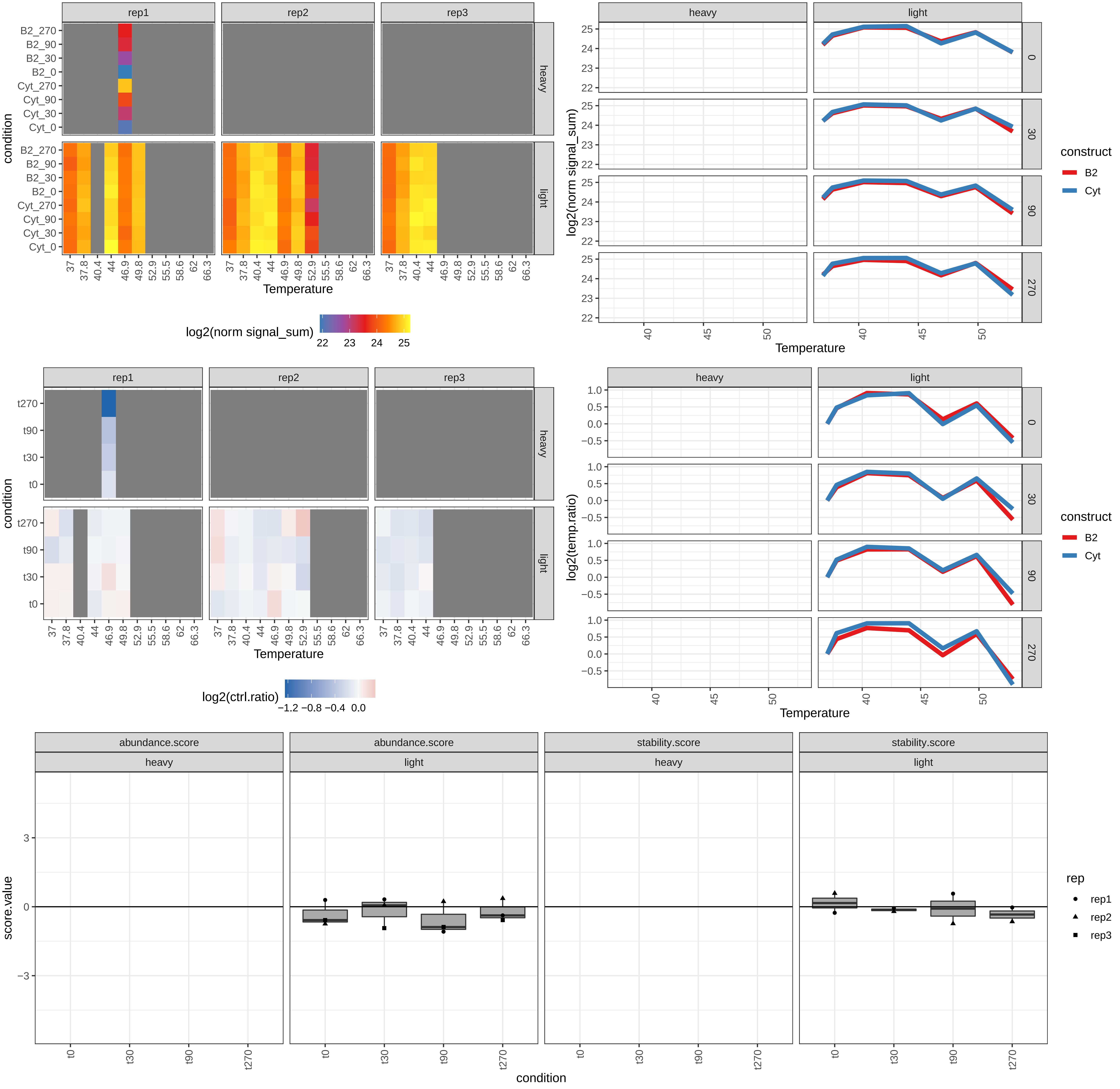

P40157 − Vacuolar import and degradation protein 27 OS=Saccharomyces cerevisiae (strain ATCC 204508 / S288c) GN=VID27 PE=1 SV=1

VID28 − average.top3: 6.72140913043478 − max.qupm: 10

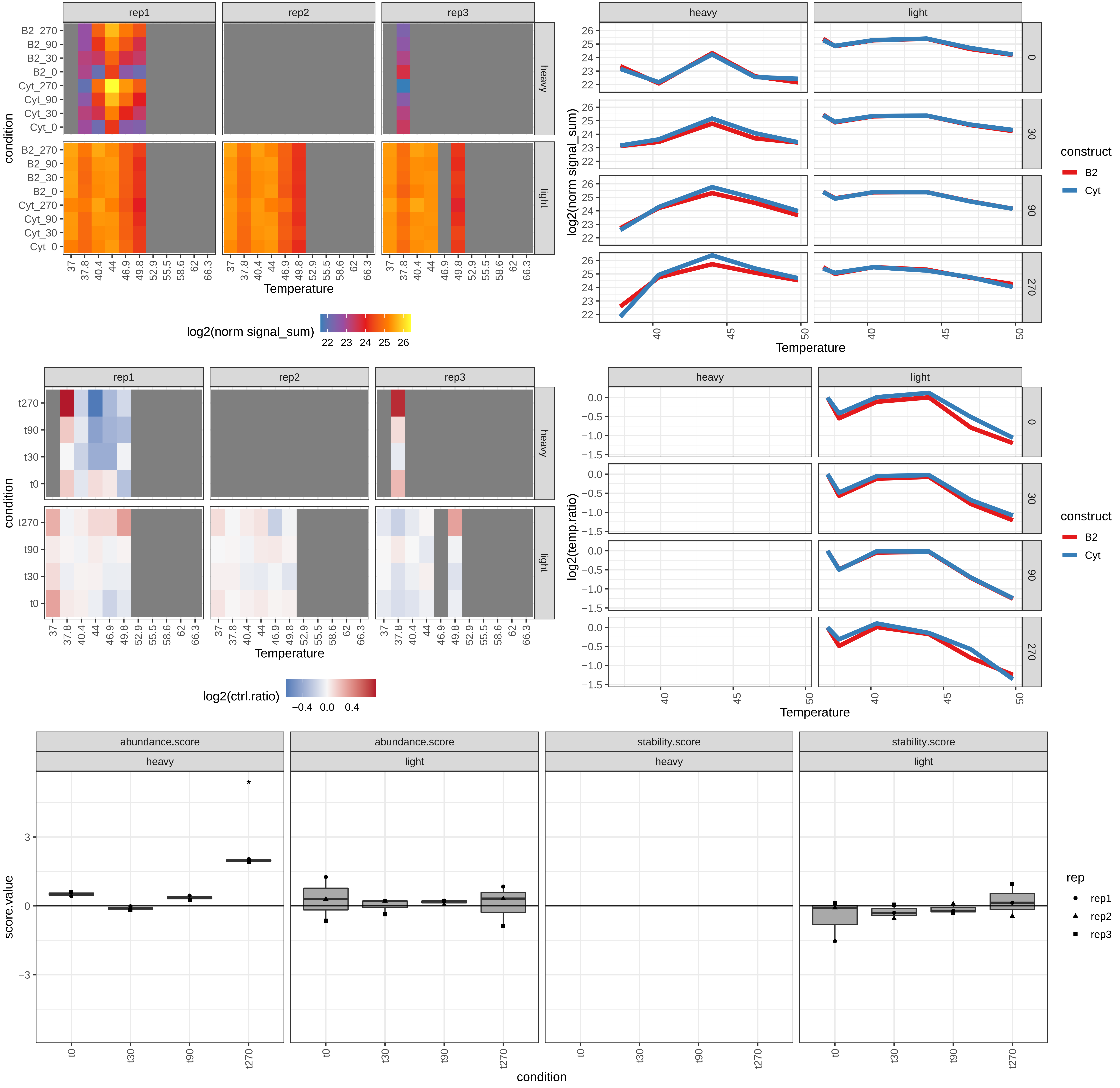

P40547 − Vacuolar import and degradation protein 28 OS=Saccharomyces cerevisiae (strain ATCC 204508 / S288c) GN=VID28 PE=1 SV=1

VID30 − average.top3: 6.31688611111111 − max.qupm: 7

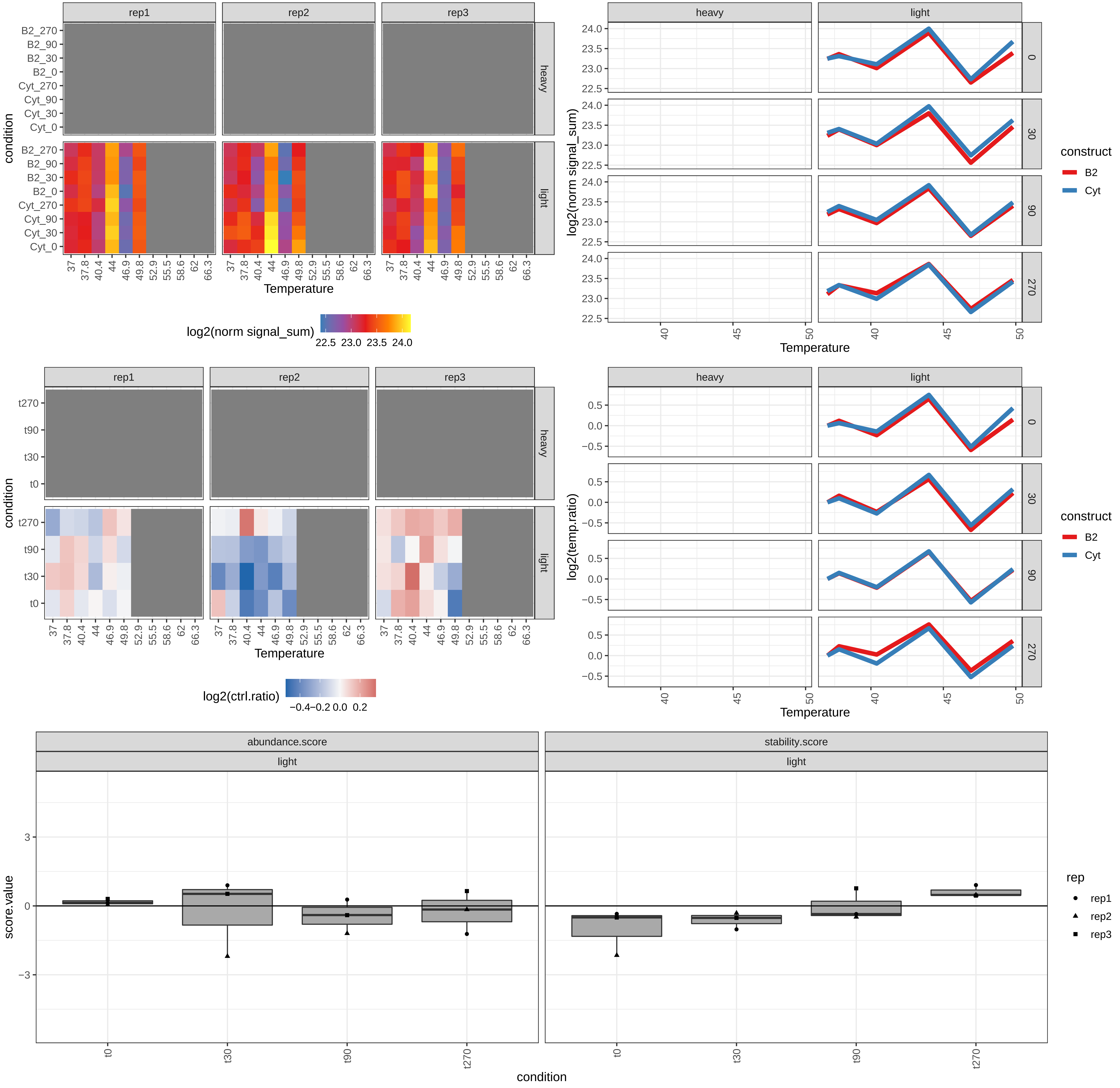

P53076 − Vacuolar import and degradation protein 30 OS=Saccharomyces cerevisiae (strain ATCC 204508 / S288c) GN=VID30 PE=1 SV=1

VIP1 − average.top3: 6.88762909090909 − max.qupm: 20

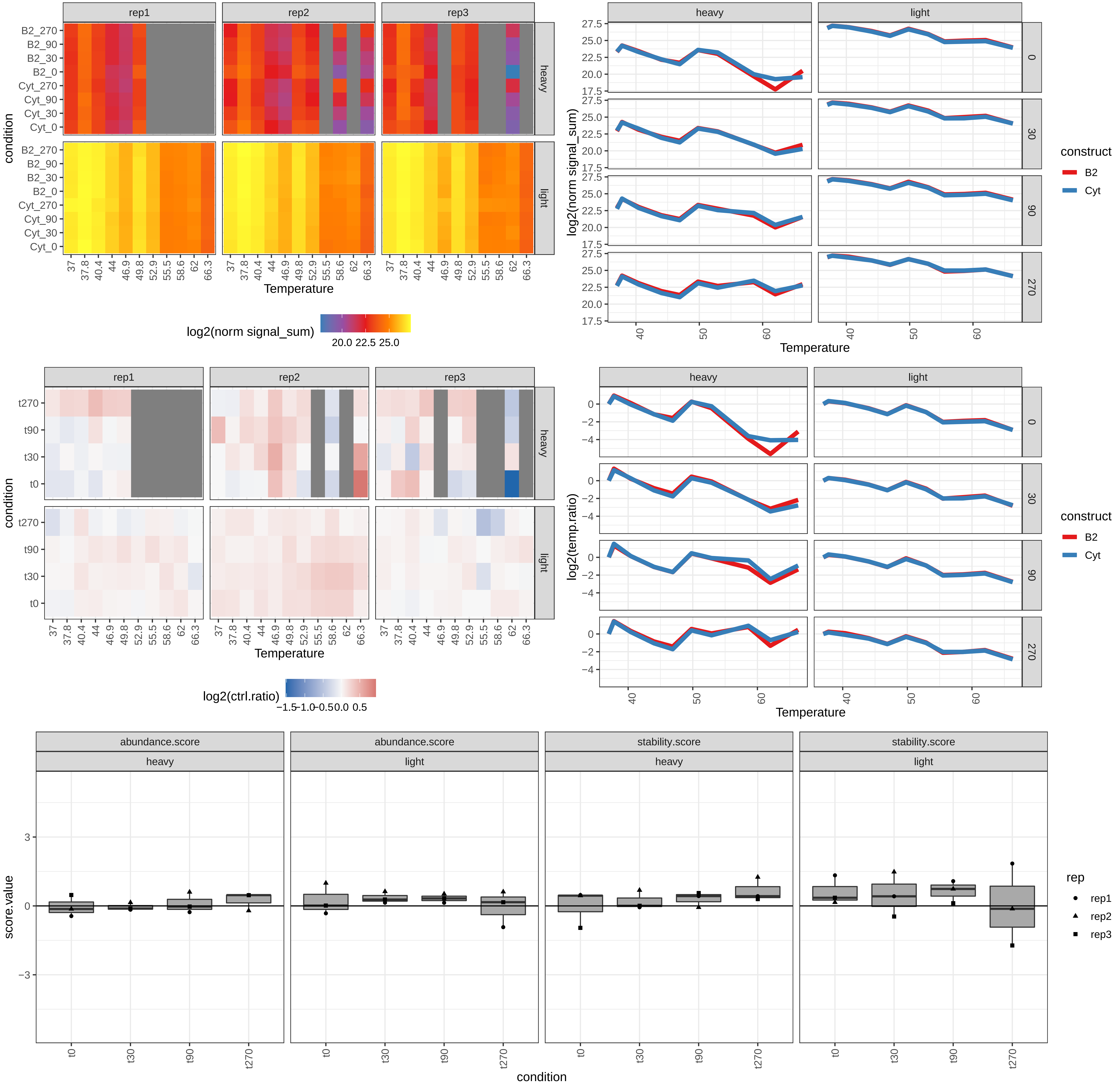

Q06685 − Inositol hexakisphosphate and diphosphoinositol−pentakisphosphate kinase OS=Saccharomyces cerevisiae (strain ATCC 204508 / S288c) GN=VIP1 PE=1 SV=1

VMA1 − average.top3: 8.06268030769231 − max.qupm: 44

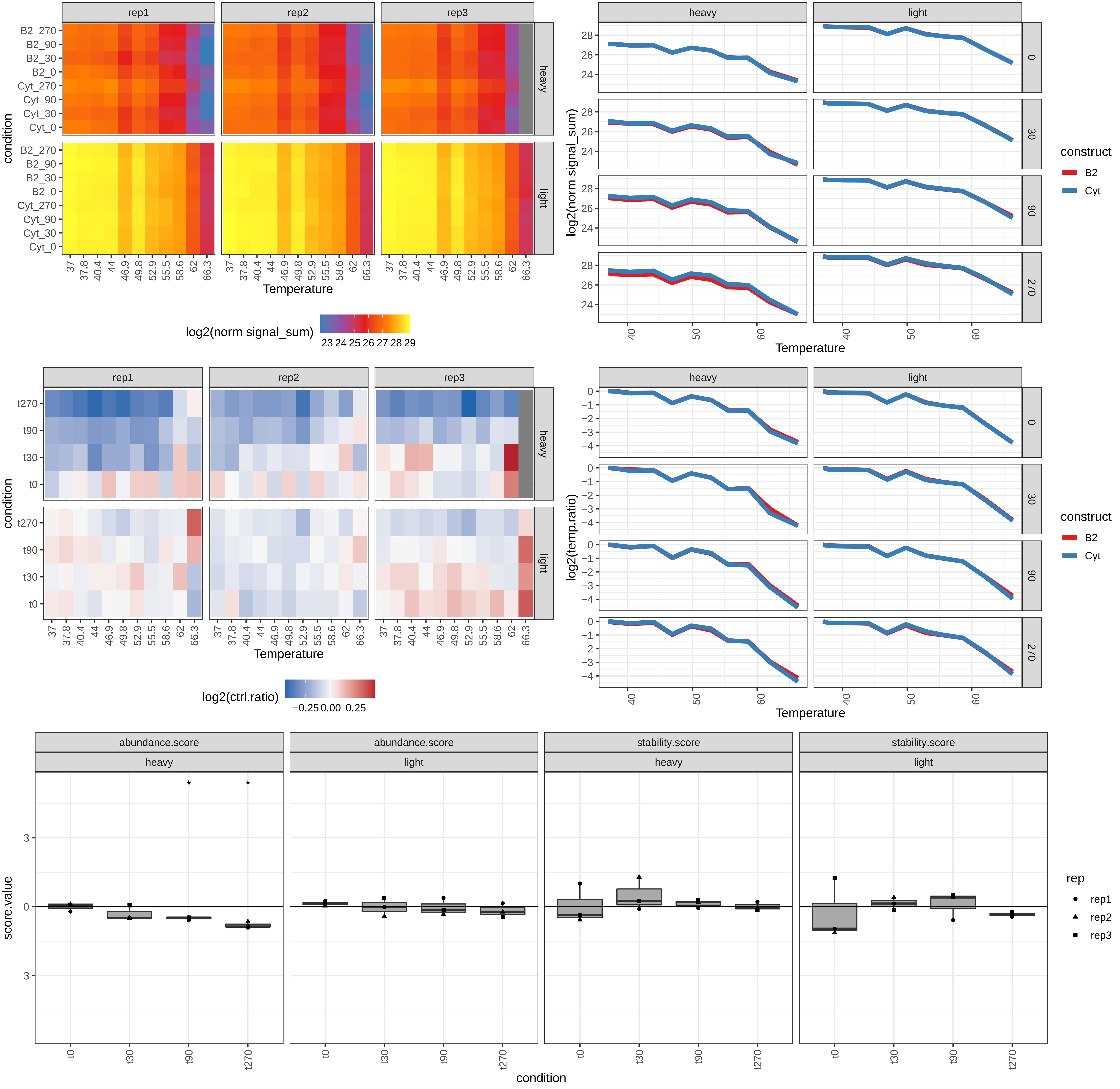

P17255 − V−type proton ATPase catalytic subunit A OS=Saccharomyces cerevisiae (strain ATCC 204508 / S288c) GN=VMA1 PE=1 SV=3

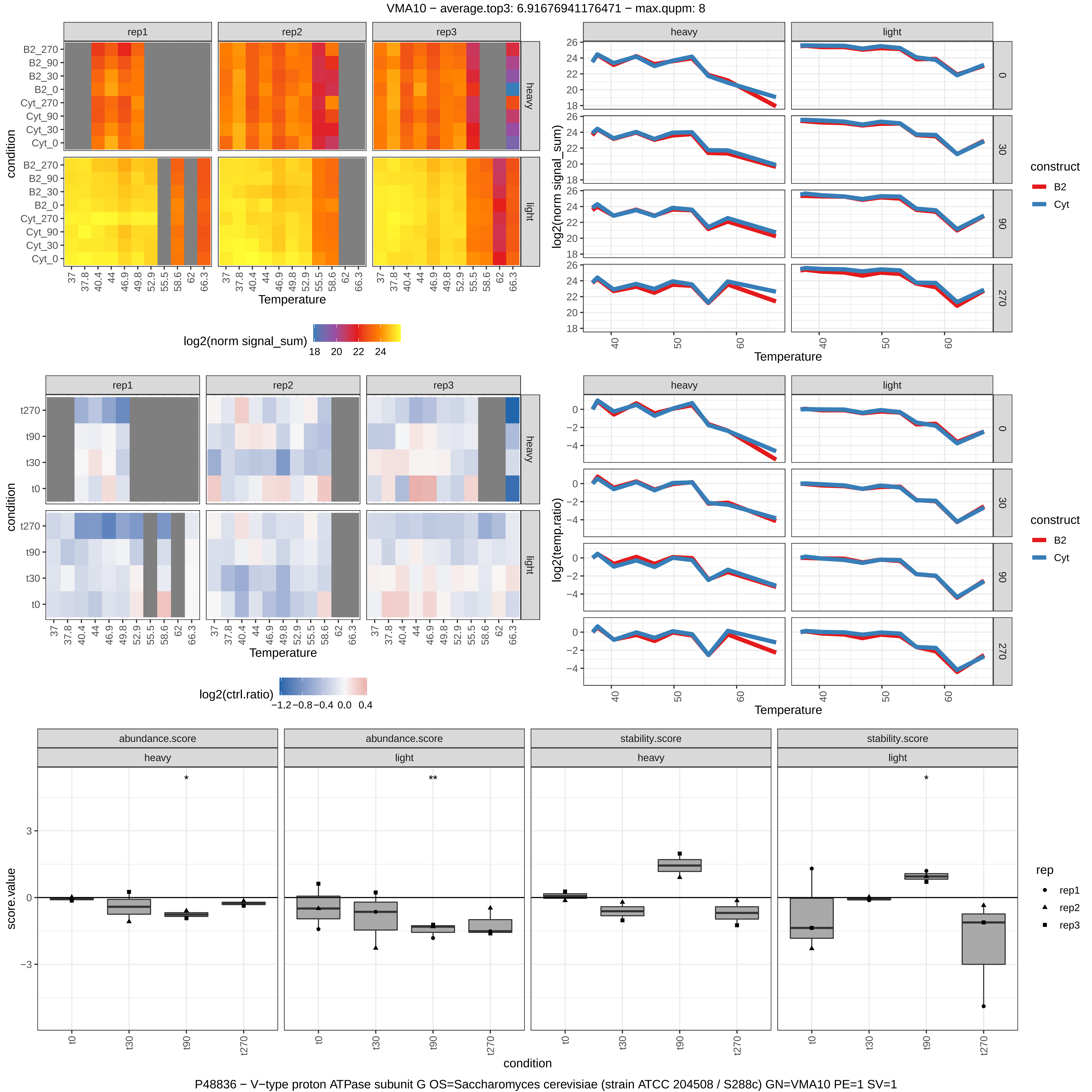

VMA10 − average.top3: 6.91676941176471 − max.qupm: 8
rep1
rep2
rep3
heavy
light
condition
Temperature
log2(norm signal_sum)
log2(temp.ratio)
log2(ctrl.ratio)
construct
B2
Cyt
abundance.score
stability.score
score.value
rep
rep1
rep2
rep3
P48836 − V−type proton ATPase subunit G OS=Saccharomyces cerevisiae (strain ATCC 204508 / S288c) GN=VMA10 PE=1 SV=1

VMA13 − average.top3: 7.65909659574468 − max.qupm: 18

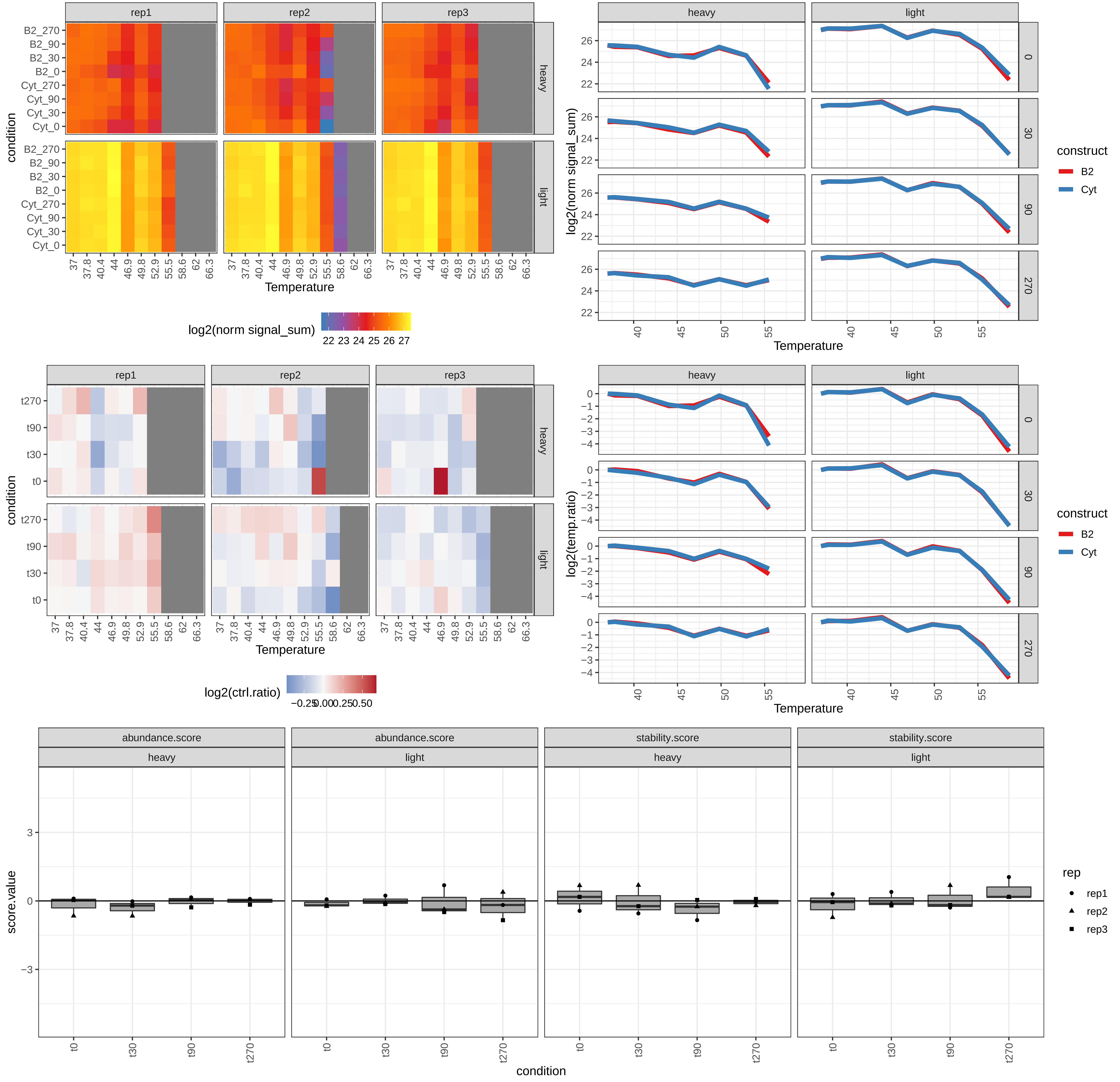

P41807 − V−type proton ATPase subunit H OS=Saccharomyces cerevisiae (strain ATCC 204508 / S288c) GN=VMA13 PE=1 SV=1

VMA16 − average.top3: 6.82838647058824 − max.qupm: 3

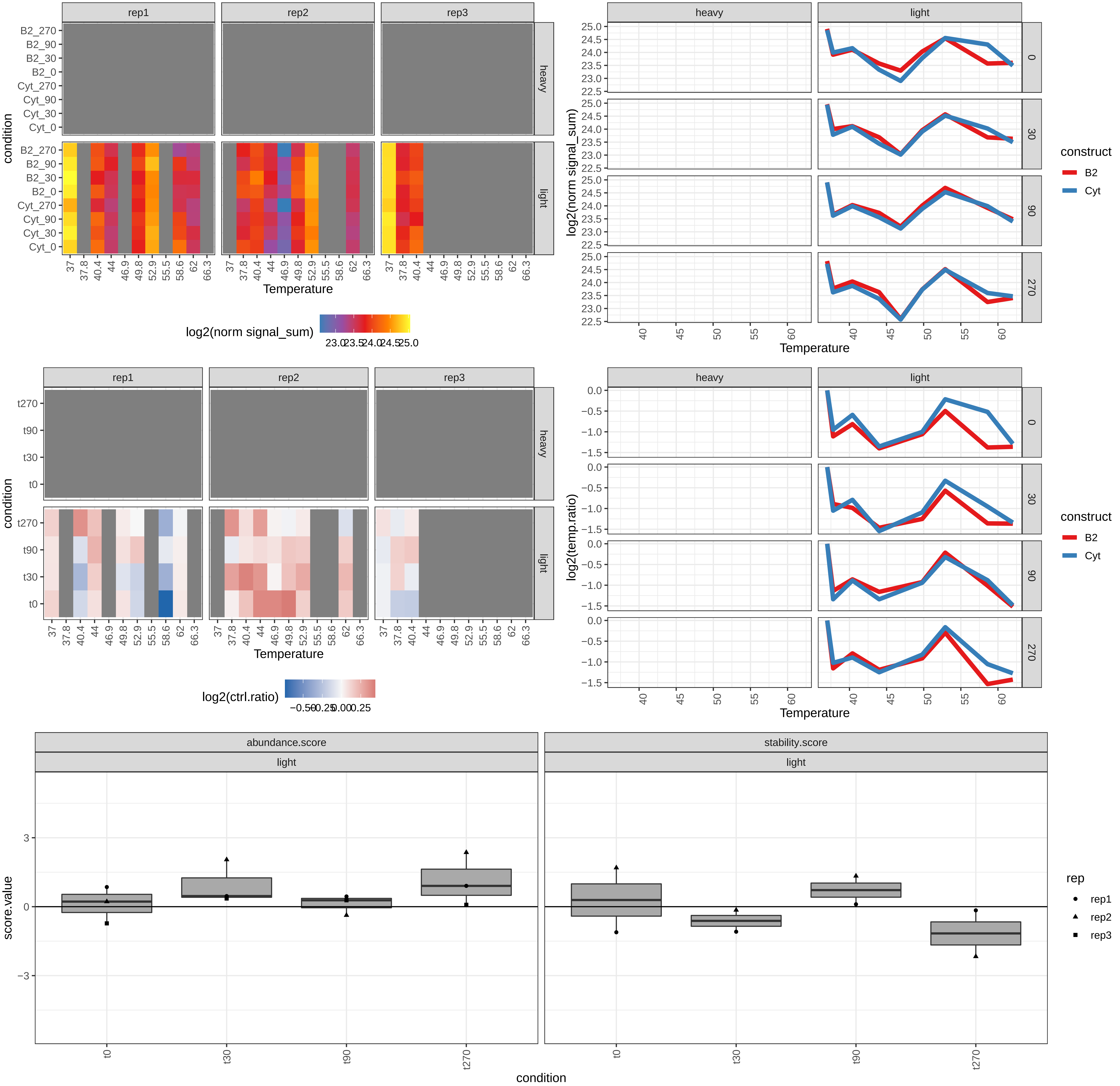

P23968 − V−type proton ATPase subunit c" OS=Saccharomyces cerevisiae (strain ATCC 204508 / S288c) GN=VMA16 PE=1 SV=1

VMA2 − average.top3: 7.77531225806452 − max.qupm: 29

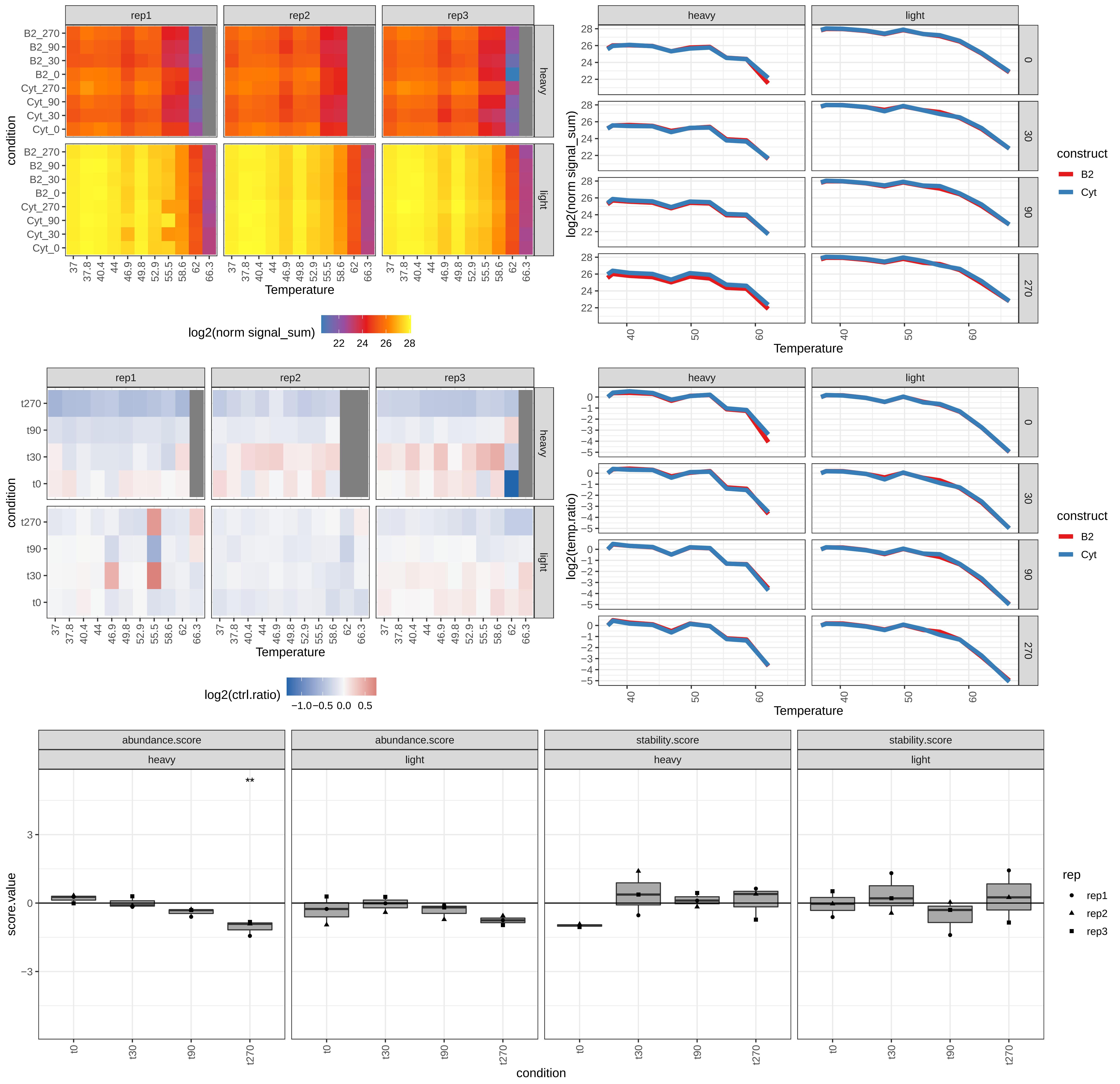

P16140 − V−type proton ATPase subunit B OS=Saccharomyces cerevisiae (strain ATCC 204508 / S288c) GN=VMA2 PE=1 SV=2

VMA3 − average.top3: 7.382147 − max.qupm: 2

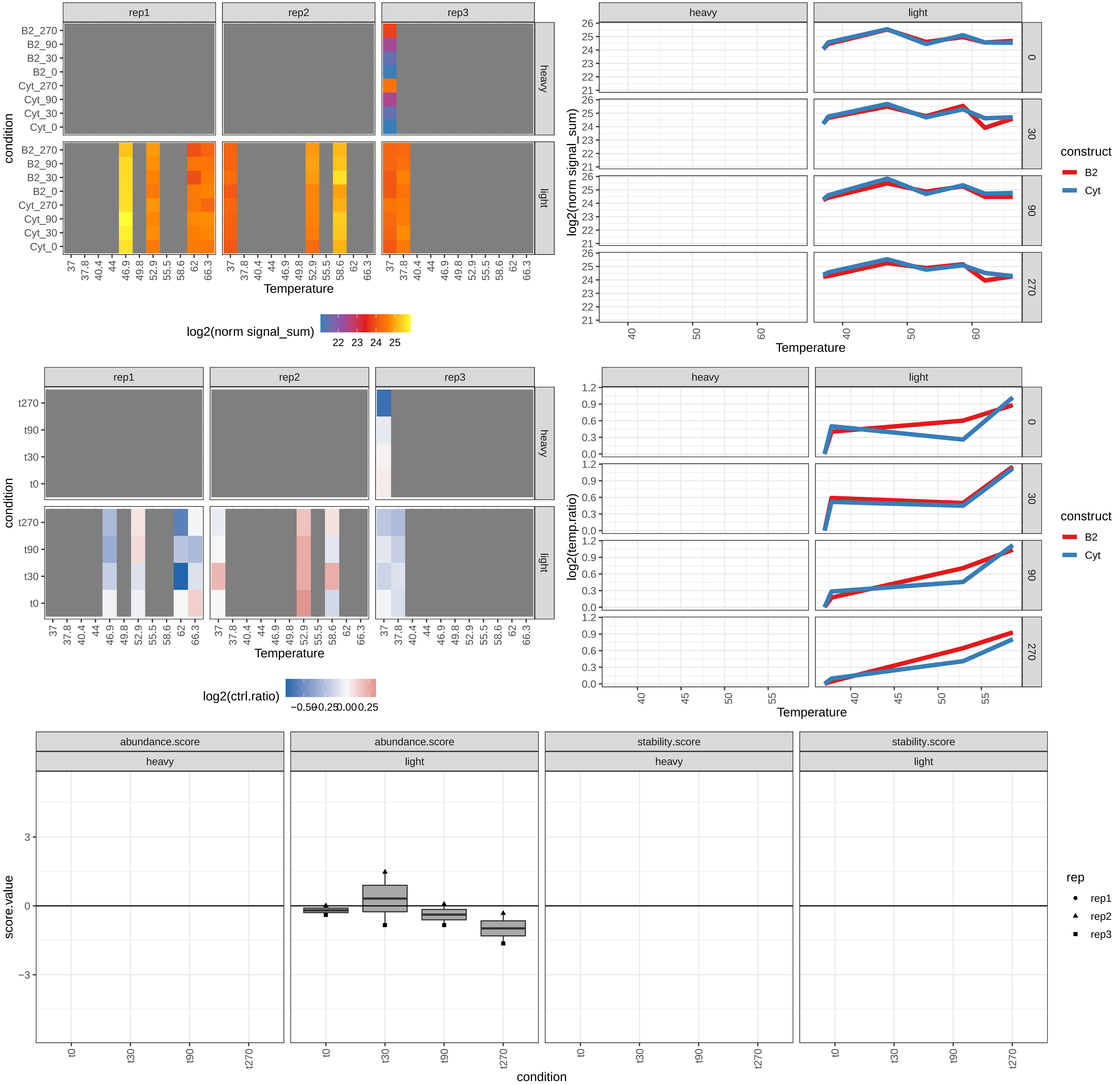

P25515 − V−type proton ATPase subunit c OS=Saccharomyces cerevisiae (strain ATCC 204508 / S288c) GN=VMA3 PE=1 SV=1

VMA4 − average.top3: 7.52036259259259 − max.qupm: 13

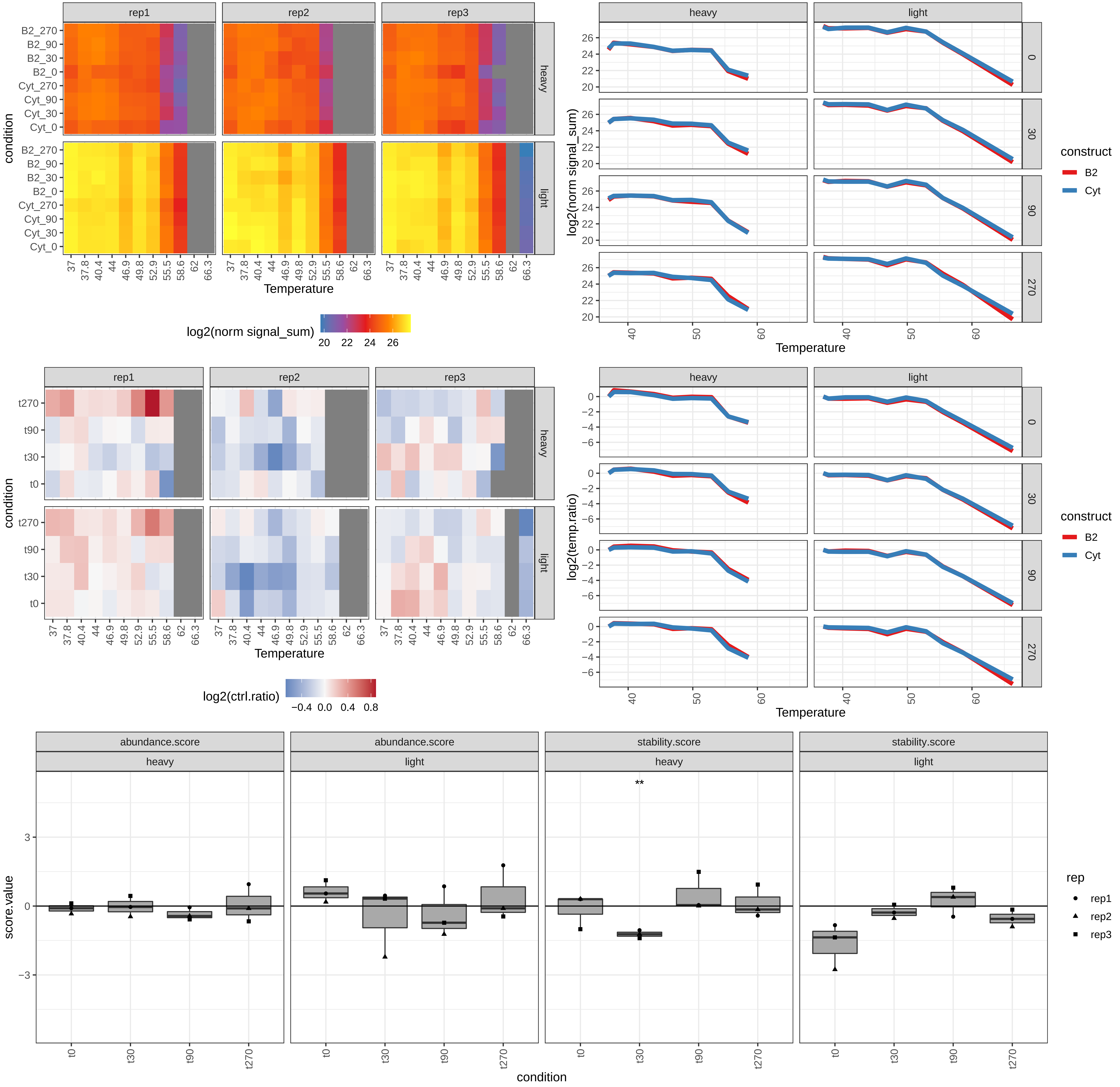

P22203 − V−type proton ATPase subunit E OS=Saccharomyces cerevisiae (strain ATCC 204508 / S288c) GN=VMA4 PE=1 SV=4

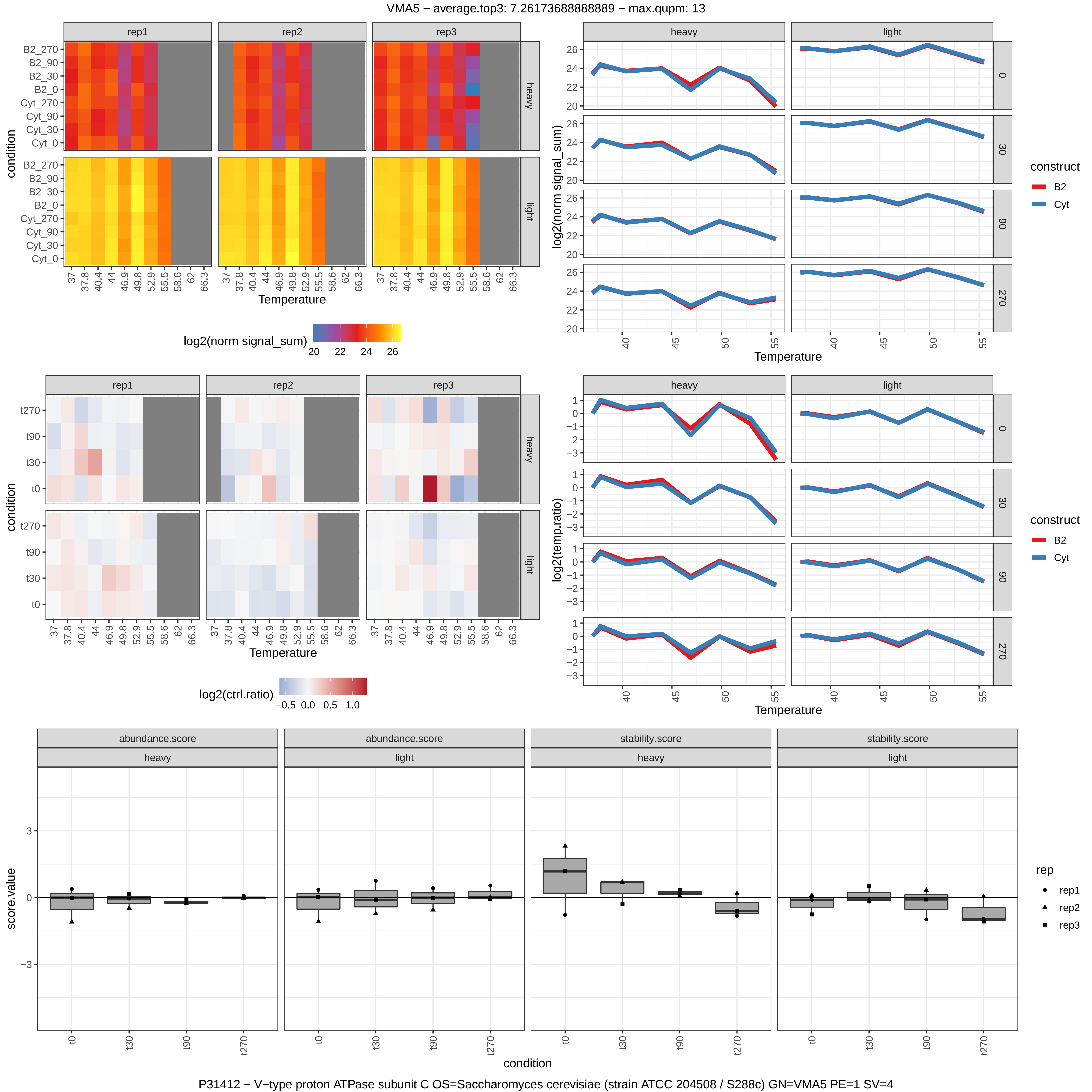

VMA5 − average.top3: 7.26173688888889 − max.qupm: 13
rep1
rep2
rep3
heavy
light
condition
Temperature
log2(norm signal_sum)
log2(ctrl.ratio)
log2(temp.ratio)
construct
B2
Cyt
abundance.score
stability.score
score.value
rep
P31412 − V−type proton ATPase subunit C OS=Saccharomyces cerevisiae (strain ATCC 204508 / S288c) GN=VMA5 PE=1 SV=4

VMA6 − average.top3: 7.32379594594595 − max.qupm: 6

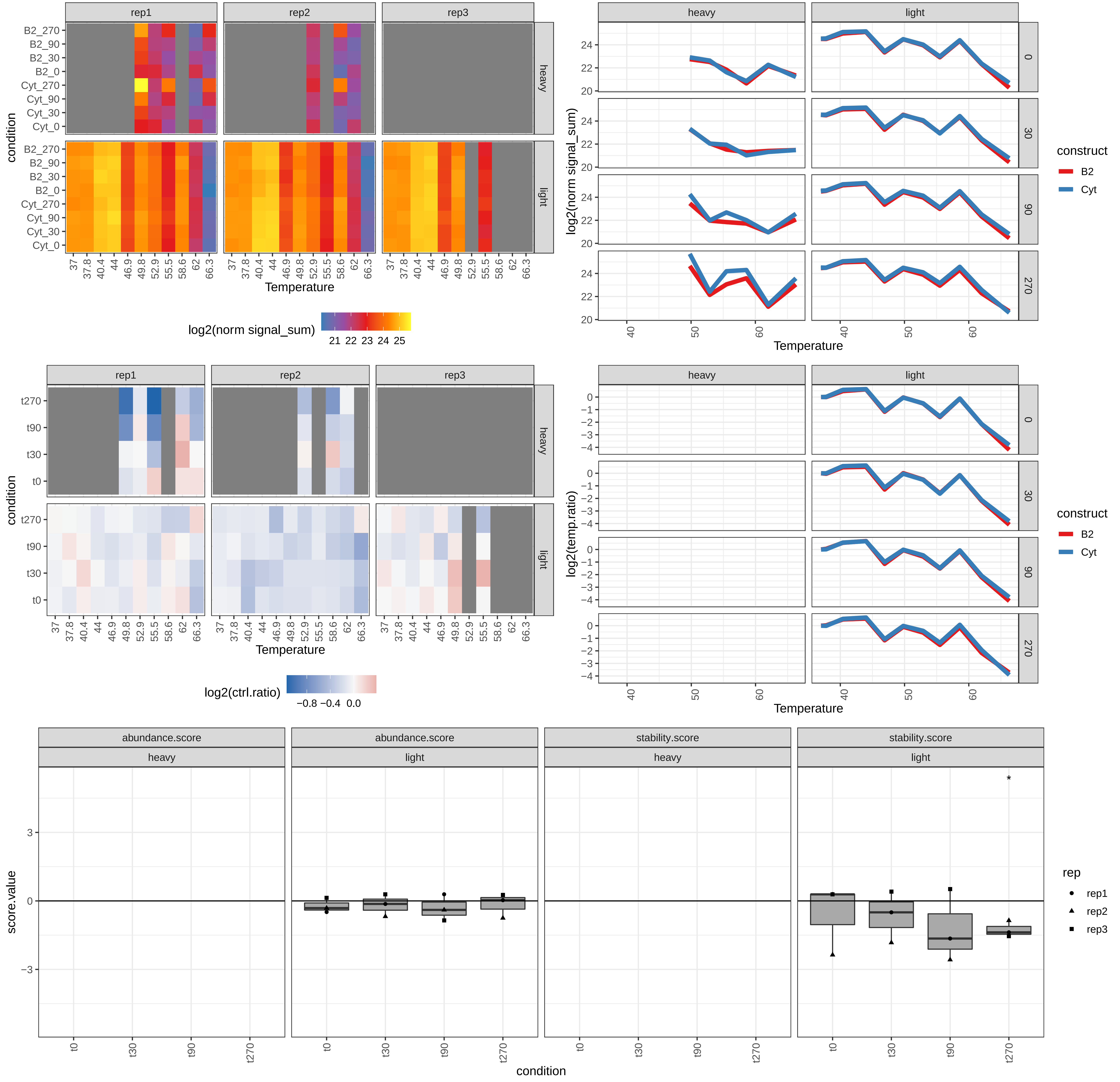

P32366 − V−type proton ATPase subunit d OS=Saccharomyces cerevisiae (strain ATCC 204508 / S288c) GN=VMA6 PE=1 SV=2

VMA7 − average.top3: 7.218171 − max.qupm: 7

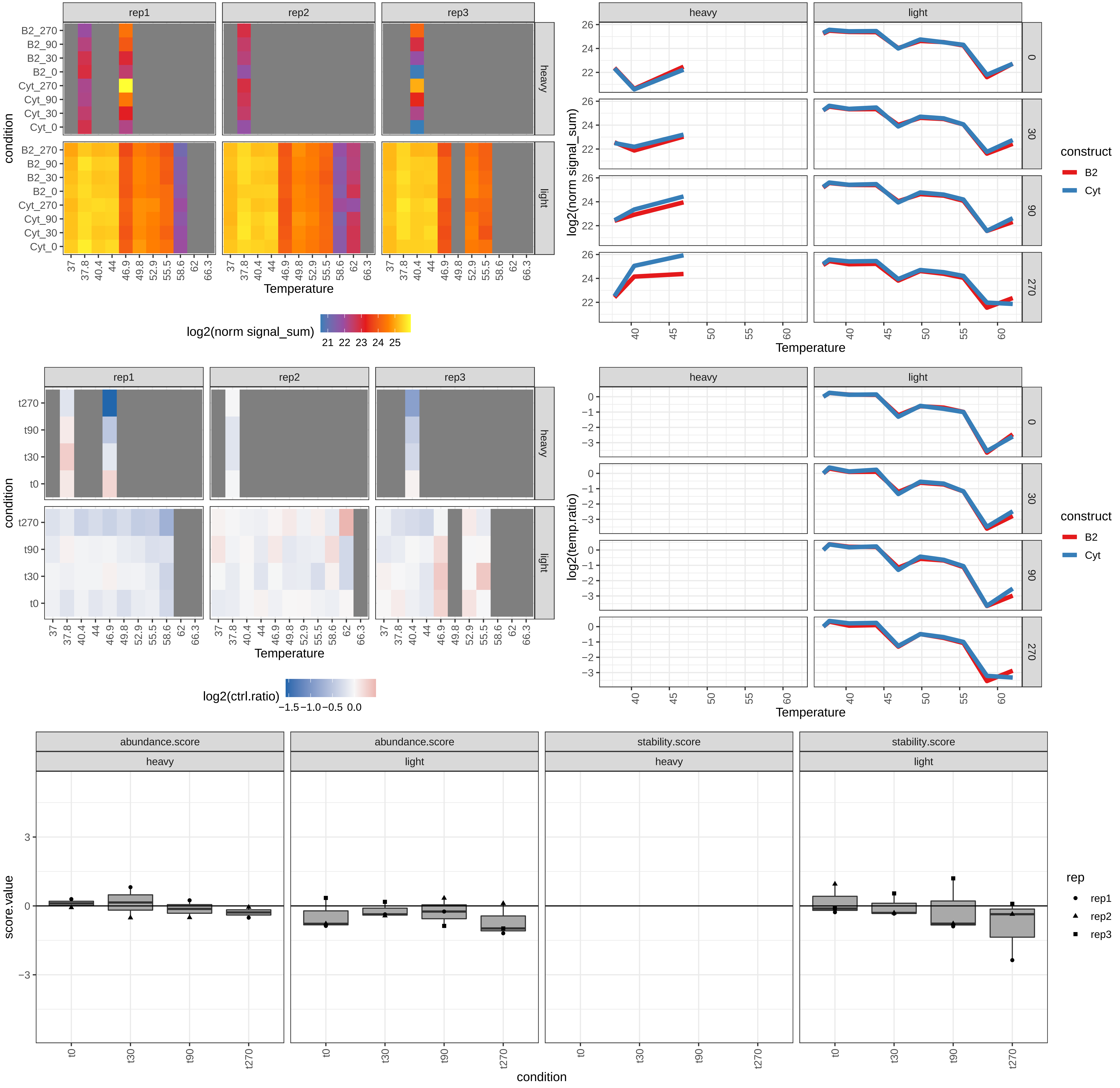

P39111 − V−type proton ATPase subunit F OS=Saccharomyces cerevisiae (strain ATCC 204508 / S288c) GN=VMA7 PE=1 SV=1

VMA8 − average.top3: 7.35844540540541 − max.qupm: 6

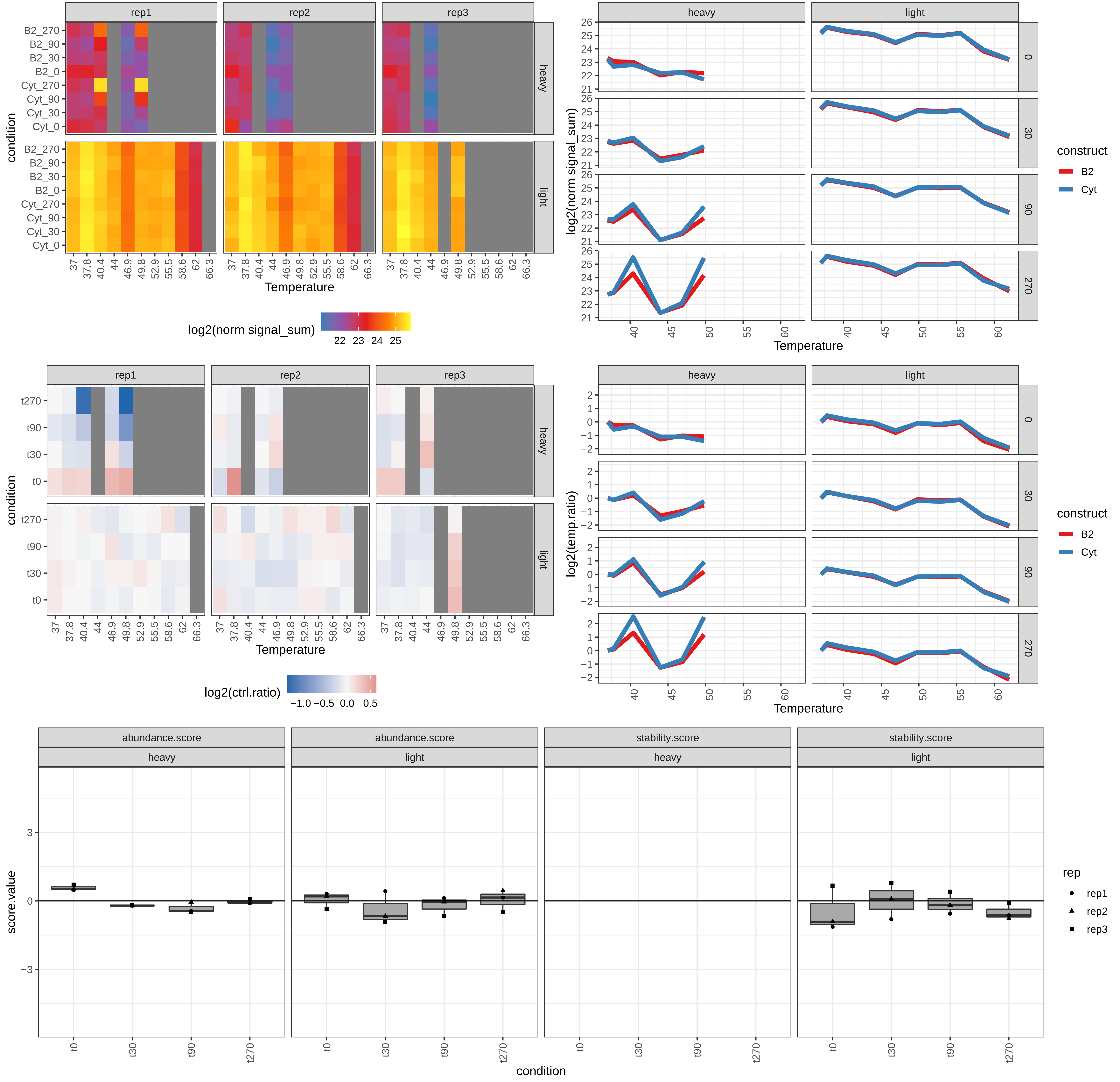

P32610 − V−type proton ATPase subunit D OS=Saccharomyces cerevisiae (strain ATCC 204508 / S288c) GN=VMA8 PE=1 SV=1

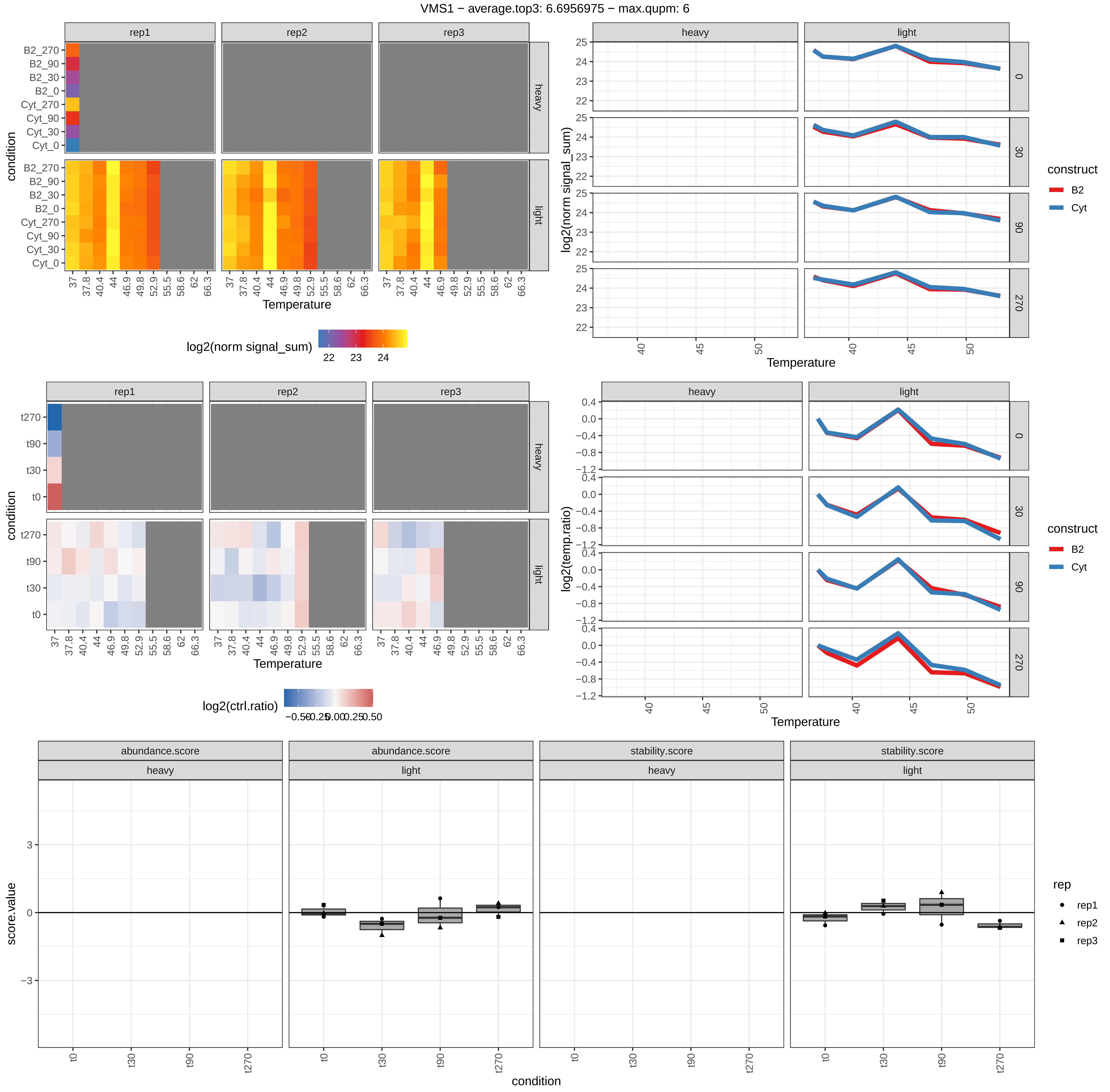

Q04311 − Protein VMS1 OS=Saccharomyces cerevisiae (strain ATCC 204508 / S288c) GN=VMS1 PE=1 SV=1

VOA1 − average.top3: 7.02814466666667 − max.qupm: 3

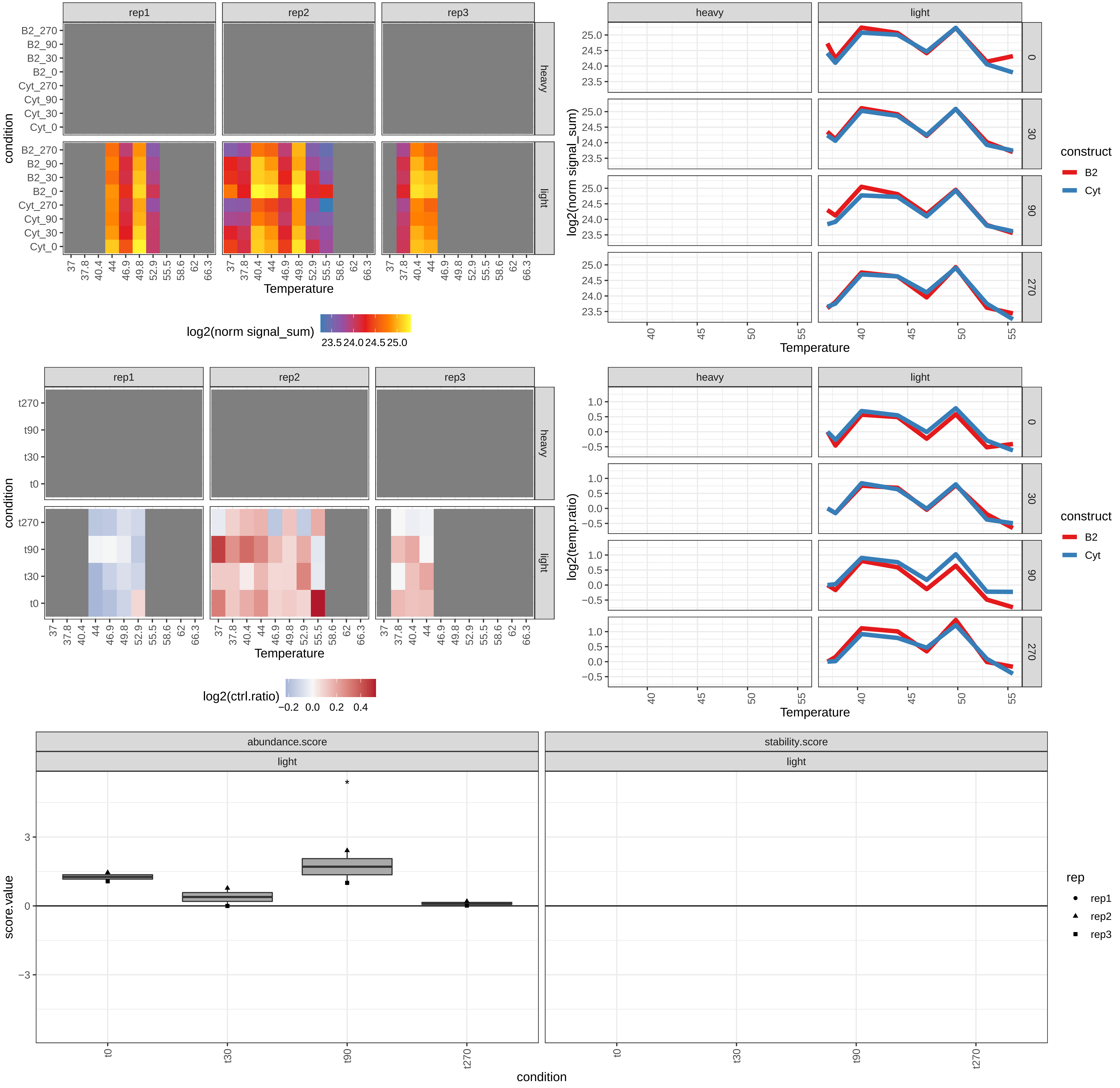

P53262 − V0 assembly protein 1 OS=Saccharomyces cerevisiae (strain ATCC 204508 / S288c) GN=VOA1 PE=1 SV=1

VPH1 − average.top3: 7.38035873015873 − max.qupm: 22

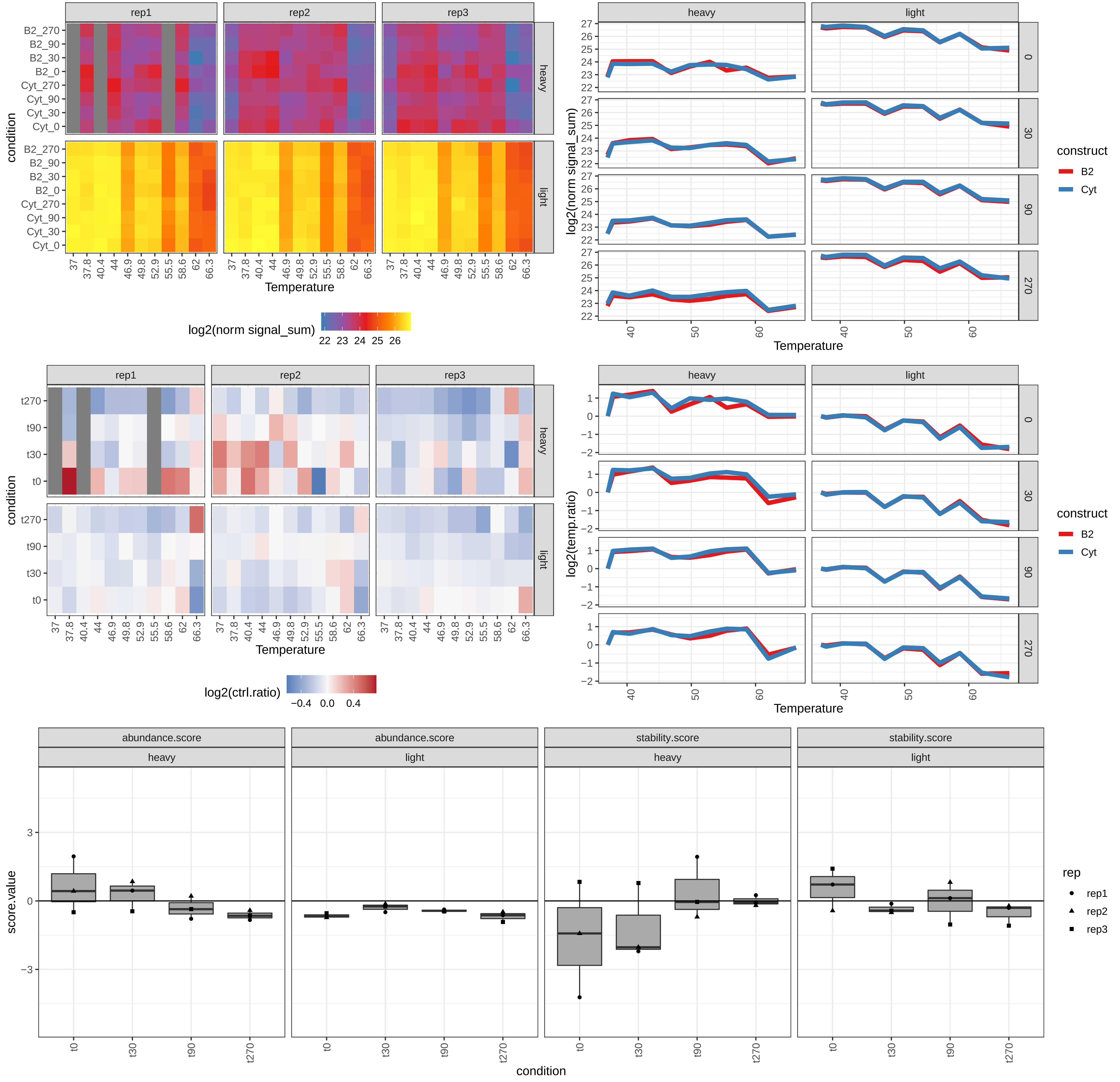

P32563 − V−type proton ATPase subunit a, vacuolar isoform OS=Saccharomyces cerevisiae (strain ATCC 204508 / S288c) GN=VPH1 PE=1 SV=3

VPS1 − average.top3: 7.28238224489796 − max.qupm: 28

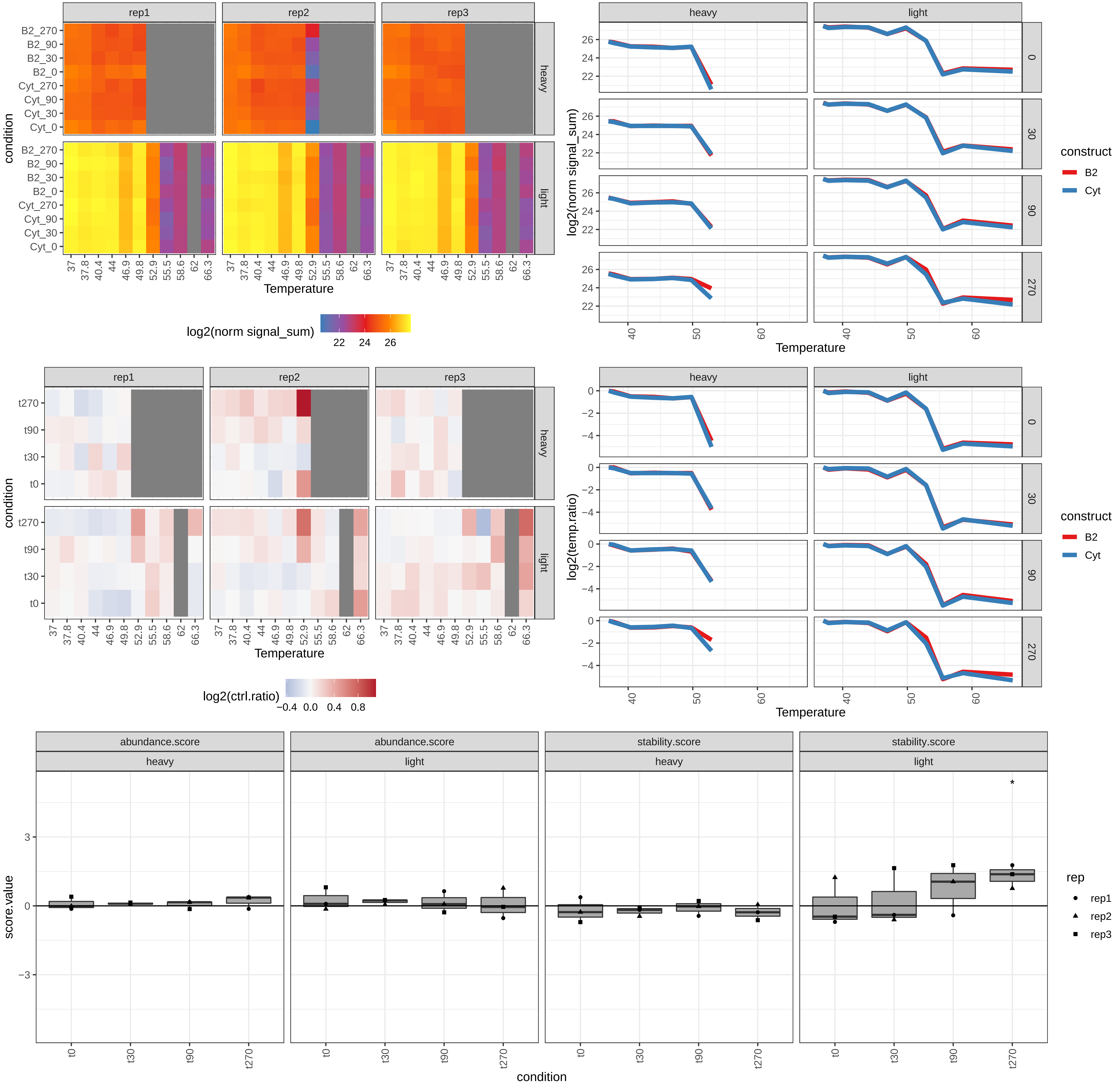

P21576 − Vacuolar protein sorting−associated protein 1 OS=Saccharomyces cerevisiae (strain ATCC 204508 / S288c) GN=VPS1 PE=1 SV=2

VPS13 − average.top3: 6.83442591836735 − max.qupm: 55

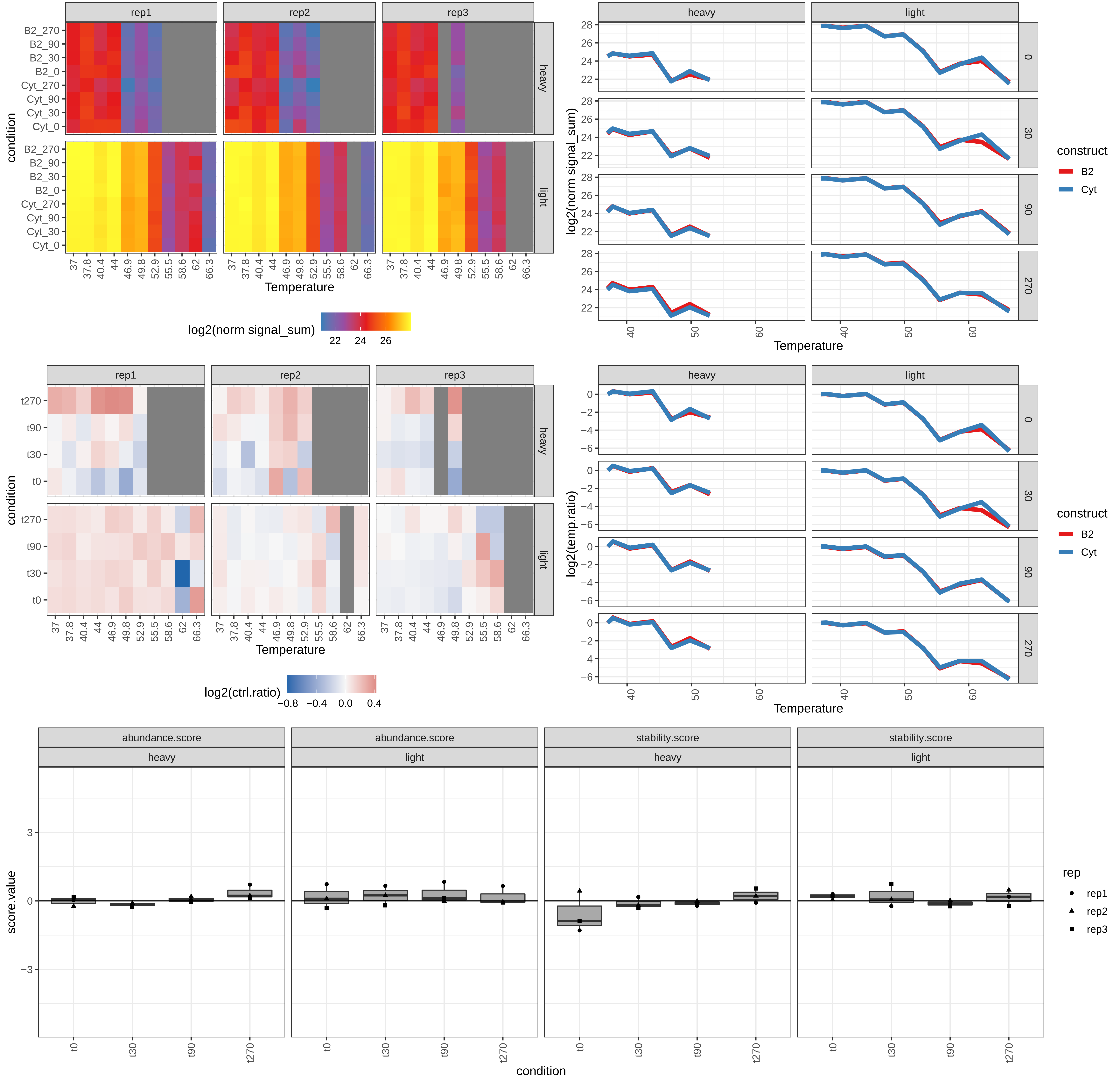

Q07878 − Vacuolar protein sorting−associated protein 13 OS=Saccharomyces cerevisiae (strain ATCC 204508 / S288c) GN=VPS13 PE=1 SV=1

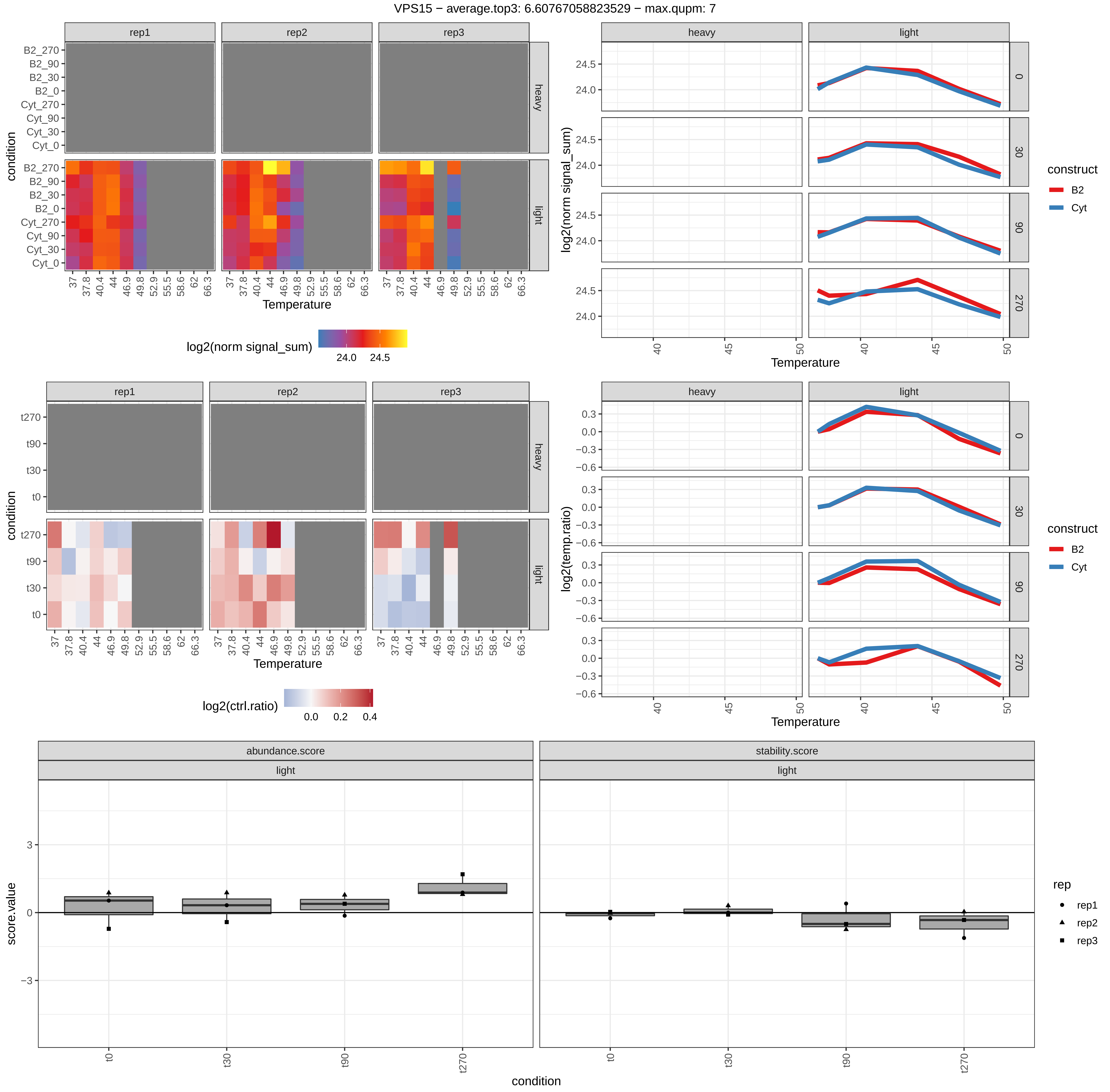

P22219 − Serine/threonine−protein kinase VPS15 OS=Saccharomyces cerevisiae (strain ATCC 204508 / S288c) GN=VPS15 PE=1 SV=4

VPS16 − average.top3: 6.45815642857143 − max.qupm: 6

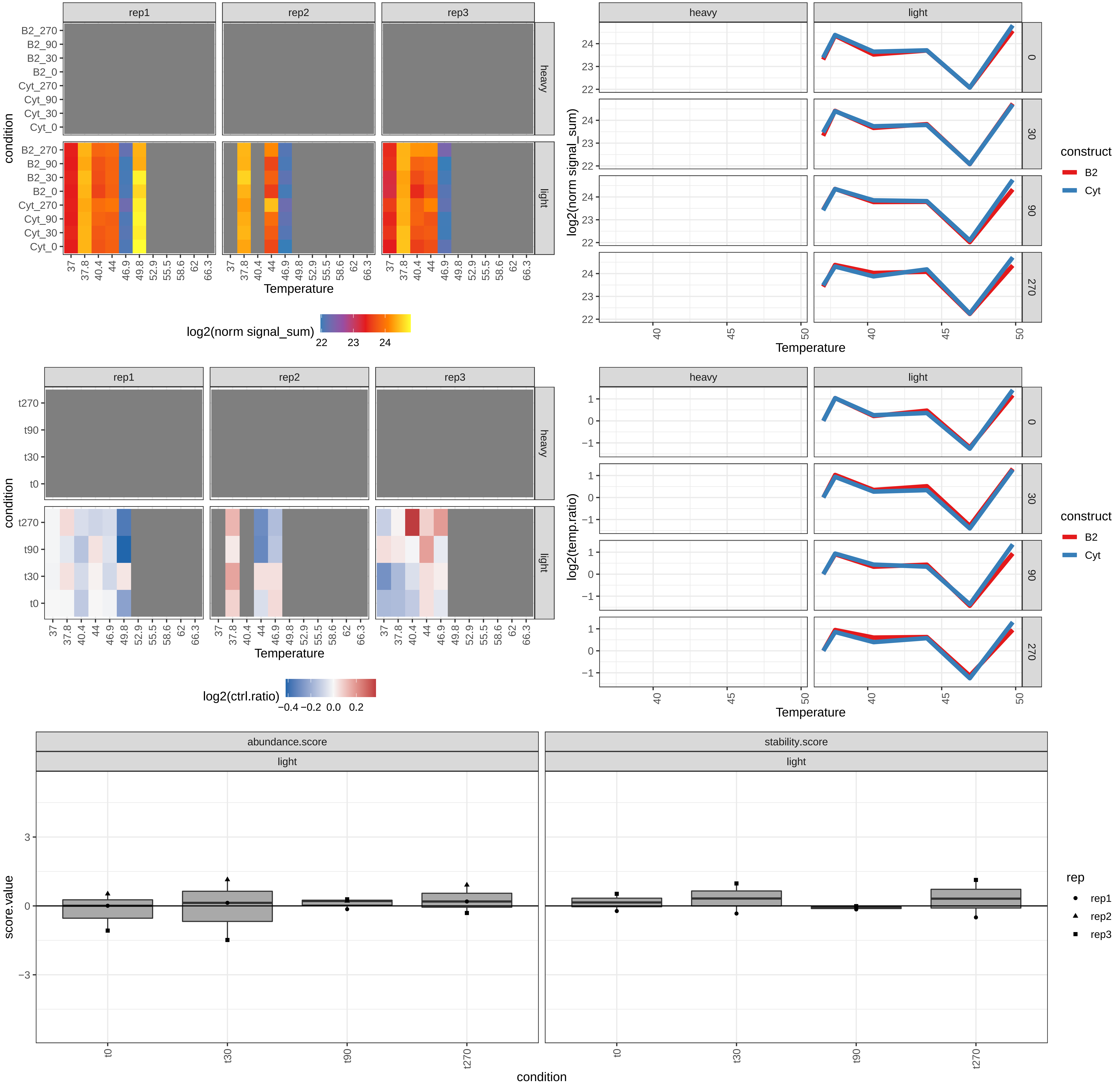

Q03308 − Vacuolar protein sorting−associated protein 16 OS=Saccharomyces cerevisiae (strain ATCC 204508 / S288c) GN=VPS16 PE=1 SV=2

VPS17 − average.top3: 7.109079 − max.qupm: 8

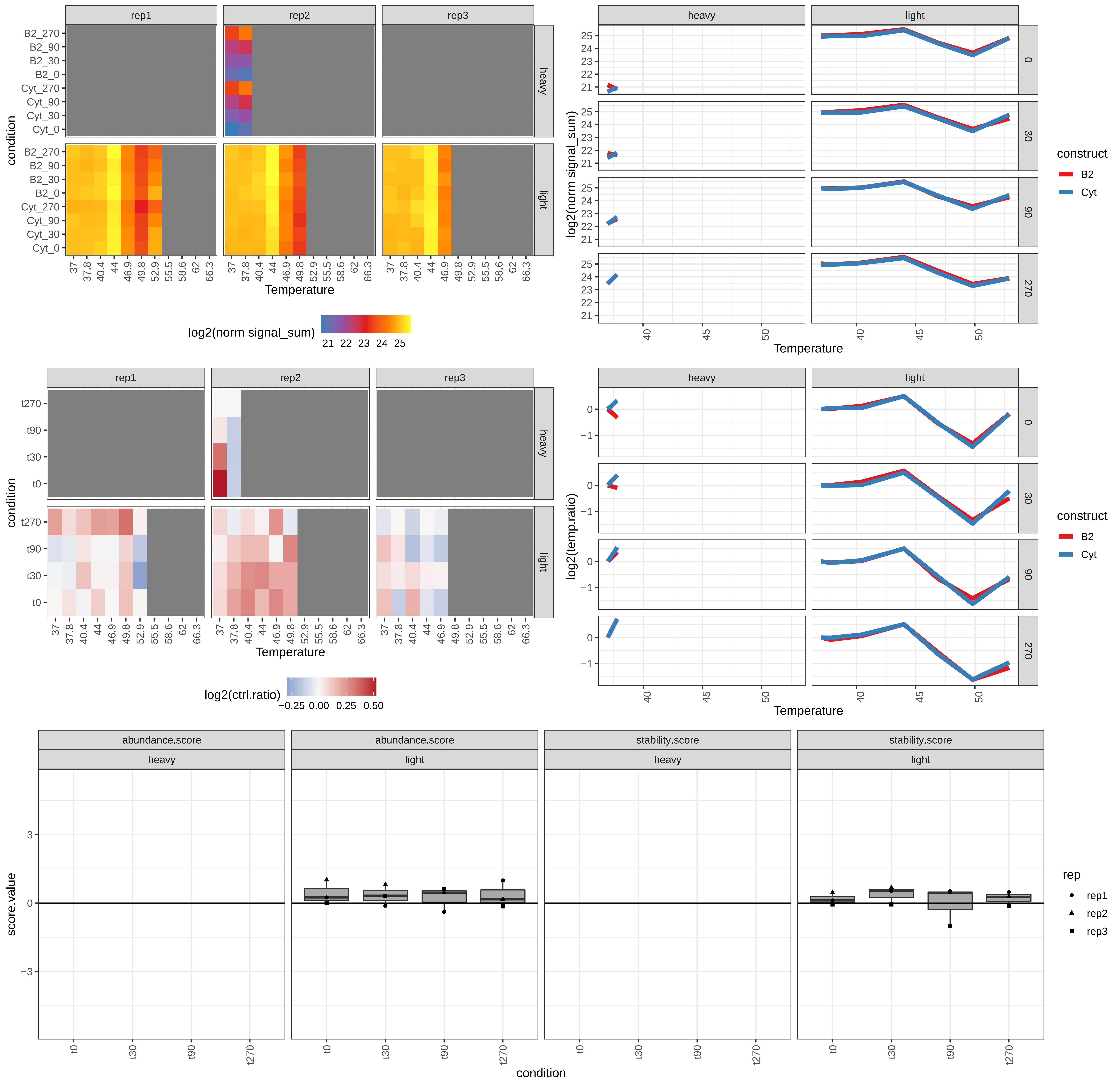

P32913 − Vacuolar protein sorting−associated protein 17 OS=Saccharomyces cerevisiae (strain ATCC 204508 / S288c) GN=VPS17 PE=1 SV=2

VPS20 − average.top3: 6.24883 − max.qupm: 2

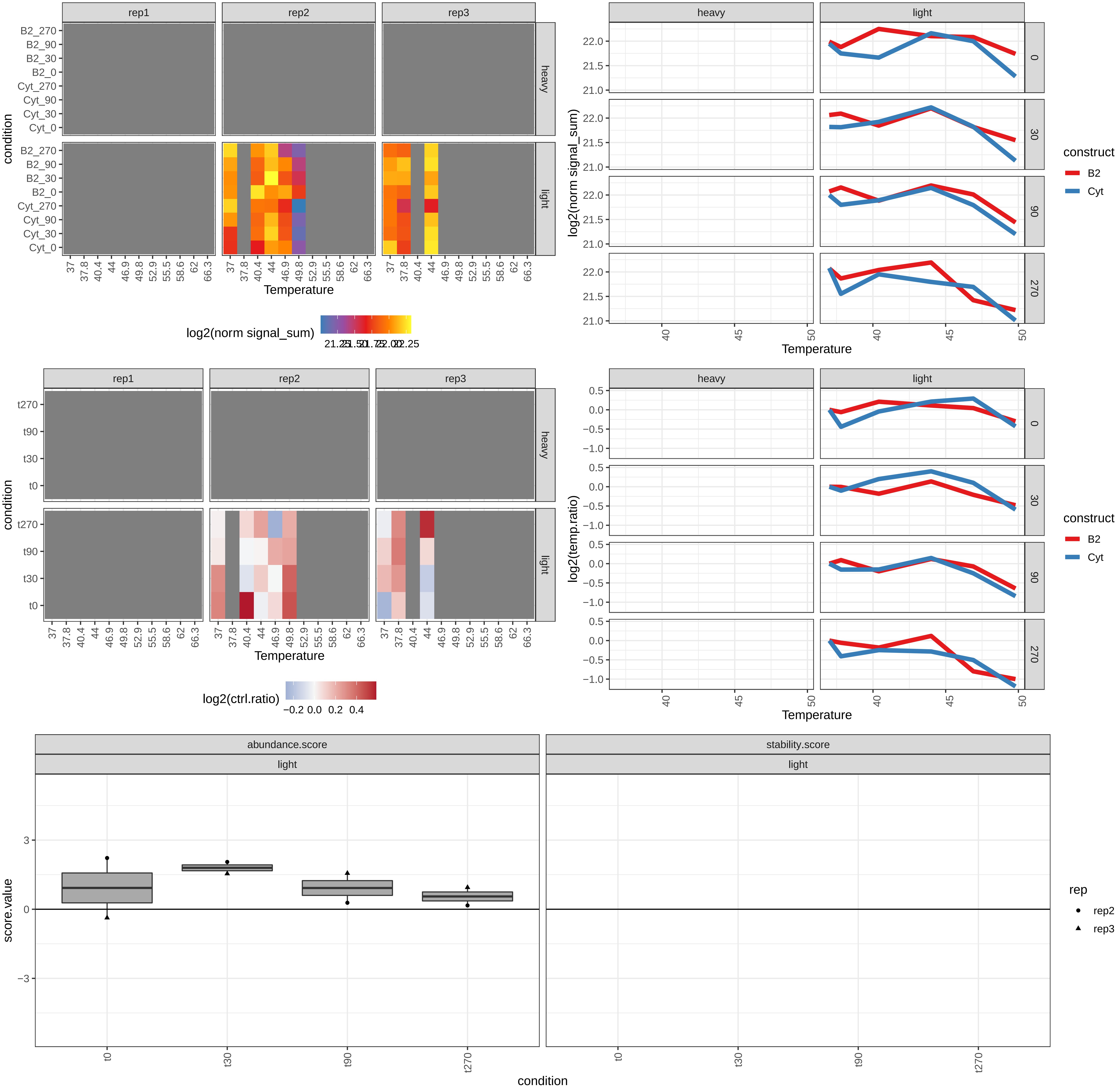

Q04272 − Vacuolar protein sorting−associated protein 20 OS=Saccharomyces cerevisiae (strain ATCC 204508 / S288c) GN=VPS20 PE=1 SV=3

VPS21 − average.top3: 6.77750924528302 − max.qupm: 9

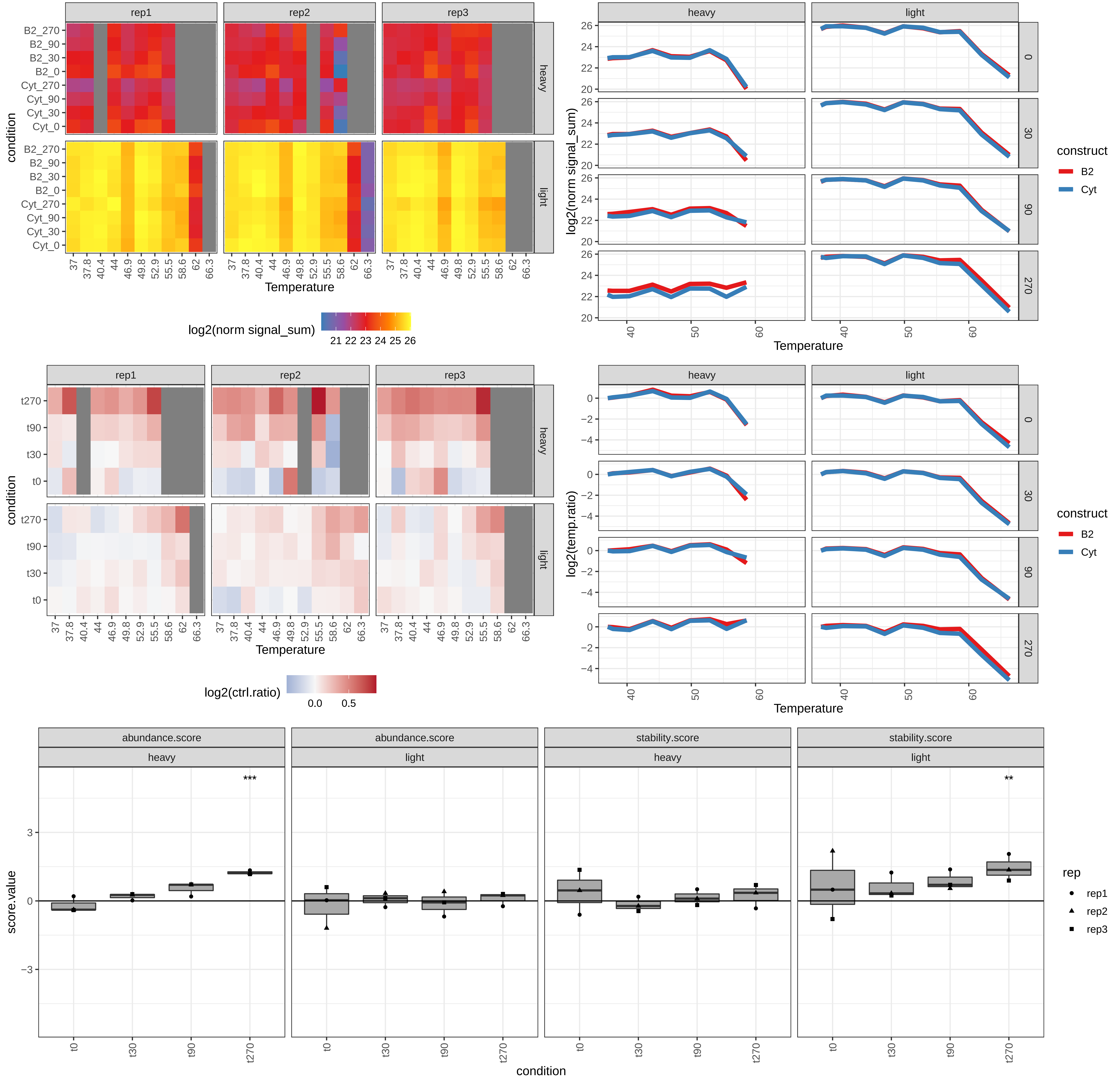

P36017 − Vacuolar protein sorting−associated protein 21 OS=Saccharomyces cerevisiae (strain ATCC 204508 / S288c) GN=VPS21 PE=1 SV=1

VPS24 − average.top3: 7.18757545454545 − max.qupm: 6

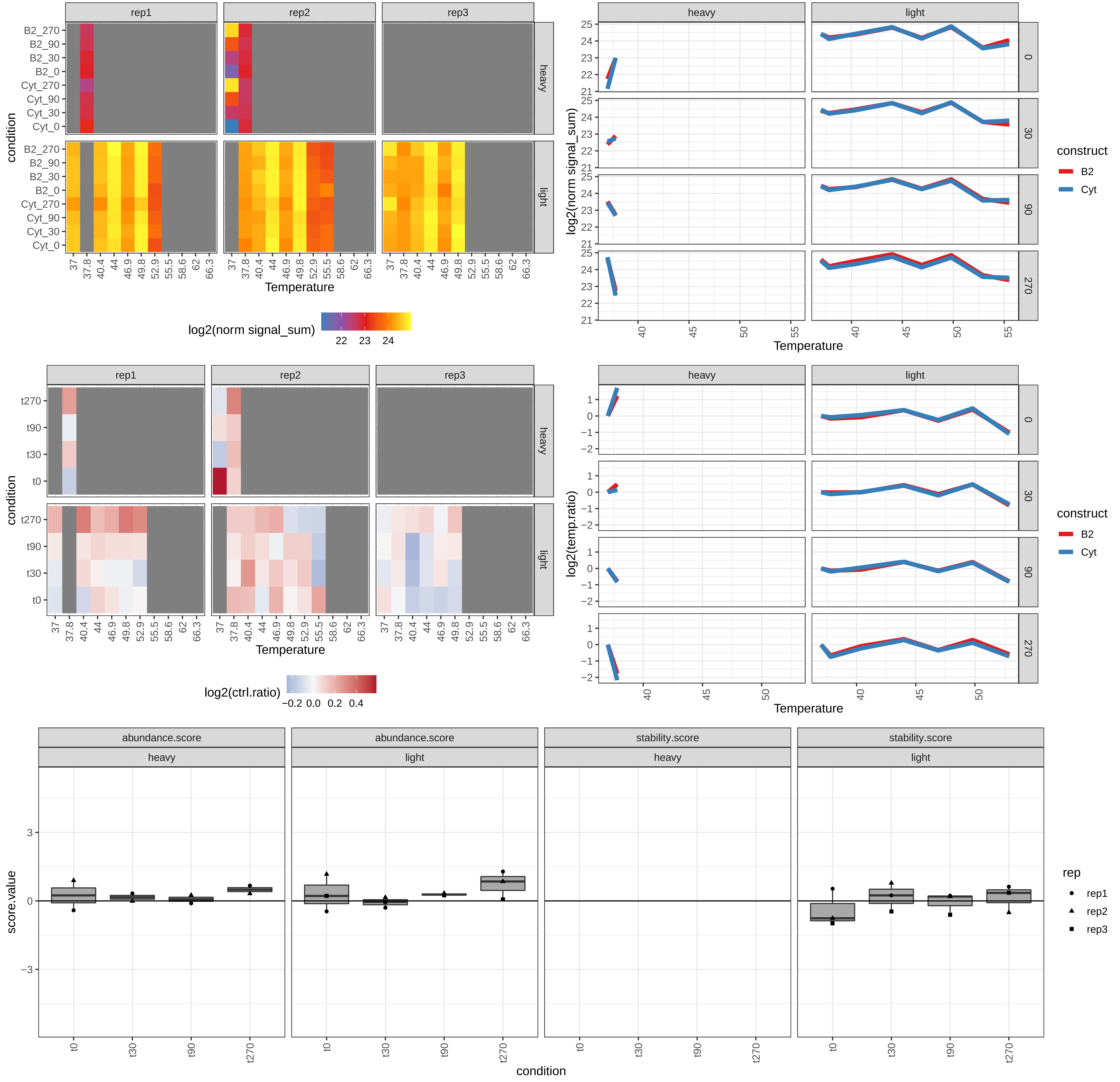

P36095 − Vacuolar protein−sorting−associated protein 24 OS=Saccharomyces cerevisiae (strain ATCC 204508 / S288c) GN=VPS24 PE=1 SV=1

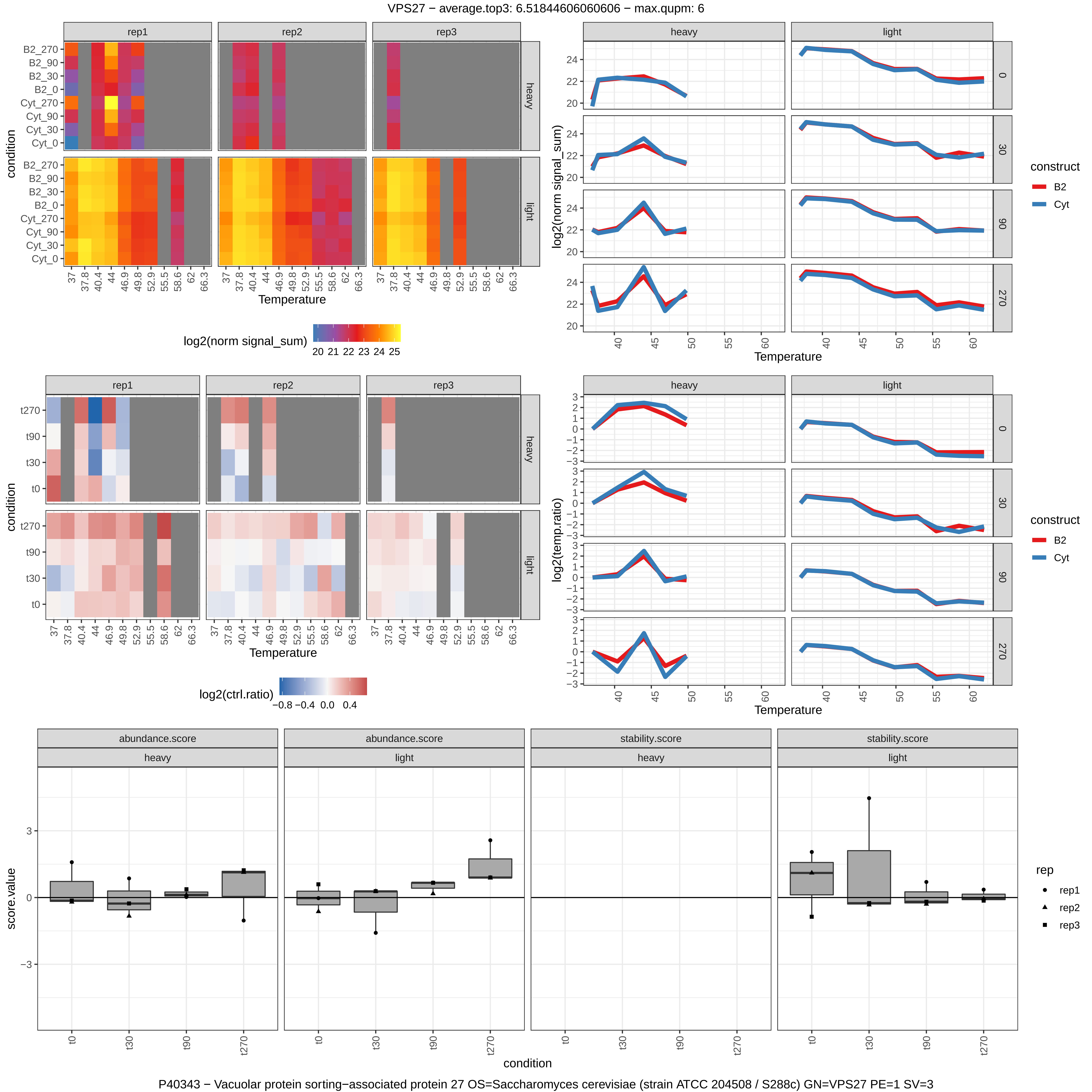
VPS27 − average.top3: 6.51844606060606 − max.qupm: 6
rep1
rep2
rep3
heavy
light
condition
Temperature
log2(norm signal_sum)
construct
B2
Cyt
log2(ctrl.ratio)
log2(temp.ratio)
abundance.score
stability.score
score.value
rep
P40343 − Vacuolar protein sorting−associated protein 27 OS=Saccharomyces cerevisiae (strain ATCC 204508 / S288c) GN=VPS27 PE=1 SV=3

VPS29 − average.top3: 6.78089181818182 − max.qupm: 7

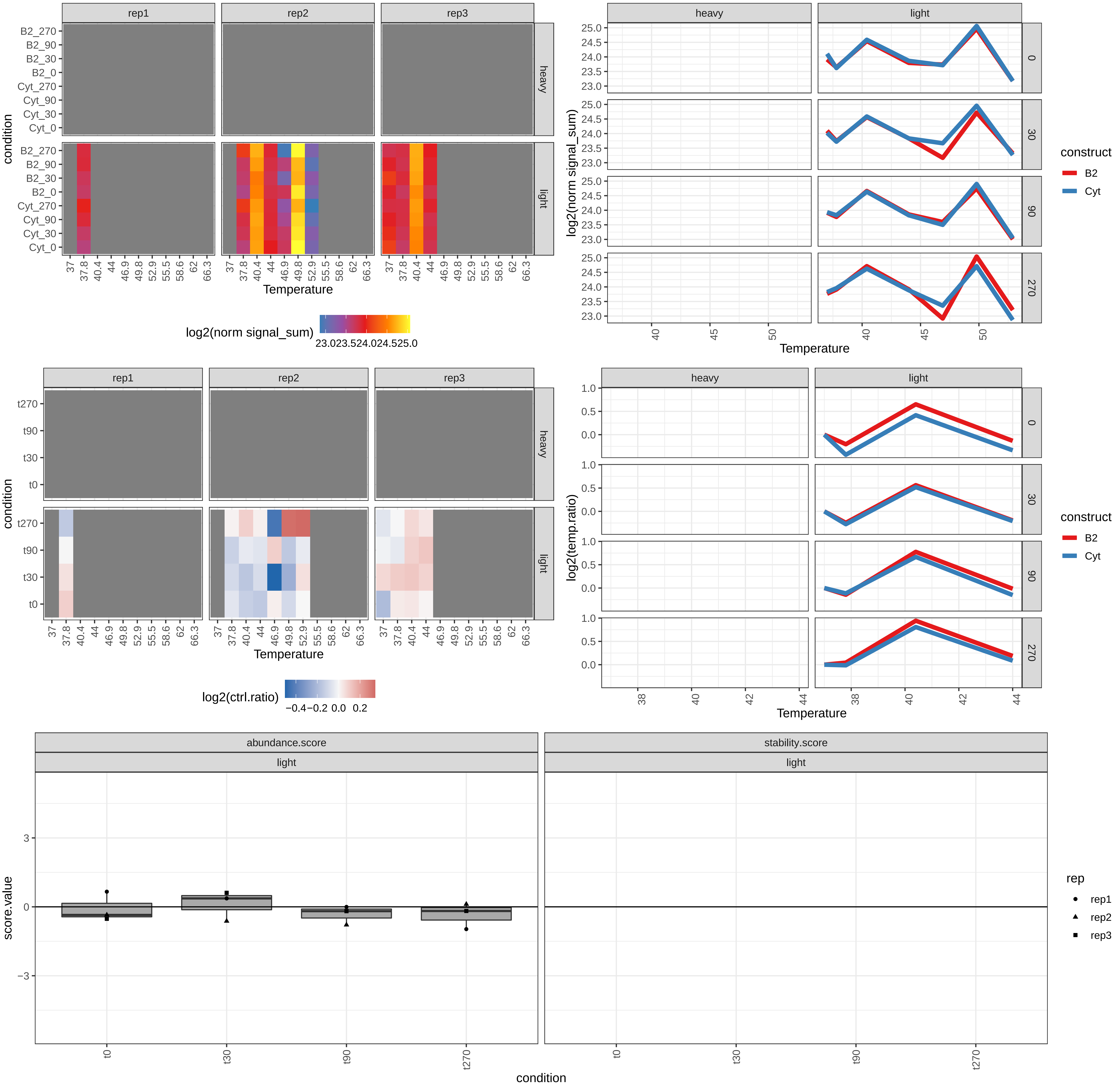

P38759 − Vacuolar protein sorting−associated protein 29 OS=Saccharomyces cerevisiae (strain ATCC 204508 / S288c) GN=VPS29 PE=1 SV=1

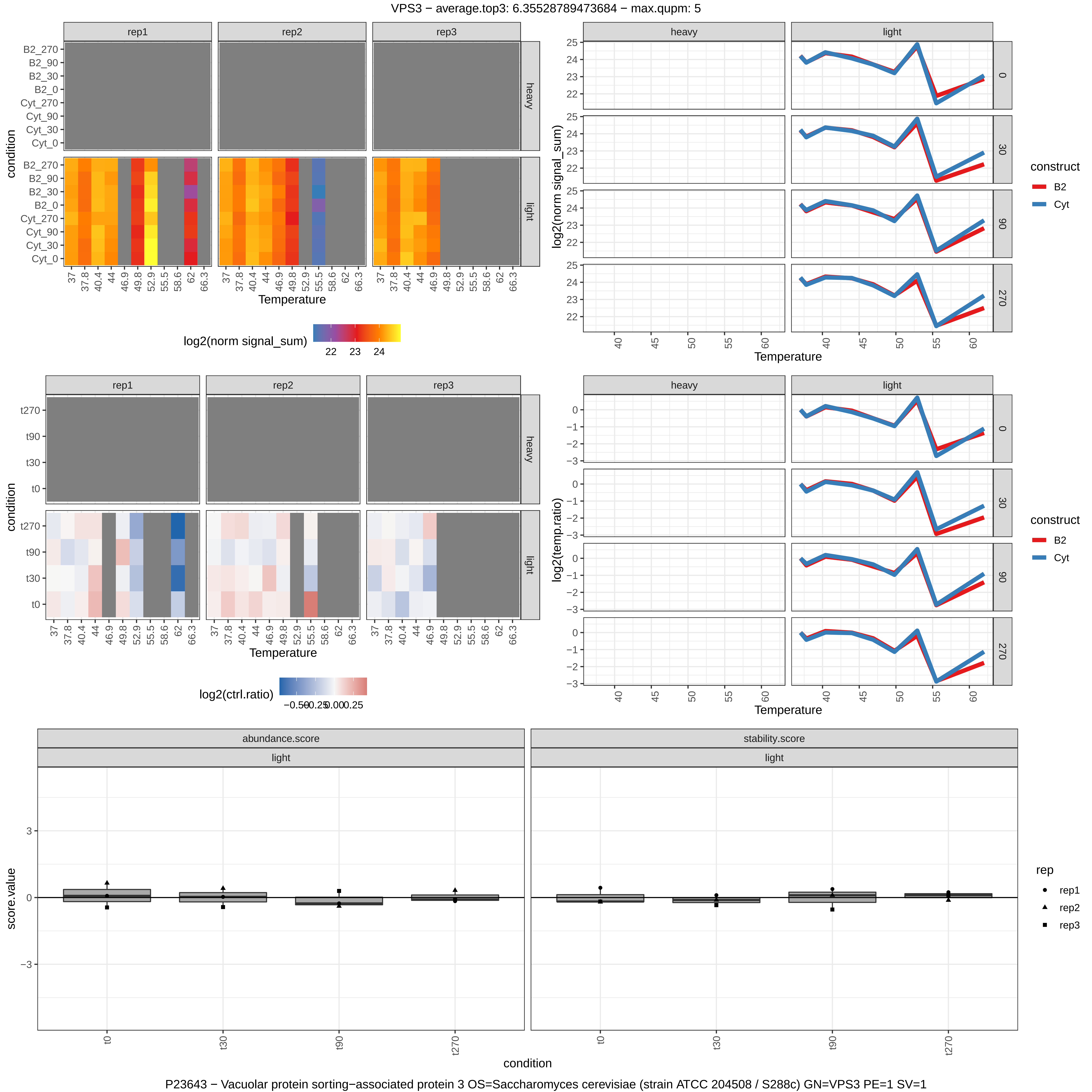

VPS3 − average.top3: 6.35528789473684 − max.qupm: 5
rep1
rep2
rep3
heavy
light
condition
Temperature
log2(norm signal_sum)
log2(ctrl.ratio)
log2(temp.ratio)
construct
B2
Cyt
abundance.score
stability.score
score.value
rep
rep1
rep2
rep3
P23643 − Vacuolar protein sorting−associated protein 3 OS=Saccharomyces cerevisiae (strain ATCC 204508 / S288c) GN=VPS3 PE=1 SV=1

VPS33 − average.top3: 6.46311375 − max.qupm: 4

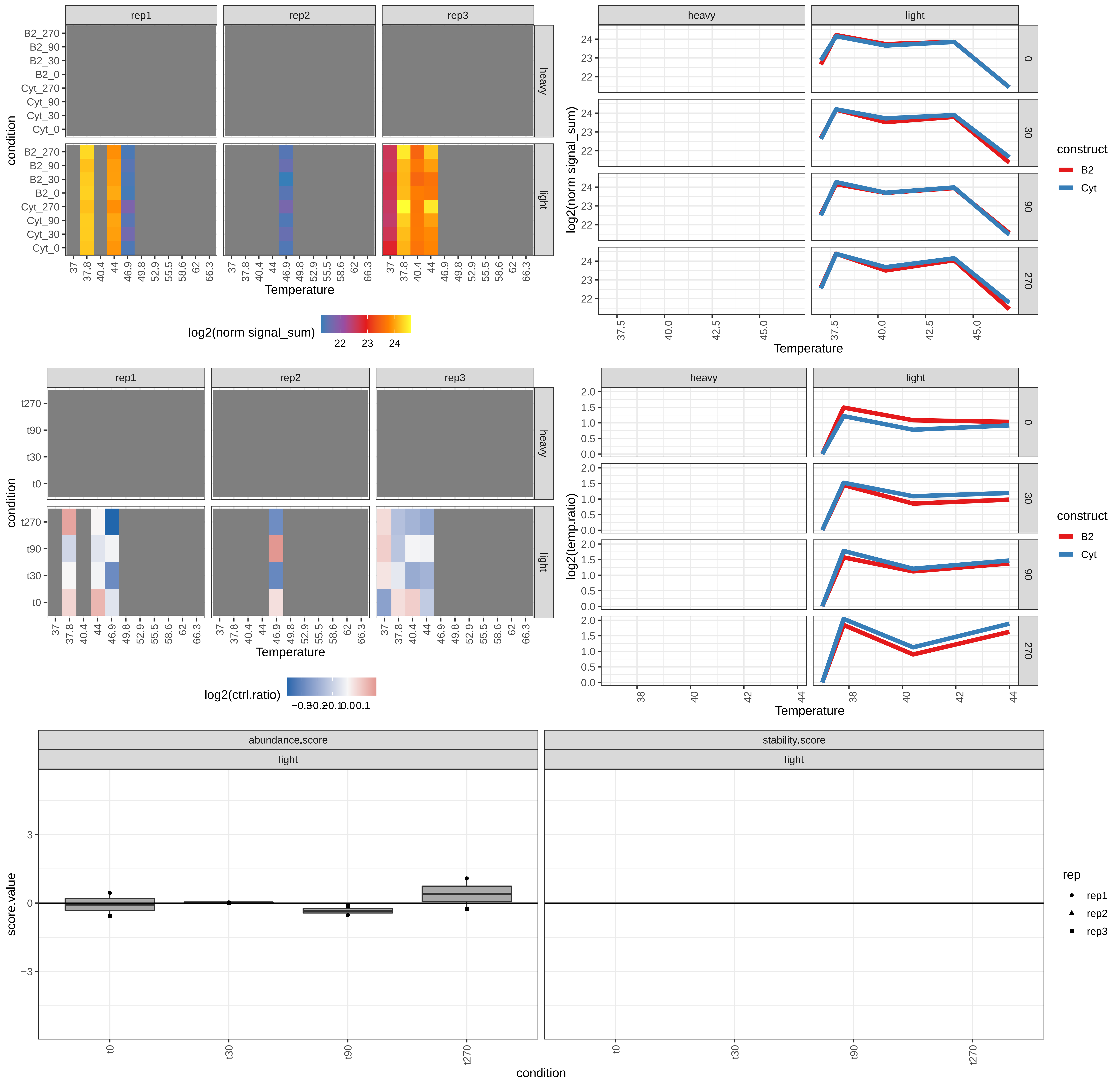

P20795 − Vacuolar protein sorting−associated protein 33 OS=Saccharomyces cerevisiae (strain ATCC 204508 / S288c) GN=VPS33 PE=1 SV=1

VPS35 − average.top3: 6.69271086956522 − max.qupm: 6

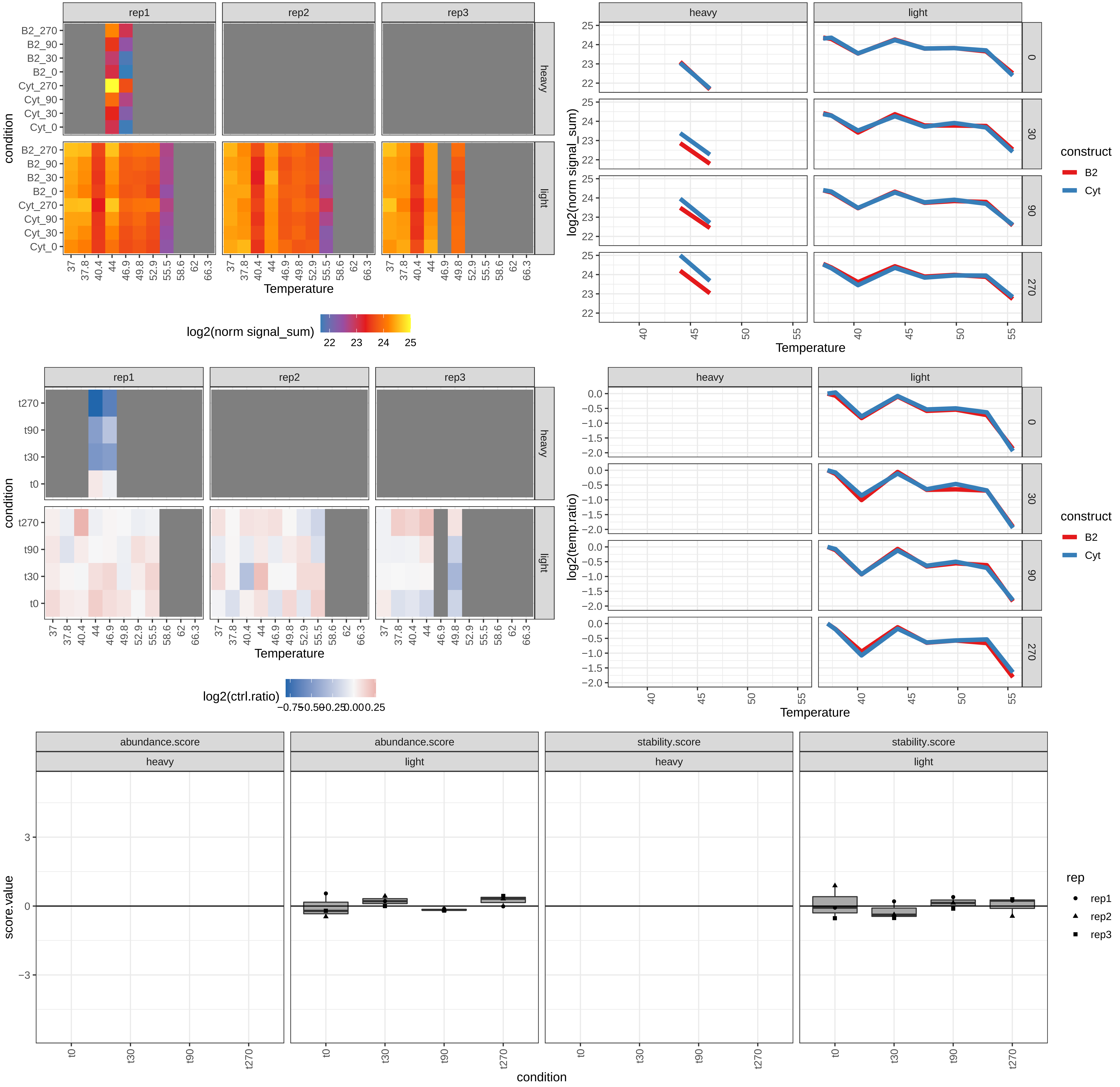

P34110 − Vacuolar protein sorting−associated protein 35 OS=Saccharomyces cerevisiae (strain ATCC 204508 / S288c) GN=VPS35 PE=1 SV=2

VPS36 − average.top3: 6.408780625 − max.qupm: 5

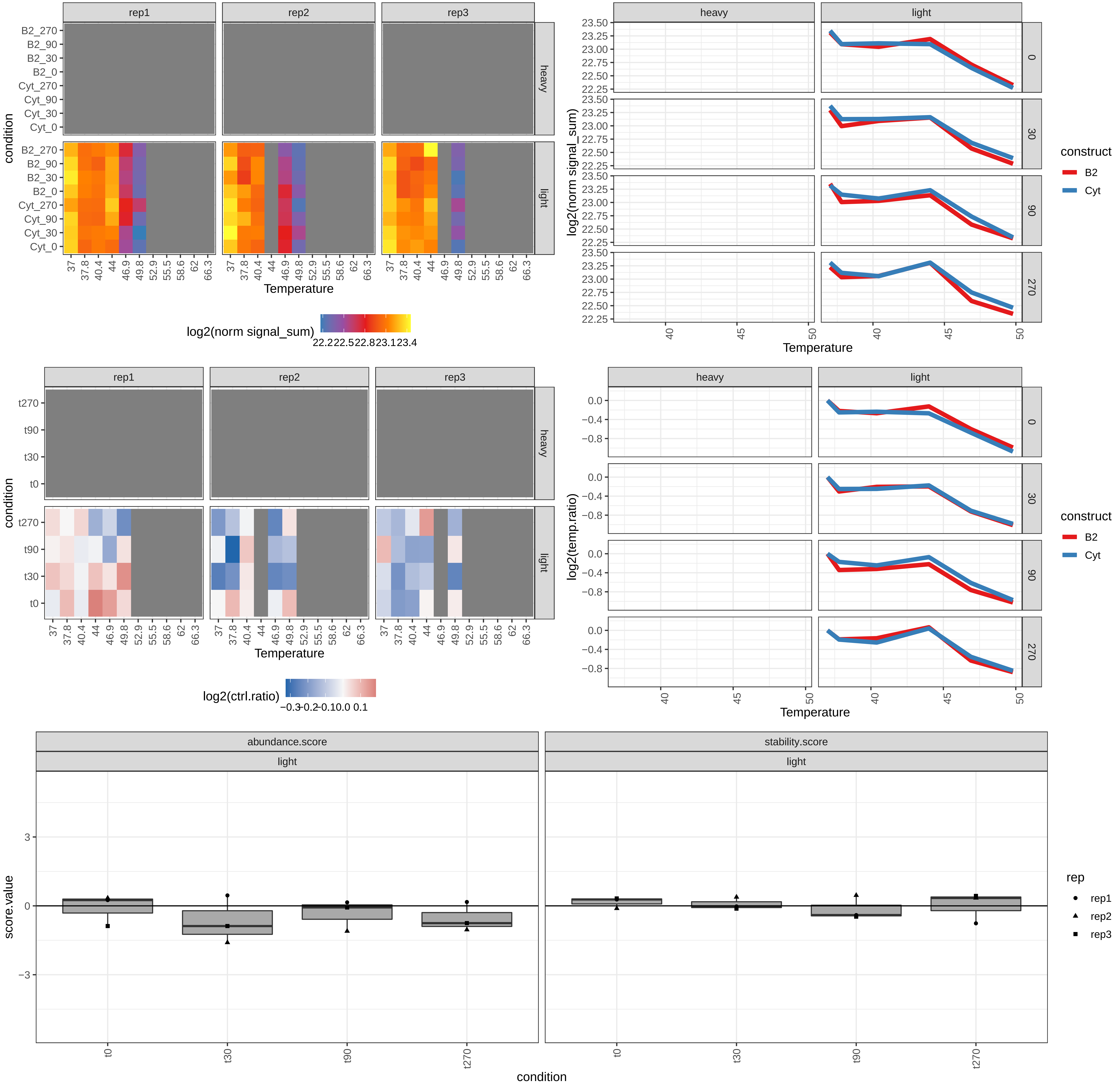

Q06696 − Vacuolar protein−sorting−associated protein 36 OS=Saccharomyces cerevisiae (strain ATCC 204508 / S288c) GN=VPS36 PE=1 SV=1

VPS4 − average.top3: 6.75183357142857 − max.qupm: 6

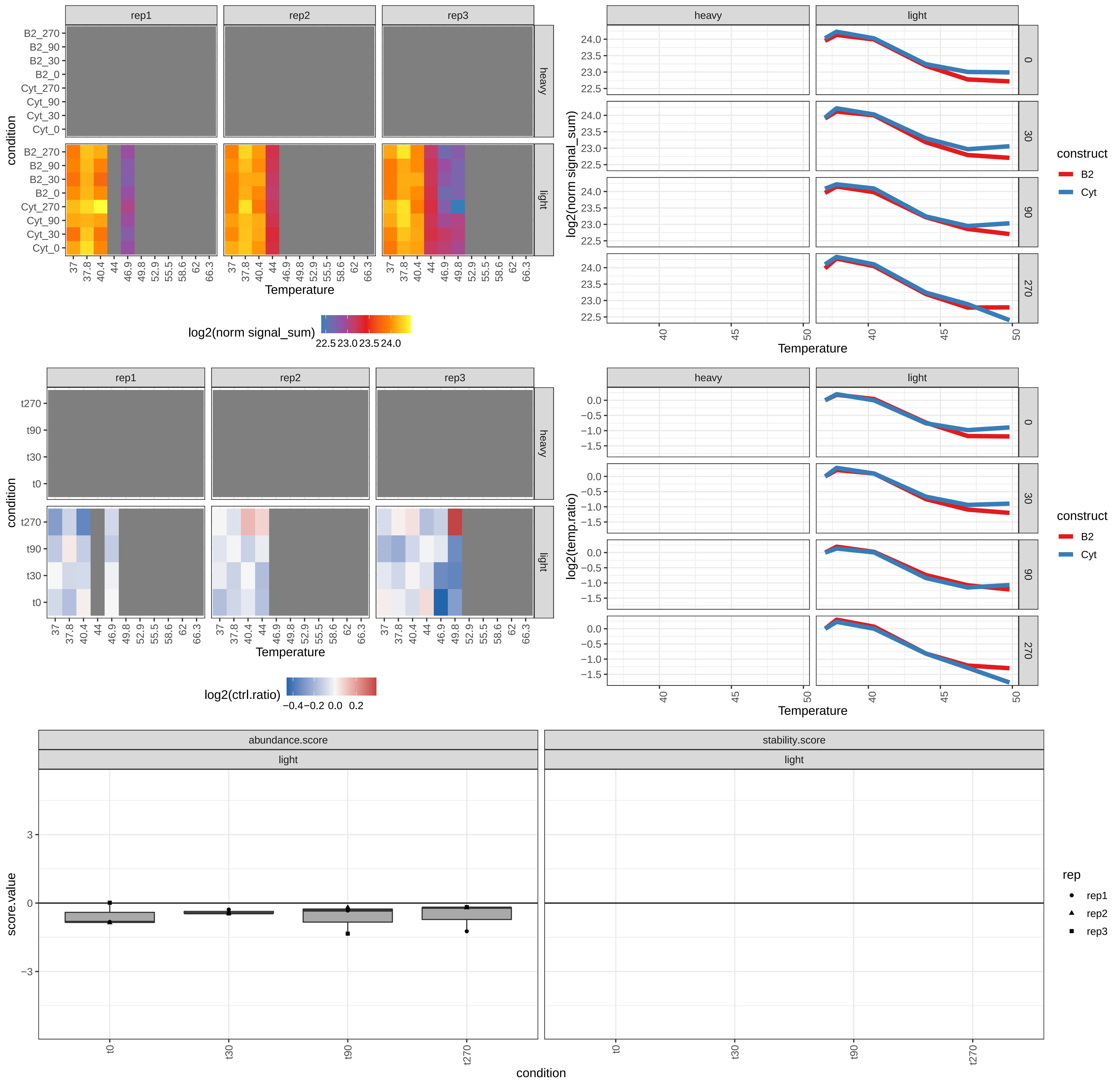

P52917 − Vacuolar protein sorting−associated protein 4 OS=Saccharomyces cerevisiae (strain ATCC 204508 / S288c) GN=VPS4 PE=1 SV=1

VPS41 − average.top3: 6.69065461538462 − max.qupm: 5

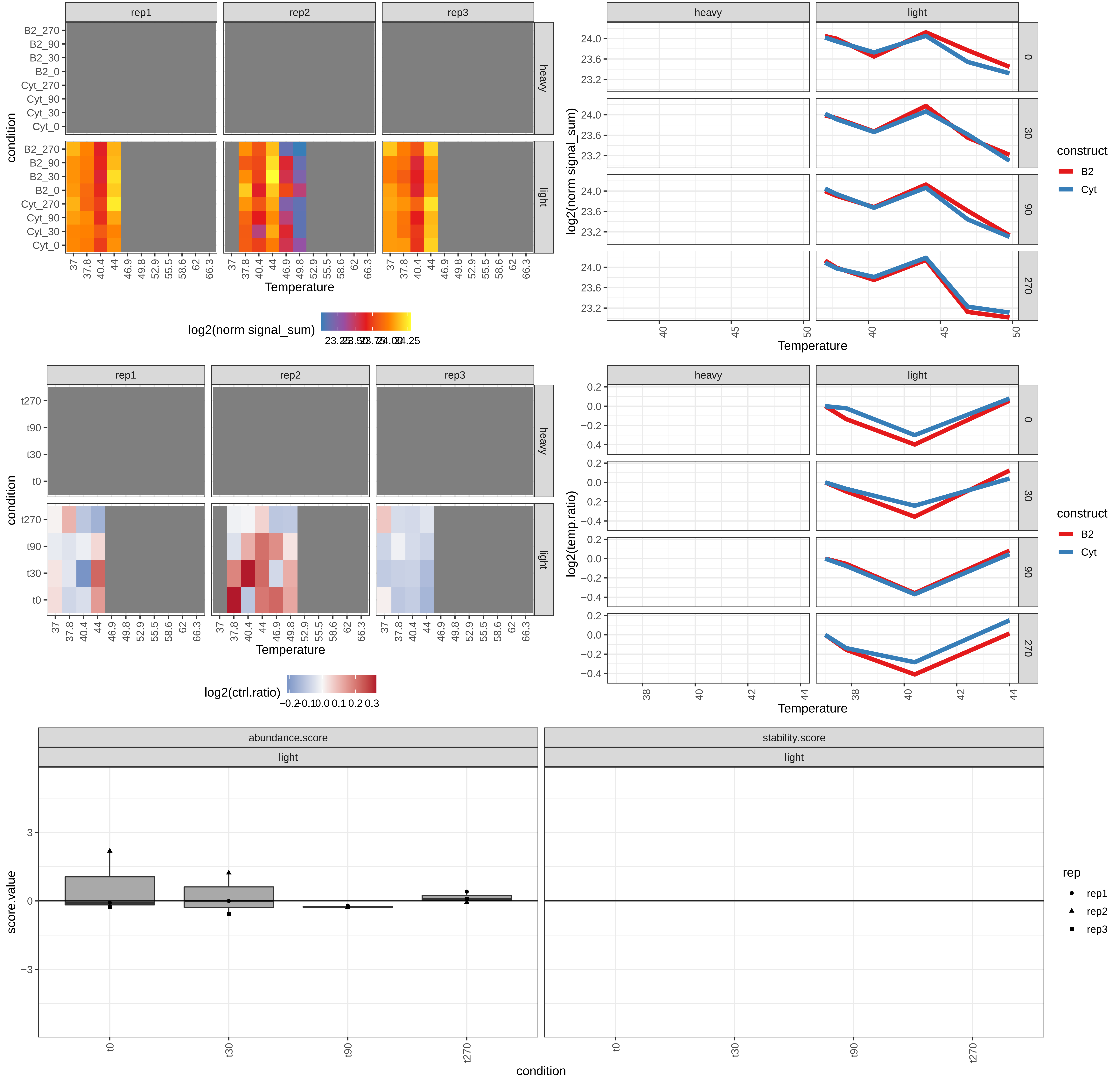

P38959 − Vacuolar protein sorting−associated protein 41 OS=Saccharomyces cerevisiae (strain ATCC 204508 / S288c) GN=VPS41 PE=1 SV=2

VPS45 − average.top3: 6.80891136363636 − max.qupm: 11

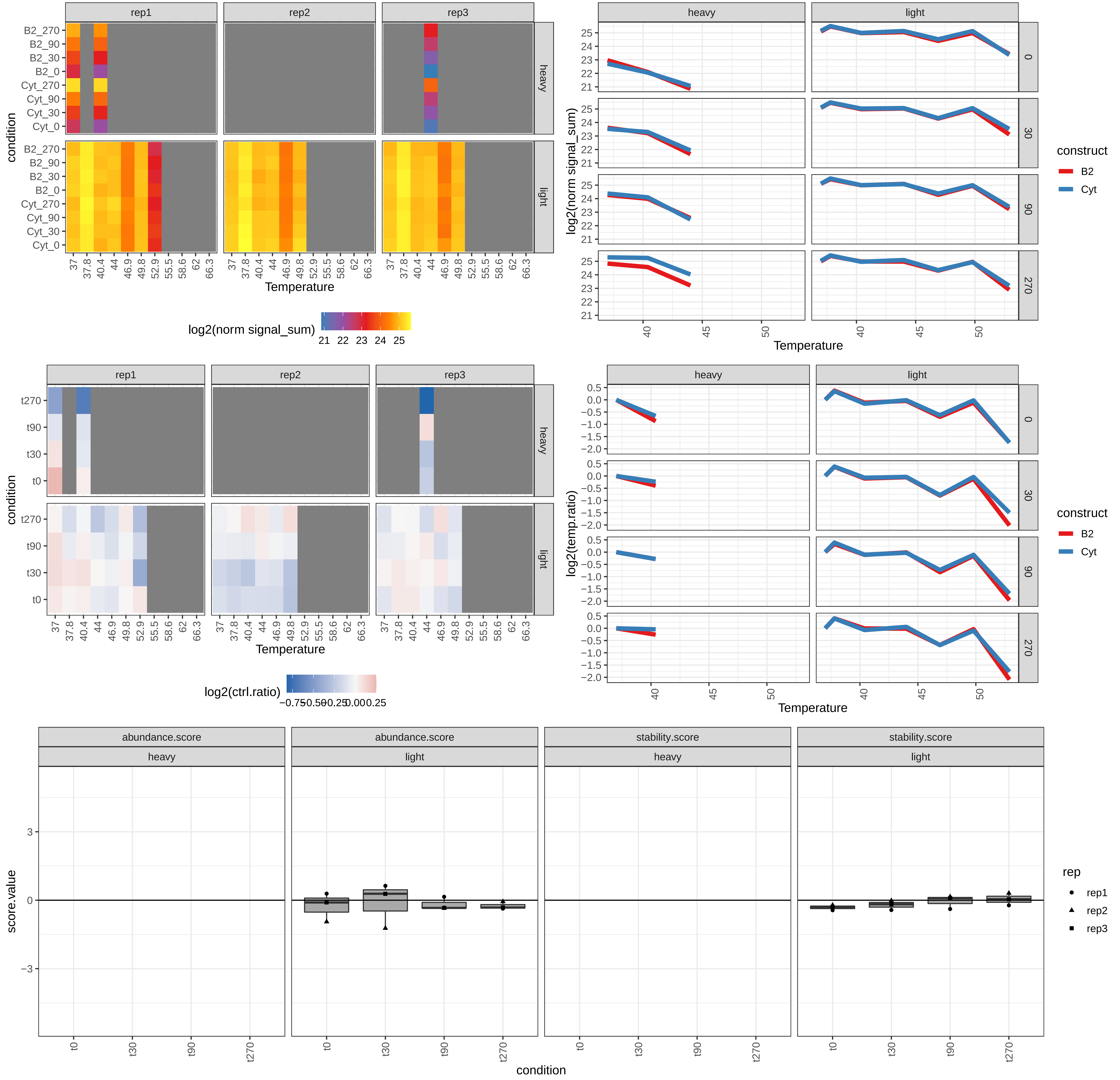

P38932 − Vacuolar protein sorting−associated protein 45 OS=Saccharomyces cerevisiae (strain ATCC 204508 / S288c) GN=VPS45 PE=1 SV=2

VPS5 − average.top3: 7.12970526315789 − max.qupm: 7

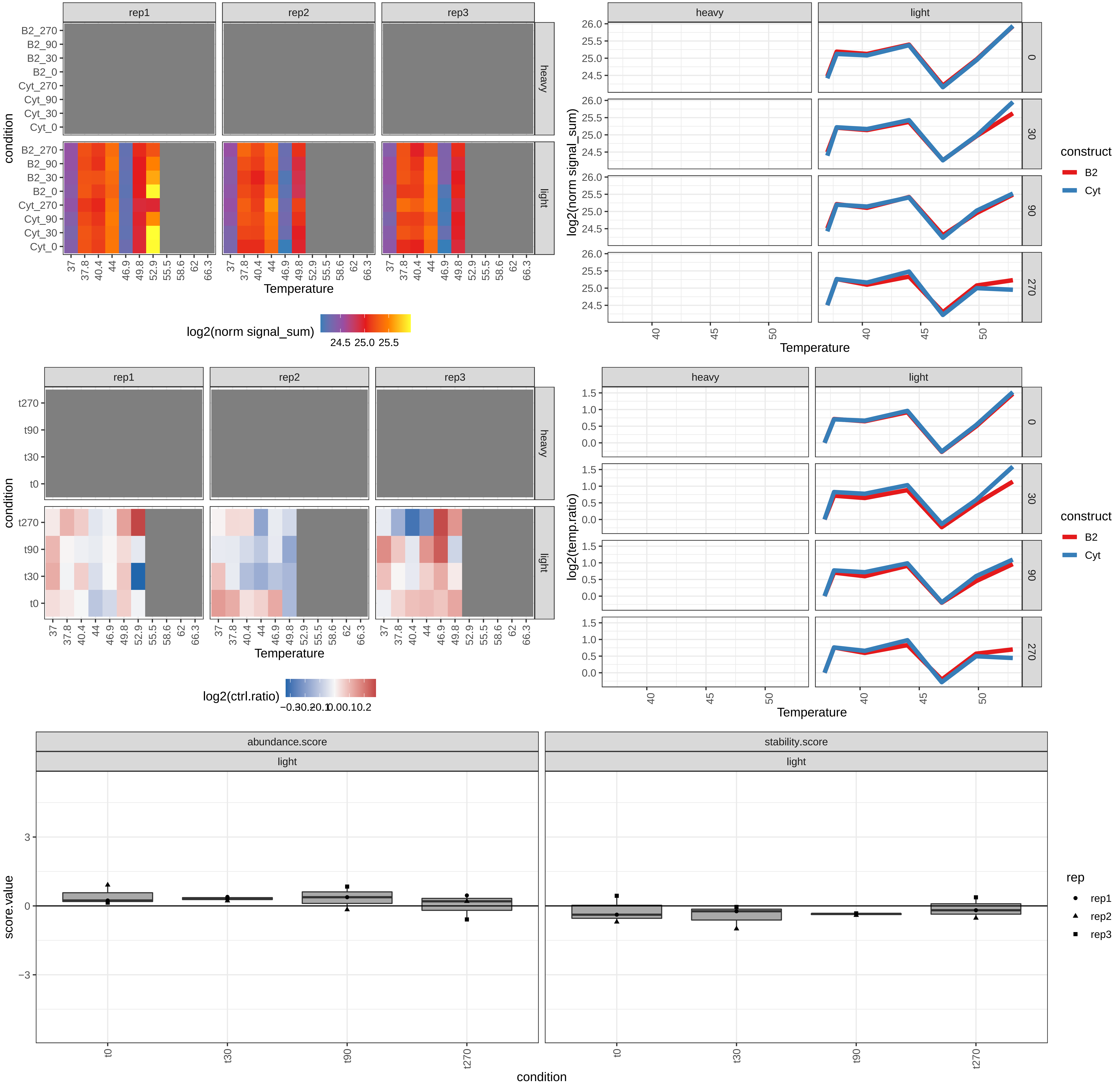

Q92331 − Vacuolar protein sorting−associated protein 5 OS=Saccharomyces cerevisiae (strain ATCC 204508 / S288c) GN=VPS5 PE=1 SV=1

VPS53 − average.top3: 6.55088611111111 − max.qupm: 5

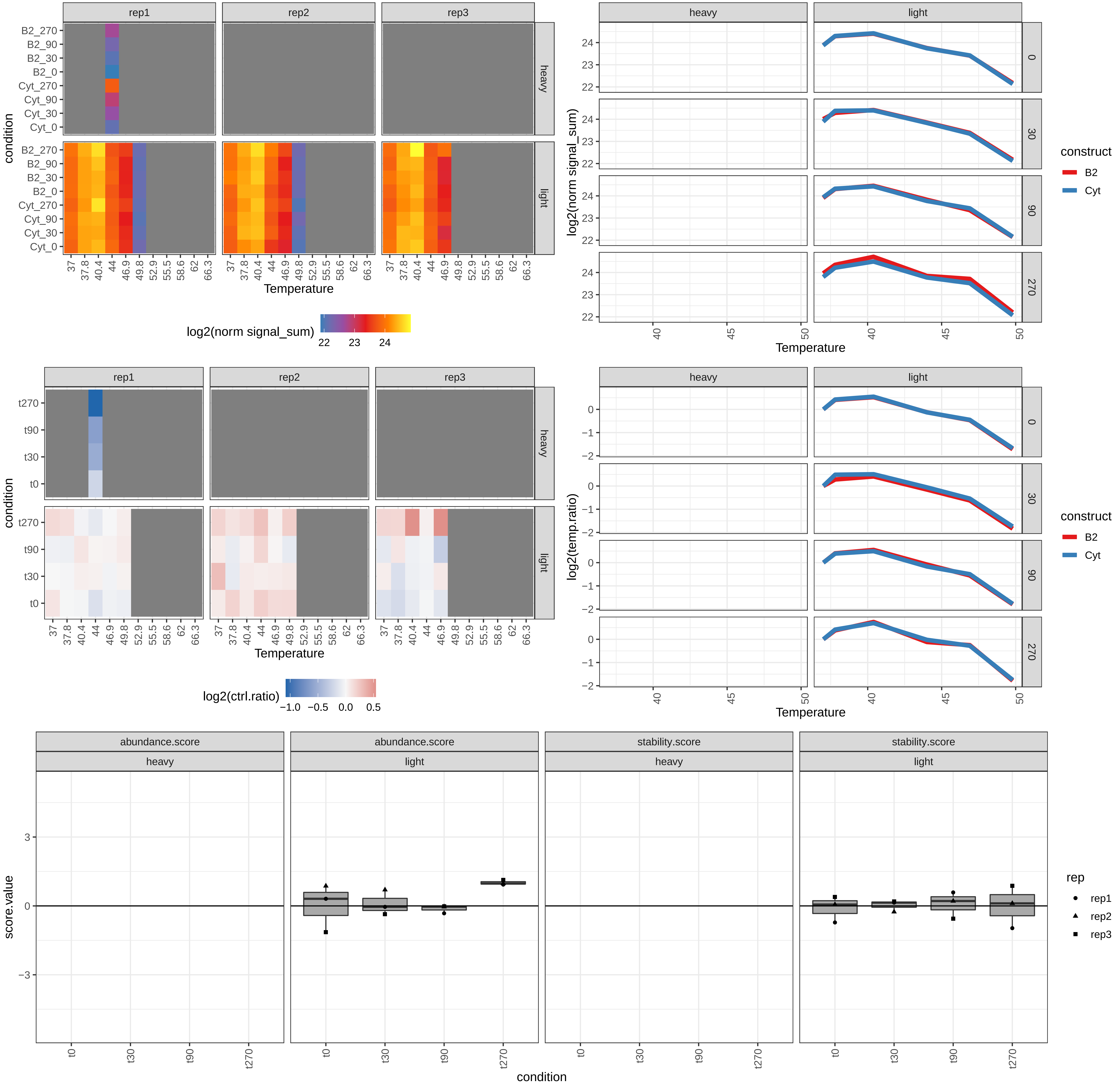

P47061 − Vacuolar protein sorting−associated protein 53 OS=Saccharomyces cerevisiae (strain ATCC 204508 / S288c) GN=VPS53 PE=1 SV=1

VPS54 − average.top3: 6.80052266666667 − max.qupm: 7

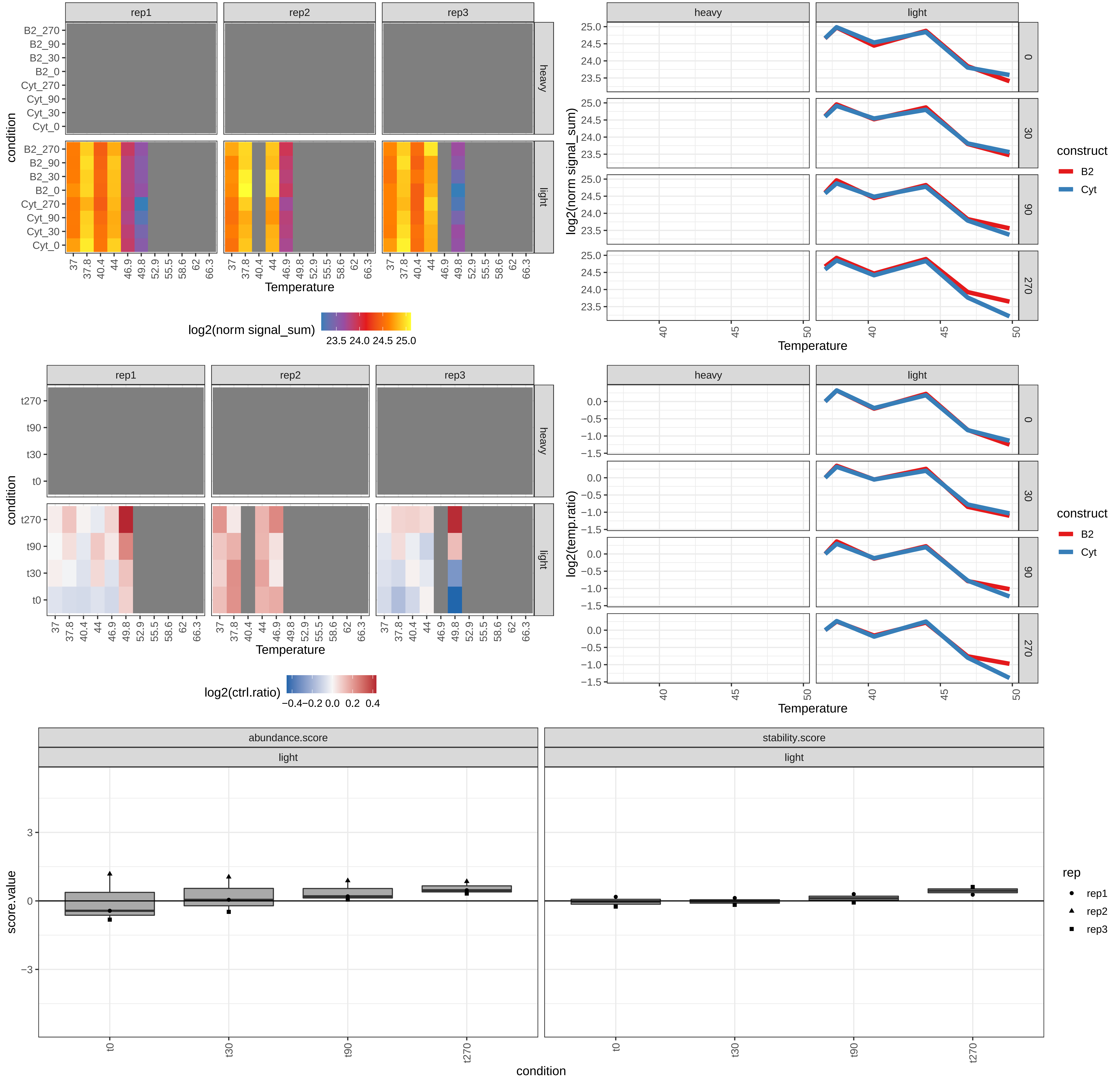

Q12071 − Vacuolar protein sorting−associated protein 54 OS=Saccharomyces cerevisiae (strain ATCC 204508 / S288c) GN=VPS54 PE=1 SV=1

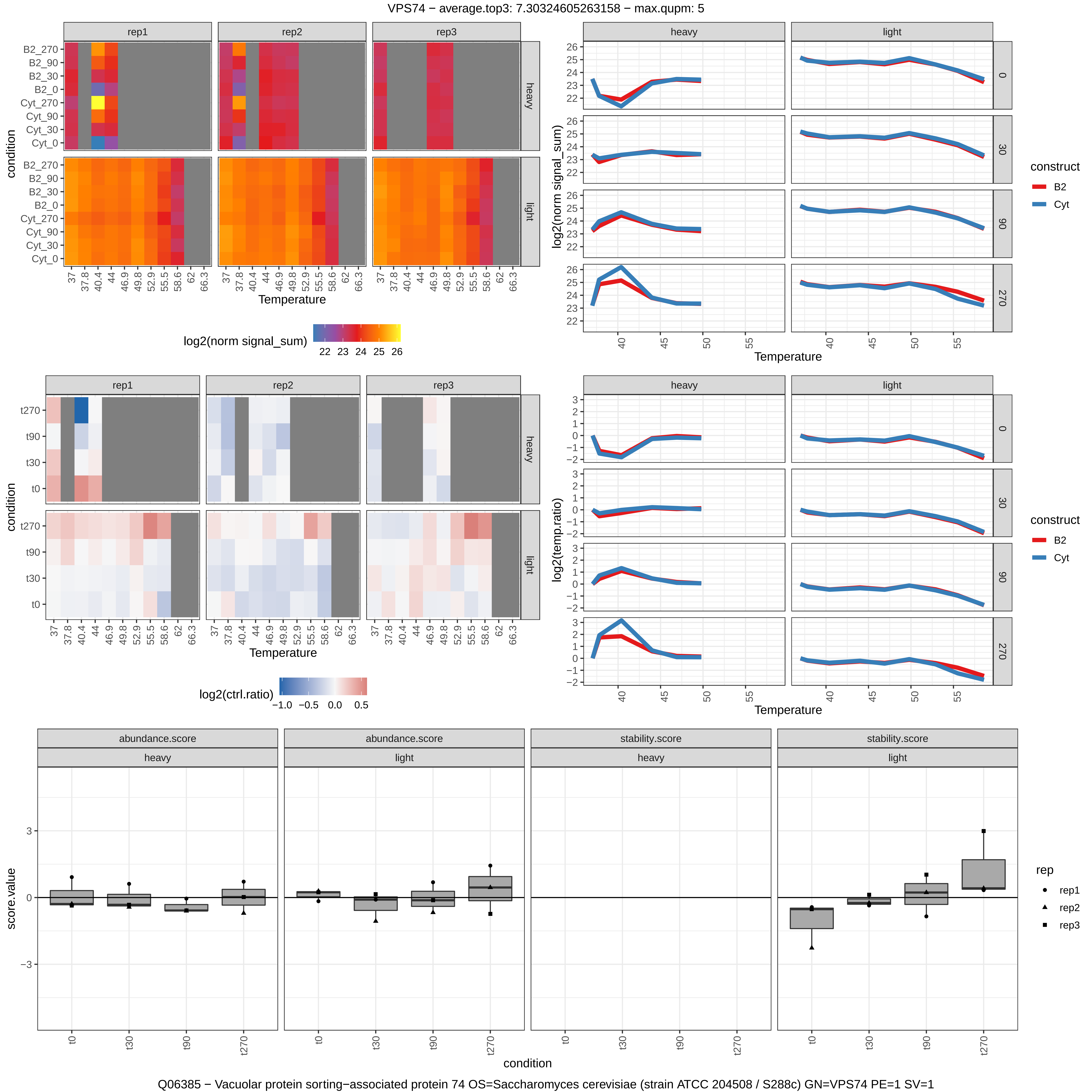

VPS74 − average.top3: 7.30324605263158 − max.qupm: 5
rep1
rep2
rep3
heavy
light
condition
Temperature
log2(norm signal_sum)
construct
B2
Cyt
log2(temp.ratio)
log2(ctrl.ratio)
abundance.score
stability.score
score.value
rep
rep1
rep2
rep3
Q06385 − Vacuolar protein sorting−associated protein 74 OS=Saccharomyces cerevisiae (strain ATCC 204508 / S288c) GN=VPS74 PE=1 SV=1

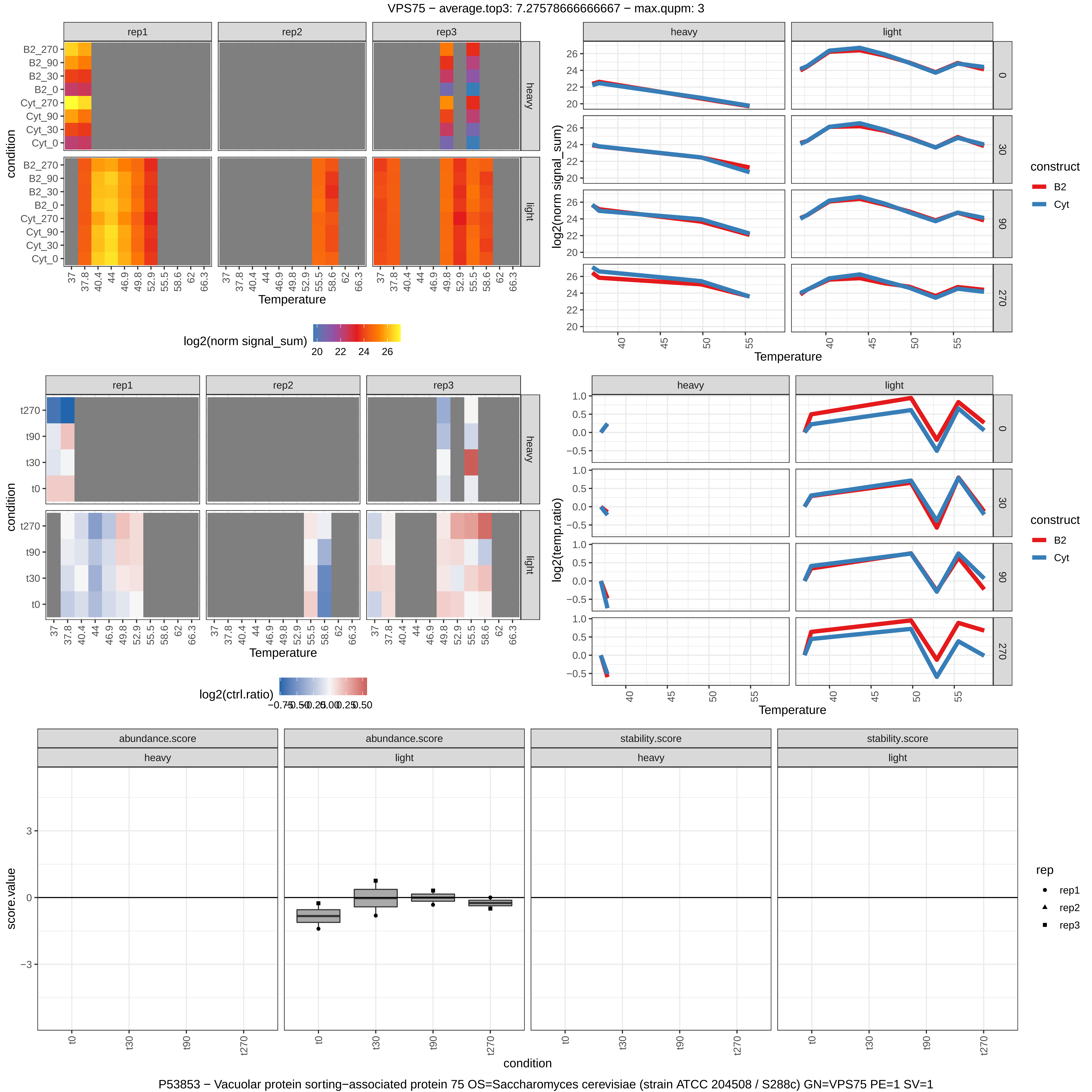
VPS75 − average.top3: 7.27578666666667 − max.qupm: 3
rep1
rep2
rep3
heavy
light
condition
Temperature
log2(norm signal_sum)
construct
B2
Cyt
log2(ctrl.ratio)
log2(temp.ratio)
abundance.score
stability.score
score.value
rep
rep1
rep2
rep3
P53853 − Vacuolar protein sorting−associated protein 75 OS=Saccharomyces cerevisiae (strain ATCC 204508 / S288c) GN=VPS75 PE=1 SV=1

VPS9 − average.top3: 6.64877125 − max.qupm: 7

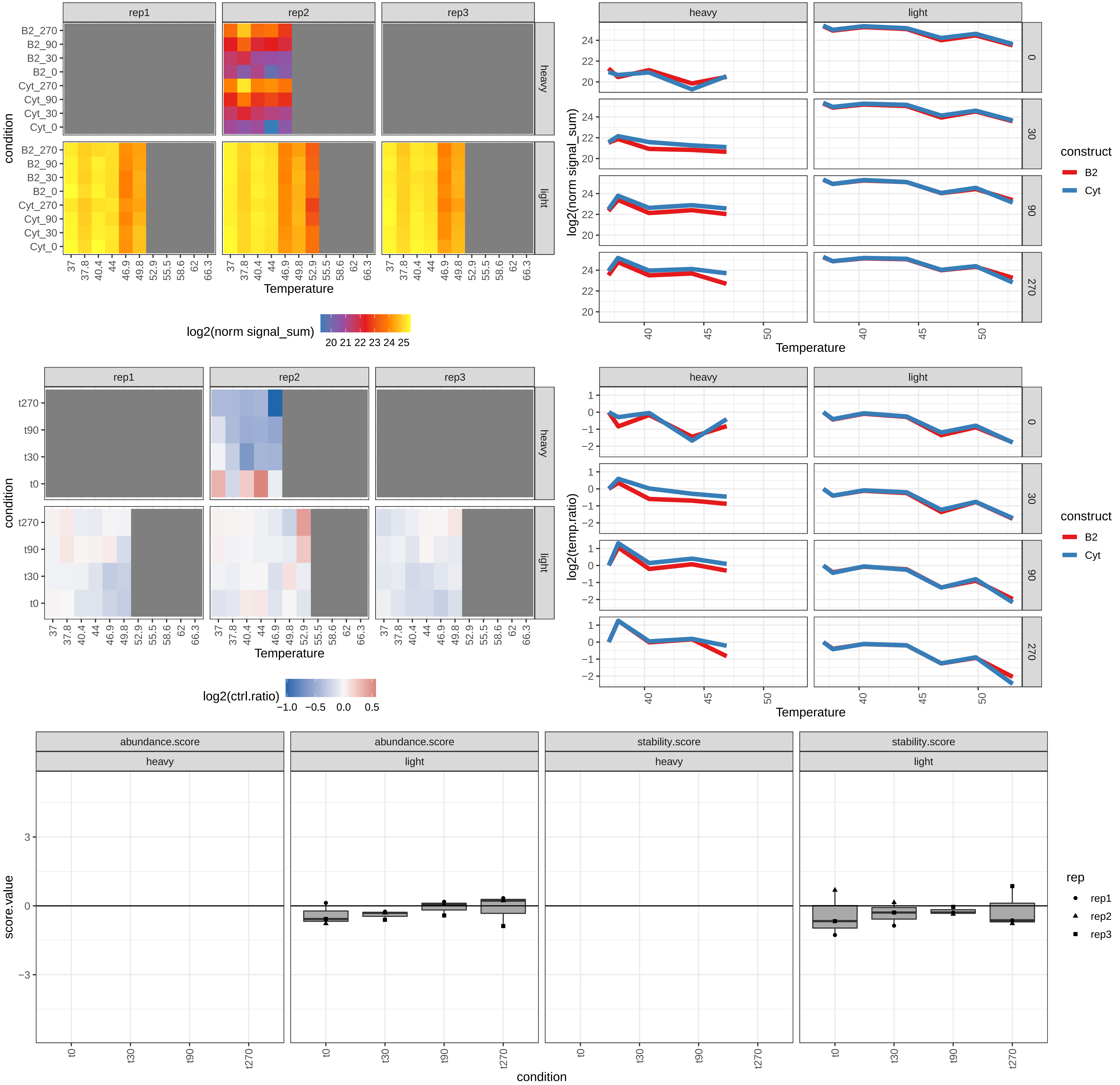

P54787 − Vacuolar protein sorting−associated protein 9 OS=Saccharomyces cerevisiae (strain ATCC 204508 / S288c) GN=VPS9 PE=1 SV=2

VRP1 − average.top3: 7.09671933333333 − max.qupm: 3

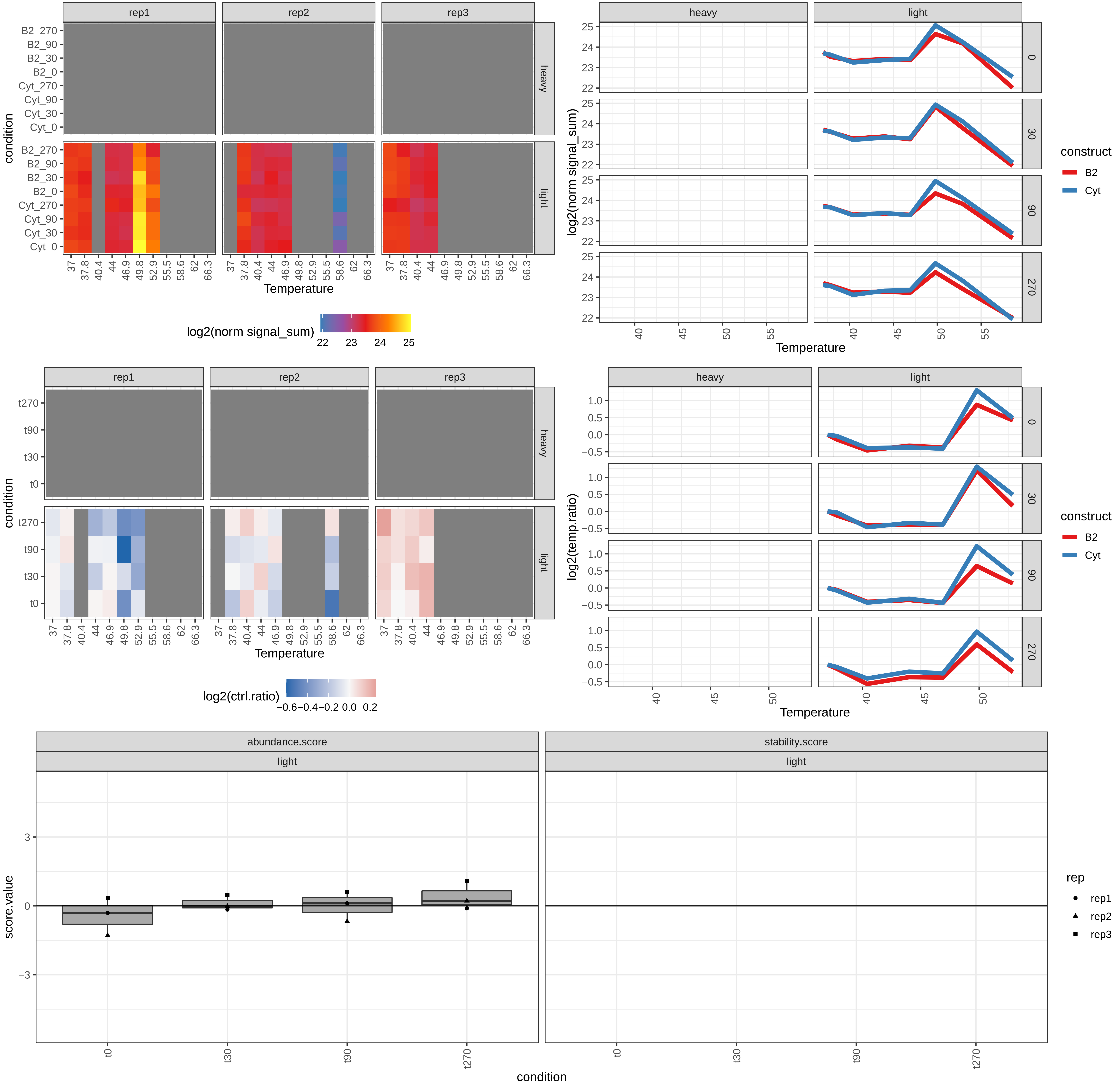

P37370 − Verprolin OS=Saccharomyces cerevisiae (strain ATCC 204508 / S288c) GN=VRP1 PE=1 SV=2

VTA1 − average.top3: 6.77582909090909 − max.qupm: 3

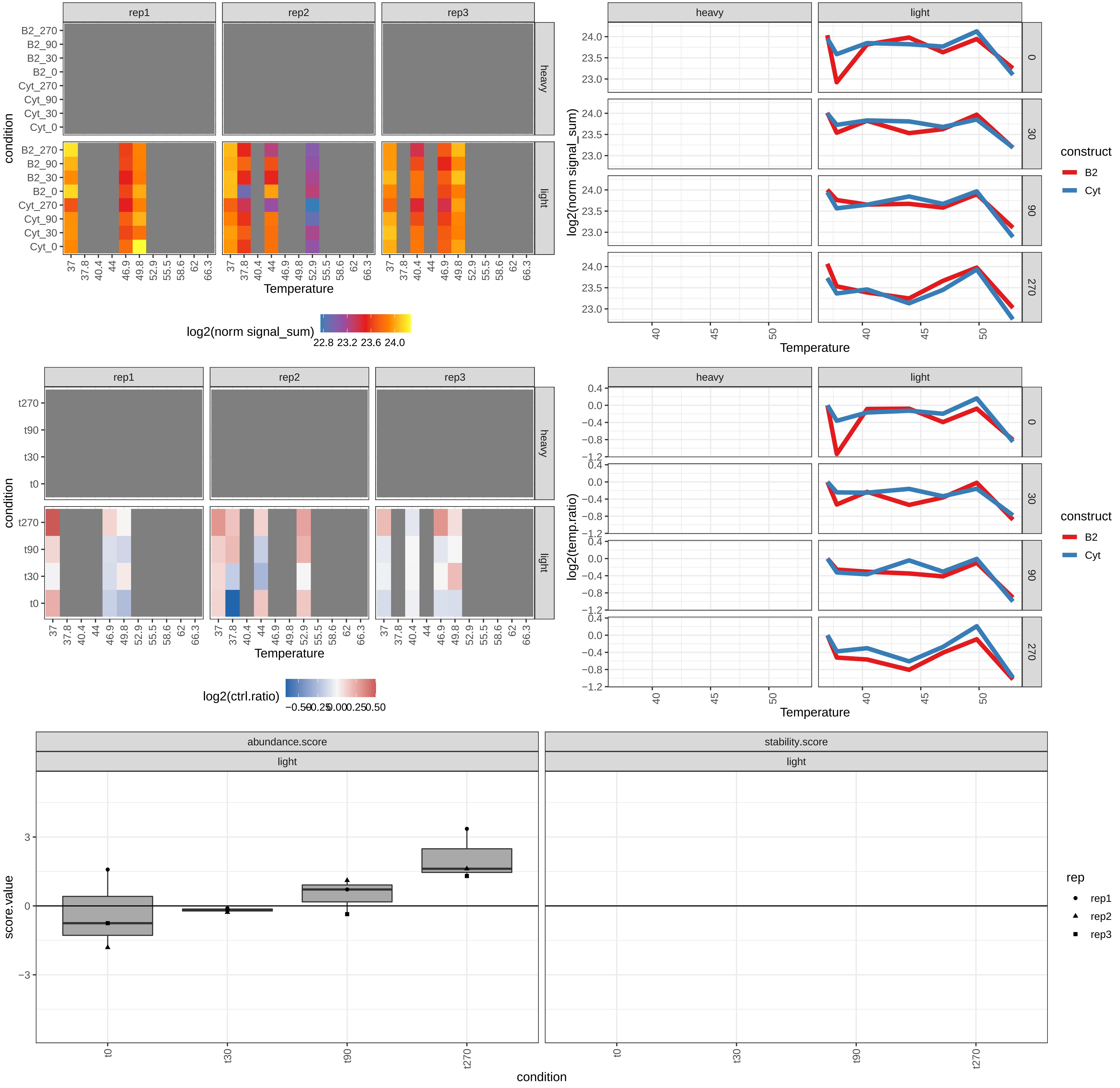

Q06263 − Vacuolar protein sorting−associated protein VTA1 OS=Saccharomyces cerevisiae (strain ATCC 204508 / S288c) GN=VTA1 PE=1 SV=1

VTC1 − average.top3: 7.13596206896552 − max.qupm: 4

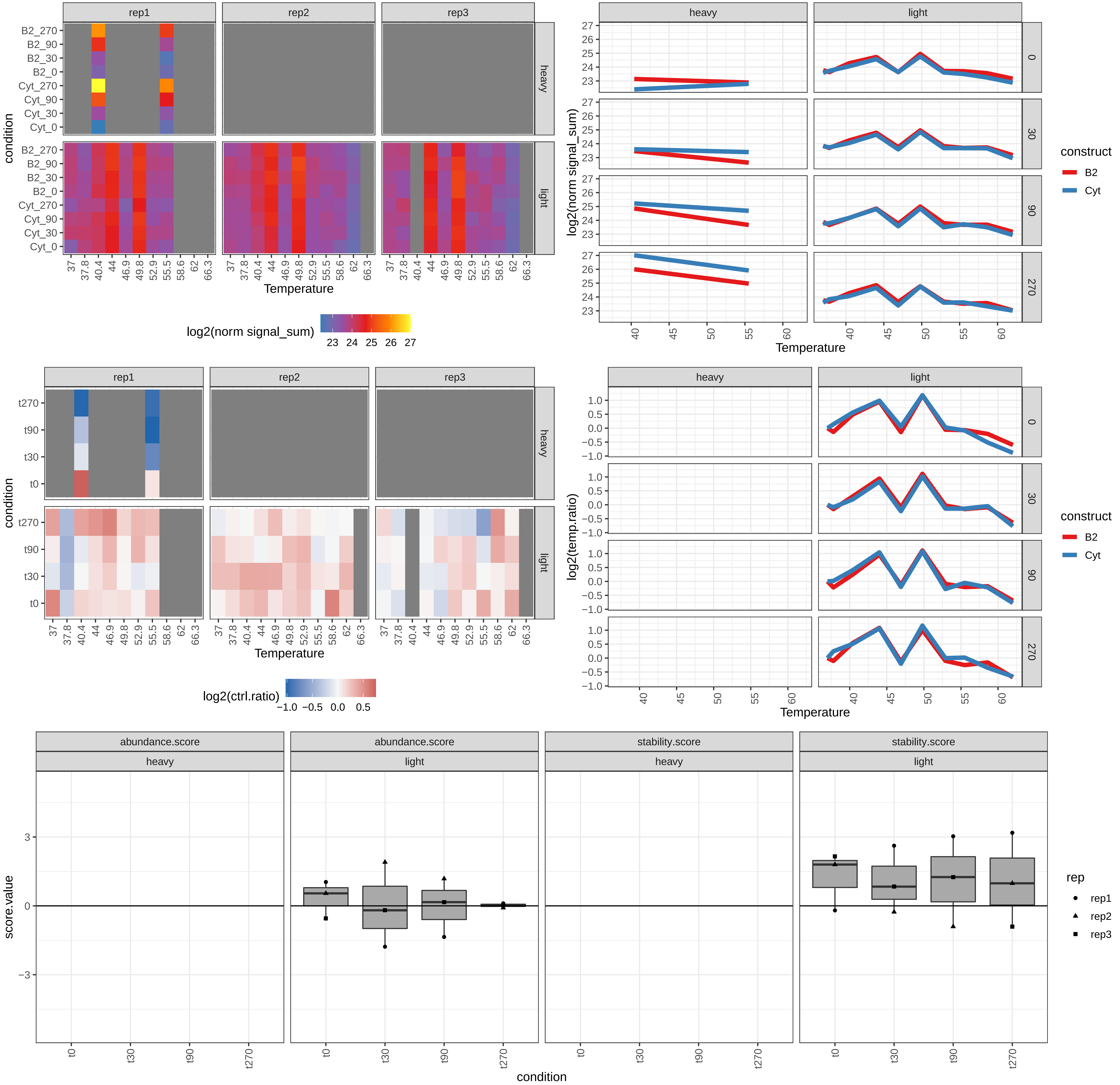

P40046 − Vacuolar transporter chaperone 1 OS=Saccharomyces cerevisiae (strain ATCC 204508 / S288c) GN=VTC1 PE=1 SV=3

VTC2 − average.top3: 7.00231395348837 − max.qupm: 18

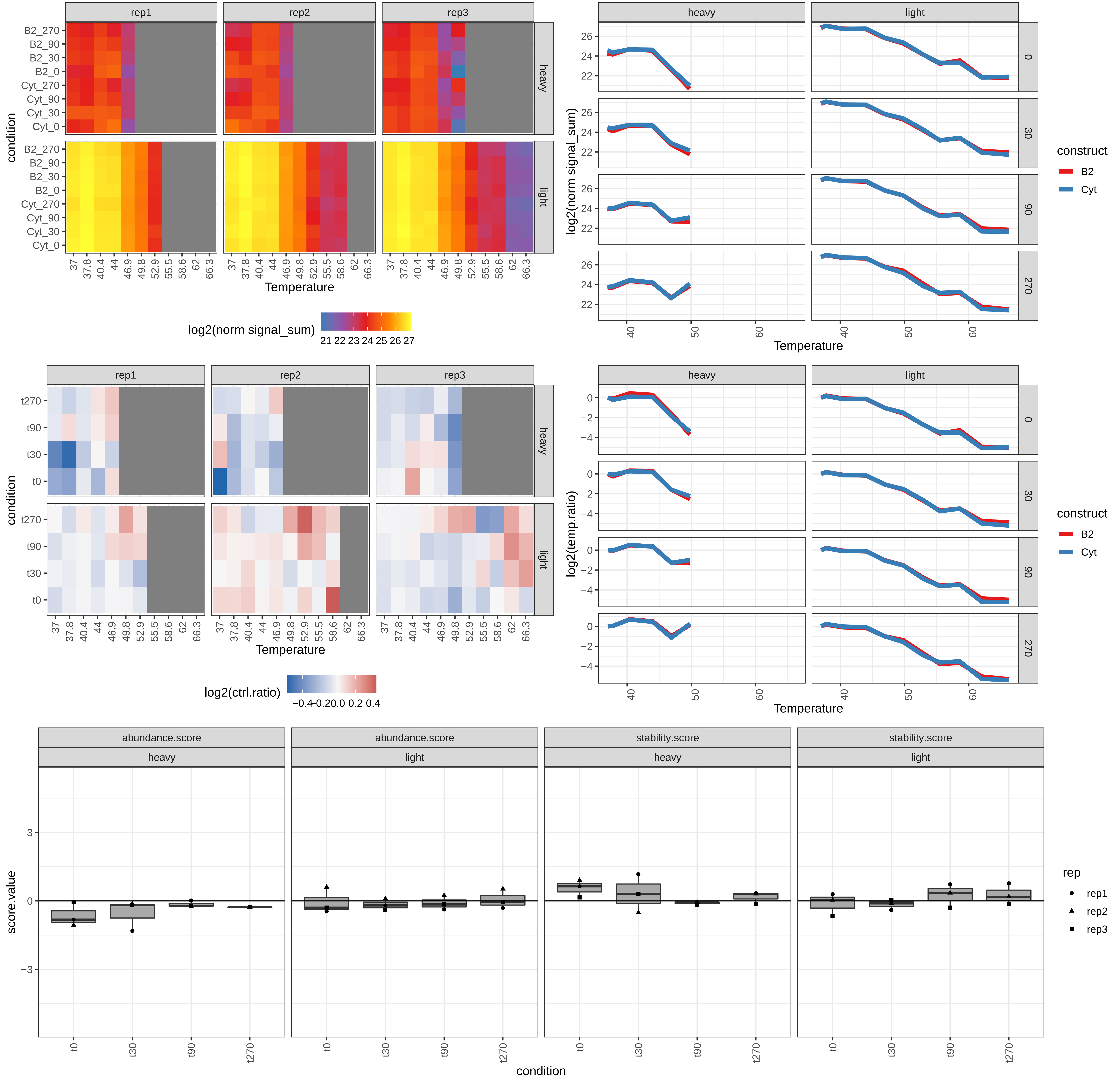

P43585 − Vacuolar transporter chaperone 2 OS=Saccharomyces cerevisiae (strain ATCC 204508 / S288c) GN=VTC2 PE=1 SV=1

VTC3 − average.top3: 6.78207136363636 − max.qupm: 12

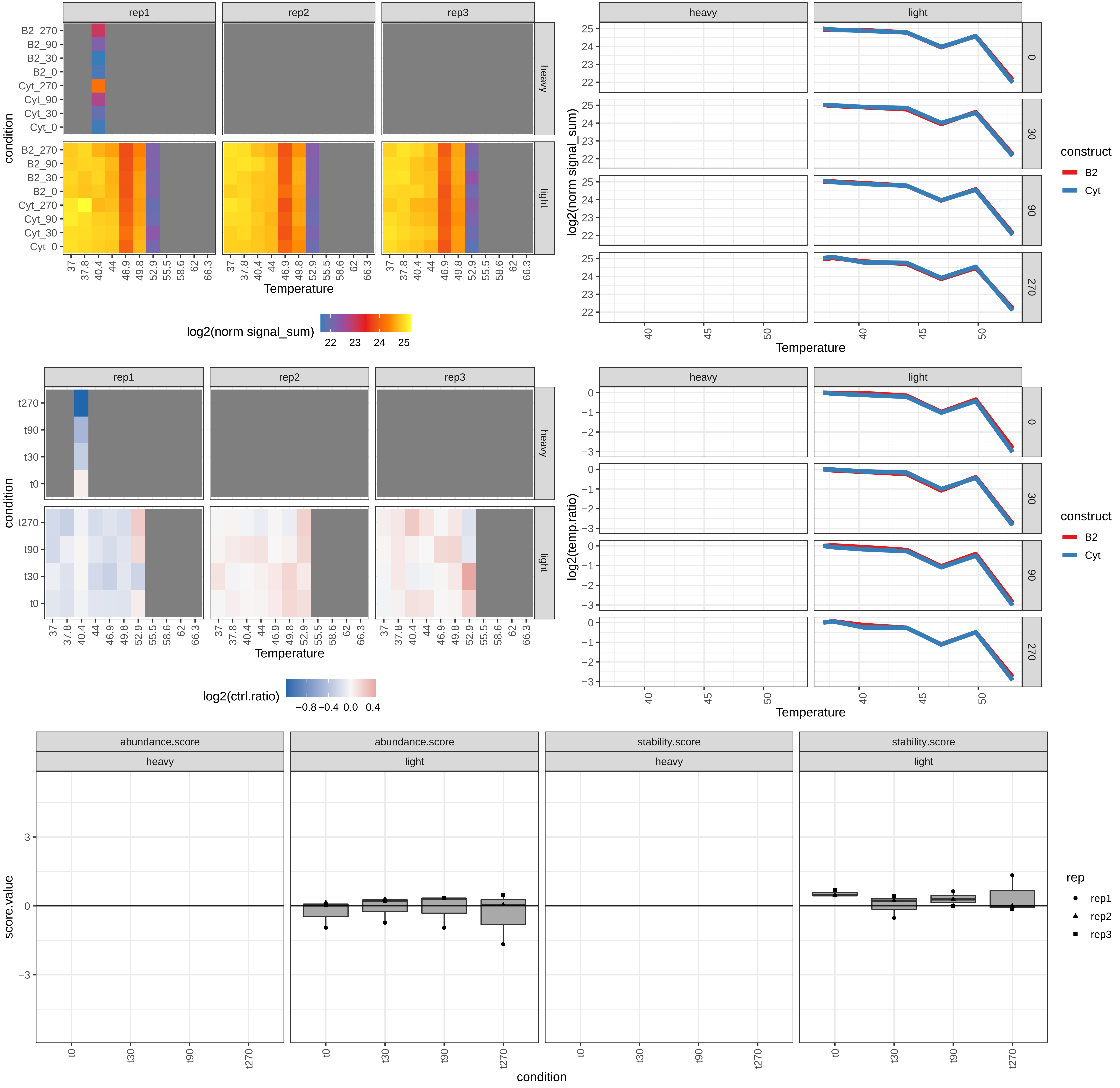

Q02725 − Vacuolar transporter chaperone 3 OS=Saccharomyces cerevisiae (strain ATCC 204508 / S288c) GN=VTC3 PE=1 SV=1

VTC4 − average.top3: 7.0877688372093 − max.qupm: 20

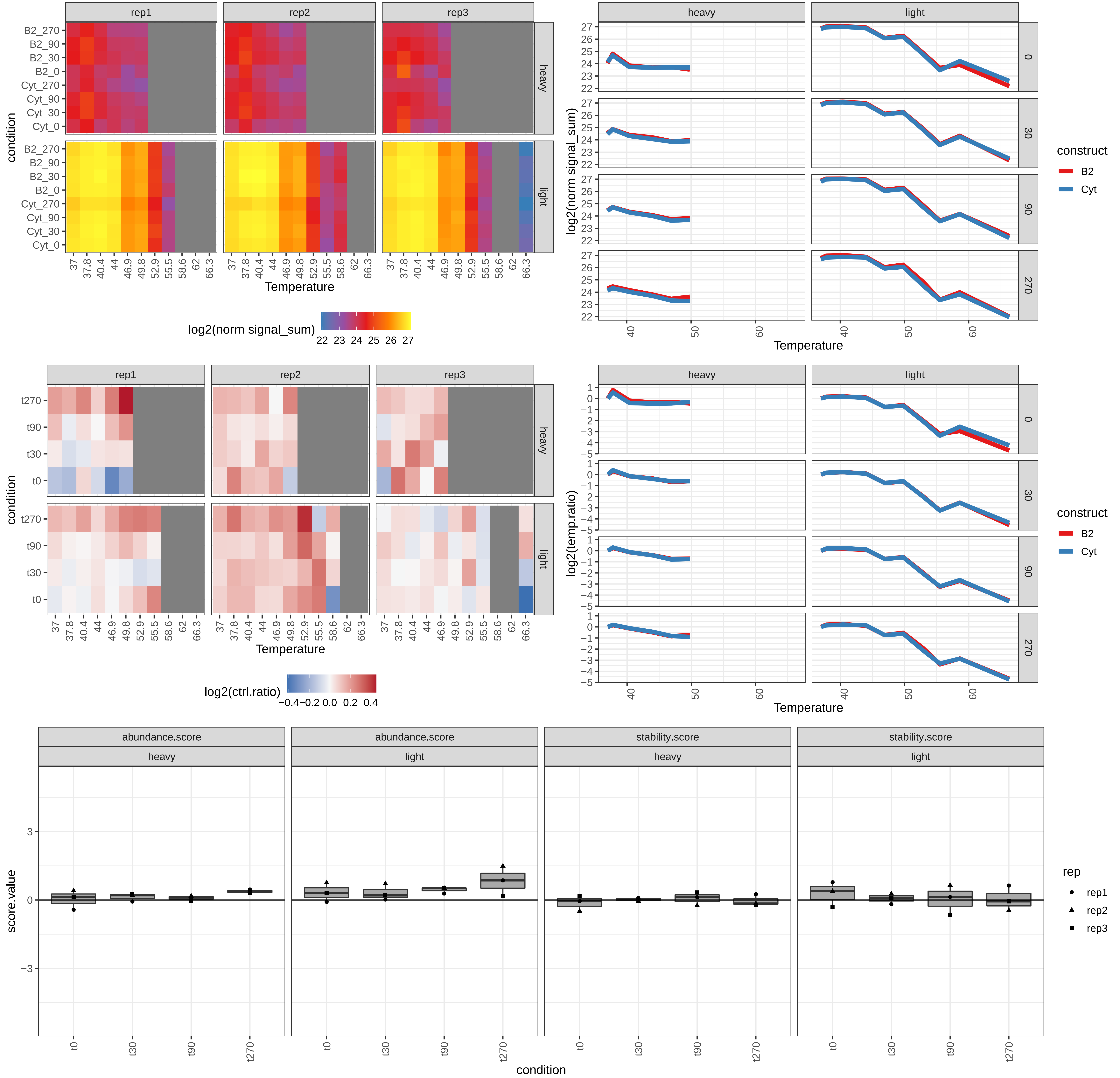

P47075 − Vacuolar transporter chaperone 4 OS=Saccharomyces cerevisiae (strain ATCC 204508 / S288c) GN=VTC4 PE=1 SV=2

VTI1 − average.top3: 6.77615578947368 − max.qupm: 5

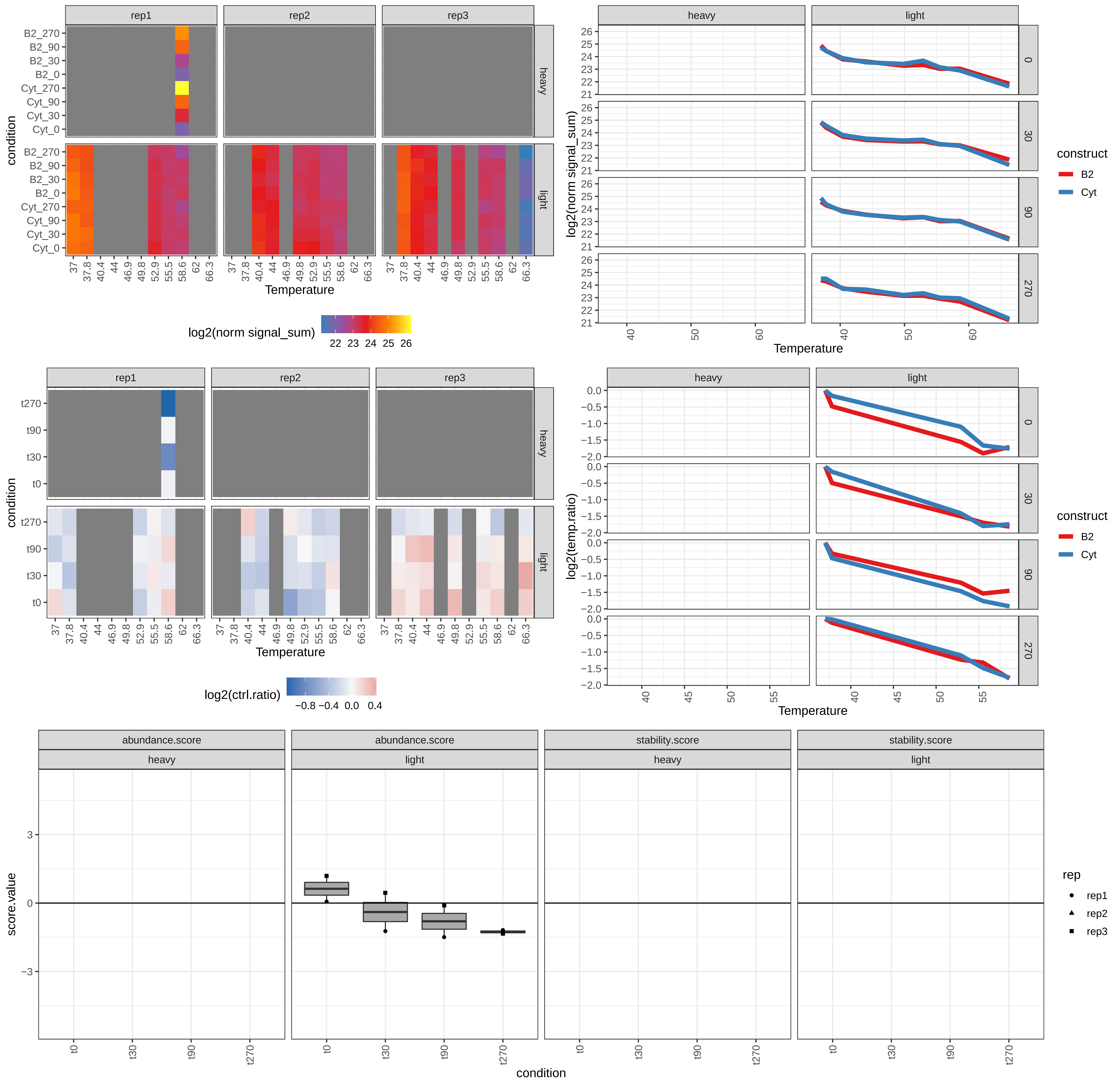

Q04338 − t−SNARE VTI1 OS=Saccharomyces cerevisiae (strain ATCC 204508 / S288c) GN=VTI1 PE=1 SV=3

WAR1 − average.top3: 6.2617745 − max.qupm: 5

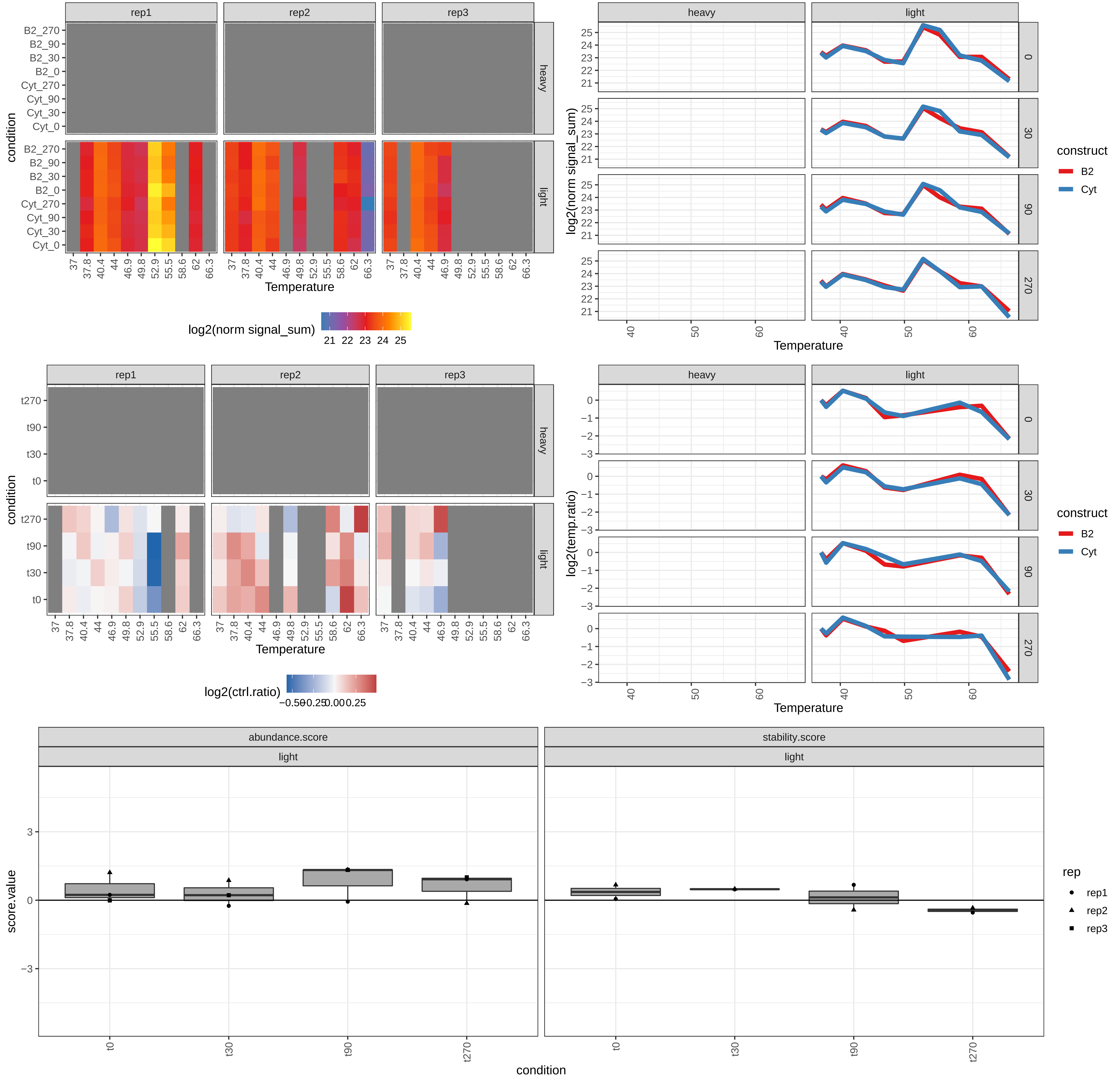

Q03631 − Weak acid resistance protein 1 OS=Saccharomyces cerevisiae (strain ATCC 204508 / S288c) GN=WAR1 PE=1 SV=1

WBP1 − average.top3: 7.15337613636364 − max.qupm: 10

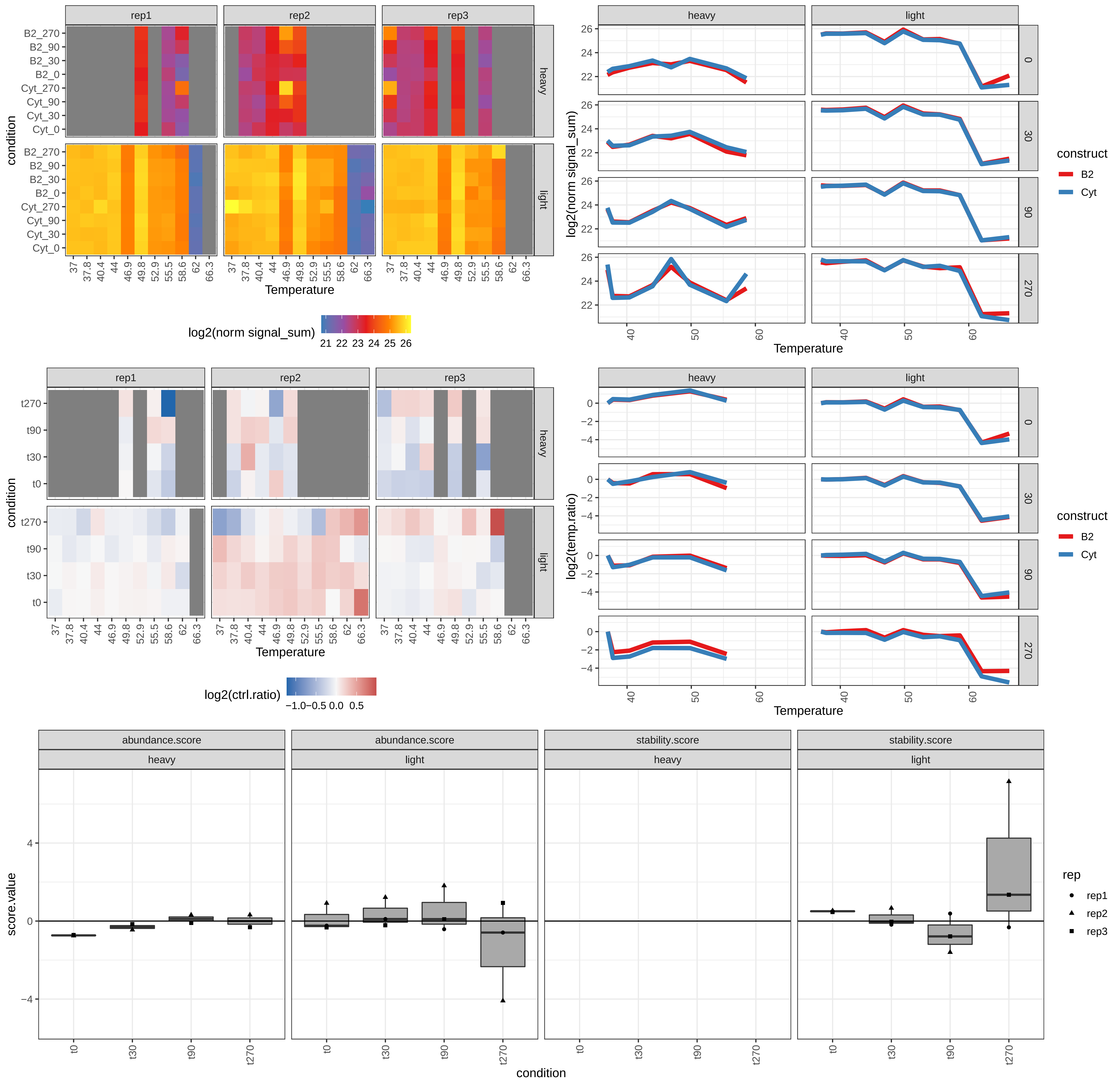

P33767 − Dolichyl−diphosphooligosaccharide−−protein glycosyltransferase subunit WBP1 OS=Saccharomyces cerevisiae (strain ATCC 204508 / S288c) GN=WBP1 PE=1 SV=1

WHI2 − average.top3: 6.81769 − max.qupm: 6

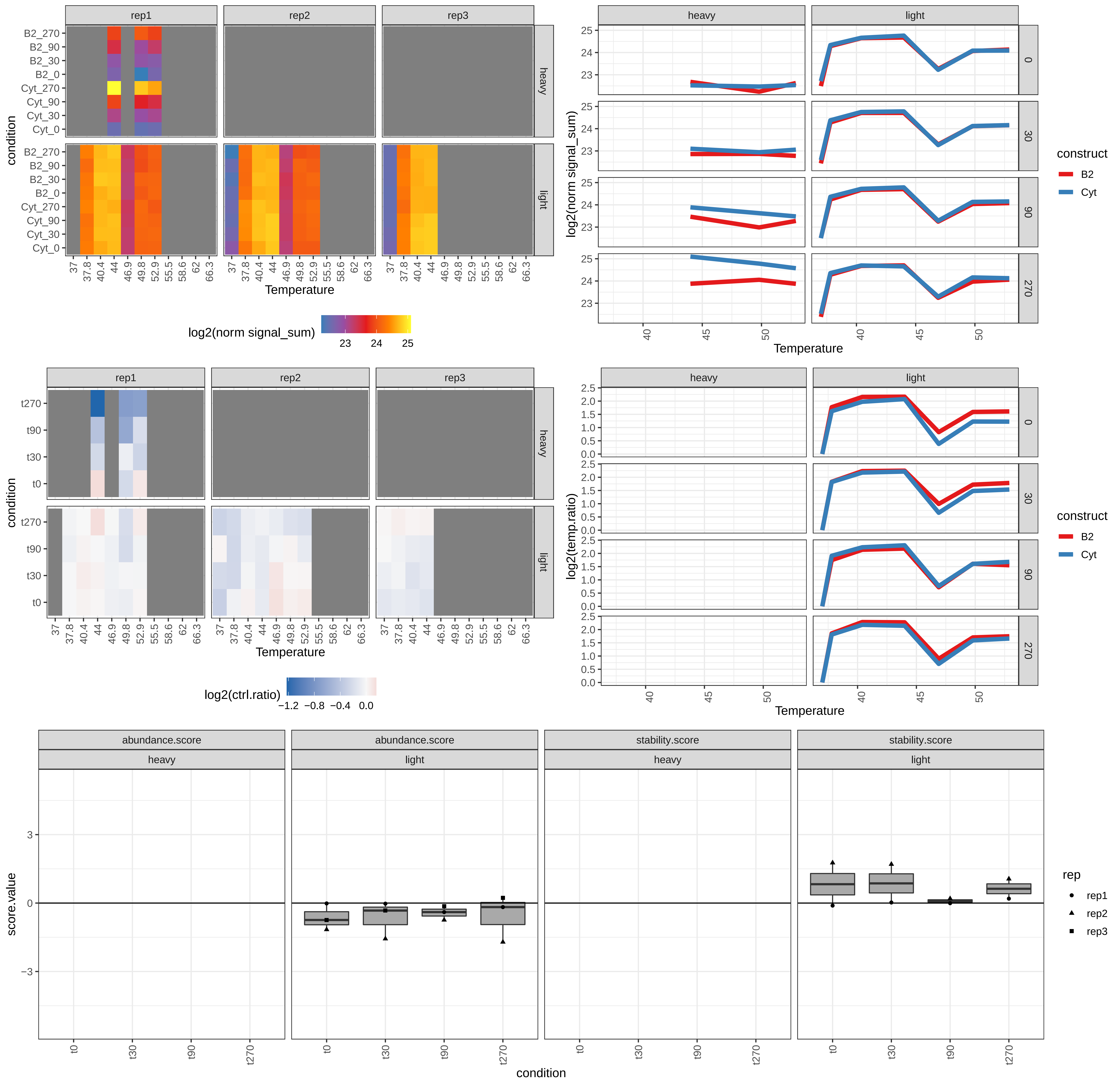

P12611 − Growth regulation protein OS=Saccharomyces cerevisiae (strain ATCC 204508 / S288c) GN=WHI2 PE=1 SV=2

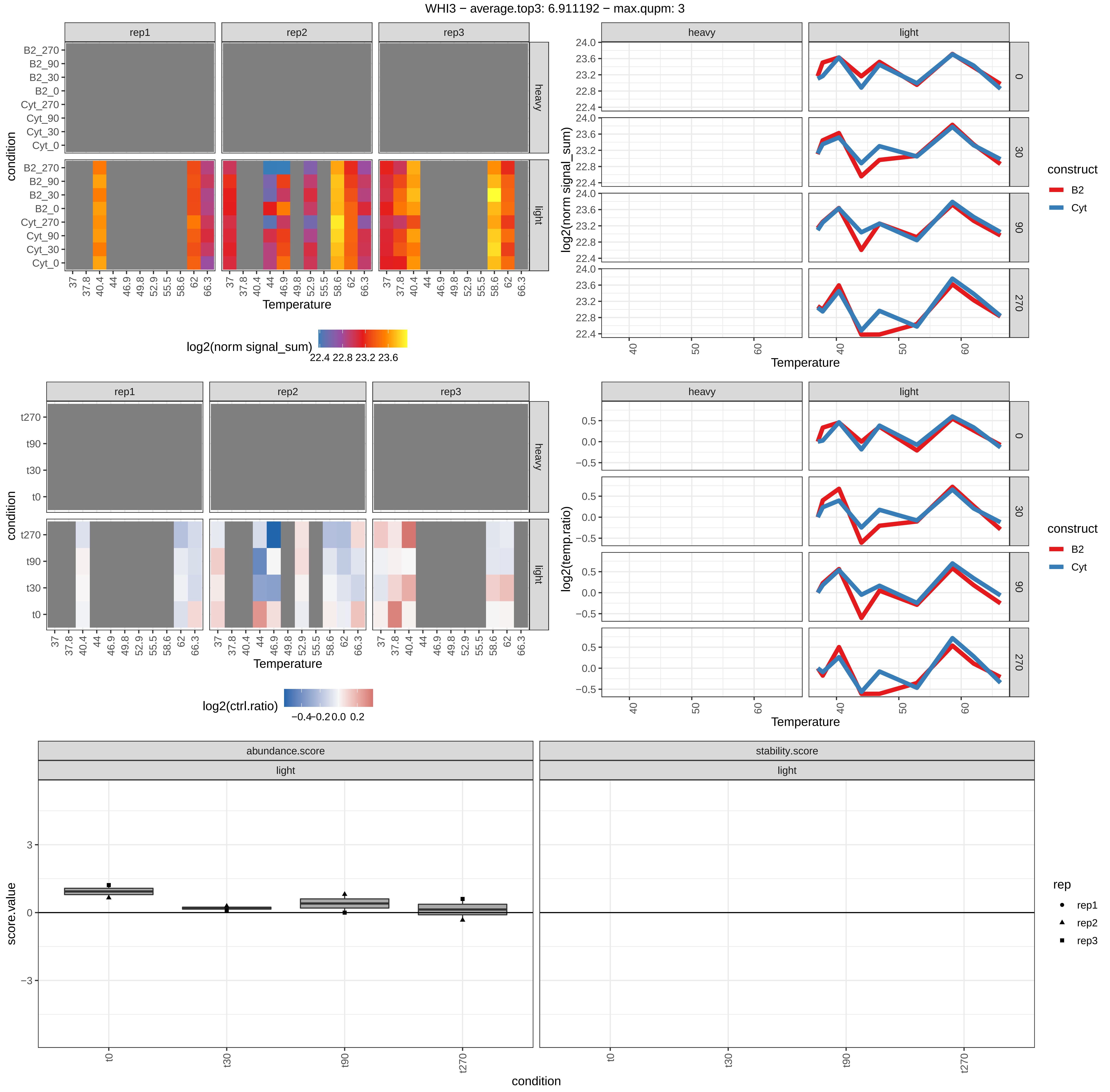

P34761 − Protein WHI3 OS=Saccharomyces cerevisiae (strain ATCC 204508 / S288c) GN=WHI3 PE=1 SV=1

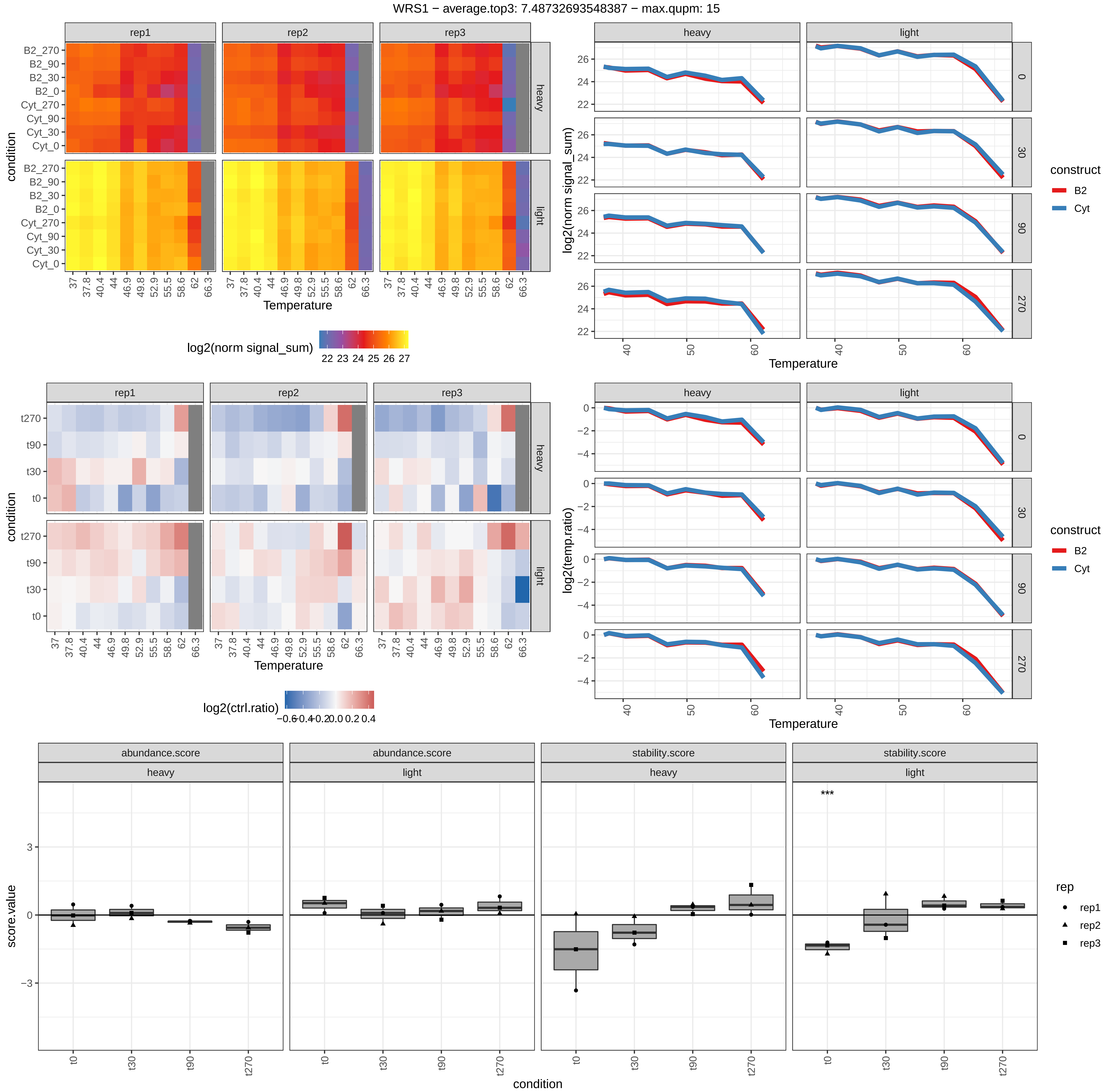

Q12109 − Tryptophan−−tRNA ligase, cytoplasmic OS=Saccharomyces cerevisiae (strain ATCC 204508 / S288c) GN=WRS1 PE=1 SV=1

WTM1 − average.top3: 7.56680288135593 − max.qupm: 14

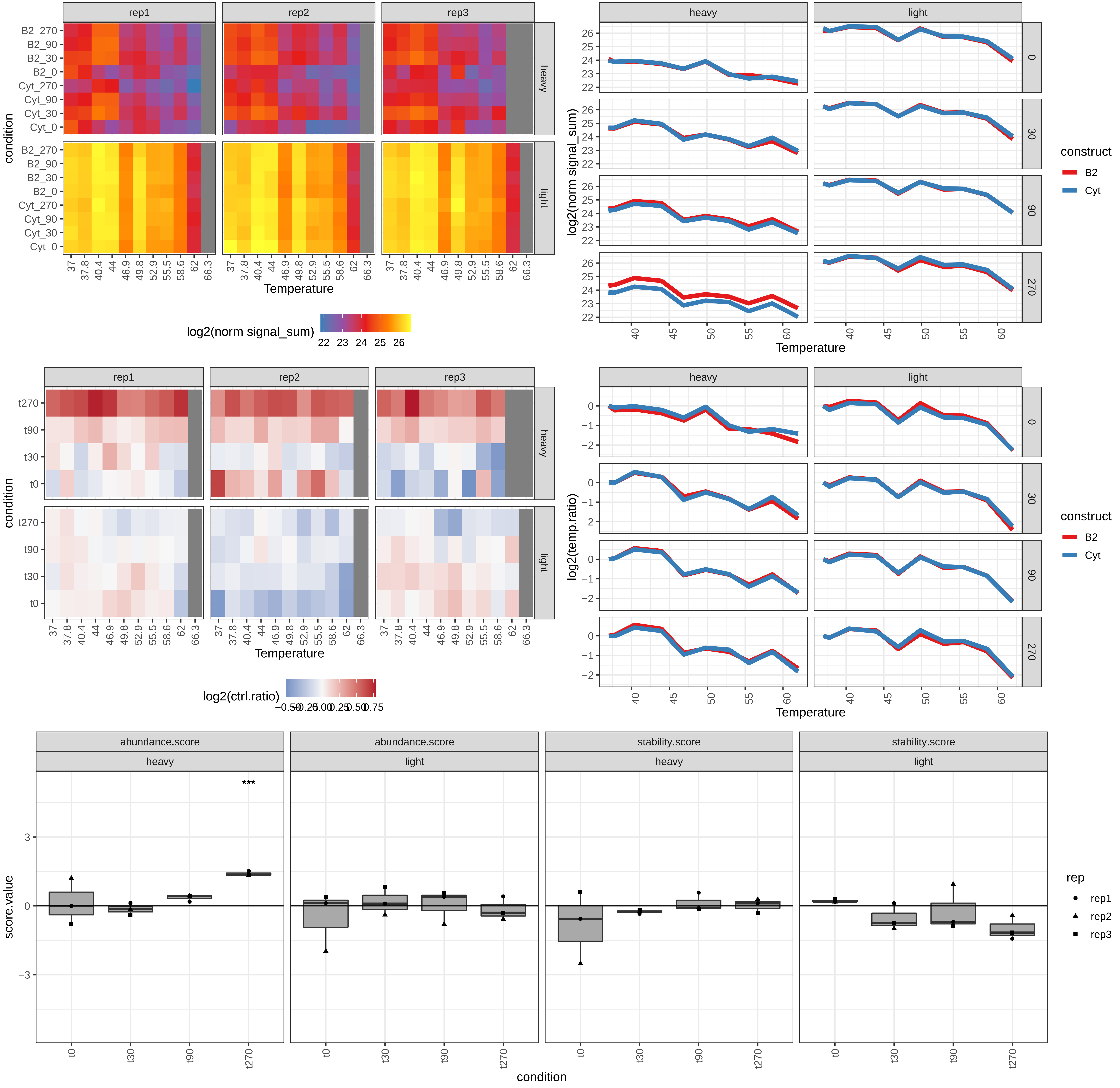

Q12363 − Transcriptional modulator WTM1 OS=Saccharomyces cerevisiae (strain ATCC 204508 / S288c) GN=WTM1 PE=1 SV=1

WTM2 − average.top3: 6.58701384615385 − max.qupm: 7

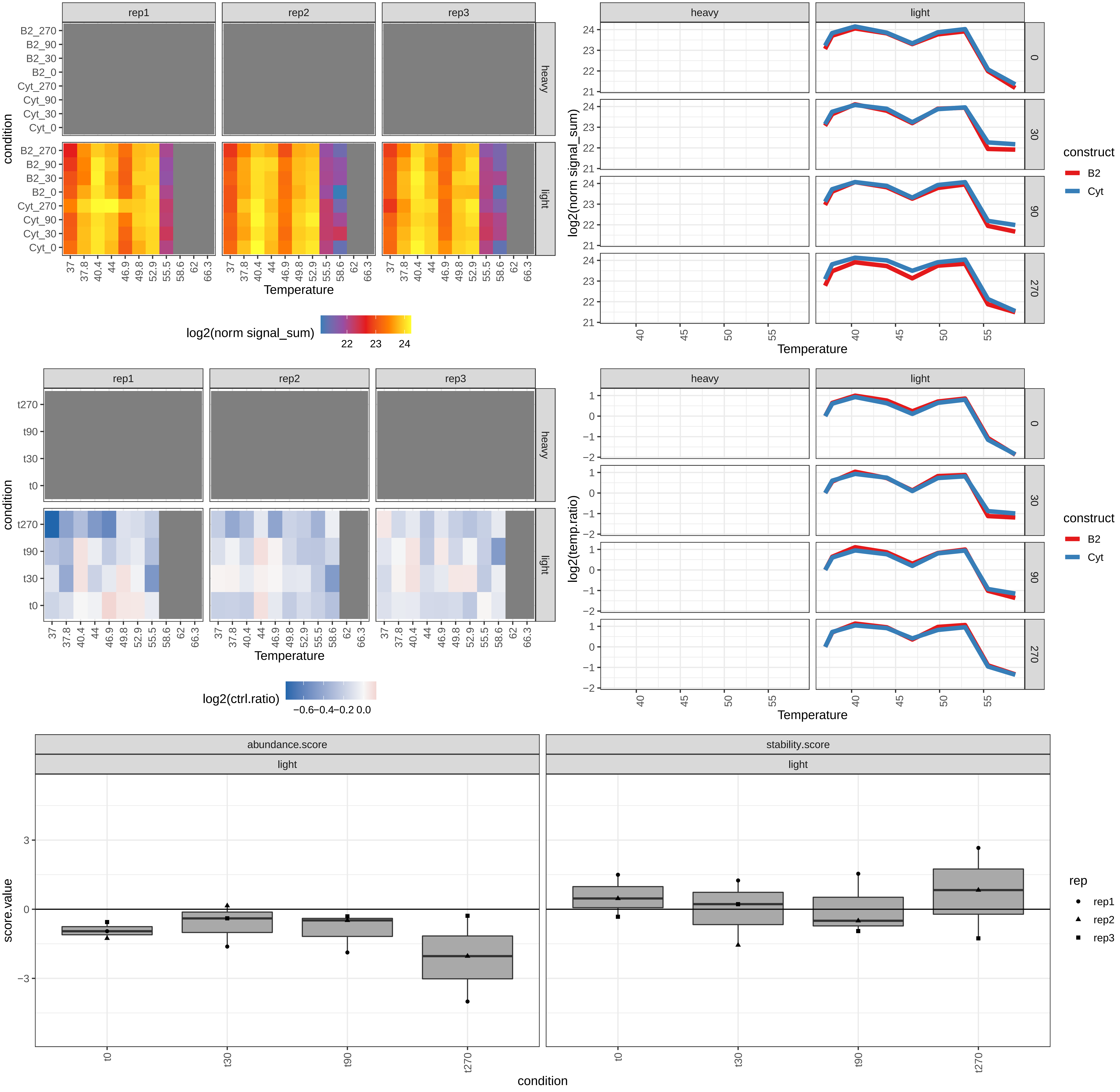

Q12206 − Transcriptional modulator WTM2 OS=Saccharomyces cerevisiae (strain ATCC 204508 / S288c) GN=WTM2 PE=1 SV=1

XKS1 − average.top3: 7.02244666666667 − max.qupm: 10

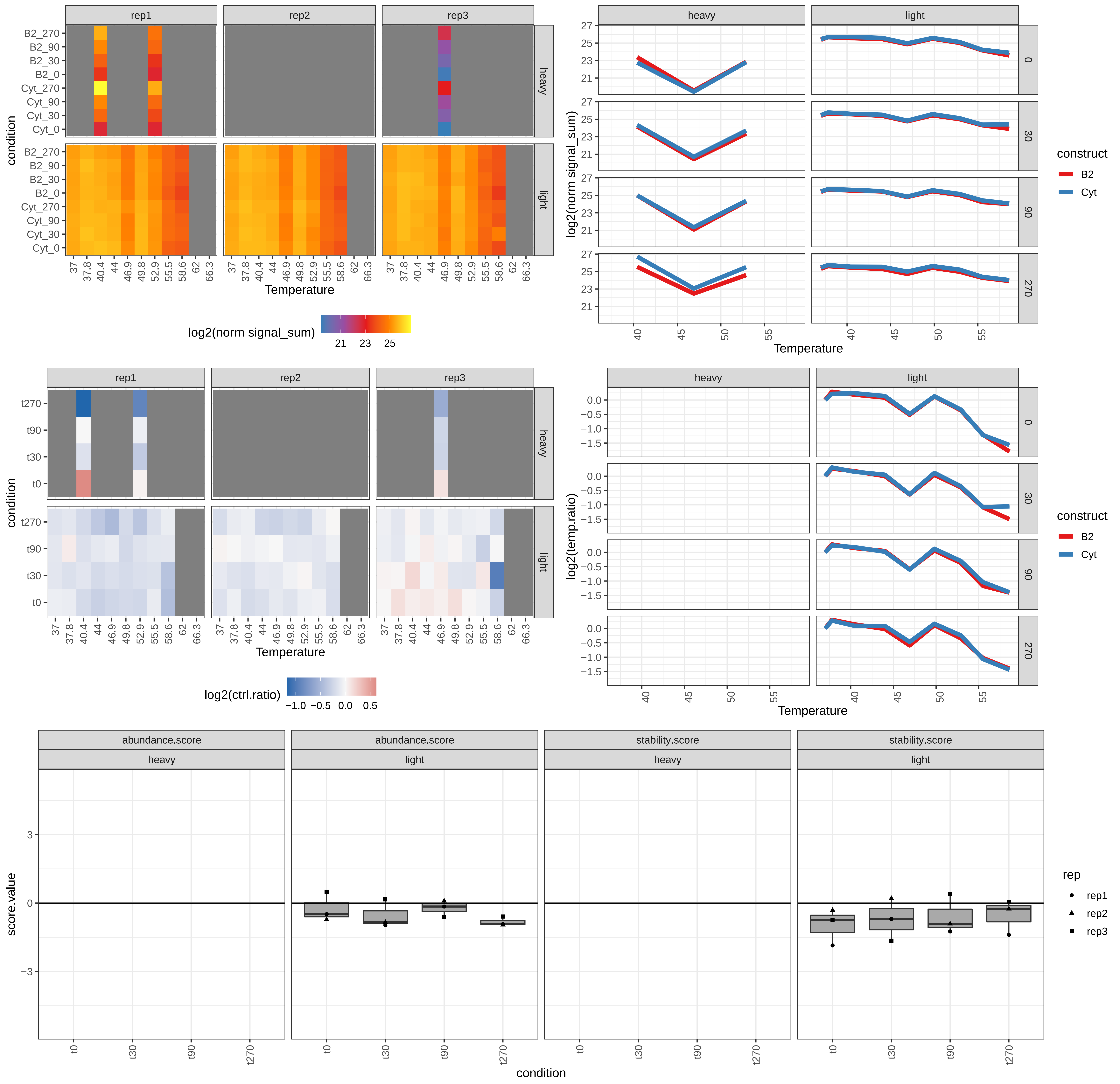

P42826 − Xylulose kinase OS=Saccharomyces cerevisiae (strain ATCC 204508 / S288c) GN=XKS1 PE=1 SV=2

XPT1 − average.top3: 7.08445594594595 − max.qupm: 7

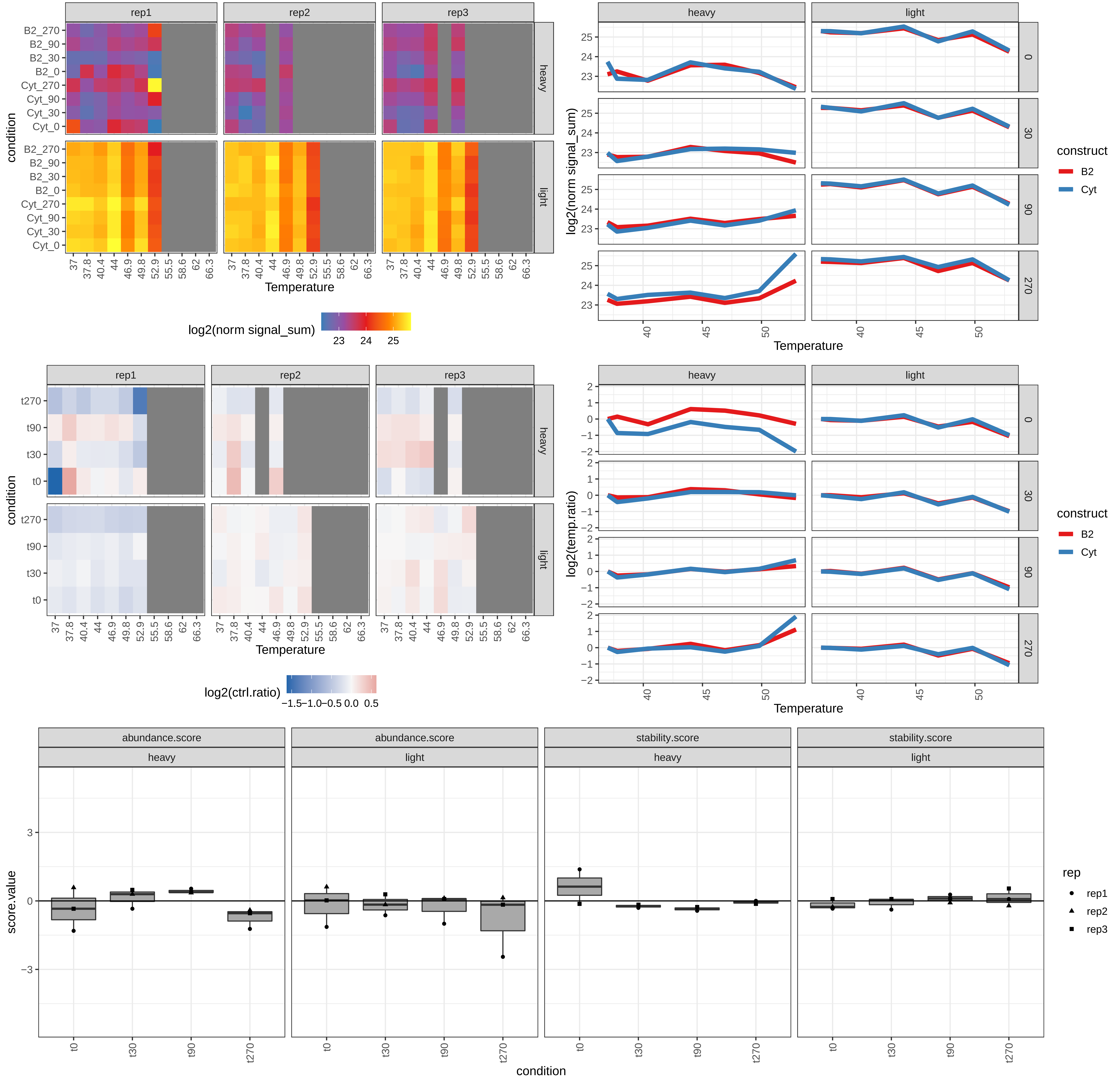

P47165 − Xanthine phosphoribosyltransferase 1 OS=Saccharomyces cerevisiae (strain ATCC 204508 / S288c) GN=XPT1 PE=1 SV=1

XRN1 − average.top3: 7.28249846153846 − max.qupm: 44

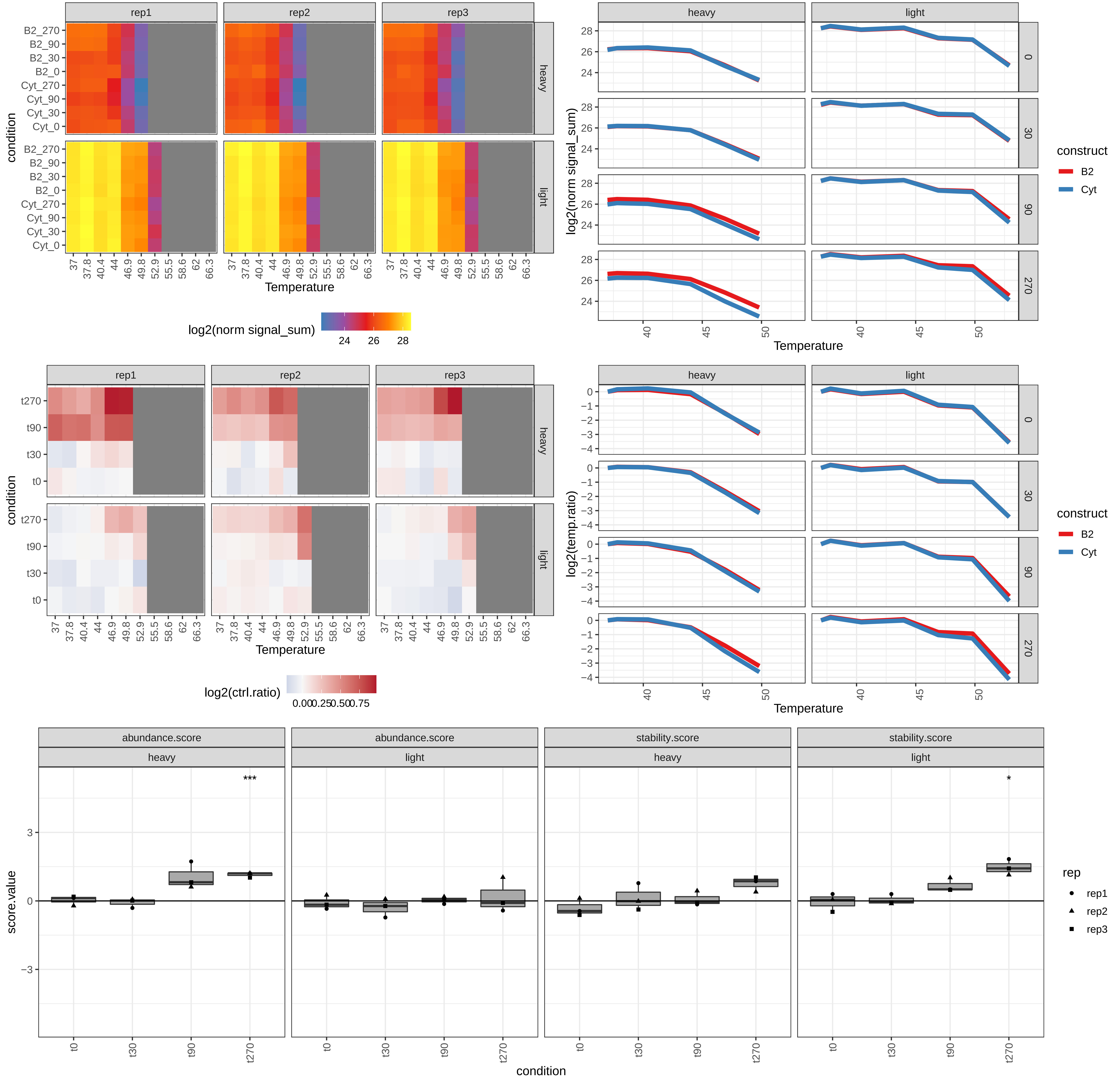

P22147 − 5'−3' exoribonuclease 1 OS=Saccharomyces cerevisiae (strain ATCC 204508 / S288c) GN=XRN1 PE=1 SV=1

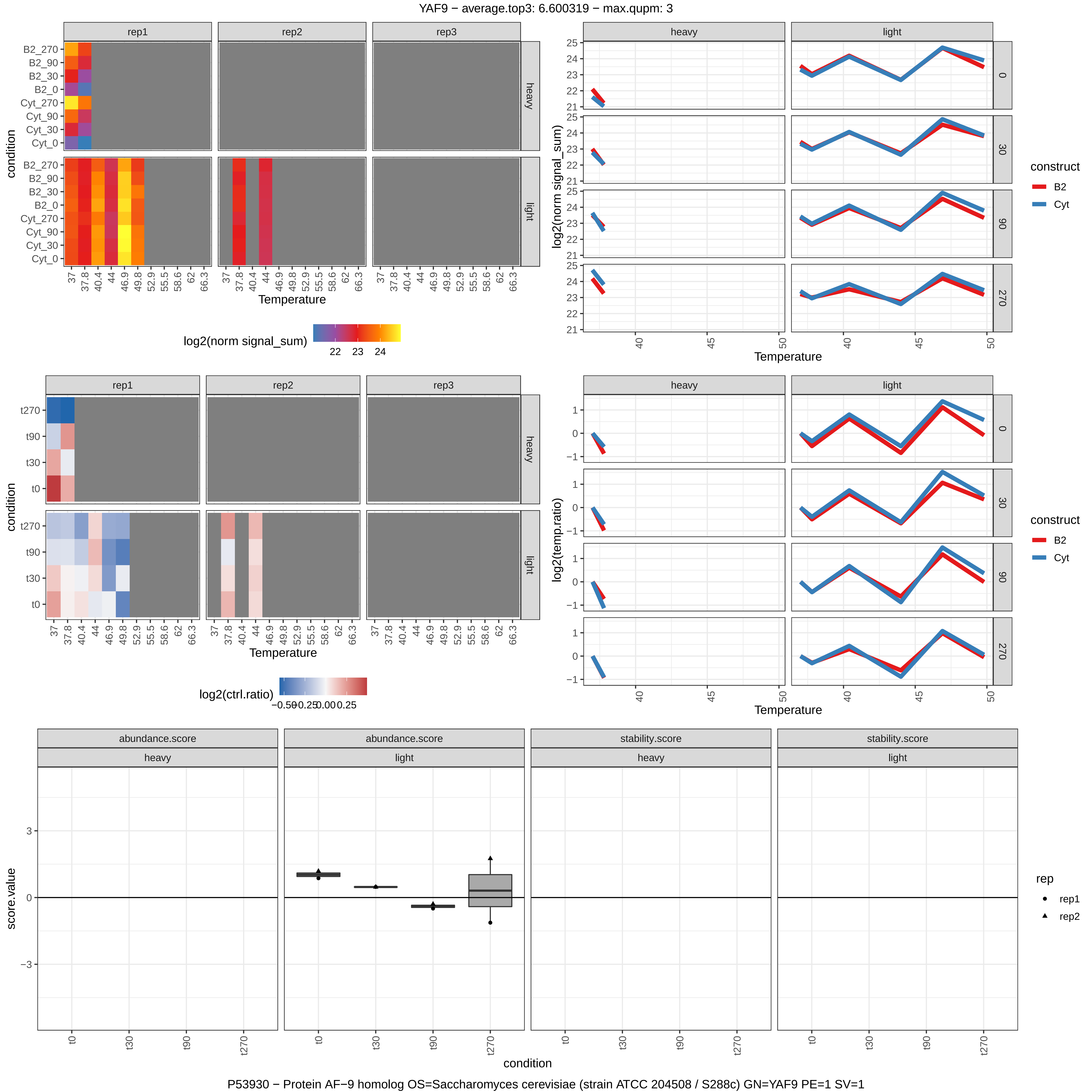

YAF9 − average.top3: 6.600319 − max.qupm: 3
rep1
rep2
rep3
heavy
light
condition
Temperature
log2(norm signal_sum)
log2(ctrl.ratio)
log2(temp.ratio)
construct
B2
Cyt
abundance.score
stability.score
score.value
rep
rep1
rep2
P53930 − Protein AF−9 homolog OS=Saccharomyces cerevisiae (strain ATCC 204508 / S288c) GN=YAF9 PE=1 SV=1

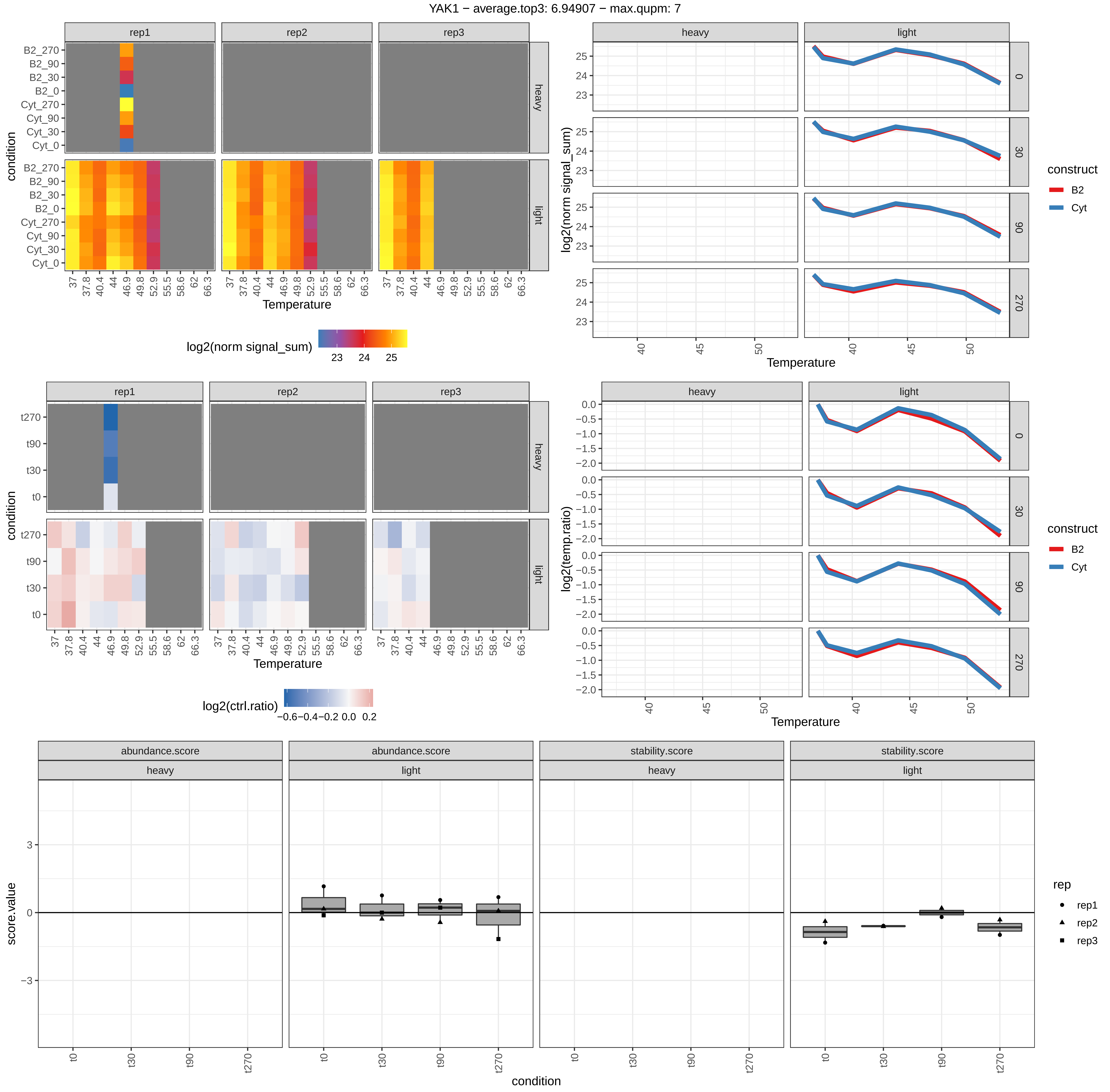

P14680 − Dual specificity protein kinase YAK1 OS=Saccharomyces cerevisiae (strain ATCC 204508 / S288c) GN=YAK1 PE=1 SV=1

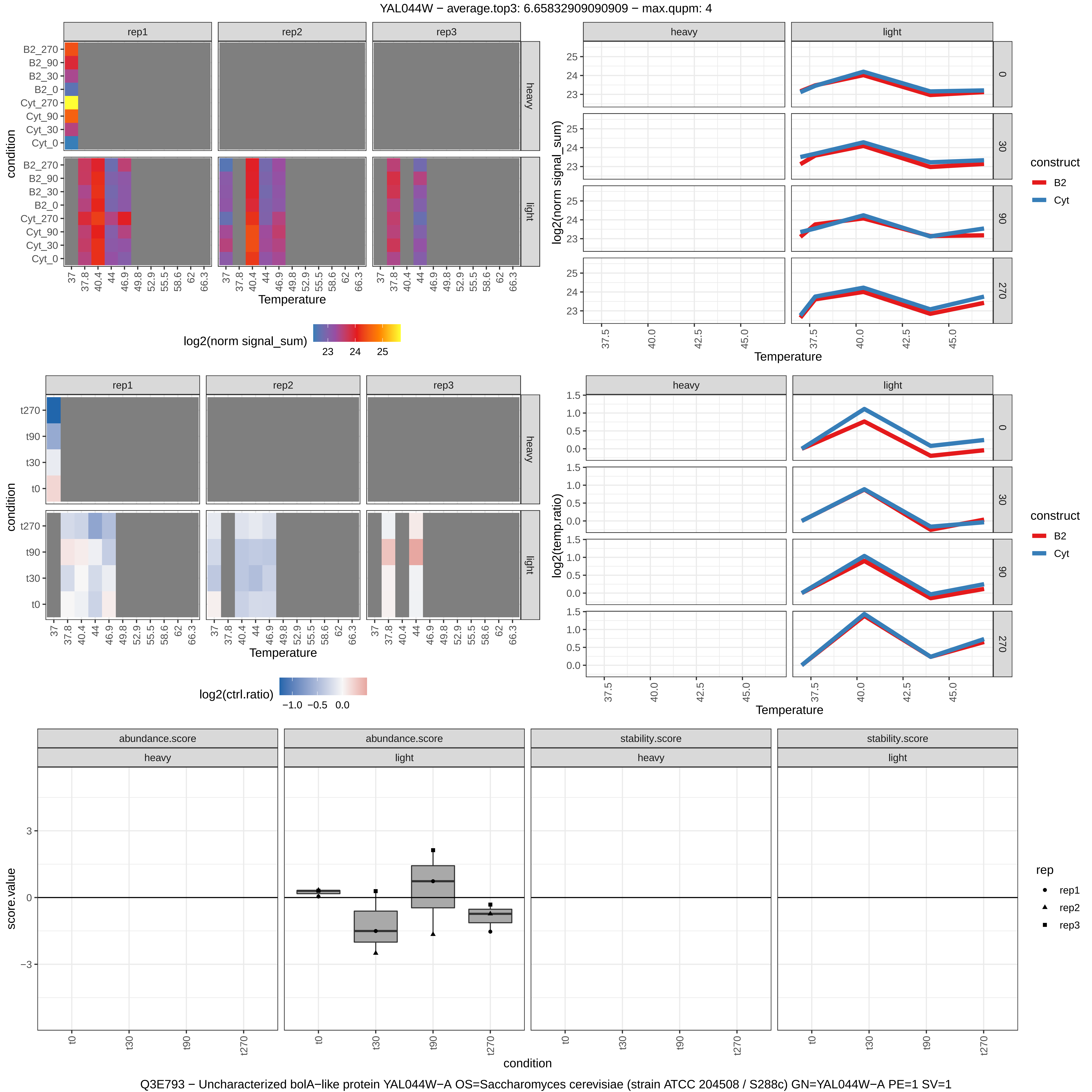
YAL044W − average.top3: 6.65832909090909 − max.qupm: 4
rep1
rep2
rep3
heavy
light
condition
Temperature
log2(norm signal_sum)
log2(ctrl.ratio)
log2(temp.ratio)
construct
B2
Cyt
abundance.score
stability.score
score.value
rep
condition
Q3E793 − Uncharacterized bolA−like protein YAL044W−A OS=Saccharomyces cerevisiae (strain ATCC 204508 / S288c) GN=YAL044W−A PE=1 SV=1

YAP1801 − average.top3: 6.568215 − max.qupm: 4

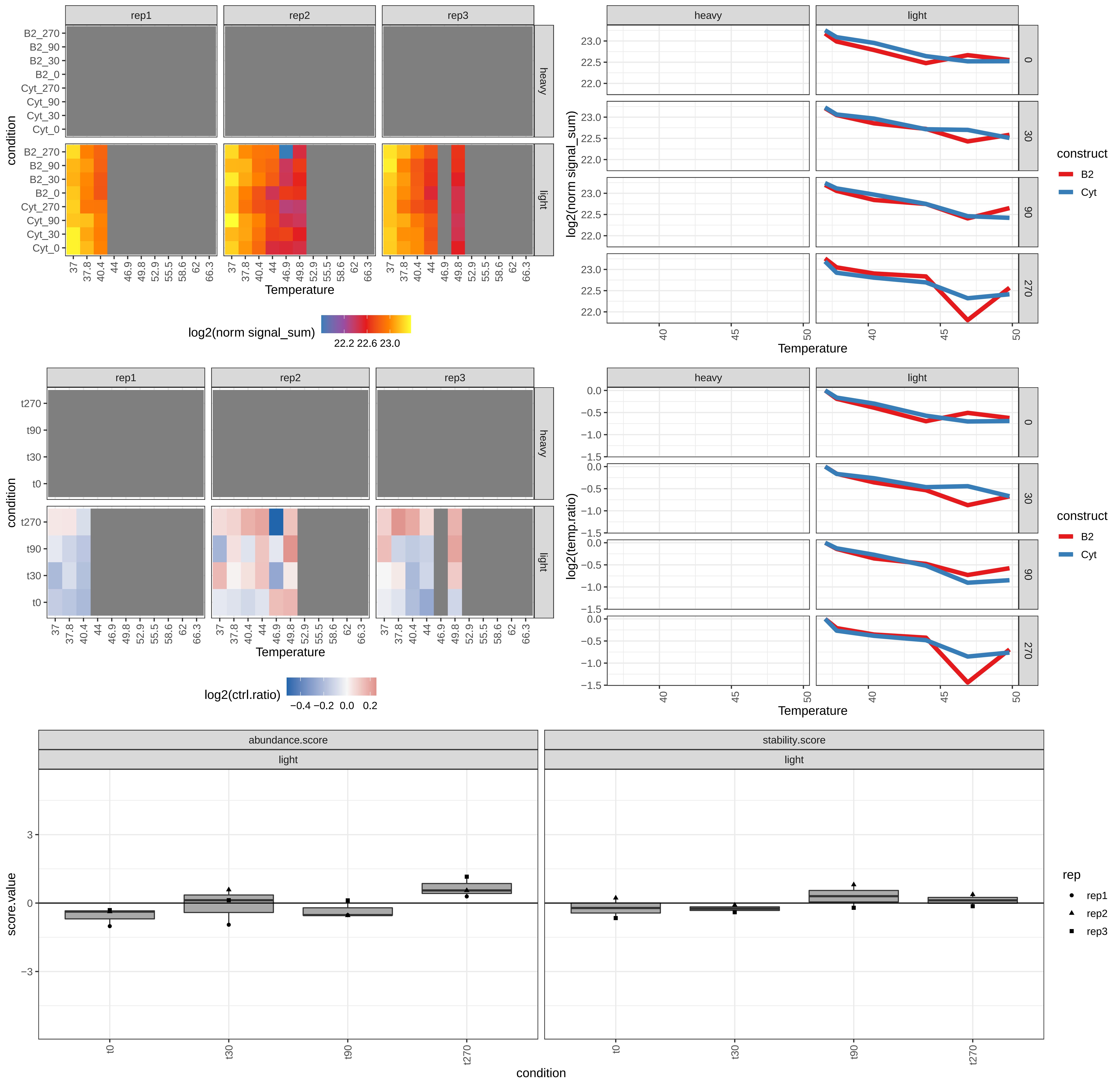

P38856 − Clathrin coat assembly protein AP180A OS=Saccharomyces cerevisiae (strain ATCC 204508 / S288c) GN=YAP1801 PE=1 SV=1

YAP1802 − average.top3: 6.64793894736842 − max.qupm: 7

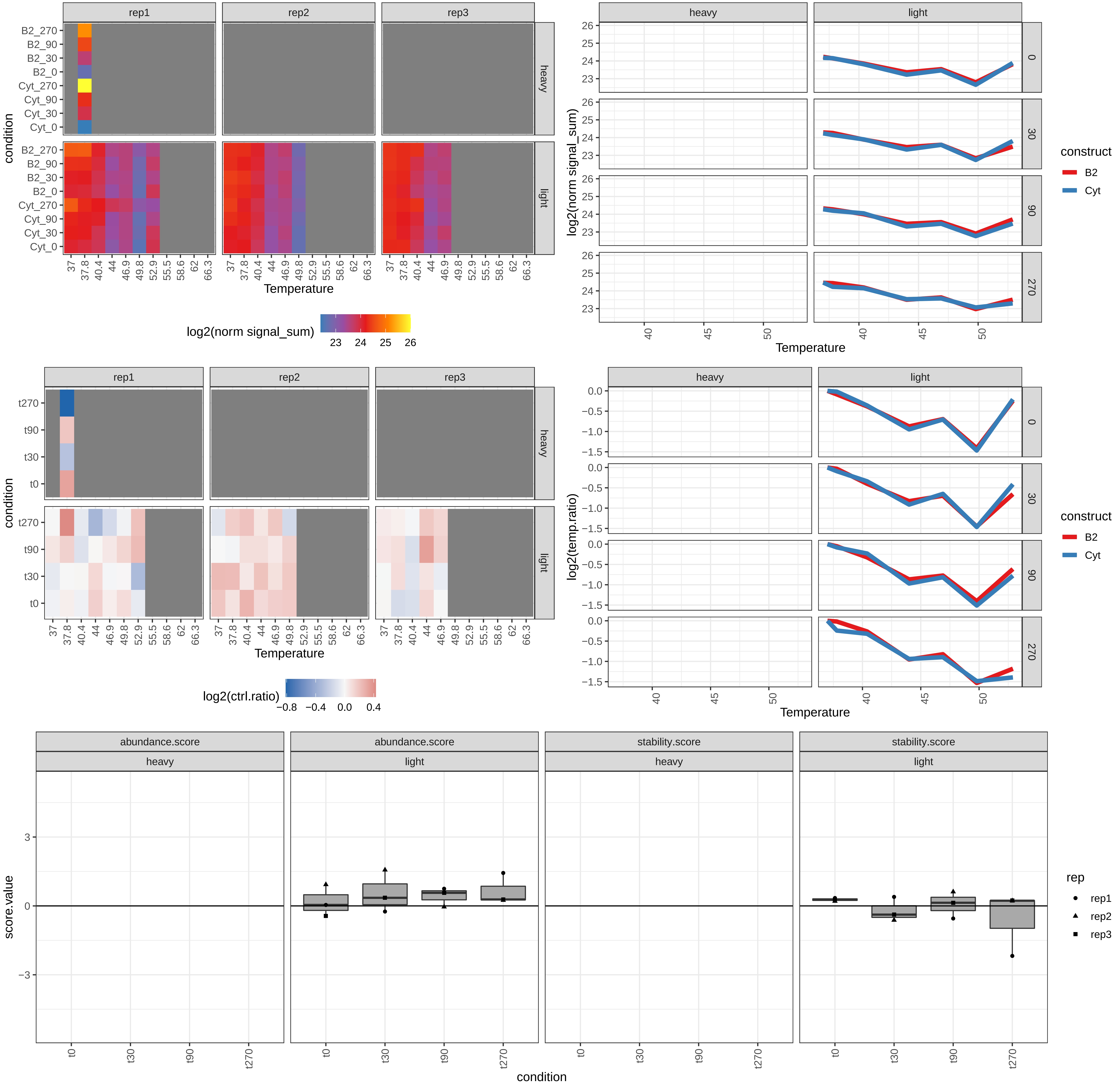

P53309 − Clathrin coat assembly protein AP180B OS=Saccharomyces cerevisiae (strain ATCC 204508 / S288c) GN=YAP1802 PE=1 SV=1

YAR1 − average.top3: 6.95960388888889 − max.qupm: 5

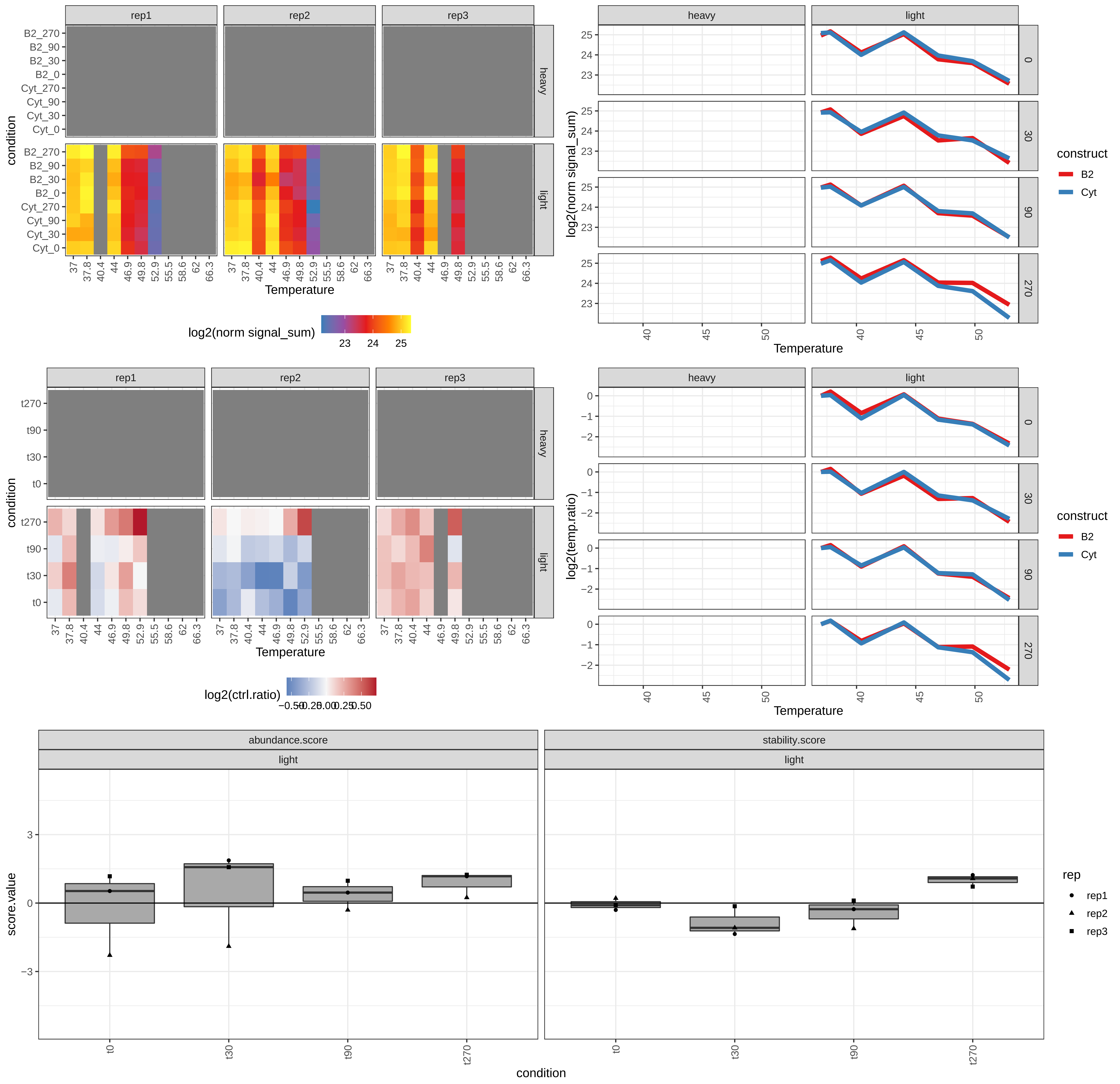

P46683 − Ankyrin repeat−containing protein YAR1 OS=Saccharomyces cerevisiae (strain ATCC 204508 / S288c) GN=YAR1 PE=1 SV=1

YAT1 − average.top3: 7.17117472222222 − max.qupm: 18

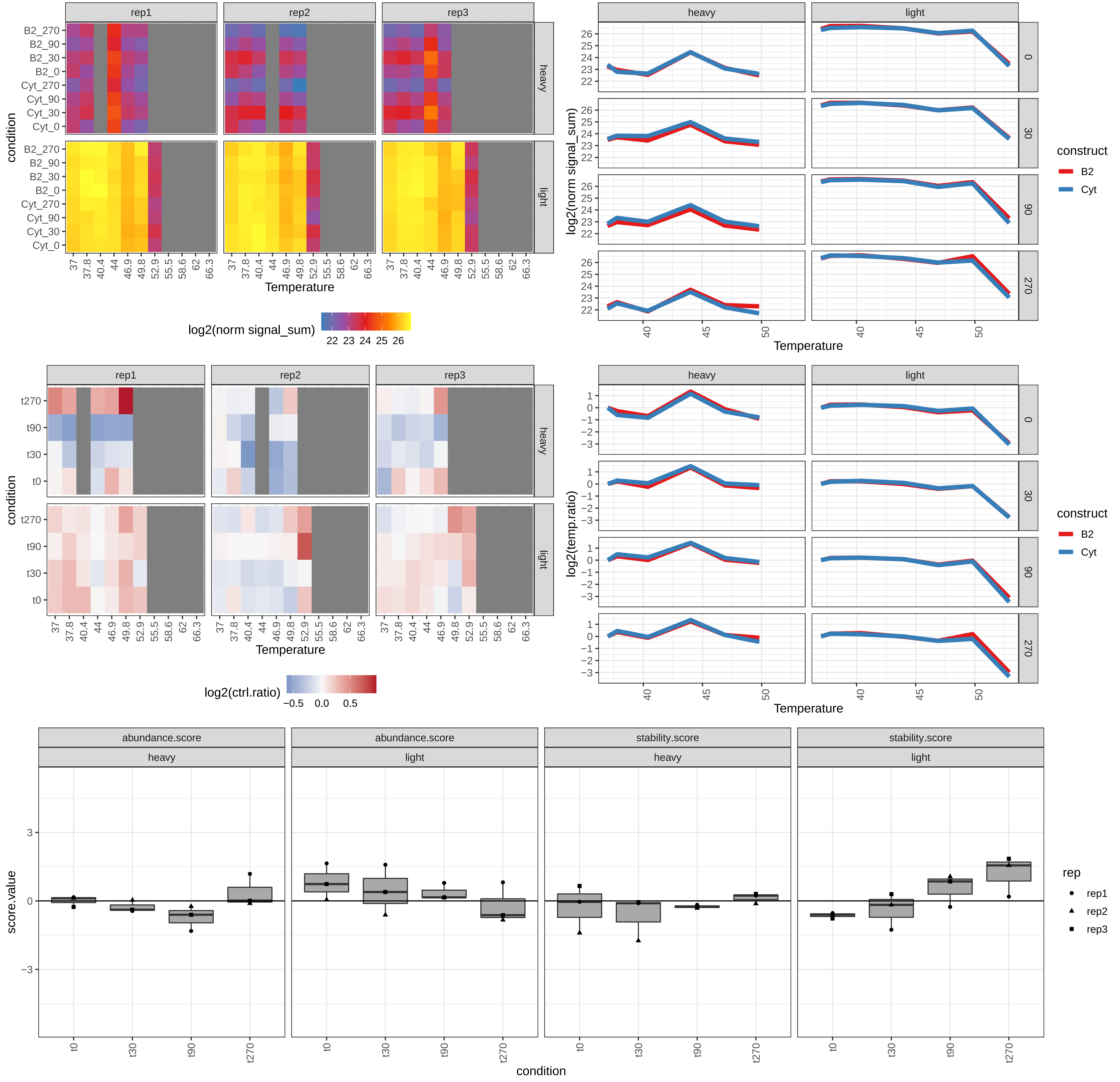

P80235 − Putative mitochondrial carnitine O−acetyltransferase OS=Saccharomyces cerevisiae (strain ATCC 204508 / S288c) GN=YAT1 PE=1 SV=2

YAT2 − average.top3: 7.38099421052632 − max.qupm: 20

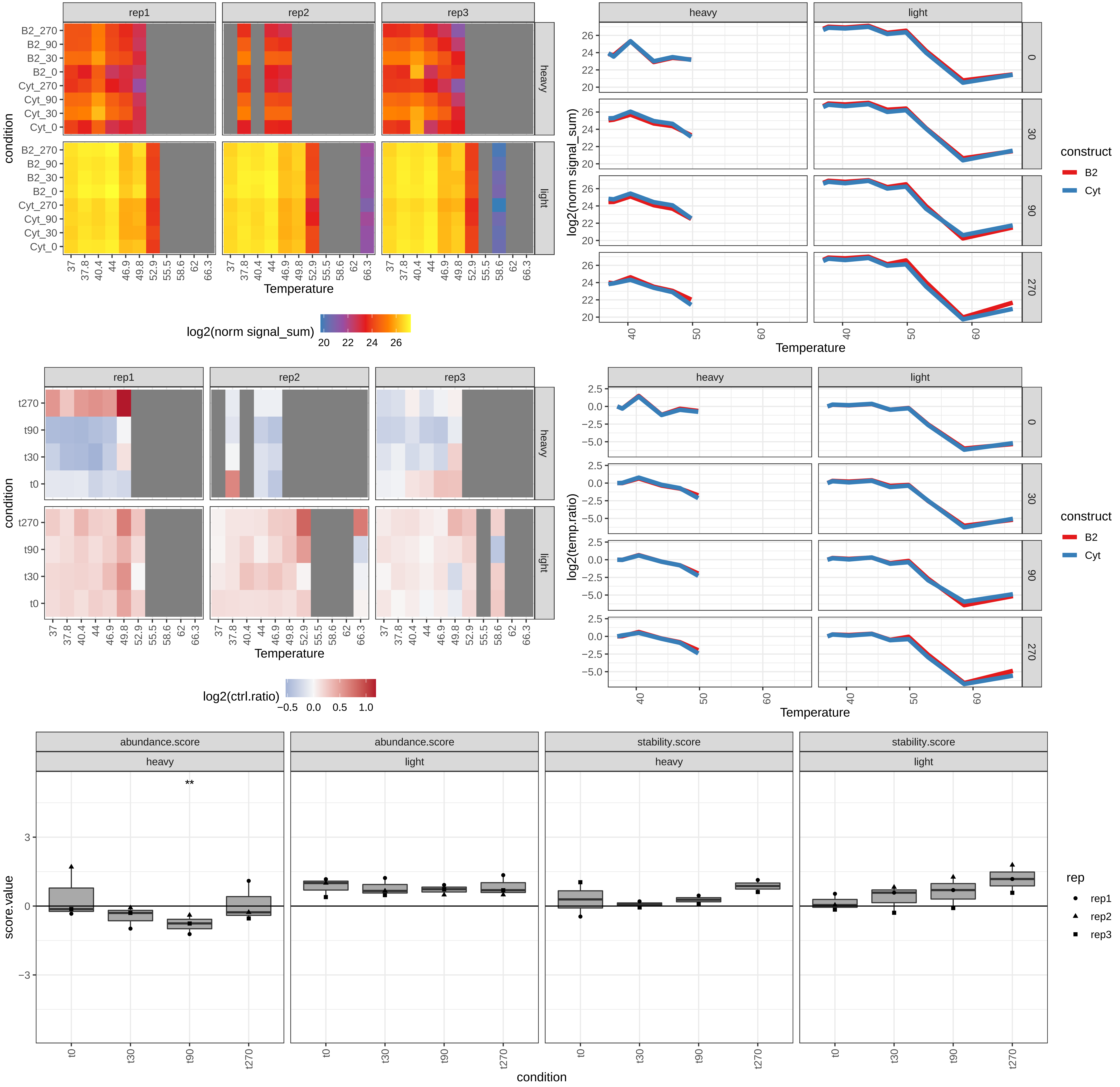

P40017 − Carnitine O−acetyltransferase YAT2 OS=Saccharomyces cerevisiae (strain ATCC 204508 / S288c) GN=YAT2 PE=1 SV=1

YBL029C − average.top3: 7.02789766666667 − max.qupm: 4

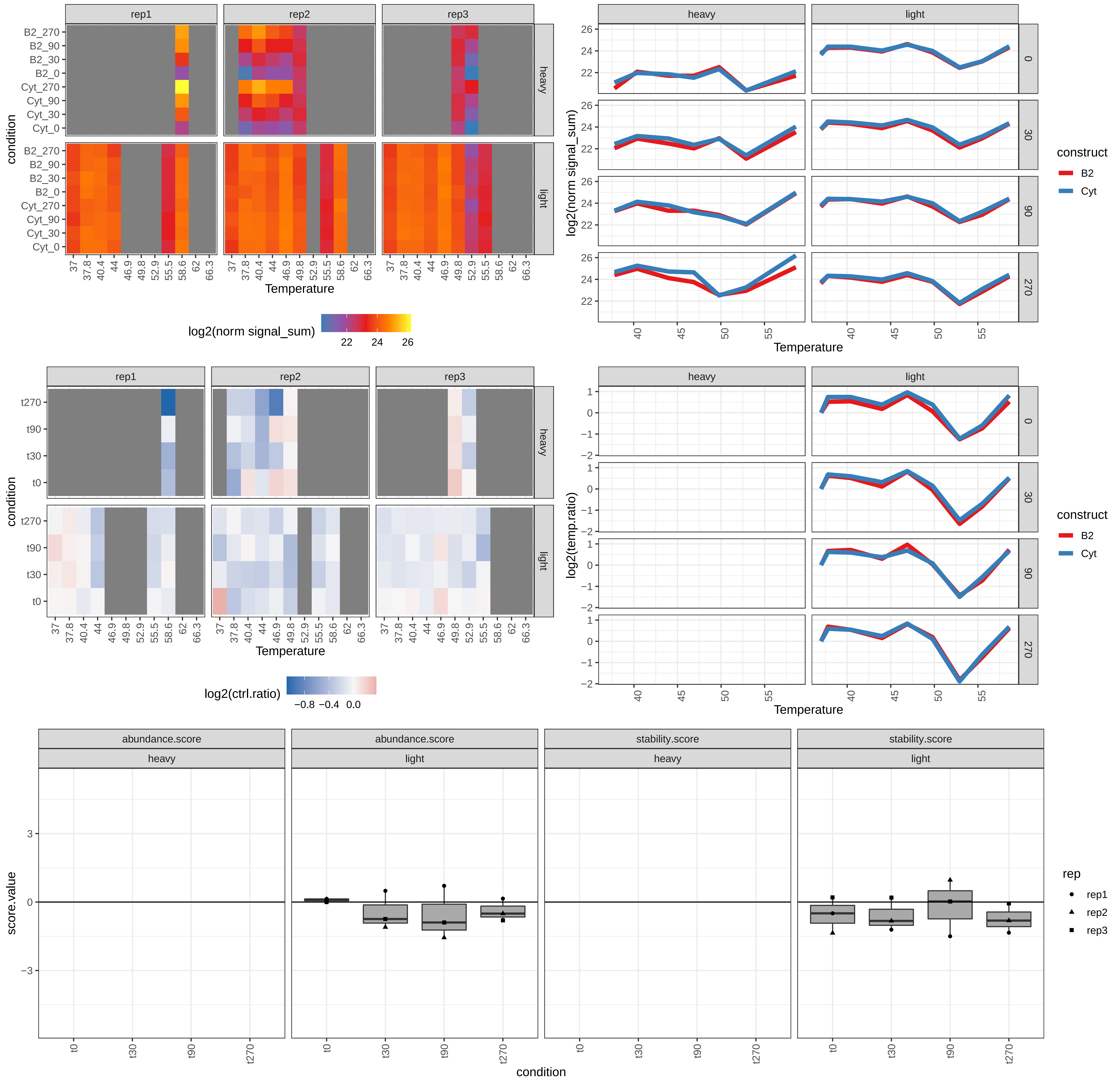

Q3E756 − UPF0768 protein YBL029C−A OS=Saccharomyces cerevisiae (strain ATCC 204508 / S288c) GN=YBL029C−A PE=1 SV=1

YBL036C − average.top3: 7.19145653061225 − max.qupm: 12

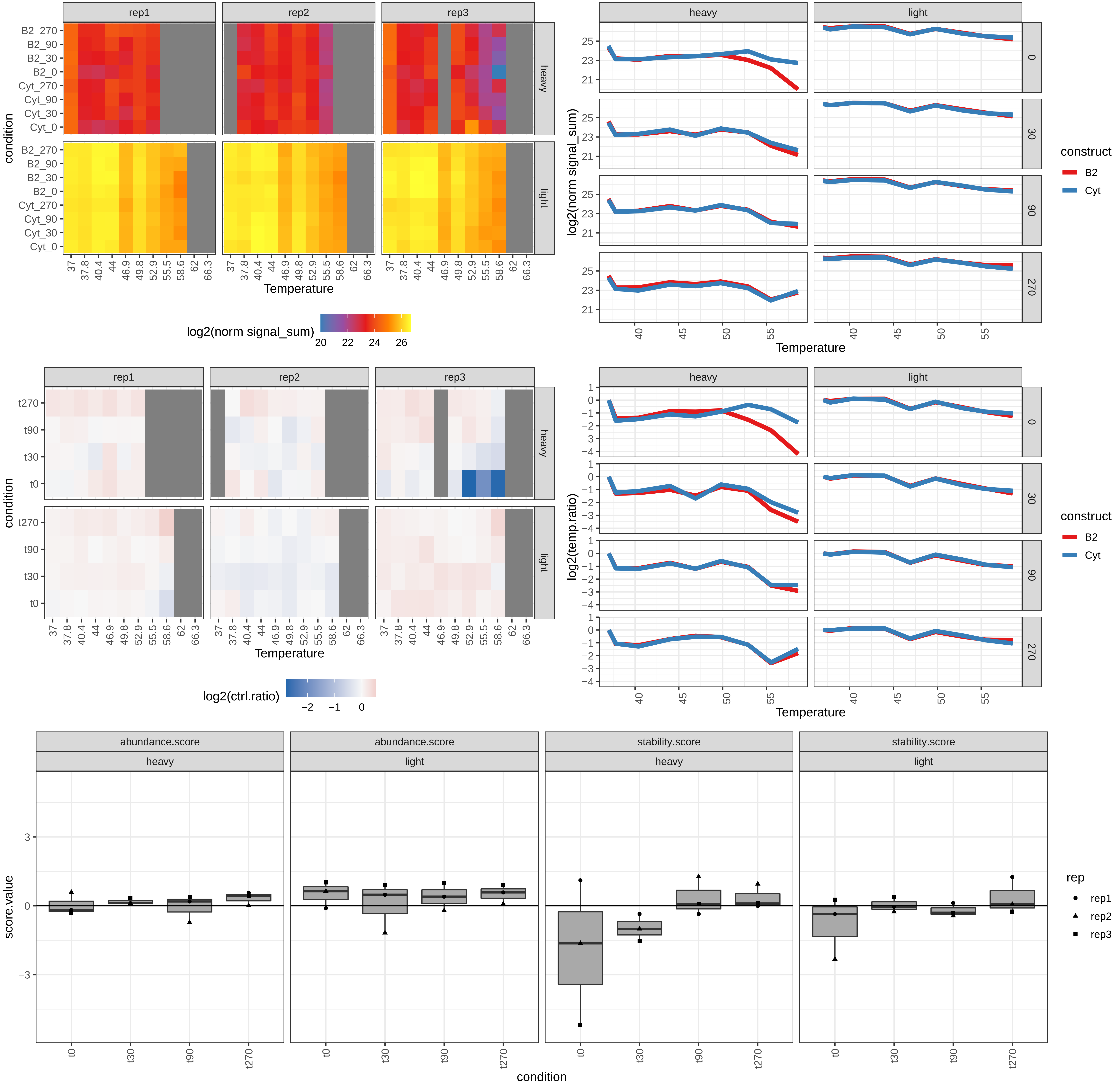

P38197 − UPF0001 protein YBL036C OS=Saccharomyces cerevisiae (strain ATCC 204508 / S288c) GN=YBL036C PE=1 SV=1

YBL055C − average.top3: 6.821417 − max.qupm: 11

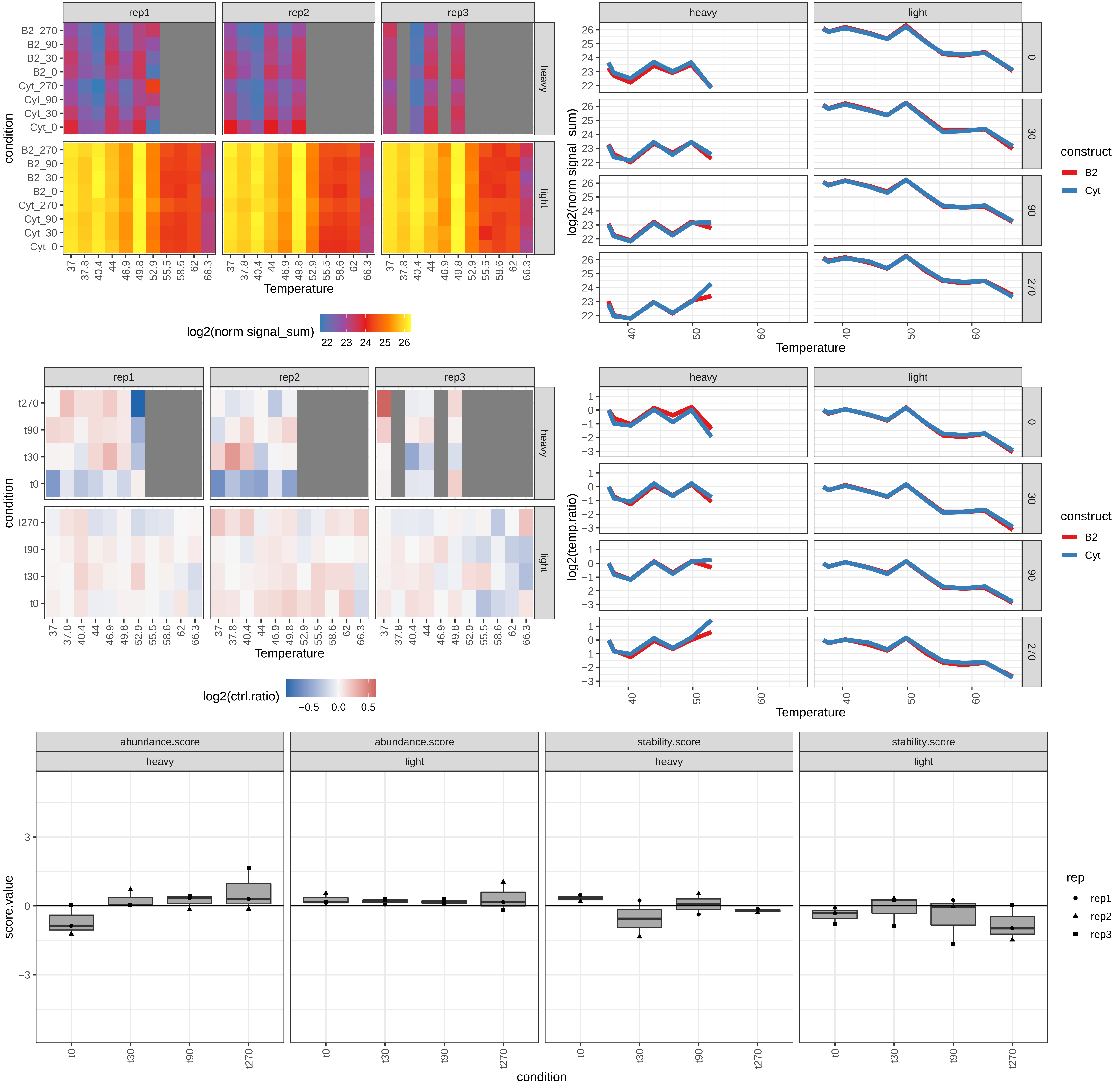

P34220 − Deoxyribonuclease Tat−D OS=Saccharomyces cerevisiae (strain ATCC 204508 / S288c) GN=YBL055C PE=1 SV=1

YBP1 − average.top3: 6.80834736842105 − max.qupm: 7

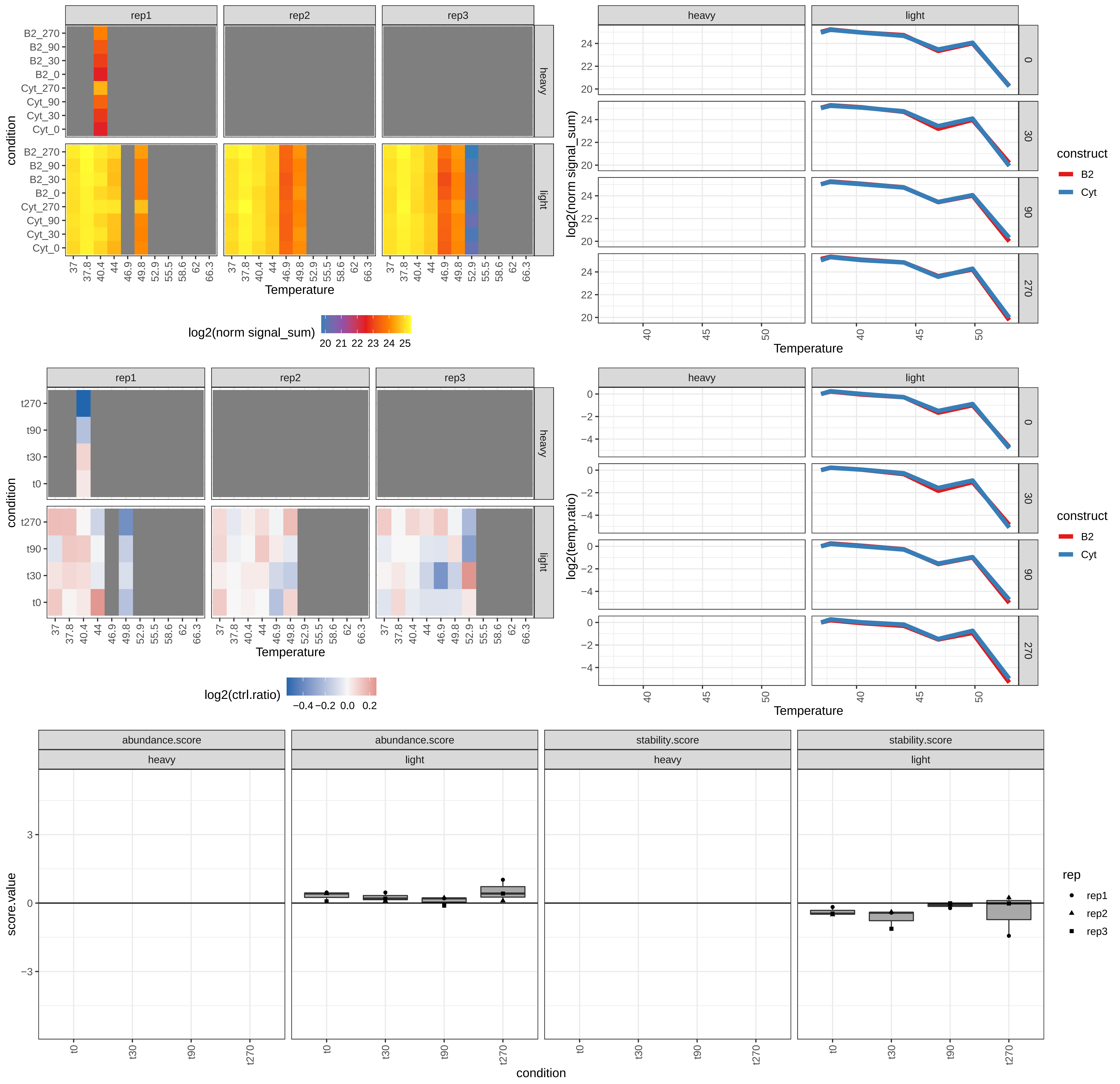

P38315 − YAP1−binding protein 1 OS=Saccharomyces cerevisiae (strain ATCC 204508 / S288c) GN=YBP1 PE=1 SV=1

YBP2 − average.top3: 6.42976615384615 − max.qupm: 4

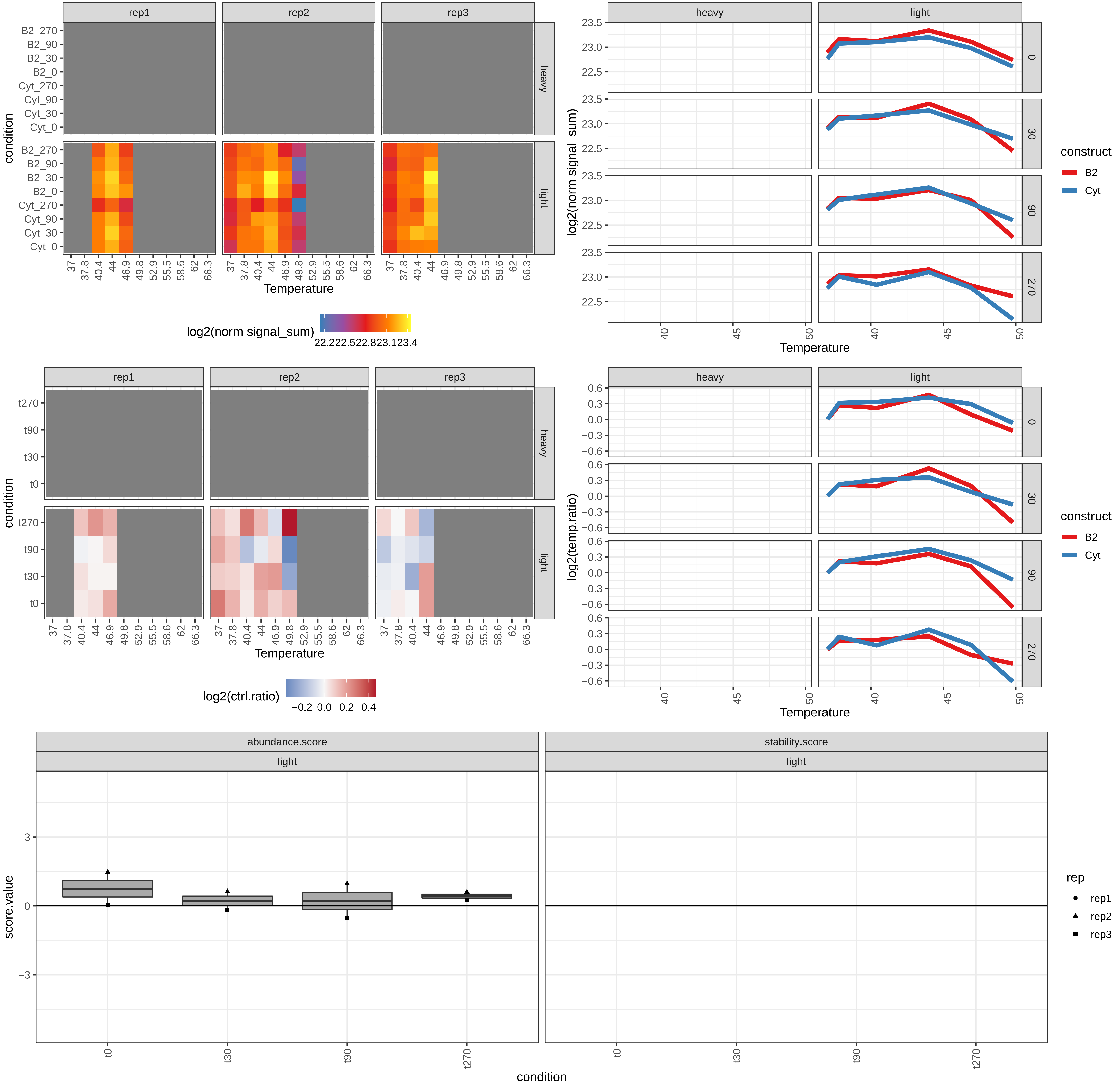

P53169 − YAP1−binding protein 2 OS=Saccharomyces cerevisiae (strain ATCC 204508 / S288c) GN=YBP2 PE=1 SV=1

YBR053C − average.top3: 7.2481992 − max.qupm: 13

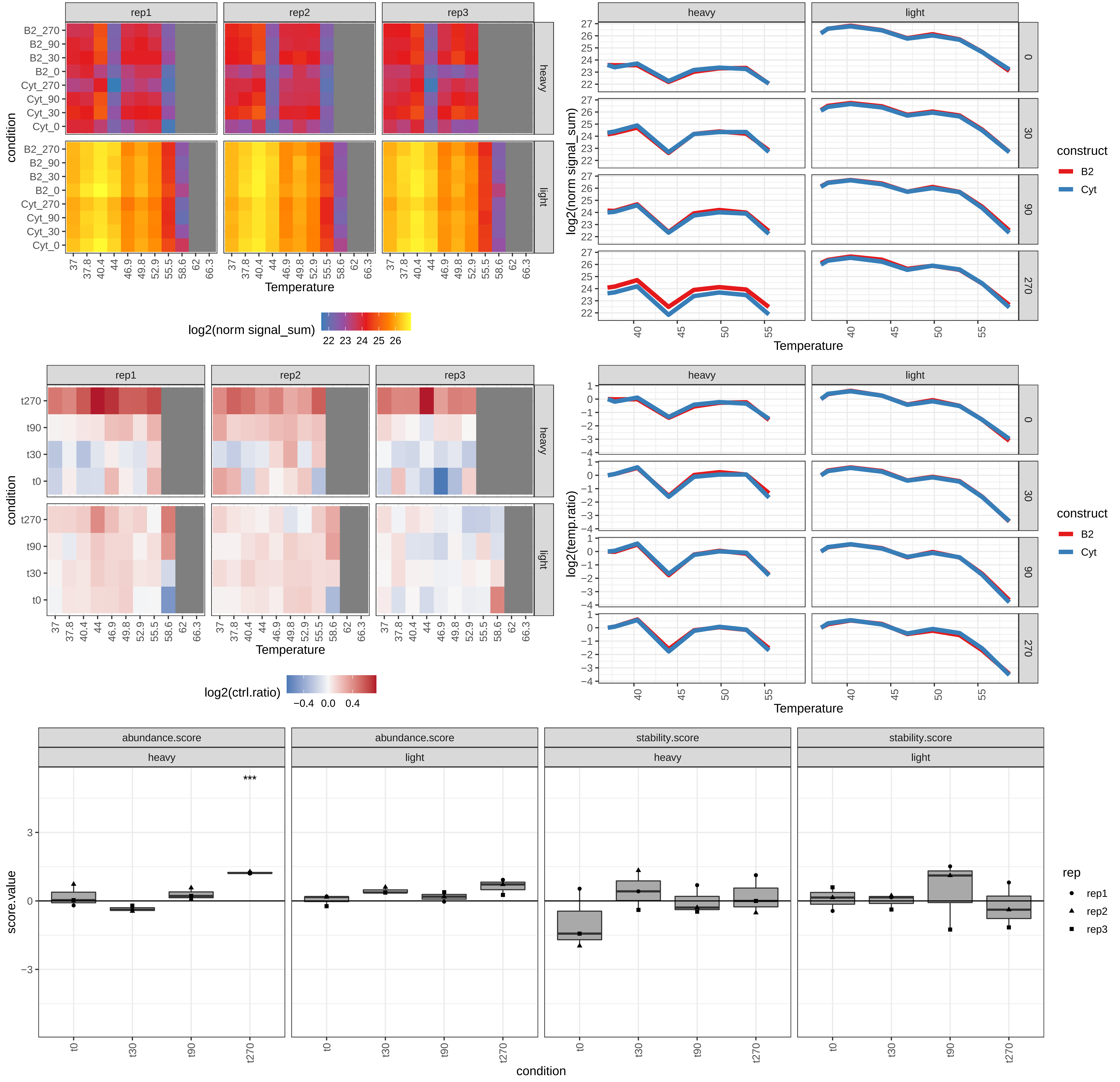

P38235 − Uncharacterized protein YBR053C OS=Saccharomyces cerevisiae (strain ATCC 204508 / S288c) GN=YBR053C PE=1 SV=1

YBR056W − average.top3: 7.29478435897436 − max.qupm: 18

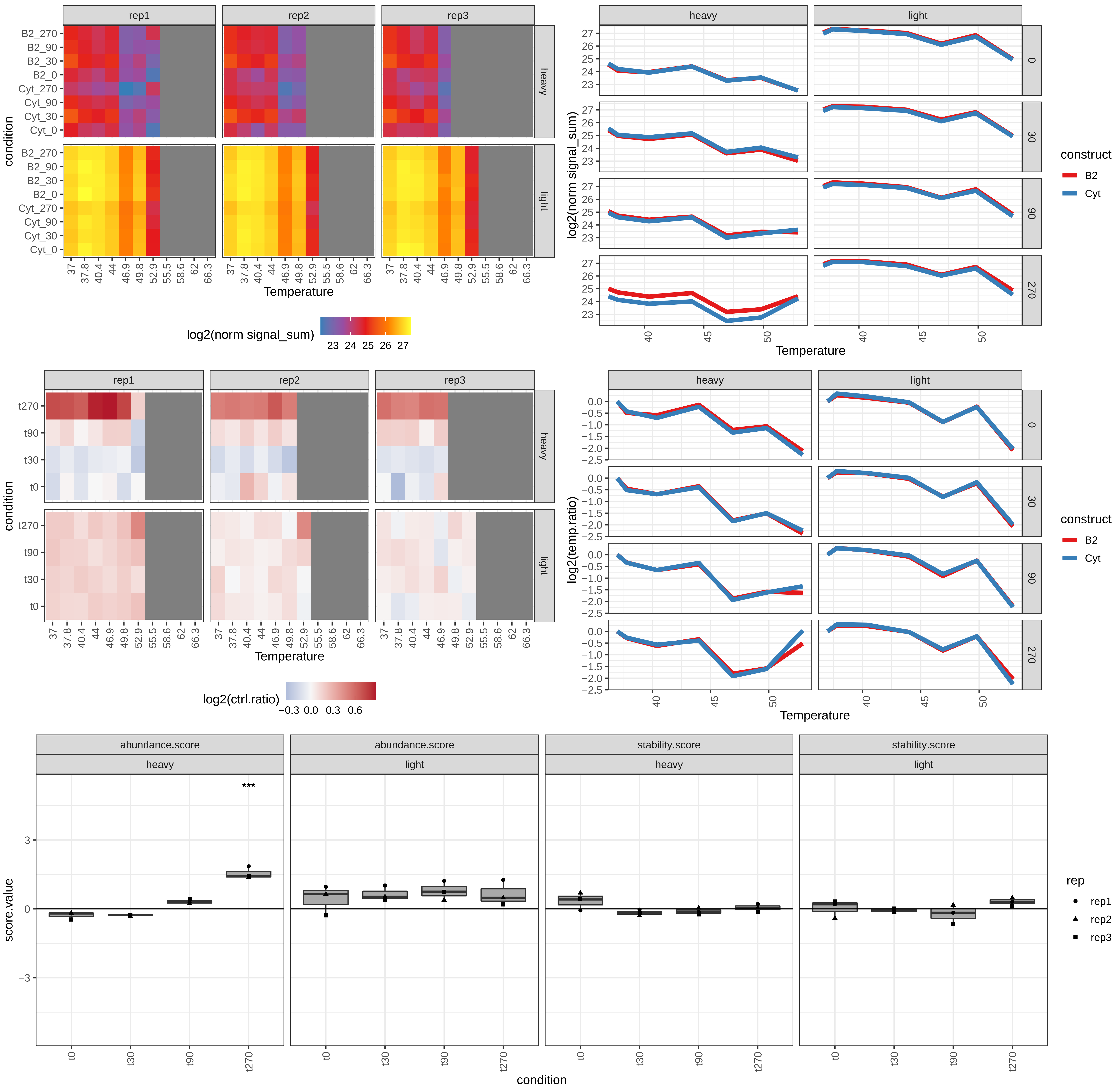

P38081 − Uncharacterized glycosyl hydrolase YBR056W OS=Saccharomyces cerevisiae (strain ATCC 204508 / S288c) GN=YBR056W PE=1 SV=1

YBR085C − average.top3: 5.748278 − max.qupm: 4

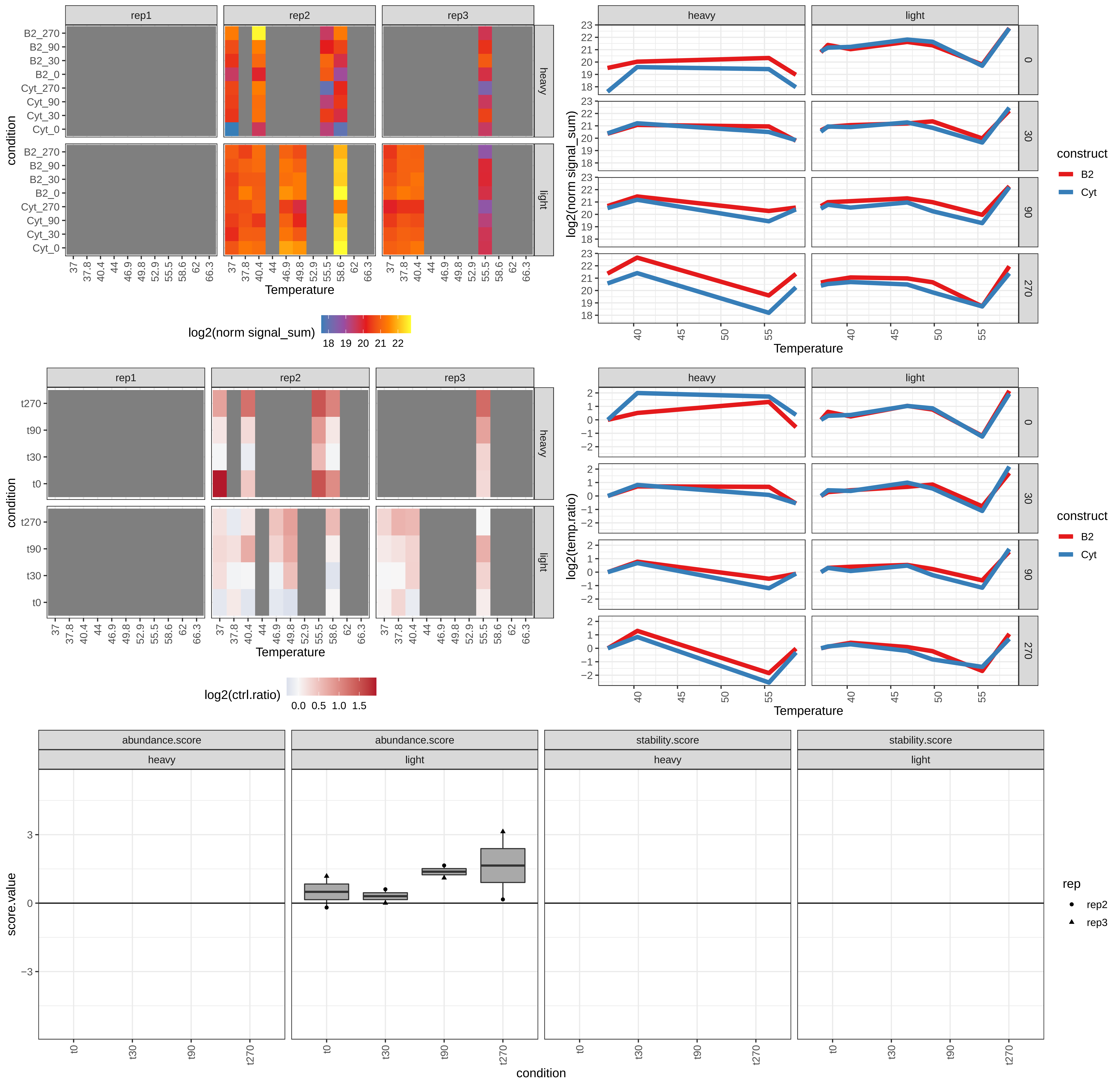

O43137 − Uncharacterized protein YBR085C−A OS=Saccharomyces cerevisiae (strain ATCC 204508 / S288c) GN=YBR085C−A PE=1 SV=1

YBR096W − average.top3: 6.88799846153846 − max.qupm: 6

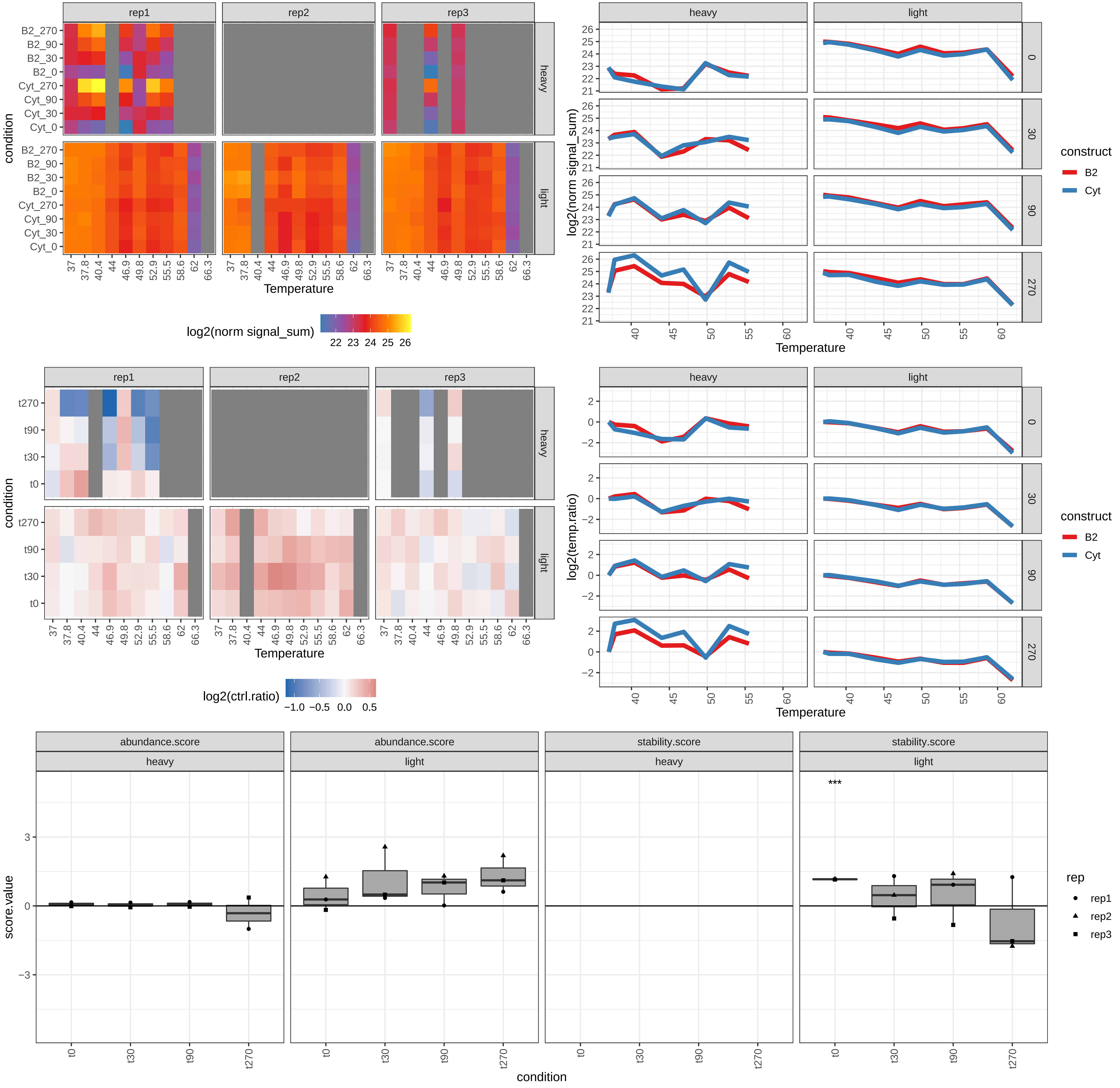

P38256 − Uncharacterized protein YBR096W OS=Saccharomyces cerevisiae (strain ATCC 204508 / S288c) GN=YBR096W PE=1 SV=1

YBR137W − average.top3: 6.88133033333333 − max.qupm: 5

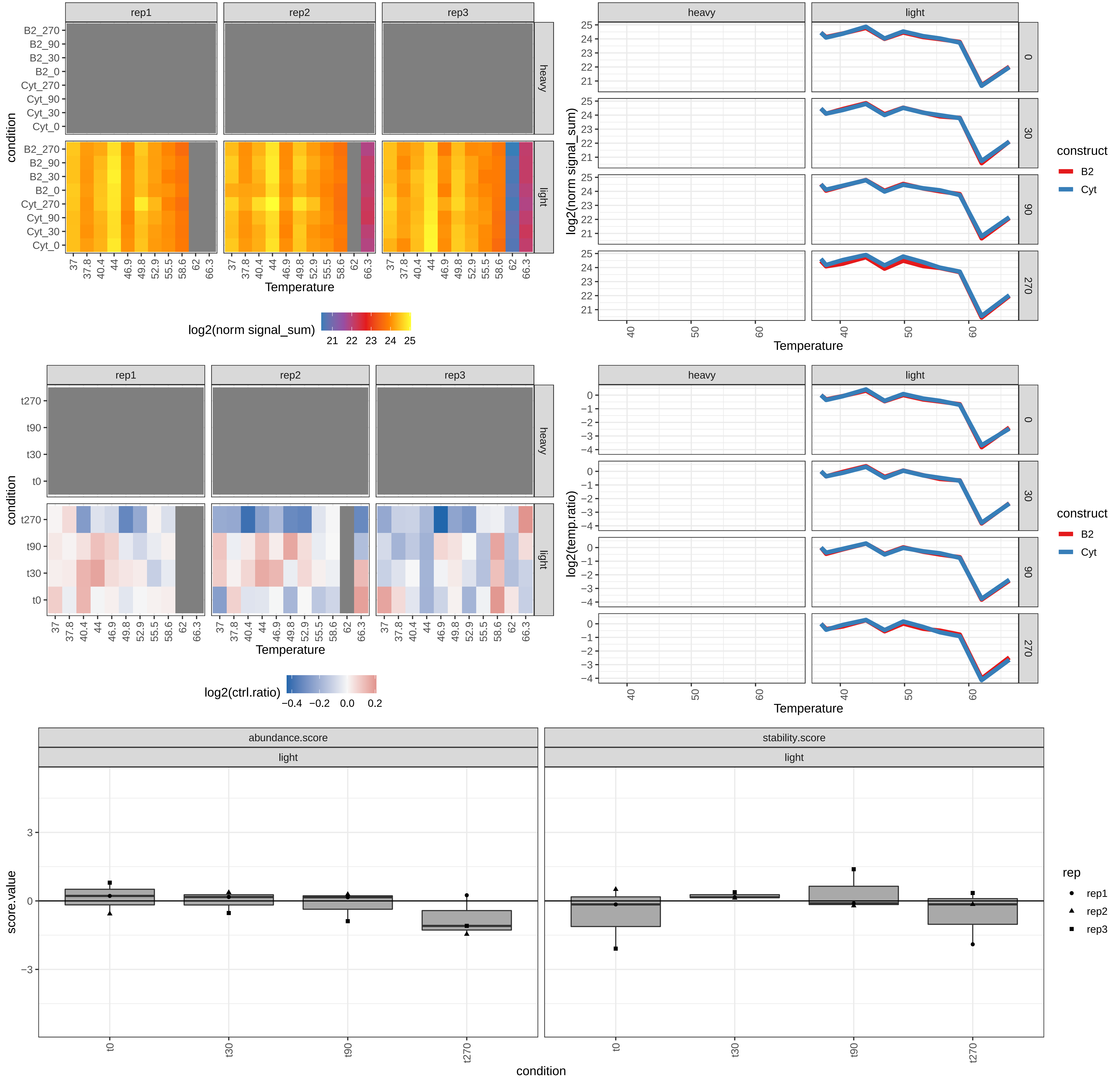

P38276 − UPF0303 protein YBR137W OS=Saccharomyces cerevisiae (strain ATCC 204508 / S288c) GN=YBR137W PE=1 SV=4

YBR139W − average.top3: 7.22687393939394 − max.qupm: 7

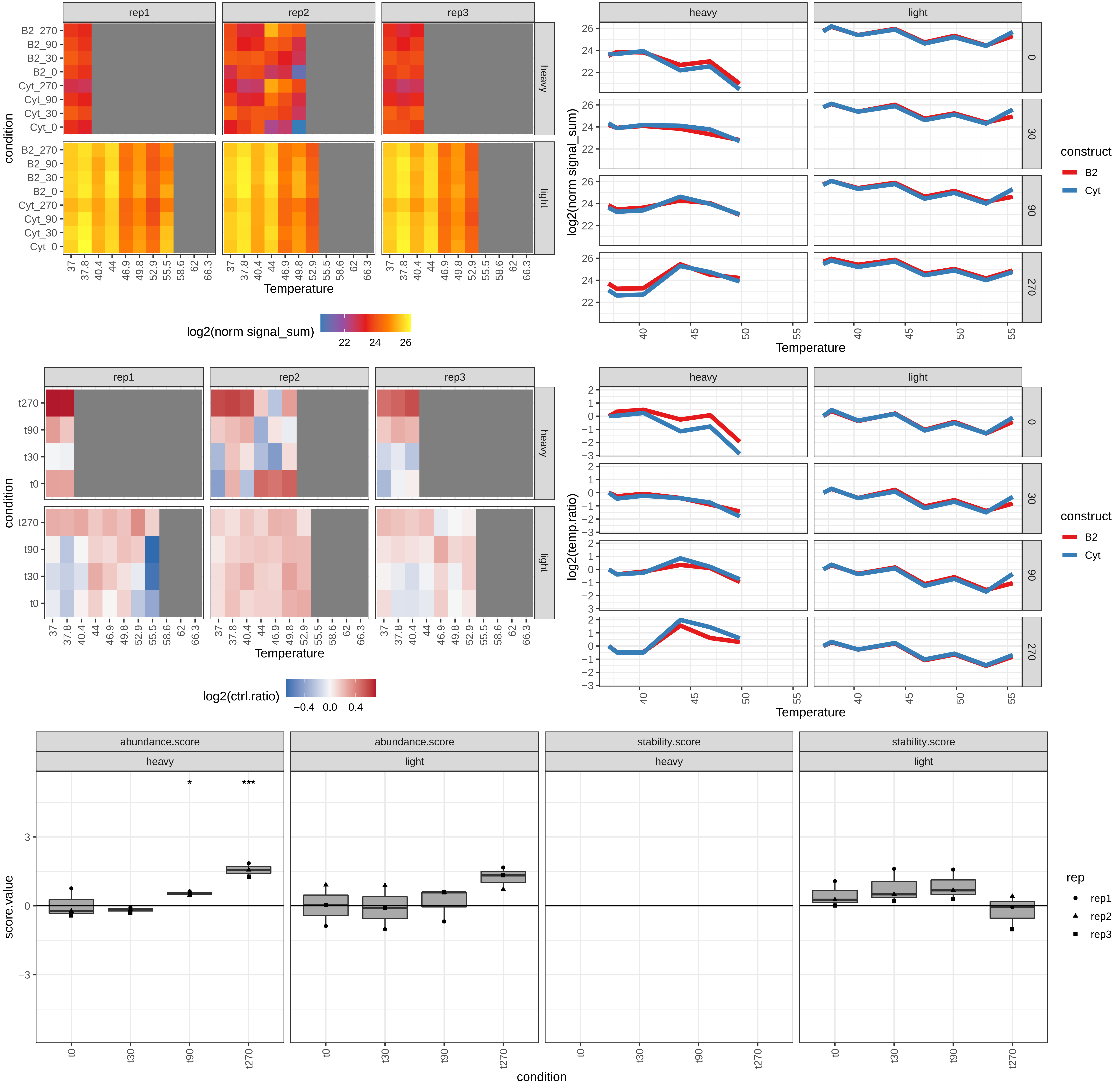

P38109 − Putative serine carboxypeptidase YBR139W OS=Saccharomyces cerevisiae (strain ATCC 204508 / S288c) GN=YBR139W PE=1 SV=1

YBR225W − average.top3: 6.81796208333333 − max.qupm: 7

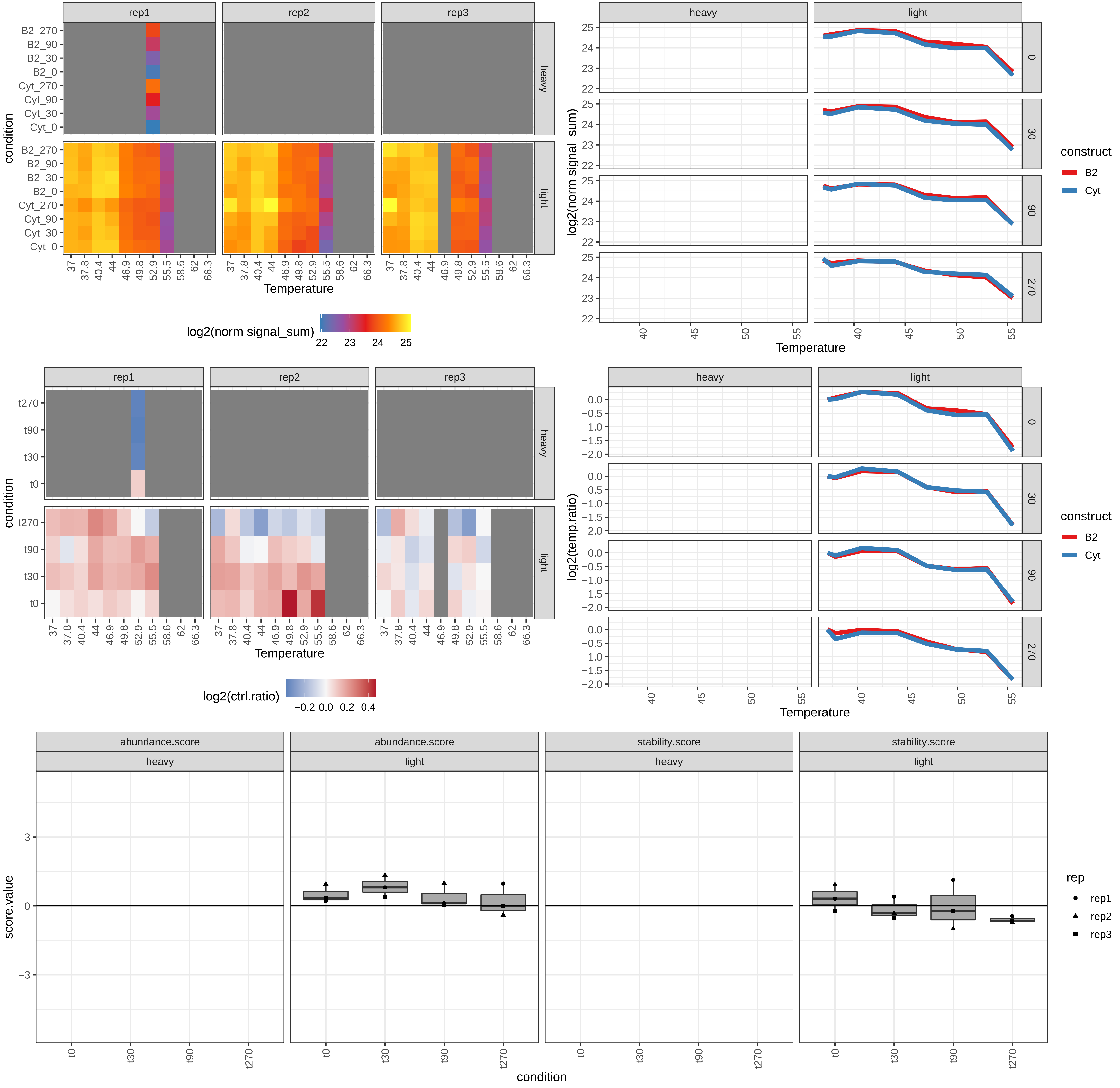

P38321 − Uncharacterized protein YBR225W OS=Saccharomyces cerevisiae (strain ATCC 204508 / S288c) GN=YBR225W PE=1 SV=1

YBT1 − average.top3: 7.1125341509434 − max.qupm: 27

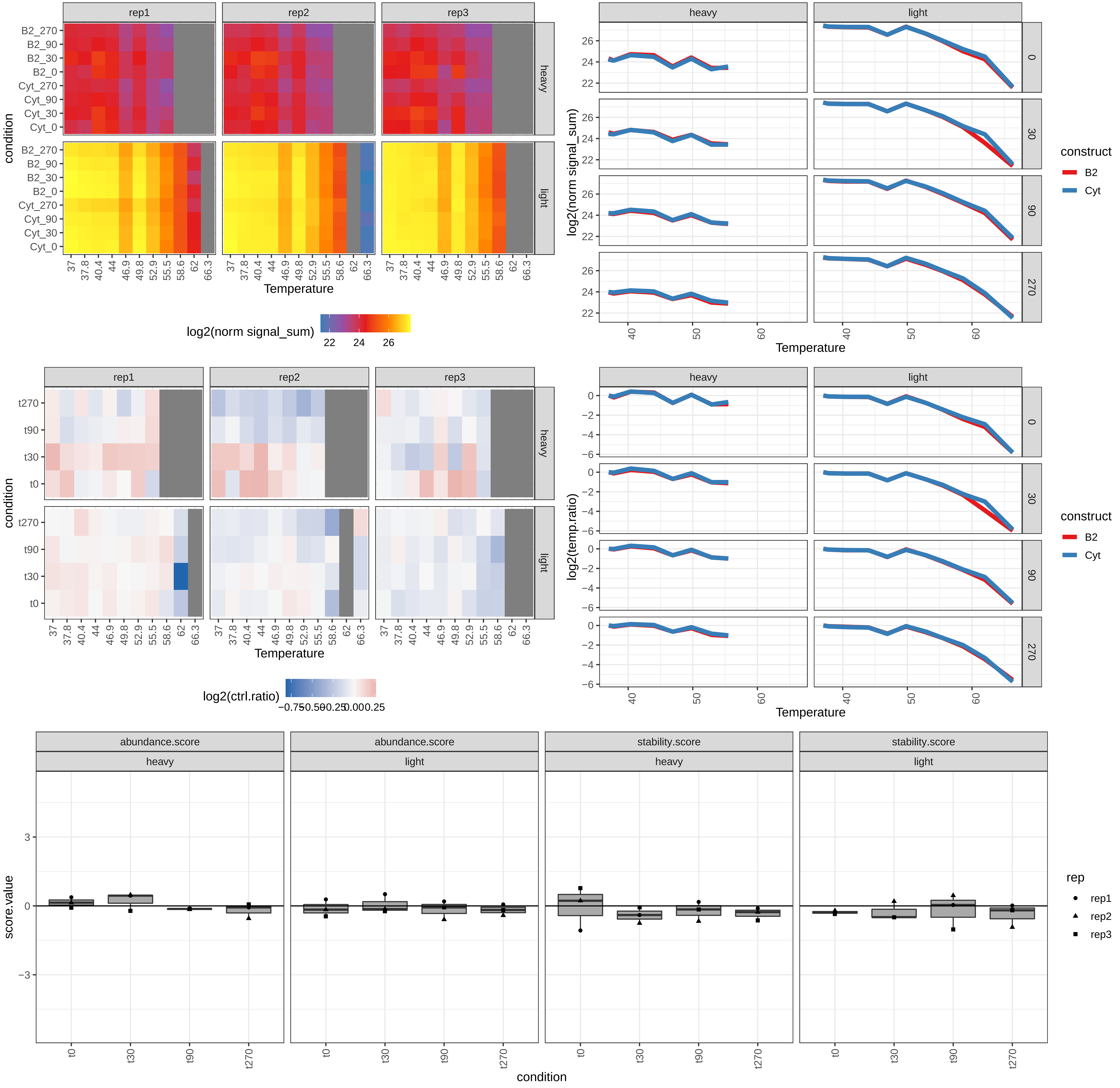

P32386 − ATP−dependent bile acid permease OS=Saccharomyces cerevisiae (strain ATCC 204508 / S288c) GN=YBT1 PE=1 SV=2

YCF1 − average.top3: 6.86514545454545 − max.qupm: 23

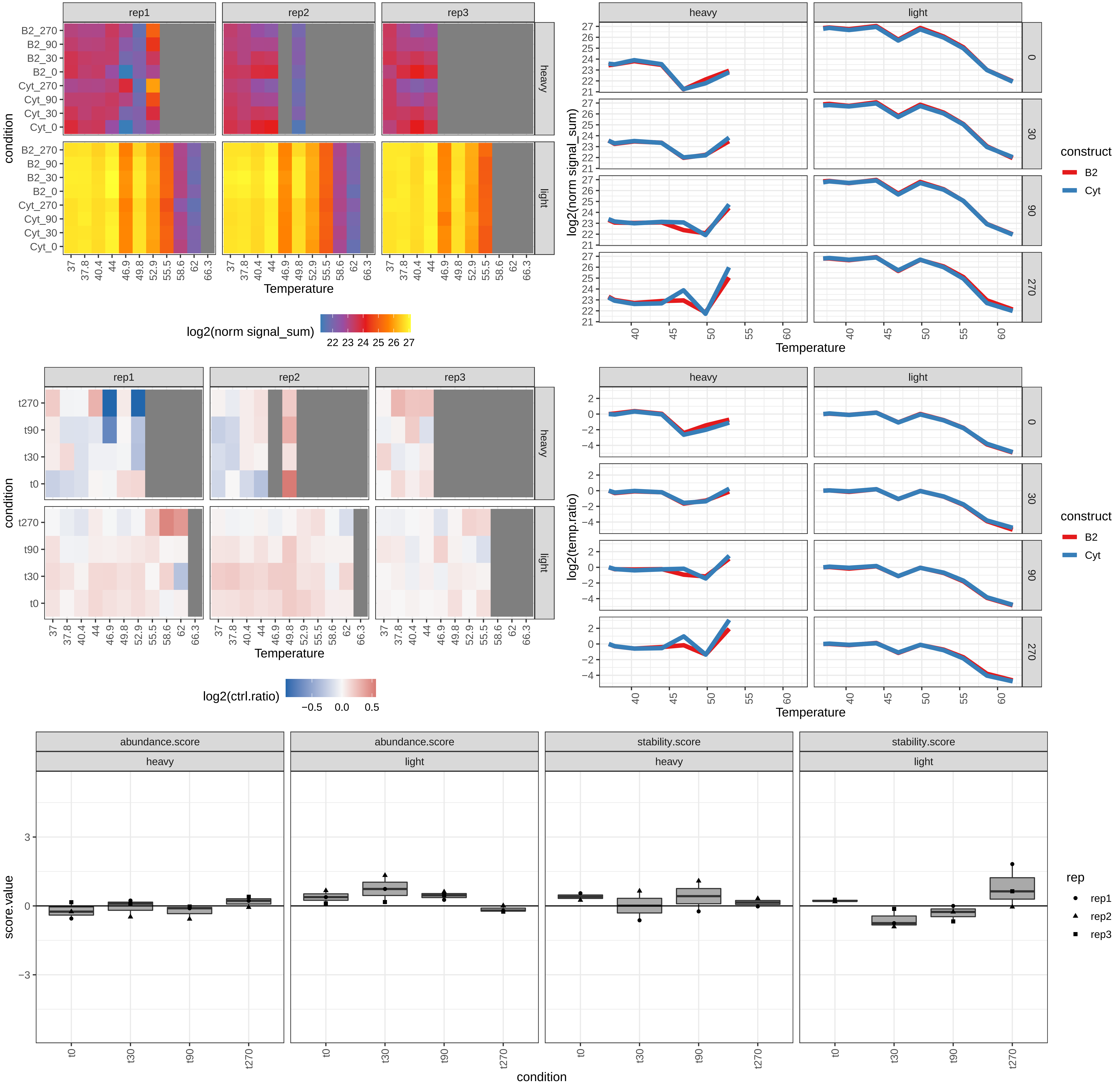

P39109 − Metal resistance protein YCF1 OS=Saccharomyces cerevisiae (strain ATCC 204508 / S288c) GN=YCF1 PE=1 SV=2

YCH1 − average.top3: 7.2286019047619 − max.qupm: 6

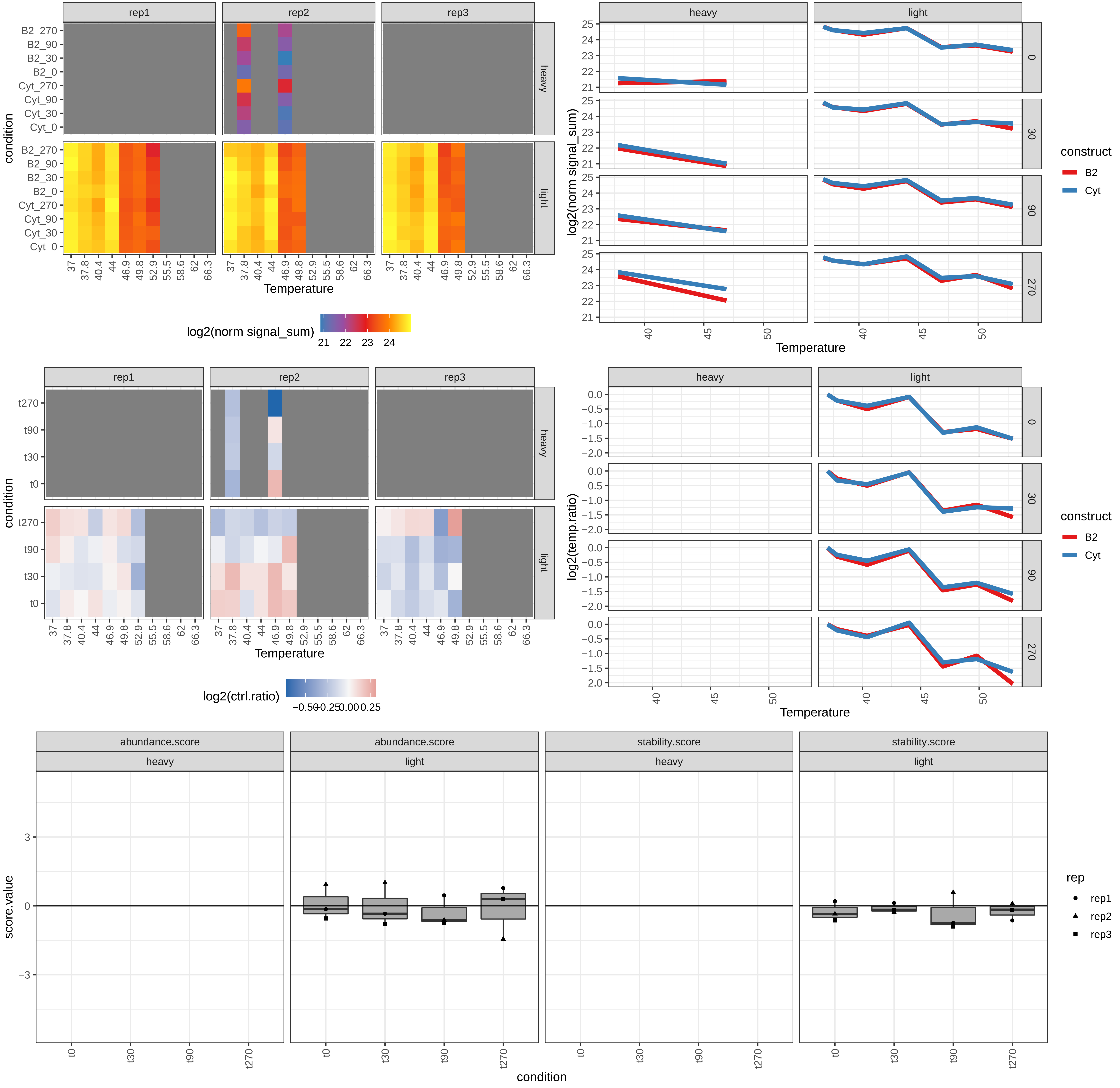

P42937 − CDC25−like phosphatase YCH1 OS=Saccharomyces cerevisiae (strain ATCC 204508 / S288c) GN=YCH1 PE=1 SV=1

YCK1 − average.top3: 6.63808363636364 − max.qupm: 6

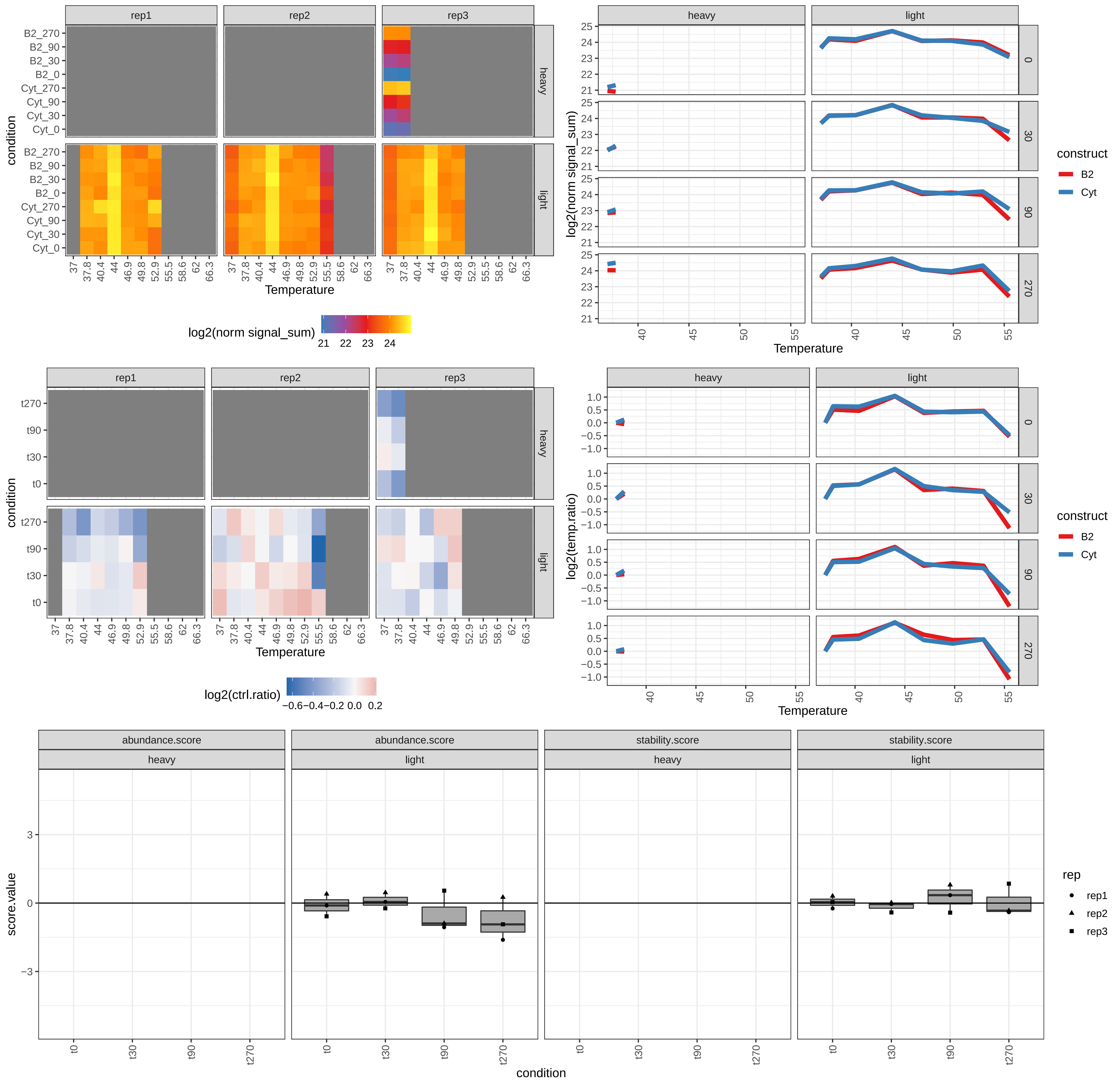

P23291 − Casein kinase I homolog 1 OS=Saccharomyces cerevisiae (strain ATCC 204508 / S288c) GN=YCK1 PE=1 SV=1

YCK2 − average.top3: 6.94147814814815 − max.qupm: 6

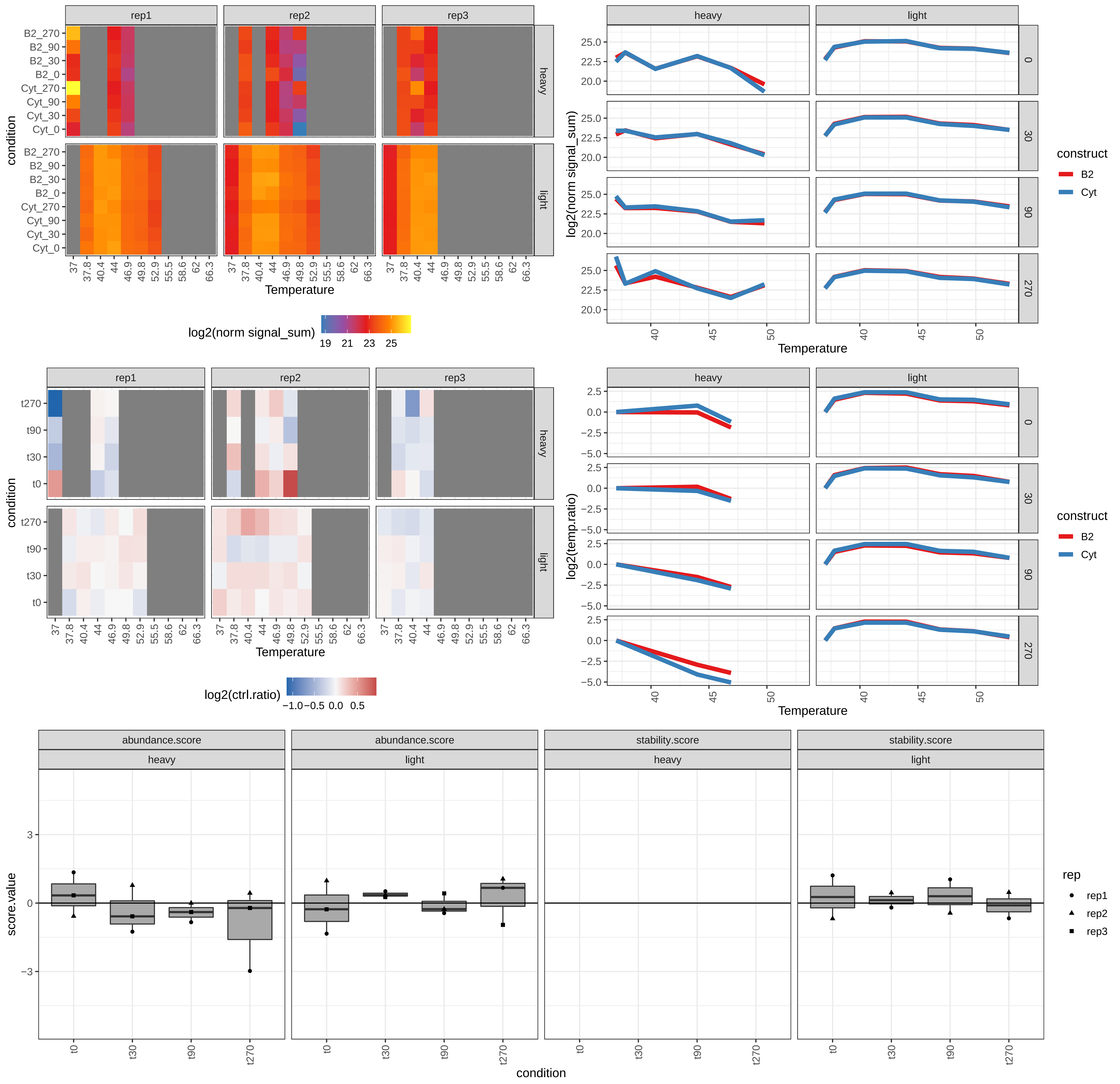

P23292 − Casein kinase I homolog 2 OS=Saccharomyces cerevisiae (strain ATCC 204508 / S288c) GN=YCK2 PE=1 SV=1

YCK3 − average.top3: 6.74747166666667 − max.qupm: 5

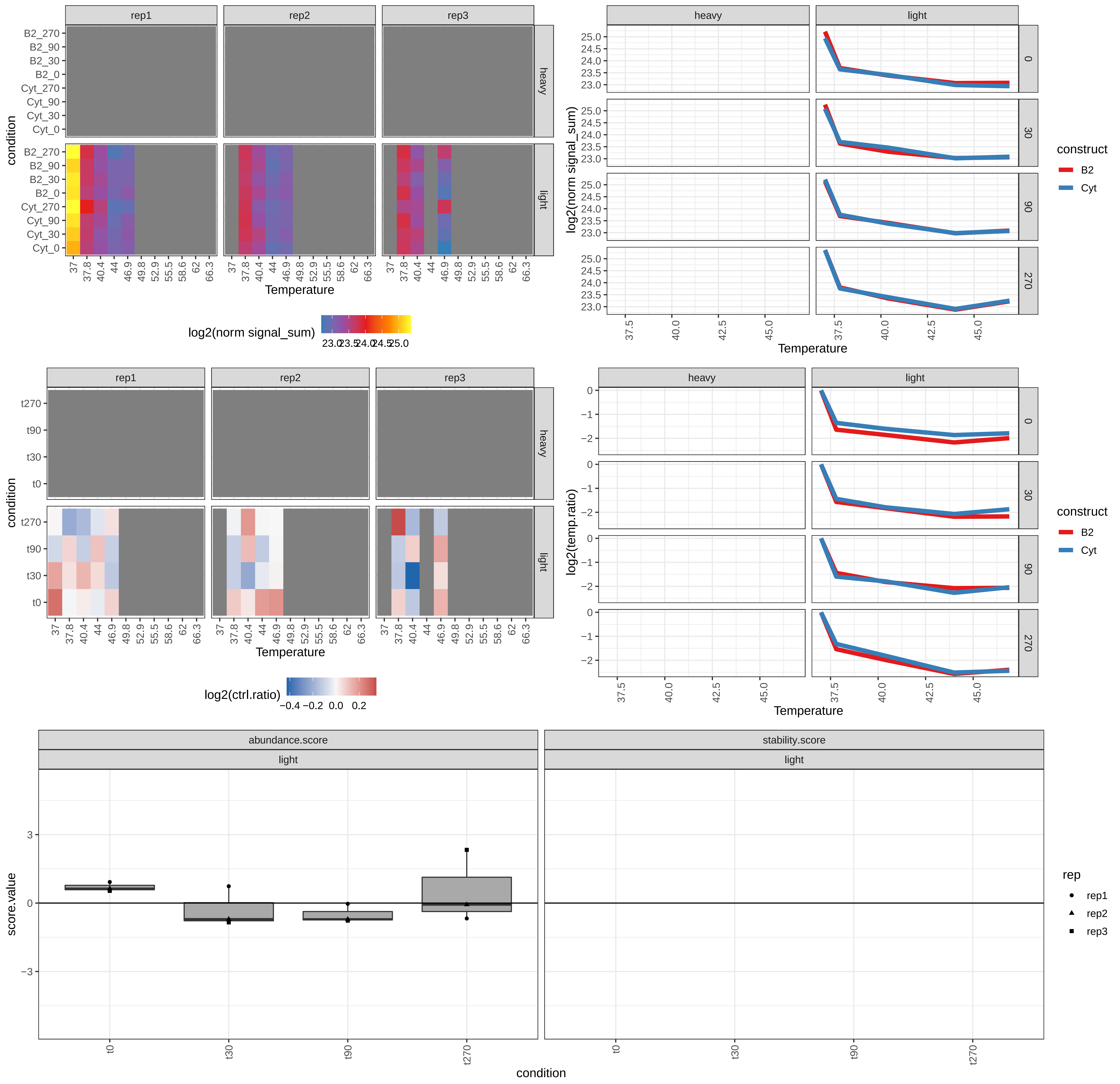

P39962 − Casein kinase I homolog 3 OS=Saccharomyces cerevisiae (strain ATCC 204508 / S288c) GN=YCK3 PE=1 SV=2

YCP4 − average.top3: 7.64406133333333 − max.qupm: 10

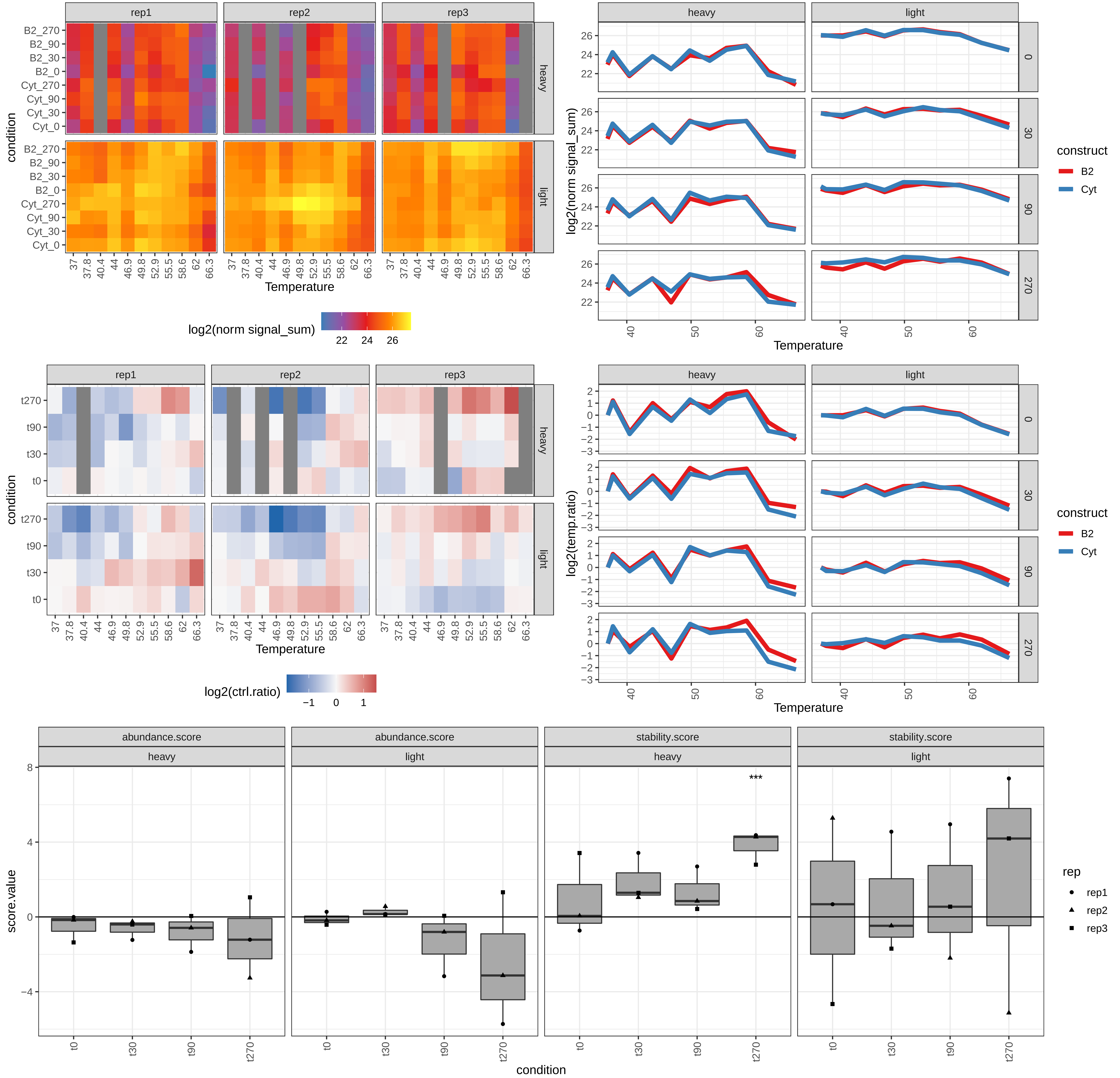

P25349 − Flavoprotein−like protein YCP4 OS=Saccharomyces cerevisiae (strain ATCC 204508 / S288c) GN=YCP4 PE=1 SV=1

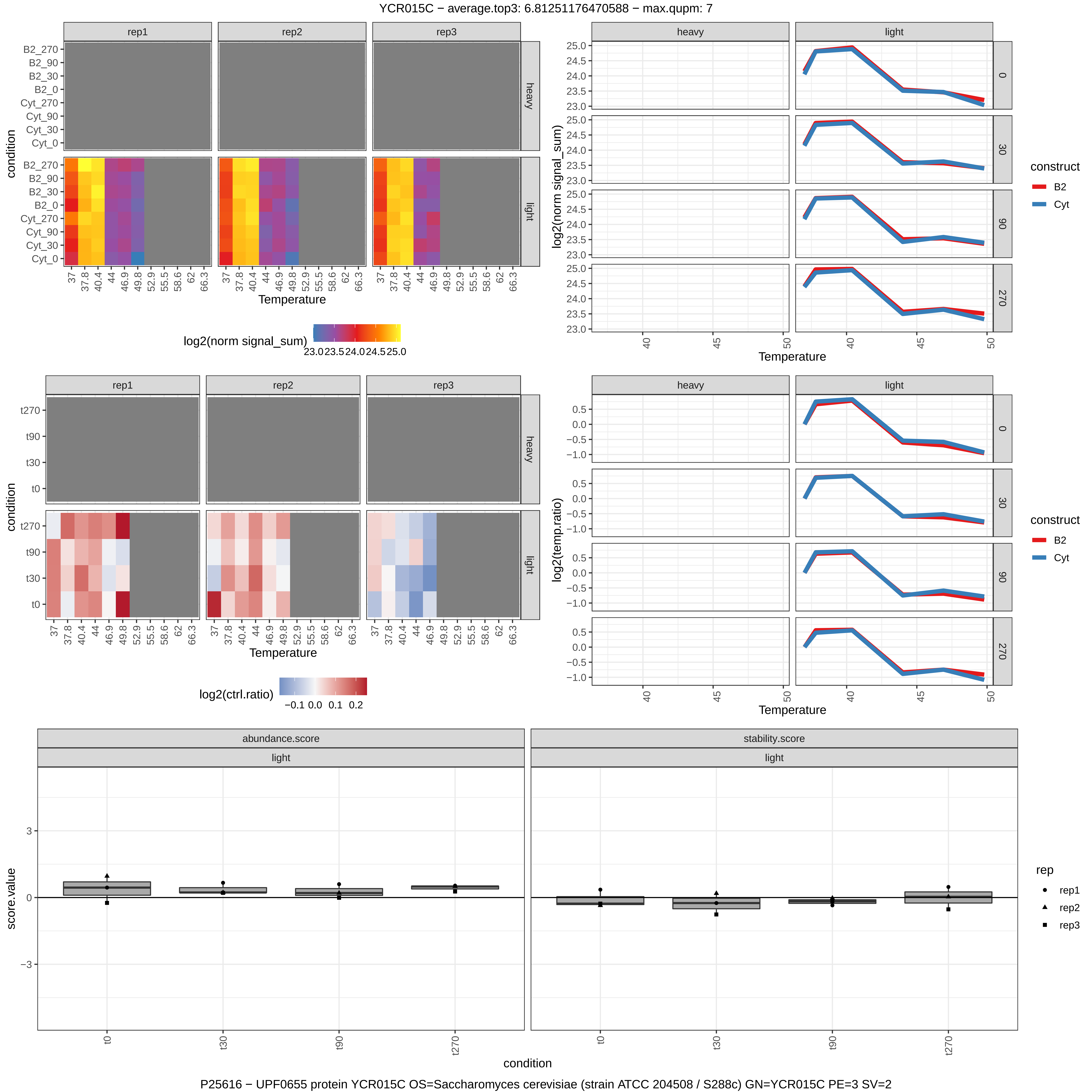
YCR015C − average.top3: 6.81251176470588 − max.qupm: 7
rep1
rep2
rep3
heavy
light
condition
Temperature
log2(norm signal_sum)
log2(norm signal_sum)
construct
B2
Cyt
log2(ctrl.ratio)
log2(temp.ratio)
abundance.score
stability.score
score.value
rep
rep1
rep2
rep3
P25616 − UPF0655 protein YCR015C OS=Saccharomyces cerevisiae (strain ATCC 204508 / S288c) GN=YCR015C PE=3 SV=2

YCR043C − average.top3: 6.62136666666667 − max.qupm: 2

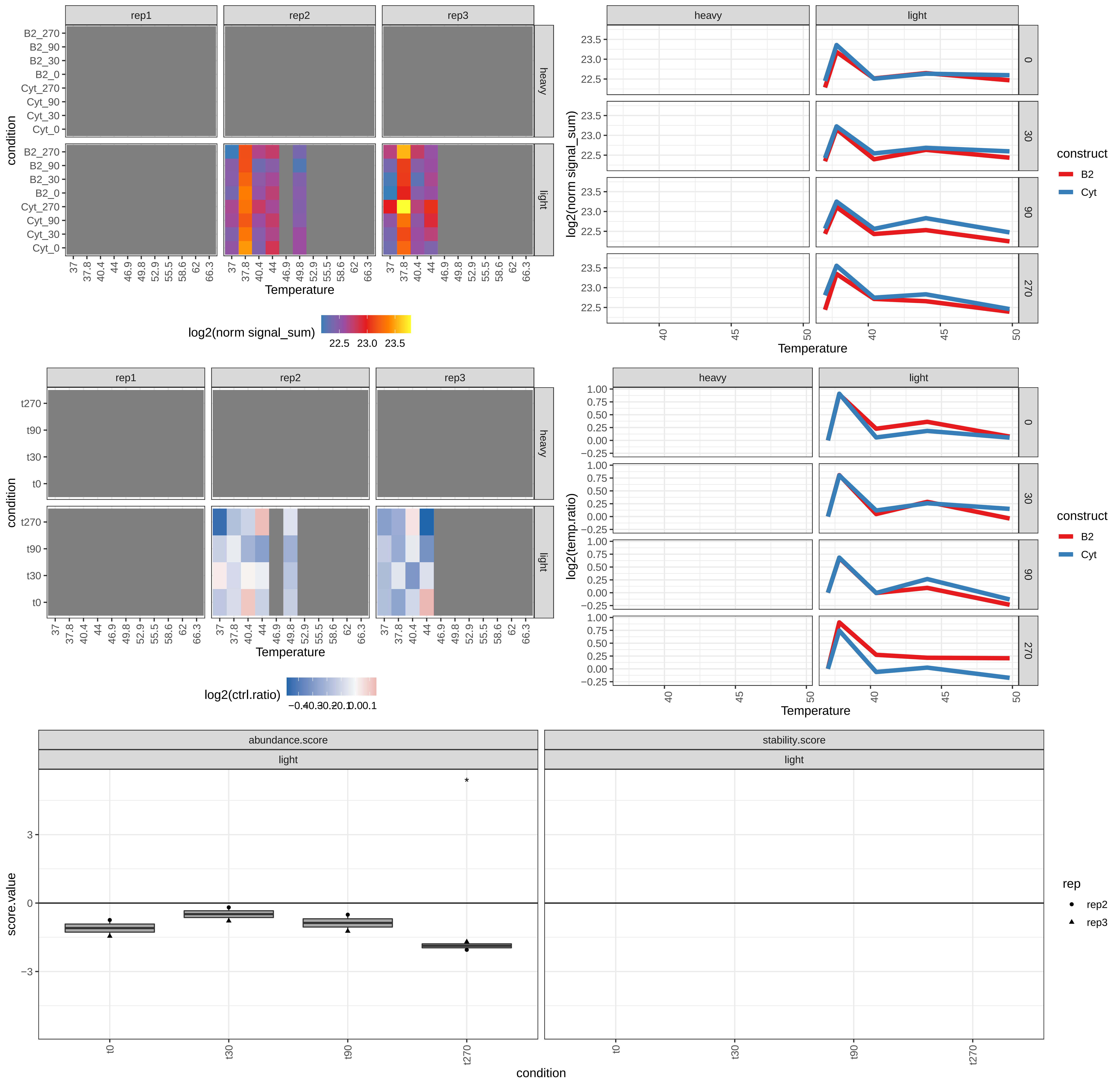

P25361 − Uncharacterized protein YCR043C OS=Saccharomyces cerevisiae (strain ATCC 204508 / S288c) GN=YCR043C PE=1 SV=1

YCR051W − average.top3: 6.37731058823529 − max.qupm: 4

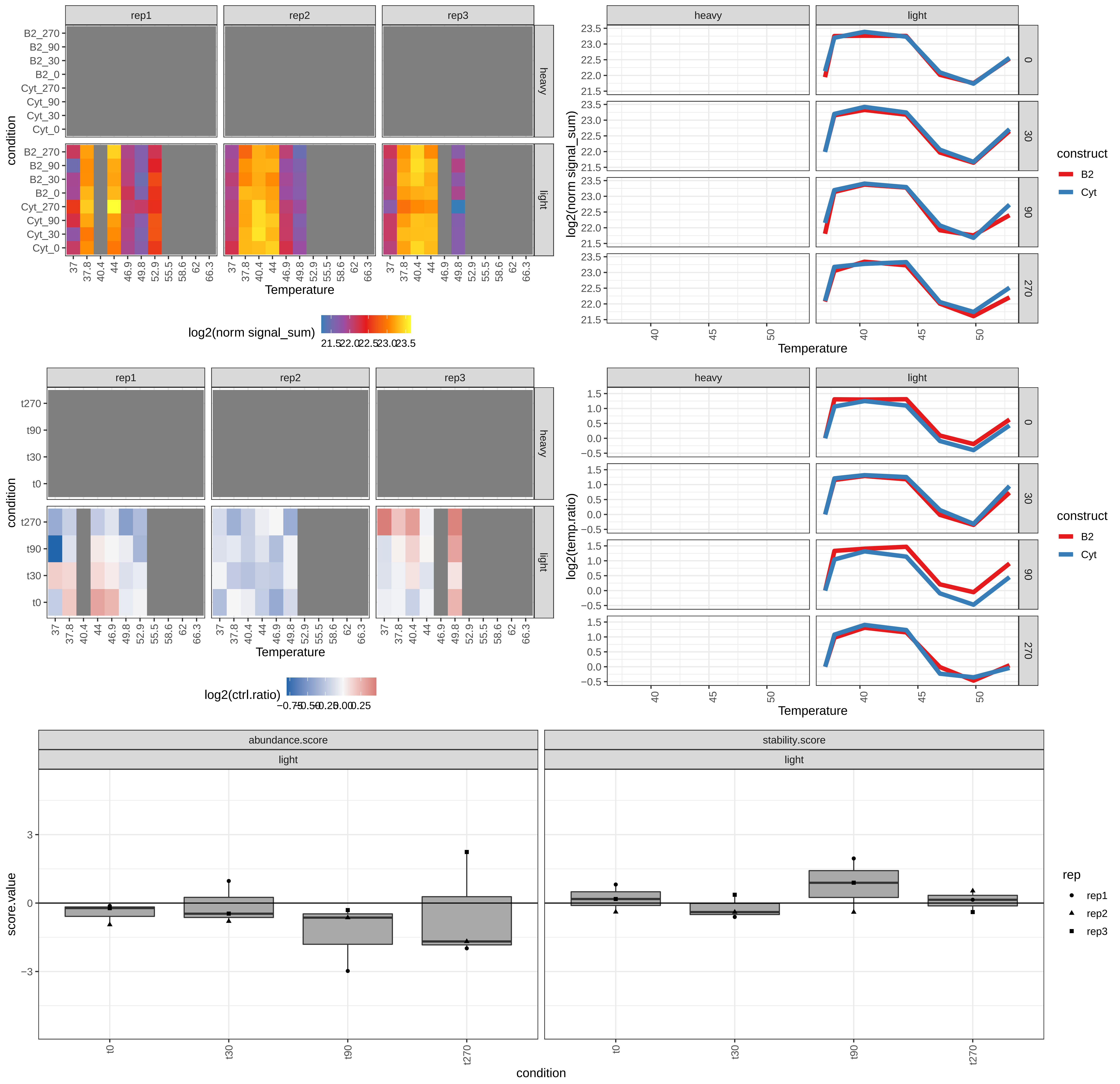

P25631 − Ankyrin repeat−containing protein YCR051W OS=Saccharomyces cerevisiae (strain ATCC 204508 / S288c) GN=YCR051W PE=1 SV=1

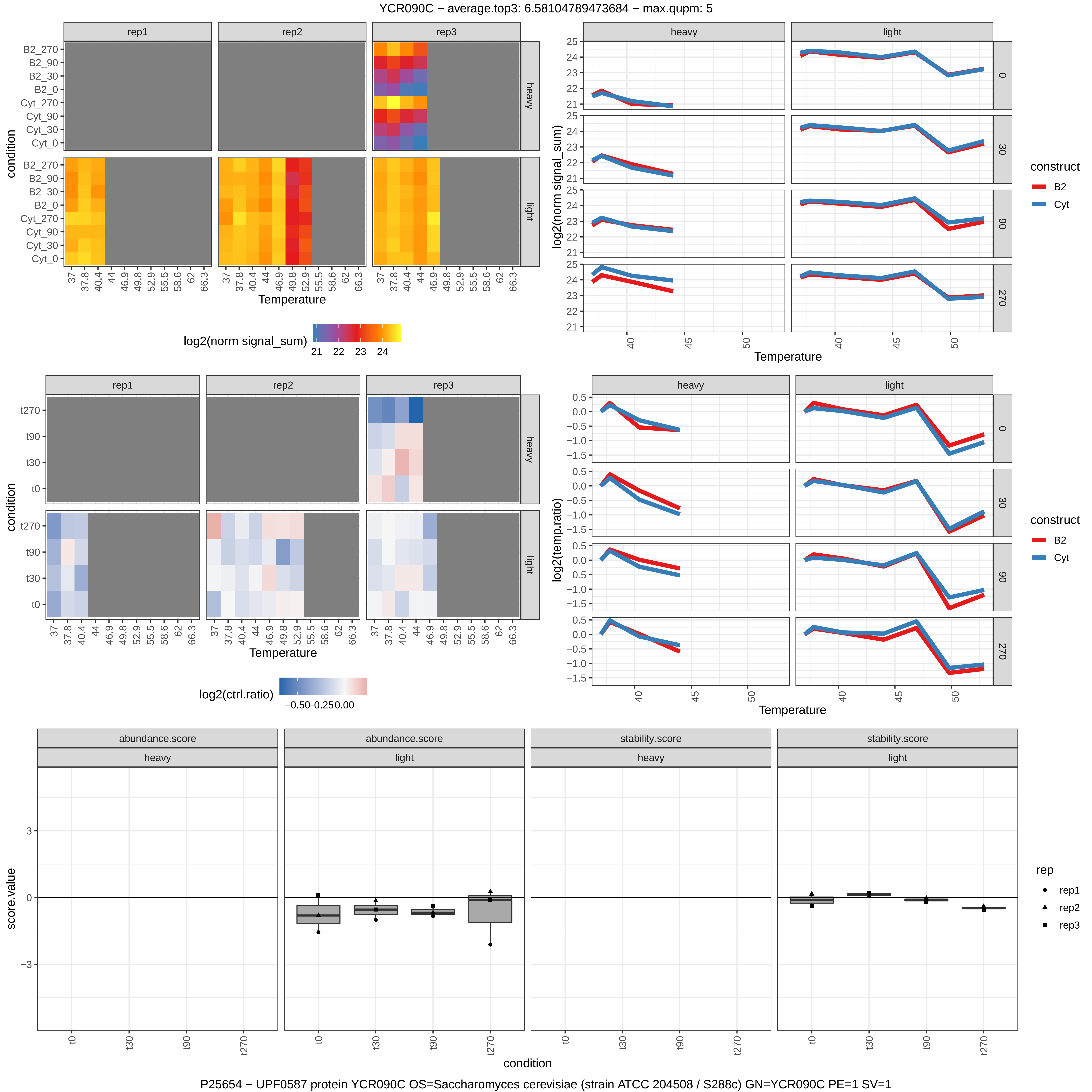

YCR090C − average.top3: 6.58104789473684 − max.qupm: 5
rep1
rep2
rep3
heavy
light
condition
Temperature
log2(norm signal_sum)
construct
B2
Cyt
log2(ctrl.ratio)
log2(temp.ratio)
abundance.score
stability.score
score.value
rep
rep1
rep2
rep3
P25654 − UPF0587 protein YCR090C OS=Saccharomyces cerevisiae (strain ATCC 204508 / S288c) GN=YCR090C PE=1 SV=1

YDJ1 − average.top3: 7.09763448275862 − max.qupm: 13

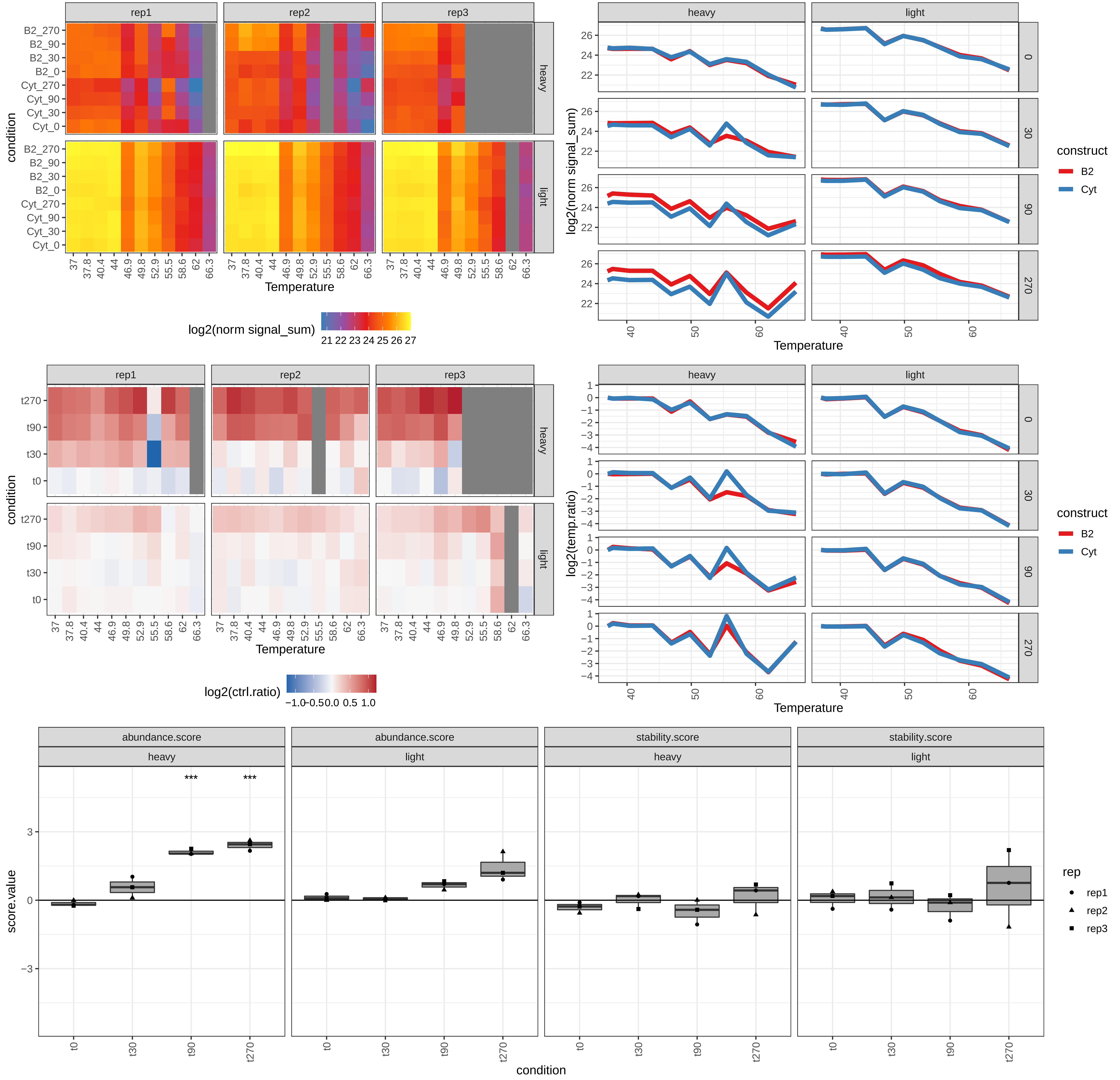

P25491 − Mitochondrial protein import protein MAS5 OS=Saccharomyces cerevisiae (strain ATCC 204508 / S288c) GN=YDJ1 PE=1 SV=1

YDL085C − average.top3: 7.02605842105263 − max.qupm: 3

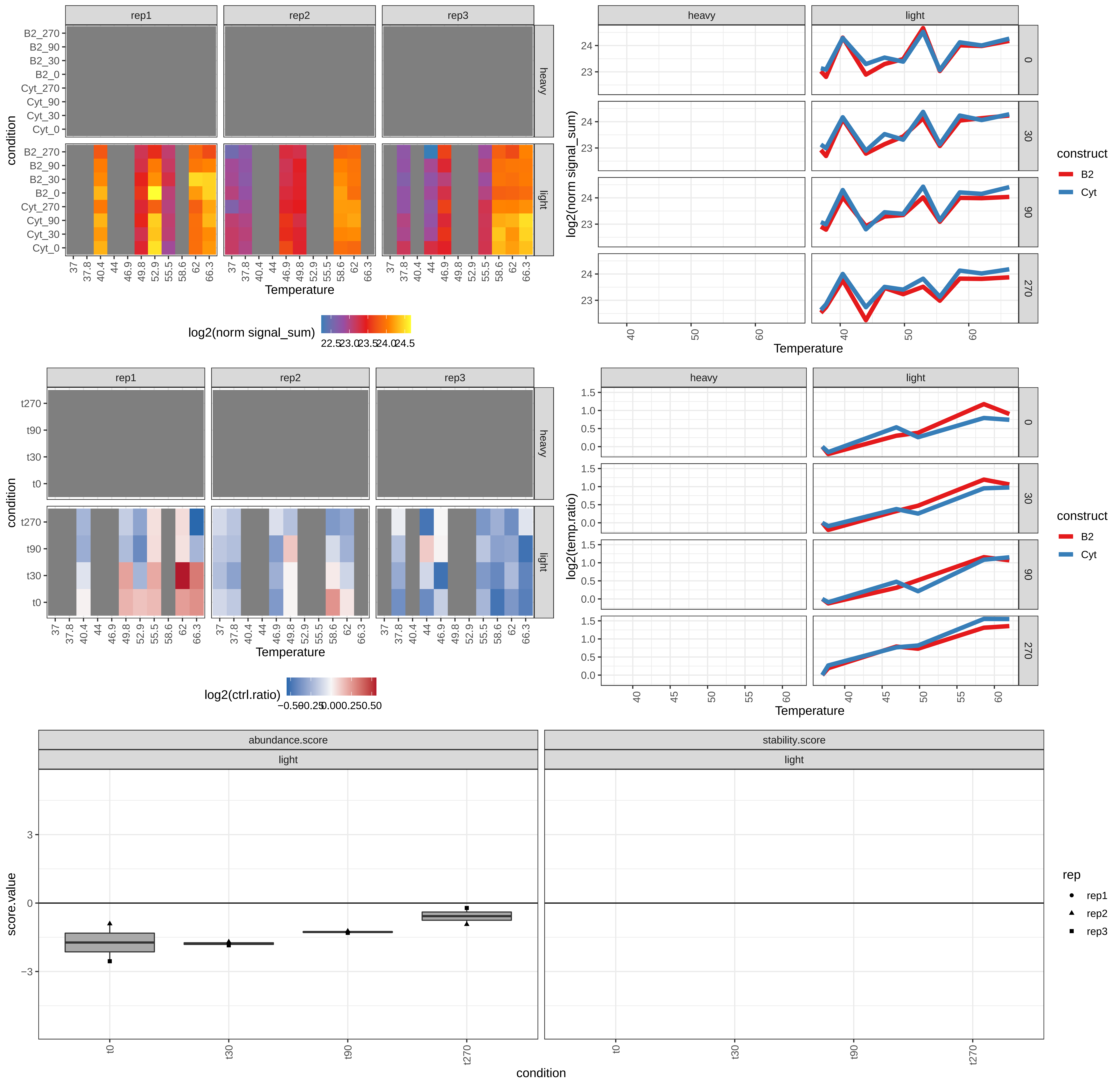

Q3E7B7 − SERF−like protein YDL085C−A OS=Saccharomyces cerevisiae (strain ATCC 204508 / S288c) GN=YDL085C−A PE=1 SV=1

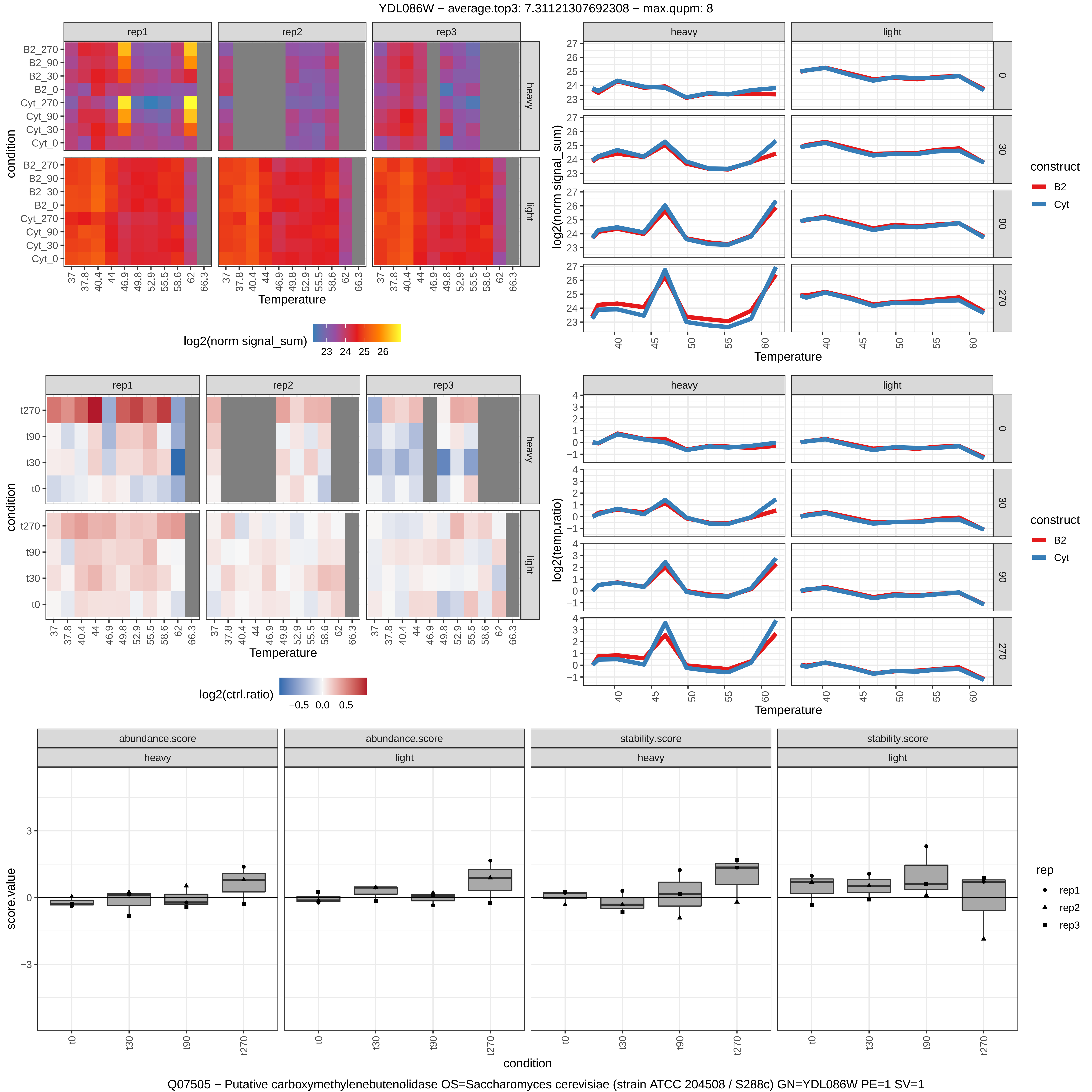
YDL086W − average.top3: 7.31121307692308 − max.qupm: 8
rep1
rep2
rep3
heavy
light
condition
Temperature
log2(norm signal_sum)
construct
B2
Cyt
log2(ctrl.ratio)
log2(temp.ratio)
abundance.score
stability.score
score.value
rep
rep1
rep2
rep3
Q07505 − Putative carboxymethylenebutenolidase OS=Saccharomyces cerevisiae (strain ATCC 204508 / S288c) GN=YDL086W PE=1 SV=1

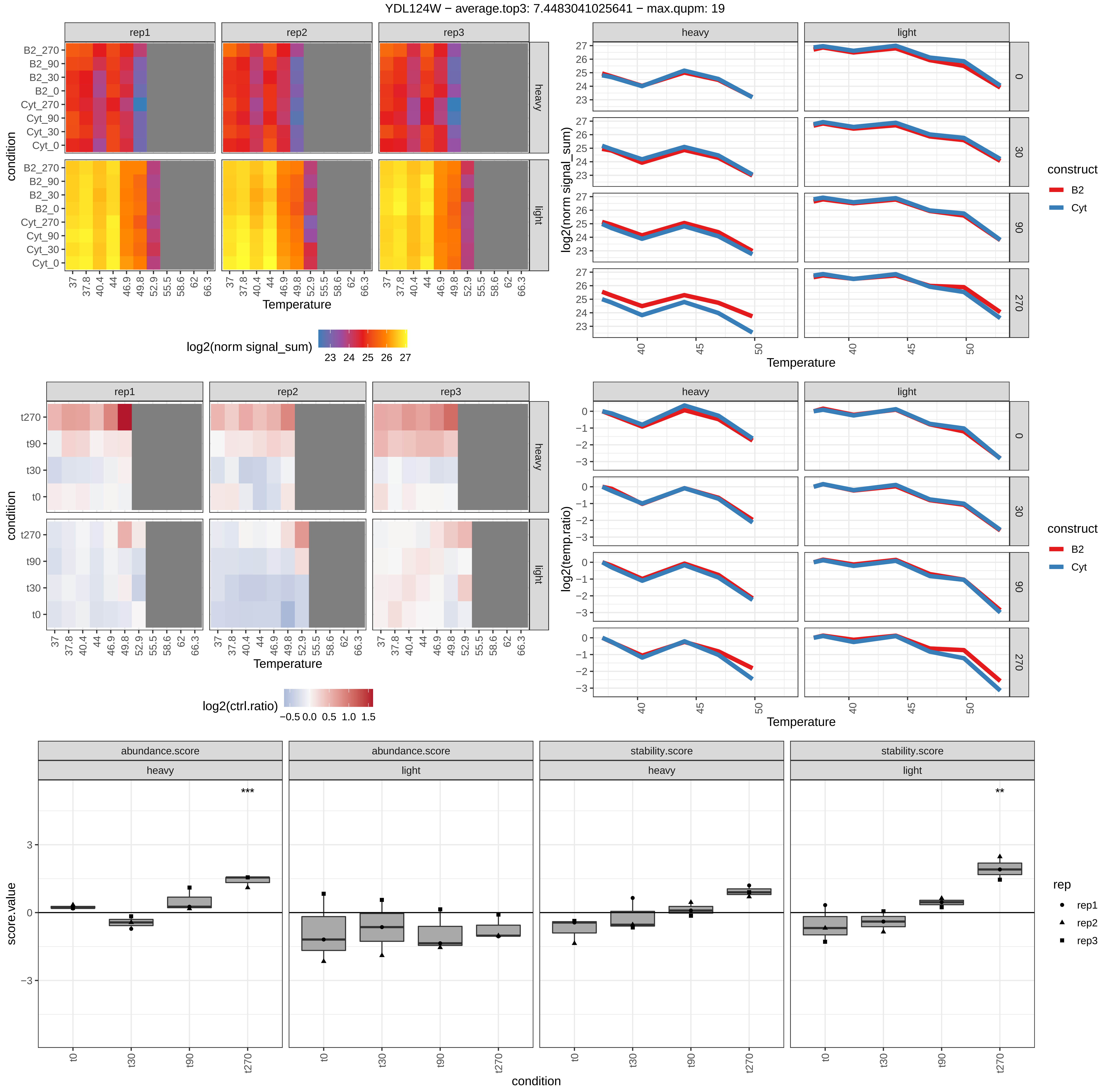

Q07551 − NADPH−dependent alpha−keto amide reductase OS=Saccharomyces cerevisiae (strain ATCC 204508 / S288c) GN=YDL124W PE=1 SV=1

YDL144C − average.top3: 7.10419625 − max.qupm: 7

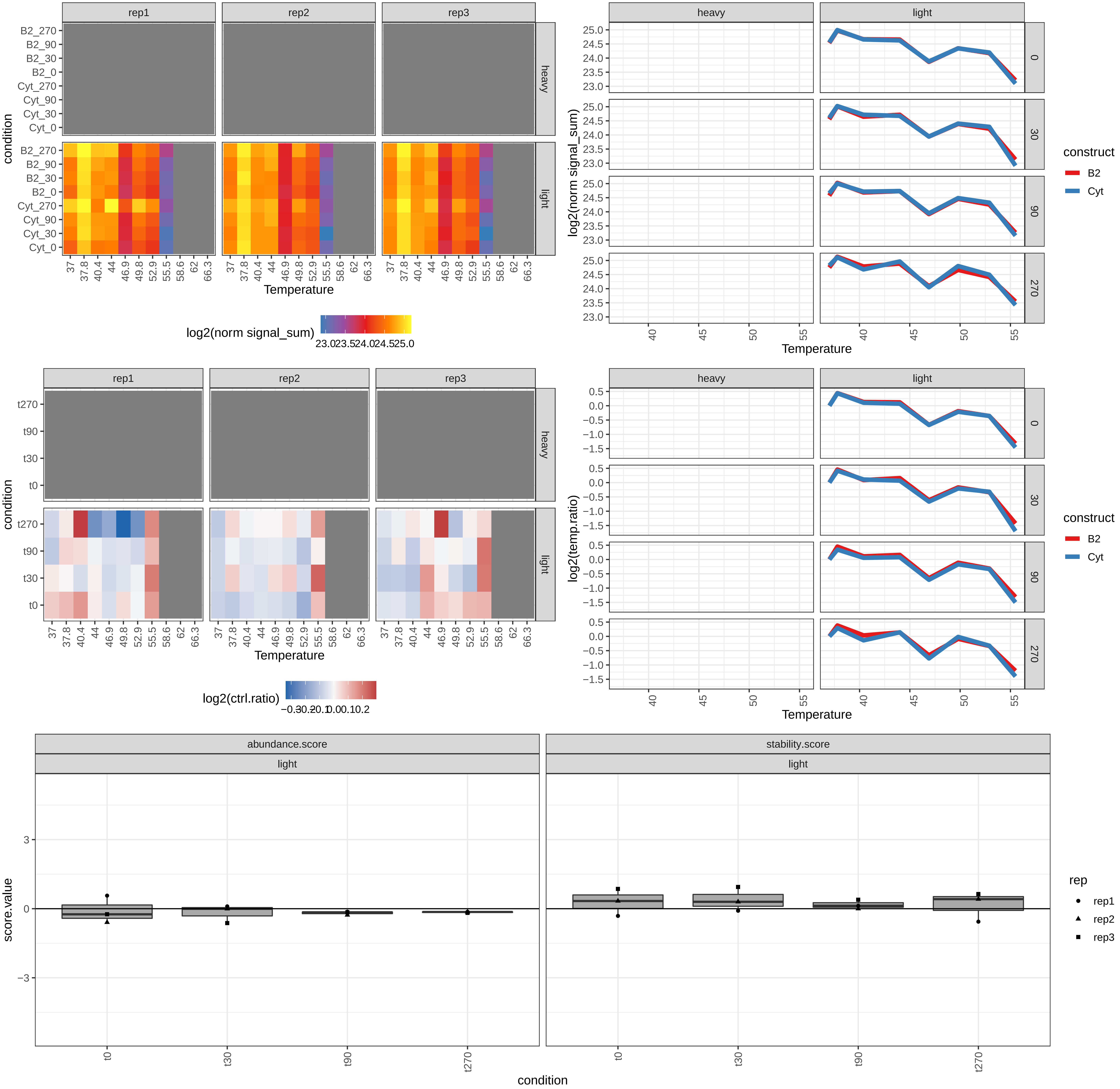

Q07589 − Uncharacterized protein YDL144C OS=Saccharomyces cerevisiae (strain ATCC 204508 / S288c) GN=YDL144C PE=1 SV=2

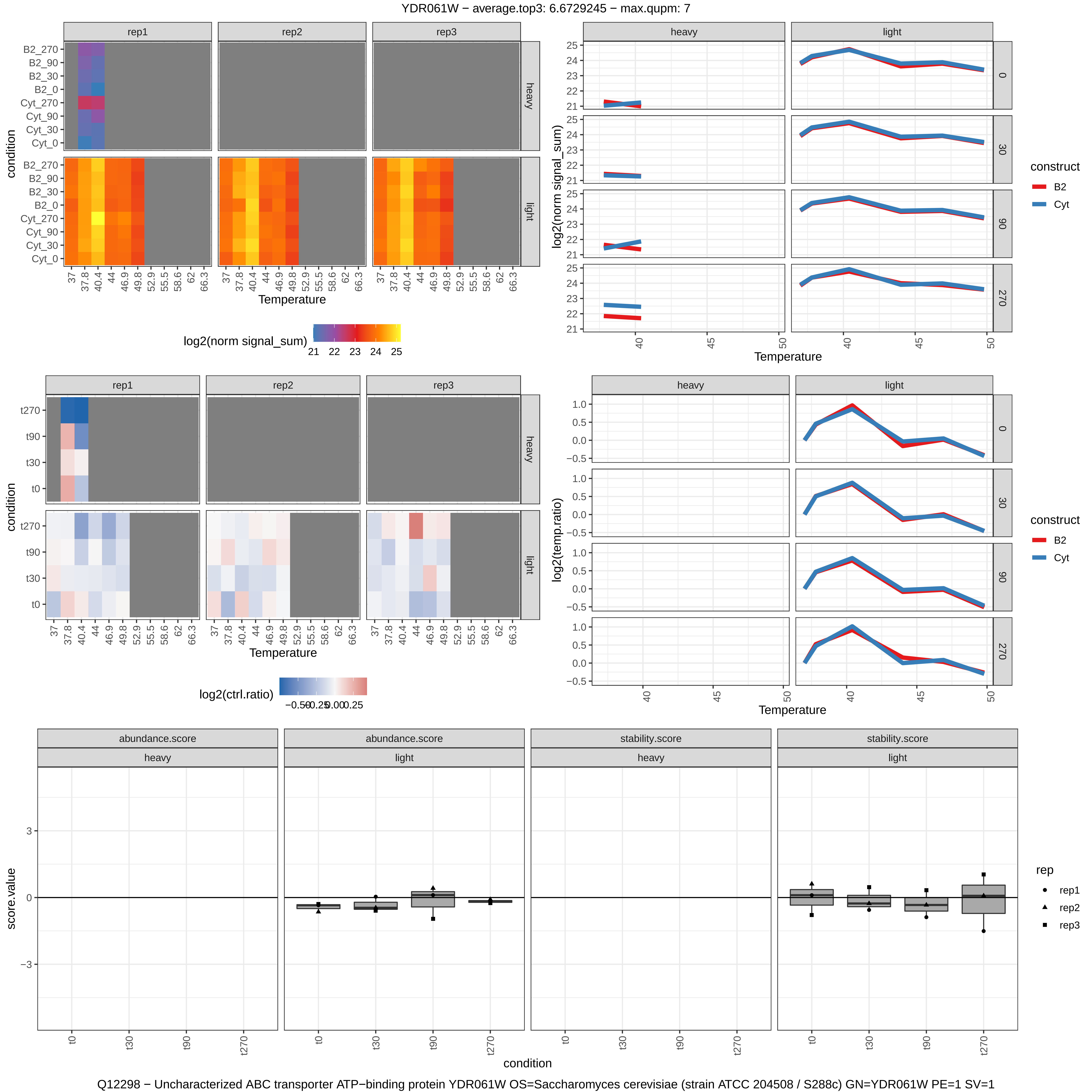
YDR061W − average.top3: 6.6729245 − max.qupm: 7
rep1
rep2
rep3
heavy
light
condition
B2_270
B2_90
B2_30
B2_0
Cyt_270
Cyt_90
Cyt_30
Cyt_0
Temperature
log2(norm signal_sum)
log2(norm signal_sum)
construct
B2
Cyt
t270
t90
t30
t0
log2(ctrl.ratio)
log2(temp.ratio)
abundance.score
stability.score
score.value
condition
rep
rep1
rep2
rep3
Q12298 − Uncharacterized ABC transporter ATP−binding protein YDR061W OS=Saccharomyces cerevisiae (strain ATCC 204508 / S288c) GN=YDR061W PE=1 SV=1

YDR109C − average.top3: 6.39804809523809 − max.qupm: 6

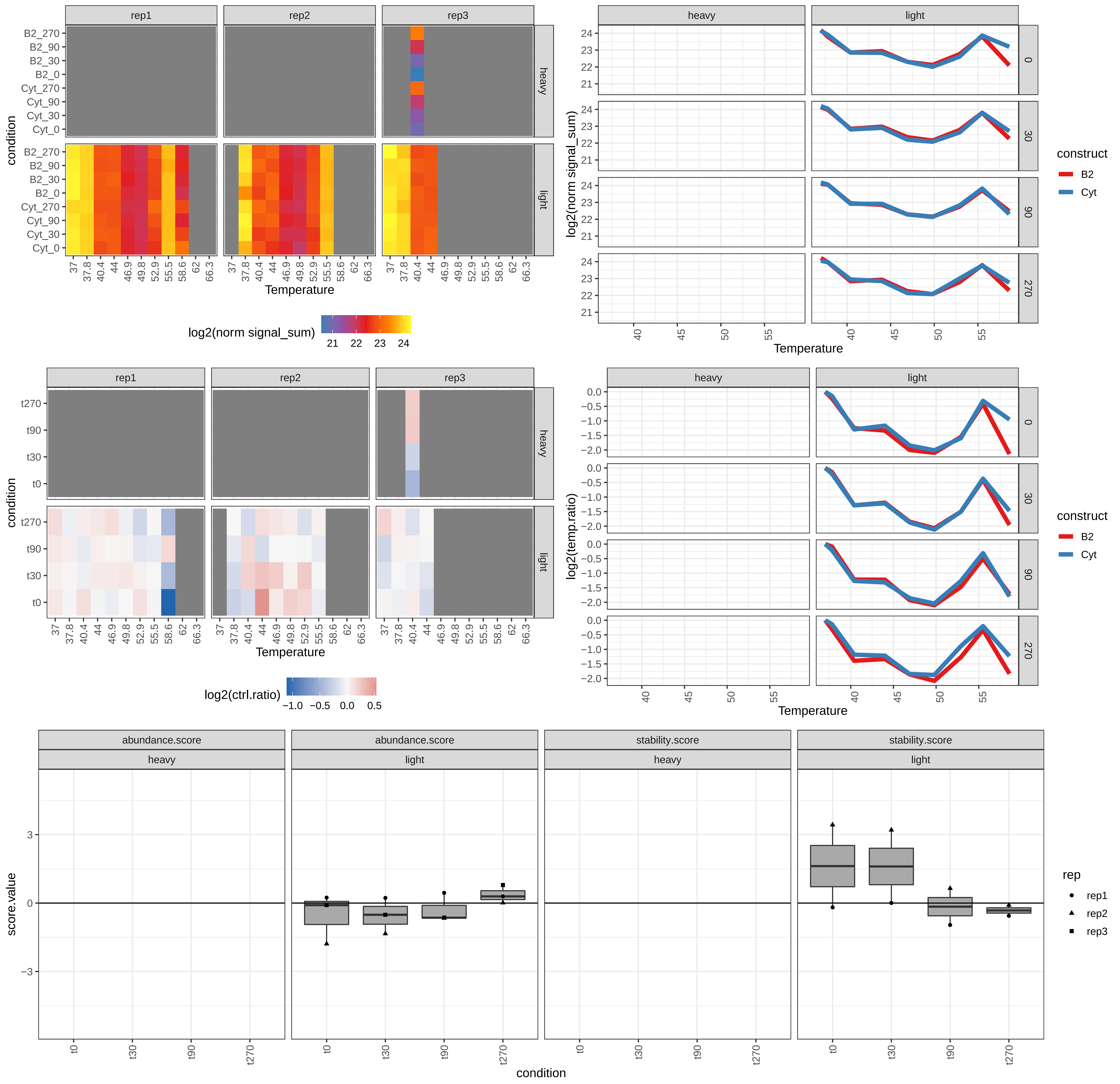

Q04585 − Uncharacterized sugar kinase YDR109C OS=Saccharomyces cerevisiae (strain ATCC 204508 / S288c) GN=YDR109C PE=1 SV=1

YDR248C − average.top3: 6.930728 − max.qupm: 6

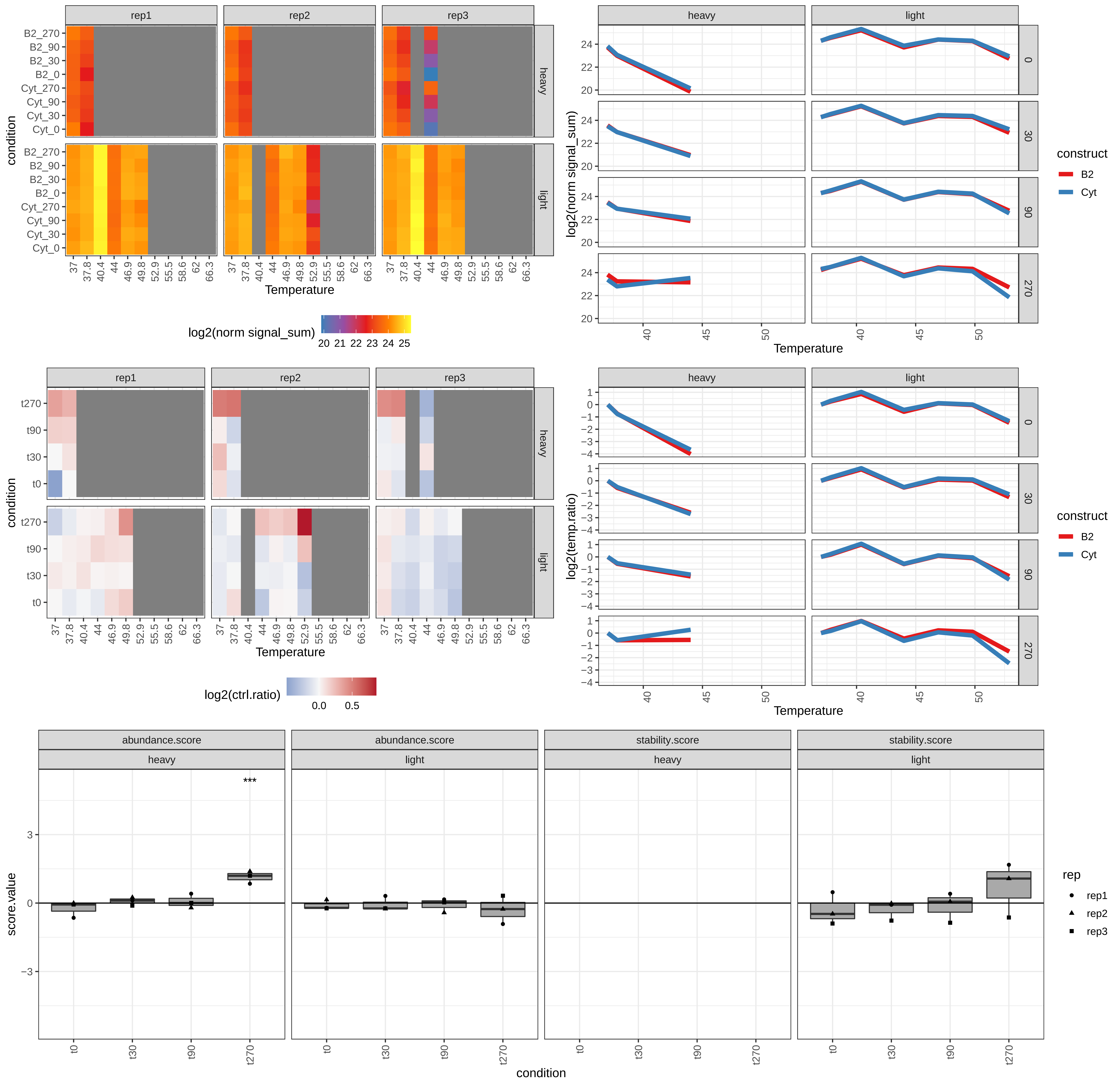

Q03786 − Probable gluconokinase OS=Saccharomyces cerevisiae (strain ATCC 204508 / S288c) GN=YDR248C PE=1 SV=1

YDR341C − average.top3: 7.63607924528302 − max.qupm: 33

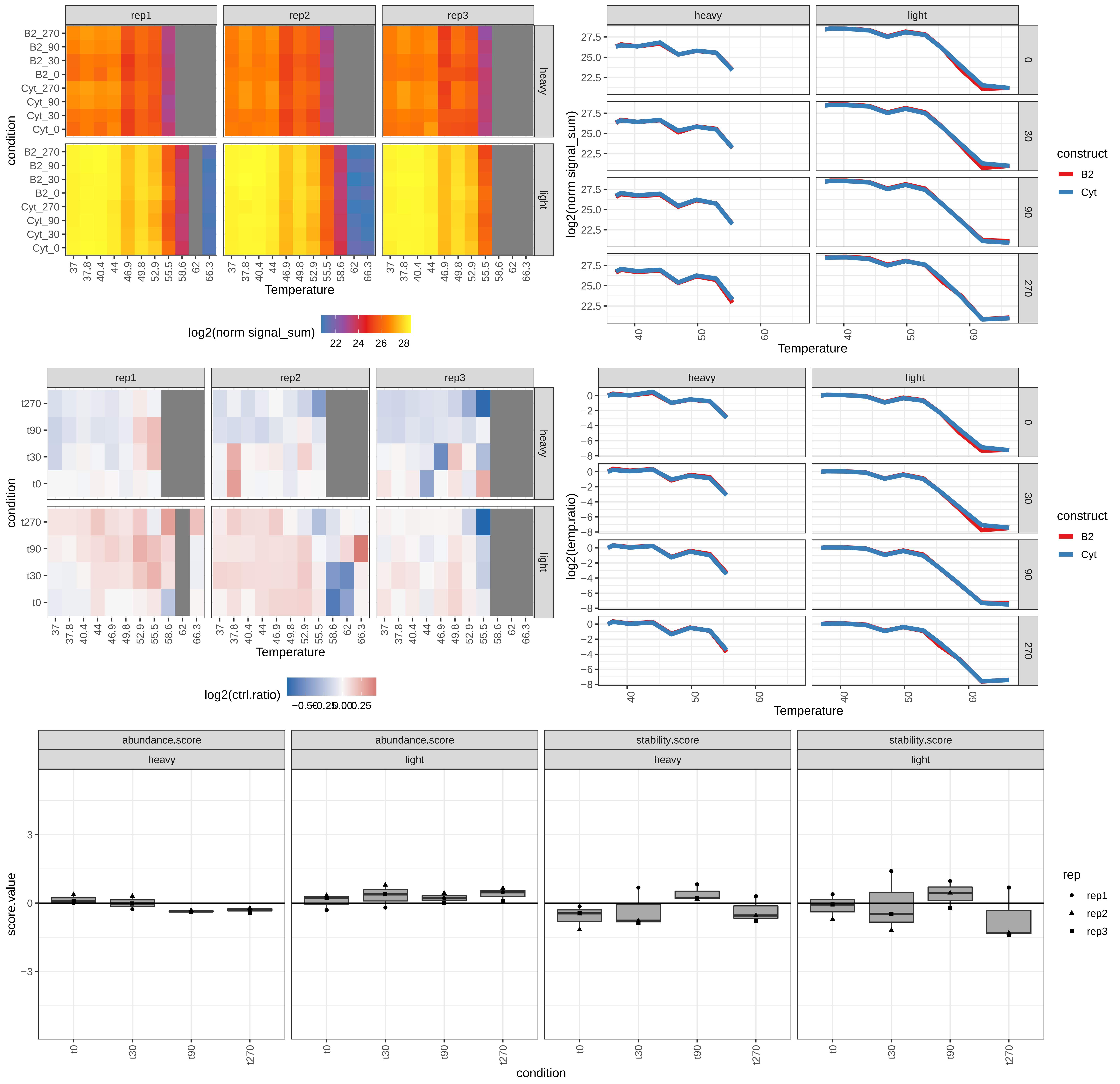

Q05506 − Arginine−−tRNA ligase, cytoplasmic OS=Saccharomyces cerevisiae (strain ATCC 204508 / S288c) GN=YDR341C PE=1 SV=1

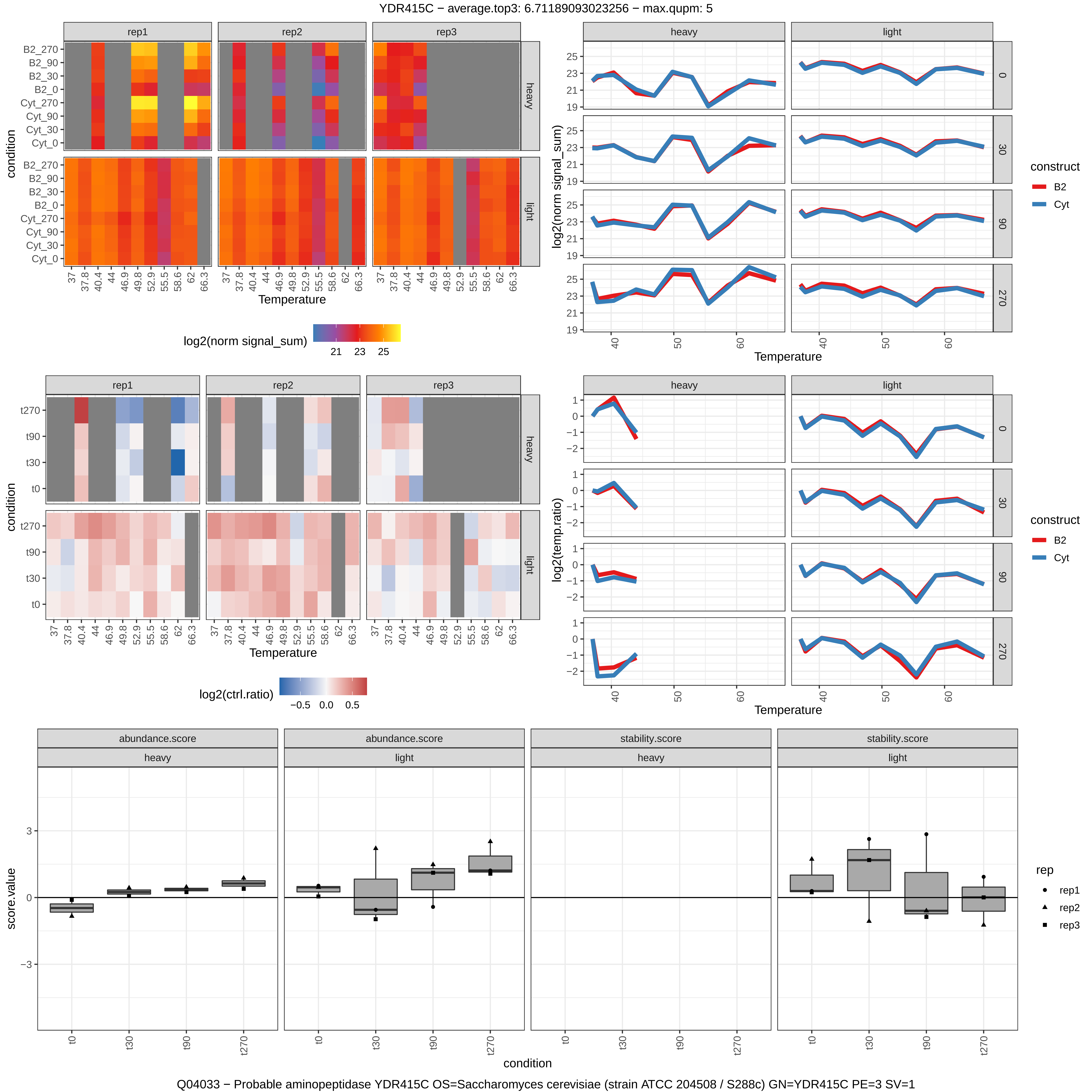
YDR415C − average.top3: 6.71189093023256 − max.qupm: 5
rep1
rep2
rep3
heavy
light
condition
Temperature
log2(norm signal_sum)
construct
B2
Cyt
log2(ctrl.ratio)
log2(temp.ratio)
abundance.score
stability.score
score.value
rep
condition
Q04033 − Probable aminopeptidase YDR415C OS=Saccharomyces cerevisiae (strain ATCC 204508 / S288c) GN=YDR415C PE=3 SV=1

YDR476C − average.top3: 6.59420866666667 − max.qupm: 3

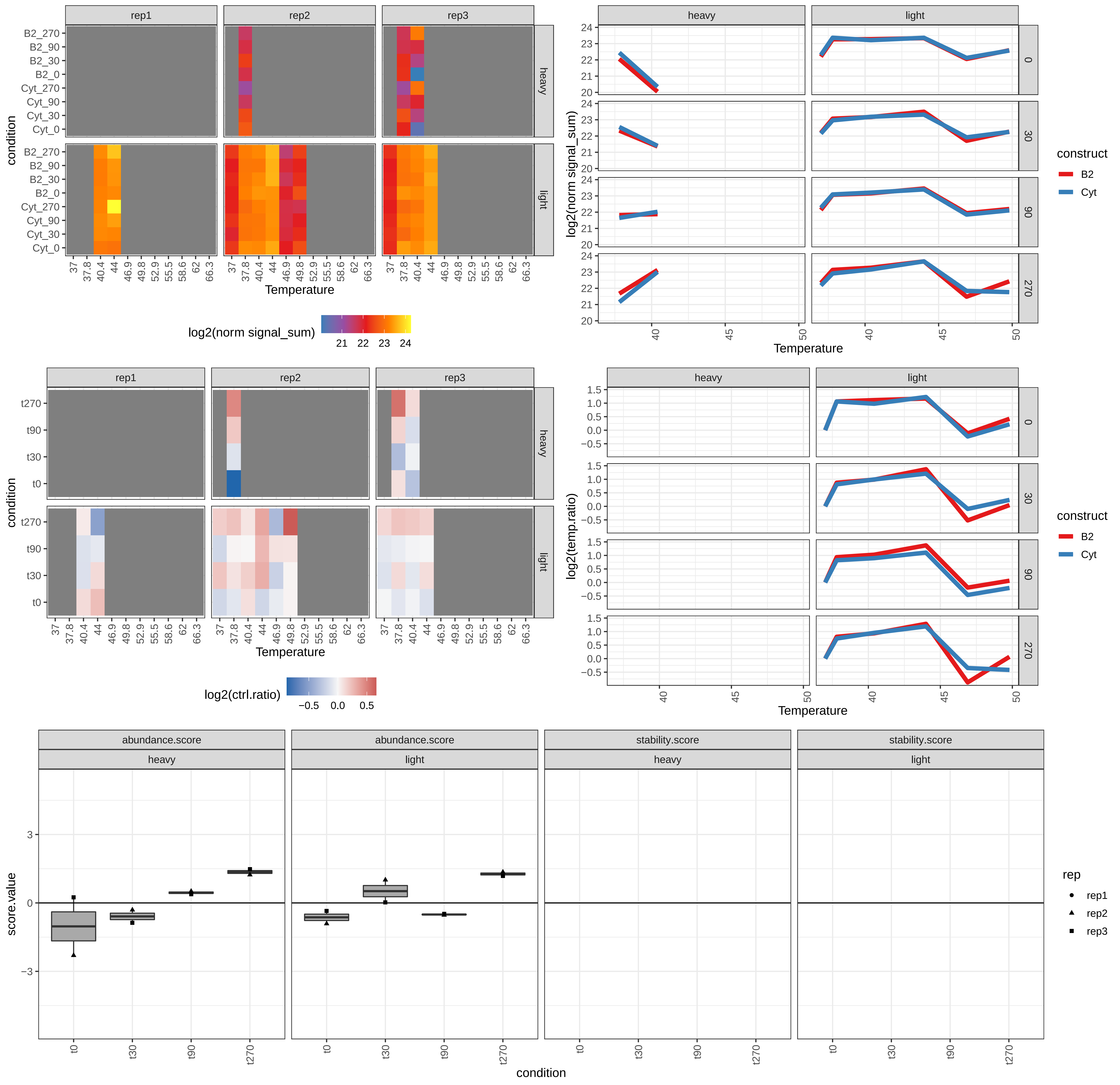

Q03362 − Uncharacterized protein YDR476C OS=Saccharomyces cerevisiae (strain ATCC 204508 / S288c) GN=YDR476C PE=1 SV=1

YDR514C − average.top3: 6.42622666666667 − max.qupm: 5

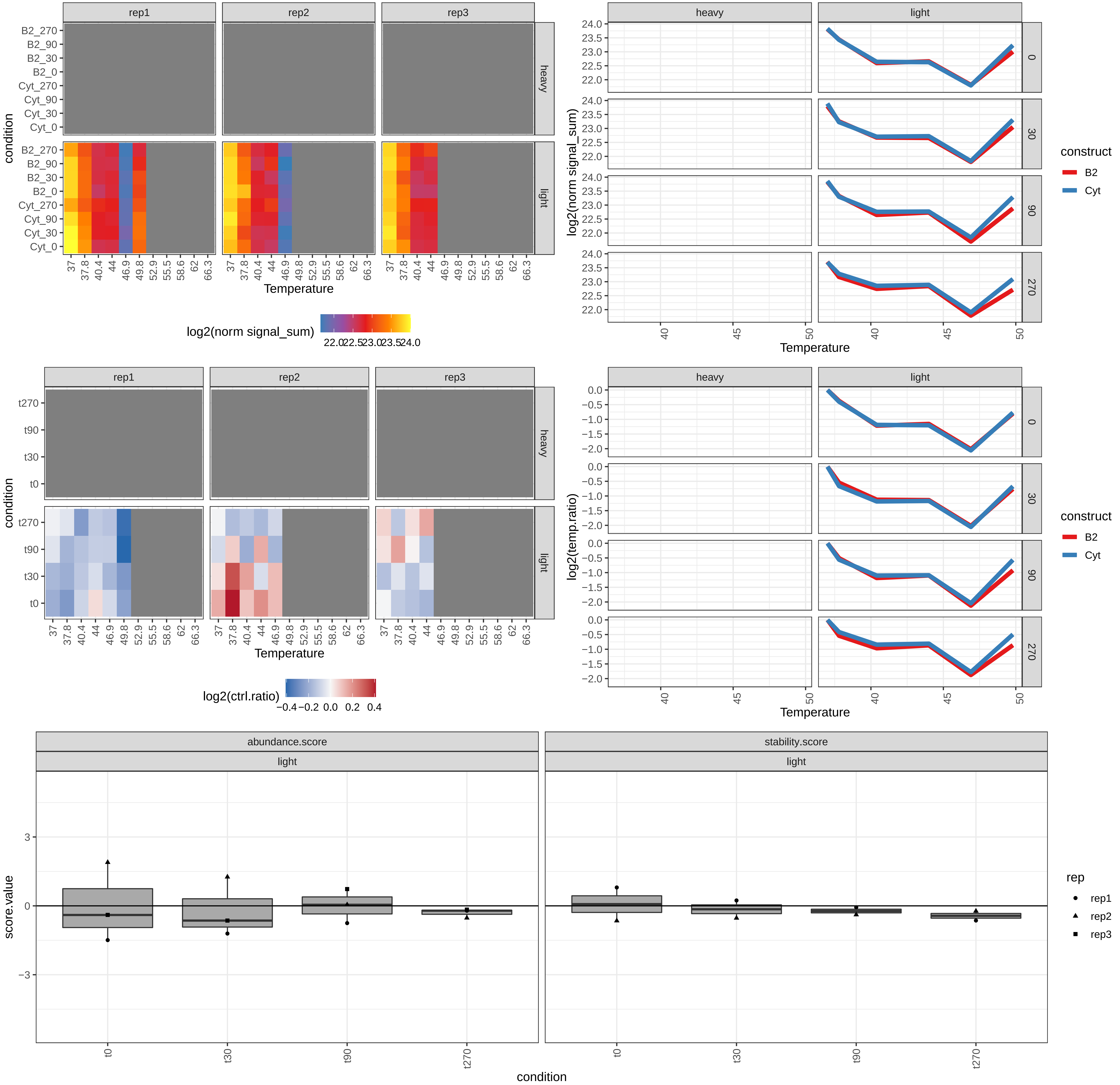

Q04408 − Uncharacterized protein YDR514C OS=Saccharomyces cerevisiae (strain ATCC 204508 / S288c) GN=YDR514C PE=1 SV=1

YEF3 − average.top3: 7.83225509433962 − max.qupm: 28

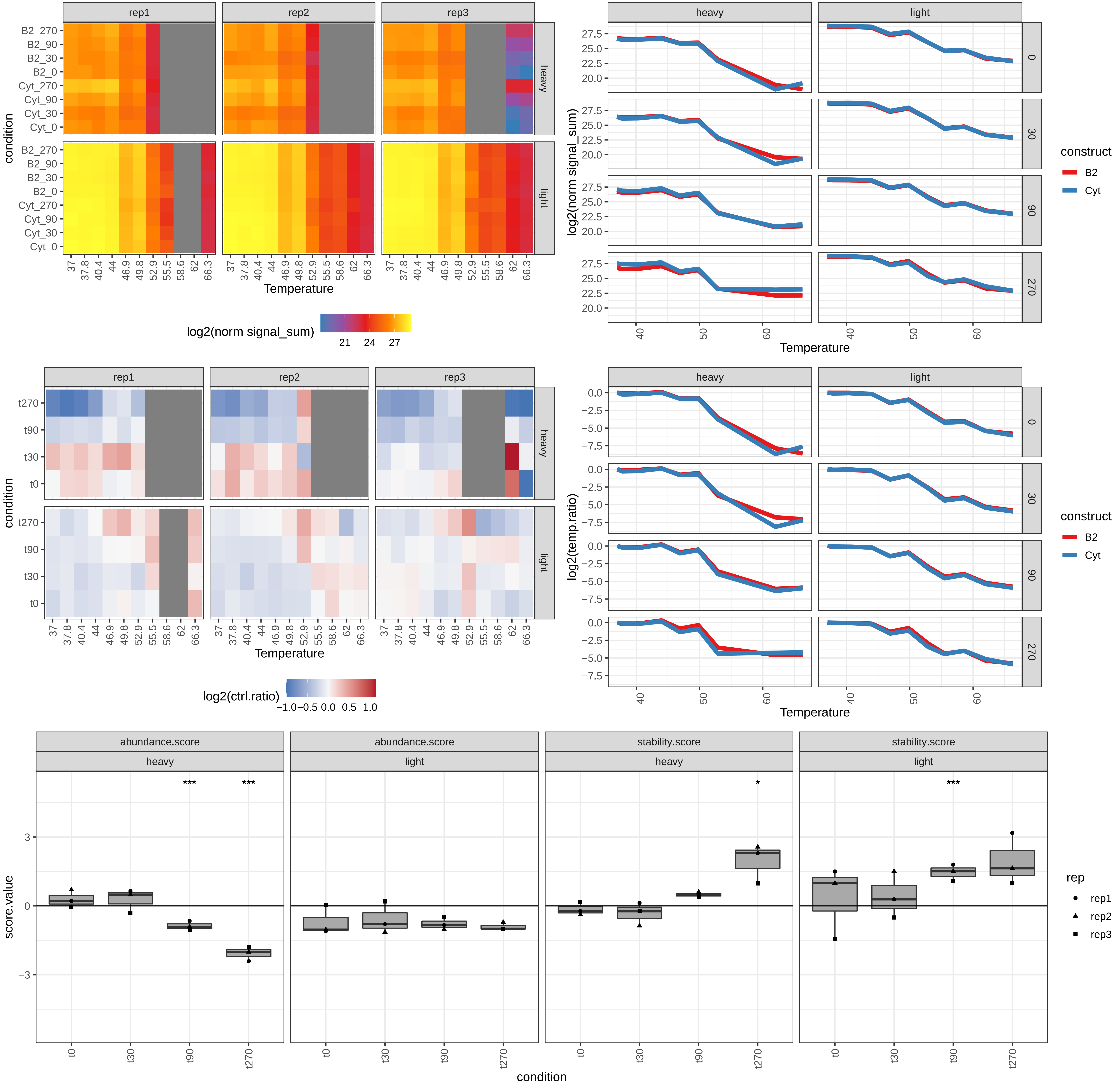

P16521 − Elongation factor 3A OS=Saccharomyces cerevisiae (strain ATCC 204508 / S288c) GN=YEF3 PE=1 SV=4

YEL020C − average.top3: 6.662225 − max.qupm: 4

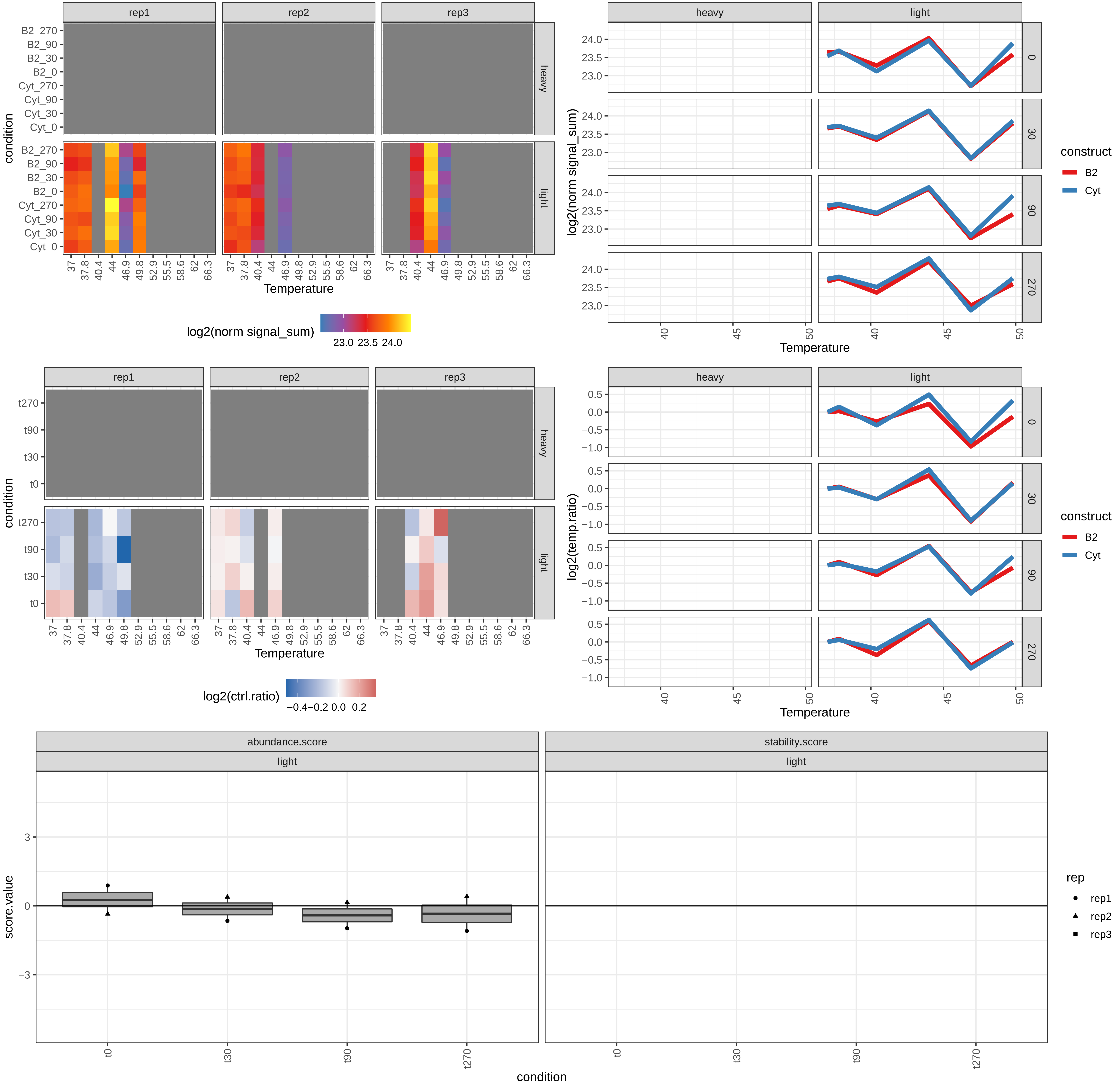

P39994 − Putative 2−hydroxyacyl−CoA lyase OS=Saccharomyces cerevisiae (strain ATCC 204508 / S288c) GN=YEL020C PE=1 SV=1

YER010C − average.top3: 7.19160085714286 − max.qupm: 7

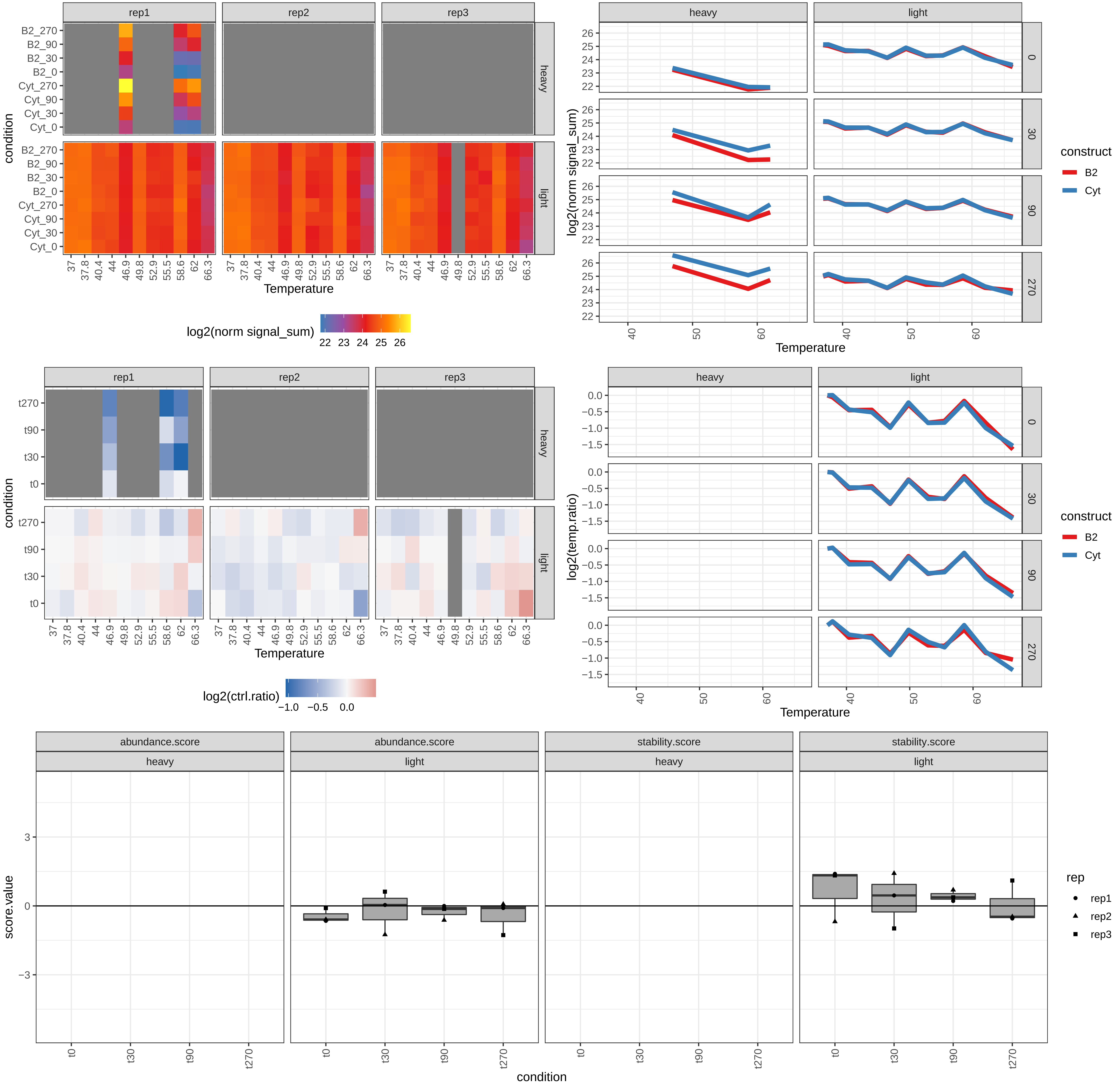

P40011 − 4−hydroxy−4−methyl−2−oxoglutarate aldolase OS=Saccharomyces cerevisiae (strain ATCC 204508 / S288c) GN=YER010C PE=1 SV=1

YER134C − average.top3: 7.01991074074074 − max.qupm: 8

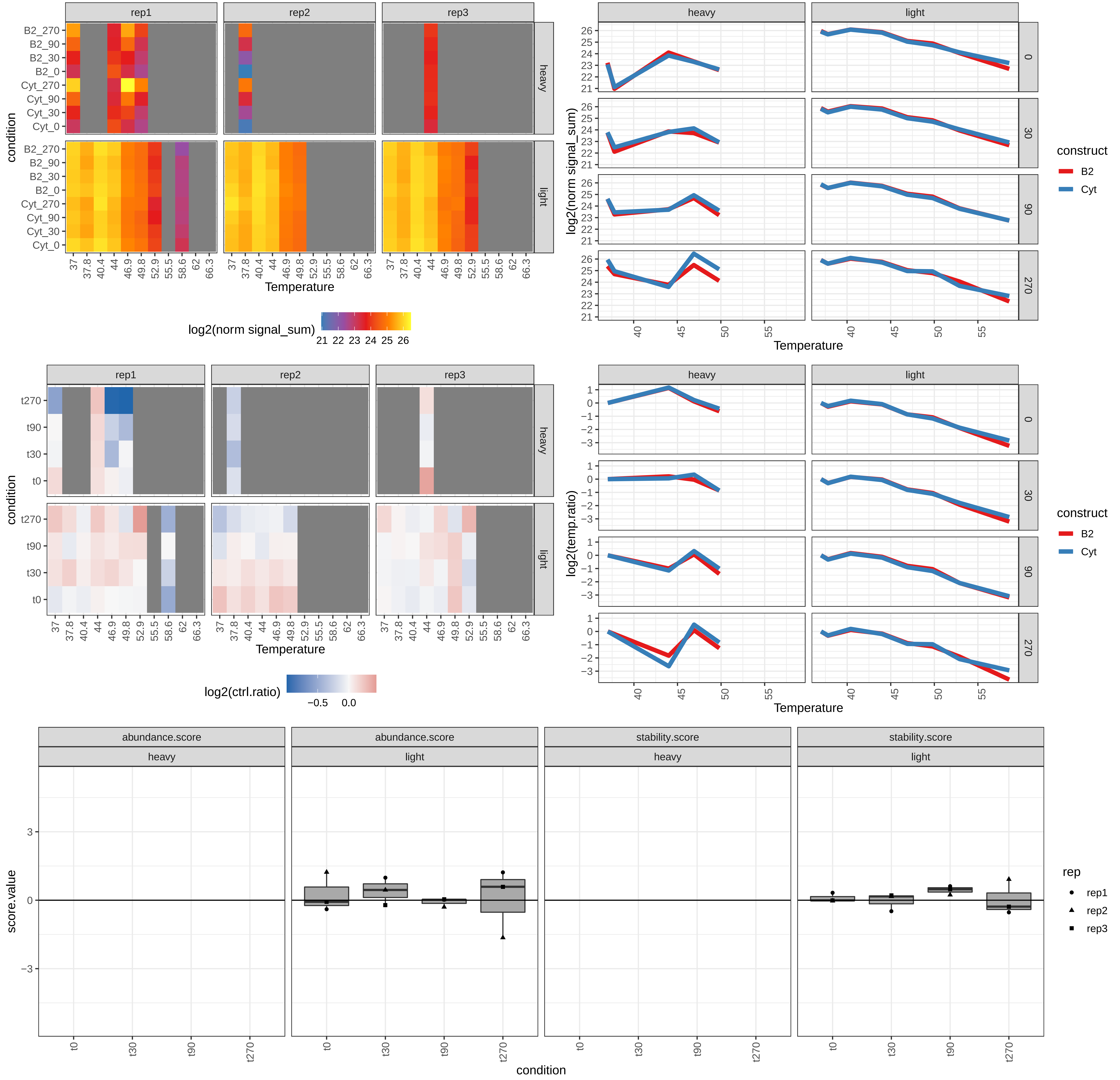

P40081 − Putative magnesium−dependent phosphatase YER134C OS=Saccharomyces cerevisiae (strain ATCC 204508 / S288c) GN=YER134C PE=1 SV=1

YER152C − average.top3: 6.62863 − max.qupm: 5

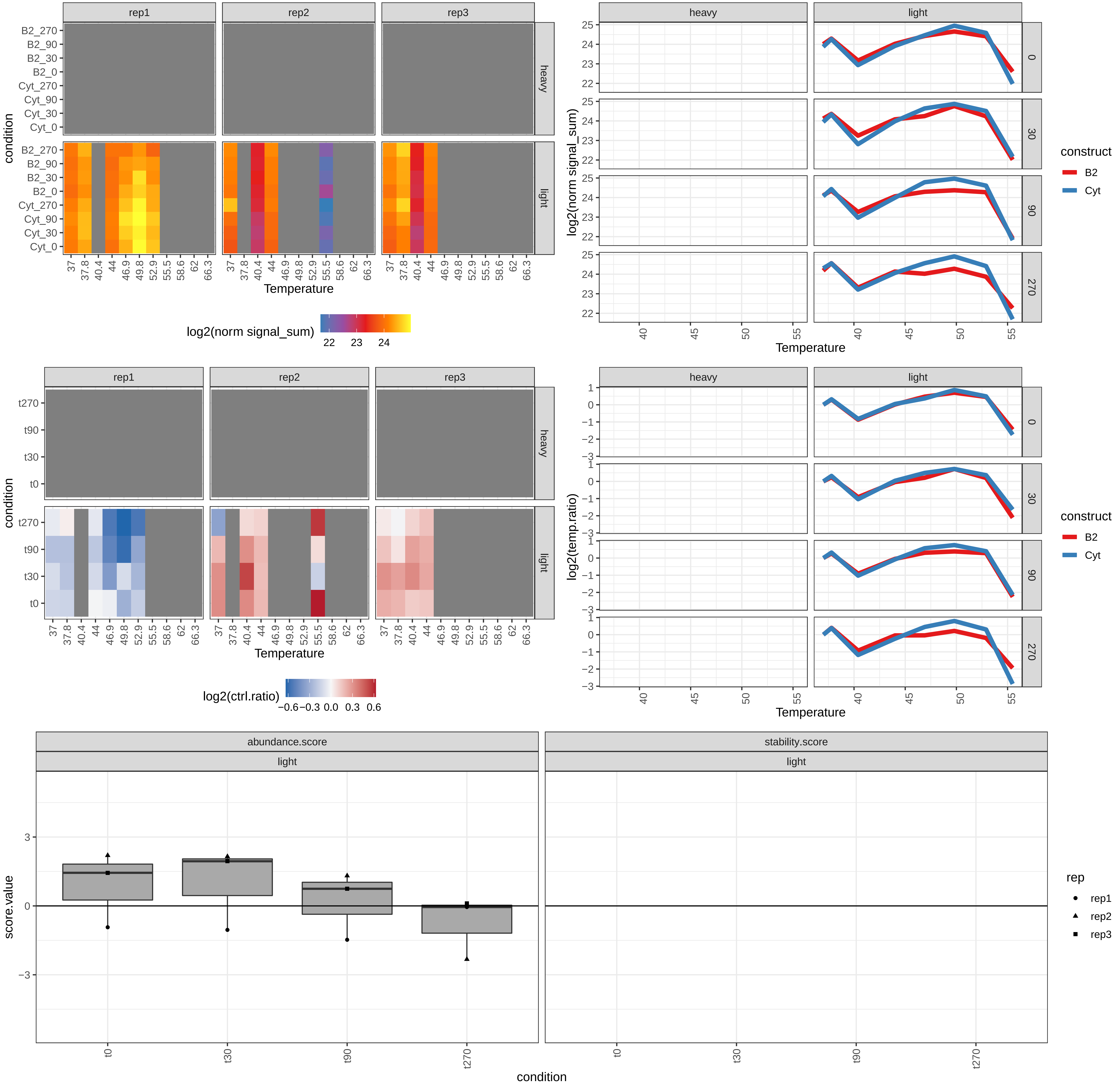

P10356 − Uncharacterized protein YER152C OS=Saccharomyces cerevisiae (strain ATCC 204508 / S288c) GN=YER152C PE=1 SV=2

YER156C − average.top3: 7.18865363636364 − max.qupm: 12

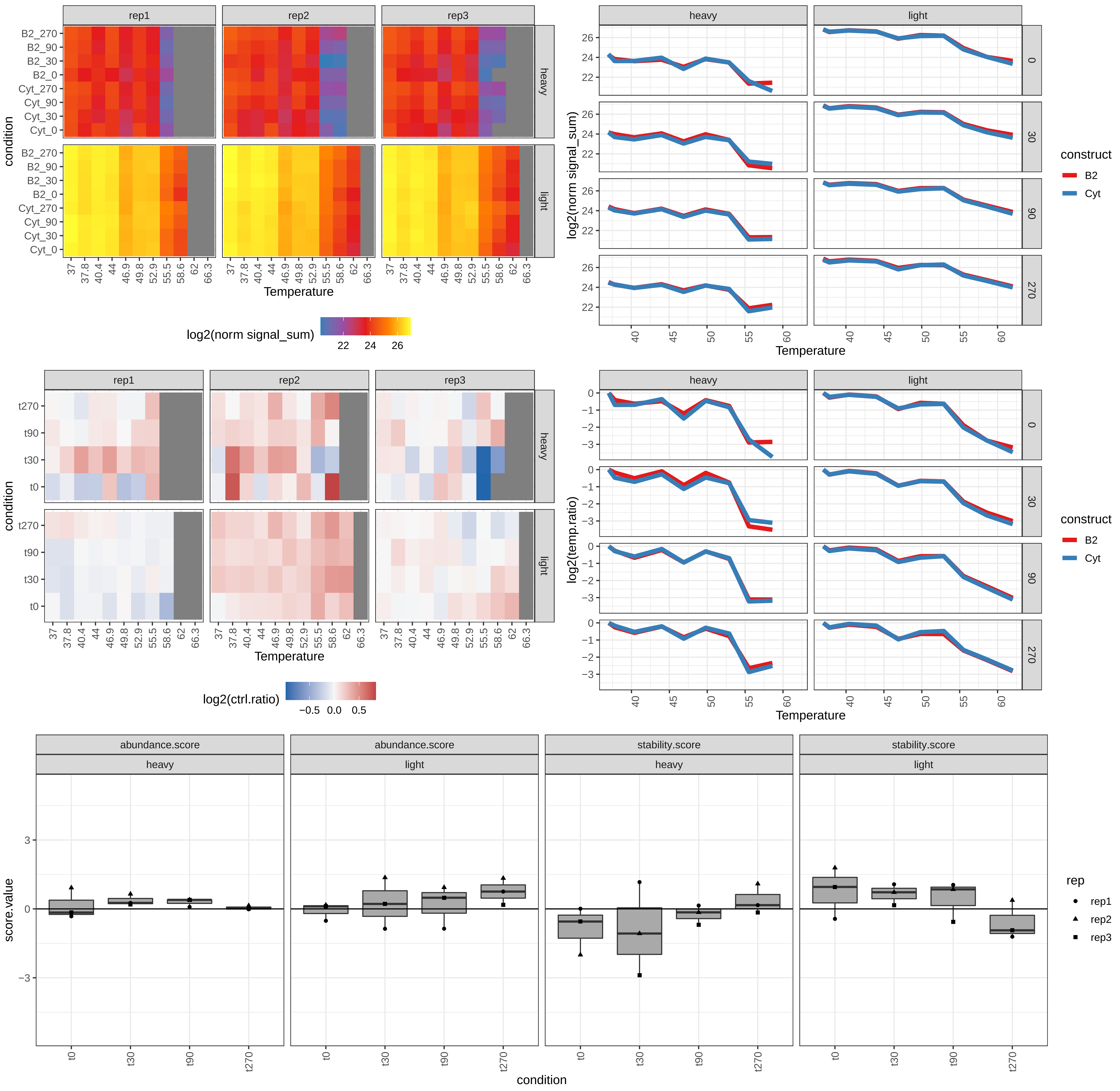

P40093 − UPF0160 protein YER156C OS=Saccharomyces cerevisiae (strain ATCC 204508 / S288c) GN=YER156C PE=1 SV=1

YET1 − average.top3: 7.24204483333333 − max.qupm: 8

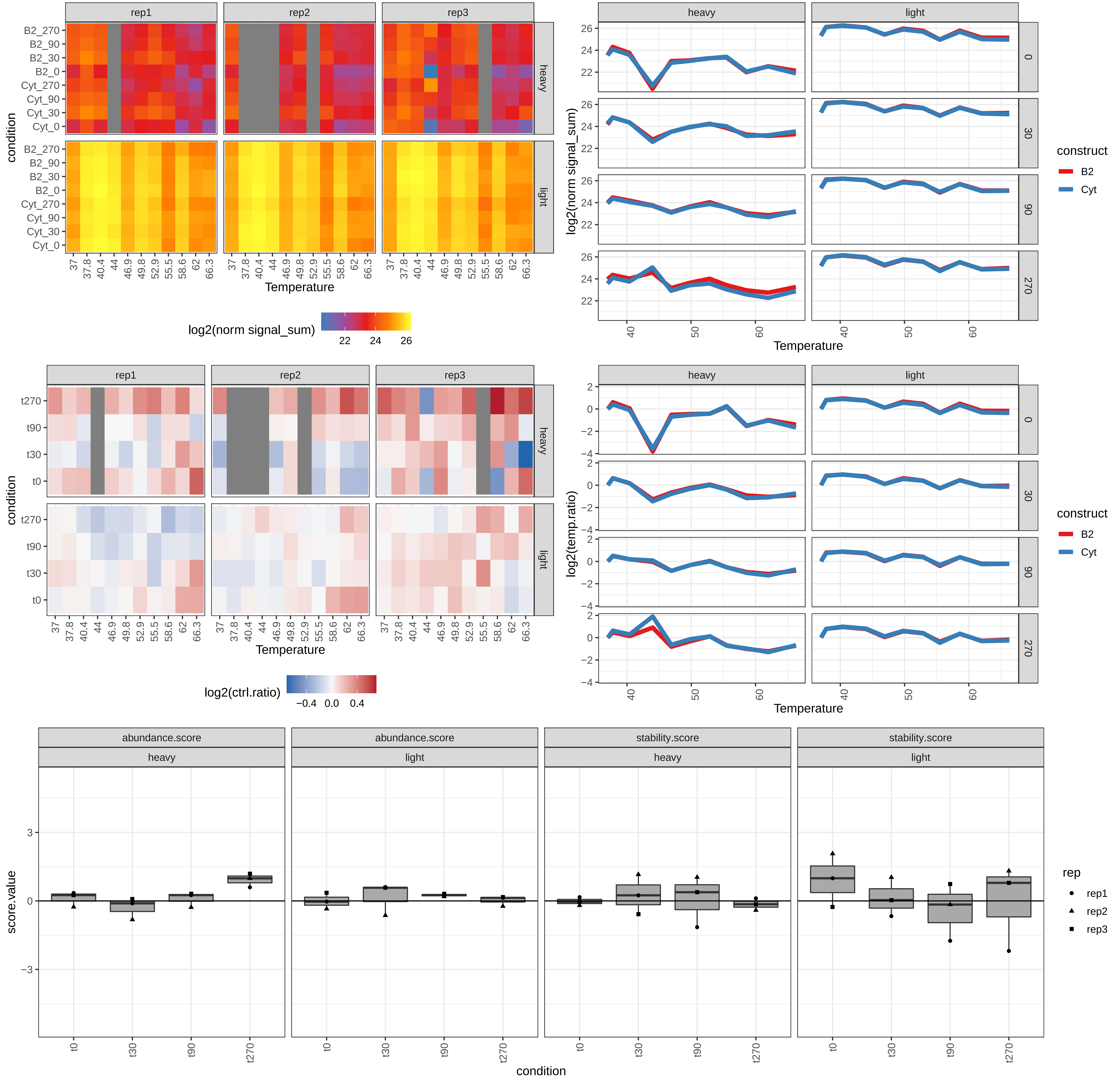

P35723 − Endoplasmic reticulum transmembrane protein 1 OS=Saccharomyces cerevisiae (strain ATCC 204508 / S288c) GN=YET1 PE=1 SV=2

YET3 − average.top3: 6.971535 − max.qupm: 3

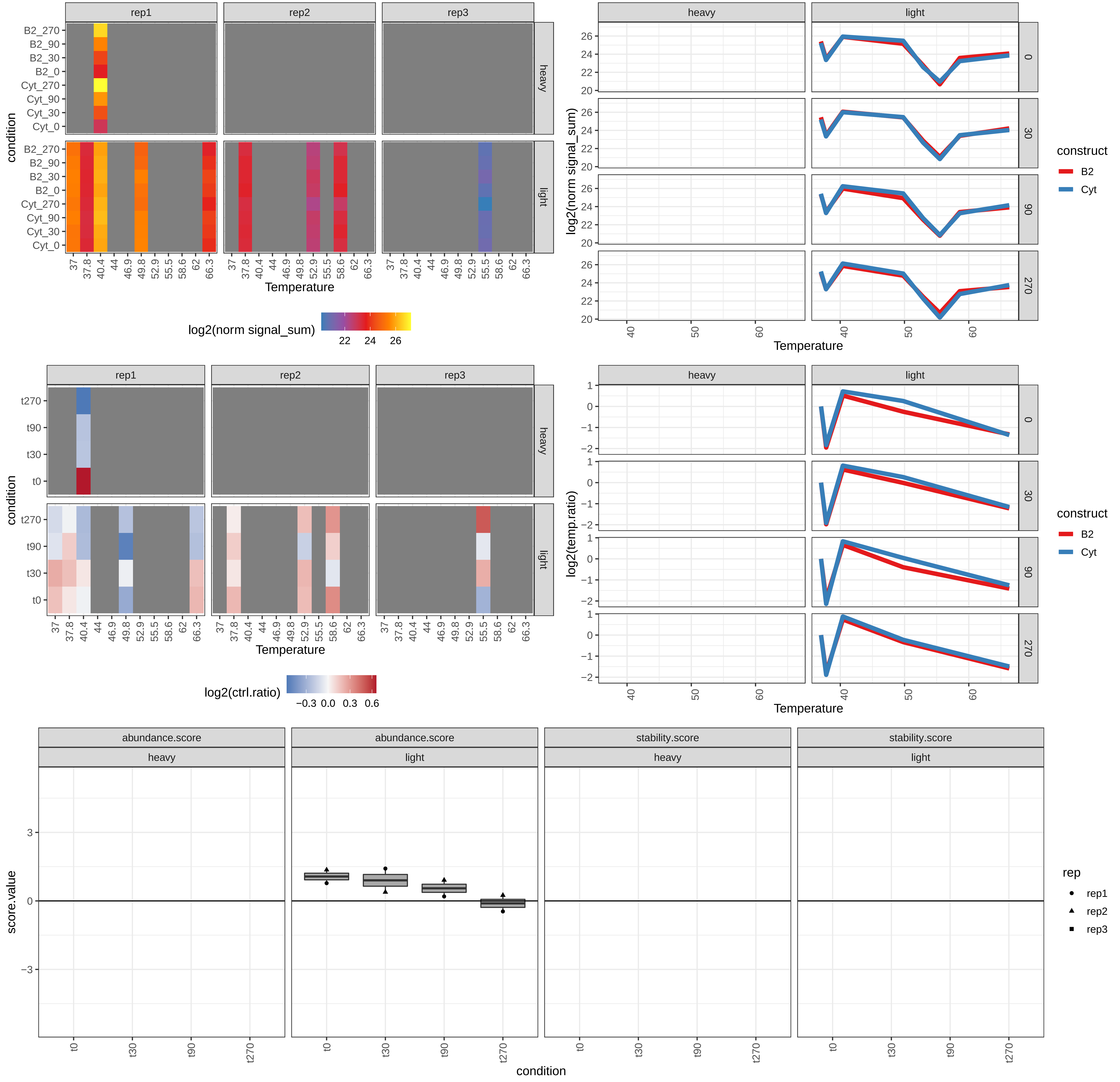

Q07451 − Endoplasmic reticulum transmembrane protein 3 OS=Saccharomyces cerevisiae (strain ATCC 204508 / S288c) GN=YET3 PE=1 SV=1

YFH7 − average.top3: 6.542558125 − max.qupm: 4

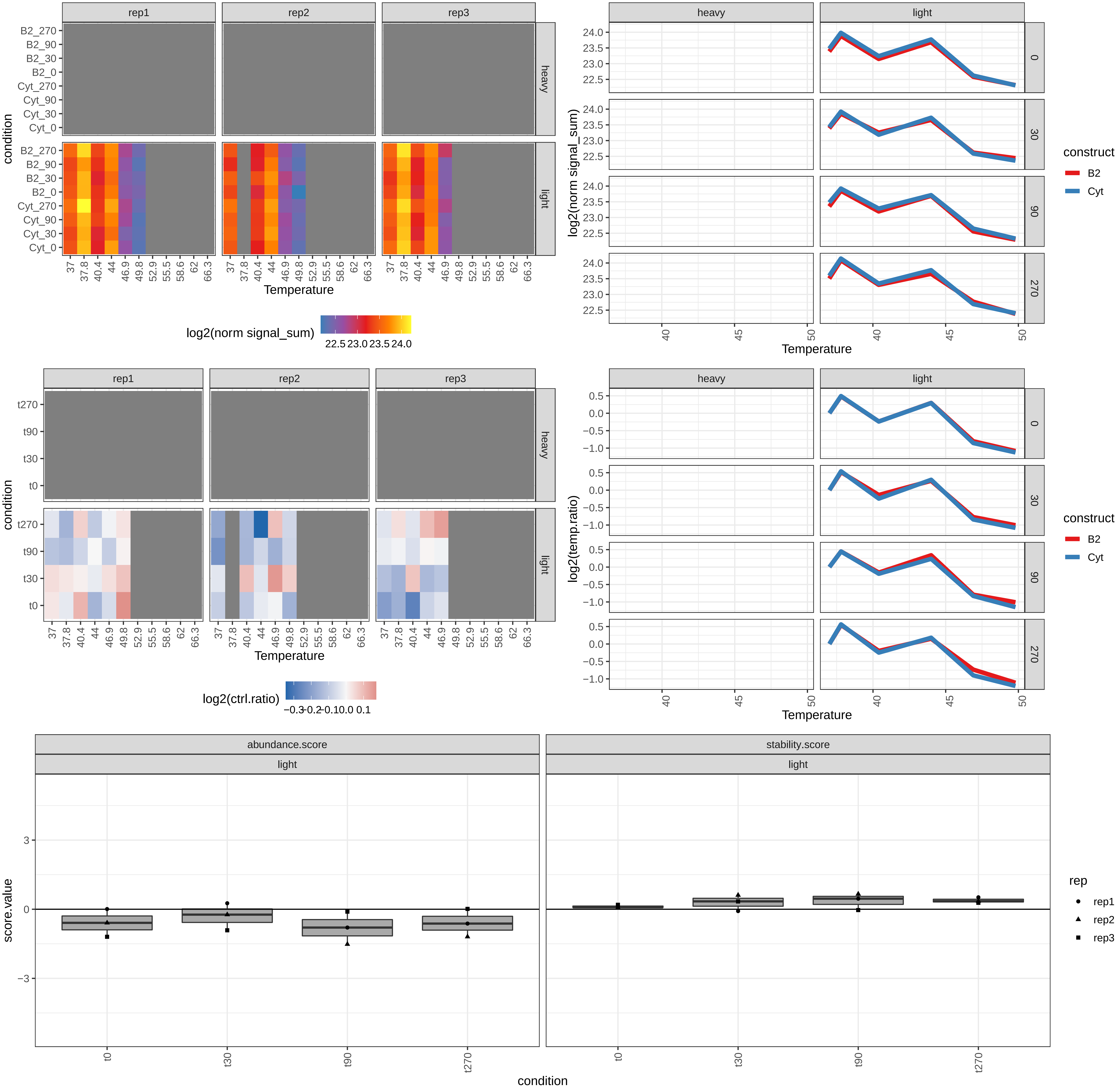

P43591 − ATP−dependent kinase YFH7 OS=Saccharomyces cerevisiae (strain ATCC 204508 / S288c) GN=YFH7 PE=1 SV=1

YFR006W − average.top3: 7.23518092307692 − max.qupm: 15

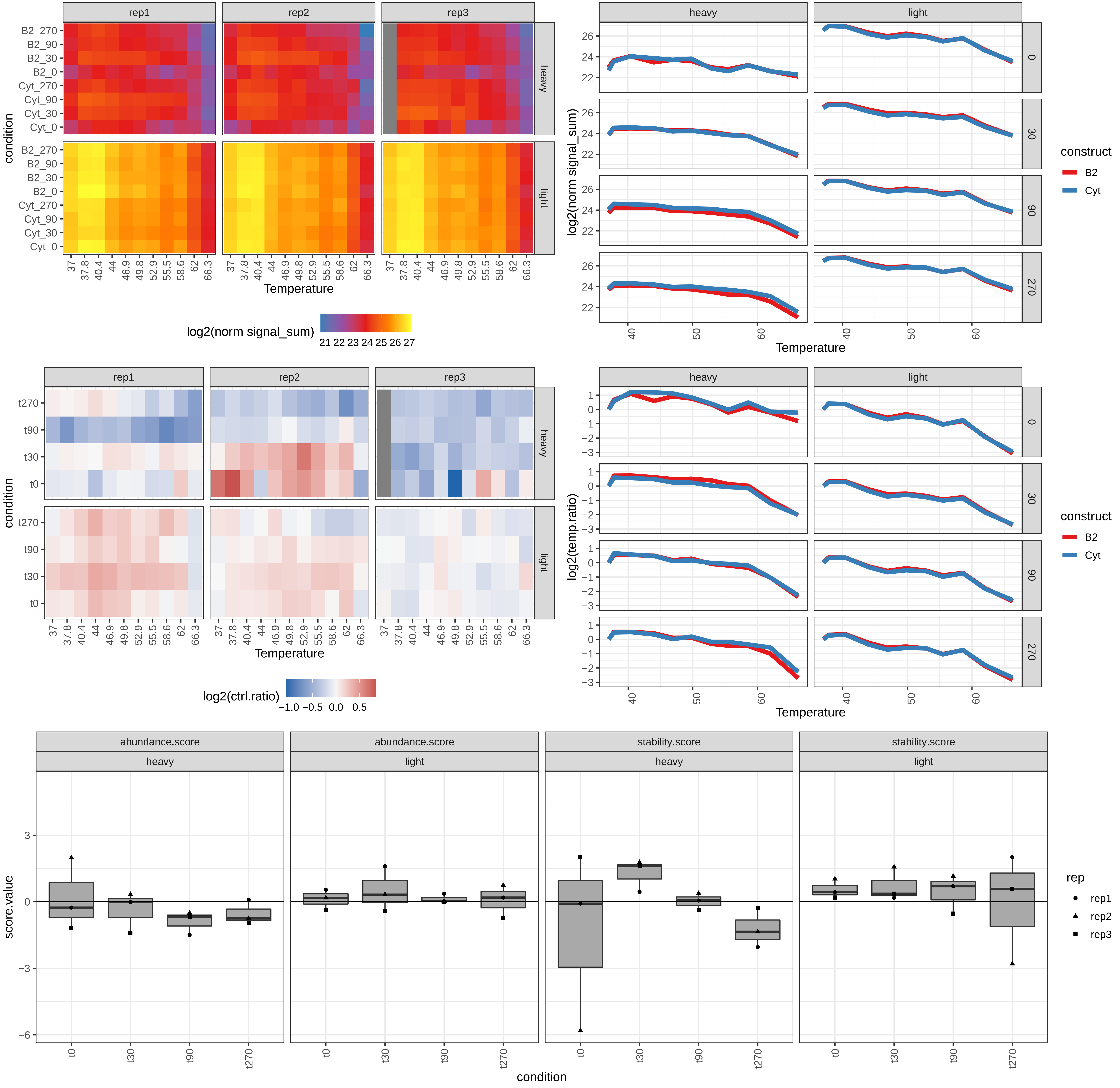

P43590 − Uncharacterized peptidase YFR006W OS=Saccharomyces cerevisiae (strain ATCC 204508 / S288c) GN=YFR006W PE=1 SV=1

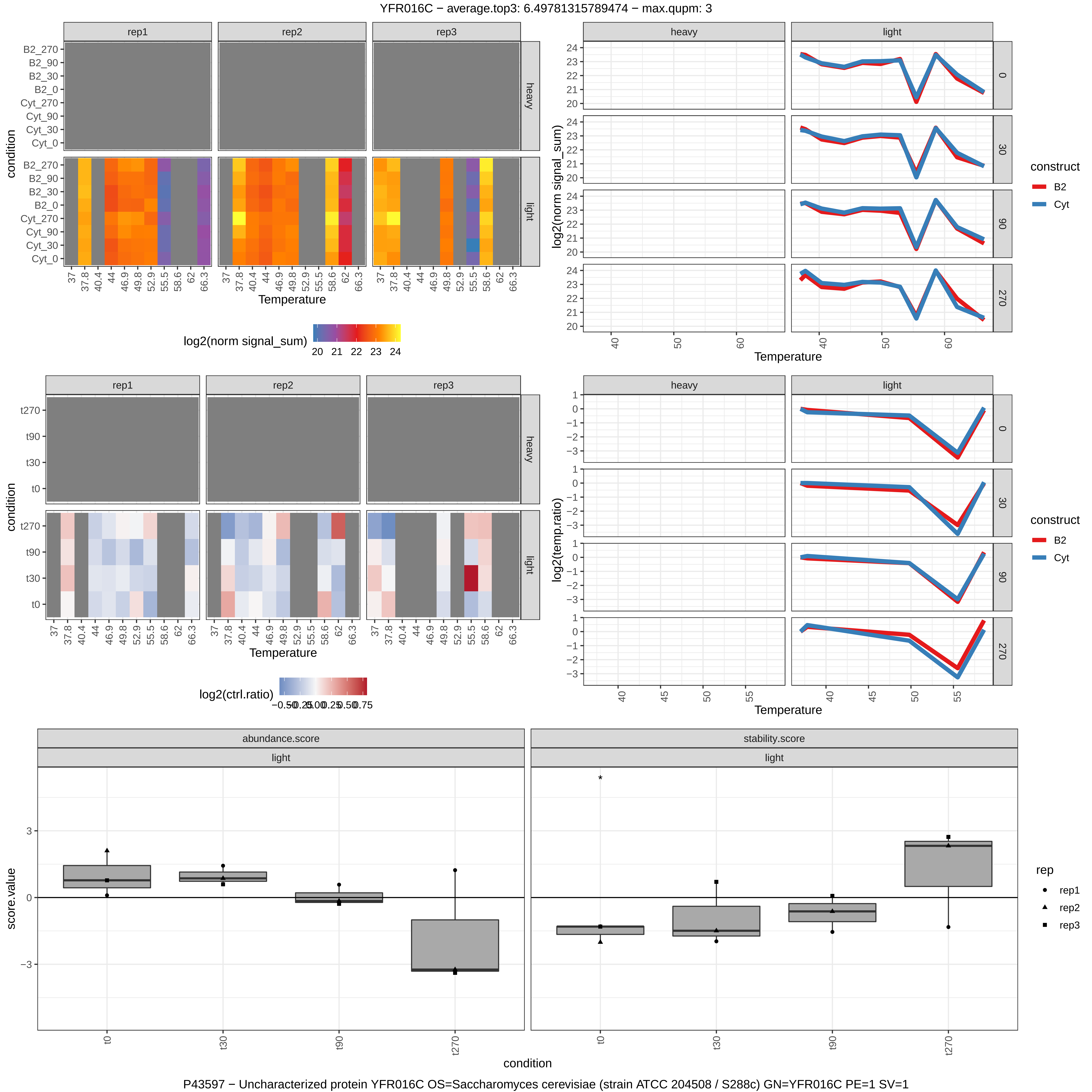
YFR016C − average.top3: 6.49781315789474 − max.qupm: 3
rep1
rep2
rep3
heavy
light
condition
Temperature
log2(norm signal_sum)
log2(ctrl.ratio)
log2(temp.ratio)
construct
B2
Cyt
abundance.score
stability.score
score.value
rep
rep1
rep2
rep3
P43597 − Uncharacterized protein YFR016C OS=Saccharomyces cerevisiae (strain ATCC 204508 / S288c) GN=YFR016C PE=1 SV=1

YFR045W − average.top3: 6.751746 − max.qupm: 3

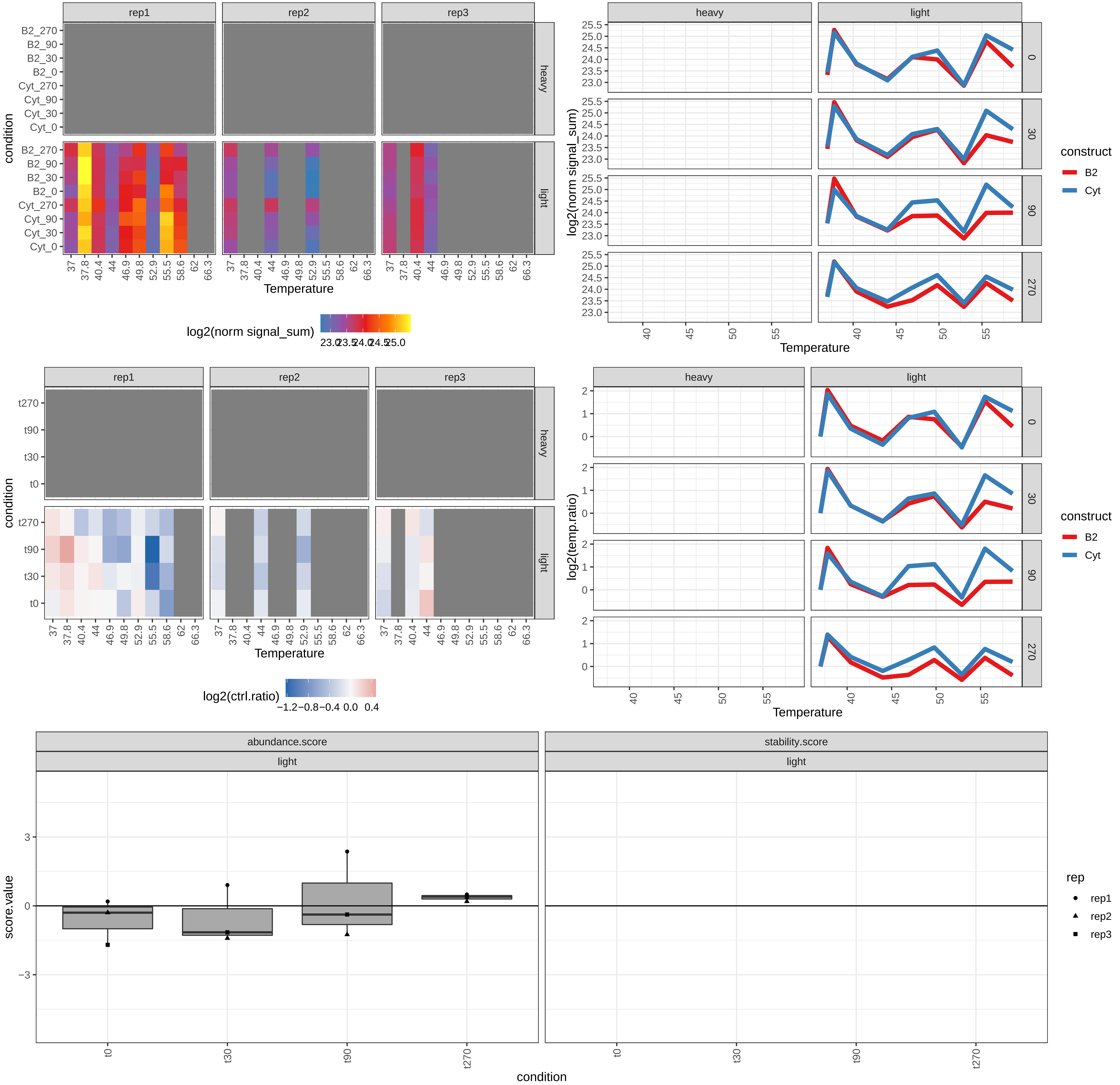

P43617 − Uncharacterized mitochondrial carrier YFR045W OS=Saccharomyces cerevisiae (strain ATCC 204508 / S288c) GN=YFR045W PE=3 SV=3

YGL039W − average.top3: 6.498871875 − max.qupm: 5

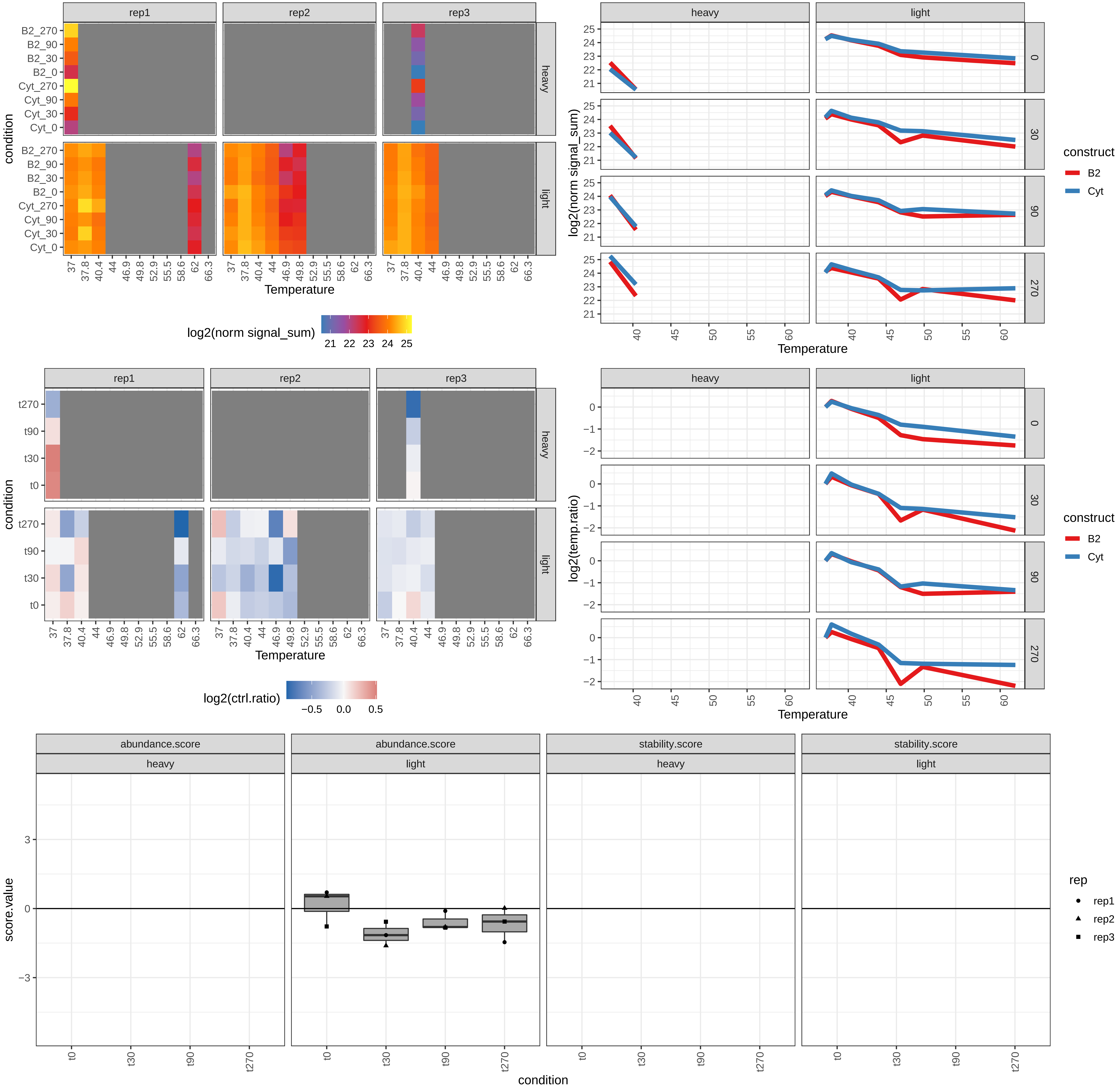

P53183 − Putative uncharacterized oxidoreductase YGL039W OS=Saccharomyces cerevisiae (strain ATCC 204508 / S288c) GN=YGL039W PE=1 SV=1

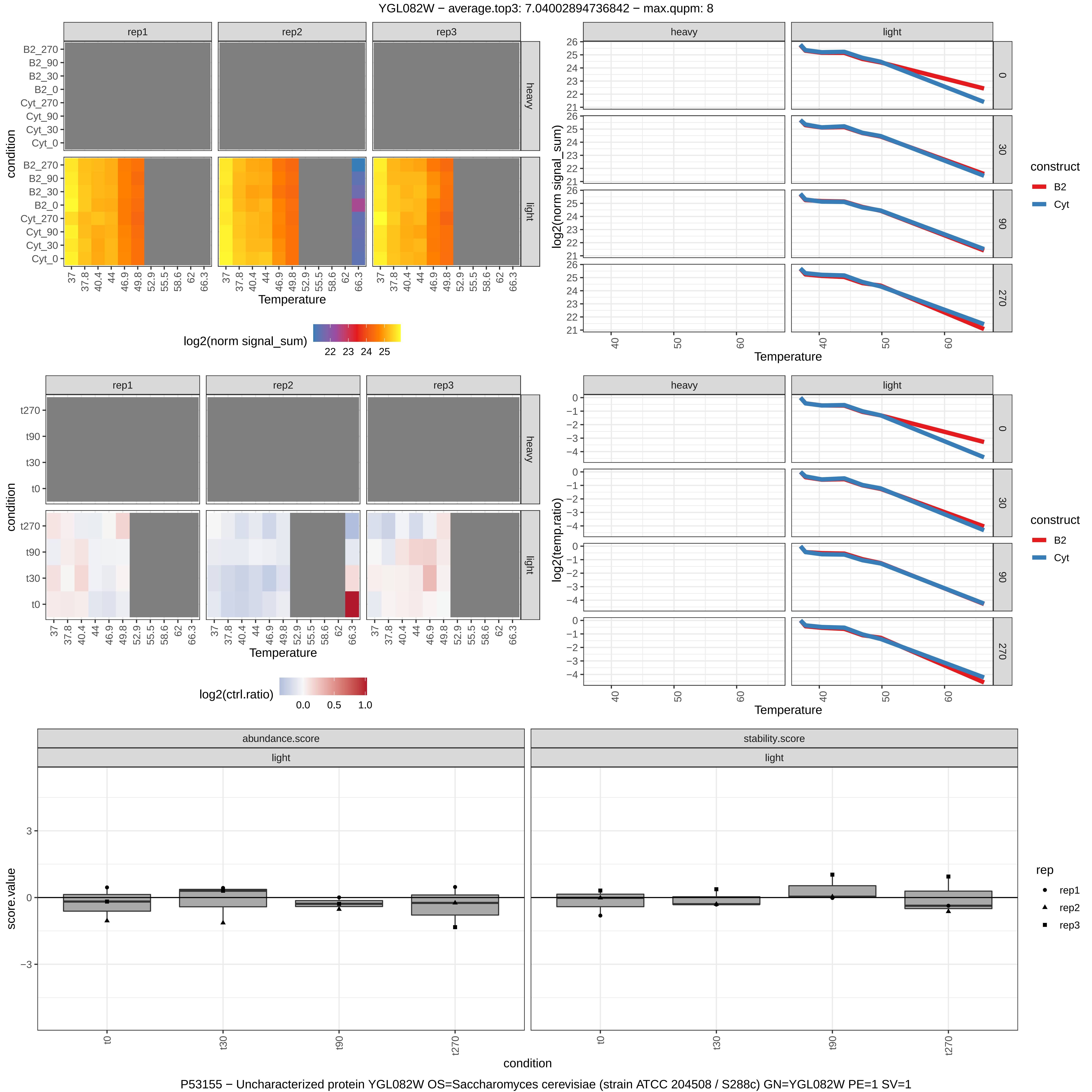

YGL082W − average.top3: 7.04002894736842 − max.qupm: 8
rep1
rep2
rep3
heavy
light
condition
Temperature
log2(norm signal_sum)
log2(temp.ratio)
log2(ctrl.ratio)
construct
B2
Cyt
abundance.score
stability.score
score.value
rep
rep1
rep2
rep3
P53155 − Uncharacterized protein YGL082W OS=Saccharomyces cerevisiae (strain ATCC 204508 / S288c) GN=YGL082W PE=1 SV=1

YGL101W − average.top3: 6.507429 − max.qupm: 3

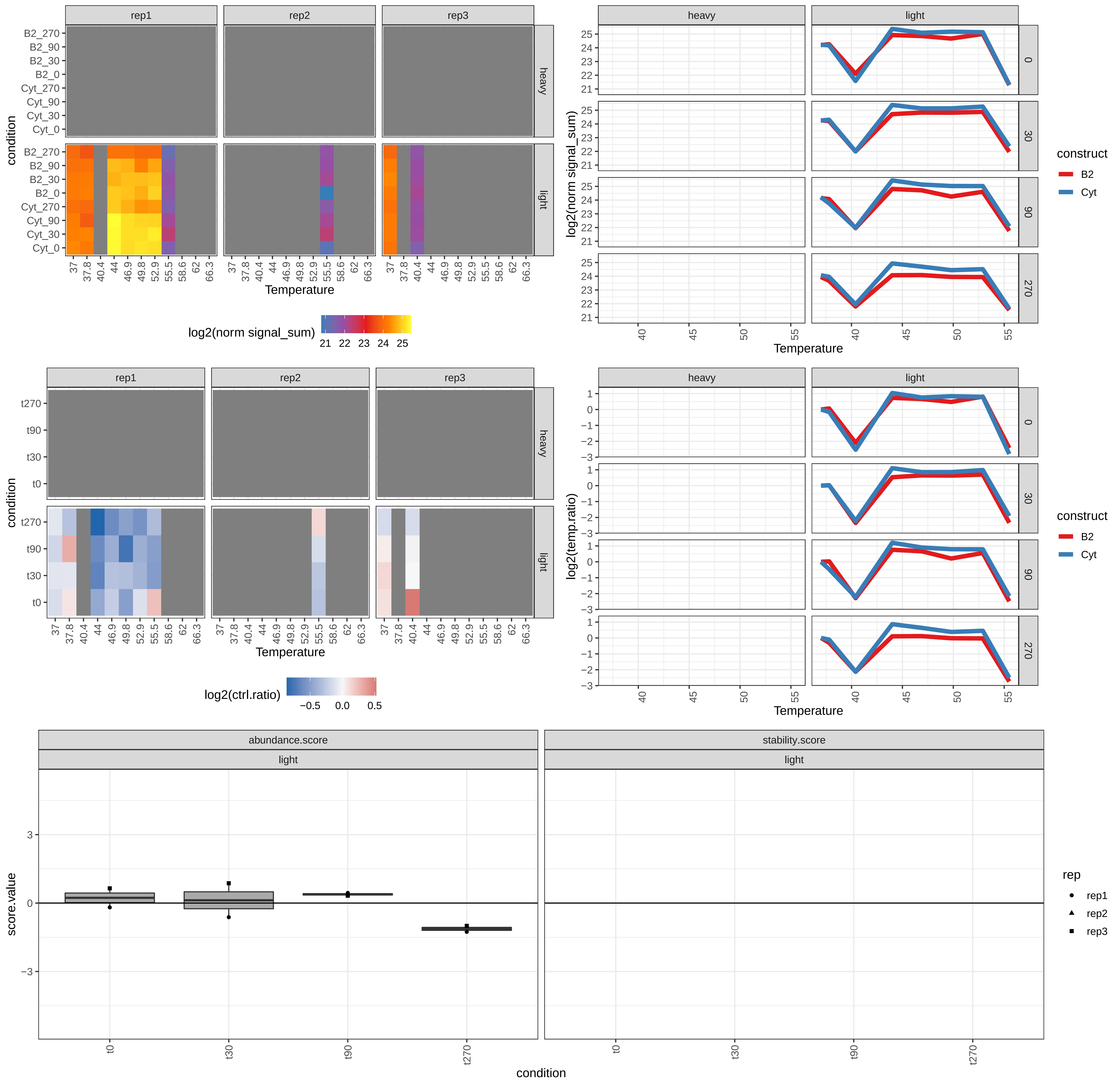

P53144 − HD domain−containing protein YGL101W OS=Saccharomyces cerevisiae (strain ATCC 204508 / S288c) GN=YGL101W PE=1 SV=1

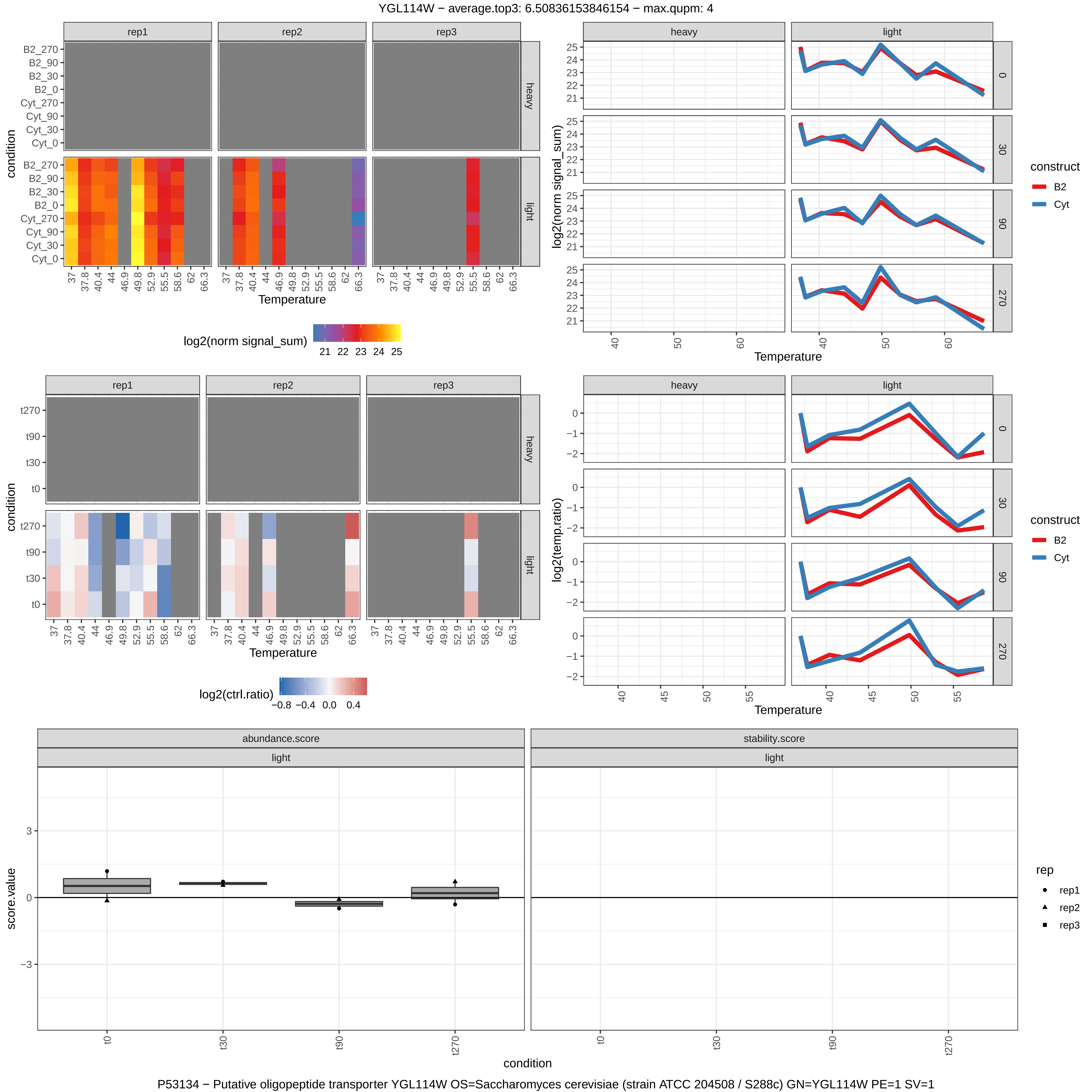

YGL114W − average.top3: 6.50836153846154 − max.qupm: 4
rep1
rep2
rep3
heavy
light
condition
Temperature
log2(norm signal_sum)
construct
B2
Cyt
log2(temp.ratio)
log2(ctrl.ratio)
abundance.score
stability.score
score.value
rep
P53134 − Putative oligopeptide transporter YGL114W OS=Saccharomyces cerevisiae (strain ATCC 204508 / S288c) GN=YGL114W PE=1 SV=1

YGL117W − average.top3: 6.57713375 − max.qupm: 4

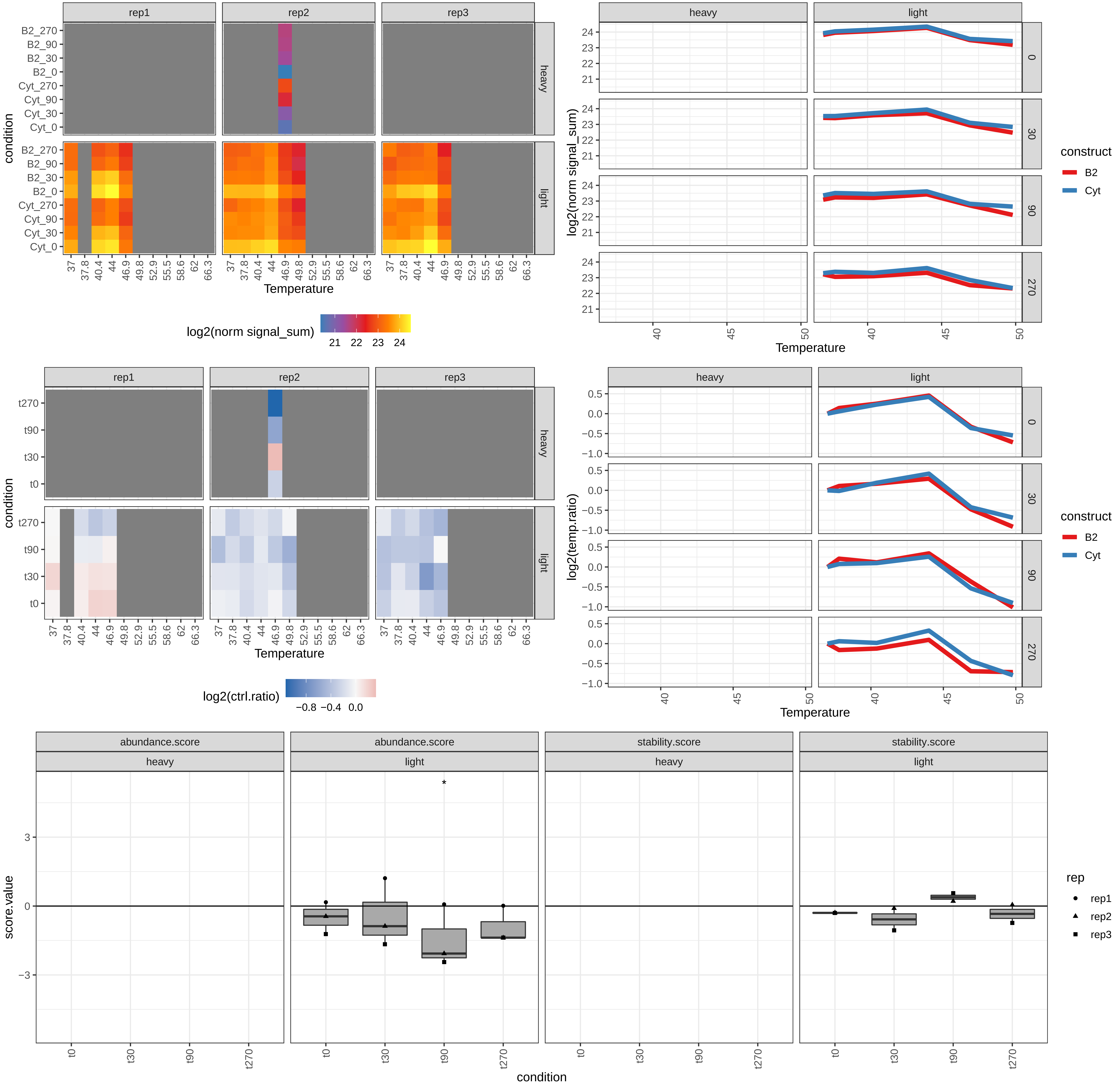

P53133 − Uncharacterized protein YGL117W OS=Saccharomyces cerevisiae (strain ATCC 204508 / S288c) GN=YGL117W PE=1 SV=1

YGL140C − average.top3: 6.43562214285714 − max.qupm: 3

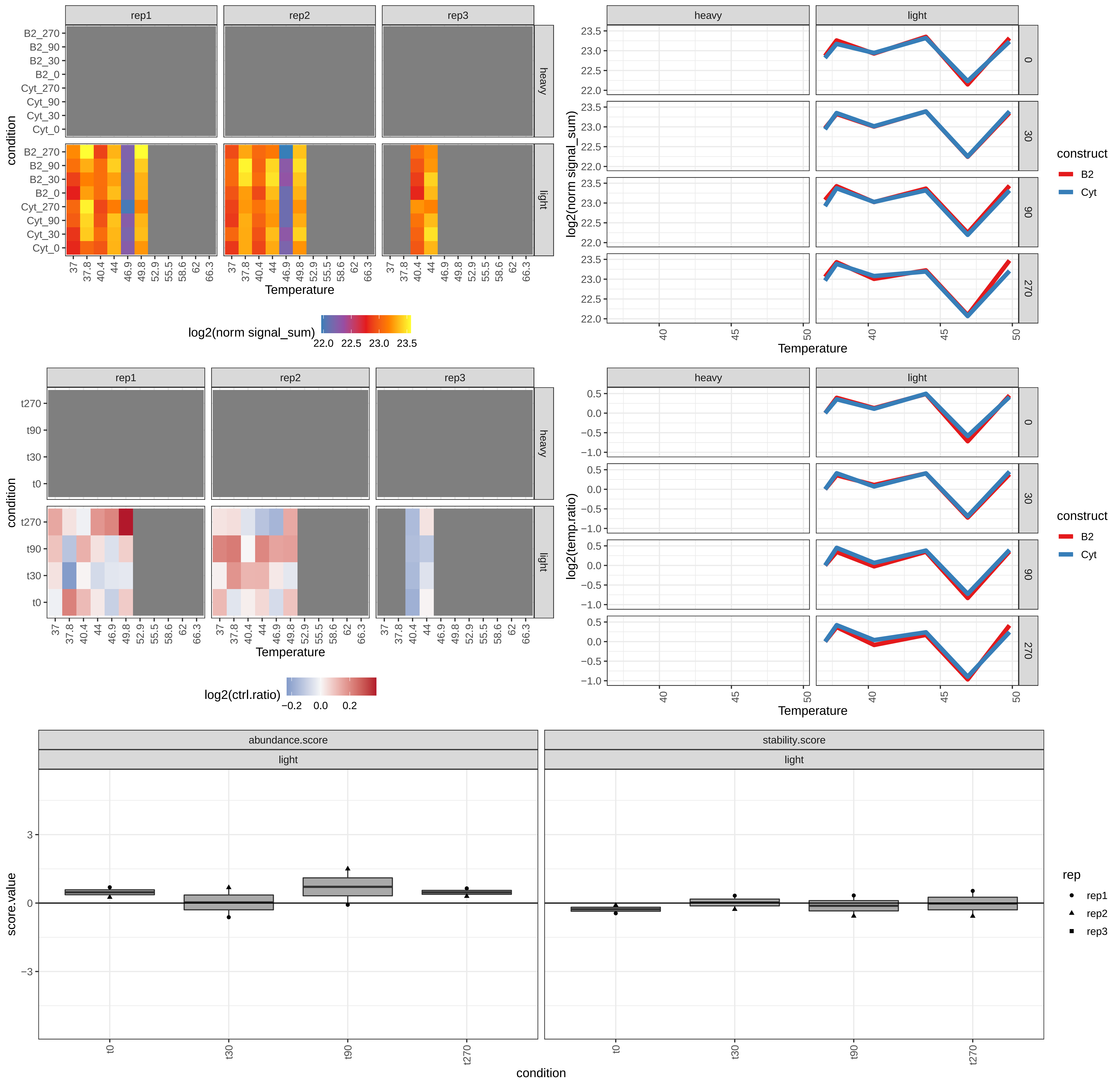

P53120 − Uncharacterized membrane protein YGL140C OS=Saccharomyces cerevisiae (strain ATCC 204508 / S288c) GN=YGL140C PE=1 SV=1

YGL159W − average.top3: 6.8389656097561 − max.qupm: 8

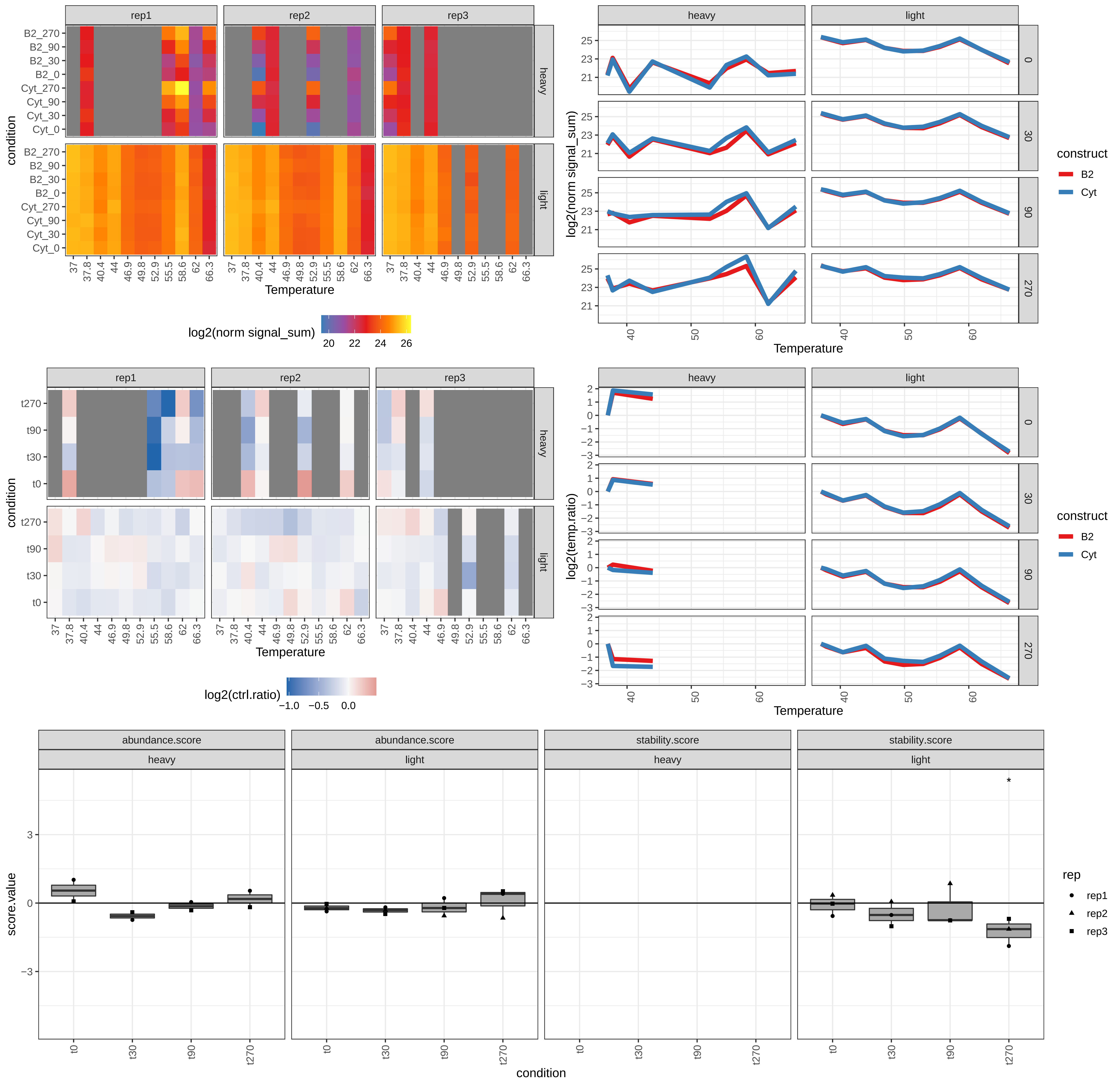

P53110 − Uncharacterized protein YGL159W OS=Saccharomyces cerevisiae (strain ATCC 204508 / S288c) GN=YGL159W PE=1 SV=1

YGL185C − average.top3: 6.69873136363636 − max.qupm: 8

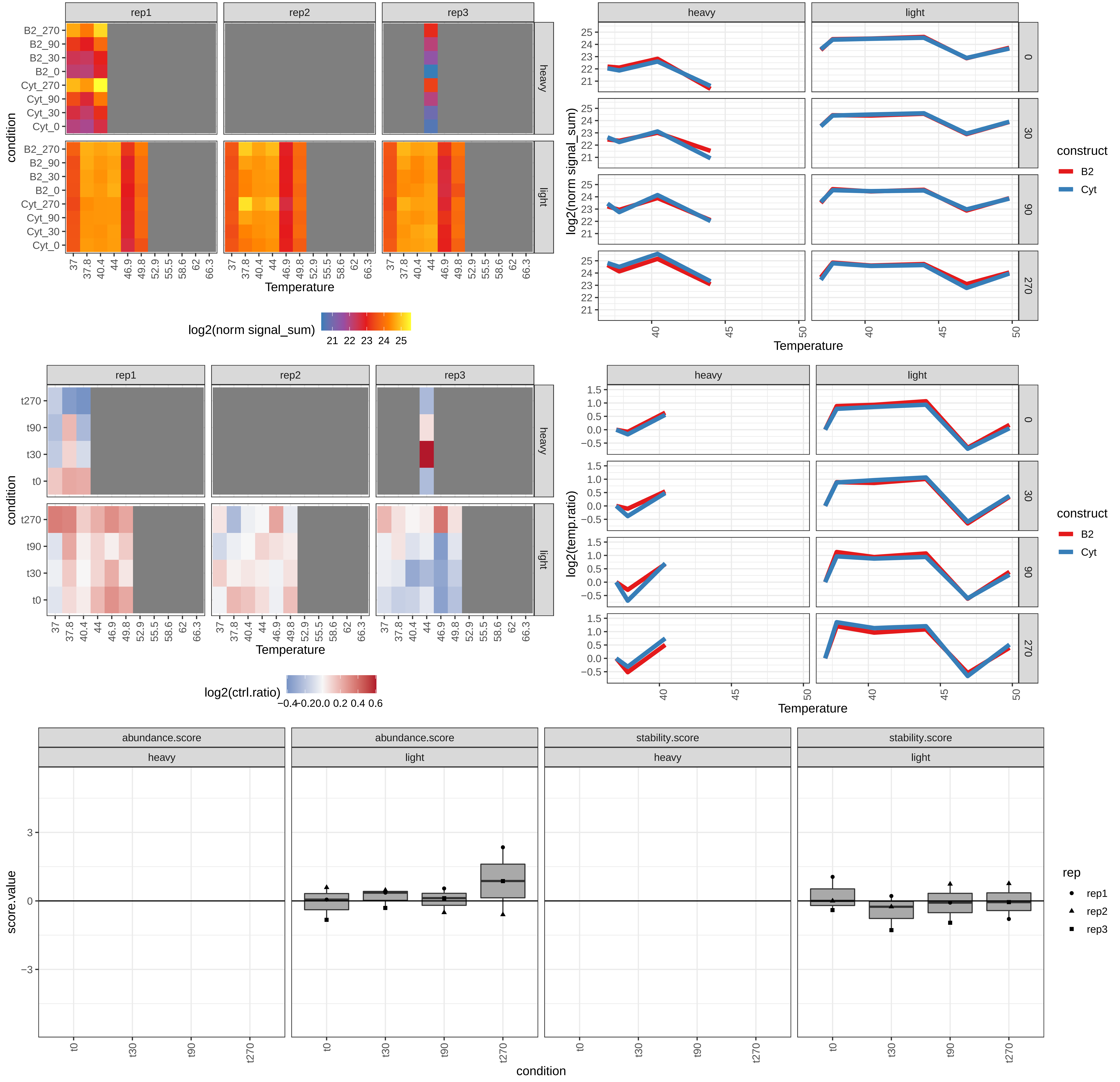

P53100 − Putative 2−hydroxyacid dehydrogenase YGL185C OS=Saccharomyces cerevisiae (strain ATCC 204508 / S288c) GN=YGL185C PE=1 SV=1

YGP1 − average.top3: 7.50762476190476 − max.qupm: 4

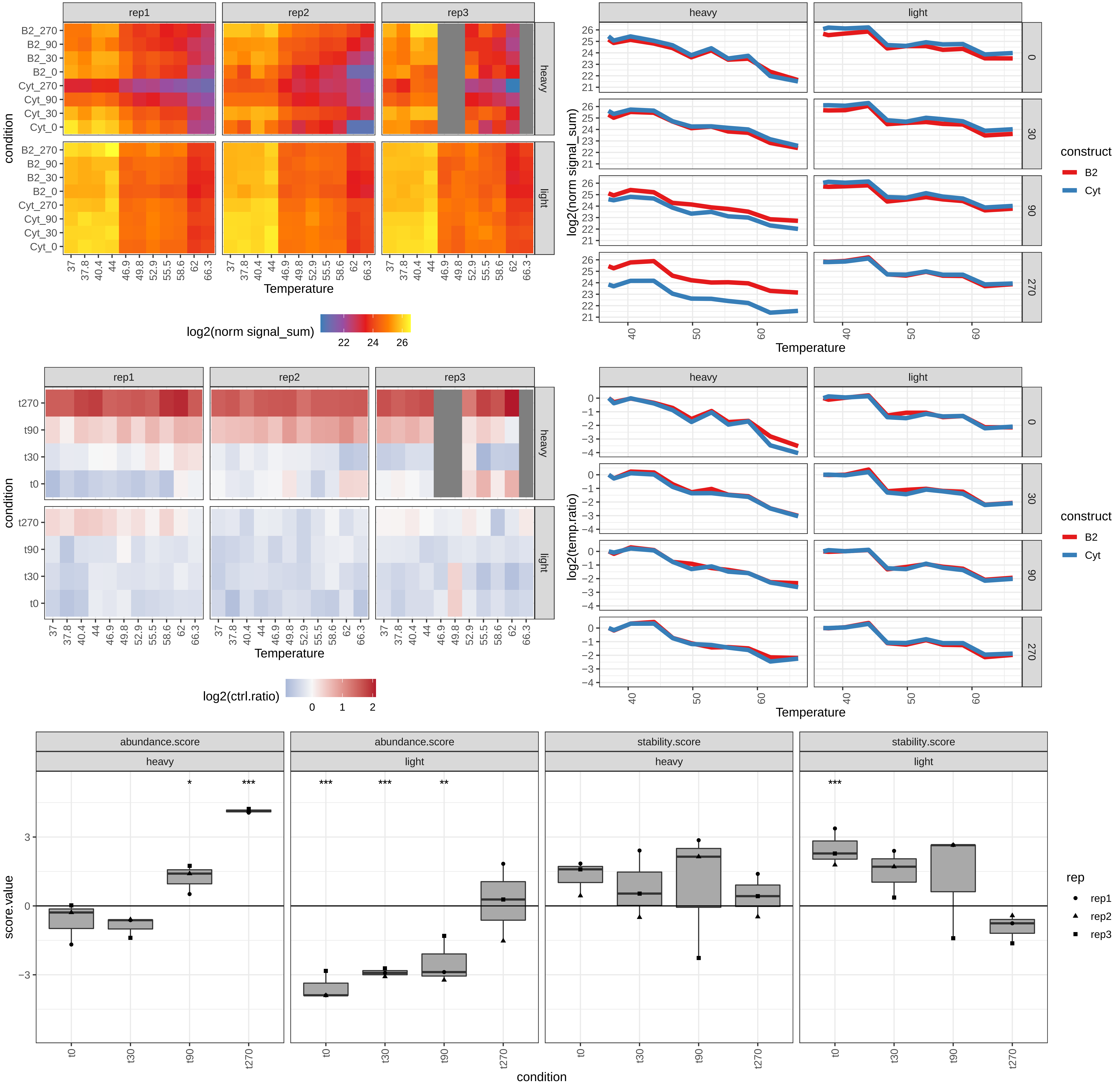

P38616 − Protein YGP1 OS=Saccharomyces cerevisiae (strain ATCC 204508 / S288c) GN=YGP1 PE=1 SV=2

YGR012W − average.top3: 6.97617148936 17 − max.qupm: 9

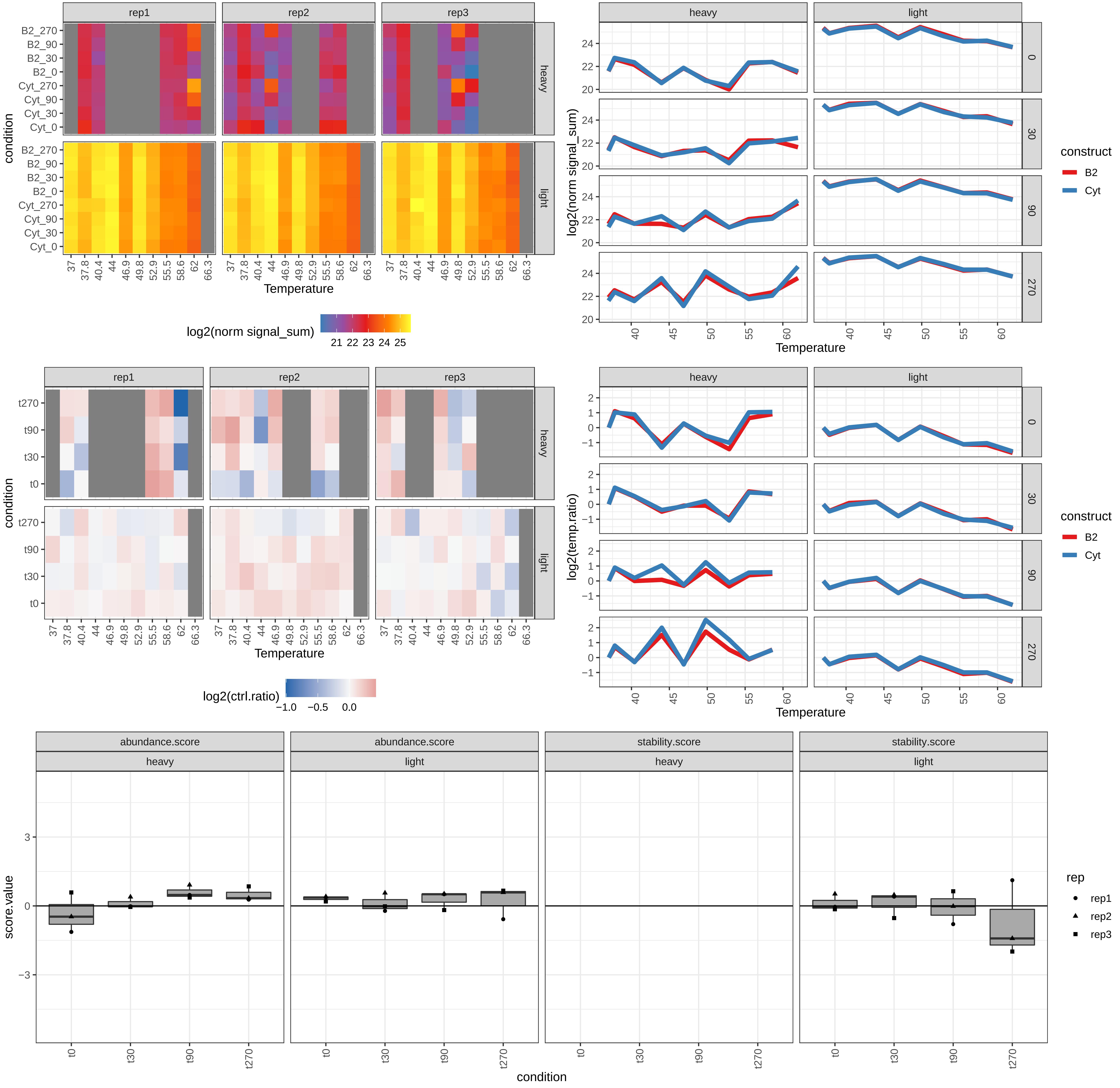

P53206 − Putative cysteine synthase OS=Saccharomyces cerevisiae (strain ATCC 204508 / S288c) GN=YGR012W PE=1 SV=1

YGR017W − average.top3: 6.46835857142857 − max.qupm: 4

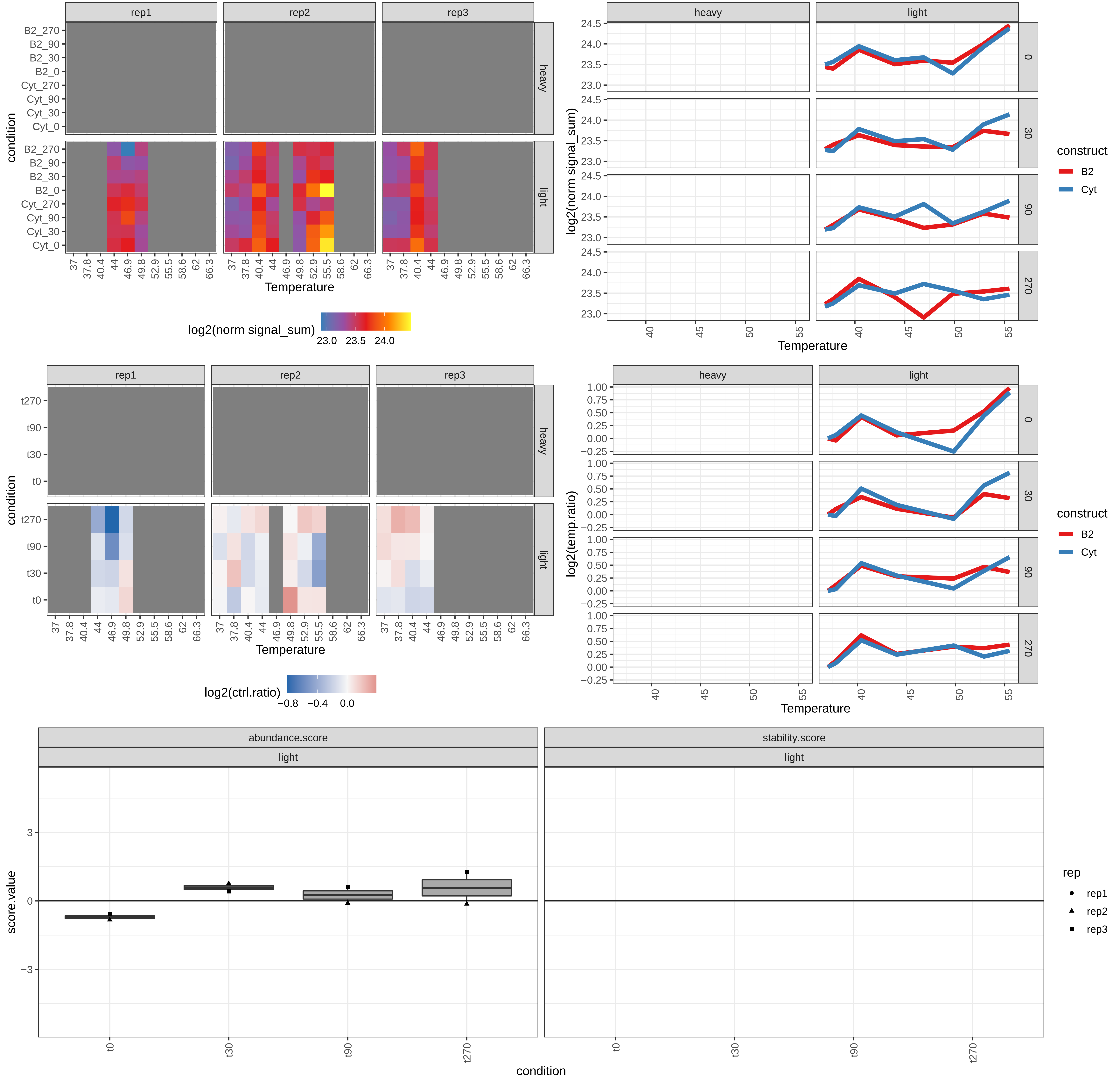

P53210 − Uncharacterized protein YGR017W OS=Saccharomyces cerevisiae (strain ATCC 204508 / S288c) GN=YGR017W PE=1 SV=1

YGR026W − average.top3: 7.04401810810811 − max.qupm: 6

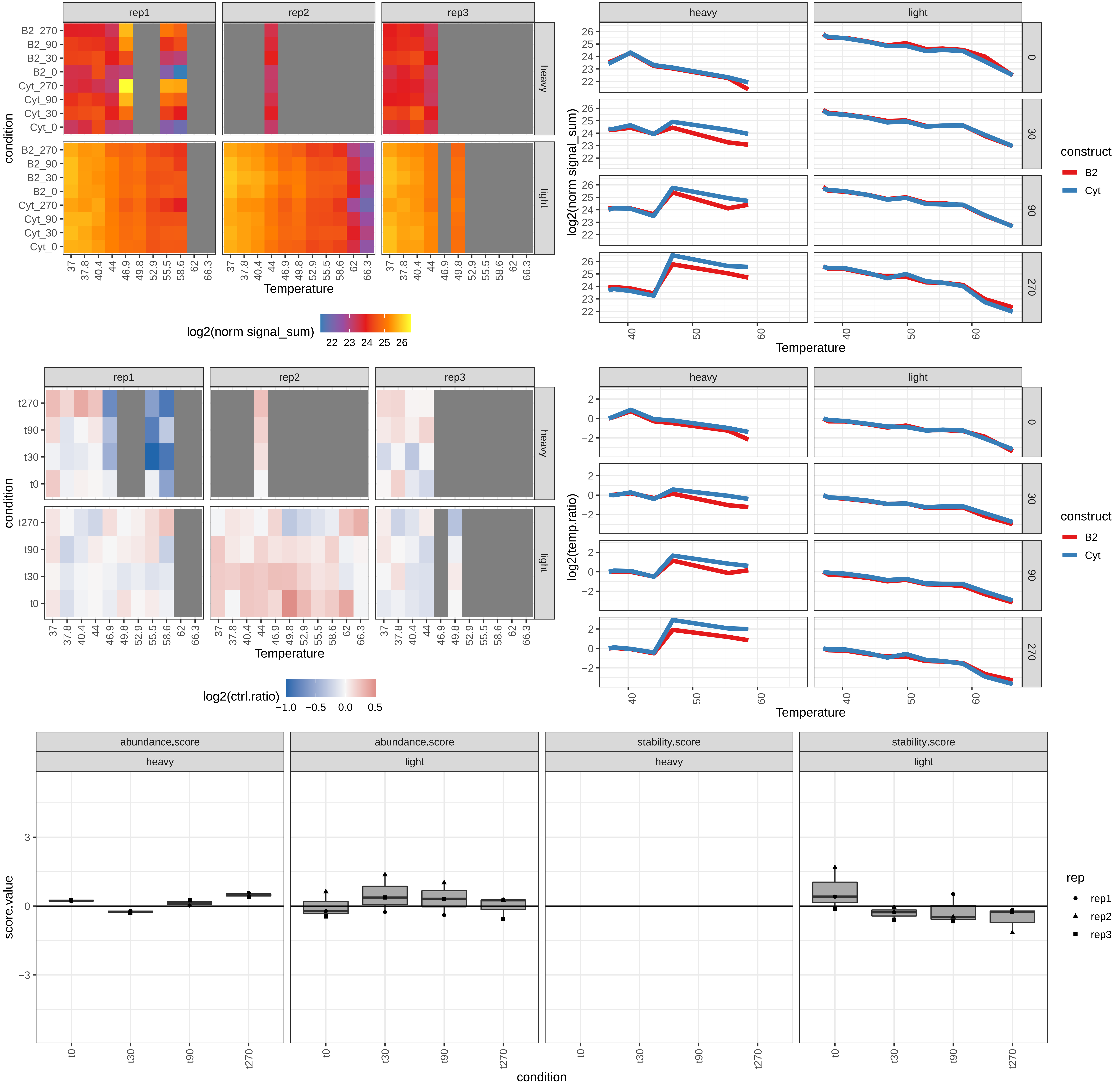

P53217 − Uncharacterized membrane protein YGR026W OS=Saccharomyces cerevisiae (strain ATCC 204508 / S288c) GN=YGR026W PE=1 SV=1

YGR054W − average.top3: 7.21076341463415 − max.qupm: 9

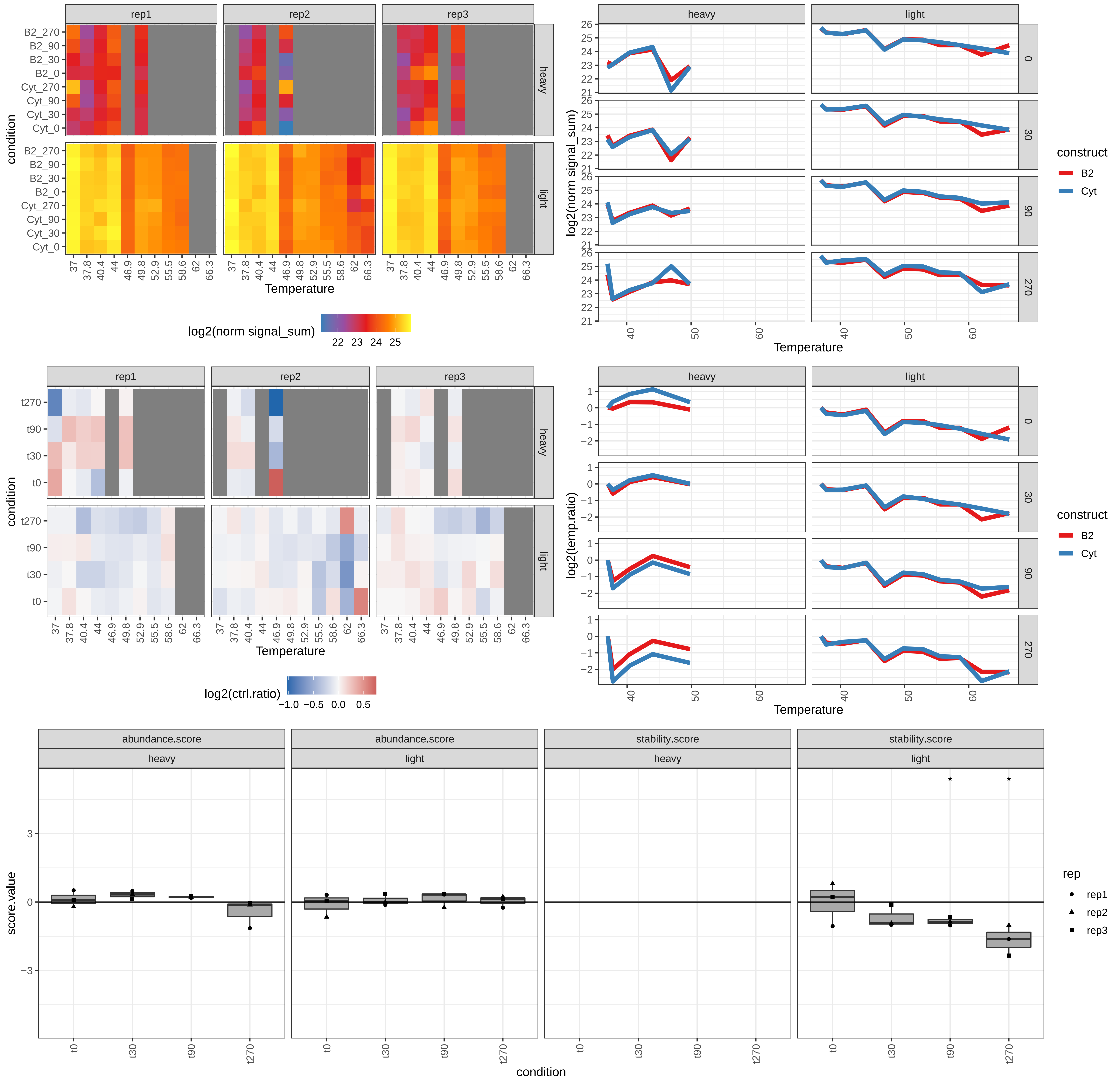

P53235 − Eukaryotic translation initiation factor 2A OS=Saccharomyces cerevisiae (strain ATCC 204508 / S288c) GN=YGR054W PE=1 SV=1

YGR111W − average.top3: 7.07374303030303 − max.qupm: 14

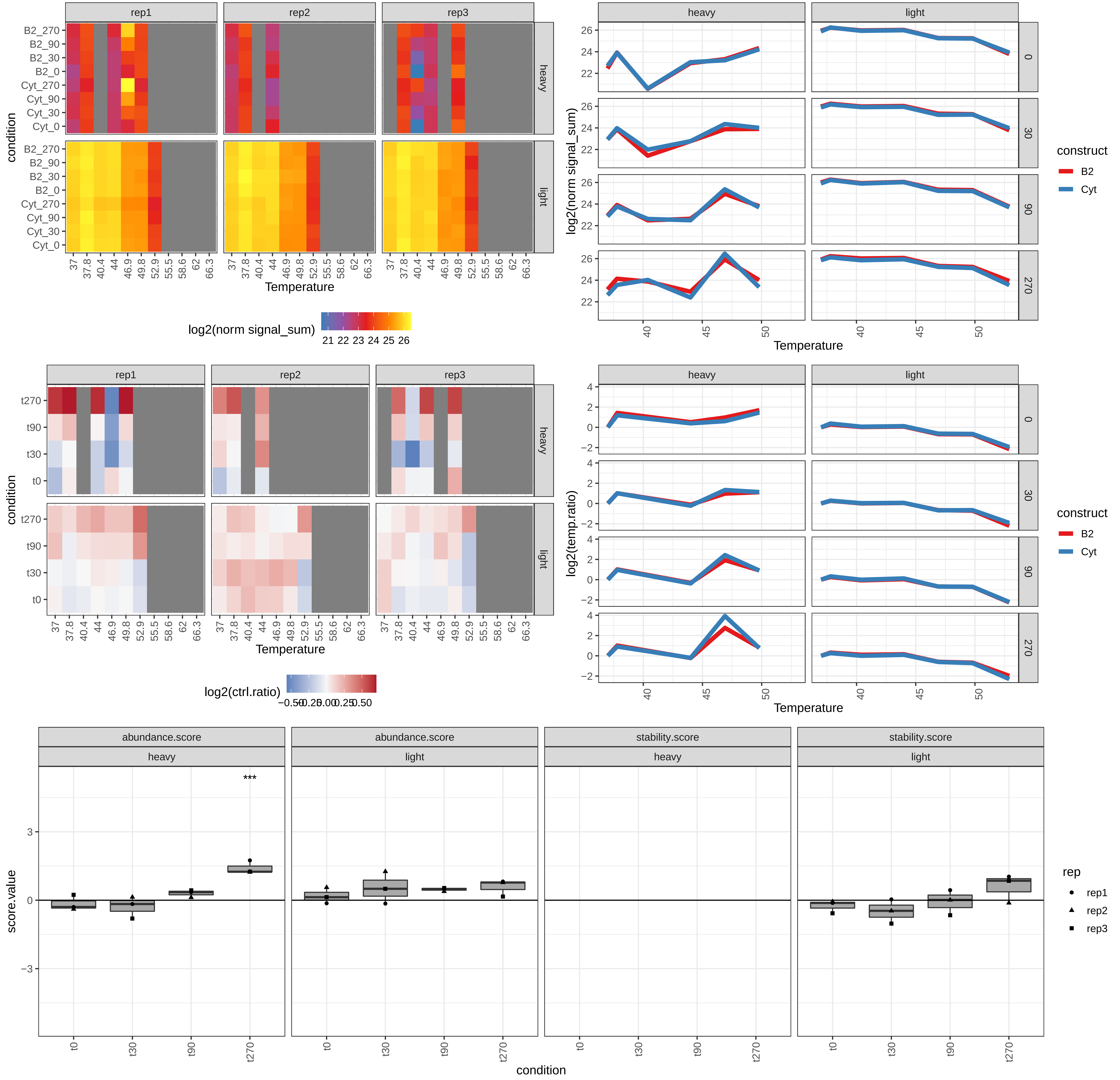

P53265 − Uncharacterized protein YGR111W OS=Saccharomyces cerevisiae (strain ATCC 204508 / S288c) GN=YGR111W PE=1 SV=1

YGR117C − average.top3: 6.75605666666667 − max.qupm: 5

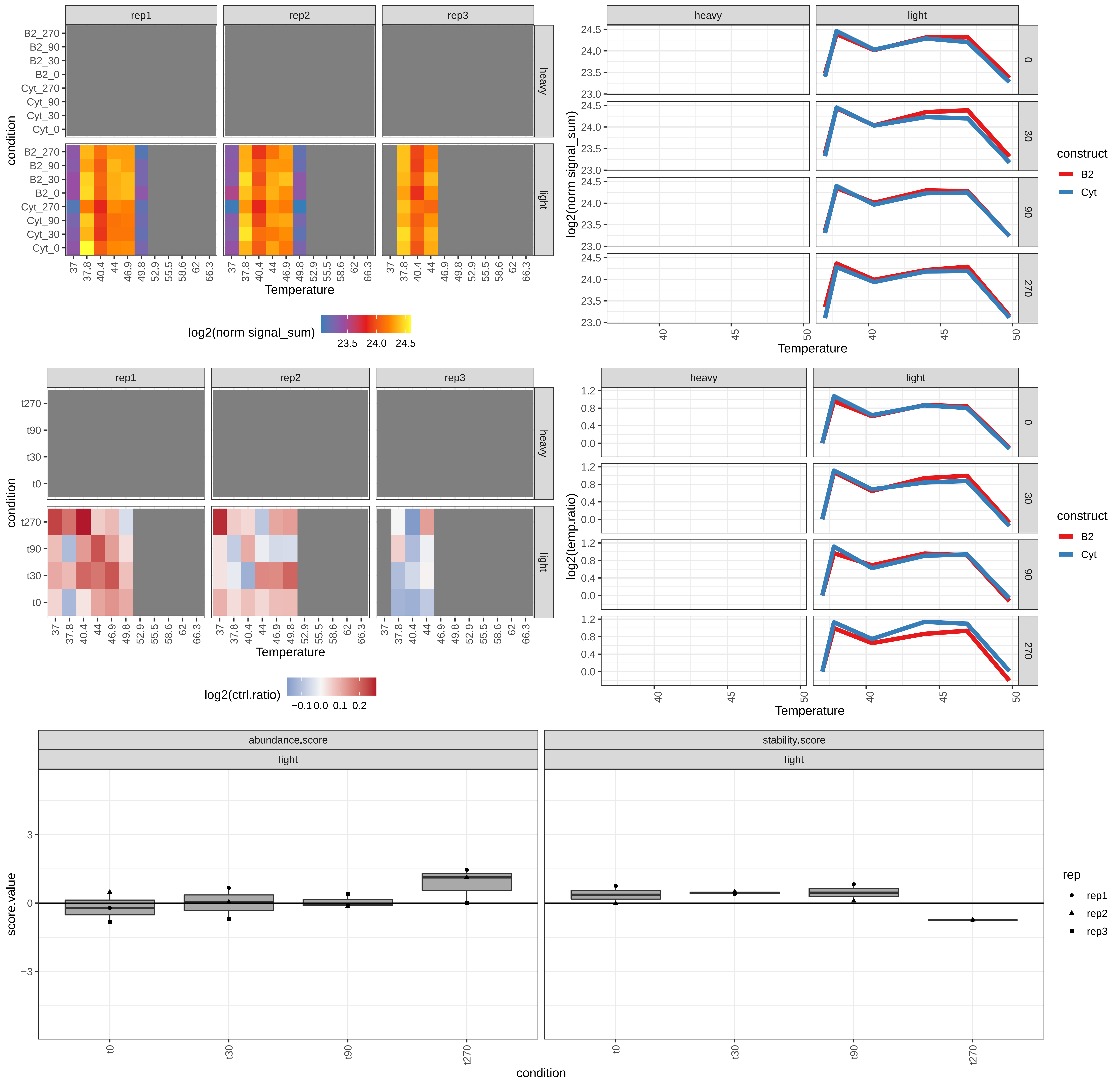

P53270 − Uncharacterized protein YGR117C OS=Saccharomyces cerevisiae (strain ATCC 204508 / S288c) GN=YGR117C PE=1 SV=1

YGR125W − average.top3: 6.68573166666667 − max.qupm: 5

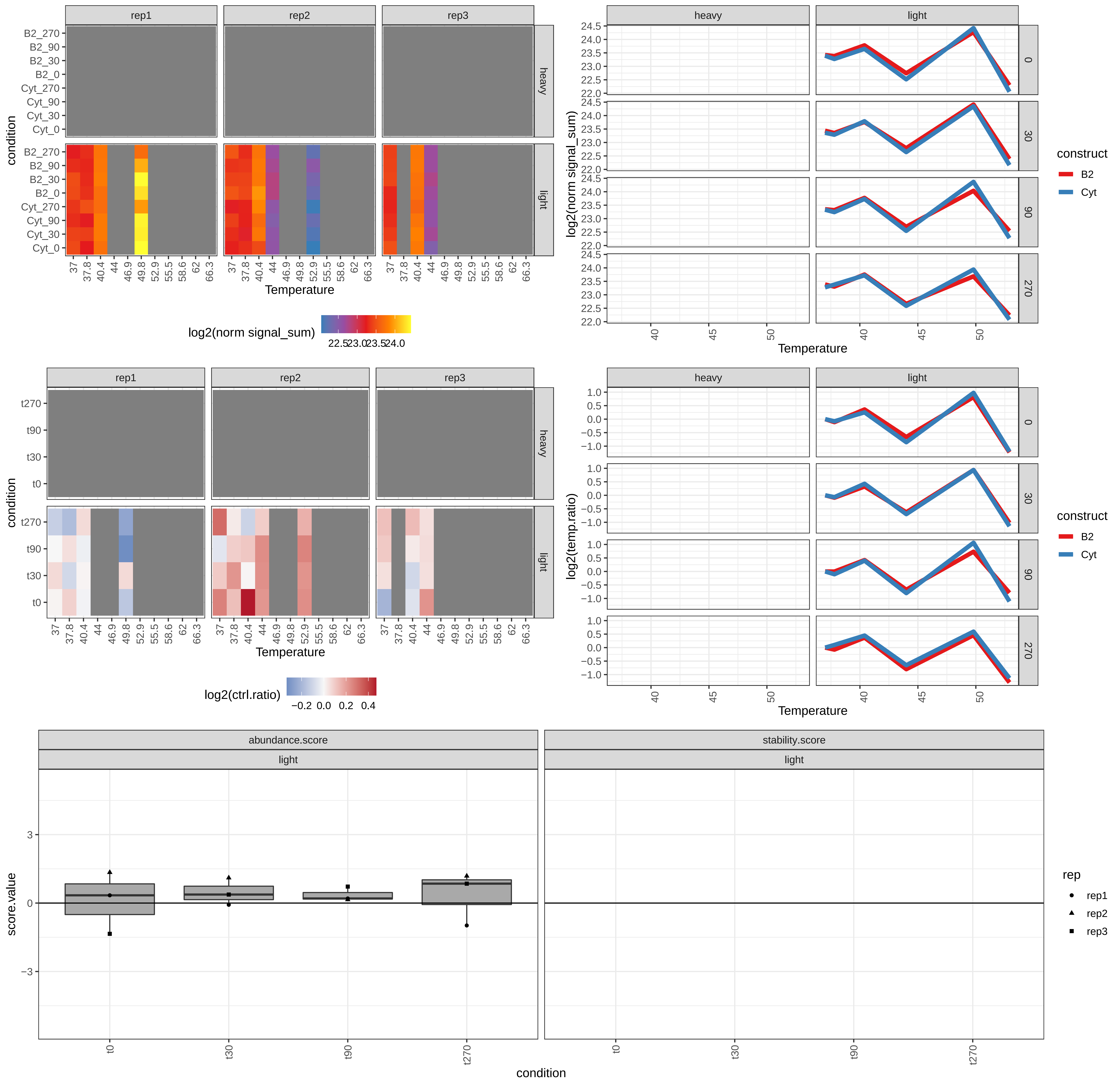

P53273 − Uncharacterized vacuolar membrane protein YGR125W OS=Saccharomyces cerevisiae (strain ATCC 204508 / S288c) GN=YGR125W PE=1 SV=1

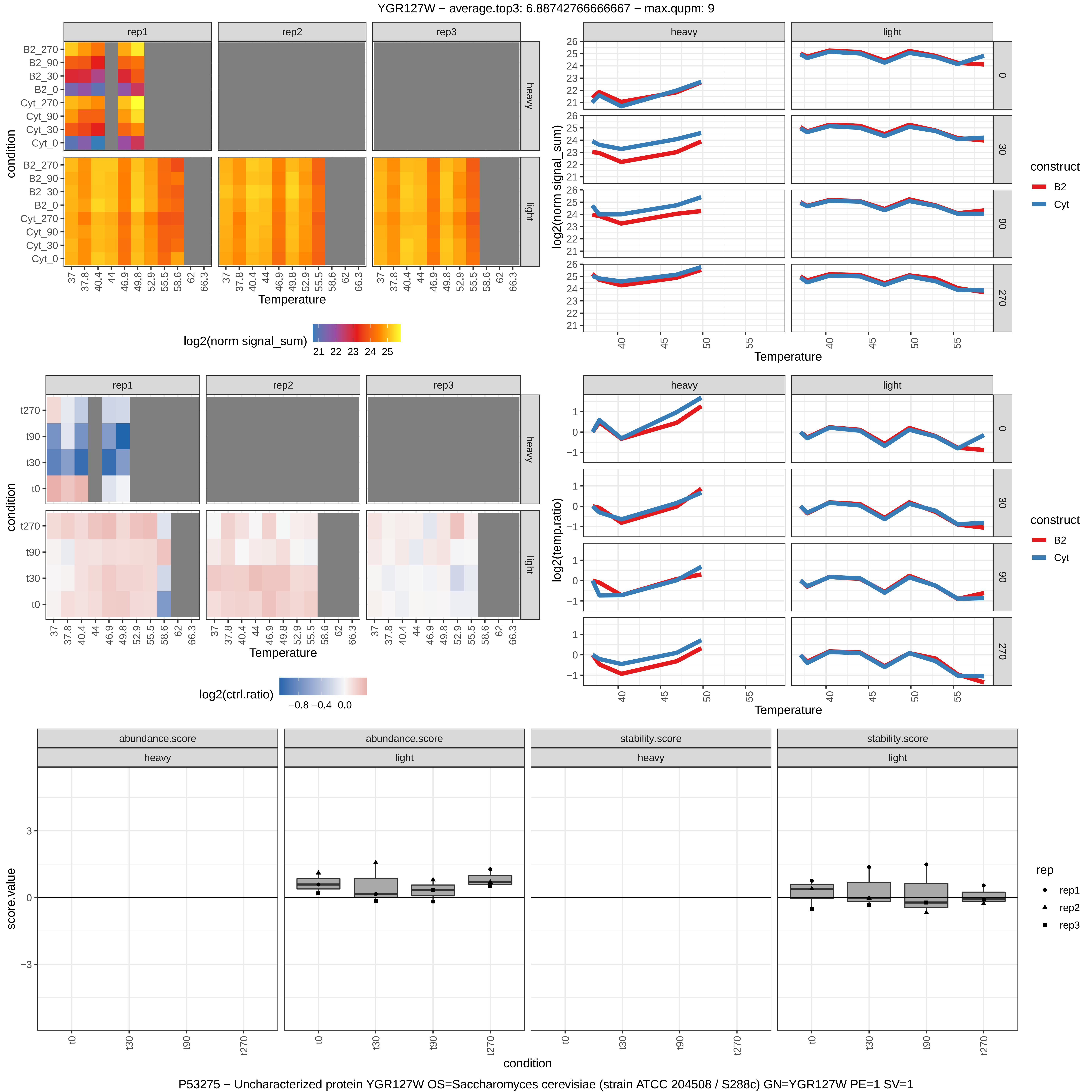

YGR127W − average.top3: 6.88742766666667 − max.qupm: 9
rep1
rep2
rep3
heavy
light
condition
Temperature
log2(norm signal_sum)
log2(temp.ratio)
log2(ctrl.ratio)
construct
B2
Cyt
abundance.score
stability.score
score.value
rep
P53275 − Uncharacterized protein YGR127W OS=Saccharomyces cerevisiae (strain ATCC 204508 / S288c) GN=YGR127W PE=1 SV=1

YGR130C − average.top3: 6.81937117647059 − max.qupm: 7

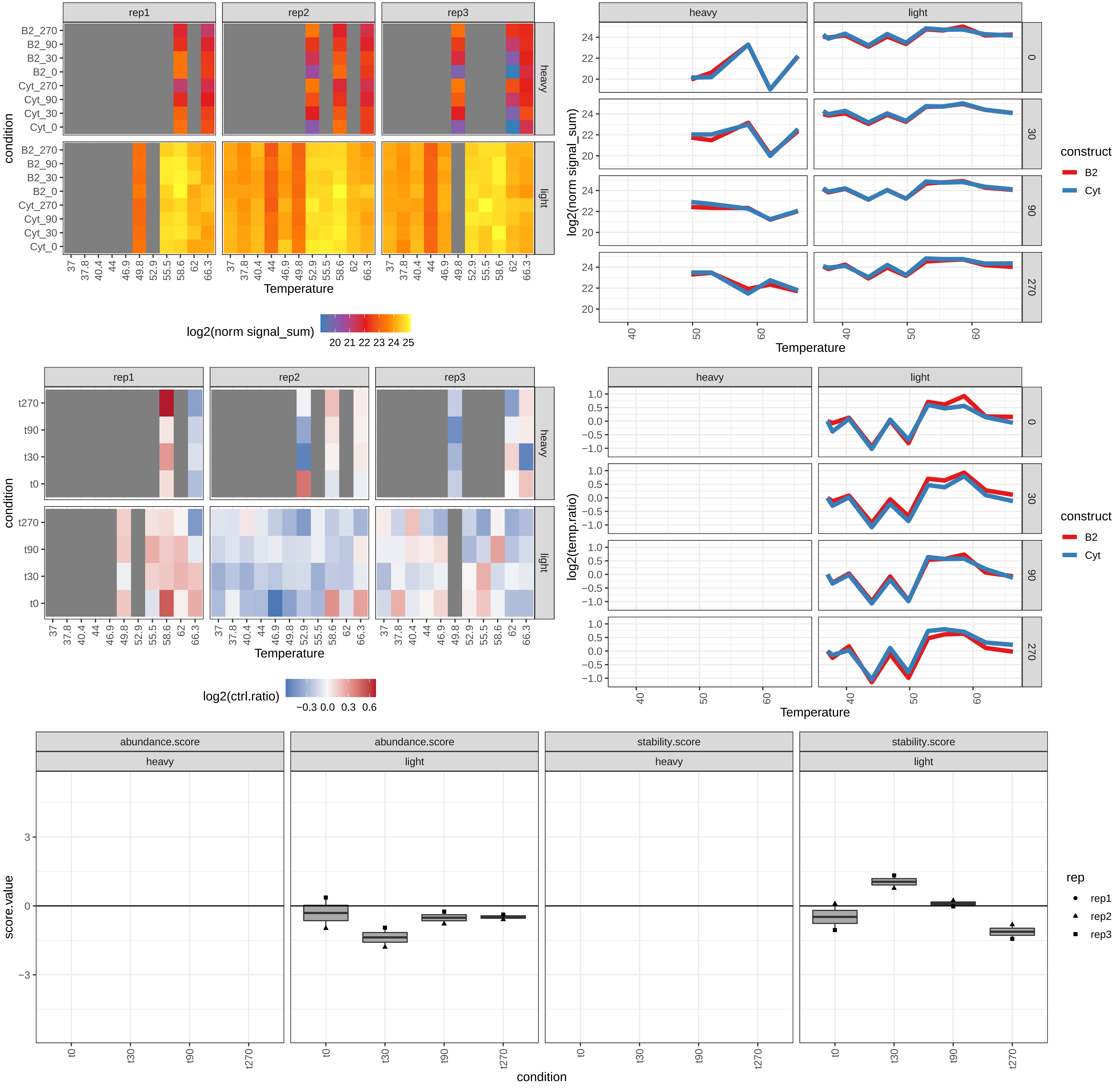

P53278 − Uncharacterized protein YGR130C OS=Saccharomyces cerevisiae (strain ATCC 204508 / S288c) GN=YGR130C PE=1 SV=1

YGR201C − average.top3: 6.232158 − max.qupm: 5

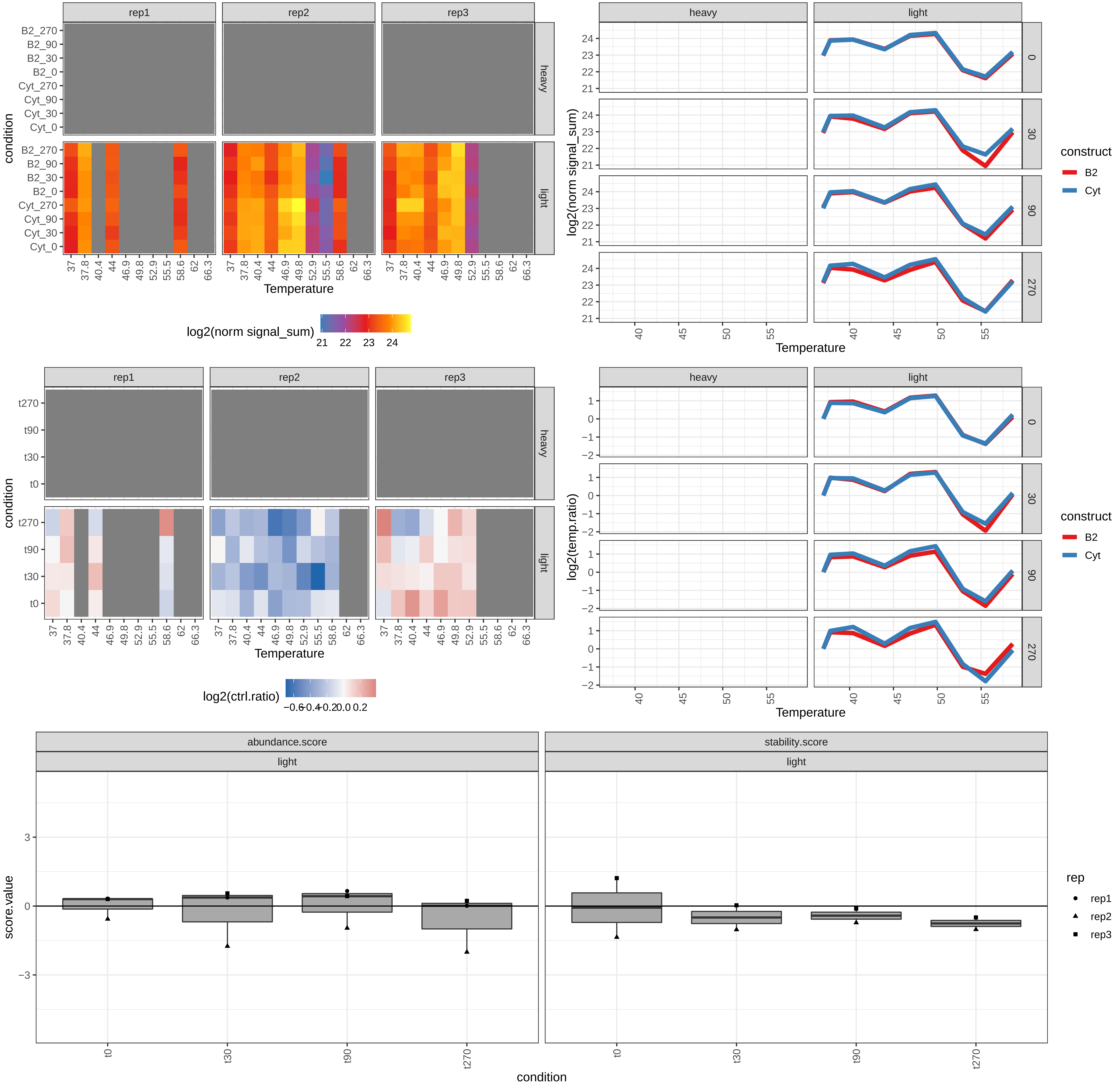

P42936 − Putative elongation factor 1 gamma homolog OS=Saccharomyces cerevisiae (strain ATCC 204508 / S288c) GN=YGR201C PE=4 SV=3

YGR237C − average.top3: 6.72449772727273 − max.qupm: 7

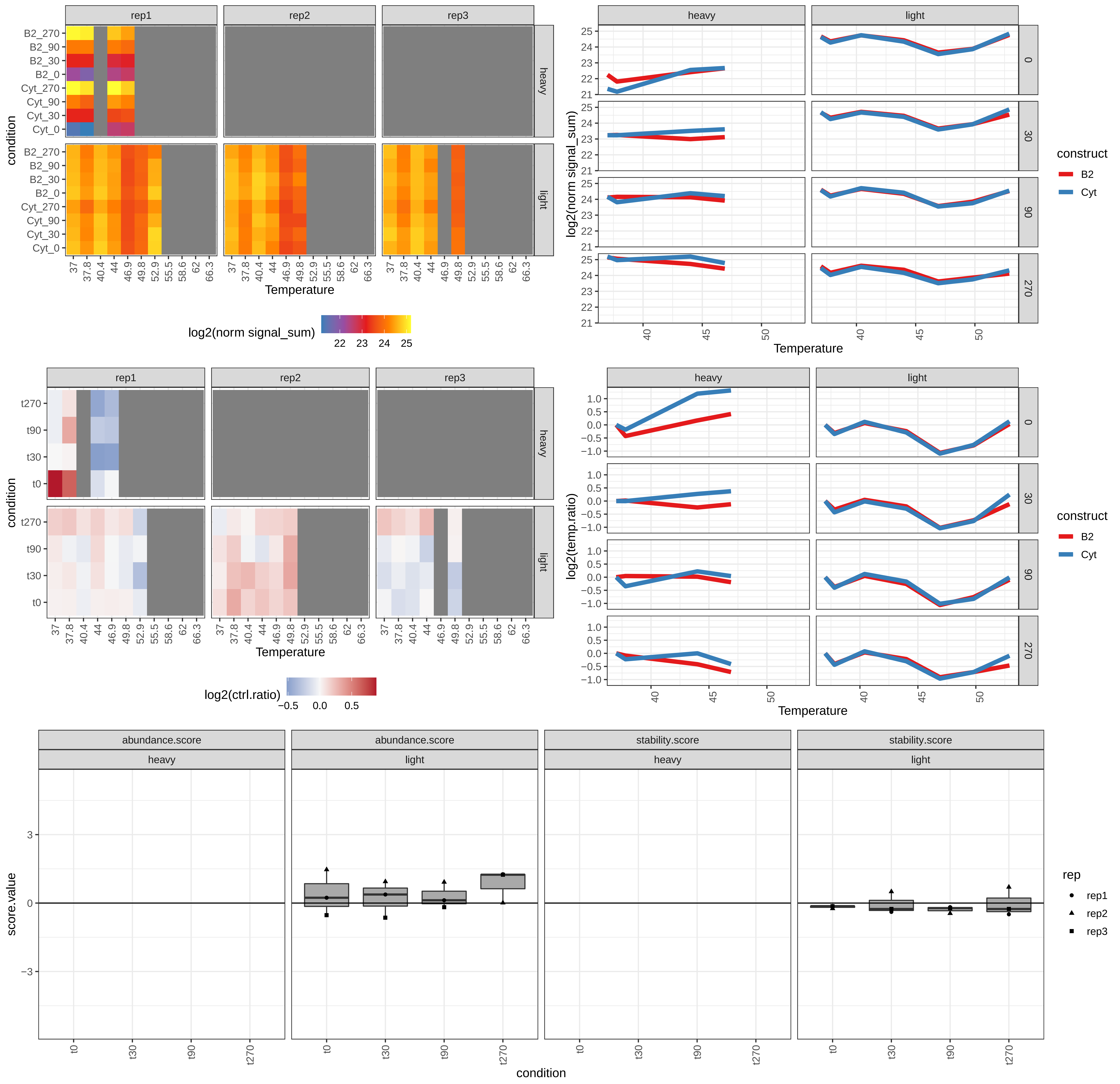

P50089 − Uncharacterized protein YGR237C OS=Saccharomyces cerevisiae (strain ATCC 204508 / S288c) GN=YGR237C PE=1 SV=1

YGR250C − average.top3: 6.90657378378378 − max.qupm: 14

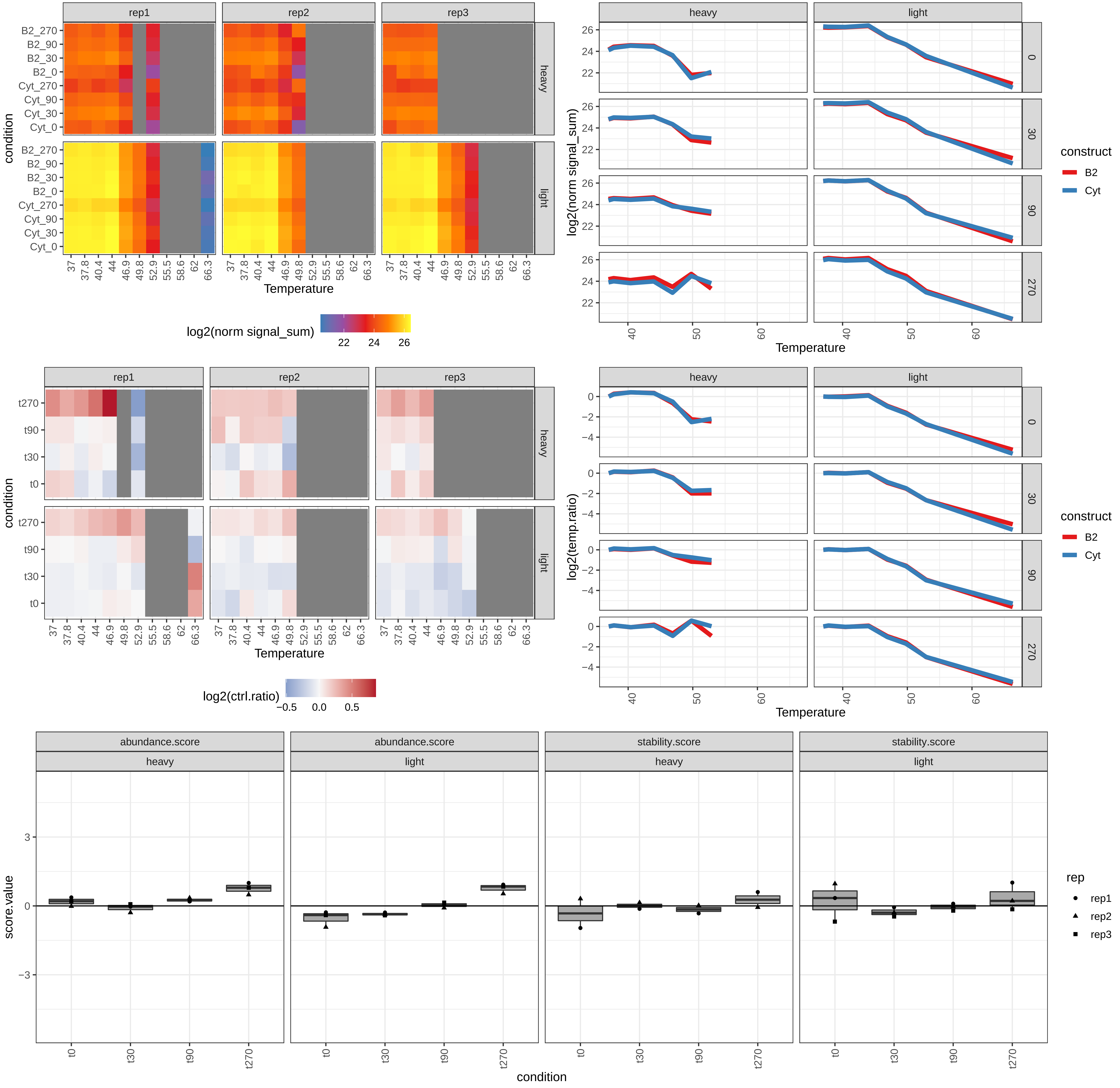

P53316 − Uncharacterized RNA−binding protein YGR250C OS=Saccharomyces cerevisiae (strain ATCC 204508 / S288c) GN=YGR250C PE=1 SV=1

YGR266W − average.top3: 7.09152511627907 − max.qupm: 18

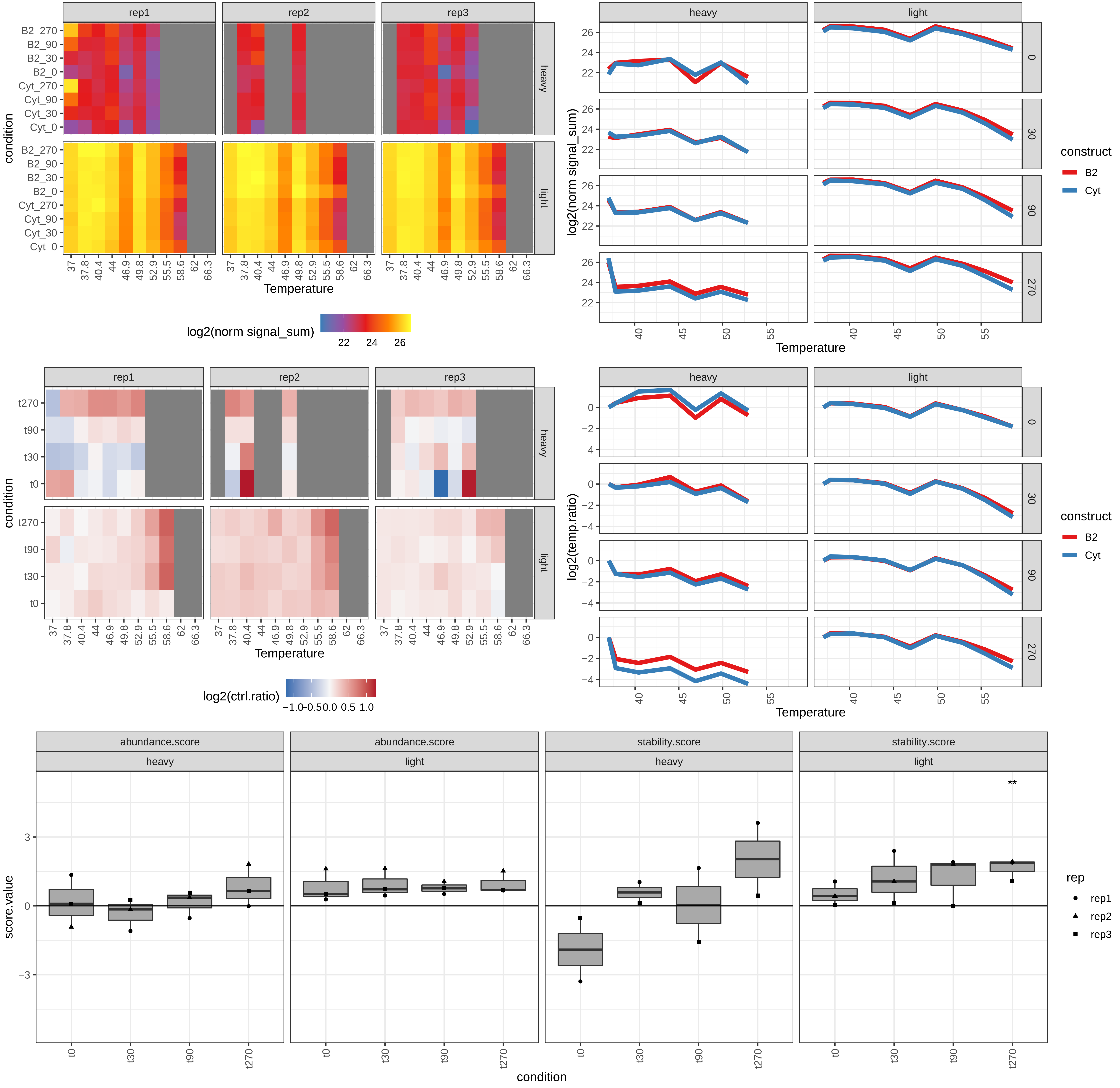

P53326 − Uncharacterized protein YGR266W OS=Saccharomyces cerevisiae (strain ATCC 204508 / S288c) GN=YGR266W PE=1 SV=1

YHB1 − average.top3: 7.3609174 − max.qupm: 13

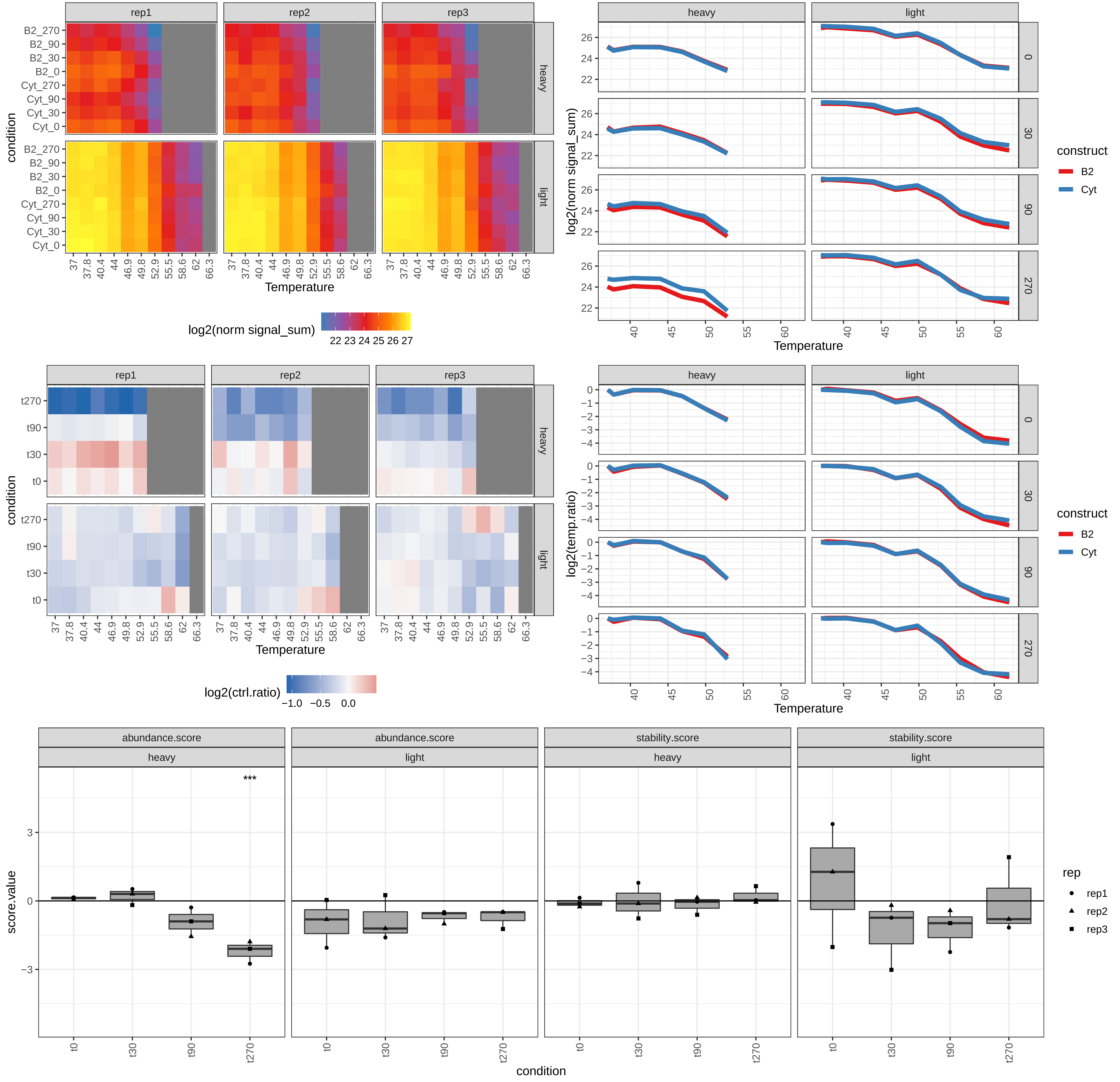

P39676 − Flavohemoprotein OS=Saccharomyces cerevisiae (strain ATCC 204508 / S288c) GN=YHB1 PE=1 SV=2

YHI9 − average.top3: 6.83292142857143 − max.qupm: 8

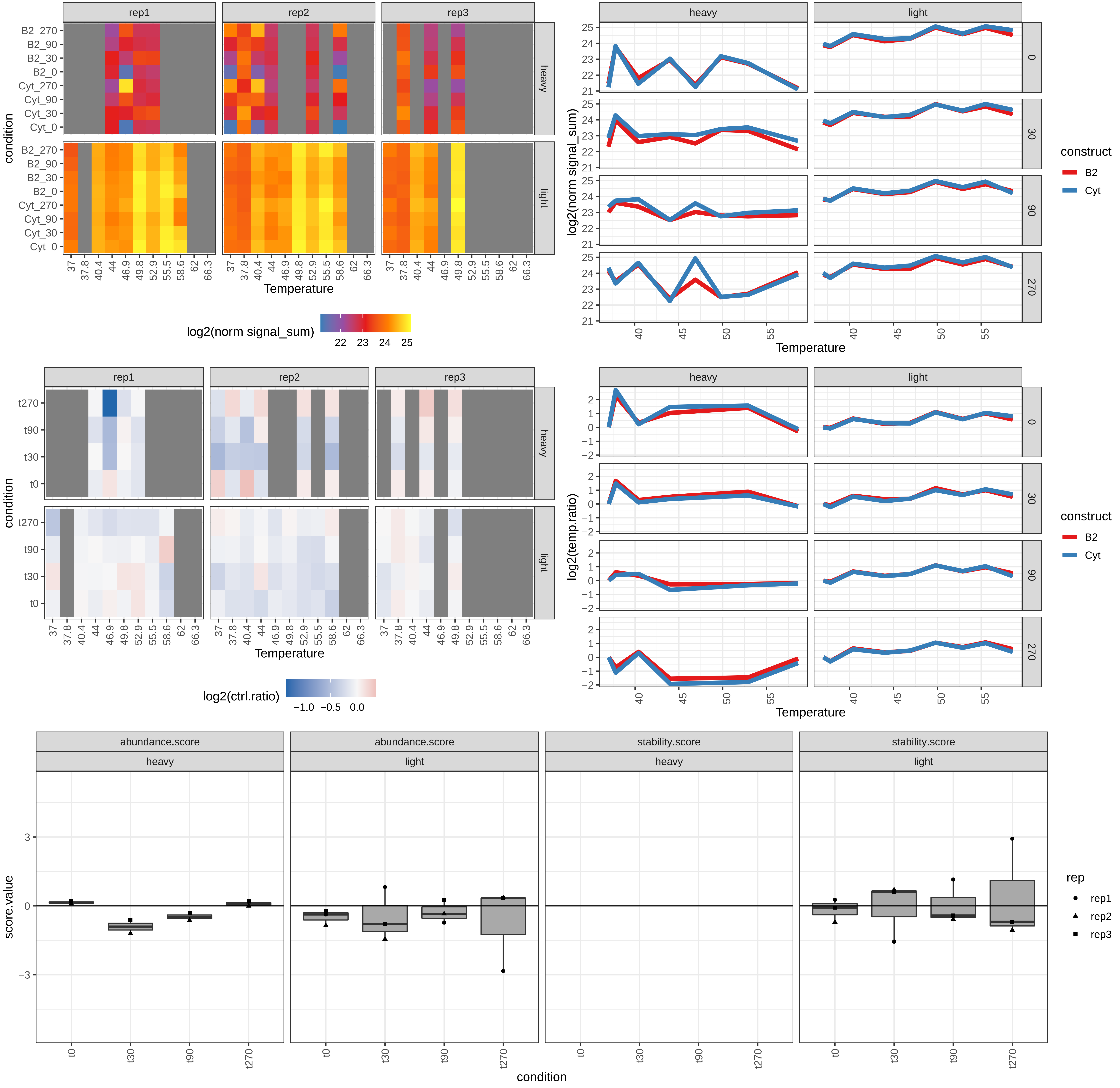

P38765 − Uncharacterized isomerase YHI9 OS=Saccharomyces cerevisiae (strain ATCC 204508 / S288c) GN=YHI9 PE=1 SV=1

YHL018W − average.top3: 6.63113818181818 − max.qupm: 3

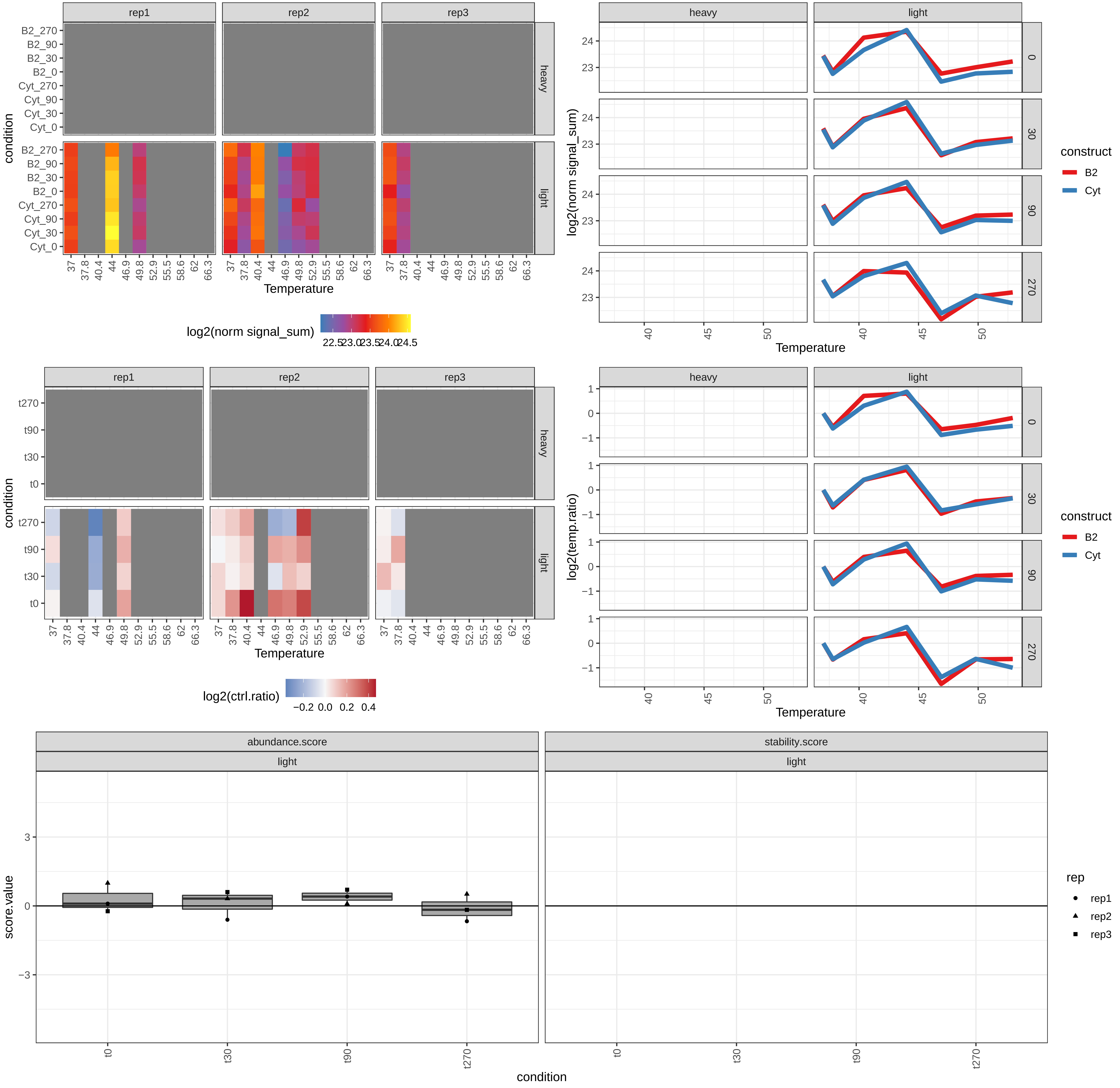

P38744 − Putative pterin−4−alpha−carbinolamine dehydratase OS=Saccharomyces cerevisiae (strain ATCC 204508 / S288c) GN=YHL018W PE=1 SV=1

YHM2 − average.top3: 7.18578285714286 − max.qupm: 8

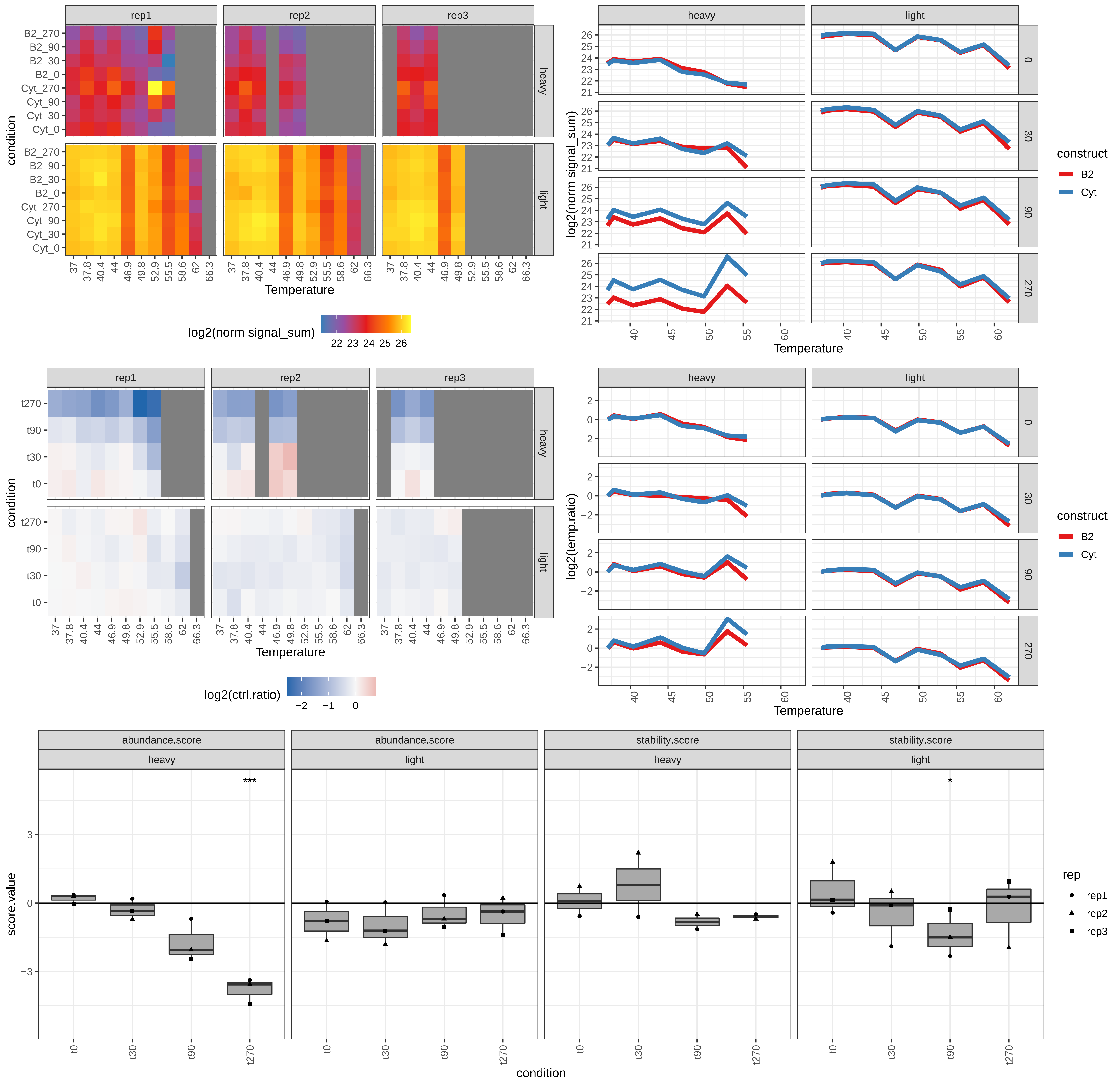

Q04013 − Citrate/oxoglutarate carrier protein OS=Saccharomyces cerevisiae (strain ATCC 204508 / S288c) GN=YHM2 PE=1 SV=1

YHR020W − average.top3: 7.05020872727273 − max.qupm: 14

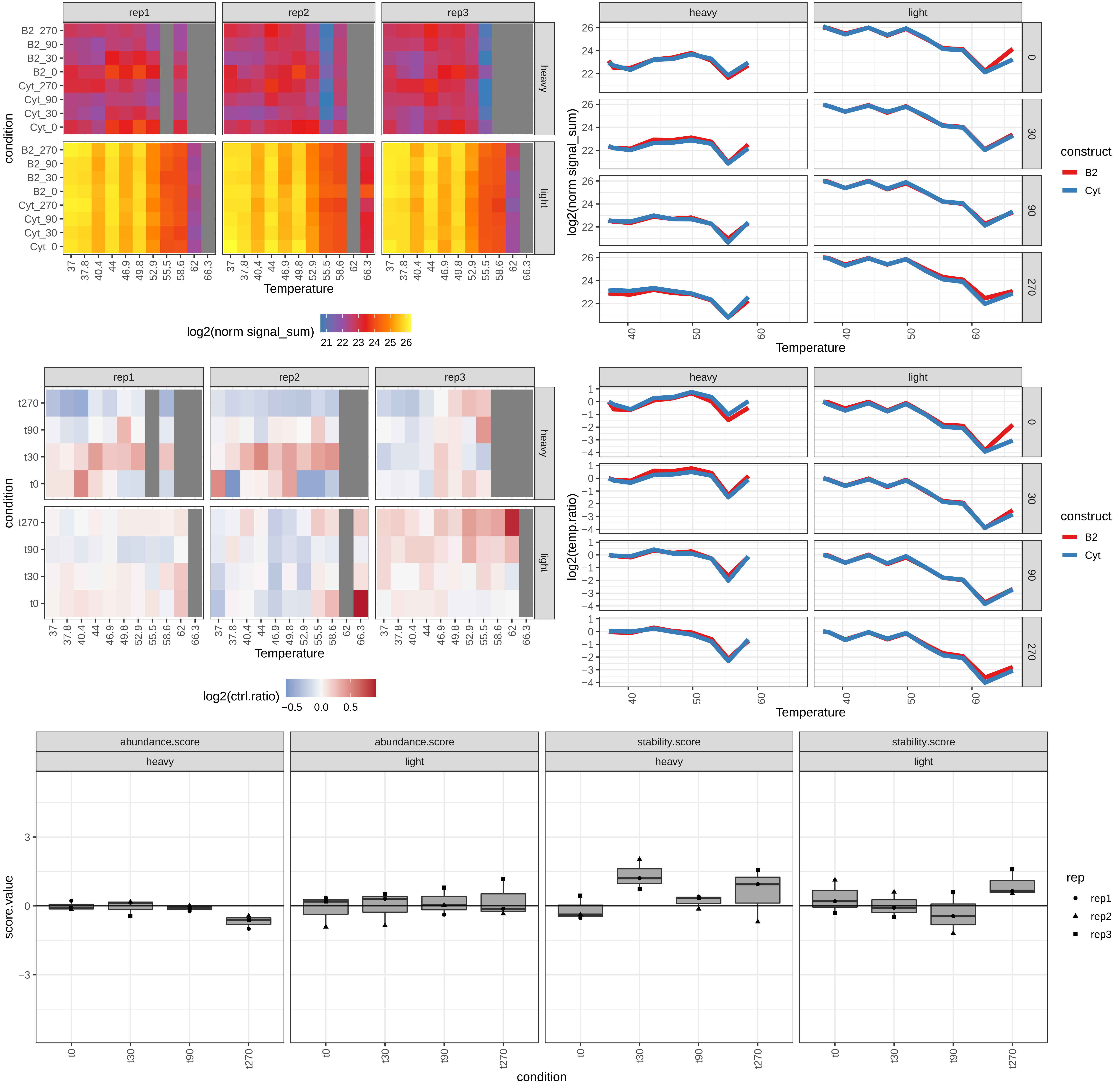

P38708 − Putative proline−−tRNA ligase YHR020W OS=Saccharomyces cerevisiae (strain ATCC 204508 / S288c) GN=YHR020W PE=1 SV=1

YHR033W − average.top3: 6.73330648648649 − max.qupm: 9

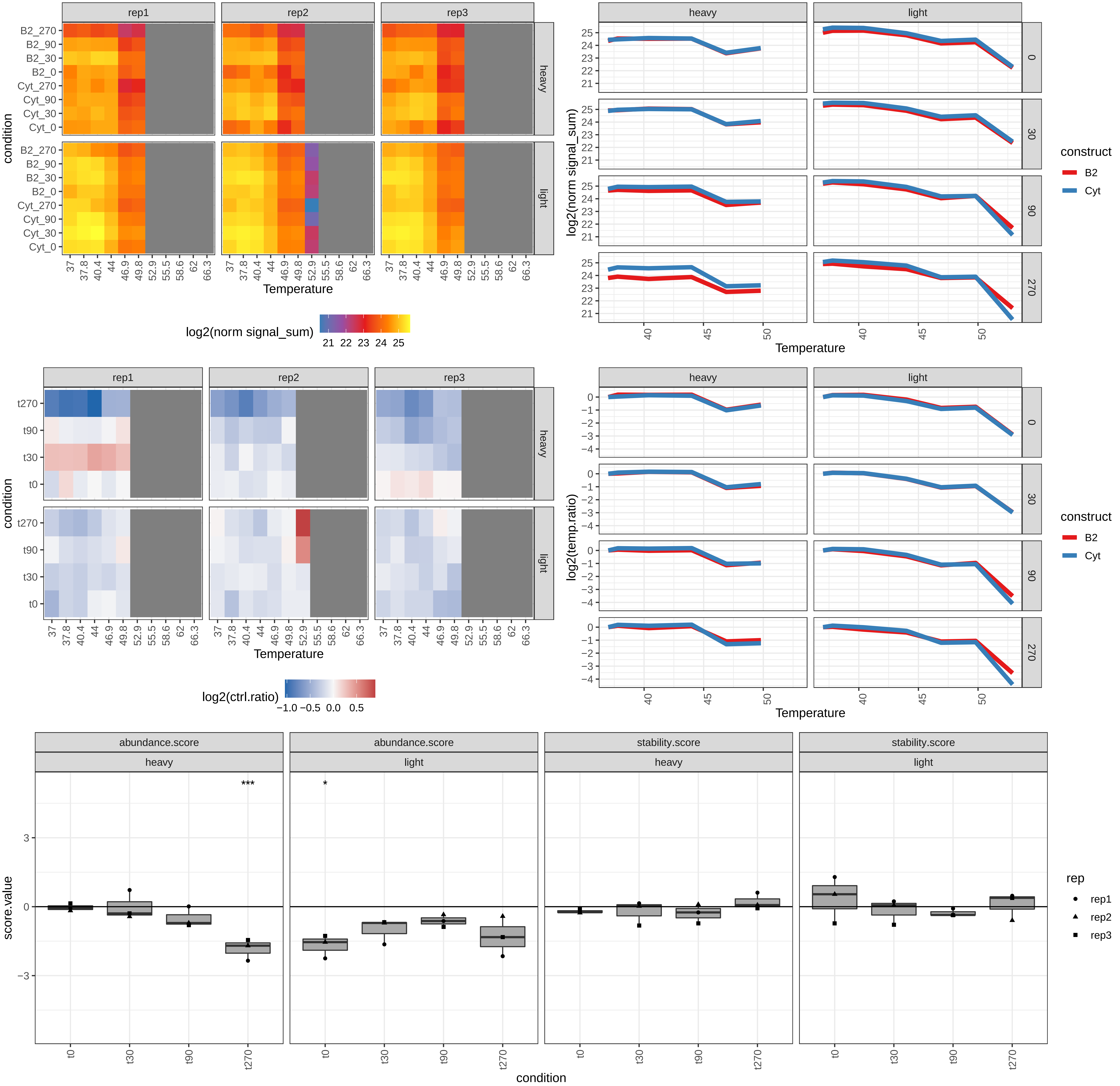

P38690 − Uncharacterized protein YHR033W OS=Saccharomyces cerevisiae (strain ATCC 204508 / S288c) GN=YHR033W PE=3 SV=1

YHR045W − average.top3: 7.08584210526316 − max.qupm: 11

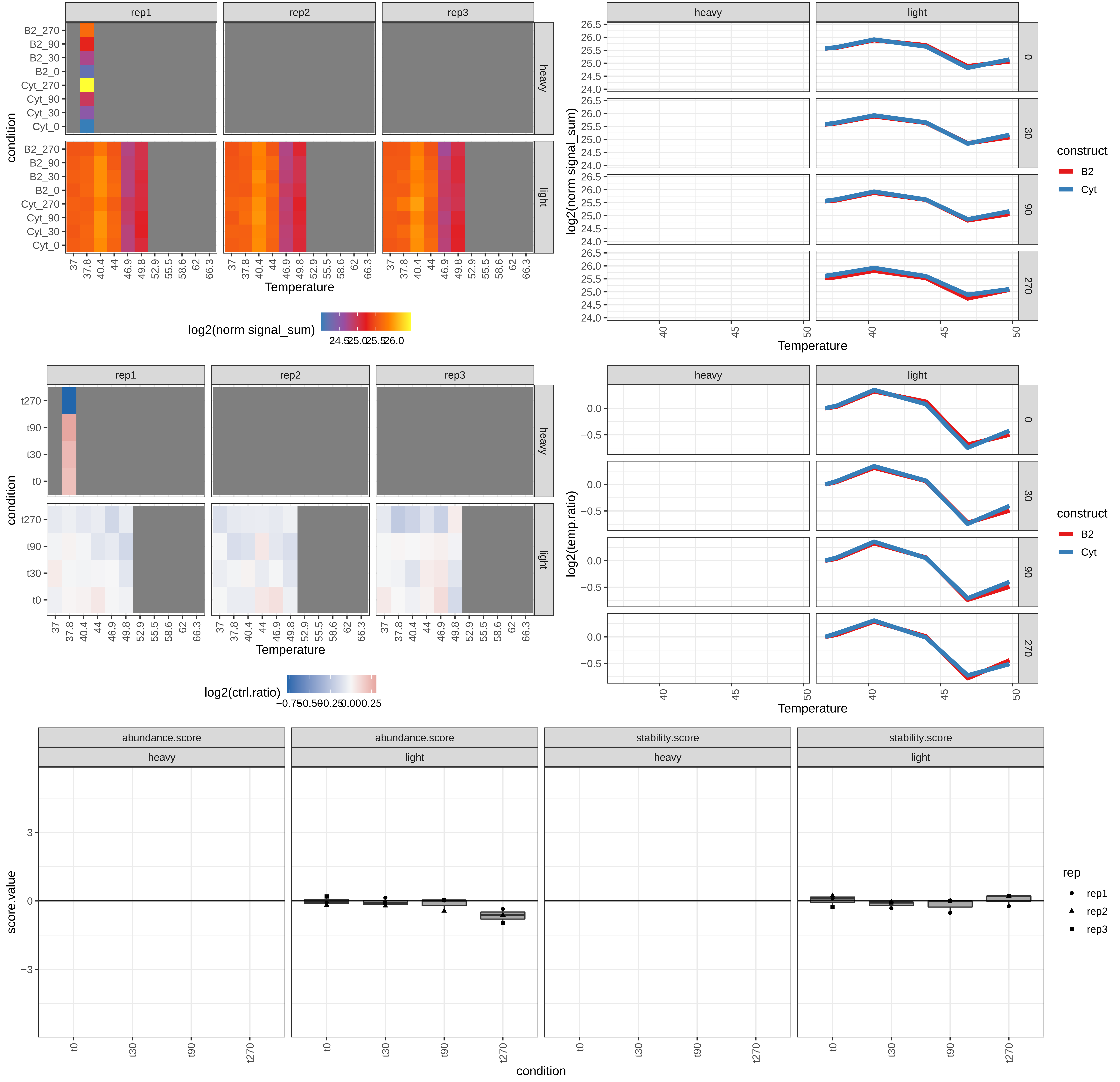

P38775 − Putative uncharacterized protein YHR045W OS=Saccharomyces cerevisiae (strain ATCC 204508 / S288c) GN=YHR045W PE=4 SV=1

YHR078W − average.top3: 6.0969275 − max.qupm: 3

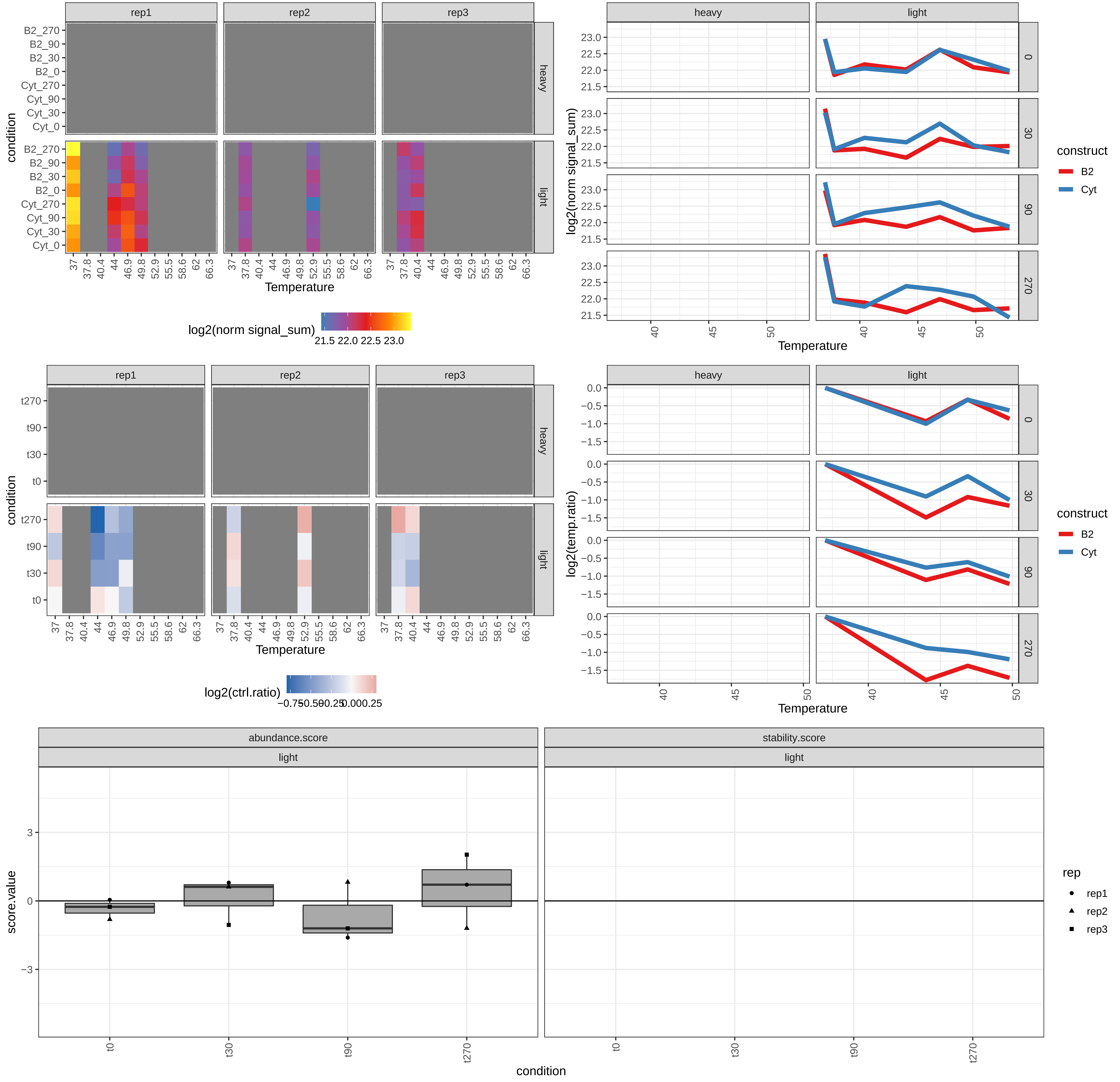

P38799 − Uncharacterized protein YHR078W OS=Saccharomyces cerevisiae (strain ATCC 204508 / S288c) GN=YHR078W PE=4 SV=1

YHR112C − average.top3: 6.82348862745098 − max.qupm: 10

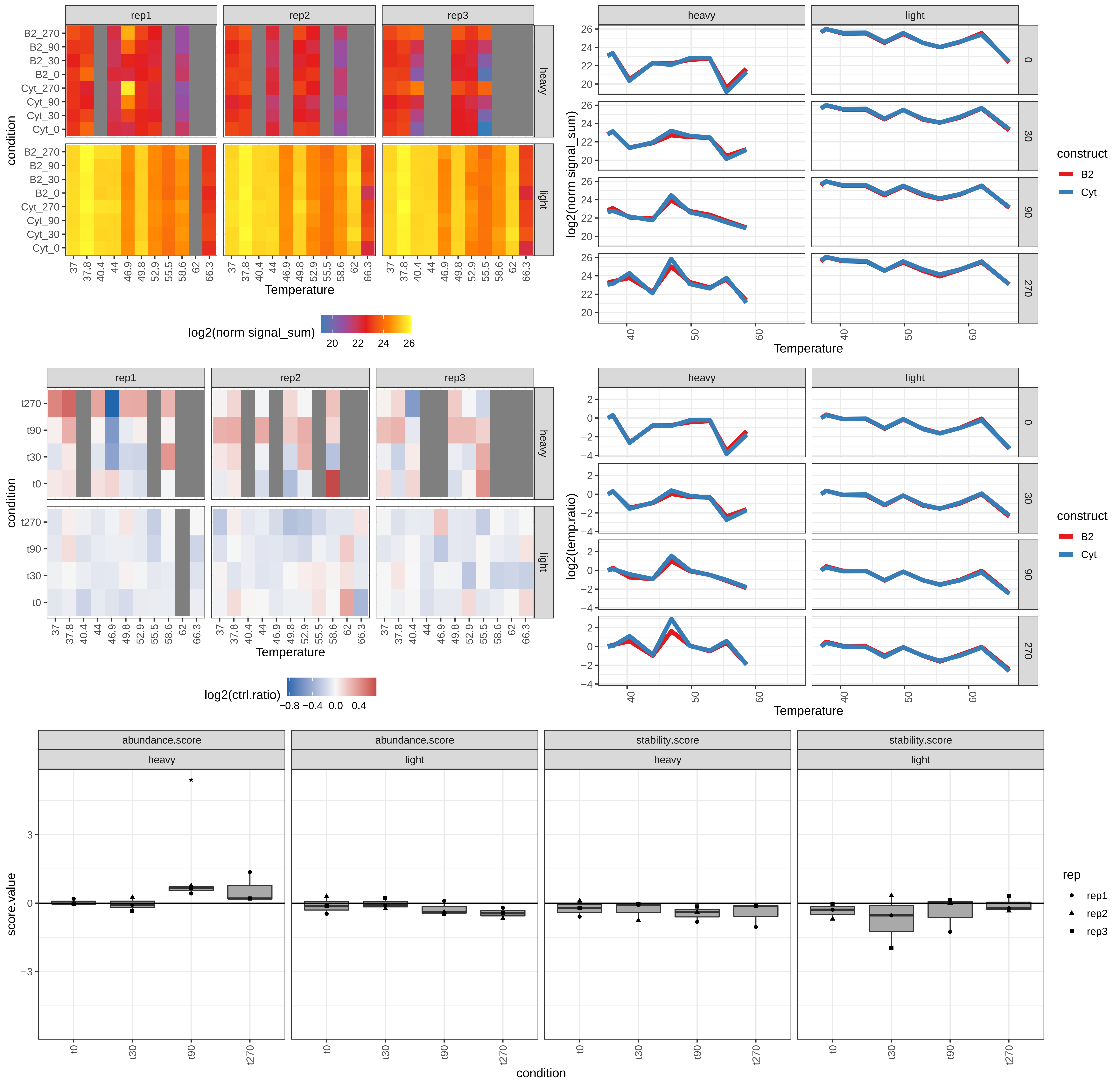

P38716 − Uncharacterized trans−sulfuration enzyme YHR112C OS=Saccharomyces cerevisiae (strain ATCC 204508 / S288c) GN=YHR112C PE=1 SV=1

YHR138C − average.top3: 6.57049782608696 − max.qupm: 4

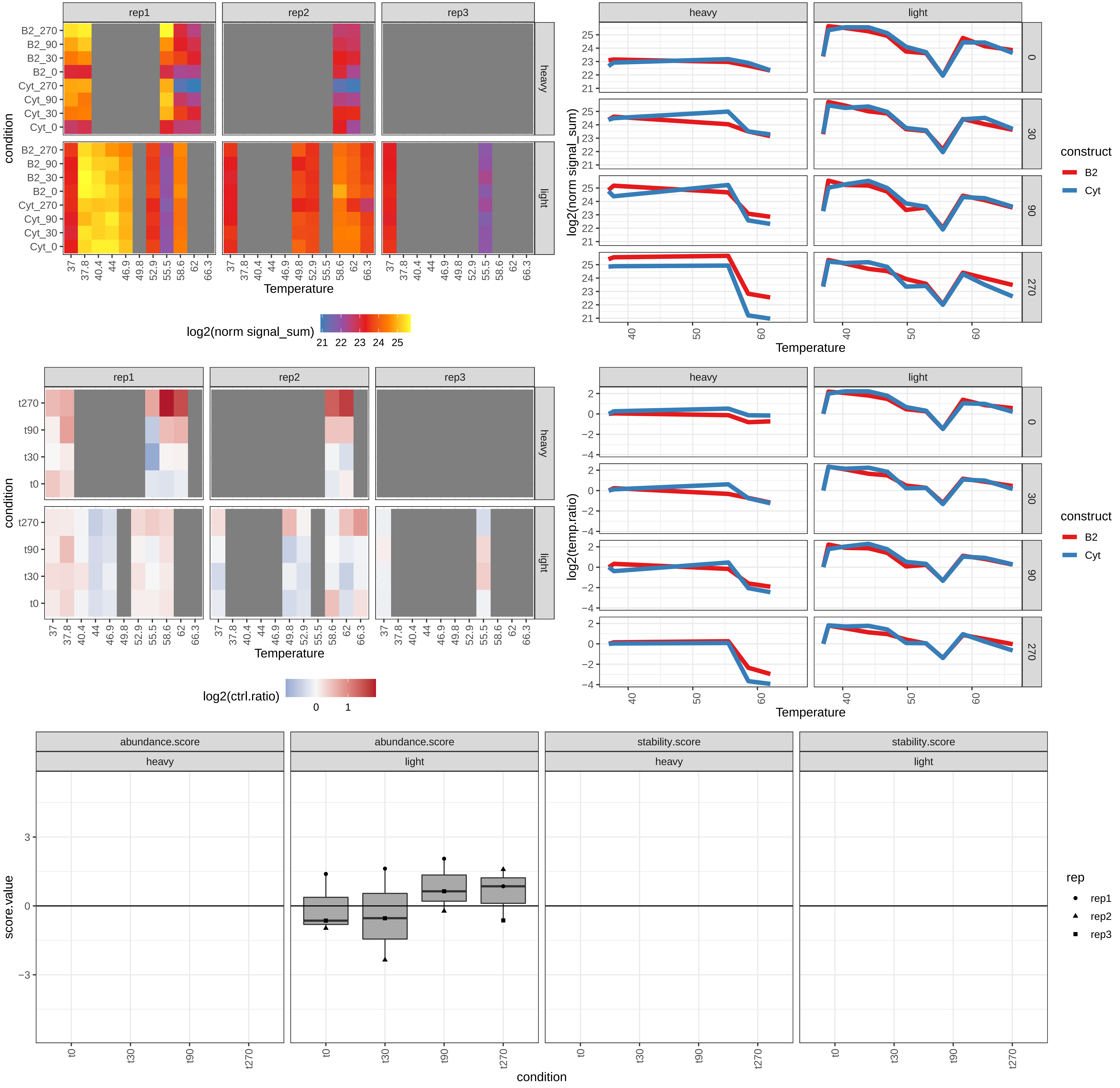

P38841 − Uncharacterized protein YHR138C OS=Saccharomyces cerevisiae (strain ATCC 204508 / S288c) GN=YHR138C PE=1 SV=1

YHR202W − average.top3: 6.70828458333333 − max.qupm: 7

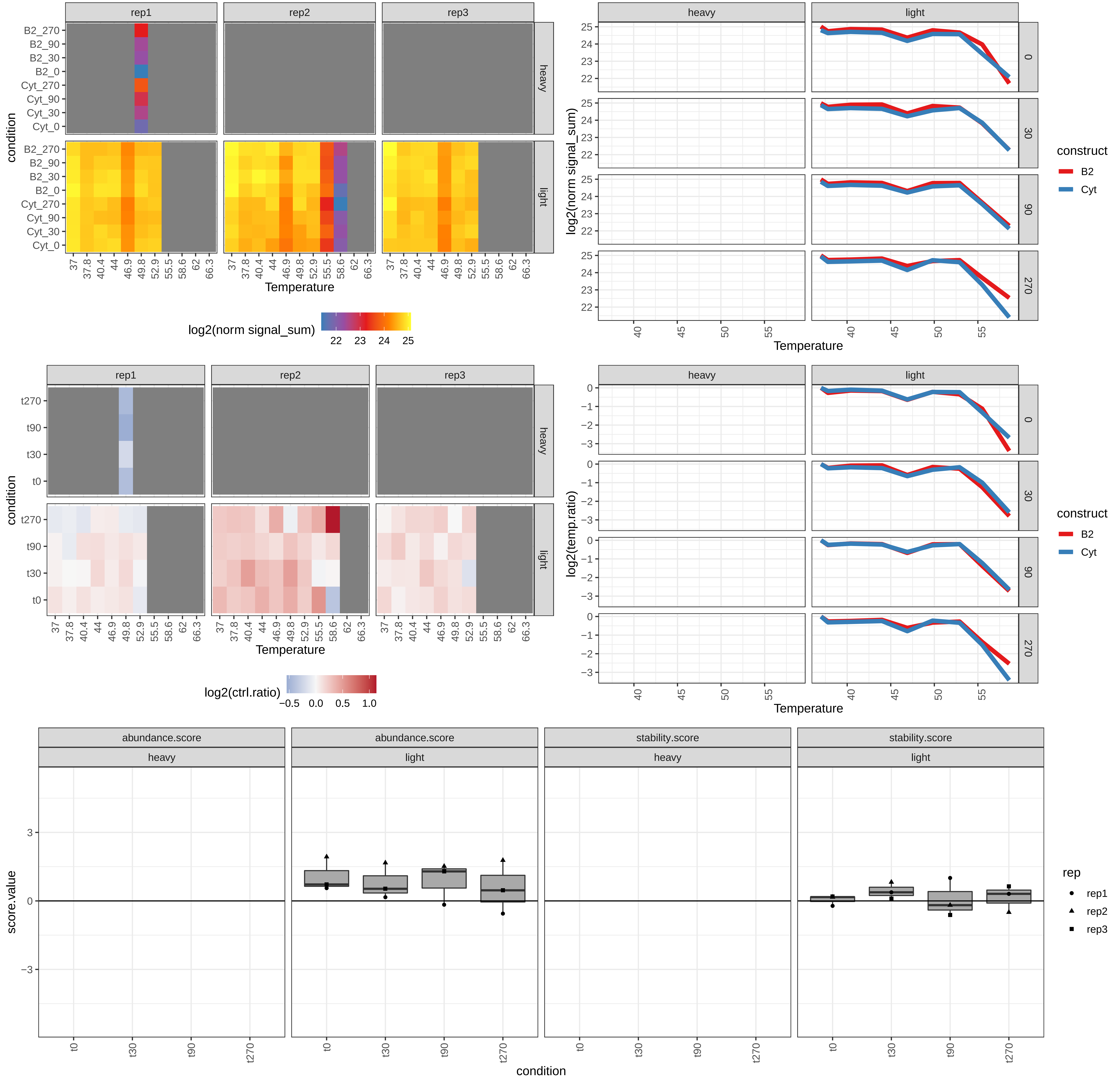

P38887 − Uncharacterized protein YHR202W OS=Saccharomyces cerevisiae (strain ATCC 204508 / S288c) GN=YHR202W PE=1 SV=1

YIF1 − average.top3: 6.49652 − max.qupm: 3

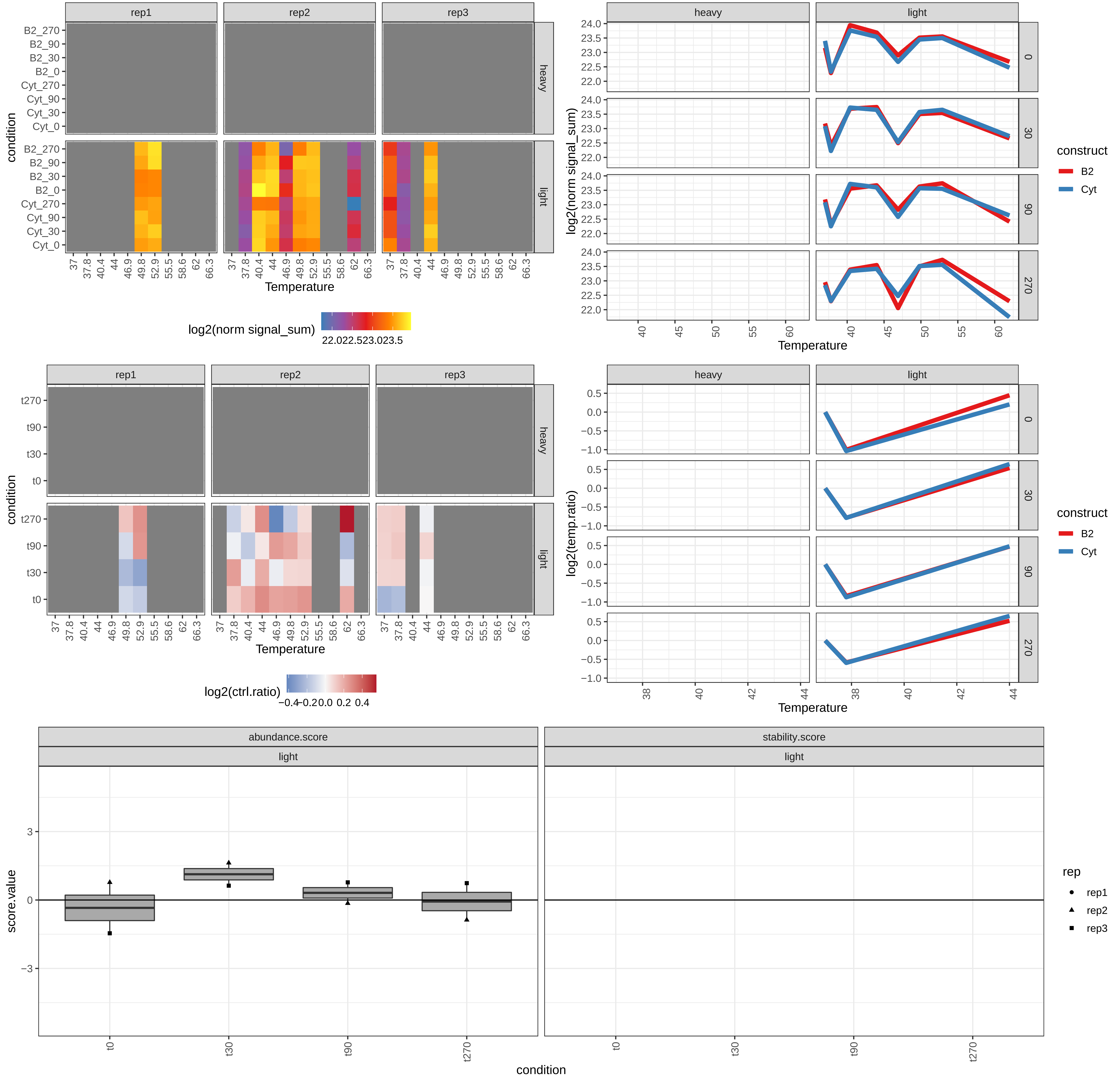

P53845 − Protein transport protein YIF1 OS=Saccharomyces cerevisiae (strain ATCC 204508 / S288c) GN=YIF1 PE=1 SV=1

YIL055C − average.top3: 6.56322043478261 − max.qupm: 3

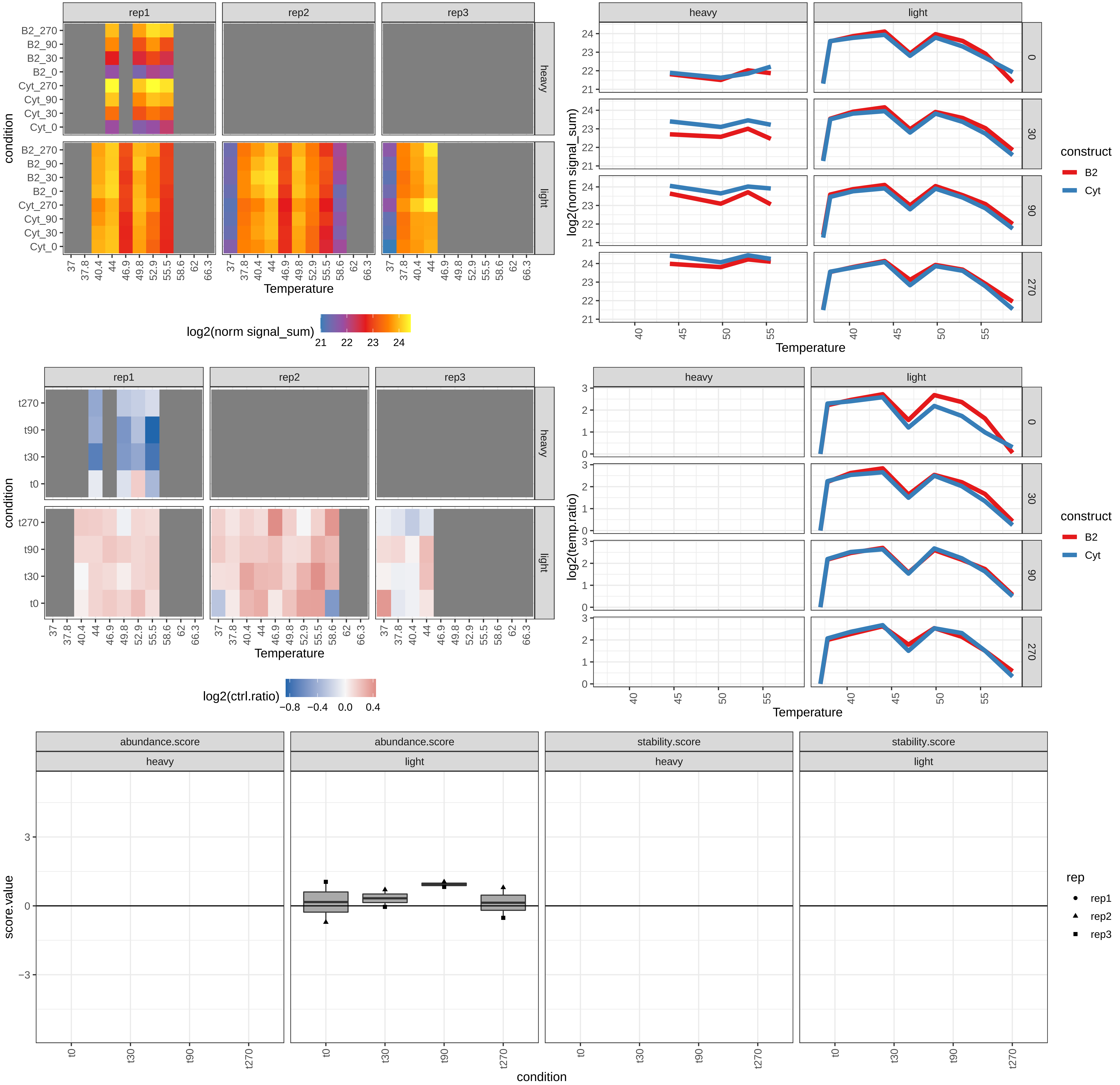

P40523 − Uncharacterized protein YIL055C OS=Saccharomyces cerevisiae (strain ATCC 204508 / S288c) GN=YIL055C PE=1 SV=1

YIL108W − average.top3: 6.8452430952381 − max.qupm: 17

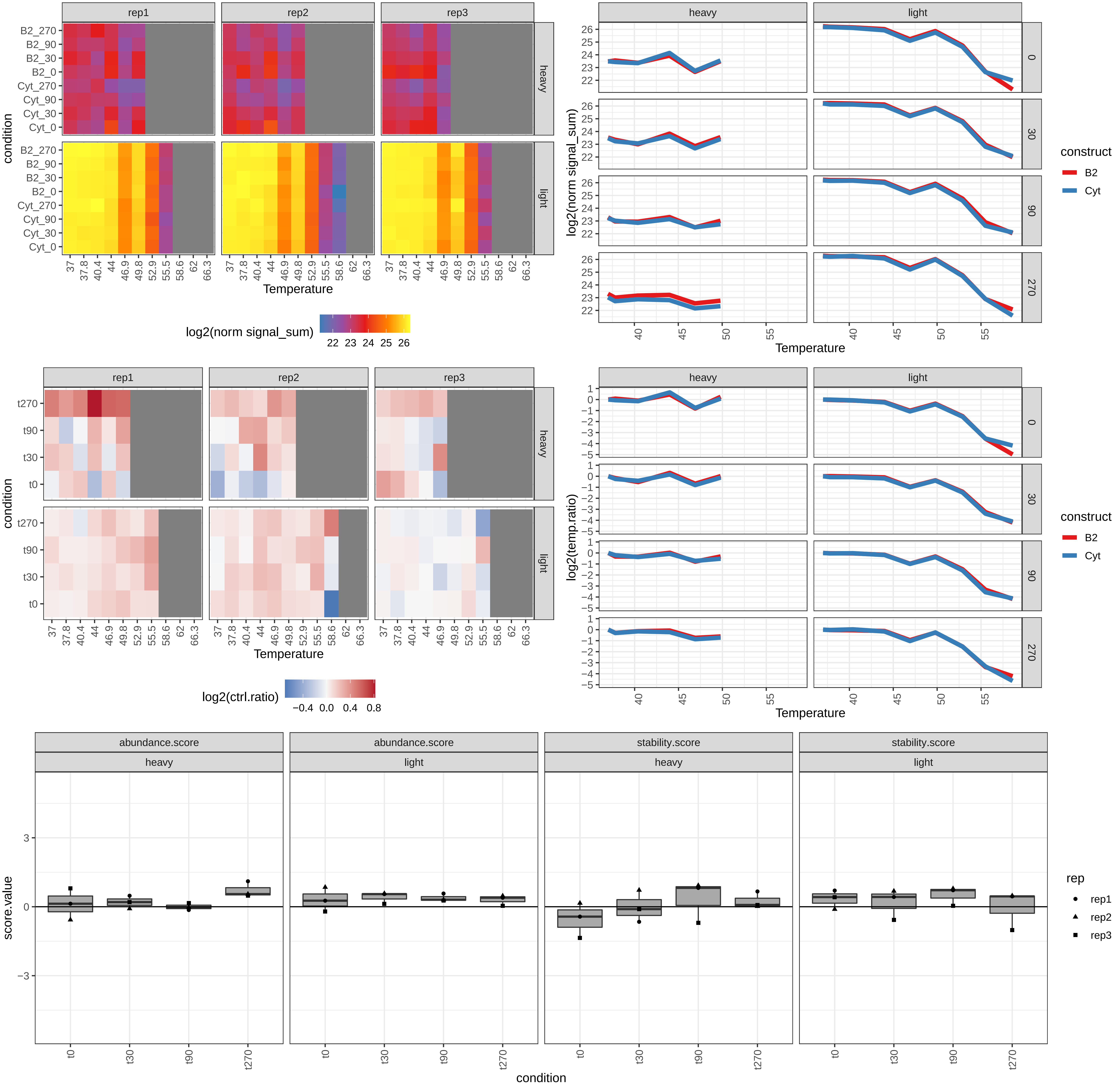

P40483 − Putative zinc metalloproteinase YIL108W OS=Saccharomyces cerevisiae (strain ATCC 204508 / S288c) GN=YIL108W PE=1 SV=1

YIM1 − average.top3: 6.91502297297297 − max.qupm: 9

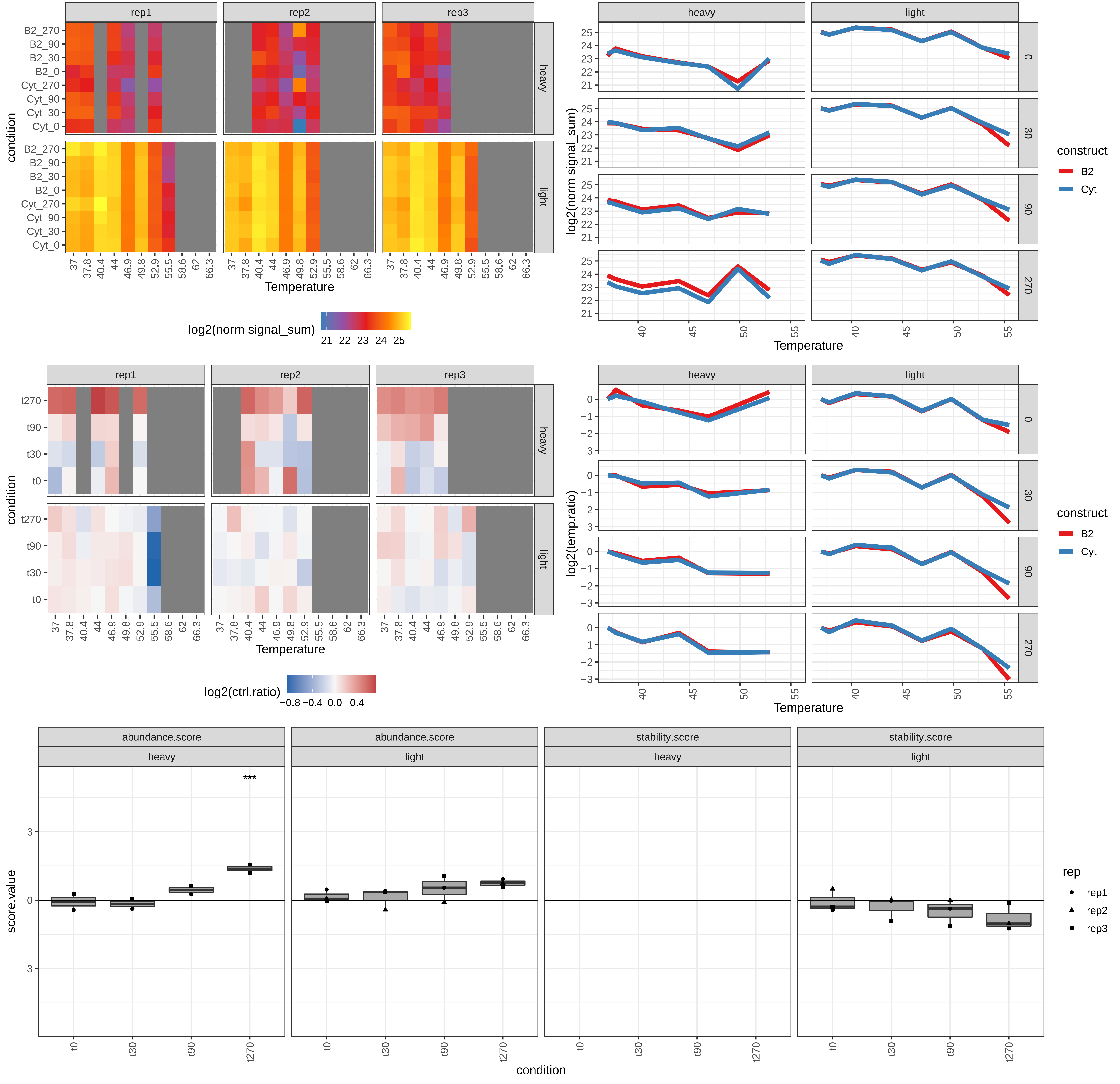

P28625 − Protein YIM1 OS=Saccharomyces cerevisiae (strain ATCC 204508 / S288c) GN=YIM1 PE=1 SV=2

YIP3 − average.top3: 7.2774235483871 − max.qupm: 6

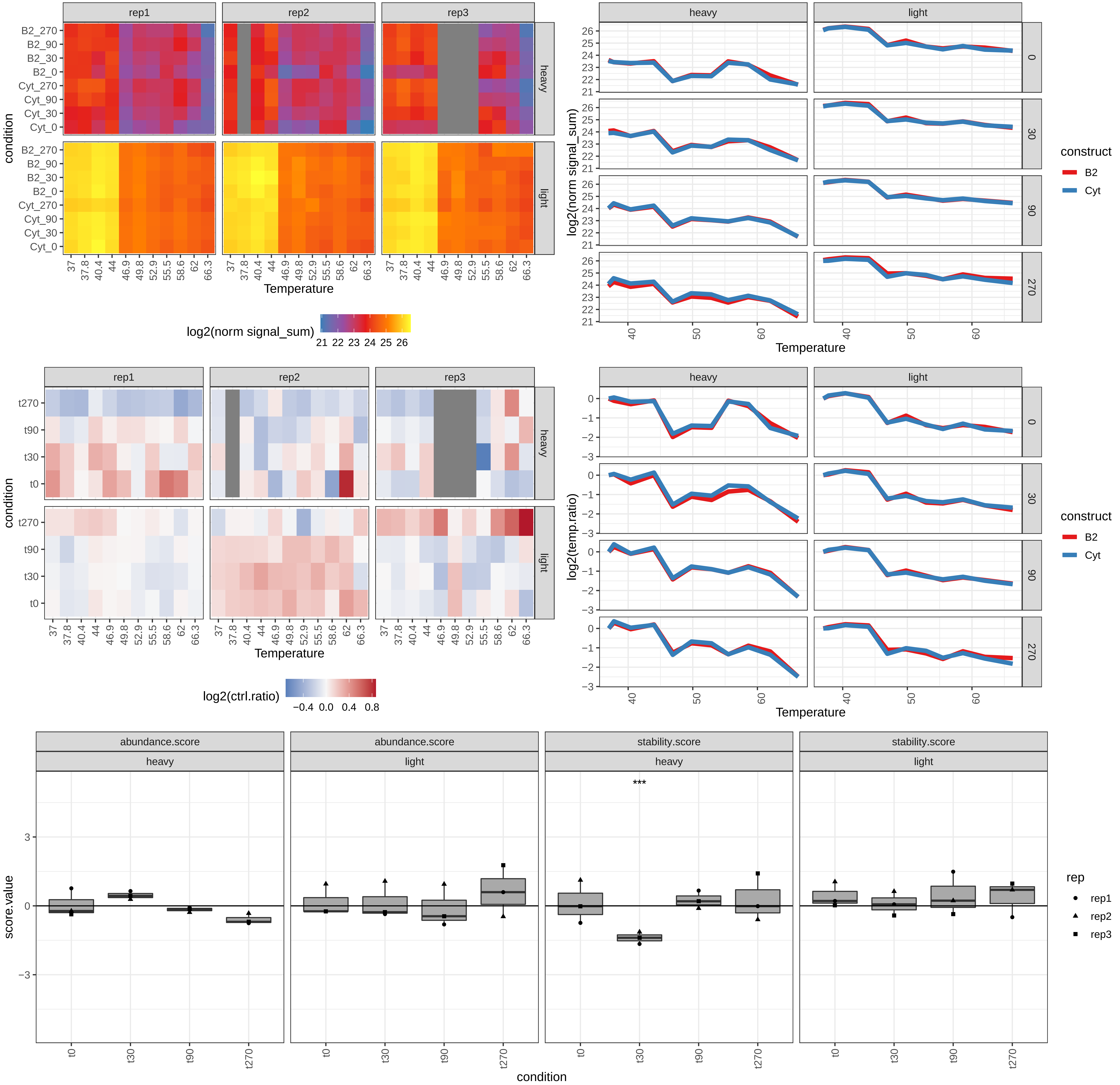

P53633 − Prenylated Rab acceptor 1 OS=Saccharomyces cerevisiae (strain ATCC 204508 / S288c) GN=YIP3 PE=1 SV=2

YIP4 − average.top3: 7.05117052631579 − max.qupm: 2

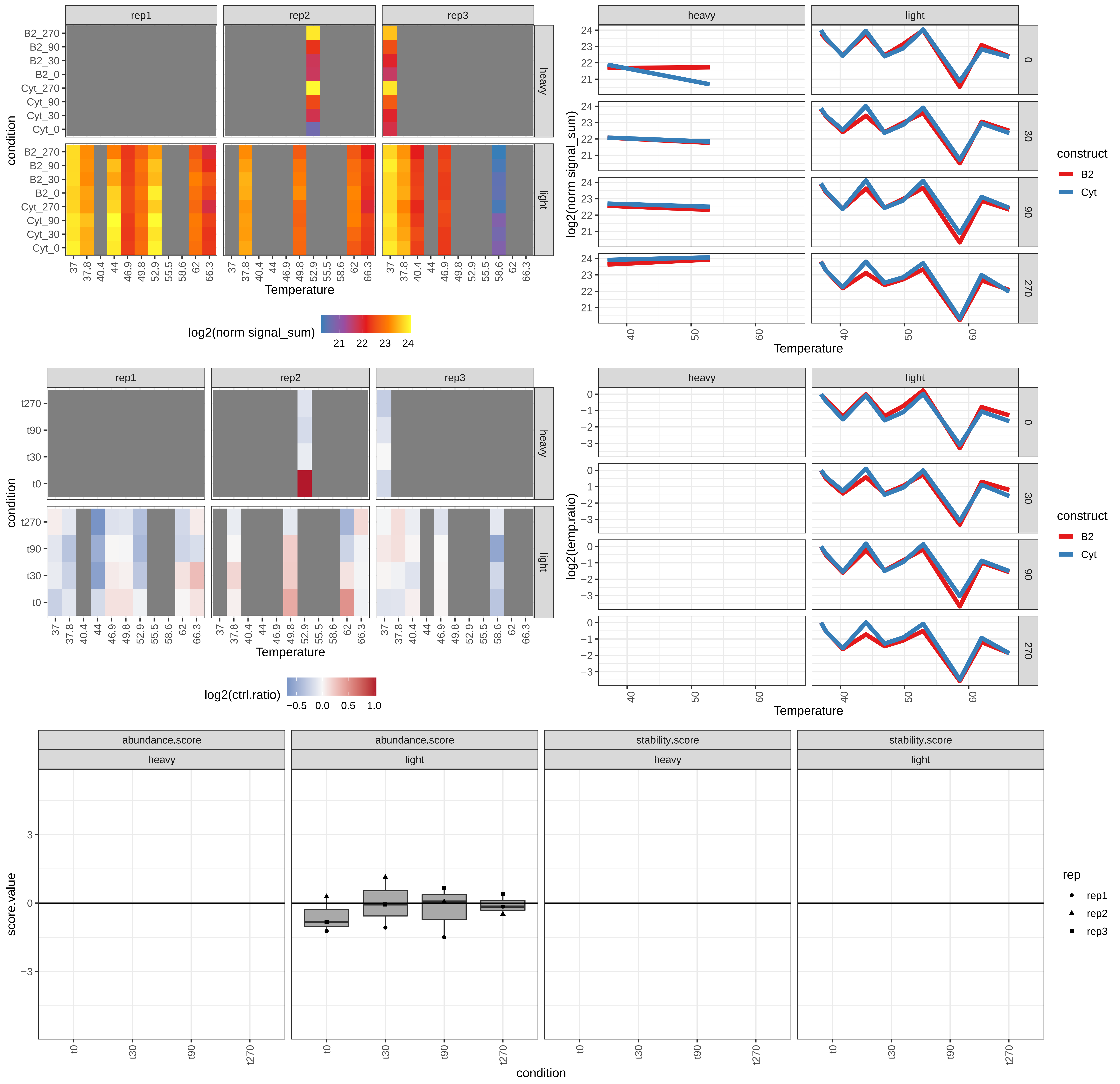

P53093 − Protein YIP4 OS=Saccharomyces cerevisiae (strain ATCC 204508 / S288c) GN=YIP4 PE=1 SV=2

YIR007W − average.top3: 6.78582371428571 − max.qupm: 8

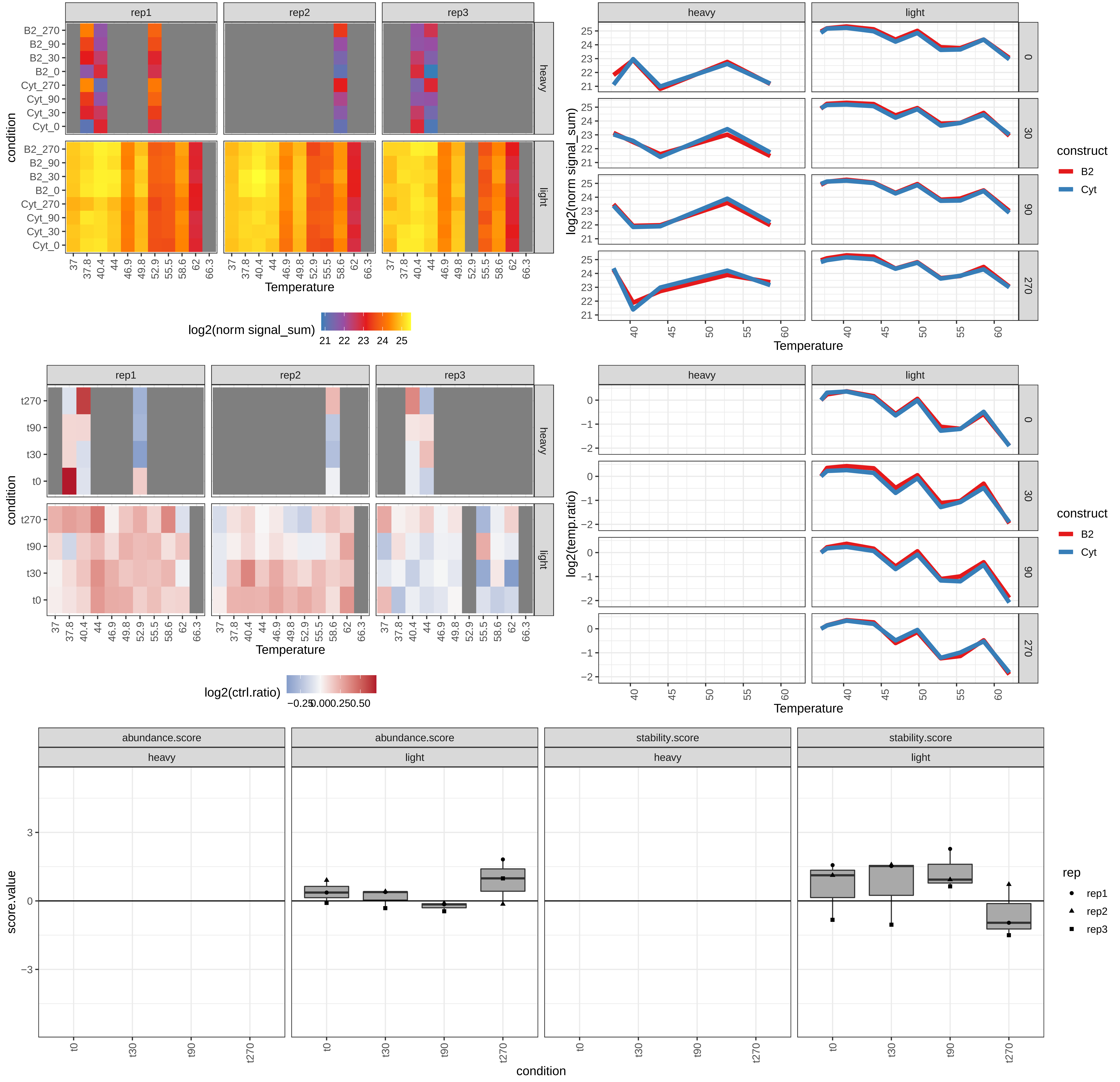

P40566 − Uncharacterized glycosyl hydrolase YIR007W OS=Saccharomyces cerevisiae (strain ATCC 204508 / S288c) GN=YIR007W PE=1 SV=1

YIR035C − average.top3: 7.1621125 − max.qupm: 3

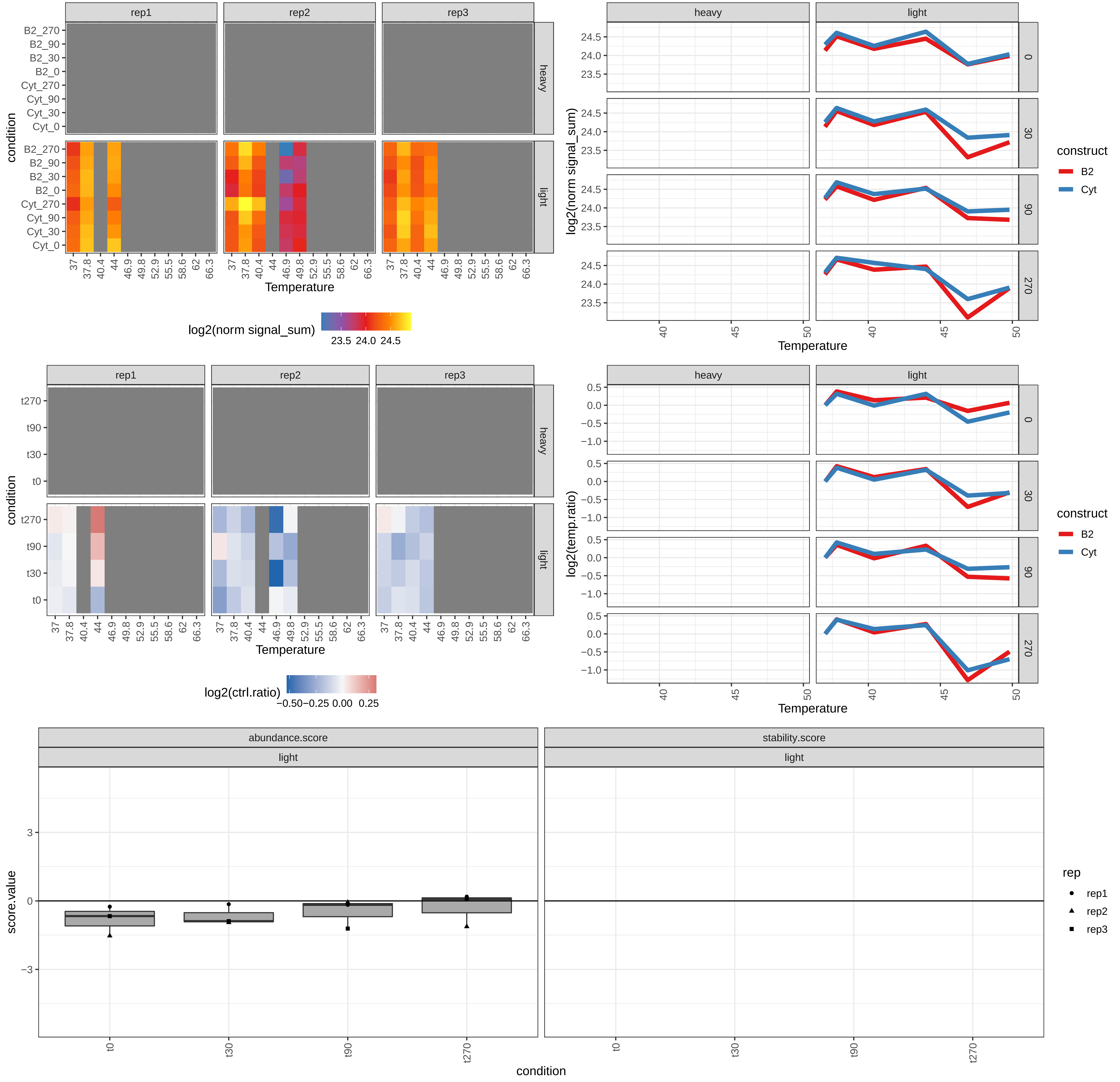

P40579 − Uncharacterized oxidoreductase YIR035C OS=Saccharomyces cerevisiae (strain ATCC 204508 / S288c) GN=YIR035C PE=1 SV=1

YJL045W − average.top3: 6.27335 − max.qupm: 2

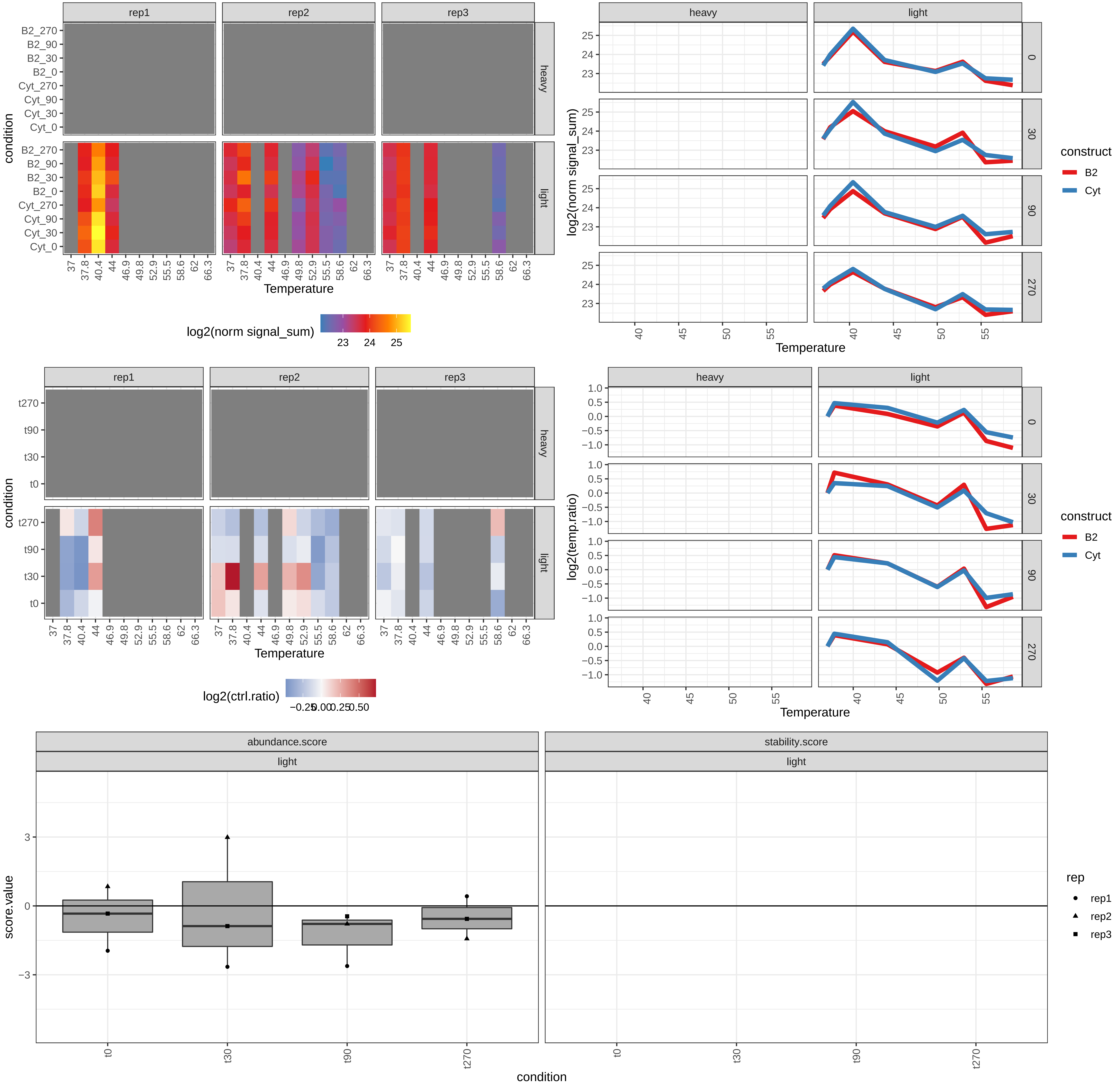

P47052 − Succinate dehydrogenase [ubiquinone] flavoprotein subunit 2, mitochondrial OS=Saccharomyces cerevisiae (strain ATCC 204508 / S288c) GN=YJL045W PE=3 SV=1

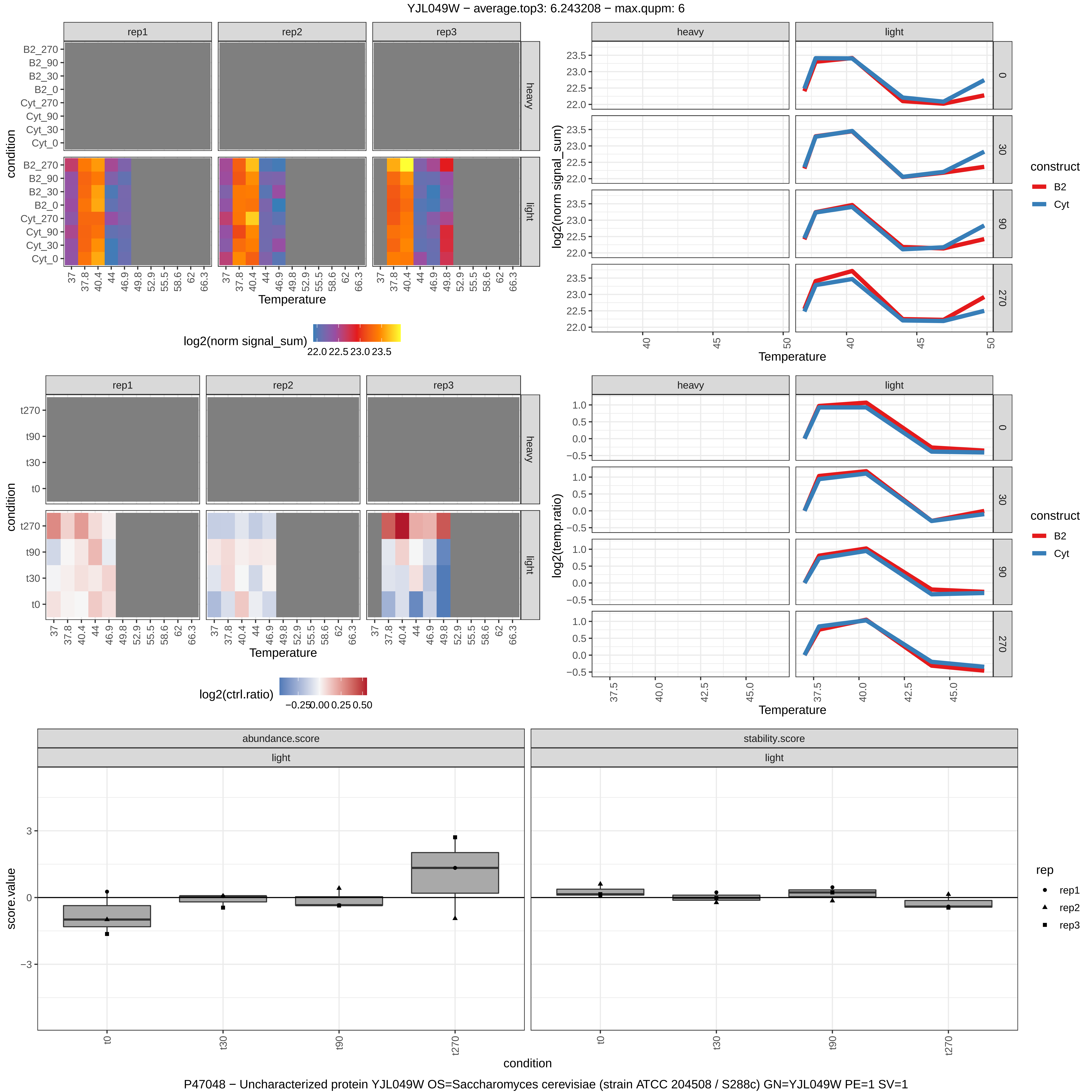

YJL049W − average.top3: 6.243208 − max.qupm: 6
rep1
rep2
rep3
heavy
light
condition
B2_270
B2_90
B2_30
B2_0
Cyt_270
Cyt_90
Cyt_30
Cyt_0
37
37.8
40.4
44
46.9
49.8
52.9
55.5
58.6
62
66.3
Temperature
log2(norm signal_sum)
22.0 22.5 23.0 23.5
log2(norm signal_sum)
0
30
90
270
40
45
50
construct
B2
Cyt
t270
t90
t30
t0
log2(ctrl.ratio)
−0.25 0.00 0.25 0.50
log2(temp.ratio)
1.0
0.5
0.0
−0.5
37.5
40.0
42.5
45.0
abundance.score
stability.score
score.value
3
0
−3
rep
rep1
rep2
rep3
P47048 − Uncharacterized protein YJL049W OS=Saccharomyces cerevisiae (strain ATCC 204508 / S288c) GN=YJL049W PE=1 SV=1

YJL055W − average.top3: 7.07410625 − max.qupm: 8

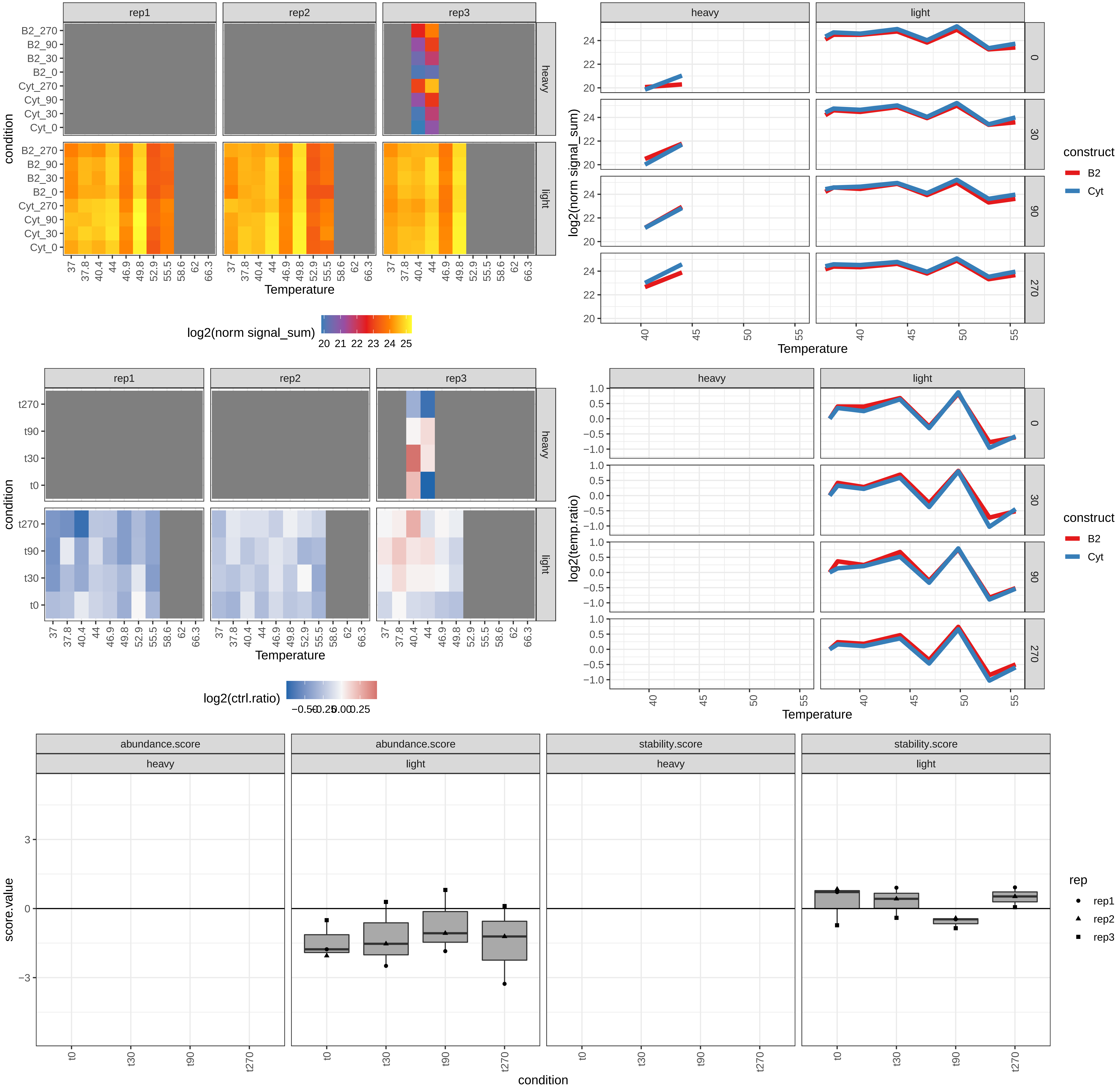

P47044 − LOG family protein YJL055W OS=Saccharomyces cerevisiae (strain ATCC 204508 / S288c) GN=YJL055W PE=1 SV=1

YJL068C − average.top3: 7.14348557692308 − max.qupm: 10

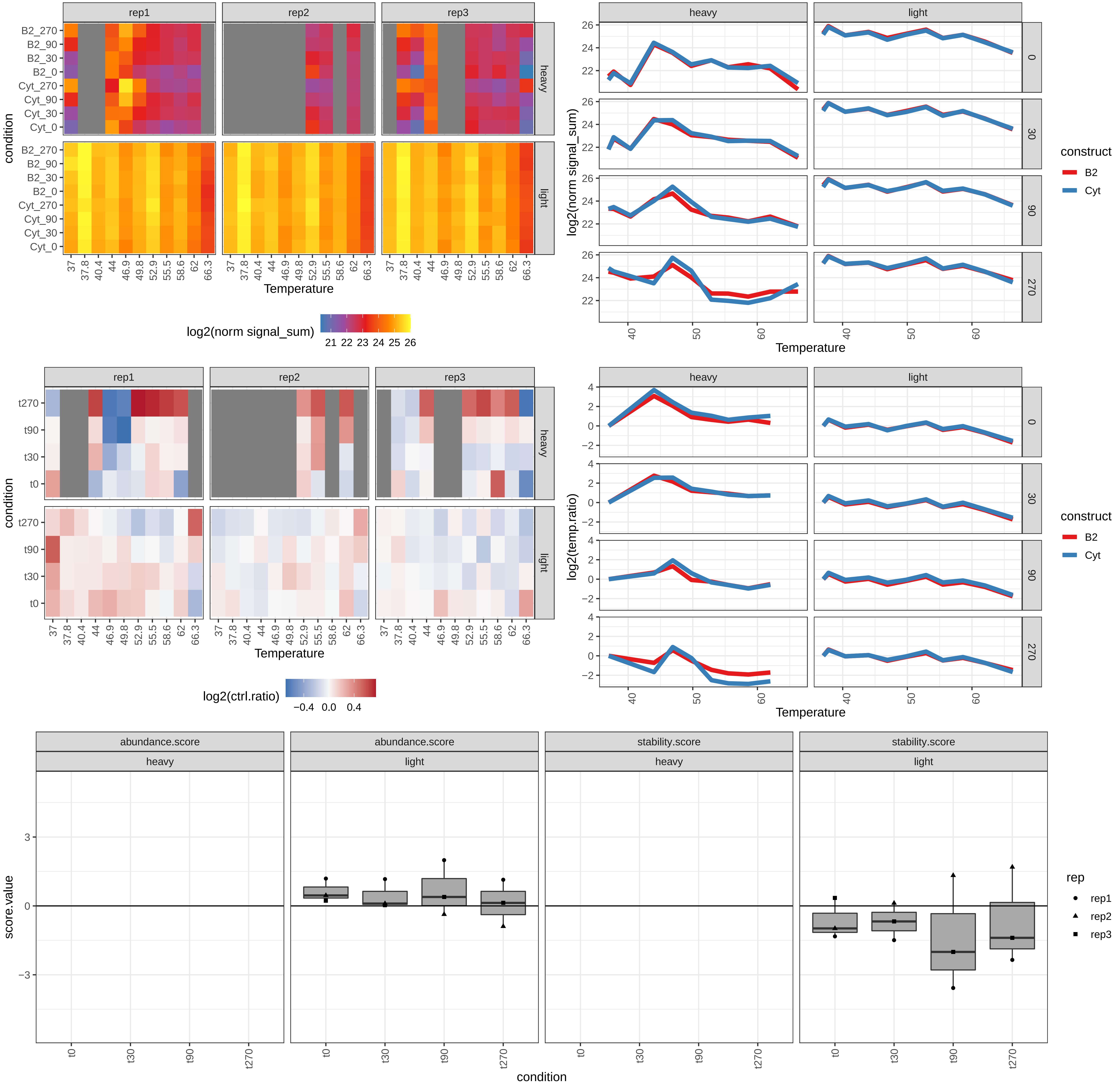

P40363 − S−formylglutathione hydrolase OS=Saccharomyces cerevisiae (strain ATCC 204508 / S288c) GN=YJL068C PE=1 SV=1

YJL070C − average.top3: 6.70953619047619 − max.qupm: 7

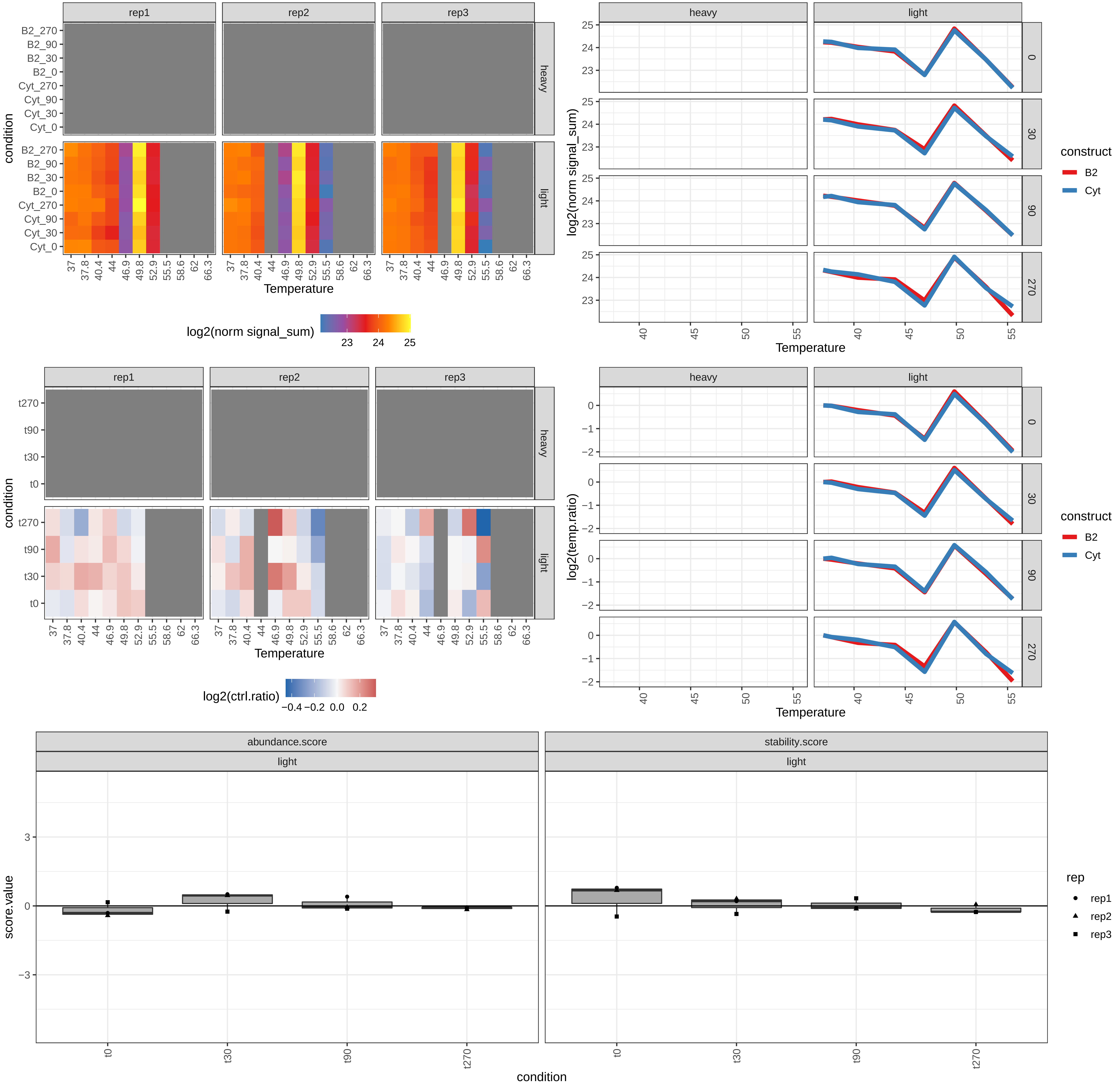

P40361 − Inactive deaminase YJL070C OS=Saccharomyces cerevisiae (strain ATCC 204508 / S288c) GN=YJL070C PE=1 SV=1

YJL132W − average.top3: 6.45811263157895 − max.qupm: 7

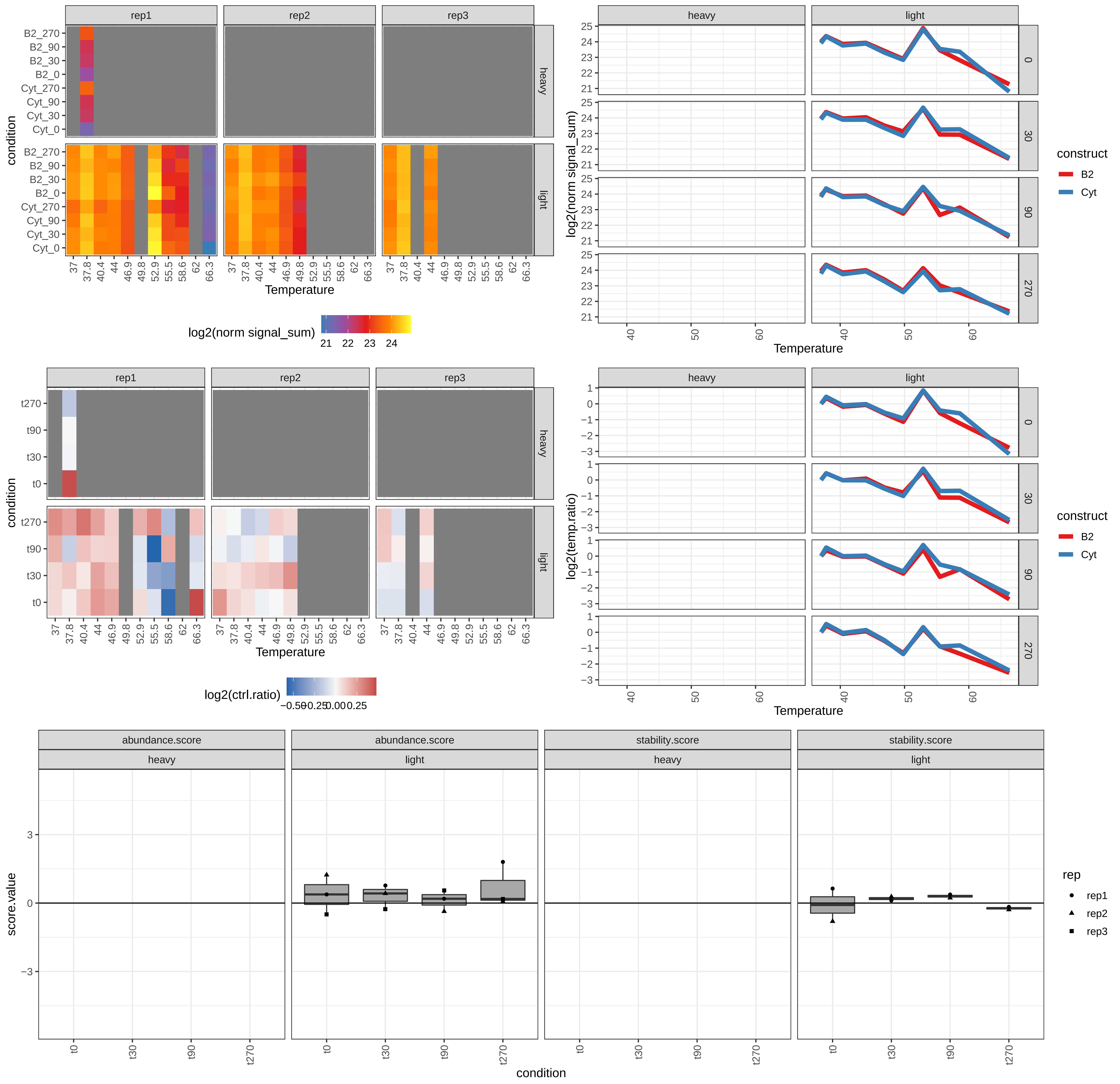

P47014 − Uncharacterized protein YJL132W OS=Saccharomyces cerevisiae (strain ATCC 204508 / S288c) GN=YJL132W PE=4 SV=1

YJL171C − average.top3: 7.11424 − max.qupm: 5

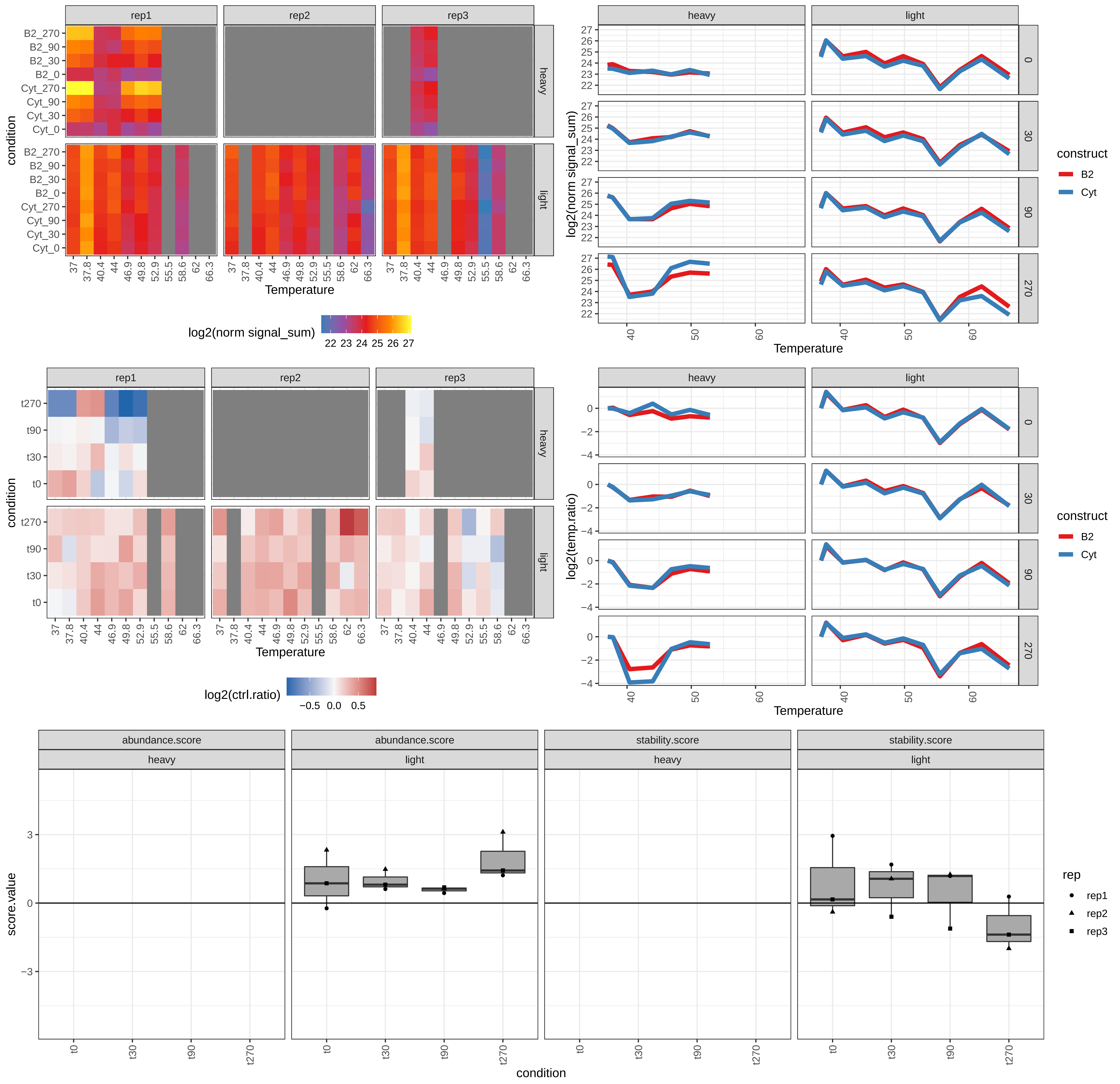

P46992 − Cell wall protein YJL171C OS=Saccharomyces cerevisiae (strain ATCC 204508 / S288c) GN=YJL171C PE=1 SV=2

YJL218W − average.top3: 6.612053 − max.qupm: 4

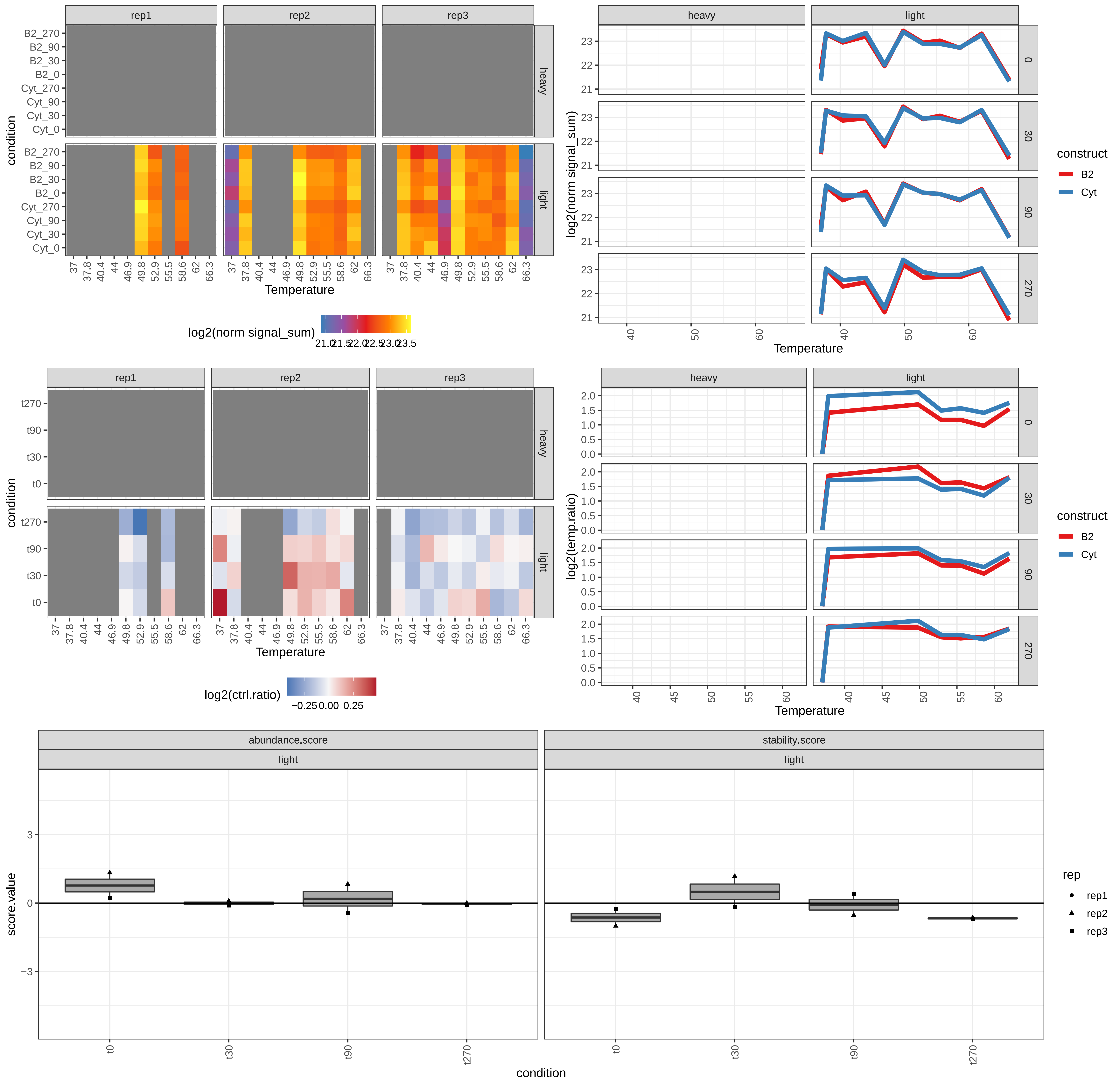

P40892 − Putative acetyltransferase YJL218W OS=Saccharomyces cerevisiae (strain ATCC 204508 / S288c) GN=YJL218W PE=1 SV=1

YJR008W − average.top3: 7.31013575757576 − max.qupm: 8

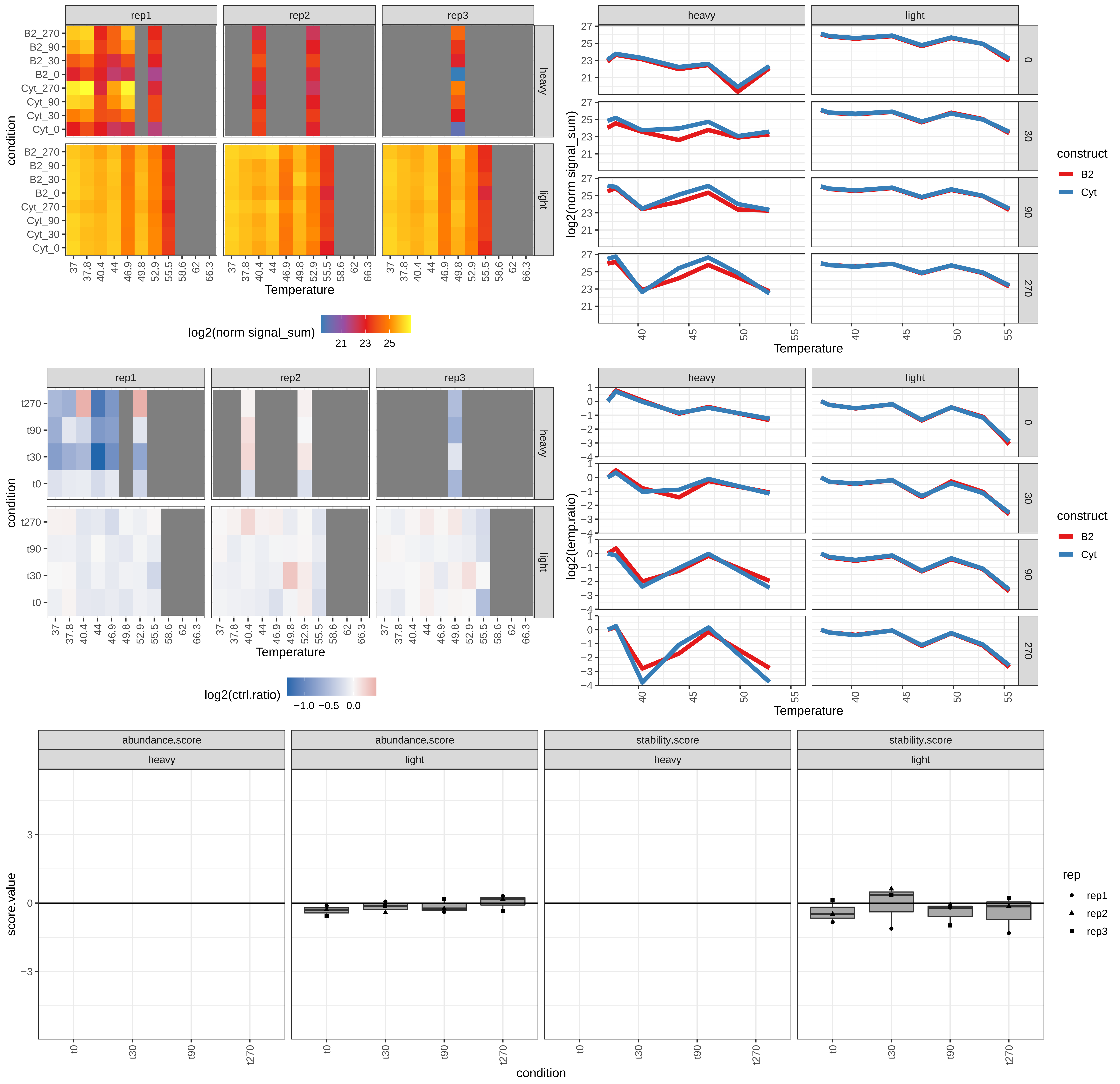

P47085 − MEMO1 family protein YJR008W OS=Saccharomyces cerevisiae (strain ATCC 204508 / S288c) GN=YJR008W PE=1 SV=1

YJR096W − average.top3: 6.87987 − max.qupm: 9

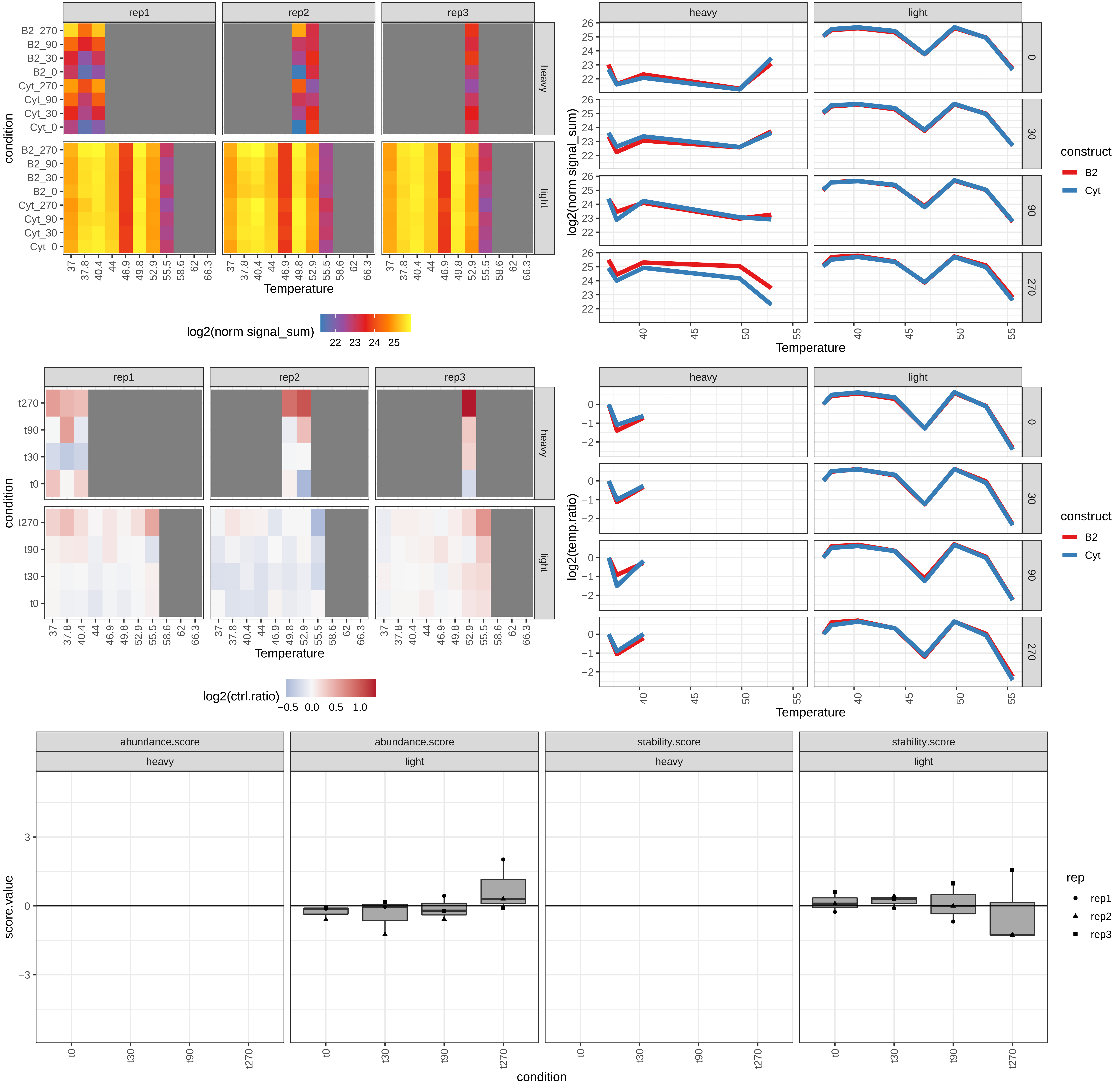

P47137 − Uncharacterized oxidoreductase YJR096W OS=Saccharomyces cerevisiae (strain ATCC 204508 / S288c) GN=YJR096W PE=1 SV=1

YJR098C − average.top3: 6.326977 − max.qupm: 4

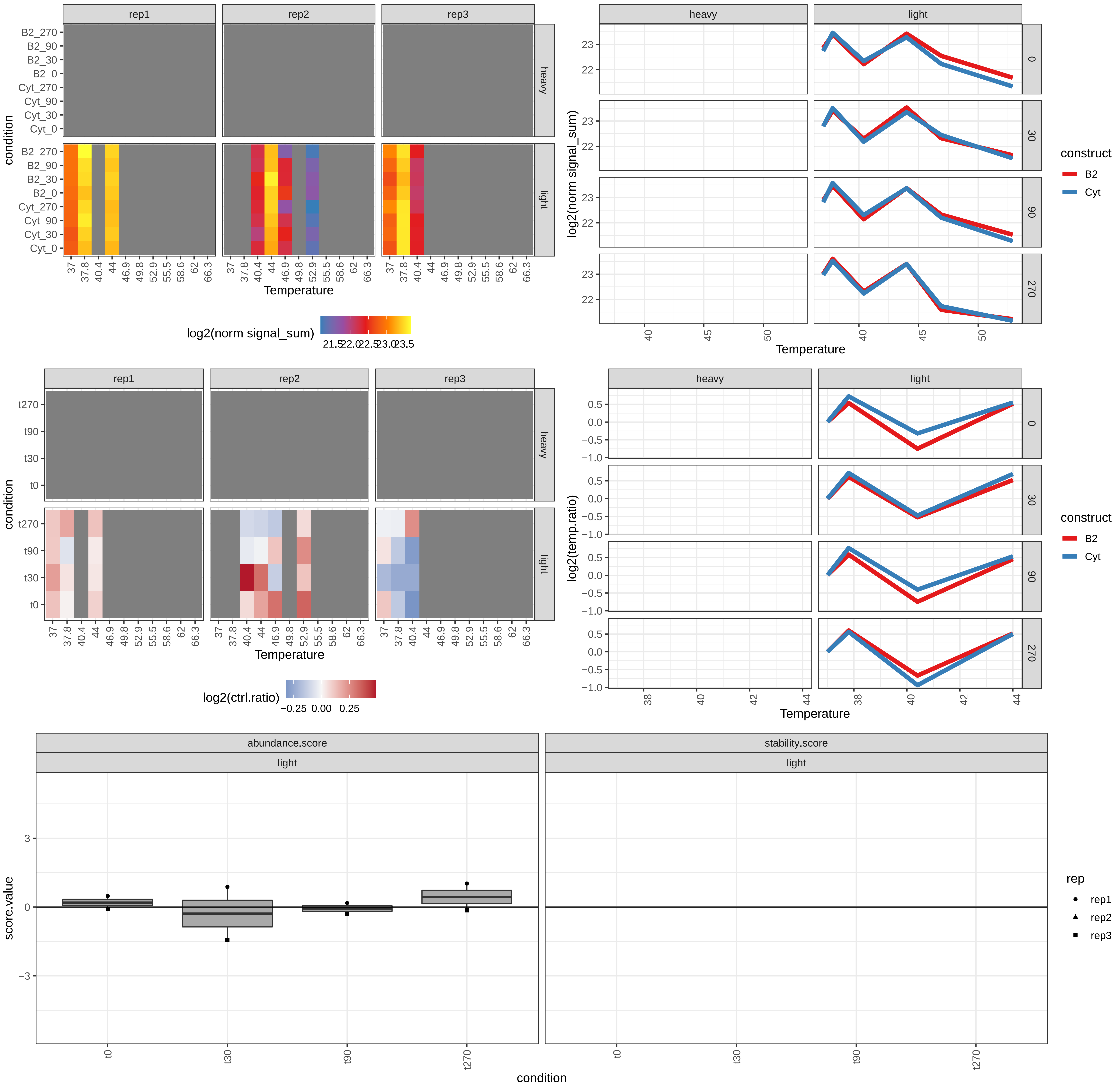

P47139 − Uncharacterized protein YJR098C OS=Saccharomyces cerevisiae (strain ATCC 204508 / S288c) GN=YJR098C PE=1 SV=2

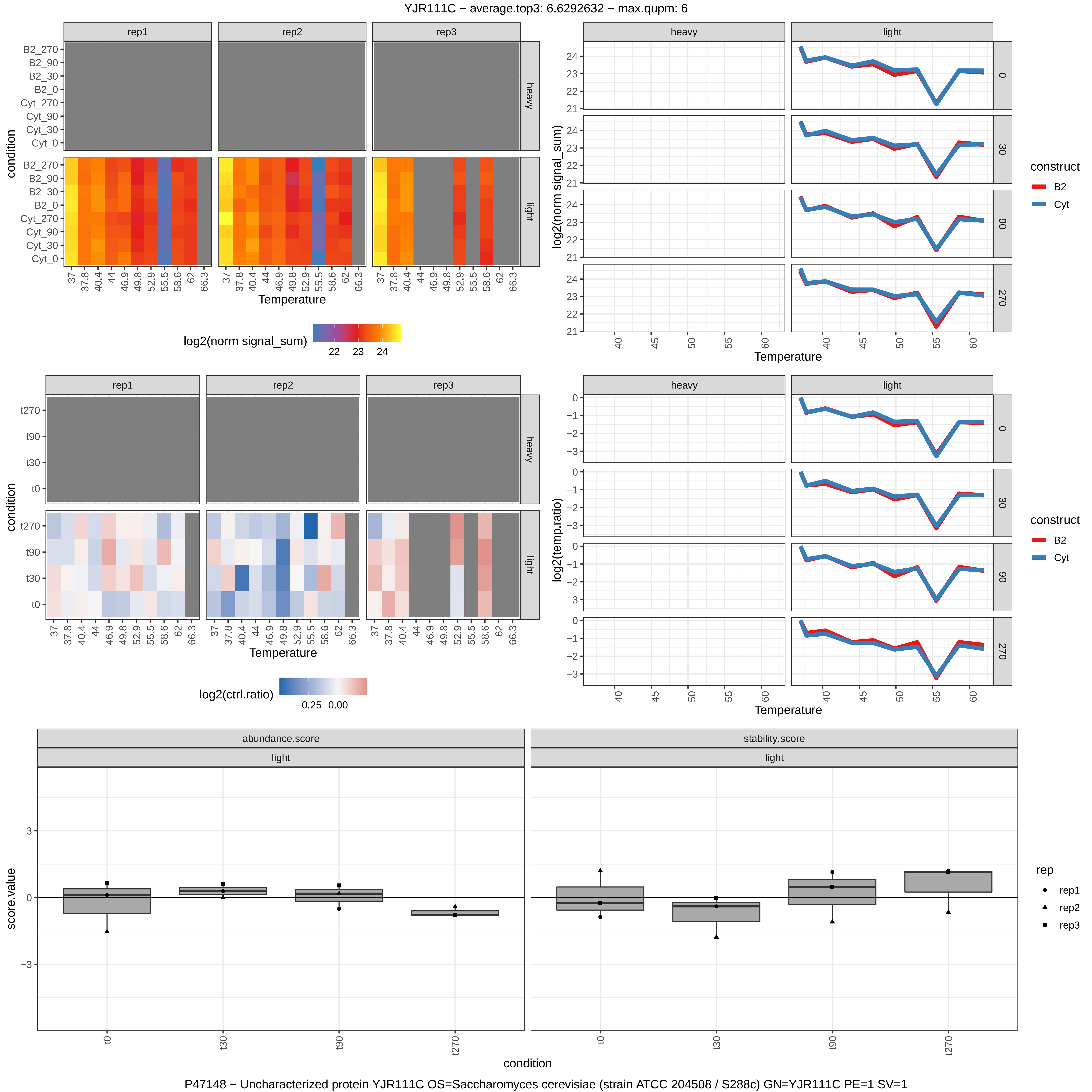
YJR111C − average.top3: 6.6292632 − max.qupm: 6
rep1
rep2
rep3
heavy
light
condition
Temperature
log2(norm signal_sum)
log2(norm signal_sum)
construct
B2
Cyt
log2(ctrl.ratio)
log2(temp.ratio)
abundance.score
stability.score
score.value
rep
rep1
rep2
rep3
P47148 − Uncharacterized protein YJR111C OS=Saccharomyces cerevisiae (strain ATCC 204508 / S288c) GN=YJR111C PE=1 SV=1

YJR142W − average.top3: 7.07260926829268 − max.qupm: 10

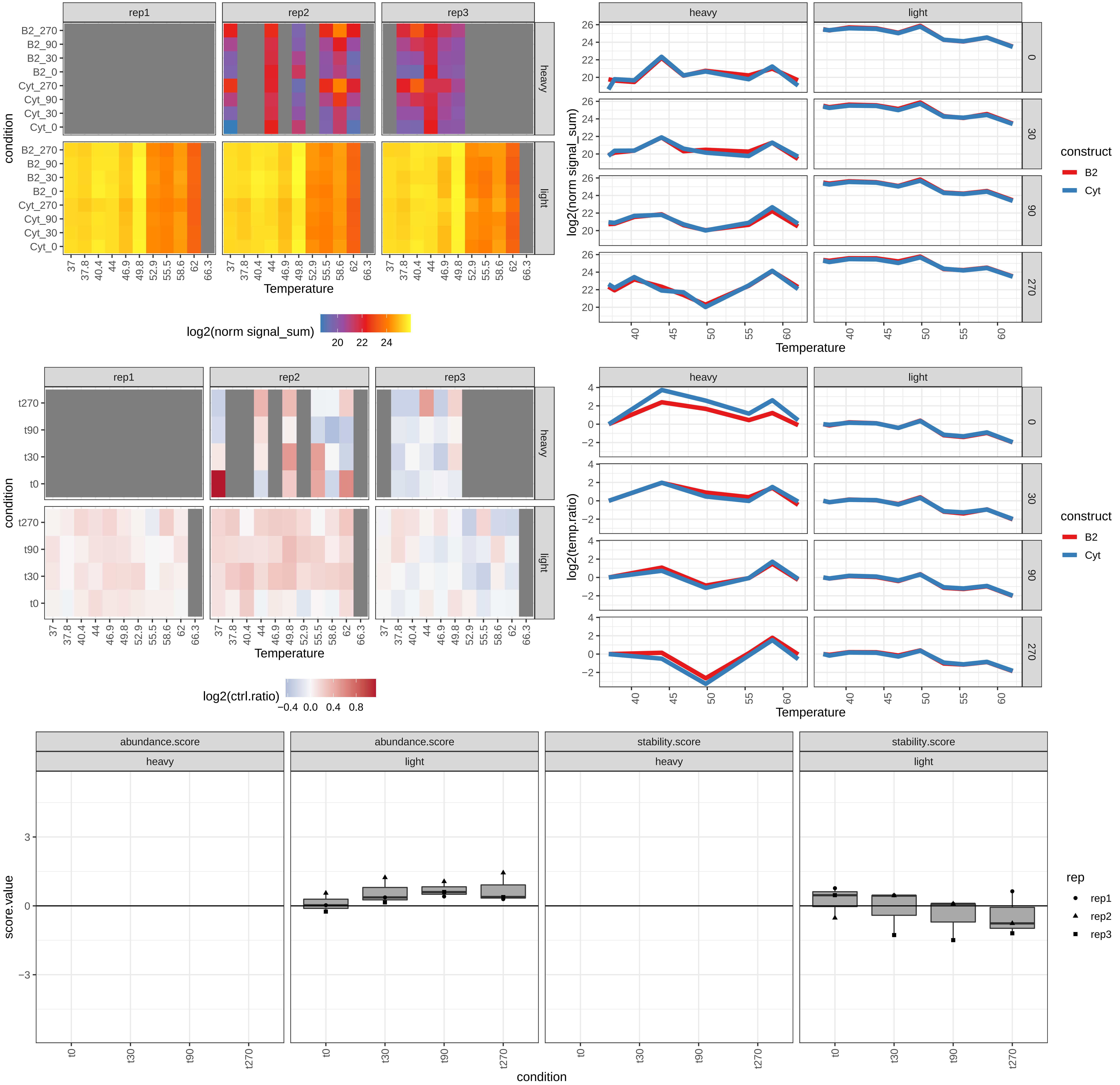

P47173 − Uncharacterized protein YJR142W OS=Saccharomyces cerevisiae (strain ATCC 204508 / S288c) GN=YJR142W PE=1 SV=1

YJR149W − average.top3: 6.75433090909091 − max.qupm: 6

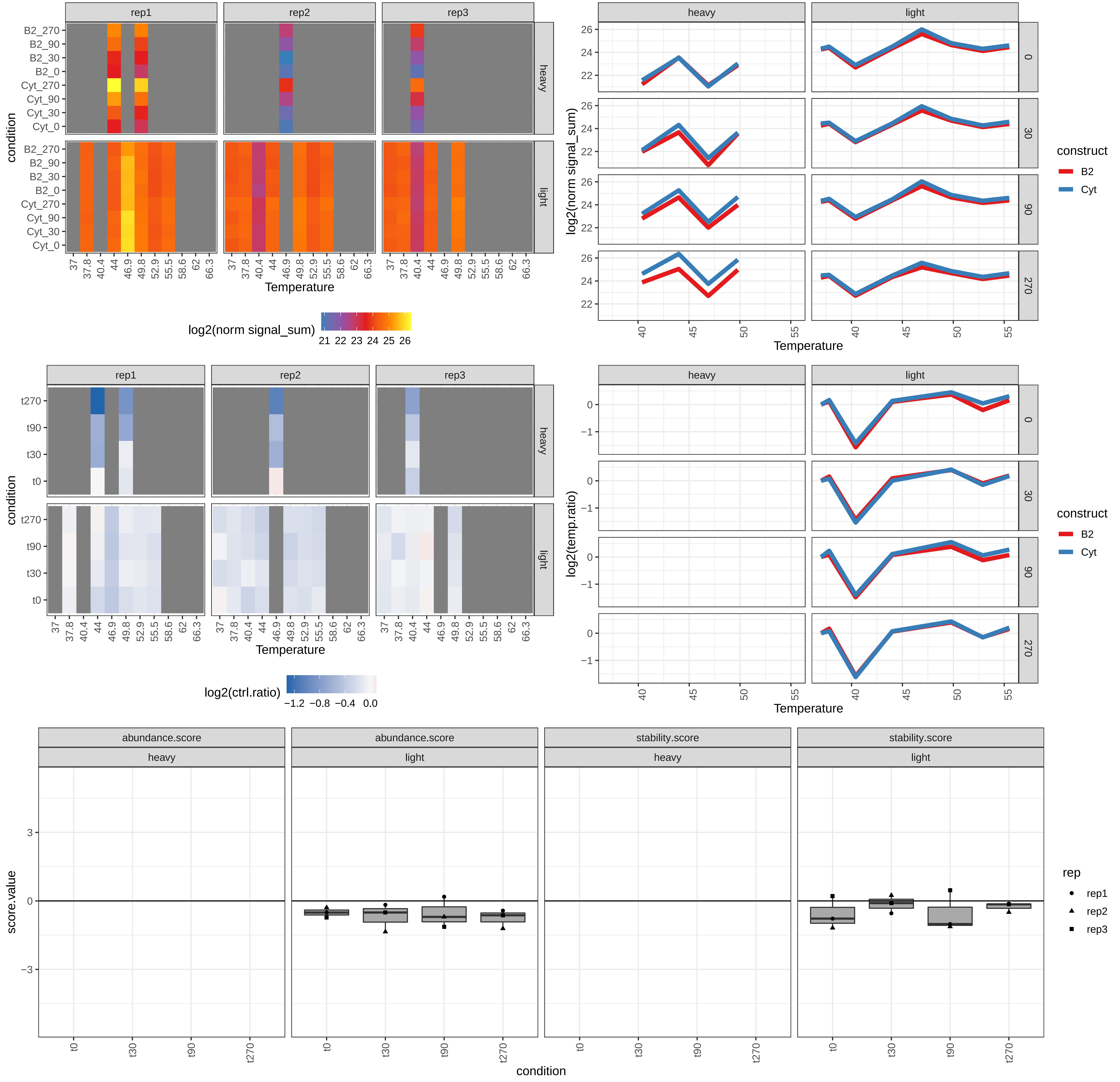

P47177 − Putative nitronate monooxygenase OS=Saccharomyces cerevisiae (strain ATCC 204508 / S288c) GN=YJR149W PE=1 SV=2

YJU3 − average.top3: 7.06785666666667 − max.qupm: 7

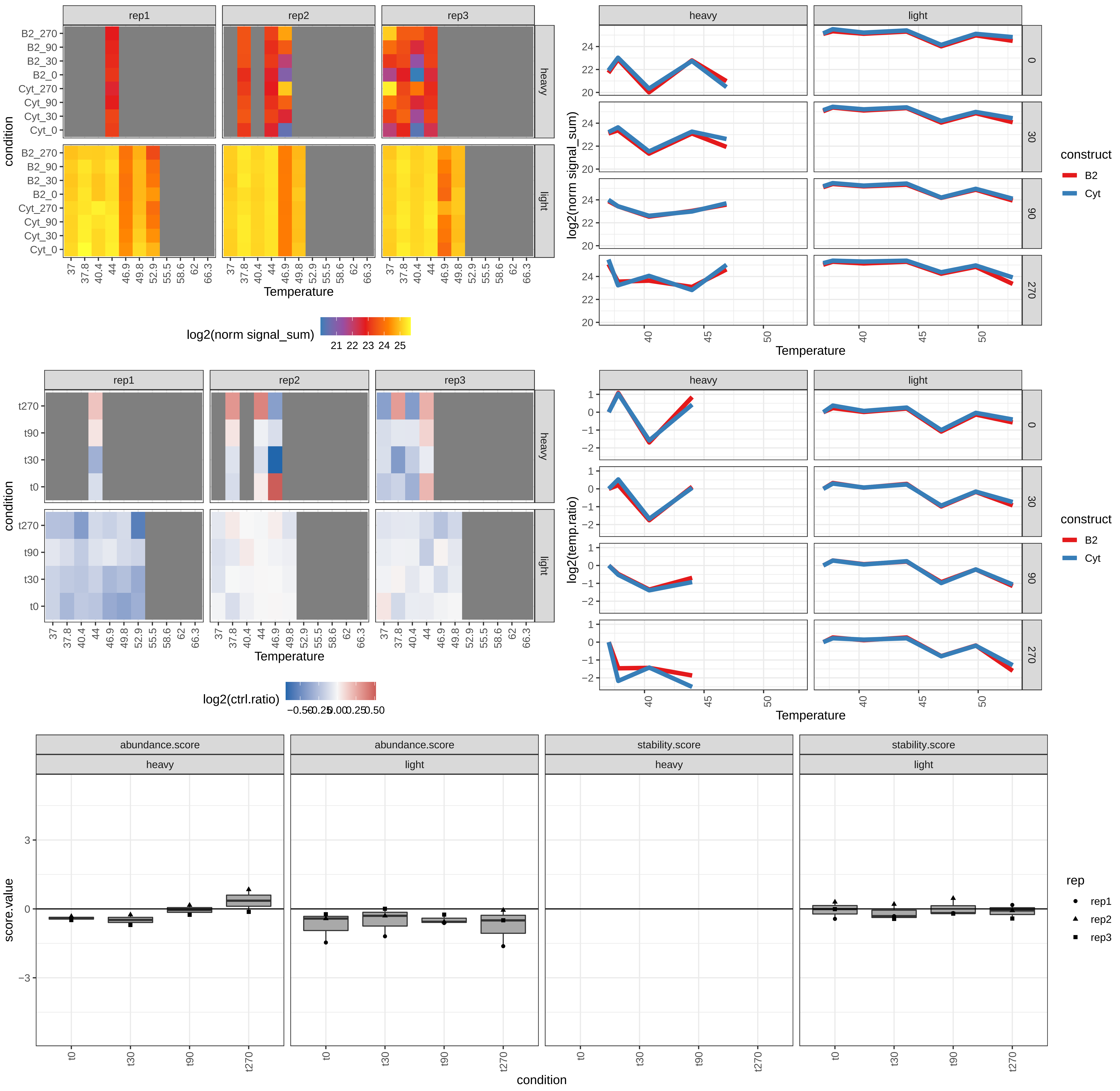

P28321 − Monoglyceride lipase OS=Saccharomyces cerevisiae (strain ATCC 204508 / S288c) GN=YJU3 PE=1 SV=2

YKL018C − average.top3: 7.0793428125 − max.qupm: 5

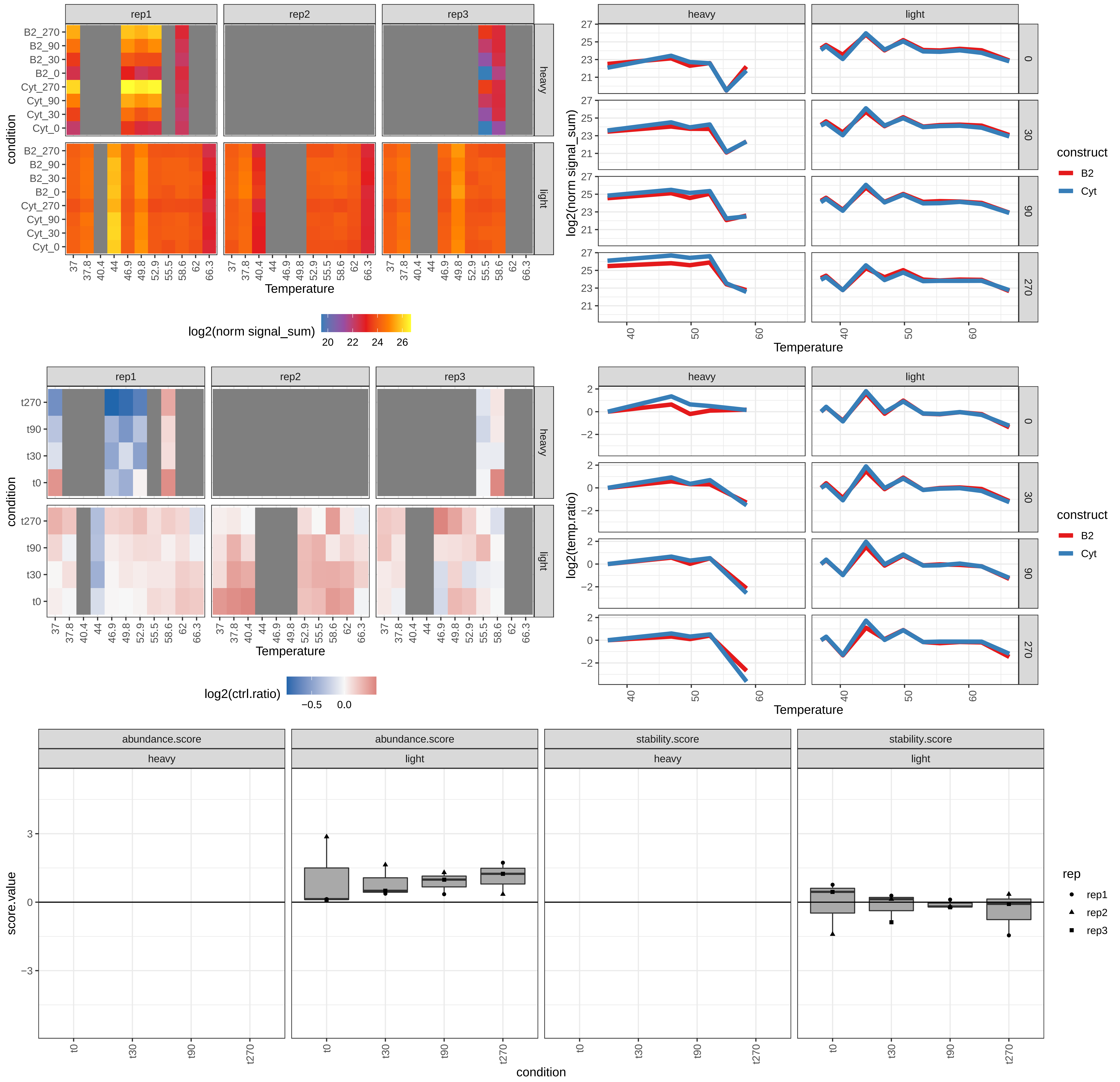

Q3E7A7 − Uncharacterized protein YKL018C−A OS=Saccharomyces cerevisiae (strain ATCC 204508 / S288c) GN=YKL018C−A PE=1 SV=1

YKL023W − average.top3: 7.000664 − max.qupm: 5

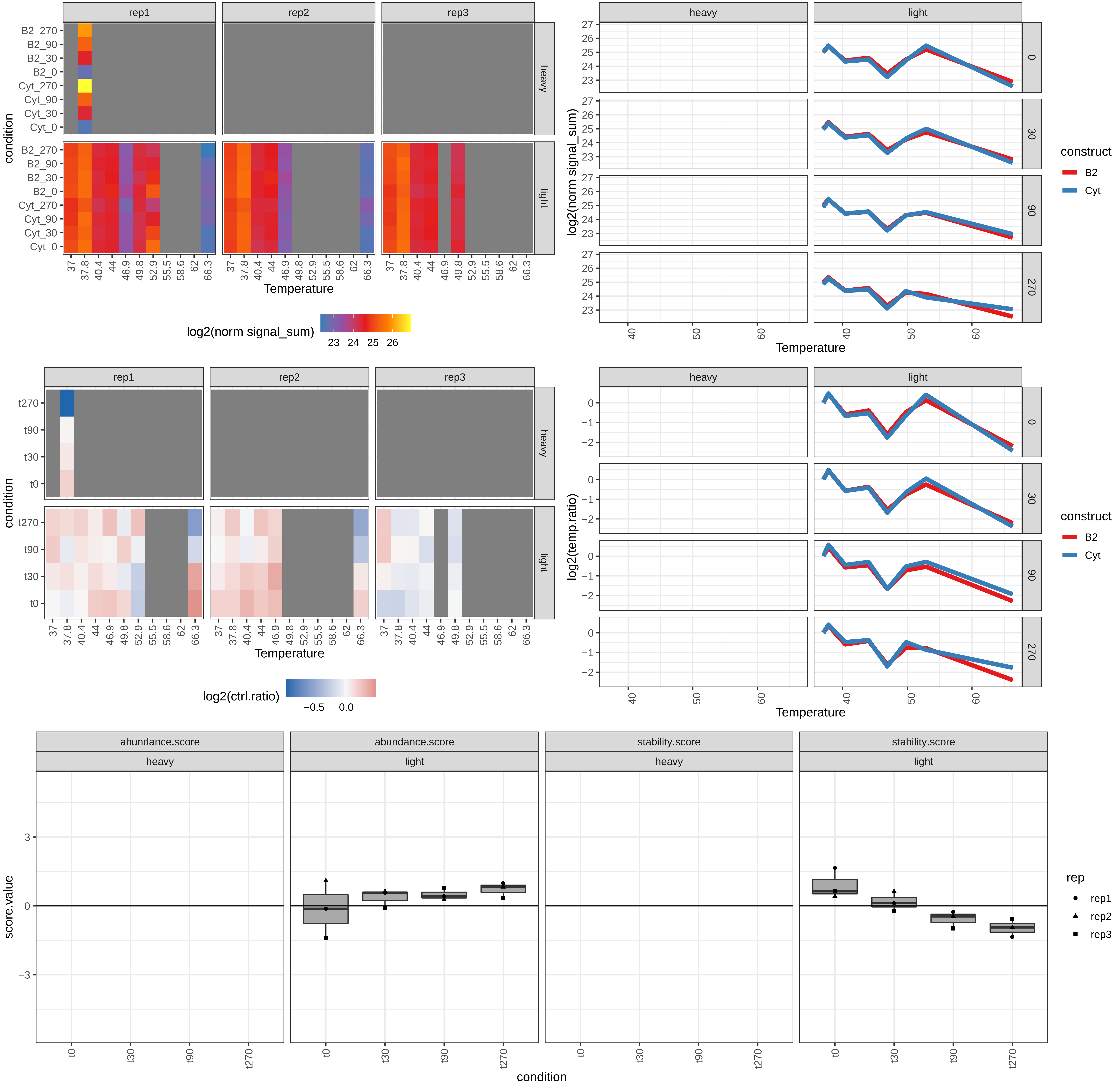

P36103 − Uncharacterized protein YKL023W OS=Saccharomyces cerevisiae (strain ATCC 204508 / S288c) GN=YKL023W PE=1 SV=2

YKL033W − average.top3: 7.08935322580645 − max.qupm: 8

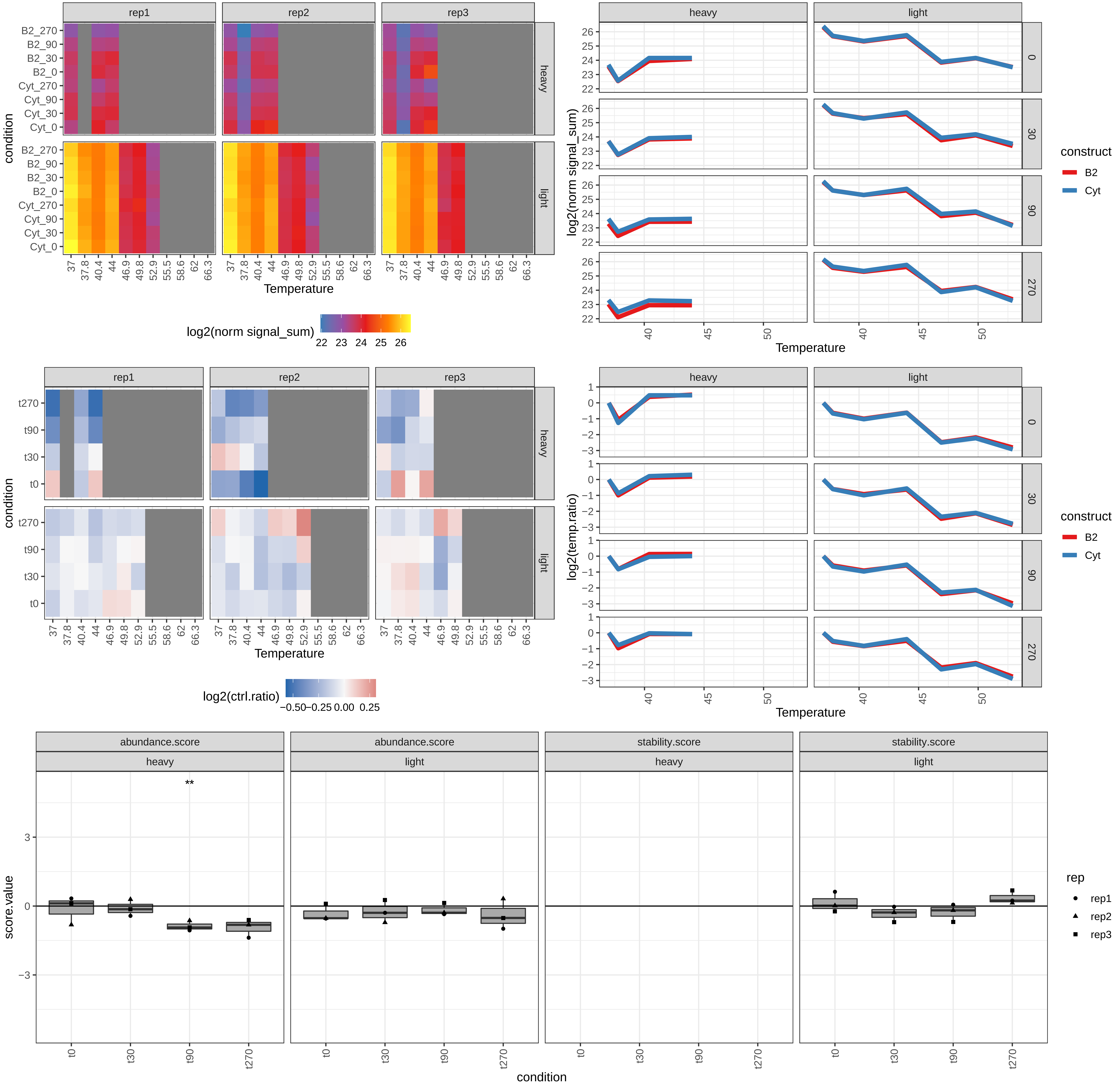

Q86ZR7 − Putative uncharacterized hydrolase YKL033W−A OS=Saccharomyces cerevisiae (strain ATCC 204508 / S288c) GN=YKL033W−A PE=3 SV=2

YKL069W − average.top3: 6.694818 − max.qupm: 5

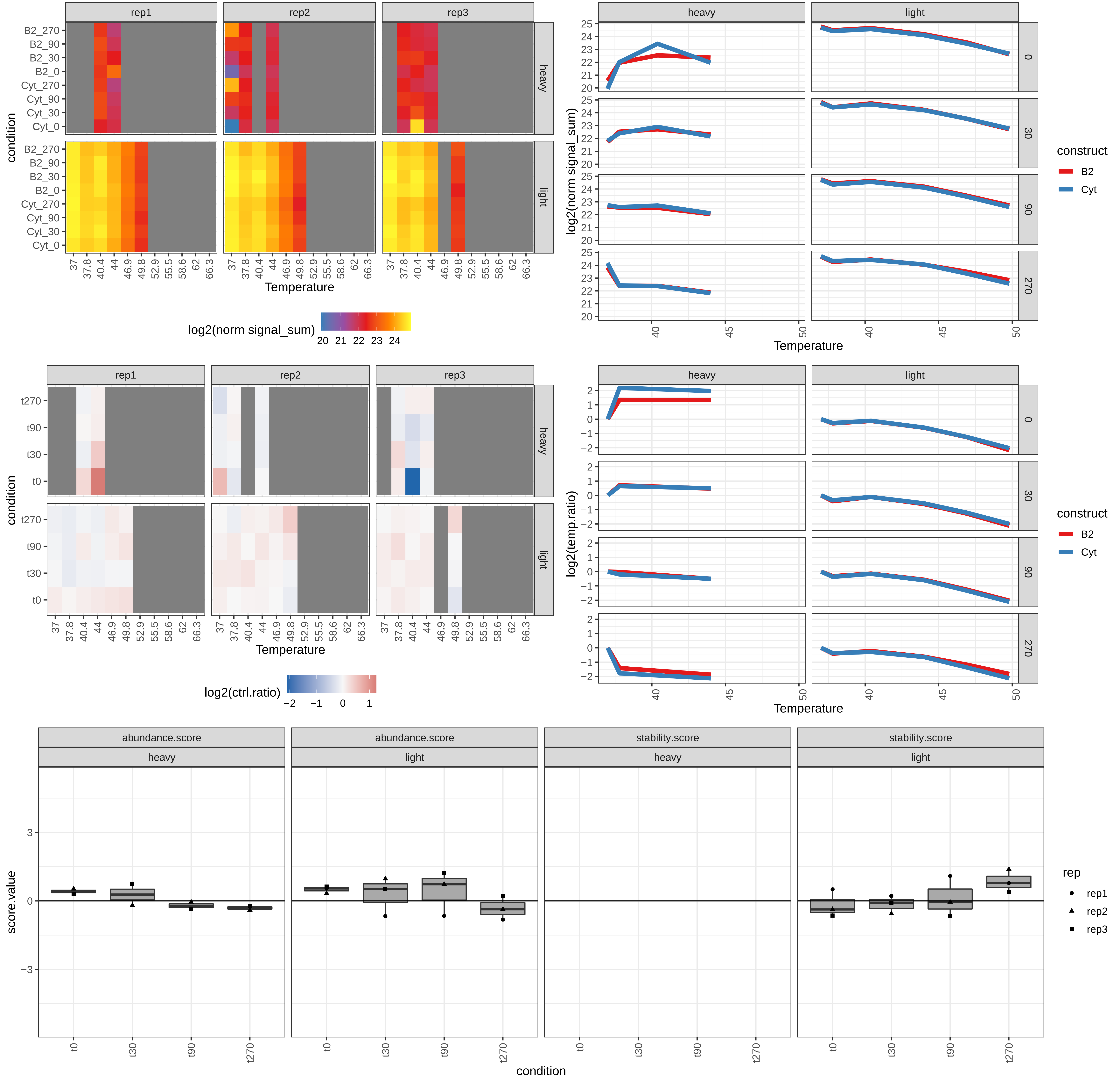

P36088 − Free methionine−R−sulfoxide reductase OS=Saccharomyces cerevisiae (strain ATCC 204508 / S288c) GN=YKL069W PE=1 SV=1

YKL077W − average.top3: 7.14305380952381 − max.qupm: 7

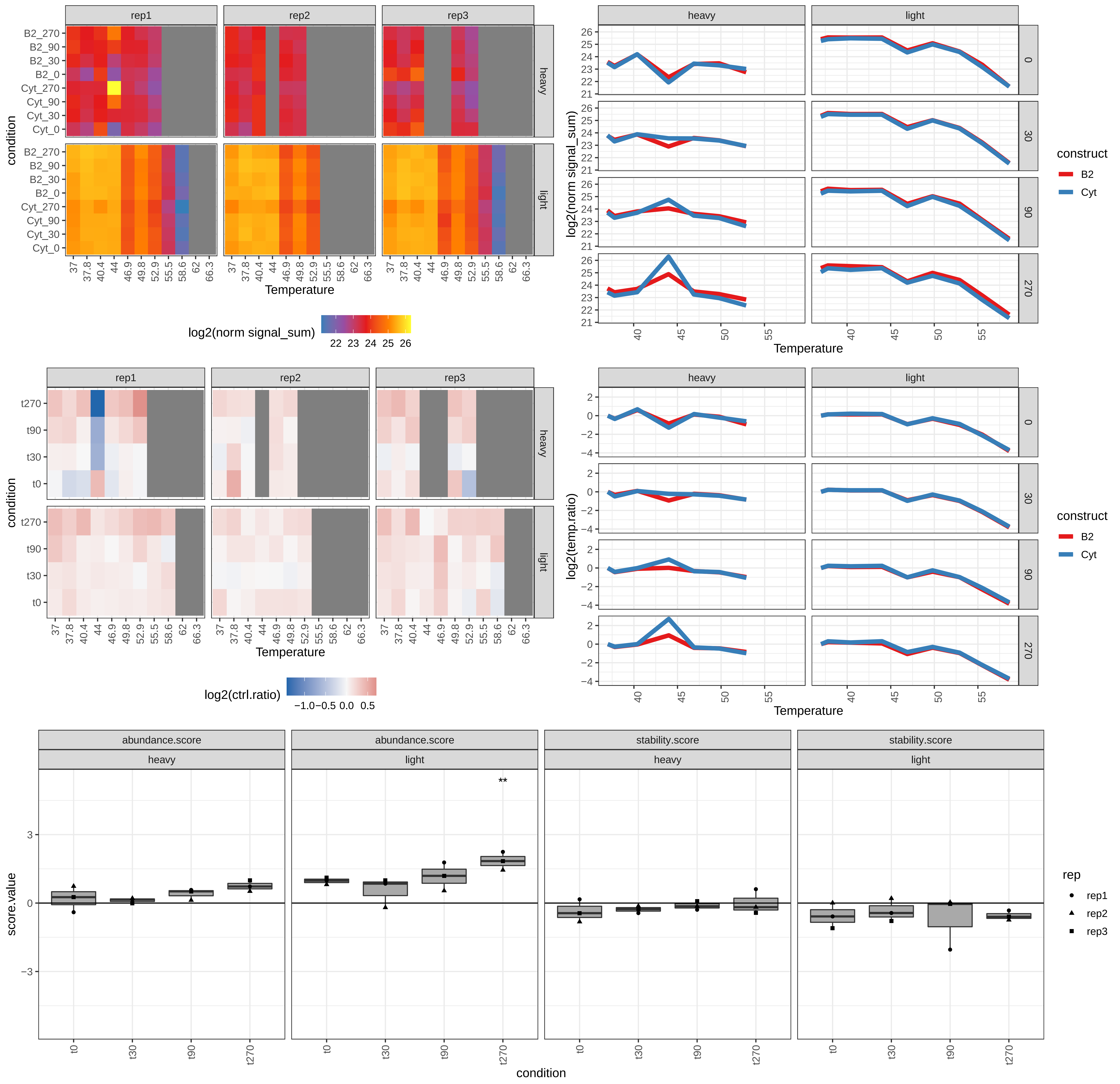

P36081 − Uncharacterized protein YKL077W OS=Saccharomyces cerevisiae (strain ATCC 204508 / S288c) GN=YKL077W PE=1 SV=1

YKL091C − average.top3: 6.90919454545455 − max.qupm: 8

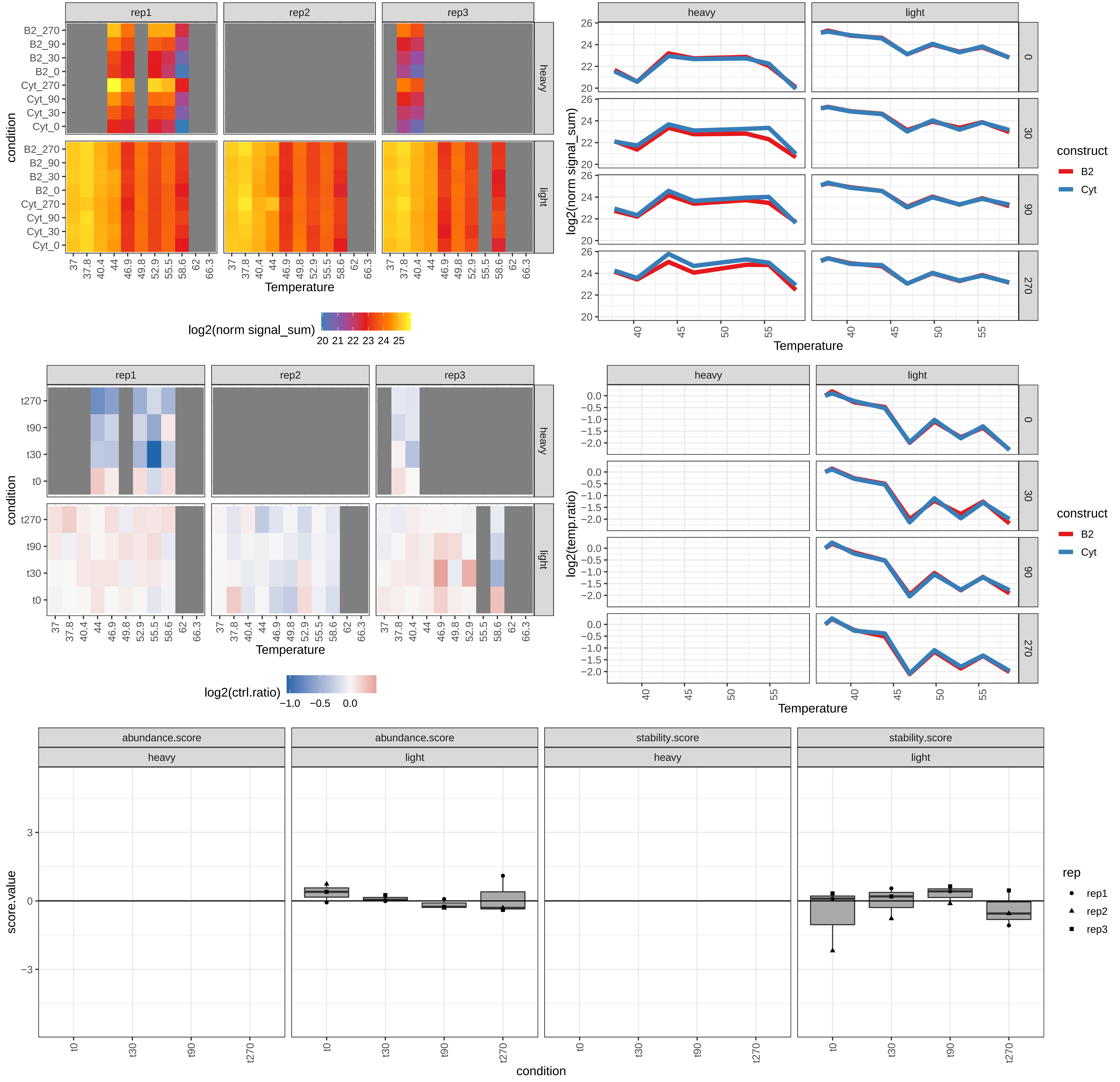

P33324 − CRAL−TRIO domain−containing protein YKL091C OS=Saccharomyces cerevisiae (strain ATCC 204508 / S288c) GN=YKL091C PE=1 SV=2

YKL100C − average.top3: 6.93981583333333 − max.qupm: 6

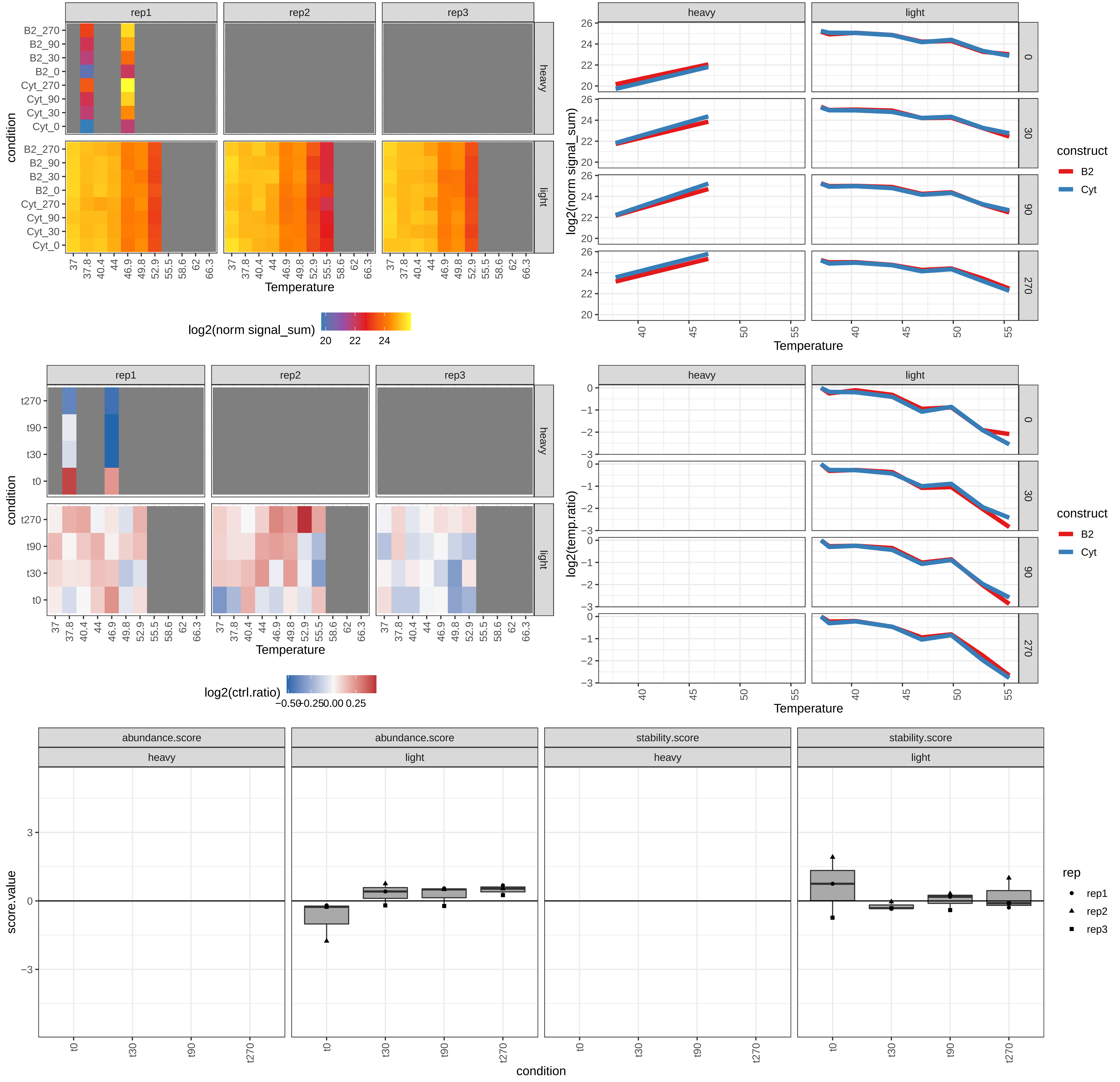

P34248 − Probable intramembrane protease YKL100C OS=Saccharomyces cerevisiae (strain ATCC 204508 / S288c) GN=YKL100C PE=1 SV=1

YKL151C − average.top3: 7.09776309090909 − max.qupm: 10

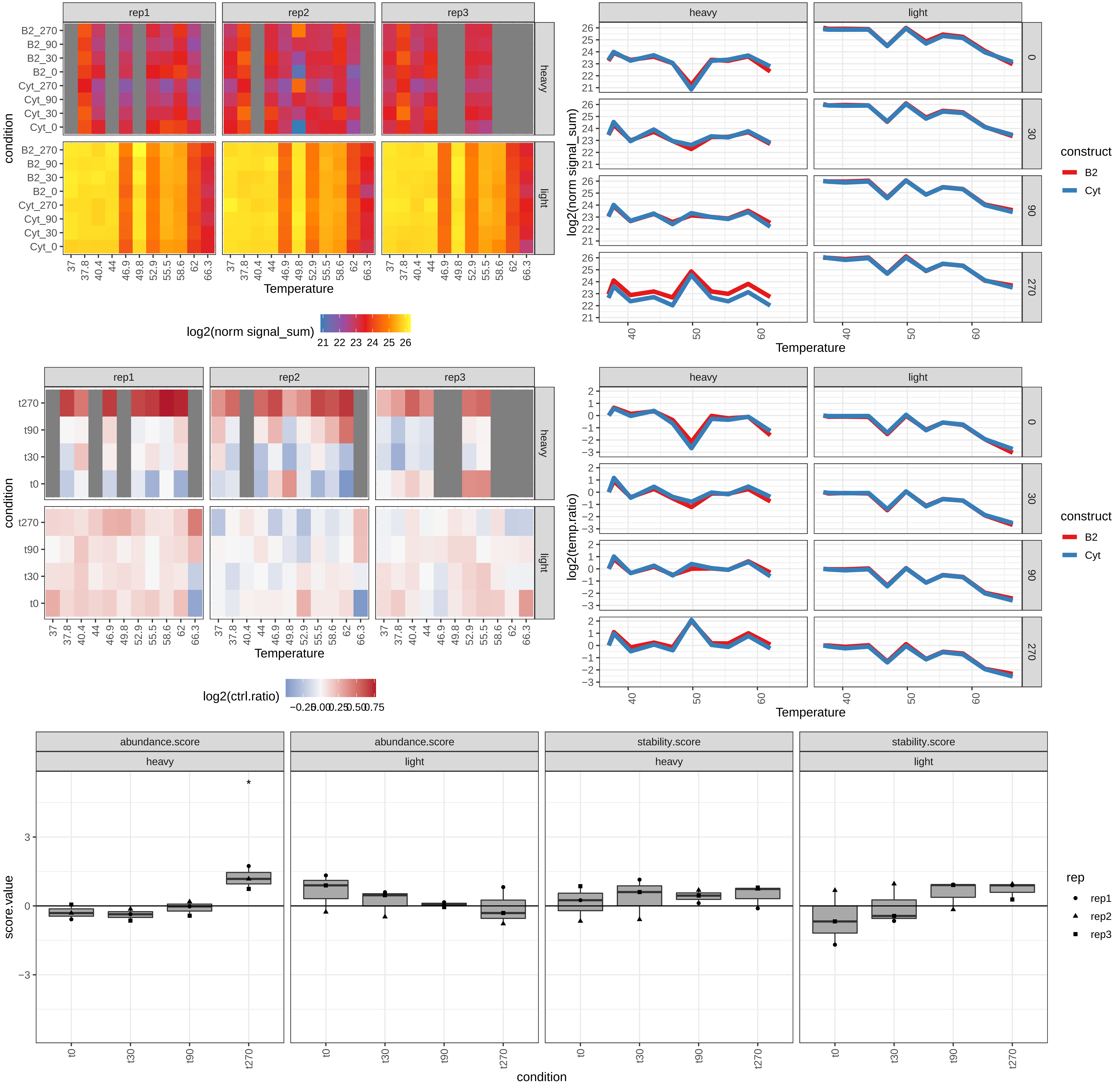

P36059 − ATP−dependent (S)−NAD(P)H−hydrate dehydratase OS=Saccharomyces cerevisiae (strain ATCC 204508 / S288c) GN=YKL151C PE=1 SV=1

YKL187C − average.top3: 6.60844394736842 − max.qupm: 5

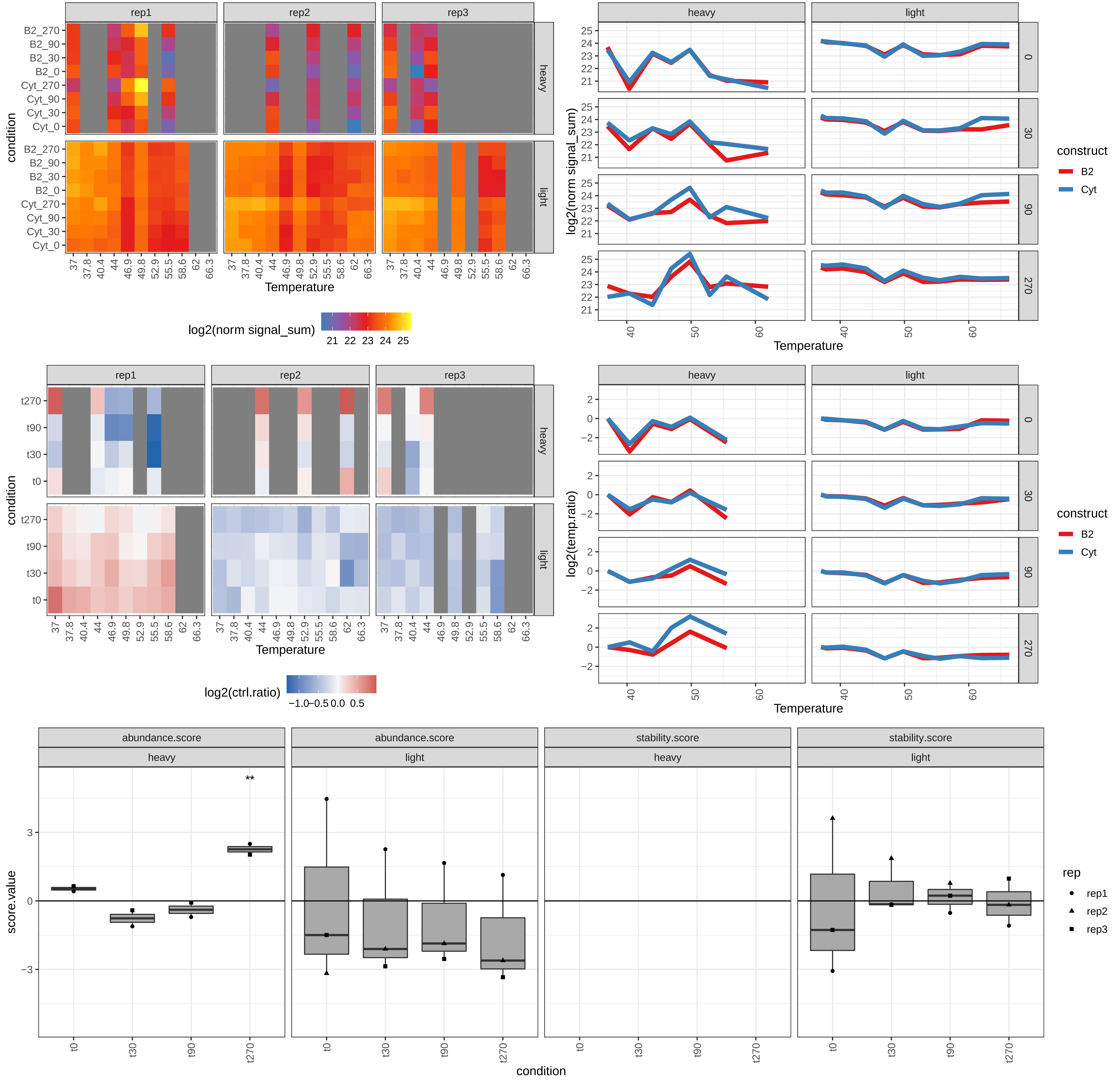

P34231 − Uncharacterized protein YKL187C OS=Saccharomyces cerevisiae (strain ATCC 204508 / S288c) GN=YKL187C PE=1 SV=1

YKR018C − average.top3: 7.04713975 − max.qupm: 9

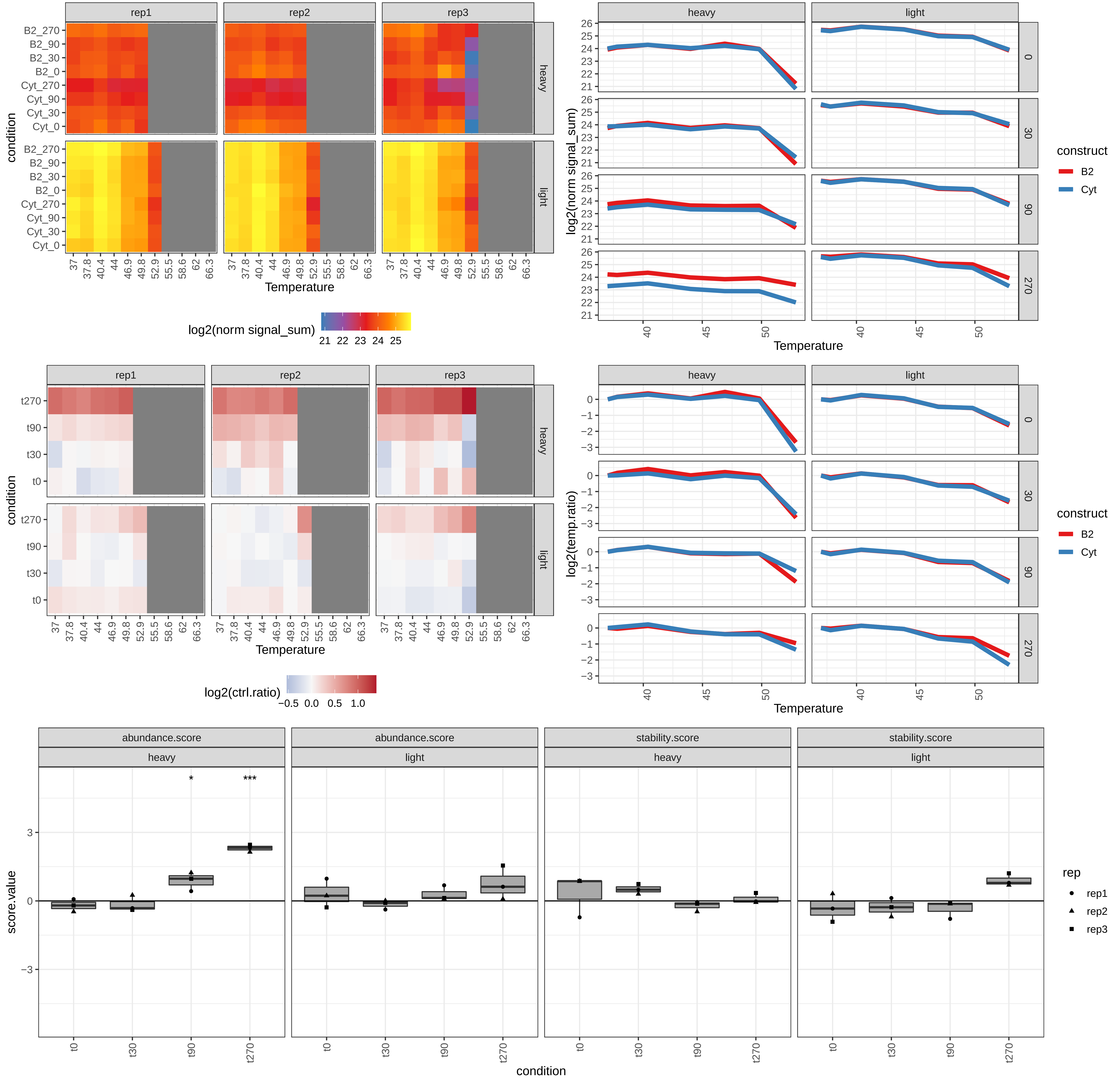

P36114 − Mitochondrial outer membrane protein YKR018C OS=Saccharomyces cerevisiae (strain ATCC 204508 / S288c) GN=YKR018C PE=1 SV=1

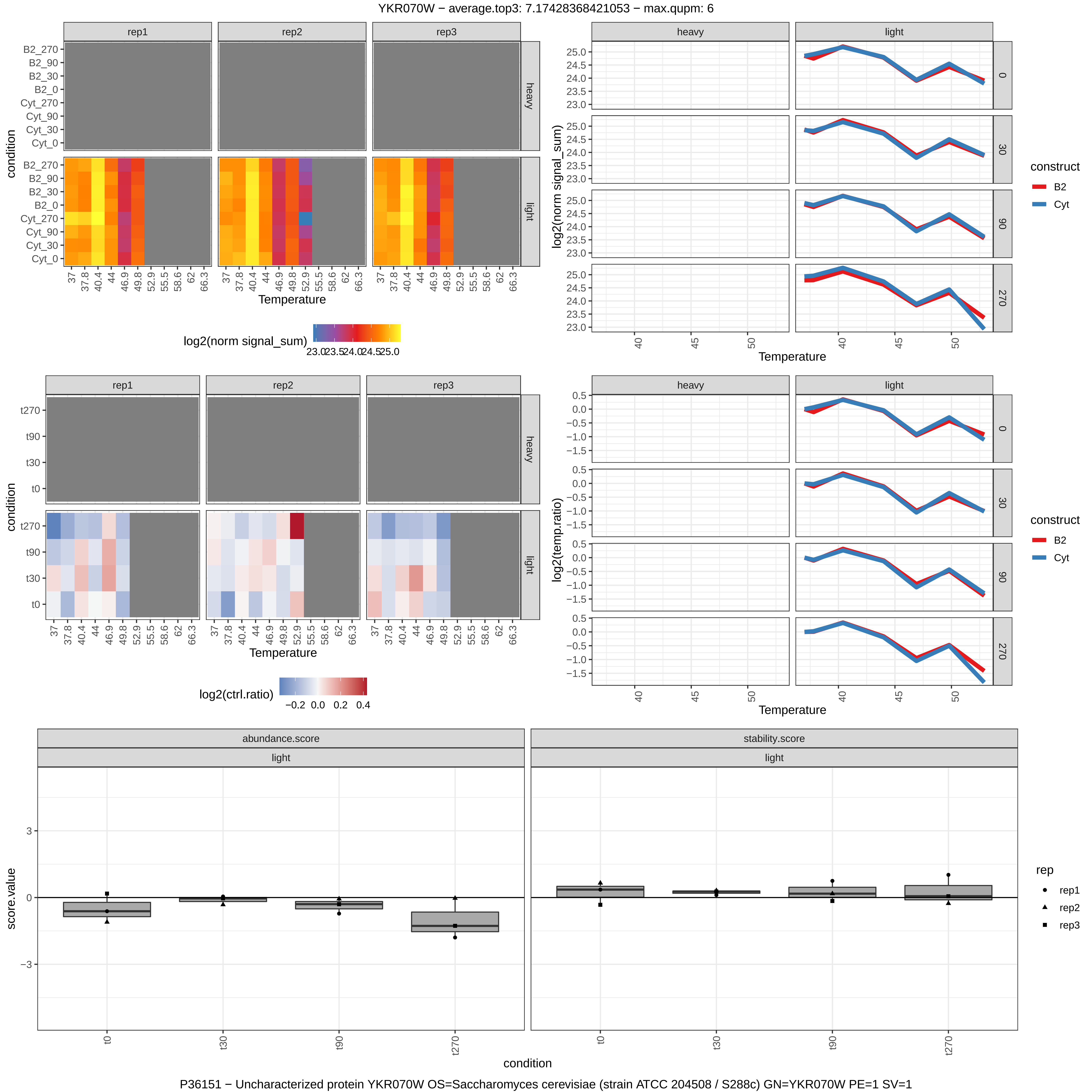
YKR070W − average.top3: 7.17428368421053 − max.qupm: 6
rep1
rep2
rep3
heavy
light
B2_270
B2_90
B2_30
B2_0
Cyt_270
Cyt_90
Cyt_30
Cyt_0
condition
37
37.8
40.4
44
46.9
49.8
52.9
55.5
58.6
62
66.3
Temperature
log2(norm signal_sum)
23.0
23.5
24.0
24.5
25.0
log2(norm signal_sum)
0
30
90
270
construct
B2
Cyt
40
45
50
t270
t90
t30
t0
log2(ctrl.ratio)
−0.2
0.0
0.2
0.4
log2(temp.ratio)
0.5
0.0
−0.5
−1.0
−1.5
abundance.score
stability.score
score.value
3
0
−3
rep
rep1
rep2
rep3
condition
P36151 − Uncharacterized protein YKR070W OS=Saccharomyces cerevisiae (strain ATCC 204508 / S288c) GN=YKR070W PE=1 SV=1

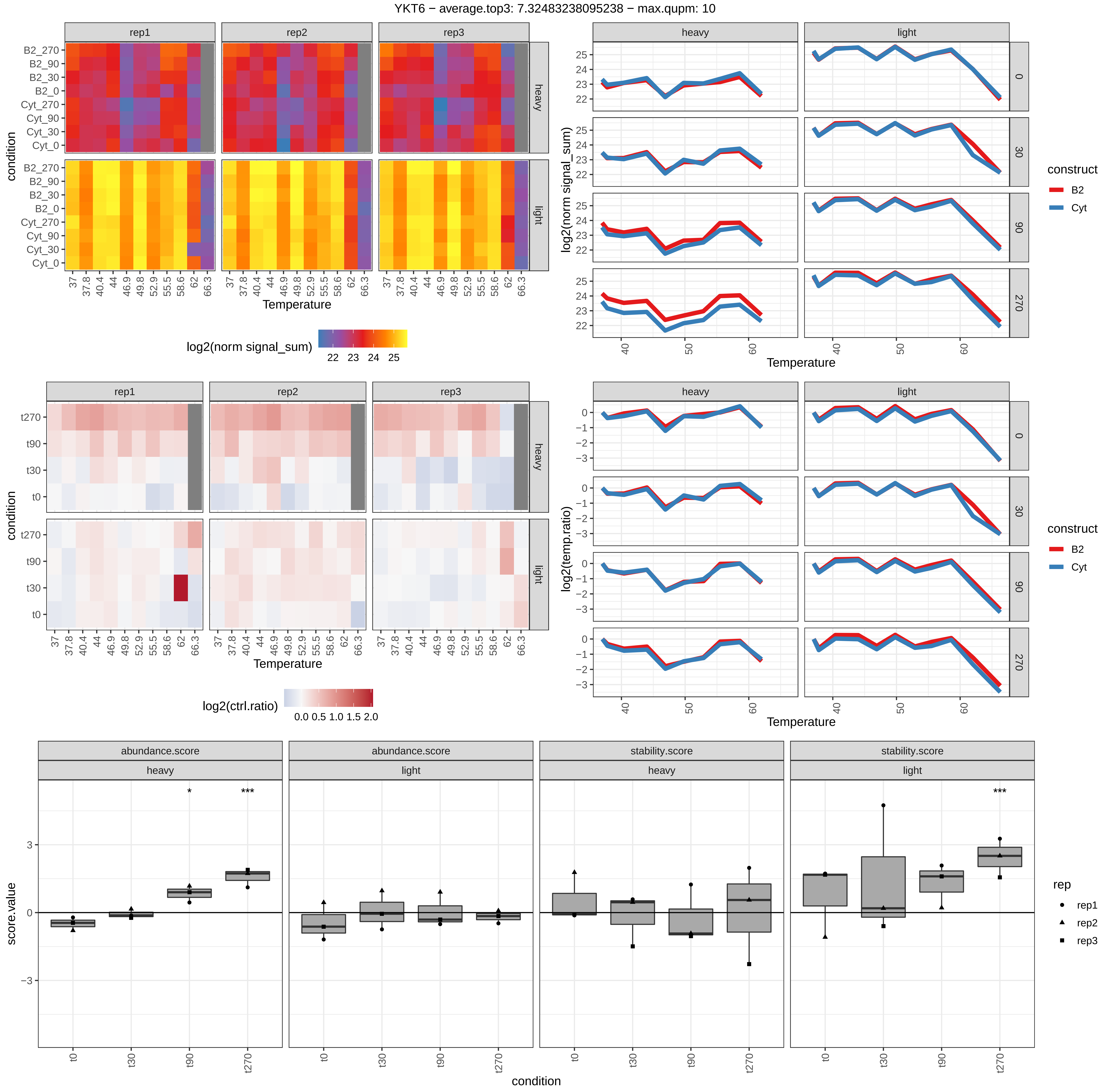

P36015 − Synaptobrevin homolog YKT6 OS=Saccharomyces cerevisiae (strain ATCC 204508 / S288c) GN=YKT6 PE=1 SV=1

YKU70 − average.top3: 6.16401545454545 − max.qupm: 6

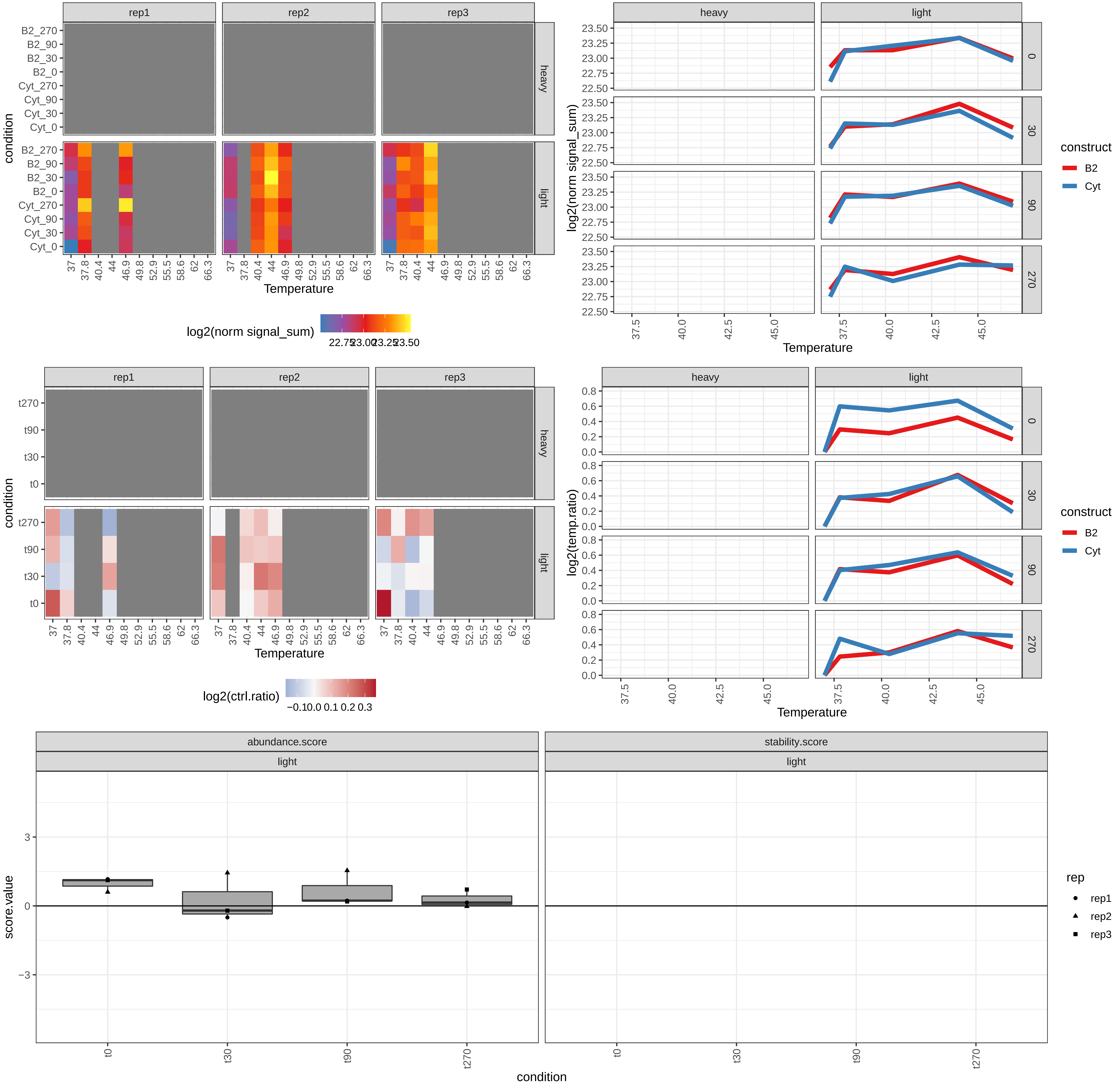

P32807 − ATP−dependent DNA helicase II subunit 1 OS=Saccharomyces cerevisiae (strain ATCC 204508 / S288c) GN=YKU70 PE=1 SV=2

YLF2 − average.top3: 6.00761444444444 − max.qupm: 3

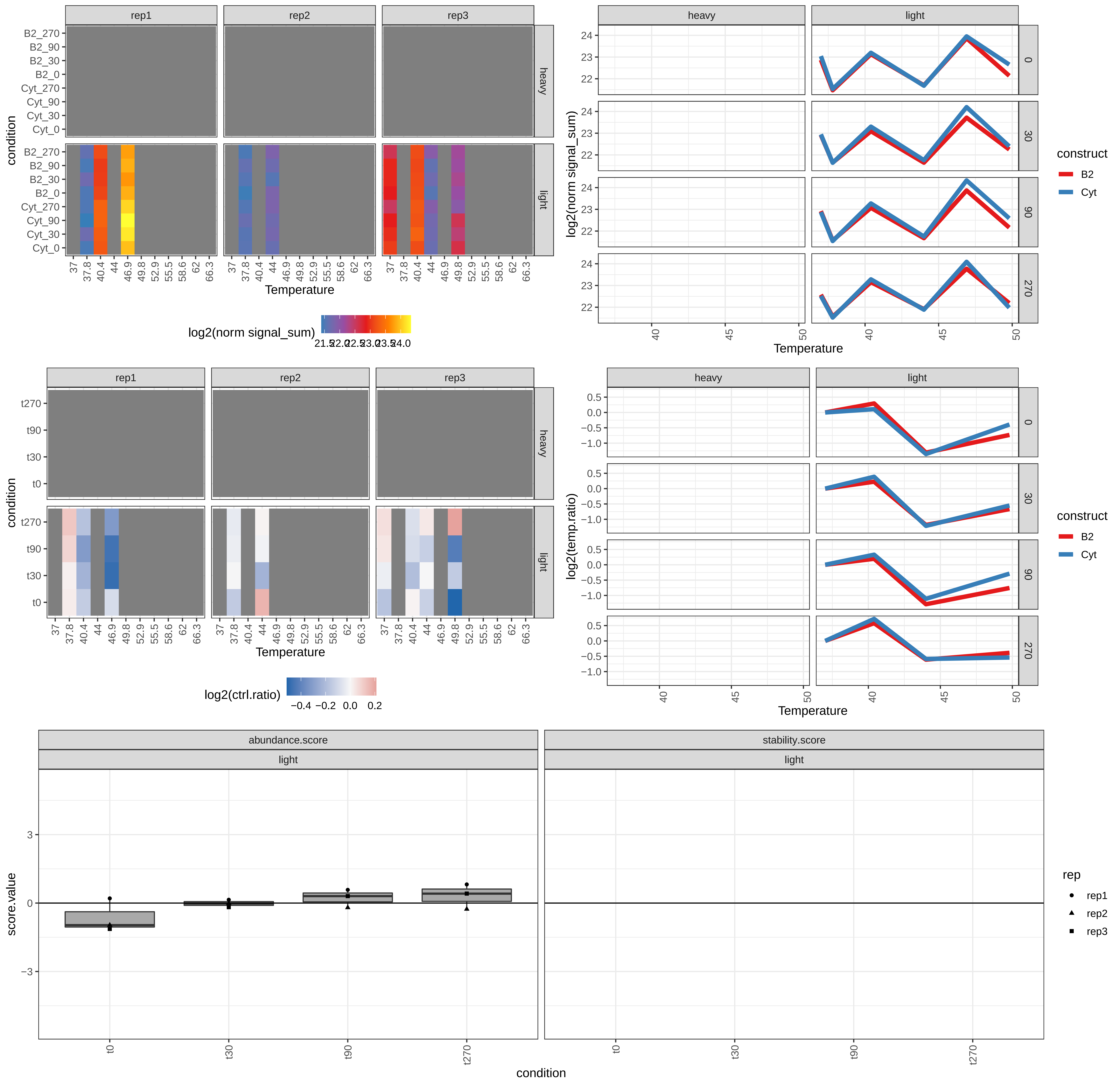

P38746 − Obg−like ATPase homolog OS=Saccharomyces cerevisiae (strain ATCC 204508 / S288c) GN=YLF2 PE=1 SV=1

YLH47 − average.top3: 7.0573615 − max.qupm: 6

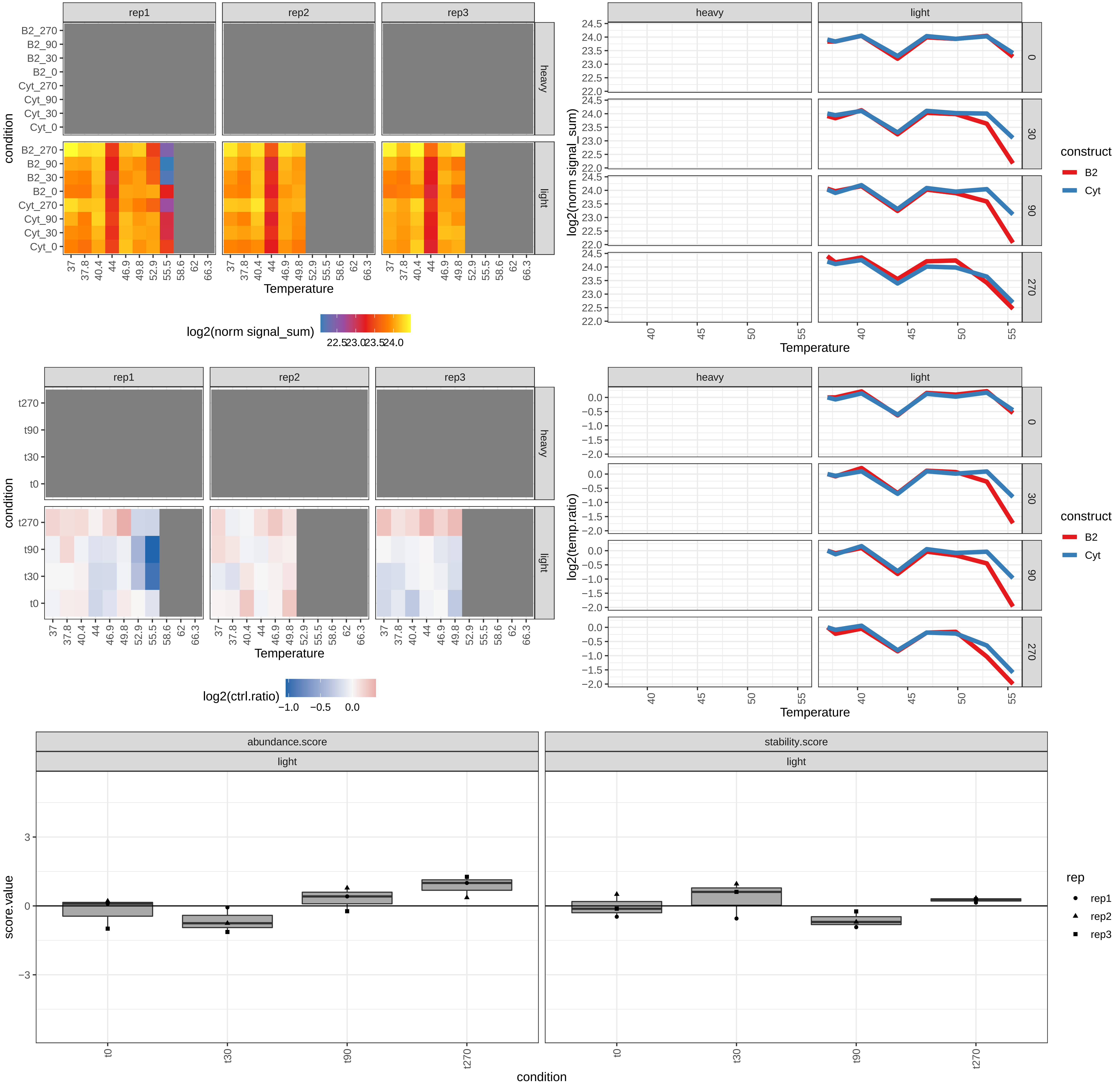

Q06493 − LETM1 domain−containing protein YLH47, mitochondrial OS=Saccharomyces cerevisiae (strain ATCC 204508 / S288c) GN=YLH47 PE=1 SV=1

YLL007C − average.top3: 6.3722975 − max.qupm: 5

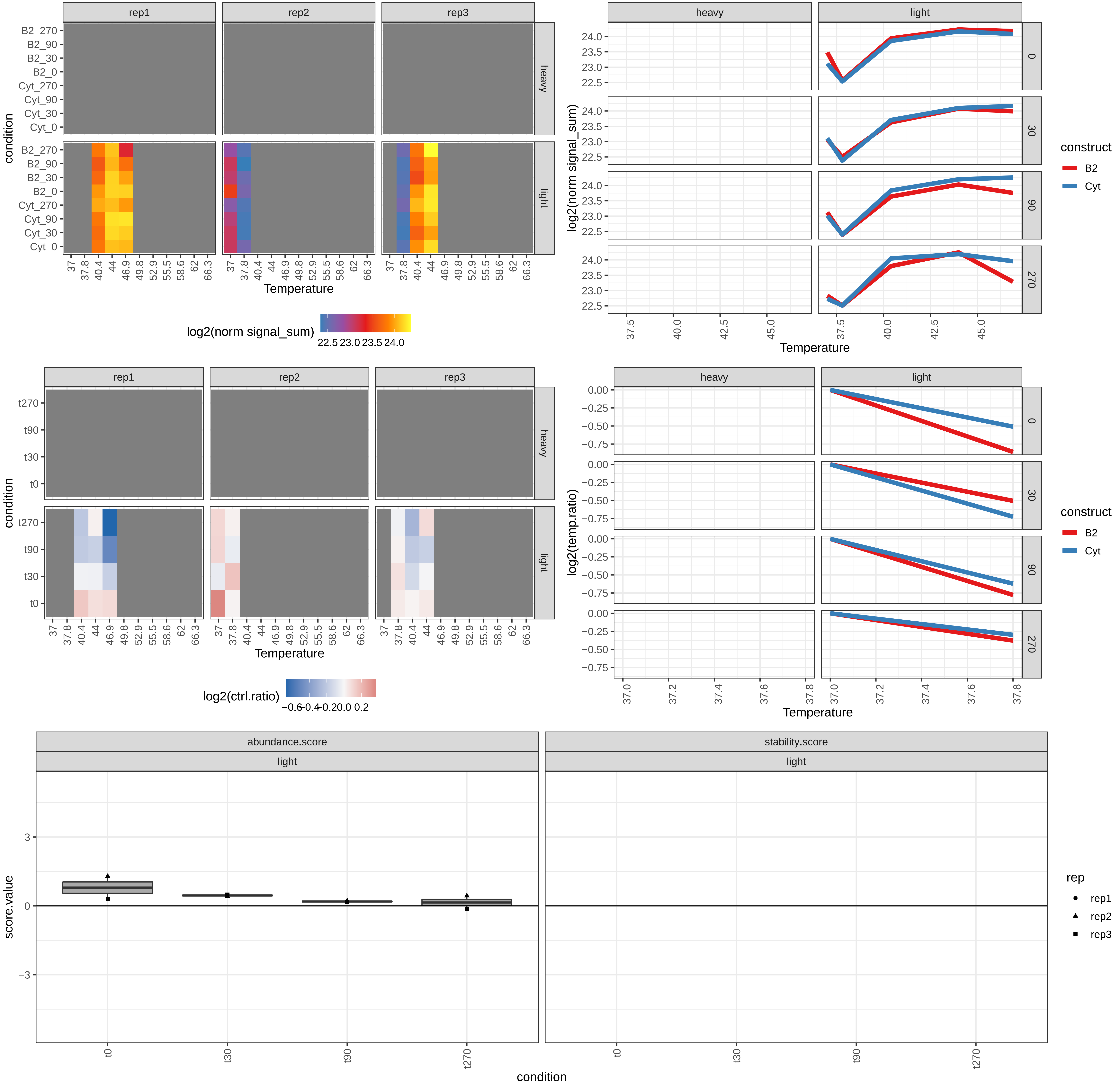

Q07799 − Uncharacterized protein YLL007C OS=Saccharomyces cerevisiae (strain ATCC 204508 / S288c) GN=YLL007C PE=1 SV=1

YLL032C − average.top3: 6.83166125 − max.qupm: 6

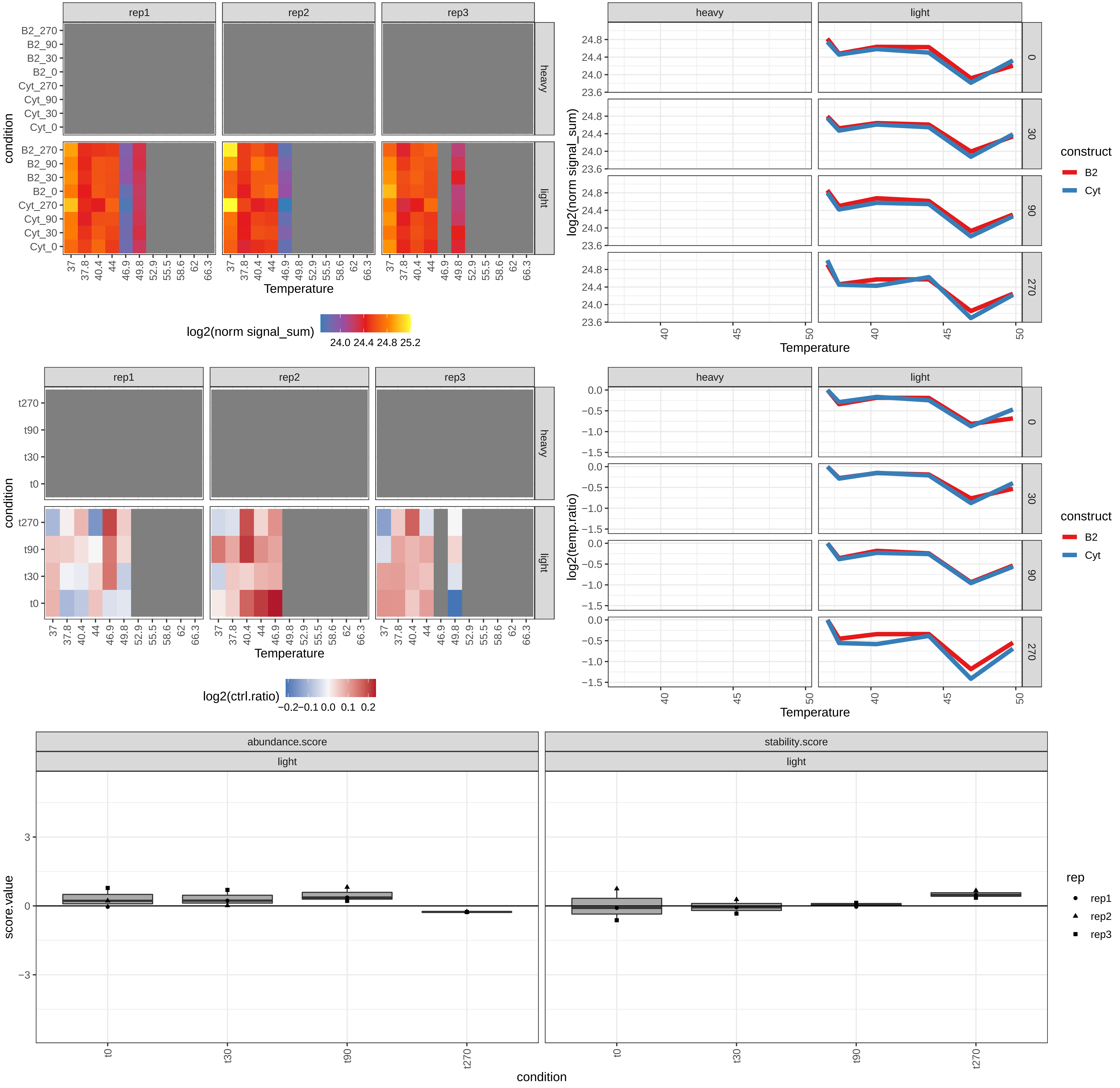

Q07834 − KH domain−containing protein YLL032C OS=Saccharomyces cerevisiae (strain ATCC 204508 / S288c) GN=YLL032C PE=1 SV=1

YLR118C − average.top3: 6.41221772727273 − max.qupm: 4

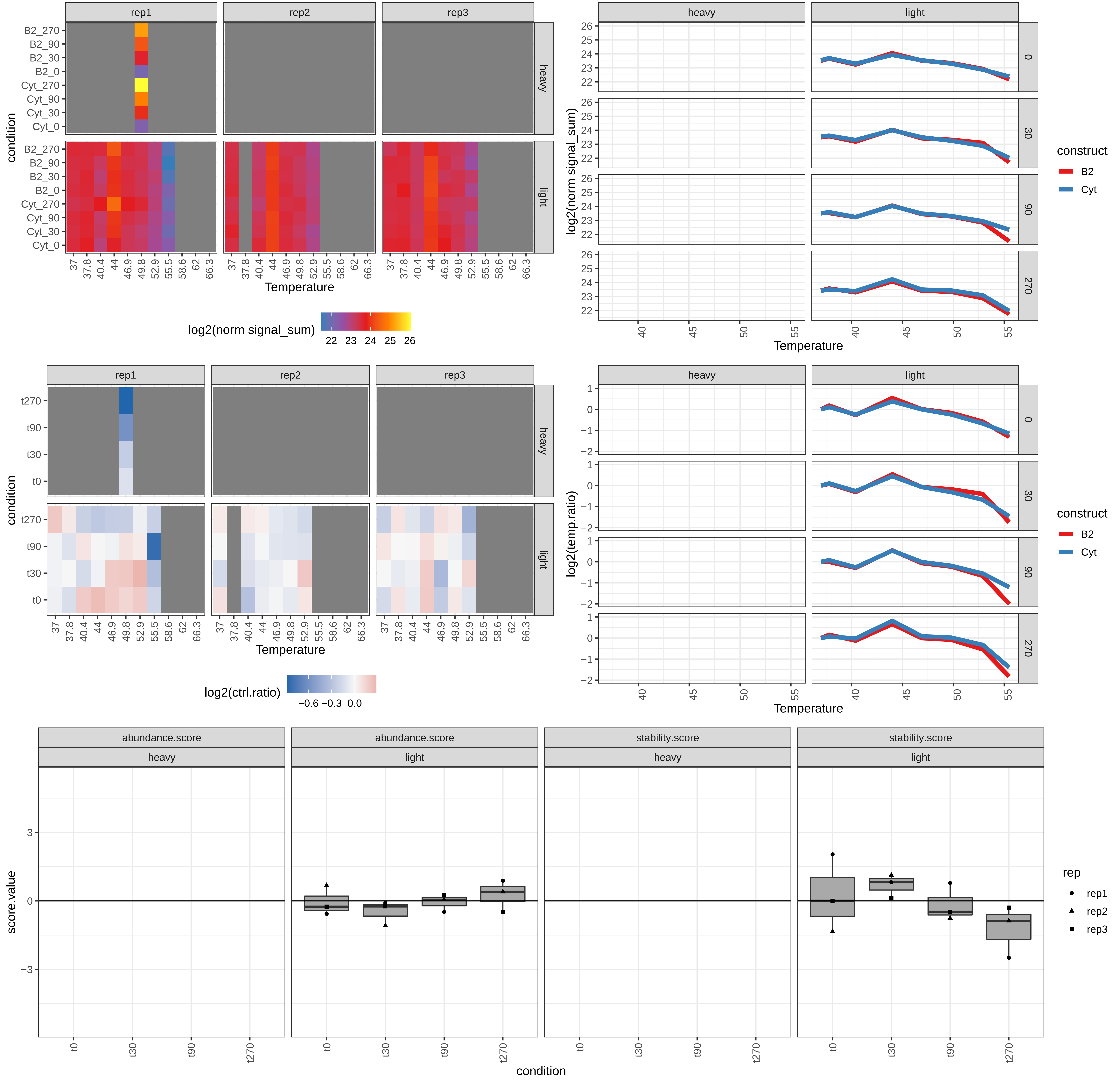

Q12354 − Acyl−protein thioesterase 1 OS=Saccharomyces cerevisiae (strain ATCC 204508 / S288c) GN=YLR118C PE=1 SV=1

YLR126C − average.top3: 6.68413071428571 − max.qupm: 5

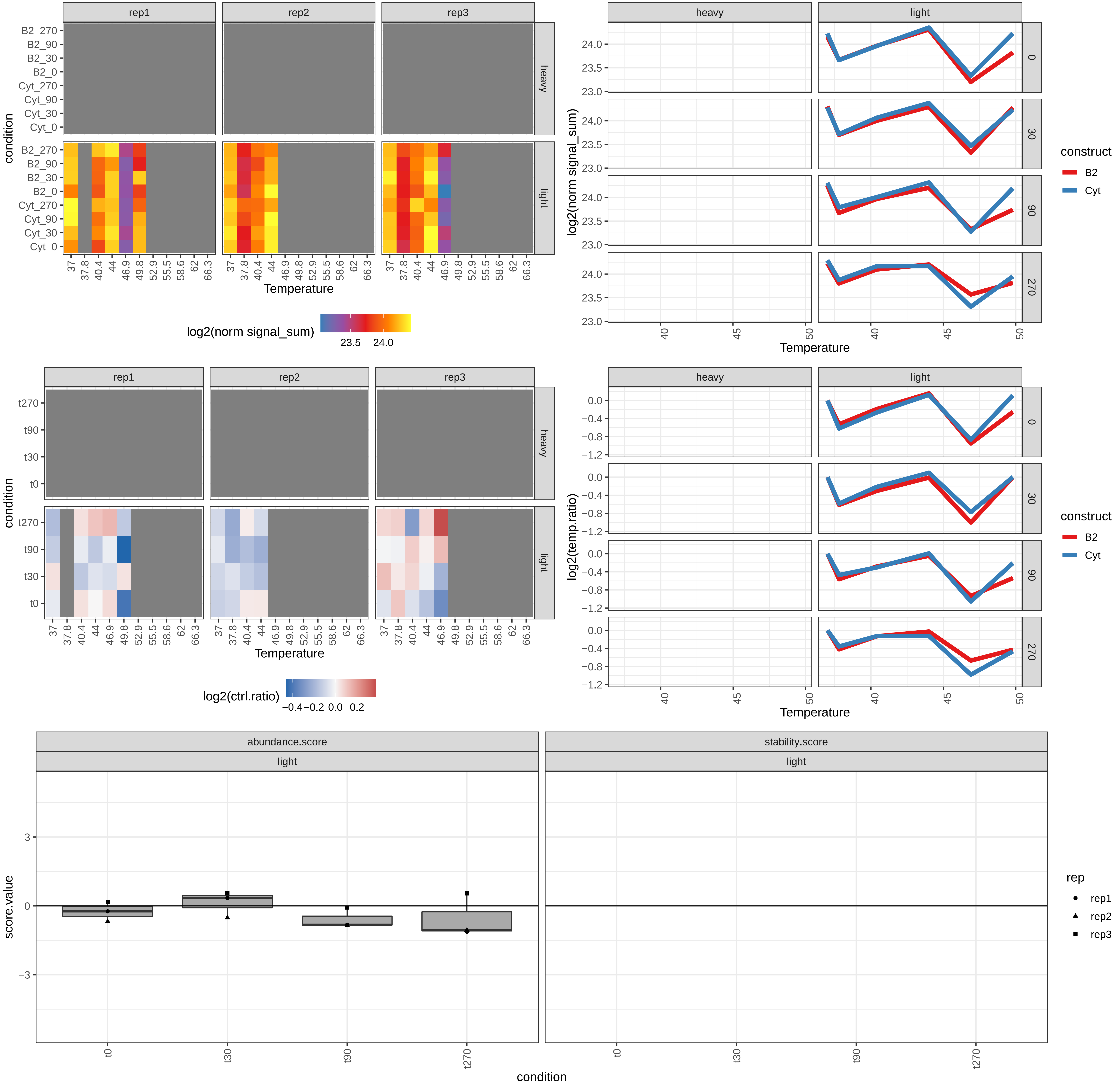

Q12288 − Putative glutamine amidotransferase YLR126C OS=Saccharomyces cerevisiae (strain ATCC 204508 / S288c) GN=YLR126C PE=1 SV=1

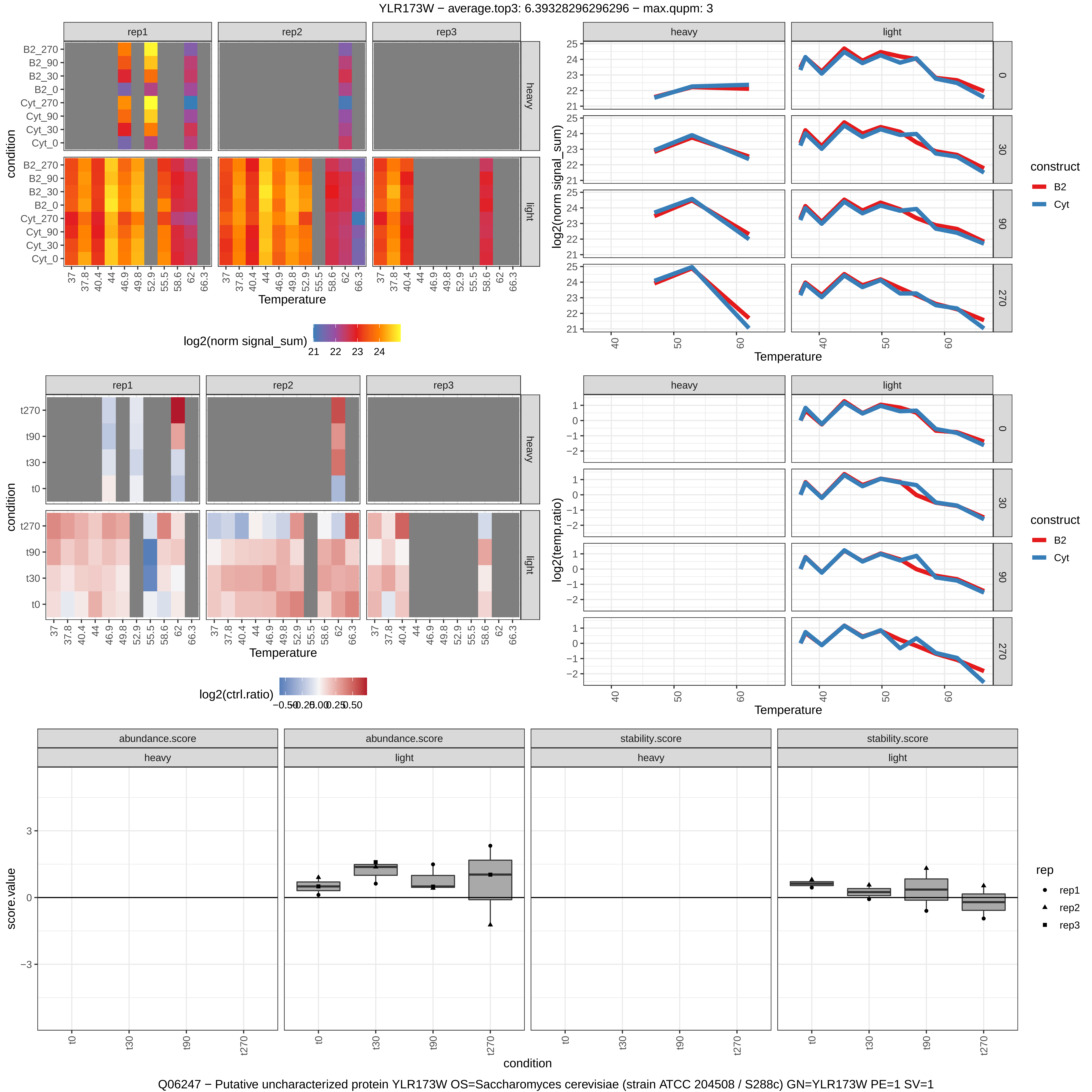
YLR173W − average.top3: 6.39328296296296 − max.qupm: 3
rep1
rep2
rep3
heavy
light
condition
Temperature
log2(norm signal_sum)
log2(norm signal_sum)
construct
B2
Cyt
log2(ctrl.ratio)
log2(temp.ratio)
abundance.score
stability.score
score.value
rep
rep1
rep2
rep3
Q06247 − Putative uncharacterized protein YLR173W OS=Saccharomyces cerevisiae (strain ATCC 204508 / S288c) GN=YLR173W PE=1 SV=1

YLR179C − average.top3: 7.15367918918919 − max.qupm: 4

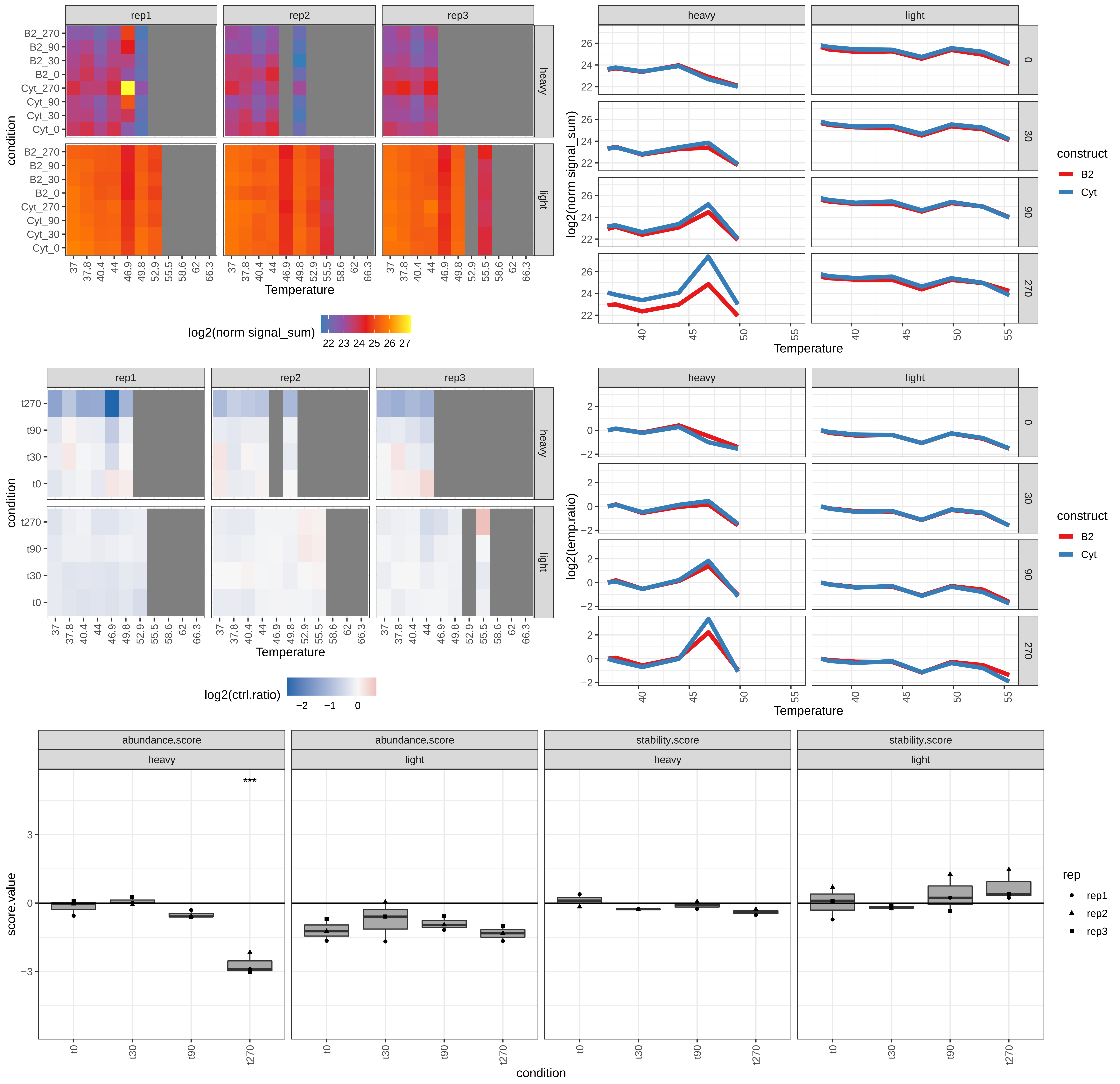

Q06252 − Uncharacterized protein YLR179C OS=Saccharomyces cerevisiae (strain ATCC 204508 / S288c) GN=YLR179C PE=1 SV=1

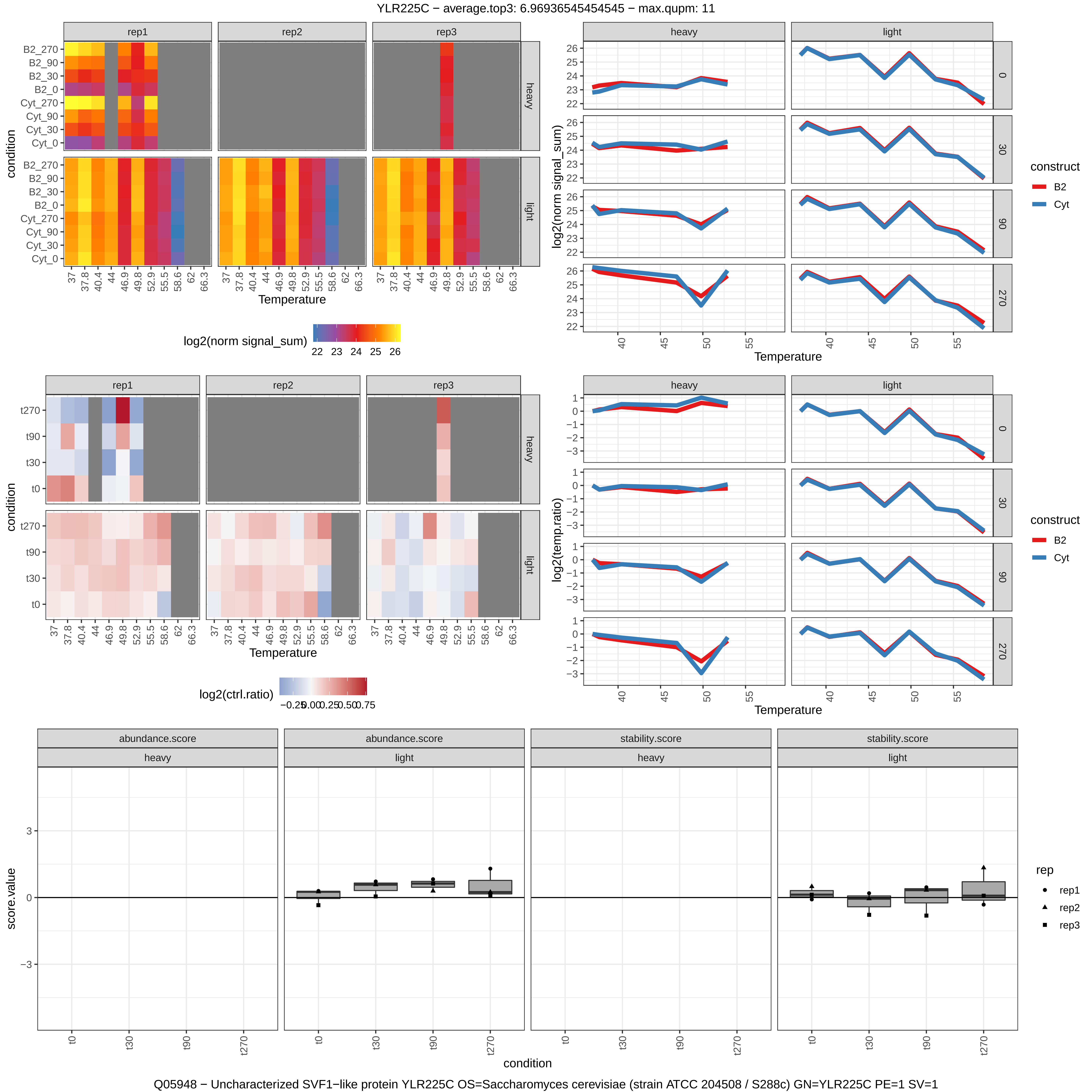

YLR225C − average.top3: 6.96936545454545 − max.qupm: 11
log2(norm signal_sum)
log2(ctrl.ratio)
log2(temp.ratio)
Temperature
condition
construct
B2
Cyt
abundance.score
stability.score
heavy
light
rep1
rep2
rep3
score.value
Q05948 − Uncharacterized SVF1−like protein YLR225C OS=Saccharomyces cerevisiae (strain ATCC 204508 / S288c) GN=YLR225C PE=1 SV=1

YLR283W − average.top3: 6.6095744 − max.qupm: 6

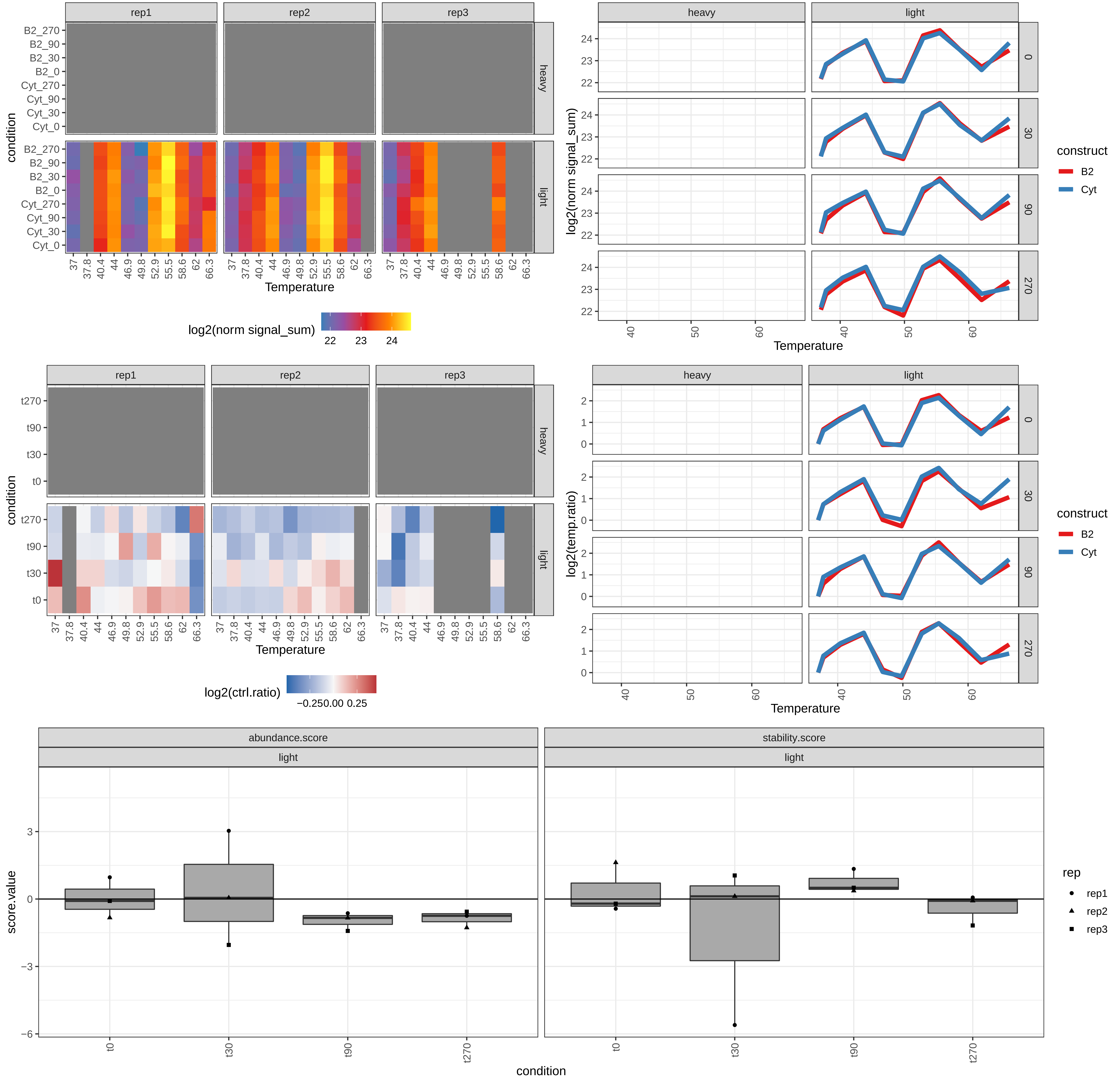

Q05867 − Uncharacterized protein YLR283W, mitochondrial OS=Saccharomyces cerevisiae (strain ATCC 204508 / S288c) GN=YLR283W PE=1 SV=1

YLR345W − average.top3: 6.95397 − max.qupm: 7

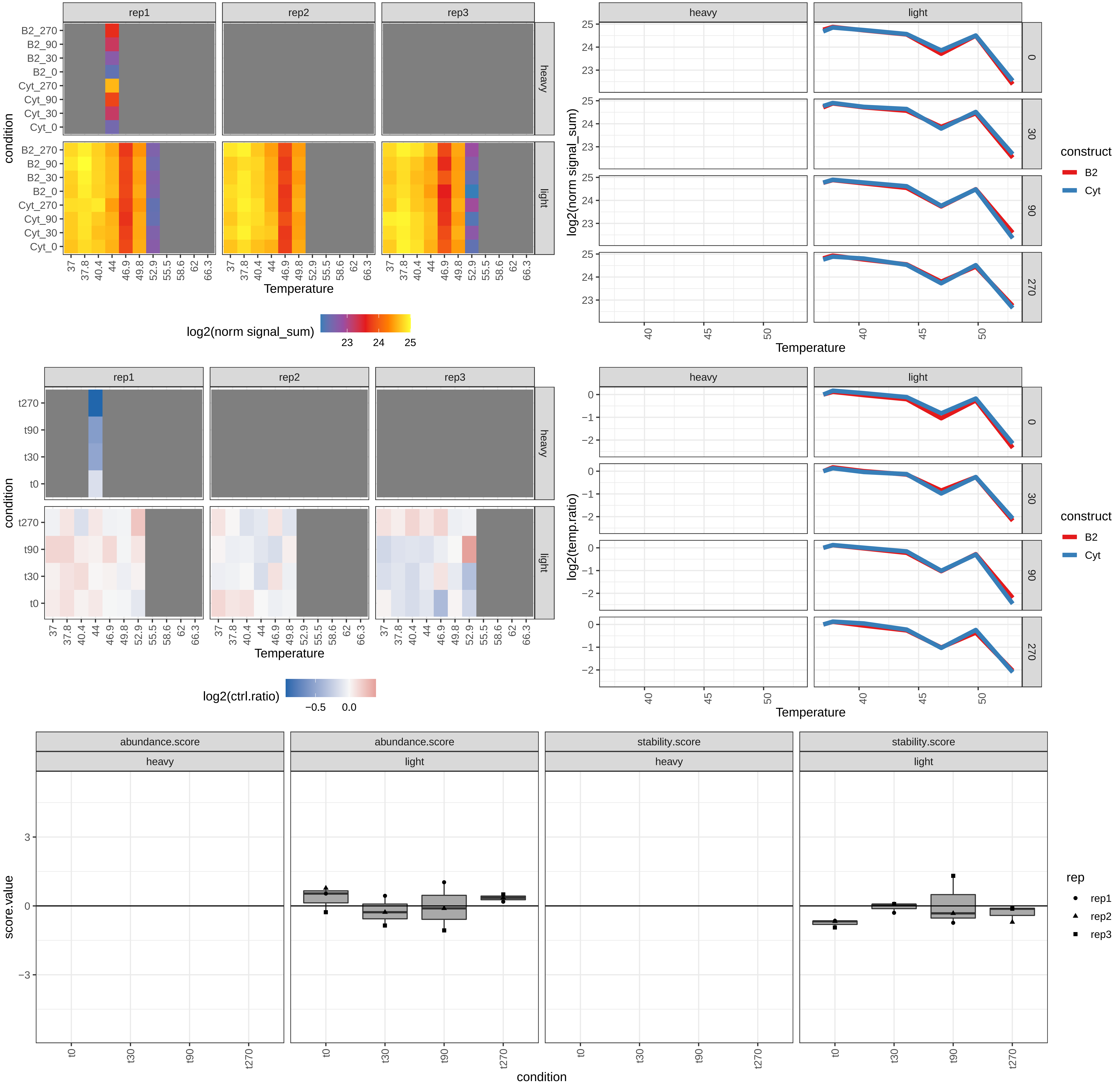

Q06137 − Putative 6−phosphofructo−2−kinase/fructose−2,6−bisphosphatase YLR345W OS=Saccharomyces cerevisiae (strain ATCC 204508 / S288c) GN=YLR345W PE=1 SV=1

YLR352W − average.top3: 6.36615473684211 − max.qupm: 6

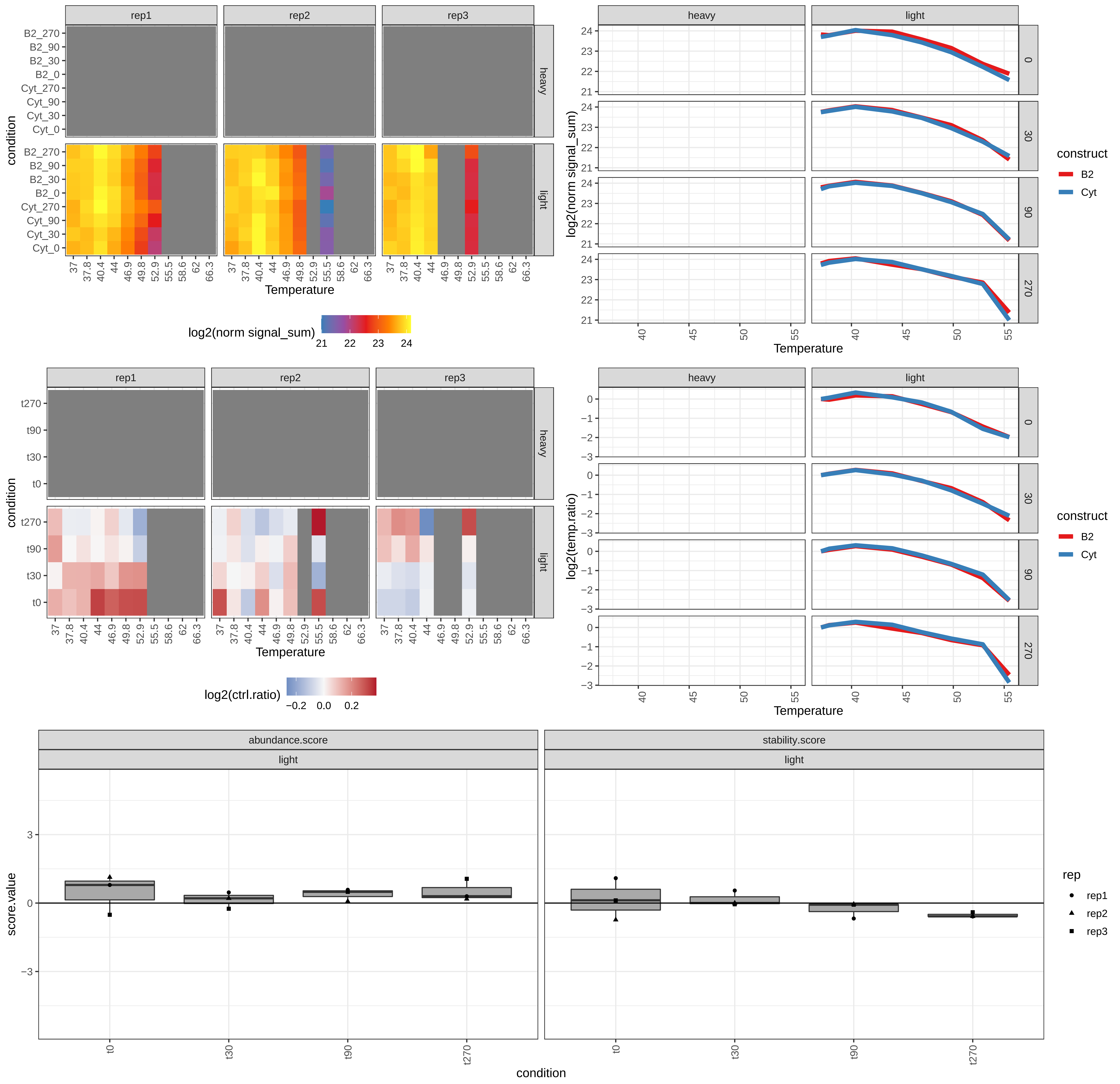

Q06479 − F−box protein YLR352W OS=Saccharomyces cerevisiae (strain ATCC 204508 / S288c) GN=YLR352W PE=1 SV=1

YLR419W − average.top3: 6.71403909090909 − max.qupm: 14

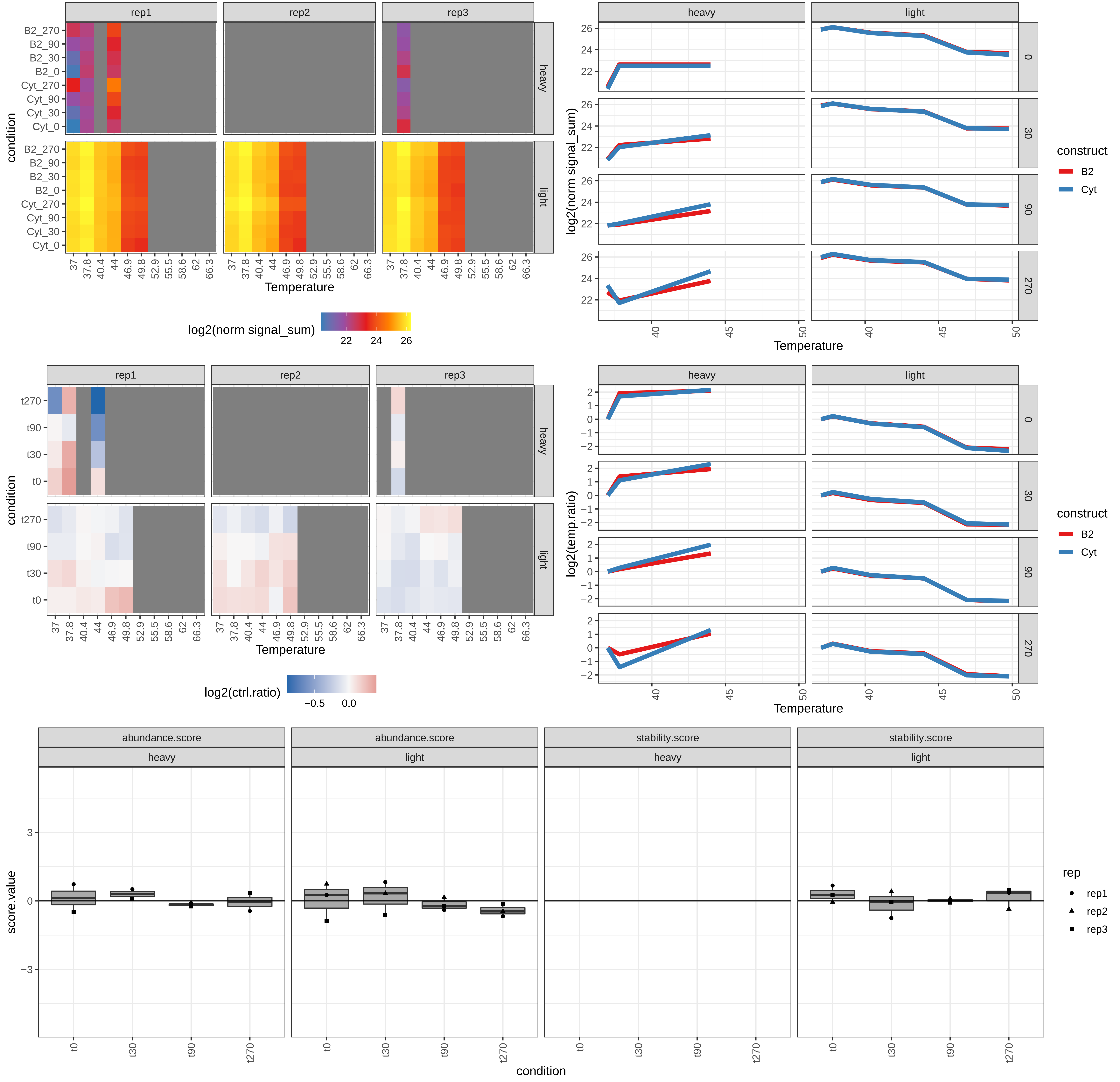

Q06698 − Putative ATP−dependent RNA helicase YLR419W OS=Saccharomyces cerevisiae (strain ATCC 204508 / S288c) GN=YLR419W PE=1 SV=1

YLR422W − average.top3: 6.42788411764706 − max.qupm: 8

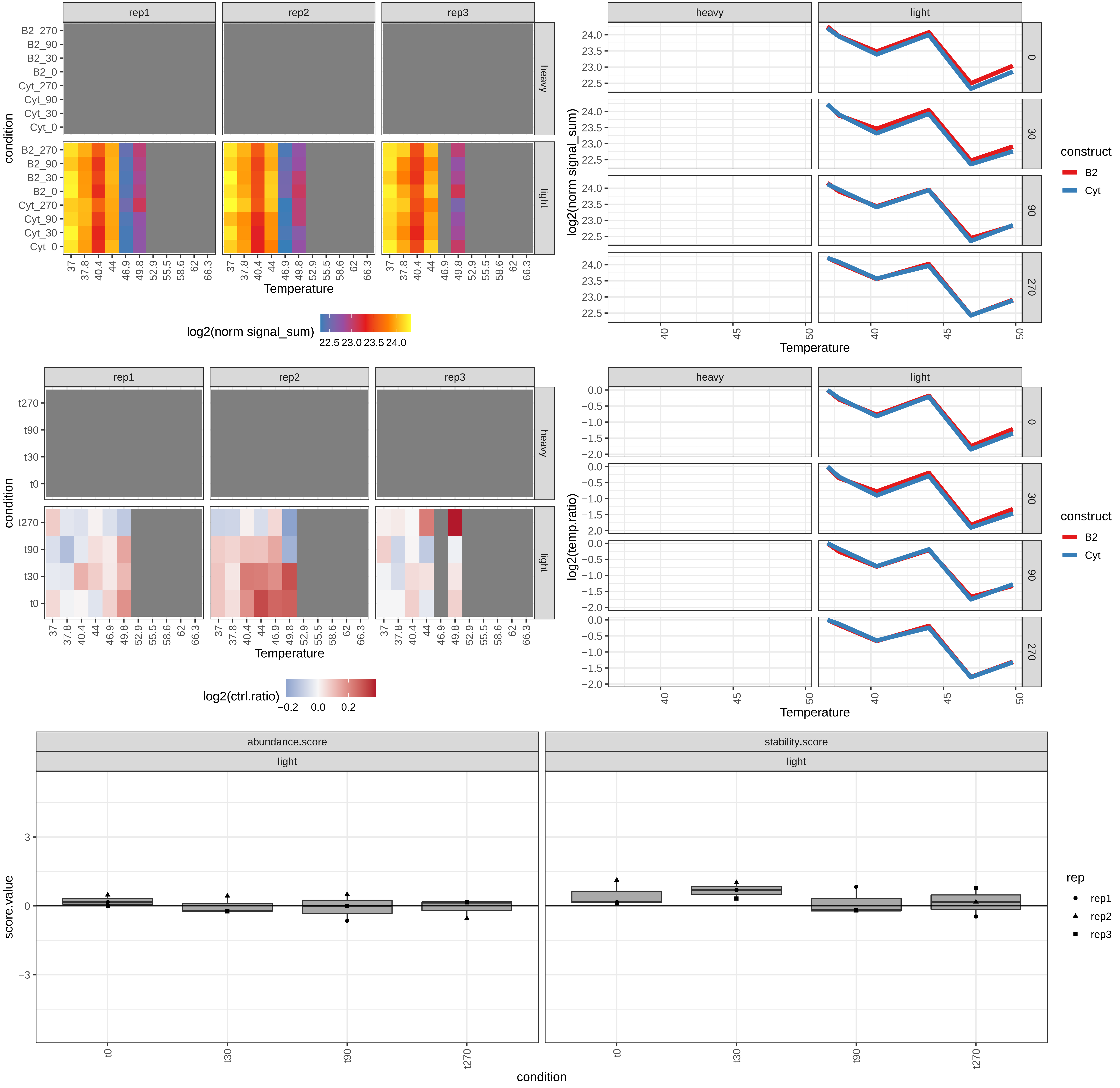

Q06409 − DOCK−like protein YLR422W OS=Saccharomyces cerevisiae (strain ATCC 204508 / S288c) GN=YLR422W PE=1 SV=1

YMC1 − average.top3: 7.11326411764706 − max.qupm: 6

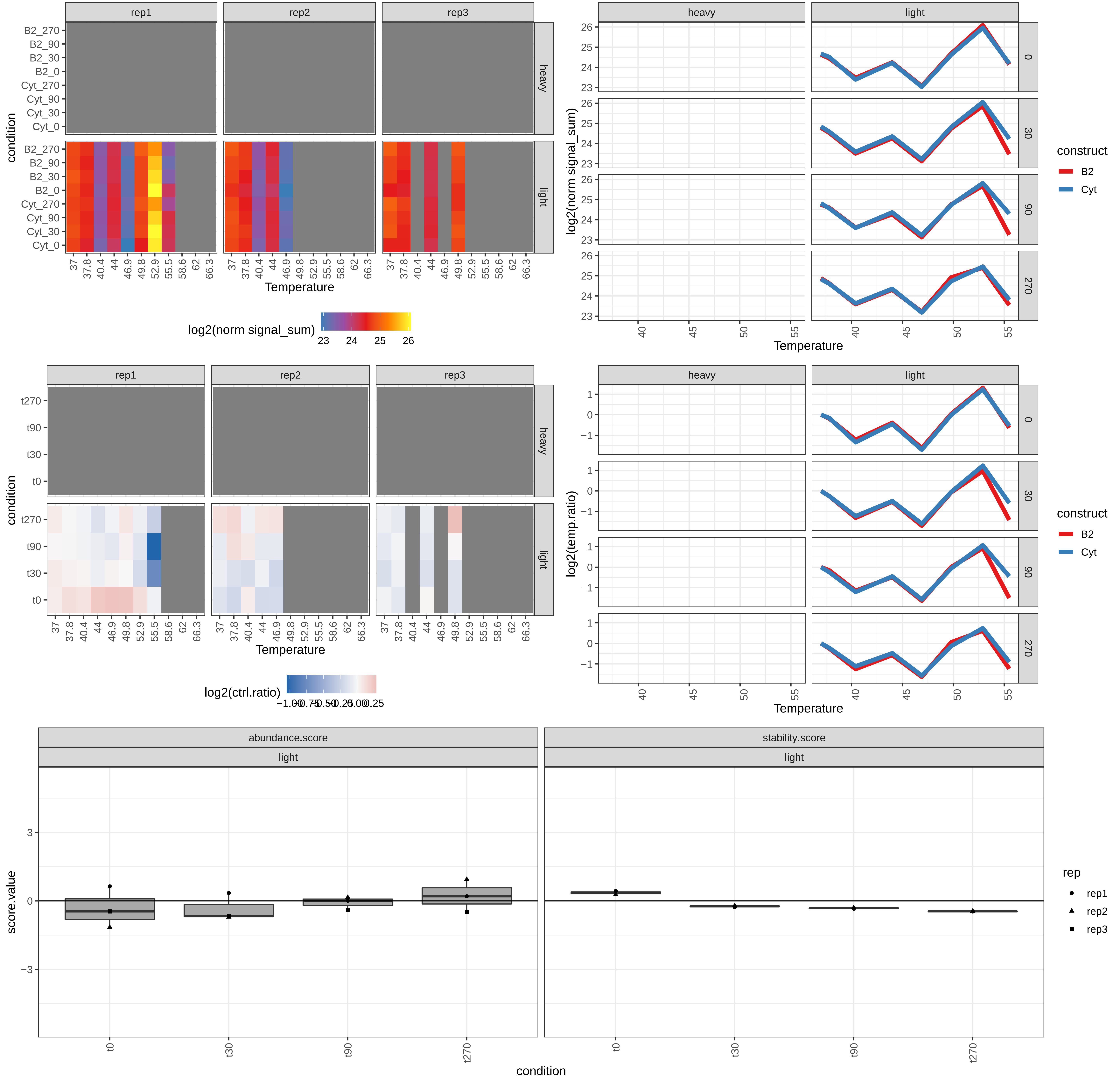

P32331 − Carrier protein YMC1, mitochondrial OS=Saccharomyces cerevisiae (strain ATCC 204508 / S288c) GN=YMC1 PE=3 SV=2

YME1 − average.top3: 6.77986782608696 − max.qupm: 12

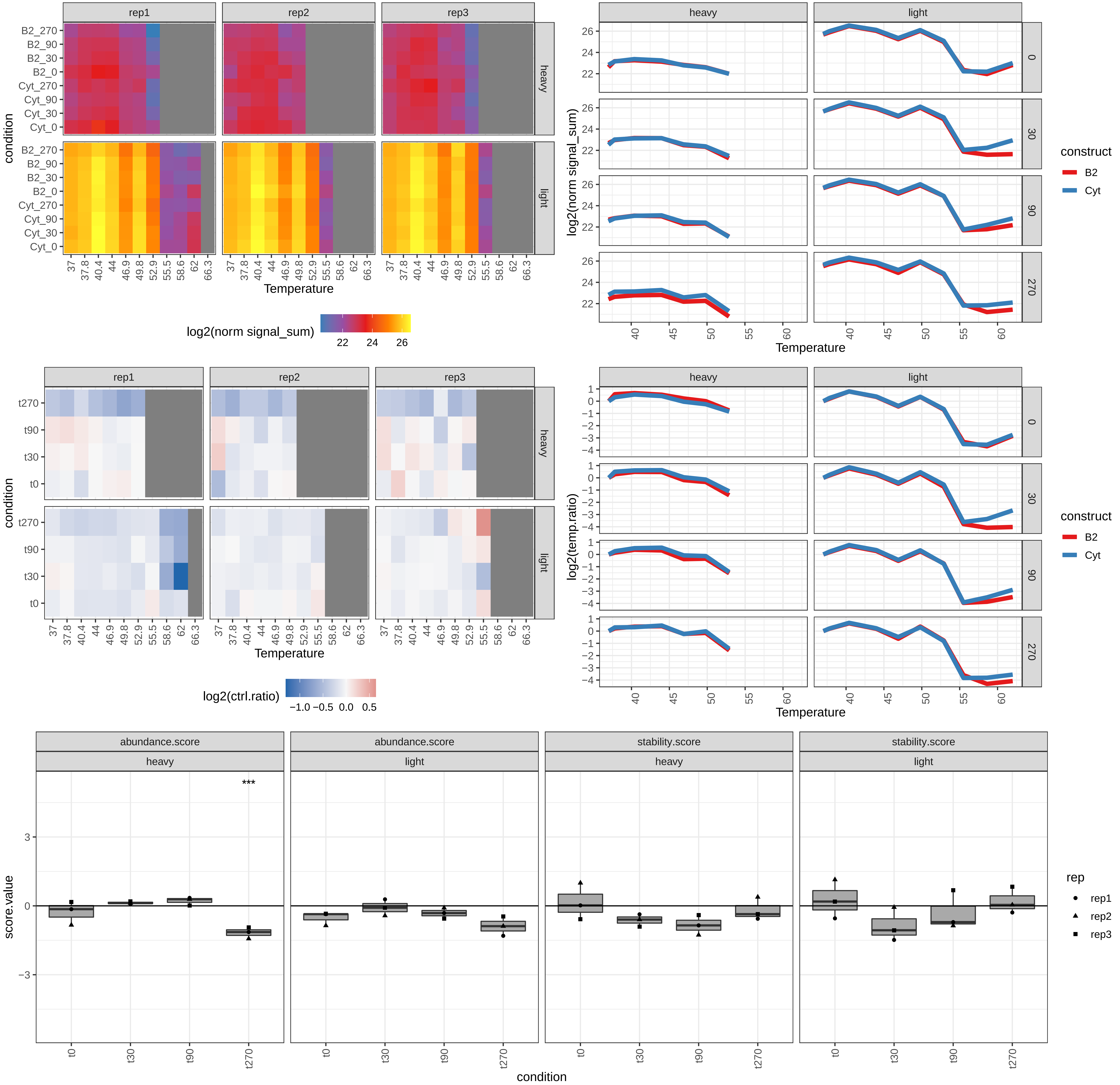

P32795 − Mitochondrial inner membrane i−AAA protease supercomplex subunit YME1 OS=Saccharomyces cerevisiae (strain ATCC 204508 / S288c) GN=YME1 PE=1 SV=1

YME2 − average.top3: 6.98607633333333 − max.qupm: 14

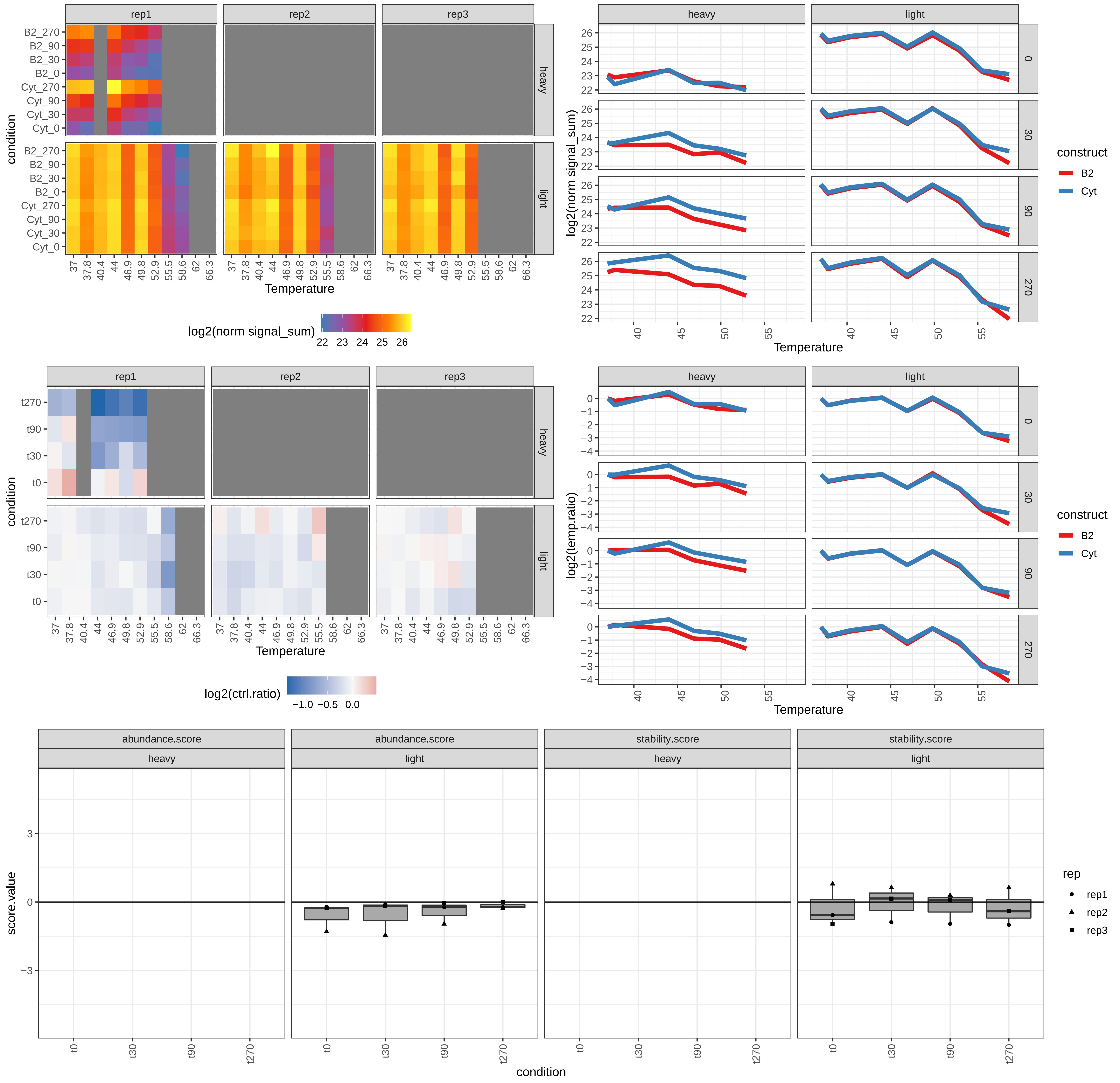

P32843 − Mitochondrial escape protein 2 OS=Saccharomyces cerevisiae (strain ATCC 204508 / S288c) GN=YME2 PE=1 SV=1

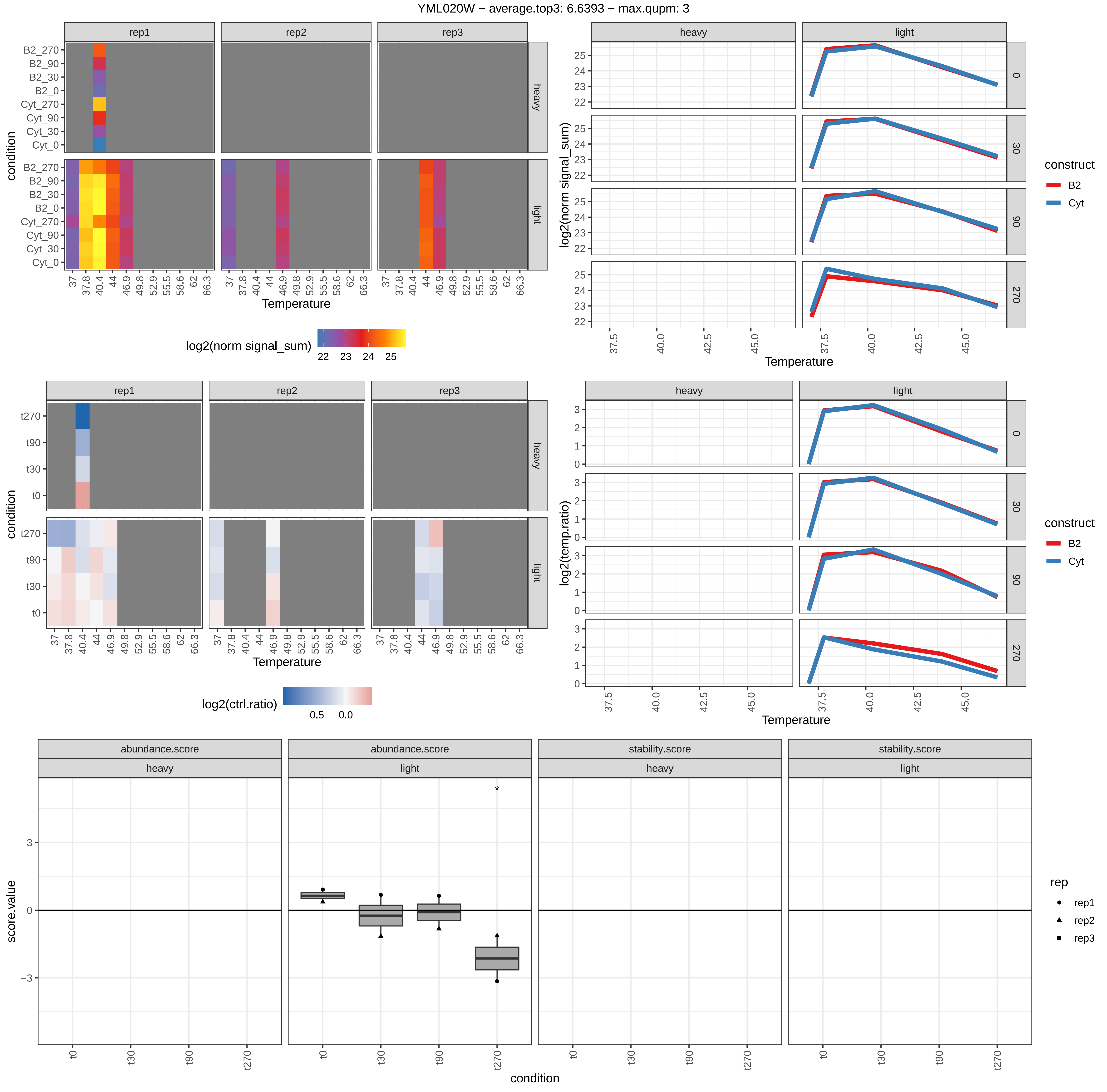

Q03722 − Uncharacterized protein YML020W OS=Saccharomyces cerevisiae (strain ATCC 204508 / S288c) GN=YML020W PE=1 SV=1

YML079W − average.top3: 6.8981303030303 − max.qupm: 6

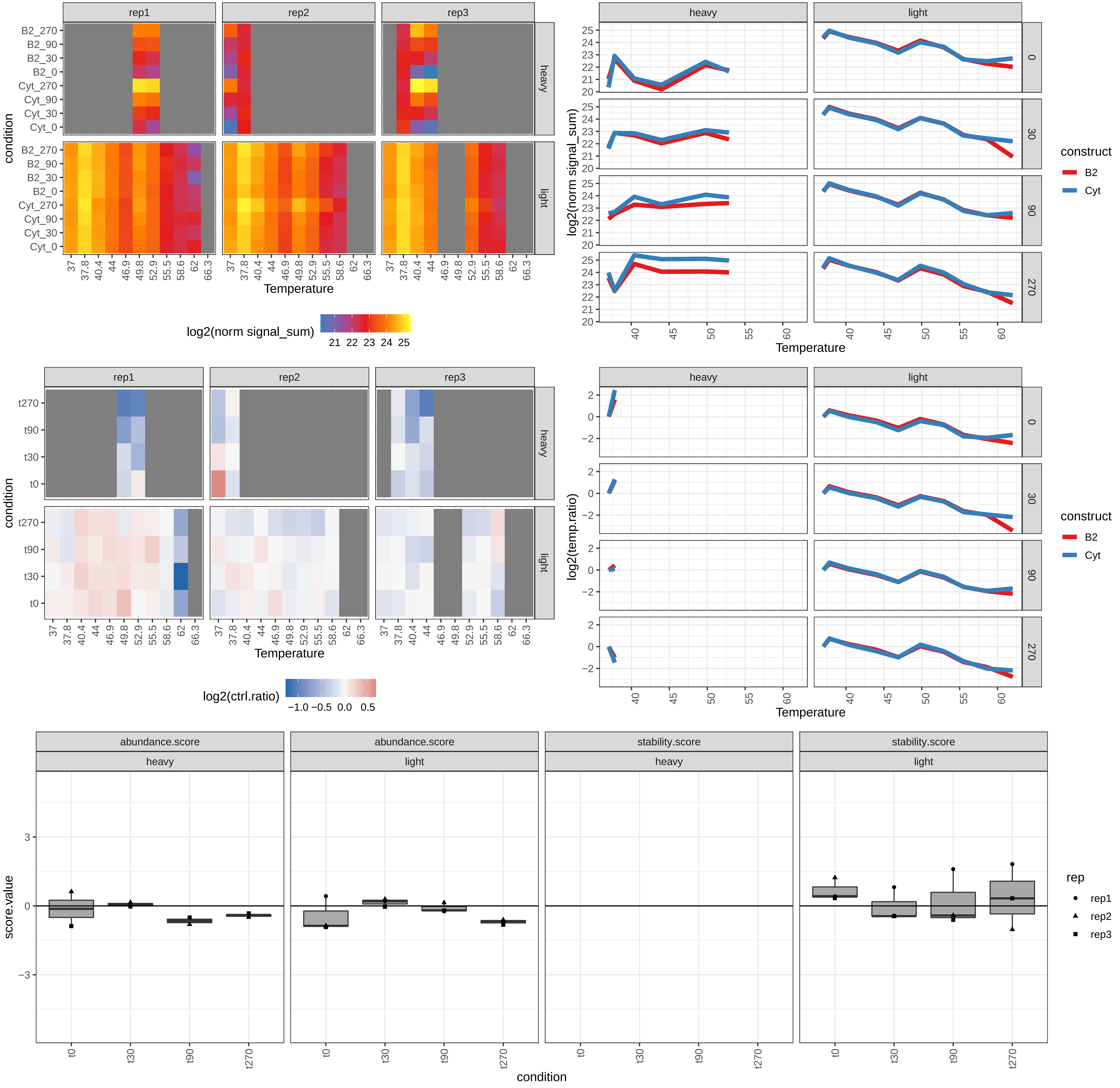

Q03629 − Uncharacterized protein YML079W OS=Saccharomyces cerevisiae (strain ATCC 204508 / S288c) GN=YML079W PE=1 SV=1

YML082W − average.top3: 6.72015310344828 − max.qupm: 8

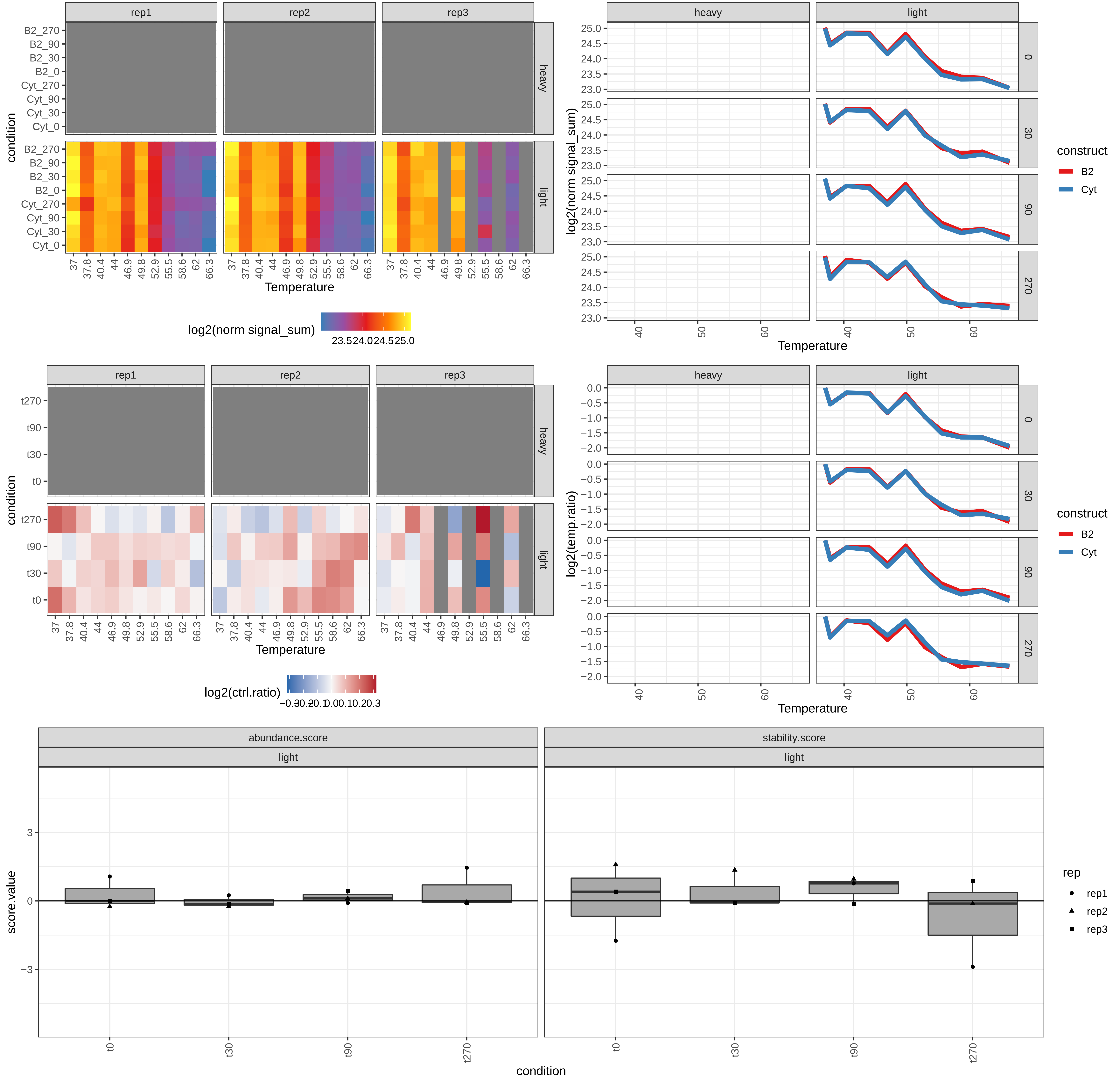

Q04533 − Putative cystathionine gamma−synthase YML082W OS=Saccharomyces cerevisiae (strain ATCC 204508 / S288c) GN=YML082W PE=1 SV=1

YML096W − average.top3: 6.386429375 − max.qupm: 4

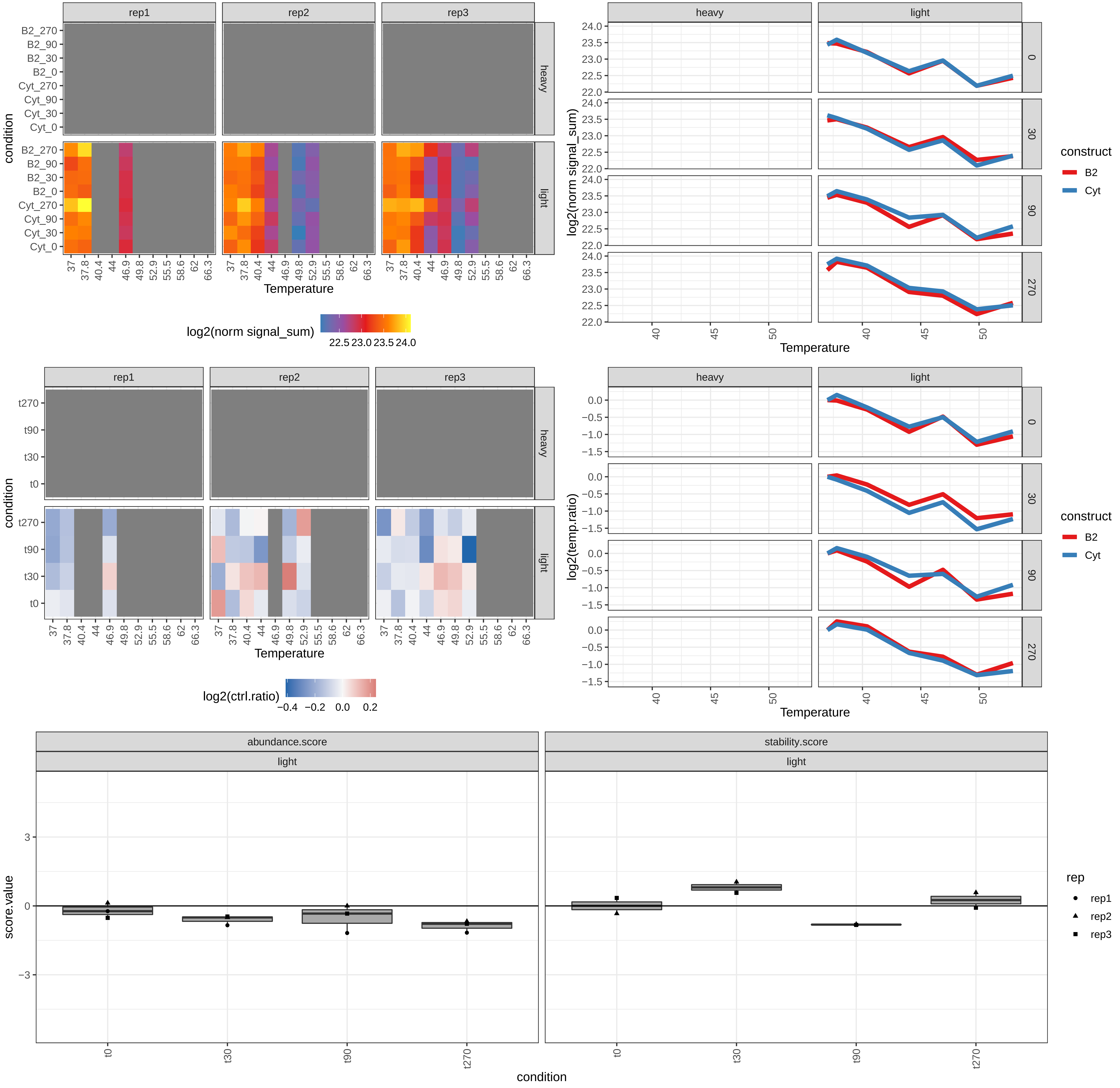

Q04489 − Asparagine synthetase domain−containing protein YML096W OS=Saccharomyces cerevisiae (strain ATCC 204508 / S288c) GN=YML096W PE=1 SV=1

YML131W − average.top3: 7.46051934782609 − max.qupm: 11

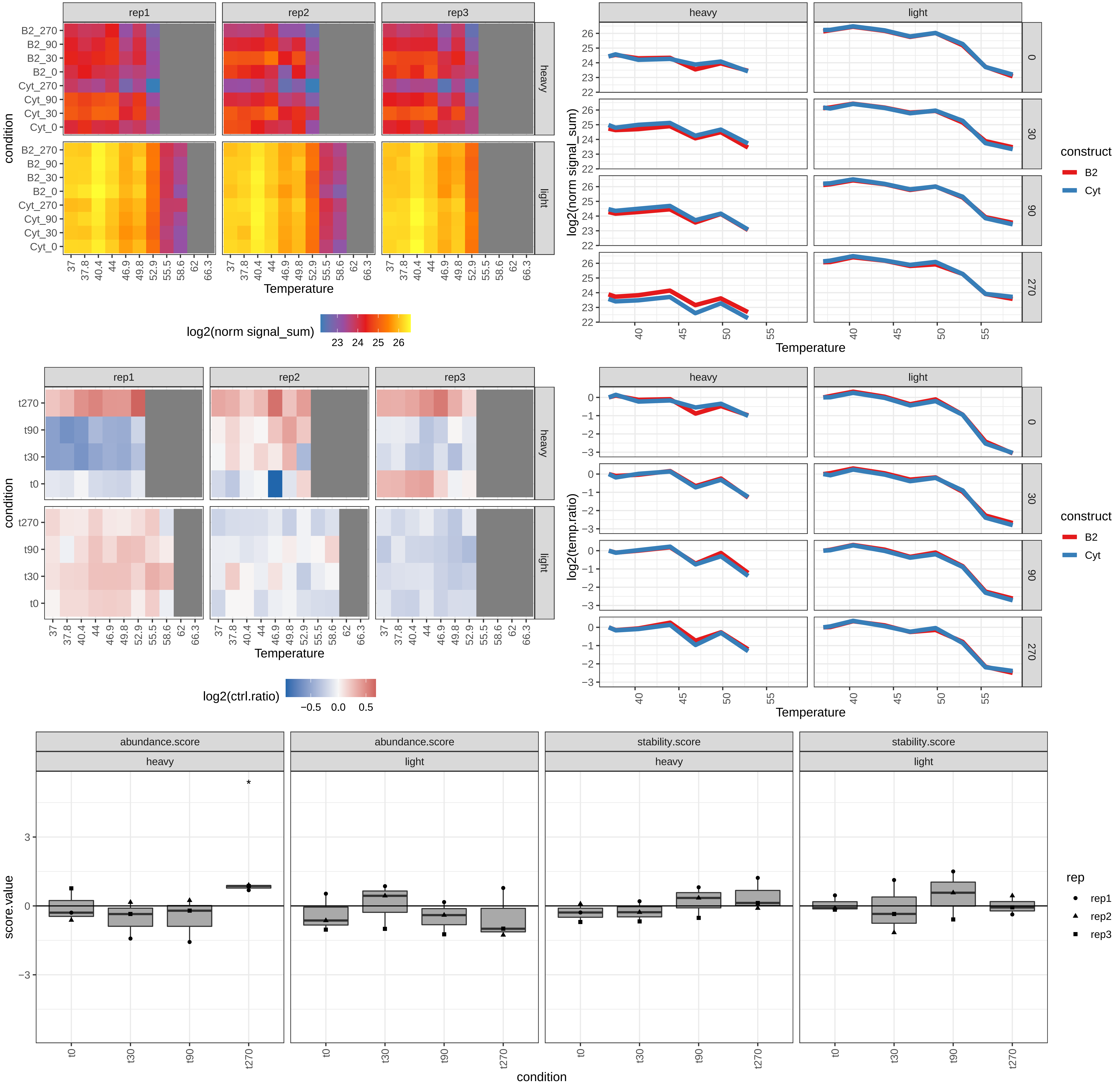

Q03102 − Uncharacterized membrane protein YML131W OS=Saccharomyces cerevisiae (strain ATCC 204508 / S288c) GN=YML131W PE=1 SV=1

YML6 − average.top3: 6.72460161290323 − max.qupm: 10

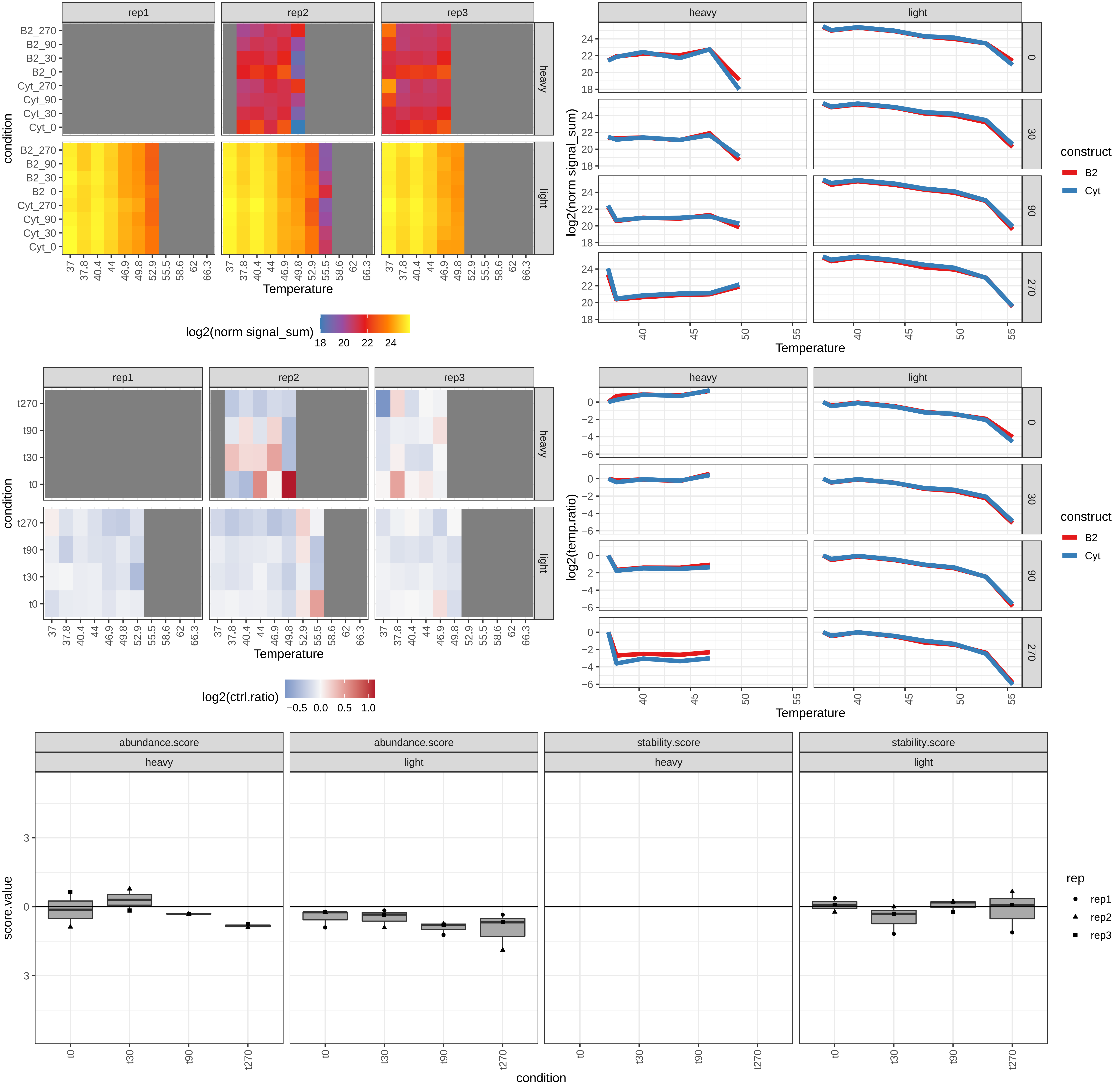

P51998 − 54S ribosomal protein YmL6, mitochondrial OS=Saccharomyces cerevisiae (strain ATCC 204508 / S288c) GN=YML6 PE=1 SV=1

YMR027W − average.top3: 7.21125411764706 − max.qupm: 16

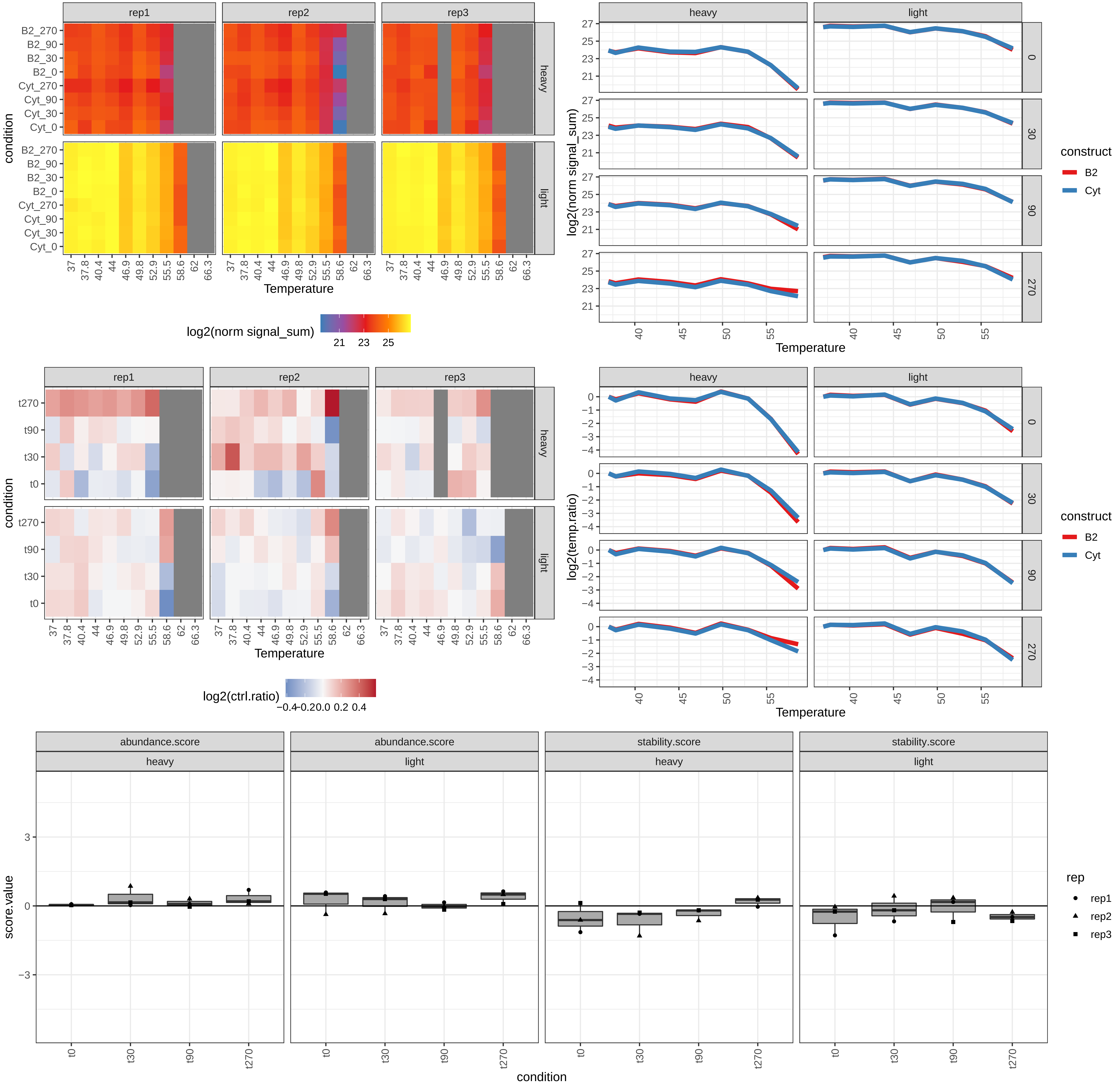

Q04371 − Protein−glutamate O−methyltransferase OS=Saccharomyces cerevisiae (strain ATCC 204508 / S288c) GN=YMR027W PE=1 SV=1

YMR074C − average.top3: 6.7772887804878 − max.qupm: 5

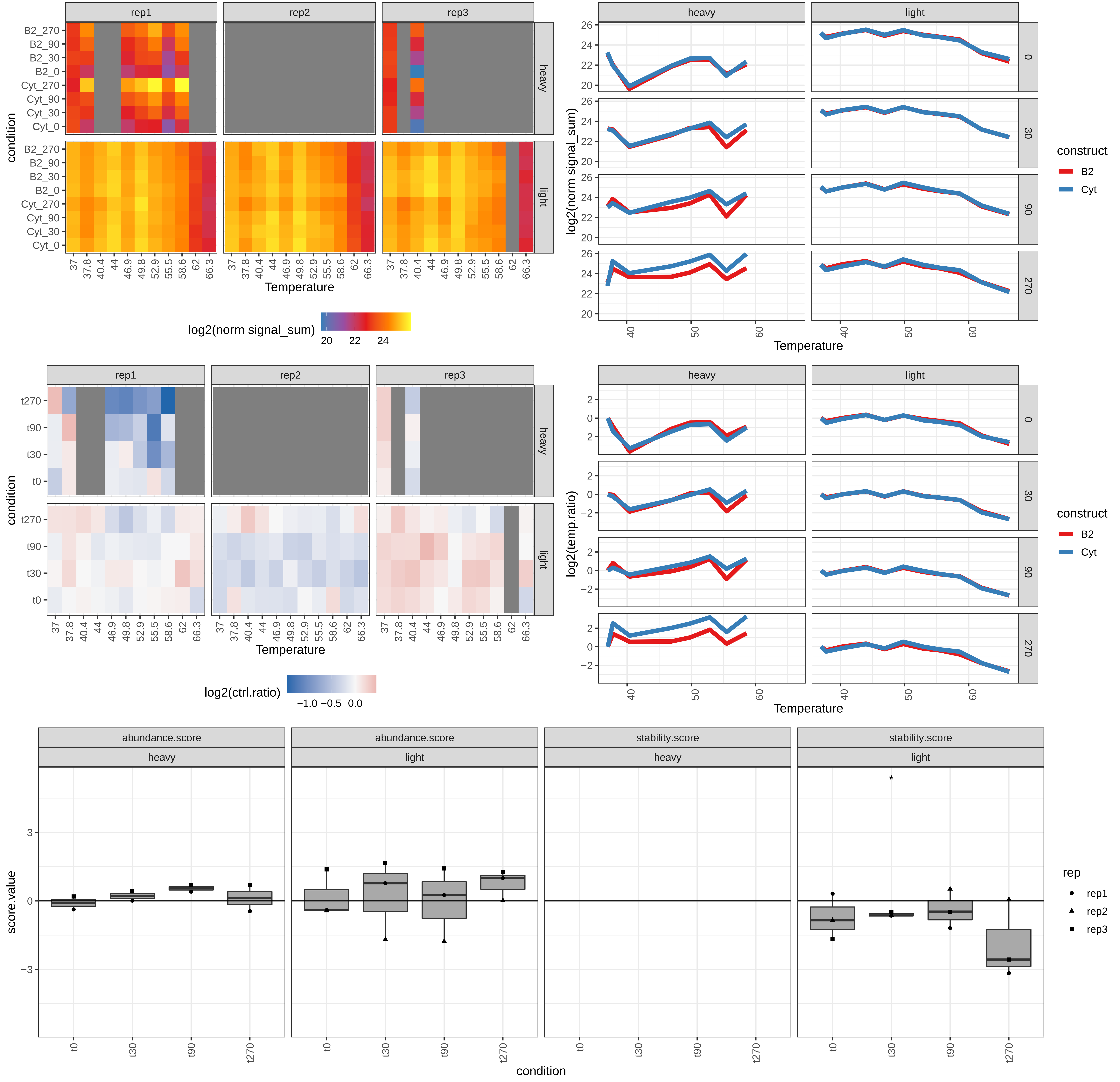

Q04773 − Uncharacterized protein YMR074C OS=Saccharomyces cerevisiae (strain ATCC 204508 / S288c) GN=YMR074C PE=1 SV=1

YMR090W − average.top3: 6.888162 − max.qupm: 9

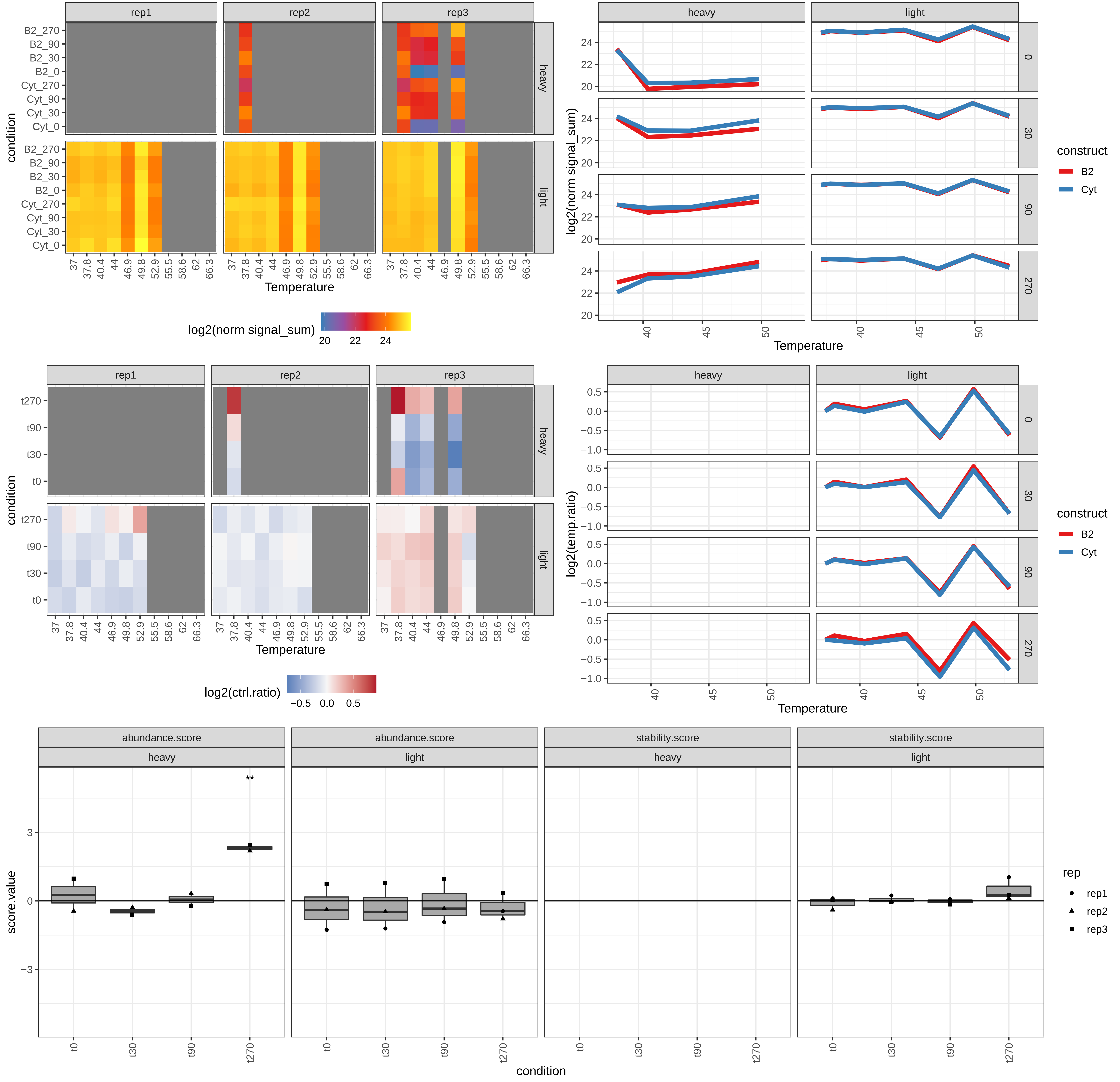

Q04304 − UPF0659 protein YMR090W OS=Saccharomyces cerevisiae (strain ATCC 204508 / S288c) GN=YMR090W PE=1 SV=1

YMR099C − average.top3: 7.30854057692308 − max.qupm: 12

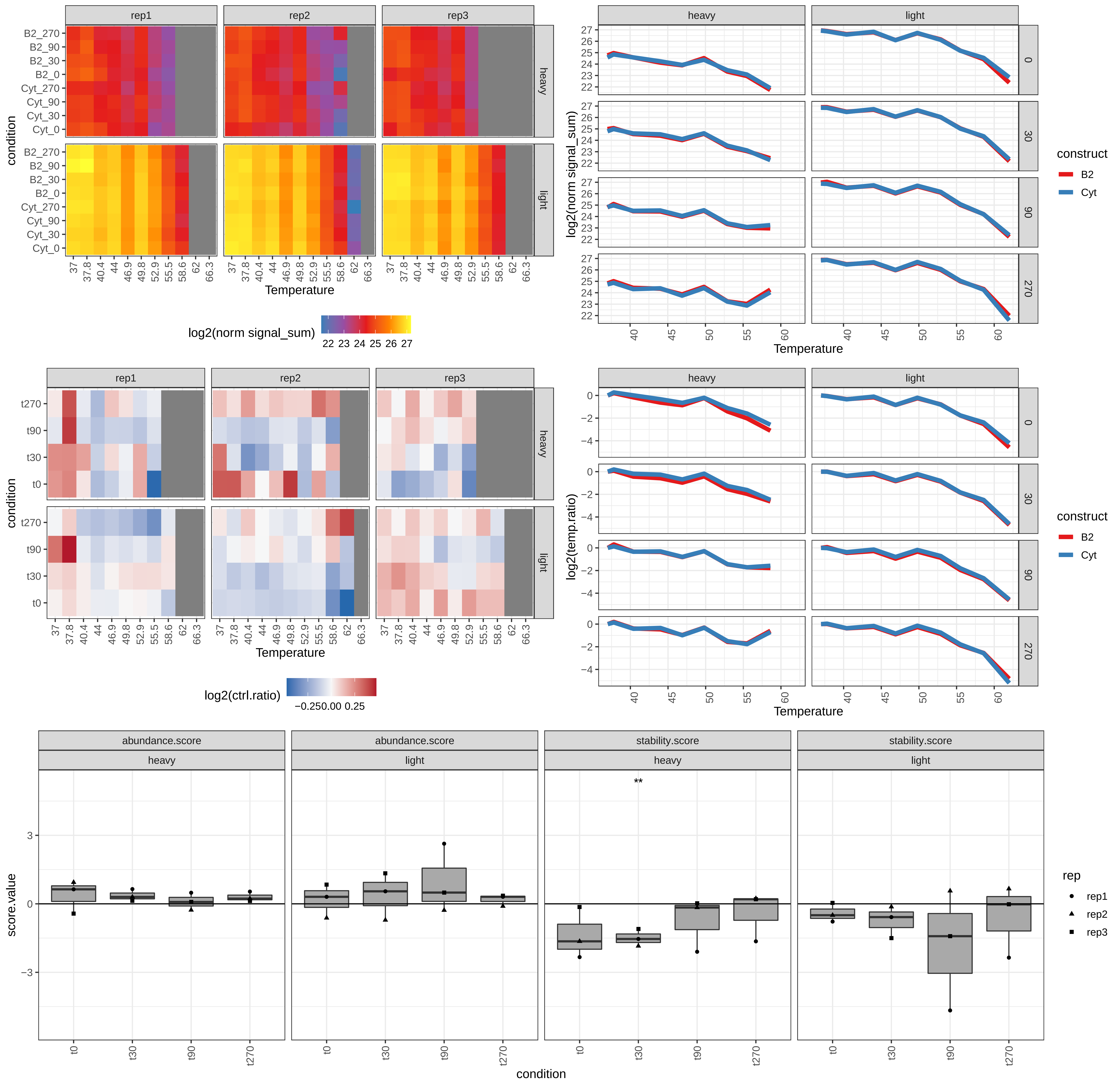

Q03161 − Glucose−6−phosphate 1−epimerase OS=Saccharomyces cerevisiae (strain ATCC 204508 / S288c) GN=YMR099C PE=1 SV=1

YMR1 − average.top3: 6.561988 − max.qupm: 3

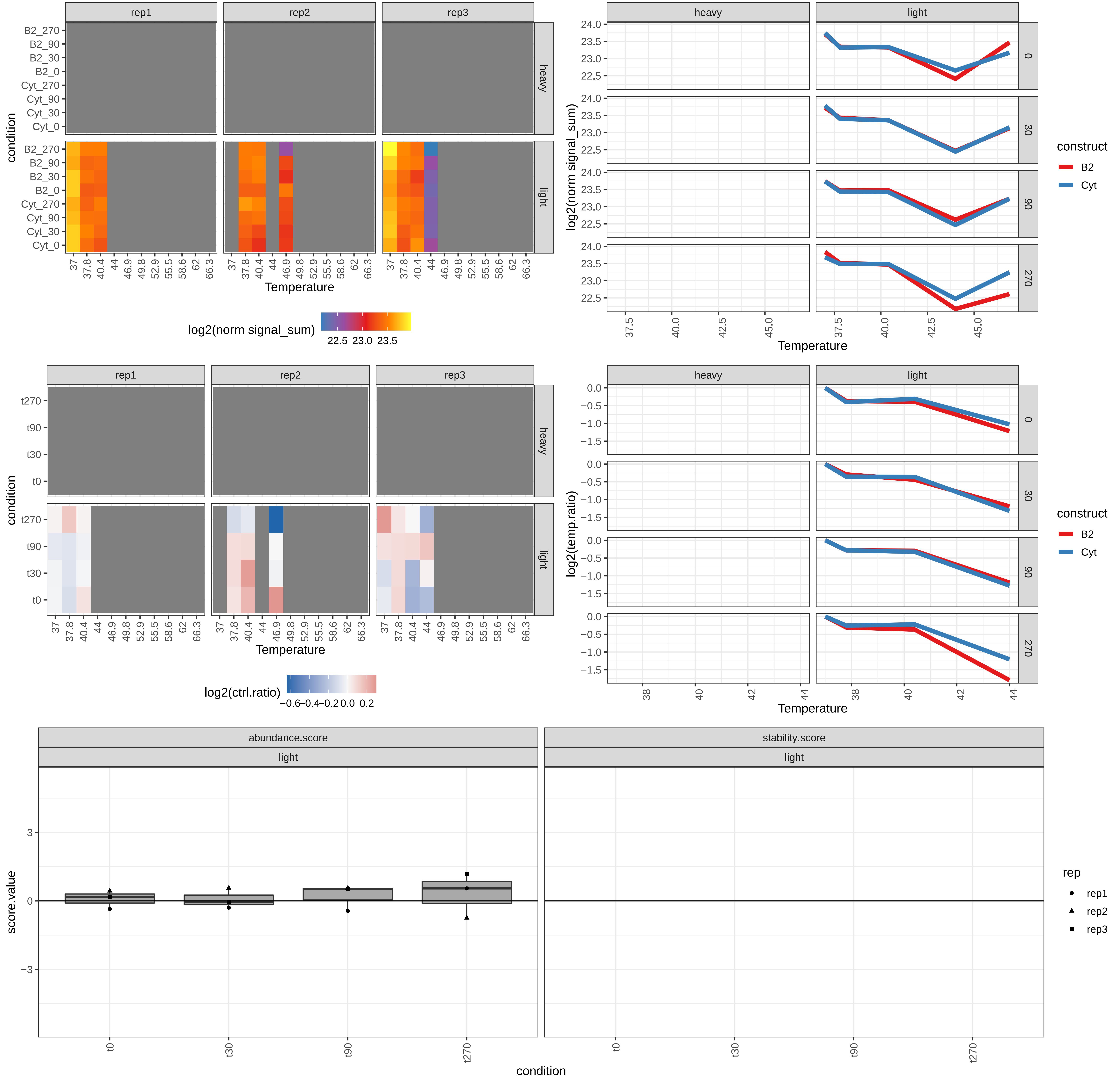

P47147 − Phosphoinositide 3−phosphatase OS=Saccharomyces cerevisiae (strain ATCC 204508 / S288c) GN=YMR1 PE=1 SV=1

YMR114C − average.top3: 6.68211264705882 − max.qupm: 8

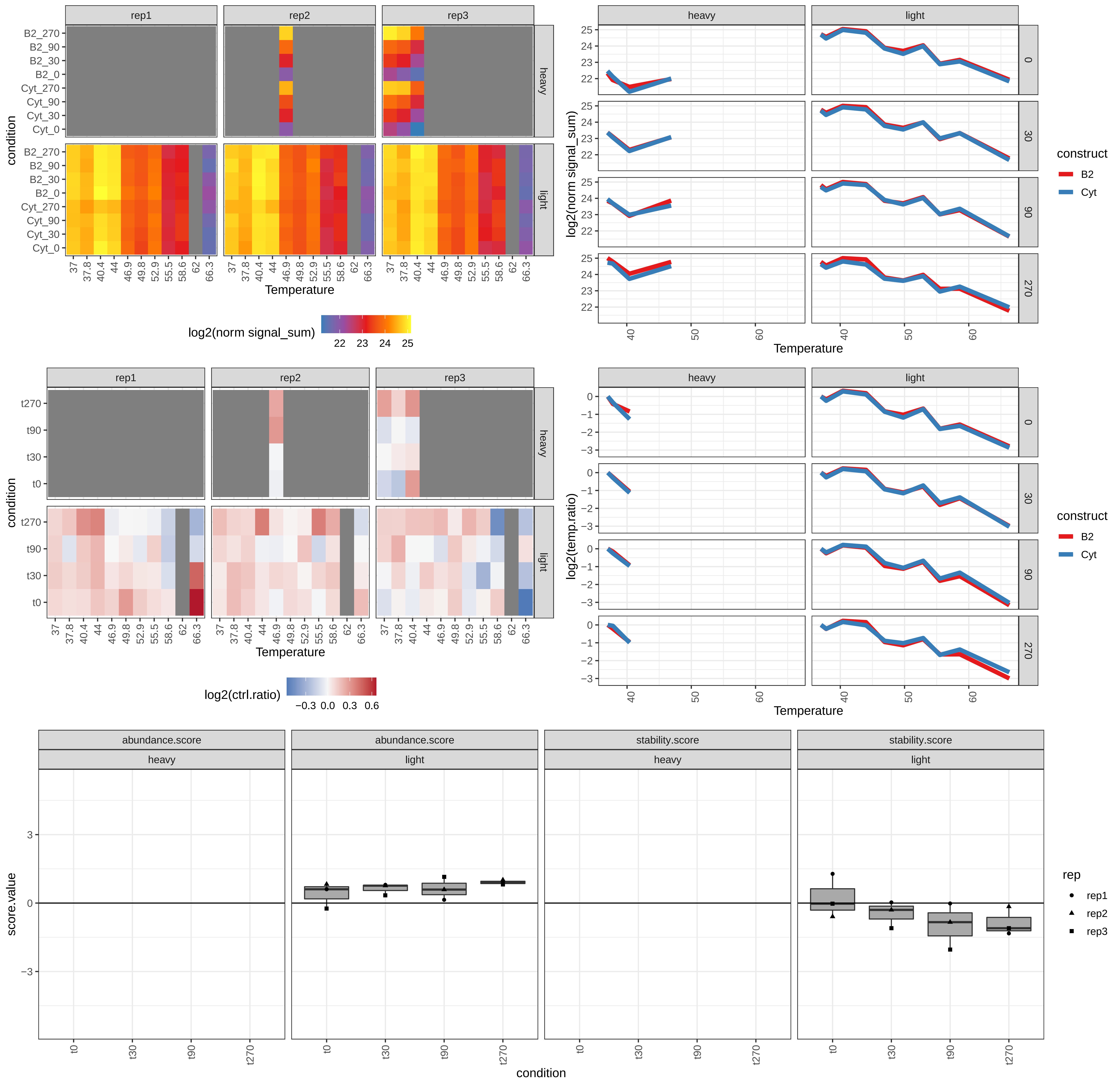

Q04471 − Putative peptidase YMR114C OS=Saccharomyces cerevisiae (strain ATCC 204508 / S288c) GN=YMR114C PE=1 SV=1

YMR134W − average.top3: 6.890194375 − max.qupm: 6

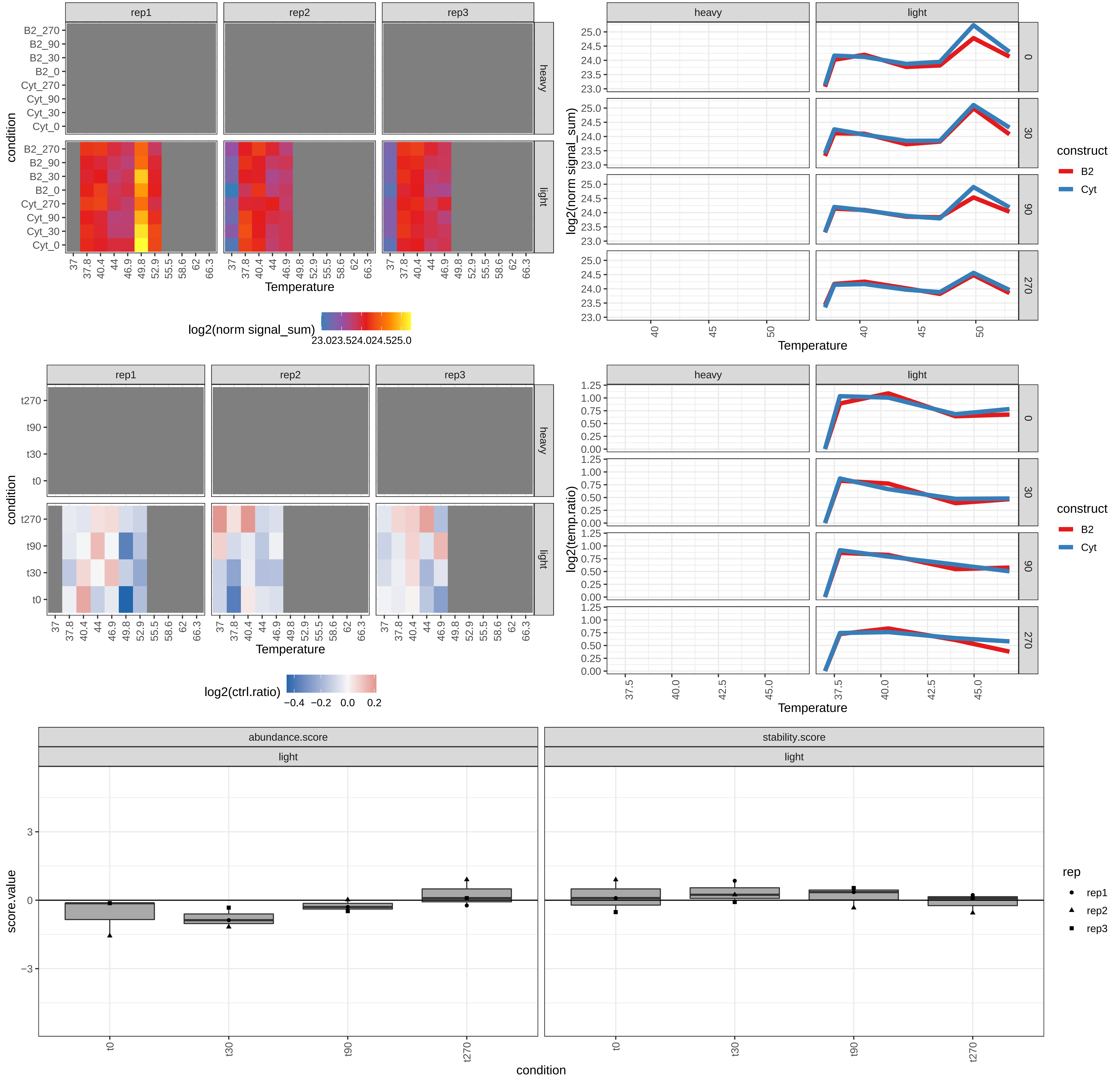

P40207 − Uncharacterized protein YMR134W OS=Saccharomyces cerevisiae (strain ATCC 204508 / S288c) GN=YMR134W PE=1 SV=1

YMR178W − average.top3: 6.93524666666667 − max.qupm: 8

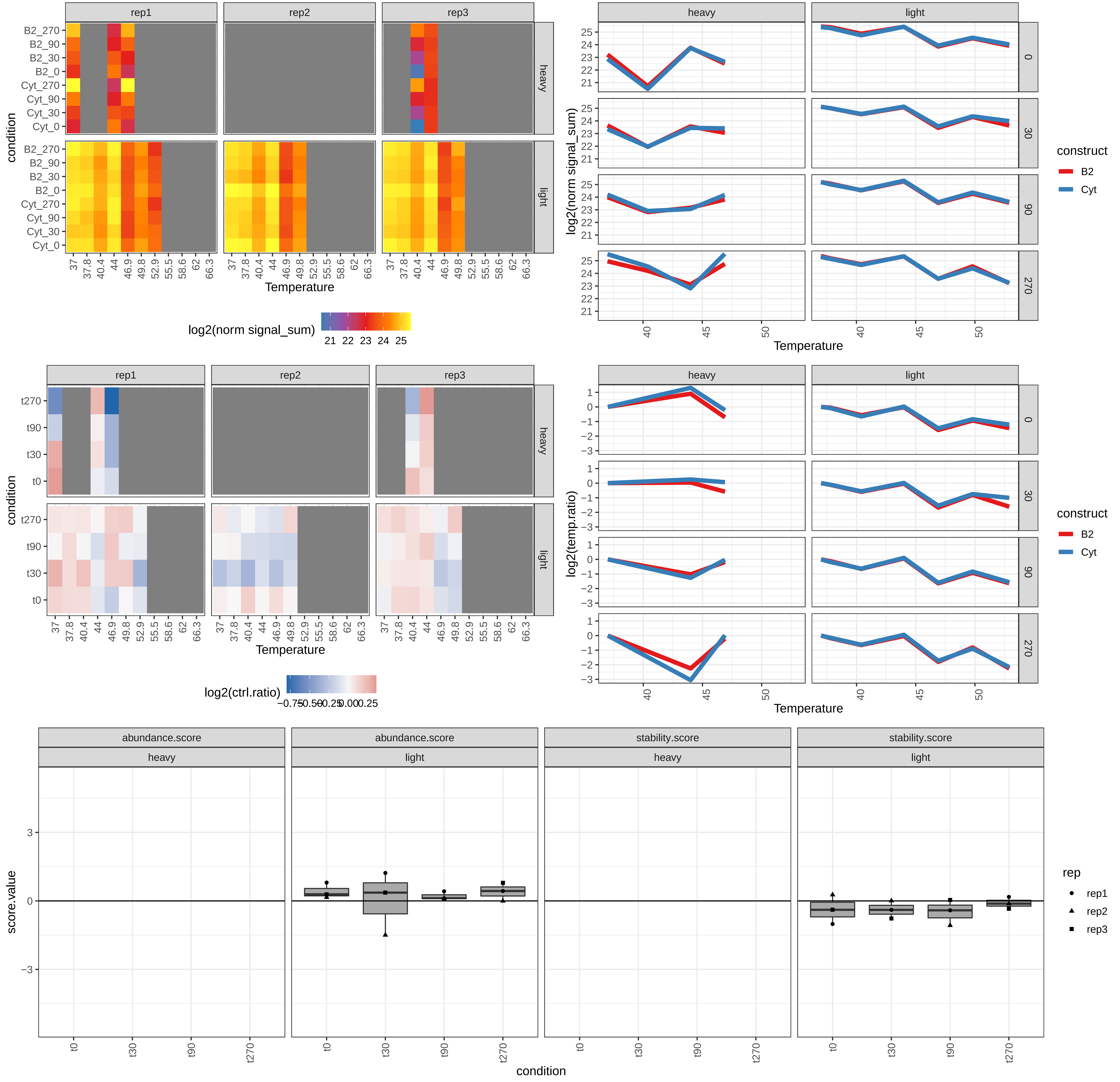

Q03219 − Uncharacterized protein YMR178W OS=Saccharomyces cerevisiae (strain ATCC 204508 / S288c) GN=YMR178W PE=1 SV=1

YMR196W − average.top3: 7.20179511111111 − max.qupm: 22

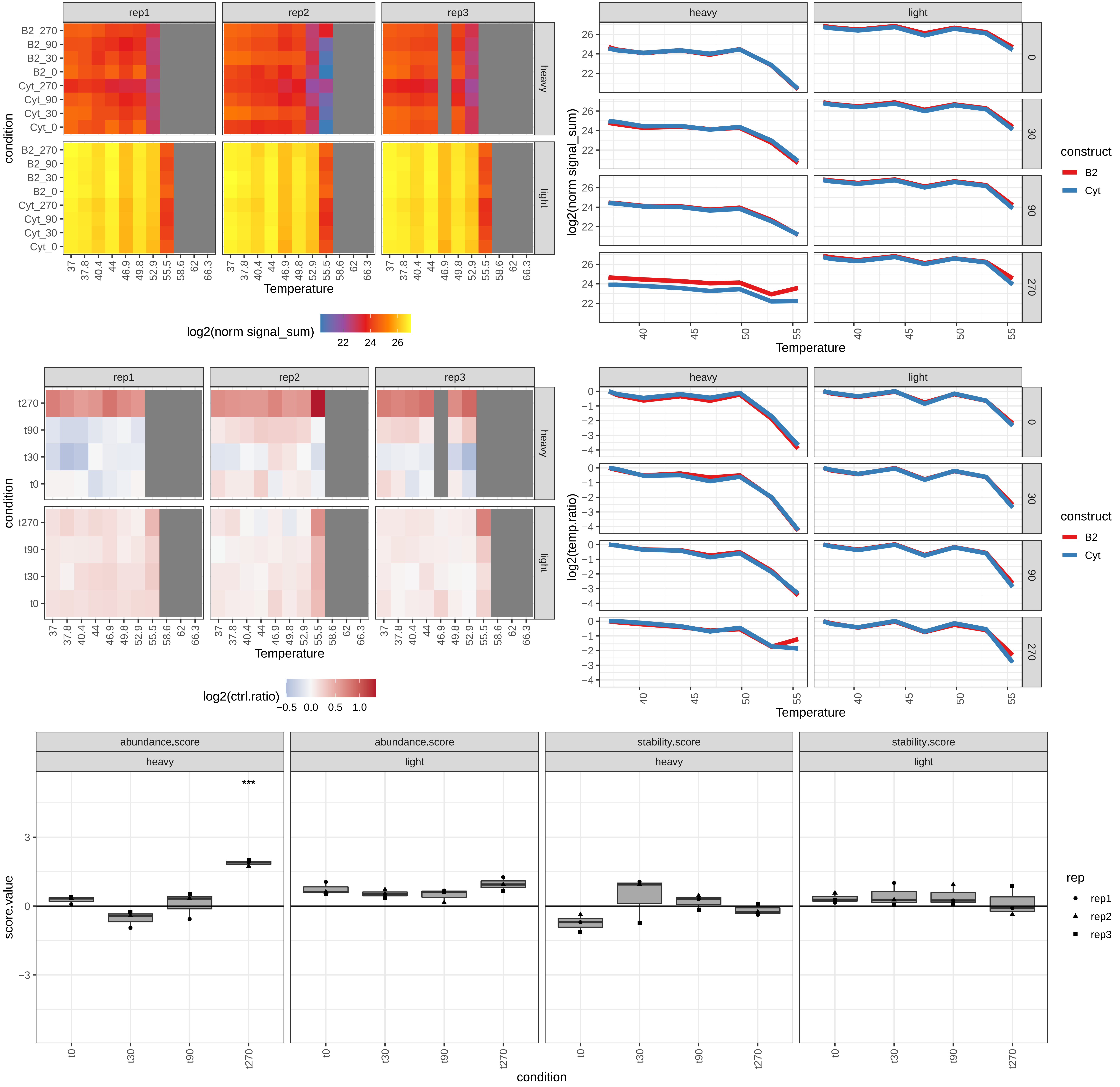

Q04336 − Uncharacterized protein YMR196W OS=Saccharomyces cerevisiae (strain ATCC 204508 / S288c) GN=YMR196W PE=1 SV=1

YMR209C − average.top3: 6.78721029411765 − max.qupm: 11

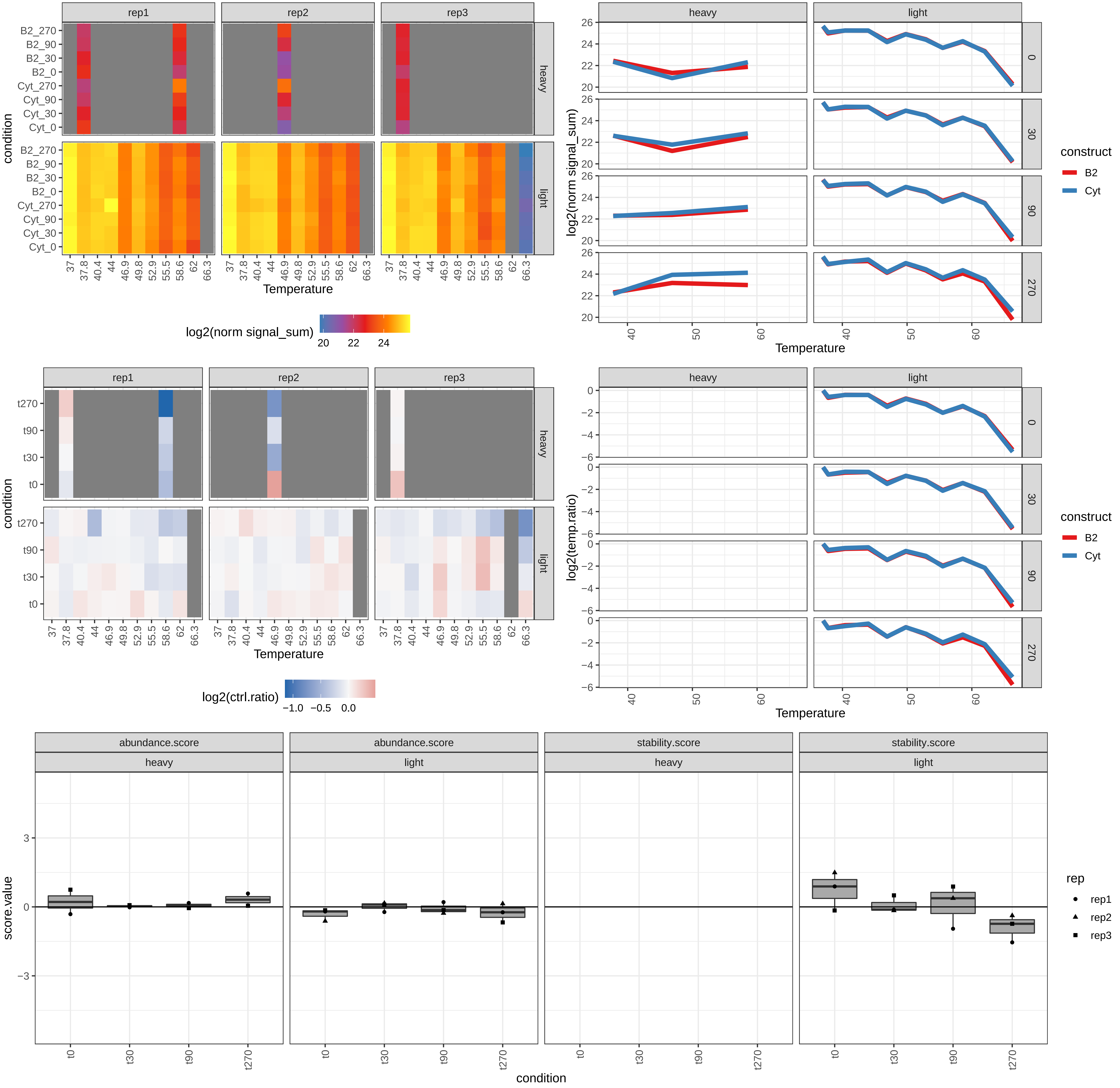

Q03648 − Uncharacterized protein YMR209C OS=Saccharomyces cerevisiae (strain ATCC 204508 / S288c) GN=YMR209C PE=1 SV=1

YMR226C − average.top3: 7.57954586206897 − max.qupm: 12

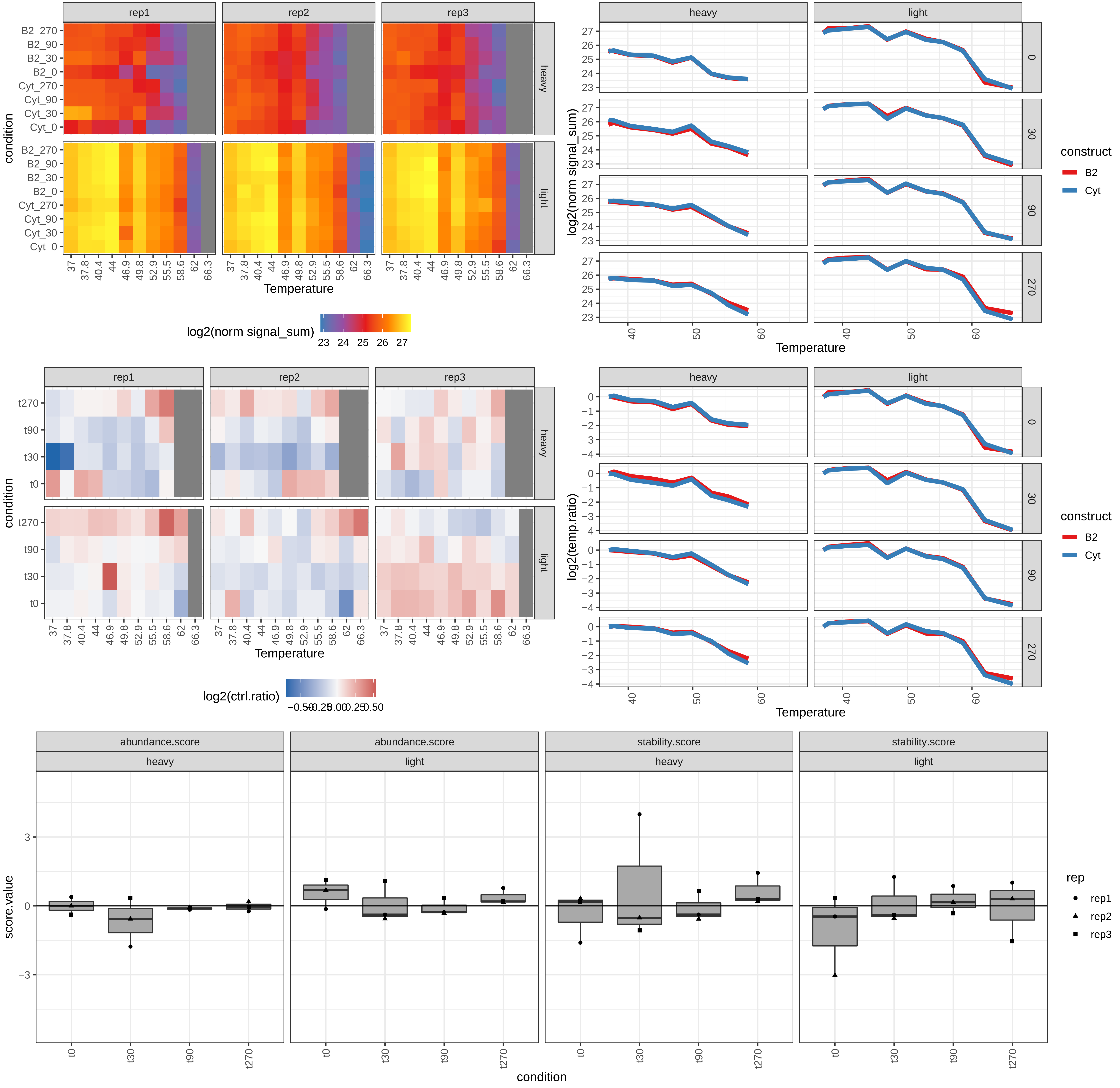

Q05016 − NADP−dependent 3−hydroxy acid dehydrogenase OS=Saccharomyces cerevisiae (strain ATCC 204508 / S288c) GN=YMR226C PE=1 SV=1

YMR262W − average.top3: 6.72420296296296 − max.qupm: 8

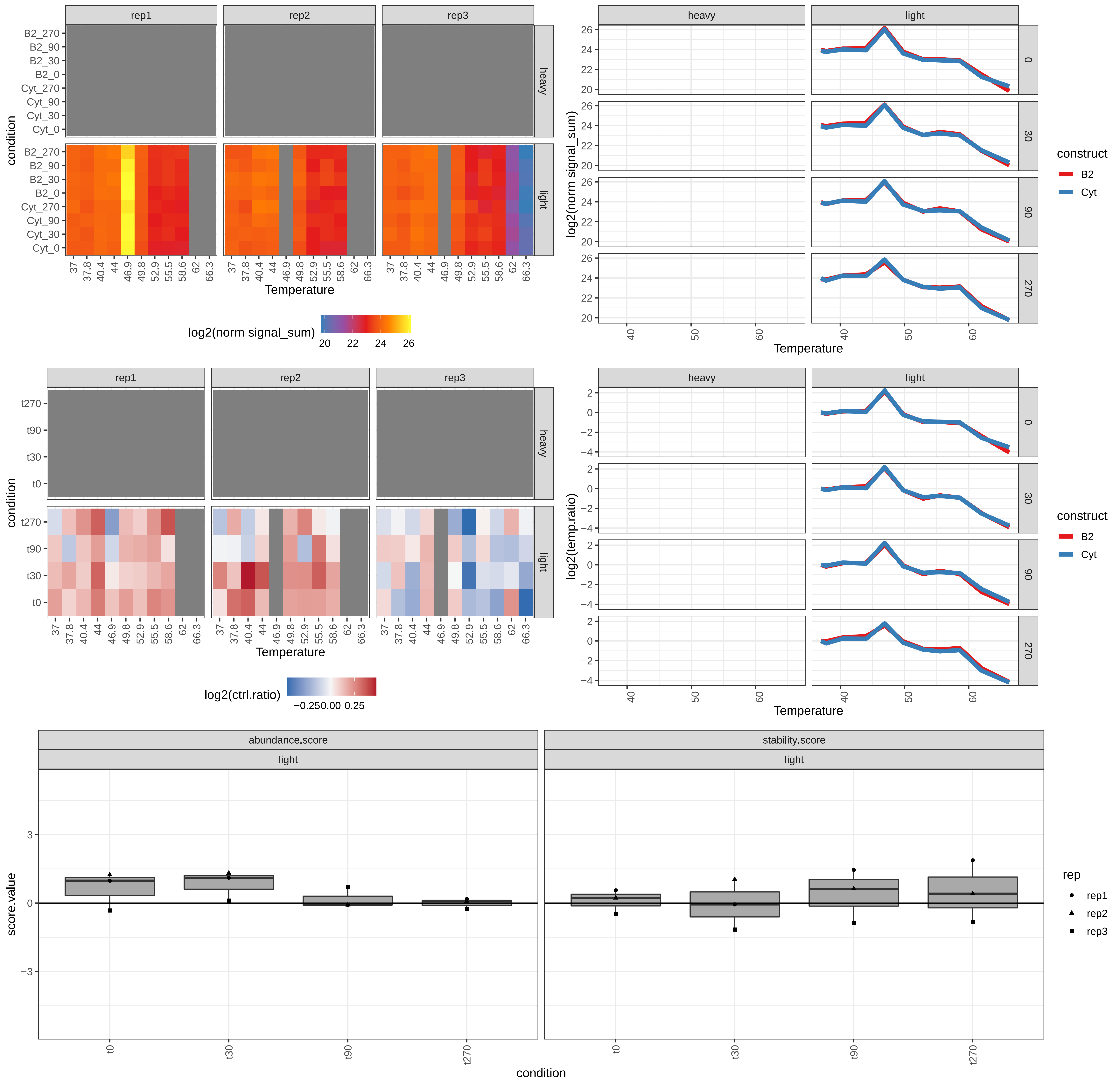

P38430 − Uncharacterized deoxyribonuclease YMR262W OS=Saccharomyces cerevisiae (strain ATCC 204508 / S288c) GN=YMR262W PE=1 SV=3

YMR315W − average.top3: 7.13267034482759 − max.qupm: 9

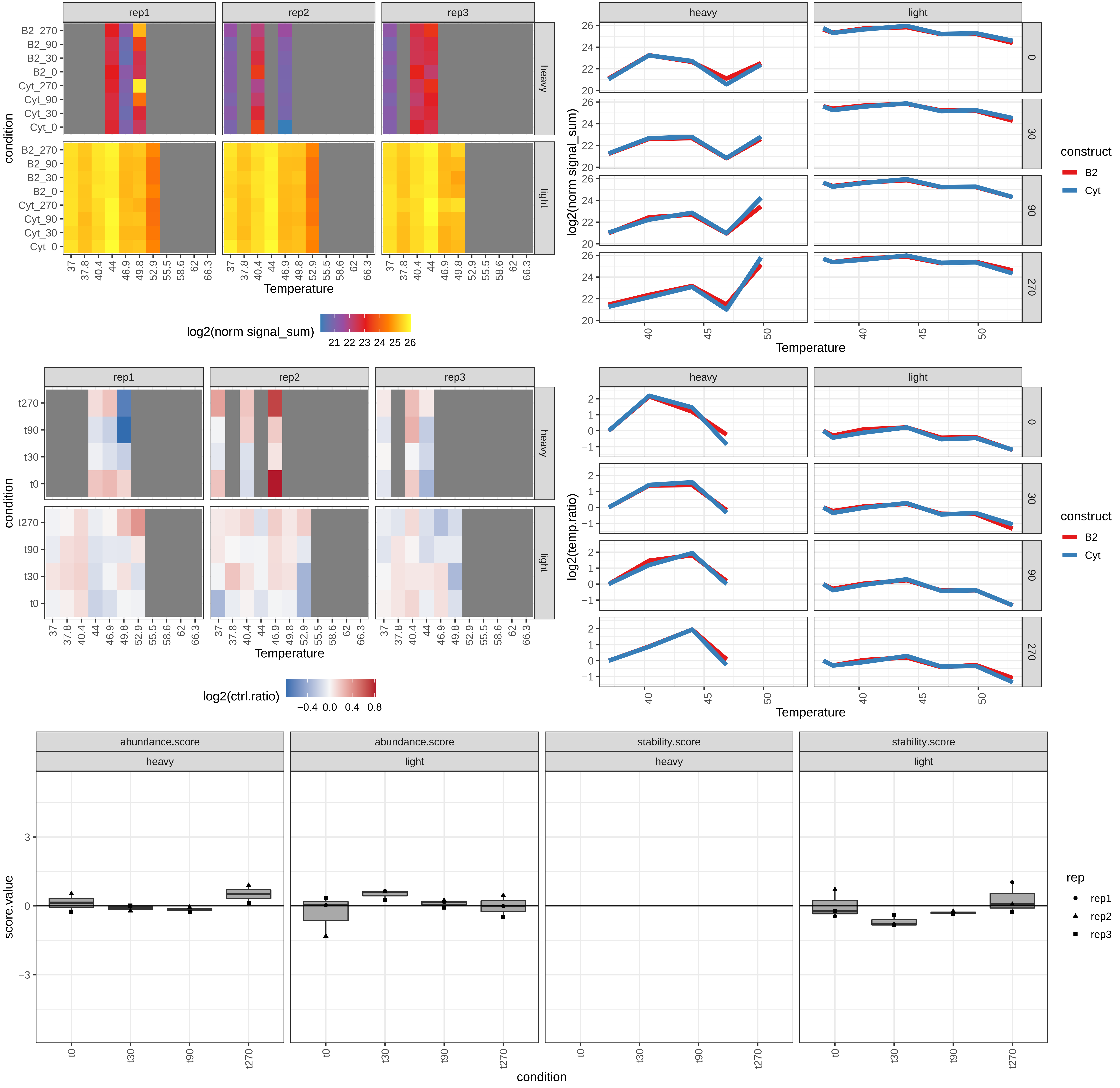

Q04869 − Uncharacterized protein YMR315W OS=Saccharomyces cerevisiae (strain ATCC 204508 / S288c) GN=YMR315W PE=1 SV=1

YNK1 − average.top3: 7.77533454545455 − max.qupm: 10

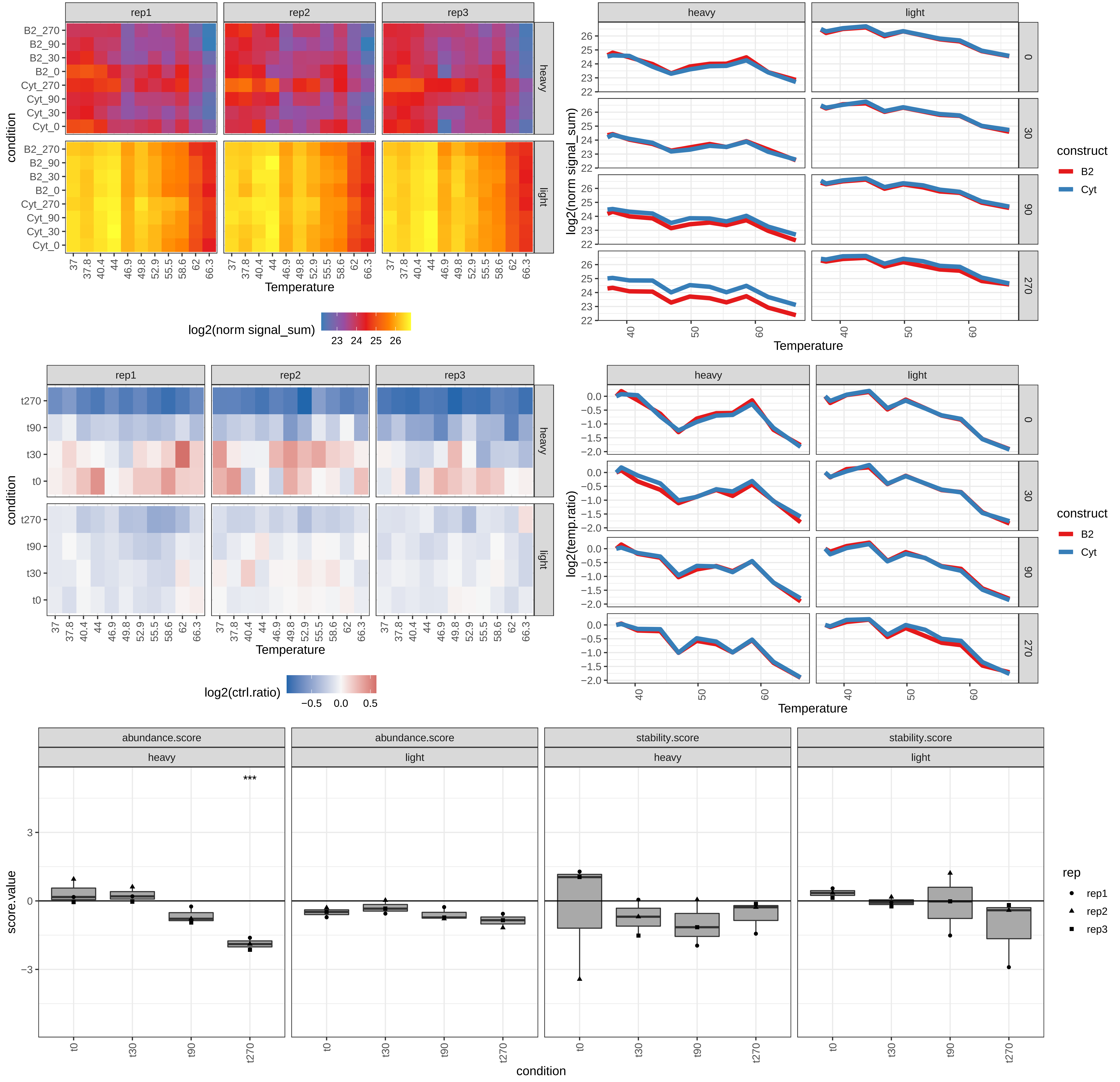

P36010 − Nucleoside diphosphate kinase OS=Saccharomyces cerevisiae (strain ATCC 204508 / S288c) GN=YNK1 PE=1 SV=1

YNL010W − average.top3: 7.6346356097561 − max.qupm: 16

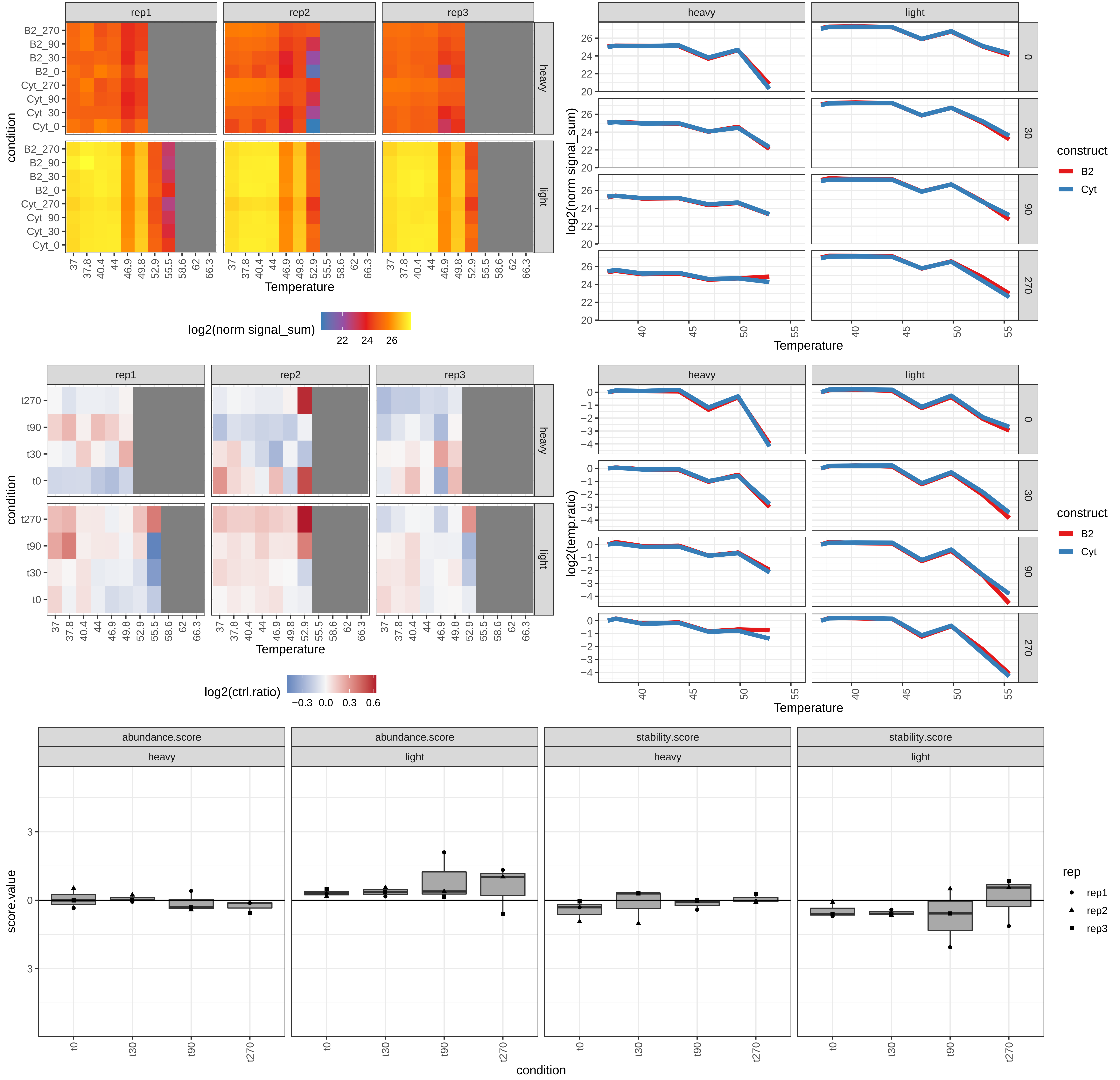

P53981 − Uncharacterized phosphatase YNL010W OS=Saccharomyces cerevisiae (strain ATCC 204508 / S288c) GN=YNL010W PE=1 SV=1

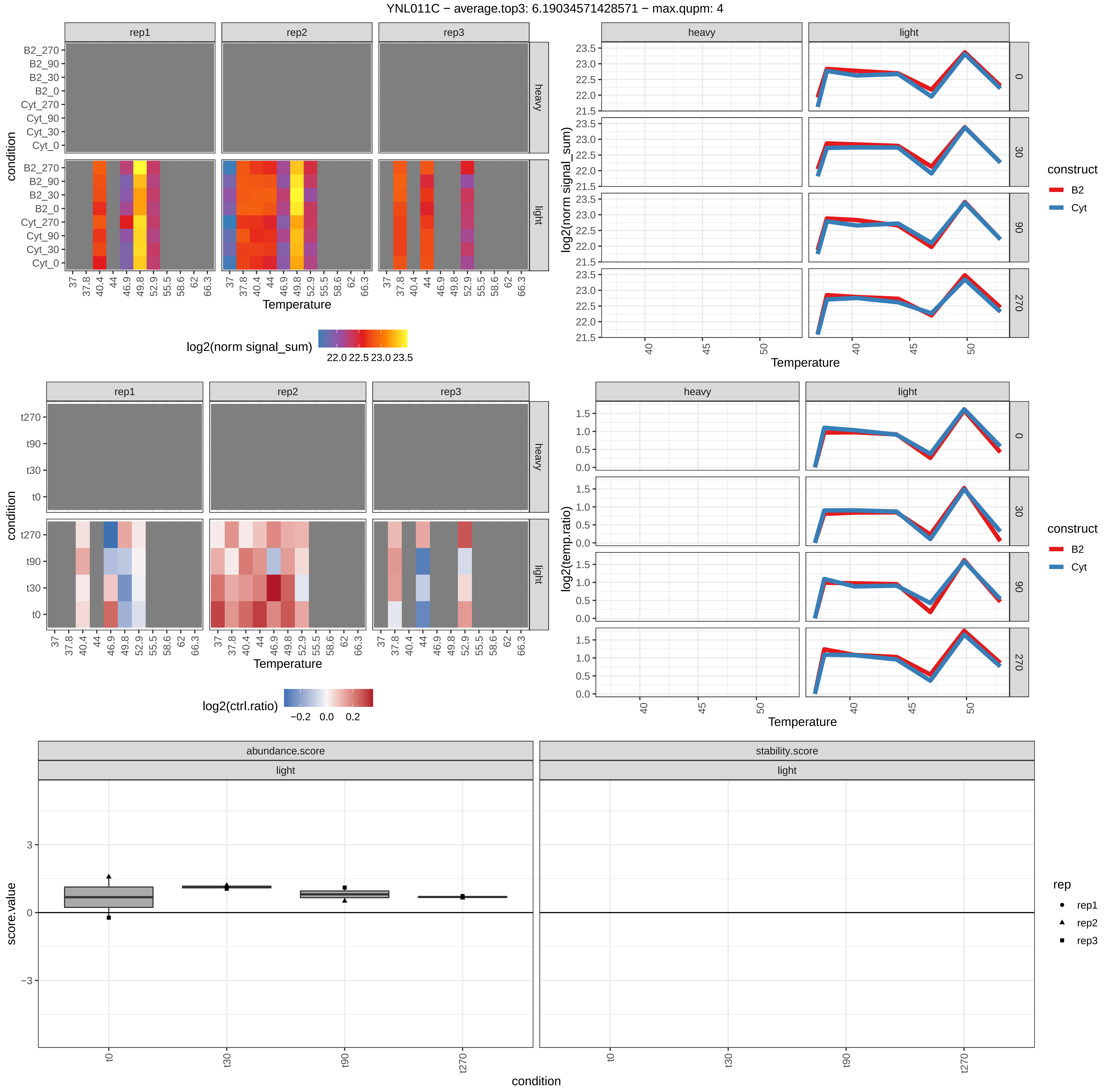

P53980 − Uncharacterized protein YNL011C OS=Saccharomyces cerevisiae (strain ATCC 204508 / S288c) GN=YNL011C PE=1 SV=1

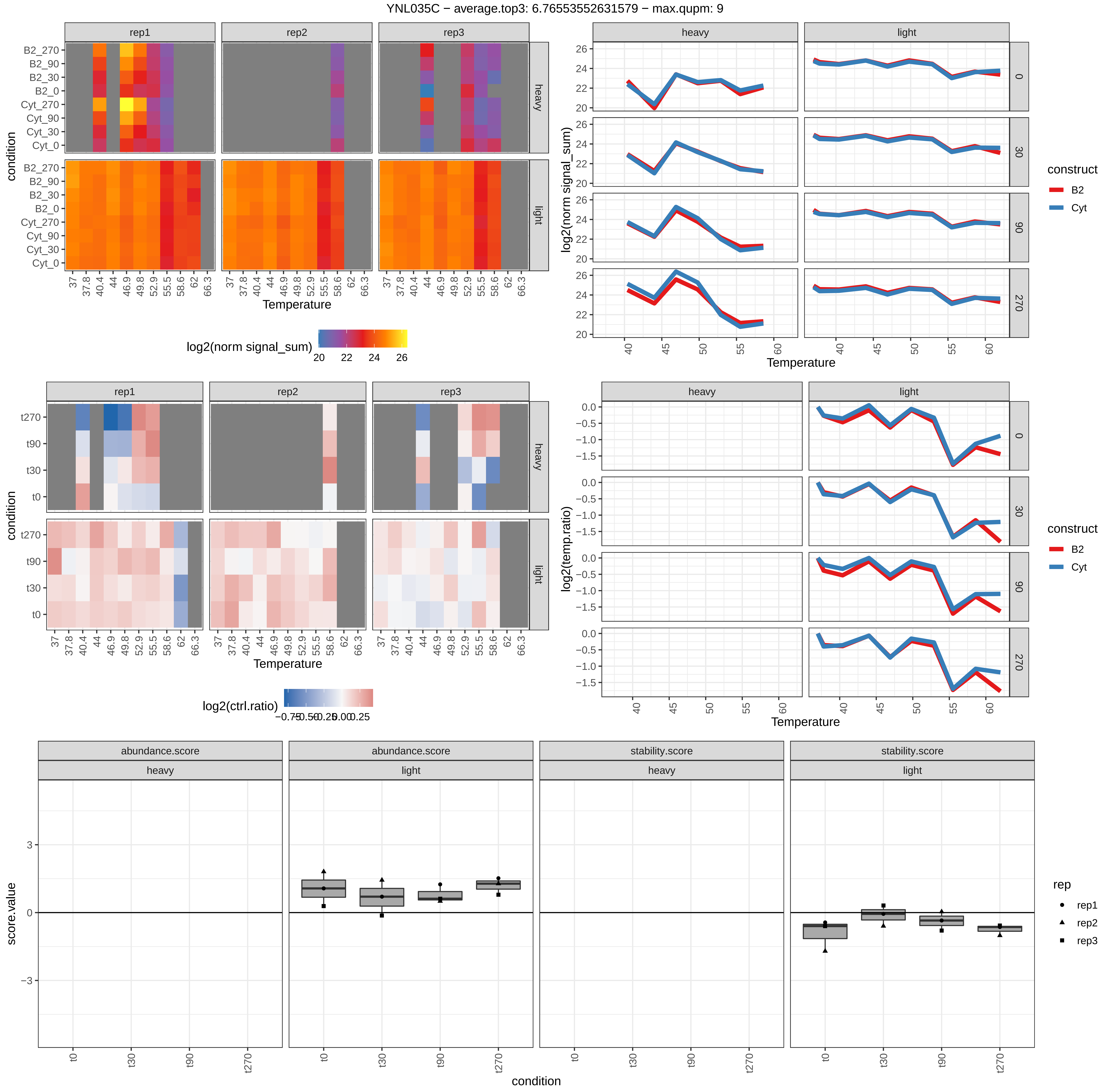

P53962 − Uncharacterized WD repeat−containing protein YNL035C OS=Saccharomyces cerevisiae (strain ATCC 204508 / S288c) GN=YNL035C PE=1 SV=1

YNL040W − average.top3: 6.7958372 − max.qupm: 9

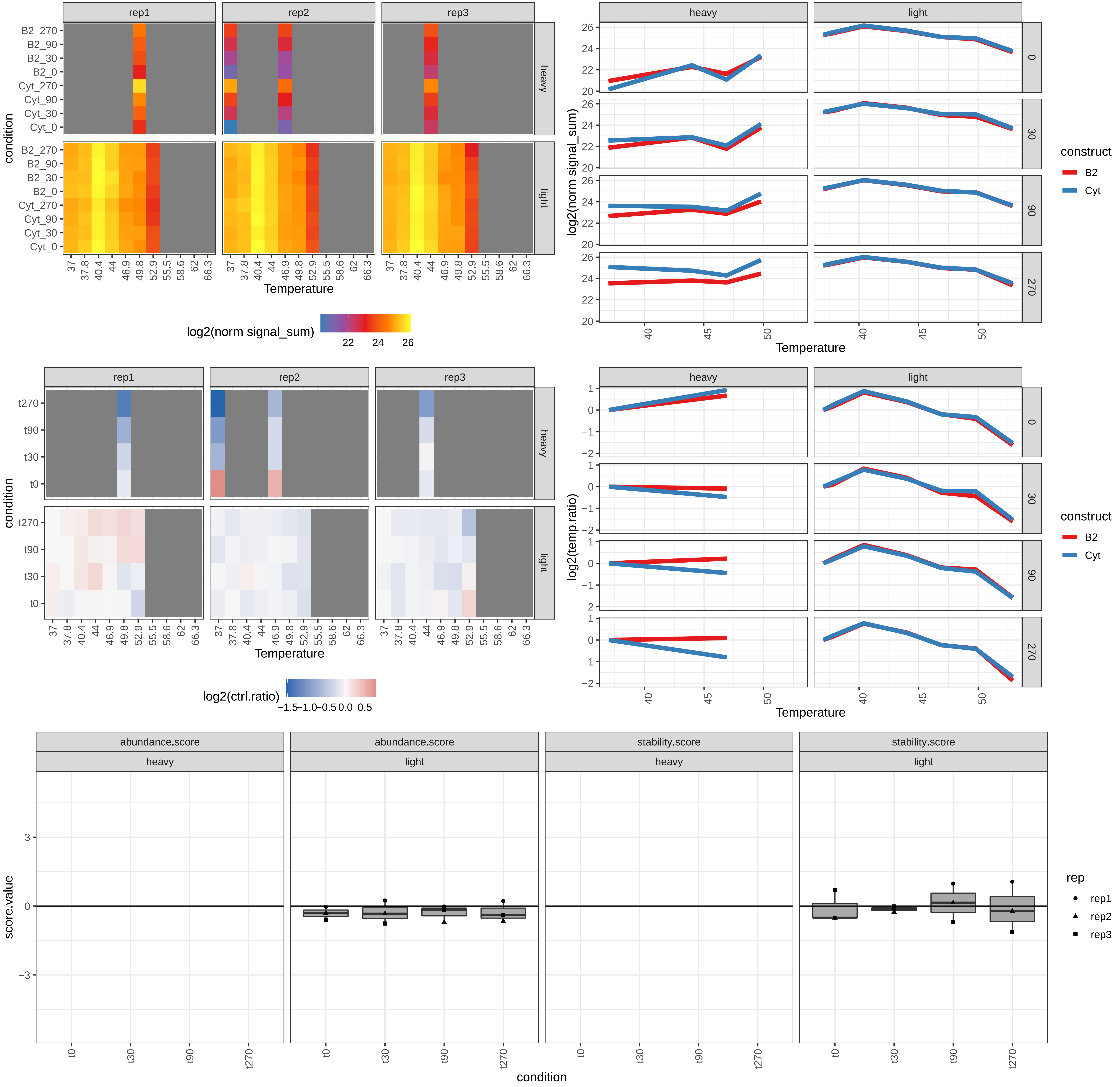

P53960 − Putative alanyl−tRNA editing protein alaX OS=Saccharomyces cerevisiae (strain ATCC 204508 / S288c) GN=YNL040W PE=1 SV=1

YNL115C − average.top3: 7.31418921875 − max.qupm: 12

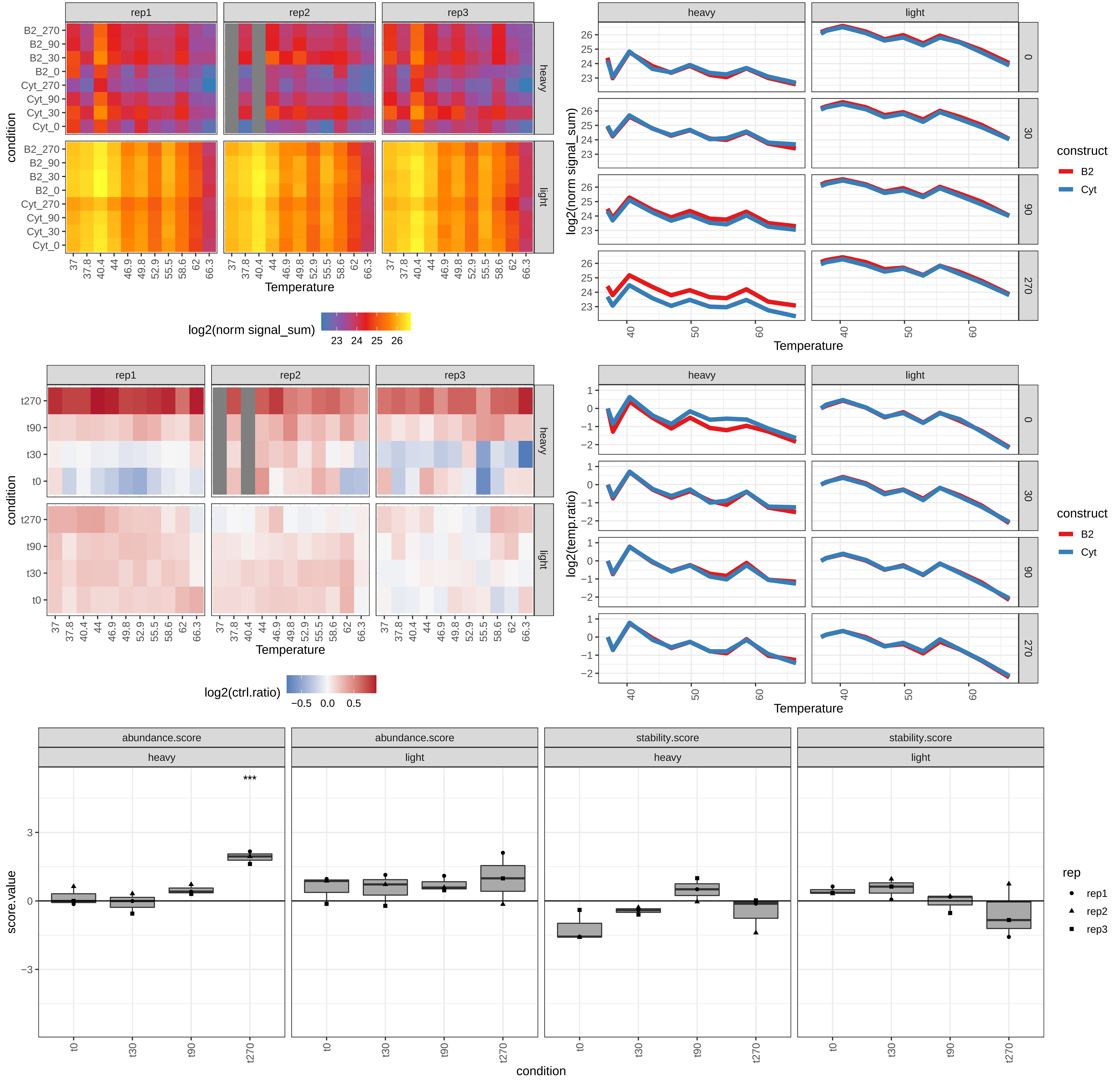

P53925 − Uncharacterized vacuolar membrane protein YNL115C OS=Saccharomyces cerevisiae (strain ATCC 204508 / S288c) GN=YNL115C PE=1 SV=1

YNL134C − average.top3: 7.46510366666667 − max.qupm: 12

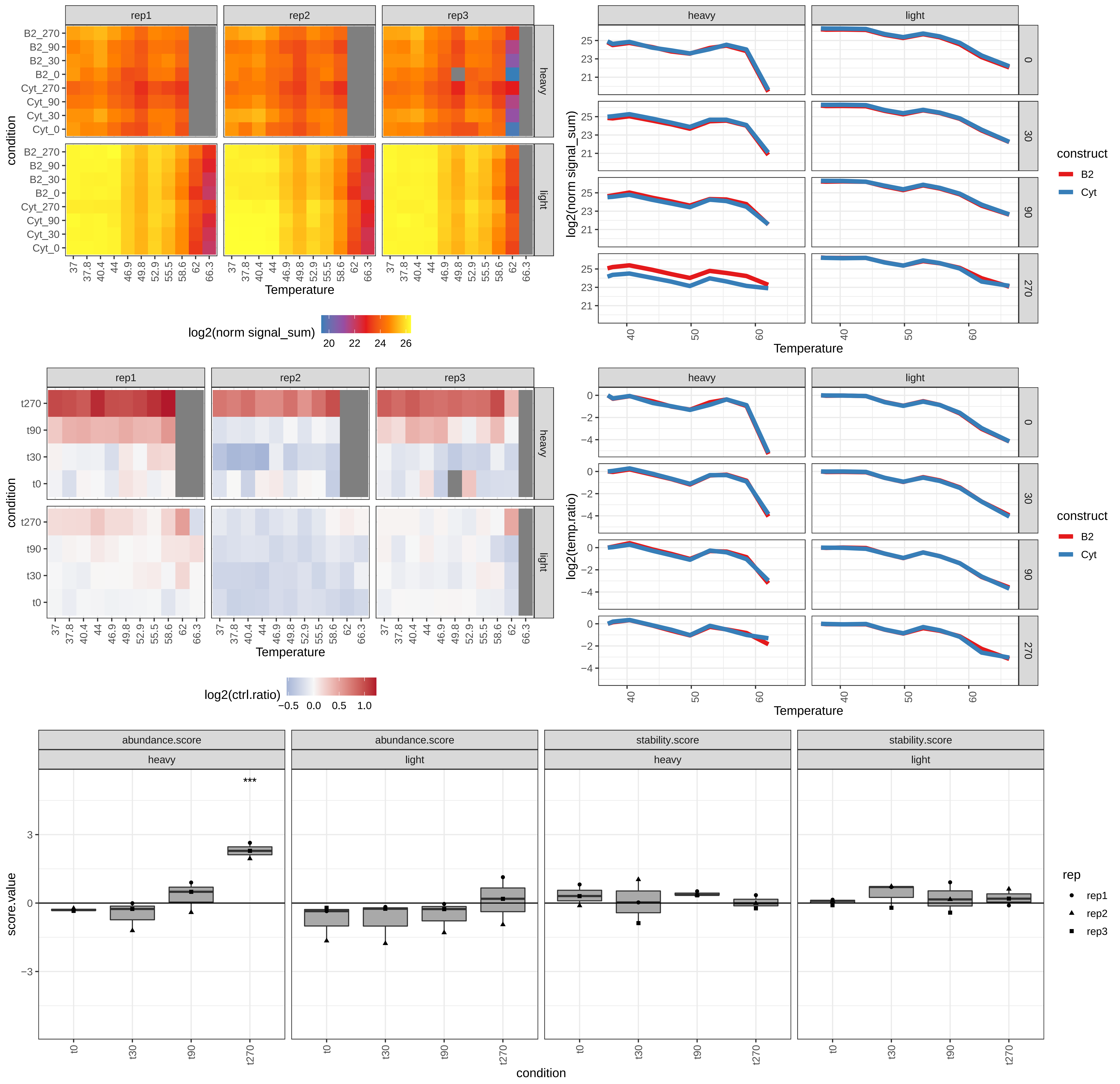

P53912 − Uncharacterized protein YNL134C OS=Saccharomyces cerevisiae (strain ATCC 204508 / S288c) GN=YNL134C PE=1 SV=1

YNL181W − average.top3: 7.01755177777778 − max.qupm: 13

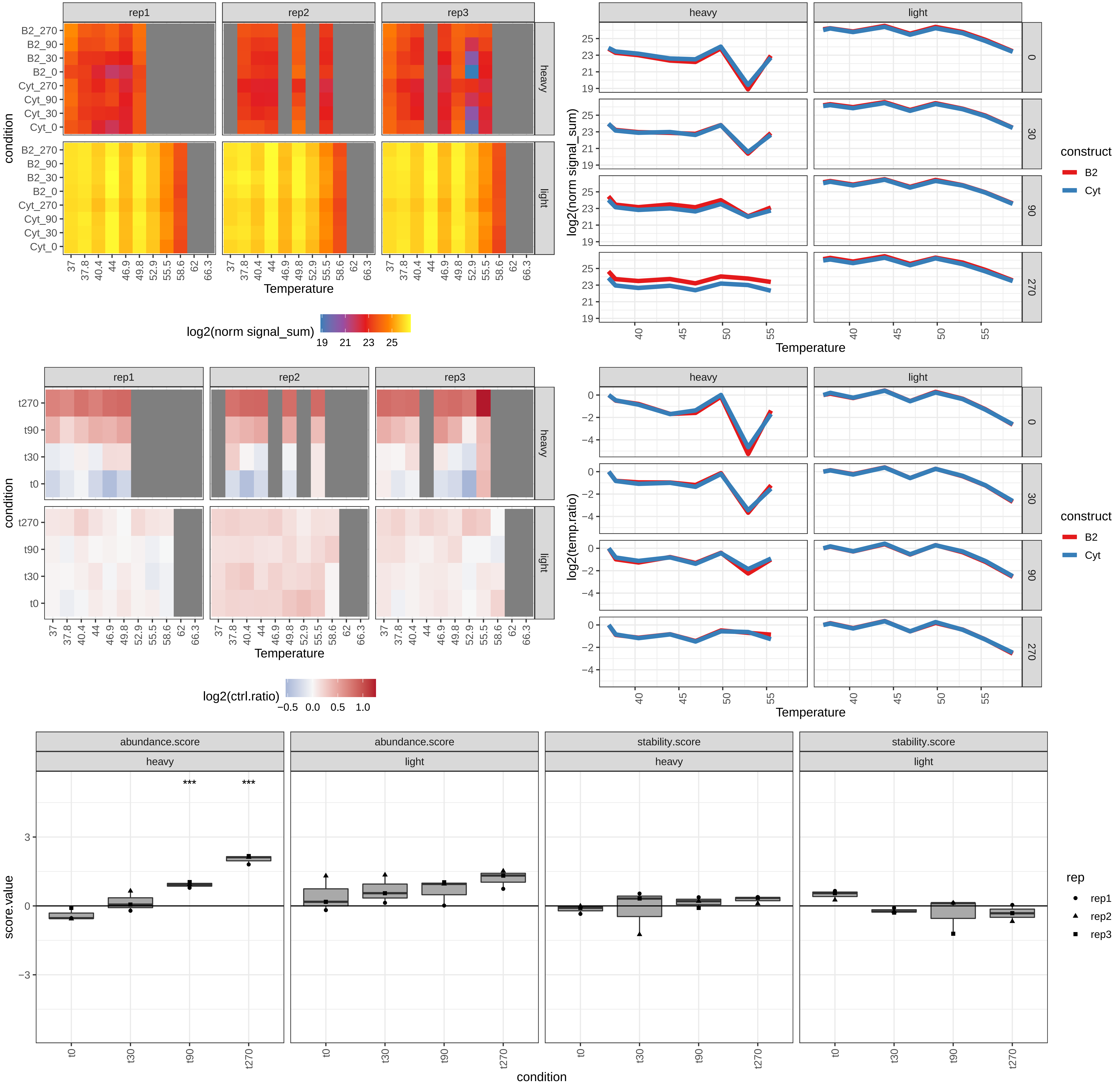

P53878 − Uncharacterized oxidoreductase YNL181W OS=Saccharomyces cerevisiae (strain ATCC 204508 / S288c) GN=YNL181W PE=1 SV=2

YNL194C − average.top3: 6.87768666666667 − max.qupm: 5

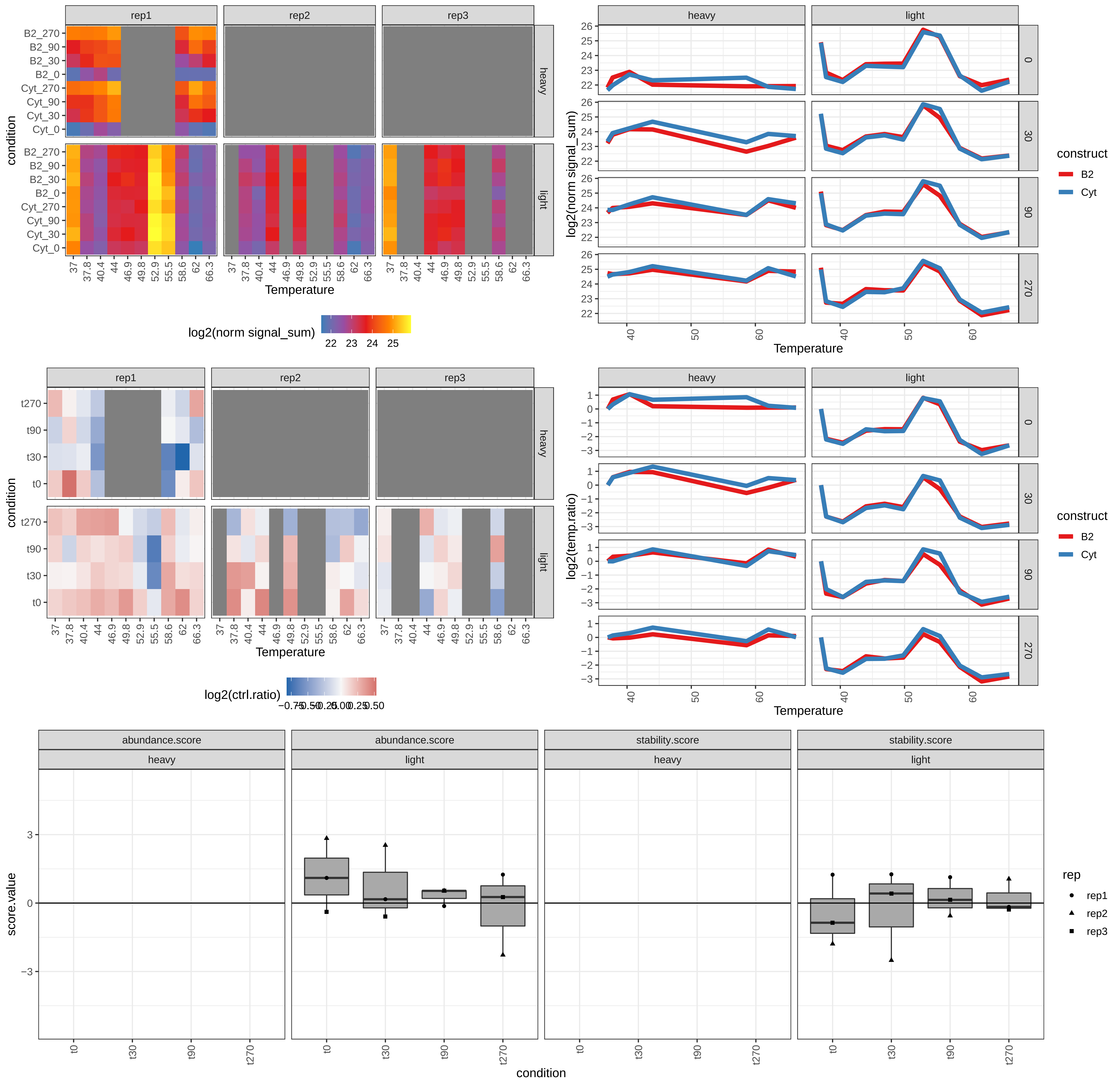

P40169 − Uncharacterized plasma membrane protein YNL194C OS=Saccharomyces cerevisiae (strain ATCC 204508 / S288c) GN=YNL194C PE=1 SV=1

YNL200C − average.top3: 7.19075148148148 − max.qupm: 3

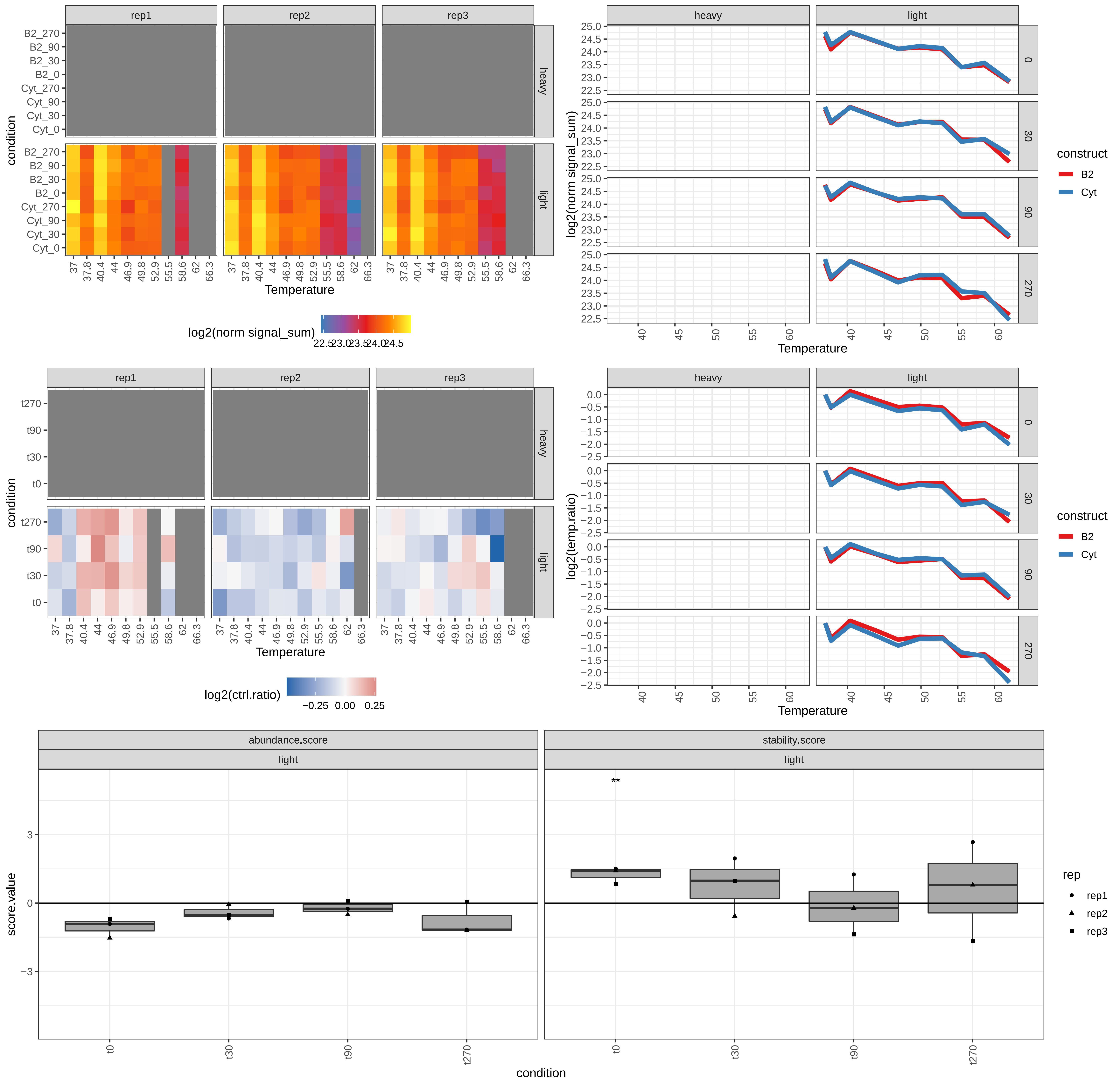

P40165 − NAD(P)H−hydrate epimerase OS=Saccharomyces cerevisiae (strain ATCC 204508 / S288c) GN=YNL200C PE=1 SV=1

YNL208W − average.top3: 6.60462454545455 − max.qupm: 3

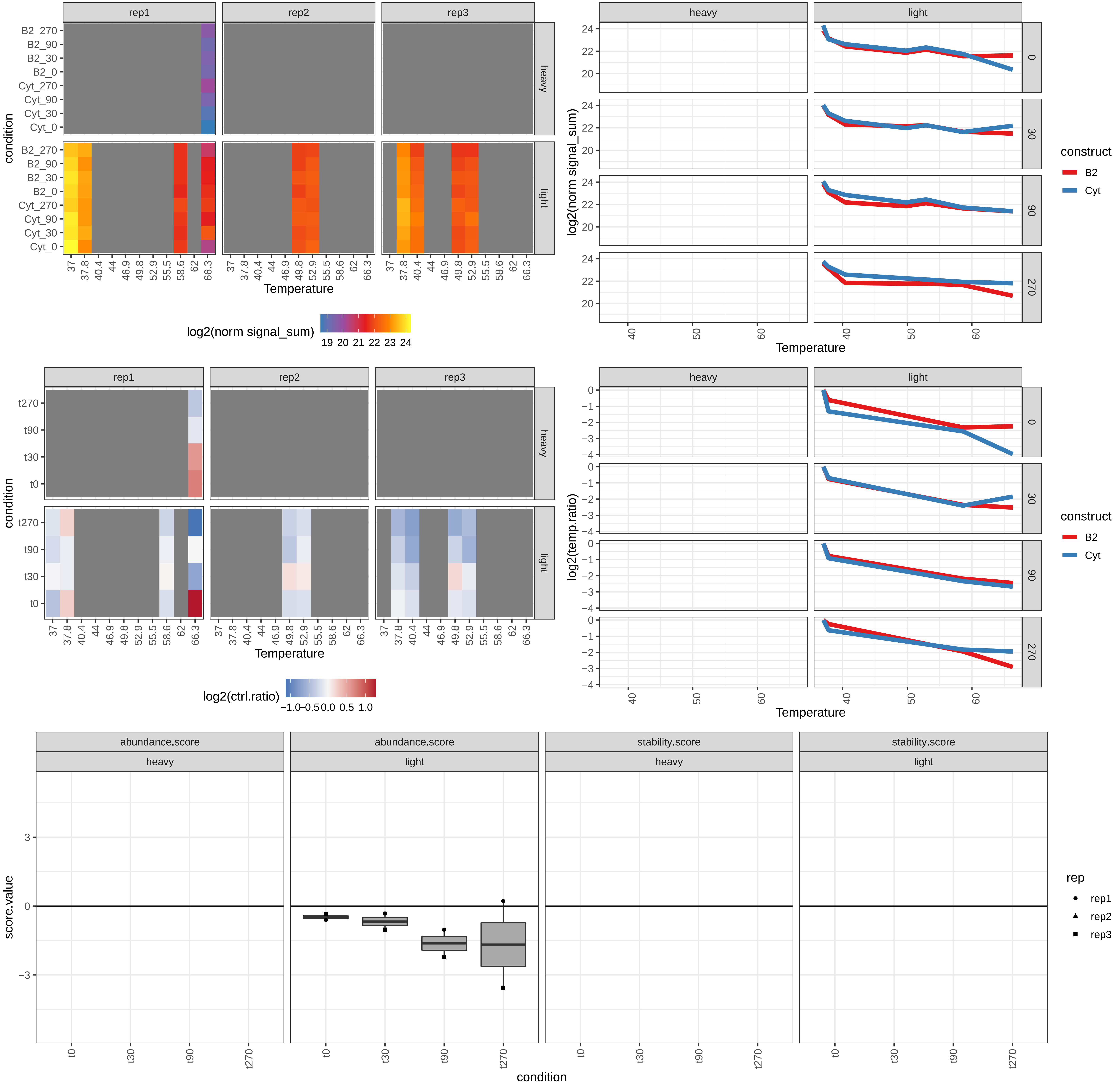

P40159 − Uncharacterized protein YNL208W OS=Saccharomyces cerevisiae (strain ATCC 204508 / S288c) GN=YNL208W PE=1 SV=2

YNL247W − average.top3: 7.18175533333333 − max.qupm: 28

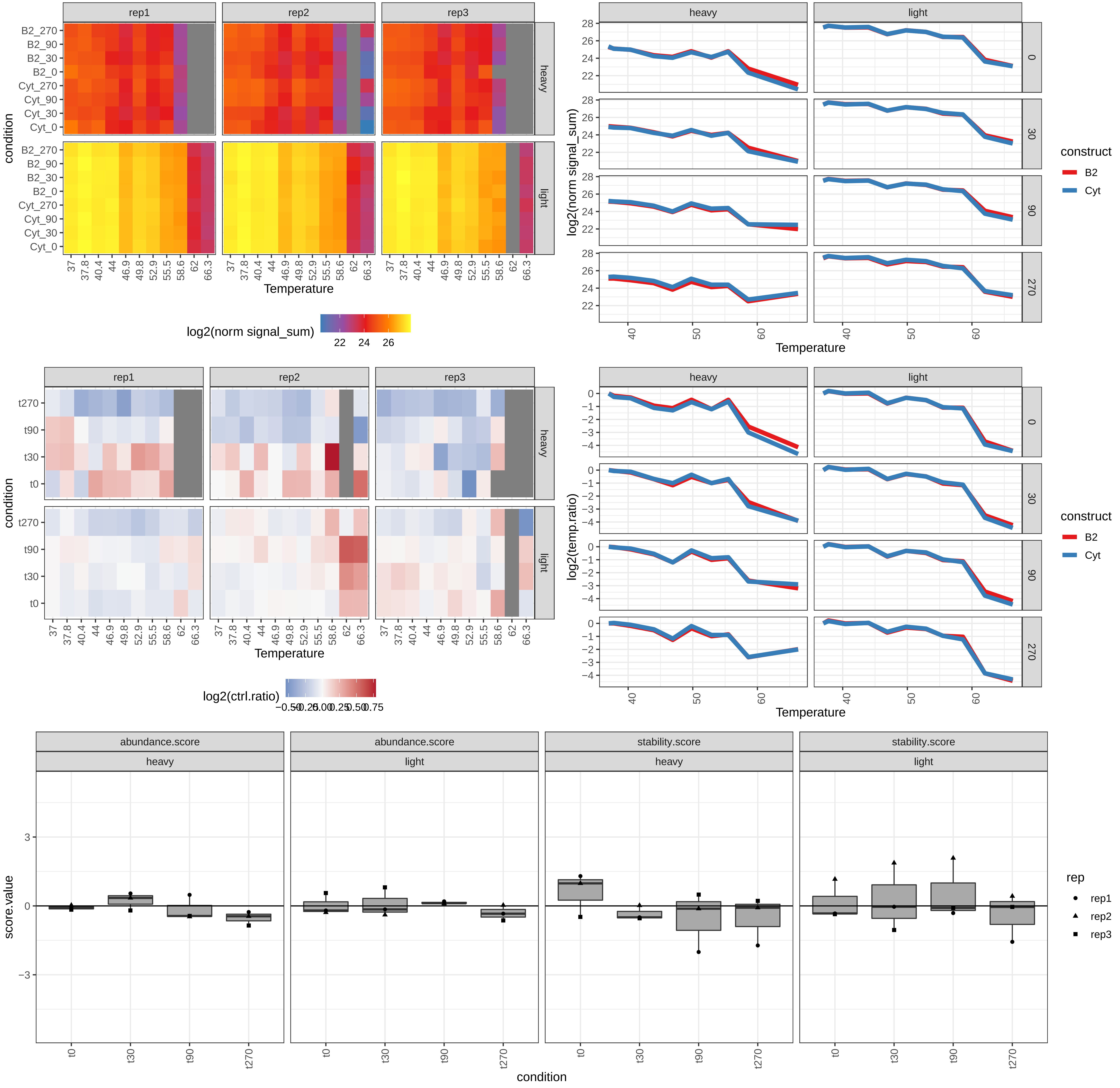

P53852 − Cysteine−−tRNA ligase OS=Saccharomyces cerevisiae (strain ATCC 204508 / S288c) GN=YNL247W PE=1 SV=1

YNL320W − average.top3: 6.796440625 − max.qupm: 7

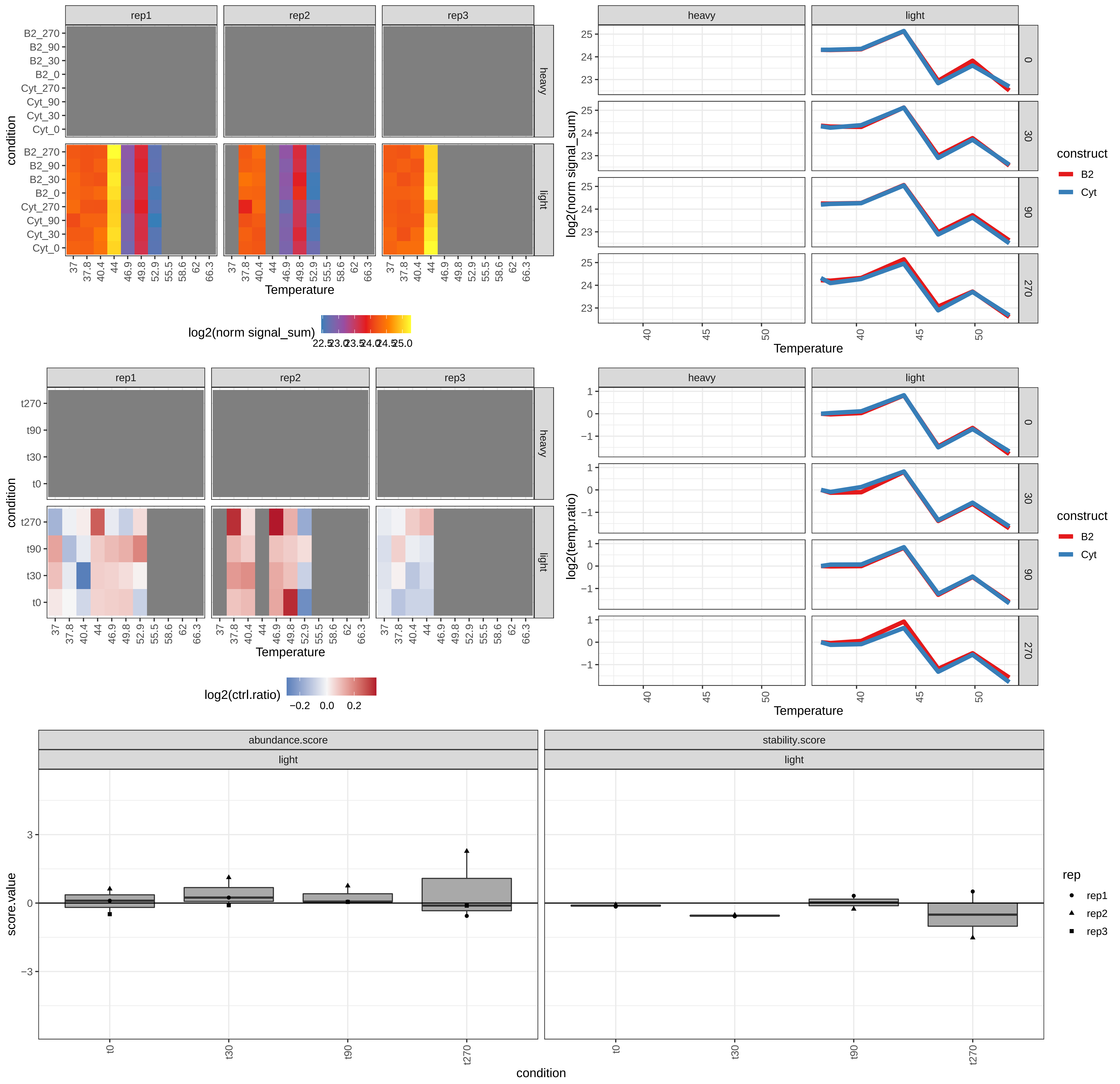

P42840 − Uncharacterized membrane protein YNL320W OS=Saccharomyces cerevisiae (strain ATCC 204508 / S288c) GN=YNL320W PE=1 SV=1

YNR021W − average.top3: 7.14849903225806 − max.qupm: 8

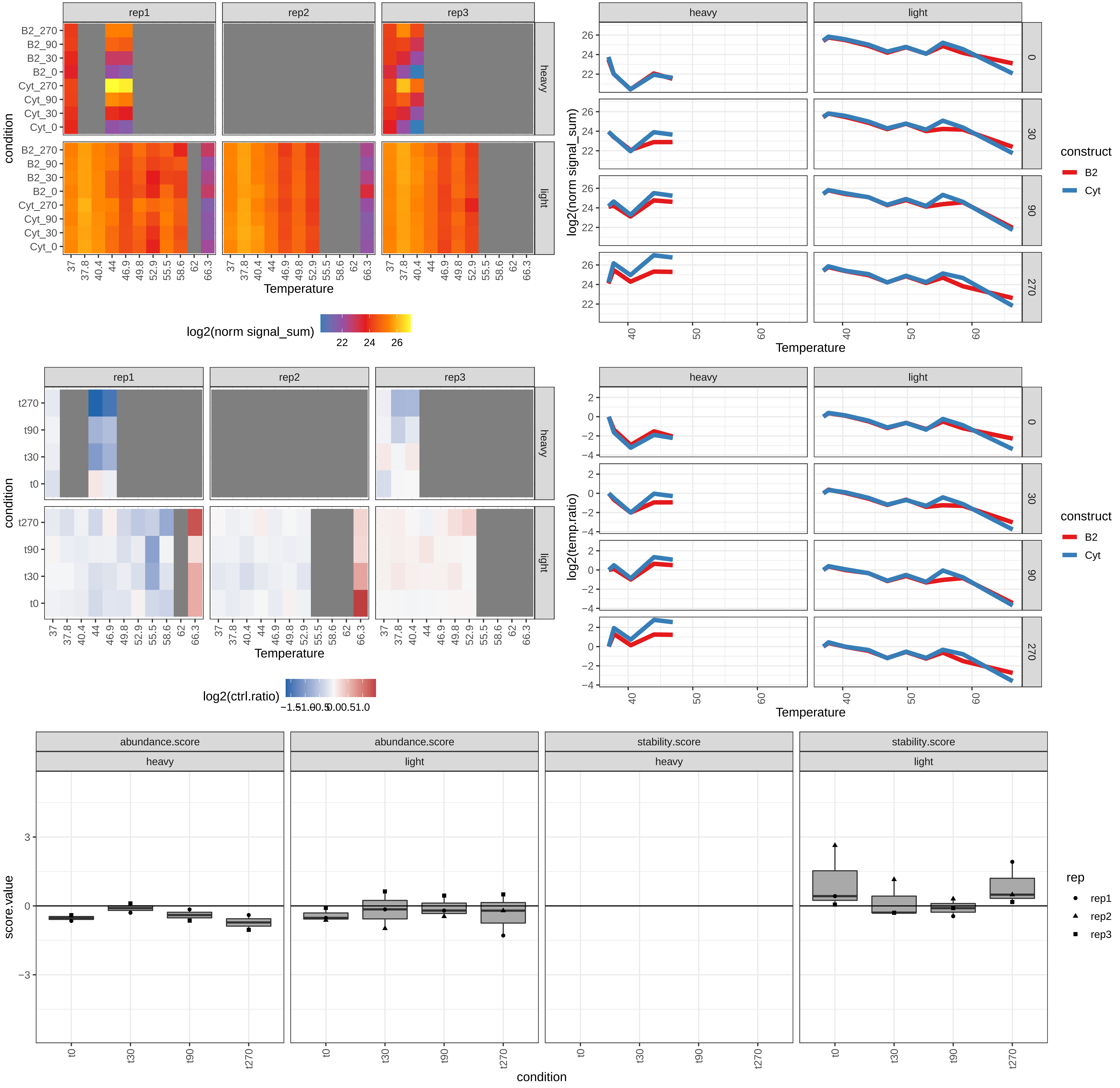

P53723 − UPF0674 endoplasmic reticulum membrane protein YNR021W OS=Saccharomyces cerevisiae (strain ATCC 204508 / S288c) GN=YNR021W PE=1 SV=3

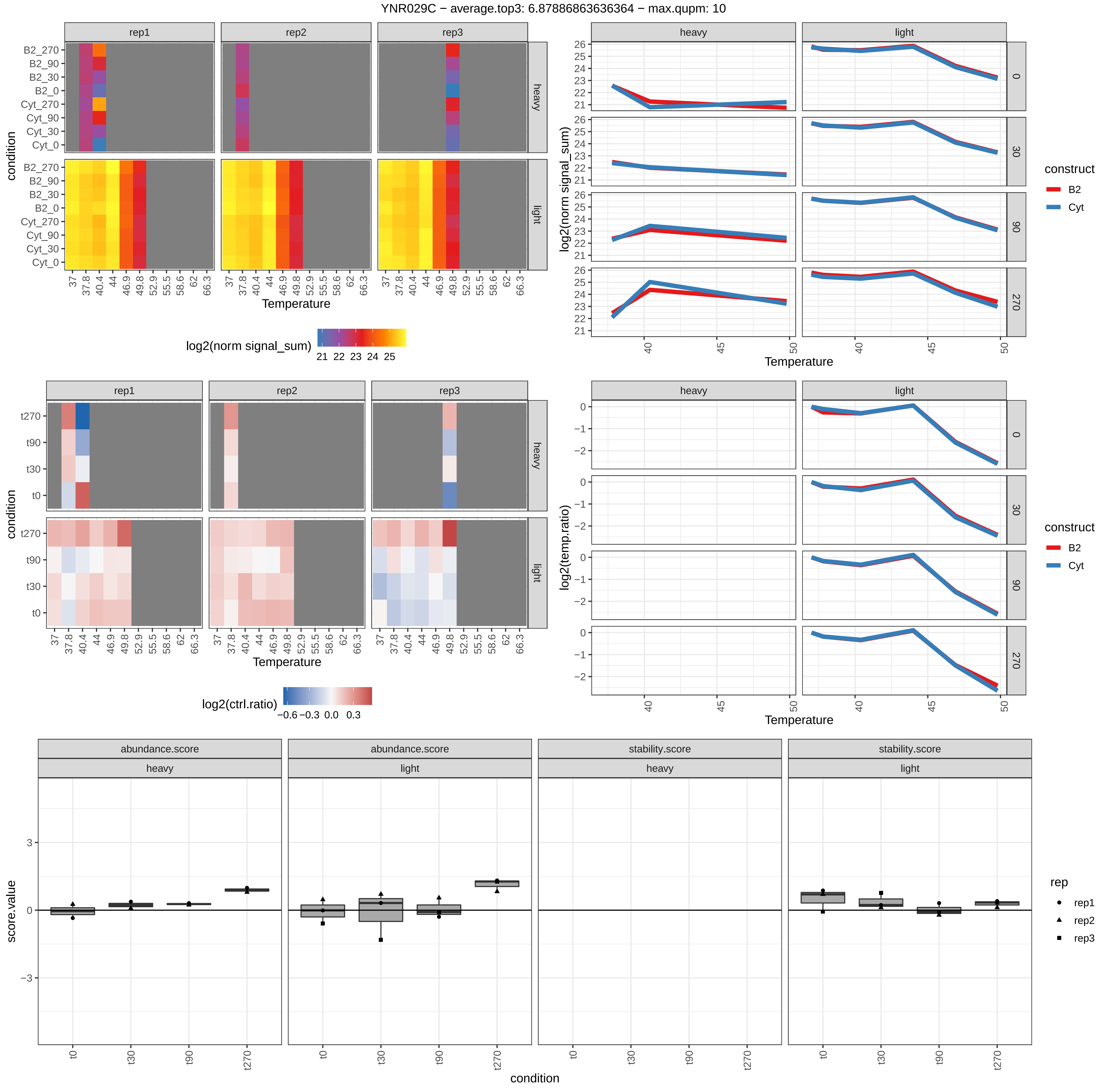

P53729 − Uncharacterized protein YNR029C OS=Saccharomyces cerevisiae (strain ATCC 204508 / S288c) GN=YNR029C PE=1 SV=1

YNR034W − average.top3: 6.84082470588235 − max.qupm: 5

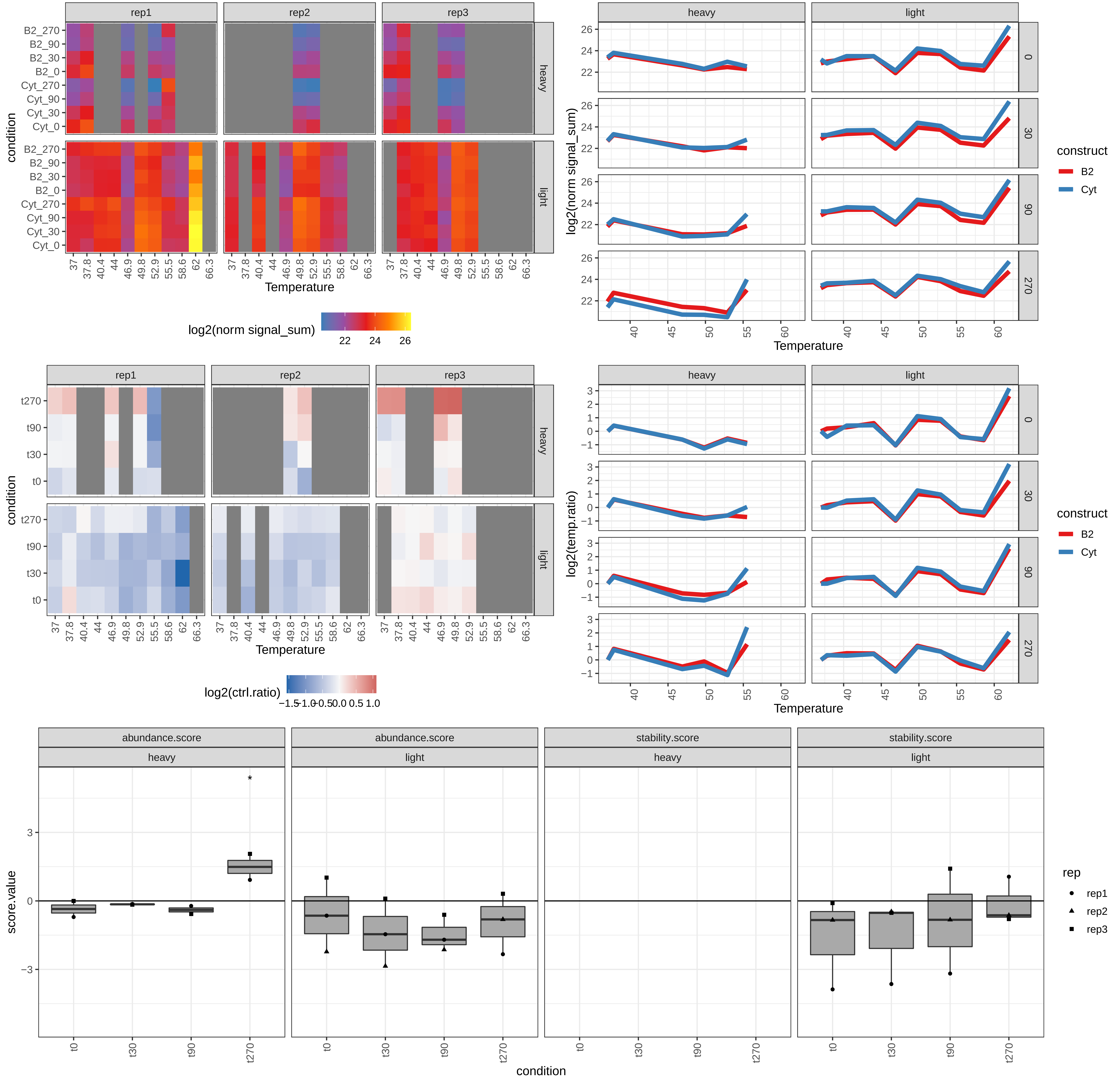

Q3E841 − Uncharacterized protein YNR034W−A OS=Saccharomyces cerevisiae (strain ATCC 204508 / S288c) GN=YNR034W−A PE=1 SV=1

YNR048W − average.top3: 6.31638846153846 − max.qupm: 3

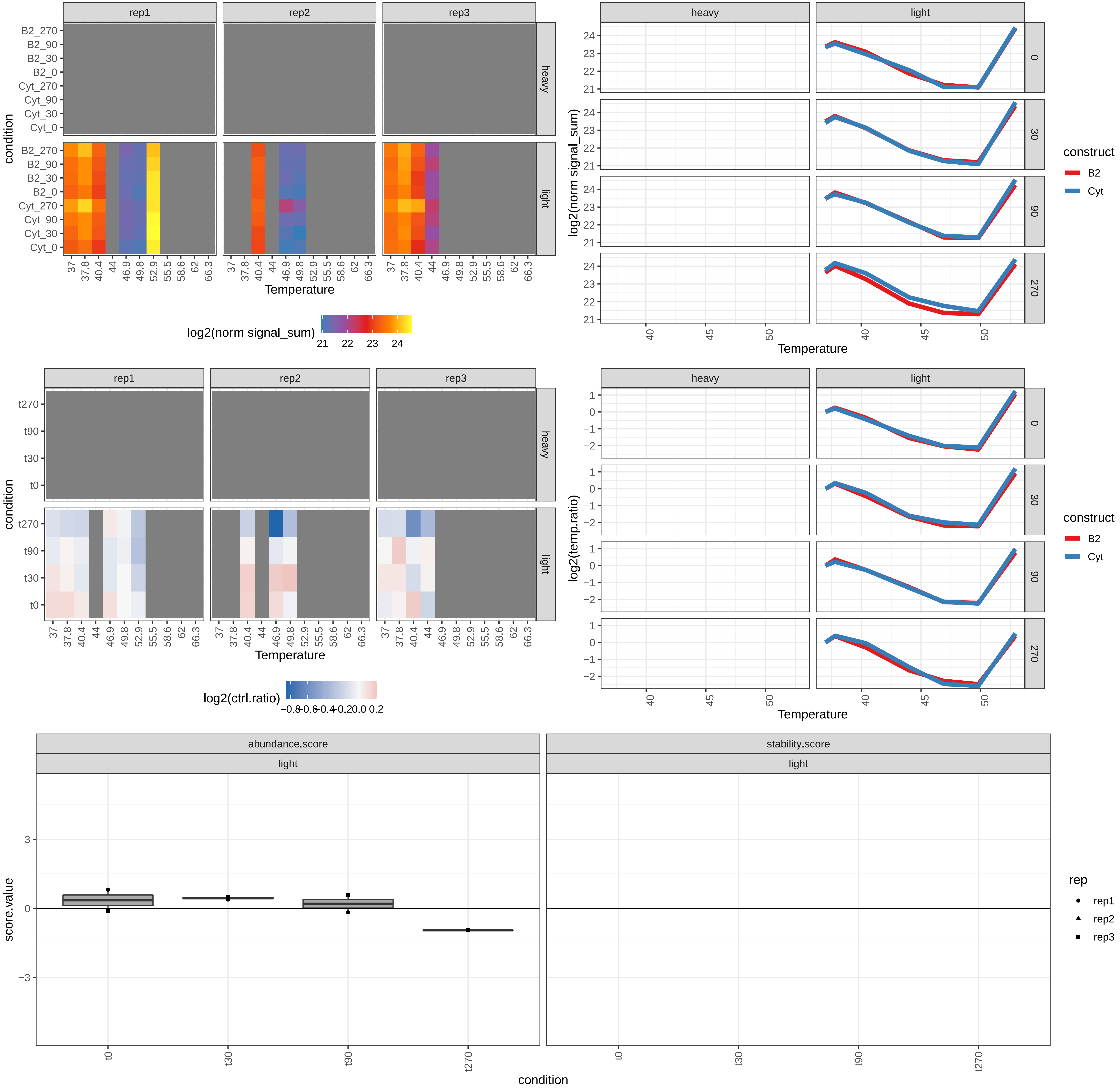

P53740 − Uncharacterized protein YNR048W OS=Saccharomyces cerevisiae (strain ATCC 204508 / S288c) GN=YNR048W PE=1 SV=1

YOL057W − average.top3: 6.84253217391304 − max.qupm: 13

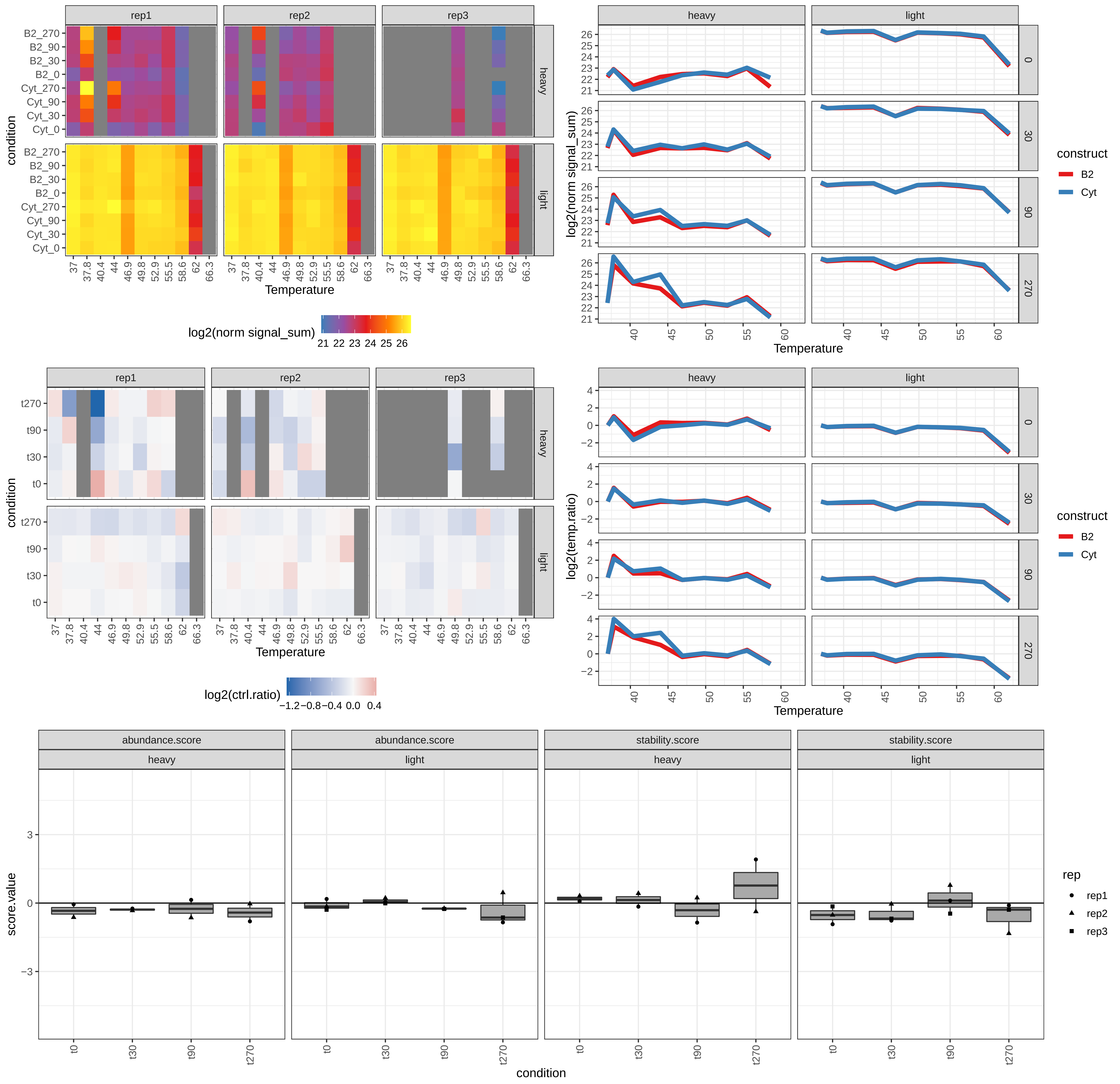

Q08225 − Probable dipeptidyl peptidase 3 OS=Saccharomyces cerevisiae (strain ATCC 204508 / S288c) GN=YOL057W PE=1 SV=1

YOL087C − average.top3: 7.11770761904762 − max.qupm: 8

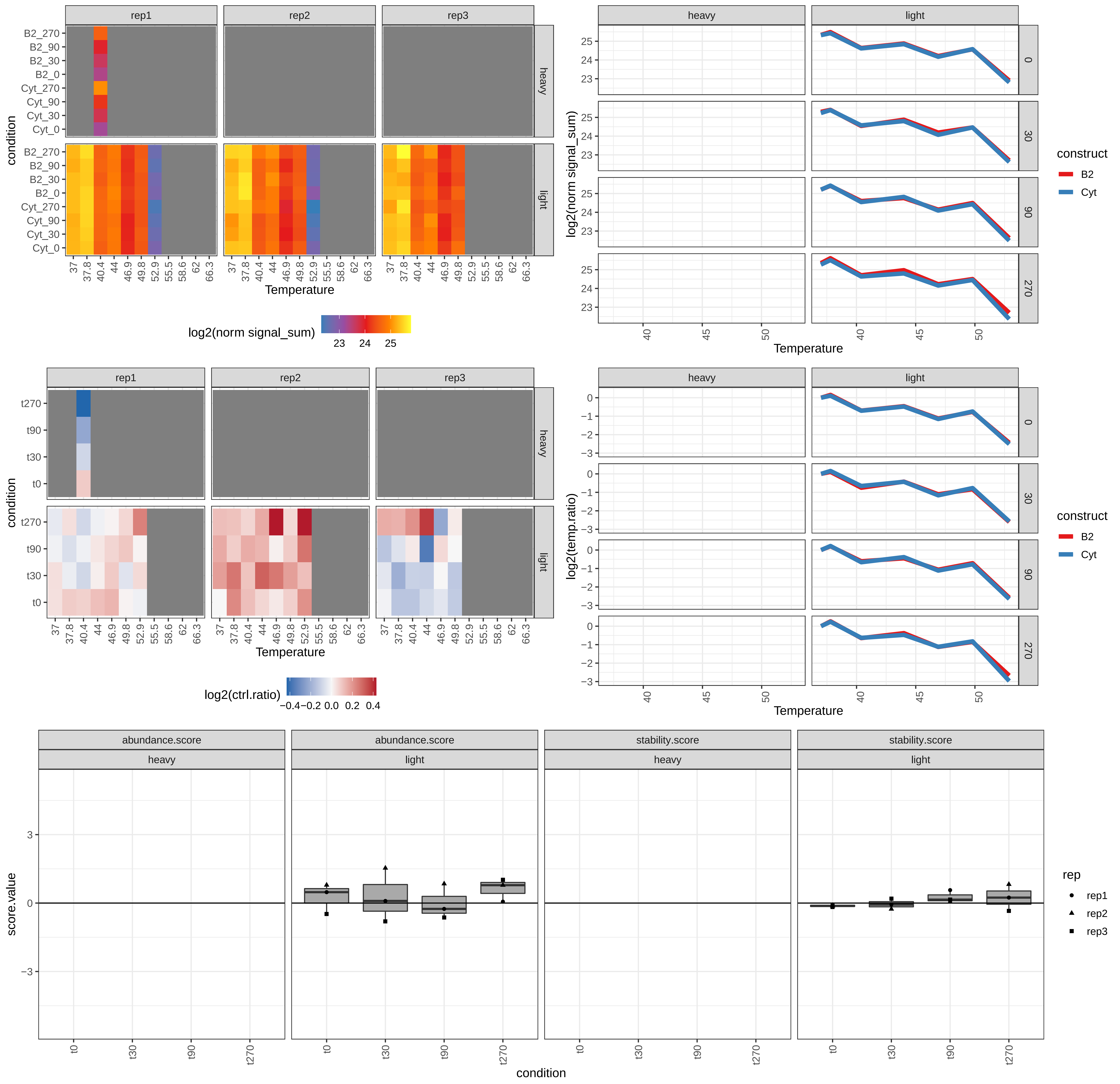

Q99247 − Uncharacterized WD repeat−containing protein YOL087C OS=Saccharomyces cerevisiae (strain ATCC 204508 / S288c) GN=YOL087C PE=1 SV=1

YOL098C − average.top3: 7.0019475 − max.qupm: 19

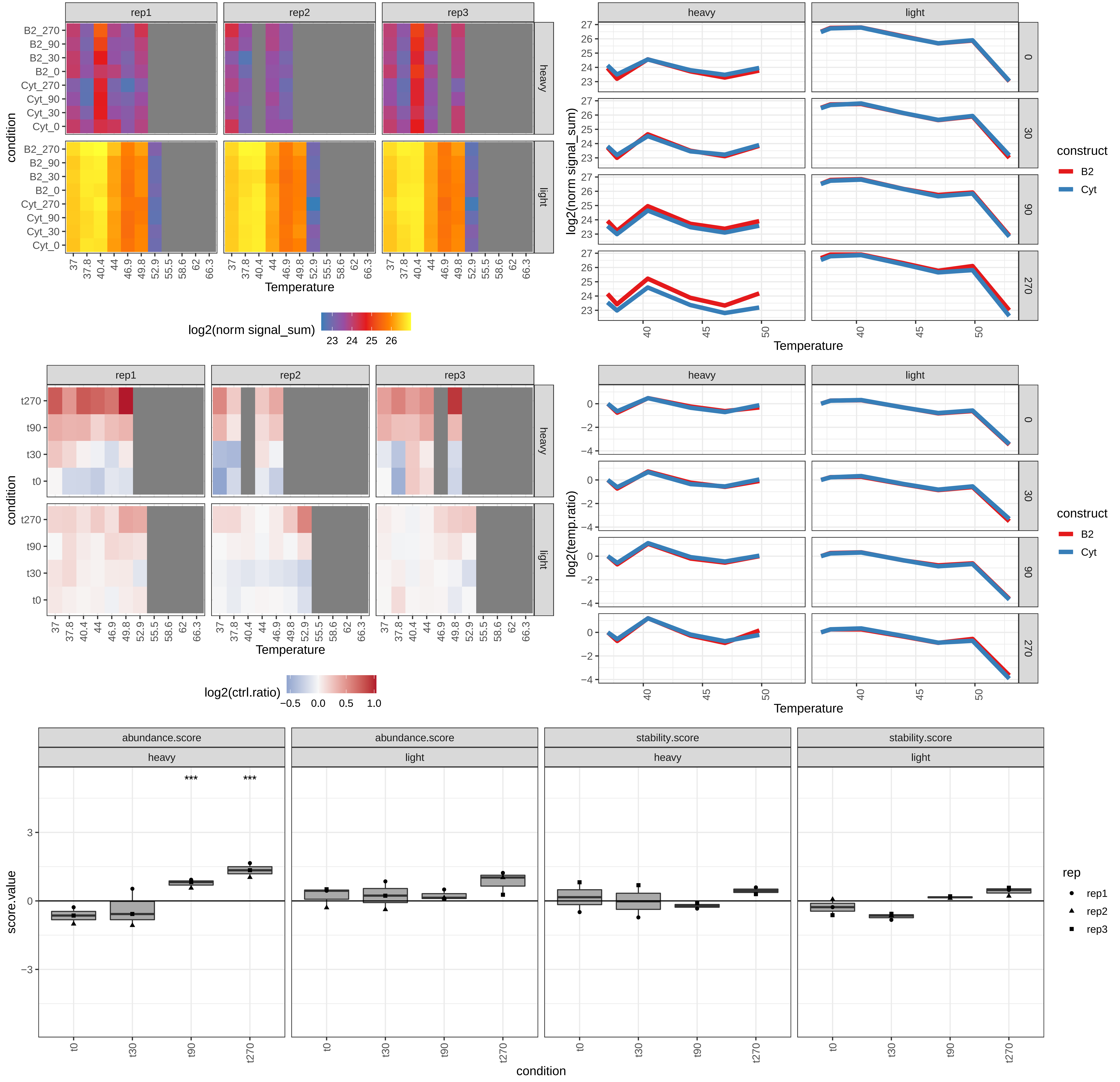

Q12496 − Uncharacterized protein YOL098C OS=Saccharomyces cerevisiae (strain ATCC 204508 / S288c) GN=YOL098C PE=1 SV=1

YOP1 − average.top3: 7.90049605263158 − max.qupm: 3

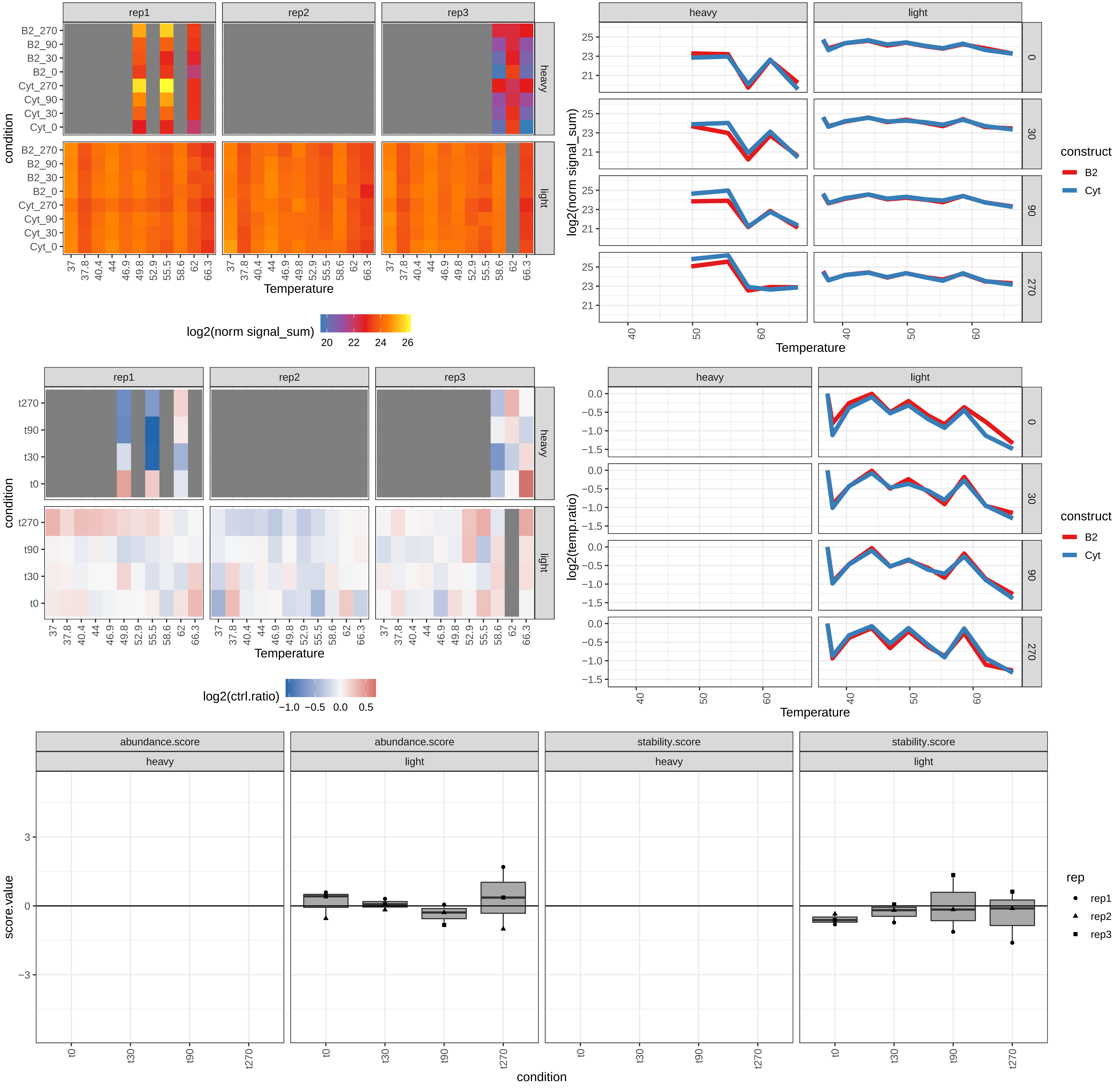

Q12402 − Protein YOP1 OS=Saccharomyces cerevisiae (strain ATCC 204508 / S288c) GN=YOP1 PE=1 SV=3

YOR022C − average.top3: 6.38758842105263 − max.qupm: 5

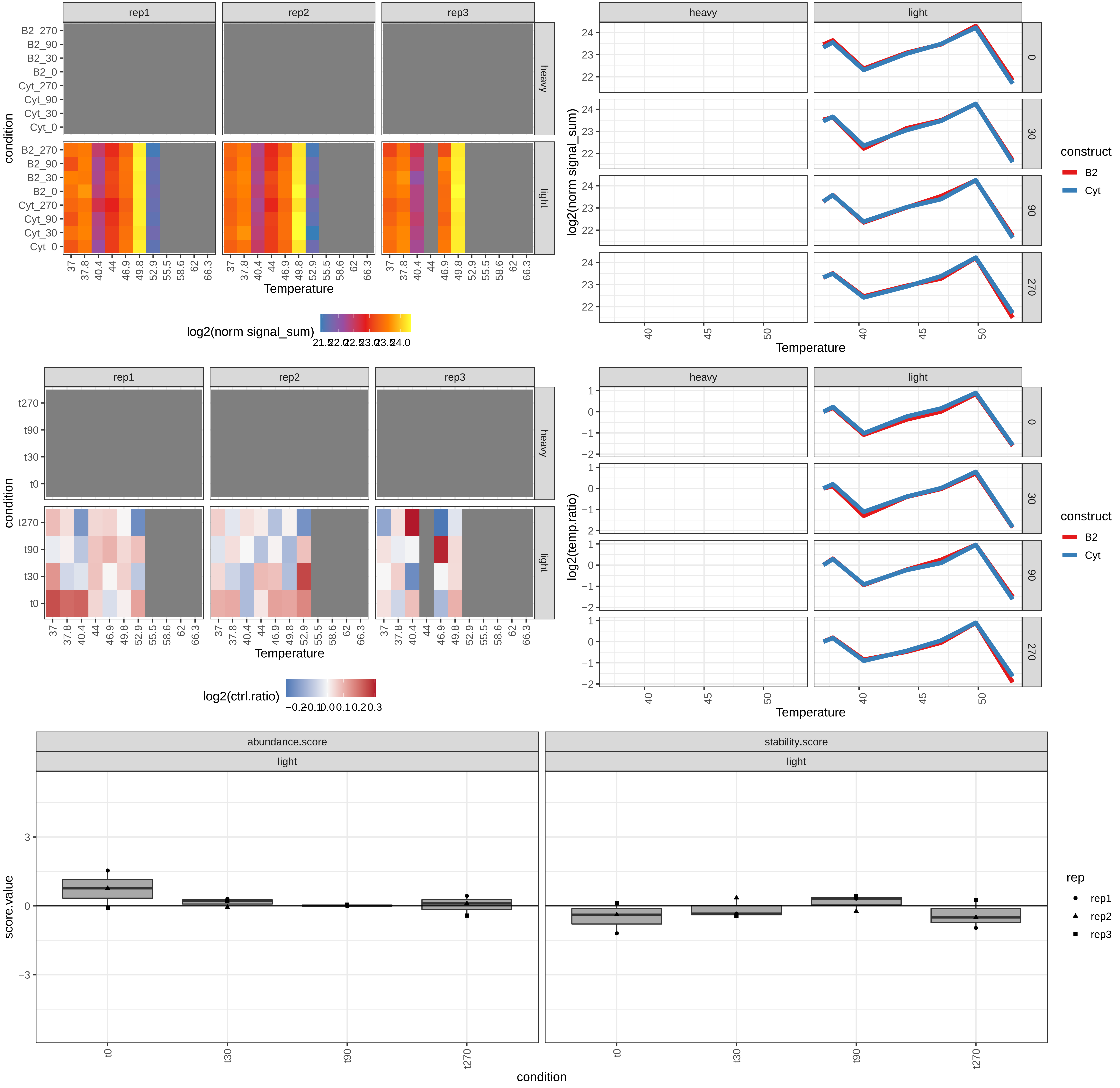

Q12204 − Probable phospholipase YOR022C, mitochondrial OS=Saccharomyces cerevisiae (strain ATCC 204508 / S288c) GN=YOR022C PE=1 SV=1

YOR093C − average.top3: 6.24365230769231 − max.qupm: 5

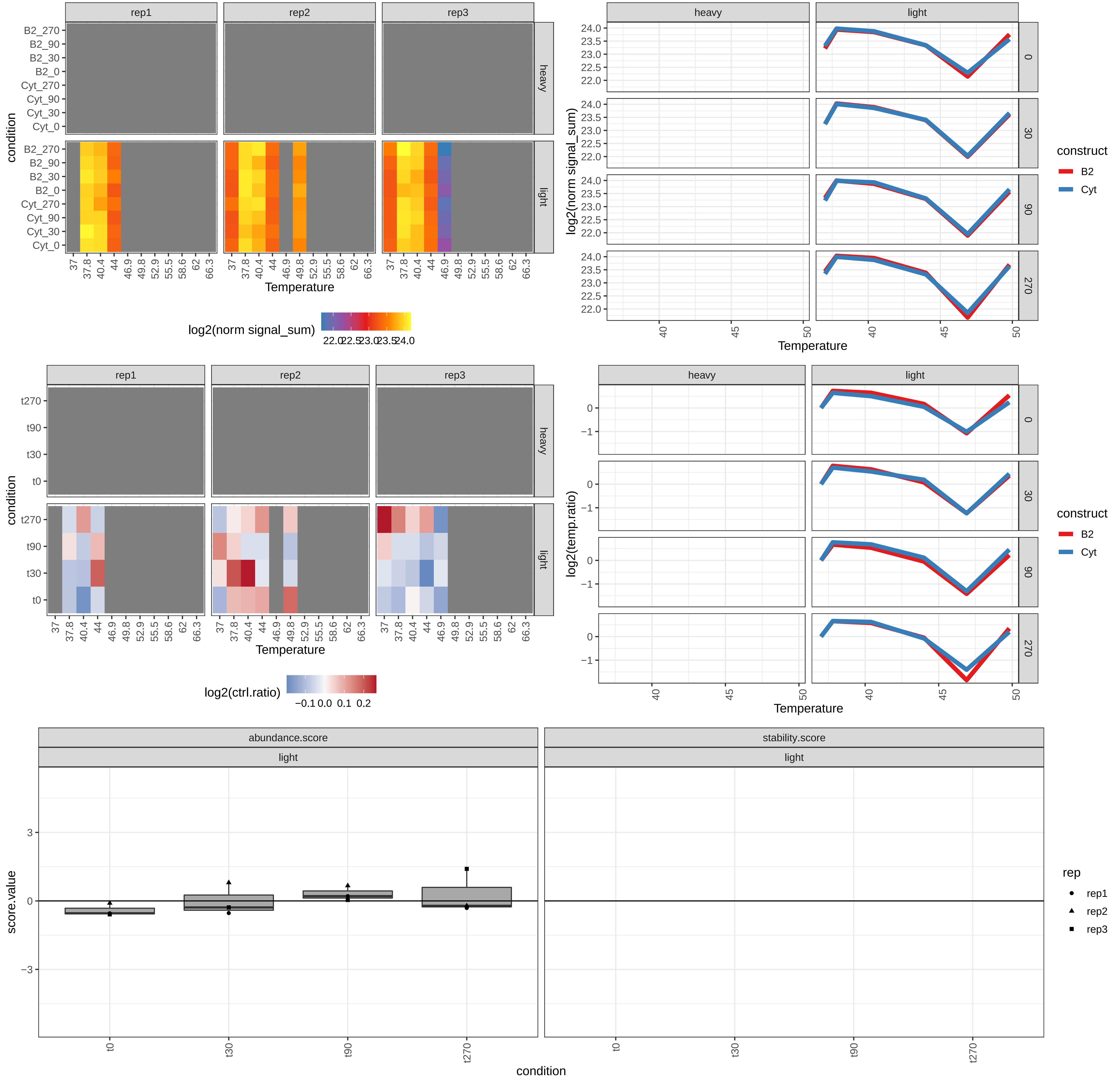

Q12275 − Uncharacterized protein YOR093C OS=Saccharomyces cerevisiae (strain ATCC 204508 / S288c) GN=YOR093C PE=1 SV=1

YOR1 − average.top3: 6.43557375 − max.qupm: 10

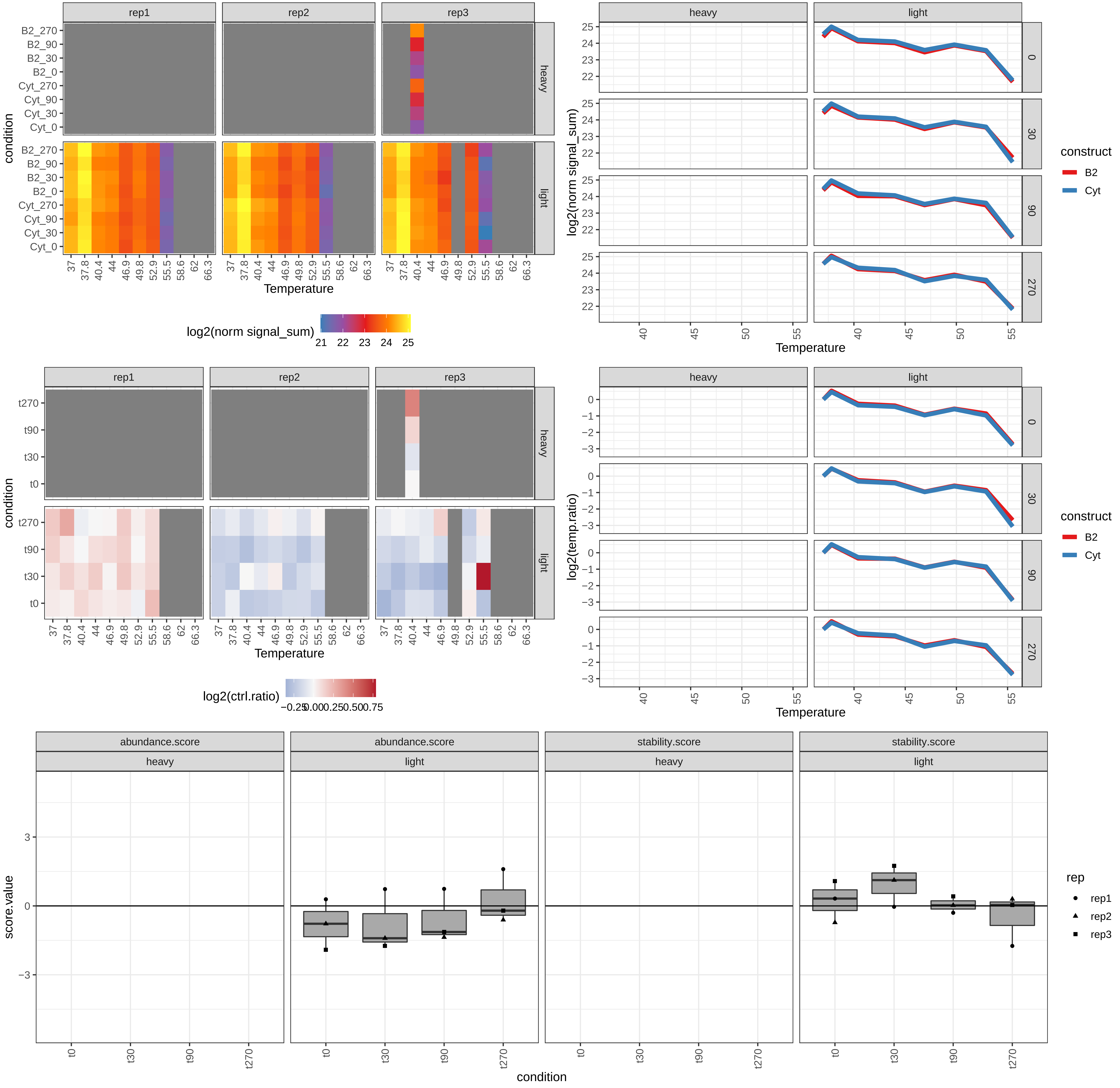

P53049 − Oligomycin resistance ATP−dependent permease YOR1 OS=Saccharomyces cerevisiae (strain ATCC 204508 / S288c) GN=YOR1 PE=1 SV=1

YOR111W − average.top3: 6.81826428571429 − max.qupm: 3

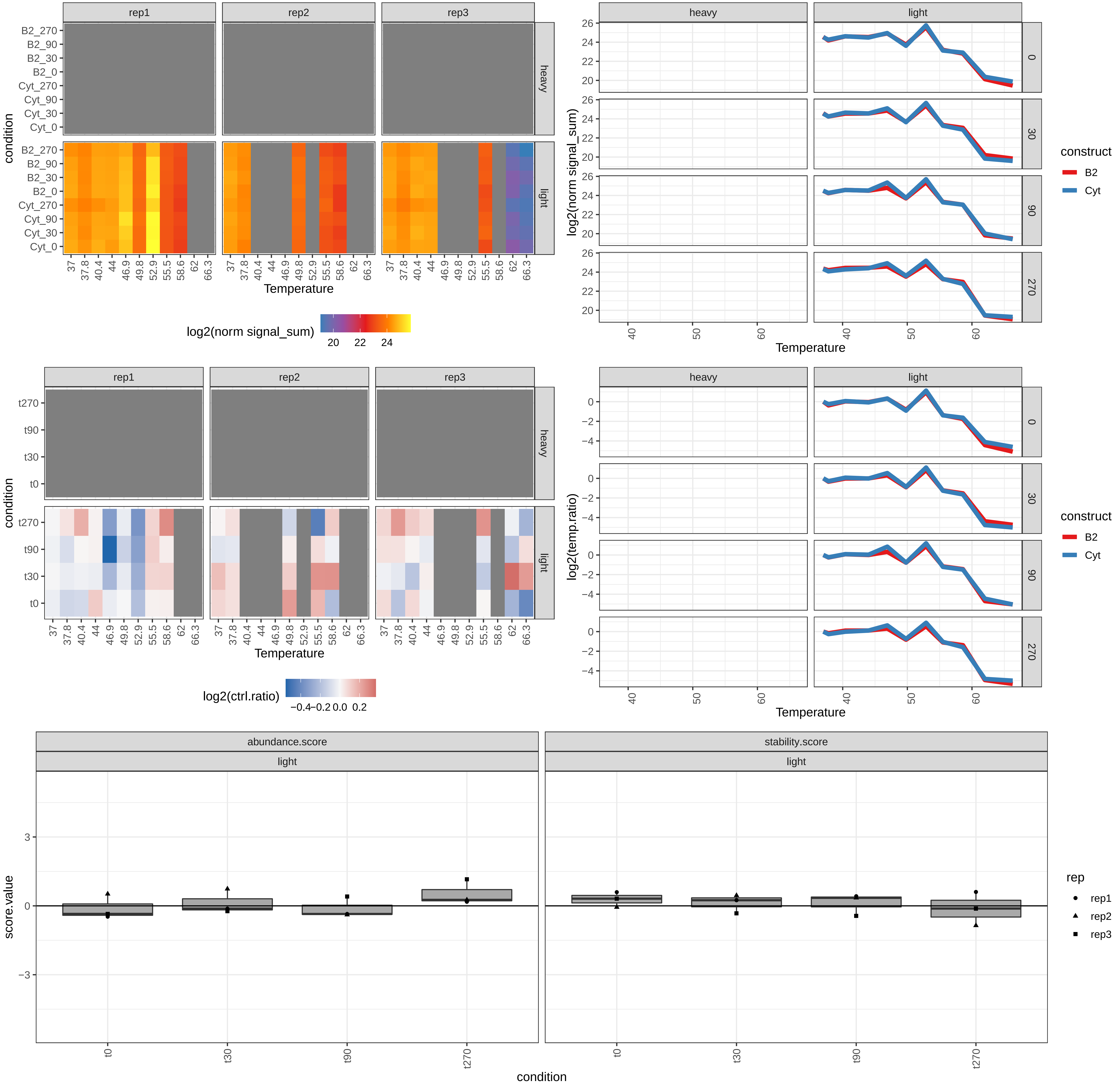

Q99210 − Maf−like protein YOR111W OS=Saccharomyces cerevisiae (strain ATCC 204508 / S288c) GN=YOR111W PE=1 SV=1

YOR223W − average.top3: 6.63752454545455 − max.qupm: 3

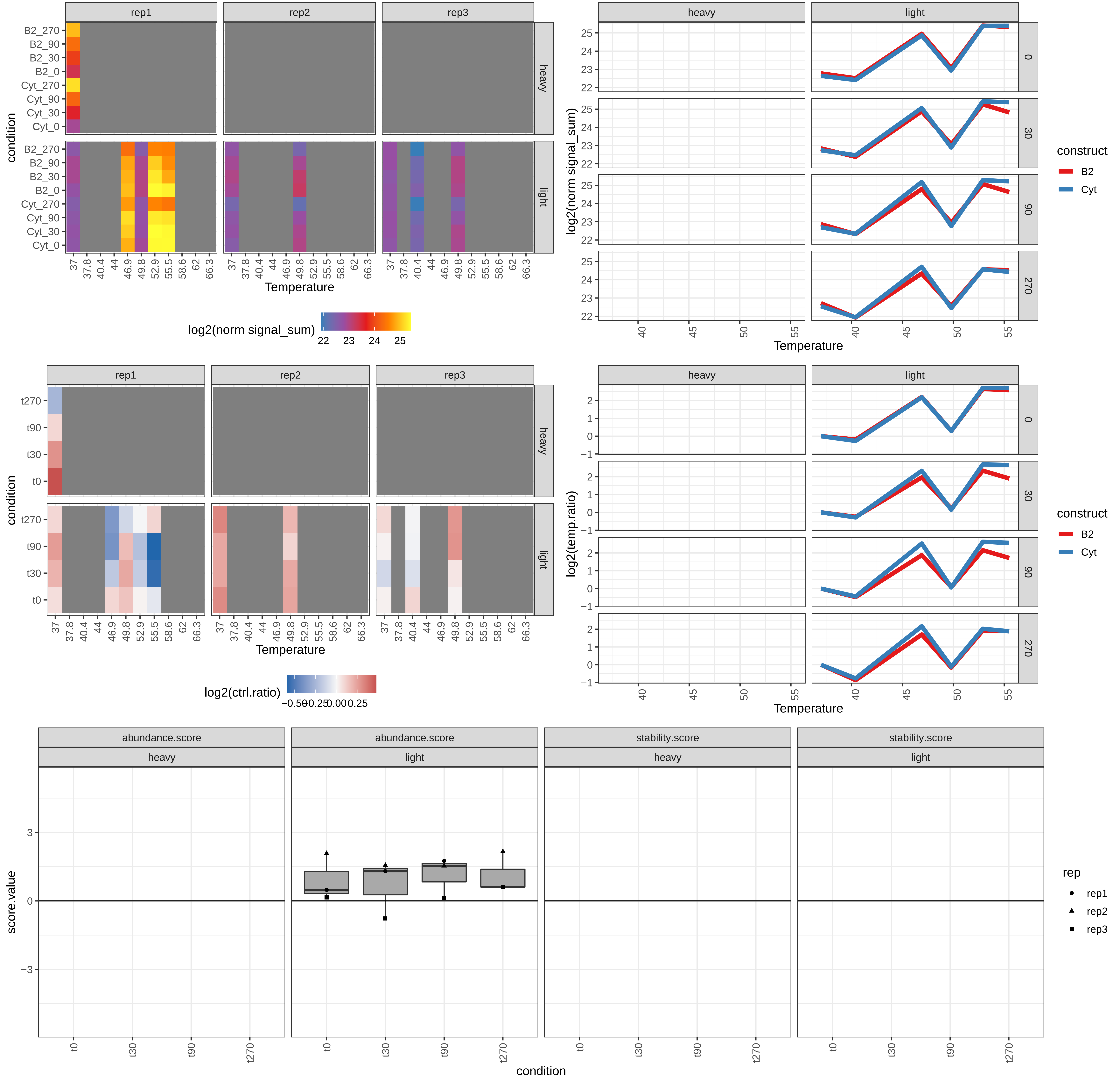

Q12015 − Transmembrane protein YOR223W OS=Saccharomyces cerevisiae (strain ATCC 204508 / S288c) GN=YOR223W PE=1 SV=1

YOR283W − average.top3: 6.85513857142857 − max.qupm: 7

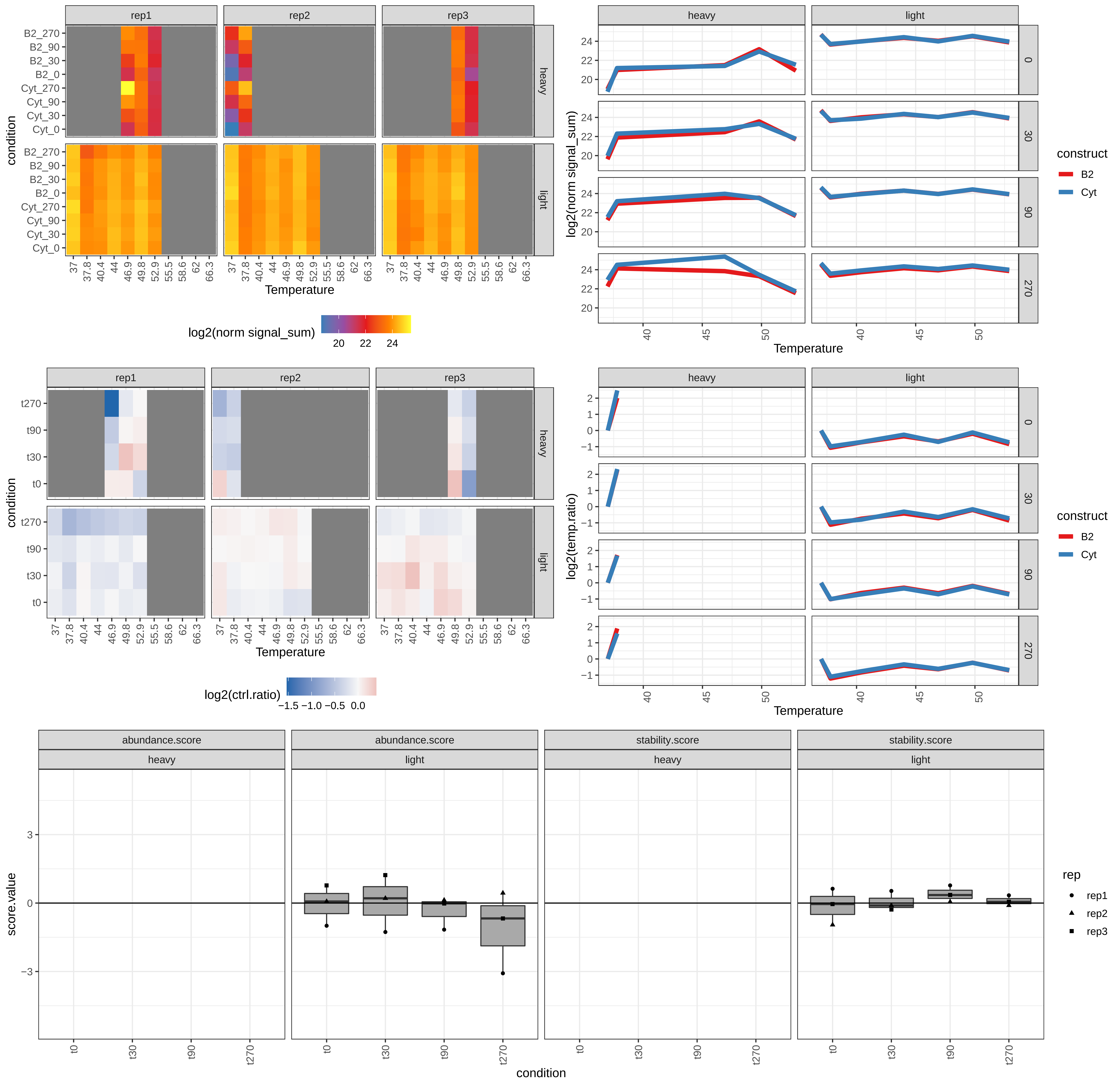

Q12040 − Broad−specificity phosphatase YOR283W OS=Saccharomyces cerevisiae (strain ATCC 204508 / S288c) GN=YOR283W PE=1 SV=1

YOR289W − average.top3: 7.11541909090909 − max.qupm: 10

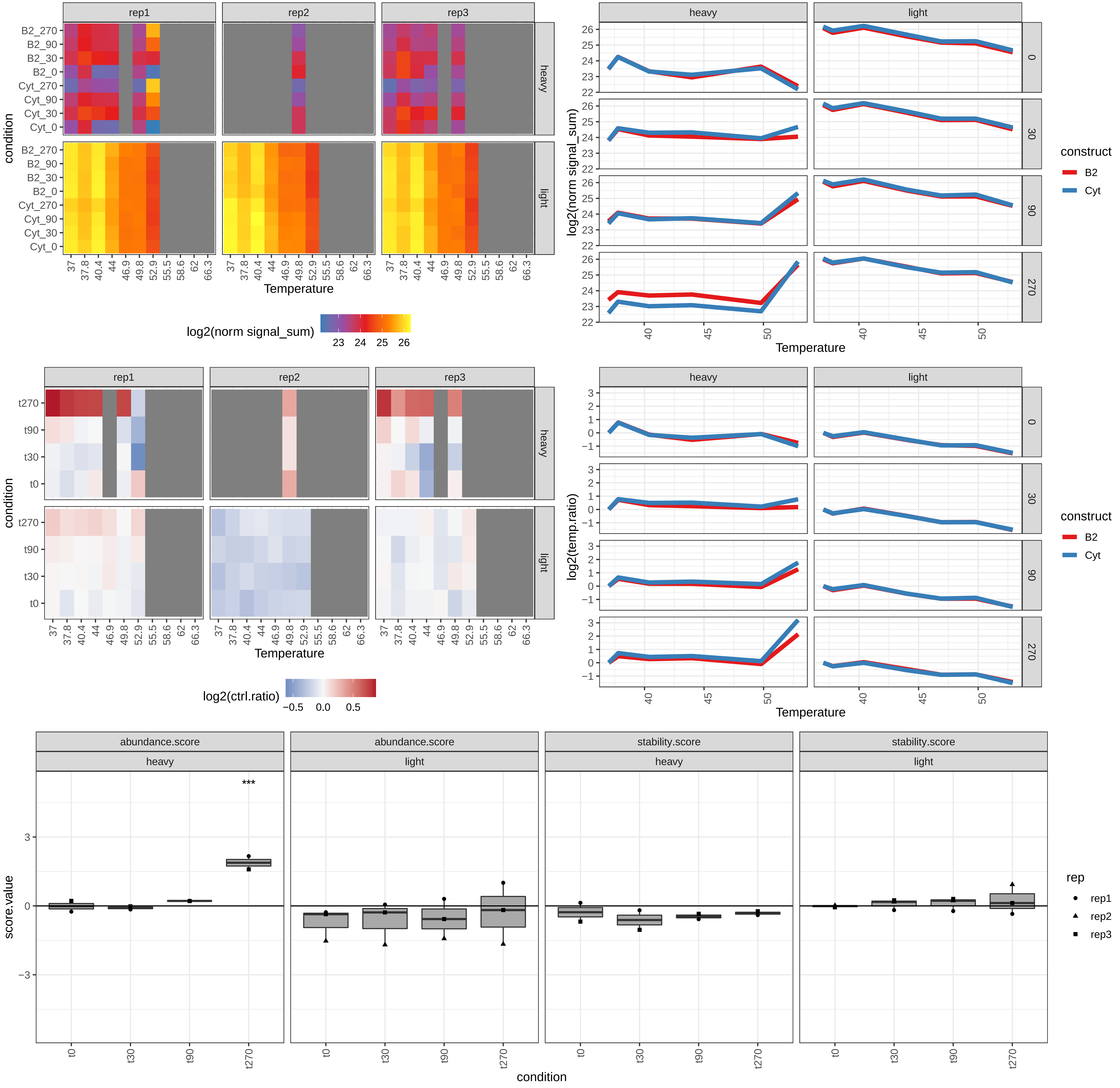

Q12012 − Uncharacterized protein YOR289W OS=Saccharomyces cerevisiae (strain ATCC 204508 / S288c) GN=YOR289W PE=1 SV=1

YOS9 − average.top3: 6.70161 − max.qupm: 4

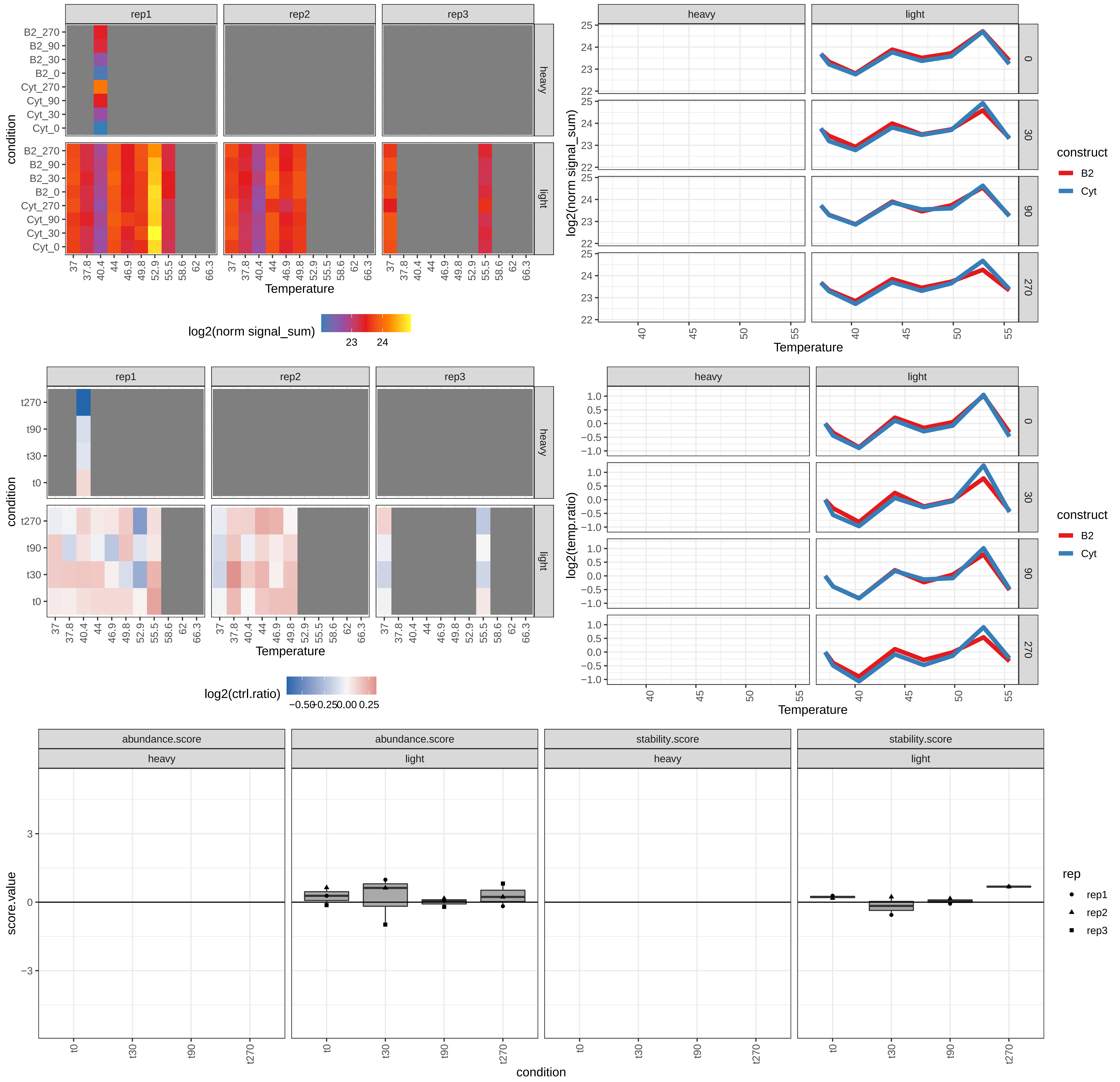

Q99220 − Protein OS−9 homolog OS=Saccharomyces cerevisiae (strain ATCC 204508 / S288c) GN=YOS9 PE=1 SV=1

YPI1 − average.top3: 6.57905538461538 − max.qupm: 3

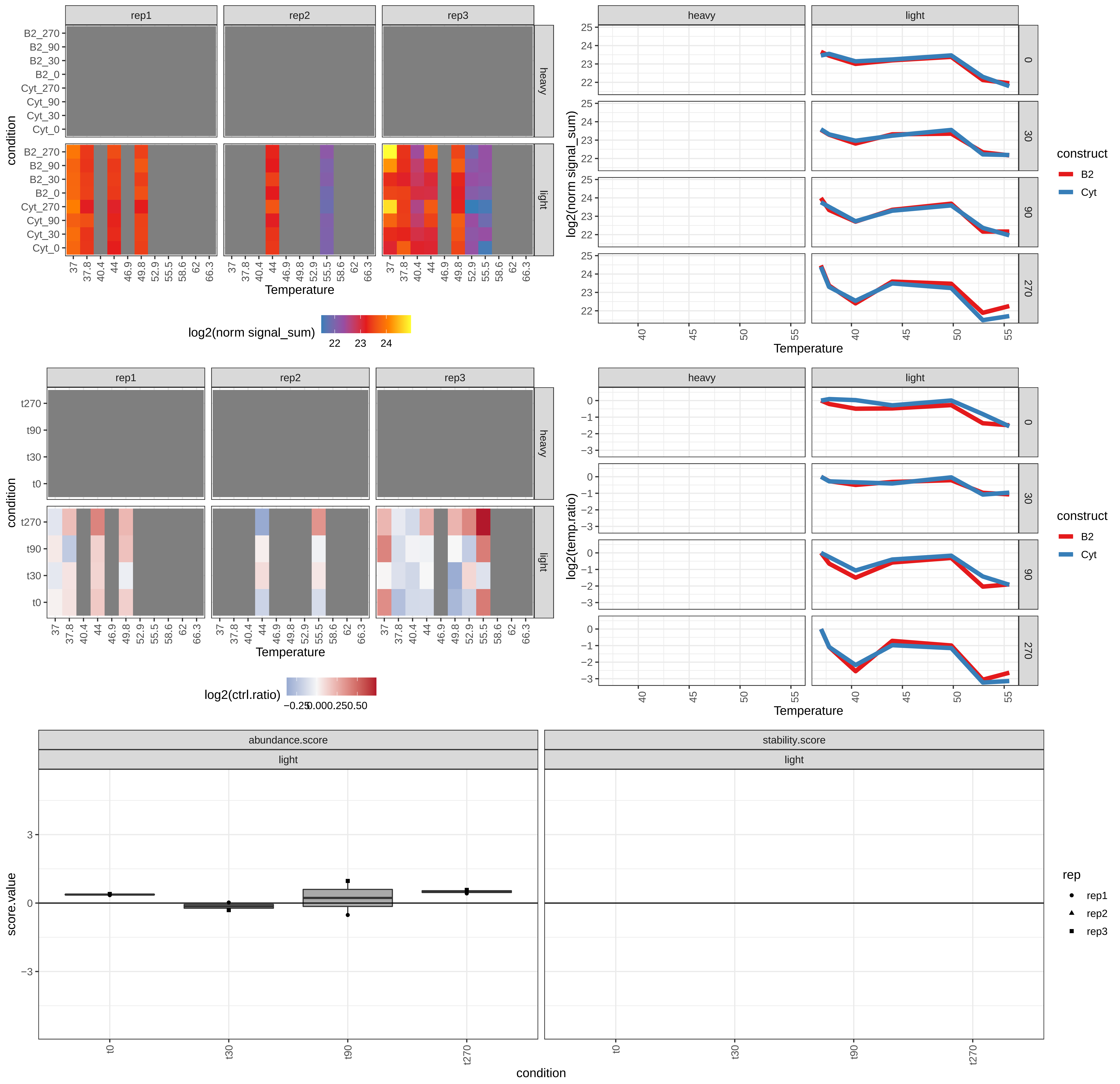

P43587 − Type 1 phosphatases regulator YPI1 OS=Saccharomyces cerevisiae (strain ATCC 204508 / S288c) GN=YPI1 PE=1 SV=1

YPK1 − average.top3: 6.290539 − max.qupm: 4

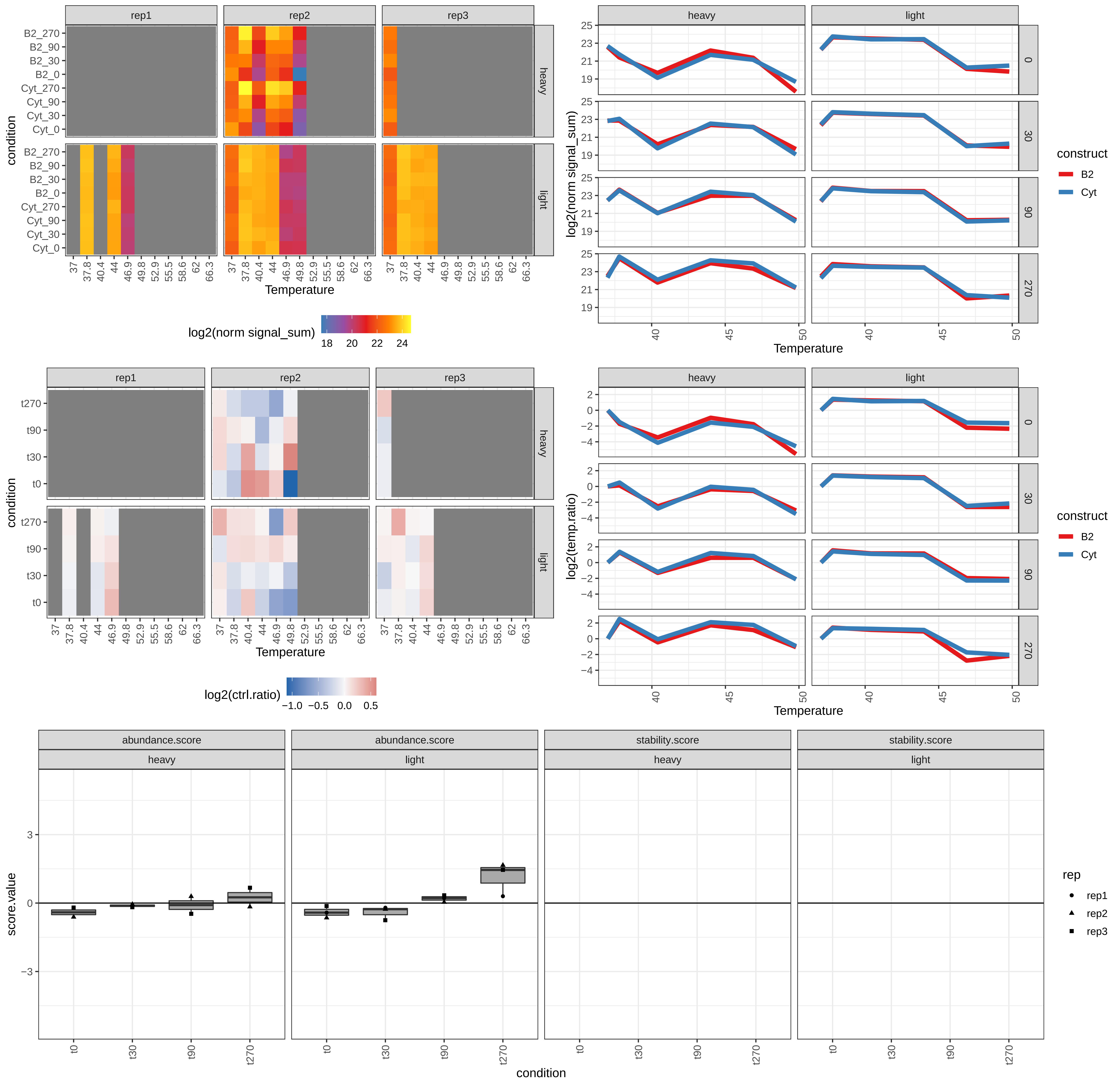

P12688 − Serine/threonine−protein kinase YPK1 OS=Saccharomyces cerevisiae (strain ATCC 204508 / S288c) GN=YPK1 PE=1 SV=2

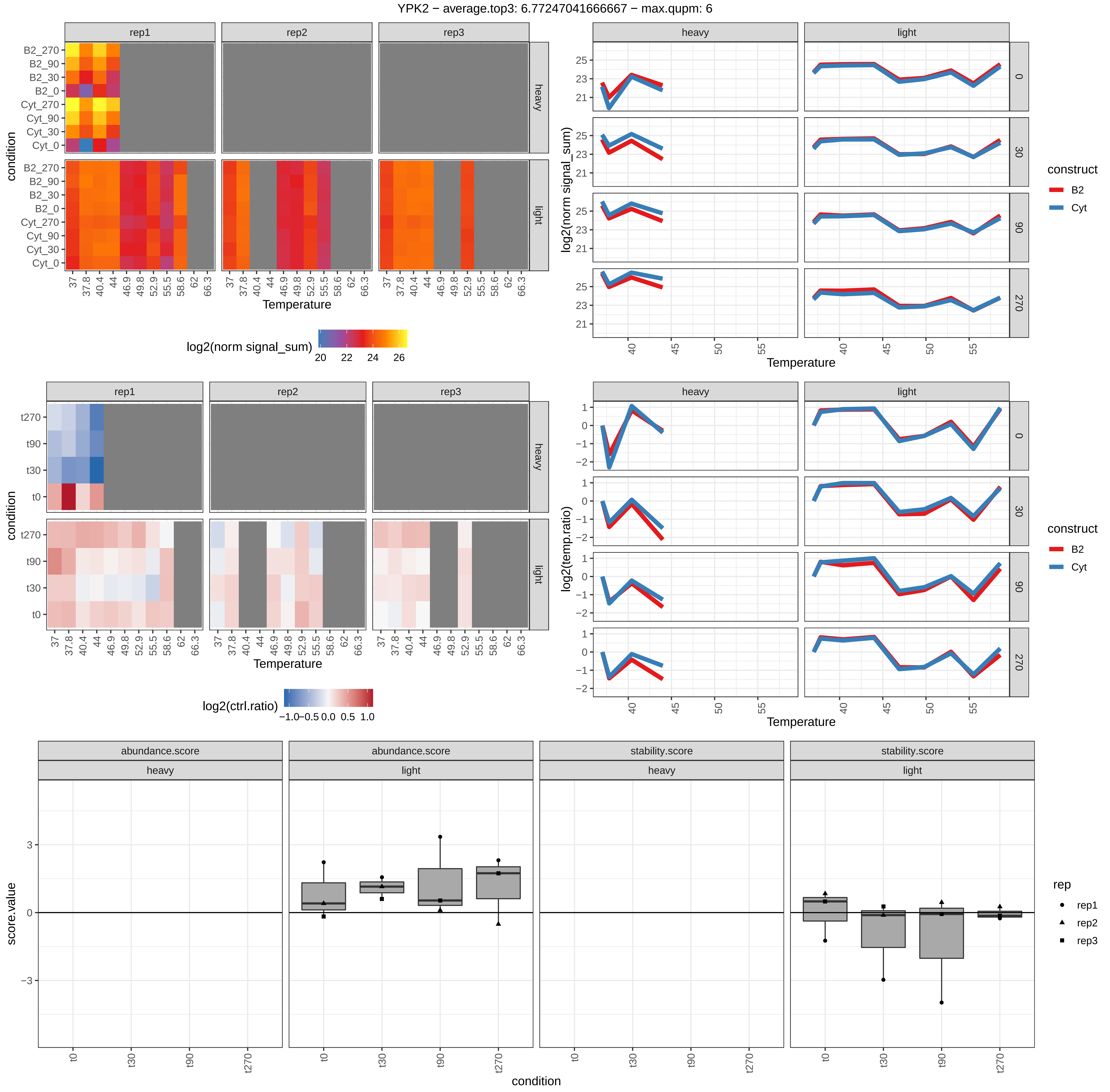

P18961 − Serine/threonine−protein kinase YPK2/YKR2 OS=Saccharomyces cerevisiae (strain ATCC 204508 / S288c) GN=YPK2 PE=1 SV=1

YPK9 − average.top3: 6.3895032 − max.qupm: 10

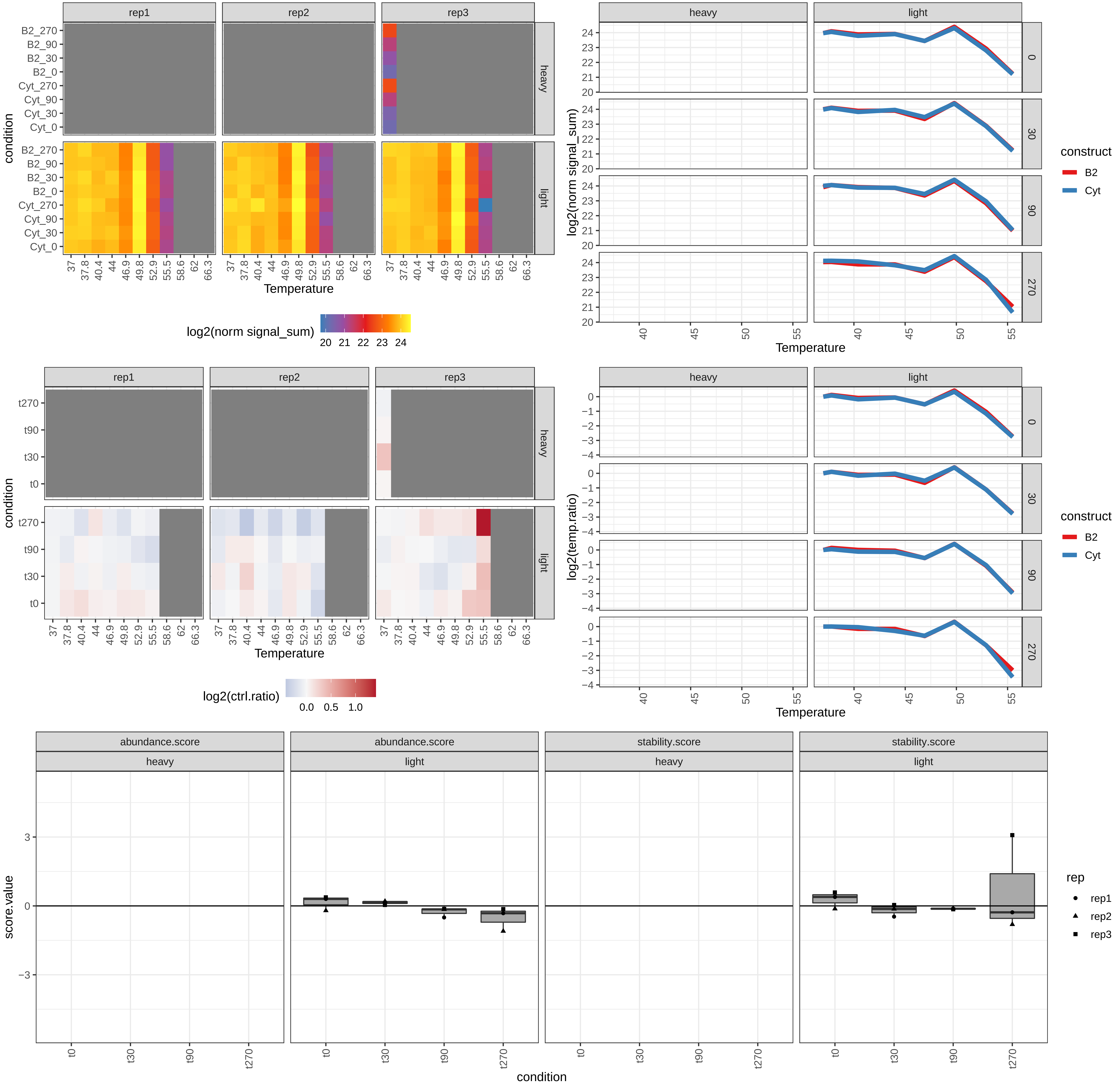

Q12697 − Vacuolar cation−transporting ATPase YPK9 OS=Saccharomyces cerevisiae (strain ATCC 204508 / S288c) GN=YPK9 PE=1 SV=1

YPL067C − average.top3: 6.756476875 − max.qupm: 4

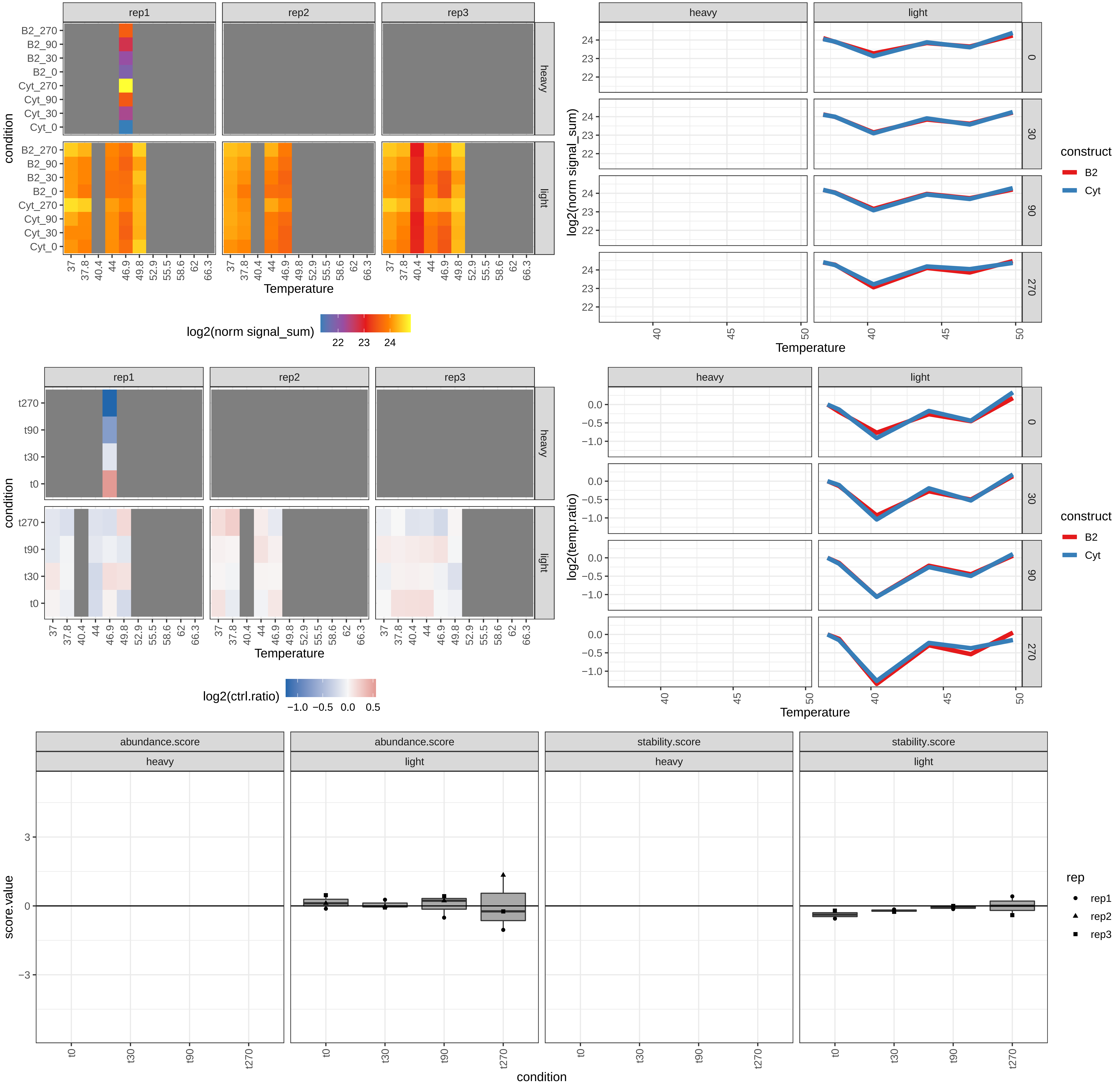

Q02754 − Uncharacterized protein YPL067C OS=Saccharomyces cerevisiae (strain ATCC 204508 / S288c) GN=YPL067C PE=1 SV=1

YPL109C − average.top3: 6.51755833333333 − max.qupm: 4

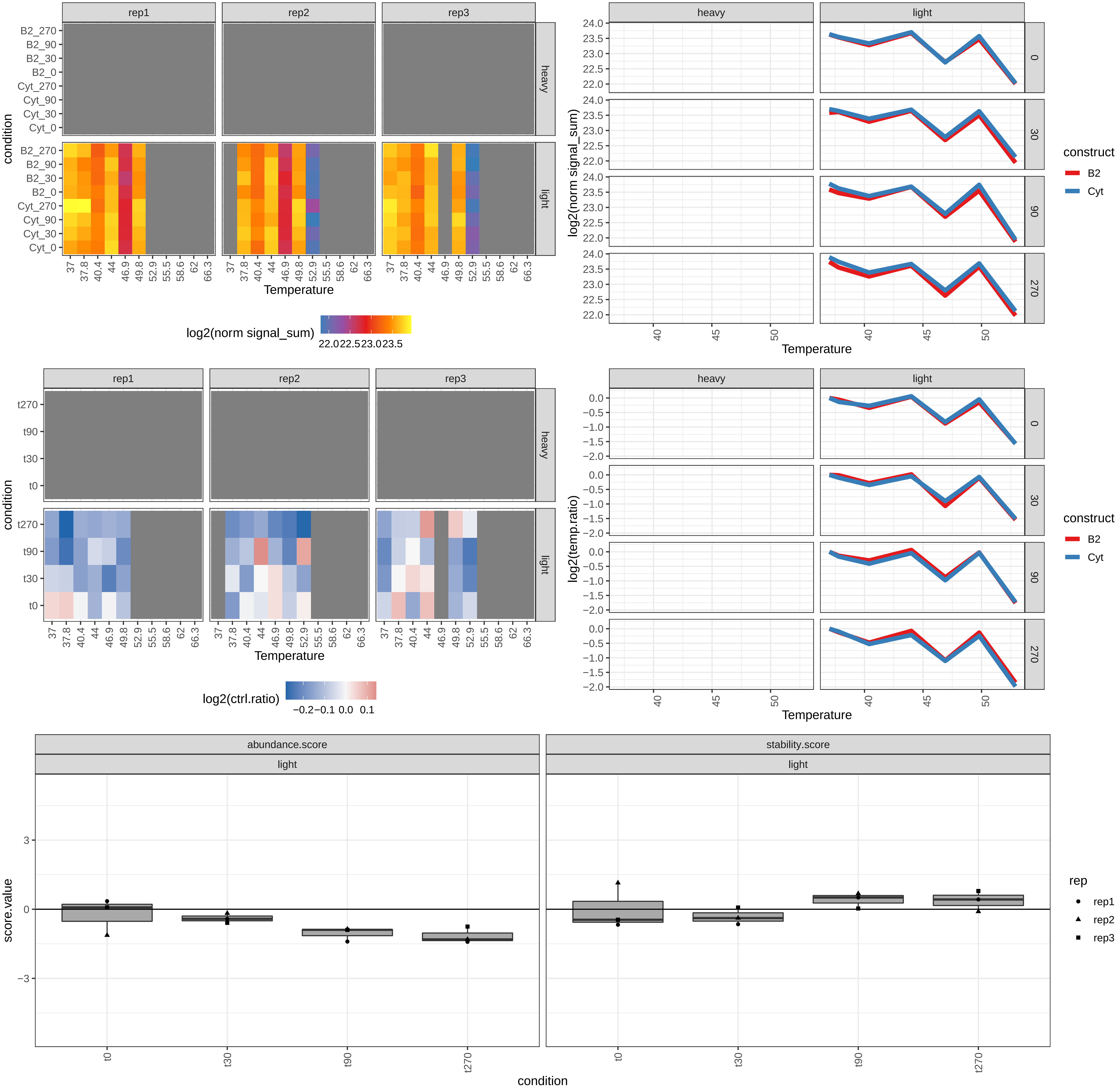

Q02981 − ABC1 family protein YPL109C, mitochondrial OS=Saccharomyces cerevisiae (strain ATCC 204508 / S288c) GN=YPL109C PE=1 SV=2

YPL113C − average.top3: 6.91908947368421 − max.qupm: 6

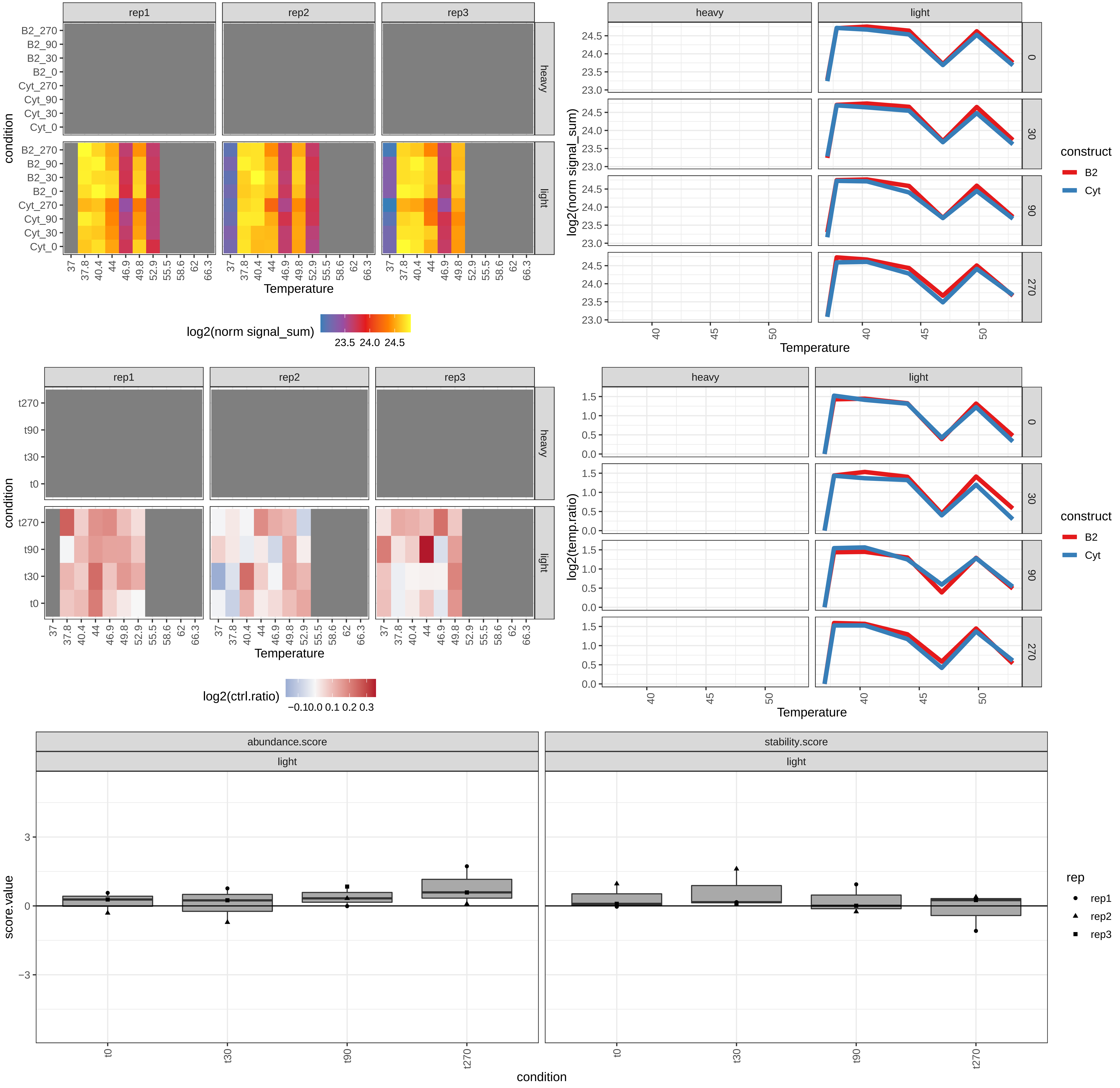

Q02961 − Putative 2−hydroxyacid dehydrogenase YPL113C OS=Saccharomyces cerevisiae (strain ATCC 204508 / S288c) GN=YPL113C PE=2 SV=1

YPL150W − average.top3: 6.42753214285714 − max.qupm: 6

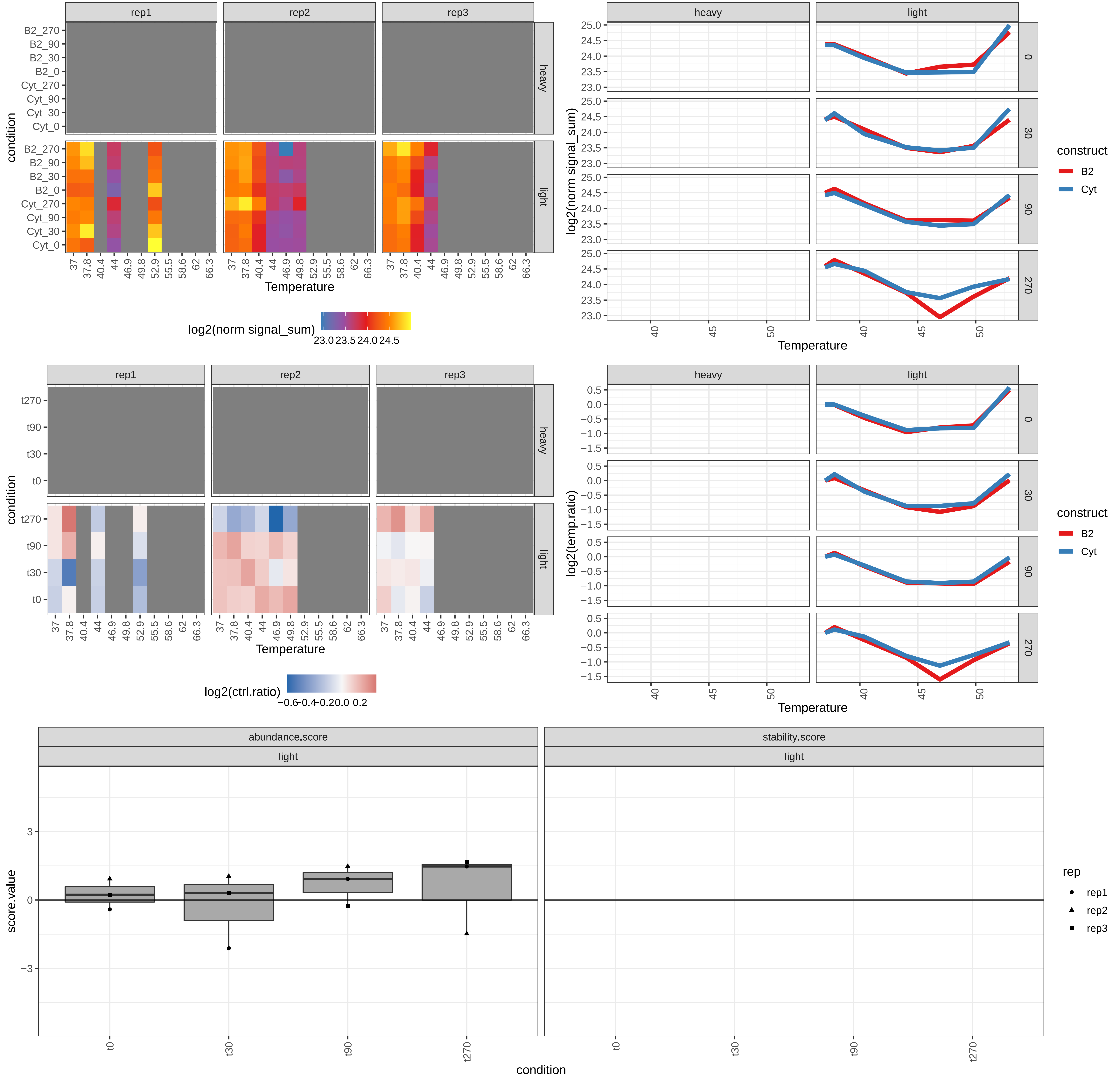

Q12152 − Putative serine/threonine−protein kinase YPL150W OS=Saccharomyces cerevisiae (strain ATCC 204508 / S288c) GN=YPL150W PE=1 SV=1

YPL225W − average.top3: 7.25882705882353 − max.qupm: 10

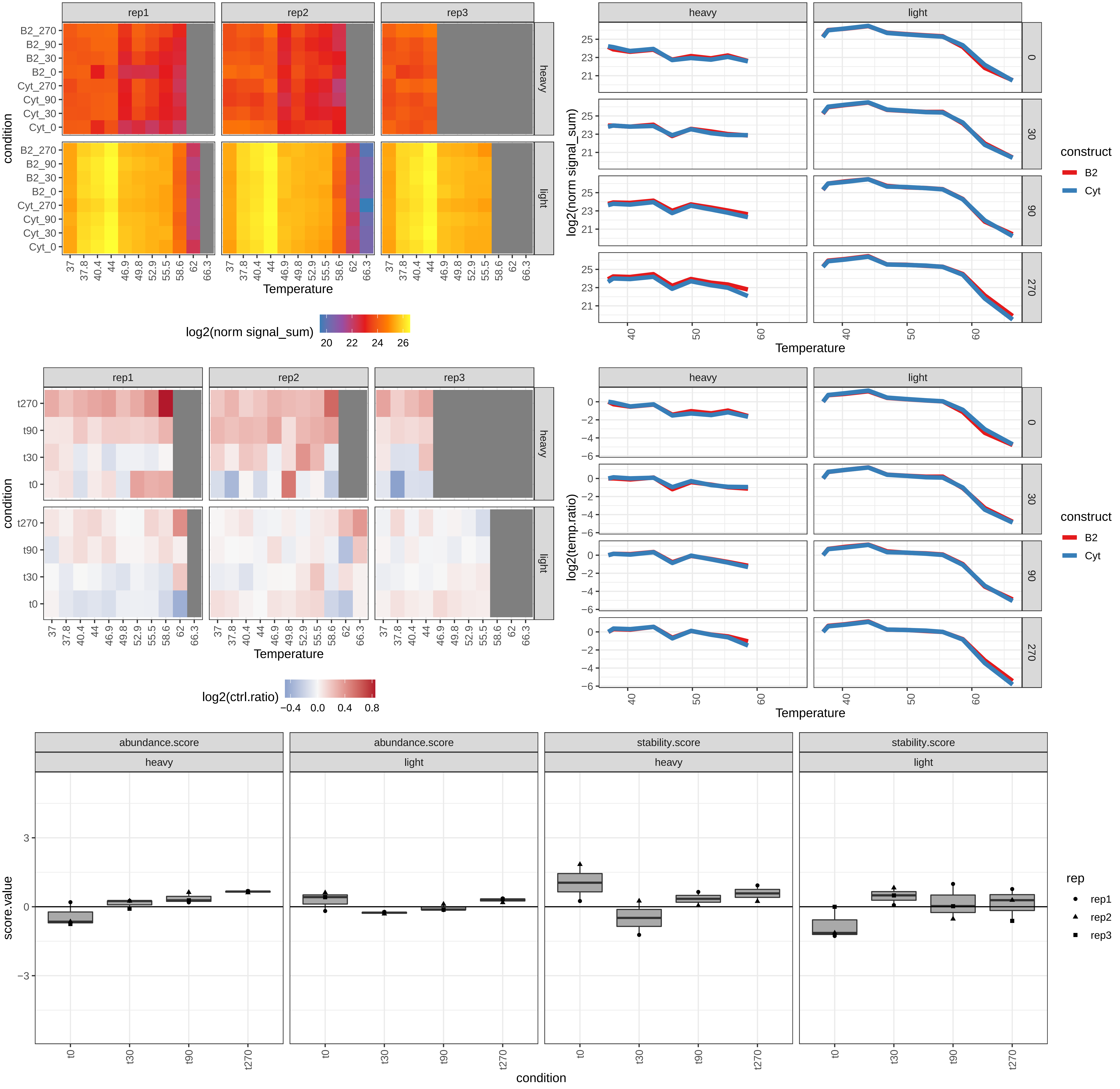

Q08971 − Protein PBDC1 homolog OS=Saccharomyces cerevisiae (strain ATCC 204508 / S288c) GN=YPL225W PE=1 SV=1

YPL247C − average.top3: 6.44159157894737 − max.qupm: 3

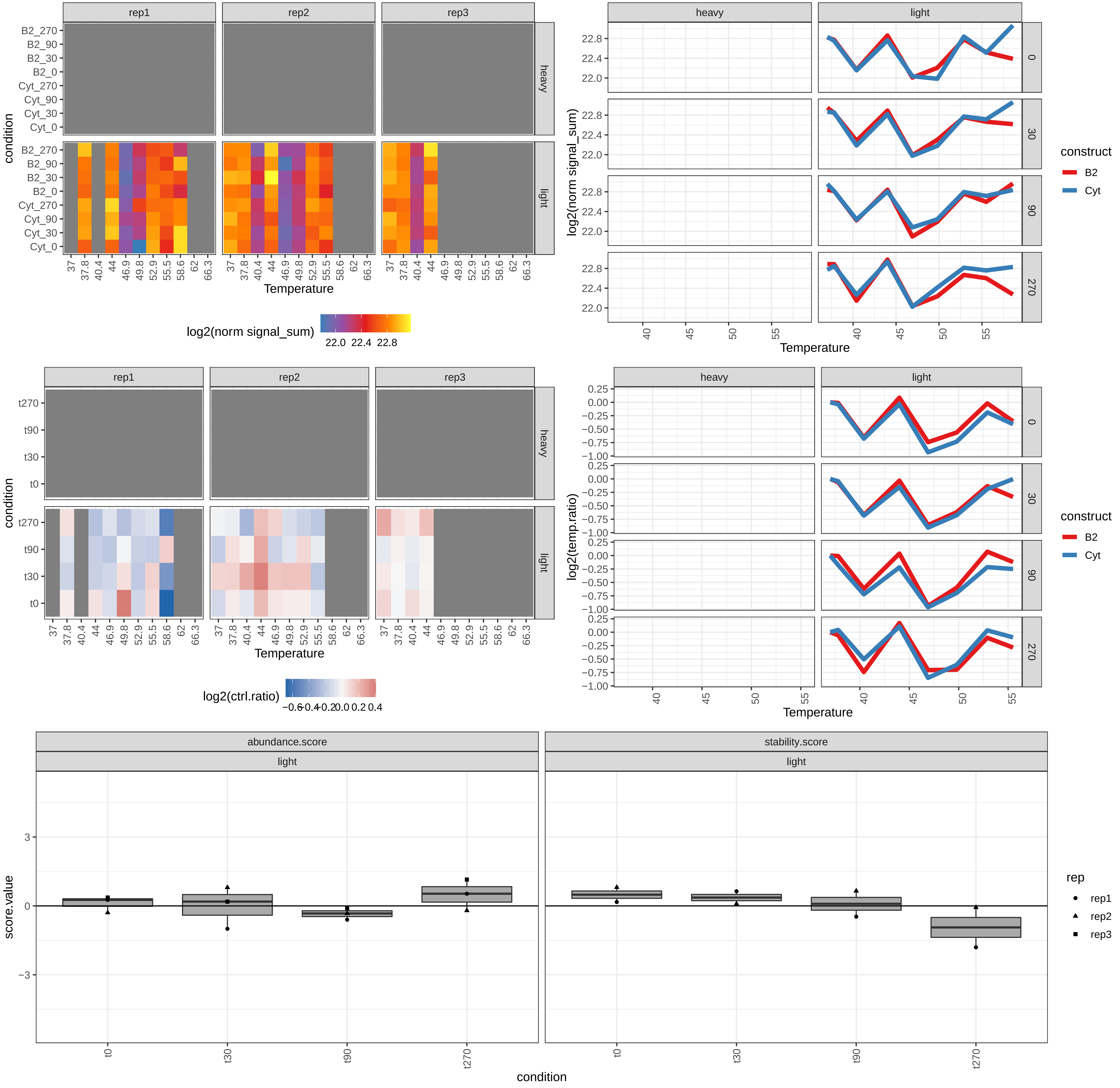

Q12523 − WD repeat−containing protein YPL247C OS=Saccharomyces cerevisiae (strain ATCC 204508 / S288c) GN=YPL247C PE=1 SV=1

YPL260W − average.top3: 7.18978980392157 − max.qupm: 19

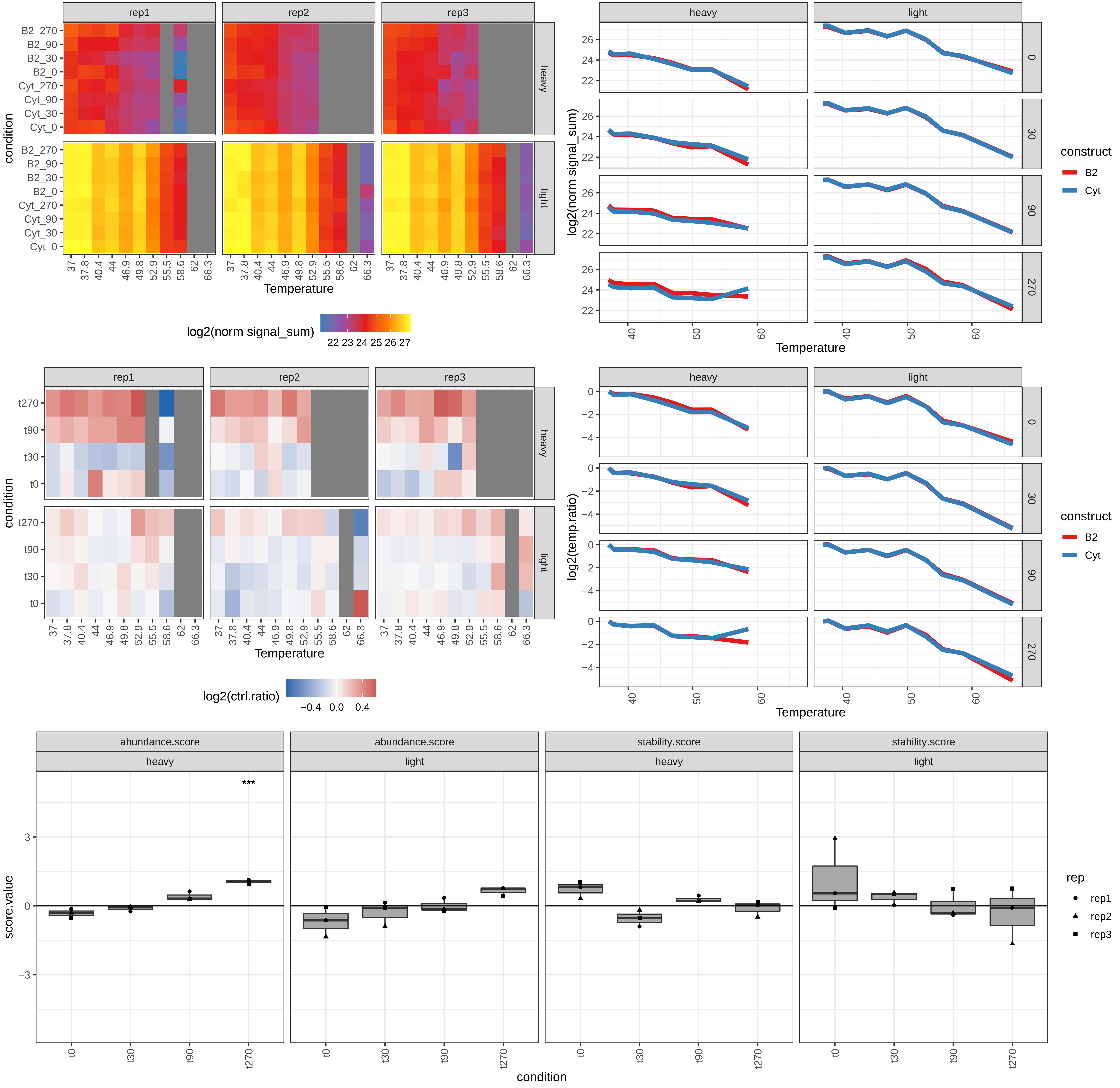

Q08977 − UPF0662 protein YPL260W OS=Saccharomyces cerevisiae (strain ATCC 204508 / S288c) GN=YPL260W PE=1 SV=1

YPP1 − average.top3: 6.613009375 − max.qupm: 10

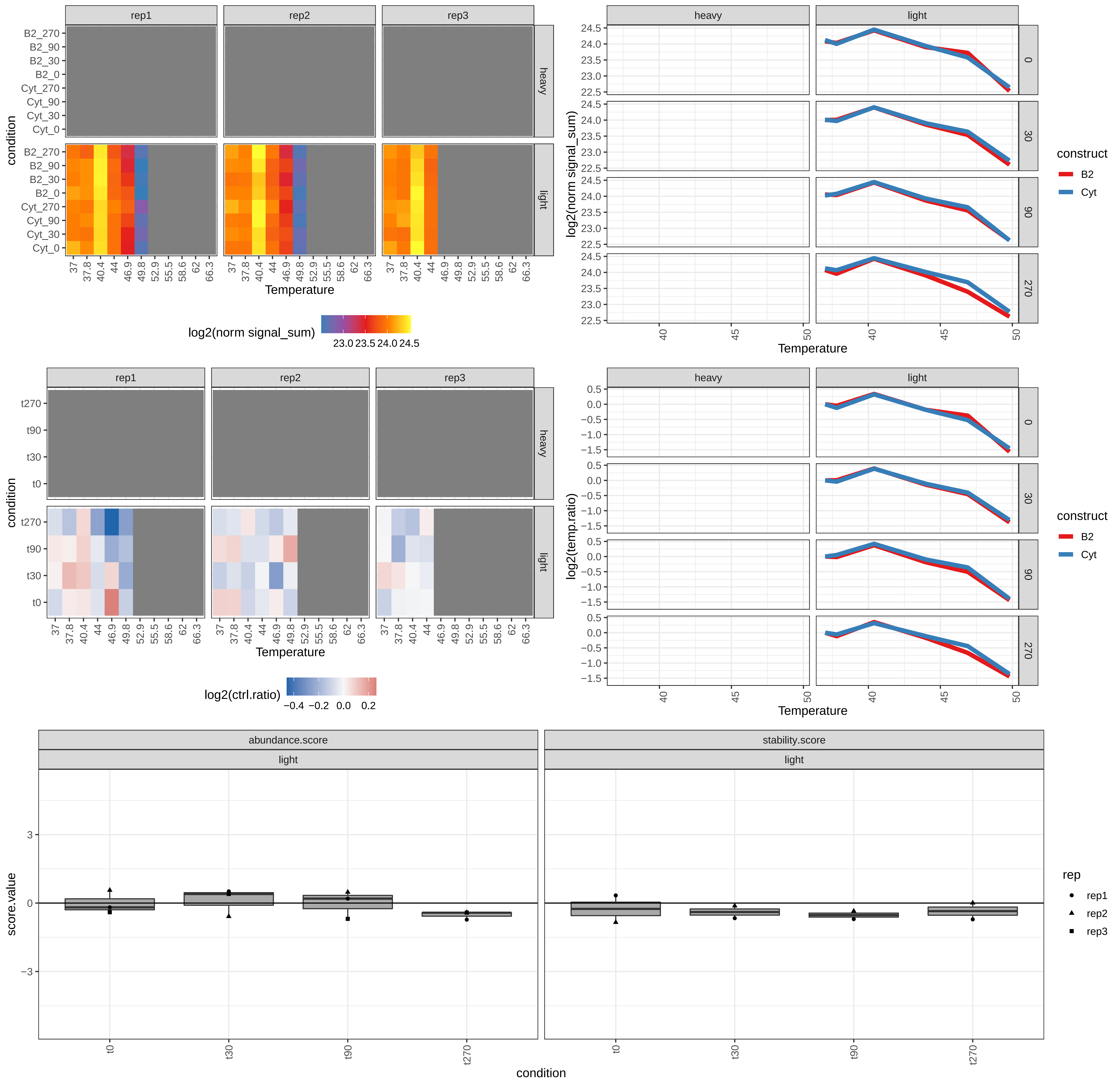

P46951 − Cargo−transport protein YPP1 OS=Saccharomyces cerevisiae (strain ATCC 204508 / S288c) GN=YPP1 PE=1 SV=1

YPQ1 − average.top3: 6.90163818181818 − max.qupm: 3

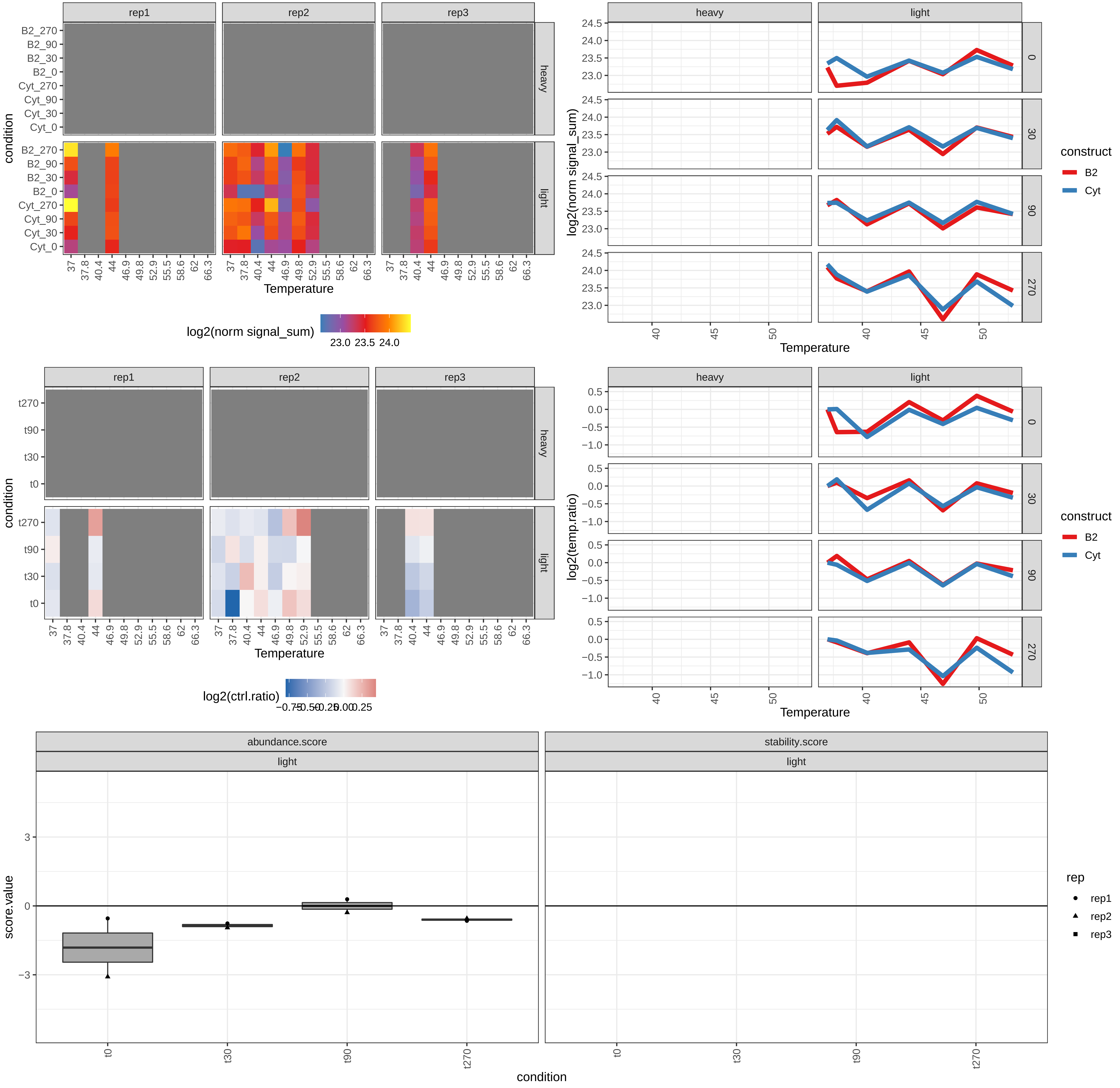

Q12010 − Probable vacuolar amino acid transporter YPQ1 OS=Saccharomyces cerevisiae (strain ATCC 204508 / S288c) GN=YPQ1 PE=1 SV=1

YPR089W − average.top3: 6.57272571428571 − max.qupm: 5

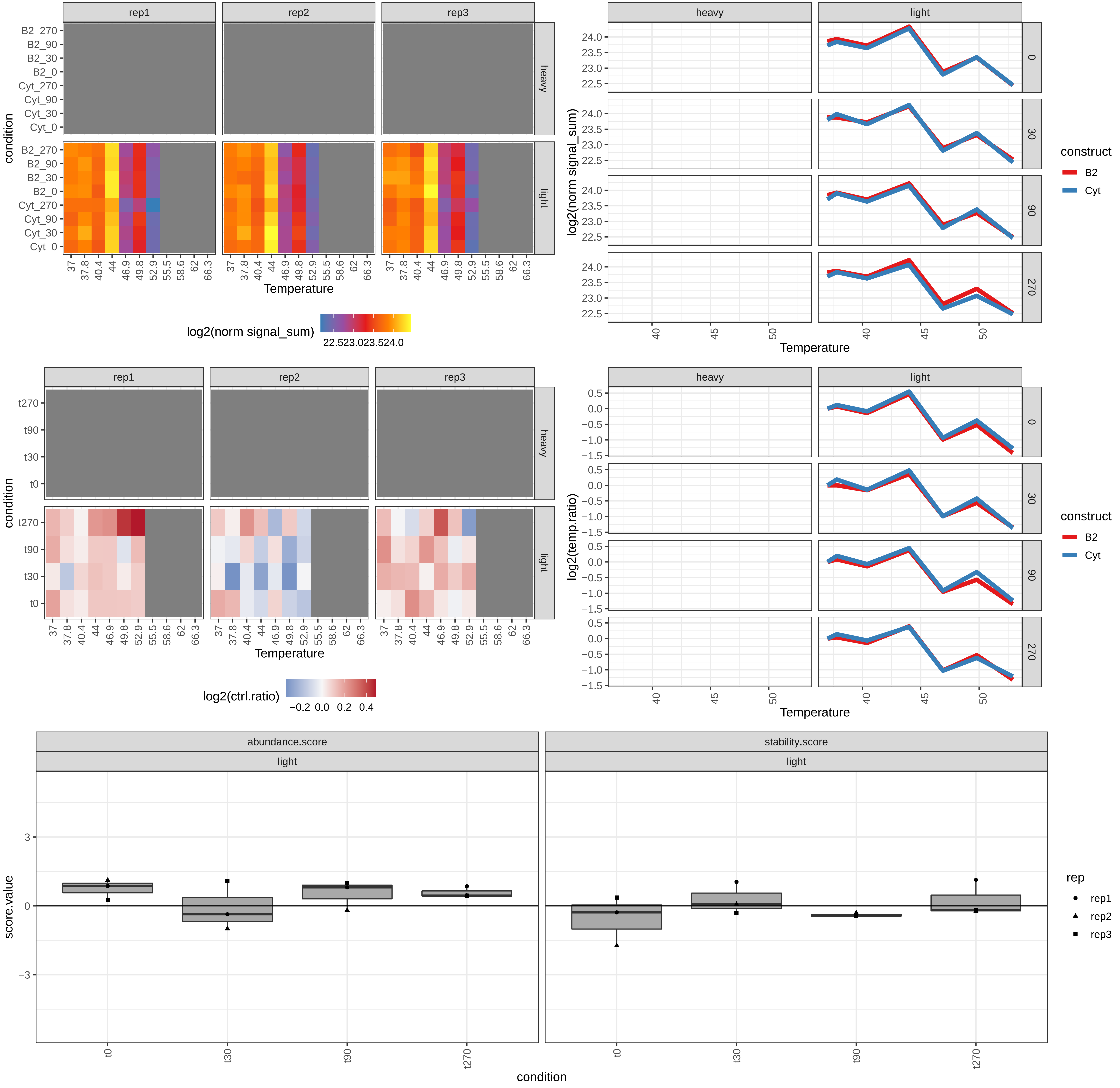

O13585 − Dilute domain−containing protein YPR089W OS=Saccharomyces cerevisiae (strain ATCC 204508 / S288c) GN=YPR089W PE=1 SV=2

YPR091C − average.top3: 6.44862866666667 − max.qupm: 5

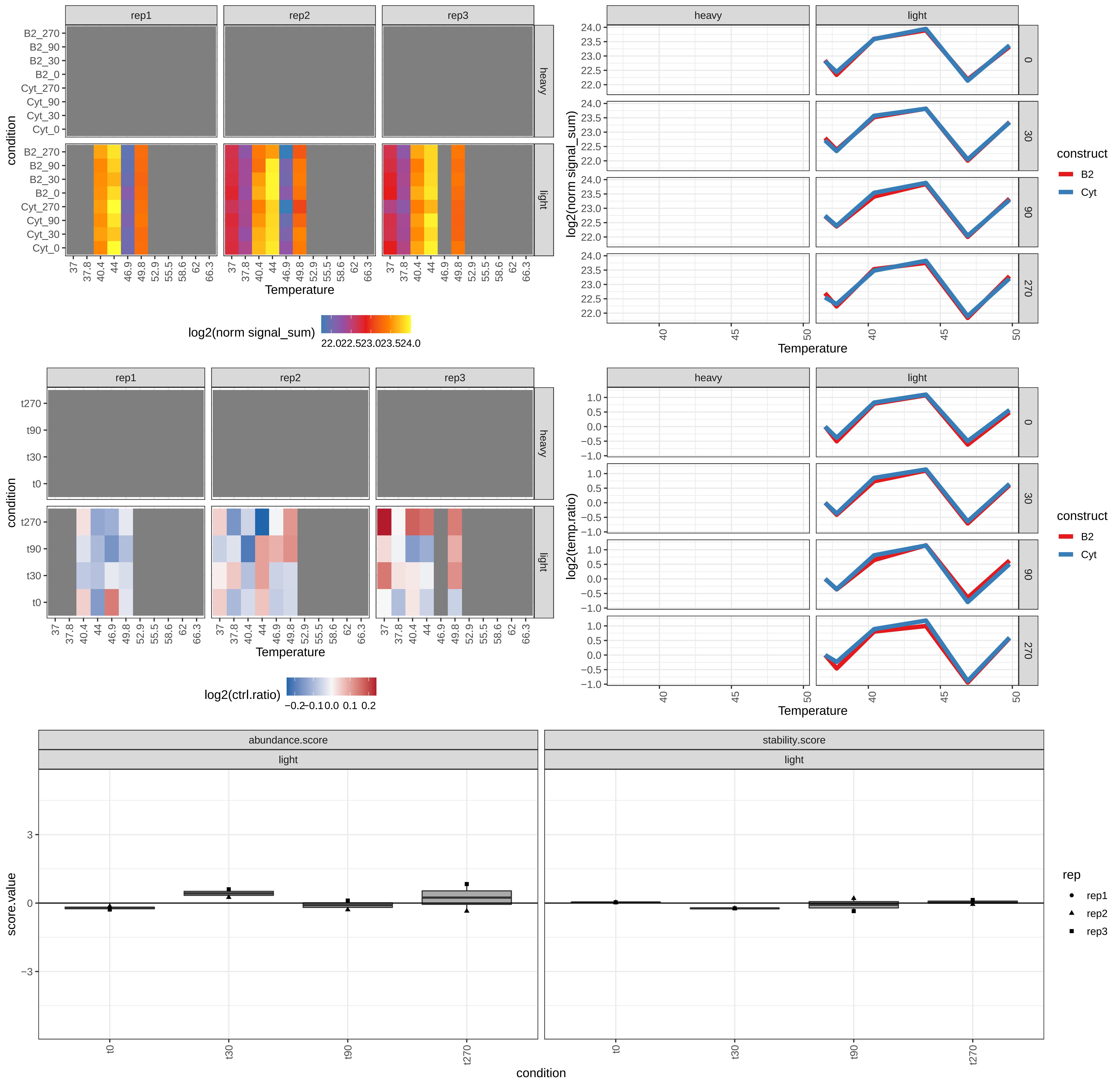

Q06833 − Uncharacterized PH domain−containing protein YPR091C OS=Saccharomyces cerevisiae (strain ATCC 204508 / S288c) GN=YPR091C PE=1 SV=1

YPR097W − average.top3: 6.57224529411765 − max.qupm: 7

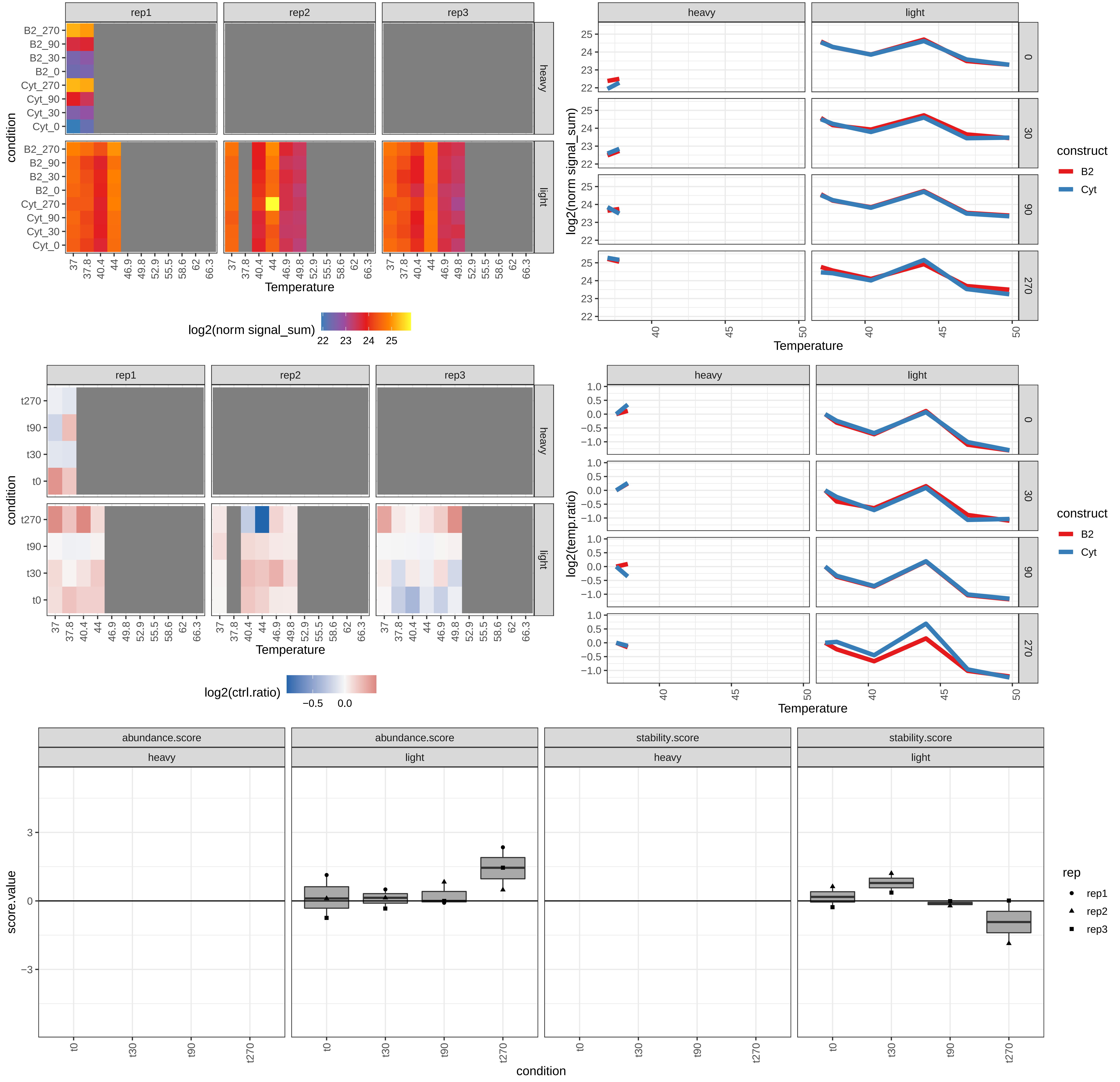

Q06839 − PX domain−containing protein YPR097W OS=Saccharomyces cerevisiae (strain ATCC 204508 / S288c) GN=YPR097W PE=1 SV=2

YPR098C − average.top3: 7.35328552631579 − max.qupm: 4

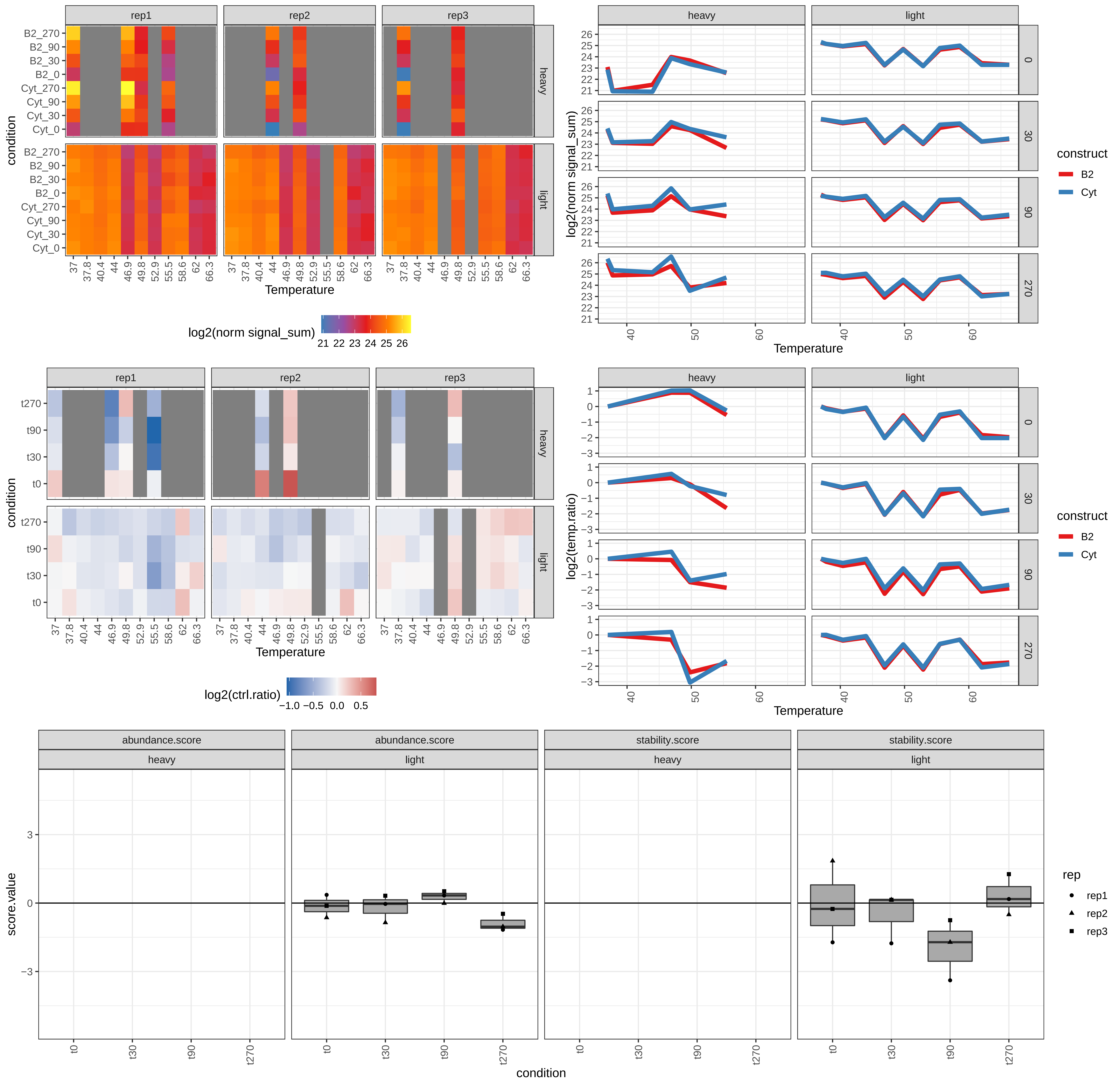

Q06089 − Uncharacterized mitochondrial outer membrane protein YPR098C OS=Saccharomyces cerevisiae (strain ATCC 204508 / S288c) GN=YPR098C PE=1 SV=2

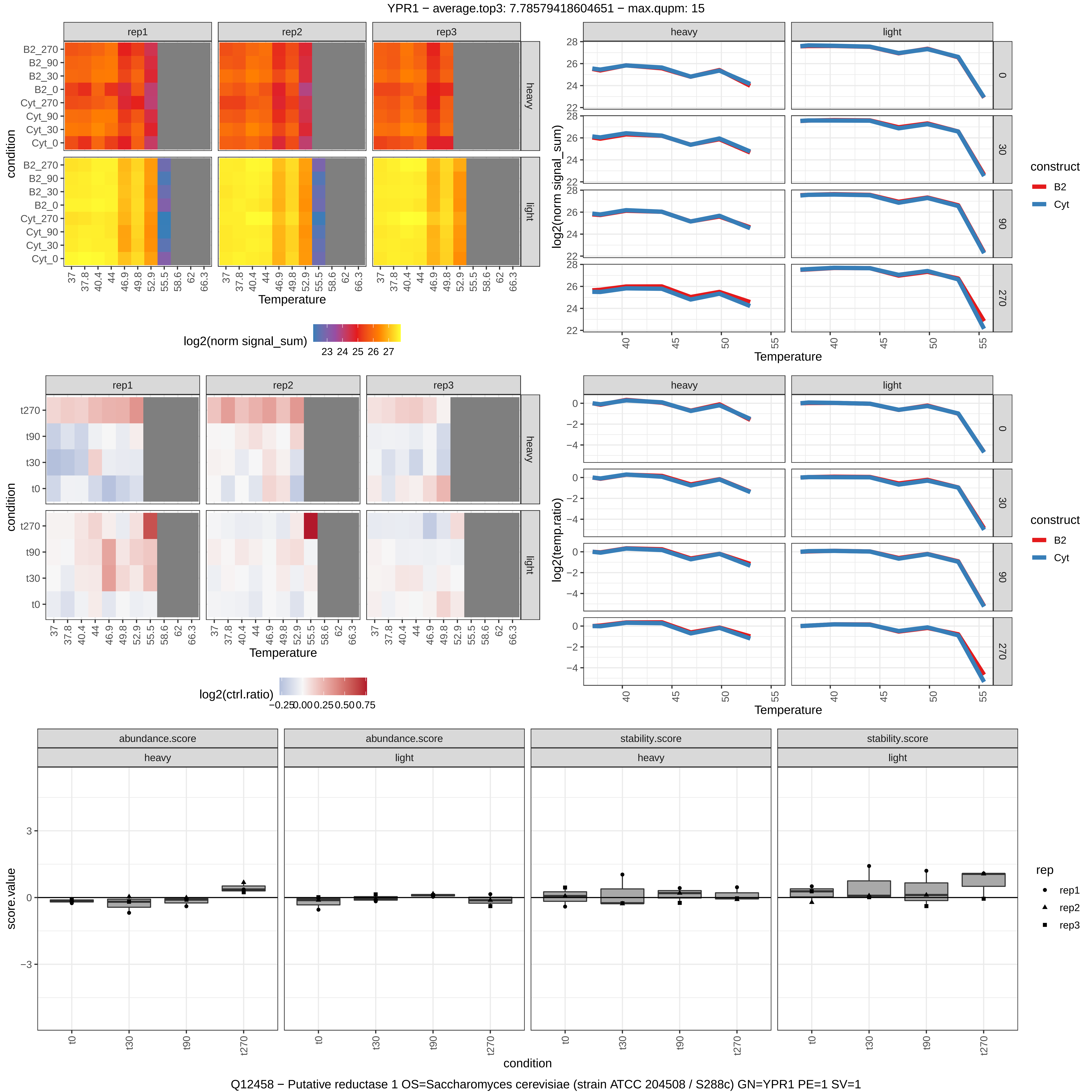

YPR1 − average.top3: 7.78579418604651 − max.qupm: 15
rep1
rep2
rep3
heavy
light
condition
Temperature
log2(norm signal_sum)
log2(ctrl.ratio)
log2(temp.ratio)
construct
B2
Cyt
abundance.score
stability.score
score.value
rep
rep1
rep2
rep3
Q12458 − Putative reductase 1 OS=Saccharomyces cerevisiae (strain ATCC 204508 / S288c) GN=YPR1 PE=1 SV=1

YPR114W − average.top3: 6.73160611111111 − max.qupm: 3

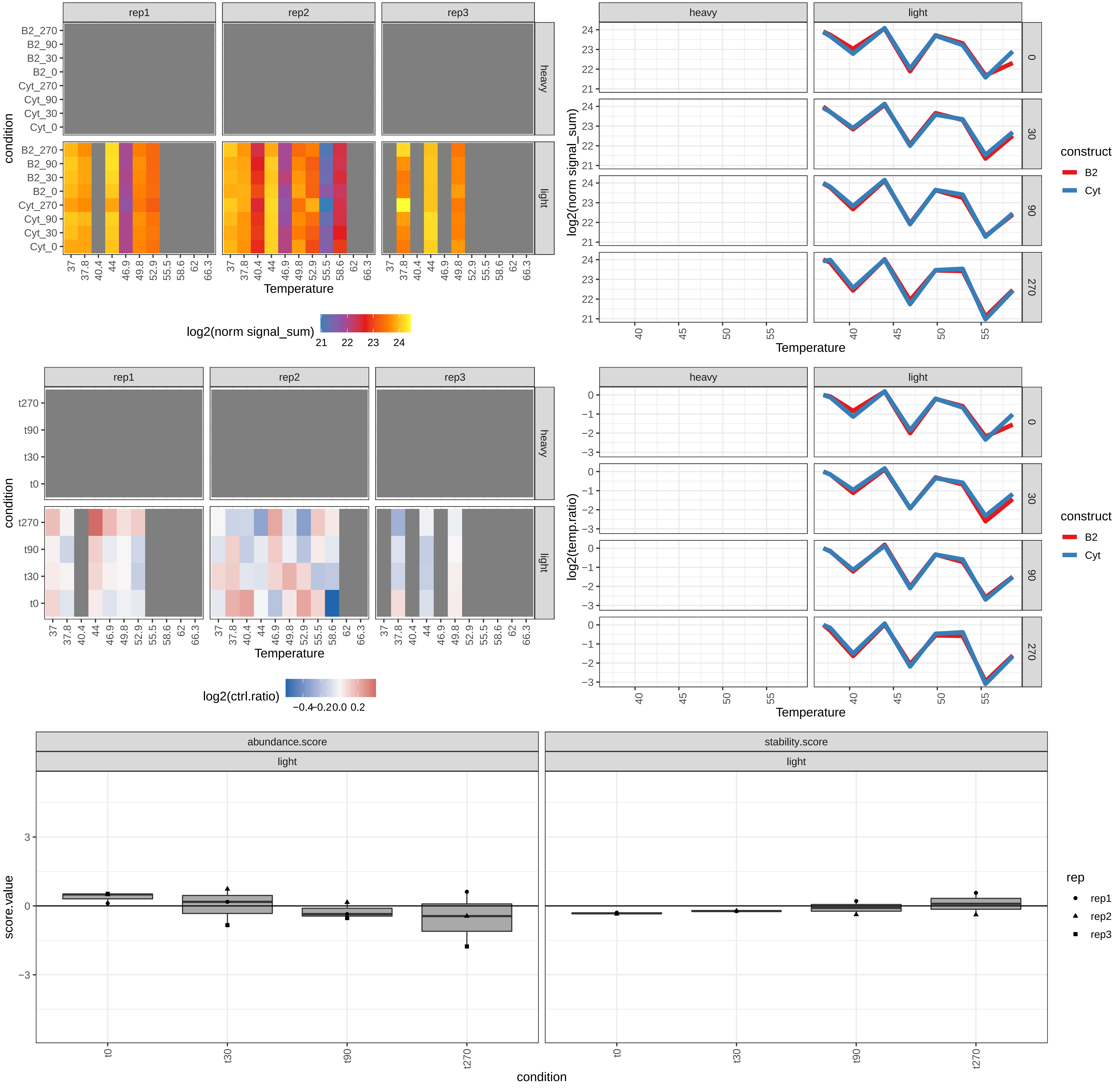

Q06107 − Uncharacterized TLC domain−containing protein YPR114W OS=Saccharomyces cerevisiae (strain ATCC 204508 / S288c) GN=YPR114W PE=1 SV=1

YPR127W − average.top3: 7.02852347826087 − max.qupm: 11

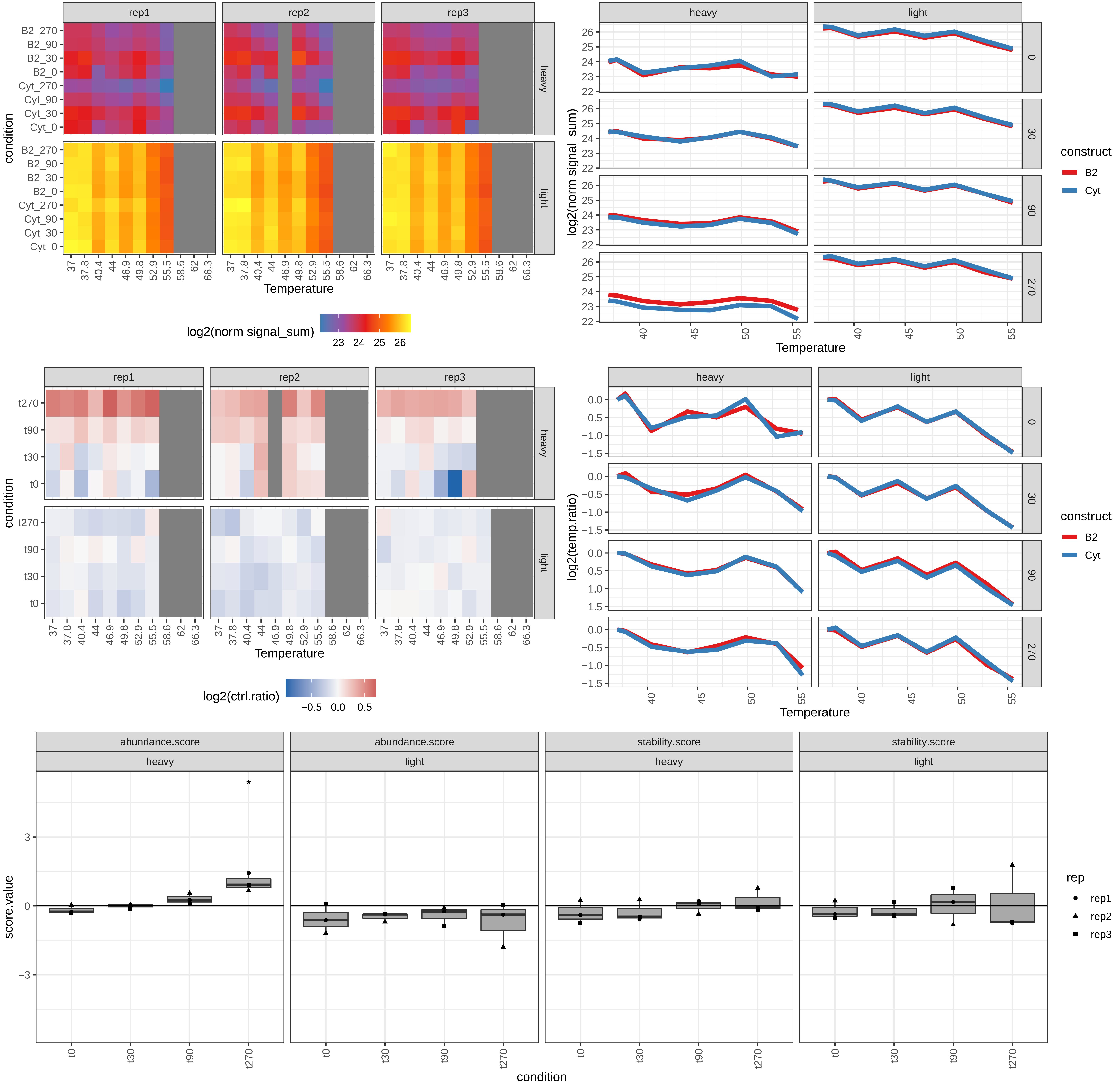

Q06494 − Putative pyridoxal reductase OS=Saccharomyces cerevisiae (strain ATCC 204508 / S288c) GN=YPR127W PE=1 SV=1

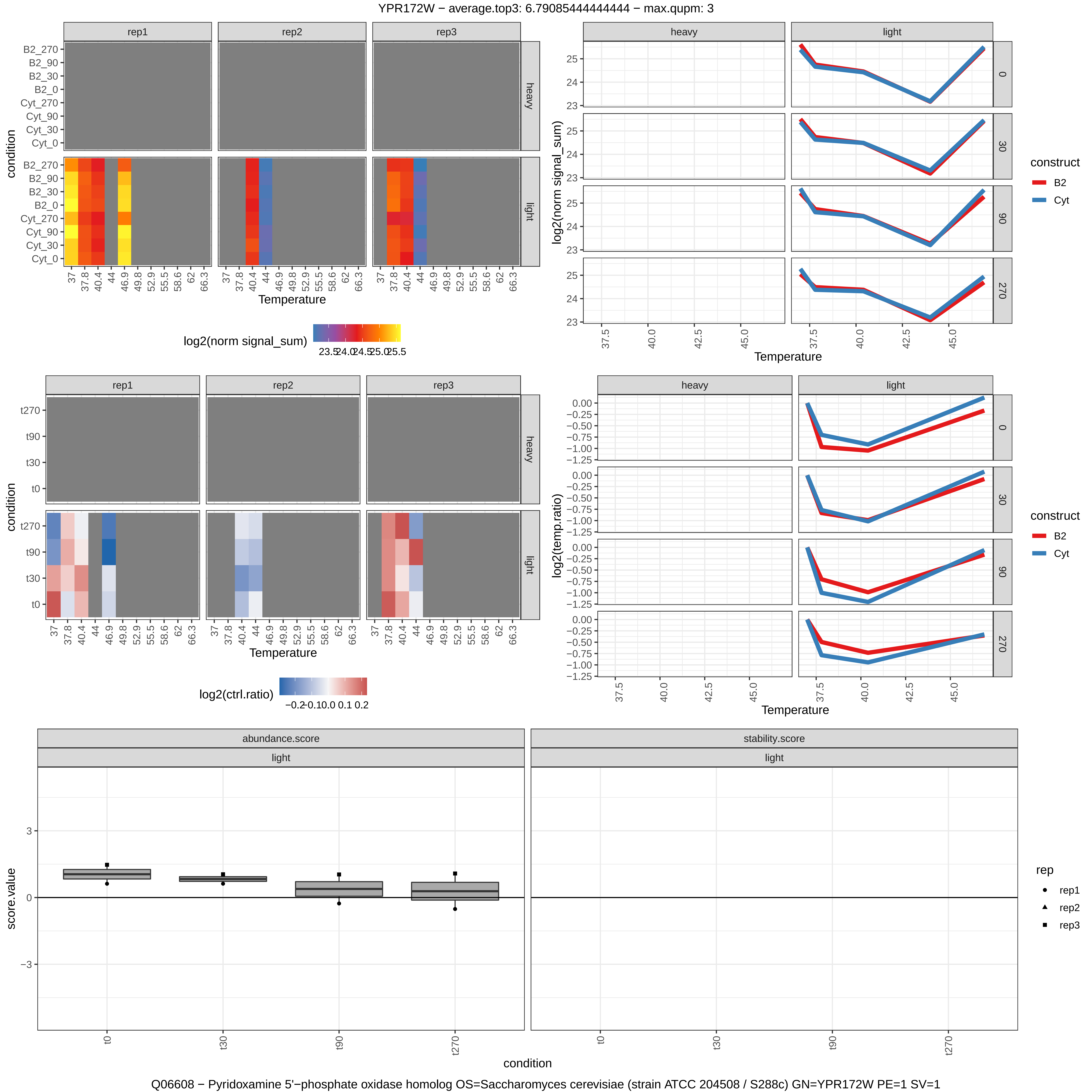
YPR172W − average.top3: 6.79085444444444 − max.qupm: 3
log2(norm signal_sum)
log2(ctrl.ratio)
log2(temp.ratio)
construct
B2
Cyt
abundance.score
stability.score
score.value
condition
Temperature
rep
rep1
rep2
rep3
Q06608 − Pyridoxamine 5'−phosphate oxidase homolog OS=Saccharomyces cerevisiae (strain ATCC 204508 / S288c) GN=YPR172W PE=1 SV=1

YPS1 − average.top3: 7.41778421052632 − max.qupm: 3

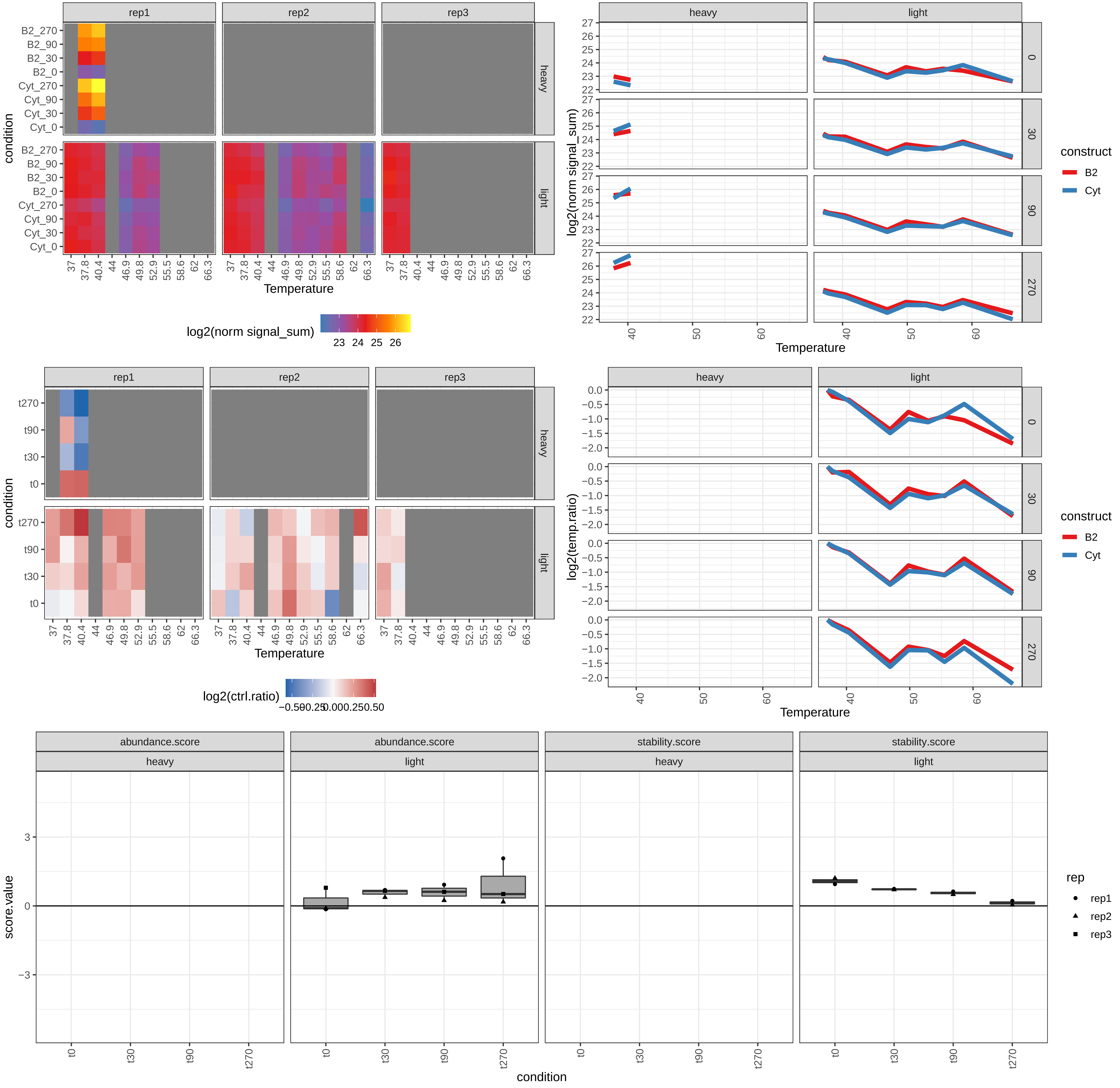

P32329 − Aspartic proteinase 3 OS=Saccharomyces cerevisiae (strain ATCC 204508 / S288c) GN=YPS1 PE=1 SV=2

YPT1 − average.top3: 7.71276876923077 − max.qupm: 12

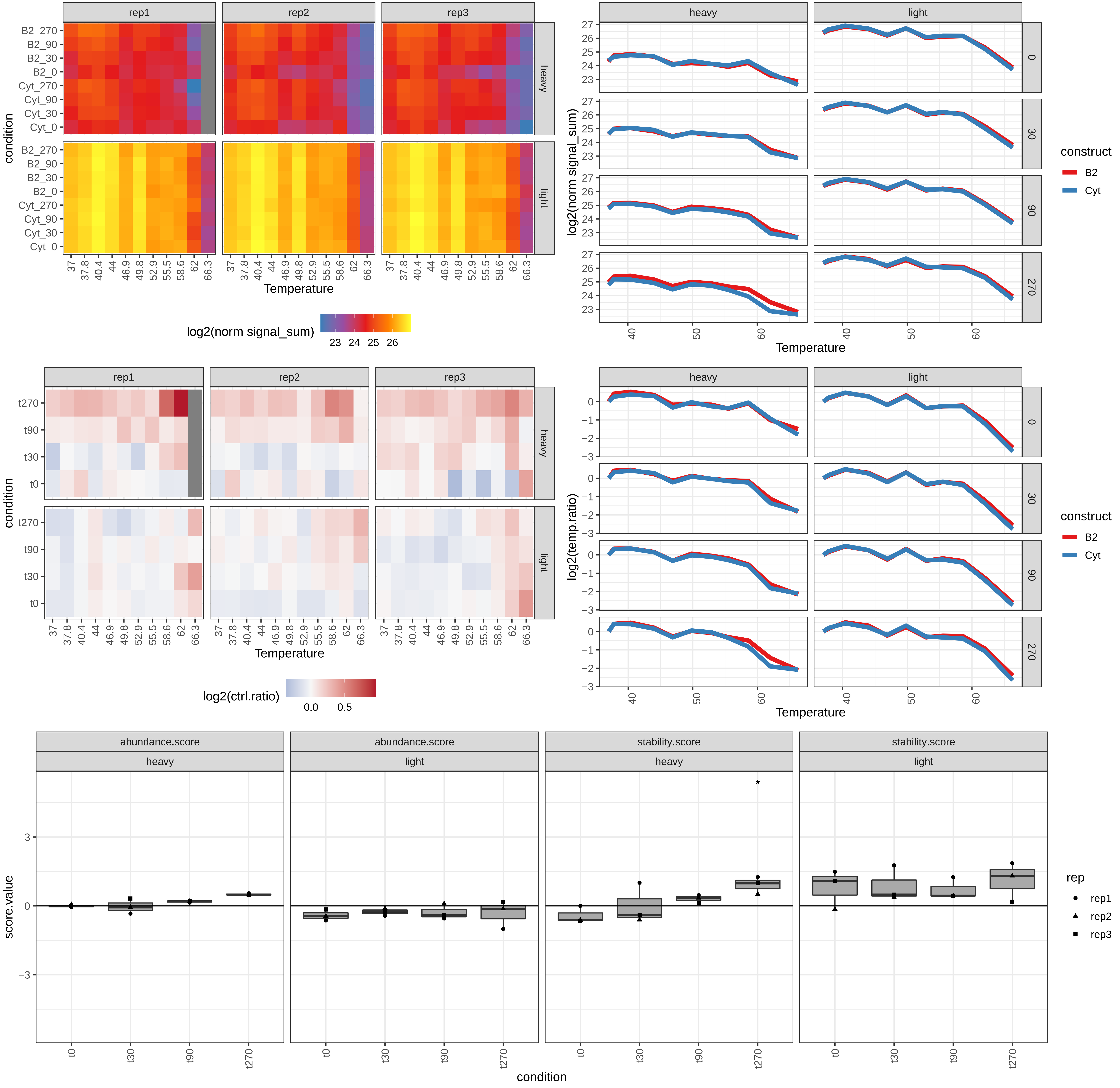

P01123 − GTP−binding protein YPT1 OS=Saccharomyces cerevisiae (strain ATCC 204508 / S288c) GN=YPT1 PE=1 SV=2

YPT10 − average.top3: 6.494624 − max.qupm: 4

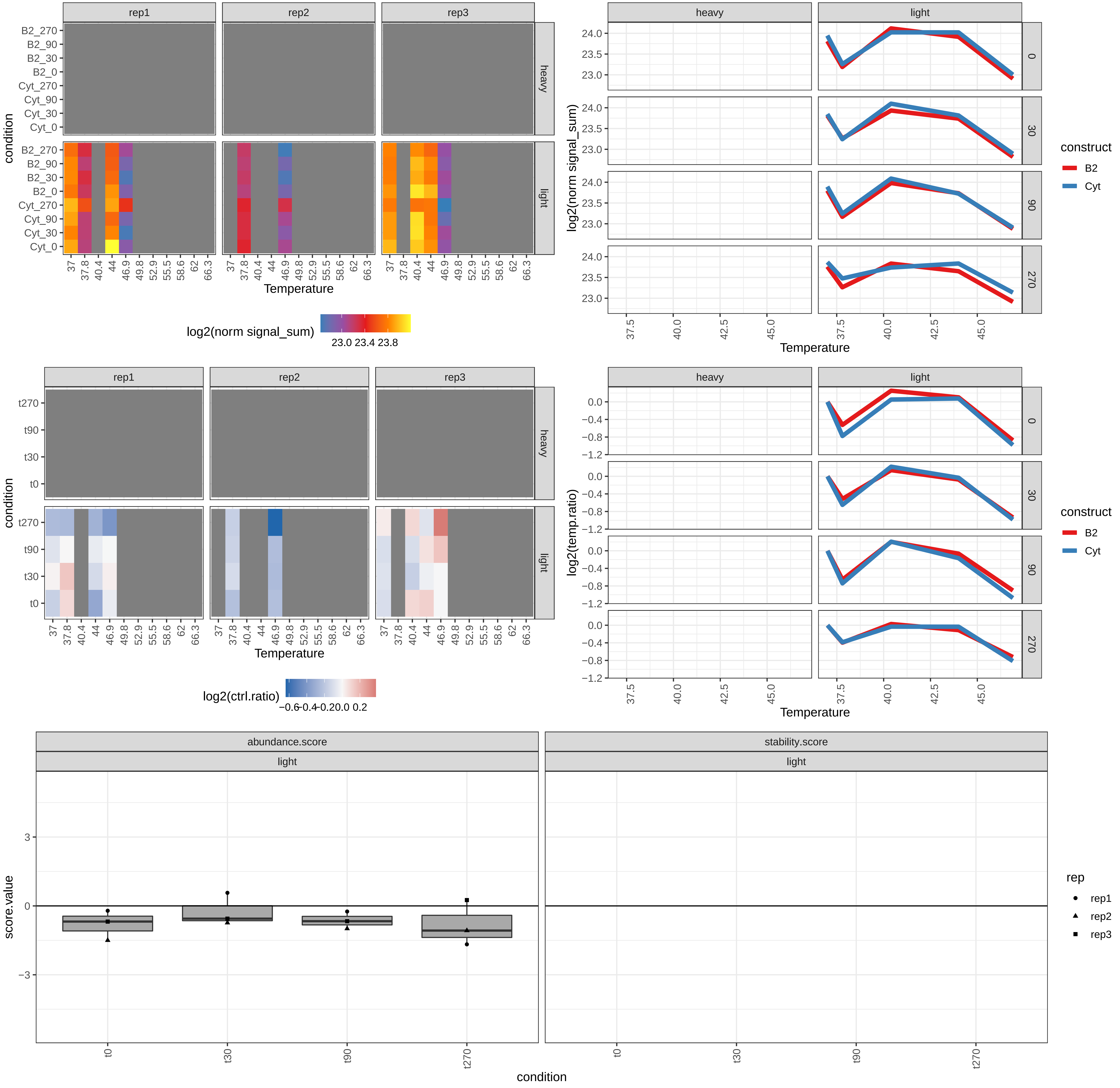

P38146 − GTP−binding protein YPT10 OS=Saccharomyces cerevisiae (strain ATCC 204508 / S288c) GN=YPT10 PE=1 SV=2

YPT31 − average.top3: 7.35352194444444 − max.qupm: 6

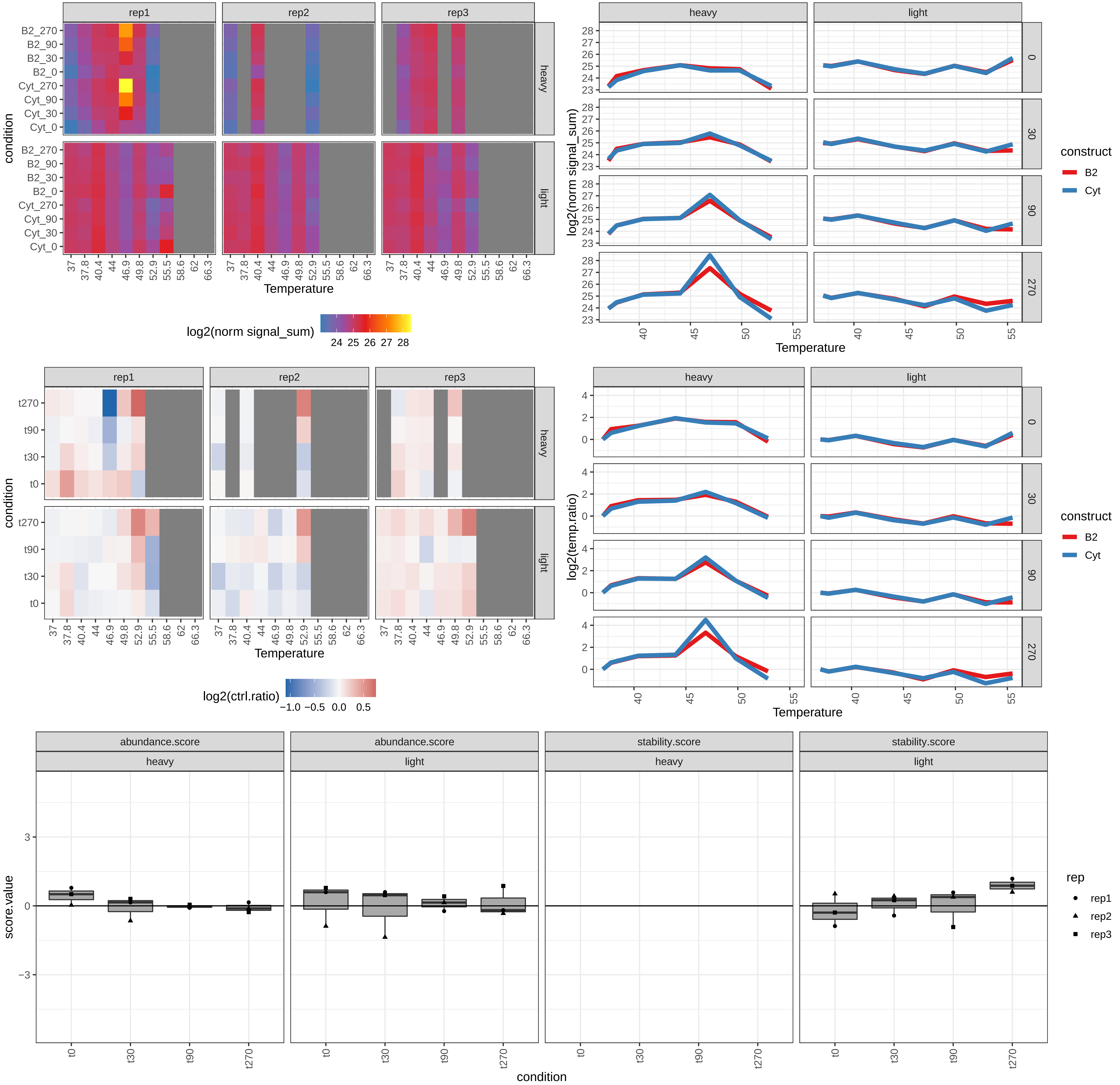

P38555 − GTP−binding protein YPT31/YPT8 OS=Saccharomyces cerevisiae (strain ATCC 204508 / S288c) GN=YPT31 PE=1 SV=3

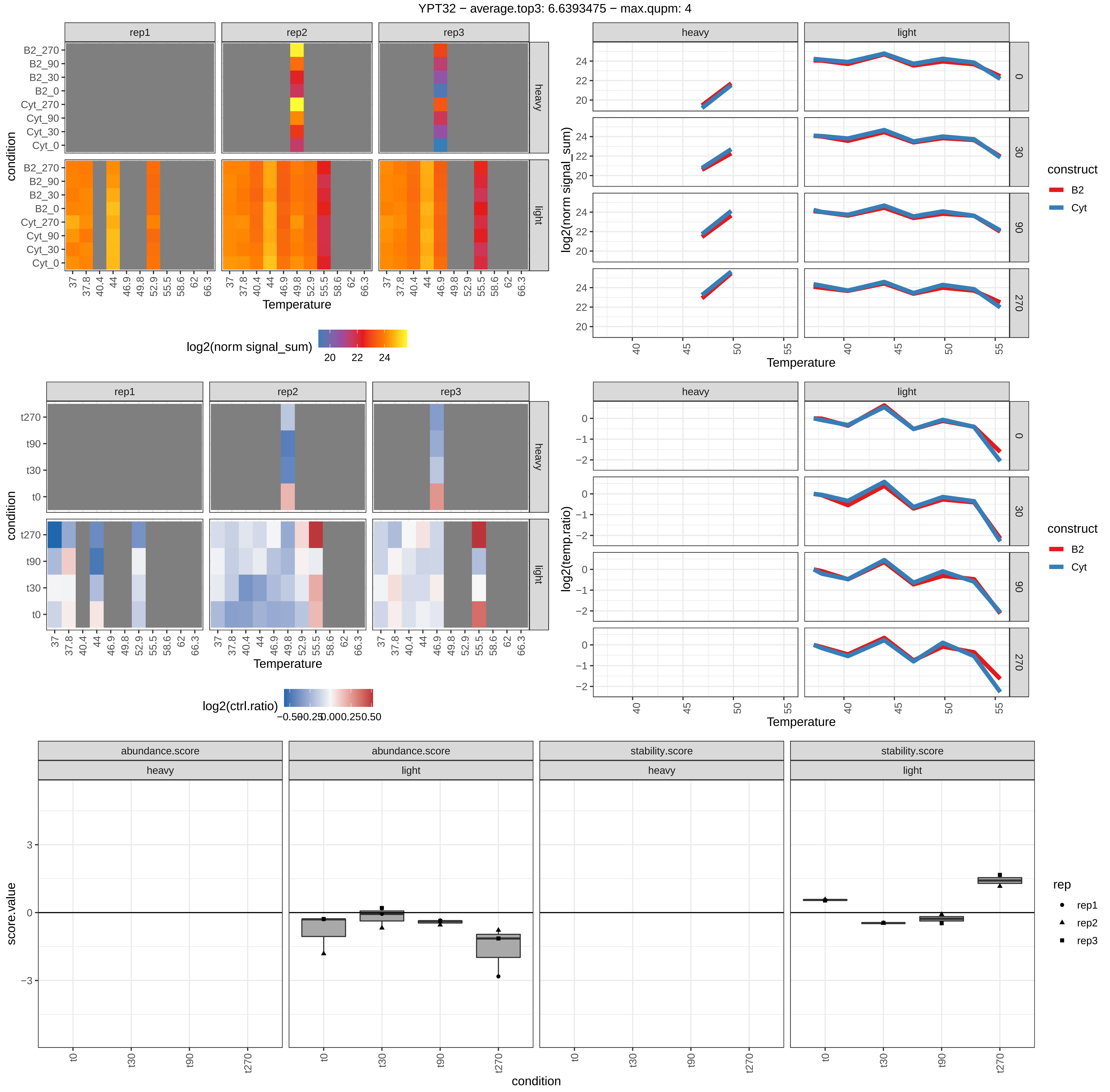

P51996 − GTP−binding protein YPT32/YPT11 OS=Saccharomyces cerevisiae (strain ATCC 204508 / S288c) GN=YPT32 PE=1 SV=3

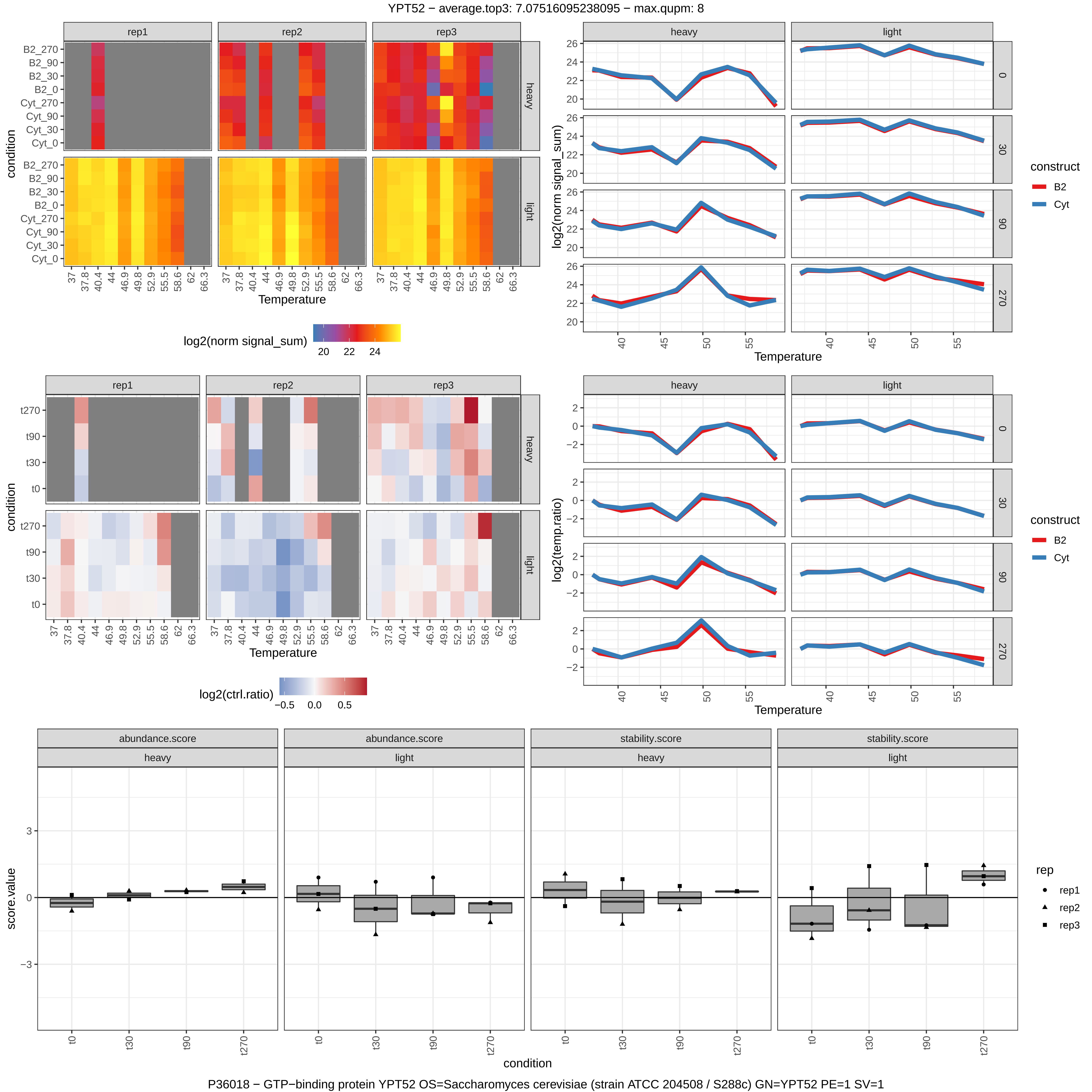
YPT52 − average.top3: 7.07516095238095 − max.qupm: 8
rep1
rep2
rep3
heavy
light
condition
B2_270
B2_90
B2_30
B2_0
Cyt_270
Cyt_90
Cyt_30
Cyt_0
37
37.8
40.4
44
46.9
49.8
52.9
55.5
58.6
62
66.3
Temperature
log2(norm signal_sum)
20
22
24
26
0
30
90
270
40
45
50
55
construct
B2
Cyt
t270
t90
t30
t0
log2(ctrl.ratio)
−0.5
0.0
0.5
log2(temp.ratio)
2
0
−2
abundance.score
stability.score
score.value
3
−3
rep
rep1
rep2
rep3
P36018 − GTP−binding protein YPT52 OS=Saccharomyces cerevisiae (strain ATCC 204508 / S288c) GN=YPT52 PE=1 SV=1

YPT6 − average.top3: 6.98361536585366 − max.qupm: 11

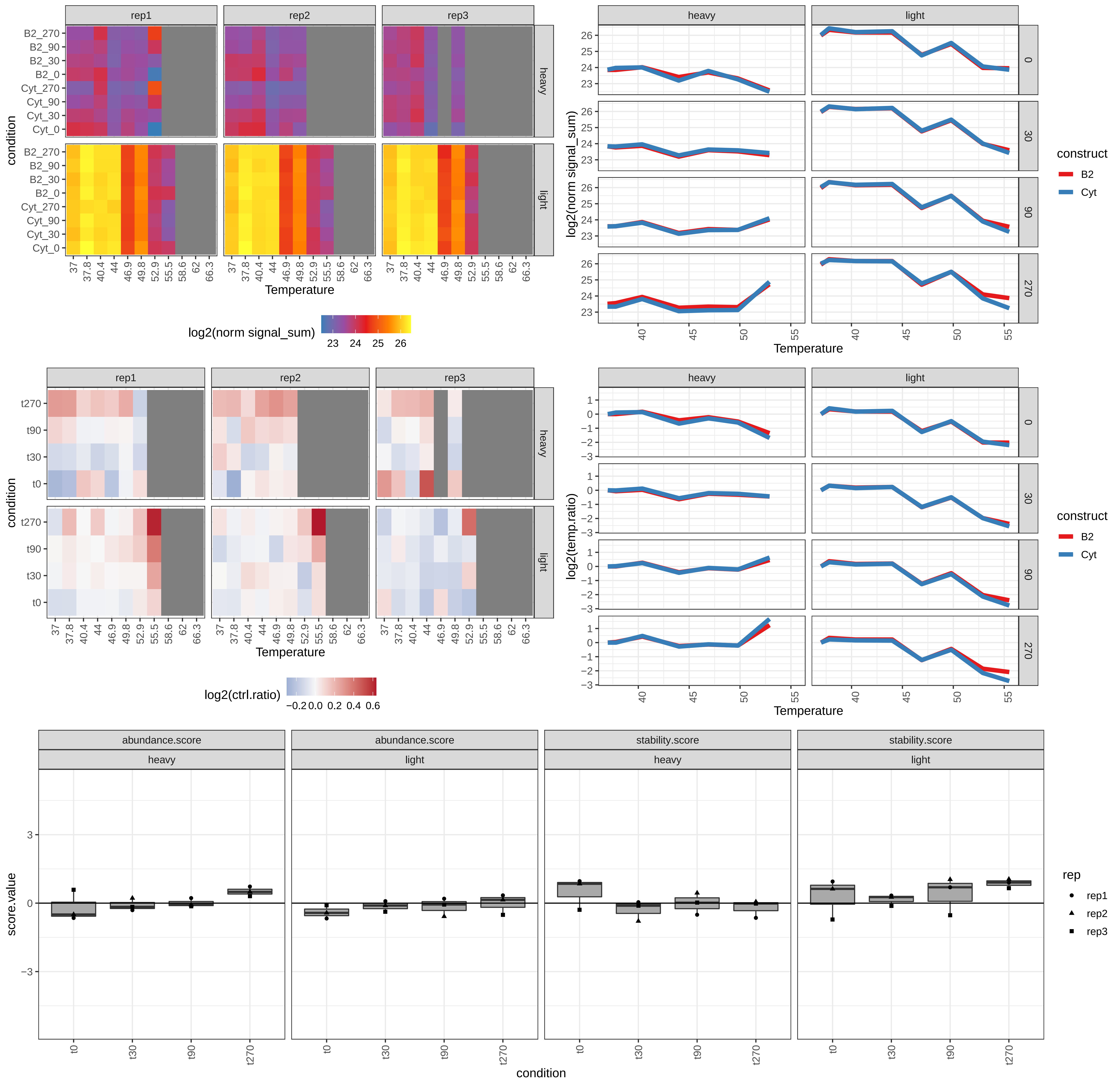

Q99260 − GTP−binding protein YPT6 OS=Saccharomyces cerevisiae (strain ATCC 204508 / S288c) GN=YPT6 PE=1 SV=1

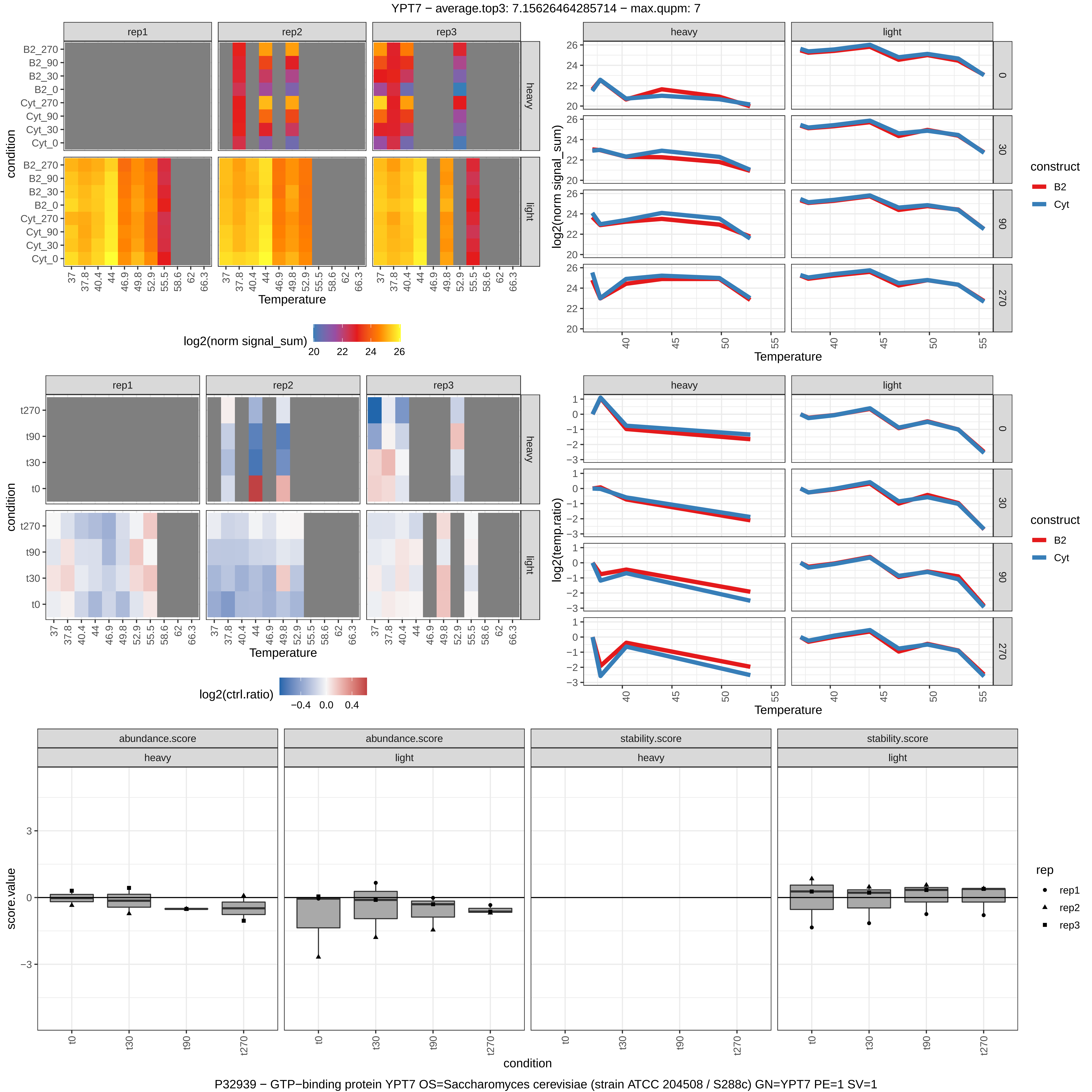
YPT7 − average.top3: 7.15626464285714 − max.qupm: 7
rep1
rep2
rep3
heavy
light
condition
Temperature
log2(norm signal_sum)
construct
B2
Cyt
log2(temp.ratio)
log2(ctrl.ratio)
abundance.score
stability.score
score.value
rep
rep1
rep2
rep3
P32939 − GTP−binding protein YPT7 OS=Saccharomyces cerevisiae (strain ATCC 204508 / S288c) GN=YPT7 PE=1 SV=1

YRA1 − average.top3: 7.49734938461538 − max.qupm: 10

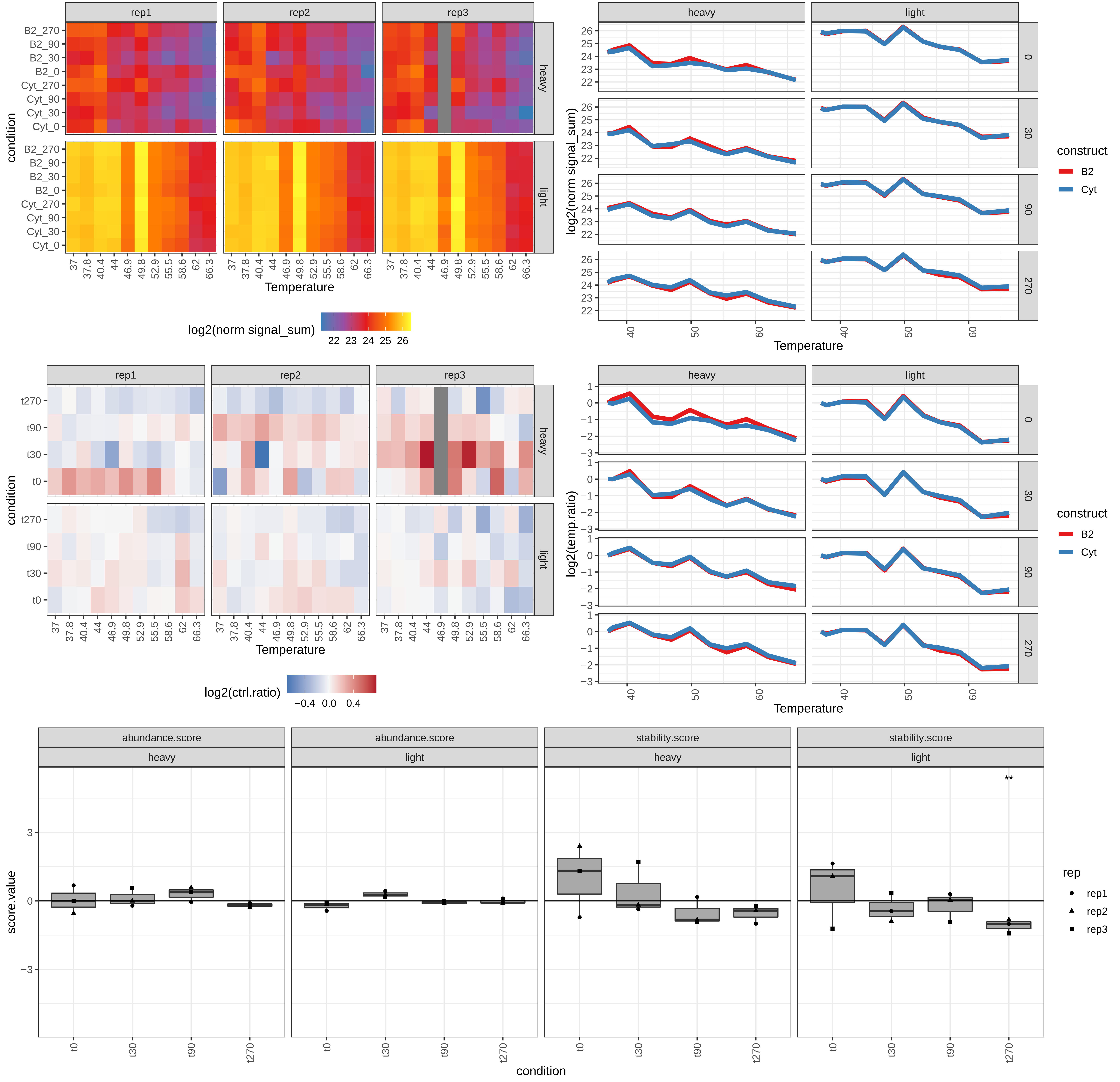

Q12159 − RNA annealing protein YRA1 OS=Saccharomyces cerevisiae (strain ATCC 204508 / S288c) GN=YRA1 PE=1 SV=2

YRA2 − average.top3: 6.36414548387097 − max.qupm: 7

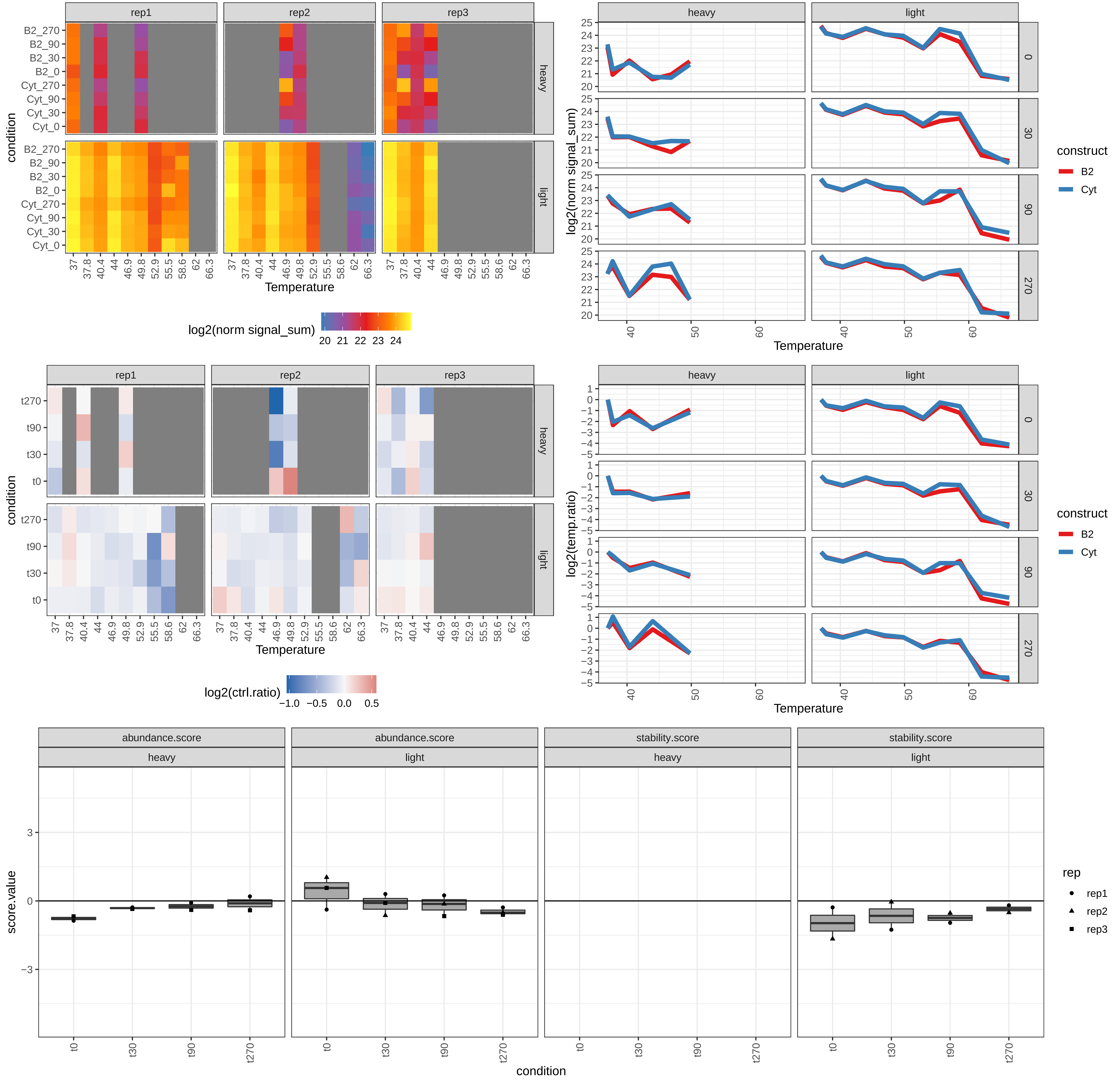

P36036 − RNA annealing protein YRA2 OS=Saccharomyces cerevisiae (strain ATCC 204508 / S288c) GN=YRA2 PE=1 SV=1

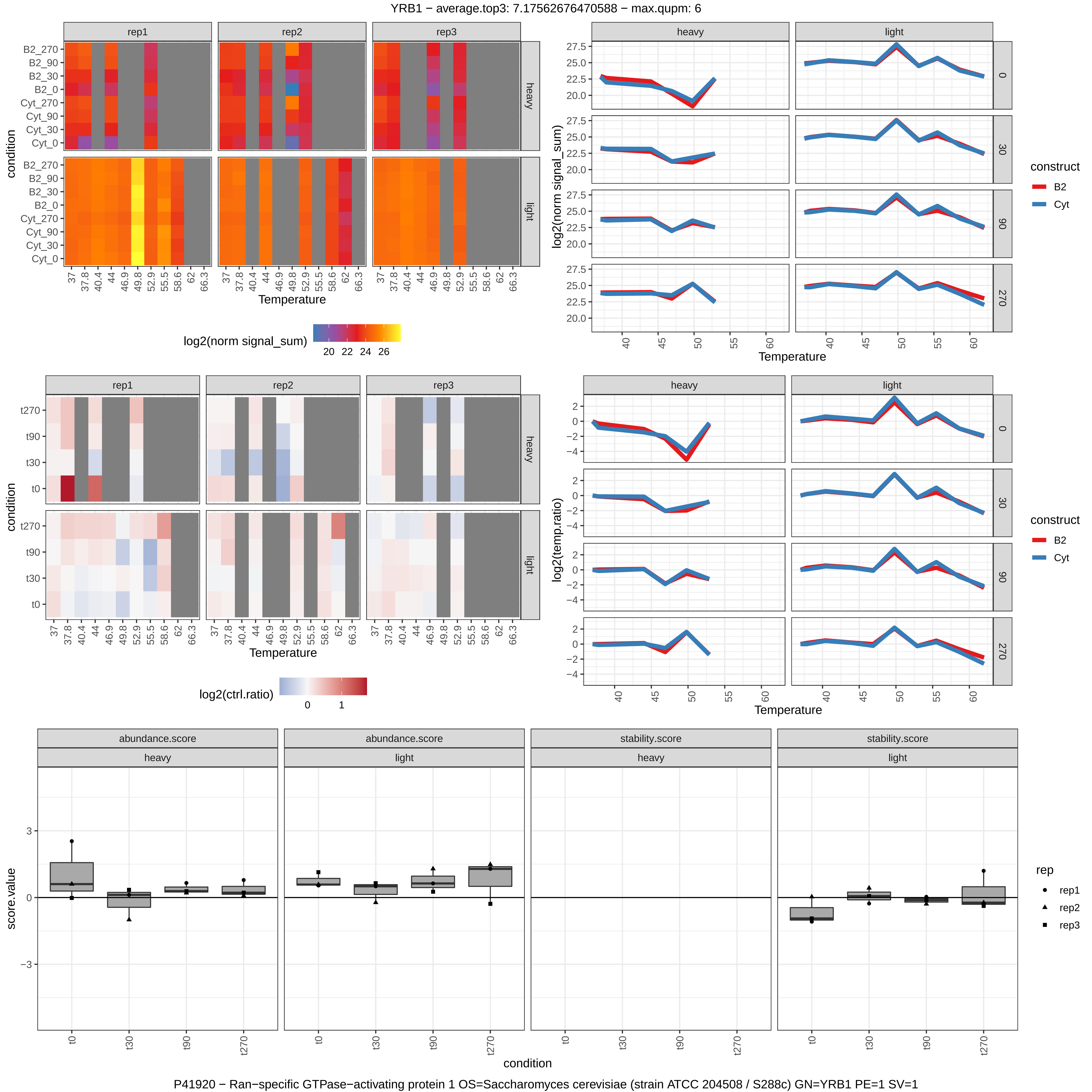
YRB1 − average.top3: 7.17562676470588 − max.qupm: 6
rep1
rep2
rep3
heavy
light
condition
Temperature
log2(norm signal_sum)
log2(ctrl.ratio)
log2(temp.ratio)
construct
B2
Cyt
abundance.score
stability.score
score.value
rep
rep1
rep2
rep3
P41920 − Ran−specific GTPase−activating protein 1 OS=Saccharomyces cerevisiae (strain ATCC 204508 / S288c) GN=YRB1 PE=1 SV=1

YRB2 − average.top3: 6.55836766666667 − max.qupm: 5

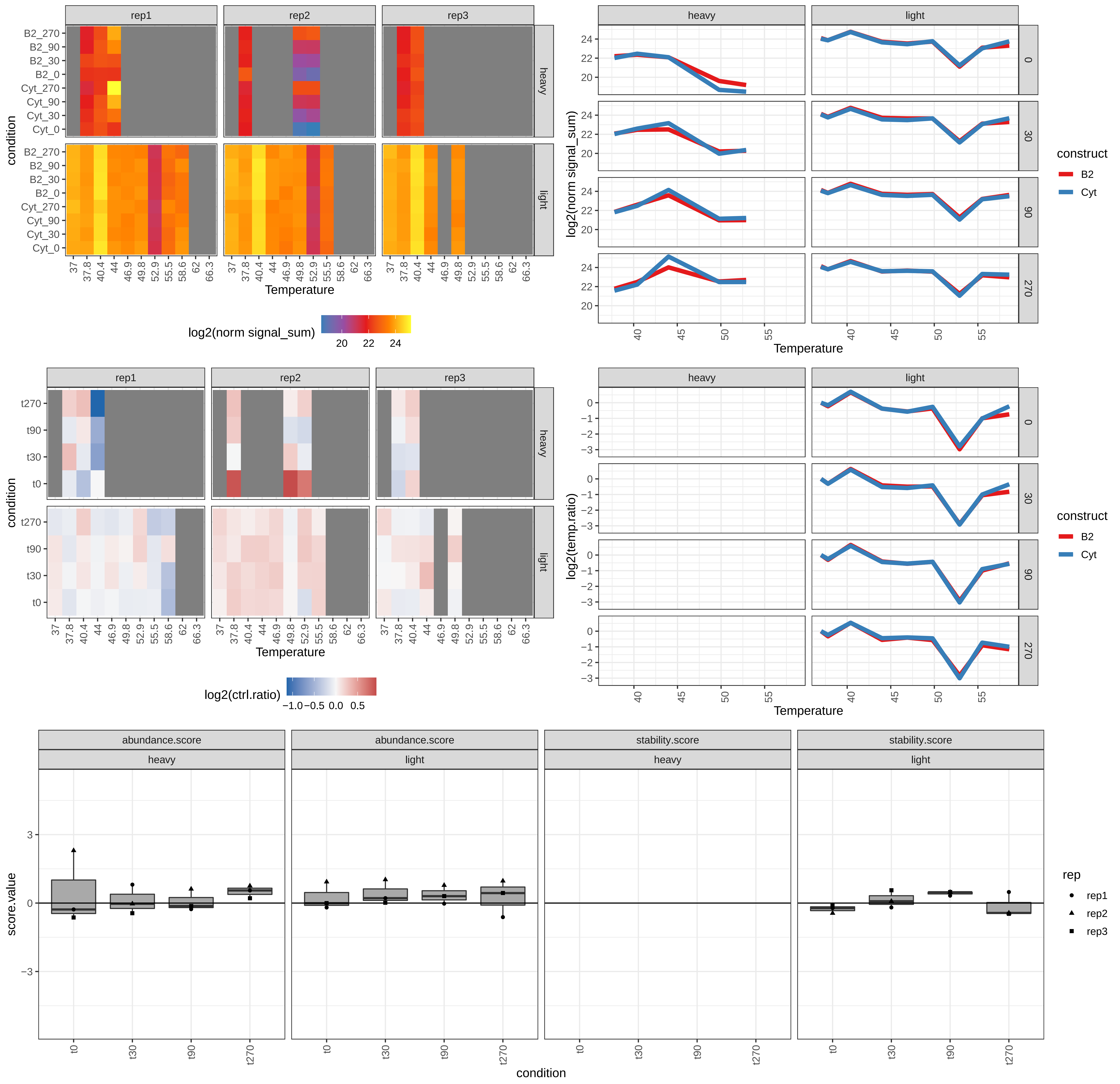

P40517 − Ran−specific GTPase−activating protein 2 OS=Saccharomyces cerevisiae (strain ATCC 204508 / S288c) GN=YRB2 PE=1 SV=1

YRB30 − average.top3: 6.83617774193548 − max.qupm: 8

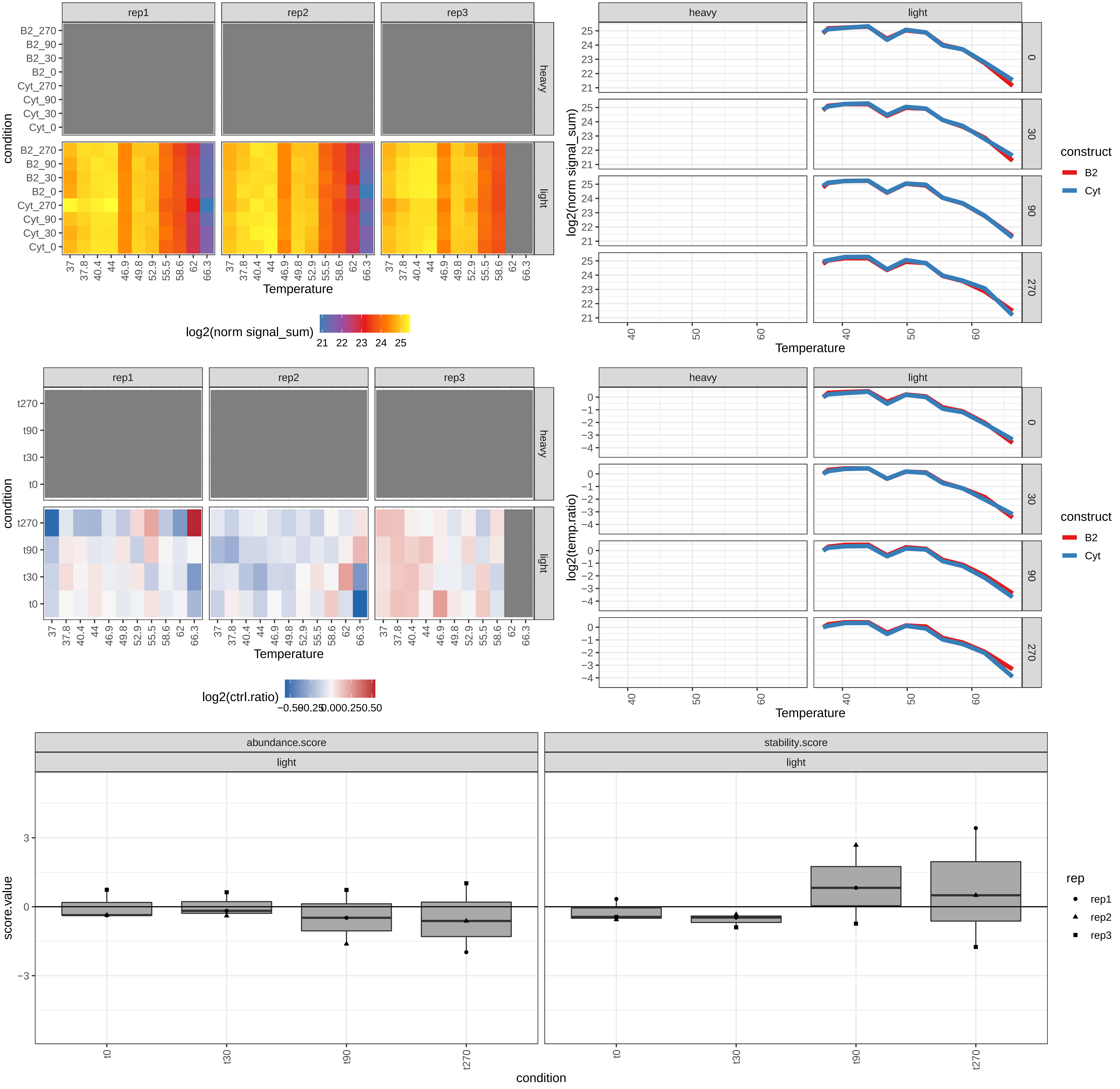

P53107 − Ran−specific GTPase−activating protein 30 OS=Saccharomyces cerevisiae (strain ATCC 204508 / S288c) GN=YRB30 PE=1 SV=1

YRO2 − average.top3: 7.2557901754386 − max.qupm: 6

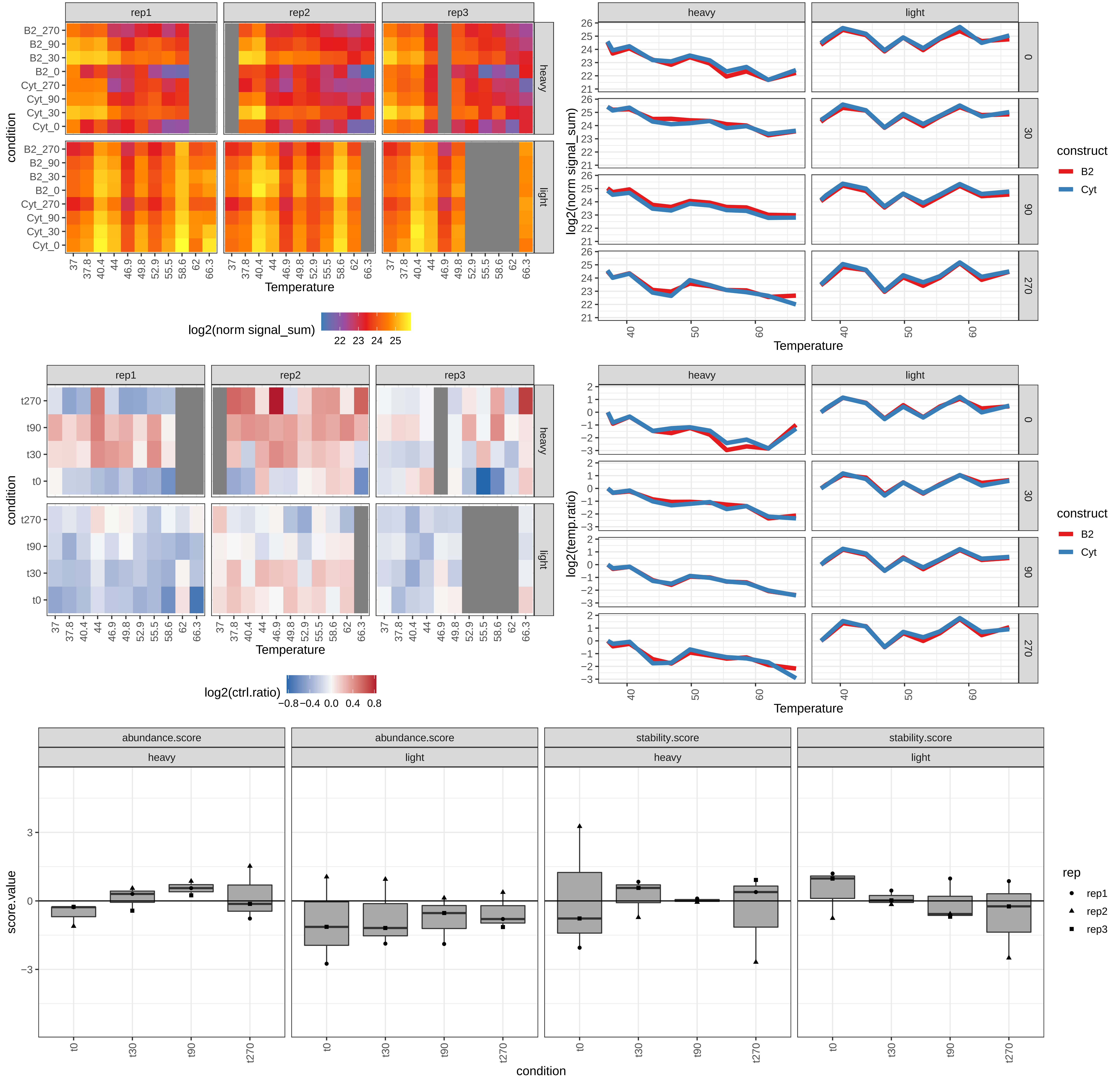

P38079 − Protein YRO2 OS=Saccharomyces cerevisiae (strain ATCC 204508 / S288c) GN=YRO2 PE=1 SV=1

YSA1 − average.top3: 7.13335846153846 − max.qupm: 7

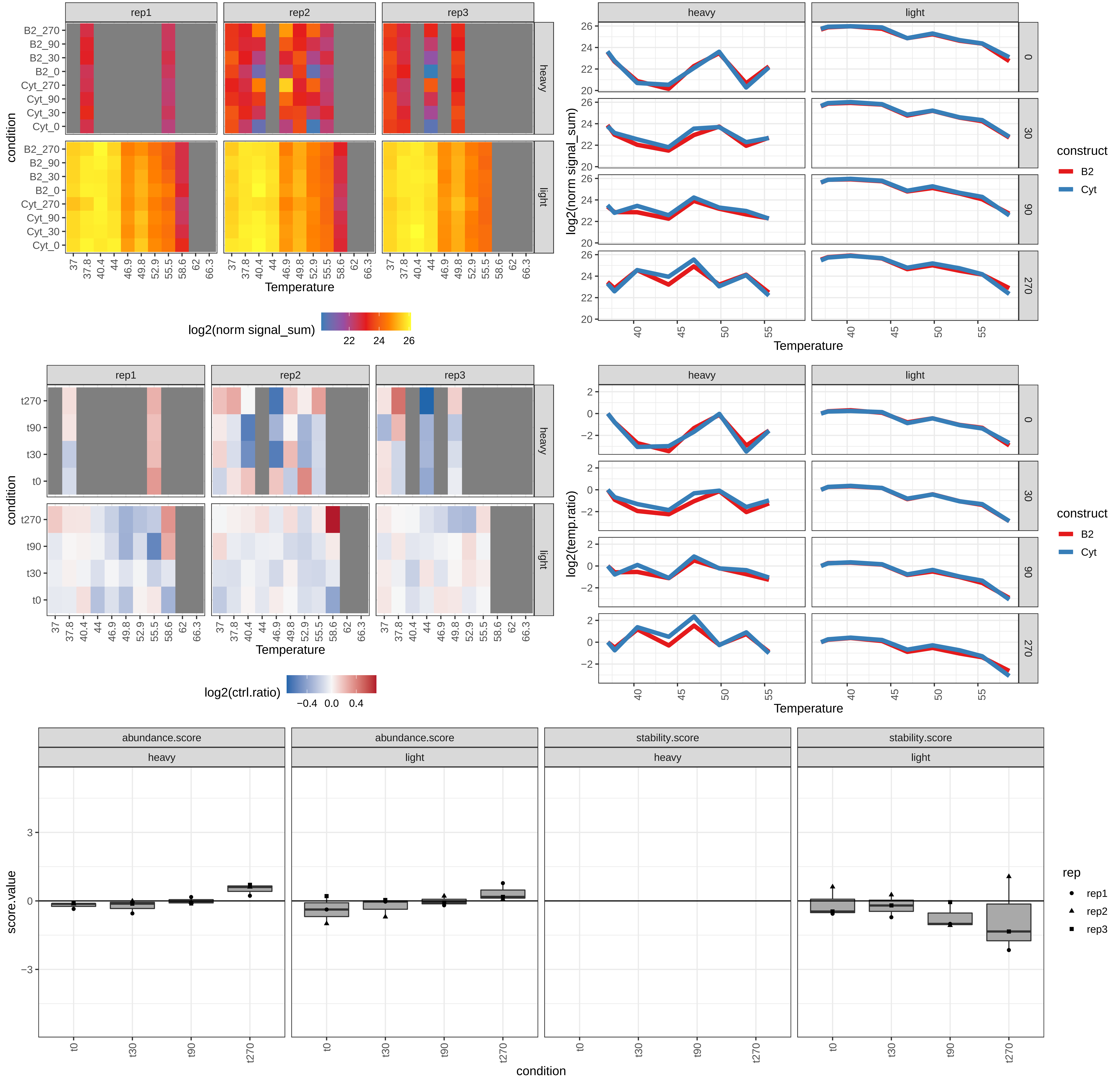

P−ribose pyrophosphatase OS=Saccharomyces cerevisiae (strain ATCC 204508 / S288c) GN=YSA1 PE=1 SV=2|Isoform 2 of ADP−ribose pyrophosphatase OS=Saccharomyces cerevisiae (strain ATCC 20

YSC83 − average.top3: 6.51277304347826 − max.qupm: 5

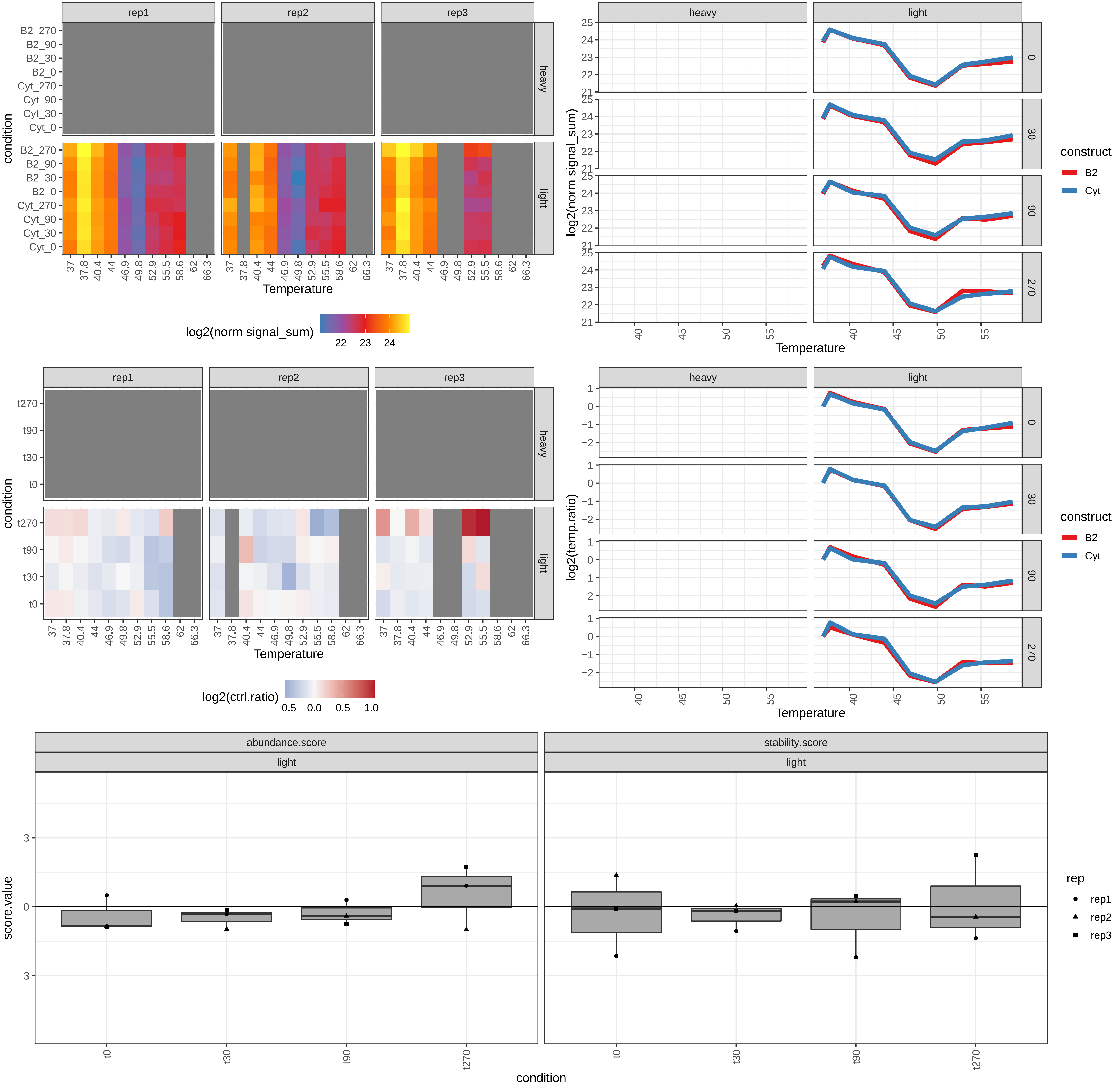

P32792 − UPF0744 protein YSC83 OS=Saccharomyces cerevisiae (strain ATCC 204508 / S288c) GN=YSC83 PE=1 SV=1

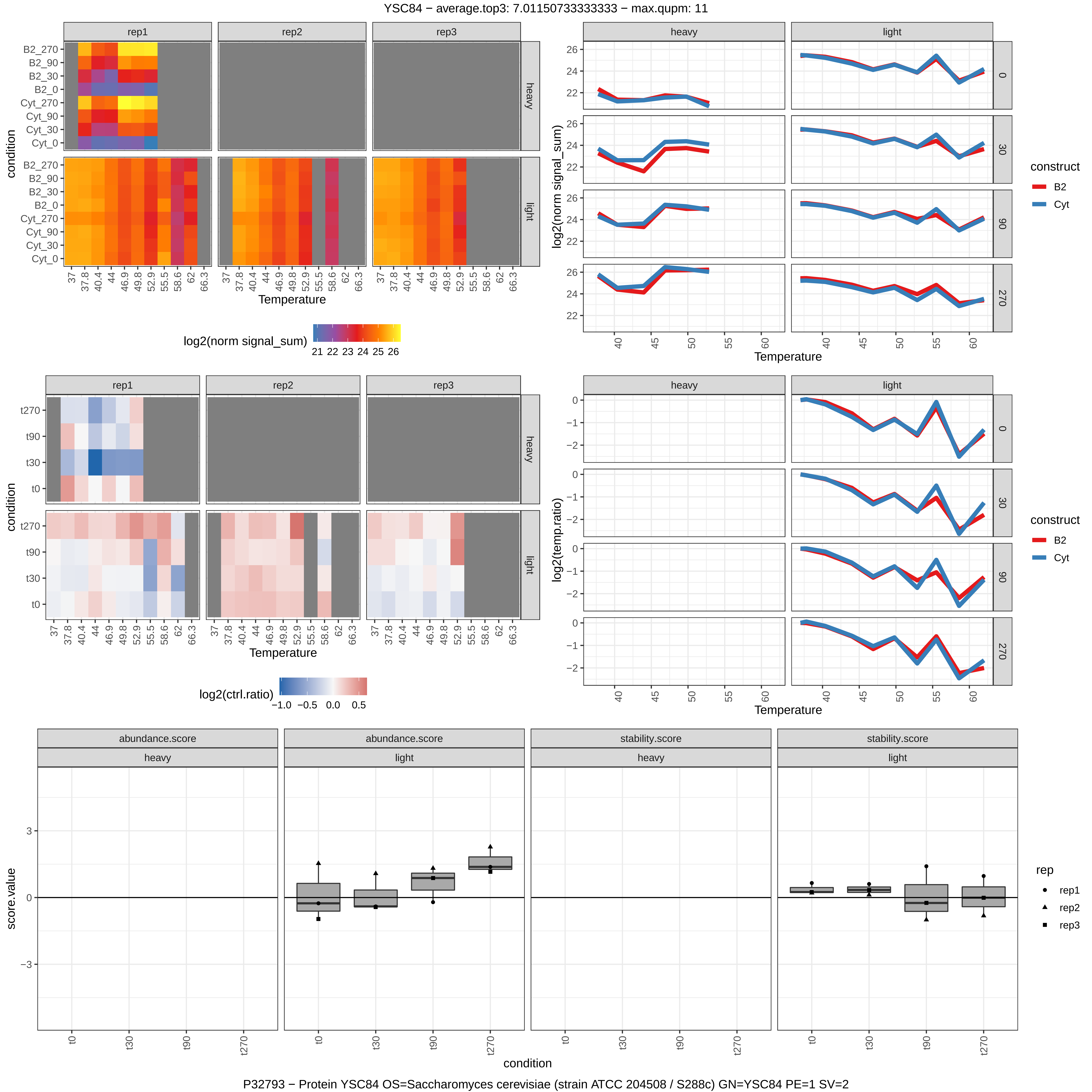
YSC84 − average.top3: 7.01150733333333 − max.qupm: 11
rep1
rep2
rep3
heavy
light
condition
Temperature
log2(norm signal_sum)
construct
B2
Cyt
log2(temp.ratio)
log2(ctrl.ratio)
abundance.score
stability.score
score.value
rep
rep1
rep2
rep3
P32793 − Protein YSC84 OS=Saccharomyces cerevisiae (strain ATCC 204508 / S288c) GN=YSC84 PE=1 SV=2

YSH1 − average.top3: 6.64491291666667 − max.qupm: 5

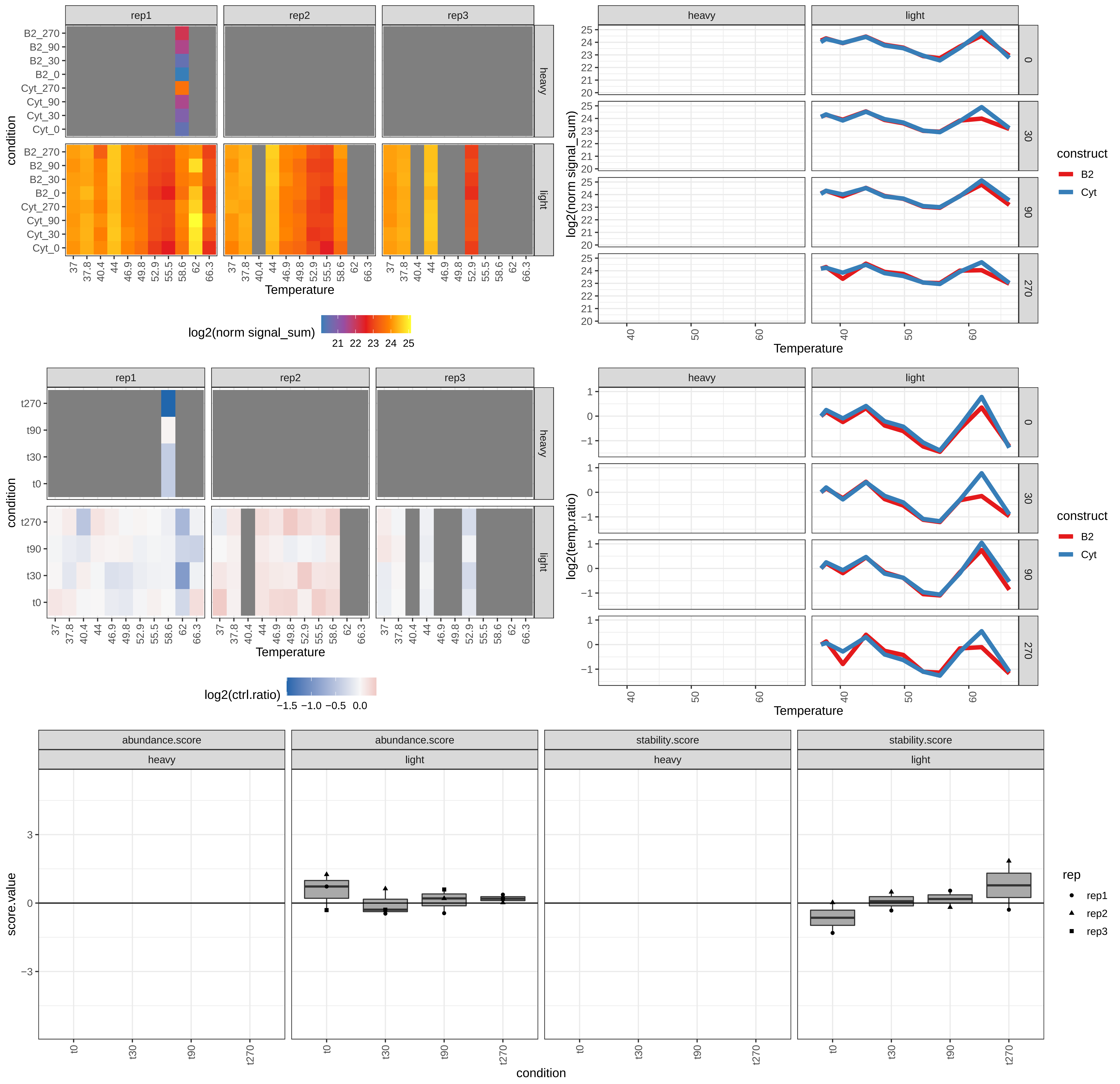

Q06224 − Endoribonuclease YSH1 OS=Saccharomyces cerevisiae (strain ATCC 204508 / S288c) GN=YSH1 PE=1 SV=1

YSP3 − average.top3: 6.7642424 − max.qupm: 6

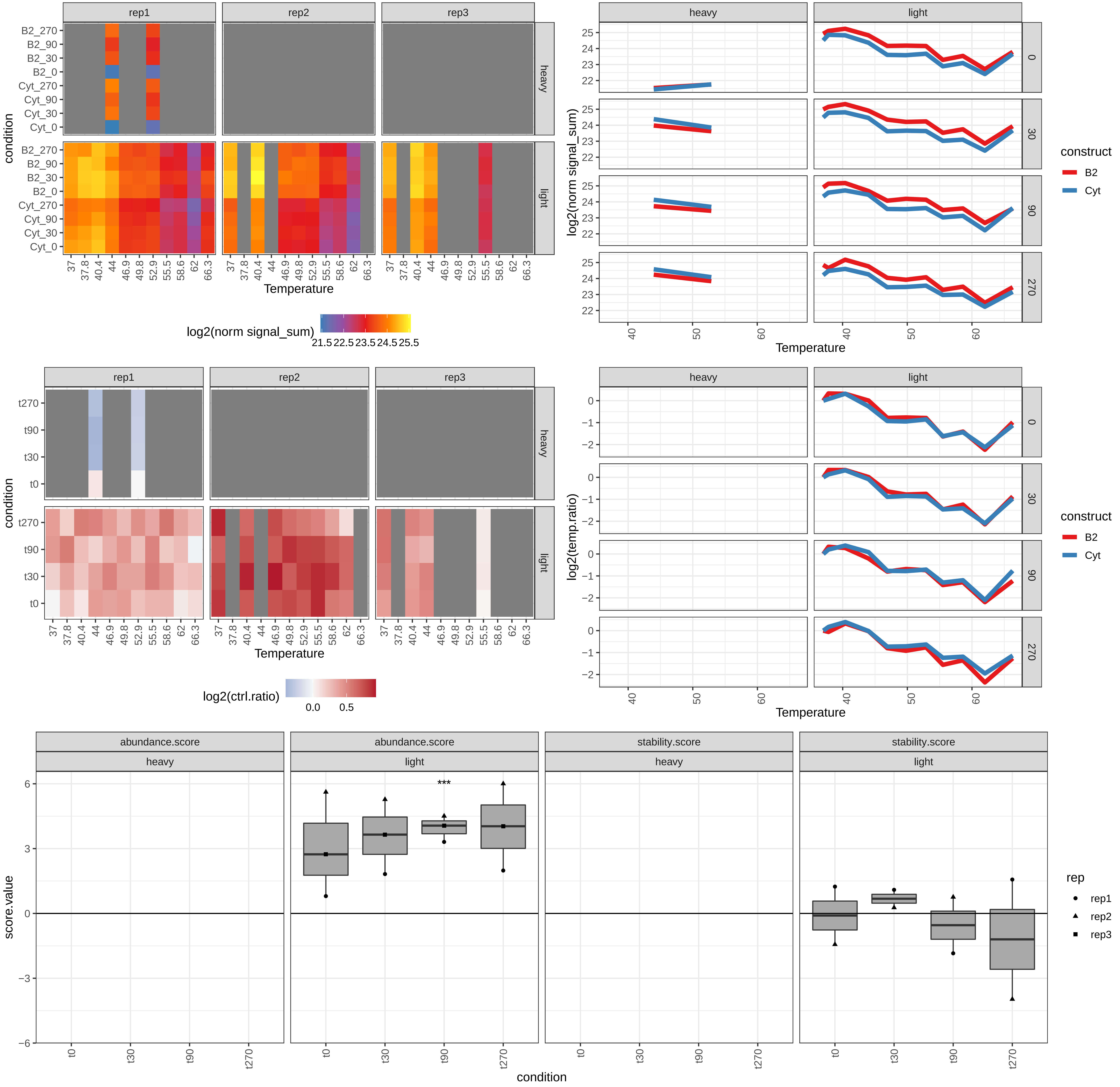

P25036 − Subtilisin−like protease 3 OS=Saccharomyces cerevisiae (strain ATCC 204508 / S288c) GN=YSP3 PE=2 SV=2

YTA12 − average.top3: 6.74010066666667 − max.qupm: 12

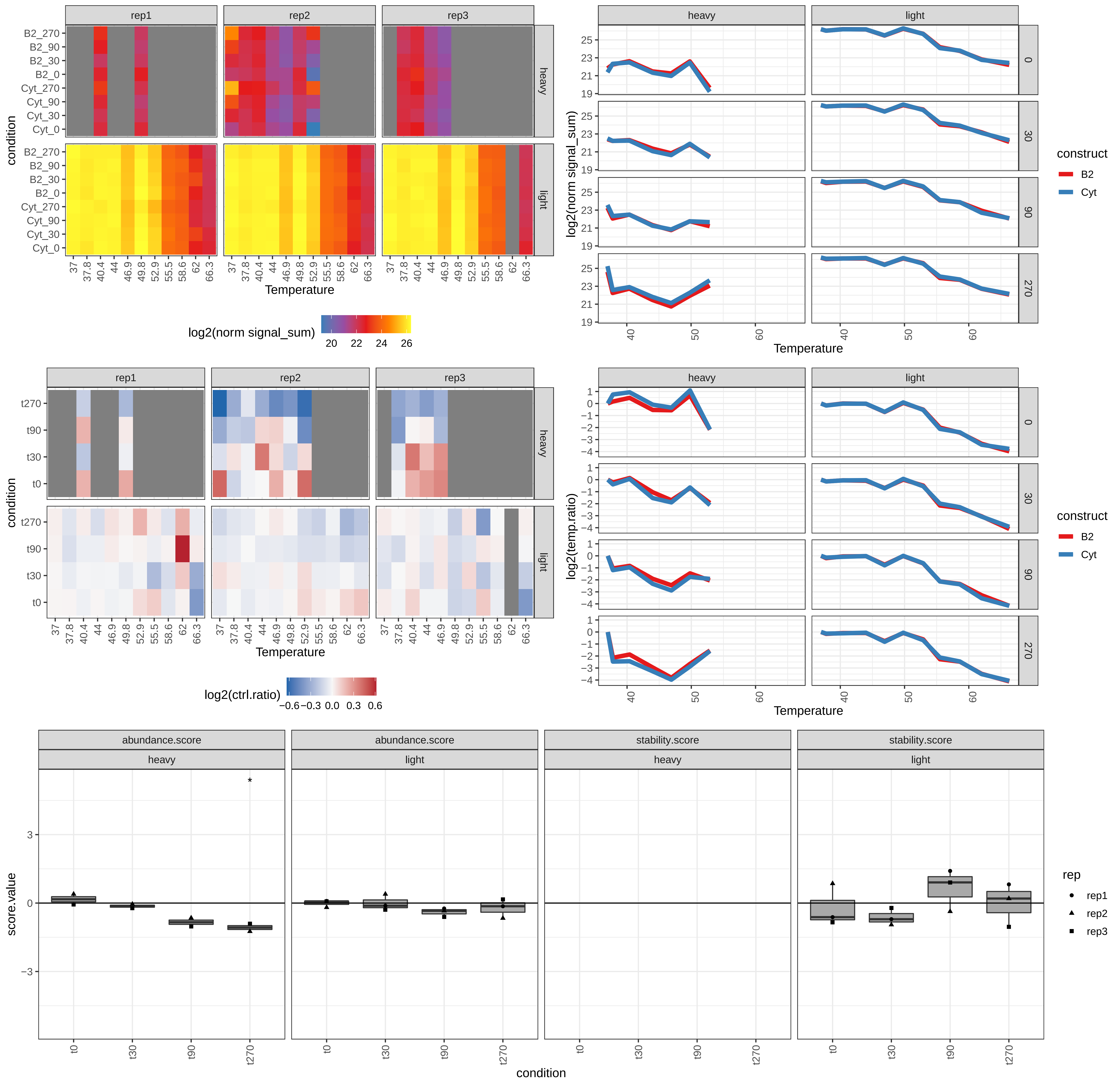

P40341 − Mitochondrial respiratory chain complexes assembly protein YTA12 OS=Saccharomyces cerevisiae (strain ATCC 204508 / S288c) GN=YTA12 PE=1 SV=2

YTA7 − average.top3: 6.73892307692308 − max.qupm: 8

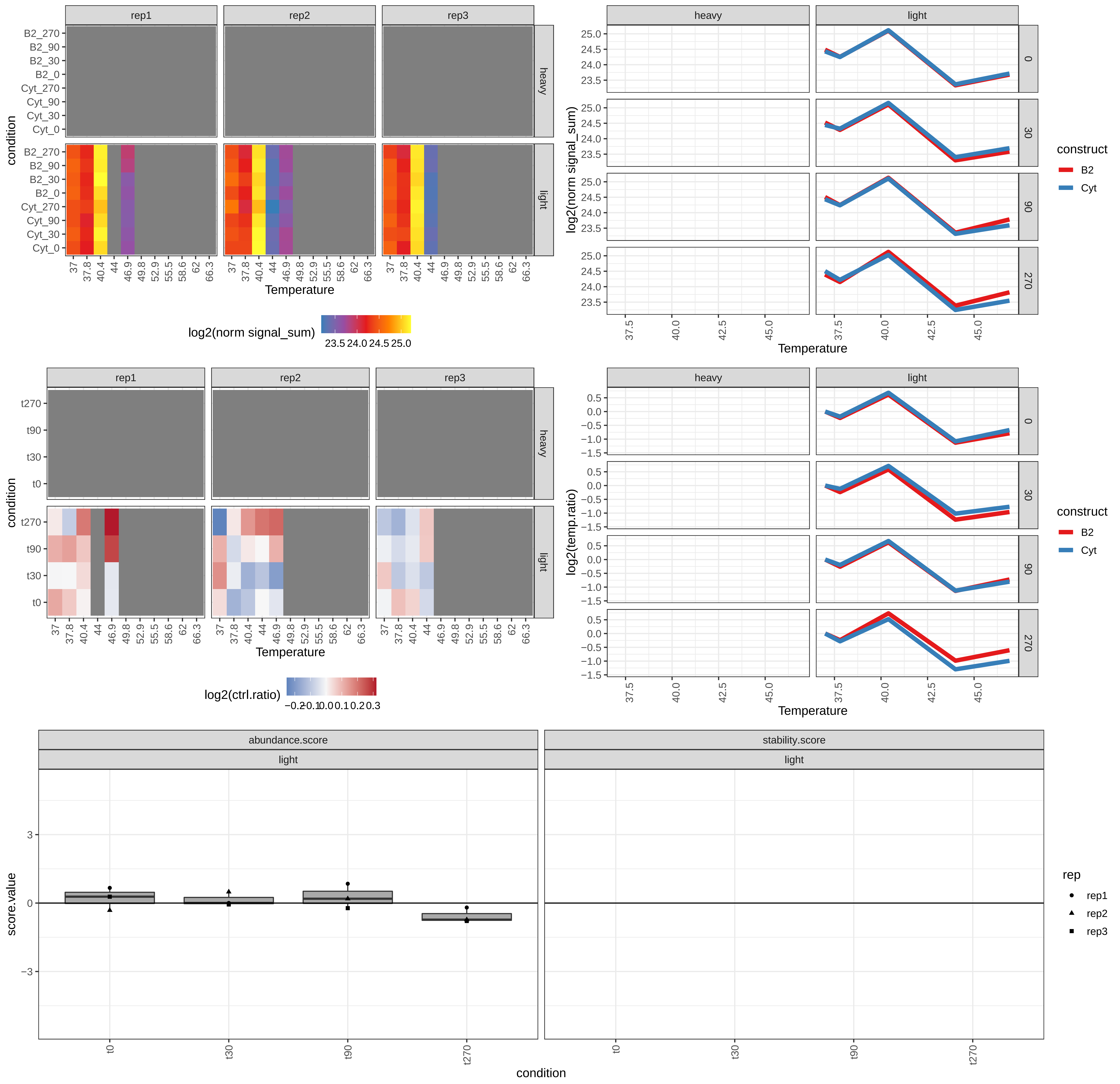

P40340 − Tat−binding homolog 7 OS=Saccharomyces cerevisiae (strain ATCC 204508 / S288c) GN=YTA7 PE=1 SV=2

YTM1 − average.top3: 6.98616827586207 − max.qupm: 10

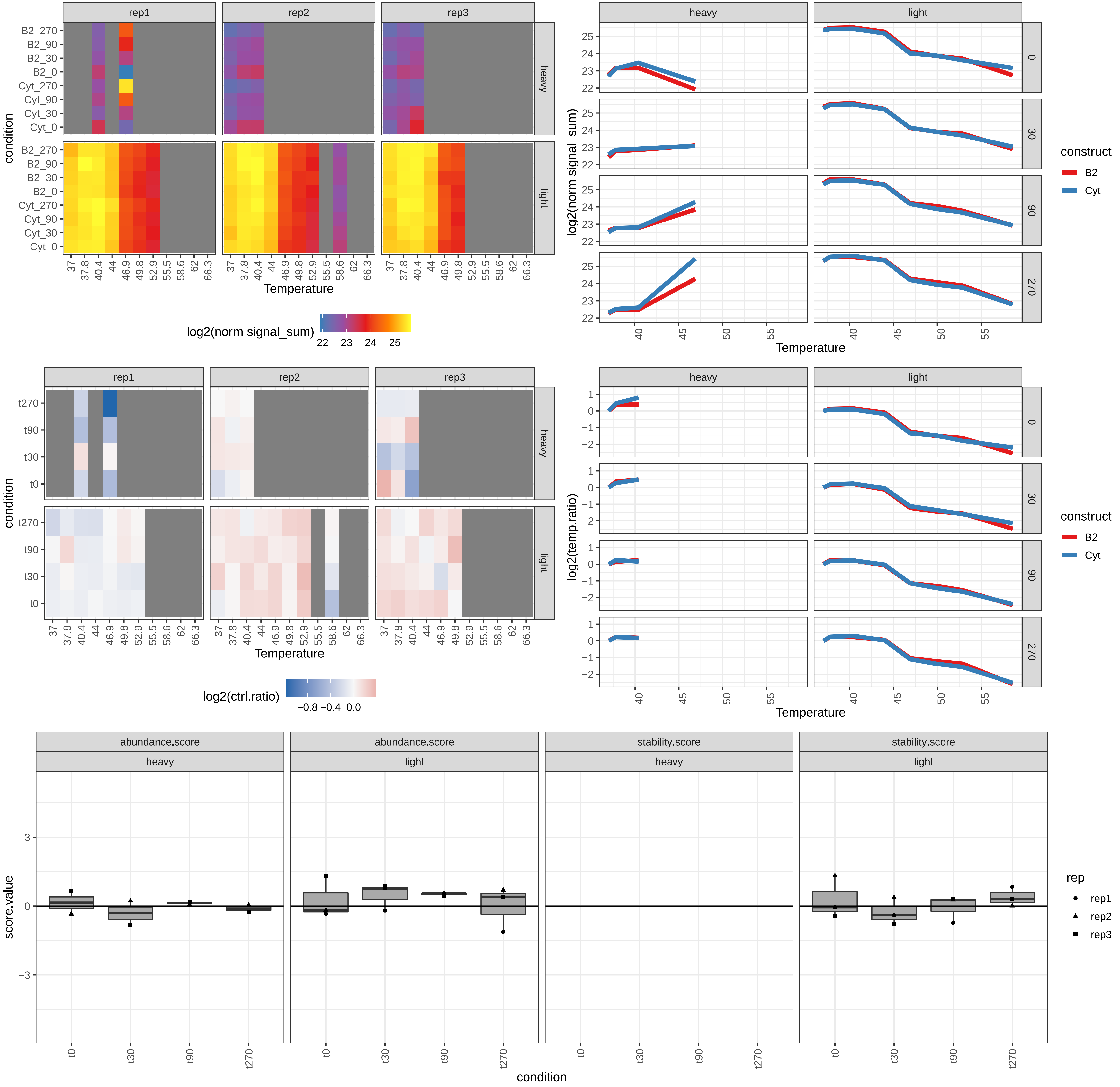

Q12024 − Ribosome biogenesis protein YTM1 OS=Saccharomyces cerevisiae (strain ATCC 204508 / S288c) GN=YTM1 PE=1 SV=1

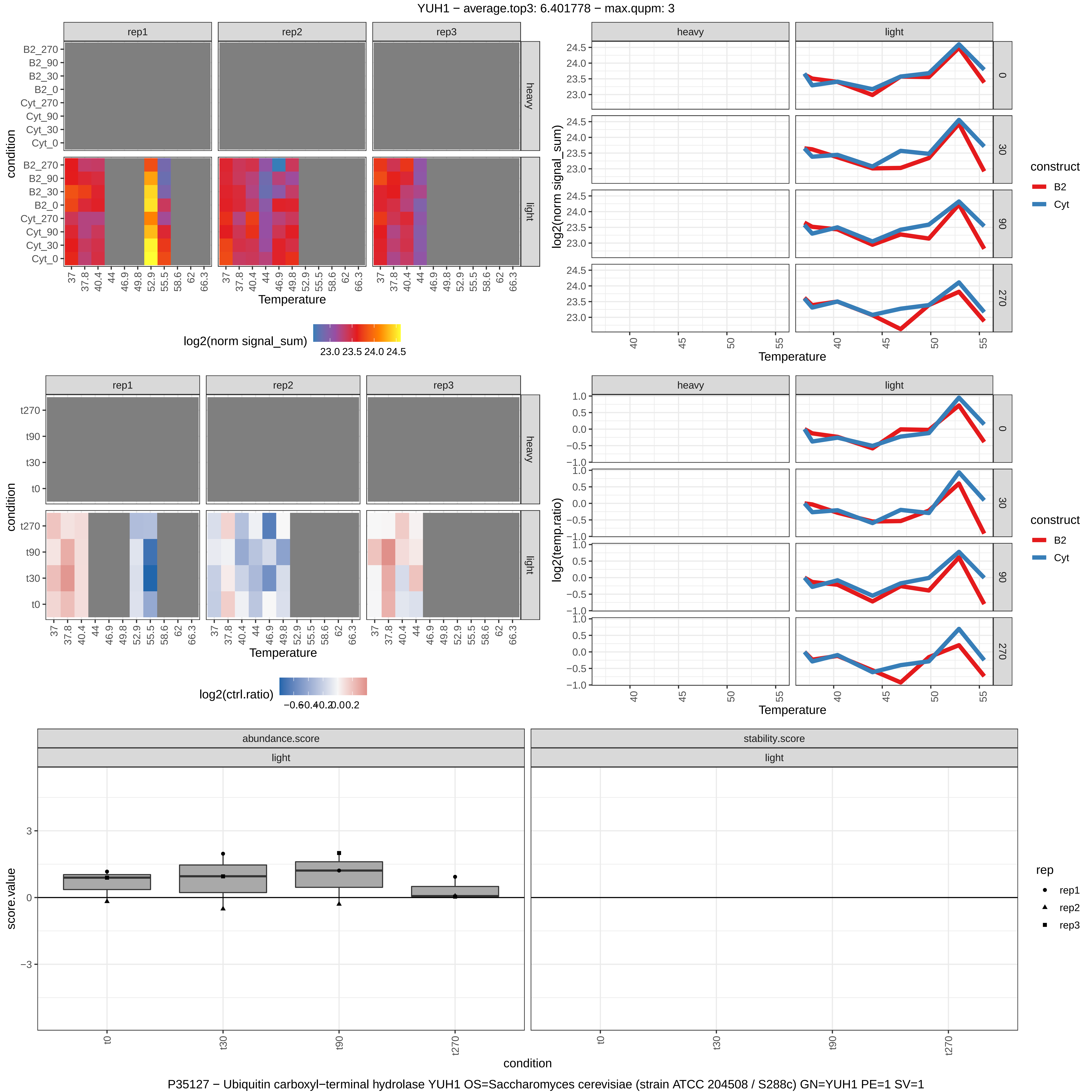
YUH1 − average.top3: 6.401778 − max.qupm: 3
rep1
rep2
rep3
heavy
light
condition
B2_270
B2_90
B2_30
B2_0
Cyt_270
Cyt_90
Cyt_30
Cyt_0
Temperature
log2(norm signal_sum)
23.0 23.5 24.0 24.5
log2(norm signal_sum)
construct
B2
Cyt
t270
t90
t30
t0
log2(ctrl.ratio)
−0.6 −0.4 −0.2 0.0 0.2
log2(temp.ratio)
abundance.score
stability.score
score.value
rep
rep1
rep2
rep3
P35127 − Ubiquitin carboxyl−terminal hydrolase YUH1 OS=Saccharomyces cerevisiae (strain ATCC 204508 / S288c) GN=YUH1 PE=1 SV=1

YUR1 − average.top3: 6.14830923076923 − max.qupm: 5

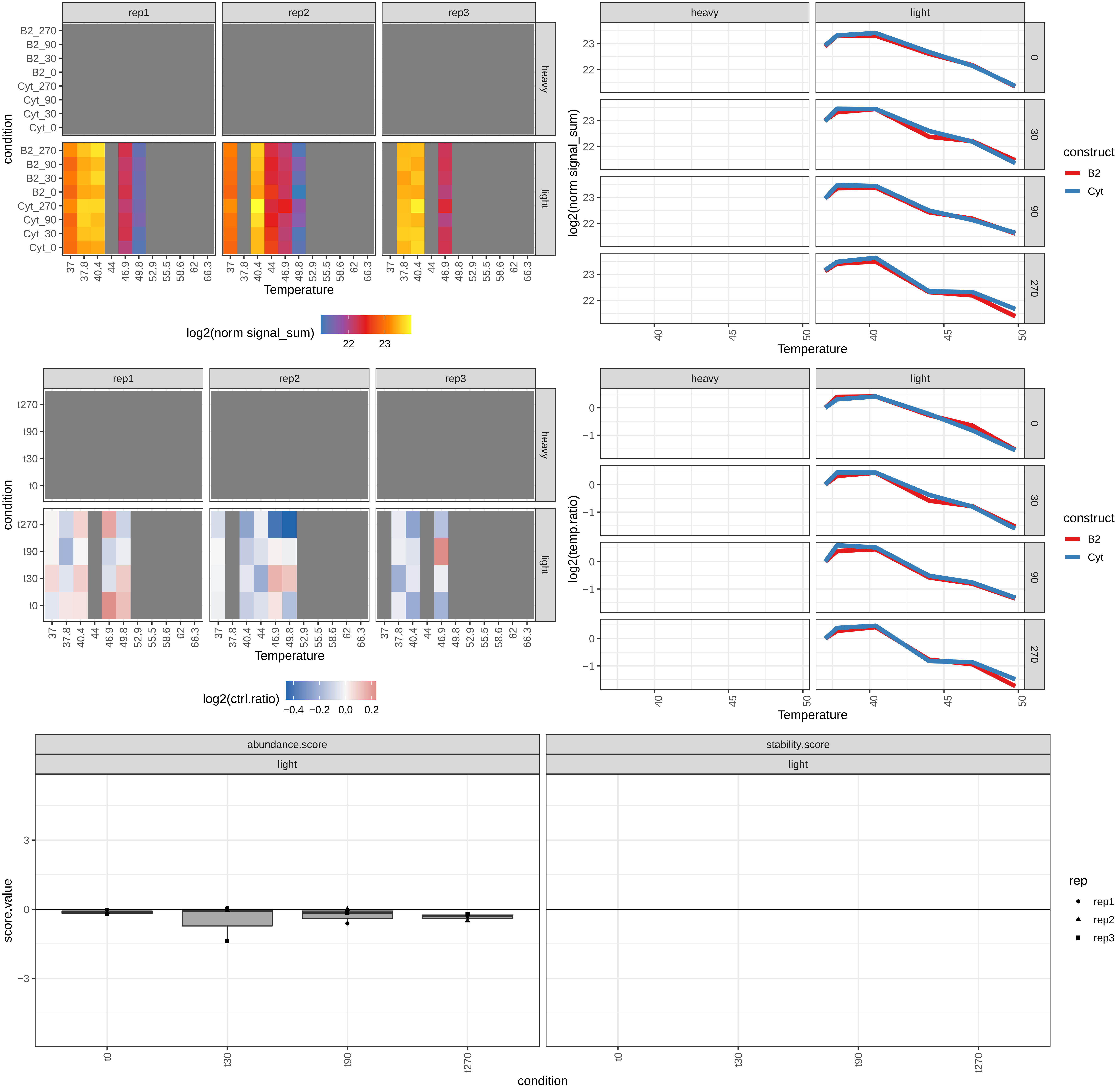

P26725 − Probable mannosyltransferase YUR1 OS=Saccharomyces cerevisiae (strain ATCC 204508 / S288c) GN=YUR1 PE=1 SV=1

YVC1 − average.top3: 6.43414444444444 − max.qupm: 3

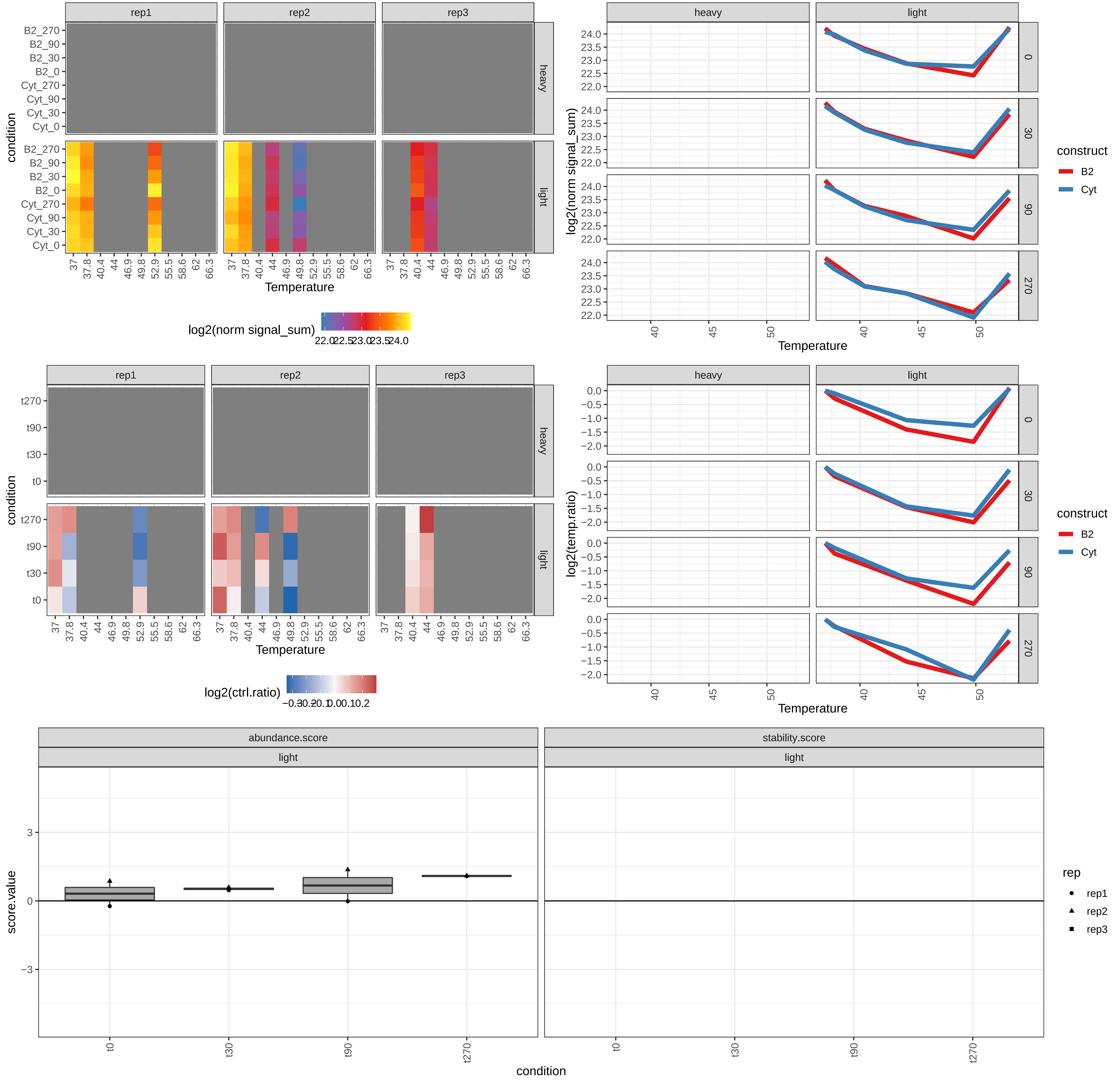

Q12324 − Calcium channel YVC1 OS=Saccharomyces cerevisiae (strain ATCC 204508 / S288c) GN=YVC1 PE=1 SV=2

YVH1 − average.top3: 6.35775933333333 − max.qupm: 5

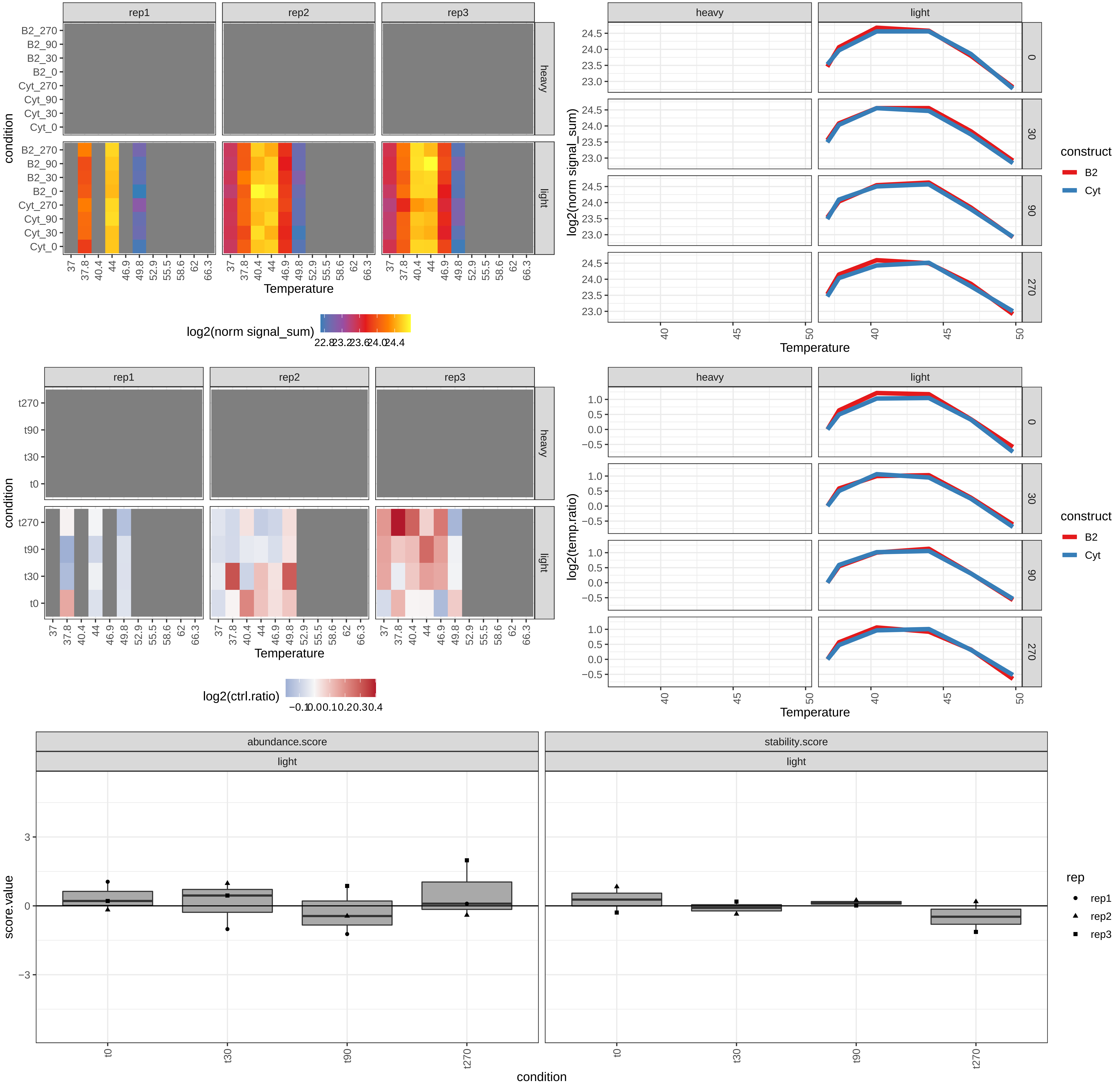

Q02256 − Tyrosine−protein phosphatase YVH1 OS=Saccharomyces cerevisiae (strain ATCC 204508 / S288c) GN=YVH1 PE=1 SV=1

ZEO1 − average.top3: 6.74250575757576 − max.qupm: 4

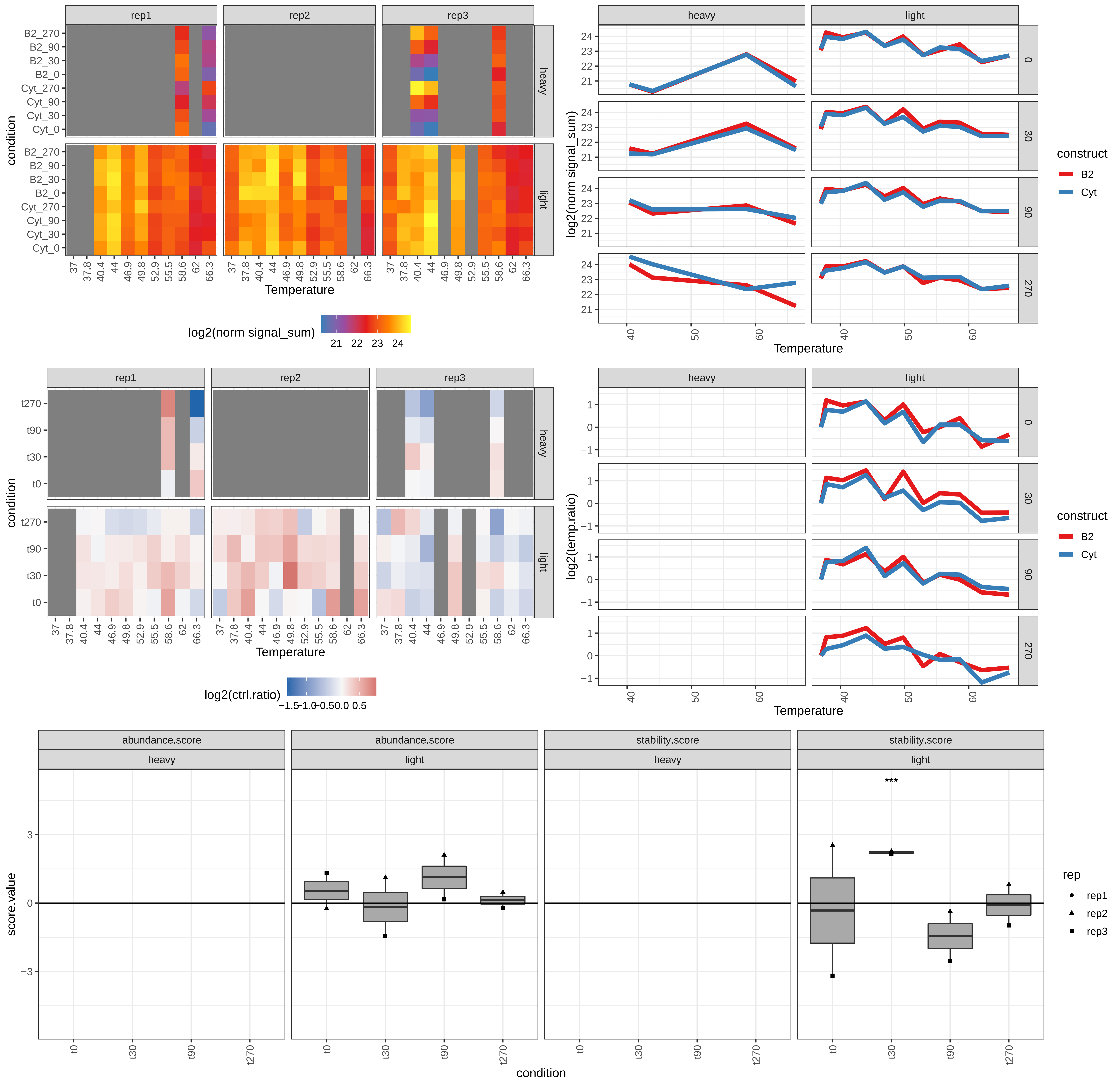

Q08245 − Protein ZEO1 OS=Saccharomyces cerevisiae (strain ATCC 204508 / S288c) GN=ZEO1 PE=1 SV=3

ZPR1 − average.top3: 6.84163977272727 − max.qupm: 10

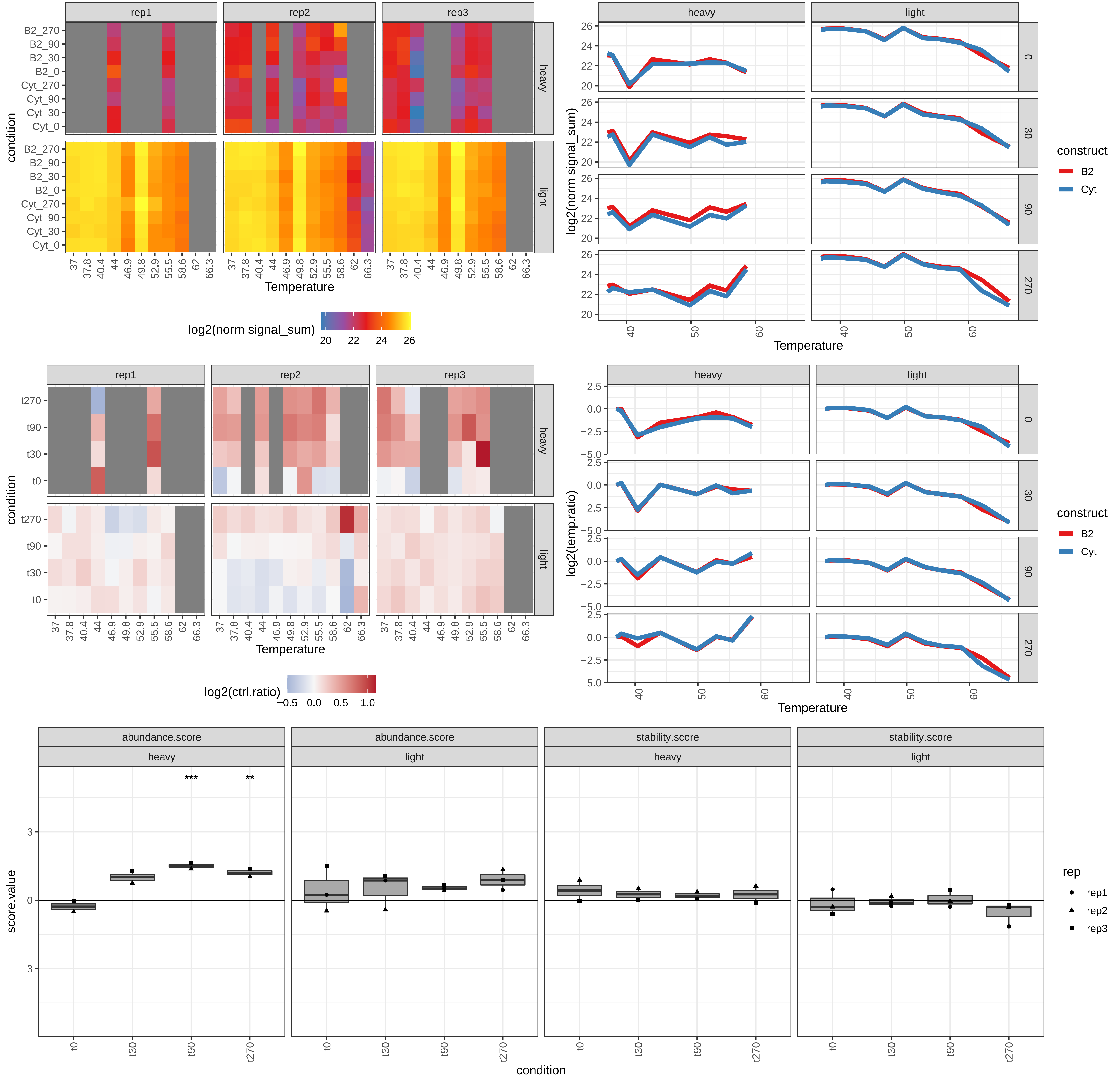

P53303 − Zinc finger protein ZPR1 OS=Saccharomyces cerevisiae (strain ATCC 204508 / S288c) GN=ZPR1 PE=1 SV=1

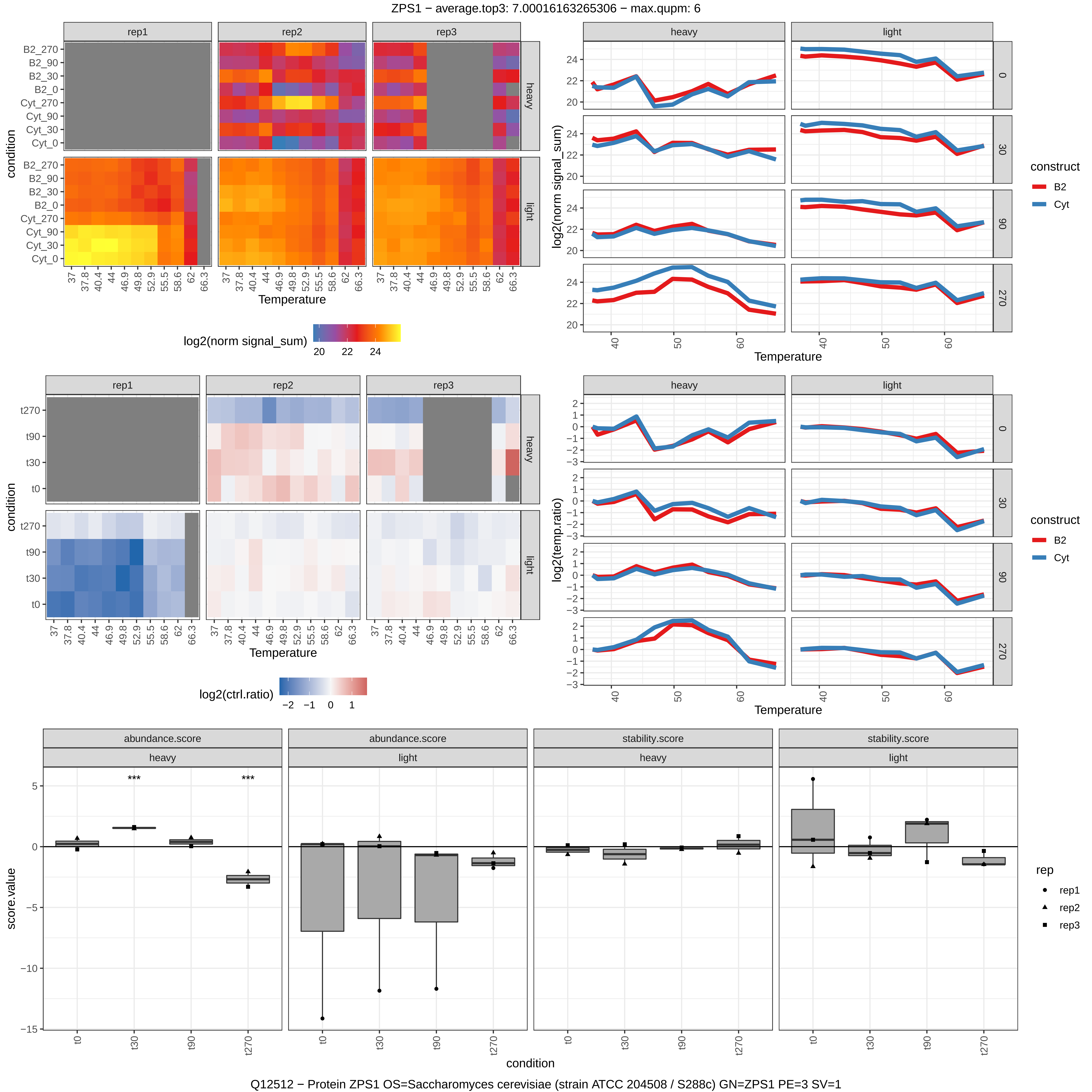
ZPS1 − average.top3: 7.00016163265306 − max.qupm: 6
rep1
rep2
rep3
heavy
light
condition
Temperature
log2(norm signal_sum)
log2(ctrl.ratio)
log2(temp.ratio)
construct
B2
Cyt
abundance.score
stability.score
score.value
rep
rep1
rep2
rep3
***
***
Q12512 − Protein ZPS1 OS=Saccharomyces cerevisiae (strain ATCC 204508 / S288c) GN=ZPS1 PE=3 SV=1

ZRT1 − average.top3: 7.278817 − max.qupm: 7

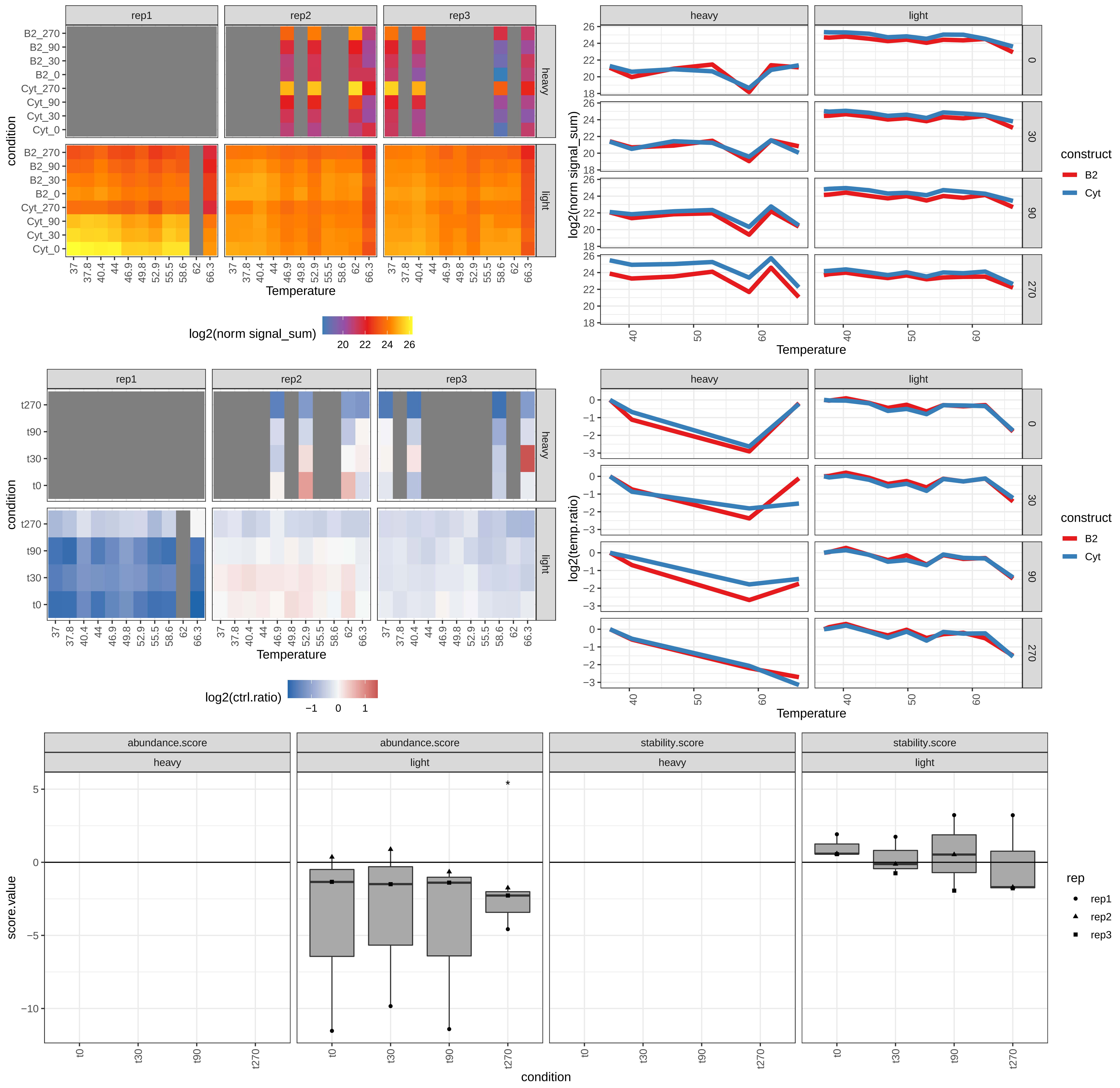

P32804 − Zinc−regulated transporter 1 OS=Saccharomyces cerevisiae (strain ATCC 204508 / S288c) GN=ZRT1 PE=1 SV=1

ZRT3 − average.top3: 6.24925809523809 − max.qupm: 6

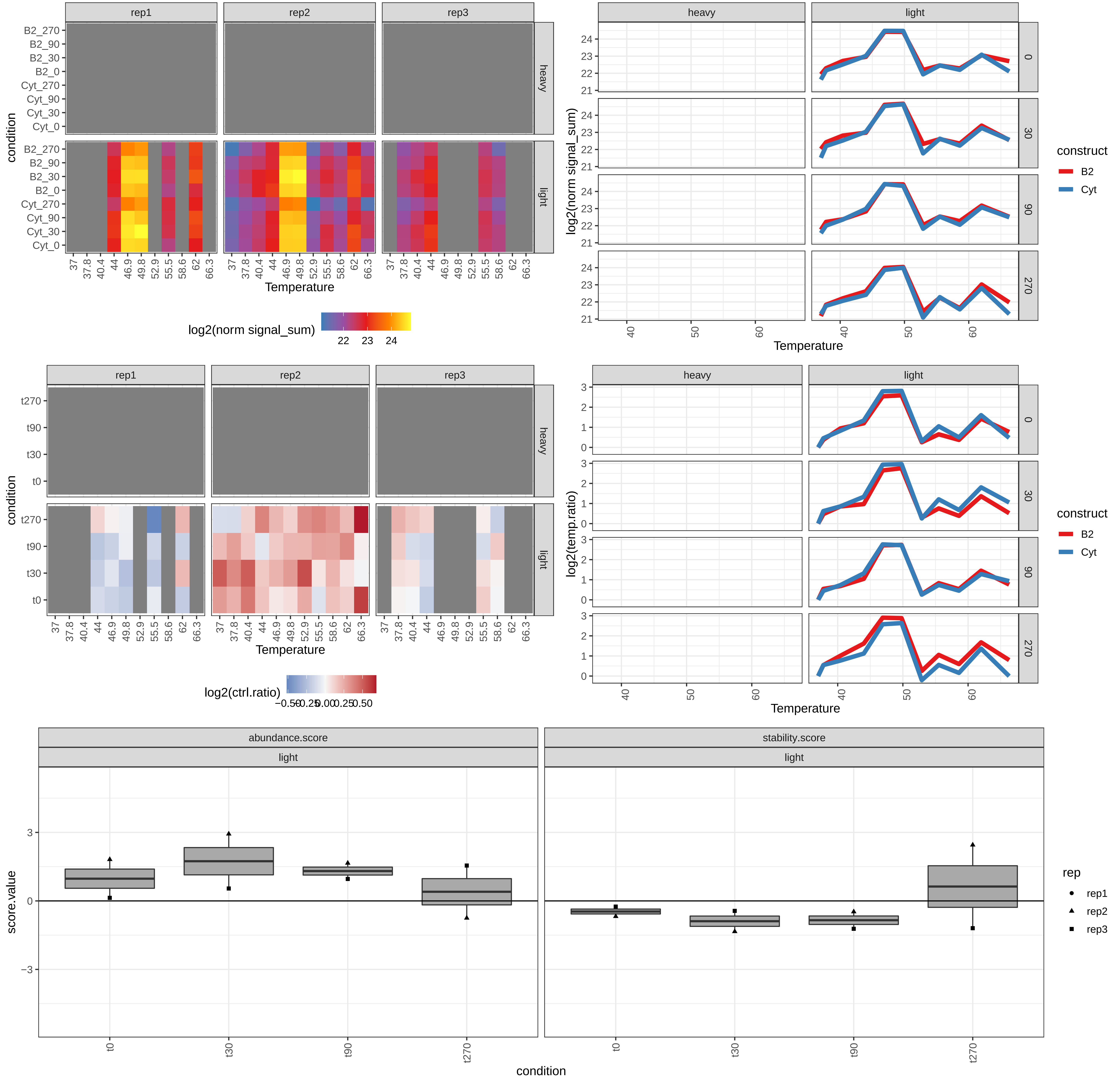

P34240 − Zinc−regulated transporter 3 OS=Saccharomyces cerevisiae (strain ATCC 204508 / S288c) GN=ZRT3 PE=1 SV=2

ZTA1 − average.top3: 7.35942770833333 − max.qupm: 14

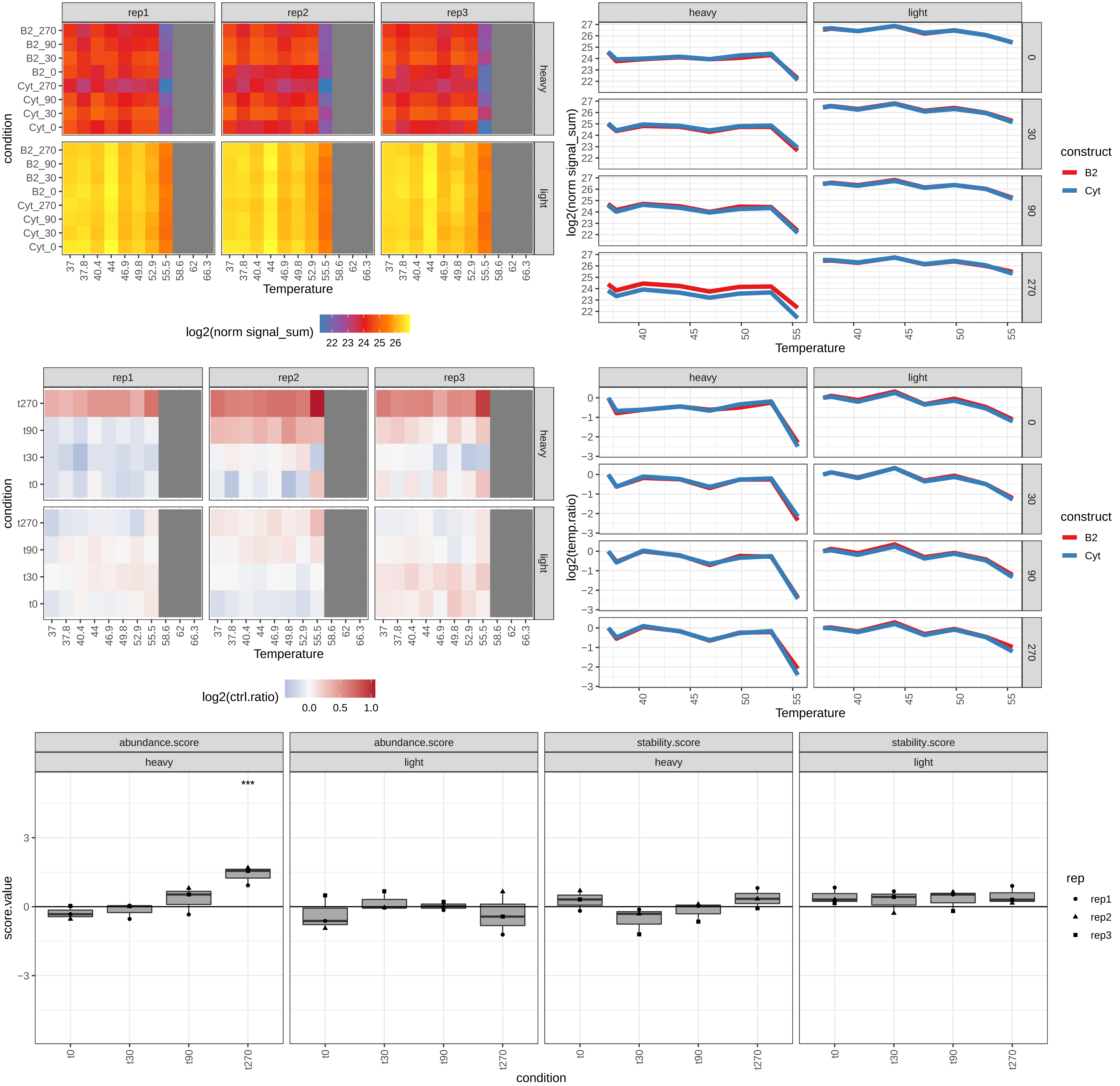

P38230 − Probable quinone oxidoreductase OS=Saccharomyces cerevisiae (strain ATCC 204508 / S288c) GN=ZTA1 PE=1 SV=1

ZUO1 − average.top3: 7.5644275862069 − max.qupm: 16

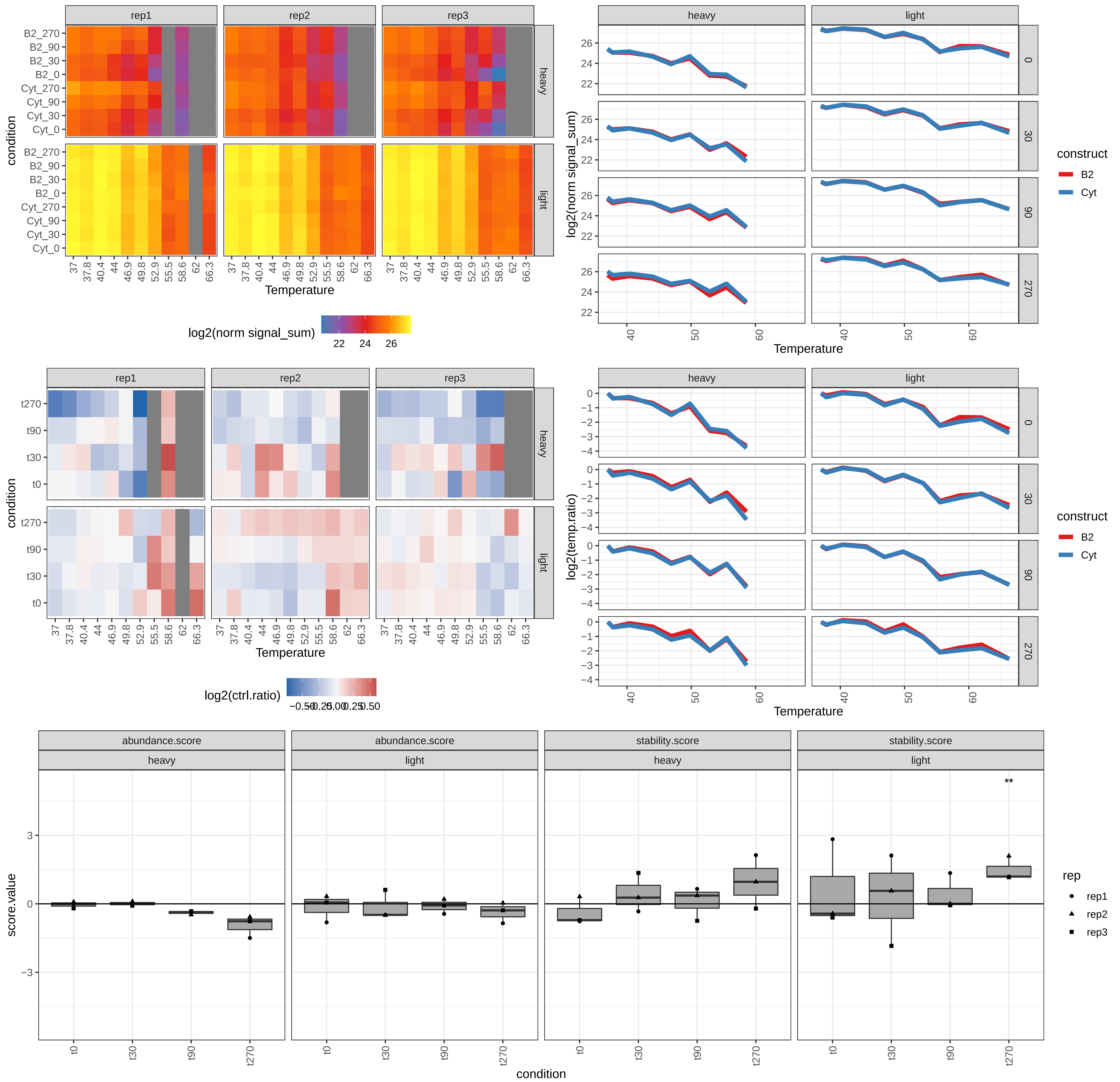

P32527 − Zuotin OS=Saccharomyces cerevisiae (strain ATCC 204508 / S288c) GN=ZUO1 PE=1 SV=1

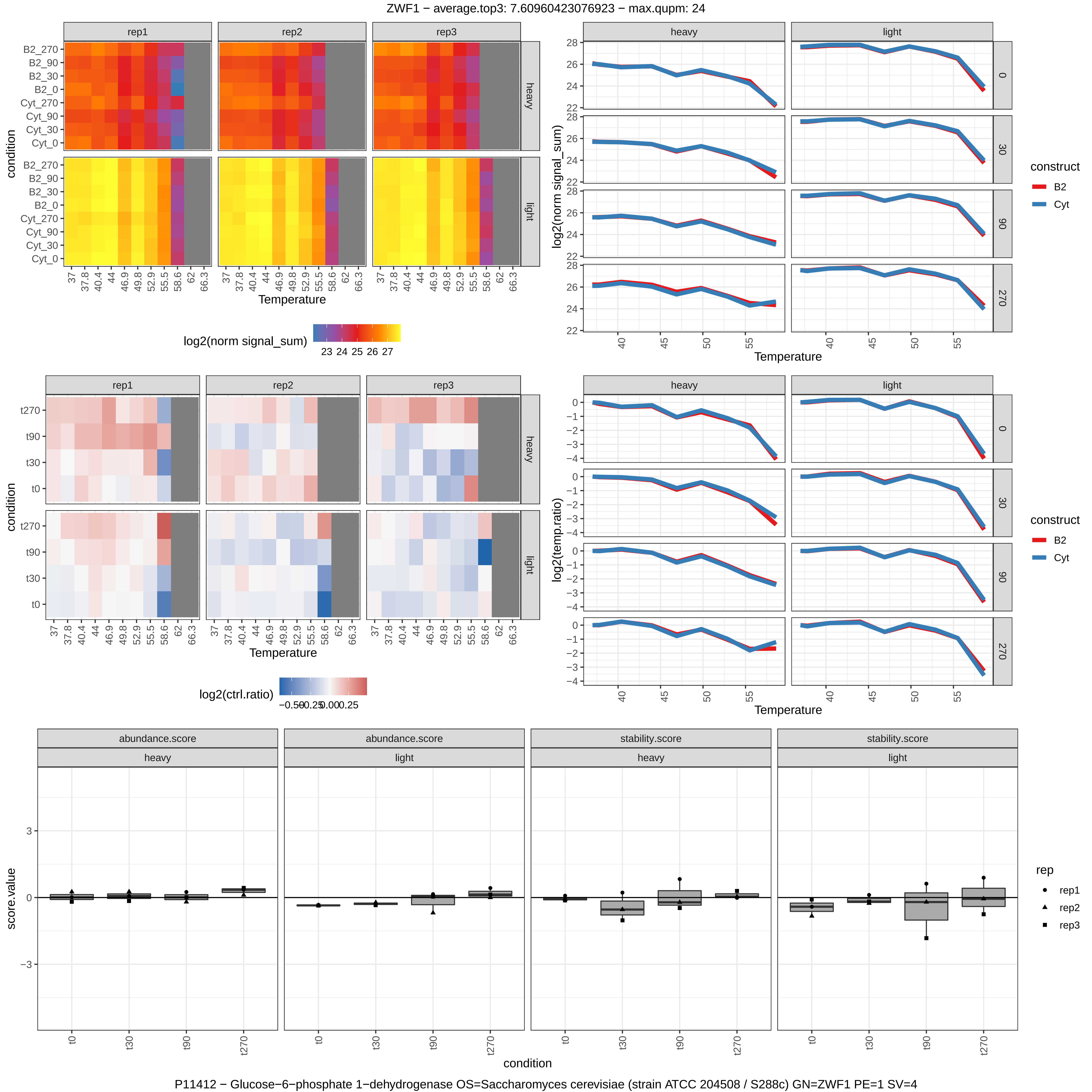
ZWF1 − average.top3: 7.60960423076923 − max.qupm: 24
rep1
rep2
rep3
heavy
light
condition
B2_270
B2_90
B2_30
B2_0
Cyt_270
Cyt_90
Cyt_30
Cyt_0
37
37.8
40.4
44
46.9
49.8
52.9
55.5
58.6
62
66.3
Temperature
log2(norm signal_sum)
23 24 25 26 27
log2(norm signal_sum)
0
30
90
270
construct
B2
Cyt
t270
t90
t30
t0
log2(ctrl.ratio)
−0.50 −0.25 0.00 0.25
log2(temp.ratio)
abundance.score
stability.score
score.value
rep
rep1
rep2
rep3
condition
P11412 − Glucose−6−phosphate 1−dehydrogenase OS=Saccharomyces cerevisiae (strain ATCC 204508 / S288c) GN=ZWF1 PE=1 SV=4
